# Systematic review with meta-analysis of the epidemiological evidence in the 1900s relating smoking to lung cancer

**DOI:** 10.1186/1471-2407-12-385

**Published:** 2012-09-03

**Authors:** Peter N Lee, Barbara A Forey, Katharine J Coombs

**Affiliations:** 1 P N Lee Statistics and Computing Ltd, Sutton, Surrey, United Kingdom

## Abstract

**Background:**

Smoking is a known lung cancer cause, but no detailed quantitative systematic review exists. We summarize evidence for various indices.

**Methods:**

Papers published before 2000 describing epidemiological studies involving 100+ lung cancer cases were obtained from Medline and other sources. Studies were classified as principal, or subsidiary where cases overlapped with principal studies. Data were extracted on design, exposures, histological types and confounder adjustment. RRs/ORs and 95% CIs were extracted for ever, current and ex smoking of cigarettes, pipes and cigars and indices of cigarette type and dose–response. Meta-analyses and meta-regressions investigated how relationships varied by study and RR characteristics, mainly for outcomes exactly or closely equivalent to all lung cancer, squamous cell carcinoma (“squamous”) and adenocarcinoma (“adeno”).

**Results:**

287 studies (20 subsidiary) were identified. Although RR estimates were markedly heterogeneous, the meta-analyses demonstrated a relationship of smoking with lung cancer risk, clearly seen for ever smoking (random-effects RR 5.50, CI 5.07-5.96) current smoking (8.43, 7.63-9.31), ex smoking (4.30, 3.93-4.71) and pipe/cigar only smoking (2.92, 2.38-3.57). It was stronger for squamous (current smoking RR 16.91, 13.14-21.76) than adeno (4.21, 3.32-5.34), and evident in both sexes (RRs somewhat higher in males), all continents (RRs highest for North America and lowest for Asia, particularly China), and both study types (RRs higher for prospective studies). Relationships were somewhat stronger in later starting and larger studies. RR estimates were similar in cigarette only and mixed smokers, and similar in smokers of pipes/cigars only, pipes only and cigars only. Exceptionally no increase in adeno risk was seen for pipe/cigar only smokers (0.93, 0.62-1.40). RRs were unrelated to mentholation, and higher for non-filter and handrolled cigarettes. RRs increased with amount smoked, duration, earlier starting age, tar level and fraction smoked and decreased with time quit. Relationships were strongest for small and squamous cell, intermediate for large cell and weakest for adenocarcinoma. Covariate-adjustment little affected RR estimates.

**Conclusions:**

The association of lung cancer with smoking is strong, evident for all lung cancer types, dose-related and insensitive to covariate-adjustment. This emphasises the causal nature of the relationship. Our results quantify the relationships more precisely than previously.

## Background

It has been known for many years that smoking causes lung cancer. An association was clearly documented in case–control studies conducted in Germany in the 1930s [1], and in the United States and Great Britain [2,3] in the 1950s, and was strengthened by surveys of large cohorts. This led the US Surgeon General to conclude in 1964 [4] that “cigarette smoking is a cause of lung cancer in men, and a suspected cause of lung cancer in women”. Further reports [5,6] have defined the relationship in more detail, and it has been estimated that, in the United States, 90% of male lung cancer deaths and 75%-80% of female lung cancer deaths are caused by smoking [7].

While some meta-analyses of the evidence have been published in recent years [8-10] none consider more than a relatively small fraction of the published evidence. We attempt to rectify this omission, though the sheer extent of the available data, and resources available, has meant limiting attention to papers published in the last century and studies involving over 100 lung cancer cases. As will be seen, this still gives us an extensive database involving almost 300 studies.

Because the relationship of smoking to the two major types of lung cancer (squamous cell carcinoma and adenocarcinoma) is known to vary [5,6], we present detailed results relating, not only to total lung cancer risk, but also to these two histological types of lung cancer. We also present some more limited results for other lung cancer types. To provide a broad description of the relationship of smoking to lung cancer, we do not concentrate on a single primary analysis, but quantify the relationships to each of a range of indices of smoking, investigating how these relationships vary according to characteristics such as sex, age, location, study design, period considered, definition of exposure and extent of confounder adjustment. The style of this systematic review is similar to one we have recently published for smoking and COPD, chronic bronchitis and emphysema [11].

## Methods

Full details of the methods used are described in Additional file 1: Methods, and are summarized below. Throughout this paper, we use the term relative risk (RR) to include its various estimators, including the odds ratio and the hazard ratio.

### Inclusion and exclusion criteria

Attention was restricted to epidemiological prospective or case–control studies published up to and including 1999, which involved 100 lung cancers or more, and which provided RR estimates for one or more defined major, cigarette-type or dose-related smoking indices. The “major indices” compare ever, current or ex smoking with never or non-current smoking, and refer to smoking of any product, cigarettes, pipes, cigars and combinations, or of specific types of cigarette. The “cigarette type indices” compare smokers of different types of cigarette – filter with plain, manufactured with handrolled and mentholated with non-mentholated. The “dose-related indices” concern amount smoked, age of starting to smoke, duration of smoking, duration of quitting, tar level, butt length or fraction smoked. Pack-years was not considered as it was felt more important to separate effects of extent and duration of exposure. Uncontrolled case studies were not included. There were no further exclusion criteria.

### Literature searching

Between 1997 and 2001 potentially relevant papers were sought from Medline and Emtree searches, from British Library monthly bulletins, from files on smoking and health accumulated over many years by P N Lee Statistics and Computing Ltd, and from references cited in papers obtained, until ultimately no paper examined cited a paper of possible relevance not previously examined.

### Identification of studies

Relevant papers were allocated to studies, noting multiple papers on the same study, and papers reporting on multiple studies. Each study was given a unique reference code (REF) of up to 6 characters (e.g. COMSTO or LUBIN2), based on the principal author’s name and distinguishing multiple studies by the same author.

Some studies were noted as having overlaps with other studies. To minimize problems in meta-analysis arising from double-counting of cases, overlapping studies were divided into two categories, as shown in Additional file 2: Studies. The first category involved minor overlap, which could not be disentangled, and which it was decided to ignore. The second category contains sets of studies which probably or definitely overlap. Here the set member containing the most comprehensive data (e.g. largest number of cases or longest follow-up) was called the ‘principal study’, other members being ‘subsidiary studies’ only considered in meta-analyses where the required RR was unavailable from the principal study.

### Data recorded

Relevant information was entered onto a study database and two linked RR databases. Data entry was carried out in two stages. In 1997–2002, data were entered on the first RR database for the major smoking indices, cigarette type indices, and amount smoked. In 2009–2010, data were entered on the second RR database for the remaining dose-related indices.

The study database contains a record for each study, describing the following aspects: relevant publications; study title; study design; sexes considered; age range, race(s) and other details of the population studied; location; timing and length of follow-up; whether principal or subsidiary, with details of overlaps or links with other studies; number of cases and extent of histological confirmation; number of controls or subjects at risk; types of controls and matching factors used in case–control studies; use of proxy respondents, interview setting and response rates; confounding variables considered; availability of results by histological types; and availability of results for all smoking indices (including those indices not considered here, such as pack-years).

The RR databases hold the detailed results, typically containing multiple records for each study. Each record is linked to the relevant study and refers to a specific RR, recording the comparison made and the results. This record includes the sex, age range, race, lung cancer type, and (for prospective studies) the follow-up period. The smoking exposure of the numerator of the RR is defined by the smoking status (ever, current or ex), smoking product (e.g. any, cigarettes, cigarettes only, pipes only) and cigarette type (e.g. any, mainly hand-rolled cigarettes, filter cigarettes only, mentholated cigarettes). Similar information is recorded about the denominator of the RR. For dose-related indices, the level of exposure is recorded. The source of the RR is also recorded, as are details on adjustment variables. Results recorded include numbers of cases for the numerator and denominator, and, for unadjusted results, numbers of controls, persons at risk or person-years at risk. The RR itself and its lower and upper 95% confidence limits (LCL and UCL) are always recorded. These may be as reported, or derived by various means (see below), with the method of derivation noted.

### Identifying which RRs to enter

RRs were entered relating to defined combinations of lung cancer type, smoking index (major, cigarette type or dose-related), confounders adjusted for, and strata, as described below.

#### Lung cancer type

Results were entered for all lung cancer, for Kreyberg I (as originally presented, or by combining squamous, small and large cell carcinoma) and Kreyberg II (as originally presented, or by combining adenocarcinoma and others not in Kreyberg I), and for squamous, small, and large cell carcinoma and for adenocarcinoma separately. Additionally, the following groups were constructed if not originally presented: all lung cancer or nearest equivalent, but at least squamous cell carcinoma and adenocarcinoma; squamous cell carcinoma or nearest equivalent; adenocarcinoma or nearest equivalent.

#### Major and cigarette type smoking indices

The intention was to enter RRs comparing current smokers, ever smokers or ex smokers with never or non smokers. Near-equivalent definitions were accepted when stricter definitions were unavailable, so that, for example, never smokers could include occasional smokers (or exceptionally, light smokers), while current smokers could include, and ex-smokers exclude, recent quitters. RRs were to be entered relating to smoking of defined products and, when the product related to cigarette smoking, to defined cigarette types (see also Additional file 1: Methods). If available, results (for each of current, ex and ever smoking) were entered for five comparisons: any product vs. never any product, cigarettes vs. never any product, cigarettes only vs. never any product, cigarettes vs. never cigarettes, and cigarettes only vs. never cigarettes (and also for five equivalent comparisons for current vs non smoking). Here “cigarettes” ignores whether other products (i.e. pipes and cigars) are also smoked, while “cigarettes only” excludes mixed smokers. Additionally, when the numerator related to the smoking of filter, handrolled or mentholated cigarettes, RRs were entered with the denominator defined as relating to plain, manufactured or non-mentholated smokers respectively.

#### Dose-related smoking indices

RRs were entered for seven measures: amount smoked, age of starting, duration of smoking, duration of quitting, tar level, butt length and fraction smoked. RRs were expressed relative to never smokers (or near equivalent), if available, or relative to non smokers otherwise. For duration of quitting, RRs were also expressed relative to current smokers. Except for amount smoked, further RRs were entered, restricted to smokers, and expressed relative to the level expected to have the lowest risk (e.g. shortest duration or latest age started).

#### Confounders adjusted for

For case–control studies, results were entered adjusted for the greatest number of potential confounding variables for which results were available, and also unadjusted (or adjusted for the smallest number of confounders). For prospective studies, results were entered adjusted for age and the greatest number of confounders, and for age only or age and the smallest number of confounders, with unadjusted results entered only if no age-adjusted results were available. These alternative RRs are subsequently referred to as “most-adjusted” and “least-adjusted”. For dose-related RRs restricted to smokers, results with “most adjustment” but without adjustment for other aspects of smoking were also entered if available.

#### Strata

Three strata were considered – sex, age and race. Results were entered for males and females separately when available, with combined sex results only entered when sex-specific results were not available. Results were entered for all ages combined and for individual age groups, and for all races and for individual racial groups.

### Derivation of RRs

Adjusted RRs and their 95% CIs were entered as provided, when available. Unadjusted RRs and CIs were calculated from their 2 × 2 table, using standard methods (e.g. [12]), noting any discrepancies between calculated values and those provided by the author. Sometimes the 2 × 2 table was constructed by summing over groups (e.g. adding current and ex smokers to obtain ever smokers) or from a percentage distribution. Various other methods were used as required to provide estimates of the RR and CI. Some more commonly used methods are summarized below, fuller details being given in Additional file 1: Methods.

#### Correction for zero cell

If the 2 × 2 table has a zero cell, 0.5 was added to each cell, and the standard formulae applied.

#### Combining independent RRs

RRs were combined over ℓ strata (e.g. from a 2 × 2 × ℓ table) using fixed-effect meta-analysis [13], giving an estimate adjusted for the stratifying variable.

#### Combining non-independent RRs

The Hamling et al. method [14] was used (e.g. to derive an adjusted RR for ever smokers from available adjusted RRs for current and ex smokers, each relative to never smokers, or to combine adjusted RRs for several histological types, each relative to a single control group).

#### Estimating CI from crude numbers

If an adjusted RR lacked a CI or p-value but the corresponding 2 × 2 table was available, the CI was estimated assuming that the ratio UCL/LCL was the same as for the equivalent unadjusted RR.

### Data entry and checking

Master copies of all the papers in the study file were read closely, with relevant information highlighted to facilitate checking. Where multiple papers are available for a study, a principal publication was identified, although details described only in other publications were also recorded. Preliminary calculations and data entry were carried out by one author and checked by another, and automated checks of completeness and consistency were also conducted. RR/CIs underwent validation checks [15].

### Meta-analyses conducted – overview

A pre-planned series of meta-analyses was conducted for various smoking indices for each of the three main outcomes (all lung cancer, squamous cell carcinoma, and adenocarcinoma) and also for some indices for two other outcomes (large cell carcinoma and small cell carcinoma). Nearest equivalent definitions are allowed for the three main outcomes, with the terms “squamous” and “adeno” used subsequently to distinguish these results from those specifically for these cell types. Each meta-analysis was repeated, based on most-adjusted RRs and on least-adjusted RRs. For each meta-analysis conducted, combined estimates were made first for all the RRs selected, then for RRs subdivided by level of various characteristics, testing for heterogeneity between levels.

### Selecting RRs for the meta-analyses

All meta-analyses are restricted to records with available RR and CI values. The process of selecting RRs for inclusion in a meta-analysis must try to include all relevant data and to avoid double-counting. For a given analysis (e.g. of current cigarette smoking), several definitions of RR may be acceptable (e.g. cigarette smoking, or cigarette only smoking), so, for studies with multiple RRs, the one to be used is determined by a preference order defined for the meta-analysis. Preference orders may be required for smoking status, smoking product, the unexposed base, and extent of confounder adjustment. As the definitions of RR available may differ by sex (e.g. a study may provide RRs for any product smoking for males, but only for cigarette smoking for females), the RRs chosen for each sex may not necessarily have the same definition. Sexes combined results are only considered where sex-specific results are not available. Similarly RRs from a subsidiary study are only used where eligible RRs are unavailable from the principal study. When multiple preference orders are involved, the sequence of implementation may affect the selection, so preferences for the most important aspects, usually concerning smoking, are implemented first.

### Carrying out the meta-analyses

Fixed-effect and random-effects meta-analyses were conducted using the method of Fleiss and Gross [13], with heterogeneity quantified by H, the ratio of the heterogeneity chisquared to its degrees of freedom, which is directly related to the statistic I^2^[16] by the formula I^2^ = 100 (H-1)/H. For all meta-analyses, Egger’s test of publication bias [17] was also included.

Meta-analyses were conducted in various sets (A to N) corresponding to the sub-sections of the results section of the paper. A full list of the analyses is given in Additional file 1: Methods.

#### The major smoking indices

For the major smoking indices, the first four sets of meta-analyses relate to: A ever smoking, B current smoking, C ever smoking (but with current smoking used if ever smoking not available), referred to subsequently as “ever/current” smoking, and D ex smoking. In what is referred to as the main analysis in each set, smoking of any product is preferred by selecting RRs in the following preference order: 1. smoking of any product vs. never smoked any product; 2. smoking of cigarettes vs. never smoked any product, 3. smoking of cigarettes only vs. never smoked any product; 4. smoking of cigarettes vs. never smoked cigarettes; 5. smoking of cigarettes only vs. never smoked cigarettes; with options 6–10 the same as options 1–5 except that “never smoked” is replaced by “never smoked near equivalent”. A variant analysis prefers cigarette smoking (by changing the preference order to 4, 5, 2, 3, 1, 9, 10, 7, 8, 6). In meta-analyses of type C, a further variant analysis reverses the preference so current smoking results are preferred to those for ever smoking, referred to subsequently as “current/ever” smoking. Other variant analyses are based on RRs for specified age ranges.

A further set of meta-analyses, E, concerns smoking of pipes and/or cigars (but not cigarettes), referred to subsequently as smoking of “pipes/cigars only”, smokers of pipes only, smokers of cigars only, and smokers of cigarettes and pipes/cigars (“mixed” smokers). Separate meta-analyses were conducted for ever smoking, current smoking, ever/current smoking, current/ever smoking and ex smoking.

#### The cigarette type indices

Meta-analyses were conducted, in set F, for only filter vs. only plain, ever filter vs. only plain, only filter vs. ever plain, handrolled vs. manufactured, and mentholated vs. non-mentholated. These were only conducted for ever/current smoking, and preferring RRs for cigarettes over RRs for cigarettes only. The analyses with only filter as the numerator used the preference order of filter only, always, mainly, both, equally, and ever, while the analyses with ever filter as the numerator used the reverse preference. Similar preference orders applied to the denominators. The analyses of handrolled vs. manufactured cigarettes used the preference order of any, both, mainly, and only for handrolled, and only ever, only current, any and ever for manufactured.

#### The dose-related smoking indices

For the dose-related indices, sets of meta-analyses were conducted for: G amount smoked, H age of starting to smoke, I duration of smoking, J duration of quitting compared to never smokers (or long-term ex smokers), K duration of quitting compared to current smokers (or short-term quitters), L tar level, and M butt length or fraction smoked (taking short butt length as being equivalent to a large fraction smoked). For any measure, a study typically provides a set of non-independent RRs for each dose-category, expressed relative to a common base. To avoid double-counting only one was included in any one meta-analysis. Two approaches were adopted. The first involves specifying a scheme with a number of levels of exposure (“key values”), then carrying out meta-analyses for each level in turn, expressed relative to never smokers. For an RR to be allocated to a key value, its dose-category has to include that key-value and no other. Schemes with a few, widely spaced, key values tend to involve more studies, whereas schemes with more key values, closely spaced, involve RRs from fewer studies, but ones with dose categories more closely clustered around the key value. The sets of key values used (with 999 indicating an open-ended category) were 5, 20, 45 and 1, 10, 20, 30, 40, 999 for amount smoked; 26, 18, 14 and 30, 26, 22, 18, 14, 10 for age of starting to smoke; 20, 35, 50 and 5, 20, 30, 40, 50, 999 for duration of smoking; 12, 7, 3 and 20, 12, 3 for duration of quitting vs. never; and 3, 7, 12 and 3, 12, 20 for duration of quitting vs. current. No key value analysis was conducted for tar level, or for butt length/fraction smoked. The second approach (not conducted for amount smoked) involves meta-analysing of RRs for the highest compared with the lowest categories of exposure within smokers available for each study.

### Meta-regression analyses

While full multivariable analysis of the data is considered beyond the scope of this report, meta-regression analyses were also carried out using the sets of RRs selected for the main meta-analyses for ever smoking and for current smoking. Following preliminary meta-regressions (not shown), a “fixed model” was fitted to examine the effect on the results of six different categorical variables (sex, location, start year of study, major study type, number of lung cancer cases and number of adjustment factors). Note that the number of lung cancer cases (in the study as a whole), which is referred to subsequently as “number of cases”, is used as an indicator of study size. The significance of each of these variables was estimated by an F-test based on the increase in deviance resulting from its exclusion from the basic model. A list of secondary variables was also defined (relating to more detailed aspects of location, outcome, study type and confounder adjustment, national cigarette tobacco type, the product smoked, the denominator used in the RR, use of proxy respondents, whether the study required 100% histological confirmation of lung cancer, whether the population studied worked in risky occupations, the age of the subjects, and the derivation of the RR) with the significance of adding each characteristic to the fixed model estimated by an F-test based on the increase in deviance. Fuller details are given in Additional file 1: Methods.

### Additional analyses

Additional tests of the relationship of lung cancer risk to various characteristics of interest were based on corresponding pairs of RR and CI estimates within the same study for the same definition of outcome and exposure, and deriving the ratio of the two RRs. Where the pairs involved independent sets of subjects, the variance of the ratio was also derived, and meta-analyses of the ratio were conducted. Where the pairs involved non-independent sets of subjects the numbers of ratios greater and less than 1 were compared using the sign test. Tests of independent pairs related to sex (males vs. females), age (oldest vs. youngest age group) and race (white people vs. non-white or black people). Tests of non-independent pairs related to level of adjustment (most-adjusted vs. least-adjusted), and to comparisons of product smoked (mixed smokers vs. cigarette only smokers, and vs. smokers of pipes/cigars only). Tests were always carried out for all lung cancer and ever/current smoking. For sex, additional analyses were conducted for current and for ever smoking, for squamous and adeno, and also within level of amount smoked. For level of adjustment, two sets of analyses were run. The first, relating to RRs for ever/current smoking were based on the most-adjusted/least-adjusted ratio, while the second, for highest vs. lowest RRs for age of starting to smoke, duration, years quit and tar level, compared RRs that were most- or least-adjusted for other aspects of smoking.

### Software

All data entry and most statistical analyses were carried out using ROELEE version 3.1 (available from P.N. Lee Statistics and Computing Ltd, 17 Cedar Road, Sutton, Surrey SM2 5DA, UK). Some analyses were conducted using Quattro Pro 9 or Excel 2003.

## Results

### Studies identified

A total of 5,993 potentially relevant papers were identified, providing information on 287 eligible studies (Table 1).

**Table 1:** Literature searching and study identification

	**N**	**(%)**
**Papers**		
Identified as potentially relevant	5993	100%
Not obtainable	244	4.1%
Obtained	5749	95.9%
Did not provide relevant data at all	4901	81.8%
Satisfied inclusion criteria except less than 100 lung cancer cases	175	2.9%
Satisfied all inclusion criteria	673	11.2%
**Studies**		
Identified	287	100.0%
Principal	267	93.0%
Subsidiary	20	7.0%

Table 2 presents selected details of the 287 studies while Table 3 gives the distribution of their major characteristics. Additional file 2: Studies gives fuller descriptions of the studies.

**Table 2:** Selected details of the 287 studies of lung cancer

**Study REF**	**Main ref**	**Other refs**	**Brief study description**^**a**^	**Outcome(s)**^**b**^	**Cases**^**c**^	**Full Hist**^**d**^	**Princ REF**^**e**^
ABELIN	[29]	[30]	Switzerland rural CC 1941-64	All	118	No	
ABRAHA	[31]	[32]	Hungary Budapest cohort 1975-94	All,Sq,Ad	571	No	
AGUDO	[33]		Spain Barcelona area CC 1989-92	All	103	No	
AKIBA	[34]	[35-37]	Japan atomic bomb survivors cohort 1963-87	All	610	No	
ALDERS^f^	[38]	[39,40]	UK CC 1977-82	All,Sq,Ad	1448	No	
AMANDU	[41]		US metal miner cohort 1959-75	All	132	No	
AMES	[42]	[43]	US 4 NIOSH coal miner cohorts nested CC 1959-75	All	317	No	
ANDERS	[44]	[45-47]	US Iowa Women's Health cohort 1986-94	All,Sq,Ad	343	No	
ARCHER	[48]	[49-53]	US uranium miners cohort 1950-74	All	146	No	
ARMADA	[54]		Spain CC 1986-90	All	325	Yes	
AUSTIN	[55]	[56]	US Ohio foundry workers CC 1970-86	All	166	No	
AUVINE	[57]	[58-60]	Finland radon CC 1986-92	All	517	No	
AXELSO	[61]		Sweden radon CC 1960-81	All	152	No	
AXELSS	[62]	[63-65]	Sweden 26 municipality CC 1989-93	All	436	No	
BAND	[66]		Canada occupational CC 1983-90	All,Sq,Ad	2831	Yes	
BARBON	[67]	[68-72]	Italy Trieste CC 1979-86	All,Sq,Ad	755	Yes	
BECHER	[73]	[74,75]	Germany pilot for BIPS CC 1985-86	All,Sq,Ad	194	Yes	
BENHAM	[76]	[77-81]	France CC 1976-80	All,Sq,Ad	1625	Yes	LUBIN2
BENSHL	[82]	[83-85]	UK Whitehall civil servants cohort 1967-87	All	486	No	
BERRIN	[86]	[87]	Italy CC 1977-80	All	1101	Yes	LUBIN2
BEST	[88]	[89-91]	Canada war veteran pensioners cohort 1955-62	All	381	No	
BLOHMK	[92]		Germany Heidelberg personality CC	All	888	Yes	
BLOT1	[93]		US Georgia CC 1970-76	All	458	No	
BLOT3	[94]	[95,96]	US Florida CC 1976-79	All	321	No	
BLOT4	[97]	[96]	US Pennsylvania CC 1974-77	All	335	No	
BOFFET	[98]		West Europe pipe and cigar CC 1988-94	All	5621	No	
BOUCHA	[99]		France Paris CC 1988-92	Sq	150	Yes	
BOUCOT	[100]	[101-103]	US Philadelphia LC Research cohort 1951-65	All,Sq,Ad	121	No	
BRESLO	[104]	[105]	US California CC 1949-52	All,Sq,Ad	518	Yes	
BRETT	[106]		UK X-ray volunteers cohort 1960-63	All	150	No	
BROCKM	[107]		Germany Berlin CC study 1990-92	All	117	Yes	
BROSS	[108]	[109]	US Roswell Park Memorial CC study 1960-66	All	974	No	
BROWN1	[110]		US Colorado adenocarcinoma CC 1979-82	Ad	102	Yes	
BROWN2	[111]		US Missouri CC 1984-90	All,Sq,Ad	14596	Yes	
BUELL	[112]		US California American Legion cohort 1957-62	All	304	No	
BUFFLE	[113]	[114-118]	US Texas 6 counties CC 1976-80	All,Sq,Ad	943	No	
BYERS1	[119]		US Roswell Park Memorial CC 1957-65	Sq,Ad	1002	No	BROSS, GRAHAM
BYERS2	[120]		US Western New York Diet CC 1980-84	All	448	Yes	
CARPEN	[121]	[122-126]	US California Genetics CC 1991-94	All	356	No	
CASCO2	[127]		Germany Berlin NAT2 Genotyping CC 1991-94	All	155	No	
CASCOR	[127]		Germany Berlin NAT2 Phenotyping CC	All	389	Yes	
CEDERL	[128]	[129-131]	Sweden cohort 1963-89	All	491	No	
CHAN	[132]		Hong Kong 5 hospital CC 1976-77	All,Sq,Ad	397	No	
CHANG	[133]		US California cholesterol cohort 1972-91	All	136	No	
CHATZI	[134]		Greece Athens CC 1987-88	All	282	Yes	
CHEN	[135]		Taiwan Taipai CC	Sq,Ad	323	Yes	
CHEN2	[136]	[137]	China Guangzhou CC	All	193	No	
CHEN3	[137]	[138,139]	China Zhengzhou CC	All	254	No	
CHIAZZ	[140]	[141]	US Owens-Corning Fiberglass Newark CC 1940-82	All	144	No	
CHOI	[142]		South Korea Cancer Centre CC 1985-88	All,Sq,Ad	375	No	
CHOW	[143]		US Lutheran Brotherhood Ins. cohort 1966-86	All	219	No	
CHYOU	[144]	[145,146]	US Hawaii Oahu Japanese cohort 1965-90	All,Sq,Ad	227	Yes	
COMSTO	[147]		US Washington Co Serum Bank nested CC 1975-93	All,Sq,Ad	258	No	
COOKSO	[148]		Zimbabwe-Rhodesia Harare CC 1961-72	All	234	Yes	
CORREA	[149]	[95,96,150-152]	US Louisiana CC 1979-82	All,Sq,Ad	1359	No	
CPSI^g^	[153]	[154-178]	US ACS million person CPSI cohort 1959-72	All,Sq,Ad	5138	No	
CPSII	[179]	[6,175-178,180-188]	US 2nd ACS cancer prevention cohort 1982-94	All,Sq,Ad	3229	No	
DAMBER	[189]	[190-196]	Sweden North CC 1972-77	All,Sq,Ad	579	No	
DARBY	[197]		UK SW England radon CC 1988-93	All	982	No	
DAVEYS	[198]	[198-201]	Germany Thuringia (Schairer&Schoniger) CC 1930-41	All	109	No	
DEAN	[202]	[203]	South Africa CC 1947-56	All	603	No	
DEAN2	[204]	[205]	UK/N Ireland CC 1960-62	All	954	No	
DEAN3^h^	[206]	[207]	UK Cleveland Co CC 1969-73	All	766	No	
DEKLER	[208]	[209,210]	Australia Kalgoorlie miners cohort 1961-93	All	138	No	
DESTE2	[211]	[211-217]	Uruguay Montevideo CC 1993-96	All,Sq,Ad	463	No	
DESTEF	[218]	[219-221]	Uruguay Montevideo CC 1988-94	All,Sq,Ad	497	Yes	
DOCKER	[222]	[223]	US Harvard six cities cohort 1974-91	All	120	No	
DOLL	[3]	[224-227]	UK original Doll and Hill CC 1948-52	All,Sq,Ad	1465	No	
DOLL2^i^	[228]	[229-239]	UK British Doctors cohort 1951-91	All	920	No	
DORANT	[240]	[241-243]	Netherlands case-cohort 1986-89	All	550	Yes	
DORGAN	[244]	[95,96,245-250]	US New Jersey CC 1980-83	All,Sq,Ad	2026	Yes	
DORN	[251]	[252-260]	US Veterans cohort 1954-80	All,Sq,Ad	5097	No	
DOSEME	[261]		Turkey Istanbul CC 1979-84	All,Sq,Ad	1210	No	
DROSTE	[262]		Belgium Antwerp CC 1995-97	All	478	Yes	
DU	[263]	[264]	China Guangzhou CC 1985	All	849	No	
DUNN	[265]		US California 9 occupations cohort 1954-58	All	139	No	
EBELIN	[266]		Germany Berlin (Lichtenberg) CC 1980-85	All	130	No	
ENGELA	[267]	[268-270]	Norway cohort 1964-93	All,Sq,Ad	435	No	
ENSTRO	[271]		US California CPSI cohort 1959-97	All	2879	No	
ESAKI	[272]		Japan Omuta and Arao CC 1961-71	All	245	No	
FAN	[273]		China Sino-MONICA-Beijing Project CC 1990-91	All,Sq,Ad	403	No	
GAO	[274]	[137,139,275-281]	China Shanghai CC 1984-86	All,Sq,Ad	1405	No	
GAO2	[282]	[283]	Japan Tokai diet CC 1988-91	All	282	No	
GARCIA	[284]	[285-287]	US Boston genetics CC 1992-96	All	416	Yes	
GARDIN	[288]	[289]	UK/Scotland Airdrie avian CC 1988-92	All	143	No	
GARSHI	[290]		US railroad workers diesel CC 1981-82	All	1081	No	
GENG	[291]	[292]	China Tianjin CC	All	292	No	
GER	[293]	[294]	Taiwan Tri-Service General Hospital 1990-91	All,Sq,Ad	141	No	
GILLIS	[295]	[87]	UK/Scotland West CC 1977-81	All,Sq,Ad	656	No	LUBIN2
GODLEY	[296]	[297]	US 1966–68 NMFS & 1967 current pop survey CC	All	1986	No	
GOLLED	[298]	[299]	UK Teeside CC 1952-62	All	443	No	
GOODMA	[300]	[301]	US Hawaii CC 1983-85	All	326	Yes	
GRAHAM	[302]		US Roswell Park Memorial CC 1956-60	All	685	No	
GREGOR	[303]		UK Brompton Hospital vitamin A CC 1976-77	All	104	Yes	
GSELL	[304]	[305]	Switzerland St Gallen CC 1937-54	All	150	Yes	
GUO	[306]		China Quanshan county Jiangsu CC 1984-86	All	196	No	
HAENSZ	[307]		US Multiple hospital CC 1955-57	All,Sq,Ad	158	Yes	
HAMMO2	[308]	[309-312]	US & Canada asbestos workers cohort 1967-76	All	450	No	
HAMMON	[313]	[314-320]	US 9 state cohort 1952-55	All,Sq,Ad	448	No	
HANSEN	[321]	[322]	Denmark welding companies cohort 1968-86	All	105	No	
HEGMAN	[323]	[324]	US Utah radon CC 1989-91	All,Sq,Ad	282	Yes	
HEIN	[325]		Denmark Copenhagen Male cohort 1970-88	All	144	No	
HENNEK	[326]	[327]	US doctors betacarotene trial cohort 1982-95	All	169	No	
HINDS	[328]		US Hawaii CC 1968-78	All,Sq,Ad	292	No	
HIRAY2	[329]	[330]	Japan Tokyo CC 1950-52	All	145	No	
HIRAYA^j^	[331]	[330,332-345]	Japan 6 prefecture cohort 1965-82	All	1917	No	
HITOSU	[346]		Japan Amagaski and Nishinomiya CC 1960-66	All	216	No	
HOLE^k^	[347]	[348-351]	UK/Scotland Renfrew & Paisley cohort 1972-85	All	225	No	
HOROWI	[352]		Canada Montreal CC 1956-67	All	236	No	
HORWIT	[353]		US Yale/New Haven CC 1977-82	All	112	No	
HU	[354]		China Heilongjiang 5 hospital CC study 1985-87	All	227	Yes	
HU2	[355]	[137,139,356]	China Harbin CC study 1977-79	All	523	No	
HUANG	[357]		China Sichuan CC 1990-91	All	135	No	
HUMBLE	[358]	[359-362]	US New Mexico statewide CC 1980-82	All	521	No	
ISHIMA	[363]	[364]	Japan A bomb survivors CC 1961-70	All,Sq,Ad	180	Yes	AKIBA
JAHN	[365]	[366-369]	Germany BIPS CC 1988-93	All,Sq,Ad	1004	No	BOFFET
JAIN	[370]	[371,372]	Canada Ontario CC 1981-85	All,Sq,Ad	845	No	
JARUP	[373]	[374,375]	Sweden smelter workers CC 1928-81	All	102	No	
JARVHO	[376]		Sweden Goteborg asbestos CC 1983-84	All	147	No	
JEDRYC	[377]	[378-382]	Poland CC 1980-87	All,Sq,Ad	1630	No	
JIANG	[383]	[137,139]	China Nanchang CC 1984	All,Sq,Ad	125	No	
JOLY	[384]		Cuba Havana CC 1978-80	All,Sq,Ad	826	No	
JUSSAW	[385]		India Greater Bombay CC 1964-73	All,Sq,Ad	792	No	
KAISE2	[386]	[387,388]	US California Kaiser cohort 1979-91	All	318	No	
KAISER	[389]	[390]	US California Kaiser cohort 1964-80	All	714	No	
KANELL	[391]	[391,392]	Greece Hellenic Anticancer Inst CC 1950-62	All	862	No	
KATSOU	[393]		Greece Athens CC 1987-89	All,Sq,Ad	101	No	
KAUFMA	[394]		US & Canada tar level CC 1981-86	All	881	No	
KELLER	[395]		US Illinois CC 1985-87	All	15038	No	
KHUDER	[396]		US Philadelphia 15 hospital CC 1985-87	All,Sq,Ad	482	Yes	
KIHARA	[397]	[398]	Japan Kanagawa genetic CC 1991-98	All,Sq,Ad	440	No	
KINLEN	[399]		UK tea drinking cohort 1967-86	All	718	No	
KJUUS	[400]	[401,402]	Norway Telemark and Vestfold CC 1979-83	All	176	No	
KNEKT	[403]	[404-407]	Finland Mobile Clinic Health cohort 1966-91	All	515	No	
KO	[408]		Taiwan Kaohsiung CC 1992-93	All	117	Yes	
KOHLME	[409]		Germany Berlin pet birds CC 1990	All	239	No	
KOO	[410]	[411-414]	Hong Kong 8 hospital CC 1981-83	All,Sq,Ad	200	No	
KOULUM	[415]		Finland Helsinki CC 1936-52	All	812	No	
KREUZE	[416]	[417,418]	Germany radon CC 1990-96	All	2260	No	BOFFET
KREYBE	[419]	[420,421]	Norway CC 1948-53	All,Sq,Ad	300	Yes	
KUBIK	[422]	[423]	Czechoslovakia Kolin district cohort 1965-71	All	108	No	
LAMTH	[424]	[411]	Hong Kong 8 hospital CC 1983-86	All,Sq,Ad	445	No	
LAMWK	[425]	[411]	Hong Kong Queen Mary Hospital CC 1981-84	All,Sq,Ad	163	No	
LAMWK2	[426]	[132,413]	Hong Kong Queen Mary Hospital CC 1976-80	All,Sq,Ad	480	No	
LANGE	[427]	[428-430]	Denmark Copenhagen City Heart cohort 1976-89	All	268	No	
LAURIL	[431]	[432,433]	Finland ATBC nested CC 1988-93	All	230	No	
LAUSSM	[434]		Germany Aue/Saxony uranium miners CC 1982-89	All	432	No	
LEI	[435]		China Guangzhou CC 1986	All	792	No	
LEMARC	[436]		US Hawaii Oahu genotyping CC 1992-97	All	341	Yes	
LETOUR	[437]		Canada Winnipeg radon CC 1983-90	All	738	Yes	
LEVIN	[438]	[302,439,440]	US Roswell Park Memorial CC 1938-52	All	475	No	
LIAW	[441]		Taiwan 12 township cohort 1982-94	All	127	No	
LICKIN	[442]	[443,444]	Germany West CC	All	224	No	
LIDDEL	[445]	[446-449]	Canada Quebec chrysotile mine cohort 1970-88	All	304	No	
LIU	[450]		China Shun Yi CC 1980-86	All	229	No	
LIU2	[451]		China Guangzhou CC 1983-84	All	316	No	
LIU3	[452]	[137,453,454]	China Xuanwei farmers CC 1985-86	All	110	No	
LIU4	[455]		China million deaths 1986-88	All	100000	No	
LIU5	[456]	[137]	China Wuhan CC 1978-79	All	111	No	
LOMBA2	[457]	[458]	US Boston CC 1960-67	All,Sq,Ad	225	No	
LOMBAR	[458]	[457,459]	US Boston CC 1951-64	All	1040	No	
LUBIN	[460]	[460-462]	China Yunnan tin miners CC 1984-88	All,Sq,Ad	427	No	XIANGZ
LUBIN2	[463]	[464-469]	West Europe CC 1976-80	All,Sq,Ad	7804	Yes	
LUO	[470]		China Fuzhou CC 1990-91	All,Sq,Ad	102	Yes	
MACLEN	[471]		Singapore CC 1972-73	All	233	No	
MAGNUS	[472]	[473]	Norway nickel workers cohort 1953-93	All	203	No	
MARSH	[474]		US Arizona 6 smelter town CC 1979-90	All	150	No	
MARSH2	[475]		US Arizona 4 smelter town CC 1979-90	All	114	No	
MARTIS	[476]		UK Tyneside asbestos CC 1972-73	All	201	No	
MASTRA	[477]		Italy silica CC 1973-80	All	309	No	
MATOS	[478]		Argentina Buenos Aires CC 1994-96	All,Sq,Ad	200	No	
MATSUD	[479]	[330]	Japan Osaka CC 1965	All,Sq,Ad	179	No	
MCCONN	[480]		UK Liverpool CC 1946-49	All	100	Yes	
MCDUFF	[481]		Canada Saskatchewan CC 1979-83	All	165	No	
MCLAUG	[482]		China 5 region silica workers CC 1972-89	All	316	No	
MIGRAN	[483]	[484,485]	UK British part of migrant cohort 1964-77	All	259	No	
MILLER	[486]		US Erie County CC 1972-84	All	168	No	
MILLS	[487]		US Ohio CC 1940-47	All	444	No	
MOLLO	[488]		Italy Turin CC 1982-92	All,Sq,Ad	145	Yes	
MRFIT	[489]	[490]	US MRFIT initial screening cohort 1973-82	All	2004	No	
MRFITR	[489]	[490-492]	US MRFIT randomized subjects cohort 1973-85	All	119	No	MRFIT
MURATA	[493]		Japan Chiba gastric screen nested CC 1984-93	All	107	No	
MZILEN	[494]		South Africa Northern Province black people CC	All	374	No	
NAM	[495]		US National Mortality Followback 1986	All	1199	No	
NOTAN2	[496]	[497]	India Tata Memorial Hospital CC 1963-71	All	683	No	
NOTANI	[498]		India Tata Memorial Hospital CC 1986-90	All	246	No	
NOU	[499]		Sweden Uppsala CC 1971-76	All,Sq,Ad	273	No	
ODRISC	[500]	[501]	UK Salford Bronchoscopy Database CC	All	446	No	
ORMOS	[502]		Hungary Szeged CC 1947-59	All,Sq,Ad	119	Yes	
OSANN	[503]		US Orange Co. Cancer Surveillance CC 1984-86	All,Sq,Ad	1986	No	
OSANN2	[504]	[505]	US California Kaiser nested CC 1969-77	All,Sq,Ad	217	Yes	KAISER
PARKIN	[506]		Zimbabwe-Rhodesia Bulawayo CC 1963-77	All	877	No	
PASTOR	[507]		Italy Lombardy CC 1976-79	All	204	No	
PAWLEG	[508]		Poland Cracow CC 1992-94	All	176	Yes	
PERNU	[509]		Finland CC 1944-58	All	1606	No	
PERSH2	[510]		Sweden 109 municipality CC 1980-84	All	1022	No	
PETO	[511]		UK FEV cohort 1954-81	All	103	No	
PEZZO2	[512]		Argentina Rosario CC 1992-98	All	367	Yes	
PEZZOT	[513]		Argentina Rosario CC 1987-91	All,Sq,Ad	215	Yes	
PIKE	[514]		US California LA County air poll. CC 1972-75	All	731	No	
PISANI	[515]	[87]	Italy Lombardy diet CC 1980-81	All	417	No	LUBIN2
POFFIJ	[516]	[517]	West Europe Ardennes-Eifel radon CC 1990-95	All	971	No	
POLEDN	[518]		US Toxic waste dumpsite CC 1978-81	All	209	No	
PRESCO	[519]	[520]	Denmark 3 Copenhagen cohort studies pooled 1964-93	All,Sq,Ad	867	No	HEIN, LANGE
QIAO	[521]	[522,523]	China Yunnan tin miners CC 1985	All	107	No	XIANGZ
QIAO2	[524]		China Yunnan tin miners cohort 1992-95	All	241	No	
RACHTA	[525]		Poland Cracow CC 1991-94	All	118	Yes	
RADZIK	[526]		Poland lung cancer relatives CC 1986-87	All	189	No	
RANDIG	[527]		Germany Berlin CC 1951-54	All	448	No	
REN	[137]		China CC	All	244	No	
RESTRE	[528]		Colombia CC 1978-80	All	102	No	
RIMING	[529]		UK Mass radiography cohort 1970-76	All	104	No	
RONCO	[530]		Italy Turin CC 1976-80	All	126	No	
ROOTS	[531]		Germany Berlin debrisoquine CC	All	270	Yes	CASCOR
ROTHSC	[532]		US Southern Louisiana CC 1971-77	All	284	No	
SAARIK	[533]		Finland genetics CC 1988-96	All,Sq,Ad	205	Yes	
SADOWS	[534]		US National Cancer Institute CC 1938-43	All	477	No	
SANKAR	[535]		India Trivandrum diet CC 1990	All	281	No	
SCHWAR	[536]	[537]	US Michigan CC 1984-87	All,Sq,Ad	5588	Yes	
SEGI	[538]		Japan nationwide CC 1948-52	All	159	No	
SEGI2	[539]		Japan Tokyo and Sendai CC 1962-70	All,Sq,Ad	378	No	
SEOW	[540]		Singapore NAT2 CC 1997-98	All,Sq,Ad	153	Yes	
SHAW	[541]		US & Canada Bethesda/Quebec debrisoquine CC 1988-92	All	335	Yes	
SHIMIZ	[542]		Japan Sendai Kosei Hospital CC 1977-82	All,Sq,Ad	751	No	
SIEMIA	[543]	[544-547]	Canada Montreal occupational CC 1979-85	All,Sq,Ad	857	Yes	
SIMARA	[548]		Thailand Chiang Mai CC 1971-72	All	115	No	
SITAS	[549]		South Africa Johannesburg black people CC −1997	All	*	No	
SOBUE	[550]	[551,552]	Japan Osaka CC 1986-88	All,Sq,Ad	1376	Yes	
SOBUE2	[553]		Japan Osaka CC 1965-83	All,Sq,Ad	2083	No	
SPEIZE	[554]	[555]	US Nurses' Health cohort 1976-92	All	593	Yes	
SPITZ	[556]	[557-566]	US Texas University Genetics	All	177	Yes	
STASZE	[567]		Poland Gliwice CC 1954-58	All,Sq,Ad	281	Yes	
STAYNE	[568]	[569]	US Third National Cancer Survey CC 1969-71	All,Sq,Ad	420	No	
STOCKS	[570]	[571,572]	UK British Empire Cancer Campaign CC 1952-55	All	2932	No	
STOCKW	[573]		US Florida phosphate mining area CC 1981-83	All	22161	No	
STUCKE	[574]		France GSTM1 CC 1989-92	All	247	Yes	
SUN	[575]		China Liaoning genetics CC 1992-94	All	207	Yes	
SUZUK2	[576]	[577]	Brazil Rio de Janeiro CC 1991-92	All,Sq,Ad	123	Yes	
SUZUKI	[578]		Japan Osaka CC 1978-86	Ad	238	Yes	
SVENSS	[579]	[580,581]	Sweden Stockholm County CC 1983-86	All,Sq,Ad	210	No	
TANG	[582]	[583]	US Columbia Presbyterian genetics CC	All	119	Yes	
TANG2	[584]		UK 4 cohort studies pooled 1967-90	All	836	No	BENSHL, HOLE, WALD
TAO	[585]		China Shanghai CC 1988-90	All	723	No	
TENKAN	[586]	[587-591]	Finland part Finland/Norway cohort 1962-87	All	242	No	
TIZZAN	[592]		Italy CC 1959-61	All,Sq,Ad	1358	No	
TOKARS	[593]	[594-597]	Russia Nuclear Workers nested CC 1966-91	All,Sq,Ad	162	Yes	
TOUSEY	[598]		US Duval County CC 1993-96	All	507	Yes	
TSUGAN	[599]		Japan National Cancer Centre CC 1976-85	All,Sq,Ad	134	Yes	
TULINI	[600]		Iceland Rejkjavik cohort 1967-95	All	472	No	
TVERDA	[601]		Norway cohort 1972-88	All	238	No	
ULMER	[602]		Germany Bochum CC 1971-75	All	726	No	
VEIERO	[603]		Norway Health Screening cohort 1977-91	All	153	No	TVERDA
VUTUC	[604]	[604-620]	Austria CC 1976-80	All,Sq,Ad	1877	No	LUBIN2
WAKAI	[621]		Japan Okinawa CC 1988-91	All,Sq,Ad	333	Yes	
WALD	[622]		UK BUPA cohort 1975-93	All	102	No	
WANG	[623]		China Guangdong CC 1990-93	All,Sq,Ad	390	Yes	
WANG2	[624]	[137,139]	China Tai Yuan CC 1980-82	All	103	No	
WANG3	[137]	[139,625]	China Nanjing CC	All	293	No	
WANG4	[626]		China Xuanwei farmers cohort 1976-96	All	1170	No	
WARSIN	[442]		Netherlands CC	All	134	No	
WATSON	[627]	[628]	US New York Memorial Hospital CC 1950-52	All	301	Yes	
WICKLU	[629]		US Washington County orchardists CC 1968-80	All	155	No	
WIGLE	[630]		Canada Alberta CC 1971-73	All	728	No	
WILKIN	[631]		UK London Chest Hospital CC 1992-93	All	271	No	
WU	[632]		US California LA County CC 1981-82	All,Sq,Ad	220	Yes	
WU2	[633]		US California LA County CC 1983-86	Ad	336	Yes	
WUNSCH	[634]	[635]	Brazil Sao Paulo CC study 1990-91	All	398	No	
WUWILL	[636]	[137,139,637-640]	China Shenyang and Harbin CC study 1985-87	All,Sq,Ad	965	No	
WYNDE2	[641]	[642-644]	US New York CC study 1962-64	All,Sq,Ad	404	Yes	
WYNDE3	[645]		US New York Memorial CC study 1966-69	All,Sq,Ad	350	Yes	
WYNDE4	[2]	[646]	US 8 state CC study 1948-50	All,Sq,Ad	684	No	
WYNDE5	[647]	[648]	US 4 city CC study 1969-76	All,Sq,Ad	1365	Yes	WYNDE6
WYNDE6^l^	[649]	[644,650-677]	US 4 city CC study 1969-96	All,Sq,Ad	4423	Yes	
WYNDE7	[676]		US 6 city CC study 1977-84	All,Sq,Ad	2085	Yes	WYNDE6
WYNDE8	[677]		US 4 city CC study 1985-90	All,Sq,Ad	1044	Yes	WYNDE6
WYNDER	[678]		Cuba Havana CC study 1956-57	All	120	No	
XIANGZ	[679]		China Yunnan tin miners cohort study 1976-87	All	983	No	
XU	[637]	[137,139,638,639]	China Shenyang CC study 1985-87	All,Sq,Ad	729	No	
XU2	[680]	[639]	China Anshan Iron-Steel workers CC study 1987-93	All	610	No	
XU3	[681]	[137]	China Tianjin CC study 1981	All, Sq, Ad	135	No	
XU4	[137]	[682]	China 26 city air pollution CC study	All	206	No	
YAMAGU	[683]		Japan occupational CC study 1989-90	All	144	Yes	
YONG	[684]	[260]	US NHANES I - NHEFS cohort study 1971-92	All	216	No	
YUAN	[685]		China Shanghai cohort study 1986-93	All	142	No	
ZHANG	[686]	[137]	China Jinzhou CC study 1988-89	All	100	No	
ZHENG	[687]	[137,139,688]	China Shanghai CC study 1982-84	All,Sq,Ad	540	Yes	
ZHOU	[689]		China Medical Univ CC study 1978-94	All,Sq,Ad	1360	No	

^a^ CC = case–control, date range for cohort study is from start at baseline interview to end of follow-up.

^b^ Indicates whether the study provided data for all lung cancer (All), squamous cell carcinoma (Sq) or adenocarcinoma (Ad), or a near equivalent definition (see Methods).

^c^ Number of lung cancer cases.

^d^ Whether or not full histological confirmation of cases was carried out.

^e^ For subsidiary studies, this column shows the relevant principal study.

^f^ Additional sources were two unpublished reports made available by personal communication from P N Lee.

^g^ Additional sources were two unpublished reports made available by personal communication from Dr E C Hammond.

^h^ An additional source was an unpublished report made available by personal communication from Dr G Dean.

^i^ An additional source was an unpublished report made available by personal communication from Dr J Peto.

^j^ An additional source was an unpublished report made available by personal communication from Dr T Hirayama.

^k^ An additional source was an unpublished report made available by personal communication from Dr V Hawthorne.

^l^ An additional source was an unpublished report made available by personal communication from Dr G C Kabat.

**Table 3:** Distribution of the main characteristics of the 287 studies of lung cancer

**Characteristic**	**Level**	**Principal studies**						**Subsidiary studies**	**All studies**
		**Outcome**^**a**^			**Study type**^**b**^			**Total**	**Total**
		**All**	**Squamous**	**Adeno**	**CC**	**prosp**	**Total**		
Study status	Principal	262	84	86	209	58	267	-	267
	Subsidiary	(19)	(12)	(12)	(15)	(5)	(20)	20	20
Study type	Case–control	204	73	75	209	-	209	15	224
	Prospective	52	9	9	-	52	52	4	56
	Other	6	2	2	-	6	6	1	7
Study sex	Both	154	53	54	133	25	158	11	169
	Male	90	19	19	59	31	90	8	98
	Female	18	12	13	17	2	19	1	20
Lowest age^c^	<20 or unlimited	177	56	57	165	16	181	13	194
	20-29	15	3	3	9	6	15	2	17
	30-39	39	15	16	26	14	40	5	45
	40+	31	10	10	9	22	31	0	31
Highest age	<60	16	2	2	10	6	16	3	19
	60-69	21	6	6	6	15	21	1	22
	70-79	29	11	12	23	7	30	3	33
	80+ or unlimited	196	65	66	170	30	200	13	213
Location	North America	87	29	31	63	26	89	6	95
	United Kingdom	22	2	2	13	9	22	2	24
	Scandinavia	25	6	6	14	11	25	2	27
	Other Europe	42	13	12	39	4	43	7	50
	China	37	10	10	34	3	37	2	39
	Japan	17	8	9	15	3	18	1	19
	Other Asia	16	10	10	16	1	17	0	17
	Other	16	6	6	15	1	16	0	16
Start year of study	<1960	47	12	12	33	14	47	1	48
	1960-69	40	13	13	21	19	40	6	46
	1970-79	59	17	19	45	16	61	7	68
	1980-89	83	33	33	78	8	86	5	91
	1990+	33	9	9	32	1	33	1	34
Number of cases^d^	100-249	109	25	26	84	28	112	5	117
	250-499	70	25	26	57	15	72	3	75
	500-999	47	14	14	38	9	47	3	50
	1000+	36	20	20	30	6	36	9	45
Risky occupational population	No	244	83	85	203	46	249	18	267
	Mining	7	0	0	0	7	7	2	9
	Other risky	11	1	1	6	5	11	0	11
National cigarette tobacco type	Virginia	42	6	6	30	12	42	2	44
	Blended	180	66	68	142	42	184	16	200
	Other^e^	40	12	12	37	4	41	2	43
Any proxy use	None/unknown	189	66	67	137	56	193	17	210
	Yes	73	18	19	72	2	74	3	77
Full histological confirmation	No	199	49	49	145	54	199	12	211
	Yes	63	35	37	64	4	68	8	76
Main outcome(s)^a^	All	262	82	82	204	58	262	19	281
	Squamous	82	84	83	73	11	84	12	96
	Adeno	82	83	86	75	11	86	12	98
	All only	180	-	-	133	47	180	8	188
	Squamous only	-	1	-	1	0	1	0	1
	Adeno only	-	-	3	3	0	3	0	3
	more than one	82	83	83	72	11	83	12	95

^a^ Indicates whether the study provided data for all lung cancer, squamous cell carcinoma or adenocarcinoma, or their near equivalent definitions (see Methods). For squamous, the near equivalent definitions included are: Kreyberg I; squamous cell or small carcinoma; squamous cell or undifferentiated carcinoma; and not adenocarcinoma. For adeno, the near equivalent definitions included are: Kreyberg II; adenocarcinoma or large cell carcinoma; adenocarcinoma, alveolar or bronchioloalveolar carcinoma; not squamous cell or small cell carcinoma; and not squamous cell or undifferentiated carcinoma.

^b^ CC = case–control, prosp = prospective.

^c^ At start of study.

^d^ In study as a whole.

^e^ Indicates China and Taiwan.

Of the 287 studies, 267 are classified as principal, 209 (78.3%) of these being case–control studies, 52 (19.5%) prospective, 5 (1.9%) nested case–control and 1 (0.4%) case-cohort. Note that the last three study designs, where exposure was determined before diagnosis, are combined into one category in Table 3 (and the text below based on it). The other 20 studies are classified as subsidiary. Of the principal studies, 262 provide data for all lung cancer, 84 for squamous and 86 for adeno. Only rarely did these studies provide data only for squamous (1 study) or adeno (3 studies). The data come less often from case–control designs for all lung cancer (77.9%) than for squamous (86.9%) and adeno (87.2%).

Of the 267 principal studies, 158 (59.2%) provide results for both sexes, 90 (33.7%) for males only, and 19 (7.1%) for females only. One hundred and ninety-six (73.4%) of the studies included subjects who are under 30 years old (or allowed their inclusion by having no age restriction), while only 31 (11.6%) were restricted to subjects aged 40 or more. Subjects aged 80 years or more were included by 200 (74.9%), while only 16 (6.0%) were restricted to subjects aged 60 or less. Prospective studies were much more likely than case–control studies to specify age restrictions, e.g. 62.1% vs. 16.7% for age 30 years or more, and 48.3% vs. 18.7% for age less than 80 years. Eighty-nine (33.3%) principal studies were conducted in USA or Canada, with 22 (8.2%) in the UK, 25 (9.4%) in Scandinavia, 43 (16.1%) in other parts of Europe, 37 (13.9%) in China, 18 (6.7%) in Japan, 17 (6.4%) in the rest of Asia and 16 (6.0%) elsewhere – in South or Central America, Africa or Australia. Of the 58 prospective studies, all but 12 were conducted in North America, UK or Scandinavia. Of the principal studies, 42 (15.7%) were conducted in countries where at least 75% of cigarettes smoked are made from Virginia tobacco, with 184 (68.9%) carried out where at least 75% of cigarettes are from blended tobaccos. Forty seven (17.6%) started before 1960. Studies starting after 1979 were predominantly (92.4%) case–control. Thirty-six (13.5%) involved at least 1,000 lung cancer cases. Seven (2.6%) were conducted in miners, with a further 11 (4.1%) conducted in other occupational groups with a known relationship with lung cancer. Proxy respondents were used for some subjects in 74 (27.7%), with full histological confirmation of cases reported to be carried out in 68 (25.5%).

Most study groups (i.e. a principal study or one of its subsidiaries) provide some results for the major indices compared to never smokers, 240 (89.9%) for ever smokers, 134 (50.2%) for current smokers and 127 (47.6%) for ex smokers. Many studies provide results for smoking of any product (162 studies, 60.7%) or for cigarettes (147, 55.1%), but less do so for cigarette only smoking (55, 20.6%), smoking of pipes/cigars only (62, 23.2%), mixed smoking (29, 10.9%), or for the cigarette type indices filter/plain cigarette smoking (38, 14.2%), hand-rolled cigarette smoking (15, 5.6%), or mentholated cigarette smoking (3, 1.1%). Though dose–response data are most commonly available by amount smoked (162, 60.7%), many studies provide data by age of starting to smoke (62, 23.2%), duration (77, 28.8%), and time quit (58, 21.7%). Few studies provide data on tar level (11 studies, 4.1%), fraction smoked (9 studies, 3.4%), or butt length (2 studies, 0.7%).

### Relative risks

A total of 16,616 RRs were entered, the number recorded per study varying from 1 to 1,029. Of these, 1,266 relate to subsidiary studies. Table 4 summarizes the distribution of various characteristics of the RRs by outcome, sex, study type and location.

**Table 4:** **Distribution of the main characteristics of the relative risks**^**a**^

**Smoking index**^**b**^	**Dose response RRs**^**b,c**^	**Total**	**Principal**^**d**^	**By lung cancer type**^**a**^			**By sex**		
				**All**	**Squamous**	**Adeno**	**Combined**	**Male**	**Female**
All		16616	15350	11316	2268	1698	1031	11202	4383
Any product or cigarettes vs. never/non		3614	3366	2359	488	432	342	2065	1207
Cig only smoking vs. never/non		678	621	535	72	56	18	513	147
Pipe/Cigar/Mixed vs. never/non		769	678	644	61	53	22	717	30
Handrolled vs. manufactured		120	104	74	22	14	8	93	19
Filter vs. Plain		303	264	167	74	37	9	182	112
Menthol vs. non-menthol		25	16	20	2	1	5	10	10
Amount smoked	All RRs	3627	3509	2708	412	310	198	2459	970
	Sets vs. never/non	1145	1104	858	123	100	64	741	340
Age of starting	All RRs	1442	1344	1052	167	128	67	970	405
	Sets vs. never/non	256	242	188	29	24	13	159	84
	Sets vs. low	301	285	197	44	35	14	196	91
	Non-categorical RRs	14	14	6	2	2	2	7	5
Duration of smoking	All RRs	2337	2129	1544	342	240	202	1470	665
	Sets vs. never/non	374	337	248	54	40	37	221	116
	Sets vs. low	434	384	261	75	56	38	260	136
	Non-categorical RRs	72	70	32	19	20	1	39	32
Years quit (vs. never)	All RRs	1665	1504	991	276	198	64	1241	360
	Sets vs. never/non	207	194	124	35	26	11	146	50
	Sets vs. low	255	234	157	39	27	8	190	57
	Non-categorical RRs	3	3	3	0	0	2	1	0
Years quit (vs. current)	All RRs	1421	1248	867	221	145	55	1076	290
	Sets vs. current	251	231	155	38	25	11	183	57
	Sets vs. low	244	223	158	33	23	10	177	57
	Non-categorical RRs	0	0	0	0	0	0	0	0
Tar	All RRs	222	198	156	33	33	18	127	77
	Sets vs. never/non	22	18	16	3	3	4	13	5
	Sets vs. low	41	36	33	4	4	3	24	14
	Non-categorical RRs	55	55	19	18	18	0	27	28
Butt length	All RRs	15	15	15	0	0	0	6	9
	Sets vs. never/non	3	3	3	0	0	0	1	2
	Sets vs. low	2	2	2	0	0	0	1	1
	Non-categorical RRs	2	2	2	0	0	0	1	1
Fraction smoked	All RRs	192	192	42	70	40	5	140	47
	Sets vs. never/non	40	40	10	14	8	1	29	10
	Sets vs. low	50	50	12	18	10	1	34	15
	Non-categorical RRs	0	0	0	0	0	0	0	0
Lung cancer type (near equivalent definitions)^a^	All	11316	10553	11316	0	0	760	7700	2856
	Squamous	2268	2038	0	2268	0	113	1538	617
	Adeno	1698	1532	0	0	1698	68	1098	532
Lung cancer type (exact definitions)	All lung cancer	10980	10247	10980	0	0	671	7571	2738
	Squamous cell carcinoma	1064	1004	0	1064	0	66	710	288
	Adenocarcinoma	1172	1112	0	0	1172	64	726	382
	Large cell carcinoma	393	392	0	0	0	13	266	114
	Small cell carcinoma	718	662	0	0	0	29	497	192
	Other	223	173	0	0	0	48	103	72
Adjustment	None	10113	9266	6740	1362	1086	651	6613	2849
	Sex and/or age only	3099	3053	2423	264	234	132	2307	660
	Other (but not sex or age)	701	686	442	142	63	51	418	232
	Sex and/or age plus other	2703	2345	1711	365	315	197	1864	642
Derivation	Original	1853	1705	1088	261	254	163	1157	533
	Standard calculations	9803	8983	6701	1371	996	663	6376	2764
	Other methods	4960	4662	3527	638	448	205	3669	1086
			**Continent**				**Study type**		
		**Total**	**North America**	**Europe**	**Asia**	**Other**	**Case–control**	**Other**	
All		16616	6676	6122	2770	1048	11945	4671	
Any product or cigarettes vs. never/non		3614	1493	1163	771	187	2716	898	
Cigarette only smoking vs. never/non		678	284	340	27	27	345	333	
Pipe/Cigar/Mixed vs. never/non		769	380	327	39	23	428	341	
Handrolled vs. manufactured		120	0	68	30	22	90	30	
Filter vs. Plain		303	74	178	27	24	268	35	
Menthol vs. non-menthol		25	25	0	0	0	13	12	
Amount smoked	All RRs	3627	1601	1288	612	126	2240	1387	
	Sets vs. never/non	1145	507	404	189	45	712	433	
Age of starting	All RRs	1442	496	512	301	133	1017	425	
	Sets vs. never/non	256	82	93	58	23	186	70	
	Sets vs. low	301	93	105	71	32	224	77	
	Non-categorical RRs	14	10	1	3	0	14	0	
Duration of smoking	All RRs	2337	939	706	462	230	1816	521	
	Sets vs. never/non	374	148	111	80	35	287	87	
	Sets vs. low	434	154	141	94	45	353	81	
	Non-categorical RRs	72	60	9	3	0	70	2	
Years quit (vs. never)	All RRs	1665	668	657	196	144	1339	326	
	Sets vs. never/non	207	89	75	23	20	170	37	
	Sets vs. low	255	86	111	33	25	208	47	
	Non-categorical RRs	3	3	0	0	0	3	0	
Years quit (vs. current)	All RRs	1421	457	674	177	113	1155	266	
	Sets vs. current	251	76	112	36	27	213	38	
	Sets vs. low	244	84	102	31	27	198	46	
	Non-categorical RRs	0	0	0	0	0	0	0	
Tar	All RRs	222	158	64	0	0	182	40	
	Sets vs. never/non	22	16	6	0	0	19	3	
	Sets vs. low	41	25	16	0	0	26	15	
	Non-categorical RRs	55	55	0	0	0	54	1	
Butt length	All RRs	15	0	13	0	2	15	0	
	Sets vs. never/non	3	0	3	0	0	3	0	
	Sets vs. low	2	0	2	0	0	2	0	
	Non-categorical RRs	2	0	0	0	2	2	0	
Fraction smoked	All RRs	192	32	56	99	5	190	2	
	Sets vs. never/non	40	10	8	21	1	40	0	
	Sets vs. low	50	12	16	21	1	48	2	
	Non-categorical RRs	0	0	0	0	0	0	0	
Lung cancer type (near equivalent)^a^	All	11316	4879	3955	1765	717	6942	4374	
	Squamous	2268	773	931	445	119	2131	137	
	Adeno	1698	615	618	346	119	1601	97	
Lung cancer type (exact definitions)	All lung cancer	10980	4654	3923	1686	717	6608	4372	
	Squamous carcinoma	1064	205	433	307	119	1008	56	
	Adenocarcinoma	1172	308	435	310	119	1097	75	
	Large cell carcinoma	393	208	94	88	3	379	14	
	Small cell carcinoma	718	148	427	108	35	669	49	
	Other	223	53	97	18	55	223	0	
Adjustment	None	10113	4105	3489	1847	672	8126	1987	
	Sex and/or age only	3099	1515	1153	399	32	1216	1883	
	Other (but not sex or age)	701	177	409	79	36	579	122	
	Sex and/or age plus other	2703	879	1071	445	308	2024	679	
Derivation	Original	1853	649	649	379	176	1436	417	
	Standard calculations	9803	3865	3519	1811	608	7703	2100	
	Other methods	4960	2162	1954	580	264	2806	2154	

^a^ RRs relating to all lung cancer (all), squamous cell carcinoma (squamous) or adenocarcinoma (adeno) or to a near equivalent definition.

^b^ “never/non” indicates never smoker or non smoker.

^c^ Sets are dose response results for an identical definition of smoking product, strata and confounding variables. Sets vs. low are sets where the RR has been calculated compared to the level with the lowest expected risk, i.e. the smallest amount smoked, or the latest age of starting. Non-categorical RRs are typically where RRs and CIs are not available, but some other information was provided.

^d^ RRs from principal studies.

Of the total of 16,616 RRs, 71.9% relate to case–control studies, and 93.8% are sex-specific. 40.2% come from North American studies, 36.8% from Europe, 16.7% from Asia, and 6.3% from other continents. 60.9% are unadjusted for potential confounding variables and 18.7% are adjusted for sex and/or age only. 70.1% are given directly or are calculated by standard methods, the rest being derived by more complex methods.

Of the total RRs, 5,061 relate to the major smoking indices, where the denominator is never or non smoking, with 3,614 of these relating to smoking of any product or cigarettes (regardless of pipe or cigar smoking), 678 to cigarette only smoking and 769 to pipe, cigar or mixed smoking. Four hundred and forty-eight relate to cigarette type comparisons, most commonly (303 RRs) to the filter vs. plain comparison. All the 25 RRs for the mentholated/non-mentholated comparison come from North American studies, while none of those for the handrolled/manufactured comparison do. There are 10,921 RRs for dose-related indices, based mainly on 3,625 sets, 2,047 vs. never or non smoking, 1,327 vs. the low level, and 251 vs. current smoking. There are most sets for amount smoked (1,145) and least for butt length (5). For amount smoked, age of starting, duration of smoking, years quit (vs. never and vs. current) there are sufficient numbers of dose–response sets to study variation in RR by sex, study type and continent.

None of the RRs included in the meta-analyses and meta-regressions show more than minor failures of the validation tests used, attributable to rounding errors or small imprecisions or uncertainties in estimating the RRs and CIs. Additional file 3: RRs provides further detail.

For dose-related indices, Additional file 4: Dose Not Meta gives results originally presented in forms unsuitable for meta-analysis.

### The meta-analyses and meta-regressions

The main findings are summarized in the following sections, with tables and forest plots. Additional file 5: Detailed Analysis Tables fully presents all the meta-analyses and meta-regressions conducted. The interested reader should first see Additional file 1: Methods, which lists the other files, and describes their content and structure.

Findings are generally presented for three outcomes, referred to as “all lung cancer”, “squamous” or “adeno”. These outcomes are defined in the Methods section, and also in the footnotes to the tables, and allow the inclusion of results based on alternative similar definitions. (Note that the terms “squamous cell carcinoma” and “adenocarcinoma” are only used when reference is made to results specifically for the particular cell type).

#### A. Risk from ever smoking

**Figure 1:**
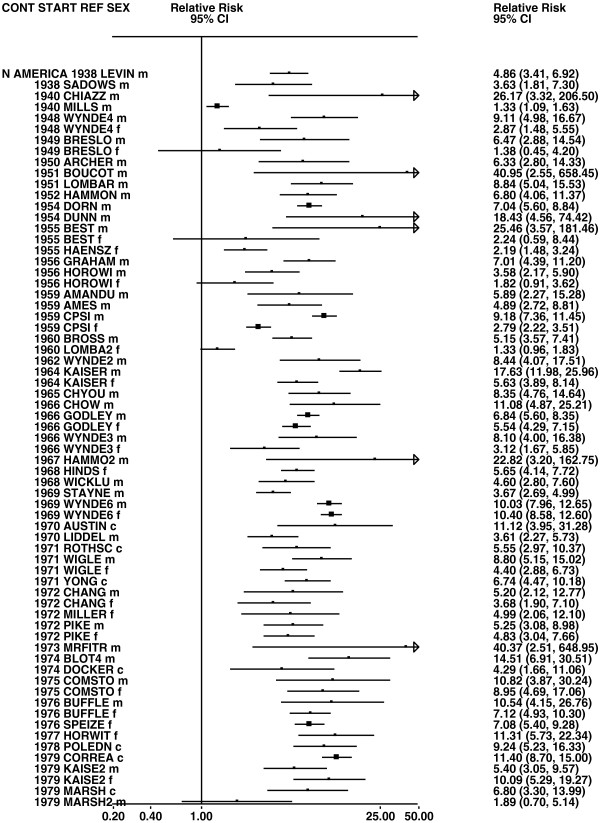
**Forest plot of ever smoking of any product and all lung cancer – part 1.** Table 5 presents the results of a main meta-analysis for all lung cancer based on 328 relative risk (RR) and 95% confidence interval (CI) estimates for ever smoking of any product (or cigarettes if any product not available). The individual study estimates are shown numerically and graphically on a logarithmic scale in Figures 1, 2, 3, 4, 5. The studies are sorted in order of sex within study reference (REF) within start year of study (START) within continent (CONT), with the exception of study LIU4 shown at the end of Figure 5. In the graphical representation individual RRs are indicated by a solid square, with the area of the square proportional to the weight (inverse-variance of log RR). Arrows indicate where the CI extends outside the range allocated.

**Figure 2:**
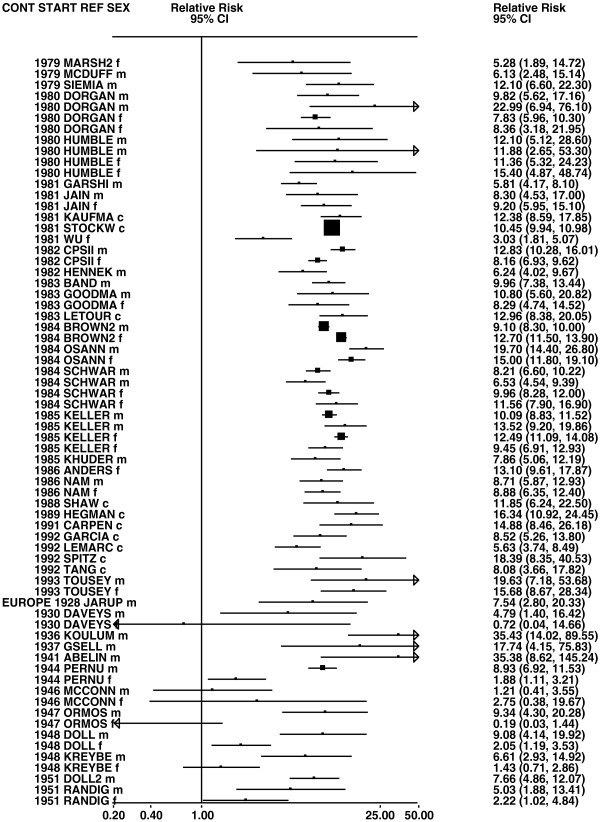
**Forest plot of ever smoking of any product and all lung cancer – part 2.** This is a continuation of Figure 1, presenting further individual study data included in the main meta-analysis for all lung cancer shown in Table 5. For study DORGAN separate estimates, within sex, are shown for whites then blacks. For study HUMBLE they are shown for non-hispanic whites then hispanics, and for study KELLER for whites then non-whites.

**Figure 3:**
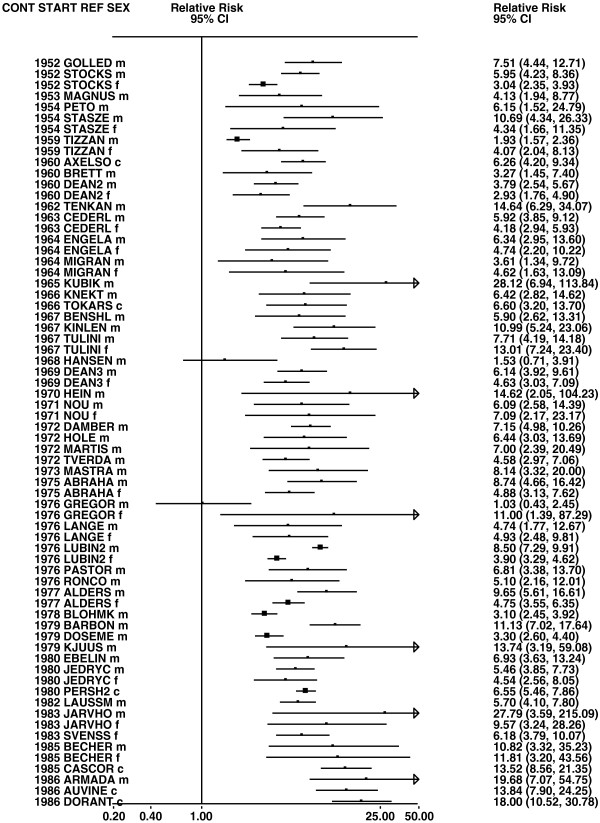
**Forest plot of ever smoking of any product and all lung cancer – part 3.** This is a continuation of Figure 2, presenting further individual study data included in the main meta-analysis for all lung cancer shown in Table 5.

**Figure 4:**
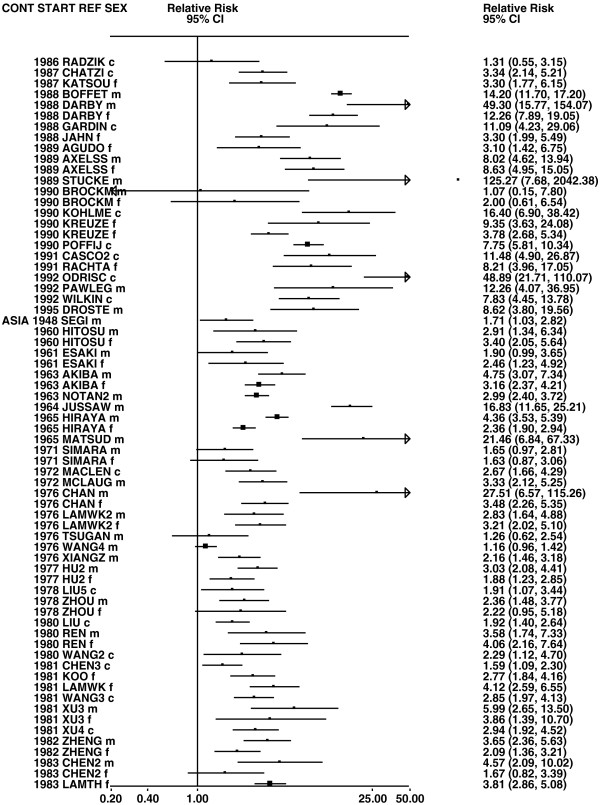
**Forest plot of ever smoking of any product and all lung cancer – part 4.** This is a continuation of Figure 3, presenting further individual study data included in the main meta-analysis for all lung cancer shown in Table 5.

**Figure 5:**
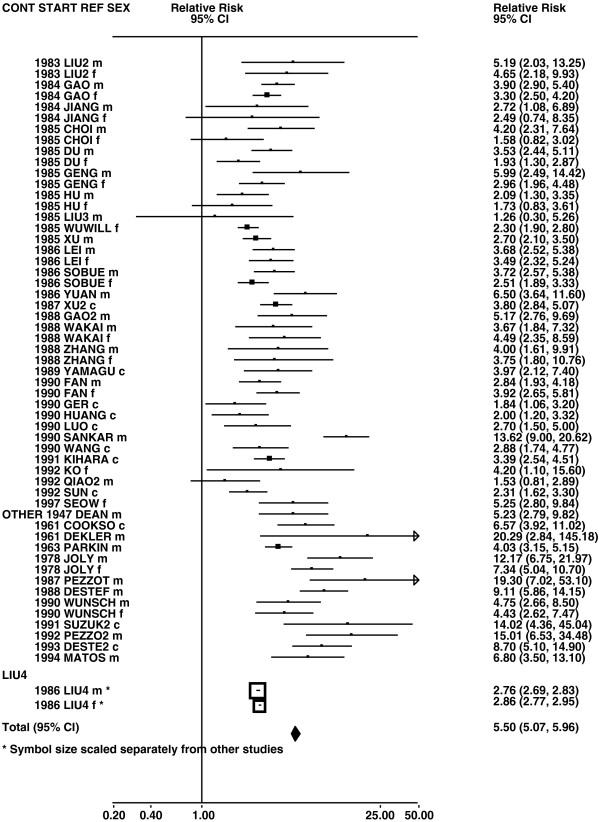
**Forest plot of ever smoking of any product and all lung cancer – part 5.** This is a continuation of Figure 4, presenting the remaining individual study data included in the main meta-analysis for all lung cancer shown in Table 5. Also shown are the combined random-effect estimates. These are represented by a diamond of standard height, with the width indicating the 95% CI. Note that the sizes of the squares for the two estimates from study LIU4 indicate the relative weight of the male and female data, but are not comparable with the sizes of the squares for the other estimates.

**Figure 6:**
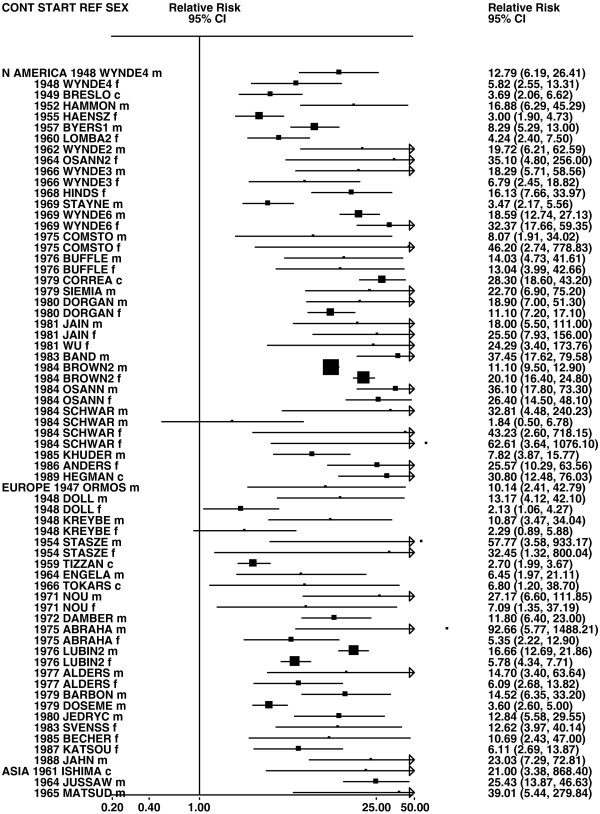
**Forest plot of ever smoking of any product and squamous – part 1.** Table 5 presents the results of a main meta-analysis for squamous based on 102 relative risk (RR) and 95% confidence interval (CI) estimates for ever smoking of any product (or cigarettes if any product not available). The individual study estimates are shown numerically and graphically on a logarithmic scale in Figures 6, 7. The studies are sorted in order of sex within study reference (REF) within start year of study (START) within continent (CONT). In the graphical representation individual RRs are indicated by a solid square, with the area of the square proportional to the weight (inverse-variance of log RR). Arrows indicate where the CI extends outside the range allocated. For study SCHWAR separate estimates, within sex, are shown for whites then blacks.

**Figure 7:**
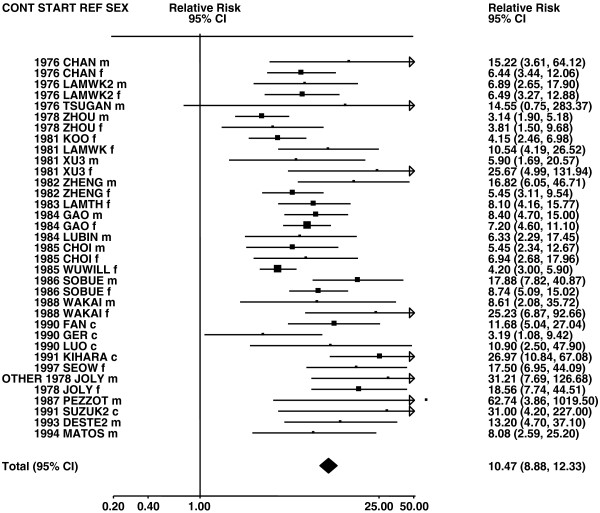
**Forest plot of ever smoking of any product and squamous – part 2.** This is a continuation of Figure 6, presenting the remaining individual study data included in the main meta-analysis for squamous shown in Table 5. Also shown are the combined random-effect estimates. These are represented by a diamond of standard height, with the width indicating the 95% CI.

**Figure 8:**
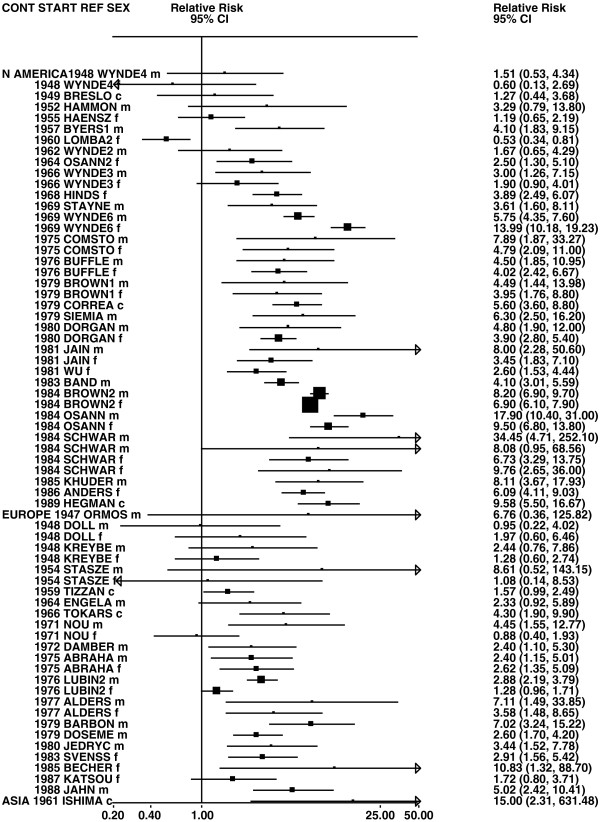
**Forest plot of ever smoking of any product and adeno – part 1.** Table 5 presents the results of a main meta-analysis for adeno based on 107 relative risk (RR) and 95% confidence interval (CI) estimates for ever smoking of any product (or cigarettes if any product not available). The individual study estimates are shown numerically and graphically on a logarithmic scale in Figures 8, 9. The studies are sorted in order of sex within study reference (REF) within start year of study (START) within continent (CONT). In the graphical representation individual RRs are indicated by a solid square, with the area of the square proportional to the weight (inverse-variance of log RR). Arrows indicate where the CI extends outside the range allocated. For study SCHWAR separate estimates, within sex, are shown for whites then blacks.

Figures 1, 2, 3, 4 and 5 (all lung cancer), Figures 6, 7 (squamous) and Figure 8, 9 (adeno) present the results of the main meta-analyses for ever smoking any product (or cigarette smoking for studies without RRs for any product), based on most-adjusted RRs. Table 5 presents additional results subdivided by level of certain characteristics, while Table 6 presents results of some alternative meta-analyses of ever smoking. From these findings, various observations can be made.

**Figure 9:**
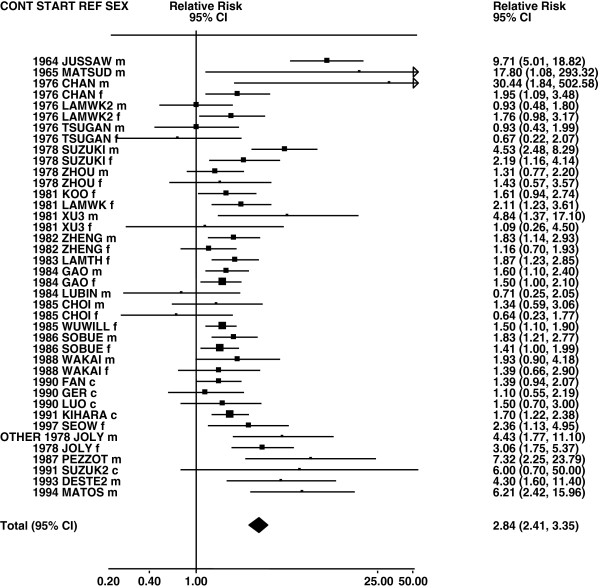
**Forest plot of ever smoking of any product and adeno – part 2.** This is a continuation of Figure 8, presenting the remaining individual study data included in the main meta-analysis for adeno shown in Table 5. Also shown are the combined random-effect estimates. These are represented by a diamond of standard height, with the width indicating the 95% CI.

**Table 5:** **Main meta-analyses for ever smoking of any product (or cigarettes if any product not available)**^**a**^

**Characteristic**	**Level**	**Statistic**^**b**^	**All lung cancer**^**c**^	**Squamous**^**d**^	**Adeno**^**e**^
All	All	n	328	102	107
		F	4.22 (4.16-4.28)	9.52 (8.94-10.13)	3.44 (3.27-3.61)
		R	5.50 (5.07-5.96)	10.47 (8.88-12.33)	2.84 (2.41-3.35)
		H, P_H_	22.84, p < 0.001	5.17, p < 0.001	8.78, p < 0.001
		P_E_	p < 0.001	NS	p < 0.01
Sex	Male	n	171	49	51
		F	3.74 (3.67-3.82)	10.74 (9.82-11.74)	4.09 (3.76-4.44)
		R	6.18 (5.49-6.95)	11.98 (9.68-14.82)	3.55 (2.83-4.45)
	Female	n	108	42	45
		F	3.95 (3.86-4.05)	8.86 (8.05-9.75)	3.27 (3.05-3.50)
		R	4.43 (3.84-5.10)	8.97 (6.95-11.57)	2.32 (1.78-3.02)
	Combined	n	49	11	11
		F	8.26 (7.94-8.60)	7.04 (5.76-8.60)	2.29 (1.93-2.71)
		R	6.09 (4.98-7.44)	10.70 (4.89-23.40)	2.52 (1.56-4.08)
	Between levels	P_B_	<0.01	NS	<0.05
Location	North America	n	116	38	40
		F	8.86 (8.63-9.10)	12.69 (11.63-13.84)	5.75 (5.36-6.16)
		R	7.49 (6.78-8.27)	13.42 (10.45-17.24)	4.37 (3.48-5.48)
	United Kingdom	n	29	4	4
		F	5.22 (4.72-5.78)	4.75 (3.00-7.51)	2.74 (1.52-4.94)
		R	5.83 (4.54-7.49)	6.27 (2.49-15.83)	2.68 (1.31-5.47)
	Scandinavia	n	32	7	7
		F	6.43 (5.88-7.04)	8.67 (5.88-12.78)	2.04 (1.49-2.78)
		R	6.39 (5.29-7.71)	8.62 (4.81-15.43)	2.05 (1.37-3.06)
	Other Europe	n	50	15	15
		F	5.55 (5.22-5.90)	6.56 (5.72-7.52)	2.30 (1.99-2.65)
		R	6.09 (4.95-7.51)	8.87 (5.50-14.31)	2.72 (2.01-3.68)
	China	n	51	12	12
		F	2.77 (2.72-2.83)	5.65 (4.71-6.78)	1.48 (1.28-1.70)
		R	2.69 (2.50-2.88)	6.39 (4.73-8.63)	1.48 (1.28-1.70)
	Japan	N	18	8	11
		F	3.19 (2.91-3.49)	13.94 (9.71-20.00)	1.74 (1.47-2.06)
		R	3.21 (2.68-3.85)	14.54 (9.78-21.62)	1.80 (1.34-2.41)
	Other Asia	N	18	12	12
		F	3.79 (3.41-4.20)	7.87 (6.31-9.81)	1.88 (1.56-2.26)
		R	3.76 (2.72-5.18)	8.02 (5.54-11.60)	1.87 (1.29-2.71)
	Other or multiregion	n	14	6	6
		F	6.25 (5.45-7.16)	16.70 (10.07-27.70)	4.20 (2.91-6.06)
		R	7.41 (5.72-9.60)	16.70 (10.07-27.70)	4.20 (2.91-6.06)
	Between levels	P_B_	<0.001	<0.01	<0.001
Start year of study	Before 1960	n	54	14	14
		F	3.96 (3.71-4.23)	4.35 (3.66-5.18)	1.64 (1.27-2.10)
		R	4.67 (3.75-5.82)	5.89 (3.89-8.92)	1.64 (1.27-2.10)
	1960-69	n	52	14	14
		F	5.16 (4.88-5.45)	11.87 (9.75-14.44)	4.38 (3.78-5.08)
		R	5.45 (4.60-6.46)	12.88 (7.71-21.52)	3.62 (2.00-6.54)
	1970-79	n	71	26	31
		F	4.58 (4.34-4.82)	8.76 (7.74-9.92)	2.47 (2.21-2.76)
		R	5.05 (4.27-5.96)	10.08 (7.21-14.09)	2.69 (2.14-3.37)
	1980-89	n	114	40	40
		F	4.11 (4.04-4.17)	11.42 (10.46-12.47)	4.17 (3.90-4.45)
		R	5.95 (5.18-6.83)	11.74 (9.40-14.66)	3.23 (2.48-4.20)
	1990 or later	n	37	8	8
		F	5.28 (4.83-5.77)	12.39 (8.51-18.05)	1.76 (1.44-2.17)
		R	6.22 (4.89-7.92)	12.21 (7.56-19.72)	1.99 (1.40-2.83)
	Between levels	P_B_	NS	<0.1	<0.01
Study type	Case–control	n	262	93	98
		F	4.12 (4.06-4.18)	9.46 (8.89-10.08)	3.41 (3.24-3.59)
		R	5.32 (4.87-5.82)	10.31 (8.70-12.22)	2.78 (2.33-3.32)
	Prospective^f^	n	66	9	9
		F	6.01 (5.69-6.34)	12.23 (8.00-18.69)	3.95 (3.11-5.01)
		R	6.24 (5.34-7.28)	12.78 (7.29-22.41)	3.66 (2.67-5.03)
	Between levels	P_B_	<0.1	NS	NS
National cigarette tobacco type	Virginia	n	50	9	9
		F	5.60 (5.22-6.00)	12.69 (9.36-17.20)	4.36 (3.48-5.48)
		R	6.24 (5.16-7.54)	13.80 (6.53-29.17)	4.37 (3.01-6.34)
	Blended	n	225	80	85
		F	7.48 (7.32-7.65)	10.12 (9.46-10.83)	3.90 (3.69-4.12)
		R	6.30 (5.79-6.87)	11.07 (9.21-13.31)	3.07 (2.55-3.69)
	Other	n	53	13	13
		F	2.77 (2.72-2.83)	5.56 (4.65-6.66)	1.46 (1.27-1.67)
		R	2.68 (2.49-2.87)	6.15 (4.60-8.23)	1.46 (1.27-1.67)
	Between levels	P_B_	<0.001	<0.01	<0.001
Any proxy use	No^g^	n	227	76	79
		F	6.87 (6.72-7.03)	8.95 (8.37-9.56)	3.41 (3.23-3.61)
		R	5.51 (5.02-6.04)	9.64 (7.99-11.64)	2.63 (2.15-3.20)
	Yes	n	101	26	28
		F	3.15 (3.09-3.20)	14.65 (12.30-17.46)	3.56 (3.13-4.04)
		R	5.39 (4.84-6.02)	13.82 (10.45-18.27)	3.61 (2.73-4.76)
	Between levels	P_B_	NS	<0.05	<0.1
Full histological confirmation	No	n	245	59	59
		F	3.86 (3.80-3.92)	7.21 (6.57-7.90)	2.43 (2.24-2.63)
		R	5.25 (4.80-5.74)	9.39 (7.54-11.68)	2.56 (2.09-3.15)
	Yes	n	83	43	48
		F	7.67 (7.38-7.98)	11.97 (11.01-13.02)	4.37 (4.09-4.66)
		R	6.30 (5.47-7.25)	12.32 (9.78-15.52)	3.22 (2.53-4.10)
	Between levels	P_B_	<0.05	<0.1	NS
Number of cases^h^	100-249	n	115	22	27
		F	3.74 (3.51-3.99)	6.02 (4.86-7.45)	1.93 (1.67-2.24)
		R	4.43 (3.86-5.09)	8.39 (5.91-11.92)	2.31 (1.71-3.12)
	250-499	n	86	31	31
		F	4.85 (4.59-5.12)	8.89 (7.51-10.52)	2.37 (2.10-2.68)
		R	5.75 (4.95-6.69)	10.17 (7.81-13.22)	2.37 (1.81-3.09)
	500-999	n	64	18	18
		F	4.93 (4.68-5.19)	8.26 (6.92-9.85)	2.40 (2.07-2.77)
		R	6.17 (5.25-7.25)	11.35 (7.79-16.53)	2.86 (2.08-3.92)
	1000+	n	63	31	31
		F	4.15 (4.08-4.21)	10.51 (9.74-11.35)	4.61 (4.32-4.92)
		R	6.14 (5.15-7.32)	10.88 (8.16-14.51)	3.77 (2.82-5.02)
	Between levels	P_B_	<0.01	NS	<0.1
Smoking product	Any	n	205	54	55
		F	3.79 (3.73-3.85)	6.86 (6.16-7.65)	2.42 (2.20-2.66)
		R	5.41 (4.90-5.97	8.94 (7.02-11.39)	2.27 (1.87-2.76)
	Cigarettes (ignoring	n	114	46	50
	other products)	F	6.52 (6.31-6.73)	10.99 (10.18-11.85)	3.94 (3.71-4.19)
		R	5.54 (4.87-6.30)	11.86 (9.57-14.71)	3.46 (2.70-4.44)
	Cigarettes only	n	9	2	2
		F	8.11 (7.11-9.26)	38.79 (18.74-80.31)	4.26 (3.15-5.74)
		R	6.83 (4.63-10.08)	38.79 (18.74-80.31)	4.26 (3.15-5.74)
	Between levels	P_B_	NS	<0.001	<0.001
Unexposed base	Never any product	n	236	64	67
		F	3.84 (3.78-3.90)	8.06 (7.37-8.82)	2.57 (2.38-2.77)
		R	5.31 (4.84-5.81)	9.68 (7.81-12.01)	2.46 (2.09-2.89)
	Never cigarettes	n	92	38	40
		F	7.01 (6.77-7.26)	11.04 (10.13-12.02)	4.30 (4.02-4.60)
		R	5.96 (5.17-6.88)	11.92 (9.24-15.38)	3.56 (2.66-4.78)
	Between levels	P_B_	NS	NS	<0.05
Number of adjustment factors	0	n	164	53	54
		F	7.12 (6.92-7.33)	7.93 (7.20-8.74)	2.40 (2.21-2.60)
		R	5.44 (4.86-6.09)	9.86 (7.79-12.49)	2.61 (2.11-3.23)
	1	n	69	18	20
		F	5.13 (4.90-5.37)	12.59 (10.37-15.30)	3.24 (2.81-3.73)
		R	5.48 (4.72-6.37)	11.34 (7.36-17.47)	2.66 (1.74-4.06)
	2+	n	95	31	33
		F	3.40 (3.34-3.46)	10.48 (9.59-11.45)	4.61 (4.29-4.95)
		R	5.56 (4.87-6.34)	11.02 (8.35-14.54)	3.30 (2.50-4.36)
	Between levels	P_B_	NS	NS	NS

^a^ Within each study, results for ever smokers are selected in the following preference order, within each sex, for:

smoking product – any, cigarettes (ignoring other products), cigarettes only;

cigarette type – any, manufactured (with or without handrolled), manufactured only;

unexposed group – never any product, never cigarettes, near equivalent (see Methods);

follow-up period – longest available;

lung cancer type – see notes c to e;

race – all or nearest available, otherwise by race;

overlapping studies – principal, subsidiary;

age – whole study, widest available age group.

Results are then selected for:

sex – single sex results, combined sex results;

adjustment for potential confounders – most available.

^b^ n = number of estimates combined, F = fixed-effect meta-analysis RR (95% CI), R = random-effects meta-analysis RR (95% CI), H = heterogeneity chisquared per degree of freedom, P_H_ = probability value for heterogeneity expressed as p < 0.001, p < 0.01, p < 0.05, p < 0.1 or NS (p ≥ 0.1), P_E_ = probability value for Egger’s test of publication bias similarly expressed, P_B_ = probability value for between levels (see Methods) similarly expressed.

^c^ All or nearest available, must include at least squamous cell carcinoma and adenocarcinoma.

^d^ Squamous cell carcinoma or nearest available, but not including adenocarcinoma.

^e^ Adenocarcinoma or nearest available, but not including squamous cell carcinoma.

^f^ Or nested case–control or case-cohort in the case of 5 estimates for all lung cancer, 4 for squamous and 4 for adeno.

^g^ Or not known in the case of 10 estimates for all lung cancer, 2 for squamous, and 2 for adeno.

^h^ In the study as a whole.

**Table 6:** **Some alternative meta-analyses for ever smoking compared to those in Table**5

**Analysis description**	**Statistic**^**b**^	**All lung cancer**^**c**^	**Squamous**^**d**^	**Adeno**^**e**^
As Table 5^a^	n	328	102	107
	F	4.22 (4.16-4.28)	9.52 (8.94-10.13)	3.44 (3.27-3.61)
	R	5.50 (5.07-5.96)	10.47 (8.88-12.33)	2.84 (2.41-3.35)
	H, P_H_	22.84, p < 0.001	5.17, p < 0.001	8.78, p < 0.001
Using more precise outcome definition^f^	n	317	74	87
	F	4.23 (4.17-4.29)	10.43 (9.72-11.20)	3.58 (3.39-3.78)
	R	5.59 (5.15-6.07)	11.56 (9.68-13.81)	2.99 (2.49-3.58)
	H, P_H_	23.48, p < 0.001	4.18, p < 0.001	9.18, p < 0.001
Using least rather than most adjusted estimates	n	331	102	107
	F	4.23 (4.17-4.29)	9.49 (8.93-10.09)	3.38 (3.21-3.55)
	R	5.48 (5.06-5.93)	10.37 (8.83-12.18)	2.83 (2.39-3.34)
	H, P_H_	23.36, p < 0.001	5.23, p < 0.001	9.57, p < 0.001
Omitting study LIU4	n	326	NA^g^	NA^g^
	F	6.47 (6.34-6.60)		
	R	5.52 (5.10-5.97)		
	H, P_H_	12.54, p < 0.001		
Preferring results for cigarettes to results for any product	n	327	102	107
	F	4.73 (4.66-4.80)	9.48 (8.91-10.08)	3.43 (3.27-3.61)
	R	5.49 (5.10-5.92)	10.44 (8.88-12.29)	2.82 (2.39-3.32)
	H, P_H_	19.39, p < 0.001	5.12, p < 0.001	8.72, p < 0.001
Selecting results for cigarettes only^h^	n	54	11	11
	F	4.37 (4.28-4.47)	9.05 (7.84-10.45)	1.93 (1.70-2.18)
	R	6.45 (5.41-7.70)	11.50 (7.47-17.69)	2.87 (1.49-5.55)
	H, P_H_	22.68, p < 0.001	4.57, p < 0.001	16.64, p < 0.001
Selecting results specific for age <56 years	n	38	10	10
	F	5.79 (5.15-6.51)	11.04 (7.08-17.20)	4.94 (3.73-6.56)
	R	6.57 (4.94-8.74)	14.73 (6.83-31.76)	4.17 (1.86-9.35)
	H, P_H_	4.17, p < 0.001	2.35, p < 0.05	7.00, p < 0.001
Selecting results specific for age 50–70 years	n	31	6	4
	F	5.77 (5.29-6.29)	16.32 (11.11-23.95)	5.62 (3.91-8.07)
	R	6.46 (4.99-8.35)	17.30 (10.78-27.74)	5.31 (3.20-8.79)
	H, P_H_	7.67, p < 0.001	1.37, NS	1.14, NS
Selecting results specific for age 65+ years	n	37	2	2
	F	2.88 (2.78-3.00)	15.00 (6.46-34.80)	1.73 (0.99-3.02)
	R	5.48 (4.59-6.55)	15.00 (6.46-34.80)	1.73 (0.99-3.02)
	H, P_H_	9.66, p < 0.001	0.02, NS	0.19, NS

^a^ See Table 5 for details of the definition of this analysis.

^b^ n = number of estimates combined, F = fixed-effect meta-analysis RR (95% CI), R = random-effects meta-analysis RR (95% CI), H = heterogeneity chisquared per degree of freedom, P_H_ = probability value for heterogeneity expressed as p < 0.001, p < 0.01, p < 0.05, p < 0.1 or NS (p ≥ 0.1), P_B_ = probability value for between levels (see methods) similarly expressed.

^c^ All or nearest available, must include at least squamous cell carcinoma and adenocarcinoma.

^d^ Squamous cell carcinoma or nearest available, but not including adenocarcinoma.

^e^ Adenocarcinoma or nearest available, but not including squamous cell carcinoma.

^f^ Only including results for all lung cancer, squamous cell carcinoma specifically, or adenocarcinoma specifically.

^g^ Not applicable as results by histological type were not available for study LIU4.

^h^ See also Table 13 (footnote c).

First, the RRs for all three outcomes are markedly heterogeneous. As shown in Table 5, H is estimated as 22.84 for all lung cancer, 5.17 for squamous and 8.78 for adeno (p < 0.001). Individual RRs vary up to 125.27 for all lung cancer (study STUCKE for males), 92.66 for squamous (ABRAHA/males), and 34.45 for adeno (SCHWAR/males). Based on random-effects estimates, a positive association is seen, strongest for squamous (RR 10.47, 95% CI 8.88-12.33, based on 102 RRs), but also clearly evident for all lung cancer (5.50, 5.07-5.96, n = 328) and adeno (2.84, 2.41-3.35, n = 107). Although the strength of association varies markedly by study, the consistency of direction is clear, with only two of the all lung cancer RRs, none of the 102 squamous RRs, and nine of the 107 adeno RRs below 1.0.

As shown in Table 6, the overall estimates for each outcome were virtually unchanged by using least-adjusted rather than most-adjusted estimates. They were slightly increased by restricting attention to estimates using a more precise outcome definition, the random-effects estimates changing to 5.59 (5.15-6.07) for the 317 estimates specifically for all lung cancer, 11.56 (9.68-13.81) for the 74 estimates specifically for squamous cell carcinoma, and 2.99 (2.49-3.58) for the 87 estimates specifically for adenocarcinoma. The overall estimates for each outcome were virtually unchanged when RRs for ever smoking cigarettes were preferred to RRs for ever smoking any product. This is partly due to many studies providing only one type of RR, so that for all lung cancer, for example, 250 of the 328 RRs are common to both meta-analyses. A much smaller number of estimates were available for cigarette only smoking; RRs from these were slightly higher: 6.45 (5.41-7.70, n = 54) for all lung cancer, 11.50 (7.47-17.69, n = 11) for squamous, and 2.87 (1.49-5.55, n = 11) for adeno. Estimates were also extracted specifically for populations of age <56, 50–70 or 65+ years (with age determined at baseline for prospective studies). As shown in Table 6, data were rather limited for squamous and adeno, particularly for older populations. For all lung cancer, the three RRs: 6.57 (4.94-8.74, n = 38) for age <56 years, 6.46 (4.99-8.35, n = 31) for age 50–70 years, and 5.48 (4.59-6.55, n = 37) for age 65+ years were all consistent with the overall RR of 5.50, with no clear trend.

Returning to the main meta-analysis (most-adjusted and preferring ever smoking any product), there is a large variation between RRs in the weight they contribute to the analysis. This is very marked for all lung cancer. Here the 328 estimates provided a combined weight of 19,346 (mean 59.0), but the male and female estimates from study LIU4 together contributed a weight of 9,846, 50.9% of the total. Omitting these two estimates substantially reduced the heterogeneity, H falling from 22.84 to 12.54. The next largest weights were 1,550 in study STOCKW (sexes combined), 443 in BROWN2 (males) and 428 in BROWN2 (females). For squamous, the total weight was 1,000 for the 102 RRs (mean 9.8). The largest contributors to this were 164 for BROWN2/males, 90 for BROWN2/females, 52 for LUBIN2/males and 47 for LUBIN2/females, together contributing 35% of the total weight. For adeno, the total weight was 1,514 for the 107 RRs (mean 14.1). Again, BROWN2 and LUBIN2 were the largest contributors, providing, respectively, 24% and 6% of the total weight.

In investigating sources of heterogeneity, variation was studied firstly using a univariable approach, the results for the characteristics considered in Table 5 being summarized below, based on the random-effects estimates.

#### Sex

For all three outcomes, RRs were always somewhat lower for females than for males or for sexes combined, though the variation by sex was not significant (p ≥ 0.1) for squamous.

#### Location

For all three outcomes, RRs were lower from studies conducted in Europe and Asia than from studies conducted in North America. While for all lung cancer and adeno RRs were noticeably lower in Asia than in Europe, this difference was not evident for squamous. The difference in RRs by continent was very marked and highly significant (p < 0.001) for all lung cancer and adeno, but less marked, though still significant (p < 0.01) for squamous.

#### Start year of study

For all lung cancer and squamous, variation by start year was not significant (p ≥ 0.05) although there was some tendency for RRs to be higher in more recent studies. For adeno, the variation was significant (p < 0.01) but there was no clear trend.

#### Study type

For all three outcomes, RRs were somewhat lower for case–control studies than for prospective studies (or other study designs where the smoking data were collected before lung cancer diagnosis). However, the difference was never statistically significant (p ≥ 0.05).

#### National cigarette tobacco type

For all three outcomes, there was significant (p < 0.01 or< 0.001) variation. This was mainly due to low estimates in the “other” group, which mainly included results from China. For all lung cancer, RRs for Virginia (6.24, 5.16-7.54, n = 50) and blended (6.30, 5.79-6.87) were quite similar. For squamous and adeno, there were limited results for Virginia, and no clear difference from blended was evident.

#### Any proxy use

There was some evidence that RRs were higher where proxy respondents were used for squamous (p < 0.05) and adeno (p < 0.1), but not for all lung cancer.

#### Full histological confirmation

RR estimates were somewhat higher where full histological confirmation of diagnosis was a study requirement, but this was only significant at p < 0.05 for all lung cancer.

#### Number of cases

Some tendency for RRs to increase with increasing number of cases was evident for all three outcomes, but variation in number of cases was only significant for all lung cancer (p < 0.01).

#### Smoking product

The analyses in Table 5 are based on a preference order of any product, cigarettes (ignoring other products) and cigarettes only. For all lung cancer, where 205 of the 328 estimates were for any product, 114 were for cigarettes and 9 for cigarettes only, there was no evidence that the RRs included varied by smoking product. For squamous and adeno (both p < 0.001), however, RRs were lowest for smoking any product, intermediate for cigarettes, and highest for cigarettes only (though based on only two RRs for cigarettes only for each outcome).

#### Unexposed base

RRs were somewhat higher where the unexposed base group was never cigarettes than when it was never any product, though this was only significant (p < 0.05) for adeno. This result is somewhat counter-intuitive, as lower RRs might be expected where the base (never cigarettes) includes some smokers (pipe/cigar only), and probably arises from the strong correlation between the definitions of smoking product and unexposed base. Two combinations – any product vs. never any product (n = 203) and cigarettes vs. never cigarettes (n = 90) – form a large proportion of the total RRs (with any product vs never cigarettes not a valid possibility).

#### Number of adjustment factors

There was no evidence that RR estimates varied by whether they were adjusted for 0, 1 or 2+ potential confounding variables.

The full meta-analysis (see Additional file 5: Detailed Analysis Tables) also includes results by levels of some other characteristics. In an attempt to evaluate the independent role of a whole range of characteristics, preliminary meta-regression analyses were conducted for each outcome (results not shown). As a result, it was decided to present findings for a fixed model involving six major characteristics (see Table 7), test the effect of each by deleting each of the six individually from the fixed model (and also by allowing each to enter a step-wise model in order of significance), and test the effect of a range of other characteristics by adding each individually into the fixed model (see Additional file 5: Detailed Analysis Tables). The main conclusions to be drawn from these analyses are summarized below.

**Table 7:** **Meta-regression results for ever smoking of any product (or cigarettes if any product not available)**^**a**^

**Characteristic**	**Level**	**All lung cancer**		**Squamous**		**Adeno**	
		**Estimate**^**b**^**(SE**^**c**^**)**	**p**^**d**^	**Estimate**^**b**^**(SE**^**c**^**)**	**p**^**d**^	**Estimate**^**b**^**(SE**^**c**^**)**	**p**^**d**^
Constant		+1.280 (0.052)		+1.281 (0.174)		+0.368 (0.178)	
Sex	Male	Base	NS	Base	NS	Base	<0.05
	Female	−0.040 (0.016)		−0.046 (0.072)		−0.275 (0.058)	
	Combined	+0.018 (0.029)		−0.130 (0.141)		+0.102 (0.138)	
Location	North America	Base	<0.001	Base	<0.001	Base	<0.001
	United Kingdom	−0.108 (0.057)		−0.861 (0.266)		−0.535 (0.324)	
	Scandinavia	−0.095 (0.051)		−0.484 (0.241)		−0.630 (0.190)	
	Other Europe	−0.319 (0.039)		−0.609 (0.114)		−0.731 (0.107)	
	China	−1.320 (0.024)		−1.028 (0.120)		−1.356 (0.094)	
	Japan	−0.858 (0.053)		−0.562 (0.226)		−1.158 (0.119)	
	Other Asia	−0.646 (0.060)		−0.390 (0.158)		−0.557 (0.127)	
	Other or multiregion	−0.173 (0.076)		−0.031 (0.301)		−0.024 (0.213)	
Start year of study	Before 1960	Base	<0.001	Base	<0.001	Base	<0.05
	1960-69	+0.384 (0.047)		+0.819 (0.154)		+0.750 (0.166)	
	1970-79	+0.430 (0.045)		+0.802 (0.130)		+0.672 (0.150)	
	1980-89	+0.783 (0.042)		+0.990 (0.134)		+0.857 (0.155)	
	1990 or later	+0.872 (0.063)		+1.788 (0.263)		+0.892 (0.200)	
Study type	Case–control	Base	<0.01	Base	NS	Base	NS
	Prospective^e^	+0.238 (0.036)		+0.395 (0.233)		+0.352 (0.140)	
Number of cases^f^	100-249	Base	<0.001	Base	<0.05	Base	<0.05
	250-499	+0.101 (0.044)		+0.251 (0.152)		+0.265 (0.107)	
	500-999	+0.293 (0.043)		+0.729 (0.162)		+0.550 (0.121)	
	1000+	+0.290 (0.038)		+0.655 (0.145)		+0.594 (0.100)	
Number of adjustment factors	0	Base	<0.05	Base	<0.05	Base	<0.1
	1	−0.171 (0.034)		+0.477 (0.147)		+0.391 (0.110)	
	2+	+0.017 (0.025)		−0.226 (0.097)		+0.272 (0.078)	

^a^ Based on the same data as for Table 5. See that table for further definition of RRs selected for analysis, and numbers of estimates of each characteristic level.

^b^ Estimates are of log RR. The predicted RR of a given estimate can be calculated by adding the constant to the values for the level of each characteristic applicable to the estimate (taking the value for a base level as zero) and taking the exponential of the result.

^c^ SE = standard error.

^d^ The p value is estimated from the drop in deviance from removing the characteristic from the fixed model using an F-test. It is expressed as p < 0.001, p < 0.01, p < 0.05, p < 0.1 or NS (p ≥ 0.1).

^e^ Or nested case–control or case-cohort in the case of 5 estimates for all lung cancer, 4 for squamous and 4 for adeno.

^f^ In the study as a whole.

Note For the forward stepwise analyses, deviances, degrees of freedom and p values for inclusion in order of inclusion are as follows:

**All lung cancer**: Constant 7470.075 on 327 d.f. (p < 0.001), location 2247.358 on 320 d.f. (p < 0.001), start year of study 1599.339 on 316 d.f. (p < 0.001), number of cases 1523.722 on 313 d.f. (p < 0.01), study type 1497.854 on 312 d.f. (p < 0.05) and number of adjustment variables 1463.482 on 310 d.f. (p < 0.05). Fixed model deviance 1454.810 on 308 d.f.

**Squamous**: Constant 522.221 on 101 d.f. (p < 0.001), start year of study 419.586 on 97 d.f. (p < 0.001) and location 308.715 on 90 d.f. (p < 0.001). Fixed model deviance 260.432 on 82 d.f.

**Adeno**: Constant 930.356 on 106 d.f. (p < 0.001), location 431.955 on 99 d.f. (p < 0.001), number of cases 369.648 on 96 d.f. (p <0.01), start year of study 328.896 on 92 d.f. (p < 0.05) and sex 307.336 on 90 d.f. (p < 0.05). Fixed model deviance 284.147 on 87 d.f.

For all lung cancer, by far the strongest source of variation was location, with the overall heterogeneity reduced from 22.84 per d.f. to 7.02 per d.f. after including location only into the model. As noted earlier this was mainly due to relatively high RRs in North America and low RRs in Asia. Other clear effects were also associated with start year of study (p < 0.001, higher risks in later studies, much more clearly evident than in the univariable analyses in Table 5), study type (p < 0.01, higher risks in prospective studies) and number of cases (p < 0.001, higher risks in larger studies). There was no significant effect of sex, and the weakly significant (p < 0.05) effect for number of adjustment factors was associated with an erratic pattern, with lower RRs where the number of factors was 1, and higher RRs where it was 0 or 2+. The heterogeneity for the fixed model including all the six characteristics included in Table 7 was 4.72 per d.f., with the model explaining 80.5% of the overall variation between the RRs. Inspection of standardized residuals revealed eight estimates where the value was outside the range +/− 2.5 SEs : MILLS/males (RR 1.33, fitted 3.35), LOMBA2/females (RR 1.33, fitted 5.07), TIZZAN/males (RR 1.93, fitted 3.50), WANG4/males (RR 1.16, fitted 2.01), PERNU/males (RR 8.93, fitted 4.37), LUBIN2/males (RR 8.50, fitted 5.47), BOFFET/males (RR 14.20, fitted 7.78) and JUSSAW/males (RR 16.83, fitted 3.77). Only two other characteristics studied significantly (p < 0.05) improved the fit of the model, both related to study location. One was a variable subdividing “Other Europe” (i.e. other than UK and Scandinavia) into five smaller regions, with risk relatively low in the Balkans (Greek and Turkish studies) and relatively high in multiregional studies compared with the rest, and the other a variable subdividing “Other Asia” (i.e. other than China or Japan) into three smaller regions, with risk higher in India compared to Hong Kong and the rest of Asia (Taiwan, Thailand, Singapore and South Korea). No independent effect was evident for national cigarette tobacco type. Additional analysis (data not shown) confirmed the strong independent effect of start year of study separately within studies conducted in North America, Europe and Asia, though the tendency for higher RRs in more recent studies was stronger in North America than in Europe, and the pattern of variation was more erratic for Asia. It also confirmed the strong independent effects of location and start year of study separately for males and for females.

For squamous, start year of study was the most important factor, on its own reducing the heterogeneity from 5.17 to 4.33 per d.f. (p < 0.001). Other significant characteristics included location (p < 0.001), with RRs high in North America and low in China, and number of cases (p < 0.05), with higher RRs in larger studies. Number of adjustment factors was also significant (p < 0.05), but the pattern was erratic and not the same as for all lung cancer. Though the pattern of results by study type was similar to that for all lung cancer, this characteristic did not contribute significantly to the model. The heterogeneity for the fixed model (Table 7) was 3.18 per d.f., the model explaining 49.9% of the overall variation. Two standardized residuals were outside the range +/− 2.5 SEs : STAYNE/males (RR 3.47, fitted 10.50) and LUBIN2/males (RR 16.66, fitted 8.41). Two other characteristics significantly improved the model fit. One was national cigarette tobacco type, with RRs higher where flue-cured Virginia tobacco was smoked, than where blended tobacco was smoked. Also, RRs were higher (p < 0.01) where they had been derived by a relatively complex method (see Methods) than where they were as reported originally, or derived by more standard methods.

For adeno, location was the most important factor, on its own reducing the heterogeneity from 8.78 to 4.36 per d.f. (p < 0.001), with the pattern of results (RRs high in North America and low in Asia) similar to that for all lung cancer. As for all lung cancer, there was variation by start year of study (p < 0.05) and number of cases (p < 0.05), with RRs higher for recent and larger studies. RRs were again higher for prospective studies, but here the difference was not significant (p ≥ 0.05). Here, variation by sex was significant (p < 0.05) with RRs higher for males than females, but number of adjustment factors was not (p ≥ 0.05). The heterogeneity for the fixed model (Table 7) was 3.27 per d.f., the model explaining 69.5% of the overall variation. Two standardized residuals were outside the range +/− 2.5 SEs : LOMBA2/females (RR 0.53, fitted 2.32) and WYNDER6/females (RR 13.99 fitted 6.22). Four other characteristics significantly improved the model fit. One was “Other Asia” (p < 0.05) where RRs were high in India (based on a single RR from JUSSAW) and relatively low in Hong Kong, Taiwan, Thailand, Singapore and South Korea. National cigarette tobacco type was also significant (p < 0.05), with RRs for blended higher than for Virginia, opposite to the finding for squamous. RRs were also lower where there was any use of proxy respondents (p < 0.05). Also, RRs varied (p < 0.001) by the detailed definition of adenocarcinoma used. This appeared to be mainly because of a low RR for “not squamous or undifferentiated”, a definition used only for LOMBA2/females, where the standardized residual of −3.721 SEs was the largest for any RR (see also above).

The fixed model (Table 7) considered how RR estimates varied by six main characteristics and additional analyses (see Additional file 5: Detailed Analysis Tables) tested whether adding in further characteristics improved the model fit. Characteristics which did not improve the fit for any of the three outcomes considered included whether there was adjustment for specific factors (such as age), the age of the subjects studied, the definition of smoking product, the definition of the unexposed base, whether the study was conducted in a population working in a risky occupation, and whether the study procedures required full histological confirmation.

#### B. Risk from current smoking

**Figure 10:**
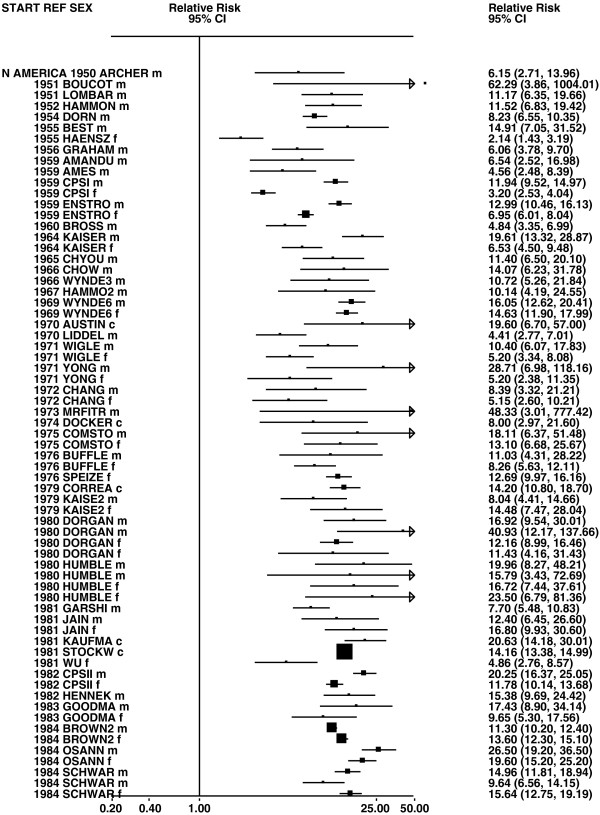
**Forest plot of current smoking of any product and all lung cancer – part 1.** Table 8 presents the results of a main meta-analysis for all lung cancer based on 195 relative risk (RR) and 95% confidence interval (CI) estimates for current smoking of any product (or cigarettes if any product not available). The individual study estimates are shown numerically and graphically on a logarithmic scale in Figures 10, 11, 12. The studies are sorted in order of sex within study reference (REF) within start year of study (START) within continent (CONT). In the graphical representation individual RRs are indicated by a solid square, with the area of the square proportional to the weight (inverse-variance of log RR). Arrows indicate where the CI extends outside the range allocated. For study DORGAN separate estimates, within sex, are shown for whites then blacks. For study HUMBLE they are shown for non-hispanic whites then hispanics, and for study SCHWAR for whites then non-whites.

**Figure 11:**
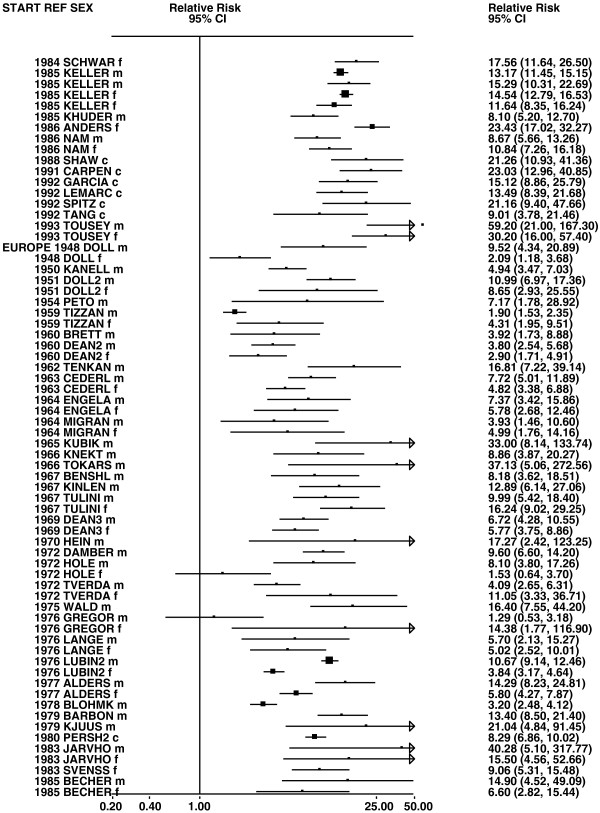
**Forest plot of current smoking of any product and all lung cancer – part 2.** This is a continuation of Figure 10, presenting further individual study data included in the main meta-analysis for all lung cancer shown in Table 8. For study KELLER separate estimates, within sex, are shown for whites then non-whites.

**Figure 12:**
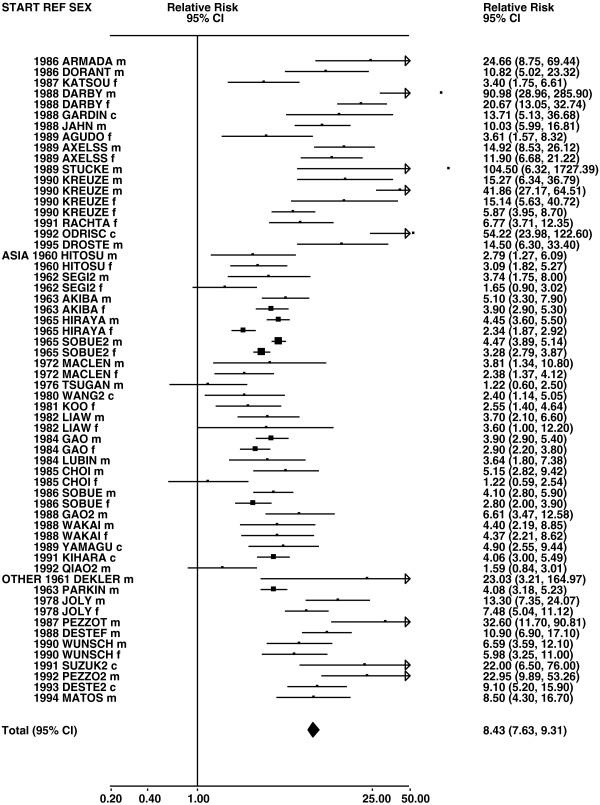
**Forest plot of current smoking of any product and all lung cancer – part 3.** This is a continuation of Figure 11, presenting the remaining individual study data included in the main meta-analysis for all lung cancer shown in Table 8. Also shown are the combined random-effect estimates. These are represented by a diamond of standard height, with the width indicating the 95% CI. For study KREUZE separate estimates, within sex, are shown for age ≤ 45 and 55–69.

**Figure 13:**
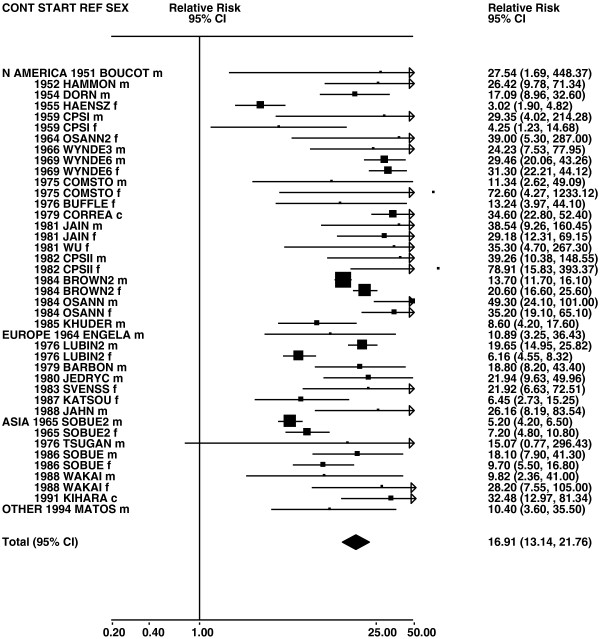
**Forest plot of current smoking of any product and squamous.** Table 8 presents the results of a main meta-analysis for squamous based on 41 relative risk (RR) and 95% confidence interval (CI) estimates for current smoking of any product (or cigarettes if any product not available). The individual study estimates are shown numerically and graphically on a logarithmic scale. The studies are sorted in order of sex within study reference (REF) within start year of study (START) within continent (CONT). In the graphical representation individual RRs are indicated by a solid square, with the area of the square proportional to the weight (inverse-variance of log RR). Arrows indicate where the CI extends outside the range allocated. Also shown are the combined random-effect estimates. These are represented by a diamond of standard height, with the width indicating the 95% CI.

**Figure 14:**
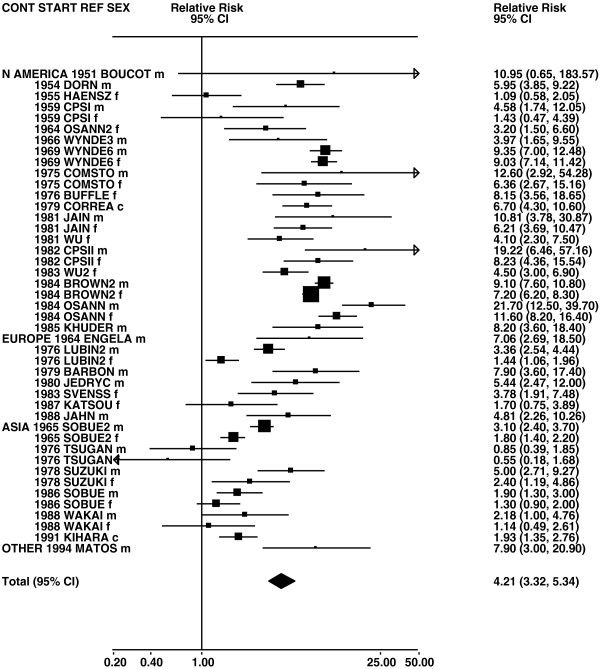
**Forest plot of current smoking of any product and adeno.** Table 8 presents the results of a main meta-analysis for adeno based on 44 relative risk (RR) and 95% confidence interval (CI) estimates for current smoking of any product (or cigarettes if any product not available). The individual study estimates are shown numerically and graphically on a logarithmic scale. The studies are sorted in order of sex within study reference (REF) within start year of study (START) within continent (CONT). In the graphical representation individual RRs are indicated by a solid square, with the area of the square proportional to the weight (inverse-variance of log RR). Arrows indicate where the CI extends outside the range allocated. Also shown are the combined random-effect estimates. These are represented by a diamond of standard height, with the width indicating the 95% CI.

Figures 10, 11, 12 (all lung cancer), Figure 13 (squamous) and Figure 14 (adeno) present the results of the main meta-analyses for current smoking of any product. As before, RRs for smoking of cigarettes are used if RRs for any product smoking are not available, and RRs are most-adjusted. For prospective studies, current smoking refers to smoking status as at baseline. Table 8 presents additional results by level of the same set of characteristics considered in Table 5, while Table 9 presents results of alternative meta-analyses of current smoking.

**Table 8:** **Main meta-analyses for current smoking of any product (or cigarettes, if any product not available)**^**a**^

**Characteristic**	**Level**	**Statistic**^**b**^	**All lung cancer**^**c**^	**Squamous**^**d**^	**Adeno**^**e**^
All	All	n	195	41	44
		F	9.29 (9.07-9.52)	13.77 (12.75-14.87)	4.77 (4.49-5.07)
		R	8.43 (7.63-9.31)	16.91 (13.14-21.76)	4.21 (3.32-5.34)
		H, P_H_	13.76, p < 0.001	7.22, p < 0.001	12.28, p < 0.001
		P_E_	p < 0.05	NS	NS
Sex	Male	n	108	22	22
		F	8.68 (8.36-9.02)	13.11 (11.85-14.51)	5.54 (5.04-6.09)
		R	9.16 (8.00-10.49)	17.73 (12.78-24.59)	5.56 (4.06-7.61)
	Female	n	68	17	20
		F	8.06 (7.75-8.38)	13.44 (11.86-15.23)	4.40 (4.05-7.79)
		R	6.76 (5.65-8.08)	14.77 (9.42-23.17)	3.20 (2.18-4.71)
	Combined	n	19	2	2
		F	13.19 (12.55-13.86)	34.23 (23.43-50.00)	3.11 (2.35-4.11)
		R	12.09 (9.38-15.60)	34.23 (23.43-50.00)	3.57 (1.06-12.07)
	Between levels	P_B_	<0.001	<0.01	<0.1
Location	North America	n	84	24	24
		F	12.45 (12.09-12.82)	17.99 (16.29-19.85)	7.63 (7.05-8.25)
		R	11.68 (10.61-12.85)	20.95 (15.41-28.48)	6.72 (5.50-8.21)
	United Kingdom	n	25	0	0
		F	6.90 (6.10-7.81)		
		R	7.53 (5.40-10.50)		
	Scandinavia	n	21	2	2
		F	8.16 (7.30-9.12)	15.51 (6.63-36.28)	4.66 (2.67-8.13)
		R	8.68 (7.14-10.54)	15.51 (6.63-36.28)	4.69 (2.62-8.40)
	Other Europe	n	23	6	6
		F	5.88 (5.43-6.38)	12.22 (10.16-14.69)	2.65 (2.20-3.18)
		R	8.65 (5.98-12.51)	13.66 (7.38-25.30)	3.30 (1.93-5.65)
	China	n	5	0	0
		F	3.07 (2.56-3.68)		
		R	2.94 (2.23-3.88)		
	Japan	n	18	8	11
		F	3.68 (3.43-3.95)	6.85 (5.77-8.13)	2.11 (1.87-2.37)
		R	3.55 (3.05-4.14)	11.25 (6.89-18.35)	1.87 (1.42-2.47)
	Other Asia	n	7	0	0
		F	2.91 (2.26-3.76)		
		R	2.90 (2.04-4.13)		
	Other or multiregion	n	12	1	1
		F	7.09 (6.10-8.24)	10.40 (3.31-32.66)	7.90 (2.99-20.85)
		R	9.88 (6.89-14.17)	10.40 (3.31-32.66)	7.90 (2.99-20.85)
	Between levels	P_B_	<0.001	NS	<0.001
Start year of study	Before 1960	n	22	6	5
		F	6.13 (5.70-6.59)	6.87 (4.93-9.08)	3.35 (2.43-4.62)
		R	6.39 (4.70-8.69)	11.05 (4.07-30.00)	2.92 (1.17-7.28)
	1960-69	n	40	7	7
		F	5.43 (5.12-5.75)	10.55 (9.07-12.26)	4.22 (3.76-4.74)
		R	6.44 (5.21-7.95)	15.48 (7.08-33.83)	4.55 (2.50-8.30)
	1970-79	n	41	8	11
		F	7.27 (6.79-7.79)	14.53 (12.21-17.29)	2.99 (2.55-3.50)
		R	7.34 (5.94-9.06)	16.43 (8.67-31.14)	3.41 (2.10-5.53)
	1980-89	n	70	18	19
		F	12.35 (11.97-12.75)	16.70 (14.93-18.67)	6.38 (5.84-6.96)
		R	10.18 (9.02-11.49)	19.69 (15.08-25.71)	5.06 (3.65-7.02)
	1990 or later	n	22	2	2
		F	10.32 (9.10-11.70)	20.79 (10.16-42.55)	2.28 (1.63-3.19)
		R	12.81 (8.70-18.85)	19.39 (6.38-58.88)	3.62 (0.92-14.29)
	Between levels	P_B_	<0.001	NS	NS
Study type	Case–control	n	128	30	34
		F	9.69 (9.43-9.95)	13.59 (12.56-14.70)	4.71 (4.42-5.01)
		R	8.56 (7.58-9.68)	16.21 (12.23-21.50)	3.88 (2.96-5.07)
	Prospective^f^	n	67	11	10
		F	8.04 (7.65-8.46)	18.87 (12.90-27.59)	5.93 (4.59-7.66)
		R	8.15 (6.87-9.67)	19.63 (12.21-31.56)	5.95 (4.07-8.69)
	Between levels	P_B_	NS	NS	p < 0.1
National cigarette tobacco type	Virginia	n	34	2	2
		F	6.75 (6.12-7.44)	31.44 (15.03-65.78)	6.93 (4.35-11.07)
		R	8.01 (6.16-10.41)	31.44 (15.03-65.78)	6.93 (4.35-11.07)
	Blended	n	154	39	42
		F	9.70 (9.46-9.95)	13.65 (12.63-14.75)	4.74 (4.45-5.04)
		R	8.92 (8.01-9.94)	16.41 (12.68-21.24)	4.10 (3.21-5.23)
	Other	n	7	0	0
		F	3.13 (2.64-3.72)		
		R	3.09 (2.50-3.83)		
	Between levels	P_B_	<0.001	NS	p < 0.1
Any proxy use	No^g^	n	156	35	38
		F	9.24 (9.00-9.48)	13.09 (12.09-14.18)	4.67 (4.39-4.98)
		R	8.04 (7.17-9.01)	15.78 (12.00-20.74)	3.88 (2.99-5.03)
	Yes	n	39	6	6
		F	9.68 (9.05-10.36)	27.96 (20.74-37.69)	6.88 (5.27-8.97)
		R	10.03 (8.32-12.09)	27.96 (20.74-37.69)	6.88 (5.27-8.97)
	Between levels	P_B_	<0.05	<0.01	p < 0.01
Full histological confirmation	No	n	144	24	23
		F	8.87 (8.62-9.12)	11.53 (10.05-13.23)	3.99 (3.59-4.43)
		R	7.91 (7.00-8.93)	19.20 (12.62-29.22)	5.59 (3.98-7.84)
	Yes	n	51	17	21
		F	10.45 (9.99-10.92)	14.94 (13.61-16.40)	5.25 (4.87-5.67)
		R	10.08 (8.46-12.01)	14.77 (10.61-20.57)	3.20 (2.28-4.49)
	Between levels	P_B_	<0.05	NS	p < 0.05
Number of cases^h^	100-249	n	56	8	11
		F	5.50 (4.96-6.11)	5.62 (3.96-7.98)	2.54 (2.02-3.19)
		R	6.90 (5.46-8.72)	11.28 (5.08-25.04)	2.45 (1.54-3.90)
	250-499	n	48	9	9
		F	8.47 (7.79-9.22)	16.67 (11.49-24.18)	3.20 (2.59-3.95)
		R	8.60 (7.02-10.52)	17.09 (11.28-25.91)	3.83 (2.41-6.09)
	500-999	n	38	4	4
		F	7.56 (7.04-8.13)	22.44 (13.58-37.07)	7.35 (5.12-10.55)
		R	9.54 (7.65-11.89)	22.44 (13.58-37.07)	7.35 (5.12-10.55)
	1000+	n	53	20	20
		F	10.01 (9.74-10.29)	14.15 (13.03-15.35)	5.18 (4.84-5.54)
		R	9.03 (7.63-10.69)	17.87 (12.92-24.70)	4.97 (3.54-6.97)
	Between levels	P_B_	NS	NS	p <0.01
Smoking product	Any	n	85	9	11
		F	8.00 (7.64-8.37)	17.58 (11.99-25.77)	2.61 (2.16-3.14)
		R	7.57 (6.47-8.84)	17.76 (11.75-26.86)	2.40 (1.59-3.61)
	Cigarettes (ignoring	n	95	26	28
	other products)	F	9.84 (9.56-10.14)	13.51 (12.47-14.64)	5.11 (4.79-5.46)
		R	8.95 (7.76-10.33)	16.43 (12.08-22.33)	5.02 (3.76-6.70)
	Cigarettes only	n	15	6	5
		F	9.77 (8.92-10.69)	17.80 (11.39-27.83)	5.69 (4.00-8.09)
		R	10.51 (7.70-14.34)	18.04 (9.79-33.27)	5.56 (2.61-11.85)
	Between levels	P_B_	NS	NS	p < 0.05
Unexposed base	Never any product	n	134	24	27
		F	8.80 (8.55-9.06)	9.80 (8.75-10.98)	3.01 (2.75-3.29)
		R	8.24 (7.28-9.34)	14.05 (9.75-20.23)	3.10 (2.37-4.07)
	Never cigarettes	n	61	17	17
		F	10.43 (10.00-10.88)	18.44 (16.60-20.48)	7.17 (6.59-7.80)
		R	8.89 (7.52-10.51)	21.71 (16.38-28.76)	6.61 (4.89-8.95)
	Between levels	P_B_	NS	<0.1	<0.001
Number of adjustment variables	0	n	86	16	17
		F	10.84 (10.46-11.23)	14.93 (13.02-17.12)	4.28 (3.83-4.79)
		R	9.40 (8.02-11.03)	17.15 (10.78-27.27)	3.90 (2.54-6.00)
	1	n	62	15	14
		F	7.80 (7.43-8.19)	19.11 (15.33-23.83)	3.25 (2.76-3.83)
		R	7.28 (6.14-8.63)	18.27 (12.55-26.60)	3.46 (2.27-5.29)
	2+	n	47	10	13
		F	8.49 (8.13-8.87)	12.24 (11.04-13.57)	5.58 (5.13-6.05)
		R	8.54 (7.02-10.39)	15.20 (9.40-24.58)	5.68 (3.80-8.49)
	Between levels	P_B_	p < 0.1	NS	NS

^a^ Within each study, results for current smokers are selected in the following preference order, within each sex, for:

smoking product – any, cigarettes (ignoring other products), cigarettes only;

cigarette type – any, manufactured (with or without handrolled), manufactured only;

unexposed group – never any product, never cigarettes, near equivalent (see Methods);

follow-up period – longest available;

lung cancer type – see notes c to e;

race – all or nearest available, otherwise by race;

overlapping studies – principal, subsidiary;

age – whole study, widest available age group.

Results are then selected for:

sex – single sex results, combined sex results;

adjustment for potential confounders – most available.

^b^ n = number of estimates combined, F = fixed-effect meta-analysis RR (95% CI), R = random-effects meta-analysis RR (95% CI), H = heterogeneity chisquared per degree of freedom, P_H_ = probability value for heterogeneity expressed as p < 0.001, p < 0.05, p < 0.1 or NS (p ≥ 0.1), P_E_ = probability value for Egger’s test of publication bias similarly expressed, P_B_ = probability value for between levels (see Methods) similarly expressed.

^c^ All or nearest available, must include at least squamous cell carcinoma and adenocarcinoma.

^d^ Squamous cell carcinoma or nearest available, but not including adenocarcinoma.

^e^ Adenocarcinoma or nearest available, but not including squamous cell carcinoma.

^f^ Or nested case–control or case-cohort in the case of 5 estimates for all lung cancer, 3 for squamous and 3 for adeno.

^g^ Including not known.

^h^ In the study as a whole.

**Table 9:** **Some alternative meta-analyses for current smoking compared to those in Table**8

**Analysis description**	**Statistic**^**b**^	**All lung cancer**^**c**^	**Squamous**^**d**^	**Adeno**^**e**^
As Table 8^a^	n	195	41	44
	F	9.29 (9.07-9.52)	13.77 (12.75-14.87)	4.77 (4.49-5.07)
	R	8.43 (7.63-9.31)	16.91 (13.14-21.76)	4.21 (3.32-5.34)
	H, P_H_	13.76, p < 0.001	7.22, p < 0.001	12.28, p < 0.001
Using more precise outcome definition^f^	n	187	33	40
	F	9.93 (9.68-10.17)	12.74 (11.71-13.87)	4.40 (4.12-4.70)
	R	8.79 (7.97-9.68)	16.43 (12.66-21.32)	4.05 (3.15-5.22)
	H, P_H_	11.96, p < 0.001	5.70, p < 0.001	12.28, p < 0.001
Using least rather than most adjusted estimates	n	195	41	44
	F	9.12 (8.91-9.34)	13.81 (12.79-14.92)	4.82 (4.53-5.12)
	R	8.26 (7.49-9.12)	16.83 (13.12-21.60)	4.27 (3.37-5.40)
	H, P_H_	13.91, p < 0.001	7.17, p < 0.001	12.33, p < 0.001
Denominator non-smoker rather than never smoker	n	188	36	38
	F	3.99 (3.92-4.05)	3.59 (3.42-3.77)	2.65 (250–2.80)
	R	3.75 (3.48-4.03)	4.71 (3.84-5.79)	2.46 (2.07-2.93)
	H, P_H_	15.67, p < 0.001	12.71, p < 0.001	7.64, p < 0.001
Preferring results for cigarettes to results for any product	n	195	41	44
	F	9.47 (9.25-9.69)	13.77 (12.75-14.87)	4.77 (4.49-5.07)
	R	8.64 (7.83-9.54)	16.91 (13.14-21.76)	4.21 (3.32-5.34)
	H, P_H_	13.72, p < 0.001	7.22, p < 0.001	12.28, p < 0.001
Selecting results for cigarettes only^g^	n	38	8	7
	F	9.25 (8.81-9.71)	21.49 (16.73-27.61)	7.18 (5.71-9.04)
	R	9.52 (7.89-11.49)	20.85 (14.84-29.29)	6.05 (3.69-9.92)
	H, P_H_	10.61, p < 0.001	1.38, NS	2.98, p < 0.1
Selecting results specific for age <56 years	n	25	2	3
	F	7.64 (6.69-8.73)	6.48 (2.90-14.52)	0.74 (0.42-1.32)
	R	6.57 (4.68-9.23)	6.48 (2.90-14.52)	0.74 (0.42-1.32)
	H, P_H_	4.17, p < 0.001	0.33, NS	0.21, NS
Selecting results specific for age 50–70 years	n	24	1	0
	F	10.45 (9.65-11.31)	26.42 (9.78-71.36)	
	R	9.62 (7.10-13.05)	26.42 (9.78-71.36)	
	H, P_H_	9.99, p < 0.001	NA	
Selecting results specific for age 65+ years	n	27	0	0
	F	8.79 (8.06-9.58)		
	R	9.07 (6.83-12.04)		
	H, P_H_	8.37, p < 0.001)		

^a^ See Table 8 for details of the definition of this analysis.

^b^ n = number of estimates combined, F = fixed-effect meta-analysis RR (95% CI), R = random-effects meta-analysis RR (95% CI), H = heterogeneity chisquared per degree of freedom, P_H_ = probability value for heterogeneity expressed as p < 0.001, p < 0.05, p < 0.1 or NS (p ≥ 0.1), P_B_ = probability value for between levels (see Methods) similarly expressed.

^c^ All or nearest available, must include at least squamous cell carcinoma and adenocarcinoma.

^d^ Squamous cell carcinoma or nearest available, but not including adenocarcinoma.

^e^ Adenocarcinoma or nearest available, but not including squamous cell carcinoma.

^f^ Only including results for all lung cancer, squamous cell carcinoma specifically, or adenocarcinoma specifically.

^g^ See also Table 13 (footnote c).

As for ever smoking, the RRs for all three outcomes are heterogeneous (p < 0.001), with the largest estimates seen being 104.50 for all lung cancer (STUCKE/males), 78.91 for squamous (CPSII/females), and 21.70 for adeno (OSANN/males). The random-effects estimates (all lung cancer 8.43, 95% CI 7.63-9.31, n = 195; squamous 16.91, 13.14-21.76, n = 41; adeno 4.21, 3.32-5.34, n = 44) are all clearly positive, larger than the corresponding estimates for ever smoking, and also show a stronger relationship with squamous than adeno. Similarly to ever smoking, the individual RRs are virtually all above 1.0, though varying substantially. The estimates are again little affected (Table 9) by preferring least, rather than most, adjusted RRs, by restricting to a more precise outcome definition, or by preferring RRs for current smoking of cigarettes to those for current smoking of any product. Again estimates based specifically on cigarette only smoking were slightly higher than those shown in Table 8 – 9.52 (7.89-11.49, n = 38) for all lung cancer, 20.85 (14.84-29.29, n = 8) for squamous, and 6.05 (3.69-9.92, n = 7) for adeno. More so than in Table 6, data by age were rather limited for squamous and adeno. For all lung cancer estimates were 6.57 (4.68-9.23, n = 25) for age <56 years, 9.62 (7.10-13.05, n = 24) for age 50–70 years, and 9.07 (6.83-12.04, n = 27) for age 65+ years, no clear trend being evident. Table 9 also includes results for the comparison current vs. non-current smokers. The RRs here (3.75, 3.48-4.03 for all lung cancer; 4.71, 3.84-5.79 for squamous; 2.46, 2.07-2.93 for adeno) were markedly lower than the corresponding estimates for current vs. never smokers, reflecting the increased risk in ex-smokers described later (see section D below).

For the main meta-analysis, the studies contributing most to the total weight for current smoking for all lung cancer were STOCKW/sexes combined (17.8% of the total of 6,750) followed by BROWNS/males (6.0%) and BROWNS/females (5.4%). BROWNS was the major contributor for both squamous and adeno, with the two sex-specific results contributing 36.0% of the total weight of 646 for squamous, and 30.0% of the total weight of 1,017 for adeno. The huge LIU4 study did not provide results for current smoking.

For the characteristics considered in Table 8, the pattern of variation has a number of similarities to that for ever smoking in Table 5. Thus, as for ever smoking, RRs for all three outcomes tend to be higher for males, for North American studies, and where the unexposed base is never cigarettes, and smaller for older studies and smaller studies, with no clear variation by extent of adjustment. A tendency for RRs to be higher where data may be reported by proxy respondents seems somewhat stronger for current smoking, although based on few estimates for squamous and adeno. A tendency for RRs to be higher where the smoking product is cigarettes or cigarettes only than when it is any product is also evident, though not for squamous, whereas it was seen most clearly in squamous for ever smoking. There is also some indication that RRs are higher in prospective studies, though interestingly not for all lung cancer. Whereas for ever smoking, RRs for studies requiring full histological confirmation were higher than for those that did not for all three outcomes, the tendency was in the reverse direction for squamous and adeno for current smoking. For national cigarette tobacco type, current smoking RRs for squamous and adeno are virtually all for blended, so are unhelpful. For all lung cancer, RRs are quite similar for Virginia and blended, the significant (p < 0.001) variation shown in Table 8 arising because of the low RRs in the “Other” group, mainly for China.

As for ever smoking, meta-regression analyses were conducted to give further insight, the results from the same fixed model including six characteristics being summarized in Table 10. Based on these results and those for other characteristics in Additional file 5: Detailed Analysis Tables various conclusions can be drawn.

**Table 10:** **Meta-regression analyses for current smoking of any product (or cigarettes if any product not available)**^**a**^

**Characteristic**	**Level**	**All lung cancer**		**Squamous**		**Adeno**	
		**Estimate**^**b**^**(SE**^**c**^**)**	**p**^**d**^	**Estimate**^**b**^**(SE**^**c**^**)**	**p**^**d**^	**Estimate**^**b**^**(SE**^**c**^**)**	**p**^**d**^
Constant		+1.566 (0.081)		+1.193 (0.239)		+0.816 (0.239)	
Sex	Male	Base	<0.01	Base	NS	Base	<0.001
	Female	−0.204 (0.029)		−0.002 (0.088)		−0.440 (0.067)	
	Combined	−0.043 (0.039)		+0.224 (0.459)		−0.612 (0.378)	
Location	North America	Base	<0.001	Base	<0.05	Base	<0.001
	United Kingdom	−0.345 (0.071)		No data		No data	
	Scandinavia	−0.417 (0.066)		−0.177 (0.513)		−0.298 (0.320)	
	Other Europe	−0.602 (0.057)		−0.440 (0.266)		−1.157 (0.178)	
	China	−1.528 (0.097)		No data		No data	
	Japan	−1.174 (0.056)		−1.174 (0.193)		−1.525 (0.136)	
	Other Asia	−1.221 (0.139)		No data		No data	
	Other or multiregion	−0.593 (0.088)		−1.221 (1.045)		−1.014 (0.754)	
Start year of study	Before 1960	Base	<0.001	Base	<0.1	Base	<0.05
	1960-69	+0.538 (0.063)		+1.374 (0.321)		+1.179 (0.231)	
	1970-79	+0.571 (0.058)		+0.896 (0.418)		+1.142 (0.258)	
	1980-89	+0.788 (0.051)		+1.276 (0.325)		+0.957 (0.224)	
	1990 or later	+1.169 (0.089)		+2.817 (0.823)		+1.982 (0.575)	
Study type	Case–control	Base	<0.1	Base	NS	Base	NS
	Prospective^e^	+0.182 (0.047)		+0.404 (0.371)		+0.178 (0.224)	
Number of cases^f^	100-249	Base	<0.1	Base	NS	Base	NS
	250-499	+0.222 (0.071)		+0.420 (0.384)		−0.002 (0.195)	
	500-999	+0.266 (0.070)		+0.852 (0.400)		+0.646 (0.232)	
	1000+	+0.340 (0.062)		+0.803 (0.298)		+0.362 (0.149)	
Number of adjustment factors	0	Base	NS	Base	<0.05	Base	NS
	1	+0.002 (0.047)		+0.428 (0.277)		+0.195 (0.213)	
	2+	+0.069 (0.034)		−0.447 (0.175)		+0.283 (0.125)	

^a^ Based on the same data as for Table 8. See that table for further definition of RRs selected for analysis, and numbers of estimates of each characteristic level.

^b^ Estimates are of log RR. The predicted RR of a given estimate can be calculated by adding the constant to the values for the level of each characteristic applicable to the estimate (taking the value for a base level as zero) and taking the exponential of the result.

^c^ SE = standard error.

^d^ The p value is estimated from the drop in deviance from removing the characteristic from the fixed model using an F-test. It is expressed as p < 0.001, p < 0.01, p < 0.05, p < 0.1 or NS (p ≥ 0.1).

^e^ Or nested case–control or case-cohort in the case of 5 estimates for all lung cancer, 3 for squamous and 3 for adeno.

^f^ In the study as a whole.

Note For the forward stepwise analyses, deviances, degrees of freedom and p values for inclusion in order of inclusion are as follows:

**All lung cancer**: Constant 2669.216 on 194 d.f. (p < 0.001), location 1257.714 on 187 d.f. (p < 0.001), start year of study 918.536 on 183 d.f. (p < 0.001) and sex 869.479 on 181 d.f. (p < 0.01). Fixed model deviance 819.856 on 175 d.f.

**Squamous**: Constant 288.688 on 40 d.f. (p < 0.001), location 194.753 on 36 d.f. (p < 0.01), start year of study 143.741 on 32 d.f. (p < 0.05) and number of adjustment variables 100.916 on 30 d.f. (p < 0.01). Fixed model deviance 86.049 on 24 d.f.

**Adeno**: Constant 528.128 on 43 d.f. (p < 0.001), location 170.181 on 39 d.f. (p < 0.001), sex 128.397 on 37 d.f. (p < 0.01) and start year of study 89.243 on 33 d.f. (p < 0.05). Fixed model deviance 65.727 on 27 d.f.

For all lung cancer, as was the case for ever smoking RRs, by far the strongest source of variation in current smoking RRs was location with relatively high risks in North America and low risks in Asia. The overall heterogeneity reduced from 13.76 per d.f. to 6.73 per d.f. after including location only into the model. Higher risks were also seen in the fixed model in more recent studies (p < 0.001) and for males than females (p < 0.01). There was some evidence (p < 0.1) of higher RRs in larger studies and in prospective studies, but no association was seen with the number of adjustment factors. The heterogeneity for the fixed model shown in Table 10 was 4.68 per d.f., with the model explaining 69.3% of the overall variation between the current smoking RRs. Four standardized residuals were outside the range +/− 2.5 SEs : BROWN2/males (RR 11.30, fitted 15.86), TIZZAN/males (RR 1.90, fitted 3.68), CPSI/females (RR 3.20, fitted 6.59) and KREUZE/males aged 55–69 (RR 41.86, fitted 11.85). No other characteristic significantly improved the fit when added to the fixed model. Additional analysis (data not shown) confirmed the effect of start year of study separately for North America and Europe (though no such relationship was seen in Asia) and also confirmed that the effects of location and start year of study were evident separately for males and for females.

For squamous and adeno, numbers of current smoking RRs (41 and 44 respectively) were much lower than those for all lung cancer, with no data for China or the United Kingdom, or for national cigarette type “other”. For squamous, only two characteristics in the fixed model (Table 10) were significant, and then only at p < 0.05, and one of these was number of adjustment factors, where the pattern of response was erratic. Location was the other, with RRs again highest in North America and lowest in Asia. There were no estimates with large standardized residuals, and no other characteristic improved the model fit.

For adeno, three of the characteristics considered in Table 10 contributed significantly to the model, sex (p < 0.001), location (p < 0.001) and start year of study (p < 0.05), with the direction of effect similar to that noted earlier for ever smoking. There were no large standardized residuals, and the only additional characteristic which improved the model fit (p < 0.05) related to somewhat lower RRs being seen for studies with full histological confirmation.

For none of the three outcomes did characteristics associated with detailed location, national cigarette tobacco type, the precise definition of the outcome, adjustment for specific factors, the definitions of smoking product or of the unexposed base, whether the study was conducted in a population working in a risky occupation or whether proxy respondents were used, add significantly to the model.

#### C. Risk from ever or current smoking

In an attempt to incorporate data from a greater number of studies, additional analyses were carried out for ever/current smoking and for current/ever smoking. The meta-analysis RRs are shown in Table 11. The number of studies included increased from 236 to 242 for all lung cancer, from 73 to 78 for squamous and from 75 to 81 for adeno, compared with Table 5. Note that the slightly higher number of RR estimates in the current/ever analysis arises from inclusion there of more sex-specific results.

**Table 11:** **Main meta-analyses for current or ever smoking of any product (or cigarettes, if not available)**^**a**^

**Preference**	**Statistic**^**b**^	**All lung cancer**^**c**^	**Squamous**^**d**^	**Adeno**^**e**^
Ever smoking to current smoking	n	342	110	116
	F	4.25 (4.20-4.31)	9.15 (8.63-9.70)	3.38 (3.23-3.55)
	R	5.48 (5.07-5.93)	10.58 (9.04-12.37)	2.94 (2.52-3.43)
	H, P_H_	22.46, p < 0.001	5.22, p < 0.001	8.61, p < 0.001
Current smoking to ever smoking	n	344	110	116
	F	4.45 (4.39-4.51)	10.08 (9.51-10.69)	3.58 (3.41-3.76)
	R	6.20 (5.68-6.77)	11.53 (9.73-13.66)	3.13 (2.67-3.67)
	H, P_H_	28.33, p < 0.001	6.30, p < 0.001	8.99, p < 0.001

^a^ Within each study, results are selected in the following preference order, within each sex, for:

smoking status – ever, current or current, ever according to analysis;

smoking product – any, cigarettes (ignoring other products), cigarettes only;

cigarette type – any, manufactured (with or without handrolled), manufactured only;

unexposed group – never any product, never cigarettes, near equivalent (see Methods);

follow-up period – longest available;

lung cancer type – see notes c to e;

race – all or nearest available, otherwise by race;

overlapping studies – principal, subsidiary;

age – whole study, widest available age group.

Results are then selected for:

sex – single sex results, combined sex results;

adjustment for potential confounders – most available.

^b^ n = number of estimates combined, F = fixed-effect meta-analysis RR (95% CI), R = random-effects meta-analysis RR (95% CI), H = heterogeneity chisquared per degree of freedom, P_H_ = probability value for heterogeneity expressed as p < 0.001, p < 0.05, p < 0.1 or NS (p ≥ 0.1), P_B_ = probability value for between levels (see Methods) similarly expressed.

^c^ All or nearest available, must include at least squamous cell carcinoma and adenocarcinoma.

^d^ Squamous cell carcinoma or nearest available, but not including adenocarcinoma.

^e^ Adenocarcinoma or nearest available, but not including squamous cell carcinoma.

As many of the RRs are common between the specific ever smoking analyses in Table 5 and the ever/current smoking analyses in Table 11, the meta-analysis RRs tend to be quite similar. However those for current/ever smoking are intermediate between those specifically for ever smoking (Table 5) and those specifically for current smoking (Table 8). For example, for all lung cancer, random-effects estimates are 5.50 (95% CI 5.07-5.96, n = 328) for ever smoking, 5.48 (5.07-5.93, n = 342) for ever/current smoking, 6.20 (5.68-6.77, n = 344) for current/ever smoking, and 8.43 (7.63-9.31, n = 195) for current smoking. The pattern of RRs by level of the characteristics studied for both ever/current and current/ever smoking tends to be quite similar to that for the specific analyses. Results for ever or current smoking by level of selected characteristics are therefore only presented in Additional file 5: Detailed Analysis Tables.

#### D. Risk from ex smoking

**Figure 15:**
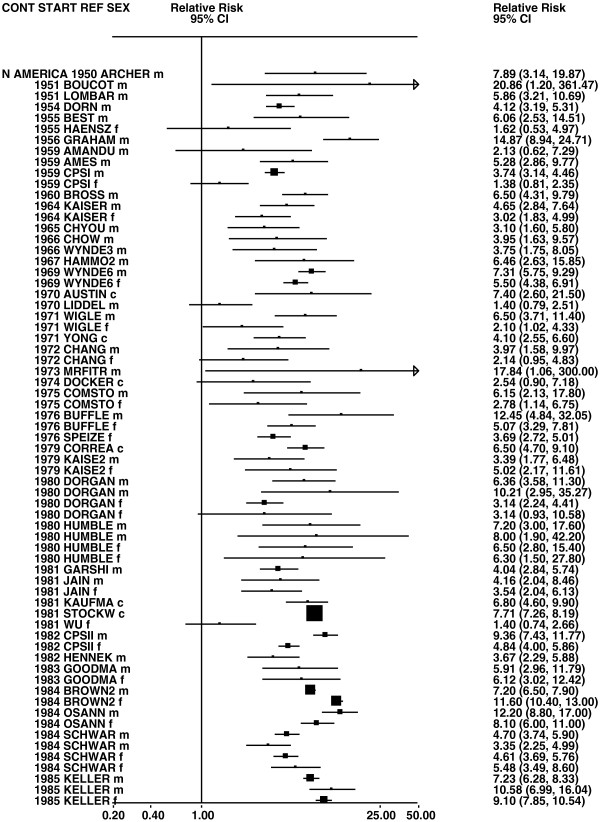
**Forest plot of ex smoking of any product and all lung cancer – part 1.** Table 12 presents the results of a main meta-analysis for all lung cancer based on 182 relative risk (RR) and 95% confidence interval (CI) estimates for ex smoking of any product (or cigarettes if any product not available). The individual study estimates are shown numerically and graphically on a logarithmic scale in Figures 15, 16, 17. The studies are sorted in order of sex within study reference (REF) within start year of study (START) within continent (CONT). In the graphical representation individual RRs are indicated by a solid square, with the area of the square proportional to the weight (inverse-variance of log RR). Arrows indicate where the CI extends outside the range allocated. For studies DORGAN and KELLER separate estimates, within sex, are shown for whites then blacks. For study HUMBLE they are shown for non-hispanic whites then Hispanics. For study KELLER the estimate shown for females is for whites.

**Figure 16:**
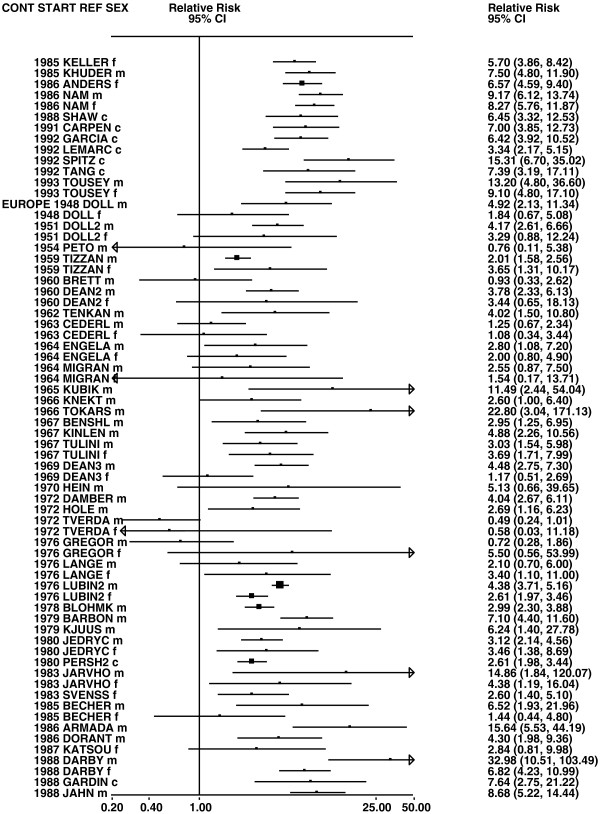
**Forest plot of ex smoking of any product and all lung cancer – part 2.** This is a continuation of Figure 15, presenting further individual study data included in the main meta-analysis for all lung cancer shown in Table 12. For study KELLER the estimate shown for females is for non-whites.

**Figure 17:**
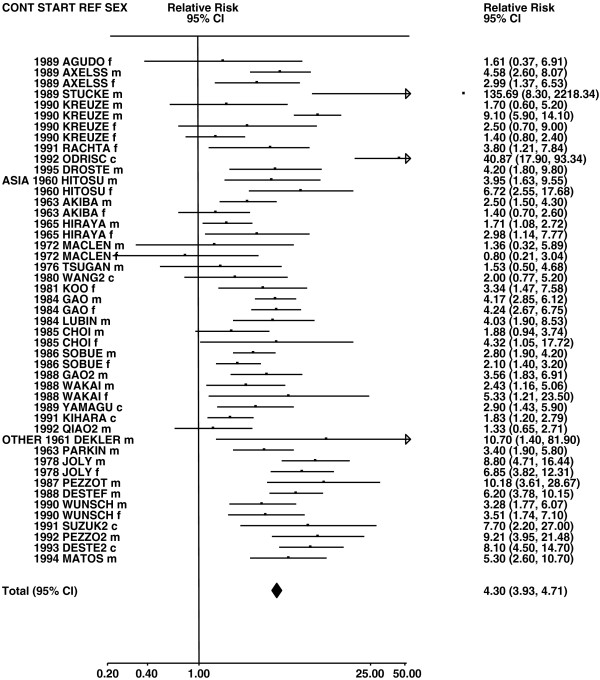
**Forest plot of ex smoking of any product and all lung cancer – part 3.** This is a continuation of Figure 16, presenting the remaining individual study data included in the main meta-analysis for all lung cancer shown in Table 12. Also shown are the combined random-effect estimates. These are represented by a diamond of standard height, with the width indicating the 95% CI. For study KREUZE separate estimates, within sex, are shown for age ≤ 45 and 55–69.

**Figure 18:**
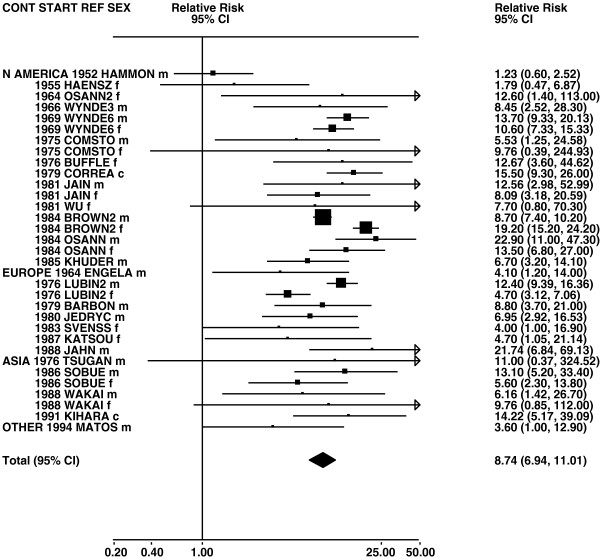
**Forest plot of ex smoking of any product and squamous.** Table 12 presents the results of a main meta-analysis for squamous based on 33 relative risk (RR) and 95% confidence interval (CI) estimates for ex smoking of any product (or cigarettes if any product not available). The individual study estimates are shown numerically and graphically on a logarithmic scale. The studies are sorted in order of sex within study reference (REF) within start year of study (START) within continent (CONT). In the graphical representation individual RRs are indicated by a solid square, with the area of the square proportional to the weight (inverse-variance of log RR). Arrows indicate where the CI extends outside the range allocated. Also shown are the combined random-effect estimates. These are represented by a diamond of standard height, with the width indicating the 95% CI.

**Figure 19:**
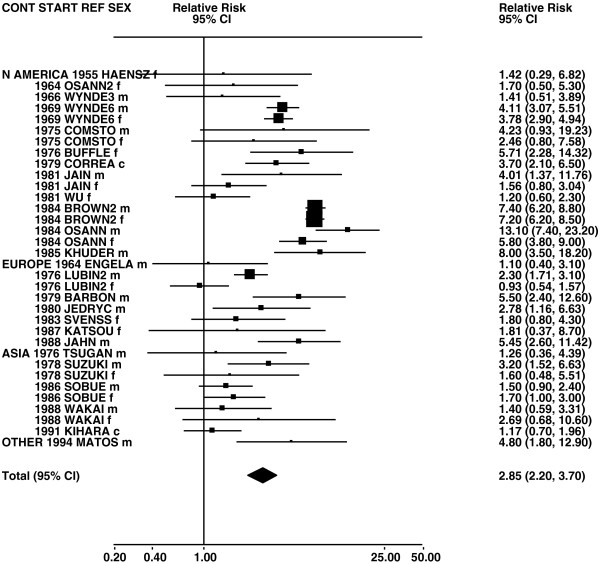
**Forest plot of ex smoking of any product and adeno.** Table 5 presents the results of a main meta-analysis for adeno based on 34 relative risk (RR) and 95% confidence interval (CI) estimates for ex smoking of any product (or cigarettes if any product not available). The individual study estimates are shown numerically and graphically on a logarithmic scale. The studies are sorted in order of sex within study reference (REF) within start year of study (START) within continent (CONT). In the graphical representation individual RRs are indicated by a solid square, with the area of the square proportional to the weight (inverse-variance of log RR). Arrows indicate where the CI extends outside the range allocated. Also shown are the combined random-effect estimates. These are represented by a diamond of standard height, with the width indicating the 95% CI.

Figures 15, 16, 17 (all lung cancer), Figure 18 (squamous) and Figure 19 (adeno) present the results of the main meta-analyses for ex smoking of any product (or cigarettes if any product was not available), based on most-adjusted RRs. Some results by levels of characteristics are shown in Table 12.

**Table 12:** **Main meta-analyses for ex smoking of any product (or cigarettes, if any product not available)**^**a**^

**Characteristic**	**Level**	**Statistic**^**b**^	**All lung cancer**^**c**^	**Squamous**^**d**^	**Adeno**^**e**^
All	All	n	182	33	34
		F	5.80 (5.63-5.96)	10.25 (9.34-11.24)	4.38 (4.04-4.73)
		R	4.30 (3.93-4.71)	8.74 (6.94-11.01)	2.85 (2.20-3.70)
		H, P_H_	6.98, <0.001	3.54, <0.001	7.91, <0.001
		P_E_	p < 0.001	NS	p < 0.001
Sex	Male	n	100	17	17
		F	5.15 (4.93-5.37)	9.31 (8.27-10.47)	4.59 (4.10-5.15)
		R	4.48 (3.98-5.06)	8.41 (6.18-11.45)	3.45 (2.40-4.96)
	Female	n	62	14	15
		F	5.81 (5.51-6.14)	11.66 (9.94-13.68)	4.48 (4.00-5.02)
		R	3.58 (3.00-4.29)	8.03 (5.18-12.45)	2.39 (1.55-3.69)
	Combined	n	20	2	2
		F	6.98 (6.61-7.37)	15.23 (9.63-24.08)	1.97 (1.35-2.88)
		R	5.58 (4.23-7.36)	15.23 (9.63-24.08)	2.07 (0.67-6.38)
	Between levels	P_B_	<0.05	<0.1	NS
Location	North America	n	80	18	17
		F	6.79 (6.57-7.01)	10.83 (9.73-12.05)	5.69 (5.20-6.23)
		R	5.44 (4.91-6.03)	9.43 (6.84-13.01)	4.08 (3.08-5.39)
	United Kingdom	n	21	0	0
		F	4.14 (3.48-4.92)		
		R	3.72 (2.51-5.50)		
	Scandinavia	n	21	2	2
		F	2.67 (2.29-3.11)	4.06 (1.61-10.25)	1.48 (0.77-2.83)
		R	2.62 (2.01-3.42)	4.06 (1.61-10.25)	1.48 (0.77-2.83)
	Other Europe	n	24	6	6
		F	3.57 (3.26-3.92)	9.10 (7.38-11.22)	2.27 (1.82-2.84)
		R	3.96 (3.07-5.12)	8.42 (5.03-14.10)	2.58 (1.47-4.52)
	China	n	5	0	0
		F	3.46 (2.70-4.44)		
		R	3.09 (2.02-4.74)		
	Japan	n	14	6	8
		F	2.35 (2.01-2.76)	9.32 (5.69-15.28)	1.60 (1.25-2.05)
		R	2.40 (1.99-2.89)	9.32 (5.69-15.28)	1.60 (1.25-2.05)
	Other Asia	n	5	0	0
		F	2.13 (1.37-3.32)		
		R	2.12 (1.29-3.47)		
	Other or multiregion	n	12	1	1
		F	5.77 (4.73-7.03)	3.60 (1.00-12.93)	4.80 (1.79-12.85)
		R	5.84 (4.60-7.41)	3.60 (1.00-12.93)	4.80 (1.79-12.85)
	Between levels	P_B_	<0.001	NS	<0.001
Start year of study	Before 1960	n	18	2	1
		F	3.55 (3.19-3.94)	1.34 (0.71-2.52)	1.42 (0.29-6.82)
		R	3.81 (2.84-5.11)	1.34 (0.71-2.52)	1.42 (0.29-6.82)
	1960-69	n	36	5	5
		F	4.06 (3.68-4.48)	11.29 (8.77-14.54)	3.56 (2.95-4.29)
		R	3.32 (2.71-4.08)	11.29 (8.77-14.54)	2.80 (1.86-4.21)
	1970-79	n	36	8	10
		F	3.81 (3.50-4.14)	9.85 (8.08-12.02)	2.38 (1.95-2.91)
		R	3.48 (2.85-4.25)	9.39 (6.00-14.68)	2.59 (1.75-3.84)
	1980-89	n	70	16	16
		F	6.91 (6.68-7.15)	10.97 (9.75-12.36)	5.67 (5.13-6.26)
		R	5.22 (4.65-5.86)	10.35 (7.57-14.15)	3.37 (2.35-4.85)
	1990 or later	n	22	2	2
		F	4.73 (4.11-5.43)	8.38 (3.79-18.53)	1.58 (1.00-2.49)
		R	5.17 (3.66-7.31)	7.58 (1.98-29.00)	2.22 (0.56-8.79)
	Between levels	P_B_	<0.001	<0.001	NS
Study type	Case–control	n	123	28	30
		F	6.28 (6.09-6.48)	10.71 (9.74-11.76)	4.44 (4.10-4.81)
		R	4.88 (4.41-5.41)	9.94 (8.05-12.28)	2.96 (2.26-3.87)
	Prospective^f^	n	59	5	4
		F	3.84 (3.58-4.12)	2.34 (1.35-4.03)	1.87 (1.04-3.36)
		R	3.18 (2.70-3.74)	3.54 (1.38-9.09)	1.87 (1.04-3.36)
	Between levels	P_B_	<0.001	<0.05	NS
National cigarette tobacco type	Virginia	n	30	2	2
		F	3.95 (3.45-4.53)	9.22 (4.21-20.18)	2.03 (1.15-3.57)
		R	3.79 (2.85-5.04)	9.22 (4.21-20.18)	2.27 (0.92-5.59)
	Blended	n	147	31	32
		F	5.94 (5.77-6.12)	10.26 (9.35-11.27)	4.44 (4.10-4.81)
		R	4.45 (4.04-4.91)	8.68 (6.83-11.04)	2.89 (2.22-3.77)
	Other	n	5	0	0
		F	3.46 (2.70-4.44)		
		R	3.09 (2.02-4.74)		
	Between levels	P_B_	NS	NS	NS
Any proxy use	No^g^	n	141	27	28
		F	6.02 (5.84-6.21)	10.17 (9.23-11.20)	4.46 (4.11-4.84)
		R	4.12 (3.70-4.59)	8.33 (6.38-10.89)	2.73 (2.04-3.65)
	Yes	n	41	6	6
		F	4.60 (4.26-4.96)	11.24 (8.01-15.78)	3.30 (2.42-4.52)
		R	4.79 (4.20-5.47)	11.24 (8.01-15.78)	3.39 (2.25-5.11)
	Between levels	P_B_	<0.1	NS	NS
Full histological confirmation	No	n	131	16	15
		F	5.73 (5.53-5.93)	8.91 (7.03-11.30)	3.50 (2.89-4.23)
		R	4.11 (3.68-4.60)	8.11 (5.05-13.01)	3.24 (2.13-4.94)
	Yes	n	51	17	19
		F	5.93 (5.65-6.23)	10.51 (9.50-11.62)	4.59 (4.21-5.00)
		R	4.76 (4.02-5.63)	9.28 (7.08-12.15)	2.60 (1.85-3.65)
	Between levels	P_B_	NS	NS	NS
Number of cases^g^	100-249	n	51	7	9
		F	3.18 (2.79-3.64)	4.03 (2.17-7.49)	1.95 (1.40-2.70)
		R	3.36 (2.65-4.25)	4.03 (2.17-7.49)	1.95 (1.40-2.70)
	250-499	n	45	9	8
		F	4.21 (3.80-4.66)	4.59 (3.17-6.65)	1.86 (1.36-2.54)
		R	4.13 (3.42-4.99)	5.48 (2.84-10.58)	2.10 (1.21-3.64)
	500-999	n	37	4	4
		F	4.61 (4.21-5.04)	9.58 (5.65-16.25)	3.23 (2.13-4.90)
		R	4.86 (4.07-5.80)	9.58 (5.65-16.25)	3.58 (1.79-7.13)
	1000+	n	49	13	13
		F	6.39 (6.19-6.60)	11.13 (10.08-12.28)	5.00 (4.59-5.45)
		R	4.76 (4.13-5.48)	11.53 (8.87-14.99)	3.69 (2.60-5.22)
	Between levels	P_B_	<0.05	<0.01	<0.05
Smoking product	Any	n	81	9	9
		F	4.76 (4.51-5.02)	8.13 (5.16-12.79)	1.60 (1.21-2.12)
		R	4.09 (3.53-4.74)	8.13 (5.16-12.79)	1.68 (1.17-2.40)
	Cigarettes (ignoring	n	90	23	25
	other products)	F	6.38 (6.16-6.60)	10.75 (9.77-11.83)	4.77 (4.40-5.18)
		R	4.49 (3.98-5.06)	9.99 (7.93-12.58)	3.35 (2.54-4.41)
	Cigarettes only	n	11	1	0
		F	4.96 (4.36-5.64)	1.23 (0.60-2.52)	
		R	4.18 (2.63-6.66)	1.23 (0.60-2.52)	
	Between levels	P_B_	NS	<0.001	<0.01
Unexposed base	Never any product	n	121	17	18
		F	5.64 (5.44-5.85)	8.41 (7.12-9.94)	2.38 (2.06-2.76)
		R	4.35 (3.89-4.88)	6.63 (4.39-10.02)	2.17 (1.61-2.94)
	Never cigarettes	n	61	16	16
		F	6.08 (5.80-6.37)	11.19 (10.00-12.51)	5.60 (5.10-6.15)
		R	4.18 (3.56-4.91)	11.13 (8.48-14.60)	3.83 (2.78-5.27)
	Between levels	P_B_	NS	<0.05	<0.05
Number of adjustment factors	0	n	86	16	16
		F	6.10 (5.87-6.34)	9.81 (8.41-11.45)	2.82 (2.47-3.22)
		R	4.84 (4.26-5.50)	9.11 (6.95-11.94)	2.61 (1.91-3.58)
	1	n	48	10	9
		F	4.40 (4.11-4.71)	7.03 (5.21-9.47)	2.13 (1.66-2.73)
		R	3.96 (3.37-4.65)	6.43 (3.31-12.47)	2.17 (1.56-3.03)
	2+	n	48	7	9
		F	6.23 (5.91-6.58)	11.28 (9.94-12.80)	6.64 (5.97-7.39)
		R	3.81 (3.11-4.66)	11.09 (6.78-18.13)	4.42 (3.05-6.40)
	Between levels	P_B_	<0.1	NS	<0.05

^a^ Within each study, results for ex smokers are selected in the following preference order, within each sex, for:

smoking product – any, cigarettes (ignoring other products), cigarettes only;

cigarette type – any, manufactured (with or without handrolled), manufactured only;

unexposed group – never any product, never cigarettes, near equivalent (see Methods);

follow-up period – longest available;

lung cancer type – see notes c to e;

race – all or nearest available, otherwise by race;

overlapping studies – principal, subsidiary;

age – whole study, widest available age group.

Results are then selected for:

sex – single sex results, combined sex results;

adjustment for potential confounders – most available.

^b^ n = number of estimates combined, F = fixed-effect meta-analysis RR (95% CI), R = random-effects meta-analysis RR (95% CI), H = heterogeneity chisquared per degree of freedom, P_H_ = probability value for heterogeneity expressed as p < 0.001, p < 0.05, p < 0.1 or NS (p ≥ 0.1), P_E_ = probability value for Egger’s test of publication bias similarly expressed, P_B_ = probability value for between levels (see Methods) similarly expressed.

^c^ All or nearest available, must include at least squamous cell carcinoma and adenocarcinoma.

^d^ Squamous cell carcinoma or nearest available, but not including adenocarcinoma.

^e^ Adenocarcinoma or nearest available, but not including squamous cell carcinoma.

^f^ Or nested case–control or case-cohort in the case of 5 estimates for all lung cancer, 3 for squamous and 3 for adenocarcinoma.

^g^ In the study as a whole.

Again the RRs are markedly heterogeneous (p < 0.001 for all three outcomes), ranging up to 135.69 for all lung cancer (STUCKE/males), 22.90 for squamous (OSANN/males) and 13.10 for adeno (OSANN/males). The random-effects estimates (all lung cancer 4.30, 95% CI 3.93-4.71, n = 182, squamous 8.74, 6.94-11.01, n = 33, and adeno 2.85, 2.20-3.70, n = 34), though all clearly positive, are smaller than the corresponding estimates for current smoking. Individual RRs are only very occasionally below 1.0 and never significantly so. Estimates are little affected by using the more specific definition of each outcome, preferring least-adjusted RRs to most-adjusted RRs, or preferring RRs for ever smoking cigarettes to those for ever smoking any product. RRs for ever smoking cigarettes only were too few for useful analysis for squamous and adeno, but for all lung cancer were similar to those for ever smoking any product. Fuller details are given in the Additional file 5: Detailed Analysis Tables.

For the main meta-analysis of ex smoking, the studies contributing most to the total weight for all lung cancer were STOCKW/sexes combined (22.4% of the total of 4,739), followed by BROWNS/males (8.5%) and BROWNS/females (6.5%). BROWNS was the major contributor for both squamous and adeno, with the two sex-specific results contributing 49.4% of the total weight of 446 for squamous, and 45.2% of the total weight of 619 for adeno.

For the characteristics considered in Table 12 the sources of variation for all lung cancer are generally quite similar to those seen for ever smoking in Table 5 and for current smoking in Table 8. Thus, RRs are higher for males, for North America, for more recent studies and for larger studies. Interestingly RRs are clearly lower for prospective than for case–control studies. Numbers of ex smoking RRs are less for squamous (33) and for adeno (34) than for all lung cancer (182), but nevertheless some associations are evident in relation to location for adeno, to study type for squamous, to number of adjustment factors for adeno, and to number of cases, smoking product and unexposed base for both squamous and adeno. Meta-regression analyses were not attempted for ex smoking.

#### E. Risk from smoking specific products compared to smoking of any product

**Figure 20:**
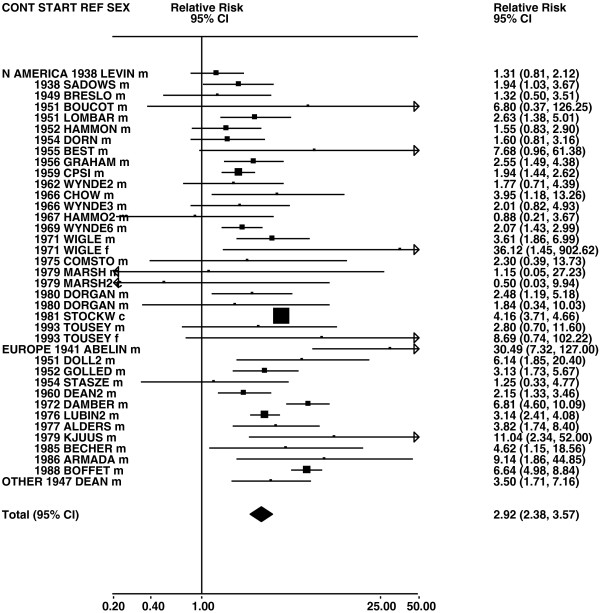
**Forest plot of ever pipe and/or cigar smoking and all lung cancer.** Table 13 presents the results of a meta-analysis for all lung cancer based on 56 relative risk (RR) and 95% confidence interval (CI) estimates for ever pipe and/or cigar smoking. The individual study estimates are shown numerically and graphically on a logarithmic scale. The studies are sorted in order of sex within study reference (REF) within start year of study (START) within continent (CONT). In the graphical representation individual RRs are indicated by a solid square, with the area of the square proportional to the weight (inverse-variance of log RR). Arrows indicate where the CI extends outside the range allocated. Also shown are the combined random-effects estimates. These are represented by a diamond of standard height, with the width indicating the 95% CI.

Table 13 summarizes the results of meta-analyses for all lung cancer for cigarette only smokers, smokers of pipes/cigars only, smokers of pipes only, and smokers of cigars only. In each analysis, the base is never smokers of any product. The results for ever smoking of pipes/cigars only are also shown in Figure 20.

**Table 13:** **Meta-analyses for smoking of cigarettes, cigars and pipes (all lung cancer)**^**a**^

**Product smoked**	**Statistic**^**b**^	**Ever smoking**	**Current smoking**	**Ex smoking**
Cigarettes only	n	53^c^	35^c^	21
	F	4.37 (4.27-4.47)	9.32 (8.87-9.80)	4.36 (4.05-4.69)
	R	6.36 (5.33-7.59)	9.57 (7.90-11.59)	4.22 (3.29-5.40)
	H, P_H_	22.95, p < 0.001	10.91, p < 0.001	7.96, p < 0.001
Pipes/cigars only	n	38	26	7
	F	3.46 (3.20-3.73)	3.74 (3.29-4.26)	2.00 (1.50-2.65)
	R	2.92 (2.38-3.57)	4.76 (3.44-6.59)	2.00 (1.50-2.65)
	H, P_H_	3.60, p < 0.001	4.18, p < 0.001	0.46, NS
Pipes only	n	23	12	5
	F	3.36 (2.95-3.81)	5.28 (4.55-6.13)	3.32 (2.42-4.55)
	R	3.31 (2.51-4.35)	5.20 (3.50-7.73)	2.69 (1.53-4.72)
	H, P_H_	3.49, p < 0.001	5.43, p < 0.001	2.66, p < 0.05
Cigars only	n	15	15	5
	F	2.73 (2.32-3.21)	4.05 (3.61-4.54)	3.27 (2.36-4.52)
	R	2.95 (1.91-4.56)	4.67 (3.49-6.25)	2.85 (1.45-5.61)
	H, P_H_	5.68, p < 0.001	4.27, p < 0.001	4.11, p < 0.01
Mixed	n	27	9	7
	F	7.87 (7.22-8.59)	9.63 (8.46-10.96)	4.79 (4.10-5.60)
	R	7.37 (5.97-9.11)	9.60 (8.37-11.00)	5.51 (3.88-7.82)
	H, P_H_	4.09, p < 0.001	1.05, NS	3.72, p < 0.01

^a^ Within each study, results for the relevant smoking product and smoking status are selected in the following preference order, within each sex, for:

unexposed group – never any product, near equivalent (see Methods);

follow-up period – longest available;

lung cancer type – all or nearest available, must include at least squamous cell carcinoma and adenocarcinoma;

race – all or nearest available, otherwise by race;

overlapping studies – principal, subsidiary;

age – whole study, widest available age group;

sex – single sex results, combined sex results;

adjustment for potential confounders – most available.

^b^ n = number of estimates combined, F = fixed-effect meta-analysis RR (95% CI), R = random-effects meta-analysis RR (95% CI), H = heterogeneity chisquared per degree of freedom, P_H_ = probability value for heterogeneity expressed as p < 0.001, p < 0.05, p < 0.1 or NS (p ≥ 0.1), P_B_ = probability value for between levels (see Methods) similarly expressed.

^c^ Results differ from those shown earlier (Tables 6, 9) because RRs with unexposed group “never cigarettes” are excluded here.

For ever smoking, current smoking and ex smoking the random-effects RRs are similarly elevated for pipes/cigars, pipes only and cigars only, but to a markedly lesser extent than for cigarettes only. As for cigarette smoking, RRs for pipe and cigar smoking are clearly higher for current smokers than for ex smokers.

Available results for squamous and adeno are limited, and mainly for ever smoking. For pipe and/or cigar smoking, the RR for squamous (3.72, 95% CI 1.95-7.10, n = 8) is somewhat higher than that for all lung cancer (2.92, 2.38-3.57, n = 38), but the RR for adeno is not elevated (0.93, 0.62-1.40, n = 7). The lack of association of adeno with pipe and cigar smoking is also evident in the RRs for pipes only (0.50, 0.23-1.10, n = 4) and for cigars only (0.55, 0.11-2.88, n = 3).

The results for pipe and cigar smoking mainly apply to males, as the few available estimates for females have wide variability. The increased risk in smokers of pipes and cigars is evident in each location studied, though data for Asia are extremely sparse. Unlike for cigarettes, higher RRs are seen for Scandinavia (7.02, 4.72-10.44, n = 6) and for Other Europe (5.17, 2.91-9.19, n = 8) than for North America (2.27, 1.79-2.89, n = 26) or the UK (4.32, 2.73-6.84, n = 11). These results are for ever/current smoking, with the full results given in Additional file 5: Detailed Analysis Tables.

Table 13 also shows results for lung cancer for mixed smokers. For ever, current and ex smoking, the random-effects RRs are slightly, but not significantly, higher than those for smokers of cigarettes only. Available results for squamous and adeno are again limited, and mainly for ever smokers. The RRs for squamous (9.78, 4.94-19.35, n = 6) and for adeno (2.48, 1.25-4.95, n = 6) do not clearly differ from the RRs for squamous (11.09, 7.19-17.09, n = 10) and for adeno (2.63, 1.32-5.24, n = 10) for smokers of cigarettes only.

#### F. Risk by type of cigarette smoked

**Figure 21:**
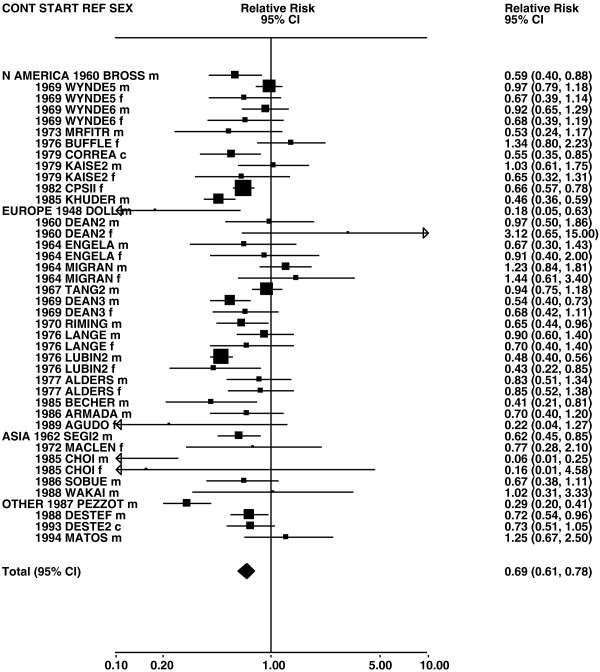
**Forest plot of only filter vs. only plain cigarette smoking and all lung cancer.** Table 14 presents the results of a meta-analysis for all lung cancer based on 42 relative risk (RR) and 95% confidence interval (CI) estimates for only filter vs. only plain cigarette smoking. The individual study estimates are shown numerically and graphically on a logarithmic scale. The studies are sorted in order of sex within study reference (REF) within start year of study (START) within continent (CONT). In the graphical representation individual RRs are indicated by a solid square, with the area of the square proportional to the weight (inverse-variance of log RR). Arrows indicate where the CI extends outside the range allocated. Also shown are the combined random-effects estimates. These are represented by a diamond of standard height, with the width indicating the 95% CI.

**Figure 22:**
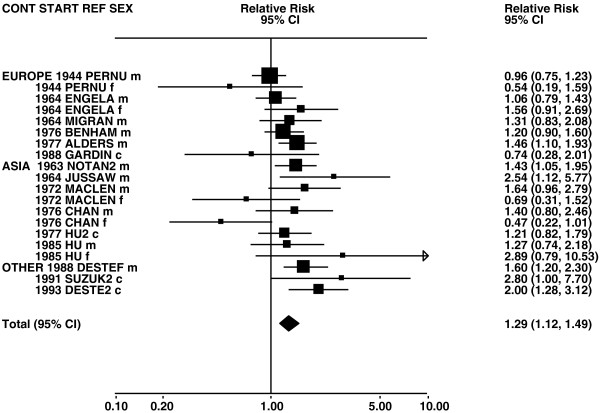
**Forest plot of handrolled vs. manufactured cigarette smoking and all lung cancer.** Table 14 presents the results of a meta-analysis for all lung cancer based on 20 relative risk (RR) and 95% confidence interval (CI) estimates for handrolled vs. manufactured cigarette smoking. The individual study estimates are shown numerically and graphically on a logarithmic scale. The studies are sorted on sex within study reference (REF) within start year of study (START) within continent (CONT). In the graphical representation individual RRs are indicated by a solid square, with the area of the square proportional to the weight (inverse-variance of log RR). Arrows indicate where the CI extends outside the range allocated. Also shown are the combined random-effects estimates. These are represented by a diamond of standard height, with the width indicating the 95% CI.

**Figure 23:**
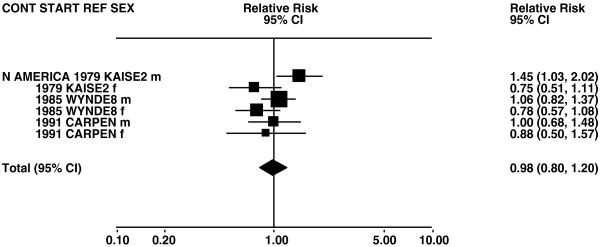
**Forest plot of mentholated vs. non-mentholated cigarette smoking of any product and all lung cancer.** Table 14 presents the results of a meta-analysis for all lung cancer based on six relative risk (RR) and 95% confidence interval (CI) estimates for mentholated vs. non-mentholated cigarette smoking. The individual study estimates are shown numerically and graphically on a logarithmic scale sorted on sex within study reference (REF) within start year of study (START) within continent (CONT). The studies are sorted in order of sex within study reference (REF). In the graphical representation individual RRs are indicated by a solid square, with the area of the square proportional to the weight (inverse-variance of log RR). Arrows indicate where the CI extends outside the range allocated. Also shown are the combined random-effects estimates. These are represented by a diamond of standard height, with the width indicating the 95% CI.

Table 14 summarizes results by type of cigarette smoked. For filter and plain cigarette smoking results are shown for three comparisons, including, for studies where there is a choice, the nearest available equivalents to only filter vs. only plain (with results for all lung cancer also shown in Figure 21), ever filter vs. only plain, and only filter vs. ever plain. Results are also shown for the comparison of handrolled and manufactured cigarette smoking, and for mentholated vs. non-mentholated cigarette smoking, with results for all lung cancer also shown in Figures 22 and 23.

**Table 14:** **Meta-analyses by type of cigarette smoked**^**a**^

**Type of cigarette smoked**	**Statistic**^**b**^	**All lung cancer**^**c**^	**Squamous**^**d**^	**Adeno**^**e**^
Only filter vs. only plain^f^	n	42	13	10
	F	0.67 (0.64-0.72)	0.58 (0.52-0.64)	0.88 (0.75-1.03)
	R	0.69 (0.61-0.78)	0.52 (0.40-0.68)	0.84 (0.66-1.08)
	H, P_H_	3.18, p < 0.001	4.73, p < 0.001	1.90, p < 0.05
Ever filter vs. only plain^g^	n	42	11	10
	F	0.79 (0.75-0.83)	0.85 (0.79-0.91)	1.00 (0.89-1.12)
	R	0.73 (0.65-0.82)	0.55 (0.41-0.74)	0.99 (0.84-1.16)
	H, P_H_	3.60, p < 0.001	7.73, p < 0.001	1.35, NS
Only filter vs. ever plain^h^	n	42	13	10
	F	0.67 (0.63-0.71)	0.87 (0.81-0.92)	1.05 (0.93-1.18)
	R	0.70 (0.62-0.78)	0.69 (0.57-0.83)	0.98 (0.80-1.21)
	H, P_H_	3.30, p < 0.001	3.15, p < 0.001	1.88, p < 0.05
Handrolled vs. manufactured^i^	n	20	5	4
	F	1.27 (1.16-1.40)	1.47 (1.24-1.76)	2.12 (1.53-2.96)
	R	1.29 (1.12-1.49)	1.62 (1.18-2.21)	2.09 (0.83-5.25)
	H, P_H_	1.81, p < 0.05	2.61, p < 0.05	6.54, p < 0.001
Mentholated vs. non-mentholated	n	6	1	1
	F	0.99 (0.86-1.14)	1.04 (0.75-1.44)	0.96 (0.73-1.27)
	R	0.98 (0.80-1.20)	1.04 (0.75-1.44)	0.96 (0.73-1.27)
	H, P_H_	1.89, p < 0.1	NA	NA

^a^ Within each study, results for the relevant smoking product and smoking status are selected in the following preference order, within each sex, for:

cigarette type – see notes f to i;

unexposed group – see notes f to i;

smoking product – any, cigarettes (ignoring other products), cigarettes only;

smoking status – ever, current;

lung cancer type – see notes c to e;

race – all or nearest available, otherwise by race;

follow-up period – longest available;

overlapping studies – principal, subsidiary;

age – whole study, widest available age group;

Results are then selected for:

sex – single sex results, combined sex results;

adjustment for potential confounders – most available.

^b^ n = number of estimates combined, F = fixed-effect meta-analysis RR (95% CI), R = random-effects meta-analysis RR (95% CI), H = heterogeneity chisquared per degree of freedom, P_H_ = probability value for heterogeneity expressed as p < 0.001, p < 0.05, p < 0.1 or NS (p ≥ 0.1), P_B_ = probability value for between levels (see Methods) similarly expressed.

^c^ All or nearest available, must include at least squamous cell carcinoma and adenocarcinoma.

^d^ Squamous cell carcinoma or nearest available, but not including adenocarcinoma.

^e^ Adenocarcinoma or nearest available, but not including squamous cell carcinoma.

^f^ Or nearest available.

Preference order for cigarette type – filter only/NOS, always, mainly, both, equally, ever.

Preference order for comparison group – plain only/NOS, always, mainly, ever.

^g^ Or nearest available.

Preference order for cigarette type – filter ever, equally, both, mainly, always, only/NOS.

Preference order for comparison group – plain only/NOS, always, mainly, ever.

^h^ Or nearest available.

Preference order for cigarette type – filter only/NOS, always, mainly, both, equally, ever.

Preference order for comparison group – plain ever, mainly, always, only/NOS.

^i^ Preference order for cigarette type – handrolled any, both, mainly, only.

Preference order for comparison group – manufactured only ever, only current, any, ever.

The random-effects RRs show a reduction in risk for only filter vs. only plain cigarette smoking that is significant for all lung cancer (RR 0.69, 95% CI 0.61-0.78, n = 42), and squamous (0.52, 0.40-0.68, n = 13), though not for adeno (0.84, 0.66-1.08, n = 10). The alternative comparisons for filter and plain, where only a third to a half of the RRs included actually differ, show clear reductions for all lung cancer and squamous associated with filter cigarette smoking, though no difference for adeno (see Table 14). The reductions for all lung cancer and squamous are evident in both sexes and all continents (see Additional file 5: Detailed Analysis Tables).

The risk associated with handrolled smoking is greater than that with manufactured cigarette smoking, with RRs of 1.29 (1.12-1.49, n = 20) for all lung cancer and 1.62 (1.18-2.21, n = 5) for squamous. The RR of 2.09 (0.83-5.25, n = 4) for adeno is based on very heterogeneous estimates, varying from 0.43 to 8.76, and allows no clear conclusion. As results for females are limited, and have wide variability, the conclusions mainly apply to males. The estimated RR for all lung cancer is greater than 1 in all locations studied, though not always statistically significant. However, there are no data from North America.

Data on mentholated cigarette smoking are limited, particularly by histological type. For all lung cancer, the RR of 0.98 (0.80-1.20, n = 6) is consistent with no effect of mentholation on risk, five RR estimates close to or below 1.0, counterbalancing one reported significant increase in males for study KAISER of 1.45 (1.03-2.02). There is some evidence (p < 0.05) of heterogeneity by sex with estimates of 1.15 (0.93-1.43, n = 3) for males, and 0.78 (0.63-0.98, n = 3) for females.

#### G. Risk by amount smoked

Table 15 summarizes the results of meta-analyses using RRs categorized by number of cigarettes (or cigarette equivalents) smoked per day and based on data for ever/current smoking and for smoking of any product (or cigarettes if not available). These are based on those 140 studies for all lung cancer, 36 for squamous, and 34 for adeno which provided data that could be used in the meta-analyses. For all three outcomes, results are shown for one of the sets of “key values” (see Methods). For all lung cancer, squamous and adeno, a clear increase is seen for RRs for categories including 5, but not 20, cigarettes/day, with the meta-analysis RR increasing monotonically with increasing amount smoked. Random-effects estimates for categories including 45, but not 20 cigarettes/day, are 13.69 (11.80-15.89, n = 128) for all lung cancer, 27.65 (20.42-37.44, n = 37) for squamous and 4.80 (3.29-7.01, n = 34) for adeno. The increase with amount smoked is also clearly evident when an alternative set of key values (1, 10, 20, 30, 40, 999) is used, though numbers of available RRs are quite sparse for the higher key values, when least-adjusted RRs are considered, and in both sexes (see Additional file 5: Detailed Analysis Tables). The key value analyses do not use results for all the dose–response data available, as a number of the studies use broad dose–response categories (such as 1–20 or 20+ cigs/day) which span more than one of the key values. Additional file 5: Detailed Analysis Tables also includes results for alternative definitions of smoking status and product smoked, which show a similarly clear dose–response. For example, for current smoking of any product, the RRs for squamous rise from 9.92 (7.41-13.28, n = 8) for key value 5 cigs/day to 39.16 (23.67-64.79, n = 12) for key value 45 cigs/day. Additional file 4: Dose Not Meta also includes available results for some other studies which present dose–response data in a form that cannot readily be included in the meta-analyses (e.g. where the only available comparison is with an inappropriate base group). These results do not appear inconsistent with those summarized in Table 15.

**Table 15:** **Meta-analyses for number of cigarettes smoked**^**a**^

**Amount smoked**	**Statistic**^**b**^	**All lung cancer**^**c**^	**Squamous**^**d**^	**Adeno**^**e**^
Number of sets^f^		190	48	46
About 5 cigs/day^g^	n	174	41	39
	F	3.25 (3.17-3.34)	6.07 (5.50-6.70)	2.70 (2.47-2.96)
	R	3.49 (3.13-3.89)	4.98 (3.93-6.31)	1.83 (1.40-2.39)
	H, P_H_	10.77, p < 0.001	3.85, p < 0.001	6.19, p < 0.001
About 20 cigs/day^h^	n	113	30	28
	F	5.30 (5.18-5.43)	12.36 (10.89-14.03)	3.58 (3.18-4.04)
	R	7.33 (6.29-8.54)	11.86 (8.92-15.76)	2.73 (2.06-3.61)
	H, P_H_	22.45, p < 0.001	3.72, p < 0.001	4.27, p < 0.001
About 45 cigs/day^i^	n	128	37	34
	F	10.17 (9.89-10.45)	28.95 (25.42-32.98)	7.20 (6.38-8.13)
	R	13.69 (11.80-15.89)	27.65 (20.42-37.44)	4.80 (3.29-7.01)
	H, P_H_	14.89, p < 0.001	3.98, p < 0.001	7.69, p < 0.001

^a^ Within each study, results are selected in the following preference order, within each sex, for:

smoking status – ever, current;

smoking product – any, cigarettes (ignoring other products), cigarettes only;

cigarette type – any, manufactured (with or without handrolled), manufactured only;

unexposed group – never any product, never cigarettes, near equivalent (see Methods);

follow-up period – longest available;

lung cancer type – see notes c to e;

race – all or nearest available, otherwise by race;

overlapping studies – principal, subsidiary;

age – whole study, widest available age group;

Results are then selected for:

sex – single sex results, combined sex results;

adjustment for potential confounders – most available.

Results are by number of cigarettes or cigarette equivalents.

^b^ n = number of estimates combined, F = fixed-effect meta-analysis RR (95% CI), R = random-effects meta-analysis RR (95% CI), H = heterogeneity chisquared per degree of freedom, P_H_ = probability value for heterogeneity expressed as p < 0.001, p < 0.05, p < 0.1 or NS (p ≥ 0.1).

^c^ All or nearest available, must include at least squamous cell carcinoma and adenocarcinoma.

^d^ Squamous cell carcinoma or nearest available, but not including adenocarcinoma.

^e^ Adenocarcinoma or nearest available, but not including squamous cell carcinoma.

^f^ Number of sets of RRs available for the key value analysis, where base for comparison is never smoked.

^g^ Category for which results are provided includes 5 cigs/day but does not include 20 cigs/day.

^h^ Category for which results are provided includes 20 cigs/day but does not include 5 or 45 cigs/day.

^i^ Category for which results are provided includes 45 cigs/day but does not include 20 cigs/day.

Dose–response by amount smoked was investigated for pipe and cigar smoking, but the number of estimates available was small, and referred only to males. However, there was some evidence of dose–response. Thus for all lung cancer, one can compare RRs for cigar only smoking for the highest (8.21, 4.36-15.49, n = 6) and lowest exposure groups (1.84, 1.22-2.79, n = 5), and can also compare RRs for pipe only smoking for the highest (5.99, 3.57-10.04, n = 9) and lowest exposure groups (3.68, 2.75-4.93, n = 8).

#### H. Risk by age of starting to smoke

Table 16 summarizes meta-analysis results for age of starting to smoke based on data for ever/current smoking and for smoking of any product (or cigarettes if not available). Random-effects RRs for earliest compared to latest starting, and selecting results least-adjusted for other aspects of smoking, are significantly elevated for all lung cancer (2.35, 2.08-2.65, n = 73), squamous (2.23, 1.66-2.98, n = 18) and adeno (1.99, 1.48-2.67, n = 17). Alternatively selecting results most-adjusted for other aspects of smoking, the RR for all lung cancer is 2.20 (1.96-2.47, n = 73). The increase in risk with earlier starting is consistent with the results of the key value analyses, with, for example, random-effects estimates relative to never smokers for squamous rising from 11.06 (6.87-17.81, n = 14) for categories including 26 years but not including 18 years to 31.07 (17.93-53.85, n = 6) for categories including 14, but not 18 years. As seen in Additional file 5: Detailed Analysis Tables, a similar pattern is generally seen for other definitions of smoking status and product smoked, although data for smokers of pipes and/or cigars are very limited.

**Table 16:** **Meta-analyses for age started to smoke**^**a**^

**Age started**	**Statistic**^**b**^	**All lung cancer**^**c**^	**Squamous**^**d**^	**Adeno**^**e**^
Number of sets^f^		69	15	14
About age 26 years^g^	n	60	14	13
	F	2.70 (2.62-2.79)	10.42 (8.25-13.15)	3.79 (3.12-4.60)
	R	3.89 (3.33-4.56)	11.06 (6.87-17.81)	3.21 (2.12-4.87)
	H, P_H_	8.73, p < 0.001	3.36, p < 0.001	4.09, p < 0.001
About age 18 years^h^	n	29	6	5
	F	7.75 (7.18-8.37)	20.15 (15.26-26.61)	9.74 (7.63-12.43)
	R	7.48 (5.94-9.42)	20.28 (13.92-29.53)	8.84 (6.14-12.73)
	H, P_H_	7.44, p < 0.001	1.60, NS	1.80, NS
About age 14 years^i^	n	35	6	5
	F	11.11 (10.23-12.06)	29.91 (22.62-39.54)	13.62 (10.78-17.22)
	R	10.32 (8.04-13.26)	31.07 (17.93-53.85)	12.34 (7.23-21.08)
	H, P_H_	7.42, p < 0.001	3.28, p < 0.01	3.76, p < 0.01
Earliest vs. latest^j^	n	73	18	17
	F	1.69 (1.64-1.74)	1.99 (1.72-2.29)	1.94 (1.64-2.29)
	R	2.35 (2.08-2.65)	2.23 (1.66-2.98)	1.99 (1.48-2.67)
	H, P_H_	4.52, p < 0.001	3.19, p < 0.001	2.62, p < 0.001

^a^ Within each study, results are selected in the following preference order, within each sex, for:

smoking status – ever, current;

smoking product – any, cigarettes (ignoring other products), cigarettes only;

cigarette type – any, manufactured (with or without handrolled), manufactured only;

unexposed group – never any product, never cigarettes, near equivalent (see Methods), but see also footnote j;

follow-up period – longest available;

lung cancer type – see notes c to e;

race – all or nearest available, otherwise by race;

overlapping studies – principal, subsidiary;

age – whole study, widest available age group;

Results are then selected for:

sex – single sex results, combined sex results;

adjustment for potential confounders – most available.

^b^ n = number of estimates combined, F = fixed-effect meta-analysis RR (95% CI), R = random-effects meta-analysis RR (95% CI), H = heterogeneity chisquared per degree of freedom, P_H_ = probability value for heterogeneity expressed as p < 0.001, p < 0.05, p < 0.1 or NS (p ≥ 0.1).

^c^ All or nearest available, must include at least squamous cell carcinoma and adenocarcinoma.

^d^ Squamous cell carcinoma or nearest available, but not including adenocarcinoma.

^e^ Adenocarcinoma or nearest available, but not including squamous cell carcinoma.

^f^ Number of sets of RRs available for the key value analysis, where base for comparison is never smoked.

^g^ Category for which results are provided includes 26 years but does not include 18 years.

^h^ Category for which results are provided includes 18 years but does not include 14 or 26 years.

^i^ Category for which results are provided includes 14 years but does not include 18 years.

^j^ For this analysis only, the exposed and unexposed group have the same smoking status, product and cigarette type. There is an additional preference to select the results with least adjustment for other aspects of smoking, followed by a preference to select the results for the earliest (=exposed) and latest (=unexposed) starters. Alternatively preferring results with most adjustment for other aspects of smoking gives n = 73, F = 1.67 (1.62-1.72), R = 2.20 (1.96-2.47), H, P_H_ = 4.01, p < 0.001 for all lung cancer.

#### I. Risk by duration of smoking

Table 17 is laid out similarly to Table 16 and also presents results for ever/current smoking. Random-effects RRs for longest compared to shortest duration of smoking, and selecting results least adjusted for other aspects of smoking, are significantly elevated for all lung cancer (3.56, 2.90-4.35, n = 76), squamous (3.93, 3.10-4.97, n = 27) and adeno (2.64, 2.04-3.43, n = 23). Alternatively selecting results most adjusted for other aspects of smoking, the RR for all lung cancer is 3.00 (2.57-3.49, n = 77). The increase in risk with longer duration is consistent with the results of the key value analyses, with, for example, random-effects estimates for all lung cancer rising from 2.48 (2.09-2.95, n = 55) for categories including 20 years but not including 35 years to 10.13 (7.66-13.39, n = 45) for categories including 50, but not 35 years. A clear trend of risk with increasing duration is also seen for other definitions of smoking status and product smoked (see Additional file 5: Detailed Analysis Tables). Data for pipe and cigar smoking are limited, though even so there is some evidence of a trend. Thus, for all lung cancer longest to shortest RRs are elevated, both in smokers of pipes only (4.32, 1.57-11.89, n = 5) and smokers of cigars only (2.43, 1.02-5.79, n = 3).

**Table 17:** **Meta-analyses for duration of smoking**^**a**^

**Duration of smoking**	**Statistic**^**b**^	**All lung cancer**^**c**^	**Squamous**^**d**^	**Adeno**^**e**^
Number of sets^f^		72	26	23
About 20 years^g^	n	55	23	21
	F	2.46 (2.31-2.63)	6.46 (5.60-7.45)	2.33 (2.04-2.66)
	R	2.48 (2.09-2.95)	4.66 (3.03-7.16)	1.72 (1.20-2.46)
	H, P_H_	5.70, p < 0.001	6.52, p < 0.001	6.04, p < 0.001
About 35 years^h^	n	39	15	12
	F	6.17 (5.80-6.55)	18.25 (15.61-21.34)	5.13 (4.43-5.95)
	R	5.90 (4.75-7.32)	14.06 (7.45-26.52)	3.53 (1.81-6.88)
	H, P_H_	10.39, p < 0.001	11.37, p < 0.001	15.91, p < 0.001
About 50 years^i^	n	45	16	13
	F	13.46 (12.61-14.36)	26.27 (22.20-31.09)	5.24 (4.41-6.23)
	R	10.13 (7.66-13.39)	27.18 (13.36-55.28)	5.25 (2.70-10.20)
	H, P_H_	15.58, p < 0.001	13.13, p < 0.001	12.82, p < 0.001
Longest vs. shortest^j^	n	76	27	23
	F	3.81 (3.62-4.02)	3.55 (3.25-3.89)	2.39 (2.09-2.72)
	R	3.56 (2.90-4.35)	3.93 (3.10-4.97)	2.64 (2.04-3.43)
	H, P_H_	11.48, p < 0.001	4.23, p < 0.001	3.00, p < 0.001

^a^ Within each study, results are selected in the following preference order, within each sex, for:

smoking status – ever, current;

smoking product – any, cigarettes (ignoring other products), cigarettes only;

cigarette type – any, manufactured (with or without handrolled), manufactured only;

unexposed group – never any product, never cigarettes, near equivalent (see Methods), but see also footnote j;

follow-up period – longest available;

lung cancer type – see notes c to e;

race – all or nearest available, otherwise by race;

overlapping studies – principal, subsidiary;

age – whole study, widest available age group;

Results are then selected for:

sex - single sex results, combined sex results;

adjustment for potential confounders – most available.

^b^ n = number of estimates combined, F = fixed-effect meta-analysis RR (95% CI), R = random-effects meta-analysis RR (95% CI), H = heterogeneity chisquared per degree of freedom, P_H_ = probability value for heterogeneity expressed as p < 0.001, p < 0.05, p < 0.1 or NS (p ≥ 0.1).

^c^ All or nearest available, must include at least squamous cell carcinoma and adenocarcinoma.

^d^ Squamous cell carcinoma or nearest available, but not including adenocarcinoma.

^e^ Adenocarcinoma or nearest available, but not including squamous cell carcinoma.

^f^ Number of sets of RRs available for the key value analysis, where base for comparison is never smoked.

^g^ Category for which results are provided includes 20 years but does not include 35 years.

^h^ Category for which results are provided includes 35 years but does not include 20 or 50 years.

^i^ Category for which results are provided includes 50 years but does not include 35 years.

^j^ For this analysis only, the exposed and unexposed group have the same smoking status, product and cigarette type. There is an additional preference to select the results with least adjustment for other aspects of smoking, followed by a preference to select the results for the longest (=exposed) and shortest (=unexposed) duration smokers. Alternatively preferring results with most adjustment for other aspects of smoking gives n = 77, F = 2.67 (2.53-2.82), R = 3.00 (2.57-3.49), H, P_H_ = 5.82, p < 0.001 for all lung cancer.

#### J. Risk by duration of quitting (vs. never smoking)

Table 18 presents results for duration of quitting (vs. never smoking) based on results for smoking of any product (or cigarettes if not available). Random-effects RRs for shortest compared to longest duration of quitting, selecting results least adjusted for other aspects of smoking, are significantly elevated for all lung cancer (3.97, 3.32-4.75, n = 65), squamous (6.22, 3.75-10.30, n = 14) and adeno (3.32, 1.98-5.58, n = 14). Alternatively selecting results most adjusted for other aspects of smoking, the RR for all lung cancer is 3.61 (3.04-4.28, n = 65). The increase in risk with shorter duration of quitting is consistent with the results of the key value analyses, with, for example, random-effects estimates relative to never smokers for adeno rising from 2.10 (1.49-2.94, n = 12) for categories including 12 years but not including 7 years to 6.73 (3.46-13.12, n = 6) for categories including 3, but not 7 years. A clear trend of risk with increasing duration of quitting is also seen for cigarette smoking (or any product if not available), and for cigarette only smoking (see Additional file 5: Detailed Analysis Tables). Data for pipe and cigar smoking were too limited for reliable conclusions.

**Table 18:** **Meta-analyses for duration of quitting (vs. never smoked)**^**a**^

**Duration of quitting**	**Statistic**^**b**^	**All lung cancer**^**c**^	**Squamous**^**d**^	**Adeno**^**e**^
Number of sets^f^		68	16	16
About 12 years^g^	n	53	13	12
	F	3.62 (3.34-3.93)	7.76 (6.07-9.91)	2.13 (1.64-2.76)
	R	2.97 (2.48-3.55)	5.89 (3.85-9.08)	2.10 (1.49-2.94)
	H, P_H_	3.93, p < 0.001	1.69, p < 0.1	1.45, NS
About 7 years^h^	n	33	6	6
	F	6.07 (5.56-6.63)	14.34 (11.10-18.54)	3.29 (2.47-4.39)
	R	5.08 (4.24-6.10)	14.34 (11.10-18.54)	3.74 (2.23-6.25)
	H, P_H_	3.20, p < 0.001	0.49, NS	2.33, p < 0.05
About 3 years^i^	n	43	6	6
	F	9.69 (8.96-10.47)	24.95 (19.45-31.99)	5.05 (3.85-6.64)
	R	8.60 (7.22-10.23)	26.22 (17.19-39.98)	6.73 (3.46-13.12)
	H, P_H_	3.93, p < 0.001	1.46, NS	3.86, p < 0.01
Shortest vs. longest^j^	n	65	14	14
	F	3.94 (3.68-4.22)	5.09 (4.33-5.99)	2.63 (2.11-3.28)
	R	3.97 (3.32-4.75)	6.22 (3.75-10.30)	3.32 (1.98-5.58)
	H, P_H_	5.47, p < 0.001	6.00, p < 0.001	4.41, p < 0.001

^a^ Within each study, results for ex smokers are selected in the following preference order, within each sex, for:

smoking product – any, cigarettes (ignoring other products), cigarettes only;

cigarette type – any, manufactured (with or without handrolled), manufactured only;

unexposed group – never any product, never cigarettes, near equivalent (see Methods) but see also footnote j;

follow-up period – longest available;

lung cancer type – see notes c to e;

race – all or nearest available, otherwise by race;

overlapping studies – principal, subsidiary;

age – whole study, widest available age group;

Results are then selected for:

sex – single sex results, combined sex results;

adjustment for potential confounders – most available.

^b^ n = number of estimates combined, F = fixed-effect meta-analysis RR (95% CI), R = random-effects meta-analysis RR (95% CI), H = heterogeneity chisquared per degree of freedom, P_H_ = probability value for heterogeneity expressed as p < 0.001, p < 0.05, p < 0.1 or NS (p ≥ 0.1).

^c^ All or nearest available, must include at least squamous cell carcinoma and adenocarcinoma.

^d^ Squamous cell carcinoma or nearest available, but not including adenocarcinoma.

^e^ Adenocarcinoma or nearest available, but not including squamous cell carcinoma.

^f^ Number of sets of RRs available for the key value analysis, where base for comparison is never smoked.

^g^ Category for which results are provided includes 12 years but does not include 7 years.

^h^ Category for which results are provided includes 7 years but does not include 3 or 12 years.

^i^ Category for which results are provided includes 3 years but does not include 7 years.

^j^ For this analysis only, the exposed and unexposed group have the same smoking status, product and cigarette type. There is an additional preference to select the results with least adjustment for other aspects of smoking, followed by a preference to select the results for the shortest (=exposed) and longest (=unexposed) duration quitters. Alternatively preferring results with most adjustment for other aspects of smoking gives n = 65, F = 3.59 (3.35-3.85), R = 3.61 (3.04-4.28), H, P_H_ = 4.79, p < 0.001 for all lung cancer. Note that the “shortest” group, as reported by some studies, may omit (unlimited) recent quitters.

#### K. Risk by duration of quitting (vs. current smoking)

For duration of quitting compared to current smoking the number of data sets available are somewhat less than the corresponding number for duration of quitting compared to never smoking. Results included in the longest vs. shortest analysis shown in Table 19 are generally the inverse of those in the shortest vs. longest analysis in Table 18 (exceptions arising for studies which combined current smokers and recent quitters of more than 2 years). While the key value analyses shown in Table 19 echo the trends shown in Table 18, they also show that for shorter term quitting (categories including 3 but not 7 years) there is no evidence of a decline in risk from quitting. Thus the RRs for all lung cancer (0.95, 0.84-1.08, n = 41) and adeno (1.02, 0.85-1.22, n = 6) are close to 1.00, and the RR for squamous (1.15, 1.03-1.28, n = 6) is slightly elevated. Longer quit durations are, however, clearly associated with a reduction in risk. For all lung cancer, almost 40% of the RRs used in the key value analyses included short-term quitters (of up to 2 years) in the current smoker base. No difference was seen between those RRs and those with a more precisely defined current smoker base.

**Table 19:** **Meta-analyses for duration of quitting (vs. current smoking)**^**a**^

**Duration of quitting**	**Statistic**^**b**^	**All lung cancer**^**c**^	**Squamous**^**d**^	**Adeno**^**e**^
Number of sets^f^		58	11	11
About 3 years^g^	n	41	6	6
	F	0.98 (0.93-1.04)	1.15 (1.03-1.28)	1.02 (0.85-1.22)
	R	0.95 (0.84-1.08)	1.15 (1.03-1.28)	1.02 (0.85-1.22)
	H, P_H_	3.55 p < 0.001	0.51, NS	0.74, NS
About 7 years^h^	n	29	4	4
	F	0.60 (0.56-0.64)	0.74 (0.65-0.85)	0.73 (0.58-0.92)
	R	0.57 (0.50-0.64)	0.74 (0.65-0.85)	0.73 (0.58-0.92)
	H, P_H_	2.09, p < 0.001	0.09, NS	0.59, NS
About 12 years^i^	n	48	9	9
	F	0.32 (0.30-0.34)	0.40 (0.35-0.47)	0.50 (0.41-0.60)
	R	0.28 (0.24-0.32)	0.27 (0.18-0.40)	0.39 (0.26-0.58)
	H, P_H_	4.22, p < 0.001	4.07, p < 0.001	3.68, p < 0.001
Longest vs. shortest^j^	n	61	12	12
	F	0.23 (0.21-0.25)	0.19 (0.16-0.22)	0.30 (0.23-0.37)
	R	0.24 (0.20-0.29)	0.14 (0.08-0.25)	0.21 (0.10-0.46)
	H, P_H_	5.21, p < 0.001	6.68 p < 0.001	9.44, p < 0.001

^a^ Within each study, results for ex smokers are selected in the following preference order, within each sex, for:

smoking product – any, cigarettes (ignoring other products), cigarettes only;

cigarette type – any, manufactured (with or without handrolled), manufactured only;

adjustment for other aspects of smoking – least available;

comparison group – current smokers, current and recent smokers (up to 2 years);

follow-up period – longest available;

lung cancer type – see notes c to e;

race – all or nearest available, otherwise by race;

overlapping studies – principal, subsidiary;

age – whole study, widest available age group;

Results are then selected for:

sex –- single sex results, combined sex results;

adjustment for potential confounders – most available.

^b^ n = number of estimates combined, F = fixed-effect meta-analysis RR (95% CI), R = random-effects meta-analysis RR (95% CI), H = heterogeneity chisquared per degree of freedom, P_H_ = probability value for heterogeneity expressed as p < 0.001, p < 0.05, p < 0.1 or NS (p ≥ 0.1).

^c^ All or nearest available, must include at least squamous cell carcinoma and adenocarcinoma.

^d^ Squamous cell carcinoma or nearest available, but not including adenocarcinoma.

^e^ Adenocarcinoma or nearest available, but not including squamous cell carcinoma.

^f^ Number of sets of RRs available for the key value analysis, where base for comparison is never smoked.

^g^ Category for which results are provided includes 3 years but does not include 7 years.

^h^ Category for which results are provided includes 7 years but does not include 3 or 12 years.

^i^ Category for which results are provided includes 12 years but does not include 7 years.

^j^ For this analysis only, the exposed and unexposed group have the same smoking status (i.e. ex-smokers), product and cigarette type. There is a preference (instead of that for comparison group) to select the results for the longest (=exposed) and shortest (=unexposed) duration quitters. Note that (unlike the inverse results shown in Table 18), the “shortest” quitters here may omit recent quitters, but subject to a limit of no more than two years.

#### L. Risk by tar level

Due to the variety of different methods of quantifying tar levels, only highest vs. lowest analyses have been carried out. No data were available by histological type, and all data relate to cigarette smoking. For all lung cancer and for ever/current smoking of cigarettes the 14 available estimates, from 9 studies, showed some evidence of heterogeneity (H = 2.29, p < 0.01). However, 12 of the estimates showed a higher risk in the higher tar group, and the random-effect estimate (1.42, 1.18-1.71) confirmed the relationship between risk and tar level. The increase was evident for males (1.29, 1.08-1.53, n = 7) and females (1.48, 1.05-2.09, n = 6). There was no evidence of heterogeneity by any specific characteristic, including extent of adjustment, 7 of the 14 estimates being adjusted for one or more of aspects of smoking. These results are based on RRs that are selected as being least adjusted for other aspects of smoking. Alternatively, using RRs selected as most adjusted for other aspects of smoking, the overall estimate was 1.34 (1.16-1.56, n = 14).

#### M. Risk by butt length and fraction smoked

All the available data relate to cigarette smoking. As the number of available estimates were quite limited, particularly for butt length, they have been combined into a single analysis including RRs for shortest vs. longest butt lengths and for greatest vs. smallest fraction smoked, and including results for ever smoking and current smoking. The combined estimates were 1.43 (1.14-1.79, n = 11) for all lung cancer, 1.39 (1.04-1.86, n = 7) for squamous, and 1.30 (1.07-1.58, n = 6) for adeno. There was some evidence of heterogeneity for all lung cancer (H = 2.29, p < 0.05) and for squamous (H = 2.96, p <0.01), though not for adeno (H = 0.75), but a clear majority (18/24 = 75.0%) of the estimates indicated a higher risk associated with smoking more of the cigarette.

#### N. Further analyses by histological type

The results so far have been restricted to all lung cancer, squamous or adeno. Table 20 gives results for ever, current and ever/current smoking of any product (or cigarettes if not available) for small cell carcinoma and large cell carcinoma, with corresponding results also shown for all lung cancer, squamous cell carcinoma and for adenocarcinoma. For ever/current smoking, the RR for large cell carcinoma (5.33, 4.02-7.07, n = 29) is quite similar to that for all lung cancer (5.48, 5.07-5.93, n = 342), while the RR for small cell carcinoma (11.14, 8.59-14.46, n = 61) is markedly higher, and similar to that for squamous cell carcinoma (11.62, 9.80-13.78, n = 82). This pattern is also true for current smoking, where RR estimates are higher than for ever/current smoking, and for ever smoking. Additional file 5: Detailed Analysis Tables gives results by level of the various characteristics studied. As for all lung cancer, squamous and adeno, RRs for small cell and large cell carcinoma varied substantially by location, with RRs much higher in North America than in China, and no clear pattern for the other regions, some of which have sparse data. There was also a tendency for RRs to be higher where there was 100% histological confirmation. For ever/current smoking RRs and for small cell carcinoma, the RRs were 9.84 (7.19-13.45, n = 42) without such confirmation, and 14.62 (9.38-22.80, n = 19) with it (p < 0.01). For large cell carcinoma, the corresponding RRs were 3.90 (2.90-5.24, n = 19) without confirmation and 8.28 (5.89-11.65, n = 10) with it (p < 0.01). There was also some evidence for small cell carcinoma only that RRs were higher from more recent studies.

**Table 20:** **Meta-analyses for additional lung cancer types (all lung cancer)**^**a**^

**Lung cancer type**	**Statistic**^**b**^	**Ever smoking**	**Current smoking**	**Ever/current smoking**
All lung cancer^c^	n	328	195	342
	F	4.22 (4.16-4.28)	9.29 (9.07-9.52)	4.25 (4.20-4.31)
	R	5.50 (5.07-5.96)	8.43 (7.63-9.31)	5.48 (5.07-5.93)
	H, P_H_	22.84, p < 0.001	13.76, p < 0.001	22.46, p < 0.001
Squamous cell carcinoma	n	74	33	82
	F	10.43 (9.72-11.20)	12.74 (11.71-13.87)	9.80 (9.18-10.47)
	R	11.56 (9.68-13.81)	16.43 (12.66-21.32)	11.62 (9.80-13.78)
	H, P_H_	4.18, p < 0.001	5.70, p < 0.001	4.44, p < 0.001
Adenocarcinoma	n	87	40	96
	F	3.58 (3.39-3.78)	4.40 (4.12-4.70)	3.50 (3.33-3.67)
	R	2.99 (2.49-3.58)	4.05 (3.15-5.22)	3.10 (2.52-3.65)
	H, P_H_	9.18, p < 0.001	12.28, p < 0.001	8.96, p < 0.001
Large cell carcinoma	n	26	15	29
	F	6.11 (4.78-7.81)	8.41 (6.36-11.12)	5.57 (4.48-6.92)
	R	5.59 (4.05-7.72)	8.56 (5.29-13.86)	5.33 (4.02-7.07)
	H, P_H_	1.47, p < 0.1	2.13, p < 0.01	1.40, p < 0.1
Small cell carcinoma	n	54	27	61
	F	9.88 (8.94-10.02)	15.31 (13.64-17.19)	9.99 (9.09-10.97)
	R	10.98 (8.25-14.61)	18.17 (12.92-25.56)	11.14 (8.59-14.46)
	H, P_H_	6.08, p < 0.001	5.67, p < 0.001	5.70, p < 0.001

^a^ Within each study, results for the relevant lung cancer type and smoking status are selected in the following preference order, within each sex, for:

smoking product – any, cigarettes (ignoring other products), cigarettes only;

cigarette type – any, manufactured (with or without handrolled), manufactured only;

unexposed group – never any product, never cigarettes, other;

follow-up period – longest available;

race – all or nearest available, otherwise by race;

overlapping studies – principal, subsidiary;

age – whole study, widest available age group;

Results are then selected for:

sex – single sex results, combined sex results;

adjustment for potential confounders – most available.

^b^ n = number of estimates combined, F = fixed-effect meta-analysis RR (95% CI), R = random-effects meta-analysis RR (95% CI), H = heterogeneity chisquared per degree of freedom, P_H_ = probability value for heterogeneity expressed as p < 0.001, p < 0.05, p < 0.1 or NS (p ≥ 0.1), P_B_ = probability value for between levels (see Methods) similarly expressed.

^c^ All or nearest available, must include at least squamous cell carcinoma and adenocarcinoma.

#### O. Further analyses based on independent pairs of relative risks

Some studies provide independent RRs for males and females for the same definition of outcome and exposure. Random-effects meta-analysis of the male/female sex ratio confirms the impression already gained from the analyses shown in earlier Tables that RRs tend to be somewhat higher for males, although estimates are heterogeneous. For ever/current smoking, the sex ratio is 1.38 (1.23-1.54) for all lung cancer, based on 93 ratios, 64 higher in males; 1.31 (0.91-1.90) for squamous, based on 30 ratios, 18 higher in males, and 1.43 (1.14-1.78) for adeno, based on 33 ratios, 27 higher in males.

As sex differences may reflect greater cigarette consumption in males, meta-analysis estimates of the sex ratio for ever/current smokers and for all lung cancer were also calculated within levels of amount smoked (as defined in section G). The sex ratio is 1.33 (1.05-1.68) for smokers of about 5 cigs/day, based on 46 ratios, 26 higher in males, 1.59 (1.25-2.01) for smokers of about 20 cigs/day, based on 25 ratios, 20 higher in males, and 1.21 (0.99-1.49) for smokers of about 45 cigs/day, based on 26 ratios, 17 higher in males.

A number of studies provide RR estimates for ever/current smoking separately by age, and random-effects meta-analysis were conducted, based on the ratio of the estimate for the oldest age group for which data were available compared to that for the youngest. Despite only 22 of the 45 (48.9%) of the ratios showing a greater risk in the oldest age group, the meta-analysis showed a significantly higher risk in the oldest age group (ratio 1.17, 95% CI 1.10-1.25), the seven ratios with most weight all being greater than 1.0.

There were also eight studies, all conducted in the US, which provide comparable sex-specific results for ever/current smoking separately for white people and black people (or non-white people). Random-effects meta-analyses of the white/black race ratio showed no difference between the races (1.05, 0.90-1.23, n = 14).

#### P. Further analyses based on non-independent pairs of relative risks

Some studies also provide separate non-independent least-adjusted and most-adjusted RRs for the same definition of exposure. There is little evidence that adjustment reduces the RR for ever/current smoking. Using the same preferences as in Table 11, the most-adjusted estimate is lower than the least-adjusted estimate for 57 of the 126 (45.2%) pairs for all lung cancer, for 14 of the 36 (38.9%) pairs for squamous, and for 21 of the 41 (51.2%) pairs for adeno. In no case do the percentages differ from 50% (at p < 0.05), and in each case the random-effects meta-analysis estimate based on the most-adjusted pair members is similar to the corresponding estimate based on the least-adjusted pair members (data not shown).

RRs for a dose-related index of smoking may be adjusted for other such indices. For all lung cancer, and for four dose-related indices of smoking, pairs of otherwise similar highest vs lowest RRs were identified in which one of the pair was adjusted for the most available other aspects of smoking, and the other had no such adjustment. Both were also chosen as adjusted for the most possible other variables (although those other variables may differ between the pair). There was a clear tendency for the additional adjustment for other aspects of smoking, typically including amount smoked, to produce lower RR estimates. This was true for 18/22 (81.8%, p < 0.01) of the pairs of estimates for age of starting to smoke, 12/15 (80.0%, p < 0.05) of the pairs for duration of smoking, all 17 (100%, p < 0.001) of those for years quit, and 5/7 (71.4%, NS) of those for tar level.

Based on results for ever/current smoking and for all lung cancer, RRs for mixed smokers were compared with those for smokers of cigarettes only. For 22 of the 34 (64.7%) pairs, the RR was lower for mixed smokers, but this tendency was not significant (p = 0.12). RRs for mixed smokers were also compared with those for smokers of pipes/cigars only. Here 23 of the 24 (95.8%, p < 0.001) pairs showed a lower risk in the smokers of pipes/cigars only.

#### Q. Publication bias

**Figure 24:**
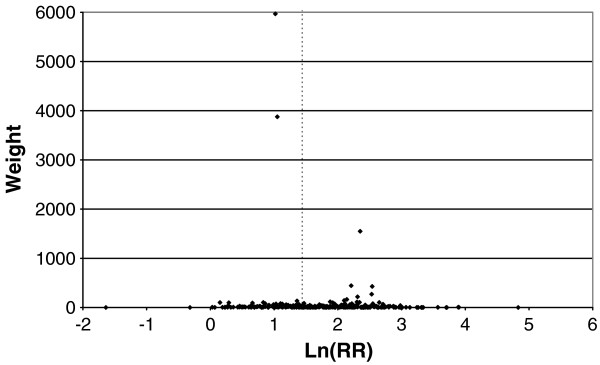
**Funnel plot for ever smoking and all lung cancer.** Funnel plot of the 328 relative risk estimates for ever smoking and all lung cancer included in the main meta-analysis in Table 5 against their weight (inverse-variance of log RR). The dotted vertical line indicates the fixed-effect meta-analysis estimate.

**Figure 25:**
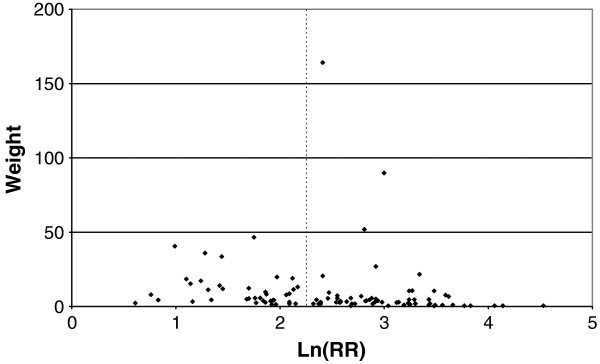
**Funnel plot for ever smoking and squamous.** Funnel plot of the 102 relative risk estimates for ever smoking and squamous included in the main meta-analysis in Table 5 against their weight (inverse-variance of log RR). The dotted vertical line indicates the fixed-effect meta-analysis estimate.

**Figure 26:**
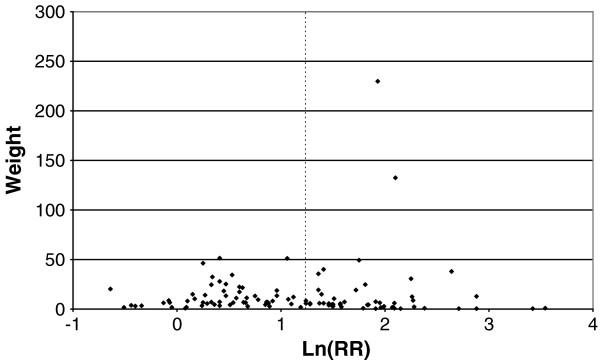
**Funnel plot for ever smoking and adeno.** Funnel plot of the 107 relative risk estimates for ever smoking and adeno included in the main meta-analysis in Table 5 against their weight (inverse-variance of log RR). The dotted vertical line indicates the fixed-effect meta-analysis estimate.

Some results of Egger’s test [17] for publication bias are presented in Tables 5, 8 and 12, with further results given in Additional file 5: Detailed Analysis Tables, but have not previously been referred to in the text. For ever smoking there is evidence of publication bias for all lung cancer (p < 0.001) and adeno (p < 0.01), but not for squamous (p ≥ 0.1). For current smoking, some evidence of publication bias is seen for all lung cancer (p < 0.05), but not for squamous or adeno (p ≥ 0.1). For ex smoking, there is again evidence of bias for all lung cancer and for adeno (p < 0.001) but not for squamous. Figure 24 (all lung cancer), Figure 25 (squamous) and Figure 26 (adeno) show funnel plots for ever smoking. Where asymmetry is seen, this in the direction of there being more higher-weight RRs above the mean. This is consistent with the evidence in Table 5 of higher RRs for larger studies. Inspection of a funnel plot for ex-smoking for all lung cancer (data not shown) also showed the high weight RRs tended to be above the mean.

## Discussion

### Evidence of a relationship

The meta-analyses carried out demonstrate a clear relationship of smoking to overall lung cancer risk. This is evident for ever, current and ex smoking, for pipes and cigars, and for all types of cigarette studied. The increased risk in smokers is evident in both sexes, in younger and older subjects, in all continents studied and in prospective and case–control studies. That this relationship is causal is supported by the evidence of a dose–response, risk increasing with increasing amount smoked, duration of smoking, tar level and fraction smoked, and with earlier age of starting to smoke, and decreasing with duration of quitting. It is also supported by the similarity of results based on most-adjusted and least-adjusted RRs (though adjustment for amount smoked reduces the association with other dose–response indices of smoking). The association is clearly evident with each of the major histological types of lung cancer studied, being stronger for squamous and small cell carcinoma, intermediate for large cell carcinoma, and weakest for adenocarcinoma. Exceptionally, no relationship is seen between adenocarcinoma and pipe or cigar smoking.

### Heterogeneity

The studies are remarkably consistent in reporting an increased risk in ever smokers. Only two of the 328 all lung cancer RRs, none of the 102 squamous RRs, and nine of the 107 adeno RRs considered in Figures 1, 2, 3, 4, 5, 6, 7, 8, 9 are less than 1.0. However, studies also vary markedly in the magnitude of the estimated RR, as illustrated by the high values of H seen in the meta-analysis of the major smoking indices, which often exceed 5 and sometimes exceed 20. (H values of 5, 10 and 20 are the same as I^2^ values [16] of 80%, 90% and 95%). This heterogeneity is perhaps unsurprising given the many sources of variation involved, including sex, location, timing, study design and populations, definition of outcome and type of product smoked, and extent of confounder adjustment.

Using univariable and multivariable (meta-regression) methods, we investigated variation in risk by a number of characteristics of the study and the RR for the outcomes all lung cancer, squamous and adeno. While our “fixed” multivariable models involving six characteristics (sex, location, start year of study, study type, number of cases and number of adjustment factors) explained a substantial proportion of the variation (e.g. reducing H from 22.84 to 4.72 for all lung cancer for ever smoking), there was always substantial residual heterogeneity (with H varying from 2.43 to 4.72 in the six analyses in Tables 7 and 10). Of the six characteristics studied, location was generally the most important characteristic, with RR estimates for ever and for current smoking and for all three outcomes always highest in North America, and lowest in China, and (with the exception of ever smoking for squamous) lower in the rest of Asia than in Europe, with no consistent differences seen between results for the United Kingdom, Scandinavia and the rest of Europe. Another consistently seen relationship was the tendency for RRs to vary by start year of study, with higher RRs seen in more recent studies. Three other tendencies were generally seen, though the level of significance varied according to the analysis. One was the tendency for RRs to vary by number of cases, with the lowest estimates always seen for the smaller studies, (involving 100 to 249 cases), another was the tendency for RRs to be higher in prospective studies than in case–control studies, and the third was the tendency for RRs to be somewhat higher in males than females. The final characteristic included in the fixed model, number of adjustment factors, showed no clear relationship with the RR, with significance either not present or weak (0.01 < p < 0.05), and the direction of effect inconsistent.

We also tested for the effect of a number of other characteristics on the estimated RR. A number of relationships were seen in the univariable models that were significant. However, these mainly became non-significant in the multivariable models, presumably due to correlations between the characteristics. Where a characteristic was significant, this tended to be only in one of the six analyses, so not providing convincing evidence of a true effect. It would have been possible, for each of the six combinations of smoking status and outcome we considered, to present analyses of “best” models, based on forward stepwise regression, that each included a different set of predictive characteristics. However we felt that the regressions we presented based on a fixed model were more useful. Sources of variation are discussed further in the following paragraphs.

### Sex

If possible, sex-specific results are included in the meta-analyses, with combined sex results included only if not. Though variation by sex was not significant in all the main analyses, risk estimates generally tended to be higher for males than females. This is supported by additional analyses comparing RRs within study for the same outcome and exposure definition. Somewhat higher RRs were found in males even in analyses where comparisons were made within the same levels of daily cigarette consumption (about 5, 20 or 45 cigs/day). Even so, the existence of somewhat higher RRs for males does not necessarily indicate any greater susceptibility, as it may reflect their increased exposure to occupational carcinogens, or other differences in smoking history such as greater duration of smoking or increased use of plain and higher tar cigarettes. It should be noted however that in prospective studies where smoking habits were determined at baseline, the greater tendency of males to quit during follow-up may cause bias in the reverse direction. It should also be noted that comparison of smoker/never smoker RRs for men and women does not take account of possible differences in risk between male and female never smokers, the base groups for these comparisons. A detailed overall assessment of this aspect is beyond the scope of this paper, and ideally would involve direct comparison of risk in male and female smokers, with detailed adjustment for age, smoking characteristics and major potential confounding variables. We note that Bain et al. [18] concluded, based on analysis of two large prospective studies and review of results from six other such studies, that “women do not appear to have a greater susceptibility to lung cancer than men, given equal smoking exposure”.

### Age

While it is clear that absolute risk of lung cancer rises markedly with age, both in smokers and never smokers, it is far less clear whether the smoker/never smoker RR also does. Predictions based on the multistage model [19] suggest that there should be a modest rise, but there is difficulty in establishing this, especially when the great majority of the studies do not give results by age. Possible effects of age were investigated in two ways. The first method (see Tables 6 and 9) was to compare RRs which were specific to subjects in specific age groups. Data here were limited for squamous and adeno, and for all lung cancer suggested a possible increase in RR with age for current smoking, but not for ever smoking. More reliable are the comparisons (described in results section O), of RRs for the highest and lowest age groups within study for ever/current smoking; between-study differences are automatically controlled for under this approach. These showed a 17% greater risk for the highest age group (95% CI 10% to 25%). Whether or not a RR was adjusted for age was considered as a characteristic in the meta-regression analyses, but it never added significantly to the fixed model for either ever or current smoking for any of the three outcomes.

### Race

Although RRs were entered onto the database, if available, there were few studies that provided such data. For eight studies which provided pairs of comparable RRs for ever/current smoking, there was no indication that RRs for white people differed systematically from those for black people (or non-white people). This, of course, does not rule out the possibility that absolute risks for white people and black people with similar smoking habits may differ. As our concern was only with RRs for smoking, and whether these vary by other characteristics, we have not attempted to collect data comparing absolute risk according to these characteristics, such as white/black RRs within never smokers, or within smokers. Detailed analysis and discussion of racial differences in lung cancer risk between black people and white people is therefore beyond the scope of this paper. Elsewhere Lee [20] points out that in the USA black men have a higher risk of lung cancer than do white men. However, interpretation of this difference in terms of effects of smoking is not straightforward for various reasons. Thus Lee notes that though black people are more often current smokers, are less likely to quit smoking, smoke cigarettes with a higher tar level, and have higher cotinine levels, all characteristics predictive of a higher risk of lung cancer, they are also less likely to have ever smoked, smoke fewer cigarettes a day and start to smoke later, all characteristics predictive of a lower risk. Also little or no difference in lung cancer rate is seen between black and white women. Black people are much more likely than white people to use mentholated cigarettes, but no evidence of a difference in lung cancer risk associated with mentholation was found, either in the present analysis or in other reviews [20,21].

### Location and national cigarette tobacco type

A consistent tendency in our meta-analyses was for RRs to be highest in studies in North America, intermediate in Europe and lowest in Asia, particularly in China. There was no very clear evidence of a difference between European countries, or between other countries in Asia, though some of the analyses suggested relatively lower RRs in Greece and Turkey than in the rest of Europe, and higher RRs in India than in the rest of Asia. In an attempt to study a possible explanation for this difference we divided countries into three groups by national cigarette tobacco type. One was the countries (Australia, Canada, India, South Africa, UK and Zimbabwe) which typically use flue-cured Virginia tobacco, another was the countries (all except those in the other two groups) which typically use blended tobacco, and the third included Taiwan and China (countries which used both types quite commonly or where we lacked confirmed information). Including this variable into the meta-analyses did not consistently improve the prediction of our model, a finding which is consistent with the conclusions of other analyses we have conducted based on national data on lung cancer rates and smoking frequency [22]. There are, of course other possible explanations of the clear differences in lung cancer RRs between continents, including genetic differences, and differences in baseline rates of the disease.

### Study timing

Our meta-regressions generally showed a tendency for RRs to be lower in studies which started earlier. There may be a number of reasons for this, such as changes in the relative use of cigarettes and pipes or cigars, and improvement of study quality, with better standardization of questionnaires and definition of products smoked. However we consider the most plausible reason to be changes in patterns of uptake of smoking, with smokers in earlier born cohorts being less likely to have a lengthy smoking career than smokers in later born cohorts.

### Study type

Though this was only clearly significant in the analyses of ever smoking for all lung cancer, there was a consistent tendency for RRs to be somewhat higher from prospective studies than from case–control studies. If this is a true effect, the explanation for it is unclear.

### Number of cases

In order to limit the considerable amount of work needed, we limited attention to studies involving at least 100 lung cancer cases. Given that smaller studies would have contributed much less weight to the meta-analyses than would the studies that were included, we consider that this restriction unlikely to have any material effect on our conclusions. The meta-regression analyses did show a consistent tendency for RRs to be higher in larger studies, though this was only significant for ever smoking (all lung cancer p < 0.001), squamous and adeno p < 0.05). This tendency is in the opposite direction to that predicted from publication bias. The explanation is unclear.

### Adjustment for other factors

Generally our analyses showed that adjustment for age and other factors had very little effect on the meta-analysis estimates of smoking-related RR, whether one considered the total number of adjustment factors, or the effect of specific factors. This conclusion of a minimal effect of confounding is consistent with that of a detailed analysis of data from the huge CPSII prospective study [23], and means that though the main results we report are based on most-adjusted estimates, this decision had little or no effect on our conclusions or on the magnitude of our estimates.

Adjustment for other aspects of smoking is, however, important when considering the dose-related variables. Though studies rarely, if ever, present results to allow detailed analysis of the effect of adjustment for one specific aspect of smoking on RRs for another aspect, we have shown that adjustment for other aspects of smoking (which typically includes amount smoked) consistently tends to reduce associations with age of starting to smoke, duration of smoking, years quit and tar level. This is presumably due to the tendency for earlier starters and high tar smokers to smoke more heavily than do later starters and low tar smokers, and for lighter smokers to be more ready to quit smoking. Below, we further discuss the effect of adjustment on results for type of cigarette.

### Product smoked

There was consistent evidence that risk of lung cancer was higher for cigarette only smokers than for smokers of any product, and substantially higher than for smokers of pipes only, cigars only or pipes/cigars only. For current smokers, for example, RRs were 9.57 (7.90-11.59) for cigarettes only, as compared to 4.76 (3.44-6.59) for pipes/cigars only. Mixed smokers tended to have similar risks to cigarette only smokers. Interpretation of this finding is difficult as mixed smokers and cigarette only smokers may have a different total exposure to tobacco, as well as a different cigarette consumption. Data on the types of cigars or pipes smoked have not been recorded on the database, but the increased risk is evident in each continent. The results for pipes and cigars mainly apply to males and to RRs for all lung cancer. Though there are only limited results by histological type, it is interesting that there is no indication of an increased risk of adenocarcinoma for pipe and cigar smokers.

### Type of cigarette smoked

The conclusions drawn from the results in Table 14 are consistent with those drawn by one of us in a review of the relationship between lung cancer and type of cigarette conducted in 2001 [24]. This is unsurprising, because the data sets considered are very similar. The conclusions are also very similar to those of a review by Kabat carried out in 2003 [25].

Comparisons between filter and plain smoking are made more difficult by the variety of ways in which different reports present their results, but based on the index most closely equivalent to only filter vs. only plain, the present report shows a reduction in risk that is significant for all lung cancer (0.69, 95% CI 0.61-0.78) and for squamous (0.52, 0.40-0.68), though not for adeno (0.84, 0.66-1.08). Significant reductions in risk for all lung cancer and squamous, but not for adeno were also evident for the alternative comparisons ever filter vs. only plain, and only filter vs. ever plain. Our analyses were based on most-adjusted RR estimates, with many of the estimates adjusted for other aspects of smoking, such as number of cigarettes smoked. In 2001, a National Cancer Institute monograph [26] claimed that apparent benefits of filter vs. plain and of low tar vs. high tar cigarettes may be illusory if RRs are adjusted for daily consumption, as switching to cigarettes with a lower machine-smoked delivery of tar and nicotine leads to “compensation” for the reduced nicotine intake by increasing numbers of cigarettes smoked. Lee and Sanders [27] investigated this claim in detail by comparing RRs for all lung cancer adjusted and unadjusted specifically for daily cigarette consumption, and concluded that “whether or not relative risk estimates are adjusted for cigarette consumption is not crucial to the conclusion of a clear advantage to filter cigarettes and tar reduction”. This analysis is more precise than that used in this report, but its conclusions are similar, as we also found adjustment not to affect our overall conclusion that filter vs. plain cigarette smoking was associated with a lower risk of all lung cancer and of squamous. It should be noted that although no significant reduction in risk for filter cigarette smoking was seen for adeno, there was also no evidence of an increase. This would seem to argue against the claim often made that the observed rise over time in the incidence of adenocarcinoma relative to squamous cell carcinoma seen in many countries is due to changes in cigarette design increasing the risk of smoking-related adenocarcinoma. In this context, it should be noted that though our database contains evidence by histological type for filter vs. plain cigarette smoking, no such data were found relating to tar level.

Our conclusions of a higher RR in handrolled vs. manufactured cigarette smokers is consistent with that of the 2001 review [24], with the increased risk evident, despite the limited amount of data, for squamous and adeno as well as for all lung cancer.

Our review also found no difference in risk between smokers of mentholated and non mentholated cigarette smokers, though based on data from only three studies, only one of which provided results by histological type. Though no more recent studies have reported results by histological type, five further studies have reported results for all lung cancer, and a recently published systematic review [20] confirms the lack of apparent effect of cigarette mentholation on the lung carcinogenicity of cigarettes.

### Dose–response relationships

We have investigated the relationship of lung cancer risk to various indices of the dose–response relationship. We did not record data on our database for pack-years, as we wished to investigate the separate roles of daily amount smoked and duration of smoking. Indeed, previous work (e.g. [19,28]) has in fact suggested that pack-years is not a valid measure, as for example, smokers of 20 cigs/day for 40 years and smokers of 40 cigs/day for 20 years have very different smoking RRs despite their identical pack-years. For those indices that we did consider where there were substantial amounts of data – daily amount smoked, duration, age of starting to smoke, and time of quit (relative both to current smoking and to never smoking) – there was very clear evidence that greater exposure leads to greater risk, not only for all lung cancer, but also for squamous and adeno. The results by time of quit extend the observation that RRs in ex smokers are intermediate between those of never smokers and current smokers. Because dose–response results are expressed in categories of exposure which vary from study to study, there are difficulties in combining the evidence over studies. We have used two approaches. One is to consider the RR for the highest vs. lowest level of exposure (where highest and lowest refer to expected risk, so that early ages of starting, for example, are considered highest). The other is the key value approach where we consider categories including a specified level of exposure and not including another specified level. Both approaches have limitations. The highest vs. lowest approach will vary between study in the ratio of exposures considered, while the key value approach, although combining results relating to different exposures in different studies to a lesser extent, necessarily omits results from studies with broader categories while somewhat arbitrarily selecting or discarding RRs from studies with narrow categories. Work is ongoing on a third approach to fit a dose–response curve to the RRs and estimated dose mid-points of the categories for each study. This approach is complex, and was considered outside the scope of the current paper, which was more intended to summarize major features of the data. However, a future paper is planned which will describe the shape of these dose–response relationships including characteristics of the curves, such as the estimated time after quitting by which half the excess risk associated with continued smoking has disappeared. We note that, when considering RR for time of quitting, the problem of “reverse causation” needs to be taken into account, as evidenced by the data in Table 19 showing no decrease in risk compared to current smokers for quitters of about 3 years. Our analyses also showed that for all lung cancer, risk increased with increasing tar level and with increasing fraction smoked (or equivalently short butt length), data here being more limited and non existent by histological type. As noted earlier, when discussing cigarette type, the relationship with tar level is not an artefact of inappropriate adjustment for amount smoked [27], as has been claimed [26].

### Derivation of RRs

Almost a third of RRs used in meta-analyses were not directly available from the source or calculated directly from cross-tables of exposure by outcome, and required more complex methods to derive the required RR. It was reassuring that whether or not the RR was derived did not (with one minor exception) add predictive power to the main meta-regression models, suggesting that our extensive use of derived RRs caused no material bias.

### Effect of studies with high RRs or large weight

The statistical analyses investigated the role of various characteristics on the estimated risk of all lung cancer, squamous and adeno in relation to ever and current smoking, but generally did not formally test the effect of exclusion of specific studies with extreme RRs or large weights. An exception was the case of study LIU4 for ever smoking and all lung cancer, this study not giving data for current smoking or by histological type. The two sex-specific RRs for this study together contributed 50.9% of the weight for the 328 available RRs from all the studies, and its exclusion increased the overall fixed-effect RR from 4.22 (95% CI 4.16-4.28) to 6.47 (95% 6.34-6.60). However there was little difference in the random-effects estimates, and in the meta-regression analysis the two LIU4 RRs did not produce unusual standardized residuals, suggesting that the relatively low RRs from this study (2.76, 2.69-2.83 for males, and 2.86, 2.77-2.95 for females), were due to the characteristics of the study included in the model (in particular that it was conducted in China) and not due to its unusual results. While there are other large studies, none involved nearly as many lung cancer cases as LIU4, and we feel it unlikely that excluding other specific studies would have had a major effect on our meta-analysis estimates or on our conclusions as to how RRs varied by exposure, outcome and study and RR characteristics.

### Representativeness

We did not exclude studies on the basis of the population studied. However, most studies include subjects broadly representative of the general population. A small number of studies were conducted in miners or in other occupations with a known or suspected lung cancer risk, such as welding or foundry working. Risky occupation was considered as a characteristic in the meta-regression models but was never found to be an independent predictor of RRs associated with ever or current smoking.

### Publication bias

It is well known that researchers are more likely to wish to publish, and editors more likely to accept for publication, studies finding a statistically significant association between exposure and disease. The published literature may therefore overstate any true association or produce a false-positive relationship. As part of each meta-analysis we have carried out Egger’s test of publication bias, though results are generally shown only in the detailed tables. While evidence for such bias generally is mixed, the results for all lung cancer suggest that, where significant bias is seen, it is not in the direction of smaller studies with lower-weight RRs producing higher RRs. Rather it is, as noted above, the larger studies that tend to produce higher RRs. The reason for this finding is unclear. It should also be noted that our analyses are based only on those studies satisfying the inclusion criteria, and that one of these criteria restricted attention to studies with at least 100 lung cancer cases.

We have not attempted to try to correct for publication bias for four reasons. Firstly, we feel that evidence for its existence is not strong. Second, any adjustment for it seems unlikely to affect our main conclusions. Third, any adjustment for it would be complicated by the restriction on study size. Finally, any correction for publication bias would be open to question, as it inevitably involves assumptions that are impossible to verify.

### Bias due to misclassification of smoking status

Another source of bias is misclassification of smoking status. Random misclassification would dilute the association, as would any tendency for cases to deny or understate their smoking more than for the general population. Any tendency for current smokers to claim to be ex-smokers, as might happen in a study conducted in a clinical setting or where patients have been advised to stop smoking, would tend to inflate the risk for ex smoking. Adjustment for misclassification would be difficult, as denial rates are likely to vary by aspects of the study design, the way questions are asked, and also by sex, age, location and other demographic variables.

### Limitations

This review has various limitations, many unavoidable. Lack of access to individual subject data limits the ability to carry out meta-analyses using similar exposure indices and confounder adjustment throughout, but obtaining such data was not feasible given many studies were conducted years ago. Obtaining a reliable definition of outcome and exposure is often hindered by incomplete information in the source papers. We do not consider that limiting attention to studies of 100 cases or more is of particular importance as results from smaller studies would contribute little weight to the overall meta-analyses. Limiting attention to studies conducted up to 1999 may be more relevant for some exposures and issues (particularly the trend in RR over time), though we feel that our consideration of data from 287 published studies should give a very reliable overall picture. The problem is that the procedures conducted for this review were extremely time-consuming and it would take some years to update the database and include smaller and more recent studies.

It may also be argued that the analyses presented here do not make full use of all the data collected. This is inevitable, given the extensive amount of information collected and the need to present the findings in a paper of reasonable length. As noted, when discussing dose–response, we do plan further analyses. We would also be willing to make the database available to *bona fide* researchers for further analysis.

## Conclusions

After excluding studies involving less than 100 lung cancer cases, we identified 287 epidemiological studies of lung cancer which provided information on risk in relation to one or more of a defined list of smoking indices [2,3,6,29-689]. Of the 267 independent principal studies, 262 provided RRs relating to all lung cancer, 84 provided RRs relating to squamous cell carcinoma, and 86 provided RRs relating to adenocarcinoma (or to outcomes that are closely equivalent). One major conclusion is that for each outcome the RRs for all major smoking indices were markedly heterogeneous.

Another conclusion is that RR estimates for ever, current or ex smoking of any product (or cigarettes if not available) are clearly elevated for all three outcomes. Individual study RRs virtually all exceed 1.0, and based on random-effects meta-analyses of most-adjusted RRs, increases were seen for ever smoking (all lung cancer 5.50, CI 5.07-5.96, n = 328 RRs; squamous, 10.47, 8.88-12.33, n = 102; adeno 2.84, 2.41-3.35, n = 107), current smoking (all lung cancer 8.43, 7.63-9.31; squamous 16.91, 13.14-21.76; adeno 4.21, 3.32-5.34) and ex smoking (all lung cancer 4.30, 3.93-4.71; squamous 8.74, 6.94-11.01; adeno 2.85, 2.20-3.70). For all lung cancer, RRs were also elevated for cigarettes only smokers (ever smoking 6.36, 5.33-7.59) and mixed smokers of cigarettes and pipes/cigars (7.37, 5.97-9.11), though lower for smokers of pipes/cigars only (2.92, 2.38-3.57), pipes only (3.31, 2.51-4.35) and cigars only (2.95, 1.91-4.56). While pipe and cigar smoking is associated with an increased risk for squamous, there is no increase for adeno. The consistency and strength of the relationships are consistent with a causal relationship (except for pipe and cigar smoking and adenocarcinoma). A causal relationship is also supported by the fact that estimates are generally not materially affected by adjustment for confounding variables, and by the strong evidence of a dose–response relationship, with RRs for all outcomes clearly increasing with amount smoked, duration and earlier starting age, and decreasing with time quit, and for all lung cancer increasing with tar level and fraction smoked. Relationships were also clearly seen between smoking and RRs for the other major histological types, small cell carcinoma and large cell carcinoma.

Our review also provides evidence that risk varied by type of cigarette smoked, with filter cigarette smokers having lower risks than plain cigarette smokers (a conclusion not explained by “over-adjustment” for amount smoked), and that handrolled cigarette smokers have higher risks than manufactured cigarette smokers, though mentholation of cigarettes seems unrelated to risk. It also shows that various characteristics of the study and of the RR affect risk estimates. Thus RRs were generally highest for studies in North America and lowest for Asia, particularly in China, and higher in later starting, larger and prospective studies. RRs were also somewhat higher in males than in females, though this may be related to differences in their detailed smoking habits. There is no clear tendency for the smoking/lung cancer relationship to vary with age.

This comprehensive review provides further insight into the relationship of smoking to lung cancer and its major histological types.

## Abbreviations

CI: Confidence Interval; d.f: Degrees of Freedom; H: Heterogeneity Statistic (Ratio of deviance to degrees of freedom); LCL: Lower Confidence Limit; n: number of estimates included in meta-analysis; OR: Odds Ratio; REF: 6 character Reference code used to identify a study; RR: Relative Risk (used also to indicate any effect estimate including Odds Ratio and Hazard Ratio); UCL: Upper Confidence Limit.

## Competing interests

PNL, founder of P.N.Lee Statistics and Computing Ltd., is an independent consultant in statistics and an advisor in the fields of epidemiology and toxicology to a number of tobacco, pharmaceutical and chemical companies. This includes Philip Morris Products S.A., the sponsor of this study. BAF and KJC are employees of P.N.Lee Statistics and Computing Ltd.

## Authors’ contributions

BAF and PNL were responsible for planning the study. Literature searches were carried out by KJC with the assistance of PNL and BAF. Data entry was either carried out by KJC and checked by BAF, or carried out by BAF and checked by PNL. Where appropriate, difficulties in interpreting published data or in the appropriate methods for derivation of RRs were discussed by BAF and PNL. The statistical analyses were conducted by BAF along lines discussed and agreed with PNL. PNL and BAF jointly drafted the paper, which was then critically reviewed by KJC. All authors read and approved the final manuscript.

## Pre-publication history

The pre-publication history for this paper can be accessed here:

http://www.biomedcentral.com/1471-2407/12/385/prepub

## Supplementary Material

Additional file 1 **Methods [**[690-692]**].** Click here for file

Additional file 2 Studies. Click here for file

Additional file 3 RRs. Click here for file

Additional file 4 Dose-response data, not eligible for inclusion in meta-analysis. Click here for file

Additional file 5 **Detailed Analysis Tables (Individual file names as described in Additional file ****1****: Methods, Table**1). Click here for file
