# Supplementary material for: Systematic review with meta-analysis of the epidemiological evidence in the 1900s relating smoking to lung cancer
Source: BMC Cancer. 2012 Sep 3;12:385. doi: 10.1186/1471-2407-12-385 (PMC3505152; doi:10.1186/1471-2407-12-385)
Supplement: Additional file 1 — Methods [[690-692]]. [file 1471-2407-12-385-S1.doc]

# Additional files

### Additional file 1 – Methods

.DOC file giving a fuller version of the Methods section than in the paper. Particular topics described in more detail include the following:

- the methods by which RRs and CIs were derived, where required, from the data presented in the source papers.
- the statistical analyses conducted. It does not include any results itself, but describes the content and structure of the data in Additional file 5 : DetailedAnalysisTables that does provide detailed statistical results.

### Additional file 2 – Studies

.DOC file concerning the 287 studies included on the database. This gives fuller details of overlapping studies, some further description of the studies than shown in Table 2, details of the control groups used in each case-control study, and lists which studies provided data for which outcome and for which smoking indices.

### Additional file 3 – RRs

.DOC file concerning the RRs included on the database. This gives details of the numbers of RRs per study, and the results of checking RRs for apparent errors.

### Additional file 4 – DoseNotMeta

.DOC file summarizing the results of the dose-related data that could not be included in the dose-related meta-analyses.

### Additional file 5 – DetailedAnalysisTables

A mini website giving the full statistical output for all the meta-analyses and meta-regressions conducted. File names are as described in Additional file 1 : Methods (Table 1).

**Systematic review with meta-analysis of the epidemiological evidence in the 1900s relating smoking to lung cancer**

Peter N Lee, Barbara A Forey and Katharine J Coombs

**Additional file 1: Methods**

Contents

[Inclusion and exclusion criteria 5](#__RefHeading___Toc313356336)

[Literature searching 6](#__RefHeading___Toc313356337)

[Identification of studies 7](#__RefHeading___Toc313356338)

[Data recorded 8](#__RefHeading___Toc313356339)

[Identifying which RRs to enter 10](#__RefHeading___Toc313356340)

[Major and cigarette type smoking indices 12](#__RefHeading___Toc313356341)

[Dose-related smoking indices 17](#__RefHeading___Toc313356342)

[Confounders adjusted for 20](#__RefHeading___Toc313356343)

[Strata 21](#__RefHeading___Toc313356344)

[Derivation of RRs 21](#__RefHeading___Toc313356345)

[1 Using 2 x 2 tables 22](#__RefHeading___Toc313356346)

[2 Correction for zero cell 23](#__RefHeading___Toc313356347)

[3 Combining independent RRs 23](#__RefHeading___Toc313356348)

[4 Combining non-independent RRs 24](#__RefHeading___Toc313356349)

[5 Ratio of rates 24](#__RefHeading___Toc313356350)

[6 CI estimated from crude numbers 24](#__RefHeading___Toc313356351)

[7 Converting CI from a different confidence level 25](#__RefHeading___Toc313356352)

[8 Inverting from a different denominator 25](#__RefHeading___Toc313356353)

[9 Using symmetry of the CI 26](#__RefHeading___Toc313356354)

[10 Using SMRs, or expected values 26](#__RefHeading___Toc313356355)

[Data entry and checking 26](#__RefHeading___Toc313356356)

[Meta-analyses 27](#__RefHeading___Toc313356357)

[Overview 27](#__RefHeading___Toc313356358)

[Selecting RRs for the meta-analyses 27](#__RefHeading___Toc313356359)

[Carrying out the meta-analyses 28](#__RefHeading___Toc313356360)

[The major smoking indices 29](#__RefHeading___Toc313356361)

[The dose-related smoking indices 30](#__RefHeading___Toc313356362)

[The meta-analysis tables 32](#__RefHeading___Toc313356363)

[*Numbering system for the tables* 32](#__RefHeading___Toc313356364)

[*Layout of the tables* 33](#__RefHeading___Toc313356365)

[*Notation* 36](#__RefHeading___Toc313356366)

[*Characteristics considered* 39](#__RefHeading___Toc313356367)

[*Dose-related “overview” tables* 43](#__RefHeading___Toc313356368)

[Forest plots 43](#__RefHeading___Toc313356369)

[Funnel plots 44](#__RefHeading___Toc313356370)

[Meta-regression 44](#__RefHeading___Toc313356371)

[Other dose-related data 47](#__RefHeading___Toc313356372)

[Additional analyses 47](#__RefHeading___Toc313356373)

[Software 48](#__RefHeading___Toc313356374)

[Table 1 Meta-analysis tables 50](#__RefHeading___Toc313356375)

[Main and variant analyses for major smoking indices 50](#__RefHeading___Toc313356376)

[Analyses for cigarette type indices 51](#__RefHeading___Toc313356377)

[Analyses for dose-related indices 52](#__RefHeading___Toc313356378)

[Table 2 Abbreviations used in listings 57](#__RefHeading___Toc313356379)

References – see main paper

# Inclusion and exclusion criteria

The objective was to identify epidemiological studies of prospective or case-control design which involved 100 lung cancers or more. Attention was restricted to papers published before 2000,[[1]](#footnote-2)a but no restriction was made on language. Attention was also restricted to studies where relative risk (RR) estimates were available, could be derived, or a statement on their statistical significance provided, for one or more of

the following:

**major indices** – ever, current or ex smoking compared with never or non-current smoking of any product, cigarettes, pipes, cigars and combinations, or specific types of cigarettes (filter, plain, manufactured, handrolled, mentholated, non-mentholated);

**cigarette type indices** – where the comparison is within smokers and the comparison is between smokers of different types of cigarette (filter vs. plain, manufactured vs. handrolled, mentholated vs. non-mentholated); and

**dose-related indices** – where exposure was categorized according to amount smoked, age of starting to smoke, duration of smoking, duration of quitting, tar level, butt length or fraction smoked.

Uncontrolled case studies were not included, as RRs cannot be calculated. There were no further exclusion criteria.

Note that in this paper the term RR is used generically to include various estimators of it, including the odds ratio and the hazard ratio.

# Literature searching

To obtain papers satisfying the inclusion criteria, literature searching was carried out starting in April 1997, and updated at intervals up to December 2001. Techniques used included Medline searches using the terms “(smoking /adverse effects /epidemiology /mortality /pathology or tobacco /adverse effects) and (lung neoplasm /epidemiology /mortality /etiology)”, Emtree searches using the terms “(smoking or tobacco) and lung neoplasm”, inspection of monthly bulletins on smoking and lung cancer from the British Library, and inspection of the extensive files on smoking and health accumulated over many years by P N Lee Statistics and Computing Ltd (PNLSC). Papers at all likely to contain material of interest for the project were examined to see if they either provided relevant information and/or cited other apparently relevant papers not so far obtained. These cited papers were then obtained, and examined as above. Ultimately, a position was reached whereby no paper which had been examined cited a paper of possible relevance that had not previously been examined. Where necessary (principally Chinese and Japanese papers), English translations were obtained, although as far as possible dictionaries were used to identify key information from non-English papers.

Overall, 5,993 papers were identified, of which 5,749 could be obtained and examined. Of these, 673 contained data relevant to the project, 175 described studies which were not relevant because the number of lung cancers considered was less than 100, and the remaining 4,901 did not provide relevant data at all.

# Identification of studies

Relevant papers were classified into distinct studies, identifying multiple papers referring to the same study (with one being designated as the principal paper), and multiple studies reported within a paper. Studies were named by a unique alphanumeric reference code (REF) of up to 6 characters (e.g. COMSTO or LUBIN2), based on the name of the principal author and distinguishing multiple studies with the same author, and they were then entered on a database (see also next section).

Some studies were noted as having overlaps with other studies. In theory, RRs being meta-analysed should come from independent studies involving distinct lung cancer cases, otherwise cases featured in more than one study will be "double-counted". In practice, avoidance of such double-counting is difficult and not necessarily the most desirable solution. Thus, if study A describes a case-control study conducted in 1970-80 involving all hospital cases in town X admitted with lung cancer, while study B describes a similar case-control study in the same town conducted in 1978-88, including results from both studies would involve some double-counting, of deaths in 1978-80, but avoiding this would require totally ignoring results from one study (or both), with a substantial loss of power. This would seem less desirable than allowing some double-counting. Even if study B had been conducted in 1975-77 (totally within the period for study A) omitting it may still not be appropriate, if the paper describing study B reports results for some exposure indices not considered in the paper describing study A. While one would not want to include results from both studies in an analysis (and would omit study B if RR estimates were available from both studies), one might want to use data from either study if only one provides the required RR. There are other possibilities too; for example, studies of overlapping regions, or studies which incompletely describe where or when they were conducted and may overlap other studies.

To minimize problems in meta-analysis arising from double-counting of cases, overlapping studies were divided into two categories, as described more fully in Additional file 2 : Studies. The first category involved minor overlap, which could not be disentangled, and which it was decided to ignore. The second category contains sets of studies which probably or definitely overlap. Here the set member containing the most valuable data (e.g. largest study size or longest follow-up) was called the ‘principal study’, other members being ‘subsidiary studies’ only considered in meta-analyses where the required RR was unavailable from the principal study. Note that the allocation as principal or subsidiary study did not take study quality into account.

# Data recorded

For each study, relevant information was entered onto a study database and two linked RR databases.

Data entry was carried out in two stages. In 1997-2002, data were entered on the first RR database for the major smoking indices, cigarette type indices, and amount smoked. In 2009-2010, data were entered on the second RR database for the remaining dose-related indices.

The study database contains a single record for each study, describing the following:

- relevant publications;
- study title;
- study design;
- sexes considered;
- age range, race(s) and other details of the population studied;
- location;
- timing and length of follow-up;
- whether principal or subsidiary, with details of overlaps or links with other studies;
- number of cases and extent of histological confirmation;
- number of controls or subjects at risk;
- types of controls and matching factors used in case-control studies;
- use of proxy respondents, interview setting and response rates;
- confounding variables considered; and
- availability of results by histological types and for each of the smoking indices considered.

Details were also recorded of whether data were available (but not entered) for various other diseases, or for other indices of tobacco exposure not considered, including rarely studied indices such as smoking before breakfast, and pack-years (not included because it was considered more important to separate effects of extent and duration of exposure). Fuller details of the study database structure are available on request.

The RR databases holds the detailed results, typically containing multiple records for each study. Each record refers to a specific RR, describing the comparison made and the actual results, as well as the study REF to link it to the relevant study. The descriptive information includes the sex, age range, race, lung cancer type, and (for prospective studies) the follow-up period. The smoking exposure of the numerator of the RR is defined by the smoking status (ever, current or ex) and smoking product (e.g. any product, cigarettes irrespective of other products, cigarettes only, pipes only), the cigarette type (e.g. any, manufactured cigarettes, manufactured +/- handrolled cigarettes, filter cigarettes only, mentholated cigarettes). For the dose-related indices, the level of exposure is recorded. Similar information is recorded in relation to the denominator of the RR (e.g. never smoker, nonsmoker, never cigarettes, ever plain, non-mentholated cigarettes, low duration smoker). The source of the RR (publication, table and page numbers) is also recorded, as are details on adjustment variables, including, for the dose-related indices, adjustment for other aspects of smoking. The results recorded include the numbers of cases for the numerator and denominator, and, for unadjusted results, either the numbers of controls, persons at risk, or person-years at risk. The estimate of the RR and its lower and upper 95% confidence limits (LCL and UCL) are always recorded. For case-control studies, the estimate is generally the odds ratio [OR]. For an incidence analysis, relative risks (or hazard ratios if provided) were chosen in preference to ORs, which were used only where RRs were not available (typically when estimated from a multiple logistic regression). Data may be as provided in the source, or derived by various means, with the method of derivation noted, possible methods of derivation being described below. Fuller details of the RR database structure are also available on request.

# Identifying which RRs to enter

RRs were entered relating to defined combinations of lung cancer type, smoking index (major, cigarette type or dose-related), confounders adjusted for, and strata, as described below.

**Lung cancer type**

Results were entered for the following lung cancer types[[2]](#footnote-3):

1. all lung cancers (as originally presented, or by combining all types for which results are available provided that the great majority were included),
2. Kreyberg I (as originally presented, or by combining squamous cell carcinoma, small cell carcinoma and large cell carcinoma),
3. Kreyberg II (as originally presented, or by combining adenocarcinoma and “other” – i.e. lung cancer type known but not squamous cell carcinoma, small cell carcinoma or large cell carcinoma),
4. squamous cell carcinoma,
5. small (or oat) cell carcinoma,
6. large cell carcinoma and
7. adenocarcinoma.
8. In addition, if results were not available for types 1, 4 or 7 above, results for the nearest equivalents were entered (as originally presented, or by combining as necessary). These were defined as
   1. nearest possible to all, including at least squamous cell carcinoma and adenocarcinoma,
   2. nearest possible to squamous cell carcinoma, but not including adenocarcinoma,
   3. nearest possible to adenocarcinoma but not including squamous cell carcinoma.

Throughout the rest of this report, the term “all lung cancer” is used to refer to all lung cancer (1) or nearest available (8a), “squamous” to squamous cell carcinoma (4) or nearest available (8b), and “adeno” to adenocarcinoma (7) or nearest available (8c).

## Major and cigarette type smoking indices

The intention was to enter RRs comparing current smokers, ever smokers or ex smokers with never or non smokers. However various near equivalents were accepted, depending on the definition used in the source. Results were accepted whether treating occasional smokers as smokers or as non smokers. Eight further rules were adopted:

1. If never smokers are combined with smokers of a low amount, the limit is set at 5 cigarettes per day.

2. If never smokers are combined with long-term ex-smokers, the limit is set at 20 years.

3. If never smokers are combined with short duration smokers, the limit is set at 5 years.

4. If current smokers are combined with recent quitters, the limit is set at 5 years.

5. If ex-smokers omit recent quitters, the limit is set at 5 years..

6. For the cigarette type indices (but not the major indices), if current smokers are combined with recent quitters of more than 5 years duration, and no better data are available, the data are classified as referring to ever smokers, with no comparisons with non-current considered.

7. For the major indices, complementary restrictions apply to the smoking group (e.g. complementary to restriction 1, if smokers exclude smokers of a low amount, the limit is set at 5 cigarettes per day). For the cigarette type indices, results with similarly restricted smoking groups would be preferred, but other data are accepted if no better available (e.g. cigarette type indices restricted to smokers of 20 years duration).

8. Other restrictions on aspects of smoking are not permitted (e.g. results restricted to smokers who inhale). However, results for ex and ever smokers omitting those who quit on doctor’s orders are retained in study DORN.

When identifying the numerator of the RR, smoking status was defined as current smoker, ex smoker or ever smoker. If smoking status was not clearly defined by the original author as current or ever smoking, we assumed that it was ever.

Product was defined as one of nine levels:

- all products
- cigarettes (with or without other products)
- other products but not cigarettes
- cigarettes only
- both cigarettes and others
- other products (with or without cigarettes)
- pipe only
- cigar only
- both pipe and cigar (but not cigarettes)

Where the product related to cigarette smokers, one of the following levels additionally indicated cigarette type:

- all types
- manufactured (with or without hand-rolled)
- hand-rolled (with or without manufactured)
- manufactured only
- hand-rolled only
- both hand-rolled and manufactured
- mainly manufactured
- mainly hand-rolled
- mentholated
- filter only
- plain only
- mainly filter
- mainly plain
- always filter
- always plain
- ever filter
- ever plain
- both plain and filter
- plain and filter equally

When identifying the denominator, attention was usually restricted to just four groups:

- never smokers,
- non smokers,
- never smoked cigarettes (of any type) and
- non smokers of cigarettes.

Other denominators such as “never smoked or smoked a low amount” or “never smoked or gave up a long time ago” were used only if none of the four main denominators were available (and were subject to the eight rules mentioned at the start of this section). Note that the denominator “never cigarettes” includes smokers who have only ever smoked pipe and/or cigars).

If available, results (for each of current, ex and ever smoking) were entered for five comparisons:

- any product vs never any product,
- cigarettes vs. never any product,
- cigarettes only vs. never any product,
- cigarettes vs. never cigarettes, and
- cigarettes only vs. never cigarettes.

Here “cigarettes” is irrespective of whether other products (i.e. pipes and cigars) are also smoked, while “cigarettes only” excludes mixed smokers of other products. For current smoking, results were also entered for five equivalent “vs non” comparisons.

Additionally, when the numerator related to the smoking of filter, hand-rolled or mentholated cigarettes, results for further comparisons were entered with the denominator relating to plain, manufactured or non-mentholated smokers respectively. These are referred to as the “cigarette type indices”.

It may be useful to note some examples of combinations that have not been entered: mixed products versus cigarettes only; pipe smoker versus non smoker of pipes. Note also that ever smoker versus non smoker would not be valid, as ex smokers would be counted both in the numerator and denominator. Similarly, pipe smokers versus never cigarette smokers would not be valid because pipe only smokers would be counted twice.

For some studies the smoking product was poorly defined. This often arose for studies conducted in countries where cigarettes were the predominant product, so that papers (and also the original questionnaires used in the studies) refer only to “smoking” and, without specific local and historical knowledge, it is difficult to know whether this should be interpreted as meaning cigarettes only, or all products, or that these are in any case the same thing.

In addition, for studies where it was clearly stated that there were no smokers of other products, three of the product levels (all products, cigarettes and cigarettes only) are identical, as are certain smoking status levels for the denominator (never smoked and never smoked cigarettes; and non smoker and non smoker of cigarettes). Thus each basic result should in principle be entered under six separate definitions. However, this was considered to represent excessive duplication[[3]](#footnote-4) and instead the policy adopted was as follows:

1. If only “smoking” is referred to (even if tables are subdivided by numbers of cigarettes or pack-years) then define RRs as relating to any product (numerator) vs never/non smoker (denominator).
2. If the terms “cigarette smoking” and “smoking” are used as if synonymous with no mention at all of other products, then define RRs as relating to cigarettes vs never/non cigarettes,
3. But if there is any hint that other products were asked about in the questionnaire, then define the RRs as relating to cigarettes, vs either never/non smoked if the smokers of other products have been excluded from the analysis, or never/non smoked cigarettes if they have been included with the unexposed group (e.g. if pipe/cigar only smokers have been combined with the never smokers to form the denominator).
4. Only enter RRs using the “cigarettes only” level if mixed smokers have specifically been excluded (i.e. do not use it when there were no mixed smokers).
5. When there are no smokers of other products, enter as all products vs never/non smokers – with the exception that when there are male but no female smokers of other products, at least the main results for females should be entered twice, both for all products and for cigarettes.

## Dose-related smoking indices

Results were entered for seven measures of smoking-related dose:

- amount smoked,
- age of starting to smoke,
- duration of smoking,
- duration of quitting,
- tar level,
- butt length and
- fraction smoked.

RRs were expressed relative to never smokers (or near equivalent), if available or relative to nonsmokers otherwise. For duration of quitting, RRs were also expressed relative to current smokers.

For amount smoked, exposure was usually measured as number of cigarettes per day, but results including smoking of other products expressed as cigarette-equivalents, or as grams of tobacco were also accepted.

RRs are often available for each of a set of dose categories (e.g. 1-10, 11-20, 21-30 etc cigarettes/day) compared with a common base of never smokers, and were entered provided the set included at least two dose categories. For amount smoked, duration of smoking, tar level, and fraction smoked, risk was expected to rise as values increased, whereas for age of starting to smoke, duration of quitting and butt length, risk was expected to rise as values decreased (e.g. categories for age of starting might be 25+, 20-24, 16-19, under 16).

Near equivalents for never smoking were accepted as described previously (rules 1-3 above), and, failing that, a base of non-smoking (or near equivalent) was accepted (e.g. current smokers of 1-10, 11-20 etc compared with never smokers and ex smokers combined).

Further RRs were entered, restricted to smokers. For amount smoked, duration of smoking, tar level, and fraction smoked, these were expressed relative to smokers with a low value as the common base (e.g. comparing 11-20, 21-30, 31+ years smoked with 1-10 years smoked). For age of starting to smoke, duration of quitting and butt length, where (as explained in the preceding paragraph) risk was expected to rise as values decreased, the RRs were expressed relative to smokers with a high value as the common base. This base is subsequently referred to as “low”, meaning low smoking exposure, irrespective of whether the actual values of the measure are low or high. For amount smoked, these RRs were entered only when no RRs relative to a never or non-smoking base were available (and they were not included in any meta-analysis). For duration of quitting, additional RRs were entered relative to a base of current smokers. Whatever base was used, sets of RRs were entered for all the combinations of smoking status and product described in the previous section, except that duration of quitting refers only to ex smokers. The restrictions on acceptable definitions of smokers were as described previously (rules 4-8), with tar, butt length and fraction smoked treated as for the cigarette type indices.

For the dose-related measures other than amount smoked, if the categories reported did not cover the full range of possible values for the dose measure, a “dummy” relative risk was entered on the database indicating a gap in the sequence of dose categories.

Sometimes, the only available results had a base group combining never smokers with a low exposure group, beyond the limit we had defined as acceptable for the major smoking indices. For example, where the combined group of never smokers and smokers of up to 10 cigs/day was compared with smokers of 11-20, 21-30, 31+ cigs/day. These results were entered compared with the base as given (but such data were not considered eligible for meta-analysis, and further comparisons within smokers, e.g. 21-30 relative to 11-20 cigs/day, were not entered).

Except for amount smoked, where results for the dose-related smoking indices were provided, but not in the form of categorical data, a comment was recorded in the database. This includes results from regression analyses expressed as risk per unit dose, differences in mean dose between subjects with and without disease, and general statements that risk was or was not related to level of exposure.

Availability of results for interactions between dose measures was noted on the database, but not entered.

## Confounders adjusted for

For case-control studies, results were entered adjusted for the greatest number of potential confounding variables for which results were available, and also unadjusted (or adjusted for the smallest number of confounders). For prospective studies, only age-adjusted results were usually considered, with results entered adjusted for age and the greatest number of confounders, and for age only or age and the smallest number of confounders; unadjusted results were entered only if no age-adjusted results were available. The alternative RRs are subsequently referred to as “most-adjusted” and “least-adjusted”.

For RRs restricted to smokers, adjustment for other smoking variables is possible (e.g. adjustment for amount smoked when studying age of starting to smoke), which is not possible in an analysis with never smokers as the base group[[4]](#footnote-5). For dose-related RRs restricted to smokers, any such adjusting variables for aspects of smoking were recorded separately from other adjusting variables. Where the adjusting variables for the most-adjusted RR included another aspect of smoking, an additional RR was entered (if available) with the same adjusting variables, but without adjustment for the other aspect of smoking. Similarly, if a RR was available with the same adjusting variables as the least-adjusted, but additionally adjusted for another aspect of smoking, then that RR was also entered.

## Strata

Three strata were considered – sex, age and race. Results were entered for males and females separately when available. Combined sex results were only entered when the equivalent results (i.e. for the same smoking indices, confounders, age and race) were not available. Results were entered both for individual age groups and for all ages combined, as presented by the original authors. The age groups used vary considerably from study to study, and have been entered as found, except that adjacent groups may have been combined together in exceptional circumstances, for instance to avoid very small number of cases. Similarly, results were entered both for individual racial groups and for all races.. For the second RR database only (i.e. for dose-related indices other than amount smoked), results for combined ages and races were also derived and entered where these had not been presented by the original authors.

# Derivation of RRs

Adjusted RRs and their 95% CIs were entered as given when available. A variety of methods were used to provide estimates of the RR and CI in other circumstances. The main methods are described below. Calculations were mainly carried out using Quattro Pro or Excel spreadsheets. Where a RR was given for which no CI was available (and could not be estimated), the RR was entered with the CI marked as missing.

## 1 Using 2 x 2 tables

Unadjusted RRs were calculated from their 2 × 2 table, if available using standard methods (e.g. [12]) and otherwise they were entered as given. If the numbers of cases are denoted by *a_i_* and the numbers of controls (or the disease-free population in a cross-sectional study) by *b_i_*, where the subscript *i* = 0 refers to the unexposed group and *i* = 1 refers to the exposed group, then the RR and its 95% confidence limits LCL and UCL (as estimated by the OR) are calculated by:

RR = (*a*_1_ *b*_0_) / (*a*_0_ *b*_1_)

LCL = RR / *φ*

UCL = RR *φ*

where *φ*, a factor based on the variance of the RR, is given by:

ln( *φ* ) = N_95_$\sqrt{((1/a_{0})+(1/a_{1})+(1/b_{0})+(1/b_{1}))}$

Here N_95_ denotes the inverse standard normal value for 95% confidence (i.e. very close to 1.96).

For an incidence analysis, b_i_ denotes the at-risk population, and the formulae to calculate the RR and its CI are the same, except that

ln( *φ* ) = N_95_$\sqrt{((1/a_{0})+(1/a_{1})-(1/b_{0})-(1/b_{1}))}$

If both a 2 × 2 table and an unadjusted RR/CI were provided by the author, then the RR/CI calculated as above was used, any discrepancy from that originally given being noted in the database.

The 2 × 2 table may be constructed by summing groups (e.g. adding current and ex smokers to obtain ever smokers, or adding over stratifying factors), or from a percentage distribution.

Where the original authors presented the results as a 2 × 2 table of matched pairs, the RR and CI were calculated using CIA (Confidence Interval Analysis) [12].

## 2 Correction for zero cell

If the 2 × 2 table has one cell with value zero, the unadjusted RR and CI cannot be calculated by the usual formulae. The method used was to add a correction of 0.5 to each of the four cells, and then apply the formulae. [No calculation is made if a table has two zero cells. Usually such RRs have not been entered at all; for instance if they occur in a breakdown to very narrow age groups, then adjacent age groups would simply be combined. However they have been entered on a very few occasions, for instance if a product code such as pipe smoking has been entered for males but has no exposure for females.]

## 3 Combining independent RRs

Combining RRs over strata was carried out using fixed-effect meta-analysis [13]. The resulting estimate is adjusted for the stratifying variable. When this combined RR is subsequently used in a fixed-effects meta-analysis, the end result will be exactly the same as if all the original RRs had been included. This method is also appropriate for combining RRs for individual disease groups, provided they are independent estimates (i.e. each disease group has a separate control group).

When combining strata from data available as a 2 × 2 × ℓ table (i.e. ℓ levels of the stratifying variable), adjacent levels were combined if necessary to avoid any empty cells, or if that was not possible, then the correction for zero cell (as described above) was carried out within strata.

## 4 Combining non-independent RRs

When non-independent RRs were to be combined, for instance where adjusted RRs are available for current and ex smokers, each versus never smokers, then the method of Hamling *et al* [14] was used to provide a combined estimate for ever smokers. This method starts from a source table giving adjusted RRs and CIs for *n* exposed groups relative to a single non-exposed base group. The hypothetical underlying 2 × (*n* + 1) table of numbers of ‘adjusted cases and controls’ is estimated, these then being summed to give the required groups for the numerator and denominator, and the resulting 2 × 2 table used with the usual formulae to estimate the adjusted RR and CI. A variation of the method allows non-independent disease groups to be combined. Thus when RRs for individual histological types were available, each relative to a single shared control group, the disease groups can be combined.

## 5 Ratio of rates

Results from prospective studies are often presented as mortality rates rather than as RRs. If they are presented separately for the exposed and unexposed groups (R1 and R0), then the RR is calculated by:

RR = R1 / R0

## 6 CI estimated from crude numbers

When an adjusted RR was presented originally without a CI or p-value, but the corresponding 2 × 2 table (or numbers of exposed and unexposed cases from a prospective study) was available, then the original RR was used and its CI estimated by assuming its width is the same as the width of the interval for the equivalent unadjusted RR. In fact, the estimated interval will be narrower than the true one (since adjustment widens the interval [15]) and thus this method will increase the weight that the estimate is given when entered into a meta-analysis. However this will usually have a very small effect, the only alternative being to omit the RR altogether from all meta-analyses. The same method is used for a RR calculated as a ratio of rates if no CIs were presented for the rates.

## 7 Converting CI from a different confidence level

Where the RR and CI were originally presented with a different confidence level *c* the 95% CIs were calculated from the c% CI using the formulae from section 1 with:

ln ( *φ* ) = N_95_ (ln (UCL*c*) – ln (LCL*c*) ) / 2N*c*

## 8 Inverting from a different denominator

If the RR and CI were originally presented with the exposed and unexposed groups reversed from those required, then the required values were calculated by:

RR = 1 / RR*O*

LCL = 1 / UCL*O*

UCL = 1 / LCL*O*

where the subscript *O* indicates the values as originally presented.

If RR/CIs for separate levels of an exposure variable are given relative to a common base level, then RR/CIs each relative to another of the levels can be estimated by the method described above for “Combining non-independent RRs”.

## 9 Using symmetry of the CI

When only two of the RR, LCL and UCL are given, then the third can be calculated to give a CI symmetrical about the RR. For instance if UCL is missing, then the formula from section 1 is used with:

*φ* = RR / LCL, i.e.

UCL = RR^2^ / LCL

## 10 Using SMRs, or expected values

When the observed numbers of cases were given together with SMRs or expected values relative to a standard (e.g. national) population, then the ‘ratio of two standardised ratios’ was calculated as described by Altman *et al* [690] using the program CIA (Confidence Interval Analysis) [12].

# Data entry and checking

Master copies of all the papers in the study file were read closely. The information to be entered for a study was identified and highlighted on the papers (with notes made as necessary) to facilitate later checking. Where more than one paper was available for the study, a principal publication to provide most of the information was selected, though details of interest described only in other publications were also identified. Occasionally, descriptions of some study aspects conflicted between papers. Where necessary, the most likely version was determined by discussion between the authors of this report, sometimes after attempts to contact the original authors, with notes on the problem being recorded. Preliminary calculations and data entry were carried out by KJC and checked by BAF, or carried out by BAF and checked by PNL, and an automatic program investigated the completeness and consistency of the data. RR/CIs underwent validation checks ([15]).

Detailed instructions used for data extraction and entry onto the databases, along with full details of the automated checks carried out, are available on request.

# Meta-analyses

## Overview

A pre-planned series of meta-analyses was conducted for various smoking indices for each of the three main outcomes (all lung cancer, squamous, and adeno – see *Lung cancer type* above for definitions) and also for some indices for two other outcomes (large cell carcinoma and small cell carcinoma). Each meta-analysis was repeated, based on most-adjusted RRs and on least-adjusted RRs. For each meta-analysis conducted, combined estimates were made first for all the RRs selected, then for RRs subdivided by level of various characteristics, testing for heterogeneity between levels. Results are presented in Tables (both full and summary) and plots. Further detail is given in the following sections.

## Selecting RRs for the meta-analyses

All meta-analyses are restricted to records where values are available for both the RR and the CI. The process of selecting those RRs to include in an analysis is quite complex as it must simultaneously address the two main objectives of inclusion of all relevant data and avoidance of double-counting.

When defining relevant data for an analysis (e.g. of current cigarette smoking), a single specific value of a smoking index may be chosen, and studies with no such RRs are excluded. However on occasion, a number of values of a smoking index may be acceptable in an analysis, (e.g. any product smoking, cigarette smoking or cigarette only smoking), and if a study has more than one acceptable RR, the one to be used in the meta-analysis has to be determined by a preference order defined specifically for that meta-analysis. Similarly a preference order may be required for the unexposed base. The choice of most-adjusted or least-adjusted RRs is handled similarly (noting that if a study has only one RR eligible for an analysis, it will be selected for both the most- and least- adjusted analysis, whether it is unadjusted or adjusted) When multiple preference orders are specified, the sequence of implementation may affect the selection, so preferences for the most important aspects of the analysis, usually concerning smoking, are implemented first, with the demographic aspects (e.g. widest available age and racial groups) considered later. As smoking results may differ between the sexes (e.g. a study may provide RRs for smoking of any product for males but RRs for smoking of cigarettes for females, or may provide unadjusted results for separate sexes, but adjusted results only for sexes combined), care is taken to ensure that the most appropriate RR is chosen within each sex stratum, with a preference for single sex to sexes-combined results implemented later. Finally, RRs from a subsidiary study are retained only where no eligible RRs are available from its principal study.

## Carrying out the meta-analyses

The method used to carry out meta-analyses of selected RRs is that described by Fleiss and Gross [13]. Both fixed-effect and random-effects meta-analysis have been conducted to produce combined estimates. Fixed-effect meta-analysis assumes a common underlying RR estimate and only takes into account within-study variability, whereas random-effects meta-analysis also takes into account between-study variability. Where there is no evidence of heterogeneity, the two analyses give the same results. Heterogeneity has been quantified by H, the ratio of the heterogeneity chisquared to its degrees of freedom. If required, the statistic I^2^ [16] can be calculated directly from H using the formula I^2^ = 100 (H-1)/H.

For all the meta-analyses conducted, a test of publication bias using Egger’s test [17] was also included.

Meta-analyses were conducted in various sets (A to N) corresponding to the sub-sections of the Results section of the paper.

## The major smoking indices

For the major smoking indices, the first four sets of meta-analyses relate to: A ever smoking, B current smoking, C ever smoking (but with current smoking used if ever smoking not available), subsequently referred to as “ever/current”, and D ex smoking. In what is referred to as the main analysis in each set, smoking of any product is preferred by selecting RRs in the following preference order:

(1) smoking of any product vs. never smoked any product,

(2) smoking of cigarettes vs. never smoked any product,

(3) smoking of cigarettes only vs. never smoked any product,

(4) smoking of cigarettes vs. never smoked cigarettes,

(5) smoking of cigarettes only vs. never smoked cigarettes,

(6-10) as options 1-5 except “never smoked” is replaced by “never smoked near equivalent”.

A variant analysis prefers cigarette smoking (by changing the preference order to 4, 5, 2, 3, 1, 9, 10, 7, 8, 6). In meta-analyses of type C (ever smoking), a further variant analysis reverses the preference so current smoking results are preferred to those for ever smoking. Other variant analyses are based on RRs where the age of the subjects lies within specified age ranges (<56, 50-70 and 65+ years). Note that in theory it is possible for RRs for some age groups (e.g. 50-54 or 65-69 years) to fall into two of these age categories. However use of non-overlapping categories (e.g. <50, 50-70 and >70 years would have reduced the number of RRs that could be used even more dramatically.

A further set of meta-analyses, E, concerns smokers of pipes and/or cigars (but not cigarettes), smokers of pipes only, smokers of cigars only, and mixed smokers (pipe and/or cigars, and cigarettes). Separate meta-analyses were conducted for ever smoking, current smoking, ever smoking (or current if not available), current smoking (or ever if not available) and ex smoking.

**The cigarette type indices**

Meta-analyses were conducted, in set F, for only filter vs. only plain, ever filter vs. only plain, only filter vs. ever plain, handrolled vs. manufactured, and mentholated vs. non-mentholated. These meta-analyses were only conducted for ever smoking (or current if not available), and with a preference for RRs for cigarettes over RRs for cigarettes only (though there was only rarely a choice). The analyses with only filter as the numerator used the preference order of filter only, always, mainly, both, equally, and ever, while the analyses with ever filter as the numerator used the reverse preference. A similar preference order applied to the denominator. The analyses of handrolled vs. manufactured cigarettes used the preference order of any, both, mainly, and only for handrolled, and only ever, only current, any and ever for manufactured.

## The dose-related smoking indices

For the dose-related indices, sets of meta-analyses were conducted for: G amount smoked, H age of starting to smoke, I duration of smoking, J duration of quitting compared to never smokers (or long-term ex smokers), K duration of quitting compared to current smokers (or short-term quitters), L tar level, and M butt length or fraction smoked (with short butt length being taken as equivalent to a large fraction smoked). For any measure of exposure, the data for a study typically consist of an RR for each of a set of dose-categories expressed relative to a common base. Within each set, the RRs are not independent, and to avoid “double-counting” only one RR was included in any one meta-analysis. Two approaches were adopted. The first involves defining a number of levels of exposure, then carrying out meta-analyses for each level in turn, based on the RR from each sex within study that compares that level of exposure with never smokers, if such an RR is available. The second approach involves conducting a meta-analysis of RRs for the highest compared with the lowest categories of exposure available for each study.

For the first approach, the levels were defined by a scheme of “key values”. A category was allocated to the level whose key value it included, providing it did not also include another key value. Thus categories which included no key value, or more than one, were excluded. For example, if key values for number smoked were defined as 5, 20 and 45 cigs/day, and a study provided RRs for 1-9, 10-19, 20-29, 30-39 and 40+ cigs/day relative to never smokers, the RR for 1-9 cigs/day would be allocated to level 1, that for 20-29 cigs/day would be allocated to level 2, and that for 40+ cigs/day to level 3, with the RRs for 10-19 and 30-39 cigs/day not being used; while if another study provided RRs for 1-19 and 20+, the RR for 1-19 would be allocated to level 1 with that for 20+ unused. Note that a scheme with a few key values, widely spaced, is likely to involve RRs from more studies, whereas a scheme with more key values, closely spaced, will involve RRs from fewer studies, but ones with dose categories more closely clustered around the key value. For most of the measures two schemes were used; for amount smoked, age of starting to smoke and duration of smoking, one scheme had broader and the other closer spaced key values; for duration of quitting one scheme focused more on shorter-term and the other on longer-term quitting. The sets of key values used (with 999 indicating an open-ended category) were as follows:

- for amount smoked : 5, 20, 45 and 1, 10, 20, 30, 40, 999,
- for age of starting to smoke : 26, 18, 14 and 30, 26, 22, 18, 14, 10,
- for duration of smoking : 20, 35, 50 and 5, 20, 30, 40, 50, 999,
- for duration of quitting vs. never : 12, 7, 3 and 20, 12, 3 and
- for duration of quitting vs. current : 3, 7, 12 and 3, 12, 20.

Note also that although the second approach will generally include RRs from all studies and the first approach will not, the “highest” and “lowest” categories being compared under the second approach may vary considerably between studies.

Generally, analyses of the dose-related indices are grouped in sets of five tables – four relating to the first approach (an “overview” table, then a table relating to each of the three levels for the first key scheme, referred to as “low”, “mid” and “high”), and the fifth relating to the second approach, referred to as the “highest vs lowest” analysis. No “highest vs lowest” analysis was conducted for amount smoked, and no key value analysis was conducted for tar level, and for butt length and fraction smoked.

## The meta-analysis tables

For each meta-analysis, a full output is available comprising eight Sections. Further detail is given below.

### *Numbering system for the tables*

Tables are numbered by a four part code such as Table 1C7 – 5.

The first part identifies the outcome (1 = all lung cancer, 2 = squamous, 3 = adenocarcinoma – and for some additional analyses 4 = large cell carcinoma, 5 = small cell carcinoma).

The second part identifies the smoking index (A = ever smoking, B = current smoking, C = ever/current smoking, D = ex smoking, E = pipe and cigar smoking, F = cigarette type, G = amount smoked, H = age started, I = duration of smoking, J = years quit vs. never, K = years quit vs. current, L = tar level, M = butt length/fraction smoked.

The third part of the code identifies the analysis carried out. This varies by smoking index and is explained more fully in Table 1A for the major smoking indices, Table 1B for the cigarette type indices, and in Table 1C for the dose-related indices. For analyses of the major smoking indices Tables with a third-part code of 1 are the main analyses, with codes of 2 or greater indicating variant analyses, as described above. For analyses of the cigarette type indices, this part of the code discriminates the types of product being compared. For analyses of the dose-related smoking indices, it discriminates overview analyses and analyses for different key values or for “highest vs lowest” comparisons.

The fourth part of the code identifies the section of table.

### *Layout of the tables*

The full detailed output for all the smoking indices is shown in Additional file 5 : DetailedAnalysisTables. Each table comes in nine sections preceded by a cover page. All the pages for the meta-analysis have the same first three-part code in the Table number and the same main heading (describing the analysis), with the fourth part (the section number) blank for the cover page and 1 to 9 for the specific section. For sections 1 to 3, from each study, the RR(s) adjusted for the most potential confounders are chosen, referred to as the ‘most-adjusted analysis’. Sections 4 to 6 concern ‘least-adjusted’ data, i.e. with RRs adjusted for the least potential confounders chosen from each study, recalling that age-adjusted data if available have already been selected in preference to unadjusted data for prospective studies (see *Identifying which RRs to enter – confounders* above).

The content of each section is as follows:

| Cover page : | This shows  (i) restrictions on the data included,  (ii) the preference order for selecting RRs to be included,  (iii) a short description of the contents of the table, and  (iv) relevant abbreviations used in the table |
| --- | --- |
|  |  |
| Section 1 : | For all the most-adjusted RRs selected, a listing of their relevant characteristics. This includes the values of certain variables used to select the RR and used as ‘characteristics’ in Section 3, as well as the two key identifiers of the RR: the study 6-character reference (REF) and the number of the RR within that study (NRR). It also may indicate where RRs differ from those in another table – for example, where Table 1A1-1 presents analyses preferring smoking of any product and Table 1A2-1 an analysis preferring smoking of cigarettes, a character "x" in a column headed "1A1" in the output for Table 1A2-1 indicates those studies where the RRs in the two tables actually differ. |
|  |  |
| Section 2 : | For each most-adjusted RR selected, the output shows in the first part of the section the sex, the number of potential confounding variables adjusted for, the numbers of cases and controls (“cont”) for unadjusted RRs where available, the RR with its 95% confidence interval (CI), and in the second part of the section Y_s_, W_s_, Q_s_ and P_s_ (as defined in *Notation* below). Where multiple independent RRs are available for a study (typically different sexes, ages or races), combined results are also shown for the study as “Subtotal”. Note that the 2 × 2 table is headed “exposed/non-exposed” × “case/cont”. Exposed and non-exposed are as defined in the cover page. Cont will be numbers at risk or person-years for prospective studies. Unadjusted RRs calculated using the “correction for zero cell” method are indicated by a tilde (~). Section 2 ends with the results of a meta-analysis of the overall data, similar in style to that described below in section 3, except that it includes coded P values for Egger’s test of publication bias (Asymm P) [17]. |
|  |  |
| Section 3 : | This gives the results of fixed-effect and random-effects meta-analyses of the most-adjusted data. For the data subdivided by sex, and for data subdivided by levels of various other characteristics, the output indicates, for each level, the number of RRs combined (N), the number of studies from which these RRs come (NS), the combined weight for the studies combined (Wt), the chisquared (Het Chi), degrees of freedom (Het df) and coded P values (Het P), testing for heterogeneity within each level of the characteristic, as well as the RRs and CIs themselves (RR, RRl, RRu) for the fixed-effect and random-effects meta-analyses. Finally, at the foot of the total column, the results of testing for heterogeneity between levels of the characteristic are shown (Between Chi, Between df, Between p, Btwn (F) p and Btwn (R) p), as described in *Notation* below, which also describes the coding of the P values. Levels of the characteristics are as described in *Characteristics considered* below. |
|  |  |
| Sections 4 to 6 : | As for Sections 1 to 3 but for least-adjusted data. In a column headed X in the section 4 output, those RRs that differ from the corresponding most-adjusted RRs are indicated by “x”. Typically, an x will not be entered where a study only has one relevant RR available, adjusted or unadjusted. |
|  |  |
| Section 7 : | This lists the studies excluded from consideration, together with information on the stage at which they were excluded, the stages being numbered as on the cover page. A study is excluded when no RR can be found to satisfy the criteria required. |
|  |  |
| Section 8 : | This lists potentially overlapping studies for which data have been included (about which more information is given in Additional File 2 : DetailedAnalysisTables). |
|  |  |
| Section 9 : | This lists any results which would have been included in preference except that they had incomplete data (typically a RR with no CI). |
|  |  |

Note that the main results are given in Sections 3 and 6, while Sections 1, 2, 4, 5, 7, 8 and 9 mainly provide detailed information only required when one wants to see the individual RRs or to check that the program is correctly selecting the data.

### *Notation*

The notation used in some of the output (particularly sections 2, 3, 5, 6) is the same, where relevant, as that used by Fleiss and Gross [13]. Thus, we have:

N the number of RRs being combined

NS the number of studies from which the RRs are taken, (except that when the analysis is subdivided into levels of characteristics, NS in the Total column is the sum of the values in the individual columns, i.e. the number of study × characteristic levels from which the RRs are taken)

s the individual RR estimate being combined (s = 1, …N)

Y_s_ the logarithm of the RR estimate s

W_s_ the associated weight, calculated as the inverse of the variance of the logarithm of the RR

Wt the total weight for all the RRs being combined

Fixed RR the fixed-effect RR estimate, calculated by
exp ((∑ W_s_Y_s_)/( ∑ W_s_)) = exp ($\overline{Y}$) summation being over s = 1, … N

Fixed RRl the lower 95% confidence limit (CL) of the fixed-effect RR estimate, calculated by exp($\overline{Y}$-1.96/$\sqrt{\sum W_{s}}$)

Fixed RRu the upper 95% CL of the fixed-effect RR estimate, calculated by exp($\overline{Y}$+1.96/$\sqrt{\sum W_{s}}$)

Fixed P the probability value associated with the fixed-effect RR estimate, given in coded form as +++, --- p<0.001; ++, -- p<0.01; +, - p<0.05; (+), (-) p<0.1; N.S. (not significant) p>0.1. Plus signs indicate the RR is significantly greater than 1.0, minus signs that it is significantly less

Q_s_ the study’s contribution to the heterogeneity estimate, calculated by $W_{s}(Y_{s}-\overline{Y})^{2}$. Where N is large, this can be regarded approximately as a chisquared on 1 d.f.

P_s_ the associated probability value, used to indicate outliers, coded as for Fixed P

Het Chi (or Q in Fleiss and Gross notation) the heterogeneity chisquared on N-1 d.f., calculated by ∑ Q_s_. If Q < N-1, the random-effects and fixed-effect estimates are the same, but if Q > N-1 they differ.

Het df the degrees of freedom corresponding to Het Chi (= N-1)

(Note that Het Chi divided by Het df is referred to as H in the tables in the paper)

Het P the probability value associated with Het Chi and Het df, coded as for Fixed P

Random RR,

Random RRl,

Random RRu The random-effects RR estimate and its lower and upper 95% CLs. The method for deriving this, originally described by DerSimonian and Laird [691], is most conveniently given by Fleiss and Gross [13].

Random P the probability value associated with the random-effects RR estimate, coded as for Fixed P

Asymm P the probability value associated with Egger's test of publication bias, [17] coded as *** p<0.001; ** p<0.01; * p<0.05; (*) p<0.1; N.S. (not significant) p>0.1.. Only presented for analyses not subdivided by levels of different characteristics

Between Chi where the meta-analysis is subdivided by levels of a characteristic, this is the chisquared value for the difference between the fixed-effect RR estimates for the levels of the characteristics

Between df the degrees of freedom corresponding to Between Chi, equal to the number of levels of the characteristic minus 1

Between P the probability value associated with Between Chi and Between df, coded as for Fixed P

Btwn (F) P the coded probability value associated with the residual variation. This is an alternative test for the difference between the fixed effect estimates (F) for the characteristic levels, and may be the more appropriate test when there is substantial unexplained heterogeneity. The residual heterogeneity chisquared is calculated by subtracting the Between Chi value for the characteristic from the total Het Chi, and the residual df is calculated by subtracting the Between df from the total Het df. The ratio of the heterogeneity chisquared per degree of freedom for the characteristic to that for the residual is then tested according to the F distribution.

Btwn (R) P this is the chisquared value for the difference between the random-effects RR estimates for the levels of the characteristics. (Note that Btwn (R) P is referred to as P_H_ in the tables in the paper)

### *Characteristics considered*

The meta-analysis output in Sections 3 and 6 first gives results of an analysis subdivided by the characteristic **sex,** with RRs compared for combined sex results and those specifically for males and females (recalling that sexes-combined results are only entered on the database when equivalent sex-specific results are not available).

With the exception of the dose-related “overview” tables (see below), further analyses may show results for the various characteristics described below. The most-adjusted analysis of the first table for each major smoking index gives results for the full list of characteristics, but the least-adjusted and variant analyses, and the analyses of dose-related indices, may include a shorter list of characteristics, or no characteristics. The analysis by characteristic are omitted from tables involving less than 10 RRs.

**Lung cancer type** The levels are all and other (includes q+a, q+s+a, q+s+l+a, not s, not alv) for all lung cancer, q, q+s, q+u, KI, and not a for squamous, and a, a+l, a+al+br, KII, not q+u and not q+s for adeno. Abbreviations used are a=adenocarcinoma, al=alveolar carcinoma, br=bronchioalveolar carcinoma, KI=Kreyberg I, KII=Kreyberg II, l=large cell carcinoma, q=squamous cell carcinoma, s=small cell carcinoma, and u=undifferentiated carcinoma.

**Location** The levels are: NAmer (=North America); UK (=United Kingdom), Scand (=Scandinavia including Iceland), othEur (=other Europe), China (which is defined as excluding Hong Kong), Japan, othAs (=other Asia) and other.

**Detailed Country in “other Europe”**  The levels are: multi (=multicountry), Germany, oth West (=Austria, Belgium, France, Italy, Netherlands, Spain, Switzerland), East (=Czechoslovakia, Hungary, Poland, Russia)and Balkans (Greece, Turkey).

**Detailed Country in “other Asia”** The levels are: India, Hong Kong and other (Taiwan, Thailand, Singapore, South Korea).

**Detailed other continent**  The levels are SCAmer (=Argentina, Brazil, Colombia, Cuba, Uruguay), Auslia (=Australia) and Africa (=South Africa, Zimbabwe/Rhodesia).

**Start year of study** The levels are: <1960; 1960-69, 1970-79, 1980-89; and 1990+. For retrospective studies, this refers to the earliest observations.

**Study type (1)** The levels are: CC (=case-control) and other.

**Study type (2)** The levels are: CC (=case-control); prosp (=prospective); and other (=nested case-control or case-cohort)

**Study size (number of LC cases)** The levels are 100-249; 250-499; 500-999; and 1000+ lung cancer cases.

**Risky occupational population** The levels are no; mining; and othrisky (=other risky i.e. working with asbestos, fibreglass, iron-steel, nickel, silica or welding, or at a foundry, nuclear plant, railroad, or smelter).

**National cigarette tobacco type** The levels are Virginia (=Australia, Canada, India, UK, South Africa, Zimbabwe), Blended (=all other countries [see above under *Location*] except China and Taiwan), and other (=China, Taiwan). The multi-country studies were allocated to the category having the majority of cases. This classification was based on data supplied by PMI on 2^nd^ September 2009, using a criteria of at least 75% market share and assuming that the cigarette type had not varied over time.. For Taiwan the market includes at least 25% of each type, while for China no data were supplied. Though we understand that China uses predominantly Virginia, it was felt better to keep it separate due to its different tobacco types and curing practices.

**Any proxy use**  The levels are No/nk (=no or not known) and Yes.

**Full histological information** The levels are No and Yes.

**Number of adjustment variables (1)** The levels are 0, 1 and 2+/+nk (= 2+ or not known).

**Number of adjustment variables (2)** The levels are 0, 1, 2, 3-5 and 6+/+nk (= 6+ or not known).

**Smoking product** The levels are: all/unsp (=any product or unspecified); cigs+/-ot (=cigarettes irrespective of smoking of other products); and cig only (=cigarettes only).

**Smoking status** For the Tables for smoking indices ever/current and current/ever (Tables C) only, the levels are: ever; and current.

**Cigarette type** For analyses of filter (f) vs. plain (p) smoking, the levels are only f, always f, mainly f, equl p&f (=equal p and f), both p&f, and ever f. For analyses of handrolled (hr) vs. manufactured (mc) cigarette smoking , the levels are only hr, mainly hr, both m&h, and any hr

**Denominator** The levels are nev any (=never smoked any product or near equivalent); and nev cig (=never smoked cigarettes or near equivalent). (See *Major and cigarette type smoking indices* above for definitions of “near equivalent”). For analyses of filter/plain smoking, the levels are ever p (p=plain), mainly p, p NOS (not otherwise specified), and always p. For analyses of handrolled vs. manufactured cigarette smoking , the levels are ever mc, any mc, cu onlym (=cigarettes usually only manufactured), and only mc.

**Derivation of RR/CI** The levels are: Orig (=as given by original author); StdCalc (=Standard calculations – see *Derivations of RRs* subsections 1,3,5,7,8); and Other (=other methods – including those described in *Derivations of RRs* subsections 2,4,6,9,10, or combinations of methods)

**Study LIU4** The levels are LIU4 and others, to allow one to see the effect of excluding a study with very large weight.

### *Dose-related “overview” tables*

The layout of the dose-related “overview” tables is generally the same as the other tables, with the following exceptions.

In Sections -1 and -4, columns headed *S1* and *S2* show which levels of the two key schemes the RR matches, if any. Sections -2 and -5 include subtotals, but as the RRs for each study share a common base and are not independent, these subtotals should be ignored. Sections -3 and -6 show meta-analyses corresponding to the both key schemes, with a column corresponding to each level of the key scheme. All RRs that do not match a level of the key scheme are grouped in the column labelled “absent”, and again, these RRs and those in the “Total” column are not independent and those columns in the meta-analysis output should be ignored. The meta-analyses are first shown irrespective of sex, followed by analyses for males and then females.

Rarely, there may be differences in the RRs selected for the first key scheme in the overview table and the subsequent “low” “mid” and “high” tables. This occurs where a study provides more than one set of results eligible for the analysis, but with differing categories. The choice of set for the overview table is based on the preference order, and does not take account of whether a match to the key levels is provided. However this is taken into account for the specific low, mid or high tables, and another set (with lower preference) may provide a match for the level when the highest preference set did not.

## Forest plots

For each RR included, referenced by the study REF and sex, the RR is shown as a rectangle, the area of which is proportional to its weight. The CI is indicated by a horizontal line. The RRs and CIs are plotted on a logarithmic scale so that the RR is centred in the CI. Where the lower or upper CL is outside the range shown, this is indicated by an arrow on the end of the line. Rarely, when the RR itself is greater than the range, its rectangle is shown to the right of the line. Also shown are the values of each RR and CI and the weight as a percentage of the total. Results from the random-effects meta-analysis are shown at the bottom of the plot. The combined estimate is presented as a diamond with the width corresponding to the CI and the RR as the centre of the diamond.

## Funnel plots

The logarithm of the RR is plotted against its weight. A dotted vertical line corresponds to the fixed-effect RR estimate.

# Meta-regression

While full multivariable analysis of the data was considered beyond the scope of this report, meta-regression analyses were carried out using the sets of RRs selected for the main meta-analyses for ever smoking and for current smoking, preferring the most-adjusted data. Full results are presented in Additional file 5 : DetailedAnalysisTables. The meta-regression output comes in six tables, Tables 1A1R, 1B1R, 2A1R, 2B1R, 3A1R and 3B1R. As before, the first part of the code relates to outcome (1=All lung cancer, 2=Squamous, 3=Adeno), the second to smoking index (A=ever smoking, B=current smoking), and the third to smoking product (here always 1=any product, or cigarettes if all product not available). The letter R refers to it being a regression analysis. The final number, after the dash, relates to the section of the meta-regression output.

For each RR included, lists of the individual values of the characteristics used in the analysis, the values of the RR and CI, and the distribution of each characteristic considered, can be found in sections 1 - 3 of the corresponding meta-analysis table.

The first section of the meta-regression table gives results for a basic model including those characteristics selected as likely to be relevant. These characteristics, sex, location, start year of study, study type (1), study size (number of LC cases), and number of adjustment variables (1) (all with levels as defined in *Characteristics considered*) were selected, following preliminary meta-regression analyses using alternative lists of characteristics (not shown) and the desire to avoid inclusion of highly correlated variables in the same model. First the model is built up, by including the most significant factors in turn, subject to significance of at least p < 0.05, and then the model (the “fixed model”) is shown including all the selected characteristics. The deviance and degrees of freedom (DF) for Model 1 (including the constant only) is the same as that given in the main meta-analysis output. (Heterogeneity chisquared). This can be compared with the deviance for other models to see the proportion of deviance explained. Thus, for 1A1R, the deviance of 7518.629 on 328 d.f.for Model 1 given at the start of section - 1 is the same as that given at the end of Table 1A1 -2, and can be compared with the deviances given later in section -1 of 2189.033 on 321 d.f for Model 2 (including only location) and with the deviance of 1444.829 on 309 d.f. for Model 7 (the fixed model including all six characteristics). For each level of each characteristic, the output for each model shows the estimates, standard errors and p-values. The p values are coded as +++, --- p<0.001, ++, -- p<0.01, +, - p<0.05, (+), (-) p<0.1, and N.S. (not significant) p≥0.1, with the signs indicating the direction of difference. The output also shows the RR and 95% CI estimated from the weighted least-squares means and SEs, equivalent to using the SAS OM (observed marginals) technique [692].

Section -2, is headed “Effect of removing characteristics”. Starting with the fixed model, each characteristic in the basic list is removed in turn, indicated on the output by e.g. “Omit location”, with the output shown for the model omitting the given characteristic laid out similarly to before. The drop in deviance (Drop Dev) compared to the fixed model including all the basic characteristics is also shown, together with its p-value using an F-test, coded as above. This is the p-value shown in Tables 7 and 10 of the main paper. Note that when omitting characteristics, the deviance increases, so that the drop shown is negative.

Section -3, “Study outliers” shows fitted values and residuals for the fixed model. The output shows, for each RR, the study REF (see Table 2 of the main paper), the number of the RR on the relative risk database, the number of the RR within the study (NRR), the logarithm of the RR (LOGRR), the fitted value (FITVAL), its standard error (SEFITV) and the standardized residual (STDRES).

The RRs are sorted in order of increasing standardized residual.

Section -4, “Effect of additional characteristics” then shows the effect of adding to the fixed model each of a list of secondary characteristics. These include all other characteristics considered in *Characteristics Considered* that are not already in the fixed model, as well as the folllowing:

**RR adjusted for or study matched on age** The levels are Yes and No

**RR adjusted for or study matched on factor other than sex, age** The levels are Yes and No.

**Lowest age in RR** The levels are <25/unlim (=less than 25 years or no minimum age); 25-39; 40+; and unknown.

**Highest age in RR** The levels are <65; 65-74; 75-84; 85+/unlim (=at least 85 years or no maximum age); and unknown.

**Midpoint age** The midpoint of the age range (taking the lower limit as 15 if no minimum age or simply referred to as “adults”, taking the upper limit as 99 if no maximum age, or, if unknown, assuming 60 for case-control studies and 40 for a prospective study of miners).

**Derivation of RR/CI**  The levels are Orig/2x2 and Other, being a two level version of the 3 level characteristic described in *Characteristics Considered.*

For each of these meta-regressions, the drop in deviance compared to the fixed model is that shown at the start of the output for the characteristic, with the fitted estimates for the introduced characteristic at the end.

# Other dose-related data

Dose-related data not eligible for either the “key-value” or the “highest vs lowest” meta-analyses are shown in Additional File 4: DoseNotMeta.

# Additional analyses

For the three outcomes, and for ever smoking, current smoking and ever/current smoking of any product (or nearest equivalent) pairs of corresponding RR and CI estimates within the same study for males and for females, were identified and used to carry out meta-analyses of the male/female sex ratio. Meta-analyses of the sex ratio were also conducted for ever/current smokers within levels of amount smoked, defined using key value scheme 1. For all lung cancer only, meta-analyses of within-study ratios were also carried out to compare RRs for the oldest and youngest age group for which data were available, and to compare RRs for white people to black people (or non-white people).

While these additional analyses for sex, age and race compared independent pairs of pairs of corresponding RRs within the same study, further additional analyses were carried out for non-independent pairs. Here meta-analyses of the ratios were not conducted as the variance of the ratios could not readily be calculated. Instead the numbers of pairs where the ratio did or did not exceed 1 were counted and compared by the sign test, with meta-analyses also conducted separately for the numerators and denominators of the selected ratios. This technique was used to compare, for all three outcomes, most-adjusted and least-adjusted RRs for ever/current smoking, and, for all lung cancer, to compare highest vs. lowest RRs for four dose-related indices of smoking (age of starting to smoke, duration, years quit, and tar level) that were either adjusted for the most possible other variables including other aspects of smoking, with RRs that were otherwise similar but not adjusted for other aspects of smoking. members of the pairs. Similar methods were also used, for ever/current smoking and for all lung cancer, to compare pairs of RRs for mixed smokers of cigarettes and pipes/cigars and for smokers of cigarettes only, and pairs of RRs for smokers of pipes/cigars only and for smokers of cigarettes only.

# Software

All data entry and most statistical analysis were carried out using ROELEE version 3.1 (available from P.N.Lee Statistics and Computing Ltd, 17 Cedar Road, Sutton, Surrey SM2 5DA, UK). Preliminary calculations were carried out in Quattro Pro, Excel or CIA [12]. Some additional analyses were carried out using Excel 2003.

# Table 1 Meta-analysis tables

## Main and variant analyses for major smoking indices

| Major smoking  index | Smoking product preference |  | Table number | |  |
| --- | --- | --- | --- | --- | --- |
|  | (denominator never smoking unless stated) |  | All lung cancer | Squamous | Adeno |
|  |  |  |  |  |  |
| A Ever smoking | Any product^a,b,c^ |  | 1A1 | 2A1 | 3A1^d^ |
|  | Cigarettes^e^ |  | 1A2 | 2A2 | 3A2 |
|  | Cigarettes only |  | 1A3 | 2A3 | 3A3 |
|  | Any product^b^ (age <56) |  | 1A4 | 2A4 | 3A4 |
|  | Any product^b^ (age 50-70) |  | 1A5 | 2A5 | 3A5 |
|  | Any product^b^ (age 65+) |  | 1A6 | 2A6 | 3A6 |
|  | Cigarettes^e^ (age <56) |  | 1A7 | 2A7 | 3A7 |
|  | Cigarettes^e^ (age 50-70) |  | 1A8 | 2A8 | 3A8 |
|  | Cigarettes^e^ (age 65+) |  | 1A9 | 2A9 | 3A9 |
|  | Cigarettes only (age <56) |  | 1A10 | 2A10 | 3A10 |
|  | Cigarettes only (age 50-70) |  | 1A11 | 2A11 | 3A11 |
|  | Cigarettes only (age 65+) |  | 1A12 | 2A12 | 3A12 |
|  |  |  |  |  |  |
| B Current smoking | Any product^a,b,c^ |  | 1B1 | 2B1 | 3B1^d^ |
|  | Cigarettes^e^ |  | 1B2 | 2B2 | 3B2 |
|  | Cigarettes only |  | 1B3 | 2B3 | 3B3 |
|  | Any product^b^ (age <56) |  | 1B4 | 2B4 | 3B4 |
|  | Any product^b^ (age 50-70) |  | 1B5 | 2B5 | 3B5 |
|  | Any product^b^ (age 65+) |  | 1B6 | 2B6 | 3B6 |
|  | Cigarettes^e^ (age <56) |  | 1B7 | 2B7 | 3B7 |
|  | Cigarettes^e^ (age 50-70) |  | 1B8 | 2B8 | 3B8 |
|  | Cigarettes^e^ (age 65+) |  | 1B9 | 2B9 | 3B9 |
|  | Cigarettes only (age <56) |  | 1B10 | 2B10 | 3B10 |
|  | Cigarettes only (age 50-70) |  | 1B11 | 2B11 | 3B11 |
|  | Cigarettes only (age 65+) |  | 1B12 | 2B12 | 3B12 |
|  | Any product^b^ (vs. non-current smokers) |  | 1B13 | 2B13 | 3B13 |
|  | Cigarettes^e^ (vs. non-current smokers) |  | 1B14 | 2B14 | 3B14 |
|  | Cigarettes only (vs. non-current smokers) |  | 1B15 | 2B15 | 3B15 |
|  |  |  |  |  |  |
| C Ever/current smoking | Any product^a,b^ (Prefer ever to current smoking) |  | 1C1 | 2C1 | 3C1^d^ |
|  | Any product^b^ (Prefer current to ever smoking) |  | 1C2 | 2C2 | 3C2 |
|  | Cigarettes^e^ (Prefer ever to current smoking) |  | 1C3 | 2C3 | 3C3 |
|  | Cigarettes^e^ (Prefer current to ever smoking) |  | 1C4 | 2C4 | 3C4 |
|  | Cigarettes only (Prefer ever to current smoking) |  | 1C5 | 2C5 | 3C5 |
|  | Cigarettes only (Prefer current to ever smoking) |  | 1C6 | 2C6 | 3C6 |
|  |  |  |  |  |  |
| D Ex smoking | Any product^a,b^ |  | 1D1 | 2D1 | 3D1^d^ |
|  | Cigarettes^e^ |  | 1D2 | 2D2 | 3D2 |
|  | Cigarettes only |  | 1D3 | 2D3 | 3D3 |
|  |  |  |  |  |  |
| E Pipe and cigar smoking | Pipes and/or cigars (not cigs) – Ever |  | 1E1 | 2E1 | 3E1 |
|  | Pipes and/or cigars (not cigs) – Current |  | 1E2 | 2E2 | 3E2 |
|  | Pipes and/or cigars (not cigs) – Ever/current |  | 1E3 | 2E3 | 3E3 |
|  | Pipes and/or cigars (not cigs) – Current/ever |  | 1E4 | 2E4 | 3E4 |
|  | Pipes and/or cigars (not cigs) – Ex |  | 1E5 | 2E5 | 3E5 |
|  | Pipes only – Ever |  | 1E6 | 2E6 | 3E6 |
|  | Pipes only – Current |  | 1E7 | 2E7 | 3E7 |
|  | Pipes only – Ever/current |  | 1E8 | 2E8 | 3E8 |
|  | Pipes only – Current/ever |  | 1E9 | 2E9 | 3E9 |
|  | Pipes only – Ex |  | 1E10 | 2E10 | 3E10 |
|  | Cigars only – Ever |  | 1E11 | 2E11 | 3E11 |
|  | Cigars only – Current |  | 1E12 | 2E12 | 3E12 |
|  | Cigars only – Ever/current |  | 1E13 | 2E13 | 3E13 |
|  | Cigars only – Current/ever |  | 1E14 | 2E14 | 3E14 |
|  | Cigars only – Ex |  | 1E15 | 2E15 | 3E15 |
|  | Mixed smokers^f^ – Ever |  | 1E16 | 2E16 | 3E16 |
|  | Mixed smokers^f^ – Current |  | 1E17 | 2E17 | 3E17 |
|  | Mixed smokers^f^ – Ever/current |  | 1E18 | 2E18 | 3E18 |
|  | Mixed smokers^f^ – Current/ever |  | 1E19 | 2E19 | 3E19 |
|  | Mixed smokers^f^ – Ex |  | 1E20 | 2E20 | 3E20 |
|  |  |  |  |  |  |
| ^a^ Main analysis  ^b^ or cigarettes if all product not available, see *The major smoking indices* for detail  ^c^ Also regression analysis  ^d^ Analyses also conducted for large cell carcinoma (first code 4) and for small cell carcinoma (first code 5)  ^e^ or all product if cigarettes not available, see *The major smoking indices* for detail  ^f^ Mixed smokers of cigarettes together with pipes and/or cigars | | | | | |
|  | | | | | |

## Analyses for cigarette type indices

| Numerator | Denominator |  | Table number | |  |
| --- | --- | --- | --- | --- | --- |
|  |  |  | All lung cancer | Squamous | Adeno |
|  |  |  |  |  |  |
| Only Filter^a^ | Only Plain^b^ |  | 1F1 | 2F1 | 3F1 |
| Ever Filter^c^ | Only Plain^b^ |  | 1F2 | 2F2 | 3F2 |
| Only Filter^a^ | Ever Plain^d^ |  | 1F3 | 2F3 | 3F3 |
| Handrolled^e^ | Manufactured^f^ |  | 1F4 | 2F4 | 3F4 |
| Menthol | Non-menthol |  | 1F5 | 2F5 | 3F5 |
|  |  |  |  |  |  |
| ^a^ Preference filter only/NOS, always, mainly, both, equally, ever  ^b^ Preference plain only/NOS, always. mainly, ever  ^c^ Preference filter ever, equally, both, mainly, always, only/NOS  ^d^ Preference plain ever, mainly, always, only/NOS  ^e^ Preference handrolled any, both, mainly, only  ^f^ Preference manufactured only ever, only current, any, ever | | | | | |

## Analyses for dose-related indices

| **Dose-related index** | **Table type** | **Key value** | **Max. range** | **Table number** | | |
| --- | --- | --- | --- | --- | --- | --- |
|  |  |  |  | **All lung cancer** | **Squamous** | **Adeno** |
| **G Amount smoked** |  |  |  |  |  |  |
| **Any product^a^ , ever smoking** | **Overview** |  |  | **1G1** | **2G1** | **3G1** |
|  | **Low** | **5 cigs/day** | **1-19** | **1G2** | **2G2** | **3G2** |
|  | **Mid** | **20** | **6-44** | **1G3** | **2G3** | **3G3** |
|  | **High** | **45** | **21+** | **1G4** | **2G4** | **3G4** |
|  | **Highest v Lowest** |  |  | **NA** | **NA** | **NA** |
| **Any product^a^ , current smoking** | **Overview** |  |  | **1G6** | **2G6** | **3G6** |
|  | **Low** | **5 cigs/day** | **1-19** | **1G7** | **2G7** | **3G7** |
|  | **Mid** | **20** | **6-44** | **1G8** | **2G8** | **3G8** |
|  | **High** | **45** | **21+** | **1G9** | **2G9** | **3G9** |
|  | **Highest v Lowest** |  |  | **NA** | **NA** | **NA** |
| **Any product^a^ , ever/current smoking** | **Overview** |  |  | **1G11** | **2G11** | **3G11** |
|  | **Low** | **5 cigs/day** | **1-19** | **1G12** | **2G12** | **3G12** |
|  | **Mid** | **20** | **6-44** | **1G13** | **2G13** | **3G13** |
|  | **High** | **45** | **21+** | **1G14** | **2G14** | **3G14** |
|  | **Highest v Lowest** |  |  | **NA** | **NA** | **NA** |
| **Cigarettes^b^ , ever/current smoking** | **Overview** |  |  | **1G16** | **2G16** | **3G16** |
|  | **Low** | **5 cigs/day** | **1-19** | **1G17** | **2G17** | **3G17** |
|  | **Mid** | **20** | **6-44** | **1G18** | **2G18** | **3G18** |
|  | **High** | **45** | **21+** | **1G19** | **2G19** | **3G19** |
|  | **Highest v Lowest** |  |  | **NA** | **NA** | **NA** |
| **Cigarettes only, ever/current smoking** | **Overview** |  |  | **1G21** | **2G21** | **3G21** |
|  | **Low** | **5 cigs/day** | **1-19** | **1G22** | **2G22** | **3G22** |
|  | **Mid** | **20** | **6-44** | **1G23** | **2G23** | **3G23** |
|  | **High** | **45** | **21+** | **1G24** | **2G24** | **3G24** |
|  | **Highest v Lowest** |  |  | **NA** | **NA** | **NA** |
| **Pipes and/or cigars^c^ , ever smoking** | **Overview** |  |  | **1G26** | **2G26** | **3G26** |
| **Pipes and/or cigars^c^ , current smoking** | **Overview** |  |  | **1G28** | **2G28** | **3G28** |
| **Pipesand/or cigars^c^ , ever/current smoking** | **Overview** |  |  | **1G30** | **2G30** | **3G30** |
| **Pipes only, ever/current smoking** | **Overview** |  |  | **1G32** | **2G32** | **3G32** |
| **Cigars only, ever/current smoking** | **Overview** |  |  | **1G34** | **2G34** | **3G34** |
| **Mixed smokers^d^ , ever/current smoking** | **Overview** |  |  | **1G36** | **2G36** | **3G36** |
|  |  |  |  |  |  |  |
| **H Age started** |  |  |  |  |  |  |
| **Any product^a^ , ever smoking** | **Overview** |  |  | **1H1** | **2H1** | **3H1** |
|  | **Low** | **26 years** | **19-26** | **1H2** | **2H2** | **3H2** |
|  | **Mid** | **18** | **15-25** | **1H3** | **2H3** | **3H3** |
|  | **High** | **14** | **1-17** | **1H4** | **2H4** | **3H4** |
|  | **Highest v Lowest** |  |  | **1H5** | **2H5** | **3H5** |
| **Any product^a^ , current smoking** | **Overview** |  |  | **1H6** | **2H6** | **3H6** |
|  | **Low** | **26 years** | **19-26** | **1H7** | **2H7** | **3H7** |
|  | **Mid** | **18** | **15-25** | **1H8** | **2H8** | **3H8** |
|  | **High** | **14** | **1-17** | **1H9** | **2H9** | **3H9** |
|  | **Highest v Lowest** |  |  | **1H10** | **2H10** | **3H10** |
| **Any product^a^ , ever/current smoking** | **Overview** |  |  | **1H11** | **2H11** | **3H11** |
|  | **Low** | **26 years** | **19-26** | **1H12** | **2H12** | **3H12** |
|  | **Mid** | **18** | **15-25** | **1H13** | **2H13** | **3H13** |
|  | **High** | **14** | **1-17** | **1H14** | **2H14** | **3H14** |
|  | **Highest v Lowest** |  |  | **1H15** | **2H15** | **3H15** |
| **Cigarettes^b^ , ever/current smoking** | **Overview** |  |  | **1H16** | **2H16** | **3H16** |
|  | **Low** | **26 years** | **19-26** | **1H17** | **2H17** | **3H17** |
|  | **Mid** | **18** | **15-25** | **1H18** | **2H18** | **3H18** |
|  | **High** | **14** | **1-17** | **1H19** | **2H19** | **3H19** |
|  | **Highest v Lowest** |  |  | **1H20** | **2H20** | **3H20** |
| **Cigarettes only, ever/current smoking** | **Overview** |  |  | **1H21** | **2H21** | **3H21** |
|  | **Low** | **26 years** | **19-26** | **1H22** | **2H22** | **3H22** |
|  | **Mid** | **18** | **15-25** | **1H23** | **2H23** | **3H23** |
|  | **High** | **14** | **1-17** | **1H24** | **2H24** | **3H24** |
|  | **Highest v Lowest** |  |  | **1H25** | **2H25** | **3H25** |
| **Pipes and/or cigars^c^ , ever smoking** | **Overview** |  |  | **1H26** | **NA** | **NA** |
|  | **Highest v Lowest** |  |  | **1H27** | **NA** | **NA** |
| **Pipes and/or cigars^c^ , current smoking** | **Overview** |  |  | **1H28** | **NA** | **NA** |
|  | **Highest v Lowest** |  |  | **1H29** | **NA** | **NA** |
| **Pipesand/or cigars^c^ , ever/current smoking** | **Overview** |  |  | **1H30** | **NA** | **NA** |
|  | **Highest v Lowest** |  |  | **1H31** | **NA** | **NA** |
| **Pipes only, ever/current smoking** | **Overview** |  |  | **1H32** | **NA** | **NA** |
|  | **Highest v Lowest** |  |  | **1H33** | **NA** | **NA** |
| **Cigars only, ever/current smoking** | **Overview** |  |  | **1H34** | **NA** | **NA** |
|  | **Highest v Lowest** |  |  | **1H35** | **NA** | **NA** |
| **Mixed smokers^d^ , ever/current smoking** | **Overview** |  |  | **1H36** | **NA** | **NA** |
|  | **Highest v Lowest** |  |  | **1H37** | **NA** | **NA** |
|  |  |  |  |  |  |  |
| **I Duration** |  |  |  |  |  |  |
| **Any product^a^ , ever smoking** | **Overview** |  |  | **1I1** | **2I1** | **3I1** |
|  | **Low** | **20 years** | **1-34** | **1I2** | **2I2** | **3I2** |
|  | **Mid** | **35** | **21-49** | **1I3** | **2I3** | **3I3** |
|  | **High** | **50** | **36+** | **1I4** | **2I4** | **3I4** |
|  | **Highest v Lowest** |  |  | **1I5** | **2I5** | **3I5** |
| **Any product^a^ , current smoking** | **Overview** |  |  | **1I6** | **2I6** | **3I6** |
|  | **Low** | **20 years** | **1-34** | **1I7** | **2I7** | **3I7** |
|  | **Mid** | **35** | **21-49** | **1I8** | **2I8** | **3I8** |
|  | **High** | **50** | **36+** | **1I9** | **2I9** | **3I9** |
|  | **Highest v Lowest** |  |  | **1I10** | **2I10** | **3I10** |
| **Any product^a^ , ever/current smoking** | **Overview** |  |  | **1I11** | **2I11** | **3I11** |
|  | **Low** | **20 years** | **1-34** | **1I12** | **2I12** | **3I12** |
|  | **Mid** | **35** | **21-49** | **1I13** | **2I13** | **3I13** |
|  | **High** | **50** | **36+** | **1I14** | **2I14** | **3I14** |
|  | **Highest v Lowest** |  |  | **1I15** | **2I15** | **3I15** |
| **Cigarettes^b^ , ever/current smoking** | **Overview** |  |  | **1I16** | **2I16** | **3I16** |
|  | **Low** | **20 years** | **1-34** | **1I17** | **2I17** | **3I17** |
|  | **Mid** | **35** | **21-49** | **1I18** | **2I18** | **3I18** |
|  | **High** | **50** | **36+** | **1I19** | **2I19** | **3I19** |
|  | **Highest v Lowest** |  |  | **1I20** | **2I20** | **3I20** |
| **Cigarettes only, ever/current smoking** | **Overview** |  |  | **1I21** | **2I21** | **3I21** |
|  | **Low** | **20 years** | **1-34** | **1I22** | **2I22** | **3I22** |
|  | **Mid** | **35** | **21-49** | **1I23** | **2I23** | **3I23** |
|  | **High** | **50** | **36+** | **1I24** | **2I24** | **3I24** |
|  | **Highest v Lowest** |  |  | **1I25** | **2I25** | **3I25** |
| **Pipes and/or cigars^c^ , ever smoking** | **Overview** |  |  | **1I26** | **NA** | **NA** |
|  | **Highest v Lowest** |  |  | **1I27** | **NA** | **NA** |
| **Pipes and/or cigars^c^ , current smoking** | **Overview** |  |  | **1I28** | **NA** | **NA** |
|  | **Highest v Lowest** |  |  | **1I29** | **NA** | **NA** |
| **Pipesand/or cigars^c^ , ever/current smoking** | **Overview** |  |  | **1I30** | **NA** | **NA** |
|  | **Highest v Lowest** |  |  | **1I31** | **NA** | **NA** |
| **Pipes only, ever/current smoking** | **Overview** |  |  | **1I32** | **NA** | **NA** |
|  | **Highest v Lowest** |  |  | **1I33** | **NA** | **NA** |
| **Cigars only, ever/current smoking** | **Overview** |  |  | **1I34** | **NA** | **NA** |
|  | **Highest v Lowest** |  |  | **1I35** | **NA** | **NA** |
| **Mixed smokers^d^ , ever/current smoking** | **Overview** |  |  | **1I36** | **NA** | **NA** |
|  | **Highest v Lowest** |  |  | **1I37** | **NA** | **NA** |
|  |  |  |  |  |  |  |
| **J Years quit vs. never** | |  |  |  |  |  |
| **Any product^a^** | **Overview** |  |  | **1J1** | **2J1** | **3J1** |
|  | **Low** | **12 years** | **8+** | **1J2** | **2J2** | **3J2** |
|  | **Mid** | **7** | **4-11** | **1J3** | **2J3** | **3J3** |
|  | **High** | **3** | **1-6** | **1J4** | **2J4** | **3J4** |
|  | **Highest v Lowest** |  |  | **1J5** | **2J5** | **3J5** |
| **Cigarettes^b^** | **Overview** |  |  | **1J6** | **2J6** | **3J6** |
|  | **Low** | **12 years** | **8+** | **1J7** | **2J7** | **3J7** |
|  | **Mid** | **7** | **4-11** | **1J8** | **2J8** | **3J8** |
|  | **High** | **3** | **1-6** | **1J9** | **2J9** | **3J9** |
|  | **Highest v Lowest** |  |  | **1J10** | **2J10** | **3J10** |
| **Cigarettes only** | **Overview** |  |  | **1J11** | **2J11** | **3J11** |
|  | **Low** | **12 years** | **8+** | **1J12** | **2J12** | **3J12** |
|  | **Mid** | **7** | **4-11** | **1J13** | **2J13** | **3J13** |
|  | **High** | **3** | **1-6** | **1J14** | **2J14** | **3J14** |
|  | **Highest v Lowest** |  |  | **1J15** | **2J15** | **3J15** |
| **Pipes and/or cigars^c^** | **Overview** |  |  | **1J16** | **NA** | **NA** |
|  | **Highest v Lowest** |  |  | **1J17** | **NA** | **NA** |
| **Pipes only** | **Overview** |  |  | **1J18** | **NA** | **NA** |
|  | **Highest v Lowest** |  |  | **1J19** | **NA** | **NA** |
| **Cigars only** | **Overview** |  |  | **1J20** | **NA** | **NA** |
|  | **Highest v Lowest** |  |  | **1J21** | **NA** | **NA** |
| **Mixed smokers^d^** | **Overview** |  |  | **1J22** | **NA** | **NA** |
|  | **Highest v Lowest** |  |  | **1J23** | **NA** | **NA** |
|  |  |  |  |  |  |  |
| **K Years quit vs. current** | |  |  |  |  |  |
| **Any product^a^** | **Overview** |  |  | **1K1** | **2K1** | **3K1** |
|  | **Low** | **3 years** | **1-6** | **1K2** | **2K2** | **3K2** |
|  | **Mid** | **7** | **4-11** | **1K3** | **2K3** | **3K3** |
|  | **High** | **12** | **8+** | **1K4** | **2K4** | **3K4** |
|  | **Highest v Lowest** |  |  | **1K5** | **2K5** | **3K5** |
| **Cigarettes^b^** | **Overview** |  |  | **1K6** | **2K6** | **3K6** |
|  | **Low** | **3 years** | **1-6** | **1K7** | **2K7** | **3K7** |
|  | **Mid** | **7** | **4-11** | **1K8** | **2K8** | **3K8** |
|  | **High** | **12** | **8+** | **1K9** | **2K9** | **3K9** |
|  | **Highest v Lowest** |  |  | **1K10** | **2K10** | **3K10** |
| **Cigarettes only** | **Overview** |  |  | **1K11** | **2K11** | **3K11** |
|  | **Low** | **3 years** | **1-6** | **1K12** | **2K12** | **3K12** |
|  | **Mid** | **7** | **4-11** | **1K13** | **2K13** | **3K13** |
|  | **High** | **12** | **8+** | **1K14** | **2K14** | **3K14** |
|  | **Highest v Lowest** |  |  | **1K15** | **2K15** | **3K15** |
| **Pipes and/or cigars^c^** | **Overview** |  |  | **1K16** | **NA** | **NA** |
|  | **Highest v Lowest** |  |  | **1K17** | **NA** | **NA** |
|  |  |  |  |  |  | **/continued** |
| **Pipes only** | **Overview** |  |  | **1K18** | **NA** | **NA** |
|  | **Highest v Lowest** |  |  | **1K19** | **NA** | **NA** |
| **Cigars only** | **Overview** |  |  | **1K20** | **NA** | **NA** |
|  | **Highest v Lowest** |  |  | **1K21** | **NA** | **NA** |
| **Mixed smokers^d^** | **Overview** |  |  | **1K22** | **NA** | **NA** |
|  | **Highest v Lowest** |  |  | **1K23** | **NA** | **NA** |
|  |  |  |  |  |  |  |
| **L Tar level** |  |  |  |  |  |  |
| **Cigarettes^a^ - ever smoking** | **Highest v Lowest** |  |  | **1L1** | **2L1** | **3L1** |
| **Cigarettes^a^ - current smoking** | **Highest v Lowest** |  |  | **1L2** | **2L2** | **3L2** |
| **Cigarettes^a^ - ever/current smoking** | **Highest v Lowest** |  |  | **1L3** | **2L3** | **3L3** |
| **Cigarettes only - ever smoking** | **Highest v Lowest** |  |  | **1L4** | **2L4** | **3L4** |
| **Cigarettes only- current smoking** | **Highest v Lowest** |  |  | **1L5** | **2L5** | **3L5** |
| **Cigarettes only -ever/current smoking** | **Highest v Lowest** |  |  | **1L6** | **2L6** | **3L6** |
|  |  |  |  |  |  |  |
| **M Butt length and fraction smoked** |  |  |  |  |  |  |
| **Cigarettes^a^ - ever smoking** | **Highest v Lowest** |  |  | **1M1** | **2M1** | **3M1** |
| **Cigarettes^a^ - current smoking** | **Highest v Lowest** |  |  | **1M2** | **2M2** | **3M2** |
| **Cigarettes^a^ - ever/current smoking** | **Highest v Lowest** |  |  | **1M3** | **2M3** | **3M3** |
| **Cigarettes only - ever smoking** | **Highest v Lowest** |  |  | **1M4** | **2M4** | **3M4** |
| **Cigarettes only- current smoking** | **Highest v Lowest** |  |  | **1M5** | **2M5** | **3M5** |
| **Cigarettes only -ever/current smoking** | **Highest v Lowest** |  |  | **1M6** | **2M6** | **3M6** |
| ^a^ or cigarettes if any product not available  ^b^ or any product if cigarettes not available  ^c^ not cigarettes  ^d^ mixed smokers of cigarettes together with pipes and/or cigars | | | | | | |

# Table 2 Abbreviations used in listings

| Variable name | Meaning | Level abbreviation^a^ | Level meaning |
| --- | --- | --- | --- |
| REF | 6-character study reference |  |  |
| NRR | number of the RR on the database within the study |  |  |
| e.g. 1A1 | Comparison with another table (e.g. 1A1 for comparison with Table 1A1) | x | The RR does not appear in the comparison table |
| X | In section 4 (least-adjusted analysis), comparison with most-adjusted analysis (section 1 of same table) | x | The RR does not appear in the most-adjusted analysis |
| SEX | Sex of the RR | m | Male |
|  |  | f | Female |
|  |  | b | Both |
| AGEL, AGEH | Lower and higher limits of age range of RR |  |  |
| RACE | Race | All | All or nearest available |
|  |  | As | Asian |
|  |  | b or bl | Black |
|  |  | ch | Chinese |
|  |  | haw | Hawaiian |
|  |  | hi | Hispanic |
|  |  | jap | Japanese |
|  |  | nonw | Non-white |
|  |  | o | Oriental |
|  |  | sca | Scandinavian |
|  |  | wh or w | White |
|  |  | w+b | White+Black |
|  |  | w-hi | White-Hispanic |
| YF | Years of follow-up (for prospective studies) |  |  |
| LC | LC type | a | Adenocarcinoma |
|  |  | alv | Alveolar carcinoma |
|  |  | br | Broncioalveolar carcinoma |
|  |  | l | Large cell carcinoma |
|  |  | o | Other carcinoma |
|  |  | q | Squamous cell carcinoma |
|  |  | s | Small cell carcinoma |
|  |  | u | Undifferentiated carcinoma |
| LOC | Location | NAMer | North America |
|  |  | Eu:UK | UK |
|  |  | Eu:Scand | Scandinavia |
|  |  | Eu:Ger | Germany |
|  |  | Eu:mul | Europe – multicountry |
|  |  | Eu:wst | Western Europe (not UK, Scandinavia or Germany) |
|  |  | Eu:est | Eastern Europe (not Balkans) |
|  |  | Eu:bal | The Balkans |
|  |  | As:Chi | China |
|  |  | As:HK | Hong Kong |
|  |  | As:India | India |
|  |  | As:Jap | Japan |
|  |  | As:oth | Asia (not China, Hong Kong, India, Japan) |
|  |  | Auslia | Australasia |
|  |  | Africa | Africa |
|  |  | Multi | More than one of the above regions |
| START | Start year of study | * | Unknown |
| ST | Study type | CC | Case-control |
|  |  | Pr or prosp | Prospective |
|  |  | ot | Other |
| NLC | Number of lung cancers in study as a whole |  |  |
| R | Risky occupation | n | No |
|  |  | m | Mining |
|  |  | o | Other risky occupation |
| VB | Virginia/blended | V | Virginia (at least 75%) |
|  |  | bl | Blended (at least 75%) |
|  |  | ot | Other (mixed or China) |
| P | Any proxy use | y | Yes |
|  |  | n | No |
|  |  | * | Not known |
| H | 100% histological confirmation | y | Yes |
|  |  | n | No |
| AD | Number of adjustment variables in RR |  |  |
| PRODUCT | Smoking product | all/unsp | Smoked any product, or smoked unspecified product |
|  |  | cig+/-ot | Smoked cigarettes (irrespective of whether also smoked other products) |
|  |  | cig only | Smoked cigarettes only |
| CIGTYPE | Cigarette type | f | Filter |
|  |  | p | Plain |
|  |  | h or hr | Handrolled |
|  |  | m or mc | Manufactured |
| DENOM | Definition of denominator | nev any | Never smoked any product |
|  |  | nev cigs | Never smoked cigarettes |
|  |  | nev + 1 | Never smoked any product or long term quitter |
|  |  | nev + 2 | Never smoked any product or smoked an unknown amount |
|  |  | nev + 3 | Never smoked cigarettes or long term quitter |
|  |  | neva + lo | Never smoked any product or low exposure (in terms of the dose measure in the relevant table^b^) |
|  |  | nevc + lo | Never smoked cigarettes or low exposure (in terms of the dose measure in the relevant table^b^) |
|  |  | Cur any | Current smoker of any product |
|  |  | Cur cigs | Current smoker of cigarettes |
| De | Derivation of RR/CI | or | Original |
|  |  | ot | Other |
|  |  | st | Standard method |
| exL | For a dose-response analysis analysis, the lower exposure limit. For a “highest vs lowest analysis” the lower exposure limit for the highest exposure^b^ |  |  |
| exH | For a dose-response analysis analysis, the higher exposure limit. For a “highest vs. lowest analysis, the higher exposure limit for the highest exposure^b^ |  |  |
| unexL | For a “highest vs. lowest” analysis, the lower exposure limit for the lowest exposure^b^ |  |  |
| unexH | For a “highest vs. lowest” analysis, the higher exposure limit for the lowest exposure^b^ |  |  |

^a^ Only abbreviated levels are shown, except that unabbreviated levels are also shown where necessary to define an “other” level.

^b^ For age started smoking and years quit (vs never) higher ages of starting or more years quit correspond to lower exposure. The units are in terms of the measure of exposure (e.g number of cigarettes for amount smoked, years of age for age start).

1. ^a^ Exceptionally, a 2000 conference paper of which the abstract had been published in 1997, and a 2001 reprint of a 1943 paper were included. [↑](#footnote-ref-2)
2. At the start of the project, results were also entered for some other types or combinations, but this was discontinued and no analyses are presented of those data. [↑](#footnote-ref-3)
3. Readers of our recent paper [11] may note that in that paper, the policy of duplicating the data entry was adopted. [↑](#footnote-ref-4)
4. Exceptionally, an analysis of cigarette smoking vs never cigarette smoking could be adjusted for pipe smoking, but this was rarely encountered. [↑](#footnote-ref-5)
