# Supplementary material for: Systematic review with meta-analysis of the epidemiological evidence in the 1900s relating smoking to lung cancer
Source: BMC Cancer. 2012 Sep 3;12:385. doi: 10.1186/1471-2407-12-385 (PMC3505152; doi:10.1186/1471-2407-12-385)
Supplement: Additional file 2 — Studies. [file 1471-2407-12-385-S2.doc]

**Systematic review with meta-analysis of the epidemiological evidence in the 1900s relating smoking to lung cancer**

Peter N Lee, Barbara A Forey, and Katharine J Coombs

**Additional File 2 : Studies**

**Contents**

[Overlapping studies 3](#__RefHeading___Toc311643225)

[Table 1 Study description 8](#__RefHeading___Toc311643226)

[Table 2 Details of control groups (case-control and nested case-control studies) 15](#__RefHeading___Toc311643227)

[Table 3 Outcomes available in each study^a^ 21](#__RefHeading___Toc311643228)

[Table 4 Smoking results available from each study 27](#__RefHeading___Toc311643229)

### Overlapping studies

To minimize problems in meta-analysis arising from double-counting of cases, studies were divided into two categories, as already discussed in Additional file 1: Methods. The first category involved those with a modest degree of overlap, which cannot be disentangled and which it was decided to ignore. They are entered on the database as principal studies, each with a separate value of REFGP (REF group). These sets of studies are described briefly below.

1. CHAN contains data from a case-control study in five hospitals in Hong Kong in 1976-77, involving 397 cases, while the LAMWK2 case-control study contains data involving 480 cases from one of these hospitals for 1976-80.
2. KOO contains data from a case-control study in eight hospitals in Hong Kong in 1981-83 involving 120 lung cancer cases, LAMWK contains data from a case-control study in one of these hospitals for 1981-84 involving 163 cases, while LAMTH contains data from another case-control study in 8 hospitals in Hong Kong in 1983-86 involving 445 cases, apparently not from the hospital used by LAMWK. [Note that KOO and LAMTH clearly overlap little (1983) and that, unlike LAMTH and LAMWK which appeared to involve all the available cases, KOO only involved a proportion.]
3. SOBUE2 contains data from a case-control study conducted in Osaka in 1965-83 involving 2083 cases diagnosed at one Center, while MATSUD contains data from a case-control study conducted in certain regions of, and companies in, Osaka in 1965.
4. GOODMA contains data from a case-control study conducted in Oahu, Hawaii in 1983-85 involving 326 cases with no restriction on race or sex, while CHYOU contains data from a prospective study also conducted in Oahu where 7961 Japanese-American men were interviewed in 1965-68 and followed until 1990.
5. CPSI contains 12 year follow up data from all the US states participating in the million person study, while ENSTRO contains 28 year follow up data only from Californian participants in this study.

The second category contains sets of studies which clearly do overlap, where one or more members of the set (allocated as “principal studies”) contain the most appropriate data (and do not themselves overlap) and where, for other members (“subsidiary studies”), RRs should only be included in meta-analyses if equivalent results are not available from the principal studies. On the database, all studies in the group have a common value for REFGP. Description of these sets of studies are as follows:

1. XIANGZ (principal study) is a cohort study of Yunnan tin miners followed from 1976-87, including 983 cases while QIAO and LUBIN (subsidiary studies) are case-control studies of Yunnan tin miners involving, respectively, 107 cases occurring in 1976-84 and 427 cases occurring in 1984-88.
2. MRFIT (principal study) involves 9 year follow up of all 361662 men screened in this study, while MRFITR (subsidiary study) involves 12 year follow up of only those 12866 men taking part in the intervention trial proper.
3. LUBIN2 (principal study) contains combined results from a large multicentre case-control study in seven centres in five West European countries whilst VUTUC - Austria, BENHAM - France, BERRIN and PISANI - Italy and GILLIS - Scotland (subsidiary studies) present results from individual countries not including Germany.
4. BOFFET (principal study) contains combined results from a multicentre case-control study in seven centres in Germany, Sweden and Italy, whilst JAHN and KREUZE (subsidiary studies) present results from one German centre each.
5. AKIBA (principal study) contains data from the Atomic bomb survivors cohort study, whilst ISHIMA (subsidiary study) presents data from a case-control study from within this cohort.
6. CASCOR (principal study) is a case-control study in Berlin involving 389 cases, 220 of which were already considered in ROOTS (subsidiary study), another Berlin case-control study involving 270 cases in all.
7. KAISER (principal study) is a cohort study of Kaiser health check attendees in California involving men and women interviewed in 1964-73 and followed up to 1980, by which time 714 had lung cancer, whilst OSANN2 (subsidiary study) is a nested case-control study within the Kaiser population involving 217 female cases enrolled in 1964-77 and diagnosed in 1969-77.
8. TVERDA (principal study) contains results from a Norwegian cohort study involving 44,290 men and 24,535 women aged 35-49 examined in five areas of Norway between 1972 and 1978 and followed up until 1988. VEIERO (subsidiary study) contains results of follow up until 1983 of 25,956 men and 25,496 women in three of these areas examined a second time starting in 1977.
9. HEIN and LANGE (principal studies) contain results from, respectively, the Copenhagen Male Cohort study population, interviewed in 1970-71 and followed to 1988 and the Copenhagen City Heart Cohort study population, interviewed in 1976-78 and followed to 1989. PRESCO (subsidiary study) contains results from a combined analysis, with follow up to 1993, of data from three cohort studies in Copenhagen including the two referred to above. [Note that, because of the overlap, only data by age and by amount smoked, not available for the principal studies, were entered on the database for PRESCO.]
10. WALD, BENSHL and HOLE (principal studies) contain results from follow up of three UK cohort studies, the BUPA study, the Whitehall study and the Paisley-Renfrew study. TANG2 (subsidiary study) contains results from a combined analysis, specifically with respect to the filter/plain comparison, of 4 UK cohort studies including the three referred to above.
11. GRAHAM and BROSS (principal studies) contains results from case-control studies conducted at the Roswell Park Memorial Institute in, respectively, 1956-60 and 1960-66. BYERS1 (subsidiary study) contains results from cases at the same institute interviewed in 1957-65. [Note that, because of the overlap, only data on histological type of lung cancer, not considered in the two principal studies, were entered on the database for BYERS1.]
12. WYNDER6 (principal study) contains results from a case-control study continuously ongoing in various US hospitals for 1969-96, whilst WYNDER5, WYNDER7 and WYNDER8 (subsidiary studies) contains results from the same study for interviews conducted in, respectively, 1969-76, 1977-84 and 1985-90.

[Note that there were an extremely large number of papers covering results from various periods in various sets of hospitals and that results were selected to choose what seemed the most appropriate analyses, whilst attempting to avoid overlap. Note also that, when considering results for filter/plain, one can include results from both WYNDER5 and WYNDER6, without overlap.]

Note that for sets 9 and 10, the individual studies are chosen to be the principal studies as they were originally intended to be separate and the combined analysis occurred later. For sets 3 and 4, the combined studies are chosen to be the principal studies as they were planned as multicentre studies.

##

### Table 1 Study description

| **REF** | **Principal study (if subsidiary)** | **Brief study description** | **Age range** | | | **Races included** | **Eligible population** |
| --- | --- | --- | --- | --- | --- | --- | --- |
|  |  |  | **Low** | **High** | |  |  |
|  |  |  |  | **Baseline** | **Final** |  |  |
| ABELIN |  | Switzerland rural CC study 1941-64 | 1 | 99 | - | all (in country) | general population |
| ABRAHA |  | Hungary Budapest cohort study 1975-94 | 40 | 77 | 93 | all (in country) | general population |
| AGUDO |  | Spain Barcelona area CC study 1989-92 | 1 | 99 | - | all (in country) | general population |
| AKIBA |  | Japan atomic bomb survivors cohort study 1963-87 | 1 | 99 | 99 | all (in country) | a bomb survivors + others |
| ALDERS |  | UK CC Study 1977-82 | 35 | 74 | - | all (in country) | general population |
| AMANDU |  | US metal miner cohort study 1959-75 | 15 | 99 | 99 | whites (inc hispanic) | employed-specific industry/ies |
| AMES |  | US 4 NIOSH coal miner cohorts nested CC 1959-75 | 15 | 99 | 99 | whites (inc hispanic) | employed-specific industry/ies |
| ANDERS |  | US Iowa Women's Health cohort study 1986-94 | 55 | 69 | 77 | all (in country) | holding valid driving licence |
| ARCHER |  | US uranium miners cohort study 1950-74 | 15 | 99 | 99 | all (in country) | employed-specific industry/ies |
| ARMADA |  | Spain CC study 1986-90 | 1 | 80 | - | all (in country) | general population |
| AUSTIN |  | US Ohio foundry workers CC study 1970-86 | 15 | 99 | - | all (in country) | employed-specific industry/ies |
| AUVINE |  | Finland radon CC study 1986-92 | 1 | 99 | - | all (in country) | gen pop long term resident |
| AXELSO |  | Sweden radon CC study 1960-81 | 30 | 84 | - | all (in country) | gen pop long term resident |
| AXELSS |  | Sweden 26 municipality CC study 1989-93 | 1 | 75 | - | scandinavian | general population |
| BAND |  | Canada occupational CC study 1983-90 | 20 | 99 | - | all (in country) | general population |
| BARBON |  | Italy Trieste CC study 1979-86 | 1 | 99 | - | all (in country) | general population |
| BECHER |  | Germany pilot for BIPS CC study 1985-86 | 1 | 99 | - | all (in country) | general population |
| BENHAM | LUBIN2 | France CC study 1976-80 | 1 | 99 | - | all (in country) | general population |
| BENSHL |  | UK Whitehall civil servants cohort study 1967-87 | 40 | 69 | 89 | all (in country) | employed-specific industry/ies |
| BERRIN | LUBIN2 | Italy CC study 1977-80 | * | * | - | all (in country) | general population |
| BEST |  | Canada war veteran pensioners cohort study 1955-62 | 15 | 99 | 99 | all (in country) | Canadian veteran pensioners |
| BLOHMK |  | Germany Heidelberg personality CC study | 1 | 99 | - | all (in country) | general population |
| BLOT1 |  | US Georgia CC study 1970-76 | 1 | 99 | - | all (in country) | gen pop long term resident |
| BLOT3 |  | US Florida CC study 1976-79 | 1 | 99 | - | all (in country) | gen pop long term resident |
| BLOT4 |  | US Pennsylvania CC study 1974-77 | 30 | 79 | - | whites (inc hispanic) | general population |
| BOFFET |  | West Europe pipe and cigar CC study 1988-94 | 1 | 75 | - | all (in country) | general population |
| BOUCHA |  | France Paris CC study 1988-92 | 1 | 99 | - | whites (inc hispanic) | general population |
| BOUCOT |  | US Philadelphia LC Research cohort study 1951-65 | 45 | 99 | 99 | all (in country) | participants long term screening |
| BRESLO |  | US California CC study 1949-52 | 1 | 99 | - | all (in country) | general population |
| BRETT |  | UK X-ray volunteers cohort study 1960-63 | 40 | 99 | 99 | all (in country) | industrial workers |
| BROCKM |  | Germany Berlin CC study 1990-92 | 1 | 84 | - | whites (inc hispanic) | general population |
| BROSS |  | US Roswell Park Memorial CC study 1960-66 | 1 | 99 | - | whites (inc hispanic) | general population |
| BROWN1 |  | US Colorado adenocarcinoma CC study 1979-82 | 1 | 99 | - | whites (inc hispanic) | general population |
| BROWN2 |  | US Missouri CC study 1984-90 | 20 | 99 | - | whites (inc hispanic) | general population |
| BUELL |  | US California American Legion cohort study 1957-62 | 25 | 99 | 99 | all (in country) | American legion mag subscriber |
| BUFFLE |  | US Texas 6 counties CC study 1976-80 | 30 | 79 | - | whites (inc hispanic) | general population |
| BYERS1 | BROSS, GRAHAM | US Roswell Park Memorial CC study 1957-65 | 35 | 79 | - | whites (inc hispanic) | general population |
| BYERS2 |  | US Western New York Diet CC study 1980-84 | 35 | 79 | - | whites (inc hispanic) | general population |
| CARPEN |  | US California Genetics CC study 1991-94 | 40 | 84 | - | whites and blacks | general population |
| CASCO2 |  | Germany Berlin NAT2 Genotyping CC study 1991-94 | 1 | 99 | - | whites (inc hispanic) | general population |
| CASCOR |  | Germany Berlin NAT2 Phenotyping CC study | 1 | 99 | - | whites (inc hispanic) | general population |
| CEDERL |  | Sweden cohort study 1963-89 | 18 | 69 | 95 | all (in country) | general population |
| CHAN |  | Hong Kong 5 hospital CC study 1976-77 | 1 | 99 | - | all (in country) | general population |
| CHANG |  | US California cholesterol cohort study 1972-91 | 40 | 89 | * | all (in country) | resident of elderly community |
| CHATZI |  | Greece Athens CC study 1987-88 | 1 | 99 | - | all (in country) | general population |
| CHEN |  | Taiwan Taipai CC study | 1 | 99 | - | all (in country) | general population |
| CHEN2 |  | China Guangzhou CC study | 1 | 99 | - | all (in country) | gen pop long term resident |
| CHEN3 |  | China Zhengzhou CC study | * | * | - | all (in country) | * |
| CHIAZZ |  | US Owens-Corning Fiberglass Newark CC study 1940-82 | 15 | 99 | - | all (in country) | employed-specific industry/ies |
| CHOI |  | South Korea Cancer Centre CC study 1985-88 | 1 | 99 | - | all (in country) | general population |
| CHOW |  | US Lutheran Brotherhood Ins. cohort study 1966-86 | 35 | 99 | 99 | whites (inc hispanic) | Lutheran Insurance holders |
| CHYOU |  | US Hawaii Oahu Japanese cohort study 1965-90 | 46 | 68 | 90 | japanese | volunteers |
| COMSTO |  | US Washington Co Serum Bank nested CC study 1975-93 | 1 | 99 | 99 | all (in country) | general population |
| COOKSO |  | Zimbabwe-Rhodesia Harare CC study 1961-72 | 1 | 99 | - | blacks | general population |
| CORREA |  | US Louisiana CC study 1979-82 | 1 | 99 | - | all (in country) | general population |
| CPSI |  | US ACS million person CPSI cohort study 1959-72 | 30 | 99 | 99 | all (in country) | household with person>45, no migrant workers |
| CPSII |  | US 2nd ACS cancer prevention cohort study 1982-94 | 30 | 99 | 99 | all (in country) | general population |
| DAMBER |  | Sweden North CC study 1972-77 | 1 | 99 | - | all (in country) | general population |
| DARBY |  | UK SW England radon CC study 1988-93 | 1 | 74 | - | whites (inc hispanic) | gen pop long term resident |
| DAVEYS |  | Germany Thuringia (Schairer&Schoniger) CC 1930-41 | 1 | 99 | - | all (in country) | general population |
| DEAN |  | South Africa CC study 1947-56 | 1 | 99 | - | whites (inc hispanic) | general population |
| DEAN2 |  | UK/N Ireland CC study 1960-62 | 35 | 99 | - | all (in country) | general population |
| DEAN3 |  | UK Cleveland Co CC study 1969-73 | 35 | 99 | - | all (in country) | general population |
| DEKLER |  | Australia Kalgoorlie miners cohort study 1961-93 | * | * | * | all (in country) | employed-specific industry/ies |
| DESTE2 |  | Uruguay Montevideo CC study 1993-96 | 30 | 89 | - | all (in country) | general population |
| DESTEF |  | Uruguay Montevideo CC study 1988-94 | 25 | 84 | - | all (in country) | general population |
| DOCKER |  | US Harvard six cities cohort study 1974-91 | 25 | 74 | 91 | whites (inc hispanic) | general population |
| DOLL |  | UK original Doll and Hill CC study 1948-52 | 1 | 74 | - | all (in country) | general population |
| DOLL2 |  | UK British Doctors cohort study 1951-91 | 20 | 99 | 99 | all (in country) | employed-specific industry/ies |
| DORANT |  | Netherlands case-cohort study 1986-89 | 55 | 69 | 73 | all (in country) | general population |
| DORGAN |  | US New Jersey CC study 1980-83 | 1 | 99 | - | all (in country) | general population |
| DORN |  | US Veterans cohort study 1954-80 | 30 | 84 | 99 | whites (inc hispanic) | US war veteran pensioners |
| DOSEME |  | Turkey Istanbul CC study 1979-84 | 1 | 99 | - | all (in country) | industrial workers |
| DROSTE |  | Belgium Antwerp CC study 1995-97 | 1 | 99 | - | all (in country) | gen pop long term resident |
| DU |  | China Guangzhou CC study 1985 | 1 | 99 | - | all (in country) | gen pop long term resident |
| DUNN |  | US California 9 occupations cohort study 1954-58 | 35 | 64 | 68 | all (in country) | employed-specific industry/ies |
| EBELIN |  | Germany Berlin (Lichtenberg) CC study 1980-85 | 1 | 69 | - | all (in country) | general population |
| ENGELA |  | Norway cohort study 1964-93 | 32 | 72 | 99 | all (in country) | gen pop + migrant siblings |
| ENSTRO |  | US California CPSI cohort study 1959-97 | 30 | 99 | 99 | all (in country) | volunteer interviewer's friend |
| ESAKI |  | Japan Omuta and Arao CC study 1961-71 | 1 | 99 | - | all (in country) | general population |
| FAN |  | China Sino-MONICA-Beijing Project CC study 1990-91 | 18 | 80 | - | all (in country) | general population |
| GAO |  | China Shanghai CC study 1984-86 | 35 | 69 | - | all (in country) | general population |
| GAO2 |  | Japan Tokai diet CC study 1988-91 | 1 | 99 | - | all (in country) | general population |
| GARCIA |  | US Boston genetics CC study 1992-96 | 1 | 99 | - | all (in country) | general population |
| GARDIN |  | UK/Scotland Airdrie avian CC study 1988-92 | 1 | 99 | - | all (in country) | general population |
| GARSHI |  | US railroad workers diesel CC study 1981-82 | 25 | 82 | - | all (in country) | employed-specific industry/ies |
| GENG |  | China Tianjin CC study | 1 | 99 | - | all (in country) | gen pop long term resident |
| GER |  | Taiwan Tri-Service General Hospital 1990-91 | 1 | 99 | - | all (in country) | general population |
| GILLIS | LUBIN2 | UK/Scotland West CC study 1977-81 | 1 | 99 | - | all (in country) | general population |
| GODLEY |  | US 1966-68 NMFS & 1967 current pop survey CC study | 45 | 79 | - | all (in country) | general population |
| GOLLED |  | UK Teeside CC study 1952-62 | 1 | 99 | - | all (in country) | general population |
| GOODMA |  | US Hawaii CC study 1983-85 | 30 | 84 | - | 9 | general population |
| GRAHAM |  | US Roswell Park Memorial CC study 1956-60 | 1 | 99 | - | whites (inc hispanic) | general population |
| GREGOR |  | UK Brompton Hospital vitamin A CC study 1976-77 | 1 | 99 | - | all (in country) | general population |
| GSELL |  | Switzerland St Gallen CC study 1937-54 | 1 | 99 | - | all (in country) | general population |
| GUO |  | China Quanshan county Jiangsu CC study 1984-86 | 1 | 99 | - | all (in country) | general population |
| HAENSZ |  | US Multiple hospital CC study 1955-57 | 1 | 99 | - | all (in country) | general population |
| HAMMO2 |  | US & Canada asbestos workers cohort study 1967-76 | 15 | 99 | 99 | all (in country) | employed-specific industry/ies |
| HAMMON |  | US 9 state cohort study 1952-55 | 50 | 69 | 73 | whites (inc hispanic) | volunteer interviewer's friend |
| HANSEN |  | Denmark welding companies cohort study 1968-86 | 15 | 99 | 99 | all (in country) | employed-specific industry/ies |
| HEGMAN |  | US Utah radon CC study 1989-91 | 40 | 79 | - | all (in country) | general population |
| HEIN |  | Denmark Copenhagen Male cohort study 1970-88 | 40 | 59 | 76 | all (in country) | employed persons |
| HENNEK |  | US doctors betacarotene trial cohort study 1982-95 | 40 | 84 | 98 | all (in country) | employed-specific industry/ies |
| HINDS |  | US Hawaii CC study 1968-78 | 1 | 99 | - | 8 | general population |
| HIRAY2 |  | Japan Tokyo CC study 1950-52 | 1 | 99 | - | all (in country) | * |
| HIRAYA |  | Japan 6 prefecture cohort study 1965-82 | 40 | 99 | 99 | all (in country) | general population |
| HITOSU |  | Japan Amagaski and Nishinomiya CC study 1960-66 | 1 | 99 | - | all (in country) | general population |
| HOLE |  | UK/Scotland Renfrew & Paisley cohort study 1972-85 | 45 | 64 | 77 | all (in country) | general population |
| HOROWI |  | Canada Montreal CC study 1956-67 | 1 | 99 | - | all (in country) | general population |
| HORWIT |  | US Yale/New Haven CC study 1977-82 | 1 | 99 | - | all (in country) | general population |
| HU |  | China Heilongjiang 5 hospital CC study 1985-87 | 1 | 99 | - | all (in country) | general population |
| HU2 |  | China Harbin CC study 1977-79 | 1 | 99 | - | all (in country) | general population |
| HUANG |  | China Sichuan CC study 1990-91 | 1 | 99 | - | all (in country) | general population |
| HUMBLE |  | US New Mexico statewide CC study 1980-82 | 25 | 84 | - | whites (inc hispanic) | general population |
| ISHIMA | AKIBA | Japan A bomb survivors CC study 1961-70 | 1 | 99 | - | all (in country) | a bomb survivors + others |
| JAHN | BOFFET | Germany BIPS CC study 1988-93 | 1 | 80 | - | all (in country) | gen pop - nationals only |
| JAIN |  | Canada Ontario CC study 1981-85 | 30 | 79 | - | all (in country) | general population |
| JARUP |  | Sweden smelter workers CC study 1928-81 | 15 | 99 | - | all (in country) | employed-specific industry/ies |
| JARVHO |  | Sweden Goteborg asbestos CC study 1983-84 | 1 | 75 | - | all (in country) | general population |
| JEDRYC |  | Poland CC study 1980-87 | 1 | 99 | - | all (in country) | general population |
| JIANG |  | China Nanchang CC study 1984 | 1 | 99 | - | all (in country) | general population |
| JOLY |  | Cuba Havana CC study 1978-80 | 1 | 99 | - | all (in country) | general population |
| JUSSAW |  | India Greater Bombay CC study 1964-73 | 1 | 99 | - | all (in country) | general population |
| KAISE2 |  | US California Kaiser cohort study 1979-91 | 30 | 99 | 99 | all (in country) | persons attending screening |
| KAISER |  | US California Kaiser cohort study 1964-80 | 1 | 99 | 99 | all (in country) | persons attending screening |
| KANELL |  | Greece Hellenic Anticancer Inst CC study 1950-62 | 1 | 99 | - | all (in country) | general population |
| KATSOU |  | Greece Athens CC study 1987-89 | 1 | 99 | - | all (in country) | general population |
| KAUFMA |  | US & Canada tar level CC study 1981-86 | 40 | 69 | - | all (in country) | general population |
| KELLER |  | US Illinois CC study 1985-87 | 1 | 99 | - | all (in country) | general population |
| KHUDER |  | US Philadelphia 15 hospital CC study 1985-87 | 1 | 99 | - | all (in country) | general population |
| KIHARA |  | Japan Kanagawa genetic CC study 1991-98 | 1 | 99 | - | japanese | general population |
| KINLEN |  | UK tea drinking cohort study 1967-86 | 45 | 60 | 80 | all (in country) | general population |
| KJUUS |  | Norway Telemark and Vestfold CC study 1979-83 | 1 | 79 | - | all (in country) | employed persons |
| KNEKT |  | Finland Mobile Clinic Health cohort study 1966-91 | 15 | 99 | 99 | all (in country) | general population |
| KO |  | Taiwan Kaohsiung CC study 1992-93 | 1 | 99 | - | all (in country) | general population |
| KOHLME |  | Germany Berlin pet birds CC study 1990 | 1 | 65 | - | all (in country) | gen pop - nationals only |
| KOO |  | Hong Kong 8 hospital CC study 1981-83 | 1 | 99 | - | all (in country) | general population |
| KOULUM |  | Finland Helsinki CC study 1936-52 | 1 | 99 | - | all (in country) | general population |
| KREUZE | BOFFET | Germany radon CC study 1990-96 | 1 | 69 | - | all (in country) | general population |
| KREYBE |  | Norway CC study 1948-53 | 1 | 99 | - | all (in country) | patients/doctors/industrial workers |
| KUBIK |  | Czechoslovakia Kolin district cohort study 1965-71 | 40 | 64 | 70 | all (in country) | gen pop excl abnormal Xray |
| LAMTH |  | Hong Kong 8 hospital CC study 1983-86 | 1 | 99 | - | chinese | general population |
| LAMWK |  | Hong Kong Queen Mary Hospital CC study 1981-84 | 1 | 99 | - | chinese | general population |
| LAMWK2 |  | Hong Kong Queen Mary Hospital CC study 1976-80 | 1 | 99 | - | all (in country) | general population |
| LANGE |  | Denmark Copenhagen City Heart cohort study 1976-89 | 20 | 99 | 99 | all (in country) | general population |
| LAURIL |  | Finland ATBC nested CC study 1988-93 | 53 | 72 | 77 | all (in country) | participants supplements trial |
| LAUSSM |  | Germany Aue/Saxony uranium miners CC study 1982-89 | 1 | 99 | - | all (in country) | general population |
| LEI |  | China Guangzhou CC study 1986 | 1 | 99 | - | all (in country) | gen pop long term resident |
| LEMARC |  | US Hawaii Oahu genotyping CC study 1992-97 | 26 | 79 | - | white, japanese & hawaiian | general population |
| LETOUR |  | Canada Winnipeg radon CC study 1983-90 | 35 | 80 | - | all (in country) | general population |
| LEVIN |  | US Roswell Park Memorial CC study 1938-52 | 1 | 99 | - | all (in country) | general population |
| LIAW |  | Taiwan 12 township cohort study 1982-94 | 40 | 99 | 99 | all (in country) | general population |
| LICKIN |  | Germany West CC study | * | * | - | * | * |
| LIDDEL |  | Canada Quebec chrysotile mine cohort study 1970-88 | 50 | 79 | 97 | all (in country) | employed-specific industry/ies |
| LIU |  | China Shun Yi CC study 1980-86 | 1 | 99 | - | all (in country) | general population |
| LIU2 |  | China Guangzhou CC study 1983-84 | 1 | 99 | - | all (in country) | general population |
| LIU3 |  | China Xuanwei farmers CC study 1985-86 | 1 | 99 | - | all (in country) | employed-specific industry/ies |
| LIU4 |  | China million deaths study 1986-88 | 35 | 99 | - | all (in country) | general population |
| LIU5 |  | China Wuhan CC study 1978-79 | 1 | 99 | - | all (in country) | general population |
| LOMBA2 |  | US Boston CC study 1960-67 | 1 | 99 | - | all (in country) | general population |
| LOMBAR |  | US Boston CC study 1951-64 | 1 | 99 | - | all (in country) | general population |
| LUBIN | XIANGZ | China Yunnan tin miners CC study 1984-88 | 35 | 75 | - | all (in country) | gen pop plus specific industry |
| LUBIN2 |  | West Europe CC study 1976-80 | 1 | 99 | - | all (in country) | general population |
| LUO |  | China Fuzhou CC study 1990-91 | 1 | 99 | - | all (in country) | general population |
| MACLEN |  | Singapore CC study 1972-73 | 1 | 99 | - | chinese | general population |
| MAGNUS |  | Norway nickel workers cohort study 1953-93 | 15 | 99 | 99 | all (in country) | employed-specific industry/ies |
| MARSH |  | US Arizona 6 smelter town CC study 1979-90 | 1 | 99 | - | all (in country) | general population |
| MARSH2 |  | US Arizona 4 smelter town CC study 1979-90 | 1 | 99 | - | all (in country) | general population |
| MARTIS |  | UK Tyneside asbestos CC study 1972-73 | 1 | 99 | - | all (in country) | general population |
| MASTRA |  | Italy silica CC study 1973-80 | 1 | 99 | - | all (in country) | general population |
| MATOS |  | Argentina Buenos Aires CC study 1994-96 | 1 | 99 | - | all (in country) | general population |
| MATSUD |  | Japan Osaka CC study 1965 | 40 | 99 | - | all (in country) | general population |
| MCCONN |  | UK Liverpool CC study 1946-49 | 1 | 99 | - | all (in country) | general population |
| MCDUFF |  | Canada Saskatchewan CC study 1979-83 | 1 | 99 | - | all (in country) | same-sex sibling pairs |
| MCLAUG |  | China 5 region silica workers CC study 1972-89 | 15 | 99 | - | all (in country) | employed-specific industry/ies |
| MIGRAN |  | UK British part of migrant cohort study 1964-77 | 35 | 69 | 81 | all (in country) | gen pop + migrant siblings |
| MILLER |  | US Erie County CC study 1972-84 | 30 | 99 | - | all (in country) | general population |
| MILLS |  | US Ohio CC study 1940-47 | 1 | 99 | - | whites (inc hispanic) | general population |
| MOLLO |  | Italy Turin CC study 1982-92 | 1 | 99 | - | all (in country) | general population |
| MRFIT |  | US MRFIT initial screening cohort study 1973-82 | 35 | 57 | 69 | all (in country) | persons attending screening |
| MRFITR | MRFIT | US MRFIT randomized subjects cohort study 1973-85 | 35 | 57 | 69 | all (in country) | high coronary risk |
| MURATA |  | Japan Chiba gastric screen nested CC study 1984-93 | * | * | * | all (in country) | persons attending screening |
| MZILEN |  | South Africa Northern Province blacks CC study | * | * | - | blacks | general population |
| NAM |  | US National Mortality Followback Study 1986 | 25 | 99 | - | all (in country) | general population |
| NOTAN2 |  | India Tata Memorial Hospital CC study 1963-71 | 30 | 99 | - | all (in country) | general population |
| NOTANI |  | India Tata Memorial Hospital CC study 1986-90 | 1 | 99 | - | all (in country) | general population |
| NOU |  | Sweden Uppsala CC study 1971-76 | 1 | 99 | - | all (in country) | general population |
| ODRISC |  | UK Salford Bronchoscopy Database CC study | 1 | 75 | - | all (in country) | general population |
| ORMOS |  | Hungary Szeged CC study 1947-59 | 1 | 99 | - | all (in country) | general population |
| OSANN |  | US Orange Co. Cancer Surveillance CC study 1984-86 | 1 | 99 | - | all (in country) | general population |
| OSANN2 | KAISER | US California Kaiser nested CC study 1969-77 | 1 | 99 | 99 | all (in country) | persons attending screening |
| PARKIN |  | Zimbabwe-Rhodesia Bulawayo CC study 1963-77 | 1 | 99 | - | blacks | general population |
| PASTOR |  | Italy Lombardy CC study 1976-79 | 1 | 99 | - | all (in country) | general population |
| PAWLEG |  | Poland Cracow CC study 1992-94 | 1 | 99 | - | all (in country) | general population |
| PERNU |  | Finland CC study 1944-58 | 1 | 99 | - | all (in country) | general population |
| PERSH2 |  | Sweden 109 municipality CC study 1980-84 | 35 | 74 | - | all (in country) | gen pop long term resident |
| PETO |  | UK FEV cohort study 1954-81 | 25 | 64 | 89 | all (in country) | gen pop plus specific industry |
| PEZZO2 |  | Argentina Rosario CC study 1992-98 | 1 | 99 | - | all (in country) | gen pop - <3 lifetime occupations |
| PEZZOT |  | Argentina Rosario CC study 1987-91 | 1 | 99 | - | all (in country) | general population |
| PIKE |  | US California LA County air poll. CC study 1972-75 | 1 | 99 | - | whites excluding hispanics | general population |
| PISANI | LUBIN2 | Italy Lombardy diet CC study 1980-81 | 1 | 99 | - | all (in country) | general population |
| POFFIJ |  | West Europe Ardennes-Eifel radon CC study 1990-95 | 40 | 75 | - | all (in country) | gen pop long term resident |
| POLEDN |  | US Toxic waste dumpsite CC study 1978-81 | 1 | 99 | - | all (in country) | general population |
| PRESCO | HEIN, LANGE | Denmark 3 Copenhagen cohort studies pooled 1964-93 | 20 | 99 | 99 | all (in country) | general population |
| QIAO | XIANGZ | China Yunnan tin miners CC study 1985 | 35 | 80 | - | all (in country) | employed-specific industry/ies |
| QIAO2 |  | China Yunnan tin miners cohort study 1992-95 | 40 | 99 | 99 | all (in country) | employed-specific industry/ies |
| RACHTA |  | Poland Cracow CC study 1991-94 | 1 | 99 | - | all (in country) | next-of-kin of other patient |
| RADZIK |  | Poland lung cancer relatives CC study 1986-87 | 1 | 99 | - | all (in country) | general population |
| RANDIG |  | Germany Berlin CC study 1951-54 | 1 | 99 | - | all (in country) | general population |
| REN |  | China CC study | * | * | - | all (in country) | * |
| RESTRE |  | Colombia CC study 1978-80 | 1 | 99 | - | all (in country) | general population |
| RIMING |  | UK Mass radiography cohort study 1970-76 | 40 | 99 | 99 | all (in country) | volunteers mass radiography |
| RONCO |  | Italy Turin CC study 1976-80 | 1 | 99 | - | all (in country) | general population |
| ROOTS | CASCOR | Germany Berlin debrisoquine CC study | 1 | 80 | - | whites (inc hispanic) | general population |
| ROTHSC |  | US Southern Louisiana CC study 1971-77 | 1 | 99 | - | all (in country) | general population |
| SAARIK |  | Finland genetics CC study 1988-96 | 1 | 99 | - | whites (inc hispanic) | general population |
| SADOWS |  | US National Cancer Institute CC study 1938-43 | 1 | 99 | - | whites (inc hispanic) | general population |
| SANKAR |  | India Trivandrum diet CC study 1990 | 1 | 99 | - | all (in country) | general population |
| SCHWAR |  | US Michigan CC study 1984-87 | 40 | 84 | - | whites and blacks | general population |
| SEGI |  | Japan nationwide CC study 1948-52 | 30 | 99 | - | all (in country) | general population |
| SEGI2 |  | Japan Tokyo and Sendai CC study 1962-70 | 1 | 99 | - | all (in country) | general population |
| SEOW |  | Singapore NAT2 CC study 1997-98 | 1 | 99 | - | chinese | gen pop long term resident |
| SHAW |  | US & Canada Bethesda/Quebec debrisoquine CC 1988-92 | 1 | 99 | - | whites (inc hispanic) | general population |
| SHIMIZ |  | Japan Sendai Kosei Hospital CC study 1977-82 | 40 | 99 | - | all (in country) | general population |
| SIEMIA |  | Canada Montreal occupational CC study 1979-85 | 35 | 70 | - | all (in country) | general population |
| SIMARA |  | Thailand Chiang Mai CC study 1971-72 | 1 | 99 | - | all (in country) | general population |
| SITAS |  | South Africa Johannesburg blacks CC study -1997 | 1 | 99 | - | blacks | general population |
| SOBUE |  | Japan Osaka CC study 1986-88 | 1 | 99 | - | all (in country) | general population |
| SOBUE2 |  | Japan Osaka CC study 1965-83 | 1 | 99 | - | all (in country) | general population |
| SPEIZE |  | US Nurses' Health cohort study 1976-92 | 30 | 55 | 71 | all (in country) | employed-specific industry/ies |
| SPITZ |  | US Texas University Genetics Study | 1 | 99 | - | blacks and hispanics | general population |
| STASZE |  | Poland Gliwice CC study 1954-58 | 1 | 99 | - | all (in country) | general population |
| STAYNE |  | US Third National Cancer Survey CC Study 1969-71 | 30 | 84 | - | all (in country) | general population |
| STOCKS |  | UK British Empire Cancer Campaign CC study 1952-55 | 1 | 99 | - | all (in country) | general population |
| STOCKW |  | US Florida phosphate mining area CC study 1981-83 | 1 | 99 | - | all (in country) | general population |
| STUCKE |  | France GSTM1 CC study 1989-92 | 1 | 74 | - | all (in country) | general population |
| SUN |  | China Liaoning genetics CC study 1992-94 | 1 | 99 | - | all (in country) | general population |
| SUZUK2 |  | Brazil Rio de Janeiro CC study 1991-92 | 1 | 99 | - | all (in country) | general population |
| SUZUKI |  | Japan Osaka CC study 1978-86 | 1 | 99 | - | all (in country) | general population |
| SVENSS |  | Sweden Stockholm County CC study 1983-86 | 1 | 99 | - | all (in country) | general population |
| TANG |  | US Columbia Presbyterian genetics CC study | 1 | 99 | - | all (in country) | general population |
| TANG2 | BENSHL, HOLE, WALD | UK 4 cohort studies pooled 1967-90 | 35 | 78 | 98 | all (in country) | professional/civil service/factory/residents |
| TAO |  | China Shanghai CC study 1988-90 | 1 | 99 | - | all (in country) | general population |
| TENKAN |  | Finland part Finland/Norway cohort study 1962-87 | 45 | 64 | 89 | all (in country) | general population |
| TIZZAN |  | Italy CC study 1959-61 | 1 | 99 | - | all (in country) | general population |
| TOKARS |  | Russia Nuclear Workers nested CC study 1966-91 | 15 | 70 | 99 | all (in country) | employed-specific industry/ies |
| TOUSEY |  | US Duval County CC study 1993-96 | 30 | 84 | - | all (in country) | general population |
| TSUGAN |  | Japan National Cancer Centre CC study 1976-85 | 30 | 49 | - | all (in country) | general population |
| TULINI |  | Iceland Rejkjavik cohort study 1967-95 | 32 | 84 | 88 | all (in country) | general population |
| TVERDA |  | Norway cohort study 1972-88 | 35 | 49 | 63 | all (in country) | persons attending screening |
| ULMER |  | Germany Bochum CC study 1971-75 | 1 | 99 | - | all (in country) | general population |
| VEIERO | TVERDA | Norway Health Screening cohort study 1977-91 | 16 | 56 | 70 | all (in country) | general population |
| VUTUC | LUBIN2 | Austria CC study 1976-80 | 1 | 99 | - | all (in country) | general population |
| WAKAI |  | Japan Okinawa CC study 1988-91 | 40 | 89 | - | all (in country) | general population |
| WALD |  | UK BUPA cohort study 1975-93 | 35 | 64 | 75 | all (in country) | professional and business men |
| WANG |  | China Guangdong CC study 1990-93 | 32 | 78 | - | all (in country) | gen pop long term resident |
| WANG2 |  | China Tai Yuan CC study 1980-82 | 1 | 99 | - | all (in country) | general population |
| WANG3 |  | China Nanjing CC study | * | * | - | all (in country) | * |
| WANG4 |  | China Xuanwei farmers cohort study 1976-96 | 25 | 59 | - | all (in country) | employed-specific industry/ies |
| WARSIN |  | Netherlands CC study | * | * | - | * | * |
| WATSON |  | US New York Memorial Hospital CC study 1950-52 | 1 | 99 | - | all (in country) | general population |
| WICKLU |  | US Washington County orchardists CC study 1968-80 | 1 | 99 | - | whites (inc hispanic) | employed-specific industry/ies |
| WIGLE |  | Canada Alberta CC study 1971-73 | 25 | 99 | - | all (in country) | general population |
| WILKIN |  | UK London Chest Hospital CC study 1992-93 | 1 | 99 | - | all (in country) | general population |
| WU |  | US California LA County CC study 1981-82 | 1 | 75 | - | whites (inc hispanic) | gen pop - English speaking |
| WU2 |  | US California LA County CC study 1983-86 | 30 | 75 | - | all (in country) | general population |
| WUNSCH |  | Brazil Sao Paulo CC study 1990-91 | 1 | 99 | - | all (in country) | general population |
| WUWILL |  | China Shenyang and Harbin CC study 1985-87 | 30 | 69 | - | all (in country) | general population |
| WYNDE2 |  | US New York CC study 1962-64 | 1 | 99 | - | all (in country) | general population |
| WYNDE3 |  | US New York Memorial CC study 1966-69 | 1 | 99 | - | all (in country) | general population |
| WYNDE4 |  | US 8 state CC study 1948-50 | 1 | 99 | - | all (in country) | general population |
| WYNDE5 | WYNDE6 | US 4 city CC study 1969-76 | 1 | 99 | - | all (in country) | general population |
| WYNDE6 |  | US 4 city CC study 1969-96 | 1 | 99 | - | all (in country) | general population |
| WYNDE7 | WYNDE6 | US 6 city CC study 1977-84 | 20 | 80 | - | all (in country) | general population |
| WYNDE8 | WYNDE6 | US 4 city CC study 1985-90 | 1 | 99 | - | all (in country) | general population |
| WYNDER |  | Cuba Havana CC study 1956-57 | 1 | 99 | - | all (in country) | persons receiving free care |
| XIANGZ |  | China Yunnan tin miners cohort study 1976-87 | 1 | 99 | 99 | all (in country) | employed-specific industry/ies |
| XU |  | China Shenyang CC study 1985-87 | 30 | 69 | - | all (in country) | general population |
| XU2 |  | China Anshan Iron-Steel workers CC study 1987-93 | 30 | 70 | - | all (in country) | employed-specific industry/ies |
| XU3 |  | China Tianjin CC study 1981 | 1 | 99 | - | all (in country) | general population |
| XU4 |  | China 26 city air pollution CC study | * | * | - | all (in country) | * |
| YAMAGU |  | Japan occupational CC study 1989-90 | 1 | 99 | - | all (in country) | general population |
| YONG |  | US NHANES I - NHEFS cohort study 1971-92 | 25 | 74 | 95 | all (in country) | general population |
| YUAN |  | China Shanghai cohort study 1986-93 | 45 | 64 | 72 | all (in country) | general population |
| ZHANG |  | China Jinzhou CC study 1988-89 | 1 | 99 | - | all (in country) | general population |
| ZHENG |  | China Shanghai CC study 1982-84 | * | * | - | all (in country) | general population |
| ZHOU |  | China Medical Univ CC study 1978-94 | 1 | 99 | - | all (in country) | general population |

### Table 2 Details of control groups (case-control and nested case-control studies)

| **REF** | **Principal study (if subsidiary)** | **Type of control** | **Diseased/hospital controls include:** | | | | | | |
| --- | --- | --- | --- | --- | --- | --- | --- | --- | --- |
|  |  |  | **smoking related cancer** | **respiratory  disease** | **heart  disease** | **other smoking related disease** | **non smoking related cancer** | **orthopaedic/ trauma patients** | **other non smoking related disease** |
| ABELIN |  | healthy | - | - | - | - | - | - | - |
| AGUDO |  | diseased/hospital |  |  |  |  | All | All | All |
| ALDERS |  | diseased/hospital |  |  |  |  | All | All | All |
| AMES |  | decedents |  | All | All | All |  | Some | All |
| ARMADA |  | diseased/hospital |  |  |  |  | All | Some | All |
| AUSTIN |  | healthy | - | - | - | - | - | - | - |
| AUVINE |  | healthy | - | - | - | - | - | - | - |
| AXELSO |  | decedents |  | All | All | All |  | All | All |
| AXELSS |  | healthy | - | - | - | - | - | - | - |
| BAND |  | diseased/hospital |  |  |  |  | All |  |  |
| BARBON |  | decedents | All |  | All | All | All | All | All |
| BECHER |  | healthy + diseased |  |  |  |  | All | All | All |
| BENHAM | LUBIN2 | diseased/hospital |  |  |  |  | All | All | Some |
| BERRIN | LUBIN2 | diseased/hospital |  |  |  |  | All | All | All |
| BLOHMK |  | healthy | - | - | - | - | - | - | - |
| BLOT1 |  | diseased + decedents | Some |  | All | All | All | All | All |
| BLOT3 |  | diseased + decedents | Some |  | All | All | All | All | Some |
| BLOT4 |  | decedents | Some |  | All | All | All | Some | All |
| BOFFET |  | healthy + diseased |  |  |  |  | All | All | All |
| BOUCHA |  | diseased/hospital |  | All | All | All |  | All | All |
| BRESLO |  | diseased/hospital |  |  | All | All |  | All | All |
| BROCKM |  | diseased/hospital |  | All | All | All |  | All | All |
| BROSS |  | diseased/hospital |  | All | All | All |  | All | All |
| BROWN1 |  | diseased + decedents |  |  |  |  | Some |  |  |
| BROWN2 |  | diseased/hospital |  |  |  |  | All |  |  |
| BUFFLE |  | healthy + decedents | Some |  | All | All | All | Some | All |
| BYERS1 | BROSS, GRAHAM | diseased/hospital |  |  | All | All |  | All | Some |
| BYERS2 |  | healthy | - | - | - | - | - | - | - |
| CARPEN |  | healthy | - | - | - | - | - | - | - |
| CASCO2 |  | diseased/hospital |  | All |  |  |  |  |  |
| CASCOR |  | diseased/hospital |  | All | All | All |  | All | All |
| CHAN |  | diseased/hospital |  |  |  |  |  | Some |  |
| CHATZI |  | healthy + diseased |  |  |  |  |  | Some | Some |
| CHEN |  | diseased/hospital |  |  |  |  |  |  | Some |
| CHEN2 |  | decedents | Some |  | All | All | All | All | All |
| CHEN3 |  | healthy | - | - | - | - | - | - | - |
| CHIAZZ |  | healthy + decedents | Some |  | All | All | All | Some | All |
| CHOI |  | diseased/hospital |  |  |  |  | All | All | All |
| COMSTO |  | healthy | - | - | - | - | - | - | - |
| COOKSO |  | diseased/hospital |  |  |  |  |  | Some |  |
| CORREA |  | diseased/hospital |  |  | All | All | All | All | All |
| DAMBER |  | decedents | All | All | All | All | All | Some | All |
| DARBY |  | diseased/hospital |  |  |  |  | All | All | All |
| DAVEYS |  | diseased/hospital |  |  |  |  | Some |  |  |
| DEAN |  | decedents | All | All | All | All | All | All | All |
| DEAN2 |  | decedents | Some |  | All | All | All | All | All |
| DEAN3 |  | healthy | - | - | - | - | - | - | - |
| DESTE2 |  | diseased/hospital |  | All | All | All |  | All | All |
| DESTEF |  | diseased/hospital | Some | All | All | All | All | All | All |
| DOLL |  | diseased/hospital | Some | All | All | All | All | All | All |
| DORGAN |  | healthy + decedents | All |  | All | All | All | All | All |
| DOSEME |  | diseased/hospital |  | All | All | All | All | All | All |
| DROSTE |  | diseased/hospital |  |  | All | All |  | All | All |
| DU |  | decedents |  | All | All | All |  | All | All |
| EBELIN |  | diseased/hospital |  |  | Some | Some |  | Some | Some |
| ESAKI |  | decedents | Some | All | All | All | Some | All | All |
| FAN |  | healthy | - | - | - | - | - | - | - |
| GAO |  | healthy | - | - | - | - | - | - | - |
| GAO2 |  | diseased/hospital |  |  | All | All |  | All | All |
| GARCIA |  | healthy | - | - | - | - | - | - | - |
| GARDIN |  | diseased/hospital |  |  |  |  |  | Some |  |
| GARSHI |  | decedents |  | All | All | All |  | Some | All |
| GENG |  | unstated | * | * | * | * | * | * | * |
| GER |  | healthy + diseased |  |  |  |  |  |  | Some |
| GILLIS | LUBIN2 | diseased/hospital |  |  |  |  | All | All | All |
| GODLEY |  | healthy | - | - | - | - | - | - | - |
| GOLLED |  | healthy | - | - | - | - | - | - | - |
| GOODMA |  | healthy | - | - | - | - | - | - | - |
| GRAHAM |  | diseased/hospital |  |  |  |  | All | All | All |
| GREGOR |  | diseased/hospital |  | Some | Some | All |  | Some | All |
| GSELL |  | diseased/hospital | All | All | All | All | All | All | All |
| GUO |  | healthy | - | - | - | - | - | - | - |
| HAENSZ |  | diseased/hospital | All | All | All | All | All | All | All |
| HEGMAN |  | healthy | - | - | - | - | - | - | - |
| HINDS |  | healthy | - | - | - | - | - | - | - |
| HIRAY2 |  | unstated | * | * | * | * | * | * | * |
| HITOSU |  | healthy | - | - | - | - | - | - | - |
| HOROWI |  | healthy | - | - | - | - | - | - | - |
| HORWIT |  | diseased/hospital | All | All | All | All | All | All | All |
| HU |  | diseased/hospital |  |  | All | All |  | All | All |
| HU2 |  | decedents | Some |  | All | All | All | All | All |
| HUANG |  | healthy | - | - | - | - | - | - | - |
| HUMBLE |  | healthy | - | - | - | - | - | - | - |
| ISHIMA | AKIBA | decedents |  | All | All | All |  | All | All |
| JAHN | BOFFET | healthy | - | - | - | - | - | - | - |
| JAIN |  | healthy | - | - | - | - | - | - | - |
| JARUP |  | decedents |  |  |  |  | All | All | All |
| JARVHO |  | healthy | - | - | - | - | - | - | - |
| JEDRYC |  | decedents |  |  | All | All | All | All | All |
| JIANG |  | diseased/hospital |  |  |  |  |  | All | All |
| JOLY |  | healthy + diseased |  |  |  |  | All | All | All |
| JUSSAW |  | healthy | - | - | - | - | - | - | - |
| KANELL |  | diseased/hospital | Some |  |  |  | All |  |  |
| KATSOU |  | diseased/hospital |  |  |  |  |  | All |  |
| KAUFMA |  | diseased/hospital |  |  |  |  | All | All | All |
| KELLER |  | diseased/hospital |  |  |  |  | Some |  |  |
| KHUDER |  | healthy | - | - | - | - | - | - | - |
| KIHARA |  | healthy | - | - | - | - | - | - | - |
| KJUUS |  | diseased/hospital | All | Some | All | All | All | All | All |
| KO |  | diseased/hospital |  |  |  |  |  |  | Some |
| KOHLME |  | healthy | - | - | - | - | - | - | - |
| KOO |  | healthy | - | - | - | - | - | - | - |
| KOULUM |  | diseased/hospital |  | All | All | All |  | All | All |
| KREUZE | BOFFET | healthy | - | - | - | - | - | - | - |
| KREYBE |  | healthy + diseased | Some | All | All | All | All | All | Some |
| LAMTH |  | healthy | - | - | - | - | - | - | - |
| LAMWK |  | diseased/hospital |  |  |  |  |  | Some |  |
| LAMWK2 |  | diseased/hospital |  |  |  |  |  | Some |  |
| LAURIL |  | healthy | - | - | - | - | - | - | - |
| LAUSSM |  | diseased/hospital | All |  |  |  | Some |  |  |
| LEI |  | decedents |  |  | All | All |  | All | All |
| LEMARC |  | healthy | - | - | - | - | - | - | - |
| LETOUR |  | healthy | - | - | - | - | - | - | - |
| LEVIN |  | diseased/hospital |  | All | All | All |  | All | All |
| LICKIN |  | unstated | * | * | * | * | * | * | * |
| LIU |  | healthy | - | - | - | - | - | - | - |
| LIU2 |  | diseased/hospital |  | Some | Some | All |  | All | All |
| LIU3 |  | healthy | - | - | - | - | - | - | - |
| LIU4 |  | decedents |  |  |  | All |  | All | All |
| LIU5 |  | healthy | - | - | - | - | - | - | - |
| LOMBA2 |  | healthy | - | - | - | - | - | - | - |
| LOMBAR |  | healthy | - | - | - | - | - | - | - |
| LUBIN | XIANGZ | healthy | - | - | - | - | - | - | - |
| LUBIN2 |  | diseased/hospital |  |  |  |  | All | All | All |
| LUO |  | healthy | - | - | - | - | - | - | - |
| MACLEN |  | diseased/hospital |  |  |  |  | All | All | All |
| MARSH |  | decedents | Some | All | All | All | Some | All | All |
| MARSH2 |  | decedents | Some | All | All | All | Some | All | All |
| MARTIS |  | diseased/hospital | All | All | All | All | All | All | All |
| MASTRA |  | diseased/hospital |  | Some |  |  |  |  |  |
| MATOS |  | diseased/hospital |  |  |  |  | All | All | All |
| MATSUD |  | healthy | - | - | - | - | - | - | - |
| MCCONN |  | diseased/hospital |  | All | All | All |  | All | All |
| MCDUFF |  | healthy | - | - | - | - | - | - | - |
| MCLAUG |  | healthy | - | - | - | - | - | - | - |
| MILLER |  | decedents | All | All | All | All | All | Some | All |
| MILLS |  | healthy | - | - | - | - | - | - | - |
| MOLLO |  | decedents | Some | Some | All | All | All | All | All |
| MURATA |  | healthy | - | - | - | - | - | - | - |
| MZILEN |  | diseased/hospital |  |  |  |  | All |  |  |
| NAM |  | decedents |  |  |  | All |  | All | All |
| NOTAN2 |  | diseased/hospital |  |  | All | All |  | All | All |
| NOTANI |  | healthy | - | - | - | - | - | - | - |
| NOU |  | healthy | - | - | - | - | - | - | - |
| ODRISC |  | diseased/hospital |  |  |  |  | All | All | All |
| ORMOS |  | healthy | - | - | - | - | - | - | - |
| OSANN |  | decedents |  |  |  |  | All |  |  |
| OSANN2 | KAISER | healthy | - | - | - | - | - | - | - |
| PARKIN |  | diseased/hospital |  |  |  |  | All |  |  |
| PASTOR |  | healthy | - | - | - | - | - | - | - |
| PAWLEG |  | healthy | - | - | - | - | - | - | - |
| PERNU |  | healthy | - | - | - | - | - | - | - |
| PERSH2 |  | healthy + decedents |  |  |  |  | All | All | All |
| PEZZO2 |  | diseased/hospital |  |  |  |  | All | All | All |
| PEZZOT |  | diseased/hospital |  |  |  |  | All | All | All |
| PIKE |  | healthy | - | - | - | - | - | - | - |
| PISANI | LUBIN2 | diseased/hospital |  |  |  |  | All | All | All |
| POFFIJ |  | healthy + diseased |  |  |  |  | All | All | All |
| POLEDN |  | decedents | Some |  | All | All | All | All | All |
| QIAO | XIANGZ | healthy | - | - | - | - | - | - | - |
| RACHTA |  | healthy | - | - | - | - | - | - | - |
| RADZIK |  | diseased/hospital |  |  |  |  |  | Some |  |
| RANDIG |  | diseased/hospital |  | Some | Some | Some |  | All | All |
| REN |  | unstated | * | * | * | * | * | * | * |
| RESTRE |  | healthy + diseased |  |  |  |  | All | All | All |
| RONCO |  | decedents |  |  | All | All | All | All | All |
| ROOTS | CASCOR | diseased/hospital |  | All | All | All |  | All | All |
| ROTHSC |  | decedents | All | All | All | All | All | All | All |
| SAARIK |  | healthy | - | - | - | - | - | - | - |
| SADOWS |  | diseased/hospital |  | All | All | All |  | All | All |
| SANKAR |  | healthy | - | - | - | - | - | - | - |
| SCHWAR |  | healthy | - | - | - | - | - | - | - |
| SEGI |  | diseased/hospital |  | All | All | All |  | All | All |
| SEGI2 |  | diseased/hospital | All | All | All | All | All | All | All |
| SEOW |  | diseased/hospital |  |  | All | All |  | All | All |
| SHAW |  | diseased/hospital |  | All | All | All |  | All | All |
| SHIMIZ |  | healthy | - | - | - | - | - | - | - |
| SIEMIA |  | healthy + diseased |  |  |  |  | All |  |  |
| SIMARA |  | diseased/hospital | All |  |  |  | All |  |  |
| SITAS |  | diseased/hospital |  |  |  |  | All |  |  |
| SOBUE |  | diseased/hospital |  |  |  |  | All | All | All |
| SOBUE2 |  | healthy | - | - | - | - | - | - | - |
| SPITZ |  | healthy | - | - | - | - | - | - | - |
| STASZE |  | diseased/hospital |  | All | All | All | All | All | All |
| STAYNE |  | diseased/hospital |  |  |  |  | Some |  |  |
| STOCKS |  | diseased + decedents |  | All | All | All | Some | All | All |
| STOCKW |  | diseased/hospital |  |  |  |  | Some |  |  |
| STUCKE |  | diseased/hospital |  |  | All | All |  | All | All |
| SUN |  | healthy | - | - | - | - | - | - | - |
| SUZUK2 |  | diseased/hospital |  |  | All | All |  | All | All |
| SUZUKI |  | diseased/hospital |  |  |  |  | All | All | All |
| SVENSS |  | healthy | - | - | - | - | - | - | - |
| TANG |  | diseased/hospital |  |  |  |  |  | Some |  |
| TAO |  | healthy | - | - | - | - | - | - | - |
| TIZZAN |  | diseased/hospital |  |  |  |  | All | All | All |
| TOKARS |  | healthy | - | - | - | - | - | - | - |
| TOUSEY |  | healthy | - | - | - | - | - | - | - |
| TSUGAN |  | diseased/hospital |  | All | All | All |  | All | All |
| ULMER |  | healthy | - | - | - | - | - | - | - |
| VUTUC | LUBIN2 | diseased/hospital |  |  |  |  | All | All | Some |
| WAKAI |  | healthy | - | - | - | - | - | - | - |
| WANG |  | diseased/hospital |  |  | All | All |  | All | All |
| WANG2 |  | healthy + diseased |  |  |  |  | All |  |  |
| WANG3 |  | healthy | - | - | - | - | - | - | - |
| WANG4 |  | healthy | - | - | - | - | - | - | - |
| WARSIN |  | unstated | * | * | * | * | * | * | * |
| WATSON |  | diseased/hospital | Some | All |  |  |  |  |  |
| WICKLU |  | decedents | Some | All | All | All | All | All | All |
| WIGLE |  | diseased/hospital |  |  |  |  | Some |  |  |
| WILKIN |  | diseased/hospital |  | All | All |  |  |  |  |
| WU |  | healthy | - | - | - | - | - | - | - |
| WU2 |  | healthy | - | - | - | - | - | - | - |
| WUNSCH |  | diseased/hospital |  | Some | All | All | All | All | All |
| WUWILL |  | healthy | - | - | - | - | - | - | - |
| WYNDE2 |  | diseased/hospital | Some |  |  |  | Some |  |  |
| WYNDE3 |  | diseased/hospital |  |  |  |  | All | All | All |
| WYNDE4 |  | diseased/hospital | All | All | All | All | All | All | All |
| WYNDE5 | WYNDE6 | diseased/hospital |  |  |  |  | All | All | All |
| WYNDE6 |  | diseased/hospital |  |  |  |  | All | All | All |
| WYNDE7 | WYNDE6 | diseased/hospital | All | All | All | All | All | All | All |
| WYNDE8 | WYNDE6 | diseased/hospital |  |  |  |  | All | All | All |
| WYNDER |  | diseased/hospital | Some | All | All | All | All | All | All |
| XU |  | healthy | - | - | - | - | - | - | - |
| XU2 |  | healthy + decedents | All | All | All | All | All | All | All |
| XU3 |  | diseased/hospital | Some |  | Some | Some | Some | Some | Some |
| XU4 |  | unstated | * | * | * | * | * | * | * |
| YAMAGU |  | diseased/hospital | All | All | All | All | All | All | All |
| ZHANG |  | diseased/hospital |  |  |  |  | All | All | All |
| ZHENG |  | diseased/hospital |  |  |  |  | All | All | All |
| ZHOU |  | diseased/hospital |  | All | All | All |  | All | All |

### Table 3 Outcomes available in each studya

| **REF** | **Principal study (if subsidiary)** | **All LC^b^** | **Squamous^b^** | **Adeno^b^** | **large** | **small** |
| --- | --- | --- | --- | --- | --- | --- |
| ABELIN |  | allLC |  |  |  |  |
| ABRAHA |  | sq+sm+ad | sq | adeno |  | small |
| AGUDO |  | allLC |  |  |  |  |
| AKIBA |  | allLC |  |  |  |  |
| ALDERS |  | allLC | sq; sq+sm | adeno; la+ad+other; not sm,sq |  | small |
| AMANDU |  | allLC |  |  |  |  |
| AMES |  | allLC |  |  |  |  |
| ANDERS |  | allLC | sq; KI | adeno | large | small |
| ARCHER |  | allLC |  |  |  |  |
| ARMADA |  | allLC |  |  |  |  |
| AUSTIN |  | allLC |  |  |  |  |
| AUVINE |  | allLC |  |  |  |  |
| AXELSO |  | allLC |  |  |  |  |
| AXELSS |  | allLC |  |  |  |  |
| BAND |  | allLC | sq; KI | adeno | large | small |
| BARBON |  | allLC | sq; KI | adeno | large | small |
| BECHER |  | allLC | sq+sm | not sm,sq |  |  |
| BENHAM | LUBIN2 | allLC; KI+KII exc mixed/unspec | KI | KII |  |  |
| BENSHL |  | allLC |  |  |  |  |
| BERRIN | LUBIN2 | allLC |  |  |  |  |
| BEST |  | allLC |  |  |  |  |
| BLOHMK |  | allLC |  |  |  |  |
| BLOT1 |  | allLC |  |  |  |  |
| BLOT3 |  | allLC |  |  |  |  |
| BLOT4 |  | allLC |  |  |  |  |
| BOFFET |  | allLC |  |  |  |  |
| BOUCHA |  |  | sq+sm |  |  |  |
| BOUCOT |  | allLC | sq; KI | adeno; KII | large | small |
| BRESLO |  | allLC | not ad | adeno |  |  |
| BRETT |  | allLC |  |  |  |  |
| BROCKM |  | allLC |  |  |  |  |
| BROSS |  | allLC |  |  |  |  |
| BROWN1 |  |  |  | adeno |  |  |
| BROWN2 |  | allLC | sq | adeno |  | small |
| BUELL |  | allLC |  |  |  |  |
| BUFFLE |  | allLC | sq; KI; not ad | adeno; KII | large | small |
| BYERS1 | BROSS, GRAHAM |  | sq | adeno |  | small |
| BYERS2 |  | allLC |  |  |  |  |
| CARPEN |  | allLC |  |  |  |  |
| CASCO2 |  | allLC |  |  |  |  |
| CASCOR |  | allLC |  |  |  |  |
| CEDERL |  | allLC |  |  |  |  |
| CHAN |  | allLC | sq+sm | ad+la |  |  |
| CHANG |  | allLC |  |  |  |  |
| CHATZI |  | allLC |  |  |  |  |
| CHEN |  |  | sq | adeno |  | small |
| CHEN2 |  | allLC |  |  |  |  |
| CHEN3 |  | allLC |  |  |  |  |
| CHIAZZ |  | allLC |  |  |  |  |
| CHOI |  | allLC | sq | adeno |  | small |
| CHOW |  | allLC |  |  |  |  |
| CHYOU |  | allLC | sq+sm | adeno |  |  |
| COMSTO |  | allLC | sq; KI | adeno | large | small |
| COOKSO |  | allLC |  |  |  |  |
| CORREA |  | allLC | sq+sm | adeno |  |  |
| CPSI |  | allLC | sq | adeno |  | small |
| CPSII |  | allLC | sq | adeno |  | small |
| DAMBER |  | allLC | sq | ad+alveolar+bronchiolar |  | small |
| DARBY |  | allLC |  |  |  |  |
| DAVEYS |  | allLC |  |  |  |  |
| DEAN |  | allLC |  |  |  |  |
| DEAN2 |  | allLC |  |  |  |  |
| DEAN3 |  | allLC |  |  |  |  |
| DEKLER |  | allLC |  |  |  |  |
| DESTE2 |  | allLC | sq | adeno |  |  |
| DESTEF |  | allLC | sq | adeno | large | small |
| DOCKER |  | allLC |  |  |  |  |
| DOLL |  | allLC | KI | KII |  |  |
| DOLL2 |  | allLC |  |  |  |  |
| DORANT |  | allLC |  |  |  |  |
| DORGAN |  | allLC | sq | adeno |  | small |
| DORN |  | allLC | sq; KI | adeno; KII |  | small |
| DOSEME |  | allLC | sq | not sm,sq |  | small |
| DROSTE |  | allLC |  |  |  |  |
| DU |  | allLC |  |  |  |  |
| DUNN |  | allLC |  |  |  |  |
| EBELIN |  | allLC |  |  |  |  |
| ENGELA |  | allLC | sq | adeno |  | small |
| ENSTRO |  | allLC |  |  |  |  |
| ESAKI |  | allLC |  |  |  |  |
| FAN |  | allLC | sq | adeno |  | small |
| GAO |  | allLC | sq | adeno |  | small |
| GAO2 |  | allLC |  |  |  |  |
| GARCIA |  | allLC |  |  |  |  |
| GARDIN |  | allLC |  |  |  |  |
| GARSHI |  | allLC |  |  |  |  |
| GENG |  | allLC |  |  |  |  |
| GER |  | allLC | sq+sm | adeno |  | small |
| GILLIS | LUBIN2 | allLC | sq | adeno |  | small |
| GODLEY |  | allLC |  |  |  |  |
| GOLLED |  | allLC |  |  |  |  |
| GOODMA |  | allLC |  |  |  |  |
| GRAHAM |  | allLC |  |  |  |  |
| GREGOR |  | allLC |  |  |  |  |
| GSELL |  | allLC |  |  |  |  |
| GUO |  | allLC |  |  |  |  |
| HAENSZ |  | all except alveolar | sq+undiff | adeno |  | small |
| HAMMO2 |  | allLC |  |  |  |  |
| HAMMON |  | allLC | not ad | adeno |  |  |
| HANSEN |  | allLC |  |  |  |  |
| HEGMAN |  | allLC | sq; KI | adeno | large | small |
| HEIN |  | allLC |  |  |  |  |
| HENNEK |  | allLC |  |  |  |  |
| HINDS |  | allLC | sq+sm | adeno | large |  |
| HIRAY2 |  | allLC |  |  |  |  |
| HIRAYA |  | allLC |  |  |  |  |
| HITOSU |  | allLC |  |  |  |  |
| HOLE |  | allLC |  |  |  |  |
| HOROWI |  | allLC |  |  |  |  |
| HORWIT |  | allLC |  |  |  |  |
| HU |  | allLC |  |  |  |  |
| HU2 |  | allLC |  |  |  |  |
| HUANG |  | allLC |  |  |  |  |
| HUMBLE |  | allLC; all except alveolar |  |  |  |  |
| ISHIMA | AKIBA | allLC | sq | adeno |  | small |
| JAHN | BOFFET | allLC | sq | adeno |  | small |
| JAIN |  | allLC | sq; KI | adeno | large | small |
| JARUP |  | allLC |  |  |  |  |
| JARVHO |  | allLC |  |  |  |  |
| JEDRYC |  | allLC | sq; KI | adeno |  | small |
| JIANG |  | allLC | sq | adeno |  |  |
| JOLY |  | allLC | sq | adeno |  | small |
| JUSSAW |  | allLC | KI | KII |  |  |
| KAISE2 |  | allLC |  |  |  |  |
| KAISER |  | allLC |  |  |  |  |
| KANELL |  | allLC |  |  |  |  |
| KATSOU |  | allLC | KI; not ad | adeno |  | small |
| KAUFMA |  | allLC |  |  |  |  |
| KELLER |  | allLC |  |  |  |  |
| KHUDER |  | allLC | sq; KI | adeno; KII | large | small |
| KIHARA |  | allLC | sq; KI | adeno; KII | large | small |
| KINLEN |  | allLC |  |  |  |  |
| KJUUS |  | allLC |  |  |  |  |
| KNEKT |  | allLC |  |  |  |  |
| KO |  | allLC |  |  |  |  |
| KOHLME |  | allLC |  |  |  |  |
| KOO |  | allLC | sq; sq+sm | adeno; ad+la | large | small |
| KOULUM |  | allLC |  |  |  |  |
| KREUZE | BOFFET | allLC |  |  |  |  |
| KREYBE |  | allLC | KI | KII |  |  |
| KUBIK |  | allLC |  |  |  |  |
| LAMTH |  | allLC | sq; KI | adeno; KII | large | small |
| LAMWK |  | allLC | sq; KI | adeno | large | small |
| LAMWK2 |  | sq+sm+la+ad | sq; KI | adeno | large | small |
| LANGE |  | allLC |  |  |  |  |
| LAURIL |  | allLC |  |  |  |  |
| LAUSSM |  | allLC |  |  |  |  |
| LEI |  | allLC |  |  |  |  |
| LEMARC |  | allLC |  |  |  |  |
| LETOUR |  | allLC |  |  |  |  |
| LEVIN |  | allLC |  |  |  |  |
| LIAW |  | allLC |  |  |  |  |
| LICKIN |  | allLC |  |  |  |  |
| LIDDEL |  | allLC |  |  |  |  |
| LIU |  | allLC |  |  |  |  |
| LIU2 |  | allLC |  |  |  |  |
| LIU3 |  | allLC |  |  |  |  |
| LIU4 |  | allLC |  |  |  |  |
| LIU5 |  | allLC |  |  |  |  |
| LOMBA2 |  | allLC | sq+undiff | not sq,undiff |  |  |
| LOMBAR |  | allLC |  |  |  |  |
| LUBIN | XIANGZ | allLC | KI | KII |  |  |
| LUBIN2 |  | allLC | sq; KI | adeno |  | small |
| LUO |  | allLC | sq | adeno |  |  |
| MACLEN |  | allLC |  |  |  |  |
| MAGNUS |  | allLC |  |  |  |  |
| MARSH |  | allLC |  |  |  |  |
| MARSH2 |  | allLC |  |  |  |  |
| MARTIS |  | allLC |  |  |  |  |
| MASTRA |  | allLC |  |  |  |  |
| MATOS |  | allLC | sq | adeno |  | small |
| MATSUD |  | allLC | sq | adeno |  |  |
| MCCONN |  | allLC |  |  |  |  |
| MCDUFF |  | allLC |  |  |  |  |
| MCLAUG |  | allLC |  |  |  |  |
| MIGRAN |  | allLC |  |  |  |  |
| MILLER |  | allLC |  |  |  |  |
| MILLS |  | allLC |  |  |  |  |
| MOLLO |  | allLC | not ad | adeno |  |  |
| MRFIT |  | allLC |  |  |  |  |
| MRFITR | MRFIT | allLC |  |  |  |  |
| MURATA |  | allLC |  |  |  |  |
| MZILEN |  | allLC |  |  |  |  |
| NAM |  | allLC |  |  |  |  |
| NOTAN2 |  | allLC |  |  |  |  |
| NOTANI |  | allLC |  |  |  |  |
| NOU |  | allLC | sq; KI | adeno | large | small |
| ODRISC |  | allLC |  |  |  |  |
| ORMOS |  | allLC | sq; KI | adeno; KII | large | small |
| OSANN |  | allLC | sq | adeno |  | small |
| OSANN2 | KAISER | allLC | KI | KII |  |  |
| PARKIN |  | allLC |  |  |  |  |
| PASTOR |  | allLC |  |  |  |  |
| PAWLEG |  | allLC |  |  |  |  |
| PERNU |  | allLC |  |  |  |  |
| PERSH2 |  | allLC |  |  |  |  |
| PETO |  | allLC |  |  |  |  |
| PEZZO2 |  | allLC |  |  |  |  |
| PEZZOT |  | allLC | sq | adeno |  | small |
| PIKE |  | allLC |  |  |  |  |
| PISANI | LUBIN2 | allLC |  |  |  |  |
| POFFIJ |  | allLC |  |  |  |  |
| POLEDN |  | allLC |  |  |  |  |
| PRESCO | HEIN, LANGE | allLC | sq |  |  | small |
| QIAO | XIANGZ | allLC |  |  |  |  |
| QIAO2 |  | allLC |  |  |  |  |
| RACHTA |  | allLC |  |  |  |  |
| RADZIK |  | allLC |  |  |  |  |
| RANDIG |  | allLC |  |  |  |  |
| REN |  | allLC |  |  |  |  |
| RESTRE |  | allLC |  |  |  |  |
| RIMING |  | allLC |  |  |  |  |
| RONCO |  | allLC |  |  |  |  |
| ROOTS | CASCOR | allLC |  |  |  |  |
| ROTHSC |  | allLC |  |  |  |  |
| SAARIK |  | allLC | sq | adeno |  |  |
| SADOWS |  | allLC |  |  |  |  |
| SANKAR |  | allLC |  |  |  |  |
| SCHWAR |  | allLC | sq | adeno |  |  |
| SEGI |  | allLC |  |  |  |  |
| SEGI2 |  | allLC | sq | adeno |  |  |
| SEOW |  | sq+sm+la+ad | sq; KI | adeno | large | small |
| SHAW |  | allLC |  |  |  |  |
| SHIMIZ |  | allLC | sq; KI | adeno | large | small |
| SIEMIA |  | allLC | sq | adeno |  | small |
| SIMARA |  | allLC |  |  |  |  |
| SITAS |  | allLC |  |  |  |  |
| SOBUE |  | allLC; sq+sm+la+ad | sq; KI | adeno | large | small |
| SOBUE2 |  | sq+sm+la+ad | sq; KI | adeno | large | small |
| SPEIZE |  | allLC |  |  |  |  |
| SPITZ |  | allLC |  |  |  |  |
| STASZE |  | allLC | sq | adeno |  | small |
| STAYNE |  | allLC | sq | adeno |  | small |
| STOCKS |  | allLC |  |  |  |  |
| STOCKW |  | allLC |  |  |  |  |
| STUCKE |  | allLC |  |  |  |  |
| SUN |  | allLC |  |  |  |  |
| SUZUK2 |  | allLC | sq | adeno |  |  |
| SUZUKI |  |  |  | adeno |  |  |
| SVENSS |  | allLC | sq | adeno |  | small |
| TANG |  | non small |  |  |  |  |
| TANG2 | BENSHL, HOLE | allLC |  |  |  |  |
| TAO |  | allLC |  |  |  |  |
| TENKAN |  | allLC |  |  |  |  |
| TIZZAN |  | allLC | sq+undiff | adeno |  | small |
| TOKARS |  | allLC | sq | adeno |  |  |
| TOUSEY |  | allLC |  |  |  |  |
| TSUGAN |  |  | sq | adeno |  |  |
| TULINI |  | allLC |  |  |  |  |
| TVERDA |  | allLC |  |  |  |  |
| ULMER |  | allLC |  |  |  |  |
| VEIERO | TVERDA | allLC |  |  |  |  |
| VUTUC | LUBIN2 | allLC | KI | KII |  |  |
| WAKAI |  | allLC | sq | adeno |  | small |
| WALD |  | allLC |  |  |  |  |
| WANG |  | allLC | sq | adeno |  |  |
| WANG2 |  | allLC |  |  |  |  |
| WANG3 |  | allLC |  |  |  |  |
| WANG4 |  | allLC |  |  |  |  |
| WARSIN |  | allLC |  |  |  |  |
| WATSON |  | allLC |  |  |  |  |
| WICKLU |  | allLC |  |  |  |  |
| WIGLE |  | allLC |  |  |  |  |
| WILKIN |  | allLC |  |  |  |  |
| WU |  | sq+ad | sq | adeno |  | small |
| WU2 |  |  |  | adeno |  | small |
| WUNSCH |  | allLC |  |  |  |  |
| WUWILL |  | allLC | sq; sq+sm | adeno |  | small |
| WYNDE2 |  | allLC | KI | KII |  |  |
| WYNDE3 |  | allLC | KI | KII |  |  |
| WYNDE4 |  | allLC | not ad | adeno |  |  |
| WYNDE5 | WYNDE6 | allLC | KI | KII |  |  |
| WYNDE6 |  | allLC; sq+sm+ad; sq+ad | sq; KI | adeno; KII | large | small |
| WYNDE7 | WYNDE6 | allLC | KI | KII |  |  |
| WYNDE8 | WYNDE6 | allLC | sq; KI | adeno | large | small |
| WYNDER |  | allLC |  |  |  |  |
| XIANGZ |  | allLC |  |  |  |  |
| XU |  | allLC | sq+sm |  |  | small |
| XU2 |  | allLC |  |  |  |  |
| XU3 |  | allLC | KI | KII |  |  |
| XU4 |  | allLC |  |  |  |  |
| YAMAGU |  | allLC |  |  |  |  |
| YONG |  | allLC |  |  |  |  |
| YUAN |  | allLC |  |  |  |  |
| ZHANG |  | allLC |  |  |  |  |
| ZHENG |  | allLC | sq | adeno |  | small |
| ZHOU |  | allLC | sq; KI | adeno | large | small |

^a^ At the start of the project, RRs were also entered for some other histological types or combinations, but this was discontinued and they are not shown in this table.

^b^ Includes outcomes which were the nearest possible to the named outcome. Where more than one outcome definition is listed in a column, they may be relevant to RRs with differing exposure or strata definitions. See Additional file 1: Methods , section *Identifying which RRs to enter*.

Abbreviations : sq = squamous cell carcinoma, sm = small cell carcinoma, ad or adeno = adenocarcinoma, KI = Kreyberg I, la = large cell carcinoma, KII = Kreyberg II, undiff = undifferentiated

### Table 4 Smoking results available from each study

| **REF** | **Principal study (if subsidiary)** | **Major indices ^a^** | | | | | | | | | |  | **Cigarette type indices** | | |  | **Dose-related indices** | | | | | | | |
| --- | --- | --- | --- | --- | --- | --- | --- | --- | --- | --- | --- | --- | --- | --- | --- | --- | --- | --- | --- | --- | --- | --- | --- | --- |
|  |  | **Ever** | **Current** | **Ex** | **Current vs non** | **Any/ Cig/ Cig only^b^** | **Any Product** | **Cigarettes^c^** | **Cigarettes only** | **Pipe/ Cigar^d^** | **Mixed^e^** |  | **MC v HR** | **F v P** | **Menthol v non-** |  | **Amount** | **Age start** | **Duration** | **Yrs quit (v never)** | **Yrs quit (v current)** | **Tar** | **Butt length** | **Fraction smoked** |
| ABELIN |  | yes |  |  |  | yes | yes | yes | yes |  | yes |  |  |  |  |  |  |  |  |  |  |  |  |  |
| ABRAHA |  | yes |  |  |  | yes | yes |  |  |  |  |  |  |  |  |  |  |  |  |  |  |  |  |  |
| AGUDO |  | yes | yes | yes | yes | yes |  |  | yes |  |  |  |  | yes |  |  | yes | yes | yes |  |  |  |  |  |
| AKIBA |  | yes | yes | yes | yes | yes |  | yes |  |  |  |  |  |  |  |  | yes |  | yes |  |  |  |  |  |
| ALDERS |  | yes | yes |  |  | yes | yes | yes | yes |  | yes |  | yes | yes |  |  | yes | yes |  | yes | yes | yes |  |  |
| AMANDU |  | yes | yes | yes | yes | yes |  | yes |  |  |  |  |  |  |  |  |  |  | yes |  |  |  |  |  |
| AMES |  | yes | yes | yes | yes | yes | yes |  |  |  |  |  |  |  |  |  |  |  | yes |  |  |  |  |  |
| ANDERS |  | yes | yes | yes | yes | yes |  | yes |  |  |  |  |  |  |  |  |  |  |  |  |  |  |  |  |
| ARCHER |  | yes | yes | yes | yes | yes |  | yes |  |  |  |  |  |  |  |  | yes |  |  |  |  |  |  |  |
| ARMADA |  | yes | yes | yes | yes | yes | yes | yes | yes |  | yes |  |  | yes |  |  | yes | yes | yes | yes | yes |  |  |  |
| AUSTIN |  | yes | yes | yes | yes | yes |  | yes |  |  |  |  |  |  |  |  |  |  |  |  |  |  |  |  |
| AUVINE |  | yes |  |  |  | yes |  | yes |  |  |  |  |  |  |  |  | yes | yes | yes | yes |  |  |  |  |
| AXELSO |  | yes |  |  |  | yes | yes |  |  |  |  |  |  |  |  |  |  |  |  |  |  |  |  |  |
| AXELSS |  | yes | yes | yes | yes | yes | yes |  |  |  |  |  |  |  |  |  | yes |  | yes |  |  |  |  |  |
| BAND |  | yes |  |  |  | yes |  |  | yes |  |  |  |  |  |  |  |  |  |  |  |  |  |  |  |
| BARBON |  | yes | yes | yes | yes | yes | yes |  |  |  |  |  |  |  |  |  | yes | yes | yes | yes | yes |  |  |  |
| BECHER |  | yes | yes | yes | yes | yes | yes | yes |  |  |  |  |  | yes |  |  |  |  |  | yes | yes |  |  |  |
| BENHAM | LUBIN2 | yes | yes | yes |  | yes |  | yes | yes |  |  |  | yes | yes |  |  | yes | yes | yes | yes | yes |  |  |  |
| BENSHL |  | yes | yes | yes | yes | yes | yes | yes |  |  |  |  |  |  |  |  | yes |  | yes | yes |  | yes |  |  |
| BERRIN | LUBIN2 |  |  |  |  |  |  |  |  |  |  |  |  | yes |  |  |  |  |  |  |  |  |  |  |
| BEST |  | yes | yes | yes |  | yes | yes | yes | yes |  | yes |  |  |  |  |  | yes |  | yes |  |  |  |  |  |
| BLOHMK |  | yes | yes | yes | yes | yes | yes |  |  |  |  |  |  |  |  |  |  |  |  |  |  |  |  |  |
| BLOT1 |  |  |  |  |  |  |  |  |  |  |  |  |  |  |  |  | yes |  |  | yes |  |  |  |  |
| BLOT3 |  |  |  |  |  |  |  |  |  |  |  |  |  |  |  |  | yes |  |  |  |  |  |  |  |
| BLOT4 |  | yes |  |  |  | yes |  | yes |  |  |  |  |  |  |  |  |  |  |  |  |  |  |  |  |
| BOFFET |  | yes | yes | yes |  | yes | yes | yes | yes |  | yes |  |  |  |  |  | yes | yes | yes | yes | yes |  |  |  |
| BOUCHA |  |  |  |  |  |  |  |  |  |  |  |  |  |  |  |  |  |  | yes |  |  |  |  |  |
| BOUCOT |  | yes | yes | yes |  | yes | yes | yes | yes |  | yes |  |  |  |  |  | yes |  | yes |  |  |  |  |  |
| BRESLO |  | yes |  |  |  | yes | yes | yes | yes | yes | yes |  |  |  |  |  |  | yes |  |  |  |  |  |  |
| BRETT |  | yes | yes | yes | yes | yes |  | yes |  |  |  |  |  |  |  |  | yes |  |  |  |  |  |  |  |
| BROCKM |  | yes |  |  |  | yes |  | yes |  |  |  |  |  |  |  |  |  |  |  |  |  |  |  |  |
| BROSS |  | yes | yes | yes | yes | yes | yes | yes |  |  |  |  |  | yes |  |  | yes |  | yes | yes | yes |  |  |  |
| BROWN1 |  | yes |  |  |  | yes |  | yes |  |  |  |  |  |  |  |  |  |  |  |  |  |  |  |  |
| BROWN2 |  | yes | yes | yes |  | yes |  | yes |  |  |  |  |  |  |  |  | yes |  |  |  |  |  |  |  |
| BUELL |  |  |  |  |  |  |  |  |  |  |  |  |  |  |  |  | yes |  |  |  |  |  |  |  |
| BUFFLE |  | yes | yes | yes | yes | yes | yes | yes |  |  |  |  |  | yes |  |  | yes | yes | yes |  |  |  |  |  |
| BYERS1 | BROSS, GRAHAM | yes |  |  |  | yes |  | yes |  |  |  |  |  |  |  |  |  |  |  |  |  |  |  |  |
| BYERS2 |  |  |  |  | yes |  |  |  |  |  |  |  |  |  |  |  |  |  |  |  |  |  |  |  |
| CARPEN |  | yes | yes | yes | yes | yes |  | yes |  |  |  |  |  |  | yes |  |  |  |  | yes | yes |  |  |  |
| CASCO2 |  | yes |  |  |  | yes | yes |  |  |  |  |  |  |  |  |  |  |  |  |  |  |  |  |  |
| CASCOR |  | yes |  |  |  | yes | yes |  |  |  |  |  |  |  |  |  |  |  |  |  |  |  |  |  |
| CEDERL |  | yes | yes | yes | yes | yes | yes | yes | yes | yes |  |  |  |  |  |  | yes | yes | yes | yes | yes |  |  |  |
| CHAN |  | yes |  |  |  | yes | yes | yes |  |  |  |  | yes |  |  |  |  |  |  |  |  |  |  |  |
| CHANG |  | yes | yes | yes | yes | yes |  | yes |  |  |  |  |  |  |  |  | yes |  |  |  |  |  |  |  |
| CHATZI |  | yes |  |  |  | yes | yes |  |  |  |  |  |  |  |  |  | yes |  |  |  |  |  |  |  |
| CHEN |  | yes |  |  |  | yes |  | yes |  |  |  |  |  |  |  |  | yes |  | yes |  |  |  |  |  |
| CHEN2 |  | yes |  |  |  | yes | yes |  |  |  |  |  |  |  |  |  | yes | yes | yes |  |  |  |  |  |
| CHEN3 |  | yes |  |  |  | yes | yes |  |  |  |  |  |  |  |  |  |  |  |  |  |  |  |  |  |
| CHIAZZ |  | yes |  |  |  | yes |  | yes |  |  |  |  |  |  |  |  |  | yes |  |  |  |  |  |  |
| CHOI |  | yes | yes | yes | yes | yes |  | yes |  |  |  |  |  | yes |  |  | yes | yes | yes | yes | yes |  |  | yes |
| CHOW |  | yes | yes | yes | yes | yes | yes | yes | yes | yes | yes |  |  |  |  |  | yes |  |  |  |  |  |  |  |
| CHYOU |  | yes | yes | yes | yes | yes |  | yes |  |  |  |  |  |  |  |  |  |  |  | yes | yes |  |  |  |
| COMSTO |  | yes | yes | yes | yes | yes | yes | yes |  |  |  |  |  |  |  |  | yes |  |  |  |  |  |  |  |
| COOKSO |  | yes |  |  |  | yes | yes | yes |  |  |  |  |  |  |  |  | yes |  |  |  |  |  |  |  |
| CORREA |  | yes | yes | yes | yes | yes |  | yes |  |  |  |  |  | yes |  |  | yes | yes | yes | yes | yes |  |  |  |
| CPSI |  | yes | yes | yes | yes | yes |  | yes | yes |  | yes |  |  |  |  |  | yes | yes | yes | yes | yes | yes |  |  |
| CPSII |  | yes | yes | yes | yes | yes |  | yes | yes |  |  |  |  | yes |  |  | yes |  | yes | yes | yes | yes |  |  |
| DAMBER |  | yes | yes | yes |  | yes | yes | yes | yes |  |  |  |  |  |  |  | yes | yes | yes | yes | yes |  |  |  |
| DARBY |  | yes | yes | yes | yes | yes | yes | yes |  | yes |  |  |  |  |  |  | yes |  |  | yes | yes |  |  |  |
| DAVEYS |  | yes |  |  |  | yes | yes |  |  |  |  |  |  |  |  |  | yes |  |  |  |  |  |  |  |
| DEAN |  | yes |  |  |  | yes | yes | yes | yes |  | yes |  |  |  |  |  | yes |  |  |  |  |  |  |  |
| DEAN2 |  | yes | yes | yes | yes | yes | yes | yes | yes |  | yes |  |  | yes |  |  | yes |  | yes |  |  |  |  |  |
| DEAN3 |  | yes | yes | yes | yes | yes | yes | yes | yes | yes |  |  |  | yes |  |  | yes | yes |  | yes | yes |  |  |  |
| DEKLER |  | yes | yes | yes | yes | yes | yes | yes |  |  |  |  |  |  |  |  | yes |  |  |  |  |  |  |  |
| DESTE2 |  | yes | yes | yes | yes | yes | yes | yes |  |  |  |  | yes | yes |  |  |  |  |  |  |  |  |  |  |
| DESTEF |  | yes | yes | yes | yes | yes | yes | yes |  |  |  |  | yes | yes |  |  | yes |  | yes | yes | yes |  |  |  |
| DOCKER |  | yes | yes | yes | yes | yes |  | yes |  |  |  |  |  |  |  |  |  |  |  |  |  |  |  |  |
| DOLL |  | yes | yes | yes | yes | yes | yes | yes | yes |  |  |  |  | yes |  |  | yes | yes | yes | yes | yes |  |  |  |
| DOLL2 |  | yes | yes | yes | yes | yes | yes | yes | yes | yes | yes |  |  |  |  |  | yes |  |  | yes | yes |  |  |  |
| DORANT |  | yes | yes | yes | yes | yes | yes | yes |  |  |  |  |  |  |  |  | yes |  |  |  |  |  |  |  |
| DORGAN |  | yes | yes | yes | yes | yes | yes | yes |  |  |  |  |  |  |  |  | yes |  | yes | yes | yes | yes |  |  |
| DORN |  | yes | yes | yes | yes | yes | yes | yes | yes | yes | yes |  |  |  |  |  | yes | yes |  | yes | yes |  |  |  |
| DOSEME |  | yes |  |  |  | yes |  | yes |  |  |  |  |  |  |  |  | yes |  | yes |  |  |  |  |  |
| DROSTE |  | yes | yes | yes | yes | yes | yes |  |  |  |  |  |  |  |  |  |  |  |  |  |  |  |  |  |
| DU |  | yes |  |  |  | yes | yes |  |  |  |  |  |  |  |  |  |  |  |  |  |  |  |  |  |
| DUNN |  | yes |  |  |  | yes |  | yes |  |  |  |  |  |  |  |  | yes |  |  |  |  |  |  |  |
| EBELIN |  | yes |  |  |  | yes | yes |  |  |  |  |  |  |  |  |  | yes |  |  |  |  |  |  |  |
| ENGELA |  | yes | yes | yes | yes | yes | yes | yes | yes | yes |  |  | yes | yes |  |  | yes | yes |  |  |  |  |  |  |
| ENSTRO |  |  | yes |  |  | yes |  |  | yes |  |  |  |  |  |  |  | yes |  |  |  |  |  |  |  |
| ESAKI |  | yes |  |  |  | yes |  | yes |  |  |  |  |  |  |  |  | yes |  |  |  |  |  |  |  |
| FAN |  | yes |  |  |  | yes |  | yes |  |  |  |  |  |  |  |  | yes |  | yes |  |  |  |  |  |
| GAO |  | yes | yes | yes | yes | yes |  | yes |  |  |  |  |  |  |  |  | yes | yes | yes | yes | yes |  |  |  |
| GAO2 |  | yes | yes | yes | yes | yes |  | yes |  |  |  |  |  |  |  |  | yes | yes |  | yes | yes |  |  |  |
| GARCIA |  | yes | yes | yes | yes | yes |  | yes |  |  |  |  |  |  |  |  |  |  |  | yes | yes |  |  |  |
| GARDIN |  | yes | yes | yes | yes | yes | yes |  | yes | yes |  |  | yes |  |  |  |  |  |  |  |  |  |  |  |
| GARSHI |  | yes | yes | yes | yes | yes | yes |  |  |  |  |  |  |  |  |  | yes |  | yes | yes | yes |  |  |  |
| GENG |  | yes |  |  |  | yes |  | yes |  |  |  |  |  |  |  |  |  | yes | yes |  |  |  |  |  |
| GER |  | yes |  |  |  | yes | yes |  |  |  |  |  |  |  |  |  | yes |  | yes |  |  |  |  |  |
| GILLIS | LUBIN2 | yes | yes | yes |  | yes |  | yes |  |  |  |  |  |  |  |  | yes |  |  | yes | yes | yes |  |  |
| GODLEY |  | yes |  |  |  | yes |  | yes |  |  |  |  |  |  |  |  |  |  |  |  |  |  |  |  |
| GOLLED |  | yes |  |  |  | yes |  | yes | yes |  | yes |  |  |  |  |  | yes |  |  |  |  |  |  |  |
| GOODMA |  | yes | yes | yes |  | yes |  | yes |  |  |  |  |  |  |  |  |  |  |  |  |  |  |  |  |
| GRAHAM |  | yes | yes | yes | yes | yes | yes | yes | yes | yes | yes |  |  |  |  |  |  |  | yes | yes | yes |  |  |  |
| GREGOR |  | yes | yes | yes | yes | yes | yes | yes |  |  |  |  |  |  |  |  |  |  |  |  |  |  |  |  |
| GSELL |  | yes |  |  |  | yes | yes | yes |  |  |  |  |  |  |  |  | yes |  |  |  |  |  |  |  |
| GUO |  |  |  |  |  |  |  |  |  |  |  |  |  |  |  |  |  | yes |  |  |  |  |  |  |
| HAENSZ |  | yes | yes | yes | yes | yes | yes | yes |  |  |  |  |  |  |  |  | yes | yes | yes |  |  |  |  |  |
| HAMMO2 |  | yes | yes | yes | yes | yes | yes | yes |  |  |  |  |  |  |  |  | yes |  |  | yes | yes |  |  |  |
| HAMMON |  | yes | yes | yes |  | yes | yes | yes | yes |  | yes |  |  |  |  |  | yes |  | yes | yes | yes |  |  |  |
| HANSEN |  | yes |  |  |  | yes | yes |  |  |  |  |  |  |  |  |  | yes |  |  |  |  |  |  |  |
| HEGMAN |  | yes |  |  |  | yes | yes |  |  |  |  |  |  |  |  |  |  | yes |  |  |  |  |  |  |
| HEIN |  | yes | yes | yes | yes | yes | yes |  | yes | yes |  |  |  |  |  |  |  |  |  |  |  |  |  |  |
| HENNEK |  | yes | yes | yes | yes | yes | yes |  |  |  |  |  |  |  |  |  |  |  |  |  |  |  |  |  |
| HINDS |  | yes |  |  |  | yes | yes |  |  |  |  |  |  |  |  |  |  |  |  |  |  |  |  |  |
| HIRAY2 |  |  |  |  | yes |  |  |  |  | yes | yes |  |  |  |  |  | yes |  |  |  |  |  |  |  |
| HIRAYA |  | yes | yes | yes | yes | yes |  | yes |  |  |  |  |  | yes |  |  | yes | yes |  | yes | yes |  |  |  |
| HITOSU |  | yes | yes | yes | yes | yes | yes |  |  |  |  |  |  |  |  |  | yes |  |  |  |  |  |  |  |
| HOLE |  | yes | yes | yes | yes | yes | yes | yes |  | yes |  |  |  |  |  |  | yes | yes |  |  |  |  |  |  |
| HOROWI |  | yes |  |  |  | yes |  | yes |  |  |  |  |  |  |  |  |  |  |  |  |  |  |  |  |
| HORWIT |  | yes |  |  |  | yes |  | yes |  |  |  |  |  |  |  |  |  |  |  |  |  |  |  |  |
| HU |  | yes |  |  |  | yes |  | yes |  |  |  |  | yes |  |  |  | yes | yes | yes |  |  |  |  |  |
| HU2 |  | yes |  |  |  | yes |  | yes |  |  |  |  | yes |  |  |  | yes | yes | yes |  |  |  |  |  |
| HUANG |  | yes |  |  |  | yes | yes |  |  |  |  |  |  |  |  |  |  |  |  |  |  |  |  |  |
| HUMBLE |  | yes | yes | yes | yes | yes |  | yes |  |  |  |  |  | yes |  |  | yes |  | yes |  | yes |  |  |  |
| ISHIMA | AKIBA | yes |  |  |  | yes | yes |  |  |  |  |  |  |  |  |  |  |  |  |  |  |  |  |  |
| JAHN | BOFFET | yes | yes | yes | yes | yes | yes | yes |  |  |  |  |  |  |  |  |  |  |  | yes | yes |  |  |  |
| JAIN |  | yes | yes | yes | yes | yes |  | yes |  |  |  |  |  |  |  |  |  |  |  | yes | yes |  |  |  |
| JARUP |  | yes |  |  |  | yes | yes |  |  |  |  |  |  |  |  |  | yes |  |  |  |  |  |  |  |
| JARVHO |  | yes | yes | yes | yes | yes | yes |  |  |  |  |  |  |  |  |  |  |  |  |  |  |  |  |  |
| JEDRYC |  | yes | yes | yes | yes | yes |  | yes |  |  |  |  |  |  |  |  | yes | yes | yes | yes | yes |  |  |  |
| JIANG |  | yes |  |  |  | yes | yes |  |  |  |  |  |  |  |  |  |  |  |  |  |  |  |  |  |
| JOLY |  | yes | yes | yes | yes | yes | yes | yes | yes | yes | yes |  |  | yes |  |  | yes | yes | yes | yes | yes |  | yes |  |
| JUSSAW |  | yes |  |  |  | yes | yes |  | yes |  |  |  | yes |  |  |  | yes |  | yes |  |  |  |  |  |
| KAISE2 |  | yes | yes | yes | yes | yes |  |  | yes |  |  |  |  | yes | yes |  | yes |  | yes | yes | yes | yes |  | yes |
| KAISER |  | yes | yes | yes | yes | yes |  | yes |  |  |  |  |  |  |  |  | yes |  |  |  |  |  |  |  |
| KANELL |  |  | yes |  |  | yes | yes |  |  |  |  |  |  |  |  |  | yes |  |  |  |  |  |  |  |
| KATSOU |  | yes | yes | yes | yes | yes | yes |  |  |  |  |  |  |  |  |  | yes |  | yes |  |  |  |  |  |
| KAUFMA |  | yes | yes | yes | yes | yes |  | yes |  |  |  |  |  |  |  |  | yes |  |  |  |  | yes |  |  |
| KELLER |  | yes | yes | yes | yes | yes | yes |  |  |  |  |  |  |  |  |  |  |  |  |  |  |  |  |  |
| KHUDER |  | yes | yes | yes | yes | yes |  | yes |  |  |  |  |  | yes |  |  | yes | yes | yes | yes | yes |  |  |  |
| KIHARA |  | yes | yes | yes | yes | yes | yes |  |  |  |  |  |  |  |  |  |  |  |  |  |  |  |  |  |
| KINLEN |  | yes | yes | yes | yes | yes | yes | yes |  |  |  |  |  |  |  |  | yes |  |  |  |  |  |  |  |
| KJUUS |  | yes | yes | yes | yes | yes | yes |  | yes |  |  |  |  |  |  |  |  |  |  |  |  |  |  |  |
| KNEKT |  | yes | yes | yes | yes | yes | yes | yes |  | yes |  |  |  |  |  |  | yes |  |  |  |  |  |  |  |
| KO |  | yes |  |  |  | yes |  | yes |  |  |  |  |  |  |  |  |  |  |  |  |  |  |  |  |
| KOHLME |  | yes |  |  |  | yes | yes |  |  |  |  |  |  |  |  |  |  |  |  |  |  |  |  |  |
| KOO |  | yes | yes | yes | yes | yes | yes |  |  |  |  |  |  |  |  |  | yes |  |  |  |  |  |  | yes |
| KOULUM |  | yes |  |  |  | yes | yes |  | yes |  |  |  |  |  |  |  | yes | yes |  |  |  |  |  |  |
| KREUZE | BOFFET | yes | yes | yes | yes | yes | yes | yes |  | yes |  |  |  |  |  |  | yes |  | yes |  |  |  |  |  |
| KREYBE |  | yes |  |  |  | yes | yes |  |  |  |  |  |  |  |  |  | yes |  |  |  |  |  |  |  |
| KUBIK |  | yes | yes | yes | yes | yes | yes | yes |  |  |  |  |  |  |  |  |  |  |  |  |  |  |  |  |
| LAMTH |  | yes |  |  |  | yes | yes |  |  |  |  |  |  |  |  |  | yes |  |  |  |  |  |  |  |
| LAMWK |  | yes |  |  |  | yes | yes |  |  |  |  |  |  |  |  |  |  |  |  |  |  |  |  |  |
| LAMWK2 |  | yes |  |  |  | yes | yes |  |  |  |  |  |  |  |  |  |  |  |  |  |  |  |  |  |
| LANGE |  | yes | yes | yes | yes | yes | yes |  | yes |  |  |  |  | yes |  |  |  |  | yes |  |  |  |  |  |
| LAURIL |  |  |  |  |  |  |  |  |  |  |  |  |  |  |  |  |  |  |  |  |  |  |  |  |
| LAUSSM |  | yes |  |  |  | yes | yes |  |  |  |  |  |  |  |  |  | yes |  |  | yes |  |  |  |  |
| LEI |  | yes |  |  |  | yes | yes |  |  |  |  |  |  |  |  |  |  |  |  |  |  |  |  |  |
| LEMARC |  | yes | yes | yes | yes | yes | yes |  |  |  |  |  |  |  |  |  |  |  |  |  |  |  |  |  |
| LETOUR |  | yes |  |  |  | yes |  | yes |  |  |  |  |  |  |  |  | yes | yes | yes |  |  |  |  |  |
| LEVIN |  | yes |  |  |  | yes | yes | yes |  |  |  |  |  |  |  |  |  |  | yes |  |  |  |  |  |
| LIAW |  |  | yes |  |  | yes | yes |  |  |  |  |  |  |  |  |  | yes | yes | yes |  |  |  |  |  |
| LICKIN |  |  |  |  | yes |  |  |  |  |  |  |  |  |  |  |  | yes |  |  |  |  |  |  |  |
| LIDDEL |  | yes | yes | yes | yes | yes |  | yes |  |  |  |  |  |  |  |  | yes |  |  |  |  |  |  |  |
| LIU |  | yes |  |  |  | yes | yes |  |  |  |  |  |  |  |  |  |  |  |  |  |  |  |  |  |
| LIU2 |  | yes |  |  |  | yes | yes |  |  |  |  |  |  |  |  |  | yes |  |  |  |  |  |  |  |
| LIU3 |  | yes |  |  |  | yes | yes |  |  |  |  |  |  |  |  |  | yes | yes | yes |  |  |  |  |  |
| LIU4 |  | yes |  |  |  | yes | yes |  | yes |  |  |  |  |  |  |  | yes | yes |  |  |  |  |  |  |
| LIU5 |  | yes |  |  |  | yes | yes |  |  |  |  |  |  |  |  |  | yes | yes | yes |  |  |  |  |  |
| LOMBA2 |  | yes |  |  |  | yes |  | yes |  |  |  |  |  |  |  |  |  |  |  |  |  |  |  |  |
| LOMBAR |  | yes | yes | yes | yes | yes | yes | yes | yes |  | yes |  |  |  |  |  |  |  |  |  |  |  |  |  |
| LUBIN | XIANGZ | yes | yes | yes |  | yes | yes | yes | yes |  |  |  |  |  |  |  | yes | yes | yes | yes | yes |  |  |  |
| LUBIN2 |  | yes | yes | yes | yes | yes | yes | yes | yes | yes | yes |  |  | yes |  |  | yes | yes | yes | yes | yes | yes |  | yes |
| LUO |  | yes |  |  |  | yes |  | yes |  |  |  |  |  |  |  |  | yes | yes | yes | yes | yes |  |  |  |
| MACLEN |  | yes | yes | yes | yes | yes |  | yes |  |  |  |  | yes | yes |  |  | yes |  |  |  |  |  |  |  |
| MAGNUS |  | yes |  |  |  | yes | yes |  |  |  |  |  |  |  |  |  |  |  |  |  |  |  |  |  |
| MARSH |  | yes |  |  |  | yes | yes | yes |  |  |  |  |  |  |  |  |  |  |  |  |  |  |  |  |
| MARSH2 |  | yes |  |  |  | yes | yes | yes |  |  |  |  |  |  |  |  |  |  |  |  |  |  |  |  |
| MARTIS |  | yes |  |  |  | yes |  | yes |  |  |  |  |  |  |  |  | yes |  |  |  |  |  |  |  |
| MASTRA |  | yes |  |  |  | yes | yes |  |  |  |  |  |  |  |  |  | yes |  |  |  |  |  |  |  |
| MATOS |  | yes | yes | yes | yes | yes |  | yes |  |  |  |  |  | yes |  |  | yes | yes | yes | yes | yes |  |  |  |
| MATSUD |  | yes |  |  |  | yes |  | yes |  |  |  |  |  |  |  |  | yes |  |  |  |  |  |  |  |
| MCCONN |  | yes |  |  |  | yes | yes | yes | yes |  | yes |  |  |  |  |  | yes |  | yes |  |  |  |  |  |
| MCDUFF |  | yes |  |  |  | yes |  | yes |  |  |  |  |  |  |  |  |  | yes | yes | yes |  |  |  |  |
| MCLAUG |  | yes |  |  |  | yes | yes |  |  |  |  |  |  |  |  |  |  |  |  |  |  |  |  |  |
| MIGRAN |  | yes | yes | yes | yes | yes | yes | yes | yes | yes | yes |  | yes | yes |  |  | yes | yes |  |  |  |  |  |  |
| MILLER |  | yes |  |  |  | yes |  | yes |  |  |  |  |  |  |  |  |  |  |  |  |  |  |  |  |
| MILLS |  | yes |  |  |  | yes | yes |  | yes |  |  |  |  |  |  |  |  |  |  |  |  |  |  |  |
| MOLLO |  |  |  |  |  |  |  |  |  |  |  |  |  |  |  |  | yes |  |  |  |  |  |  |  |
| MRFIT |  |  |  |  | yes |  |  |  |  |  |  |  |  |  |  |  | yes |  |  |  |  |  |  |  |
| MRFITR | MRFIT | yes | yes | yes | yes | yes |  | yes |  |  |  |  |  | yes |  |  | yes | yes |  |  |  | yes |  |  |
| MURATA |  |  |  |  | yes |  |  |  |  |  |  |  |  |  |  |  | yes |  |  |  |  |  |  |  |
| MZILEN |  |  |  |  |  |  |  |  |  |  |  |  |  |  |  |  | yes |  |  |  |  |  |  |  |
| NAM |  | yes | yes | yes | yes | yes |  | yes |  |  |  |  |  |  |  |  | yes |  |  |  |  |  |  |  |
| NOTAN2 |  | yes |  |  |  | yes | yes |  | yes |  |  |  | yes |  |  |  | yes |  | yes |  |  |  |  |  |
| NOTANI |  |  |  |  | yes |  |  |  |  |  |  |  |  |  |  |  |  |  |  |  |  |  |  |  |
| NOU |  | yes |  |  |  | yes | yes |  |  |  |  |  |  |  |  |  |  |  |  |  |  |  |  |  |
| ODRISC |  | yes | yes | yes | yes | yes | yes |  |  |  |  |  |  |  |  |  |  |  |  |  |  |  |  |  |
| ORMOS |  | yes |  |  |  | yes |  | yes |  |  |  |  |  |  |  |  | yes |  |  |  |  |  |  |  |
| OSANN |  | yes | yes | yes | yes | yes |  | yes |  |  |  |  |  |  |  |  | yes |  |  |  |  |  |  |  |
| OSANN2 | KAISER | yes | yes | yes | yes | yes |  | yes |  |  |  |  |  |  |  |  | yes |  | yes |  |  |  |  |  |
| PARKIN |  | yes | yes | yes | yes | yes | yes | yes |  |  |  |  |  |  |  |  | yes |  |  |  |  |  |  |  |
| PASTOR |  | yes |  |  |  | yes | yes |  |  |  |  |  |  |  |  |  | yes |  |  |  |  |  |  |  |
| PAWLEG |  | yes |  |  |  | yes | yes |  |  |  |  |  |  |  |  |  |  |  |  |  |  |  |  |  |
| PERNU |  | yes |  |  |  | yes | yes |  | yes |  |  |  | yes |  |  |  | yes | yes |  |  |  |  |  |  |
| PERSH2 |  | yes | yes | yes | yes | yes | yes |  |  |  |  |  |  |  |  |  | yes |  |  |  |  |  |  |  |
| PETO |  | yes | yes | yes | yes | yes | yes |  |  |  |  |  |  |  |  |  | yes |  |  |  |  |  |  |  |
| PEZZO2 |  | yes | yes | yes | yes | yes |  | yes |  |  |  |  |  |  |  |  | yes |  | yes | yes | yes |  |  |  |
| PEZZOT |  | yes | yes | yes | yes | yes |  |  | yes |  |  |  |  | yes |  |  | yes | yes | yes | yes | yes |  |  |  |
| PIKE |  | yes |  |  |  | yes | yes |  |  |  |  |  |  |  |  |  | yes |  |  |  |  |  |  |  |
| PISANI | LUBIN2 | yes | yes | yes | yes | yes | yes |  |  |  |  |  |  |  |  |  |  |  | yes |  |  |  |  |  |
| POFFIJ |  | yes |  |  |  | yes | yes |  |  |  |  |  |  |  |  |  |  |  |  |  |  |  |  |  |
| POLEDN |  | yes |  |  |  | yes |  | yes |  |  |  |  |  |  |  |  | yes |  |  |  |  |  |  |  |
| PRESCO | HEIN, LANGE | yes |  | yes |  | yes | yes |  |  |  |  |  |  |  |  |  | yes |  | yes |  |  |  |  |  |
| QIAO | XIANGZ | yes |  |  |  | yes | yes | yes | yes |  |  |  |  |  |  |  |  |  |  | yes | yes |  |  |  |
| QIAO2 |  | yes | yes | yes | yes | yes | yes | yes |  |  |  |  |  |  |  |  |  | yes | yes |  |  |  |  |  |
| RACHTA |  | yes | yes | yes | yes | yes |  | yes |  |  |  |  |  |  |  |  | yes | yes | yes |  |  |  |  |  |
| RADZIK |  | yes |  |  |  | yes | yes |  |  |  |  |  |  |  |  |  |  |  |  |  |  |  |  |  |
| RANDIG |  | yes |  |  |  | yes | yes | yes |  |  |  |  |  |  |  |  | yes |  |  |  |  |  |  |  |
| REN |  | yes |  |  |  | yes | yes |  |  |  |  |  |  |  |  |  |  |  |  |  |  |  |  |  |
| RESTRE |  |  | yes | yes |  | yes |  | yes |  |  |  |  |  |  |  |  | yes |  | yes |  |  |  |  | yes |
| RIMING |  |  |  |  |  |  |  |  |  |  |  |  |  | yes |  |  |  |  |  |  |  |  |  |  |
| RONCO |  | yes |  |  |  | yes | yes |  | yes |  |  |  |  |  |  |  |  |  |  |  |  |  |  |  |
| ROOTS | CASCOR | yes |  |  |  | yes | yes |  |  |  |  |  |  |  |  |  |  |  |  |  |  |  |  |  |
| ROTHSC |  | yes |  |  |  | yes | yes |  |  |  |  |  |  |  |  |  |  |  |  |  |  |  |  |  |
| SAARIK |  |  |  |  | yes |  |  |  |  |  |  |  |  |  |  |  |  |  |  |  |  |  |  |  |
| SADOWS |  | yes |  |  |  | yes | yes | yes | yes |  | yes |  |  |  |  |  | yes |  | yes |  |  |  |  |  |
| SANKAR |  | yes |  |  |  | yes | yes |  |  |  |  |  |  |  |  |  |  |  |  |  |  |  |  |  |
| SCHWAR |  | yes | yes | yes | yes | yes |  | yes |  |  |  |  |  |  |  |  |  |  |  |  |  |  |  |  |
| SEGI |  | yes |  |  |  | yes | yes |  |  |  |  |  |  |  |  |  |  |  |  |  |  |  |  |  |
| SEGI2 |  |  | yes |  | yes | yes |  | yes |  |  |  |  |  | yes |  |  | yes | yes |  |  |  |  |  |  |
| SEOW |  | yes |  |  |  | yes |  | yes |  |  |  |  |  |  |  |  |  |  |  |  |  |  |  |  |
| SHAW |  | yes | yes | yes | yes | yes | yes |  |  |  |  |  |  |  |  |  | yes |  |  |  |  |  |  |  |
| SHIMIZ |  |  |  |  | yes |  |  |  |  |  |  |  |  |  |  |  |  |  |  |  |  |  |  |  |
| SIEMIA |  | yes |  |  |  | yes |  | yes |  |  |  |  |  |  |  |  | yes |  |  |  |  |  |  |  |
| SIMARA |  | yes |  |  |  | yes |  | yes |  |  |  |  |  |  |  |  |  |  |  |  |  |  |  |  |
| SITAS |  |  |  |  | yes |  |  |  |  |  |  |  |  |  |  |  |  |  |  |  |  |  |  |  |
| SOBUE |  | yes | yes | yes | yes | yes |  | yes |  |  |  |  |  | yes |  |  | yes | yes | yes | yes | yes |  |  | yes |
| SOBUE2 |  |  | yes |  |  | yes |  | yes |  |  |  |  |  |  |  |  |  |  |  |  |  |  |  |  |
| SPEIZE |  | yes | yes | yes | yes | yes |  | yes |  |  |  |  |  |  |  |  | yes | yes |  | yes | yes | yes |  |  |
| SPITZ |  | yes | yes | yes | yes | yes |  | yes |  |  |  |  |  |  |  |  | yes | yes | yes | yes |  |  |  |  |
| STASZE |  | yes |  |  |  | yes | yes | yes | yes |  | yes |  |  |  |  |  |  | yes | yes |  |  |  |  |  |
| STAYNE |  | yes |  |  | yes | yes | yes |  |  |  |  |  |  |  |  |  |  |  |  |  |  |  |  |  |
| STOCKS |  | yes |  |  |  | yes | yes | yes |  |  |  |  |  |  |  |  | yes |  |  |  |  |  |  |  |
| STOCKW |  | yes | yes | yes | yes | yes | yes | yes |  |  |  |  |  |  |  |  | yes |  |  |  |  |  |  |  |
| STUCKE |  | yes | yes | yes | yes | yes | yes |  |  |  |  |  |  |  |  |  |  |  |  |  |  |  |  |  |
| SUN |  | yes |  |  |  | yes | yes |  |  |  |  |  |  |  |  |  |  |  |  |  |  |  |  |  |
| SUZUK2 |  | yes | yes | yes | yes | yes | yes |  | yes |  |  |  | yes |  |  |  |  | yes |  | yes | yes |  |  |  |
| SUZUKI |  | yes | yes | yes | yes | yes |  | yes |  |  |  |  |  |  |  |  |  |  |  |  |  |  |  |  |
| SVENSS |  | yes | yes | yes | yes | yes | yes |  |  |  |  |  |  |  |  |  | yes | yes |  | yes | yes |  |  |  |
| TANG |  | yes | yes | yes | yes | yes |  | yes |  |  |  |  |  |  |  |  |  |  |  |  |  |  |  |  |
| TANG2 | BENSHL, HOLE, WALD |  | yes |  | yes | yes |  |  | yes |  |  |  |  | yes |  |  |  |  |  |  |  |  |  |  |
| TAO |  | yes |  |  |  | yes | yes |  |  |  |  |  |  |  |  |  |  |  |  |  |  |  |  |  |
| TENKAN |  | yes | yes | yes | yes | yes | yes |  |  |  |  |  |  |  |  |  | yes |  |  |  |  |  |  |  |
| TIZZAN |  | yes | yes | yes | yes | yes | yes |  | yes |  |  |  |  |  |  |  | yes | yes | yes |  |  |  | yes |  |
| TOKARS |  | yes | yes | yes | yes | yes | yes |  |  |  |  |  |  |  |  |  |  |  |  |  |  |  |  |  |
| TOUSEY |  | yes | yes | yes | yes | yes | yes | yes |  |  |  |  |  |  |  |  |  |  |  |  |  |  |  |  |
| TSUGAN |  | yes | yes | yes | yes | yes | yes |  |  |  |  |  |  |  |  |  | yes |  |  |  |  |  |  |  |
| TULINI |  | yes | yes | yes | yes | yes | yes | yes |  | yes |  |  |  |  |  |  | yes |  |  |  |  |  |  |  |
| TVERDA |  | yes | yes | yes | yes | yes |  | yes | yes | yes | yes |  |  |  |  |  | yes |  |  | yes | yes |  |  |  |
| ULMER |  | yes |  |  |  | yes | yes |  |  |  |  |  |  |  |  |  |  |  |  |  |  |  |  |  |
| VEIERO | TVERDA | yes | yes | yes | yes | yes | yes |  | yes |  |  |  |  |  |  |  |  |  |  |  |  |  |  |  |
| VUTUC | LUBIN2 | yes | yes | yes | yes | yes |  | yes |  |  |  |  |  |  |  |  |  |  |  |  |  | yes |  |  |
| WAKAI |  | yes | yes | yes | yes | yes | yes | yes |  |  |  |  |  | yes |  |  | yes | yes |  | yes | yes |  |  | yes |
| WALD |  |  | yes |  |  | yes |  |  | yes |  |  |  |  |  |  |  |  |  |  |  |  |  |  |  |
| WANG |  | yes |  |  |  | yes | yes |  |  |  |  |  |  |  |  |  |  |  |  |  |  |  |  |  |
| WANG2 |  | yes | yes | yes | yes | yes |  | yes |  |  |  |  |  |  |  |  | yes |  | yes | yes | yes |  |  |  |
| WANG3 |  | yes |  |  |  | yes | yes |  |  |  |  |  |  |  |  |  |  |  |  |  |  |  |  |  |
| WANG4 |  | yes |  |  |  | yes | yes |  |  |  |  |  |  |  |  |  |  |  |  |  |  |  |  |  |
| WARSIN |  |  |  |  | yes |  |  |  |  |  |  |  |  |  |  |  | yes |  |  |  |  |  |  |  |
| WATSON |  |  |  |  | yes |  |  |  |  |  |  |  |  |  |  |  | yes |  | yes |  |  |  |  |  |
| WICKLU |  | yes |  |  |  | yes |  | yes |  |  |  |  |  |  |  |  |  |  |  |  |  |  |  |  |
| WIGLE |  | yes | yes | yes | yes | yes | yes | yes | yes | yes |  |  |  |  |  |  |  |  |  | yes | yes |  |  |  |
| WILKIN |  | yes |  |  |  | yes |  | yes |  |  |  |  |  |  |  |  |  |  |  |  |  |  |  |  |
| WU |  | yes | yes | yes | yes | yes | yes |  |  |  |  |  |  |  |  |  | yes | yes |  |  |  |  |  |  |
| WU2 |  |  | yes |  |  | yes | yes |  |  |  |  |  |  |  |  |  |  |  | yes | yes |  |  |  |  |
| WUNSCH |  | yes | yes | yes | yes | yes |  | yes |  |  |  |  |  |  |  |  |  |  |  |  |  |  |  |  |
| WUWILL |  | yes |  |  |  | yes |  | yes |  |  |  |  |  |  |  |  | yes |  | yes |  |  |  |  |  |
| WYNDE2 |  | yes |  |  |  | yes | yes | yes |  |  |  |  |  |  |  |  | yes |  | yes |  |  |  |  | yes |
| WYNDE3 |  | yes | yes | yes | yes | yes | yes | yes |  |  |  |  |  | yes |  |  | yes |  |  | yes | yes |  |  |  |
| WYNDE4 |  | yes |  |  |  | yes | yes | yes |  |  |  |  |  |  |  |  | yes |  |  |  |  |  |  |  |
| WYNDE5 | WYNDE6 |  | yes |  | yes | yes |  | yes |  |  |  |  |  | yes |  |  |  |  |  |  |  |  |  |  |
| WYNDE6 |  | yes | yes | yes | yes | yes | yes | yes |  |  |  |  |  | yes |  |  | yes | yes | yes | yes | yes | yes |  | yes |
| WYNDE7 | WYNDE6 | yes | yes | yes |  | yes | yes |  | yes |  |  |  |  |  |  |  | yes | yes | yes |  |  |  |  |  |
| WYNDE8 | WYNDE6 |  |  |  |  |  |  |  |  |  |  |  |  |  | yes |  |  |  | yes |  |  |  |  |  |
| WYNDER |  |  |  |  | yes |  |  |  |  | yes | yes |  |  |  |  |  | yes |  |  |  |  |  |  |  |
| XIANGZ |  | yes |  |  |  | yes | yes | yes | yes |  | yes |  |  |  |  |  |  |  |  |  |  |  |  |  |
| XU |  | yes |  |  |  | yes | yes |  |  |  |  |  |  |  |  |  |  |  | yes |  |  |  |  |  |
| XU2 |  | yes |  |  |  | yes | yes |  |  |  |  |  |  |  |  |  |  |  |  |  |  |  |  |  |
| XU3 |  | yes |  |  |  | yes | yes |  |  |  |  |  |  |  |  |  | yes |  |  |  |  |  |  |  |
| XU4 |  | yes |  |  |  | yes | yes |  |  |  |  |  |  |  |  |  |  |  |  |  |  |  |  |  |
| YAMAGU |  | yes | yes | yes | yes | yes | yes |  |  |  |  |  |  |  |  |  | yes |  |  |  |  |  |  |  |
| YONG |  | yes | yes | yes | yes | yes |  | yes |  |  |  |  |  |  |  |  |  |  |  |  |  |  |  |  |
| YUAN |  | yes |  |  |  | yes |  | yes |  |  |  |  |  |  |  |  | yes | yes |  |  |  |  |  |  |
| ZHANG |  | yes |  |  |  | yes | yes |  |  |  |  |  |  |  |  |  |  | yes | yes |  |  |  |  |  |
| ZHENG |  | yes |  |  |  | yes |  | yes |  |  |  |  |  |  |  |  | yes | yes | yes |  |  |  |  |  |
| ZHOU |  | yes |  |  |  | yes | yes |  |  |  |  |  |  |  |  |  | yes |  | yes |  |  |  |  |  |

^a^ vs never smokers or never cigarette smokers (or near equivalent) except where specified as vs non (=non-current smoker or non-current cigarette smoker)

^b^ Any product, cigarettes (irrespective of pipe/cigar) or cigarettes only

^c^ irrespective of pipe/cigar

^d^ pipe and/or cigar, pipe only or cigar only – not cigarettes

^e^ pipe/cigar **and** cigarettes
