# Supplementary material for: Systematic review with meta-analysis of the epidemiological evidence in the 1900s relating smoking to lung cancer
Source: BMC Cancer. 2012 Sep 3;12:385. doi: 10.1186/1471-2407-12-385 (PMC3505152; doi:10.1186/1471-2407-12-385)
Supplement: Additional file 3 — RRs. [file 1471-2407-12-385-S3.doc]

**Systematic review with meta-analysis of the epidemiological evidence in the 1900s relating smoking to lung cancer**

Peter N Lee, Barbara A Forey, and Katharine J Coombs

**Additional File 3 : RRs**

Contents

[Table 1 Numbers of relative risks per study 3](#__RefHeading___Toc311560957)

[Table 2 Relative risks with apparent errors 4](#__RefHeading___Toc311560958)

[Table 2A RR non-central in CI 4](#__RefHeading___Toc311560959)

[Table 2B Number of subjects less than minimum implied by CI 6](#__RefHeading___Toc311560960)

[Table 2C Number of cases less than minimum implied by CI 6](#__RefHeading___Toc311560961)

[Table 2D Number in any cell of 2x2 table less than minimum implied by CI 7](#__RefHeading___Toc311560962)

[Table 2E RRs omitted because of apparent erroneous adjustment 8](#__RefHeading___Toc311560963)

[Table 2F Other 9](#__RefHeading___Toc311560964)

References – see main paper

## Table 1 Numbers of relative risks per study

|  |  | **Study Type** | | | |
| --- | --- | --- | --- | --- | --- |
|  |  | **Principal** | | **Subsid** | **Total** |
|  |  | **CC** | **Prosp** |  |  |
|  |  |  |  |  |  |
| **Number of relative risks (overall)** |  |  |  |  |  |
|  | **1-2** | 36 | 0 | 1 | 37 |
|  | **3-4** | 20 | 7 | 2 | 29 |
|  | **5-6** | 16 | 4 | 0 | 20 |
|  | **7-8** | 10 | 3 | 1 | 14 |
|  | **9-10** | 3 | 5 | 2 | 10 |
|  | **11-20** | 28 | 11 | 3 | 42 |
|  | **21-50** | 48 | 11 | 3 | 62 |
|  | **51-100** | 23 | 5 | 4 | 32 |
|  | **>100** | 25 | 12 | 4 | 41 |
|  | Total | 209 | 58 | 20 | 287 |
|  | Median | 16.00 | 19.00 | 39.00 | 17.00 |
|  | Range | 1:1029 | 3:833 | 1:248 | 1:1029 |
|  |  |  |  |  |  |
| **Number of relative risks for All LC^a^** |  |  |  |  |  |
|  | **0** | 5 | 0 | 1 | 6 |
|  | **1-2** | 49 | 1 | 2 | 52 |
|  | **3-4** | 18 | 7 | 1 | 26 |
|  | **5-6** | 14 | 5 | 0 | 19 |
|  | **7-8** | 10 | 2 | 1 | 13 |
|  | **9-10** | 5 | 4 | 3 | 12 |
|  | **11-20** | 30 | 11 | 3 | 44 |
|  | **21-50** | 46 | 11 | 4 | 61 |
|  | **51-100** | 20 | 6 | 3 | 29 |
|  | **>100** | 12 | 11 | 2 | 25 |
|  | Total^b^ | 204 | 58 | 19 | 281 |
|  | Median^b^ | 12.00 | 19.00 | 18.00 | 14.00 |
|  | Range^b^ | 1:505 | 2:828 | 1:157 | 1:828 |
|  |  |  |  |  |  |
| **Number of relative risks for Squamous^c^** |  |  |  |  |  |
|  | **0** | 136 | 47 | 8 | 191 |
|  | **1-2** | 15 | 7 | 3 | 25 |
|  | **3-4** | 14 | 0 | 2 | 16 |
|  | **5-6** | 2 | 0 | 0 | 2 |
|  | **7-8** | 5 | 0 | 0 | 5 |
|  | **9-10** | 5 | 1 | 1 | 7 |
|  | **11-20** | 11 | 1 | 2 | 14 |
|  | **21-50** | 11 | 2 | 3 | 16 |
|  | **51-100** | 6 | 0 | 1 | 7 |
|  | **>100** | 4 | 0 | 0 | 4 |
|  | Total^b^ | 73 | 11 | 12 | 96 |
|  | Median^b^ | 9.00 | 2.00 | 12.00 | 8.50 |
|  | Range^b^ | 1:262 | 1:49 | 1:78 | 1:262 |
|  |  |  |  |  |  |
| **Number of relative risks for Adeno^d^** |  |  |  |  |  |
|  | **0** | 134 | 47 | 8 | 189 |
|  | **1-2** | 20 | 7 | 3 | 30 |
|  | **3-4** | 13 | 0 | 2 | 15 |
|  | **5-6** | 2 | 0 | 0 | 2 |
|  | **7-8** | 5 | 1 | 0 | 6 |
|  | **9-10** | 5 | 1 | 2 | 8 |
|  | **11-20** | 12 | 0 | 2 | 14 |
|  | **21-50** | 10 | 2 | 3 | 15 |
|  | **51-100** | 6 | 0 | 0 | 6 |
|  | **>100** | 2 | 0 | 0 | 2 |
|  | Total^b^ | 75 | 11 | 12 | 98 |
|  | Median^b^ | 8.00 | 2.00 | 12.00 | 8.00 |
|  | Range^b^ | 0:262 | 1:49 | 1:78 | 0:262 |

^a^ Lung cancer type includes at least squamous and adeno

^b^ Excluding zero category

^c^ Lung cancer type includes squamous but not adeno

^d^ Lung cancer type includes adeno but not squamous

## Table 2 Relative risks with apparent errors

## Table 2A RR non-central in CI

This listing shows the statistic (CENTR) calculated as (RR*RR)/(RRL*RRU), which should have the value 1.0. This listing shows those outside the range 0.9 to 1.2, arbitrarily chosen to exclude those where the apparent non-centrality is probably due to rounding error. However when RRL is very low and given only to 1 or 2 decimal places, values of CENTR may well fall outside that range, so entry in this list is not necessarily indicative of an error of real importance.

Some of the fields defining the RR are shown, although not all due to lack of space. Footnotes are given after Table 2E.

| **REF** | **NRR^a^** | **LCtype^b^** | **Sex** | **Smoking status** | **Adj^c^** | **Dose measure** | **CENTR** | **RR^d^** | **RRL^d^** | **RRU^d^** | **DERIVE^e^** |
| --- | --- | --- | --- | --- | --- | --- | --- | --- | --- | --- | --- |
| BARBON | 574 | sq | m | ever | 1 | age started | 0.90 | 9.40 | 4.20 | 23.40 | original |
| BARBON | 840 | all | m | ex | 3 | yrs quit (v cur) | 0.88 | 1.00 | 0.60 | 1.90 | original |
| BARBON | 852 | sq | m | ex | 3 | yrs quit (v cur) | 0.89 | 0.40 | 0.20 | 0.90 | original |
| BARBON | 853 | sq | m | ex | 3 | yrs quit (v cur) | 0.50 | 0.10 | 0.05 | 0.40 | original |
| BARBON | 87 | la | m | ever | 1 | amount | 0.90 | 1.10 | 0.30 | 4.50 | original |
| BARBON | 896 | KI | m | ex | 3 | yrs quit (v cur) | 0.89 | 0.16 | 0.07 | 0.41 | derived |
| BENHAM | 584 | all | f | ever | 3 | age started | 0.85 | 8.16 | 3.99 | 19.64 | original |
| BENHAM | 635 | all | m | ever | 1 | duration | 0.63 | 1.04 | 0.63 | 2.74 | original |
| BOFFET | 553 | all | m | ex | 2 | yrs quit (v cur) | 0.89 | 0.11 | 0.04 | 0.34 | derived |
| BOUCOT | 124 | all | m | 2 | 2 | amount | 0.75 | 54.09 | 3.86 | 1013.32 | derived |
| BOUCOT | 128 | all | m | 2 | 1 | amount | 0.81 | 39.49 | 2.69 | 714.44 | derived |
| BOUCOT | 131 | all | m | 2 | 1 | amount | 0.64 | 8.48 | 0.62 | 180.86 | derived |
| BOUCOT | 142 | sq | m | 2 | 2 | amount | 0.85 | 21.41 | 1.40 | 385.13 | derived |
| BOUCOT | 145 | la | m | 2 | 2 | amount | 0.74 | 4.35 | 0.27 | 94.69 | derived |
| BOUCOT | 148 | ad | m | 2 | 2 | amount | 0.72 | 8.22 | 0.56 | 166.83 | derived |
| BOUCOT | 151 | sm | m | 2 | 2 | amount | 0.76 | 3.06 | 0.18 | 68.23 | derived |
| CPSI | 917 | all | m | ex | 1 | yrs quit (v cur) | 0.89 | 0.04 | 0.02 | 0.09 | derived |
| CPSI | 923 | all | m | ex | 2 | yrs quit (v cur) | 0.89 | 0.04 | 0.02 | 0.09 | derived |
| CPSII | 702 | all | m | ex | 2 | yrs quit (v cur) | 0.90 | 0.11 | 0.09 | 0.15 | derived |
| DAMBER | 31 | la | m | ever | 1 |  | 0.80 | 13.80 | 5.20 | 45.60 | original |
| DAMBER | 34 | other | m | ever | 1 |  | 0.82 | 7.30 | 2.00 | 32.50 | original |
| DAMBER | 523 | all | m | ex | 1 | yrs quit (v nev) | 0.88 | 2.60 | 1.70 | 4.50 | derived |
| DAMBER | 534 | la | m | ever | 1 | duration | 0.84 | 10.50 | 3.40 | 38.40 | original |
| DAMBER | 535 | la | m | ever | 1 | duration | 0.85 | 19.60 | 6.50 | 69.30 | original |
| DAMBER | 536 | la | m | ever | 1 | duration | 0.86 | 25.10 | 8.20 | 89.00 | original |
| DESTEF | 548 | all | m | ex | 4 | yrs quit (v cur) | 0.89 | 0.50 | 0.20 | 1.40 | original |
| DESTEF | 550 | all | m | ex | 4 | yrs quit (v cur) | 0.83 | 0.50 | 0.20 | 1.50 | original |
| DESTEF | 565 | all | m | ex | 4 | yrs quit (v cur) | 0.83 | 0.50 | 0.30 | 1.00 | original |
| DOLL2 | 119 | all | m | cur | 1 |  | 1.26 | 3.63 | 2.06 | 5.08 | derived |
| DOLL2 | 537 | all | m | ex | 2 | yrs quit (v cur) | 0.89 | 0.28 | 0.06 | 1.46 | derived |
| DOSEME | 516 | othAd | m | ever | 2 | duration | 0.85 | 0.80 | 0.30 | 2.50 | original |
| DOSEME | 517 | othAd | m | ever | 2 | duration | 0.66 | 3.30 | 2.20 | 7.50 | original |
| ENGELA | 193 | ad | m | 2 | 7 |  | 0.75 | 0.30 | 0.10 | 1.20 | original |
| ENGELA | 197 | la | m | 2 | 7 |  | 0.69 | 0.30 | 0.10 | 1.30 | original |
| ENGELA | 31 | all | m | 2 | 7 | amount | 0.88 | 1.40 | 0.60 | 3.70 | original |
| GRAHAM | 544 | all | m | ex | 1 | yrs quit (v cur) | 0.82 | 0.07 | 0.04 | 0.15 | derived |
| HANSEN | 1 | all | m | ever | 2 | amount | 0.84 | 1.37 | 0.63 | 3.54 | derived |
| HANSEN | 2 | all | m | ever | 2 | amount | 0.89 | 2.90 | 1.14 | 8.27 | derived |
| HANSEN | 3 | all | m | ever | 2 |  | 0.84 | 1.53 | 0.71 | 3.91 | derived |
| HIRAYA | 91 | all | m | 2 | 1 | amount | 0.90 | 4.10 | 2.20 | 8.50 | original |
| HIRAYA | 92 | all | m | 2 | 1 | amount | 0.89 | 4.60 | 2.50 | 9.50 | original |
| HIRAYA | 94 | all | m | cur | 1 | amount | 1.67 | 3.80 | 0.60 | 14.40 | original |
| HUMBLE | 12 | all | f | 2 | 1 | amount | 0.83 | 36.90 | 7.60 | 217.10 | original |
| HUMBLE | 4 | all | m | ex | 1 |  | 0.80 | 8.00 | 1.90 | 42.20 | original |
| HUMBLE | 5 | all | m | 2 | 1 | amount | 0.81 | 11.60 | 2.70 | 61.50 | original |
| HUMBLE | 6 | all | m | 2 | 1 | amount | 0.83 | 26.10 | 5.60 | 146.60 | original |
| ISHIMA | 6 | sq | c | ever | 5 |  | 0.15 | 21.00 | 3.38 | 868.40 | derived |
| ISHIMA | 7 | la | c | ever | 5 |  | 0.70 | 3.00 | 0.75 | 17.23 | derived |
| ISHIMA | 8 | ad | c | ever | 5 |  | 0.15 | 15.00 | 2.31 | 631.48 | derived |
| JAHN | 591 | la | m | ex | 3 | yrs quit (v cur) | 0.67 | 0.02 | 0.01 | 0.06 | derived |
| JAHN | 682 | ad | m | ex | 3 | yrs quit (v cur) | 0.73 | 0.04 | 0.02 | 0.11 | derived |
| JAHN | 684 | ad | m | ex | 3 | yrs quit (v cur) | 0.67 | 0.02 | 0.01 | 0.06 | derived |
| JAHN | 727 | other | m | ex | 3 | yrs quit (v cur) | 0.78 | 0.05 | 0.02 | 0.16 | derived |
| JAHN | 729 | other | m | ex | 3 | yrs quit (v cur) | 0.89 | 0.05 | 0.02 | 0.14 | derived |
| JAIN | 43 | sq | f | ever | 2 |  | 0.53 | 25.50 | 7.93 | 156.00 | original |
| JAIN | 44 | la | f | ever | 2 |  | 0.26 | 48.00 | 10.50 | 849.00 | original |
| JAIN | 45 | sm | f | ever | 2 |  | 0.56 | 6.50 | 1.80 | 41.60 | original |
| JAIN | 46 | all | m | ever | 2 |  | 0.89 | 8.30 | 4.53 | 17.00 | original |
| JAIN | 47 | ad | m | ever | 2 |  | 0.55 | 8.00 | 2.28 | 50.60 | original |
| JAIN | 48 | sq | m | ever | 2 |  | 0.53 | 18.00 | 5.50 | 111.00 | original |
| JAIN | 49 | la | m | ever | 2 |  | 0.69 | 6.33 | 2.16 | 27.00 | original |
| JAIN | 50 | sm | m | ever | 2 |  | 0.31 | 6.00 | 1.02 | 113.00 | original |
| JAIN | 52 | all | m | cur | 2 |  | 0.90 | 12.40 | 6.45 | 26.60 | original |
| JARUP | 4 | all | m | ever | 2 | amount | 0.87 | 6.90 | 2.40 | 22.70 | original |
| JARUP | 5 | all | m | ever | 2 | amount | 0.86 | 8.00 | 3.00 | 24.80 | original |
| JEDRYC | 49 | all | f | ever | 4 | amount | 0.71 | 2.38 | 1.17 | 6.86 | original |
| JUSSAW | 33 | all | m | ever | 2 | amount | 0.61 | 56.67 | 19.07 | 277.52 | derived |
| JUSSAW | 34 | all | m | ever | 2 | amount | 0.85 | 5.57 | 2.46 | 14.76 | derived |
| JUSSAW | 35 | all | m | ever | 2 | amount | 0.71 | 14.00 | 5.17 | 53.16 | derived |
| KAISE2 | 49 | all | f | cur | 1 |  | 1.28 | 23.50 | 2.80 | 154.00 | derived |
| KAISE2 | 73 | all | m | cur | 0 |  | 0.87 | 2.20 | 0.50 | 11.10 | original |
| KAISE2 | 77 | all | f | cur | 0 |  | 0.56 | 0.30 | 0.10 | 1.60 | original |
| KAISER | 8 | all | m | cur | 2 | amount | 1.23 | 20.91 | 12.78 | 27.73 | original |
| KREUZE | 25 | all | f | ever | 3 | amount | 1.22 | 5.70 | 1.60 | 16.60 | original |
| KREUZE | 26 | all | f | ever | 3 | amount | 1.37 | 11.80 | 3.50 | 29.00 | original |
| LANGE | 13 | all | f | cur | 1 |  | 0.67 | 4.90 | 3.00 | 12.00 | original |
| LANGE | 16 | all | m | cur | 1 |  | 0.86 | 6.00 | 2.20 | 19.00 | original |
| LANGE | 19 | all | f | cur | 2 |  | 0.88 | 0.70 | 0.40 | 1.40 | original |
| LIAW | 501 | all | c | cur | 2 | duration | 0.87 | 0.90 | 0.30 | 3.10 | original |
| LUBIN2 | 14 | all | m | ex | 2 |  | 0.88 | 1.54 | 0.60 | 4.50 | original |
| LUO | 10 | sq | c | ever | 20 | amount | 1.44 | 1.20 | 0.10 | 10.00 | original |
| LUO | 512 | sq | c | ever | 20 | age started | 0.80 | 3.10 | 0.30 | 40.20 | original |
| LUO | 522 | sq | c | ex | 20 | yrs quit (v nev) | 0.87 | 2.00 | 0.20 | 23.10 | original |
| MATOS | 25 | all | m | ex | 4 |  | 0.89 | 3.33 | 1.25 | 10.00 | derived |
| MATOS | 39 | sq | m | cur | 2 |  | 0.85 | 10.40 | 3.60 | 35.50 | original |
| MATOS | 573 | all | m | ex | 2 | age started | 0.16 | 4.90 | 2.20 | 69.90 | original |
| MATOS | 598 | all | m | ex | 2 | yrs quit (v cur) | 0.75 | 0.30 | 0.20 | 0.60 | original |
| MATOS | 678 | ad | m | ex | 2 | yrs quit (v cur) | 0.64 | 0.30 | 0.20 | 0.70 | original |
| QIAO | 7 | all | m | ever | 1 |  | 1.50 | 0.90 | 0.10 | 5.40 | original |
| RACHTA | 8 | all | f | ex | 1 |  | 1.52 | 3.80 | 1.21 | 7.84 | original |
| SIEMIA | 14 | all | m | ever | 0 | amount | 0.85 | 4.50 | 1.80 | 13.20 | original |
| SIEMIA | 15 | all | m | ever | 0 | amount | 0.86 | 7.90 | 3.00 | 24.10 | original |
| SOBUE | 49 | sm | f | ex | 1 |  | 0.37 | 4.10 | 1.40 | 32.80 | original |
| SOBUE | 635 | la | m | cur | 5 | fraction smoked | 0.88 | 1.00 | 0.60 | 1.90 | original |
| SPEIZE | 1 | all | f | cur | 1 | amount | 1.50 | 2.70 | 0.90 | 5.40 | derived |
| SPEIZE | 513 | all | f | ex | 2 | yrs quit (v cur) | 0.89 | 0.50 | 0.40 | 0.70 | original |
| SPEIZE | 515 | all | f | ex | 2 | yrs quit (v cur) | 0.25 | 0.10 | 0.10 | 0.40 | original |
| SPEIZE | 523 | all | f | ex | 3 | yrs quit (v cur) | 0.33 | 0.10 | 0.10 | 0.30 | original |
| SPEIZE | 524 | all | f | ex | 3 | yrs quit (v cur) | 0.50 | 0.10 | 0.10 | 0.20 | original |
| SUZUK2 | 518 | all | c | ex | 4 | yrs quit (v cur) | 0.89 | 0.50 | 0.20 | 1.40 | original |
| SUZUK2 | 519 | all | c | ex | 4 | yrs quit (v cur) | 0.83 | 0.50 | 0.20 | 1.50 | original |
| SUZUK2 | 520 | all | c | ex | 4 | yrs quit (v cur) | 0.67 | 0.20 | 0.10 | 0.60 | original |
| SUZUK2 | 525 | all | c | ex | 3 | yrs quit (v cur) | 0.89 | 0.50 | 0.20 | 1.40 | original |
| SUZUK2 | 526 | all | c | ex | 3 | yrs quit (v cur) | 0.25 | 0.10 | 0.10 | 0.40 | original |
| SVENSS | 10 | other | f | cur | 1 | amount | 0.88 | 3.60 | 1.10 | 13.40 | original |
| SVENSS | 12 | sq | f | cur | 1 | amount | 0.65 | 36.20 | 12.00 | 168.90 | original |
| SVENSS | 3 | la | f | ex | 1 |  | 0.85 | 9.10 | 1.40 | 69.70 | original |
| SVENSS | 546 | la | f | cur | 1 | age started | 0.86 | 1.30 | 0.40 | 4.90 | original |
| SVENSS | 588 | ad | f | ex | 2 | yrs quit (v cur) | 0.83 | 0.50 | 0.20 | 1.50 | original |
| SVENSS | 589 | other | f | ex | 2 | yrs quit (v cur) | 0.77 | 0.70 | 0.20 | 3.20 | original |
| SVENSS | 7 | sq | f | cur | 1 | amount | 0.71 | 9.70 | 2.90 | 45.90 | original |
| SVENSS | 8 | la | f | cur | 1 | amount | 0.62 | 33.70 | 6.90 | 265.30 | original |
| SVENSS | 9 | ad | f | cur | 1 | amount | 0.83 | 2.20 | 1.00 | 5.80 | original |
| WALD | 4 | all | m | cur | 1 |  | 0.81 | 16.40 | 7.55 | 44.20 | original |
| WYNDE6 | 381 | sm | m | ever | 2 |  | 0.88 | 0.90 | 0.20 | 4.60 | original |
| WYNDE6 | 727 | all | m | ex | 5 | yrs quit (v cur) | 0.80 | 0.20 | 0.10 | 0.50 | original |
| WYNDE6 | 734 | all | f | ex | 5 | yrs quit (v cur) | 0.67 | 0.20 | 0.20 | 0.30 | original |
| WYNDE6 | 831 | KII | m | ex | 2 | yrs quit (v cur) | 0.80 | 0.02 | 0.01 | 0.05 | derived |
| XIANGZ | 16 | all | m | ever | 2 |  | 0.44 | 0.66 | 0.55 | 1.79 | derived |
| ZHANG | 3 | all | f | ever | 7 |  | 0.73 | 3.75 | 1.80 | 10.76 | original |

## Table 2B Number of subjects less than minimum implied by CI

This listing shows RRs which fail the check shown in formula 7 of Lee (1999) [15]. The statistic (R7) is calculated as 245.86 / Q^2^ (where Q=ln (rru/rrl)), and is compared with the total number of subjects in the study. Some minor failures of this check may be attributable to estimation of the CI from crude numbers, as explained in Additional File 1 : Methods (*Derivation of RRs – 6)*. Note also this check is not relevant to prospective studies (see section 3.3 of [15]).

| **REF** | **NRR^a^** | **LCtype^b^** | **Sex** | **Smoking status** | **Adj^c^** | **Dose measure^d^** | **Total subjects** | **RR^e^** | **RRL^e^** | **RRU^e^** | **R7** | **Q** | **DERIVE** |
| --- | --- | --- | --- | --- | --- | --- | --- | --- | --- | --- | --- | --- | --- |
| AMES | 5 | all | m | 2 | 0 |  | 317 | 1.21 | 0.88 | 1.65 | 619.16 | 0.63 | derived |
| COMSTO | 90 | all | c | 2 | 1 |  | 515 | 6.34 | 4.49 | 8.94 | 518.38 | 0.69 | derived |
| LAURIL | 1 | all | m | 2 | 0 | amount | 230 | 2.33 | 1.40 | 3.87 | 239.29 | 1.01 | derived |
| LAURIL | 4 | all | m | 2 | 0 | amount | 230 | 2.30 | 1.43 | 3.69 | 273.90 | 0.95 | derived |
| LIU | 2 | all | c | ever | 2 |  | 458 | 1.92 | 1.40 | 2.64 | 611.07 | 0.63 | original |
| MILLS | 1 | all | m | ever | 1 |  | 874 | 1.27 | 1.01 | 1.61 | 1143.67 | 0.46 | derived |
| MILLS | 3 | all | m | ever | 1 |  | 874 | 1.33 | 1.09 | 1.63 | 1546.01 | 0.40 | derived |
| MURATA | 4 | all | m | 2 | 0 |  | 214 | 3.19 | 1.94 | 5.25 | 248.07 | 1.00 | derived |
| OSANN2 | 1 | all | f | ever | 0 |  | 213 | 5.84 | 3.70 | 9.24 | 293.44 | 0.92 | derived |
| OSANN2 | 2 | all | f | 2 | 0 |  | 213 | 7.57 | 4.69 | 12.21 | 269.25 | 0.96 | derived |
| OSANN2 | 5 | all | f | ever | 0 | amount | 213 | 10.47 | 6.29 | 17.42 | 237.17 | 1.02 | derived |
| OSANN2 | 6 | all | f | 2 | 0 |  | 213 | 6.21 | 4.08 | 9.47 | 345.79 | 0.84 | derived |
| AMES | 501 | all | m | 2 | 2 | duration | 317 | 2.28 | 1.58 | 3.29 | 457.02 | 0.73 | original |
| OSANN2 | 502 | all | f | ever | 0 | duration | 213 | 9.33 | 5.70 | 15.27 | 253.04 | 0.99 | derived |

## Table 2C Number of cases less than minimum implied by CI

This listing shows RRs which fail the check shown in formula 9 of Lee (1999) [15]. The statistic (R9) is calculated as 61.47 / Q^2^ (where Q=ln (rru/rrl)), and is compared with the number of cases. Some minor failures of this check may be attributable to estimation of the CI from crude numbers, as explained in Additional File 1 : Methods (*Derivation of RRs – 6)*. Note that this check has not been modified for prospective studies (St type = p), and should be regarded as only approximate (see formula 16 of [15]).

| **REF** | **NRR** | **LCtype** | **SEX** | **SMKSTA** | **Adj^c^** | **SMK meas^d^** | **RR^e^** | **RRL^e^** | **RRU^e^** | **Total cases** | **R9** | **Q** | **DERIVE** | ST type |
| --- | --- | --- | --- | --- | --- | --- | --- | --- | --- | --- | --- | --- | --- | --- |
| HENNEK | 4 | all | m | 2 | 0 |  | 7.05 | 5.23 | 9.51 | 169 | 171.63 | 0.60 | derived | p |
| HIRAYA | 108 | all | m | 2 | 1 |  | 3.75 | 3.44 | 4.09 | 1917 | 2052.12 | 0.17 | derived | p |
| RIMING | 4 | all | m | 2 | 0 |  | 0.60 | 0.41 | 0.88 | 104 | 104.11 | 0.77 | derived | p |
| XIANGZ | 7 | all | m | ever | 0 |  | 0.32 | 0.28 | 0.36 | 983 | 994.07 | 0.25 | derived | p |

## Table 2D Number in any cell of 2x2 table less than minimum implied by CI

This listing shows RRs which fail the check shown in formula 10 of Lee (1999) [15]. The statistic (R10) is calculated as 15.3664 / Q^2^ (where Q=ln (rru/rrl)), and is compared with each of the exposed and unexposed cases (CA1, CA0) for all RRs, and also with the exposed and unexposed controls/disease-free (CO1,CO0) for unadjusted RRs from case-control studies. Some minor failures of this check may be attributable to estimation of the CI from crude numbers, as explained in Additional File 1 : Methods (*Derivation of RRs – 6)*. Note that this check is relevant only to the number of cases in prospective studies (St type = p) (see section 3.5 of [15].

| **REF** | **NRR** | **LCtype** | **SEX** | **SMKSTA** | **Adj^c^** | **Dose measure^d^** | **CA1** | **CA0** | **CO1** | **CO0** | **RR^e^** | **RRL^e^** | **RRU^e^** | **R10** | **Q** | **DERIVE** | **St Type** |
| --- | --- | --- | --- | --- | --- | --- | --- | --- | --- | --- | --- | --- | --- | --- | --- | --- | --- |
| BENSHL | 502 | all | m | ex | 2 | duration | **5** | 10 | - | - | 1.27 | 0.74 | 2.20 | 12.94 | 1.09 | original | p |
| BEST | 513 | all | m | 2 | 1 | duration | 137 | **1** | - | - | 8.88 | 1.25 | 62.98 | 1.00 | 3.92 | derived | p |
| CEDERL | 522 | all | m | 2 | 1 | age st | 16 | **4** | - | - | 3.19 | 1.20 | 8.47 | 4.02 | 1.95 | derived | p |
| CEDERL | 543 | all | m | ex | 1 | duration | 12 | **1** | - | - | 4.32 | 0.69 | 26.96 | 1.14 | 3.67 | derived | p |
| CPSI | 818 | all | m | ex | 1 | yq v cur | **15** | 844 | - | - | 0.09 | 0.06 | 0.15 | 18.30 | 0.92 | derived | p |
| CPSI | 917 | all | m | ex | 1 | yq v cur | **6** | 36 | - | - | 0.04 | 0.02 | 0.09 | 6.79 | 1.50 | derived | p |
| CPSI | 923 | all | m | ex | 2 | yq v cur | **6** | 36 | - | - | 0.04 | 0.02 | 0.09 | 6.79 | 1.50 | derived | p |
| CPSII | 646 | all | f | ex | 1 | yq v cur | **50** | 530 | - | - | 0.14 | 0.11 | 0.19 | 51.44 | 0.55 | derived | p |
| DEAN3 | 560 | all | f | ex | 1 | yq v cur | **1** | 102 | - | - | 0.20 | 0.03 | 1.42 | 1.03 | 3.86 | derived | c |
| DOLL2 | 534 | all | m | ex | 2 | yq v cur | **2** | 124 | - | - | 0.15 | 0.04 | 0.60 | 2.10 | 2.71 | derived | p |
| DORN | 504 | all | m | ex | 2 | age st | **91** | 325 | - | - | 5.20 | 4.10 | 6.10 | 97.35 | 0.40 | original | p |
| DORN | 553 | all | m | 4 | 1 | yq v nev | 704 | **41** | - | - | 10.29 | 7.72 | 13.71 | 46.59 | 0.57 | original | p |
| DORN | 577 | all | m | ex | 2 | yq v cur | **115** | 2609 | - | - | 0.44 | 0.37 | 0.53 | 118.98 | 0.36 | derived | p |
| DORN | 578 | all | m | ex | 2 | yq v cur | **123** | 2609 | - | - | 0.19 | 0.16 | 0.22 | 151.52 | 0.32 | derived | p |
| DORN | 858 | all | m | ex | 1 | yq v cur | **6** | 379 | - | - | 0.13 | 0.06 | 0.29 | 6.19 | 1.58 | derived | p |
| DORN | 870 | all | m | ex | 1 | yq v cur | **13** | 299 | - | - | 0.22 | 0.13 | 0.38 | 13.36 | 1.07 | derived | p |
| DU | 1 | all | m | ever | 0 |  | 538 | **28** | * | * | 3.53 | 2.44 | 5.11 | 28.12 | 0.74 | original | **c** |
| ENGELA | 546 | all | f | cur | 4 | age st | 18 | **10** | - | - | 10.00 | 5.77 | 17.32 | 12.72 | 1.10 | derived | p |
| HAMMO2 | 511 | all | m | ex | 1 | yq v cur | **11** | 209 | - | - | 0.39 | 0.22 | 0.71 | 11.19 | 1.17 | derived | p |
| JAHN | 587 | la | m | ex | 3 | yq v cur | **2** | 86 | - | - | 0.07 | 0.02 | 0.27 | 2.27 | 2.60 | derived | c |
| JEDRYC | 581 | ad | m | ex | 5 | yq v cur | **9** | 68 | - | - | 1.08 | 0.57 | 2.04 | 9.45 | 1.28 | original | c |
| JEDRYC | 609 | all | m | ever | 5 | age st | 239 | **49** | - | - | 1.30 | 1.00 | 1.68 | 57.09 | 0.52 | original | c |
| KAISE2 | 577 | all | f | ex | 1 | yq v cur | **4** | 50 | - | - | 0.34 | 0.13 | 0.91 | 4.06 | 1.95 | derived | p |
| KHUDER | 529 | all | m | ever | 5 | duration | 443 | **16** | - | - | 2.20 | 1.50 | 3.30 | 24.72 | 0.79 | original | c |
| LUBIN2 | 824 | la | m | ex | 2 | yq v cur | **28** | 826 | - | - | 0.20 | 0.14 | 0.29 | 28.98 | 0.73 | derived | c |
| LUO | 518 | sq | c | ex | 20 | yq v cur | **1** | 33 | - | - | 0.13 | 0.02 | 0.82 | 1.11 | 3.71 | derived | c |
| MRFITR | 506 | all | m | cur | 4 | tar | 48 | **12** | - | - | 0.81 | 0.49 | 1.33 | 15.41 | 1.00 | original | p |
| MRFITR | 507 | all | m | cur | 4 | tar | 35 | **12** | - | - | 1.14 | 0.67 | 1.94 | 13.59 | 1.06 | original | p |
| PEZZOT | 550 | sq | m | ever | 3 | age st | 45 | **10** | - | - | 2.17 | 1.31 | 3.59 | 15.12 | 1.01 | derived | c |
| SPEIZE | 513 | all | f | ex | 2 | yq v cur | **41** | 319 | - | - | 0.50 | 0.40 | 0.70 | 49.07 | 0.56 | original | p |
| SPEIZE | 524 | all | f | ex | 3 | yq v cur | **28** | 391 | - | - | 0.10 | 0.10 | 0.20 | 31.98 | 0.69 | original | p |
| XIANGZ | 8 | all | m | ever | 0 |  | 907 | **25** | 13037 | 974 | 2.71 | 1.83 | 4.01 | 25.00 | 0.78 | derived | p |

## Table 2E RRs omitted because of apparent erroneous adjustment

This listing shows studies for which RRs were omitted because they had a “never smoker” base but were stated to be adjusted for some aspect(s) of smoking – which cannot be a valid analysis.

| BENHAM |
| --- |
| CHOI |
| CORREA |
| GUO |
| JAHN |
| WEIR |

Note that for study CORREA, some results assumed to be validly analysed but described as “adjusted for pack-years”, and for studies ENGELA and MAGNUS some results which were not clearly defined, the results have been retained, with data entry using the most consistent possible assumptions.

## Table 2F Other

This listing shows comments on the second RR database indicating other problems.

Some of the fields defining the RR are shown, although not all due to lack of space.

| **REF** | **NRR^a^** | **LCTYPE^b^** | **SEX** | **SMKSTA** | **AD^c^** | **Dose measure^d^** | **Source** | **RR** | **Comment** |
| --- | --- | --- | --- | --- | --- | --- | --- | --- | --- |
| CHOI | 533 | all | m | ex | 0 | yrs quit (v nev) | Table 3 page 70 and Table 2 page 69 CHOI1989 | 1.54 | Numbers for ex and current do not agree with those in Table 2 page 69 CHOI1989 |
| CHOI | 534 | all | m | ex | 0 | yrs quit (v nev) | Table 3 page 70 and Table 2 page 69 CHOI1989 | 1.27 | Numbers for ex and current do not agree with those in Table 2 page 69 CHOI1989 |
| CHOI | 535 | all | m | ex | 0 | yrs quit (v nev) | Table 3 page 70 and Table 2 page 69 CHOI1989 | 1.22 | Numbers for ex and current do not agree with those in Table 2 page 69 CHOI1989 |
| CHOI | 536 | all | m | ex | 0 | yrs quit (v nev) | Table 3 page 70 and Table 2 page 69 CHOI1989 | 2.85 | Numbers for ex and current do not agree with those in Table 2 page 69 CHOI1989 |
| CHOI | 539 | all | m | ex | 0 | yrs quit (v nev) | Table 3 page 70 CHOI1989 | 0.83 | Numbers for ex and current do not agree with those in Table 2 page 69 CHOI1989 |
| CHOI | 540 | all | m | ex | 0 | yrs quit (v nev) | Table 3 page 70 CHOI1989 | 0.79 | Numbers for ex and current do not agree with those in Table 2 page 69 CHOI1989 |
| CHOI | 541 | all | m | ex | 0 | yrs quit (v nev) | Table 3 page 70 CHOI1989 | 1.86 | Numbers for ex and current do not agree with those in Table 2 page 69 CHOI1989 |
| CHOI | 543 | all | m | ex | 0 | yrs quit (v cur) | Table 3 page 70 CHOI1989 | 0.56 | Numbers for ex and current do not agree with those in Table 2 page 69 CHOI1989 |
| CHOI | 544 | all | m | ex | 0 | yrs quit (v cur) | Table 3 page 70 CHOI1989 | 0.24 | Numbers for ex and current do not agree with those in Table 2 page 69 CHOI1989 |
| CHOI | 545 | all | m | ex | 0 | yrs quit (v cur) | Table 3 page 70 CHOI1989 | 0.25 | Numbers for ex and current do not agree with those in Table 2 page 69 CHOI1989 |
| CHOI | 546 | all | m | ex | 0 | yrs quit (v cur) | Table 3 page 70 CHOI1989 | 0.30 | Numbers for ex and current do not agree with those in Table 2 page 69 CHOI1989 |
| CHOI | 547 | all | m | ex | 0 | yrs quit (v cur) | Table 3 page 70 CHOI1989 | 0.43 | Numbers for ex and current do not agree with those in Table 2 page 69 CHOI1989 |
| CHOI | 548 | all | m | ex | 0 | yrs quit (v cur) | Table 3 page 70 CHOI1989 | 0.45 | Numbers for ex and current do not agree with those in Table 2 page 69 CHOI1989 |
| CHOI | 549 | all | m | ex | 0 | yrs quit (v cur) | Table 3 page 70 CHOI1989 | 0.54 | Numbers for ex and current do not agree with those in Table 2 page 69 CHOI1989 |
| CHOI | 550 | all | f | ex | 0 | yrs quit (v nev) | Table 3 page 70 and Table 2 page 69 CHOI1989 | 10.75 | Numbers for ex and current do not agree with those in Table 2 page 69 CHOI1989 |
| CHOI | 551 | all | f | ex | 0 | yrs quit (v nev) | Table 3 page 70 and Table 2 page 69 CHOI1989 | 3.24 | Numbers for ex and current do not agree with those in Table 2 page 69 CHOI1989 |
| CHOI | 554 | all | f | ex | 0 | yrs quit (v nev) | Table 3 page 70 CHOI1989 | 0.28 | Numbers for ex and current do not agree with those in Table 2 page 69 CHOI1989 |
| CHOI | 556 | all | f | ex | 0 | yrs quit (v cur) | Table 3 page 70 CHOI1989 | 2.88 | Numbers for ex and current do not agree with those in Table 2 page 69 CHOI1989 |
| CHOI | 557 | all | f | ex | 0 | yrs quit (v cur) | Table 3 page 70 CHOI1989 | 9.44 | Numbers for ex and current do not agree with those in Table 2 page 69 CHOI1989 |
| CHOI | 558 | all | f | ex | 0 | yrs quit (v cur) | Table 3 page 70 CHOI1989 | 3.57 | Numbers for ex and current do not agree with those in Table 2 page 69 CHOI1989 |
| DOLL2 | 502 | all | m | ex | 1 | yrs quit (v nev) | Table IX & Table X p1531 DOLL1976 | 5.30 | When one man who stopped smoking after he developed the disease was excluded the RR reduced to 4.7 |
| DOLL2 | 504 | all | m | ex | 1 | yrs quit (v nev) | Table IX & Table X p1531 DOLL1976 | 16.00 | When five mwn who stopped smoking after they developed the disease were excluded the RR reduced to 10.7 |
| DOLL2 | 509 | all | m | ex | 1 | yrs quit (v cur) | TIX & TX p1531 DOLL1976 and personal communication | 1.02 | When five men who stopped smoking after they developed lung cancer were excluded the RR reduced to 0.68 |
| DOLL2 | 511 | all | m | ex | 1 | yrs quit (v cur) | TIX & TX p1531 DOLL1976 and personal communication | 0.28 | When one man who stopped smoking after he developed the disease was excluded theRR reduced to 0.25 |
| FAN | 503 | all | m | ever | 0 | duration | Table 6 page 5 FAN1994 | 3.89 | Original RR inaccurate (no original CI given) |
| FAN | 508 | all | f | ever | 0 | duration | Table 6 page 5 FAN1994 | 4.32 | Original RR inaccurate (no original CI given) |
| GENG | 533 | all | f | ever | 1 | age started | Table 4 page 484 GENG1988 | 1.55 | Table 4 page 484 GENG1988 gives a value of 1.59 without CI. The same value is also given in GENG1994 Table 7 as referring to sexes-combined, but that is assumed to be erroneous |
| GENG | 534 | all | f | ever | 1 | age started | Table 4 page 484 GENG1988 | 2.95 | Table 4 page 484 GENG1988 gives a value of 3.10 without CI. The same value is also given in GENG1994 Table 7 as referring to sexes-combined, but that is assumed to be erroneous |
| GENG | 535 | all | f | ever | 1 | age started | Table 4 page 484 GENG1988 | 6.24 | Table 4 page 484 GENG1988 gives a value of 6.3 without CI. The same value is also given in GENG1994 Table 7 as referring to sexes-combined but that is assumed to be erroneous |
| GILLIS | 520 | all | m | ex | 3 | yrs quit (v cur) | Figure 3 page 41 and Table 2 page 39 GILLIS1988 | 0.91 | Results for current derived from Table 2 do not agree completly with results read from graph in Figure 3 for current smokers even though they are stated to be the same (GILLIS1988 page 41 columns 1 and 2) |
| GILLIS | 521 | all | m | ex | 3 | yrs quit (v cur) | Figure 3 page 41 and Table 2 page 39 GILLIS1988 | 0.65 | Results for current derived from Table 2 do not agree completly with results read from graph in Figure 3 for current smokers even though they are stated to be the same (GILLIS1988 page 41 columns 1 and 2) |
| GILLIS | 522 | all | m | ex | 3 | yrs quit (v cur) | Figure 3 page 41 and Table 2 page 39 GILLIS1988 | 0.53 | Results for current derived from Table 2 do not agree completly with results read from graph in Figure 3 for current smokers even though they are stated to be the same (GILLIS1988 page 41 columns 1 and 2) |
| GILLIS | 523 | all | m | ex | 3 | yrs quit (v cur) | Figure 3 page 41 and Table 2 page 39 GILLIS1988 | 0.33 | Results for current derived from Table 2 do not agree completly with results read from graph in Figure 3 for current smokers even though they are stated to be the same (GILLIS1988 page 41 columns 1 and 2) |
| GILLIS | 524 | all | m | ex | 3 | yrs quit (v cur) | Figure 3 page 41 and Table 2 page 39 GILLIS1988 | 0.18 | Results for current derived from Table 2 do not agree completly with results read from graph in Figure 3 for current smokers even though they are stated to be the same (GILLIS1988 page 41 columns 1 and 2) |
| HUMBLE | 530 | other | c | current | 2 | duration | Table 3 page 600 PATHAK1986 | 2.98 | Given as 3.0 in Table 4 page 600 PATHAK1986 |
| HUMBLE | 531 | other | c | current | 2 | duration | Table 3 page 600 PATHAK1986 | 4.44 | Given as 4.8 in Table 4 page 600 PATHAK1986 |
| HUMBLE | 532 | other | c | current | 2 | duration | Table 3 page 600 PATHAK1986 | 7.09 | Given as 7.9 in Table 4 page 600 PATHAK1986 |
| HUMBLE | 538 | other | c | current | 2 | duration | Table 3 page 600 PATHAK1986 | 0.29 | Given as 0.3 in Table 4 page 600 PATHAK1986 |
| HUMBLE | 539 | other | c | current | 2 | duration | Table 3 page 600 PATHAK1986 | 0.51 | Given as 0.6 in Table 4 page 600 PATHAK1986 |
| HUMBLE | 540 | other | c | current | 2 | duration | Table 3 page 600 PATHAK1986 | 0.70 | Given as 1.8 in Table 4 page 600 PATHAK1986. This is assumed to be a typo for 0.8 |
| HUMBLE | 541 | other | c | current | 2 | duration | Table 3 page 600 PATHAK1986 | 0.52 | Given as 0.4 in Table 4 page 600 PATHAK1986 |
| JOLY | 612 | sq | f | ever | 0 | duration | T2 p1034, T4 p1035 & T8 p1037 JOLY1983 | 9.83 | Value of 9.4 given in Table 7 page 1036 JOLY1983 |
| JOLY | 618 | other | f | ever | 0 | duration | T2 p1034, T4 p1035 & T8 p1037 JOLY1983 | 5.24 | Value of 3.9 given in Table 7 page 1036 JOLY1983 |
| JOLY | 619 | other | f | ever | 0 | duration | T2 p1034, T4 p1035 & T8 p1037 JOLY1983 | 16.21 | Value of 15.6 given in Table 7 page 1036 JOLY1983 |
| JOLY | 626 | other | f | ever | 0 | duration | T2 p1034, T4 p1035 & T8 p1037 JOLY1983 | 8.16 | Value of 7.8 given in Table 7 page 1036 JOLY1983 |
| JOLY | 633 | adeno | f | ever | 0 | duration | T2 p1034, T4 p1035 & T8 p1037 JOLY1983 | 2.83 | Value of 2.7 given in Table 7 page 1036 JOLY1983 |
| JOLY | 646 | other | m | ever | 0 | duration | T2 p1034, T4 p1035 & T8 p1037 JOLY1983 | 53.88 | Value of 26.0 given in Table 7 page 1036 JOLY1983. Calculated with 1 replacing 0 nonsmoking cases |
| JOLY | 647 | other | m | ever | 0 | duration | T2 p1034, T4 p1035 & T8 p1037 JOLY1983 | 54.13 | Value of 26.4 given in Table 7 page 1036 JOLY1983. Calculated with 1 replacing 0 nonsmoking cases |
| JOLY | 648 | other | m | ever | 0 | duration | T2 p1034, T4 p1035 & T8 p1037 JOLY1983 | 82.61 | Value of 40.7 given in Table 7 page 1036 JOLY1983. Calculated with 1 replacing 0 nonsmoking cases |
| JOLY | 649 | other | m | ever | 0 | duration | T2 p1034, T4 p1035 & T8 p1037 JOLY1983 | 100.85 | Value of 50.0 given in Table 7 page 1036 JOLY1983. Calculated with 1 replacing 0 nonsmoking cases |
| KAISE2 | 517 | all | f | current | 1 | duration | Table 5 page 488 FRIEDM1997 | 30.41 | Given as 27.5 in Table 5 page 488 FRIEDA1997 |
| KAISE2 | 566 | all | f | ex | 1 | yrs quit (v nev) | Table 7 page 491 FRIEDM1997 | 6.29 | Originally given as 4.4 in Table 7 page 491 FRIEDM1997 |
| KAISE2 | 567 | all | f | ex | 1 | yrs quit (v nev) | Table 7 page 491 FRIEDM1997 | 4.37 | Originally given as 3.8 in Table 7 page 491 FRIEDM1997 |
| KAISE2 | 568 | all | f | ex | 1 | yrs quit (v nev) | Table 7 page 491 FRIEDM1997 | 7.95 | Originally given as 8.4 in Table 7 page 491 FRIEDM1997 |
| KAISE2 | 597 | all | m | current | 1 | duration | Table 6 page 489 FRIEDM1997 | 15.64 | Originally given as 11.4 in Table 6 page 489 FRIEDM1997 |
| KAISE2 | 646 | all | m | ex | 1 | yrs quit (v nev) | Table 8 page 492 FRIEDM1997 | 1.94 | Originally given as 1.6 in Table 8 page 492 FRIEDM1997 |
| KAISE2 | 647 | all | m | ex | 1 | yrs quit (v nev) | Table 8 page 492 FRIEDM1997 | 3.14 | Originally given as 2.7 in Table 8 page 492 FRIEDM1997 |
| KAISE2 | 648 | all | m | ex | 1 | yrs quit (v nev) | Table 8 page 492 FRIEDM1997 | 8.26 | Originally given as 8.5 in Table 8 page 492 FRIEDM1997 |
| SVENSS | 551 | all | f | ex | 0 | yrs quit (v nev) | Ex/Current T5 page 627 never T3 p626 SVENSS1989 | 1.84 | Current smoking controls assumed to be 53 not 52 as given in Table 5 page 627 |
| SVENSS | 552 | all | f | ex | 0 | yrs quit (v nev) | Ex/Current T5 page 627 never T3 p626 SVENSS1989 | 3.89 | Current smoking controls assumed to be 53 not 52 as given in Table 5 page 627 |
| SVENSS | 553 | all | f | ex | 0 | yrs quit (v nev) | Ex/Current T5 page 627 never T3 p626 SVENSS1989 | 2.11 | Current smoking controls assumed to be 53 not 52 as given in Table 5 page 627 |
| SVENSS | 554 | all | f | ex | 0 | yrs quit (v cur) | Ex/Current T5 page 627 never T3 p626 SVENSS1989 | 0.46 | Current smoking controls assumed to be 53 not 52 as given in Table 5 page 627 |
| SVENSS | 555 | all | f | ex | 0 | yrs quit (v cur) | Ex/Current T5 page 627 never T3 p626 SVENSS1989 | 0.22 | Current smoking controls assumed to be 53 not 52 as given in Table 5 page 627 |
| SVENSS | 556 | all | f | ex | 0 | yrs quit (v cur) | Ex/Current T5 page 627 never T3 p626 SVENSS1989 | 0.47 | Current smoking controls assumed to be 53 not 52 as given in Table 5 page 627 |
| SVENSS | 557 | sq | f | ex | 0 | yrs quit (v nev) | Ex/Current T5 page 627 never T3 p626 SVENSS1989 | 1.00 | Current smoking controls assumed to be 53 not 52 as given in Table 5 page 627 |
| SVENSS | 558 | sq | f | ex | 0 | yrs quit (v nev) | Ex/Current T5 page 627 never T3 p626 SVENSS1989 | 9.23 | Current smoking controls assumed to be 53 not 52 as given in Table 5 page 627 |
| SVENSS | 559 | sq | f | ex | 0 | yrs quit (v nev) | Ex/Current T5 page 627 never T3 p626 SVENSS1989 | 9.23 | Current smoking controls assumed to be 53 not 52 as given in Table 5 page 627 |
| SVENSS | 560 | sq | f | ex | 0 | yrs quit (v cur) | Ex/Current T5 page 627 never T3 p626 SVENSS1989 | 0.49 | Current smoking controls assumed to be 53 not 52 as given in Table 5 page 627 |
| SVENSS | 561 | sq | f | ex | 0 | yrs quit (v cur) | Ex/Current T5 page 627 never T3 p626 SVENSS1989 | 0.05 | Current smoking controls assumed to be 53 not 52 as given in Table 5 page 627 |
| SVENSS | 562 | sq | f | ex | 0 | yrs quit (v cur) | Ex/Current T5 page 627 never T3 p626 SVENSS1989 | 0.11 | Current smoking controls assumed to be 53 not 52 as given in Table 5 page 627 |
| SVENSS | 563 | sm | f | ex | 0 | yrs quit (v nev) | Ex/Current T5 page 627 never T3 p626 SVENSS1989 | 7.50 | Current smoking controls assumed to be 53 not 52 as given in Table 5 page 627 |
| SVENSS | 564 | sm | f | ex | 0 | yrs quit (v nev) | Ex/Current T5 page 627 never T3 p626 SVENSS1989 | 9.23 | Current smoking controls assumed to be 53 not 52 as given in Table 5 page 627 |
| SVENSS | 565 | sm | f | ex | 0 | yrs quit (v nev) | Ex/Current T5 page 627 never T3 p626 SVENSS1989 | 1.23 | Current smoking controls assumed to be 53 not 52 as given in Table 5 page 627 |
| SVENSS | 566 | sm | f | ex | 0 | yrs quit (v cur) | Ex/Current T5 page 627 never T3 p626 SVENSS1989 | 0.21 | Current smoking controls assumed to be 53 not 52 as given in Table 5 page 627 |
| SVENSS | 567 | sm | f | ex | 0 | yrs quit (v cur) | Ex/Current T5 page 627 never T3 p626 SVENSS1989 | 0.17 | Current smoking controls assumed to be 53 not 52 as given in Table 5 page 627 |
| SVENSS | 568 | sm | f | ex | 0 | yrs quit (v cur) | Ex/Current T5 page 627 never T3 p626 SVENSS1989 | 0.81 | Current smoking controls assumed to be 53 not 52 as given in Table 5 page 627 |
| SVENSS | 569 | adeno | f | ex | 0 | yrs quit (v nev) | Ex/Current T5 page 627 never T3 p626 SVENSS1989 | 1.59 | Current smoking controls assumed to be 53 not 52 as given in Table 5 page 627 |
| SVENSS | 570 | adeno | f | ex | 0 | yrs quit (v nev) | Ex/Current T5 page 627 never T3 p626 SVENSS1989 | 2.10 | Current smoking controls assumed to be 53 not 52 as given in Table 5 page 627 |
| SVENSS | 571 | adeno | f | ex | 0 | yrs quit (v nev) | Ex/Current T5 page 627 never T3 p626 SVENSS1989 | 1.32 | Current smoking controls assumed to be 53 not 52 as given in Table 5 page 627 |
| SVENSS | 572 | adeno | f | ex | 0 | yrs quit (v cur) | Ex/Current T5 page 627 never T3 p626 SVENSS1989 | 0.54 | Current smoking controls assumed to be 53 not 52 as given in Table 5 page 627 |
| SVENSS | 573 | adeno | f | ex | 0 | yrs quit (v cur) | Ex/Current T5 page 627 never T3 p626 SVENSS1989 | 0.41 | Current smoking controls assumed to be 53 not 52 as given in Table 5 page 627 |
| SVENSS | 574 | adeno | f | ex | 0 | yrs quit (v cur) | Ex/Current T5 page 627 never T3 p626 SVENSS1989 | 0.76 | Current smoking controls assumed to be 53 not 52 as given in Table 5 page 627 |
| SVENSS | 575 | othAll | f | ex | 0 | yrs quit (v nev) | Ex/Current T5 page 627 never T3 p626 SVENSS1989 | 1.67 | Current smoking controls assumed to be 53 not 52 as given in Table 5 page 627 |
| SVENSS | 576 | othAll | f | ex | 0 | yrs quit (v nev) | Ex/Current T5 page 627 never T3 p626 SVENSS1989 | 4.10 | Current smoking controls assumed to be 53 not 52 as given in Table 5 page 627 |
| SVENSS | 577 | othAll | f | ex | 0 | yrs quit (v nev) | Ex/Current T5 page 627 never T3 p626 SVENSS1989 | 2.46 | Current smoking controls assumed to be 53 not 52 as given in Table 5 page 627 |
| SVENSS | 578 | othAll | f | ex | 0 | yrs quit (v cur) | Ex/Current T5 page 627 never T3 p626 SVENSS1989 | 0.68 | Current smoking controls assumed to be 53 not 52 as given in Table 5 page 627 |
| SVENSS | 579 | othAll | f | ex | 0 | yrs quit (v cur) | Ex/Current T5 page 627 never T3 p626 SVENSS1989 | 0.28 | Current smoking controls assumed to be 53 not 52 as given in Table 5 page 627 |
| SVENSS | 580 | othAll | f | ex | 0 | yrs quit (v cur) | Ex/Current T5 page 627 never T3 p626 SVENSS1989 | 0.41 | Current smoking controls assumed to be 53 not 52 as given in Table 5 page 627 |
| SVENSS | 591 | all | f | ex | 0 | yrs quit (v nev) | Ex/Current T5 page 627 never T3 p626 SVENSS1989 | * | Current smoking controls assumed to be 53 not 52 as given in Table 5 page 627 |
| SVENSS | 592 | all | f | ex | 0 | yrs quit (v nev) | Ex/Current T5 page 627 never T3 p626 SVENSS1989 | * | Current smoking controls assumed to be 53 not 52 as given in Table 5 page 627 |
| SVENSS | 593 | sq | f | ex | 0 | yrs quit (v nev) | Ex/Current T5 page 627 never T3 p626 SVENSS1989 | * | Current smoking controls assumed to be 53 not 52 as given in Table 5 page 627 |
| SVENSS | 594 | sq | f | ex | 0 | yrs quit (v nev) | Ex/Current T5 page 627 never T3 p626 SVENSS1989 | * | Current smoking controls assumed to be 53 not 52 as given in Table 5 page 627 |
| SVENSS | 595 | sm | f | ex | 0 | yrs quit (v nev) | Ex/Current T5 page 627 never T3 p626 SVENSS1989 | * | Current smoking controls assumed to be 53 not 52 as given in Table 5 page 627 |
| SVENSS | 596 | sm | f | ex | 0 | yrs quit (v nev) | Ex/Current T5 page 627 never T3 p626 SVENSS1989 | * | Current smoking controls assumed to be 53 not 52 as given in Table 5 page 627 |
| SVENSS | 597 | adeno | f | ex | 0 | yrs quit (v nev) | Ex/Current T5 page 627 never T3 p626 SVENSS1989 | * | Current smoking controls assumed to be 53 not 52 as given in Table 5 page 627 |
| SVENSS | 598 | adeno | f | ex | 0 | yrs quit (v nev) | Ex/Current T5 page 627 never T3 p626 SVENSS1989 | * | Current smoking controls assumed to be 53 not 52 as given in Table 5 page 627 |
| SVENSS | 599 | othAll | f | ex | 0 | yrs quit (v nev) | Ex/Current T5 page 627 never T3 p626 SVENSS1989 | * | Current smoking controls assumed to be 53 not 52 as given in Table 5 page 627 |
| SVENSS | 600 | othAll | f | ex | 0 | yrs quit (v nev) | Ex/Current T5 page 627 never T3 p626 SVENSS1989 | * | Current smoking controls assumed to be 53 not 52 as given in Table 5 page 627 |
| SVENSS | 601 | all | f | 5 | 0 | yrs quit (v nev) | Ex/Current T5 page 627 never T3 p626 SVENSS1989 | 8.46 | Current smoking controls assumed to be 53 not 52 as given in Table 5 page 627 |
| SVENSS | 602 | sq | f | 5 | 0 | yrs quit (v nev) | Ex/Current T5 page 627 never T3 p626 SVENSS1989 | 19.02 | Current smoking controls assumed to be 53 not 52 as given in Table 5 page 627 |
| SVENSS | 603 | sm | f | 5 | 0 | yrs quit (v nev) | Ex/Current T5 page 627 never T3 p626 SVENSS1989 | 43.02 | Current smoking controls assumed to be 53 not 52 as given in Table 5 page 627 |
| SVENSS | 604 | adeno | f | 5 | 0 | yrs quit (v nev) | Ex/Current T5 page 627 never T3 p626 SVENSS1989 | 3.91 | Current smoking controls assumed to be 53 not 52 as given in Table 5 page 627 |
| SVENSS | 605 | othAll | f | 5 | 0 | yrs quit (v nev) | Ex/Current T5 page 627 never T3 p626 SVENSS1989 | 6.04 | Current smoking controls assumed to be 53 not 52 as given in Table 5 page 627 |
| SVENSS | 606 | all | f | ex | 2 | yrs quit (v nev) | T5 page 627 SVENSS1989 | 2.00 | Current smoking controls assumed to be 53 not 52 as given in Table 5 page 627 |
| SVENSS | 607 | all | f | ex | 2 | yrs quit (v nev) | T5 page 627 SVENSS1989 | * | Current smoking controls assumed to be 53 not 52 as given in Table 5 page 627 |
| SVENSS | 608 | all | f | ex | 2 | yrs quit (v cur) | T5 page 627 SVENSS1989 | 0.50 | Current smoking controls assumed to be 53 not 52 as given in Table 5 page 627 |
| SVENSS | 615 | adeno | f | ex | 2 | yrs quit (v nev) | T5 page 627 SVENSS1989 | 1.00 | Current smoking controls assumed to be 53 not 52 as given in Table 5 page 627 |
| SVENSS | 616 | adeno | f | ex | 2 | yrs quit (v nev) | T5 page 627 SVENSS1989 | * | Current smoking controls assumed to be 53 not 52 as given in Table 5 page 627 |
| SVENSS | 617 | adeno | f | ex | 2 | yrs quit (v cur) | T5 page 627 SVENSS1989 | 1.00 | Current smoking controls assumed to be 53 not 52 as given in Table 5 page 627 |
| SVENSS | 618 | othAll | f | ex | 2 | yrs quit (v nev) | T5 page 627 SVENSS1989 | 1.75 | Current smoking controls assumed to be 53 not 52 as given in Table 5 page 627 |
| SVENSS | 619 | othAll | f | ex | 2 | yrs quit (v nev) | T5 page 627 SVENSS1989 | * | Current smoking controls assumed to be 53 not 52 as given in Table 5 page 627 |
| SVENSS | 620 | othAll | f | ex | 2 | yrs quit (v cur) | T5 page 627 SVENSS1989 | 0.57 | Current smoking controls assumed to be 53 not 52 as given in Table 5 page 627 |
| TIZZAN | 530 | all | f | ever | 0 | duration | T2 page 111 and T11 page 120 TIZZAN1965 | 0.28 | Results using female data as given in Table 12 page 121 were 0.07 (0.02-0.30) |
| TIZZAN | 533 | all | f | ever | 0 | duration | T2 page 111 and T11 page 120 TIZZAN1965 | 0.28 | Results using female data as given in Table 12 page 121 were 0.07 (0.02-0.30) |
| WYNDE6 | 772 | KI | m | ex | 0 | yrs quit (v nev) | Table 9 page 4614 WYNDER1977A | 7.47 | Slightly different values are given in WYNDER1984A Figure2 |
| WYNDE6 | 773 | KI | m | ex | 0 | yrs quit (v nev) | Table 9 page 4614 WYNDER1977A | 17.70 | Slightly different values are given in WYNDER1984A Figure2 |
| WYNDE6 | 774 | KI | m | ex | 0 | yrs quit (v nev) | Table 9 page 4614 WYNDER1977A | 22.06 | Slightly different values are given in WYNDER1984A Figure2 |
| WYNDE6 | 775 | KI | m | ex | 0 | yrs quit (v nev) | Table 9 page 4614 WYNDER1977A | 28.56 | Slightly different values are given in WYNDER1984A Figure2 |
| WYNDE6 | 776 | KI | m | ex | 0 | yrs quit (v nev) | Table 9 page 4614 WYNDER1977A | 54.30 | Slightly different values are given in WYNDER1984A Figure2 |
| WYNDE6 | 777 | KI | m | ex | 0 | yrs quit (v nev) | Table 9 page 4614 WYNDER1977A | * | Slightly different values are given in WYNDER1984A Figure2 |
| WYNDE6 | 778 | KI | m | ex | 0 | yrs quit (v nev) | Table 9 page 4614 WYNDER1977A | 2.37 | Slightly different values are given in WYNDER1984A Figure2 |
| WYNDE6 | 779 | KI | m | ex | 0 | yrs quit (v nev) | Table 9 page 4614 WYNDER1977A | 2.95 | Slightly different values are given in WYNDER1984A Figure2 |
| WYNDE6 | 780 | KI | m | ex | 0 | yrs quit (v nev) | Table 9 page 4614 WYNDER1977A | 3.82 | Slightly different values are given in WYNDER1984A Figure2 |
| WYNDE6 | 781 | KI | m | ex | 0 | yrs quit (v nev) | Table 9 page 4614 WYNDER1977A | 7.27 | Slightly different values are given in WYNDER1984A Figure2 |
| WYNDE6 | 782 | KI | m | ex | 0 | yrs quit (v nev) | Table 9 page 4614 WYNDER1977A | * | Slightly different values are given in WYNDER1984A Figure2 |
| WYNDE6 | 783 | KI | m | ex | 0 | yrs quit (v cur) | Table 9 page 4614 WYNDER1977A | 0.53 | Slightly different values are given in WYNDER1984A Figure2 |
| WYNDE6 | 784 | KI | m | ex | 0 | yrs quit (v cur) | Table 9 page 4614 WYNDER1977A | 0.41 | Slightly different values are given in WYNDER1984A Figure2 |
| WYNDE6 | 785 | KI | m | ex | 0 | yrs quit (v cur) | Table 9 page 4614 WYNDER1977A | 0.33 | Slightly different values are given in WYNDER1984A Figure2 |
| WYNDE6 | 786 | KI | m | ex | 0 | yrs quit (v cur) | Table 9 page 4614 WYNDER1977A | 0.14 | Slightly different values are given in WYNDER1984A Figure2 |

^a^ Number of relative risk on the RR databases (Numbers over 500 are from the second database)

^b^ Lung cancer type (ad= adenocarcinoma, KI=Kreyberg type I, KII=Kreyberg type II, la=large cell carcinoma, othAd=other adeno [at least adeno but not squamous], othAll=other all [at least squamous and adeno], sm=small cell carcinoma, sq=squamous cell carcinoma)

^c^ Number of adjusting variables.

^d^ Blank indicate a major or cigarette type index.

^e^ RR and 95% CI .
