# Supplementary material for: Systematic review with meta-analysis of the epidemiological evidence in the 1900s relating smoking to lung cancer
Source: BMC Cancer. 2012 Sep 3;12:385. doi: 10.1186/1471-2407-12-385 (PMC3505152; doi:10.1186/1471-2407-12-385)
Supplement: Additional file 4 — Dose-response data, not eligible for inclusion in meta-analysis. [file 1471-2407-12-385-S4.doc]

**Systematic review with meta-analysis of the epidemiological evidence in the 1900s relating smoking to lung cancer**

Peter N Lee, Barbara A Forey and Katharine J Coombs

**Additional file 4:** Dose-response data, not eligible for inclusion in meta-analysis

This file shows dose-related results from the RR databases which were not eligible for the main meta-analysis tables. The numbering style of the tables reflects that of the main tables – the number indicates the lung cancer type (1 = all lung cancer or near equivalent, 2 = squamous or near equivalent [“squamous”], 3 = adenocarcinoma or near equivalent [“adeno”], 4 = large, 5 = small) and the letter indicates the dose measure (G=amount, H=age started, I=duration, J=years quit (vs never or long-term ex), K=year quit (vs current or recent quitters), L=tar, M=butt length or fraction smoked)

The rules for data entry differed between amount smoked (first database, data entry 1997-2002) and the other measures (second database, data entry 2009-2010). See also Additional file 1 : Methods.

The following categories of results are shown:

- **Regression** Results expressed on a “per unit dose” basis, usually from a multiple logistic regression (not available for amount smoked.)
- **Base “low”** Results relative to a “low” base – included here only for amount smoked, where such data were entered on the database only where no results relative to “never” or “non” base were available, and not included in any of the meta-analyses.
- **Base includes ex-smokers** Results for current smokers relative to a non-smoker base (i.e. the base includes ex smokers). Although always entered on the database, these results are included here only where there were no results relative to never smokers.
- **Base includes low smokers beyond limit defined as acceptable** Results relative to a base of “never smokers or low exposure”, where the level of exposure in the base exceeded that defined as an acceptable equivalent to never smokers – See Additional file Methods (*Identifying which RRs to enter).*
- **Other** Other results recorded as comments on the database (not available for amount smoked).

Results which would be eligible for meta-analysis except that the RR or CI values are missing are not included in this file. They are shown in section -9 of each meta-analysis unless there is another result with higher preference order for that analysis.

Abbreviations used in the tables are as follows:

AD: number of potential confounders adjusted for

ADOSMK: number of other smoking variables adjusted for

CIGT: cigarette type

EXPLO, EXPHI: range of exposure for the numerator of the RR

LC TYPE: lung cancer type (q = squamous, s = small, l = large, a =adeno, mix = mixed, alv = alveolar)

NCIGLO, NCIGHI: range of number of cigarettes/day

NRR: number of the RR on the database within the study

REF: 6-character study reference

RR: relative risk (or odds ratio)

RRL, RRU: lower and upper 95% confidence limits of the RR

SMKSTA: smoking status

UNEXLO, UNEXHI: range of exposure for the denominator of the RR

Horizontal lines in the tables separate dose-response sets of RRs.

**Tables**

[Table G Amount 5](#__RefHeading___Toc312145463)

[1G All LC – Base "low" 5](#__RefHeading___Toc312145464)

[1G All LC – Base includes ex-smokers 6](#__RefHeading___Toc312145465)

[1G All LC – Base includes low smokers beyond limit defined as acceptable 7](#__RefHeading___Toc312145466)

[2G Squamous – Base "low" 7](#__RefHeading___Toc312145467)

[2G Squamous – Base includes ex-smokers 7](#__RefHeading___Toc312145468)

[2G Squamous – Base includes low smokers beyond limit defined as acceptable 7](#__RefHeading___Toc312145469)

[3G Adeno – Base "low" 8](#__RefHeading___Toc312145470)

[3G Adeno – Base includes ex-smokers 8](#__RefHeading___Toc312145471)

[3G Adeno – Base includes low smokers beyond limit defined as acceptable 8](#__RefHeading___Toc312145472)

[4G Large – Base "low" 8](#__RefHeading___Toc312145473)

[G5 Small – Base "low" 8](#__RefHeading___Toc312145474)

[Table H Age started 9](#__RefHeading___Toc312145475)

[1H All LC – Regression 9](#__RefHeading___Toc312145476)

[1H All LC – Other 9](#__RefHeading___Toc312145477)

[2H Squamous – Other 9](#__RefHeading___Toc312145478)

[3H Adeno – Other 9](#__RefHeading___Toc312145479)

[4H Large – Other 9](#__RefHeading___Toc312145480)

[5H Small – Other 9](#__RefHeading___Toc312145481)

[Table I Duration 10](#__RefHeading___Toc312145482)

[1I All LC – Regression 10](#__RefHeading___Toc312145483)

[1I All LC – Base includes ex-smokers 11](#__RefHeading___Toc312145484)

[1I All LC – Other 11](#__RefHeading___Toc312145485)

[1I All LC – Other 12](#__RefHeading___Toc312145486)

[1I Squamous – Other 13](#__RefHeading___Toc312145487)

[3I Adeno – Other 14](#__RefHeading___Toc312145488)

[5I Small –Other 15](#__RefHeading___Toc312145489)

[Table J Years quit (vs never) 16](#__RefHeading___Toc312145490)

[1J All LC – Regression 16](#__RefHeading___Toc312145491)

[1J All LC – Other 16](#__RefHeading___Toc312145492)

[Table K Years quit (vs current) 17](#__RefHeading___Toc312145493)

[1K All LC – Base includes recent smokers beyond limit defined as acceptable 17](#__RefHeading___Toc312145494)

[2K Squamous – Base includes recent smokers beyond limit defined as acceptable 18](#__RefHeading___Toc312145495)

[3K Adeno – Base includes recent smokers beyond limit defined as acceptable 18](#__RefHeading___Toc312145496)

[5K Small – Base includes recent smokers beyond limit defined as acceptable 19](#__RefHeading___Toc312145497)

[L Tar 20](#__RefHeading___Toc312145498)

[1L All LC – Other 20](#__RefHeading___Toc312145499)

[2L Squamous – Other 21](#__RefHeading___Toc312145500)

[3L Adeno – Other 22](#__RefHeading___Toc312145501)

[M Butt length or Fraction smoked 23](#__RefHeading___Toc312145502)

[1M All LC – Other 23](#__RefHeading___Toc312145503)

# Table G Amount

### 1G All LC – Base "low"

| **REF** | **NRR** | **SEX** | **LC TYPE** | **SMKSTA** | **PRODUCT** | **CIGT** | **AD** | **NCIGLO** | **NCIGHI** | **RR** | **RRL** | **RRU** | **DERIVE** | **COMMENT** |
| --- | --- | --- | --- | --- | --- | --- | --- | --- | --- | --- | --- | --- | --- | --- |
|  |  |  |  |  |  |  |  |  |  |  |  |  |  |  |
| DESTEF | 19 | m | all | ever | cig+/-ot | HR | 0 | 15 | 20 | 2.04 | 1.30 | 3.19 | derived |  |
| DESTEF | 20 | m | all | ever | cig+/-ot | HR | 0 | 21 | 40 | 2.80 | 1.79 | 4.38 | derived |  |
| DESTEF | 21 | m | all | ever | cig+/-ot | HR | 0 | 41 | 99 | 3.86 | 2.37 | 6.28 | derived |  |
| DESTEF | 22 | m | all | ever | cig+/-ot | HR | 4 | 15 | 20 | 1.90 | 1.20 | 3.00 | original |  |
| DESTEF | 23 | m | all | ever | cig+/-ot | HR | 4 | 21 | 40 | 2.40 | 1.50 | 3.90 | original |  |
| DESTEF | 24 | m | all | ever | cig+/-ot | HR | 4 | 41 | 99 | 4.10 | 2.40 | 6.80 | original |  |
| DESTEF | 42 | m | all | current | cig+/-ot | MC only | 0 | 15 | 20 | 3.93 | 1.84 | 8.37 | derived |  |
| DESTEF | 43 | m | all | current | cig+/-ot | MC only | 0 | 21 | 40 | 6.10 | 2.84 | 13.09 | derived |  |
| DESTEF | 44 | m | all | current | cig+/-ot | MC only | 0 | 41 | 99 | 15.55 | 5.49 | 44.05 | derived |  |
| DESTEF | 45 | m | all | current | cig+/-ot | MC only | 4 | 15 | 20 | 3.30 | 1.40 | 7.70 | original |  |
| DESTEF | 46 | m | all | current | cig+/-ot | MC only | 4 | 21 | 40 | 4.40 | 1.80 | 10.60 | original |  |
| DESTEF | 47 | m | all | current | cig+/-ot | MC only | 4 | 41 | 99 | 11.90 | 3.70 | 38.60 | original |  |
| KHUDER | 20 | m | all | ever | cig+/-ot | all | 5 | 40 | 99 | 6.10 | 4.20 | 8.70 | original |  |
| LAURIL | 1 | m | all | current | cig+/-ot | all | 0 | 15 | 24 | 2.33 | 1.40 | 3.87 | derived |  |
| LAURIL | 2 | m | all | current | cig+/-ot | all | 0 | 25 | 34 | 2.43 | 1.40 | 4.23 | derived |  |
| LAURIL | 3 | m | all | current | cig+/-ot | all | 0 | 35 | 99 | 1.72 | 0.78 | 3.82 | derived |  |
| LAURIL | 4 | m | all | current | cig+/-ot | all | 0 | 15 | 99 | 2.30 | 1.43 | 3.69 | derived |  |
| MCCONN | 12 | c | all | ever | cig only | all | 0 | 10 | 20 | 2.11 | 0.80 | 5.55 | derived |  |
| MCCONN | 11 | c | all | ever | cig only | all | 0 | 21 | 99 | 4.69 | 1.71 | 12.85 | derived |  |
| MCCONN | 7 | c | all | ever | cig only | all | 0 | 10 | 20 | 0.43 | 0.02 | 9.36 | derived |  |
| MCCONN | 3 | c | all | ever | cig only | all | 0 | 21 | 99 | 4.50 | 0.25 | 80.57 | derived |  |
| MCCONN | 8 | c | all | ever | cig only | all | 0 | 10 | 20 | 3.39 | 0.38 | 30.09 | derived |  |
| MCCONN | 4 | c | all | ever | cig only | all | 0 | 21 | 99 | 6.42 | 0.68 | 60.84 | derived |  |
| MCCONN | 9 | c | all | ever | cig only | all | 0 | 10 | 20 | 6.36 | 0.73 | 55.30 | derived |  |
| MCCONN | 5 | c | all | ever | cig only | all | 0 | 21 | 99 | 12.00 | 1.37 | 105.13 | derived |  |
| MCCONN | 10 | c | all | ever | cig only | all | 0 | 10 | 20 | 0.75 | 0.14 | 4.00 | derived |  |
| MCCONN | 6 | c | all | ever | cig only | all | 0 | 21 | 99 | 2.00 | 0.28 | 14.20 | derived |  |
| PISANI | 7 | c | all | current | all/unsp | - | 10 | 10 | 19 | 3.80 | * | * | original |  |
| PISANI | 8 | c | all | current | all/unsp | - | 10 | 20 | 29 | 7.10 | * | * | original |  |
| PISANI | 9 | c | all | current | all/unsp | - | 10 | 30 | 39 | 7.90 | * | * | original |  |
| PISANI | 4 | c | all | ex | all/unsp | - | 10 | 10 | 19 | 3.40 | * | * | original |  |
| PISANI | 5 | c | all | ex | all/unsp | - | 10 | 20 | 29 | 3.90 | * | * | original |  |
| PISANI | 6 | c | all | ex | all/unsp | - | 10 | 30 | 99 | 6.80 | * | * | original |  |
| SOBUE | 123 | m | q+s+l+a | current | cig+/-ot | all | 5 | 20 | 29 | 1.30 | 1.00 | 1.80 | original |  |
| SOBUE | 124 | m | q+s+l+a | current | cig+/-ot | all | 5 | 30 | 99 | 1.70 | 1.20 | 2.30 | original |  |

### 1G All LC – Base includes ex-smokers

| **REF** | **NRR** | **SEX** | **LC TYPE** | **SMKSTA** | **PRODUCT** | **CIGT** | **AD** | **NCIGLO** | **NCIGHI** | **RR** | **RRL** | **RRU** | **DERIVE** | **COMMENT** |
| --- | --- | --- | --- | --- | --- | --- | --- | --- | --- | --- | --- | --- | --- | --- |
| HIRAY2 | 3 | m | all | current | cig only | all | 1 | 1 | 9 | 0.82 | 0.24 | 2.81 | derived |  |
| HIRAY2 | 2 | m | all | current | cig only | all | 1 | 10 | 20 | 2.41 | 1.14 | 5.08 | derived |  |
| HIRAY2 | 1 | m | all | current | cig only | all | 1 | 21 | 99 | 7.66 | 3.65 | 16.06 | derived |  |
| HIRAY2 | 13 | m | all | current | cig only | all | 1 | 1 | 9 | 0.39 | 0.13 | 1.13 | derived |  |
| HIRAY2 | 12 | m | all | current | cig only | all | 1 | 10 | 20 | 1.13 | 0.72 | 1.76 | derived |  |
| HIRAY2 | 11 | m | all | current | cig only | all | 1 | 21 | 99 | 3.59 | 2.33 | 5.55 | derived |  |
| HIRAY2 | 6 | m | all | current | p only | - | 1 | 1 | 9 | 1.49 | 0.28 | 7.83 | derived |  |
| HIRAY2 | 5 | m | all | current | p only | - | 1 | 10 | 20 | 3.20 | 1.44 | 7.11 | derived |  |
| HIRAY2 | 4 | m | all | current | p only | - | 1 | 21 | 99 | 3.83 | 1.27 | 11.53 | derived |  |
| LICKIN | 3 | m | all | current | all/unsp | - | 0 | 1 | 19 | 3.93 | 1.40 | 11.01 | derived |  |
| LICKIN | 2 | m | all | current | all/unsp | - | 0 | 20 | 99 | 22.90 | 8.34 | 62.89 | derived |  |
| MRFIT | 18 | m | all | current | cig+/-ot | all | 0 | 1 | 15 | 2.34 | 1.93 | 2.84 | derived |  |
| MRFIT | 19 | m | all | current | cig+/-ot | all | 0 | 16 | 25 | 5.35 | 4.71 | 6.08 | derived |  |
| MRFIT | 20 | m | all | current | cig+/-ot | all | 0 | 26 | 35 | 6.47 | 5.65 | 7.42 | derived |  |
| MRFIT | 21 | m | all | current | cig+/-ot | all | 0 | 36 | 45 | 8.80 | 7.71 | 10.05 | derived |  |
| MRFIT | 22 | m | all | current | cig+/-ot | all | 0 | 46 | 99 | 9.49 | 7.93 | 11.36 | derived |  |
| MRFIT | 24 | m | all | current | cig+/-ot | all | 0 | 1 | 19 | 2.14 | 1.61 | 2.85 | derived |  |
| MRFIT | 25 | m | all | current | cig+/-ot | all | 0 | 20 | 39 | 4.69 | 3.95 | 5.57 | derived |  |
| MRFIT | 26 | m | all | current | cig+/-ot | all | 0 | 40 | 99 | 7.63 | 6.35 | 9.18 | derived |  |
| MRFIT | 1 | m | all | current | cig+/-ot | all | 1 | 1 | 15 | 2.58 | 2.12 | 3.13 | derived |  |
| MRFIT | 2 | m | all | current | cig+/-ot | all | 1 | 16 | 25 | 5.82 | 5.12 | 6.62 | derived |  |
| MRFIT | 3 | m | all | current | cig+/-ot | all | 1 | 26 | 35 | 7.31 | 6.38 | 8.39 | derived |  |
| MRFIT | 4 | m | all | current | cig+/-ot | all | 1 | 36 | 45 | 9.84 | 8.62 | 11.25 | derived |  |
| MRFIT | 5 | m | all | current | cig+/-ot | all | 1 | 46 | 99 | 10.68 | 8.91 | 12.81 | derived |  |
| MRFIT | 14 | m | all | current | cig+/-ot | all | 1 | 1 | 19 | 2.36 | 1.77 | 3.14 | derived |  |
| MRFIT | 15 | m | all | current | cig+/-ot | all | 1 | 20 | 39 | 5.20 | 4.38 | 6.18 | derived |  |
| MRFIT | 16 | m | all | current | cig+/-ot | all | 1 | 40 | 99 | 8.56 | 7.12 | 10.31 | derived |  |
| MURATA | 1 | m | all | current | cig+/-ot | all | 0 | 1 | 10 | 1.41 | 0.60 | 3.31 | derived |  |
| MURATA | 2 | m | all | current | cig+/-ot | all | 0 | 11 | 20 | 3.60 | 2.06 | 6.29 | derived |  |
| MURATA | 3 | m | all | current | cig+/-ot | all | 0 | 21 | 99 | 4.59 | 2.15 | 9.79 | derived |  |
| WARSIN | 3 | m | all | current | all/unsp | - | 0 | 1 | 19 | 3.37 | 1.18 | 9.63 | derived |  |
| WARSIN | 2 | m | all | current | all/unsp | - | 0 | 20 | 99 | 14.80 | 4.89 | 44.76 | derived |  |
| WATSON | 1 | m | all | current | all/unsp | - | 0 | 1 | 19 | 3.50 | 1.29 | 9.52 | derived |  |
| WATSON | 2 | m | all | current | all/unsp | - | 0 | 20 | 99 | 7.17 | 2.70 | 19.09 | derived |  |
| WATSON | 3 | f | all | current | all/unsp | - | 0 | 1 | 19 | 3.18 | 1.46 | 6.94 | derived |  |
| WATSON | 4 | f | all | current | all/unsp | - | 0 | 20 | 99 | 3.52 | 0.31 | 40.57 | derived |  |
| WYNDER | 1 | m | all | current | all/unsp | - | 0 | 1 | 15 | 44.70 | 2.41 | 829.04 | derived |  |
| WYNDER | 2 | m | all | current | all/unsp | - | 0 | 16 | 20 | 19.14 | 1.11 | 329.90 | derived |  |
| WYNDER | 3 | m | all | current | all/unsp | - | 0 | 21 | 34 | 28.26 | 1.65 | 482.66 | derived |  |
| WYNDER | 4 | m | all | current | all/unsp | - | 0 | 35 | 99 | 40.18 | 2.39 | 674.49 | derived |  |
| WYNDER | 4 | f | all | current | all/unsp | - | 0 | 1 | 15 | 31.33 | 6.89 | 142.53 | derived |  |
| WYNDER | 6 | f | all | current | all/unsp | - | 0 | 16 | 20 | 57.07 | 12.29 | 264.94 | derived |  |
| WYNDER | 7 | f | all | current | all/unsp | - | 0 | 21 | 34 | 47.00 | 7.14 | 309.18 | derived |  |
| WYNDER | 8 | f | all | current | all/unsp | - | 0 | 35 | 99 | 35.25 | 3.92 | 317.34 | derived |  |

### 1G All LC – Base includes low smokers beyond limit defined as acceptable

| **REF** | **NRR** | **SEX** | **LC TYPE** | **SMKSTA** | **PRODUCT** | **CIGT** | **AD** | **NCIGLO** | **NCIGHI** | **RR** | **RRL** | **RRU** | **DERIVE** | **COMMENT** |
| --- | --- | --- | --- | --- | --- | --- | --- | --- | --- | --- | --- | --- | --- | --- |
|  |  |  |  |  |  |  |  |  |  |  |  |  |  |  |
| BLOT3 | 1 | m | all | ever | cig+/-ot | all | 0 | 10 | 20 | 5.16 | 3.12 | 8.54 | derived |  |
| BLOT3 | 2 | m | all | ever | cig+/-ot | all | 0 | 21 | 99 | 6.57 | 3.90 | 11.05 | derived |  |
| MOLLO | 1 | m | all | ever | cig+/-ot | all | 0 | 11 | 20 | 3.68 | 1.85 | 7.33 | derived |  |
| MOLLO | 2 | m | all | ever | cig+/-ot | all | 0 | 21 | 99 | 3.07 | 1.54 | 6.10 | derived |  |

### 2G Squamous – Base "low"

| BOUCHA | 1 | c | q+s | ever | all/unsp | - | 0 | 21 | 30 | 1.13 | 0.64 | 2.00 | derived |  |
| --- | --- | --- | --- | --- | --- | --- | --- | --- | --- | --- | --- | --- | --- | --- |
| BOUCHA | 2 | c | q+s | ever | all/unsp | - | 0 | 30 | 99 | 1.13 | 0.67 | 1.90 | derived |  |
| KHUDER | 21 | m | q | ever | cig+/-ot | all | 5 | 40 | 99 | 8.70 | 4.80 | 15.70 | original |  |
| LUBIN | 21 | m | KI | ever | cig only | all | 5 | 7 | 14 | 1.60 | * | * | original |  |
| LUBIN | 22 | m | KI | ever | cig only | all | 5 | 15 | 19 | 11.50 | * | * | original |  |
| LUBIN | 23 | m | KI | ever | cig only | all | 5 | 20 | 99 | 8.17 | * | * | original |  |
| SOBUE | 77 | m | q | current | cig+/-ot | all | 5 | 20 | 29 | 1.50 | 1.00 | 2.30 | original |  |
| SOBUE | 78 | m | q | current | cig+/-ot | all | 5 | 30 | 99 | 1.90 | 1.20 | 2.90 | original |  |
| SOBUE | 177 | m | KI | current | cig+/-ot | all | 5 | 20 | 29 | 1.36 | 0.95 | 1.96 | derived |  |
| SOBUE | 178 | m | KI | current | cig+/-ot | all | 5 | 30 | 99 | 2.08 | 1.43 | 3.02 | derived |  |

### 2G Squamous – Base includes ex-smokers

| SEGI2 | 37 | m | q | current | cig+/-ot | all | 0 | 1 | 19 | 2.13 | 0.96 | 4.72 | derived |  |
| --- | --- | --- | --- | --- | --- | --- | --- | --- | --- | --- | --- | --- | --- | --- |
| SEGI2 | 39 | m | q | current | cig+/-ot | all | 0 | 20 | 99 | 3.32 | 1.54 | 7.17 | derived |  |
| SEGI2 | 38 | m | q | current | cig+/-ot | all | 1 | 1 | 19 | 2.10 | 0.95 | 4.66 | derived |  |
| SEGI2 | 40 | m | q | current | cig+/-ot | all | 1 | 20 | 99 | 3.60 | 1.67 | 7.76 | derived |  |

### 2G Squamous – Base includes low smokers beyond limit defined as acceptable

| MOLLO | 5 | m | not a | ever | cig+/-ot | all | 0 | 11 | 20 | 4.17 | 1.92 | 9.03 | derived |  |
| --- | --- | --- | --- | --- | --- | --- | --- | --- | --- | --- | --- | --- | --- | --- |
| MOLLO | 6 | m | not a | ever | cig+/-ot | all | 0 | 21 | 99 | 3.34 | 1.54 | 7.24 | derived |  |

### 3G Adeno – Base "low"

| **REF** | **NRR** | **SEX** | **LC TYPE** | **SMKSTA** | **PRODUCT** | **CIGT** | **AD** | **NCIGLO** | **NCIGHI** | **RR** | **RRL** | **RRU** | **DERIVE** | **COMMENT** |
| --- | --- | --- | --- | --- | --- | --- | --- | --- | --- | --- | --- | --- | --- | --- |
|  |  |  |  |  |  |  |  |  |  |  |  |  |  |  |
| KHUDER | 23 | m | a | ever | cig+/-ot | all | 5 | 40 | 99 | 3.50 | 1.90 | 6.40 | original |  |
| LUBIN | 24 | m | KII | ever | cig only | all | 5 | 7 | 14 | 1.06 | * | * | original |  |
| LUBIN | 25 | m | KII | ever | cig only | all | 5 | 15 | 19 | 13.10 | * | * | original |  |
| LUBIN | 26 | m | KII | ever | cig only | all | 5 | 20 | 99 | 9.34 | * | * | original |  |
| SOBUE | 79 | m | a | current | cig+/-ot | all | 5 | 20 | 29 | 1.20 | 0.80 | 1.80 | original |  |
| SOBUE | 80 | m | a | current | cig+/-ot | all | 5 | 30 | 99 | 1.20 | 0.80 | 1.90 | original |  |

### 3G Adeno – Base includes ex-smokers

| SEGI2 | 29 | m | a | current | cig+/-ot | all | 0 | 1 | 19 | 0.94 | 0.41 | 2.16 | derived |  |
| --- | --- | --- | --- | --- | --- | --- | --- | --- | --- | --- | --- | --- | --- | --- |
| SEGI2 | 31 | m | a | current | cig+/-ot | all | 0 | 20 | 99 | 1.50 | 0.69 | 3.24 | derived |  |
| SEGI2 | 30 | m | a | current | cig+/-ot | all | 1 | 1 | 19 | 0.91 | 0.40 | 2.08 | derived |  |
| SEGI2 | 32 | m | a | current | cig+/-ot | all | 1 | 20 | 99 | 1.43 | 0.66 | 3.09 | derived |  |

### 3G Adeno – Base includes low smokers beyond limit defined as acceptable

| MOLLO | 3 | m | a | ever | cig+/-ot | all | 0 | 11 | 20 | 2.46 | 0.75 | 8.04 | derived |  |
| --- | --- | --- | --- | --- | --- | --- | --- | --- | --- | --- | --- | --- | --- | --- |
| MOLLO | 4 | m | a | ever | cig+/-ot | all | 0 | 21 | 99 | 2.38 | 0.74 | 7.69 | derived |  |

### 4G Large – Base "low"

| SOBUE | 83 | m | 1 | current | cig+/-ot | all | 5 | 20 | 29 | 2.10 | 0.80 | 5.30 | original |  |
| --- | --- | --- | --- | --- | --- | --- | --- | --- | --- | --- | --- | --- | --- | --- |
| SOBUE | 84 | m | 1 | current | cig+/-ot | all | 5 | 30 | 99 | 2.60 | 1.00 | 6.60 | original |  |

### G5 Small – Base "low"

| KHUDER | 22 | m | 1 | ever | cig+/-ot | all | 5 | 40 | 99 | 11.50 | 3.90 | 34.40 | original |  |
| --- | --- | --- | --- | --- | --- | --- | --- | --- | --- | --- | --- | --- | --- | --- |
| SOBUE | 81 | m | 1 | current | cig+/-ot | all | 5 | 20 | 29 | 0.80 | 0.40 | 1.50 | original |  |
| SOBUE | 82 | m | 1 | current | cig+/-ot | all | 5 | 30 | 99 | 2.30 | 1.30 | 4.20 | original |  |

# Table H Age started

### 1H All LC – Regression

| **REF** | **NRR** | **SEX** | **LC TYPE** | **SMKSTA** | **PRODUCT** | **CIGT** | **AD** | **ADOSMK** | **EXPLO** | **EXPHI** | **UNEXLO** | **UNEXHI** | **RR** | **RRL** | **RRU** | **DERIVE** | **COMMENT** |
| --- | --- | --- | --- | --- | --- | --- | --- | --- | --- | --- | --- | --- | --- | --- | --- | --- | --- |
|  |  |  |  |  |  |  |  |  |  |  |  |  |  |  |  |  |  |
| GUO | 501 | c | all | ever | cig+/-ot | all | 4 | 0 | - | - | - | - | * | * | * | original | Stated to be significant but flawed model as appears to include pack years as well |
| ZHANG | 501 | m | all | ever | all/unsp | - | 3 | 0 | - | - | - | - | 1.02 | 0.98 | 1.06 | original |  |
| ZHANG | 502 | f | all | ever | all/unsp | - | 4 | 0 | - | - | - | - | 1.02 | 0.97 | 1.07 | original |  |

### 1H All LC – Other

| MCDUFF | 501 | m | all | current | cig+/-ot | all | 0 | 0 | - | - | - | - | * | * | * | original | A greater percentage of cases (43.4% v 27.8% of controls) began smoking before the age of 15, and the mean age of commencement was earlier for cases than controls |
| --- | --- | --- | --- | --- | --- | --- | --- | --- | --- | --- | --- | --- | --- | --- | --- | --- | --- |
| SPITZ | 503 | c | all | ever | cig+/-ot | all | 0 | 0 | - | - | - | - | * | * | * | derived | Mean age started was 17.8 for cases and 19.3 for controls (derived from similar separate data by ethnic group) |
| STASZE | 505 | m | all | ever | all/unsp | - | 0 | 0 | - | - | - | - | * | * | * | derived | Individuals with lung cancer began smoking on the average 1.8 years earlier than those in the control group |

### 2H Squamous – Other

| WYNDE6 | 940 | m | q | current | cig only | all | 0 | 0 | - | - | - | - | * | * | * | original | Mean age of start was 16.0 in cases and 18.0 in controls |
| --- | --- | --- | --- | --- | --- | --- | --- | --- | --- | --- | --- | --- | --- | --- | --- | --- | --- |
| WYNDE6 | 944 | f | q | current | cig only | all | 0 | 0 | - | - | - | - | * | * | * | original | Mean age of start was 19.1 in cases and 21.5 in controls |

### 3H Adeno – Other

| WYNDE6 | 943 | m | a | current | cig only | all | 0 | 0 | - | - | - | - | * | * | * | original | Mean age of start was 16.6 in cases and 18.0 in controls |
| --- | --- | --- | --- | --- | --- | --- | --- | --- | --- | --- | --- | --- | --- | --- | --- | --- | --- |
| WYNDE6 | 947 | f | a | current | cig only | all | 0 | 0 | - | - | - | - | * | * | * | original | Mean age of start was 18.4 in cases and 21.5 in controls |

### 4H Large – Other

| WYNDE6 | 942 | m | 1 | current | cig only | all | 0 | 0 | - | - | - | - | * | * | * | original | Mean age of start was 15.4 in cases and 18.0 in controls |
| --- | --- | --- | --- | --- | --- | --- | --- | --- | --- | --- | --- | --- | --- | --- | --- | --- | --- |
| WYNDE6 | 946 | f | 1 | current | cig only | all | 0 | 0 | - | - | - | - | * | * | * | original | Mean age of start was 18.6 in cases and 21.5 in controls |

### 5H Small – Other

| WYNDE6 | 941 | m | 1 | current | cig only | all | 0 | 0 | - | - | - | - | * | * | * | original | Mean age of start was 17.5 in cases and 18.0 in controls |
| --- | --- | --- | --- | --- | --- | --- | --- | --- | --- | --- | --- | --- | --- | --- | --- | --- | --- |
| WYNDE6 | 945 | f | 1 | current | cig only | all | 0 | 0 | - | - | - | - | * | * | * | original | Mean age of start was 18.4 in cases and 21.5 in controls |

# Table I Duration

### 1I All LC – Regression

| **REF** | **NRR** | | **SEX** | | **LC TYPE** | **SMKSTA** | **PRODUCT** | **CIGT** | **AD** | **ADOSMK** | **EXPLO** | **EXPHI** | **UNEXLO** | **UNEXHI** | **RR** | **RRL** | **RRU** | **DERIVE** | **COMMENT** |  |
| --- | --- | --- | --- | --- | --- | --- | --- | --- | --- | --- | --- | --- | --- | --- | --- | --- | --- | --- | --- | --- |
|  |  | |  | |  |  |  |  |  |  |  |  |  |  |  |  |  |  |  |  |
| BOFFET | 561 | | m | | all | ever | cig only | all | 3 | 1 # | - | - | - | - | 1.08 | 1.08 | 1.09 | original | RR presented is the increase in risk for 1 year. Originally presented as 1.084(1.078-1.091) |  |
| BOFFET | 563 | | m | | all | ever | p only | - | 3 | 1 # | - | - | - | - | 1.09 | 1.05 | 1.14 | original | RR presented is the increase in risk for 1 year. Originally given as 1.091(1.047-1.137) |  |
| BOFFET | 562 | | m | | all | ever | cgr only | - | 3 | 1 # | - | - | - | - | 1.06 | 1.02 | 1.10 | original | RR presented is the increase in risk for 1 year. Originally given as 1.058(1.019-1.098) |  |
| GENG | 538 | | f | | all | ever | cig+/-ot | all | 0 | 0 | - | - | - | - | * | * | * | original | The RR/CI for 1- years smoking is given as 1.73 (1.38-2.18), for 20- years as 3.00 (2.17-4.16) and for 40- years as 5.20 (3.49-7.75). This is assumed to be a fitted model |  |
| WYNDE8 | 505 | | m | | all | current | cig+/-ot | all | 6 | 3 # | - | - | - | - | * | * | * | original | The adjusted regression for duration of smoking was 1.07 (1.04-1.09) |  |
| WYNDE8 | 506 | | f | | all | current | cig+/-ot | all | 6 | 3 # | - | - | - | - | * | * | * | original | The adjusted regression for duration of smoking was 1.08 (1.06-1.11) |  |
| ZHANG | 503 | | m | | all | ever | all/unsp | - | 3 | 0 | - | - | - | - | 1.07 | 1.03 | 1.11 | original |  |  |
| ZHANG | 504 | | f | | all | ever | all/unsp | - | 4 | 0 | - | - | - | - | 1.12 | 1.04 | 1.20 | original |  |  |
|  | |  | |  | | | | | | | | | | | | | | | | |
|  | |  | | **Comments on values in listings** | | | | | | | | | | | | | | | | |
|  | |  | |  | | | | | | | | | | | | | | | | |
| **BOFFET** | | **ADOSMK** | | Age at start of smoking | | | | | | | | | | | | | | | | |
| **WYNDE8** | | **ADOSMK** | | Menthol, inhalation and CPD (of the current brand) | | | | | | | | | | | | | | | | |

### 1I All LC – Base includes ex-smokers

| **REF** | **NRR** | **SEX** | **LC TYPE** | **SMKSTA** | **PRODUCT** | **CIGT** | **AD** | **ADOSMK** | **EXPLO** | **EXPHI** | **UNEXLO** | **UNEXHI** | **RR** | **RRL** | **RRU** | **DERIVE** | **COMMENT** |
| --- | --- | --- | --- | --- | --- | --- | --- | --- | --- | --- | --- | --- | --- | --- | --- | --- | --- |
|  |  |  |  |  |  |  |  |  |  |  |  |  |  |  |  |  |  |
| AKIBA | 501 | c | all | current | cig+/-ot | all | 5 | 0 | 5.00 | 19.00 | - | 4.00 | * | * | * | derived |  |
| AKIBA | 502 | c | all | current | cig+/-ot | all | 5 | 0 | 20.00 | 999.00 | - | 4.00 | * | * | * | other | RRs calculated as exponential of regression coefficient, CIs (where possible) from P values. Calculated as 1.53 (0.86-2.75) for 5 cigs/day, 2.10 (1.27-3.49) for 10 cigs/day, 3.34 for 20 cigs per day and 4.60 for 30 cigs/day |
| WATSON | 501 | m | all | current | all/unsp | - | 0 | 0 | 1.00 | 19.00 | - | - | 2.16 | 0.61 | 7.60 | derived |  |
| WATSON | 502 | m | all | current | all/unsp | - | 0 | 0 | 20.00 | 999.00 | - | - | 5.89 | 2.23 | 15.55 | derived |  |
| WATSON | 504 | f | all | current | all/unsp | - | 0 | 0 | 1.00 | 19.00 | - | - | 1.76 | 0.60 | 5.20 | derived |  |
| WATSON | 505 | f | all | current | all/unsp | - | 0 | 0 | 20.00 | 999.00 | - | - | 5.42 | 2.11 | 13.91 | derived |  |

### 1I All LC – Other

| LANGE | 508 | m | all | ever | all/unsp | - | 0 | 0 | - | - | - | - | * | * | * | original | Men who did not have lung cancer had an average duration of smoking of 28.2 years +-16.4 years (SD). Those who had lung cancer had an average duration of 43.3 years of smoking +-11.5 years (SD). |
| --- | --- | --- | --- | --- | --- | --- | --- | --- | --- | --- | --- | --- | --- | --- | --- | --- | --- |
| LANGE | 507 | f | all | ever | all/unsp | - | 0 | 0 | - | - | - | - | * | * | * | original | Women who did not have lung cancer had an average duration of smoking of 17.3 years +- 14.8 years (SD). Those who had lung cancer had an average duration of 28.9 years of smoking +- 12.6 years (SD). |
| MCDUFF | 502 | m | all | current | cig+/-ot | all | 0 | 0 | - | - | - | - | * | * | * | signif | The distribution of years smoked was significantly different between male patients and their current smoking siblings. More patients than siblings (controls) smoked for more than 50 years. |
| MCDUFF | 504 | m | all | ex | cig+/-ot | all | 0 | 0 | - | - | - | - | * | * | * | signif | The frequency distribution of the number of years smoked was highly significantly different. Patients smoked longer (>75% for more than 30 years, figures not stated for controls) |
| SPITZ | 504 | c | all | current | cig+/-ot | all | 0 | 0 | - | - | - | - | * | * | * | derived | Mean duration (years) was 36.2 for cases and 16.4 for controls (derived from similar separate data by ethnic group). Results based on smaller numbers of subjects, but not clearly stated if current or ever smokers, were similar from WU1998D but differed substantially from WU1998 |
| STASZE | 504 | m | all | ever | all/unsp | - | 0 | 0 | - | - | - | - | * | * | * | original | Cases smoked for an average of 37.1 years while controls smoked for an average of 32.2 years. Found not significant by original author. Lung cancer patients were two years older than controls |

### 1I All LC – Other

| **REF** | **NRR** | **SEX** | **LC TYPE** | **SMKSTA** | **PRODUCT** | **CIGT** | **AD** | **ADOSMK** | **EXPLO** | **EXPHI** | **UNEXLO** | **UNEXHI** | **RR** | **RRL** | **RRU** | **DERIVE** | **COMMENT** |
| --- | --- | --- | --- | --- | --- | --- | --- | --- | --- | --- | --- | --- | --- | --- | --- | --- | --- |
|  |  |  |  |  |  |  |  |  |  |  |  |  |  |  |  |  |  |
| WYNDE6 | 926 | m^1^ | all | ever | cig only | all | 0 | 0 | - | - | - | - | * | * | * | derived | Mean duration of smoking (years) was 37.9 in cases and 30.1 in controls |
| WYNDE6 | 932 | m^1^ | all | current | cig only | all | 0 | 0 | - | - | - | - | * | * | * | derived | Mean duration of smoking (years) was 40.3 in cases and 37.1 in controls |
| WYNDE6 | 938 | m^1^ | all | ex | cig only | all | 0 | 0 | - | - | - | - | * | * | * | derived | Mean duration of smoking (years) was 33.8 in cases and 25 in controls |
| WYNDE6 | 922 | m^2^ | all | ever | cig only | all | 0 | 0 | - | - | - | - | * | * | * | derived | Mean duration of smoking (years) was 37.9 in cases and 30 in controls |
| WYNDE6 | 928 | m^2^ | all | current | cig only | all | 0 | 0 | - | - | - | - | * | * | * | derived | Mean duration of smoking (years) was 40.2 in cases and 37 in controls |
| WYNDE6 | 934 | m^2^ | all | ex | cig only | all | 0 | 0 | - | - | - | - | * | * | * | derived | Mean duration of smoking (years) was 33.9 in cases and 25 in controls |
| WYNDE6 | 923 | m^3^ | all | ever | cig only | all | 0 | 0 | - | - | - | - | * | * | * | derived | Mean duration of smoking (years) was 38.6 in cases and 32 in controls |
| WYNDE6 | 929 | m^3^ | all | current | cig only | all | 0 | 0 | - | - | - | - | * | * | * | derived | Mean duration of smoking (years) was 40.6 in cases and 38 in controls |
| WYNDE6 | 935 | m^3^ | all | ex | cig only | all | 0 | 0 | - | - | - | - | * | * | * | derived | Mean duration of smoking (years) was 32.6 in cases and 25 in controls |
| WYNDE6 | 927 | f^1^ | all | ever | cig only | all | 0 | 0 | - | - | - | - | * | * | * | derived | Mean duration of smoking (years) was 36.5 in cases and 28.9 in controls |
| WYNDE6 | 933 | f^1^ | all | current | cig only | all | 0 | 0 | - | - | - | - | * | * | * | derived | Mean duration of smoking (years) was 38 in cases and 33.8 in controls |
| WYNDE6 | 939 | f^1^ | all | ex | cig only | all | 0 | 0 | - | - | - | - | * | * | * | derived | Mean duration of smoking (years) was 32.1 in cases and 23 in controls |
| WYNDE6 | 924 | f^2^ | all | ever | cig only | all | 0 | 0 | - | - | - | - | * | * | * | derived | Mean duration of smoking (years) was 36.6 in cases and 29 in controls |
| WYNDE6 | 930 | f^2^ | all | current | cig only | all | 0 | 0 | - | - | - | - | * | * | * | derived | Mean duration of smoking (years) was 38.1 in cases and 34 in controls |
| WYNDE6 | 936 | f^2^ | all | ex | cig only | all | 0 | 0 | - | - | - | - | * | * | * | derived | Mean duration of smoking (years) was 32.4 in cases and 23 in controls |
| WYNDE6 | 925 | f^3^ | all | ever | cig only | all | 0 | 0 | - | - | - | - | * | * | * | derived | Mean duration of smoking (years) was 35.2 in cases and 28 in controls |
| WYNDE6 | 931 | f^3^ | all | current | cig only | all | 0 | 0 | - | - | - | - | * | * | * | derived | Mean duration of smoking (years) was 37.2 in cases and 32 in controls |
| WYNDE6 | 937 | f^3^ | all | ex | cig only | all | 0 | 0 | - | - | - | - | * | * | * | derived | Mean duration of smoking (years) was 28.1 in cases and 23 in controls |

^1^ All races

^2^ White

^3^ Black

### 1I Squamous – Other

| **REF** | **NRR** | **SEX** | **LC TYPE** | **SMKSTA** | **PRODUCT** | **CIGT** | **AD** | **ADOSMK** | **EXPLO** | **EXPHI** | **UNEXLO** | **UNEXHI** | **RR** | **RRL** | **RRU** | **DERIVE** | **COMMENT** |
| --- | --- | --- | --- | --- | --- | --- | --- | --- | --- | --- | --- | --- | --- | --- | --- | --- | --- |
|  |  |  |  |  |  |  |  |  |  |  |  |  |  |  |  |  |  |
| STASZE | 501 | m | q | ever | all/unsp | - | 0 | 0 | - | - | - | - | * | * | * | original | Cases smoked for an average of 38.7 years while controls smoked for an average of 32.2 years. Found not significant by original author. Lung cancer patients were 2 years older than controls |
| WYNDE6 | 890 | m^1^ | KI | ever | cig only | all | 0 | 0 | - | - | - | - | * | * | * | derived | Mean duration of smoking (years) was 39 in cases and 30.1 in controls |
| WYNDE6 | 896 | m^1^ | KI | current | cig only | all | 0 | 0 | - | - | - | - | * | * | * | derived | Mean duration of smoking (years) was 41 in cases and 37.1 in controls |
| WYNDE6 | 902 | m^1^ | KI | ex | cig only | all | 0 | 0 | - | - | - | - | * | * | * | derived | Mean duration of smoking (years) was 34.9 in cases and 25 in controls |
| WYNDE6 | 886 | m^2^ | KI | ever | cig only | all | 0 | 0 | - | - | - | - | * | * | * | original | Mean duration of smoking (years) was 39 in cases and 30 in controls |
| WYNDE6 | 892 | m^2^ | KI | current | cig only | all | 0 | 0 | - | - | - | - | * | * | * | original | Mean duration of smoking (years) was 41 in cases and 37 in controls |
| WYNDE6 | 898 | m^2^ | KI | ex | cig only | all | 0 | 0 | - | - | - | - | * | * | * | original | Mean duration of smoking (years) was 35 in cases and 25 in controls |
| WYNDE6 | 887 | m^3^ | KI | ever | cig only | all | 0 | 0 | - | - | - | - | * | * | * | original | Mean duration of smoking (years) was 39 in cases and 32 in controls |
| WYNDE6 | 893 | m^3^ | KI | current | cig only | all | 0 | 0 | - | - | - | - | * | * | * | original | Mean duration of smoking (years) was 41 in cases and 38 in controls |
| WYNDE6 | 899 | m^3^ | KI | ex | cig only | all | 0 | 0 | - | - | - | - | * | * | * | original | Mean duration of smoking (years) was 33 in cases and 25 in controls |
| WYNDE6 | 891 | f^1^ | KI | ever | cig only | all | 0 | 0 | - | - | - | - | * | * | * | derived | Mean duration of smoking (years) was 37.9 in cases and 28.9 in controls |
| WYNDE6 | 897 | f^1^ | KI | current | cig only | all | 0 | 0 | - | - | - | - | * | * | * | derived | Mean duration of smoking (years) was 39 in cases and 33.8 in controls |
| WYNDE6 | 903 | f^1^ | KI | ex | cig only | all | 0 | 0 | - | - | - | - | * | * | * | derived | Mean duration of smoking (years) was 34.6 in cases and 23 in controls |
| WYNDE6 | 888 | f^2^ | KI | ever | cig only | all | 0 | 0 | - | - | - | - | * | * | * | original | Mean duration of smoking (years) was 38 in cases and 29 in controls |
| WYNDE6 | 894 | f^2^ | KI | current | cig only | all | 0 | 0 | - | - | - | - | * | * | * | original | Mean duration of smoking (years) was 39 in cases and 34 in controls |
| WYNDE6 | 900 | f^2^ | KI | ex | cig only | all | 0 | 0 | - | - | - | - | * | * | * | original | Mean duration of smoking (years) was 35 in cases and 23 in controls |
| WYNDE6 | 889 | f^3^ | KI | ever | cig only | all | 0 | 0 | - | - | - | - | * | * | * | original | Mean duration of smoking (years) was 37 in cases and 28 in controls |
| WYNDE6 | 895 | f^3^ | KI | current | cig only | all | 0 | 0 | - | - | - | - | * | * | * | original | Mean duration of smoking (years) was 39 in cases and 32 in controls |
| WYNDE6 | 901 | f^3^ | KI | ex | cig only | all | 0 | 0 | - | - | - | - | * | * | * | original | Mean duration of smoking (years) was 29 in cases and 23 in controls |

^1^ All races

^2^ White

^3^ Black

### 3I Adeno – Other

| **REF** | **NRR** | **SEX** | **LC TYPE** | **SMKSTA** | **PRODUCT** | **CIGT** | | **AD** | | **ADOSMK** | | | **EXPLO** | | **EXPHI** | | **UNEXLO** | | **UNEXHI** | | **RR** | | **RRL** | | **RRU** | | **DERIVE** | | **COMMENT** | |
| --- | --- | --- | --- | --- | --- | --- | --- | --- | --- | --- | --- | --- | --- | --- | --- | --- | --- | --- | --- | --- | --- | --- | --- | --- | --- | --- | --- | --- | --- | --- |
|  |  |  |  |  |  |  | |  | |  | | |  | |  | |  | |  | |  | |  | |  | |  | |  | |
| STASZE | 502 | m | a | ever | all/unsp | - | | 0 | | 0 | | | - | | - | | - | | - | | * | | * | | * | | original | | Cases smoked for an average of 32.9 years while controls smoked for an average of 32.2 years. Found not significant by original author. Lung cancer patients were 2 years older than controls | |
| WU2 | 503 | f | a | ever | all/unsp | - | | 2 | | 0 | | | - | | - | | - | | - | | * | | * | | * | | original | | A significant trend in risk with years of smoking was seen | |
| WYNDE6 | 908 | m^1^ | KII | ever | cig only | all | | 0 | | 0 | | | - | | - | | - | | - | | * | | * | | * | | derived | | Mean duration of smoking (years) was 36.2 in cases and 30.1 in controls | |
| WYNDE6 | 914 | m^1^ | KII | current | cig only | all | | 0 | | 0 | | | - | | - | | - | | - | | * | | * | | * | | derived | | Mean duration of smoking (years) was 39.1 in cases and 37.1 in controls | |
| WYNDE6 | 920 | m^1^ | KII | ex | cig only | all | | 0 | | 0 | | | - | | - | | - | | - | | * | | * | | * | | derived | | Mean duration of smoking (years) was 32 in cases and 25 in controls | |
| WYNDE6 | 904 | m^2^ | KII | ever | cig only | all | | 0 | | 0 | | | - | | - | | - | | - | | * | | * | | * | | original | | Mean duration of smoking (years) was 36 in cases and 30 in controls | |
| WYNDE6 | 910 | m^2^ | KII | current | cig only | all | | 0 | | 0 | | | - | | - | | - | | - | | * | | * | | * | | original | | Mean duration of smoking (years) was 39 in cases and 37 in controls | |
| WYNDE6 | 916 | m^2^ | KII | ex | cig only | all | | 0 | | 0 | | | - | | - | | - | | - | | * | | * | | * | | original | | Mean duration of smoking (years) was 32 in cases and 25 in controls | |
| WYNDE6 | 905 | m^3^ | KII | ever | cig only | all | | 0 | | 0 | | | - | | - | | - | | - | | * | | * | | * | | original | | Mean duration of smoking (years) was 38 in cases and 32 in controls | |
| WYNDE6 | 911 | m^3^ | KII | current | cig only | all | | 0 | | 0 | | | - | | - | | - | | - | | * | | * | | * | | original | | Mean duration of smoking (years) was 40 in cases and 38 in controls | |
| WYNDE6 | 917 | m^3^ | KII | ex | cig only | all | | 0 | | 0 | | | - | | - | | - | | - | | * | | * | | * | | original | | Mean duration of smoking (years) was 32 in cases and 25 in controls | |
| WYNDE6 | 909 | f^1^ | KII | ever | cig only | all | | 0 | | 0 | | | - | | - | | - | | - | | * | | * | | * | | derived | | Mean duration of smoking (years) was 34.9 in cases and 28.9 in controls | |
| WYNDE6 | 915 | f^1^ | KII | current | cig only | all | | 0 | | 0 | | | - | | - | | - | | - | | * | | * | | * | | derived | | Mean duration of smoking (years) was 36.9 in cases and 33.8 in controls | |
| WYNDE6 | 921 | f^1^ | KII | ex | cig only | all | | 0 | | 0 | | | - | | - | | - | | - | | * | | * | | * | | derived | | Mean duration of smoking (years) was 29.8 in cases and 23 in controls | |
| WYNDE6 | 906 | f^2^ | KII | ever | cig only | all | | 0 | | 0 | | | - | | - | | - | | - | | * | | * | | * | | original | | Mean duration of smoking (years) was 35 in cases and 29 in controls | |
| WYNDE6 | 912 | f^2^ | KII | current | cig only | all | | 0 | | 0 | | | - | | - | | - | | - | | * | | * | | * | | original | | Mean duration of smoking (years) was 37 in cases and 34 in controls | |
| WYNDE6 | 918 | f^2^ | KII | ex | cig only | all | | 0 | | 0 | | | - | | - | | - | | - | | * | | * | | * | | original | | Mean duration of smoking (years) was 30 in cases and 23 in controls | |
| WYNDE6 | 907 | f^3^ | KII | ever | cig only | all | | 0 | | 0 | | | - | | - | | - | | - | | * | | * | | * | | original | | Mean duration of smoking (years) was 33 in cases and 28 in controls | |
| WYNDE6 | 913 | f^3^ | KII | current | cig only | | all | | 0 | | 0 | - | | - | | - | | - | | * | | * | | * | | original | | Mean duration of smoking (years) was 35 in cases and 32 in controls | |  |
| WYNDE6 | 919 | f^3^ | KII | ex | cig only | | all | | 0 | | 0 | - | | - | | - | | - | | * | | * | | * | | original | | Mean duration of smoking (years) was 27 in cases and 23 in controls | |  |

^1^ All races

^2^ White

^3^ Black

### 5I Small –Other

| **REF** | **NRR** | **SEX** | **LC TYPE** | **SMKSTA** | **PRODUCT** | **CIGT** | **AD** | **ADOSMK** | **EXPLO** | **EXPHI** | **UNEXLO** | **UNEXHI** | **RR** | **RRL** | **RRU** | **DERIVE** | **COMMENT** |
| --- | --- | --- | --- | --- | --- | --- | --- | --- | --- | --- | --- | --- | --- | --- | --- | --- | --- |
|  |  |  |  |  |  |  |  |  |  |  |  |  |  |  |  |  |  |
| STASZE | 503 | m | 1 | ever | all/unsp | - | 0 | 0 | - | - | - | - | * | * | * | original | Cases smoked for an average of 31.0 years while controls smoked for an average of 32.2 years. Found not significant by original author. Lung cancer patients were two years older than controls |

# Table J Years quit (vs never)

### 1J All LC – Regression

| **REF** | | **NRR** | **SEX** | | **LC TYPE** | **SMKSTA** | **PRODUCT** | **CIGT** | **AD** | **ADOSMK** | **EXPLO** | **EXPHI** | **UNEXLO** | **UNEXHI** | **RR** | **RRL** | **RRU** | **DERIVE** | **COMMENT** |  |
| --- | --- | --- | --- | --- | --- | --- | --- | --- | --- | --- | --- | --- | --- | --- | --- | --- | --- | --- | --- | --- |
|  | |  |  | |  |  |  |  |  |  |  |  |  |  |  |  |  |  |  |  |
| SPITZ | | 502 | c | | all | ex | cig+/-ot | all | 5 | 1 # | - | - | - | - | * | * | * | original | OR 0.77 (0.66-0.90) per decade of cessation |  |
|  |  | | |  | | | | | | | | | | | | | | | | |
|  |  | | | **Comments on values in listings** | | | | | | | | | | | | | | | | |
|  |  | | |  | | | | | | | | | | | | | | | | |
| **SPITZ** | **ADOSMK** | | | pack years | | | | | | | | | | | | | | | | |

### 1J All LC – Other

| **REF** | **NRR** | **SEX** | **LC TYPE** | **SMKSTA** | **PRODUCT** | **CIGT** | **AD** | **ADOSMK** | **EXPLO** | **EXPHI** | **UNEXLO** | **UNEXHI** | **RR** | **RRL** | **RRU** | **DERIVE** | **COMMENT** |
| --- | --- | --- | --- | --- | --- | --- | --- | --- | --- | --- | --- | --- | --- | --- | --- | --- | --- |
|  |  |  |  |  |  |  |  |  |  |  |  |  |  |  |  |  |  |
| MCDUFF | 503 | m | all | ex | cig+/-ot | all | 0 | 0 | - | - | - | - | * | * | * | signif | The frequency distribution of the number of years refrained from smoking was highly significantly different. The majority of patients (64%) refrained for less than 5 years (figures not stated for controls) |
| SPITZ | 501 | c | all | ex | cig+/-ot | all | 0 | 0 | - | - | - | - | * | * | * | original | Mean years quit was 7.8 for cases and 17.9 for controls (p<0.001). Results in African-Americans and Mexican-Americans, based on smaller numbers of subjects reported by other papers, were similar (WU1997B, WU1998, WU1998D) |

# Table K Years quit (vs current)

### 1K All LC – Base includes recent smokers beyond limit defined as acceptable

| **REF** | **NRR** | | **SEX** | | **LC TYPE** | **SMKSTA** | **PRODUCT** | **CIGT** | **AD** | **ADOSMK** | **EXPLO** | **EXPHI** | **UNEXLO** | **UNEXHI** | **RR** | **RRL** | **RRU** | **DERIVE** | **COMMENT** |  |
| --- | --- | --- | --- | --- | --- | --- | --- | --- | --- | --- | --- | --- | --- | --- | --- | --- | --- | --- | --- | --- |
|  |  | |  | |  |  |  |  |  |  |  |  |  |  |  |  |  |  |  |  |
| GARSHI | 520 | | m | | all | ex | all/unsp | - | 0 | 0 | 5.00 | 14.00 | - | 4.00 | 0.68 | 0.55 | 0.85 | derived |  |  |
| GARSHI | 521 | | m | | all | ex | all/unsp | - | 0 | 0 | 15.00 | 999.00 | - | 4.00 | 0.43 | 0.34 | 0.55 | derived |  |  |
| GARSHI | 527 | | m | | all | ex | all/unsp | - | 1 | 0 | 5.00 | 14.00 | - | 4.00 | 0.65 | 0.52 | 0.81 | derived |  |  |
| GARSHI | 528 | | m | | all | ex | all/unsp | - | 1 | 0 | 15.00 | 999.00 | - | 4.00 | 0.41 | 0.32 | 0.52 | derived |  |  |
| GARSHI | 506 | | m | | all | ex | all/unsp | - | 0 | 0 | 5.00 | 14.00 | - | 4.00 | 0.67 | 0.42 | 1.07 | derived |  |  |
| GARSHI | 507 | | m | | all | ex | all/unsp | - | 0 | 0 | 15.00 | 999.00 | - | 4.00 | 0.39 | 0.22 | 0.69 | derived |  |  |
| GARSHI | 513 | | m | | all | ex | all/unsp | - | 0 | 0 | 5.00 | 14.00 | - | 4.00 | 0.65 | 0.50 | 0.83 | derived |  |  |
| GARSHI | 514 | | m | | all | ex | all/unsp | - | 0 | 0 | 15.00 | 999.00 | - | 4.00 | 0.41 | 0.32 | 0.53 | derived |  |  |
| JEDRYC | 616 | | m | | all | ex | cig+/-ot | all | 5 | 2 # | 5.00 | 9.00 | - | 4.00 | 0.66 | 0.45 | 0.98 | original |  |  |
| JEDRYC | 617 | | m | | all | ex | cig+/-ot | all | 5 | 2 # | 10.00 | 999.00 | - | 4.00 | 0.40 | 0.29 | 0.56 | original |  |  |
| WAKAI | 527 | | m | | all | ex | cig+/-ot | all | 0 | 0 | 5.00 | 9.00 | - | 4.00 | 0.63 | 0.36 | 1.10 | derived |  |  |
| WAKAI | 528 | | m | | all | ex | cig+/-ot | all | 0 | 0 | 10.00 | 19.00 | - | 4.00 | 0.97 | 0.58 | 1.63 | derived |  |  |
| WAKAI | 529 | | m | | all | ex | cig+/-ot | all | 0 | 0 | 20.00 | 999.00 | - | 4.00 | 0.24 | 0.10 | 0.53 | derived |  |  |
| WAKAI | 535 | | m | | all | ex | cig+/-ot | all | 2 | 0 | 5.00 | 9.00 | - | 4.00 | 0.61 | 0.34 | 1.09 | derived |  |  |
| WAKAI | 536 | | m | | all | ex | cig+/-ot | all | 2 | 0 | 10.00 | 19.00 | - | 4.00 | 0.90 | 0.53 | 1.53 | derived |  |  |
| WAKAI | 537 | | m | | all | ex | cig+/-ot | all | 2 | 0 | 20.00 | 999.00 | - | 4.00 | 0.25 | 0.11 | 0.56 | derived |  |  |
|  | |  | |  | | | | | | | | | | | | | | | | |
|  | |  | | **Comments on values in listings** | | | | | | | | | | | | | | | | |
|  | |  | |  | | | | | | | | | | | | | | | | |
| **JEDRYC** | | **ADOSMK** | | Number of cigs per day & age of start | | | | | | | | | | | | | | | | |

### 2K Squamous – Base includes recent smokers beyond limit defined as acceptable

| **REF** | **NRR** | | **SEX** | | **LC TYPE** | **SMKSTA** | **PRODUCT** | **CIGT** | **AD** | **ADOSMK** | **EXPLO** | **EXPHI** | **UNEXLO** | **UNEXHI** | **RR** | **RRL** | **RRU** | **DERIVE** | **COMMENT** |  |
| --- | --- | --- | --- | --- | --- | --- | --- | --- | --- | --- | --- | --- | --- | --- | --- | --- | --- | --- | --- | --- |
|  |  | |  | |  |  |  |  |  |  |  |  |  |  |  |  |  |  |  |  |
| JEDRYC | 549 | | m | | q | ex | cig+/-ot | all | 0 | 0 | 5.00 | 9.00 | - | 4.00 | 0.59 | 0.36 | 0.97 | derived |  |  |
| JEDRYC | 550 | | m | | q | ex | cig+/-ot | all | 0 | 0 | 10.00 | 999.00 | - | 4.00 | 0.22 | 0.14 | 0.35 | derived |  |  |
| JEDRYC | 654 | | m | | KI | ex | cig+/-ot | all | 0 | 0 | 5.00 | 9.00 | - | 4.00 | 0.61 | 0.40 | 0.94 | derived |  |  |
| JEDRYC | 655 | | m | | KI | ex | cig+/-ot | all | 0 | 0 | 10.00 | 999.00 | - | 4.00 | 0.26 | 0.18 | 0.38 | derived |  |  |
| JEDRYC | 573 | | m | | q | ex | cig+/-ot | all | 5 | 2 # | 5.00 | 9.00 | - | 4.00 | 0.58 | 0.36 | 0.94 | original |  |  |
| JEDRYC | 574 | | m | | q | ex | cig+/-ot | all | 5 | 2 # | 10.00 | 999.00 | - | 4.00 | 0.31 | 0.19 | 0.52 | original |  |  |
| JEDRYC | 602 | | m | | KI | ex | cig+/-ot | all | 5 | 2 # | 5.00 | 9.00 | - | 4.00 | 0.63 | 0.42 | 0.95 | original |  |  |
| JEDRYC | 603 | | m | | KI | ex | cig+/-ot | all | 5 | 2 # | 10.00 | 999.00 | - | 4.00 | 0.38 | 0.26 | 0.58 | original |  |  |
| WAKAI | 543 | | m | | q | ex | cig+/-ot | all | 0 | 0 | 5.00 | 9.00 | - | 4.00 | 0.75 | 0.37 | 1.51 | derived |  |  |
| WAKAI | 544 | | m | | q | ex | cig+/-ot | all | 0 | 0 | 10.00 | 19.00 | - | 4.00 | 0.89 | 0.45 | 1.77 | derived |  |  |
| WAKAI | 545 | | m | | q | ex | cig+/-ot | all | 0 | 0 | 20.00 | 999.00 | - | 4.00 | 0.21 | 0.06 | 0.69 | derived |  |  |
| WAKAI | 551 | | m | | q | ex | cig+/-ot | all | 1 | 0 | 5.00 | 9.00 | - | 4.00 | 0.74 | 0.37 | 1.48 | derived |  |  |
| WAKAI | 552 | | m | | q | ex | cig+/-ot | all | 1 | 0 | 10.00 | 19.00 | - | 4.00 | 0.88 | 0.45 | 1.74 | derived |  |  |
| WAKAI | 553 | | m | | q | ex | cig+/-ot | all | 1 | 0 | 20.00 | 999.00 | - | 4.00 | 0.20 | 0.06 | 0.66 | derived |  |  |
|  | |  | |  | | | | | | | | | | | | | | | | |
|  | |  | | **Comments on values in listings** | | | | | | | | | | | | | | | | |
|  | |  | |  | | | | | | | | | | | | | | | | |
| **JEDRYC** | | **ADOSMK** | | Number of cigarettes per day & age of start | | | | | | | | | | | | | | | | |

### 3K Adeno – Base includes recent smokers beyond limit defined as acceptable

| **REF** | **NRR** | | **SEX** | | **LC TYPE** | **SMKSTA** | **PRODUCT** | **CIGT** | **AD** | **ADOSMK** | **EXPLO** | **EXPHI** | **UNEXLO** | **UNEXHI** | **RR** | **RRL** | **RRU** | **DERIVE** | **COMMENT** |  |
| --- | --- | --- | --- | --- | --- | --- | --- | --- | --- | --- | --- | --- | --- | --- | --- | --- | --- | --- | --- | --- |
|  |  | |  | |  |  |  |  |  |  |  |  |  |  |  |  |  |  |  |  |
| JEDRYC | 565 | | m | | a | ex | cig+/-ot | all | 0 | 0 | 5.00 | 9.00 | - | 4.00 | 0.83 | 0.40 | 1.73 | derived |  |  |
| JEDRYC | 566 | | m | | a | ex | cig+/-ot | all | 0 | 0 | 10.00 | 999.00 | - | 4.00 | 0.40 | 0.21 | 0.75 | derived |  |  |
| JEDRYC | 581 | | m | | a | ex | cig+/-ot | all | 5 | 2 # | 5.00 | 9.00 | - | 4.00 | 1.08 | 0.57 | 2.04 | original |  |  |
| JEDRYC | 582 | | m | | a | ex | cig+/-ot | all | 5 | 2 # | 10.00 | 999.00 | - | 4.00 | 0.42 | 0.19 | 0.90 | original |  |  |
| WAKAI | 559 | | m | | a | ex | cig+/-ot | all | 0 | 0 | 5.00 | 9.00 | - | 4.00 | 0.56 | 0.24 | 1.30 | derived |  |  |
| WAKAI | 560 | | m | | a | ex | cig+/-ot | all | 0 | 0 | 10.00 | 19.00 | - | 4.00 | 1.14 | 0.58 | 2.23 | derived |  |  |
| WAKAI | 561 | | m | | a | ex | cig+/-ot | all | 0 | 0 | 20.00 | 999.00 | - | 4.00 | 0.25 | 0.07 | 0.81 | derived |  |  |
| WAKAI | 567 | | m | | a | ex | cig+/-ot | all | 1 | 0 | 5.00 | 9.00 | - | 4.00 | 0.57 | 0.25 | 1.31 | derived |  |  |
| WAKAI | 568 | | m | | a | ex | cig+/-ot | all | 1 | 0 | 10.00 | 19.00 | - | 4.00 | 1.16 | 0.59 | 2.26 | derived |  |  |
| WAKAI | 569 | | m | | a | ex | cig+/-ot | all | 1 | 0 | 20.00 | 999.00 | - | 4.00 | 0.25 | 0.08 | 0.82 | derived |  |  |
|  | |  | |  | | | | | | | | | | | | | | | | |
|  | |  | | **Comments on values in listings** | | | | | | | | | | | | | | | | |
|  | |  | |  | | | | | | | | | | | | | | | | |
| **JEDRYC** | | **ADOSMK** | | Number of cigarettes per day & age of start | | | | | | | | | | | | | | | | |

### 5K Small – Base includes recent smokers beyond limit defined as acceptable

| **REF** | **NRR** | | **SEX** | | **LC TYPE** | **SMKSTA** | **PRODUCT** | **CIGT** | **AD** | **ADOSMK** | **EXPLO** | **EXPHI** | **UNEXLO** | **UNEXHI** | **RR** | **RRL** | **RRU** | **DERIVE** | **COMMENT** |  |
| --- | --- | --- | --- | --- | --- | --- | --- | --- | --- | --- | --- | --- | --- | --- | --- | --- | --- | --- | --- | --- |
|  |  | |  | |  |  |  |  |  |  |  |  |  |  |  |  |  |  |  |  |
| JEDRYC | 557 | | m | | 1 | ex | cig+/-ot | all | 0 | 0 | 5.00 | 9.00 | - | 4.00 | 0.66 | 0.34 | 1.28 | derived |  |  |
| JEDRYC | 558 | | m | | 1 | ex | cig+/-ot | all | 0 | 0 | 10.00 | 999.00 | - | 4.00 | 0.36 | 0.21 | 0.62 | derived |  |  |
| JEDRYC | 577 | | m | | 1 | ex | cig+/-ot | all | 5 | 2 # | 5.00 | 9.00 | - | 4.00 | 0.81 | 0.44 | 1.49 | original |  |  |
| JEDRYC | 578 | | m | | 1 | ex | cig+/-ot | all | 5 | 2 # | 10.00 | 999.00 | - | 4.00 | 0.52 | 0.28 | 0.94 | original |  |  |
|  | |  | |  | | | | | | | | | | | | | | | | |
|  | |  | | **Comments on values in listings** | | | | | | | | | | | | | | | | |
|  | |  | |  | | | | | | | | | | | | | | | | |
| **JEDRYC** | | **ADOSMK** | | Number of cigarettes per day & age of start | | | | | | | | | | | | | | | | |

# L Tar

### 1L All LC – Other

| **REF** | | **NRR** | **SEX** | | **LC TYPE** | **SMKSTA** | **PRODUCT** | **CIGT** | **AD** | **ADOSMK** | **EXPLO** | **EXPHI** | **UNEXLO** | **UNEXHI** | **RR** | **RRL** | **RRU** | **DERIVE** | **COMMENT** |  |
| --- | --- | --- | --- | --- | --- | --- | --- | --- | --- | --- | --- | --- | --- | --- | --- | --- | --- | --- | --- | --- |
|  | |  |  | |  |  |  |  |  |  |  |  |  |  |  |  |  |  |  |  |
| CPSII | | 703 | f | | all | current | cig+/-ot | 1 | 3 | 2 # | - | - | - | - | * | * | * | original | In the model given the exponentiated coefficient for tar yield was 1.031 (p<0.01) but the model includes never smokers as well as ncigs and inhalation so is difficult to interpret |  |
| WYNDE6 | | 872 | m | | all | ever | cig only | all | 0 | 0 | - | - | - | - | * | * | * | derived | Mean tar level (mg/cig) was 19.6 in cases and 20 in controls |  |
| WYNDE6 | | 878 | m | | all | current | cig only | all | 0 | 0 | - | - | - | - | * | * | * | derived | Mean tar level (mg/cig) was 19.6 in cases and 19.1 in controls |  |
| WYNDE6 | | 884 | m | | all | ex | cig only | all | 0 | 0 | - | - | - | - | * | * | * | derived | Mean tar level (mg/cig) was 20 in cases and 20 in controls |  |
| WYNDE6 | | 868 | m | | all | ever | cig only | all | 0 | 0 | - | - | - | - | * | * | * | derived | Mean tar level (mg/cig) was 19.6 in cases and 20 in controls |  |
| WYNDE6 | | 874 | m | | all | current | cig only | all | 0 | 0 | - | - | - | - | * | * | * | derived | Mean tar level (mg/cig) was 19.6 in cases and 19 in controls |  |
| WYNDE6 | | 880 | m | | all | ex | cig only | all | 0 | 0 | - | - | - | - | * | * | * | derived | Mean tar level (mg/cig) was 20 in cases and 20 in controls |  |
| WYNDE6 | | 869 | m | | all | ever | cig only | all | 0 | 0 | - | - | - | - | * | * | * | derived | Mean tar level (mg/cig) was 19.6 in cases and 20 in controls |  |
| WYNDE6 | | 875 | m | | all | current | cig only | all | 0 | 0 | - | - | - | - | * | * | * | derived | Mean tar level (mg/cig) was 19.6 in cases and 20 in controls |  |
| WYNDE6 | | 881 | m | | all | ex | cig only | all | 0 | 0 | - | - | - | - | * | * | * | derived | Mean tar level (mg/cig) was 19.6 in cases and 20 in controls |  |
| WYNDE6 | | 873 | f | | all | ever | cig only | all | 0 | 0 | - | - | - | - | * | * | * | derived | Mean tar level (mg/cig) was 17.1 in cases and 17 in controls |  |
| WYNDE6 | | 878 | f | | all | current | cig only | all | 0 | 0 | - | - | - | - | * | * | * | derived | Mean tar level (mg/cig) was 17.2 in cases and 17.1 in controls |  |
| WYNDE6 | | 885 | f | | all | ex | cig only | all | 0 | 0 | - | - | - | - | * | * | * | derived | Mean tar level (mg/cig) was 17.1 in cases and 17.9 in controls |  |
| WYNDE6 | | 870 | f | | all | ever | cig only | all | 0 | 0 | - | - | - | - | * | * | * | derived | Mean tar level (mg/cig) was 17 in cases and 17 in controls |  |
| WYNDE6 | | 876 | f | | all | current | cig only | all | 0 | 0 | - | - | - | - | * | * | * | derived | Mean tar level (mg/cig) was 17 in cases and 17 in controls |  |
| WYNDE6 | | 882 | f | | all | ex | cig only | all | 0 | 0 | - | - | - | - | * | * | * | derived | Mean tar level (mg/cig) was 17 in cases and 18 in controls |  |
| WYNDE6 | | 871 | f | | all | ever | cig only | all | 0 | 0 | - | - | - | - | * | * | * | derived | Mean tar level (mg/cig) was 19 in cases and 17 in controls |  |
| WYNDE6 | | 877 | f | | all | current | cig only | all | 0 | 0 | - | - | - | - | * | * | * | derived | Mean tar level (mg/cig) was 19 in cases and 18 in controls |  |
| WYNDE6 | | 883 | f | | all | ex | cig only | all | 0 | 0 | - | - | - | - | * | * | * | derived | Mean tar level (mg/cig) was 18.5 in cases and 17 in controls |  |
|  |  | | |  | | | | | | | | | | | | | | | | |
|  |  | | | **Comments on values in listings** | | | | | | | | | | | | | | | | |
|  |  | | |  | | | | | | | | | | | | | | | | |
| **CPSII** | **ADOSMK** | | | Number per day and inhalation | | | | | | | | | | | | | | | | |

### 2L Squamous – Other

| **REF** | **NRR** | **SEX** | **LC TYPE** | **SMKSTA** | **PRODUCT** | **CIGT** | **AD** | **ADOSMK** | **EXPLO** | **EXPHI** | **UNEXLO** | **UNEXHI** | **RR** | **RRL** | **RRU** | **DERIVE** | **COMMENT** |
| --- | --- | --- | --- | --- | --- | --- | --- | --- | --- | --- | --- | --- | --- | --- | --- | --- | --- |
|  |  |  |  |  |  |  |  |  |  |  |  |  |  |  |  |  |  |
| WYNDE6 | 836 | m | KI | ever | cig only | all | 0 | 0 | - | - | - | - | * | * | * | derived | Mean tar level (mg/cig) was 20 in cases and 20 in controls |
| WYNDE6 | 842 | m | KI | current | cig only | all | 0 | 0 | - | - | - | - | * | * | * | derived | Mean tar level (mg/cig) was 20 in cases and 19.1 in controls |
| WYNDE6 | 848 | m | KI | ex | cig only | all | 0 | 0 | - | - | - | - | * | * | * | derived | Mean tar level (mg/cig) was 20 in cases and 20 in controls |
| WYNDE6 | 832 | m | KI | ever | cig only | all | 0 | 0 | - | - | - | - | * | * | * | original | Mean tar level (mg/cig) was 20 in cases and 20 in controls |
| WYNDE6 | 838 | m | KI | current | cig only | all | 0 | 0 | - | - | - | - | * | * | * | original | Mean tar level (mg/cig) was 20 in cases and 19 in controls |
| WYNDE6 | 844 | m | KI | ex | cig only | all | 0 | 0 | - | - | - | - | * | * | * | original | Mean tar level (mg/cig) was 20 in cases and 20 in controls |
| WYNDE6 | 833 | m | KI | ever | cig only | all | 0 | 0 | - | - | - | - | * | * | * | original | Mean tar level (mg/cig) was 20 in cases and 20 in controls |
| WYNDE6 | 839 | m | KI | current | cig only | all | 0 | 0 | - | - | - | - | * | * | * | original | Mean tar level (mg/cig) was 20 in cases and 20 in controls |
| WYNDE6 | 845 | m | KI | ex | cig only | all | 0 | 0 | - | - | - | - | * | * | * | original | Mean tar level (mg/cig) was 20 in cases and 20 in controls |
| WYNDE6 | 837 | f | KI | ever | cig only | all | 0 | 0 | - | - | - | - | * | * | * | derived | Mean tar level (mg/cig) was 17.2 in cases and 17 in controls |
| WYNDE6 | 843 | f | KI | current | cig only | all | 0 | 0 | - | - | - | - | * | * | * | derived | Mean tar level (mg/cig) was 17.2 in cases and 17.1 in controls |
| WYNDE6 | 849 | f | KI | ex | cig only | all | 0 | 0 | - | - | - | - | * | * | * | derived | Mean tar level (mg/cig) was 17.1 in cases and 17.9 in controls |
| WYNDE6 | 834 | f | KI | ever | cig only | all | 0 | 0 | - | - | - | - | * | * | * | original | Mean tar level (mg/cig) was 17 in cases and 17 in controls |
| WYNDE6 | 840 | f | KI | current | cig only | all | 0 | 0 | - | - | - | - | * | * | * | original | Mean tar level (mg/cig) was 17 in cases and 17 in controls |
| WYNDE6 | 846 | f | KI | ex | cig only | all | 0 | 0 | - | - | - | - | * | * | * | original | Mean tar level (mg/cig) was 17 in cases and 18 in controls |
| WYNDE6 | 835 | f | KI | ever | cig only | all | 0 | 0 | - | - | - | - | * | * | * | original | Mean tar level (mg/cig) was 19 in cases and 17 in controls |
| WYNDE6 | 841 | f | KI | current | cig only | all | 0 | 0 | - | - | - | - | * | * | * | original | Mean tar level (mg/cig) was 19 in cases and 18 in controls |
| WYNDE6 | 847 | f | KI | ex | cig only | all | 0 | 0 | - | - | - | - | * | * | * | original | Mean tar level (mg/cig) was 19 in cases and 17 in controls |

### 3L Adeno – Other

| **REF** | **NRR** | **SEX** | **LC TYPE** | **SMKSTA** | **PRODUCT** | **CIGT** | **AD** | **ADOSMK** | **EXPLO** | **EXPHI** | **UNEXLO** | **UNEXHI** | **RR** | **RRL** | **RRU** | **DERIVE** | **COMMENT** |
| --- | --- | --- | --- | --- | --- | --- | --- | --- | --- | --- | --- | --- | --- | --- | --- | --- | --- |
|  |  |  |  |  |  |  |  |  |  |  |  |  |  |  |  |  |  |
| WYNDE6 | 854 | m | KII | ever | cig only | all | 0 | 0 | - | - | - | - | * | * | * | derived | Mean tar level (mg/cig) was 19 in cases and 20 in controls |
| WYNDE6 | 860 | m | KII | current | cig only | all | 0 | 0 | - | - | - | - | * | * | * | derived | Mean tar level (mg/cig) was 19 in cases and 19.1 in controls |
| WYNDE6 | 866 | m | KII | ex | cig only | all | 0 | 0 | - | - | - | - | * | * | * | derived | Mean tar level (mg/cig) was 19.9 in cases and 20 in controls |
| WYNDE6 | 850 | m | KII | ever | cig only | all | 0 | 0 | - | - | - | - | * | * | * | original | Mean tar level (mg/cig) was 19 in cases and 20 in controls |
| WYNDE6 | 856 | m | KII | current | cig only | all | 0 | 0 | - | - | - | - | * | * | * | original | Mean tar level (mg/cig) was 19 in cases and 19 in controls |
| WYNDE6 | 862 | m | KII | ex | cig only | all | 0 | 0 | - | - | - | - | * | * | * | original | Mean tar level (mg/cig) was 20 in cases and 20 in controls |
| WYNDE6 | 851 | m | KII | ever | cig only | all | 0 | 0 | - | - | - | - | * | * | * | original | Mean tar level (mg/cig) was 19 in cases and 20 in controls |
| WYNDE6 | 857 | m | KII | current | cig only | all | 0 | 0 | - | - | - | - | * | * | * | original | Mean tar level (mg/cig) was 19 in cases and 20 in controls |
| WYNDE6 | 863 | m | KII | ex | cig only | all | 0 | 0 | - | - | - | - | * | * | * | original | Mean tar level (mg/cig) was 19 in cases and 20 in controls |
| WYNDE6 | 855 | f | KII | ever | cig only | all | 0 | 0 | - | - | - | - | * | * | * | derived | Mean tar level (mg/cig) was 17.1 in cases and 17 in controls |
| WYNDE6 | 861 | f | KII | current | cig only | all | 0 | 0 | - | - | - | - | * | * | * | derived | Mean tar level (mg/cig) was 17.1 in cases and 17.1 in controls |
| WYNDE6 | 867 | f | KII | ex | cig only | all | 0 | 0 | - | - | - | - | * | * | * | derived | Mean tar level (mg/cig) was 17.1 in cases and 17.9 in controls |
| WYNDE6 | 852 | f | KII | ever | cig only | all | 0 | 0 | - | - | - | - | * | * | * | original | Mean tar level (mg/cig) was 17 in cases and 17 in controls |
| WYNDE6 | 858 | f | KII | current | cig only | all | 0 | 0 | - | - | - | - | * | * | * | original | Mean tar level (mg/cig) was 17 in cases and 17 in controls |
| WYNDE6 | 864 | f | KII | ex | cig only | all | 0 | 0 | - | - | - | - | * | * | * | original | Mean tar level (mg/cig) was 17 in cases and 18 in controls |
| WYNDE6 | 853 | f | KII | ever | cig only | all | 0 | 0 | - | - | - | - | * | * | * | original | Mean tar level (mg/cig) was 19 in cases and 17 in controls |
| WYNDE6 | 859 | f | KII | current | cig only | all | 0 | 0 | - | - | - | - | * | * | * | original | Mean tar level (mg/cig) was 19 in cases and 18 in controls |
| WYNDE6 | 865 | f | KII | ex | cig only | all | 0 | 0 | - | - | - | - | * | * | * | original | Mean tar level (mg/cig) was 18 in cases and 17 in controls |

# M Butt length or Fraction smoked

### 1M All LC – Other

| **REF** | **NRR** | **SEX** | **LC TYPE** | **SMKSTA** | **PRODUCT** | **CIGT** | **AD** | **ADOSMK** | **EXPLO** | **EXPHI** | **UNEXLO** | **UNEXHI** | **RR** | **RRL** | **RRU** | **DERIVE** | **COMMENT** |
| --- | --- | --- | --- | --- | --- | --- | --- | --- | --- | --- | --- | --- | --- | --- | --- | --- | --- |
|  |  |  |  |  |  |  |  |  |  |  |  |  |  |  |  |  |  |
| JOLY | 697 | m | all | ever | cig+/-ot | all | 0 | 0 | - | - | - | - | * | * | * | original | No significant differences were noted between cases and controls with respect to butt length, most smokers consumed at least 3/4 of the cigarette |
| JOLY | 698 | f | all | ever | cig+/-ot | all | 0 | 0 | - | - | - | - | * | * | * | original | No significant differences were noted between cases and controls with respect to butt length, most smokers consumed at least 3/4 of the cigarette |
