# Supplementary material for: Systematic review with meta-analysis of the epidemiological evidence in the 1900s relating smoking to lung cancer
Source: BMC Cancer. 2012 Sep 3;12:385. doi: 10.1186/1471-2407-12-385 (PMC3505152; doi:10.1186/1471-2407-12-385)
Supplement: Additional file 5 — Detailed Analysis Tables (Individual file names as described in Additional file 1: Methods, Table1). [file 1471-2407-12-385-S5.zip › PDF/4ABCD.pdf]

Table 4A1 -

IESLC - Meta-analysis of Ever Smoking, Any product (or Cigarettes if Any not available)
Large

This analysis is restricted to results for:
1) Non-dose-response data
2) Ever smokers
3) Results complete enough for use in metaanalysis

Within each study, results are then selected (in the following order of preference, within each sex) for:
4) PRODUCT: all/unspec, cigarettes regardless of other products, cigarettes only
5) CIGTYPE: all/unspecified, MC regardless of HR, MC only
6) DENOM: never smoked anything, never smoked cigarettes, (never +1 = +long term ex, +2 = +amount unknown, +3 = never cigs+long term ex)
7) Followup period (YF, prospective studies): whole study (coded as 0) or longest available
8) LCtype: large (specifically)

9) Race: all or nearest available, otherwise by race (wh or w = white, bl or b = black, hi = hispanic ch = chinese, jap = japanese, haw = hawaiian, w+o = white + oriental, sca = scandinavian, as = asian)
10) For overlapping studies: principal rather than subsidiary studies
Finally by Age: whole study (coded as 0) if available, otherwise by widest available age group
and then for single sex results (m, f) in preference to combined sex results (c).

Results adjusted (AD) for the most potential confounders are then chosen in Sections -1 to -3
and results adjusted for the least confounders in Sections -4 to -6. (Those least adjusted results which
actually differ from the most adjusted as marked 'x' in column X in Section -4)
(Results adjusted for an unknown number of confounder(s) are coded as 20.)

Section -7 shows excluded studies, together with the stage (as above) at which no qualifying
results were found.

Section -8 lists the potentially overlapping studies which have been included (1=principal, 2=subsidiary).

Section -9 lists any results which would have been included in preference except that they had data not complete
enough for use in meta-analysis, with their significance (yes/no), if known, and any further comment as entered
on the database.

In addition to those mentioned above, the following fields, levels and abbreviations are used:
* or nk = not known, n = no, y = yes, ot = other
nev = never
all/unspec = all or unspecified, cig+/-ot = cigarettes irrespective of other products (cigar, pipe etc)
MC = manufactured cigarettes, HR = hand-rolled cigarettes
REF: 6-character study reference
NRR: number of the RR on the database within the study
ST : study type (CC = case control, pr or prosp = prospective)
NLC: number of lung cancer cases in whole study
R : risky occupational population (n = no, m = mining, o = other risky)
VB : national cigarette type (V = at least 75% Virginia, bl = at least 75% blended, ot = other)
P : any proxy use
H : full histological confirmation
De : derivation of RR/CI (or = original, st = standard method, ot = other method of estimation)

Table 4A1 - 1

IESLC - Meta-analysis of Ever Smoking, Any product (or Cigarettes if Any not available)
Large
Most adjusted

REF	NRR	SEX	AGEL	AGEH	RACE	YF	LC TYPE	LOC	START	ST	NLC	R	VB	P	H	AD	PRODUCT	DENOM	De
ANDERS	11	f	0	0	all	0	large	NAmer	1986	pr	343	n	bl	n	n	0	cig+/-ot	nev cigs	st
BAND	4	m	0	0	all	-	large	NAmer	1983	CC	2831	n	V	y	y	2	cig only	nev any	ot
BARBON	129	m	0	0	all	-	large	Eu:wst	1979	CC	755	n	bl	y	y	3	all/unsp	nev any	ot
BUFFLE	74	f	0	0	w-hi	-	large	NAmer	1976	CC	943	n	bl	y	n	0	cig+/-ot	nev cigs	ot
COMSTO	68	m	0	0	all	-	large	NAmer	1975	ot	258	n	bl	n	n	0	cig+/-ot	nev cigs	ot
COMSTO	80	f	0	0	all	-	large	NAmer	1975	ot	258	n	bl	n	n	0	cig+/-ot	nev cigs	ot
HEGMAN	5	c	0	0	all	-	large	NAmer	1989	CC	282	n	bl	y	y	0	all/unsp	nev any	st
HINDS	25	f	0	0	o	-	large	NAmer	1968	CC	292	n	bl	n	n	3	all/unsp	nev any	st
JAIN	50	m	0	0	all	-	large	NAmer	1981	CC	845	n	V	y	n	2	cig+/-ot	nev cigs	or
JAIN	45	f	0	0	all	-	large	NAmer	1981	CC	845	n	V	y	n	2	cig+/-ot	nev cigs	or
KHUDER	26	m	0	0	all	-	large	NAmer	1985	CC	482	n	bl	n	y	0	cig+/-ot	nev cigs	ot
KIHARA	28	c	0	0	jap	-	large	As:Jap	1991	CC	440	n	bl	n	n	0	all/unsp	nev any	st
LAMTH	4	f	0	0	ch	-	large	As:HK	1983	CC	445	n	bl	n	n	0	all/unsp	nev any	or
LAMWK	5	f	0	0	ch	-	large	As:HK	1981	CC	163	n	bl	n	n	0	all/unsp	nev any	st
LAMWK2	4	m	0	0	all	-	large	As:HK	1976	CC	480	n	bl	n	n	0	all/unsp	nev any	st
LAMWK2	8	f	0	0	all	-	large	As:HK	1976	CC	480	n	bl	n	n	0	all/unsp	nev any	st
NOU	4	m	0	0	all	-	large	Eu:Sca	1971	CC	273	n	bl	y	n	0	all/unsp	nev any	st
NOU	9	f	0	0	all	-	large	Eu:Sca	1971	CC	273	n	bl	y	n	0	all/unsp	nev any	st
ORMOS	16	m	0	0	all	-	large	Eu:est	1947	CC	119	n	bl	y	y	0	cig+/-ot	nev any	st
SEOW	5	f	0	0	ch	-	large	As:oth	1997	CC	153	n	bl	n	y	0	cig+/-ot	nev cigs	st
SOBUE	103	m	0	0	all	-	large	As:Jap	1986	CC	1376	n	bl	n	y	1	cig+/-ot	nev cigs	ot
SOBUE	113	f	0	0	all	-	large	As:Jap	1986	CC	1376	n	bl	n	y	1	cig+/-ot	nev cigs	ot
WYNDE6	389	m	0	0	all	-	large	NAmer	1969	CC	4423	n	bl	n	y	2	all/unsp	nev any	ot
WYNDE6	404	f	0	0	all	-	large	NAmer	1969	CC	4423	n	bl	n	y	2	all/unsp	nev any	ot
ZHOU	20	m	0	0	all	-	large	As:Chi	1978	CC	1360	n	ot	n	n	0	all/unsp	nev any	st
ZHOU	21	f	0	0	all	-	large	As:Chi	1978	CC	1360	n	ot	n	n	0	all/unsp	nev any	st

Cigarette type is all/unspec for all RRs

Table 4A1 - 2

IESLC - Meta-analysis of Ever Smoking, Any product (or Cigarettes if Any not available)
Large
Most adjusted

REF	NRR	SEX	AD	Number Exposed Case	Number Exposed Cont	Non-exposed Case	Non-exposed Cont	RR	95.00%CI
*ANDERS	11	f	0	20	96164	1	195158	40.59	(5.45- 302.43)
BAND	4	m	2	-	-	-	-	12.94	(4.08- 41.06)
BARBON	129	m	3	-	-	-	-	30.43	(4.19- 221.06)
BUFFLE	74	f	0	11	166	0	112	15.54~	(0.91- 266.39)
COMSTO	68	m	0	16	229	0	84	12.15~	(0.72- 204.77)
COMSTO	80	f	0	7	87	0	115	19.80~	(1.12- 351.37)
Subtotal COMSTO								15.44	(2.06- 115.86)
HEGMAN	5	c	0	18	1202	4	2080	7.79	(2.63- 23.06)
HINDS	25	f	3	-	-	-	-	4.71	(1.99- 11.11)
JAIN	50	m	2	-	-	-	-	6.00	(1.02- 113.00)
JAIN	45	f	2	-	-	-	-	6.50	(1.80- 41.60)
Subtotal JAIN								6.34	(1.72- 23.41)
KHUDER	26	m	0	23	-	2	-	5.90	(1.24- 28.10)
KIHARA	28	c	0	4	232	1	237	4.09	(0.45- 36.83)
LAMTH	4	f	0	11	3	9	17	6.93	(1.53- 31.38)
LAMWK	5	f	0	2	41	5	144	1.40	(0.26- 7.51)
LAMWK2	4	m	0	24	161	2	43	3.20	(0.73- 14.10)
LAMWK2	8	f	0	2	50	5	139	1.11	(0.21- 5.92)
Subtotal LAMWK2								2.01	(0.66- 6.10)
NOU	4	m	0	15	247	2	122	3.70	(0.83- 16.46)
NOU	9	f	0	2	92	7	261	0.81	(0.17- 3.97)
Subtotal NOU								1.82	(0.61- 5.40)
ORMOS	16	m	0	7	1034	1	777	5.26	(0.65- 42.84)
SEOW	5	f	0	8	15	13	125	5.13	(1.83- 14.38)
SOBUE	103	m	1	-	-	-	-	3.64	(1.15- 11.53)
SOBUE	113	f	1	-	-	-	-	3.81	(1.30- 11.15)
Subtotal SOBUE								3.73	(1.70- 8.19)
WYNDE6	389	m	2	-	-	-	-	9.72	(4.57- 20.70)
WYNDE6	404	f	2	-	-	-	-	12.83	(7.17- 22.93)
Subtotal WYNDE6								11.57	(7.30- 18.34)
ZHOU	20	m	0	29	41	8	36	3.18	(1.29- 7.84)
ZHOU	21	f	0	3	7	6	32	2.29	(0.46- 11.43)
Subtotal ZHOU								2.94	(1.34- 6.46)
Partial Totals				202	99771	66	199482		

*prospective study ~ With 0.5 adjustment for zero

REF	NRR	SEX	AD	Ys	Ws	Qs	Ps
*ANDERS	11	f	0	3.70	0.95	3.42	0.0003
BAND	4	m	2	2.56	2.88	1.62	0.0000
BARBON	129	m	3	3.42	0.98	2.52	0.0007
BUFFLE	74	f	0	2.74	0.48	0.41	0.0584
COMSTO	68	m	0	2.50	0.48	0.23	0.0831
COMSTO	80	f	0	2.99	0.46	0.64	0.0419
Subtotal COMSTO				2.74	0.95	0.87	
HEGMAN	5	c	0	2.05	3.26	0.19	0.0002
HINDS	25	f	3	1.55	5.20	0.35	0.0004
JAIN	50	m	2	1.79	0.69	0.00	0.1357
JAIN	45	f	2	1.87	1.56	0.01	0.0195
Subtotal JAIN				1.85	2.25	0.01	
KHUDER	26	m	0	1.77	1.58	0.00	0.0258
KIHARA	28	c	0	1.41	0.79	0.13	0.2096
LAMTH	4	f	0	1.94	1.68	0.03	0.0121
LAMWK	5	f	0	0.34	1.37	2.95	0.6910
LAMWK2	4	m	0	1.16	1.75	0.73	0.1233
LAMWK2	8	f	0	0.11	1.38	3.99	0.9009
Subtotal LAMWK2				0.70	3.13	4.72	
NOU	4	m	0	1.31	1.73	0.43	0.0852
NOU	9	f	0	-0.21	1.52	6.20	0.7956
Subtotal NOU				0.60	3.25	6.64	
ORMOS	16	m	0	1.66	0.87	0.02	0.1208
SEOW	5	f	0	1.63	3.62	0.11	0.0019
SOBUE	103	m	1	1.29	2.89	0.77	0.0280
SOBUE	113	f	1	1.34	3.33	0.74	0.0147
Subtotal SOBUE				1.32	6.22	1.52	
WYNDE6	389	m	2	2.27	6.73	1.45	0.0000
WYNDE6	404	f	2	2.55	11.37	6.26	0.0000
Subtotal WYNDE6				2.45	18.10	7.71	
ZHOU	20	m	0	1.16	4.72	2.01	0.0118
ZHOU	21	f	0	0.83	1.48	1.43	0.3140
Subtotal ZHOU				1.08	6.21	3.44	

Table 4A1 - 2

IESLC - Meta-analysis of Ever Smoking, Any product (or Cigarettes if Any not available)
Large
Most adjusted

	N	26
	NS	19
	Wt	63.75
Het	Chi	36.66
Het	df	25
Het	P	(*)
Fixed	RR	6.11
	RRl	4.78
	RRu	7.81
	P	+++
Random	RR	5.59
	RRl	4.05
	RRu	7.72
	P	+++
Asymm	P	N.S.

Table 4A1 - 3

IESLC - Meta-analysis of Ever Smoking, Any product (or Cigarettes if Any not available)

Large

Most adjusted

	Sex			
	combined	male	female	Total
N	2	11	13	26
NS	2	11	13	26
Wt	4.05	25.31	34.39	63.75
Het Chi	0.27	9.78	26.53	36.66
Het df	1	10	12	25
Het P	N.S.	N.S.	**	(*)
Fixed RR	6.86	6.21	5.95	6.11
RRl	2.59	4.21	4.26	4.78
RRu	18.17	9.17	8.31	7.81
P	+++	+++	+++	+++
Random RR	6.86	6.21	4.82	5.59
RRl	2.59	4.21	2.74	4.05
RRu	18.17	9.17	8.45	7.72
P	+++	+++	+++	+++
Between Chi				0.09
Between df				2
Between P				N.S.
Btwn(F) P				N.S.
Btwn(R) P				N.S.

	Lung cancer type	
	large	Total
N	26	26
NS	19	19
Wt	63.75	63.75
Het Chi	36.66	36.66
Het df	25	25
Het P	(*)	(*)
Fixed RR	6.11	6.11
RRl	4.78	4.78
RRu	7.81	7.81
P	+++	+++
Random RR	5.59	5.59
RRl	4.05	4.05
RRu	7.72	7.72
P	+++	+++
Between Chi		
Between df		
Between P		N.S.
Btwn(F) P		N.S.
Btwn(R) P		N.S.

	Location								
	NAmer	UK	Scand	othEur	China	Japan	othAs	other	Total
N	12		2	2	2	3	5		26
NS	9		1	2	1	2	4		19
Wt	35.64		3.25	1.85	6.21	7.01	9.79		63.75
Het Chi	7.11		1.87	1.42	0.12	0.01	4.25		36.66
Het df	11		1	1	1	2	4		25
Het P	N.S.		N.S.	N.S.	N.S.	N.S.	N.S.		(*)
Fixed RR	9.66		1.82	13.29	2.94	3.77	3.34		6.11
RRl	6.95		0.61	3.15	1.34	1.80	1.79		4.78
RRu	13.41		5.40	56.14	6.46	7.90	6.25		7.81
P	+++		N.S.	+++	++	+++	+++		+++
Random RR	9.66		1.78	13.10	2.94	3.77	3.30		5.59
RRl	6.95		0.40	2.35	1.34	1.80	1.72		4.05
RRu	13.41		7.88	73.06	6.46	7.90	6.33		7.72
P	+++		N.S.	++	++	+++	+++		+++
Between Chi									21.87
Between df									5
Between P									***
Btwn(F) P									**
Btwn(R) P									**

Table 4A1 - 3

IESLC - Meta-analysis of Ever Smoking, Any product (or Cigarettes if Any not available)

Large

Most adjusted

Detailed Country in "other Europe"

	multi	Germany	othWest	East	Balkans	Total
N			1	1		2
NS			1	1		2
Wt			0.98	0.87		1.85
Het Chi			0.00	0.00		1.42
Het df			0	0		1
Het P			N.S.	N.S.		N.S.
Fixed RR			30.43	5.26		13.29
RRl			4.19	0.65		3.15
RRu			221.03	42.84		56.14
P			+++	N.S.		+++
Random RR			30.43	5.26		13.10
RRl			4.19	0.65		2.35
RRu			221.03	42.84		73.06
P			+++	N.S.		++
Between Chi						1.42
Between df						1
Between P						N.S.
Btwn(F) P						N.S.
Btwn(R) P						N.S.

Detailed Country in "other Asia"

	India	HongKong	other	Total
N		4	1	5
NS		3	1	4
Wt		6.18	3.62	9.79
Het Chi		3.20	0.00	4.25
Het df		3	0	4
Het P		N.S.	N.S.	N.S.
Fixed RR		2.60	5.13	3.34
RRl		1.18	1.83	1.79
RRu		5.73	14.38	6.25
P		+	++	+++
Random RR		2.59	5.13	3.30
RRl		1.15	1.83	1.72
RRu		5.85	14.38	6.33
P		+	++	+++
Between Chi				1.05
Between df				1
Between P				N.S.
Btwn(F) P				N.S.
Btwn(R) P				N.S.

Detailed other continent

	SCAmer	Auslia	Africa	Total
N				
NS				
Wt				
Het Chi				
Het df				
Het P				N.S.
Fixed RR				
RRl				
RRu				
P				+++
Random RR				
RRl				
RRu				
P				+++
Between Chi				
Between df				
Between P				N.S.
Btwn(F) P				N.S.
Btwn(R) P				N.S.

Table 4A1 - 3

IESLC - Meta-analysis of Ever Smoking, Any product (or Cigarettes if Any not available)
Large
Most adjusted

	Start year of study					
	<1960	1960-69	1970-79	1980-89	1990+	Total
N	1	3	10	10	2	26
NS	1	2	6	8	2	19
Wt	0.87	23.30	14.98	20.19	4.41	63.75
Het Chi	0.00	3.59	13.13	9.74	0.03	36.66
Het df	0	2	9	9	1	25
Het P	N.S.	N.S.	N.S.	N.S.	N.S.	(*)
Fixed RR	5.26	9.47	3.34	6.08	4.92	6.11
RRl	0.65	6.31	2.01	3.93	1.94	4.78
RRu	42.84	14.21	5.54	9.41	12.52	7.81
P	N.S.	+++	+++	+++	+++	+++
Random RR	5.26	8.97	3.59	6.10	4.92	5.59
RRl	0.65	5.13	1.87	3.86	1.94	4.05
RRu	42.84	15.67	6.90	9.64	12.52	7.72
P	N.S.	+++	+++	+++	+++	+++
Between Chi						10.18
Between df						4
Between P						*
Btwn(F) P						N.S.
Btwn(R) P						N.S.

	Study type (1)		
	CC	other	Total
N	23	3	26
NS	17	2	19
Wt	61.86	1.90	63.75
Het Chi	32.26	0.50	36.66
Het df	22	2	25
Het P	(*)	N.S.	(*)
Fixed RR	5.85	25.08	6.11
RRl	4.56	6.05	4.78
RRu	7.50	104.01	7.81
P	+++	+++	+++
Random RR	5.23	25.08	5.59
RRl	3.78	6.05	4.05
RRu	7.24	104.01	7.72
P	+++	+++	+++
Between Chi			3.90
Between df			1
Between P			*
Btwn(F) P			N.S.
Btwn(R) P			*

	Study type (2)			
	CC	prosp	other	Total
N	23	1	2	26
NS	17	1	1	19
Wt	61.86	0.95	0.95	63.75
Het Chi	32.26	0.00	0.06	36.66
Het df	22	0	1	25
Het P	(*)	N.S.	N.S.	(*)
Fixed RR	5.85	40.59	15.44	6.11
RRl	4.56	5.45	2.06	4.78
RRu	7.50	302.43	115.86	7.81
P	+++	+++	++	+++
Random RR	5.23	40.59	15.44	5.59
RRl	3.78	5.45	2.06	4.05
RRu	7.24	302.43	115.86	7.72
P	+++	+++	++	+++
Between Chi				4.35
Between df				2
Between P				N.S.
Btwn(F) P				N.S.
Btwn(R) P				(*)

Table 4A1 - 3

IESLC - Meta-analysis of Ever Smoking, Any product (or Cigarettes if Any not available)

Large

Most adjusted

	Study size (number of LC cases)				
	100-249	250-499	500-999	1000+	Total
N	3	12	4	7	26
NS	3	9	3	4	19
Wt	5.86	20.78	3.70	33.41	63.75
Het Chi	1.77	14.91	1.75	13.17	36.66
Het df	2	11	3	6	25
Het P	N.S.	N.S.	N.S.	*	(*)
Fixed RR	3.80	4.70	10.76	7.34	6.11
RRl	1.69	3.06	3.89	5.23	4.78
RRu	8.55	7.22	29.79	10.30	7.81
P	++	+++	+++	+++	+++
Random RR	3.80	4.71	10.76	6.27	5.59
RRl	1.69	2.78	3.89	3.66	4.05
RRu	8.55	7.97	29.79	10.72	7.72
P	++	+++	+++	+++	+++
Between Chi					5.05
Between df					3
Between P					N.S.
Btwn(F) P					N.S.
Btwn(R) P					N.S.

	Risky occupational population			
	no	mining	othRisky	Total
N	26			26
NS	19			19
Wt	63.75			63.75
Het Chi	36.66			36.66
Het df	25			25
Het P	(*)			(*)
Fixed RR	6.11			6.11
RRl	4.78			4.78
RRu	7.81			7.81
P	+++			+++
Random RR	5.59			5.59
RRl	4.05			4.05
RRu	7.72			7.72
P	+++			+++
Between Chi				
Between df				
Between P				N.S.
Btwn(F) P				N.S.
Btwn(R) P				N.S.

	National cigarette tobacco type			
	Virginia	blended	other	Total
N	3	21	2	26
NS	2	16	1	19
Wt	5.13	52.41	6.21	63.75
Het Chi	0.65	31.49	0.12	36.66
Het df	2	20	1	25
Het P	N.S.	*	N.S.	(*)
Fixed RR	9.46	6.38	2.94	6.11
RRl	3.98	4.87	1.34	4.78
RRu	22.48	8.37	6.46	7.81
P	+++	+++	++	+++
Random RR	9.46	5.66	2.94	5.59
RRl	3.98	3.89	1.34	4.05
RRu	22.48	8.24	6.46	7.72
P	+++	+++	++	+++
Between Chi				4.40
Between df				2
Between P				N.S.
Btwn(F) P				N.S.
Btwn(R) P				N.S.

Table 4A1 - 3

IESLC - Meta-analysis of Ever Smoking, Any product (or Cigarettes if Any not available)
Large
Most adjusted

	Any proxy use		
	No/nk	Yes	Total
N	17	9	26
NS	12	7	19
Wt	49.79	13.97	63.75
Het Chi	25.23	11.34	36.66
Het df	16	8	25
Het P	(*)	N.S.	(*)
Fixed RR	5.99	6.56	6.11
RRl	4.54	3.88	4.78
RRu	7.91	11.08	7.81
P	+++	+++	+++
Random RR	5.28	6.42	5.59
RRl	3.60	3.35	4.05
RRu	7.75	12.30	7.72
P	+++	+++	+++
Between Chi			0.09
Between df			1
Between P			N.S.
Btwn(F) P			N.S.
Btwn(R) P			N.S.

	Full histological confirmation		
	No	Yes	Total
N	16	10	26
NS	11	8	19
Wt	26.25	37.51	63.75
Het Chi	17.29	9.73	36.66
Het df	15	9	25
Het P	N.S.	N.S.	(*)
Fixed RR	3.84	8.46	6.11
RRl	2.62	6.14	4.78
RRu	5.63	11.65	7.81
P	+++	+++	+++
Random RR	3.87	8.28	5.59
RRl	2.53	5.89	4.05
RRu	5.93	11.65	7.72
P	+++	+++	+++
Between Chi			9.65
Between df			1
Between P			**
Btwn(F) P			**
Btwn(R) P			**

	Number of adjustment variables (1)			
	0	1	2+/+nk	Total
N	17	2	7	26
NS	13	1	5	19
Wt	28.13	6.22	29.41	63.75
Het Chi	18.65	0.00	5.52	36.66
Het df	16	1	6	25
Het P	N.S.	N.S.	N.S.	(*)
Fixed RR	4.14	3.73	9.84	6.11
RRl	2.86	1.70	6.86	4.78
RRu	5.99	8.19	14.13	7.81
P	+++	++	+++	+++
Random RR	4.16	3.73	9.84	5.59
RRl	2.76	1.70	6.86	4.05
RRu	6.27	8.19	14.13	7.72
P	+++	++	+++	+++
Between Chi				12.48
Between df				2
Between P				**
Btwn(F) P				**
Btwn(R) P				**

Table 4A1 - 3

IESLC - Meta-analysis of Ever Smoking, Any product (or Cigarettes if Any not available)
Large
Most adjusted

	Number of adjustment variables (2) 0	1	2	3-5	6+/+nk	Total
N	17	2	5	2		26
NS	13	1	3	2		19
Wt	28.13	6.22	23.24	6.17		63.75
Het Chi	18.65	0.00	1.13	2.86		36.66
Het df	16	1	4	1		25
Het P	N.S.	N.S.	N.S.	(*)		(*)
Fixed RR	4.14	3.73	11.07	6.33		6.11
RRl	2.86	1.70	7.37	2.88		4.78
RRu	5.99	8.19	16.62	13.93		7.81
P	+++	++	+++	+++		+++
Random RR	4.16	3.73	11.07	9.58		5.59
RRl	2.76	1.70	7.37	1.62		4.05
RRu	6.27	8.19	16.62	56.56		7.72
P	+++	++	+++	+		+++
Between Chi						14.01
Between df						3
Between P						**
Btwn(F) P						*
Btwn(R) P						**

	Product all/unsp	cig+/-ot	cig only	Total
N	14	11	1	26
NS	10	8	1	19
Wt	43.96	16.91	2.88	63.75
Het Chi	28.63	6.33	0.00	36.66
Het df	13	10	0	25
Het P	**	N.S.	N.S.	(*)
Fixed RR	5.91	5.87	12.94	6.11
RRl	4.39	3.64	4.08	4.78
RRu	7.94	9.45	41.05	7.81
P	+++	+++	+++	+++
Random RR	4.63	5.87	12.94	5.59
RRl	2.85	3.64	4.08	4.05
RRu	7.50	9.45	41.05	7.72
P	+++	+++	+++	+++
Between Chi				1.70
Between df				2
Between P				N.S.
Btwn(F) P				N.S.
Btwn(R) P				N.S.

	Denominator nev any	nev cigs	Total
N	16	10	26
NS	12	7	19
Wt	47.72	16.04	63.75
Het Chi	30.32	6.32	36.66
Het df	15	9	25
Het P	*	N.S.	(*)
Fixed RR	6.18	5.90	6.11
RRl	4.65	3.62	4.78
RRu	8.21	9.63	7.81
P	+++	+++	+++
Random RR	5.05	5.90	5.59
RRl	3.24	3.62	4.05
RRu	7.86	9.63	7.72
P	+++	+++	+++
Between Chi			0.02
Between df			1
Between P			N.S.
Btwn(F) P			N.S.
Btwn(R) P			N.S.

Table 4A1 - 3

IESLC - Meta-analysis of Ever Smoking, Any product (or Cigarettes if Any not available)

Large
Most adjusted

	Derivation of RR/CI			
	Orig	StdCalc	Other	Total
N	3	13	10	26
NS	2	10	7	19
Wt	3.93	28.64	31.18	63.75
Het Chi	0.01	15.32	8.82	36.66
Het df	2	12	9	25
Het P	N.S.	N.S.	N.S.	(*)
Fixed RR	6.59	3.77	9.42	6.11
RRl	2.45	2.62	6.63	4.78
RRu	17.69	5.44	13.38	7.81
P	+++	+++	+++	+++
Random RR	6.59	3.69	9.42	5.59
RRl	2.45	2.40	6.63	4.05
RRu	17.69	5.67	13.38	7.72
P	+++	+++	+++	+++
Between Chi				12.51
Between df				2
Between P				**
Btwn(F) P				**
Btwn(R) P				**

Table 4A1 - 4

IESLC - Meta-analysis of Ever Smoking, Any product (or Cigarettes if Any not available)
Large
Least adjusted

REF	NRR	X	SEX	AGEL	AGEH	RACE	YF	LC TYPE	LOC	START	ST	NLC	R	VB	P	H	AD	PRODUCT	DENOM	De
ANDERS	11		f	0	0	all	0	large	NAmer	1986	pr	343	n	bl	n	n	0	cig+/-ot	nev cigs	st
BAND	4		m	0	0	all	-	large	NAmer	1983	CC	2831	n	V	y	y	2	cig only	nev any	ot
BARBON	118	x	m	0	0	all	-	large	Eu:wst	1979	CC	755	n	bl	y	y	0	all/unsp	nev any	st
BUFFLE	74		f	0	0	w-hi	-	large	NAmer	1976	CC	943	n	bl	y	n	0	cig+/-ot	nev cigs	ot
COMSTO	68		m	0	0	all	-	large	NAmer	1975	ot	258	n	bl	n	n	0	cig+/-ot	nev cigs	ot
COMSTO	80		f	0	0	all	-	large	NAmer	1975	ot	258	n	bl	n	n	0	cig+/-ot	nev cigs	ot
HEGMAN	5		c	0	0	all	-	large	NAmer	1989	CC	282	n	bl	y	y	0	all/unsp	nev any	st
HINDS	25		f	0	0	o	-	large	NAmer	1968	CC	292	n	bl	n	n	3	all/unsp	nev any	st
JAIN	10	x	m	0	0	all	-	large	NAmer	1981	CC	845	n	V	y	n	0	cig+/-ot	nev cigs	st
JAIN	5	x	f	0	0	all	-	large	NAmer	1981	CC	845	n	V	y	n	0	cig+/-ot	nev cigs	st
KHUDER	26		m	0	0	all	-	large	NAmer	1985	CC	482	n	bl	n	y	0	cig+/-ot	nev cigs	ot
KIHARA	28		c	0	0	jap	-	large	As:Jap	1991	CC	440	n	bl	n	n	0	all/unsp	nev any	st
LAMTH	4		f	0	0	ch	-	large	As:HK	1983	CC	445	n	bl	n	n	0	all/unsp	nev any	or
LAMWK	5		f	0	0	ch	-	large	As:HK	1981	CC	163	n	bl	n	n	0	all/unsp	nev any	st
LAMWK2	4		m	0	0	all	-	large	As:HK	1976	CC	480	n	bl	n	n	0	all/unsp	nev any	st
LAMWK2	8		f	0	0	all	-	large	As:HK	1976	CC	480	n	bl	n	n	0	all/unsp	nev any	st
NOU	4		m	0	0	all	-	large	Eu:Sca	1971	CC	273	n	bl	y	n	0	all/unsp	nev any	st
NOU	9		f	0	0	all	-	large	Eu:Sca	1971	CC	273	n	bl	y	n	0	all/unsp	nev any	st
ORMOS	16		m	0	0	all	-	large	Eu:est	1947	CC	119	n	bl	y	y	0	cig+/-ot	nev any	st
SEOW	5		f	0	0	ch	-	large	As:oth	1997	CC	153	n	bl	n	y	0	cig+/-ot	nev cigs	st
SOBUE	15	x	m	0	0	all	-	large	As:Jap	1986	CC	1376	n	bl	n	y	0	cig+/-ot	nev cigs	st
SOBUE	31	x	f	0	0	all	-	large	As:Jap	1986	CC	1376	n	bl	n	y	0	cig+/-ot	nev cigs	st
WYNDE6	343	x	m	0	0	all	-	large	NAmer	1969	CC	4423	n	bl	n	y	0	all/unsp	nev any	st
WYNDE6	373	x	f	0	0	all	-	large	NAmer	1969	CC	4423	n	bl	n	y	0	all/unsp	nev any	st
ZHOU	20		m	0	0	all	-	large	As:Chi	1978	CC	1360	n	ot	n	n	0	all/unsp	nev any	st
ZHOU	21		f	0	0	all	-	large	As:Chi	1978	CC	1360	n	ot	n	n	0	all/unsp	nev any	st

Cigarette type is all/unspec for all RRs

Table 4A1 - 5

IESLC - Meta-analysis of Ever Smoking, Any product (or Cigarettes if Any not available)
Large
Least adjusted

REF	NRR	SEX	AD	Number Exposed Case	Number Exposed Cont	Non-exposed Case	Non-exposed Cont	RR	95.00%CI
*ANDERS	11	f	0	20	96164	1	195158	40.59	(5.45- 302.43)
BAND	4	m	2	-	-	-	-	12.94	(4.08- 41.06)
BARBON	118	m	0	89	567	1	188	29.51	(4.08- 213.27)
BUFFLE	74	f	0	11	166	0	112	15.54~	(0.91- 266.39)
COMSTO	68	m	0	16	229	0	84	12.15~	(0.72- 204.77)
COMSTO	80	f	0	7	87	0	115	19.80~	(1.12- 351.37)
Subtotal COMSTO								15.44	(2.06- 115.86)
HEGMAN	5	c	0	18	1202	4	2080	7.79	(2.63- 23.06)
HINDS	25	f	3	-	-	-	-	4.71	(1.99- 11.11)
JAIN	10	m	0	28	277	1	85	8.59	(1.15- 64.09)
JAIN	5	f	0	28	196	4	214	7.64	(2.63- 22.18)
Subtotal JAIN								7.84	(3.06- 20.10)
KHUDER	26	m	0	23	-	2	-	5.90	(1.24- 28.10)
KIHARA	28	c	0	4	232	1	237	4.09	(0.45- 36.83)
LAMTH	4	f	0	11	3	9	17	6.93	(1.53- 31.38)
LAMWK	5	f	0	2	41	5	144	1.40	(0.26- 7.51)
LAMWK2	4	m	0	24	161	2	43	3.20	(0.73- 14.10)
LAMWK2	8	f	0	2	50	5	139	1.11	(0.21- 5.92)
Subtotal LAMWK2								2.01	(0.66- 6.10)
NOU	4	m	0	15	247	2	122	3.70	(0.83- 16.46)
NOU	9	f	0	2	92	7	261	0.81	(0.17- 3.97)
Subtotal NOU								1.82	(0.61- 5.40)
ORMOS	16	m	0	7	1034	1	777	5.26	(0.65- 42.84)
SEOW	5	f	0	8	15	13	125	5.13	(1.83- 14.38)
SOBUE	15	m	0	78	1013	3	128	3.29	(1.02- 10.56)
SOBUE	31	f	0	7	232	7	857	3.69	(1.28- 10.64)
Subtotal SOBUE								3.50	(1.60- 7.67)
WYNDE6	343	m	0	221	1895	7	650	10.83	(5.08- 23.10)
WYNDE6	373	f	0	141	779	13	936	13.03	(7.33- 23.18)
Subtotal WYNDE6								12.18	(7.70- 19.26)
ZHOU	20	m	0	29	41	8	36	3.18	(1.29- 7.84)
ZHOU	21	f	0	3	7	6	32	2.29	(0.46- 11.43)
Subtotal ZHOU								2.94	(1.34- 6.46)
Partial Totals				794	104730	102	202540		

*prospective study ~ With 0.5 adjustment for zero

REF	NRR	SEX	AD	Ys	Ws	Qs	Ps
*ANDERS	11	f	0	3.70	0.95	3.33	0.0003
BAND	4	m	2	2.56	2.88	1.52	0.0000
BARBON	118	m	0	3.38	0.98	2.36	0.0008
BUFFLE	74	f	0	2.74	0.48	0.39	0.0584
COMSTO	68	m	0	2.50	0.48	0.21	0.0831
COMSTO	80	f	0	2.99	0.46	0.62	0.0419
Subtotal COMSTO				2.74	0.95	0.83	
HEGMAN	5	c	0	2.05	3.26	0.16	0.0002
HINDS	25	f	3	1.55	5.20	0.42	0.0004
JAIN	10	m	0	2.15	0.95	0.10	0.0359
JAIN	5	f	0	2.03	3.38	0.14	0.0002
Subtotal JAIN				2.06	4.34	0.23	
KHUDER	26	m	0	1.77	1.58	0.01	0.0258
KIHARA	28	c	0	1.41	0.79	0.14	0.2096
LAMTH	4	f	0	1.94	1.68	0.02	0.0121
LAMWK	5	f	0	0.34	1.37	3.05	0.6910
LAMWK2	4	m	0	1.16	1.75	0.78	0.1233
LAMWK2	8	f	0	0.11	1.38	4.11	0.9009
Subtotal LAMWK2				0.70	3.13	4.89	
NOU	4	m	0	1.31	1.73	0.48	0.0852
NOU	9	f	0	-0.21	1.52	6.35	0.7956
Subtotal NOU				0.60	3.25	6.83	
ORMOS	16	m	0	1.66	0.87	0.03	0.1208
SEOW	5	f	0	1.63	3.62	0.14	0.0019
SOBUE	15	m	0	1.19	2.82	1.17	0.0459
SOBUE	31	f	0	1.31	3.43	0.95	0.0155
Subtotal SOBUE				1.25	6.25	2.13	
WYNDE6	343	m	0	2.38	6.69	2.01	0.0000
WYNDE6	373	f	0	2.57	11.58	6.23	0.0000
Subtotal WYNDE6				2.50	18.27	8.24	
ZHOU	20	m	0	1.16	4.72	2.16	0.0118
ZHOU	21	f	0	0.83	1.48	1.51	0.3140
Subtotal ZHOU				1.08	6.21	3.67	

Table 4A1 - 5

IESLC - Meta-analysis of Ever Smoking, Any product (or Cigarettes if Any not available)
Large
Least adjusted

	N	26
	NS	19
	Wt	66.04
Het	Chi	38.38
Het	df	25
Het	P	*
Fixed	RR	6.26
	RRl	4.92
	RRu	7.97
	P	+++
Random	RR	5.70
	RRl	4.12
	RRu	7.88
	P	+++
Asymm	P	N.S.

Table 4A1 - 6

IESLC - Meta-analysis of Ever Smoking, Any product (or Cigarettes if Any not available)
Large
Least adjusted

	Sex			
	combined	male	female	Total
N	2	11	13	26
NS	2	11	13	26
Wt	4.05	25.46	36.53	66.04
Het Chi	0.27	10.81	27.23	38.38
Het df	1	10	12	25
Het P	N.S.	N.S.	**	*
Fixed RR	6.86	6.40	6.10	6.26
RRl	2.59	4.34	4.41	4.92
RRu	18.17	9.44	8.44	7.97
P	+++	+++	+++	+++
Random RR	6.86	6.36	4.94	5.70
RRl	2.59	4.21	2.85	4.12
RRu	18.17	9.61	8.56	7.88
P	+++	+++	+++	+++
Between Chi				0.07
Between df				2
Between P				N.S.
Btwn(F) P				N.S.
Btwn(R) P				N.S.

	Lung cancer type	
	large	Total
N	26	26
NS	19	19
Wt	66.04	66.04
Het Chi	38.38	38.38
Het df	25	25
Het P	*	*
Fixed RR	6.26	6.26
RRl	4.92	4.92
RRu	7.97	7.97
P	+++	+++
Random RR	5.70	5.70
RRl	4.12	4.12
RRu	7.88	7.88
P	+++	+++
Between Chi		
Between df		
Between P		N.S.
Btwn(F) P		N.S.
Btwn(R) P		N.S.

	Location								
	NAmer	UK	Scand	othEur	China	Japan	othAs	other	Total
N	12		2	2	2	3	5		26
NS	9		1	2	1	2	4		19
Wt	37.89		3.25	1.86	6.21	7.05	9.79		66.04
Het Chi	7.09		1.87	1.37	0.12	0.04	4.25		38.38
Het df	11		1	1	1	2	4		25
Het P	N.S.		N.S.	N.S.	N.S.	N.S.	N.S.		*
Fixed RR	9.92		1.82	13.10	2.94	3.57	3.34		6.26
RRl	7.21		0.61	3.11	1.34	1.70	1.79		4.92
RRu	13.63		5.40	55.25	6.46	7.46	6.25		7.97
P	+++		N.S.	+++	++	+++	+++		+++
Random RR	9.92		1.78	12.93	2.94	3.57	3.30		5.70
RRl	7.21		0.40	2.39	1.34	1.70	1.72		4.12
RRu	13.63		7.88	69.94	6.46	7.46	6.33		7.88
P	+++		N.S.	++	++	+++	+++		+++
Between Chi									23.63
Between df									5
Between P									***
Btwn(F) P									**
Btwn(R) P									***

Table 4A1 - 6

IESLC - Meta-analysis of Ever Smoking, Any product (or Cigarettes if Any not available)
Large
Least adjusted

	Detailed Country in "other Europe"					
	multi	Germany	othWest	East	Balkans	Total
N			1	1		2
NS			1	1		2
Wt			0.98	0.87		1.86
Het Chi			0.00	0.00		1.37
Het df			0	0		1
Het P			N.S.	N.S.		N.S.
Fixed RR			29.51	5.26		13.10
RRl			4.08	0.65		3.11
RRu			213.27	42.84		55.25
P			+++	N.S.		+++
Random RR			29.51	5.26		12.93
RRl			4.08	0.65		2.39
RRu			213.27	42.84		69.94
P			+++	N.S.		++
Between Chi						1.37
Between df						1
Between P						N.S.
Btwn(F) P						N.S.
Btwn(R) P						N.S.

	Detailed Country in "other Asia"			
	India	HongKong	other	Total
N		4	1	5
NS		3	1	4
Wt		6.18	3.62	9.79
Het Chi		3.20	0.00	4.25
Het df		3	0	4
Het P		N.S.	N.S.	N.S.
Fixed RR		2.60	5.13	3.34
RRl		1.18	1.83	1.79
RRu		5.73	14.38	6.25
P		+	++	+++
Random RR		2.59	5.13	3.30
RRl		1.15	1.83	1.72
RRu		5.85	14.38	6.33
P		+	++	+++
Between Chi				1.05
Between df				1
Between P				N.S.
Btwn(F) P				N.S.
Btwn(R) P				N.S.

	Detailed other continent			
	SCAmer	Auslia	Africa	Total
N				
NS				
Wt				
Het Chi				
Het df				
Het P				N.S.
Fixed RR				
RRl				
RRu				
P				+++
Random RR				
RRl				
RRu				
P				+++
Between Chi				
Between df				
Between P				N.S.
Btwn(F) P				N.S.
Btwn(R) P				N.S.

Table 4A1 - 6

IESLC - Meta-analysis of Ever Smoking, Any product (or Cigarettes if Any not available)
Large
Least adjusted

	Start year of study					
	<1960	1960-69	1970-79	1980-89	1990+	Total
N	1	3	10	10	2	26
NS	1	2	6	8	2	19
Wt	0.87	23.47	14.99	22.31	4.41	66.04
Het Chi	0.00	3.80	13.02	10.44	0.03	38.38
Het df	0	2	9	9	1	25
Het P	N.S.	N.S.	N.S.	N.S.	N.S.	*
Fixed RR	5.26	9.87	3.33	6.25	4.92	6.26
RRl	0.65	6.58	2.01	4.13	1.94	4.92
RRu	42.84	14.79	5.53	9.47	12.52	7.97
P	N.S.	+++	+++	+++	+++	+++
Random RR	5.26	9.31	3.58	6.28	4.92	5.70
RRl	0.65	5.24	1.87	3.99	1.94	4.12
RRu	42.84	16.54	6.85	9.87	12.52	7.88
P	N.S.	+++	+++	+++	+++	+++
Between Chi						11.09
Between df						4
Between P						*
Btwn(F) P						N.S.
Btwn(R) P						N.S.

	Study type (1)		
	CC	other	Total
N	23	3	26
NS	17	2	19
Wt	64.15	1.90	66.04
Het Chi	34.11	0.50	38.38
Het df	22	2	25
Het P	*	N.S.	*
Fixed RR	6.01	25.08	6.26
RRl	4.70	6.05	4.92
RRu	7.67	104.01	7.97
P	+++	+++	+++
Random RR	5.34	25.08	5.70
RRl	3.84	6.05	4.12
RRu	7.41	104.01	7.88
P	+++	+++	+++
Between Chi			3.76
Between df			1
Between P			(*)
Btwn(F) P			N.S.
Btwn(R) P			*

	Study type (2)			
	CC	prosp	other	Total
N	23	1	2	26
NS	17	1	1	19
Wt	64.15	0.95	0.95	66.04
Het Chi	34.11	0.00	0.06	38.38
Het df	22	0	1	25
Het P	*	N.S.	N.S.	*
Fixed RR	6.01	40.59	15.44	6.26
RRl	4.70	5.45	2.06	4.92
RRu	7.67	302.43	115.86	7.97
P	+++	+++	++	+++
Random RR	5.34	40.59	15.44	5.70
RRl	3.84	5.45	2.06	4.12
RRu	7.41	302.43	115.86	7.88
P	+++	+++	++	+++
Between Chi				4.21
Between df				2
Between P				N.S.
Btwn(F) P				N.S.
Btwn(R) P				(*)

Table 4A1 - 6

IESLC - Meta-analysis of Ever Smoking, Any product (or Cigarettes if Any not available)

Large

Least adjusted

	Study size (number of LC cases)				
	100-249	250-499	500-999	1000+	Total
N	3	12	4	7	26
NS	3	9	3	4	19
Wt	5.86	20.78	5.79	33.61	66.04
Het Chi	1.77	14.91	1.50	14.50	38.38
Het df	2	11	3	6	25
Het P	N.S.	N.S.	N.S.	*	*
Fixed RR	3.80	4.70	10.38	7.47	6.26
RRl	1.69	3.06	4.60	5.33	4.92
RRu	8.55	7.22	23.44	10.47	7.97
P	++	+++	+++	+++	+++
Random RR	3.80	4.71	10.38	6.24	5.70
RRl	1.69	2.78	4.60	3.56	4.12
RRu	8.55	7.97	23.44	10.95	7.88
P	++	+++	+++	+++	+++
Between Chi					5.69
Between df					3
Between P					N.S.
Btwn(F) P					N.S.
Btwn(R) P					N.S.

	Risky occupational population			
	no	mining	othRisky	Total
N	26			26
NS	19			19
Wt	66.04			66.04
Het Chi	38.38			38.38
Het df	25			25
Het P	*			*
Fixed RR	6.26			6.26
RRl	4.92			4.92
RRu	7.97			7.97
P	+++			+++
Random RR	5.70			5.70
RRl	4.12			4.12
RRu	7.88			7.88
P	+++			+++
Between Chi				
Between df				
Between P				N.S.
Btwn(F) P				N.S.
Btwn(R) P				N.S.

	National cigarette tobacco type			
	Virginia	blended	other	Total
N	3	21	2	26
NS	2	16	1	19
Wt	7.22	52.62	6.21	66.04
Het Chi	0.44	32.91	0.12	38.38
Het df	2	20	1	25
Het P	N.S.	*	N.S.	*
Fixed RR	9.58	6.45	2.94	6.26
RRl	4.62	4.93	1.34	4.92
RRu	19.87	8.46	6.46	7.97
P	+++	+++	++	+++
Random RR	9.58	5.66	2.94	5.70
RRl	4.62	3.85	1.34	4.12
RRu	19.87	8.30	6.46	7.88
P	+++	+++	++	+++
Between Chi				4.90
Between df				2
Between P				(*)
Btwn(F) P				N.S.
Btwn(R) P				(*)

Table 4A1 - 6

IESLC - Meta-analysis of Ever Smoking, Any product (or Cigarettes if Any not available)
Large
Least adjusted

	Any proxy use No/nk	Yes	Total
N	17	9	26
NS	12	7	19
Wt	49.99	16.06	66.04
Het Chi	26.81	11.36	38.38
Het df	16	8	25
Het P	*	N.S.	*
Fixed RR	6.07	6.90	6.26
RRl	4.60	4.23	4.92
RRu	8.00	11.26	7.97
P	+++	+++	+++
Random RR	5.27	6.78	5.70
RRl	3.55	3.69	4.12
RRu	7.83	12.46	7.88
P	+++	+++	+++
Between Chi			0.20
Between df			1
Between P			N.S.
Btwn(F) P			N.S.
Btwn(R) P			N.S.

	Full histological confirmation No	Yes	Total
N	16	10	26
NS	11	8	19
Wt	28.33	37.71	66.04
Het Chi	18.80	10.82	38.38
Het df	15	9	25
Het P	N.S.	N.S.	*
Fixed RR	4.11	8.58	6.26
RRl	2.85	6.24	4.92
RRu	5.94	11.81	7.97
P	+++	+++	+++
Random RR	4.14	8.19	5.70
RRl	2.68	5.67	4.12
RRu	6.38	11.83	7.88
P	+++	+++	+++
Between Chi			8.75
Between df			1
Between P			**
Btwn(F) P			*
Btwn(R) P			*

	Number of adjustment variables (1) 0	1	2+/+nk	Total
N	24		2	26
NS	17		2	19
Wt	57.97		8.08	66.04
Het Chi	36.43		1.89	38.38
Het df	23		1	25
Het P	*		N.S.	*
Fixed RR	6.19		6.76	6.26
RRl	4.79		3.39	4.92
RRu	8.01		13.46	7.97
P	+++		+++	+++
Random RR	5.50		7.23	5.70
RRl	3.86		2.72	4.12
RRu	7.84		19.25	7.88
P	+++		+++	+++
Between Chi				0.05
Between df				1
Between P				N.S.
Btwn(F) P				N.S.
Btwn(R) P				N.S.

Table 4A1 - 6

IESLC - Meta-analysis of Ever Smoking, Any product (or Cigarettes if Any not available)
Large
Least adjusted

	Number of adjustment variables (2) 0	1	2	3-5	6+/+nk	Total
N	24		1	1		26
NS	17		1	1		19
Wt	57.97		2.88	5.20		66.04
Het Chi	36.43		0.00	0.00		38.38
Het df	23		0	0		25
Het P	*		N.S.	N.S.		*
Fixed RR	6.19		12.94	4.71		6.26
RRl	4.79		4.08	1.99		4.92
RRu	8.01		41.05	11.13		7.97
P	+++		+++	+++		+++
Random RR	5.50		12.94	4.71		5.70
RRl	3.86		4.08	1.99		4.12
RRu	7.84		41.05	11.13		7.88
P	+++		+++	+++		+++
Between Chi						1.95
Between df						2
Between P						N.S.
Btwn(F) P						N.S.
Btwn(R) P						N.S.

	Product all/unsp	cig+/-ot	cig only	Total
N	14	11	1	26
NS	10	8	1	19
Wt	44.13	19.03	2.88	66.04
Het Chi	29.72	7.07	0.00	38.38
Het df	13	10	0	25
Het P	**	N.S.	N.S.	*
Fixed RR	6.04	6.08	12.94	6.26
RRl	4.50	3.88	4.08	4.92
RRu	8.12	9.53	41.05	7.97
P	+++	+++	+++	+++
Random RR	4.66	6.08	12.94	5.70
RRl	2.85	3.88	4.08	4.12
RRu	7.63	9.53	41.05	7.88
P	+++	+++	+++	+++
Between Chi				1.59
Between df				2
Between P				N.S.
Btwn(F) P				N.S.
Btwn(R) P				N.S.

	Denominator nev any	nev cigs	Total
N	16	10	26
NS	12	7	19
Wt	47.89	18.15	66.04
Het Chi	31.32	7.05	38.38
Het df	15	9	25
Het P	**	N.S.	*
Fixed RR	6.31	6.12	6.26
RRl	4.75	3.87	4.92
RRu	8.38	9.70	7.97
P	+++	+++	+++
Random RR	5.08	6.12	5.70
RRl	3.24	3.87	4.12
RRu	7.97	9.70	7.88
P	+++	+++	+++
Between Chi			0.01
Between df			1
Between P			N.S.
Btwn(F) P			N.S.
Btwn(R) P			N.S.

Table 4A1 - 6

IESLC - Meta-analysis of Ever Smoking, Any product (or Cigarettes if Any not available)

Large
Least adjusted

	Derivation of RR/CI Orig	StdCalc	Other	Total
N	1	20	5	26
NS	1	14	4	19
Wt	1.68	58.48	5.88	66.04
Het Chi	0.00	35.41	0.91	38.38
Het df	0	19	4	25
Het P	N.S.	*	N.S.	*
Fixed RR	6.93	5.90	10.94	6.26
RRl	1.53	4.57	4.88	4.92
RRu	31.38	7.62	24.56	7.97
P	+	+++	+++	+++
Random RR	6.93	5.08	10.94	5.70
RRl	1.53	3.48	4.88	4.12
RRu	31.38	7.43	24.56	7.88
P	+	+++	+++	+++
Between Chi				2.06
Between df				2
Between P				N.S.
Btwn(F) P				N.S.
Btwn(R) P				N.S.

Table 4A1 - 7

IESLC - Meta-analysis of Ever Smoking, Any product (or Cigarettes if Any not available)
Large
Excluded studies (and stage at which they were excluded)

Stage	Studies
1	BOUCHA BUELL LAURIL MZILEN
2	BYERS2 ENSTRO HIRAY2 KANELL LIAW LICKIN MRFIT MURATA NOTANI RESTRE RIMING SAARIK SEGI2 SHIMIZ SITAS SOBUE2 TANG2 WALD WARSIN WATSON WU2 WYNDE5 WYNDE8 WYNDER
3	BERRIN CHEN TAO ULMER
6	BLOT1 BLOT2 BLOT3 HIRAOK JONES MOLLO PERSHA SCHWA2 VANDER
8	ABELIN ABRAHA AGUDO AKIBA ALDERS AMANDU AMES ARCHER ARMADA AUSTIN AUVINE AXELSO AXELSS BECHER BENHAM BENSHL BEST BLOHMK BLOT4 BOFFET BOUCOT BRESLO BRETT BROCKM BROSS BROWN1 BROWN2 BYERS1 CARPEN CASCO2 CASCOR CEDERL CHAN CHANG CHATZI CHEN2 CHEN3 CHIAZZ CHOI CHOW CHYOU COOKSO CORREA CPSI CPSII DAMBER DARBY DAVEYS DEAN DEAN2 DEAN3 DEKLER DESTE2 DESTEF DOCKER DOLL DOLL2 DORANT DORGAN DORN DOSEME DROSTE DU DUNN EBELIN ENGELA ESAKI FAN GAO GAO2 GARCIA GARDIN GARSHI GENG GER GILLIS GODLEY GOLLED GOODMA GRAHAM GREGOR GSELL HAENSZ HAMMO2 HAMMON HANSEN HEIN HENNEK HIRAYA HITOSU HOLE HOROWI HORWIT HU HU2 HUANG HUMBLE ISHIMA JAHN JARUP JARVHO JEDRYC JIANG JOLY JUSSAW KAISE2 KAISER KATSOU KAUFMA KELLER KINLEN KJUUS KNEKT KO KOHLME KOO KOULUM KREUZE KREYBE KUBIK LANGE LAUSSM LEI LEMARC LETOUR LEVIN LIDDEL LIU LIU2 LIU3 LIU4 LIU5 LOMBA2 LOMBAR LUBIN LUBIN2 LUO MACLEN MAGNUS MARSH MARSH2 MARTIS MASTRA MATOS MATSUD MCCONN MCDUFF MCLAUG MIGRAN MILLER MILLS MRFITR NAM NOTAN2 ODRISC OSANN OSANN2 PARKIN PASTOR PAWLEG PERNU PERSH2 PETO PEZZO2 PEZZOT PIKE PISANI POFFIJ POLEDN PRESCO QIAO QIAO2 RACHTA RADZIK RANDIG REN RONCO ROOTS ROTHSC SADOWS SANKAR SCHWAR SEGI SHAW SIEMIA SIMARA SIMONA SPEIZE SPITZ STASZE STAYNE STOCKS STOCKW STUCKE SUN SUZUK2 SUZUKI SVENSS TANG TENKAN TIZZAN TOKARS TOUSEY TSUGAN TULINI TVERDA VEIERO VUTUC WAKAI WANG WANG2 WANG3 WANG4 WICKLU WIGLE WILKIN WU WUNSCH WUWILL WYNDE2 WYNDE3 WYNDE4 WYNDE7 XIANGZ XU XU2 XU3 XU4 YAMAGU YONG YUAN ZHANG ZHENG

Table 4A1 - 8

Potentially overlapping studies

REF	REFGP	PRINC	OVERLAP/LINK
LAMWK2	LAMWK2	1	Chan/LamWK2
LAMTH	LAMTH	1	KOO/LAMTH/LAMWK
LAMWK	LAMWK	1	KOO/LAMTH/LAMWK
WYNDE6	WYNDE6	1	WYNDE5/6/7/8

Table 4A1 - 9

Most adjusted - insufficient data for metaanalysis

REF	NRR	SEX	AGEL	AGEH	RACE	YF	LC TYPE	LOC	START	ST	NLC	R	VB	P	H	AD	PRODUCT	DENOM	De
KOO	5	f	0	0	all	-	large	As:HK	1981	CC	200	n	bl	n	n	0	all/unsp	nev any	or

REF	NRR	RR	SIG	RRDATA	comment
KOO	5	0.48			0

Table 4B1 -

IESLC - Meta-analysis of Current Smoking (vs never smoking), Any product (or Cigarettes if Any not available)
Large

This analysis is restricted to results for:
1) Non-dose-response data
2) Current smokers
3) Results complete enough for use in metaanalysis

Within each study, results are then selected (in the following order of preference, within each sex) for:
4) PRODUCT: all/unspec, cigarettes regardless of other products, cigarettes only
5) CIGTYPE: all/unspecified, MC regardless of HR, MC only
6) DENOM: never smoked anything, never smoked cigarettes, (never +1 = +long term ex, +2 = +amount unknown,
+3 = never cigs+long term ex)
7) Followup period (YF, prospective studies): whole study (coded as 0) or longest available
8) LCtype: large (specifically)

9) Race: all or nearest available, otherwise by race (wh or w = white, bl or b = black, hi = hispanic
ch = chinese, jap = japanese, haw = hawaiian, w+o = white + oriental, sca = scandinavian, as = asian)
10) For overlapping studies: principal rather than subsidiary studies
Finally by Age: whole study (coded as 0) if available, otherwise by widest available age group
and then for single sex results (m, f) in preference to combined sex results (c).

Results adjusted (AD) for the most potential confounders are then chosen in Sections -1 to -3
and results adjusted for the least confounders in Sections -4 to -6. (Those least adjusted results which
actually differ from the most adjusted as marked 'x' in column X in Section -4)
(Results adjusted for an unknown number of confounder(s) are coded as 20.)

Section -7 shows excluded studies, together with the stage (as above) at which no qualifying
results were found.

Section -8 lists the potentially overlapping studies which have been included (1=principal, 2=subsidiary).

Section -9 lists any results which would have been included in preference except that they had data not complete
enough for use in meta-analysis, with their significance (yes/no), if known, and any further comment as entered
on the database.

In addition to those mentioned above, the following fields, levels and abbreviations are used:
* or nk = not known, n = no, y = yes, ot = other
nev = never
all/unspec = all or unspecified, cig+/-ot = cigarettes irrespective of other products (cigar, pipe etc)
MC = manufactured cigarettes, HR = hand-rolled cigarettes
REF: 6-character study reference
NRR: number of the RR on the database within the study
ST : study type (CC = case control, pr or prosp = prospective)
NLC: number of lung cancer cases in whole study
R : risky occupational population (n = no, m = mining, o = other risky)
VB : national cigarette type (V = at least 75% Virginia, bl = at least 75% blended, ot = other)
P : any proxy use
H : full histological confirmation
De : derivation of RR/CI (or = original, st = standard method, ot = other method of estimation)

Table 4B1 - 1

IESLC - Meta-analysis of Current Smoking (vs never smoking), Any product (or Cigarettes if Any not available)
Large
Most adjusted

REF	NRR	SEX	AGEL	AGEH	RACE	YF	LC TYPE	LOC	START	ST	NLC	R	VB	P	H	AD	PRODUCT	DENOM	De
BARBON	93	m	0	0	all	-	large	Eu:wst	1979	CC	755	n	bl	y	y	1	all/unsp	nev any	or
BOUCOT	150	m	0	0	all	0	large	NAmer	1951	pr	121	n	bl	n	n	2	cig only	nev any	ot
BUFFLE	75	f	0	0	w-hi	-	large	NAmer	1976	CC	943	n	bl	y	n	0	cig+/-ot	nev cigs	ot
COMSTO	25	m	0	0	all	-	large	NAmer	1975	ot	258	n	bl	n	n	0	cig+/-ot	nev cigs	ot
COMSTO	32	f	0	0	all	-	large	NAmer	1975	ot	258	n	bl	n	n	0	cig+/-ot	nev cigs	ot
JAIN	20	m	0	0	all	-	large	NAmer	1981	CC	845	n	V	y	n	0	cig+/-ot	nev cigs	st
JAIN	15	f	0	0	all	-	large	NAmer	1981	CC	845	n	V	y	n	0	cig+/-ot	nev cigs	st
KHUDER	16	m	0	0	all	-	large	NAmer	1985	CC	482	n	bl	n	y	0	cig+/-ot	nev cigs	or
KIHARA	4	c	0	0	jap	-	large	As:Jap	1991	CC	440	n	bl	n	n	0	all/unsp	nev any	st
SOBUE	40	m	0	0	all	-	large	As:Jap	1986	CC	1376	n	bl	n	y	1	cig+/-ot	nev cigs	or
SOBUE	50	f	0	0	all	-	large	As:Jap	1986	CC	1376	n	bl	n	y	1	cig+/-ot	nev cigs	or
SOBUE2	4	m	0	0	all	-	large	As:Jap	1965	CC	2083	n	bl	n	n	2	cig+/-ot	nev any	or
SOBUE2	8	f	0	0	all	-	large	As:Jap	1965	CC	2083	n	bl	n	n	2	cig+/-ot	nev any	or
WYNDE6	380	m	0	0	all	-	large	NAmer	1969	CC	4423	n	bl	n	y	2	cig+/-ot	nev any	or
WYNDE6	395	f	0	0	all	-	large	NAmer	1969	CC	4423	n	bl	n	y	2	cig+/-ot	nev any	or

Cigarette type is all/unspec for all RRs

Table 4B1 - 2

IESLC - Meta-analysis of Current Smoking (vs never smoking), Any product (or Cigarettes if Any not available)
Large
Most adjusted

REF	NRR	SEX	AD	Number Exposed Case	Cont	Non-exposed Case	Cont	RR	95.00%CI
BARBON	93	m	1	-	-	-	-	34.30	(4.70- 250.00)
*BOUCOT	150	m	2	-	-	-	-	3.40	(0.18- 63.12)
BUFFLE	75	f	0	8	110	0	112	17.31~	(0.99- 303.50)
COMSTO	25	m	0	12	100	0	84	21.02~	(1.23- 360.30)
COMSTO	32	f	0	7	52	0	115	33.00~	(1.85- 588.60)
Subtotal COMSTO								26.25	(3.47- 198.54)
JAIN	20	m	0	21	118	1	85	15.13	(2.00- 114.65)
JAIN	15	f	0	21	99	4	214	11.35	(3.79- 33.94)
Subtotal JAIN								12.11	(4.62- 31.74)
KHUDER	16	m	0	13	-	2	-	5.90	(1.20- 29.50)
KIHARA	4	c	0	4	162	1	237	5.85	(0.65- 52.83)
SOBUE	40	m	1	-	-	-	-	3.80	(1.20- 12.10)
SOBUE	50	f	1	-	-	-	-	3.70	(1.10- 11.70)
Subtotal SOBUE								3.75	(1.64- 8.57)
SOBUE2	4	m	2	-	-	-	-	4.10	(2.40- 7.20)
SOBUE2	8	f	2	-	-	-	-	3.80	(1.50- 9.30)
Subtotal SOBUE2								4.02	(2.51- 6.43)
WYNDE6	380	m	2	-	-	-	-	18.00	(8.30- 39.00)
WYNDE6	395	f	2	-	-	-	-	20.20	(11.10- 36.70)
Subtotal WYNDE6								19.35	(12.05- 31.05)
Partial Totals				86	641	8	847		

*prospective study ~ With 0.5 adjustment for zero

REF	NRR	SEX	AD	Ys	Ws	Qs	Ps
BARBON	93	m	1	3.54	0.97	1.92	0.0005
*BOUCOT	150	m	2	1.22	0.45	0.37	0.4130
BUFFLE	75	f	0	2.85	0.47	0.24	0.0511
COMSTO	25	m	0	3.05	0.48	0.40	0.0357
COMSTO	32	f	0	3.50	0.46	0.86	0.0174
Subtotal COMSTO				3.27	0.94	1.26	
JAIN	20	m	0	2.72	0.94	0.32	0.0086
JAIN	15	f	0	2.43	3.20	0.29	0.0000
Subtotal JAIN				2.49	4.14	0.61	
KHUDER	16	m	0	1.77	1.50	0.19	0.0298
KIHARA	4	c	0	1.77	0.79	0.10	0.1156
SOBUE	40	m	1	1.34	2.88	1.82	0.0235
SOBUE	50	f	1	1.31	2.75	1.85	0.0301
Subtotal SOBUE				1.32	5.63	3.67	
SOBUE2	4	m	2	1.41	12.73	6.57	0.0000
SOBUE2	8	f	2	1.34	4.62	2.91	0.0041
Subtotal SOBUE2				1.39	17.35	9.49	
WYNDE6	380	m	2	2.89	6.42	3.72	0.0000
WYNDE6	395	f	2	3.01	10.75	8.25	0.0000
Subtotal WYNDE6				2.96	17.16	11.97	

	N	15
	NS	10
	Wt	49.39
Het	Chi	29.82
Het	df	14
Het	P	**
Fixed	RR	8.41
	RRl	6.36
	RRu	11.12
	P	+++
Random	RR	8.56
	RRl	5.29
	RRu	13.86
	P	+++
Asymm	P	N.S.

Table 4B1 - 3

IESLC - Meta-analysis of Current Smoking (vs never smoking), Any product (or Cigarettes if Any not available)
Large
Most adjusted

		Sex		
	combined	male	female	Total
N	1	8	6	15
NS	1	8	6	15
Wt	0.79	26.36	22.24	49.39
Het Chi	0.00	14.30	13.09	29.82
Het df	0	7	5	14
Het P	N.S.	*	*	**
Fixed RR	5.85	6.92	10.73	8.41
RRl	0.65	4.73	7.08	6.36
RRu	52.83	10.14	16.26	11.12
P	N.S.	+++	+++	+++
Random RR	5.85	8.29	9.23	8.56
RRl	0.65	4.20	4.15	5.29
RRu	52.83	16.37	20.55	13.86
P	N.S.	+++	+++	+++
Between Chi				2.42
Between df				2
Between P				N.S.
Btwn(F) P				N.S.
Btwn(R) P				N.S.

	Lung cancer type	
	large	Total
N	15	15
NS	10	10
Wt	49.39	49.39
Het Chi	29.82	29.82
Het df	14	14
Het P	**	**
Fixed RR	8.41	8.41
RRl	6.36	6.36
RRu	11.12	11.12
P	+++	+++
Random RR	8.56	8.56
RRl	5.29	5.29
RRu	13.86	13.86
P	+++	+++
Between Chi		
Between df		
Between P		N.S.
Btwn(F) P		N.S.
Btwn(R) P		N.S.

				Location					
	NAmer	UK	Scand	othEur	China	Japan	othAs	other	Total
N	9			1		5			15
NS	6			1		3			10
Wt	24.65			0.97		23.77			49.39
Het Chi	3.89			0.00		0.16			29.82
Het df	8			0		4			14
Het P	N.S.			N.S.		N.S.			**
Fixed RR	16.28			34.30		4.00			8.41
RRl	10.97			4.70		2.68			6.36
RRu	24.15			250.16		5.98			11.12
P	+++			+++		+++			+++
Random RR	16.28			34.30		4.00			8.56
RRl	10.97			4.70		2.68			5.29
RRu	24.15			250.16		5.98			13.86
P	+++			+++		+++			+++
Between Chi									25.77
Between df									2
Between P									***
Btwn(F) P									***
Btwn(R) P									***

Table 4B1 - 3

IESLC - Meta-analysis of Current Smoking (vs never smoking), Any product (or Cigarettes if Any not available)
Large
Most adjusted

	Detailed Country in "other Europe"					
	multi	Germany	othWest	East	Balkans	Total
N			1			1
NS			1			1
Wt			0.97			0.97
Het Chi			0.00			0.00
Het df			0			0
Het P			N.S.			N.S.
Fixed RR			34.30			34.30
RRl			4.70			4.70
RRu			250.16			250.16
P			+++			+++
Random RR			34.30			34.30
RRl			4.70			4.70
RRu			250.16			250.16
P			+++			+++
Between Chi						
Between df						
Between P						N.S.
Btwn(F) P						N.S.
Btwn(R) P						N.S.

	Detailed Country in "other Asia"			
	India	HongKong	other	Total
N				
NS				
Wt				
Het Chi				
Het df				
Het P				
Fixed RR				
RRl				
RRu				
P				
Random RR				
RRl				
RRu				
P				
Between Chi				
Between df				
Between P				N.S.
Btwn(F) P				N.S.
Btwn(R) P				N.S.

	Detailed other continent			
	SCAmer	Auslia	Africa	Total
N				
NS				
Wt				
Het Chi				
Het df				
Het P				
Fixed RR				
RRl				
RRu				
P				
Random RR				
RRl				
RRu				
P				
Between Chi				
Between df				
Between P				N.S.
Btwn(F) P				N.S.
Btwn(R) P				N.S.

Table 4B1 - 3

IESLC - Meta-analysis of Current Smoking (vs never smoking), Any product (or Cigarettes if Any not available)
Large
Most adjusted

	<1960	Start year of study 1960-69	1970-79	1980-89	1990+	Total
N	1	4	4	5	1	15
NS	1	2	3	3	1	10
Wt	0.45	34.51	2.38	11.26	0.79	49.39
Het Chi	0.00	21.39	0.20	3.34	0.00	29.82
Het df	0	3	3	4	0	14
Het P	N.S.	***	N.S.	N.S.	N.S.	**
Fixed RR	3.40	8.78	26.98	6.13	5.85	8.41
RRl	0.18	6.29	7.57	3.42	0.65	6.36
RRu	63.67	12.26	96.13	10.99	52.83	11.12
P	N.S.	+++	+++	+++	N.S.	+++
Random RR	3.40	8.77	26.98	6.13	5.85	8.56
RRl	0.18	3.50	7.57	3.42	0.65	5.29
RRu	63.67	21.98	96.13	10.99	52.83	13.86
P	N.S.	+++	+++	+++	N.S.	+++
Between Chi						4.90
Between df						4
Between P						N.S.
Btwn(F) P						N.S.
Btwn(R) P						N.S.

	Study type (1) CC	other	Total
N	12	3	15
NS	8	2	10
Wt	48.01	1.39	49.39
Het Chi	28.18	1.31	29.82
Het df	11	2	14
Het P	**	N.S.	**
Fixed RR	8.29	13.57	8.41
RRl	6.25	2.57	6.36
RRu	11.01	71.72	11.12
P	+++	++	+++
Random RR	8.28	13.57	8.56
RRl	4.94	2.57	5.29
RRu	13.89	71.72	13.86
P	+++	++	+++
Between Chi			0.33
Between df			1
Between P			N.S.
Btwn(F) P			N.S.
Btwn(R) P			N.S.

	Study type (2) CC	prosp	other	Total
N	12	1	2	15
NS	8	1	1	10
Wt	48.01	0.45	0.94	49.39
Het Chi	28.18	0.00	0.05	29.82
Het df	11	0	1	14
Het P	**	N.S.	N.S.	**
Fixed RR	8.29	3.40	26.25	8.41
RRl	6.25	0.18	3.47	6.36
RRu	11.01	63.67	198.54	11.12
P	+++	N.S.	++	+++
Random RR	8.28	3.40	26.25	8.56
RRl	4.94	0.18	3.47	5.29
RRu	13.89	63.67	198.54	13.86
P	+++	N.S.	++	+++
Between Chi				1.59
Between df				2
Between P				N.S.
Btwn(F) P				N.S.
Btwn(R) P				N.S.

Table 4B1 - 3

IESLC - Meta-analysis of Current Smoking (vs never smoking), Any product (or Cigarettes if Any not available)

	Study size (number of LC cases) 100-249	250-499	500-999	Large Most adjusted 1000+	Total
N	1	4	4	6	15
NS	1	3	3	3	10
Wt	0.45	3.23	5.58	40.14	49.39
Het Chi	0.00	1.54	0.92	24.89	29.82
Het df	0	3	3	5	14
Het P	N.S.	N.S.	N.S.	***	**
Fixed RR	3.40	9.09	14.96	7.79	8.41
RRl	0.18	3.05	6.53	5.72	6.36
RRu	63.67	27.03	34.31	10.62	11.12
P	N.S.	+++	+++	+++	+++
Random RR	3.40	9.09	14.96	6.93	8.56
RRl	0.18	3.05	6.53	3.34	5.29
RRu	63.67	27.03	34.31	14.36	13.86
P	N.S.	+++	+++	+++	+++
Between Chi					2.47
Between df					3
Between P					N.S.
Btwn(F) P					N.S.
Btwn(R) P					N.S.

	Risky occupational population no	mining	othRisky	Total
N	15			15
NS	10			10
Wt	49.39			49.39
Het Chi	29.82			29.82
Het df	14			14
Het P	**			**
Fixed RR	8.41			8.41
RRl	6.36			6.36
RRu	11.12			11.12
P	+++			+++
Random RR	8.56			8.56
RRl	5.29			5.29
RRu	13.86			13.86
P	+++			+++
Between Chi				
Between df				
Between P				N.S.
Btwn(F) P				N.S.
Btwn(R) P				N.S.

	National cigarette tobacco type Virginia	blended	other	Total
N	2	13		15
NS	1	9		10
Wt	4.14	45.26		49.39
Het Chi	0.06	29.16		29.82
Het df	1	12		14
Het P	N.S.	**		**
Fixed RR	12.11	8.13		8.41
RRl	4.62	6.08		6.36
RRu	31.74	10.89		11.12
P	+++	+++		+++
Random RR	12.11	8.14		8.56
RRl	4.62	4.71		5.29
RRu	31.74	14.05		13.86
P	+++	+++		+++
Between Chi				0.60
Between df				1
Between P				N.S.
Btwn(F) P				N.S.
Btwn(R) P				N.S.

Table 4B1 - 3

IESLC - Meta-analysis of Current Smoking (vs never smoking), Any product (or Cigarettes if Any not available)
Large
Most adjusted

	Any proxy use No/nk	Yes	Total
N	11	4	15
NS	7	3	10
Wt	43.81	5.58	49.39
Het Chi	26.81	0.92	29.82
Het df	10	3	14
Het P	**	N.S.	**
Fixed RR	7.82	14.96	8.41
RRl	5.81	6.53	6.36
RRu	10.51	34.31	11.12
P	+++	+++	+++
Random RR	7.31	14.96	8.56
RRl	4.12	6.53	5.29
RRu	12.99	34.31	13.86
P	+++	+++	+++
Between Chi			2.09
Between df			1
Between P			N.S.
Btwn(F) P			N.S.
Btwn(R) P			N.S.

	Full histological confirmation No	Yes	Total
N	9	6	15
NS	6	4	10
Wt	24.13	25.26	49.39
Het Chi	7.43	13.31	29.82
Het df	8	5	14
Het P	N.S.	*	**
Fixed RR	5.42	12.79	8.41
RRl	3.64	8.66	6.36
RRu	8.08	18.89	11.12
P	+++	+++	+++
Random RR	5.42	10.25	8.56
RRl	3.64	5.01	5.29
RRu	8.08	20.97	13.86
P	+++	+++	+++
Between Chi			9.09
Between df			1
Between P			**
Btwn(F) P			*
Btwn(R) P			N.S.

	Number of adjustment variables (1) 0	1	2+/+nk	Total
N	7	3	5	15
NS	5	2	3	10
Wt	7.84	6.60	34.96	49.39
Het Chi	1.85	4.06	21.78	29.82
Het df	6	2	4	14
Het P	N.S.	N.S.	***	**
Fixed RR	10.99	5.20	8.67	8.41
RRl	5.46	2.42	6.23	6.36
RRu	22.13	11.15	12.08	11.12
P	+++	+++	+++	+++
Random RR	10.99	6.10	8.24	8.56
RRl	5.46	1.95	3.46	5.29
RRu	22.13	19.03	19.60	13.86
P	+++	++	+++	+++
Between Chi				2.12
Between df				2
Between P				N.S.
Btwn(F) P				N.S.
Btwn(R) P				N.S.

Table 4B1 - 3

IESLC - Meta-analysis of Current Smoking (vs never smoking), Any product (or Cigarettes if Any not available)
Large
Most adjusted

	Number of adjustment variables (2) 0	1	2	3-5	6+/+nk	Total
N	7	3	5			15
NS	5	2	3			10
Wt	7.84	6.60	34.96			49.39
Het Chi	1.85	4.06	21.78			29.82
Het df	6	2	4			14
Het P	N.S.	N.S.	***			**
Fixed RR	10.99	5.20	8.67			8.41
RRl	5.46	2.42	6.23			6.36
RRu	22.13	11.15	12.08			11.12
P	+++	+++	+++			+++
Random RR	10.99	6.10	8.24			8.56
RRl	5.46	1.95	3.46			5.29
RRu	22.13	19.03	19.60			13.86
P	+++	++	+++			+++
Between Chi						2.12
Between df						2
Between P						N.S.
Btwn(F) P						N.S.
Btwn(R) P						N.S.

	Product all/unsp	cig+/-ot	cig only	Total
N	2	12	1	15
NS	2	7	1	10
Wt	1.77	47.18	0.45	49.39
Het Chi	1.37	27.42	0.00	29.82
Het df	1	11	0	14
Het P	N.S.	**	N.S.	**
Fixed RR	15.50	8.29	3.40	8.41
RRl	3.55	6.23	0.18	6.36
RRu	67.73	11.03	63.67	11.12
P	+++	+++	N.S.	+++
Random RR	15.13	8.36	3.40	8.56
RRl	2.69	4.95	0.18	5.29
RRu	85.19	14.10	63.67	13.86
P	++	+++	N.S.	+++
Between Chi				1.04
Between df				2
Between P				N.S.
Btwn(F) P				N.S.
Btwn(R) P				N.S.

	Denominator nev any	nev cigs	Total
N	7	8	15
NS	5	5	10
Wt	36.72	12.67	49.39
Het Chi	23.72	5.61	29.82
Het df	6	7	14
Het P	***	N.S.	**
Fixed RR	8.92	7.09	8.41
RRl	6.45	4.09	6.36
RRu	12.33	12.30	11.12
P	+++	+++	+++
Random RR	9.15	7.09	8.56
RRl	4.27	4.09	5.29
RRu	19.61	12.30	13.86
P	+++	+++	+++
Between Chi			0.49
Between df			1
Between P			N.S.
Btwn(F) P			N.S.
Btwn(R) P			N.S.

Table 4B1 - 3

IESLC - Meta-analysis of Current Smoking (vs never smoking), Any product (or Cigarettes if Any not available)

Large
Most adjusted

	Derivation of RR/CI Orig	StdCalc	Other	Total
N	8	3	4	15
NS	5	2	3	10
Wt	42.61	4.93	1.85	49.39
Het Chi	27.12	0.41	1.33	29.82
Het df	7	2	3	14
Het P	***	N.S.	N.S.	**
Fixed RR	7.98	10.77	14.43	8.41
RRl	5.91	4.46	3.42	6.36
RRu	10.78	26.04	60.87	11.12
P	+++	+++	+++	+++
Random RR	7.63	10.77	14.43	8.56
RRl	3.98	4.46	3.42	5.29
RRu	14.63	26.04	60.87	13.86
P	+++	+++	+++	+++
Between Chi				0.96
Between df				2
Between P				N.S.
Btwn(F) P				N.S.
Btwn(R) P				N.S.

Table 4B1 - 4

IESLC - Meta-analysis of Current Smoking (vs never smoking), Any product (or Cigarettes if Any not available)
Large
Least adjusted

REF	NRR	X	SEX	AGEL	AGEH	RACE	YF	LC TYPE	LOC	START	ST	NLC	R	VB	P	H	AD	PRODUCT	DENOM	De
BARBON	37	x	m	0	0	all	-	large	Eu:wst	1979	CC	755	n	bl	y	y	0	all/unsp	nev any	st
BOUCOT	73	x	m	0	0	all	0	large	NAmer	1951	pr	121	n	bl	n	n	0	cig only	nev any	ot
BUFFLE	75		f	0	0	w-hi	-	large	NAmer	1976	CC	943	n	bl	y	n	0	cig+/-ot	nev cigs	ot
COMSTO	25		m	0	0	all	-	large	NAmer	1975	ot	258	n	bl	n	n	0	cig+/-ot	nev cigs	ot
COMSTO	32		f	0	0	all	-	large	NAmer	1975	ot	258	n	bl	n	n	0	cig+/-ot	nev cigs	ot
JAIN	20		m	0	0	all	-	large	NAmer	1981	CC	845	n	V	y	n	0	cig+/-ot	nev cigs	st
JAIN	15		f	0	0	all	-	large	NAmer	1981	CC	845	n	V	y	n	0	cig+/-ot	nev cigs	st
KHUDER	16		m	0	0	all	-	large	NAmer	1985	CC	482	n	bl	n	y	0	cig+/-ot	nev cigs	or
KIHARA	4		c	0	0	jap	-	large	As:Jap	1991	CC	440	n	bl	n	n	0	all/unsp	nev any	st
SOBUE	14	x	m	0	0	all	-	large	As:Jap	1986	CC	1376	n	bl	n	y	0	cig+/-ot	nev cigs	st
SOBUE	30	x	f	0	0	all	-	large	As:Jap	1986	CC	1376	n	bl	n	y	0	cig+/-ot	nev cigs	st
SOBUE2	4		m	0	0	all	-	large	As:Jap	1965	CC	2083	n	bl	n	n	2	cig+/-ot	nev any	or
SOBUE2	8		f	0	0	all	-	large	As:Jap	1965	CC	2083	n	bl	n	n	2	cig+/-ot	nev any	or
WYNDE6	331	x	m	0	0	all	-	large	NAmer	1969	CC	4423	n	bl	n	y	0	cig+/-ot	nev any	st
WYNDE6	361	x	f	0	0	all	-	large	NAmer	1969	CC	4423	n	bl	n	y	0	cig+/-ot	nev any	st

Cigarette type is all/unspec for all RRs

Table 4B1 - 5

IESLC - Meta-analysis of Current Smoking (vs never smoking), Any product (or Cigarettes if Any not available)
Large
Least adjusted

REF	NRR	SEX	AD	Number Exposed Case	Cont	Non-exposed Case	Cont	RR	95.00%CI
BARBON	37	m	0	68	362	1	188	35.31	(4.87- 256.33)
*BOUCOT	73	m	0	4	22177	0	7551	3.06~	(0.17- 56.91)
BUFFLE	75	f	0	8	110	0	112	17.31~	(0.99- 303.50)
COMSTO	25	m	0	12	100	0	84	21.02~	(1.23- 360.30)
COMSTO	32	f	0	7	52	0	115	33.00~	(1.85- 588.60)
Subtotal COMSTO								26.25	(3.47- 198.54)
JAIN	20	m	0	21	118	1	85	15.13	(2.00- 114.65)
JAIN	15	f	0	21	99	4	214	11.35	(3.79- 33.94)
Subtotal JAIN								12.11	(4.62- 31.74)
KHUDER	16	m	0	13	-	2	-	5.90	(1.20- 29.50)
KIHARA	4	c	0	4	162	1	237	5.85	(0.65- 52.83)
SOBUE	14	m	0	57	650	3	128	3.74	(1.15- 12.13)
SOBUE	30	f	0	5	168	7	857	3.64	(1.14- 11.62)
Subtotal SOBUE								3.69	(1.62- 8.43)
SOBUE2	4	m	2	-	-	-	-	4.10	(2.40- 7.20)
SOBUE2	8	f	2	-	-	-	-	3.80	(1.50- 9.30)
Subtotal SOBUE2								4.02	(2.51- 6.43)
WYNDE6	331	m	0	120	593	7	650	18.79	(8.70- 40.59)
WYNDE6	361	f	0	100	348	13	936	20.69	(11.46- 37.35)
Subtotal WYNDE6								19.96	(12.49- 31.90)
Partial Totals				440	24939	39	11157		

*prospective study ~ With 0.5 adjustment for zero

REF	NRR	SEX	AD	Ys	Ws	Qs	Ps
BARBON	37	m	0	3.56	0.98	1.97	0.0004
*BOUCOT	73	m	0	1.12	0.45	0.47	0.4525
BUFFLE	75	f	0	2.85	0.47	0.23	0.0511
COMSTO	25	m	0	3.05	0.48	0.39	0.0357
COMSTO	32	f	0	3.50	0.46	0.85	0.0174
Subtotal COMSTO				3.27	0.94	1.23	
JAIN	20	m	0	2.72	0.94	0.31	0.0086
JAIN	15	f	0	2.43	3.20	0.26	0.0000
Subtotal JAIN				2.49	4.14	0.57	
KHUDER	16	m	0	1.77	1.50	0.20	0.0298
KIHARA	4	c	0	1.77	0.79	0.11	0.1156
SOBUE	14	m	0	1.32	2.78	1.89	0.0279
SOBUE	30	f	0	1.29	2.86	2.07	0.0288
Subtotal SOBUE				1.31	5.63	3.95	
SOBUE2	4	m	2	1.41	12.73	6.83	0.0000
SOBUE2	8	f	2	1.34	4.62	3.02	0.0041
Subtotal SOBUE2				1.39	17.35	9.85	
WYNDE6	331	m	0	2.93	6.48	4.04	0.0000
WYNDE6	361	f	0	3.03	11.01	8.64	0.0000
Subtotal WYNDE6				2.99	17.48	12.68	

	N	15
	NS	10
	Wt	49.73
Het	Chi	31.28
Het	df	14
Het	P	**
Fixed	RR	8.53
	RRl	6.46
	RRu	11.26
	P	+++
Random	RR	8.62
	RRl	5.27
	RRu	14.10
	P	+++
Asymm	P	N.S.

Table 4B1 - 6

IESLC - Meta-analysis of Current Smoking (vs never smoking), Any product (or Cigarettes if Any not available)
Large
Least adjusted

	Sex			
	combined	male	female	Total
N	1	8	6	15
NS	1	8	6	15
Wt	0.79	26.32	22.61	49.73
Het Chi	0.00	15.09	13.75	31.28
Het df	0	7	5	14
Het P	N.S.	*	*	**
Fixed RR	5.85	7.01	10.86	8.53
RRl	0.65	4.79	7.19	6.46
RRu	52.83	10.27	16.40	11.26
P	N.S.	+++	+++	+++
Random RR	5.85	8.40	9.25	8.62
RRl	0.65	4.17	4.09	5.27
RRu	52.83	16.94	20.92	14.10
P	N.S.	+++	+++	+++
Between Chi				2.44
Between df				2
Between P				N.S.
Btwn(F) P				N.S.
Btwn(R) P				N.S.

	Lung cancer type	
	large	Total
N	15	15
NS	10	10
Wt	49.73	49.73
Het Chi	31.28	31.28
Het df	14	14
Het P	**	**
Fixed RR	8.53	8.53
RRl	6.46	6.46
RRu	11.26	11.26
P	+++	+++
Random RR	8.62	8.62
RRl	5.27	5.27
RRu	14.10	14.10
P	+++	+++
Between Chi		
Between df		
Between P		N.S.
Btwn(F) P		N.S.
Btwn(R) P		N.S.

	Location								
	NAmer	UK	Scand	othEur	China	Japan	othAs	other	Total
N	9			1		5			15
NS	6			1		3			10
Wt	24.97			0.98		23.77			49.73
Het Chi	4.24			0.00		0.17			31.28
Het df	8			0		4			14
Het P	N.S.			N.S.		N.S.			**
Fixed RR	16.64			35.31		3.99			8.53
RRl	11.24			4.87		2.67			6.46
RRu	24.63			256.33		5.96			11.26
P	+++			+++		+++			+++
Random RR	16.64			35.31		3.99			8.62
RRl	11.24			4.87		2.67			5.27
RRu	24.63			256.33		5.96			14.10
P	+++			+++		+++			+++
Between Chi									26.87
Between df									2
Between P									***
Btwn(F) P									***
Btwn(R) P									***

Table 4B1 - 6

IESLC - Meta-analysis of Current Smoking (vs never smoking), Any product (or Cigarettes if Any not available)
Large
Least adjusted

	Detailed Country in "other Europe"					
	multi	Germany	othWest	East	Balkans	Total
N			1			1
NS			1			1
Wt			0.98			0.98
Het Chi			0.00			0.00
Het df			0			0
Het P			N.S.			N.S.
Fixed RR			35.31			35.31
RRl			4.87			4.87
RRu			256.33			256.33
P			+++			+++
Random RR			35.31			35.31
RRl			4.87			4.87
RRu			256.33			256.33
P			+++			+++
Between Chi						
Between df						
Between P						N.S.
Btwn(F) P						N.S.
Btwn(R) P						N.S.

	Detailed Country in "other Asia"			
	India	HongKong	other	Total
N				
NS				
Wt				
Het Chi				
Het df				
Het P				
Fixed RR				
RRl				
RRu				
P				
Random RR				
RRl				
RRu				
P				
Between Chi				
Between df				
Between P				N.S.
Btwn(F) P				N.S.
Btwn(R) P				N.S.

	Detailed other continent			
	SCAmer	Auslia	Africa	Total
N				
NS				
Wt				
Het Chi				
Het df				
Het P				
Fixed RR				
RRl				
RRu				
P				
Random RR				
RRl				
RRu				
P				
Between Chi				
Between df				
Between P				N.S.
Btwn(F) P				N.S.
Btwn(R) P				N.S.

Table 4B1 - 6

IESLC - Meta-analysis of Current Smoking (vs never smoking), Any product (or Cigarettes if Any not available)

Large

Least adjusted

	Start year of study <1960	1960-69	1970-79	1980-89	1990+	Total
N	1	4	4	5	1	15
NS	1	2	3	3	1	10
Wt	0.45	34.83	2.38	11.27	0.79	49.73
Het Chi	0.00	22.44	0.21	3.43	0.00	31.28
Het df	0	3	3	4	0	14
Het P	N.S.	***	N.S.	N.S.	N.S.	**
Fixed RR	3.06	8.98	27.32	6.08	5.85	8.53
RRl	0.17	6.45	7.68	3.39	0.65	6.46
RRu	56.91	12.52	97.21	10.90	52.83	11.26
P	N.S.	+++	+++	+++	N.S.	+++
Random RR	3.06	8.92	27.32	6.08	5.85	8.62
RRl	0.17	3.50	7.68	3.39	0.65	5.27
RRu	56.91	22.76	97.21	10.90	52.83	14.10
P	N.S.	+++	+++	+++	N.S.	+++
Between Chi						5.20
Between df						4
Between P						N.S.
Btwn(F) P						N.S.
Btwn(R) P						N.S.

	Study type (1) CC	other	Total
N	12	3	15
NS	8	2	10
Wt	48.34	1.39	49.73
Het Chi	29.57	1.45	31.28
Het df	11	2	14
Het P	**	N.S.	**
Fixed RR	8.43	13.09	8.53
RRl	6.36	2.48	6.46
RRu	11.17	69.06	11.26
P	+++	++	+++
Random RR	8.35	13.09	8.62
RRl	4.92	2.48	5.27
RRu	14.17	69.06	14.10
P	+++	++	+++
Between Chi			0.26
Between df			1
Between P			N.S.
Btwn(F) P			N.S.
Btwn(R) P			N.S.

	Study type (2) CC	prosp	other	Total
N	12	1	2	15
NS	8	1	1	10
Wt	48.34	0.45	0.94	49.73
Het Chi	29.57	0.00	0.05	31.28
Het df	11	0	1	14
Het P	**	N.S.	N.S.	**
Fixed RR	8.43	3.06	26.25	8.53
RRl	6.36	0.17	3.47	6.46
RRu	11.17	56.91	198.54	11.26
P	+++	N.S.	++	+++
Random RR	8.35	3.06	26.25	8.62
RRl	4.92	0.17	3.47	5.27
RRu	14.17	56.91	198.54	14.10
P	+++	N.S.	++	+++
Between Chi				1.67
Between df				2
Between P				N.S.
Btwn(F) P				N.S.
Btwn(R) P				N.S.

Table 4B1 - 6

IESLC - Meta-analysis of Current Smoking (vs never smoking), Any product (or Cigarettes if Any not available)
Large
Least adjusted

	Study size (number of LC cases)				
	100-249	250-499	500-999	1000+	Total
N	1	4	4	6	15
NS	1	3	3	3	10
Wt	0.45	3.23	5.58	40.46	49.73
Het Chi	0.00	1.54	0.98	26.27	31.28
Het df	0	3	3	5	14
Het P	N.S.	N.S.	N.S.	***	**
Fixed RR	3.06	9.09	15.05	7.94	8.53
RRl	0.17	3.05	6.57	5.83	6.46
RRu	56.91	27.03	34.50	10.80	11.26
P	N.S.	+++	+++	+++	+++
Random RR	3.06	9.09	15.05	6.97	8.62
RRl	0.17	3.05	6.57	3.30	5.27
RRu	56.91	27.03	34.50	14.69	14.10
P	N.S.	+++	+++	+++	+++
Between Chi					2.49
Between df					3
Between P					N.S.
Btwn(F) P					N.S.
Btwn(R) P					N.S.

	Risky occupational population			
	no	mining	othRisky	Total
N	15			15
NS	10			10
Wt	49.73			49.73
Het Chi	31.28			31.28
Het df	14			14
Het P	**			**
Fixed RR	8.53			8.53
RRl	6.46			6.46
RRu	11.26			11.26
P	+++			+++
Random RR	8.62			8.62
RRl	5.27			5.27
RRu	14.10			14.10
P	+++			+++
Between Chi				
Between df				
Between P				N.S.
Btwn(F) P				N.S.
Btwn(R) P				N.S.

	National cigarette tobacco type			
	Virginia	blended	other	Total
N	2	13		15
NS	1	9		10
Wt	4.14	45.59		49.73
Het Chi	0.06	30.67		31.28
Het df	1	12		14
Het P	N.S.	**		**
Fixed RR	12.11	8.26		8.53
RRl	4.62	6.18		6.46
RRu	31.74	11.05		11.26
P	+++	+++		+++
Random RR	12.11	8.19		8.62
RRl	4.62	4.69		5.27
RRu	31.74	14.32		14.10
P	+++	+++		+++
Between Chi				0.55
Between df				1
Between P				N.S.
Btwn(F) P				N.S.
Btwn(R) P				N.S.

Table 4B1 - 6

IESLC - Meta-analysis of Current Smoking (vs never smoking), Any product (or Cigarettes if Any not available)
Large
Least adjusted

	Any proxy use No/nk	Yes	Total
N	11	4	15
NS	7	3	10
Wt	44.14	5.58	49.73
Het Chi	28.28	0.98	31.28
Het df	10	3	14
Het P	**	N.S.	**
Fixed RR	7.94	15.05	8.53
RRl	5.91	6.57	6.46
RRu	10.66	34.50	11.26
P	+++	+++	+++
Random RR	7.33	15.05	8.62
RRl	4.07	6.57	5.27
RRu	13.20	34.50	14.10
P	+++	+++	+++
Between Chi			2.03
Between df			1
Between P			N.S.
Btwn(F) P			N.S.
Btwn(R) P			N.S.

	Full histological confirmation No	Yes	Total
N	9	6	15
NS	6	4	10
Wt	24.13	25.59	49.73
Het Chi	7.47	14.09	31.28
Het df	8	5	14
Het P	N.S.	*	**
Fixed RR	5.41	13.10	8.53
RRl	3.63	8.89	6.46
RRu	8.06	19.30	11.26
P	+++	+++	+++
Random RR	5.41	10.35	8.62
RRl	3.63	4.97	5.27
RRu	8.06	21.55	14.10
P	+++	+++	+++
Between Chi			9.71
Between df			1
Between P			**
Btwn(F) P			*
Btwn(R) P			N.S.

	Number of adjustment variables (1) 0	1	2+/+nk	Total
N	13		2	15
NS	9		1	10
Wt	32.38		17.35	49.73
Het Chi	16.16		0.02	31.28
Het df	12		1	14
Het P	N.S.		N.S.	**
Fixed RR	12.77		4.02	8.53
RRl	9.05		2.51	6.46
RRu	18.02		6.43	11.26
P	+++		+++	+++
Random RR	11.32		4.02	8.62
RRl	7.18		2.51	5.27
RRu	17.84		6.43	14.10
P	+++		+++	+++
Between Chi				15.10
Between df				1
Between P				***
Btwn(F) P				**
Btwn(R) P				**

Table 4B1 - 6

IESLC - Meta-analysis of Current Smoking (vs never smoking), Any product (or Cigarettes if Any not available)
Large
Least adjusted

	Number of adjustment variables (2)					
	0	1	2	3-5	6+/+nk	Total
N	13		2			15
NS	9		1			10
Wt	32.38		17.35			49.73
Het Chi	16.16		0.02			31.28
Het df	12		1			14
Het P	N.S.		N.S.			**
Fixed RR	12.77		4.02			8.53
RRl	9.05		2.51			6.46
RRu	18.02		6.43			11.26
P	+++		+++			+++
Random RR	11.32		4.02			8.62
RRl	7.18		2.51			5.27
RRu	17.84		6.43			14.10
P	+++		+++			+++
Between Chi						15.10
Between df						1
Between P						***
Btwn(F) P						**
Btwn(R) P						**

	Product			
	all/unsp	cig+/-ot	cig only	Total
N	2	12	1	15
NS	2	7	1	10
Wt	1.77	47.50	0.45	49.73
Het Chi	1.42	28.71	0.00	31.28
Het df	1	11	0	14
Het P	N.S.	**	N.S.	**
Fixed RR	15.79	8.42	3.06	8.53
RRl	3.62	6.33	0.17	6.46
RRu	68.84	11.19	56.91	11.26
P	+++	+++	N.S.	+++
Random RR	15.36	8.42	3.06	8.62
RRl	2.65	4.94	0.17	5.27
RRu	88.98	14.37	56.91	14.10
P	++	+++	N.S.	+++
Between Chi				1.15
Between df				2
Between P				N.S.
Btwn(F) P				N.S.
Btwn(R) P				N.S.

	Denominator		
	nev any	nev cigs	Total
N	7	8	15
NS	5	5	10
Wt	37.05	12.68	49.73
Het Chi	24.93	5.73	31.28
Het df	6	7	14
Het P	***	N.S.	**
Fixed RR	9.11	7.04	8.53
RRl	6.60	4.06	6.46
RRu	12.57	12.21	11.26
P	+++	+++	+++
Random RR	9.25	7.04	8.62
RRl	4.25	4.06	5.27
RRu	20.14	12.21	14.10
P	+++	+++	+++
Between Chi			0.63
Between df			1
Between P			N.S.
Btwn(F) P			N.S.
Btwn(R) P			N.S.

Table 4B1 - 6

IESLC - Meta-analysis of Current Smoking (vs never smoking), Any product (or Cigarettes if Any not available)

Large
Least adjusted

	Derivation of RR/CI			
	Orig	StdCalc	Other	Total
N	3	8	4	15
NS	2	5	3	10
Wt	18.85	29.02	1.86	49.73
Het Chi	0.22	13.74	1.48	31.28
Het df	2	7	3	14
Het P	N.S.	(*)	N.S.	**
Fixed RR	4.14	13.21	14.04	8.53
RRl	2.64	9.18	3.33	6.46
RRu	6.51	19.00	59.18	11.26
P	+++	+++	+++	+++
Random RR	4.14	11.21	14.04	8.62
RRl	2.64	6.30	3.33	5.27
RRu	6.51	19.95	59.18	14.10
P	+++	+++	+++	+++
Between Chi				15.84
Between df				2
Between P				***
Btwn(F) P				*
Btwn(R) P				*

Table 4B1 - 7

IESLC - Meta-analysis of Current Smoking (vs never smoking), Any product (or Cigarettes if Any not available)
Large
Excluded studies (and stage at which they were excluded)

1 BOUCHA BUELL LAURIL MZILEN

2 ABELIN ABRAHA AUVINE AXELSO BAND BERRIN BLOT1 BLOT2 BLOT3 BLOT4 BRESLO BROCKM BROWN1 BYERS1 CASCO2 CASCOR
CHAN CHATZI CHEN CHEN2 CHEN3 CHIAZZ COOKSO DAVEYS DEAN DOSEME DU DUNN EBELIN ESAKI FAN GENG
GER GODLEY GOLLED GSELL HANSEN HEGMAN HINDS HIRAOK HOROWI HORWIT HU HU2 HUANG ISHIMA JARUP JIANG
JONES JUSSAW KO KOHLME KOULUM KREYBE LAMTH LAMWK LAMWK2 LAUSSM LEI LETOUR LEVIN LIU LIU2 LIU3
LIU4 LIU5 LOMBA2 LUO MAGNUS MARSH MARSH2 MARTIS MASTRA MATSUD MCCONN MCDUFF MCLAUG MILLER MILLS MOLLO
NOTAN2 NOU ORMOS PASTOR PAWLEG PERNU PERSHA PIKE POFFIJ POLEDN PRESCO QIAO RADZIK RANDIG REN RONCO
ROOTS ROTHSC SADOWS SANKAR SCHWA2 SEGI SEOW SIEMIA SIMARA STASZE STOCKS SUN TAO ULMER VANDER WANG
WANG3 WANG4 WICKLU WILKIN WUWILL WYNDE2 WYNDE4 XIANGZ XU XU2 XU3 XU4 YUAN ZHANG ZHENG ZHOU

3 RESTRE

4 BOFFET

5 RIMING TANG2 WYNDE5 WYNDE8

6 BYERS2 HIRAY2 LICKIN MRFIT MURATA NOTANI SAARIK SHIMIZ SITAS STAYNE WARSIN WATSON WYNDER

8 AGUDO AKIBA ALDERS AMANDU AMES ANDERS ARCHER ARMADA AUSTIN AXELSS BECHER BENHAM BENSHL BEST BLOHMK BRETT
BROSS BROWN2 CARPEN CEDERL CHANG CHOI CHOW CHYOU CORREA CPSI CPSII DAMBER DARBY DEAN2 DEAN3 DEKLER
DESTE2 DESTEF DOCKER DOLL DOLL2 DORANT DORGAN DORN DROSTE ENGELA ENSTRO GAO GAO2 GARCIA GARDIN GARSHI
GILLIS GOODMA GRAHAM GREGOR HAENSZ HAMMO2 HAMMON HEIN HENNEK HIRAYA HITOSU HOLE HUMBLE JAHN JARVHO JEDRYC
JOLY KAISE2 KAISER KANELL KATSOU KAUFMA KELLER KINLEN KJUUS KNEKT KOO KREUZE KUBIK LANGE LEMARC LIAW
LIDDEL LOMBAR LUBIN LUBIN2 MACLEN MATOS MIGRAN MRFITR NAM ODRISC OSANN OSANN2 PARKIN PERSH2 PETO PEZZO2
PEZZOT PISANI QIAO2 RACHTA SCHWAR SEGI2 SHAW SIMONA SPEIZE SPITZ STOCKW STUCKE SUZUK2 SUZUKI SVENSS TANG
TENKAN TIZZAN TOKARS TOUSEY TSUGAN TULINI TVERDA VEIERO VUTUC WAKAI WALD WANG2 WIGLE WU WU2 WUNSCH
WYNDE3 WYNDE7 YAMAGU YONG

Table 4B1 - 8
Potentially overlapping studies

REF	REFGP	PRINC	.	OVERLAP/LINK
WYNDE6	WYNDE6	1		WYNDE5/6/7/8
SOBUE2	SOBUE2	1		SOBUE2/MATSUD

Table 4C1 -

IESLC - Meta-anal of Ever Smoking (or Current if Ever not available), Any prod (or Cigs if Any not avail)
Large

This analysis is restricted to results for:
1) Non-dose-response data
2) Results complete enough for use in metaanalysis

Within each study, results are then selected (in the following order of preference, within each sex) for:
3) SMKSTA: ever smokers, current smokers
4) PRODUCT: all/unspec, cigarettes regardless of other products, cigarettes only
5) CIGTYPE: all/unspecified, MC regardless of HR, MC only
6) DENOM: never smoked anything, never smoked cigarettes, (never +1 = +long term ex, +2 = +amount unknown,
+3 = never cigs+long term ex)
7) Followup period (YF, prospective studies): whole study (coded as 0) or longest available
8) LCtype: large (specifically)

9) Race: all or nearest available, otherwise by race (wh or w = white, bl or b = black, hi = hispanic
ch = chinese, jap = japanese, haw = hawaiian, w+o = white + oriental, sca = scandinavian, as = asian)
10) For overlapping studies: principal rather than subsidiary studies
Finally by Age: whole study (coded as 0) if available, otherwise by widest available age group
and then for single sex results (m, f) in preference to combined sex results (c).

Results adjusted (AD) for the most potential confounders are then chosen in Sections -1 to -3
and results adjusted for the least confounders in Sections -4 to -6. (Those least adjusted results which
actually differ from the most adjusted as marked 'x' in column X in Section -4)
(Results adjusted for an unknown number of confounder(s) are coded as 20.)

Section -7 shows excluded studies, together with the stage (as above) at which no qualifying
results were found.

Section -8 lists the potentially overlapping studies which have been included (1=principal, 2=subsidiary).

Section -9 lists any results which would have been included in preference except that they had data not complete
enough for use in meta-analysis, with their significance (yes/no), if known, and any further comment as entered
on the database.

In addition to those mentioned above, the following fields, levels and abbreviations are used:
* or nk = not known, n = no, y = yes, ot = other
ev = ever, cu = current, nev = never
all/unspec = all or unspecified, cig+/-ot = cigarettes irrespective of other products (cigar, pipe etc)
MC = manufactured cigarettes, HR = hand-rolled cigarettes
REF: 6-character study reference
NRR: number of the RR on the database within the study
ST : study type (CC = case control, pr or prosp = prospective)
NLC: number of lung cancer cases in whole study
R : risky occupational population (n = no, m = mining, o = other risky)
VB : national cigarette type (V = at least 75% Virginia, bl = at least 75% blended, ot = other)
P : any proxy use
H : full histological confirmation
De : derivation of RR/CI (or = original, st = standard method, ot = other method of estimation)

Table 4C1 - 1

IESLC - Meta-anal of Ever Smoking (or Current if Ever not available), Any prod (or Cigs if Any not avail)
Large
Most adjusted

REF	NRR	SEX	AGEL	AGEH	RACE	YF	LC TYPE	LOC	START	ST	NLC	R	VB	P	H	AD	SM	PRODUCT	DENOM	De
ANDERS	11	f	0	0	all	0	large	NAmer	1986	pr	343	n	bl	n	n	0	ev	cig+/-ot	nev cigs	st
BAND	4	m	0	0	all	-	large	NAmer	1983	CC	2831	n	V	y	y	2	ev	cig only	nev any	ot
BARBON	129	m	0	0	all	-	large	Eu:wst	1979	CC	755	n	bl	y	y	3	ev	all/unsp	nev any	ot
BOUCOT	150	m	0	0	all	0	large	NAmer	1951	pr	121	n	bl	n	n	2	cu	cig only	nev any	ot
BUFFLE	74	f	0	0	w-hi	-	large	NAmer	1976	CC	943	n	bl	y	n	0	ev	cig+/-ot	nev cigs	ot
COMSTO	68	m	0	0	all	-	large	NAmer	1975	ot	258	n	bl	n	n	0	ev	cig+/-ot	nev cigs	ot
COMSTO	80	f	0	0	all	-	large	NAmer	1975	ot	258	n	bl	n	n	0	ev	cig+/-ot	nev cigs	ot
HEGMAN	5	c	0	0	all	-	large	NAmer	1989	CC	282	n	bl	y	y	0	ev	all/unsp	nev any	st
HINDS	25	f	0	0	o	-	large	NAmer	1968	CC	292	n	bl	n	n	3	ev	all/unsp	nev any	st
JAIN	50	m	0	0	all	-	large	NAmer	1981	CC	845	n	V	y	n	2	ev	cig+/-ot	nev cigs	or
JAIN	45	f	0	0	all	-	large	NAmer	1981	CC	845	n	V	y	n	2	ev	cig+/-ot	nev cigs	or
KHUDER	26	m	0	0	all	-	large	NAmer	1985	CC	482	n	bl	n	y	0	ev	cig+/-ot	nev cigs	ot
KIHARA	28	c	0	0	jap	-	large	As:Jap	1991	CC	440	n	bl	n	n	0	ev	all/unsp	nev any	st
LAMTH	4	f	0	0	ch	-	large	As:HK	1983	CC	445	n	bl	n	n	0	ev	all/unsp	nev any	or
LAMWK	5	f	0	0	ch	-	large	As:HK	1981	CC	163	n	bl	n	n	0	ev	all/unsp	nev any	st
LAMWK2	4	m	0	0	all	-	large	As:HK	1976	CC	480	n	bl	n	n	0	ev	all/unsp	nev any	st
LAMWK2	8	f	0	0	all	-	large	As:HK	1976	CC	480	n	bl	n	n	0	ev	all/unsp	nev any	st
NOU	4	m	0	0	all	-	large	Eu:Sca	1971	CC	273	n	bl	y	n	0	ev	all/unsp	nev any	st
NOU	9	f	0	0	all	-	large	Eu:Sca	1971	CC	273	n	bl	y	n	0	ev	all/unsp	nev any	st
ORMOS	16	m	0	0	all	-	large	Eu:est	1947	CC	119	n	bl	y	y	0	ev	cig+/-ot	nev any	st
SEOW	5	f	0	0	ch	-	large	As:oth	1997	CC	153	n	bl	n	y	0	ev	cig+/-ot	nev cigs	st
SOBUE	103	m	0	0	all	-	large	As:Jap	1986	CC	1376	n	bl	n	y	1	ev	cig+/-ot	nev cigs	ot
SOBUE	113	f	0	0	all	-	large	As:Jap	1986	CC	1376	n	bl	n	y	1	ev	cig+/-ot	nev cigs	ot
SOBUE2	4	m	0	0	all	-	large	As:Jap	1965	CC	2083	n	bl	n	n	2	cu	cig+/-ot	nev any	or
SOBUE2	8	f	0	0	all	-	large	As:Jap	1965	CC	2083	n	bl	n	n	2	cu	cig+/-ot	nev any	or
WYNDE6	389	m	0	0	all	-	large	NAmer	1969	CC	4423	n	bl	n	y	2	ev	all/unsp	nev any	ot
WYNDE6	404	f	0	0	all	-	large	NAmer	1969	CC	4423	n	bl	n	y	2	ev	all/unsp	nev any	ot
ZHOU	20	m	0	0	all	-	large	As:Chi	1978	CC	1360	n	ot	n	n	0	ev	all/unsp	nev any	st
ZHOU	21	f	0	0	all	-	large	As:Chi	1978	CC	1360	n	ot	n	n	0	ev	all/unsp	nev any	st

Cigarette type is all/unspec for all RRs

Table 4C1 - 2

IESLC - Meta-anal of Ever Smoking (or Current if Ever not available), Any prod (or Cigs if Any not avail)
Large
Most adjusted

REF	NRR	SEX	AD	Number Exposed Case	Number Exposed Cont	Non-exposed Case	Non-exposed Cont	RR	95.00%CI
*ANDERS	11	f	0	20	96164	1	195158	40.59	(5.45- 302.43)
BAND	4	m	2	-	-	-	-	12.94	(4.08- 41.06)
BARBON	129	m	3	-	-	-	-	30.43	(4.19- 221.06)
*BOUCOT	150	m	2	-	-	-	-	3.40	(0.18- 63.12)
BUFFLE	74	f	0	11	166	0	112	15.54~	(0.91- 266.39)
COMSTO	68	m	0	16	229	0	84	12.15~	(0.72- 204.77)
COMSTO	80	f	0	7	87	0	115	19.80~	(1.12- 351.37)
Subtotal COMSTO								15.44	(2.06- 115.86)
HEGMAN	5	c	0	18	1202	4	2080	7.79	(2.63- 23.06)
HINDS	25	f	3	-	-	-	-	4.71	(1.99- 11.11)
JAIN	50	m	2	-	-	-	-	6.00	(1.02- 113.00)
JAIN	45	f	2	-	-	-	-	6.50	(1.80- 41.60)
Subtotal JAIN								6.34	(1.72- 23.41)
KHUDER	26	m	0	23	-	2	-	5.90	(1.24- 28.10)
KIHARA	28	c	0	4	232	1	237	4.09	(0.45- 36.83)
LAMTH	4	f	0	11	3	9	17	6.93	(1.53- 31.38)
LAMWK	5	f	0	2	41	5	144	1.40	(0.26- 7.51)
LAMWK2	4	m	0	24	161	2	43	3.20	(0.73- 14.10)
LAMWK2	8	f	0	2	50	5	139	1.11	(0.21- 5.92)
Subtotal LAMWK2								2.01	(0.66- 6.10)
NOU	4	m	0	15	247	2	122	3.70	(0.83- 16.46)
NOU	9	f	0	2	92	7	261	0.81	(0.17- 3.97)
Subtotal NOU								1.82	(0.61- 5.40)
ORMOS	16	m	0	7	1034	1	777	5.26	(0.65- 42.84)
SEOW	5	f	0	8	15	13	125	5.13	(1.83- 14.38)
SOBUE	103	m	1	-	-	-	-	3.64	(1.15- 11.53)
SOBUE	113	f	1	-	-	-	-	3.81	(1.30- 11.15)
Subtotal SOBUE								3.73	(1.70- 8.19)
SOBUE2	4	m	2	-	-	-	-	4.10	(2.40- 7.20)
SOBUE2	8	f	2	-	-	-	-	3.80	(1.50- 9.30)
Subtotal SOBUE2								4.02	(2.51- 6.43)
WYNDE6	389	m	2	-	-	-	-	9.72	(4.57- 20.70)
WYNDE6	404	f	2	-	-	-	-	12.83	(7.17- 22.93)
Subtotal WYNDE6								11.57	(7.30- 18.34)
ZHOU	20	m	0	29	41	8	36	3.18	(1.29- 7.84)
ZHOU	21	f	0	3	7	6	32	2.29	(0.46- 11.43)
Subtotal ZHOU								2.94	(1.34- 6.46)
Partial Totals				202	99771	66	199482		

*prospective study ~ With 0.5 adjustment for zero

REF	NRR	SEX	AD	Ys	Ws	Qs	Ps
*ANDERS	11	f	0	3.70	0.95	3.76	0.0003
BAND	4	m	2	2.56	2.88	2.05	0.0000
BARBON	129	m	3	3.42	0.98	2.82	0.0007
*BOUCOT	150	m	2	1.22	0.45	0.11	0.4130
BUFFLE	74	f	0	2.74	0.48	0.50	0.0584
COMSTO	68	m	0	2.50	0.48	0.29	0.0831
COMSTO	80	f	0	2.99	0.46	0.75	0.0419
Subtotal COMSTO				2.74	0.95	1.04	
HEGMAN	5	c	0	2.05	3.26	0.37	0.0002
HINDS	25	f	3	1.55	5.20	0.15	0.0004
JAIN	50	m	2	1.79	0.69	0.00	0.1357
JAIN	45	f	2	1.87	1.56	0.04	0.0195
Subtotal JAIN				1.85	2.25	0.04	
KHUDER	26	m	0	1.77	1.58	0.01	0.0258
KIHARA	28	c	0	1.41	0.79	0.08	0.2096
LAMTH	4	f	0	1.94	1.68	0.08	0.0121
LAMWK	5	f	0	0.34	1.37	2.59	0.6910
LAMWK2	4	m	0	1.16	1.75	0.53	0.1233
LAMWK2	8	f	0	0.11	1.38	3.57	0.9009
Subtotal LAMWK2				0.70	3.13	4.10	
NOU	4	m	0	1.31	1.73	0.29	0.0852
NOU	9	f	0	-0.21	1.52	5.65	0.7956
Subtotal NOU				0.60	3.25	5.94	
ORMOS	16	m	0	1.66	0.87	0.00	0.1208
SEOW	5	f	0	1.63	3.62	0.02	0.0019
SOBUE	103	m	1	1.29	2.89	0.52	0.0280
SOBUE	113	f	1	1.34	3.33	0.48	0.0147
Subtotal SOBUE				1.32	6.22	1.00	
SOBUE2	4	m	2	1.41	12.73	1.19	0.0000

Table 4C1 - 2

IESLC - Meta-anal of Ever Smoking (or Current if Ever not available), Any prod (or Cigs if Any not avail)
Large
Most adjusted

REF	NRR	SEX	AD	Ys	Ws	Qs	Ps
SOBUE2	8	f	2	1.34	4.62	0.67	0.0041
Subtotal SOBUE2				1.39	17.35	1.87	
WYNDE6	389	m	2	2.27	6.73	2.09	0.0000
WYNDE6	404	f	2	2.55	11.37	7.92	0.0000
Subtotal WYNDE6				2.45	18.10	10.00	
ZHOU	20	m	0	1.16	4.72	1.48	0.0118
ZHOU	21	f	0	0.83	1.48	1.18	0.3140
Subtotal ZHOU				1.08	6.21	2.66	

	N	29
	NS	21
	Wt	81.55
Het	Chi	39.18
Het	df	28
Het	P	(*)
Fixed	RR	5.57
	RRl	4.48
	RRu	6.92
	P	+++
Random	RR	5.33
	RRl	4.02
	RRu	7.07
	P	+++
Asymm	P	N.S.

Table 4C1 - 3

IESLC - Meta-anal of Ever Smoking (or Current if Ever not available), Any prod (or Cigs if Any not avail)

Large

Most adjusted

	Sex combined	male	female	Total
N	2	13	14	29
NS	2	13	14	29
Wt	4.05	38.49	39.00	81.55
Het Chi	0.27	11.34	27.35	39.18
Het df	1	12	13	28
Het P	N.S.	N.S.	*	(*)
Fixed RR	6.86	5.38	5.64	5.57
RRl	2.59	3.92	4.12	4.48
RRu	18.17	7.37	7.72	6.92
P	+++	+++	+++	+++
Random RR	6.86	5.38	4.72	5.33
RRl	2.59	3.92	2.84	4.02
RRu	18.17	7.37	7.86	7.07
P	+++	+++	+++	+++
Between Chi				0.23
Between df				2
Between P				N.S.
Btwn(F) P				N.S.
Btwn(R) P				N.S.

	Lung cancer type large	Total
N	29	29
NS	21	21
Wt	81.55	81.55
Het Chi	39.18	39.18
Het df	28	28
Het P	(*)	(*)
Fixed RR	5.57	5.57
RRl	4.48	4.48
RRu	6.92	6.92
P	+++	+++
Random RR	5.33	5.33
RRl	4.02	4.02
RRu	7.07	7.07
P	+++	+++
Between Chi		
Between df		
Between P		N.S.
Btwn(F) P		N.S.
Btwn(R) P		N.S.

	Location NAmer	UK	Scand	othEur	China	Japan	othAs	other	Total
N	13		2	2	2	5	5		29
NS	10		1	2	1	3	4		21
Wt	36.09		3.25	1.85	6.21	24.36	9.79		81.55
Het Chi	7.60		1.87	1.42	0.12	0.05	4.25		39.18
Het df	12		1	1	1	4	4		28
Het P	N.S.		N.S.	N.S.	N.S.	N.S.	N.S.		(*)
Fixed RR	9.53		1.82	13.29	2.94	3.94	3.34		5.57
RRl	6.88		0.61	3.15	1.34	2.65	1.79		4.48
RRu	13.21		5.40	56.14	6.46	5.87	6.25		6.92
P	+++		N.S.	+++	++	+++	+++		+++
Random RR	9.53		1.78	13.10	2.94	3.94	3.30		5.33
RRl	6.88		0.40	2.35	1.34	2.65	1.72		4.02
RRu	13.21		7.88	73.06	6.46	5.87	6.33		7.07
P	+++		N.S.	++	++	+++	+++		+++
Between Chi									23.87
Between df									5
Between P									***
Btwn(F) P									***
Btwn(R) P									***

Table 4C1 - 3

IESLC - Meta-anal of Ever Smoking (or Current if Ever not available), Any prod (or Cigs if Any not avail)
Large
Most adjusted

Detailed Country in "other Europe"

	multi	Germany	othWest	East	Balkans	Total
N			1	1		2
NS			1	1		2
Wt			0.98	0.87		1.85
Het Chi			0.00	0.00		1.42
Het df			0	0		1
Het P			N.S.	N.S.		N.S.
Fixed RR			30.43	5.26		13.29
RRl			4.19	0.65		3.15
RRu			221.03	42.84		56.14
P			+++	N.S.		+++
Random RR			30.43	5.26		13.10
RRl			4.19	0.65		2.35
RRu			221.03	42.84		73.06
P			+++	N.S.		++
Between Chi						1.42
Between df						1
Between P						N.S.
Btwn(F) P						N.S.
Btwn(R) P						N.S.

Detailed Country in "other Asia"

	India	HongKong	other	Total
N		4	1	5
NS		3	1	4
Wt		6.18	3.62	9.79
Het Chi		3.20	0.00	4.25
Het df		3	0	4
Het P		N.S.	N.S.	N.S.
Fixed RR		2.60	5.13	3.34
RRl		1.18	1.83	1.79
RRu		5.73	14.38	6.25
P		+	++	+++
Random RR		2.59	5.13	3.30
RRl		1.15	1.83	1.72
RRu		5.85	14.38	6.33
P		+	++	+++
Between Chi				1.05
Between df				1
Between P				N.S.
Btwn(F) P				N.S.
Btwn(R) P				N.S.

Detailed other continent

	SCAmer	Auslia	Africa	Total
N				
NS				
Wt				
Het Chi				
Het df				
Het P				N.S.
Fixed RR				
RRl				
RRu				
P				+++
Random RR				
RRl				
RRu				
P				+++
Between Chi				
Between df				
Between P				N.S.
Btwn(F) P				N.S.
Btwn(R) P				N.S.

Table 4C1 - 3

IESLC - Meta-anal of Ever Smoking (or Current if Ever not available), Any prod (or Cigs if Any not avail)
Large
Most adjusted

	Start year of study <1960	1960-69	1970-79	1980-89	1990+	Total
N	2	5	10	10	2	29
NS	2	3	6	8	2	21
Wt	1.32	40.65	14.98	20.19	4.41	81.55
Het Chi	0.06	10.92	13.13	9.74	0.03	39.18
Het df	1	4	9	9	1	28
Het P	N.S.	*	N.S.	N.S.	N.S.	(*)
Fixed RR	4.54	6.57	3.34	6.08	4.92	5.57
RRl	0.82	4.83	2.01	3.93	1.94	4.48
RRu	24.97	8.93	5.54	9.41	12.52	6.92
P	(+)	+++	+++	+++	+++	+++
Random RR	4.54	6.38	3.59	6.10	4.92	5.33
RRl	0.82	3.78	1.87	3.86	1.94	4.02
RRu	24.97	10.79	6.90	9.64	12.52	7.07
P	(+)	+++	+++	+++	+++	+++
Between Chi						5.31
Between df						4
Between P						N.S.
Btwn(F) P						N.S.
Btwn(R) P						N.S.

	Study type (1) CC	other	Total
N	25	4	29
NS	18	3	21
Wt	79.20	2.35	81.55
Het Chi	34.19	1.95	39.18
Het df	24	3	28
Het P	(*)	N.S.	(*)
Fixed RR	5.39	17.13	5.57
RRl	4.32	4.76	4.48
RRu	6.71	61.59	6.92
P	+++	+++	+++
Random RR	5.07	17.13	5.33
RRl	3.81	4.76	4.02
RRu	6.74	61.59	7.07
P	+++	+++	+++
Between Chi			3.05
Between df			1
Between P			(*)
Btwn(F) P			N.S.
Btwn(R) P			(*)

	Study type (2) CC	prosp	other	Total
N	25	2	2	29
NS	18	2	1	21
Wt	79.20	1.40	0.95	81.55
Het Chi	34.19	1.87	0.06	39.18
Het df	24	1	1	28
Het P	(*)	N.S.	N.S.	(*)
Fixed RR	5.39	18.37	15.44	5.57
RRl	4.32	3.51	2.06	4.48
RRu	6.71	96.29	115.86	6.92
P	+++	+++	++	+++
Random RR	5.07	14.92	15.44	5.33
RRl	3.81	1.37	2.06	4.02
RRu	6.74	161.91	115.86	7.07
P	+++	+	++	+++
Between Chi				3.07
Between df				2
Between P				N.S.
Btwn(F) P				N.S.
Btwn(R) P				N.S.

Table 4C1 - 3

IESLC - Meta-anal of Ever Smoking (or Current if Ever not available), Any prod (or Cigs if Any not avail)

	Study size (number of LC cases) 100-249	250-499	500-999	Large Most adjusted 1000+	Total
N	4	12	4	9	29
NS	4	9	3	5	21
Wt	6.30	20.78	3.70	50.76	81.55
Het Chi	1.78	14.91	1.75	17.33	39.18
Het df	3	11	3	8	28
Het P	N.S.	N.S.	N.S.	*	(*)
Fixed RR	3.77	4.70	10.76	5.97	5.57
RRl	1.73	3.06	3.89	4.54	4.48
RRu	8.24	7.22	29.79	7.86	6.92
P	+++	+++	+++	+++	+++
Random RR	3.77	4.71	10.76	5.58	5.33
RRl	1.73	2.78	3.89	3.62	4.02
RRu	8.24	7.97	29.79	8.60	7.07
P	+++	+++	+++	+++	+++
Between Chi					3.41
Between df					3
Between P					N.S.
Btwn(F) P					N.S.
Btwn(R) P					N.S.

	Risky occupational population no	mining	othRisky	Total
N	29			29
NS	21			21
Wt	81.55			81.55
Het Chi	39.18			39.18
Het df	28			28
Het P	(*)			(*)
Fixed RR	5.57			5.57
RRl	4.48			4.48
RRu	6.92			6.92
P	+++			+++
Random RR	5.33			5.33
RRl	4.02			4.02
RRu	7.07			7.07
P	+++			+++
Between Chi				
Between df				
Between P				N.S.
Btwn(F) P				N.S.
Btwn(R) P				N.S.

	National cigarette tobacco type Virginia	blended	other	Total
N	3	24	2	29
NS	2	18	1	21
Wt	5.13	70.21	6.21	81.55
Het Chi	0.65	34.42	0.12	39.18
Het df	2	23	1	28
Het P	N.S.	(*)	N.S.	(*)
Fixed RR	9.46	5.67	2.94	5.57
RRl	3.98	4.49	1.34	4.48
RRu	22.48	7.16	6.46	6.92
P	+++	+++	++	+++
Random RR	9.46	5.35	2.94	5.33
RRl	3.98	3.89	1.34	4.02
RRu	22.48	7.37	6.46	7.07
P	+++	+++	++	+++
Between Chi				4.00
Between df				2
Between P				N.S.
Btwn(F) P				N.S.
Btwn(R) P				N.S.

Table 4C1 - 3

IESLC - Meta-anal of Ever Smoking (or Current if Ever not available), Any prod (or Cigs if Any not avail)
Large
Most adjusted

	Any proxy use No/nk	Yes	Total
N	20	9	29
NS	14	7	21
Wt	67.58	13.97	81.55
Het Chi	27.39	11.34	39.18
Het df	19	8	28
Het P	(*)	N.S.	(*)
Fixed RR	5.39	6.56	5.57
RRl	4.24	3.88	4.48
RRu	6.84	11.08	6.92
P	+++	+++	+++
Random RR	5.06	6.42	5.33
RRl	3.69	3.35	4.02
RRu	6.94	12.30	7.07
P	+++	+++	+++
Between Chi			0.45
Between df			1
Between P			N.S.
Btwn(F) P			N.S.
Btwn(R) P			N.S.

	Full histological confirmation No	Yes	Total
N	19	10	29
NS	13	8	21
Wt	44.04	37.51	81.55
Het Chi	17.34	9.73	39.18
Het df	18	9	28
Het P	N.S.	N.S.	(*)
Fixed RR	3.90	8.46	5.57
RRl	2.90	6.14	4.48
RRu	5.24	11.65	6.92
P	+++	+++	+++
Random RR	3.90	8.28	5.33
RRl	2.90	5.89	4.02
RRu	5.24	11.65	7.07
P	+++	+++	+++
Between Chi			12.12
Between df			1
Between P			***
Btwn(F) P			**
Btwn(R) P			**

	Number of adjustment variables (1) 0	1	2+/+nk	Total
N	17	2	10	29
NS	13	1	7	21
Wt	28.13	6.22	47.20	81.55
Het Chi	18.65	0.00	14.54	39.18
Het df	16	1	9	28
Het P	N.S.	N.S.	N.S.	(*)
Fixed RR	4.14	3.73	7.01	5.57
RRl	2.86	1.70	5.27	4.48
RRu	5.99	8.19	9.32	6.92
P	+++	++	+++	+++
Random RR	4.16	3.73	7.14	5.33
RRl	2.76	1.70	4.76	4.02
RRu	6.27	8.19	10.72	7.07
P	+++	++	+++	+++
Between Chi				5.99
Between df				2
Between P				(*)
Btwn(F) P				N.S.
Btwn(R) P				N.S.

Table 4C1 - 3

IESLC - Meta-anal of Ever Smoking (or Current if Ever not available), Any prod (or Cigs if Any not avail)

Large
Most adjusted

	Number of adjustment variables (2) 0	1	2	3-5	6+/+nk	Total
N	17	2	8	2		29
NS	13	1	5	2		21
Wt	28.13	6.22	41.03	6.17		81.55
Het Chi	18.65	0.00	11.60	2.86		39.18
Het df	16	1	7	1		28
Het P	N.S.	N.S.	N.S.	(*)		(*)
Fixed RR	4.14	3.73	7.12	6.33		5.57
RRl	2.86	1.70	5.24	2.88		4.48
RRu	5.99	8.19	9.67	13.93		6.92
P	+++	++	+++	+++		+++
Random RR	4.16	3.73	7.14	9.58		5.33
RRl	2.76	1.70	4.58	1.62		4.02
RRu	6.27	8.19	11.15	56.56		7.07
P	+++	++	+++	+		+++
Between Chi						6.06
Between df						3
Between P						N.S.
Btwn(F) P						N.S.
Btwn(R) P						N.S.

	Product all/unsp	cig+/-ot	cig only	Total
N	14	13	2	29
NS	10	9	2	21
Wt	43.96	34.26	3.33	81.55
Het Chi	28.63	7.58	0.69	39.18
Het df	13	12	1	28
Het P	**	N.S.	N.S.	(*)
Fixed RR	5.91	4.84	10.81	5.57
RRl	4.39	3.47	3.69	4.48
RRu	7.94	6.77	31.65	6.92
P	+++	+++	+++	+++
Random RR	4.63	4.84	10.81	5.33
RRl	2.85	3.47	3.69	4.02
RRu	7.50	6.77	31.65	7.07
P	+++	+++	+++	+++
Between Chi				2.28
Between df				2
Between P				N.S.
Btwn(F) P				N.S.
Btwn(R) P				N.S.

	Denominator nev any	nev cigs	Total
N	19	10	29
NS	14	7	21
Wt	65.51	16.04	81.55
Het Chi	32.80	6.32	39.18
Het df	18	9	28
Het P	*	N.S.	(*)
Fixed RR	5.49	5.90	5.57
RRl	4.31	3.62	4.48
RRu	7.00	9.63	6.92
P	+++	+++	+++
Random RR	4.91	5.90	5.33
RRl	3.41	3.62	4.02
RRu	7.08	9.63	7.07
P	+++	+++	+++
Between Chi			0.07
Between df			1
Between P			N.S.
Btwn(F) P			N.S.
Btwn(R) P			N.S.

Table 4C1 - 3

IESLC - Meta-anal of Ever Smoking (or Current if Ever not available), Any prod (or Cigs if Any not avail)

Large
Most adjusted

	Derivation of RR/CI			
	Orig	StdCalc	Other	Total
N	5	13	11	29
NS	3	10	8	21
Wt	21.28	28.64	31.63	81.55
Het Chi	0.81	15.32	9.28	39.18
Het df	4	12	10	28
Het P	N.S.	N.S.	N.S.	(*)
Fixed RR	4.40	3.77	9.28	5.57
RRl	2.88	2.62	6.55	4.48
RRu	6.73	5.44	13.15	6.92
P	+++	+++	+++	+++
Random RR	4.40	3.69	9.28	5.33
RRl	2.88	2.40	6.55	4.02
RRu	6.73	5.67	13.15	7.07
P	+++	+++	+++	+++
Between Chi				13.77
Between df				2
Between P				**
Btwn(F) P				**
Btwn(R) P				**

	Smoking status		
	ever	current	Total
N	26	3	29
NS	19	2	21
Wt	63.75	17.79	81.55
Het Chi	36.66	0.03	39.18
Het df	25	2	28
Het P	(*)	N.S.	(*)
Fixed RR	6.11	4.00	5.57
RRl	4.78	2.51	4.48
RRu	7.81	6.37	6.92
P	+++	+++	+++
Random RR	5.59	4.00	5.33
RRl	4.05	2.51	4.02
RRu	7.72	6.37	7.07
P	+++	+++	+++
Between Chi			2.49
Between df			1
Between P			N.S.
Btwn(F) P			N.S.
Btwn(R) P			N.S.

Table 4C1 - 4

IESLC - Meta-anal of Ever Smoking (or Current if Ever not available), Any prod (or Cigs if Any not avail)
Large
Least adjusted

REF	NRR	X	SEX	AGEL	AGEH	RACE	YF	LC TYPE	LOC	START	ST	NLC	R	VB	P	H	AD	SM	PRODUCT	DENOM	De
ANDERS	11		f	0	0	all	0	large	NAmer	1986	pr	343	n	bl	n	n	0	ev	cig+/-ot	nev cigs	st
BAND	4		m	0	0	all	-	large	NAmer	1983	CC	2831	n	V	y	y	2	ev	cig only	nev any	ot
BARBON	118	x	m	0	0	all	-	large	Eu:wst	1979	CC	755	n	bl	y	y	0	ev	all/unsp	nev any	st
BOUCOT	73	x	m	0	0	all	0	large	NAmer	1951	pr	121	n	bl	n	n	0	cu	cig only	nev any	ot
BUFFLE	74		f	0	0	w-hi	-	large	NAmer	1976	CC	943	n	bl	y	n	0	ev	cig+/-ot	nev cigs	ot
COMSTO	68		m	0	0	all	-	large	NAmer	1975	ot	258	n	bl	n	n	0	ev	cig+/-ot	nev cigs	ot
COMSTO	80		f	0	0	all	-	large	NAmer	1975	ot	258	n	bl	n	n	0	ev	cig+/-ot	nev cigs	ot
HEGMAN	5		c	0	0	all	-	large	NAmer	1989	CC	282	n	bl	y	y	0	ev	all/unsp	nev any	st
HINDS	25		f	0	0	o	-	large	NAmer	1968	CC	292	n	bl	n	n	3	ev	all/unsp	nev any	st
JAIN	10	x	m	0	0	all	-	large	NAmer	1981	CC	845	n	V	y	n	0	ev	cig+/-ot	nev cigs	st
JAIN	5	x	f	0	0	all	-	large	NAmer	1981	CC	845	n	V	y	n	0	ev	cig+/-ot	nev cigs	st
KHUDER	26		m	0	0	all	-	large	NAmer	1985	CC	482	n	bl	n	y	0	ev	cig+/-ot	nev cigs	ot
KIHARA	28		c	0	0	jap	-	large	As:Jap	1991	CC	440	n	bl	n	n	0	ev	all/unsp	nev any	st
LAMTH	4		f	0	0	ch	-	large	As:HK	1983	CC	445	n	bl	n	n	0	ev	all/unsp	nev any	or
LAMWK	5		f	0	0	ch	-	large	As:HK	1981	CC	163	n	bl	n	n	0	ev	all/unsp	nev any	st
LAMWK2	4		m	0	0	all	-	large	As:HK	1976	CC	480	n	bl	n	n	0	ev	all/unsp	nev any	st
LAMWK2	8		f	0	0	all	-	large	As:HK	1976	CC	480	n	bl	n	n	0	ev	all/unsp	nev any	st
NOU	4		m	0	0	all	-	large	Eu:Sca	1971	CC	273	n	bl	y	n	0	ev	all/unsp	nev any	st
NOU	9		f	0	0	all	-	large	Eu:Sca	1971	CC	273	n	bl	y	n	0	ev	all/unsp	nev any	st
ORMOS	16		m	0	0	all	-	large	Eu:est	1947	CC	119	n	bl	y	y	0	ev	cig+/-ot	nev any	st
SEOW	5		f	0	0	ch	-	large	As:oth	1997	CC	153	n	bl	n	y	0	ev	cig+/-ot	nev cigs	st
SOBUE	15	x	m	0	0	all	-	large	As:Jap	1986	CC	1376	n	bl	n	y	0	ev	cig+/-ot	nev cigs	st
SOBUE	31	x	f	0	0	all	-	large	As:Jap	1986	CC	1376	n	bl	n	y	0	ev	cig+/-ot	nev cigs	st
SOBUE2	4		m	0	0	all	-	large	As:Jap	1965	CC	2083	n	bl	n	n	2	cu	cig+/-ot	nev any	or
SOBUE2	8		f	0	0	all	-	large	As:Jap	1965	CC	2083	n	bl	n	n	2	cu	cig+/-ot	nev any	or
WYNDE6	343	x	m	0	0	all	-	large	NAmer	1969	CC	4423	n	bl	n	y	0	ev	all/unsp	nev any	st
WYNDE6	373	x	f	0	0	all	-	large	NAmer	1969	CC	4423	n	bl	n	y	0	ev	all/unsp	nev any	st
ZHOU	20		m	0	0	all	-	large	As:Chi	1978	CC	1360	n	ot	n	n	0	ev	all/unsp	nev any	st
ZHOU	21		f	0	0	all	-	large	As:Chi	1978	CC	1360	n	ot	n	n	0	ev	all/unsp	nev any	st

Cigarette type is all/unspec for all RRs

Table 4C1 - 5

IESLC - Meta-anal of Ever Smoking (or Current if Ever not available), Any prod (or Cigs if Any not avail)
Large
Least adjusted

REF	NRR	SEX	AD	Number Exposed Case	Number Exposed Cont	Non-exposed Case	Non-exposed Cont	RR	95.00%CI
*ANDERS	11	f	0	20	96164	1	195158	40.59	(5.45- 302.43)
BAND	4	m	2	-	-	-	-	12.94	(4.08- 41.06)
BARBON	118	m	0	89	567	1	188	29.51	(4.08- 213.27)
*BOUCOT	73	m	0	4	22177	0	7551	3.06~	(0.17- 56.91)
BUFFLE	74	f	0	11	166	0	112	15.54~	(0.91- 266.39)
COMSTO	68	m	0	16	229	0	84	12.15~	(0.72- 204.77)
COMSTO	80	f	0	7	87	0	115	19.80~	(1.12- 351.37)
Subtotal COMSTO								15.44	(2.06- 115.86)
HEGMAN	5	c	0	18	1202	4	2080	7.79	(2.63- 23.06)
HINDS	25	f	3	-	-	-	-	4.71	(1.99- 11.11)
JAIN	10	m	0	28	277	1	85	8.59	(1.15- 64.09)
JAIN	5	f	0	28	196	4	214	7.64	(2.63- 22.18)
Subtotal JAIN								7.84	(3.06- 20.10)
KHUDER	26	m	0	23	-	2	-	5.90	(1.24- 28.10)
KIHARA	28	c	0	4	232	1	237	4.09	(0.45- 36.83)
LAMTH	4	f	0	11	3	9	17	6.93	(1.53- 31.38)
LAMWK	5	f	0	2	41	5	144	1.40	(0.26- 7.51)
LAMWK2	4	m	0	24	161	2	43	3.20	(0.73- 14.10)
LAMWK2	8	f	0	2	50	5	139	1.11	(0.21- 5.92)
Subtotal LAMWK2								2.01	(0.66- 6.10)
NOU	4	m	0	15	247	2	122	3.70	(0.83- 16.46)
NOU	9	f	0	2	92	7	261	0.81	(0.17- 3.97)
Subtotal NOU								1.82	(0.61- 5.40)
ORMOS	16	m	0	7	1034	1	777	5.26	(0.65- 42.84)
SEOW	5	f	0	8	15	13	125	5.13	(1.83- 14.38)
SOBUE	15	m	0	78	1013	3	128	3.29	(1.02- 10.56)
SOBUE	31	f	0	7	232	7	857	3.69	(1.28- 10.64)
Subtotal SOBUE								3.50	(1.60- 7.67)
SOBUE2	4	m	2	-	-	-	-	4.10	(2.40- 7.20)
SOBUE2	8	f	2	-	-	-	-	3.80	(1.50- 9.30)
Subtotal SOBUE2								4.02	(2.51- 6.43)
WYNDE6	343	m	0	221	1895	7	650	10.83	(5.08- 23.10)
WYNDE6	373	f	0	141	779	13	936	13.03	(7.33- 23.18)
Subtotal WYNDE6								12.18	(7.70- 19.26)
ZHOU	20	m	0	29	41	8	36	3.18	(1.29- 7.84)
ZHOU	21	f	0	3	7	6	32	2.29	(0.46- 11.43)
Subtotal ZHOU								2.94	(1.34- 6.46)
Partial Totals				798	126907	102	210091		

*prospective study ~ With 0.5 adjustment for zero

REF	NRR	SEX	AD	Ys	Ws	Qs	Ps
*ANDERS	11	f	0	3.70	0.95	3.68	0.0003
BAND	4	m	2	2.56	2.88	1.95	0.0000
BARBON	118	m	0	3.38	0.98	2.66	0.0008
*BOUCOT	73	m	0	1.12	0.45	0.17	0.4525
BUFFLE	74	f	0	2.74	0.48	0.48	0.0584
COMSTO	68	m	0	2.50	0.48	0.28	0.0831
COMSTO	80	f	0	2.99	0.46	0.72	0.0419
Subtotal COMSTO				2.74	0.95	1.00	
HEGMAN	5	c	0	2.05	3.26	0.32	0.0002
HINDS	25	f	3	1.55	5.20	0.19	0.0004
JAIN	10	m	0	2.15	0.95	0.16	0.0359
JAIN	5	f	0	2.03	3.38	0.30	0.0002
Subtotal JAIN				2.06	4.34	0.46	
KHUDER	26	m	0	1.77	1.58	0.00	0.0258
KIHARA	28	c	0	1.41	0.79	0.09	0.2096
LAMTH	4	f	0	1.94	1.68	0.07	0.0121
LAMWK	5	f	0	0.34	1.37	2.67	0.6910
LAMWK2	4	m	0	1.16	1.75	0.58	0.1233
LAMWK2	8	f	0	0.11	1.38	3.66	0.9009
Subtotal LAMWK2				0.70	3.13	4.24	
NOU	4	m	0	1.31	1.73	0.32	0.0852
NOU	9	f	0	-0.21	1.52	5.77	0.7956
Subtotal NOU				0.60	3.25	6.09	
ORMOS	16	m	0	1.66	0.87	0.01	0.1208
SEOW	5	f	0	1.63	3.62	0.04	0.0019
SOBUE	15	m	0	1.19	2.82	0.85	0.0459
SOBUE	31	f	0	1.31	3.43	0.64	0.0155
Subtotal SOBUE				1.25	6.25	1.49	
SOBUE2	4	m	2	1.41	12.73	1.37	0.0000

Table 4C1 - 5

IESLC - Meta-anal of Ever Smoking (or Current if Ever not available), Any prod (or Cigs if Any not avail)
Large
Least adjusted

REF	NRR	SEX	AD	Ys	Ws	Qs	Ps
SOBUE2	8	f	2	1.34	4.62	0.75	0.0041
Subtotal SOBUE2				1.39	17.35	2.12	
WYNDE6	343	m	0	2.38	6.69	2.77	0.0000
WYNDE6	373	f	0	2.57	11.58	7.96	0.0000
Subtotal WYNDE6				2.50	18.27	10.73	
ZHOU	20	m	0	1.16	4.72	1.59	0.0118
ZHOU	21	f	0	0.83	1.48	1.23	0.3140
Subtotal ZHOU				1.08	6.21	2.83	

	N	29
	NS	21
	Wt	83.84
Het	Chi	41.27
Het	df	28
Het	P	(*)
Fixed	RR	5.69
	RRl	4.59
	RRu	7.05
	P	+++
Random	RR	5.42
	RRl	4.08
	RRu	7.22
	P	+++
Asymm	P	N.S.

Table 4C1 - 6

IESLC - Meta-anal of Ever Smoking (or Current if Ever not available), Any prod (or Cigs if Any not avail)

Large

Least adjusted

	combined	Sex male	female	Total
N	2	13	14	29
NS	2	13	14	29
Wt	4.05	38.64	41.15	83.84
Het Chi	0.27	12.65	28.15	41.27
Het df	1	12	13	28
Het P	N.S.	N.S.	**	(*)
Fixed RR	6.86	5.48	5.78	5.69
RRl	2.59	4.00	4.26	4.59
RRu	18.17	7.51	7.85	7.05
P	+++	+++	+++	+++
Random RR	6.86	5.54	4.83	5.42
RRl	2.59	3.97	2.93	4.08
RRu	18.17	7.75	7.97	7.22
P	+++	+++	+++	+++
Between Chi				0.21
Between df				2
Between P				N.S.
Btwn(F) P				N.S.
Btwn(R) P				N.S.

	Lung cancer type large	Total
N	29	29
NS	21	21
Wt	83.84	83.84
Het Chi	41.27	41.27
Het df	28	28
Het P	(*)	(*)
Fixed RR	5.69	5.69
RRl	4.59	4.59
RRu	7.05	7.05
P	+++	+++
Random RR	5.42	5.42
RRl	4.08	4.08
RRu	7.22	7.22
P	+++	+++
Between Chi		
Between df		
Between P		N.S.
Btwn(F) P		N.S.
Btwn(R) P		N.S.

	NAmer	UK	Scand	Location othEur	China	Japan	othAs	other	Total
N	13		2	2	2	5	5		29
NS	10		1	2	1	3	4		21
Wt	38.34		3.25	1.86	6.21	24.39	9.79		83.84
Het Chi	7.71		1.87	1.37	0.12	0.13	4.25		41.27
Het df	12		1	1	1	4	4		28
Het P	N.S.		N.S.	N.S.	N.S.	N.S.	N.S.		(*)
Fixed RR	9.78		1.82	13.10	2.94	3.88	3.34		5.69
RRl	7.13		0.61	3.11	1.34	2.61	1.79		4.59
RRu	13.42		5.40	55.25	6.46	5.77	6.25		7.05
P	+++		N.S.	+++	++	+++	+++		+++
Random RR	9.78		1.78	12.93	2.94	3.88	3.30		5.42
RRl	7.13		0.40	2.39	1.34	2.61	1.72		4.08
RRu	13.42		7.88	69.94	6.46	5.77	6.33		7.22
P	+++		N.S.	++	++	+++	+++		+++
Between Chi									25.81
Between df									5
Between P									***
Btwn(F) P									***
Btwn(R) P									***

Table 4C1 - 6

IESLC - Meta-anal of Ever Smoking (or Current if Ever not available), Any prod (or Cigs if Any not avail)
Large
Least adjusted

	Detailed Country in "other Europe"					
	multi	Germany	othWest	East	Balkans	Total
N			1	1		2
NS			1	1		2
Wt			0.98	0.87		1.86
Het Chi			0.00	0.00		1.37
Het df			0	0		1
Het P			N.S.	N.S.		N.S.
Fixed RR			29.51	5.26		13.10
RRl			4.08	0.65		3.11
RRu			213.27	42.84		55.25
P			+++	N.S.		+++
Random RR			29.51	5.26		12.93
RRl			4.08	0.65		2.39
RRu			213.27	42.84		69.94
P			+++	N.S.		++
Between Chi						1.37
Between df						1
Between P						N.S.
Btwn(F) P						N.S.
Btwn(R) P						N.S.

	Detailed Country in "other Asia"			
	India	HongKong	other	Total
N		4	1	5
NS		3	1	4
Wt		6.18	3.62	9.79
Het Chi		3.20	0.00	4.25
Het df		3	0	4
Het P		N.S.	N.S.	N.S.
Fixed RR		2.60	5.13	3.34
RRl		1.18	1.83	1.79
RRu		5.73	14.38	6.25
P		+	++	+++
Random RR		2.59	5.13	3.30
RRl		1.15	1.83	1.72
RRu		5.85	14.38	6.33
P		+	++	+++
Between Chi				1.05
Between df				1
Between P				N.S.
Btwn(F) P				N.S.
Btwn(R) P				N.S.

	Detailed other continent			
	SCAmer	Auslia	Africa	Total
N				
NS				
Wt				
Het Chi				
Het df				
Het P				N.S.
Fixed RR				
RRl				
RRu				
P				+++
Random RR				
RRl				
RRu				
P				+++
Between Chi				
Between df				
Between P				N.S.
Btwn(F) P				N.S.
Btwn(R) P				N.S.

Table 4C1 - 6

IESLC - Meta-anal of Ever Smoking (or Current if Ever not available), Any prod (or Cigs if Any not avail)
Large
Least adjusted

	Start year of study <1960	1960-69	1970-79	1980-89	1990+	Total
N	2	5	10	10	2	29
NS	2	3	6	8	2	21
Wt	1.32	40.81	14.99	22.31	4.41	83.84
Het Chi	0.09	11.87	13.02	10.44	0.03	41.27
Het df	1	4	9	9	1	28
Het P	N.S.	*	N.S.	N.S.	N.S.	(*)
Fixed RR	4.38	6.74	3.33	6.25	4.92	5.69
RRl	0.80	4.96	2.01	4.13	1.94	4.59
RRu	24.05	9.15	5.53	9.47	12.52	7.05
P	(+)	+++	+++	+++	+++	+++
Random RR	4.38	6.53	3.58	6.28	4.92	5.42
RRl	0.80	3.78	1.87	3.99	1.94	4.08
RRu	24.05	11.28	6.85	9.87	12.52	7.22
P	(+)	+++	+++	+++	+++	+++
Between Chi						5.83
Between df						4
Between P						N.S.
Btwn(F) P						N.S.
Btwn(R) P						N.S.

	Study type (1) CC	other	Total
N	25	4	29
NS	18	3	21
Wt	81.49	2.35	83.84
Het Chi	36.34	2.11	41.27
Het df	24	3	28
Het P	(*)	N.S.	(*)
Fixed RR	5.51	16.76	5.69
RRl	4.44	4.67	4.59
RRu	6.85	60.23	7.05
P	+++	+++	+++
Random RR	5.16	16.76	5.42
RRl	3.86	4.67	4.08
RRu	6.89	60.23	7.22
P	+++	+++	+++
Between Chi			2.82
Between df			1
Between P			(*)
Btwn(F) P			N.S.
Btwn(R) P			(*)

	Study type (2) CC	prosp	other	Total
N	25	2	2	29
NS	18	2	1	21
Wt	81.49	1.40	0.95	83.84
Het Chi	36.34	2.04	0.06	41.27
Het df	24	1	1	28
Het P	(*)	N.S.	N.S.	(*)
Fixed RR	5.51	17.72	15.44	5.69
RRl	4.44	3.39	2.06	4.59
RRu	6.85	92.71	115.86	7.05
P	+++	+++	++	+++
Random RR	5.16	13.99	15.44	5.42
RRl	3.86	1.16	2.06	4.08
RRu	6.89	169.20	115.86	7.22
P	+++	+	++	+++
Between Chi				2.83
Between df				2
Between P				N.S.
Btwn(F) P				N.S.
Btwn(R) P				N.S.

Table 4C1 - 6

IESLC - Meta-anal of Ever Smoking (or Current if Ever not available), Any prod (or Cigs if Any not avail)

Large
Least adjusted

	Study size (number of LC cases)				
	100-249	250-499	500-999	1000+	Total
N	4	12	4	9	29
NS	4	9	3	5	21
Wt	6.31	20.78	5.79	50.96	83.84
Het Chi	1.79	14.91	1.50	18.92	41.27
Het df	3	11	3	8	28
Het P	N.S.	N.S.	N.S.	*	(*)
Fixed RR	3.75	4.70	10.38	6.05	5.69
RRl	1.72	3.06	4.60	4.60	4.59
RRu	8.18	7.22	23.44	7.96	7.05
P	+++	+++	+++	+++	+++
Random RR	3.75	4.71	10.38	5.58	5.42
RRl	1.72	2.78	4.60	3.55	4.08
RRu	8.18	7.97	23.44	8.76	7.22
P	+++	+++	+++	+++	+++
Between Chi					4.15
Between df					3
Between P					N.S.
Btwn(F) P					N.S.
Btwn(R) P					N.S.

	Risky occupational population			
	no	mining	othRisky	Total
N	29			29
NS	21			21
Wt	83.84			83.84
Het Chi	41.27			41.27
Het df	28			28
Het P	(*)			(*)
Fixed RR	5.69			5.69
RRl	4.59			4.59
RRu	7.05			7.05
P	+++			+++
Random RR	5.42			5.42
RRl	4.08			4.08
RRu	7.22			7.22
P	+++			+++
Between Chi				
Between df				
Between P				N.S.
Btwn(F) P				N.S.
Btwn(R) P				N.S.

	National cigarette tobacco type			
	Virginia	blended	other	Total
N	3	24	2	29
NS	2	18	1	21
Wt	7.22	70.41	6.21	83.84
Het Chi	0.44	36.04	0.12	41.27
Het df	2	23	1	28
Het P	N.S.	*	N.S.	(*)
Fixed RR	9.58	5.72	2.94	5.69
RRl	4.62	4.53	1.34	4.59
RRu	19.87	7.22	6.46	7.05
P	+++	+++	++	+++
Random RR	9.58	5.35	2.94	5.42
RRl	4.62	3.85	1.34	4.08
RRu	19.87	7.42	6.46	7.22
P	+++	+++	++	+++
Between Chi				4.66
Between df				2
Between P				(*)
Btwn(F) P				N.S.
Btwn(R) P				(*)

Table 4C1 - 6

IESLC - Meta-anal of Ever Smoking (or Current if Ever not available), Any prod (or Cigs if Any not avail)
Large
Least adjusted

	Any proxy use No/nk	Yes	Total
N	20	9	29
NS	14	7	21
Wt	67.78	16.06	83.84
Het Chi	29.16	11.36	41.27
Het df	19	8	28
Het P	(*)	N.S.	(*)
Fixed RR	5.43	6.90	5.69
RRl	4.28	4.23	4.59
RRu	6.89	11.26	7.05
P	+++	+++	+++
Random RR	5.04	6.78	5.42
RRl	3.63	3.69	4.08
RRu	7.00	12.46	7.22
P	+++	+++	+++
Between Chi			0.74
Between df			1
Between P			N.S.
Btwn(F) P			N.S.
Btwn(R) P			N.S.

	Full histological confirmation No	Yes	Total
N	19	10	29
NS	13	8	21
Wt	46.13	37.71	83.84
Het Chi	18.87	10.82	41.27
Het df	18	9	28
Het P	N.S.	N.S.	(*)
Fixed RR	4.07	8.58	5.69
RRl	3.05	6.24	4.59
RRu	5.43	11.81	7.05
P	+++	+++	+++
Random RR	4.07	8.19	5.42
RRl	3.00	5.67	4.08
RRu	5.51	11.83	7.22
P	+++	+++	+++
Between Chi			11.58
Between df			1
Between P			***
Btwn(F) P			**
Btwn(R) P			**

	Number of adjustment variables (1) 0	1	2+/+nk	Total
N	25		4	29
NS	18		3	21
Wt	58.42		25.42	83.84
Het Chi	36.65		3.40	41.27
Het df	24		3	28
Het P	*		N.S.	(*)
Fixed RR	6.16		4.74	5.69
RRl	4.77		3.21	4.59
RRu	7.96		6.99	7.05
P	+++		+++	+++
Random RR	5.47		4.82	5.42
RRl	3.86		3.15	4.08
RRu	7.75		7.37	7.22
P	+++		+++	+++
Between Chi				1.22
Between df				1
Between P				N.S.
Btwn(F) P				N.S.
Btwn(R) P				N.S.

Table 4C1 - 6

IESLC - Meta-anal of Ever Smoking (or Current if Ever not available), Any prod (or Cigs if Any not avail)

Large

Least adjusted

	Number of adjustment variables (2)					
	0	1	2	3-5	6+/+nk	Total
N	25		3	1		29
NS	18		2	1		21
Wt	58.42		20.23	5.20		83.84
Het Chi	36.65		3.40	0.00		41.27
Het df	24		2	0		28
Het P	*		N.S.	N.S.		(*)
Fixed RR	6.16		4.75	4.71		5.69
RRl	4.77		3.07	1.99		4.59
RRu	7.96		7.34	11.13		7.05
P	+++		+++	+++		+++
Random RR	5.47		5.13	4.71		5.42
RRl	3.86		2.74	1.99		4.08
RRu	7.75		9.60	11.13		7.22
P	+++		+++	+++		+++
Between Chi						1.22
Between df						2
Between P						N.S.
Btwn(F) P						N.S.
Btwn(R) P						N.S.

	Product			
	all/unsp	cig+/-ot	cig only	Total
N	14	13	2	29
NS	10	9	2	21
Wt	44.13	36.37	3.33	83.84
Het Chi	29.72	8.64	0.81	41.27
Het df	13	12	1	28
Het P	**	N.S.	N.S.	(*)
Fixed RR	6.04	4.99	10.65	5.69
RRl	4.50	3.61	3.64	4.59
RRu	8.12	6.91	31.17	7.05
P	+++	+++	+++	+++
Random RR	4.66	4.99	10.65	5.42
RRl	2.85	3.61	3.64	4.08
RRu	7.63	6.91	31.17	7.22
P	+++	+++	+++	+++
Between Chi				2.10
Between df				2
Between P				N.S.
Btwn(F) P				N.S.
Btwn(R) P				N.S.

	Denominator		
	nev any	nev cigs	Total
N	19	10	29
NS	14	7	21
Wt	65.69	18.15	83.84
Het Chi	34.10	7.05	41.27
Het df	18	9	28
Het P	*	N.S.	(*)
Fixed RR	5.57	6.12	5.69
RRl	4.38	3.87	4.59
RRu	7.10	9.70	7.05
P	+++	+++	+++
Random RR	4.93	6.12	5.42
RRl	3.40	3.87	4.08
RRu	7.16	9.70	7.22
P	+++	+++	+++
Between Chi			0.13
Between df			1
Between P			N.S.
Btwn(F) P			N.S.
Btwn(R) P			N.S.

Table 4C1 - 6

IESLC - Meta-anal of Ever Smoking (or Current if Ever not available), Any prod (or Cigs if Any not avail)

Large
Least adjusted

	Derivation of RR/CI			
	Orig	StdCalc	Other	Total
N	3	20	6	29
NS	2	14	5	21
Wt	19.03	58.48	6.33	83.84
Het Chi	0.47	35.41	1.59	41.27
Het df	2	19	5	28
Het P	N.S.	*	N.S.	(*)
Fixed RR	4.22	5.90	10.00	5.69
RRl	2.69	4.57	4.59	4.59
RRu	6.61	7.62	21.79	7.05
P	+++	+++	+++	+++
Random RR	4.22	5.08	10.00	5.42
RRl	2.69	3.48	4.59	4.08
RRu	6.61	7.43	21.79	7.22
P	+++	+++	+++	+++
Between Chi				3.80
Between df				2
Between P				N.S.
Btwn(F) P				N.S.
Btwn(R) P				N.S.

	Smoking status		
	ever	current	Total
N	26	3	29
NS	19	2	21
Wt	66.04	17.80	83.84
Het Chi	38.38	0.05	41.27
Het df	25	2	28
Het P	*	N.S.	(*)
Fixed RR	6.26	3.99	5.69
RRl	4.92	2.51	4.59
RRu	7.97	6.35	7.05
P	+++	+++	+++
Random RR	5.70	3.99	5.42
RRl	4.12	2.51	4.08
RRu	7.88	6.35	7.22
P	+++	+++	+++
Between Chi			2.84
Between df			1
Between P			(*)
Btwn(F) P			N.S.
Btwn(R) P			N.S.

Table 4C1 - 7

IESLC - Meta-anal of Ever Smoking (or Current if Ever not available), Any prod (or Cigs if Any not avail)
Large
Excluded studies (and stage at which they were excluded)

1	BOUCHA BUELL LAURIL MZILEN
2	BERRIN CHEN RESTRE TAO ULMER
5	RIMING TANG2 WYNDE5 WYNDE8
6	BLOT1 BLOT2 BLOT3 BYERS2 HIRAOK HIRAY2 JONES LICKIN MOLLO MRFIT MURATA NOTANI PERSHA SAARIK SCHWA2 SHIMIZ SITAS VANDER WARSIN WATSON WYNDER
8	ABELIN ABRAHA AGUDO AKIBA ALDERS AMANDU AMES ARCHER ARMADA AUSTIN AUVINE AXELSO AXELSS BECHER BENHAM BENSHL BEST BLOHMK BLOT4 BOFFET BRESLO BRETT BROCKM BROSS BROWN1 BROWN2 BYERS1 CARPEN CASCO2 CASCOR CEDERL CHAN CHANG CHATZI CHEN2 CHEN3 CHIAZZ CHOI CHOW CHYOU COOKSO CORREA CPSI CPSII DAMBER DARBY DAVEYS DEAN DEAN2 DEAN3 DEKLER DESTE2 DESTEF DOCKER DOLL DOLL2 DORANT DORGAN DORN DOSEME DROSTE DU DUNN EBELIN ENGELA ENSTRO ESAKI FAN GAO GAO2 GARCIA GARDIN GARSHI GENG GER GILLIS GODLEY GOLLED GOODMA GRAHAM GREGOR GSELL HAENSZ HAMMO2 HAMMON HANSEN HEIN HENNEK HIRAYA HITOSU HOLE HOROWI HORWIT HU HU2 HUANG HUMBLE ISHIMA JAHN JARUP JARVHO JEDRYC JIANG JOLY JUSSAW KAISE2 KAISER KANELL KATSOU KAUFMA KELLER KINLEN KJUUS KNEKT KO KOHLME KOO KOULUM KREUZE KREYBE KUBIK LANGE LAUSSM LEI LEMARC LETOUR LEVIN LIAW LIDDEL LIU LIU2 LIU3 LIU4 LIU5 LOMBA2 LOMBAR LUBIN LUBIN2 LUO MACLEN MAGNUS MARSH MARSH2 MARTIS MASTRA MATOS MATSUD MCCONN MCDUFF MCLAUG MIGRAN MILLER MILLS MRFITR NAM NOTAN2 ODRISC OSANN OSANN2 PARKIN PASTOR PAWLEG PERNU PERSH2 PETO PEZZO2 PEZZOT PIKE PISANI POFFIJ POLEDN PRESCO QIAO QIAO2 RACHTA RADZIK RANDIG REN RONCO ROOTS ROTHSC SADOWS SANKAR SCHWAR SEGI SEGI2 SHAW SIEMIA SIMARA SIMONA SPEIZE SPITZ STASZE STAYNE STOCKS STOCKW STUCKE SUN SUZUK2 SUZUKI SVENSS TANG TENKAN TIZZAN TOKARS TOUSEY TSUGAN TULINI TVERDA VEIERO VUTUC WAKAI WALD WANG WANG2 WANG3 WANG4 WICKLU WIGLE WILKIN WU WU2 WUNSCH WUWILL WYNDE2 WYNDE3 WYNDE4 WYNDE7 XIANGZ XU XU2 XU3 XU4 YAMAGU YONG YUAN ZHANG ZHENG

Table 4C1 - 8
Potentially overlapping studies

REF	REFGP	PRINC	.	OVERLAP/LINK
LAMWK2	LAMWK2	1		Chan/LamWK2
LAMTH	LAMTH	1		KOO/LAMTH/LAMWK
LAMWK	LAMWK	1		KOO/LAMTH/LAMWK
WYNDE6	WYNDE6	1		WYNDE5/6/7/8
SOBUE2	SOBUE2	1		SOBUE2/MATSUD

Table 4C1 - 9
Most adjusted - insufficient data for metaanalysis

REF	NRR	SEX	AGEL	AGEH	RACE	YF	LC TYPE	LOC	START	ST	NLC	R	VB	P	H	AD	SM	PRODUCT	DENOM	De
KOO	5	f	0	0	all	-	large	As:HK	1981	CC	200	n	bl	n	n	0	ev	all/unsp	nev any	or

REF	NRR	RR	SIG	RRDATA comment
KOO	5	0.48		0

Table 4D1 -

IESLC - Meta-analysis of Ex Smoking, Any product (or Cigarettes if Any not available)
Large

This analysis is restricted to results for:
1) Non-dose-response data
2) Ex smokers
3) Results complete enough for use in metaanalysis

Within each study, results are then selected (in the following order of preference, within each sex) for:
4) PRODUCT: all/unspec, cigarettes regardless of other products, cigarettes only
5) CIGTYPE: all/unspecified, MC regardless of HR, MC only
6) DENOM: never smoked anything, never smoked cigarettes, (never +1 = +long term ex, +2 = +amount unknown, +3 = never cigs+long term ex)
7) Followup period (YF, prospective studies): whole study (coded as 0) or longest available
8) LCtype: large (specifically)

9) Race: all or nearest available, otherwise by race (wh or w = white, bl or b = black, hi = hispanic ch = chinese, jap = japanese, haw = hawaiian, w+o = white + oriental, sca = scandinavian, as = asian)
10) For overlapping studies: principal rather than subsidiary studies
Finally by Age: whole study (coded as 0) if available, otherwise by widest available age group
and then for single sex results (m, f) in preference to combined sex results (c).

Results adjusted (AD) for the most potential confounders are then chosen in Sections -1 to -3 and results adjusted for the least confounders in Sections -4 to -6. (Those least adjusted results which actually differ from the most adjusted as marked 'x' in column X in Section -4)
(Results adjusted for an unknown number of confounder(s) are coded as 20.)

Section -7 shows excluded studies, together with the stage (as above) at which no qualifying results were found.

Section -8 lists the potentially overlapping studies which have been included (1=principal, 2=subsidiary).

Section -9 lists any results which would have been included in preference except that they had data not complete enough for use in meta-analysis, with their significance (yes/no), if known, and any further comment as entered on the database.

In addition to those mentioned above, the following fields, levels and abbreviations are used:
* or nk = not known, n = no, y = yes, ot = other
nev = never
all/unspec = all or unspecified, cig+/-ot = cigarettes irrespective of other products (cigar, pipe etc)
MC = manufactured cigarettes, HR = hand-rolled cigarettes
REF: 6-character study reference
NRR: number of the RR on the database within the study
ST : study type (CC = case control, pr or prosp = prospective)
NLC: number of lung cancer cases in whole study
R : risky occupational population (n = no, m = mining, o = other risky)
VB : national cigarette type (V = at least 75% Virginia, bl = at least 75% blended, ot = other)
P : any proxy use
H : full histological confirmation
De : derivation of RR/CI (or = original, st = standard method, ot = other method of estimation)

Table 4D1 - 1

IESLC - Meta-analysis of Ex Smoking, Any product (or Cigarettes if Any not available)
Large
Most adjusted

REF	NRR	SEX	AGEL	AGEH	RACE	YF	LC TYPE	LOC	START	ST	NLC	R	VB	P	H	AD	PRODUCT	DENOM	De
BARBON	92	m	0	0	all	-	large	Eu:wst	1979	CC	755	n	bl	y	y	1	all/unsp	nev any	or
BUFFLE	76	f	0	0	w-hi	-	large	NAmer	1976	CC	943	n	bl	y	n	0	cig+/-ot	nev cigs	ot
COMSTO	21	m	0	0	all	-	large	NAmer	1975	ot	258	n	bl	n	n	0	cig+/-ot	nev cigs	ot
JAIN	30	m	0	0	all	-	large	NAmer	1981	CC	845	n	V	y	n	0	cig+/-ot	nev cigs	st
JAIN	25	f	0	0	all	-	large	NAmer	1981	CC	845	n	V	y	n	0	cig+/-ot	nev cigs	st
KHUDER	10	m	0	0	all	-	large	NAmer	1985	CC	482	n	bl	n	y	0	cig+/-ot	nev cigs	or
KIHARA	12	c	0	0	jap	-	large	As:Jap	1991	CC	440	n	bl	n	n	0	all/unsp	nev any	ot
SOBUE	39	m	0	0	all	-	large	As:Jap	1986	CC	1376	n	bl	n	y	1	cig+/-ot	nev cigs	or
SOBUE	49	f	0	0	all	-	large	As:Jap	1986	CC	1376	n	bl	n	y	1	cig+/-ot	nev cigs	or
WYNDE6	379	m	0	0	all	-	large	NAmer	1969	CC	4423	n	bl	n	y	2	cig+/-ot	nev any	or
WYNDE6	394	f	0	0	all	-	large	NAmer	1969	CC	4423	n	bl	n	y	2	cig+/-ot	nev any	or

Cigarette type is all/unspec for all RRs

Table 4D1 - 2

IESLC - Meta-analysis of Ex Smoking, Any product (or Cigarettes if Any not available)
Large
Most adjusted

REF	NRR	SEX	AD	Number Case	Exposed Cont	Non-exposed Case	Cont	RR	95.00%CI
BARBON	92	m	1	-	-	-	-	19.40	(2.60- 145.00)
BUFFLE	76	f	0	3	56	0	112	13.94~	(0.71- 274.51)
COMSTO	21	m	0	4	129	0	84	5.87~	(0.31- 110.48)
JAIN	30	m	0	7	159	1	85	3.74	(0.45- 30.92)
JAIN	25	f	0	7	97	4	214	3.86	(1.10- 13.50)
Subtotal JAIN								3.83	(1.30- 11.24)
KHUDER	10	m	0	10	-	2	-	5.90	(1.10- 31.10)
KIHARA	12	c	0	0	70	1	237	1.12~	(0.05- 27.87)
SOBUE	39	m	1	-	-	-	-	2.60	(0.70- 10.00)
SOBUE	49	f	1	-	-	-	-	4.10	(1.40- 32.80)
Subtotal SOBUE								3.14	(1.14- 8.68)
WYNDE6	379	m	2	-	-	-	-	7.90	(3.70- 17.20)
WYNDE6	394	f	2	-	-	-	-	7.20	(3.80- 13.50)
Subtotal WYNDE6								7.48	(4.58- 12.19)
Partial Totals				31	511	8	732		

*prospective study ~ With 0.5 adjustment for zero

REF	NRR	SEX	AD	Ys	Ws	Qs	Ps
BARBON	92	m	1	2.97	0.95	1.27	0.0038
BUFFLE	76	f	0	2.63	0.43	0.29	0.0832
COMSTO	21	m	0	1.77	0.45	0.00	0.2371
JAIN	30	m	0	1.32	0.86	0.21	0.2207
JAIN	25	f	0	1.35	2.45	0.51	0.0344
Subtotal JAIN				1.34	3.31	0.72	
KHUDER	10	m	0	1.77	1.38	0.00	0.0373
KIHARA	12	c	0	0.12	0.37	1.07	0.9436
SOBUE	39	m	1	0.96	2.17	1.58	0.1590
SOBUE	49	f	1	1.41	1.54	0.24	0.0795
Subtotal SOBUE				1.14	3.72	1.83	
WYNDE6	379	m	2	2.07	6.51	0.43	0.0000
WYNDE6	394	f	2	1.97	9.56	0.26	0.0000
Subtotal WYNDE6				2.01	16.07	0.69	

	N	11
	NS	8
	Wt	26.68
Het	Chi	5.88
Het	df	10
Het	P	N.S.
Fixed	RR	6.11
	RRl	4.18
	RRu	8.92
	P	+++
Random	RR	6.11
	RRl	4.18
	RRu	8.92
	P	+++
Asymm	P	N.S.

Table 4D1 - 3

IESLC - Meta-analysis of Ex Smoking, Any product (or Cigarettes if Any not available)
Large
Most adjusted

	Sex			
	combined	male	female	Total
N	1	6	4	11
NS	1	6	4	11
Wt	0.37	12.31	13.99	26.68
Het Chi	0.00	3.48	1.31	5.88
Het df	0	5	3	10
Het P	N.S.	N.S.	N.S.	N.S.
Fixed RR	1.12	6.32	6.19	6.11
RRl	0.05	3.62	3.67	4.18
RRu	27.87	11.06	10.46	8.92
P	N.S.	+++	+++	+++
Random RR	1.12	6.32	6.19	6.11
RRl	0.05	3.62	3.67	4.18
RRu	27.87	11.06	10.46	8.92
P	N.S.	+++	+++	+++
Between Chi				1.09
Between df				2
Between P				N.S.
Btwn(F) P				N.S.
Btwn(R) P				N.S.

	Lung cancer type	
	large	Total
N	11	11
NS	8	8
Wt	26.68	26.68
Het Chi	5.88	5.88
Het df	10	10
Het P	N.S.	N.S.
Fixed RR	6.11	6.11
RRl	4.18	4.18
RRu	8.92	8.92
P	+++	+++
Random RR	6.11	6.11
RRl	4.18	4.18
RRu	8.92	8.92
P	+++	+++
Between Chi		
Between df		
Between P		N.S.
Btwn(F) P		N.S.
Btwn(R) P		N.S.

	Location								
	NAmer	UK	Scand	othEur	China	Japan	othAs	other	Total
N	7			1		3			11
NS	5			1		2			8
Wt	21.64			0.95		4.09			26.68
Het Chi	1.53			0.00		0.55			5.88
Het df	6			0		2			10
Het P	N.S.			N.S.		N.S.			N.S.
Fixed RR	6.70			19.40		2.86			6.11
RRl	4.39			2.60		1.09			4.18
RRu	10.21			144.88		7.54			8.92
P	+++			++		+			+++
Random RR	6.70			19.40		2.86			6.11
RRl	4.39			2.60		1.09			4.18
RRu	10.21			144.88		7.54			8.92
P	+++			++		+			+++
Between Chi									3.81
Between df									2
Between P									N.S.
Btwn(F) P									*
Btwn(R) P									N.S.

Table 4D1 - 3

IESLC - Meta-analysis of Ex Smoking, Any product (or Cigarettes if Any not available)
Large
Most adjusted

	Detailed Country in "other Europe"					
	multi	Germany	othWest	East	Balkans	Total
N			1			1
NS			1			1
Wt			0.95			0.95
Het Chi			0.00			0.00
Het df			0			0
Het P			N.S.			N.S.
Fixed RR			19.40			19.40
RRl			2.60			2.60
RRu			144.88			144.88
P			++			++
Random RR			19.40			19.40
RRl			2.60			2.60
RRu			144.88			144.88
P			++			++
Between Chi						
Between df						
Between P						N.S.
Btwn(F) P						N.S.
Btwn(R) P						N.S.

	Detailed Country in "other Asia"			
	India	HongKong	other	Total
N				
NS				
Wt				
Het Chi				
Het df				
Het P				
Fixed RR				
RRl				
RRu				
P				
Random RR				
RRl				
RRu				
P				
Between Chi				
Between df				
Between P				N.S.
Btwn(F) P				N.S.
Btwn(R) P				N.S.

	Detailed other continent			
	SCAmer	Auslia	Africa	Total
N				
NS				
Wt				
Het Chi				
Het df				
Het P				
Fixed RR				
RRl				
RRu				
P				
Random RR				
RRl				
RRu				
P				
Between Chi				
Between df				
Between P				N.S.
Btwn(F) P				N.S.
Btwn(R) P				N.S.

Table 4D1 - 3

IESLC - Meta-analysis of Ex Smoking, Any product (or Cigarettes if Any not available)
Large
Most adjusted

	Start year of study <1960	1960-69	1970-79	1980-89	1990+	Total
N		2	3	5	1	11
NS		1	3	3	1	8
Wt		16.07	1.83	8.41	0.37	26.68
Het Chi		0.03	0.43	0.59	0.00	5.88
Het df		1	2	4	0	10
Het P		N.S.	N.S.	N.S.	N.S.	N.S.
Fixed RR		7.48	13.40	3.77	1.12	6.11
RRl		4.58	3.15	1.92	0.05	4.18
RRu		12.19	57.11	7.40	27.87	8.92
P		+++	+++	+++	N.S.	+++
Random RR		7.48	13.40	3.77	1.12	6.11
RRl		4.58	3.15	1.92	0.05	4.18
RRu		12.19	57.11	7.40	27.87	8.92
P		+++	+++	+++	N.S.	+++
Between Chi						4.82
Between df						3
Between P						N.S.
Btwn(F) P						**
Btwn(R) P						N.S.

	Study type (1) CC	other	Total
N	10	1	11
NS	7	1	8
Wt	26.23	0.45	26.68
Het Chi	5.88	0.00	5.88
Het df	9	0	10
Het P	N.S.	N.S.	N.S.
Fixed RR	6.11	5.87	6.11
RRl	4.17	0.31	4.18
RRu	8.96	110.48	8.92
P	+++	N.S.	+++
Random RR	6.11	5.87	6.11
RRl	4.17	0.31	4.18
RRu	8.96	110.48	8.92
P	+++	N.S.	+++
Between Chi			0.00
Between df			1
Between P			N.S.
Btwn(F) P			N.S.
Btwn(R) P			N.S.

	Study type (2) CC	prosp	other	Total
N	10		1	11
NS	7		1	8
Wt	26.23		0.45	26.68
Het Chi	5.88		0.00	5.88
Het df	9		0	10
Het P	N.S.		N.S.	N.S.
Fixed RR	6.11		5.87	6.11
RRl	4.17		0.31	4.18
RRu	8.96		110.48	8.92
P	+++		N.S.	+++
Random RR	6.11		5.87	6.11
RRl	4.17		0.31	4.18
RRu	8.96		110.48	8.92
P	+++		N.S.	+++
Between Chi				0.00
Between df				1
Between P				N.S.
Btwn(F) P				N.S.
Btwn(R) P				N.S.

Table 4D1 - 3

IESLC - Meta-analysis of Ex Smoking, Any product (or Cigarettes if Any not available)

Large

Most adjusted

	Study size (number of LC cases)				
	100-249	250-499	500-999	1000+	Total
N		3	4	4	11
NS		3	3	2	8
Wt		2.19	4.70	19.79	26.68
Het Chi		0.85	2.28	2.49	5.88
Het df		2	3	3	10
Het P		N.S.	N.S.	N.S.	N.S.
Fixed RR		4.45	5.99	6.35	6.11
RRl		1.18	2.42	4.09	4.18
RRu		16.70	14.80	9.87	8.92
P		+	+++	+++	+++
Random RR		4.45	5.99	6.35	6.11
RRl		1.18	2.42	4.09	4.18
RRu		16.70	14.80	9.87	8.92
P		+	+++	+++	+++
Between Chi					0.25
Between df					2
Between P					N.S.
Btwn(F) P					N.S.
Btwn(R) P					N.S.

	Risky occupational population			
	no	mining	othRisky	Total
N	11			11
NS	8			8
Wt	26.68			26.68
Het Chi	5.88			5.88
Het df	10			10
Het P	N.S.			N.S.
Fixed RR	6.11			6.11
RRl	4.18			4.18
RRu	8.92			8.92
P	+++			+++
Random RR	6.11			6.11
RRl	4.18			4.18
RRu	8.92			8.92
P	+++			+++
Between Chi				
Between df				
Between P				N.S.
Btwn(F) P				N.S.
Btwn(R) P				N.S.

	National cigarette tobacco type			
	Virginia	blended	other	Total
N	2	9		11
NS	1	7		8
Wt	3.31	23.36		26.68
Het Chi	0.00	5.05		5.88
Het df	1	8		10
Het P	N.S.	N.S.		N.S.
Fixed RR	3.83	6.52		6.11
RRl	1.30	4.35		4.18
RRu	11.24	9.78		8.92
P	+	+++		+++
Random RR	3.83	6.52		6.11
RRl	1.30	4.35		4.18
RRu	11.24	9.78		8.92
P	+	+++		+++
Between Chi				0.82
Between df				1
Between P				N.S.
Btwn(F) P				N.S.
Btwn(R) P				N.S.

Table 4D1 - 3

IESLC - Meta-analysis of Ex Smoking, Any product (or Cigarettes if Any not available)
Large
Most adjusted

	Any proxy use No/nk	Yes	Total
N	7	4	11
NS	5	3	8
Wt	21.98	4.70	26.68
Het Chi	3.59	2.28	5.88
Het df	6	3	10
Het P	N.S.	N.S.	N.S.
Fixed RR	6.13	5.99	6.11
RRl	4.04	2.42	4.18
RRu	9.31	14.80	8.92
P	+++	+++	+++
Random RR	6.13	5.99	6.11
RRl	4.04	2.42	4.18
RRu	9.31	14.80	8.92
P	+++	+++	+++
Between Chi			0.00
Between df			1
Between P			N.S.
Btwn(F) P			N.S.
Btwn(R) P			N.S.

	Full histological confirmation No	Yes	Total
N	5	6	11
NS	4	4	8
Wt	4.56	22.11	26.68
Het Chi	1.35	3.64	5.88
Het df	4	5	10
Het P	N.S.	N.S.	N.S.
Fixed RR	4.08	6.63	6.11
RRl	1.63	4.37	4.18
RRu	10.22	10.06	8.92
P	++	+++	+++
Random RR	4.08	6.63	6.11
RRl	1.63	4.37	4.18
RRu	10.22	10.06	8.92
P	++	+++	+++
Between Chi			0.89
Between df			1
Between P			N.S.
Btwn(F) P			N.S.
Btwn(R) P			N.S.

	Number of adjustment variables (1) 0	1	2+/+nk	Total
N	6	3	2	11
NS	5	2	1	8
Wt	5.94	4.67	16.07	26.68
Het Chi	1.49	2.70	0.03	5.88
Het df	5	2	1	10
Het P	N.S.	N.S.	N.S.	N.S.
Fixed RR	4.45	4.55	7.48	6.11
RRl	1.99	1.84	4.58	4.18
RRu	9.94	11.27	12.19	8.92
P	+++	++	+++	+++
Random RR	4.45	4.84	7.48	6.11
RRl	1.99	1.66	4.58	4.18
RRu	9.94	14.12	12.19	8.92
P	+++	++	+++	+++
Between Chi				1.66
Between df				2
Between P				N.S.
Btwn(F) P				N.S.
Btwn(R) P				N.S.

Table 4D1 - 3

IESLC - Meta-analysis of Ex Smoking, Any product (or Cigarettes if Any not available)
Large
Most adjusted

	Number of adjustment variables (2) 0	1	2	3-5	6+/+nk	Total
N	6	3	2			11
NS	5	2	1			8
Wt	5.94	4.67	16.07			26.68
Het Chi	1.49	2.70	0.03			5.88
Het df	5	2	1			10
Het P	N.S.	N.S.	N.S.			N.S.
Fixed RR	4.45	4.55	7.48			6.11
RRl	1.99	1.84	4.58			4.18
RRu	9.94	11.27	12.19			8.92
P	+++	++	+++			+++
Random RR	4.45	4.84	7.48			6.11
RRl	1.99	1.66	4.58			4.18
RRu	9.94	14.12	12.19			8.92
P	+++	++	+++			+++
Between Chi						1.66
Between df						2
Between P						N.S.
Btwn(F) P						N.S.
Btwn(R) P						N.S.

	Product all/unsp	cig+/-ot	cig only	Total
N	2	9		11
NS	2	6		8
Wt	1.32	25.35		26.68
Het Chi	2.17	3.53		5.88
Het df	1	8		10
Het P	N.S.	N.S.		N.S.
Fixed RR	8.70	5.99		6.11
RRl	1.58	4.06		4.18
RRu	47.81	8.85		8.92
P	+	+++		+++
Random RR	6.22	5.99		6.11
RRl	0.40	4.06		4.18
RRu	95.81	8.85		8.92
P	N.S.	+++		+++
Between Chi				0.17
Between df				1
Between P				N.S.
Btwn(F) P				N.S.
Btwn(R) P				N.S.

	Denominator nev any	nev cigs	Total
N	4	7	11
NS	3	5	8
Wt	17.39	9.29	26.68
Het Chi	2.23	1.35	5.88
Het df	3	6	10
Het P	N.S.	N.S.	N.S.
Fixed RR	7.56	4.09	6.11
RRl	4.73	2.15	4.18
RRu	12.10	7.78	8.92
P	+++	+++	+++
Random RR	7.56	4.09	6.11
RRl	4.73	2.15	4.18
RRu	12.10	7.78	8.92
P	+++	+++	+++
Between Chi			2.29
Between df			1
Between P			N.S.
Btwn(F) P			*
Btwn(R) P			N.S.

Table 4D1 - 3

IESLC - Meta-analysis of Ex Smoking, Any product (or Cigarettes if Any not available)

Large

Most adjusted

	Derivation of RR/CI			
	Orig	StdCalc	Other	Total
N	6	2	3	11
NS	4	1	3	8
Wt	22.11	3.31	1.25	26.68
Het Chi	3.64	0.00	1.30	5.88
Het df	5	1	2	10
Het P	N.S.	N.S.	N.S.	N.S.
Fixed RR	6.63	3.83	4.84	6.11
RRl	4.37	1.30	0.84	4.18
RRu	10.06	11.24	27.91	8.92
P	+++	+	(+)	+++
Random RR	6.63	3.83	4.84	6.11
RRl	4.37	1.30	0.84	4.18
RRu	10.06	11.24	27.91	8.92
P	+++	+	(+)	+++
Between Chi				0.94
Between df				2
Between P				N.S.
Btwn(F) P				N.S.
Btwn(R) P				N.S.

Table 4D1 - 4

IESLC - Meta-analysis of Ex Smoking, Any product (or Cigarettes if Any not available)
Large
Least adjusted

REF	NRR	X	SEX	AGEL	AGEH	RACE	YF	LC TYPE	LOC	START	ST	NLC	R	VB	P	H	AD	PRODUCT	DENOM	De
BARBON	36	x	m	0	0	all	-	large	Eu:wst	1979	CC	755	n	bl	y	y	0	all/unsp	nev any	st
BUFFLE	76		f	0	0	w-hi	-	large	NAmer	1976	CC	943	n	bl	y	n	0	cig+/-ot	nev cigs	ot
COMSTO	21		m	0	0	all	-	large	NAmer	1975	ot	258	n	bl	n	n	0	cig+/-ot	nev cigs	ot
JAIN	30		m	0	0	all	-	large	NAmer	1981	CC	845	n	V	y	n	0	cig+/-ot	nev cigs	st
JAIN	25		f	0	0	all	-	large	NAmer	1981	CC	845	n	V	y	n	0	cig+/-ot	nev cigs	st
KHUDER	10		m	0	0	all	-	large	NAmer	1985	CC	482	n	bl	n	y	0	cig+/-ot	nev cigs	or
KIHARA	12		c	0	0	jap	-	large	As:Jap	1991	CC	440	n	bl	n	n	0	all/unsp	nev any	ot
SOBUE	13	x	m	0	0	all	-	large	As:Jap	1986	CC	1376	n	bl	n	y	0	cig+/-ot	nev cigs	st
SOBUE	29	x	f	0	0	all	-	large	As:Jap	1986	CC	1376	n	bl	n	y	0	cig+/-ot	nev cigs	st
WYNDE6	330	x	m	0	0	all	-	large	NAmer	1969	CC	4423	n	bl	n	y	0	cig+/-ot	nev any	st
WYNDE6	360	x	f	0	0	all	-	large	NAmer	1969	CC	4423	n	bl	n	y	0	cig+/-ot	nev any	st

Cigarette type is all/unspec for all RRs

Table 4D1 - 5

IESLC - Meta-analysis of Ex Smoking, Any product (or Cigarettes if Any not available)
Large
Least adjusted

REF	NRR	SEX	AD	Number Exposed Case	Exposed Cont	Non-exposed Case	Non-exposed Cont	RR	95.00%CI
BARBON	36	m	0	21	205	1	188	19.26	(2.57- 144.57)
BUFFLE	76	f	0	3	56	0	112	13.94~	(0.71- 274.51)
COMSTO	21	m	0	4	129	0	84	5.87~	(0.31- 110.48)
JAIN	30	m	0	7	159	1	85	3.74	(0.45- 30.92)
JAIN	25	f	0	7	97	4	214	3.86	(1.10- 13.50)
Subtotal JAIN								3.83	(1.30- 11.24)
KHUDER	10	m	0	10	-	2	-	5.90	(1.10- 31.10)
KIHARA	12	c	0	0	70	1	237	1.12~	(0.05- 27.87)
SOBUE	13	m	0	21	363	3	128	2.47	(0.72- 8.41)
SOBUE	29	f	0	2	64	7	857	3.83	(0.78- 18.80)
Subtotal SOBUE								2.91	(1.10- 7.68)
WYNDE6	330	m	0	99	1113	7	650	8.26	(3.81- 17.89)
WYNDE6	360	f	0	41	431	13	936	6.85	(3.63- 12.91)
Subtotal WYNDE6								7.39	(4.52- 12.06)
Partial Totals				215	2687	39	3491		

*prospective study ~ With 0.5 adjustment for zero

REF	NRR	SEX	AD	Ys	Ws	Qs	Ps
BARBON	36	m	0	2.96	0.95	1.31	0.0040
BUFFLE	76	f	0	2.63	0.43	0.32	0.0832
COMSTO	21	m	0	1.77	0.45	0.00	0.2371
JAIN	30	m	0	1.32	0.86	0.18	0.2207
JAIN	25	f	0	1.35	2.45	0.45	0.0344
Subtotal JAIN				1.34	3.31	0.64	
KHUDER	10	m	0	1.77	1.38	0.00	0.0373
KIHARA	12	c	0	0.12	0.37	1.03	0.9436
SOBUE	13	m	0	0.90	2.55	1.96	0.1487
SOBUE	29	f	0	1.34	1.52	0.29	0.0985
Subtotal SOBUE				1.07	4.07	2.26	
WYNDE6	330	m	0	2.11	6.44	0.71	0.0000
WYNDE6	360	f	0	1.92	9.55	0.20	0.0000
Subtotal WYNDE6				2.00	15.99	0.90	

	N	11
	NS	8
	Wt	26.94
Het	Chi	6.45
Het	df	10
Het	P	N.S.
Fixed	RR	5.93
	RRl	4.07
	RRu	8.65
	P	+++
Random	RR	5.93
	RRl	4.07
	RRu	8.65
	P	+++
Asymm	P	N.S.

Table 4D1 - 6

IESLC - Meta-analysis of Ex Smoking, Any product (or Cigarettes if Any not available)

Large

Least adjusted

	Sex			
	combined	male	female	Total
N	1	6	4	11
NS	1	6	4	11
Wt	0.37	12.62	13.95	26.94
Het Chi	0.00	4.13	1.26	6.45
Het df	0	5	3	10
Het P	N.S.	N.S.	N.S.	N.S.
Fixed RR	1.12	6.22	5.94	5.93
RRl	0.05	3.58	3.52	4.07
RRu	27.87	10.80	10.04	8.65
P	N.S.	+++	+++	+++
Random RR	1.12	6.22	5.94	5.93
RRl	0.05	3.58	3.52	4.07
RRu	27.87	10.80	10.04	8.65
P	N.S.	+++	+++	+++
Between Chi				1.06
Between df				2
Between P				N.S.
Btwn(F) P				N.S.
Btwn(R) P				N.S.

	Lung cancer type	
	large	Total
N	11	11
NS	8	8
Wt	26.94	26.94
Het Chi	6.45	6.45
Het df	10	10
Het P	N.S.	N.S.
Fixed RR	5.93	5.93
RRl	4.07	4.07
RRu	8.65	8.65
P	+++	+++
Random RR	5.93	5.93
RRl	4.07	4.07
RRu	8.65	8.65
P	+++	+++
Between Chi		
Between df		
Between P		N.S.
Btwn(F) P		N.S.
Btwn(R) P		N.S.

	Location								
	NAmer	UK	Scand	othEur	China	Japan	othAs	other	Total
N	7			1		3			11
NS	5			1		2			8
Wt	21.55			0.95		4.44			26.94
Het Chi	1.58			0.00		0.49			6.45
Het df	6			0		2			10
Het P	N.S.			N.S.		N.S.			N.S.
Fixed RR	6.63			19.26		2.68			5.93
RRl	4.35			2.57		1.06			4.07
RRu	10.12			144.57		6.80			8.65
P	+++			++		+			+++
Random RR	6.63			19.26		2.68			5.93
RRl	4.35			2.57		1.06			4.07
RRu	10.12			144.57		6.80			8.65
P	+++			++		+			+++
Between Chi									4.38
Between df									2
Between P									N.S.
Btwn(F) P									*
Btwn(R) P									N.S.

Table 4D1 - 6

IESLC - Meta-analysis of Ex Smoking, Any product (or Cigarettes if Any not available)
Large
Least adjusted

	Detailed Country in "other Europe" multi	Germany	othWest	East	Balkans	Total
N			1			1
NS			1			1
Wt			0.95			0.95
Het Chi			0.00			0.00
Het df			0			0
Het P			N.S.			N.S.
Fixed RR			19.26			19.26
RRl			2.57			2.57
RRu			144.57			144.57
P			++			++
Random RR			19.26			19.26
RRl			2.57			2.57
RRu			144.57			144.57
P			++			++
Between Chi						
Between df						
Between P						N.S.
Btwn(F) P						N.S.
Btwn(R) P						N.S.

	Detailed Country in "other Asia" India	HongKong	other	Total
N				
NS				
Wt				
Het Chi				
Het df				
Het P				
Fixed RR				
RRl				
RRu				
P				
Random RR				
RRl				
RRu				
P				
Between Chi				
Between df				
Between P				N.S.
Btwn(F) P				N.S.
Btwn(R) P				N.S.

	Detailed other continent SCAmer	Auslia	Africa	Total
N				
NS				
Wt				
Het Chi				
Het df				
Het P				
Fixed RR				
RRl				
RRu				
P				
Random RR				
RRl				
RRu				
P				
Between Chi				
Between df				
Between P				N.S.
Btwn(F) P				N.S.
Btwn(R) P				N.S.

Table 4D1 - 6

IESLC - Meta-analysis of Ex Smoking, Any product (or Cigarettes if Any not available)
Large
Least adjusted

	Start year of study <1960	1960-69	1970-79	1980-89	1990+	Total
N		2	3	5	1	11
NS		1	3	3	1	8
Wt		15.99	1.82	8.76	0.37	26.94
Het Chi		0.13	0.43	0.72	0.00	6.45
Het df		1	2	4	0	10
Het P		N.S.	N.S.	N.S.	N.S.	N.S.
Fixed RR		7.39	13.34	3.61	1.12	5.93
RRl		4.52	3.13	1.86	0.05	4.07
RRu		12.06	56.95	6.99	27.87	8.65
P		+++	+++	+++	N.S.	+++
Random RR		7.39	13.34	3.61	1.12	5.93
RRl		4.52	3.13	1.86	0.05	4.07
RRu		12.06	56.95	6.99	27.87	8.65
P		+++	+++	+++	N.S.	+++
Between Chi						5.17
Between df						3
Between P						N.S.
Btwn(F) P						**
Btwn(R) P						N.S.

	Study type (1) CC	other	Total
N	10	1	11
NS	7	1	8
Wt	26.50	0.45	26.94
Het Chi	6.45	0.00	6.45
Het df	9	0	10
Het P	N.S.	N.S.	N.S.
Fixed RR	5.93	5.87	5.93
RRl	4.05	0.31	4.07
RRu	8.68	110.48	8.65
P	+++	N.S.	+++
Random RR	5.93	5.87	5.93
RRl	4.05	0.31	4.07
RRu	8.68	110.48	8.65
P	+++	N.S.	+++
Between Chi			0.00
Between df			1
Between P			N.S.
Btwn(F) P			N.S.
Btwn(R) P			N.S.

	Study type (2) CC	prosp	other	Total
N	10		1	11
NS	7		1	8
Wt	26.50		0.45	26.94
Het Chi	6.45		0.00	6.45
Het df	9		0	10
Het P	N.S.		N.S.	N.S.
Fixed RR	5.93		5.87	5.93
RRl	4.05		0.31	4.07
RRu	8.68		110.48	8.65
P	+++		N.S.	+++
Random RR	5.93		5.87	5.93
RRl	4.05		0.31	4.07
RRu	8.68		110.48	8.65
P	+++		N.S.	+++
Between Chi				0.00
Between df				1
Between P				N.S.
Btwn(F) P				N.S.
Btwn(R) P				N.S.

Table 4D1 - 6

IESLC - Meta-analysis of Ex Smoking, Any product (or Cigarettes if Any not available)
Large
Least adjusted

	Study size (number of LC cases)				
	100-249	250-499	500-999	1000+	Total
N		3	4	4	11
NS		3	3	2	8
Wt		2.19	4.69	20.06	26.94
Het Chi		0.85	2.26	3.14	6.45
Het df		2	3	3	10
Het P		N.S.	N.S.	N.S.	N.S.
Fixed RR		4.45	5.97	6.11	5.93
RRl		1.18	2.42	3.95	4.07
RRu		16.70	14.77	9.47	8.65
P		+	+++	+++	+++
Random RR		4.45	5.97	6.06	5.93
RRl		1.18	2.42	3.85	4.07
RRu		16.70	14.77	9.54	8.65
P		+	+++	+++	+++
Between Chi					0.20
Between df					2
Between P					N.S.
Btwn(F) P					N.S.
Btwn(R) P					N.S.

	Risky occupational population			
	no	mining	othRisky	Total
N	11			11
NS	8			8
Wt	26.94			26.94
Het Chi	6.45			6.45
Het df	10			10
Het P	N.S.			N.S.
Fixed RR	5.93			5.93
RRl	4.07			4.07
RRu	8.65			8.65
P	+++			+++
Random RR	5.93			5.93
RRl	4.07			4.07
RRu	8.65			8.65
P	+++			+++
Between Chi				
Between df				
Between P				N.S.
Btwn(F) P				N.S.
Btwn(R) P				N.S.

	National cigarette tobacco type			
	Virginia	blended	other	Total
N	2	9		11
NS	1	7		8
Wt	3.31	23.63		26.94
Het Chi	0.00	5.73		6.45
Het df	1	8		10
Het P	N.S.	N.S.		N.S.
Fixed RR	3.83	6.31		5.93
RRl	1.30	4.21		4.07
RRu	11.24	9.44		8.65
P	+	+++		+++
Random RR	3.83	6.31		5.93
RRl	1.30	4.21		4.07
RRu	11.24	9.44		8.65
P	+	+++		+++
Between Chi				0.72
Between df				1
Between P				N.S.
Btwn(F) P				N.S.
Btwn(R) P				N.S.

Table 4D1 - 6

IESLC - Meta-analysis of Ex Smoking, Any product (or Cigarettes if Any not available)
Large
Least adjusted

	Any proxy use No/nk	Yes	Total
N	7	4	11
NS	5	3	8
Wt	22.25	4.69	26.94
Het Chi	4.19	2.26	6.45
Het df	6	3	10
Het P	N.S.	N.S.	N.S.
Fixed RR	5.92	5.97	5.93
RRl	3.91	2.42	4.07
RRu	8.97	14.77	8.65
P	+++	+++	+++
Random RR	5.92	5.97	5.93
RRl	3.91	2.42	4.07
RRu	8.97	14.77	8.65
P	+++	+++	+++
Between Chi			0.00
Between df			1
Between P			N.S.
Btwn(F) P			N.S.
Btwn(R) P			N.S.

	Full histological confirmation No	Yes	Total
N	5	6	11
NS	4	4	8
Wt	4.56	22.38	26.94
Het Chi	1.35	4.34	6.45
Het df	4	5	10
Het P	N.S.	N.S.	N.S.
Fixed RR	4.08	6.40	5.93
RRl	1.63	4.23	4.07
RRu	10.22	9.69	8.65
P	++	+++	+++
Random RR	4.08	6.40	5.93
RRl	1.63	4.23	4.07
RRu	10.22	9.69	8.65
P	++	+++	+++
Between Chi			0.77
Between df			1
Between P			N.S.
Btwn(F) P			N.S.
Btwn(R) P			N.S.

	Number of adjustment variables (1) 0	1	2+/+nk	Total
N	11			11
NS	8			8
Wt	26.94			26.94
Het Chi	6.45			6.45
Het df	10			10
Het P	N.S.			N.S.
Fixed RR	5.93			5.93
RRl	4.07			4.07
RRu	8.65			8.65
P	+++			+++
Random RR	5.93			5.93
RRl	4.07			4.07
RRu	8.65			8.65
P	+++			+++
Between Chi				
Between df				
Between P				N.S.
Btwn(F) P				N.S.
Btwn(R) P				N.S.

Table 4D1 - 6

IESLC - Meta-analysis of Ex Smoking, Any product (or Cigarettes if Any not available)
Large
Least adjusted

	Number of adjustment variables (2) 0	1	2	3-5	6+/+nk	Total
N	11					11
NS	8					8
Wt	26.94					26.94
Het Chi	6.45					6.45
Het df	10					10
Het P	N.S.					N.S.
Fixed RR	5.93					5.93
RRl	4.07					4.07
RRu	8.65					8.65
P	+++					+++
Random RR	5.93					5.93
RRl	4.07					4.07
RRu	8.65					8.65
P	+++					+++
Between Chi						
Between df						
Between P						N.S.
Btwn(F) P						N.S.
Btwn(R) P						N.S.

	Product all/unsp	cig+/-ot	cig only	Total
N	2	9		11
NS	2	6		8
Wt	1.32	25.62		26.94
Het Chi	2.16	4.10		6.45
Het df	1	8		10
Het P	N.S.	N.S.		N.S.
Fixed RR	8.63	5.82		5.93
RRl	1.56	3.95		4.07
RRu	47.57	8.57		8.65
P	+	+++		+++
Random RR	6.19	5.82		5.93
RRl	0.40	3.95		4.07
RRu	94.75	8.57		8.65
P	N.S.	+++		+++
Between Chi				0.19
Between df				1
Between P				N.S.
Btwn(F) P				N.S.
Btwn(R) P				N.S.

	Denominator nev any	nev cigs	Total
N	4	7	11
NS	3	5	8
Wt	17.30	9.64	26.94
Het Chi	2.32	1.55	6.45
Het df	3	6	10
Het P	N.S.	N.S.	N.S.
Fixed RR	7.47	3.92	5.93
RRl	4.67	2.08	4.07
RRu	11.97	7.37	8.65
P	+++	+++	+++
Random RR	7.47	3.92	5.93
RRl	4.67	2.08	4.07
RRu	11.97	7.37	8.65
P	+++	+++	+++
Between Chi			2.58
Between df			1
Between P			N.S.
Btwn(F) P			*
Btwn(R) P			N.S.

Table 4D1 - 6

IESLC - Meta-analysis of Ex Smoking, Any product (or Cigarettes if Any not available)

Large
Least adjusted

	Derivation of RR/CI			
	Orig	StdCalc	Other	Total
N	1	7	3	11
NS	1	4	3	8
Wt	1.38	24.31	1.25	26.94
Het Chi	0.00	5.10	1.30	6.45
Het df	0	6	2	10
Het P	N.S.	N.S.	N.S.	N.S.
Fixed RR	5.90	6.00	4.84	5.93
RRl	1.11	4.03	0.84	4.07
RRu	31.37	8.92	27.91	8.65
P	+	+++	(+)	+++
Random RR	5.90	6.00	4.84	5.93
RRl	1.11	4.03	0.84	4.07
RRu	31.37	8.92	27.91	8.65
P	+	+++	(+)	+++
Between Chi				0.05
Between df				2
Between P				N.S.
Btwn(F) P				N.S.
Btwn(R) P				N.S.

Table 4D1 - 7

IESLC - Meta-analysis of Ex Smoking, Any product (or Cigarettes if Any not available)
Large
Excluded studies (and stage at which they were excluded)

1	BOUCHA BUELL LAURIL MZILEN
2	ABELIN ABRAHA ALDERS AUVINE AXELSO BAND BERRIN BLOT1 BLOT2 BLOT3 BLOT4 BRESLO BROCKM BROWN1 BYERS1 BYERS2
	CASCO2 CASCOR CHAN CHATZI CHEN CHEN2 CHEN3 CHIAZZ COOKSO DAVEYS DEAN DOSEME DU DUNN EBELIN ENSTRO
	ESAKI FAN GENG GER GODLEY GOLLED GSELL HANSEN HEGMAN HINDS HIRAOK HIRAY2 HOROWI HORWIT HU HU2
	HUANG ISHIMA JARUP JIANG JONES JUSSAW KANELL KO KOHLME KOULUM KREYBE LAMTH LAMWK LAMWK2 LAUSSM LEI
	LETOUR LEVIN LIAW LICKIN LIU LIU2 LIU3 LIU4 LIU5 LOMBA2 LUO MAGNUS MARSH MARSH2 MARTIS MASTRA
	MATSUD MCCONN MCDUFF MCLAUG MILLER MILLS MOLLO MRFIT MURATA NOTAN2 NOTANI NOU ORMOS PASTOR PAWLEG PERNU
	PERSHA PIKE POFFIJ POLEDN QIAO RADZIK RANDIG REN RIMING RONCO ROOTS ROTHSC SAARIK SADOWS SANKAR SCHWA2
	SEGI SEGI2 SEOW SHIMIZ SIEMIA SIMARA SITAS SOBUE2 STASZE STAYNE STOCKS SUN TANG2 TAO ULMER VANDER
	WALD WANG WANG3 WANG4 WARSIN WATSON WICKLU WILKIN WU2 WUWILL WYNDE2 WYNDE4 WYNDE5 WYNDE8 WYNDER XIANGZ
	XU XU2 XU3 XU4 YUAN ZHANG ZHENG ZHOU
3	RESTRE
4	BOFFET
8	AGUDO AKIBA AMANDU AMES ANDERS ARCHER ARMADA AUSTIN AXELSS BECHER BENHAM BENSHL BEST BLOHMK BOUCOT BRETT
	BROSS BROWN2 CARPEN CEDERL CHANG CHOI CHOW CHYOU CORREA CPSI CPSII DAMBER DARBY DEAN2 DEAN3 DEKLER
	DESTE2 DESTEF DOCKER DOLL DOLL2 DORANT DORGAN DORN DROSTE ENGELA GAO GAO2 GARCIA GARDIN GARSHI GILLIS
	GOODMA GRAHAM GREGOR HAENSZ HAMMO2 HAMMON HEIN HENNEK HIRAYA HITOSU HOLE HUMBLE JAHN JARVHO JEDRYC JOLY
	KAISE2 KAISER KATSOU KAUFMA KELLER KINLEN KJUUS KNEKT KOO KREUZE KUBIK LANGE LEMARC LIDDEL LOMBAR LUBIN
	LUBIN2 MACLEN MATOS MIGRAN MRFITR NAM ODRISC OSANN OSANN2 PARKIN PERSH2 PETO PEZZO2 PEZZOT PISANI PRESCO
	QIAO2 RACHTA SCHWAR SHAW SIMONA SPEIZE SPITZ STOCKW STUCKE SUZUK2 SUZUKI SVENSS TANG TENKAN TIZZAN TOKARS
	TOUSEY TSUGAN TULINI TVERDA VEIERO VUTUC WAKAI WANG2 WIGLE WU WUNSCH WYNDE3 WYNDE7 YAMAGU YONG

Table 4D1 - 8

Potentially overlapping studies

REF	REFGP	PRINC	.	OVERLAP/LINK
WYNDE6	WYNDE6	1		WYNDE5/6/7/8
