# Supplementary material for: Systematic review with meta-analysis of the epidemiological evidence in the 1900s relating smoking to lung cancer
Source: BMC Cancer. 2012 Sep 3;12:385. doi: 10.1186/1471-2407-12-385 (PMC3505152; doi:10.1186/1471-2407-12-385)
Supplement: Additional file 5 — Detailed Analysis Tables (Individual file names as described in Additional file 1: Methods, Table1). [file 1471-2407-12-385-S5.zip › PDF/2A.pdf]

Table 2A1 -

IESLC - Meta-analysis of Ever Smoking, Any product (or Cigarettes if Any not available)
Squamous

This analysis is restricted to results for:
1) Non-dose-response data
2) Ever smokers
3) Results complete enough for use in metaanalysis

Within each study, results are then selected (in the following order of preference, within each sex) for:
4) PRODUCT: all/unspec, cigarettes regardless of other products, cigarettes only
5) CIGTYPE: all/unspecified, MC regardless of HR, MC only
6) DENOM: never smoked anything, never smoked cigarettes, (never +1 = +long term ex, +2 = +amount unknown,
+3 = never cigs+long term ex)
7) Followup period (YF, prospective studies): whole study (coded as 0) or longest available
8) LCtype: squamous or nearest available, but not adeno. (q = squamous, s = small,
a = adeno, KI = Kreyberg I, u = undifferentiated)
9) Race: all or nearest available, otherwise by race (wh or w = white, bl or b = black, hi = hispanic
ch = chinese, jap = japanese, haw = hawaiian, w+o = white + oriental, sca = scandinavian, as = asian)
10) For overlapping studies: principal rather than subsidiary studies
Finally by Age: whole study (coded as 0) if available, otherwise by widest available age group
and then for single sex results (m, f) in preference to combined sex results (c).

Results adjusted (AD) for the most potential confounders are then chosen in Sections -1 to -3
and results adjusted for the least confounders in Sections -4 to -6. (Those least adjusted results which
actually differ from the most adjusted as marked 'x' in column X in Section -4)
(Results adjusted for an unknown number of confounder(s) are coded as 20.)

Section -7 shows excluded studies, together with the stage (as above) at which no qualifying
results were found.

Section -8 lists the potentially overlapping studies which have been included (1=principal, 2=subsidiary).

Section -9 lists any results which would have been included in preference except that they had data not complete
enough for use in meta-analysis, with their significance (yes/no), if known, and any further comment as entered
on the database.

In addition to those mentioned above, the following fields, levels and abbreviations are used:
* or nk = not known, n = no, y = yes, ot = other
nev = never
all/unspec = all or unspecified, cig+/-ot = cigarettes irrespective of other products (cigar, pipe etc)
MC = manufactured cigarettes, HR = hand-rolled cigarettes
REF: 6-character study reference
NRR: number of the RR on the database within the study
ST : study type (CC = case control, pr or prosp = prospective)
NLC: number of lung cancer cases in whole study
R : risky occupational population (n = no, m = mining, o = other risky)
VB : national cigarette type (V = at least 75% Virginia, bl = at least 75% blended, ot = other)
P : any proxy use
H : full histological confirmation
De : derivation of RR/CI (or = original, st = standard method, ot = other method of estimation)

Table 2A1 - 1

IESLC - Meta-analysis of Ever Smoking, Any product (or Cigarettes if Any not available)
Squamous
Most adjusted

REF	NRR	SEX	AGEL	AGEH	RACE	YF	LC TYPE	LOC	START	ST	NLC	R	VB	P	H	AD	PRODUCT	DENOM	De
ABRAHA	1	m	0	0	all	0	q	Eu:est	1975	pr	571	n	bl	n	n	0	all/unsp	nev any	ot
ABRAHA	4	f	0	0	all	0	q	Eu:est	1975	pr	571	n	bl	n	n	0	all/unsp	nev any	ot
ALDERS	52	m	0	0	all	-	q	Eu:UK	1977	CC	1448	n	V	n	n	2	all/unsp	nev any	or
ALDERS	55	f	0	0	all	-	q	Eu:UK	1977	CC	1448	n	V	n	n	2	all/unsp	nev any	or
ANDERS	10	f	0	0	all	0	q	NAmer	1986	pr	343	n	bl	n	n	0	cig+/-ot	nev cigs	st
BAND	5	m	0	0	all	-	q	NAmer	1983	CC	2831	n	V	y	y	2	cig only	nev any	ot
BARBON	127	m	0	0	all	-	q	Eu:wst	1979	CC	755	n	bl	y	y	3	all/unsp	nev any	ot
BECHER	11	f	0	0	all	-	q+s	Eu:Ger	1985	CC	194	n	bl	n	y	1	all/unsp	nev any	or
BRESLO	36	c	0	0	all	-	not a	NAmer	1949	CC	518	n	bl	n	y	0	all/unsp	nev+1	st
BROWN2	6	m	0	0	wh	-	q	NAmer	1984	CC	14596	n	bl	n	y	2	cig+/-ot	nev cigs	or
BROWN2	5	f	0	0	wh	-	q	NAmer	1984	CC	14596	n	bl	n	y	2	cig+/-ot	nev cigs	or
BUFFLE	49	m	0	0	wh	-	q	NAmer	1976	CC	943	n	bl	y	n	0	cig+/-ot	nev cigs	ot
BUFFLE	62	f	0	0	w-hi	-	q	NAmer	1976	CC	943	n	bl	y	n	0	cig+/-ot	nev cigs	st
BYERS1	1	m	0	0	wh	-	q	NAmer	1957	CC	1002	n	bl	n	n	0	cig+/-ot	nev cigs	st
CHAN	11	m	0	0	all	-	q+s	As:HK	1976	CC	397	n	bl	n	n	0	all/unsp	nev any	st
CHAN	15	f	0	0	all	-	q+s	As:HK	1976	CC	397	n	bl	n	n	0	all/unsp	nev any	st
CHOI	62	m	0	0	all	-	q	As:oth	1985	CC	375	n	bl	n	n	0	cig+/-ot	nev cigs	st
CHOI	64	f	0	0	all	-	q	As:oth	1985	CC	375	n	bl	n	n	0	cig+/-ot	nev cigs	st
COMSTO	66	m	0	0	all	-	q	NAmer	1975	ot	258	n	bl	n	n	0	cig+/-ot	nev cigs	st
COMSTO	78	f	0	0	all	-	q	NAmer	1975	ot	258	n	bl	n	n	0	cig+/-ot	nev cigs	ot
CORREA	35	c	0	0	all	-	q+s	NAmer	1979	CC	1359	n	bl	y	n	1	cig+/-ot	nev cigs	or
DAMBER	33	m	0	0	all	-	q	Eu:Sca	1972	CC	579	n	bl	y	n	1	all/unsp	nev any	or
DESTE2	16	m	0	0	all	-	q	SCAmer	1993	CC	463	n	bl	n	n	2	all/unsp	nev any	or
DOLL	86	m	0	0	all	-	KI	Eu:UK	1948	CC	1465	n	V	n	n	1	all/unsp	nev any	ot
DOLL	88	f	0	0	all	-	KI	Eu:UK	1948	CC	1465	n	V	n	n	1	all/unsp	nev any	ot
DORGAN	113	m	0	0	wh	-	q	NAmer	1980	CC	2026	n	bl	y	y	2	cig+/-ot	nev any	or
DORGAN	98	f	0	0	all	-	q	NAmer	1980	CC	2026	n	bl	y	y	3	cig+/-ot	nev any	or
DOSEME	3	m	0	0	all	-	q	Eu:bal	1979	CC	1210	n	bl	n	n	2	cig+/-ot	nev cigs	or
ENGELA	62	m	0	0	all	0	q	Eu:Sca	1964	pr	435	n	bl	n	n	7	cig+/-ot	nev cigs	ot
FAN	3	c	0	0	all	-	q	As:Chi	1990	CC	403	n	ot	y	n	0	cig+/-ot	nev cigs	ot
GAO	2	m	0	0	all	-	q	As:Chi	1984	CC	1405	n	ot	n	n	2	cig+/-ot	nev cigs	or
GAO	12	f	0	0	all	-	q	As:Chi	1984	CC	1405	n	ot	n	n	2	cig+/-ot	nev cigs	or
GER	13	c	0	0	all	-	q+s	As:oth	1990	CC	141	n	ot	y	n	10	all/unsp	nev any	ot
HAENSZ	1	f	0	0	all	-	q+u	NAmer	1955	CC	158	n	bl	n	y	2	all/unsp	nev any	ot
HAMMON	60	m	0	0	wh	0	not a	NAmer	1952	pr	448	n	bl	n	n	1	all/unsp	nev any	ot
HEGMAN	2	c	0	0	all	-	q	NAmer	1989	CC	282	n	bl	y	y	0	all/unsp	nev any	st
HINDS	23	f	0	0	o	-	q+s	NAmer	1968	CC	292	n	bl	n	n	3	all/unsp	nev any	st
ISHIMA	6	c	0	0	all	-	q	As:Jap	1961	CC	180	n	bl	y	y	5	all/unsp	nev any	st
JAHN	46	m	0	0	all	-	q	Eu:Ger	1988	CC	1004	n	bl	n	n	0	all/unsp	nev any	st
JAIN	48	m	0	0	all	-	q	NAmer	1981	CC	845	n	V	y	n	2	cig+/-ot	nev cigs	or
JAIN	43	f	0	0	all	-	q	NAmer	1981	CC	845	n	V	y	n	2	cig+/-ot	nev cigs	or
JEDRYC	54	m	0	0	all	-	q	Eu:est	1980	CC	1630	n	bl	y	n	3	cig+/-ot	nev any	ot
JOLY	54	m	0	0	all	-	q	SCAmer	1978	CC	826	n	bl	n	n	0	cig+/-ot	nev any	st
JOLY	52	f	0	0	all	-	q	SCAmer	1978	CC	826	n	bl	n	n	0	cig+/-ot	nev any	st
JUSSAW	23	m	0	0	all	-	KI	As:Ind	1964	CC	792	n	V	n	n	0	all/unsp	nev any	st
KATSOU	37	f	0	0	all	-	KI	Eu:bal	1987	CC	101	n	bl	n	n	1	all/unsp	nev any	ot
KHUDER	24	m	0	0	all	-	q	NAmer	1985	CC	482	n	bl	n	y	0	cig+/-ot	nev cigs	ot
KIHARA	26	c	0	0	jap	-	q	As:Jap	1991	CC	440	n	bl	n	n	0	all/unsp	nev any	st
KOO	6	f	0	0	all	-	q+s	As:HK	1981	CC	200	n	bl	n	n	0	all/unsp	nev any	st
KREYBE	4	m	0	0	all	-	KI	Eu:Sca	1948	CC	300	n	bl	n	y	1	all/unsp	nev any	ot
KREYBE	25	f	0	0	all	-	KI	Eu:Sca	1948	CC	300	n	bl	n	y	1	all/unsp	nev any	ot
LAMTH	1	f	0	0	ch	-	q	As:HK	1983	CC	445	n	bl	n	n	0	all/unsp	nev any	or
LAMWK	2	f	0	0	ch	-	q	As:HK	1981	CC	163	n	bl	n	n	0	all/unsp	nev any	st
LAMWK2	1	m	0	0	all	-	q	As:HK	1976	CC	480	n	bl	n	n	0	all/unsp	nev any	st
LAMWK2	5	f	0	0	all	-	q	As:HK	1976	CC	480	n	bl	n	n	0	all/unsp	nev any	st
LOMBA2	2	f	0	0	all	-	q+u	NAmer	1960	CC	225	n	bl	n	n	0	cig+/-ot	nev cigs	st
LUBIN	33	m	0	0	all	-	KI	As:Chi	1984	CC	427	m	ot	y	n	0	all/unsp	nev any	st
LUBIN2	145	m	0	0	all	-	q	Eu:mul	1976	CC	7804	n	bl	n	y	0	cig+/-ot	nev any	st
LUBIN2	165	f	0	0	all	-	q	Eu:mul	1976	CC	7804	n	bl	n	y	0	cig+/-ot	nev any	st
LUO	8	c	0	0	all	-	q	As:Chi	1990	CC	102	n	ot	n	y	20	cig+/-ot	nev cigs	or
MATOS	67	m	0	0	all	-	q	SCAmer	1994	CC	200	n	bl	n	n	2	cig+/-ot	nev any	ot
MATSUD	11	m	0	0	all	-	q	As:Jap	1965	CC	179	n	bl	n	n	0	cig+/-ot	nev cigs	st
NOU	1	m	0	0	all	-	q	Eu:Sca	1971	CC	273	n	bl	y	n	0	all/unsp	nev any	st
NOU	6	f	0	0	all	-	q	Eu:Sca	1971	CC	273	n	bl	y	n	0	all/unsp	nev any	st
ORMOS	8	m	0	0	all	-	q	Eu:est	1947	CC	119	n	bl	y	y	0	cig+/-ot	nev any	st
OSANN	43	m	0	0	all	-	q	NAmer	1984	CC	1986	n	bl	n	n	2	cig+/-ot	nev cigs	or
OSANN	44	f	0	0	all	-	q	NAmer	1984	CC	1986	n	bl	n	n	2	cig+/-ot	nev cigs	or
OSANN2	25	f	0	0	all	-	KI	NAmer	1964	ot	217	n	bl	n	y	1	cig+/-ot	nev cigs	or
PEZZOT	6	m	0	0	all	-	q	SCAmer	1987	CC	215	n	bl	n	y	0	cig only	nev cigs	ot
SCHWAR	10	m	40	54	wh	-	q	NAmer	1984	CC	5588	n	bl	y	y	0	cig+/-ot	nev cigs	st
SCHWAR	9	m	40	54	bl	-	q	NAmer	1984	CC	5588	n	bl	y	y	0	cig+/-ot	nev cigs	st
SCHWAR	18	f	40	54	wh	-	q	NAmer	1984	CC	5588	n	bl	y	y	0	cig+/-ot	nev cigs	ot

Table 2A1 - 1

IESLC - Meta-analysis of Ever Smoking, Any product (or Cigarettes if Any not available)
Squamous
Most adjusted

REF	NRR	SEX	AGEL	AGEH	RACE	YF	LC TYPE	LOC	START	ST	NLC	R	VB	P	H	AD	PRODUCT	DENOM	De
SCHWAR	17	f	40	54	bl	-	q	NAmer	1984	CC	5588	n	bl	y	y	0	cig+/-ot	nev cigs	ot
SEOW	3	f	0	0	ch	-	q	As:oth	1997	CC	153	n	bl	n	y	0	cig+/-ot	nev cigs	st
SIEMIA	7	m	0	0	all	-	q	NAmer	1979	CC	857	n	V	y	y	7	cig+/-ot	nev cigs	or
SOBUE	97	m	0	0	all	-	q	As:Jap	1986	CC	1376	n	bl	n	y	1	cig+/-ot	nev cigs	ot
SOBUE	107	f	0	0	all	-	q	As:Jap	1986	CC	1376	n	bl	n	y	1	cig+/-ot	nev cigs	ot
STASZE	12	m	0	0	all	-	q	Eu:est	1954	CC	281	n	bl	n	y	0	all/unsp	nev any	ot
STASZE	38	f	0	0	all	-	q	Eu:est	1954	CC	281	n	bl	n	y	0	all/unsp	nev any	ot
STAYNE	3	m	0	0	all	-	q	NAmer	1969	CC	420	n	bl	n	n	0	all/unsp	nev any	st
SUZUK2	15	c	0	0	all	-	q	SCAmer	1991	CC	123	n	bl	n	y	3	all/unsp	nev any	or
SVENSS	72	f	0	0	all	-	q	Eu:Sca	1983	CC	210	n	bl	n	n	1	all/unsp	nev any	ot
TIZZAN	18	c	0	0	all	-	q+u	Eu:wst	1959	CC	1358	n	bl	n	n	0	all/unsp	nev any	st
TOKARS	10	c	0	0	all	-	q	Eu:est	1966	ot	162	o	bl	n	y	3	all/unsp	nev any	or
TSUGAN	13	m	0	0	all	-	q	As:Jap	1976	CC	134	n	bl	n	y	0	all/unsp	nev any	ot
WAKAI	74	m	0	0	all	-	q	As:Jap	1988	CC	333	n	bl	n	y	1	all/unsp	nev any	ot
WAKAI	80	f	0	0	all	-	q	As:Jap	1988	CC	333	n	bl	n	y	1	all/unsp	nev any	ot
WU	32	f	0	0	wh	-	q	NAmer	1981	CC	220	n	bl	n	y	2	all/unsp	nev any	ot
WUWILL	9	f	0	0	all	-	q	As:Chi	1985	CC	965	n	ot	n	n	3	cig+/-ot	nev cigs	or
WYNDE2	7	m	0	0	all	-	KI	NAmer	1962	CC	404	n	bl	n	y	0	all/unsp	nev any	st
WYNDE3	9	m	0	0	all	-	KI	NAmer	1966	CC	350	n	bl	n	y	0	all/unsp	nev any	st
WYNDE3	132	f	0	0	all	-	KI	NAmer	1966	CC	350	n	bl	n	y	0	all/unsp	nev any	st
WYNDE4	68	m	0	0	all	-	not a	NAmer	1948	CC	684	n	bl	y	n	2	all/unsp	nev any	ot
WYNDE4	54	f	0	0	all	-	not a	NAmer	1948	CC	684	n	bl	y	n	2	all/unsp	nev any	ot
WYNDE6	66	m	0	0	all	-	KI	NAmer	1969	CC	4423	n	bl	n	y	0	all/unsp	nev any	st
WYNDE6	412	f	0	0	wh	-	q	NAmer	1969	CC	4423	n	bl	n	y	1	cig+/-ot	nev cigs	ot
XU3	20	m	0	0	all	-	KI	As:Chi	1981	CC	135	n	ot	n	n	1	all/unsp	nev any	ot
XU3	24	f	0	0	all	-	KI	As:Chi	1981	CC	135	n	ot	n	n	1	all/unsp	nev any	ot
ZHENG	5	m	0	0	all	-	q	As:Chi	1982	CC	540	n	ot	*	y	0	cig+/-ot	nev cigs	st
ZHENG	18	f	0	0	all	-	q	As:Chi	1982	CC	540	n	ot	*	y	0	cig+/-ot	nev cigs	st
ZHOU	8	m	0	0	all	-	q	As:Chi	1978	CC	1360	n	ot	n	n	0	all/unsp	nev any	st
ZHOU	9	f	0	0	all	-	q	As:Chi	1978	CC	1360	n	ot	n	n	0	all/unsp	nev any	st

Cigarette type is all/unspec for all RRs

Table 2A1 - 2

IESLC - Meta-analysis of Ever Smoking, Any product (or Cigarettes if Any not available)
Squamous
Most adjusted

REF	NRR	SEX	AD	Number Exposed Case	Cont	Non-exposed Case	Cont	RR	95.00%CI
*ABRAHA	1	m	0	142	10351	0	3365	92.66~	(5.77-1488.21)
*ABRAHA	4	f	0	17	5256	7	11589	5.35	(2.22- 12.90)
Subtotal ABRAHA								6.95	(3.00- 16.06)
ALDERS	52	m	2	-	-	-	-	14.70	(3.40- 63.64)
ALDERS	55	f	2	-	-	-	-	6.09	(2.68- 13.82)
Subtotal ALDERS								7.52	(3.67- 15.37)
*ANDERS	10	f	0	63	96164	5	195158	25.57	(10.29- 63.56)
BAND	5	m	2	-	-	-	-	37.45	(17.62- 79.58)
BARBON	127	m	3	-	-	-	-	14.52	(6.35- 33.20)
BECHER	11	f	1	-	-	-	-	10.69	(2.43- 47.00)
BRESLO	36	c	0	457	462	15	56	3.69	(2.06- 6.62)
BROWN2	6	m	2	-	-	-	-	11.10	(9.50- 12.90)
BROWN2	5	f	2	-	-	-	-	20.10	(16.40- 24.80)
Subtotal BROWN2								13.69	(12.11- 15.49)
BUFFLE	49	m	0	-	-	-	-	14.03	(4.73- 41.61)
BUFFLE	62	f	0	58	166	3	112	13.04	(3.99- 42.66)
Subtotal BUFFLE								13.57	(6.09- 30.24)
BYERS1	1	m	0	299	695	22	424	8.29	(5.29- 13.00)
CHAN	11	m	0	114	161	2	43	15.22	(3.61- 64.12)
CHAN	15	f	0	44	50	19	139	6.44	(3.44- 12.06)
Subtotal CHAN								7.39	(4.16- 13.13)
CHOI	62	m	0	160	465	6	95	5.45	(2.34- 12.67)
CHOI	64	f	0	11	26	10	164	6.94	(2.68- 17.96)
Subtotal CHOI								6.06	(3.22- 11.40)
COMSTO	66	m	0	44	229	2	84	8.07	(1.91- 34.02)
COMSTO	78	f	0	17	87	0	115	46.20~	(2.74- 778.83)
Subtotal COMSTO								11.56	(3.21- 41.67)
CORREA	35	c	1	-	-	-	-	28.30	(18.60- 43.20)
DAMBER	33	m	1	-	-	-	-	11.80	(6.40- 23.00)
DESTE2	16	m	2	-	-	-	-	13.20	(4.70- 37.10)
DOLL	86	m	1	-	-	-	-	13.17	(4.12- 42.10)
DOLL	88	f	1	-	-	-	-	2.13	(1.06- 4.27)
Subtotal DOLL								3.45	(1.90- 6.27)
DORGAN	113	m	2	-	-	-	-	18.90	(7.00- 51.30)
DORGAN	98	f	3	-	-	-	-	11.10	(7.20- 17.10)
Subtotal DORGAN								12.08	(8.12- 17.96)
DOSEME	3	m	2	-	-	-	-	3.60	(2.60- 5.00)
*ENGELA	62	m	7	-	-	-	-	6.45	(1.97- 21.11)
FAN	3	c	0	75	595	6	556	11.68	(5.04- 27.04)
GAO	2	m	2	-	-	-	-	8.40	(4.70- 15.00)
GAO	12	f	2	-	-	-	-	7.20	(4.60- 11.10)
Subtotal GAO								7.62	(5.36- 10.82)
GER	13	c	10	-	-	-	-	3.19	(1.08- 9.42)
HAENSZ	1	f	2	-	-	-	-	3.00	(1.90- 4.73)
*HAMMON	60	m	1	-	-	-	-	16.88	(6.29- 45.29)
HEGMAN	2	c	0	89	1202	5	2080	30.80	(12.48- 76.03)
HINDS	23	f	3	-	-	-	-	16.13	(7.66- 33.97)
ISHIMA	6	c	5	-	-	-	-	21.00	(3.38- 868.40)
JAHN	46	m	0	351	701	3	138	23.03	(7.29- 72.81)
JAIN	48	m	2	-	-	-	-	18.00	(5.50- 111.00)
JAIN	43	f	2	-	-	-	-	25.50	(7.93- 156.00)
Subtotal JAIN								21.46	(7.45- 61.79)
JEDRYC	54	m	3	-	-	-	-	12.84	(5.58- 29.55)
JOLY	54	m	0	203	709	2	218	31.21	(7.69- 126.68)
JOLY	52	f	0	48	122	6	283	18.56	(7.74- 44.51)
Subtotal JOLY								21.47	(10.22- 45.09)
JUSSAW	23	m	0	89	168	13	624	25.43	(13.87- 46.63)
KATSOU	37	f	1	-	-	-	-	6.11	(2.69- 13.87)
KHUDER	24	m	0	176	-	9	-	7.82	(3.87- 15.77)
KIHARA	26	c	0	132	232	5	237	26.97	(10.84- 67.08)
KOO	6	f	0	61	63	32	137	4.15	(2.46- 6.98)
KREYBE	4	m	1	-	-	-	-	10.87	(3.47- 34.04)
KREYBE	25	f	1	-	-	-	-	2.29	(0.89- 5.88)
Subtotal KREYBE								4.31	(2.08- 8.92)
LAMTH	1	f	0	63	20	28	72	8.10	(4.16- 15.77)
LAMWK	2	f	0	21	41	7	144	10.54	(4.19- 26.52)
LAMWK2	1	m	0	129	161	5	43	6.89	(2.65- 17.90)
LAMWK2	5	f	0	35	50	15	139	6.49	(3.27- 12.88)
Subtotal LAMWK2								6.62	(3.79- 11.56)
LOMBA2	2	f	0	94	353	15	239	4.24	(2.40- 7.50)
LUBIN	33	m	0	330	939	4	72	6.33	(2.29- 17.45)

Table 2A1 - 2

IESLC - Meta-analysis of Ever Smoking, Any product (or Cigarettes if Any not available)
Squamous
Most adjusted

REF	NRR	SEX	AD	Number Exposed Case	Number Exposed Cont	Non-exposed Case	Non-exposed Cont	RR	95.00%CI
LUBIN2	145	m	0	3587	10433	54	2616	16.66	(12.69- 21.86)
LUBIN2	165	f	0	200	567	72	1180	5.78	(4.34- 7.71)
Subtotal LUBIN2								10.10	(8.29- 12.31)
LUO	8	c	20	-	-	-	-	10.90	(2.50- 47.90)
MATOS	67	m	2	-	-	-	-	8.08	(2.59- 25.20)
MATSUD	11	m	0	103	3314	1	1255	39.01	(5.44- 279.84)
NOU	1	m	0	110	247	2	122	27.17	(6.60- 111.85)
NOU	6	f	0	5	92	2	261	7.09	(1.35- 37.19)
Subtotal NOU								15.42	(5.26- 45.22)
ORMOS	8	m	0	27	1034	2	777	10.14	(2.41- 42.79)
OSANN	43	m	2	-	-	-	-	36.10	(17.80- 73.30)
OSANN	44	f	2	-	-	-	-	26.40	(14.50- 48.10)
Subtotal OSANN								30.09	(19.04- 47.54)
OSANN2	25	f	1	-	-	-	-	35.10	(4.80- 256.00)
PEZZOT	6	m	0	85	317	0	116	62.74~	(3.86-1019.50)
SCHWAR	10	m	0	80	178	1	73	32.81	(4.48- 240.23)
SCHWAR	9	m	0	41	39	4	7	1.84	(0.50- 6.78)
SCHWAR	18	f	0	29	108	0	79	43.23~	(2.60- 718.15)
SCHWAR	17	f	0	21	28	0	41	62.61~	(3.64-1076.10)
Subtotal SCHWAR								7.71	(2.96- 20.10)
SEOW	3	f	0	21	15	10	125	17.50	(6.95- 44.09)
SIEMIA	7	m	7	-	-	-	-	22.70	(6.90- 75.20)
SOBUE	97	m	1	-	-	-	-	17.88	(7.82- 40.87)
SOBUE	107	f	1	-	-	-	-	8.74	(5.09- 15.02)
Subtotal SOBUE								10.83	(6.89- 17.03)
STASZE	12	m	0	137	754	0	158	57.77~	(3.58- 933.17)
STASZE	38	f	0	1	153	0	1660	32.45~	(1.32- 800.04)
Subtotal STASZE								45.09	(5.52- 368.55)
STAYNE	3	m	0	130	567	22	333	3.47	(2.17- 5.56)
SUZUK2	15	c	3	-	-	-	-	31.00	(4.20- 227.00)
SVENSS	72	f	1	-	-	-	-	12.62	(3.97- 40.14)
TIZZAN	18	c	0	333	939	55	419	2.70	(1.99- 3.67)
TOKARS	10	c	3	-	-	-	-	6.80	(1.20- 38.70)
TSUGAN	13	m	0	20	15	0	5	14.55~	(0.75- 283.37)
WAKAI	74	m	1	-	-	-	-	8.61	(2.08- 35.72)
WAKAI	80	f	1	-	-	-	-	25.23	(6.87- 92.66)
Subtotal WAKAI								15.46	(5.92- 40.36)
WU	32	f	2	-	-	-	-	24.29	(3.40- 173.76)
WUWILL	9	f	3	-	-	-	-	4.20	(3.00- 5.90)
WYNDE2	7	m	0	347	616	3	105	19.72	(6.21- 62.59)
WYNDE3	9	m	0	207	332	3	88	18.29	(5.71- 58.56)
WYNDE3	132	f	0	25	56	5	76	6.79	(2.45- 18.82)
Subtotal WYNDE3								10.44	(4.85- 22.49)
WYNDE4	68	m	2	-	-	-	-	12.79	(6.19- 26.41)
WYNDE4	54	f	2	-	-	-	-	5.82	(2.55- 13.31)
Subtotal WYNDE4								9.08	(5.26- 15.66)
WYNDE6	66	m	0	1744	1996	29	617	18.59	(12.74- 27.13)
WYNDE6	412	f	1	-	-	-	-	32.37	(17.66- 59.35)
Subtotal WYNDE6								21.71	(15.76- 29.92)
XU3	20	m	1	-	-	-	-	5.90	(1.69- 20.57)
XU3	24	f	1	-	-	-	-	25.67	(4.99- 131.94)
Subtotal XU3								10.14	(3.75- 27.37)
ZHENG	5	m	0	156	218	4	94	16.82	(6.05- 46.71)
ZHENG	18	f	0	43	44	33	184	5.45	(3.11- 9.54)
Subtotal ZHENG								7.07	(4.33- 11.56)
ZHOU	8	m	0	343	41	96	36	3.14	(1.90- 5.18)
ZHOU	9	f	0	35	7	42	32	3.81	(1.50- 9.68)
Subtotal ZHOU								3.28	(2.11- 5.10)
Partial Totals				11152	141578	686	226785		

*prospective study ~ With 0.5 adjustment for zero

Table 2A1 - 2

IESLC - Meta-analysis of Ever Smoking, Any product (or Cigarettes if Any not available)
Squamous
Most adjusted

REF	NRR	SEX	AD	Ys	Ws	Qs	Ps
*ABRAHA	1	m	0	4.53	0.50	2.58	0.0014
*ABRAHA	4	f	0	1.68	4.97	1.64	0.0002
Subtotal ABRAHA				1.94	5.46	4.22	
ALDERS	52	m	2	2.69	1.79	0.34	0.0003
ALDERS	55	f	2	1.81	5.71	1.14	0.0000
Subtotal ALDERS				2.02	7.50	1.48	
*ANDERS	10	f	0	3.24	4.63	4.53	0.0000
BAND	5	m	2	3.62	6.76	12.69	0.0000
BARBON	127	m	3	2.68	5.62	1.00	0.0000
BECHER	11	f	1	2.37	1.75	0.02	0.0017
BRESLO	36	c	0	1.31	11.25	10.08	0.0000
BROWN2	6	m	2	2.41	164.17	3.89	0.0000
BROWN2	5	f	2	3.00	89.84	50.22	0.0000
Subtotal BROWN2				2.62	254.01	54.11	
BUFFLE	49	m	0	2.64	3.25	0.49	0.0000
BUFFLE	62	f	0	2.57	2.74	0.27	0.0000
Subtotal BUFFLE				2.61	5.99	0.76	
BYERS1	1	m	0	2.12	19.01	0.36	0.0000
CHAN	11	m	0	2.72	1.86	0.41	0.0002
CHAN	15	f	0	1.86	9.75	1.49	0.0000
Subtotal CHAN				2.00	11.61	1.90	
CHOI	62	m	0	1.70	5.39	1.68	0.0001
CHOI	64	f	0	1.94	4.25	0.42	0.0001
Subtotal CHOI				1.80	9.63	2.10	
COMSTO	66	m	0	2.09	1.86	0.05	0.0045
COMSTO	78	f	0	3.83	0.48	1.20	0.0078
Subtotal COMSTO				2.45	2.34	1.25	
CORREA	35	c	1	3.34	21.64	25.70	0.0000
DAMBER	33	m	1	2.47	9.39	0.43	0.0000
DESTE2	16	m	2	2.58	3.60	0.39	0.0000
DOLL	86	m	1	2.58	2.84	0.30	0.0000
DOLL	88	f	1	0.76	7.91	17.73	0.0334
Subtotal DOLL				1.24	10.76	18.04	
DORGAN	113	m	2	2.94	3.87	1.82	0.0000
DORGAN	98	f	3	2.41	20.54	0.49	0.0000
Subtotal DORGAN				2.49	24.41	2.31	
DOSEME	3	m	2	1.28	35.93	33.96	0.0000
*ENGELA	62	m	7	1.86	2.73	0.41	0.0021
FAN	3	c	0	2.46	5.45	0.23	0.0000
GAO	2	m	2	2.13	11.41	0.18	0.0000
GAO	12	f	2	1.97	19.80	1.54	0.0000
Subtotal GAO				2.03	31.21	1.72	
GER	13	c	10	1.16	3.28	3.91	0.0358
HAENSZ	1	f	2	1.10	18.47	24.62	0.0000
*HAMMON	60	m	1	2.83	3.94	1.29	0.0000
HEGMAN	2	c	0	3.43	4.70	6.49	0.0000
HINDS	23	f	3	2.78	6.93	1.93	0.0000
ISHIMA	6	c	5	3.04	0.50	0.31	0.0315
JAHN	46	m	0	3.14	2.90	2.27	0.0000
JAIN	48	m	2	2.89	1.70	0.69	0.0002
JAIN	43	f	2	3.24	1.73	1.68	0.0000
Subtotal JAIN				3.07	3.43	2.37	
JEDRYC	54	m	3	2.55	5.53	0.50	0.0000
JOLY	54	m	0	3.44	1.96	2.76	0.0000
JOLY	52	f	0	2.92	5.02	2.24	0.0000
Subtotal JOLY				3.07	6.98	5.00	
JUSSAW	23	m	0	3.24	10.45	10.09	0.0000
KATSOU	37	f	1	1.81	5.71	1.12	0.0000
KHUDER	24	m	0	2.06	7.79	0.30	0.0000
KIHARA	26	c	0	3.29	4.63	5.02	0.0000
KOO	6	f	0	1.42	14.12	9.75	0.0000
KREYBE	4	m	1	2.39	2.95	0.05	0.0000
KREYBE	25	f	1	0.83	4.31	8.75	0.0854
Subtotal KREYBE				1.46	7.26	8.80	
LAMTH	1	f	0	2.09	8.66	0.22	0.0000
LAMWK	2	f	0	2.35	4.51	0.05	0.0000
LAMWK2	1	m	0	1.93	4.22	0.44	0.0001
LAMWK2	5	f	0	1.87	8.17	1.20	0.0000
Subtotal LAMWK2				1.89	12.38	1.64	
LOMBA2	2	f	0	1.45	11.86	7.74	0.0000
LUBIN	33	m	0	1.84	3.73	0.62	0.0004

Table 2A1 - 2

IESLC - Meta-analysis of Ever Smoking, Any product (or Cigarettes if Any not available)
Squamous
Most adjusted

REF	NRR	SEX	AD	Ys	Ws	Qs	Ps
LUBIN2	145	m	0	2.81	51.88	16.25	0.0000
LUBIN2	165	f	0	1.75	46.51	11.56	0.0000
Subtotal LUBIN2				2.31	98.39	27.81	
LUO	8	c	20	2.39	1.76	0.03	0.0015
MATOS	67	m	2	2.09	2.97	0.08	0.0003
MATSUD	11	m	0	3.66	0.99	1.97	0.0003
NOU	1	m	0	3.30	1.92	2.11	0.0000
NOU	6	f	0	1.96	1.40	0.12	0.0205
Subtotal NOU				2.74	3.32	2.23	
ORMOS	8	m	0	2.32	1.85	0.01	0.0016
OSANN	43	m	2	3.59	7.67	13.63	0.0000
OSANN	44	f	2	3.27	10.69	11.13	0.0000
Subtotal OSANN				3.40	18.36	24.76	
OSANN2	25	f	1	3.56	0.97	1.66	0.0005
PEZZOT	6	m	0	4.14	0.49	1.76	0.0036
SCHWAR	10	m	0	3.49	0.97	1.48	0.0006
SCHWAR	9	m	0	0.61	2.26	6.10	0.3596
SCHWAR	18	f	0	3.77	0.49	1.11	0.0086
SCHWAR	17	f	0	4.14	0.47	1.69	0.0044
Subtotal SCHWAR				2.04	4.19	10.38	
SEOW	3	f	0	2.86	4.50	1.67	0.0000
SIEMIA	7	m	7	3.12	2.69	2.04	0.0000
SOBUE	97	m	1	2.88	5.62	2.23	0.0000
SOBUE	107	f	1	2.17	13.12	0.10	0.0000
Subtotal SOBUE				2.38	18.74	2.33	
STASZE	12	m	0	4.06	0.50	1.61	0.0043
STASZE	38	f	0	3.48	0.37	0.56	0.0333
Subtotal STASZE				3.81	0.87	2.18	
STAYNE	3	m	0	1.24	17.27	17.57	0.0000
SUZUK2	15	c	3	3.43	0.97	1.35	0.0007
SVENSS	72	f	1	2.54	2.87	0.23	0.0000
TIZZAN	18	c	0	0.99	40.59	64.36	0.0000
TOKARS	10	c	3	1.92	1.27	0.14	0.0305
TSUGAN	13	m	0	2.68	0.44	0.08	0.0772
WAKAI	74	m	1	2.15	1.90	0.02	0.0030
WAKAI	80	f	1	3.23	2.27	2.16	0.0000
Subtotal WAKAI				2.74	4.17	2.18	
WU	32	f	2	3.19	0.99	0.87	0.0015
WUWILL	9	f	3	1.44	33.59	22.47	0.0000
WYNDE2	7	m	0	2.98	2.88	1.53	0.0000
WYNDE3	9	m	0	2.91	2.84	1.21	0.0000
WYNDE3	132	f	0	1.91	3.69	0.42	0.0002
Subtotal WYNDE3				2.35	6.53	1.63	
WYNDE4	68	m	2	2.55	7.30	0.64	0.0000
WYNDE4	54	f	2	1.76	5.63	1.36	0.0000
Subtotal WYNDE4				2.21	12.93	2.00	
WYNDE6	66	m	0	2.92	26.90	12.06	0.0000
WYNDE6	412	f	1	3.48	10.46	15.67	0.0000
Subtotal WYNDE6				3.08	37.36	27.73	
XU3	20	m	1	1.77	2.46	0.56	0.0054
XU3	24	f	1	3.25	1.43	1.41	0.0001
Subtotal XU3				2.32	3.89	1.97	
ZHENG	5	m	0	2.82	3.68	1.19	0.0000
ZHENG	18	f	0	1.70	12.24	3.80	0.0000
Subtotal ZHENG				1.96	15.92	5.00	
ZHOU	8	m	0	1.14	15.27	18.80	0.0000
ZHOU	9	f	0	1.34	4.42	3.70	0.0049
Subtotal ZHOU				1.19	19.68	22.50	

Table 2A1 - 2

IESLC - Meta-analysis of Ever Smoking, Any product (or Cigarettes if Any not available)
Squamous
Most adjusted

	N	102
	NS	73
	Wt	999.88
Het	Chi	522.25
Het	df	101
Het	P	***
Fixed	RR	9.52
	RRl	8.94
	RRu	10.13
	P	+++
Random	RR	10.47
	RRl	8.88
	RRu	12.33
	P	+++
Asymm	P	N.S.

Table 2A1 - 3

IESLC - Meta-analysis of Ever Smoking, Any product (or Cigarettes if Any not available)
Squamous
Most adjusted

		combined	Sex: male	female	Total
	N	11	49	42	102
	NS	11	48	41	100
	Wt	96.04	485.88	417.97	999.88
Het	Chi	108.90	175.84	219.57	522.25
Het	df	10	48	41	101
Het	P	***	***	***	***
Fixed	RR	7.04	10.74	8.86	9.52
	RRl	5.76	9.82	8.05	8.94
	RRu	8.60	11.74	9.75	10.13
	P	+++	+++	+++	+++
Random	RR	10.70	11.98	8.97	10.47
	RRl	4.89	9.68	6.95	8.88
	RRu	23.40	14.82	11.57	12.33
	P	+++	+++	+++	+++
Between	Chi				17.93
Between	df				2
Between	P				***
Btwn(F)	P				N.S.
Btwn(R)	P				N.S.

		q	Lung cancer type: q+s	q+u	KI	not a	Total
	N	74	7	3	14	4	102
	NS	52	6	3	10	3	74
	Wt	762.44	59.32	70.92	79.08	28.12	999.88
Het	Chi	304.99	41.80	1.87	55.77	10.50	522.25
Het	df	73	6	2	13	3	101
Het	P	***	***	N.S.	***	*	***
Fixed	RR	10.43	11.11	2.99	11.04	6.91	9.52
	RRl	9.72	8.61	2.37	8.86	4.78	8.94
	RRu	11.20	14.33	3.78	13.77	10.00	10.13
	P	+++	+++	+++	+++	+++	+++
Random	RR	11.56	9.39	2.99	9.71	7.85	10.47
	RRl	9.68	4.47	2.37	5.85	3.84	8.88
	RRu	13.81	19.74	3.78	16.11	16.05	12.33
	P	+++	+++	+++	+++	+++	+++
Between	Chi						107.33
Between	df						4
Between	P						***
Btwn(F)	P						***
Btwn(R)	P						***

		NAmer	UK	Scand	othEur	Location: China	Japan	othAs	other	Total
	N	38	4	7	15	12	8	12	6	102
	NS	26	2	5	12	8	6	9	5	73
	Wt	511.32	18.26	25.57	205.89	115.24	29.46	79.14	15.00	999.88
Het	Chi	204.40	10.69	11.88	109.09	23.25	7.60	28.49	3.82	522.25
Het	df	37	3	6	14	11	7	11	5	101
Het	P	***	*	(*)	***	*	N.S.	**	N.S.	***
Fixed	RR	12.69	4.75	8.67	6.56	5.65	13.94	7.87	16.70	9.52
	RRl	11.63	3.00	5.88	5.72	4.71	9.71	6.31	10.07	8.94
	RRu	13.84	7.51	12.78	7.52	6.78	20.00	9.81	27.70	10.13
	P	+++	+++	+++	+++	+++	+++	+++	+++	+++
Random	RR	13.42	6.27	8.62	8.87	6.39	14.54	8.02	16.70	10.47
	RRl	10.45	2.49	4.81	5.50	4.73	9.78	5.54	10.07	8.88
	RRu	17.24	15.83	15.43	14.31	8.63	21.62	11.60	27.70	12.33
	P	+++	+++	+++	+++	+++	+++	+++	+++	+++
Between	Chi									123.03
Between	df									7
Between	P									***
Btwn(F)	P									***
Btwn(R)	P									**

Table 2A1 - 3

IESLC - Meta-analysis of Ever Smoking, Any product (or Cigarettes if Any not available)
Squamous
Most adjusted

	Detailed Country in "other Europe"					
	multi	Germany	othWest	East	Balkans	Total
N	2	2	2	7	2	15
NS	1	2	2	5	2	12
Wt	98.39	4.65	46.21	14.99	41.65	205.89
Het Chi	27.46	0.64	13.95	6.98	1.38	109.09
Het df	1	1	1	6	1	14
Het P	***	N.S.	***	N.S.	N.S.	***
Fixed RR	10.10	17.25	3.31	10.16	3.87	6.56
RRl	8.29	6.95	2.48	6.12	2.86	5.72
RRu	12.31	42.81	4.42	16.85	5.24	7.52
P	+++	+++	+++	+++	+++	+++
Random RR	9.82	17.25	5.98	10.63	4.08	8.87
RRl	3.48	6.95	1.15	5.91	2.63	5.50
RRu	27.71	42.81	31.02	19.11	6.34	14.31
P	+++	+++	+	+++	+++	+++
Between Chi						58.67
Between df						4
Between P						***
Btwn(F) P						(*)
Btwn(R) P						*

	Detailed Country in "other Asia"			
	India	HongKong	other	Total
N	1	7	4	12
NS	1	5	3	9
Wt	10.45	51.28	17.41	79.14
Het Chi	0.00	5.68	6.14	28.49
Het df	0	6	3	11
Het P	N.S.	N.S.	N.S.	**
Fixed RR	25.43	6.43	7.06	7.87
RRl	13.87	4.89	4.42	6.31
RRu	46.63	8.45	11.30	9.81
P	+++	+++	+++	+++
Random RR	25.43	6.43	6.94	8.02
RRl	13.87	4.89	3.53	5.54
RRu	46.63	8.45	13.65	11.60
P	+++	+++	+++	+++
Between Chi				16.67
Between df				2
Between P				***
Btwn(F) P				*
Btwn(R) P				***

	Detailed other continent			
	SCAmer	Auslia	Africa	Total
N	6			6
NS	5			5
Wt	15.00			15.00
Het Chi	3.82			3.82
Het df	5			5
Het P	N.S.			N.S.
Fixed RR	16.70			16.70
RRl	10.07			10.07
RRu	27.70			27.70
P	+++			+++
Random RR	16.70			16.70
RRl	10.07			10.07
RRu	27.70			27.70
P	+++			+++
Between Chi				
Between df				
Between P				N.S.
Btwn(F) P				N.S.
Btwn(R) P				N.S.

Table 2A1 - 3

IESLC - Meta-analysis of Ever Smoking, Any product (or Cigarettes if Any not available)
Squamous
Most adjusted

	Start year of study <1960	1960-69	1970-79	1980-89	1990+	Total
N	14	14	26	40	8	102
NS	10	12	16	27	8	73
Wt	126.94	99.73	249.35	496.71	27.15	999.88
Het Chi	54.12	67.86	130.30	156.56	10.78	522.25
Het df	13	13	25	39	7	101
Het P	***	***	***	***	N.S.	***
Fixed RR	4.35	11.87	8.76	11.42	12.39	9.52
RRl	3.66	9.75	7.74	10.46	8.51	8.94
RRu	5.18	14.44	9.92	12.47	18.05	10.13
P	+++	+++	+++	+++	+++	+++
Random RR	5.89	12.88	10.08	11.74	12.21	10.47
RRl	3.89	7.71	7.21	9.40	7.56	8.88
RRu	8.92	21.52	14.09	14.66	19.72	12.33
P	+++	+++	+++	+++	+++	+++
Between Chi						102.63
Between df						4
Between P						***
Btwn(F) P						***
Btwn(R) P						(*)

	Study type (1) CC	other	Total
N	93	9	102
NS	66	7	73
Wt	978.53	21.35	999.88
Het Chi	508.71	12.17	522.25
Het df	92	8	101
Het P	***	N.S.	***
Fixed RR	9.46	12.23	9.52
RRl	8.89	8.00	8.94
RRu	10.08	18.69	10.13
P	+++	+++	+++
Random RR	10.31	12.78	10.47
RRl	8.70	7.29	8.88
RRu	12.22	22.41	12.33
P	+++	+++	+++
Between Chi			1.37
Between df			1
Between P			N.S.
Btwn(F) P			N.S.
Btwn(R) P			N.S.

	Study type (2) CC	prosp	other	Total
N	93	5	4	102
NS	66	4	3	73
Wt	978.53	16.77	4.58	999.88
Het Chi	508.71	9.48	2.69	522.25
Het df	92	4	3	101
Het P	***	(*)	N.S.	***
Fixed RR	9.46	12.12	12.62	9.52
RRl	8.89	7.51	5.05	8.94
RRu	10.08	19.56	31.54	10.13
P	+++	+++	+++	+++
Random RR	10.31	12.90	12.62	10.47
RRl	8.70	5.88	5.05	8.88
RRu	12.22	28.29	31.54	12.33
P	+++	+++	+++	+++
Between Chi				1.38
Between df				2
Between P				N.S.
Btwn(F) P				N.S.
Btwn(R) P				N.S.

Table 2A1 - 3

IESLC - Meta-analysis of Ever Smoking, Any product (or Cigarettes if Any not available)
Squamous
Most adjusted

	Study size (number of LC cases) 100-249	250-499	500-999	1000+	Total
N	22	31	18	31	102
NS	21	22	12	18	73
Wt	84.17	135.04	123.69	656.97	999.88
Het Chi	41.64	63.82	62.97	326.49	522.25
Het df	21	30	17	30	101
Het P	**	***	***	***	***
Fixed RR	6.02	8.89	8.26	10.51	9.52
RRl	4.86	7.51	6.92	9.74	8.94
RRu	7.45	10.52	9.85	11.35	10.13
P	+++	+++	+++	+++	+++
Random RR	8.39	10.17	11.35	10.88	10.47
RRl	5.91	7.81	7.79	8.16	8.88
RRu	11.92	13.22	16.53	14.51	12.33
P	+++	+++	+++	+++	+++
Between Chi					27.34
Between df					3
Between P					***
Btwn(F) P					N.S.
Btwn(R) P					N.S.

	Risky occupational population no	mining	othRisky	Total
N	100	1	1	102
NS	71	1	1	73
Wt	994.88	3.73	1.27	999.88
Het Chi	521.48	0.00	0.00	522.25
Het df	99	0	0	101
Het P	***	N.S.	N.S.	***
Fixed RR	9.54	6.33	6.80	9.52
RRl	8.96	2.29	1.20	8.94
RRu	10.15	17.45	38.62	10.13
P	+++	+++	+	+++
Random RR	10.55	6.33	6.80	10.47
RRl	8.94	2.29	1.20	8.88
RRu	12.45	17.45	38.62	12.33
P	+++	+++	+	+++
Between Chi				0.77
Between df				2
Between P				N.S.
Btwn(F) P				N.S.
Btwn(R) P				N.S.

	National cigarette tobacco type Virginia	blended	other	Total
N	9	80	13	102
NS	6	58	9	73
Wt	41.59	839.77	118.52	999.88
Het Chi	43.26	413.91	24.29	522.25
Het df	8	79	12	101
Het P	***	***	*	***
Fixed RR	12.69	10.12	5.56	9.52
RRl	9.36	9.46	4.65	8.94
RRu	17.20	10.83	6.66	10.13
P	+++	+++	+++	+++
Random RR	13.80	11.07	6.15	10.47
RRl	6.53	9.21	4.60	8.88
RRu	29.17	13.31	8.23	12.33
P	+++	+++	+++	+++
Between Chi				40.79
Between df				2
Between P				***
Btwn(F) P				*
Btwn(R) P				**

Table 2A1 - 3

IESLC - Meta-analysis of Ever Smoking, Any product (or Cigarettes if Any not available)
Squamous
Most adjusted

	Any proxy use No/nk	Yes	Total
N	76	26	102
NS	55	18	73
Wt	874.48	125.41	999.88
Het Chi	444.91	50.62	522.25
Het df	75	25	101
Het P	***	**	***
Fixed RR	8.95	14.65	9.52
RRl	8.37	12.30	8.94
RRu	9.56	17.46	10.13
P	+++	+++	+++
Random RR	9.64	13.82	10.47
RRl	7.99	10.45	8.88
RRu	11.64	18.27	12.33
P	+++	+++	+++
Between Chi			26.72
Between df			1
Between P			***
Btwn(F) P			*
Btwn(R) P			*

	Full histological confirmation No	Yes	Total
N	59	43	102
NS	43	30	73
Wt	452.38	547.50	999.88
Het Chi	276.16	182.20	522.25
Het df	58	42	101
Het P	***	***	***
Fixed RR	7.21	11.97	9.52
RRl	6.57	11.01	8.94
RRu	7.90	13.02	10.13
P	+++	+++	+++
Random RR	9.39	12.32	10.47
RRl	7.54	9.78	8.88
RRu	11.68	15.52	12.33
P	+++	+++	+++
Between Chi			63.89
Between df			1
Between P			***
Btwn(F) P			***
Btwn(R) P			(*)

	Number of adjustment variables (1) 0	1	2+/+nk	Total
N	53	18	31	102
NS	37	13	24	74
Wt	412.88	101.56	485.44	999.88
Het Chi	233.67	71.48	190.94	522.25
Het df	52	17	30	101
Het P	***	***	***	***
Fixed RR	7.93	12.59	10.48	9.52
RRl	7.20	10.37	9.59	8.94
RRu	8.74	15.30	11.45	10.13
P	+++	+++	+++	+++
Random RR	9.86	11.34	11.02	10.47
RRl	7.79	7.36	8.35	8.88
RRu	12.49	17.47	14.54	12.33
P	+++	+++	+++	+++
Between Chi				26.15
Between df				2
Between P				***
Btwn(F) P				(*)
Btwn(R) P				N.S.

Table 2A1 - 3

IESLC - Meta-analysis of Ever Smoking, Any product (or Cigarettes if Any not available)
Squamous
Most adjusted

	Number of adjustment variables (2) 0	1	2	3-5	6+/+nk	Total
N	53	18	19	8	4	102
NS	37	13	13	8	4	75
Wt	412.88	101.56	400.04	74.94	10.46	999.88
Het Chi	233.67	71.48	150.52	24.97	5.99	522.25
Het df	52	17	18	7	3	101
Het P	***	***	***	***	N.S.	***
Fixed RR	7.93	12.59	11.18	7.74	7.81	9.52
RRl	7.20	10.37	10.13	6.17	4.26	8.94
RRu	8.74	15.30	12.33	9.70	14.32	10.13
P	+++	+++	+++	+++	+++	+++
Random RR	9.86	11.34	11.66	10.71	8.13	10.47
RRl	7.79	7.36	8.20	6.25	3.43	8.88
RRu	12.49	17.47	16.59	18.36	19.30	12.33
P	+++	+++	+++	+++	+++	+++
Between Chi						35.61
Between df						4
Between P						***
Btwn(F) P						N.S.
Btwn(R) P						N.S.

	Product all/unsp	cig+/-ot	cig only	Total
N	54	46	2	102
NS	41	31	2	74
Wt	324.67	667.96	7.25	999.88
Het Chi	213.86	245.44	0.12	522.25
Het df	53	45	1	101
Het P	***	***	N.S.	***
Fixed RR	6.86	10.99	38.79	9.52
RRl	6.16	10.18	18.74	8.94
RRu	7.65	11.85	80.31	10.13
P	+++	+++	+++	+++
Random RR	8.94	11.86	38.79	10.47
RRl	7.02	9.57	18.74	8.88
RRu	11.39	14.71	80.31	12.33
P	+++	+++	+++	+++
Between Chi				62.82
Between df				2
Between P				***
Btwn(F) P				**
Btwn(R) P				***

	Denominator nev any	nev cigs	Total
N	64	38	102
NS	48	26	74
Wt	471.56	528.32	999.88
Het Chi	283.98	213.69	522.25
Het df	63	37	101
Het P	***	***	***
Fixed RR	8.06	11.04	9.52
RRl	7.37	10.13	8.94
RRu	8.82	12.02	10.13
P	+++	+++	+++
Random RR	9.68	11.92	10.47
RRl	7.81	9.24	8.88
RRu	12.01	15.38	12.33
P	+++	+++	+++
Between Chi			24.58
Between df			1
Between P			***
Btwn(F) P			*
Btwn(R) P			N.S.

Table 2A1 - 3

IESLC - Meta-analysis of Ever Smoking, Any product (or Cigarettes if Any not available)
Squamous
Most adjusted

	Derivation of RR/CI			
	Orig	StdCalc	Other	Total
N	24	42	36	102
NS	18	32	27	77
Wt	461.15	386.46	152.27	999.88
Het Chi	160.86	222.05	110.60	522.25
Het df	23	41	35	101
Het P	***	***	***	***
Fixed RR	11.35	7.86	9.06	9.52
RRl	10.36	7.12	7.73	8.94
RRu	12.43	8.69	10.62	10.13
P	+++	+++	+++	+++
Random RR	12.31	9.46	10.71	10.47
RRl	9.10	7.30	7.84	8.88
RRu	16.65	12.26	14.64	12.33
P	+++	+++	+++	+++
Between Chi				28.73
Between df				2
Between P				***
Btwn(F) P				(*)
Btwn(R) P				N.S.

Table 2A1 - 4

IESLC - Meta-analysis of Ever Smoking, Any product (or Cigarettes if Any not available)
Squamous
Least adjusted

REF	NRR	X	SEX	AGEL	AGEH	RACE	YF	LC TYPE	LOC	START	ST	NLC	R	VB	P	H	AD	PRODUCT	DENOM	De
ABRAHA	1		m	0	0	all	0	q	Eu:est	1975	pr	571	n	bl	n	n	0	all/unsp	nev any	ot
ABRAHA	4		f	0	0	all	0	q	Eu:est	1975	pr	571	n	bl	n	n	0	all/unsp	nev any	ot
ALDERS	52		m	0	0	all	-	q	Eu:UK	1977	CC	1448	n	V	n	n	2	all/unsp	nev any	or
ALDERS	55		f	0	0	all	-	q	Eu:UK	1977	CC	1448	n	V	n	n	2	all/unsp	nev any	or
ANDERS	10		f	0	0	all	0	q	NAmer	1986	pr	343	n	bl	n	n	0	cig+/-ot	nev cigs	st
BAND	5		m	0	0	all	-	q	NAmer	1983	CC	2831	n	V	y	y	2	cig only	nev any	ot
BARBON	110	x	m	0	0	all	-	q	Eu:wst	1979	CC	755	n	bl	y	y	0	all/unsp	nev any	st
BECHER	11		f	0	0	all	-	q+s	Eu:Ger	1985	CC	194	n	bl	n	y	1	all/unsp	nev any	or
BRESLO	36		c	0	0	all	-	not a	NAmer	1949	CC	518	n	bl	n	y	0	all/unsp	nev+1	st
BROWN2	6		m	0	0	wh	-	q	NAmer	1984	CC	14596	n	bl	n	y	2	cig+/-ot	nev cigs	or
BROWN2	5		f	0	0	wh	-	q	NAmer	1984	CC	14596	n	bl	n	y	2	cig+/-ot	nev cigs	or
BUFFLE	49		m	0	0	wh	-	q	NAmer	1976	CC	943	n	bl	y	n	0	cig+/-ot	nev cigs	ot
BUFFLE	62		f	0	0	w-hi	-	q	NAmer	1976	CC	943	n	bl	y	n	0	cig+/-ot	nev cigs	st
BYERS1	1		m	0	0	wh	-	q	NAmer	1957	CC	1002	n	bl	n	n	0	cig+/-ot	nev cigs	st
CHAN	11		m	0	0	all	-	q+s	As:HK	1976	CC	397	n	bl	n	n	0	all/unsp	nev any	st
CHAN	15		f	0	0	all	-	q+s	As:HK	1976	CC	397	n	bl	n	n	0	all/unsp	nev any	st
CHOI	62		m	0	0	all	-	q	As:oth	1985	CC	375	n	bl	n	n	0	cig+/-ot	nev cigs	st
CHOI	64		f	0	0	all	-	q	As:oth	1985	CC	375	n	bl	n	n	0	cig+/-ot	nev cigs	st
COMSTO	66		m	0	0	all	-	q	NAmer	1975	ot	258	n	bl	n	n	0	cig+/-ot	nev cigs	st
COMSTO	78		f	0	0	all	-	q	NAmer	1975	ot	258	n	bl	n	n	0	cig+/-ot	nev cigs	ot
CORREA	35		c	0	0	all	-	q+s	NAmer	1979	CC	1359	n	bl	y	n	1	cig+/-ot	nev cigs	or
DAMBER	12	x	m	0	0	all	-	q	Eu:Sca	1972	CC	579	n	bl	y	n	0	all/unsp	nev any	st
DESTE2	16		m	0	0	all	-	q	SCAmer	1993	CC	463	n	bl	n	n	2	all/unsp	nev any	or
DOLL	82	x	m	0	0	all	-	KI	Eu:UK	1948	CC	1465	n	V	n	n	0	all/unsp	nev any	st
DOLL	84	x	f	0	0	all	-	KI	Eu:UK	1948	CC	1465	n	V	n	n	0	all/unsp	nev any	st
DORGAN	113		m	0	0	wh	-	q	NAmer	1980	CC	2026	n	bl	y	y	2	cig+/-ot	nev any	or
DORGAN	98		f	0	0	all	-	q	NAmer	1980	CC	2026	n	bl	y	y	3	cig+/-ot	nev any	or
DOSEME	19	x	m	0	0	all	-	q	Eu:bal	1979	CC	1210	n	bl	n	n	0	cig+/-ot	nev cigs	st
ENGELA	62		m	0	0	all	0	q	Eu:Sca	1964	pr	435	n	bl	n	n	7	cig+/-ot	nev cigs	ot
FAN	3		c	0	0	all	-	q	As:Chi	1990	CC	403	n	ot	y	n	0	cig+/-ot	nev cigs	ot
GAO	7	x	m	0	0	all	-	q	As:Chi	1984	CC	1405	n	ot	n	n	0	cig+/-ot	nev cigs	st
GAO	17	x	f	0	0	all	-	q	As:Chi	1984	CC	1405	n	ot	n	n	0	cig+/-ot	nev cigs	st
GER	5	x	c	0	0	all	-	q+s	As:oth	1990	CC	141	n	ot	y	n	0	all/unsp	nev any	st
HAENSZ	16	x	f	0	0	all	-	q+u	NAmer	1955	CC	158	n	bl	n	y	0	all/unsp	nev any	or
HAMMON	73	x	m	0	0	wh	0	not a	NAmer	1952	pr	448	n	bl	n	n	0	all/unsp	nev any	st
HEGMAN	2		c	0	0	all	-	q	NAmer	1989	CC	282	n	bl	y	y	0	all/unsp	nev any	st
HINDS	23		f	0	0	o	-	q+s	NAmer	1968	CC	292	n	bl	n	n	3	all/unsp	nev any	st
ISHIMA	1	x	c	0	0	all	-	q	As:Jap	1961	CC	180	n	bl	y	y	0	all/unsp	nev any	st
JAHN	46		m	0	0	all	-	q	Eu:Ger	1988	CC	1004	n	bl	n	n	0	all/unsp	nev any	st
JAIN	8	x	m	0	0	all	-	q	NAmer	1981	CC	845	n	V	y	n	0	cig+/-ot	nev cigs	st
JAIN	3	x	f	0	0	all	-	q	NAmer	1981	CC	845	n	V	y	n	0	cig+/-ot	nev cigs	st
JEDRYC	7	x	m	0	0	all	-	q	Eu:est	1980	CC	1630	n	bl	y	n	0	cig+/-ot	nev any	st
JOLY	54		m	0	0	all	-	q	SCAmer	1978	CC	826	n	bl	n	n	0	cig+/-ot	nev any	st
JOLY	52		f	0	0	all	-	q	SCAmer	1978	CC	826	n	bl	n	n	0	cig+/-ot	nev any	st
JUSSAW	23		m	0	0	all	-	KI	As:Ind	1964	CC	792	n	V	n	n	0	all/unsp	nev any	st
KATSOU	35	x	f	0	0	all	-	KI	Eu:bal	1987	CC	101	n	bl	n	n	0	all/unsp	nev any	st
KHUDER	24		m	0	0	all	-	q	NAmer	1985	CC	482	n	bl	n	y	0	cig+/-ot	nev cigs	ot
KIHARA	26		c	0	0	jap	-	q	As:Jap	1991	CC	440	n	bl	n	n	0	all/unsp	nev any	st
KOO	6		f	0	0	all	-	q+s	As:HK	1981	CC	200	n	bl	n	n	0	all/unsp	nev any	st
KREYBE	16	x	m	0	0	all	-	KI	Eu:Sca	1948	CC	300	n	bl	n	y	0	all/unsp	nev any	st
KREYBE	33	x	f	0	0	all	-	KI	Eu:Sca	1948	CC	300	n	bl	n	y	0	all/unsp	nev any	st
LAMTH	1		f	0	0	ch	-	q	As:HK	1983	CC	445	n	bl	n	n	0	all/unsp	nev any	or
LAMWK	2		f	0	0	ch	-	q	As:HK	1981	CC	163	n	bl	n	n	0	all/unsp	nev any	st
LAMWK2	1		m	0	0	all	-	q	As:HK	1976	CC	480	n	bl	n	n	0	all/unsp	nev any	st
LAMWK2	5		f	0	0	all	-	q	As:HK	1976	CC	480	n	bl	n	n	0	all/unsp	nev any	st
LOMBA2	2		f	0	0	all	-	q+u	NAmer	1960	CC	225	n	bl	n	n	0	cig+/-ot	nev cigs	st
LUBIN	33		m	0	0	all	-	KI	As:Chi	1984	CC	427	m	ot	y	n	0	all/unsp	nev any	st
LUBIN2	145		m	0	0	all	-	q	Eu:mul	1976	CC	7804	n	bl	n	y	0	cig+/-ot	nev any	st
LUBIN2	165		f	0	0	all	-	q	Eu:mul	1976	CC	7804	n	bl	n	y	0	cig+/-ot	nev any	st
LUO	2	x	c	0	0	all	-	q	As:Chi	1990	CC	102	n	ot	n	y	0	cig+/-ot	nev cigs	st
MATOS	66	x	m	0	0	all	-	q	SCAmer	1994	CC	200	n	bl	n	n	0	cig+/-ot	nev any	st
MATSUD	11		m	0	0	all	-	q	As:Jap	1965	CC	179	n	bl	n	n	0	cig+/-ot	nev cigs	st
NOU	1		m	0	0	all	-	q	Eu:Sca	1971	CC	273	n	bl	y	n	0	all/unsp	nev any	st
NOU	6		f	0	0	all	-	q	Eu:Sca	1971	CC	273	n	bl	y	n	0	all/unsp	nev any	st
ORMOS	8		m	0	0	all	-	q	Eu:est	1947	CC	119	n	bl	y	y	0	cig+/-ot	nev any	st
OSANN	18	x	m	0	0	all	-	q	NAmer	1984	CC	1986	n	bl	n	n	0	cig+/-ot	nev cigs	st
OSANN	22	x	f	0	0	all	-	q	NAmer	1984	CC	1986	n	bl	n	n	0	cig+/-ot	nev cigs	st
OSANN2	7	x	f	0	0	all	-	KI	NAmer	1964	ot	217	n	bl	n	y	0	cig+/-ot	nev cigs	st
PEZZOT	6		m	0	0	all	-	q	SCAmer	1987	CC	215	n	bl	n	y	0	cig only	nev cigs	ot
SCHWAR	10		m	40	54	wh	-	q	NAmer	1984	CC	5588	n	bl	y	y	0	cig+/-ot	nev cigs	st
SCHWAR	9		m	40	54	bl	-	q	NAmer	1984	CC	5588	n	bl	y	y	0	cig+/-ot	nev cigs	st
SCHWAR	18		f	40	54	wh	-	q	NAmer	1984	CC	5588	n	bl	y	y	0	cig+/-ot	nev cigs	ot

Table 2A1 - 4

IESLC - Meta-analysis of Ever Smoking, Any product (or Cigarettes if Any not available)
Squamous
Least adjusted

REF	NRR	X	SEX	AGEL	AGEH	RACE	YF	LC TYPE	LOC	START	ST	NLC	R	VB	P	H	AD	PRODUCT	DENOM	De
SCHWAR	17		f	40	54	bl	-	q	NAmer	1984	CC	5588	n	bl	y	y	0	cig+/-ot	nev cigs	ot
SEOW	3		f	0	0	ch	-	q	As:oth	1997	CC	153	n	bl	n	y	0	cig+/-ot	nev cigs	st
SIEMIA	11	x	m	0	0	all	-	q	NAmer	1979	CC	857	n	V	y	y	0	cig+/-ot	nev cigs	st
SOBUE	3	x	m	0	0	all	-	q	As:Jap	1986	CC	1376	n	bl	n	y	0	cig+/-ot	nev cigs	st
SOBUE	19	x	f	0	0	all	-	q	As:Jap	1986	CC	1376	n	bl	n	y	0	cig+/-ot	nev cigs	st
STASZE	12		m	0	0	all	-	q	Eu:est	1954	CC	281	n	bl	n	y	0	all/unsp	nev any	ot
STASZE	38		f	0	0	all	-	q	Eu:est	1954	CC	281	n	bl	n	y	0	all/unsp	nev any	ot
STAYNE	3		m	0	0	all	-	q	NAmer	1969	CC	420	n	bl	n	n	0	all/unsp	nev any	st
SUZUK2	12	x	c	0	0	all	-	q	SCAmer	1991	CC	123	n	bl	n	y	0	all/unsp	nev any	st
SVENSS	57	x	f	0	0	all	-	q	Eu:Sca	1983	CC	210	n	bl	n	n	0	all/unsp	nev any	st
TIZZAN	18		c	0	0	all	-	q+u	Eu:wst	1959	CC	1358	n	bl	n	n	0	all/unsp	nev any	st
TOKARS	9	x	c	0	0	all	-	q	Eu:est	1966	ot	162	o	bl	n	y	0	all/unsp	nev any	st
TSUGAN	13		m	0	0	all	-	q	As:Jap	1976	CC	134	n	bl	n	y	0	all/unsp	nev any	ot
WAKAI	15	x	m	0	0	all	-	q	As:Jap	1988	CC	333	n	bl	n	y	0	all/unsp	nev any	st
WAKAI	33	x	f	0	0	all	-	q	As:Jap	1988	CC	333	n	bl	n	y	0	all/unsp	nev any	st
WU	14	x	f	0	0	wh	-	q	NAmer	1981	CC	220	n	bl	n	y	0	all/unsp	nev any	st
WUWILL	9		f	0	0	all	-	q	As:Chi	1985	CC	965	n	ot	n	n	3	cig+/-ot	nev cigs	or
WYNDE2	7		m	0	0	all	-	KI	NAmer	1962	CC	404	n	bl	n	y	0	all/unsp	nev any	st
WYNDE3	9		m	0	0	all	-	KI	NAmer	1966	CC	350	n	bl	n	y	0	all/unsp	nev any	st
WYNDE3	132		f	0	0	all	-	KI	NAmer	1966	CC	350	n	bl	n	y	0	all/unsp	nev any	st
WYNDE4	35	x	m	0	0	all	-	not a	NAmer	1948	CC	684	n	bl	y	n	0	all/unsp	nev any	st
WYNDE4	54		f	0	0	all	-	not a	NAmer	1948	CC	684	n	bl	y	n	2	all/unsp	nev any	ot
WYNDE6	66		m	0	0	all	-	KI	NAmer	1969	CC	4423	n	bl	n	y	0	all/unsp	nev any	st
WYNDE6	411	x	f	0	0	wh	-	q	NAmer	1969	CC	4423	n	bl	n	y	0	cig+/-ot	nev cigs	st
XU3	19	x	m	0	0	all	-	KI	As:Chi	1981	CC	135	n	ot	n	n	0	all/unsp	nev any	st
XU3	23	x	f	0	0	all	-	KI	As:Chi	1981	CC	135	n	ot	n	n	0	all/unsp	nev any	st
ZHENG	5		m	0	0	all	-	q	As:Chi	1982	CC	540	n	ot	*	y	0	cig+/-ot	nev cigs	st
ZHENG	18		f	0	0	all	-	q	As:Chi	1982	CC	540	n	ot	*	y	0	cig+/-ot	nev cigs	st
ZHOU	8		m	0	0	all	-	q	As:Chi	1978	CC	1360	n	ot	n	n	0	all/unsp	nev any	st
ZHOU	9		f	0	0	all	-	q	As:Chi	1978	CC	1360	n	ot	n	n	0	all/unsp	nev any	st

Cigarette type is all/unspec for all RRs

Table 2A1 - 5

IESLC - Meta-analysis of Ever Smoking, Any product (or Cigarettes if Any not available)
Squamous
Least adjusted

REF	NRR	SEX	AD	Number Exposed Case	Cont	Non-exposed Case	Cont	RR	95.00%CI
*ABRAHA	1	m	0	142	10351	0	3365	92.66~	(5.77-1488.21)
*ABRAHA	4	f	0	17	5256	7	11589	5.35	(2.22- 12.90)
Subtotal ABRAHA								6.95	(3.00- 16.06)
ALDERS	52	m	2	-	-	-	-	14.70	(3.40- 63.64)
ALDERS	55	f	2	-	-	-	-	6.09	(2.68- 13.82)
Subtotal ALDERS								7.52	(3.67- 15.37)
*ANDERS	10	f	0	63	96164	5	195158	25.57	(10.29- 63.56)
BAND	5	m	2	-	-	-	-	37.45	(17.62- 79.58)
BARBON	110	m	0	261	567	6	188	14.42	(6.31- 32.94)
BECHER	11	f	1	-	-	-	-	10.69	(2.43- 47.00)
BRESLO	36	c	0	457	462	15	56	3.69	(2.06- 6.62)
BROWN2	6	m	2	-	-	-	-	11.10	(9.50- 12.90)
BROWN2	5	f	2	-	-	-	-	20.10	(16.40- 24.80)
Subtotal BROWN2								13.69	(12.11- 15.49)
BUFFLE	49	m	0	-	-	-	-	14.03	(4.73- 41.61)
BUFFLE	62	f	0	58	166	3	112	13.04	(3.99- 42.66)
Subtotal BUFFLE								13.57	(6.09- 30.24)
BYERS1	1	m	0	299	695	22	424	8.29	(5.29- 13.00)
CHAN	11	m	0	114	161	2	43	15.22	(3.61- 64.12)
CHAN	15	f	0	44	50	19	139	6.44	(3.44- 12.06)
Subtotal CHAN								7.39	(4.16- 13.13)
CHOI	62	m	0	160	465	6	95	5.45	(2.34- 12.67)
CHOI	64	f	0	11	26	10	164	6.94	(2.68- 17.96)
Subtotal CHOI								6.06	(3.22- 11.40)
COMSTO	66	m	0	44	229	2	84	8.07	(1.91- 34.02)
COMSTO	78	f	0	17	87	0	115	46.20~	(2.74- 778.83)
Subtotal COMSTO								11.56	(3.21- 41.67)
CORREA	35	c	1	-	-	-	-	28.30	(18.60- 43.20)
DAMBER	12	m	0	271	169	14	103	11.80	(6.54- 21.29)
DESTE2	16	m	2	-	-	-	-	13.20	(4.70- 37.10)
DOLL	82	m	0	829	1296	3	61	13.01	(4.07- 41.59)
DOLL	84	f	0	32	49	16	59	2.41	(1.18- 4.90)
Subtotal DOLL								3.81	(2.08- 6.98)
DORGAN	113	m	2	-	-	-	-	18.90	(7.00- 51.30)
DORGAN	98	f	3	-	-	-	-	11.10	(7.20- 17.10)
Subtotal DORGAN								12.08	(8.12- 17.96)
DOSEME	19	m	0	434	536	58	293	4.09	(3.00- 5.57)
*ENGELA	62	m	7	-	-	-	-	6.45	(1.97- 21.11)
FAN	3	c	0	75	595	6	556	11.68	(5.04- 27.04)
GAO	7	m	0	314	558	13	202	8.74	(4.91- 15.58)
GAO	17	f	0	66	130	53	605	5.80	(3.85- 8.72)
Subtotal GAO								6.65	(4.76- 9.28)
GER	5	c	0	48	156	11	80	2.24	(1.10- 4.54)
HAENSZ	16	f	0	58	103	44	236	3.02	(1.92- 4.76)
*HAMMON	73	m	0	286	510108	4	115884	16.24	(6.05- 43.57)
HEGMAN	2	c	0	89	1202	5	2080	30.80	(12.48- 76.03)
HINDS	23	f	3	-	-	-	-	16.13	(7.66- 33.97)
ISHIMA	1	c	0	53	33	5	25	8.03	(2.80- 23.04)
JAHN	46	m	0	351	701	3	138	23.03	(7.29- 72.81)
JAIN	8	m	0	154	277	2	85	23.63	(5.73- 97.35)
JAIN	3	f	0	103	196	6	214	18.74	(8.05- 43.66)
Subtotal JAIN								19.92	(9.64- 41.17)
JEDRYC	7	m	0	337	1054	6	289	15.40	(6.80- 34.89)
JOLY	54	m	0	203	709	2	218	31.21	(7.69- 126.68)
JOLY	52	f	0	48	122	6	283	18.56	(7.74- 44.51)
Subtotal JOLY								21.47	(10.22- 45.09)
JUSSAW	23	m	0	89	168	13	624	25.43	(13.87- 46.63)
KATSOU	35	f	0	28	22	14	67	6.09	(2.73- 13.59)
KHUDER	24	m	0	176	-	9	-	7.82	(3.87- 15.77)
KIHARA	26	c	0	132	232	5	237	26.97	(10.84- 67.08)
KOO	6	f	0	61	63	32	137	4.15	(2.46- 6.98)
KREYBE	16	m	0	210	3514	3	644	12.83	(4.09- 40.22)
KREYBE	33	f	0	2	328	3	657	1.34	(0.22- 8.03)
Subtotal KREYBE								6.68	(2.55- 17.51)
LAMTH	1	f	0	63	20	28	72	8.10	(4.16- 15.77)
LAMWK	2	f	0	21	41	7	144	10.54	(4.19- 26.52)
LAMWK2	1	m	0	129	161	5	43	6.89	(2.65- 17.90)
LAMWK2	5	f	0	35	50	15	139	6.49	(3.27- 12.88)
Subtotal LAMWK2								6.62	(3.79- 11.56)
LOMBA2	2	f	0	94	353	15	239	4.24	(2.40- 7.50)
LUBIN	33	m	0	330	939	4	72	6.33	(2.29- 17.45)

Table 2A1 - 5

IESLC - Meta-analysis of Ever Smoking, Any product (or Cigarettes if Any not available)
Squamous
Least adjusted

REF	NRR	SEX	AD	Number Exposed Case	Number Exposed Cont	Non-exposed Case	Non-exposed Cont	RR	95.00%CI
LUBIN2	145	m	0	3587	10433	54	2616	16.66	(12.69- 21.86)
LUBIN2	165	f	0	200	567	72	1180	5.78	(4.34- 7.71)
Subtotal LUBIN2								10.10	(8.29- 12.31)
LUO	2	c	0	34	146	5	160	7.45	(2.84- 19.56)
MATOS	66	m	0	47	283	3	110	6.09	(1.86- 19.97)
MATSUD	11	m	0	103	3314	1	1255	39.01	(5.44- 279.84)
NOU	1	m	0	110	247	2	122	27.17	(6.60- 111.85)
NOU	6	f	0	5	92	2	261	7.09	(1.35- 37.19)
Subtotal NOU								15.42	(5.26- 45.22)
ORMOS	8	m	0	27	1034	2	777	10.14	(2.41- 42.79)
OSANN	18	m	0	352	1018	8	833	36.00	(17.76- 72.99)
OSANN	22	f	0	159	563	12	1093	25.72	(14.18- 46.66)
Subtotal OSANN								29.58	(18.76- 46.64)
OSANN2	7	f	0	112	61	7	58	15.21	(6.54- 35.38)
PEZZOT	6	m	0	85	317	0	116	62.74~	(3.86-1019.50)
SCHWAR	10	m	0	80	178	1	73	32.81	(4.48- 240.23)
SCHWAR	9	m	0	41	39	4	7	1.84	(0.50- 6.78)
SCHWAR	18	f	0	29	108	0	79	43.23~	(2.60- 718.15)
SCHWAR	17	f	0	21	28	0	41	62.61~	(3.64-1076.10)
Subtotal SCHWAR								7.71	(2.96- 20.10)
SEOW	3	f	0	21	15	10	125	17.50	(6.95- 44.09)
SIEMIA	11	m	0	356	428	3	105	29.11	(9.16- 92.52)
SOBUE	3	m	0	422	1013	3	128	17.77	(5.63- 56.16)
SOBUE	19	f	0	36	232	14	857	9.50	(5.04- 17.91)
Subtotal SOBUE								10.99	(6.31- 19.15)
STASZE	12	m	0	137	754	0	158	57.77~	(3.58- 933.17)
STASZE	38	f	0	1	153	0	1660	32.45~	(1.32- 800.04)
Subtotal STASZE								45.09	(5.52- 368.55)
STAYNE	3	m	0	130	567	22	333	3.47	(2.17- 5.56)
SUZUK2	12	c	0	75	36	5	44	18.33	(6.70- 50.17)
SVENSS	57	f	0	48	89	5	120	12.94	(4.95- 33.84)
TIZZAN	18	c	0	333	939	55	419	2.70	(1.99- 3.67)
TOKARS	9	c	0	45	77	2	19	5.55	(1.24- 24.95)
TSUGAN	13	m	0	20	15	0	5	14.55~	(0.75- 283.37)
WAKAI	15	m	0	113	424	2	65	8.66	(2.09- 35.92)
WAKAI	33	f	0	16	31	3	145	24.95	(6.85- 90.87)
Subtotal WAKAI								15.46	(5.94- 40.24)
WU	14	f	0	69	41	2	30	25.24	(5.73- 111.19)
WUWILL	9	f	3	-	-	-	-	4.20	(3.00- 5.90)
WYNDE2	7	m	0	347	616	3	105	19.72	(6.21- 62.59)
WYNDE3	9	m	0	207	332	3	88	18.29	(5.71- 58.56)
WYNDE3	132	f	0	25	56	5	76	6.79	(2.45- 18.82)
Subtotal WYNDE3								10.44	(4.85- 22.49)
WYNDE4	35	m	0	597	665	8	115	12.91	(6.25- 26.65)
WYNDE4	54	f	2	-	-	-	-	5.82	(2.55- 13.31)
Subtotal WYNDE4								9.13	(5.29- 15.74)
WYNDE6	66	m	0	1744	1996	29	617	18.59	(12.74- 27.13)
WYNDE6	411	f	0	153	275	12	673	31.20	(17.05- 57.09)
Subtotal WYNDE6								21.51	(15.61- 29.63)
XU3	19	m	0	39	68	3	31	5.93	(1.70- 20.66)
XU3	23	f	0	15	11	2	25	17.05	(3.32- 87.61)
Subtotal XU3								8.74	(3.24- 23.59)
ZHENG	5	m	0	156	218	4	94	16.82	(6.05- 46.71)
ZHENG	18	f	0	43	44	33	184	5.45	(3.11- 9.54)
Subtotal ZHENG								7.07	(4.33- 11.56)
ZHOU	8	m	0	343	41	96	36	3.14	(1.90- 5.18)
ZHOU	9	f	0	35	7	42	32	3.81	(1.50- 9.68)
Subtotal ZHOU								3.28	(2.11- 5.10)
Partial Totals				17326	666135	1046	351088		

*prospective study ~ With 0.5 adjustment for zero

Table 2A1 - 5

IESLC - Meta-analysis of Ever Smoking, Any product (or Cigarettes if Any not available)
Squamous
Least adjusted

REF	NRR	SEX	AD	Ys	Ws	Qs	Ps
*ABRAHA	1	m	0	4.53	0.50	2.59	0.0014
*ABRAHA	4	f	0	1.68	4.97	1.63	0.0002
Subtotal ABRAHA				1.94	5.46	4.21	
ALDERS	52	m	2	2.69	1.79	0.34	0.0003
ALDERS	55	f	2	1.81	5.71	1.12	0.0000
Subtotal ALDERS				2.02	7.50	1.47	
*ANDERS	10	f	0	3.24	4.63	4.55	0.0000
BAND	5	m	2	3.62	6.76	12.74	0.0000
BARBON	110	m	0	2.67	5.63	0.99	0.0000
BECHER	11	f	1	2.37	1.75	0.02	0.0017
BRESLO	36	c	0	1.31	11.25	10.02	0.0000
BROWN2	6	m	2	2.41	164.17	4.04	0.0000
BROWN2	5	f	2	3.00	89.84	50.61	0.0000
Subtotal BROWN2				2.62	254.01	54.65	
BUFFLE	49	m	0	2.64	3.25	0.50	0.0000
BUFFLE	62	f	0	2.57	2.74	0.28	0.0000
Subtotal BUFFLE				2.61	5.99	0.77	
BYERS1	1	m	0	2.12	19.01	0.35	0.0000
CHAN	11	m	0	2.72	1.86	0.42	0.0002
CHAN	15	f	0	1.86	9.75	1.47	0.0000
Subtotal CHAN				2.00	11.61	1.88	
CHOI	62	m	0	1.70	5.39	1.66	0.0001
CHOI	64	f	0	1.94	4.25	0.42	0.0001
Subtotal CHOI				1.80	9.63	2.08	
COMSTO	66	m	0	2.09	1.86	0.05	0.0045
COMSTO	78	f	0	3.83	0.48	1.21	0.0078
Subtotal COMSTO				2.45	2.34	1.25	
CORREA	35	c	1	3.34	21.64	25.84	0.0000
DAMBER	12	m	0	2.47	11.02	0.52	0.0000
DESTE2	16	m	2	2.58	3.60	0.39	0.0000
DOLL	82	m	0	2.57	2.84	0.28	0.0000
DOLL	84	f	0	0.88	7.63	14.34	0.0152
Subtotal DOLL				1.34	10.47	14.63	
DORGAN	113	m	2	2.94	3.87	1.84	0.0000
DORGAN	98	f	3	2.41	20.54	0.50	0.0000
Subtotal DORGAN				2.49	24.41	2.34	
DOSEME	19	m	0	1.41	40.28	28.53	0.0000
*ENGELA	62	m	7	1.86	2.73	0.41	0.0021
FAN	3	c	0	2.46	5.45	0.24	0.0000
GAO	7	m	0	2.17	11.51	0.08	0.0000
GAO	17	f	0	1.76	23.06	5.61	0.0000
Subtotal GAO				1.89	34.57	5.68	
GER	5	c	0	0.81	7.65	15.97	0.0259
HAENSZ	16	f	0	1.11	18.55	24.31	0.0000
*HAMMON	73	m	0	2.79	3.94	1.14	0.0000
HEGMAN	2	c	0	3.43	4.70	6.52	0.0000
HINDS	23	f	3	2.78	6.93	1.95	0.0000
ISHIMA	1	c	0	2.08	3.46	0.10	0.0001
JAHN	46	m	0	3.14	2.90	2.28	0.0000
JAIN	8	m	0	3.16	1.92	1.59	0.0000
JAIN	3	f	0	2.93	5.37	2.49	0.0000
Subtotal JAIN				2.99	7.29	4.08	
JEDRYC	7	m	0	2.73	5.75	1.35	0.0000
JOLY	54	m	0	3.44	1.96	2.77	0.0000
JOLY	52	f	0	2.92	5.02	2.26	0.0000
Subtotal JOLY				3.07	6.98	5.03	
JUSSAW	23	m	0	3.24	10.45	10.15	0.0000
KATSOU	35	f	0	1.81	5.97	1.17	0.0000
KHUDER	24	m	0	2.06	7.79	0.29	0.0000
KIHARA	26	c	0	3.29	4.63	5.05	0.0000
KOO	6	f	0	1.42	14.12	9.69	0.0000
KREYBE	16	m	0	2.55	2.94	0.27	0.0000
KREYBE	33	f	0	0.29	1.19	4.59	0.7520
Subtotal KREYBE				1.90	4.14	4.86	
LAMTH	1	f	0	2.09	8.66	0.22	0.0000
LAMWK	2	f	0	2.35	4.51	0.05	0.0000
LAMWK2	1	m	0	1.93	4.22	0.43	0.0001
LAMWK2	5	f	0	1.87	8.17	1.18	0.0000
Subtotal LAMWK2				1.89	12.38	1.61	
LOMBA2	2	f	0	1.45	11.86	7.68	0.0000
LUBIN	33	m	0	1.84	3.73	0.61	0.0004

Table 2A1 - 5

IESLC - Meta-analysis of Ever Smoking, Any product (or Cigarettes if Any not available)
Squamous
Least adjusted

REF	NRR	SEX	AD	Ys	Ws	Qs	Ps
LUBIN2	145	m	0	2.81	51.88	16.42	0.0000
LUBIN2	165	f	0	1.75	46.51	11.42	0.0000
Subtotal LUBIN2				2.31	98.39	27.84	
LUO	2	c	0	2.01	4.12	0.24	0.0000
MATOS	66	m	0	1.81	2.72	0.54	0.0029
MATSUD	11	m	0	3.66	0.99	1.98	0.0003
NOU	1	m	0	3.30	1.92	2.12	0.0000
NOU	6	f	0	1.96	1.40	0.12	0.0205
Subtotal NOU				2.74	3.32	2.24	
ORMOS	8	m	0	2.32	1.85	0.01	0.0016
OSANN	18	m	0	3.58	7.69	13.68	0.0000
OSANN	22	f	0	3.25	10.83	10.77	0.0000
Subtotal OSANN				3.39	18.52	24.45	
OSANN2	7	f	0	2.72	5.39	1.20	0.0000
PEZZOT	6	m	0	4.14	0.49	1.76	0.0036
SCHWAR	10	m	0	3.49	0.97	1.49	0.0006
SCHWAR	9	m	0	0.61	2.26	6.08	0.3596
SCHWAR	18	f	0	3.77	0.49	1.12	0.0086
SCHWAR	17	f	0	4.14	0.47	1.69	0.0044
Subtotal SCHWAR				2.04	4.19	10.38	
SEOW	3	f	0	2.86	4.50	1.69	0.0000
SIEMIA	11	m	0	3.37	2.87	3.61	0.0000
SOBUE	3	m	0	2.88	2.90	1.14	0.0000
SOBUE	19	f	0	2.25	9.55	0.00	0.0000
Subtotal SOBUE				2.40	12.46	1.14	
STASZE	12	m	0	4.06	0.50	1.62	0.0043
STASZE	38	f	0	3.48	0.37	0.57	0.0333
Subtotal STASZE				3.81	0.87	2.18	
STAYNE	3	m	0	1.24	17.27	17.47	0.0000
SUZUK2	12	c	0	2.91	3.79	1.64	0.0000
SVENSS	57	f	0	2.56	4.16	0.40	0.0000
TIZZAN	18	c	0	0.99	40.59	64.06	0.0000
TOKARS	9	c	0	1.71	1.70	0.49	0.0254
TSUGAN	13	m	0	2.68	0.44	0.08	0.0772
WAKAI	15	m	0	2.16	1.90	0.02	0.0029
WAKAI	33	f	0	3.22	2.30	2.15	0.0000
Subtotal WAKAI				2.74	4.20	2.16	
WU	14	f	0	3.23	1.75	1.67	0.0000
WUWILL	9	f	3	1.44	33.59	22.32	0.0000
WYNDE2	7	m	0	2.98	2.88	1.54	0.0000
WYNDE3	9	m	0	2.91	2.84	1.22	0.0000
WYNDE3	132	f	0	1.91	3.69	0.41	0.0002
Subtotal WYNDE3				2.35	6.53	1.64	
WYNDE4	35	m	0	2.56	7.31	0.69	0.0000
WYNDE4	54	f	2	1.76	5.63	1.34	0.0000
Subtotal WYNDE4				2.21	12.93	2.04	
WYNDE6	66	m	0	2.92	26.90	12.16	0.0000
WYNDE6	411	f	0	3.44	10.53	14.92	0.0000
Subtotal WYNDE6				3.07	37.42	27.08	
XU3	19	m	0	1.78	2.46	0.55	0.0052
XU3	23	f	0	2.84	1.43	0.49	0.0007
Subtotal XU3				2.17	3.90	1.04	
ZHENG	5	m	0	2.82	3.68	1.21	0.0000
ZHENG	18	f	0	1.70	12.24	3.77	0.0000
Subtotal ZHENG				1.96	15.92	4.97	
ZHOU	8	m	0	1.14	15.27	18.70	0.0000
ZHOU	9	f	0	1.34	4.42	3.68	0.0049
Subtotal ZHOU				1.19	19.68	22.38	

Table 2A1 - 5

IESLC - Meta-analysis of Ever Smoking, Any product (or Cigarettes if Any not available)
Squamous
Least adjusted

	N	102
	NS	73
	Wt	1023.57
Het	Chi	527.92
Het	df	101
Het	P	***
Fixed	RR	9.49
	RRl	8.93
	RRu	10.09
	P	+++
Random	RR	10.37
	RRl	8.83
	RRu	12.18
	P	+++
Asymm	P	N.S.

Table 2A1 - 6

IESLC - Meta-analysis of Ever Smoking, Any product (or Cigarettes if Any not available)
Squamous
Least adjusted

	Sex			
	combined	male	female	Total
N	11	49	42	102
NS	11	48	41	100
Wt	108.99	489.65	424.94	1023.57
Het Chi	117.26	172.74	215.62	527.92
Het df	10	48	41	101
Het P	***	***	***	***
Fixed RR	6.73	10.78	8.95	9.49
RRl	5.57	9.86	8.14	8.93
RRu	8.12	11.78	9.84	10.09
P	+++	+++	+++	+++
Random RR	9.05	12.13	9.01	10.37
RRl	4.46	9.82	7.03	8.83
RRu	18.35	14.98	11.56	12.18
P	+++	+++	+++	+++
Between Chi				22.30
Between df				2
Between P				***
Btwn(F) P				N.S.
Btwn(R) P				N.S.

	Lung cancer type					
	q	q+s	q+u	KI	not a	Total
N	74	7	3	14	4	102
NS	52	6	3	10	3	74
Wt	780.40	63.70	71.00	80.35	28.13	1023.57
Het Chi	307.90	55.30	1.87	44.89	10.32	527.92
Het df	73	6	2	13	3	101
Het P	***	***	N.S.	***	*	***
Fixed RR	10.39	9.77	3.00	11.90	6.89	9.49
RRl	9.69	7.64	2.38	9.56	4.76	8.93
RRu	11.14	12.49	3.78	14.80	9.97	10.09
P	+++	+++	+++	+++	+++	+++
Random RR	11.47	8.69	3.00	9.86	7.80	10.37
RRl	9.64	3.88	2.38	6.27	3.84	8.83
RRu	13.65	19.47	3.78	15.49	15.84	12.18
P	+++	+++	+++	+++	+++	+++
Between Chi						107.64
Between df						4
Between P						***
Btwn(F) P						***
Btwn(R) P						***

	Location								
	NAmer	UK	Scand	othEur	China	Japan	othAs	other	Total
N	38	4	7	15	12	8	12	6	102
NS	26	2	5	12	8	6	9	5	73
Wt	520.85	17.97	25.36	211.15	120.97	26.16	83.52	17.58	1023.57
Het Chi	204.28	8.97	8.12	106.77	20.37	6.90	37.39	4.65	527.92
Het df	37	3	6	14	11	7	11	5	101
Het P	***	*	N.S.	***	*	N.S.	***	N.S.	***
Fixed RR	12.74	5.06	10.59	6.66	5.45	13.76	7.27	15.93	9.49
RRl	11.69	3.18	7.18	5.82	4.56	9.38	5.86	9.98	8.93
RRu	13.89	8.03	15.63	7.63	6.52	20.18	9.01	25.42	10.09
P	+++	+++	+++	+++	+++	+++	+++	+++	+++
Random RR	13.45	6.38	10.14	8.94	6.02	13.76	7.61	15.93	10.37
RRl	10.54	2.72	6.21	5.60	4.59	9.38	5.06	9.98	8.83
RRu	17.17	14.96	16.57	14.26	7.90	20.18	11.45	25.42	12.18
P	+++	+++	+++	+++	+++	+++	+++	+++	+++
Between Chi									130.47
Between df									7
Between P									***
Btwn(F) P									***
Btwn(R) P									***

Table 2A1 - 6

IESLC - Meta-analysis of Ever Smoking, Any product (or Cigarettes if Any not available)
Squamous
Least adjusted

	Detailed Country in "other Europe"					
	multi	Germany	othWest	East	Balkans	Total
N	2	2	2	7	2	15
NS	1	2	2	5	2	12
Wt	98.39	4.65	46.22	15.63	46.25	211.15
Het Chi	27.46	0.64	13.87	8.07	0.82	106.77
Het df	1	1	1	6	1	14
Het P	***	N.S.	***	N.S.	N.S.	***
Fixed RR	10.10	17.25	3.31	10.54	4.31	6.66
RRl	8.29	6.95	2.48	6.42	3.23	5.82
RRu	12.31	42.81	4.42	17.31	5.74	7.63
P	+++	+++	+++	+++	+++	+++
Random RR	9.82	17.25	5.96	11.20	4.31	8.94
RRl	3.48	6.95	1.16	5.91	3.23	5.60
RRu	27.71	42.81	30.71	21.22	5.74	14.26
P	+++	+++	+	+++	+++	+++
Between Chi						55.90
Between df						4
Between P						***
Btwn(F) P						(*)
Btwn(R) P						**

	Detailed Country in "other Asia"			
	India	HongKong	other	Total
N	1	7	4	12
NS	1	5	3	9
Wt	10.45	51.28	21.79	83.52
Het Chi	0.00	5.68	12.42	37.39
Het df	0	6	3	11
Het P	N.S.	N.S.	**	***
Fixed RR	25.43	6.43	5.32	7.27
RRl	13.87	4.89	3.49	5.86
RRu	46.63	8.45	8.09	9.01
P	+++	+++	+++	+++
Random RR	25.43	6.43	6.00	7.61
RRl	13.87	4.89	2.53	5.06
RRu	46.63	8.45	14.26	11.45
P	+++	+++	+++	+++
Between Chi				19.29
Between df				2
Between P				***
Btwn(F) P				*
Btwn(R) P				***

	Detailed other continent			
	SCAmer	Auslia	Africa	Total
N	6			6
NS	5			5
Wt	17.58			17.58
Het Chi	4.65			4.65
Het df	5			5
Het P	N.S.			N.S.
Fixed RR	15.93			15.93
RRl	9.98			9.98
RRu	25.42			25.42
P	+++			+++
Random RR	15.93			15.93
RRl	9.98			9.98
RRu	25.42			25.42
P	+++			+++
Between Chi				
Between df				
Between P				N.S.
Btwn(F) P				N.S.
Btwn(R) P				N.S.

Table 2A1 - 6

IESLC - Meta-analysis of Ever Smoking, Any product (or Cigarettes if Any not available)
Squamous
Least adjusted

	Start year of study <1960	1960-69	1970-79	1980-89	1990+	Total
N	14	14	26	40	8	102
NS	10	12	16	27	8	73
Wt	123.61	107.60	255.53	500.36	36.47	1023.57
Het Chi	53.12	67.26	127.03	162.38	25.74	527.92
Het df	13	13	25	39	7	101
Het P	***	***	***	***	***	***
Fixed RR	4.46	11.62	8.85	11.36	9.27	9.49
RRl	3.74	9.62	7.83	10.41	6.70	8.93
RRu	5.32	14.04	10.01	12.40	12.82	10.09
P	+++	+++	+++	+++	+++	+++
Random RR	6.11	11.81	10.21	11.79	10.21	10.37
RRl	4.00	7.38	7.35	9.43	5.44	8.83
RRu	9.33	18.91	14.17	14.74	19.18	12.18
P	+++	+++	+++	+++	+++	+++
Between Chi						92.40
Between df						4
Between P						***
Btwn(F) P						***
Btwn(R) P						N.S.

	Study type (1) CC	other	Total
N	93	9	102
NS	66	7	73
Wt	997.37	26.20	1023.57
Het Chi	514.62	11.86	527.92
Het df	92	8	101
Het P	***	N.S.	***
Fixed RR	9.43	11.95	9.49
RRl	8.86	8.15	8.93
RRu	10.04	17.53	10.09
P	+++	+++	+++
Random RR	10.26	11.98	10.37
RRl	8.67	7.32	8.83
RRu	12.13	19.61	12.18
P	+++	+++	+++
Between Chi			1.43
Between df			1
Between P			N.S.
Btwn(F) P			N.S.
Btwn(R) P			N.S.

	Study type (2) CC	prosp	other	Total
N	93	5	4	102
NS	66	4	3	73
Wt	997.37	16.77	9.43	1023.57
Het Chi	514.62	9.38	2.48	527.92
Het df	92	4	3	101
Het P	***	(*)	N.S.	***
Fixed RR	9.43	12.01	11.85	9.49
RRl	8.86	7.44	6.26	8.93
RRu	10.04	19.38	22.43	10.09
P	+++	+++	+++	+++
Random RR	10.26	12.78	11.85	10.37
RRl	8.67	5.85	6.26	8.83
RRu	12.13	27.90	22.43	12.18
P	+++	+++	+++	+++
Between Chi				1.43
Between df				2
Between P				N.S.
Btwn(F) P				N.S.
Btwn(R) P				N.S.

Table 2A1 - 6

IESLC - Meta-analysis of Ever Smoking, Any product (or Cigarettes if Any not available)
Squamous
Least adjusted

	Study size (number of LC cases)				
	100-249	250-499	500-999	1000+	Total
N	22	31	18	31	102
NS	21	22	12	18	73
Wt	103.68	131.95	129.38	658.56	1023.57
Het Chi	51.26	60.12	67.34	320.94	527.92
Het df	21	30	17	30	101
Het P	***	***	***	***	***
Fixed RR	6.08	9.16	8.56	10.46	9.49
RRl	5.02	7.72	7.20	9.69	8.93
RRu	7.38	10.86	10.17	11.29	10.09
P	+++	+++	+++	+++	+++
Random RR	7.97	10.39	11.66	10.94	10.37
RRl	5.71	8.01	8.01	8.22	8.83
RRu	11.12	13.46	16.98	14.56	12.18
P	+++	+++	+++	+++	+++
Between Chi					28.26
Between df					3
Between P					***
Btwn(F) P					N.S.
Btwn(R) P					N.S.

	Risky occupational population			
	no	mining	othRisky	Total
N	100	1	1	102
NS	71	1	1	73
Wt	1018.14	3.73	1.70	1023.57
Het Chi	526.81	0.00	0.00	527.92
Het df	99	0	0	101
Het P	***	N.S.	N.S.	***
Fixed RR	9.51	6.33	5.55	9.49
RRl	8.95	2.29	1.24	8.93
RRu	10.11	17.45	24.95	10.09
P	+++	+++	+	+++
Random RR	10.47	6.33	5.55	10.37
RRl	8.90	2.29	1.24	8.83
RRu	12.32	17.45	24.95	12.18
P	+++	+++	+	+++
Between Chi				1.11
Between df				2
Between P				N.S.
Btwn(F) P				N.S.
Btwn(R) P				N.S.

	National cigarette tobacco type			
	Virginia	blended	other	Total
N	9	80	13	102
NS	6	58	9	73
Wt	45.34	849.61	128.62	1023.57
Het Chi	40.38	403.38	26.08	527.92
Het df	8	79	12	101
Het P	***	***	*	***
Fixed RR	13.77	10.20	5.17	9.49
RRl	10.29	9.53	4.35	8.93
RRu	18.43	10.91	6.15	10.09
P	+++	+++	+++	+++
Random RR	14.45	11.06	5.64	10.37
RRl	7.30	9.24	4.24	8.83
RRu	28.60	13.23	7.49	12.18
P	+++	+++	+++	+++
Between Chi				58.07
Between df				2
Between P				***
Btwn(F) P				**
Btwn(R) P				***

Table 2A1 - 6

IESLC - Meta-analysis of Ever Smoking, Any product (or Cigarettes if Any not available)
Squamous
Least adjusted

	Any proxy use No/nk	Yes	Total
N	76	26	102
NS	55	18	73
Wt	884.93	138.64	1023.57
Het Chi	434.63	71.59	527.92
Het df	75	25	101
Het P	***	***	***
Fixed RR	8.96	13.71	9.49
RRl	8.39	11.61	8.93
RRu	9.57	16.19	10.09
P	+++	+++	+++
Random RR	9.58	13.26	10.37
RRl	7.97	9.73	8.83
RRu	11.52	18.08	12.18
P	+++	+++	+++
Between Chi			21.70
Between df			1
Between P			***
Btwn(F) P			*
Btwn(R) P			(*)

	Full histological confirmation No	Yes	Total
N	59	43	102
NS	43	30	73
Wt	471.36	552.21	1023.57
Het Chi	282.62	176.48	527.92
Het df	58	42	101
Het P	***	***	***
Fixed RR	7.17	12.06	9.49
RRl	6.55	11.09	8.93
RRu	7.84	13.11	10.09
P	+++	+++	+++
Random RR	9.28	12.26	10.37
RRl	7.48	9.82	8.83
RRu	11.52	15.30	12.18
P	+++	+++	+++
Between Chi			68.82
Between df			1
Between P			***
Btwn(F) P			***
Btwn(R) P			(*)

	Number of adjustment variables (1) 0	1	2+/+nk	Total
N	88	2	12	102
NS	63	2	9	74
Wt	655.02	23.39	345.16	1023.57
Het Chi	387.90	1.54	78.94	527.92
Het df	87	1	11	101
Het P	***	N.S.	***	***
Fixed RR	8.09	26.31	11.97	9.49
RRl	7.50	17.54	10.77	8.93
RRu	8.74	39.46	13.31	10.09
P	+++	+++	+++	+++
Random RR	10.09	22.77	11.37	10.37
RRl	8.42	10.29	7.87	8.83
RRu	12.08	50.40	16.43	12.18
P	+++	+++	+++	+++
Between Chi				59.54
Between df				2
Between P				***
Btwn(F) P				**
Btwn(R) P				N.S.

Table 2A1 - 6

IESLC - Meta-analysis of Ever Smoking, Any product (or Cigarettes if Any not available)
Squamous
Least adjusted

	Number of adjustment variables (2) 0	1	2	3-5	6+/+nk	Total
N	88	2	8	3	1	102
NS	63	2	6	3	1	75
Wt	655.02	23.39	281.37	61.05	2.73	1023.57
Het Chi	387.90	1.54	35.59	17.90	0.00	527.92
Het df	87	1	7	2	0	101
Het P	***	N.S.	***	***	N.S.	***
Fixed RR	8.09	26.31	13.63	6.78	6.45	9.49
RRl	7.50	17.54	12.12	5.28	1.97	8.93
RRu	8.74	39.46	15.31	8.72	21.11	10.09
P	+++	+++	+++	+++	++	+++
Random RR	10.09	22.77	13.70	8.72	6.45	10.37
RRl	8.42	10.29	9.25	3.84	1.97	8.83
RRu	12.08	50.40	20.31	19.80	21.11	12.18
P	+++	+++	+++	+++	++	+++
Between Chi						84.99
Between df						4
Between P						***
Btwn(F) P						**
Btwn(R) P						N.S.

	Product all/unsp	cig+/-ot	cig only	Total
N	54	46	2	102
NS	41	31	2	74
Wt	335.91	680.41	7.25	1023.57
Het Chi	219.32	246.80	0.12	527.92
Het df	53	45	1	101
Het P	***	***	N.S.	***
Fixed RR	6.91	10.93	38.79	9.49
RRl	6.21	10.14	18.74	8.93
RRu	7.69	11.78	80.31	10.09
P	+++	+++	+++	+++
Random RR	8.86	11.78	38.79	10.37
RRl	6.98	9.54	18.74	8.83
RRu	11.24	14.55	80.31	12.18
P	+++	+++	+++	+++
Between Chi				61.67
Between df				2
Between P				***
Btwn(F) P				**
Btwn(R) P				***

	Denominator nev any	nev cigs	Total
N	64	38	102
NS	48	26	74
Wt	482.77	540.80	1023.57
Het Chi	290.37	213.89	527.92
Het df	63	37	101
Het P	***	***	***
Fixed RR	8.08	10.96	9.49
RRl	7.39	10.07	8.93
RRu	8.83	11.92	10.09
P	+++	+++	+++
Random RR	9.59	11.80	10.37
RRl	7.75	9.21	8.83
RRu	11.87	15.13	12.18
P	+++	+++	+++
Between Chi			23.66
Between df			1
Between P			***
Btwn(F) P			*
Btwn(R) P			N.S.

Table 2A1 - 6

IESLC - Meta-analysis of Ever Smoking, Any product (or Cigarettes if Any not available)
Squamous
Least adjusted

	Derivation of RR/CI			
	Orig	StdCalc	Other	Total
N	12	75	15	102
NS	9	56	12	77
Wt	373.71	609.55	40.31	1023.57
Het Chi	117.54	357.59	25.52	527.92
Het df	11	74	14	101
Het P	***	***	*	***
Fixed RR	11.52	8.30	12.02	9.49
RRl	10.41	7.66	8.83	8.93
RRu	12.75	8.98	16.37	10.09
P	+++	+++	+++	+++
Random RR	10.19	10.04	13.75	10.37
RRl	6.78	8.29	8.47	8.83
RRu	15.32	12.15	22.33	12.18
P	+++	+++	+++	+++
Between Chi				27.28
Between df				2
Between P				***
Btwn(F) P				(*)
Btwn(R) P				N.S.

Table 2A1 - 7

IESLC - Meta-analysis of Ever Smoking, Any product (or Cigarettes if Any not available)
Squamous
Excluded studies (and stage at which they were excluded)

1	BOUCHA BUELL LAURIL MZILEN
2	BYERS2 ENSTRO HIRAY2 KANELL LIAW LICKIN MRFIT MURATA NOTANI RESTRE RIMING SAARIK SEGI2 SHIMIZ SITAS SOBUE2 TANG2 WALD WARSIN WATSON WU2 WYNDE5 WYNDE8 WYNDER
3	BERRIN CHEN TAO ULMER
6	BLOT1 BLOT2 BLOT3 HIRAOK JONES MOLLO PERSHA SCHWA2 VANDER
8	ABELIN AGUDO AKIBA AMANDU AMES ARCHER ARMADA AUSTIN AUVINE AXELSO AXELSS BENSHL BEST BLOHMK BLOT4 BOFFET BOUCOT BRETT BROCKM BROSS BROWN1 CARPEN CASCO2 CASCOR CEDERL CHANG CHATZI CHEN2 CHEN3 CHIAZZ CHOW CHYOU COOKSO CPSI CPSII DARBY DAVEYS DEAN DEAN2 DEAN3 DEKLER DESTEF DOCKER DOLL2 DORANT DORN DROSTE DU DUNN EBELIN ESAKI GAO2 GARCIA GARDIN GARSHI GENG GILLIS GODLEY GOLLED GOODMA GRAHAM GREGOR GSELL HAMMO2 HANSEN HEIN HENNEK HIRAYA HITOSU HOLE HOROWI HORWIT HU HU2 HUANG HUMBLE JARUP JARVHO JIANG KAISE2 KAISER KAUFMA KELLER KINLEN KJUUS KNEKT KO KOHLME KOULUM KREUZE KUBIK LANGE LAUSSM LEI LEMARC LETOUR LEVIN LIDDEL LIU LIU2 LIU3 LIU4 LIU5 LOMBAR MACLEN MAGNUS MARSH MARSH2 MARTIS MASTRA MCCONN MCDUFF MCLAUG MIGRAN MILLER MILLS MRFITR NAM NOTAN2 ODRISC PARKIN PASTOR PAWLEG PERNU PERSH2 PETO PEZZO2 PIKE PISANI POFFIJ POLEDN PRESCO QIAO QIAO2 RACHTA RADZIK RANDIG REN RONCO ROOTS ROTHSC SADOWS SANKAR SEGI SHAW SIMARA SIMONA SPEIZE SPITZ STOCKS STOCKW STUCKE SUN SUZUKI TANG TENKAN TOUSEY TULINI TVERDA VEIERO WANG WANG2 WANG3 WANG4 WICKLU WIGLE WILKIN WUNSCH XIANGZ XU XU2 XU4 YAMAGU YONG YUAN ZHANG
10	BENHAM VUTUC WYNDE7

Table 2A1 - 8
Potentially overlapping studies

REF	REFGP	PRINC	. OVERLAP/LINK
LUBIN2	LUBIN2	1	Lubin-combined
CHAN	CHAN	1	Chan/LamWK2
LAMWK2	LAMWK2	1	Chan/LamWK2
LAMTH	LAMTH	1	KOO/LAMTH/LAMWK
KOO	KOO	1	KOO/LAMTH/LAMWK
LAMWK	LAMWK	1	KOO/LAMTH/LAMWK
ISHIMA	AKIBA	2	AKIBA/ISHIMA
BYERS1	BYERS1	2	GRAHAM/BROSS/BYERS1
OSANN2	KAISER	2	KAISER/OSANN2
WYNDE6	WYNDE6	1	WYNDE5/6/7/8
MATSUD	MATSUD	1	SOBUE2/MATSUD
JAHN	BOFFET	2	Subset of BOFFET
LUBIN	XIANGZ	2	LUBIN/XIANGZ/QIAO

Table 2A1 - 9
Most adjusted - insufficient data for metaanalysis

REF	NRR	SEX	AGEL	AGEH	RACE	YF	LC TYPE	LOC	START	ST	NLC	R	VB	P	H	AD	PRODUCT	DENOM	De
BUFFLE	47	f	0	0	wh	-	q	NAmer	1976	CC	943	n	bl	y	n	0	cig+/-ot	nev cigs	or
CHEN	1	c	0	0	all	-	q	As:oth	1987	CC	323	n	ot	n	y	2	cig+/-ot	nev cigs	ot
JIANG	3	m	0	0	all	-	q	As:Chi	1984	CC	125	n	ot	n	n	0	all/unsp	nev any	or
JIANG	4	f	0	0	all	-	q	As:Chi	1984	CC	125	n	ot	n	n	0	all/unsp	nev any	ot
KOO	2	f	0	0	all	-	q	As:HK	1981	CC	200	n	bl	n	n	0	all/unsp	nev any	or
WANG	3	c	0	0	all	-	q	As:Chi	1990	CC	390	n	ot	*	y	0	all/unsp	nev any	ot

REF	NRR	RR	SIG	RRDATA comment
BUFFLE	47	8.30		0
CHEN	1	6.66	y	P < 0.001
JIANG	3	12.78		0
JIANG	4	*	n	0
KOO	2	3.62		0
WANG	3	4.66	y	p<0.01

Table 2A2 -

IESLC - Meta-analysis of Ever Smoking, Cigarettes (or Any Product if Cigarettes not available)
Squamous

This analysis is restricted to results for:
1) Non-dose-response data
2) Ever smokers
3) Results complete enough for use in metaanalysis

Within each study, results are then selected (in the following order of preference, within each sex) for:
4) PRODUCT: cigarettes regardless of other products, cigarettes only, all/unspec
5) CIGTYPE: all/unspecified, MC regardless of HR, MC only
6) DENOM: never smoked anything, never smoked cigarettes, (never +1 = +long term ex, +2 = +amount unknown, +3 = never cigs+long term ex)
7) Followup period (YF, prospective studies): whole study (coded as 0) or longest available
8) LCtype: squamous or nearest available, but not adeno. (q = squamous, s = small, a = adeno, KI = Kreyberg I, u = undifferentiated)
9) Race: all or nearest available, otherwise by race (wh or w = white, bl or b = black, hi = hispanic ch = chinese, jap = japanese, haw = hawaiian, w+o = white + oriental, sca = scandinavian, as = asian)
10) For overlapping studies: principal rather than subsidiary studies
Finally by Age: whole study (coded as 0) if available, otherwise by widest available age group
and then for single sex results (m, f) in preference to combined sex results (c).

Results adjusted (AD) for the most potential confounders are then chosen in Sections -1 to -3
(and those which actually differ from the adjusted results in Table 2A1 - 1 are marked 'x' in Section -1)
and results adjusted for the least confounders in Sections -4 to -6. (Those least adjusted results which
actually differ from the most adjusted as marked 'x' in column X in Section -4)
(Results adjusted for an unknown number of confounder(s) are coded as 20.)

Section -7 shows excluded studies, together with the stage (as above) at which no qualifying
results were found.

Section -8 lists the potentially overlapping studies which have been included (1=principal, 2=subsidiary).

Section -9 lists any results which would have been included in preference except that they had data not complete
enough for use in meta-analysis, with their significance (yes/no), if known, and any further comment as entered
on the database.

In addition to those mentioned above, the following fields, levels and abbreviations are used:
* or nk = not known, n = no, y = yes, ot = other
nev = never
all/unspec = all or unspecified, cig+/-ot = cigarettes irrespective of other products (cigar, pipe etc)
MC = manufactured cigarettes, HR = hand-rolled cigarettes
REF: 6-character study reference
NRR: number of the RR on the database within the study
ST : study type (CC = case control, pr or prosp = prospective)
NLC: number of lung cancer cases in whole study
R : risky occupational population (n = no, m = mining, o = other risky)
VB : national cigarette type (V = at least 75% Virginia, bl = at least 75% blended, ot = other)
P : any proxy use
H : full histological confirmation
De : derivation of RR/CI (or = original, st = standard method, ot = other method of estimation)

Table 2A2 - 1

IESLC - Meta-analysis of Ever Smoking, Cigarettes (or Any Product if Cigarettes not available)
Squamous
Most adjusted

REF	NRR	2A1	SEX	AGEL	AGEH	RACE	YF	LC TYPE	LOC	START	ST	NLC	R	VB	P	H	AD	PRODUCT	DENOM	De
ABRAHA	1		m	0	0	all	0	q	Eu:est	1975	pr	571	n	bl	n	n	0	all/unsp	nev any	ot
ABRAHA	4		f	0	0	all	0	q	Eu:est	1975	pr	571	n	bl	n	n	0	all/unsp	nev any	ot
ALDERS	75	x	m	0	0	all	-	q+s	Eu:UK	1977	CC	1448	n	V	n	n	1	cig+/-ot	nev any	ot
ALDERS	33	x	f	0	0	all	-	q+s	Eu:UK	1977	CC	1448	n	V	n	n	1	cig only	nev any	ot
ANDERS	10		f	0	0	all	0	q	NAmer	1986	pr	343	n	bl	n	n	0	cig+/-ot	nev cigs	st
BAND	5		m	0	0	all	-	q	NAmer	1983	CC	2831	n	V	y	y	2	cig only	nev any	ot
BARBON	127		m	0	0	all	-	q	Eu:wst	1979	CC	755	n	bl	y	y	3	all/unsp	nev any	ot
BECHER	11		f	0	0	all	-	q+s	Eu:Ger	1985	CC	194	n	bl	n	y	1	all/unsp	nev any	or
BRESLO	7	x	c	0	0	all	-	not a	NAmer	1949	CC	518	n	bl	n	y	0	cig+/-ot	nev+1	st
BROWN2	6		m	0	0	wh	-	q	NAmer	1984	CC	14596	n	bl	n	y	2	cig+/-ot	nev cigs	or
BROWN2	5		f	0	0	wh	-	q	NAmer	1984	CC	14596	n	bl	n	y	2	cig+/-ot	nev cigs	or
BUFFLE	49		m	0	0	wh	-	q	NAmer	1976	CC	943	n	bl	y	n	0	cig+/-ot	nev cigs	ot
BUFFLE	62		f	0	0	w-hi	-	q	NAmer	1976	CC	943	n	bl	y	n	0	cig+/-ot	nev cigs	st
BYERS1	1		m	0	0	wh	-	q	NAmer	1957	CC	1002	n	bl	n	n	0	cig+/-ot	nev cigs	st
CHAN	18	x	m	0	0	all	-	q+s	As:HK	1976	CC	397	n	bl	n	n	0	cig+/-ot	nev any	st
CHAN	22	x	f	0	0	all	-	q+s	As:HK	1976	CC	397	n	bl	n	n	0	cig+/-ot	nev any	st
CHOI	62		m	0	0	all	-	q	As:oth	1985	CC	375	n	bl	n	n	0	cig+/-ot	nev cigs	st
CHOI	64		f	0	0	all	-	q	As:oth	1985	CC	375	n	bl	n	n	0	cig+/-ot	nev cigs	st
COMSTO	66		m	0	0	all	-	q	NAmer	1975	ot	258	n	bl	n	n	0	cig+/-ot	nev cigs	st
COMSTO	78		f	0	0	all	-	q	NAmer	1975	ot	258	n	bl	n	n	0	cig+/-ot	nev cigs	ot
CORREA	35		c	0	0	all	-	q+s	NAmer	1979	CC	1359	n	bl	y	n	1	cig+/-ot	nev cigs	or
DAMBER	33		m	0	0	all	-	q	Eu:Sca	1972	CC	579	n	bl	y	n	1	all/unsp	nev any	or
DESTE2	16		m	0	0	all	-	q	SCAmer	1993	CC	463	n	bl	n	n	2	all/unsp	nev any	or
DOLL	86		m	0	0	all	-	KI	Eu:UK	1948	CC	1465	n	V	n	n	1	all/unsp	nev any	ot
DOLL	88		f	0	0	all	-	KI	Eu:UK	1948	CC	1465	n	V	n	n	1	all/unsp	nev any	ot
DORGAN	113		m	0	0	wh	-	q	NAmer	1980	CC	2026	n	bl	y	y	2	cig+/-ot	nev any	or
DORGAN	98		f	0	0	all	-	q	NAmer	1980	CC	2026	n	bl	y	y	3	cig+/-ot	nev any	or
DOSEME	3		m	0	0	all	-	q	Eu:bal	1979	CC	1210	n	bl	n	n	2	cig+/-ot	nev cigs	or
ENGELA	62		m	0	0	all	0	q	Eu:Sca	1964	pr	435	n	bl	n	n	7	cig+/-ot	nev cigs	ot
FAN	3		c	0	0	all	-	q	As:Chi	1990	CC	403	n	ot	y	n	0	cig+/-ot	nev cigs	ot
GAO	2		m	0	0	all	-	q	As:Chi	1984	CC	1405	n	ot	n	n	2	cig+/-ot	nev cigs	or
GAO	12		f	0	0	all	-	q	As:Chi	1984	CC	1405	n	ot	n	n	2	cig+/-ot	nev cigs	or
GER	13		c	0	0	all	-	q+s	As:oth	1990	CC	141	n	ot	y	n	10	all/unsp	nev any	ot
HAENSZ	22	x	f	0	0	all	-	q+u	NAmer	1955	CC	158	n	bl	n	y	0	cig+/-ot	nev any	st
HAMMON	59	x	m	0	0	wh	0	not a	NAmer	1952	pr	448	n	bl	n	n	1	cig+/-ot	nev any	ot
HEGMAN	2		c	0	0	all	-	q	NAmer	1989	CC	282	n	bl	y	y	0	all/unsp	nev any	st
HINDS	23		f	0	0	o	-	q+s	NAmer	1968	CC	292	n	bl	n	n	3	all/unsp	nev any	st
ISHIMA	6		c	0	0	all	-	q	As:Jap	1961	CC	180	n	bl	y	y	5	all/unsp	nev any	st
JAHN	42	x	m	0	0	all	-	q	Eu:Ger	1988	CC	1004	n	bl	n	n	0	cig+/-ot	nev any	st
JAIN	48		m	0	0	all	-	q	NAmer	1981	CC	845	n	V	y	n	2	cig+/-ot	nev cigs	or
JAIN	43		f	0	0	all	-	q	NAmer	1981	CC	845	n	V	y	n	2	cig+/-ot	nev cigs	or
JEDRYC	54		m	0	0	all	-	q	Eu:est	1980	CC	1630	n	bl	y	n	3	cig+/-ot	nev any	ot
JOLY	54		m	0	0	all	-	q	SCAmer	1978	CC	826	n	bl	n	n	0	cig+/-ot	nev any	st
JOLY	52		f	0	0	all	-	q	SCAmer	1978	CC	826	n	bl	n	n	0	cig+/-ot	nev any	st
JUSSAW	25	x	m	0	0	all	-	KI	As:Ind	1964	CC	792	n	V	n	n	0	cig only	nev any	st
KATSOU	37		f	0	0	all	-	KI	Eu:bal	1987	CC	101	n	bl	n	n	1	all/unsp	nev any	ot
KHUDER	24		m	0	0	all	-	q	NAmer	1985	CC	482	n	bl	n	y	0	cig+/-ot	nev cigs	ot
KIHARA	26		c	0	0	jap	-	q	As:Jap	1991	CC	440	n	bl	n	n	0	all/unsp	nev any	st
KOO	6		f	0	0	all	-	q+s	As:HK	1981	CC	200	n	bl	n	n	0	all/unsp	nev any	st
KREYBE	4		m	0	0	all	-	KI	Eu:Sca	1948	CC	300	n	bl	n	y	1	all/unsp	nev any	ot
KREYBE	25		f	0	0	all	-	KI	Eu:Sca	1948	CC	300	n	bl	n	y	1	all/unsp	nev any	ot
LAMTH	1		f	0	0	ch	-	q	As:HK	1983	CC	445	n	bl	n	n	0	all/unsp	nev any	or
LAMWK	2		f	0	0	ch	-	q	As:HK	1981	CC	163	n	bl	n	n	0	all/unsp	nev any	st
LAMWK2	1		m	0	0	all	-	q	As:HK	1976	CC	480	n	bl	n	n	0	all/unsp	nev any	st
LAMWK2	5		f	0	0	all	-	q	As:HK	1976	CC	480	n	bl	n	n	0	all/unsp	nev any	st
LOMBA2	2		f	0	0	all	-	q+u	NAmer	1960	CC	225	n	bl	n	n	0	cig+/-ot	nev cigs	st
LUBIN	34	x	m	0	0	all	-	KI	As:Chi	1984	CC	427	m	ot	y	n	0	cig+/-ot	nev any	st
LUBIN2	145		m	0	0	all	-	q	Eu:mul	1976	CC	7804	n	bl	n	y	0	cig+/-ot	nev any	st
LUBIN2	165		f	0	0	all	-	q	Eu:mul	1976	CC	7804	n	bl	n	y	0	cig+/-ot	nev any	st
LUO	8		c	0	0	all	-	q	As:Chi	1990	CC	102	n	ot	n	y	20	cig+/-ot	nev cigs	or
MATOS	67		m	0	0	all	-	q	SCAmer	1994	CC	200	n	bl	n	n	2	cig+/-ot	nev any	ot
MATSUD	11		m	0	0	all	-	q	As:Jap	1965	CC	179	n	bl	n	n	0	cig+/-ot	nev cigs	st
NOU	1		m	0	0	all	-	q	Eu:Sca	1971	CC	273	n	bl	y	n	0	all/unsp	nev any	st
NOU	6		f	0	0	all	-	q	Eu:Sca	1971	CC	273	n	bl	y	n	0	all/unsp	nev any	st
ORMOS	8		m	0	0	all	-	q	Eu:est	1947	CC	119	n	bl	y	y	0	cig+/-ot	nev any	st
OSANN	43		m	0	0	all	-	q	NAmer	1984	CC	1986	n	bl	n	n	2	cig+/-ot	nev cigs	or
OSANN	44		f	0	0	all	-	q	NAmer	1984	CC	1986	n	bl	n	n	2	cig+/-ot	nev cigs	or
OSANN2	25		f	0	0	all	-	KI	NAmer	1964	ot	217	n	bl	n	y	1	cig+/-ot	nev cigs	or
PEZZOT	6		m	0	0	all	-	q	SCAmer	1987	CC	215	n	bl	n	y	0	cig only	nev cigs	ot
SCHWAR	10		m	40	54	wh	-	q	NAmer	1984	CC	5588	n	bl	y	y	0	cig+/-ot	nev cigs	st
SCHWAR	9		m	40	54	bl	-	q	NAmer	1984	CC	5588	n	bl	y	y	0	cig+/-ot	nev cigs	st
SCHWAR	18		f	40	54	wh	-	q	NAmer	1984	CC	5588	n	bl	y	y	0	cig+/-ot	nev cigs	ot

Table 2A2 - 1

IESLC - Meta-analysis of Ever Smoking, Cigarettes (or Any Product if Cigarettes not available)
Squamous
Most adjusted

REF	NRR	2A1	SEX	AGEL	AGEH	RACE	YF	LC TYPE	LOC	START	ST	NLC	R	VB	P	H	AD	PRODUCT	DENOM	De
SCHWAR	17		f	40	54	bl	-	q	NAmer	1984	CC	5588	n	bl	y	y	0	cig+/-ot	nev cigs	ot
SEOW	3		f	0	0	ch	-	q	As:oth	1997	CC	153	n	bl	n	y	0	cig+/-ot	nev cigs	st
SIEMIA	7		m	0	0	all	-	q	NAmer	1979	CC	857	n	V	y	y	7	cig+/-ot	nev cigs	or
SOBUE	97		m	0	0	all	-	q	As:Jap	1986	CC	1376	n	bl	n	y	1	cig+/-ot	nev cigs	ot
SOBUE	107		f	0	0	all	-	q	As:Jap	1986	CC	1376	n	bl	n	y	1	cig+/-ot	nev cigs	ot
STASZE	16	x	m	0	0	all	-	q	Eu:est	1954	CC	281	n	bl	n	y	0	cig+/-ot	nev any	ot
STASZE	38		f	0	0	all	-	q	Eu:est	1954	CC	281	n	bl	n	y	0	all/unsp	nev any	ot
STAYNE	3		m	0	0	all	-	q	NAmer	1969	CC	420	n	bl	n	n	0	all/unsp	nev any	st
SUZUK2	15		c	0	0	all	-	q	SCAmer	1991	CC	123	n	bl	n	y	3	all/unsp	nev any	or
SVENSS	72		f	0	0	all	-	q	Eu:Sca	1983	CC	210	n	bl	n	n	1	all/unsp	nev any	ot
TIZZAN	18		c	0	0	all	-	q+u	Eu:wst	1959	CC	1358	n	bl	n	n	0	all/unsp	nev any	st
TOKARS	10		c	0	0	all	-	q	Eu:est	1966	ot	162	o	bl	n	y	3	all/unsp	nev any	or
TSUGAN	13		m	0	0	all	-	q	As:Jap	1976	CC	134	n	bl	n	y	0	all/unsp	nev any	ot
WAKAI	74		m	0	0	all	-	q	As:Jap	1988	CC	333	n	bl	n	y	1	all/unsp	nev any	ot
WAKAI	80		f	0	0	all	-	q	As:Jap	1988	CC	333	n	bl	n	y	1	all/unsp	nev any	ot
WU	32		f	0	0	wh	-	q	NAmer	1981	CC	220	n	bl	n	y	2	all/unsp	nev any	ot
WUWILL	9		f	0	0	all	-	q	As:Chi	1985	CC	965	n	ot	n	n	3	cig+/-ot	nev cigs	or
WYNDE2	2	x	m	0	0	all	-	KI	NAmer	1962	CC	404	n	bl	n	y	0	cig+/-ot	nev any	st
WYNDE3	8	x	m	0	0	all	-	KI	NAmer	1966	CC	350	n	bl	n	y	0	cig+/-ot	nev any	st
WYNDE3	67	x	f	0	0	all	-	KI	NAmer	1966	CC	350	n	bl	n	y	0	cig+/-ot	nev any	st
WYNDE4	69	x	m	0	0	all	-	not a	NAmer	1948	CC	684	n	bl	y	n	2	cig+/-ot	nev any	ot
WYNDE4	54		f	0	0	all	-	not a	NAmer	1948	CC	684	n	bl	y	n	2	all/unsp	nev any	ot
WYNDE6	75	x	m	0	0	all	-	KI	NAmer	1969	CC	4423	n	bl	n	y	0	cig+/-ot	nev any	st
WYNDE6	412		f	0	0	wh	-	q	NAmer	1969	CC	4423	n	bl	n	y	1	cig+/-ot	nev cigs	ot
XU3	20		m	0	0	all	-	KI	As:Chi	1981	CC	135	n	ot	n	n	1	all/unsp	nev any	ot
XU3	24		f	0	0	all	-	KI	As:Chi	1981	CC	135	n	ot	n	n	1	all/unsp	nev any	ot
ZHENG	5		m	0	0	all	-	q	As:Chi	1982	CC	540	n	ot	*	y	0	cig+/-ot	nev cigs	st
ZHENG	18		f	0	0	all	-	q	As:Chi	1982	CC	540	n	ot	*	y	0	cig+/-ot	nev cigs	st
ZHOU	8		m	0	0	all	-	q	As:Chi	1978	CC	1360	n	ot	n	n	0	all/unsp	nev any	st
ZHOU	9		f	0	0	all	-	q	As:Chi	1978	CC	1360	n	ot	n	n	0	all/unsp	nev any	st

Cigarette type is all/unspec for all RRs

except for the following:

REF	NRR	CIGTYPE
ALDERS	33	MC only
CHAN	18	MC+-HR
CHAN	22	MC+-HR
JUSSAW	25	MC only

Table 2A2 - 2

IESLC - Meta-analysis of Ever Smoking, Cigarettes (or Any Product if Cigarettes not available)
Squamous
Most adjusted

REF	NRR	SEX	AD	Number Exposed Case	Cont	Non-exposed Case	Cont	RR	95.00%CI
*ABRAHA	1	m	0	142	10351	0	3365	92.66~	(5.77-1488.21)
*ABRAHA	4	f	0	17	5256	7	11589	5.35	(2.22- 12.90)
Subtotal ABRAHA								6.95	(3.00- 16.06)
ALDERS	75	m	1	-	-	-	-	10.23	(3.78- 27.72)
ALDERS	33	f	1	-	-	-	-	6.70	(3.92- 11.46)
Subtotal ALDERS								7.37	(4.59- 11.82)
*ANDERS	10	f	0	63	96164	5	195158	25.57	(10.29- 63.56)
BAND	5	m	2	-	-	-	-	37.45	(17.62- 79.58)
BARBON	127	m	3	-	-	-	-	14.52	(6.35- 33.20)
BECHER	11	f	1	-	-	-	-	10.69	(2.43- 47.00)
BRESLO	7	c	0	444	394	15	56	4.21	(2.34- 7.56)
BROWN2	6	m	2	-	-	-	-	11.10	(9.50- 12.90)
BROWN2	5	f	2	-	-	-	-	20.10	(16.40- 24.80)
Subtotal BROWN2								13.69	(12.11- 15.49)
BUFFLE	49	m	0	-	-	-	-	14.03	(4.73- 41.61)
BUFFLE	62	f	0	58	166	3	112	13.04	(3.99- 42.66)
Subtotal BUFFLE								13.57	(6.09- 30.24)
BYERS1	1	m	0	299	695	22	424	8.29	(5.29- 13.00)
CHAN	18	m	0	112	160	2	43	15.05	(3.57- 63.41)
CHAN	22	f	0	37	38	19	139	7.12	(3.68- 13.77)
Subtotal CHAN								8.11	(4.45- 14.77)
CHOI	62	m	0	160	465	6	95	5.45	(2.34- 12.67)
CHOI	64	f	0	11	26	10	164	6.94	(2.68- 17.96)
Subtotal CHOI								6.06	(3.22- 11.40)
COMSTO	66	m	0	44	229	2	84	8.07	(1.91- 34.02)
COMSTO	78	f	0	17	87	0	115	46.20~	(2.74- 778.83)
Subtotal COMSTO								11.56	(3.21- 41.67)
CORREA	35	c	1	-	-	-	-	28.30	(18.60- 43.20)
DAMBER	33	m	1	-	-	-	-	11.80	(6.40- 23.00)
DESTE2	16	m	2	-	-	-	-	13.20	(4.70- 37.10)
DOLL	86	m	1	-	-	-	-	13.17	(4.12- 42.10)
DOLL	88	f	1	-	-	-	-	2.13	(1.06- 4.27)
Subtotal DOLL								3.45	(1.90- 6.27)
DORGAN	113	m	2	-	-	-	-	18.90	(7.00- 51.30)
DORGAN	98	f	3	-	-	-	-	11.10	(7.20- 17.10)
Subtotal DORGAN								12.08	(8.12- 17.96)
DOSEME	3	m	2	-	-	-	-	3.60	(2.60- 5.00)
*ENGELA	62	m	7	-	-	-	-	6.45	(1.97- 21.11)
FAN	3	c	0	75	595	6	556	11.68	(5.04- 27.04)
GAO	2	m	2	-	-	-	-	8.40	(4.70- 15.00)
GAO	12	f	2	-	-	-	-	7.20	(4.60- 11.10)
Subtotal GAO								7.62	(5.36- 10.82)
GER	13	c	10	-	-	-	-	3.19	(1.08- 9.42)
HAENSZ	22	f	0	56	103	44	236	2.92	(1.85- 4.61)
*HAMMON	59	m	1	-	-	-	-	21.66	(8.07- 58.14)
HEGMAN	2	c	0	89	1202	5	2080	30.80	(12.48- 76.03)
HINDS	23	f	3	-	-	-	-	16.13	(7.66- 33.97)
ISHIMA	6	c	5	-	-	-	-	21.00	(3.38- 868.40)
JAHN	42	m	0	343	671	3	138	23.51	(7.44- 74.35)
JAIN	48	m	2	-	-	-	-	18.00	(5.50- 111.00)
JAIN	43	f	2	-	-	-	-	25.50	(7.93- 156.00)
Subtotal JAIN								21.46	(7.45- 61.79)
JEDRYC	54	m	3	-	-	-	-	12.84	(5.58- 29.55)
JOLY	54	m	0	203	709	2	218	31.21	(7.69- 126.68)
JOLY	52	f	0	48	122	6	283	18.56	(7.74- 44.51)
Subtotal JOLY								21.47	(10.22- 45.09)
JUSSAW	25	m	0	17	77	13	624	10.60	(4.96- 22.66)
KATSOU	37	f	1	-	-	-	-	6.11	(2.69- 13.87)
KHUDER	24	m	0	176	-	9	-	7.82	(3.87- 15.77)
KIHARA	26	c	0	132	232	5	237	26.97	(10.84- 67.08)
KOO	6	f	0	61	63	32	137	4.15	(2.46- 6.98)
KREYBE	4	m	1	-	-	-	-	10.87	(3.47- 34.04)
KREYBE	25	f	1	-	-	-	-	2.29	(0.89- 5.88)
Subtotal KREYBE								4.31	(2.08- 8.92)
LAMTH	1	f	0	63	20	28	72	8.10	(4.16- 15.77)
LAMWK	2	f	0	21	41	7	144	10.54	(4.19- 26.52)
LAMWK2	1	m	0	129	161	5	43	6.89	(2.65- 17.90)
LAMWK2	5	f	0	35	50	15	139	6.49	(3.27- 12.88)
Subtotal LAMWK2								6.62	(3.79- 11.56)
LOMBA2	2	f	0	94	353	15	239	4.24	(2.40- 7.50)
LUBIN	34	m	0	291	788	4	72	6.65	(2.41- 18.36)

Table 2A2 - 2

IESLC - Meta-analysis of Ever Smoking, Cigarettes (or Any Product if Cigarettes not available)
Squamous
Most adjusted

REF	NRR	SEX	AD	Number Exposed Case	Number Exposed Cont	Non-exposed Case	Non-exposed Cont	RR	95.00%CI
LUBIN2	145	m	0	3587	10433	54	2616	16.66	(12.69- 21.86)
LUBIN2	165	f	0	200	567	72	1180	5.78	(4.34- 7.71)
Subtotal LUBIN2								10.10	(8.29- 12.31)
LUO	8	c	20	-	-	-	-	10.90	(2.50- 47.90)
MATOS	67	m	2	-	-	-	-	8.08	(2.59- 25.20)
MATSUD	11	m	0	103	3314	1	1255	39.01	(5.44- 279.84)
NOU	1	m	0	110	247	2	122	27.17	(6.60- 111.85)
NOU	6	f	0	5	92	2	261	7.09	(1.35- 37.19)
Subtotal NOU								15.42	(5.26- 45.22)
ORMOS	8	m	0	27	1034	2	777	10.14	(2.41- 42.79)
OSANN	43	m	2	-	-	-	-	36.10	(17.80- 73.30)
OSANN	44	f	2	-	-	-	-	26.40	(14.50- 48.10)
Subtotal OSANN								30.09	(19.04- 47.54)
OSANN2	25	f	1	-	-	-	-	35.10	(4.80- 256.00)
PEZZOT	6	m	0	85	317	0	116	62.74~	(3.86-1019.50)
SCHWAR	10	m	0	80	178	1	73	32.81	(4.48- 240.23)
SCHWAR	9	m	0	41	39	4	7	1.84	(0.50- 6.78)
SCHWAR	18	f	0	29	108	0	79	43.23~	(2.60- 718.15)
SCHWAR	17	f	0	21	28	0	41	62.61~	(3.64-1076.10)
Subtotal SCHWAR								7.71	(2.96- 20.10)
SEOW	3	f	0	21	15	10	125	17.50	(6.95- 44.09)
SIEMIA	7	m	7	-	-	-	-	22.70	(6.90- 75.20)
SOBUE	97	m	1	-	-	-	-	17.88	(7.82- 40.87)
SOBUE	107	f	1	-	-	-	-	8.74	(5.09- 15.02)
Subtotal SOBUE								10.83	(6.89- 17.03)
STASZE	16	m	0	135	653	0	158	65.73~	(4.07-1061.95)
STASZE	38	f	0	1	153	0	1660	32.45~	(1.32- 800.04)
Subtotal STASZE								48.53	(5.94- 396.73)
STAYNE	3	m	0	130	567	22	333	3.47	(2.17- 5.56)
SUZUK2	15	c	3	-	-	-	-	31.00	(4.20- 227.00)
SVENSS	72	f	1	-	-	-	-	12.62	(3.97- 40.14)
TIZZAN	18	c	0	333	939	55	419	2.70	(1.99- 3.67)
TOKARS	10	c	3	-	-	-	-	6.80	(1.20- 38.70)
TSUGAN	13	m	0	20	15	0	5	14.55~	(0.75- 283.37)
WAKAI	74	m	1	-	-	-	-	8.61	(2.08- 35.72)
WAKAI	80	f	1	-	-	-	-	25.23	(6.87- 92.66)
Subtotal WAKAI								15.46	(5.92- 40.36)
WU	32	f	2	-	-	-	-	24.29	(3.40- 173.76)
WUWILL	9	f	3	-	-	-	-	4.20	(3.00- 5.90)
WYNDE2	2	m	0	336	512	3	105	22.97	(7.23- 72.97)
WYNDE3	8	m	0	197	264	3	88	21.89	(6.82- 70.20)
WYNDE3	67	f	0	25	56	5	76	6.79	(2.45- 18.82)
Subtotal WYNDE3								11.28	(5.23- 24.31)
WYNDE4	69	m	2	-	-	-	-	15.45	(7.47- 31.96)
WYNDE4	54	f	2	-	-	-	-	5.82	(2.55- 13.31)
Subtotal WYNDE4								10.09	(5.85- 17.42)
WYNDE6	75	m	0	1706	1797	29	617	20.20	(13.84- 29.48)
WYNDE6	412	f	1	-	-	-	-	32.37	(17.66- 59.35)
Subtotal WYNDE6								23.05	(16.73- 31.78)
XU3	20	m	1	-	-	-	-	5.90	(1.69- 20.57)
XU3	24	f	1	-	-	-	-	25.67	(4.99- 131.94)
Subtotal XU3								10.14	(3.75- 27.37)
ZHENG	5	m	0	156	218	4	94	16.82	(6.05- 46.71)
ZHENG	18	f	0	43	44	33	184	5.45	(3.11- 9.54)
Subtotal ZHENG								7.07	(4.33- 11.56)
ZHOU	8	m	0	343	41	96	36	3.14	(1.90- 5.18)
ZHOU	9	f	0	35	7	42	32	3.81	(1.50- 9.68)
Subtotal ZHOU								3.28	(2.11- 5.10)
Partial Totals				11006	140856	730	227021		

*prospective study ~ With 0.5 adjustment for zero

Table 2A2 - 2

IESLC - Meta-analysis of Ever Smoking, Cigarettes (or Any Product if Cigarettes not available)
Squamous
Most adjusted

REF	NRR	SEX	AD	Ys	Ws	Qs	Ps
*ABRAHA	1	m	0	4.53	0.50	2.59	0.0014
*ABRAHA	4	f	0	1.68	4.97	1.62	0.0002
Subtotal ABRAHA				1.94	5.46	4.21	
ALDERS	75	m	1	2.33	3.87	0.02	0.0000
ALDERS	33	f	1	1.90	13.35	1.61	0.0000
Subtotal ALDERS				2.00	17.22	1.63	
*ANDERS	10	f	0	3.24	4.63	4.56	0.0000
BAND	5	m	2	3.62	6.76	12.76	0.0000
BARBON	127	m	3	2.68	5.62	1.02	0.0000
BECHER	11	f	1	2.37	1.75	0.03	0.0017
BRESLO	7	c	0	1.44	11.20	7.39	0.0000
BROWN2	6	m	2	2.41	164.17	4.09	0.0000
BROWN2	5	f	2	3.00	89.84	50.76	0.0000
Subtotal BROWN2				2.62	254.01	54.86	
BUFFLE	49	m	0	2.64	3.25	0.50	0.0000
BUFFLE	62	f	0	2.57	2.74	0.28	0.0000
Subtotal BUFFLE				2.61	5.99	0.78	
BYERS1	1	m	0	2.12	19.01	0.34	0.0000
CHAN	18	m	0	2.71	1.86	0.40	0.0002
CHAN	22	f	0	1.96	8.84	0.72	0.0000
Subtotal CHAN				2.09	10.69	1.12	
CHOI	62	m	0	1.70	5.39	1.65	0.0001
CHOI	64	f	0	1.94	4.25	0.41	0.0001
Subtotal CHOI				1.80	9.63	2.07	
COMSTO	66	m	0	2.09	1.86	0.05	0.0045
COMSTO	78	f	0	3.83	0.48	1.21	0.0078
Subtotal COMSTO				2.45	2.34	1.26	
CORREA	35	c	1	3.34	21.64	25.89	0.0000
DAMBER	33	m	1	2.47	9.39	0.45	0.0000
DESTE2	16	m	2	2.58	3.60	0.39	0.0000
DOLL	86	m	1	2.58	2.84	0.31	0.0000
DOLL	88	f	1	0.76	7.91	17.64	0.0334
Subtotal DOLL				1.24	10.76	17.95	
DORGAN	113	m	2	2.94	3.87	1.84	0.0000
DORGAN	98	f	3	2.41	20.54	0.51	0.0000
Subtotal DORGAN				2.49	24.41	2.36	
DOSEME	3	m	2	1.28	35.93	33.68	0.0000
*ENGELA	62	m	7	1.86	2.73	0.40	0.0021
FAN	3	c	0	2.46	5.45	0.24	0.0000
GAO	2	m	2	2.13	11.41	0.17	0.0000
GAO	12	f	2	1.97	19.80	1.50	0.0000
Subtotal GAO				2.03	31.21	1.66	
GER	13	c	10	1.16	3.28	3.88	0.0358
HAENSZ	22	f	0	1.07	18.34	25.48	0.0000
*HAMMON	59	m	1	3.08	3.94	2.69	0.0000
HEGMAN	2	c	0	3.43	4.70	6.53	0.0000
HINDS	23	f	3	2.78	6.93	1.96	0.0000
ISHIMA	6	c	5	3.04	0.50	0.32	0.0315
JAHN	42	m	0	3.16	2.90	2.39	0.0000
JAIN	48	m	2	2.89	1.70	0.70	0.0002
JAIN	43	f	2	3.24	1.73	1.70	0.0000
Subtotal JAIN				3.07	3.43	2.40	
JEDRYC	54	m	3	2.55	5.53	0.51	0.0000
JOLY	54	m	0	3.44	1.96	2.78	0.0000
JOLY	52	f	0	2.92	5.02	2.27	0.0000
Subtotal JOLY				3.07	6.98	5.04	
JUSSAW	25	m	0	2.36	6.65	0.08	0.0000
KATSOU	37	f	1	1.81	5.71	1.10	0.0000
KHUDER	24	m	0	2.06	7.79	0.29	0.0000
KIHARA	26	c	0	3.29	4.63	5.06	0.0000
KOO	6	f	0	1.42	14.12	9.66	0.0000
KREYBE	4	m	1	2.39	2.95	0.06	0.0000
KREYBE	25	f	1	0.83	4.31	8.70	0.0854
Subtotal KREYBE				1.46	7.26	8.75	
LAMTH	1	f	0	2.09	8.66	0.21	0.0000
LAMWK	2	f	0	2.35	4.51	0.05	0.0000
LAMWK2	1	m	0	1.93	4.22	0.43	0.0001
LAMWK2	5	f	0	1.87	8.17	1.17	0.0000
Subtotal LAMWK2				1.89	12.38	1.60	
LOMBA2	2	f	0	1.45	11.86	7.66	0.0000
LUBIN	34	m	0	1.89	3.72	0.47	0.0003

Table 2A2 - 2

IESLC - Meta-analysis of Ever Smoking, Cigarettes (or Any Product if Cigarettes not available)
Squamous
Most adjusted

REF	NRR	SEX	AD	Ys	Ws	Qs	Ps
LUBIN2	145	m	0	2.81	51.88	16.49	0.0000
LUBIN2	165	f	0	1.75	46.51	11.37	0.0000
Subtotal LUBIN2				2.31	98.39	27.86	
LUO	8	c	20	2.39	1.76	0.03	0.0015
MATOS	67	m	2	2.09	2.97	0.08	0.0003
MATSUD	11	m	0	3.66	0.99	1.98	0.0003
NOU	1	m	0	3.30	1.92	2.13	0.0000
NOU	6	f	0	1.96	1.40	0.12	0.0205
Subtotal NOU				2.74	3.32	2.24	
ORMOS	8	m	0	2.32	1.85	0.01	0.0016
OSANN	43	m	2	3.59	7.67	13.72	0.0000
OSANN	44	f	2	3.27	10.69	11.21	0.0000
Subtotal OSANN				3.40	18.36	24.93	
OSANN2	25	f	1	3.56	0.97	1.67	0.0005
PEZZOT	6	m	0	4.14	0.49	1.77	0.0036
SCHWAR	10	m	0	3.49	0.97	1.49	0.0006
SCHWAR	9	m	0	0.61	2.26	6.07	0.3596
SCHWAR	18	f	0	3.77	0.49	1.12	0.0086
SCHWAR	17	f	0	4.14	0.47	1.69	0.0044
Subtotal SCHWAR				2.04	4.19	10.38	
SEOW	3	f	0	2.86	4.50	1.69	0.0000
SIEMIA	7	m	7	3.12	2.69	2.05	0.0000
SOBUE	97	m	1	2.88	5.62	2.26	0.0000
SOBUE	107	f	1	2.17	13.12	0.09	0.0000
Subtotal SOBUE				2.38	18.74	2.35	
STASZE	16	m	0	4.19	0.50	1.86	0.0032
STASZE	38	f	0	3.48	0.37	0.57	0.0333
Subtotal STASZE				3.88	0.87	2.43	
STAYNE	3	m	0	1.24	17.27	17.43	0.0000
SUZUK2	15	c	3	3.43	0.97	1.36	0.0007
SVENSS	72	f	1	2.54	2.87	0.24	0.0000
TIZZAN	18	c	0	0.99	40.59	63.95	0.0000
TOKARS	10	c	3	1.92	1.27	0.14	0.0305
TSUGAN	13	m	0	2.68	0.44	0.08	0.0772
WAKAI	74	m	1	2.15	1.90	0.02	0.0030
WAKAI	80	f	1	3.23	2.27	2.18	0.0000
Subtotal WAKAI				2.74	4.17	2.19	
WU	32	f	2	3.19	0.99	0.88	0.0015
WUWILL	9	f	3	1.44	33.59	22.25	0.0000
WYNDE2	2	m	0	3.13	2.88	2.25	0.0000
WYNDE3	8	m	0	3.09	2.83	1.98	0.0000
WYNDE3	67	f	0	1.91	3.69	0.41	0.0002
Subtotal WYNDE3				2.42	6.52	2.39	
WYNDE4	69	m	2	2.74	7.27	1.74	0.0000
WYNDE4	54	f	2	1.76	5.63	1.34	0.0000
Subtotal WYNDE4				2.31	12.90	3.07	
WYNDE6	75	m	0	3.01	26.85	15.37	0.0000
WYNDE6	412	f	1	3.48	10.46	15.78	0.0000
Subtotal WYNDE6				3.14	37.31	31.14	
XU3	20	m	1	1.77	2.46	0.55	0.0054
XU3	24	f	1	3.25	1.43	1.42	0.0001
Subtotal XU3				2.32	3.89	1.98	
ZHENG	5	m	0	2.82	3.68	1.21	0.0000
ZHENG	18	f	0	1.70	12.24	3.75	0.0000
Subtotal ZHENG				1.96	15.92	4.96	
ZHOU	8	m	0	1.14	15.27	18.67	0.0000
ZHOU	9	f	0	1.34	4.42	3.67	0.0049
Subtotal ZHOU				1.19	19.68	22.33	

Table 2A2 - 2

IESLC - Meta-analysis of Ever Smoking, Cigarettes (or Any Product if Cigarettes not available)
Squamous
Most adjusted

	N	102
	NS	73
	Wt	1004.60
Het	Chi	517.30
Het	df	101
Het	P	***
Fixed	RR	9.48
	RRl	8.91
	RRu	10.08
	P	+++
Random	RR	10.44
	RRl	8.88
	RRu	12.29
	P	+++
Asymm	P	N.S.

Table 2A2 - 3

IESLC - Meta-analysis of Ever Smoking, Cigarettes (or Any Product if Cigarettes not available)
Squamous
Most adjusted

		Sex		
	combined	male	female	Total
N	11	49	42	102
NS	11	48	41	100
Wt	95.98	484.06	424.56	1004.60
Het Chi	107.16	173.71	220.22	517.30
Het df	10	48	41	101
Het P	***	***	***	***
Fixed RR	7.15	10.65	8.85	9.48
RRl	5.85	9.74	8.04	8.91
RRu	8.73	11.64	9.73	10.08
P	+++	+++	+++	+++
Random RR	10.84	11.85	8.99	10.44
RRl	4.98	9.59	6.98	8.88
RRu	23.59	14.64	11.57	12.29
P	+++	+++	+++	+++
Between Chi				16.21
Between df				2
Between P				***
Btwn(F) P				N.S.
Btwn(R) P				N.S.

		Lung cancer type				
	q	q+s	q+u	KI	not a	Total
N	72	9	3	14	4	102
NS	51	7	3	10	3	74
Wt	754.94	75.63	70.79	75.21	28.04	1004.60
Het Chi	303.44	43.64	1.88	51.55	12.25	517.30
Het df	71	8	2	13	3	101
Het P	***	***	N.S.	***	**	***
Fixed RR	10.47	10.30	2.97	10.24	7.92	9.48
RRl	9.75	8.22	2.35	8.17	5.47	8.91
RRu	11.24	12.91	3.75	12.84	11.47	10.08
P	+++	+++	+++	+++	+++	+++
Random RR	11.68	9.19	2.97	9.26	9.10	10.44
RRl	9.75	5.18	2.35	5.62	4.20	8.88
RRu	13.99	16.31	3.75	15.26	19.69	12.29
P	+++	+++	+++	+++	+++	+++
Between Chi						104.54
Between df						4
Between P						***
Btwn(F) P						***
Btwn(R) P						***

				Location					
	NAmer	UK	Scand	othEur	China	Japan	othAs	other	Total
N	38	4	7	15	12	8	12	6	102
NS	26	2	5	12	8	6	9	5	73
Wt	511.04	27.98	25.57	205.88	115.23	29.46	74.43	15.00	1004.60
Het Chi	205.94	11.30	11.88	109.52	23.30	7.60	13.23	3.82	517.30
Het df	37	3	6	14	11	7	11	5	101
Het P	***	*	(*)	***	*	N.S.	N.S.	N.S.	***
Fixed RR	12.86	5.50	8.67	6.56	5.66	13.94	6.95	16.70	9.48
RRl	11.79	3.80	5.88	5.73	4.72	9.71	5.54	10.07	8.91
RRu	14.02	7.97	12.78	7.52	6.79	20.00	8.73	27.70	10.08
P	+++	+++	+++	+++	+++	+++	+++	+++	+++
Random RR	13.78	6.14	8.62	8.91	6.41	14.54	7.10	16.70	10.44
RRl	10.72	2.82	4.81	5.52	4.74	9.78	5.50	10.07	8.88
RRu	17.72	13.39	15.43	14.39	8.66	21.62	9.15	27.70	12.29
P	+++	+++	+++	+++	+++	+++	+++	+++	+++
Between Chi									130.70
Between df									7
Between P									***
Btwn(F) P									***
Btwn(R) P									***

Table 2A2 - 3

IESLC - Meta-analysis of Ever Smoking, Cigarettes (or Any Product if Cigarettes not available)
Squamous
Most adjusted

Detailed Country in "other Europe"

	multi	Germany	othWest	East	Balkans	Total
N	2	2	2	7	2	15
NS	1	2	2	5	2	12
Wt	98.39	4.65	46.21	14.99	41.65	205.88
Het Chi	27.46	0.68	13.95	7.21	1.38	109.52
Het df	1	1	1	6	1	14
Het P	***	N.S.	***	N.S.	N.S.	***
Fixed RR	10.10	17.47	3.31	10.20	3.87	6.56
RRl	8.29	7.04	2.48	6.15	2.86	5.73
RRu	12.31	43.37	4.42	16.93	5.24	7.52
P	+++	+++	+++	+++	+++	+++
Random RR	9.82	17.47	5.98	10.79	4.08	8.91
RRl	3.48	7.04	1.15	5.91	2.63	5.52
RRu	27.71	43.37	31.02	19.72	6.34	14.39
P	+++	+++	+	+++	+++	+++
Between Chi						58.84
Between df						4
Between P						***
Btwn(F) P						(*)
Btwn(R) P						*

Detailed Country in "other Asia"

	India	HongKong	other	Total
N	1	7	4	12
NS	1	5	3	9
Wt	6.65	50.37	17.41	74.43
Het Chi	0.00	5.72	6.14	13.23
Het df	0	6	3	11
Het P	N.S.	N.S.	N.S.	N.S.
Fixed RR	10.60	6.54	7.06	6.95
RRl	4.96	4.96	4.42	5.54
RRu	22.66	8.62	11.30	8.73
P	+++	+++	+++	+++
Random RR	10.60	6.54	6.94	7.10
RRl	4.96	4.96	3.53	5.50
RRu	22.66	8.62	13.65	9.15
P	+++	+++	+++	+++
Between Chi				1.37
Between df				2
Between P				N.S.
Btwn(F) P				N.S.
Btwn(R) P				N.S.

Detailed other continent

	SCAmer	Auslia	Africa	Total
N	6			6
NS	5			5
Wt	15.00			15.00
Het Chi	3.82			3.82
Het df	5			5
Het P	N.S.			N.S.
Fixed RR	16.70			16.70
RRl	10.07			10.07
RRu	27.70			27.70
P	+++			+++
Random RR	16.70			16.70
RRl	10.07			10.07
RRu	27.70			27.70
P	+++			+++
Between Chi				
Between df				
Between P				N.S.
Btwn(F) P				N.S.
Btwn(R) P				N.S.

Table 2A2 - 3

IESLC - Meta-analysis of Ever Smoking, Cigarettes (or Any Product if Cigarettes not available)
Squamous
Most adjusted

	Start year of study <1960	1960-69	1970-79	1980-89	1990+	Total
N	14	14	26	40	8	102
NS	10	12	16	27	8	73
Wt	126.72	95.87	258.16	496.70	27.15	1004.60
Het Chi	60.55	64.77	129.54	156.43	10.78	517.30
Het df	13	13	25	39	7	101
Het P	***	***	***	***	N.S.	***
Fixed RR	4.47	11.20	8.74	11.43	12.39	9.48
RRl	3.76	9.17	7.73	10.47	8.51	8.91
RRu	5.32	13.68	9.87	12.48	18.05	10.08
P	+++	+++	+++	+++	+++	+++
Random RR	6.25	12.26	10.01	11.76	12.21	10.44
RRl	4.04	7.34	7.22	9.42	7.56	8.88
RRu	9.68	20.46	13.87	14.68	19.72	12.29
P	+++	+++	+++	+++	+++	+++
Between Chi						95.21
Between df						4
Between P						***
Btwn(F) P						***
Btwn(R) P						N.S.

	Study type (1) CC	other	Total
N	93	9	102
NS	66	7	73
Wt	983.25	21.35	1004.60
Het Chi	502.32	13.00	517.30
Het df	92	8	101
Het P	***	N.S.	***
Fixed RR	9.42	12.80	9.48
RRl	8.85	8.38	8.91
RRu	10.02	19.57	10.08
P	+++	+++	+++
Random RR	10.26	13.42	10.44
RRl	8.67	7.49	8.88
RRu	12.14	24.05	12.29
P	+++	+++	+++
Between Chi			1.97
Between df			1
Between P			N.S.
Btwn(F) P			N.S.
Btwn(R) P			N.S.

	Study type (2) CC	prosp	other	Total
N	93	5	4	102
NS	66	4	3	73
Wt	983.25	16.77	4.58	1004.60
Het Chi	502.32	10.32	2.69	517.30
Het df	92	4	3	101
Het P	***	*	N.S.	***
Fixed RR	9.42	12.85	12.62	9.48
RRl	8.85	7.96	5.05	8.91
RRu	10.02	20.74	31.54	10.08
P	+++	+++	+++	+++
Random RR	10.26	13.78	12.62	10.44
RRl	8.67	6.06	5.05	8.88
RRu	12.14	31.31	31.54	12.29
P	+++	+++	+++	+++
Between Chi				1.97
Between df				2
Between P				N.S.
Btwn(F) P				N.S.
Btwn(R) P				N.S.

Table 2A2 - 3

IESLC - Meta-analysis of Ever Smoking, Cigarettes (or Any Product if Cigarettes not available)
Squamous
Most adjusted

	Study size (number of LC cases) 100-249	250-499	500-999	1000+	Total
N	22	31	18	31	102
NS	21	22	12	18	73
Wt	84.03	134.11	119.82	666.64	1004.60
Het Chi	42.31	66.35	48.99	330.10	517.30
Het df	21	30	17	30	101
Het P	**	***	***	***	***
Fixed RR	5.99	9.11	7.77	10.49	9.48
RRl	4.83	7.70	6.50	9.72	8.91
RRu	7.41	10.80	9.30	11.32	10.08
P	+++	+++	+++	+++	+++
Random RR	8.40	10.48	10.60	10.84	10.44
RRl	5.90	8.00	7.55	8.15	8.88
RRu	11.97	13.71	14.87	14.41	12.29
P	+++	+++	+++	+++	+++
Between Chi					29.55
Between df					3
Between P					***
Btwn(F) P					N.S.
Btwn(R) P					N.S.

	Risky occupational population no	mining	othRisky	Total
N	100	1	1	102
NS	71	1	1	73
Wt	999.60	3.72	1.27	1004.60
Het Chi	516.68	0.00	0.00	517.30
Het df	99	0	0	101
Het P	***	N.S.	N.S.	***
Fixed RR	9.50	6.65	6.80	9.48
RRl	8.92	2.41	1.20	8.91
RRu	10.10	18.36	38.62	10.08
P	+++	+++	+	+++
Random RR	10.52	6.65	6.80	10.44
RRl	8.93	2.41	1.20	8.88
RRu	12.40	18.36	38.62	12.29
P	+++	+++	+	+++
Between Chi				0.61
Between df				2
Between P				N.S.
Btwn(F) P				N.S.
Btwn(R) P				N.S.

	National cigarette tobacco type Virginia	blended	other	Total
N	9	80	13	102
NS	6	58	9	73
Wt	47.52	838.57	118.51	1004.60
Het Chi	36.87	417.88	24.35	517.30
Het df	8	79	12	101
Het P	***	***	*	***
Fixed RR	9.47	10.22	5.57	9.48
RRl	7.13	9.55	4.65	8.91
RRu	12.59	10.93	6.67	10.08
P	+++	+++	+++	+++
Random RR	11.73	11.25	6.17	10.44
RRl	6.13	9.35	4.61	8.88
RRu	22.47	13.54	8.26	12.29
P	+++	+++	+++	+++
Between Chi				38.20
Between df				2
Between P				***
Btwn(F) P				*
Btwn(R) P				**

Table 2A2 - 3

IESLC - Meta-analysis of Ever Smoking, Cigarettes (or Any Product if Cigarettes not available)
Squamous
Most adjusted

	Any proxy use No/nk	Yes	Total
N	76	26	102
NS	55	18	73
Wt	879.23	125.37	1004.60
Het Chi	438.34	50.18	517.30
Het df	75	25	101
Het P	***	**	***
Fixed RR	8.89	14.84	9.48
RRl	8.32	12.46	8.91
RRu	9.50	17.68	10.08
P	+++	+++	+++
Random RR	9.58	14.00	10.44
RRl	7.95	10.60	8.88
RRu	11.54	18.48	12.29
P	+++	+++	+++
Between Chi			28.77
Between df			1
Between P			***
Btwn(F) P			*
Btwn(R) P			*

	Full histological confirmation No	Yes	Total
N	59	43	102
NS	43	30	73
Wt	457.35	547.25	1004.60
Het Chi	263.60	183.41	517.30
Het df	58	42	101
Het P	***	***	***
Fixed RR	7.10	12.07	9.48
RRl	6.48	11.10	8.91
RRu	7.78	13.13	10.08
P	+++	+++	+++
Random RR	9.25	12.51	10.44
RRl	7.48	9.93	8.88
RRu	11.44	15.77	12.29
P	+++	+++	+++
Between Chi			70.29
Between df			1
Between P			***
Btwn(F) P			***
Btwn(R) P			(*)

	Number of adjustment variables (1) 0	1	2+/+nk	Total
N	54	20	28	102
NS	38	14	22	74
Wt	426.38	118.78	459.44	1004.60
Het Chi	240.85	77.21	159.39	517.30
Het df	53	19	27	101
Het P	***	***	***	***
Fixed RR	7.52	11.75	11.11	9.48
RRl	6.84	9.81	10.14	8.91
RRu	8.27	14.06	12.18	10.08
P	+++	+++	+++	+++
Random RR	9.48	11.03	11.99	10.44
RRl	7.51	7.45	9.06	8.88
RRu	11.97	16.32	15.87	12.29
P	+++	+++	+++	+++
Between Chi				39.84
Between df				2
Between P				***
Btwn(F) P				*
Btwn(R) P				N.S.

Table 2A2 - 3

IESLC - Meta-analysis of Ever Smoking, Cigarettes (or Any Product if Cigarettes not available)
Squamous
Most adjusted

	Number of adjustment variables (2) 0	1	2	3-5	6+/+nk	Total
N	54	20	16	8	4	102
NS	38	14	11	8	4	75
Wt	426.38	118.78	374.04	74.94	10.46	1004.60
Het Chi	240.85	77.21	114.76	24.97	5.99	517.30
Het df	53	19	15	7	3	101
Het P	***	***	***	***	N.S.	***
Fixed RR	7.52	11.75	12.07	7.74	7.81	9.48
RRl	6.84	9.81	10.90	6.17	4.26	8.91
RRu	8.27	14.06	13.35	9.70	14.32	10.08
P	+++	+++	+++	+++	+++	+++
Random RR	9.48	11.03	13.47	10.71	8.13	10.44
RRl	7.51	7.45	9.42	6.25	3.43	8.88
RRu	11.97	16.32	19.26	18.36	19.30	12.29
P	+++	+++	+++	+++	+++	+++
Between Chi						53.51
Between df						4
Between P						***
Btwn(F) P						*
Btwn(R) P						N.S.

	Product all/unsp	cig+/-ot	cig only	Total
N	38	60	4	102
NS	30	42	4	76
Wt	210.72	766.63	27.26	1004.60
Het Chi	124.61	310.23	14.75	517.30
Het df	37	59	3	101
Het P	***	***	**	***
Fixed RR	5.73	10.79	11.96	9.48
RRl	5.01	10.06	8.21	8.91
RRu	6.56	11.59	17.40	10.08
P	+++	+++	+++	+++
Random RR	8.14	11.63	15.54	10.44
RRl	6.15	9.59	5.93	8.88
RRu	10.75	14.11	40.76	12.29
P	+++	+++	+++	+++
Between Chi				67.71
Between df				2
Between P				***
Btwn(F) P				***
Btwn(R) P				(*)

	Denominator nev any	nev cigs	Total
N	64	38	102
NS	48	26	74
Wt	476.28	528.32	1004.60
Het Chi	277.82	213.69	517.30
Het df	63	37	101
Het P	***	***	***
Fixed RR	8.01	11.04	9.48
RRl	7.32	10.13	8.91
RRu	8.76	12.02	10.08
P	+++	+++	+++
Random RR	9.65	11.92	10.44
RRl	7.81	9.24	8.88
RRu	11.92	15.38	12.29
P	+++	+++	+++
Between Chi			25.79
Between df			1
Between P			***
Btwn(F) P			*
Btwn(R) P			N.S.

Table 2A2 - 3

IESLC - Meta-analysis of Ever Smoking, Cigarettes (or Any Product if Cigarettes not available)
Squamous
Most adjusted

	Derivation of RR/CI			
	Orig	StdCalc	Other	Total
N	22	43	37	102
NS	17	33	27	77
Wt	453.65	399.96	150.99	1004.60
Het Chi	158.51	228.51	89.76	517.30
Het df	21	42	36	101
Het P	***	***	***	***
Fixed RR	11.43	7.43	10.29	9.48
RRl	10.42	6.74	8.78	8.91
RRu	12.53	8.20	12.07	10.08
P	+++	+++	+++	+++
Random RR	12.67	9.05	11.04	10.44
RRl	9.25	7.02	8.37	8.88
RRu	17.37	11.67	14.56	12.29
P	+++	+++	+++	+++
Between Chi				40.51
Between df				2
Between P				***
Btwn(F) P				*
Btwn(R) P				N.S.

Table 2A2 - 4

IESLC - Meta-analysis of Ever Smoking, Cigarettes (or Any Product if Cigarettes not available)
Squamous
Least adjusted

REF	NRR	X	SEX	AGEL	AGEH	RACE	YF	LC TYPE	LOC	START	ST	NLC	R	VB	P	H	AD	PRODUCT	DENOM	De
ABRAHA	1		m	0	0	all	0	q	Eu:est	1975	pr	571	n	bl	n	n	0	all/unsp	nev any	ot
ABRAHA	4		f	0	0	all	0	q	Eu:est	1975	pr	571	n	bl	n	n	0	all/unsp	nev any	ot
ALDERS	88	x	m	0	0	all	-	q+s	Eu:UK	1977	CC	1448	n	V	n	n	0	cig+/-ot	nev any	st
ALDERS	84	x	f	0	0	all	-	q+s	Eu:UK	1977	CC	1448	n	V	n	n	0	cig only	nev any	st
ANDERS	10		f	0	0	all	0	q	NAmer	1986	pr	343	n	bl	n	n	0	cig+/-ot	nev cigs	st
BAND	5		m	0	0	all	-	q	NAmer	1983	CC	2831	n	V	y	y	2	cig only	nev any	ot
BARBON	110	x	m	0	0	all	-	q	Eu:wst	1979	CC	755	n	bl	y	y	0	all/unsp	nev any	st
BECHER	11		f	0	0	all	-	q+s	Eu:Ger	1985	CC	194	n	bl	n	y	1	all/unsp	nev any	or
BRESLO	7		c	0	0	all	-	not a	NAmer	1949	CC	518	n	bl	n	y	0	cig+/-ot	nev+1	st
BROWN2	6		m	0	0	wh	-	q	NAmer	1984	CC	14596	n	bl	n	y	2	cig+/-ot	nev cigs	or
BROWN2	5		f	0	0	wh	-	q	NAmer	1984	CC	14596	n	bl	n	y	2	cig+/-ot	nev cigs	or
BUFFLE	49		m	0	0	wh	-	q	NAmer	1976	CC	943	n	bl	y	n	0	cig+/-ot	nev cigs	ot
BUFFLE	62		f	0	0	w-hi	-	q	NAmer	1976	CC	943	n	bl	y	n	0	cig+/-ot	nev cigs	st
BYERS1	1		m	0	0	wh	-	q	NAmer	1957	CC	1002	n	bl	n	n	0	cig+/-ot	nev cigs	st
CHAN	18		m	0	0	all	-	q+s	As:HK	1976	CC	397	n	bl	n	n	0	cig+/-ot	nev any	st
CHAN	22		f	0	0	all	-	q+s	As:HK	1976	CC	397	n	bl	n	n	0	cig+/-ot	nev any	st
CHOI	62		m	0	0	all	-	q	As:oth	1985	CC	375	n	bl	n	n	0	cig+/-ot	nev cigs	st
CHOI	64		f	0	0	all	-	q	As:oth	1985	CC	375	n	bl	n	n	0	cig+/-ot	nev cigs	st
COMSTO	66		m	0	0	all	-	q	NAmer	1975	ot	258	n	bl	n	n	0	cig+/-ot	nev cigs	st
COMSTO	78		f	0	0	all	-	q	NAmer	1975	ot	258	n	bl	n	n	0	cig+/-ot	nev cigs	ot
CORREA	35		c	0	0	all	-	q+s	NAmer	1979	CC	1359	n	bl	y	n	1	cig+/-ot	nev cigs	or
DAMBER	12	x	m	0	0	all	-	q	Eu:Sca	1972	CC	579	n	bl	y	n	0	all/unsp	nev any	st
DESTE2	16		m	0	0	all	-	q	SCAmer	1993	CC	463	n	bl	n	n	2	all/unsp	nev any	or
DOLL	82	x	m	0	0	all	-	KI	Eu:UK	1948	CC	1465	n	V	n	n	0	all/unsp	nev any	st
DOLL	84	x	f	0	0	all	-	KI	Eu:UK	1948	CC	1465	n	V	n	n	0	all/unsp	nev any	st
DORGAN	113		m	0	0	wh	-	q	NAmer	1980	CC	2026	n	bl	y	y	2	cig+/-ot	nev any	or
DORGAN	98		f	0	0	all	-	q	NAmer	1980	CC	2026	n	bl	y	y	3	cig+/-ot	nev any	or
DOSEME	19	x	m	0	0	all	-	q	Eu:bal	1979	CC	1210	n	bl	n	n	0	cig+/-ot	nev cigs	st
ENGELA	62		m	0	0	all	0	q	Eu:Sca	1964	pr	435	n	bl	n	n	7	cig+/-ot	nev cigs	ot
FAN	3		c	0	0	all	-	q	As:Chi	1990	CC	403	n	ot	y	n	0	cig+/-ot	nev cigs	ot
GAO	7	x	m	0	0	all	-	q	As:Chi	1984	CC	1405	n	ot	n	n	0	cig+/-ot	nev cigs	st
GAO	17	x	f	0	0	all	-	q	As:Chi	1984	CC	1405	n	ot	n	n	0	cig+/-ot	nev cigs	st
GER	5	x	c	0	0	all	-	q+s	As:oth	1990	CC	141	n	ot	y	n	0	all/unsp	nev any	st
HAENSZ	22		f	0	0	all	-	q+u	NAmer	1955	CC	158	n	bl	n	y	0	cig+/-ot	nev any	st
HAMMON	72	x	m	0	0	wh	0	not a	NAmer	1952	pr	448	n	bl	n	n	0	cig+/-ot	nev any	st
HEGMAN	2		c	0	0	all	-	q	NAmer	1989	CC	282	n	bl	y	y	0	all/unsp	nev any	st
HINDS	23		f	0	0	o	-	q+s	NAmer	1968	CC	292	n	bl	n	n	3	all/unsp	nev any	st
ISHIMA	1	x	c	0	0	all	-	q	As:Jap	1961	CC	180	n	bl	y	y	0	all/unsp	nev any	st
JAHN	42		m	0	0	all	-	q	Eu:Ger	1988	CC	1004	n	bl	n	n	0	cig+/-ot	nev any	st
JAIN	8	x	m	0	0	all	-	q	NAmer	1981	CC	845	n	V	y	n	0	cig+/-ot	nev cigs	st
JAIN	3	x	f	0	0	all	-	q	NAmer	1981	CC	845	n	V	y	n	0	cig+/-ot	nev cigs	st
JEDRYC	7	x	m	0	0	all	-	q	Eu:est	1980	CC	1630	n	bl	y	n	0	cig+/-ot	nev any	st
JOLY	54		m	0	0	all	-	q	SCAmer	1978	CC	826	n	bl	n	n	0	cig+/-ot	nev any	st
JOLY	52		f	0	0	all	-	q	SCAmer	1978	CC	826	n	bl	n	n	0	cig+/-ot	nev any	st
JUSSAW	25		m	0	0	all	-	KI	As:Ind	1964	CC	792	n	V	n	n	0	cig only	nev any	st
KATSOU	35	x	f	0	0	all	-	KI	Eu:bal	1987	CC	101	n	bl	n	n	0	all/unsp	nev any	st
KHUDER	24		m	0	0	all	-	q	NAmer	1985	CC	482	n	bl	n	y	0	cig+/-ot	nev cigs	ot
KIHARA	26		c	0	0	jap	-	q	As:Jap	1991	CC	440	n	bl	n	n	0	all/unsp	nev any	st
KOO	6		f	0	0	all	-	q+s	As:HK	1981	CC	200	n	bl	n	n	0	all/unsp	nev any	st
KREYBE	16	x	m	0	0	all	-	KI	Eu:Sca	1948	CC	300	n	bl	n	y	0	all/unsp	nev any	st
KREYBE	33	x	f	0	0	all	-	KI	Eu:Sca	1948	CC	300	n	bl	n	y	0	all/unsp	nev any	st
LAMTH	1		f	0	0	ch	-	q	As:HK	1983	CC	445	n	bl	n	n	0	all/unsp	nev any	or
LAMWK	2		f	0	0	ch	-	q	As:HK	1981	CC	163	n	bl	n	n	0	all/unsp	nev any	st
LAMWK2	1		m	0	0	all	-	q	As:HK	1976	CC	480	n	bl	n	n	0	all/unsp	nev any	st
LAMWK2	5		f	0	0	all	-	q	As:HK	1976	CC	480	n	bl	n	n	0	all/unsp	nev any	st
LOMBA2	2		f	0	0	all	-	q+u	NAmer	1960	CC	225	n	bl	n	n	0	cig+/-ot	nev cigs	st
LUBIN	34		m	0	0	all	-	KI	As:Chi	1984	CC	427	m	ot	y	n	0	cig+/-ot	nev any	st
LUBIN2	145		m	0	0	all	-	q	Eu:mul	1976	CC	7804	n	bl	n	y	0	cig+/-ot	nev any	st
LUBIN2	165		f	0	0	all	-	q	Eu:mul	1976	CC	7804	n	bl	n	y	0	cig+/-ot	nev any	st
LUO	2	x	c	0	0	all	-	q	As:Chi	1990	CC	102	n	ot	n	y	0	cig+/-ot	nev cigs	st
MATOS	66	x	m	0	0	all	-	q	SCAmer	1994	CC	200	n	bl	n	n	0	cig+/-ot	nev any	st
MATSUD	11		m	0	0	all	-	q	As:Jap	1965	CC	179	n	bl	n	n	0	cig+/-ot	nev cigs	st
NOU	1		m	0	0	all	-	q	Eu:Sca	1971	CC	273	n	bl	y	n	0	all/unsp	nev any	st
NOU	6		f	0	0	all	-	q	Eu:Sca	1971	CC	273	n	bl	y	n	0	all/unsp	nev any	st
ORMOS	8		m	0	0	all	-	q	Eu:est	1947	CC	119	n	bl	y	y	0	cig+/-ot	nev any	st
OSANN	18	x	m	0	0	all	-	q	NAmer	1984	CC	1986	n	bl	n	n	0	cig+/-ot	nev cigs	st
OSANN	22	x	f	0	0	all	-	q	NAmer	1984	CC	1986	n	bl	n	n	0	cig+/-ot	nev cigs	st
OSANN2	7	x	f	0	0	all	-	KI	NAmer	1964	ot	217	n	bl	n	y	0	cig+/-ot	nev cigs	st
PEZZOT	6		m	0	0	all	-	q	SCAmer	1987	CC	215	n	bl	n	y	0	cig only	nev cigs	ot
SCHWAR	10		m	40	54	wh	-	q	NAmer	1984	CC	5588	n	bl	y	y	0	cig+/-ot	nev cigs	st
SCHWAR	9		m	40	54	bl	-	q	NAmer	1984	CC	5588	n	bl	y	y	0	cig+/-ot	nev cigs	st
SCHWAR	18		f	40	54	wh	-	q	NAmer	1984	CC	5588	n	bl	y	y	0	cig+/-ot	nev cigs	ot

Table 2A2 - 4

IESLC - Meta-analysis of Ever Smoking, Cigarettes (or Any Product if Cigarettes not available)
Squamous
Least adjusted

REF	NRR	X	SEX	AGEL	AGEH	RACE	YF	LC TYPE	LOC	START	ST	NLC	R	VB	P	H	AD	PRODUCT	DENOM	De
SCHWAR	17		f	40	54	bl	-	q	NAmer	1984	CC	5588	n	bl	y	y	0	cig+/-ot	nev cigs	ot
SEOW	3		f	0	0	ch	-	q	As:oth	1997	CC	153	n	bl	n	y	0	cig+/-ot	nev cigs	st
SIEMIA	11	x	m	0	0	all	-	q	NAmer	1979	CC	857	n	V	y	y	0	cig+/-ot	nev cigs	st
SOBUE	3	x	m	0	0	all	-	q	As:Jap	1986	CC	1376	n	bl	n	y	0	cig+/-ot	nev cigs	st
SOBUE	19	x	f	0	0	all	-	q	As:Jap	1986	CC	1376	n	bl	n	y	0	cig+/-ot	nev cigs	st
STASZE	16		m	0	0	all	-	q	Eu:est	1954	CC	281	n	bl	n	y	0	cig+/-ot	nev any	ot
STASZE	38		f	0	0	all	-	q	Eu:est	1954	CC	281	n	bl	n	y	0	all/unsp	nev any	ot
STAYNE	3		m	0	0	all	-	q	NAmer	1969	CC	420	n	bl	n	n	0	all/unsp	nev any	st
SUZUK2	12	x	c	0	0	all	-	q	SCAmer	1991	CC	123	n	bl	n	y	0	all/unsp	nev any	st
SVENSS	57	x	f	0	0	all	-	q	Eu:Sca	1983	CC	210	n	bl	n	n	0	all/unsp	nev any	st
TIZZAN	18		c	0	0	all	-	q+u	Eu:wst	1959	CC	1358	n	bl	n	n	0	all/unsp	nev any	st
TOKARS	9	x	c	0	0	all	-	q	Eu:est	1966	ot	162	o	bl	n	y	0	all/unsp	nev any	st
TSUGAN	13		m	0	0	all	-	q	As:Jap	1976	CC	134	n	bl	n	y	0	all/unsp	nev any	ot
WAKAI	15	x	m	0	0	all	-	q	As:Jap	1988	CC	333	n	bl	n	y	0	all/unsp	nev any	st
WAKAI	33	x	f	0	0	all	-	q	As:Jap	1988	CC	333	n	bl	n	y	0	all/unsp	nev any	st
WU	14	x	f	0	0	wh	-	q	NAmer	1981	CC	220	n	bl	n	y	0	all/unsp	nev any	st
WUWILL	9		f	0	0	all	-	q	As:Chi	1985	CC	965	n	ot	n	n	3	cig+/-ot	nev cigs	or
WYNDE2	2		m	0	0	all	-	KI	NAmer	1962	CC	404	n	bl	n	y	0	cig+/-ot	nev any	st
WYNDE3	8		m	0	0	all	-	KI	NAmer	1966	CC	350	n	bl	n	y	0	cig+/-ot	nev any	st
WYNDE3	67		f	0	0	all	-	KI	NAmer	1966	CC	350	n	bl	n	y	0	cig+/-ot	nev any	st
WYNDE4	69		m	0	0	all	-	not a	NAmer	1948	CC	684	n	bl	y	n	2	cig+/-ot	nev any	ot
WYNDE4	54		f	0	0	all	-	not a	NAmer	1948	CC	684	n	bl	y	n	2	all/unsp	nev any	ot
WYNDE6	75		m	0	0	all	-	KI	NAmer	1969	CC	4423	n	bl	n	y	0	cig+/-ot	nev any	st
WYNDE6	411	x	f	0	0	wh	-	q	NAmer	1969	CC	4423	n	bl	n	y	0	cig+/-ot	nev cigs	st
XU3	19	x	m	0	0	all	-	KI	As:Chi	1981	CC	135	n	ot	n	n	0	all/unsp	nev any	st
XU3	23	x	f	0	0	all	-	KI	As:Chi	1981	CC	135	n	ot	n	n	0	all/unsp	nev any	st
ZHENG	5		m	0	0	all	-	q	As:Chi	1982	CC	540	n	ot	*	y	0	cig+/-ot	nev cigs	st
ZHENG	18		f	0	0	all	-	q	As:Chi	1982	CC	540	n	ot	*	y	0	cig+/-ot	nev cigs	st
ZHOU	8		m	0	0	all	-	q	As:Chi	1978	CC	1360	n	ot	n	n	0	all/unsp	nev any	st
ZHOU	9		f	0	0	all	-	q	As:Chi	1978	CC	1360	n	ot	n	n	0	all/unsp	nev any	st

Cigarette type is all/unspec for all RRs

except for the following:

REF	NRR	CIGTYPE
ALDERS	84	MC only
CHAN	18	MC+-HR
CHAN	22	MC+-HR
JUSSAW	25	MC only

Table 2A2 - 5

IESLC - Meta-analysis of Ever Smoking, Cigarettes (or Any Product if Cigarettes not available)
Squamous
Least adjusted

REF	NRR	SEX	AD	Number Exposed Case	Number Exposed Cont	Non-exposed Case	Non-exposed Cont	RR	95.00%CI
*ABRAHA	1	m	0	142	10351	0	3365	92.66~	(5.77-1488.21)
*ABRAHA	4	f	0	17	5256	7	11589	5.35	(2.22- 12.90)
Subtotal ABRAHA								6.95	(3.00- 16.06)
ALDERS	88	m	0	277	641	4	133	14.37	(5.26- 39.24)
ALDERS	84	f	0	176	371	16	243	7.20	(4.21- 12.32)
Subtotal ALDERS								8.40	(5.23- 13.48)
*ANDERS	10	f	0	63	96164	5	195158	25.57	(10.29- 63.56)
BAND	5	m	2	-	-	-	-	37.45	(17.62- 79.58)
BARBON	110	m	0	261	567	6	188	14.42	(6.31- 32.94)
BECHER	11	f	1	-	-	-	-	10.69	(2.43- 47.00)
BRESLO	7	c	0	444	394	15	56	4.21	(2.34- 7.56)
BROWN2	6	m	2	-	-	-	-	11.10	(9.50- 12.90)
BROWN2	5	f	2	-	-	-	-	20.10	(16.40- 24.80)
Subtotal BROWN2								13.69	(12.11- 15.49)
BUFFLE	49	m	0	-	-	-	-	14.03	(4.73- 41.61)
BUFFLE	62	f	0	58	166	3	112	13.04	(3.99- 42.66)
Subtotal BUFFLE								13.57	(6.09- 30.24)
BYERS1	1	m	0	299	695	22	424	8.29	(5.29- 13.00)
CHAN	18	m	0	112	160	2	43	15.05	(3.57- 63.41)
CHAN	22	f	0	37	38	19	139	7.12	(3.68- 13.77)
Subtotal CHAN								8.11	(4.45- 14.77)
CHOI	62	m	0	160	465	6	95	5.45	(2.34- 12.67)
CHOI	64	f	0	11	26	10	164	6.94	(2.68- 17.96)
Subtotal CHOI								6.06	(3.22- 11.40)
COMSTO	66	m	0	44	229	2	84	8.07	(1.91- 34.02)
COMSTO	78	f	0	17	87	0	115	46.20~	(2.74- 778.83)
Subtotal COMSTO								11.56	(3.21- 41.67)
CORREA	35	c	1	-	-	-	-	28.30	(18.60- 43.20)
DAMBER	12	m	0	271	169	14	103	11.80	(6.54- 21.29)
DESTE2	16	m	2	-	-	-	-	13.20	(4.70- 37.10)
DOLL	82	m	0	829	1296	3	61	13.01	(4.07- 41.59)
DOLL	84	f	0	32	49	16	59	2.41	(1.18- 4.90)
Subtotal DOLL								3.81	(2.08- 6.98)
DORGAN	113	m	2	-	-	-	-	18.90	(7.00- 51.30)
DORGAN	98	f	3	-	-	-	-	11.10	(7.20- 17.10)
Subtotal DORGAN								12.08	(8.12- 17.96)
DOSEME	19	m	0	434	536	58	293	4.09	(3.00- 5.57)
*ENGELA	62	m	7	-	-	-	-	6.45	(1.97- 21.11)
FAN	3	c	0	75	595	6	556	11.68	(5.04- 27.04)
GAO	7	m	0	314	558	13	202	8.74	(4.91- 15.58)
GAO	17	f	0	66	130	53	605	5.80	(3.85- 8.72)
Subtotal GAO								6.65	(4.76- 9.28)
GER	5	c	0	48	156	11	80	2.24	(1.10- 4.54)
HAENSZ	22	f	0	56	103	44	236	2.92	(1.85- 4.61)
*HAMMON	72	m	0	265	382338	4	115884	20.08	(7.48- 53.90)
HEGMAN	2	c	0	89	1202	5	2080	30.80	(12.48- 76.03)
HINDS	23	f	3	-	-	-	-	16.13	(7.66- 33.97)
ISHIMA	1	c	0	53	33	5	25	8.03	(2.80- 23.04)
JAHN	42	m	0	343	671	3	138	23.51	(7.44- 74.35)
JAIN	8	m	0	154	277	2	85	23.63	(5.73- 97.35)
JAIN	3	f	0	103	196	6	214	18.74	(8.05- 43.66)
Subtotal JAIN								19.92	(9.64- 41.17)
JEDRYC	7	m	0	337	1054	6	289	15.40	(6.80- 34.89)
JOLY	54	m	0	203	709	2	218	31.21	(7.69- 126.68)
JOLY	52	f	0	48	122	6	283	18.56	(7.74- 44.51)
Subtotal JOLY								21.47	(10.22- 45.09)
JUSSAW	25	m	0	17	77	13	624	10.60	(4.96- 22.66)
KATSOU	35	f	0	28	22	14	67	6.09	(2.73- 13.59)
KHUDER	24	m	0	176	-	9	-	7.82	(3.87- 15.77)
KIHARA	26	c	0	132	232	5	237	26.97	(10.84- 67.08)
KOO	6	f	0	61	63	32	137	4.15	(2.46- 6.98)
KREYBE	16	m	0	210	3514	3	644	12.83	(4.09- 40.22)
KREYBE	33	f	0	2	328	3	657	1.34	(0.22- 8.03)
Subtotal KREYBE								6.68	(2.55- 17.51)
LAMTH	1	f	0	63	20	28	72	8.10	(4.16- 15.77)
LAMWK	2	f	0	21	41	7	144	10.54	(4.19- 26.52)
LAMWK2	1	m	0	129	161	5	43	6.89	(2.65- 17.90)
LAMWK2	5	f	0	35	50	15	139	6.49	(3.27- 12.88)
Subtotal LAMWK2								6.62	(3.79- 11.56)
LOMBA2	2	f	0	94	353	15	239	4.24	(2.40- 7.50)
LUBIN	34	m	0	291	788	4	72	6.65	(2.41- 18.36)

Table 2A2 - 5

IESLC - Meta-analysis of Ever Smoking, Cigarettes (or Any Product if Cigarettes not available)
Squamous
Least adjusted

REF	NRR	SEX	AD	Number Exposed Case	Number Exposed Cont	Non-exposed Case	Non-exposed Cont	RR	95.00%CI
LUBIN2	145	m	0	3587	10433	54	2616	16.66	(12.69- 21.86)
LUBIN2	165	f	0	200	567	72	1180	5.78	(4.34- 7.71)
Subtotal LUBIN2								10.10	(8.29- 12.31)
LUO	2	c	0	34	146	5	160	7.45	(2.84- 19.56)
MATOS	66	m	0	47	283	3	110	6.09	(1.86- 19.97)
MATSUD	11	m	0	103	3314	1	1255	39.01	(5.44- 279.84)
NOU	1	m	0	110	247	2	122	27.17	(6.60- 111.85)
NOU	6	f	0	5	92	2	261	7.09	(1.35- 37.19)
Subtotal NOU								15.42	(5.26- 45.22)
ORMOS	8	m	0	27	1034	2	777	10.14	(2.41- 42.79)
OSANN	18	m	0	352	1018	8	833	36.00	(17.76- 72.99)
OSANN	22	f	0	159	563	12	1093	25.72	(14.18- 46.66)
Subtotal OSANN								29.58	(18.76- 46.64)
OSANN2	7	f	0	112	61	7	58	15.21	(6.54- 35.38)
PEZZOT	6	m	0	85	317	0	116	62.74~	(3.86-1019.50)
SCHWAR	10	m	0	80	178	1	73	32.81	(4.48- 240.23)
SCHWAR	9	m	0	41	39	4	7	1.84	(0.50- 6.78)
SCHWAR	18	f	0	29	108	0	79	43.23~	(2.60- 718.15)
SCHWAR	17	f	0	21	28	0	41	62.61~	(3.64-1076.10)
Subtotal SCHWAR								7.71	(2.96- 20.10)
SEOW	3	f	0	21	15	10	125	17.50	(6.95- 44.09)
SIEMIA	11	m	0	356	428	3	105	29.11	(9.16- 92.52)
SOBUE	3	m	0	422	1013	3	128	17.77	(5.63- 56.16)
SOBUE	19	f	0	36	232	14	857	9.50	(5.04- 17.91)
Subtotal SOBUE								10.99	(6.31- 19.15)
STASZE	16	m	0	135	653	0	158	65.73~	(4.07-1061.95)
STASZE	38	f	0	1	153	0	1660	32.45~	(1.32- 800.04)
Subtotal STASZE								48.53	(5.94- 396.73)
STAYNE	3	m	0	130	567	22	333	3.47	(2.17- 5.56)
SUZUK2	12	c	0	75	36	5	44	18.33	(6.70- 50.17)
SVENSS	57	f	0	48	89	5	120	12.94	(4.95- 33.84)
TIZZAN	18	c	0	333	939	55	419	2.70	(1.99- 3.67)
TOKARS	9	c	0	45	77	2	19	5.55	(1.24- 24.95)
TSUGAN	13	m	0	20	15	0	5	14.55~	(0.75- 283.37)
WAKAI	15	m	0	113	424	2	65	8.66	(2.09- 35.92)
WAKAI	33	f	0	16	31	3	145	24.95	(6.85- 90.87)
Subtotal WAKAI								15.46	(5.94- 40.24)
WU	14	f	0	69	41	2	30	25.24	(5.73- 111.19)
WUWILL	9	f	3	-	-	-	-	4.20	(3.00- 5.90)
WYNDE2	2	m	0	336	512	3	105	22.97	(7.23- 72.97)
WYNDE3	8	m	0	197	264	3	88	21.89	(6.82- 70.20)
WYNDE3	67	f	0	25	56	5	76	6.79	(2.45- 18.82)
Subtotal WYNDE3								11.28	(5.23- 24.31)
WYNDE4	69	m	2	-	-	-	-	15.45	(7.47- 31.96)
WYNDE4	54	f	2	-	-	-	-	5.82	(2.55- 13.31)
Subtotal WYNDE4								10.09	(5.85- 17.42)
WYNDE6	75	m	0	1706	1797	29	617	20.20	(13.84- 29.48)
WYNDE6	411	f	0	153	275	12	673	31.20	(17.05- 57.09)
Subtotal WYNDE6								22.83	(16.57- 31.46)
XU3	19	m	0	39	68	3	31	5.93	(1.70- 20.66)
XU3	23	f	0	15	11	2	25	17.05	(3.32- 87.61)
Subtotal XU3								8.74	(3.24- 23.59)
ZHENG	5	m	0	156	218	4	94	16.82	(6.05- 46.71)
ZHENG	18	f	0	43	44	33	184	5.45	(3.11- 9.54)
Subtotal ZHENG								7.07	(4.33- 11.56)
ZHOU	8	m	0	343	41	96	36	3.14	(1.90- 5.18)
ZHOU	9	f	0	35	7	42	32	3.81	(1.50- 9.68)
Subtotal ZHOU								3.28	(2.11- 5.10)
Partial Totals				16957	537887	1058	351349		

*prospective study ~ With 0.5 adjustment for zero

Table 2A2 - 5

IESLC - Meta-analysis of Ever Smoking, Cigarettes (or Any Product if Cigarettes not available)
Squamous
Least adjusted

REF	NRR	SEX	AD	Ys	Ws	Qs	Ps
*ABRAHA	1	m	0	4.53	0.50	2.59	0.0014
*ABRAHA	4	f	0	1.68	4.97	1.61	0.0002
Subtotal ABRAHA				1.94	5.46	4.21	
ALDERS	88	m	0	2.67	3.81	0.66	0.0000
ALDERS	84	f	0	1.97	13.33	1.00	0.0000
Subtotal ALDERS				2.13	17.14	1.66	
*ANDERS	10	f	0	3.24	4.63	4.57	0.0000
BAND	5	m	2	3.62	6.76	12.78	0.0000
BARBON	110	m	0	2.67	5.63	1.00	0.0000
BECHER	11	f	1	2.37	1.75	0.03	0.0017
BRESLO	7	c	0	1.44	11.20	7.37	0.0000
BROWN2	6	m	2	2.41	164.17	4.14	0.0000
BROWN2	5	f	2	3.00	89.84	50.87	0.0000
Subtotal BROWN2				2.62	254.01	55.01	
BUFFLE	49	m	0	2.64	3.25	0.50	0.0000
BUFFLE	62	f	0	2.57	2.74	0.28	0.0000
Subtotal BUFFLE				2.61	5.99	0.78	
BYERS1	1	m	0	2.12	19.01	0.34	0.0000
CHAN	18	m	0	2.71	1.86	0.40	0.0002
CHAN	22	f	0	1.96	8.84	0.72	0.0000
Subtotal CHAN				2.09	10.69	1.12	
CHOI	62	m	0	1.70	5.39	1.65	0.0001
CHOI	64	f	0	1.94	4.25	0.41	0.0001
Subtotal CHOI				1.80	9.63	2.06	
COMSTO	66	m	0	2.09	1.86	0.05	0.0045
COMSTO	78	f	0	3.83	0.48	1.21	0.0078
Subtotal COMSTO				2.45	2.34	1.26	
CORREA	35	c	1	3.34	21.64	25.93	0.0000
DAMBER	12	m	0	2.47	11.02	0.53	0.0000
DESTE2	16	m	2	2.58	3.60	0.40	0.0000
DOLL	82	m	0	2.57	2.84	0.29	0.0000
DOLL	84	f	0	0.88	7.63	14.30	0.0152
Subtotal DOLL				1.34	10.47	14.59	
DORGAN	113	m	2	2.94	3.87	1.85	0.0000
DORGAN	98	f	3	2.41	20.54	0.52	0.0000
Subtotal DORGAN				2.49	24.41	2.37	
DOSEME	19	m	0	1.41	40.28	28.40	0.0000
*ENGELA	62	m	7	1.86	2.73	0.40	0.0021
FAN	3	c	0	2.46	5.45	0.24	0.0000
GAO	7	m	0	2.17	11.51	0.07	0.0000
GAO	17	f	0	1.76	23.06	5.56	0.0000
Subtotal GAO				1.89	34.57	5.64	
GER	5	c	0	0.81	7.65	15.93	0.0259
HAENSZ	22	f	0	1.07	18.34	25.45	0.0000
*HAMMON	72	m	0	3.00	3.94	2.23	0.0000
HEGMAN	2	c	0	3.43	4.70	6.54	0.0000
HINDS	23	f	3	2.78	6.93	1.96	0.0000
ISHIMA	1	c	0	2.08	3.46	0.09	0.0001
JAHN	42	m	0	3.16	2.90	2.40	0.0000
JAIN	8	m	0	3.16	1.92	1.60	0.0000
JAIN	3	f	0	2.93	5.37	2.50	0.0000
Subtotal JAIN				2.99	7.29	4.10	
JEDRYC	7	m	0	2.73	5.75	1.36	0.0000
JOLY	54	m	0	3.44	1.96	2.78	0.0000
JOLY	52	f	0	2.92	5.02	2.27	0.0000
Subtotal JOLY				3.07	6.98	5.05	
JUSSAW	25	m	0	2.36	6.65	0.08	0.0000
KATSOU	35	f	0	1.81	5.97	1.16	0.0000
KHUDER	24	m	0	2.06	7.79	0.29	0.0000
KIHARA	26	c	0	3.29	4.63	5.07	0.0000
KOO	6	f	0	1.42	14.12	9.64	0.0000
KREYBE	16	m	0	2.55	2.94	0.27	0.0000
KREYBE	33	f	0	0.29	1.19	4.58	0.7520
Subtotal KREYBE				1.90	4.14	4.85	
LAMTH	1	f	0	2.09	8.66	0.21	0.0000
LAMWK	2	f	0	2.35	4.51	0.05	0.0000
LAMWK2	1	m	0	1.93	4.22	0.43	0.0001
LAMWK2	5	f	0	1.87	8.17	1.17	0.0000
Subtotal LAMWK2				1.89	12.38	1.60	
LOMBA2	2	f	0	1.45	11.86	7.65	0.0000
LUBIN	34	m	0	1.89	3.72	0.47	0.0003

Table 2A2 - 5

IESLC - Meta-analysis of Ever Smoking, Cigarettes (or Any Product if Cigarettes not available)
Squamous
Least adjusted

REF	NRR	SEX	AD	Ys	Ws	Qs	Ps
LUBIN2	145	m	0	2.81	51.88	16.53	0.0000
LUBIN2	165	f	0	1.75	46.51	11.33	0.0000
Subtotal LUBIN2				2.31	98.39	27.87	
LUO	2	c	0	2.01	4.12	0.24	0.0000
MATOS	66	m	0	1.81	2.72	0.53	0.0029
MATSUD	11	m	0	3.66	0.99	1.98	0.0003
NOU	1	m	0	3.30	1.92	2.13	0.0000
NOU	6	f	0	1.96	1.40	0.12	0.0205
Subtotal NOU				2.74	3.32	2.25	
ORMOS	8	m	0	2.32	1.85	0.01	0.0016
OSANN	18	m	0	3.58	7.69	13.72	0.0000
OSANN	22	f	0	3.25	10.83	10.81	0.0000
Subtotal OSANN				3.39	18.52	24.53	
OSANN2	7	f	0	2.72	5.39	1.21	0.0000
PEZZOT	6	m	0	4.14	0.49	1.77	0.0036
SCHWAR	10	m	0	3.49	0.97	1.50	0.0006
SCHWAR	9	m	0	0.61	2.26	6.06	0.3596
SCHWAR	18	f	0	3.77	0.49	1.12	0.0086
SCHWAR	17	f	0	4.14	0.47	1.69	0.0044
Subtotal SCHWAR				2.04	4.19	10.37	
SEOW	3	f	0	2.86	4.50	1.70	0.0000
SIEMIA	11	m	0	3.37	2.87	3.62	0.0000
SOBUE	3	m	0	2.88	2.90	1.15	0.0000
SOBUE	19	f	0	2.25	9.55	0.00	0.0000
Subtotal SOBUE				2.40	12.46	1.15	
STASZE	16	m	0	4.19	0.50	1.86	0.0032
STASZE	38	f	0	3.48	0.37	0.57	0.0333
Subtotal STASZE				3.88	0.87	2.43	
STAYNE	3	m	0	1.24	17.27	17.40	0.0000
SUZUK2	12	c	0	2.91	3.79	1.65	0.0000
SVENSS	57	f	0	2.56	4.16	0.41	0.0000
TIZZAN	18	c	0	0.99	40.59	63.86	0.0000
TOKARS	9	c	0	1.71	1.70	0.49	0.0254
TSUGAN	13	m	0	2.68	0.44	0.08	0.0772
WAKAI	15	m	0	2.16	1.90	0.02	0.0029
WAKAI	33	f	0	3.22	2.30	2.16	0.0000
Subtotal WAKAI				2.74	4.20	2.17	
WU	14	f	0	3.23	1.75	1.68	0.0000
WUWILL	9	f	3	1.44	33.59	22.21	0.0000
WYNDE2	2	m	0	3.13	2.88	2.26	0.0000
WYNDE3	8	m	0	3.09	2.83	1.99	0.0000
WYNDE3	67	f	0	1.91	3.69	0.41	0.0002
Subtotal WYNDE3				2.42	6.52	2.40	
WYNDE4	69	m	2	2.74	7.27	1.74	0.0000
WYNDE4	54	f	2	1.76	5.63	1.33	0.0000
Subtotal WYNDE4				2.31	12.90	3.08	
WYNDE6	75	m	0	3.01	26.85	15.40	0.0000
WYNDE6	411	f	0	3.44	10.53	14.97	0.0000
Subtotal WYNDE6				3.13	37.38	30.37	
XU3	19	m	0	1.78	2.46	0.54	0.0052
XU3	23	f	0	2.84	1.43	0.50	0.0007
Subtotal XU3				2.17	3.90	1.04	
ZHENG	5	m	0	2.82	3.68	1.21	0.0000
ZHENG	18	f	0	1.70	12.24	3.74	0.0000
Subtotal ZHENG				1.96	15.92	4.95	
ZHOU	8	m	0	1.14	15.27	18.64	0.0000
ZHOU	9	f	0	1.34	4.42	3.66	0.0049
Subtotal ZHOU				1.19	19.68	22.30	

Table 2A2 - 5

IESLC - Meta-analysis of Ever Smoking, Cigarettes (or Any Product if Cigarettes not available)
Squamous
Least adjusted

	N	102
	NS	73
	Wt	1028.13
Het	Chi	522.83
Het	df	101
Het	P	***
Fixed	RR	9.47
	RRl	8.91
	RRu	10.07
	P	+++
Random	RR	10.39
	RRl	8.85
	RRu	12.19
	P	+++
Asymm	P	N.S.

Table 2A2 - 6

IESLC - Meta-analysis of Ever Smoking, Cigarettes (or Any Product if Cigarettes not available)
Squamous
Least adjusted

		Sex		
	combined	male	female	Total
N	11	49	42	102
NS	11	48	41	100
Wt	108.93	487.76	431.43	1028.13
Het Chi	115.66	170.67	215.97	522.83
Het df	10	48	41	101
Het P	***	***	***	***
Fixed RR	6.82	10.71	8.95	9.47
RRl	5.65	9.80	8.15	8.91
RRu	8.23	11.71	9.84	10.07
P	+++	+++	+++	+++
Random RR	9.17	12.07	9.06	10.39
RRl	4.54	9.79	7.08	8.85
RRu	18.50	14.89	11.59	12.19
P	+++	+++	+++	+++
Between Chi				20.53
Between df				2
Between P				***
Btwn(F) P				N.S.
Btwn(R) P				N.S.

Table 2A2 - 7

IESLC - Meta-analysis of Ever Smoking, Cigarettes (or Any Product if Cigarettes not available)
Squamous
Excluded studies (and stage at which they were excluded)

Stage	Studies
1	BOUCHA BUELL LAURIL MZILEN
2	BYERS2 ENSTRO HIRAY2 KANELL LIAW LICKIN MRFIT MURATA NOTANI RESTRE RIMING SAARIK SEGI2 SHIMIZ SITAS SOBUE2 TANG2 WALD WARSIN WATSON WU2 WYNDE5 WYNDE8 WYNDER
3	BERRIN CHEN TAO ULMER
6	BLOT1 BLOT2 BLOT3 HIRAOK JONES MOLLO PERSHA SCHWA2 VANDER
8	ABELIN AGUDO AKIBA AMANDU AMES ARCHER ARMADA AUSTIN AUVINE AXELSO AXELSS BENSHL BEST BLOHMK BLOT4 BOFFET BOUCOT BRETT BROCKM BROSS BROWN1 CARPEN CASCO2 CASCOR CEDERL CHANG CHATZI CHEN2 CHEN3 CHIAZZ CHOW CHYOU COOKSO CPSI CPSII DARBY DAVEYS DEAN DEAN2 DEAN3 DEKLER DESTEF DOCKER DOLL2 DORANT DORN DROSTE DU DUNN EBELIN ESAKI GAO2 GARCIA GARDIN GARSHI GENG GILLIS GODLEY GOLLED GOODMA GRAHAM GREGOR GSELL HAMMO2 HANSEN HEIN HENNEK HIRAYA HITOSU HOLE HOROWI HORWIT HU HU2 HUANG HUMBLE JARUP JARVHO JIANG KAISE2 KAISER KAUFMA KELLER KINLEN KJUUS KNEKT KO KOHLME KOULUM KREUZE KUBIK LANGE LAUSSM LEI LEMARC LETOUR LEVIN LIDDEL LIU LIU2 LIU3 LIU4 LIU5 LOMBAR MACLEN MAGNUS MARSH MARSH2 MARTIS MASTRA MCCONN MCDUFF MCLAUG MIGRAN MILLER MILLS MRFITR NAM NOTAN2 ODRISC PARKIN PASTOR PAWLEG PERNU PERSH2 PETO PEZZO2 PIKE PISANI POFFIJ POLEDN PRESCO QIAO QIAO2 RACHTA RADZIK RANDIG REN RONCO ROOTS ROTHSC SADOWS SANKAR SEGI SHAW SIMARA SIMONA SPEIZE SPITZ STOCKS STOCKW STUCKE SUN SUZUKI TANG TENKAN TOUSEY TULINI TVERDA VEIERO WANG WANG2 WANG3 WANG4 WICKLU WIGLE WILKIN WUNSCH XIANGZ XU XU2 XU4 YAMAGU YONG YUAN ZHANG
10	BENHAM VUTUC WYNDE7

Table 2A2 - 8
Potentially overlapping studies

REF	REFGP	PRINC	OVERLAP/LINK
LUBIN2	LUBIN2	1	Lubin-combined
CHAN	CHAN	1	Chan/LamWK2
LAMWK2	LAMWK2	1	Chan/LamWK2
LAMTH	LAMTH	1	KOO/LAMTH/LAMWK
KOO	KOO	1	KOO/LAMTH/LAMWK
LAMWK	LAMWK	1	KOO/LAMTH/LAMWK
ISHIMA	AKIBA	2	AKIBA/ISHIMA
BYERS1	BYERS1	2	GRAHAM/BROSS/BYERS1
OSANN2	KAISER	2	KAISER/OSANN2
WYNDE6	WYNDE6	1	WYNDE5/6/7/8
MATSUD	MATSUD	1	SOBUE2/MATSUD
JAHN	BOFFET	2	Subset of BOFFET
LUBIN	XIANGZ	2	LUBIN/XIANGZ/QIAO

Table 2A2 - 9
Most adjusted - insufficient data for metaanalysis

REF	NRR	SEX	AGEL	AGEH	RACE	YF	LC TYPE	LOC	START	ST	NLC	R	VB	P	H	AD	PRODUCT	DENOM	De
BUFFLE	47	f	0	0	wh	-	q	NAmer	1976	CC	943	n	bl	y	n	0	cig+/-ot	nev cigs	or
CHEN	1	c	0	0	all	-	q	As:oth	1987	CC	323	n	ot	n	y	2	cig+/-ot	nev cigs	ot
JIANG	3	m	0	0	all	-	q	As:Chi	1984	CC	125	n	ot	n	n	0	all/unsp	nev any	or
JIANG	4	f	0	0	all	-	q	As:Chi	1984	CC	125	n	ot	n	n	0	all/unsp	nev any	ot
KOO	2	f	0	0	all	-	q	As:HK	1981	CC	200	n	bl	n	n	0	all/unsp	nev any	or
WANG	3	c	0	0	all	-	q	As:Chi	1990	CC	390	n	ot	*	y	0	all/unsp	nev any	ot

REF	NRR	RR	SIG	RRDATA comment
BUFFLE	47	8.30		0
CHEN	1	6.66	y	P < 0.001
JIANG	3	12.78		0
JIANG	4	*	n	0
KOO	2	3.62		0
WANG	3	4.66	y	p<0.01

Table 2A3 -

IESLC - Meta-analysis of Ever Smoking, Cigarettes only
Squamous

This analysis is restricted to results for:
1) Non-dose-response data
2) Ever smokers
3) Results complete enough for use in metaanalysis

Within each study, results are then selected (in the following order of preference, within each sex) for:
4) PRODUCT: cigarettes only
5) CIGTYPE: all/unspecified, MC regardless of HR, MC only
6) DENOM: never smoked anything, never smoked cigarettes, (never +1 = +long term ex, +2 = +amount unknown,
+3 = never cigs+long term ex)
7) Followup period (YF, prospective studies): whole study (coded as 0) or longest available
8) LCtype: squamous or nearest available, but not adeno. (q = squamous, s = small,
a = adeno, KI = Kreyberg I, u = undifferentiated)
9) Race: all or nearest available, otherwise by race (wh or w = white, bl or b = black, hi = hispanic
ch = chinese, jap = japanese, haw = hawaiian, w+o = white + oriental, sca = scandinavian, as = asian)
10) For overlapping studies: principal rather than subsidiary studies
Finally by Age: whole study (coded as 0) if available, otherwise by widest available age group
and then for single sex results (m, f) in preference to combined sex results (c).

Results adjusted (AD) for the most potential confounders are then chosen in Sections -1 to -3
(and those which actually differ from the adjusted results in Table 2A1 - 1 are marked 'x' in Section -1)
and results adjusted for the least confounders in Sections -4 to -6. (Those least adjusted results which
actually differ from the most adjusted as marked 'x' in column X in Section -4)
(Results adjusted for an unknown number of confounder(s) are coded as 20.)

Section -7 shows excluded studies, together with the stage (as above) at which no qualifying
results were found.

Section -8 lists the potentially overlapping studies which have been included (1=principal, 2=subsidiary).

Section -9 lists any results which would have been included in preference except that they had data not complete
enough for use in meta-analysis, with their significance (yes/no), if known, and any further comment as entered
on the database.

In addition to those mentioned above, the following fields, levels and abbreviations are used:
* or nk = not known, n = no, y = yes, ot = other
nev = never
all/unspec = all or unspecified, MC = manufactured cigarettes, HR = hand-rolled cigarettes
REF: 6-character study reference
NRR: number of the RR on the database within the study
ST : study type (CC = case control, pr or prosp = prospective)
NLC: number of lung cancer cases in whole study
R : risky occupational population (n = no, m = mining, o = other risky)
VB : national cigarette type (V = at least 75% Virginia, bl = at least 75% blended, ot = other)
P : any proxy use
H : full histological confirmation
De : derivation of RR/CI (or = original, st = standard method, ot = other method of estimation)

Table 2A3 - 1

IESLC - Meta-analysis of Ever Smoking, Cigarettes only
Squamous
Most adjusted

REF	NRR	2A1	SEX	AGEL	AGEH	RACE	YF	LC TYPE	LOC	START	ST	NLC	R	VB	P	H	AD	PRODUCT	DENOM	De
ALDERS	74	x	m	0	0	all	-	q+s	Eu:UK	1977	CC	1448	n	V	n	n	1	cig only	nev any	ot
ALDERS	33	x	f	0	0	all	-	q+s	Eu:UK	1977	CC	1448	n	V	n	n	1	cig only	nev any	ot
BAND	5		m	0	0	all	-	q	NAmer	1983	CC	2831	n	V	y	y	2	cig only	nev any	ot
BRESLO	8	x	c	0	0	all	-	not a	NAmer	1949	CC	518	n	bl	n	y	0	cig only	nev+1	st
HAMMON	58	x	m	0	0	wh	0	not a	NAmer	1952	pr	448	n	bl	n	n	1	cig only	nev any	ot
JUSSAW	25	x	m	0	0	all	-	KI	As:Ind	1964	CC	792	n	V	n	n	0	cig only	nev any	st
LUBIN	15	x	m	0	0	all	-	KI	As:Chi	1984	CC	427	m	ot	y	n	0	cig only	nev any	st
LUBIN2	141	x	m	0	0	all	-	q	Eu:mul	1976	CC	7804	n	bl	n	y	0	cig only	nev any	st
PEZZOT	6		m	0	0	all	-	q	SCAmer	1987	CC	215	n	bl	n	y	0	cig only	nev cigs	ot
STASZE	13	x	m	0	0	all	-	q	Eu:est	1954	CC	281	n	bl	n	y	0	cig only	nev any	ot
WYNDE7	59	x	m	0	0	all	-	KI	NAmer	1977	CC	2085	n	bl	n	y	0	cig only	nev any	st

Cigarette type is all/unspec for all RRs

except for the following:

REF	NRR	CIGTYPE
ALDERS	33	MC only
JUSSAW	25	MC only

Table 2A3 - 2

IESLC - Meta-analysis of Ever Smoking, Cigarettes only
Squamous
Most adjusted

REF	NRR	SEX	AD	Number Exposed Case	Number Exposed Cont	Non-exposed Case	Non-exposed Cont	RR	95.00%CI
ALDERS	74	m	1	-	-	-	-	11.58	(4.25- 31.54)
ALDERS	33	f	1	-	-	-	-	6.70	(3.92- 11.46)
Subtotal ALDERS								7.57	(4.72- 12.14)
BAND	5	m	2	-	-	-	-	37.45	(17.62- 79.58)
BRESLO	8	c	0	298	240	15	56	4.64	(2.56- 8.40)
*HAMMON	58	m	1	-	-	-	-	23.12	(8.57- 62.34)
JUSSAW	25	m	0	17	77	13	624	10.60	(4.96- 22.66)
LUBIN	15	m	0	42	191	4	72	3.96	(1.37- 11.43)
LUBIN2	141	m	0	3474	9345	127	2616	7.66	(6.38- 9.19)
PEZZOT	6	m	0	85	317	0	116	62.74~	(3.86-1019.50)
STASZE	13	m	0	117	552	0	158	67.42~	(4.17-1090.28)
WYNDE7	59	m	0	1000	2108	22	918	19.79	(12.88- 30.41)
Partial Totals				5033	12830	181	4560		

*prospective study ~ With 0.5 adjustment for zero

REF	NRR	SEX	AD	Ys	Ws	Qs	Ps
ALDERS	74	m	1	2.45	3.82	0.23	0.0000
ALDERS	33	f	1	1.90	13.35	1.21	0.0000
Subtotal ALDERS				2.02	17.18	1.44	
BAND	5	m	2	3.62	6.76	13.63	0.0000
BRESLO	8	c	0	1.53	10.86	4.87	0.0000
*HAMMON	58	m	1	3.14	3.90	3.43	0.0000
JUSSAW	25	m	0	2.36	6.65	0.16	0.0000
LUBIN	15	m	0	1.38	3.41	2.34	0.0110
LUBIN2	141	m	0	2.04	115.59	3.24	0.0000
PEZZOT	6	m	0	4.14	0.49	1.85	0.0036
STASZE	13	m	0	4.21	0.50	2.00	0.0030
WYNDE7	59	m	0	2.99	20.83	12.74	0.0000

	N	11
	NS	10
	Wt	186.18
Het	Chi	45.71
Het	df	10
Het	P	***
Fixed	RR	9.05
	RRl	7.84
	RRu	10.45
	P	+++
Random	RR	11.50
	RRl	7.47
	RRu	17.69
	P	+++
Asymm	P	N.S.

Table 2A3 - 3

IESLC - Meta-analysis of Ever Smoking, Cigarettes only
Squamous
Most adjusted

	Sex			
	combined	male	female	Total
N	1	9	1	11
NS	1	9	1	11
Wt	10.86	161.96	13.35	186.18
Het Chi	0.00	38.84	0.00	45.71
Het df	0	8	0	10
Het P	N.S.	***	N.S.	***
Fixed RR	4.64	9.71	6.70	9.05
RRl	2.56	8.32	3.92	7.84
RRu	8.40	11.32	11.46	10.45
P	+++	+++	+++	+++
Random RR	4.64	14.55	6.70	11.50
RRl	2.56	8.53	3.92	7.47
RRu	8.40	24.81	11.46	17.69
P	+++	+++	+++	+++
Between Chi				6.87
Between df				2
Between P				*
Btwn(F) P				N.S.
Btwn(R) P				*

	Lung cancer type					
	q	q+s	q+u	KI	not a	Total
N	4	2		3	2	11
NS	4	1		3	2	10
Wt	123.34	17.18		30.89	14.77	186.18
Het Chi	20.23	0.89		8.43	7.41	45.71
Het df	3	1		2	1	10
Het P	***	N.S.		*	**	***
Fixed RR	8.50	7.57		14.48	7.09	9.05
RRl	7.12	4.72		10.18	4.26	7.84
RRu	10.14	12.14		20.61	11.80	10.45
P	+++	+++		+++	+++	+++
Random RR	23.96	7.57		10.49	9.84	11.50
RRl	6.44	4.72		4.45	2.04	7.47
RRu	89.16	12.14		24.73	47.36	17.69
P	+++	+++		+++	++	+++
Between Chi						8.75
Between df						3
Between P						*
Btwn(F) P						N.S.
Btwn(R) P						N.S.

	Location								
	NAmer	UK	Scand	othEur	China	Japan	othAs	other	Total
N	4	2		2	1		1	1	11
NS	4	1		2	1		1	1	10
Wt	42.35	17.18		116.09	3.41		6.65	0.49	186.18
Het Chi	22.95	0.89		2.34	0.00		0.00	0.00	45.71
Het df	3	1		1	0		0	0	10
Het P	***	N.S.		N.S.	N.S.		N.S.	N.S.	***
Fixed RR	15.32	7.57		7.73	3.96		10.60	62.74	9.05
RRl	11.34	4.72		6.44	1.37		4.96	3.86	7.84
RRu	20.70	12.14		9.27	11.43		22.66	1019.50	10.45
P	+++	+++		+++	+		+++	++	+++
Random RR	16.37	7.57		14.32	3.96		10.60	62.74	11.50
RRl	6.65	4.72		2.08	1.37		4.96	3.86	7.47
RRu	40.29	12.14		98.68	11.43		22.66	1019.50	17.69
P	+++	+++		++	+		+++	++	+++
Between Chi									19.53
Between df									5
Between P									**
Btwn(F) P									N.S.
Btwn(R) P									N.S.

Table 2A3 - 3

IESLC - Meta-analysis of Ever Smoking, Cigarettes only
Squamous
Most adjusted

Detailed Country in "other Europe"

	multi	Germany	othWest	East	Balkans	Total
N	1			1		2
NS	1			1		2
Wt	115.59			0.50		116.09
Het Chi	0.00			0.00		2.34
Het df	0			0		1
Het P	N.S.			N.S.		N.S.
Fixed RR	7.66			67.42		7.73
RRl	6.38			4.17		6.44
RRu	9.19			1090.28		9.27
P	+++			++		+++
Random RR	7.66			67.42		14.32
RRl	6.38			4.17		2.08
RRu	9.19			1090.28		98.68
P	+++			++		++
Between Chi						2.34
Between df						1
Between P						N.S.
Btwn(F) P						N.S.
Btwn(R) P						N.S.

Detailed Country in "other Asia"

	India	HongKong	other	Total
N	1			1
NS	1			1
Wt	6.65			6.65
Het Chi	0.00			0.00
Het df	0			0
Het P	N.S.			N.S.
Fixed RR	10.60			10.60
RRl	4.96			4.96
RRu	22.66			22.66
P	+++			+++
Random RR	10.60			10.60
RRl	4.96			4.96
RRu	22.66			22.66
P	+++			+++
Between Chi				
Between df				
Between P				N.S.
Btwn(F) P				N.S.
Btwn(R) P				N.S.

Detailed other continent

	SCAmer	Auslia	Africa	Total
N	1			1
NS	1			1
Wt	0.49			0.49
Het Chi	0.00			0.00
Het df	0			0
Het P	N.S.			N.S.
Fixed RR	62.74			62.74
RRl	3.86			3.86
RRu	1019.50			1019.50
P	++			++
Random RR	62.74			62.74
RRl	3.86			3.86
RRu	1019.50			1019.50
P	++			++
Between Chi				
Between df				
Between P				N.S.
Btwn(F) P				N.S.
Btwn(R) P				N.S.

Table 2A3 - 3

IESLC - Meta-analysis of Ever Smoking, Cigarettes only
Squamous
Most adjusted

	Start year of study					
	<1960	1960-69	1970-79	1980-89	1990+	Total
N	3	1	4	3		11
NS	3	1	3	3		10
Wt	15.26	6.65	153.59	10.67		186.18
Het Chi	9.85	0.00	17.18	12.22		45.71
Het df	2	0	3	2		10
Het P	**	N.S.	***	**		***
Fixed RR	7.63	10.60	8.70	18.69		9.05
RRl	4.62	4.96	7.43	10.25		7.84
RRu	12.59	22.66	10.19	34.05		10.45
P	+++	+++	+++	+++		+++
Random RR	13.83	10.60	10.25	17.81		11.50
RRl	3.17	4.96	5.98	2.93		7.47
RRu	60.45	22.66	17.56	108.24		17.69
P	+++	+++	+++	++		+++
Between Chi						6.46
Between df						3
Between P						(*)
Btwn(F) P						N.S.
Btwn(R) P						N.S.

	Study type (1)		
	CC	other	Total
N	10	1	11
NS	9	1	10
Wt	182.27	3.90	186.18
Het Chi	42.20	0.00	45.71
Het df	9	0	10
Het P	***	N.S.	***
Fixed RR	8.87	23.12	9.05
RRl	7.68	8.57	7.84
RRu	10.26	62.36	10.45
P	+++	+++	+++
Random RR	10.78	23.12	11.50
RRl	6.90	8.57	7.47
RRu	16.85	62.36	17.69
P	+++	+++	+++
Between Chi			3.50
Between df			1
Between P			(*)
Btwn(F) P			N.S.
Btwn(R) P			N.S.

	Study type (2)			
	CC	prosp	other	Total
N	10	1		11
NS	9	1		10
Wt	182.27	3.90		186.18
Het Chi	42.20	0.00		45.71
Het df	9	0		10
Het P	***	N.S.		***
Fixed RR	8.87	23.12		9.05
RRl	7.68	8.57		7.84
RRu	10.26	62.36		10.45
P	+++	+++		+++
Random RR	10.78	23.12		11.50
RRl	6.90	8.57		7.47
RRu	16.85	62.36		17.69
P	+++	+++		+++
Between Chi				3.50
Between df				1
Between P				(*)
Btwn(F) P				N.S.
Btwn(R) P				N.S.

Table 2A3 - 3

IESLC - Meta-analysis of Ever Smoking, Cigarettes only
Squamous
Most adjusted

	Study size (number of LC cases)				
	100-249	250-499	500-999	1000+	Total
N	1	3	2	5	11
NS	1	3	2	4	10
Wt	0.49	7.81	17.52	160.35	186.18
Het Chi	0.00	7.34	2.82	30.98	45.71
Het df	0	2	1	4	10
Het P	N.S.	*	(*)	***	***
Fixed RR	62.74	11.44	6.35	9.25	9.05
RRl	3.86	5.68	3.97	7.92	7.84
RRu	1019.50	23.07	10.14	10.80	10.45
P	++	+++	+++	+++	+++
Random RR	62.74	13.93	6.77	12.97	11.50
RRl	3.86	3.01	3.02	7.14	7.47
RRu	1019.50	64.45	15.17	23.56	17.69
P	++	+++	+++	+++	+++
Between Chi					4.57
Between df					3
Between P					N.S.
Btwn(F) P					N.S.
Btwn(R) P					N.S.

	Risky occupational population			
	no	mining	othRisky	Total
N	10	1		11
NS	9	1		10
Wt	182.76	3.41		186.18
Het Chi	43.32	0.00		45.71
Het df	9	0		10
Het P	***	N.S.		***
Fixed RR	9.20	3.96		9.05
RRl	7.95	1.37		7.84
RRu	10.63	11.43		10.45
P	+++	+		+++
Random RR	12.59	3.96		11.50
RRl	8.03	1.37		7.47
RRu	19.74	11.43		17.69
P	+++	+		+++
Between Chi				2.38
Between df				1
Between P				N.S.
Btwn(F) P				N.S.
Btwn(R) P				*

	National cigarette tobacco type			
	Virginia	blended	other	Total
N	4	6	1	11
NS	3	6	1	10
Wt	30.59	152.17	3.41	186.18
Het Chi	13.36	27.99	0.00	45.71
Het df	3	5	0	10
Het P	**	***	N.S.	***
Fixed RR	11.59	8.78	3.96	9.05
RRl	8.13	7.49	1.37	7.84
RRu	16.53	10.29	11.43	10.45
P	+++	+++	+	+++
Random RR	13.02	12.57	3.96	11.50
RRl	5.96	6.64	1.37	7.47
RRu	28.48	23.77	11.43	17.69
P	+++	+++	+	+++
Between Chi				4.36
Between df				2
Between P				N.S.
Btwn(F) P				N.S.
Btwn(R) P				N.S.

Table 2A3 - 3

IESLC - Meta-analysis of Ever Smoking, Cigarettes only
Squamous
Most adjusted

	Any proxy use No/nk	Yes	Total
N	9	2	11
NS	8	2	10
Wt	176.00	10.17	186.18
Het Chi	29.48	11.45	45.71
Het df	8	1	10
Het P	***	***	***
Fixed RR	8.71	17.62	9.05
RRl	7.52	9.53	7.84
RRu	10.10	32.57	10.45
P	+++	+++	+++
Random RR	10.68	12.57	11.50
RRl	7.04	1.39	7.47
RRu	16.21	113.64	17.69
P	+++	+	+++
Between Chi			4.77
Between df			1
Between P			*
Btwn(F) P			N.S.
Btwn(R) P			N.S.

	Full histological confirmation No	Yes	Total
N	5	6	11
NS	4	6	10
Wt	31.14	155.03	186.18
Het Chi	7.33	38.32	45.71
Het df	4	5	10
Het P	N.S.	***	***
Fixed RR	8.71	9.12	9.05
RRl	6.13	7.80	7.84
RRu	12.38	10.68	10.45
P	+++	+++	+++
Random RR	9.19	14.63	11.50
RRl	5.55	7.20	7.47
RRu	15.22	29.70	17.69
P	+++	+++	+++
Between Chi			0.06
Between df			1
Between P			N.S.
Btwn(F) P			N.S.
Btwn(R) P			N.S.

	Number of adjustment variables (1) 0	1	2+/+nk	Total
N	7	3	1	11
NS	7	2	1	10
Wt	158.34	21.08	6.76	186.18
Het Chi	26.55	4.86	0.00	45.71
Het df	6	2	0	10
Het P	***	(*)	N.S.	***
Fixed RR	8.49	9.31	37.45	9.05
RRl	7.27	6.07	17.62	7.84
RRu	9.92	14.26	79.59	10.45
P	+++	+++	+++	+++
Random RR	9.49	11.08	37.45	11.50
RRl	5.59	5.26	17.62	7.47
RRu	16.13	23.33	79.59	17.69
P	+++	+++	+++	+++
Between Chi				14.30
Between df				2
Between P				***
Btwn(F) P				N.S.
Btwn(R) P				*

Table 2A3 - 3

IESLC - Meta-analysis of Ever Smoking, Cigarettes only
Squamous
Most adjusted

Number of adjustment variables (2)

	0	1	2	3-5	6+/+nk	Total
N	7	3	1			11
NS	7	2	1			10
Wt	158.34	21.08	6.76			186.18
Het Chi	26.55	4.86	0.00			45.71
Het df	6	2	0			10
Het P	***	(*)	N.S.			***
Fixed RR	8.49	9.31	37.45			9.05
RRl	7.27	6.07	17.62			7.84
RRu	9.92	14.26	79.59			10.45
P	+++	+++	+++			+++
Random RR	9.49	11.08	37.45			11.50
RRl	5.59	5.26	17.62			7.47
RRu	16.13	23.33	79.59			17.69
P	+++	+++	+++			+++
Between Chi						14.30
Between df						2
Between P						***
Btwn(F) P						N.S.
Btwn(R) P						*

Product

	all/unsp	cig+/-ot	cig only	Total
N			11	11
NS			10	10
Wt			186.18	186.18
Het Chi			45.71	45.71
Het df			10	10
Het P			***	***
Fixed RR			9.05	9.05
RRl			7.84	7.84
RRu			10.45	10.45
P			+++	+++
Random RR			11.50	11.50
RRl			7.47	7.47
RRu			17.69	17.69
P			+++	+++
Between Chi				
Between df				
Between P				N.S.
Btwn(F) P				N.S.
Btwn(R) P				N.S.

Denominator

	nev any	nev cigs	Total
N	10	1	11
NS	9	1	10
Wt	185.68	0.49	186.18
Het Chi	43.85	0.00	45.71
Het df	9	0	10
Het P	***	N.S.	***
Fixed RR	9.01	62.74	9.05
RRl	7.80	3.86	7.84
RRu	10.40	1019.50	10.45
P	+++	++	+++
Random RR	11.09	62.74	11.50
RRl	7.19	3.86	7.47
RRu	17.09	1019.50	17.69
P	+++	++	+++
Between Chi			1.86
Between df			1
Between P			N.S.
Btwn(F) P			N.S.
Btwn(R) P			N.S.

Table 2A3 - 3

IESLC - Meta-analysis of Ever Smoking, Cigarettes only
Squamous
Most adjusted

	Derivation of RR/CI			
	Orig	StdCalc	Other	Total
N		5	6	11
NS		5	5	10
Wt		157.35	28.83	186.18
Het Chi		22.42	17.25	45.71
Het df		4	5	10
Het P		***	**	***
Fixed RR		8.38	13.79	9.05
RRl		7.17	9.57	7.84
RRu		9.80	19.87	10.45
P		+++	+++	+++
Random RR		8.36	18.90	11.50
RRl		4.93	8.39	7.47
RRu		14.20	42.57	17.69
P		+++	+++	+++
Between Chi				6.04
Between df				1
Between P				*
Btwn(F) P				N.S.
Btwn(R) P				(*)

Table 2A3 - 4

IESLC - Meta-analysis of Ever Smoking, Cigarettes only
Squamous
Least adjusted

REF	NRR	X	SEX	AGEL	AGEH	RACE	YF	LC TYPE	LOC	START	ST	NLC	R	VB	P	H	AD	PRODUCT	DENOM	De
ALDERS	87	x	m	0	0	all	-	q+s	Eu:UK	1977	CC	1448	n	V	n	n	0	cig only	nev any	st
ALDERS	84	x	f	0	0	all	-	q+s	Eu:UK	1977	CC	1448	n	V	n	n	0	cig only	nev any	st
BAND	5		m	0	0	all	-	q	NAmer	1983	CC	2831	n	V	y	y	2	cig only	nev any	ot
BRESLO	8		c	0	0	all	-	not a	NAmer	1949	CC	518	n	bl	n	y	0	cig only	nev+1	st
HAMMON	71	x	m	0	0	wh	0	not a	NAmer	1952	pr	448	n	bl	n	n	0	cig only	nev any	st
JUSSAW	25		m	0	0	all	-	KI	As:Ind	1964	CC	792	n	V	n	n	0	cig only	nev any	st
LUBIN	15		m	0	0	all	-	KI	As:Chi	1984	CC	427	m	ot	y	n	0	cig only	nev any	st
LUBIN2	141		m	0	0	all	-	q	Eu:mul	1976	CC	7804	n	bl	n	y	0	cig only	nev any	st
PEZZOT	6		m	0	0	all	-	q	SCAmer	1987	CC	215	n	bl	n	y	0	cig only	nev cigs	ot
STASZE	13		m	0	0	all	-	q	Eu:est	1954	CC	281	n	bl	n	y	0	cig only	nev any	ot
WYNDE7	59		m	0	0	all	-	KI	NAmer	1977	CC	2085	n	bl	n	y	0	cig only	nev any	st

Cigarette type is all/unspec for all RRs

except for the following:

REF	NRR	CIGTYPE
ALDERS	84	MC only
JUSSAW	25	MC only

Table 2A3 - 5

IESLC - Meta-analysis of Ever Smoking, Cigarettes only
Squamous
Least adjusted

REF	NRR	SEX	AD	Number Exposed Case	Cont	Non-exposed Case	Cont	RR	95.00%CI
ALDERS	87	m	0	207	462	4	133	14.90	(5.44- 40.82)
ALDERS	84	f	0	176	371	16	243	7.20	(4.21- 12.32)
Subtotal ALDERS								8.46	(5.27- 13.59)
BAND	5	m	2	-	-	-	-	37.45	(17.62- 79.58)
BRESLO	8	c	0	298	240	15	56	4.64	(2.56- 8.40)
*HAMMON	71	m	0	162	225565	4	115884	20.81	(7.72- 56.11)
JUSSAW	25	m	0	17	77	13	624	10.60	(4.96- 22.66)
LUBIN	15	m	0	42	191	4	72	3.96	(1.37- 11.43)
LUBIN2	141	m	0	3474	9345	127	2616	7.66	(6.38- 9.19)
PEZZOT	6	m	0	85	317	0	116	62.74~	(3.86-1019.50)
STASZE	13	m	0	117	552	0	158	67.42~	(4.17-1090.28)
WYNDE7	59	m	0	1000	2108	22	918	19.79	(12.88- 30.41)
Partial Totals				5578	239228	205	120820		

*prospective study ~ With 0.5 adjustment for zero

REF	NRR	SEX	AD	Ys	Ws	Qs	Ps
ALDERS	87	m	0	2.70	3.78	0.91	0.0000
ALDERS	84	f	0	1.97	13.33	0.75	0.0000
Subtotal ALDERS				2.14	17.12	1.65	
BAND	5	m	2	3.62	6.76	13.47	0.0000
BRESLO	8	c	0	1.53	10.86	4.99	0.0000
*HAMMON	71	m	0	3.04	3.90	2.65	0.0000
JUSSAW	25	m	0	2.36	6.65	0.15	0.0000
LUBIN	15	m	0	1.38	3.41	2.38	0.0110
LUBIN2	141	m	0	2.04	115.59	3.57	0.0000
PEZZOT	6	m	0	4.14	0.49	1.84	0.0036
STASZE	13	m	0	4.21	0.50	1.98	0.0030
WYNDE7	59	m	0	2.99	20.83	12.48	0.0000

	N	11
	NS	10
	Wt	186.12
Het	Chi	45.16
Het	df	10
Het	P	***
Fixed	RR	9.13
	RRl	7.91
	RRu	10.54
	P	+++
Random	RR	11.73
	RRl	7.64
	RRu	18.01
	P	+++
Asymm	P	N.S.

Table 2A3 - 6

IESLC - Meta-analysis of Ever Smoking, Cigarettes only
Squamous
Least adjusted

		Sex		
	combined	male	female	Total
N	1	9	1	11
NS	1	9	1	11
Wt	10.86	161.92	13.33	186.12
Het Chi	0.00	38.74	0.00	45.16
Het df	0	8	0	10
Het P	N.S.	***	N.S.	***
Fixed RR	4.64	9.74	7.20	9.13
RRl	2.56	8.35	4.21	7.91
RRu	8.40	11.36	12.32	10.54
P	+++	+++	+++	+++
Random RR	4.64	14.78	7.20	11.73
RRl	2.56	8.67	4.21	7.64
RRu	8.40	25.20	12.32	18.01
P	+++	+++	+++	+++
Between Chi				6.42
Between df				2
Between P				*
Btwn(F) P				N.S.
Btwn(R) P				*

Table 2A3 - 7

IESLC - Meta-analysis of Ever Smoking, Cigarettes only
Squamous
Excluded studies (and stage at which they were excluded)

Stage	Studies
1	BOUCHA BUELL LAURIL MZILEN
2	BYERS2 ENSTRO HIRAY2 KANELL LIAW LICKIN MRFIT MURATA NOTANI RESTRE RIMING SAARIK SEGI2 SHIMIZ SITAS SOBUE2 TANG2 WALD WARSIN WATSON WU2 WYNDE5 WYNDE8 WYNDER
3	BERRIN CHEN TAO ULMER
4	ABRAHA AKIBA AMANDU AMES ANDERS ARCHER AUSTIN AUVINE AXELSO AXELSS BARBON BECHER BENSHL BLOHMK BLOT1 BLOT2 BLOT3 BLOT4 BRETT BROCKM BROSS BROWN1 BROWN2 BUFFLE BYERS1 CARPEN CASCO2 CASCOR CEDERL CHAN CHANG CHATZI CHEN2 CHEN3 CHIAZZ CHOI CHYOU COMSTO COOKSO CORREA DARBY DAVEYS DEKLER DESTE2 DESTEF DOCKER DORANT DORGAN DOSEME DROSTE DU DUNN EBELIN ENGELA ESAKI FAN GAO GAO2 GARCIA GARDIN GARSHI GENG GER GILLIS GODLEY GOODMA GREGOR GSELL HAENSZ HAMMO2 HANSEN HEGMAN HEIN HENNEK HINDS HIRAOK HIRAYA HITOSU HOLE HOROWI HORWIT HU HU2 HUANG HUMBLE ISHIMA JAHN JAIN JARUP JARVHO JEDRYC JIANG JONES KAISER KATSOU KAUFMA KELLER KHUDER KIHARA KINLEN KNEKT KO KOHLME KOO KREUZE KREYBE KUBIK LAMTH LAMWK LAMWK2 LANGE LAUSSM LEI LEMARC LETOUR LEVIN LIDDEL LIU LIU2 LIU3 LIU5 LOMBA2 LUO MACLEN MAGNUS MARSH MARSH2 MARTIS MASTRA MATOS MATSUD MCDUFF MCLAUG MIGRAN MILLER MOLLO MRFITR NAM NOU ODRISC ORMOS OSANN OSANN2 PARKIN PASTOR PAWLEG PERSH2 PERSHA PETO PEZZO2 PIKE PISANI POFFIJ POLEDN PRESCO QIAO2 RACHTA RADZIK RANDIG REN ROOTS ROTHSC SANKAR SCHWAR SEGI SEOW SHAW SIEMIA SIMARA SIMONA SOBUE SPEIZE SPITZ STAYNE STOCKS STOCKW STUCKE SUN SUZUKI SVENSS TANG TENKAN TOKARS TOUSEY TSUGAN TULINI TVERDA VANDER VEIERO VUTUC WAKAI WANG WANG2 WANG3 WANG4 WICKLU WILKIN WU WUNSCH WUWILL WYNDE2 WYNDE3 WYNDE4 WYNDE6 XU XU2 XU3 XU4 YAMAGU YONG YUAN ZHANG ZHENG ZHOU
6	SCHWA2
8	ABELIN AGUDO ARMADA BEST BOFFET BOUCOT CHOW CPSI CPSII DAMBER DEAN DEAN2 DEAN3 DOLL DOLL2 DORN GOLLED GRAHAM JOLY KAISE2 KJUUS KOULUM LIU4 LOMBAR MCCONN MILLS NOTAN2 PERNU QIAO RONCO SADOWS SUZUK2 TIZZAN WIGLE XIANGZ
10	BENHAM

Table 2A3 - 8

Potentially overlapping studies

REF	REFGP	PRINC	OVERLAP/LINK
LUBIN2	LUBIN2	1	Lubin-combined
WYNDE7	WYNDE6	2	WYNDE5/6/7/8
LUBIN	XIANGZ	2	LUBIN/XIANGZ/QIAO

Table 2A4 -

IESLC - Meta-analysis of Ever Smoking, Any product (or Cigarettes if Any not available), Age <56
Squamous

This analysis is restricted to results for:
1) Non-dose-response data
2) Ever smokers
3) Age <56
4) Results complete enough for use in metaanalysis

Within each study, results are then selected (in the following order of preference, within each sex) for:
5) PRODUCT: all/unspec, cigarettes regardless of other products, cigarettes only
6) CIGTYPE: all/unspecified, MC regardless of HR, MC only
7) DENOM: never smoked anything, never smoked cigarettes, (never +1 = +long term ex, +2 = +amount unknown,
+3 = never cigs+long term ex)
8) Followup period (YF, prospective studies): whole study (coded as 0) or longest available
9) LCtype: all or nearest available, at least Squamous and Adeno. (q = squamous, s = small,
a = adeno, KI = Kreyberg I, u = undifferentiated)
10) Race: all or nearest available, otherwise by race (wh or w = white, bl or b = black, hi = hispanic
ch = chinese, jap = japanese, haw = hawaiian, w+o = white + oriental, sca = scandinavian, as = asian)
11) For overlapping studies: principal rather than subsidiary studies
Finally by Age: whole study (actual age shown) if available, otherwise by widest available age group
and then for single sex results (m, f) in preference to combined sex results (c).

Results adjusted (AD) for the most potential confounders are then chosen in Sections -1 to -3
(and those which actually differ from the adjusted results in Table 2A1 - 1 are marked 'x' in Section -1)
and results adjusted for the least confounders in Sections -4 to -6. (Those least adjusted results which
actually differ from the most adjusted as marked 'x' in column X in Section -4)
(Results adjusted for an unknown number of confounder(s) are coded as 20.)

Section -7 shows excluded studies, together with the stage (as above) at which no qualifying
results were found.

Section -8 lists the potentially overlapping studies which have been included (1=principal, 2=subsidiary).

Section -9 lists any results which would have been included in preference except that they had data not complete
enough for use in meta-analysis, with their significance (yes/no), if known, and any further comment as entered
on the database.

In addition to those mentioned above, the following fields, levels and abbreviations are used:
* or nk = not known, n = no, y = yes, ot = other
nev = never
all/unspec = all or unspecified, cig+/-ot = cigarettes irrespective of other products (cigar, pipe etc)
MC = manufactured cigarettes, HR = hand-rolled cigarettes
REF: 6-character study reference
NRR: number of the RR on the database within the study
ST : study type (CC = case control, pr or prosp = prospective)
NLC: number of lung cancer cases in whole study
R : risky occupational population (n = no, m = mining, o = other risky)
VB : national cigarette type (V = at least 75% Virginia, bl = at least 75% blended, ot = other)
P : any proxy use
H : full histological confirmation
De : derivation of RR/CI (or = original, st = standard method, ot = other method of estimation)

Table 2A4 - 1

IESLC - Meta-analysis of Ever Smoking, Any product (or Cigarettes if Any not available), Age <56
Squamous
Most adjusted

REF	NRR	2A1	SEX	AGEL	AGEH	RACE	YF	LC TYPE	LOC	START	ST	NLC	R	VB	P	H	AD	PRODUCT	DENOM	De
BENHAM	2	x	m	1	49	all	-	KI	Eu:wst	1976	CC	1625	n	bl	n	y	0	cig only	nev any	st
SCHWAR	10		m	40	54	wh	-	q	NAmer	1984	CC	5588	n	bl	y	y	0	cig+/-ot	nev cigs	st
SCHWAR	9		m	40	54	bl	-	q	NAmer	1984	CC	5588	n	bl	y	y	0	cig+/-ot	nev cigs	st
SCHWAR	18		f	40	54	wh	-	q	NAmer	1984	CC	5588	n	bl	y	y	0	cig+/-ot	nev cigs	ot
SCHWAR	17		f	40	54	bl	-	q	NAmer	1984	CC	5588	n	bl	y	y	0	cig+/-ot	nev cigs	ot
TSUGAN	13		m	30	49	all	-	q	As:Jap	1976	CC	134	n	bl	n	y	0	all/unsp	nev any	ot
VUTUC	19	x	m	41	50	all	-	KI	Eu:wst	1976	CC	1877	n	bl	n	n	0	cig+/-ot	nev cigs	st
WYNDE4	31	x	m	30	49	all	-	not a	NAmer	1948	CC	684	n	bl	y	n	0	all/unsp	nev any	st
WYNDE6	322	x	m	1	54	wh	-	q	NAmer	1969	CC	4423	n	bl	n	y	0	cig+/-ot	nev cigs	st
WYNDE6	326	x	f	1	54	wh	-	q	NAmer	1969	CC	4423	n	bl	n	y	0	cig+/-ot	nev cigs	st

Cigarette type is all/unspec for all RRs

Table 2A4 - 2

IESLC - Meta-analysis of Ever Smoking, Any product (or Cigarettes if Any not available), Age <56
Squamous
Most adjusted

REF	NRR	SEX	AD	Number Exposed Case	Cont	Non-exposed Case	Cont	RR	95.00%CI
BENHAM	2	m	0	190	264	5	89	12.81	(5.11- 32.14)
SCHWAR	10	m	0	80	178	1	73	32.81	(4.48- 240.23)
SCHWAR	9	m	0	41	39	4	7	1.84	(0.50- 6.78)
SCHWAR	18	f	0	29	108	0	79	43.23~	(2.60- 718.15)
SCHWAR	17	f	0	21	28	0	41	62.61~	(3.64-1076.10)
Subtotal SCHWAR								7.71	(2.96- 20.10)
TSUGAN	13	m	0	20	15	0	5	14.55~	(0.75- 283.37)
VUTUC	19	m	0	89	157	7	64	5.18	(2.28- 11.80)
WYNDE4	31	m	0	118	274	1	36	15.50	(2.10- 114.41)
WYNDE6	322	m	0	113	177	1	150	95.76	(13.21- 694.00)
WYNDE6	326	f	0	53	115	3	183	28.11	(8.58- 92.07)
Subtotal WYNDE6								38.86	(14.04- 107.51)
Totals				754	1355	22	727		

*prospective study ~ With 0.5 adjustment for zero

REF	NRR	SEX	AD	Ys	Ws	Qs	Ps
BENHAM	2	m	0	2.55	4.54	0.10	0.0000
SCHWAR	10	m	0	3.49	0.97	1.15	0.0006
SCHWAR	9	m	0	0.61	2.26	7.25	0.3596
SCHWAR	18	f	0	3.77	0.49	0.91	0.0086
SCHWAR	17	f	0	4.14	0.47	1.43	0.0044
Subtotal SCHWAR				2.04	4.19	10.74	
TSUGAN	13	m	0	2.68	0.44	0.03	0.0772
VUTUC	19	m	0	1.65	5.68	3.24	0.0001
WYNDE4	31	m	0	2.74	0.96	0.11	0.0072
WYNDE6	322	m	0	4.56	0.98	4.57	0.0000
WYNDE6	326	f	0	3.34	2.73	2.39	0.0000
Subtotal WYNDE6				3.66	3.71	6.96	

	N	10
	NS	6
	Wt	19.51
Het	Chi	21.18
Het	df	9
Het	P	*
Fixed	RR	11.04
	RRl	7.08
	RRu	17.20
	P	+++
Random	RR	14.73
	RRl	6.83
	RRu	31.76
	P	+++
Asymm	P	N.S.

Table 2A4 - 3

IESLC - Meta-analysis of Ever Smoking, Any product (or Cigarettes if Any not available), Age <56
Squamous
Most adjusted

	Sex combined	male	female	Total
N		7	3	10
NS		6	2	8
Wt		15.82	3.69	19.51
Het Chi		15.43	0.30	21.18
Het df		6	2	9
Het P		*	N.S.	*
Fixed RR		8.55	32.98	11.04
RRl		5.22	11.89	7.08
RRu		13.99	91.48	17.20
P		+++	+++	+++
Random RR		10.98	32.98	14.73
RRl		4.50	11.89	6.83
RRu		26.83	91.48	31.76
P		+++	+++	+++
Between Chi				5.46
Between df				1
Between P				*
Btwn(F) P				N.S.
Btwn(R) P				N.S.

Table 2A4 - 4

IESLC - Meta-analysis of Ever Smoking, Any product (or Cigarettes if Any not available), Age <56
Squamous
Least adjusted

REF	NRR	X	SEX	AGEL	AGEH	RACE	YF	LC TYPE	LOC	START	ST	NLC	R	VB	P	H	AD	PRODUCT	DENOM	De
BENHAM	2		m	1	49	all	-	KI	Eu:wst	1976	CC	1625	n	bl	n	y	0	cig only	nev any	st
SCHWAR	10		m	40	54	wh	-	q	NAmer	1984	CC	5588	n	bl	y	y	0	cig+/-ot	nev cigs	st
SCHWAR	9		m	40	54	bl	-	q	NAmer	1984	CC	5588	n	bl	y	y	0	cig+/-ot	nev cigs	st
SCHWAR	18		f	40	54	wh	-	q	NAmer	1984	CC	5588	n	bl	y	y	0	cig+/-ot	nev cigs	ot
SCHWAR	17		f	40	54	bl	-	q	NAmer	1984	CC	5588	n	bl	y	y	0	cig+/-ot	nev cigs	ot
TSUGAN	13		m	30	49	all	-	q	As:Jap	1976	CC	134	n	bl	n	y	0	all/unsp	nev any	ot
VUTUC	19		m	41	50	all	-	KI	Eu:wst	1976	CC	1877	n	bl	n	n	0	cig+/-ot	nev cigs	st
WYNDE4	31		m	30	49	all	-	not a	NAmer	1948	CC	684	n	bl	y	n	0	all/unsp	nev any	st
WYNDE6	322		m	1	54	wh	-	q	NAmer	1969	CC	4423	n	bl	n	y	0	cig+/-ot	nev cigs	st
WYNDE6	326		f	1	54	wh	-	q	NAmer	1969	CC	4423	n	bl	n	y	0	cig+/-ot	nev cigs	st

Cigarette type is all/unspec for all RRs

Table 2A4 - 5

IESLC - Meta-analysis of Ever Smoking, Any product (or Cigarettes if Any not available), Age <56
Squamous
Least adjusted

REF	NRR	SEX	AD	Number Exposed Case	Cont	Non-exposed Case	Cont	RR	95.00%CI
BENHAM	2	m	0	190	264	5	89	12.81	(5.11- 32.14)
SCHWAR	10	m	0	80	178	1	73	32.81	(4.48- 240.23)
SCHWAR	9	m	0	41	39	4	7	1.84	(0.50- 6.78)
SCHWAR	18	f	0	29	108	0	79	43.23~	(2.60- 718.15)
SCHWAR	17	f	0	21	28	0	41	62.61~	(3.64-1076.10)
Subtotal SCHWAR								7.71	(2.96- 20.10)
TSUGAN	13	m	0	20	15	0	5	14.55~	(0.75- 283.37)
VUTUC	19	m	0	89	157	7	64	5.18	(2.28- 11.80)
WYNDE4	31	m	0	118	274	1	36	15.50	(2.10- 114.41)
WYNDE6	322	m	0	113	177	1	150	95.76	(13.21- 694.00)
WYNDE6	326	f	0	53	115	3	183	28.11	(8.58- 92.07)
Subtotal WYNDE6								38.86	(14.04- 107.51)
Totals				754	1355	22	727		

*prospective study ~ With 0.5 adjustment for zero

REF	NRR	SEX	AD	Ys	Ws	Qs	Ps
BENHAM	2	m	0	2.55	4.54	0.10	0.0000
SCHWAR	10	m	0	3.49	0.97	1.15	0.0006
SCHWAR	9	m	0	0.61	2.26	7.25	0.3596
SCHWAR	18	f	0	3.77	0.49	0.91	0.0086
SCHWAR	17	f	0	4.14	0.47	1.43	0.0044
Subtotal SCHWAR				2.04	4.19	10.74	
TSUGAN	13	m	0	2.68	0.44	0.03	0.0772
VUTUC	19	m	0	1.65	5.68	3.24	0.0001
WYNDE4	31	m	0	2.74	0.96	0.11	0.0072
WYNDE6	322	m	0	4.56	0.98	4.57	0.0000
WYNDE6	326	f	0	3.34	2.73	2.39	0.0000
Subtotal WYNDE6				3.66	3.71	6.96	

	N	10
	NS	6
	Wt	19.51
Het	Chi	21.18
Het	df	9
Het	P	*
Fixed	RR	11.04
	RRl	7.08
	RRu	17.20
	P	+++
Random	RR	14.73
	RRl	6.83
	RRu	31.76
	P	+++
Asymm	P	N.S.

Table 2A4 - 6

IESLC - Meta-analysis of Ever Smoking, Any product (or Cigarettes if Any not available), Age <56
Squamous
Least adjusted

Sex combined	male	female	Total
N	7	3	10
NS	6	2	8
Wt	15.82	3.69	19.51
Het Chi	15.43	0.30	21.18
Het df	6	2	9
Het P	*	N.S.	*
Fixed RR	8.55	32.98	11.04
RRl	5.22	11.89	7.08
RRu	13.99	91.48	17.20
P	+++	+++	+++
Random RR	10.98	32.98	14.73
RRl	4.50	11.89	6.83
RRu	26.83	91.48	31.76
P	+++	+++	+++
Between Chi			5.46
Between df			1
Between P			*
Btwn(F) P			N.S.
Btwn(R) P			N.S.

Table 2A4 - 7

IESLC - Meta-analysis of Ever Smoking, Any product (or Cigarettes if Any not available), Age <56
Squamous
Excluded studies (and stage at which they were excluded)

1	BOUCHA BUELL LAURIL MZILEN
2	BYERS2 ENSTRO HIRAY2 KANELL LIAW LICKIN MRFIT MURATA NOTANI RESTRE RIMING SAARIK SEGI2 SHIMIZ SITAS SOBUE2 TANG2 WALD WARSIN WATSON WU2 WYNDE5 WYNDE8 WYNDER
3	ABELIN ABRAHA AGUDO AKIBA ALDERS AMANDU AMES ANDERS ARCHER ARMADA AUSTIN AUVINE AXELSO AXELSS BAND BARBON BECHER BENSHL BERRIN BEST BLOHMK BLOT1 BLOT2 BLOT3 BLOT4 BOFFET BOUCOT BRESLO BRETT BROCKM BROSS BROWN1 BROWN2 BUFFLE BYERS1 CARPEN CASCO2 CASCOR CEDERL CHAN CHANG CHATZI CHEN CHEN2 CHEN3 CHIAZZ CHOI CHOW CHYOU COOKSO CORREA CPSII DAMBER DARBY DAVEYS DEAN DEAN2 DEKLER DESTE2 DESTEF DOCKER DOLL DOLL2 DORANT DORGAN DORN DOSEME DROSTE DU DUNN EBELIN ENGELA ESAKI FAN GAO GAO2 GARCIA GARDIN GARSHI GENG GER GILLIS GODLEY GOLLED GOODMA GRAHAM GREGOR GSELL HAENSZ HAMMO2 HANSEN HEGMAN HEIN HENNEK HIRAOK HIRAYA HOLE HOROWI HORWIT HU HU2 HUANG HUMBLE ISHIMA JAHN JAIN JARUP JARVHO JEDRYC JIANG JOLY JONES JUSSAW KAISE2 KAISER KATSOU KAUFMA KELLER KHUDER KIHARA KINLEN KJUUS KNEKT KO KOHLME KOO KOULUM KREYBE LAMTH LAMWK LAMWK2 LANGE LAUSSM LEI LEMARC LETOUR LIDDEL LIU LIU2 LIU3 LIU4 LIU5 LOMBA2 LOMBAR LUBIN LUBIN2 LUO MACLEN MAGNUS MARSH MARSH2 MARTIS MASTRA MATOS MATSUD MCCONN MCDUFF MCLAUG MIGRAN MILLER MILLS MOLLO MRFITR NOTAN2 NOU ODRISC ORMOS OSANN OSANN2 PARKIN PASTOR PAWLEG PERNU PERSH2 PERSHA PETO PEZZO2 PEZZOT PIKE PISANI POFFIJ POLEDN QIAO QIAO2 RACHTA RADZIK RANDIG REN RONCO ROOTS ROTHSC SANKAR SCHWA2 SEGI SEOW SHAW SIEMIA SIMARA SIMONA SOBUE SPITZ STASZE STAYNE STOCKW STUCKE SUN SUZUK2 SUZUKI SVENSS TANG TAO TENKAN TIZZAN TOKARS TOUSEY TULINI ULMER VANDER VEIERO WAKAI WANG WANG2 WANG3 WANG4 WICKLU WIGLE WILKIN WU WUNSCH WYNDE2 WYNDE3 WYNDE7 XIANGZ XU XU2 XU3 XU4 YAMAGU YONG YUAN ZHANG ZHENG ZHOU
4	SADOWS
9	COMSTO CPSI DEAN3 HAMMON HINDS HITOSU KREUZE KUBIK LEVIN NAM PRESCO SPEIZE STOCKS TVERDA WUWILL

Table 2A4 - 8

Potentially overlapping studies

REF	REFGP	PRINC	OVERLAP/LINK
VUTUC	LUBIN2	2	Subset of Lubin2
BENHAM	LUBIN2	2	Subset of Lubin2
WYNDE6	WYNDE6	1	WYNDE5/6/7/8

Table 2A5 -

IESLC - Meta-analysis of Ever Smoking, Any product (or Cigarettes if Any not available), Age 50-70
Squamous

This analysis is restricted to results for:
1) Non-dose-response data
2) Ever smokers
3) Maximum age range 50-70
4) Results complete enough for use in metaanalysis

Within each study, results are then selected (in the following order of preference, within each sex) for:
5) PRODUCT: all/unspec, cigarettes regardless of other products, cigarettes only
6) CIGTYPE: all/unspecified, MC regardless of HR, MC only
7) DENOM: never smoked anything, never smoked cigarettes, (never +1 = +long term ex, +2 = +amount unknown,
+3 = never cigs+long term ex)
8) Followup period (YF, prospective studies): whole study (coded as 0) or longest available
9) LCtype: all or nearest available, at least Squamous and Adeno. (q = squamous, s = small,
a = adeno, KI = Kreyberg I, u = undifferentiated)
10) Race: all or nearest available, otherwise by race (wh or w = white, bl or b = black, hi = hispanic
ch = chinese, jap = japanese, haw = hawaiian, w+o = white + oriental, sca = scandinavian, as = asian)
11) For overlapping studies: principal rather than subsidiary studies
Finally by Age: whole study (actual age shown) if available, otherwise by widest available age group
and then for single sex results (m, f) in preference to combined sex results (c).

Results adjusted (AD) for the most potential confounders are then chosen in Sections -1 to -3
(and those which actually differ from the adjusted results in Table 2A1 - 1 are marked 'x' in Section -1)
and results adjusted for the least confounders in Sections -4 to -6. (Those least adjusted results which
actually differ from the most adjusted as marked 'x' in column X in Section -4)
(Results adjusted for an unknown number of confounder(s) are coded as 20.)

Section -7 shows excluded studies, together with the stage (as above) at which no qualifying
results were found.

Section -8 lists the potentially overlapping studies which have been included (1=principal, 2=subsidiary).

Section -9 lists any results which would have been included in preference except that they had data not complete
enough for use in meta-analysis, with their significance (yes/no), if known, and any further comment as entered
on the database.

In addition to those mentioned above, the following fields, levels and abbreviations are used:
* or nk = not known, n = no, y = yes, ot = other
nev = never
all/unspec = all or unspecified, cig+/-ot = cigarettes irrespective of other products (cigar, pipe etc)
MC = manufactured cigarettes, HR = hand-rolled cigarettes
REF: 6-character study reference
NRR: number of the RR on the database within the study
ST : study type (CC = case control, pr or prosp = prospective)
NLC: number of lung cancer cases in whole study
R : risky occupational population (n = no, m = mining, o = other risky)
VB : national cigarette type (V = at least 75% Virginia, bl = at least 75% blended, ot = other)
P : any proxy use
H : full histological confirmation
De : derivation of RR/CI (or = original, st = standard method, ot = other method of estimation)

Table 2A5 - 1

IESLC - Meta-analysis of Ever Smoking, Any product (or Cigarettes if Any not available), Age 50-70
Squamous
Most adjusted

REF	NRR	2A1	SEX	AGEL	AGEH	RACE	YF	LC TYPE	LOC	START	ST	NLC	R	VB	P	H	AD	PRODUCT	DENOM	De
ANDERS	10		f	55	69	all	0	q	NAmer	1986	pr	343	n	bl	n	n	0	cig+/-ot	nev cigs	st
BENHAM	3	x	m	50	59	all	-	KI	Eu:wst	1976	CC	1625	n	bl	n	y	0	cig only	nev any	st
BENHAM	4	x	m	60	69	all	-	KI	Eu:wst	1976	CC	1625	n	bl	n	y	0	cig only	nev any	st
HAMMON	60		m	50	69	wh	0	not a	NAmer	1952	pr	448	n	bl	n	n	1	all/unsp	nev any	ot
WYNDE4	32	x	m	50	59	all	-	not a	NAmer	1948	CC	684	n	bl	y	n	0	all/unsp	nev any	st
WYNDE4	33	x	m	60	69	all	-	not a	NAmer	1948	CC	684	n	bl	y	n	0	all/unsp	nev any	st

Cigarette type is all/unspec for all RRs

Table 2A5 - 2

IESLC - Meta-analysis of Ever Smoking, Any product (or Cigarettes if Any not available), Age 50-70
Squamous
Most adjusted

REF	NRR	SEX	AD	Number Exposed Case	Number Exposed Cont	Non-exposed Case	Non-exposed Cont	RR	95.00%CI
*ANDERS	10	f	0	63	96164	5	195158	25.57	(10.29- 63.56)
BENHAM	3	m	0	379	476	2	174	69.27	(17.08- 281.01)
BENHAM	4	m	0	341	319	12	129	11.49	(6.24- 21.17)
Subtotal BENHAM								15.31	(8.75- 26.81)
*HAMMON	60	m	1	-	-	-	-	16.88	(6.29- 45.29)
WYNDE4	32	m	0	254	179	4	31	11.00	(3.81- 31.70)
WYNDE4	33	m	0	185	137	2	23	15.53	(3.60- 66.98)
Subtotal WYNDE4								12.38	(5.25- 29.19)
Partial Totals				1222	97275	25	195515		

*prospective study

REF	NRR	SEX	AD	Ys	Ws	Qs	Ps
*ANDERS	10	f	0	3.24	4.63	0.94	0.0000
BENHAM	3	m	0	4.24	1.96	4.10	0.0000
BENHAM	4	m	0	2.44	10.29	1.26	0.0000
Subtotal BENHAM				2.73	12.25	5.36	
*HAMMON	60	m	1	2.83	3.94	0.00	0.0000
WYNDE4	32	m	0	2.40	3.43	0.53	0.0000
WYNDE4	33	m	0	2.74	1.80	0.00	0.0002
Subtotal WYNDE4				2.52	5.23	0.54	

	N	6
	NS	4
	Wt	26.05
Het	Chi	6.84
Het	df	5
Het	P	N.S.
Fixed	RR	16.32
	RRl	11.11
	RRu	23.95
	P	+++
Random	RR	17.30
	RRl	10.78
	RRu	27.74
	P	+++
Asymm	P	N.S.

Table 2A5 - 3

IESLC - Meta-analysis of Ever Smoking, Any product (or Cigarettes if Any not available), Age 50-70
Squamous
Most adjusted

	Sex combined	male	female	Total
N		5	1	6
NS		3	1	4
Wt		21.42	4.63	26.05
Het Chi		5.70	0.00	6.84
Het df		4	0	5
Het P		N.S.	N.S.	N.S.
Fixed RR		14.80	25.57	16.32
RRl		9.69	10.29	11.11
RRu		22.61	63.56	23.95
P		+++	+++	+++
Random RR		15.95	25.57	17.30
RRl		9.26	10.29	10.78
RRu		27.47	63.56	27.74
P		+++	+++	+++
Between Chi				1.14
Between df				1
Between P				N.S.
Btwn(F) P				N.S.
Btwn(R) P				N.S.

Too few RRs for analysis by factor

Table 2A5 - 4

IESLC - Meta-analysis of Ever Smoking, Any product (or Cigarettes if Any not available), Age 50-70
Squamous
Least adjusted

REF	NRR	X	SEX	AGEL	AGEH	RACE	YF	LC TYPE	LOC	START	ST	NLC	R	VB	P	H	AD	PRODUCT	DENOM	De
ANDERS	10		f	55	69	all	0	q	NAmer	1986	pr	343	n	bl	n	n	0	cig+/-ot	nev cigs	st
BENHAM	3		m	50	59	all	-	KI	Eu:wst	1976	CC	1625	n	bl	n	y	0	cig only	nev any	st
BENHAM	4		m	60	69	all	-	KI	Eu:wst	1976	CC	1625	n	bl	n	y	0	cig only	nev any	st
HAMMON	73	x	m	50	69	wh	0	not a	NAmer	1952	pr	448	n	bl	n	n	0	all/unsp	nev any	st
WYNDE4	32		m	50	59	all	-	not a	NAmer	1948	CC	684	n	bl	y	n	0	all/unsp	nev any	st
WYNDE4	33		m	60	69	all	-	not a	NAmer	1948	CC	684	n	bl	y	n	0	all/unsp	nev any	st

Cigarette type is all/unspec for all RRs

Table 2A5 - 5

IESLC - Meta-analysis of Ever Smoking, Any product (or Cigarettes if Any not available), Age 50-70
Squamous
Least adjusted

REF	NRR	SEX	AD	Number Exposed Case	Number Exposed Cont	Non-exposed Case	Non-exposed Cont	RR	95.00%CI
*ANDERS	10	f	0	63	96164	5	195158	25.57	(10.29- 63.56)
BENHAM	3	m	0	379	476	2	174	69.27	(17.08- 281.01)
BENHAM	4	m	0	341	319	12	129	11.49	(6.24- 21.17)
Subtotal BENHAM								15.31	(8.75- 26.81)
*HAMMON	73	m	0	286	510108	4	115884	16.24	(6.05- 43.57)
WYNDE4	32	m	0	254	179	4	31	11.00	(3.81- 31.70)
WYNDE4	33	m	0	185	137	2	23	15.53	(3.60- 66.98)
Subtotal WYNDE4								12.38	(5.25- 29.19)
Totals				1508	607383	29	311399		

*prospective study

REF	NRR	SEX	AD	Ys	Ws	Qs	Ps
*ANDERS	10	f	0	3.24	4.63	0.96	0.0000
BENHAM	3	m	0	4.24	1.96	4.13	0.0000
BENHAM	4	m	0	2.44	10.29	1.22	0.0000
Subtotal BENHAM				2.73	12.25	5.35	
*HAMMON	73	m	0	2.79	3.94	0.00	0.0000
WYNDE4	32	m	0	2.40	3.43	0.52	0.0000
WYNDE4	33	m	0	2.74	1.80	0.00	0.0002
Subtotal WYNDE4				2.52	5.23	0.52	

	N	6
	NS	4
	Wt	26.05
Het	Chi	6.83
Het	df	5
Het	P	N.S.
Fixed	RR	16.22
	RRl	11.05
	RRu	23.81
	P	+++
Random	RR	17.18
	RRl	10.71
	RRu	27.55
	P	+++
Asymm	P	N.S.

Table 2A5 - 6

IESLC - Meta-analysis of Ever Smoking, Any product (or Cigarettes if Any not available), Age 50-70

Squamous

Least adjusted

	Sex combined	male	female	Total
N		5	1	6
NS		3	1	4
Wt		21.42	4.63	26.05
Het Chi		5.66	0.00	6.83
Het df		4	0	5
Het P		N.S.	N.S.	N.S.
Fixed RR		14.70	25.57	16.22
RRl		9.63	10.29	11.05
RRu		22.45	63.56	23.81
P		+++	+++	+++
Random RR		15.80	25.57	17.18
RRl		9.20	10.29	10.71
RRu		27.16	63.56	27.55
P		+++	+++	+++
Between Chi				1.17
Between df				1
Between P				N.S.
Btwn(F) P				N.S.
Btwn(R) P				N.S.

Table 2A5 - 7

IESLC - Meta-analysis of Ever Smoking, Any product (or Cigarettes if Any not available), Age 50-70
Squamous
Excluded studies (and stage at which they were excluded)

Stage	Studies
1	BOUCHA BUELL LAURIL MZILEN
2	BYERS2 ENSTRO HIRAY2 KANELL LIAW LICKIN MRFIT MURATA NOTANI RESTRE RIMING SAARIK SEGI2 SHIMIZ SITAS SOBUE2 TANG2 WALD WARSIN WATSON WU2 WYNDE5 WYNDE8 WYNDER
3	ABELIN ABRAHA AGUDO AKIBA ALDERS AMANDU AMES ARCHER ARMADA AUSTIN AUVINE AXELSO AXELSS BAND BARBON BECHER BENSHL BERRIN BEST BLOHMK BLOT1 BLOT2 BLOT3 BLOT4 BOFFET BOUCOT BRESLO BRETT BROCKM BROSS BROWN1 BYERS1 CARPEN CASCO2 CASCOR CEDERL CHAN CHANG CHATZI CHEN CHEN2 CHEN3 CHIAZZ CHOI CHOW CHYOU COMSTO COOKSO CORREA CPSII DARBY DAVEYS DEAN DEAN2 DEKLER DESTE2 DESTEF DOCKER DOLL DOLL2 DORGAN DOSEME DROSTE DU DUNN EBELIN ENGELA ESAKI FAN GAO GAO2 GARCIA GARDIN GARSHI GENG GER GILLIS GODLEY GOLLED GOODMA GRAHAM GREGOR GSELL HAENSZ HAMMO2 HANSEN HEGMAN HEIN HENNEK HIRAOK HIRAYA HOLE HOROWI HORWIT HU HU2 HUANG HUMBLE ISHIMA JAHN JAIN JARUP JARVHO JEDRYC JIANG JOLY JONES JUSSAW KAISE2 KAISER KATSOU KAUFMA KELLER KHUDER KINLEN KJUUS KNEKT KO KOHLME KOO KOULUM KREYBE LAMTH LAMWK LAMWK2 LANGE LAUSSM LEI LEMARC LETOUR LIDDEL LIU LIU2 LIU3 LIU4 LIU5 LOMBA2 LOMBAR LUBIN LUBIN2 LUO MACLEN MAGNUS MARSH MARSH2 MARTIS MASTRA MATOS MATSUD MCCONN MCDUFF MCLAUG MIGRAN MILLER MILLS MOLLO MRFITR NOTAN2 NOU ODRISC ORMOS OSANN OSANN2 PARKIN PASTOR PAWLEG PERNU PERSH2 PERSHA PETO PEZZO2 PEZZOT PIKE PISANI POFFIJ POLEDN QIAO QIAO2 RACHTA RADZIK RANDIG REN RONCO ROOTS ROTHSC SANKAR SCHWA2 SCHWAR SEGI SEOW SHAW SIEMIA SIMARA SIMONA SOBUE SPEIZE SPITZ STASZE STAYNE STOCKW STUCKE SUN SUZUK2 SUZUKI SVENSS TANG TAO TENKAN TIZZAN TOKARS TOUSEY TSUGAN TULINI TVERDA ULMER VANDER VEIERO WAKAI WANG WANG2 WANG3 WANG4 WICKLU WIGLE WILKIN WU WUNSCH WYNDE2 WYNDE3 WYNDE6 WYNDE7 XIANGZ XU XU2 XU3 XU4 YAMAGU YONG YUAN ZHANG ZHENG ZHOU
4	BROWN2 SADOWS
9	BUFFLE CPSI DAMBER DEAN3 DORANT DORN HINDS HITOSU KIHARA KREUZE KUBIK LEVIN NAM PRESCO STOCKS VUTUC WUWILL

Table 2A5 - 8
Potentially overlapping studies

REF	REFGP	PRINC	.	OVERLAP/LINK
BENHAM	LUBIN2	2		Subset of Lubin2

Table 2A6 -

IESLC - Meta-analysis of Ever Smoking, Any product (or Cigarettes if Any not available), Age 65+
Squamous

This analysis is restricted to results for:
1) Non-dose-response data
2) Ever smokers
3) Age 65+
4) Results complete enough for use in metaanalysis

Within each study, results are then selected (in the following order of preference, within each sex) for:
5) PRODUCT: all/unspec, cigarettes regardless of other products, cigarettes only
6) CIGTYPE: all/unspecified, MC regardless of HR, MC only
7) DENOM: never smoked anything, never smoked cigarettes, (never +1 = +long term ex, +2 = +amount unknown,
+3 = never cigs+long term ex)
8) Followup period (YF, prospective studies): whole study (coded as 0) or longest available
9) LCtype: all or nearest available, at least Squamous and Adeno. (q = squamous, s = small,
a = adeno, KI = Kreyberg I, u = undifferentiated)
10) Race: all or nearest available, otherwise by race (wh or w = white, bl or b = black, hi = hispanic
ch = chinese, jap = japanese, haw = hawaiian, w+o = white + oriental, sca = scandinavian, as = asian)
11) For overlapping studies: principal rather than subsidiary studies
Finally by Age: whole study (actual age shown) if available, otherwise by widest available age group
and then for single sex results (m, f) in preference to combined sex results (c).

Results adjusted (AD) for the most potential confounders are then chosen in Sections -1 to -3
(and those which actually differ from the adjusted results in Table 2A1 - 1 are marked 'x' in Section -1)
and results adjusted for the least confounders in Sections -4 to -6. (Those least adjusted results which
actually differ from the most adjusted as marked 'x' in column X in Section -4)
(Results adjusted for an unknown number of confounder(s) are coded as 20.)

Section -7 shows excluded studies, together with the stage (as above) at which no qualifying
results were found.

Section -8 lists the potentially overlapping studies which have been included (1=principal, 2=subsidiary).

Section -9 lists any results which would have been included in preference except that they had data not complete
enough for use in meta-analysis, with their significance (yes/no), if known, and any further comment as entered
on the database.

In addition to those mentioned above, the following fields, levels and abbreviations are used:
* or nk = not known, n = no, y = yes, ot = other
nev = never
all/unspec = all or unspecified, cig+/-ot = cigarettes irrespective of other products (cigar, pipe etc)
MC = manufactured cigarettes, HR = hand-rolled cigarettes
REF: 6-character study reference
NRR: number of the RR on the database within the study
ST : study type (CC = case control, pr or prosp = prospective)
NLC: number of lung cancer cases in whole study
R : risky occupational population (n = no, m = mining, o = other risky)
VB : national cigarette type (V = at least 75% Virginia, bl = at least 75% blended, ot = other)
P : any proxy use
H : full histological confirmation
De : derivation of RR/CI (or = original, st = standard method, ot = other method of estimation)

Table 2A6 - 1

IESLC - Meta-analysis of Ever Smoking, Any product (or Cigarettes if Any not available), Age 65+
Squamous
Most adjusted

REF	NRR	2A1	SEX	AGEL	AGEH	RACE	YF	LC TYPE	LOC	START	ST	NLC	R	VB	P	H	AD	PRODUCT	DENOM	De
BENHAM	5	x	m	70	99	all	-	KI	Eu:wst	1976	CC	1625	n	bl	n	y	0	cig only	nev any	st
WYNDE4	34	x	m	70	79	all	-	not a	NAmer	1948	CC	684	n	bl	y	n	0	all/unsp	nev any	st

Cigarette type is all/unspec for all RRs

Table 2A6 - 2

IESLC - Meta-analysis of Ever Smoking, Any product (or Cigarettes if Any not available), Age 65+
Squamous
Most adjusted

REF	NRR	SEX	AD	Number Exposed Case	Cont	Non-exposed Case	Cont	RR	95.00%CI
BENHAM	5	m	0	164	190	5	89	15.36	(6.09- 38.74)
WYNDE4	34	m	0	40	75	1	25	13.33	(1.74- 102.06)
Totals				204	265	6	114		

*prospective study

REF	NRR	SEX	AD	Ys	Ws	Qs	Ps
BENHAM	5	m	0	2.73	4.49	0.00	0.0000
WYNDE4	34	m	0	2.59	0.93	0.01	0.0126

	N	2
	NS	2
	Wt	5.42
Het	Chi	0.02
Het	df	1
Het	P	N.S.
Fixed	RR	15.00
	RRl	6.46
	RRu	34.80
	P	+++
Random	RR	15.00
	RRl	6.46
	RRu	34.80
	P	+++
Asymm	P	

Table 2A6 - 3

IESLC - Meta-analysis of Ever Smoking, Any product (or Cigarettes if Any not available), Age 65+
Squamous
Most adjusted

	Sex combined	male	female	Total
N		2		2
NS		2		2
Wt		5.42		5.42
Het Chi		0.02		0.02
Het df		1		1
Het P		N.S.		N.S.
Fixed RR		15.00		15.00
RRl		6.46		6.46
RRu		34.80		34.80
P		+++		+++
Random RR		15.00		15.00
RRl		6.46		6.46
RRu		34.80		34.80
P		+++		+++
Between Chi				
Between df				
Between P				N.S.
Btwn(F) P				N.S.
Btwn(R) P				N.S.

Too few RRs for analysis by factor

Table 2A6 - 4

IESLC - Meta-analysis of Ever Smoking, Any product (or Cigarettes if Any not available), Age 65+
Squamous
Least adjusted

REF	NRR	X	SEX	AGEL	AGEH	RACE	YF	LC TYPE	LOC	START	ST	NLC	R	VB	P	H	AD	PRODUCT	DENOM	De
BENHAM	5		m	70	99	all	-	KI	Eu:wst	1976	CC	1625	n	bl	n	y	0	cig only	nev any	st
WYNDE4	34		m	70	79	all	-	not a	NAmer	1948	CC	684	n	bl	y	n	0	all/unsp	nev any	st

Cigarette type is all/unspec for all RRs

Table 2A6 - 5

IESLC - Meta-analysis of Ever Smoking, Any product (or Cigarettes if Any not available), Age 65+
Squamous
Least adjusted

REF	NRR	SEX	AD	Number Exposed Case	Cont	Non-exposed Case	Cont	RR	95.00%CI
BENHAM	5	m	0	164	190	5	89	15.36	(6.09- 38.74)
WYNDE4	34	m	0	40	75	1	25	13.33	(1.74- 102.06)
Totals				204	265	6	114		

*prospective study

REF	NRR	SEX	AD	Ys	Ws	Qs	Ps
BENHAM	5	m	0	2.73	4.49	0.00	0.0000
WYNDE4	34	m	0	2.59	0.93	0.01	0.0126

	N	2
	NS	2
	Wt	5.42
Het	Chi	0.02
Het	df	1
Het	P	N.S.
Fixed	RR	15.00
	RRl	6.46
	RRu	34.80
	P	+++
Random	RR	15.00
	RRl	6.46
	RRu	34.80
	P	+++
Asymm	P	

Table 2A6 - 6

IESLC - Meta-analysis of Ever Smoking, Any product (or Cigarettes if Any not available), Age 65+
Squamous
Least adjusted

	Sex combined	male	female	Total
N		2		2
NS		2		2
Wt		5.42		5.42
Het Chi		0.02		0.02
Het df		1		1
Het P		N.S.		N.S.
Fixed RR		15.00		15.00
RRl		6.46		6.46
RRu		34.80		34.80
P		+++		+++
Random RR		15.00		15.00
RRl		6.46		6.46
RRu		34.80		34.80
P		+++		+++
Between Chi				
Between df				
Between P				N.S.
Btwn(F) P				N.S.
Btwn(R) P				N.S.

Table 2A6 - 7

IESLC - Meta-analysis of Ever Smoking, Any product (or Cigarettes if Any not available), Age 65+
Squamous
Excluded studies (and stage at which they were excluded)

1 BOUCHA BUELL LAURIL MZILEN

2 BYERS2 ENSTRO HIRAY2 KANELL LIAW LICKIN MRFIT MURATA NOTANI RESTRE RIMING SAARIK SEGI2 SHIMIZ SITAS SOBUE2
TANG2 WALD WARSIN WATSON WU2 WYNDE5 WYNDE8 WYNDER

3 ABELIN ABRAHA AGUDO AKIBA ALDERS AMANDU AMES ANDERS ARCHER ARMADA AUSTIN AUVINE AXELSO AXELSS BAND BARBON
BECHER BENSHL BERRIN BEST BLOHMK BLOT1 BLOT2 BLOT3 BLOT4 BOFFET BOUCOT BRESLO BRETT BROCKM BROSS BROWN1
BYERS1 CARPEN CASCO2 CASCOR CEDERL CHAN CHANG CHATZI CHEN CHEN2 CHEN3 CHIAZZ CHOI CHOW CHYOU COOKSO
CORREA CPSII DARBY DAVEYS DEAN DEAN2 DEKLER DESTE2 DESTEF DOCKER DOLL DOLL2 DORANT DOSEME DROSTE DU
DUNN EBELIN ENGELA ESAKI FAN GAO GAO2 GARCIA GARDIN GENG GER GILLIS GOLLED GOODMA GRAHAM GREGOR
GSELL HAENSZ HAMMO2 HANSEN HEGMAN HEIN HENNEK HIRAOK HIRAYA HITOSU HOLE HOROWI HORWIT HU HU2 HUANG
ISHIMA JAHN JAIN JARUP JARVHO JEDRYC JIANG JOLY JONES JUSSAW KAISER KATSOU KAUFMA KELLER KHUDER KIHARA
KINLEN KJUUS KNEKT KO KOHLME KOO KOULUM KREUZE KREYBE KUBIK LAMTH LAMWK LAMWK2 LANGE LAUSSM LEI
LEMARC LETOUR LIDDEL LIU LIU2 LIU3 LIU5 LOMBA2 LOMBAR LUBIN LUBIN2 LUO MACLEN MAGNUS MARSH MARSH2
MARTIS MASTRA MATOS MATSUD MCCONN MCDUFF MCLAUG MIGRAN MILLER MILLS MOLLO MRFITR NOTAN2 NOU ODRISC ORMOS
OSANN OSANN2 PARKIN PASTOR PAWLEG PERNU PERSH2 PETO PEZZO2 PEZZOT PIKE PISANI POFFIJ POLEDN QIAO QIAO2
RACHTA RADZIK RANDIG REN RONCO ROOTS ROTHSC SANKAR SCHWA2 SCHWAR SEGI SEOW SHAW SIEMIA SIMARA SIMONA
SOBUE SPEIZE SPITZ STASZE STAYNE STOCKW STUCKE SUN SUZUK2 SUZUKI SVENSS TANG TAO TENKAN TIZZAN TOKARS
TOUSEY TSUGAN TULINI TVERDA ULMER VANDER VEIERO VUTUC WAKAI WANG WANG2 WANG3 WANG4 WICKLU WIGLE WILKIN
WU WUNSCH WYNDE2 WYNDE3 WYNDE6 WYNDE7 XIANGZ XU XU2 XU3 XU4 YAMAGU YONG YUAN ZHANG ZHENG
ZHOU

4 BROWN2 SADOWS

7 PERSHA

9 BUFFLE COMSTO CPSI DAMBER DEAN3 DORGAN DORN GARSHI GODLEY HAMMON HINDS HUMBLE KAISE2 LEVIN LIU4 NAM
PRESCO STOCKS WUWILL

Table 2A6 - 8
Potentially overlapping studies

REF	REFGP	PRINC	.	OVERLAP/LINK
BENHAM	LUBIN2	2		Subset of Lubin2

Table 2A7 -

IESLC - Meta-analysis of Ever Smoking, Cigarettes (or Any Product if Cigarettes not available), Age <56
Squamous

This analysis is restricted to results for:
1) Non-dose-response data
2) Ever smokers
3) Age <56
4) Results complete enough for use in metaanalysis

Within each study, results are then selected (in the following order of preference, within each sex) for:
5) PRODUCT: cigarettes regardless of other products, cigarettes only, all/unspec
6) CIGTYPE: all/unspecified, MC regardless of HR, MC only
7) DENOM: never smoked anything, never smoked cigarettes, (never +1 = +long term ex, +2 = +amount unknown,
+3 = never cigs+long term ex)
8) Followup period (YF, prospective studies): whole study (coded as 0) or longest available
9) LCtype: all or nearest available, at least Squamous and Adeno. (q = squamous, s = small,
a = adeno, KI = Kreyberg I, u = undifferentiated)
10) Race: all or nearest available, otherwise by race (wh or w = white, bl or b = black, hi = hispanic
ch = chinese, jap = japanese, haw = hawaiian, w+o = white + oriental, sca = scandinavian, as = asian)
11) For overlapping studies: principal rather than subsidiary studies
Finally by Age: whole study (actual age shown) if available, otherwise by widest available age group
and then for single sex results (m, f) in preference to combined sex results (c).

Results adjusted (AD) for the most potential confounders are then chosen in Sections -1 to -3
(and those which actually differ from the adjusted results in Table 1A2 - 1 are marked 'x' in Section -1)
and results adjusted for the least confounders in Sections -4 to -6. (Those least adjusted results which
actually differ from the most adjusted as marked 'x' in column X in Section -4)
(Results adjusted for an unknown number of confounder(s) are coded as 20.)

Section -7 shows excluded studies, together with the stage (as above) at which no qualifying
results were found.

Section -8 lists the potentially overlapping studies which have been included (1=principal, 2=subsidiary).

Section -9 lists any results which would have been included in preference except that they had data not complete
enough for use in meta-analysis, with their significance (yes/no), if known, and any further comment as entered
on the database.

In addition to those mentioned above, the following fields, levels and abbreviations are used:
* or nk = not known, n = no, y = yes, ot = other
nev = never
all/unspec = all or unspecified, cig+/-ot = cigarettes irrespective of other products (cigar, pipe etc)
MC = manufactured cigarettes, HR = hand-rolled cigarettes
REF: 6-character study reference
NRR: number of the RR on the database within the study
ST : study type (CC = case control, pr or prosp = prospective)
NLC: number of lung cancer cases in whole study
R : risky occupational population (n = no, m = mining, o = other risky)
VB : national cigarette type (V = at least 75% Virginia, bl = at least 75% blended, ot = other)
P : any proxy use
H : full histological confirmation
De : derivation of RR/CI (or = original, st = standard method, ot = other method of estimation)

Table 2A7 - 1

IESLC - Meta-analysis of Ever Smoking, Cigarettes (or Any Product if Cigarettes not available), Age <56
Squamous
Most adjusted

REF	NRR	2A2	SEX	AGEL	AGEH	RACE	YF	LC TYPE	LOC	START	ST	NLC	R	VB	P	H	AD	PRODUCT	DENOM	De
BENHAM	2	x	m	1	49	all	-	KI	Eu:wst	1976	CC	1625	n	bl	n	y	0	cig only	nev any	st
SCHWAR	10		m	40	54	wh	-	q	NAmer	1984	CC	5588	n	bl	y	y	0	cig+/-ot	nev cigs	st
SCHWAR	9		m	40	54	bl	-	q	NAmer	1984	CC	5588	n	bl	y	y	0	cig+/-ot	nev cigs	st
SCHWAR	18		f	40	54	wh	-	q	NAmer	1984	CC	5588	n	bl	y	y	0	cig+/-ot	nev cigs	ot
SCHWAR	17		f	40	54	bl	-	q	NAmer	1984	CC	5588	n	bl	y	y	0	cig+/-ot	nev cigs	ot
TSUGAN	13		m	30	49	all	-	q	As:Jap	1976	CC	134	n	bl	n	y	0	all/unsp	nev any	ot
VUTUC	19	x	m	41	50	all	-	KI	Eu:wst	1976	CC	1877	n	bl	n	n	0	cig+/-ot	nev cigs	st
WYNDE4	31	x	m	30	49	all	-	not a	NAmer	1948	CC	684	n	bl	y	n	0	all/unsp	nev any	st
WYNDE6	322	x	m	1	54	wh	-	q	NAmer	1969	CC	4423	n	bl	n	y	0	cig+/-ot	nev cigs	st
WYNDE6	326	x	f	1	54	wh	-	q	NAmer	1969	CC	4423	n	bl	n	y	0	cig+/-ot	nev cigs	st

Cigarette type is all/unspec for all RRs

Table 2A7 - 2

IESLC - Meta-analysis of Ever Smoking, Cigarettes (or Any Product if Cigarettes not available), Age <56
Squamous
Most adjusted

REF	NRR	SEX	AD	Number Exposed Case	Cont	Non-exposed Case	Cont	RR	95.00%CI
BENHAM	2	m	0	190	264	5	89	12.81	(5.11- 32.14)
SCHWAR	10	m	0	80	178	1	73	32.81	(4.48- 240.23)
SCHWAR	9	m	0	41	39	4	7	1.84	(0.50- 6.78)
SCHWAR	18	f	0	29	108	0	79	43.23~	(2.60- 718.15)
SCHWAR	17	f	0	21	28	0	41	62.61~	(3.64-1076.10)
Subtotal SCHWAR								7.71	(2.96- 20.10)
TSUGAN	13	m	0	20	15	0	5	14.55~	(0.75- 283.37)
VUTUC	19	m	0	89	157	7	64	5.18	(2.28- 11.80)
WYNDE4	31	m	0	118	274	1	36	15.50	(2.10- 114.41)
WYNDE6	322	m	0	113	177	1	150	95.76	(13.21- 694.00)
WYNDE6	326	f	0	53	115	3	183	28.11	(8.58- 92.07)
Subtotal WYNDE6								38.86	(14.04- 107.51)
Totals				754	1355	22	727		

*prospective study ~ With 0.5 adjustment for zero

REF	NRR	SEX	AD	Ys	Ws	Qs	Ps
BENHAM	2	m	0	2.55	4.54	0.10	0.0000
SCHWAR	10	m	0	3.49	0.97	1.15	0.0006
SCHWAR	9	m	0	0.61	2.26	7.25	0.3596
SCHWAR	18	f	0	3.77	0.49	0.91	0.0086
SCHWAR	17	f	0	4.14	0.47	1.43	0.0044
Subtotal SCHWAR				2.04	4.19	10.74	
TSUGAN	13	m	0	2.68	0.44	0.03	0.0772
VUTUC	19	m	0	1.65	5.68	3.24	0.0001
WYNDE4	31	m	0	2.74	0.96	0.11	0.0072
WYNDE6	322	m	0	4.56	0.98	4.57	0.0000
WYNDE6	326	f	0	3.34	2.73	2.39	0.0000
Subtotal WYNDE6				3.66	3.71	6.96	

	N	10
	NS	6
	Wt	19.51
Het	Chi	21.18
Het	df	9
Het	P	*
Fixed	RR	11.04
	RRl	7.08
	RRu	17.20
	P	+++
Random	RR	14.73
	RRl	6.83
	RRu	31.76
	P	+++
Asymm	P	N.S.

Table 2A7 - 3

IESLC - Meta-analysis of Ever Smoking, Cigarettes (or Any Product if Cigarettes not available), Age <56

Squamous

Most adjusted

	combined	Sex male	female	Total
N		7	3	10
NS		6	2	8
Wt		15.82	3.69	19.51
Het Chi		15.43	0.30	21.18
Het df		6	2	9
Het P		*	N.S.	*
Fixed RR		8.55	32.98	11.04
RRl		5.22	11.89	7.08
RRu		13.99	91.48	17.20
P		+++	+++	+++
Random RR		10.98	32.98	14.73
RRl		4.50	11.89	6.83
RRu		26.83	91.48	31.76
P		+++	+++	+++
Between Chi				5.46
Between df				1
Between P				*
Btwn(F) P				N.S.
Btwn(R) P				N.S.

Table 2A7 - 4

IESLC - Meta-analysis of Ever Smoking, Cigarettes (or Any Product if Cigarettes not available), Age <56
Squamous
Least adjusted

REF	NRR	X	SEX	AGEL	AGEH	RACE	YF	LC TYPE	LOC	START	ST	NLC	R	VB	P	H	AD	PRODUCT	DENOM	De
BENHAM	2		m	1	49	all	-	KI	Eu:wst	1976	CC	1625	n	bl	n	y	0	cig only	nev any	st
SCHWAR	10		m	40	54	wh	-	q	NAmer	1984	CC	5588	n	bl	y	y	0	cig+/-ot	nev cigs	st
SCHWAR	9		m	40	54	bl	-	q	NAmer	1984	CC	5588	n	bl	y	y	0	cig+/-ot	nev cigs	st
SCHWAR	18		f	40	54	wh	-	q	NAmer	1984	CC	5588	n	bl	y	y	0	cig+/-ot	nev cigs	ot
SCHWAR	17		f	40	54	bl	-	q	NAmer	1984	CC	5588	n	bl	y	y	0	cig+/-ot	nev cigs	ot
TSUGAN	13		m	30	49	all	-	q	As:Jap	1976	CC	134	n	bl	n	y	0	all/unsp	nev any	ot
VUTUC	19		m	41	50	all	-	KI	Eu:wst	1976	CC	1877	n	bl	n	n	0	cig+/-ot	nev cigs	st
WYNDE4	31		m	30	49	all	-	not a	NAmer	1948	CC	684	n	bl	y	n	0	all/unsp	nev any	st
WYNDE6	322		m	1	54	wh	-	q	NAmer	1969	CC	4423	n	bl	n	y	0	cig+/-ot	nev cigs	st
WYNDE6	326		f	1	54	wh	-	q	NAmer	1969	CC	4423	n	bl	n	y	0	cig+/-ot	nev cigs	st

Cigarette type is all/unspec for all RRs

Table 2A7 - 5

IESLC - Meta-analysis of Ever Smoking, Cigarettes (or Any Product if Cigarettes not available), Age <56
Squamous
Least adjusted

REF	NRR	SEX	AD	Number Exposed Case	Number Exposed Cont	Non-exposed Case	Non-exposed Cont	RR	95.00%CI
BENHAM	2	m	0	190	264	5	89	12.81	(5.11- 32.14)
SCHWAR	10	m	0	80	178	1	73	32.81	(4.48- 240.23)
SCHWAR	9	m	0	41	39	4	7	1.84	(0.50- 6.78)
SCHWAR	18	f	0	29	108	0	79	43.23~	(2.60- 718.15)
SCHWAR	17	f	0	21	28	0	41	62.61~	(3.64-1076.10)
Subtotal SCHWAR								7.71	(2.96- 20.10)
TSUGAN	13	m	0	20	15	0	5	14.55~	(0.75- 283.37)
VUTUC	19	m	0	89	157	7	64	5.18	(2.28- 11.80)
WYNDE4	31	m	0	118	274	1	36	15.50	(2.10- 114.41)
WYNDE6	322	m	0	113	177	1	150	95.76	(13.21- 694.00)
WYNDE6	326	f	0	53	115	3	183	28.11	(8.58- 92.07)
Subtotal WYNDE6								38.86	(14.04- 107.51)
Totals				754	1355	22	727		

*prospective study ~ With 0.5 adjustment for zero

REF	NRR	SEX	AD	Ys	Ws	Qs	Ps
BENHAM	2	m	0	2.55	4.54	0.10	0.0000
SCHWAR	10	m	0	3.49	0.97	1.15	0.0006
SCHWAR	9	m	0	0.61	2.26	7.25	0.3596
SCHWAR	18	f	0	3.77	0.49	0.91	0.0086
SCHWAR	17	f	0	4.14	0.47	1.43	0.0044
Subtotal SCHWAR				2.04	4.19	10.74	
TSUGAN	13	m	0	2.68	0.44	0.03	0.0772
VUTUC	19	m	0	1.65	5.68	3.24	0.0001
WYNDE4	31	m	0	2.74	0.96	0.11	0.0072
WYNDE6	322	m	0	4.56	0.98	4.57	0.0000
WYNDE6	326	f	0	3.34	2.73	2.39	0.0000
Subtotal WYNDE6				3.66	3.71	6.96	

	N	10
	NS	6
	Wt	19.51
Het	Chi	21.18
Het	df	9
Het	P	*
Fixed	RR	11.04
	RRl	7.08
	RRu	17.20
	P	+++
Random	RR	14.73
	RRl	6.83
	RRu	31.76
	P	+++
Asymm	P	N.S.

Table 2A7 - 6

IESLC - Meta-analysis of Ever Smoking, Cigarettes (or Any Product if Cigarettes not available), Age <56

Squamous

Least adjusted

	combined	Sex male	female	Total
N		7	3	10
NS		6	2	8
Wt		15.82	3.69	19.51
Het Chi		15.43	0.30	21.18
Het df		6	2	9
Het P		*	N.S.	*
Fixed RR		8.55	32.98	11.04
RRl		5.22	11.89	7.08
RRu		13.99	91.48	17.20
P		+++	+++	+++
Random RR		10.98	32.98	14.73
RRl		4.50	11.89	6.83
RRu		26.83	91.48	31.76
P		+++	+++	+++
Between Chi				5.46
Between df				1
Between P				*
Btwn(F) P				N.S.
Btwn(R) P				N.S.

Table 2A7 - 7

IESLC - Meta-analysis of Ever Smoking, Cigarettes (or Any Product if Cigarettes not available), Age <56
Squamous
Excluded studies (and stage at which they were excluded)

1	BOUCHA BUELL LAURIL MZILEN
2	BYERS2 ENSTRO HIRAY2 KANELL LIAW LICKIN MRFIT MURATA NOTANI RESTRE RIMING SAARIK SEGI2 SHIMIZ SITAS SOBUE2 TANG2 WALD WARSIN WATSON WU2 WYNDE5 WYNDE8 WYNDER
3	ABELIN ABRAHA AGUDO AKIBA ALDERS AMANDU AMES ANDERS ARCHER ARMADA AUSTIN AUVINE AXELSO AXELSS BAND BARBON BECHER BENSHL BERRIN BEST BLOHMK BLOT1 BLOT2 BLOT3 BLOT4 BOFFET BOUCOT BRESLO BRETT BROCKM BROSS BROWN1 BROWN2 BUFFLE BYERS1 CARPEN CASCO2 CASCOR CEDERL CHAN CHANG CHATZI CHEN CHEN2 CHEN3 CHIAZZ CHOI CHOW CHYOU COOKSO CORREA CPSII DAMBER DARBY DAVEYS DEAN DEAN2 DEKLER DESTE2 DESTEF DOCKER DOLL DOLL2 DORANT DORGAN DORN DOSEME DROSTE DU DUNN EBELIN ENGELA ESAKI FAN GAO GAO2 GARCIA GARDIN GARSHI GENG GER GILLIS GODLEY GOLLED GOODMA GRAHAM GREGOR GSELL HAENSZ HAMMO2 HANSEN HEGMAN HEIN HENNEK HIRAOK HIRAYA HOLE HOROWI HORWIT HU HU2 HUANG HUMBLE ISHIMA JAHN JAIN JARUP JARVHO JEDRYC JIANG JOLY JONES JUSSAW KAISE2 KAISER KATSOU KAUFMA KELLER KHUDER KIHARA KINLEN KJUUS KNEKT KO KOHLME KOO KOULUM KREYBE LAMTH LAMWK LAMWK2 LANGE LAUSSM LEI LEMARC LETOUR LIDDEL LIU LIU2 LIU3 LIU4 LIU5 LOMBA2 LOMBAR LUBIN LUBIN2 LUO MACLEN MAGNUS MARSH MARSH2 MARTIS MASTRA MATOS MATSUD MCCONN MCDUFF MCLAUG MIGRAN MILLER MILLS MOLLO MRFITR NOTAN2 NOU ODRISC ORMOS OSANN OSANN2 PARKIN PASTOR PAWLEG PERNU PERSH2 PERSHA PETO PEZZO2 PEZZOT PIKE PISANI POFFIJ POLEDN QIAO QIAO2 RACHTA RADZIK RANDIG REN RONCO ROOTS ROTHSC SANKAR SCHWA2 SEGI SEOW SHAW SIEMIA SIMARA SIMONA SOBUE SPITZ STASZE STAYNE STOCKW STUCKE SUN SUZUK2 SUZUKI SVENSS TANG TAO TENKAN TIZZAN TOKARS TOUSEY TULINI ULMER VANDER VEIERO WAKAI WANG WANG2 WANG3 WANG4 WICKLU WIGLE WILKIN WU WUNSCH WYNDE2 WYNDE3 WYNDE7 XIANGZ XU XU2 XU3 XU4 YAMAGU YONG YUAN ZHANG ZHENG ZHOU
4	SADOWS
9	COMSTO CPSI DEAN3 HAMMON HINDS HITOSU KREUZE KUBIK LEVIN NAM PRESCO SPEIZE STOCKS TVERDA WUWILL

Table 2A7 - 8
Potentially overlapping studies

REF	REFGP	PRINC	OVERLAP/LINK
VUTUC	LUBIN2	2	Subset of Lubin2
BENHAM	LUBIN2	2	Subset of Lubin2
WYNDE6	WYNDE6	1	WYNDE5/6/7/8

Table 2A8 -

IESLC - Meta-analysis of Ever Smoking, Cigarettes (or Any Product if Cigarettes not available), Age 50-70
Squamous

This analysis is restricted to results for:
1) Non-dose-response data
2) Ever smokers
3) Maximum age range 50-70
4) Results complete enough for use in metaanalysis

Within each study, results are then selected (in the following order of preference, within each sex) for:
5) PRODUCT: cigarettes regardless of other products, cigarettes only, all/unspec
6) CIGTYPE: all/unspecified, MC regardless of HR, MC only
7) DENOM: never smoked anything, never smoked cigarettes, (never +1 = +long term ex, +2 = +amount unknown,
+3 = never cigs+long term ex)
8) Followup period (YF, prospective studies): whole study (coded as 0) or longest available
9) LCtype: all or nearest available, at least Squamous and Adeno. (q = squamous, s = small,
a = adeno, KI = Kreyberg I, u = undifferentiated)
10) Race: all or nearest available, otherwise by race (wh or w = white, bl or b = black, hi = hispanic
ch = chinese, jap = japanese, haw = hawaiian, w+o = white + oriental, sca = scandinavian, as = asian)
11) For overlapping studies: principal rather than subsidiary studies
Finally by Age: whole study (actual age shown) if available, otherwise by widest available age group
and then for single sex results (m, f) in preference to combined sex results (c).

Results adjusted (AD) for the most potential confounders are then chosen in Sections -1 to -3
(and those which actually differ from the adjusted results in Table 1A2 - 1 are marked 'x' in Section -1)
and results adjusted for the least confounders in Sections -4 to -6. (Those least adjusted results which
actually differ from the most adjusted as marked 'x' in column X in Section -4)
(Results adjusted for an unknown number of confounder(s) are coded as 20.)

Section -7 shows excluded studies, together with the stage (as above) at which no qualifying
results were found.

Section -8 lists the potentially overlapping studies which have been included (1=principal, 2=subsidiary).

Section -9 lists any results which would have been included in preference except that they had data not complete
enough for use in meta-analysis, with their significance (yes/no), if known, and any further comment as entered
on the database.

In addition to those mentioned above, the following fields, levels and abbreviations are used:
* or nk = not known, n = no, y = yes, ot = other
nev = never
all/unspec = all or unspecified, cig+/-ot = cigarettes irrespective of other products (cigar, pipe etc)
MC = manufactured cigarettes, HR = hand-rolled cigarettes
REF: 6-character study reference
NRR: number of the RR on the database within the study
ST : study type (CC = case control, pr or prosp = prospective)
NLC: number of lung cancer cases in whole study
R : risky occupational population (n = no, m = mining, o = other risky)
VB : national cigarette type (V = at least 75% Virginia, bl = at least 75% blended, ot = other)
P : any proxy use
H : full histological confirmation
De : derivation of RR/CI (or = original, st = standard method, ot = other method of estimation)

Table 2A8 - 1

IESLC - Meta-analysis of Ever Smoking, Cigarettes (or Any Product if Cigarettes not available), Age 50-70
Squamous
Most adjusted

REF	NRR	2A2	SEX	AGEL	AGEH	RACE	YF	LC TYPE	LOC	START	ST	NLC	R	VB	P	H	AD	PRODUCT	DENOM	De
ANDERS	10		f	55	69	all	0	q	NAmer	1986	pr	343	n	bl	n	n	0	cig+/-ot	nev cigs	st
BENHAM	3	x	m	50	59	all	-	KI	Eu:wst	1976	CC	1625	n	bl	n	y	0	cig only	nev any	st
BENHAM	4	x	m	60	69	all	-	KI	Eu:wst	1976	CC	1625	n	bl	n	y	0	cig only	nev any	st
HAMMON	59		m	50	69	wh	0	not a	NAmer	1952	pr	448	n	bl	n	n	1	cig+/-ot	nev any	ot
WYNDE4	32	x	m	50	59	all	-	not a	NAmer	1948	CC	684	n	bl	y	n	0	all/unsp	nev any	st
WYNDE4	33	x	m	60	69	all	-	not a	NAmer	1948	CC	684	n	bl	y	n	0	all/unsp	nev any	st

Cigarette type is all/unspec for all RRs

Table 2A8 - 2

IESLC - Meta-analysis of Ever Smoking, Cigarettes (or Any Product if Cigarettes not available), Age 50-70
Squamous
Most adjusted

REF	NRR	SEX	AD	Number Exposed Case	Number Exposed Cont	Non-exposed Case	Non-exposed Cont	RR	95.00%CI
*ANDERS	10	f	0	63	96164	5	195158	25.57	(10.29- 63.56)
BENHAM	3	m	0	379	476	2	174	69.27	(17.08- 281.01)
BENHAM	4	m	0	341	319	12	129	11.49	(6.24- 21.17)
Subtotal BENHAM								15.31	(8.75- 26.81)
*HAMMON	59	m	1	-	-	-	-	21.66	(8.07- 58.14)
WYNDE4	32	m	0	254	179	4	31	11.00	(3.81- 31.70)
WYNDE4	33	m	0	185	137	2	23	15.53	(3.60- 66.98)
Subtotal WYNDE4								12.38	(5.25- 29.19)
Partial Totals				1222	97275	25	195515		

*prospective study

REF	NRR	SEX	AD	Ys	Ws	Qs	Ps
*ANDERS	10	f	0	3.24	4.63	0.79	0.0000
BENHAM	3	m	0	4.24	1.96	3.88	0.0000
BENHAM	4	m	0	2.44	10.29	1.55	0.0000
Subtotal BENHAM				2.73	12.25	5.44	
*HAMMON	59	m	1	3.08	3.94	0.24	0.0000
WYNDE4	32	m	0	2.40	3.43	0.64	0.0000
WYNDE4	33	m	0	2.74	1.80	0.01	0.0002
Subtotal WYNDE4				2.52	5.23	0.65	

	N	6
	NS	4
	Wt	26.05
Het	Chi	7.11
Het	df	5
Het	P	N.S.
Fixed	RR	16.94
	RRl	11.54
	RRu	24.87
	P	+++
Random	RR	18.14
	RRl	11.18
	RRu	29.41
	P	+++
Asymm	P	N.S.

Table 2A8 - 3

IESLC - Meta-analysis of Ever Smoking, Cigarettes (or Any Product if Cigarettes not available), Age 50-70
Squamous
Most adjusted

	Sex			
	combined	male	female	Total
N		5	1	6
NS		3	1	4
Wt		21.42	4.63	26.05
Het Chi		6.16	0.00	7.11
Het df		4	0	5
Het P		N.S.	N.S.	N.S.
Fixed RR		15.50	25.57	16.94
RRl		10.15	10.29	11.54
RRu		23.67	63.56	24.87
P		+++	+++	+++
Random RR		17.01	25.57	18.14
RRl		9.63	10.29	11.18
RRu		30.04	63.56	29.41
P		+++	+++	+++
Between Chi				0.95
Between df				1
Between P				N.S.
Btwn(F) P				N.S.
Btwn(R) P				N.S.

Too few RRs for analysis by factor

Table 2A8 - 4

IESLC - Meta-analysis of Ever Smoking, Cigarettes (or Any Product if Cigarettes not available), Age 50-70
Squamous
Least adjusted

REF	NRR	X	SEX	AGEL	AGEH	RACE	YF	LC TYPE	LOC	START	ST	NLC	R	VB	P	H	AD	PRODUCT	DENOM	De
ANDERS	10		f	55	69	all	0	q	NAmer	1986	pr	343	n	bl	n	n	0	cig+/-ot	nev cigs	st
BENHAM	3		m	50	59	all	-	KI	Eu:wst	1976	CC	1625	n	bl	n	y	0	cig only	nev any	st
BENHAM	4		m	60	69	all	-	KI	Eu:wst	1976	CC	1625	n	bl	n	y	0	cig only	nev any	st
HAMMON	72	x	m	50	69	wh	0	not a	NAmer	1952	pr	448	n	bl	n	n	0	cig+/-ot	nev any	st
WYNDE4	32		m	50	59	all	-	not a	NAmer	1948	CC	684	n	bl	y	n	0	all/unsp	nev any	st
WYNDE4	33		m	60	69	all	-	not a	NAmer	1948	CC	684	n	bl	y	n	0	all/unsp	nev any	st

Cigarette type is all/unspec for all RRs

Table 2A8 - 5

IESLC - Meta-analysis of Ever Smoking, Cigarettes (or Any Product if Cigarettes not available), Age 50-70
Squamous
Least adjusted

REF	NRR	SEX	AD	Number Exposed Case	Cont	Non-exposed Case	Cont	RR	95.00%CI
*ANDERS	10	f	0	63	96164	5	195158	25.57	(10.29- 63.56)
BENHAM	3	m	0	379	476	2	174	69.27	(17.08- 281.01)
BENHAM	4	m	0	341	319	12	129	11.49	(6.24- 21.17)
Subtotal BENHAM								15.31	(8.75- 26.81)
*HAMMON	72	m	0	265	382338	4	115884	20.08	(7.48- 53.90)
WYNDE4	32	m	0	254	179	4	31	11.00	(3.81- 31.70)
WYNDE4	33	m	0	185	137	2	23	15.53	(3.60- 66.98)
Subtotal WYNDE4								12.38	(5.25- 29.19)
Totals				1487	479613	29	311399		

*prospective study

REF	NRR	SEX	AD	Ys	Ws	Qs	Ps
*ANDERS	10	f	0	3.24	4.63	0.83	0.0000
BENHAM	3	m	0	4.24	1.96	3.95	0.0000
BENHAM	4	m	0	2.44	10.29	1.46	0.0000
Subtotal BENHAM				2.73	12.25	5.41	
*HAMMON	72	m	0	3.00	3.94	0.13	0.0000
WYNDE4	32	m	0	2.40	3.43	0.61	0.0000
WYNDE4	33	m	0	2.74	1.80	0.01	0.0002
Subtotal WYNDE4				2.52	5.23	0.62	

	N	6
	NS	4
	Wt	26.05
Het	Chi	6.99
Het	df	5
Het	P	N.S.
Fixed	RR	16.75
	RRl	11.41
	RRu	24.59
	P	+++
Random	RR	17.86
	RRl	11.07
	RRu	28.82
	P	+++
Asymm	P	N.S.

Table 2A8 - 6

IESLC - Meta-analysis of Ever Smoking, Cigarettes (or Any Product if Cigarettes not available), Age 50-70
Squamous
Least adjusted

	Sex		
combined	male	female	Total
N	5	1	6
NS	3	1	4
Wt	21.42	4.63	26.05
Het Chi	5.98	0.00	6.99
Het df	4	0	5
Het P	N.S.	N.S.	N.S.
Fixed RR	15.28	25.57	16.75
RRl	10.01	10.29	11.41
RRu	23.34	63.56	24.59
P	+++	+++	+++
Random RR	16.66	25.57	17.86
RRl	9.53	10.29	11.07
RRu	29.15	63.56	28.82
P	+++	+++	+++
Between Chi			1.01
Between df			1
Between P			N.S.
Btwn(F) P			N.S.
Btwn(R) P			N.S.

Table 2A8 - 7

IESLC - Meta-analysis of Ever Smoking, Cigarettes (or Any Product if Cigarettes not available), Age 50-70
Squamous
Excluded studies (and stage at which they were excluded)

Stage	Studies
1	BOUCHA BUELL LAURIL MZILEN
2	BYERS2 ENSTRO HIRAY2 KANELL LIAW LICKIN MRFIT MURATA NOTANI RESTRE RIMING SAARIK SEGI2 SHIMIZ SITAS SOBUE2 TANG2 WALD WARSIN WATSON WU2 WYNDE5 WYNDE8 WYNDER
3	ABELIN ABRAHA AGUDO AKIBA ALDERS AMANDU AMES ARCHER ARMADA AUSTIN AUVINE AXELSO AXELSS BAND BARBON BECHER BENSHL BERRIN BEST BLOHMK BLOT1 BLOT2 BLOT3 BLOT4 BOFFET BOUCOT BRESLO BRETT BROCKM BROSS BROWN1 BYERS1 CARPEN CASCO2 CASCOR CEDERL CHAN CHANG CHATZI CHEN CHEN2 CHEN3 CHIAZZ CHOI CHOW CHYOU COMSTO COOKSO CORREA CPSII DARBY DAVEYS DEAN DEAN2 DEKLER DESTE2 DESTEF DOCKER DOLL DOLL2 DORGAN DOSEME DROSTE DU DUNN EBELIN ENGELA ESAKI FAN GAO GAO2 GARCIA GARDIN GARSHI GENG GER GILLIS GODLEY GOLLED GOODMA GRAHAM GREGOR GSELL HAENSZ HAMMO2 HANSEN HEGMAN HEIN HENNEK HIRAOK HIRAYA HOLE HOROWI HORWIT HU HU2 HUANG HUMBLE ISHIMA JAHN JAIN JARUP JARVHO JEDRYC JIANG JOLY JONES JUSSAW KAISE2 KAISER KATSOU KAUFMA KELLER KHUDER KINLEN KJUUS KNEKT KO KOHLME KOO KOULUM KREYBE LAMTH LAMWK LAMWK2 LANGE LAUSSM LEI LEMARC LETOUR LIDDEL LIU LIU2 LIU3 LIU4 LIU5 LOMBA2 LOMBAR LUBIN LUBIN2 LUO MACLEN MAGNUS MARSH MARSH2 MARTIS MASTRA MATOS MATSUD MCCONN MCDUFF MCLAUG MIGRAN MILLER MILLS MOLLO MRFITR NOTAN2 NOU ODRISC ORMOS OSANN OSANN2 PARKIN PASTOR PAWLEG PERNU PERSH2 PERSHA PETO PEZZO2 PEZZOT PIKE PISANI POFFIJ POLEDN QIAO QIAO2 RACHTA RADZIK RANDIG REN RONCO ROOTS ROTHSC SANKAR SCHWA2 SCHWAR SEGI SEOW SHAW SIEMIA SIMARA SIMONA SOBUE SPEIZE SPITZ STASZE STAYNE STOCKW STUCKE SUN SUZUK2 SUZUKI SVENSS TANG TAO TENKAN TIZZAN TOKARS TOUSEY TSUGAN TULINI TVERDA ULMER VANDER VEIERO WAKAI WANG WANG2 WANG3 WANG4 WICKLU WIGLE WILKIN WU WUNSCH WYNDE2 WYNDE3 WYNDE6 WYNDE7 XIANGZ XU XU2 XU3 XU4 YAMAGU YONG YUAN ZHANG ZHENG ZHOU
4	BROWN2 SADOWS
9	BUFFLE CPSI DAMBER DEAN3 DORANT DORN HINDS HITOSU KIHARA KREUZE KUBIK LEVIN NAM PRESCO STOCKS VUTUC WUWILL

Table 2A8 - 8
Potentially overlapping studies

REF	REFGP	PRINC	.	OVERLAP/LINK
BENHAM	LUBIN2	2		Subset of Lubin2

Table 2A9 -

IESLC - Meta-analysis of Ever Smoking, Cigarettes (or Any Product if Cigarettes not available), Age 65+
Squamous

This analysis is restricted to results for:
1) Non-dose-response data
2) Ever smokers
3) Age 65+
4) Results complete enough for use in metaanalysis

Within each study, results are then selected (in the following order of preference, within each sex) for:
5) PRODUCT: cigarettes regardless of other products, cigarettes only, all/unspec
6) CIGTYPE: all/unspecified, MC regardless of HR, MC only
7) DENOM: never smoked anything, never smoked cigarettes, (never +1 = +long term ex, +2 = +amount unknown,
+3 = never cigs+long term ex)
8) Followup period (YF, prospective studies): whole study (coded as 0) or longest available
9) LCtype: all or nearest available, at least Squamous and Adeno. (q = squamous, s = small,
a = adeno, KI = Kreyberg I, u = undifferentiated)
10) Race: all or nearest available, otherwise by race (wh or w = white, bl or b = black, hi = hispanic
ch = chinese, jap = japanese, haw = hawaiian, w+o = white + oriental, sca = scandinavian, as = asian)
11) For overlapping studies: principal rather than subsidiary studies
Finally by Age: whole study (actual age shown) if available, otherwise by widest available age group
and then for single sex results (m, f) in preference to combined sex results (c).

Results adjusted (AD) for the most potential confounders are then chosen in Sections -1 to -3
(and those which actually differ from the adjusted results in Table 1A2 - 1 are marked 'x' in Section -1)
and results adjusted for the least confounders in Sections -4 to -6. (Those least adjusted results which
actually differ from the most adjusted as marked 'x' in column X in Section -4)
(Results adjusted for an unknown number of confounder(s) are coded as 20.)

Section -7 shows excluded studies, together with the stage (as above) at which no qualifying
results were found.

Section -8 lists the potentially overlapping studies which have been included (1=principal, 2=subsidiary).

Section -9 lists any results which would have been included in preference except that they had data not complete
enough for use in meta-analysis, with their significance (yes/no), if known, and any further comment as entered
on the database.

In addition to those mentioned above, the following fields, levels and abbreviations are used:
* or nk = not known, n = no, y = yes, ot = other
nev = never
all/unspec = all or unspecified, cig+/-ot = cigarettes irrespective of other products (cigar, pipe etc)
MC = manufactured cigarettes, HR = hand-rolled cigarettes
REF: 6-character study reference
NRR: number of the RR on the database within the study
ST : study type (CC = case control, pr or prosp = prospective)
NLC: number of lung cancer cases in whole study
R : risky occupational population (n = no, m = mining, o = other risky)
VB : national cigarette type (V = at least 75% Virginia, bl = at least 75% blended, ot = other)
P : any proxy use
H : full histological confirmation
De : derivation of RR/CI (or = original, st = standard method, ot = other method of estimation)

Table 2A9 - 1

IESLC - Meta-analysis of Ever Smoking, Cigarettes (or Any Product if Cigarettes not available), Age 65+
Squamous
Most adjusted

REF	NRR	2A2	SEX	AGEL	AGEH	RACE	YF	LC TYPE	LOC	START	ST	NLC	R	VB	P	H	AD	PRODUCT	DENOM	De
BENHAM	5	x	m	70	99	all	-	KI	Eu:wst	1976	CC	1625	n	bl	n	y	0	cig only	nev any	st
WYNDE4	34	x	m	70	79	all	-	not a	NAmer	1948	CC	684	n	bl	y	n	0	all/unsp	nev any	st

Cigarette type is all/unspec for all RRs

Table 2A9 - 2

IESLC - Meta-analysis of Ever Smoking, Cigarettes (or Any Product if Cigarettes not available), Age 65+
Squamous
Most adjusted

REF	NRR	SEX	AD	Number Exposed Case	Cont	Non-exposed Case	Cont	RR	95.00%CI
BENHAM	5	m	0	164	190	5	89	15.36	(6.09- 38.74)
WYNDE4	34	m	0	40	75	1	25	13.33	(1.74- 102.06)
Totals				204	265	6	114		

*prospective study

REF	NRR	SEX	AD	Ys	Ws	Qs	Ps
BENHAM	5	m	0	2.73	4.49	0.00	0.0000
WYNDE4	34	m	0	2.59	0.93	0.01	0.0126

	N	2
	NS	2
	Wt	5.42
Het	Chi	0.02
Het	df	1
Het	P	N.S.
Fixed	RR	15.00
	RRl	6.46
	RRu	34.80
	P	+++
Random	RR	15.00
	RRl	6.46
	RRu	34.80
	P	+++
Asymm	P	

Table 2A9 - 3

IESLC - Meta-analysis of Ever Smoking, Cigarettes (or Any Product if Cigarettes not available), Age 65+
Squamous
Most adjusted

	Sex combined	male	female	Total
N		2		2
NS		2		2
Wt		5.42		5.42
Het Chi		0.02		0.02
Het df		1		1
Het P		N.S.		N.S.
Fixed RR		15.00		15.00
RRl		6.46		6.46
RRu		34.80		34.80
P		+++		+++
Random RR		15.00		15.00
RRl		6.46		6.46
RRu		34.80		34.80
P		+++		+++
Between Chi				
Between df				
Between P				N.S.
Btwn(F) P				N.S.
Btwn(R) P				N.S.

Too few RRs for analysis by factor

Table 2A9 - 4

IESLC - Meta-analysis of Ever Smoking, Cigarettes (or Any Product if Cigarettes not available), Age 65+
Squamous
Least adjusted

REF	NRR	X	SEX	AGEL	AGEH	RACE	YF	LC TYPE	LOC	START	ST	NLC	R	VB	P	H	AD	PRODUCT	DENOM	De
BENHAM	5		m	70	99	all	-	KI	Eu:wst	1976	CC	1625	n	bl	n	y	0	cig only	nev	any st
WYNDE4	34		m	70	79	all	-	not a	NAmer	1948	CC	684	n	bl	y	n	0	all/unsp	nev	any st

Cigarette type is all/unspec for all RRs

Table 2A9 - 5

IESLC - Meta-analysis of Ever Smoking, Cigarettes (or Any Product if Cigarettes not available), Age 65+
Squamous
Least adjusted

REF	NRR	SEX	AD	Number Exposed Case	Exposed Cont	Non-exposed Case	Non-exposed Cont	RR	95.00%CI
BENHAM	5	m	0	164	190	5	89	15.36	(6.09- 38.74)
WYNDE4	34	m	0	40	75	1	25	13.33	(1.74- 102.06)
Totals				204	265	6	114		

*prospective study

REF	NRR	SEX	AD	Ys	Ws	Qs	Ps
BENHAM	5	m	0	2.73	4.49	0.00	0.0000
WYNDE4	34	m	0	2.59	0.93	0.01	0.0126

	N	2
	NS	2
	Wt	5.42
Het	Chi	0.02
Het	df	1
Het	P	N.S.
Fixed	RR	15.00
	RRl	6.46
	RRu	34.80
	P	+++
Random	RR	15.00
	RRl	6.46
	RRu	34.80
	P	+++
Asymm	P	

Table 2A9 - 6

IESLC - Meta-analysis of Ever Smoking, Cigarettes (or Any Product if Cigarettes not available), Age 65+
Squamous
Least adjusted

	Sex			
	combined	male	female	Total
N		2		2
NS		2		2
Wt		5.42		5.42
Het Chi		0.02		0.02
Het df		1		1
Het P		N.S.		N.S.
Fixed RR		15.00		15.00
RRl		6.46		6.46
RRu		34.80		34.80
P		+++		+++
Random RR		15.00		15.00
RRl		6.46		6.46
RRu		34.80		34.80
P		+++		+++
Between Chi				
Between df				
Between P				N.S.
Btwn(F) P				N.S.
Btwn(R) P				N.S.

Table 2A9 - 7

IESLC - Meta-analysis of Ever Smoking, Cigarettes (or Any Product if Cigarettes not available), Age 65+
Squamous
Excluded studies (and stage at which they were excluded)

1 BOUCHA BUELL LAURIL MZILEN

2 BYERS2 ENSTRO HIRAY2 KANELL LIAW LICKIN MRFIT MURATA NOTANI RESTRE RIMING SAARIK SEGI2 SHIMIZ SITAS SOBUE2
TANG2 WALD WARSIN WATSON WU2 WYNDE5 WYNDE8 WYNDER

3 ABELIN ABRAHA AGUDO AKIBA ALDERS AMANDU AMES ANDERS ARCHER ARMADA AUSTIN AUVINE AXELSO AXELSS BAND BARBON
BECHER BENSHL BERRIN BEST BLOHMK BLOT1 BLOT2 BLOT3 BLOT4 BOFFET BOUCOT BRESLO BRETT BROCKM BROSS BROWN1
BYERS1 CARPEN CASCO2 CASCOR CEDERL CHAN CHANG CHATZI CHEN CHEN2 CHEN3 CHIAZZ CHOI CHOW CHYOU COOKSO
CORREA CPSII DARBY DAVEYS DEAN DEAN2 DEKLER DESTE2 DESTEF DOCKER DOLL DOLL2 DORANT DOSEME DROSTE DU
DUNN EBELIN ENGELA ESAKI FAN GAO GAO2 GARCIA GARDIN GENG GER GILLIS GOLLED GOODMA GRAHAM GREGOR
GSELL HAENSZ HAMMO2 HANSEN HEGMAN HEIN HENNEK HIRAOK HIRAYA HITOSU HOLE HOROWI HORWIT HU HU2 HUANG
ISHIMA JAHN JAIN JARUP JARVHO JEDRYC JIANG JOLY JONES JUSSAW KAISER KATSOU KAUFMA KELLER KHUDER KIHARA
KINLEN KJUUS KNEKT KO KOHLME KOO KOULUM KREUZE KREYBE KUBIK LAMTH LAMWK LAMWK2 LANGE LAUSSM LEI
LEMARC LETOUR LIDDEL LIU LIU2 LIU3 LIU5 LOMBA2 LOMBAR LUBIN LUBIN2 LUO MACLEN MAGNUS MARSH MARSH2
MARTIS MASTRA MATOS MATSUD MCCONN MCDUFF MCLAUG MIGRAN MILLER MILLS MOLLO MRFITR NOTAN2 NOU ODRISC ORMOS
OSANN OSANN2 PARKIN PASTOR PAWLEG PERNU PERSH2 PETO PEZZO2 PEZZOT PIKE PISANI POFFIJ POLEDN QIAO QIAO2
RACHTA RADZIK RANDIG REN RONCO ROOTS ROTHSC SANKAR SCHWA2 SCHWAR SEGI SEOW SHAW SIEMIA SIMARA SIMONA
SOBUE SPEIZE SPITZ STASZE STAYNE STOCKW STUCKE SUN SUZUK2 SUZUKI SVENSS TANG TAO TENKAN TIZZAN TOKARS
TOUSEY TSUGAN TULINI TVERDA ULMER VANDER VEIERO VUTUC WAKAI WANG WANG2 WANG3 WANG4 WICKLU WIGLE WILKIN
WU WUNSCH WYNDE2 WYNDE3 WYNDE6 WYNDE7 XIANGZ XU XU2 XU3 XU4 YAMAGU YONG YUAN ZHANG ZHENG
ZHOU

4 BROWN2 SADOWS

7 PERSHA

9 BUFFLE COMSTO CPSI DAMBER DEAN3 DORGAN DORN GARSHI GODLEY HAMMON HINDS HUMBLE KAISE2 LEVIN LIU4 NAM
PRESCO STOCKS WUWILL

Table 2A9 - 8
Potentially overlapping studies

REF	REFGP	PRINC	.	OVERLAP/LINK
BENHAM	LUBIN2	2		Subset of Lubin2

Table 2A10 -

IESLC - Meta-analysis of Ever Smoking, Cigarettes only, Age <56
Squamous

This analysis is restricted to results for:
1) Non-dose-response data
2) Ever smokers
3) Age <56
4) Results complete enough for use in metaanalysis

Within each study, results are then selected (in the following order of preference, within each sex) for:
5) PRODUCT: cigarettes only
6) CIGTYPE: all/unspecified, MC regardless of HR, MC only
7) DENOM: never smoked anything, never smoked cigarettes, (never +1 = +long term ex, +2 = +amount unknown,
+3 = never cigs+long term ex)
8) Followup period (YF, prospective studies): whole study (coded as 0) or longest available
9) LCtype: all or nearest available, at least Squamous and Adeno. (q = squamous, s = small,
a = adeno, KI = Kreyberg I, u = undifferentiated)
10) Race: all or nearest available, otherwise by race (wh or w = white, bl or b = black, hi = hispanic
ch = chinese, jap = japanese, haw = hawaiian, w+o = white + oriental, sca = scandinavian, as = asian)
11) For overlapping studies: principal rather than subsidiary studies
Finally by Age: whole study (actual age shown) if available, otherwise by widest available age group
and then for single sex results (m, f) in preference to combined sex results (c).

Results adjusted (AD) for the most potential confounders are then chosen in Sections -1 to -3
(and those which actually differ from the adjusted results in Table 2A3 - 1 are marked 'x' in Section -1)
and results adjusted for the least confounders in Sections -4 to -6. (Those least adjusted results which
actually differ from the most adjusted as marked 'x' in column X in Section -4)
(Results adjusted for an unknown number of confounder(s) are coded as 20.)

Section -7 shows excluded studies, together with the stage (as above) at which no qualifying
results were found.

Section -8 lists the potentially overlapping studies which have been included (1=principal, 2=subsidiary).

Section -9 lists any results which would have been included in preference except that they had data not complete
enough for use in meta-analysis, with their significance (yes/no), if known, and any further comment as entered
on the database.

In addition to those mentioned above, the following fields, levels and abbreviations are used:
* or nk = not known, n = no, y = yes, ot = other
nev = never
all/unspec = all or unspecified, MC = manufactured cigarettes, HR = hand-rolled cigarettes
REF: 6-character study reference
NRR: number of the RR on the database within the study
ST : study type (CC = case control, pr or prosp = prospective)
NLC: number of lung cancer cases in whole study
R : risky occupational population (n = no, m = mining, o = other risky)
VB : national cigarette type (V = at least 75% Virginia, bl = at least 75% blended, ot = other)
P : any proxy use
H : full histological confirmation
De : derivation of RR/CI (or = original, st = standard method, ot = other method of estimation)

Table 2A10 - 1

IESLC - Meta-analysis of Ever Smoking, Cigarettes only, Age <56
Squamous
Most adjusted

REF	NRR	2A3	SEX	AGEL	AGEH	RACE	YF	LC TYPE	LOC	START	ST	NLC	R	VB	P	H	AD	PRODUCT	DENOM	De
BENHAM	2	x	m	1	49	all	-	KI	Eu:wst	1976	CC	1625	n	bl	n	y	0	cig only	nev any	st

Cigarette type is all/unspec for all RRs

Table 2A10 - 2

IESLC - Meta-analysis of Ever Smoking, Cigarettes only, Age <56
Squamous
Most adjusted

REF	NRR	SEX	AD	Number Exposed Case	Number Exposed Cont	Non-exposed Case	Non-exposed Cont	RR	95.00%CI
BENHAM	2	m	0	190	264	5	89	12.81	(5.11- 32.14)
Totals				190	264	5	89		

*prospective study

REF	NRR	SEX	AD	Ys	Ws	Qs	Ps
BENHAM	2	m	0	2.55	4.54	0.00	0.0000

N	1
NS	1
Wt	4.54
Het Chi	0.00
Het df	0
Het P	N.S.
Fixed RR	12.81
RRl	5.11
RRu	32.14
P	+++
Random RR	12.81
RRl	5.11
RRu	32.14
P	+++
Asymm P	

Table 2A10 - 3

IESLC - Meta-analysis of Ever Smoking, Cigarettes only, Age <56
Squamous
Most adjusted

	Sex			
	combined	male	female	Total
N		1		1
NS		1		1
Wt		4.54		4.54
Het Chi		0.00		0.00
Het df		0		0
Het P		N.S.		N.S.
Fixed RR		12.81		12.81
RRl		5.11		5.11
RRu		32.14		32.14
P		+++		+++
Random RR		12.81		12.81
RRl		5.11		5.11
RRu		32.14		32.14
P		+++		+++
Between Chi				
Between df				
Between P				N.S.
Btwn(F) P				N.S.
Btwn(R) P				N.S.

Too few RRs for analysis by factor

Table 2A10 - 4

IESLC - Meta-analysis of Ever Smoking, Cigarettes only, Age <56
Squamous
Least adjusted

REF	NRR	X	SEX	AGEL	AGEH	RACE	YF	LC TYPE	LOC	START	ST	NLC	R	VB	P	H	AD	PRODUCT	DENOM	De
BENHAM	2		m	1	49	all	-	KI	Eu:wst	1976	CC	1625	n	bl	n	y	0	cig only	nev any	st

Cigarette type is all/unspec for all RRs

Table 2A10 - 5

IESLC - Meta-analysis of Ever Smoking, Cigarettes only, Age <56
Squamous
Least adjusted

REF	NRR	SEX	AD	Number Exposed Case	Cont	Non-exposed Case	Cont	RR	95.00%CI
BENHAM	2	m	0	190	264	5	89	12.81	(5.11- 32.14)
Totals				190	264	5	89		

*prospective study

REF	NRR	SEX	AD	Ys	Ws	Qs	Ps
BENHAM	2	m	0	2.55	4.54	0.00	0.0000

	N	1
	NS	1
	Wt	4.54
Het	Chi	0.00
Het	df	0
Het	P	N.S.
Fixed	RR	12.81
	RRl	5.11
	RRu	32.14
	P	+++
Random	RR	12.81
	RRl	5.11
	RRu	32.14
	P	+++
Asymm	P	

Table 2A10 - 6

IESLC - Meta-analysis of Ever Smoking, Cigarettes only, Age <56
Squamous
Least adjusted

	Sex combined	male	female	Total
N		1		1
NS		1		1
Wt		4.54		4.54
Het Chi		0.00		0.00
Het df		0		0
Het P		N.S.		N.S.
Fixed RR		12.81		12.81
RRl		5.11		5.11
RRu		32.14		32.14
P		+++		+++
Random RR		12.81		12.81
RRl		5.11		5.11
RRu		32.14		32.14
P		+++		+++
Between Chi				
Between df				
Between P				N.S.
Btwn(F) P				N.S.
Btwn(R) P				N.S.

Table 2A10 - 7

IESLC - Meta-analysis of Ever Smoking, Cigarettes only, Age <56
Squamous
Excluded studies (and stage at which they were excluded)

1 BOUCHA BUELL LAURIL MZILEN

2 BYERS2 ENSTRO HIRAY2 KANELL LIAW LICKIN MRFIT MURATA NOTANI RESTRE RIMING SAARIK SEGI2 SHIMIZ SITAS SOBUE2 TANG2 WALD WARSIN WATSON WU2 WYNDE5 WYNDE8 WYNDER

3 ABELIN ABRAHA AGUDO AKIBA ALDERS AMANDU AMES ANDERS ARCHER ARMADA AUSTIN AUVINE AXELSO AXELSS BAND BARBON BECHER BENSHL BERRIN BEST BLOHMK BLOT1 BLOT2 BLOT3 BLOT4 BOFFET BOUCOT BRESLO BRETT BROCKM BROSS BROWN1 BROWN2 BUFFLE BYERS1 CARPEN CASCO2 CASCOR CEDERL CHAN CHANG CHATZI CHEN CHEN2 CHEN3 CHIAZZ CHOI CHOW CHYOU COOKSO CORREA CPSII DAMBER DARBY DAVEYS DEAN DEAN2 DEKLER DESTE2 DESTEF DOCKER DOLL DOLL2 DORANT DORGAN DORN DOSEME DROSTE DU DUNN EBELIN ENGELA ESAKI FAN GAO GAO2 GARCIA GARDIN GARSHI GENG GER GILLIS GODLEY GOLLED GOODMA GRAHAM GREGOR GSELL HAENSZ HAMMO2 HANSEN HEGMAN HEIN HENNEK HIRAOK HIRAYA HOLE HOROWI HORWIT HU HU2 HUANG HUMBLE ISHIMA JAHN JAIN JARUP JARVHO JEDRYC JIANG JOLY JONES JUSSAW KAISE2 KAISER KATSOU KAUFMA KELLER KHUDER KIHARA KINLEN KJUUS KNEKT KO KOHLME KOO KOULUM KREYBE LAMTH LAMWK LAMWK2 LANGE LAUSSM LEI LEMARC LETOUR LIDDEL LIU LIU2 LIU3 LIU4 LIU5 LOMBA2 LOMBAR LUBIN LUBIN2 LUO MACLEN MAGNUS MARSH MARSH2 MARTIS MASTRA MATOS MATSUD MCCONN MCDUFF MCLAUG MIGRAN MILLER MILLS MOLLO MRFITR NOTAN2 NOU ODRISC ORMOS OSANN OSANN2 PARKIN PASTOR PAWLEG PERNU PERSH2 PERSHA PETO PEZZO2 PEZZOT PIKE PISANI POFFIJ POLEDN QIAO QIAO2 RACHTA RADZIK RANDIG REN RONCO ROOTS ROTHSC SANKAR SCHWA2 SEGI SEOW SHAW SIEMIA SIMARA SIMONA SOBUE SPITZ STASZE STAYNE STOCKW STUCKE SUN SUZUK2 SUZUKI SVENSS TANG TAO TENKAN TIZZAN TOKARS TOUSEY TULINI ULMER VANDER VEIERO WAKAI WANG WANG2 WANG3 WANG4 WICKLU WIGLE WILKIN WU WUNSCH WYNDE2 WYNDE3 WYNDE7 XIANGZ XU XU2 XU3 XU4 YAMAGU YONG YUAN ZHANG ZHENG ZHOU

4 SADOWS

5 COMSTO HINDS HITOSU KREUZE KUBIK LEVIN NAM PRESCO SCHWAR SPEIZE STOCKS TSUGAN TVERDA VUTUC WUWILL WYNDE4 WYNDE6

9 CPSI DEAN3 HAMMON

Table 2A10 - 8

Potentially overlapping studies

REF	REFGP	PRINC	. OVERLAP/LINK
BENHAM	LUBIN2	2	Subset of Lubin2

Table 2A11 -

IESLC - Meta-analysis of Ever Smoking, Cigarettes only, Age 50-70
Squamous

This analysis is restricted to results for:
1) Non-dose-response data
2) Ever smokers
3) Maximum age range 50-70
4) Results complete enough for use in metaanalysis

Within each study, results are then selected (in the following order of preference, within each sex) for:
5) PRODUCT: cigarettes only
6) CIGTYPE: all/unspecified, MC regardless of HR, MC only
7) DENOM: never smoked anything, never smoked cigarettes, (never +1 = +long term ex, +2 = +amount unknown, +3 = never cigs+long term ex)
8) Followup period (YF, prospective studies): whole study (coded as 0) or longest available
9) LCtype: all or nearest available, at least Squamous and Adeno. (q = squamous, s = small, a = adeno, KI = Kreyberg I, u = undifferentiated)
10) Race: all or nearest available, otherwise by race (wh or w = white, bl or b = black, hi = hispanic ch = chinese, jap = japanese, haw = hawaiian, w+o = white + oriental, sca = scandinavian, as = asian)
11) For overlapping studies: principal rather than subsidiary studies
Finally by Age: whole study (actual age shown) if available, otherwise by widest available age group and then for single sex results (m, f) in preference to combined sex results (c).

Results adjusted (AD) for the most potential confounders are then chosen in Sections -1 to -3 (and those which actually differ from the adjusted results in Table 2A3 - 1 are marked 'x' in Section -1) and results adjusted for the least confounders in Sections -4 to -6. (Those least adjusted results which actually differ from the most adjusted as marked 'x' in column X in Section -4)
(Results adjusted for an unknown number of confounder(s) are coded as 20.)

Section -7 shows excluded studies, together with the stage (as above) at which no qualifying results were found.

Section -8 lists the potentially overlapping studies which have been included (1=principal, 2=subsidiary).

Section -9 lists any results which would have been included in preference except that they had data not complete enough for use in meta-analysis, with their significance (yes/no), if known, and any further comment as entered on the database.

In addition to those mentioned above, the following fields, levels and abbreviations are used:
* or nk = not known, n = no, y = yes, ot = other
nev = never
all/unspec = all or unspecified, MC = manufactured cigarettes, HR = hand-rolled cigarettes
REF: 6-character study reference
NRR: number of the RR on the database within the study
ST : study type (CC = case control, pr or prosp = prospective)
NLC: number of lung cancer cases in whole study
R : risky occupational population (n = no, m = mining, o = other risky)
VB : national cigarette type (V = at least 75% Virginia, bl = at least 75% blended, ot = other)
P : any proxy use
H : full histological confirmation
De : derivation of RR/CI (or = original, st = standard method, ot = other method of estimation)

Table 2A11 - 1

IESLC - Meta-analysis of Ever Smoking, Cigarettes only, Age 50-70
Squamous
Most adjusted

REF	NRR	2A3	SEX	AGEL	AGEH	RACE	YF	LC TYPE	LOC	START	ST	NLC	R	VB	P	H	AD	PRODUCT	DENOM	De
BENHAM	3	x	m	50	59	all	-	KI	Eu:wst	1976	CC	1625	n	bl	n	y	0	cig only	nev any	st
BENHAM	4	x	m	60	69	all	-	KI	Eu:wst	1976	CC	1625	n	bl	n	y	0	cig only	nev any	st
HAMMON	58		m	50	69	wh	0	not a	NAmer	1952	pr	448	n	bl	n	n	1	cig only	nev any	ot

Cigarette type is all/unspec for all RRs

Table 2A11 - 2

IESLC - Meta-analysis of Ever Smoking, Cigarettes only, Age 50-70
Squamous
Most adjusted

REF	NRR	SEX	AD	Number Exposed Case	Cont	Non-exposed Case	Cont	RR	95.00%CI
BENHAM	3	m	0	379	476	2	174	69.27	(17.08- 281.01)
BENHAM	4	m	0	341	319	12	129	11.49	(6.24- 21.17)
Subtotal BENHAM								15.31	(8.75- 26.81)
*HAMMON	58	m	1	-	-	-	-	23.12	(8.57- 62.34)
Partial Totals				720	795	14	303		

*prospective study

REF	NRR	SEX	AD	Ys	Ws	Qs	Ps
BENHAM	3	m	0	4.24	1.96	3.89	0.0000
BENHAM	4	m	0	2.44	10.29	1.54	0.0000
Subtotal BENHAM				2.73	12.25	5.43	
*HAMMON	58	m	1	3.14	3.90	0.38	0.0000

	N	3
	NS	2
	Wt	16.15
Het	Chi	5.81
Het	df	2
Het	P	(*)
Fixed	RR	16.92
	RRl	10.39
	RRu	27.55
	P	+++
Random	RR	22.42
	RRl	8.68
	RRu	57.91
	P	+++
Asymm	P	(*)

Table 2A11 - 3

IESLC - Meta-analysis of Ever Smoking, Cigarettes only, Age 50-70
Squamous
Most adjusted

	Sex combined	male	female	Total
N		3		3
NS		2		2
Wt		16.15		16.15
Het Chi		5.81		5.81
Het df		2		2
Het P		(*)		(*)
Fixed RR		16.92		16.92
RRl		10.39		10.39
RRu		27.55		27.55
P		+++		+++
Random RR		22.42		22.42
RRl		8.68		8.68
RRu		57.91		57.91
P		+++		+++
Between Chi				
Between df				
Between P				N.S.
Btwn(F) P				N.S.
Btwn(R) P				N.S.

Too few RRs for analysis by factor

Table 2A11 - 4

IESLC - Meta-analysis of Ever Smoking, Cigarettes only, Age 50-70
Squamous
Least adjusted

REF	NRR	X	SEX	AGEL	AGEH	RACE	YF	LC TYPE	LOC	START	ST	NLC	R	VB	P	H	AD	PRODUCT	DENOM	De
BENHAM	3		m	50	59	all	-	KI	Eu:wst	1976	CC	1625	n	bl	n	y	0	cig only	nev any	st
BENHAM	4		m	60	69	all	-	KI	Eu:wst	1976	CC	1625	n	bl	n	y	0	cig only	nev any	st
HAMMON	71	x	m	50	69	wh	0	not a	NAmer	1952	pr	448	n	bl	n	n	0	cig only	nev any	st

Cigarette type is all/unspec for all RRs

Table 2A11 - 5

IESLC - Meta-analysis of Ever Smoking, Cigarettes only, Age 50-70
Squamous
Least adjusted

				Number Exposed		Non-exposed			
REF	NRR	SEX	AD	Case	Cont	Case	Cont	RR	95.00%CI
BENHAM	3	m	0	379	476	2	174	69.27	(17.08- 281.01)
BENHAM	4	m	0	341	319	12	129	11.49	(6.24- 21.17)
Subtotal BENHAM								15.31	(8.75- 26.81)
*HAMMON	71	m	0	162	225565	4	115884	20.81	(7.72- 56.11)
Totals				882	226360	18	116187		

*prospective study

REF	NRR	SEX	AD	Ys	Ws	Qs	Ps
BENHAM	3	m	0	4.24	1.96	4.03	0.0000
BENHAM	4	m	0	2.44	10.29	1.34	0.0000
Subtotal BENHAM				2.73	12.25	5.38	
*HAMMON	71	m	0	3.04	3.90	0.21	0.0000

	N	3
	NS	2
	Wt	16.16
Het	Chi	5.59
Het	df	2
Het	P	(*)
Fixed	RR	16.49
	RRl	10.13
	RRu	26.86
	P	+++
Random	RR	21.52
	RRl	8.49
	RRu	54.54
	P	+++
Asymm	P	N.S.

Table 2A11 - 6

IESLC - Meta-analysis of Ever Smoking, Cigarettes only, Age 50-70
Squamous
Least adjusted

	Sex combined	male	female	Total
N		3		3
NS		2		2
Wt		16.16		16.16
Het Chi		5.59		5.59
Het df		2		2
Het P		(*)		(*)
Fixed RR		16.49		16.49
RRl		10.13		10.13
RRu		26.86		26.86
P		+++		+++
Random RR		21.52		21.52
RRl		8.49		8.49
RRu		54.54		54.54
P		+++		+++
Between Chi				
Between df				
Between P				N.S.
Btwn(F) P				N.S.
Btwn(R) P				N.S.

Table 2A11 - 7

IESLC - Meta-analysis of Ever Smoking, Cigarettes only, Age 50-70
Squamous
Excluded studies (and stage at which they were excluded)

Stage	Studies
1	BOUCHA BUELL LAURIL MZILEN
2	BYERS2 ENSTRO HIRAY2 KANELL LIAW LICKIN MRFIT MURATA NOTANI RESTRE RIMING SAARIK SEGI2 SHIMIZ SITAS SOBUE2 TANG2 WALD WARSIN WATSON WU2 WYNDE5 WYNDE8 WYNDER
3	ABELIN ABRAHA AGUDO AKIBA ALDERS AMANDU AMES ARCHER ARMADA AUSTIN AUVINE AXELSO AXELSS BAND BARBON BECHER BENSHL BERRIN BEST BLOHMK BLOT1 BLOT2 BLOT3 BLOT4 BOFFET BOUCOT BRESLO BRETT BROCKM BROSS BROWN1 BYERS1 CARPEN CASCO2 CASCOR CEDERL CHAN CHANG CHATZI CHEN CHEN2 CHEN3 CHIAZZ CHOI CHOW CHYOU COMSTO COOKSO CORREA CPSII DARBY DAVEYS DEAN DEAN2 DEKLER DESTE2 DESTEF DOCKER DOLL DOLL2 DORGAN DOSEME DROSTE DU DUNN EBELIN ENGELA ESAKI FAN GAO GAO2 GARCIA GARDIN GARSHI GENG GER GILLIS GODLEY GOLLED GOODMA GRAHAM GREGOR GSELL HAENSZ HAMMO2 HANSEN HEGMAN HEIN HENNEK HIRAOK HIRAYA HOLE HOROWI HORWIT HU HU2 HUANG HUMBLE ISHIMA JAHN JAIN JARUP JARVHO JEDRYC JIANG JOLY JONES JUSSAW KAISE2 KAISER KATSOU KAUFMA KELLER KHUDER KINLEN KJUUS KNEKT KO KOHLME KOO KOULUM KREYBE LAMTH LAMWK LAMWK2 LANGE LAUSSM LEI LEMARC LETOUR LIDDEL LIU LIU2 LIU3 LIU4 LIU5 LOMBA2 LOMBAR LUBIN LUBIN2 LUO MACLEN MAGNUS MARSH MARSH2 MARTIS MASTRA MATOS MATSUD MCCONN MCDUFF MCLAUG MIGRAN MILLER MILLS MOLLO MRFITR NOTAN2 NOU ODRISC ORMOS OSANN OSANN2 PARKIN PASTOR PAWLEG PERNU PERSH2 PERSHA PETO PEZZO2 PEZZOT PIKE PISANI POFFIJ POLEDN QIAO QIAO2 RACHTA RADZIK RANDIG REN RONCO ROOTS ROTHSC SANKAR SCHWA2 SCHWAR SEGI SEOW SHAW SIEMIA SIMARA SIMONA SOBUE SPEIZE SPITZ STASZE STAYNE STOCKW STUCKE SUN SUZUK2 SUZUKI SVENSS TANG TAO TENKAN TIZZAN TOKARS TOUSEY TSUGAN TULINI TVERDA ULMER VANDER VEIERO WAKAI WANG WANG2 WANG3 WANG4 WICKLU WIGLE WILKIN WU WUNSCH WYNDE2 WYNDE3 WYNDE6 WYNDE7 XIANGZ XU XU2 XU3 XU4 YAMAGU YONG YUAN ZHANG ZHENG ZHOU
4	BROWN2 SADOWS
5	ANDERS BUFFLE DAMBER DORANT DORN HINDS HITOSU KIHARA KREUZE KUBIK LEVIN NAM PRESCO STOCKS VUTUC WUWILL WYNDE4
9	CPSI DEAN3

Table 2A11 - 8

Potentially overlapping studies

REF	REFGP	PRINC	OVERLAP/LINK
BENHAM	LUBIN2	2	Subset of Lubin2

Table 2A12 -

IESLC - Meta-analysis of Ever Smoking, Cigarettes only, Age 65+
Squamous

This analysis is restricted to results for:
1) Non-dose-response data
2) Ever smokers
3) Age 65+
4) Results complete enough for use in metaanalysis

Within each study, results are then selected (in the following order of preference, within each sex) for:
5) PRODUCT: cigarettes only
6) CIGTYPE: all/unspecified, MC regardless of HR, MC only
7) DENOM: never smoked anything, never smoked cigarettes, (never +1 = +long term ex, +2 = +amount unknown,
+3 = never cigs+long term ex)
8) Followup period (YF, prospective studies): whole study (coded as 0) or longest available
9) LCtype: all or nearest available, at least Squamous and Adeno. (q = squamous, s = small,
a = adeno, KI = Kreyberg I, u = undifferentiated)
10) Race: all or nearest available, otherwise by race (wh or w = white, bl or b = black, hi = hispanic
ch = chinese, jap = japanese, haw = hawaiian, w+o = white + oriental, sca = scandinavian, as = asian)
11) For overlapping studies: principal rather than subsidiary studies
Finally by Age: whole study (actual age shown) if available, otherwise by widest available age group
and then for single sex results (m, f) in preference to combined sex results (c).

Results adjusted (AD) for the most potential confounders are then chosen in Sections -1 to -3
(and those which actually differ from the adjusted results in Table 2A3 - 1 are marked 'x' in Section -1)
and results adjusted for the least confounders in Sections -4 to -6. (Those least adjusted results which
actually differ from the most adjusted as marked 'x' in column X in Section -4)
(Results adjusted for an unknown number of confounder(s) are coded as 20.)

Section -7 shows excluded studies, together with the stage (as above) at which no qualifying
results were found.

Section -8 lists the potentially overlapping studies which have been included (1=principal, 2=subsidiary).

Section -9 lists any results which would have been included in preference except that they had data not complete
enough for use in meta-analysis, with their significance (yes/no), if known, and any further comment as entered
on the database.

In addition to those mentioned above, the following fields, levels and abbreviations are used:
* or nk = not known, n = no, y = yes, ot = other
nev = never
all/unspec = all or unspecified, MC = manufactured cigarettes, HR = hand-rolled cigarettes
REF: 6-character study reference
NRR: number of the RR on the database within the study
ST : study type (CC = case control, pr or prosp = prospective)
NLC: number of lung cancer cases in whole study
R : risky occupational population (n = no, m = mining, o = other risky)
VB : national cigarette type (V = at least 75% Virginia, bl = at least 75% blended, ot = other)
P : any proxy use
H : full histological confirmation
De : derivation of RR/CI (or = original, st = standard method, ot = other method of estimation)

Table 2A12 - 1

IESLC - Meta-analysis of Ever Smoking, Cigarettes only, Age 65+
Squamous
Most adjusted

REF	NRR	2A3	SEX	AGEL	AGEH	RACE	YF	LC TYPE	LOC	START	ST	NLC	R	VB	P	H	AD	PRODUCT	DENOM	De
BENHAM	5	x	m	70	99	all	-	KI	Eu:wst	1976	CC	1625	n	bl	n	y	0	cig only	nev	any st

Cigarette type is all/unspec for all RRs

Table 2A12 - 2

IESLC - Meta-analysis of Ever Smoking, Cigarettes only, Age 65+
Squamous
Most adjusted

REF	NRR	SEX	AD	Number Exposed Case	Cont	Non-exposed Case	Cont	RR	95.00%CI
BENHAM	5	m	0	164	190	5	89	15.36	(6.09- 38.74)
Totals				164	190	5	89		

*prospective study

REF	NRR	SEX	AD	Ys	Ws	Qs	Ps
BENHAM	5	m	0	2.73	4.49	0.00	0.0000

	N	1
	NS	1
	Wt	4.49
Het	Chi	0.00
Het	df	0
Het	P	N.S.
Fixed	RR	15.36
	RRl	6.09
	RRu	38.74
	P	+++
Random	RR	15.36
	RRl	6.09
	RRu	38.74
	P	+++
Asymm	P	

Table 2A12 - 3

IESLC - Meta-analysis of Ever Smoking, Cigarettes only, Age 65+
Squamous
Most adjusted

	Sex combined	male	female	Total
N		1		1
NS		1		1
Wt		4.49		4.49
Het Chi		0.00		0.00
Het df		0		0
Het P		N.S.		N.S.
Fixed RR		15.36		15.36
RRl		6.09		6.09
RRu		38.74		38.74
P		+++		+++
Random RR		15.36		15.36
RRl		6.09		6.09
RRu		38.74		38.74
P		+++		+++
Between Chi				
Between df				
Between P				N.S.
Btwn(F) P				N.S.
Btwn(R) P				N.S.

Too few RRs for analysis by factor

Table 2A12 - 4

IESLC - Meta-analysis of Ever Smoking, Cigarettes only, Age 65+
Squamous
Least adjusted

REF	NRR	X	SEX	AGEL	AGEH	RACE	YF	LC TYPE	LOC	START	ST	NLC	R	VB	P	H	AD	PRODUCT	DENOM	De
BENHAM	5		m	70	99	all	-	KI	Eu:wst	1976	CC	1625	n	bl	n	y	0	cig only	nev any	st

Cigarette type is all/unspec for all RRs

Table 2A12 - 5

IESLC - Meta-analysis of Ever Smoking, Cigarettes only, Age 65+
Squamous
Least adjusted

REF	NRR	SEX	AD	Number Case	Exposed Cont	Non-exposed Case	Cont	RR	95.00%CI
BENHAM	5	m	0	164	190	5	89	15.36	(6.09- 38.74)
Totals				164	190	5	89		

*prospective study

REF	NRR	SEX	AD	Ys	Ws	Qs	Ps
BENHAM	5	m	0	2.73	4.49	0.00	0.0000

	N	1
	NS	1
	Wt	4.49
Het	Chi	0.00
Het	df	0
Het	P	N.S.
Fixed	RR	15.36
	RRl	6.09
	RRu	38.74
	P	+++
Random	RR	15.36
	RRl	6.09
	RRu	38.74
	P	+++
Asymm	P	

Table 2A12 - 6

IESLC - Meta-analysis of Ever Smoking, Cigarettes only, Age 65+
Squamous
Least adjusted

	Sex combined	male	female	Total
N		1		1
NS		1		1
Wt		4.49		4.49
Het Chi		0.00		0.00
Het df		0		0
Het P		N.S.		N.S.
Fixed RR		15.36		15.36
RRl		6.09		6.09
RRu		38.74		38.74
P		+++		+++
Random RR		15.36		15.36
RRl		6.09		6.09
RRu		38.74		38.74
P		+++		+++
Between Chi				
Between df				
Between P				N.S.
Btwn(F) P				N.S.
Btwn(R) P				N.S.

Table 2A12 - 7

IESLC - Meta-analysis of Ever Smoking, Cigarettes only, Age 65+
Squamous
Excluded studies (and stage at which they were excluded)

Stage	Excluded studies
1	BOUCHA BUELL LAURIL MZILEN
2	BYERS2 ENSTRO HIRAY2 KANELL LIAW LICKIN MRFIT MURATA NOTANI RESTRE RIMING SAARIK SEGI2 SHIMIZ SITAS SOBUE2 TANG2 WALD WARSIN WATSON WU2 WYNDE5 WYNDE8 WYNDER
3	ABELIN ABRAHA AGUDO AKIBA ALDERS AMANDU AMES ANDERS ARCHER ARMADA AUSTIN AUVINE AXELSO AXELSS BAND BARBON BECHER BENSHL BERRIN BEST BLOHMK BLOT1 BLOT2 BLOT3 BLOT4 BOFFET BOUCOT BRESLO BRETT BROCKM BROSS BROWN1 BYERS1 CARPEN CASCO2 CASCOR CEDERL CHAN CHANG CHATZI CHEN CHEN2 CHEN3 CHIAZZ CHOI CHOW CHYOU COOKSO CORREA CPSII DARBY DAVEYS DEAN DEAN2 DEKLER DESTE2 DESTEF DOCKER DOLL DOLL2 DORANT DOSEME DROSTE DU DUNN EBELIN ENGELA ESAKI FAN GAO GAO2 GARCIA GARDIN GENG GER GILLIS GOLLED GOODMA GRAHAM GREGOR GSELL HAENSZ HAMMO2 HANSEN HEGMAN HEIN HENNEK HIRAOK HIRAYA HITOSU HOLE HOROWI HORWIT HU HU2 HUANG ISHIMA JAHN JAIN JARUP JARVHO JEDRYC JIANG JOLY JONES JUSSAW KAISER KATSOU KAUFMA KELLER KHUDER KIHARA KINLEN KJUUS KNEKT KO KOHLME KOO KOULUM KREUZE KREYBE KUBIK LAMTH LAMWK LAMWK2 LANGE LAUSSM LEI LEMARC LETOUR LIDDEL LIU LIU2 LIU3 LIU5 LOMBA2 LOMBAR LUBIN LUBIN2 LUO MACLEN MAGNUS MARSH MARSH2 MARTIS MASTRA MATOS MATSUD MCCONN MCDUFF MCLAUG MIGRAN MILLER MILLS MOLLO MRFITR NOTAN2 NOU ODRISC ORMOS OSANN OSANN2 PARKIN PASTOR PAWLEG PERNU PERSH2 PETO PEZZO2 PEZZOT PIKE PISANI POFFIJ POLEDN QIAO QIAO2 RACHTA RADZIK RANDIG REN RONCO ROOTS ROTHSC SANKAR SCHWA2 SCHWAR SEGI SEOW SHAW SIEMIA SIMARA SIMONA SOBUE SPEIZE SPITZ STASZE STAYNE STOCKW STUCKE SUN SUZUK2 SUZUKI SVENSS TANG TAO TENKAN TIZZAN TOKARS TOUSEY TSUGAN TULINI TVERDA ULMER VANDER VEIERO VUTUC WAKAI WANG WANG2 WANG3 WANG4 WICKLU WIGLE WILKIN WU WUNSCH WYNDE2 WYNDE3 WYNDE6 WYNDE7 XIANGZ XU XU2 XU3 XU4 YAMAGU YONG YUAN ZHANG ZHENG ZHOU
4	BROWN2 SADOWS
5	BUFFLE COMSTO DAMBER DORGAN DORN GARSHI GODLEY HINDS HUMBLE LEVIN LIU4 NAM PERSHA PRESCO STOCKS WUWILL WYNDE4
9	CPSI DEAN3 HAMMON KAISE2

Table 2A12 - 8
Potentially overlapping studies

REF	REFGP	PRINC	.	OVERLAP/LINK
BENHAM	LUBIN2	2		Subset of Lubin2
