# Supplementary material for: Systematic review with meta-analysis of the epidemiological evidence in the 1900s relating smoking to lung cancer
Source: BMC Cancer. 2012 Sep 3;12:385. doi: 10.1186/1471-2407-12-385 (PMC3505152; doi:10.1186/1471-2407-12-385)
Supplement: Additional file 5 — Detailed Analysis Tables (Individual file names as described in Additional file 1: Methods, Table1). [file 1471-2407-12-385-S5.zip › PDF/3I.pdf]

Table 3I1 -

IESLC - Meta-analysis of Ever Smoking by Duration, Overview
Adenocarcinoma, Any Product (or Cigarettes if Any not available)

This analysis is restricted to results for:
1) Ever smokers
2) Results by Duration
3) Categorical results by Duration
Results by Duration are grouped under 2 schemes (S1, S2). Each scheme has a set of "key values". An interval is allocated to the category whose key value it includes, and intervals which include none or more than one of the key values are excluded. (Open-ended intervals are coded as 999)

S1	key value	maximum range
1	20	1-34
2	35	21-49
3	50	36+

S2	key value	maximum range
1	5	1-19
2	20	6-29
3	30	21-39
4	40	31-49
5	50	41-998
6	999	51+

4) Adenocarcinoma (or near equivalent)
5) Results complete enough for use in metaanalysis

Within each study, results are then selected (in the following order of preference, within each sex) for:
6) (not applicable)
7) PRODUCT: all/unspec, cigarettes regardless of other products, cigarettes only
8) CIGTYPE: all/unspecified, MC regardless of HR, MC only
9) (not applicable)
10) DENOM: never smoked anything, never smoked cigarettes, never any + low, never cigs + low
11) Followup period (YF, prospective studies): whole study (coded as 0) or longest available
12) LCtype: adeno or nearest available, but not squamous. (q = squamous, s = small,
a = adeno, l = large, KII = Kreyberg II, al = alveolar, br = bronchiolar, u = undifferentiated)
13) Race: all or nearest available, otherwise by race (wh or w = white, bl or b = black, hi = hispanic
ch = chinese, jap = japanese, haw = hawaiian, w+o = white + oriental, sca = scandinavian, as = asian)
14) For overlapping studies: principal rather than subsidiary studies
Finally by Age: whole study (coded as 0) if available, otherwise by widest available age group
and then for single sex results (m, f) in preference to results for both sexes combined (c).

Results adjusted (AD) for the most potential confounders are then chosen in Sections -1 to -3
and results adjusted for the least confounders in Sections -4 to -6. (Those least adjusted results which actually differ from the most adjusted are marked 'x' in column X in Section -4)

Section -7 shows excluded studies, together with the stage (as above) at which no qualifying results were found.

Section -8 lists the potentially overlapping studies which have been included (1=principal, 2=subsidiary).

Section -9 lists any results which would have been included in preference except that they had data not complete enough for use in meta-analysis, with their significance (yes/no), if known, and any further comment as entered on the database. It also lists as "gap" any categories for which no data were presented by the original authors.

In addition to those mentioned above, the following fields, levels and abbreviations are used:
* or nk = not known, n = no, y = yes, ot = other
nev = never
all/unspec = all or unspecified, cig+/-ot = cigarettes irrespective of other products (cigar, pipe etc)
MC = manufactured cigarettes, HR = hand-rolled cigarettes
exL, exH = range of exposure (low and high) in the smoking group, in terms of Duration
REF: 6-character study reference
NRR: number of the RR on the database within the study
ST : study type (CC = case control, pr or prosp = prospective)
NLC: number of lung cancer cases in whole study
R : risky occupational population (n = no, m = mining, o = other risky)
VB : national cigarette type (V = at least 75% Virginia, bl = at least 75% blended, ot = other)
P : any proxy use
H : full histological confirmation
De : derivation of RR/CI (or = original, st = standard method, ot = other method of estimation)

Table 3I1 - 1

IESLC - Meta-analysis of Ever Smoking by Duration, Overview
Adenocarcinoma, Any Product (or Cigarettes if Any not available)
Most adjusted

REF	NRR	SEX	AGEL	AGEH	RACE	YF	LC TYPE	LOC	START	ST	NLC	R	VB	P	H	AD	PRODUCT	exL	exH	S1	S2	DENOM	De
BARBON	724	m	0	0	all	-	a	Eu:wst	1979	CC	755	n	bl	y	y	1	all/unsp	1	29	1	0	nev any	or
BARBON	725	m	0	0	all	-	a	Eu:wst	1979	CC	755	n	bl	y	y	1	all/unsp	30	39	2	3	nev any	or
BARBON	726	m	0	0	all	-	a	Eu:wst	1979	CC	755	n	bl	y	y	1	all/unsp	40	49	0	4	nev any	or
BARBON	727	m	0	0	all	-	a	Eu:wst	1979	CC	755	n	bl	y	y	1	all/unsp	50	999	3	0	nev any	or
BUFFLE	509	m	0	0	wh	-	a	NAmer	1976	CC	943	n	bl	y	n	0	cig+/-ot	1	33	1	0	nev cigs	or
BUFFLE	510	m	0	0	wh	-	a	NAmer	1976	CC	943	n	bl	y	n	0	cig+/-ot	34	43	2	4	nev cigs	or
BUFFLE	511	m	0	0	wh	-	a	NAmer	1976	CC	943	n	bl	y	n	0	cig+/-ot	44	49	0	0	nev cigs	or
BUFFLE	512	m	0	0	wh	-	a	NAmer	1976	CC	943	n	bl	y	n	0	cig+/-ot	50	999	3	0	nev cigs	or
CHOI	566	m	0	0	all	-	a	As:oth	1985	CC	375	n	bl	n	n	0	cig+/-ot	1	29	1	0	nev cigs	st
CHOI	567	m	0	0	all	-	a	As:oth	1985	CC	375	n	bl	n	n	0	cig+/-ot	30	39	2	3	nev cigs	st
CHOI	568	m	0	0	all	-	a	As:oth	1985	CC	375	n	bl	n	n	0	cig+/-ot	40	49	0	4	nev cigs	st
CHOI	569	m	0	0	all	-	a	As:oth	1985	CC	375	n	bl	n	n	0	cig+/-ot	50	999	3	0	nev cigs	st
CHOI	578	f	0	0	all	-	a	As:oth	1985	CC	375	n	bl	n	n	0	cig+/-ot	1	29	1	0	nev cigs	st
CHOI	579	f	0	0	all	-	a	As:oth	1985	CC	375	n	bl	n	n	0	cig+/-ot	30	999	0	0	nev cigs	st
DAMBER	540	m	0	0	all	-	a+al+br	Eu:Sca	1972	CC	579	n	bl	y	n	1	all/unsp	1	30	1	0	nev any	or
DAMBER	541	m	0	0	all	-	a+al+br	Eu:Sca	1972	CC	579	n	bl	y	n	1	all/unsp	31	40	2	4	nev any	or
DAMBER	542	m	0	0	all	-	a+al+br	Eu:Sca	1972	CC	579	n	bl	y	n	1	all/unsp	41	50	3	5	nev any	or
DAMBER	543	m	0	0	all	-	a+al+br	Eu:Sca	1972	CC	579	n	bl	y	n	1	all/unsp	51	999	0	6	nev any	or
DORGAN	576	m	0	0	wh	-	a	NAmer	1980	CC	2026	n	bl	y	y	2	cig+/-ot	1	34	1	0	nev any	ot
DORGAN	577	m	0	0	wh	-	a	NAmer	1980	CC	2026	n	bl	y	y	2	cig+/-ot	35	999	0	0	nev any	ot
DORGAN	568	f	0	0	all	-	a	NAmer	1980	CC	2026	n	bl	y	y	3	cig+/-ot	1	34	1	0	nev any	ot
DORGAN	569	f	0	0	all	-	a	NAmer	1980	CC	2026	n	bl	y	y	3	cig+/-ot	35	999	0	0	nev any	ot
DOSEME	516	m	0	0	all	-	not q+s	Eu:bal	1979	CC	1210	n	bl	n	n	2	cig+/-ot	1	10	0	1	nev cigs	or
DOSEME	517	m	0	0	all	-	not q+s	Eu:bal	1979	CC	1210	n	bl	n	n	2	cig+/-ot	11	20	1	2	nev cigs	or
DOSEME	518	m	0	0	all	-	not q+s	Eu:bal	1979	CC	1210	n	bl	n	n	2	cig+/-ot	21	999	0	0	nev cigs	or
GER	507	c	0	0	all	-	a	As:oth	1990	CC	141	n	ot	y	n	5	all/unsp	1	30	1	0	nev any	ot
GER	508	c	0	0	all	-	a	As:oth	1990	CC	141	n	ot	y	n	5	all/unsp	31	999	0	0	nev any	ot
HAENSZ	525	f	0	0	all	-	a	NAmer	1955	CC	158	n	bl	n	y	0	cig+/-ot	1	14	0	1	nev any	st
HAENSZ	526	f	0	0	all	-	a	NAmer	1955	CC	158	n	bl	n	y	0	cig+/-ot	15	999	0	0	nev any	st
JEDRYC	595	m	0	0	all	-	a	Eu:est	1980	CC	1630	n	bl	y	n	3	cig+/-ot	1	19	0	1	nev any	or
JEDRYC	596	m	0	0	all	-	a	Eu:est	1980	CC	1630	n	bl	y	n	3	cig+/-ot	20	39	0	0	nev any	or
JEDRYC	597	m	0	0	all	-	a	Eu:est	1980	CC	1630	n	bl	y	n	3	cig+/-ot	40	999	3	0	nev any	or
JOLY	660	m	0	0	all	-	a	SCAmer	1978	CC	826	n	bl	n	n	0	cig+/-ot	1	29	1	0	nev any	st
JOLY	661	m	0	0	all	-	a	SCAmer	1978	CC	826	n	bl	n	n	0	cig+/-ot	30	39	2	3	nev any	st
JOLY	662	m	0	0	all	-	a	SCAmer	1978	CC	826	n	bl	n	n	0	cig+/-ot	40	49	0	4	nev any	st
JOLY	663	m	0	0	all	-	a	SCAmer	1978	CC	826	n	bl	n	n	0	cig+/-ot	50	999	3	0	nev any	st
JOLY	632	f	0	0	all	-	a	SCAmer	1978	CC	826	n	bl	n	n	0	cig+/-ot	1	29	1	0	nev any	st
JOLY	633	f	0	0	all	-	a	SCAmer	1978	CC	826	n	bl	n	n	0	cig+/-ot	30	39	2	3	nev any	st
JOLY	634	f	0	0	all	-	a	SCAmer	1978	CC	826	n	bl	n	n	0	cig+/-ot	40	49	0	4	nev any	st
JOLY	635	f	0	0	all	-	a	SCAmer	1978	CC	826	n	bl	n	n	0	cig+/-ot	50	999	3	0	nev any	st
LUBIN2	700	m	0	0	all	-	a	Eu:mul	1976	CC	7804	n	bl	n	y	0	cig+/-ot	1	29	1	0	nev any	st
LUBIN2	701	m	0	0	all	-	a	Eu:mul	1976	CC	7804	n	bl	n	y	0	cig+/-ot	30	39	2	3	nev any	st
LUBIN2	702	m	0	0	all	-	a	Eu:mul	1976	CC	7804	n	bl	n	y	0	cig+/-ot	40	49	0	4	nev any	st
LUBIN2	703	m	0	0	all	-	a	Eu:mul	1976	CC	7804	n	bl	n	y	0	cig+/-ot	50	999	3	0	nev any	st
LUBIN2	752	f	0	0	all	-	a	Eu:mul	1976	CC	7804	n	bl	n	y	0	cig+/-ot	1	29	1	0	nev any	st
LUBIN2	753	f	0	0	all	-	a	Eu:mul	1976	CC	7804	n	bl	n	y	0	cig+/-ot	30	39	2	3	nev any	st
LUBIN2	754	f	0	0	all	-	a	Eu:mul	1976	CC	7804	n	bl	n	y	0	cig+/-ot	40	49	0	4	nev any	st
LUBIN2	755	f	0	0	all	-	a	Eu:mul	1976	CC	7804	n	bl	n	y	0	cig+/-ot	50	999	3	0	nev any	st
MATOS	646	m	0	0	all	-	a	SCAmer	1994	CC	200	n	bl	n	n	2	cig+/-ot	1	24	1	0	nev any	or
MATOS	647	m	0	0	all	-	a	SCAmer	1994	CC	200	n	bl	n	n	2	cig+/-ot	25	39	2	3	nev any	or
MATOS	648	m	0	0	all	-	a	SCAmer	1994	CC	200	n	bl	n	n	2	cig+/-ot	40	70	3	0	nev any	or
OSANN2	516	f	0	0	all	-	KII	NAmer	1964	ot	217	n	bl	n	y	1	cig+/-ot	1	20	1	0	nev cigs	or
OSANN2	517	f	0	0	all	-	KII	NAmer	1964	ot	217	n	bl	n	y	1	cig+/-ot	21	999	0	0	nev cigs	or
PEZZOT	516	m	0	0	all	-	a	SCAmer	1987	CC	215	n	bl	n	y	0	cig only	1	30	1	0	nev cigs	st
PEZZOT	517	m	0	0	all	-	a	SCAmer	1987	CC	215	n	bl	n	y	0	cig only	31	40	2	4	nev cigs	st
PEZZOT	518	m	0	0	all	-	a	SCAmer	1987	CC	215	n	bl	n	y	0	cig only	41	999	3	0	nev cigs	st
WUWILL	526	f	0	0	all	-	a	As:Chi	1985	CC	965	n	ot	n	n	3	cig+/-ot	1	29	1	0	nev cigs	ot
WUWILL	527	f	0	0	all	-	a	As:Chi	1985	CC	965	n	ot	n	n	3	cig+/-ot	30	39	2	3	nev cigs	ot
WUWILL	528	f	0	0	all	-	a	As:Chi	1985	CC	965	n	ot	n	n	3	cig+/-ot	40	999	3	0	nev cigs	ot
ZHENG	512	m	0	0	all	-	a	As:Chi	1982	CC	540	n	ot	*	y	0	cig+/-ot	1	29	1	0	nev cigs	or
ZHENG	513	m	0	0	all	-	a	As:Chi	1982	CC	540	n	ot	*	y	0	cig+/-ot	30	999	0	0	nev cigs	st
ZHENG	516	f	0	0	all	-	a	As:Chi	1982	CC	540	n	ot	*	y	0	cig+/-ot	1	29	1	0	nev cigs	or
ZHENG	517	f	0	0	all	-	a	As:Chi	1982	CC	540	n	ot	*	y	0	cig+/-ot	30	999	0	0	nev cigs	st

Cigarette type is all/unspec for all RRs

In this overview table, subtotals and Qs values may be invalid and should be ignored

Table 3I1 - 2

IESLC - Meta-analysis of Ever Smoking by Duration, Overview
Adenocarcinoma, Any Product (or Cigarettes if Any not available)
Most adjusted

REF	NRR	SEX	AD	Number Exposed Case	Cont	Non-exposed Case	Cont	RR	95.00%CI
BARBON	724	m	1	15	-	7	-	3.70	(1.40- 9.70)
BARBON	725	m	1	23	-	7	-	5.10	(2.10- 12.50)
BARBON	726	m	1	46	-	7	-	8.20	(3.60- 18.70)
BARBON	727	m	1	67	-	7	-	8.30	(3.70- 18.70)
Subtotal BARBON								6.27	(4.06- 9.67)
BUFFLE	509	m	0	-	-	-	-	4.40	(1.70- 11.50)
BUFFLE	510	m	0	-	-	-	-	5.70	(2.20- 14.80)
BUFFLE	511	m	0	-	-	-	-	4.30	(1.60- 11.40)
BUFFLE	512	m	0	-	-	-	-	3.50	(1.30- 9.70)
Subtotal BUFFLE								4.44	(2.73- 7.22)
CHOI	566	m	0	17	221	7	95	1.04	(0.42- 2.60)
CHOI	567	m	0	13	160	7	65	0.75	(0.29- 1.98)
CHOI	568	m	0	12	64	7	95	2.54	(0.95- 6.81)
CHOI	569	m	0	4	20	7	95	2.71	(0.73- 10.16)
CHOI	578	f	0	2	23	48	164	0.30	(0.07- 1.31)
CHOI	579	f	0	2	3	48	164	2.28	(0.37- 14.03)
Subtotal CHOI								1.23	(0.78- 1.96)
DAMBER	540	m	1	-	-	16	-	1.80	(0.60- 5.40)
DAMBER	541	m	1	-	-	16	-	1.20	(0.20- 6.00)
DAMBER	542	m	1	-	-	16	-	3.40	(1.30- 9.10)
DAMBER	543	m	1	-	-	16	-	2.50	(0.90- 6.70)
Subtotal DAMBER								2.35	(1.35- 4.10)
DORGAN	576	m	2	-	-	-	-	2.14	(0.78- 5.84)
DORGAN	577	m	2	-	-	-	-	6.73	(2.66- 17.03)
DORGAN	568	f	3	-	-	-	-	2.43	(1.65- 3.59)
DORGAN	569	f	3	-	-	-	-	5.14	(3.63- 7.28)
Subtotal DORGAN								3.72	(2.92- 4.74)
DOSEME	516	m	2	5	-	24	-	0.80	(0.30- 2.50)
DOSEME	517	m	2	26	-	24	-	3.30	(2.20- 7.50)
DOSEME	518	m	2	69	-	24	-	4.10	(2.20- 7.50)
Subtotal DOSEME								2.96	(1.98- 4.42)
GER	507	c	5	14	-	37	-	1.40	(0.62- 3.19)
GER	508	c	5	21	-	37	-	0.85	(0.39- 1.86)
Subtotal GER								1.08	(0.61- 1.90)
HAENSZ	525	f	0	2	26	37	236	0.49	(0.11- 2.15)
HAENSZ	526	f	0	16	77	37	236	1.33	(0.70- 2.51)
Subtotal HAENSZ								1.13	(0.63- 2.04)
JEDRYC	595	m	3	2	-	7	-	1.10	(0.20- 5.95)
JEDRYC	596	m	3	43	-	7	-	3.47	(1.40- 8.58)
JEDRYC	597	m	3	47	-	7	-	4.41	(1.86- 10.45)
Subtotal JEDRYC								3.38	(1.88- 6.07)
JOLY	660	m	0	5	109	5	218	2.00	(0.57- 7.06)
JOLY	661	m	0	12	165	5	218	3.17	(1.10- 9.18)
JOLY	662	m	0	22	182	5	218	5.27	(1.96- 14.19)
JOLY	663	m	0	33	253	5	218	5.69	(2.18- 14.82)
JOLY	632	f	0	10	54	25	283	2.10	(0.95- 4.61)
JOLY	633	f	0	6	24	25	283	2.83	(1.06- 7.57)
JOLY	634	f	0	10	24	25	283	4.72	(2.03- 10.96)
JOLY	635	f	0	7	20	25	283	3.96	(1.53- 10.27)
Subtotal JOLY								3.51	(2.50- 4.92)
LUBIN2	700	m	0	131	2964	57	2616	2.03	(1.48- 2.78)
LUBIN2	701	m	0	242	3473	57	2616	3.20	(2.39- 4.29)
LUBIN2	702	m	0	208	2540	57	2616	3.76	(2.79- 5.06)
LUBIN2	703	m	0	90	1460	57	2616	2.83	(2.02- 3.97)
LUBIN2	752	f	0	174	229	138	1180	6.50	(4.99- 8.46)
LUBIN2	753	f	0	383	186	138	1180	17.61	(13.73- 22.58)
LUBIN2	754	f	0	284	118	138	1180	20.58	(15.59- 27.17)
LUBIN2	755	f	0	181	34	138	1180	45.52	(30.31- 68.36)
Subtotal LUBIN2								7.21	(6.49- 8.00)
MATOS	646	m	2	7	-	5	-	1.70	(0.50- 5.50)
MATOS	647	m	2	39	-	5	-	7.30	(2.70- 19.70)
MATOS	648	m	2	33	-	5	-	10.70	(3.80- 29.90)
Subtotal MATOS								5.70	(3.08- 10.54)
OSANN2	516	f	1	10	-	22	-	0.70	(0.20- 1.90)
OSANN2	517	f	1	51	-	22	-	4.10	(1.80- 9.40)
Subtotal OSANN2								2.21	(1.13- 4.30)
PEZZOT	516	m	0	11	134	3	116	3.17	(0.86- 11.65)
PEZZOT	517	m	0	22	82	3	116	10.37	(3.01- 35.81)
PEZZOT	518	m	0	27	101	3	116	10.34	(3.04- 35.09)
Subtotal PEZZOT								7.18	(3.49- 14.81)
WUWILL	526	f	3	34	-	172	-	0.80	(0.51- 1.25)

Table 3I1 - 2

IESLC - Meta-analysis of Ever Smoking by Duration, Overview
Adenocarcinoma, Any Product (or Cigarettes if Any not available)
Most adjusted

REF	NRR	SEX	AD	Number Exposed Case	Number Exposed Cont	Non-exposed Case	Non-exposed Cont	RR	95.00%CI
WUWILL	527	f	3	52	-	172	-	1.99	(1.37- 2.89)
WUWILL	528	f	3	52	-	172	-	2.09	(1.40- 3.10)
Subtotal WUWILL								1.58	(1.25- 2.00)
ZHENG	512	m	0	24	75	29	94	1.04	(0.56- 1.93)
ZHENG	513	m	0	99	143	29	94	2.24	(1.38- 3.66)
ZHENG	516	f	0	9	17	119	184	0.82	(0.35- 1.90)
ZHENG	517	f	0	24	27	119	184	1.37	(0.76- 2.49)
Subtotal ZHENG								1.45	(1.07- 1.96)
Partial Totals				2738	13008	2237	19242		

*prospective study

REF	NRR	SEX	AD	Ys	Ws	Qs	Ps
BARBON	724	m	1	1.31	4.10	0.07	0.0081
BARBON	725	m	1	1.63	4.83	0.18	0.0003
BARBON	726	m	1	2.10	5.66	2.53	0.0000
BARBON	727	m	1	2.12	5.85	2.71	0.0000
Subtotal BARBON				1.84	20.44	5.48	
BUFFLE	509	m	0	1.48	4.20	0.01	0.0024
BUFFLE	510	m	0	1.74	4.23	0.39	0.0003
BUFFLE	511	m	0	1.46	3.99	0.00	0.0036
BUFFLE	512	m	0	1.25	3.80	0.13	0.0145
Subtotal BUFFLE				1.49	16.22	0.53	
CHOI	566	m	0	0.04	4.61	8.95	0.9264
CHOI	567	m	0	-0.28	4.14	12.22	0.5664
CHOI	568	m	0	0.93	3.96	1.00	0.0630
CHOI	569	m	0	1.00	2.21	0.42	0.1381
CHOI	578	f	0	-1.21	1.75	12.31	0.1081
CHOI	579	f	0	0.82	1.16	0.44	0.3748
Subtotal CHOI				0.21	17.84	35.34	
DAMBER	540	m	1	0.59	3.18	2.29	0.2943
DAMBER	541	m	1	0.18	1.33	2.09	0.8336
DAMBER	542	m	1	1.22	4.06	0.18	0.0137
DAMBER	543	m	1	0.92	3.81	1.03	0.0736
Subtotal DAMBER				0.85	12.38	5.59	
DORGAN	576	m	2	0.76	3.79	1.73	0.1385
DORGAN	577	m	2	1.91	4.46	0.99	0.0001
DORGAN	568	f	3	0.89	25.43	7.64	0.0000
DORGAN	569	f	3	1.64	31.73	1.28	0.0000
Subtotal DORGAN				1.31	65.41	11.64	
DOSEME	516	m	2	-0.22	3.42	9.41	0.6799
DOSEME	517	m	2	1.19	10.22	0.60	0.0001
DOSEME	518	m	2	1.41	10.22	0.01	0.0000
Subtotal DOSEME				1.08	23.85	10.01	
GER	507	c	5	0.34	5.73	6.92	0.4207
GER	508	c	5	-0.16	6.30	16.09	0.6834
Subtotal GER				0.08	12.02	23.01	
HAENSZ	525	f	0	-0.71	1.76	8.10	0.3455
HAENSZ	526	f	0	0.28	9.37	12.48	0.3886
Subtotal HAENSZ				0.12	11.12	20.58	
JEDRYC	595	m	3	0.10	1.33	2.40	0.9123
JEDRYC	596	m	3	1.24	4.67	0.17	0.0071
JEDRYC	597	m	3	1.48	5.16	0.01	0.0008
Subtotal JEDRYC				1.22	11.17	2.58	
JOLY	660	m	0	0.69	2.42	1.33	0.2812
JOLY	661	m	0	1.15	3.40	0.27	0.0333
JOLY	662	m	0	1.66	3.91	0.20	0.0010
JOLY	663	m	0	1.74	4.19	0.38	0.0004
JOLY	632	f	0	0.74	6.17	2.99	0.0660
JOLY	633	f	0	1.04	3.97	0.62	0.0382
JOLY	634	f	0	1.55	5.40	0.07	0.0003
JOLY	635	f	0	1.38	4.23	0.01	0.0046
Subtotal JOLY				1.25	33.69	5.88	
LUBIN2	700	m	0	0.71	38.61	20.51	0.0000
LUBIN2	701	m	0	1.16	44.75	3.35	0.0000
LUBIN2	702	m	0	1.32	43.24	0.54	0.0000
LUBIN2	703	m	0	1.04	33.64	5.28	0.0000
LUBIN2	752	f	0	1.87	54.92	10.41	0.0000
LUBIN2	753	f	0	2.87	62.18	127.57	0.0000
LUBIN2	754	f	0	3.02	49.78	125.57	0.0000
LUBIN2	755	f	0	3.82	23.24	131.87	0.0000

Table 3I1 - 2

IESLC - Meta-analysis of Ever Smoking by Duration, Overview
Adenocarcinoma, Any Product (or Cigarettes if Any not available)
Most adjusted

REF	NRR	SEX	AD	Ys	Ws	Qs	Ps
Subtotal LUBIN2				1.98	350.37	425.10	
MATOS	646	m	2	0.53	2.67	2.19	0.3857
MATOS	647	m	2	1.99	3.89	1.18	0.0001
MATOS	648	m	2	2.37	3.61	3.15	0.0000
Subtotal MATOS				1.74	10.17	6.53	
OSANN2	516	f	1	-0.36	3.03	9.74	0.5346
OSANN2	517	f	1	1.41	5.62	0.00	0.0008
Subtotal OSANN2				0.79	8.66	9.75	
PEZZOT	516	m	0	1.16	2.27	0.18	0.0817
PEZZOT	517	m	0	2.34	2.50	2.04	0.0002
PEZZOT	518	m	0	2.34	2.57	2.08	0.0002
Subtotal PEZZOT				1.97	7.34	4.30	
WUWILL	526	f	3	-0.22	19.12	52.63	0.3292
WUWILL	527	f	3	0.69	27.58	15.42	0.0003
WUWILL	528	f	3	0.74	24.32	11.88	0.0003
Subtotal WUWILL				0.46	71.01	79.93	
ZHENG	512	m	0	0.04	9.99	19.56	0.9080
ZHENG	513	m	0	0.81	16.07	6.33	0.0012
ZHENG	516	f	0	-0.20	5.44	14.57	0.6405
ZHENG	517	f	0	0.32	10.81	13.51	0.2958
Subtotal ZHENG				0.37	42.31	53.97	

N	63
NS	16

Table 3I1 - 3

IESLC - Meta-analysis of Ever Smoking by Duration, Overview
Adenocarcinoma, Any Product (or Cigarettes if Any not available)
Most adjusted

	Sex			
	combined	male	female	Total
N	2	40	21	63
NS	1	12	8	21

In this overview table, other than the "N" rows, entries in the "absent" and "Total" columns may be invalid and should be ignored

	Duration of smoking (broad categories)				
	absent	1-34k20	21-49k35	36+k50	Total
N	21	19	11	12	63
NS	13	14	9	10	46
Wt	226.67	207.66	162.81	116.88	714.01
Het Chi	198.74	113.37	149.04	148.54	700.23
Het df	20	18	10	11	62
Het P	***	***	***	***	***
Fixed RR	4.75	2.44	5.77	5.60	4.20
RRl	4.17	2.13	4.95	4.67	3.91
RRu	5.41	2.80	6.73	6.71	4.52
P	+++	+++	+++	+++	+++
Random RR	3.00	1.80	3.87	5.56	3.01
RRl	1.92	1.23	1.94	2.69	2.32
RRu	4.71	2.64	7.72	11.45	3.92
P	+++	++	+++	+++	+++

	Duration of smoking (narrow categories)							
	absent	1-19k1	6-29k20	21-39k30	31-49k40	41-998k50	51+k999	Total
N	40	3	1	8	9	1	1	63
NS	16	3	1	6	7	1	1	34
Wt	414.66	6.51	10.22	154.75	120.01	4.06	3.81	714.01
Het Chi	352.74	0.53	0.00	144.90	81.91	0.00	0.00	700.23
Het df	39	2	0	7	8	0	0	62
Het P	***	N.S.	N.S.	***	***	N.S.	N.S.	***
Fixed RR	3.21	0.75	3.30	5.79	8.15	3.40	2.50	4.20
RRl	2.91	0.35	1.79	4.95	6.81	1.29	0.92	3.91
RRu	3.53	1.61	6.09	6.78	9.74	9.00	6.82	4.52
P	+++	N.S.	+++	+++	+++	+	(+)	+++
Random RR	2.74	0.75	3.30	3.68	5.60	3.40	2.50	3.01
RRl	2.01	0.35	1.79	1.62	2.81	1.29	0.92	2.32
RRu	3.73	1.61	6.09	8.33	11.17	9.00	6.82	3.92
P	+++	N.S.	+++	++	+++	+	(+)	+++

Table 3I1 - 3

IESLC - Meta-analysis of Ever Smoking by Duration, Overview
Adenocarcinoma, Any Product (or Cigarettes if Any not available)
Most adjusted

MALES

	Duration of smoking (broad categories) absent	1-34k20	21-49k35	36+k50	Total
N	12	11	8	9	40
NS	10	11	8	9	38
Wt	104.75	86.07	69.07	65.09	324.99
Het Chi	20.53	13.53	18.32	14.01	85.99
Het df	11	10	7	8	39
Het P	*	N.S.	*	(*)	***
Fixed RR	3.45	2.06	3.37	3.91	3.07
RRl	2.85	1.67	2.66	3.07	2.75
RRu	4.18	2.54	4.26	4.99	3.42
P	+++	+++	+++	+++	+++
Random RR	3.38	2.09	3.55	4.71	3.22
RRl	2.49	1.57	2.14	3.22	2.68
RRu	4.58	2.77	5.91	6.89	3.88
P	+++	+++	+++	+++	+++

	Duration of smoking (narrow categories) absent	1-19k1	6-29k20	21-39k30	31-49k40	41-998k50	51+k999	Total
N	23	2	1	5	7	1	1	40
NS	12	2	1	5	7	1	1	28
Wt	176.30	4.75	10.22	61.01	64.84	4.06	3.81	324.99
Het Chi	49.20	0.10	0.00	12.34	8.79	0.00	0.00	85.99
Het df	22	1	0	4	6	0	0	39
Het P	***	N.S.	N.S.	*	N.S.	N.S.	N.S.	***
Fixed RR	2.80	0.87	3.30	3.17	4.19	3.40	2.50	3.07
RRl	2.41	0.36	1.79	2.47	3.28	1.29	0.92	2.75
RRu	3.24	2.15	6.09	4.07	5.34	9.00	6.82	3.42
P	+++	N.S.	+++	+++	+++	+	(+)	+++
Random RR	3.15	0.87	3.30	3.11	4.54	3.40	2.50	3.22
RRl	2.46	0.36	1.79	1.69	3.09	1.29	0.92	2.68
RRu	4.05	2.15	6.09	5.75	6.68	9.00	6.82	3.88
P	+++	N.S.	+++	+++	+++	+	(+)	+++

FEMALES

	Duration of smoking (broad categories) absent	1-34k20	21-49k35	36+k50	Total
N	8	7	3	3	21
NS	7	7	3	3	20
Wt	115.62	115.86	93.73	51.79	377.00
Het Chi	131.81	92.79	95.91	115.71	521.87
Het df	7	6	2	2	20
Het P	***	***	***	***	***
Fixed RR	6.98	2.85	8.58	8.78	5.76
RRl	5.81	2.38	7.01	6.68	5.20
RRu	8.37	3.42	10.51	11.52	6.37
P	+++	+++	+++	+++	+++
Random RR	3.05	1.37	4.72	7.29	2.83
RRl	1.23	0.60	0.87	0.77	1.64
RRu	7.58	3.13	25.49	69.06	4.90
P	+	N.S.	(+)	(+)	+++

Table 3I1 - 3

IESLC - Meta-analysis of Ever Smoking by Duration, Overview
Adenocarcinoma, Any Product (or Cigarettes if Any not available)
Most adjusted

FEMALES

		Duration of smoking (narrow categories)						
	absent	1-19k1	6-29k20	21-39k30	31-49k40	41-998k50	51+k999	Total
N	15	1		3	2			21
NS	8	1		3	2			14
Wt	226.34	1.76		93.73	55.18			377.00
Het Chi	279.16	0.00		95.91	10.57			521.87
Het df	14	0		2	1			20
Het P	***	N.S.		***	**			***
Fixed RR	3.78	0.49		8.58	17.82			5.76
RRl	3.32	0.11		7.01	13.68			5.20
RRu	4.30	2.15		10.51	23.20			6.37
P	+++	N.S.		+++	+++			+++
Random RR	2.34	0.49		4.72	10.42			2.83
RRl	1.26	0.11		0.87	2.47			1.64
RRu	4.36	2.15		25.49	43.96			4.90
P	++	N.S.		(+)	++			+++

Table 3I1 - 4

IESLC - Meta-analysis of Ever Smoking by Duration, Overview
Adenocarcinoma, Any Product (or Cigarettes if Any not available)
Least adjusted

REF	NRR	X	SEX	AGEL	AGEH	RACE	YF	LC TYPE	LOC	START	ST	NLC	R	VB	P	H	AD	PRODUCT	exL	exH	S1	S2	DENOM	De
BARBON	717	x	m	0	0	all	-	a	Eu:wst	1979	CC	755	n	bl	y	y	0	all/unsp	1	29	1	0	nev any	st
BARBON	718	x	m	0	0	all	-	a	Eu:wst	1979	CC	755	n	bl	y	y	0	all/unsp	30	39	2	3	nev any	st
BARBON	719	x	m	0	0	all	-	a	Eu:wst	1979	CC	755	n	bl	y	y	0	all/unsp	40	49	0	4	nev any	st
BARBON	720	x	m	0	0	all	-	a	Eu:wst	1979	CC	755	n	bl	y	y	0	all/unsp	50	999	3	0	nev any	st
BUFFLE	509		m	0	0	wh	-	a	NAmer	1976	CC	943	n	bl	y	n	0	cig+/-ot	1	33	1	0	nev cigs	or
BUFFLE	510		m	0	0	wh	-	a	NAmer	1976	CC	943	n	bl	y	n	0	cig+/-ot	34	43	2	4	nev cigs	or
BUFFLE	511		m	0	0	wh	-	a	NAmer	1976	CC	943	n	bl	y	n	0	cig+/-ot	44	49	0	0	nev cigs	or
BUFFLE	512		m	0	0	wh	-	a	NAmer	1976	CC	943	n	bl	y	n	0	cig+/-ot	50	999	3	0	nev cigs	or
CHOI	566		m	0	0	all	-	a	As:oth	1985	CC	375	n	bl	n	n	0	cig+/-ot	1	29	1	0	nev cigs	st
CHOI	567		m	0	0	all	-	a	As:oth	1985	CC	375	n	bl	n	n	0	cig+/-ot	30	39	2	3	nev cigs	st
CHOI	568		m	0	0	all	-	a	As:oth	1985	CC	375	n	bl	n	n	0	cig+/-ot	40	49	0	4	nev cigs	st
CHOI	569		m	0	0	all	-	a	As:oth	1985	CC	375	n	bl	n	n	0	cig+/-ot	50	999	3	0	nev cigs	st
CHOI	578		f	0	0	all	-	a	As:oth	1985	CC	375	n	bl	n	n	0	cig+/-ot	1	29	1	0	nev cigs	st
CHOI	579		f	0	0	all	-	a	As:oth	1985	CC	375	n	bl	n	n	0	cig+/-ot	30	999	0	0	nev cigs	st
DAMBER	540		m	0	0	all	-	a+al+br	Eu:Sca	1972	CC	579	n	bl	y	n	1	all/unsp	1	30	1	0	nev any	or
DAMBER	541		m	0	0	all	-	a+al+br	Eu:Sca	1972	CC	579	n	bl	y	n	1	all/unsp	31	40	2	4	nev any	or
DAMBER	542		m	0	0	all	-	a+al+br	Eu:Sca	1972	CC	579	n	bl	y	n	1	all/unsp	41	50	3	5	nev any	or
DAMBER	543		m	0	0	all	-	a+al+br	Eu:Sca	1972	CC	579	n	bl	y	n	1	all/unsp	51	999	0	6	nev any	or
DORGAN	576		m	0	0	wh	-	a	NAmer	1980	CC	2026	n	bl	y	y	2	cig+/-ot	1	34	1	0	nev any	ot
DORGAN	577		m	0	0	wh	-	a	NAmer	1980	CC	2026	n	bl	y	y	2	cig+/-ot	35	999	0	0	nev any	ot
DORGAN	568		f	0	0	all	-	a	NAmer	1980	CC	2026	n	bl	y	y	3	cig+/-ot	1	34	1	0	nev any	ot
DORGAN	569		f	0	0	all	-	a	NAmer	1980	CC	2026	n	bl	y	y	3	cig+/-ot	35	999	0	0	nev any	ot
DOSEME	516		m	0	0	all	-	not q+s	Eu:bal	1979	CC	1210	n	bl	n	n	2	cig+/-ot	1	10	0	1	nev cigs	or
DOSEME	517		m	0	0	all	-	not q+s	Eu:bal	1979	CC	1210	n	bl	n	n	2	cig+/-ot	11	20	1	2	nev cigs	or
DOSEME	518		m	0	0	all	-	not q+s	Eu:bal	1979	CC	1210	n	bl	n	n	2	cig+/-ot	21	999	0	0	nev cigs	or
GER	501	x	c	0	0	all	-	a	As:oth	1990	CC	141	n	ot	y	n	0	all/unsp	1	30	1	0	nev any	st
GER	502	x	c	0	0	all	-	a	As:oth	1990	CC	141	n	ot	y	n	0	all/unsp	31	999	0	0	nev any	st
HAENSZ	525		f	0	0	all	-	a	NAmer	1955	CC	158	n	bl	n	y	0	cig+/-ot	1	14	0	1	nev any	st
HAENSZ	526		f	0	0	all	-	a	NAmer	1955	CC	158	n	bl	n	y	0	cig+/-ot	15	999	0	0	nev any	st
JEDRYC	519	x	m	0	0	all	-	a	Eu:est	1980	CC	1630	n	bl	y	n	0	cig+/-ot	1	19	0	1	nev any	st
JEDRYC	520	x	m	0	0	all	-	a	Eu:est	1980	CC	1630	n	bl	y	n	0	cig+/-ot	20	29	1	2	nev any	st
JEDRYC	521	x	m	0	0	all	-	a	Eu:est	1980	CC	1630	n	bl	y	n	0	cig+/-ot	30	39	2	3	nev any	st
JEDRYC	522	x	m	0	0	all	-	a	Eu:est	1980	CC	1630	n	bl	y	n	0	cig+/-ot	40	49	0	4	nev any	st
JEDRYC	523	x	m	0	0	all	-	a	Eu:est	1980	CC	1630	n	bl	y	n	0	cig+/-ot	50	999	3	0	nev any	st
JOLY	660		m	0	0	all	-	a	SCAmer	1978	CC	826	n	bl	n	n	0	cig+/-ot	1	29	1	0	nev any	st
JOLY	661		m	0	0	all	-	a	SCAmer	1978	CC	826	n	bl	n	n	0	cig+/-ot	30	39	2	3	nev any	st
JOLY	662		m	0	0	all	-	a	SCAmer	1978	CC	826	n	bl	n	n	0	cig+/-ot	40	49	0	4	nev any	st
JOLY	663		m	0	0	all	-	a	SCAmer	1978	CC	826	n	bl	n	n	0	cig+/-ot	50	999	3	0	nev any	st
JOLY	632		f	0	0	all	-	a	SCAmer	1978	CC	826	n	bl	n	n	0	cig+/-ot	1	29	1	0	nev any	st
JOLY	633		f	0	0	all	-	a	SCAmer	1978	CC	826	n	bl	n	n	0	cig+/-ot	30	39	2	3	nev any	st
JOLY	634		f	0	0	all	-	a	SCAmer	1978	CC	826	n	bl	n	n	0	cig+/-ot	40	49	0	4	nev any	st
JOLY	635		f	0	0	all	-	a	SCAmer	1978	CC	826	n	bl	n	n	0	cig+/-ot	50	999	3	0	nev any	st
LUBIN2	700		m	0	0	all	-	a	Eu:mul	1976	CC	7804	n	bl	n	y	0	cig+/-ot	1	29	1	0	nev any	st
LUBIN2	701		m	0	0	all	-	a	Eu:mul	1976	CC	7804	n	bl	n	y	0	cig+/-ot	30	39	2	3	nev any	st
LUBIN2	702		m	0	0	all	-	a	Eu:mul	1976	CC	7804	n	bl	n	y	0	cig+/-ot	40	49	0	4	nev any	st
LUBIN2	703		m	0	0	all	-	a	Eu:mul	1976	CC	7804	n	bl	n	y	0	cig+/-ot	50	999	3	0	nev any	st
LUBIN2	752		f	0	0	all	-	a	Eu:mul	1976	CC	7804	n	bl	n	y	0	cig+/-ot	1	29	1	0	nev any	st
LUBIN2	753		f	0	0	all	-	a	Eu:mul	1976	CC	7804	n	bl	n	y	0	cig+/-ot	30	39	2	3	nev any	st
LUBIN2	754		f	0	0	all	-	a	Eu:mul	1976	CC	7804	n	bl	n	y	0	cig+/-ot	40	49	0	4	nev any	st
LUBIN2	755		f	0	0	all	-	a	Eu:mul	1976	CC	7804	n	bl	n	y	0	cig+/-ot	50	999	3	0	nev any	st
MATOS	641	x	m	0	0	all	-	a	SCAmer	1994	CC	200	n	bl	n	n	0	cig+/-ot	1	24	1	0	nev any	st
MATOS	642	x	m	0	0	all	-	a	SCAmer	1994	CC	200	n	bl	n	n	0	cig+/-ot	25	39	2	3	nev any	st
MATOS	643	x	m	0	0	all	-	a	SCAmer	1994	CC	200	n	bl	n	n	0	cig+/-ot	40	70	3	0	nev any	st
OSANN2	513	x	f	0	0	all	-	KII	NAmer	1964	ot	217	n	bl	n	y	0	cig+/-ot	1	20	1	0	nev cigs	st
OSANN2	514	x	f	0	0	all	-	KII	NAmer	1964	ot	217	n	bl	n	y	0	cig+/-ot	21	999	0	0	nev cigs	st
PEZZOT	516		m	0	0	all	-	a	SCAmer	1987	CC	215	n	bl	n	y	0	cig only	1	30	1	0	nev cigs	st
PEZZOT	517		m	0	0	all	-	a	SCAmer	1987	CC	215	n	bl	n	y	0	cig only	31	40	2	4	nev cigs	st
PEZZOT	518		m	0	0	all	-	a	SCAmer	1987	CC	215	n	bl	n	y	0	cig only	41	999	3	0	nev cigs	st
WUWILL	511	x	f	0	0	all	-	a	As:Chi	1985	CC	965	n	ot	n	n	0	cig+/-ot	1	29	1	0	nev cigs	st
WUWILL	512	x	f	0	0	all	-	a	As:Chi	1985	CC	965	n	ot	n	n	0	cig+/-ot	30	39	2	3	nev cigs	st
WUWILL	513	x	f	0	0	all	-	a	As:Chi	1985	CC	965	n	ot	n	n	0	cig+/-ot	40	999	3	0	nev cigs	st
ZHENG	512		m	0	0	all	-	a	As:Chi	1982	CC	540	n	ot	*	y	0	cig+/-ot	1	29	1	0	nev cigs	or
ZHENG	513		m	0	0	all	-	a	As:Chi	1982	CC	540	n	ot	*	y	0	cig+/-ot	30	999	0	0	nev cigs	st
ZHENG	516		f	0	0	all	-	a	As:Chi	1982	CC	540	n	ot	*	y	0	cig+/-ot	1	29	1	0	nev cigs	or
ZHENG	517		f	0	0	all	-	a	As:Chi	1982	CC	540	n	ot	*	y	0	cig+/-ot	30	999	0	0	nev cigs	st

Cigarette type is all/unspec for all RRs

Table 3I1 - 4

IESLC - Meta-analysis of Ever Smoking by Duration, Overview
Adenocarcinoma, Any Product (or Cigarettes if Any not available)
Least adjusted

In this overview table, subtotals and Qs values may be invalid and should be ignored

Table 3I1 - 5

IESLC - Meta-analysis of Ever Smoking by Duration, Overview
Adenocarcinoma, Any Product (or Cigarettes if Any not available)
Least adjusted

				Number Exposed		Non-exposed			
REF	NRR	SEX	AD	Case	Cont	Case	Cont	RR	95.00%CI
BARBON	717	m	0	15	91	7	188	4.43	(1.74- 11.24)
BARBON	718	m	0	23	102	7	188	6.06	(2.51- 14.60)
BARBON	719	m	0	46	139	7	188	8.89	(3.90- 20.28)
BARBON	720	m	0	67	235	7	188	7.66	(3.43- 17.07)
Subtotal BARBON								6.72	(4.38- 10.30)
BUFFLE	509	m	0	-	-	-	-	4.40	(1.70- 11.50)
BUFFLE	510	m	0	-	-	-	-	5.70	(2.20- 14.80)
BUFFLE	511	m	0	-	-	-	-	4.30	(1.60- 11.40)
BUFFLE	512	m	0	-	-	-	-	3.50	(1.30- 9.70)
Subtotal BUFFLE								4.44	(2.73- 7.22)
CHOI	566	m	0	17	221	7	95	1.04	(0.42- 2.60)
CHOI	567	m	0	13	160	7	65	0.75	(0.29- 1.98)
CHOI	568	m	0	12	64	7	95	2.54	(0.95- 6.81)
CHOI	569	m	0	4	20	7	95	2.71	(0.73- 10.16)
CHOI	578	f	0	2	23	48	164	0.30	(0.07- 1.31)
CHOI	579	f	0	2	3	48	164	2.28	(0.37- 14.03)
Subtotal CHOI								1.23	(0.78- 1.96)
DAMBER	540	m	1	-	-	16	-	1.80	(0.60- 5.40)
DAMBER	541	m	1	-	-	16	-	1.20	(0.20- 6.00)
DAMBER	542	m	1	-	-	16	-	3.40	(1.30- 9.10)
DAMBER	543	m	1	-	-	16	-	2.50	(0.90- 6.70)
Subtotal DAMBER								2.35	(1.35- 4.10)
DORGAN	576	m	2	-	-	-	-	2.14	(0.78- 5.84)
DORGAN	577	m	2	-	-	-	-	6.73	(2.66- 17.03)
DORGAN	568	f	3	-	-	-	-	2.43	(1.65- 3.59)
DORGAN	569	f	3	-	-	-	-	5.14	(3.63- 7.28)
Subtotal DORGAN								3.72	(2.92- 4.74)
DOSEME	516	m	2	5	-	24	-	0.80	(0.30- 2.50)
DOSEME	517	m	2	26	-	24	-	3.30	(2.20- 7.50)
DOSEME	518	m	2	69	-	24	-	4.10	(2.20- 7.50)
Subtotal DOSEME								2.96	(1.98- 4.42)
GER	501	c	0	14	51	37	149	1.11	(0.55- 2.21)
GER	502	c	0	21	98	37	149	0.86	(0.48- 1.56)
Subtotal GER								0.96	(0.61- 1.50)
HAENSZ	525	f	0	2	26	37	236	0.49	(0.11- 2.15)
HAENSZ	526	f	0	16	77	37	236	1.33	(0.70- 2.51)
Subtotal HAENSZ								1.13	(0.63- 2.04)
JEDRYC	519	m	0	2	68	7	289	1.21	(0.25- 5.98)
JEDRYC	520	m	0	15	160	7	289	3.87	(1.55- 9.69)
JEDRYC	521	m	0	28	231	7	289	5.00	(2.15- 11.66)
JEDRYC	522	m	0	30	223	7	289	5.55	(2.40- 12.88)
JEDRYC	523	m	0	17	214	7	289	3.28	(1.34- 8.05)
Subtotal JEDRYC								4.02	(2.63- 6.12)
JOLY	660	m	0	5	109	5	218	2.00	(0.57- 7.06)
JOLY	661	m	0	12	165	5	218	3.17	(1.10- 9.18)
JOLY	662	m	0	22	182	5	218	5.27	(1.96- 14.19)
JOLY	663	m	0	33	253	5	218	5.69	(2.18- 14.82)
JOLY	632	f	0	10	54	25	283	2.10	(0.95- 4.61)
JOLY	633	f	0	6	24	25	283	2.83	(1.06- 7.57)
JOLY	634	f	0	10	24	25	283	4.72	(2.03- 10.96)
JOLY	635	f	0	7	20	25	283	3.96	(1.53- 10.27)
Subtotal JOLY								3.51	(2.50- 4.92)
LUBIN2	700	m	0	131	2964	57	2616	2.03	(1.48- 2.78)
LUBIN2	701	m	0	242	3473	57	2616	3.20	(2.39- 4.29)
LUBIN2	702	m	0	208	2540	57	2616	3.76	(2.79- 5.06)
LUBIN2	703	m	0	90	1460	57	2616	2.83	(2.02- 3.97)
LUBIN2	752	f	0	174	229	138	1180	6.50	(4.99- 8.46)
LUBIN2	753	f	0	383	186	138	1180	17.61	(13.73- 22.58)
LUBIN2	754	f	0	284	118	138	1180	20.58	(15.59- 27.17)
LUBIN2	755	f	0	181	34	138	1180	45.52	(30.31- 68.36)
Subtotal LUBIN2								7.21	(6.49- 8.00)
MATOS	641	m	0	7	84	5	110	1.83	(0.56- 5.98)
MATOS	642	m	0	39	110	5	110	7.80	(2.96- 20.53)
MATOS	643	m	0	33	89	5	110	8.16	(3.06- 21.76)
Subtotal MATOS								5.49	(3.03- 9.96)
OSANN2	513	f	0	10	20	22	43	0.98	(0.39- 2.44)
OSANN2	514	f	0	51	20	22	43	4.98	(2.40- 10.33)
Subtotal OSANN2								2.65	(1.50- 4.69)
PEZZOT	516	m	0	11	134	3	116	3.17	(0.86- 11.65)
PEZZOT	517	m	0	22	82	3	116	10.37	(3.01- 35.81)
PEZZOT	518	m	0	27	101	3	116	10.34	(3.04- 35.09)

Table 3I1 - 5

IESLC - Meta-analysis of Ever Smoking by Duration, Overview
Adenocarcinoma, Any Product (or Cigarettes if Any not available)
Least adjusted

REF	NRR	SEX	AD	Number Exposed Case	Number Exposed Cont	Non-exposed Case	Non-exposed Cont	RR	95.00%CI
Subtotal PEZZOT								7.18	(3.49- 14.81)
WUWILL	511	f	0	34	139	172	601	0.85	(0.57- 1.29)
WUWILL	512	f	0	52	98	172	601	1.85	(1.27- 2.70)
WUWILL	513	f	0	52	114	172	601	1.59	(1.10- 2.31)
Subtotal WUWILL								1.40	(1.12- 1.75)
ZHENG	512	m	0	24	75	29	94	1.04	(0.56- 1.93)
ZHENG	513	m	0	99	143	29	94	2.24	(1.38- 3.66)
ZHENG	516	f	0	9	17	119	184	0.82	(0.35- 1.90)
ZHENG	517	f	0	24	27	119	184	1.37	(0.76- 2.49)
Subtotal ZHENG								1.45	(1.07- 1.96)
Partial Totals				2738	15294	2251	23956		

*prospective study

REF	NRR	SEX	AD	Ys	Ws	Qs	Ps
BARBON	717	m	0	1.49	4.43	0.03	0.0017
BARBON	718	m	0	1.80	4.96	0.79	0.0001
BARBON	719	m	0	2.18	5.65	3.45	0.0000
BARBON	720	m	0	2.04	5.98	2.40	0.0000
Subtotal BARBON				1.90	21.01	6.67	
BUFFLE	509	m	0	1.48	4.20	0.03	0.0024
BUFFLE	510	m	0	1.74	4.23	0.48	0.0003
BUFFLE	511	m	0	1.46	3.99	0.01	0.0036
BUFFLE	512	m	0	1.25	3.80	0.09	0.0145
Subtotal BUFFLE				1.49	16.22	0.61	
CHOI	566	m	0	0.04	4.61	8.53	0.9264
CHOI	567	m	0	-0.28	4.14	11.75	0.5664
CHOI	568	m	0	0.93	3.96	0.87	0.0630
CHOI	569	m	0	1.00	2.21	0.36	0.1381
CHOI	578	f	0	-1.21	1.75	12.00	0.1081
CHOI	579	f	0	0.82	1.16	0.39	0.3748
Subtotal CHOI				0.21	17.84	33.90	
DAMBER	540	m	1	0.59	3.18	2.11	0.2943
DAMBER	541	m	1	0.18	1.33	1.98	0.8336
DAMBER	542	m	1	1.22	4.06	0.13	0.0137
DAMBER	543	m	1	0.92	3.81	0.90	0.0736
Subtotal DAMBER				0.85	12.38	5.12	
DORGAN	576	m	2	0.76	3.79	1.56	0.1385
DORGAN	577	m	2	1.91	4.46	1.13	0.0001
DORGAN	568	f	3	0.89	25.43	6.73	0.0000
DORGAN	569	f	3	1.64	31.73	1.75	0.0000
Subtotal DORGAN				1.31	65.41	11.17	
DOSEME	516	m	2	-0.22	3.42	9.03	0.6799
DOSEME	517	m	2	1.19	10.22	0.44	0.0001
DOSEME	518	m	2	1.41	10.22	0.00	0.0000
Subtotal DOSEME				1.08	23.85	9.48	
GER	501	c	0	0.10	8.01	13.59	0.7765
GER	502	c	0	-0.15	10.92	26.24	0.6261
Subtotal GER				-0.04	18.94	39.83	
HAENSZ	525	f	0	-0.71	1.76	7.85	0.3455
HAENSZ	526	f	0	0.28	9.37	11.77	0.3886
Subtotal HAENSZ				0.12	11.12	19.62	
JEDRYC	519	m	0	0.19	1.51	2.21	0.8113
JEDRYC	520	m	0	1.35	4.56	0.01	0.0038
JEDRYC	521	m	0	1.61	5.37	0.23	0.0002
JEDRYC	522	m	0	1.71	5.43	0.53	0.0001
JEDRYC	523	m	0	1.19	4.77	0.22	0.0095
Subtotal JEDRYC				1.39	21.64	3.20	
JOLY	660	m	0	0.69	2.42	1.22	0.2812
JOLY	661	m	0	1.15	3.40	0.21	0.0333
JOLY	662	m	0	1.66	3.91	0.26	0.0010
JOLY	663	m	0	1.74	4.19	0.47	0.0004
JOLY	632	f	0	0.74	6.17	2.71	0.0660
JOLY	633	f	0	1.04	3.97	0.52	0.0382
JOLY	634	f	0	1.55	5.40	0.12	0.0003
JOLY	635	f	0	1.38	4.23	0.00	0.0046
Subtotal JOLY				1.25	33.69	5.51	
LUBIN2	700	m	0	0.71	38.61	18.66	0.0000
LUBIN2	701	m	0	1.16	44.75	2.58	0.0000
LUBIN2	702	m	0	1.32	43.24	0.27	0.0000
LUBIN2	703	m	0	1.04	33.64	4.42	0.0000

Table 3I1 - 5

IESLC - Meta-analysis of Ever Smoking by Duration, Overview
Adenocarcinoma, Any Product (or Cigarettes if Any not available)
Least adjusted

REF	NRR	SEX	AD	Ys	Ws	Qs	Ps
LUBIN2	752	f	0	1.87	54.92	12.07	0.0000
LUBIN2	753	f	0	2.87	62.18	133.61	0.0000
LUBIN2	754	f	0	3.02	49.78	130.93	0.0000
LUBIN2	755	f	0	3.82	23.24	135.61	0.0000
Subtotal LUBIN2				1.98	350.37	438.15	
MATOS	641	m	0	0.61	2.75	1.74	0.3150
MATOS	642	m	0	2.05	4.10	1.74	0.0000
MATOS	643	m	0	2.10	3.99	1.94	0.0000
Subtotal MATOS				1.70	10.84	5.42	
OSANN2	513	f	0	-0.02	4.57	9.29	0.9608
OSANN2	514	f	0	1.61	7.23	0.30	0.0000
Subtotal OSANN2				0.98	11.80	9.59	
PEZZOT	516	m	0	1.16	2.27	0.14	0.0817
PEZZOT	517	m	0	2.34	2.50	2.20	0.0002
PEZZOT	518	m	0	2.34	2.57	2.24	0.0002
Subtotal PEZZOT				1.97	7.34	4.57	
WUWILL	511	f	0	-0.16	22.68	55.17	0.4546
WUWILL	512	f	0	0.62	27.09	16.70	0.0013
WUWILL	513	f	0	0.47	28.18	24.71	0.0133
Subtotal WUWILL				0.34	77.96	96.58	
ZHENG	512	m	0	0.04	9.99	18.64	0.9080
ZHENG	513	m	0	0.81	16.07	5.68	0.0012
ZHENG	516	f	0	-0.20	5.44	13.98	0.6405
ZHENG	517	f	0	0.32	10.81	12.71	0.2958
Subtotal ZHENG				0.37	42.31	51.00	

N	65
NS	16

Table 3I1 - 6

IESLC - Meta-analysis of Ever Smoking by Duration, Overview
Adenocarcinoma, Any Product (or Cigarettes if Any not available)
Least adjusted

	Sex			
	combined	male	female	Total
N	2	42	21	65
NS	1	12	8	21

In this overview table, other than the "N" rows, entries in the "absent" and "Total" columns may be invalid and should be ignored

	Duration of smoking (broad categories)				
	absent	1-34k20	21-49k35	36+k50	Total
N	21	20	12	12	65
NS	13	15	10	10	48
Wt	233.82	220.02	168.03	120.86	742.72
Het Chi	211.86	118.59	152.90	167.58	740.41
Het df	20	19	11	11	64
Het P	***	***	***	***	***
Fixed RR	4.67	2.41	5.73	4.98	4.07
RRl	4.11	2.11	4.93	4.17	3.78
RRu	5.31	2.75	6.67	5.96	4.37
P	+++	+++	+++	+++	+++
Random RR	3.11	1.89	4.01	5.14	3.06
RRl	1.97	1.31	2.09	2.41	2.36
RRu	4.89	2.73	7.70	10.94	3.97
P	+++	+++	+++	+++	+++

	Duration of smoking (narrow categories)							
	absent	1-19k1	6-29k20	21-39k30	31-49k40	41-998k50	51+k999	Total
N	39	3	2	9	10	1	1	65
NS	16	3	2	7	8	1	1	37
Wt	427.99	6.69	14.78	159.97	125.43	4.06	3.81	742.72
Het Chi	374.01	0.68	0.08	148.77	82.72	0.00	0.00	740.41
Het df	38	2	1	8	9	0	0	64
Het P	***	N.S.	N.S.	***	***	N.S.	N.S.	***
Fixed RR	3.03	0.77	3.47	5.76	8.04	3.40	2.50	4.07
RRl	2.76	0.36	2.08	4.93	6.75	1.29	0.92	3.78
RRu	3.34	1.65	5.77	6.72	9.58	9.00	6.82	4.37
P	+++	N.S.	+++	+++	+++	+	(+)	+++
Random RR	2.68	0.77	3.47	3.87	5.66	3.40	2.50	3.06
RRl	1.95	0.36	2.08	1.81	3.01	1.29	0.92	2.36
RRu	3.68	1.65	5.77	8.27	10.66	9.00	6.82	3.97
P	+++	N.S.	+++	+++	+++	+	(+)	+++

Table 3I1 - 6

IESLC - Meta-analysis of Ever Smoking by Duration, Overview
Adenocarcinoma, Any Product (or Cigarettes if Any not available)
Least adjusted

MALES

Duration of smoking (broad categories)

	absent	1-34k20	21-49k35	36+k50	Total
N	12	12	9	9	42
NS	10	12	9	9	40
Wt	105.67	91.04	74.78	65.20	336.69
Het Chi	22.40	16.29	20.44	11.87	88.71
Het df	11	11	8	8	41
Het P	*	N.S.	**	N.S.	***
Fixed RR	3.55	2.15	3.53	3.76	3.13
RRl	2.93	1.75	2.81	2.95	2.81
RRu	4.29	2.64	4.42	4.80	3.48
P	+++	+++	+++	+++	+++
Random RR	3.52	2.23	3.82	4.32	3.31
RRl	2.57	1.67	2.40	3.06	2.77
RRu	4.83	2.97	6.07	6.11	3.96
P	+++	+++	+++	+++	+++

Duration of smoking (narrow categories)

	absent	1-19k1	6-29k20	21-39k30	31-49k40	41-998k50	51+k999	Total
N	22	2	2	6	8	1	1	42
NS	12	2	2	6	8	1	1	31
Wt	172.13	4.93	14.78	66.73	70.25	4.06	3.81	336.69
Het Chi	45.65	0.18	0.08	14.85	9.81	0.00	0.00	88.71
Het df	21	1	1	5	7	0	0	41
Het P	**	N.S.	N.S.	*	N.S.	N.S.	N.S.	***
Fixed RR	2.76	0.91	3.47	3.36	4.31	3.40	2.50	3.13
RRl	2.37	0.38	2.08	2.64	3.41	1.29	0.92	2.81
RRu	3.20	2.20	5.77	4.27	5.44	9.00	6.82	3.48
P	+++	N.S.	+++	+++	+++	+	(+)	+++
Random RR	3.09	0.91	3.47	3.51	4.68	3.40	2.50	3.31
RRl	2.40	0.38	2.08	2.04	3.31	1.29	0.92	2.77
RRu	3.96	2.20	5.77	6.02	6.62	9.00	6.82	3.96
P	+++	N.S.	+++	+++	+++	+	(+)	+++

FEMALES

Duration of smoking (broad categories)

	absent	1-34k20	21-49k35	36+k50	Total
N	8	7	3	3	21
NS	7	7	3	3	20
Wt	117.23	120.97	93.25	55.65	387.10
Het Chi	131.04	93.98	100.59	144.54	554.56
Het df	7	6	2	2	20
Het P	***	***	***	***	***
Fixed RR	7.01	2.77	8.47	6.92	5.48
RRl	5.85	2.31	6.91	5.32	4.96
RRu	8.40	3.30	10.38	9.00	6.05
P	+++	+++	+++	+++	+++
Random RR	3.14	1.44	4.60	6.64	2.85
RRl	1.28	0.64	0.81	0.58	1.63
RRu	7.69	3.22	26.10	76.18	4.96
P	+	N.S.	(+)	N.S.	+++

Table 3I1 - 6

IESLC - Meta-analysis of Ever Smoking by Duration, Overview
Adenocarcinoma, Any Product (or Cigarettes if Any not available)
Least adjusted

FEMALES

Duration of smoking (narrow categories)

	absent	1-19k1	6-29k20	21-39k30	31-49k40	41-998k50	51+k999	Total
N	15	1		3	2			21
NS	8	1		3	2			14
Wt	236.92	1.76		93.25	55.18			387.10
Het Chi	295.14	0.00		100.59	10.57			554.56
Het df	14	0		2	1			20
Het P	***	N.S.		***	**			***
Fixed RR	3.57	0.49		8.47	17.82			5.48
RRl	3.14	0.11		6.91	13.68			4.96
RRu	4.05	2.15		10.38	23.20			6.05
P	+++	N.S.		+++	+++			+++
Random RR	2.38	0.49		4.60	10.42			2.85
RRl	1.28	0.11		0.81	2.47			1.63
RRu	4.43	2.15		26.10	43.96			4.96
P	++	N.S.		(+)	++			+++

Table 3I1 - 7

<u>IESLC - Meta-analysis of Ever Smoking by Duration, Overview</u>
Adenocarcinoma, Any Product (or Cigarettes if Any not available)
Excluded studies (and stage at which they were excluded)

	1	2	3	4	5	6	7	8	9	10	11	12	13	14	15	16
1	AKIBA	AMANDU	AMES	BECHER	BENSHL	BEST	BLOT1	BROSS	BROWN3	CARPEN	CEDERL	CHYOU	CPSI	CPSII	DARBY	DEAN2
	DEAN3	DOLL2	ENGELA	GAO2	GARCIA	GILLIS	GRAHAM	GURSEL	HAMMO2	HIRAYA	HOLE	HUMBLE	JAHN	JAIN	KAISE2	KATSOU
	KAUFMA	LAUSSM	LIAW	MCDUFF	MIGRAN	MRFITR	PEZZO2	PISANI	PRESCO	QIAO	SEGI2	SPEIZE	SVENSS	TVERDA	WAKAI	WATSON
	WIGLE	WU	WYNDE3	WYNDE8												
2	ALDERS	BRESLO	CHIAZZ	DORN	GUO	HEGMAN	KOO	KOULUM	LIU4	PERNU	SOBUE	SPITZ	SUZUK2	VUTUC	YUAN	
3	GENG	STASZE	WU2	ZHANG												
4	AGUDO	ARMADA	AUVINE	AXELSS	BOFFET	BOUCHA	BOUCOT	CHEN2	CORREA	DESTEF	DOLL	FAN	GAO	GARSHI	HAMMON	HU
	HU2	JUSSAW	KREUZE	LETOUR	LEVIN	LIU3	LIU5	LUO	MCCONN	NOTAN2	QIAO2	RACHTA	RESTRE	SADOWS	TIZZAN	WANG2
	WYNDE2	WYNDE6	WYNDE7	ZHOU												
5	CHEN	LUBIN	XU													
10	KHUDER															
14	BENHAM															

Table 3I1 - 8
Potentially overlapping studies

REF	REFGP	PRINC	.	OVERLAP/LINK
LUBIN2	LUBIN2	1		Lubin-combined
OSANN2	KAISER	2		KAISER/OSANN2

Table 3I1 - 9
Most adjusted - insufficient data for meta-analysis

REF	NRR	SEX	AGEL	AGEH	RACE	YF	LC TYPE	LOC	START	ST	NLC	R	VB	P	H	AD	PRODUCT	exL	exH	S1	S2	DENOM	De
CHEN	509	c	0	0	all	-	a	As:oth	1987	CC	323	n	ot	n	y	2	cig+/-ot	1	20	1	0	nev cigs	ot
CHEN	510	c	0	0	all	-	a	As:oth	1987	CC	323	n	ot	n	y	2	cig+/-ot	21	30	0	3	nev cigs	ot
CHEN	511	c	0	0	all	-	a	As:oth	1987	CC	323	n	ot	n	y	2	cig+/-ot	31	40	2	4	nev cigs	ot
CHEN	512	c	0	0	all	-	a	As:oth	1987	CC	323	n	ot	n	y	2	cig+/-ot	41	999	3	0	nev cigs	ot
XU	506	m	0	0	all	-	a	As:Chi	1985	CC	729	n	ot	n	n	2	all/unsp	1	29	1	0	nev any	or
XU	507	m	0	0	all	-	a	As:Chi	1985	CC	729	n	ot	n	n	2	all/unsp	30	39	2	3	nev any	or
XU	508	m	0	0	all	-	a	As:Chi	1985	CC	729	n	ot	n	n	2	all/unsp	40	999	3	0	nev any	or

REF	NRR	RR	SIG	RRDATA comment
CHEN	509	1.23	n	0
CHEN	510	1.52	n	0
CHEN	511	1.60	n	0
CHEN	512	3.79	y	p<0.001
XU	506		*	RR for 1-19/day is 1.4(NS), for 20-29/ day is 0.7(p<0.05) and for >=30/day is 5.4(p<0.05)
XU	507		*	RR for 1-19/day is 2.2(p<0.05), for 20-29/day is 1.5(NS) and for >=30/day is 3.2(p<0.05)
XU	508		*	RR for 1-19/day is 2.6(p<0.05), for 20-29/day is 3.6(p<0.05) and for >=30/ day is 11.8(p<0.05)

Table 3I2 -

IESLC - Meta-analysis of Ever Smoking, Duration, "Low"
Adenocarcinoma, Any Product (or Cigarettes if Any not available)

This analysis is restricted to results for:
1) Ever smokers
2) Results by Duration
3) Categorical results by Duration
4) Adenocarcinoma (or near equivalent)
5) Results complete enough for use in metaanalysis

Within each study, results are then selected (in the following order of preference, within each sex) for:
6) (not applicable)
7) PRODUCT: all/unspec, cigarettes regardless of other products, cigarettes only
8) CIGTYPE: all/unspecified, MC regardless of HR, MC only
9) (not applicable)
10) DENOM: never smoked anything, never smoked cigarettes, never any + low, never cigs + low
11) Followup period (YF, prospective studies): whole study (coded as 0) or longest available
12) LCtype: adeno or nearest available, but not squamous. (q = squamous, s = small,
a = adeno, l = large, KII = Kreyberg II, al = alveolar, br = bronchiolar, u = undifferentiated)
13) Race: all or nearest available, otherwise by race (wh or w = white, bl or b = black, hi = hispanic
ch = chinese, jap = japanese, haw = hawaiian, w+o = white + oriental, sca = scandinavian, as = asian)
14) Duration "low" in key scheme 1 (key value 20, maximum range 1-34)
15) For overlapping studies: principal rather than subsidiary studies
Finally by Age: whole study (coded as 0) if available, otherwise by widest available age group
and then for single sex results (m, f) in preference to results for both sexes combined (c).

Results adjusted (AD) for the most potential confounders are then chosen in Sections -1 to -3
and results adjusted for the least confounders in Sections -4 to -6. (Those least adjusted results which
actually differ from the most adjusted are marked 'x' in column X in Section -4)

Section -7 shows excluded studies, together with the stage (as above) at which no qualifying
results were found.

Section -8 lists the potentially overlapping studies which have been included (1=principal, 2=subsidiary).

Section -9 lists any results which would have been included in preference except that they had data not complete
enough for use in meta-analysis, with their significance (yes/no), if known, and any further comment as entered
on the database. It also lists as "gap" any categories for which no data were presented by the original authors.

In addition to those mentioned above, the following fields, levels and abbreviations are used:
* or nk = not known, n = no, y = yes, ot = other
nev = never
all/unspec = all or unspecified, cig+/-ot = cigarettes irrespective of other products (cigar, pipe etc)
MC = manufactured cigarettes, HR = hand-rolled cigarettes
exL, exH = range of exposure (low and high) in the smoking group, in terms of Duration
REF: 6-character study reference
NRR: number of the RR on the database within the study
ST : study type (CC = case control, pr or prosp = prospective)
NLC: number of lung cancer cases in whole study
R : risky occupational population (n = no, m = mining, o = other risky)
VB : national cigarette type (V = at least 75% Virginia, bl = at least 75% blended, ot = other)
P : any proxy use
H : full histological confirmation
De : derivation of RR/CI (or = original, st = standard method, ot = other method of estimation)

Table 3I2 - 1

IESLC - Meta-analysis of Ever Smoking, Duration, "Low"
Adenocarcinoma, Any Product (or Cigarettes if Any not available)
Most adjusted

REF	NRR	SEX	AGEL	AGEH	RACE	YF	LC TYPE	LOC	START	ST	NLC	R	VB	P	H	AD	PRODUCT	exL	exH	DENOM	De
BARBON	724	m	0	0	all	-	a	Eu:wst	1979	CC	755	n	bl	y	y	1	all/unsp	1	29	nev any	or
BUFFLE	509	m	0	0	wh	-	a	NAmer	1976	CC	943	n	bl	y	n	0	cig+/-ot	1	33	nev cigs	or
CHOI	566	m	0	0	all	-	a	As:oth	1985	CC	375	n	bl	n	n	0	cig+/-ot	1	29	nev cigs	st
CHOI	578	f	0	0	all	-	a	As:oth	1985	CC	375	n	bl	n	n	0	cig+/-ot	1	29	nev cigs	st
DAMBER	540	m	0	0	all	-	a+al+br	Eu:Sca	1972	CC	579	n	bl	y	n	1	all/unsp	1	30	nev any	or
DORGAN	576	m	0	0	wh	-	a	NAmer	1980	CC	2026	n	bl	y	y	2	cig+/-ot	1	34	nev any	ot
DORGAN	568	f	0	0	all	-	a	NAmer	1980	CC	2026	n	bl	y	y	3	cig+/-ot	1	34	nev any	ot
DOSEME	517	m	0	0	all	-	not q+s	Eu:bal	1979	CC	1210	n	bl	n	n	2	cig+/-ot	11	20	nev cigs	or
GER	507	c	0	0	all	-	a	As:oth	1990	CC	141	n	ot	y	n	5	all/unsp	1	30	nev any	ot
JEDRYC	520	m	0	0	all	-	a	Eu:est	1980	CC	1630	n	bl	y	n	0	cig+/-ot	20	29	nev any	st
JOLY	660	m	0	0	all	-	a	SCAmer	1978	CC	826	n	bl	n	n	0	cig+/-ot	1	29	nev any	st
JOLY	632	f	0	0	all	-	a	SCAmer	1978	CC	826	n	bl	n	n	0	cig+/-ot	1	29	nev any	st
LUBIN2	700	m	0	0	all	-	a	Eu:mul	1976	CC	7804	n	bl	n	y	0	cig+/-ot	1	29	nev any	st
LUBIN2	752	f	0	0	all	-	a	Eu:mul	1976	CC	7804	n	bl	n	y	0	cig+/-ot	1	29	nev any	st
MATOS	646	m	0	0	all	-	a	SCAmer	1994	CC	200	n	bl	n	n	2	cig+/-ot	1	24	nev any	or
OSANN2	516	f	0	0	all	-	KII	NAmer	1964	ot	217	n	bl	n	y	1	cig+/-ot	1	20	nev cigs	or
PEZZOT	516	m	0	0	all	-	a	SCAmer	1987	CC	215	n	bl	n	y	0	cig only	1	30	nev cigs	st
WUWILL	526	f	0	0	all	-	a	As:Chi	1985	CC	965	n	ot	n	n	3	cig+/-ot	1	29	nev cigs	ot
ZHENG	512	m	0	0	all	-	a	As:Chi	1982	CC	540	n	ot	*	y	0	cig+/-ot	1	29	nev cigs	or
ZHENG	516	f	0	0	all	-	a	As:Chi	1982	CC	540	n	ot	*	y	0	cig+/-ot	1	29	nev cigs	or

Cigarette type is all/unspec for all RRs

Table 3I2 - 2

IESLC - Meta-analysis of Ever Smoking, Duration, "Low"
Adenocarcinoma, Any Product (or Cigarettes if Any not available)
Most adjusted

REF	NRR	SEX	AD	Number Exposed Case	Number Exposed Cont	Non-exposed Case	Non-exposed Cont	RR	95.00%CI
BARBON	724	m	1	15	-	7	-	3.70	(1.40- 9.70)
BUFFLE	509	m	0	-	-	-	-	4.40	(1.70- 11.50)
CHOI	566	m	0	17	221	7	95	1.04	(0.42- 2.60)
CHOI	578	f	0	2	23	48	164	0.30	(0.07- 1.31)
Subtotal CHOI								0.74	(0.34- 1.61)
DAMBER	540	m	1	-	-	16	-	1.80	(0.60- 5.40)
DORGAN	576	m	2	-	-	-	-	2.14	(0.78- 5.84)
DORGAN	568	f	3	-	-	-	-	2.43	(1.65- 3.59)
Subtotal DORGAN								2.39	(1.66- 3.43)
DOSEME	517	m	2	26	-	24	-	3.30	(2.20- 7.50)
GER	507	c	5	14	-	37	-	1.40	(0.62- 3.19)
JEDRYC	520	m	0	15	160	7	289	3.87	(1.55- 9.69)
JOLY	660	m	0	5	109	5	218	2.00	(0.57- 7.06)
JOLY	632	f	0	10	54	25	283	2.10	(0.95- 4.61)
Subtotal JOLY								2.07	(1.06- 4.04)
LUBIN2	700	m	0	131	2964	57	2616	2.03	(1.48- 2.78)
LUBIN2	752	f	0	174	229	138	1180	6.50	(4.99- 8.46)
Subtotal LUBIN2								4.02	(3.28- 4.92)
MATOS	646	m	2	7	-	5	-	1.70	(0.50- 5.50)
OSANN2	516	f	1	10	-	22	-	0.70	(0.20- 1.90)
PEZZOT	516	m	0	11	134	3	116	3.17	(0.86- 11.65)
WUWILL	526	f	3	34	-	172	-	0.80	(0.51- 1.25)
ZHENG	512	m	0	24	75	29	94	1.04	(0.56- 1.93)
ZHENG	516	f	0	9	17	119	184	0.82	(0.35- 1.90)
Subtotal ZHENG								0.95	(0.58- 1.57)
Partial Totals				504	3986	721	5239		

*prospective study

REF	NRR	SEX	AD	Ys	Ws	Qs	Ps
BARBON	724	m	1	1.31	4.10	0.67	0.0081
BUFFLE	509	m	0	1.48	4.20	1.41	0.0024
CHOI	566	m	0	0.04	4.61	3.41	0.9264
CHOI	578	f	0	-1.21	1.75	7.85	0.1081
Subtotal CHOI				-0.30	6.37	11.26	
DAMBER	540	m	1	0.59	3.18	0.32	0.2943
DORGAN	576	m	2	0.76	3.79	0.08	0.1385
DORGAN	568	f	3	0.89	25.43	0.01	0.0000
Subtotal DORGAN				0.87	29.22	0.08	
DOSEME	517	m	2	1.19	10.22	0.87	0.0001
GER	507	c	5	0.34	5.73	1.84	0.4207
JEDRYC	520	m	0	1.35	4.56	0.93	0.0038
JOLY	660	m	0	0.69	2.42	0.11	0.2812
JOLY	632	f	0	0.74	6.17	0.16	0.0660
Subtotal JOLY				0.73	8.59	0.27	
LUBIN2	700	m	0	0.71	38.61	1.48	0.0000
LUBIN2	752	f	0	1.87	54.92	51.52	0.0000
Subtotal LUBIN2				1.39	93.54	53.00	
MATOS	646	m	2	0.53	2.67	0.37	0.3857
OSANN2	516	f	1	-0.36	3.03	4.81	0.5346
PEZZOT	516	m	0	1.16	2.27	0.14	0.0817
WUWILL	526	f	3	-0.22	19.12	24.24	0.3292
ZHENG	512	m	0	0.04	9.99	7.49	0.9080
ZHENG	516	f	0	-0.20	5.44	6.62	0.6405
Subtotal ZHENG				-0.05	15.43	14.11	

Table 3I2 - 2

IESLC - Meta-analysis of Ever Smoking, Duration, "Low"
Adenocarcinoma, Any Product (or Cigarettes if Any not available)
Most adjusted

	N	20
	NS	15
	Wt	212.22
Het	Chi	114.32
Het	df	19
Het	P	***
Fixed	RR	2.47
	RRl	2.16
	RRu	2.82
	P	+++
Random	RR	1.87
	RRl	1.29
	RRu	2.71
	P	+++
Asymm	P	(*)

Table 3I2 - 3

IESLC - Meta-analysis of Ever Smoking, Duration, "Low"
Adenocarcinoma, Any Product (or Cigarettes if Any not available)
Most adjusted

	Sex: combined	male	female	Total
N	1	12	7	20
NS	1	12	7	20
Wt	5.73	90.63	115.86	212.22
Het Chi	0.00	15.26	92.79	114.32
Het df	0	11	6	19
Het P	N.S.	N.S.	***	***
Fixed RR	1.40	2.12	2.85	2.47
RRl	0.62	1.73	2.38	2.16
RRu	3.18	2.61	3.42	2.82
P	N.S.	+++	+++	+++
Random RR	1.40	2.18	1.37	1.87
RRl	0.62	1.66	0.60	1.29
RRu	3.18	2.88	3.13	2.71
P	N.S.	+++	N.S.	+++
Between Chi				6.27
Between df				2
Between P				*
Btwn(F) P				N.S.
Btwn(R) P				N.S.

Table 3I2 - 4

IESLC - Meta-analysis of Ever Smoking, Duration, "Low"
Adenocarcinoma, Any Product (or Cigarettes if Any not available)
Least adjusted

REF	NRR	X	SEX	AGEL	AGEH	RACE	YF	LC TYPE	LOC	START	ST	NLC	R	VB	P	H	AD	PRODUCT	exL	exH	DENOM	De
BARBON	717	x	m	0	0	all	-	a	Eu:wst	1979	CC	755	n	bl	y	y	0	all/unsp	1	29	nev any	st
BUFFLE	509		m	0	0	wh	-	a	NAmer	1976	CC	943	n	bl	y	n	0	cig+/-ot	1	33	nev cigs	or
CHOI	566		m	0	0	all	-	a	As:oth	1985	CC	375	n	bl	n	n	0	cig+/-ot	1	29	nev cigs	st
CHOI	578		f	0	0	all	-	a	As:oth	1985	CC	375	n	bl	n	n	0	cig+/-ot	1	29	nev cigs	st
DAMBER	540		m	0	0	all	-	a+al+br	Eu:Sca	1972	CC	579	n	bl	y	n	1	all/unsp	1	30	nev any	or
DORGAN	576		m	0	0	wh	-	a	NAmer	1980	CC	2026	n	bl	y	y	2	cig+/-ot	1	34	nev any	ot
DORGAN	568		f	0	0	all	-	a	NAmer	1980	CC	2026	n	bl	y	y	3	cig+/-ot	1	34	nev any	ot
DOSEME	517		m	0	0	all	-	not q+s	Eu:bal	1979	CC	1210	n	bl	n	n	2	cig+/-ot	11	20	nev cigs	or
GER	501	x	c	0	0	all	-	a	As:oth	1990	CC	141	n	ot	y	n	0	all/unsp	1	30	nev any	st
JEDRYC	520		m	0	0	all	-	a	Eu:est	1980	CC	1630	n	bl	y	n	0	cig+/-ot	20	29	nev any	st
JOLY	660		m	0	0	all	-	a	SCAmer	1978	CC	826	n	bl	n	n	0	cig+/-ot	1	29	nev any	st
JOLY	632		f	0	0	all	-	a	SCAmer	1978	CC	826	n	bl	n	n	0	cig+/-ot	1	29	nev any	st
LUBIN2	700		m	0	0	all	-	a	Eu:mul	1976	CC	7804	n	bl	n	y	0	cig+/-ot	1	29	nev any	st
LUBIN2	752		f	0	0	all	-	a	Eu:mul	1976	CC	7804	n	bl	n	y	0	cig+/-ot	1	29	nev any	st
MATOS	641	x	m	0	0	all	-	a	SCAmer	1994	CC	200	n	bl	n	n	0	cig+/-ot	1	24	nev any	st
OSANN2	513	x	f	0	0	all	-	KII	NAmer	1964	ot	217	n	bl	n	y	0	cig+/-ot	1	20	nev cigs	st
PEZZOT	516		m	0	0	all	-	a	SCAmer	1987	CC	215	n	bl	n	y	0	cig only	1	30	nev cigs	st
WUWILL	511	x	f	0	0	all	-	a	As:Chi	1985	CC	965	n	ot	n	n	0	cig+/-ot	1	29	nev cigs	st
ZHENG	512		m	0	0	all	-	a	As:Chi	1982	CC	540	n	ot	*	y	0	cig+/-ot	1	29	nev cigs	or
ZHENG	516		f	0	0	all	-	a	As:Chi	1982	CC	540	n	ot	*	y	0	cig+/-ot	1	29	nev cigs	or

Cigarette type is all/unspec for all RRs

Table 3I2 - 5

IESLC - Meta-analysis of Ever Smoking, Duration, "Low"
Adenocarcinoma, Any Product (or Cigarettes if Any not available)
Least adjusted

REF	NRR	SEX	AD	Number Exposed Case	Exposed Cont	Non-exposed Case	Non-exposed Cont	RR	95.00%CI
BARBON	717	m	0	15	91	7	188	4.43	(1.74- 11.24)
BUFFLE	509	m	0	-	-	-	-	4.40	(1.70- 11.50)
CHOI	566	m	0	17	221	7	95	1.04	(0.42- 2.60)
CHOI	578	f	0	2	23	48	164	0.30	(0.07- 1.31)
Subtotal CHOI								0.74	(0.34- 1.61)
DAMBER	540	m	1	-	-	16	-	1.80	(0.60- 5.40)
DORGAN	576	m	2	-	-	-	-	2.14	(0.78- 5.84)
DORGAN	568	f	3	-	-	-	-	2.43	(1.65- 3.59)
Subtotal DORGAN								2.39	(1.66- 3.43)
DOSEME	517	m	2	26	-	24	-	3.30	(2.20- 7.50)
GER	501	c	0	14	51	37	149	1.11	(0.55- 2.21)
JEDRYC	520	m	0	15	160	7	289	3.87	(1.55- 9.69)
JOLY	660	m	0	5	109	5	218	2.00	(0.57- 7.06)
JOLY	632	f	0	10	54	25	283	2.10	(0.95- 4.61)
Subtotal JOLY								2.07	(1.06- 4.04)
LUBIN2	700	m	0	131	2964	57	2616	2.03	(1.48- 2.78)
LUBIN2	752	f	0	174	229	138	1180	6.50	(4.99- 8.46)
Subtotal LUBIN2								4.02	(3.28- 4.92)
MATOS	641	m	0	7	84	5	110	1.83	(0.56- 5.98)
OSANN2	513	f	0	10	20	22	43	0.98	(0.39- 2.44)
PEZZOT	516	m	0	11	134	3	116	3.17	(0.86- 11.65)
WUWILL	511	f	0	34	139	172	601	0.85	(0.57- 1.29)
ZHENG	512	m	0	24	75	29	94	1.04	(0.56- 1.93)
ZHENG	516	f	0	9	17	119	184	0.82	(0.35- 1.90)
Subtotal ZHENG								0.95	(0.58- 1.57)
Partial Totals				504	4371	721	6330		

*prospective study

REF	NRR	SEX	AD	Ys	Ws	Qs	Ps
BARBON	717	m	0	1.49	4.43	1.64	0.0017
BUFFLE	509	m	0	1.48	4.20	1.52	0.0024
CHOI	566	m	0	0.04	4.61	3.23	0.9264
CHOI	578	f	0	-1.21	1.75	7.68	0.1081
Subtotal CHOI				-0.30	6.37	10.92	
DAMBER	540	m	1	0.59	3.18	0.27	0.2943
DORGAN	576	m	2	0.76	3.79	0.05	0.1385
DORGAN	568	f	3	0.89	25.43	0.00	0.0000
Subtotal DORGAN				0.87	29.22	0.06	
DOSEME	517	m	2	1.19	10.22	1.01	0.0001
GER	501	c	0	0.10	8.01	4.87	0.7765
JEDRYC	520	m	0	1.35	4.56	1.02	0.0038
JOLY	660	m	0	0.69	2.42	0.08	0.2812
JOLY	632	f	0	0.74	6.17	0.12	0.0660
Subtotal JOLY				0.73	8.59	0.21	
LUBIN2	700	m	0	0.71	38.61	1.15	0.0000
LUBIN2	752	f	0	1.87	54.92	53.97	0.0000
Subtotal LUBIN2				1.39	93.54	55.13	
MATOS	641	m	0	0.61	2.75	0.21	0.3150
OSANN2	513	f	0	-0.02	4.57	3.73	0.9608
PEZZOT	516	m	0	1.16	2.27	0.17	0.0817
WUWILL	511	f	0	-0.16	22.68	24.39	0.4546
ZHENG	512	m	0	0.04	9.99	7.11	0.9080
ZHENG	516	f	0	-0.20	5.44	6.35	0.6405
Subtotal ZHENG				-0.05	15.43	13.45	

Table 3I2 - 5

IESLC - Meta-analysis of Ever Smoking, Duration, "Low"
Adenocarcinoma, Any Product (or Cigarettes if Any not available)
Least adjusted

	N	20
	NS	15
	Wt	220.02
Het	Chi	118.59
Het	df	19
Het	P	***
Fixed	RR	2.41
	RRl	2.11
	RRu	2.75
	P	+++
Random	RR	1.89
	RRl	1.31
	RRu	2.73
	P	+++
Asymm	P	(*)

Table 3I2 - 6

IESLC - Meta-analysis of Ever Smoking, Duration, "Low"
Adenocarcinoma, Any Product (or Cigarettes if Any not available)
Least adjusted

	Sex			
	combined	male	female	Total
N	1	12	7	20
NS	1	12	7	20
Wt	8.01	91.04	120.97	220.02
Het Chi	0.00	16.29	93.98	118.59
Het df	0	11	6	19
Het P	N.S.	N.S.	***	***
Fixed RR	1.11	2.15	2.77	2.41
RRl	0.55	1.75	2.31	2.11
RRu	2.21	2.64	3.30	2.75
P	N.S.	+++	+++	+++
Random RR	1.11	2.23	1.44	1.89
RRl	0.55	1.67	0.64	1.31
RRu	2.21	2.97	3.22	2.73
P	N.S.	+++	N.S.	+++
Between Chi				8.32
Between df				2
Between P				*
Btwn(F) P				N.S.
Btwn(R) P				N.S.

Table 3I2 - 7

IESLC - Meta-analysis of Ever Smoking, Duration, "Low"
Adenocarcinoma, Any Product (or Cigarettes if Any not available)
Excluded studies (and stage at which they were excluded)

	1	2	3	4	5	6	7	8	9	10	11	12	13	14	15	16
1	AKIBA	AMANDU	AMES	BECHER	BENSHL	BEST	BLOT1	BROSS	BROWN3	CARPEN	CEDERL	CHYOU	CPSI	CPSII	DARBY	DEAN2
	DEAN3	DOLL2	ENGELA	GAO2	GARCIA	GILLIS	GRAHAM	GURSEL	HAMMO2	HIRAYA	HOLE	HUMBLE	JAHN	JAIN	KAISE2	KATSOU
	KAUFMA	LAUSSM	LIAW	MCDUFF	MIGRAN	MRFITR	PEZZO2	PISANI	PRESCO	QIAO	SEGI2	SPEIZE	SVENSS	TVERDA	WAKAI	WATSON
	WIGLE	WU	WYNDE3	WYNDE8												
2	ALDERS	BRESLO	CHIAZZ	DORN	GUO	HEGMAN	KOO	KOULUM	LIU4	PERNU	SOBUE	SPITZ	SUZUK2	VUTUC	YUAN	
3	GENG	STASZE	WU2	ZHANG												
4	AGUDO	ARMADA	AUVINE	AXELSS	BOFFET	BOUCHA	BOUCOT	CHEN2	CORREA	DESTEF	DOLL	FAN	GAO	GARSHI	HAMMON	HU
	HU2	JUSSAW	KREUZE	LETOUR	LEVIN	LIU3	LIU5	LUO	MCCONN	NOTAN2	QIAO2	RACHTA	RESTRE	SADOWS	TIZZAN	WANG2
	WYNDE2	WYNDE6	WYNDE7	ZHOU												
5	CHEN	LUBIN	XU													
10	KHUDER															
14	BENHAM	HAENSZ														

Table 3I2 - 8
Potentially overlapping studies

REF	REFGP	PRINC	.	OVERLAP/LINK
LUBIN2	LUBIN2	1		Lubin-combined
OSANN2	KAISER	2		KAISER/OSANN2

Table 3I2 - 9
Most adjusted - insufficient data for meta-analysis

REF	NRR	SEX	AGEL	AGEH	RACE	YF	LC TYPE	LOC	START	ST	NLC	R	VB	P	H	AD	PRODUCT	exL	exH	DENOM	De
CHEN	509	c	0	0	all	-	a	As:oth	1987	CC	323	n	ot	n	y	2	cig+/-ot	1	20	nev cigs	ot
XU	506	m	0	0	all	-	a	As:Chi	1985	CC	729	n	ot	n	n	2	all/unsp	1	29	nev any	or

REF	NRR	RR	SIG	RRDATA comment
CHEN	509	1.23	n	0
XU	506	*		RR for 1-19/day is 1.4(NS), for 20-29/ day is 0.7(p<0.05) and for >=30/day is 5.4(p<0.05)

Table 3I3 -

IESLC - Meta-analysis of Ever Smoking, Duration, "Mid"
Adenocarcinoma, Any Product (or Cigarettes if Any not available)

This analysis is restricted to results for:
1) Ever smokers
2) Results by Duration
3) Categorical results by Duration
4) Adenocarcinoma (or near equivalent)
5) Results complete enough for use in metaanalysis

Within each study, results are then selected (in the following order of preference, within each sex) for:
6) (not applicable)
7) PRODUCT: all/unspec, cigarettes regardless of other products, cigarettes only
8) CIGTYPE: all/unspecified, MC regardless of HR, MC only
9) (not applicable)
10) DENOM: never smoked anything, never smoked cigarettes, never any + low, never cigs + low
11) Followup period (YF, prospective studies): whole study (coded as 0) or longest available
12) LCtype: adeno or nearest available, but not squamous. (q = squamous, s = small,
a = adeno, l = large, KII = Kreyberg II, al = alveolar, br = bronchiolar, u = undifferentiated)
13) Race: all or nearest available, otherwise by race (wh or w = white, bl or b = black, hi = hispanic
ch = chinese, jap = japanese, haw = hawaiian, w+o = white + oriental, sca = scandinavian, as = asian)
14) Duration "mid" in key scheme 1 (key value 35, maximum range 21-49)
15) For overlapping studies: principal rather than subsidiary studies
Finally by Age: whole study (coded as 0) if available, otherwise by widest available age group
and then for single sex results (m, f) in preference to results for both sexes combined (c).

Results adjusted (AD) for the most potential confounders are then chosen in Sections -1 to -3
and results adjusted for the least confounders in Sections -4 to -6. (Those least adjusted results which
actually differ from the most adjusted are marked 'x' in column X in Section -4)

Section -7 shows excluded studies, together with the stage (as above) at which no qualifying
results were found.

Section -8 lists the potentially overlapping studies which have been included (1=principal, 2=subsidiary).

Section -9 lists any results which would have been included in preference except that they had data not complete
enough for use in meta-analysis, with their significance (yes/no), if known, and any further comment as entered
on the database. It also lists as "gap" any categories for which no data were presented by the original authors.

In addition to those mentioned above, the following fields, levels and abbreviations are used:
* or nk = not known, n = no, y = yes, ot = other
nev = never
all/unspec = all or unspecified, cig+/-ot = cigarettes irrespective of other products (cigar, pipe etc)
MC = manufactured cigarettes, HR = hand-rolled cigarettes
exL, exH = range of exposure (low and high) in the smoking group, in terms of Duration
REF: 6-character study reference
NRR: number of the RR on the database within the study
ST : study type (CC = case control, pr or prosp = prospective)
NLC: number of lung cancer cases in whole study
R : risky occupational population (n = no, m = mining, o = other risky)
VB : national cigarette type (V = at least 75% Virginia, bl = at least 75% blended, ot = other)
P : any proxy use
H : full histological confirmation
De : derivation of RR/CI (or = original, st = standard method, ot = other method of estimation)

Table 3I3 - 1

IESLC - Meta-analysis of Ever Smoking, Duration, "Mid"
Adenocarcinoma, Any Product (or Cigarettes if Any not available)
Most adjusted

REF	NRR	SEX	AGEL	AGEH	RACE	YF	LC TYPE	LOC	START	ST	NLC	R	VB	P	H	AD	PRODUCT	exL	exH	DENOM	De
BARBON	725	m	0	0	all	-	a	Eu:wst	1979	CC	755	n	bl	y	y	1	all/unsp	30	39	nev any	or
BUFFLE	510	m	0	0	wh	-	a	NAmer	1976	CC	943	n	bl	y	n	0	cig+/-ot	34	43	nev cigs	or
CHOI	567	m	0	0	all	-	a	As:oth	1985	CC	375	n	bl	n	n	0	cig+/-ot	30	39	nev cigs	st
DAMBER	541	m	0	0	all	-	a+al+br	Eu:Sca	1972	CC	579	n	bl	y	n	1	all/unsp	31	40	nev any	or
JEDRYC	521	m	0	0	all	-	a	Eu:est	1980	CC	1630	n	bl	y	n	0	cig+/-ot	30	39	nev any	st
JOLY	661	m	0	0	all	-	a	SCAmer	1978	CC	826	n	bl	n	n	0	cig+/-ot	30	39	nev any	st
JOLY	633	f	0	0	all	-	a	SCAmer	1978	CC	826	n	bl	n	n	0	cig+/-ot	30	39	nev any	st
LUBIN2	701	m	0	0	all	-	a	Eu:mul	1976	CC	7804	n	bl	n	y	0	cig+/-ot	30	39	nev any	st
LUBIN2	753	f	0	0	all	-	a	Eu:mul	1976	CC	7804	n	bl	n	y	0	cig+/-ot	30	39	nev any	st
MATOS	647	m	0	0	all	-	a	SCAmer	1994	CC	200	n	bl	n	n	2	cig+/-ot	25	39	nev any	or
PEZZOT	517	m	0	0	all	-	a	SCAmer	1987	CC	215	n	bl	n	y	0	cig only	31	40	nev cigs	st
WUWILL	527	f	0	0	all	-	a	As:Chi	1985	CC	965	n	ot	n	n	3	cig+/-ot	30	39	nev cigs	ot

Cigarette type is all/unspec for all RRs

Table 3I3 - 2

IESLC - Meta-analysis of Ever Smoking, Duration, "Mid"
Adenocarcinoma, Any Product (or Cigarettes if Any not available)
Most adjusted

REF	NRR	SEX	AD	Number Exposed Case	Cont	Non-exposed Case	Cont	RR	95.00%CI
BARBON	725	m	1	23	-	7	-	5.10	(2.10- 12.50)
BUFFLE	510	m	0	-	-	-	-	5.70	(2.20- 14.80)
CHOI	567	m	0	13	160	7	65	0.75	(0.29- 1.98)
DAMBER	541	m	1	-	-	16	-	1.20	(0.20- 6.00)
JEDRYC	521	m	0	28	231	7	289	5.00	(2.15- 11.66)
JOLY	661	m	0	12	165	5	218	3.17	(1.10- 9.18)
JOLY	633	f	0	6	24	25	283	2.83	(1.06- 7.57)
Subtotal JOLY								2.98	(1.45- 6.14)
LUBIN2	701	m	0	242	3473	57	2616	3.20	(2.39- 4.29)
LUBIN2	753	f	0	383	186	138	1180	17.61	(13.73- 22.58)
Subtotal LUBIN2								8.62	(7.13- 10.42)
MATOS	647	m	2	39	-	5	-	7.30	(2.70- 19.70)
PEZZOT	517	m	0	22	82	3	116	10.37	(3.01- 35.81)
WUWILL	527	f	3	52	-	172	-	1.99	(1.37- 2.89)
Partial Totals				820	4321	442	4767		

*prospective study

REF	NRR	SEX	AD	Ys	Ws	Qs	Ps
BARBON	725	m	1	1.63	4.83	0.07	0.0003
BUFFLE	510	m	0	1.74	4.23	0.00	0.0003
CHOI	567	m	0	-0.28	4.14	17.06	0.5664
DAMBER	541	m	1	0.18	1.33	3.26	0.8336
JEDRYC	521	m	0	1.61	5.37	0.10	0.0002
JOLY	661	m	0	1.15	3.40	1.20	0.0333
JOLY	633	f	0	1.04	3.97	1.99	0.0382
Subtotal JOLY				1.09	7.37	3.19	
LUBIN2	701	m	0	1.16	44.75	15.34	0.0000
LUBIN2	753	f	0	2.87	62.18	78.06	0.0000
Subtotal LUBIN2				2.15	106.93	93.39	
MATOS	647	m	2	1.99	3.89	0.22	0.0001
PEZZOT	517	m	0	2.34	2.50	0.88	0.0002
WUWILL	527	f	3	0.69	27.58	30.97	0.0003

	N	12
	NS	10
	Wt	168.17
Het	Chi	149.14
Het	df	11
Het	P	***
Fixed	RR	5.74
	RRl	4.94
	RRu	6.68
	P	+++
Random	RR	3.96
	RRl	2.08
	RRu	7.54
	P	+++
Asymm	P	N.S.

Table 3I3 - 3

IESLC - Meta-analysis of Ever Smoking, Duration, "Mid"
Adenocarcinoma, Any Product (or Cigarettes if Any not available)
Most adjusted

	Sex combined	male	female	Total
N		9	3	12
NS		9	3	12
Wt		74.44	93.73	168.17
Het Chi		19.10	95.91	149.14
Het df		8	2	11
Het P		*	***	***
Fixed RR		3.46	8.58	5.74
RRl		2.76	7.01	4.94
RRu		4.35	10.51	6.68
P		+++	+++	+++
Random RR		3.71	4.72	3.96
RRl		2.36	0.87	2.08
RRu		5.81	25.49	7.54
P		+++	(+)	+++
Between Chi				34.14
Between df				1
Between P				***
Btwn(F) P				N.S.
Btwn(R) P				N.S.

Table 3I3 - 4

IESLC - Meta-analysis of Ever Smoking, Duration, "Mid"
Adenocarcinoma, Any Product (or Cigarettes if Any not available)
Least adjusted

REF	NRR	X	SEX	AGEL	AGEH	RACE	YF	LC TYPE	LOC	START	ST	NLC	R	VB	P	H	AD	PRODUCT	exL	exH	DENOM	De
BARBON	718	x	m	0	0	all	-	a	Eu:wst	1979	CC	755	n	bl	y	y	0	all/unsp	30	39	nev any	st
BUFFLE	510		m	0	0	wh	-	a	NAmer	1976	CC	943	n	bl	y	n	0	cig+/-ot	34	43	nev cigs	or
CHOI	567		m	0	0	all	-	a	As:oth	1985	CC	375	n	bl	n	n	0	cig+/-ot	30	39	nev cigs	st
DAMBER	541		m	0	0	all	-	a+al+br	Eu:Sca	1972	CC	579	n	bl	y	n	1	all/unsp	31	40	nev any	or
JEDRYC	521		m	0	0	all	-	a	Eu:est	1980	CC	1630	n	bl	y	n	0	cig+/-ot	30	39	nev any	st
JOLY	661		m	0	0	all	-	a	SCAmer	1978	CC	826	n	bl	n	n	0	cig+/-ot	30	39	nev any	st
JOLY	633		f	0	0	all	-	a	SCAmer	1978	CC	826	n	bl	n	n	0	cig+/-ot	30	39	nev any	st
LUBIN2	701		m	0	0	all	-	a	Eu:mul	1976	CC	7804	n	bl	n	y	0	cig+/-ot	30	39	nev any	st
LUBIN2	753		f	0	0	all	-	a	Eu:mul	1976	CC	7804	n	bl	n	y	0	cig+/-ot	30	39	nev any	st
MATOS	642	x	m	0	0	all	-	a	SCAmer	1994	CC	200	n	bl	n	n	0	cig+/-ot	25	39	nev any	st
PEZZOT	517		m	0	0	all	-	a	SCAmer	1987	CC	215	n	bl	n	y	0	cig only	31	40	nev cigs	st
WUWILL	512	x	f	0	0	all	-	a	As:Chi	1985	CC	965	n	ot	n	n	0	cig+/-ot	30	39	nev cigs	st

Cigarette type is all/unspec for all RRs

Table 3I3 - 5

IESLC - Meta-analysis of Ever Smoking, Duration, "Mid"
Adenocarcinoma, Any Product (or Cigarettes if Any not available)
Least adjusted

REF	NRR	SEX	AD	Number Exposed Case	Cont	Non-exposed Case	Cont	RR	95.00%CI
BARBON	718	m	0	23	102	7	188	6.06	(2.51- 14.60)
BUFFLE	510	m	0	-	-	-	-	5.70	(2.20- 14.80)
CHOI	567	m	0	13	160	7	65	0.75	(0.29- 1.98)
DAMBER	541	m	1	-	-	16	-	1.20	(0.20- 6.00)
JEDRYC	521	m	0	28	231	7	289	5.00	(2.15- 11.66)
JOLY	661	m	0	12	165	5	218	3.17	(1.10- 9.18)
JOLY	633	f	0	6	24	25	283	2.83	(1.06- 7.57)
Subtotal JOLY								2.98	(1.45- 6.14)
LUBIN2	701	m	0	242	3473	57	2616	3.20	(2.39- 4.29)
LUBIN2	753	f	0	383	186	138	1180	17.61	(13.73- 22.58)
Subtotal LUBIN2								8.62	(7.13- 10.42)
MATOS	642	m	0	39	110	5	110	7.80	(2.96- 20.53)
PEZZOT	517	m	0	22	82	3	116	10.37	(3.01- 35.81)
WUWILL	512	f	0	52	98	172	601	1.85	(1.27- 2.70)
Partial Totals				820	4631	442	5666		

*prospective study

REF	NRR	SEX	AD	Ys	Ws	Qs	Ps
BARBON	718	m	0	1.80	4.96	0.01	0.0001
BUFFLE	510	m	0	1.74	4.23	0.00	0.0003
CHOI	567	m	0	-0.28	4.14	17.04	0.5664
DAMBER	541	m	1	0.18	1.33	3.25	0.8336
JEDRYC	521	m	0	1.61	5.37	0.10	0.0002
JOLY	661	m	0	1.15	3.40	1.19	0.0333
JOLY	633	f	0	1.04	3.97	1.98	0.0382
Subtotal JOLY				1.09	7.37	3.17	
LUBIN2	701	m	0	1.16	44.75	15.26	0.0000
LUBIN2	753	f	0	2.87	62.18	78.26	0.0000
Subtotal LUBIN2				2.15	106.93	93.52	
MATOS	642	m	0	2.05	4.10	0.39	0.0000
PEZZOT	517	m	0	2.34	2.50	0.88	0.0002
WUWILL	512	f	0	0.62	27.09	34.54	0.0013

	N	12
	NS	10
	Wt	168.03
Het	Chi	152.90
Het	df	11
Het	P	***
Fixed	RR	5.73
	RRl	4.93
	RRu	6.67
	P	+++
Random	RR	4.01
	RRl	2.09
	RRu	7.70
	P	+++
Asymm	P	N.S.

Table 3I3 - 6

IESLC - Meta-analysis of Ever Smoking, Duration, "Mid"
Adenocarcinoma, Any Product (or Cigarettes if Any not available)
Least adjusted

	Sex combined	male	female	Total
N		9	3	12
NS		9	3	12
Wt		74.78	93.25	168.03
Het Chi		20.44	100.59	152.90
Het df		8	2	11
Het P		**	***	***
Fixed RR		3.53	8.47	5.73
RRl		2.81	6.91	4.93
RRu		4.42	10.38	6.67
P		+++	+++	+++
Random RR		3.82	4.60	4.01
RRl		2.40	0.81	2.09
RRu		6.07	26.10	7.70
P		+++	(+)	+++
Between Chi				31.87
Between df				1
Between P				***
Btwn(F) P				N.S.
Btwn(R) P				N.S.

Table 3I3 - 7

IESLC - Meta-analysis of Ever Smoking, Duration, "Mid"
Adenocarcinoma, Any Product (or Cigarettes if Any not available)
Excluded studies (and stage at which they were excluded)

	1	2	3	4	5	6	7	8	9	10	11	12	13	14	15	16
1	AKIBA	AMANDU	AMES	BECHER	BENSHL	BEST	BLOT1	BROSS	BROWN3	CARPEN	CEDERL	CHYOU	CPSI	CPSII	DARBY	DEAN2
	DEAN3	DOLL2	ENGELA	GAO2	GARCIA	GILLIS	GRAHAM	GURSEL	HAMMO2	HIRAYA	HOLE	HUMBLE	JAHN	JAIN	KAISE2	KATSOU
	KAUFMA	LAUSSM	LIAW	MCDUFF	MIGRAN	MRFITR	PEZZO2	PISANI	PRESCO	QIAO	SEGI2	SPEIZE	SVENSS	TVERDA	WAKAI	WATSON
	WIGLE	WU	WYNDE3	WYNDE8												
2	ALDERS	BRESLO	CHIAZZ	DORN	GUO	HEGMAN	KOO	KOULUM	LIU4	PERNU	SOBUE	SPITZ	SUZUK2	VUTUC	YUAN	
3	GENG	STASZE	WU2	ZHANG												
4	AGUDO	ARMADA	AUVINE	AXELSS	BOFFET	BOUCHA	BOUCOT	CHEN2	CORREA	DESTEF	DOLL	FAN	GAO	GARSHI	HAMMON	HU
	HU2	JUSSAW	KREUZE	LETOUR	LEVIN	LIU3	LIU5	LUO	MCCONN	NOTAN2	QIAO2	RACHTA	RESTRE	SADOWS	TIZZAN	WANG2
	WYNDE2	WYNDE6	WYNDE7	ZHOU												
5	CHEN	LUBIN	XU													
10	KHUDER															
14	BENHAM	DORGAN	DOSEME	GER	HAENSZ	OSANN2	ZHENG									

Table 3I3 - 8

Potentially overlapping studies

REF	REFGP	PRINC	.	OVERLAP/LINK
LUBIN2	LUBIN2	1		Lubin-combined

Table 3I3 - 9

Most adjusted - insufficient data for meta-analysis

REF	NRR	SEX	AGEL	AGEH	RACE	YF	LC TYPE	LOC	START	ST	NLC	R	VB	P	H	AD	PRODUCT	exL	exH	DENOM	De
CHEN	511	c	0	0	all	-	a	As:oth	1987	CC	323	n	ot	n	y	2	cig+/-ot	31	40	nev cigs	ot
XU	507	m	0	0	all	-	a	As:Chi	1985	CC	729	n	ot	n	n	2	all/unsp	30	39	nev any	or

REF	NRR	RR	SIG	RRDATA comment
CHEN	511	1.60	n	0
XU	507	*		RR for 1-19/day is 2.2(p<0.05), for 20-29/day is 1.5(NS) and for >=30/day is 3.2(p<0.05)

Table 3I4 -

IESLC - Meta-analysis of Ever Smoking, Duration, "High"
Adenocarcinoma, Any Product (or Cigarettes if Any not available)

This analysis is restricted to results for:
1) Ever smokers
2) Results by Duration
3) Categorical results by Duration
4) Adenocarcinoma (or near equivalent)
5) Results complete enough for use in metaanalysis

Within each study, results are then selected (in the following order of preference, within each sex) for:
6) PRODUCT: all/unspec, cigarettes regardless of other products, cigarettes only
7) CIGTYPE: all/unspecified, MC regardless of HR, MC only
8) (not applicable)
9) DENOM: never smoked anything, never smoked cigarettes, never any + low, never cigs + low
10) Followup period (YF, prospective studies): whole study (coded as 0) or longest available
11) LCtype: adeno or nearest available, but not squamous. (q = squamous, s = small,
a = adeno, l = large, KII = Kreyberg II, al = alveolar, br = bronchiolar, u = undifferentiated)
12) Race: all or nearest available, otherwise by race (wh or w = white, bl or b = black, hi = hispanic
ch = chinese, jap = japanese, haw = hawaiian, w+o = white + oriental, sca = scandinavian, as = asian)
13) Duration "high" in key scheme 1 (key value 50, maximum range 36+)
14) For overlapping studies: principal rather than subsidiary studies
Finally by Age: whole study (coded as 0) if available, otherwise by widest available age group
and then for single sex results (m, f) in preference to results for both sexes combined (c).

Results adjusted (AD) for the most potential confounders are then chosen in Sections -1 to -3
and results adjusted for the least confounders in Sections -4 to -6. (Those least adjusted results which
actually differ from the most adjusted are marked 'x' in column X in Section -4)

Section -7 shows excluded studies, together with the stage (as above) at which no qualifying
results were found.

Section -8 lists the potentially overlapping studies which have been included (1=principal, 2=subsidiary).

Section -9 lists any results which would have been included in preference except that they had data not complete
enough for use in meta-analysis, with their significance (yes/no), if known, and any further comment as entered
on the database. It also lists as "gap" any categories for which no data were presented by the original authors.

In addition to those mentioned above, the following fields, levels and abbreviations are used:
* or nk = not known, n = no, y = yes, ot = other
nev = never
all/unspec = all or unspecified, cig+/-ot = cigarettes irrespective of other products (cigar, pipe etc)
MC = manufactured cigarettes, HR = hand-rolled cigarettes
exL, exH = range of exposure (low and high) in the smoking group, in terms of Duration
REF: 6-character study reference
NRR: number of the RR on the database within the study
ST : study type (CC = case control, pr or prosp = prospective)
NLC: number of lung cancer cases in whole study
R : risky occupational population (n = no, m = mining, o = other risky)
VB : national cigarette type (V = at least 75% Virginia, bl = at least 75% blended, ot = other)
P : any proxy use
H : full histological confirmation
De : derivation of RR/CI (or = original, st = standard method, ot = other method of estimation)

Table 3I4 - 1

IESLC - Meta-analysis of Ever Smoking, Duration, "High"
Adenocarcinoma, Any Product (or Cigarettes if Any not available)
Most adjusted

REF	NRR	SEX	AGEL	AGEH	RACE	YF	LC TYPE	LOC	START	ST	NLC	R	VB	P	H	AD	PRODUCT	exL	exH	DENOM	De
BARBON	727	m	0	0	all	-	a	Eu:wst	1979	CC	755	n	bl	y	y	1	all/unsp	50	999	nev any	or
BUFFLE	512	m	0	0	wh	-	a	NAmer	1976	CC	943	n	bl	y	n	0	cig+/-ot	50	999	nev cigs	or
CHOI	569	m	0	0	all	-	a	As:oth	1985	CC	375	n	bl	n	n	0	cig+/-ot	50	999	nev cigs	st
DAMBER	542	m	0	0	all	-	a+al+br	Eu:Sca	1972	CC	579	n	bl	y	n	1	all/unsp	41	50	nev any	or
JEDRYC	597	m	0	0	all	-	a	Eu:est	1980	CC	1630	n	bl	y	n	3	cig+/-ot	40	999	nev any	or
JOLY	663	m	0	0	all	-	a	SCAmer	1978	CC	826	n	bl	n	n	0	cig+/-ot	50	999	nev any	st
JOLY	635	f	0	0	all	-	a	SCAmer	1978	CC	826	n	bl	n	n	0	cig+/-ot	50	999	nev any	st
LUBIN2	703	m	0	0	all	-	a	Eu:mul	1976	CC	7804	n	bl	n	y	0	cig+/-ot	50	999	nev any	st
LUBIN2	755	f	0	0	all	-	a	Eu:mul	1976	CC	7804	n	bl	n	y	0	cig+/-ot	50	999	nev any	st
MATOS	648	m	0	0	all	-	a	SCAmer	1994	CC	200	n	bl	n	n	2	cig+/-ot	40	70	nev any	or
PEZZOT	518	m	0	0	all	-	a	SCAmer	1987	CC	215	n	bl	n	y	0	cig only	41	999	nev cigs	st
WUWILL	528	f	0	0	all	-	a	As:Chi	1985	CC	965	n	ot	n	n	3	cig+/-ot	40	999	nev cigs	ot

Cigarette type is all/unspec for all RRs

Table 3I4 - 2

IESLC - Meta-analysis of Ever Smoking, Duration, "High"
Adenocarcinoma, Any Product (or Cigarettes if Any not available)
Most adjusted

REF	NRR	SEX	AD	Number Exposed Case	Number Exposed Cont	Non-exposed Case	Non-exposed Cont	RR	95.00%CI
BARBON	727	m	1	67	-	7	-	8.30	(3.70- 18.70)
BUFFLE	512	m	0	-	-	-	-	3.50	(1.30- 9.70)
CHOI	569	m	0	4	20	7	95	2.71	(0.73- 10.16)
DAMBER	542	m	1	-	-	16	-	3.40	(1.30- 9.10)
JEDRYC	597	m	3	47	-	7	-	4.41	(1.86- 10.45)
JOLY	663	m	0	33	253	5	218	5.69	(2.18- 14.82)
JOLY	635	f	0	7	20	25	283	3.96	(1.53- 10.27)
Subtotal JOLY								4.74	(2.41- 9.32)
LUBIN2	703	m	0	90	1460	57	2616	2.83	(2.02- 3.97)
LUBIN2	755	f	0	181	34	138	1180	45.52	(30.31- 68.36)
Subtotal LUBIN2								8.80	(6.79- 11.41)
MATOS	648	m	2	33	-	5	-	10.70	(3.80- 29.90)
PEZZOT	518	m	0	27	101	3	116	10.34	(3.04- 35.09)
WUWILL	528	f	3	52	-	172	-	2.09	(1.40- 3.10)
Partial Totals				541	1888	442	4508		

*prospective study

REF	NRR	SEX	AD	Ys	Ws	Qs	Ps
BARBON	727	m	1	2.12	5.85	0.91	0.0000
BUFFLE	512	m	0	1.25	3.80	0.84	0.0145
CHOI	569	m	0	1.00	2.21	1.16	0.1381
DAMBER	542	m	1	1.22	4.06	1.01	0.0137
JEDRYC	597	m	3	1.48	5.16	0.29	0.0008
JOLY	663	m	0	1.74	4.19	0.00	0.0004
JOLY	635	f	0	1.38	4.23	0.50	0.0046
Subtotal JOLY				1.56	8.42	0.51	
LUBIN2	703	m	0	1.04	33.64	15.66	0.0000
LUBIN2	755	f	0	3.82	23.24	102.09	0.0000
Subtotal LUBIN2				2.17	56.88	117.75	
MATOS	648	m	2	2.37	3.61	1.52	0.0000
PEZZOT	518	m	0	2.34	2.57	0.97	0.0002
WUWILL	528	f	3	0.74	24.32	23.59	0.0003

	N	12
	NS	10
	Wt	116.88
Het	Chi	148.54
Het	df	11
Het	P	***
Fixed	RR	5.60
	RRl	4.67
	RRu	6.71
	P	+++
Random	RR	5.56
	RRl	2.69
	RRu	11.45
	P	+++
Asymm	P	N.S.

Table 3I4 - 3

IESLC - Meta-analysis of Ever Smoking, Duration, "High"
Adenocarcinoma, Any Product (or Cigarettes if Any not available)
Most adjusted

	combined	Sex<br>male	female	Total
N		9	3	12
NS		9	3	12
Wt		65.09	51.79	116.88
Het Chi		14.01	115.71	148.54
Het df		8	2	11
Het P		(*)	***	***
Fixed RR		3.91	8.78	5.60
RRl		3.07	6.68	4.67
RRu		4.99	11.52	6.71
P		+++	+++	+++
Random RR		4.71	7.29	5.56
RRl		3.22	0.77	2.69
RRu		6.89	69.06	11.45
P		+++	(+)	+++
Between Chi				18.82
Between df				1
Between P				***
Btwn(F) P				N.S.
Btwn(R) P				N.S.

Table 3I4 - 4

IESLC - Meta-analysis of Ever Smoking, Duration, "High"
Adenocarcinoma, Any Product (or Cigarettes if Any not available)
Least adjusted

REF	NRR	X	SEX	AGEL	AGEH	RACE	YF	LC TYPE	LOC	START	ST	NLC	R	VB	P	H	AD	PRODUCT	exL	exH	DENOM	De
BARBON	720	x	m	0	0	all	-	a	Eu:wst	1979	CC	755	n	bl	y	y	0	all/unsp	50	999	nev any	st
BUFFLE	512		m	0	0	wh	-	a	NAmer	1976	CC	943	n	bl	y	n	0	cig+/-ot	50	999	nev cigs	or
CHOI	569		m	0	0	all	-	a	As:oth	1985	CC	375	n	bl	n	n	0	cig+/-ot	50	999	nev cigs	st
DAMBER	542		m	0	0	all	-	a+al+br	Eu:Sca	1972	CC	579	n	bl	y	n	1	all/unsp	41	50	nev any	or
JEDRYC	523	x	m	0	0	all	-	a	Eu:est	1980	CC	1630	n	bl	y	n	0	cig+/-ot	50	999	nev any	st
JOLY	663		m	0	0	all	-	a	SCAmer	1978	CC	826	n	bl	n	n	0	cig+/-ot	50	999	nev any	st
JOLY	635		f	0	0	all	-	a	SCAmer	1978	CC	826	n	bl	n	n	0	cig+/-ot	50	999	nev any	st
LUBIN2	703		m	0	0	all	-	a	Eu:mul	1976	CC	7804	n	bl	n	y	0	cig+/-ot	50	999	nev any	st
LUBIN2	755		f	0	0	all	-	a	Eu:mul	1976	CC	7804	n	bl	n	y	0	cig+/-ot	50	999	nev any	st
MATOS	643	x	m	0	0	all	-	a	SCAmer	1994	CC	200	n	bl	n	n	0	cig+/-ot	40	70	nev any	st
PEZZOT	518		m	0	0	all	-	a	SCAmer	1987	CC	215	n	bl	n	y	0	cig only	41	999	nev cigs	st
WUWILL	513	x	f	0	0	all	-	a	As:Chi	1985	CC	965	n	ot	n	n	0	cig+/-ot	40	999	nev cigs	st

Cigarette type is all/unspec for all RRs

Table 3I4 - 5

IESLC - Meta-analysis of Ever Smoking, Duration, "High"
Adenocarcinoma, Any Product (or Cigarettes if Any not available)
Least adjusted

REF	NRR	SEX	AD	Number Exposed Case	Cont	Non-exposed Case	Cont	RR	95.00%CI
BARBON	720	m	0	67	235	7	188	7.66	(3.43- 17.07)
BUFFLE	512	m	0	-	-	-	-	3.50	(1.30- 9.70)
CHOI	569	m	0	4	20	7	95	2.71	(0.73- 10.16)
DAMBER	542	m	1	-	-	16	-	3.40	(1.30- 9.10)
JEDRYC	523	m	0	17	214	7	289	3.28	(1.34- 8.05)
JOLY	663	m	0	33	253	5	218	5.69	(2.18- 14.82)
JOLY	635	f	0	7	20	25	283	3.96	(1.53- 10.27)
Subtotal JOLY								4.74	(2.41- 9.32)
LUBIN2	703	m	0	90	1460	57	2616	2.83	(2.02- 3.97)
LUBIN2	755	f	0	181	34	138	1180	45.52	(30.31- 68.36)
Subtotal LUBIN2								8.80	(6.79- 11.41)
MATOS	643	m	0	33	89	5	110	8.16	(3.06- 21.76)
PEZZOT	518	m	0	27	101	3	116	10.34	(3.04- 35.09)
WUWILL	513	f	0	52	114	172	601	1.59	(1.10- 2.31)
Partial Totals				511	2540	442	5696		

*prospective study

REF	NRR	SEX	AD	Ys	Ws	Qs	Ps
BARBON	720	m	0	2.04	5.98	1.10	0.0000
BUFFLE	512	m	0	1.25	3.80	0.47	0.0145
CHOI	569	m	0	1.00	2.21	0.81	0.1381
DAMBER	542	m	1	1.22	4.06	0.59	0.0137
JEDRYC	523	m	0	1.19	4.77	0.83	0.0095
JOLY	663	m	0	1.74	4.19	0.07	0.0004
JOLY	635	f	0	1.38	4.23	0.22	0.0046
Subtotal JOLY				1.56	8.42	0.30	
LUBIN2	703	m	0	1.04	33.64	10.78	0.0000
LUBIN2	755	f	0	3.82	23.24	113.72	0.0000
Subtotal LUBIN2				2.17	56.88	124.50	
MATOS	643	m	0	2.10	3.99	0.97	0.0000
PEZZOT	518	m	0	2.34	2.57	1.37	0.0002
WUWILL	513	f	0	0.47	28.18	36.62	0.0133

	N	12
	NS	10
	Wt	120.86
Het	Chi	167.58
Het	df	11
Het	P	***
Fixed	RR	4.98
	RRl	4.17
	RRu	5.96
	P	+++
Random	RR	5.14
	RRl	2.41
	RRu	10.94
	P	+++
Asymm	P	N.S.

Table 3I4 - 6

IESLC - Meta-analysis of Ever Smoking, Duration, "High"
Adenocarcinoma, Any Product (or Cigarettes if Any not available)
Least adjusted

	Sex combined	male	female	Total
N		9	3	12
NS		9	3	12
Wt		65.20	55.65	120.86
Het Chi		11.87	144.54	167.58
Het df		8	2	11
Het P		N.S.	***	***
Fixed RR		3.76	6.92	4.98
RRl		2.95	5.32	4.17
RRu		4.80	9.00	5.96
P		+++	+++	+++
Random RR		4.32	6.64	5.14
RRl		3.06	0.58	2.41
RRu		6.11	76.18	10.94
P		+++	N.S.	+++
Between Chi				11.17
Between df				1
Between P				***
Btwn(F) P				N.S.
Btwn(R) P				N.S.

Table 3I4 - 7

IESLC - Meta-analysis of Ever Smoking, Duration, "High"
Adenocarcinoma, Any Product (or Cigarettes if Any not available)
Excluded studies (and stage at which they were excluded)

	1	2	3	4	5	6	7	8	9	10	11	12	13	14	15	16
1	AKIBA	AMANDU	AMES	BECHER	BENSHL	BEST	BLOT1	BROSS	BROWN3	CARPEN	CEDERL	CHYOU	CPSI	CPSII	DARBY	DEAN2
	DEAN3	DOLL2	ENGELA	GAO2	GARCIA	GILLIS	GRAHAM	GURSEL	HAMMO2	HIRAYA	HOLE	HUMBLE	JAHN	JAIN	KAISE2	KATSOU
	KAUFMA	LAUSSM	LIAW	MCDUFF	MIGRAN	MRFITR	PEZZO2	PISANI	PRESCO	QIAO	SEGI2	SPEIZE	SVENSS	TVERDA	WAKAI	WATSON
	WIGLE	WU	WYNDE3	WYNDE8												
2	ALDERS	BRESLO	CHIAZZ	DORN	GUO	HEGMAN	KOO	KOULUM	LIU4	PERNU	SOBUE	SPITZ	SUZUK2	VUTUC	YUAN	
3	GENG	STASZE	WU2	ZHANG												
4	AGUDO	ARMADA	AUVINE	AXELSS	BOFFET	BOUCHA	BOUCOT	CHEN2	CORREA	DESTEF	DOLL	FAN	GAO	GARSHI	HAMMON	HU
	HU2	JUSSAW	KREUZE	LETOUR	LEVIN	LIU3	LIU5	LUO	MCCONN	NOTAN2	QIAO2	RACHTA	RESTRE	SADOWS	TIZZAN	WANG2
	WYNDE2	WYNDE6	WYNDE7	ZHOU												
5	CHEN	LUBIN	XU													
10	KHUDER															
14	DORGAN	DOSEME	GER	HAENSZ	OSANN2	ZHENG										
15	BENHAM															

Table 3I4 - 8
Potentially overlapping studies

REF	REFGP	PRINC	.	OVERLAP/LINK
LUBIN2	LUBIN2	1		Lubin-combined

Table 3I4 - 9
Most adjusted - insufficient data for meta-analysis

REF	NRR	SEX	AGEL	AGEH	RACE	YF	LC TYPE	LOC	START	ST	NLC	R	VB	P	H	AD	PRODUCT	exL	exH	DENOM	De
CHEN	512	c	0	0	all	-	a	As:oth	1987	CC	323	n	ot	n	y	2	cig+/-ot	41	999	nev cigs	ot
XU	508	m	0	0	all	-	a	As:Chi	1985	CC	729	n	ot	n	n	2	all/unsp	40	999	nev any	or

REF	NRR	RR	SIG	RRDATA comment
CHEN	512	3.79	y	p<0.001
XU	508	*		RR for 1-19/day is 2.6(p<0.05), for 20-29/day is 3.6(p<0.05) and for >=30/ day is 11.8(p<0.05)

Table 3I5 -

IESLC - Meta-analysis of Ever Smoking, Duration, "Highest vs lowest"
Adenocarcinoma, Any Product (or Cigarettes if Any not available)

This analysis is restricted to results for:
1) Ever smokers
2) Results by Duration
3) Categorical results by Duration
4) Denominator (unexposed) = "low"
5) Adenocarcinoma (or near equivalent)
6) Results complete enough for use in metaanalysis

Within each study, results are then selected (in the following order of preference, within each sex) for:
7) (not applicable)
8) PRODUCT: all/unspec, cigarettes regardless of other products, cigarettes only
9) CIGTYPE: all/unspecified, MC regardless of HR, MC only
10) Results with least adjustment for other aspects of smoking (ADOS)
11) The highest vs lowest category
12) Followup period (YF, prospective studies): whole study (coded as 0) or longest available
13) LCtype: adeno or nearest available, but not squamous. (q = squamous, s = small,
a = adeno, l = large, KII = Kreyberg II, al = alveolar, br = bronchiolar, u = undifferentiated)
14) Race: all or nearest available, otherwise by race (wh or w = white, bl or b = black, hi = hispanic
ch = chinese, jap = japanese, haw = hawaiian, w+o = white + oriental, sca = scandinavian, as = asian)
15) For overlapping studies: principal rather than subsidiary studies
Finally by Age: whole study (coded as 0) if available, otherwise by widest available age group
and then for single sex results (m, f) in preference to results for both sexes combined (c).

Results adjusted (AD) for the most potential confounders are then chosen in Sections -1 to -3
and results adjusted for the least confounders in Sections -4 to -6. (Those least adjusted results which
actually differ from the most adjusted are marked 'x' in column X in Section -4)

Section -7 shows excluded studies, together with the stage (as above) at which no qualifying
results were found.

Section -8 lists the potentially overlapping studies which have been included (1=principal, 2=subsidiary).

Section -9 lists any results which would have been included in preference except that they had data not complete
enough for use in meta-analysis, with their significance (yes/no), if known, and any further comment as entered
on the database. It also lists as "gap" any categories for which no data were presented by the original authors.

In addition to those mentioned above, the following fields, levels and abbreviations are used:
* or nk = not known, n = no, y = yes, ot = other
all/unspec = all or unspecified, cig+/-ot = cigarettes irrespective of other products (cigar, pipe etc)
MC = manufactured cigarettes, HR = hand-rolled cigarettes
exL, exH = range of exposure (low and high) in the "highest" group, in terms of Duration
unexL, unexH = range of exposure (low and high) in the "lowest" group, in terms of Duration
REF: 6-character study reference
NRR: number of the RR on the database within the study
ST : study type (CC = case control, pr or prosp = prospective)
NLC: number of lung cancer cases in whole study
R : risky occupational population (n = no, m = mining, o = other risky)
VB : national cigarette type (V = at least 75% Virginia, bl = at least 75% blended, ot = other)
P : any proxy use
H : full histological confirmation
De : derivation of RR/CI (or = original, st = standard method, ot = other method of estimation)

Table 3I5 - 1

IESLC - Meta-analysis of Ever Smoking, Duration, "Highest vs lowest"
Adenocarcinoma, Any Product (or Cigarettes if Any not available)
Most adjusted

REF	NRR	SEX	AGEL	AGEH	RACE	YF	LC TYPE	LOC	START	ST	NLC	R	VB	P	H	AD	ADOS	PRODUCT	exL	exH	unexL	unexH	De
BARBON	730	m	0	0	all	-	a	Eu:wst	1979	CC	755	n	bl	y	y	1	0	all/unsp	50	999	1	29	ot
CHOI	572	m	0	0	all	-	a	As:oth	1985	CC	375	n	bl	n	n	0	0	cig+/-ot	50	999	1	29	st
CHOI	580	f	0	0	all	-	a	As:oth	1985	CC	375	n	bl	n	n	0	0	cig+/-ot	30	999	1	29	st
DAMBER	546	m	0	0	all	-	a+al+br	Eu:Sca	1972	CC	579	n	bl	y	n	1	0	all/unsp	51	999	1	30	ot
DORGAN	529	m	0	0	wh	-	a	NAmer	1980	CC	2026	n	bl	y	y	2	0	cig+/-ot	35	999	1	34	ot
DORGAN	525	f	0	0	all	-	a	NAmer	1980	CC	2026	n	bl	y	y	3	0	cig+/-ot	35	999	1	34	ot
DOSEME	520	m	0	0	all	-	not q+s	Eu:bal	1979	CC	1210	n	bl	n	n	2	0	cig+/-ot	21	999	1	10	ot
GER	509	c	0	0	all	-	a	As:oth	1990	CC	141	n	ot	y	n	5	0	all/unsp	31	999	1	30	ot
HAENSZ	536	f	0	0	all	-	a	NAmer	1955	CC	158	n	bl	n	y	1	0	cig+/-ot	15	999	1	14	ot
JEDRYC	527	m	0	0	all	-	a	Eu:est	1980	CC	1630	n	bl	y	n	0	0	cig+/-ot	50	999	1	19	st
JOLY	666	m	0	0	all	-	a	SCAmer	1978	CC	826	n	bl	n	n	0	0	cig+/-ot	50	999	1	29	st
JOLY	638	f	0	0	all	-	a	SCAmer	1978	CC	826	n	bl	n	n	0	0	cig+/-ot	50	999	1	29	st
KHUDER	532	m	0	0	all	-	a	NAmer	1985	CC	482	n	bl	n	y	5	3#	cig+/-ot	30	999	1	29	or
LUBIN2	706	m	0	0	all	-	a	Eu:mul	1976	CC	7804	n	bl	n	y	0	0	cig+/-ot	50	999	1	29	st
LUBIN2	758	f	0	0	all	-	a	Eu:mul	1976	CC	7804	n	bl	n	y	0	0	cig+/-ot	50	999	1	29	st
MATOS	650	m	0	0	all	-	a	SCAmer	1994	CC	200	n	bl	n	n	2	0	cig+/-ot	40	70	1	24	ot
OSANN2	518	f	0	0	all	-	KII	NAmer	1964	ot	217	n	bl	n	y	1	0	cig+/-ot	21	999	1	20	ot
PEZZOT	522	m	0	0	all	-	a	SCAmer	1987	CC	215	n	bl	n	y	2	0	cig only	41	999	1	30	ot
WUWILL	515	f	0	0	all	-	a	As:Chi	1985	CC	965	n	ot	n	n	0	0	cig+/-ot	40	999	1	29	st
ZHENG	515	m	0	0	all	-	a	As:Chi	1982	CC	540	n	ot	*	y	1	0	cig+/-ot	30	999	1	29	ot
ZHENG	519	f	0	0	all	-	a	As:Chi	1982	CC	540	n	ot	*	y	1	0	cig+/-ot	30	999	1	29	ot

Comments on values in listings

KHUDER ADOS Age at starting smoking, No of cigarettes per day, Quitted smoking

Cigarette type is all/unspec for all RRs

Table 3I5 - 2

IESLC - Meta-analysis of Ever Smoking, Duration, "Highest vs lowest"
Adenocarcinoma, Any Product (or Cigarettes if Any not available)
Most adjusted

REF	NRR	SEX	AD	Number Exposed Case	Cont	Non-exposed Case	Cont	RR	95.00%CI
BARBON	730	m	1	67	-	15	-	2.24	(1.15- 4.36)
CHOI	572	m	0	4	20	17	221	2.60	(0.80- 8.47)
CHOI	580	f	0	2	3	2	23	7.67	(0.77- 76.45)
Subtotal CHOI								3.26	(1.14- 9.32)
DAMBER	546	m	1	-	-	-	-	1.39	(0.45- 4.30)
DORGAN	529	m	2	-	-	-	-	3.15	(1.90- 5.23)
DORGAN	525	f	3	-	-	-	-	2.11	(1.50- 2.98)
Subtotal DORGAN								2.39	(1.80- 3.18)
DOSEME	520	m	2	69	-	5	-	5.13	(1.81- 14.51)
GER	509	c	5	21	-	14	-	0.61	(0.24- 1.52)
HAENSZ	536	f	1	16	-	2	-	2.69	(0.57- 12.56)
JEDRYC	527	m	0	17	214	2	68	2.70	(0.61- 11.99)
JOLY	666	m	0	33	253	5	109	2.84	(1.08- 7.48)
JOLY	638	f	0	7	20	10	54	1.89	(0.63- 5.64)
Subtotal JOLY								2.38	(1.15- 4.91)
KHUDER	532	m	5	-	-	-	-	2.70	(1.40- 5.30)
LUBIN2	706	m	0	90	1460	131	2964	1.39	(1.06- 1.84)
LUBIN2	758	f	0	181	34	174	229	7.01	(4.62- 10.62)
Subtotal LUBIN2								2.28	(1.81- 2.87)
MATOS	650	m	2	33	-	7	-	6.29	(2.59- 15.31)
OSANN2	518	f	1	51	-	10	-	5.86	(1.88- 18.26)
PEZZOT	522	m	2	27	-	11	-	4.60	(1.95- 10.82)
WUWILL	515	f	0	52	114	34	139	1.86	(1.13- 3.07)
ZHENG	515	m	1	99	-	24	-	2.42	(1.43- 4.10)
ZHENG	519	f	1	24	-	9	-	2.08	(0.78- 5.53)
Subtotal ZHENG								2.34	(1.47- 3.72)
Partial Totals				793	2118	472	3807		

*prospective study

REF	NRR	SEX	AD	Ys	Ws	Qs	Ps
BARBON	730	m	1	0.81	8.65	0.04	0.0177
CHOI	572	m	0	0.96	2.75	0.02	0.1129
CHOI	580	f	0	2.04	0.73	0.99	0.0826
Subtotal CHOI				1.18	3.48	1.00	
DAMBER	546	m	1	0.33	3.02	0.89	0.5674
DORGAN	529	m	2	1.15	14.99	1.14	0.0000
DORGAN	525	f	3	0.75	32.61	0.51	0.0000
Subtotal DORGAN				0.87	47.60	1.65	
DOSEME	520	m	2	1.64	3.55	2.07	0.0021
GER	509	c	5	-0.49	4.51	8.42	0.2938
HAENSZ	536	f	1	0.99	1.61	0.02	0.2098
JEDRYC	527	m	0	0.99	1.73	0.03	0.1913
JOLY	666	m	0	1.05	4.11	0.12	0.0342
JOLY	638	f	0	0.64	3.21	0.18	0.2540
Subtotal JOLY				0.87	7.32	0.30	
KHUDER	532	m	5	0.99	8.67	0.13	0.0034
LUBIN2	706	m	0	0.33	50.59	14.71	0.0180
LUBIN2	758	f	0	1.95	22.20	25.65	0.0000
Subtotal LUBIN2				0.82	72.79	40.35	
MATOS	650	m	2	1.84	4.87	4.55	0.0000
OSANN2	518	f	1	1.77	2.97	2.39	0.0023
PEZZOT	522	m	2	1.53	5.23	2.24	0.0005
WUWILL	515	f	0	0.62	15.48	0.96	0.0142
ZHENG	515	m	1	0.88	13.85	0.00	0.0010
ZHENG	519	f	1	0.73	4.01	0.08	0.1427
Subtotal ZHENG				0.85	17.86	0.08	

Table 3I5 - 2

IESLC - Meta-analysis of Ever Smoking, Duration, "Highest vs lowest"
Adenocarcinoma, Any Product (or Cigarettes if Any not available)
Most adjusted

	N	21
	NS	16
	Wt	209.32
Het	Chi	65.11
Het	df	20
Het	P	***
Fixed	RR	2.39
	RRl	2.09
	RRu	2.74
	P	+++
Random	RR	2.65
	RRl	2.00
	RRu	3.51
	P	+++
Asymm	P	N.S.

Table 3I5 - 3

IESLC - Meta-analysis of Ever Smoking, Duration, "Highest vs lowest"
Adenocarcinoma, Any Product (or Cigarettes if Any not available)
Most adjusted

	Sex			
	combined	male	female	Total
N	1	12	8	21
NS	1	12	8	21
Wt	4.51	122.00	82.81	209.32
Het Chi	0.00	24.72	26.72	65.11
Het df	0	11	7	20
Het P	N.S.	*	***	***
Fixed RR	0.61	2.16	2.98	2.39
RRl	0.24	1.81	2.41	2.09
RRu	1.54	2.59	3.70	2.74
P	N.S.	+++	+++	+++
Random RR	0.61	2.68	3.03	2.65
RRl	0.24	1.96	1.82	2.00
RRu	1.54	3.66	5.07	3.51
P	N.S.	+++	+++	+++
Between Chi				13.68
Between df				2
Between P				**
Btwn(F) P				N.S.
Btwn(R) P				**

	Lung cancer type						
	a	a+l	a+al+br	KII	not q+u	not q+s	Total
N	18		1	1		1	21
NS	13		1	1		1	16
Wt	199.78		3.02	2.97		3.55	209.32
Het Chi	59.70		0.00	0.00		0.00	65.11
Het df	17		0	0		0	20
Het P	***		N.S.	N.S.		N.S.	***
Fixed RR	2.35		1.39	5.86		5.13	2.39
RRl	2.04		0.45	1.88		1.81	2.09
RRu	2.70		4.30	18.26		14.52	2.74
P	+++		N.S.	++		++	+++
Random RR	2.56		1.39	5.86		5.13	2.65
RRl	1.90		0.45	1.88		1.81	2.00
RRu	3.45		4.30	18.26		14.52	3.51
P	+++		N.S.	++		++	+++
Between Chi							5.41
Between df							3
Between P							N.S.
Btwn(F) P							N.S.
Btwn(R) P							N.S.

	Location								
	NAmer	UK	Scand	othEur	China	Japan	othAs	other	Total
N	5		1	5	3		3	4	21
NS	4		1	4	2		2	3	16
Wt	60.85		3.02	86.71	33.33		7.99	17.42	209.32
Het Chi	3.93		0.00	42.47	0.50		6.19	3.34	65.11
Het df	4		0	4	2		2	3	20
Het P	N.S.		N.S.	***	N.S.		*	N.S.	***
Fixed RR	2.55		1.39	2.36	2.11		1.27	3.80	2.39
RRl	1.98		0.45	1.91	1.50		0.63	2.38	2.09
RRu	3.28		4.30	2.92	2.96		2.53	6.08	2.74
P	+++		N.S.	+++	+++		N.S.	+++	+++
Random RR	2.55		1.39	3.10	2.11		1.78	3.78	2.65
RRl	1.98		0.45	1.33	1.50		0.45	2.30	2.00
RRu	3.28		4.30	7.23	2.96		7.00	6.21	3.51
P	+++		N.S.	++	+++		N.S.	+++	+++
Between Chi									8.69
Between df									5
Between P									N.S.
Btwn(F) P									N.S.
Btwn(R) P									N.S.

Table 3I5 - 3

IESLC - Meta-analysis of Ever Smoking, Duration, "Highest vs lowest"
Adenocarcinoma, Any Product (or Cigarettes if Any not available)
Most adjusted

Detailed Country in "other Europe"

	multi	Germany	othWest	East	Balkans	Total
N	2		1	1	1	5
NS	1		1	1	1	4
Wt	72.79		8.65	1.73	3.55	86.71
Het Chi	40.19		0.00	0.00	0.00	42.47
Het df	1		0	0	0	4
Het P	***		N.S.	N.S.	N.S.	***
Fixed RR	2.28		2.24	2.70	5.13	2.36
RRl	1.81		1.15	0.61	1.81	1.91
RRu	2.87		4.36	11.99	14.52	2.92
P	+++		+	N.S.	++	+++
Random RR	3.10		2.24	2.70	5.13	3.10
RRl	0.64		1.15	0.61	1.81	1.33
RRu	15.08		4.36	11.99	14.52	7.23
P	N.S.		+	N.S.	++	++
Between Chi						2.28
Between df						3
Between P						N.S.
Btwn(F) P						N.S.
Btwn(R) P						N.S.

Detailed Country in "other Asia"

	India	HongKong	other	Total
N			3	3
NS			2	2
Wt			7.99	7.99
Het Chi			6.19	6.19
Het df			2	2
Het P			*	*
Fixed RR			1.27	1.27
RRl			0.63	0.63
RRu			2.53	2.53
P			N.S.	N.S.
Random RR			1.78	1.78
RRl			0.45	0.45
RRu			7.00	7.00
P			N.S.	N.S.
Between Chi				
Between df				
Between P				N.S.
Btwn(F) P				N.S.
Btwn(R) P				N.S.

Detailed other continent

	SCAmer	Total
N	4	4
NS	3	3
Wt	17.42	17.42
Het Chi	3.34	3.34
Het df	3	3
Het P	N.S.	N.S.
Fixed RR	3.80	3.80
RRl	2.38	2.38
RRu	6.08	6.08
P	+++	+++
Random RR	3.78	3.78
RRl	2.30	2.30
RRu	6.21	6.21
P	+++	+++
Between Chi		
Between df		
Between P		N.S.
Btwn(F) P		N.S.
Btwn(R) P		N.S.

Table 3I5 - 3

IESLC - Meta-analysis of Ever Smoking, Duration, "Highest vs lowest"
Adenocarcinoma, Any Product (or Cigarettes if Any not available)
Most adjusted

	Start year of study					
	<1960	1960-69	1970-79	1980-89	1990+	Total
N	1	1	7	10	2	21
NS	1	1	5	7	2	16
Wt	1.61	2.97	95.32	100.04	9.38	209.32
Het Chi	0.00	0.00	43.55	6.06	12.74	65.11
Het df	0	0	6	9	1	20
Het P	N.S.	N.S.	***	N.S.	***	***
Fixed RR	2.69	5.86	2.32	2.43	2.05	2.39
RRl	0.57	1.88	1.90	2.00	1.08	2.09
RRu	12.63	18.26	2.83	2.95	3.88	2.74
P	N.S.	++	+++	+++	+	+++
Random RR	2.69	5.86	2.65	2.43	1.97	2.65
RRl	0.57	1.88	1.38	2.00	0.20	2.00
RRu	12.63	18.26	5.10	2.95	19.34	3.51
P	N.S.	++	++	+++	N.S.	+++
Between Chi						2.75
Between df						4
Between P						N.S.
Btwn(F) P						N.S.
Btwn(R) P						N.S.

	Study type (1)		
	CC	other	Total
N	20	1	21
NS	15	1	16
Wt	206.34	2.97	209.32
Het Chi	62.69	0.00	65.11
Het df	19	0	20
Het P	***	N.S.	***
Fixed RR	2.36	5.86	2.39
RRl	2.06	1.88	2.09
RRu	2.71	18.26	2.74
P	+++	++	+++
Random RR	2.58	5.86	2.65
RRl	1.94	1.88	2.00
RRu	3.43	18.26	3.51
P	+++	++	+++
Between Chi			2.42
Between df			1
Between P			N.S.
Btwn(F) P			N.S.
Btwn(R) P			N.S.

	Study type (2)			
	CC	prosp	other	Total
N	20		1	21
NS	15		1	16
Wt	206.34		2.97	209.32
Het Chi	62.69		0.00	65.11
Het df	19		0	20
Het P	***		N.S.	***
Fixed RR	2.36		5.86	2.39
RRl	2.06		1.88	2.09
RRu	2.71		18.26	2.74
P	+++		++	+++
Random RR	2.58		5.86	2.65
RRl	1.94		1.88	2.00
RRu	3.43		18.26	3.51
P	+++		++	+++
Between Chi				2.42
Between df				1
Between P				N.S.
Btwn(F) P				N.S.
Btwn(R) P				N.S.

Table 3I5 - 3

IESLC - Meta-analysis of Ever Smoking, Duration, "Highest vs lowest"
Adenocarcinoma, Any Product (or Cigarettes if Any not available)
Most adjusted

	Study size (number of LC cases)				
	100-249	250-499	500-999	1000+	Total
N	5	3	7	6	21
NS	5	2	5	4	16
Wt	19.19	12.15	52.32	125.66	209.32
Het Chi	16.41	0.76	1.46	44.09	65.11
Het df	4	2	6	5	20
Het P	**	N.S.	N.S.	***	***
Fixed RR	3.07	2.85	2.11	2.38	2.39
RRl	1.97	1.62	1.61	2.00	2.09
RRu	4.81	5.00	2.77	2.84	2.74
P	+++	+++	+++	+++	+++
Random RR	3.08	2.85	2.11	3.02	2.65
RRl	1.21	1.62	1.61	1.67	2.00
RRu	7.82	5.00	2.77	5.46	3.51
P	+	+++	+++	+++	+++
Between Chi					2.39
Between df					3
Between P					N.S.
Btwn(F) P					N.S.
Btwn(R) P					N.S.

	Risky occupational population			
	no	mining	othRisky	Total
N	21			21
NS	16			16
Wt	209.32			209.32
Het Chi	65.11			65.11
Het df	20			20
Het P	***			***
Fixed RR	2.39			2.39
RRl	2.09			2.09
RRu	2.74			2.74
P	+++			+++
Random RR	2.65			2.65
RRl	2.00			2.00
RRu	3.51			3.51
P	+++			+++
Between Chi				
Between df				
Between P				N.S.
Btwn(F) P				N.S.
Btwn(R) P				N.S.

	National cigarette tobacco type			
	Virginia	blended	other	Total
N		17	4	21
NS		13	3	16
Wt		171.47	37.84	209.32
Het Chi		55.02	6.59	65.11
Het df		16	3	20
Het P		***	(*)	***
Fixed RR		2.54	1.82	2.39
RRl		2.19	1.32	2.09
RRu		2.95	2.50	2.74
P		+++	+++	+++
Random RR		3.04	1.68	2.65
RRl		2.19	1.01	2.00
RRu		4.22	2.79	3.51
P		+++	+	+++
Between Chi				3.49
Between df				1
Between P				(*)
Btwn(F) P				N.S.
Btwn(R) P				(*)

Table 3I5 - 3

IESLC - Meta-analysis of Ever Smoking, Duration, "Highest vs lowest"
Adenocarcinoma, Any Product (or Cigarettes if Any not available)
Most adjusted

	Any proxy use		
	No/nk	Yes	Total
N	15	6	21
NS	11	5	16
Wt	143.81	65.50	209.32
Het Chi	53.64	10.01	65.11
Het df	14	5	20
Het P	***	(*)	***
Fixed RR	2.53	2.11	2.39
RRl	2.15	1.66	2.09
RRu	2.98	2.69	2.74
P	+++	+++	+++
Random RR	3.11	1.95	2.65
RRl	2.14	1.31	2.00
RRu	4.51	2.93	3.51
P	+++	++	+++
Between Chi			1.46
Between df			1
Between P			N.S.
Btwn(F) P			N.S.
Btwn(R) P			(*)

	Full histological confirmation		
	No	Yes	Total
N	10	11	21
NS	8	8	16
Wt	43.94	165.37	209.32
Het Chi	18.01	46.84	65.11
Het df	9	10	20
Het P	*	***	***
Fixed RR	2.23	2.44	2.39
RRl	1.66	2.09	2.09
RRu	3.00	2.84	2.74
P	+++	+++	+++
Random RR	2.37	2.85	2.65
RRl	1.50	1.97	2.00
RRu	3.75	4.13	3.51
P	+++	+++	+++
Between Chi			0.26
Between df			1
Between P			N.S.
Btwn(F) P			N.S.
Btwn(R) P			N.S.

	Number of adjustment variables (1)			
	0	1	2+/+nk	Total
N	8	6	7	21
NS	5	5	6	16
Wt	100.79	34.10	74.42	209.32
Het Chi	42.28	3.41	18.60	65.11
Het df	7	5	6	20
Het P	***	N.S.	**	***
Fixed RR	2.25	2.41	2.58	2.39
RRl	1.85	1.72	2.06	2.09
RRu	2.74	3.37	3.24	2.74
P	+++	+++	+++	+++
Random RR	2.69	2.41	2.81	2.65
RRl	1.47	1.72	1.79	2.00
RRu	4.95	3.37	4.40	3.51
P	++	+++	+++	+++
Between Chi				0.81
Between df				2
Between P				N.S.
Btwn(F) P				N.S.
Btwn(R) P				N.S.

Table 3I5 - 3

IESLC - Meta-analysis of Ever Smoking, Duration, "Highest vs lowest"
Adenocarcinoma, Any Product (or Cigarettes if Any not available)
Most adjusted

	Number of adjustment variables (2) 0	1	2	3-5	6+/+nk	Total
N	8	6	4	3		21
NS	5	5	4	3		17
Wt	100.79	34.10	28.63	45.79		209.32
Het Chi	42.28	3.41	2.17	7.21		65.11
Het df	7	5	3	2		20
Het P	***	N.S.	N.S.	*		***
Fixed RR	2.25	2.41	4.03	1.96		2.39
RRl	1.85	1.72	2.80	1.46		2.09
RRu	2.74	3.37	5.82	2.61		2.74
P	+++	+++	+++	+++		+++
Random RR	2.69	2.41	4.03	1.67		2.65
RRl	1.47	1.72	2.80	0.85		2.00
RRu	4.95	3.37	5.82	3.30		3.51
P	++	+++	+++	N.S.		+++
Between Chi						10.03
Between df						3
Between P						*
Btwn(F) P						N.S.
Btwn(R) P						(*)

	Product all/unsp	cig+/-ot	cig only	Total
N	3	17	1	21
NS	3	12	1	16
Wt	16.18	187.91	5.23	209.32
Het Chi	5.02	53.40	0.00	65.11
Het df	2	16	0	20
Het P	(*)	***	N.S.	***
Fixed RR	1.43	2.46	4.60	2.39
RRl	0.88	2.13	1.95	2.09
RRu	2.32	2.83	10.84	2.74
P	N.S.	+++	+++	+++
Random RR	1.29	2.89	4.60	2.65
RRl	0.57	2.13	1.95	2.00
RRu	2.92	3.93	10.84	3.51
P	N.S.	+++	+++	+++
Between Chi				6.69
Between df				2
Between P				*
Btwn(F) P				N.S.
Btwn(R) P				(*)

	Derivation of RR/CI Orig	StdCalc	Other	Total
N	1	8	12	21
NS	1	5	10	16
Wt	8.67	100.79	99.85	209.32
Het Chi	0.00	42.28	22.09	65.11
Het df	0	7	11	20
Het P	N.S.	***	*	***
Fixed RR	2.70	2.25	2.51	2.39
RRl	1.39	1.85	2.07	2.09
RRu	5.25	2.74	3.06	2.74
P	++	+++	+++	+++
Random RR	2.70	2.69	2.63	2.65
RRl	1.39	1.47	1.93	2.00
RRu	5.25	4.95	3.60	3.51
P	++	++	+++	+++
Between Chi				0.74
Between df				2
Between P				N.S.
Btwn(F) P				N.S.
Btwn(R) P				N.S.

Table 3I5 - 4

IESLC - Meta-analysis of Ever Smoking, Duration, "Highest vs lowest"
Adenocarcinoma, Any Product (or Cigarettes if Any not available)
Least adjusted

REF	NRR	X	SEX	AGEL	AGEH	RACE	YF	LC TYPE	LOC	START	ST	NLC	R	VB	P	H	AD	ADOS	PRODUCT	exL	exH	unexL	unexH	De
BARBON	723	x	m	0	0	all	-	a	Eu:wst	1979	CC	755	n	bl	y	y	0	0	all/unsp	50	999	1	29	st
CHOI	572		m	0	0	all	-	a	As:oth	1985	CC	375	n	bl	n	n	0	0	cig+/-ot	50	999	1	29	st
CHOI	580		f	0	0	all	-	a	As:oth	1985	CC	375	n	bl	n	n	0	0	cig+/-ot	30	999	1	29	st
DAMBER	546		m	0	0	all	-	a+al+br	Eu:Sca	1972	CC	579	n	bl	y	n	1	0	all/unsp	51	999	1	30	ot
DORGAN	529		m	0	0	wh	-	a	NAmer	1980	CC	2026	n	bl	y	y	2	0	cig+/-ot	35	999	1	34	ot
DORGAN	525		f	0	0	all	-	a	NAmer	1980	CC	2026	n	bl	y	y	3	0	cig+/-ot	35	999	1	34	ot
DOSEME	520		m	0	0	all	-	not q+s	Eu:bal	1979	CC	1210	n	bl	n	n	2	0	cig+/-ot	21	999	1	10	ot
GER	503	x	c	0	0	all	-	a	As:oth	1990	CC	141	n	ot	y	n	0	0	all/unsp	31	999	1	30	st
HAENSZ	529	x	f	0	0	all	-	a	NAmer	1955	CC	158	n	bl	n	y	0	0	cig+/-ot	15	999	1	14	st
JEDRYC	527		m	0	0	all	-	a	Eu:est	1980	CC	1630	n	bl	y	n	0	0	cig+/-ot	50	999	1	19	st
JOLY	666		m	0	0	all	-	a	SCAmer	1978	CC	826	n	bl	n	n	0	0	cig+/-ot	50	999	1	29	st
JOLY	638		f	0	0	all	-	a	SCAmer	1978	CC	826	n	bl	n	n	0	0	cig+/-ot	50	999	1	29	st
KHUDER	532		m	0	0	all	-	a	NAmer	1985	CC	482	n	bl	n	y	5	3#	cig+/-ot	30	999	1	29	or
LUBIN2	706		m	0	0	all	-	a	Eu:mul	1976	CC	7804	n	bl	n	y	0	0	cig+/-ot	50	999	1	29	st
LUBIN2	758		f	0	0	all	-	a	Eu:mul	1976	CC	7804	n	bl	n	y	0	0	cig+/-ot	50	999	1	29	st
MATOS	645	x	m	0	0	all	-	a	SCAmer	1994	CC	200	n	bl	n	n	0	0	cig+/-ot	40	70	1	24	st
OSANN2	515	x	f	0	0	all	-	KII	NAmer	1964	ot	217	n	bl	n	y	0	0	cig+/-ot	21	999	1	20	st
PEZZOT	520	x	m	0	0	all	-	a	SCAmer	1987	CC	215	n	bl	n	y	0	0	cig only	41	999	1	30	st
WUWILL	515		f	0	0	all	-	a	As:Chi	1985	CC	965	n	ot	n	n	0	0	cig+/-ot	40	999	1	29	st
ZHENG	514	x	m	0	0	all	-	a	As:Chi	1982	CC	540	n	ot	*	y	0	0	cig+/-ot	30	999	1	29	st
ZHENG	518	x	f	0	0	all	-	a	As:Chi	1982	CC	540	n	ot	*	y	0	0	cig+/-ot	30	999	1	29	st

Comments on values in listings

KHUDER ADOS Age at starting smoking, No of cigarettes per day, Quitted smoking

Cigarette type is all/unspec for all RRs

Table 3I5 - 5

IESLC - Meta-analysis of Ever Smoking, Duration, "Highest vs lowest"
Adenocarcinoma, Any Product (or Cigarettes if Any not available)
Least adjusted

REF	NRR	SEX	AD	Number Exposed Case	Number Exposed Cont	Non-exposed Case	Non-exposed Cont	RR	95.00%CI
BARBON	723	m	0	67	235	15	91	1.73	(0.94- 3.18)
CHOI	572	m	0	4	20	17	221	2.60	(0.80- 8.47)
CHOI	580	f	0	2	3	2	23	7.67	(0.77- 76.45)
Subtotal CHOI								3.26	(1.14- 9.32)
DAMBER	546	m	1	-	-	-	-	1.39	(0.45- 4.30)
DORGAN	529	m	2	-	-	-	-	3.15	(1.90- 5.23)
DORGAN	525	f	3	-	-	-	-	2.11	(1.50- 2.98)
Subtotal DORGAN								2.39	(1.80- 3.18)
DOSEME	520	m	2	69	-	5	-	5.13	(1.81- 14.51)
GER	503	c	0	21	98	14	51	0.78	(0.37- 1.66)
HAENSZ	529	f	0	16	77	2	26	2.70	(0.58- 12.55)
JEDRYC	527	m	0	17	214	2	68	2.70	(0.61- 11.99)
JOLY	666	m	0	33	253	5	109	2.84	(1.08- 7.48)
JOLY	638	f	0	7	20	10	54	1.89	(0.63- 5.64)
Subtotal JOLY								2.38	(1.15- 4.91)
KHUDER	532	m	5	-	-	-	-	2.70	(1.40- 5.30)
LUBIN2	706	m	0	90	1460	131	2964	1.39	(1.06- 1.84)
LUBIN2	758	f	0	181	34	174	229	7.01	(4.62- 10.62)
Subtotal LUBIN2								2.28	(1.81- 2.87)
MATOS	645	m	0	33	89	7	84	4.45	(1.87- 10.60)
OSANN2	515	f	0	51	20	10	20	5.10	(2.04- 12.78)
PEZZOT	520	m	0	27	27	11	39	3.55	(1.51- 8.34)
WUWILL	515	f	0	52	114	34	139	1.86	(1.13- 3.07)
ZHENG	514	m	0	99	143	24	75	2.16	(1.28- 3.66)
ZHENG	518	f	0	24	27	9	17	1.68	(0.63- 4.46)
Subtotal ZHENG								2.04	(1.29- 3.25)
Partial Totals				793	2834	472	4210		

*prospective study

REF	NRR	SEX	AD	Ys	Ws	Qs	Ps
BARBON	723	m	0	0.55	10.33	0.83	0.0783
CHOI	572	m	0	0.96	2.75	0.04	0.1129
CHOI	580	f	0	2.04	0.73	1.06	0.0826
Subtotal CHOI				1.18	3.48	1.10	
DAMBER	546	m	1	0.33	3.02	0.76	0.5674
DORGAN	529	m	2	1.15	14.99	1.49	0.0000
DORGAN	525	f	3	0.75	32.61	0.24	0.0000
Subtotal DORGAN				0.87	47.60	1.73	
DOSEME	520	m	2	1.64	3.55	2.29	0.0021
GER	503	c	0	-0.25	6.72	7.83	0.5209
HAENSZ	529	f	0	0.99	1.63	0.04	0.2047
JEDRYC	527	m	0	0.99	1.73	0.05	0.1913
JOLY	666	m	0	1.05	4.11	0.19	0.0342
JOLY	638	f	0	0.64	3.21	0.12	0.2540
Subtotal JOLY				0.87	7.32	0.31	
KHUDER	532	m	5	0.99	8.67	0.23	0.0034
LUBIN2	706	m	0	0.33	50.59	12.59	0.0180
LUBIN2	758	f	0	1.95	22.20	27.60	0.0000
Subtotal LUBIN2				0.82	72.79	40.20	
MATOS	645	m	0	1.49	5.09	2.23	0.0008
OSANN2	515	f	0	1.63	4.55	2.90	0.0005
PEZZOT	520	m	0	1.27	5.25	0.99	0.0037
WUWILL	515	f	0	0.62	15.48	0.67	0.0142
ZHENG	514	m	0	0.77	13.87	0.05	0.0041
ZHENG	518	f	0	0.52	4.02	0.40	0.2987
Subtotal ZHENG				0.71	17.89	0.44	

Table 3I5 - 5

IESLC - Meta-analysis of Ever Smoking, Duration, "Highest vs lowest"
Adenocarcinoma, Any Product (or Cigarettes if Any not available)
Least adjusted

	N	21
	NS	16
	Wt	215.08
Het	Chi	62.58
Het	df	20
Het	P	***
Fixed	RR	2.30
	RRl	2.01
	RRu	2.63
	P	+++
Random	RR	2.50
	RRl	1.91
	RRu	3.28
	P	+++
Asymm	P	N.S.

Table 3I5 - 6

IESLC - Meta-analysis of Ever Smoking, Duration, "Highest vs lowest"
Adenocarcinoma, Any Product (or Cigarettes if Any not available)
Least adjusted

	Sex			
	combined	male	female	Total
N	1	12	8	21
NS	1	12	8	21
Wt	6.72	123.94	84.43	215.08
Het Chi	0.00	20.06	27.48	62.58
Het df	0	11	7	20
Het P	N.S.	*	***	***
Fixed RR	0.78	2.05	2.97	2.30
RRl	0.37	1.72	2.40	2.01
RRu	1.66	2.44	3.67	2.63
P	N.S.	+++	+++	+++
Random RR	0.78	2.41	2.95	2.50
RRl	0.37	1.83	1.77	1.91
RRu	1.66	3.18	4.91	3.28
P	N.S.	+++	+++	+++
Between Chi				15.04
Between df				2
Between P				***
Btwn(F) P				(*)
Btwn(R) P				*

Table 3I5 - 7

IESLC - Meta-analysis of Ever Smoking, Duration, "Highest vs lowest"
Adenocarcinoma, Any Product (or Cigarettes if Any not available)
Excluded studies (and stage at which they were excluded)

	1	2	3	4	5	6	7	8	9	10	11	12	13	14	15	16
1	AKIBA	AMANDU	AMES	BECHER	BENSHL	BEST	BLOT1	BROSS	BROWN3	CARPEN	CEDERL	CHYOU	CPSI	CPSII	DARBY	DEAN2
	DEAN3	DOLL2	ENGELA	GAO2	GARCIA	GILLIS	GRAHAM	GURSEL	HAMMO2	HIRAYA	HOLE	HUMBLE	JAHN	JAIN	KAISE2	KATSOU
	KAUFMA	LAUSSM	LIAW	MCDUFF	MIGRAN	MRFITR	PEZZO2	PISANI	PRESCO	QIAO	SEGI2	SPEIZE	SVENSS	TVERDA	WAKAI	WATSON
	WIGLE	WU	WYNDE3	WYNDE8												
2	ALDERS	BRESLO	CHIAZZ	DORN	GUO	HEGMAN	KOO	KOULUM	LIU4	PERNU	SOBUE	SPITZ	SUZUK2	VUTUC	YUAN	
3	GENG	STASZE	WU2	ZHANG												
4	GARSHI															
5	AGUDO	ARMADA	AUVINE	AXELSS	BOFFET	BOUCHA	BOUCOT	CHEN2	CORREA	DESTEF	DOLL	FAN	GAO	HAMMON	HU	HU2
	JUSSAW	KREUZE	LETOUR	LEVIN	LIU3	LIU5	LUO	MCCONN	NOTAN2	QIAO2	RACHTA	RESTRE	SADOWS	TIZZAN	WANG2	WYNDE2
	WYNDE6	WYNDE7	ZHOU													
6	BUFFLE	CHEN	LUBIN	XU												
15	BENHAM															

Table 3I5 - 8
Potentially overlapping studies

REF	REFGP	PRINC	.	OVERLAP/LINK
LUBIN2	LUBIN2	1		Lubin-combined
OSANN2	KAISER	2		KAISER/OSANN2

Table 3I5 - 9
Most adjusted - insufficient data for meta-analysis

REF	NRR	SEX	AGEL	AGEH	RACE	YF	LC TYPE	LOC	START	ST	NLC	R	VB	P	H	AD	ADOS	PRODUCT	exL	exH	unexL	unexH	De
BUFFLE	554	m	0	0	wh	-	a	NAmer	1976	CC	943	n	bl	y	n	0	0	cig+/-ot	50	999	1	33	st
CHEN	521	c	0	0	all	-	a	As:oth	1987	CC	323	n	ot	n	y	2	0	cig+/-ot	41	999	1	20	st
LUBIN	620	m	0	0	all	-	KII	As:Chi	1984	CC	427	m	ot	y	n	5	1#	cig+/-ot	50	999	1	29	or
XU	510	m	0	0	all	-	a	As:Chi	1985	CC	729	n	ot	n	n	2	0	all/unsp	40	999	1	29	st

Comments on values in listings

LUBIN ADOS Duration of pipe use

REF	NRR	RR	SIG	RRDATA comment
BUFFLE	554	0.80		0
CHEN	521	3.08		0
LUBIN	620	2.51		0
XU	510	*		RR for 1-19/day is 1.9, for 20-29/day is 5.1 and for >=30/day is 2.2

Table 3I6 -

IESLC - Meta-analysis of Current Smoking by Duration, Overview
Adenocarcinoma, Any Product (or Cigarettes if Any not available)

This analysis is restricted to results for:
1) Current smokers
2) Results by Duration
3) Categorical results by Duration
Results by Duration are grouped under 2 schemes (S1, S2). Each scheme has a set of "key values". An interval is allocated to the category whose key value it includes, and intervals which include none or more than one of the key values are excluded. (Open-ended intervals are coded as 999)

S1	key value	maximum range
1	20	1-34
2	35	21-49
3	50	36+

S2	key value	maximum range
1	5	1-19
2	20	6-29
3	30	21-39
4	40	31-49
5	50	41-998
6	999	51+

4) Adenocarcinoma (or near equivalent)
5) Results complete enough for use in metaanalysis

Within each study, results are then selected (in the following order of preference, within each sex) for:
6) (not applicable)
7) PRODUCT: all/unspec, cigarettes regardless of other products, cigarettes only
8) CIGTYPE: all/unspecified, MC regardless of HR, MC only
9) (not applicable)
10) DENOM: never smoked anything, never smoked cigarettes, never any + low, never cigs + low
11) Followup period (YF, prospective studies): whole study (coded as 0) or longest available
12) LCtype: adeno or nearest available, but not squamous. (q = squamous, s = small,
a = adeno, l = large, KII = Kreyberg II, al = alveolar, br = bronchiolar, u = undifferentiated)
13) Race: all or nearest available, otherwise by race (wh or w = white, bl or b = black, hi = hispanic
ch = chinese, jap = japanese, haw = hawaiian, w+o = white + oriental, sca = scandinavian, as = asian)
14) For overlapping studies: principal rather than subsidiary studies
Finally by Age: whole study (coded as 0) if available, otherwise by widest available age group
and then for single sex results (m, f) in preference to results for both sexes combined (c).

Results adjusted (AD) for the most potential confounders are then chosen in Sections -1 to -3
and results adjusted for the least confounders in Sections -4 to -6. (Those least adjusted results which
actually differ from the most adjusted are marked 'x' in column X in Section -4)

Section -7 shows excluded studies, together with the stage (as above) at which no qualifying
results were found.

Section -8 lists the potentially overlapping studies which have been included (1=principal, 2=subsidiary).

Section -9 lists any results which would have been included in preference except that they had data not complete
enough for use in meta-analysis, with their significance (yes/no), if known, and any further comment as entered
on the database. It also lists as "gap" any categories for which no data were presented by the original authors.

In addition to those mentioned above, the following fields, levels and abbreviations are used:
* or nk = not known, n = no, y = yes, ot = other
nev = never
all/unspec = all or unspecified, cig+/-ot = cigarettes irrespective of other products (cigar, pipe etc)
MC = manufactured cigarettes, HR = hand-rolled cigarettes
exL, exH = range of exposure (low and high) in the smoking group, in terms of Duration
REF: 6-character study reference
NRR: number of the RR on the database within the study
ST : study type (CC = case control, pr or prosp = prospective)
NLC: number of lung cancer cases in whole study
R : risky occupational population (n = no, m = mining, o = other risky)
VB : national cigarette type (V = at least 75% Virginia, bl = at least 75% blended, ot = other)
P : any proxy use
H : full histological confirmation
De : derivation of RR/CI (or = original, st = standard method, ot = other method of estimation)

Table 3I6 - 1

IESLC - Meta-analysis of Current Smoking by Duration, Overview
Adenocarcinoma, Any Product (or Cigarettes if Any not available)
Most adjusted

REF	NRR	SEX	AGEL	AGEH	RACE	YF	LC TYPE	LOC	START	ST	NLC	R	VB	P	H	AD	PRODUCT	exL	exH	S1	S2	DENOM	De
KATSOU	520	f	0	0	all	-	a	Eu:bal	1987	CC	101	n	bl	n	n	1	all/unsp	1	29	1	0	nev any	or
KATSOU	521	f	0	0	all	-	a	Eu:bal	1987	CC	101	n	bl	n	n	1	all/unsp	30	999	0	0	nev any	or
SOBUE	513	m	0	0	all	-	a	As:Jap	1986	CC	1376	n	bl	n	y	0	cig+/-ot	1	29	1	0	nev cigs	st
SOBUE	514	m	0	0	all	-	a	As:Jap	1986	CC	1376	n	bl	n	y	0	cig+/-ot	30	39	2	3	nev cigs	st
SOBUE	515	m	0	0	all	-	a	As:Jap	1986	CC	1376	n	bl	n	y	0	cig+/-ot	40	49	0	4	nev cigs	st
SOBUE	516	m	0	0	all	-	a	As:Jap	1986	CC	1376	n	bl	n	y	0	cig+/-ot	50	999	3	0	nev cigs	st

Cigarette type is all/unspec for all RRs

In this overview table, subtotals and Qs values may be invalid and should be ignored

Table 3I6 - 2

IESLC - Meta-analysis of Current Smoking by Duration, Overview
Adenocarcinoma, Any Product (or Cigarettes if Any not available)
Most adjusted

REF	NRR	SEX	AD	Number Exposed Case	Number Exposed Cont	Non-exposed Case	Non-exposed Cont	RR	95.00%CI
KATSOU	520	f	1	6	-	30	-	0.84	(0.27- 2.58)
KATSOU	521	f	1	9	-	30	-	3.61	(1.17- 11.17)
Subtotal KATSOU								1.74	(0.78- 3.87)
SOBUE	513	m	0	33	119	27	128	1.31	(0.75- 2.32)
SOBUE	514	m	0	62	200	27	128	1.47	(0.89- 2.43)
SOBUE	515	m	0	96	174	27	128	2.62	(1.61- 4.24)
SOBUE	516	m	0	43	73	27	128	2.79	(1.59- 4.89)
Subtotal SOBUE								1.96	(1.51- 2.54)
Partial Totals				249	566	168	512		

*prospective study

REF	NRR	SEX	AD	Ys	Ws	Qs	Ps
KATSOU	520	f	1	-0.17	3.02	2.10	0.7620
KATSOU	521	f	1	1.28	3.02	1.17	0.0257
Subtotal KATSOU				0.55	6.03	3.27	
SOBUE	513	m	0	0.27	11.97	1.79	0.3439
SOBUE	514	m	0	0.39	15.16	1.15	0.1339
SOBUE	515	m	0	0.96	16.39	1.49	0.0001
SOBUE	516	m	0	1.03	12.22	1.65	0.0003
Subtotal SOBUE				0.67	55.74	6.07	

N	6
NS	2

Table 3I6 - 3

IESLC - Meta-analysis of Current Smoking by Duration, Overview
Adenocarcinoma, Any Product (or Cigarettes if Any not available)
Most adjusted

	Sex			
	combined	male	female	Total
N		4	2	6
NS		1	1	2

In this overview table, other than the "N" rows, entries in the "absent" and "Total" columns may be invalid and should be ignored

	Duration of smoking (broad categories)				
	absent	1-34k20	21-49k35	36+k50	Total
N	2	2	1	1	6
NS	2	2	1	1	4
Wt	19.41	14.98	15.16	12.22	61.77
Het Chi	0.26	0.48	0.00	0.00	9.34
Het df	1	1	0	0	5
Het P	N.S.	N.S.	N.S.	N.S.	(*)
Fixed RR	2.75	1.20	1.47	2.79	1.93
RRl	1.76	0.72	0.89	1.59	1.51
RRu	4.29	1.99	2.43	4.89	2.48
P	+++	N.S.	N.S.	+++	+++
Random RR	2.75	1.20	1.47	2.79	1.91
RRl	1.76	0.72	0.89	1.59	1.33
RRu	4.29	1.99	2.43	4.89	2.74
P	+++	N.S.	N.S.	+++	+++

	Duration of smoking (narrow categories)							
	absent	1-19k1	6-29k20	21-39k30	31-49k40	41-998k50	51+k999	Total
N	4			1	1			6
NS	2			1	1			2
Wt	30.23			15.16	16.39			61.77
Het Chi	6.69			0.00	0.00			9.34
Het df	3			0	0			5
Het P	(*)			N.S.	N.S.			(*)
Fixed RR	1.89			1.47	2.62			1.93
RRl	1.32			0.89	1.61			1.51
RRu	2.69			2.43	4.24			2.48
P	+++			N.S.	+++			+++
Random RR	1.86			1.47	2.62			1.91
RRl	1.04			0.89	1.61			1.33
RRu	3.32			2.43	4.24			2.74
P	+			N.S.	+++			+++

Table 3I6 - 3

IESLC - Meta-analysis of Current Smoking by Duration, Overview
Adenocarcinoma, Any Product (or Cigarettes if Any not available)
Most adjusted

MALES

	Duration of smoking (broad categories)				
	absent	1-34k20	21-49k35	36+k50	Total
N	1	1	1	1	4
NS	1	1	1	1	1
Wt	16.39	11.97	15.16	12.22	55.74
Het Chi	0.00	0.00	0.00	0.00	6.06
Het df	0	0	0	0	3
Het P	N.S.	N.S.	N.S.	N.S.	N.S.
Fixed RR	2.62	1.31	1.47	2.79	1.96
RRl	1.61	0.75	0.89	1.59	1.51
RRu	4.24	2.32	2.43	4.89	2.54
P	+++	N.S.	N.S.	+++	+++
Random RR	2.62	1.31	1.47	2.79	1.95
RRl	1.61	0.75	0.89	1.59	1.34
RRu	4.24	2.32	2.43	4.89	2.83
P	+++	N.S.	N.S.	+++	+++

	Duration of smoking (narrow categories)							
	absent	1-19k1	6-29k20	21-39k30	31-49k40	41-998k50	51+k999	Total
N	2			1	1			4
NS	1			1	1			1
Wt	24.19			15.16	16.39			55.74
Het Chi	3.43			0.00	0.00			6.06
Het df	1			0	0			3
Het P	(*)			N.S.	N.S.			N.S.
Fixed RR	1.92			1.47	2.62			1.96
RRl	1.29			0.89	1.61			1.51
RRu	2.87			2.43	4.24			2.54
P	++			N.S.	+++			+++
Random RR	1.92			1.47	2.62			1.95
RRl	0.92			0.89	1.61			1.34
RRu	4.01			2.43	4.24			2.83
P	(+)			N.S.	+++			+++

FEMALES

	Duration of smoking (broad categories)				
	absent	1-34k20	21-49k35	36+k50	Total
N	1	1			2
NS	1	1			1
Wt	3.02	3.02			6.03
Het Chi	0.00	0.00			3.21
Het df	0	0			1
Het P	N.S.	N.S.			(*)
Fixed RR	3.61	0.84			1.74
RRl	1.17	0.27			0.78
RRu	11.15	2.60			3.87
P	+	N.S.			N.S.
Random RR	3.61	0.84			1.74
RRl	1.17	0.27			0.42
RRu	11.15	2.60			7.27
P	+	N.S.			N.S.

Table 3I6 - 3

IESLC - Meta-analysis of Current Smoking by Duration, Overview
Adenocarcinoma, Any Product (or Cigarettes if Any not available)
Most adjusted

FEMALES

		Duration of smoking (narrow categories)							
		absent	1-19k1	6-29k20	21-39k30	31-49k40	41-998k50	51+k999	Total
	N	2							2
	NS	1							1
	Wt	6.03							6.03
Het	Chi	3.21							3.21
Het	df	1							1
Het	P	(*)							(*)
Fixed	RR	1.74							1.74
	RRl	0.78							0.78
	RRu	3.87							3.87
	P	N.S.							N.S.
Random	RR	1.74							1.74
	RRl	0.42							0.42
	RRu	7.27							7.27
	P	N.S.							N.S.

Table 3I6 - 4

IESLC - Meta-analysis of Current Smoking by Duration, Overview
Adenocarcinoma, Any Product (or Cigarettes if Any not available)
Least adjusted

REF	NRR	X	SEX	AGEL	AGEH	RACE	YF	LC TYPE	LOC	START	ST	NLC	R	VB	P	H	AD	PRODUCT	exL	exH	S1	S2	DENOM	De
KATSOU	515	x	f	0	0	all	-	a	Eu:bal	1987	CC	101	n	bl	n	n	0	all/unsp	1	29	1	0	nev any	st
KATSOU	516	x	f	0	0	all	-	a	Eu:bal	1987	CC	101	n	bl	n	n	0	all/unsp	30	999	0	0	nev any	st
SOBUE	513		m	0	0	all	-	a	As:Jap	1986	CC	1376	n	bl	n	y	0	cig+/-ot	1	29	1	0	nev cigs	st
SOBUE	514		m	0	0	all	-	a	As:Jap	1986	CC	1376	n	bl	n	y	0	cig+/-ot	30	39	2	3	nev cigs	st
SOBUE	515		m	0	0	all	-	a	As:Jap	1986	CC	1376	n	bl	n	y	0	cig+/-ot	40	49	0	4	nev cigs	st
SOBUE	516		m	0	0	all	-	a	As:Jap	1986	CC	1376	n	bl	n	y	0	cig+/-ot	50	999	3	0	nev cigs	st

Cigarette type is all/unspec for all RRs

In this overview table, subtotals and Qs values may be invalid and should be ignored

Table 3I6 - 5

IESLC - Meta-analysis of Current Smoking by Duration, Overview
Adenocarcinoma, Any Product (or Cigarettes if Any not available)
Least adjusted

REF	NRR	SEX	AD	Number Case	Exposed Cont	Non-exposed Case	Cont	RR	95.00%CI
KATSOU	515	f	0	6	12	30	67	1.12	(0.38- 3.26)
KATSOU	516	f	0	9	6	30	67	3.35	(1.09- 10.26)
Subtotal KATSOU								1.89	(0.87- 4.09)
SOBUE	513	m	0	33	119	27	128	1.31	(0.75- 2.32)
SOBUE	514	m	0	62	200	27	128	1.47	(0.89- 2.43)
SOBUE	515	m	0	96	174	27	128	2.62	(1.61- 4.24)
SOBUE	516	m	0	43	73	27	128	2.79	(1.59- 4.89)
Subtotal SOBUE								1.96	(1.51- 2.54)
Totals				249	584	168	646		

*prospective study

REF	NRR	SEX	AD	Ys	Ws	Qs	Ps
KATSOU	515	f	0	0.11	3.35	1.04	0.8399
KATSOU	516	f	0	1.21	3.07	0.90	0.0342
Subtotal KATSOU				0.64	6.42	1.94	
SOBUE	513	m	0	0.27	11.97	1.86	0.3439
SOBUE	514	m	0	0.39	15.16	1.21	0.1339
SOBUE	515	m	0	0.96	16.39	1.42	0.0001
SOBUE	516	m	0	1.03	12.22	1.58	0.0003
Subtotal SOBUE				0.67	55.74	6.06	

N	6
NS	2

Table 3I6 - 6

IESLC - Meta-analysis of Current Smoking by Duration, Overview
Adenocarcinoma, Any Product (or Cigarettes if Any not available)
Least adjusted

	Sex: combined	male	female	Total
N		4	2	6
NS		1	1	2

In this overview table, other than the "N" rows, entries in the "absent" and "Total" columns may be invalid and should be ignored

	Duration of smoking (broad categories): absent	1-34k20	21-49k35	36+k50	Total
N	2	2	1	1	6
NS	2	2	1	1	4
Wt	19.46	15.32	15.16	12.22	62.16
Het Chi	0.16	0.07	0.00	0.00	8.00
Het df	1	1	0	0	5
Het P	N.S.	N.S.	N.S.	N.S.	N.S.
Fixed RR	2.72	1.27	1.47	2.79	1.95
RRl	1.74	0.77	0.89	1.59	1.52
RRu	4.24	2.09	2.43	4.89	2.50
P	+++	N.S.	N.S.	+++	+++
Random RR	2.72	1.27	1.47	2.79	1.94
RRl	1.74	0.77	0.89	1.59	1.39
RRu	4.24	2.09	2.43	4.89	2.70
P	+++	N.S.	N.S.	+++	+++

	Duration of smoking (narrow categories): absent	1-19k1	6-29k20	21-39k30	31-49k40	41-998k50	51+k999	Total
N	4			1	1			6
NS	2			1	1			2
Wt	30.61			15.16	16.39			62.16
Het Chi	5.37			0.00	0.00			8.00
Het df	3			0	0			5
Het P	N.S.			N.S.	N.S.			N.S.
Fixed RR	1.92			1.47	2.62			1.95
RRl	1.34			0.89	1.61			1.52
RRu	2.73			2.43	4.24			2.50
P	+++			N.S.	+++			+++
Random RR	1.91			1.47	2.62			1.94
RRl	1.15			0.89	1.61			1.39
RRu	3.19			2.43	4.24			2.70
P	+			N.S.	+++			+++

Table 3I6 - 6

IESLC - Meta-analysis of Current Smoking by Duration, Overview
Adenocarcinoma, Any Product (or Cigarettes if Any not available)
Least adjusted

MALES

	Duration of smoking (broad categories)				
	absent	1-34k20	21-49k35	36+k50	Total
N	1	1	1	1	4
NS	1	1	1	1	1
Wt	16.39	11.97	15.16	12.22	55.74
Het Chi	0.00	0.00	0.00	0.00	6.06
Het df	0	0	0	0	3
Het P	N.S.	N.S.	N.S.	N.S.	N.S.
Fixed RR	2.62	1.31	1.47	2.79	1.96
RRl	1.61	0.75	0.89	1.59	1.51
RRu	4.24	2.32	2.43	4.89	2.54
P	+++	N.S.	N.S.	+++	+++
Random RR	2.62	1.31	1.47	2.79	1.95
RRl	1.61	0.75	0.89	1.59	1.34
RRu	4.24	2.32	2.43	4.89	2.83
P	+++	N.S.	N.S.	+++	+++

	Duration of smoking (narrow categories)							
	absent	1-19k1	6-29k20	21-39k30	31-49k40	41-998k50	51+k999	Total
N	2			1	1			4
NS	1			1	1			1
Wt	24.19			15.16	16.39			55.74
Het Chi	3.43			0.00	0.00			6.06
Het df	1			0	0			3
Het P	(*)			N.S.	N.S.			N.S.
Fixed RR	1.92			1.47	2.62			1.96
RRl	1.29			0.89	1.61			1.51
RRu	2.87			2.43	4.24			2.54
P	++			N.S.	+++			+++
Random RR	1.92			1.47	2.62			1.95
RRl	0.92			0.89	1.61			1.34
RRu	4.01			2.43	4.24			2.83
P	(+)			N.S.	+++			+++

FEMALES

	Duration of smoking (broad categories)				
	absent	1-34k20	21-49k35	36+k50	Total
N	1	1			2
NS	1	1			1
Wt	3.07	3.35			6.42
Het Chi	0.00	0.00			1.93
Het df	0	0			1
Het P	N.S.	N.S.			N.S.
Fixed RR	3.35	1.12			1.89
RRl	1.09	0.38			0.87
RRu	10.26	3.26			4.09
P	+	N.S.			N.S.
Random RR	3.35	1.12			1.91
RRl	1.09	0.38			0.65
RRu	10.26	3.26			5.60
P	+	N.S.			N.S.

Table 3I6 - 6

IESLC - Meta-analysis of Current Smoking by Duration, Overview
Adenocarcinoma, Any Product (or Cigarettes if Any not available)
Least adjusted

FEMALES

	Duration of smoking (narrow categories)							
	absent	1-19k1	6-29k20	21-39k30	31-49k40	41-998k50	51+k999	Total
N	2							2
NS	1							1
Wt	6.42							6.42
Het Chi	1.93							1.93
Het df	1							1
Het P	N.S.							N.S.
Fixed RR	1.89							1.89
RRl	0.87							0.87
RRu	4.09							4.09
P	N.S.							N.S.
Random RR	1.91							1.91
RRl	0.65							0.65
RRu	5.60							5.60
P	N.S.							N.S.

Table 3I6 - 7

IESLC - Meta-analysis of Current Smoking by Duration, Overview
Adenocarcinoma, Any Product (or Cigarettes if Any not available)
Excluded studies (and stage at which they were excluded)

	1	2	3	4	5	6	7	8	9	10	11	12	13	14	15	16
1	AGUDO	ALDERS	ARMADA	AUVINE	AXELSS	BARBON	BECHER	BENHAM	BLOT1	BOFFET	BOUCHA	BRESLO	BROWN3	CARPEN	CHEN	CHEN2
	CHIAZZ	CHOI	CHYOU	CORREA	DAMBER	DARBY	DESTEF	DOLL	DOLL2	DORGAN	DOSEME	FAN	GAO	GARCIA	GARSHI	GENG
	GER	GRAHAM	GUO	GURSEL	HAENSZ	HAMMO2	HAMMON	HEGMAN	HU	HU2	JAHN	JAIN	JEDRYC	JOLY	JUSSAW	KHUDER
	KOO	KOULUM	KREUZE	LAUSSM	LETOUR	LEVIN	LIU3	LIU4	LIU5	LUBIN	LUBIN2	LUO	MCCONN	NOTAN2	OSANN2	PERNU
	PEZZOT	PRESCO	QIAO	QIAO2	RACHTA	RESTRE	SADOWS	STASZE	SUZUK2	TIZZAN	TVERDA	VUTUC	WANG2	WIGLE	WU2	WUWILL
	WYNDE2	WYNDE3	XU	YUAN	ZHANG	ZHENG	ZHOU									
2	BENSHL	DEAN3	DORN	ENGELA	GAO2	GILLIS	HIRAYA	HOLE	KAUFMA	MIGRAN	MRFITR	SEGI2	SPEIZE	SVENSS	WAKAI	WU
3	MCDUFF	SPITZ	WYNDE6													
4	AKIBA	AMANDU	AMES	BEST	BOUCOT	BROSS	BUFFLE	CEDERL	CPSI	CPSII	DEAN2	HUMBLE	KAISE2	LIAW	MATOS	PEZZO2
	PISANI	WATSON	WYNDE7	WYNDE8												

Table 3I7 -

IESLC - Meta-analysis of Current Smoking, Duration, "Low"
Adenocarcinoma, Any Product (or Cigarettes if Any not available)

This analysis is restricted to results for:
1) Current smokers
2) Results by Duration
3) Categorical results by Duration
4) Adenocarcinoma (or near equivalent)
5) Results complete enough for use in metaanalysis

Within each study, results are then selected (in the following order of preference, within each sex) for:
6) (not applicable)
7) PRODUCT: all/unspec, cigarettes regardless of other products, cigarettes only
8) CIGTYPE: all/unspecified, MC regardless of HR, MC only
9) (not applicable)
10) DENOM: never smoked anything, never smoked cigarettes, never any + low, never cigs + low
11) Followup period (YF, prospective studies): whole study (coded as 0) or longest available
12) LCtype: adeno or nearest available, but not squamous. (q = squamous, s = small,
a = adeno, l = large, KII = Kreyberg II, al = alveolar, br = bronchiolar, u = undifferentiated)
13) Race: all or nearest available, otherwise by race (wh or w = white, bl or b = black, hi = hispanic
ch = chinese, jap = japanese, haw = hawaiian, w+o = white + oriental, sca = scandinavian, as = asian)
14) Duration "low" in key scheme 1 (key value 20, maximum range 1-34)
15) For overlapping studies: principal rather than subsidiary studies
Finally by Age: whole study (coded as 0) if available, otherwise by widest available age group
and then for single sex results (m, f) in preference to results for both sexes combined (c).

Results adjusted (AD) for the most potential confounders are then chosen in Sections -1 to -3
and results adjusted for the least confounders in Sections -4 to -6. (Those least adjusted results which
actually differ from the most adjusted are marked 'x' in column X in Section -4)

Section -7 shows excluded studies, together with the stage (as above) at which no qualifying
results were found.

Section -8 lists the potentially overlapping studies which have been included (1=principal, 2=subsidiary).

Section -9 lists any results which would have been included in preference except that they had data not complete
enough for use in meta-analysis, with their significance (yes/no), if known, and any further comment as entered
on the database. It also lists as "gap" any categories for which no data were presented by the original authors.

In addition to those mentioned above, the following fields, levels and abbreviations are used:
* or nk = not known, n = no, y = yes, ot = other
nev = never
all/unspec = all or unspecified, cig+/-ot = cigarettes irrespective of other products (cigar, pipe etc)
MC = manufactured cigarettes, HR = hand-rolled cigarettes
exL, exH = range of exposure (low and high) in the smoking group, in terms of Duration
REF: 6-character study reference
NRR: number of the RR on the database within the study
ST : study type (CC = case control, pr or prosp = prospective)
NLC: number of lung cancer cases in whole study
R : risky occupational population (n = no, m = mining, o = other risky)
VB : national cigarette type (V = at least 75% Virginia, bl = at least 75% blended, ot = other)
P : any proxy use
H : full histological confirmation
De : derivation of RR/CI (or = original, st = standard method, ot = other method of estimation)

Table 3I7 - 1

IESLC - Meta-analysis of Current Smoking, Duration, "Low"
Adenocarcinoma, Any Product (or Cigarettes if Any not available)
Most adjusted

REF	NRR	SEX	AGEL	AGEH	RACE	YF	LC TYPE	LOC	START	ST	NLC	R	VB	P	H	AD	PRODUCT	exL	exH	DENOM	De
KATSOU	520	f	0	0	all	-	a	Eu:bal	1987	CC	101	n	bl	n	n	1	all/unsp	1	29	nev any	or
SOBUE	513	m	0	0	all	-	a	As:Jap	1986	CC	1376	n	bl	n	y	0	cig+/-ot	1	29	nev cigs	st

Cigarette type is all/unspec for all RRs

Table 3I7 - 2

IESLC - Meta-analysis of Current Smoking, Duration, "Low"
Adenocarcinoma, Any Product (or Cigarettes if Any not available)
Most adjusted

REF	NRR	SEX	AD	Number Exposed Case	Number Exposed Cont	Non-exposed Case	Non-exposed Cont	RR	95.00%CI
KATSOU	520	f	1	6	-	30	-	0.84	(0.27- 2.58)
SOBUE	513	m	0	33	119	27	128	1.31	(0.75- 2.32)
Partial Totals				39	119	57	128		

*prospective study

REF	NRR	SEX	AD	Ys	Ws	Qs	Ps
KATSOU	520	f	1	-0.17	3.02	0.39	0.7620
SOBUE	513	m	0	0.27	11.97	0.10	0.3439

	N	2
	NS	2
	Wt	14.98
Het	Chi	0.48
Het	df	1
Het	P	N.S.
Fixed	RR	1.20
	RRl	0.72
	RRu	1.99
	P	N.S.
Random	RR	1.20
	RRl	0.72
	RRu	1.99
	P	N.S.
Asymm	P	

Table 3I7 - 3

IESLC - Meta-analysis of Current Smoking, Duration, "Low"
Adenocarcinoma, Any Product (or Cigarettes if Any not available)
Most adjusted

	Sex combined	male	female	Total
N		1	1	2
NS		1	1	2
Wt		11.97	3.02	14.98
Het Chi		0.00	0.00	0.48
Het df		0	0	1
Het P		N.S.	N.S.	N.S.
Fixed RR		1.31	0.84	1.20
RRl		0.75	0.27	0.72
RRu		2.32	2.60	1.99
P		N.S.	N.S.	N.S.
Random RR		1.31	0.84	1.20
RRl		0.75	0.27	0.72
RRu		2.32	2.60	1.99
P		N.S.	N.S.	N.S.
Between Chi				0.48
Between df				1
Between P				N.S.
Btwn(F) P				N.S.
Btwn(R) P				N.S.

Too few RRs for analysis by factor

Table 3I7 - 4

IESLC - Meta-analysis of Current Smoking, Duration, "Low"
Adenocarcinoma, Any Product (or Cigarettes if Any not available)
Least adjusted

REF	NRR	X	SEX	AGEL	AGEH	RACE	YF	LC TYPE	LOC	START	ST	NLC	R	VB	P	H	AD	PRODUCT	exL	exH	DENOM	De
KATSOU	515	x	f	0	0	all	-	a	Eu:bal	1987	CC	101	n	bl	n	n	0	all/unsp	1	29	nev any	st
SOBUE	513		m	0	0	all	-	a	As:Jap	1986	CC	1376	n	bl	n	y	0	cig+/-ot	1	29	nev cigs	st

Cigarette type is all/unspec for all RRs

Table 3I7 - 5

IESLC - Meta-analysis of Current Smoking, Duration, "Low"
Adenocarcinoma, Any Product (or Cigarettes if Any not available)
Least adjusted

REF	NRR	SEX	AD	Number Exposed Case	Number Exposed Cont	Non-exposed Case	Non-exposed Cont	RR	95.00%CI
KATSOU	515	f	0	6	12	30	67	1.12	(0.38- 3.26)
SOBUE	513	m	0	33	119	27	128	1.31	(0.75- 2.32)
Totals				39	131	57	195		

*prospective study

REF	NRR	SEX	AD	Ys	Ws	Qs	Ps
KATSOU	515	f	0	0.11	3.35	0.05	0.8399
SOBUE	513	m	0	0.27	11.97	0.02	0.3439

	N	2
	NS	2
	Wt	15.32
Het	Chi	0.07
Het	df	1
Het	P	N.S.
Fixed	RR	1.27
	RRl	0.77
	RRu	2.09
	P	N.S.
Random	RR	1.27
	RRl	0.77
	RRu	2.09
	P	N.S.
Asymm	P	

Table 3I7 - 6

IESLC - Meta-analysis of Current Smoking, Duration, "Low"
Adenocarcinoma, Any Product (or Cigarettes if Any not available)
Least adjusted

	Sex		
combined	male	female	Total
N	1	1	2
NS	1	1	2
Wt	11.97	3.35	15.32
Het Chi	0.00	0.00	0.07
Het df	0	0	1
Het P	N.S.	N.S.	N.S.
Fixed RR	1.31	1.12	1.27
RRl	0.75	0.38	0.77
RRu	2.32	3.26	2.09
P	N.S.	N.S.	N.S.
Random RR	1.31	1.12	1.27
RRl	0.75	0.38	0.77
RRu	2.32	3.26	2.09
P	N.S.	N.S.	N.S.
Between Chi			0.07
Between df			1
Between P			N.S.
Btwn(F) P			N.S.
Btwn(R) P			N.S.

Table 3I7 - 7

IESLC - Meta-analysis of Current Smoking, Duration, "Low"
Adenocarcinoma, Any Product (or Cigarettes if Any not available)
Excluded studies (and stage at which they were excluded)

	1	2	3	4	5	6	7	8	9	10	11	12	13	14	15	16
1	AGUDO	ALDERS	ARMADA	AUVINE	AXELSS	BARBON	BECHER	BENHAM	BLOT1	BOFFET	BOUCHA	BRESLO	BROWN3	CARPEN	CHEN	CHEN2
	CHIAZZ	CHOI	CHYOU	CORREA	DAMBER	DARBY	DESTEF	DOLL	DOLL2	DORGAN	DOSEME	FAN	GAO	GARCIA	GARSHI	GENG
	GER	GRAHAM	GUO	GURSEL	HAENSZ	HAMMO2	HAMMON	HEGMAN	HU	HU2	JAHN	JAIN	JEDRYC	JOLY	JUSSAW	KHUDER
	KOO	KOULUM	KREUZE	LAUSSM	LETOUR	LEVIN	LIU3	LIU4	LIU5	LUBIN	LUBIN2	LUO	MCCONN	NOTAN2	OSANN2	PERNU
	PEZZOT	PRESCO	QIAO	QIAO2	RACHTA	RESTRE	SADOWS	STASZE	SUZUK2	TIZZAN	TVERDA	VUTUC	WANG2	WIGLE	WU2	WUWILL
	WYNDE2	WYNDE3	XU	YUAN	ZHANG	ZHENG	ZHOU									
2	BENSHL	DEAN3	DORN	ENGELA	GAO2	GILLIS	HIRAYA	HOLE	KAUFMA	MIGRAN	MRFITR	SEGI2	SPEIZE	SVENSS	WAKAI	WU
3	MCDUFF	SPITZ	WYNDE6													
4	AKIBA	AMANDU	AMES	BEST	BOUCOT	BROSS	BUFFLE	CEDERL	CPSI	CPSII	DEAN2	HUMBLE	KAISE2	LIAW	MATOS	PEZZO2
	PISANI	WATSON	WYNDE7	WYNDE8												

Table 3I8 -

IESLC - Meta-analysis of Current Smoking, Duration, "Mid"
Adenocarcinoma, Any Product (or Cigarettes if Any not available)

This analysis is restricted to results for:
1) Current smokers
2) Results by Duration
3) Categorical results by Duration
4) Adenocarcinoma (or near equivalent)
5) Results complete enough for use in metaanalysis

Within each study, results are then selected (in the following order of preference, within each sex) for:
6) (not applicable)
7) PRODUCT: all/unspec, cigarettes regardless of other products, cigarettes only
8) CIGTYPE: all/unspecified, MC regardless of HR, MC only
9) (not applicable)
10) DENOM: never smoked anything, never smoked cigarettes, never any + low, never cigs + low
11) Followup period (YF, prospective studies): whole study (coded as 0) or longest available
12) LCtype: adeno or nearest available, but not squamous. (q = squamous, s = small,
a = adeno, l = large, KII = Kreyberg II, al = alveolar, br = bronchiolar, u = undifferentiated)
13) Race: all or nearest available, otherwise by race (wh or w = white, bl or b = black, hi = hispanic
ch = chinese, jap = japanese, haw = hawaiian, w+o = white + oriental, sca = scandinavian, as = asian)
14) Duration "mid" in key scheme 1 (key value 35, maximum range 21-49)
15) For overlapping studies: principal rather than subsidiary studies
Finally by Age: whole study (coded as 0) if available, otherwise by widest available age group
and then for single sex results (m, f) in preference to results for both sexes combined (c).

Results adjusted (AD) for the most potential confounders are then chosen in Sections -1 to -3
and results adjusted for the least confounders in Sections -4 to -6. (Those least adjusted results which
actually differ from the most adjusted are marked 'x' in column X in Section -4)

Section -7 shows excluded studies, together with the stage (as above) at which no qualifying
results were found.

Section -8 lists the potentially overlapping studies which have been included (1=principal, 2=subsidiary).

Section -9 lists any results which would have been included in preference except that they had data not complete
enough for use in meta-analysis, with their significance (yes/no), if known, and any further comment as entered
on the database. It also lists as "gap" any categories for which no data were presented by the original authors.

In addition to those mentioned above, the following fields, levels and abbreviations are used:
* or nk = not known, n = no, y = yes, ot = other
nev = never
all/unspec = all or unspecified, cig+/-ot = cigarettes irrespective of other products (cigar, pipe etc)
MC = manufactured cigarettes, HR = hand-rolled cigarettes
exL, exH = range of exposure (low and high) in the smoking group, in terms of Duration
REF: 6-character study reference
NRR: number of the RR on the database within the study
ST : study type (CC = case control, pr or prosp = prospective)
NLC: number of lung cancer cases in whole study
R : risky occupational population (n = no, m = mining, o = other risky)
VB : national cigarette type (V = at least 75% Virginia, bl = at least 75% blended, ot = other)
P : any proxy use
H : full histological confirmation
De : derivation of RR/CI (or = original, st = standard method, ot = other method of estimation)

Table 3I8 - 1

IESLC - Meta-analysis of Current Smoking, Duration, "Mid"
Adenocarcinoma, Any Product (or Cigarettes if Any not available)
Most adjusted

REF	NRR	SEX	AGEL	AGEH	RACE	YF	LC TYPE	LOC	START	ST	NLC	R	VB	P	H	AD	PRODUCT	exL	exH	DENOM	De
SOBUE	514	m	0	0	all	-	a	As:Jap	1986	CC	1376	n	bl	n	y	0	cig+/-ot	30	39	nev cigs	st

Cigarette type is all/unspec for all RRs

Table 3I8 - 2

IESLC - Meta-analysis of Current Smoking, Duration, "Mid"
Adenocarcinoma, Any Product (or Cigarettes if Any not available)
Most adjusted

REF	NRR	SEX	AD	Number Case	Exposed Cont	Non-exposed Case	Cont	RR	95.00%CI
SOBUE	514	m	0	62	200	27	128	1.47	(0.89- 2.43)
Totals				62	200	27	128		

*prospective study

REF	NRR	SEX	AD	Ys	Ws	Qs	Ps
SOBUE	514	m	0	0.39	15.16	0.00	0.1339

	N	1
	NS	1
	Wt	15.16
Het	Chi	0.00
Het	df	0
Het	P	N.S.
Fixed	RR	1.47
	RRl	0.89
	RRu	2.43
	P	N.S.
Random	RR	1.47
	RRl	0.89
	RRu	2.43
	P	N.S.
Asymm	P	

Table 3I8 - 3

IESLC - Meta-analysis of Current Smoking, Duration, "Mid"
Adenocarcinoma, Any Product (or Cigarettes if Any not available)
Most adjusted

	Sex: combined	male	female	Total
N		1		1
NS		1		1
Wt		15.16		15.16
Het Chi		0.00		0.00
Het df		0		0
Het P		N.S.		N.S.
Fixed RR		1.47		1.47
RRl		0.89		0.89
RRu		2.43		2.43
P		N.S.		N.S.
Random RR		1.47		1.47
RRl		0.89		0.89
RRu		2.43		2.43
P		N.S.		N.S.
Between Chi				
Between df				
Between P				N.S.
Btwn(F) P				N.S.
Btwn(R) P				N.S.

Too few RRs for analysis by factor

Table 3I8 - 4

IESLC - Meta-analysis of Current Smoking, Duration, "Mid"
Adenocarcinoma, Any Product (or Cigarettes if Any not available)
Least adjusted

REF	NRR	X	SEX	AGEL	AGEH	RACE	YF	LC TYPE	LOC	START	ST	NLC	R	VB	P	H	AD	PRODUCT	exL	exH	DENOM	De
SOBUE	514		m	0	0	all	-	a	As:Jap	1986	CC	1376	n	bl	n	y	0	cig+/-ot	30	39	nev cigs	st

Cigarette type is all/unspec for all RRs

Table 3I8 - 5

IESLC - Meta-analysis of Current Smoking, Duration, "Mid"
Adenocarcinoma, Any Product (or Cigarettes if Any not available)
Least adjusted

REF	NRR	SEX	AD	Number Exposed Case	Cont	Non-exposed Case	Cont	RR	95.00%CI
SOBUE	514	m	0	62	200	27	128	1.47	(0.89- 2.43)
Totals				62	200	27	128		

*prospective study

REF	NRR	SEX	AD	Ys	Ws	Qs	Ps
SOBUE	514	m	0	0.39	15.16	0.00	0.1339

	N	1
	NS	1
	Wt	15.16
Het	Chi	0.00
Het	df	0
Het	P	N.S.
Fixed	RR	1.47
	RRl	0.89
	RRu	2.43
	P	N.S.
Random	RR	1.47
	RRl	0.89
	RRu	2.43
	P	N.S.
Asymm	P	

Table 3I8 - 6

IESLC - Meta-analysis of Current Smoking, Duration, "Mid"
Adenocarcinoma, Any Product (or Cigarettes if Any not available)
Least adjusted

	Sex: combined	male	female	Total
N		1		1
NS		1		1
Wt		15.16		15.16
Het Chi		0.00		0.00
Het df		0		0
Het P		N.S.		N.S.
Fixed RR		1.47		1.47
RRl		0.89		0.89
RRu		2.43		2.43
P		N.S.		N.S.
Random RR		1.47		1.47
RRl		0.89		0.89
RRu		2.43		2.43
P		N.S.		N.S.
Between Chi				
Between df				
Between P				N.S.
Btwn(F) P				N.S.
Btwn(R) P				N.S.

Table 3I8 - 7

IESLC - Meta-analysis of Current Smoking, Duration, "Mid"
Adenocarcinoma, Any Product (or Cigarettes if Any not available)
Excluded studies (and stage at which they were excluded)

	1	2	3	4	5	6	7	8	9	10	11	12	13	14	15	16
1	AGUDO	ALDERS	ARMADA	AUVINE	AXELSS	BARBON	BECHER	BENHAM	BLOT1	BOFFET	BOUCHA	BRESLO	BROWN3	CARPEN	CHEN	CHEN2
	CHIAZZ	CHOI	CHYOU	CORREA	DAMBER	DARBY	DESTEF	DOLL	DOLL2	DORGAN	DOSEME	FAN	GAO	GARCIA	GARSHI	GENG
	GER	GRAHAM	GUO	GURSEL	HAENSZ	HAMMO2	HAMMON	HEGMAN	HU	HU2	JAHN	JAIN	JEDRYC	JOLY	JUSSAW	KHUDER
	KOO	KOULUM	KREUZE	LAUSSM	LETOUR	LEVIN	LIU3	LIU4	LIU5	LUBIN	LUBIN2	LUO	MCCONN	NOTAN2	OSANN2	PERNU
	PEZZOT	PRESCO	QIAO	QIAO2	RACHTA	RESTRE	SADOWS	STASZE	SUZUK2	TIZZAN	TVERDA	VUTUC	WANG2	WIGLE	WU2	WUWILL
	WYNDE2	WYNDE3	XU	YUAN	ZHANG	ZHENG	ZHOU									
2	BENSHL	DEAN3	DORN	ENGELA	GAO2	GILLIS	HIRAYA	HOLE	KAUFMA	MIGRAN	MRFITR	SEGI2	SPEIZE	SVENSS	WAKAI	WU
3	MCDUFF	SPITZ	WYNDE6													
4	AKIBA	AMANDU	AMES	BEST	BOUCOT	BROSS	BUFFLE	CEDERL	CPSI	CPSII	DEAN2	HUMBLE	KAISE2	LIAW	MATOS	PEZZO2
	PISANI	WATSON	WYNDE7	WYNDE8												
14	KATSOU															

Table 3I9 -

IESLC - Meta-analysis of Current Smoking, Duration, "High"
Adenocarcinoma, Any Product (or Cigarettes if Any not available)

This analysis is restricted to results for:
1) Current smokers
2) Results by Duration
3) Categorical results by Duration
4) Adenocarcinoma (or near equivalent)
5) Results complete enough for use in metaanalysis

Within each study, results are then selected (in the following order of preference, within each sex) for:
6) PRODUCT: all/unspec, cigarettes regardless of other products, cigarettes only
7) CIGTYPE: all/unspecified, MC regardless of HR, MC only
8) (not applicable)
9) DENOM: never smoked anything, never smoked cigarettes, never any + low, never cigs + low
10) Followup period (YF, prospective studies): whole study (coded as 0) or longest available
11) LCtype: adeno or nearest available, but not squamous. (q = squamous, s = small,
a = adeno, l = large, KII = Kreyberg II, al = alveolar, br = bronchiolar, u = undifferentiated)
12) Race: all or nearest available, otherwise by race (wh or w = white, bl or b = black, hi = hispanic
ch = chinese, jap = japanese, haw = hawaiian, w+o = white + oriental, sca = scandinavian, as = asian)
13) Duration "high" in key scheme 1 (key value 50, maximum range 36+)
14) For overlapping studies: principal rather than subsidiary studies
Finally by Age: whole study (coded as 0) if available, otherwise by widest available age group
and then for single sex results (m, f) in preference to results for both sexes combined (c).

Results adjusted (AD) for the most potential confounders are then chosen in Sections -1 to -3
and results adjusted for the least confounders in Sections -4 to -6. (Those least adjusted results which
actually differ from the most adjusted are marked 'x' in column X in Section -4)

Section -7 shows excluded studies, together with the stage (as above) at which no qualifying
results were found.

Section -8 lists the potentially overlapping studies which have been included (1=principal, 2=subsidiary).

Section -9 lists any results which would have been included in preference except that they had data not complete
enough for use in meta-analysis, with their significance (yes/no), if known, and any further comment as entered
on the database. It also lists as "gap" any categories for which no data were presented by the original authors.

In addition to those mentioned above, the following fields, levels and abbreviations are used:
* or nk = not known, n = no, y = yes, ot = other
nev = never
all/unspec = all or unspecified, cig+/-ot = cigarettes irrespective of other products (cigar, pipe etc)
MC = manufactured cigarettes, HR = hand-rolled cigarettes
exL, exH = range of exposure (low and high) in the smoking group, in terms of Duration
REF: 6-character study reference
NRR: number of the RR on the database within the study
ST : study type (CC = case control, pr or prosp = prospective)
NLC: number of lung cancer cases in whole study
R : risky occupational population (n = no, m = mining, o = other risky)
VB : national cigarette type (V = at least 75% Virginia, bl = at least 75% blended, ot = other)
P : any proxy use
H : full histological confirmation
De : derivation of RR/CI (or = original, st = standard method, ot = other method of estimation)

Table 3I9 - 1

IESLC - Meta-analysis of Current Smoking, Duration, "High"
Adenocarcinoma, Any Product (or Cigarettes if Any not available)
Most adjusted

REF	NRR	SEX	AGEL	AGEH	RACE	YF	LC TYPE	LOC	START	ST	NLC	R	VB	P	H	AD	PRODUCT	exL	exH	DENOM	De
SOBUE	516	m	0	0	all	-	a	As:Jap	1986	CC	1376	n	bl	n	y	0	cig+/-ot	50	999	nev cigs	st

Cigarette type is all/unspec for all RRs

Table 3I9 - 2

IESLC - Meta-analysis of Current Smoking, Duration, "High"
Adenocarcinoma, Any Product (or Cigarettes if Any not available)
Most adjusted

REF	NRR	SEX	AD	Number Exposed Case	Number Exposed Cont	Non-exposed Case	Non-exposed Cont	RR	95.00%CI
SOBUE	516	m	0	43	73	27	128	2.79	(1.59- 4.89)
Totals				43	73	27	128		

*prospective study

REF	NRR	SEX	AD	Ys	Ws	Qs	Ps
SOBUE	516	m	0	1.03	12.22	0.00	0.0003

	N	1
	NS	1
	Wt	12.22
Het	Chi	0.00
Het	df	0
Het	P	N.S.
Fixed	RR	2.79
	RRl	1.59
	RRu	4.89
	P	+++
Random	RR	2.79
	RRl	1.59
	RRu	4.89
	P	+++
Asymm	P	

Table 3I9 - 3

IESLC - Meta-analysis of Current Smoking, Duration, "High"
Adenocarcinoma, Any Product (or Cigarettes if Any not available)
Most adjusted

	Sex combined	male	female	Total
N		1		1
NS		1		1
Wt		12.22		12.22
Het Chi		0.00		0.00
Het df		0		0
Het P		N.S.		N.S.
Fixed RR		2.79		2.79
RRl		1.59		1.59
RRu		4.89		4.89
P		+++		+++
Random RR		2.79		2.79
RRl		1.59		1.59
RRu		4.89		4.89
P		+++		+++
Between Chi				
Between df				
Between P				N.S.
Btwn(F) P				N.S.
Btwn(R) P				N.S.

Too few RRs for analysis by factor

Table 3I9 - 4

IESLC - Meta-analysis of Current Smoking, Duration, "High"
Adenocarcinoma, Any Product (or Cigarettes if Any not available)
Least adjusted

REF	NRR	X	SEX	AGEL	AGEH	RACE	YF	LC TYPE	LOC	START	ST	NLC	R	VB	P	H	AD	PRODUCT	exL	exH	DENOM	De
SOBUE	516		m	0	0	all	-	a	As:Jap	1986	CC	1376	n	bl	n	y	0	cig+/-ot	50	999	nev cigs	st

Cigarette type is all/unspec for all RRs

Table 3I9 - 5

IESLC - Meta-analysis of Current Smoking, Duration, "High"
Adenocarcinoma, Any Product (or Cigarettes if Any not available)
Least adjusted

REF	NRR	SEX	AD	Number Exposed Case	Number Exposed Cont	Non-exposed Case	Non-exposed Cont	RR	95.00%CI
SOBUE	516	m	0	43	73	27	128	2.79	(1.59- 4.89)
Totals				43	73	27	128		

*prospective study

REF	NRR	SEX	AD	Ys	Ws	Qs	Ps
SOBUE	516	m	0	1.03	12.22	0.00	0.0003

	N	1
	NS	1
	Wt	12.22
Het	Chi	0.00
Het	df	0
Het	P	N.S.
Fixed	RR	2.79
	RRl	1.59
	RRu	4.89
	P	+++
Random	RR	2.79
	RRl	1.59
	RRu	4.89
	P	+++
Asymm	P	

Table 3I9 - 6

IESLC - Meta-analysis of Current Smoking, Duration, "High"
Adenocarcinoma, Any Product (or Cigarettes if Any not available)
Least adjusted

	combined	Sex: male	female	Total
N		1		1
NS		1		1
Wt		12.22		12.22
Het Chi		0.00		0.00
Het df		0		0
Het P		N.S.		N.S.
Fixed RR		2.79		2.79
RRl		1.59		1.59
RRu		4.89		4.89
P		+++		+++
Random RR		2.79		2.79
RRl		1.59		1.59
RRu		4.89		4.89
P		+++		+++
Between Chi				
Between df				
Between P				N.S.
Btwn(F) P				N.S.
Btwn(R) P				N.S.

Table 3I9 - 7

IESLC - Meta-analysis of Current Smoking, Duration, "High"
Adenocarcinoma, Any Product (or Cigarettes if Any not available)
Excluded studies (and stage at which they were excluded)

	1	2	3	4	5	6	7	8	9	10	11	12	13	14	15	16
1	AGUDO	ALDERS	ARMADA	AUVINE	AXELSS	BARBON	BECHER	BENHAM	BLOT1	BOFFET	BOUCHA	BRESLO	BROWN3	CARPEN	CHEN	CHEN2
	CHIAZZ	CHOI	CHYOU	CORREA	DAMBER	DARBY	DESTEF	DOLL	DOLL2	DORGAN	DOSEME	FAN	GAO	GARCIA	GARSHI	GENG
	GER	GRAHAM	GUO	GURSEL	HAENSZ	HAMMO2	HAMMON	HEGMAN	HU	HU2	JAHN	JAIN	JEDRYC	JOLY	JUSSAW	KHUDER
	KOO	KOULUM	KREUZE	LAUSSM	LETOUR	LEVIN	LIU3	LIU4	LIU5	LUBIN	LUBIN2	LUO	MCCONN	NOTAN2	OSANN2	PERNU
	PEZZOT	PRESCO	QIAO	QIAO2	RACHTA	RESTRE	SADOWS	STASZE	SUZUK2	TIZZAN	TVERDA	VUTUC	WANG2	WIGLE	WU2	WUWILL
	WYNDE2	WYNDE3	XU	YUAN	ZHANG	ZHENG	ZHOU									
2	BENSHL	DEAN3	DORN	ENGELA	GAO2	GILLIS	HIRAYA	HOLE	KAUFMA	MIGRAN	MRFITR	SEGI2	SPEIZE	SVENSS	WAKAI	WU
3	MCDUFF	SPITZ	WYNDE6													
4	AKIBA	AMANDU	AMES	BEST	BOUCOT	BROSS	BUFFLE	CEDERL	CPSI	CPSII	DEAN2	HUMBLE	KAISE2	LIAW	MATOS	PEZZO2
	PISANI	WATSON	WYNDE7	WYNDE8												
14	KATSOU															

Table 3I10 -

IESLC - Meta-analysis of Current Smoking, Duration, "Highest vs lowest"
Adenocarcinoma, Any Product (or Cigarettes if Any not available)

This analysis is restricted to results for:
1) Current smokers
2) Results by Duration
3) Categorical results by Duration
4) Denominator (unexposed) = "low"
5) Adenocarcinoma (or near equivalent)
6) Results complete enough for use in metaanalysis

Within each study, results are then selected (in the following order of preference, within each sex) for:
7) (not applicable)
8) PRODUCT: all/unspec, cigarettes regardless of other products, cigarettes only
9) CIGTYPE: all/unspecified, MC regardless of HR, MC only
10) Results with least adjustment for other aspects of smoking (ADOS)
11) The highest vs lowest category
12) Followup period (YF, prospective studies): whole study (coded as 0) or longest available
13) LCtype: adeno or nearest available, but not squamous. (q = squamous, s = small,
a = adeno, l = large, KII = Kreyberg II, al = alveolar, br = bronchiolar, u = undifferentiated)
14) Race: all or nearest available, otherwise by race (wh or w = white, bl or b = black, hi = hispanic
ch = chinese, jap = japanese, haw = hawaiian, w+o = white + oriental, sca = scandinavian, as = asian)
15) For overlapping studies: principal rather than subsidiary studies
Finally by Age: whole study (coded as 0) if available, otherwise by widest available age group
and then for single sex results (m, f) in preference to results for both sexes combined (c).

Results adjusted (AD) for the most potential confounders are then chosen in Sections -1 to -3
and results adjusted for the least confounders in Sections -4 to -6. (Those least adjusted results which
actually differ from the most adjusted are marked 'x' in column X in Section -4)

Section -7 shows excluded studies, together with the stage (as above) at which no qualifying
results were found.

Section -8 lists the potentially overlapping studies which have been included (1=principal, 2=subsidiary).

Section -9 lists any results which would have been included in preference except that they had data not complete
enough for use in meta-analysis, with their significance (yes/no), if known, and any further comment as entered
on the database. It also lists as "gap" any categories for which no data were presented by the original authors.

In addition to those mentioned above, the following fields, levels and abbreviations are used:
* or nk = not known, n = no, y = yes, ot = other
all/unspec = all or unspecified, cig+/-ot = cigarettes irrespective of other products (cigar, pipe etc)
MC = manufactured cigarettes, HR = hand-rolled cigarettes
exL, exH = range of exposure (low and high) in the "highest" group, in terms of Duration
unexL, unexH = range of exposure (low and high) in the "lowest" group, in terms of Duration
REF: 6-character study reference
NRR: number of the RR on the database within the study
ST : study type (CC = case control, pr or prosp = prospective)
NLC: number of lung cancer cases in whole study
R : risky occupational population (n = no, m = mining, o = other risky)
VB : national cigarette type (V = at least 75% Virginia, bl = at least 75% blended, ot = other)
P : any proxy use
H : full histological confirmation
De : derivation of RR/CI (or = original, st = standard method, ot = other method of estimation)

Table 3I10 - 1

IESLC - Meta-analysis of Current Smoking, Duration, "Highest vs lowest"
Adenocarcinoma, Any Product (or Cigarettes if Any not available)
Most adjusted

REF	NRR	SEX	AGEL	AGEH	RACE	YF	LC TYPE	LOC	START	ST	NLC	R	VB	P	H	AD	ADOS	PRODUCT	exL	exH	unexL	unexH	De
KATSOU	522	f	0	0	all	-	a	Eu:bal	1987	CC	101	n	bl	n	n	1	0	all/unsp	30	999	1	29	ot
SOBUE	519	m	0	0	all	-	a	As:Jap	1986	CC	1376	n	bl	n	y	0	0	cig+/-ot	50	999	1	29	st

Cigarette type is all/unspec for all RRs

Table 3I10 - 2

IESLC - Meta-analysis of Current Smoking, Duration, "Highest vs lowest"
Adenocarcinoma, Any Product (or Cigarettes if Any not available)
Most adjusted

REF	NRR	SEX	AD	Number Case	Exposed Cont	Non-exposed Case	Cont	RR	95.00%CI
KATSOU	522	f	1	9	-	6	-	4.30	(0.98- 18.82)
SOBUE	519	m	0	43	73	33	119	2.12	(1.24- 3.64)
Partial Totals				52	73	39	119		

*prospective study

REF	NRR	SEX	AD	Ys	Ws	Qs	Ps
KATSOU	522	f	1	1.46	1.76	0.68	0.0530
SOBUE	519	m	0	0.75	13.22	0.09	0.0062

	N	2
	NS	2
	Wt	14.98
Het	Chi	0.77
Het	df	1
Het	P	N.S.
Fixed	RR	2.31
	RRl	1.39
	RRu	3.83
	P	++
Random	RR	2.31
	RRl	1.39
	RRu	3.83
	P	++
Asymm	P	

Table 3I10 - 3

IESLC - Meta-analysis of Current Smoking, Duration, "Highest vs lowest"
Adenocarcinoma, Any Product (or Cigarettes if Any not available)
Most adjusted

	Sex combined	male	female	Total
N		1	1	2
NS		1	1	2
Wt		13.22	1.76	14.98
Het Chi		0.00	0.00	0.77
Het df		0	0	1
Het P		N.S.	N.S.	N.S.
Fixed RR		2.12	4.30	2.31
RRl		1.24	0.98	1.39
RRu		3.64	18.84	3.83
P		++	(+)	++
Random RR		2.12	4.30	2.31
RRl		1.24	0.98	1.39
RRu		3.64	18.84	3.83
P		++	(+)	++
Between Chi				0.77
Between df				1
Between P				N.S.
Btwn(F) P				N.S.
Btwn(R) P				N.S.

Too few RRs for analysis by factor

Table 3I10 - 4

IESLC - Meta-analysis of Current Smoking, Duration, "Highest vs lowest"
Adenocarcinoma, Any Product (or Cigarettes if Any not available)
Least adjusted

REF	NRR	X	SEX	AGEL	AGEH	RACE	YF	LC TYPE	LOC	START	ST	NLC	R	VB	P	H	AD	ADOS	PRODUCT	exL	exH	unexL	unexH	De
KATSOU	517	x	f	0	0	all	-	a	Eu:bal	1987	CC	101	n	bl	n	n	0	0	all/unsp	30	999	1	29	st
SOBUE	519		m	0	0	all	-	a	As:Jap	1986	CC	1376	n	bl	n	y	0	0	cig+/-ot	50	999	1	29	st

Cigarette type is all/unspec for all RRs

Table 3I10 - 5

IESLC - Meta-analysis of Current Smoking, Duration, "Highest vs lowest"
Adenocarcinoma, Any Product (or Cigarettes if Any not available)
Least adjusted

REF	NRR	SEX	AD	Number Case	Exposed Cont	Non-exposed Case	Cont	RR	95.00%CI
KATSOU	517	f	0	9	6	6	12	3.00	(0.72- 12.46)
SOBUE	519	m	0	43	73	33	119	2.12	(1.24- 3.64)
Totals				52	79	39	131		

*prospective study

REF	NRR	SEX	AD	Ys	Ws	Qs	Ps
KATSOU	517	f	0	1.10	1.89	0.17	0.1305
SOBUE	519	m	0	0.75	13.22	0.02	0.0062

	N	2
	NS	2
	Wt	15.11
Het	Chi	0.20
Het	df	1
Het	P	N.S.
Fixed	RR	2.22
	RRl	1.34
	RRu	3.67
	P	++
Random	RR	2.22
	RRl	1.34
	RRu	3.67
	P	++
Asymm	P	

Table 3I10 - 6

IESLC - Meta-analysis of Current Smoking, Duration, "Highest vs lowest"
Adenocarcinoma, Any Product (or Cigarettes if Any not available)
Least adjusted

	Sex combined	male	female	Total
N		1	1	2
NS		1	1	2
Wt		13.22	1.89	15.11
Het Chi		0.00	0.00	0.20
Het df		0	0	1
Het P		N.S.	N.S.	N.S.
Fixed RR		2.12	3.00	2.22
RRl		1.24	0.72	1.34
RRu		3.64	12.46	3.67
P		++	N.S.	++
Random RR		2.12	3.00	2.22
RRl		1.24	0.72	1.34
RRu		3.64	12.46	3.67
P		++	N.S.	++
Between Chi				0.20
Between df				1
Between P				N.S.
Btwn(F) P				N.S.
Btwn(R) P				N.S.

Table 3I10 - 7

IESLC - Meta-analysis of Current Smoking, Duration, "Highest vs lowest"
Adenocarcinoma, Any Product (or Cigarettes if Any not available)
Excluded studies (and stage at which they were excluded)

	1	2	3	4	5	6	7	8	9	10	11	12	13	14	15	16
1	AGUDO	ALDERS	ARMADA	AUVINE	AXELSS	BARBON	BECHER	BENHAM	BLOT1	BOFFET	BOUCHA	BRESLO	BROWN3	CARPEN	CHEN	CHEN2
	CHIAZZ	CHOI	CHYOU	CORREA	DAMBER	DARBY	DESTEF	DOLL	DOLL2	DORGAN	DOSEME	FAN	GAO	GARCIA	GARSHI	GENG
	GER	GRAHAM	GUO	GURSEL	HAENSZ	HAMMO2	HAMMON	HEGMAN	HU	HU2	JAHN	JAIN	JEDRYC	JOLY	JUSSAW	KHUDER
	KOO	KOULUM	KREUZE	LAUSSM	LETOUR	LEVIN	LIU3	LIU4	LIU5	LUBIN	LUBIN2	LUO	MCCONN	NOTAN2	OSANN2	PERNU
	PEZZOT	PRESCO	QIAO	QIAO2	RACHTA	RESTRE	SADOWS	STASZE	SUZUK2	TIZZAN	TVERDA	VUTUC	WANG2	WIGLE	WU2	WUWILL
	WYNDE2	WYNDE3	XU	YUAN	ZHANG	ZHENG	ZHOU									
2	BENSHL	DEAN3	DORN	ENGELA	GAO2	GILLIS	HIRAYA	HOLE	KAUFMA	MIGRAN	MRFITR	SEGI2	SPEIZE	SVENSS	WAKAI	WU
3	MCDUFF	SPITZ	WYNDE6													
4	AKIBA															
5	AMANDU	AMES	BEST	BOUCOT	BROSS	BUFFLE	CEDERL	CPSI	CPSII	DEAN2	HUMBLE	KAISE2	LIAW	MATOS	PEZZO2	PISANI
	WATSON	WYNDE7	WYNDE8													

Table 3I11 -

IESLC - Meta-analysis of Ever/current Smoking by Duration, Overview
Adenocarcinoma, Any Product (or Cigarettes if Any not available)

This analysis is restricted to results for:
1) Ever/current smokers
2) Results by Duration
3) Categorical results by Duration
Results by Duration are grouped under 2 schemes (S1, S2). Each scheme has a set of "key values". An interval is allocated to the category whose key value it includes, and intervals which include none or more than one of the key values are excluded. (Open-ended intervals are coded as 999)

S1	key value	maximum range
1	20	1-34
2	35	21-49
3	50	36+

S2	key value	maximum range
1	5	1-19
2	20	6-29
3	30	21-39
4	40	31-49
5	50	41-998
6	999	51+

4) Adenocarcinoma (or near equivalent)
5) Results complete enough for use in metaanalysis

Within each study, results are then selected (in the following order of preference, within each sex) for:
6) SMKSTA: ever, current
7) PRODUCT: all/unspec, cigarettes regardless of other products, cigarettes only
8) CIGTYPE: all/unspecified, MC regardless of HR, MC only
9) (not applicable)
10) DENOM: never smoked anything, never smoked cigarettes, never any + low, never cigs + low
11) Followup period (YF, prospective studies): whole study (coded as 0) or longest available
12) LCtype: adeno or nearest available, but not squamous. (q = squamous, s = small,
a = adeno, l = large, KII = Kreyberg II, al = alveolar, br = bronchiolar, u = undifferentiated)
13) Race: all or nearest available, otherwise by race (wh or w = white, bl or b = black, hi = hispanic
ch = chinese, jap = japanese, haw = hawaiian, w+o = white + oriental, sca = scandinavian, as = asian)
14) For overlapping studies: principal rather than subsidiary studies
Finally by Age: whole study (coded as 0) if available, otherwise by widest available age group
and then for single sex results (m, f) in preference to results for both sexes combined (c).

Results adjusted (AD) for the most potential confounders are then chosen in Sections -1 to -3
and results adjusted for the least confounders in Sections -4 to -6. (Those least adjusted results which actually differ from the most adjusted are marked 'x' in column X in Section -4)

Section -7 shows excluded studies, together with the stage (as above) at which no qualifying results were found.

Section -8 lists the potentially overlapping studies which have been included (1=principal, 2=subsidiary).

Section -9 lists any results which would have been included in preference except that they had data not complete enough for use in meta-analysis, with their significance (yes/no), if known, and any further comment as entered on the database. It also lists as "gap" any categories for which no data were presented by the original authors.

In addition to those mentioned above, the following fields, levels and abbreviations are used:
* or nk = not known, n = no, y = yes, ot = other
ev = ever, cu = current, nev = never
all/unspec = all or unspecified, cig+/-ot = cigarettes irrespective of other products (cigar, pipe etc)
MC = manufactured cigarettes, HR = hand-rolled cigarettes
exL, exH = range of exposure (low and high) in the smoking group, in terms of Duration
REF: 6-character study reference
NRR: number of the RR on the database within the study
ST : study type (CC = case control, pr or prosp = prospective)
NLC: number of lung cancer cases in whole study
R : risky occupational population (n = no, m = mining, o = other risky)
VB : national cigarette type (V = at least 75% Virginia, bl = at least 75% blended, ot = other)
P : any proxy use
H : full histological confirmation
De : derivation of RR/CI (or = original, st = standard method, ot = other method of estimation)

Table 3I11 - 1

IESLC - Meta-analysis of Ever/current Smoking by Duration, Overview
Adenocarcinoma, Any Product (or Cigarettes if Any not available)
Most adjusted

REF	NRR	SEX	AGEL	AGEH	RACE	YF	LC TYPE	LOC	START	ST	NLC	R	VB	P	H	AD	SM	PRODUCT	exL	exH	S1	S2	DENOM	De
BARBON	724	m	0	0	all	-	a	Eu:wst	1979	CC	755	n	bl	y	y	1	ev	all/unsp	1	29	1	0	nev any	or
BARBON	725	m	0	0	all	-	a	Eu:wst	1979	CC	755	n	bl	y	y	1	ev	all/unsp	30	39	2	3	nev any	or
BARBON	726	m	0	0	all	-	a	Eu:wst	1979	CC	755	n	bl	y	y	1	ev	all/unsp	40	49	0	4	nev any	or
BARBON	727	m	0	0	all	-	a	Eu:wst	1979	CC	755	n	bl	y	y	1	ev	all/unsp	50	999	3	0	nev any	or
BUFFLE	509	m	0	0	wh	-	a	NAmer	1976	CC	943	n	bl	y	n	0	ev	cig+/-ot	1	33	1	0	nev cigs	or
BUFFLE	510	m	0	0	wh	-	a	NAmer	1976	CC	943	n	bl	y	n	0	ev	cig+/-ot	34	43	2	4	nev cigs	or
BUFFLE	511	m	0	0	wh	-	a	NAmer	1976	CC	943	n	bl	y	n	0	ev	cig+/-ot	44	49	0	0	nev cigs	or
BUFFLE	512	m	0	0	wh	-	a	NAmer	1976	CC	943	n	bl	y	n	0	ev	cig+/-ot	50	999	3	0	nev cigs	or
CHOI	566	m	0	0	all	-	a	As:oth	1985	CC	375	n	bl	n	n	0	ev	cig+/-ot	1	29	1	0	nev cigs	st
CHOI	567	m	0	0	all	-	a	As:oth	1985	CC	375	n	bl	n	n	0	ev	cig+/-ot	30	39	2	3	nev cigs	st
CHOI	568	m	0	0	all	-	a	As:oth	1985	CC	375	n	bl	n	n	0	ev	cig+/-ot	40	49	0	4	nev cigs	st
CHOI	569	m	0	0	all	-	a	As:oth	1985	CC	375	n	bl	n	n	0	ev	cig+/-ot	50	999	3	0	nev cigs	st
CHOI	578	f	0	0	all	-	a	As:oth	1985	CC	375	n	bl	n	n	0	ev	cig+/-ot	1	29	1	0	nev cigs	st
CHOI	579	f	0	0	all	-	a	As:oth	1985	CC	375	n	bl	n	n	0	ev	cig+/-ot	30	999	0	0	nev cigs	st
DAMBER	540	m	0	0	all	-	a+al+br	Eu:Sca	1972	CC	579	n	bl	y	n	1	ev	all/unsp	1	30	1	0	nev any	or
DAMBER	541	m	0	0	all	-	a+al+br	Eu:Sca	1972	CC	579	n	bl	y	n	1	ev	all/unsp	31	40	2	4	nev any	or
DAMBER	542	m	0	0	all	-	a+al+br	Eu:Sca	1972	CC	579	n	bl	y	n	1	ev	all/unsp	41	50	3	5	nev any	or
DAMBER	543	m	0	0	all	-	a+al+br	Eu:Sca	1972	CC	579	n	bl	y	n	1	ev	all/unsp	51	999	0	6	nev any	or
DORGAN	576	m	0	0	wh	-	a	NAmer	1980	CC	2026	n	bl	y	y	2	ev	cig+/-ot	1	34	1	0	nev any	ot
DORGAN	577	m	0	0	wh	-	a	NAmer	1980	CC	2026	n	bl	y	y	2	ev	cig+/-ot	35	999	0	0	nev any	ot
DORGAN	568	f	0	0	all	-	a	NAmer	1980	CC	2026	n	bl	y	y	3	ev	cig+/-ot	1	34	1	0	nev any	ot
DORGAN	569	f	0	0	all	-	a	NAmer	1980	CC	2026	n	bl	y	y	3	ev	cig+/-ot	35	999	0	0	nev any	ot
DOSEME	516	m	0	0	all	-	not q+s	Eu:bal	1979	CC	1210	n	bl	n	n	2	ev	cig+/-ot	1	10	0	1	nev cigs	or
DOSEME	517	m	0	0	all	-	not q+s	Eu:bal	1979	CC	1210	n	bl	n	n	2	ev	cig+/-ot	11	20	1	2	nev cigs	or
DOSEME	518	m	0	0	all	-	not q+s	Eu:bal	1979	CC	1210	n	bl	n	n	2	ev	cig+/-ot	21	999	0	0	nev cigs	or
GER	507	c	0	0	all	-	a	As:oth	1990	CC	141	n	ot	y	n	5	ev	all/unsp	1	30	1	0	nev any	ot
GER	508	c	0	0	all	-	a	As:oth	1990	CC	141	n	ot	y	n	5	ev	all/unsp	31	999	0	0	nev any	ot
HAENSZ	525	f	0	0	all	-	a	NAmer	1955	CC	158	n	bl	n	y	0	ev	cig+/-ot	1	14	0	1	nev any	st
HAENSZ	526	f	0	0	all	-	a	NAmer	1955	CC	158	n	bl	n	y	0	ev	cig+/-ot	15	999	0	0	nev any	st
JEDRYC	595	m	0	0	all	-	a	Eu:est	1980	CC	1630	n	bl	y	n	3	ev	cig+/-ot	1	19	0	1	nev any	or
JEDRYC	596	m	0	0	all	-	a	Eu:est	1980	CC	1630	n	bl	y	n	3	ev	cig+/-ot	20	39	0	0	nev any	or
JEDRYC	597	m	0	0	all	-	a	Eu:est	1980	CC	1630	n	bl	y	n	3	ev	cig+/-ot	40	999	3	0	nev any	or
JOLY	660	m	0	0	all	-	a	SCAmer	1978	CC	826	n	bl	n	n	0	ev	cig+/-ot	1	29	1	0	nev any	st
JOLY	661	m	0	0	all	-	a	SCAmer	1978	CC	826	n	bl	n	n	0	ev	cig+/-ot	30	39	2	3	nev any	st
JOLY	662	m	0	0	all	-	a	SCAmer	1978	CC	826	n	bl	n	n	0	ev	cig+/-ot	40	49	0	4	nev any	st
JOLY	663	m	0	0	all	-	a	SCAmer	1978	CC	826	n	bl	n	n	0	ev	cig+/-ot	50	999	3	0	nev any	st
JOLY	632	f	0	0	all	-	a	SCAmer	1978	CC	826	n	bl	n	n	0	ev	cig+/-ot	1	29	1	0	nev any	st
JOLY	633	f	0	0	all	-	a	SCAmer	1978	CC	826	n	bl	n	n	0	ev	cig+/-ot	30	39	2	3	nev any	st
JOLY	634	f	0	0	all	-	a	SCAmer	1978	CC	826	n	bl	n	n	0	ev	cig+/-ot	40	49	0	4	nev any	st
JOLY	635	f	0	0	all	-	a	SCAmer	1978	CC	826	n	bl	n	n	0	ev	cig+/-ot	50	999	3	0	nev any	st
KATSOU	520	f	0	0	all	-	a	Eu:bal	1987	CC	101	n	bl	n	n	1	cu	all/unsp	1	29	1	0	nev any	or
KATSOU	521	f	0	0	all	-	a	Eu:bal	1987	CC	101	n	bl	n	n	1	cu	all/unsp	30	999	0	0	nev any	or
LUBIN2	700	m	0	0	all	-	a	Eu:mul	1976	CC	7804	n	bl	n	y	0	ev	cig+/-ot	1	29	1	0	nev any	st
LUBIN2	701	m	0	0	all	-	a	Eu:mul	1976	CC	7804	n	bl	n	y	0	ev	cig+/-ot	30	39	2	3	nev any	st
LUBIN2	702	m	0	0	all	-	a	Eu:mul	1976	CC	7804	n	bl	n	y	0	ev	cig+/-ot	40	49	0	4	nev any	st
LUBIN2	703	m	0	0	all	-	a	Eu:mul	1976	CC	7804	n	bl	n	y	0	ev	cig+/-ot	50	999	3	0	nev any	st
LUBIN2	752	f	0	0	all	-	a	Eu:mul	1976	CC	7804	n	bl	n	y	0	ev	cig+/-ot	1	29	1	0	nev any	st
LUBIN2	753	f	0	0	all	-	a	Eu:mul	1976	CC	7804	n	bl	n	y	0	ev	cig+/-ot	30	39	2	3	nev any	st
LUBIN2	754	f	0	0	all	-	a	Eu:mul	1976	CC	7804	n	bl	n	y	0	ev	cig+/-ot	40	49	0	4	nev any	st
LUBIN2	755	f	0	0	all	-	a	Eu:mul	1976	CC	7804	n	bl	n	y	0	ev	cig+/-ot	50	999	3	0	nev any	st
MATOS	646	m	0	0	all	-	a	SCAmer	1994	CC	200	n	bl	n	n	2	ev	cig+/-ot	1	24	1	0	nev any	or
MATOS	647	m	0	0	all	-	a	SCAmer	1994	CC	200	n	bl	n	n	2	ev	cig+/-ot	25	39	2	3	nev any	or
MATOS	648	m	0	0	all	-	a	SCAmer	1994	CC	200	n	bl	n	n	2	ev	cig+/-ot	40	70	3	0	nev any	or
OSANN2	516	f	0	0	all	-	KII	NAmer	1964	ot	217	n	bl	n	y	1	ev	cig+/-ot	1	20	1	0	nev cigs	or
OSANN2	517	f	0	0	all	-	KII	NAmer	1964	ot	217	n	bl	n	y	1	ev	cig+/-ot	21	999	0	0	nev cigs	or
PEZZOT	516	m	0	0	all	-	a	SCAmer	1987	CC	215	n	bl	n	y	0	ev	cig only	1	30	1	0	nev cigs	st
PEZZOT	517	m	0	0	all	-	a	SCAmer	1987	CC	215	n	bl	n	y	0	ev	cig only	31	40	2	4	nev cigs	st
PEZZOT	518	m	0	0	all	-	a	SCAmer	1987	CC	215	n	bl	n	y	0	ev	cig only	41	999	3	0	nev cigs	st
SOBUE	513	m	0	0	all	-	a	As:Jap	1986	CC	1376	n	bl	n	y	0	cu	cig+/-ot	1	29	1	0	nev cigs	st
SOBUE	514	m	0	0	all	-	a	As:Jap	1986	CC	1376	n	bl	n	y	0	cu	cig+/-ot	30	39	2	3	nev cigs	st
SOBUE	515	m	0	0	all	-	a	As:Jap	1986	CC	1376	n	bl	n	y	0	cu	cig+/-ot	40	49	0	4	nev cigs	st
SOBUE	516	m	0	0	all	-	a	As:Jap	1986	CC	1376	n	bl	n	y	0	cu	cig+/-ot	50	999	3	0	nev cigs	st
WUWILL	526	f	0	0	all	-	a	As:Chi	1985	CC	965	n	ot	n	n	3	ev	cig+/-ot	1	29	1	0	nev cigs	ot
WUWILL	527	f	0	0	all	-	a	As:Chi	1985	CC	965	n	ot	n	n	3	ev	cig+/-ot	30	39	2	3	nev cigs	ot
WUWILL	528	f	0	0	all	-	a	As:Chi	1985	CC	965	n	ot	n	n	3	ev	cig+/-ot	40	999	3	0	nev cigs	ot
ZHENG	512	m	0	0	all	-	a	As:Chi	1982	CC	540	n	ot	*	y	0	ev	cig+/-ot	1	29	1	0	nev cigs	or
ZHENG	513	m	0	0	all	-	a	As:Chi	1982	CC	540	n	ot	*	y	0	ev	cig+/-ot	30	999	0	0	nev cigs	st
ZHENG	516	f	0	0	all	-	a	As:Chi	1982	CC	540	n	ot	*	y	0	ev	cig+/-ot	1	29	1	0	nev cigs	or
ZHENG	517	f	0	0	all	-	a	As:Chi	1982	CC	540	n	ot	*	y	0	ev	cig+/-ot	30	999	0	0	nev cigs	st

Table 3I11 - 1

IESLC - Meta-analysis of Ever/current Smoking by Duration, Overview
Adenocarcinoma, Any Product (or Cigarettes if Any not available)
Most adjusted

Cigarette type is all/unspec for all RRs

In this overview table, subtotals and Qs values may be invalid and should be ignored

Table 3I11 - 2

IESLC - Meta-analysis of Ever/current Smoking by Duration, Overview
Adenocarcinoma, Any Product (or Cigarettes if Any not available)
Most adjusted

REF	NRR	SEX	AD	Number Exposed Case	Cont	Non-exposed Case	Cont	RR	95.00%CI
BARBON	724	m	1	15	-	7	-	3.70	(1.40- 9.70)
BARBON	725	m	1	23	-	7	-	5.10	(2.10- 12.50)
BARBON	726	m	1	46	-	7	-	8.20	(3.60- 18.70)
BARBON	727	m	1	67	-	7	-	8.30	(3.70- 18.70)
Subtotal BARBON								6.27	(4.06- 9.67)
BUFFLE	509	m	0	-	-	-	-	4.40	(1.70- 11.50)
BUFFLE	510	m	0	-	-	-	-	5.70	(2.20- 14.80)
BUFFLE	511	m	0	-	-	-	-	4.30	(1.60- 11.40)
BUFFLE	512	m	0	-	-	-	-	3.50	(1.30- 9.70)
Subtotal BUFFLE								4.44	(2.73- 7.22)
CHOI	566	m	0	17	221	7	95	1.04	(0.42- 2.60)
CHOI	567	m	0	13	160	7	65	0.75	(0.29- 1.98)
CHOI	568	m	0	12	64	7	95	2.54	(0.95- 6.81)
CHOI	569	m	0	4	20	7	95	2.71	(0.73- 10.16)
CHOI	578	f	0	2	23	48	164	0.30	(0.07- 1.31)
CHOI	579	f	0	2	3	48	164	2.28	(0.37- 14.03)
Subtotal CHOI								1.23	(0.78- 1.96)
DAMBER	540	m	1	-	-	16	-	1.80	(0.60- 5.40)
DAMBER	541	m	1	-	-	16	-	1.20	(0.20- 6.00)
DAMBER	542	m	1	-	-	16	-	3.40	(1.30- 9.10)
DAMBER	543	m	1	-	-	16	-	2.50	(0.90- 6.70)
Subtotal DAMBER								2.35	(1.35- 4.10)
DORGAN	576	m	2	-	-	-	-	2.14	(0.78- 5.84)
DORGAN	577	m	2	-	-	-	-	6.73	(2.66- 17.03)
DORGAN	568	f	3	-	-	-	-	2.43	(1.65- 3.59)
DORGAN	569	f	3	-	-	-	-	5.14	(3.63- 7.28)
Subtotal DORGAN								3.72	(2.92- 4.74)
DOSEME	516	m	2	5	-	24	-	0.80	(0.30- 2.50)
DOSEME	517	m	2	26	-	24	-	3.30	(2.20- 7.50)
DOSEME	518	m	2	69	-	24	-	4.10	(2.20- 7.50)
Subtotal DOSEME								2.96	(1.98- 4.42)
GER	507	c	5	14	-	37	-	1.40	(0.62- 3.19)
GER	508	c	5	21	-	37	-	0.85	(0.39- 1.86)
Subtotal GER								1.08	(0.61- 1.90)
HAENSZ	525	f	0	2	26	37	236	0.49	(0.11- 2.15)
HAENSZ	526	f	0	16	77	37	236	1.33	(0.70- 2.51)
Subtotal HAENSZ								1.13	(0.63- 2.04)
JEDRYC	595	m	3	2	-	7	-	1.10	(0.20- 5.95)
JEDRYC	596	m	3	43	-	7	-	3.47	(1.40- 8.58)
JEDRYC	597	m	3	47	-	7	-	4.41	(1.86- 10.45)
Subtotal JEDRYC								3.38	(1.88- 6.07)
JOLY	660	m	0	5	109	5	218	2.00	(0.57- 7.06)
JOLY	661	m	0	12	165	5	218	3.17	(1.10- 9.18)
JOLY	662	m	0	22	182	5	218	5.27	(1.96- 14.19)
JOLY	663	m	0	33	253	5	218	5.69	(2.18- 14.82)
JOLY	632	f	0	10	54	25	283	2.10	(0.95- 4.61)
JOLY	633	f	0	6	24	25	283	2.83	(1.06- 7.57)
JOLY	634	f	0	10	24	25	283	4.72	(2.03- 10.96)
JOLY	635	f	0	7	20	25	283	3.96	(1.53- 10.27)
Subtotal JOLY								3.51	(2.50- 4.92)
KATSOU	520	f	1	6	-	30	-	0.84	(0.27- 2.58)
KATSOU	521	f	1	9	-	30	-	3.61	(1.17- 11.17)
Subtotal KATSOU								1.74	(0.78- 3.87)
LUBIN2	700	m	0	131	2964	57	2616	2.03	(1.48- 2.78)
LUBIN2	701	m	0	242	3473	57	2616	3.20	(2.39- 4.29)
LUBIN2	702	m	0	208	2540	57	2616	3.76	(2.79- 5.06)
LUBIN2	703	m	0	90	1460	57	2616	2.83	(2.02- 3.97)
LUBIN2	752	f	0	174	229	138	1180	6.50	(4.99- 8.46)
LUBIN2	753	f	0	383	186	138	1180	17.61	(13.73- 22.58)
LUBIN2	754	f	0	284	118	138	1180	20.58	(15.59- 27.17)
LUBIN2	755	f	0	181	34	138	1180	45.52	(30.31- 68.36)
Subtotal LUBIN2								7.21	(6.49- 8.00)
MATOS	646	m	2	7	-	5	-	1.70	(0.50- 5.50)
MATOS	647	m	2	39	-	5	-	7.30	(2.70- 19.70)
MATOS	648	m	2	33	-	5	-	10.70	(3.80- 29.90)
Subtotal MATOS								5.70	(3.08- 10.54)
OSANN2	516	f	1	10	-	22	-	0.70	(0.20- 1.90)
OSANN2	517	f	1	51	-	22	-	4.10	(1.80- 9.40)
Subtotal OSANN2								2.21	(1.13- 4.30)
PEZZOT	516	m	0	11	134	3	116	3.17	(0.86- 11.65)
PEZZOT	517	m	0	22	82	3	116	10.37	(3.01- 35.81)

Table 3I11 - 2

IESLC - Meta-analysis of Ever/current Smoking by Duration, Overview
Adenocarcinoma, Any Product (or Cigarettes if Any not available)
Most adjusted

REF	NRR	SEX	AD	Number Exposed Case	Cont	Non-exposed Case	Cont	RR	95.00%CI
PEZZOT	518	m	0	27	101	3	116	10.34	(3.04- 35.09)
Subtotal PEZZOT								7.18	(3.49- 14.81)
SOBUE	513	m	0	33	119	27	128	1.31	(0.75- 2.32)
SOBUE	514	m	0	62	200	27	128	1.47	(0.89- 2.43)
SOBUE	515	m	0	96	174	27	128	2.62	(1.61- 4.24)
SOBUE	516	m	0	43	73	27	128	2.79	(1.59- 4.89)
Subtotal SOBUE								1.96	(1.51- 2.54)
WUWILL	526	f	3	34	-	172	-	0.80	(0.51- 1.25)
WUWILL	527	f	3	52	-	172	-	1.99	(1.37- 2.89)
WUWILL	528	f	3	52	-	172	-	2.09	(1.40- 3.10)
Subtotal WUWILL								1.58	(1.25- 2.00)
ZHENG	512	m	0	24	75	29	94	1.04	(0.56- 1.93)
ZHENG	513	m	0	99	143	29	94	2.24	(1.38- 3.66)
ZHENG	516	f	0	9	17	119	184	0.82	(0.35- 1.90)
ZHENG	517	f	0	24	27	119	184	1.37	(0.76- 2.49)
Subtotal ZHENG								1.45	(1.07- 1.96)
Partial Totals				2987	13574	2405	19754		

*prospective study

REF	NRR	SEX	AD	Ys	Ws	Qs	Ps
BARBON	724	m	1	1.31	4.10	0.02	0.0081
BARBON	725	m	1	1.63	4.83	0.31	0.0003
BARBON	726	m	1	2.10	5.66	3.02	0.0000
BARBON	727	m	1	2.12	5.85	3.22	0.0000
Subtotal BARBON				1.84	20.44	6.57	
BUFFLE	509	m	0	1.48	4.20	0.05	0.0024
BUFFLE	510	m	0	1.74	4.23	0.57	0.0003
BUFFLE	511	m	0	1.46	3.99	0.03	0.0036
BUFFLE	512	m	0	1.25	3.80	0.06	0.0145
Subtotal BUFFLE				1.49	16.22	0.70	
CHOI	566	m	0	0.04	4.61	8.18	0.9264
CHOI	567	m	0	-0.28	4.14	11.36	0.5664
CHOI	568	m	0	0.93	3.96	0.77	0.0630
CHOI	569	m	0	1.00	2.21	0.31	0.1381
CHOI	578	f	0	-1.21	1.75	11.74	0.1081
CHOI	579	f	0	0.82	1.16	0.35	0.3748
Subtotal CHOI				0.21	17.84	32.71	
DAMBER	540	m	1	0.59	3.18	1.97	0.2943
DAMBER	541	m	1	0.18	1.33	1.89	0.8336
DAMBER	542	m	1	1.22	4.06	0.09	0.0137
DAMBER	543	m	1	0.92	3.81	0.80	0.0736
Subtotal DAMBER				0.85	12.38	4.75	
DORGAN	576	m	2	0.76	3.79	1.43	0.1385
DORGAN	577	m	2	1.91	4.46	1.26	0.0001
DORGAN	568	f	3	0.89	25.43	6.01	0.0000
DORGAN	569	f	3	1.64	31.73	2.19	0.0000
Subtotal DORGAN				1.31	65.41	10.90	
DOSEME	516	m	2	-0.22	3.42	8.72	0.6799
DOSEME	517	m	2	1.19	10.22	0.33	0.0001
DOSEME	518	m	2	1.41	10.22	0.01	0.0000
Subtotal DOSEME				1.08	23.85	9.07	
GER	507	c	5	0.34	5.73	6.17	0.4207
GER	508	c	5	-0.16	6.30	14.87	0.6834
Subtotal GER				0.08	12.02	21.04	
HAENSZ	525	f	0	-0.71	1.76	7.64	0.3455
HAENSZ	526	f	0	0.28	9.37	11.18	0.3886
Subtotal HAENSZ				0.12	11.12	18.82	
JEDRYC	595	m	3	0.10	1.33	2.18	0.9123
JEDRYC	596	m	3	1.24	4.67	0.08	0.0071
JEDRYC	597	m	3	1.48	5.16	0.06	0.0008
Subtotal JEDRYC				1.22	11.17	2.32	
JOLY	660	m	0	0.69	2.42	1.12	0.2812
JOLY	661	m	0	1.15	3.40	0.16	0.0333
JOLY	662	m	0	1.66	3.91	0.32	0.0010
JOLY	663	m	0	1.74	4.19	0.55	0.0004
JOLY	632	f	0	0.74	6.17	2.48	0.0660
JOLY	633	f	0	1.04	3.97	0.44	0.0382
JOLY	634	f	0	1.55	5.40	0.17	0.0003
JOLY	635	f	0	1.38	4.23	0.00	0.0046
Subtotal JOLY				1.25	33.69	5.26	

Table 3I11 - 2

IESLC - Meta-analysis of Ever/current Smoking by Duration, Overview
Adenocarcinoma, Any Product (or Cigarettes if Any not available)
Most adjusted

REF	NRR	SEX	AD	Ys	Ws	Qs	Ps
KATSOU	520	f	1	-0.17	3.02	7.23	0.7620
KATSOU	521	f	1	1.28	3.02	0.02	0.0257
Subtotal KATSOU				0.55	6.03	7.26	
LUBIN2	700	m	0	0.71	38.61	17.18	0.0000
LUBIN2	701	m	0	1.16	44.75	2.01	0.0000
LUBIN2	702	m	0	1.32	43.24	0.11	0.0000
LUBIN2	703	m	0	1.04	33.64	3.76	0.0000
LUBIN2	752	f	0	1.87	54.92	13.57	0.0000
LUBIN2	753	f	0	2.87	62.18	138.81	0.0000
LUBIN2	754	f	0	3.02	49.78	135.53	0.0000
LUBIN2	755	f	0	3.82	23.24	138.80	0.0000
Subtotal LUBIN2				1.98	350.37	449.78	
MATOS	646	m	2	0.53	2.67	1.90	0.3857
MATOS	647	m	2	1.99	3.89	1.47	0.0001
MATOS	648	m	2	2.37	3.61	3.58	0.0000
Subtotal MATOS				1.74	10.17	6.95	
OSANN2	516	f	1	-0.36	3.03	9.08	0.5346
OSANN2	517	f	1	1.41	5.62	0.01	0.0008
Subtotal OSANN2				0.79	8.66	9.09	
PEZZOT	516	m	0	1.16	2.27	0.11	0.0817
PEZZOT	517	m	0	2.34	2.50	2.33	0.0002
PEZZOT	518	m	0	2.34	2.57	2.38	0.0002
Subtotal PEZZOT				1.97	7.34	4.82	
SOBUE	513	m	0	0.27	11.97	14.50	0.3439
SOBUE	514	m	0	0.39	15.16	14.83	0.1339
SOBUE	515	m	0	0.96	16.39	2.79	0.0001
SOBUE	516	m	0	1.03	12.22	1.47	0.0003
Subtotal SOBUE				0.67	55.74	33.60	
WUWILL	526	f	3	-0.22	19.12	48.78	0.3292
WUWILL	527	f	3	0.69	27.58	12.98	0.0003
WUWILL	528	f	3	0.74	24.32	9.87	0.0003
Subtotal WUWILL				0.46	71.01	71.63	
ZHENG	512	m	0	0.04	9.99	17.87	0.9080
ZHENG	513	m	0	0.81	16.07	5.15	0.0012
ZHENG	516	f	0	-0.20	5.44	13.49	0.6405
ZHENG	517	f	0	0.32	10.81	12.05	0.2958
Subtotal ZHENG				0.37	42.31	48.56	

N	69
NS	18

Table 3I11 - 3

IESLC - Meta-analysis of Ever/current Smoking by Duration, Overview
Adenocarcinoma, Any Product (or Cigarettes if Any not available)
Most adjusted

	Sex: combined	male	female	Total
N	2	44	23	69
NS	1	13	9	23

In this overview table, other than the "N" rows, entries in the "absent" and "Total" columns may be invalid and should be ignored

	Duration of smoking (broad categories): absent	1-34k20	21-49k35	36+k50	Total
N	23	21	12	13	69
NS	15	16	10	11	52
Wt	246.07	222.65	177.96	129.10	775.79
Het Chi	204.35	120.89	174.97	153.89	743.81
Het df	22	20	11	12	68
Het P	***	***	***	***	***
Fixed RR	4.55	2.33	5.13	5.24	3.95
RRl	4.02	2.04	4.43	4.41	3.68
RRu	5.16	2.66	5.95	6.23	4.24
P	+++	+++	+++	+++	+++
Random RR	3.01	1.72	3.53	5.25	2.89
RRl	1.99	1.20	1.81	2.70	2.25
RRu	4.57	2.46	6.88	10.20	3.70
P	+++	++	+++	+++	+++

	Duration of smoking (narrow categories): absent	1-19k1	6-29k20	21-39k30	31-49k40	41-998k50	51+k999	Total
N	44	3	1	9	10	1	1	69
NS	18	3	1	7	8	1	1	38
Wt	444.89	6.51	10.22	169.90	136.40	4.06	3.81	775.79
Het Chi	367.35	0.53	0.00	170.88	100.52	0.00	0.00	743.81
Het df	43	2	0	8	9	0	0	68
Het P	***	N.S.	N.S.	***	***	N.S.	N.S.	***
Fixed RR	3.09	0.75	3.30	5.13	7.11	3.40	2.50	3.95
RRl	2.82	0.35	1.79	4.41	6.01	1.29	0.92	3.68
RRu	3.39	1.61	6.09	5.96	8.41	9.00	6.82	4.24
P	+++	N.S.	+++	+++	+++	+	(+)	+++
Random RR	2.64	0.75	3.30	3.30	5.13	3.40	2.50	2.89
RRl	1.98	0.35	1.79	1.52	2.67	1.29	0.92	2.25
RRu	3.53	1.61	6.09	7.16	9.84	9.00	6.82	3.70
P	+++	N.S.	+++	++	+++	+	(+)	+++

Table 3I11 - 3

IESLC - Meta-analysis of Ever/current Smoking by Duration, Overview
Adenocarcinoma, Any Product (or Cigarettes if Any not available)
Most adjusted

MALES

Duration of smoking (broad categories)

		absent	1-34k20	21-49k35	36+k50	Total
	N	13	12	9	10	44
	NS	11	12	9	10	42
	Wt	121.14	98.04	84.23	77.32	380.72
Het	Chi	21.62	15.64	26.85	15.18	101.70
Het	df	12	11	8	9	43
Het	P	*	N.S.	***	(*)	***
Fixed	RR	3.32	1.95	2.90	3.71	2.87
	RRl	2.78	1.60	2.34	2.97	2.60
	RRu	3.97	2.38	3.59	4.64	3.18
	P	+++	+++	+++	+++	+++
Random	RR	3.28	1.97	3.10	4.30	3.01
	RRl	2.49	1.51	1.91	3.08	2.53
	RRu	4.31	2.57	5.04	5.99	3.59
	P	+++	+++	+++	+++	+++

Duration of smoking (narrow categories)

		absent	1-19k1	6-29k20	21-39k30	31-49k40	41-998k50	51+k999	Total
	N	25	2	1	6	8	1	1	44
	NS	13	2	1	6	8	1	1	31
	Wt	200.49	4.75	10.22	76.17	81.22	4.06	3.81	380.72
Het	Chi	55.61	0.10	0.00	19.51	11.68	0.00	0.00	101.70
Het	df	24	1	0	5	7	0	0	43
Het	P	***	N.S.	N.S.	**	N.S.	N.S.	N.S.	***
Fixed	RR	2.67	0.87	3.30	2.72	3.81	3.40	2.50	2.87
	RRl	2.33	0.36	1.79	2.17	3.06	1.29	0.92	2.60
	RRu	3.07	2.15	6.09	3.40	4.73	9.00	6.82	3.18
	P	+++	N.S.	+++	+++	+++	+	(+)	+++
Random	RR	2.98	0.87	3.30	2.67	4.05	3.40	2.50	3.01
	RRl	2.36	0.36	1.79	1.54	2.86	1.29	0.92	2.53
	RRu	3.77	2.15	6.09	4.62	5.73	9.00	6.82	3.59
	P	+++	N.S.	+++	+++	+++	+	(+)	+++

FEMALES

Duration of smoking (broad categories)

		absent	1-34k20	21-49k35	36+k50	Total
	N	9	8	3	3	23
	NS	8	8	3	3	22
	Wt	118.64	118.88	93.73	51.79	383.04
Het	Chi	133.09	97.18	95.91	115.71	533.56
Het	df	8	7	2	2	22
Het	P	***	***	***	***	***
Fixed	RR	6.86	2.76	8.58	8.78	5.65
	RRl	5.73	2.31	7.01	6.68	5.11
	RRu	8.21	3.31	10.51	11.52	6.24
	P	+++	+++	+++	+++	+++
Random	RR	3.11	1.29	4.72	7.29	2.72
	RRl	1.34	0.60	0.87	0.77	1.61
	RRu	7.24	2.82	25.49	69.06	4.61
	P	++	N.S.	(+)	(+)	+++

Table 3I11 - 3

IESLC - Meta-analysis of Ever/current Smoking by Duration, Overview
Adenocarcinoma, Any Product (or Cigarettes if Any not available)
Most adjusted

FEMALES

	absent	1-19k1	6-29k20	21-39k30	31-49k40	41-998k50	51+k999	Total
		Duration of smoking (narrow categories)						
N	17	1		3	2			23
NS	9	1		3	2			15
Wt	232.37	1.76		93.73	55.18			383.04
Het Chi	285.88	0.00		95.91	10.57			533.56
Het df	16	0		2	1			22
Het P	***	N.S.		***	**			***
Fixed RR	3.70	0.49		8.58	17.82			5.65
RRl	3.25	0.11		7.01	13.68			5.11
RRu	4.21	2.15		10.51	23.20			6.24
P	+++	N.S.		+++	+++			+++
Random RR	2.27	0.49		4.72	10.42			2.72
RRl	1.27	0.11		0.87	2.47			1.61
RRu	4.07	2.15		25.49	43.96			4.61
P	++	N.S.		(+)	++			+++

Table 3I11 - 4

IESLC - Meta-analysis of Ever/current Smoking by Duration, Overview
Adenocarcinoma, Any Product (or Cigarettes if Any not available)
Least adjusted

REF	NRR	X	SEX	AGEL	AGEH	RACE	YF	LC TYPE	LOC	START	ST	NLC	R	VB	P	H	AD	SM	PRODUCT	exL	exH	S1	S2	DENOM	De
BARBON	717	x	m	0	0	all	-	a	Eu:wst	1979	CC	755	n	bl	y	y	0	ev	all/unsp	1	29	1	0	nev any	st
BARBON	718	x	m	0	0	all	-	a	Eu:wst	1979	CC	755	n	bl	y	y	0	ev	all/unsp	30	39	2	3	nev any	st
BARBON	719	x	m	0	0	all	-	a	Eu:wst	1979	CC	755	n	bl	y	y	0	ev	all/unsp	40	49	0	4	nev any	st
BARBON	720	x	m	0	0	all	-	a	Eu:wst	1979	CC	755	n	bl	y	y	0	ev	all/unsp	50	999	3	0	nev any	st
BUFFLE	509		m	0	0	wh	-	a	NAmer	1976	CC	943	n	bl	y	n	0	ev	cig+/-ot	1	33	1	0	nev cigs	or
BUFFLE	510		m	0	0	wh	-	a	NAmer	1976	CC	943	n	bl	y	n	0	ev	cig+/-ot	34	43	2	4	nev cigs	or
BUFFLE	511		m	0	0	wh	-	a	NAmer	1976	CC	943	n	bl	y	n	0	ev	cig+/-ot	44	49	0	0	nev cigs	or
BUFFLE	512		m	0	0	wh	-	a	NAmer	1976	CC	943	n	bl	y	n	0	ev	cig+/-ot	50	999	3	0	nev cigs	or
CHOI	566		m	0	0	all	-	a	As:oth	1985	CC	375	n	bl	n	n	0	ev	cig+/-ot	1	29	1	0	nev cigs	st
CHOI	567		m	0	0	all	-	a	As:oth	1985	CC	375	n	bl	n	n	0	ev	cig+/-ot	30	39	2	3	nev cigs	st
CHOI	568		m	0	0	all	-	a	As:oth	1985	CC	375	n	bl	n	n	0	ev	cig+/-ot	40	49	0	4	nev cigs	st
CHOI	569		m	0	0	all	-	a	As:oth	1985	CC	375	n	bl	n	n	0	ev	cig+/-ot	50	999	3	0	nev cigs	st
CHOI	578		f	0	0	all	-	a	As:oth	1985	CC	375	n	bl	n	n	0	ev	cig+/-ot	1	29	1	0	nev cigs	st
CHOI	579		f	0	0	all	-	a	As:oth	1985	CC	375	n	bl	n	n	0	ev	cig+/-ot	30	999	0	0	nev cigs	st
DAMBER	540		m	0	0	all	-	a+al+br	Eu:Sca	1972	CC	579	n	bl	y	n	1	ev	all/unsp	1	30	1	0	nev any	or
DAMBER	541		m	0	0	all	-	a+al+br	Eu:Sca	1972	CC	579	n	bl	y	n	1	ev	all/unsp	31	40	2	4	nev any	or
DAMBER	542		m	0	0	all	-	a+al+br	Eu:Sca	1972	CC	579	n	bl	y	n	1	ev	all/unsp	41	50	3	5	nev any	or
DAMBER	543		m	0	0	all	-	a+al+br	Eu:Sca	1972	CC	579	n	bl	y	n	1	ev	all/unsp	51	999	0	6	nev any	or
DORGAN	576		m	0	0	wh	-	a	NAmer	1980	CC	2026	n	bl	y	y	2	ev	cig+/-ot	1	34	1	0	nev any	ot
DORGAN	577		m	0	0	wh	-	a	NAmer	1980	CC	2026	n	bl	y	y	2	ev	cig+/-ot	35	999	0	0	nev any	ot
DORGAN	568		f	0	0	all	-	a	NAmer	1980	CC	2026	n	bl	y	y	3	ev	cig+/-ot	1	34	1	0	nev any	ot
DORGAN	569		f	0	0	all	-	a	NAmer	1980	CC	2026	n	bl	y	y	3	ev	cig+/-ot	35	999	0	0	nev any	ot
DOSEME	516		m	0	0	all	-	not q+s	Eu:bal	1979	CC	1210	n	bl	n	n	2	ev	cig+/-ot	1	10	0	1	nev cigs	or
DOSEME	517		m	0	0	all	-	not q+s	Eu:bal	1979	CC	1210	n	bl	n	n	2	ev	cig+/-ot	11	20	1	2	nev cigs	or
DOSEME	518		m	0	0	all	-	not q+s	Eu:bal	1979	CC	1210	n	bl	n	n	2	ev	cig+/-ot	21	999	0	0	nev cigs	or
GER	501	x	c	0	0	all	-	a	As:oth	1990	CC	141	n	ot	y	n	0	ev	all/unsp	1	30	1	0	nev any	st
GER	502	x	c	0	0	all	-	a	As:oth	1990	CC	141	n	ot	y	n	0	ev	all/unsp	31	999	0	0	nev any	st
HAENSZ	525		f	0	0	all	-	a	NAmer	1955	CC	158	n	bl	n	y	0	ev	cig+/-ot	1	14	0	1	nev any	st
HAENSZ	526		f	0	0	all	-	a	NAmer	1955	CC	158	n	bl	n	y	0	ev	cig+/-ot	15	999	0	0	nev any	st
JEDRYC	519	x	m	0	0	all	-	a	Eu:est	1980	CC	1630	n	bl	y	n	0	ev	cig+/-ot	1	19	0	1	nev any	st
JEDRYC	520	x	m	0	0	all	-	a	Eu:est	1980	CC	1630	n	bl	y	n	0	ev	cig+/-ot	20	29	1	2	nev any	st
JEDRYC	521	x	m	0	0	all	-	a	Eu:est	1980	CC	1630	n	bl	y	n	0	ev	cig+/-ot	30	39	2	3	nev any	st
JEDRYC	522	x	m	0	0	all	-	a	Eu:est	1980	CC	1630	n	bl	y	n	0	ev	cig+/-ot	40	49	0	4	nev any	st
JEDRYC	523	x	m	0	0	all	-	a	Eu:est	1980	CC	1630	n	bl	y	n	0	ev	cig+/-ot	50	999	3	0	nev any	st
JOLY	660		m	0	0	all	-	a	SCAmer	1978	CC	826	n	bl	n	n	0	ev	cig+/-ot	1	29	1	0	nev any	st
JOLY	661		m	0	0	all	-	a	SCAmer	1978	CC	826	n	bl	n	n	0	ev	cig+/-ot	30	39	2	3	nev any	st
JOLY	662		m	0	0	all	-	a	SCAmer	1978	CC	826	n	bl	n	n	0	ev	cig+/-ot	40	49	0	4	nev any	st
JOLY	663		m	0	0	all	-	a	SCAmer	1978	CC	826	n	bl	n	n	0	ev	cig+/-ot	50	999	3	0	nev any	st
JOLY	632		f	0	0	all	-	a	SCAmer	1978	CC	826	n	bl	n	n	0	ev	cig+/-ot	1	29	1	0	nev any	st
JOLY	633		f	0	0	all	-	a	SCAmer	1978	CC	826	n	bl	n	n	0	ev	cig+/-ot	30	39	2	3	nev any	st
JOLY	634		f	0	0	all	-	a	SCAmer	1978	CC	826	n	bl	n	n	0	ev	cig+/-ot	40	49	0	4	nev any	st
JOLY	635		f	0	0	all	-	a	SCAmer	1978	CC	826	n	bl	n	n	0	ev	cig+/-ot	50	999	3	0	nev any	st
KATSOU	515	x	f	0	0	all	-	a	Eu:bal	1987	CC	101	n	bl	n	n	0	cu	all/unsp	1	29	1	0	nev any	st
KATSOU	516	x	f	0	0	all	-	a	Eu:bal	1987	CC	101	n	bl	n	n	0	cu	all/unsp	30	999	0	0	nev any	st
LUBIN2	700		m	0	0	all	-	a	Eu:mul	1976	CC	7804	n	bl	n	y	0	ev	cig+/-ot	1	29	1	0	nev any	st
LUBIN2	701		m	0	0	all	-	a	Eu:mul	1976	CC	7804	n	bl	n	y	0	ev	cig+/-ot	30	39	2	3	nev any	st
LUBIN2	702		m	0	0	all	-	a	Eu:mul	1976	CC	7804	n	bl	n	y	0	ev	cig+/-ot	40	49	0	4	nev any	st
LUBIN2	703		m	0	0	all	-	a	Eu:mul	1976	CC	7804	n	bl	n	y	0	ev	cig+/-ot	50	999	3	0	nev any	st
LUBIN2	752		f	0	0	all	-	a	Eu:mul	1976	CC	7804	n	bl	n	y	0	ev	cig+/-ot	1	29	1	0	nev any	st
LUBIN2	753		f	0	0	all	-	a	Eu:mul	1976	CC	7804	n	bl	n	y	0	ev	cig+/-ot	30	39	2	3	nev any	st
LUBIN2	754		f	0	0	all	-	a	Eu:mul	1976	CC	7804	n	bl	n	y	0	ev	cig+/-ot	40	49	0	4	nev any	st
LUBIN2	755		f	0	0	all	-	a	Eu:mul	1976	CC	7804	n	bl	n	y	0	ev	cig+/-ot	50	999	3	0	nev any	st
MATOS	641	x	m	0	0	all	-	a	SCAmer	1994	CC	200	n	bl	n	n	0	ev	cig+/-ot	1	24	1	0	nev any	st
MATOS	642	x	m	0	0	all	-	a	SCAmer	1994	CC	200	n	bl	n	n	0	ev	cig+/-ot	25	39	2	3	nev any	st
MATOS	643	x	m	0	0	all	-	a	SCAmer	1994	CC	200	n	bl	n	n	0	ev	cig+/-ot	40	70	3	0	nev any	st
OSANN2	513	x	f	0	0	all	-	KII	NAmer	1964	ot	217	n	bl	n	y	0	ev	cig+/-ot	1	20	1	0	nev cigs	st
OSANN2	514	x	f	0	0	all	-	KII	NAmer	1964	ot	217	n	bl	n	y	0	ev	cig+/-ot	21	999	0	0	nev cigs	st
PEZZOT	516		m	0	0	all	-	a	SCAmer	1987	CC	215	n	bl	n	y	0	ev	cig only	1	30	1	0	nev cigs	st
PEZZOT	517		m	0	0	all	-	a	SCAmer	1987	CC	215	n	bl	n	y	0	ev	cig only	31	40	2	4	nev cigs	st
PEZZOT	518		m	0	0	all	-	a	SCAmer	1987	CC	215	n	bl	n	y	0	ev	cig only	41	999	3	0	nev cigs	st
SOBUE	513		m	0	0	all	-	a	As:Jap	1986	CC	1376	n	bl	n	y	0	cu	cig+/-ot	1	29	1	0	nev cigs	st
SOBUE	514		m	0	0	all	-	a	As:Jap	1986	CC	1376	n	bl	n	y	0	cu	cig+/-ot	30	39	2	3	nev cigs	st
SOBUE	515		m	0	0	all	-	a	As:Jap	1986	CC	1376	n	bl	n	y	0	cu	cig+/-ot	40	49	0	4	nev cigs	st
SOBUE	516		m	0	0	all	-	a	As:Jap	1986	CC	1376	n	bl	n	y	0	cu	cig+/-ot	50	999	3	0	nev cigs	st
WUWILL	511	x	f	0	0	all	-	a	As:Chi	1985	CC	965	n	ot	n	n	0	ev	cig+/-ot	1	29	1	0	nev cigs	st
WUWILL	512	x	f	0	0	all	-	a	As:Chi	1985	CC	965	n	ot	n	n	0	ev	cig+/-ot	30	39	2	3	nev cigs	st
WUWILL	513	x	f	0	0	all	-	a	As:Chi	1985	CC	965	n	ot	n	n	0	ev	cig+/-ot	40	999	3	0	nev cigs	st
ZHENG	512		m	0	0	all	-	a	As:Chi	1982	CC	540	n	ot	*	y	0	ev	cig+/-ot	1	29	1	0	nev cigs	or
ZHENG	513		m	0	0	all	-	a	As:Chi	1982	CC	540	n	ot	*	y	0	ev	cig+/-ot	30	999	0	0	nev cigs	st
ZHENG	516		f	0	0	all	-	a	As:Chi	1982	CC	540	n	ot	*	y	0	ev	cig+/-ot	1	29	1	0	nev cigs	or
ZHENG	517		f	0	0	all	-	a	As:Chi	1982	CC	540	n	ot	*	y	0	ev	cig+/-ot	30	999	0	0	nev cigs	st

Table 3I11 - 4

IESLC - Meta-analysis of Ever/current Smoking by Duration, Overview
Adenocarcinoma, Any Product (or Cigarettes if Any not available)
Least adjusted

Cigarette type is all/unspec for all RRs

In this overview table, subtotals and Qs values may be invalid and should be ignored

Table 3I11 - 5

IESLC - Meta-analysis of Ever/current Smoking by Duration, Overview
Adenocarcinoma, Any Product (or Cigarettes if Any not available)
Least adjusted

REF	NRR	SEX	AD	Number Exposed Case	Cont	Non-exposed Case	Cont	RR	95.00%CI
BARBON	717	m	0	15	91	7	188	4.43	(1.74- 11.24)
BARBON	718	m	0	23	102	7	188	6.06	(2.51- 14.60)
BARBON	719	m	0	46	139	7	188	8.89	(3.90- 20.28)
BARBON	720	m	0	67	235	7	188	7.66	(3.43- 17.07)
Subtotal BARBON								6.72	(4.38- 10.30)
BUFFLE	509	m	0	-	-	-	-	4.40	(1.70- 11.50)
BUFFLE	510	m	0	-	-	-	-	5.70	(2.20- 14.80)
BUFFLE	511	m	0	-	-	-	-	4.30	(1.60- 11.40)
BUFFLE	512	m	0	-	-	-	-	3.50	(1.30- 9.70)
Subtotal BUFFLE								4.44	(2.73- 7.22)
CHOI	566	m	0	17	221	7	95	1.04	(0.42- 2.60)
CHOI	567	m	0	13	160	7	65	0.75	(0.29- 1.98)
CHOI	568	m	0	12	64	7	95	2.54	(0.95- 6.81)
CHOI	569	m	0	4	20	7	95	2.71	(0.73- 10.16)
CHOI	578	f	0	2	23	48	164	0.30	(0.07- 1.31)
CHOI	579	f	0	2	3	48	164	2.28	(0.37- 14.03)
Subtotal CHOI								1.23	(0.78- 1.96)
DAMBER	540	m	1	-	-	16	-	1.80	(0.60- 5.40)
DAMBER	541	m	1	-	-	16	-	1.20	(0.20- 6.00)
DAMBER	542	m	1	-	-	16	-	3.40	(1.30- 9.10)
DAMBER	543	m	1	-	-	16	-	2.50	(0.90- 6.70)
Subtotal DAMBER								2.35	(1.35- 4.10)
DORGAN	576	m	2	-	-	-	-	2.14	(0.78- 5.84)
DORGAN	577	m	2	-	-	-	-	6.73	(2.66- 17.03)
DORGAN	568	f	3	-	-	-	-	2.43	(1.65- 3.59)
DORGAN	569	f	3	-	-	-	-	5.14	(3.63- 7.28)
Subtotal DORGAN								3.72	(2.92- 4.74)
DOSEME	516	m	2	5	-	24	-	0.80	(0.30- 2.50)
DOSEME	517	m	2	26	-	24	-	3.30	(2.20- 7.50)
DOSEME	518	m	2	69	-	24	-	4.10	(2.20- 7.50)
Subtotal DOSEME								2.96	(1.98- 4.42)
GER	501	c	0	14	51	37	149	1.11	(0.55- 2.21)
GER	502	c	0	21	98	37	149	0.86	(0.48- 1.56)
Subtotal GER								0.96	(0.61- 1.50)
HAENSZ	525	f	0	2	26	37	236	0.49	(0.11- 2.15)
HAENSZ	526	f	0	16	77	37	236	1.33	(0.70- 2.51)
Subtotal HAENSZ								1.13	(0.63- 2.04)
JEDRYC	519	m	0	2	68	7	289	1.21	(0.25- 5.98)
JEDRYC	520	m	0	15	160	7	289	3.87	(1.55- 9.69)
JEDRYC	521	m	0	28	231	7	289	5.00	(2.15- 11.66)
JEDRYC	522	m	0	30	223	7	289	5.55	(2.40- 12.88)
JEDRYC	523	m	0	17	214	7	289	3.28	(1.34- 8.05)
Subtotal JEDRYC								4.02	(2.63- 6.12)
JOLY	660	m	0	5	109	5	218	2.00	(0.57- 7.06)
JOLY	661	m	0	12	165	5	218	3.17	(1.10- 9.18)
JOLY	662	m	0	22	182	5	218	5.27	(1.96- 14.19)
JOLY	663	m	0	33	253	5	218	5.69	(2.18- 14.82)
JOLY	632	f	0	10	54	25	283	2.10	(0.95- 4.61)
JOLY	633	f	0	6	24	25	283	2.83	(1.06- 7.57)
JOLY	634	f	0	10	24	25	283	4.72	(2.03- 10.96)
JOLY	635	f	0	7	20	25	283	3.96	(1.53- 10.27)
Subtotal JOLY								3.51	(2.50- 4.92)
KATSOU	515	f	0	6	12	30	67	1.12	(0.38- 3.26)
KATSOU	516	f	0	9	6	30	67	3.35	(1.09- 10.26)
Subtotal KATSOU								1.89	(0.87- 4.09)
LUBIN2	700	m	0	131	2964	57	2616	2.03	(1.48- 2.78)
LUBIN2	701	m	0	242	3473	57	2616	3.20	(2.39- 4.29)
LUBIN2	702	m	0	208	2540	57	2616	3.76	(2.79- 5.06)
LUBIN2	703	m	0	90	1460	57	2616	2.83	(2.02- 3.97)
LUBIN2	752	f	0	174	229	138	1180	6.50	(4.99- 8.46)
LUBIN2	753	f	0	383	186	138	1180	17.61	(13.73- 22.58)
LUBIN2	754	f	0	284	118	138	1180	20.58	(15.59- 27.17)
LUBIN2	755	f	0	181	34	138	1180	45.52	(30.31- 68.36)
Subtotal LUBIN2								7.21	(6.49- 8.00)
MATOS	641	m	0	7	84	5	110	1.83	(0.56- 5.98)
MATOS	642	m	0	39	110	5	110	7.80	(2.96- 20.53)
MATOS	643	m	0	33	89	5	110	8.16	(3.06- 21.76)
Subtotal MATOS								5.49	(3.03- 9.96)
OSANN2	513	f	0	10	20	22	43	0.98	(0.39- 2.44)
OSANN2	514	f	0	51	20	22	43	4.98	(2.40- 10.33)
Subtotal OSANN2								2.65	(1.50- 4.69)

Table 3I11 - 5

IESLC - Meta-analysis of Ever/current Smoking by Duration, Overview
Adenocarcinoma, Any Product (or Cigarettes if Any not available)
Least adjusted

REF	NRR	SEX	AD	Number Exposed Case	Cont	Non-exposed Case	Cont	RR	95.00%CI
PEZZOT	516	m	0	11	134	3	116	3.17	(0.86- 11.65)
PEZZOT	517	m	0	22	82	3	116	10.37	(3.01- 35.81)
PEZZOT	518	m	0	27	101	3	116	10.34	(3.04- 35.09)
Subtotal PEZZOT								7.18	(3.49- 14.81)
SOBUE	513	m	0	33	119	27	128	1.31	(0.75- 2.32)
SOBUE	514	m	0	62	200	27	128	1.47	(0.89- 2.43)
SOBUE	515	m	0	96	174	27	128	2.62	(1.61- 4.24)
SOBUE	516	m	0	43	73	27	128	2.79	(1.59- 4.89)
Subtotal SOBUE								1.96	(1.51- 2.54)
WUWILL	511	f	0	34	139	172	601	0.85	(0.57- 1.29)
WUWILL	512	f	0	52	98	172	601	1.85	(1.27- 2.70)
WUWILL	513	f	0	52	114	172	601	1.59	(1.10- 2.31)
Subtotal WUWILL								1.40	(1.12- 1.75)
ZHENG	512	m	0	24	75	29	94	1.04	(0.56- 1.93)
ZHENG	513	m	0	99	143	29	94	2.24	(1.38- 3.66)
ZHENG	516	f	0	9	17	119	184	0.82	(0.35- 1.90)
ZHENG	517	f	0	24	27	119	184	1.37	(0.76- 2.49)
Subtotal ZHENG								1.45	(1.07- 1.96)
Partial Totals				2987	15878	2419	24602		

*prospective study

REF	NRR	SEX	AD	Ys	Ws	Qs	Ps
BARBON	717	m	0	1.49	4.43	0.09	0.0017
BARBON	718	m	0	1.80	4.96	1.03	0.0001
BARBON	719	m	0	2.18	5.65	3.97	0.0000
BARBON	720	m	0	2.04	5.98	2.84	0.0000
Subtotal BARBON				1.90	21.01	7.94	
BUFFLE	509	m	0	1.48	4.20	0.08	0.0024
BUFFLE	510	m	0	1.74	4.23	0.66	0.0003
BUFFLE	511	m	0	1.46	3.99	0.05	0.0036
BUFFLE	512	m	0	1.25	3.80	0.03	0.0145
Subtotal BUFFLE				1.49	16.22	0.82	
CHOI	566	m	0	0.04	4.61	7.83	0.9264
CHOI	567	m	0	-0.28	4.14	10.97	0.5664
CHOI	568	m	0	0.93	3.96	0.67	0.0630
CHOI	569	m	0	1.00	2.21	0.27	0.1381
CHOI	578	f	0	-1.21	1.75	11.48	0.1081
CHOI	579	f	0	0.82	1.16	0.32	0.3748
Subtotal CHOI				0.21	17.84	31.54	
DAMBER	540	m	1	0.59	3.18	1.83	0.2943
DAMBER	541	m	1	0.18	1.33	1.80	0.8336
DAMBER	542	m	1	1.22	4.06	0.06	0.0137
DAMBER	543	m	1	0.92	3.81	0.70	0.0736
Subtotal DAMBER				0.85	12.38	4.39	
DORGAN	576	m	2	0.76	3.79	1.30	0.1385
DORGAN	577	m	2	1.91	4.46	1.40	0.0001
DORGAN	568	f	3	0.89	25.43	5.33	0.0000
DORGAN	569	f	3	1.64	31.73	2.69	0.0000
Subtotal DORGAN				1.31	65.41	10.72	
DOSEME	516	m	2	-0.22	3.42	8.41	0.6799
DOSEME	517	m	2	1.19	10.22	0.24	0.0001
DOSEME	518	m	2	1.41	10.22	0.04	0.0000
Subtotal DOSEME				1.08	23.85	8.69	
GER	501	c	0	0.10	8.01	12.43	0.7765
GER	502	c	0	-0.15	10.92	24.35	0.6261
Subtotal GER				-0.04	18.94	36.78	
HAENSZ	525	f	0	-0.71	1.76	7.43	0.3455
HAENSZ	526	f	0	0.28	9.37	10.61	0.3886
Subtotal HAENSZ				0.12	11.12	18.04	
JEDRYC	519	m	0	0.19	1.51	2.01	0.8113
JEDRYC	520	m	0	1.35	4.56	0.00	0.0038
JEDRYC	521	m	0	1.61	5.37	0.38	0.0002
JEDRYC	522	m	0	1.71	5.43	0.74	0.0001
JEDRYC	523	m	0	1.19	4.77	0.12	0.0095
Subtotal JEDRYC				1.39	21.64	3.24	
JOLY	660	m	0	0.69	2.42	1.03	0.2812
JOLY	661	m	0	1.15	3.40	0.13	0.0333
JOLY	662	m	0	1.66	3.91	0.39	0.0010
JOLY	663	m	0	1.74	4.19	0.64	0.0004
JOLY	632	f	0	0.74	6.17	2.26	0.0660

Table 3I11 - 5

IESLC - Meta-analysis of Ever/current Smoking by Duration, Overview
Adenocarcinoma, Any Product (or Cigarettes if Any not available)
Least adjusted

REF	NRR	SEX	AD	Ys	Ws	Qs	Ps
JOLY	633	f	0	1.04	3.97	0.37	0.0382
JOLY	634	f	0	1.55	5.40	0.23	0.0003
JOLY	635	f	0	1.38	4.23	0.00	0.0046
Subtotal JOLY				1.25	33.69	5.06	
KATSOU	515	f	0	0.11	3.35	5.12	0.8399
KATSOU	516	f	0	1.21	3.07	0.06	0.0342
Subtotal KATSOU				0.64	6.42	5.17	
LUBIN2	700	m	0	0.71	38.61	15.74	0.0000
LUBIN2	701	m	0	1.16	44.75	1.50	0.0000
LUBIN2	702	m	0	1.32	43.24	0.02	0.0000
LUBIN2	703	m	0	1.04	33.64	3.15	0.0000
LUBIN2	752	f	0	1.87	54.92	15.17	0.0000
LUBIN2	753	f	0	2.87	62.18	144.15	0.0000
LUBIN2	754	f	0	3.02	49.78	140.25	0.0000
LUBIN2	755	f	0	3.82	23.24	142.06	0.0000
Subtotal LUBIN2				1.98	350.37	462.05	
MATOS	641	m	0	0.61	2.75	1.50	0.3150
MATOS	642	m	0	2.05	4.10	2.06	0.0000
MATOS	643	m	0	2.10	3.99	2.26	0.0000
Subtotal MATOS				1.70	10.84	5.82	
OSANN2	513	f	0	-0.02	4.57	8.57	0.9608
OSANN2	514	f	0	1.61	7.23	0.49	0.0000
Subtotal OSANN2				0.98	11.80	9.06	
PEZZOT	516	m	0	1.16	2.27	0.08	0.0817
PEZZOT	517	m	0	2.34	2.50	2.47	0.0002
PEZZOT	518	m	0	2.34	2.57	2.52	0.0002
Subtotal PEZZOT				1.97	7.34	5.07	
SOBUE	513	m	0	0.27	11.97	13.76	0.3439
SOBUE	514	m	0	0.39	15.16	13.99	0.1339
SOBUE	515	m	0	0.96	16.39	2.42	0.0001
SOBUE	516	m	0	1.03	12.22	1.24	0.0003
Subtotal SOBUE				0.67	55.74	31.41	
WUWILL	511	f	0	-0.16	22.68	51.23	0.4546
WUWILL	512	f	0	0.62	27.09	14.37	0.0013
WUWILL	513	f	0	0.47	28.18	21.81	0.0133
Subtotal WUWILL				0.34	77.96	87.41	
ZHENG	512	m	0	0.04	9.99	17.12	0.9080
ZHENG	513	m	0	0.81	16.07	4.64	0.0012
ZHENG	516	f	0	-0.20	5.44	13.00	0.6405
ZHENG	517	f	0	0.32	10.81	11.41	0.2958
Subtotal ZHENG				0.37	42.31	46.18	

N	71
NS	18

Table 3I11 - 6

IESLC - Meta-analysis of Ever/current Smoking by Duration, Overview
Adenocarcinoma, Any Product (or Cigarettes if Any not available)
Least adjusted

	Sex			
	combined	male	female	Total
N	2	46	23	71
NS	1	13	9	23

In this overview table, other than the "N" rows, entries in the "absent" and "Total" columns may be invalid and should be ignored

	Duration of smoking (broad categories)				
	absent	1-34k20	21-49k35	36+k50	Total
N	23	22	13	13	71
NS	15	17	11	11	54
Wt	253.27	235.34	183.19	133.08	804.88
Het Chi	217.28	124.57	178.67	171.30	779.39
Het df	22	21	12	12	70
Het P	***	***	***	***	***
Fixed RR	4.48	2.31	5.12	4.72	3.84
RRl	3.96	2.03	4.43	3.99	3.58
RRu	5.07	2.63	5.92	5.60	4.12
P	+++	+++	+++	+++	+++
Random RR	3.10	1.82	3.68	4.89	2.94
RRl	2.04	1.29	1.95	2.45	2.30
RRu	4.71	2.57	6.92	9.75	3.75
P	+++	+++	+++	+++	+++

	Duration of smoking (narrow categories)							
	absent	1-19k1	6-29k20	21-39k30	31-49k40	41-998k50	51+k999	Total
N	43	3	2	10	11	1	1	71
NS	18	3	2	8	9	1	1	41
Wt	458.60	6.69	14.78	175.13	141.82	4.06	3.81	804.88
Het Chi	385.42	0.68	0.08	174.58	101.01	0.00	0.00	779.39
Het df	42	2	1	9	10	0	0	70
Het P	***	N.S.	N.S.	***	***	N.S.	N.S.	***
Fixed RR	2.94	0.77	3.47	5.12	7.06	3.40	2.50	3.84
RRl	2.69	0.36	2.08	4.41	5.99	1.29	0.92	3.58
RRu	3.22	1.65	5.77	5.93	8.33	9.00	6.82	4.12
P	+++	N.S.	+++	+++	+++	+	(+)	+++
Random RR	2.60	0.77	3.47	3.49	5.22	3.40	2.50	2.94
RRl	1.94	0.36	2.08	1.69	2.85	1.29	0.92	2.30
RRu	3.49	1.65	5.77	7.21	9.54	9.00	6.82	3.75
P	+++	N.S.	+++	+++	+++	+	(+)	+++

Table 3I11 - 6

IESLC - Meta-analysis of Ever/current Smoking by Duration, Overview
Adenocarcinoma, Any Product (or Cigarettes if Any not available)
Least adjusted

MALES

	Duration of smoking (broad categories)				
	absent	1-34k20	21-49k35	36+k50	Total
N	13	13	10	10	46
NS	11	13	10	10	44
Wt	122.06	103.00	89.94	77.43	392.43
Het Chi	23.72	18.86	30.09	12.78	105.33
Het df	12	12	9	9	45
Het P	*	(*)	***	N.S.	***
Fixed RR	3.41	2.03	3.04	3.59	2.93
RRl	2.85	1.68	2.47	2.87	2.65
RRu	4.07	2.47	3.74	4.49	3.23
P	+++	+++	+++	+++	+++
Random RR	3.40	2.10	3.35	3.98	3.09
RRl	2.56	1.60	2.12	2.94	2.61
RRu	4.53	2.76	5.30	5.37	3.67
P	+++	+++	+++	+++	+++

	Duration of smoking (narrow categories)							
	absent	1-19k1	6-29k20	21-39k30	31-49k40	41-998k50	51+k999	Total
N	24	2	2	7	9	1	1	46
NS	13	2	2	7	9	1	1	34
Wt	196.33	4.93	14.78	81.88	86.64	4.06	3.81	392.43
Het Chi	51.82	0.18	0.08	23.27	13.11	0.00	0.00	105.33
Het df	23	1	1	6	8	0	0	45
Het P	***	N.S.	N.S.	***	N.S.	N.S.	N.S.	***
Fixed RR	2.64	0.91	3.47	2.88	3.92	3.40	2.50	2.93
RRl	2.29	0.38	2.08	2.32	3.17	1.29	0.92	2.65
RRu	3.03	2.20	5.77	3.58	4.84	9.00	6.82	3.23
P	+++	N.S.	+++	+++	+++	+	(+)	+++
Random RR	2.92	0.91	3.47	3.01	4.21	3.40	2.50	3.09
RRl	2.31	0.38	2.08	1.80	3.04	1.29	0.92	2.61
RRu	3.69	2.20	5.77	5.03	5.84	9.00	6.82	3.67
P	+++	N.S.	+++	+++	+++	+	(+)	+++

FEMALES

	Duration of smoking (broad categories)				
	absent	1-34k20	21-49k35	36+k50	Total
N	9	8	3	3	23
NS	8	8	3	3	22
Wt	120.29	124.32	93.25	55.65	393.52
Het Chi	132.66	96.66	100.59	144.54	563.66
Het df	8	7	2	2	22
Het P	***	***	***	***	***
Fixed RR	6.88	2.70	8.47	6.92	5.38
RRl	5.75	2.26	6.91	5.32	4.88
RRu	8.22	3.22	10.38	9.00	5.94
P	+++	+++	+++	+++	+++
Random RR	3.17	1.40	4.60	6.64	2.76
RRl	1.38	0.66	0.81	0.58	1.62
RRu	7.29	2.96	26.10	76.18	4.69
P	++	N.S.	(+)	N.S.	+++

Table 3I11 - 6

IESLC - Meta-analysis of Ever/current Smoking by Duration, Overview
Adenocarcinoma, Any Product (or Cigarettes if Any not available)
Least adjusted

FEMALES

		Duration of smoking (narrow categories)						
	absent	1-19k1	6-29k20	21-39k30	31-49k40	41-998k50	51+k999	Total
N	17	1		3	2			23
NS	9	1		3	2			15
Wt	243.34	1.76		93.25	55.18			393.52
Het Chi	299.61	0.00		100.59	10.57			563.66
Het df	16	0		2	1			22
Het P	***	N.S.		***	**			***
Fixed RR	3.51	0.49		8.47	17.82			5.38
RRl	3.09	0.11		6.91	13.68			4.88
RRu	3.98	2.15		10.38	23.20			5.94
P	+++	N.S.		+++	+++			+++
Random RR	2.33	0.49		4.60	10.42			2.76
RRl	1.30	0.11		0.81	2.47			1.62
RRu	4.16	2.15		26.10	43.96			4.69
P	++	N.S.		(+)	++			+++

Table 3I11 - 7

IESLC - Meta-analysis of Ever/current Smoking by Duration, Overview
Adenocarcinoma, Any Product (or Cigarettes if Any not available)
Excluded studies (and stage at which they were excluded)

1	BECHER BLOT1 BROWN3 CARPEN CHYOU DARBY DOLL2 GARCIA GRAHAM GURSEL HAMMO2 JAHN JAIN LAUSSM PRESCO QIAO TVERDA WIGLE WYNDE3
2	ALDERS BENSHL BRESLO CHIAZZ DEAN3 DORN ENGELA GAO2 GILLIS GUO HEGMAN HIRAYA HOLE KAUFMA KOO KOULUM LIU4 MIGRAN MRFITR PERNU SEGI2 SPEIZE SUZUK2 SVENSS VUTUC WAKAI WU YUAN
3	GENG MCDUFF SPITZ STASZE WU2 ZHANG
4	AGUDO AKIBA AMANDU AMES ARMADA AUVINE AXELSS BEST BOFFET BOUCHA BOUCOT BROSS CEDERL CHEN2 CORREA CPSI CPSII DEAN2 DESTEF DOLL FAN GAO GARSHI HAMMON HU HU2 HUMBLE JUSSAW KAISE2 KREUZE LETOUR LEVIN LIAW LIU3 LIU5 LUO MCCONN NOTAN2 PEZZO2 PISANI QIAO2 RACHTA RESTRE SADOWS TIZZAN WANG2 WATSON WYNDE2 WYNDE6 WYNDE7 WYNDE8 ZHOU
5	CHEN LUBIN XU
10	KHUDER
14	BENHAM

Table 3I11 - 8
Potentially overlapping studies

REF	REFGP	PRINC	. OVERLAP/LINK
LUBIN2	LUBIN2	1	Lubin-combined
OSANN2	KAISER	2	KAISER/OSANN2

Table 3I11 - 9
Most adjusted - insufficient data for meta-analysis

REF	NRR	SEX	AGEL	AGEH	RACE	YF	LC TYPE	LOC	START	ST	NLC	R	VB	P	H	AD	SM	PRODUCT	exL	exH	S1	S2	DENOM	De
CHEN	509	c	0	0	all	-	a	As:oth	1987	CC	323	n	ot	n	y	2	ev	cig+/-ot	1	20	1	0	nev cigs	ot
CHEN	510	c	0	0	all	-	a	As:oth	1987	CC	323	n	ot	n	y	2	ev	cig+/-ot	21	30	0	3	nev cigs	ot
CHEN	511	c	0	0	all	-	a	As:oth	1987	CC	323	n	ot	n	y	2	ev	cig+/-ot	31	40	2	4	nev cigs	ot
CHEN	512	c	0	0	all	-	a	As:oth	1987	CC	323	n	ot	n	y	2	ev	cig+/-ot	41	999	3	0	nev cigs	ot
XU	506	m	0	0	all	-	a	As:Chi	1985	CC	729	n	ot	n	n	2	ev	all/unsp	1	29	1	0	nev any	or
XU	507	m	0	0	all	-	a	As:Chi	1985	CC	729	n	ot	n	n	2	ev	all/unsp	30	39	2	3	nev any	or
XU	508	m	0	0	all	-	a	As:Chi	1985	CC	729	n	ot	n	n	2	ev	all/unsp	40	999	3	0	nev any	or

REF	NRR	RR	SIG	RRDATA comment
CHEN	509	1.23	n	0
CHEN	510	1.52	n	0
CHEN	511	1.60	n	0
CHEN	512	3.79	y	p<0.001
XU	506	*		RR for 1-19/day is 1.4(NS), for 20-29/ day is 0.7(p<0.05) and for >=30/day is 5.4(p<0.05)
XU	507	*		RR for 1-19/day is 2.2(p<0.05), for 20-29/day is 1.5(NS) and for >=30/day is 3.2(p<0.05)
XU	508	*		RR for 1-19/day is 2.6(p<0.05), for 20-29/day is 3.6(p<0.05) and for >=30/ day is 11.8(p<0.05)

Table 3I12 -

IESLC - Meta-analysis of Ever/current Smoking, Duration, "Low"
Adenocarcinoma, Any Product (or Cigarettes if Any not available)

This analysis is restricted to results for:
1) Ever/current smokers
2) Results by Duration
3) Categorical results by Duration
4) Adenocarcinoma (or near equivalent)
5) Results complete enough for use in metaanalysis

Within each study, results are then selected (in the following order of preference, within each sex) for:
6) SMKSTA: ever, current
7) PRODUCT: all/unspec, cigarettes regardless of other products, cigarettes only
8) CIGTYPE: all/unspecified, MC regardless of HR, MC only
9) (not applicable)
10) DENOM: never smoked anything, never smoked cigarettes, never any + low, never cigs + low
11) Followup period (YF, prospective studies): whole study (coded as 0) or longest available
12) LCtype: adeno or nearest available, but not squamous. (q = squamous, s = small,
a = adeno, l = large, KII = Kreyberg II, al = alveolar, br = bronchiolar, u = undifferentiated)
13) Race: all or nearest available, otherwise by race (wh or w = white, bl or b = black, hi = hispanic
ch = chinese, jap = japanese, haw = hawaiian, w+o = white + oriental, sca = scandinavian, as = asian)
14) Duration "low" in key scheme 1 (key value 20, maximum range 1-34)
15) For overlapping studies: principal rather than subsidiary studies
Finally by Age: whole study (coded as 0) if available, otherwise by widest available age group
and then for single sex results (m, f) in preference to results for both sexes combined (c).

Results adjusted (AD) for the most potential confounders are then chosen in Sections -1 to -3
and results adjusted for the least confounders in Sections -4 to -6. (Those least adjusted results which
actually differ from the most adjusted are marked 'x' in column X in Section -4)

Section -7 shows excluded studies, together with the stage (as above) at which no qualifying
results were found.

Section -8 lists the potentially overlapping studies which have been included (1=principal, 2=subsidiary).

Section -9 lists any results which would have been included in preference except that they had data not complete
enough for use in meta-analysis, with their significance (yes/no), if known, and any further comment as entered
on the database. It also lists as "gap" any categories for which no data were presented by the original authors.

In addition to those mentioned above, the following fields, levels and abbreviations are used:
* or nk = not known, n = no, y = yes, ot = other
ev = ever, cu = current, nev = never
all/unspec = all or unspecified, cig+/-ot = cigarettes irrespective of other products (cigar, pipe etc)
MC = manufactured cigarettes, HR = hand-rolled cigarettes
exL, exH = range of exposure (low and high) in the smoking group, in terms of Duration
REF: 6-character study reference
NRR: number of the RR on the database within the study
ST : study type (CC = case control, pr or prosp = prospective)
NLC: number of lung cancer cases in whole study
R : risky occupational population (n = no, m = mining, o = other risky)
VB : national cigarette type (V = at least 75% Virginia, bl = at least 75% blended, ot = other)
P : any proxy use
H : full histological confirmation
De : derivation of RR/CI (or = original, st = standard method, ot = other method of estimation)

Table 3I12 - 1

IESLC - Meta-analysis of Ever/current Smoking, Duration, "Low"
Adenocarcinoma, Any Product (or Cigarettes if Any not available)
Most adjusted

REF	NRR	SEX	AGEL	AGEH	RACE	YF	LC TYPE	LOC	START	ST	NLC	R	VB	P	H	AD	SM	PRODUCT	exL	exH	DENOM	De
BARBON	724	m	0	0	all	-	a	Eu:wst	1979	CC	755	n	bl	y	y	1	ev	all/unsp	1	29	nev any	or
BUFFLE	509	m	0	0	wh	-	a	NAmer	1976	CC	943	n	bl	y	n	0	ev	cig+/-ot	1	33	nev cigs	or
CHOI	566	m	0	0	all	-	a	As:oth	1985	CC	375	n	bl	n	n	0	ev	cig+/-ot	1	29	nev cigs	st
CHOI	578	f	0	0	all	-	a	As:oth	1985	CC	375	n	bl	n	n	0	ev	cig+/-ot	1	29	nev cigs	st
DAMBER	540	m	0	0	all	-	a+al+br	Eu:Sca	1972	CC	579	n	bl	y	n	1	ev	all/unsp	1	30	nev any	or
DORGAN	576	m	0	0	wh	-	a	NAmer	1980	CC	2026	n	bl	y	y	2	ev	cig+/-ot	1	34	nev any	ot
DORGAN	568	f	0	0	all	-	a	NAmer	1980	CC	2026	n	bl	y	y	3	ev	cig+/-ot	1	34	nev any	ot
DOSEME	517	m	0	0	all	-	not q+s	Eu:bal	1979	CC	1210	n	bl	n	n	2	ev	cig+/-ot	11	20	nev cigs	or
GER	507	c	0	0	all	-	a	As:oth	1990	CC	141	n	ot	y	n	5	ev	all/unsp	1	30	nev any	ot
JEDRYC	520	m	0	0	all	-	a	Eu:est	1980	CC	1630	n	bl	y	n	0	ev	cig+/-ot	20	29	nev any	st
JOLY	660	m	0	0	all	-	a	SCAmer	1978	CC	826	n	bl	n	n	0	ev	cig+/-ot	1	29	nev any	st
JOLY	632	f	0	0	all	-	a	SCAmer	1978	CC	826	n	bl	n	n	0	ev	cig+/-ot	1	29	nev any	st
KATSOU	520	f	0	0	all	-	a	Eu:bal	1987	CC	101	n	bl	n	n	1	cu	all/unsp	1	29	nev any	or
LUBIN2	700	m	0	0	all	-	a	Eu:mul	1976	CC	7804	n	bl	n	y	0	ev	cig+/-ot	1	29	nev any	st
LUBIN2	752	f	0	0	all	-	a	Eu:mul	1976	CC	7804	n	bl	n	y	0	ev	cig+/-ot	1	29	nev any	st
MATOS	646	m	0	0	all	-	a	SCAmer	1994	CC	200	n	bl	n	n	2	ev	cig+/-ot	1	24	nev any	or
OSANN2	516	f	0	0	all	-	KII	NAmer	1964	ot	217	n	bl	n	y	1	ev	cig+/-ot	1	20	nev cigs	or
PEZZOT	516	m	0	0	all	-	a	SCAmer	1987	CC	215	n	bl	n	y	0	ev	cig only	1	30	nev cigs	st
SOBUE	513	m	0	0	all	-	a	As:Jap	1986	CC	1376	n	bl	n	y	0	cu	cig+/-ot	1	29	nev cigs	st
WUWILL	526	f	0	0	all	-	a	As:Chi	1985	CC	965	n	ot	n	n	3	ev	cig+/-ot	1	29	nev cigs	ot
ZHENG	512	m	0	0	all	-	a	As:Chi	1982	CC	540	n	ot	*	y	0	ev	cig+/-ot	1	29	nev cigs	or
ZHENG	516	f	0	0	all	-	a	As:Chi	1982	CC	540	n	ot	*	y	0	ev	cig+/-ot	1	29	nev cigs	or

Cigarette type is all/unspec for all RRs

Table 3I12 - 2

IESLC - Meta-analysis of Ever/current Smoking, Duration, "Low"
Adenocarcinoma, Any Product (or Cigarettes if Any not available)
Most adjusted

REF	NRR	SEX	AD	Number Exposed Case	Cont	Non-exposed Case	Cont	RR	95.00%CI
BARBON	724	m	1	15	-	7	-	3.70	(1.40- 9.70)
BUFFLE	509	m	0	-	-	-	-	4.40	(1.70- 11.50)
CHOI	566	m	0	17	221	7	95	1.04	(0.42- 2.60)
CHOI	578	f	0	2	23	48	164	0.30	(0.07- 1.31)
Subtotal CHOI								0.74	(0.34- 1.61)
DAMBER	540	m	1	-	-	16	-	1.80	(0.60- 5.40)
DORGAN	576	m	2	-	-	-	-	2.14	(0.78- 5.84)
DORGAN	568	f	3	-	-	-	-	2.43	(1.65- 3.59)
Subtotal DORGAN								2.39	(1.66- 3.43)
DOSEME	517	m	2	26	-	24	-	3.30	(2.20- 7.50)
GER	507	c	5	14	-	37	-	1.40	(0.62- 3.19)
JEDRYC	520	m	0	15	160	7	289	3.87	(1.55- 9.69)
JOLY	660	m	0	5	109	5	218	2.00	(0.57- 7.06)
JOLY	632	f	0	10	54	25	283	2.10	(0.95- 4.61)
Subtotal JOLY								2.07	(1.06- 4.04)
KATSOU	520	f	1	6	-	30	-	0.84	(0.27- 2.58)
LUBIN2	700	m	0	131	2964	57	2616	2.03	(1.48- 2.78)
LUBIN2	752	f	0	174	229	138	1180	6.50	(4.99- 8.46)
Subtotal LUBIN2								4.02	(3.28- 4.92)
MATOS	646	m	2	7	-	5	-	1.70	(0.50- 5.50)
OSANN2	516	f	1	10	-	22	-	0.70	(0.20- 1.90)
PEZZOT	516	m	0	11	134	3	116	3.17	(0.86- 11.65)
SOBUE	513	m	0	33	119	27	128	1.31	(0.75- 2.32)
WUWILL	526	f	3	34	-	172	-	0.80	(0.51- 1.25)
ZHENG	512	m	0	24	75	29	94	1.04	(0.56- 1.93)
ZHENG	516	f	0	9	17	119	184	0.82	(0.35- 1.90)
Subtotal ZHENG								0.95	(0.58- 1.57)
Partial Totals				543	4105	778	5367		

*prospective study

REF	NRR	SEX	AD	Ys	Ws	Qs	Ps
BARBON	724	m	1	1.31	4.10	0.84	0.0081
BUFFLE	509	m	0	1.48	4.20	1.65	0.0024
CHOI	566	m	0	0.04	4.61	3.04	0.9264
CHOI	578	f	0	-1.21	1.75	7.50	0.1081
Subtotal CHOI				-0.30	6.37	10.55	
DAMBER	540	m	1	0.59	3.18	0.23	0.2943
DORGAN	576	m	2	0.76	3.79	0.03	0.1385
DORGAN	568	f	3	0.89	25.43	0.03	0.0000
Subtotal DORGAN				0.87	29.22	0.06	
DOSEME	517	m	2	1.19	10.22	1.17	0.0001
GER	507	c	5	0.34	5.73	1.54	0.4207
JEDRYC	520	m	0	1.35	4.56	1.13	0.0038
JOLY	660	m	0	0.69	2.42	0.06	0.2812
JOLY	632	f	0	0.74	6.17	0.08	0.0660
Subtotal JOLY				0.73	8.59	0.15	
KATSOU	520	f	1	-0.17	3.02	3.20	0.7620
LUBIN2	700	m	0	0.71	38.61	0.85	0.0000
LUBIN2	752	f	0	1.87	54.92	56.69	0.0000
Subtotal LUBIN2				1.39	93.54	57.54	
MATOS	646	m	2	0.53	2.67	0.28	0.3857
OSANN2	516	f	1	-0.36	3.03	4.45	0.5346
PEZZOT	516	m	0	1.16	2.27	0.20	0.0817
SOBUE	513	m	0	0.27	11.97	4.05	0.3439
WUWILL	526	f	3	-0.22	19.12	22.24	0.3292
ZHENG	512	m	0	0.04	9.99	6.70	0.9080
ZHENG	516	f	0	-0.20	5.44	6.06	0.6405
Subtotal ZHENG				-0.05	15.43	12.76	

Table 3I12 - 2

IESLC - Meta-analysis of Ever/current Smoking, Duration, "Low"
Adenocarcinoma, Any Product (or Cigarettes if Any not available)
Most adjusted

	N	22
	NS	17
	Wt	227.21
Het	Chi	122.04
Het	df	21
Het	P	***
Fixed	RR	2.35
	RRl	2.07
	RRu	2.68
	P	+++
Random	RR	1.78
	RRl	1.26
	RRu	2.53
	P	++
Asymm	P	*

Table 3I12 - 3

IESLC - Meta-analysis of Ever/current Smoking, Duration, "Low"
Adenocarcinoma, Any Product (or Cigarettes if Any not available)
Most adjusted

		Sex		
	combined	male	female	Total
N	1	13	8	22
NS	1	13	8	22
Wt	5.73	102.60	118.88	227.21
Het Chi	0.00	17.69	97.18	122.04
Het df	0	12	7	21
Het P	N.S.	N.S.	***	***
Fixed RR	1.40	2.01	2.76	2.35
RRl	0.62	1.66	2.31	2.07
RRu	3.18	2.44	3.31	2.68
P	N.S.	+++	+++	+++
Random RR	1.40	2.06	1.29	1.78
RRl	0.62	1.58	0.60	1.26
RRu	3.18	2.68	2.82	2.53
P	N.S.	+++	N.S.	++
Between Chi				7.18
Between df				2
Between P				*
Btwn(F) P				N.S.
Btwn(R) P				N.S.

			Lung cancer type				
	a	a+l	a+al+br	KII	not q+u	not q+s	Total
N	19		1	1		1	22
NS	14		1	1		1	17
Wt	210.78		3.18	3.03		10.22	227.21
Het Chi	116.19		0.00	0.00		0.00	122.04
Het df	18		0	0		0	21
Het P	***		N.S.	N.S.		N.S.	***
Fixed RR	2.36		1.80	0.70		3.30	2.35
RRl	2.07		0.60	0.23		1.79	2.07
RRu	2.71		5.40	2.16		6.09	2.68
P	+++		N.S.	N.S.		+++	+++
Random RR	1.78		1.80	0.70		3.30	1.78
RRl	1.22		0.60	0.23		1.79	1.26
RRu	2.62		5.40	2.16		6.09	2.53
P	++		N.S.	N.S.		+++	++
Between Chi							5.86
Between df							3
Between P							N.S.
Btwn(F) P							N.S.
Btwn(R) P							N.S.

				Location					
	NAmer	UK	Scand	othEur	China	Japan	othAs	other	Total
N	4		1	6	3	1	3	4	22
NS	3		1	5	2	1	2	3	17
Wt	36.45		3.18	115.43	34.55	11.97	12.09	13.53	227.21
Het Chi	6.15		0.00	38.09	0.46	0.00	3.24	0.51	122.04
Het df	3		0	5	2	0	2	3	21
Het P	N.S.		N.S.	***	N.S.	N.S.	N.S.	N.S.	***
Fixed RR	2.32		1.80	3.77	0.87	1.31	1.00	2.14	2.35
RRl	1.67		0.60	3.14	0.62	0.75	0.57	1.26	2.07
RRu	3.20		5.40	4.53	1.21	2.32	1.76	3.64	2.68
P	+++		N.S.	+++	N.S.	N.S.	N.S.	++	+++
Random RR	2.16		1.80	3.02	0.87	1.31	0.92	2.14	1.78
RRl	1.20		0.60	1.65	0.62	0.75	0.44	1.26	1.26
RRu	3.88		5.40	5.54	1.21	2.32	1.95	3.64	2.53
P	++		N.S.	+++	N.S.	N.S.	N.S.	++	++
Between Chi									73.59
Between df									6
Between P									***
Btwn(F) P									*
Btwn(R) P									**

Table 3I12 - 3

IESLC - Meta-analysis of Ever/current Smoking, Duration, "Low"
Adenocarcinoma, Any Product (or Cigarettes if Any not available)
Most adjusted

Detailed Country in "other Europe"

	multi	Germany	othWest	East	Balkans	Total
N	2		1	1	2	6
NS	1		1	1	2	5
Wt	93.54		4.10	4.56	13.23	115.43
Het Chi	30.72		0.00	0.00	4.36	38.09
Het df	1		0	0	1	5
Het P	***		N.S.	N.S.	*	***
Fixed RR	4.02		3.70	3.87	2.42	3.77
RRl	3.28		1.41	1.55	1.41	3.14
RRu	4.92		9.74	9.69	4.14	4.53
P	+++		++	++	++	+++
Random RR	3.64		3.70	3.87	1.81	3.02
RRl	1.16		1.41	1.55	0.48	1.65
RRu	11.40		9.74	9.69	6.86	5.54
P	+		++	++	N.S.	+++
Between Chi						3.00
Between df						3
Between P						N.S.
Btwn(F) P						N.S.
Btwn(R) P						N.S.

Detailed Country in "other Asia"

	India	HongKong	other	Total
N			3	3
NS			2	2
Wt			12.09	12.09
Het Chi			3.24	3.24
Het df			2	2
Het P			N.S.	N.S.
Fixed RR			1.00	1.00
RRl			0.57	0.57
RRu			1.76	1.76
P			N.S.	N.S.
Random RR			0.92	0.92
RRl			0.44	0.44
RRu			1.95	1.95
P			N.S.	N.S.
Between Chi				
Between df				
Between P				N.S.
Btwn(F) P				N.S.
Btwn(R) P				N.S.

Detailed other continent

	SCAmer	Total
N	4	4
NS	3	3
Wt	13.53	13.53
Het Chi	0.51	0.51
Het df	3	3
Het P	N.S.	N.S.
Fixed RR	2.14	2.14
RRl	1.26	1.26
RRu	3.64	3.64
P	++	++
Random RR	2.14	2.14
RRl	1.26	1.26
RRu	3.64	3.64
P	++	++
Between Chi		
Between df		
Between P		N.S.
Btwn(F) P		N.S.
Btwn(R) P		N.S.

Table 3I12 - 3

IESLC - Meta-analysis of Ever/current Smoking, Duration, "Low"
Adenocarcinoma, Any Product (or Cigarettes if Any not available)
Most adjusted

	Start year of study <1960	1960-69	1970-79	1980-89	1990+	Total
N		1	8	11	2	22
NS		1	6	8	2	17
Wt		3.03	123.83	91.95	8.40	227.21
Het Chi		0.00	36.18	28.54	0.07	122.04
Het df		0	7	10	1	21
Het P		N.S.	***	**	N.S.	***
Fixed RR		0.70	3.70	1.39	1.49	2.35
RRl		0.23	3.10	1.13	0.76	2.07
RRu		2.16	4.41	1.70	2.93	2.68
P		N.S.	+++	++	N.S.	+++
Random RR		0.70	3.06	1.32	1.49	1.78
RRl		0.23	1.87	0.90	0.76	1.26
RRu		2.16	4.98	1.94	2.93	2.53
P		N.S.	+++	N.S.	N.S.	++
Between Chi						57.25
Between df						3
Between P						***
Btwn(F) P						**
Btwn(R) P						*

	Study type (1) CC	other	Total
N	21	1	22
NS	16	1	17
Wt	224.18	3.03	227.21
Het Chi	117.53	0.00	122.04
Het df	20	0	21
Het P	***	N.S.	***
Fixed RR	2.39	0.70	2.35
RRl	2.10	0.23	2.07
RRu	2.73	2.16	2.68
P	+++	N.S.	+++
Random RR	1.85	0.70	1.78
RRl	1.30	0.23	1.26
RRu	2.63	2.16	2.53
P	+++	N.S.	++
Between Chi			4.51
Between df			1
Between P			*
Btwn(F) P			N.S.
Btwn(R) P			N.S.

	Study type (2) CC	prosp	other	Total
N	21		1	22
NS	16		1	17
Wt	224.18		3.03	227.21
Het Chi	117.53		0.00	122.04
Het df	20		0	21
Het P	***		N.S.	***
Fixed RR	2.39		0.70	2.35
RRl	2.10		0.23	2.07
RRu	2.73		2.16	2.68
P	+++		N.S.	+++
Random RR	1.85		0.70	1.78
RRl	1.30		0.23	1.26
RRu	2.63		2.16	2.53
P	+++		N.S.	++
Between Chi				4.51
Between df				1
Between P				*
Btwn(F) P				N.S.
Btwn(R) P				N.S.

Table 3I12 - 3

IESLC - Meta-analysis of Ever/current Smoking, Duration, "Low"
Adenocarcinoma, Any Product (or Cigarettes if Any not available)
Most adjusted

	Study size (number of LC cases)				
	100-249	250-499	500-999	1000+	Total
N	5	2	8	7	22
NS	5	1	6	5	17
Wt	16.72	6.37	54.62	149.50	227.21
Het Chi	3.77	2.01	19.11	47.68	122.04
Het df	4	1	7	6	21
Het P	N.S.	N.S.	**	***	***
Fixed RR	1.30	0.74	1.31	3.27	2.35
RRl	0.80	0.34	1.00	2.79	2.07
RRu	2.10	1.61	1.71	3.84	2.68
P	N.S.	N.S.	+	+++	+++
Random RR	1.30	0.64	1.60	2.77	1.78
RRl	0.80	0.19	1.00	1.67	1.26
RRu	2.10	2.13	2.57	4.57	2.53
P	N.S.	N.S.	(+)	+++	++
Between Chi					49.47
Between df					3
Between P					***
Btwn(F) P					*
Btwn(R) P					(*)

	Risky occupational population			
	no	mining	othRisky	Total
N	22			22
NS	17			17
Wt	227.21			227.21
Het Chi	122.04			122.04
Het df	21			21
Het P	***			***
Fixed RR	2.35			2.35
RRl	2.07			2.07
RRu	2.68			2.68
P	+++			+++
Random RR	1.78			1.78
RRl	1.26			1.26
RRu	2.53			2.53
P	++			++
Between Chi				
Between df				
Between P				N.S.
Btwn(F) P				N.S.
Btwn(R) P				N.S.

	National cigarette tobacco type			
	Virginia	blended	other	Total
N		18	4	22
NS		14	3	17
Wt		186.93	40.28	227.21
Het Chi		77.98	1.60	122.04
Het df		17	3	21
Het P		***	N.S.	***
Fixed RR		2.87	0.93	2.35
RRl		2.49	0.68	2.07
RRu		3.32	1.26	2.68
P		+++	N.S.	+++
Random RR		2.10	0.93	1.78
RRl		1.47	0.68	1.26
RRu		3.00	1.26	2.53
P		+++	N.S.	++
Between Chi				42.47
Between df				1
Between P				***
Btwn(F) P				**
Btwn(R) P				***

Table 3I12 - 3

IESLC - Meta-analysis of Ever/current Smoking, Duration, "Low"
Adenocarcinoma, Any Product (or Cigarettes if Any not available)
Most adjusted

	Any proxy use No/nk	Yes	Total
N	15	7	22
NS	11	6	17
Wt	176.21	50.99	227.21
Het Chi	116.53	5.22	122.04
Het df	14	6	21
Het P	***	N.S.	***
Fixed RR	2.31	2.51	2.35
RRl	1.99	1.91	2.07
RRu	2.67	3.31	2.68
P	+++	+++	+++
Random RR	1.48	2.51	1.78
RRl	0.91	1.91	1.26
RRu	2.39	3.31	2.53
P	N.S.	+++	++
Between Chi			0.29
Between df			1
Between P			N.S.
Btwn(F) P			N.S.
Btwn(R) P			(*)

	Full histological confirmation No	Yes	Total
N	12	10	22
NS	10	7	17
Wt	67.65	159.56	227.21
Het Chi	30.10	74.81	122.04
Het df	11	9	21
Het P	**	***	***
Fixed RR	1.54	2.81	2.35
RRl	1.22	2.41	2.07
RRu	1.96	3.28	2.68
P	+++	+++	+++
Random RR	1.64	1.96	1.78
RRl	1.07	1.18	1.26
RRu	2.50	3.25	2.53
P	+	++	++
Between Chi			17.13
Between df			1
Between P			***
Btwn(F) P			(*)
Btwn(R) P			N.S.

	Number of adjustment variables (1) 0	1	2+/+nk	Total
N	12	4	6	22
NS	8	4	5	17
Wt	146.93	13.33	66.95	227.21
Het Chi	83.32	6.22	19.00	122.04
Het df	11	3	5	21
Het P	***	N.S.	**	***
Fixed RR	2.81	1.53	1.73	2.35
RRl	2.39	0.89	1.36	2.07
RRu	3.31	2.61	2.20	2.68
P	+++	N.S.	+++	+++
Random RR	1.88	1.46	1.77	1.78
RRl	1.12	0.67	1.05	1.26
RRu	3.14	3.17	2.98	2.53
P	+	N.S.	+	++
Between Chi				13.51
Between df				2
Between P				**
Btwn(F) P				N.S.
Btwn(R) P				N.S.

Table 3I12 - 3

IESLC - Meta-analysis of Ever/current Smoking, Duration, "Low"
Adenocarcinoma, Any Product (or Cigarettes if Any not available)
Most adjusted

	Number of adjustment variables (2) 0	1	2	3-5	6+/+nk	Total
N	12	4	3	3		22
NS	8	4	3	3		18
Wt	146.93	13.33	16.68	50.27		227.21
Het Chi	83.32	6.22	1.19	13.50		122.04
Het df	11	3	2	2		21
Het P	***	N.S.	N.S.	**		***
Fixed RR	2.81	1.53	2.69	1.50		2.35
RRl	2.39	0.89	1.66	1.13		2.07
RRu	3.31	2.61	4.35	1.97		2.68
P	+++	N.S.	+++	++		+++
Random RR	1.88	1.46	2.69	1.40		1.78
RRl	1.12	0.67	1.66	0.65		1.26
RRu	3.14	3.17	4.35	3.05		2.53
P	+	N.S.	+++	N.S.		++
Between Chi						17.82
Between df						3
Between P						***
Btwn(F) P						N.S.
Btwn(R) P						N.S.

	Smoking status ever	current	Total
N	20	2	22
NS	15	2	17
Wt	212.22	14.98	227.21
Het Chi	114.32	0.48	122.04
Het df	19	1	21
Het P	***	N.S.	***
Fixed RR	2.47	1.20	2.35
RRl	2.16	0.72	2.07
RRu	2.82	1.99	2.68
P	+++	N.S.	+++
Random RR	1.87	1.20	1.78
RRl	1.29	0.72	1.26
RRu	2.71	1.99	2.53
P	+++	N.S.	++
Between Chi			7.24
Between df			1
Between P			**
Btwn(F) P			N.S.
Btwn(R) P			N.S.

	Product all/unsp	cig+/-ot	cig only	Total
N	4	17	1	22
NS	4	12	1	17
Wt	16.03	208.91	2.27	227.21
Het Chi	4.20	115.94	0.00	122.04
Het df	3	16	0	21
Het P	N.S.	***	N.S.	***
Fixed RR	1.71	2.40	3.17	2.35
RRl	1.05	2.10	0.86	2.07
RRu	2.80	2.75	11.65	2.68
P	+	+++	(+)	+++
Random RR	1.71	1.75	3.17	1.78
RRl	0.95	1.17	0.86	1.26
RRu	3.07	2.63	11.65	2.53
P	(+)	++	(+)	++
Between Chi				1.90
Between df				2
Between P				N.S.
Btwn(F) P				N.S.
Btwn(R) P				N.S.

Table 3I12 - 3

IESLC - Meta-analysis of Ever/current Smoking, Duration, "Low"
Adenocarcinoma, Any Product (or Cigarettes if Any not available)
Most adjusted

		Denominator nev any	nev cigs	Total
	N	12	10	22
	NS	9	8	17
	Wt	154.60	72.61	227.21
Het	Chi	51.41	28.21	122.04
Het	df	11	9	21
Het	P	***	***	***
Fixed	RR	3.16	1.25	2.35
	RRl	2.70	0.99	2.07
	RRu	3.70	1.58	2.68
	P	+++	(+)	+++
Random	RR	2.37	1.29	1.78
	RRl	1.58	0.83	1.26
	RRu	3.56	2.00	2.53
	P	+++	N.S.	++
Between	Chi			42.42
Between	df			1
Between	P			***
Btwn(F)	P			**
Btwn(R)	P			*

		Derivation of RR/CI Orig	StdCalc	Other	Total
	N	9	9	4	22
	NS	8	6	3	17
	Wt	45.85	127.29	54.06	227.21
Het	Chi	20.01	62.52	13.95	122.04
Het	df	8	8	3	21
Het	P	*	***	**	***
Fixed	RR	1.72	3.16	1.53	2.35
	RRl	1.28	2.66	1.17	2.07
	RRu	2.29	3.76	2.00	2.68
	P	+++	+++	++	+++
Random	RR	1.67	2.03	1.52	1.78
	RRl	1.04	1.13	0.80	1.26
	RRu	2.69	3.66	2.90	2.53
	P	+	+	N.S.	++
Between	Chi				25.56
Between	df				2
Between	P				***
Btwn(F)	P				N.S.
Btwn(R)	P				N.S.

Table 3I12 - 4

IESLC - Meta-analysis of Ever/current Smoking, Duration, "Low"
Adenocarcinoma, Any Product (or Cigarettes if Any not available)
Least adjusted

REF	NRR	X	SEX	AGEL	AGEH	RACE	YF	LC TYPE	LOC	START	ST	NLC	R	VB	P	H	AD	SM	PRODUCT	exL	exH	DENOM	De
BARBON	717	x	m	0	0	all	-	a	Eu:wst	1979	CC	755	n	bl	y	y	0	ev	all/unsp	1	29	nev any	st
BUFFLE	509		m	0	0	wh	-	a	NAmer	1976	CC	943	n	bl	y	n	0	ev	cig+/-ot	1	33	nev cigs	or
CHOI	566		m	0	0	all	-	a	As:oth	1985	CC	375	n	bl	n	n	0	ev	cig+/-ot	1	29	nev cigs	st
CHOI	578		f	0	0	all	-	a	As:oth	1985	CC	375	n	bl	n	n	0	ev	cig+/-ot	1	29	nev cigs	st
DAMBER	540		m	0	0	all	-	a+al+br	Eu:Sca	1972	CC	579	n	bl	y	n	1	ev	all/unsp	1	30	nev any	or
DORGAN	576		m	0	0	wh	-	a	NAmer	1980	CC	2026	n	bl	y	y	2	ev	cig+/-ot	1	34	nev any	ot
DORGAN	568		f	0	0	all	-	a	NAmer	1980	CC	2026	n	bl	y	y	3	ev	cig+/-ot	1	34	nev any	ot
DOSEME	517		m	0	0	all	-	not q+s	Eu:bal	1979	CC	1210	n	bl	n	n	2	ev	cig+/-ot	11	20	nev cigs	or
GER	501	x	c	0	0	all	-	a	As:oth	1990	CC	141	n	ot	y	n	0	ev	all/unsp	1	30	nev any	st
JEDRYC	520		m	0	0	all	-	a	Eu:est	1980	CC	1630	n	bl	y	n	0	ev	cig+/-ot	20	29	nev any	st
JOLY	660		m	0	0	all	-	a	SCAmer	1978	CC	826	n	bl	n	n	0	ev	cig+/-ot	1	29	nev any	st
JOLY	632		f	0	0	all	-	a	SCAmer	1978	CC	826	n	bl	n	n	0	ev	cig+/-ot	1	29	nev any	st
KATSOU	515	x	f	0	0	all	-	a	Eu:bal	1987	CC	101	n	bl	n	n	0	cu	all/unsp	1	29	nev any	st
LUBIN2	700		m	0	0	all	-	a	Eu:mul	1976	CC	7804	n	bl	n	y	0	ev	cig+/-ot	1	29	nev any	st
LUBIN2	752		f	0	0	all	-	a	Eu:mul	1976	CC	7804	n	bl	n	y	0	ev	cig+/-ot	1	29	nev any	st
MATOS	641	x	m	0	0	all	-	a	SCAmer	1994	CC	200	n	bl	n	n	0	ev	cig+/-ot	1	24	nev any	st
OSANN2	513	x	f	0	0	all	-	KII	NAmer	1964	ot	217	n	bl	n	y	0	ev	cig+/-ot	1	20	nev cigs	st
PEZZOT	516		m	0	0	all	-	a	SCAmer	1987	CC	215	n	bl	n	y	0	ev	cig only	1	30	nev cigs	st
SOBUE	513		m	0	0	all	-	a	As:Jap	1986	CC	1376	n	bl	n	y	0	cu	cig+/-ot	1	29	nev cigs	st
WUWILL	511	x	f	0	0	all	-	a	As:Chi	1985	CC	965	n	ot	n	n	0	ev	cig+/-ot	1	29	nev cigs	st
ZHENG	512		m	0	0	all	-	a	As:Chi	1982	CC	540	n	ot	*	y	0	ev	cig+/-ot	1	29	nev cigs	or
ZHENG	516		f	0	0	all	-	a	As:Chi	1982	CC	540	n	ot	*	y	0	ev	cig+/-ot	1	29	nev cigs	or

Cigarette type is all/unspec for all RRs

Table 3I12 - 5

IESLC - Meta-analysis of Ever/current Smoking, Duration, "Low"
Adenocarcinoma, Any Product (or Cigarettes if Any not available)
Least adjusted

REF	NRR	SEX	AD	Number Exposed Case	Cont	Non-exposed Case	Cont	RR	95.00%CI
BARBON	717	m	0	15	91	7	188	4.43	(1.74- 11.24)
BUFFLE	509	m	0	-	-	-	-	4.40	(1.70- 11.50)
CHOI	566	m	0	17	221	7	95	1.04	(0.42- 2.60)
CHOI	578	f	0	2	23	48	164	0.30	(0.07- 1.31)
Subtotal CHOI								0.74	(0.34- 1.61)
DAMBER	540	m	1	-	-	16	-	1.80	(0.60- 5.40)
DORGAN	576	m	2	-	-	-	-	2.14	(0.78- 5.84)
DORGAN	568	f	3	-	-	-	-	2.43	(1.65- 3.59)
Subtotal DORGAN								2.39	(1.66- 3.43)
DOSEME	517	m	2	26	-	24	-	3.30	(2.20- 7.50)
GER	501	c	0	14	51	37	149	1.11	(0.55- 2.21)
JEDRYC	520	m	0	15	160	7	289	3.87	(1.55- 9.69)
JOLY	660	m	0	5	109	5	218	2.00	(0.57- 7.06)
JOLY	632	f	0	10	54	25	283	2.10	(0.95- 4.61)
Subtotal JOLY								2.07	(1.06- 4.04)
KATSOU	515	f	0	6	12	30	67	1.12	(0.38- 3.26)
LUBIN2	700	m	0	131	2964	57	2616	2.03	(1.48- 2.78)
LUBIN2	752	f	0	174	229	138	1180	6.50	(4.99- 8.46)
Subtotal LUBIN2								4.02	(3.28- 4.92)
MATOS	641	m	0	7	84	5	110	1.83	(0.56- 5.98)
OSANN2	513	f	0	10	20	22	43	0.98	(0.39- 2.44)
PEZZOT	516	m	0	11	134	3	116	3.17	(0.86- 11.65)
SOBUE	513	m	0	33	119	27	128	1.31	(0.75- 2.32)
WUWILL	511	f	0	34	139	172	601	0.85	(0.57- 1.29)
ZHENG	512	m	0	24	75	29	94	1.04	(0.56- 1.93)
ZHENG	516	f	0	9	17	119	184	0.82	(0.35- 1.90)
Subtotal ZHENG								0.95	(0.58- 1.57)
Partial Totals				543	4502	778	6525		

*prospective study

REF	NRR	SEX	AD	Ys	Ws	Qs	Ps
BARBON	717	m	0	1.49	4.43	1.87	0.0017
BUFFLE	509	m	0	1.48	4.20	1.74	0.0024
CHOI	566	m	0	0.04	4.61	2.92	0.9264
CHOI	578	f	0	-1.21	1.75	7.38	0.1081
Subtotal CHOI				-0.30	6.37	10.30	
DAMBER	540	m	1	0.59	3.18	0.20	0.2943
DORGAN	576	m	2	0.76	3.79	0.02	0.1385
DORGAN	568	f	3	0.89	25.43	0.06	0.0000
Subtotal DORGAN				0.87	29.22	0.09	
DOSEME	517	m	2	1.19	10.22	1.29	0.0001
GER	501	c	0	0.10	8.01	4.36	0.7765
JEDRYC	520	m	0	1.35	4.56	1.21	0.0038
JOLY	660	m	0	0.69	2.42	0.05	0.2812
JOLY	632	f	0	0.74	6.17	0.06	0.0660
Subtotal JOLY				0.73	8.59	0.11	
KATSOU	515	f	0	0.11	3.35	1.78	0.8399
LUBIN2	700	m	0	0.71	38.61	0.66	0.0000
LUBIN2	752	f	0	1.87	54.92	58.62	0.0000
Subtotal LUBIN2				1.39	93.54	59.28	
MATOS	641	m	0	0.61	2.75	0.15	0.3150
OSANN2	513	f	0	-0.02	4.57	3.39	0.9608
PEZZOT	516	m	0	1.16	2.27	0.23	0.0817
SOBUE	513	m	0	0.27	11.97	3.82	0.3439
WUWILL	511	f	0	-0.16	22.68	22.47	0.4546
ZHENG	512	m	0	0.04	9.99	6.42	0.9080
ZHENG	516	f	0	-0.20	5.44	5.87	0.6405
Subtotal ZHENG				-0.05	15.43	12.29	

Table 3I12 - 5

IESLC - Meta-analysis of Ever/current Smoking, Duration, "Low"
Adenocarcinoma, Any Product (or Cigarettes if Any not available)
Least adjusted

	N	22
	NS	17
	Wt	235.34
Het	Chi	124.57
Het	df	21
Het	P	***
Fixed	RR	2.31
	RRl	2.03
	RRu	2.63
	P	+++
Random	RR	1.82
	RRl	1.29
	RRu	2.57
	P	+++
Asymm	P	(*)

Table 3I12 - 6

IESLC - Meta-analysis of Ever/current Smoking, Duration, "Low"
Adenocarcinoma, Any Product (or Cigarettes if Any not available)
Least adjusted

		Sex			
		combined	male	female	Total
	N	1	13	8	22
	NS	1	13	8	22
	Wt	8.01	103.00	124.32	235.34
Het	Chi	0.00	18.86	96.66	124.57
Het	df	0	12	7	21
Het	P	N.S.	(*)	***	***
Fixed	RR	1.11	2.03	2.70	2.31
	RRl	0.55	1.68	2.26	2.03
	RRu	2.21	2.47	3.22	2.63
	P	N.S.	+++	+++	+++
Random	RR	1.11	2.10	1.40	1.82
	RRl	0.55	1.60	0.66	1.29
	RRu	2.21	2.76	2.96	2.57
	P	N.S.	+++	N.S.	+++
Between	Chi				9.04
Between	df				2
Between	P				*
Btwn(F)	P				N.S.
Btwn(R)	P				N.S.

Table 3I12 - 7

IESLC - Meta-analysis of Ever/current Smoking, Duration, "Low"
Adenocarcinoma, Any Product (or Cigarettes if Any not available)
Excluded studies (and stage at which they were excluded)

1	BECHER BLOT1 BROWN3 CARPEN CHYOU DARBY DOLL2 GARCIA GRAHAM GURSEL HAMMO2 JAHN JAIN LAUSSM PRESCO QIAO TVERDA WIGLE WYNDE3
2	ALDERS BENSHL BRESLO CHIAZZ DEAN3 DORN ENGELA GAO2 GILLIS GUO HEGMAN HIRAYA HOLE KAUFMA KOO KOULUM LIU4 MIGRAN MRFITR PERNU SEGI2 SPEIZE SUZUK2 SVENSS VUTUC WAKAI WU YUAN
3	GENG MCDUFF SPITZ STASZE WU2 ZHANG
4	AGUDO AKIBA AMANDU AMES ARMADA AUVINE AXELSS BEST BOFFET BOUCHA BOUCOT BROSS CEDERL CHEN2 CORREA CPSI CPSII DEAN2 DESTEF DOLL FAN GAO GARSHI HAMMON HU HU2 HUMBLE JUSSAW KAISE2 KREUZE LETOUR LEVIN LIAW LIU3 LIU5 LUO MCCONN NOTAN2 PEZZO2 PISANI QIAO2 RACHTA RESTRE SADOWS TIZZAN WANG2 WATSON WYNDE2 WYNDE6 WYNDE7 WYNDE8 ZHOU
5	CHEN LUBIN XU
10	KHUDER
14	BENHAM HAENSZ

Table 3I12 - 8
Potentially overlapping studies

REF	REFGP	PRINC	.	OVERLAP/LINK
LUBIN2	LUBIN2	1		Lubin-combined
OSANN2	KAISER	2		KAISER/OSANN2

Table 3I12 - 9
Most adjusted - insufficient data for meta-analysis

REF	NRR	SEX	AGEL	AGEH	RACE	YF	LC TYPE	LOC	START	ST	NLC	R	VB	P	H	AD	SM	PRODUCT	exL	exH	DENOM	De
CHEN	509	c	0	0	all	-	a	As:oth	1987	CC	323	n	ot	n	y	2	ev	cig+/-ot	1	20	nev cigs	ot
XU	506	m	0	0	all	-	a	As:Chi	1985	CC	729	n	ot	n	n	2	ev	all/unsp	1	29	nev any	or

REF	NRR	RR	SIG	RRDATA comment
CHEN	509	1.23	n	0
XU	506	*		RR for 1-19/day is 1.4(NS), for 20-29/ day is 0.7(p<0.05) and for >=30/day is 5.4(p<0.05)

Table 3I13 -

IESLC - Meta-analysis of Ever/current Smoking, Duration, "Mid"
Adenocarcinoma, Any Product (or Cigarettes if Any not available)

This analysis is restricted to results for:
1) Ever/current smokers
2) Results by Duration
3) Categorical results by Duration
4) Adenocarcinoma (or near equivalent)
5) Results complete enough for use in metaanalysis

Within each study, results are then selected (in the following order of preference, within each sex) for:
6) SMKSTA: ever, current
7) PRODUCT: all/unspec, cigarettes regardless of other products, cigarettes only
8) CIGTYPE: all/unspecified, MC regardless of HR, MC only
9) (not applicable)
10) DENOM: never smoked anything, never smoked cigarettes, never any + low, never cigs + low
11) Followup period (YF, prospective studies): whole study (coded as 0) or longest available
12) LCtype: adeno or nearest available, but not squamous. (q = squamous, s = small,
a = adeno, l = large, KII = Kreyberg II, al = alveolar, br = bronchiolar, u = undifferentiated)
13) Race: all or nearest available, otherwise by race (wh or w = white, bl or b = black, hi = hispanic
ch = chinese, jap = japanese, haw = hawaiian, w+o = white + oriental, sca = scandinavian, as = asian)
14) Duration "mid" in key scheme 1 (key value 35, maximum range 21-49)
15) For overlapping studies: principal rather than subsidiary studies
Finally by Age: whole study (coded as 0) if available, otherwise by widest available age group
and then for single sex results (m, f) in preference to results for both sexes combined (c).

Results adjusted (AD) for the most potential confounders are then chosen in Sections -1 to -3
and results adjusted for the least confounders in Sections -4 to -6. (Those least adjusted results which
actually differ from the most adjusted are marked 'x' in column X in Section -4)

Section -7 shows excluded studies, together with the stage (as above) at which no qualifying
results were found.

Section -8 lists the potentially overlapping studies which have been included (1=principal, 2=subsidiary).

Section -9 lists any results which would have been included in preference except that they had data not complete
enough for use in meta-analysis, with their significance (yes/no), if known, and any further comment as entered
on the database. It also lists as "gap" any categories for which no data were presented by the original authors.

In addition to those mentioned above, the following fields, levels and abbreviations are used:
* or nk = not known, n = no, y = yes, ot = other
ev = ever, cu = current, nev = never
all/unspec = all or unspecified, cig+/-ot = cigarettes irrespective of other products (cigar, pipe etc)
MC = manufactured cigarettes, HR = hand-rolled cigarettes
exL, exH = range of exposure (low and high) in the smoking group, in terms of Duration
REF: 6-character study reference
NRR: number of the RR on the database within the study
ST : study type (CC = case control, pr or prosp = prospective)
NLC: number of lung cancer cases in whole study
R : risky occupational population (n = no, m = mining, o = other risky)
VB : national cigarette type (V = at least 75% Virginia, bl = at least 75% blended, ot = other)
P : any proxy use
H : full histological confirmation
De : derivation of RR/CI (or = original, st = standard method, ot = other method of estimation)

Table 3I13 - 1

IESLC - Meta-analysis of Ever/current Smoking, Duration, "Mid"
Adenocarcinoma, Any Product (or Cigarettes if Any not available)
Most adjusted

REF	NRR	SEX	AGEL	AGEH	RACE	YF	LC TYPE	LOC	START	ST	NLC	R	VB	P	H	AD	SM	PRODUCT	exL	exH	DENOM	De
BARBON	725	m	0	0	all	-	a	Eu:wst	1979	CC	755	n	bl	y	y	1	ev	all/unsp	30	39	nev any	or
BUFFLE	510	m	0	0	wh	-	a	NAmer	1976	CC	943	n	bl	y	n	0	ev	cig+/-ot	34	43	nev cigs	or
CHOI	567	m	0	0	all	-	a	As:oth	1985	CC	375	n	bl	n	n	0	ev	cig+/-ot	30	39	nev cigs	st
DAMBER	541	m	0	0	all	-	a+al+br	Eu:Sca	1972	CC	579	n	bl	y	n	1	ev	all/unsp	31	40	nev any	or
JEDRYC	521	m	0	0	all	-	a	Eu:est	1980	CC	1630	n	bl	y	n	0	ev	cig+/-ot	30	39	nev any	st
JOLY	661	m	0	0	all	-	a	SCAmer	1978	CC	826	n	bl	n	n	0	ev	cig+/-ot	30	39	nev any	st
JOLY	633	f	0	0	all	-	a	SCAmer	1978	CC	826	n	bl	n	n	0	ev	cig+/-ot	30	39	nev any	st
LUBIN2	701	m	0	0	all	-	a	Eu:mul	1976	CC	7804	n	bl	n	y	0	ev	cig+/-ot	30	39	nev any	st
LUBIN2	753	f	0	0	all	-	a	Eu:mul	1976	CC	7804	n	bl	n	y	0	ev	cig+/-ot	30	39	nev any	st
MATOS	647	m	0	0	all	-	a	SCAmer	1994	CC	200	n	bl	n	n	2	ev	cig+/-ot	25	39	nev any	or
PEZZOT	517	m	0	0	all	-	a	SCAmer	1987	CC	215	n	bl	n	y	0	ev	cig only	31	40	nev cigs	st
SOBUE	514	m	0	0	all	-	a	As:Jap	1986	CC	1376	n	bl	n	y	0	cu	cig+/-ot	30	39	nev cigs	st
WUWILL	527	f	0	0	all	-	a	As:Chi	1985	CC	965	n	ot	n	n	3	ev	cig+/-ot	30	39	nev cigs	ot

Cigarette type is all/unspec for all RRs

Table 3I13 - 2

IESLC - Meta-analysis of Ever/current Smoking, Duration, "Mid"
Adenocarcinoma, Any Product (or Cigarettes if Any not available)
Most adjusted

REF	NRR	SEX	AD	Number Exposed Case	Cont	Non-exposed Case	Cont	RR	95.00%CI
BARBON	725	m	1	23	-	7	-	5.10	(2.10- 12.50)
BUFFLE	510	m	0	-	-	-	-	5.70	(2.20- 14.80)
CHOI	567	m	0	13	160	7	65	0.75	(0.29- 1.98)
DAMBER	541	m	1	-	-	16	-	1.20	(0.20- 6.00)
JEDRYC	521	m	0	28	231	7	289	5.00	(2.15- 11.66)
JOLY	661	m	0	12	165	5	218	3.17	(1.10- 9.18)
JOLY	633	f	0	6	24	25	283	2.83	(1.06- 7.57)
Subtotal JOLY								2.98	(1.45- 6.14)
LUBIN2	701	m	0	242	3473	57	2616	3.20	(2.39- 4.29)
LUBIN2	753	f	0	383	186	138	1180	17.61	(13.73- 22.58)
Subtotal LUBIN2								8.62	(7.13- 10.42)
MATOS	647	m	2	39	-	5	-	7.30	(2.70- 19.70)
PEZZOT	517	m	0	22	82	3	116	10.37	(3.01- 35.81)
SOBUE	514	m	0	62	200	27	128	1.47	(0.89- 2.43)
WUWILL	527	f	3	52	-	172	-	1.99	(1.37- 2.89)
Partial Totals				882	4521	469	4895		

*prospective study

REF	NRR	SEX	AD	Ys	Ws	Qs	Ps
BARBON	725	m	1	1.63	4.83	0.00	0.0003
BUFFLE	510	m	0	1.74	4.23	0.05	0.0003
CHOI	567	m	0	-0.28	4.14	15.22	0.5664
DAMBER	541	m	1	0.18	1.33	2.80	0.8336
JEDRYC	521	m	0	1.61	5.37	0.00	0.0002
JOLY	661	m	0	1.15	3.40	0.79	0.0333
JOLY	633	f	0	1.04	3.97	1.41	0.0382
Subtotal JOLY				1.09	7.37	2.19	
LUBIN2	701	m	0	1.16	44.75	10.00	0.0000
LUBIN2	753	f	0	2.87	62.18	94.55	0.0000
Subtotal LUBIN2				2.15	106.93	104.55	
MATOS	647	m	2	1.99	3.89	0.48	0.0001
PEZZOT	517	m	0	2.34	2.50	1.24	0.0002
SOBUE	514	m	0	0.39	15.16	23.69	0.1339
WUWILL	527	f	3	0.69	27.58	24.74	0.0003

	N	13
	NS	11
	Wt	183.33
Het	Chi	174.97
Het	df	12
Het	P	***
Fixed	RR	5.13
	RRl	4.44
	RRu	5.93
	P	+++
Random	RR	3.63
	RRl	1.94
	RRu	6.80
	P	+++
Asymm	P	N.S.

Table 3I13 - 3

IESLC - Meta-analysis of Ever/current Smoking, Duration, "Mid"
Adenocarcinoma, Any Product (or Cigarettes if Any not available)
Most adjusted

	Sex: combined	male	female	Total
N		10	3	13
NS		10	3	13
Wt		89.60	93.73	183.33
Het Chi		28.35	95.91	174.97
Het df		9	2	12
Het P		***	***	***
Fixed RR		3.00	8.58	5.13
RRl		2.44	7.01	4.44
RRu		3.69	10.51	5.93
P		+++	+++	+++
Random RR		3.26	4.72	3.63
RRl		2.08	0.87	1.94
RRu		5.09	25.49	6.80
P		+++	(+)	+++
Between Chi				50.71
Between df				1
Between P				***
Btwn(F) P				(*)
Btwn(R) P				N.S.

	Lung cancer type: a	a+l	a+al+br	KII	not q+u	not q+s	Total
N	12		1				13
NS	10		1				11
Wt	182.00		1.33				183.33
Het Chi	172.15		0.00				174.97
Het df	11		0				12
Het P	***		N.S.				***
Fixed RR	5.19		1.20				5.13
RRl	4.48		0.22				4.44
RRu	6.00		6.57				5.93
P	+++		N.S.				+++
Random RR	3.87		1.20				3.63
RRl	2.03		0.22				1.94
RRu	7.37		6.57				6.80
P	+++		N.S.				+++
Between Chi							2.82
Between df							1
Between P							(*)
Btwn(F) P							N.S.
Btwn(R) P							N.S.

	Location: NAmer	UK	Scand	othEur	China	Japan	othAs	other	Total
N	1		1	4	1	1	1	4	13
NS	1		1	3	1	1	1	3	11
Wt	4.23		1.33	117.13	27.58	15.16	4.14	13.76	183.33
Het Chi	0.00		0.00	78.39	0.00	0.00	0.00	3.86	174.97
Het df	0		0	3	0	0	0	3	12
Het P	N.S.		N.S.	***	N.S.	N.S.	N.S.	N.S.	***
Fixed RR	5.70		1.20	8.23	1.99	1.47	0.75	4.82	5.13
RRl	2.20		0.22	6.87	1.37	0.89	0.29	2.84	4.44
RRu	14.78		6.57	9.86	2.89	2.43	1.98	8.17	5.93
P	+++		N.S.	+++	+++	N.S.	N.S.	+++	+++
Random RR	5.70		1.20	6.25	1.99	1.47	0.75	4.87	3.63
RRl	2.20		0.22	2.11	1.37	0.89	0.29	2.67	1.94
RRu	14.78		6.57	18.57	2.89	2.43	1.98	8.89	6.80
P	+++		N.S.	+++	+++	N.S.	N.S.	+++	+++
Between Chi									92.72
Between df									6
Between P									***
Btwn(F) P									N.S.
Btwn(R) P									**

Table 3I13 - 3

IESLC - Meta-analysis of Ever/current Smoking, Duration, "Mid"
Adenocarcinoma, Any Product (or Cigarettes if Any not available)
Most adjusted

Detailed Country in "other Europe"

	multi	Germany	othWest	East	Balkans	Total
N	2		1	1		4
NS	1		1	1		3
Wt	106.93		4.83	5.37		117.13
Het Chi	75.72		0.00	0.00		78.39
Het df	1		0	0		3
Het P	***		N.S.	N.S.		***
Fixed RR	8.62		5.10	5.00		8.23
RRl	7.13		2.09	2.15		6.87
RRu	10.42		12.44	11.66		9.86
P	+++		+++	+++		+++
Random RR	7.52		5.10	5.00		6.25
RRl	1.41		2.09	2.15		2.11
RRu	40.00		12.44	11.66		18.57
P	+		+++	+++		+++
Between Chi						2.67
Between df						2
Between P						N.S.
Btwn(F) P						N.S.
Btwn(R) P						N.S.

Detailed Country in "other Asia"

	India	HongKong	other	Total
N			1	1
NS			1	1
Wt			4.14	4.14
Het Chi			0.00	0.00
Het df			0	0
Het P			N.S.	N.S.
Fixed RR			0.75	0.75
RRl			0.29	0.29
RRu			1.98	1.98
P			N.S.	N.S.
Random RR			0.75	0.75
RRl			0.29	0.29
RRu			1.98	1.98
P			N.S.	N.S.
Between Chi				
Between df				
Between P				N.S.
Btwn(F) P				N.S.
Btwn(R) P				N.S.

Detailed other continent

	SCAmer	Total
N	4	4
NS	3	3
Wt	13.76	13.76
Het Chi	3.86	3.86
Het df	3	3
Het P	N.S.	N.S.
Fixed RR	4.82	4.82
RRl	2.84	2.84
RRu	8.17	8.17
P	+++	+++
Random RR	4.87	4.87
RRl	2.67	2.67
RRu	8.89	8.89
P	+++	+++
Between Chi		
Between df		
Between P		N.S.
Btwn(F) P		N.S.
Btwn(R) P		N.S.

Table 3I13 - 3

IESLC - Meta-analysis of Ever/current Smoking, Duration, "Mid"
Adenocarcinoma, Any Product (or Cigarettes if Any not available)
Most adjusted

	Start year of study <1960	1960-69	1970-79	1980-89	1990+	Total
N			7	5	1	13
NS			5	5	1	11
Wt			124.69	54.74	3.89	183.33
Het Chi			89.54	16.67	0.00	174.97
Het df			6	4	0	12
Het P			***	**	N.S.	***
Fixed RR			7.66	2.01	7.30	5.13
RRl			6.43	1.54	2.70	4.44
RRu			9.13	2.62	19.72	5.93
P			+++	+++	+++	+++
Random RR			4.41	2.33	7.30	3.63
RRl			1.89	1.25	2.70	1.94
RRu			10.27	4.36	19.72	6.80
P			+++	++	+++	+++
Between Chi						68.76
Between df						2
Between P						***
Btwn(F) P						(*)
Btwn(R) P						N.S.

	Study type (1) CC	other	Total
N	13		13
NS	11		11
Wt	183.33		183.33
Het Chi	174.97		174.97
Het df	12		12
Het P	***		***
Fixed RR	5.13		5.13
RRl	4.44		4.44
RRu	5.93		5.93
P	+++		+++
Random RR	3.63		3.63
RRl	1.94		1.94
RRu	6.80		6.80
P	+++		+++
Between Chi			
Between df			
Between P			N.S.
Btwn(F) P			N.S.
Btwn(R) P			N.S.

	Study type (2) CC	prosp	other	Total
N	13			13
NS	11			11
Wt	183.33			183.33
Het Chi	174.97			174.97
Het df	12			12
Het P	***			***
Fixed RR	5.13			5.13
RRl	4.44			4.44
RRu	5.93			5.93
P	+++			+++
Random RR	3.63			3.63
RRl	1.94			1.94
RRu	6.80			6.80
P	+++			+++
Between Chi				
Between df				
Between P				N.S.
Btwn(F) P				N.S.
Btwn(R) P				N.S.

Table 3I13 - 3

IESLC - Meta-analysis of Ever/current Smoking, Duration, "Mid"
Adenocarcinoma, Any Product (or Cigarettes if Any not available)
Most adjusted

	Study size (number of LC cases) 100-249	250-499	500-999	1000+	Total
N	2	1	6	4	13
NS	2	1	5	3	11
Wt	6.39	4.14	45.34	127.46	183.33
Het Chi	0.19	0.00	7.71	117.82	174.97
Het df	1	0	5	3	12
Het P	N.S.	N.S.	N.S.	***	***
Fixed RR	8.38	0.75	2.55	6.83	5.13
RRl	3.86	0.29	1.91	5.74	4.44
RRu	18.19	1.98	3.42	8.12	5.93
P	+++	N.S.	+++	+++	+++
Random RR	8.38	0.75	2.93	4.54	3.63
RRl	3.86	0.29	1.89	1.36	1.94
RRu	18.19	1.98	4.54	15.19	6.80
P	+++	N.S.	+++	+	+++
Between Chi					49.25
Between df					3
Between P					***
Btwn(F) P					N.S.
Btwn(R) P					**

	Risky occupational population no	mining	othRisky	Total
N	13			13
NS	11			11
Wt	183.33			183.33
Het Chi	174.97			174.97
Het df	12			12
Het P	***			***
Fixed RR	5.13			5.13
RRl	4.44			4.44
RRu	5.93			5.93
P	+++			+++
Random RR	3.63			3.63
RRl	1.94			1.94
RRu	6.80			6.80
P	+++			+++
Between Chi				
Between df				
Between P				N.S.
Btwn(F) P				N.S.
Btwn(R) P				N.S.

	National cigarette tobacco type Virginia	blended	other	Total
N		12	1	13
NS		10	1	11
Wt		155.75	27.58	183.33
Het Chi		145.85	0.00	174.97
Het df		11	0	12
Het P		***	N.S.	***
Fixed RR		6.07	1.99	5.13
RRl		5.19	1.37	4.44
RRu		7.10	2.89	5.93
P		+++	+++	+++
Random RR		3.85	1.99	3.63
RRl		1.98	1.37	1.94
RRu		7.49	2.89	6.80
P		+++	+++	+++
Between Chi				29.12
Between df				1
Between P				***
Btwn(F) P				N.S.
Btwn(R) P				(*)

Table 3I13 - 3

IESLC - Meta-analysis of Ever/current Smoking, Duration, "Mid"
Adenocarcinoma, Any Product (or Cigarettes if Any not available)
Most adjusted

	Any proxy use No/nk	Yes	Total
N	9	4	13
NS	7	4	11
Wt	167.58	15.75	183.33
Het Chi	172.10	2.68	174.97
Het df	8	3	12
Het P	***	N.S.	***
Fixed RR	5.18	4.62	5.13
RRl	4.45	2.82	4.44
RRu	6.03	7.57	5.93
P	+++	+++	+++
Random RR	3.51	4.62	3.63
RRl	1.59	2.82	1.94
RRu	7.74	7.57	6.80
P	++	+++	+++
Between Chi			0.19
Between df			1
Between P			N.S.
Btwn(F) P			N.S.
Btwn(R) P			N.S.

	Full histological confirmation No	Yes	Total
N	8	5	13
NS	7	4	11
Wt	53.91	129.42	183.33
Het Chi	18.27	118.14	174.97
Het df	7	4	12
Het P	*	***	***
Fixed RR	2.52	6.90	5.13
RRl	1.93	5.81	4.44
RRu	3.29	8.19	5.93
P	+++	+++	+++
Random RR	2.84	5.25	3.63
RRl	1.71	1.78	1.94
RRu	4.71	15.46	6.80
P	+++	++	+++
Between Chi			38.56
Between df			1
Between P			***
Btwn(F) P			N.S.
Btwn(R) P			N.S.

	Number of adjustment variables (1) 0	1	2+/+nk	Total
N	9	2	2	13
NS	7	2	2	11
Wt	145.70	6.16	31.47	183.33
Het Chi	142.05	2.18	5.76	174.97
Het df	8	1	1	12
Het P	***	N.S.	*	***
Fixed RR	6.16	3.73	2.34	5.13
RRl	5.24	1.69	1.65	4.44
RRu	7.25	8.22	3.31	5.93
P	+++	++	+++	+++
Random RR	3.82	2.99	3.50	3.63
RRl	1.72	0.76	0.99	1.94
RRu	8.45	11.74	12.38	6.80
P	+++	N.S.	(+)	+++
Between Chi				24.98
Between df				2
Between P				***
Btwn(F) P				N.S.
Btwn(R) P				N.S.

Table 3I13 - 3

IESLC - Meta-analysis of Ever/current Smoking, Duration, "Mid"
Adenocarcinoma, Any Product (or Cigarettes if Any not available)
Most adjusted

	Number of adjustment variables (2) 0	1	2	3-5	6+/+nk	Total
N	9	2	1	1		13
NS	7	2	1	1		11
Wt	145.70	6.16	3.89	27.58		183.33
Het Chi	142.05	2.18	0.00	0.00		174.97
Het df	8	1	0	0		12
Het P	***	N.S.	N.S.	N.S.		***
Fixed RR	6.16	3.73	7.30	1.99		5.13
RRl	5.24	1.69	2.70	1.37		4.44
RRu	7.25	8.22	19.72	2.89		5.93
P	+++	++	+++	+++		+++
Random RR	3.82	2.99	7.30	1.99		3.63
RRl	1.72	0.76	2.70	1.37		1.94
RRu	8.45	11.74	19.72	2.89		6.80
P	+++	N.S.	+++	+++		+++
Between Chi						30.74
Between df						3
Between P						***
Btwn(F) P						N.S.
Btwn(R) P						(*)

	Smoking status ever	current	Total
N	12	1	13
NS	10	1	11
Wt	168.17	15.16	183.33
Het Chi	149.14	0.00	174.97
Het df	11	0	12
Het P	***	N.S.	***
Fixed RR	5.74	1.47	5.13
RRl	4.94	0.89	4.44
RRu	6.68	2.43	5.93
P	+++	N.S.	+++
Random RR	3.96	1.47	3.63
RRl	2.08	0.89	1.94
RRu	7.54	2.43	6.80
P	+++	N.S.	+++
Between Chi			25.83
Between df			1
Between P			***
Btwn(F) P			N.S.
Btwn(R) P			*

	Product all/unsp	cig+/-ot	cig only	Total
N	2	10	1	13
NS	2	8	1	11
Wt	6.16	174.67	2.50	183.33
Het Chi	2.18	170.93	0.00	174.97
Het df	1	9	0	12
Het P	N.S.	***	N.S.	***
Fixed RR	3.73	5.14	10.37	5.13
RRl	1.69	4.43	3.01	4.44
RRu	8.22	5.96	35.81	5.93
P	++	+++	+++	+++
Random RR	2.99	3.47	10.37	3.63
RRl	0.76	1.69	3.01	1.94
RRu	11.74	7.14	35.81	6.80
P	N.S.	+++	+++	+++
Between Chi				1.86
Between df				2
Between P				N.S.
Btwn(F) P				N.S.
Btwn(R) P				N.S.

Table 3I13 - 3

IESLC - Meta-analysis of Ever/current Smoking, Duration, "Mid"
Adenocarcinoma, Any Product (or Cigarettes if Any not available)
Most adjusted

		Denominator		
		nev any	nev cigs	Total
	N	8	5	13
	NS	6	5	11
	Wt	129.72	53.61	183.33
Het	Chi	90.14	16.79	174.97
Het	df	7	4	12
Het	P	***	**	***
Fixed	RR	7.59	1.99	5.13
	RRl	6.39	1.52	4.44
	RRu	9.02	2.60	5.93
	P	+++	+++	+++
Random	RR	4.63	2.36	3.63
	RRl	2.16	1.24	1.94
	RRu	9.93	4.49	6.80
	P	+++	++	+++
Between	Chi			68.04
Between	df			1
Between	P			***
Btwn(F)	P			*
Btwn(R)	P			N.S.

		Derivation of RR/CI			
		Orig	StdCalc	Other	Total
	N	4	8	1	13
	NS	4	6	1	11
	Wt	14.28	141.47	27.58	183.33
Het	Chi	3.33	142.02	0.00	174.97
Het	df	3	7	0	12
Het	P	N.S.	***	N.S.	***
Fixed	RR	5.08	6.18	1.99	5.13
	RRl	3.02	5.24	1.37	4.44
	RRu	8.53	7.28	2.89	5.93
	P	+++	+++	+++	+++
Random	RR	5.02	3.64	1.99	3.63
	RRl	2.89	1.53	1.37	1.94
	RRu	8.72	8.63	2.89	6.80
	P	+++	++	+++	+++
Between	Chi				29.61
Between	df				2
Between	P				***
Btwn(F)	P				N.S.
Btwn(R)	P				*

Table 3I13 - 4

IESLC - Meta-analysis of Ever/current Smoking, Duration, "Mid"
Adenocarcinoma, Any Product (or Cigarettes if Any not available)
Least adjusted

REF	NRR	X	SEX	AGEL	AGEH	RACE	YF	LC TYPE	LOC	START	ST	NLC	R	VB	P	H	AD	SM	PRODUCT	exL	exH	DENOM	De
BARBON	718	x	m	0	0	all	-	a	Eu:wst	1979	CC	755	n	bl	y	y	0	ev	all/unsp	30	39	nev any	st
BUFFLE	510		m	0	0	wh	-	a	NAmer	1976	CC	943	n	bl	y	n	0	ev	cig+/-ot	34	43	nev cigs	or
CHOI	567		m	0	0	all	-	a	As:oth	1985	CC	375	n	bl	n	n	0	ev	cig+/-ot	30	39	nev cigs	st
DAMBER	541		m	0	0	all	-	a+al+br	Eu:Sca	1972	CC	579	n	bl	y	n	1	ev	all/unsp	31	40	nev any	or
JEDRYC	521		m	0	0	all	-	a	Eu:est	1980	CC	1630	n	bl	y	n	0	ev	cig+/-ot	30	39	nev any	st
JOLY	661		m	0	0	all	-	a	SCAmer	1978	CC	826	n	bl	n	n	0	ev	cig+/-ot	30	39	nev any	st
JOLY	633		f	0	0	all	-	a	SCAmer	1978	CC	826	n	bl	n	n	0	ev	cig+/-ot	30	39	nev any	st
LUBIN2	701		m	0	0	all	-	a	Eu:mul	1976	CC	7804	n	bl	n	y	0	ev	cig+/-ot	30	39	nev any	st
LUBIN2	753		f	0	0	all	-	a	Eu:mul	1976	CC	7804	n	bl	n	y	0	ev	cig+/-ot	30	39	nev any	st
MATOS	642	x	m	0	0	all	-	a	SCAmer	1994	CC	200	n	bl	n	n	0	ev	cig+/-ot	25	39	nev any	st
PEZZOT	517		m	0	0	all	-	a	SCAmer	1987	CC	215	n	bl	n	y	0	ev	cig only	31	40	nev cigs	st
SOBUE	514		m	0	0	all	-	a	As:Jap	1986	CC	1376	n	bl	n	y	0	cu	cig+/-ot	30	39	nev cigs	st
WUWILL	512	x	f	0	0	all	-	a	As:Chi	1985	CC	965	n	ot	n	n	0	ev	cig+/-ot	30	39	nev cigs	st

Cigarette type is all/unspec for all RRs

Table 3I13 - 5

IESLC - Meta-analysis of Ever/current Smoking, Duration, "Mid"
Adenocarcinoma, Any Product (or Cigarettes if Any not available)
Least adjusted

REF	NRR	SEX	AD	Number Exposed Case	Cont	Non-exposed Case	Cont	RR	95.00%CI
BARBON	718	m	0	23	102	7	188	6.06	(2.51- 14.60)
BUFFLE	510	m	0	-	-	-	-	5.70	(2.20- 14.80)
CHOI	567	m	0	13	160	7	65	0.75	(0.29- 1.98)
DAMBER	541	m	1	-	-	16	-	1.20	(0.20- 6.00)
JEDRYC	521	m	0	28	231	7	289	5.00	(2.15- 11.66)
JOLY	661	m	0	12	165	5	218	3.17	(1.10- 9.18)
JOLY	633	f	0	6	24	25	283	2.83	(1.06- 7.57)
Subtotal JOLY								2.98	(1.45- 6.14)
LUBIN2	701	m	0	242	3473	57	2616	3.20	(2.39- 4.29)
LUBIN2	753	f	0	383	186	138	1180	17.61	(13.73- 22.58)
Subtotal LUBIN2								8.62	(7.13- 10.42)
MATOS	642	m	0	39	110	5	110	7.80	(2.96- 20.53)
PEZZOT	517	m	0	22	82	3	116	10.37	(3.01- 35.81)
SOBUE	514	m	0	62	200	27	128	1.47	(0.89- 2.43)
WUWILL	512	f	0	52	98	172	601	1.85	(1.27- 2.70)
Partial Totals				882	4831	469	5794		

*prospective study

REF	NRR	SEX	AD	Ys	Ws	Qs	Ps
BARBON	718	m	0	1.80	4.96	0.14	0.0001
BUFFLE	510	m	0	1.74	4.23	0.05	0.0003
CHOI	567	m	0	-0.28	4.14	15.20	0.5664
DAMBER	541	m	1	0.18	1.33	2.80	0.8336
JEDRYC	521	m	0	1.61	5.37	0.00	0.0002
JOLY	661	m	0	1.15	3.40	0.78	0.0333
JOLY	633	f	0	1.04	3.97	1.40	0.0382
Subtotal JOLY				1.09	7.37	2.18	
LUBIN2	701	m	0	1.16	44.75	9.94	0.0000
LUBIN2	753	f	0	2.87	62.18	94.76	0.0000
Subtotal LUBIN2				2.15	106.93	104.70	
MATOS	642	m	0	2.05	4.10	0.72	0.0000
PEZZOT	517	m	0	2.34	2.50	1.25	0.0002
SOBUE	514	m	0	0.39	15.16	23.64	0.1339
WUWILL	512	f	0	0.62	27.09	27.99	0.0013

	N	13
	NS	11
	Wt	183.19
Het	Chi	178.67
Het	df	12
Het	P	***
Fixed	RR	5.12
	RRl	4.43
	RRu	5.92
	P	+++
Random	RR	3.68
	RRl	1.95
	RRu	6.92
	P	+++
Asymm	P	N.S.

Table 3I13 - 6

IESLC - Meta-analysis of Ever/current Smoking, Duration, "Mid"
Adenocarcinoma, Any Product (or Cigarettes if Any not available)
Least adjusted

	Sex			
	combined	male	female	Total
N		10	3	13
NS		10	3	13
Wt		89.94	93.25	183.19
Het Chi		30.09	100.59	178.67
Het df		9	2	12
Het P		***	***	***
Fixed RR		3.04	8.47	5.12
RRl		2.47	6.91	4.43
RRu		3.74	10.38	5.92
P		+++	+++	+++
Random RR		3.35	4.60	3.68
RRl		2.12	0.81	1.95
RRu		5.30	26.10	6.92
P		+++	(+)	+++
Between Chi				47.99
Between df				1
Between P				***
Btwn(F) P				(*)
Btwn(R) P				N.S.

Table 3I13 - 7

IESLC - Meta-analysis of Ever/current Smoking, Duration, "Mid"
Adenocarcinoma, Any Product (or Cigarettes if Any not available)
Excluded studies (and stage at which they were excluded)

1	BECHER BLOT1 BROWN3 CARPEN CHYOU DARBY DOLL2 GARCIA GRAHAM GURSEL HAMMO2 JAHN JAIN LAUSSM PRESCO QIAO TVERDA WIGLE WYNDE3
2	ALDERS BENSHL BRESLO CHIAZZ DEAN3 DORN ENGELA GAO2 GILLIS GUO HEGMAN HIRAYA HOLE KAUFMA KOO KOULUM LIU4 MIGRAN MRFITR PERNU SEGI2 SPEIZE SUZUK2 SVENSS VUTUC WAKAI WU YUAN
3	GENG MCDUFF SPITZ STASZE WU2 ZHANG
4	AGUDO AKIBA AMANDU AMES ARMADA AUVINE AXELSS BEST BOFFET BOUCHA BOUCOT BROSS CEDERL CHEN2 CORREA CPSI CPSII DEAN2 DESTEF DOLL FAN GAO GARSHI HAMMON HU HU2 HUMBLE JUSSAW KAISE2 KREUZE LETOUR LEVIN LIAW LIU3 LIU5 LUO MCCONN NOTAN2 PEZZO2 PISANI QIAO2 RACHTA RESTRE SADOWS TIZZAN WANG2 WATSON WYNDE2 WYNDE6 WYNDE7 WYNDE8 ZHOU
5	CHEN LUBIN XU
10	KHUDER
14	BENHAM DORGAN DOSEME GER HAENSZ KATSOU OSANN2 ZHENG

Table 3I13 - 8

Potentially overlapping studies

REF	REFGP	PRINC	.	OVERLAP/LINK
LUBIN2	LUBIN2	1		Lubin-combined

Table 3I13 - 9

Most adjusted - insufficient data for meta-analysis

REF	NRR	SEX	AGEL	AGEH	RACE	YF	LC TYPE	LOC	START	ST	NLC	R	VB	P	H	AD	SM	PRODUCT	exL	exH	DENOM	De
CHEN	511	c	0	0	all	-	a	As:oth	1987	CC	323	n	ot	n	y	2	ev	cig+/-ot	31	40	nev cigs	ot
XU	507	m	0	0	all	-	a	As:Chi	1985	CC	729	n	ot	n	n	2	ev	all/unsp	30	39	nev any	or

REF	NRR	RR	SIG	RRDATA comment
CHEN	511	1.60	n	0
XU	507	*		RR for 1-19/day is 2.2(p<0.05), for 20-29/day is 1.5(NS) and for >=30/day is 3.2(p<0.05)

Table 3I14 -

IESLC - Meta-analysis of Ever/current Smoking, Duration, "High"
Adenocarcinoma, Any Product (or Cigarettes if Any not available)

This analysis is restricted to results for:
1) Ever/current smokers
2) Results by Duration
3) Categorical results by Duration
4) Adenocarcinoma (or near equivalent)
5) Results complete enough for use in metaanalysis

Within each study, results are then selected (in the following order of preference, within each sex) for:
6) PRODUCT: all/unspec, cigarettes regardless of other products, cigarettes only
7) CIGTYPE: all/unspecified, MC regardless of HR, MC only
8) (not applicable)
9) DENOM: never smoked anything, never smoked cigarettes, never any + low, never cigs + low
10) Followup period (YF, prospective studies): whole study (coded as 0) or longest available
11) LCtype: adeno or nearest available, but not squamous. (q = squamous, s = small,
a = adeno, l = large, KII = Kreyberg II, al = alveolar, br = bronchiolar, u = undifferentiated)
12) Race: all or nearest available, otherwise by race (wh or w = white, bl or b = black, hi = hispanic
ch = chinese, jap = japanese, haw = hawaiian, w+o = white + oriental, sca = scandinavian, as = asian)
13) Duration "high" in key scheme 1 (key value 50, maximum range 36+)
14) For overlapping studies: principal rather than subsidiary studies
Finally by Age: whole study (coded as 0) if available, otherwise by widest available age group
and then for single sex results (m, f) in preference to results for both sexes combined (c).

Results adjusted (AD) for the most potential confounders are then chosen in Sections -1 to -3
and results adjusted for the least confounders in Sections -4 to -6. (Those least adjusted results which
actually differ from the most adjusted are marked 'x' in column X in Section -4)

Section -7 shows excluded studies, together with the stage (as above) at which no qualifying
results were found.

Section -8 lists the potentially overlapping studies which have been included (1=principal, 2=subsidiary).

Section -9 lists any results which would have been included in preference except that they had data not complete
enough for use in meta-analysis, with their significance (yes/no), if known, and any further comment as entered
on the database. It also lists as "gap" any categories for which no data were presented by the original authors.

In addition to those mentioned above, the following fields, levels and abbreviations are used:
* or nk = not known, n = no, y = yes, ot = other
ev = ever, cu = current, nev = never
all/unspec = all or unspecified, cig+/-ot = cigarettes irrespective of other products (cigar, pipe etc)
MC = manufactured cigarettes, HR = hand-rolled cigarettes
exL, exH = range of exposure (low and high) in the smoking group, in terms of Duration
REF: 6-character study reference
NRR: number of the RR on the database within the study
ST : study type (CC = case control, pr or prosp = prospective)
NLC: number of lung cancer cases in whole study
R : risky occupational population (n = no, m = mining, o = other risky)
VB : national cigarette type (V = at least 75% Virginia, bl = at least 75% blended, ot = other)
P : any proxy use
H : full histological confirmation
De : derivation of RR/CI (or = original, st = standard method, ot = other method of estimation)

Table 3I14 - 1

IESLC - Meta-analysis of Ever/current Smoking, Duration, "High"
Adenocarcinoma, Any Product (or Cigarettes if Any not available)
Most adjusted

REF	NRR	SEX	AGEL	AGEH	RACE	YF	LC TYPE	LOC	START	ST	NLC	R	VB	P	H	AD	SM	PRODUCT	exL	exH	DENOM	De
BARBON	727	m	0	0	all	-	a	Eu:wst	1979	CC	755	n	bl	y	y	1	ev	all/unsp	50	999	nev any	or
BUFFLE	512	m	0	0	wh	-	a	NAmer	1976	CC	943	n	bl	y	n	0	ev	cig+/-ot	50	999	nev cigs	or
CHOI	569	m	0	0	all	-	a	As:oth	1985	CC	375	n	bl	n	n	0	ev	cig+/-ot	50	999	nev cigs	st
DAMBER	542	m	0	0	all	-	a+al+br	Eu:Sca	1972	CC	579	n	bl	y	n	1	ev	all/unsp	41	50	nev any	or
JEDRYC	597	m	0	0	all	-	a	Eu:est	1980	CC	1630	n	bl	y	n	3	ev	cig+/-ot	40	999	nev any	or
JOLY	663	m	0	0	all	-	a	SCAmer	1978	CC	826	n	bl	n	n	0	ev	cig+/-ot	50	999	nev any	st
JOLY	635	f	0	0	all	-	a	SCAmer	1978	CC	826	n	bl	n	n	0	ev	cig+/-ot	50	999	nev any	st
LUBIN2	703	m	0	0	all	-	a	Eu:mul	1976	CC	7804	n	bl	n	y	0	ev	cig+/-ot	50	999	nev any	st
LUBIN2	755	f	0	0	all	-	a	Eu:mul	1976	CC	7804	n	bl	n	y	0	ev	cig+/-ot	50	999	nev any	st
MATOS	648	m	0	0	all	-	a	SCAmer	1994	CC	200	n	bl	n	n	2	ev	cig+/-ot	40	70	nev any	or
PEZZOT	518	m	0	0	all	-	a	SCAmer	1987	CC	215	n	bl	n	y	0	ev	cig only	41	999	nev cigs	st
SOBUE	516	m	0	0	all	-	a	As:Jap	1986	CC	1376	n	bl	n	y	0	cu	cig+/-ot	50	999	nev cigs	st
WUWILL	528	f	0	0	all	-	a	As:Chi	1985	CC	965	n	ot	n	n	3	ev	cig+/-ot	40	999	nev cigs	ot

Cigarette type is all/unspec for all RRs

Table 3I14 - 2

IESLC - Meta-analysis of Ever/current Smoking, Duration, "High"
Adenocarcinoma, Any Product (or Cigarettes if Any not available)
Most adjusted

REF	NRR	SEX	AD	Number Exposed Case	Cont	Non-exposed Case	Cont	RR	95.00%CI
BARBON	727	m	1	67	-	7	-	8.30	(3.70- 18.70)
BUFFLE	512	m	0	-	-	-	-	3.50	(1.30- 9.70)
CHOI	569	m	0	4	20	7	95	2.71	(0.73- 10.16)
DAMBER	542	m	1	-	-	16	-	3.40	(1.30- 9.10)
JEDRYC	597	m	3	47	-	7	-	4.41	(1.86- 10.45)
JOLY	663	m	0	33	253	5	218	5.69	(2.18- 14.82)
JOLY	635	f	0	7	20	25	283	3.96	(1.53- 10.27)
Subtotal JOLY								4.74	(2.41- 9.32)
LUBIN2	703	m	0	90	1460	57	2616	2.83	(2.02- 3.97)
LUBIN2	755	f	0	181	34	138	1180	45.52	(30.31- 68.36)
Subtotal LUBIN2								8.80	(6.79- 11.41)
MATOS	648	m	2	33	-	5	-	10.70	(3.80- 29.90)
PEZZOT	518	m	0	27	101	3	116	10.34	(3.04- 35.09)
SOBUE	516	m	0	43	73	27	128	2.79	(1.59- 4.89)
WUWILL	528	f	3	52	-	172	-	2.09	(1.40- 3.10)
Partial Totals				584	1961	469	4636		

*prospective study

REF	NRR	SEX	AD	Ys	Ws	Qs	Ps
BARBON	727	m	1	2.12	5.85	1.24	0.0000
BUFFLE	512	m	0	1.25	3.80	0.62	0.0145
CHOI	569	m	0	1.00	2.21	0.95	0.1381
DAMBER	542	m	1	1.22	4.06	0.76	0.0137
JEDRYC	597	m	3	1.48	5.16	0.15	0.0008
JOLY	663	m	0	1.74	4.19	0.03	0.0004
JOLY	635	f	0	1.38	4.23	0.33	0.0046
Subtotal JOLY				1.56	8.42	0.36	
LUBIN2	703	m	0	1.04	33.64	12.78	0.0000
LUBIN2	755	f	0	3.82	23.24	108.61	0.0000
Subtotal LUBIN2				2.17	56.88	121.39	
MATOS	648	m	2	2.37	3.61	1.84	0.0000
PEZZOT	518	m	0	2.34	2.57	1.19	0.0002
SOBUE	516	m	0	1.03	12.22	4.84	0.0003
WUWILL	528	f	3	0.74	24.32	20.55	0.0003

	N	13
	NS	11
	Wt	129.10
Het	Chi	153.89
Het	df	12
Het	P	***
Fixed	RR	5.24
	RRl	4.41
	RRu	6.23
	P	+++
Random	RR	5.25
	RRl	2.70
	RRu	10.20
	P	+++
Asymm	P	N.S.

Table 3I14 - 3

IESLC - Meta-analysis of Ever/current Smoking, Duration, "High"
Adenocarcinoma, Any Product (or Cigarettes if Any not available)
Most adjusted

	combined	Sex: male	female	Total
N		10	3	13
NS		10	3	13
Wt		77.32	51.79	129.10
Het Chi		15.18	115.71	153.89
Het df		9	2	12
Het P		(*)	***	***
Fixed RR		3.71	8.78	5.24
RRl		2.97	6.68	4.41
RRu		4.64	11.52	6.23
P		+++	+++	+++
Random RR		4.30	7.29	5.25
RRl		3.08	0.77	2.70
RRu		5.99	69.06	10.20
P		+++	(+)	+++
Between Chi				22.99
Between df				1
Between P				***
Btwn(F) P				N.S.
Btwn(R) P				N.S.

	Lung cancer type: a	a+l	a+al+br	KII	not q+u	not q+s	Total
N	12		1				13
NS	10		1				11
Wt	125.05		4.06				129.10
Het Chi	153.10		0.00				153.89
Het df	11		0				12
Het P	***		N.S.				***
Fixed RR	5.31		3.40				5.24
RRl	4.46		1.29				4.41
RRu	6.33		9.00				6.23
P	+++		+				+++
Random RR	5.44		3.40				5.25
RRl	2.69		1.29				2.70
RRu	10.99		9.00				10.20
P	+++		+				+++
Between Chi							0.78
Between df							1
Between P							N.S.
Btwn(F) P							N.S.
Btwn(R) P							N.S.

	Location: NAmer	UK	Scand	othEur	China	Japan	othAs	other	Total
N	1		1	4	1	1	1	4	13
NS	1		1	3	1	1	1	3	11
Wt	3.80		4.06	67.90	24.32	12.22	2.21	14.60	129.10
Het Chi	0.00		0.00	108.35	0.00	0.00	0.00	2.55	153.89
Het df	0		0	3	0	0	0	3	12
Het P	N.S.		N.S.	***	N.S.	N.S.	N.S.	N.S.	***
Fixed RR	3.50		3.40	8.31	2.09	2.79	2.71	6.65	5.24
RRl	1.28		1.29	6.55	1.40	1.59	0.73	3.98	4.41
RRu	9.56		9.00	10.54	3.11	4.89	10.16	11.11	6.23
P	+		+	+++	+++	+++	N.S.	+++	+++
Random RR	3.50		3.40	8.35	2.09	2.79	2.71	6.65	5.25
RRl	1.28		1.29	1.72	1.40	1.59	0.73	3.98	2.70
RRu	9.56		9.00	40.46	3.11	4.89	10.16	11.11	10.20
P	+		+	++	+++	+++	N.S.	+++	+++
Between Chi									42.99
Between df									6
Between P									***
Btwn(F) P									N.S.
Btwn(R) P									*

Table 3I14 - 3

IESLC - Meta-analysis of Ever/current Smoking, Duration, "High"
Adenocarcinoma, Any Product (or Cigarettes if Any not available)
Most adjusted

Detailed Country in "other Europe"

	multi	Germany	othWest	East	Balkans	Total
N	2		1	1		4
NS	1		1	1		3
Wt	56.88		5.85	5.16		67.90
Het Chi	106.09		0.00	0.00		108.35
Het df	1		0	0		3
Het P	***		N.S.	N.S.		***
Fixed RR	8.80		8.30	4.41		8.31
RRl	6.79		3.69	1.86		6.55
RRu	11.41		18.66	10.45		10.54
P	+++		+++	+++		+++
Random RR	11.32		8.30	4.41		8.35
RRl	0.74		3.69	1.86		1.72
RRu	172.30		18.66	10.45		40.46
P	(+)		+++	+++		++
Between Chi						2.26
Between df						2
Between P						N.S.
Btwn(F) P						N.S.
Btwn(R) P						N.S.

Detailed Country in "other Asia"

	India	HongKong	other	Total
N			1	1
NS			1	1
Wt			2.21	2.21
Het Chi			0.00	0.00
Het df			0	0
Het P			N.S.	N.S.
Fixed RR			2.71	2.71
RRl			0.73	0.73
RRu			10.16	10.16
P			N.S.	N.S.
Random RR			2.71	2.71
RRl			0.73	0.73
RRu			10.16	10.16
P			N.S.	N.S.
Between Chi				
Between df				
Between P				N.S.
Btwn(F) P				N.S.
Btwn(R) P				N.S.

Detailed other continent

	SCAmer	Total
N	4	4
NS	3	3
Wt	14.60	14.60
Het Chi	2.55	2.55
Het df	3	3
Het P	N.S.	N.S.
Fixed RR	6.65	6.65
RRl	3.98	3.98
RRu	11.11	11.11
P	+++	+++
Random RR	6.65	6.65
RRl	3.98	3.98
RRu	11.11	11.11
P	+++	+++
Between Chi		
Between df		
Between P		N.S.
Btwn(F) P		N.S.
Btwn(R) P		N.S.

Table 3I14 - 3

IESLC - Meta-analysis of Ever/current Smoking, Duration, "High"
Adenocarcinoma, Any Product (or Cigarettes if Any not available)
Most adjusted

	Start year of study					
	<1960	1960-69	1970-79	1980-89	1990+	Total
N			7	5	1	13
NS			5	5	1	11
Wt			79.02	46.48	3.61	129.10
Het Chi			114.40	7.48	0.00	153.89
Het df			6	4	0	12
Het P			***	N.S.	N.S.	***
Fixed RR			7.47	2.71	10.70	5.24
RRl			6.00	2.03	3.81	4.41
RRu			9.32	3.61	30.01	6.23
P			+++	+++	+++	+++
Random RR			6.14	3.15	10.70	5.25
RRl			2.10	1.99	3.81	2.70
RRu			17.90	4.97	30.01	10.20
P			+++	+++	+++	+++
Between Chi						32.00
Between df						2
Between P						***
Btwn(F) P						N.S.
Btwn(R) P						(*)

	Study type (1)		
	CC	other	Total
N	13		13
NS	11		11
Wt	129.10		129.10
Het Chi	153.89		153.89
Het df	12		12
Het P	***		***
Fixed RR	5.24		5.24
RRl	4.41		4.41
RRu	6.23		6.23
P	+++		+++
Random RR	5.25		5.25
RRl	2.70		2.70
RRu	10.20		10.20
P	+++		+++
Between Chi			
Between df			
Between P			N.S.
Btwn(F) P			N.S.
Btwn(R) P			N.S.

	Study type (2)			
	CC	prosp	other	Total
N	13			13
NS	11			11
Wt	129.10			129.10
Het Chi	153.89			153.89
Het df	12			12
Het P	***			***
Fixed RR	5.24			5.24
RRl	4.41			4.41
RRu	6.23			6.23
P	+++			+++
Random RR	5.25			5.25
RRl	2.70			2.70
RRu	10.20			10.20
P	+++			+++
Between Chi				
Between df				
Between P				N.S.
Btwn(F) P				N.S.
Btwn(R) P				N.S.

Table 3I14 - 3

IESLC - Meta-analysis of Ever/current Smoking, Duration, "High"
Adenocarcinoma, Any Product (or Cigarettes if Any not available)
Most adjusted

	Study size (number of LC cases) 100-249	250-499	500-999	1000+	Total
N	2	1	6	4	13
NS	2	1	5	3	11
Wt	6.18	2.21	46.45	74.27	129.10
Het Chi	0.00	0.00	11.34	120.49	153.89
Het df	1	0	5	3	12
Het P	N.S.	N.S.	*	***	***
Fixed RR	10.55	2.71	3.14	6.94	5.24
RRl	4.80	0.73	2.36	5.53	4.41
RRu	23.20	10.16	4.19	8.72	6.23
P	+++	N.S.	+++	+++	+++
Random RR	10.55	2.71	3.85	6.36	5.25
RRl	4.80	0.73	2.35	1.37	2.70
RRu	23.20	10.16	6.31	29.61	10.20
P	+++	N.S.	+++	+	+++
Between Chi					22.06
Between df					3
Between P					***
Btwn(F) P					N.S.
Btwn(R) P					N.S.

	Risky occupational population no	mining	othRisky	Total
N	13			13
NS	11			11
Wt	129.10			129.10
Het Chi	153.89			153.89
Het df	12			12
Het P	***			***
Fixed RR	5.24			5.24
RRl	4.41			4.41
RRu	6.23			6.23
P	+++			+++
Random RR	5.25			5.25
RRl	2.70			2.70
RRu	10.20			10.20
P	+++			+++
Between Chi				
Between df				
Between P				N.S.
Btwn(F) P				N.S.
Btwn(R) P				N.S.

	National cigarette tobacco type Virginia	blended	other	Total
N		12	1	13
NS		10	1	11
Wt		104.79	24.32	129.10
Het Chi		128.57	0.00	153.89
Het df		11	0	12
Het P		***	N.S.	***
Fixed RR		6.49	2.09	5.24
RRl		5.36	1.40	4.41
RRu		7.85	3.11	6.23
P		+++	+++	+++
Random RR		5.72	2.09	5.25
RRl		2.81	1.40	2.70
RRu		11.64	3.11	10.20
P		+++	+++	+++
Between Chi				25.31
Between df				1
Between P				***
Btwn(F) P				N.S.
Btwn(R) P				*

Table 3I14 - 3

IESLC - Meta-analysis of Ever/current Smoking, Duration, "High"
Adenocarcinoma, Any Product (or Cigarettes if Any not available)
Most adjusted

	Any proxy use No/nk	Yes	Total
N	9	4	13
NS	7	4	11
Wt	110.23	18.87	129.10
Het Chi	151.10	2.65	153.89
Het df	8	3	12
Het P	***	N.S.	***
Fixed RR	5.31	4.84	5.24
RRl	4.41	3.08	4.41
RRu	6.40	7.60	6.23
P	+++	+++	+++
Random RR	5.56	4.84	5.25
RRl	2.30	3.08	2.70
RRu	13.41	7.60	10.20
P	+++	+++	+++
Between Chi			0.14
Between df			1
Between P			N.S.
Btwn(F) P			N.S.
Btwn(R) P			N.S.

	Full histological confirmation No	Yes	Total
N	8	5	13
NS	7	4	11
Wt	51.57	77.53	129.10
Het Chi	11.87	119.77	153.89
Het df	7	4	12
Het P	N.S.	***	***
Fixed RR	3.15	7.35	5.24
RRl	2.40	5.88	4.41
RRu	4.14	9.18	6.23
P	+++	+++	+++
Random RR	3.74	7.86	5.25
RRl	2.50	2.08	2.70
RRu	5.58	29.69	10.20
P	+++	++	+++
Between Chi			22.24
Between df			1
Between P			***
Btwn(F) P			N.S.
Btwn(R) P			N.S.

	Number of adjustment variables (1) 0	1	2+/+nk	Total
N	8	2	3	13
NS	6	2	3	11
Wt	86.11	9.91	33.09	129.10
Het Chi	124.83	1.91	9.63	153.89
Het df	7	1	2	12
Het P	***	N.S.	**	***
Fixed RR	6.59	5.76	2.81	5.24
RRl	5.33	3.09	2.00	4.41
RRu	8.14	10.73	3.95	6.23
P	+++	+++	+++	+++
Random RR	5.55	5.54	4.21	5.25
RRl	2.06	2.32	1.63	2.70
RRu	14.92	13.24	10.87	10.20
P	+++	+++	++	+++
Between Chi				17.51
Between df				2
Between P				***
Btwn(F) P				N.S.
Btwn(R) P				N.S.

Table 3I14 - 3

IESLC - Meta-analysis of Ever/current Smoking, Duration, "High"
Adenocarcinoma, Any Product (or Cigarettes if Any not available)
Most adjusted

		Number of adjustment variables (2) 0	1	2	3-5	6+/+nk	Total
	N	8	2	1	2		13
	NS	6	2	1	2		11
	Wt	86.11	9.91	3.61	29.47		129.10
Het	Chi	124.83	1.91	0.00	2.37		153.89
Het	df	7	1	0	1		12
Het	P	***	N.S.	N.S.	N.S.		***
Fixed	RR	6.59	5.76	10.70	2.38		5.24
	RRl	5.33	3.09	3.81	1.66		4.41
	RRu	8.14	10.73	30.01	3.42		6.23
	P	+++	+++	+++	+++		+++
Random	RR	5.55	5.54	10.70	2.74		5.25
	RRl	2.06	2.32	3.81	1.36		2.70
	RRu	14.92	13.24	30.01	5.54		10.20
	P	+++	+++	+++	++		+++
Between	Chi						24.77
Between	df						3
Between	P						***
Btwn(F)	P						N.S.
Btwn(R)	P						N.S.

		Smoking status ever	current	Total
	N	12	1	13
	NS	10	1	11
	Wt	116.88	12.22	129.10
Het	Chi	148.54	0.00	153.89
Het	df	11	0	12
Het	P	***	N.S.	***
Fixed	RR	5.60	2.79	5.24
	RRl	4.67	1.59	4.41
	RRu	6.71	4.89	6.23
	P	+++	+++	+++
Random	RR	5.56	2.79	5.25
	RRl	2.69	1.59	2.70
	RRu	11.45	4.89	10.20
	P	+++	+++	+++
Between	Chi			5.35
Between	df			1
Between	P			*
Btwn(F)	P			N.S.
Btwn(R)	P			N.S.

		Product all/unsp	cig+/-ot	cig only	Total
	N	2	10	1	13
	NS	2	8	1	11
	Wt	9.91	116.62	2.57	129.10
Het	Chi	1.91	150.64	0.00	153.89
Het	df	1	9	0	12
Het	P	N.S.	***	N.S.	***
Fixed	RR	5.76	5.12	10.34	5.24
	RRl	3.09	4.27	3.04	4.41
	RRu	10.73	6.14	35.09	6.23
	P	+++	+++	+++	+++
Random	RR	5.54	4.92	10.34	5.25
	RRl	2.32	2.21	3.04	2.70
	RRu	13.24	10.95	35.09	10.20
	P	+++	+++	+++	+++
Between	Chi				1.34
Between	df				2
Between	P				N.S.
Btwn(F)	P				N.S.
Btwn(R)	P				N.S.

Table 3I14 - 3

IESLC - Meta-analysis of Ever/current Smoking, Duration, "High"
Adenocarcinoma, Any Product (or Cigarettes if Any not available)
Most adjusted

	Denominator nev any	nev cigs	Total
N	8	5	13
NS	6	5	11
Wt	83.98	45.12	129.10
Het Chi	114.08	6.46	153.89
Het df	7	4	12
Het P	***	N.S.	***
Fixed RR	7.61	2.62	5.24
RRl	6.14	1.96	4.41
RRu	9.42	3.51	6.23
P	+++	+++	+++
Random RR	6.74	2.94	5.25
RRl	2.60	1.91	2.70
RRu	17.46	4.54	10.20
P	+++	+++	+++
Between Chi			33.35
Between df			1
Between P			***
Btwn(F) P			N.S.
Btwn(R) P			N.S.

	Derivation of RR/CI Orig	StdCalc	Other	Total
N	5	7	1	13
NS	5	5	1	11
Wt	22.48	82.30	24.32	129.10
Het Chi	4.56	123.24	0.00	153.89
Het df	4	6	0	12
Het P	N.S.	***	N.S.	***
Fixed RR	5.50	6.78	2.09	5.24
RRl	3.64	5.47	1.40	4.41
RRu	8.32	8.42	3.11	6.23
P	+++	+++	+++	+++
Random RR	5.48	5.91	2.09	5.25
RRl	3.52	1.98	1.40	2.70
RRu	8.54	17.57	3.11	10.20
P	+++	++	+++	+++
Between Chi				26.09
Between df				2
Between P				***
Btwn(F) P				N.S.
Btwn(R) P				**

Table 3I14 - 4

IESLC - Meta-analysis of Ever/current Smoking, Duration, "High"
Adenocarcinoma, Any Product (or Cigarettes if Any not available)
Least adjusted

REF	NRR	X	SEX	AGEL	AGEH	RACE	YF	LC TYPE	LOC	START	ST	NLC	R	VB	P	H	AD	SM	PRODUCT	exL	exH	DENOM	De
BARBON	720	x	m	0	0	all	-	a	Eu:wst	1979	CC	755	n	bl	y	y	0	ev	all/unsp	50	999	nev any	st
BUFFLE	512		m	0	0	wh	-	a	NAmer	1976	CC	943	n	bl	y	n	0	ev	cig+/-ot	50	999	nev cigs	or
CHOI	569		m	0	0	all	-	a	As:oth	1985	CC	375	n	bl	n	n	0	ev	cig+/-ot	50	999	nev cigs	st
DAMBER	542		m	0	0	all	-	a+al+br	Eu:Sca	1972	CC	579	n	bl	y	n	1	ev	all/unsp	41	50	nev any	or
JEDRYC	523	x	m	0	0	all	-	a	Eu:est	1980	CC	1630	n	bl	y	n	0	ev	cig+/-ot	50	999	nev any	st
JOLY	663		m	0	0	all	-	a	SCAmer	1978	CC	826	n	bl	n	n	0	ev	cig+/-ot	50	999	nev any	st
JOLY	635		f	0	0	all	-	a	SCAmer	1978	CC	826	n	bl	n	n	0	ev	cig+/-ot	50	999	nev any	st
LUBIN2	703		m	0	0	all	-	a	Eu:mul	1976	CC	7804	n	bl	n	y	0	ev	cig+/-ot	50	999	nev any	st
LUBIN2	755		f	0	0	all	-	a	Eu:mul	1976	CC	7804	n	bl	n	y	0	ev	cig+/-ot	50	999	nev any	st
MATOS	643	x	m	0	0	all	-	a	SCAmer	1994	CC	200	n	bl	n	n	0	ev	cig+/-ot	40	70	nev any	st
PEZZOT	518		m	0	0	all	-	a	SCAmer	1987	CC	215	n	bl	n	y	0	ev	cig only	41	999	nev cigs	st
SOBUE	516		m	0	0	all	-	a	As:Jap	1986	CC	1376	n	bl	n	y	0	cu	cig+/-ot	50	999	nev cigs	st
WUWILL	513	x	f	0	0	all	-	a	As:Chi	1985	CC	965	n	ot	n	n	0	ev	cig+/-ot	40	999	nev cigs	st

Cigarette type is all/unspec for all RRs

Table 3I14 - 5

IESLC - Meta-analysis of Ever/current Smoking, Duration, "High"
Adenocarcinoma, Any Product (or Cigarettes if Any not available)
Least adjusted

REF	NRR	SEX	AD	Number Exposed Case	Cont	Non-exposed Case	Cont	RR	95.00%CI
BARBON	720	m	0	67	235	7	188	7.66	(3.43- 17.07)
BUFFLE	512	m	0	-	-	-	-	3.50	(1.30- 9.70)
CHOI	569	m	0	4	20	7	95	2.71	(0.73- 10.16)
DAMBER	542	m	1	-	-	16	-	3.40	(1.30- 9.10)
JEDRYC	523	m	0	17	214	7	289	3.28	(1.34- 8.05)
JOLY	663	m	0	33	253	5	218	5.69	(2.18- 14.82)
JOLY	635	f	0	7	20	25	283	3.96	(1.53- 10.27)
Subtotal JOLY								4.74	(2.41- 9.32)
LUBIN2	703	m	0	90	1460	57	2616	2.83	(2.02- 3.97)
LUBIN2	755	f	0	181	34	138	1180	45.52	(30.31- 68.36)
Subtotal LUBIN2								8.80	(6.79- 11.41)
MATOS	643	m	0	33	89	5	110	8.16	(3.06- 21.76)
PEZZOT	518	m	0	27	101	3	116	10.34	(3.04- 35.09)
SOBUE	516	m	0	43	73	27	128	2.79	(1.59- 4.89)
WUWILL	513	f	0	52	114	172	601	1.59	(1.10- 2.31)
Partial Totals				554	2613	469	5824		

*prospective study

REF	NRR	SEX	AD	Ys	Ws	Qs	Ps
BARBON	720	m	0	2.04	5.98	1.39	0.0000
BUFFLE	512	m	0	1.25	3.80	0.34	0.0145
CHOI	569	m	0	1.00	2.21	0.68	0.1381
DAMBER	542	m	1	1.22	4.06	0.44	0.0137
JEDRYC	523	m	0	1.19	4.77	0.64	0.0095
JOLY	663	m	0	1.74	4.19	0.14	0.0004
JOLY	635	f	0	1.38	4.23	0.13	0.0046
Subtotal JOLY				1.56	8.42	0.27	
LUBIN2	703	m	0	1.04	33.64	8.85	0.0000
LUBIN2	755	f	0	3.82	23.24	119.26	0.0000
Subtotal LUBIN2				2.17	56.88	128.11	
MATOS	643	m	0	2.10	3.99	1.19	0.0000
PEZZOT	518	m	0	2.34	2.57	1.58	0.0002
SOBUE	516	m	0	1.03	12.22	3.38	0.0003
WUWILL	513	f	0	0.47	28.18	33.28	0.0133

	N	13
	NS	11
	Wt	133.08
Het	Chi	171.30
Het	df	12
Het	P	***
Fixed	RR	4.72
	RRl	3.99
	RRu	5.60
	P	+++
Random	RR	4.89
	RRl	2.45
	RRu	9.75
	P	+++
Asymm	P	N.S.

Table 3I14 - 6

IESLC - Meta-analysis of Ever/current Smoking, Duration, "High"
Adenocarcinoma, Any Product (or Cigarettes if Any not available)
Least adjusted

	combined	Sex<br>male	female	Total
N		10	3	13
NS		10	3	13
Wt		77.43	55.65	133.08
Het Chi		12.78	144.54	171.30
Het df		9	2	12
Het P		N.S.	***	***
Fixed RR		3.59	6.92	4.72
RRl		2.87	5.32	3.99
RRu		4.49	9.00	5.60
P		+++	+++	+++
Random RR		3.98	6.64	4.89
RRl		2.94	0.58	2.45
RRu		5.37	76.18	9.75
P		+++	N.S.	+++
Between Chi				13.97
Between df				1
Between P				***
Btwn(F) P				N.S.
Btwn(R) P				N.S.

Table 3I14 - 7

IESLC - Meta-analysis of Ever/current Smoking, Duration, "High"
Adenocarcinoma, Any Product (or Cigarettes if Any not available)
Excluded studies (and stage at which they were excluded)

1	BECHER BLOT1 BROWN3 CARPEN CHYOU DARBY DOLL2 GARCIA GRAHAM GURSEL HAMMO2 JAHN JAIN LAUSSM PRESCO QIAO TVERDA WIGLE WYNDE3
2	ALDERS BENSHL BRESLO CHIAZZ DEAN3 DORN ENGELA GAO2 GILLIS GUO HEGMAN HIRAYA HOLE KAUFMA KOO KOULUM LIU4 MIGRAN MRFITR PERNU SEGI2 SPEIZE SUZUK2 SVENSS VUTUC WAKAI WU YUAN
3	GENG MCDUFF SPITZ STASZE WU2 ZHANG
4	AGUDO AKIBA AMANDU AMES ARMADA AUVINE AXELSS BEST BOFFET BOUCHA BOUCOT BROSS CEDERL CHEN2 CORREA CPSI CPSII DEAN2 DESTEF DOLL FAN GAO GARSHI HAMMON HU HU2 HUMBLE JUSSAW KAISE2 KREUZE LETOUR LEVIN LIAW LIU3 LIU5 LUO MCCONN NOTAN2 PEZZO2 PISANI QIAO2 RACHTA RESTRE SADOWS TIZZAN WANG2 WATSON WYNDE2 WYNDE6 WYNDE7 WYNDE8 ZHOU
5	CHEN LUBIN XU
10	KHUDER
14	DORGAN DOSEME GER HAENSZ KATSOU OSANN2 ZHENG
15	BENHAM

Table 3I14 - 8
Potentially overlapping studies

REF	REFGP	PRINC .	OVERLAP/LINK
LUBIN2	LUBIN2	1	Lubin-combined

Table 3I14 - 9
Most adjusted - insufficient data for meta-analysis

REF	NRR	SEX	AGEL	AGEH	RACE	YF	LC TYPE	LOC	START	ST	NLC	R	VB	P	H	AD	SM	PRODUCT	exL	exH	DENOM	De
CHEN	512	c	0	0	all	-	a	As:oth	1987	CC	323	n	ot	n	y	2	ev	cig+/-ot	41	999	nev cigs	ot
XU	508	m	0	0	all	-	a	As:Chi	1985	CC	729	n	ot	n	n	2	ev	all/unsp	40	999	nev any	or

REF	NRR	RR	SIG	RRDATA comment
CHEN	512	3.79	y	p<0.001
XU	508	*		RR for 1-19/day is 2.6(p<0.05), for 20-29/day is 3.6(p<0.05) and for >=30/ day is 11.8(p<0.05)

Table 3I15 -

IESLC - Meta-analysis of Ever/current Smoking, Duration, "Highest vs lowest"
Adenocarcinoma, Any Product (or Cigarettes if Any not available)

This analysis is restricted to results for:
1) Ever/current smokers
2) Results by Duration
3) Categorical results by Duration
4) Denominator (unexposed) = "low"
5) Adenocarcinoma (or near equivalent)
6) Results complete enough for use in metaanalysis

Within each study, results are then selected (in the following order of preference, within each sex) for:
7) SMKSTA: ever, current
8) PRODUCT: all/unspec, cigarettes regardless of other products, cigarettes only
9) CIGTYPE: all/unspecified, MC regardless of HR, MC only
10) Results with least adjustment for other aspects of smoking (ADOS)
11) The highest vs lowest category
12) Followup period (YF, prospective studies): whole study (coded as 0) or longest available
13) LCtype: adeno or nearest available, but not squamous. (q = squamous, s = small,
a = adeno, l = large, KII = Kreyberg II, al = alveolar, br = bronchiolar, u = undifferentiated)
14) Race: all or nearest available, otherwise by race (wh or w = white, bl or b = black, hi = hispanic
ch = chinese, jap = japanese, haw = hawaiian, w+o = white + oriental, sca = scandinavian, as = asian)
15) For overlapping studies: principal rather than subsidiary studies
Finally by Age: whole study (coded as 0) if available, otherwise by widest available age group
and then for single sex results (m, f) in preference to results for both sexes combined (c).

Results adjusted (AD) for the most potential confounders are then chosen in Sections -1 to -3
and results adjusted for the least confounders in Sections -4 to -6. (Those least adjusted results which
actually differ from the most adjusted are marked 'x' in column X in Section -4)

Section -7 shows excluded studies, together with the stage (as above) at which no qualifying
results were found.

Section -8 lists the potentially overlapping studies which have been included (1=principal, 2=subsidiary).

Section -9 lists any results which would have been included in preference except that they had data not complete
enough for use in meta-analysis, with their significance (yes/no), if known, and any further comment as entered
on the database. It also lists as "gap" any categories for which no data were presented by the original authors.

In addition to those mentioned above, the following fields, levels and abbreviations are used:
* or nk = not known, n = no, y = yes, ot = other
all/unspec = all or unspecified, cig+/-ot = cigarettes irrespective of other products (cigar, pipe etc)
MC = manufactured cigarettes, HR = hand-rolled cigarettes
exL, exH = range of exposure (low and high) in the "highest" group, in terms of Duration
unexL, unexH = range of exposure (low and high) in the "lowest" group, in terms of Duration
REF: 6-character study reference
NRR: number of the RR on the database within the study
ST : study type (CC = case control, pr or prosp = prospective)
NLC: number of lung cancer cases in whole study
R : risky occupational population (n = no, m = mining, o = other risky)
VB : national cigarette type (V = at least 75% Virginia, bl = at least 75% blended, ot = other)
P : any proxy use
H : full histological confirmation
De : derivation of RR/CI (or = original, st = standard method, ot = other method of estimation)

Table 3I15 - 1

IESLC - Meta-analysis of Ever/current Smoking, Duration, "Highest vs lowest"
Adenocarcinoma, Any Product (or Cigarettes if Any not available)
Most adjusted

REF	NRR	SEX	AGEL	AGEH	RACE	YF	LC TYPE	LOC	START	ST	NLC	R	VB	P	H	AD	ADOS	SM	PRODUCT	exL	exH	unexL	unexH	De
BARBON	730	m	0	0	all	-	a	Eu:wst	1979	CC	755	n	bl	y	y	1	0	ev	all/unsp	50	999	1	29	ot
CHOI	572	m	0	0	all	-	a	As:oth	1985	CC	375	n	bl	n	n	0	0	ev	cig+/-ot	50	999	1	29	st
CHOI	580	f	0	0	all	-	a	As:oth	1985	CC	375	n	bl	n	n	0	0	ev	cig+/-ot	30	999	1	29	st
DAMBER	546	m	0	0	all	-	a+al+br	Eu:Sca	1972	CC	579	n	bl	y	n	1	0	ev	all/unsp	51	999	1	30	ot
DORGAN	529	m	0	0	wh	-	a	NAmer	1980	CC	2026	n	bl	y	y	2	0	ev	cig+/-ot	35	999	1	34	ot
DORGAN	525	f	0	0	all	-	a	NAmer	1980	CC	2026	n	bl	y	y	3	0	ev	cig+/-ot	35	999	1	34	ot
DOSEME	520	m	0	0	all	-	not q+s	Eu:bal	1979	CC	1210	n	bl	n	n	2	0	ev	cig+/-ot	21	999	1	10	ot
GER	509	c	0	0	all	-	a	As:oth	1990	CC	141	n	ot	y	n	5	0	ev	all/unsp	31	999	1	30	ot
HAENSZ	536	f	0	0	all	-	a	NAmer	1955	CC	158	n	bl	n	y	1	0	ev	cig+/-ot	15	999	1	14	ot
JEDRYC	527	m	0	0	all	-	a	Eu:est	1980	CC	1630	n	bl	y	n	0	0	ev	cig+/-ot	50	999	1	19	st
JOLY	666	m	0	0	all	-	a	SCAmer	1978	CC	826	n	bl	n	n	0	0	ev	cig+/-ot	50	999	1	29	st
JOLY	638	f	0	0	all	-	a	SCAmer	1978	CC	826	n	bl	n	n	0	0	ev	cig+/-ot	50	999	1	29	st
KATSOU	522	f	0	0	all	-	a	Eu:bal	1987	CC	101	n	bl	n	n	1	0	cu	all/unsp	30	999	1	29	ot
KHUDER	532	m	0	0	all	-	a	NAmer	1985	CC	482	n	bl	n	y	5	3#	ev	cig+/-ot	30	999	1	29	or
LUBIN2	706	m	0	0	all	-	a	Eu:mul	1976	CC	7804	n	bl	n	y	0	0	ev	cig+/-ot	50	999	1	29	st
LUBIN2	758	f	0	0	all	-	a	Eu:mul	1976	CC	7804	n	bl	n	y	0	0	ev	cig+/-ot	50	999	1	29	st
MATOS	650	m	0	0	all	-	a	SCAmer	1994	CC	200	n	bl	n	n	2	0	ev	cig+/-ot	40	70	1	24	ot
OSANN2	518	f	0	0	all	-	KII	NAmer	1964	ot	217	n	bl	n	y	1	0	ev	cig+/-ot	21	999	1	20	ot
PEZZOT	522	m	0	0	all	-	a	SCAmer	1987	CC	215	n	bl	n	y	2	0	ev	cig only	41	999	1	30	ot
SOBUE	519	m	0	0	all	-	a	As:Jap	1986	CC	1376	n	bl	n	y	0	0	cu	cig+/-ot	50	999	1	29	st
WUWILL	515	f	0	0	all	-	a	As:Chi	1985	CC	965	n	ot	n	n	0	0	ev	cig+/-ot	40	999	1	29	st
ZHENG	515	m	0	0	all	-	a	As:Chi	1982	CC	540	n	ot	*	y	1	0	ev	cig+/-ot	30	999	1	29	ot
ZHENG	519	f	0	0	all	-	a	As:Chi	1982	CC	540	n	ot	*	y	1	0	ev	cig+/-ot	30	999	1	29	ot

Comments on values in listings

KHUDER ADOS Age at starting smoking, No of cigarettes per day, Quitted smoking

Cigarette type is all/unspec for all RRs

Table 3I15 - 2

IESLC - Meta-analysis of Ever/current Smoking, Duration, "Highest vs lowest"
Adenocarcinoma, Any Product (or Cigarettes if Any not available)
Most adjusted

REF	NRR	SEX	AD	Number Exposed Case	Cont	Non-exposed Case	Cont	RR	95.00%CI
BARBON	730	m	1	67	-	15	-	2.24	(1.15- 4.36)
CHOI	572	m	0	4	20	17	221	2.60	(0.80- 8.47)
CHOI	580	f	0	2	3	2	23	7.67	(0.77- 76.45)
Subtotal CHOI								3.26	(1.14- 9.32)
DAMBER	546	m	1	-	-	-	-	1.39	(0.45- 4.30)
DORGAN	529	m	2	-	-	-	-	3.15	(1.90- 5.23)
DORGAN	525	f	3	-	-	-	-	2.11	(1.50- 2.98)
Subtotal DORGAN								2.39	(1.80- 3.18)
DOSEME	520	m	2	69	-	5	-	5.13	(1.81- 14.51)
GER	509	c	5	21	-	14	-	0.61	(0.24- 1.52)
HAENSZ	536	f	1	16	-	2	-	2.69	(0.57- 12.56)
JEDRYC	527	m	0	17	214	2	68	2.70	(0.61- 11.99)
JOLY	666	m	0	33	253	5	109	2.84	(1.08- 7.48)
JOLY	638	f	0	7	20	10	54	1.89	(0.63- 5.64)
Subtotal JOLY								2.38	(1.15- 4.91)
KATSOU	522	f	1	9	-	6	-	4.30	(0.98- 18.82)
KHUDER	532	m	5	-	-	-	-	2.70	(1.40- 5.30)
LUBIN2	706	m	0	90	1460	131	2964	1.39	(1.06- 1.84)
LUBIN2	758	f	0	181	34	174	229	7.01	(4.62- 10.62)
Subtotal LUBIN2								2.28	(1.81- 2.87)
MATOS	650	m	2	33	-	7	-	6.29	(2.59- 15.31)
OSANN2	518	f	1	51	-	10	-	5.86	(1.88- 18.26)
PEZZOT	522	m	2	27	-	11	-	4.60	(1.95- 10.82)
SOBUE	519	m	0	43	73	33	119	2.12	(1.24- 3.64)
WUWILL	515	f	0	52	114	34	139	1.86	(1.13- 3.07)
ZHENG	515	m	1	99	-	24	-	2.42	(1.43- 4.10)
ZHENG	519	f	1	24	-	9	-	2.08	(0.78- 5.53)
Subtotal ZHENG								2.34	(1.47- 3.72)
Partial Totals				845	2191	511	3926		

*prospective study

REF	NRR	SEX	AD	Ys	Ws	Qs	Ps
BARBON	730	m	1	0.81	8.65	0.03	0.0177
CHOI	572	m	0	0.96	2.75	0.02	0.1129
CHOI	580	f	0	2.04	0.73	0.99	0.0826
Subtotal CHOI				1.18	3.48	1.01	
DAMBER	546	m	1	0.33	3.02	0.88	0.5674
DORGAN	529	m	2	1.15	14.99	1.16	0.0000
DORGAN	525	f	3	0.75	32.61	0.49	0.0000
Subtotal DORGAN				0.87	47.60	1.65	
DOSEME	520	m	2	1.64	3.55	2.08	0.0021
GER	509	c	5	-0.49	4.51	8.39	0.2938
HAENSZ	536	f	1	0.99	1.61	0.02	0.2098
JEDRYC	527	m	0	0.99	1.73	0.03	0.1913
JOLY	666	m	0	1.05	4.11	0.13	0.0342
JOLY	638	f	0	0.64	3.21	0.17	0.2540
Subtotal JOLY				0.87	7.32	0.30	
KATSOU	522	f	1	1.46	1.76	0.61	0.0530
KHUDER	532	m	5	0.99	8.67	0.13	0.0034
LUBIN2	706	m	0	0.33	50.59	14.58	0.0180
LUBIN2	758	f	0	1.95	22.20	25.76	0.0000
Subtotal LUBIN2				0.82	72.79	40.34	
MATOS	650	m	2	1.84	4.87	4.57	0.0000
OSANN2	518	f	1	1.77	2.97	2.40	0.0023
PEZZOT	522	m	2	1.53	5.23	2.26	0.0005
SOBUE	519	m	0	0.75	13.22	0.18	0.0062
WUWILL	515	f	0	0.62	15.48	0.94	0.0142
ZHENG	515	m	1	0.88	13.85	0.00	0.0010
ZHENG	519	f	1	0.73	4.01	0.08	0.1427
Subtotal ZHENG				0.85	17.86	0.08	

Table 3I15 - 2

IESLC - Meta-analysis of Ever/current Smoking, Duration, "Highest vs lowest"
Adenocarcinoma, Any Product (or Cigarettes if Any not available)
Most adjusted

	N	23
	NS	18
	Wt	224.29
Het	Chi	65.90
Het	df	22
Het	P	***
Fixed	RR	2.39
	RRl	2.09
	RRu	2.72
	P	+++
Random	RR	2.64
	RRl	2.04
	RRu	3.43
	P	+++
Asymm	P	N.S.

Table 3I15 - 3

IESLC - Meta-analysis of Ever/current Smoking, Duration, "Highest vs lowest"
Adenocarcinoma, Any Product (or Cigarettes if Any not available)
Most adjusted

	combined	Sex male	female	Total
N	1	13	9	23
NS	1	13	9	23
Wt	4.51	135.22	84.57	224.29
Het Chi	0.00	24.72	26.95	65.90
Het df	0	12	8	22
Het P	N.S.	*	***	***
Fixed RR	0.61	2.16	3.01	2.39
RRl	0.24	1.83	2.43	2.09
RRu	1.54	2.56	3.72	2.72
P	N.S.	+++	+++	+++
Random RR	0.61	2.59	3.11	2.64
RRl	0.24	1.96	1.92	2.04
RRu	1.54	3.42	5.04	3.43
P	N.S.	+++	+++	+++
Between Chi				14.23
Between df				2
Between P				***
Btwn(F) P				(*)
Btwn(R) P				**

	a	a+l	Lung cancer type a+al+br	KII	not q+u	not q+s	Total
N	20		1	1		1	23
NS	15		1	1		1	18
Wt	214.76		3.02	2.97		3.55	224.29
Het Chi	60.47		0.00	0.00		0.00	65.90
Het df	19		0	0		0	22
Het P	***		N.S.	N.S.		N.S.	***
Fixed RR	2.34		1.39	5.86		5.13	2.39
RRl	2.05		0.45	1.88		1.81	2.09
RRu	2.68		4.30	18.26		14.52	2.72
P	+++		N.S.	++		++	+++
Random RR	2.56		1.39	5.86		5.13	2.64
RRl	1.95		0.45	1.88		1.81	2.04
RRu	3.37		4.30	18.26		14.52	3.43
P	+++		N.S.	++		++	+++
Between Chi							5.43
Between df							3
Between P							N.S.
Btwn(F) P							N.S.
Btwn(R) P							N.S.

	NAmer	UK	Scand	Location othEur	China	Japan	othAs	other	Total
N	5		1	6	3	1	3	4	23
NS	4		1	5	2	1	2	3	18
Wt	60.85		3.02	88.47	33.33	13.22	7.99	17.42	224.29
Het Chi	3.93		0.00	43.09	0.50	0.00	6.19	3.34	65.90
Het df	4		0	5	2	0	2	3	22
Het P	N.S.		N.S.	***	N.S.	N.S.	*	N.S.	***
Fixed RR	2.55		1.39	2.39	2.11	2.12	1.27	3.80	2.39
RRl	1.98		0.45	1.94	1.50	1.24	0.63	2.38	2.09
RRu	3.28		4.30	2.94	2.96	3.64	2.53	6.08	2.72
P	+++		N.S.	+++	+++	++	N.S.	+++	+++
Random RR	2.55		1.39	3.22	2.11	2.12	1.78	3.78	2.64
RRl	1.98		0.45	1.49	1.50	1.24	0.45	2.30	2.04
RRu	3.28		4.30	6.99	2.96	3.64	7.00	6.21	3.43
P	+++		N.S.	++	+++	++	N.S.	+++	+++
Between Chi									8.86
Between df									6
Between P									N.S.
Btwn(F) P									N.S.
Btwn(R) P									N.S.

Table 3I15 - 3

IESLC - Meta-analysis of Ever/current Smoking, Duration, "Highest vs lowest"
Adenocarcinoma, Any Product (or Cigarettes if Any not available)
Most adjusted

Detailed Country in "other Europe"

	multi	Germany	othWest	East	Balkans	Total
N	2		1	1	2	6
NS	1		1	1	2	5
Wt	72.79		8.65	1.73	5.31	88.47
Het Chi	40.19		0.00	0.00	0.04	43.09
Het df	1		0	0	1	5
Het P	***		N.S.	N.S.	N.S.	***
Fixed RR	2.28		2.24	2.70	4.84	2.39
RRl	1.81		1.15	0.61	2.07	1.94
RRu	2.87		4.36	11.99	11.33	2.94
P	+++		+	N.S.	+++	+++
Random RR	3.10		2.24	2.70	4.84	3.22
RRl	0.64		1.15	0.61	2.07	1.49
RRu	15.08		4.36	11.99	11.33	6.99
P	N.S.		+	N.S.	+++	++
Between Chi						2.86
Between df						3
Between P						N.S.
Btwn(F) P						N.S.
Btwn(R) P						N.S.

Detailed Country in "other Asia"

	India	HongKong	other	Total
N			3	3
NS			2	2
Wt			7.99	7.99
Het Chi			6.19	6.19
Het df			2	2
Het P			*	*
Fixed RR			1.27	1.27
RRl			0.63	0.63
RRu			2.53	2.53
P			N.S.	N.S.
Random RR			1.78	1.78
RRl			0.45	0.45
RRu			7.00	7.00
P			N.S.	N.S.
Between Chi				
Between df				
Between P				N.S.
Btwn(F) P				N.S.
Btwn(R) P				N.S.

Detailed other continent

	SCAmer	Total
N	4	4
NS	3	3
Wt	17.42	17.42
Het Chi	3.34	3.34
Het df	3	3
Het P	N.S.	N.S.
Fixed RR	3.80	3.80
RRl	2.38	2.38
RRu	6.08	6.08
P	+++	+++
Random RR	3.78	3.78
RRl	2.30	2.30
RRu	6.21	6.21
P	+++	+++
Between Chi		
Between df		
Between P		N.S.
Btwn(F) P		N.S.
Btwn(R) P		N.S.

Table 3I15 - 3

IESLC - Meta-analysis of Ever/current Smoking, Duration, "Highest vs lowest"
Adenocarcinoma, Any Product (or Cigarettes if Any not available)
Most adjusted

		Start year of study					
		<1960	1960-69	1970-79	1980-89	1990+	Total
	N	1	1	7	12	2	23
	NS	1	1	5	9	2	18
	Wt	1.61	2.97	95.32	115.02	9.38	224.29
Het	Chi	0.00	0.00	43.55	6.87	12.74	65.90
Het	df	0	0	6	11	1	22
Het	P	N.S.	N.S.	***	N.S.	***	***
Fixed	RR	2.69	5.86	2.32	2.41	2.05	2.39
	RRl	0.57	1.88	1.90	2.01	1.08	2.09
	RRu	12.63	18.26	2.83	2.90	3.88	2.72
	P	N.S.	++	+++	+++	+	+++
Random	RR	2.69	5.86	2.65	2.41	1.97	2.64
	RRl	0.57	1.88	1.38	2.01	0.20	2.04
	RRu	12.63	18.26	5.10	2.90	19.34	3.43
	P	N.S.	++	++	+++	N.S.	+++
Between	Chi						2.74
Between	df						4
Between	P						N.S.
Btwn(F)	P						N.S.
Btwn(R)	P						N.S.

		Study type (1)		
		CC	other	Total
	N	22	1	23
	NS	17	1	18
	Wt	221.32	2.97	224.29
Het	Chi	63.47	0.00	65.90
Het	df	21	0	22
Het	P	***	N.S.	***
Fixed	RR	2.36	5.86	2.39
	RRl	2.07	1.88	2.09
	RRu	2.69	18.26	2.72
	P	+++	++	+++
Random	RR	2.57	5.86	2.64
	RRl	1.98	1.88	2.04
	RRu	3.35	18.26	3.43
	P	+++	++	+++
Between	Chi			2.43
Between	df			1
Between	P			N.S.
Btwn(F)	P			N.S.
Btwn(R)	P			N.S.

		Study type (2)			
		CC	prosp	other	Total
	N	22		1	23
	NS	17		1	18
	Wt	221.32		2.97	224.29
Het	Chi	63.47		0.00	65.90
Het	df	21		0	22
Het	P	***		N.S.	***
Fixed	RR	2.36		5.86	2.39
	RRl	2.07		1.88	2.09
	RRu	2.69		18.26	2.72
	P	+++		++	+++
Random	RR	2.57		5.86	2.64
	RRl	1.98		1.88	2.04
	RRu	3.35		18.26	3.43
	P	+++		++	+++
Between	Chi				2.43
Between	df				1
Between	P				N.S.
Btwn(F)	P				N.S.
Btwn(R)	P				N.S.

Table 3I15 - 3

IESLC - Meta-analysis of Ever/current Smoking, Duration, "Highest vs lowest"
Adenocarcinoma, Any Product (or Cigarettes if Any not available)
Most adjusted

	Study size (number of LC cases)				
	100-249	250-499	500-999	1000+	Total
N	6	3	7	7	23
NS	6	2	5	5	18
Wt	20.95	12.15	52.32	138.87	224.29
Het Chi	16.59	0.76	1.46	44.25	65.90
Het df	5	2	6	6	22
Het P	**	N.S.	N.S.	***	***
Fixed RR	3.16	2.85	2.11	2.36	2.39
RRl	2.06	1.62	1.61	2.00	2.09
RRu	4.85	5.00	2.77	2.78	2.72
P	+++	+++	+++	+++	+++
Random RR	3.22	2.85	2.11	2.85	2.64
RRl	1.44	1.62	1.61	1.71	2.04
RRu	7.22	5.00	2.77	4.73	3.43
P	++	+++	+++	+++	+++
Between Chi					2.84
Between df					3
Between P					N.S.
Btwn(F) P					N.S.
Btwn(R) P					N.S.

	Risky occupational population			
	no	mining	othRisky	Total
N	23			23
NS	18			18
Wt	224.29			224.29
Het Chi	65.90			65.90
Het df	22			22
Het P	***			***
Fixed RR	2.39			2.39
RRl	2.09			2.09
RRu	2.72			2.72
P	+++			+++
Random RR	2.64			2.64
RRl	2.04			2.04
RRu	3.43			3.43
P	+++			+++
Between Chi				
Between df				
Between P				N.S.
Btwn(F) P				N.S.
Btwn(R) P				N.S.

	National cigarette tobacco type			
	Virginia	blended	other	Total
N		19	4	23
NS		15	3	18
Wt		186.45	37.84	224.29
Het Chi		55.92	6.59	65.90
Het df		18	3	22
Het P		***	(*)	***
Fixed RR		2.52	1.82	2.39
RRl		2.18	1.32	2.09
RRu		2.91	2.50	2.72
P		+++	+++	+++
Random RR		2.98	1.68	2.64
RRl		2.21	1.01	2.04
RRu		4.02	2.79	3.43
P		+++	+	+++
Between Chi				3.38
Between df				1
Between P				(*)
Btwn(F) P				N.S.
Btwn(R) P				(*)

Table 3I15 - 3

IESLC - Meta-analysis of Ever/current Smoking, Duration, "Highest vs lowest"
Adenocarcinoma, Any Product (or Cigarettes if Any not available)
Most adjusted

	Any proxy use No/nk	Yes	Total
N	17	6	23
NS	13	5	18
Wt	158.79	65.50	224.29
Het Chi	54.52	10.01	65.90
Het df	16	5	22
Het P	***	(*)	***
Fixed RR	2.51	2.11	2.39
RRl	2.15	1.66	2.09
RRu	2.93	2.69	2.72
P	+++	+++	+++
Random RR	3.03	1.95	2.64
RRl	2.17	1.31	2.04
RRu	4.24	2.93	3.43
P	+++	++	+++
Between Chi			1.36
Between df			1
Between P			N.S.
Btwn(F) P			N.S.
Btwn(R) P			(*)

	Full histological confirmation No	Yes	Total
N	11	12	23
NS	9	9	18
Wt	45.70	178.59	224.29
Het Chi	18.73	47.07	65.90
Het df	10	11	22
Het P	*	***	***
Fixed RR	2.29	2.41	2.39
RRl	1.71	2.08	2.09
RRu	3.06	2.79	2.72
P	+++	+++	+++
Random RR	2.46	2.76	2.64
RRl	1.59	1.97	2.04
RRu	3.79	3.88	3.43
P	+++	+++	+++
Between Chi			0.09
Between df			1
Between P			N.S.
Btwn(F) P			N.S.
Btwn(R) P			N.S.

	Number of adjustment variables (1) 0	1	2+/+nk	Total
N	9	7	7	23
NS	6	6	6	18
Wt	114.01	35.86	74.42	224.29
Het Chi	42.32	3.97	18.60	65.90
Het df	8	6	6	22
Het P	***	N.S.	**	***
Fixed RR	2.24	2.48	2.58	2.39
RRl	1.86	1.79	2.06	2.09
RRu	2.69	3.44	3.24	2.72
P	+++	+++	+++	+++
Random RR	2.59	2.48	2.81	2.64
RRl	1.55	1.79	1.79	2.04
RRu	4.34	3.44	4.40	3.43
P	+++	+++	+++	+++
Between Chi				1.00
Between df				2
Between P				N.S.
Btwn(F) P				N.S.
Btwn(R) P				N.S.

Table 3I15 - 3

IESLC - Meta-analysis of Ever/current Smoking, Duration, "Highest vs lowest"
Adenocarcinoma, Any Product (or Cigarettes if Any not available)
Most adjusted

	Number of adjustment variables (2) 0	1	2	3-5	6+/+nk	Total
N	9	7	4	3		23
NS	6	6	4	3		19
Wt	114.01	35.86	28.63	45.79		224.29
Het Chi	42.32	3.97	2.17	7.21		65.90
Het df	8	6	3	2		22
Het P	***	N.S.	N.S.	*		***
Fixed RR	2.24	2.48	4.03	1.96		2.39
RRl	1.86	1.79	2.80	1.46		2.09
RRu	2.69	3.44	5.82	2.61		2.72
P	+++	+++	+++	+++		+++
Random RR	2.59	2.48	4.03	1.67		2.64
RRl	1.55	1.79	2.80	0.85		2.04
RRu	4.34	3.44	5.82	3.30		3.43
P	+++	+++	+++	N.S.		+++
Between Chi						10.22
Between df						3
Between P						*
Btwn(F) P						N.S.
Btwn(R) P						(*)

	Smoking status ever	current	Total
N	21	2	23
NS	16	2	18
Wt	209.32	14.98	224.29
Het Chi	65.11	0.77	65.90
Het df	20	1	22
Het P	***	N.S.	***
Fixed RR	2.39	2.31	2.39
RRl	2.09	1.39	2.09
RRu	2.74	3.83	2.72
P	+++	++	+++
Random RR	2.65	2.31	2.64
RRl	2.00	1.39	2.04
RRu	3.51	3.83	3.43
P	+++	++	+++
Between Chi			0.02
Between df			1
Between P			N.S.
Btwn(F) P			N.S.
Btwn(R) P			N.S.

	Product all/unsp	cig+/-ot	cig only	Total
N	4	18	1	23
NS	4	13	1	18
Wt	17.94	201.12	5.23	224.29
Het Chi	6.95	53.66	0.00	65.90
Het df	3	17	0	22
Het P	(*)	***	N.S.	***
Fixed RR	1.59	2.43	4.60	2.39
RRl	1.00	2.12	1.95	2.09
RRu	2.52	2.79	10.84	2.72
P	+	+++	+++	+++
Random RR	1.58	2.82	4.60	2.64
RRl	0.74	2.12	1.95	2.04
RRu	3.36	3.75	10.84	3.43
P	N.S.	+++	+++	+++
Between Chi				5.29
Between df				2
Between P				(*)
Btwn(F) P				N.S.
Btwn(R) P				N.S.

Table 3I15 - 3

IESLC - Meta-analysis of Ever/current Smoking, Duration, "Highest vs lowest"
Adenocarcinoma, Any Product (or Cigarettes if Any not available)
Most adjusted

		Derivation of RR/CI			
		Orig	StdCalc	Other	Total
	N	1	9	13	23
	NS	1	6	11	18
	Wt	8.67	114.01	101.61	224.29
Het	Chi	0.00	42.32	22.59	65.90
Het	df	0	8	12	22
Het	P	N.S.	***	*	***
Fixed	RR	2.70	2.24	2.54	2.39
	RRl	1.39	1.86	2.09	2.09
	RRu	5.25	2.69	3.08	2.72
	P	++	+++	+++	+++
Random	RR	2.70	2.59	2.68	2.64
	RRl	1.39	1.55	1.98	2.04
	RRu	5.25	4.34	3.62	3.43
	P	++	+++	+++	+++
Between	Chi				0.99
Between	df				2
Between	P				N.S.
Btwn(F)	P				N.S.
Btwn(R)	P				N.S.

Table 3I15 - 4

IESLC - Meta-analysis of Ever/current Smoking, Duration, "Highest vs lowest"
Adenocarcinoma, Any Product (or Cigarettes if Any not available)
Least adjusted

REF	NRR	X	SEX	AGEL	AGEH	RACE	YF	LC TYPE	LOC	START	ST	NLC	R	VB	P	H	AD	ADOS	SM	PRODUCT	exL	exH	unexL	unexH	De
BARBON	723	x	m	0	0	all	-	a	Eu:wst	1979	CC	755	n	bl	y	y	0	0	ev	all/unsp	50	999	1	29	st
CHOI	572		m	0	0	all	-	a	As:oth	1985	CC	375	n	bl	n	n	0	0	ev	cig+/-ot	50	999	1	29	st
CHOI	580		f	0	0	all	-	a	As:oth	1985	CC	375	n	bl	n	n	0	0	ev	cig+/-ot	30	999	1	29	st
DAMBER	546		m	0	0	all	-	a+al+br	Eu:Sca	1972	CC	579	n	bl	y	n	1	0	ev	all/unsp	51	999	1	30	ot
DORGAN	529		m	0	0	wh	-	a	NAmer	1980	CC	2026	n	bl	y	y	2	0	ev	cig+/-ot	35	999	1	34	ot
DORGAN	525		f	0	0	all	-	a	NAmer	1980	CC	2026	n	bl	y	y	3	0	ev	cig+/-ot	35	999	1	34	ot
DOSEME	520		m	0	0	all	-	not q+s	Eu:bal	1979	CC	1210	n	bl	n	n	2	0	ev	cig+/-ot	21	999	1	10	ot
GER	503	x	c	0	0	all	-	a	As:oth	1990	CC	141	n	ot	y	n	0	0	ev	all/unsp	31	999	1	30	st
HAENSZ	529	x	f	0	0	all	-	a	NAmer	1955	CC	158	n	bl	n	y	0	0	ev	cig+/-ot	15	999	1	14	st
JEDRYC	527		m	0	0	all	-	a	Eu:est	1980	CC	1630	n	bl	y	n	0	0	ev	cig+/-ot	50	999	1	19	st
JOLY	666		m	0	0	all	-	a	SCAmer	1978	CC	826	n	bl	n	n	0	0	ev	cig+/-ot	50	999	1	29	st
JOLY	638		f	0	0	all	-	a	SCAmer	1978	CC	826	n	bl	n	n	0	0	ev	cig+/-ot	50	999	1	29	st
KATSOU	517	x	f	0	0	all	-	a	Eu:bal	1987	CC	101	n	bl	n	n	0	0	cu	all/unsp	30	999	1	29	st
KHUDER	532		m	0	0	all	-	a	NAmer	1985	CC	482	n	bl	n	y	5	3#	ev	cig+/-ot	30	999	1	29	or
LUBIN2	706		m	0	0	all	-	a	Eu:mul	1976	CC	7804	n	bl	n	y	0	0	ev	cig+/-ot	50	999	1	29	st
LUBIN2	758		f	0	0	all	-	a	Eu:mul	1976	CC	7804	n	bl	n	y	0	0	ev	cig+/-ot	50	999	1	29	st
MATOS	645	x	m	0	0	all	-	a	SCAmer	1994	CC	200	n	bl	n	n	0	0	ev	cig+/-ot	40	70	1	24	st
OSANN2	515	x	f	0	0	all	-	KII	NAmer	1964	ot	217	n	bl	n	y	0	0	ev	cig+/-ot	21	999	1	20	st
PEZZOT	520	x	m	0	0	all	-	a	SCAmer	1987	CC	215	n	bl	n	y	0	0	ev	cig only	41	999	1	30	st
SOBUE	519		m	0	0	all	-	a	As:Jap	1986	CC	1376	n	bl	n	y	0	0	cu	cig+/-ot	50	999	1	29	st
WUWILL	515		f	0	0	all	-	a	As:Chi	1985	CC	965	n	ot	n	n	0	0	ev	cig+/-ot	40	999	1	29	st
ZHENG	514	x	m	0	0	all	-	a	As:Chi	1982	CC	540	n	ot	*	y	0	0	ev	cig+/-ot	30	999	1	29	st
ZHENG	518	x	f	0	0	all	-	a	As:Chi	1982	CC	540	n	ot	*	y	0	0	ev	cig+/-ot	30	999	1	29	st

Comments on values in listings

KHUDER ADOS Age at starting smoking, No of cigarettes per day, Quitted smoking

Cigarette type is all/unspec for all RRs

Table 3I15 - 5

IESLC - Meta-analysis of Ever/current Smoking, Duration, "Highest vs lowest"
Adenocarcinoma, Any Product (or Cigarettes if Any not available)
Least adjusted

REF	NRR	SEX	AD	Number Exposed Case	Number Exposed Cont	Non-exposed Case	Non-exposed Cont	RR	95.00%CI
BARBON	723	m	0	67	235	15	91	1.73	(0.94- 3.18)
CHOI	572	m	0	4	20	17	221	2.60	(0.80- 8.47)
CHOI	580	f	0	2	3	2	23	7.67	(0.77- 76.45)
Subtotal CHOI								3.26	(1.14- 9.32)
DAMBER	546	m	1	-	-	-	-	1.39	(0.45- 4.30)
DORGAN	529	m	2	-	-	-	-	3.15	(1.90- 5.23)
DORGAN	525	f	3	-	-	-	-	2.11	(1.50- 2.98)
Subtotal DORGAN								2.39	(1.80- 3.18)
DOSEME	520	m	2	69	-	5	-	5.13	(1.81- 14.51)
GER	503	c	0	21	98	14	51	0.78	(0.37- 1.66)
HAENSZ	529	f	0	16	77	2	26	2.70	(0.58- 12.55)
JEDRYC	527	m	0	17	214	2	68	2.70	(0.61- 11.99)
JOLY	666	m	0	33	253	5	109	2.84	(1.08- 7.48)
JOLY	638	f	0	7	20	10	54	1.89	(0.63- 5.64)
Subtotal JOLY								2.38	(1.15- 4.91)
KATSOU	517	f	0	9	6	6	12	3.00	(0.72- 12.46)
KHUDER	532	m	5	-	-	-	-	2.70	(1.40- 5.30)
LUBIN2	706	m	0	90	1460	131	2964	1.39	(1.06- 1.84)
LUBIN2	758	f	0	181	34	174	229	7.01	(4.62- 10.62)
Subtotal LUBIN2								2.28	(1.81- 2.87)
MATOS	645	m	0	33	89	7	84	4.45	(1.87- 10.60)
OSANN2	515	f	0	51	20	10	20	5.10	(2.04- 12.78)
PEZZOT	520	m	0	27	27	11	39	3.55	(1.51- 8.34)
SOBUE	519	m	0	43	73	33	119	2.12	(1.24- 3.64)
WUWILL	515	f	0	52	114	34	139	1.86	(1.13- 3.07)
ZHENG	514	m	0	99	143	24	75	2.16	(1.28- 3.66)
ZHENG	518	f	0	24	27	9	17	1.68	(0.63- 4.46)
Subtotal ZHENG								2.04	(1.29- 3.25)
Partial Totals				845	2913	511	4341		

*prospective study

REF	NRR	SEX	AD	Ys	Ws	Qs	Ps
BARBON	723	m	0	0.55	10.33	0.82	0.0783
CHOI	572	m	0	0.96	2.75	0.04	0.1129
CHOI	580	f	0	2.04	0.73	1.06	0.0826
Subtotal CHOI				1.18	3.48	1.10	
DAMBER	546	m	1	0.33	3.02	0.75	0.5674
DORGAN	529	m	2	1.15	14.99	1.52	0.0000
DORGAN	525	f	3	0.75	32.61	0.22	0.0000
Subtotal DORGAN				0.87	47.60	1.74	
DOSEME	520	m	2	1.64	3.55	2.30	0.0021
GER	503	c	0	-0.25	6.72	7.79	0.5209
HAENSZ	529	f	0	0.99	1.63	0.04	0.2047
JEDRYC	527	m	0	0.99	1.73	0.05	0.1913
JOLY	666	m	0	1.05	4.11	0.19	0.0342
JOLY	638	f	0	0.64	3.21	0.12	0.2540
Subtotal JOLY				0.87	7.32	0.31	
KATSOU	517	f	0	1.10	1.89	0.14	0.1305
KHUDER	532	m	5	0.99	8.67	0.23	0.0034
LUBIN2	706	m	0	0.33	50.59	12.48	0.0180
LUBIN2	758	f	0	1.95	22.20	27.72	0.0000
Subtotal LUBIN2				0.82	72.79	40.20	
MATOS	645	m	0	1.49	5.09	2.24	0.0008
OSANN2	515	f	0	1.63	4.55	2.91	0.0005
PEZZOT	520	m	0	1.27	5.25	1.00	0.0037
SOBUE	519	m	0	0.75	13.22	0.08	0.0062
WUWILL	515	f	0	0.62	15.48	0.66	0.0142
ZHENG	514	m	0	0.77	13.87	0.05	0.0041
ZHENG	518	f	0	0.52	4.02	0.39	0.2987
Subtotal ZHENG				0.71	17.89	0.44	

Table 3I15 - 5

IESLC - Meta-analysis of Ever/current Smoking, Duration, "Highest vs lowest"
Adenocarcinoma, Any Product (or Cigarettes if Any not available)
Least adjusted

	N	23
	NS	18
	Wt	230.19
Het	Chi	62.80
Het	df	22
Het	P	***
Fixed	RR	2.29
	RRl	2.01
	RRu	2.61
	P	+++
Random	RR	2.48
	RRl	1.94
	RRu	3.19
	P	+++
Asymm	P	N.S.

Table 3I15 - 6

IESLC - Meta-analysis of Ever/current Smoking, Duration, "Highest vs lowest"
Adenocarcinoma, Any Product (or Cigarettes if Any not available)
Least adjusted

	Sex			
	combined	male	female	Total
N	1	13	9	23
NS	1	13	9	23
Wt	6.72	137.15	86.32	230.19
Het Chi	0.00	20.08	27.48	62.80
Het df	0	12	8	22
Het P	N.S.	(*)	***	***
Fixed RR	0.78	2.05	2.97	2.29
RRl	0.37	1.74	2.40	2.01
RRu	1.66	2.43	3.67	2.61
P	N.S.	+++	+++	+++
Random RR	0.78	2.35	2.95	2.48
RRl	0.37	1.84	1.84	1.94
RRu	1.66	3.01	4.76	3.19
P	N.S.	+++	+++	+++
Between Chi				15.24
Between df				2
Between P				***
Btwn(F) P				(*)
Btwn(R) P				*

Table 3I15 - 7

IESLC - Meta-analysis of Ever/current Smoking, Duration, "Highest vs lowest"
Adenocarcinoma, Any Product (or Cigarettes if Any not available)
Excluded studies (and stage at which they were excluded)

1	BECHER BLOT1 BROWN3 CARPEN CHYOU DARBY DOLL2 GARCIA GRAHAM GURSEL HAMMO2 JAHN JAIN LAUSSM PRESCO QIAO TVERDA WIGLE WYNDE3
2	ALDERS BENSHL BRESLO CHIAZZ DEAN3 DORN ENGELA GAO2 GILLIS GUO HEGMAN HIRAYA HOLE KAUFMA KOO KOULUM LIU4 MIGRAN MRFITR PERNU SEGI2 SPEIZE SUZUK2 SVENSS VUTUC WAKAI WU YUAN
3	GENG MCDUFF SPITZ STASZE WU2 ZHANG
4	AKIBA GARSHI
5	AGUDO AMANDU AMES ARMADA AUVINE AXELSS BEST BOFFET BOUCHA BOUCOT BROSS CEDERL CHEN2 CORREA CPSI CPSII DEAN2 DESTEF DOLL FAN GAO HAMMON HU HU2 HUMBLE JUSSAW KAISE2 KREUZE LETOUR LEVIN LIAW LIU3 LIU5 LUO MCCONN NOTAN2 PEZZO2 PISANI QIAO2 RACHTA RESTRE SADOWS TIZZAN WANG2 WATSON WYNDE2 WYNDE6 WYNDE7 WYNDE8 ZHOU
6	BUFFLE CHEN LUBIN XU
15	BENHAM

Table 3I15 - 8

Potentially overlapping studies

REF	REFGP	PRINC	.	OVERLAP/LINK
LUBIN2	LUBIN2	1		Lubin-combined
OSANN2	KAISER	2		KAISER/OSANN2

Table 3I15 - 9

Most adjusted - insufficient data for meta-analysis

REF	NRR	SEX	AGEL	AGEH	RACE	YF	LC TYPE	LOC	START	ST	NLC	R	VB	P	H	AD	ADOS	SM	PRODUCT	exL	exH	unexL	unexH	De
BUFFLE	554	m	0	0	wh	-	a	NAmer	1976	CC	943	n	bl	y	n	0	0	ev	cig+/-ot	50	999	1	33	st
CHEN	521	c	0	0	all	-	a	As:oth	1987	CC	323	n	ot	n	y	2	0	ev	cig+/-ot	41	999	1	20	st
LUBIN	620	m	0	0	all	-	KII	As:Chi	1984	CC	427	m	ot	y	n	5	1#	ev	cig+/-ot	50	999	1	29	or
XU	510	m	0	0	all	-	a	As:Chi	1985	CC	729	n	ot	n	n	2	0	ev	all/unsp	40	999	1	29	st

Comments on values in listings

LUBIN ADOS Duration of pipe use

REF	NRR	RR	SIG	RRDATA comment
BUFFLE	554	0.80		0
CHEN	521	3.08		0
LUBIN	620	2.51		0
XU	510		*	RR for 1-19/day is 1.9, for 20-29/day is 5.1 and for >=30/day is 2.2

Table 3I16 -

IESLC - Meta-analysis of Ever/current Smoking by Duration, Overview
Adenocarcinoma, Cigarettes (or Any Product if Cigarettes not available)

This analysis is restricted to results for:
1) Ever/current smokers
2) Results by Duration
3) Categorical results by Duration
Results by Duration are grouped under 2 schemes (S1, S2). Each scheme has a set of "key values". An interval is allocated to the category whose key value it includes, and intervals which include none or more than one of the key values are excluded. (Open-ended intervals are coded as 999)

S1	key value	maximum range
1	20	1-34
2	35	21-49
3	50	36+

S2	key value	maximum range
1	5	1-19
2	20	6-29
3	30	21-39
4	40	31-49
5	50	41-998
6	999	51+

4) Adenocarcinoma (or near equivalent)
5) Results complete enough for use in metaanalysis

Within each study, results are then selected (in the following order of preference, within each sex) for:
6) SMKSTA: ever, current
7) PRODUCT: cigarettes regardless of other products, cigarettes only, all/unspec
8) CIGTYPE: all/unspecified, MC regardless of HR, MC only
9) (not applicable)
10) DENOM: never smoked anything, never smoked cigarettes, never any + low, never cigs + low
11) Followup period (YF, prospective studies): whole study (coded as 0) or longest available
12) LCtype: adeno or nearest available, but not squamous. (q = squamous, s = small,
a = adeno, l = large, KII = Kreyberg II, al = alveolar, br = bronchiolar, u = undifferentiated)
13) Race: all or nearest available, otherwise by race (wh or w = white, bl or b = black, hi = hispanic
ch = chinese, jap = japanese, haw = hawaiian, w+o = white + oriental, sca = scandinavian, as = asian)
14) For overlapping studies: principal rather than subsidiary studies
Finally by Age: whole study (coded as 0) if available, otherwise by widest available age group
and then for single sex results (m, f) in preference to results for both sexes combined (c).

Results adjusted (AD) for the most potential confounders are then chosen in Sections -1 to -3
(and those which actually differ from the adjusted results in Table 3I11 - 1 are marked 'x' in Section -1)
and results adjusted for the least confounders in Sections -4 to -6. (Those least adjusted results which
actually differ from the most adjusted are marked 'x' in column X in Section -4)

Section -7 shows excluded studies, together with the stage (as above) at which no qualifying
results were found.

Section -8 lists the potentially overlapping studies which have been included (1=principal, 2=subsidiary).

Section -9 lists any results which would have been included in preference except that they had data not complete
enough for use in meta-analysis, with their significance (yes/no), if known, and any further comment as entered
on the database. It also lists as "gap" any categories for which no data were presented by the original authors.

In addition to those mentioned above, the following fields, levels and abbreviations are used:
* or nk = not known, n = no, y = yes, ot = other
ev = ever, cu = current, nev = never
all/unspec = all or unspecified, cig+/-ot = cigarettes irrespective of other products (cigar, pipe etc)
MC = manufactured cigarettes, HR = hand-rolled cigarettes
exL, exH = range of exposure (low and high) in the smoking group, in terms of Duration
REF: 6-character study reference
NRR: number of the RR on the database within the study
ST : study type (CC = case control, pr or prosp = prospective)
NLC: number of lung cancer cases in whole study
R : risky occupational population (n = no, m = mining, o = other risky)
VB : national cigarette type (V = at least 75% Virginia, bl = at least 75% blended, ot = other)
P : any proxy use
H : full histological confirmation
De : derivation of RR/CI (or = original, st = standard method, ot = other method of estimation)

Table 3I16 - 1

IESLC - Meta-analysis of Ever/current Smoking by Duration, Overview
Adenocarcinoma, Cigarettes (or Any Product if Cigarettes not available)
Most adjusted

REF	NRR	3I11	SEX	AGEL	AGEH	RACE	YF	LC TYPE	LOC	START	ST	NLC	R	VB	P	H	AD	SM	PRODUCT	exL	exH	S1	S2	DENOM	De
BARBON	724		m	0	0	all	-	a	Eu:wst	1979	CC	755	n	bl	y	y	1	ev	all/unsp	1	29	1	0	nev any	or
BARBON	725		m	0	0	all	-	a	Eu:wst	1979	CC	755	n	bl	y	y	1	ev	all/unsp	30	39	2	3	nev any	or
BARBON	726		m	0	0	all	-	a	Eu:wst	1979	CC	755	n	bl	y	y	1	ev	all/unsp	40	49	0	4	nev any	or
BARBON	727		m	0	0	all	-	a	Eu:wst	1979	CC	755	n	bl	y	y	1	ev	all/unsp	50	999	3	0	nev any	or
BUFFLE	509		m	0	0	wh	-	a	NAmer	1976	CC	943	n	bl	y	n	0	ev	cig+/-ot	1	33	1	0	nev cigs	or
BUFFLE	510		m	0	0	wh	-	a	NAmer	1976	CC	943	n	bl	y	n	0	ev	cig+/-ot	34	43	2	4	nev cigs	or
BUFFLE	511		m	0	0	wh	-	a	NAmer	1976	CC	943	n	bl	y	n	0	ev	cig+/-ot	44	49	0	0	nev cigs	or
BUFFLE	512		m	0	0	wh	-	a	NAmer	1976	CC	943	n	bl	y	n	0	ev	cig+/-ot	50	999	3	0	nev cigs	or
CHOI	566		m	0	0	all	-	a	As:oth	1985	CC	375	n	bl	n	n	0	ev	cig+/-ot	1	29	1	0	nev cigs	st
CHOI	567		m	0	0	all	-	a	As:oth	1985	CC	375	n	bl	n	n	0	ev	cig+/-ot	30	39	2	3	nev cigs	st
CHOI	568		m	0	0	all	-	a	As:oth	1985	CC	375	n	bl	n	n	0	ev	cig+/-ot	40	49	0	4	nev cigs	st
CHOI	569		m	0	0	all	-	a	As:oth	1985	CC	375	n	bl	n	n	0	ev	cig+/-ot	50	999	3	0	nev cigs	st
CHOI	578		f	0	0	all	-	a	As:oth	1985	CC	375	n	bl	n	n	0	ev	cig+/-ot	1	29	1	0	nev cigs	st
CHOI	579		f	0	0	all	-	a	As:oth	1985	CC	375	n	bl	n	n	0	ev	cig+/-ot	30	999	0	0	nev cigs	st
DAMBER	540		m	0	0	all	-	a+al+br	Eu:Sca	1972	CC	579	n	bl	y	n	1	ev	all/unsp	1	30	1	0	nev any	or
DAMBER	541		m	0	0	all	-	a+al+br	Eu:Sca	1972	CC	579	n	bl	y	n	1	ev	all/unsp	31	40	2	4	nev any	or
DAMBER	542		m	0	0	all	-	a+al+br	Eu:Sca	1972	CC	579	n	bl	y	n	1	ev	all/unsp	41	50	3	5	nev any	or
DAMBER	543		m	0	0	all	-	a+al+br	Eu:Sca	1972	CC	579	n	bl	y	n	1	ev	all/unsp	51	999	0	6	nev any	or
DORGAN	576		m	0	0	wh	-	a	NAmer	1980	CC	2026	n	bl	y	y	2	ev	cig+/-ot	1	34	1	0	nev any	ot
DORGAN	577		m	0	0	wh	-	a	NAmer	1980	CC	2026	n	bl	y	y	2	ev	cig+/-ot	35	999	0	0	nev any	ot
DORGAN	568		f	0	0	all	-	a	NAmer	1980	CC	2026	n	bl	y	y	3	ev	cig+/-ot	1	34	1	0	nev any	ot
DORGAN	569		f	0	0	all	-	a	NAmer	1980	CC	2026	n	bl	y	y	3	ev	cig+/-ot	35	999	0	0	nev any	ot
DOSEME	516		m	0	0	all	-	not q+s	Eu:bal	1979	CC	1210	n	bl	n	n	2	ev	cig+/-ot	1	10	0	1	nev cigs	or
DOSEME	517		m	0	0	all	-	not q+s	Eu:bal	1979	CC	1210	n	bl	n	n	2	ev	cig+/-ot	11	20	1	2	nev cigs	or
DOSEME	518		m	0	0	all	-	not q+s	Eu:bal	1979	CC	1210	n	bl	n	n	2	ev	cig+/-ot	21	999	0	0	nev cigs	or
GER	507		c	0	0	all	-	a	As:oth	1990	CC	141	n	ot	y	n	5	ev	all/unsp	1	30	1	0	nev any	ot
GER	508		c	0	0	all	-	a	As:oth	1990	CC	141	n	ot	y	n	5	ev	all/unsp	31	999	0	0	nev any	ot
HAENSZ	525		f	0	0	all	-	a	NAmer	1955	CC	158	n	bl	n	y	0	ev	cig+/-ot	1	14	0	1	nev any	st
HAENSZ	526		f	0	0	all	-	a	NAmer	1955	CC	158	n	bl	n	y	0	ev	cig+/-ot	15	999	0	0	nev any	st
JEDRYC	595		m	0	0	all	-	a	Eu:est	1980	CC	1630	n	bl	y	n	3	ev	cig+/-ot	1	19	0	1	nev any	or
JEDRYC	596		m	0	0	all	-	a	Eu:est	1980	CC	1630	n	bl	y	n	3	ev	cig+/-ot	20	39	0	0	nev any	or
JEDRYC	597		m	0	0	all	-	a	Eu:est	1980	CC	1630	n	bl	y	n	3	ev	cig+/-ot	40	999	3	0	nev any	or
JOLY	660		m	0	0	all	-	a	SCAmer	1978	CC	826	n	bl	n	n	0	ev	cig+/-ot	1	29	1	0	nev any	st
JOLY	661		m	0	0	all	-	a	SCAmer	1978	CC	826	n	bl	n	n	0	ev	cig+/-ot	30	39	2	3	nev any	st
JOLY	662		m	0	0	all	-	a	SCAmer	1978	CC	826	n	bl	n	n	0	ev	cig+/-ot	40	49	0	4	nev any	st
JOLY	663		m	0	0	all	-	a	SCAmer	1978	CC	826	n	bl	n	n	0	ev	cig+/-ot	50	999	3	0	nev any	st
JOLY	632		f	0	0	all	-	a	SCAmer	1978	CC	826	n	bl	n	n	0	ev	cig+/-ot	1	29	1	0	nev any	st
JOLY	633		f	0	0	all	-	a	SCAmer	1978	CC	826	n	bl	n	n	0	ev	cig+/-ot	30	39	2	3	nev any	st
JOLY	634		f	0	0	all	-	a	SCAmer	1978	CC	826	n	bl	n	n	0	ev	cig+/-ot	40	49	0	4	nev any	st
JOLY	635		f	0	0	all	-	a	SCAmer	1978	CC	826	n	bl	n	n	0	ev	cig+/-ot	50	999	3	0	nev any	st
KATSOU	520		f	0	0	all	-	a	Eu:bal	1987	CC	101	n	bl	n	n	1	cu	all/unsp	1	29	1	0	nev any	or
KATSOU	521		f	0	0	all	-	a	Eu:bal	1987	CC	101	n	bl	n	n	1	cu	all/unsp	30	999	0	0	nev any	or
LUBIN2	700		m	0	0	all	-	a	Eu:mul	1976	CC	7804	n	bl	n	y	0	ev	cig+/-ot	1	29	1	0	nev any	st
LUBIN2	701		m	0	0	all	-	a	Eu:mul	1976	CC	7804	n	bl	n	y	0	ev	cig+/-ot	30	39	2	3	nev any	st
LUBIN2	702		m	0	0	all	-	a	Eu:mul	1976	CC	7804	n	bl	n	y	0	ev	cig+/-ot	40	49	0	4	nev any	st
LUBIN2	703		m	0	0	all	-	a	Eu:mul	1976	CC	7804	n	bl	n	y	0	ev	cig+/-ot	50	999	3	0	nev any	st
LUBIN2	752		f	0	0	all	-	a	Eu:mul	1976	CC	7804	n	bl	n	y	0	ev	cig+/-ot	1	29	1	0	nev any	st
LUBIN2	753		f	0	0	all	-	a	Eu:mul	1976	CC	7804	n	bl	n	y	0	ev	cig+/-ot	30	39	2	3	nev any	st
LUBIN2	754		f	0	0	all	-	a	Eu:mul	1976	CC	7804	n	bl	n	y	0	ev	cig+/-ot	40	49	0	4	nev any	st
LUBIN2	755		f	0	0	all	-	a	Eu:mul	1976	CC	7804	n	bl	n	y	0	ev	cig+/-ot	50	999	3	0	nev any	st
MATOS	646		m	0	0	all	-	a	SCAmer	1994	CC	200	n	bl	n	n	2	ev	cig+/-ot	1	24	1	0	nev any	or
MATOS	647		m	0	0	all	-	a	SCAmer	1994	CC	200	n	bl	n	n	2	ev	cig+/-ot	25	39	2	3	nev any	or
MATOS	648		m	0	0	all	-	a	SCAmer	1994	CC	200	n	bl	n	n	2	ev	cig+/-ot	40	70	3	0	nev any	or
OSANN2	516		f	0	0	all	-	KII	NAmer	1964	ot	217	n	bl	n	y	1	ev	cig+/-ot	1	20	1	0	nev cigs	or
OSANN2	517		f	0	0	all	-	KII	NAmer	1964	ot	217	n	bl	n	y	1	ev	cig+/-ot	21	999	0	0	nev cigs	or
PEZZOT	516		m	0	0	all	-	a	SCAmer	1987	CC	215	n	bl	n	y	0	ev	cig only	1	30	1	0	nev cigs	st
PEZZOT	517		m	0	0	all	-	a	SCAmer	1987	CC	215	n	bl	n	y	0	ev	cig only	31	40	2	4	nev cigs	st
PEZZOT	518		m	0	0	all	-	a	SCAmer	1987	CC	215	n	bl	n	y	0	ev	cig only	41	999	3	0	nev cigs	st
SOBUE	513		m	0	0	all	-	a	As:Jap	1986	CC	1376	n	bl	n	y	0	cu	cig+/-ot	1	29	1	0	nev cigs	st
SOBUE	514		m	0	0	all	-	a	As:Jap	1986	CC	1376	n	bl	n	y	0	cu	cig+/-ot	30	39	2	3	nev cigs	st
SOBUE	515		m	0	0	all	-	a	As:Jap	1986	CC	1376	n	bl	n	y	0	cu	cig+/-ot	40	49	0	4	nev cigs	st
SOBUE	516		m	0	0	all	-	a	As:Jap	1986	CC	1376	n	bl	n	y	0	cu	cig+/-ot	50	999	3	0	nev cigs	st
WUWILL	526		f	0	0	all	-	a	As:Chi	1985	CC	965	n	ot	n	n	3	ev	cig+/-ot	1	29	1	0	nev cigs	ot
WUWILL	527		f	0	0	all	-	a	As:Chi	1985	CC	965	n	ot	n	n	3	ev	cig+/-ot	30	39	2	3	nev cigs	ot
WUWILL	528		f	0	0	all	-	a	As:Chi	1985	CC	965	n	ot	n	n	3	ev	cig+/-ot	40	999	3	0	nev cigs	ot
ZHENG	512		m	0	0	all	-	a	As:Chi	1982	CC	540	n	ot	*	y	0	ev	cig+/-ot	1	29	1	0	nev cigs	or
ZHENG	513		m	0	0	all	-	a	As:Chi	1982	CC	540	n	ot	*	y	0	ev	cig+/-ot	30	999	0	0	nev cigs	st
ZHENG	516		f	0	0	all	-	a	As:Chi	1982	CC	540	n	ot	*	y	0	ev	cig+/-ot	1	29	1	0	nev cigs	or
ZHENG	517		f	0	0	all	-	a	As:Chi	1982	CC	540	n	ot	*	y	0	ev	cig+/-ot	30	999	0	0	nev cigs	st

Table 3I16 - 1

IESLC - Meta-analysis of Ever/current Smoking by Duration, Overview
Adenocarcinoma, Cigarettes (or Any Product if Cigarettes not available)
Most adjusted

Cigarette type is all/unspec for all RRs

In this overview table, subtotals and Qs values may be invalid and should be ignored

Table 3I16 - 2

IESLC - Meta-analysis of Ever/current Smoking by Duration, Overview
Adenocarcinoma, Cigarettes (or Any Product if Cigarettes not available)
Most adjusted

REF	NRR	SEX	AD	Number Exposed Case	Cont	Non-exposed Case	Cont	RR	95.00%CI
BARBON	724	m	1	15	-	7	-	3.70	(1.40- 9.70)
BARBON	725	m	1	23	-	7	-	5.10	(2.10- 12.50)
BARBON	726	m	1	46	-	7	-	8.20	(3.60- 18.70)
BARBON	727	m	1	67	-	7	-	8.30	(3.70- 18.70)
Subtotal BARBON								6.27	(4.06- 9.67)
BUFFLE	509	m	0	-	-	-	-	4.40	(1.70- 11.50)
BUFFLE	510	m	0	-	-	-	-	5.70	(2.20- 14.80)
BUFFLE	511	m	0	-	-	-	-	4.30	(1.60- 11.40)
BUFFLE	512	m	0	-	-	-	-	3.50	(1.30- 9.70)
Subtotal BUFFLE								4.44	(2.73- 7.22)
CHOI	566	m	0	17	221	7	95	1.04	(0.42- 2.60)
CHOI	567	m	0	13	160	7	65	0.75	(0.29- 1.98)
CHOI	568	m	0	12	64	7	95	2.54	(0.95- 6.81)
CHOI	569	m	0	4	20	7	95	2.71	(0.73- 10.16)
CHOI	578	f	0	2	23	48	164	0.30	(0.07- 1.31)
CHOI	579	f	0	2	3	48	164	2.28	(0.37- 14.03)
Subtotal CHOI								1.23	(0.78- 1.96)
DAMBER	540	m	1	-	-	16	-	1.80	(0.60- 5.40)
DAMBER	541	m	1	-	-	16	-	1.20	(0.20- 6.00)
DAMBER	542	m	1	-	-	16	-	3.40	(1.30- 9.10)
DAMBER	543	m	1	-	-	16	-	2.50	(0.90- 6.70)
Subtotal DAMBER								2.35	(1.35- 4.10)
DORGAN	576	m	2	-	-	-	-	2.14	(0.78- 5.84)
DORGAN	577	m	2	-	-	-	-	6.73	(2.66- 17.03)
DORGAN	568	f	3	-	-	-	-	2.43	(1.65- 3.59)
DORGAN	569	f	3	-	-	-	-	5.14	(3.63- 7.28)
Subtotal DORGAN								3.72	(2.92- 4.74)
DOSEME	516	m	2	5	-	24	-	0.80	(0.30- 2.50)
DOSEME	517	m	2	26	-	24	-	3.30	(2.20- 7.50)
DOSEME	518	m	2	69	-	24	-	4.10	(2.20- 7.50)
Subtotal DOSEME								2.96	(1.98- 4.42)
GER	507	c	5	14	-	37	-	1.40	(0.62- 3.19)
GER	508	c	5	21	-	37	-	0.85	(0.39- 1.86)
Subtotal GER								1.08	(0.61- 1.90)
HAENSZ	525	f	0	2	26	37	236	0.49	(0.11- 2.15)
HAENSZ	526	f	0	16	77	37	236	1.33	(0.70- 2.51)
Subtotal HAENSZ								1.13	(0.63- 2.04)
JEDRYC	595	m	3	2	-	7	-	1.10	(0.20- 5.95)
JEDRYC	596	m	3	43	-	7	-	3.47	(1.40- 8.58)
JEDRYC	597	m	3	47	-	7	-	4.41	(1.86- 10.45)
Subtotal JEDRYC								3.38	(1.88- 6.07)
JOLY	660	m	0	5	109	5	218	2.00	(0.57- 7.06)
JOLY	661	m	0	12	165	5	218	3.17	(1.10- 9.18)
JOLY	662	m	0	22	182	5	218	5.27	(1.96- 14.19)
JOLY	663	m	0	33	253	5	218	5.69	(2.18- 14.82)
JOLY	632	f	0	10	54	25	283	2.10	(0.95- 4.61)
JOLY	633	f	0	6	24	25	283	2.83	(1.06- 7.57)
JOLY	634	f	0	10	24	25	283	4.72	(2.03- 10.96)
JOLY	635	f	0	7	20	25	283	3.96	(1.53- 10.27)
Subtotal JOLY								3.51	(2.50- 4.92)
KATSOU	520	f	1	6	-	30	-	0.84	(0.27- 2.58)
KATSOU	521	f	1	9	-	30	-	3.61	(1.17- 11.17)
Subtotal KATSOU								1.74	(0.78- 3.87)
LUBIN2	700	m	0	131	2964	57	2616	2.03	(1.48- 2.78)
LUBIN2	701	m	0	242	3473	57	2616	3.20	(2.39- 4.29)
LUBIN2	702	m	0	208	2540	57	2616	3.76	(2.79- 5.06)
LUBIN2	703	m	0	90	1460	57	2616	2.83	(2.02- 3.97)
LUBIN2	752	f	0	174	229	138	1180	6.50	(4.99- 8.46)
LUBIN2	753	f	0	383	186	138	1180	17.61	(13.73- 22.58)
LUBIN2	754	f	0	284	118	138	1180	20.58	(15.59- 27.17)
LUBIN2	755	f	0	181	34	138	1180	45.52	(30.31- 68.36)
Subtotal LUBIN2								7.21	(6.49- 8.00)
MATOS	646	m	2	7	-	5	-	1.70	(0.50- 5.50)
MATOS	647	m	2	39	-	5	-	7.30	(2.70- 19.70)
MATOS	648	m	2	33	-	5	-	10.70	(3.80- 29.90)
Subtotal MATOS								5.70	(3.08- 10.54)
OSANN2	516	f	1	10	-	22	-	0.70	(0.20- 1.90)
OSANN2	517	f	1	51	-	22	-	4.10	(1.80- 9.40)
Subtotal OSANN2								2.21	(1.13- 4.30)
PEZZOT	516	m	0	11	134	3	116	3.17	(0.86- 11.65)
PEZZOT	517	m	0	22	82	3	116	10.37	(3.01- 35.81)

Table 3I16 - 2

IESLC - Meta-analysis of Ever/current Smoking by Duration, Overview
Adenocarcinoma, Cigarettes (or Any Product if Cigarettes not available)
Most adjusted

REF	NRR	SEX	AD	Number Exposed Case	Cont	Non-exposed Case	Cont	RR	95.00%CI
PEZZOT	518	m	0	27	101	3	116	10.34	(3.04- 35.09)
Subtotal PEZZOT								7.18	(3.49- 14.81)
SOBUE	513	m	0	33	119	27	128	1.31	(0.75- 2.32)
SOBUE	514	m	0	62	200	27	128	1.47	(0.89- 2.43)
SOBUE	515	m	0	96	174	27	128	2.62	(1.61- 4.24)
SOBUE	516	m	0	43	73	27	128	2.79	(1.59- 4.89)
Subtotal SOBUE								1.96	(1.51- 2.54)
WUWILL	526	f	3	34	-	172	-	0.80	(0.51- 1.25)
WUWILL	527	f	3	52	-	172	-	1.99	(1.37- 2.89)
WUWILL	528	f	3	52	-	172	-	2.09	(1.40- 3.10)
Subtotal WUWILL								1.58	(1.25- 2.00)
ZHENG	512	m	0	24	75	29	94	1.04	(0.56- 1.93)
ZHENG	513	m	0	99	143	29	94	2.24	(1.38- 3.66)
ZHENG	516	f	0	9	17	119	184	0.82	(0.35- 1.90)
ZHENG	517	f	0	24	27	119	184	1.37	(0.76- 2.49)
Subtotal ZHENG								1.45	(1.07- 1.96)
Partial Totals				2987	13574	2405	19754		

*prospective study

REF	NRR	SEX	AD	Ys	Ws	Qs	Ps
BARBON	724	m	1	1.31	4.10	0.02	0.0081
BARBON	725	m	1	1.63	4.83	0.31	0.0003
BARBON	726	m	1	2.10	5.66	3.02	0.0000
BARBON	727	m	1	2.12	5.85	3.22	0.0000
Subtotal BARBON				1.84	20.44	6.57	
BUFFLE	509	m	0	1.48	4.20	0.05	0.0024
BUFFLE	510	m	0	1.74	4.23	0.57	0.0003
BUFFLE	511	m	0	1.46	3.99	0.03	0.0036
BUFFLE	512	m	0	1.25	3.80	0.06	0.0145
Subtotal BUFFLE				1.49	16.22	0.70	
CHOI	566	m	0	0.04	4.61	8.18	0.9264
CHOI	567	m	0	-0.28	4.14	11.36	0.5664
CHOI	568	m	0	0.93	3.96	0.77	0.0630
CHOI	569	m	0	1.00	2.21	0.31	0.1381
CHOI	578	f	0	-1.21	1.75	11.74	0.1081
CHOI	579	f	0	0.82	1.16	0.35	0.3748
Subtotal CHOI				0.21	17.84	32.71	
DAMBER	540	m	1	0.59	3.18	1.97	0.2943
DAMBER	541	m	1	0.18	1.33	1.89	0.8336
DAMBER	542	m	1	1.22	4.06	0.09	0.0137
DAMBER	543	m	1	0.92	3.81	0.80	0.0736
Subtotal DAMBER				0.85	12.38	4.75	
DORGAN	576	m	2	0.76	3.79	1.43	0.1385
DORGAN	577	m	2	1.91	4.46	1.26	0.0001
DORGAN	568	f	3	0.89	25.43	6.01	0.0000
DORGAN	569	f	3	1.64	31.73	2.19	0.0000
Subtotal DORGAN				1.31	65.41	10.90	
DOSEME	516	m	2	-0.22	3.42	8.72	0.6799
DOSEME	517	m	2	1.19	10.22	0.33	0.0001
DOSEME	518	m	2	1.41	10.22	0.01	0.0000
Subtotal DOSEME				1.08	23.85	9.07	
GER	507	c	5	0.34	5.73	6.17	0.4207
GER	508	c	5	-0.16	6.30	14.87	0.6834
Subtotal GER				0.08	12.02	21.04	
HAENSZ	525	f	0	-0.71	1.76	7.64	0.3455
HAENSZ	526	f	0	0.28	9.37	11.18	0.3886
Subtotal HAENSZ				0.12	11.12	18.82	
JEDRYC	595	m	3	0.10	1.33	2.18	0.9123
JEDRYC	596	m	3	1.24	4.67	0.08	0.0071
JEDRYC	597	m	3	1.48	5.16	0.06	0.0008
Subtotal JEDRYC				1.22	11.17	2.32	
JOLY	660	m	0	0.69	2.42	1.12	0.2812
JOLY	661	m	0	1.15	3.40	0.16	0.0333
JOLY	662	m	0	1.66	3.91	0.32	0.0010
JOLY	663	m	0	1.74	4.19	0.55	0.0004
JOLY	632	f	0	0.74	6.17	2.48	0.0660
JOLY	633	f	0	1.04	3.97	0.44	0.0382
JOLY	634	f	0	1.55	5.40	0.17	0.0003
JOLY	635	f	0	1.38	4.23	0.00	0.0046
Subtotal JOLY				1.25	33.69	5.26	

Table 3I16 - 2

IESLC - Meta-analysis of Ever/current Smoking by Duration, Overview
Adenocarcinoma, Cigarettes (or Any Product if Cigarettes not available)
Most adjusted

REF	NRR	SEX	AD	Ys	Ws	Qs	Ps
KATSOU	520	f	1	-0.17	3.02	7.23	0.7620
KATSOU	521	f	1	1.28	3.02	0.02	0.0257
Subtotal KATSOU				0.55	6.03	7.26	
LUBIN2	700	m	0	0.71	38.61	17.18	0.0000
LUBIN2	701	m	0	1.16	44.75	2.01	0.0000
LUBIN2	702	m	0	1.32	43.24	0.11	0.0000
LUBIN2	703	m	0	1.04	33.64	3.76	0.0000
LUBIN2	752	f	0	1.87	54.92	13.57	0.0000
LUBIN2	753	f	0	2.87	62.18	138.81	0.0000
LUBIN2	754	f	0	3.02	49.78	135.53	0.0000
LUBIN2	755	f	0	3.82	23.24	138.80	0.0000
Subtotal LUBIN2				1.98	350.37	449.78	
MATOS	646	m	2	0.53	2.67	1.90	0.3857
MATOS	647	m	2	1.99	3.89	1.47	0.0001
MATOS	648	m	2	2.37	3.61	3.58	0.0000
Subtotal MATOS				1.74	10.17	6.95	
OSANN2	516	f	1	-0.36	3.03	9.08	0.5346
OSANN2	517	f	1	1.41	5.62	0.01	0.0008
Subtotal OSANN2				0.79	8.66	9.09	
PEZZOT	516	m	0	1.16	2.27	0.11	0.0817
PEZZOT	517	m	0	2.34	2.50	2.33	0.0002
PEZZOT	518	m	0	2.34	2.57	2.38	0.0002
Subtotal PEZZOT				1.97	7.34	4.82	
SOBUE	513	m	0	0.27	11.97	14.50	0.3439
SOBUE	514	m	0	0.39	15.16	14.83	0.1339
SOBUE	515	m	0	0.96	16.39	2.79	0.0001
SOBUE	516	m	0	1.03	12.22	1.47	0.0003
Subtotal SOBUE				0.67	55.74	33.60	
WUWILL	526	f	3	-0.22	19.12	48.78	0.3292
WUWILL	527	f	3	0.69	27.58	12.98	0.0003
WUWILL	528	f	3	0.74	24.32	9.87	0.0003
Subtotal WUWILL				0.46	71.01	71.63	
ZHENG	512	m	0	0.04	9.99	17.87	0.9080
ZHENG	513	m	0	0.81	16.07	5.15	0.0012
ZHENG	516	f	0	-0.20	5.44	13.49	0.6405
ZHENG	517	f	0	0.32	10.81	12.05	0.2958
Subtotal ZHENG				0.37	42.31	48.56	

N	69
NS	18

Table 3I16 - 3

IESLC - Meta-analysis of Ever/current Smoking by Duration, Overview
Adenocarcinoma, Cigarettes (or Any Product if Cigarettes not available)
Most adjusted

	Sex			
	combined	male	female	Total
N	2	44	23	69
NS	1	13	9	23

In this overview table, other than the "N" rows, entries in the "absent" and "Total" columns may be invalid and should be ignored

	Duration of smoking (broad categories)				
	absent	1-34k20	21-49k35	36+k50	Total
N	23	21	12	13	69
NS	15	16	10	11	52
Wt	246.07	222.65	177.96	129.10	775.79
Het Chi	204.35	120.89	174.97	153.89	743.81
Het df	22	20	11	12	68
Het P	***	***	***	***	***
Fixed RR	4.55	2.33	5.13	5.24	3.95
RRl	4.02	2.04	4.43	4.41	3.68
RRu	5.16	2.66	5.95	6.23	4.24
P	+++	+++	+++	+++	+++
Random RR	3.01	1.72	3.53	5.25	2.89
RRl	1.99	1.20	1.81	2.70	2.25
RRu	4.57	2.46	6.88	10.20	3.70
P	+++	++	+++	+++	+++

	Duration of smoking (narrow categories)							
	absent	1-19k1	6-29k20	21-39k30	31-49k40	41-998k50	51+k999	Total
N	44	3	1	9	10	1	1	69
NS	18	3	1	7	8	1	1	38
Wt	444.89	6.51	10.22	169.90	136.40	4.06	3.81	775.79
Het Chi	367.35	0.53	0.00	170.88	100.52	0.00	0.00	743.81
Het df	43	2	0	8	9	0	0	68
Het P	***	N.S.	N.S.	***	***	N.S.	N.S.	***
Fixed RR	3.09	0.75	3.30	5.13	7.11	3.40	2.50	3.95
RRl	2.82	0.35	1.79	4.41	6.01	1.29	0.92	3.68
RRu	3.39	1.61	6.09	5.96	8.41	9.00	6.82	4.24
P	+++	N.S.	+++	+++	+++	+	(+)	+++
Random RR	2.64	0.75	3.30	3.30	5.13	3.40	2.50	2.89
RRl	1.98	0.35	1.79	1.52	2.67	1.29	0.92	2.25
RRu	3.53	1.61	6.09	7.16	9.84	9.00	6.82	3.70
P	+++	N.S.	+++	++	+++	+	(+)	+++

Table 3I16 - 3

IESLC - Meta-analysis of Ever/current Smoking by Duration, Overview
Adenocarcinoma, Cigarettes (or Any Product if Cigarettes not available)
Most adjusted

MALES

		Duration of smoking (broad categories)				
		absent	1-34k20	21-49k35	36+k50	Total
	N	13	12	9	10	44
	NS	11	12	9	10	42
	Wt	121.14	98.04	84.23	77.32	380.72
Het	Chi	21.62	15.64	26.85	15.18	101.70
Het	df	12	11	8	9	43
Het	P	*	N.S.	***	(*)	***
Fixed	RR	3.32	1.95	2.90	3.71	2.87
	RRl	2.78	1.60	2.34	2.97	2.60
	RRu	3.97	2.38	3.59	4.64	3.18
	P	+++	+++	+++	+++	+++
Random	RR	3.28	1.97	3.10	4.30	3.01
	RRl	2.49	1.51	1.91	3.08	2.53
	RRu	4.31	2.57	5.04	5.99	3.59
	P	+++	+++	+++	+++	+++

		Duration of smoking (narrow categories)							
		absent	1-19k1	6-29k20	21-39k30	31-49k40	41-998k50	51+k999	Total
	N	25	2	1	6	8	1	1	44
	NS	13	2	1	6	8	1	1	31
	Wt	200.49	4.75	10.22	76.17	81.22	4.06	3.81	380.72
Het	Chi	55.61	0.10	0.00	19.51	11.68	0.00	0.00	101.70
Het	df	24	1	0	5	7	0	0	43
Het	P	***	N.S.	N.S.	**	N.S.	N.S.	N.S.	***
Fixed	RR	2.67	0.87	3.30	2.72	3.81	3.40	2.50	2.87
	RRl	2.33	0.36	1.79	2.17	3.06	1.29	0.92	2.60
	RRu	3.07	2.15	6.09	3.40	4.73	9.00	6.82	3.18
	P	+++	N.S.	+++	+++	+++	+	(+)	+++
Random	RR	2.98	0.87	3.30	2.67	4.05	3.40	2.50	3.01
	RRl	2.36	0.36	1.79	1.54	2.86	1.29	0.92	2.53
	RRu	3.77	2.15	6.09	4.62	5.73	9.00	6.82	3.59
	P	+++	N.S.	+++	+++	+++	+	(+)	+++

FEMALES

		Duration of smoking (broad categories)				
		absent	1-34k20	21-49k35	36+k50	Total
	N	9	8	3	3	23
	NS	8	8	3	3	22
	Wt	118.64	118.88	93.73	51.79	383.04
Het	Chi	133.09	97.18	95.91	115.71	533.56
Het	df	8	7	2	2	22
Het	P	***	***	***	***	***
Fixed	RR	6.86	2.76	8.58	8.78	5.65
	RRl	5.73	2.31	7.01	6.68	5.11
	RRu	8.21	3.31	10.51	11.52	6.24
	P	+++	+++	+++	+++	+++
Random	RR	3.11	1.29	4.72	7.29	2.72
	RRl	1.34	0.60	0.87	0.77	1.61
	RRu	7.24	2.82	25.49	69.06	4.61
	P	++	N.S.	(+)	(+)	+++

Table 3I16 - 3

IESLC - Meta-analysis of Ever/current Smoking by Duration, Overview
Adenocarcinoma, Cigarettes (or Any Product if Cigarettes not available)
Most adjusted

FEMALES

	absent	1-19k1	6-29k20	21-39k30	31-49k40	41-998k50	51+k999	Total
		Duration of smoking (narrow categories)						
N	17	1		3	2			23
NS	9	1		3	2			15
Wt	232.37	1.76		93.73	55.18			383.04
Het Chi	285.88	0.00		95.91	10.57			533.56
Het df	16	0		2	1			22
Het P	***	N.S.		***	**			***
Fixed RR	3.70	0.49		8.58	17.82			5.65
RRl	3.25	0.11		7.01	13.68			5.11
RRu	4.21	2.15		10.51	23.20			6.24
P	+++	N.S.		+++	+++			+++
Random RR	2.27	0.49		4.72	10.42			2.72
RRl	1.27	0.11		0.87	2.47			1.61
RRu	4.07	2.15		25.49	43.96			4.61
P	++	N.S.		(+)	++			+++

Table 3I16 - 4

IESLC - Meta-analysis of Ever/current Smoking by Duration, Overview
Adenocarcinoma, Cigarettes (or Any Product if Cigarettes not available)
Least adjusted

REF	NRR	X	SEX	AGEL	AGEH	RACE	YF	LC TYPE	LOC	START	ST	NLC	R	VB	P	H	AD	SM	PRODUCT	exL	exH	S1	S2	DENOM	De
BARBON	717	x	m	0	0	all	-	a	Eu:wst	1979	CC	755	n	bl	y	y	0	ev	all/unsp	1	29	1	0	nev any	st
BARBON	718	x	m	0	0	all	-	a	Eu:wst	1979	CC	755	n	bl	y	y	0	ev	all/unsp	30	39	2	3	nev any	st
BARBON	719	x	m	0	0	all	-	a	Eu:wst	1979	CC	755	n	bl	y	y	0	ev	all/unsp	40	49	0	4	nev any	st
BARBON	720	x	m	0	0	all	-	a	Eu:wst	1979	CC	755	n	bl	y	y	0	ev	all/unsp	50	999	3	0	nev any	st
BUFFLE	509		m	0	0	wh	-	a	NAmer	1976	CC	943	n	bl	y	n	0	ev	cig+/-ot	1	33	1	0	nev cigs	or
BUFFLE	510		m	0	0	wh	-	a	NAmer	1976	CC	943	n	bl	y	n	0	ev	cig+/-ot	34	43	2	4	nev cigs	or
BUFFLE	511		m	0	0	wh	-	a	NAmer	1976	CC	943	n	bl	y	n	0	ev	cig+/-ot	44	49	0	0	nev cigs	or
BUFFLE	512		m	0	0	wh	-	a	NAmer	1976	CC	943	n	bl	y	n	0	ev	cig+/-ot	50	999	3	0	nev cigs	or
CHOI	566		m	0	0	all	-	a	As:oth	1985	CC	375	n	bl	n	n	0	ev	cig+/-ot	1	29	1	0	nev cigs	st
CHOI	567		m	0	0	all	-	a	As:oth	1985	CC	375	n	bl	n	n	0	ev	cig+/-ot	30	39	2	3	nev cigs	st
CHOI	568		m	0	0	all	-	a	As:oth	1985	CC	375	n	bl	n	n	0	ev	cig+/-ot	40	49	0	4	nev cigs	st
CHOI	569		m	0	0	all	-	a	As:oth	1985	CC	375	n	bl	n	n	0	ev	cig+/-ot	50	999	3	0	nev cigs	st
CHOI	578		f	0	0	all	-	a	As:oth	1985	CC	375	n	bl	n	n	0	ev	cig+/-ot	1	29	1	0	nev cigs	st
CHOI	579		f	0	0	all	-	a	As:oth	1985	CC	375	n	bl	n	n	0	ev	cig+/-ot	30	999	0	0	nev cigs	st
DAMBER	540		m	0	0	all	-	a+al+br	Eu:Sca	1972	CC	579	n	bl	y	n	1	ev	all/unsp	1	30	1	0	nev any	or
DAMBER	541		m	0	0	all	-	a+al+br	Eu:Sca	1972	CC	579	n	bl	y	n	1	ev	all/unsp	31	40	2	4	nev any	or
DAMBER	542		m	0	0	all	-	a+al+br	Eu:Sca	1972	CC	579	n	bl	y	n	1	ev	all/unsp	41	50	3	5	nev any	or
DAMBER	543		m	0	0	all	-	a+al+br	Eu:Sca	1972	CC	579	n	bl	y	n	1	ev	all/unsp	51	999	0	6	nev any	or
DORGAN	576		m	0	0	wh	-	a	NAmer	1980	CC	2026	n	bl	y	y	2	ev	cig+/-ot	1	34	1	0	nev any	ot
DORGAN	577		m	0	0	wh	-	a	NAmer	1980	CC	2026	n	bl	y	y	2	ev	cig+/-ot	35	999	0	0	nev any	ot
DORGAN	568		f	0	0	all	-	a	NAmer	1980	CC	2026	n	bl	y	y	3	ev	cig+/-ot	1	34	1	0	nev any	ot
DORGAN	569		f	0	0	all	-	a	NAmer	1980	CC	2026	n	bl	y	y	3	ev	cig+/-ot	35	999	0	0	nev any	ot
DOSEME	516		m	0	0	all	-	not q+s	Eu:bal	1979	CC	1210	n	bl	n	n	2	ev	cig+/-ot	1	10	0	1	nev cigs	or
DOSEME	517		m	0	0	all	-	not q+s	Eu:bal	1979	CC	1210	n	bl	n	n	2	ev	cig+/-ot	11	20	1	2	nev cigs	or
DOSEME	518		m	0	0	all	-	not q+s	Eu:bal	1979	CC	1210	n	bl	n	n	2	ev	cig+/-ot	21	999	0	0	nev cigs	or
GER	501	x	c	0	0	all	-	a	As:oth	1990	CC	141	n	ot	y	n	0	ev	all/unsp	1	30	1	0	nev any	st
GER	502	x	c	0	0	all	-	a	As:oth	1990	CC	141	n	ot	y	n	0	ev	all/unsp	31	999	0	0	nev any	st
HAENSZ	525		f	0	0	all	-	a	NAmer	1955	CC	158	n	bl	n	y	0	ev	cig+/-ot	1	14	0	1	nev any	st
HAENSZ	526		f	0	0	all	-	a	NAmer	1955	CC	158	n	bl	n	y	0	ev	cig+/-ot	15	999	0	0	nev any	st
JEDRYC	519	x	m	0	0	all	-	a	Eu:est	1980	CC	1630	n	bl	y	n	0	ev	cig+/-ot	1	19	0	1	nev any	st
JEDRYC	520	x	m	0	0	all	-	a	Eu:est	1980	CC	1630	n	bl	y	n	0	ev	cig+/-ot	20	29	1	2	nev any	st
JEDRYC	521	x	m	0	0	all	-	a	Eu:est	1980	CC	1630	n	bl	y	n	0	ev	cig+/-ot	30	39	2	3	nev any	st
JEDRYC	522	x	m	0	0	all	-	a	Eu:est	1980	CC	1630	n	bl	y	n	0	ev	cig+/-ot	40	49	0	4	nev any	st
JEDRYC	523	x	m	0	0	all	-	a	Eu:est	1980	CC	1630	n	bl	y	n	0	ev	cig+/-ot	50	999	3	0	nev any	st
JOLY	660		m	0	0	all	-	a	SCAmer	1978	CC	826	n	bl	n	n	0	ev	cig+/-ot	1	29	1	0	nev any	st
JOLY	661		m	0	0	all	-	a	SCAmer	1978	CC	826	n	bl	n	n	0	ev	cig+/-ot	30	39	2	3	nev any	st
JOLY	662		m	0	0	all	-	a	SCAmer	1978	CC	826	n	bl	n	n	0	ev	cig+/-ot	40	49	0	4	nev any	st
JOLY	663		m	0	0	all	-	a	SCAmer	1978	CC	826	n	bl	n	n	0	ev	cig+/-ot	50	999	3	0	nev any	st
JOLY	632		f	0	0	all	-	a	SCAmer	1978	CC	826	n	bl	n	n	0	ev	cig+/-ot	1	29	1	0	nev any	st
JOLY	633		f	0	0	all	-	a	SCAmer	1978	CC	826	n	bl	n	n	0	ev	cig+/-ot	30	39	2	3	nev any	st
JOLY	634		f	0	0	all	-	a	SCAmer	1978	CC	826	n	bl	n	n	0	ev	cig+/-ot	40	49	0	4	nev any	st
JOLY	635		f	0	0	all	-	a	SCAmer	1978	CC	826	n	bl	n	n	0	ev	cig+/-ot	50	999	3	0	nev any	st
KATSOU	515	x	f	0	0	all	-	a	Eu:bal	1987	CC	101	n	bl	n	n	0	cu	all/unsp	1	29	1	0	nev any	st
KATSOU	516	x	f	0	0	all	-	a	Eu:bal	1987	CC	101	n	bl	n	n	0	cu	all/unsp	30	999	0	0	nev any	st
LUBIN2	700		m	0	0	all	-	a	Eu:mul	1976	CC	7804	n	bl	n	y	0	ev	cig+/-ot	1	29	1	0	nev any	st
LUBIN2	701		m	0	0	all	-	a	Eu:mul	1976	CC	7804	n	bl	n	y	0	ev	cig+/-ot	30	39	2	3	nev any	st
LUBIN2	702		m	0	0	all	-	a	Eu:mul	1976	CC	7804	n	bl	n	y	0	ev	cig+/-ot	40	49	0	4	nev any	st
LUBIN2	703		m	0	0	all	-	a	Eu:mul	1976	CC	7804	n	bl	n	y	0	ev	cig+/-ot	50	999	3	0	nev any	st
LUBIN2	752		f	0	0	all	-	a	Eu:mul	1976	CC	7804	n	bl	n	y	0	ev	cig+/-ot	1	29	1	0	nev any	st
LUBIN2	753		f	0	0	all	-	a	Eu:mul	1976	CC	7804	n	bl	n	y	0	ev	cig+/-ot	30	39	2	3	nev any	st
LUBIN2	754		f	0	0	all	-	a	Eu:mul	1976	CC	7804	n	bl	n	y	0	ev	cig+/-ot	40	49	0	4	nev any	st
LUBIN2	755		f	0	0	all	-	a	Eu:mul	1976	CC	7804	n	bl	n	y	0	ev	cig+/-ot	50	999	3	0	nev any	st
MATOS	641	x	m	0	0	all	-	a	SCAmer	1994	CC	200	n	bl	n	n	0	ev	cig+/-ot	1	24	1	0	nev any	st
MATOS	642	x	m	0	0	all	-	a	SCAmer	1994	CC	200	n	bl	n	n	0	ev	cig+/-ot	25	39	2	3	nev any	st
MATOS	643	x	m	0	0	all	-	a	SCAmer	1994	CC	200	n	bl	n	n	0	ev	cig+/-ot	40	70	3	0	nev any	st
OSANN2	513	x	f	0	0	all	-	KII	NAmer	1964	ot	217	n	bl	n	y	0	ev	cig+/-ot	1	20	1	0	nev cigs	st
OSANN2	514	x	f	0	0	all	-	KII	NAmer	1964	ot	217	n	bl	n	y	0	ev	cig+/-ot	21	999	0	0	nev cigs	st
PEZZOT	516		m	0	0	all	-	a	SCAmer	1987	CC	215	n	bl	n	y	0	ev	cig only	1	30	1	0	nev cigs	st
PEZZOT	517		m	0	0	all	-	a	SCAmer	1987	CC	215	n	bl	n	y	0	ev	cig only	31	40	2	4	nev cigs	st
PEZZOT	518		m	0	0	all	-	a	SCAmer	1987	CC	215	n	bl	n	y	0	ev	cig only	41	999	3	0	nev cigs	st
SOBUE	513		m	0	0	all	-	a	As:Jap	1986	CC	1376	n	bl	n	y	0	cu	cig+/-ot	1	29	1	0	nev cigs	st
SOBUE	514		m	0	0	all	-	a	As:Jap	1986	CC	1376	n	bl	n	y	0	cu	cig+/-ot	30	39	2	3	nev cigs	st
SOBUE	515		m	0	0	all	-	a	As:Jap	1986	CC	1376	n	bl	n	y	0	cu	cig+/-ot	40	49	0	4	nev cigs	st
SOBUE	516		m	0	0	all	-	a	As:Jap	1986	CC	1376	n	bl	n	y	0	cu	cig+/-ot	50	999	3	0	nev cigs	st
WUWILL	511	x	f	0	0	all	-	a	As:Chi	1985	CC	965	n	ot	n	n	0	ev	cig+/-ot	1	29	1	0	nev cigs	st
WUWILL	512	x	f	0	0	all	-	a	As:Chi	1985	CC	965	n	ot	n	n	0	ev	cig+/-ot	30	39	2	3	nev cigs	st
WUWILL	513	x	f	0	0	all	-	a	As:Chi	1985	CC	965	n	ot	n	n	0	ev	cig+/-ot	40	999	3	0	nev cigs	st
ZHENG	512		m	0	0	all	-	a	As:Chi	1982	CC	540	n	ot	*	y	0	ev	cig+/-ot	1	29	1	0	nev cigs	or
ZHENG	513		m	0	0	all	-	a	As:Chi	1982	CC	540	n	ot	*	y	0	ev	cig+/-ot	30	999	0	0	nev cigs	st
ZHENG	516		f	0	0	all	-	a	As:Chi	1982	CC	540	n	ot	*	y	0	ev	cig+/-ot	1	29	1	0	nev cigs	or
ZHENG	517		f	0	0	all	-	a	As:Chi	1982	CC	540	n	ot	*	y	0	ev	cig+/-ot	30	999	0	0	nev cigs	st

Table 3I16 - 4

IESLC - Meta-analysis of Ever/current Smoking by Duration, Overview
Adenocarcinoma, Cigarettes (or Any Product if Cigarettes not available)
Least adjusted

Cigarette type is all/unspec for all RRs

In this overview table, subtotals and Qs values may be invalid and should be ignored

Table 3I16 - 5

IESLC - Meta-analysis of Ever/current Smoking by Duration, Overview
Adenocarcinoma, Cigarettes (or Any Product if Cigarettes not available)
Least adjusted

				Number Exposed		Non-exposed			
REF	NRR	SEX	AD	Case	Cont	Case	Cont	RR	95.00%CI
BARBON	717	m	0	15	91	7	188	4.43	(1.74- 11.24)
BARBON	718	m	0	23	102	7	188	6.06	(2.51- 14.60)
BARBON	719	m	0	46	139	7	188	8.89	(3.90- 20.28)
BARBON	720	m	0	67	235	7	188	7.66	(3.43- 17.07)
Subtotal BARBON								6.72	(4.38- 10.30)
BUFFLE	509	m	0	-	-	-	-	4.40	(1.70- 11.50)
BUFFLE	510	m	0	-	-	-	-	5.70	(2.20- 14.80)
BUFFLE	511	m	0	-	-	-	-	4.30	(1.60- 11.40)
BUFFLE	512	m	0	-	-	-	-	3.50	(1.30- 9.70)
Subtotal BUFFLE								4.44	(2.73- 7.22)
CHOI	566	m	0	17	221	7	95	1.04	(0.42- 2.60)
CHOI	567	m	0	13	160	7	65	0.75	(0.29- 1.98)
CHOI	568	m	0	12	64	7	95	2.54	(0.95- 6.81)
CHOI	569	m	0	4	20	7	95	2.71	(0.73- 10.16)
CHOI	578	f	0	2	23	48	164	0.30	(0.07- 1.31)
CHOI	579	f	0	2	3	48	164	2.28	(0.37- 14.03)
Subtotal CHOI								1.23	(0.78- 1.96)
DAMBER	540	m	1	-	-	16	-	1.80	(0.60- 5.40)
DAMBER	541	m	1	-	-	16	-	1.20	(0.20- 6.00)
DAMBER	542	m	1	-	-	16	-	3.40	(1.30- 9.10)
DAMBER	543	m	1	-	-	16	-	2.50	(0.90- 6.70)
Subtotal DAMBER								2.35	(1.35- 4.10)
DORGAN	576	m	2	-	-	-	-	2.14	(0.78- 5.84)
DORGAN	577	m	2	-	-	-	-	6.73	(2.66- 17.03)
DORGAN	568	f	3	-	-	-	-	2.43	(1.65- 3.59)
DORGAN	569	f	3	-	-	-	-	5.14	(3.63- 7.28)
Subtotal DORGAN								3.72	(2.92- 4.74)
DOSEME	516	m	2	5	-	24	-	0.80	(0.30- 2.50)
DOSEME	517	m	2	26	-	24	-	3.30	(2.20- 7.50)
DOSEME	518	m	2	69	-	24	-	4.10	(2.20- 7.50)
Subtotal DOSEME								2.96	(1.98- 4.42)
GER	501	c	0	14	51	37	149	1.11	(0.55- 2.21)
GER	502	c	0	21	98	37	149	0.86	(0.48- 1.56)
Subtotal GER								0.96	(0.61- 1.50)
HAENSZ	525	f	0	2	26	37	236	0.49	(0.11- 2.15)
HAENSZ	526	f	0	16	77	37	236	1.33	(0.70- 2.51)
Subtotal HAENSZ								1.13	(0.63- 2.04)
JEDRYC	519	m	0	2	68	7	289	1.21	(0.25- 5.98)
JEDRYC	520	m	0	15	160	7	289	3.87	(1.55- 9.69)
JEDRYC	521	m	0	28	231	7	289	5.00	(2.15- 11.66)
JEDRYC	522	m	0	30	223	7	289	5.55	(2.40- 12.88)
JEDRYC	523	m	0	17	214	7	289	3.28	(1.34- 8.05)
Subtotal JEDRYC								4.02	(2.63- 6.12)
JOLY	660	m	0	5	109	5	218	2.00	(0.57- 7.06)
JOLY	661	m	0	12	165	5	218	3.17	(1.10- 9.18)
JOLY	662	m	0	22	182	5	218	5.27	(1.96- 14.19)
JOLY	663	m	0	33	253	5	218	5.69	(2.18- 14.82)
JOLY	632	f	0	10	54	25	283	2.10	(0.95- 4.61)
JOLY	633	f	0	6	24	25	283	2.83	(1.06- 7.57)
JOLY	634	f	0	10	24	25	283	4.72	(2.03- 10.96)
JOLY	635	f	0	7	20	25	283	3.96	(1.53- 10.27)
Subtotal JOLY								3.51	(2.50- 4.92)
KATSOU	515	f	0	6	12	30	67	1.12	(0.38- 3.26)
KATSOU	516	f	0	9	6	30	67	3.35	(1.09- 10.26)
Subtotal KATSOU								1.89	(0.87- 4.09)
LUBIN2	700	m	0	131	2964	57	2616	2.03	(1.48- 2.78)
LUBIN2	701	m	0	242	3473	57	2616	3.20	(2.39- 4.29)
LUBIN2	702	m	0	208	2540	57	2616	3.76	(2.79- 5.06)
LUBIN2	703	m	0	90	1460	57	2616	2.83	(2.02- 3.97)
LUBIN2	752	f	0	174	229	138	1180	6.50	(4.99- 8.46)
LUBIN2	753	f	0	383	186	138	1180	17.61	(13.73- 22.58)
LUBIN2	754	f	0	284	118	138	1180	20.58	(15.59- 27.17)
LUBIN2	755	f	0	181	34	138	1180	45.52	(30.31- 68.36)
Subtotal LUBIN2								7.21	(6.49- 8.00)
MATOS	641	m	0	7	84	5	110	1.83	(0.56- 5.98)
MATOS	642	m	0	39	110	5	110	7.80	(2.96- 20.53)
MATOS	643	m	0	33	89	5	110	8.16	(3.06- 21.76)
Subtotal MATOS								5.49	(3.03- 9.96)
OSANN2	513	f	0	10	20	22	43	0.98	(0.39- 2.44)
OSANN2	514	f	0	51	20	22	43	4.98	(2.40- 10.33)
Subtotal OSANN2								2.65	(1.50- 4.69)

Table 3I16 - 5

IESLC - Meta-analysis of Ever/current Smoking by Duration, Overview
Adenocarcinoma, Cigarettes (or Any Product if Cigarettes not available)
Least adjusted

REF	NRR	SEX	AD	Number Exposed Case	Number Exposed Cont	Non-exposed Case	Non-exposed Cont	RR	95.00%CI
PEZZOT	516	m	0	11	134	3	116	3.17	(0.86- 11.65)
PEZZOT	517	m	0	22	82	3	116	10.37	(3.01- 35.81)
PEZZOT	518	m	0	27	101	3	116	10.34	(3.04- 35.09)
Subtotal PEZZOT								7.18	(3.49- 14.81)
SOBUE	513	m	0	33	119	27	128	1.31	(0.75- 2.32)
SOBUE	514	m	0	62	200	27	128	1.47	(0.89- 2.43)
SOBUE	515	m	0	96	174	27	128	2.62	(1.61- 4.24)
SOBUE	516	m	0	43	73	27	128	2.79	(1.59- 4.89)
Subtotal SOBUE								1.96	(1.51- 2.54)
WUWILL	511	f	0	34	139	172	601	0.85	(0.57- 1.29)
WUWILL	512	f	0	52	98	172	601	1.85	(1.27- 2.70)
WUWILL	513	f	0	52	114	172	601	1.59	(1.10- 2.31)
Subtotal WUWILL								1.40	(1.12- 1.75)
ZHENG	512	m	0	24	75	29	94	1.04	(0.56- 1.93)
ZHENG	513	m	0	99	143	29	94	2.24	(1.38- 3.66)
ZHENG	516	f	0	9	17	119	184	0.82	(0.35- 1.90)
ZHENG	517	f	0	24	27	119	184	1.37	(0.76- 2.49)
Subtotal ZHENG								1.45	(1.07- 1.96)
Partial Totals				2987	15878	2419	24602		

*prospective study

REF	NRR	SEX	AD	Ys	Ws	Qs	Ps
BARBON	717	m	0	1.49	4.43	0.09	0.0017
BARBON	718	m	0	1.80	4.96	1.03	0.0001
BARBON	719	m	0	2.18	5.65	3.97	0.0000
BARBON	720	m	0	2.04	5.98	2.84	0.0000
Subtotal BARBON				1.90	21.01	7.94	
BUFFLE	509	m	0	1.48	4.20	0.08	0.0024
BUFFLE	510	m	0	1.74	4.23	0.66	0.0003
BUFFLE	511	m	0	1.46	3.99	0.05	0.0036
BUFFLE	512	m	0	1.25	3.80	0.03	0.0145
Subtotal BUFFLE				1.49	16.22	0.82	
CHOI	566	m	0	0.04	4.61	7.83	0.9264
CHOI	567	m	0	-0.28	4.14	10.97	0.5664
CHOI	568	m	0	0.93	3.96	0.67	0.0630
CHOI	569	m	0	1.00	2.21	0.27	0.1381
CHOI	578	f	0	-1.21	1.75	11.48	0.1081
CHOI	579	f	0	0.82	1.16	0.32	0.3748
Subtotal CHOI				0.21	17.84	31.54	
DAMBER	540	m	1	0.59	3.18	1.83	0.2943
DAMBER	541	m	1	0.18	1.33	1.80	0.8336
DAMBER	542	m	1	1.22	4.06	0.06	0.0137
DAMBER	543	m	1	0.92	3.81	0.70	0.0736
Subtotal DAMBER				0.85	12.38	4.39	
DORGAN	576	m	2	0.76	3.79	1.30	0.1385
DORGAN	577	m	2	1.91	4.46	1.40	0.0001
DORGAN	568	f	3	0.89	25.43	5.33	0.0000
DORGAN	569	f	3	1.64	31.73	2.69	0.0000
Subtotal DORGAN				1.31	65.41	10.72	
DOSEME	516	m	2	-0.22	3.42	8.41	0.6799
DOSEME	517	m	2	1.19	10.22	0.24	0.0001
DOSEME	518	m	2	1.41	10.22	0.04	0.0000
Subtotal DOSEME				1.08	23.85	8.69	
GER	501	c	0	0.10	8.01	12.43	0.7765
GER	502	c	0	-0.15	10.92	24.35	0.6261
Subtotal GER				-0.04	18.94	36.78	
HAENSZ	525	f	0	-0.71	1.76	7.43	0.3455
HAENSZ	526	f	0	0.28	9.37	10.61	0.3886
Subtotal HAENSZ				0.12	11.12	18.04	
JEDRYC	519	m	0	0.19	1.51	2.01	0.8113
JEDRYC	520	m	0	1.35	4.56	0.00	0.0038
JEDRYC	521	m	0	1.61	5.37	0.38	0.0002
JEDRYC	522	m	0	1.71	5.43	0.74	0.0001
JEDRYC	523	m	0	1.19	4.77	0.12	0.0095
Subtotal JEDRYC				1.39	21.64	3.24	
JOLY	660	m	0	0.69	2.42	1.03	0.2812
JOLY	661	m	0	1.15	3.40	0.13	0.0333
JOLY	662	m	0	1.66	3.91	0.39	0.0010
JOLY	663	m	0	1.74	4.19	0.64	0.0004
JOLY	632	f	0	0.74	6.17	2.26	0.0660

Table 3I16 - 5

IESLC - Meta-analysis of Ever/current Smoking by Duration, Overview
Adenocarcinoma, Cigarettes (or Any Product if Cigarettes not available)
Least adjusted

REF	NRR	SEX	AD	Ys	Ws	Qs	Ps
JOLY	633	f	0	1.04	3.97	0.37	0.0382
JOLY	634	f	0	1.55	5.40	0.23	0.0003
JOLY	635	f	0	1.38	4.23	0.00	0.0046
Subtotal JOLY				1.25	33.69	5.06	
KATSOU	515	f	0	0.11	3.35	5.12	0.8399
KATSOU	516	f	0	1.21	3.07	0.06	0.0342
Subtotal KATSOU				0.64	6.42	5.17	
LUBIN2	700	m	0	0.71	38.61	15.74	0.0000
LUBIN2	701	m	0	1.16	44.75	1.50	0.0000
LUBIN2	702	m	0	1.32	43.24	0.02	0.0000
LUBIN2	703	m	0	1.04	33.64	3.15	0.0000
LUBIN2	752	f	0	1.87	54.92	15.17	0.0000
LUBIN2	753	f	0	2.87	62.18	144.15	0.0000
LUBIN2	754	f	0	3.02	49.78	140.25	0.0000
LUBIN2	755	f	0	3.82	23.24	142.06	0.0000
Subtotal LUBIN2				1.98	350.37	462.05	
MATOS	641	m	0	0.61	2.75	1.50	0.3150
MATOS	642	m	0	2.05	4.10	2.06	0.0000
MATOS	643	m	0	2.10	3.99	2.26	0.0000
Subtotal MATOS				1.70	10.84	5.82	
OSANN2	513	f	0	-0.02	4.57	8.57	0.9608
OSANN2	514	f	0	1.61	7.23	0.49	0.0000
Subtotal OSANN2				0.98	11.80	9.06	
PEZZOT	516	m	0	1.16	2.27	0.08	0.0817
PEZZOT	517	m	0	2.34	2.50	2.47	0.0002
PEZZOT	518	m	0	2.34	2.57	2.52	0.0002
Subtotal PEZZOT				1.97	7.34	5.07	
SOBUE	513	m	0	0.27	11.97	13.76	0.3439
SOBUE	514	m	0	0.39	15.16	13.99	0.1339
SOBUE	515	m	0	0.96	16.39	2.42	0.0001
SOBUE	516	m	0	1.03	12.22	1.24	0.0003
Subtotal SOBUE				0.67	55.74	31.41	
WUWILL	511	f	0	-0.16	22.68	51.23	0.4546
WUWILL	512	f	0	0.62	27.09	14.37	0.0013
WUWILL	513	f	0	0.47	28.18	21.81	0.0133
Subtotal WUWILL				0.34	77.96	87.41	
ZHENG	512	m	0	0.04	9.99	17.12	0.9080
ZHENG	513	m	0	0.81	16.07	4.64	0.0012
ZHENG	516	f	0	-0.20	5.44	13.00	0.6405
ZHENG	517	f	0	0.32	10.81	11.41	0.2958
Subtotal ZHENG				0.37	42.31	46.18	

N	71
NS	18

Table 3I16 - 6

IESLC - Meta-analysis of Ever/current Smoking by Duration, Overview
Adenocarcinoma, Cigarettes (or Any Product if Cigarettes not available)
Least adjusted

		Sex		
	combined	male	female	Total
N	2	46	23	71
NS	1	13	9	23

In this overview table, other than the "N" rows, entries in the "absent" and "Total" columns may be invalid and should be ignored

	Duration of smoking (broad categories)				
	absent	1-34k20	21-49k35	36+k50	Total
N	23	22	13	13	71
NS	15	17	11	11	54
Wt	253.27	235.34	183.19	133.08	804.88
Het Chi	217.28	124.57	178.67	171.30	779.39
Het df	22	21	12	12	70
Het P	***	***	***	***	***
Fixed RR	4.48	2.31	5.12	4.72	3.84
RRl	3.96	2.03	4.43	3.99	3.58
RRu	5.07	2.63	5.92	5.60	4.12
P	+++	+++	+++	+++	+++
Random RR	3.10	1.82	3.68	4.89	2.94
RRl	2.04	1.29	1.95	2.45	2.30
RRu	4.71	2.57	6.92	9.75	3.75
P	+++	+++	+++	+++	+++

		Duration of smoking (narrow categories)						
	absent	1-19k1	6-29k20	21-39k30	31-49k40	41-998k50	51+k999	Total
N	43	3	2	10	11	1	1	71
NS	18	3	2	8	9	1	1	41
Wt	458.60	6.69	14.78	175.13	141.82	4.06	3.81	804.88
Het Chi	385.42	0.68	0.08	174.58	101.01	0.00	0.00	779.39
Het df	42	2	1	9	10	0	0	70
Het P	***	N.S.	N.S.	***	***	N.S.	N.S.	***
Fixed RR	2.94	0.77	3.47	5.12	7.06	3.40	2.50	3.84
RRl	2.69	0.36	2.08	4.41	5.99	1.29	0.92	3.58
RRu	3.22	1.65	5.77	5.93	8.33	9.00	6.82	4.12
P	+++	N.S.	+++	+++	+++	+	(+)	+++
Random RR	2.60	0.77	3.47	3.49	5.22	3.40	2.50	2.94
RRl	1.94	0.36	2.08	1.69	2.85	1.29	0.92	2.30
RRu	3.49	1.65	5.77	7.21	9.54	9.00	6.82	3.75
P	+++	N.S.	+++	+++	+++	+	(+)	+++

Table 3I16 - 6

IESLC - Meta-analysis of Ever/current Smoking by Duration, Overview
Adenocarcinoma, Cigarettes (or Any Product if Cigarettes not available)
Least adjusted

MALES

	Duration of smoking (broad categories) absent	1-34k20	21-49k35	36+k50	Total
N	13	13	10	10	46
NS	11	13	10	10	44
Wt	122.06	103.00	89.94	77.43	392.43
Het Chi	23.72	18.86	30.09	12.78	105.33
Het df	12	12	9	9	45
Het P	*	(*)	***	N.S.	***
Fixed RR	3.41	2.03	3.04	3.59	2.93
RRl	2.85	1.68	2.47	2.87	2.65
RRu	4.07	2.47	3.74	4.49	3.23
P	+++	+++	+++	+++	+++
Random RR	3.40	2.10	3.35	3.98	3.09
RRl	2.56	1.60	2.12	2.94	2.61
RRu	4.53	2.76	5.30	5.37	3.67
P	+++	+++	+++	+++	+++

Duration of smoking (narrow categories)

	absent	1-19k1	6-29k20	21-39k30	31-49k40	41-998k50	51+k999	Total
N	24	2	2	7	9	1	1	46
NS	13	2	2	7	9	1	1	34
Wt	196.33	4.93	14.78	81.88	86.64	4.06	3.81	392.43
Het Chi	51.82	0.18	0.08	23.27	13.11	0.00	0.00	105.33
Het df	23	1	1	6	8	0	0	45
Het P	***	N.S.	N.S.	***	N.S.	N.S.	N.S.	***
Fixed RR	2.64	0.91	3.47	2.88	3.92	3.40	2.50	2.93
RRl	2.29	0.38	2.08	2.32	3.17	1.29	0.92	2.65
RRu	3.03	2.20	5.77	3.58	4.84	9.00	6.82	3.23
P	+++	N.S.	+++	+++	+++	+	(+)	+++
Random RR	2.92	0.91	3.47	3.01	4.21	3.40	2.50	3.09
RRl	2.31	0.38	2.08	1.80	3.04	1.29	0.92	2.61
RRu	3.69	2.20	5.77	5.03	5.84	9.00	6.82	3.67
P	+++	N.S.	+++	+++	+++	+	(+)	+++

FEMALES

	Duration of smoking (broad categories) absent	1-34k20	21-49k35	36+k50	Total
N	9	8	3	3	23
NS	8	8	3	3	22
Wt	120.29	124.32	93.25	55.65	393.52
Het Chi	132.66	96.66	100.59	144.54	563.66
Het df	8	7	2	2	22
Het P	***	***	***	***	***
Fixed RR	6.88	2.70	8.47	6.92	5.38
RRl	5.75	2.26	6.91	5.32	4.88
RRu	8.22	3.22	10.38	9.00	5.94
P	+++	+++	+++	+++	+++
Random RR	3.17	1.40	4.60	6.64	2.76
RRl	1.38	0.66	0.81	0.58	1.62
RRu	7.29	2.96	26.10	76.18	4.69
P	++	N.S.	(+)	N.S.	+++

Table 3I16 - 6

IESLC - Meta-analysis of Ever/current Smoking by Duration, Overview
Adenocarcinoma, Cigarettes (or Any Product if Cigarettes not available)
Least adjusted

FEMALES

		Duration of smoking (narrow categories)						
	absent	1-19k1	6-29k20	21-39k30	31-49k40	41-998k50	51+k999	Total
N	17	1		3	2			23
NS	9	1		3	2			15
Wt	243.34	1.76		93.25	55.18			393.52
Het Chi	299.61	0.00		100.59	10.57			563.66
Het df	16	0		2	1			22
Het P	***	N.S.		***	**			***
Fixed RR	3.51	0.49		8.47	17.82			5.38
RRl	3.09	0.11		6.91	13.68			4.88
RRu	3.98	2.15		10.38	23.20			5.94
P	+++	N.S.		+++	+++			+++
Random RR	2.33	0.49		4.60	10.42			2.76
RRl	1.30	0.11		0.81	2.47			1.62
RRu	4.16	2.15		26.10	43.96			4.69
P	++	N.S.		(+)	++			+++

Table 3I16 - 7

IESLC - Meta-analysis of Ever/current Smoking by Duration, Overview
Adenocarcinoma, Cigarettes (or Any Product if Cigarettes not available)
Excluded studies (and stage at which they were excluded)

Stage	Studies
1	BECHER BLOT1 BROWN3 CARPEN CHYOU DARBY DOLL2 GARCIA GRAHAM GURSEL HAMMO2 JAHN JAIN LAUSSM PRESCO QIAO TVERDA WIGLE WYNDE3
2	ALDERS BENSHL BRESLO CHIAZZ DEAN3 DORN ENGELA GAO2 GILLIS GUO HEGMAN HIRAYA HOLE KAUFMA KOO KOULUM LIU4 MIGRAN MRFITR PERNU SEGI2 SPEIZE SUZUK2 SVENSS VUTUC WAKAI WU YUAN
3	GENG MCDUFF SPITZ STASZE WU2 ZHANG
4	AGUDO AKIBA AMANDU AMES ARMADA AUVINE AXELSS BEST BOFFET BOUCHA BOUCOT BROSS CEDERL CHEN2 CORREA CPSI CPSII DEAN2 DESTEF DOLL FAN GAO GARSHI HAMMON HU HU2 HUMBLE JUSSAW KAISE2 KREUZE LETOUR LEVIN LIAW LIU3 LIU5 LUO MCCONN NOTAN2 PEZZO2 PISANI QIAO2 RACHTA RESTRE SADOWS TIZZAN WANG2 WATSON WYNDE2 WYNDE6 WYNDE7 WYNDE8 ZHOU
5	CHEN LUBIN XU
10	KHUDER
14	BENHAM

Table 3I16 - 8

Potentially overlapping studies

REF	REFGP	PRINC	.	OVERLAP/LINK
LUBIN2	LUBIN2	1		Lubin-combined
OSANN2	KAISER	2		KAISER/OSANN2

Table 3I16 - 9

Most adjusted - insufficient data for meta-analysis

REF	NRR	SEX	AGEL	AGEH	RACE	YF	LC TYPE	LOC	START	ST	NLC	R	VB	P	H	AD	SM	PRODUCT	exL	exH	S1	S2	DENOM	De
CHEN	509	c	0	0	all	-	a	As:oth	1987	CC	323	n	ot	n	y	2	ev	cig+/-ot	1	20	1	0	nev cigs	ot
CHEN	510	c	0	0	all	-	a	As:oth	1987	CC	323	n	ot	n	y	2	ev	cig+/-ot	21	30	0	3	nev cigs	ot
CHEN	511	c	0	0	all	-	a	As:oth	1987	CC	323	n	ot	n	y	2	ev	cig+/-ot	31	40	2	4	nev cigs	ot
CHEN	512	c	0	0	all	-	a	As:oth	1987	CC	323	n	ot	n	y	2	ev	cig+/-ot	41	999	3	0	nev cigs	ot
XU	506	m	0	0	all	-	a	As:Chi	1985	CC	729	n	ot	n	n	2	ev	all/unsp	1	29	1	0	nev any	or
XU	507	m	0	0	all	-	a	As:Chi	1985	CC	729	n	ot	n	n	2	ev	all/unsp	30	39	2	3	nev any	or
XU	508	m	0	0	all	-	a	As:Chi	1985	CC	729	n	ot	n	n	2	ev	all/unsp	40	999	3	0	nev any	or

REF	NRR	RR	SIG	RRDATA comment
CHEN	509	1.23	n	0
CHEN	510	1.52	n	0
CHEN	511	1.60	n	0
CHEN	512	3.79	y	p<0.001
XU	506	*		RR for 1-19/day is 1.4(NS), for 20-29/ day is 0.7(p<0.05) and for >=30/day is 5.4(p<0.05)
XU	507	*		RR for 1-19/day is 2.2(p<0.05), for 20-29/day is 1.5(NS) and for >=30/day is 3.2(p<0.05)
XU	508	*		RR for 1-19/day is 2.6(p<0.05), for 20-29/day is 3.6(p<0.05) and for >=30/ day is 11.8(p<0.05)

Table 3I17 -

IESLC - Meta-analysis of Ever/current Smoking, Duration, "Low"
Adenocarcinoma, Cigarettes (or Any Product if Cigarettes not available)

This analysis is restricted to results for:
1) Ever/current smokers
2) Results by Duration
3) Categorical results by Duration
4) Adenocarcinoma (or near equivalent)
5) Results complete enough for use in metaanalysis

Within each study, results are then selected (in the following order of preference, within each sex) for:
6) SMKSTA: ever, current
7) PRODUCT: cigarettes regardless of other products, cigarettes only, all/unspec
8) CIGTYPE: all/unspecified, MC regardless of HR, MC only
9) (not applicable)
10) DENOM: never smoked anything, never smoked cigarettes, never any + low, never cigs + low
11) Followup period (YF, prospective studies): whole study (coded as 0) or longest available
12) LCtype: adeno or nearest available, but not squamous. (q = squamous, s = small,
a = adeno, l = large, KII = Kreyberg II, al = alveolar, br = bronchiolar, u = undifferentiated)
13) Race: all or nearest available, otherwise by race (wh or w = white, bl or b = black, hi = hispanic
ch = chinese, jap = japanese, haw = hawaiian, w+o = white + oriental, sca = scandinavian, as = asian)
14) Duration "low" in key scheme 1 (key value 20, maximum range 1-34)
15) For overlapping studies: principal rather than subsidiary studies
Finally by Age: whole study (coded as 0) if available, otherwise by widest available age group
and then for single sex results (m, f) in preference to results for both sexes combined (c).

Results adjusted (AD) for the most potential confounders are then chosen in Sections -1 to -3
(and those which actually differ from the adjusted results in Table 3I12 - 1 are marked 'x' in Section -1)
and results adjusted for the least confounders in Sections -4 to -6. (Those least adjusted results which
actually differ from the most adjusted are marked 'x' in column X in Section -4)

Section -7 shows excluded studies, together with the stage (as above) at which no qualifying
results were found.

Section -8 lists the potentially overlapping studies which have been included (1=principal, 2=subsidiary).

Section -9 lists any results which would have been included in preference except that they had data not complete
enough for use in meta-analysis, with their significance (yes/no), if known, and any further comment as entered
on the database. It also lists as "gap" any categories for which no data were presented by the original authors.

In addition to those mentioned above, the following fields, levels and abbreviations are used:
* or nk = not known, n = no, y = yes, ot = other
ev = ever, cu = current, nev = never
all/unspec = all or unspecified, cig+/-ot = cigarettes irrespective of other products (cigar, pipe etc)
MC = manufactured cigarettes, HR = hand-rolled cigarettes
exL, exH = range of exposure (low and high) in the smoking group, in terms of Duration
REF: 6-character study reference
NRR: number of the RR on the database within the study
ST : study type (CC = case control, pr or prosp = prospective)
NLC: number of lung cancer cases in whole study
R : risky occupational population (n = no, m = mining, o = other risky)
VB : national cigarette type (V = at least 75% Virginia, bl = at least 75% blended, ot = other)
P : any proxy use
H : full histological confirmation
De : derivation of RR/CI (or = original, st = standard method, ot = other method of estimation)

Table 3I17 - 1

IESLC - Meta-analysis of Ever/current Smoking, Duration, "Low"
Adenocarcinoma, Cigarettes (or Any Product if Cigarettes not available)
Most adjusted

REF	NRR	3I12	SEX	AGEL	AGEH	RACE	YF	LC TYPE	LOC	START	ST	NLC	R	VB	P	H	AD	SM	PRODUCT	exL	exH	DENOM	De
BARBON	724		m	0	0	all	-	a	Eu:wst	1979	CC	755	n	bl	y	y	1	ev	all/unsp	1	29	nev any	or
BUFFLE	509		m	0	0	wh	-	a	NAmer	1976	CC	943	n	bl	y	n	0	ev	cig+/-ot	1	33	nev cigs	or
CHOI	566		m	0	0	all	-	a	As:oth	1985	CC	375	n	bl	n	n	0	ev	cig+/-ot	1	29	nev cigs	st
CHOI	578		f	0	0	all	-	a	As:oth	1985	CC	375	n	bl	n	n	0	ev	cig+/-ot	1	29	nev cigs	st
DAMBER	540		m	0	0	all	-	a+al+br	Eu:Sca	1972	CC	579	n	bl	y	n	1	ev	all/unsp	1	30	nev any	or
DORGAN	576		m	0	0	wh	-	a	NAmer	1980	CC	2026	n	bl	y	y	2	ev	cig+/-ot	1	34	nev any	ot
DORGAN	568		f	0	0	all	-	a	NAmer	1980	CC	2026	n	bl	y	y	3	ev	cig+/-ot	1	34	nev any	ot
DOSEME	517		m	0	0	all	-	not q+s	Eu:bal	1979	CC	1210	n	bl	n	n	2	ev	cig+/-ot	11	20	nev cigs	or
GER	507		c	0	0	all	-	a	As:oth	1990	CC	141	n	ot	y	n	5	ev	all/unsp	1	30	nev any	ot
JEDRYC	520		m	0	0	all	-	a	Eu:est	1980	CC	1630	n	bl	y	n	0	ev	cig+/-ot	20	29	nev any	st
JOLY	660		m	0	0	all	-	a	SCAmer	1978	CC	826	n	bl	n	n	0	ev	cig+/-ot	1	29	nev any	st
JOLY	632		f	0	0	all	-	a	SCAmer	1978	CC	826	n	bl	n	n	0	ev	cig+/-ot	1	29	nev any	st
KATSOU	520		f	0	0	all	-	a	Eu:bal	1987	CC	101	n	bl	n	n	1	cu	all/unsp	1	29	nev any	or
LUBIN2	700		m	0	0	all	-	a	Eu:mul	1976	CC	7804	n	bl	n	y	0	ev	cig+/-ot	1	29	nev any	st
LUBIN2	752		f	0	0	all	-	a	Eu:mul	1976	CC	7804	n	bl	n	y	0	ev	cig+/-ot	1	29	nev any	st
MATOS	646		m	0	0	all	-	a	SCAmer	1994	CC	200	n	bl	n	n	2	ev	cig+/-ot	1	24	nev any	or
OSANN2	516		f	0	0	all	-	KII	NAmer	1964	ot	217	n	bl	n	y	1	ev	cig+/-ot	1	20	nev cigs	or
PEZZOT	516		m	0	0	all	-	a	SCAmer	1987	CC	215	n	bl	n	y	0	ev	cig only	1	30	nev cigs	st
SOBUE	513		m	0	0	all	-	a	As:Jap	1986	CC	1376	n	bl	n	y	0	cu	cig+/-ot	1	29	nev cigs	st
WUWILL	526		f	0	0	all	-	a	As:Chi	1985	CC	965	n	ot	n	n	3	ev	cig+/-ot	1	29	nev cigs	ot
ZHENG	512		m	0	0	all	-	a	As:Chi	1982	CC	540	n	ot	*	y	0	ev	cig+/-ot	1	29	nev cigs	or
ZHENG	516		f	0	0	all	-	a	As:Chi	1982	CC	540	n	ot	*	y	0	ev	cig+/-ot	1	29	nev cigs	or

Cigarette type is all/unspec for all RRs

Table 3I17 - 2

IESLC - Meta-analysis of Ever/current Smoking, Duration, "Low"
Adenocarcinoma, Cigarettes (or Any Product if Cigarettes not available)
Most adjusted

REF	NRR	SEX	AD	Number Exposed Case	Cont	Non-exposed Case	Cont	RR	95.00%CI
BARBON	724	m	1	15	-	7	-	3.70	(1.40- 9.70)
BUFFLE	509	m	0	-	-	-	-	4.40	(1.70- 11.50)
CHOI	566	m	0	17	221	7	95	1.04	(0.42- 2.60)
CHOI	578	f	0	2	23	48	164	0.30	(0.07- 1.31)
Subtotal CHOI								0.74	(0.34- 1.61)
DAMBER	540	m	1	-	-	16	-	1.80	(0.60- 5.40)
DORGAN	576	m	2	-	-	-	-	2.14	(0.78- 5.84)
DORGAN	568	f	3	-	-	-	-	2.43	(1.65- 3.59)
Subtotal DORGAN								2.39	(1.66- 3.43)
DOSEME	517	m	2	26	-	24	-	3.30	(2.20- 7.50)
GER	507	c	5	14	-	37	-	1.40	(0.62- 3.19)
JEDRYC	520	m	0	15	160	7	289	3.87	(1.55- 9.69)
JOLY	660	m	0	5	109	5	218	2.00	(0.57- 7.06)
JOLY	632	f	0	10	54	25	283	2.10	(0.95- 4.61)
Subtotal JOLY								2.07	(1.06- 4.04)
KATSOU	520	f	1	6	-	30	-	0.84	(0.27- 2.58)
LUBIN2	700	m	0	131	2964	57	2616	2.03	(1.48- 2.78)
LUBIN2	752	f	0	174	229	138	1180	6.50	(4.99- 8.46)
Subtotal LUBIN2								4.02	(3.28- 4.92)
MATOS	646	m	2	7	-	5	-	1.70	(0.50- 5.50)
OSANN2	516	f	1	10	-	22	-	0.70	(0.20- 1.90)
PEZZOT	516	m	0	11	134	3	116	3.17	(0.86- 11.65)
SOBUE	513	m	0	33	119	27	128	1.31	(0.75- 2.32)
WUWILL	526	f	3	34	-	172	-	0.80	(0.51- 1.25)
ZHENG	512	m	0	24	75	29	94	1.04	(0.56- 1.93)
ZHENG	516	f	0	9	17	119	184	0.82	(0.35- 1.90)
Subtotal ZHENG								0.95	(0.58- 1.57)
Partial Totals				543	4105	778	5367		

*prospective study

REF	NRR	SEX	AD	Ys	Ws	Qs	Ps
BARBON	724	m	1	1.31	4.10	0.84	0.0081
BUFFLE	509	m	0	1.48	4.20	1.65	0.0024
CHOI	566	m	0	0.04	4.61	3.04	0.9264
CHOI	578	f	0	-1.21	1.75	7.50	0.1081
Subtotal CHOI				-0.30	6.37	10.55	
DAMBER	540	m	1	0.59	3.18	0.23	0.2943
DORGAN	576	m	2	0.76	3.79	0.03	0.1385
DORGAN	568	f	3	0.89	25.43	0.03	0.0000
Subtotal DORGAN				0.87	29.22	0.06	
DOSEME	517	m	2	1.19	10.22	1.17	0.0001
GER	507	c	5	0.34	5.73	1.54	0.4207
JEDRYC	520	m	0	1.35	4.56	1.13	0.0038
JOLY	660	m	0	0.69	2.42	0.06	0.2812
JOLY	632	f	0	0.74	6.17	0.08	0.0660
Subtotal JOLY				0.73	8.59	0.15	
KATSOU	520	f	1	-0.17	3.02	3.20	0.7620
LUBIN2	700	m	0	0.71	38.61	0.85	0.0000
LUBIN2	752	f	0	1.87	54.92	56.69	0.0000
Subtotal LUBIN2				1.39	93.54	57.54	
MATOS	646	m	2	0.53	2.67	0.28	0.3857
OSANN2	516	f	1	-0.36	3.03	4.45	0.5346
PEZZOT	516	m	0	1.16	2.27	0.20	0.0817
SOBUE	513	m	0	0.27	11.97	4.05	0.3439
WUWILL	526	f	3	-0.22	19.12	22.24	0.3292
ZHENG	512	m	0	0.04	9.99	6.70	0.9080
ZHENG	516	f	0	-0.20	5.44	6.06	0.6405
Subtotal ZHENG				-0.05	15.43	12.76	

Table 3I17 - 2

IESLC - Meta-analysis of Ever/current Smoking, Duration, "Low"
Adenocarcinoma, Cigarettes (or Any Product if Cigarettes not available)
Most adjusted

	N	22
	NS	17
	Wt	227.21
Het	Chi	122.04
Het	df	21
Het	P	***
Fixed	RR	2.35
	RRl	2.07
	RRu	2.68
	P	+++
Random	RR	1.78
	RRl	1.26
	RRu	2.53
	P	++
Asymm	P	*

Table 3I17 - 3

IESLC - Meta-analysis of Ever/current Smoking, Duration, "Low"
Adenocarcinoma, Cigarettes (or Any Product if Cigarettes not available)
Most adjusted

	Sex combined	male	female	Total
N	1	13	8	22
NS	1	13	8	22
Wt	5.73	102.60	118.88	227.21
Het Chi	0.00	17.69	97.18	122.04
Het df	0	12	7	21
Het P	N.S.	N.S.	***	***
Fixed RR	1.40	2.01	2.76	2.35
RRl	0.62	1.66	2.31	2.07
RRu	3.18	2.44	3.31	2.68
P	N.S.	+++	+++	+++
Random RR	1.40	2.06	1.29	1.78
RRl	0.62	1.58	0.60	1.26
RRu	3.18	2.68	2.82	2.53
P	N.S.	+++	N.S.	++
Between Chi				7.18
Between df				2
Between P				*
Btwn(F) P				N.S.
Btwn(R) P				N.S.

Table 3I17 - 4

IESLC - Meta-analysis of Ever/current Smoking, Duration, "Low"
Adenocarcinoma, Cigarettes (or Any Product if Cigarettes not available)
Least adjusted

REF	NRR	X	SEX	AGEL	AGEH	RACE	YF	LC TYPE	LOC	START	ST	NLC	R	VB	P	H	AD	SM	PRODUCT	exL	exH	DENOM	De
BARBON	717	x	m	0	0	all	-	a	Eu:wst	1979	CC	755	n	bl	y	y	0	ev	all/unsp	1	29	nev any	st
BUFFLE	509		m	0	0	wh	-	a	NAmer	1976	CC	943	n	bl	y	n	0	ev	cig+/-ot	1	33	nev cigs	or
CHOI	566		m	0	0	all	-	a	As:oth	1985	CC	375	n	bl	n	n	0	ev	cig+/-ot	1	29	nev cigs	st
CHOI	578		f	0	0	all	-	a	As:oth	1985	CC	375	n	bl	n	n	0	ev	cig+/-ot	1	29	nev cigs	st
DAMBER	540		m	0	0	all	-	a+al+br	Eu:Sca	1972	CC	579	n	bl	y	n	1	ev	all/unsp	1	30	nev any	or
DORGAN	576		m	0	0	wh	-	a	NAmer	1980	CC	2026	n	bl	y	y	2	ev	cig+/-ot	1	34	nev any	ot
DORGAN	568		f	0	0	all	-	a	NAmer	1980	CC	2026	n	bl	y	y	3	ev	cig+/-ot	1	34	nev any	ot
DOSEME	517		m	0	0	all	-	not q+s	Eu:bal	1979	CC	1210	n	bl	n	n	2	ev	cig+/-ot	11	20	nev cigs	or
GER	501	x	c	0	0	all	-	a	As:oth	1990	CC	141	n	ot	y	n	0	ev	all/unsp	1	30	nev any	st
JEDRYC	520		m	0	0	all	-	a	Eu:est	1980	CC	1630	n	bl	y	n	0	ev	cig+/-ot	20	29	nev any	st
JOLY	660		m	0	0	all	-	a	SCAmer	1978	CC	826	n	bl	n	n	0	ev	cig+/-ot	1	29	nev any	st
JOLY	632		f	0	0	all	-	a	SCAmer	1978	CC	826	n	bl	n	n	0	ev	cig+/-ot	1	29	nev any	st
KATSOU	515	x	f	0	0	all	-	a	Eu:bal	1987	CC	101	n	bl	n	n	0	cu	all/unsp	1	29	nev any	st
LUBIN2	700		m	0	0	all	-	a	Eu:mul	1976	CC	7804	n	bl	n	y	0	ev	cig+/-ot	1	29	nev any	st
LUBIN2	752		f	0	0	all	-	a	Eu:mul	1976	CC	7804	n	bl	n	y	0	ev	cig+/-ot	1	29	nev any	st
MATOS	641	x	m	0	0	all	-	a	SCAmer	1994	CC	200	n	bl	n	n	0	ev	cig+/-ot	1	24	nev any	st
OSANN2	513	x	f	0	0	all	-	KII	NAmer	1964	ot	217	n	bl	n	y	0	ev	cig+/-ot	1	20	nev cigs	st
PEZZOT	516		m	0	0	all	-	a	SCAmer	1987	CC	215	n	bl	n	y	0	ev	cig only	1	30	nev cigs	st
SOBUE	513		m	0	0	all	-	a	As:Jap	1986	CC	1376	n	bl	n	y	0	cu	cig+/-ot	1	29	nev cigs	st
WUWILL	511	x	f	0	0	all	-	a	As:Chi	1985	CC	965	n	ot	n	n	0	ev	cig+/-ot	1	29	nev cigs	st
ZHENG	512		m	0	0	all	-	a	As:Chi	1982	CC	540	n	ot	*	y	0	ev	cig+/-ot	1	29	nev cigs	or
ZHENG	516		f	0	0	all	-	a	As:Chi	1982	CC	540	n	ot	*	y	0	ev	cig+/-ot	1	29	nev cigs	or

Cigarette type is all/unspec for all RRs

Table 3I17 - 5

IESLC - Meta-analysis of Ever/current Smoking, Duration, "Low"
Adenocarcinoma, Cigarettes (or Any Product if Cigarettes not available)
Least adjusted

REF	NRR	SEX	AD	Number Exposed Case	Number Exposed Cont	Non-exposed Case	Non-exposed Cont	RR	95.00%CI
BARBON	717	m	0	15	91	7	188	4.43	(1.74- 11.24)
BUFFLE	509	m	0	-	-	-	-	4.40	(1.70- 11.50)
CHOI	566	m	0	17	221	7	95	1.04	(0.42- 2.60)
CHOI	578	f	0	2	23	48	164	0.30	(0.07- 1.31)
Subtotal CHOI								0.74	(0.34- 1.61)
DAMBER	540	m	1	-	-	16	-	1.80	(0.60- 5.40)
DORGAN	576	m	2	-	-	-	-	2.14	(0.78- 5.84)
DORGAN	568	f	3	-	-	-	-	2.43	(1.65- 3.59)
Subtotal DORGAN								2.39	(1.66- 3.43)
DOSEME	517	m	2	26	-	24	-	3.30	(2.20- 7.50)
GER	501	c	0	14	51	37	149	1.11	(0.55- 2.21)
JEDRYC	520	m	0	15	160	7	289	3.87	(1.55- 9.69)
JOLY	660	m	0	5	109	5	218	2.00	(0.57- 7.06)
JOLY	632	f	0	10	54	25	283	2.10	(0.95- 4.61)
Subtotal JOLY								2.07	(1.06- 4.04)
KATSOU	515	f	0	6	12	30	67	1.12	(0.38- 3.26)
LUBIN2	700	m	0	131	2964	57	2616	2.03	(1.48- 2.78)
LUBIN2	752	f	0	174	229	138	1180	6.50	(4.99- 8.46)
Subtotal LUBIN2								4.02	(3.28- 4.92)
MATOS	641	m	0	7	84	5	110	1.83	(0.56- 5.98)
OSANN2	513	f	0	10	20	22	43	0.98	(0.39- 2.44)
PEZZOT	516	m	0	11	134	3	116	3.17	(0.86- 11.65)
SOBUE	513	m	0	33	119	27	128	1.31	(0.75- 2.32)
WUWILL	511	f	0	34	139	172	601	0.85	(0.57- 1.29)
ZHENG	512	m	0	24	75	29	94	1.04	(0.56- 1.93)
ZHENG	516	f	0	9	17	119	184	0.82	(0.35- 1.90)
Subtotal ZHENG								0.95	(0.58- 1.57)
Partial Totals				543	4502	778	6525		

*prospective study

REF	NRR	SEX	AD	Ys	Ws	Qs	Ps
BARBON	717	m	0	1.49	4.43	1.87	0.0017
BUFFLE	509	m	0	1.48	4.20	1.74	0.0024
CHOI	566	m	0	0.04	4.61	2.92	0.9264
CHOI	578	f	0	-1.21	1.75	7.38	0.1081
Subtotal CHOI				-0.30	6.37	10.30	
DAMBER	540	m	1	0.59	3.18	0.20	0.2943
DORGAN	576	m	2	0.76	3.79	0.02	0.1385
DORGAN	568	f	3	0.89	25.43	0.06	0.0000
Subtotal DORGAN				0.87	29.22	0.09	
DOSEME	517	m	2	1.19	10.22	1.29	0.0001
GER	501	c	0	0.10	8.01	4.36	0.7765
JEDRYC	520	m	0	1.35	4.56	1.21	0.0038
JOLY	660	m	0	0.69	2.42	0.05	0.2812
JOLY	632	f	0	0.74	6.17	0.06	0.0660
Subtotal JOLY				0.73	8.59	0.11	
KATSOU	515	f	0	0.11	3.35	1.78	0.8399
LUBIN2	700	m	0	0.71	38.61	0.66	0.0000
LUBIN2	752	f	0	1.87	54.92	58.62	0.0000
Subtotal LUBIN2				1.39	93.54	59.28	
MATOS	641	m	0	0.61	2.75	0.15	0.3150
OSANN2	513	f	0	-0.02	4.57	3.39	0.9608
PEZZOT	516	m	0	1.16	2.27	0.23	0.0817
SOBUE	513	m	0	0.27	11.97	3.82	0.3439
WUWILL	511	f	0	-0.16	22.68	22.47	0.4546
ZHENG	512	m	0	0.04	9.99	6.42	0.9080
ZHENG	516	f	0	-0.20	5.44	5.87	0.6405
Subtotal ZHENG				-0.05	15.43	12.29	

Table 3I17 - 5

IESLC - Meta-analysis of Ever/current Smoking, Duration, "Low"
Adenocarcinoma, Cigarettes (or Any Product if Cigarettes not available)
Least adjusted

	N	22
	NS	17
	Wt	235.34
Het	Chi	124.57
Het	df	21
Het	P	***
Fixed	RR	2.31
	RRl	2.03
	RRu	2.63
	P	+++
Random	RR	1.82
	RRl	1.29
	RRu	2.57
	P	+++
Asymm	P	(*)

Table 3I17 - 6

IESLC - Meta-analysis of Ever/current Smoking, Duration, "Low"
Adenocarcinoma, Cigarettes (or Any Product if Cigarettes not available)
Least adjusted

	Sex<br>combined	male	female	Total
N	1	13	8	22
NS	1	13	8	22
Wt	8.01	103.00	124.32	235.34
Het Chi	0.00	18.86	96.66	124.57
Het df	0	12	7	21
Het P	N.S.	(*)	***	***
Fixed RR	1.11	2.03	2.70	2.31
RRl	0.55	1.68	2.26	2.03
RRu	2.21	2.47	3.22	2.63
P	N.S.	+++	+++	+++
Random RR	1.11	2.10	1.40	1.82
RRl	0.55	1.60	0.66	1.29
RRu	2.21	2.76	2.96	2.57
P	N.S.	+++	N.S.	+++
Between Chi				9.04
Between df				2
Between P				*
Btwn(F) P				N.S.
Btwn(R) P				N.S.

Table 3I17 - 7

IESLC - Meta-analysis of Ever/current Smoking, Duration, "Low"
Adenocarcinoma, Cigarettes (or Any Product if Cigarettes not available)
Excluded studies (and stage at which they were excluded)

1	BECHER BLOT1 BROWN3 CARPEN CHYOU DARBY DOLL2 GARCIA GRAHAM GURSEL HAMMO2 JAHN JAIN LAUSSM PRESCO QIAO TVERDA WIGLE WYNDE3
2	ALDERS BENSHL BRESLO CHIAZZ DEAN3 DORN ENGELA GAO2 GILLIS GUO HEGMAN HIRAYA HOLE KAUFMA KOO KOULUM LIU4 MIGRAN MRFITR PERNU SEGI2 SPEIZE SUZUK2 SVENSS VUTUC WAKAI WU YUAN
3	GENG MCDUFF SPITZ STASZE WU2 ZHANG
4	AGUDO AKIBA AMANDU AMES ARMADA AUVINE AXELSS BEST BOFFET BOUCHA BOUCOT BROSS CEDERL CHEN2 CORREA CPSI CPSII DEAN2 DESTEF DOLL FAN GAO GARSHI HAMMON HU HU2 HUMBLE JUSSAW KAISE2 KREUZE LETOUR LEVIN LIAW LIU3 LIU5 LUO MCCONN NOTAN2 PEZZO2 PISANI QIAO2 RACHTA RESTRE SADOWS TIZZAN WANG2 WATSON WYNDE2 WYNDE6 WYNDE7 WYNDE8 ZHOU
5	CHEN LUBIN XU
10	KHUDER
14	BENHAM HAENSZ

Table 3I17 - 8
Potentially overlapping studies

REF	REFGP	PRINC	.	OVERLAP/LINK
LUBIN2	LUBIN2	1		Lubin-combined
OSANN2	KAISER	2		KAISER/OSANN2

Table 3I17 - 9
Most adjusted - insufficient data for meta-analysis

REF	NRR	SEX	AGEL	AGEH	RACE	YF	LC TYPE	LOC	START	ST	NLC	R	VB	P	H	AD	SM	PRODUCT	exL	exH	DENOM	De
CHEN	509	c	0	0	all	-	a	As:oth	1987	CC	323	n	ot	n	y	2	ev	cig+/-ot	1	20	nev cigs	ot
XU	506	m	0	0	all	-	a	As:Chi	1985	CC	729	n	ot	n	n	2	ev	all/unsp	1	29	nev any	or

REF	NRR	RR	SIG	RRDATA comment
CHEN	509	1.23	n	0
XU	506	*		RR for 1-19/day is 1.4(NS), for 20-29/ day is 0.7(p<0.05) and for >=30/day is 5.4(p<0.05)

Table 3I18 -

IESLC - Meta-analysis of Ever/current Smoking, Duration, "Mid"
Adenocarcinoma, Cigarettes (or Any Product if Cigarettes not available)

This analysis is restricted to results for:
1) Ever/current smokers
2) Results by Duration
3) Categorical results by Duration
4) Adenocarcinoma (or near equivalent)
5) Results complete enough for use in metaanalysis

Within each study, results are then selected (in the following order of preference, within each sex) for:
6) SMKSTA: ever, current
7) PRODUCT: cigarettes regardless of other products, cigarettes only, all/unspec
8) CIGTYPE: all/unspecified, MC regardless of HR, MC only
9) (not applicable)
10) DENOM: never smoked anything, never smoked cigarettes, never any + low, never cigs + low
11) Followup period (YF, prospective studies): whole study (coded as 0) or longest available
12) LCtype: adeno or nearest available, but not squamous. (q = squamous, s = small,
a = adeno, l = large, KII = Kreyberg II, al = alveolar, br = bronchiolar, u = undifferentiated)
13) Race: all or nearest available, otherwise by race (wh or w = white, bl or b = black, hi = hispanic
ch = chinese, jap = japanese, haw = hawaiian, w+o = white + oriental, sca = scandinavian, as = asian)
14) Duration "mid" in key scheme 1 (key value 35, maximum range 21-49)
15) For overlapping studies: principal rather than subsidiary studies
Finally by Age: whole study (coded as 0) if available, otherwise by widest available age group
and then for single sex results (m, f) in preference to results for both sexes combined (c).

Results adjusted (AD) for the most potential confounders are then chosen in Sections -1 to -3
(and those which actually differ from the adjusted results in Table 3I13 - 1 are marked 'x' in Section -1)
and results adjusted for the least confounders in Sections -4 to -6. (Those least adjusted results which
actually differ from the most adjusted are marked 'x' in column X in Section -4)

Section -7 shows excluded studies, together with the stage (as above) at which no qualifying
results were found.

Section -8 lists the potentially overlapping studies which have been included (1=principal, 2=subsidiary).

Section -9 lists any results which would have been included in preference except that they had data not complete
enough for use in meta-analysis, with their significance (yes/no), if known, and any further comment as entered
on the database. It also lists as "gap" any categories for which no data were presented by the original authors.

In addition to those mentioned above, the following fields, levels and abbreviations are used:
* or nk = not known, n = no, y = yes, ot = other
ev = ever, cu = current, nev = never
all/unspec = all or unspecified, cig+/-ot = cigarettes irrespective of other products (cigar, pipe etc)
MC = manufactured cigarettes, HR = hand-rolled cigarettes
exL, exH = range of exposure (low and high) in the smoking group, in terms of Duration
REF: 6-character study reference
NRR: number of the RR on the database within the study
ST : study type (CC = case control, pr or prosp = prospective)
NLC: number of lung cancer cases in whole study
R : risky occupational population (n = no, m = mining, o = other risky)
VB : national cigarette type (V = at least 75% Virginia, bl = at least 75% blended, ot = other)
P : any proxy use
H : full histological confirmation
De : derivation of RR/CI (or = original, st = standard method, ot = other method of estimation)

Table 3I18 - 1

IESLC - Meta-analysis of Ever/current Smoking, Duration, "Mid"
Adenocarcinoma, Cigarettes (or Any Product if Cigarettes not available)
Most adjusted

REF	NRR	3I13	SEX	AGEL	AGEH	RACE	YF	LC TYPE	LOC	START	ST	NLC	R	VB	P	H	AD	SM	PRODUCT	exL	exH	DENOM	De
BARBON	725		m	0	0	all	-	a	Eu:wst	1979	CC	755	n	bl	y	y	1	ev	all/unsp	30	39	nev any	or
BUFFLE	510		m	0	0	wh	-	a	NAmer	1976	CC	943	n	bl	y	n	0	ev	cig+/-ot	34	43	nev cigs	or
CHOI	567		m	0	0	all	-	a	As:oth	1985	CC	375	n	bl	n	n	0	ev	cig+/-ot	30	39	nev cigs	st
DAMBER	541		m	0	0	all	-	a+al+br	Eu:Sca	1972	CC	579	n	bl	y	n	1	ev	all/unsp	31	40	nev any	or
JEDRYC	521		m	0	0	all	-	a	Eu:est	1980	CC	1630	n	bl	y	n	0	ev	cig+/-ot	30	39	nev any	st
JOLY	661		m	0	0	all	-	a	SCAmer	1978	CC	826	n	bl	n	n	0	ev	cig+/-ot	30	39	nev any	st
JOLY	633		f	0	0	all	-	a	SCAmer	1978	CC	826	n	bl	n	n	0	ev	cig+/-ot	30	39	nev any	st
LUBIN2	701		m	0	0	all	-	a	Eu:mul	1976	CC	7804	n	bl	n	y	0	ev	cig+/-ot	30	39	nev any	st
LUBIN2	753		f	0	0	all	-	a	Eu:mul	1976	CC	7804	n	bl	n	y	0	ev	cig+/-ot	30	39	nev any	st
MATOS	647		m	0	0	all	-	a	SCAmer	1994	CC	200	n	bl	n	n	2	ev	cig+/-ot	25	39	nev any	or
PEZZOT	517		m	0	0	all	-	a	SCAmer	1987	CC	215	n	bl	n	y	0	ev	cig only	31	40	nev cigs	st
SOBUE	514		m	0	0	all	-	a	As:Jap	1986	CC	1376	n	bl	n	y	0	cu	cig+/-ot	30	39	nev cigs	st
WUWILL	527		f	0	0	all	-	a	As:Chi	1985	CC	965	n	ot	n	n	3	ev	cig+/-ot	30	39	nev cigs	ot

Cigarette type is all/unspec for all RRs

Table 3I18 - 2

IESLC - Meta-analysis of Ever/current Smoking, Duration, "Mid"
Adenocarcinoma, Cigarettes (or Any Product if Cigarettes not available)
Most adjusted

REF	NRR	SEX	AD	Number Exposed Case	Cont	Non-exposed Case	Cont	RR	95.00%CI
BARBON	725	m	1	23	-	7	-	5.10	(2.10- 12.50)
BUFFLE	510	m	0	-	-	-	-	5.70	(2.20- 14.80)
CHOI	567	m	0	13	160	7	65	0.75	(0.29- 1.98)
DAMBER	541	m	1	-	-	16	-	1.20	(0.20- 6.00)
JEDRYC	521	m	0	28	231	7	289	5.00	(2.15- 11.66)
JOLY	661	m	0	12	165	5	218	3.17	(1.10- 9.18)
JOLY	633	f	0	6	24	25	283	2.83	(1.06- 7.57)
Subtotal JOLY								2.98	(1.45- 6.14)
LUBIN2	701	m	0	242	3473	57	2616	3.20	(2.39- 4.29)
LUBIN2	753	f	0	383	186	138	1180	17.61	(13.73- 22.58)
Subtotal LUBIN2								8.62	(7.13- 10.42)
MATOS	647	m	2	39	-	5	-	7.30	(2.70- 19.70)
PEZZOT	517	m	0	22	82	3	116	10.37	(3.01- 35.81)
SOBUE	514	m	0	62	200	27	128	1.47	(0.89- 2.43)
WUWILL	527	f	3	52	-	172	-	1.99	(1.37- 2.89)
Partial Totals				882	4521	469	4895		

*prospective study

REF	NRR	SEX	AD	Ys	Ws	Qs	Ps
BARBON	725	m	1	1.63	4.83	0.00	0.0003
BUFFLE	510	m	0	1.74	4.23	0.05	0.0003
CHOI	567	m	0	-0.28	4.14	15.22	0.5664
DAMBER	541	m	1	0.18	1.33	2.80	0.8336
JEDRYC	521	m	0	1.61	5.37	0.00	0.0002
JOLY	661	m	0	1.15	3.40	0.79	0.0333
JOLY	633	f	0	1.04	3.97	1.41	0.0382
Subtotal JOLY				1.09	7.37	2.19	
LUBIN2	701	m	0	1.16	44.75	10.00	0.0000
LUBIN2	753	f	0	2.87	62.18	94.55	0.0000
Subtotal LUBIN2				2.15	106.93	104.55	
MATOS	647	m	2	1.99	3.89	0.48	0.0001
PEZZOT	517	m	0	2.34	2.50	1.24	0.0002
SOBUE	514	m	0	0.39	15.16	23.69	0.1339
WUWILL	527	f	3	0.69	27.58	24.74	0.0003

	N	13
	NS	11
	Wt	183.33
Het	Chi	174.97
Het	df	12
Het	P	***
Fixed	RR	5.13
	RRl	4.44
	RRu	5.93
	P	+++
Random	RR	3.63
	RRl	1.94
	RRu	6.80
	P	+++
Asymm	P	N.S.

Table 3I18 - 3

IESLC - Meta-analysis of Ever/current Smoking, Duration, "Mid"
Adenocarcinoma, Cigarettes (or Any Product if Cigarettes not available)
Most adjusted

	combined	Sex: male	female	Total
N		10	3	13
NS		10	3	13
Wt		89.60	93.73	183.33
Het Chi		28.35	95.91	174.97
Het df		9	2	12
Het P		***	***	***
Fixed RR		3.00	8.58	5.13
RRl		2.44	7.01	4.44
RRu		3.69	10.51	5.93
P		+++	+++	+++
Random RR		3.26	4.72	3.63
RRl		2.08	0.87	1.94
RRu		5.09	25.49	6.80
P		+++	(+)	+++
Between Chi				50.71
Between df				1
Between P				***
Btwn(F) P				(*)
Btwn(R) P				N.S.

Table 3I18 - 4

IESLC - Meta-analysis of Ever/current Smoking, Duration, "Mid"
Adenocarcinoma, Cigarettes (or Any Product if Cigarettes not available)
Least adjusted

REF	NRR	X	SEX	AGEL	AGEH	RACE	YF	LC TYPE	LOC	START	ST	NLC	R	VB	P	H	AD	SM	PRODUCT	exL	exH	DENOM	De
BARBON	718	x	m	0	0	all	-	a	Eu:wst	1979	CC	755	n	bl	y	y	0	ev	all/unsp	30	39	nev any	st
BUFFLE	510		m	0	0	wh	-	a	NAmer	1976	CC	943	n	bl	y	n	0	ev	cig+/-ot	34	43	nev cigs	or
CHOI	567		m	0	0	all	-	a	As:oth	1985	CC	375	n	bl	n	n	0	ev	cig+/-ot	30	39	nev cigs	st
DAMBER	541		m	0	0	all	-	a+al+br	Eu:Sca	1972	CC	579	n	bl	y	n	1	ev	all/unsp	31	40	nev any	or
JEDRYC	521		m	0	0	all	-	a	Eu:est	1980	CC	1630	n	bl	y	n	0	ev	cig+/-ot	30	39	nev any	st
JOLY	661		m	0	0	all	-	a	SCAmer	1978	CC	826	n	bl	n	n	0	ev	cig+/-ot	30	39	nev any	st
JOLY	633		f	0	0	all	-	a	SCAmer	1978	CC	826	n	bl	n	n	0	ev	cig+/-ot	30	39	nev any	st
LUBIN2	701		m	0	0	all	-	a	Eu:mul	1976	CC	7804	n	bl	n	y	0	ev	cig+/-ot	30	39	nev any	st
LUBIN2	753		f	0	0	all	-	a	Eu:mul	1976	CC	7804	n	bl	n	y	0	ev	cig+/-ot	30	39	nev any	st
MATOS	642	x	m	0	0	all	-	a	SCAmer	1994	CC	200	n	bl	n	n	0	ev	cig+/-ot	25	39	nev any	st
PEZZOT	517		m	0	0	all	-	a	SCAmer	1987	CC	215	n	bl	n	y	0	ev	cig only	31	40	nev cigs	st
SOBUE	514		m	0	0	all	-	a	As:Jap	1986	CC	1376	n	bl	n	y	0	cu	cig+/-ot	30	39	nev cigs	st
WUWILL	512	x	f	0	0	all	-	a	As:Chi	1985	CC	965	n	ot	n	n	0	ev	cig+/-ot	30	39	nev cigs	st

Cigarette type is all/unspec for all RRs

Table 3I18 - 5

IESLC - Meta-analysis of Ever/current Smoking, Duration, "Mid"
Adenocarcinoma, Cigarettes (or Any Product if Cigarettes not available)
Least adjusted

REF	NRR	SEX	AD	Number Exposed Case	Cont	Non-exposed Case	Cont	RR	95.00%CI
BARBON	718	m	0	23	102	7	188	6.06	(2.51- 14.60)
BUFFLE	510	m	0	-	-	-	-	5.70	(2.20- 14.80)
CHOI	567	m	0	13	160	7	65	0.75	(0.29- 1.98)
DAMBER	541	m	1	-	-	16	-	1.20	(0.20- 6.00)
JEDRYC	521	m	0	28	231	7	289	5.00	(2.15- 11.66)
JOLY	661	m	0	12	165	5	218	3.17	(1.10- 9.18)
JOLY	633	f	0	6	24	25	283	2.83	(1.06- 7.57)
Subtotal JOLY								2.98	(1.45- 6.14)
LUBIN2	701	m	0	242	3473	57	2616	3.20	(2.39- 4.29)
LUBIN2	753	f	0	383	186	138	1180	17.61	(13.73- 22.58)
Subtotal LUBIN2								8.62	(7.13- 10.42)
MATOS	642	m	0	39	110	5	110	7.80	(2.96- 20.53)
PEZZOT	517	m	0	22	82	3	116	10.37	(3.01- 35.81)
SOBUE	514	m	0	62	200	27	128	1.47	(0.89- 2.43)
WUWILL	512	f	0	52	98	172	601	1.85	(1.27- 2.70)
Partial Totals				882	4831	469	5794		

*prospective study

REF	NRR	SEX	AD	Ys	Ws	Qs	Ps
BARBON	718	m	0	1.80	4.96	0.14	0.0001
BUFFLE	510	m	0	1.74	4.23	0.05	0.0003
CHOI	567	m	0	-0.28	4.14	15.20	0.5664
DAMBER	541	m	1	0.18	1.33	2.80	0.8336
JEDRYC	521	m	0	1.61	5.37	0.00	0.0002
JOLY	661	m	0	1.15	3.40	0.78	0.0333
JOLY	633	f	0	1.04	3.97	1.40	0.0382
Subtotal JOLY				1.09	7.37	2.18	
LUBIN2	701	m	0	1.16	44.75	9.94	0.0000
LUBIN2	753	f	0	2.87	62.18	94.76	0.0000
Subtotal LUBIN2				2.15	106.93	104.70	
MATOS	642	m	0	2.05	4.10	0.72	0.0000
PEZZOT	517	m	0	2.34	2.50	1.25	0.0002
SOBUE	514	m	0	0.39	15.16	23.64	0.1339
WUWILL	512	f	0	0.62	27.09	27.99	0.0013

	N	13
	NS	11
	Wt	183.19
Het	Chi	178.67
Het	df	12
Het	P	***
Fixed	RR	5.12
	RRl	4.43
	RRu	5.92
	P	+++
Random	RR	3.68
	RRl	1.95
	RRu	6.92
	P	+++
Asymm	P	N.S.

Table 3I18 - 6

IESLC - Meta-analysis of Ever/current Smoking, Duration, "Mid"
Adenocarcinoma, Cigarettes (or Any Product if Cigarettes not available)
Least adjusted

	Sex		
combined	male	female	Total
N	10	3	13
NS	10	3	13
Wt	89.94	93.25	183.19
Het Chi	30.09	100.59	178.67
Het df	9	2	12
Het P	***	***	***
Fixed RR	3.04	8.47	5.12
RRl	2.47	6.91	4.43
RRu	3.74	10.38	5.92
P	+++	+++	+++
Random RR	3.35	4.60	3.68
RRl	2.12	0.81	1.95
RRu	5.30	26.10	6.92
P	+++	(+)	+++
Between Chi			47.99
Between df			1
Between P			***
Btwn(F) P			(*)
Btwn(R) P			N.S.

Table 3I18 - 7

IESLC - Meta-analysis of Ever/current Smoking, Duration, "Mid"
Adenocarcinoma, Cigarettes (or Any Product if Cigarettes not available)
Excluded studies (and stage at which they were excluded)

1	BECHER BLOT1 BROWN3 CARPEN CHYOU DARBY DOLL2 GARCIA GRAHAM GURSEL HAMMO2 JAHN JAIN LAUSSM PRESCO QIAO TVERDA WIGLE WYNDE3
2	ALDERS BENSHL BRESLO CHIAZZ DEAN3 DORN ENGELA GAO2 GILLIS GUO HEGMAN HIRAYA HOLE KAUFMA KOO KOULUM LIU4 MIGRAN MRFITR PERNU SEGI2 SPEIZE SUZUK2 SVENSS VUTUC WAKAI WU YUAN
3	GENG MCDUFF SPITZ STASZE WU2 ZHANG
4	AGUDO AKIBA AMANDU AMES ARMADA AUVINE AXELSS BEST BOFFET BOUCHA BOUCOT BROSS CEDERL CHEN2 CORREA CPSI CPSII DEAN2 DESTEF DOLL FAN GAO GARSHI HAMMON HU HU2 HUMBLE JUSSAW KAISE2 KREUZE LETOUR LEVIN LIAW LIU3 LIU5 LUO MCCONN NOTAN2 PEZZO2 PISANI QIAO2 RACHTA RESTRE SADOWS TIZZAN WANG2 WATSON WYNDE2 WYNDE6 WYNDE7 WYNDE8 ZHOU
5	CHEN LUBIN XU
10	KHUDER
14	BENHAM DORGAN DOSEME GER HAENSZ KATSOU OSANN2 ZHENG

Table 3I18 - 8
Potentially overlapping studies

REF	REFGP	PRINC	.	OVERLAP/LINK
LUBIN2	LUBIN2	1		Lubin-combined

Table 3I18 - 9
Most adjusted - insufficient data for meta-analysis

REF	NRR	SEX	AGEL	AGEH	RACE	YF	LC TYPE	LOC	START	ST	NLC	R	VB	P	H	AD	SM	PRODUCT	exL	exH	DENOM	De
CHEN	511	c	0	0	all	-	a	As:oth	1987	CC	323	n	ot	n	y	2	ev	cig+/-ot	31	40	nev cigs	ot
XU	507	m	0	0	all	-	a	As:Chi	1985	CC	729	n	ot	n	n	2	ev	all/unsp	30	39	nev any	or

REF	NRR	RR	SIG	RRDATA comment
CHEN	511	1.60	n	0
XU	507	*		RR for 1-19/day is 2.2(p<0.05), for 20-29/day is 1.5(NS) and for >=30/day is 3.2(p<0.05)

Table 3I19 -

IESLC - Meta-analysis of Ever/current Smoking, Duration, "High"
Adenocarcinoma, Cigarettes (or Any Product if Cigarettes not available)

This analysis is restricted to results for:
1) Ever/current smokers
2) Results by Duration
3) Categorical results by Duration
4) Adenocarcinoma (or near equivalent)
5) Results complete enough for use in metaanalysis

Within each study, results are then selected (in the following order of preference, within each sex) for:
6) PRODUCT: cigarettes regardless of other products, cigarettes only, all/unspec
7) CIGTYPE: all/unspecified, MC regardless of HR, MC only
8) (not applicable)
9) DENOM: never smoked anything, never smoked cigarettes, never any + low, never cigs + low
10) Followup period (YF, prospective studies): whole study (coded as 0) or longest available
11) LCtype: adeno or nearest available, but not squamous. (q = squamous, s = small,
a = adeno, l = large, KII = Kreyberg II, al = alveolar, br = bronchiolar, u = undifferentiated)
12) Race: all or nearest available, otherwise by race (wh or w = white, bl or b = black, hi = hispanic
ch = chinese, jap = japanese, haw = hawaiian, w+o = white + oriental, sca = scandinavian, as = asian)
13) Duration "high" in key scheme 1 (key value 50, maximum range 36+)
14) For overlapping studies: principal rather than subsidiary studies
Finally by Age: whole study (coded as 0) if available, otherwise by widest available age group
and then for single sex results (m, f) in preference to results for both sexes combined (c).

Results adjusted (AD) for the most potential confounders are then chosen in Sections -1 to -3
(and those which actually differ from the adjusted results in Table 3I14 - 1 are marked 'x' in Section -1)
and results adjusted for the least confounders in Sections -4 to -6. (Those least adjusted results which
actually differ from the most adjusted are marked 'x' in column X in Section -4)

Section -7 shows excluded studies, together with the stage (as above) at which no qualifying
results were found.

Section -8 lists the potentially overlapping studies which have been included (1=principal, 2=subsidiary).

Section -9 lists any results which would have been included in preference except that they had data not complete
enough for use in meta-analysis, with their significance (yes/no), if known, and any further comment as entered
on the database. It also lists as "gap" any categories for which no data were presented by the original authors.

In addition to those mentioned above, the following fields, levels and abbreviations are used:
* or nk = not known, n = no, y = yes, ot = other
ev = ever, cu = current, nev = never
all/unspec = all or unspecified, cig+/-ot = cigarettes irrespective of other products (cigar, pipe etc)
MC = manufactured cigarettes, HR = hand-rolled cigarettes
exL, exH = range of exposure (low and high) in the smoking group, in terms of Duration
REF: 6-character study reference
NRR: number of the RR on the database within the study
ST : study type (CC = case control, pr or prosp = prospective)
NLC: number of lung cancer cases in whole study
R : risky occupational population (n = no, m = mining, o = other risky)
VB : national cigarette type (V = at least 75% Virginia, bl = at least 75% blended, ot = other)
P : any proxy use
H : full histological confirmation
De : derivation of RR/CI (or = original, st = standard method, ot = other method of estimation)

Table 3I19 - 1

IESLC - Meta-analysis of Ever/current Smoking, Duration, "High"
Adenocarcinoma, Cigarettes (or Any Product if Cigarettes not available)
Most adjusted

REF	NRR	3I14	SEX	AGEL	AGEH	RACE	YF	LC TYPE	LOC	START	ST	NLC	R	VB	P	H	AD	SM	PRODUCT	exL	exH	DENOM	De
BARBON	727		m	0	0	all	-	a	Eu:wst	1979	CC	755	n	bl	y	y	1	ev	all/unsp	50	999	nev any	or
BUFFLE	512		m	0	0	wh	-	a	NAmer	1976	CC	943	n	bl	y	n	0	ev	cig+/-ot	50	999	nev cigs	or
CHOI	569		m	0	0	all	-	a	As:oth	1985	CC	375	n	bl	n	n	0	ev	cig+/-ot	50	999	nev cigs	st
DAMBER	542		m	0	0	all	-	a+al+br	Eu:Sca	1972	CC	579	n	bl	y	n	1	ev	all/unsp	41	50	nev any	or
JEDRYC	597		m	0	0	all	-	a	Eu:est	1980	CC	1630	n	bl	y	n	3	ev	cig+/-ot	40	999	nev any	or
JOLY	663		m	0	0	all	-	a	SCAmer	1978	CC	826	n	bl	n	n	0	ev	cig+/-ot	50	999	nev any	st
JOLY	635		f	0	0	all	-	a	SCAmer	1978	CC	826	n	bl	n	n	0	ev	cig+/-ot	50	999	nev any	st
LUBIN2	703		m	0	0	all	-	a	Eu:mul	1976	CC	7804	n	bl	n	y	0	ev	cig+/-ot	50	999	nev any	st
LUBIN2	755		f	0	0	all	-	a	Eu:mul	1976	CC	7804	n	bl	n	y	0	ev	cig+/-ot	50	999	nev any	st
MATOS	648		m	0	0	all	-	a	SCAmer	1994	CC	200	n	bl	n	n	2	ev	cig+/-ot	40	70	nev any	or
PEZZOT	518		m	0	0	all	-	a	SCAmer	1987	CC	215	n	bl	n	y	0	ev	cig only	41	999	nev cigs	st
SOBUE	516		m	0	0	all	-	a	As:Jap	1986	CC	1376	n	bl	n	y	0	cu	cig+/-ot	50	999	nev cigs	st
WUWILL	528		f	0	0	all	-	a	As:Chi	1985	CC	965	n	ot	n	n	3	ev	cig+/-ot	40	999	nev cigs	ot

Cigarette type is all/unspec for all RRs

Table 3I19 - 2

IESLC - Meta-analysis of Ever/current Smoking, Duration, "High"
Adenocarcinoma, Cigarettes (or Any Product if Cigarettes not available)
Most adjusted

REF	NRR	SEX	AD	Number Exposed Case	Cont	Non-exposed Case	Cont	RR	95.00%CI
BARBON	727	m	1	67	-	7	-	8.30	(3.70- 18.70)
BUFFLE	512	m	0	-	-	-	-	3.50	(1.30- 9.70)
CHOI	569	m	0	4	20	7	95	2.71	(0.73- 10.16)
DAMBER	542	m	1	-	-	16	-	3.40	(1.30- 9.10)
JEDRYC	597	m	3	47	-	7	-	4.41	(1.86- 10.45)
JOLY	663	m	0	33	253	5	218	5.69	(2.18- 14.82)
JOLY	635	f	0	7	20	25	283	3.96	(1.53- 10.27)
Subtotal JOLY								4.74	(2.41- 9.32)
LUBIN2	703	m	0	90	1460	57	2616	2.83	(2.02- 3.97)
LUBIN2	755	f	0	181	34	138	1180	45.52	(30.31- 68.36)
Subtotal LUBIN2								8.80	(6.79- 11.41)
MATOS	648	m	2	33	-	5	-	10.70	(3.80- 29.90)
PEZZOT	518	m	0	27	101	3	116	10.34	(3.04- 35.09)
SOBUE	516	m	0	43	73	27	128	2.79	(1.59- 4.89)
WUWILL	528	f	3	52	-	172	-	2.09	(1.40- 3.10)
Partial Totals				584	1961	469	4636		

*prospective study

REF	NRR	SEX	AD	Ys	Ws	Qs	Ps
BARBON	727	m	1	2.12	5.85	1.24	0.0000
BUFFLE	512	m	0	1.25	3.80	0.62	0.0145
CHOI	569	m	0	1.00	2.21	0.95	0.1381
DAMBER	542	m	1	1.22	4.06	0.76	0.0137
JEDRYC	597	m	3	1.48	5.16	0.15	0.0008
JOLY	663	m	0	1.74	4.19	0.03	0.0004
JOLY	635	f	0	1.38	4.23	0.33	0.0046
Subtotal JOLY				1.56	8.42	0.36	
LUBIN2	703	m	0	1.04	33.64	12.78	0.0000
LUBIN2	755	f	0	3.82	23.24	108.61	0.0000
Subtotal LUBIN2				2.17	56.88	121.39	
MATOS	648	m	2	2.37	3.61	1.84	0.0000
PEZZOT	518	m	0	2.34	2.57	1.19	0.0002
SOBUE	516	m	0	1.03	12.22	4.84	0.0003
WUWILL	528	f	3	0.74	24.32	20.55	0.0003

	N	13
	NS	11
	Wt	129.10
Het	Chi	153.89
Het	df	12
Het	P	***
Fixed	RR	5.24
	RRl	4.41
	RRu	6.23
	P	+++
Random	RR	5.25
	RRl	2.70
	RRu	10.20
	P	+++
Asymm	P	N.S.

Table 3I19 - 3

IESLC - Meta-analysis of Ever/current Smoking, Duration, "High"
Adenocarcinoma, Cigarettes (or Any Product if Cigarettes not available)
Most adjusted

	Sex combined	male	female	Total
N		10	3	13
NS		10	3	13
Wt		77.32	51.79	129.10
Het Chi		15.18	115.71	153.89
Het df		9	2	12
Het P		(*)	***	***
Fixed RR		3.71	8.78	5.24
RRl		2.97	6.68	4.41
RRu		4.64	11.52	6.23
P		+++	+++	+++
Random RR		4.30	7.29	5.25
RRl		3.08	0.77	2.70
RRu		5.99	69.06	10.20
P		+++	(+)	+++
Between Chi				22.99
Between df				1
Between P				***
Btwn(F) P				N.S.
Btwn(R) P				N.S.

Table 3I19 - 4

IESLC - Meta-analysis of Ever/current Smoking, Duration, "High"
Adenocarcinoma, Cigarettes (or Any Product if Cigarettes not available)
Least adjusted

REF	NRR	X	SEX	AGEL	AGEH	RACE	YF	LC TYPE	LOC	START	ST	NLC	R	VB	P	H	AD	SM	PRODUCT	exL	exH	DENOM	De
BARBON	720	x	m	0	0	all	-	a	Eu:wst	1979	CC	755	n	bl	y	y	0	ev	all/unsp	50	999	nev any	st
BUFFLE	512		m	0	0	wh	-	a	NAmer	1976	CC	943	n	bl	y	n	0	ev	cig+/-ot	50	999	nev cigs	or
CHOI	569		m	0	0	all	-	a	As:oth	1985	CC	375	n	bl	n	n	0	ev	cig+/-ot	50	999	nev cigs	st
DAMBER	542		m	0	0	all	-	a+al+br	Eu:Sca	1972	CC	579	n	bl	y	n	1	ev	all/unsp	41	50	nev any	or
JEDRYC	523	x	m	0	0	all	-	a	Eu:est	1980	CC	1630	n	bl	y	n	0	ev	cig+/-ot	50	999	nev any	st
JOLY	663		m	0	0	all	-	a	SCAmer	1978	CC	826	n	bl	n	n	0	ev	cig+/-ot	50	999	nev any	st
JOLY	635		f	0	0	all	-	a	SCAmer	1978	CC	826	n	bl	n	n	0	ev	cig+/-ot	50	999	nev any	st
LUBIN2	703		m	0	0	all	-	a	Eu:mul	1976	CC	7804	n	bl	n	y	0	ev	cig+/-ot	50	999	nev any	st
LUBIN2	755		f	0	0	all	-	a	Eu:mul	1976	CC	7804	n	bl	n	y	0	ev	cig+/-ot	50	999	nev any	st
MATOS	643	x	m	0	0	all	-	a	SCAmer	1994	CC	200	n	bl	n	n	0	ev	cig+/-ot	40	70	nev any	st
PEZZOT	518		m	0	0	all	-	a	SCAmer	1987	CC	215	n	bl	n	y	0	ev	cig only	41	999	nev cigs	st
SOBUE	516		m	0	0	all	-	a	As:Jap	1986	CC	1376	n	bl	n	y	0	cu	cig+/-ot	50	999	nev cigs	st
WUWILL	513	x	f	0	0	all	-	a	As:Chi	1985	CC	965	n	ot	n	n	0	ev	cig+/-ot	40	999	nev cigs	st

Cigarette type is all/unspec for all RRs

Table 3I19 - 5

IESLC - Meta-analysis of Ever/current Smoking, Duration, "High"
Adenocarcinoma, Cigarettes (or Any Product if Cigarettes not available)
Least adjusted

REF	NRR	SEX	AD	Number Exposed Case	Number Exposed Cont	Non-exposed Case	Non-exposed Cont	RR	95.00%CI
BARBON	720	m	0	67	235	7	188	7.66	(3.43- 17.07)
BUFFLE	512	m	0	-	-	-	-	3.50	(1.30- 9.70)
CHOI	569	m	0	4	20	7	95	2.71	(0.73- 10.16)
DAMBER	542	m	1	-	-	16	-	3.40	(1.30- 9.10)
JEDRYC	523	m	0	17	214	7	289	3.28	(1.34- 8.05)
JOLY	663	m	0	33	253	5	218	5.69	(2.18- 14.82)
JOLY	635	f	0	7	20	25	283	3.96	(1.53- 10.27)
Subtotal JOLY								4.74	(2.41- 9.32)
LUBIN2	703	m	0	90	1460	57	2616	2.83	(2.02- 3.97)
LUBIN2	755	f	0	181	34	138	1180	45.52	(30.31- 68.36)
Subtotal LUBIN2								8.80	(6.79- 11.41)
MATOS	643	m	0	33	89	5	110	8.16	(3.06- 21.76)
PEZZOT	518	m	0	27	101	3	116	10.34	(3.04- 35.09)
SOBUE	516	m	0	43	73	27	128	2.79	(1.59- 4.89)
WUWILL	513	f	0	52	114	172	601	1.59	(1.10- 2.31)
Partial Totals				554	2613	469	5824		

*prospective study

REF	NRR	SEX	AD	Ys	Ws	Qs	Ps
BARBON	720	m	0	2.04	5.98	1.39	0.0000
BUFFLE	512	m	0	1.25	3.80	0.34	0.0145
CHOI	569	m	0	1.00	2.21	0.68	0.1381
DAMBER	542	m	1	1.22	4.06	0.44	0.0137
JEDRYC	523	m	0	1.19	4.77	0.64	0.0095
JOLY	663	m	0	1.74	4.19	0.14	0.0004
JOLY	635	f	0	1.38	4.23	0.13	0.0046
Subtotal JOLY				1.56	8.42	0.27	
LUBIN2	703	m	0	1.04	33.64	8.85	0.0000
LUBIN2	755	f	0	3.82	23.24	119.26	0.0000
Subtotal LUBIN2				2.17	56.88	128.11	
MATOS	643	m	0	2.10	3.99	1.19	0.0000
PEZZOT	518	m	0	2.34	2.57	1.58	0.0002
SOBUE	516	m	0	1.03	12.22	3.38	0.0003
WUWILL	513	f	0	0.47	28.18	33.28	0.0133

	N	13
	NS	11
	Wt	133.08
Het	Chi	171.30
Het	df	12
Het	P	***
Fixed	RR	4.72
	RRl	3.99
	RRu	5.60
	P	+++
Random	RR	4.89
	RRl	2.45
	RRu	9.75
	P	+++
Asymm	P	N.S.

Table 3I19 - 6

IESLC - Meta-analysis of Ever/current Smoking, Duration, "High"
Adenocarcinoma, Cigarettes (or Any Product if Cigarettes not available)
Least adjusted

	Sex combined	male	female	Total
N		10	3	13
NS		10	3	13
Wt		77.43	55.65	133.08
Het Chi		12.78	144.54	171.30
Het df		9	2	12
Het P		N.S.	***	***
Fixed RR		3.59	6.92	4.72
RRl		2.87	5.32	3.99
RRu		4.49	9.00	5.60
P		+++	+++	+++
Random RR		3.98	6.64	4.89
RRl		2.94	0.58	2.45
RRu		5.37	76.18	9.75
P		+++	N.S.	+++
Between Chi				13.97
Between df				1
Between P				***
Btwn(F) P				N.S.
Btwn(R) P				N.S.

Table 3I19 - 7

IESLC - Meta-analysis of Ever/current Smoking, Duration, "High"
Adenocarcinoma, Cigarettes (or Any Product if Cigarettes not available)
Excluded studies (and stage at which they were excluded)

1	BECHER BLOT1 BROWN3 CARPEN CHYOU DARBY DOLL2 GARCIA GRAHAM GURSEL HAMMO2 JAHN JAIN LAUSSM PRESCO QIAO TVERDA WIGLE WYNDE3
2	ALDERS BENSHL BRESLO CHIAZZ DEAN3 DORN ENGELA GAO2 GILLIS GUO HEGMAN HIRAYA HOLE KAUFMA KOO KOULUM LIU4 MIGRAN MRFITR PERNU SEGI2 SPEIZE SUZUK2 SVENSS VUTUC WAKAI WU YUAN
3	GENG MCDUFF SPITZ STASZE WU2 ZHANG
4	AGUDO AKIBA AMANDU AMES ARMADA AUVINE AXELSS BEST BOFFET BOUCHA BOUCOT BROSS CEDERL CHEN2 CORREA CPSI CPSII DEAN2 DESTEF DOLL FAN GAO GARSHI HAMMON HU HU2 HUMBLE JUSSAW KAISE2 KREUZE LETOUR LEVIN LIAW LIU3 LIU5 LUO MCCONN NOTAN2 PEZZO2 PISANI QIAO2 RACHTA RESTRE SADOWS TIZZAN WANG2 WATSON WYNDE2 WYNDE6 WYNDE7 WYNDE8 ZHOU
5	CHEN LUBIN XU
10	KHUDER
14	DORGAN DOSEME GER HAENSZ KATSOU OSANN2 ZHENG
15	BENHAM

Table 3I19 - 8
Potentially overlapping studies

REF	REFGP	PRINC	.	OVERLAP/LINK
LUBIN2	LUBIN2	1		Lubin-combined

Table 3I19 - 9
Most adjusted - insufficient data for meta-analysis

REF	NRR	SEX	AGEL	AGEH	RACE	YF	LC TYPE	LOC	START	ST	NLC	R	VB	P	H	AD	SM	PRODUCT	exL	exH	DENOM	De
CHEN	512	c	0	0	all	-	a	As:oth	1987	CC	323	n	ot	n	y	2	ev	cig+/-ot	41	999	nev cigs	ot
XU	508	m	0	0	all	-	a	As:Chi	1985	CC	729	n	ot	n	n	2	ev	all/unsp	40	999	nev any	or

REF	NRR	RR	SIG	RRDATA comment
CHEN	512	3.79	y	p<0.001
XU	508	*		RR for 1-19/day is 2.6(p<0.05), for 20-29/day is 3.6(p<0.05) and for >=30/ day is 11.8(p<0.05)

Table 3I20 -

IESLC - Meta-analysis of Ever/current Smoking, Duration, "Highest vs lowest"
Adenocarcinoma, Cigarettes (or Any Product if Cigarettes not available)

This analysis is restricted to results for:
1) Ever/current smokers
2) Results by Duration
3) Categorical results by Duration
4) Denominator (unexposed) = "low"
5) Adenocarcinoma (or near equivalent)
6) Results complete enough for use in metaanalysis

Within each study, results are then selected (in the following order of preference, within each sex) for:
7) SMKSTA: ever, current
8) PRODUCT: cigarettes regardless of other products, cigarettes only, all/unspec
9) CIGTYPE: all/unspecified, MC regardless of HR, MC only
10) Results with least adjustment for other aspects of smoking (ADOS)
11) The highest vs lowest category
12) Followup period (YF, prospective studies): whole study (coded as 0) or longest available
13) LCtype: adeno or nearest available, but not squamous. (q = squamous, s = small,
a = adeno, l = large, KII = Kreyberg II, al = alveolar, br = bronchiolar, u = undifferentiated)
14) Race: all or nearest available, otherwise by race (wh or w = white, bl or b = black, hi = hispanic
ch = chinese, jap = japanese, haw = hawaiian, w+o = white + oriental, sca = scandinavian, as = asian)
15) For overlapping studies: principal rather than subsidiary studies
Finally by Age: whole study (coded as 0) if available, otherwise by widest available age group
and then for single sex results (m, f) in preference to results for both sexes combined (c).

Results adjusted (AD) for the most potential confounders are then chosen in Sections -1 to -3
(and those which actually differ from the adjusted results in Table 3I15 - 1 are marked 'x' in Section -1)
and results adjusted for the least confounders in Sections -4 to -6. (Those least adjusted results which
actually differ from the most adjusted are marked 'x' in column X in Section -4)

Section -7 shows excluded studies, together with the stage (as above) at which no qualifying
results were found.

Section -8 lists the potentially overlapping studies which have been included (1=principal, 2=subsidiary).

Section -9 lists any results which would have been included in preference except that they had data not complete
enough for use in meta-analysis, with their significance (yes/no), if known, and any further comment as entered
on the database. It also lists as "gap" any categories for which no data were presented by the original authors.

In addition to those mentioned above, the following fields, levels and abbreviations are used:
* or nk = not known, n = no, y = yes, ot = other
all/unspec = all or unspecified, cig+/-ot = cigarettes irrespective of other products (cigar, pipe etc)
MC = manufactured cigarettes, HR = hand-rolled cigarettes
exL, exH = range of exposure (low and high) in the "highest" group, in terms of Duration
unexL, unexH = range of exposure (low and high) in the "lowest" group, in terms of Duration
REF: 6-character study reference
NRR: number of the RR on the database within the study
ST : study type (CC = case control, pr or prosp = prospective)
NLC: number of lung cancer cases in whole study
R : risky occupational population (n = no, m = mining, o = other risky)
VB : national cigarette type (V = at least 75% Virginia, bl = at least 75% blended, ot = other)
P : any proxy use
H : full histological confirmation
De : derivation of RR/CI (or = original, st = standard method, ot = other method of estimation)

Table 3I20 - 1

IESLC - Meta-analysis of Ever/current Smoking, Duration, "Highest vs lowest"
Adenocarcinoma, Cigarettes (or Any Product if Cigarettes not available)
Most adjusted

REF	NRR	3I15	SEX	AGEL	AGEH	RACE	YF	LC TYPE	LOC	START	ST	NLC	R	VB	P	H	AD	ADOS	SM	PRODUCT	exL	exH	unexL	unexH	De
BARBON	730		m	0	0	all	-	a	Eu:wst	1979	CC	755	n	bl	y	y	1	0	ev	all/unsp	50	999	1	29	ot
CHOI	572		m	0	0	all	-	a	As:oth	1985	CC	375	n	bl	n	n	0	0	ev	cig+/-ot	50	999	1	29	st
CHOI	580		f	0	0	all	-	a	As:oth	1985	CC	375	n	bl	n	n	0	0	ev	cig+/-ot	30	999	1	29	st
DAMBER	546		m	0	0	all	-	a+al+br	Eu:Sca	1972	CC	579	n	bl	y	n	1	0	ev	all/unsp	51	999	1	30	ot
DORGAN	529		m	0	0	wh	-	a	NAmer	1980	CC	2026	n	bl	y	y	2	0	ev	cig+/-ot	35	999	1	34	ot
DORGAN	525		f	0	0	all	-	a	NAmer	1980	CC	2026	n	bl	y	y	3	0	ev	cig+/-ot	35	999	1	34	ot
DOSEME	520		m	0	0	all	-	not q+s	Eu:bal	1979	CC	1210	n	bl	n	n	2	0	ev	cig+/-ot	21	999	1	10	ot
GER	509		c	0	0	all	-	a	As:oth	1990	CC	141	n	ot	y	n	5	0	ev	all/unsp	31	999	1	30	ot
HAENSZ	536		f	0	0	all	-	a	NAmer	1955	CC	158	n	bl	n	y	1	0	ev	cig+/-ot	15	999	1	14	ot
JEDRYC	527		m	0	0	all	-	a	Eu:est	1980	CC	1630	n	bl	y	n	0	0	ev	cig+/-ot	50	999	1	19	st
JOLY	666		m	0	0	all	-	a	SCAmer	1978	CC	826	n	bl	n	n	0	0	ev	cig+/-ot	50	999	1	29	st
JOLY	638		f	0	0	all	-	a	SCAmer	1978	CC	826	n	bl	n	n	0	0	ev	cig+/-ot	50	999	1	29	st
KATSOU	522		f	0	0	all	-	a	Eu:bal	1987	CC	101	n	bl	n	n	1	0	cu	all/unsp	30	999	1	29	ot
KHUDER	532		m	0	0	all	-	a	NAmer	1985	CC	482	n	bl	n	y	5	3#	ev	cig+/-ot	30	999	1	29	or
LUBIN2	706		m	0	0	all	-	a	Eu:mul	1976	CC	7804	n	bl	n	y	0	0	ev	cig+/-ot	50	999	1	29	st
LUBIN2	758		f	0	0	all	-	a	Eu:mul	1976	CC	7804	n	bl	n	y	0	0	ev	cig+/-ot	50	999	1	29	st
MATOS	650		m	0	0	all	-	a	SCAmer	1994	CC	200	n	bl	n	n	2	0	ev	cig+/-ot	40	70	1	24	ot
OSANN2	518		f	0	0	all	-	KII	NAmer	1964	ot	217	n	bl	n	y	1	0	ev	cig+/-ot	21	999	1	20	ot
PEZZOT	522		m	0	0	all	-	a	SCAmer	1987	CC	215	n	bl	n	y	2	0	ev	cig only	41	999	1	30	ot
SOBUE	519		m	0	0	all	-	a	As:Jap	1986	CC	1376	n	bl	n	y	0	0	cu	cig+/-ot	50	999	1	29	st
WUWILL	515		f	0	0	all	-	a	As:Chi	1985	CC	965	n	ot	n	n	0	0	ev	cig+/-ot	40	999	1	29	st
ZHENG	515		m	0	0	all	-	a	As:Chi	1982	CC	540	n	ot	*	y	1	0	ev	cig+/-ot	30	999	1	29	ot
ZHENG	519		f	0	0	all	-	a	As:Chi	1982	CC	540	n	ot	*	y	1	0	ev	cig+/-ot	30	999	1	29	ot

Comments on values in listings

KHUDER ADOS Age at starting smoking, No of cigarettes per day, Quitted smoking

Cigarette type is all/unspec for all RRs

Table 3I20 - 2

IESLC - Meta-analysis of Ever/current Smoking, Duration, "Highest vs lowest"
Adenocarcinoma, Cigarettes (or Any Product if Cigarettes not available)
Most adjusted

REF	NRR	SEX	AD	Number Exposed Case	Cont	Non-exposed Case	Cont	RR	95.00%CI
BARBON	730	m	1	67	-	15	-	2.24	(1.15- 4.36)
CHOI	572	m	0	4	20	17	221	2.60	(0.80- 8.47)
CHOI	580	f	0	2	3	2	23	7.67	(0.77- 76.45)
Subtotal CHOI								3.26	(1.14- 9.32)
DAMBER	546	m	1	-	-	-	-	1.39	(0.45- 4.30)
DORGAN	529	m	2	-	-	-	-	3.15	(1.90- 5.23)
DORGAN	525	f	3	-	-	-	-	2.11	(1.50- 2.98)
Subtotal DORGAN								2.39	(1.80- 3.18)
DOSEME	520	m	2	69	-	5	-	5.13	(1.81- 14.51)
GER	509	c	5	21	-	14	-	0.61	(0.24- 1.52)
HAENSZ	536	f	1	16	-	2	-	2.69	(0.57- 12.56)
JEDRYC	527	m	0	17	214	2	68	2.70	(0.61- 11.99)
JOLY	666	m	0	33	253	5	109	2.84	(1.08- 7.48)
JOLY	638	f	0	7	20	10	54	1.89	(0.63- 5.64)
Subtotal JOLY								2.38	(1.15- 4.91)
KATSOU	522	f	1	9	-	6	-	4.30	(0.98- 18.82)
KHUDER	532	m	5	-	-	-	-	2.70	(1.40- 5.30)
LUBIN2	706	m	0	90	1460	131	2964	1.39	(1.06- 1.84)
LUBIN2	758	f	0	181	34	174	229	7.01	(4.62- 10.62)
Subtotal LUBIN2								2.28	(1.81- 2.87)
MATOS	650	m	2	33	-	7	-	6.29	(2.59- 15.31)
OSANN2	518	f	1	51	-	10	-	5.86	(1.88- 18.26)
PEZZOT	522	m	2	27	-	11	-	4.60	(1.95- 10.82)
SOBUE	519	m	0	43	73	33	119	2.12	(1.24- 3.64)
WUWILL	515	f	0	52	114	34	139	1.86	(1.13- 3.07)
ZHENG	515	m	1	99	-	24	-	2.42	(1.43- 4.10)
ZHENG	519	f	1	24	-	9	-	2.08	(0.78- 5.53)
Subtotal ZHENG								2.34	(1.47- 3.72)
Partial Totals				845	2191	511	3926		

*prospective study

REF	NRR	SEX	AD	Ys	Ws	Qs	Ps
BARBON	730	m	1	0.81	8.65	0.03	0.0177
CHOI	572	m	0	0.96	2.75	0.02	0.1129
CHOI	580	f	0	2.04	0.73	0.99	0.0826
Subtotal CHOI				1.18	3.48	1.01	
DAMBER	546	m	1	0.33	3.02	0.88	0.5674
DORGAN	529	m	2	1.15	14.99	1.16	0.0000
DORGAN	525	f	3	0.75	32.61	0.49	0.0000
Subtotal DORGAN				0.87	47.60	1.65	
DOSEME	520	m	2	1.64	3.55	2.08	0.0021
GER	509	c	5	-0.49	4.51	8.39	0.2938
HAENSZ	536	f	1	0.99	1.61	0.02	0.2098
JEDRYC	527	m	0	0.99	1.73	0.03	0.1913
JOLY	666	m	0	1.05	4.11	0.13	0.0342
JOLY	638	f	0	0.64	3.21	0.17	0.2540
Subtotal JOLY				0.87	7.32	0.30	
KATSOU	522	f	1	1.46	1.76	0.61	0.0530
KHUDER	532	m	5	0.99	8.67	0.13	0.0034
LUBIN2	706	m	0	0.33	50.59	14.58	0.0180
LUBIN2	758	f	0	1.95	22.20	25.76	0.0000
Subtotal LUBIN2				0.82	72.79	40.34	
MATOS	650	m	2	1.84	4.87	4.57	0.0000
OSANN2	518	f	1	1.77	2.97	2.40	0.0023
PEZZOT	522	m	2	1.53	5.23	2.26	0.0005
SOBUE	519	m	0	0.75	13.22	0.18	0.0062
WUWILL	515	f	0	0.62	15.48	0.94	0.0142
ZHENG	515	m	1	0.88	13.85	0.00	0.0010
ZHENG	519	f	1	0.73	4.01	0.08	0.1427
Subtotal ZHENG				0.85	17.86	0.08	

Table 3I20 - 2

IESLC - Meta-analysis of Ever/current Smoking, Duration, "Highest vs lowest"
Adenocarcinoma, Cigarettes (or Any Product if Cigarettes not available)
Most adjusted

	N	23
	NS	18
	Wt	224.29
Het	Chi	65.90
Het	df	22
Het	P	***
Fixed	RR	2.39
	RRl	2.09
	RRu	2.72
	P	+++
Random	RR	2.64
	RRl	2.04
	RRu	3.43
	P	+++
Asymm	P	N.S.

Table 3I20 - 3

IESLC - Meta-analysis of Ever/current Smoking, Duration, "Highest vs lowest"
Adenocarcinoma, Cigarettes (or Any Product if Cigarettes not available)
Most adjusted

	combined	Sex: male	female	Total
N	1	13	9	23
NS	1	13	9	23
Wt	4.51	135.22	84.57	224.29
Het Chi	0.00	24.72	26.95	65.90
Het df	0	12	8	22
Het P	N.S.	*	***	***
Fixed RR	0.61	2.16	3.01	2.39
RRl	0.24	1.83	2.43	2.09
RRu	1.54	2.56	3.72	2.72
P	N.S.	+++	+++	+++
Random RR	0.61	2.59	3.11	2.64
RRl	0.24	1.96	1.92	2.04
RRu	1.54	3.42	5.04	3.43
P	N.S.	+++	+++	+++
Between Chi				14.23
Between df				2
Between P				***
Btwn(F) P				(*)
Btwn(R) P				**

	Lung cancer type: a	a+l	a+al+br	KII	not q+u	not q+s	Total
N	20		1	1		1	23
NS	15		1	1		1	18
Wt	214.76		3.02	2.97		3.55	224.29
Het Chi	60.47		0.00	0.00		0.00	65.90
Het df	19		0	0		0	22
Het P	***		N.S.	N.S.		N.S.	***
Fixed RR	2.34		1.39	5.86		5.13	2.39
RRl	2.05		0.45	1.88		1.81	2.09
RRu	2.68		4.30	18.26		14.52	2.72
P	+++		N.S.	++		++	+++
Random RR	2.56		1.39	5.86		5.13	2.64
RRl	1.95		0.45	1.88		1.81	2.04
RRu	3.37		4.30	18.26		14.52	3.43
P	+++		N.S.	++		++	+++
Between Chi							5.43
Between df							3
Between P							N.S.
Btwn(F) P							N.S.
Btwn(R) P							N.S.

	NAmer	UK	Scand	Location: othEur	China	Japan	othAs	other	Total
N	5		1	6	3	1	3	4	23
NS	4		1	5	2	1	2	3	18
Wt	60.85		3.02	88.47	33.33	13.22	7.99	17.42	224.29
Het Chi	3.93		0.00	43.09	0.50	0.00	6.19	3.34	65.90
Het df	4		0	5	2	0	2	3	22
Het P	N.S.		N.S.	***	N.S.	N.S.	*	N.S.	***
Fixed RR	2.55		1.39	2.39	2.11	2.12	1.27	3.80	2.39
RRl	1.98		0.45	1.94	1.50	1.24	0.63	2.38	2.09
RRu	3.28		4.30	2.94	2.96	3.64	2.53	6.08	2.72
P	+++		N.S.	+++	+++	++	N.S.	+++	+++
Random RR	2.55		1.39	3.22	2.11	2.12	1.78	3.78	2.64
RRl	1.98		0.45	1.49	1.50	1.24	0.45	2.30	2.04
RRu	3.28		4.30	6.99	2.96	3.64	7.00	6.21	3.43
P	+++		N.S.	++	+++	++	N.S.	+++	+++
Between Chi									8.86
Between df									6
Between P									N.S.
Btwn(F) P									N.S.
Btwn(R) P									N.S.

Table 3I20 - 3

IESLC - Meta-analysis of Ever/current Smoking, Duration, "Highest vs lowest"
Adenocarcinoma, Cigarettes (or Any Product if Cigarettes not available)
Most adjusted

Detailed Country in "other Europe"

	multi	Germany	othWest	East	Balkans	Total
N	2		1	1	2	6
NS	1		1	1	2	5
Wt	72.79		8.65	1.73	5.31	88.47
Het Chi	40.19		0.00	0.00	0.04	43.09
Het df	1		0	0	1	5
Het P	***		N.S.	N.S.	N.S.	***
Fixed RR	2.28		2.24	2.70	4.84	2.39
RRl	1.81		1.15	0.61	2.07	1.94
RRu	2.87		4.36	11.99	11.33	2.94
P	+++		+	N.S.	+++	+++
Random RR	3.10		2.24	2.70	4.84	3.22
RRl	0.64		1.15	0.61	2.07	1.49
RRu	15.08		4.36	11.99	11.33	6.99
P	N.S.		+	N.S.	+++	++
Between Chi						2.86
Between df						3
Between P						N.S.
Btwn(F) P						N.S.
Btwn(R) P						N.S.

Detailed Country in "other Asia"

	India	HongKong	other	Total
N			3	3
NS			2	2
Wt			7.99	7.99
Het Chi			6.19	6.19
Het df			2	2
Het P			*	*
Fixed RR			1.27	1.27
RRl			0.63	0.63
RRu			2.53	2.53
P			N.S.	N.S.
Random RR			1.78	1.78
RRl			0.45	0.45
RRu			7.00	7.00
P			N.S.	N.S.
Between Chi				
Between df				
Between P				N.S.
Btwn(F) P				N.S.
Btwn(R) P				N.S.

Detailed other continent

	SCAmer	Total
N	4	4
NS	3	3
Wt	17.42	17.42
Het Chi	3.34	3.34
Het df	3	3
Het P	N.S.	N.S.
Fixed RR	3.80	3.80
RRl	2.38	2.38
RRu	6.08	6.08
P	+++	+++
Random RR	3.78	3.78
RRl	2.30	2.30
RRu	6.21	6.21
P	+++	+++
Between Chi		
Between df		
Between P		N.S.
Btwn(F) P		N.S.
Btwn(R) P		N.S.

Table 3I20 - 3

IESLC - Meta-analysis of Ever/current Smoking, Duration, "Highest vs lowest"
Adenocarcinoma, Cigarettes (or Any Product if Cigarettes not available)
Most adjusted

	Start year of study					
	<1960	1960-69	1970-79	1980-89	1990+	Total
N	1	1	7	12	2	23
NS	1	1	5	9	2	18
Wt	1.61	2.97	95.32	115.02	9.38	224.29
Het Chi	0.00	0.00	43.55	6.87	12.74	65.90
Het df	0	0	6	11	1	22
Het P	N.S.	N.S.	***	N.S.	***	***
Fixed RR	2.69	5.86	2.32	2.41	2.05	2.39
RRl	0.57	1.88	1.90	2.01	1.08	2.09
RRu	12.63	18.26	2.83	2.90	3.88	2.72
P	N.S.	++	+++	+++	+	+++
Random RR	2.69	5.86	2.65	2.41	1.97	2.64
RRl	0.57	1.88	1.38	2.01	0.20	2.04
RRu	12.63	18.26	5.10	2.90	19.34	3.43
P	N.S.	++	++	+++	N.S.	+++
Between Chi						2.74
Between df						4
Between P						N.S.
Btwn(F) P						N.S.
Btwn(R) P						N.S.

	Study type (1)		
	CC	other	Total
N	22	1	23
NS	17	1	18
Wt	221.32	2.97	224.29
Het Chi	63.47	0.00	65.90
Het df	21	0	22
Het P	***	N.S.	***
Fixed RR	2.36	5.86	2.39
RRl	2.07	1.88	2.09
RRu	2.69	18.26	2.72
P	+++	++	+++
Random RR	2.57	5.86	2.64
RRl	1.98	1.88	2.04
RRu	3.35	18.26	3.43
P	+++	++	+++
Between Chi			2.43
Between df			1
Between P			N.S.
Btwn(F) P			N.S.
Btwn(R) P			N.S.

	Study type (2)			
	CC	prosp	other	Total
N	22		1	23
NS	17		1	18
Wt	221.32		2.97	224.29
Het Chi	63.47		0.00	65.90
Het df	21		0	22
Het P	***		N.S.	***
Fixed RR	2.36		5.86	2.39
RRl	2.07		1.88	2.09
RRu	2.69		18.26	2.72
P	+++		++	+++
Random RR	2.57		5.86	2.64
RRl	1.98		1.88	2.04
RRu	3.35		18.26	3.43
P	+++		++	+++
Between Chi				2.43
Between df				1
Between P				N.S.
Btwn(F) P				N.S.
Btwn(R) P				N.S.

Table 3I20 - 3

IESLC - Meta-analysis of Ever/current Smoking, Duration, "Highest vs lowest"
Adenocarcinoma, Cigarettes (or Any Product if Cigarettes not available)
Most adjusted

	Study size (number of LC cases)				
	100-249	250-499	500-999	1000+	Total
N	6	3	7	7	23
NS	6	2	5	5	18
Wt	20.95	12.15	52.32	138.87	224.29
Het Chi	16.59	0.76	1.46	44.25	65.90
Het df	5	2	6	6	22
Het P	**	N.S.	N.S.	***	***
Fixed RR	3.16	2.85	2.11	2.36	2.39
RRl	2.06	1.62	1.61	2.00	2.09
RRu	4.85	5.00	2.77	2.78	2.72
P	+++	+++	+++	+++	+++
Random RR	3.22	2.85	2.11	2.85	2.64
RRl	1.44	1.62	1.61	1.71	2.04
RRu	7.22	5.00	2.77	4.73	3.43
P	++	+++	+++	+++	+++
Between Chi					2.84
Between df					3
Between P					N.S.
Btwn(F) P					N.S.
Btwn(R) P					N.S.

	Risky occupational population			
	no	mining	othRisky	Total
N	23			23
NS	18			18
Wt	224.29			224.29
Het Chi	65.90			65.90
Het df	22			22
Het P	***			***
Fixed RR	2.39			2.39
RRl	2.09			2.09
RRu	2.72			2.72
P	+++			+++
Random RR	2.64			2.64
RRl	2.04			2.04
RRu	3.43			3.43
P	+++			+++
Between Chi				
Between df				
Between P				N.S.
Btwn(F) P				N.S.
Btwn(R) P				N.S.

	National cigarette tobacco type			
	Virginia	blended	other	Total
N		19	4	23
NS		15	3	18
Wt		186.45	37.84	224.29
Het Chi		55.92	6.59	65.90
Het df		18	3	22
Het P		***	(*)	***
Fixed RR		2.52	1.82	2.39
RRl		2.18	1.32	2.09
RRu		2.91	2.50	2.72
P		+++	+++	+++
Random RR		2.98	1.68	2.64
RRl		2.21	1.01	2.04
RRu		4.02	2.79	3.43
P		+++	+	+++
Between Chi				3.38
Between df				1
Between P				(*)
Btwn(F) P				N.S.
Btwn(R) P				(*)

Table 3I20 - 3

IESLC - Meta-analysis of Ever/current Smoking, Duration, "Highest vs lowest"
Adenocarcinoma, Cigarettes (or Any Product if Cigarettes not available)
Most adjusted

	Any proxy use No/nk	Yes	Total
N	17	6	23
NS	13	5	18
Wt	158.79	65.50	224.29
Het Chi	54.52	10.01	65.90
Het df	16	5	22
Het P	***	(*)	***
Fixed RR	2.51	2.11	2.39
RRl	2.15	1.66	2.09
RRu	2.93	2.69	2.72
P	+++	+++	+++
Random RR	3.03	1.95	2.64
RRl	2.17	1.31	2.04
RRu	4.24	2.93	3.43
P	+++	++	+++
Between Chi			1.36
Between df			1
Between P			N.S.
Btwn(F) P			N.S.
Btwn(R) P			(*)

	Full histological confirmation No	Yes	Total
N	11	12	23
NS	9	9	18
Wt	45.70	178.59	224.29
Het Chi	18.73	47.07	65.90
Het df	10	11	22
Het P	*	***	***
Fixed RR	2.29	2.41	2.39
RRl	1.71	2.08	2.09
RRu	3.06	2.79	2.72
P	+++	+++	+++
Random RR	2.46	2.76	2.64
RRl	1.59	1.97	2.04
RRu	3.79	3.88	3.43
P	+++	+++	+++
Between Chi			0.09
Between df			1
Between P			N.S.
Btwn(F) P			N.S.
Btwn(R) P			N.S.

	Number of adjustment variables (1) 0	1	2+/+nk	Total
N	9	7	7	23
NS	6	6	6	18
Wt	114.01	35.86	74.42	224.29
Het Chi	42.32	3.97	18.60	65.90
Het df	8	6	6	22
Het P	***	N.S.	**	***
Fixed RR	2.24	2.48	2.58	2.39
RRl	1.86	1.79	2.06	2.09
RRu	2.69	3.44	3.24	2.72
P	+++	+++	+++	+++
Random RR	2.59	2.48	2.81	2.64
RRl	1.55	1.79	1.79	2.04
RRu	4.34	3.44	4.40	3.43
P	+++	+++	+++	+++
Between Chi				1.00
Between df				2
Between P				N.S.
Btwn(F) P				N.S.
Btwn(R) P				N.S.

Table 3I20 - 3

IESLC - Meta-analysis of Ever/current Smoking, Duration, "Highest vs lowest"
Adenocarcinoma, Cigarettes (or Any Product if Cigarettes not available)
Most adjusted

	Number of adjustment variables (2)					
	0	1	2	3-5	6+/+nk	Total
N	9	7	4	3		23
NS	6	6	4	3		19
Wt	114.01	35.86	28.63	45.79		224.29
Het Chi	42.32	3.97	2.17	7.21		65.90
Het df	8	6	3	2		22
Het P	***	N.S.	N.S.	*		***
Fixed RR	2.24	2.48	4.03	1.96		2.39
RRl	1.86	1.79	2.80	1.46		2.09
RRu	2.69	3.44	5.82	2.61		2.72
P	+++	+++	+++	+++		+++
Random RR	2.59	2.48	4.03	1.67		2.64
RRl	1.55	1.79	2.80	0.85		2.04
RRu	4.34	3.44	5.82	3.30		3.43
P	+++	+++	+++	N.S.		+++
Between Chi						10.22
Between df						3
Between P						*
Btwn(F) P						N.S.
Btwn(R) P						(*)

	Smoking status		
	ever	current	Total
N	21	2	23
NS	16	2	18
Wt	209.32	14.98	224.29
Het Chi	65.11	0.77	65.90
Het df	20	1	22
Het P	***	N.S.	***
Fixed RR	2.39	2.31	2.39
RRl	2.09	1.39	2.09
RRu	2.74	3.83	2.72
P	+++	++	+++
Random RR	2.65	2.31	2.64
RRl	2.00	1.39	2.04
RRu	3.51	3.83	3.43
P	+++	++	+++
Between Chi			0.02
Between df			1
Between P			N.S.
Btwn(F) P			N.S.
Btwn(R) P			N.S.

	Product			
	all/unsp	cig+/-ot	cig only	Total
N	4	18	1	23
NS	4	13	1	18
Wt	17.94	201.12	5.23	224.29
Het Chi	6.95	53.66	0.00	65.90
Het df	3	17	0	22
Het P	(*)	***	N.S.	***
Fixed RR	1.59	2.43	4.60	2.39
RRl	1.00	2.12	1.95	2.09
RRu	2.52	2.79	10.84	2.72
P	+	+++	+++	+++
Random RR	1.58	2.82	4.60	2.64
RRl	0.74	2.12	1.95	2.04
RRu	3.36	3.75	10.84	3.43
P	N.S.	+++	+++	+++
Between Chi				5.29
Between df				2
Between P				(*)
Btwn(F) P				N.S.
Btwn(R) P				N.S.

Table 3I20 - 3

IESLC - Meta-analysis of Ever/current Smoking, Duration, "Highest vs lowest"
Adenocarcinoma, Cigarettes (or Any Product if Cigarettes not available)
Most adjusted

	Derivation of RR/CI Orig	StdCalc	Other	Total
N	1	9	13	23
NS	1	6	11	18
Wt	8.67	114.01	101.61	224.29
Het Chi	0.00	42.32	22.59	65.90
Het df	0	8	12	22
Het P	N.S.	***	*	***
Fixed RR	2.70	2.24	2.54	2.39
RRl	1.39	1.86	2.09	2.09
RRu	5.25	2.69	3.08	2.72
P	++	+++	+++	+++
Random RR	2.70	2.59	2.68	2.64
RRl	1.39	1.55	1.98	2.04
RRu	5.25	4.34	3.62	3.43
P	++	+++	+++	+++
Between Chi				0.99
Between df				2
Between P				N.S.
Btwn(F) P				N.S.
Btwn(R) P				N.S.

Table 3I20 - 4

IESLC - Meta-analysis of Ever/current Smoking, Duration, "Highest vs lowest"
Adenocarcinoma, Cigarettes (or Any Product if Cigarettes not available)
Least adjusted

REF	NRR	X	SEX	AGEL	AGEH	RACE	YF	LC TYPE	LOC	START	ST	NLC	R	VB	P	H	AD	ADOS	SM	PRODUCT	exL	exH	unexL	unexH	De
BARBON	723	x	m	0	0	all	-	a	Eu:wst	1979	CC	755	n	bl	y	y	0	0	ev	all/unsp	50	999	1	29	st
CHOI	572		m	0	0	all	-	a	As:oth	1985	CC	375	n	bl	n	n	0	0	ev	cig+/-ot	50	999	1	29	st
CHOI	580		f	0	0	all	-	a	As:oth	1985	CC	375	n	bl	n	n	0	0	ev	cig+/-ot	30	999	1	29	st
DAMBER	546		m	0	0	all	-	a+al+br	Eu:Sca	1972	CC	579	n	bl	y	n	1	0	ev	all/unsp	51	999	1	30	ot
DORGAN	529		m	0	0	wh	-	a	NAmer	1980	CC	2026	n	bl	y	y	2	0	ev	cig+/-ot	35	999	1	34	ot
DORGAN	525		f	0	0	all	-	a	NAmer	1980	CC	2026	n	bl	y	y	3	0	ev	cig+/-ot	35	999	1	34	ot
DOSEME	520		m	0	0	all	-	not q+s	Eu:bal	1979	CC	1210	n	bl	n	n	2	0	ev	cig+/-ot	21	999	1	10	ot
GER	503	x	c	0	0	all	-	a	As:oth	1990	CC	141	n	ot	y	n	0	0	ev	all/unsp	31	999	1	30	st
HAENSZ	529	x	f	0	0	all	-	a	NAmer	1955	CC	158	n	bl	n	y	0	0	ev	cig+/-ot	15	999	1	14	st
JEDRYC	527		m	0	0	all	-	a	Eu:est	1980	CC	1630	n	bl	y	n	0	0	ev	cig+/-ot	50	999	1	19	st
JOLY	666		m	0	0	all	-	a	SCAmer	1978	CC	826	n	bl	n	n	0	0	ev	cig+/-ot	50	999	1	29	st
JOLY	638		f	0	0	all	-	a	SCAmer	1978	CC	826	n	bl	n	n	0	0	ev	cig+/-ot	50	999	1	29	st
KATSOU	517	x	f	0	0	all	-	a	Eu:bal	1987	CC	101	n	bl	n	n	0	0	cu	all/unsp	30	999	1	29	st
KHUDER	532		m	0	0	all	-	a	NAmer	1985	CC	482	n	bl	n	y	5	3#	ev	cig+/-ot	30	999	1	29	or
LUBIN2	706		m	0	0	all	-	a	Eu:mul	1976	CC	7804	n	bl	n	y	0	0	ev	cig+/-ot	50	999	1	29	st
LUBIN2	758		f	0	0	all	-	a	Eu:mul	1976	CC	7804	n	bl	n	y	0	0	ev	cig+/-ot	50	999	1	29	st
MATOS	645	x	m	0	0	all	-	a	SCAmer	1994	CC	200	n	bl	n	n	0	0	ev	cig+/-ot	40	70	1	24	st
OSANN2	515	x	f	0	0	all	-	KII	NAmer	1964	ot	217	n	bl	n	y	0	0	ev	cig+/-ot	21	999	1	20	st
PEZZOT	520	x	m	0	0	all	-	a	SCAmer	1987	CC	215	n	bl	n	y	0	0	ev	cig only	41	999	1	30	st
SOBUE	519		m	0	0	all	-	a	As:Jap	1986	CC	1376	n	bl	n	y	0	0	cu	cig+/-ot	50	999	1	29	st
WUWILL	515		f	0	0	all	-	a	As:Chi	1985	CC	965	n	ot	n	n	0	0	ev	cig+/-ot	40	999	1	29	st
ZHENG	514	x	m	0	0	all	-	a	As:Chi	1982	CC	540	n	ot	*	y	0	0	ev	cig+/-ot	30	999	1	29	st
ZHENG	518	x	f	0	0	all	-	a	As:Chi	1982	CC	540	n	ot	*	y	0	0	ev	cig+/-ot	30	999	1	29	st

Comments on values in listings

KHUDER ADOS Age at starting smoking, No of cigarettes per day, Quitted smoking

Cigarette type is all/unspec for all RRs

Table 3I20 - 5

IESLC - Meta-analysis of Ever/current Smoking, Duration, "Highest vs lowest"
Adenocarcinoma, Cigarettes (or Any Product if Cigarettes not available)
Least adjusted

REF	NRR	SEX	AD	Number Exposed Case	Cont	Non-exposed Case	Cont	RR	95.00%CI
BARBON	723	m	0	67	235	15	91	1.73	(0.94- 3.18)
CHOI	572	m	0	4	20	17	221	2.60	(0.80- 8.47)
CHOI	580	f	0	2	3	2	23	7.67	(0.77- 76.45)
Subtotal CHOI								3.26	(1.14- 9.32)
DAMBER	546	m	1	-	-	-	-	1.39	(0.45- 4.30)
DORGAN	529	m	2	-	-	-	-	3.15	(1.90- 5.23)
DORGAN	525	f	3	-	-	-	-	2.11	(1.50- 2.98)
Subtotal DORGAN								2.39	(1.80- 3.18)
DOSEME	520	m	2	69	-	5	-	5.13	(1.81- 14.51)
GER	503	c	0	21	98	14	51	0.78	(0.37- 1.66)
HAENSZ	529	f	0	16	77	2	26	2.70	(0.58- 12.55)
JEDRYC	527	m	0	17	214	2	68	2.70	(0.61- 11.99)
JOLY	666	m	0	33	253	5	109	2.84	(1.08- 7.48)
JOLY	638	f	0	7	20	10	54	1.89	(0.63- 5.64)
Subtotal JOLY								2.38	(1.15- 4.91)
KATSOU	517	f	0	9	6	6	12	3.00	(0.72- 12.46)
KHUDER	532	m	5	-	-	-	-	2.70	(1.40- 5.30)
LUBIN2	706	m	0	90	1460	131	2964	1.39	(1.06- 1.84)
LUBIN2	758	f	0	181	34	174	229	7.01	(4.62- 10.62)
Subtotal LUBIN2								2.28	(1.81- 2.87)
MATOS	645	m	0	33	89	7	84	4.45	(1.87- 10.60)
OSANN2	515	f	0	51	20	10	20	5.10	(2.04- 12.78)
PEZZOT	520	m	0	27	27	11	39	3.55	(1.51- 8.34)
SOBUE	519	m	0	43	73	33	119	2.12	(1.24- 3.64)
WUWILL	515	f	0	52	114	34	139	1.86	(1.13- 3.07)
ZHENG	514	m	0	99	143	24	75	2.16	(1.28- 3.66)
ZHENG	518	f	0	24	27	9	17	1.68	(0.63- 4.46)
Subtotal ZHENG								2.04	(1.29- 3.25)
Partial Totals				845	2913	511	4341		

*prospective study

REF	NRR	SEX	AD	Ys	Ws	Qs	Ps
BARBON	723	m	0	0.55	10.33	0.82	0.0783
CHOI	572	m	0	0.96	2.75	0.04	0.1129
CHOI	580	f	0	2.04	0.73	1.06	0.0826
Subtotal CHOI				1.18	3.48	1.10	
DAMBER	546	m	1	0.33	3.02	0.75	0.5674
DORGAN	529	m	2	1.15	14.99	1.52	0.0000
DORGAN	525	f	3	0.75	32.61	0.22	0.0000
Subtotal DORGAN				0.87	47.60	1.74	
DOSEME	520	m	2	1.64	3.55	2.30	0.0021
GER	503	c	0	-0.25	6.72	7.79	0.5209
HAENSZ	529	f	0	0.99	1.63	0.04	0.2047
JEDRYC	527	m	0	0.99	1.73	0.05	0.1913
JOLY	666	m	0	1.05	4.11	0.19	0.0342
JOLY	638	f	0	0.64	3.21	0.12	0.2540
Subtotal JOLY				0.87	7.32	0.31	
KATSOU	517	f	0	1.10	1.89	0.14	0.1305
KHUDER	532	m	5	0.99	8.67	0.23	0.0034
LUBIN2	706	m	0	0.33	50.59	12.48	0.0180
LUBIN2	758	f	0	1.95	22.20	27.72	0.0000
Subtotal LUBIN2				0.82	72.79	40.20	
MATOS	645	m	0	1.49	5.09	2.24	0.0008
OSANN2	515	f	0	1.63	4.55	2.91	0.0005
PEZZOT	520	m	0	1.27	5.25	1.00	0.0037
SOBUE	519	m	0	0.75	13.22	0.08	0.0062
WUWILL	515	f	0	0.62	15.48	0.66	0.0142
ZHENG	514	m	0	0.77	13.87	0.05	0.0041
ZHENG	518	f	0	0.52	4.02	0.39	0.2987
Subtotal ZHENG				0.71	17.89	0.44	

Table 3I20 - 5

IESLC - Meta-analysis of Ever/current Smoking, Duration, "Highest vs lowest"
Adenocarcinoma, Cigarettes (or Any Product if Cigarettes not available)
Least adjusted

	N	23
	NS	18
	Wt	230.19
Het	Chi	62.80
Het	df	22
Het	P	***
Fixed	RR	2.29
	RRl	2.01
	RRu	2.61
	P	+++
Random	RR	2.48
	RRl	1.94
	RRu	3.19
	P	+++
Asymm	P	N.S.

Table 3I20 - 6

IESLC - Meta-analysis of Ever/current Smoking, Duration, "Highest vs lowest"
Adenocarcinoma, Cigarettes (or Any Product if Cigarettes not available)
Least adjusted

	Sex			
	combined	male	female	Total
N	1	13	9	23
NS	1	13	9	23
Wt	6.72	137.15	86.32	230.19
Het Chi	0.00	20.08	27.48	62.80
Het df	0	12	8	22
Het P	N.S.	(*)	***	***
Fixed RR	0.78	2.05	2.97	2.29
RRl	0.37	1.74	2.40	2.01
RRu	1.66	2.43	3.67	2.61
P	N.S.	+++	+++	+++
Random RR	0.78	2.35	2.95	2.48
RRl	0.37	1.84	1.84	1.94
RRu	1.66	3.01	4.76	3.19
P	N.S.	+++	+++	+++
Between Chi				15.24
Between df				2
Between P				***
Btwn(F) P				(*)
Btwn(R) P				*

Table 3I20 - 7

IESLC - Meta-analysis of Ever/current Smoking, Duration, "Highest vs lowest"
Adenocarcinoma, Cigarettes (or Any Product if Cigarettes not available)
Excluded studies (and stage at which they were excluded)

1	BECHER BLOT1 BROWN3 CARPEN CHYOU DARBY DOLL2 GARCIA GRAHAM GURSEL HAMMO2 JAHN JAIN LAUSSM PRESCO QIAO TVERDA WIGLE WYNDE3
2	ALDERS BENSHL BRESLO CHIAZZ DEAN3 DORN ENGELA GAO2 GILLIS GUO HEGMAN HIRAYA HOLE KAUFMA KOO KOULUM LIU4 MIGRAN MRFITR PERNU SEGI2 SPEIZE SUZUK2 SVENSS VUTUC WAKAI WU YUAN
3	GENG MCDUFF SPITZ STASZE WU2 ZHANG
4	AKIBA GARSHI
5	AGUDO AMANDU AMES ARMADA AUVINE AXELSS BEST BOFFET BOUCHA BOUCOT BROSS CEDERL CHEN2 CORREA CPSI CPSII DEAN2 DESTEF DOLL FAN GAO HAMMON HU HU2 HUMBLE JUSSAW KAISE2 KREUZE LETOUR LEVIN LIAW LIU3 LIU5 LUO MCCONN NOTAN2 PEZZO2 PISANI QIAO2 RACHTA RESTRE SADOWS TIZZAN WANG2 WATSON WYNDE2 WYNDE6 WYNDE7 WYNDE8 ZHOU
6	BUFFLE CHEN LUBIN XU
15	BENHAM

Table 3I20 - 8
Potentially overlapping studies

REF	REFGP	PRINC	.	OVERLAP/LINK
LUBIN2	LUBIN2	1		Lubin-combined
OSANN2	KAISER	2		KAISER/OSANN2

Table 3I20 - 9
Most adjusted - insufficient data for meta-analysis

REF	NRR	SEX	AGEL	AGEH	RACE	YF	LC TYPE	LOC	START	ST	NLC	R	VB	P	H	AD	ADOS	SM	PRODUCT	exL	exH	unexL	unexH	De
BUFFLE	554	m	0	0	wh	-	a	NAmer	1976	CC	943	n	bl	y	n	0	0	ev	cig+/-ot	50	999	1	33	st
CHEN	521	c	0	0	all	-	a	As:oth	1987	CC	323	n	ot	n	y	2	0	ev	cig+/-ot	41	999	1	20	st
LUBIN	620	m	0	0	all	-	KII	As:Chi	1984	CC	427	m	ot	y	n	5	1#	ev	cig+/-ot	50	999	1	29	or
XU	510	m	0	0	all	-	a	As:Chi	1985	CC	729	n	ot	n	n	2	0	ev	all/unsp	40	999	1	29	st

Comments on values in listings

LUBIN ADOS Duration of pipe use

REF	NRR	RR	SIG	RRDATA comment
BUFFLE	554	0.80		0
CHEN	521	3.08		0
LUBIN	620	2.51		0
XU	510	*		RR for 1-19/day is 1.9, for 20-29/day is 5.1 and for >=30/day is 2.2

Table 3I21 -

IESLC - Meta-analysis of Ever/current Smoking by Duration, Overview
Adenocarcinoma, Cigarettes only

This analysis is restricted to results for:
1) Ever/current smokers
2) Results by Duration
3) Categorical results by Duration
Results by Duration are grouped under 2 schemes (S1, S2). Each scheme has a set of "key values". An interval is allocated to the category whose key value it includes, and intervals which include none or more than one of the key values are excluded. (Open-ended intervals are coded as 999)

S1	key value	maximum range
1	20	1-34
2	35	21-49
3	50	36+

S2	key value	maximum range
1	5	1-19
2	20	6-29
3	30	21-39
4	40	31-49
5	50	41-998
6	999	51+

4) Adenocarcinoma (or near equivalent)
5) Results complete enough for use in metaanalysis

Within each study, results are then selected (in the following order of preference, within each sex) for:
6) SMKSTA: ever, current
7) PRODUCT: cigarettes only
8) CIGTYPE: all/unspecified, MC regardless of HR, MC only
9) (not applicable)
10) DENOM: never smoked anything, never smoked cigarettes, never any + low, never cigs + low
11) Followup period (YF, prospective studies): whole study (coded as 0) or longest available
12) LCtype: adeno or nearest available, but not squamous. (q = squamous, s = small,
a = adeno, l = large, KII = Kreyberg II, al = alveolar, br = bronchiolar, u = undifferentiated)
13) Race: all or nearest available, otherwise by race (wh or w = white, bl or b = black, hi = hispanic
ch = chinese, jap = japanese, haw = hawaiian, w+o = white + oriental, sca = scandinavian, as = asian)
14) For overlapping studies: principal rather than subsidiary studies
Finally by Age: whole study (coded as 0) if available, otherwise by widest available age group
and then for single sex results (m, f) in preference to results for both sexes combined (c).

Results adjusted (AD) for the most potential confounders are then chosen in Sections -1 to -3
(and those which actually differ from the adjusted results in Table 3I11 - 1 are marked 'x' in Section -1)
and results adjusted for the least confounders in Sections -4 to -6. (Those least adjusted results which
actually differ from the most adjusted are marked 'x' in column X in Section -4)

Section -7 shows excluded studies, together with the stage (as above) at which no qualifying
results were found.

Section -8 lists the potentially overlapping studies which have been included (1=principal, 2=subsidiary).

Section -9 lists any results which would have been included in preference except that they had data not complete
enough for use in meta-analysis, with their significance (yes/no), if known, and any further comment as entered
on the database. It also lists as "gap" any categories for which no data were presented by the original authors.

In addition to those mentioned above, the following fields, levels and abbreviations are used:
* or nk = not known, n = no, y = yes, ot = other
ev = ever, cu = current, nev = never
all/unspec = all or unspecified, MC = manufactured cigarettes, HR = hand-rolled cigarettes
exL, exH = range of exposure (low and high) in the smoking group, in terms of Duration
REF: 6-character study reference
NRR: number of the RR on the database within the study
ST : study type (CC = case control, pr or prosp = prospective)
NLC: number of lung cancer cases in whole study
R : risky occupational population (n = no, m = mining, o = other risky)
VB : national cigarette type (V = at least 75% Virginia, bl = at least 75% blended, ot = other)
P : any proxy use
H : full histological confirmation
De : derivation of RR/CI (or = original, st = standard method, ot = other method of estimation)

Table 3I21 - 1

IESLC - Meta-analysis of Ever/current Smoking by Duration, Overview
Adenocarcinoma, Cigarettes only
Most adjusted

REF	NRR	3I11	SEX	AGEL	AGEH	RACE	YF	LC TYPE	LOC	START	ST	NLC	R	VB	P	H	AD	SM	PRODUCT	exL	exH	S1	S2	DENOM	De
BENHAM	508	x	m	0	0	all	-	KII	Eu:wst	1976	CC	1625	n	bl	n	y	0	ev	cig only	1	35	0	0	nev any	st
BENHAM	509	x	m	0	0	all	-	KII	Eu:wst	1976	CC	1625	n	bl	n	y	0	ev	cig only	36	999	3	0	nev any	st
PEZZOT	516		m	0	0	all	-	a	SCAmer	1987	CC	215	n	bl	n	y	0	ev	cig only	1	30	1	0	nev cigs	st
PEZZOT	517		m	0	0	all	-	a	SCAmer	1987	CC	215	n	bl	n	y	0	ev	cig only	31	40	2	4	nev cigs	st
PEZZOT	518		m	0	0	all	-	a	SCAmer	1987	CC	215	n	bl	n	y	0	ev	cig only	41	999	3	0	nev cigs	st

Cigarette type is all/unspec for all RRs

In this overview table, subtotals and Qs values may be invalid and should be ignored

Table 3I21 - 2

IESLC - Meta-analysis of Ever/current Smoking by Duration, Overview
Adenocarcinoma, Cigarettes only
Most adjusted

REF	NRR	SEX	AD	Number Exposed Case	Number Exposed Cont	Non-exposed Case	Non-exposed Cont	RR	95.00%CI
BENHAM	508	m	0	55	93	9	42	2.76	(1.25- 6.10)
BENHAM	509	m	0	55	50	9	42	5.13	(2.27- 11.60)
Subtotal BENHAM								3.73	(2.11- 6.59)
PEZZOT	516	m	0	11	134	3	116	3.17	(0.86- 11.65)
PEZZOT	517	m	0	22	82	3	116	10.37	(3.01- 35.81)
PEZZOT	518	m	0	27	101	3	116	10.34	(3.04- 35.09)
Subtotal PEZZOT								7.18	(3.49- 14.81)
Totals				170	460	27	432		

*prospective study

REF	NRR	SEX	AD	Ys	Ws	Qs	Ps
BENHAM	508	m	0	1.02	6.10	1.86	0.0121
BENHAM	509	m	0	1.64	5.78	0.03	0.0001
Subtotal BENHAM				1.32	11.88	1.89	
PEZZOT	516	m	0	1.16	2.27	0.39	0.0817
PEZZOT	517	m	0	2.34	2.50	1.49	0.0002
PEZZOT	518	m	0	2.34	2.57	1.52	0.0002
Subtotal PEZZOT				1.97	7.34	3.40	

N	5
NS	2

Table 3I21 - 3

IESLC - Meta-analysis of Ever/current Smoking by Duration, Overview
Adenocarcinoma, Cigarettes only
Most adjusted

	Sex: combined	male	female	Total
N		5		5
NS		2		2

In this overview table, other than the "N" rows, entries in the "absent" and "Total" columns may be invalid and should be ignored

	Duration of smoking (broad categories): absent	1-34k20	21-49k35	36+k50	Total
N	1	1	1	2	5
NS	1	1	1	2	4
Wt	6.10	2.27	2.50	8.35	19.22
Het Chi	0.00	0.00	0.00	0.87	5.28
Het df	0	0	0	1	4
Het P	N.S.	N.S.	N.S.	N.S.	N.S.
Fixed RR	2.76	3.17	10.37	6.37	4.79
RRl	1.25	0.86	3.01	3.23	3.07
RRu	6.10	11.65	35.81	12.55	7.49
P	+	(+)	+++	+++	+++
Random RR	2.76	3.17	10.37	6.37	4.96
RRl	1.25	0.86	3.01	3.23	2.93
RRu	6.10	11.65	35.81	12.55	8.41
P	+	(+)	+++	+++	+++

	absent	Duration of smoking (narrow categories): 1-19k1	6-29k20	21-39k30	31-49k40	41-998k50	51+k999	Total
N	4				1			5
NS	2				1			2
Wt	16.72				2.50			19.22
Het Chi	3.57				0.00			5.28
Het df	3				0			4
Het P	N.S.				N.S.			N.S.
Fixed RR	4.27				10.37			4.79
RRl	2.64				3.01			3.07
RRu	6.90				35.81			7.49
P	+++				+++			+++
Random RR	4.33				10.37			4.96
RRl	2.54				3.01			2.93
RRu	7.37				35.81			8.41
P	+++				+++			+++

Table 3I21 - 3

IESLC - Meta-analysis of Ever/current Smoking by Duration, Overview
Adenocarcinoma, Cigarettes only
Most adjusted

MALES

		Duration of smoking (broad categories)				
		absent	1-34k20	21-49k35	36+k50	Total
	N	1	1	1	2	5
	NS	1	1	1	2	4
	Wt	6.10	2.27	2.50	8.35	19.22
Het	Chi	0.00	0.00	0.00	0.87	5.28
Het	df	0	0	0	1	4
Het	P	N.S.	N.S.	N.S.	N.S.	N.S.
Fixed	RR	2.76	3.17	10.37	6.37	4.79
	RRl	1.25	0.86	3.01	3.23	3.07
	RRu	6.10	11.65	35.81	12.55	7.49
	P	+	(+)	+++	+++	+++
Random	RR	2.76	3.17	10.37	6.37	4.96
	RRl	1.25	0.86	3.01	3.23	2.93
	RRu	6.10	11.65	35.81	12.55	8.41
	P	+	(+)	+++	+++	+++

		Duration of smoking (narrow categories)							
		absent	1-19k1	6-29k20	21-39k30	31-49k40	41-998k50	51+k999	Total
	N	4				1			5
	NS	2				1			2
	Wt	16.72				2.50			19.22
Het	Chi	3.57				0.00			5.28
Het	df	3				0			4
Het	P	N.S.				N.S.			N.S.
Fixed	RR	4.27				10.37			4.79
	RRl	2.64				3.01			3.07
	RRu	6.90				35.81			7.49
	P	+++				+++			+++
Random	RR	4.33				10.37			4.96
	RRl	2.54				3.01			2.93
	RRu	7.37				35.81			8.41
	P	+++				+++			+++

Table 3I21 - 4

IESLC - Meta-analysis of Ever/current Smoking by Duration, Overview
Adenocarcinoma, Cigarettes only
Least adjusted

REF	NRR	X	SEX	AGEL	AGEH	RACE	YF	LC TYPE	LOC	START	ST	NLC	R	VB	P	H	AD	SM	PRODUCT	exL	exH	S1	S2	DENOM	De
BENHAM	508		m	0	0	all	-	KII	Eu:wst	1976	CC	1625	n	bl	n	y	0	ev	cig only	1	35	0	0	nev any	st
BENHAM	509		m	0	0	all	-	KII	Eu:wst	1976	CC	1625	n	bl	n	y	0	ev	cig only	36	999	3	0	nev any	st
PEZZOT	516		m	0	0	all	-	a	SCAmer	1987	CC	215	n	bl	n	y	0	ev	cig only	1	30	1	0	nev cigs	st
PEZZOT	517		m	0	0	all	-	a	SCAmer	1987	CC	215	n	bl	n	y	0	ev	cig only	31	40	2	4	nev cigs	st
PEZZOT	518		m	0	0	all	-	a	SCAmer	1987	CC	215	n	bl	n	y	0	ev	cig only	41	999	3	0	nev cigs	st

Cigarette type is all/unspec for all RRs

In this overview table, subtotals and Qs values may be invalid and should be ignored

Table 3I21 - 5

IESLC - Meta-analysis of Ever/current Smoking by Duration, Overview
Adenocarcinoma, Cigarettes only
Least adjusted

REF	NRR	SEX	AD	Number Exposed Case	Exposed Cont	Non-exposed Case	Non-exposed Cont	RR	95.00%CI
BENHAM	508	m	0	55	93	9	42	2.76	(1.25- 6.10)
BENHAM	509	m	0	55	50	9	42	5.13	(2.27- 11.60)
Subtotal BENHAM								3.73	(2.11- 6.59)
PEZZOT	516	m	0	11	134	3	116	3.17	(0.86- 11.65)
PEZZOT	517	m	0	22	82	3	116	10.37	(3.01- 35.81)
PEZZOT	518	m	0	27	101	3	116	10.34	(3.04- 35.09)
Subtotal PEZZOT								7.18	(3.49- 14.81)
Totals				170	460	27	432		

*prospective study

REF	NRR	SEX	AD	Ys	Ws	Qs	Ps
BENHAM	508	m	0	1.02	6.10	1.86	0.0121
BENHAM	509	m	0	1.64	5.78	0.03	0.0001
Subtotal BENHAM				1.32	11.88	1.89	
PEZZOT	516	m	0	1.16	2.27	0.39	0.0817
PEZZOT	517	m	0	2.34	2.50	1.49	0.0002
PEZZOT	518	m	0	2.34	2.57	1.52	0.0002
Subtotal PEZZOT				1.97	7.34	3.40	

N	5
NS	2

Table 3I21 - 6

IESLC - Meta-analysis of Ever/current Smoking by Duration, Overview
Adenocarcinoma, Cigarettes only
Least adjusted

	Sex: combined	male	female	Total
N		5		5
NS		2		2

In this overview table, other than the "N" rows, entries in the "absent" and "Total" columns may be invalid and should be ignored

	Duration of smoking (broad categories): absent	1-34k20	21-49k35	36+k50	Total
N	1	1	1	2	5
NS	1	1	1	2	4
Wt	6.10	2.27	2.50	8.35	19.22
Het Chi	0.00	0.00	0.00	0.87	5.28
Het df	0	0	0	1	4
Het P	N.S.	N.S.	N.S.	N.S.	N.S.
Fixed RR	2.76	3.17	10.37	6.37	4.79
RRl	1.25	0.86	3.01	3.23	3.07
RRu	6.10	11.65	35.81	12.55	7.49
P	+	(+)	+++	+++	+++
Random RR	2.76	3.17	10.37	6.37	4.96
RRl	1.25	0.86	3.01	3.23	2.93
RRu	6.10	11.65	35.81	12.55	8.41
P	+	(+)	+++	+++	+++

	absent	Duration of smoking (narrow categories): 1-19k1	6-29k20	21-39k30	31-49k40	41-998k50	51+k999	Total
N	4				1			5
NS	2				1			2
Wt	16.72				2.50			19.22
Het Chi	3.57				0.00			5.28
Het df	3				0			4
Het P	N.S.				N.S.			N.S.
Fixed RR	4.27				10.37			4.79
RRl	2.64				3.01			3.07
RRu	6.90				35.81			7.49
P	+++				+++			+++
Random RR	4.33				10.37			4.96
RRl	2.54				3.01			2.93
RRu	7.37				35.81			8.41
P	+++				+++			+++

Table 3I21 - 6

IESLC - Meta-analysis of Ever/current Smoking by Duration, Overview
Adenocarcinoma, Cigarettes only
Least adjusted

MALES

	Duration of smoking (broad categories)				
	absent	1-34k20	21-49k35	36+k50	Total
N	1	1	1	2	5
NS	1	1	1	2	4
Wt	6.10	2.27	2.50	8.35	19.22
Het Chi	0.00	0.00	0.00	0.87	5.28
Het df	0	0	0	1	4
Het P	N.S.	N.S.	N.S.	N.S.	N.S.
Fixed RR	2.76	3.17	10.37	6.37	4.79
RRl	1.25	0.86	3.01	3.23	3.07
RRu	6.10	11.65	35.81	12.55	7.49
P	+	(+)	+++	+++	+++
Random RR	2.76	3.17	10.37	6.37	4.96
RRl	1.25	0.86	3.01	3.23	2.93
RRu	6.10	11.65	35.81	12.55	8.41
P	+	(+)	+++	+++	+++

		Duration of smoking (narrow categories)					
	absent	1-19k1	6-29k20	21-39k30	31-49k40	41-998k50	51+k999	Total
N	4				1			5
NS	2				1			2
Wt	16.72				2.50			19.22
Het Chi	3.57				0.00			5.28
Het df	3				0			4
Het P	N.S.				N.S.			N.S.
Fixed RR	4.27				10.37			4.79
RRl	2.64				3.01			3.07
RRu	6.90				35.81			7.49
P	+++				+++			+++
Random RR	4.33				10.37			4.96
RRl	2.54				3.01			2.93
RRu	7.37				35.81			8.41
P	+++				+++			+++

Table 3I21 - 7

IESLC - Meta-analysis of Ever/current Smoking by Duration, Overview
Adenocarcinoma, Cigarettes only
Excluded studies (and stage at which they were excluded)

1	BECHER BLOT1 BROWN3 CARPEN CHYOU DARBY DOLL2 GARCIA GRAHAM GURSEL HAMMO2 JAHN JAIN LAUSSM PRESCO QIAO TVERDA WIGLE WYNDE3
2	ALDERS BENSHL BRESLO CHIAZZ DEAN3 DORN ENGELA GAO2 GILLIS GUO HEGMAN HIRAYA HOLE KAUFMA KOO KOULUM LIU4 MIGRAN MRFITR PERNU SEGI2 SPEIZE SUZUK2 SVENSS VUTUC WAKAI WU YUAN
3	GENG MCDUFF SPITZ STASZE WU2 ZHANG
4	AGUDO AKIBA AMANDU AMES ARMADA AUVINE AXELSS BEST BOFFET BOUCHA BOUCOT BROSS CEDERL CHEN2 CORREA CPSI CPSII DEAN2 DESTEF DOLL FAN GAO GARSHI HAMMON HU HU2 HUMBLE JUSSAW KAISE2 KREUZE LETOUR LEVIN LIAW LIU3 LIU5 LUO MCCONN NOTAN2 PEZZO2 PISANI QIAO2 RACHTA RESTRE SADOWS TIZZAN WANG2 WATSON WYNDE2 WYNDE6 WYNDE7 WYNDE8 ZHOU
5	CHEN LUBIN XU
7	BARBON BUFFLE CHOI DAMBER DORGAN DOSEME GER HAENSZ JEDRYC JOLY KATSOU KHUDER LUBIN2 MATOS OSANN2 SOBUE WUWILL ZHENG

Table 3I21 - 8

Potentially overlapping studies

REF	REFGP	PRINC	.	OVERLAP/LINK
BENHAM	LUBIN2	2		Subset of Lubin2

Table 3I22 -

IESLC - Meta-analysis of Ever/current Smoking, Duration, "Low"
Adenocarcinoma, Cigarettes only

This analysis is restricted to results for:
1) Ever/current smokers
2) Results by Duration
3) Categorical results by Duration
4) Adenocarcinoma (or near equivalent)
5) Results complete enough for use in metaanalysis

Within each study, results are then selected (in the following order of preference, within each sex) for:
6) SMKSTA: ever, current
7) PRODUCT: cigarettes only
8) CIGTYPE: all/unspecified, MC regardless of HR, MC only
9) (not applicable)
10) DENOM: never smoked anything, never smoked cigarettes, never any + low, never cigs + low
11) Followup period (YF, prospective studies): whole study (coded as 0) or longest available
12) LCtype: adeno or nearest available, but not squamous. (q = squamous, s = small,
a = adeno, l = large, KII = Kreyberg II, al = alveolar, br = bronchiolar, u = undifferentiated)
13) Race: all or nearest available, otherwise by race (wh or w = white, bl or b = black, hi = hispanic
ch = chinese, jap = japanese, haw = hawaiian, w+o = white + oriental, sca = scandinavian, as = asian)
14) Duration "low" in key scheme 1 (key value 20, maximum range 1-34)
15) For overlapping studies: principal rather than subsidiary studies
Finally by Age: whole study (coded as 0) if available, otherwise by widest available age group
and then for single sex results (m, f) in preference to results for both sexes combined (c).

Results adjusted (AD) for the most potential confounders are then chosen in Sections -1 to -3
(and those which actually differ from the adjusted results in Table 3I12 - 1 are marked 'x' in Section -1)
and results adjusted for the least confounders in Sections -4 to -6. (Those least adjusted results which
actually differ from the most adjusted are marked 'x' in column X in Section -4)

Section -7 shows excluded studies, together with the stage (as above) at which no qualifying
results were found.

Section -8 lists the potentially overlapping studies which have been included (1=principal, 2=subsidiary).

Section -9 lists any results which would have been included in preference except that they had data not complete
enough for use in meta-analysis, with their significance (yes/no), if known, and any further comment as entered
on the database. It also lists as "gap" any categories for which no data were presented by the original authors.

In addition to those mentioned above, the following fields, levels and abbreviations are used:
* or nk = not known, n = no, y = yes, ot = other
ev = ever, cu = current, nev = never
all/unspec = all or unspecified, MC = manufactured cigarettes, HR = hand-rolled cigarettes
exL, exH = range of exposure (low and high) in the smoking group, in terms of Duration
REF: 6-character study reference
NRR: number of the RR on the database within the study
ST : study type (CC = case control, pr or prosp = prospective)
NLC: number of lung cancer cases in whole study
R : risky occupational population (n = no, m = mining, o = other risky)
VB : national cigarette type (V = at least 75% Virginia, bl = at least 75% blended, ot = other)
P : any proxy use
H : full histological confirmation
De : derivation of RR/CI (or = original, st = standard method, ot = other method of estimation)

Table 3I22 - 1

IESLC - Meta-analysis of Ever/current Smoking, Duration, "Low"
Adenocarcinoma, Cigarettes only
Most adjusted

REF	NRR	3I12	SEX	AGEL	AGEH	RACE	YF	LC TYPE	LOC	START	ST	NLC	R	VB	P	H	AD	SM	PRODUCT	exL	exH	DENOM	De
PEZZOT	516		m	0	0	all	-	a	SCAmer	1987	CC	215	n	bl	n	y	0	ev	cig only	1	30	nev cigs	st

Cigarette type is all/unspec for all RRs

Table 3I22 - 2

IESLC - Meta-analysis of Ever/current Smoking, Duration, "Low"
Adenocarcinoma, Cigarettes only
Most adjusted

REF	NRR	SEX	AD	Number Exposed Case	Number Exposed Cont	Non-exposed Case	Non-exposed Cont	RR	95.00%CI
PEZZOT	516	m	0	11	134	3	116	3.17	(0.86- 11.65)
Totals				11	134	3	116		

*prospective study

REF	NRR	SEX	AD	Ys	Ws	Qs	Ps
PEZZOT	516	m	0	1.16	2.27	0.00	0.0817

	N	1
	NS	1
	Wt	2.27
Het	Chi	0.00
Het	df	0
Het	P	N.S.
Fixed	RR	3.17
	RRl	0.86
	RRu	11.65
	P	(+)
Random	RR	3.17
	RRl	0.86
	RRu	11.65
	P	(+)
Asymm	P	

Table 3I22 - 3

IESLC - Meta-analysis of Ever/current Smoking, Duration, "Low"
Adenocarcinoma, Cigarettes only
Most adjusted

	combined	Sex: male	female	Total
N		1		1
NS		1		1
Wt		2.27		2.27
Het Chi		0.00		0.00
Het df		0		0
Het P		N.S.		N.S.
Fixed RR		3.17		3.17
RRl		0.86		0.86
RRu		11.65		11.65
P		(+)		(+)
Random RR		3.17		3.17
RRl		0.86		0.86
RRu		11.65		11.65
P		(+)		(+)
Between Chi				
Between df				
Between P				N.S.
Btwn(F) P				N.S.
Btwn(R) P				N.S.

Too few RRs for analysis by factor

Table 3I22 - 4

IESLC - Meta-analysis of Ever/current Smoking, Duration, "Low"
Adenocarcinoma, Cigarettes only
Least adjusted

REF	NRR	X	SEX	AGEL	AGEH	RACE	YF	LC TYPE	LOC	START	ST	NLC	R	VB	P	H	AD	SM	PRODUCT	exL	exH	DENOM	De
PEZZOT	516		m	0	0	all	-	a	SCAmer	1987	CC	215	n	bl	n	y	0	ev	cig only	1	30	nev cigs	st

Cigarette type is all/unspec for all RRs

Table 3I22 - 5

IESLC - Meta-analysis of Ever/current Smoking, Duration, "Low"
Adenocarcinoma, Cigarettes only
Least adjusted

REF	NRR	SEX	AD	Number Case	Exposed Cont	Non-exposed Case	Cont	RR	95.00%CI
PEZZOT	516	m	0	11	134	3	116	3.17	(0.86- 11.65)
Totals				11	134	3	116		

*prospective study

REF	NRR	SEX	AD	Ys	Ws	Qs	Ps
PEZZOT	516	m	0	1.16	2.27	0.00	0.0817

	N	1
	NS	1
	Wt	2.27
Het	Chi	0.00
Het	df	0
Het	P	N.S.
Fixed	RR	3.17
	RRl	0.86
	RRu	11.65
	P	(+)
Random	RR	3.17
	RRl	0.86
	RRu	11.65
	P	(+)
Asymm	P	

Table 3I22 - 6

IESLC - Meta-analysis of Ever/current Smoking, Duration, "Low"
Adenocarcinoma, Cigarettes only
Least adjusted

	Sex combined	male	female	Total
N		1		1
NS		1		1
Wt		2.27		2.27
Het Chi		0.00		0.00
Het df		0		0
Het P		N.S.		N.S.
Fixed RR		3.17		3.17
RRl		0.86		0.86
RRu		11.65		11.65
P		(+)		(+)
Random RR		3.17		3.17
RRl		0.86		0.86
RRu		11.65		11.65
P		(+)		(+)
Between Chi				
Between df				
Between P				N.S.
Btwn(F) P				N.S.
Btwn(R) P				N.S.

Table 3I22 - 7

IESLC - Meta-analysis of Ever/current Smoking, Duration, "Low"
Adenocarcinoma, Cigarettes only
Excluded studies (and stage at which they were excluded)

1	BECHER BLOT1 BROWN3 CARPEN CHYOU DARBY DOLL2 GARCIA GRAHAM GURSEL HAMMO2 JAHN JAIN LAUSSM PRESCO QIAO TVERDA WIGLE WYNDE3
2	ALDERS BENSHL BRESLO CHIAZZ DEAN3 DORN ENGELA GAO2 GILLIS GUO HEGMAN HIRAYA HOLE KAUFMA KOO KOULUM LIU4 MIGRAN MRFITR PERNU SEGI2 SPEIZE SUZUK2 SVENSS VUTUC WAKAI WU YUAN
3	GENG MCDUFF SPITZ STASZE WU2 ZHANG
4	AGUDO AKIBA AMANDU AMES ARMADA AUVINE AXELSS BEST BOFFET BOUCHA BOUCOT BROSS CEDERL CHEN2 CORREA CPSI CPSII DEAN2 DESTEF DOLL FAN GAO GARSHI HAMMON HU HU2 HUMBLE JUSSAW KAISE2 KREUZE LETOUR LEVIN LIAW LIU3 LIU5 LUO MCCONN NOTAN2 PEZZO2 PISANI QIAO2 RACHTA RESTRE SADOWS TIZZAN WANG2 WATSON WYNDE2 WYNDE6 WYNDE7 WYNDE8 ZHOU
5	CHEN LUBIN XU
7	BARBON BUFFLE CHOI DAMBER DORGAN DOSEME GER HAENSZ JEDRYC JOLY KATSOU KHUDER LUBIN2 MATOS OSANN2 SOBUE WUWILL ZHENG
14	BENHAM

Table 3I23 -

IESLC - Meta-analysis of Ever/current Smoking, Duration, "Mid"
Adenocarcinoma, Cigarettes only

This analysis is restricted to results for:
1) Ever/current smokers
2) Results by Duration
3) Categorical results by Duration
4) Adenocarcinoma (or near equivalent)
5) Results complete enough for use in metaanalysis

Within each study, results are then selected (in the following order of preference, within each sex) for:
6) SMKSTA: ever, current
7) PRODUCT: cigarettes only
8) CIGTYPE: all/unspecified, MC regardless of HR, MC only
9) (not applicable)
10) DENOM: never smoked anything, never smoked cigarettes, never any + low, never cigs + low
11) Followup period (YF, prospective studies): whole study (coded as 0) or longest available
12) LCtype: adeno or nearest available, but not squamous. (q = squamous, s = small,
a = adeno, l = large, KII = Kreyberg II, al = alveolar, br = bronchiolar, u = undifferentiated)
13) Race: all or nearest available, otherwise by race (wh or w = white, bl or b = black, hi = hispanic
ch = chinese, jap = japanese, haw = hawaiian, w+o = white + oriental, sca = scandinavian, as = asian)
14) Duration "mid" in key scheme 1 (key value 35, maximum range 21-49)
15) For overlapping studies: principal rather than subsidiary studies
Finally by Age: whole study (coded as 0) if available, otherwise by widest available age group
and then for single sex results (m, f) in preference to results for both sexes combined (c).

Results adjusted (AD) for the most potential confounders are then chosen in Sections -1 to -3
(and those which actually differ from the adjusted results in Table 3I13 - 1 are marked 'x' in Section -1)
and results adjusted for the least confounders in Sections -4 to -6. (Those least adjusted results which
actually differ from the most adjusted are marked 'x' in column X in Section -4)

Section -7 shows excluded studies, together with the stage (as above) at which no qualifying
results were found.

Section -8 lists the potentially overlapping studies which have been included (1=principal, 2=subsidiary).

Section -9 lists any results which would have been included in preference except that they had data not complete
enough for use in meta-analysis, with their significance (yes/no), if known, and any further comment as entered
on the database. It also lists as "gap" any categories for which no data were presented by the original authors.

In addition to those mentioned above, the following fields, levels and abbreviations are used:
* or nk = not known, n = no, y = yes, ot = other
ev = ever, cu = current, nev = never
all/unspec = all or unspecified, MC = manufactured cigarettes, HR = hand-rolled cigarettes
exL, exH = range of exposure (low and high) in the smoking group, in terms of Duration
REF: 6-character study reference
NRR: number of the RR on the database within the study
ST : study type (CC = case control, pr or prosp = prospective)
NLC: number of lung cancer cases in whole study
R : risky occupational population (n = no, m = mining, o = other risky)
VB : national cigarette type (V = at least 75% Virginia, bl = at least 75% blended, ot = other)
P : any proxy use
H : full histological confirmation
De : derivation of RR/CI (or = original, st = standard method, ot = other method of estimation)

Table 3I23 - 1

IESLC - Meta-analysis of Ever/current Smoking, Duration, "Mid"
Adenocarcinoma, Cigarettes only
Most adjusted

REF	NRR	3I13	SEX	AGEL	AGEH	RACE	YF	LC TYPE	LOC	START	ST	NLC	R	VB	P	H	AD	SM	PRODUCT	exL	exH	DENOM	De
PEZZOT	517		m	0	0	all	-	a	SCAmer	1987	CC	215	n	bl	n	y	0	ev	cig only	31	40	nev cigs	st

Cigarette type is all/unspec for all RRs

Table 3I23 - 2

IESLC - Meta-analysis of Ever/current Smoking, Duration, "Mid"
Adenocarcinoma, Cigarettes only
Most adjusted

REF	NRR	SEX	AD	Number Case	Exposed Cont	Non-exposed Case	Cont	RR	95.00%CI
PEZZOT	517	m	0	22	82	3	116	10.37	(3.01- 35.81)
Totals				22	82	3	116		

*prospective study

REF	NRR	SEX	AD	Ys	Ws	Qs	Ps
PEZZOT	517	m	0	2.34	2.50	0.00	0.0002

	N	1
	NS	1
	Wt	2.50
Het	Chi	0.00
Het	df	0
Het	P	N.S.
Fixed	RR	10.37
	RRl	3.01
	RRu	35.81
	P	+++
Random	RR	10.37
	RRl	3.01
	RRu	35.81
	P	+++
Asymm	P	

Table 3I23 - 3

IESLC - Meta-analysis of Ever/current Smoking, Duration, "Mid"
Adenocarcinoma, Cigarettes only
Most adjusted

	Sex			
	combined	male	female	Total
N		1		1
NS		1		1
Wt		2.50		2.50
Het Chi		0.00		0.00
Het df		0		0
Het P		N.S.		N.S.
Fixed RR		10.37		10.37
RRl		3.01		3.01
RRu		35.81		35.81
P		+++		+++
Random RR		10.37		10.37
RRl		3.01		3.01
RRu		35.81		35.81
P		+++		+++
Between Chi				
Between df				
Between P				N.S.
Btwn(F) P				N.S.
Btwn(R) P				N.S.

Too few RRs for analysis by factor

Table 3I23 - 4

IESLC - Meta-analysis of Ever/current Smoking, Duration, "Mid"
Adenocarcinoma, Cigarettes only
Least adjusted

REF	NRR	X	SEX	AGEL	AGEH	RACE	YF	LC TYPE	LOC	START	ST	NLC	R	VB	P	H	AD	SM	PRODUCT	exL	exH	DENOM	De
PEZZOT	517		m	0	0	all	-	a	SCAmer	1987	CC	215	n	bl	n	y	0	ev	cig only	31	40	nev cigs	st

Cigarette type is all/unspec for all RRs

Table 3I23 - 5

IESLC - Meta-analysis of Ever/current Smoking, Duration, "Mid"
Adenocarcinoma, Cigarettes only
Least adjusted

REF	NRR	SEX	AD	Number Case	Exposed Cont	Non-exposed Case	Cont	RR	95.00%CI
PEZZOT	517	m	0	22	82	3	116	10.37	(3.01- 35.81)
Totals				22	82	3	116		

*prospective study

REF	NRR	SEX	AD	Ys	Ws	Qs	Ps
PEZZOT	517	m	0	2.34	2.50	0.00	0.0002

	N	1
	NS	1
	Wt	2.50
Het	Chi	0.00
Het	df	0
Het	P	N.S.
Fixed	RR	10.37
	RRl	3.01
	RRu	35.81
	P	+++
Random	RR	10.37
	RRl	3.01
	RRu	35.81
	P	+++
Asymm	P	

Table 3I23 - 6

IESLC - Meta-analysis of Ever/current Smoking, Duration, "Mid"
Adenocarcinoma, Cigarettes only
Least adjusted

	Sex combined	male	female	Total
N		1		1
NS		1		1
Wt		2.50		2.50
Het Chi		0.00		0.00
Het df		0		0
Het P		N.S.		N.S.
Fixed RR		10.37		10.37
RRl		3.01		3.01
RRu		35.81		35.81
P		+++		+++
Random RR		10.37		10.37
RRl		3.01		3.01
RRu		35.81		35.81
P		+++		+++
Between Chi				
Between df				
Between P				N.S.
Btwn(F) P				N.S.
Btwn(R) P				N.S.

Table 3I23 - 7

IESLC - Meta-analysis of Ever/current Smoking, Duration, "Mid"
Adenocarcinoma, Cigarettes only
Excluded studies (and stage at which they were excluded)

1	BECHER BLOT1 BROWN3 CARPEN CHYOU DARBY DOLL2 GARCIA GRAHAM GURSEL HAMMO2 JAHN JAIN LAUSSM PRESCO QIAO TVERDA WIGLE WYNDE3
2	ALDERS BENSHL BRESLO CHIAZZ DEAN3 DORN ENGELA GAO2 GILLIS GUO HEGMAN HIRAYA HOLE KAUFMA KOO KOULUM LIU4 MIGRAN MRFITR PERNU SEGI2 SPEIZE SUZUK2 SVENSS VUTUC WAKAI WU YUAN
3	GENG MCDUFF SPITZ STASZE WU2 ZHANG
4	AGUDO AKIBA AMANDU AMES ARMADA AUVINE AXELSS BEST BOFFET BOUCHA BOUCOT BROSS CEDERL CHEN2 CORREA CPSI CPSII DEAN2 DESTEF DOLL FAN GAO GARSHI HAMMON HU HU2 HUMBLE JUSSAW KAISE2 KREUZE LETOUR LEVIN LIAW LIU3 LIU5 LUO MCCONN NOTAN2 PEZZO2 PISANI QIAO2 RACHTA RESTRE SADOWS TIZZAN WANG2 WATSON WYNDE2 WYNDE6 WYNDE7 WYNDE8 ZHOU
5	CHEN LUBIN XU
7	BARBON BUFFLE CHOI DAMBER DORGAN DOSEME GER HAENSZ JEDRYC JOLY KATSOU KHUDER LUBIN2 MATOS OSANN2 SOBUE WUWILL ZHENG
14	BENHAM

Table 3I24 -

IESLC - Meta-analysis of Ever/current Smoking, Duration, "High"
Adenocarcinoma, Cigarettes only

This analysis is restricted to results for:
1) Ever/current smokers
2) Results by Duration
3) Categorical results by Duration
4) Adenocarcinoma (or near equivalent)
5) Results complete enough for use in metaanalysis

Within each study, results are then selected (in the following order of preference, within each sex) for:
6) PRODUCT: cigarettes only
7) CIGTYPE: all/unspecified, MC regardless of HR, MC only
8) (not applicable)
9) DENOM: never smoked anything, never smoked cigarettes, never any + low, never cigs + low
10) Followup period (YF, prospective studies): whole study (coded as 0) or longest available
11) LCtype: adeno or nearest available, but not squamous. (q = squamous, s = small,
a = adeno, l = large, KII = Kreyberg II, al = alveolar, br = bronchiolar, u = undifferentiated)
12) Race: all or nearest available, otherwise by race (wh or w = white, bl or b = black, hi = hispanic
ch = chinese, jap = japanese, haw = hawaiian, w+o = white + oriental, sca = scandinavian, as = asian)
13) Duration "high" in key scheme 1 (key value 50, maximum range 36+)
14) For overlapping studies: principal rather than subsidiary studies
Finally by Age: whole study (coded as 0) if available, otherwise by widest available age group
and then for single sex results (m, f) in preference to results for both sexes combined (c).

Results adjusted (AD) for the most potential confounders are then chosen in Sections -1 to -3
(and those which actually differ from the adjusted results in Table 3I14 - 1 are marked 'x' in Section -1)
and results adjusted for the least confounders in Sections -4 to -6. (Those least adjusted results which
actually differ from the most adjusted are marked 'x' in column X in Section -4)

Section -7 shows excluded studies, together with the stage (as above) at which no qualifying
results were found.

Section -8 lists the potentially overlapping studies which have been included (1=principal, 2=subsidiary).

Section -9 lists any results which would have been included in preference except that they had data not complete
enough for use in meta-analysis, with their significance (yes/no), if known, and any further comment as entered
on the database. It also lists as "gap" any categories for which no data were presented by the original authors.

In addition to those mentioned above, the following fields, levels and abbreviations are used:
* or nk = not known, n = no, y = yes, ot = other
ev = ever, cu = current, nev = never
all/unspec = all or unspecified, MC = manufactured cigarettes, HR = hand-rolled cigarettes
exL, exH = range of exposure (low and high) in the smoking group, in terms of Duration
REF: 6-character study reference
NRR: number of the RR on the database within the study
ST : study type (CC = case control, pr or prosp = prospective)
NLC: number of lung cancer cases in whole study
R : risky occupational population (n = no, m = mining, o = other risky)
VB : national cigarette type (V = at least 75% Virginia, bl = at least 75% blended, ot = other)
P : any proxy use
H : full histological confirmation
De : derivation of RR/CI (or = original, st = standard method, ot = other method of estimation)

Table 3I24 - 1

IESLC - Meta-analysis of Ever/current Smoking, Duration, "High"
Adenocarcinoma, Cigarettes only
Most adjusted

REF	NRR	3I14	SEX	AGEL	AGEH	RACE	YF	LC TYPE	LOC	START	ST	NLC	R	VB	P	H	AD	SM	PRODUCT	exL	exH	DENOM	De
BENHAM	509	x	m	0	0	all	-	KII	Eu:wst	1976	CC	1625	n	bl	n	y	0	ev	cig only	36	999	nev any	st
PEZZOT	518		m	0	0	all	-	a	SCAmer	1987	CC	215	n	bl	n	y	0	ev	cig only	41	999	nev cigs	st

Cigarette type is all/unspec for all RRs

Table 3I24 - 2

IESLC - Meta-analysis of Ever/current Smoking, Duration, "High"
Adenocarcinoma, Cigarettes only
Most adjusted

REF	NRR	SEX	AD	Number Case	Exposed Cont	Non-exposed Case	Cont	RR	95.00%CI
BENHAM	509	m	0	55	50	9	42	5.13	(2.27- 11.60)
PEZZOT	518	m	0	27	101	3	116	10.34	(3.04- 35.09)
Totals				82	151	12	158		

*prospective study

REF	NRR	SEX	AD	Ys	Ws	Qs	Ps
BENHAM	509	m	0	1.64	5.78	0.27	0.0001
PEZZOT	518	m	0	2.34	2.57	0.60	0.0002

	N	2
	NS	2
	Wt	8.35
Het	Chi	0.87
Het	df	1
Het	P	N.S.
Fixed	RR	6.37
	RRl	3.23
	RRu	12.55
	P	+++
Random	RR	6.37
	RRl	3.23
	RRu	12.55
	P	+++
Asymm	P	

Table 3I24 - 3

IESLC - Meta-analysis of Ever/current Smoking, Duration, "High"
Adenocarcinoma, Cigarettes only
Most adjusted

	Sex: combined	male	female	Total
N		2		2
NS		2		2
Wt		8.35		8.35
Het Chi		0.87		0.87
Het df		1		1
Het P		N.S.		N.S.
Fixed RR		6.37		6.37
RRl		3.23		3.23
RRu		12.55		12.55
P		+++		+++
Random RR		6.37		6.37
RRl		3.23		3.23
RRu		12.55		12.55
P		+++		+++
Between Chi				
Between df				
Between P				N.S.
Btwn(F) P				N.S.
Btwn(R) P				N.S.

Too few RRs for analysis by factor

Table 3I24 - 4

IESLC - Meta-analysis of Ever/current Smoking, Duration, "High"
Adenocarcinoma, Cigarettes only
Least adjusted

REF	NRR	X	SEX	AGEL	AGEH	RACE	YF	LC TYPE	LOC	START	ST	NLC	R	VB	P	H	AD	SM	PRODUCT	exL	exH	DENOM	De
BENHAM	509		m	0	0	all	-	KII	Eu:wst	1976	CC	1625	n	bl	n	y	0	ev	cig only	36	999	nev any	st
PEZZOT	518		m	0	0	all	-	a	SCAmer	1987	CC	215	n	bl	n	y	0	ev	cig only	41	999	nev cigs	st

Cigarette type is all/unspec for all RRs

Table 3I24 - 5

IESLC - Meta-analysis of Ever/current Smoking, Duration, "High"
Adenocarcinoma, Cigarettes only
Least adjusted

REF	NRR	SEX	AD	Number Case	Exposed Cont	Non-exposed Case	Cont	RR	95.00%CI
BENHAM	509	m	0	55	50	9	42	5.13	(2.27- 11.60)
PEZZOT	518	m	0	27	101	3	116	10.34	(3.04- 35.09)
Totals				82	151	12	158		

*prospective study

REF	NRR	SEX	AD	Ys	Ws	Qs	Ps
BENHAM	509	m	0	1.64	5.78	0.27	0.0001
PEZZOT	518	m	0	2.34	2.57	0.60	0.0002

	N	2
	NS	2
	Wt	8.35
Het	Chi	0.87
Het	df	1
Het	P	N.S.
Fixed	RR	6.37
	RRl	3.23
	RRu	12.55
	P	+++
Random	RR	6.37
	RRl	3.23
	RRu	12.55
	P	+++
Asymm	P	

Table 3I24 - 6

IESLC - Meta-analysis of Ever/current Smoking, Duration, "High"
Adenocarcinoma, Cigarettes only
Least adjusted

	Sex combined	male	female	Total
N		2		2
NS		2		2
Wt		8.35		8.35
Het Chi		0.87		0.87
Het df		1		1
Het P		N.S.		N.S.
Fixed RR		6.37		6.37
RRl		3.23		3.23
RRu		12.55		12.55
P		+++		+++
Random RR		6.37		6.37
RRl		3.23		3.23
RRu		12.55		12.55
P		+++		+++
Between Chi				
Between df				
Between P				N.S.
Btwn(F) P				N.S.
Btwn(R) P				N.S.

Table 3I24 - 7

IESLC - Meta-analysis of Ever/current Smoking, Duration, "High"
Adenocarcinoma, Cigarettes only
Excluded studies (and stage at which they were excluded)

1	BECHER BLOT1 BROWN3 CARPEN CHYOU DARBY DOLL2 GARCIA GRAHAM GURSEL HAMMO2 JAHN JAIN LAUSSM PRESCO QIAO TVERDA WIGLE WYNDE3
2	ALDERS BENSHL BRESLO CHIAZZ DEAN3 DORN ENGELA GAO2 GILLIS GUO HEGMAN HIRAYA HOLE KAUFMA KOO KOULUM LIU4 MIGRAN MRFITR PERNU SEGI2 SPEIZE SUZUK2 SVENSS VUTUC WAKAI WU YUAN
3	GENG MCDUFF SPITZ STASZE WU2 ZHANG
4	AGUDO AKIBA AMANDU AMES ARMADA AUVINE AXELSS BEST BOFFET BOUCHA BOUCOT BROSS CEDERL CHEN2 CORREA CPSI CPSII DEAN2 DESTEF DOLL FAN GAO GARSHI HAMMON HU HU2 HUMBLE JUSSAW KAISE2 KREUZE LETOUR LEVIN LIAW LIU3 LIU5 LUO MCCONN NOTAN2 PEZZO2 PISANI QIAO2 RACHTA RESTRE SADOWS TIZZAN WANG2 WATSON WYNDE2 WYNDE6 WYNDE7 WYNDE8 ZHOU
5	CHEN LUBIN XU
7	BARBON BUFFLE CHOI DAMBER DORGAN DOSEME GER HAENSZ JEDRYC JOLY KATSOU KHUDER LUBIN2 MATOS OSANN2 SOBUE WUWILL ZHENG

Table 3I24 - 8

Potentially overlapping studies

REF	REFGP	PRINC	.	OVERLAP/LINK
BENHAM	LUBIN2	2		Subset of Lubin2

Table 3I25 -

IESLC - Meta-analysis of Ever/current Smoking, Duration, "Highest vs lowest"
Adenocarcinoma, Cigarettes only

This analysis is restricted to results for:
1) Ever/current smokers
2) Results by Duration
3) Categorical results by Duration
4) Denominator (unexposed) = "low"
5) Adenocarcinoma (or near equivalent)
6) Results complete enough for use in metaanalysis

Within each study, results are then selected (in the following order of preference, within each sex) for:
7) SMKSTA: ever, current
8) PRODUCT: cigarettes only
9) CIGTYPE: all/unspecified, MC regardless of HR, MC only
10) Results with least adjustment for other aspects of smoking (ADOS)
11) The highest vs lowest category
12) Followup period (YF, prospective studies): whole study (coded as 0) or longest available
13) LCtype: adeno or nearest available, but not squamous. (q = squamous, s = small,
a = adeno, l = large, KII = Kreyberg II, al = alveolar, br = bronchiolar, u = undifferentiated)
14) Race: all or nearest available, otherwise by race (wh or w = white, bl or b = black, hi = hispanic
ch = chinese, jap = japanese, haw = hawaiian, w+o = white + oriental, sca = scandinavian, as = asian)
15) For overlapping studies: principal rather than subsidiary studies
Finally by Age: whole study (coded as 0) if available, otherwise by widest available age group
and then for single sex results (m, f) in preference to results for both sexes combined (c).

Results adjusted (AD) for the most potential confounders are then chosen in Sections -1 to -3
(and those which actually differ from the adjusted results in Table 3I15 - 1 are marked 'x' in Section -1)
and results adjusted for the least confounders in Sections -4 to -6. (Those least adjusted results which
actually differ from the most adjusted are marked 'x' in column X in Section -4)

Section -7 shows excluded studies, together with the stage (as above) at which no qualifying
results were found.

Section -8 lists the potentially overlapping studies which have been included (1=principal, 2=subsidiary).

Section -9 lists any results which would have been included in preference except that they had data not complete
enough for use in meta-analysis, with their significance (yes/no), if known, and any further comment as entered
on the database. It also lists as "gap" any categories for which no data were presented by the original authors.

In addition to those mentioned above, the following fields, levels and abbreviations are used:
* or nk = not known, n = no, y = yes, ot = other
all/unspec = all or unspecified, MC = manufactured cigarettes, HR = hand-rolled cigarettes
exL, exH = range of exposure (low and high) in the "highest" group, in terms of Duration
unexL, unexH = range of exposure (low and high) in the "lowest" group, in terms of Duration
REF: 6-character study reference
NRR: number of the RR on the database within the study
ST : study type (CC = case control, pr or prosp = prospective)
NLC: number of lung cancer cases in whole study
R : risky occupational population (n = no, m = mining, o = other risky)
VB : national cigarette type (V = at least 75% Virginia, bl = at least 75% blended, ot = other)
P : any proxy use
H : full histological confirmation
De : derivation of RR/CI (or = original, st = standard method, ot = other method of estimation)

Table 3I25 - 1

IESLC - Meta-analysis of Ever/current Smoking, Duration, "Highest vs lowest"
Adenocarcinoma, Cigarettes only
Most adjusted

REF	NRR	3I15	SEX	AGEL	AGEH	RACE	YF	LC TYPE	LOC	START	ST	NLC	R	VB	P	H	AD	ADOS	SM	PRODUCT	exL	exH	unexL	unexH	De
BENHAM	510	x	m	0	0	all	-	KII	Eu:wst	1976	CC	1625	n	bl	n	y	0	0	ev	cig only	36	999	1	35	st
PEZZOT	522		m	0	0	all	-	a	SCAmer	1987	CC	215	n	bl	n	y	2	0	ev	cig only	41	999	1	30	ot

Cigarette type is all/unspec for all RRs

Table 3I25 - 2

IESLC - Meta-analysis of Ever/current Smoking, Duration, "Highest vs lowest"
Adenocarcinoma, Cigarettes only
Most adjusted

REF	NRR	SEX	AD	Number Case	Exposed Cont	Non-exposed Case	Cont	RR	95.00%CI
BENHAM	510	m	0	55	50	55	93	1.86	(1.12- 3.09)
PEZZOT	522	m	2	27	-	11	-	4.60	(1.95- 10.82)
Partial Totals				82	50	66	93		

*prospective study

REF	NRR	SEX	AD	Ys	Ws	Qs	Ps
BENHAM	510	m	0	0.62	14.90	0.83	0.0166
PEZZOT	522	m	2	1.53	5.23	2.35	0.0005

	N	2
	NS	2
	Wt	20.13
Het	Chi	3.18
Het	df	1
Het	P	(*)
Fixed	RR	2.35
	RRl	1.52
	RRu	3.64
	P	+++
Random	RR	2.73
	RRl	1.14
	RRu	6.57
	P	+
Asymm	P	

Table 3I25 - 3

IESLC - Meta-analysis of Ever/current Smoking, Duration, "Highest vs lowest"
Adenocarcinoma, Cigarettes only
Most adjusted

	Sex combined	male	female	Total
N		2		2
NS		2		2
Wt		20.13		20.13
Het Chi		3.18		3.18
Het df		1		1
Het P		(*)		(*)
Fixed RR		2.35		2.35
RRl		1.52		1.52
RRu		3.64		3.64
P		+++		+++
Random RR		2.73		2.73
RRl		1.14		1.14
RRu		6.57		6.57
P		+		+
Between Chi				
Between df				
Between P				N.S.
Btwn(F) P				N.S.
Btwn(R) P				N.S.

Too few RRs for analysis by factor

Table 3I25 - 4

IESLC - Meta-analysis of Ever/current Smoking, Duration, "Highest vs lowest"
Adenocarcinoma, Cigarettes only
Least adjusted

REF	NRR	X	SEX	AGEL	AGEH	RACE	YF	LC TYPE	LOC	START	ST	NLC	R	VB	P	H	AD	ADOS	SM	PRODUCT	exL	exH	unexL	unexH	De
BENHAM	510		m	0	0	all	-	KII	Eu:wst	1976	CC	1625	n	bl	n	y	0	0	ev	cig only	36	999	1	35	st
PEZZOT	520	x	m	0	0	all	-	a	SCAmer	1987	CC	215	n	bl	n	y	0	0	ev	cig only	41	999	1	30	st

Cigarette type is all/unspec for all RRs

Table 3I25 - 5

IESLC - Meta-analysis of Ever/current Smoking, Duration, "Highest vs lowest"
Adenocarcinoma, Cigarettes only
Least adjusted

REF	NRR	SEX	AD	Number Exposed Case	Number Exposed Cont	Non-exposed Case	Non-exposed Cont	RR	95.00%CI
BENHAM	510	m	0	55	50	55	93	1.86	(1.12- 3.09)
PEZZOT	520	m	0	27	27	11	39	3.55	(1.51- 8.34)
Totals				82	77	66	132		

*prospective study

REF	NRR	SEX	AD	Ys	Ws	Qs	Ps
BENHAM	510	m	0	0.62	14.90	0.42	0.0166
PEZZOT	520	m	0	1.27	5.25	1.19	0.0037

	N	2
	NS	2
	Wt	20.15
Het	Chi	1.61
Het	df	1
Het	P	N.S.
Fixed	RR	2.20
	RRl	1.42
	RRu	3.40
	P	+++
Random	RR	2.33
	RRl	1.28
	RRu	4.27
	P	++
Asymm	P	

Table 3I25 - 6

IESLC - Meta-analysis of Ever/current Smoking, Duration, "Highest vs lowest"
Adenocarcinoma, Cigarettes only
Least adjusted

	Sex			
	combined	male	female	Total
N		2		2
NS		2		2
Wt		20.15		20.15
Het Chi		1.61		1.61
Het df		1		1
Het P		N.S.		N.S.
Fixed RR		2.20		2.20
RRl		1.42		1.42
RRu		3.40		3.40
P		+++		+++
Random RR		2.33		2.33
RRl		1.28		1.28
RRu		4.27		4.27
P		++		++
Between Chi				
Between df				
Between P				N.S.
Btwn(F) P				N.S.
Btwn(R) P				N.S.

Table 3I25 - 7

IESLC - Meta-analysis of Ever/current Smoking, Duration, "Highest vs lowest"
Adenocarcinoma, Cigarettes only
Excluded studies (and stage at which they were excluded)

1 BECHER BLOT1 BROWN3 CARPEN CHYOU DARBY DOLL2 GARCIA GRAHAM GURSEL HAMMO2 JAHN JAIN LAUSSM PRESCO QIAO TVERDA WIGLE WYNDE3

2 ALDERS BENSHL BRESLO CHIAZZ DEAN3 DORN ENGELA GAO2 GILLIS GUO HEGMAN HIRAYA HOLE KAUFMA KOO KOULUM LIU4 MIGRAN MRFITR PERNU SEGI2 SPEIZE SUZUK2 SVENSS VUTUC WAKAI WU YUAN

3 GENG MCDUFF SPITZ STASZE WU2 ZHANG

4 AKIBA GARSHI

5 AGUDO AMANDU AMES ARMADA AUVINE AXELSS BEST BOFFET BOUCHA BOUCOT BROSS CEDERL CHEN2 CORREA CPSI CPSII DEAN2 DESTEF DOLL FAN GAO HAMMON HU HU2 HUMBLE JUSSAW KAISE2 KREUZE LETOUR LEVIN LIAW LIU3 LIU5 LUO MCCONN NOTAN2 PEZZO2 PISANI QIAO2 RACHTA RESTRE SADOWS TIZZAN WANG2 WATSON WYNDE2 WYNDE6 WYNDE7 WYNDE8 ZHOU

6 BUFFLE CHEN LUBIN XU

8 BARBON CHOI DAMBER DORGAN DOSEME GER HAENSZ JEDRYC JOLY KATSOU KHUDER LUBIN2 MATOS OSANN2 SOBUE WUWILL ZHENG

Table 3I25 - 8

Potentially overlapping studies

REF	REFGP	PRINC	. OVERLAP/LINK
BENHAM	LUBIN2	2	Subset of Lubin2
