# Supplementary material for: Systematic review with meta-analysis of the epidemiological evidence in the 1900s relating smoking to lung cancer
Source: BMC Cancer. 2012 Sep 3;12:385. doi: 10.1186/1471-2407-12-385 (PMC3505152; doi:10.1186/1471-2407-12-385)
Supplement: Additional file 5 — Detailed Analysis Tables (Individual file names as described in Additional file 1: Methods, Table1). [file 1471-2407-12-385-S5.zip › PDF/1AR.pdf]

Table 1A1R - 1

IESLC - Meta-regression of ever smoking, any product (or cigs if any not available)
Multiple regression of data from Table 1A1 (preferring adjusted RRs)
All LC types

Stepwise allowing only characteristics from the fixed model

Log Relative risk
WEIGHTED on Weight

		Deviance	(DF)				
Model 1		7470.075	(327)				
		Estimate	S.E.	P	RR	95%CIl	95%CIu
Constant		1.441	0.007	+++	4.223	4.164	4.283

		Deviance	(DF)	Drop Dev	P		
Model 2		2247.358	(320)	5222.717	***		
		Estimate	S.E.	P	RR	95%CIl	95%CIu
Constant		2.182	0.013	+++	8.864	8.634	9.100
Location							
NAmer	116	Aliased			8.864	8.267	9.505
UK	29	-0.529	0.053	---	5.223	3.992	6.835
Scand	32	-0.321	0.048	---	6.430	5.065	8.162
othEur	50	-0.469	0.034	---	5.546	4.718	6.520
China	51	-1.162	0.016	---	2.774	2.640	2.916
Japan	18	-1.023	0.049	---	3.186	2.499	4.062
othAs	18	-0.850	0.055	---	3.787	2.873	4.992
other	14	-0.349	0.071	---	6.251	4.354	8.975

		Deviance	(DF)	Drop Dev	P		
Model 3		1599.339	(316)	648.019	***		
		Estimate	S.E.	P	RR	95%CIl	95%CIu
Constant		1.473	0.036	+++	4.364	4.070	4.680
Location							
NAmer	116	Aliased			9.335	8.791	9.913
UK	29	-0.138	0.056	-	8.133	6.388	10.355
Scand	32	-0.117	0.049	-	8.303	6.743	10.224
othEur	50	-0.304	0.037	---	6.888	5.918	8.018
China	51	-1.302	0.018	---	2.538	2.424	2.657
Japan	18	-0.890	0.052	---	3.832	3.069	4.785
othAs	18	-0.763	0.056	---	4.352	3.420	5.539
other	14	-0.212	0.073	--	7.549	5.495	10.371
Start year of study							
<1960	54	Aliased			1.974	1.688	2.308
1960-69	52	0.457	0.045	+++	3.118	2.710	3.586
1970-79	71	0.448	0.044	+++	3.089	2.708	3.524
1980-89	114	0.859	0.037	+++	4.659	4.485	4.839
1990+	37	0.796	0.058	+++	4.377	3.558	5.385

		Deviance	(DF)	Drop Dev	P		
Model 4		1523.722	(313)	75.616	**		
		Estimate	S.E.	P	RR	95%CIl	95%CIu
Constant		1.278	0.048	+++	3.591	3.271	3.942
Location							
NAmer	116	Aliased			9.366	8.829	9.936
UK	29	-0.163	0.056	--	7.957	6.273	10.094
Scand	32	-0.055	0.049	N.S.	8.862	7.202	10.905
othEur	50	-0.320	0.037	---	6.803	5.861	7.897
China	51	-1.312	0.018	---	2.521	2.410	2.638
Japan	18	-0.869	0.052	---	3.928	3.158	4.885
othAs	18	-0.676	0.058	---	4.763	3.731	6.080
other	14	-0.212	0.074	--	7.573	5.522	10.386
Start year of study							
<1960	54	Aliased			2.058	1.762	2.404
1960-69	52	0.424	0.046	+++	3.146	2.731	3.624
1970-79	71	0.426	0.045	+++	3.152	2.756	3.605
1980-89	114	0.808	0.038	+++	4.616	4.443	4.796
1990+	37	0.885	0.059	+++	4.986	4.010	6.199
Study size (number of LC cases)							
100-249	115	Aliased			3.322	2.855	3.865
250-499	86	0.088	0.043	+	3.628	3.164	4.160
500-999	64	0.308	0.043	+++	4.518	3.989	5.117
1000+	63	0.260	0.037	+++	4.309	4.155	4.469

Table 1A1R - 1

IESLC - Meta-regression of ever smoking, any product (or cigs if any not available)
Multiple regression of data from Table 1A1 (preferring adjusted RRs)
All LC types

Log Relative risk
WEIGHTED on Weight

		Deviance	(DF)	Drop Dev	P		
Model 5		1497.854	(312)	25.869	*		
		Estimate	S.E.	P	RR	95%CIl	95%CIu
Constant		1.211	0.049	+++	3.356	3.046	3.697
Location							
NAmer	116	Aliased			9.288	8.755	9.853
UK	29	-0.136	0.056	-	8.110	6.400	10.276
Scand	32	-0.064	0.049	N.S.	8.708	7.083	10.705
othEur	50	-0.294	0.037	---	6.922	5.965	8.032
China	51	-1.301	0.018	---	2.530	2.418	2.646
Japan	18	-0.915	0.053	---	3.721	2.982	4.643
othAs	18	-0.635	0.058	---	4.921	3.855	6.281
other	14	-0.170	0.075	-	7.840	5.721	10.742
Start year of study							
<1960	54	Aliased			1.980	1.691	2.318
1960-69	52	0.431	0.046	+++	3.048	2.642	3.517
1970-79	71	0.444	0.045	+++	3.086	2.698	3.530
1980-89	114	0.853	0.039	+++	4.646	4.471	4.828
1990+	37	0.934	0.060	+++	5.039	4.058	6.257
Study size (number of LC cases)							
100-249	115	Aliased			3.291	2.832	3.826
250-499	86	0.088	0.043	+	3.593	3.136	4.117
500-999	64	0.306	0.043	+++	4.472	3.951	5.062
1000+	63	0.272	0.037	+++	4.319	4.165	4.478
Study type (1)							
CC	262	Aliased			4.175	4.042	4.312
other	66	0.170	0.033	+++	4.949	4.313	5.678

		Deviance	(DF)	Drop Dev	P		
Model 6		1463.482	(310)	34.372	*		
		Estimate	S.E.	P	RR	95%CIl	95%CIu
Constant		1.282	0.051	+++	3.603	3.261	3.981
Location							
NAmer	116	Aliased			9.428	8.775	10.130
UK	29	-0.116	0.057	-	8.395	6.627	10.636
Scand	32	-0.088	0.050	(-)	8.632	7.033	10.595
othEur	50	-0.320	0.038	---	6.846	5.905	7.937
China	51	-1.326	0.024	---	2.504	2.380	2.634
Japan	18	-0.859	0.053	---	3.993	3.181	5.011
othAs	18	-0.657	0.059	---	4.889	3.838	6.228
other	14	-0.172	0.075	-	7.942	5.810	10.857
Start year of study							
<1960	54	Aliased			2.107	1.790	2.479
1960-69	52	0.378	0.047	+++	3.075	2.667	3.545
1970-79	71	0.428	0.045	+++	3.232	2.816	3.708
1980-89	114	0.782	0.041	+++	4.605	4.431	4.786
1990+	37	0.880	0.061	+++	5.081	4.096	6.302
Study size (number of LC cases)							
100-249	115	Aliased			3.254	2.797	3.785
250-499	86	0.098	0.043	+	3.587	3.130	4.111
500-999	64	0.288	0.043	+++	4.340	3.827	4.922
1000+	63	0.287	0.038	+++	4.335	4.180	4.495
Study type (1)							
CC	262	Aliased			4.158	4.026	4.294
other	66	0.233	0.035	+++	5.249	4.545	6.062
Number of adjustment variables (1)							
0	164	Aliased			4.265	3.943	4.614
1	69	-0.182	0.033	---	3.556	3.122	4.051
2+/+nk	95	0.011	0.024	N.S.	4.312	4.122	4.510

Table 1A1R - 1

IESLC - Meta-regression of ever smoking, any product (or cigs if any not available)
Multiple regression of data from Table 1A1 (preferring adjusted RRs)
All LC types

Fixed model

Log Relative risk
WEIGHTED on Weight

		Deviance	(DF)				
Model 7		1454.810	(308)				
		Estimate	S.E.	P	RR	95%CIl	95%CIu
Constant		1.280	0.052	+++	3.597	3.250	3.981
Sex(RR)							
Male	171	Aliased			4.275	4.087	4.472
Female	108	-0.040	0.016	-	4.106	3.901	4.322
Combined	49	0.018	0.029	N.S.	4.354	3.913	4.845
Location							
NAmer	116	Aliased			9.393	8.726	10.110
UK	29	-0.108	0.057	(-)	8.428	6.648	10.683
Scand	32	-0.095	0.051	(-)	8.541	6.935	10.519
othEur	50	-0.319	0.039	---	6.830	5.890	7.920
China	51	-1.320	0.024	---	2.510	2.384	2.643
Japan	18	-0.858	0.053	---	3.983	3.172	5.002
othAs	18	-0.646	0.060	---	4.921	3.862	6.271
other	14	-0.173	0.076	-	7.900	5.778	10.802
Start year of study							
<1960	54	Aliased			2.104	1.779	2.488
1960-69	52	0.384	0.047	+++	3.088	2.671	3.570
1970-79	71	0.430	0.045	+++	3.234	2.817	3.713
1980-89	114	0.783	0.042	+++	4.605	4.429	4.788
1990+	37	0.872	0.063	+++	5.030	4.041	6.261
Study type (1)							
CC	262	Aliased			4.156	4.024	4.292
other	66	0.238	0.036	+++	5.274	4.565	6.093
Study size (number of LC cases)							
100-249	115	Aliased			3.243	2.785	3.775
250-499	86	0.101	0.044	+	3.588	3.130	4.113
500-999	64	0.293	0.043	+++	4.345	3.831	4.928
1000+	63	0.290	0.038	+++	4.335	4.180	4.495
Number of adjustment variables (1)							
0	164	Aliased			4.243	3.906	4.607
1	69	-0.171	0.034	---	3.576	3.138	4.075
2+/+nk	95	0.017	0.025	N.S.	4.316	4.123	4.519

Table 1A1R - 2

IESLC - Meta-regression of ever smoking, any product (or cigs if any not available)
Multiple regression of data from Table 1A1 (preferring adjusted RRs)
All LC types
Effect of removing characteristics

Log Relative risk
WEIGHTED on Weight

		Deviance	(DF)				
Model 7		1454.810	(308)				
		Estimate	S.E.	P	RR	95%CIl	95%CIu
Constant		1.280	0.052	+++	3.597	3.250	3.981
Sex(RR)							
Male	171	Aliased			4.275	4.087	4.472
Female	108	-0.040	0.016	-	4.106	3.901	4.322
Combined	49	0.018	0.029	N.S.	4.354	3.913	4.845
Location							
NAmer	116	Aliased			9.393	8.726	10.110
UK	29	-0.108	0.057	(-)	8.428	6.648	10.683
Scand	32	-0.095	0.051	(-)	8.541	6.935	10.519
othEur	50	-0.319	0.039	---	6.830	5.890	7.920
China	51	-1.320	0.024	---	2.510	2.384	2.643
Japan	18	-0.858	0.053	---	3.983	3.172	5.002
othAs	18	-0.646	0.060	---	4.921	3.862	6.271
other	14	-0.173	0.076	-	7.900	5.778	10.802
Start year of study							
<1960	54	Aliased			2.104	1.779	2.488
1960-69	52	0.384	0.047	+++	3.088	2.671	3.570
1970-79	71	0.430	0.045	+++	3.234	2.817	3.713
1980-89	114	0.783	0.042	+++	4.605	4.429	4.788
1990+	37	0.872	0.063	+++	5.030	4.041	6.261
Study type (1)							
CC	262	Aliased			4.156	4.024	4.292
other	66	0.238	0.036	+++	5.274	4.565	6.093
Study size (number of LC cases)							
100-249	115	Aliased			3.243	2.785	3.775
250-499	86	0.101	0.044	+	3.588	3.130	4.113
500-999	64	0.293	0.043	+++	4.345	3.831	4.928
1000+	63	0.290	0.038	+++	4.335	4.180	4.495
Number of adjustment variables (1)							
0	164	Aliased			4.243	3.906	4.607
1	69	-0.171	0.034	---	3.576	3.138	4.075
2+/+nk	95	0.017	0.025	N.S.	4.316	4.123	4.519

Omit Sex		Deviance	(DF)	Drop Dev	P		
Model 8		1463.482	(310)	-8.673	N.S.		
		Estimate	S.E.	P	RR	95%CIl	95%CIu
Constant		1.282	0.051	+++	3.603	3.261	3.981
Number of adjustment variables (1)							
0	164	Aliased			4.265	3.944	4.613
1	69	-0.182	0.033	---	3.556	3.123	4.050
2+/+nk	95	0.011	0.024	N.S.	4.312	4.123	4.509
Location							
NAmer	116	Aliased			9.428	8.776	10.128
UK	29	-0.116	0.057	-	8.395	6.631	10.629
Scand	32	-0.088	0.050	(-)	8.632	7.037	10.588
othEur	50	-0.320	0.038	---	6.846	5.907	7.934
China	51	-1.326	0.024	---	2.504	2.380	2.633
Japan	18	-0.859	0.053	---	3.993	3.183	5.008
othAs	18	-0.657	0.059	---	4.889	3.841	6.224
other	14	-0.172	0.075	-	7.942	5.815	10.847
Start year of study							
<1960	54	Aliased			2.107	1.791	2.478
1960-69	52	0.378	0.047	+++	3.075	2.669	3.544
1970-79	71	0.428	0.045	+++	3.232	2.818	3.707
1980-89	114	0.782	0.041	+++	4.605	4.431	4.786
1990+	37	0.880	0.061	+++	5.081	4.099	6.298
Study type (1)							
CC	262	Aliased			4.158	4.026	4.293
other	66	0.233	0.035	+++	5.249	4.547	6.059

Table 1A1R - 2

IESLC - Meta-regression of ever smoking, any product (or cigs if any not available)
Multiple regression of data from Table 1A1 (preferring adjusted RRs)
All LC types
Effect of removing characteristics

Log Relative risk
WEIGHTED on Weight

		Estimate	S.E.	P	RR	95%CIl	95%CIu
Study size (number of LC cases)							
100-249	115	Aliased			3.254	2.798	3.784
250-499	86	0.098	0.043	+	3.587	3.131	4.110
500-999	64	0.288	0.043	+++	4.340	3.829	4.921
1000+	63	0.287	0.038	+++	4.335	4.180	4.494

Omit Location		Deviance	(DF)	Drop Dev	P		
Model 8		4824.784	(315)	-3369.974	***		
		Estimate	S.E.	P	RR	95%CIl	95%CIu
Constant		1.078	0.049	+++	2.938	2.671	3.233
Number of adjustment variables (1)							
0	164	Aliased			6.665	6.198	7.168
1	69	-0.259	0.033	---	5.145	4.558	5.807
2+/+nk	95	-0.650	0.021	---	3.479	3.338	3.626
Sex(RR)							
Male	171	Aliased			4.011	3.838	4.192
Female	108	-0.008	0.016	N.S.	3.978	3.782	4.185
Combined	49	0.427	0.028	+++	6.148	5.556	6.802
Start year of study							
<1960	54	Aliased			3.129	2.674	3.663
1960-69	52	0.366	0.045	+++	4.514	3.957	5.151
1970-79	71	0.259	0.044	+++	4.054	3.577	4.595
1980-89	114	0.316	0.040	+++	4.292	4.136	4.454
1990+	37	0.296	0.061	+++	4.209	3.413	5.189
Study type (1)							
CC	262	Aliased			4.114	3.986	4.247
other	66	0.389	0.034	+++	6.069	5.288	6.966
Study size (number of LC cases)							
100-249	115	Aliased			2.715	2.344	3.144
250-499	86	0.294	0.044	+++	3.641	3.196	4.149
500-999	64	0.426	0.043	+++	4.158	3.682	4.695
1000+	63	0.481	0.037	+++	4.394	4.241	4.552

Omit Start year		Deviance	(DF)	Drop Dev	P		
Model 8		1864.392	(312)	-409.583	***		
		Estimate	S.E.	P	RR	95%CIl	95%CIu
Constant		1.800	0.040	+++	6.051	5.590	6.551
Number of adjustment variables (1)							
0	164	Aliased			4.118	3.795	4.469
1	69	-0.315	0.033	---	3.006	2.661	3.397
2+/+nk	95	0.082	0.025	++	4.472	4.277	4.677
Sex(RR)							
Male	171	Aliased			4.110	3.934	4.295
Female	108	0.004	0.016	N.S.	4.129	3.924	4.344
Combined	49	0.199	0.027	+++	5.017	4.537	5.549
Location							
NAmer	116	Aliased			9.078	8.449	9.754
UK	29	-0.356	0.055	---	6.361	5.069	7.983
Scand	32	-0.256	0.050	---	7.030	5.745	8.603
othEur	50	-0.417	0.035	---	5.982	5.230	6.841
China	51	-1.235	0.024	---	2.641	2.514	2.775
Japan	18	-0.829	0.051	---	3.962	3.201	4.905
othAs	18	-0.666	0.059	---	4.666	3.676	5.923
other	14	-0.203	0.074	--	7.413	5.461	10.063
Study type (1)							
CC	262	Aliased			4.184	4.052	4.320
other	66	0.138	0.035	+++	4.803	4.169	5.534
Study size (number of LC cases)							
100-249	115	Aliased			2.993	2.585	3.465
250-499	86	0.148	0.043	+++	3.470	3.050	3.947
500-999	64	0.283	0.043	+++	3.974	3.527	4.477
1000+	63	0.386	0.036	+++	4.404	4.251	4.562

Table 1A1R - 2

IESLC - Meta-regression of ever smoking, any product (or cigs if any not available)
Multiple regression of data from Table 1A1 (preferring adjusted RRs)
All LC types
Effect of removing characteristics

Log Relative risk
WEIGHTED on Weight

Omit Study type		Deviance	(DF)	Drop Dev	P		
Model 8		1499.802	(309)	-44.992	**		
		Estimate	S.E.	P	RR	95%CIl	95%CIu
Constant		1.343	0.051	+++	3.831	3.468	4.233
Number of adjustment variables (1)							
0	164	Aliased			4.176	3.848	4.532
1	69	-0.101	0.032	--	3.777	3.330	4.283
2+/+nk	95	0.031	0.025	N.S.	4.308	4.115	4.510
Sex(RR)							
Male	171	Aliased			4.279	4.091	4.476
Female	108	-0.039	0.016	-	4.117	3.911	4.333
Combined	49	0.006	0.029	N.S.	4.307	3.872	4.790
Location							
NAmer	116	Aliased			9.536	8.866	10.257
UK	29	-0.155	0.056	--	8.164	6.448	10.336
Scand	32	-0.081	0.050	N.S.	8.796	7.150	10.820
othEur	50	-0.350	0.038	---	6.721	5.800	7.789
China	51	-1.341	0.024	---	2.494	2.369	2.626
Japan	18	-0.823	0.053	---	4.188	3.343	5.245
othAs	18	-0.702	0.059	---	4.728	3.716	6.014
other	14	-0.237	0.075	--	7.522	5.513	10.264
Start year of study							
<1960	54	Aliased			2.157	1.826	2.549
1960-69	52	0.390	0.047	+++	3.186	2.761	3.678
1970-79	71	0.415	0.045	+++	3.267	2.846	3.749
1980-89	114	0.753	0.042	+++	4.583	4.409	4.764
1990+	37	0.838	0.063	+++	4.986	4.007	6.203
Study size (number of LC cases)							
100-249	115	Aliased			3.314	2.849	3.855
250-499	86	0.096	0.044	+	3.646	3.183	4.177
500-999	64	0.297	0.043	+++	4.461	3.939	5.053
1000+	63	0.264	0.038	+++	4.313	4.160	4.472

Omit Study size		Deviance	(DF)	Drop Dev	P		
Model 8		1533.803	(311)	-78.994	***		
		Estimate	S.E.	P	RR	95%CIl	95%CIu
Constant		1.497	0.041	+++	4.466	4.123	4.837
Number of adjustment variables (1)							
0	164	Aliased			4.145	3.825	4.492
1	69	-0.153	0.033	---	3.557	3.128	4.044
2+/+nk	95	0.050	0.025	+	4.356	4.163	4.558
Sex(RR)							
Male	171	Aliased			4.283	4.096	4.479
Female	108	-0.041	0.016	-	4.113	3.908	4.328
Combined	49	0.004	0.029	N.S.	4.302	3.870	4.782
Location							
NAmer	116	Aliased			9.525	8.858	10.243
UK	29	-0.102	0.056	(-)	8.604	6.804	10.880
Scand	32	-0.169	0.050	---	8.046	6.558	9.873
othEur	50	-0.319	0.039	---	6.926	5.979	8.024
China	51	-1.336	0.024	---	2.504	2.379	2.636
Japan	18	-0.880	0.053	---	3.952	3.150	4.957
othAs	18	-0.765	0.057	---	4.431	3.509	5.596
other	14	-0.210	0.074	--	7.719	5.682	10.486
Start year of study							
<1960	54	Aliased			2.017	1.710	2.380
1960-69	52	0.404	0.047	+++	3.022	2.626	3.477
1970-79	71	0.444	0.045	+++	3.146	2.756	3.591
1980-89	114	0.836	0.042	+++	4.656	4.483	4.835
1990+	37	0.786	0.062	+++	4.425	3.603	5.434
Study type (1)							
CC	262	Aliased			4.163	4.032	4.299
other	66	0.212	0.035	+++	5.147	4.467	5.931

Table 1A1R - 2

IESLC - Meta-regression of ever smoking, any product (or cigs if any not available)
Multiple regression of data from Table 1A1 (preferring adjusted RRs)
All LC types
Effect of removing characteristics

Log Relative risk
WEIGHTED on Weight

Omit N adjustment vars		Deviance	(DF)	Drop Dev	P		
Model 8		1486.311	(310)	-31.501	*		
		Estimate	S.E.	P	RR	95%CIl	95%CIu
Constant		1.211	0.050	+++	3.356	3.042	3.703
Study size (number of LC cases)							
100-249	115	Aliased			3.269	2.813	3.799
250-499	86	0.093	0.044	+	3.588	3.136	4.105
500-999	64	0.312	0.043	+++	4.465	3.949	5.047
1000+	63	0.279	0.037	+++	4.322	4.169	4.480
Sex(RR)							
Male	171	Aliased			4.279	4.094	4.473
Female	108	-0.045	0.016	--	4.090	3.887	4.305
Combined	49	0.025	0.027	N.S.	4.386	3.964	4.853
Location							
NAmer	116	Aliased			9.218	8.643	9.831
UK	29	-0.124	0.056	-	8.145	6.440	10.301
Scand	32	-0.071	0.049	N.S.	8.588	6.980	10.568
othEur	50	-0.290	0.038	---	6.896	5.952	7.989
China	51	-1.288	0.021	---	2.541	2.424	2.665
Japan	18	-0.909	0.053	---	3.713	2.980	4.625
othAs	18	-0.620	0.059	---	4.959	3.895	6.314
other	14	-0.166	0.075	-	7.805	5.716	10.658
Start year of study							
<1960	54	Aliased			1.984	1.690	2.329
1960-69	52	0.436	0.046	+++	3.069	2.657	3.546
1970-79	71	0.445	0.045	+++	3.095	2.709	3.537
1980-89	114	0.850	0.040	+++	4.644	4.469	4.825
1990+	37	0.918	0.063	+++	4.971	3.998	6.182
Study type (1)							
CC	262	Aliased			4.172	4.041	4.308
other	66	0.181	0.034	+++	4.999	4.358	5.734

Table 1A1R - 3

IESLC - Meta-regression of ever smoking, any product (or cigs if any not available)
Multiple regression of data from Table 1A1 (preferring adjusted RRs)
All LC types
Study outliers

Study Ref	NRR	LOGRR	FITVAL	SEFITV	STDRES
MILLS	3	0.289	1.210	0.221	-4.174
LOMBA2	1	0.283	1.624	0.356	-3.764
TIZZAN	1	0.656	1.252	0.227	-2.629
WANG4	2	0.148	0.698	0.217	-2.533
CPSI	274	1.026	1.597	0.254	-2.251
BLOHMK	3	1.132	1.684	0.259	-2.129
PERSH2	11	1.879	2.294	0.202	-2.056
KREUZE	16	1.330	2.083	0.382	-1.973
DOSEME	1	1.194	1.699	0.291	-1.733
GREGOR	3	0.030	1.602	0.962	-1.633
WU	45	1.109	2.040	0.571	-1.633
BROWN2	2	2.208	2.371	0.103	-1.576
CPSII	79	2.099	2.381	0.182	-1.548
RADZIK	1	0.272	1.763	0.967	-1.541
GER	21	0.610	1.541	0.611	-1.525
PARKIN	28	1.394	1.801	0.272	-1.498
HANSEN	3	0.425	1.824	0.944	-1.482
JAHN	22	1.194	2.012	0.562	-1.455
CHOI	5	0.455	1.478	0.721	-1.419
STAYNE	1	1.299	1.765	0.342	-1.364
PERNU	1	0.634	1.435	0.590	-1.358
CHATZI	4	1.205	1.864	0.492	-1.339
DEAN2	7	1.076	1.808	0.568	-1.288
LIU4	11	1.015	1.051	0.028	-1.283
LEMARC	3	1.729	2.271	0.455	-1.193
SCHWAR	2	1.876	2.354	0.403	-1.185
CEDERL	112	1.430	1.885	0.388	-1.171
DOLL	12	0.718	1.422	0.602	-1.170
SOBUE	115	0.920	1.285	0.314	-1.162
LIDDEL	5	1.284	1.878	0.513	-1.160
DEAN2	3	1.333	1.848	0.444	-1.159
ORMOS	26	-1.640	0.921	2.224	-1.152
GARSHI	25	1.760	2.183	0.368	-1.151
HIRAYA	150	0.859	1.123	0.242	-1.095
HAENSZ	11	0.784	1.257	0.434	-1.090
KAISER	10	1.728	2.172	0.409	-1.085
SCHWAR	1	2.105	2.354	0.242	-1.025
SIMARA	3	0.501	1.081	0.589	-0.986
BRESLO	38	0.321	1.532	1.233	-0.982
HENNEK	3	1.831	2.302	0.486	-0.969
CHEN3	1	0.464	0.863	0.414	-0.966
NOTAN2	15	1.095	1.328	0.242	-0.958
KREYBE	30	0.358	1.075	0.771	-0.930
JEDRYC	58	1.697	2.052	0.386	-0.919
WYNDE3	138	1.138	1.725	0.696	-0.843
DORGAN	53	2.058	2.313	0.303	-0.841
HOROWI	2	0.598	1.240	0.765	-0.840
WUWILL	8	0.833	1.013	0.215	-0.839
BROCKM	2	0.693	1.793	1.312	-0.838
CHANG	12	1.302	1.908	0.730	-0.830
MCCONN	1	0.194	1.172	1.189	-0.822
MARSH2	5	0.637	1.539	1.104	-0.817
SPEIZE	8	1.957	2.200	0.300	-0.812
BROCKM	1	0.071	1.833	2.195	-0.803
TSUGAN	27	0.228	0.852	0.778	-0.801
SIMARA	4	0.489	1.041	0.696	-0.793
KOO	1	1.019	1.377	0.451	-0.793
JEDRYC	59	1.513	2.012	0.635	-0.786
BROSS	12	1.639	1.957	0.405	-0.785
DU	2	0.658	0.996	0.439	-0.771
PIKE	8	1.575	1.962	0.511	-0.758
TVERDA	22	1.522	1.870	0.479	-0.728

Table 1A1R - 3

IESLC - Meta-regression of ever smoking, any product (or cigs if any not available)
Multiple regression of data from Table 1A1 (preferring adjusted RRs)
All LC types
Study outliers

Study Ref	NRR	LOGRR	FITVAL	SEFITV	STDRES
AGUDO	1	1.131	1.722	0.863	-0.684
WYNDE4	62	1.054	1.550	0.732	-0.677
BRETT	10	1.185	1.794	0.902	-0.675
QIAO2	15	0.425	0.899	0.704	-0.674
WIGLE	32	1.482	1.791	0.470	-0.659
WUNSCH	10	1.488	1.869	0.579	-0.657
DEAN3	126	1.533	1.825	0.470	-0.622
KIHARA	31	1.219	1.413	0.316	-0.613
ABRAHA	8	1.586	1.882	0.493	-0.601
LUBIN2	102	1.361	1.470	0.188	-0.582
PIKE	4	1.659	2.003	0.593	-0.579
MIGRAN	27	1.284	1.912	1.096	-0.574
WUNSCH	4	1.558	1.909	0.642	-0.547
ZHENG	24	0.738	0.996	0.475	-0.544
AKIBA	15	1.151	1.314	0.318	-0.513
DOCKER	3	1.456	1.984	1.050	-0.503
KATSOU	29	1.194	1.534	0.689	-0.493
STOCKS	50	1.112	1.251	0.285	-0.488
STOCKW	6	2.346	2.372	0.055	-0.467
LAMTH	6	1.338	1.478	0.318	-0.441
BEST	18	0.806	1.408	1.473	-0.408
XIANGZ	13	0.770	0.938	0.431	-0.390
ALDERS	6	1.558	1.681	0.322	-0.380
DAVEYS	6	-0.324	0.921	3.335	-0.374
LIU	2	0.652	0.779	0.351	-0.362
JAIN	46	2.116	2.373	0.733	-0.351
LAUSSM	11	1.740	1.863	0.356	-0.345
LIU3	2	0.231	0.761	1.587	-0.334
CEDERL	107	1.778	1.925	0.477	-0.308
KAISE2	72	1.686	1.878	0.633	-0.303
POFFIJ	1	2.048	2.144	0.319	-0.302
CHANG	6	1.649	1.948	0.995	-0.301
MIGRAN	42	1.530	1.872	1.152	-0.296
KELLER	3	2.312	2.354	0.147	-0.286
HUANG	1	0.691	0.850	0.564	-0.283
RANDIG	24	0.798	1.022	0.862	-0.261
WICKLU	1	1.526	1.664	0.553	-0.249
CHEN2	2	0.514	0.703	0.783	-0.242
GARCIA	3	2.142	2.271	0.534	-0.242
ESAKI	4	0.640	0.806	0.725	-0.229
MACLEN	73	0.982	1.099	0.525	-0.223
JAIN	41	2.219	2.333	0.516	-0.220
BAND	1	2.299	2.371	0.332	-0.218
XU	2	0.993	1.054	0.283	-0.213
KHUDER	4	2.061	2.165	0.486	-0.212
LANGE	40	1.556	1.783	1.089	-0.209
LAMWK2	9	1.040	1.165	0.603	-0.206
WILKIN	3	2.058	2.180	0.625	-0.195
LANGE	37	1.595	1.743	0.761	-0.194
KELLER	15	2.246	2.313	0.348	-0.194
HU	16	0.550	0.703	0.812	-0.189
DORGAN	76	2.123	2.313	1.070	-0.177
ENGELA	165	1.556	1.697	0.850	-0.166
CHOI	1	1.434	1.518	0.663	-0.127
DORGAN	6	2.285	2.354	0.618	-0.111
GODLEY	6	1.712	1.743	0.283	-0.109
MATOS	27	1.917	1.996	0.729	-0.109
DEAN3	49	1.815	1.865	0.496	-0.102
TANG	3	2.089	2.170	0.876	-0.092
KNEKT	87	1.859	1.929	0.911	-0.076
SCHWAR	3	2.299	2.313	0.206	-0.070
MCCONN	2	1.012	1.131	2.178	-0.055

Table 1A1R - 3

IESLC - Meta-regression of ever smoking, any product (or cigs if any not available)
Multiple regression of data from Table 1A1 (preferring adjusted RRs)
All LC types
Study outliers

Study Ref	NRR	LOGRR	FITVAL	SEFITV	STDRES
AMES	4	1.587	1.619	0.652	-0.049
NAM	77	2.164	2.183	0.438	-0.042
SUN	1	0.836	0.850	0.395	-0.037
KO	1	1.435	1.482	1.466	-0.032
WAKAI	72	1.300	1.324	0.764	-0.031
HINDS	22	1.732	1.742	0.345	-0.030
SOBUE	105	1.314	1.325	0.409	-0.028
MAGNUS	5	1.418	1.441	0.835	-0.027
HU2	10	0.631	0.642	0.465	-0.024
GOODMA	7	2.115	2.124	0.620	-0.014
HU	15	0.737	0.744	0.524	-0.013
HOROWI	1	1.274	1.280	0.555	-0.011
AXELSS	8	2.082	2.087	0.611	-0.008
BUFFLE	5	1.964	1.962	0.408	0.003
BENSHL	18	1.775	1.724	0.899	0.057
WANG2	16	0.829	0.779	0.794	0.062
LAMWK	1	1.416	1.377	0.513	0.076
ROTHSC	2	1.714	1.659	0.692	0.080
DESTE2	14	2.163	2.115	0.592	0.081
LAMWK2	10	1.166	1.124	0.512	0.082
NOU	11	1.807	1.716	0.951	0.096
SADOWS	31	1.289	1.210	0.772	0.102
MCDUFF	1	1.813	1.710	1.001	0.103
MILLER	2	1.607	1.499	0.980	0.111
NAM	93	2.184	2.142	0.371	0.112
SVENSS	71	1.821	1.757	0.540	0.119
ENGELA	159	1.847	1.737	0.846	0.130
MARSH2	6	1.664	1.499	1.137	0.145
KREUZE	14	2.235	2.083	1.047	0.145
JIANG	2	0.912	0.703	1.343	0.156
WANG	5	1.058	0.969	0.558	0.160
ZHOU	3	0.798	0.640	0.939	0.168
TULINI	38	2.043	1.925	0.675	0.174
HUMBLE	16	2.475	2.185	1.662	0.174
ESAKI	5	0.900	0.766	0.768	0.175
LUO	7	0.993	0.868	0.666	0.189
SEGI	1	0.534	0.422	0.557	0.200
AXELSS	11	2.156	2.029	0.615	0.206
MARSH	7	1.917	1.746	0.800	0.214
NOU	12	1.959	1.676	1.310	0.216
DROSTE	7	2.154	1.952	0.907	0.223
AMANDU	7	1.773	1.536	1.056	0.225
HOLE	8	1.863	1.669	0.834	0.232
YONG	2	1.908	1.796	0.456	0.247
JIANG	1	1.001	0.744	1.027	0.250
RONCO	1	1.629	1.391	0.949	0.250
TIZZAN	12	1.404	1.211	0.766	0.251
COMSTO	46	2.191	2.009	0.715	0.255
CHAN	10	1.247	1.124	0.477	0.257
FAN	1	1.044	0.933	0.428	0.259
PETO	5	1.816	1.410	1.543	0.263
EBELIN	1	1.937	1.745	0.716	0.268
JARVHO	7	2.258	1.928	1.199	0.276
MARTIS	4	1.945	1.602	1.189	0.289
COMSTO	34	2.381	2.049	1.138	0.292
GOODMA	3	2.380	2.165	0.727	0.296
WAKAI	78	1.502	1.284	0.717	0.304
KAUFMA	17	2.516	2.392	0.405	0.307
SCHWAR	4	2.447	2.313	0.421	0.318
HUMBLE	14	2.493	2.185	0.953	0.323
BRESLO	37	1.867	1.573	0.898	0.327
TOKARS	6	1.887	1.619	0.804	0.333

Table 1A1R - 3

IESLC - Meta-regression of ever smoking, any product (or cigs if any not available)
Multiple regression of data from Table 1A1 (preferring adjusted RRs)
All LC types
Study outliers

Study Ref	NRR	LOGRR	FITVAL	SEFITV	STDRES
HUMBLE	18	2.430	2.145	0.840	0.340
BUFFLE	1	2.356	2.003	1.032	0.342
ZHOU	2	0.859	0.681	0.518	0.344
ABRAHA	7	2.168	1.922	0.698	0.353
KINLEN	17	2.397	2.104	0.820	0.358
ARCHER	6	1.846	1.518	0.905	0.361
GREGOR	7	2.398	1.561	2.290	0.365
RACHTA	15	2.105	1.810	0.808	0.366
DEAN	7	1.655	1.400	0.696	0.367
LIU5	1	0.647	0.409	0.647	0.369
HEIN	7	2.682	1.853	2.173	0.381
LETOUR	1	2.562	2.374	0.483	0.389
GARDIN	7	2.406	1.973	1.065	0.406
SHAW	12	2.472	2.183	0.711	0.407
DESTEF	48	2.209	2.009	0.487	0.412
STASZE	5	1.468	1.022	1.064	0.419
WYNDE3	49	2.092	1.765	0.780	0.419
AKIBA	11	1.558	1.354	0.482	0.423
DAVEYS	5	1.567	0.962	1.365	0.444
WANG3	1	1.047	0.863	0.410	0.449
WYNDE2	21	2.133	1.765	0.808	0.455
HUMBLE	20	2.734	2.145	1.276	0.462
TOUSEY	21	2.977	2.462	1.113	0.463
YAMAGU	11	1.379	1.053	0.691	0.471
SUZUK2	20	2.640	2.014	1.290	0.485
BECHER	1	2.381	1.745	1.308	0.486
JOLY	1	1.994	1.789	0.416	0.492
HITOSU	38	1.068	0.635	0.860	0.503
RANDIG	23	1.614	1.063	1.087	0.508
BECHER	24	2.469	1.722	1.446	0.517
SEOW	6	1.658	1.294	0.695	0.524
TOUSEY	26	2.752	2.404	0.655	0.532
CPSII	104	2.552	2.421	0.245	0.533
AUVINE	19	2.628	2.297	0.621	0.533
CHOW	55	2.405	1.919	0.910	0.534
ZHENG	15	1.294	1.036	0.481	0.534
PAWLEG	2	2.506	1.850	1.221	0.537
DU	1	1.261	1.036	0.410	0.549
AXELSO	1	1.835	1.587	0.442	0.559
LEI	2	1.250	0.996	0.451	0.563
MRFITR	6	3.698	1.948	3.075	0.569
DAMBER	25	1.967	1.737	0.400	0.576
WIGLE	27	2.175	1.832	0.593	0.579
AUSTIN	7	2.409	1.746	1.146	0.579
DEKLER	6	3.010	1.746	2.173	0.582
KELLER	11	2.604	2.354	0.426	0.588
DORGAN	30	3.135	2.354	1.327	0.589
MASTRA	2	2.097	1.510	0.994	0.590
HAMMO2	4	3.128	1.832	2.176	0.595
JARVHO	3	3.325	1.968	2.265	0.599
ZHANG	3	1.322	0.721	0.991	0.607
GENG	2	1.085	0.804	0.459	0.611
ANDERS	3	2.573	2.362	0.344	0.612
DARBY	16	2.506	2.207	0.488	0.613
ZHANG	2	1.386	0.761	1.007	0.621
KJUUS	10	2.620	1.615	1.615	0.622
CASCO2	1	2.441	1.851	0.941	0.626
CHYOU	7	2.122	1.731	0.622	0.629
GODLEY	5	1.923	1.783	0.221	0.630
LEI	1	1.303	1.036	0.420	0.634
GAO	11	1.194	1.011	0.288	0.636
COOKSO	5	1.882	1.509	0.571	0.653

Table 1A1R - 3

IESLC - Meta-regression of ever smoking, any product (or cigs if any not available)
Multiple regression of data from Table 1A1 (preferring adjusted RRs)
All LC types
Study outliers

Study Ref	NRR	LOGRR	FITVAL	SEFITV	STDRES
CARPEN	12	2.700	2.288	0.625	0.658
KAISE2	64	2.312	1.838	0.716	0.662
XU4	1	1.078	0.762	0.474	0.667
REN	1	1.275	0.744	0.797	0.667
PEZZO2	10	2.709	2.080	0.919	0.684
SIEMIA	5	2.493	2.020	0.674	0.702
BOUCOT	121	3.712	1.536	3.077	0.708
XU3	4	1.351	0.532	1.130	0.724
GAO2	10	1.643	1.136	0.695	0.730
BEST	22	3.237	1.619	2.175	0.744
JARUP	6	2.020	1.202	1.098	0.745
LIU2	2	1.647	0.862	1.039	0.755
STOCKS	47	1.783	1.479	0.377	0.806
XU2	2	1.335	1.072	0.321	0.819
HAMMON	117	1.917	1.449	0.570	0.821
SPITZ	3	2.912	2.170	0.875	0.848
LIU2	4	1.537	0.822	0.840	0.851
DOLL	6	2.206	1.462	0.869	0.856
KREYBE	12	1.889	1.115	0.901	0.859
CHIAZZ	3	3.265	1.297	2.289	0.859
CHEN2	1	1.520	0.744	0.869	0.894
GAO	1	1.361	1.051	0.345	0.898
PASTOR	10	1.918	1.221	0.775	0.901
DUNN	6	2.914	1.518	1.547	0.902
ALDERS	69	2.267	1.721	0.601	0.909
LEVIN	32	1.581	1.210	0.392	0.946
WYNDE4	48	2.209	1.573	0.670	0.950
PEZZOT	25	2.960	1.890	1.118	0.957
GENG	1	1.790	0.845	0.973	0.972
LOMBAR	12	2.180	1.570	0.624	0.977
KOHLME	2	2.797	1.869	0.950	0.977
REN	2	1.401	0.703	0.700	0.997
STUCKE	3	4.830	1.745	3.091	0.998
ARMADA	29	2.980	1.846	1.133	1.000
DOLL2	56	2.036	1.532	0.503	1.002
HORWIT	1	2.426	1.670	0.754	1.003
DORANT	10	2.890	2.294	0.594	1.004
HU2	9	1.109	0.683	0.416	1.023
JOLY	14	2.499	1.830	0.653	1.026
TULINI	44	2.566	1.885	0.649	1.049
BLOT4	1	2.675	1.811	0.823	1.050
GRAHAM	27	1.947	1.402	0.519	1.052
POLEDN	1	2.224	1.557	0.630	1.057
FAN	2	1.366	0.893	0.435	1.089
TENKAN	22	2.684	1.636	0.935	1.121
HITOSU	62	1.224	0.595	0.560	1.123
KUBIK	28	3.336	1.584	1.547	1.133
LIU4	12	1.051	1.011	0.035	1.144
GSELL	8	2.876	0.962	1.610	1.189
DORN	196	1.952	1.638	0.253	1.241
STASZE	1	2.369	1.063	0.998	1.308
DARBY	15	3.898	2.248	1.261	1.309
HIRAYA	147	1.472	1.164	0.234	1.318
XU3	2	1.790	0.573	0.902	1.350
CHAN	9	3.315	1.165	1.585	1.356
YUAN	1	1.872	0.999	0.642	1.359
HEGMAN	1	2.794	2.183	0.447	1.368
WYNDE6	72	2.306	1.954	0.257	1.369
BARBON	131	2.410	1.701	0.510	1.388
OSANN	42	2.708	2.331	0.267	1.414
MCLAUG	1	1.204	0.491	0.502	1.420
CASCOR	1	2.604	1.864	0.506	1.462

Table 1A1R - 3

IESLC - Meta-regression of ever smoking, any product (or cigs if any not available)
Multiple regression of data from Table 1A1 (preferring adjusted RRs)
All LC types
Study outliers

Study Ref	NRR	LOGRR	FITVAL	SEFITV	STDRES
ORMOS	4	2.234	0.962	0.859	1.481
KAISER	13	2.870	2.212	0.428	1.536
GOLLED	7	2.016	1.102	0.582	1.571
KELLER	7	2.525	2.313	0.132	1.602
OSANN	41	2.981	2.371	0.344	1.771
ABELIN	44	3.566	0.791	1.564	1.775
MATSUD	10	3.066	0.806	1.266	1.785
ODRISC	3	3.890	2.163	0.898	1.924
CORREA	34	2.434	1.848	0.302	1.942
BROWN2	1	2.542	2.331	0.105	2.008
WYNDE6	252	2.342	1.914	0.213	2.008
KOULUM	1	3.568	1.478	1.026	2.036
SANKAR	2	2.612	1.624	0.458	2.155
CPSI	187	2.217	1.638	0.245	2.366
PERNU	2	2.190	1.475	0.282	2.529
LUBIN2	46	2.140	1.699	0.170	2.593
BOFFET	33	2.653	2.052	0.213	2.814
JUSSAW	29	2.823	1.328	0.427	3.502

Table 1A1R - 4

IESLC - Meta-regression of ever smoking, any product (or cigs if any not available)
Multiple regression of data from Table 1A1 (preferring adjusted RRs)
All LC types
Effect of additional characteristics

WEIGHTED on Weight

		Deviance	(DF)				
Log Relative risk							
Model 7		1454.810	(308)				
		Estimate	S.E.	P	RR	95%CIl	95%CIu
Constant		1.280	0.052	+++	3.597	3.250	3.981
Sex(RR)							
Male	171	Aliased			4.275	4.087	4.472
Female	108	-0.040	0.016	-	4.106	3.901	4.322
Combined	49	0.018	0.029	N.S.	4.354	3.913	4.845
Location							
NAmer	116	Aliased			9.393	8.726	10.110
UK	29	-0.108	0.057	(-)	8.428	6.648	10.683
Scand	32	-0.095	0.051	(-)	8.541	6.935	10.519
othEur	50	-0.319	0.039	---	6.830	5.890	7.920
China	51	-1.320	0.024	---	2.510	2.384	2.643
Japan	18	-0.858	0.053	---	3.983	3.172	5.002
othAs	18	-0.646	0.060	---	4.921	3.862	6.271
other	14	-0.173	0.076	-	7.900	5.778	10.802
Start year of study							
<1960	54	Aliased			2.104	1.779	2.488
1960-69	52	0.384	0.047	+++	3.088	2.671	3.570
1970-79	71	0.430	0.045	+++	3.234	2.817	3.713
1980-89	114	0.783	0.042	+++	4.605	4.429	4.788
1990+	37	0.872	0.063	+++	5.030	4.041	6.261
Study type (1)							
CC	262	Aliased			4.156	4.024	4.292
other	66	0.238	0.036	+++	5.274	4.565	6.093
Study size (number of LC cases)							
100-249	115	Aliased			3.243	2.785	3.775
250-499	86	0.101	0.044	+	3.588	3.130	4.113
500-999	64	0.293	0.043	+++	4.345	3.831	4.928
1000+	63	0.290	0.038	+++	4.335	4.180	4.495
Number of adjustment variables (1)							
0	164	Aliased			4.243	3.906	4.607
1	69	-0.171	0.034	---	3.576	3.138	4.075
2+/+nk	95	0.017	0.025	N.S.	4.316	4.123	4.519

		Deviance	(DF)	Drop Dev	P		
Model 8		1383.265	(304)	71.544	**		
		Estimate	S.E.	P	RR	95%CIl	95%CIu
Constant		1.328	0.053	+++	3.774	3.400	4.189
Sex(RR)							
Male	171	Aliased			4.282	4.097	4.476
Female	108	-0.042	0.016	--	4.104	3.903	4.316
Combined	49	0.011	0.029	N.S.	4.331	3.898	4.811
Location							
NAmer	116	Aliased			9.204	8.589	9.862
UK	29	-0.104	0.057	(-)	8.297	6.569	10.480
Scand	32	-0.102	0.051	-	8.311	6.773	10.200
othEur	50	Aliased			9.204	8.589	9.862
China	51	-1.314	0.024	---	2.475	2.349	2.607
Japan	18	-0.851	0.053	---	3.928	3.143	4.910
othAs	18	-0.659	0.060	---	4.763	3.751	6.048
other	14	-0.177	0.076	-	7.712	5.669	10.491
Start year of study							
<1960	54	Aliased			2.124	1.793	2.516
1960-69	52	0.365	0.048	+++	3.061	2.654	3.530
1970-79	71	0.387	0.047	+++	3.129	2.724	3.594
1980-89	114	0.778	0.043	+++	4.623	4.449	4.804
1990+	37	0.837	0.065	+++	4.908	3.954	6.091
Study type (1)							
CC	262	Aliased			4.153	4.024	4.287
other	66	0.249	0.036	+++	5.328	4.619	6.146

Table 1A1R - 4

IESLC - Meta-regression of ever smoking, any product (or cigs if any not available)
Multiple regression of data from Table 1A1 (preferring adjusted RRs)
All LC types
Effect of additional characteristics

WEIGHTED on Weight

		Estimate	S.E.	P	RR	95%CIl	95%CIu
Study size (number of LC cases)							
100-249	115	Aliased			3.337	2.871	3.879
250-499	86	0.105	0.044	+	3.706	3.238	4.243
500-999	64	0.279	0.043	+++	4.411	3.895	4.996
1000+	63	0.256	0.038	+++	4.310	4.158	4.467
Number of adjustment variables (1)							
0	164	Aliased			4.298	3.960	4.665
1	69	-0.202	0.034	---	3.512	3.087	3.996
2+/+nk	95	0.002	0.025	N.S.	4.307	4.117	4.506
Detailed Country in othEur							
not o E	278	Aliased			4.292	4.160	4.429
multi	4	-0.024	0.056	N.S.	4.191	3.335	5.268
Germany	17	-0.579	0.072	---	2.406	1.787	3.241
othWest	13	-0.393	0.082	---	2.898	2.061	4.075
East	13	-0.367	0.097	---	2.973	1.988	4.444
Balkans	3	-0.821	0.112	---	1.889	1.184	3.013

		Deviance	(DF)	Drop Dev	P		
Model 8		1423.143	(306)	31.667	*		
		Estimate	S.E.	P	RR	95%CIl	95%CIu
Constant		1.309	0.052	+++	3.703	3.344	4.100
Sex(RR)							
Male	171	Aliased			4.266	4.079	4.461
Female	108	-0.034	0.016	-	4.123	3.917	4.339
Combined	49	0.018	0.029	N.S.	4.342	3.903	4.830
Location							
NAmer	116	Aliased			9.264	8.621	9.956
UK	29	-0.093	0.057	N.S.	8.442	6.669	10.687
Scand	32	-0.093	0.051	(-)	8.444	6.866	10.385
othEur	50	-0.323	0.039	---	6.707	5.788	7.771
China	51	-1.318	0.024	---	2.481	2.356	2.612
Japan	18	-0.840	0.054	---	3.998	3.188	5.014
othAs	18	Aliased			9.264	8.621	9.956
other	14	-0.149	0.076	(-)	7.981	5.844	10.900
Start year of study							
<1960	54	Aliased			2.087	1.767	2.466
1960-69	52	0.354	0.047	+++	2.974	2.565	3.447
1970-79	71	0.456	0.046	+++	3.292	2.868	3.778
1980-89	114	0.794	0.042	+++	4.616	4.441	4.799
1990+	37	0.867	0.064	+++	4.969	3.988	6.192
Study type (1)							
CC	262	Aliased			4.154	4.023	4.289
other	66	0.246	0.036	+++	5.315	4.604	6.135
Study size (number of LC cases)							
100-249	115	Aliased			3.358	2.881	3.913
250-499	86	0.076	0.044	(+)	3.622	3.158	4.154
500-999	64	0.230	0.045	+++	4.226	3.721	4.799
1000+	63	0.255	0.038	+++	4.334	4.180	4.493
Number of adjustment variables (1)							
0	164	Aliased			4.272	3.929	4.644
1	69	-0.178	0.034	---	3.574	3.140	4.069
2+/+nk	95	0.008	0.026	N.S.	4.306	4.113	4.508
Detailed Country in othAsia							
not o A	310	Aliased			4.273	4.144	4.406
India	3	-0.256	0.098	--	3.307	2.185	5.006
HongKong	7	-0.739	0.091	---	2.041	1.391	2.995
othAsia	8	-1.076	0.115	---	1.457	0.897	2.367

		Deviance	(DF)	Drop Dev	P		
Model 8		1440.468	(307)	14.342	(*)		
		Estimate	S.E.	P	RR	95%CIl	95%CIu
Constant		1.300	0.052	+++	3.670	3.315	4.064

Table 1A1R - 4

IESLC - Meta-regression of ever smoking, any product (or cigs if any not available)
Multiple regression of data from Table 1A1 (preferring adjusted RRs)
All LC types
Effect of additional characteristics

WEIGHTED on Weight

		Estimate	S.E.	P	RR	95%CIl	95%CIu
Sex(RR)							
Male	171	Aliased			4.275	4.088	4.471
Female	108	-0.038	0.016	-	4.117	3.911	4.333
Combined	49	0.011	0.029	N.S.	4.322	3.884	4.809
Location							
NAmer	116	Aliased			9.408	8.742	10.124
UK	29	-0.112	0.057	-	8.408	6.638	10.650
Scand	32	-0.095	0.051	(-)	8.556	6.952	10.530
othEur	50	-0.319	0.039	---	6.836	5.898	7.923
China	51	-1.326	0.024	---	2.499	2.373	2.631
Japan	18	-0.797	0.056	---	4.239	3.343	5.375
othAs	18	-0.619	0.060	---	5.066	3.970	6.464
other	14	-0.174	0.076	-	7.904	5.786	10.795
Start year of study							
<1960	54	Aliased			2.106	1.782	2.489
1960-69	52	0.361	0.047	+++	3.023	2.611	3.500
1970-79	71	0.435	0.045	+++	3.253	2.835	3.734
1980-89	114	0.784	0.042	+++	4.611	4.436	4.793
1990+	37	0.868	0.063	+++	5.015	4.032	6.237
Study type (1)							
CC	262	Aliased			4.159	4.027	4.295
other	66	0.229	0.036	+++	5.230	4.527	6.041
Study size (number of LC cases)							
100-249	115	Aliased			3.287	2.823	3.828
250-499	86	0.088	0.044	+	3.590	3.133	4.114
500-999	64	0.282	0.043	+++	4.360	3.846	4.943
1000+	63	0.275	0.038	+++	4.330	4.176	4.490
Number of adjustment variables (1)							
0	164	Aliased			4.244	3.909	4.607
1	69	-0.167	0.034	---	3.592	3.153	4.091
2+/+nk	95	0.016	0.025	N.S.	4.313	4.121	4.515
All LC (or nearest)							
all	317	Aliased			4.236	4.108	4.368
other	11	-0.292	0.077	---	3.163	2.285	4.379

		Deviance	(DF)	Drop Dev	P		
Model 8		1440.468	(307)	14.342	(*)		
		Estimate	S.E.	P	RR	95%CIl	95%CIu
Constant		1.300	0.052	+++	3.670	3.315	4.064
Sex(RR)							
Male	171	Aliased			4.275	4.088	4.471
Female	108	-0.038	0.016	-	4.117	3.911	4.333
Combined	49	0.011	0.029	N.S.	4.322	3.884	4.809
Location							
NAmer	116	Aliased			9.408	8.742	10.124
UK	29	-0.112	0.057	-	8.408	6.638	10.650
Scand	32	-0.095	0.051	(-)	8.556	6.952	10.530
othEur	50	-0.319	0.039	---	6.836	5.898	7.923
China	51	-1.326	0.024	---	2.499	2.373	2.631
Japan	18	-0.797	0.056	---	4.239	3.343	5.375
othAs	18	-0.619	0.060	---	5.066	3.970	6.464
other	14	-0.174	0.076	-	7.904	5.786	10.795
Start year of study							
<1960	54	Aliased			2.106	1.782	2.489
1960-69	52	0.361	0.047	+++	3.023	2.611	3.500
1970-79	71	0.435	0.045	+++	3.253	2.835	3.734
1980-89	114	0.784	0.042	+++	4.611	4.436	4.793
1990+	37	0.868	0.063	+++	5.015	4.032	6.237
Study type (1)							
CC	262	Aliased			4.159	4.027	4.295
other	66	0.229	0.036	+++	5.230	4.527	6.041

Table 1A1R - 4

IESLC - Meta-regression of ever smoking, any product (or cigs if any not available)
Multiple regression of data from Table 1A1 (preferring adjusted RRs)
All LC types
Effect of additional characteristics

WEIGHTED on Weight

		Estimate	S.E.	P	RR	95%CIl	95%CIu
Study size (number of LC cases)							
100-249	115	Aliased			3.287	2.823	3.828
250-499	86	0.088	0.044	+	3.590	3.133	4.114
500-999	64	0.282	0.043	+++	4.360	3.846	4.943
1000+	63	0.275	0.038	+++	4.330	4.176	4.490
Number of adjustment variables (1)							
0	164	Aliased			4.244	3.909	4.607
1	69	-0.167	0.034	---	3.592	3.153	4.091
2+/+nk	95	0.016	0.025	N.S.	4.313	4.121	4.515
All LC (or nearest)							
all	317	Aliased			4.236	4.108	4.368
other	11	-0.292	0.077	---	3.163	2.285	4.379
		Deviance	(DF)	Drop Dev	P		
Model 8		1451.062	(307)	3.748	N.S.		
		Estimate	S.E.	P	RR	95%CIl	95%CIu
Constant		1.278	0.052	+++	3.590	3.244	3.974
Sex(RR)							
Male	171	Aliased			4.278	4.089	4.475
Female	108	-0.041	0.016	-	4.107	3.902	4.323
Combined	49	0.015	0.029	N.S.	4.342	3.901	4.832
Location							
NAmer	116	Aliased			9.402	8.735	10.121
UK	29	-0.109	0.057	(-)	8.432	6.651	10.690
Scand	32	-0.090	0.051	(-)	8.593	6.974	10.589
othEur	50	-0.326	0.039	---	6.790	5.851	7.878
China	51	-1.321	0.024	---	2.509	2.382	2.642
Japan	18	-0.855	0.053	---	4.000	3.184	5.024
othAs	18	-0.646	0.060	---	4.930	3.869	6.284
other	14	-0.174	0.076	-	7.902	5.778	10.805
Start year of study							
<1960	54	Aliased			2.100	1.776	2.484
1960-69	52	0.386	0.047	+++	3.090	2.673	3.572
1970-79	71	0.432	0.045	+++	3.235	2.817	3.714
1980-89	114	0.785	0.042	+++	4.604	4.429	4.787
1990+	37	0.879	0.063	+++	5.059	4.063	6.299
		Estimate					
Study type (1)							
CC	262	Aliased					
other	66	Aliased					
		Estimate	S.E.	P	RR	95%CIl	95%CIu
Study size (number of LC cases)							
100-249	115	Aliased			3.240	2.783	3.772
250-499	86	0.096	0.044	+	3.568	3.111	4.092
500-999	64	0.293	0.043	+++	4.343	3.829	4.926
1000+	63	0.292	0.038	+++	4.337	4.182	4.498
Number of adjustment variables (1)							
0	164	Aliased			4.238	3.902	4.603
1	69	-0.164	0.034	---	3.596	3.153	4.100
2+/+nk	95	0.018	0.025	N.S.	4.315	4.121	4.517
Study type (2)							
CC	262	Aliased			4.157	4.025	4.294
prosp	61	0.224	0.036	+++	5.199	4.484	6.027
other	5	0.525	0.152	+++	7.027	3.676	13.433
		Deviance	(DF)	Drop Dev	P		
Model 8		1450.304	(306)	4.505	N.S.		
		Estimate	S.E.	P	RR	95%CIl	95%CIu
Constant		1.276	0.052	+++	3.583	3.236	3.966
Sex(RR)							
Male	171	Aliased			4.263	4.074	4.461
Female	108	-0.038	0.016	-	4.102	3.897	4.319
Combined	49	0.035	0.030	N.S.	4.415	3.952	4.931

Table 1A1R - 4

IESLC - Meta-regression of ever smoking, any product (or cigs if any not available)
Multiple regression of data from Table 1A1 (preferring adjusted RRs)
All LC types
Effect of additional characteristics

WEIGHTED on Weight

		Estimate	S.E.	P	RR	95%CIl	95%CIu
Location							
NAmer	116	Aliased			9.436	8.761	10.165
UK	29	-0.104	0.057	(-)	8.505	6.693	10.807
Scand	32	-0.081	0.051	N.S.	8.701	7.021	10.782
othEur	50	-0.312	0.039	---	6.906	5.935	8.035
China	51	-1.330	0.025	---	2.495	2.366	2.632
Japan	18	-0.852	0.054	---	4.025	3.198	5.066
othAs	18	-0.647	0.060	---	4.942	3.874	6.304
other	14	-0.144	0.078	(-)	8.170	5.892	11.329
Start year of study							
<1960	54	Aliased			2.102	1.776	2.487
1960-69	52	0.388	0.047	+++	3.097	2.678	3.582
1970-79	71	0.424	0.045	+++	3.212	2.794	3.692
1980-89	114	0.785	0.042	+++	4.606	4.430	4.790
1990+	37	0.878	0.063	+++	5.057	4.060	6.299
Study type (1)							
CC	262	Aliased			4.157	4.024	4.293
other	66	0.236	0.036	+++	5.266	4.555	6.086
Study size (number of LC cases)							
100-249	115	Aliased			3.271	2.806	3.813
250-499	86	0.105	0.044	+	3.633	3.159	4.177
500-999	64	0.306	0.044	+++	4.441	3.886	5.075
1000+	63	0.278	0.038	+++	4.320	4.162	4.483
		Estimate					
Number of adjustment variables (1)							
0	164	Aliased					
1	69	Aliased					
2+/+nk	95	Aliased					
		Estimate	S.E.	P	RR	95%CIl	95%CIu
Number of adjustment variables (2)							
0	164	Aliased			4.185	3.837	4.566
1	69	-0.164	0.034	---	3.552	3.114	4.053
2	49	0.043	0.028	N.S.	4.369	4.147	4.604
3-5	31	-0.041	0.047	N.S.	4.019	3.311	4.877
6+/+nk	15	-0.074	0.071	N.S.	3.887	2.868	5.269

		Deviance	(DF)	Drop Dev	P		
Model 8		1454.460	(307)	0.349	N.S.		
		Estimate	S.E.	P	RR	95%CIl	95%CIu
Constant		1.289	0.054	+++	3.631	3.266	4.037
Sex(RR)							
Male	171	Aliased			4.273	4.085	4.471
Female	108	-0.041	0.016	-	4.103	3.896	4.320
Combined	49	0.023	0.030	N.S.	4.372	3.913	4.885
Location							
NAmer	116	Aliased			9.413	8.730	10.149
UK	29	-0.110	0.057	(-)	8.432	6.649	10.693
Scand	32	-0.095	0.051	(-)	8.560	6.944	10.552
othEur	50	-0.323	0.039	---	6.812	5.866	7.910
China	51	-1.323	0.025	---	2.507	2.379	2.642
Japan	18	-0.857	0.053	---	3.994	3.177	5.022
othAs	18	-0.645	0.060	---	4.937	3.869	6.300
other	14	-0.174	0.076	-	7.908	5.781	10.819
Start year of study							
<1960	54	Aliased			2.103	1.778	2.488
1960-69	52	0.381	0.047	+++	3.080	2.659	3.566
1970-79	71	0.432	0.046	+++	3.241	2.820	3.726
1980-89	114	0.784	0.042	+++	4.605	4.429	4.788
1990+	37	0.870	0.063	+++	5.022	4.033	6.255
Study type (1)							
CC	262	Aliased			4.155	4.023	4.292
other	66	0.241	0.036	+++	5.288	4.570	6.119

Table 1A1R - 4

IESLC - Meta-regression of ever smoking, any product (or cigs if any not available)
Multiple regression of data from Table 1A1 (preferring adjusted RRs)
All LC types
Effect of additional characteristics

WEIGHTED on Weight

		Estimate	S.E.	P	RR	95%CIl	95%CIu
Study size (number of LC cases)							
100-249	115	Aliased			3.231	2.768	3.771
250-499	86	0.101	0.044	+	3.575	3.111	4.109
500-999	64	0.292	0.043	+++	4.325	3.796	4.928
1000+	63	0.295	0.039	+++	4.339	4.181	4.503
Number of adjustment variables (1)							
0	164	Aliased			4.278	3.862	4.740
1	69	-0.184	0.041	---	3.558	3.107	4.075
2+/+nk	95	0.007	0.031	N.S.	4.306	4.101	4.522
RR adjusted for or study matched on age							
Yes	261	Aliased			4.237	4.075	4.405
No	67	-0.021	0.035	N.S.	4.150	3.644	4.726

		Deviance	(DF)	Drop Dev	P		
Model 8		1448.232	(307)	6.577	N.S.		
		Estimate	S.E.	P	RR	95%CIl	95%CIu
Constant		1.358	0.060	+++	3.888	3.457	4.373
Sex(RR)							
Male	171	Aliased			4.270	4.082	4.467
Female	108	-0.042	0.016	--	4.092	3.887	4.309
Combined	49	0.034	0.029	N.S.	4.417	3.960	4.928
Location							
NAmer	116	Aliased			9.477	8.792	10.215
UK	29	-0.125	0.057	-	8.362	6.595	10.602
Scand	32	-0.100	0.051	-	8.579	6.965	10.565
othEur	50	-0.331	0.039	---	6.806	5.869	7.892
China	51	-1.334	0.025	---	2.497	2.369	2.631
Japan	18	-0.847	0.054	---	4.064	3.229	5.115
othAs	18	-0.648	0.060	---	4.955	3.888	6.315
other	14	-0.180	0.076	-	7.919	5.793	10.826
Start year of study							
<1960	54	Aliased			2.146	1.809	2.545
1960-69	52	0.358	0.048	+++	3.071	2.656	3.551
1970-79	71	0.398	0.047	+++	3.194	2.778	3.672
1980-89	114	0.765	0.043	+++	4.609	4.434	4.792
1990+	37	0.835	0.065	+++	4.946	3.967	6.166
Study type (1)							
CC	262	Aliased			4.150	4.018	4.286
other	66	0.260	0.037	+++	5.384	4.642	6.246
Study size (number of LC cases)							
100-249	115	Aliased			3.225	2.769	3.755
250-499	86	0.099	0.044	+	3.560	3.104	4.083
500-999	64	0.281	0.043	+++	4.272	3.755	4.860
1000+	63	0.298	0.038	+++	4.346	4.190	4.508
Number of adjustment variables (1)							
0	164	Aliased			4.397	3.972	4.866
1	69	-0.176	0.034	---	3.687	3.205	4.242
2+/+nk	95	-0.036	0.033	N.S.	4.242	4.019	4.478
RR adjusted for or matched on factor other than sex, age							
Yes	184	Aliased			4.316	4.116	4.526
No	144	-0.088	0.034	-	3.953	3.527	4.430

		Deviance	(DF)	Drop Dev	P		
Model 8		1454.297	(306)	0.512	N.S.		
		Estimate	S.E.	P	RR	95%CIl	95%CIu
Constant		1.271	0.053	+++	3.566	3.213	3.957
Sex(RR)							
Male	171	Aliased			4.275	4.086	4.473
Female	108	-0.042	0.016	--	4.100	3.891	4.320
Combined	49	0.023	0.030	N.S.	4.374	3.911	4.892

Table 1A1R - 4

IESLC - Meta-regression of ever smoking, any product (or cigs if any not available)
Multiple regression of data from Table 1A1 (preferring adjusted RRs)
All LC types
Effect of additional characteristics

WEIGHTED on Weight

		Estimate	S.E.	P	RR	95%CIl	95%CIu
Location							
NAmer	116	Aliased			9.332	8.562	10.171
UK	29	-0.102	0.058	(-)	8.430	6.589	10.785
Scand	32	-0.088	0.052	(-)	8.549	6.936	10.537
othEur	50	-0.311	0.040	---	6.838	5.893	7.934
China	51	-1.310	0.029	---	2.518	2.378	2.667
Japan	18	-0.860	0.054	---	3.950	3.127	4.991
othAs	18	-0.639	0.061	---	4.928	3.864	6.285
other	14	-0.167	0.076	-	7.898	5.770	10.812
Start year of study							
<1960	54	Aliased			2.108	1.774	2.505
1960-69	52	0.383	0.047	+++	3.091	2.670	3.578
1970-79	71	0.428	0.046	+++	3.233	2.814	3.713
1980-89	114	0.782	0.043	+++	4.605	4.428	4.789
1990+	37	0.865	0.065	+++	5.004	4.008	6.248
Study type (1)							
CC	262	Aliased			4.156	4.024	4.293
other	66	0.237	0.036	+++	5.268	4.550	6.100
Study size (number of LC cases)							
100-249	115	Aliased			3.230	2.768	3.770
250-499	86	0.105	0.044	+	3.590	3.130	4.117
500-999	64	0.294	0.043	+++	4.335	3.817	4.922
1000+	63	0.295	0.038	+++	4.337	4.181	4.498
Number of adjustment variables (1)							
0	164	Aliased			4.254	3.907	4.632
1	69	-0.174	0.035	---	3.576	3.132	4.082
2+/+nk	95	0.014	0.026	N.S.	4.312	4.117	4.517
Product							
all/unsp	205	Aliased			4.210	4.057	4.370
cig+/-ot	114	0.017	0.025	N.S.	4.281	3.906	4.692
cig only	9	-0.008	0.075	N.S.	4.178	3.053	5.718

		Deviance	(DF)	Drop Dev	P		
Model 8		1454.809	(307)	0.001	N.S.		
		Estimate	S.E.	P	RR	95%CIl	95%CIu
Constant		1.280	0.053	+++	3.598	3.245	3.990
Sex(RR)							
Male	171	Aliased			4.275	4.087	4.472
Female	108	-0.040	0.016	-	4.106	3.898	4.326
Combined	49	0.018	0.030	N.S.	4.353	3.896	4.864
Location							
NAmer	116	Aliased			9.395	8.602	10.262
UK	29	-0.109	0.058	(-)	8.428	6.645	10.688
Scand	32	-0.095	0.052	(-)	8.541	6.932	10.523
othEur	50	-0.319	0.041	---	6.829	5.885	7.925
China	51	-1.320	0.030	---	2.509	2.371	2.656
Japan	18	-0.858	0.054	---	3.984	3.167	5.013
othAs	18	-0.647	0.061	---	4.921	3.860	6.274
other	14	-0.173	0.078	-	7.899	5.767	10.820
Start year of study							
<1960	54	Aliased			2.104	1.774	2.494
1960-69	52	0.384	0.047	+++	3.088	2.670	3.572
1970-79	71	0.430	0.046	+++	3.234	2.816	3.715
1980-89	114	0.783	0.043	+++	4.605	4.429	4.788
1990+	37	0.872	0.065	+++	5.031	4.029	6.282
Study type (1)							
CC	262	Aliased			4.156	4.024	4.293
other	66	0.238	0.036	+++	5.274	4.564	6.095
Study size (number of LC cases)							
100-249	115	Aliased			3.243	2.779	3.786
250-499	86	0.101	0.044	+	3.588	3.129	4.114
500-999	64	0.293	0.043	+++	4.346	3.823	4.939
1000+	63	0.290	0.038	+++	4.335	4.179	4.496

Table 1A1R - 4

IESLC - Meta-regression of ever smoking, any product (or cigs if any not available)
Multiple regression of data from Table 1A1 (preferring adjusted RRs)
All LC types
Effect of additional characteristics

WEIGHTED on Weight

		Estimate	S.E.	P	RR	95%CIl	95%CIu
Number of adjustment variables (1)							
0	164	Aliased			4.242	3.894	4.621
1	69	-0.171	0.034	---	3.576	3.137	4.076
2+/+nk	95	0.017	0.026	N.S.	4.317	4.119	4.523
Denominator							
nev any	236	Aliased			4.223	4.077	4.375
nev cigs	92	-0.001	0.026	N.S.	4.220	3.825	4.656

		Deviance	(DF)	Drop Dev	P		
Model 8		1444.236	(306)	10.573	N.S.		
		Estimate	S.E.	P	RR	95%CIl	95%CIu
Constant		1.313	0.077	+++	3.716	3.198	4.318
Sex(RR)							
Male	171	Aliased			4.272	4.084	4.469
Female	108	-0.040	0.016	-	4.106	3.900	4.322
Combined	49	0.022	0.029	N.S.	4.368	3.925	4.862
Location							
NAmer	116	Aliased			5.771	2.989	11.141
UK	29	-0.134	0.078	(-)	5.046	2.463	10.336
Scand	32	-0.094	0.051	(-)	5.254	2.639	10.463
othEur	50	-0.320	0.039	---	4.192	2.133	8.236
China	51	-0.455	0.273	(-)	3.660	2.204	6.076
Japan	18	-0.858	0.054	---	2.447	1.219	4.912
othAs	18	-0.619	0.063	---	3.108	1.619	5.966
other	14	-0.186	0.077	-	4.792	2.311	9.936
Start year of study							
<1960	54	Aliased			2.106	1.780	2.492
1960-69	52	0.379	0.047	+++	3.076	2.658	3.560
1970-79	71	0.428	0.045	+++	3.232	2.815	3.710
1980-89	114	0.782	0.042	+++	4.603	4.427	4.785
1990+	37	0.897	0.064	+++	5.165	4.139	6.446
Study type (1)							
CC	262	Aliased			4.155	4.023	4.291
other	66	0.241	0.036	+++	5.290	4.575	6.115
Study size (number of LC cases)							
100-249	115	Aliased			3.265	2.804	3.802
250-499	86	0.087	0.044	+	3.560	3.105	4.083
500-999	64	0.280	0.044	+++	4.321	3.793	4.923
1000+	63	0.284	0.038	+++	4.338	4.183	4.499
Number of adjustment variables (1)							
0	164	Aliased			4.231	3.890	4.602
1	69	-0.170	0.034	---	3.571	3.134	4.069
2+/+nk	95	0.021	0.026	N.S.	4.321	4.126	4.526
National cigarette tobacco type							
Virginia	50	Aliased			7.071	3.573	13.994
blended	225	-0.028	0.060	N.S.	6.874	3.560	13.274
other	53	-0.893	0.275	--	2.894	1.744	4.803

		Deviance	(DF)	Drop Dev	P		
Model 8		1443.799	(307)	11.011	N.S.		
		Estimate	S.E.	P	RR	95%CIl	95%CIu
Constant		1.289	0.052	+++	3.629	3.279	4.017
Sex(RR)							
Male	171	Aliased			4.286	4.098	4.484
Female	108	-0.043	0.016	--	4.104	3.899	4.320
Combined	49	0.007	0.029	N.S.	4.315	3.877	4.804

Table 1A1R - 4

IESLC - Meta-regression of ever smoking, any product (or cigs if any not available)
Multiple regression of data from Table 1A1 (preferring adjusted RRs)
All LC types
Effect of additional characteristics

WEIGHTED on Weight

		Estimate	S.E.	P	RR	95%CIl	95%CIu
Location							
NAmer	116	Aliased			9.110	8.383	9.900
UK	29	-0.092	0.057	N.S.	8.310	6.554	10.536
Scand	32	-0.070	0.051	N.S.	8.491	6.896	10.454
othEur	50	-0.324	0.039	---	6.592	5.648	7.694
China	51	-1.266	0.029	---	2.568	2.420	2.725
Japan	18	-0.868	0.054	---	3.823	3.027	4.827
othAs	18	-0.661	0.060	---	4.703	3.668	6.031
other	14	-0.156	0.076	-	7.795	5.702	10.656
Start year of study							
<1960	54	Aliased			2.104	1.780	2.488
1960-69	52	0.395	0.047	+++	3.123	2.700	3.612
1970-79	71	0.429	0.045	+++	3.231	2.815	3.709
1980-89	114	0.782	0.042	+++	4.601	4.426	4.783
1990+	37	0.869	0.063	+++	5.019	4.034	6.245
Study type (1)							
CC	262	Aliased			4.167	4.034	4.304
other	66	0.201	0.037	+++	5.093	4.380	5.922
Study size (number of LC cases)							
100-249	115	Aliased			3.211	2.757	3.739
250-499	86	0.105	0.044	+	3.565	3.110	4.087
500-999	64	0.301	0.043	+++	4.338	3.826	4.919
1000+	63	0.301	0.038	+++	4.340	4.185	4.501
Number of adjustment variables (1)							
0	164	Aliased			4.210	3.875	4.574
1	69	-0.146	0.035	---	3.638	3.187	4.152
2+/+nk	95	0.025	0.025	N.S.	4.318	4.125	4.520
Any proxy use							
No/nk	227	Aliased			4.447	4.134	4.784
Yes	101	-0.083	0.025	--	4.093	3.892	4.304

		Deviance	(DF)	Drop Dev	P		
Model 8		1449.174	(307)	5.635	N.S.		
		Estimate	S.E.	P	RR	95%CIl	95%CIu
Constant		1.253	0.053	+++	3.501	3.156	3.884
Sex(RR)							
Male	171	Aliased			4.267	4.078	4.464
Female	108	-0.042	0.016	--	4.092	3.886	4.309
Combined	49	0.038	0.030	N.S.	4.432	3.965	4.955
Location							
NAmer	116	Aliased			9.186	8.449	9.988
UK	29	-0.088	0.057	N.S.	8.411	6.635	10.661
Scand	32	-0.076	0.051	N.S.	8.512	6.911	10.483
othEur	50	-0.316	0.039	---	6.696	5.751	7.797
China	51	-1.284	0.029	---	2.545	2.403	2.695
Japan	18	-0.858	0.053	---	3.894	3.089	4.907
othAs	18	-0.610	0.062	---	4.992	3.912	6.369
other	14	-0.152	0.076	-	7.888	5.770	10.785
Start year of study							
<1960	54	Aliased			2.128	1.797	2.520
1960-69	52	0.379	0.047	+++	3.109	2.688	3.596
1970-79	71	0.412	0.046	+++	3.212	2.796	3.689
1980-89	114	0.772	0.043	+++	4.604	4.429	4.787
1990+	37	0.846	0.064	+++	4.959	3.979	6.181
Study type (1)							
CC	262	Aliased			4.152	4.020	4.288
other	66	0.255	0.036	+++	5.355	4.623	6.202
Study size (number of LC cases)							
100-249	115	Aliased			3.209	2.753	3.740
250-499	86	0.107	0.044	+	3.573	3.116	4.096
500-999	64	0.302	0.043	+++	4.340	3.827	4.922
1000+	63	0.302	0.038	+++	4.340	4.184	4.500

Table 1A1R - 4

IESLC - Meta-regression of ever smoking, any product (or cigs if any not available)
Multiple regression of data from Table 1A1 (preferring adjusted RRs)
All LC types
Effect of additional characteristics

WEIGHTED on Weight

		Estimate	S.E.	P	RR	95%CIl	95%CIu
Number of adjustment variables (1)							
0	164	Aliased			4.267	3.926	4.636
1	69	-0.165	0.034	---	3.619	3.170	4.131
2+/+nk	95	0.008	0.025	N.S.	4.300	4.106	4.504
Full histological confirmation							
No	245	Aliased			4.188	4.047	4.333
Yes	83	0.064	0.027	+	4.465	4.021	4.958

		Deviance	(DF)	Drop Dev	P		
Model 8		1449.138	(306)	5.671	N.S.		
		Estimate	S.E.	P	RR	95%CIl	95%CIu
Constant		1.284	0.052	+++	3.610	3.261	3.997
Sex(RR)							
Male	171	Aliased			4.279	4.091	4.476
Female	108	-0.041	0.016	--	4.106	3.900	4.323
Combined	49	0.014	0.029	N.S.	4.339	3.897	4.831
Location							
NAmer	116	Aliased			9.397	8.730	10.116
UK	29	-0.109	0.057	(-)	8.424	6.642	10.685
Scand	32	-0.103	0.051	-	8.480	6.880	10.452
othEur	50	-0.324	0.039	---	6.798	5.859	7.888
China	51	-1.320	0.024	---	2.511	2.385	2.645
Japan	18	-0.861	0.054	---	3.974	3.162	4.995
othAs	18	-0.648	0.060	---	4.917	3.856	6.270
other	14	-0.172	0.076	-	7.911	5.782	10.823
Start year of study							
<1960	54	Aliased			2.109	1.783	2.496
1960-69	52	0.378	0.047	+++	3.079	2.663	3.561
1970-79	71	0.434	0.045	+++	3.257	2.834	3.744
1980-89	114	0.780	0.042	+++	4.601	4.425	4.784
1990+	37	0.877	0.063	+++	5.068	4.069	6.313
Study type (1)							
CC	262	Aliased			4.152	4.019	4.288
other	66	0.254	0.036	+++	5.352	4.615	6.207
Study size (number of LC cases)							
100-249	115	Aliased			3.239	2.780	3.774
250-499	86	0.103	0.044	+	3.592	3.131	4.120
500-999	64	0.290	0.043	+++	4.331	3.815	4.917
1000+	63	0.292	0.038	+++	4.336	4.180	4.497
Number of adjustment variables (1)							
0	164	Aliased			4.250	3.912	4.617
1	69	-0.179	0.034	---	3.554	3.117	4.053
2+/+nk	95	0.016	0.025	N.S.	4.317	4.124	4.521
Risky occupational population							
No	310	Aliased			4.223	4.095	4.355
Mining	7	-0.228	0.122	(-)	3.361	1.999	5.650
Other risky	11	0.128	0.090	N.S.	4.800	3.277	7.031

		Deviance	(DF)	Drop Dev	P		
Model 8		1427.609	(305)	27.201	N.S.		
		Estimate	S.E.	P	RR	95%CIl	95%CIu
Constant		1.236	0.052	+++	3.442	3.105	3.814
Sex(RR)							
Male	171	Aliased			4.284	4.096	4.480
Female	108	-0.042	0.016	--	4.106	3.901	4.321
Combined	49	0.009	0.029	N.S.	4.322	3.882	4.811

Table 1A1R - 4

IESLC - Meta-regression of ever smoking, any product (or cigs if any not available)
Multiple regression of data from Table 1A1 (preferring adjusted RRs)
All LC types
Effect of additional characteristics

WEIGHTED on Weight

		Estimate	S.E.	P	RR	95%CIl	95%CIu
Location							
NAmer	116	Aliased			8.908	8.144	9.743
UK	29	-0.077	0.057	N.S.	8.249	6.507	10.456
Scand	32	-0.067	0.051	N.S.	8.332	6.758	10.272
othEur	50	-0.334	0.039	---	6.376	5.426	7.491
China	51	-1.226	0.032	---	2.614	2.450	2.788
Japan	18	-0.885	0.054	---	3.676	2.890	4.675
othAs	18	-0.622	0.060	---	4.782	3.748	6.100
other	14	-0.173	0.076	-	7.494	5.469	10.269
Start year of study							
<1960	54	Aliased			2.061	1.741	2.440
1960-69	52	0.411	0.048	+++	3.110	2.688	3.598
1970-79	71	0.442	0.045	+++	3.207	2.792	3.684
1980-89	114	0.805	0.043	+++	4.609	4.433	4.791
1990+	37	0.910	0.064	+++	5.118	4.101	6.388
Study type (1)							
CC	262	Aliased			4.141	4.010	4.277
other	66	0.292	0.037	+++	5.545	4.767	6.451
Study size (number of LC cases)							
100-249	115	Aliased			3.119	2.671	3.642
250-499	86	0.117	0.044	++	3.507	3.053	4.028
500-999	64	0.328	0.044	+++	4.328	3.814	4.910
1000+	63	0.334	0.039	+++	4.355	4.198	4.517
Number of adjustment variables (1)							
0	164	Aliased			4.153	3.811	4.526
1	69	-0.122	0.035	---	3.675	3.216	4.201
2+/+nk	95	0.042	0.026	N.S.	4.333	4.135	4.540
Lowest age in RR							
<25/unlim	214	Aliased			4.626	4.255	5.030
25-39	68	-0.144	0.030	---	4.005	3.782	4.242
40+	38	-0.093	0.038	-	4.215	3.596	4.940
unknown	8	0.010	0.094	N.S.	4.674	3.169	6.894

		Deviance	(DF)	Drop Dev	P		
Model 8		1422.479	(304)	32.331	N.S.		
		Estimate	S.E.	P	RR	95%CIl	95%CIu
Constant		0.990	0.087	+++	2.692	2.269	3.193
Sex(RR)							
Male	171	Aliased			4.258	4.070	4.455
Female	108	-0.036	0.016	-	4.108	3.904	4.324
Combined	49	0.037	0.030	N.S.	4.418	3.962	4.926
Location							
NAmer	116	Aliased			9.343	8.666	10.074
UK	29	-0.056	0.058	N.S.	8.838	6.954	11.233
Scand	32	-0.056	0.052	N.S.	8.832	7.127	10.946
othEur	50	-0.343	0.039	---	6.633	5.706	7.712
China	51	-1.313	0.025	---	2.514	2.385	2.649
Japan	18	-0.835	0.055	---	4.056	3.220	5.108
othAs	18	-0.641	0.060	---	4.924	3.859	6.282
other	14	-0.170	0.076	-	7.881	5.764	10.774
Start year of study							
<1960	54	Aliased			2.119	1.793	2.506
1960-69	52	0.362	0.047	+++	3.044	2.633	3.519
1970-79	71	0.478	0.047	+++	3.417	2.945	3.964
1980-89	114	0.772	0.042	+++	4.588	4.412	4.771
1990+	37	0.850	0.065	+++	4.960	3.965	6.204
Study type (1)							
CC	262	Aliased			4.145	4.013	4.280
other	66	0.279	0.037	+++	5.481	4.724	6.359

Table 1A1R - 4

IESLC - Meta-regression of ever smoking, any product (or cigs if any not available)
Multiple regression of data from Table 1A1 (preferring adjusted RRs)
All LC types
Effect of additional characteristics

WEIGHTED on Weight

		Estimate	S.E.	P	RR	95%CIl	95%CIu
Study size (number of LC cases)							
100-249	115	Aliased			3.256	2.795	3.792
250-499	86	0.083	0.044	(+)	3.538	3.079	4.065
500-999	64	0.290	0.044	+++	4.352	3.821	4.957
1000+	63	0.287	0.038	+++	4.338	4.182	4.500
Number of adjustment variables (1)							
0	164	Aliased			4.196	3.859	4.563
1	69	-0.194	0.035	---	3.455	3.022	3.951
2+/+nk	95	0.037	0.026	N.S.	4.355	4.157	4.562
Highest age in RR							
<65	17	Aliased			3.197	2.350	4.349
65-74	33	0.179	0.080	+	3.825	3.255	4.494
75-84	52	0.379	0.080	+++	4.672	4.086	5.341
85+/unlim	218	0.279	0.073	+++	4.226	4.087	4.369
unknown	8	0.383	0.118	++	4.688	3.178	6.916

		Deviance	(DF)	Drop Dev	P		
Model 8		1453.746	(307)	1.063	N.S.		
		Estimate	S.E.	P	RR	95%CIl	95%CIu
Constant		1.391	0.119	+++	4.020	3.181	5.081
Sex(RR)							
Male	171	Aliased			4.278	4.089	4.475
Female	108	-0.041	0.016	-	4.106	3.901	4.323
Combined	49	0.015	0.029	N.S.	4.344	3.901	4.836
Location							
NAmer	116	Aliased			9.316	8.589	10.103
UK	29	-0.107	0.057	(-)	8.367	6.586	10.630
Scand	32	-0.103	0.051	-	8.405	6.753	10.461
othEur	50	-0.319	0.039	---	6.769	5.809	7.887
China	51	-1.305	0.028	---	2.525	2.384	2.675
Japan	18	-0.853	0.054	---	3.968	3.157	4.987
othAs	18	-0.637	0.060	---	4.927	3.865	6.281
other	14	-0.168	0.076	-	7.877	5.758	10.777
Start year of study							
<1960	54	Aliased			2.100	1.775	2.484
1960-69	52	0.389	0.047	+++	3.100	2.679	3.587
1970-79	71	0.425	0.046	+++	3.212	2.790	3.699
1980-89	114	0.786	0.042	+++	4.607	4.431	4.791
1990+	37	0.871	0.063	+++	5.018	4.030	6.249
Study type (1)							
CC	262	Aliased			4.155	4.023	4.292
other	66	0.241	0.036	+++	5.286	4.573	6.111
Study size (number of LC cases)							
100-249	115	Aliased			3.220	2.757	3.760
250-499	86	0.102	0.044	+	3.567	3.104	4.098
500-999	64	0.291	0.043	+++	4.307	3.778	4.911
1000+	63	0.299	0.039	+++	4.342	4.184	4.506
Number of adjustment variables (1)							
0	164	Aliased			4.237	3.901	4.603
1	69	-0.165	0.034	---	3.591	3.147	4.098
2+/+nk	95	0.018	0.025	N.S.	4.316	4.122	4.519
Midpoint age in RR		-0.002	0.002	N.S.	4.795	2.832	8.121

		Deviance	(DF)	Drop Dev	P		
Model 8		1442.521	(307)	12.288	N.S.		
		Estimate	S.E.	P	RR	95%CIl	95%CIu
Constant		1.323	0.053	+++	3.755	3.384	4.168
Sex(RR)							
Male	171	Aliased			4.269	4.082	4.465
Female	108	-0.041	0.016	--	4.096	3.891	4.311
Combined	49	0.033	0.029	N.S.	4.411	3.960	4.912

Table 1A1R - 4

IESLC - Meta-regression of ever smoking, any product (or cigs if any not available)
Multiple regression of data from Table 1A1 (preferring adjusted RRs)
All LC types
Effect of additional characteristics

WEIGHTED on Weight

		Estimate	S.E.	P	RR	95%CIl	95%CIu
Location							
NAmer	116	Aliased			9.176	8.482	9.927
UK	29	-0.082	0.057	N.S.	8.455	6.674	10.711
Scand	32	-0.083	0.051	N.S.	8.449	6.861	10.404
othEur	50	-0.321	0.039	---	6.658	5.726	7.742
China	51	-1.281	0.027	---	2.550	2.413	2.694
Japan	18	-0.848	0.054	---	3.931	3.130	4.936
othAs	18	-0.632	0.060	---	4.875	3.827	6.210
other	14	-0.165	0.076	-	7.777	5.689	10.631
Start year of study							
<1960	54	Aliased			2.100	1.777	2.483
1960-69	52	0.408	0.047	+++	3.157	2.725	3.658
1970-79	71	0.440	0.045	+++	3.262	2.841	3.745
1980-89	114	0.783	0.042	+++	4.595	4.419	4.777
1990+	37	0.866	0.063	+++	4.991	4.012	6.210
Study type (1)							
CC	262	Aliased			4.150	4.018	4.286
other	66	0.261	0.036	+++	5.387	4.654	6.236
Study size (number of LC cases)							
100-249	115	Aliased			3.174	2.721	3.702
250-499	86	0.106	0.044	+	3.528	3.074	4.049
500-999	64	0.296	0.043	+++	4.265	3.755	4.846
1000+	63	0.316	0.039	+++	4.354	4.197	4.516
Number of adjustment variables (1)							
0	164	Aliased			4.311	3.961	4.691
1	69	-0.177	0.034	---	3.611	3.168	4.115
2+/+nk	95	-0.006	0.026	N.S.	4.286	4.091	4.490
Derivation of RR/CI							
Orig/2x2	82	Aliased			4.559	4.135	5.027
Other	246	-0.088	0.025	---	4.175	4.038	4.317

		Deviance	(DF)	Drop Dev	P		
Model 8		1429.127	(306)	25.683	(*)		
		Estimate	S.E.	P	RR	95%CIl	95%CIu
Constant		1.264	0.072	+++	3.540	3.077	4.073
Sex(RR)							
Male	171	Aliased			4.268	4.081	4.464
Female	108	-0.041	0.016	-	4.099	3.894	4.314
Combined	49	0.031	0.029	N.S.	4.405	3.957	4.903
Location							
NAmer	116	Aliased			8.883	8.143	9.690
UK	29	-0.070	0.057	N.S.	8.285	6.542	10.493
Scand	32	-0.047	0.051	N.S.	8.476	6.890	10.428
othEur	50	-0.281	0.040	---	6.706	5.770	7.793
China	51	-1.232	0.030	---	2.591	2.445	2.746
Japan	18	-0.830	0.054	---	3.875	3.085	4.866
othAs	18	-0.580	0.061	---	4.975	3.907	6.334
other	14	-0.123	0.076	N.S.	7.856	5.756	10.723
Start year of study							
<1960	54	Aliased			2.138	1.807	2.531
1960-69	52	0.393	0.047	+++	3.168	2.732	3.673
1970-79	71	0.406	0.046	+++	3.211	2.789	3.696
1980-89	114	0.765	0.043	+++	4.596	4.419	4.780
1990+	37	0.840	0.064	+++	4.952	3.981	6.161
Study type (1)							
CC	262	Aliased			4.144	4.012	4.279
other	66	0.284	0.037	+++	5.502	4.743	6.381
Study size (number of LC cases)							
100-249	115	Aliased			3.140	2.692	3.664
250-499	86	0.111	0.044	+	3.508	3.059	4.023
500-999	64	0.301	0.043	+++	4.244	3.734	4.822
1000+	63	0.328	0.039	+++	4.361	4.204	4.523

Table 1A1R - 4

IESLC - Meta-regression of ever smoking, any product (or cigs if any not available)
Multiple regression of data from Table 1A1 (preferring adjusted RRs)
All LC types
Effect of additional characteristics

WEIGHTED on Weight

		Estimate	S.E.	P	RR	95%CIl	95%CIu
Number of adjustment variables (1)							
0	164	Aliased			4.054	3.469	4.738
1	69	-0.076	0.054	N.S.	3.756	3.267	4.319
2+/+nk	95	0.072	0.048	N.S.	4.358	4.094	4.638
Derivation of RR/CI							
Orig	39	Aliased			4.694	4.144	5.316
StcCalc	170	-0.019	0.051	N.S.	4.604	3.948	5.370
Other	119	-0.154	0.032	---	4.023	3.773	4.290
