# Supplementary material for: Systematic review with meta-analysis of the epidemiological evidence in the 1900s relating smoking to lung cancer
Source: BMC Cancer. 2012 Sep 3;12:385. doi: 10.1186/1471-2407-12-385 (PMC3505152; doi:10.1186/1471-2407-12-385)
Supplement: Additional file 5 — Detailed Analysis Tables (Individual file names as described in Additional file 1: Methods, Table1). [file 1471-2407-12-385-S5.zip › PDF/1I.pdf]

Table 1I1 -

IESLC - Meta-analysis of Ever Smoking by Duration, Overview
All LC types, Any Product (or Cigarettes if Any not available)

This analysis is restricted to results for:
1) Ever smokers
2) Results by Duration
3) Categorical results by Duration
   Results by Duration are grouped under 2 schemes (S1, S2). Each scheme has a set of "key values". An interval is allocated to the category whose key value it includes, and intervals which include none or more than one of the key values are excluded. (Open-ended intervals are coded as 999)

S1	key value	maximum range
1	20	1-34
2	35	21-49
3	50	36+

S2	key value	maximum range
1	5	1-19
2	20	6-29
3	30	21-39
4	40	31-49
5	50	41-998
6	999	51+

4) All LC types (or near equivalent)
5) Results complete enough for use in metaanalysis

Within each study, results are then selected (in the following order of preference, within each sex) for:
6) (not applicable)
7) PRODUCT: all/unspec, cigarettes regardless of other products, cigarettes only
8) CIGTYPE: all/unspecified, MC regardless of HR, MC only
9) (not applicable)
10) DENOM: never smoked anything, never smoked cigarettes, never any + low, never cigs + low
11) Followup period (YF, prospective studies): whole study (coded as 0) or longest available
12) LCtype: all or nearest available, at least Squamous and Adeno. (q = squamous, s = small,
          l = large, a = adeno, mix = mixed, alv = alveolar)
13) Race: all or nearest available, otherwise by race (wh or w = white, bl or b = black, hi = hispanic
          ch = chinese, jap = japanese, haw = hawaiian, w+o = white + oriental, sca = scandinavian, as = asian)
14) For overlapping studies: principal rather than subsidiary studies
Finally by Age: whole study (coded as 0) if available, otherwise by widest available age group
and then for single sex results (m, f) in preference to results for both sexes combined (c).

Results adjusted (AD) for the most potential confounders are then chosen in Sections -1 to -3
and results adjusted for the least confounders in Sections -4 to -6. (Those least adjusted results which actually differ from the most adjusted are marked 'x' in column X in Section -4)

Section -7 shows excluded studies, together with the stage (as above) at which no qualifying results were found.

Section -8 lists the potentially overlapping studies which have been included (1=principal, 2=subsidiary).

Section -9 lists any results which would have been included in preference except that they had data not complete enough for use in meta-analysis, with their significance (yes/no), if known, and any further comment as entered on the database. It also lists as "gap" any categories for which no data were presented by the original authors.

In addition to those mentioned above, the following fields, levels and abbreviations are used:
* or nk = not known, n = no, y = yes, ot = other
nev = never
all/unspec = all or unspecified, cig+/-ot = cigarettes irrespective of other products (cigar, pipe etc)
MC = manufactured cigarettes, HR = hand-rolled cigarettes
exL, exH = range of exposure (low and high) in the smoking group, in terms of Duration
REF: 6-character study reference
NRR: number of the RR on the database within the study
ST : study type (CC = case control, pr or prosp = prospective)
NLC: number of lung cancer cases in whole study
R : risky occupational population (n = no, m = mining, o = other risky)
VB : national cigarette type (V = at least 75% Virginia, bl = at least 75% blended, ot = other)
P : any proxy use
H : full histological confirmation
De : derivation of RR/CI (or = original, st = standard method, ot = other method of estimation)

Table 1I1 - 1

IESLC - Meta-analysis of Ever Smoking by Duration, Overview
All LC types, Any Product (or Cigarettes if Any not available)
Most adjusted

REF	NRR	SEX	AGEL	AGEH	RACE	YF	LC TYPE	LOC	START	ST	NLC	R	VB	P	H	AD	PRODUCT	exL	exH	S1	S2	DENOM	De
AGUDO	510	f	0	0	all	-	all	Eu:wst	1989	CC	103	n	bl	n	n	3	cig only	1	16	0	1	nev cigs	or
AGUDO	511	f	0	0	all	-	all	Eu:wst	1989	CC	103	n	bl	n	n	3	cig only	17	999	0	0	nev cigs	or
ARMADA	506	m	0	0	all	-	all	Eu:wst	1986	CC	325	n	bl	n	y	1	cig+/-ot	1	24	1	0	nev cigs	or
ARMADA	507	m	0	0	all	-	all	Eu:wst	1986	CC	325	n	bl	n	y	1	cig+/-ot	25	49	2	0	nev cigs	or
ARMADA	508	m	0	0	all	-	all	Eu:wst	1986	CC	325	n	bl	n	y	1	cig+/-ot	50	999	3	0	nev cigs	or
AUVINE	517	c	0	0	all	-	all	Eu:Sca	1986	CC	517	n	bl	y	n	2	cig+/-ot	1	20	1	0	nev cigs	or
AUVINE	518	c	0	0	all	-	all	Eu:Sca	1986	CC	517	n	bl	y	n	2	cig+/-ot	21	40	2	0	nev cigs	or
AUVINE	519	c	0	0	all	-	all	Eu:Sca	1986	CC	517	n	bl	y	n	2	cig+/-ot	41	999	3	0	nev cigs	or
AXELSS	519	m	0	0	sca	-	all	Eu:Sca	1989	CC	436	n	bl	n	n	6	all/unsp	1	19	0	1	nev any	ot
AXELSS	520	m	0	0	sca	-	all	Eu:Sca	1989	CC	436	n	bl	n	n	6	all/unsp	20	29	1	2	nev any	ot
AXELSS	521	m	0	0	sca	-	all	Eu:Sca	1989	CC	436	n	bl	n	n	6	all/unsp	30	39	2	3	nev any	ot
AXELSS	522	m	0	0	sca	-	all	Eu:Sca	1989	CC	436	n	bl	n	n	6	all/unsp	40	49	0	4	nev any	ot
AXELSS	523	m	0	0	sca	-	all	Eu:Sca	1989	CC	436	n	bl	n	n	6	all/unsp	50	999	3	0	nev any	ot
AXELSS	510	f	0	0	sca	-	all	Eu:Sca	1989	CC	436	n	bl	n	n	0	all/unsp	1	19	0	1	nev any	st
AXELSS	511	f	0	0	sca	-	all	Eu:Sca	1989	CC	436	n	bl	n	n	0	all/unsp	20	29	1	2	nev any	st
AXELSS	512	f	0	0	sca	-	all	Eu:Sca	1989	CC	436	n	bl	n	n	0	all/unsp	30	39	2	3	nev any	st
AXELSS	513	f	0	0	sca	-	all	Eu:Sca	1989	CC	436	n	bl	n	n	0	all/unsp	40	49	0	4	nev any	st
AXELSS	514	f	0	0	sca	-	all	Eu:Sca	1989	CC	436	n	bl	n	n	0	all/unsp	50	999	3	0	nev any	st
BARBON	508	m	0	0	all	-	all	Eu:wst	1979	CC	755	n	bl	y	y	1	all/unsp	1	29	1	0	nev any	or
BARBON	509	m	0	0	all	-	all	Eu:wst	1979	CC	755	n	bl	y	y	1	all/unsp	30	39	2	3	nev any	or
BARBON	510	m	0	0	all	-	all	Eu:wst	1979	CC	755	n	bl	y	y	1	all/unsp	40	49	0	4	nev any	or
BARBON	511	m	0	0	all	-	all	Eu:wst	1979	CC	755	n	bl	y	y	1	all/unsp	50	999	3	0	nev any	or
BOUCOT	518	m	0	0	all	9	all	NAmer	1951	pr	121	n	bl	n	n	0	cig+/-ot	1	39	0	0	nev any	ot
BOUCOT	519	m	0	0	all	9	all	NAmer	1951	pr	121	n	bl	n	n	0	cig+/-ot	40	999	3	0	nev any	ot
BUFFLE	526	f	0	0	w-hi	-	all	NAmer	1976	CC	943	n	bl	y	n	0	cig+/-ot	1	30	1	0	nev cigs	or
BUFFLE	527	f	0	0	w-hi	-	all	NAmer	1976	CC	943	n	bl	y	n	0	cig+/-ot	31	40	2	4	nev cigs	or
BUFFLE	528	f	0	0	w-hi	-	all	NAmer	1976	CC	943	n	bl	y	n	0	cig+/-ot	41	999	3	0	nev cigs	or
CHEN2	501	m	0	0	all	-	all	As:Chi	1983	CC	193	n	ot	y	n	0	all/unsp	1	9	0	1	nev any	st
CHEN2	502	m	0	0	all	-	all	As:Chi	1983	CC	193	n	ot	y	n	0	all/unsp	10	20	1	2	nev any	st
CHEN2	503	m	0	0	all	-	all	As:Chi	1983	CC	193	n	ot	y	n	0	all/unsp	21	30	0	3	nev any	st
CHEN2	504	m	0	0	all	-	all	As:Chi	1983	CC	193	n	ot	y	n	0	all/unsp	31	40	2	4	nev any	st
CHEN2	505	m	0	0	all	-	all	As:Chi	1983	CC	193	n	ot	y	n	0	all/unsp	41	999	3	0	nev any	st
CHEN2	510	f	0	0	all	-	all	As:Chi	1983	CC	193	n	ot	y	n	0	all/unsp	1	20	1	0	nev any	st
CHEN2	511	f	0	0	all	-	all	As:Chi	1983	CC	193	n	ot	y	n	0	all/unsp	21	30	0	3	nev any	st
CHEN2	512	f	0	0	all	-	all	As:Chi	1983	CC	193	n	ot	y	n	0	all/unsp	31	40	2	4	nev any	st
CHEN2	513	f	0	0	all	-	all	As:Chi	1983	CC	193	n	ot	y	n	0	all/unsp	41	999	3	0	nev any	st
CHOI	501	m	0	0	all	-	all	As:oth	1985	CC	375	n	bl	n	n	0	cig+/-ot	1	19	0	1	nev cigs	st
CHOI	502	m	0	0	all	-	all	As:oth	1985	CC	375	n	bl	n	n	0	cig+/-ot	20	29	1	2	nev cigs	st
CHOI	503	m	0	0	all	-	all	As:oth	1985	CC	375	n	bl	n	n	0	cig+/-ot	30	39	2	3	nev cigs	st
CHOI	504	m	0	0	all	-	all	As:oth	1985	CC	375	n	bl	n	n	0	cig+/-ot	40	49	0	4	nev cigs	st
CHOI	505	m	0	0	all	-	all	As:oth	1985	CC	375	n	bl	n	n	0	cig+/-ot	50	999	3	0	nev cigs	st
CHOI	510	f	0	0	all	-	all	As:oth	1985	CC	375	n	bl	n	n	0	cig+/-ot	1	19	0	1	nev cigs	st
CHOI	511	f	0	0	all	-	all	As:oth	1985	CC	375	n	bl	n	n	0	cig+/-ot	20	29	1	2	nev cigs	st
CHOI	512	f	0	0	all	-	all	As:oth	1985	CC	375	n	bl	n	n	0	cig+/-ot	30	39	2	3	nev cigs	st
CHOI	513	f	0	0	all	-	all	As:oth	1985	CC	375	n	bl	n	n	0	cig+/-ot	40	999	3	0	nev cigs	st
DAMBER	506	m	0	0	all	-	all	Eu:Sca	1972	CC	579	n	bl	y	n	1	all/unsp	1	20	1	0	nev any	ot
DAMBER	507	m	0	0	all	-	all	Eu:Sca	1972	CC	579	n	bl	y	n	1	all/unsp	21	30	0	3	nev any	ot
DAMBER	508	m	0	0	all	-	all	Eu:Sca	1972	CC	579	n	bl	y	n	1	all/unsp	31	40	2	4	nev any	ot
DAMBER	509	m	0	0	all	-	all	Eu:Sca	1972	CC	579	n	bl	y	n	1	all/unsp	41	50	3	5	nev any	ot
DAMBER	510	m	0	0	all	-	all	Eu:Sca	1972	CC	579	n	bl	y	n	1	all/unsp	51	999	0	6	nev any	ot
DESTEF	508	m	0	0	all	-	all	SCAmer	1988	CC	497	n	bl	n	y	4	all/unsp	1	29	1	0	nev any	or
DESTEF	509	m	0	0	all	-	all	SCAmer	1988	CC	497	n	bl	n	y	4	all/unsp	30	39	2	3	nev any	or
DESTEF	510	m	0	0	all	-	all	SCAmer	1988	CC	497	n	bl	n	y	4	all/unsp	40	49	0	4	nev any	or
DESTEF	511	m	0	0	all	-	all	SCAmer	1988	CC	497	n	bl	n	y	4	all/unsp	50	999	3	0	nev any	or
DOLL	515	m	0	0	all	-	all	Eu:UK	1948	CC	1465	n	V	n	n	0	all/unsp	1	9	0	1	nev any	st
DOLL	516	m	0	0	all	-	all	Eu:UK	1948	CC	1465	n	V	n	n	0	all/unsp	10	19	0	0	nev any	st
DOLL	517	m	0	0	all	-	all	Eu:UK	1948	CC	1465	n	V	n	n	0	all/unsp	20	39	0	0	nev any	st
DOLL	518	m	0	0	all	-	all	Eu:UK	1948	CC	1465	n	V	n	n	0	all/unsp	40	999	3	0	nev any	st
DOLL	522	f	0	0	all	-	all	Eu:UK	1948	CC	1465	n	V	n	n	0	all/unsp	1	9	0	1	nev any	st
DOLL	523	f	0	0	all	-	all	Eu:UK	1948	CC	1465	n	V	n	n	0	all/unsp	10	19	0	0	nev any	st
DOLL	524	f	0	0	all	-	all	Eu:UK	1948	CC	1465	n	V	n	n	0	all/unsp	20	39	0	0	nev any	st
DOLL	525	f	0	0	all	-	all	Eu:UK	1948	CC	1465	n	V	n	n	0	all/unsp	40	999	3	0	nev any	st
DORGAN	570	m	0	0	wh	-	all	NAmer	1980	CC	2026	n	bl	y	y	2	cig+/-ot	1	34	1	0	nev any	ot
DORGAN	571	m	0	0	wh	-	all	NAmer	1980	CC	2026	n	bl	y	y	2	cig+/-ot	35	999	0	0	nev any	ot
DORGAN	562	f	0	0	all	-	all	NAmer	1980	CC	2026	n	bl	y	y	3	cig+/-ot	1	34	1	0	nev any	ot
DORGAN	563	f	0	0	all	-	all	NAmer	1980	CC	2026	n	bl	y	y	3	cig+/-ot	35	999	0	0	nev any	ot
DOSEME	501	m	0	0	all	-	all	Eu:bal	1979	CC	1210	n	bl	n	n	2	cig+/-ot	1	10	0	1	nev cigs	or
DOSEME	502	m	0	0	all	-	all	Eu:bal	1979	CC	1210	n	bl	n	n	2	cig+/-ot	11	20	1	2	nev cigs	or
DOSEME	503	m	0	0	all	-	all	Eu:bal	1979	CC	1210	n	bl	n	n	2	cig+/-ot	21	999	0	0	nev cigs	or
FAN	501	m	0	0	all	-	all	As:Chi	1990	CC	403	n	ot	y	n	0	cig+/-ot	1	29	1	0	nev cigs	st
FAN	502	m	0	0	all	-	all	As:Chi	1990	CC	403	n	ot	y	n	0	cig+/-ot	30	39	2	3	nev cigs	st
FAN	503	m	0	0	all	-	all	As:Chi	1990	CC	403	n	ot	y	n	0	cig+/-ot	40	999	3	0	nev cigs	st

Table 1I1 - 1

IESLC - Meta-analysis of Ever Smoking by Duration, Overview
All LC types, Any Product (or Cigarettes if Any not available)
Most adjusted

REF	NRR	SEX	AGEL	AGEH	RACE	YF	LC TYPE	LOC	START	ST	NLC	R	VB	P	H	AD	PRODUCT	exL	exH	S1	S2	DENOM	De
FAN	506	f	0	0	all	-	all	As:Chi	1990	CC	403	n	ot	y	n	0	cig+/-ot	1	29	1	0	nev cigs	st
FAN	507	f	0	0	all	-	all	As:Chi	1990	CC	403	n	ot	y	n	0	cig+/-ot	30	39	2	3	nev cigs	st
FAN	508	f	0	0	all	-	all	As:Chi	1990	CC	403	n	ot	y	n	0	cig+/-ot	40	999	3	0	nev cigs	st
GAO	564	f	0	0	all	-	all	As:Chi	1984	CC	1405	n	ot	n	n	2	cig+/-ot	1	29	1	0	nev cigs	ot
GAO	565	f	0	0	all	-	all	As:Chi	1984	CC	1405	n	ot	n	n	2	cig+/-ot	30	999	0	0	nev cigs	ot
GARSHI	536	m	0	0	all	-	all	NAmer	1981	CC	1081	o	bl	y	n	1	all/unsp	20	999	0	0	nev any	st
GER	518	c	0	0	all	-	all	As:oth	1990	CC	141	n	ot	y	n	5	all/unsp	1	20	1	0	nev any	ot
GER	519	c	0	0	all	-	all	As:oth	1990	CC	141	n	ot	y	n	5	all/unsp	21	40	2	0	nev any	ot
GER	520	c	0	0	all	-	all	As:oth	1990	CC	141	n	ot	y	n	5	all/unsp	41	999	3	0	nev any	ot
HAENSZ	542	f	0	0	all	-	not alv	NAmer	1955	CC	158	n	bl	n	y	0	cig+/-ot	1	14	0	1	nev any	st
HAENSZ	543	f	0	0	all	-	not alv	NAmer	1955	CC	158	n	bl	n	y	0	cig+/-ot	15	999	0	0	nev any	st
HU	501	m	0	0	all	-	all	As:Chi	1985	CC	227	n	ot	n	y	0	cig+/-ot	1	19	0	1	nev cigs	st
HU	502	m	0	0	all	-	all	As:Chi	1985	CC	227	n	ot	n	y	0	cig+/-ot	20	29	1	2	nev cigs	st
HU	503	m	0	0	all	-	all	As:Chi	1985	CC	227	n	ot	n	y	0	cig+/-ot	30	999	0	0	nev cigs	st
HU	506	f	0	0	all	-	all	As:Chi	1985	CC	227	n	ot	n	y	0	cig+/-ot	1	19	0	1	nev cigs	st
HU	507	f	0	0	all	-	all	As:Chi	1985	CC	227	n	ot	n	y	0	cig+/-ot	20	29	1	2	nev cigs	st
HU	508	f	0	0	all	-	all	As:Chi	1985	CC	227	n	ot	n	y	0	cig+/-ot	30	999	0	0	nev cigs	st
HU2	508	c	0	0	all	-	all	As:Chi	1977	CC	523	n	ot	y	n	0	cig+/-ot	1	19	0	1	nev cigs	ot
HU2	509	c	0	0	all	-	all	As:Chi	1977	CC	523	n	ot	y	n	0	cig+/-ot	20	29	1	2	nev cigs	or
HU2	510	c	0	0	all	-	all	As:Chi	1977	CC	523	n	ot	y	n	0	cig+/-ot	30	39	2	3	nev cigs	or
HU2	511	c	0	0	all	-	all	As:Chi	1977	CC	523	n	ot	y	n	0	cig+/-ot	40	999	3	0	nev cigs	st
JOLY	515	m	0	0	all	-	all	SCAmer	1978	CC	826	n	bl	n	n	0	cig+/-ot	1	19	0	1	nev any	st
JOLY	516	m	0	0	all	-	all	SCAmer	1978	CC	826	n	bl	n	n	0	cig+/-ot	20	29	1	2	nev any	st
JOLY	517	m	0	0	all	-	all	SCAmer	1978	CC	826	n	bl	n	n	0	cig+/-ot	30	39	2	3	nev any	st
JOLY	518	m	0	0	all	-	all	SCAmer	1978	CC	826	n	bl	n	n	0	cig+/-ot	40	49	0	4	nev any	st
JOLY	519	m	0	0	all	-	all	SCAmer	1978	CC	826	n	bl	n	n	0	cig+/-ot	50	999	3	0	nev any	st
JOLY	501	f	0	0	all	-	all	SCAmer	1978	CC	826	n	bl	n	n	0	cig+/-ot	1	19	0	1	nev any	st
JOLY	502	f	0	0	all	-	all	SCAmer	1978	CC	826	n	bl	n	n	0	cig+/-ot	20	29	1	2	nev any	st
JOLY	503	f	0	0	all	-	all	SCAmer	1978	CC	826	n	bl	n	n	0	cig+/-ot	30	39	2	3	nev any	st
JOLY	504	f	0	0	all	-	all	SCAmer	1978	CC	826	n	bl	n	n	0	cig+/-ot	40	49	0	4	nev any	st
JOLY	505	f	0	0	all	-	all	SCAmer	1978	CC	826	n	bl	n	n	0	cig+/-ot	50	999	3	0	nev any	st
JUSSAW	510	m	0	0	all	-	all	As:Ind	1964	CC	792	n	V	n	n	0	cig only	1	9	0	1	nev any	st
JUSSAW	511	m	0	0	all	-	all	As:Ind	1964	CC	792	n	V	n	n	0	cig only	10	19	0	0	nev any	st
JUSSAW	512	m	0	0	all	-	all	As:Ind	1964	CC	792	n	V	n	n	0	cig only	20	29	1	2	nev any	st
JUSSAW	513	m	0	0	all	-	all	As:Ind	1964	CC	792	n	V	n	n	0	cig only	30	39	2	3	nev any	st
JUSSAW	514	m	0	0	all	-	all	As:Ind	1964	CC	792	n	V	n	n	0	cig only	40	999	3	0	nev any	st
KHUDER	501	m	0	0	all	-	all	NAmer	1985	CC	482	n	bl	n	y	0	cig+/-ot	1	29	1	0	nev cigs	st
KHUDER	502	m	0	0	all	-	all	NAmer	1985	CC	482	n	bl	n	y	0	cig+/-ot	30	49	2	0	nev cigs	st
KHUDER	503	m	0	0	all	-	all	NAmer	1985	CC	482	n	bl	n	y	0	cig+/-ot	50	999	3	0	nev cigs	st
KREUZE	517	m	0	0	all	-	all	Eu:Ger	1990	CC	2260	n	bl	n	n	3	all/unsp	1	19	0	1	nev any	st
KREUZE	518	m	0	0	all	-	all	Eu:Ger	1990	CC	2260	n	bl	n	n	3	all/unsp	20	999	0	0	nev any	ot
KREUZE	520	f	0	0	all	-	all	Eu:Ger	1990	CC	2260	n	bl	n	n	3	all/unsp	1	19	0	1	nev any	ot
KREUZE	521	f	0	0	all	-	all	Eu:Ger	1990	CC	2260	n	bl	n	n	3	all/unsp	20	999	0	0	nev any	ot
LETOUR	506	c	0	0	all	-	all	NAmer	1983	CC	738	n	V	y	y	0	cig+/-ot	1	24	1	0	nev cigs	st
LETOUR	507	c	0	0	all	-	all	NAmer	1983	CC	738	n	V	y	y	0	cig+/-ot	25	40	2	0	nev cigs	st
LETOUR	508	c	0	0	all	-	all	NAmer	1983	CC	738	n	V	y	y	0	cig+/-ot	41	999	3	0	nev cigs	st
LEVIN	506	m	0	0	all	-	all	NAmer	1938	CC	475	n	bl	n	n	1	cig+/-ot	1	39	0	0	nev any	ot
LEVIN	507	m	0	0	all	-	all	NAmer	1938	CC	475	n	bl	n	n	1	cig+/-ot	40	999	3	0	nev any	ot
LIU3	510	m	0	0	all	-	all	As:Chi	1985	CC	110	n	ot	n	n	2	all/unsp	1	34	1	0	nev any	or
LIU3	511	m	0	0	all	-	all	As:Chi	1985	CC	110	n	ot	n	n	2	all/unsp	35	999	0	0	nev any	or
LIU5	504	c	0	0	all	-	all	As:Chi	1978	CC	111	n	ot	y	n	0	all/unsp	1	29	1	0	nev any	st
LIU5	505	c	0	0	all	-	all	As:Chi	1978	CC	111	n	ot	y	n	0	all/unsp	30	999	0	0	nev any	st
LUBIN	508	m	0	0	all	-	all	As:Chi	1984	CC	427	m	ot	y	n	0	cig+/-ot	1	29	1	0	nev any	st
LUBIN	509	m	0	0	all	-	all	As:Chi	1984	CC	427	m	ot	y	n	0	cig+/-ot	30	39	2	3	nev any	st
LUBIN	510	m	0	0	all	-	all	As:Chi	1984	CC	427	m	ot	y	n	0	cig+/-ot	40	49	0	4	nev any	st
LUBIN	511	m	0	0	all	-	all	As:Chi	1984	CC	427	m	ot	y	n	0	cig+/-ot	50	999	3	0	nev any	st
LUBIN2	531	m	0	0	all	-	all	Eu:mul	1976	CC	7804	n	bl	n	y	0	cig+/-ot	1	29	1	0	nev any	st
LUBIN2	532	m	0	0	all	-	all	Eu:mul	1976	CC	7804	n	bl	n	y	0	cig+/-ot	30	39	2	3	nev any	st
LUBIN2	533	m	0	0	all	-	all	Eu:mul	1976	CC	7804	n	bl	n	y	0	cig+/-ot	40	49	0	4	nev any	st
LUBIN2	534	m	0	0	all	-	all	Eu:mul	1976	CC	7804	n	bl	n	y	0	cig+/-ot	50	999	3	0	nev any	st
LUBIN2	574	f	0	0	all	-	all	Eu:mul	1976	CC	7804	n	bl	n	y	0	cig+/-ot	1	29	1	0	nev any	st
LUBIN2	575	f	0	0	all	-	all	Eu:mul	1976	CC	7804	n	bl	n	y	0	cig+/-ot	30	39	2	3	nev any	st
LUBIN2	576	f	0	0	all	-	all	Eu:mul	1976	CC	7804	n	bl	n	y	0	cig+/-ot	40	49	0	4	nev any	st
LUBIN2	577	f	0	0	all	-	all	Eu:mul	1976	CC	7804	n	bl	n	y	0	cig+/-ot	50	999	3	0	nev any	st
MATOS	536	m	0	0	all	-	all	SCAmer	1994	CC	200	n	bl	n	n	2	cig+/-ot	1	24	1	0	nev any	or
MATOS	537	m	0	0	all	-	all	SCAmer	1994	CC	200	n	bl	n	n	2	cig+/-ot	25	39	2	3	nev any	or
MATOS	538	m	0	0	all	-	all	SCAmer	1994	CC	200	n	bl	n	n	2	cig+/-ot	40	70	3	0	nev any	or
MCCONN	501	c	0	0	all	-	all	Eu:UK	1946	CC	100	n	V	n	y	0	all/unsp	1	9	0	1	nev any	st
MCCONN	502	c	0	0	all	-	all	Eu:UK	1946	CC	100	n	V	n	y	0	all/unsp	10	19	0	0	nev any	st
MCCONN	503	c	0	0	all	-	all	Eu:UK	1946	CC	100	n	V	n	y	0	all/unsp	20	29	1	2	nev any	st
MCCONN	504	c	0	0	all	-	all	Eu:UK	1946	CC	100	n	V	n	y	0	all/unsp	30	39	2	3	nev any	st
MCCONN	505	c	0	0	all	-	all	Eu:UK	1946	CC	100	n	V	n	y	0	all/unsp	40	999	3	0	nev any	st

Table 1I1 - 1

IESLC - Meta-analysis of Ever Smoking by Duration, Overview
All LC types, Any Product (or Cigarettes if Any not available)
Most adjusted

REF	NRR	SEX	AGEL	AGEH	RACE	YF	LC TYPE	LOC	START	ST	NLC	R	VB	P	H	AD	PRODUCT	exL	exH	S1	S2	DENOM	De
NOTAN2	513	c	0	0	all	-	all	As:Ind	1963	CC	683	n	V	n	n	0	cig only	1	10	0	1	nev any	st
NOTAN2	514	c	0	0	all	-	all	As:Ind	1963	CC	683	n	V	n	n	0	cig only	11	20	1	2	nev any	st
NOTAN2	515	c	0	0	all	-	all	As:Ind	1963	CC	683	n	V	n	n	0	cig only	21	30	0	3	nev any	st
NOTAN2	516	c	0	0	all	-	all	As:Ind	1963	CC	683	n	V	n	n	0	cig only	31	40	2	4	nev any	st
NOTAN2	517	c	0	0	all	-	all	As:Ind	1963	CC	683	n	V	n	n	0	cig only	41	999	3	0	nev any	st
OSANN2	504	f	0	0	all	-	all	NAmer	1964	ot	217	n	bl	n	y	1	cig+/-ot	1	20	1	0	nev cigs	or
OSANN2	505	f	0	0	all	-	all	NAmer	1964	ot	217	n	bl	n	y	1	cig+/-ot	21	999	0	0	nev cigs	or
PEZZOT	534	m	0	0	all	-	all	SCAmer	1987	CC	215	n	bl	n	y	0	cig only	1	30	1	0	nev cigs	st
PEZZOT	535	m	0	0	all	-	all	SCAmer	1987	CC	215	n	bl	n	y	0	cig only	31	40	2	4	nev cigs	st
PEZZOT	536	m	0	0	all	-	all	SCAmer	1987	CC	215	n	bl	n	y	0	cig only	41	999	3	0	nev cigs	st
QIAO2	516	m	0	0	all	0	all	As:Chi	1992	pr	241	m	ot	n	n	1	all/unsp	1	27	1	0	nev any	or
QIAO2	517	m	0	0	all	0	all	As:Chi	1992	pr	241	m	ot	n	n	1	all/unsp	28	41	2	0	nev any	or
QIAO2	518	m	0	0	all	0	all	As:Chi	1992	pr	241	m	ot	n	n	1	all/unsp	42	999	3	0	nev any	or
RACHTA	516	f	0	0	all	-	all	Eu:est	1991	CC	118	n	bl	n	y	1	cig+/-ot	1	20	1	0	nev cigs	or
RACHTA	517	f	0	0	all	-	all	Eu:est	1991	CC	118	n	bl	n	y	1	cig+/-ot	21	40	2	0	nev cigs	or
RACHTA	518	f	0	0	all	-	all	Eu:est	1991	CC	118	n	bl	n	y	1	cig+/-ot	41	999	3	0	nev cigs	or
TIZZAN	501	m	0	0	all	-	all	Eu:wst	1959	CC	1358	n	bl	n	n	0	cig only	1	4	0	0	nev any	st
TIZZAN	502	m	0	0	all	-	all	Eu:wst	1959	CC	1358	n	bl	n	n	0	cig only	5	10	0	1	nev any	st
TIZZAN	503	m	0	0	all	-	all	Eu:wst	1959	CC	1358	n	bl	n	n	0	cig only	11	999	0	0	nev any	st
TIZZAN	533	f	0	0	all	-	all	Eu:wst	1959	CC	1358	n	bl	n	n	0	all/unsp	1	10	0	1	nev any	st
TIZZAN	534	f	0	0	all	-	all	Eu:wst	1959	CC	1358	n	bl	n	n	0	all/unsp	11	999	0	0	nev any	st
WANG2	501	c	0	0	all	-	all	As:Chi	1980	CC	103	n	ot	n	n	0	cig+/-ot	1	19	0	1	nev cigs	st
WANG2	503	c	0	0	all	-	all	As:Chi	1980	CC	103	n	ot	n	n	0	cig+/-ot	20	29	1	2	nev cigs	st
WANG2	504	c	0	0	all	-	all	As:Chi	1980	CC	103	n	ot	n	n	0	cig+/-ot	30	39	2	3	nev cigs	st
WANG2	505	c	0	0	all	-	all	As:Chi	1980	CC	103	n	ot	n	n	0	cig+/-ot	40	49	0	4	nev cigs	st
WUWILL	516	f	0	0	all	-	all	As:Chi	1985	CC	965	n	ot	n	n	3	cig+/-ot	1	29	1	0	nev cigs	ot
WUWILL	517	f	0	0	all	-	all	As:Chi	1985	CC	965	n	ot	n	n	3	cig+/-ot	30	39	2	3	nev cigs	ot
WUWILL	518	f	0	0	all	-	all	As:Chi	1985	CC	965	n	ot	n	n	3	cig+/-ot	40	999	3	0	nev cigs	ot
ZHENG	553	m	0	0	all	-	all	As:Chi	1982	CC	540	n	ot	*	y	0	cig+/-ot	1	29	1	0	nev cigs	st
ZHENG	554	m	0	0	all	-	all	As:Chi	1982	CC	540	n	ot	*	y	0	cig+/-ot	30	999	0	0	nev cigs	st
ZHENG	558	f	0	0	all	-	all	As:Chi	1982	CC	540	n	ot	*	y	0	cig+/-ot	1	29	1	0	nev cigs	st
ZHENG	559	f	0	0	all	-	all	As:Chi	1982	CC	540	n	ot	*	y	0	cig+/-ot	30	999	0	0	nev cigs	st
ZHOU	501	c	0	0	all	-	all	As:Chi	1978	CC	1360	n	ot	n	n	0	all/unsp	1	19	0	1	nev any	st
ZHOU	502	c	0	0	all	-	all	As:Chi	1978	CC	1360	n	ot	n	n	0	all/unsp	20	999	0	0	nev any	st

Cigarette type is all/unspec for all RRs

except for the following:

REF	NRR	CIGTYPE
JUSSAW	510	MC only
JUSSAW	511	MC only
JUSSAW	512	MC only
JUSSAW	513	MC only
JUSSAW	514	MC only
NOTAN2	513	MC only
NOTAN2	514	MC only
NOTAN2	515	MC only
NOTAN2	516	MC only
NOTAN2	517	MC only

In this overview table, subtotals and Qs values may be invalid and should be ignored

Table 1I1 - 2

IESLC - Meta-analysis of Ever Smoking by Duration, Overview
All LC types, Any Product (or Cigarettes if Any not available)
Most adjusted

REF	NRR	SEX	AD	Number Exposed Case	Cont	Non-exposed Case	Cont	RR	95.00%CI
AGUDO	510	f	3	5	-	80	-	1.29	(0.40- 4.17)
AGUDO	511	f	3	18	-	80	-	5.09	(1.94- 13.35)
Subtotal AGUDO								2.92	(1.39- 6.16)
ARMADA	506	m	1	21	-	8	-	2.60	(1.00- 6.60)
ARMADA	507	m	1	219	-	8	-	11.90	(5.50- 25.50)
ARMADA	508	m	1	77	-	8	-	26.80	(11.00- 65.10)
Subtotal ARMADA								10.07	(6.14- 16.52)
AUVINE	517	c	2	26	-	44	-	20.10	(6.69- 66.00)
AUVINE	518	c	2	10	-	44	-	33.20	(14.30- 77.40)
AUVINE	519	c	2	230	-	44	-	30.40	(15.80- 58.40)
Subtotal AUVINE								29.13	(18.19- 46.66)
AXELSS	519	m	6	13	-	16	-	1.57	(0.70- 3.48)
AXELSS	520	m	6	17	-	16	-	2.23	(1.03- 4.80)
AXELSS	521	m	6	57	-	16	-	7.62	(4.01- 14.47)
AXELSS	522	m	6	104	-	16	-	11.81	(6.42- 21.73)
AXELSS	523	m	6	101	-	16	-	27.09	(13.94- 52.62)
AXELSS	510	f	0	5	24	18	154	1.78	(0.61- 5.25)
AXELSS	511	f	0	12	29	18	154	3.54	(1.54- 8.13)
AXELSS	512	f	0	29	26	18	154	9.54	(4.64- 19.61)
AXELSS	513	f	0	44	20	18	154	18.82	(9.17- 38.65)
AXELSS	514	f	0	20	10	18	154	17.11	(6.94- 42.19)
Subtotal AXELSS								7.77	(6.14- 9.83)
BARBON	508	m	1	42	-	22	-	3.20	(1.80- 5.70)
BARBON	509	m	1	118	-	22	-	7.90	(4.70- 13.50)
BARBON	510	m	1	207	-	22	-	11.40	(7.00- 18.80)
BARBON	511	m	1	366	-	22	-	14.50	(9.00- 23.30)
Subtotal BARBON								8.70	(6.73- 11.26)
*BOUCOT	518	m	0	29	2621	0	805	18.13~	(1.11- 296.36)
*BOUCOT	519	m	0	52	1563	0	805	54.09~	(3.34- 875.17)
Subtotal BOUCOT								31.38	(4.37- 225.47)
BUFFLE	526	f	0	52	57	12	112	8.51	(4.21- 17.22)
BUFFLE	527	f	0	97	62	12	112	14.60	(7.43- 28.69)
BUFFLE	528	f	0	90	42	12	112	20.00	(9.94- 40.23)
Subtotal BUFFLE								13.60	(9.12- 20.29)
CHEN2	501	m	0	2	3	9	33	2.44	(0.35- 16.93)
CHEN2	502	m	0	4	3	9	33	4.89	(0.92- 25.93)
CHEN2	503	m	0	17	24	9	33	2.60	(0.99- 6.81)
CHEN2	504	m	0	36	27	9	33	4.89	(2.01- 11.91)
CHEN2	505	m	0	62	40	9	33	5.68	(2.46- 13.13)
CHEN2	510	f	0	1	6	25	33	0.22	(0.02- 1.95)
CHEN2	511	f	0	2	2	25	33	1.32	(0.17- 10.03)
CHEN2	512	f	0	13	6	25	33	2.86	(0.95- 8.58)
CHEN2	513	f	0	21	15	25	33	1.85	(0.80- 4.29)
Subtotal CHEN2								3.01	(2.07- 4.38)
CHOI	501	m	0	19	55	13	95	2.52	(1.16- 5.51)
CHOI	502	m	0	66	166	13	95	2.91	(1.52- 5.54)
CHOI	503	m	0	102	160	13	95	4.66	(2.48- 8.75)
CHOI	504	m	0	60	64	13	95	6.85	(3.48- 13.50)
CHOI	505	m	0	20	20	13	95	7.31	(3.13- 17.07)
CHOI	510	f	0	2	9	76	164	0.48	(0.10- 2.27)
CHOI	511	f	0	8	14	76	164	1.23	(0.50- 3.06)
CHOI	512	f	0	8	2	76	164	8.63	(1.79- 41.62)
CHOI	513	f	0	1	1	76	164	2.16	(0.13- 34.96)
Subtotal CHOI								3.63	(2.73- 4.83)
DAMBER	506	m	1	-	-	42	-	1.58	(0.69- 3.66)
DAMBER	507	m	1	-	-	42	-	3.66	(2.18- 6.73)
DAMBER	508	m	1	-	-	42	-	5.15	(3.27- 8.32)
DAMBER	509	m	1	-	-	42	-	8.71	(5.84- 13.66)
DAMBER	510	m	1	-	-	42	-	11.19	(7.43- 17.33)
Subtotal DAMBER								6.42	(5.14- 8.01)
DESTEF	508	m	4	43	-	27	-	3.40	(1.70- 6.80)
DESTEF	509	m	4	78	-	27	-	5.20	(2.90- 8.90)
DESTEF	510	m	4	171	-	27	-	10.40	(6.40- 16.90)
DESTEF	511	m	4	178	-	27	-	10.80	(6.60- 17.60)
Subtotal DESTEF								7.55	(5.76- 9.90)
DOLL	515	m	0	12	15	7	61	6.97	(2.34- 20.73)
DOLL	516	m	0	34	65	7	61	4.56	(1.88- 11.05)
DOLL	517	m	0	746	725	7	61	8.97	(4.07- 19.73)
DOLL	518	m	0	558	491	7	61	9.90	(4.49- 21.85)
DOLL	522	f	0	14	18	40	59	1.15	(0.51- 2.57)
DOLL	523	f	0	12	8	40	59	2.21	(0.83- 5.90)

Table 1I1 - 2

IESLC - Meta-analysis of Ever Smoking by Duration, Overview
All LC types, Any Product (or Cigarettes if Any not available)
Most adjusted

REF	NRR	SEX	AD	Number Exposed Case	Cont	Non-exposed Case	Cont	RR	95.00%CI
DOLL	524	f	0	36	20	40	59	2.66	(1.35- 5.23)
DOLL	525	f	0	6	3	40	59	2.95	(0.70- 12.49)
Subtotal DOLL								3.93	(2.89- 5.34)
DORGAN	570	m	2	-	-	-	-	5.44	(2.97- 9.98)
DORGAN	571	m	2	-	-	-	-	16.09	(8.96- 28.88)
DORGAN	562	f	3	-	-	-	-	4.25	(3.20- 5.64)
DORGAN	563	f	3	-	-	-	-	11.73	(9.07- 15.18)
Subtotal DORGAN								7.74	(6.50- 9.20)
DOSEME	501	m	2	32	-	142	-	1.00	(0.60- 1.70)
DOSEME	502	m	2	158	-	142	-	3.80	(2.60- 5.70)
DOSEME	503	m	2	466	-	142	-	4.90	(3.50- 7.00)
Subtotal DOSEME								3.27	(2.59- 4.12)
FAN	501	m	0	29	135	36	236	1.41	(0.83- 2.40)
FAN	502	m	0	44	122	36	236	2.36	(1.45- 3.87)
FAN	503	m	0	143	241	36	236	3.89	(2.59- 5.84)
FAN	506	f	0	8	15	69	320	2.47	(1.01- 6.06)
FAN	507	f	0	19	23	69	320	3.83	(1.98- 7.42)
FAN	508	f	0	55	59	69	320	4.32	(2.76- 6.78)
Subtotal FAN								3.01	(2.43- 3.72)
GAO	564	f	2	68	-	435	-	1.89	(1.30- 2.75)
GAO	565	f	2	168	-	435	-	4.58	(3.33- 6.30)
Subtotal GAO								3.16	(2.48- 4.03)
GARSHI	536	m	1	922	-	41	-	6.28	(4.49- 8.77)
GER	518	c	5	10	-	51	-	1.30	(0.55- 3.06)
GER	519	c	5	31	-	51	-	1.56	(0.83- 2.91)
GER	520	c	5	49	-	51	-	2.14	(1.18- 3.90)
Subtotal GER								1.72	(1.17- 2.52)
HAENSZ	542	f	0	16	26	81	236	1.79	(0.92- 3.51)
HAENSZ	543	f	0	58	77	81	236	2.19	(1.44- 3.35)
Subtotal HAENSZ								2.07	(1.45- 2.97)
HU	501	m	0	41	33	41	67	2.03	(1.11- 3.70)
HU	502	m	0	60	47	41	67	2.09	(1.21- 3.60)
HU	503	m	0	19	14	41	67	2.22	(1.00- 4.90)
HU	506	f	0	11	8	40	48	1.65	(0.61- 4.50)
HU	507	f	0	11	7	40	48	1.89	(0.67- 5.32)
HU	508	f	0	4	3	40	48	1.60	(0.34- 7.57)
Subtotal HU								2.00	(1.46- 2.74)
HU2	508	c	0	21	33	121	213	1.12	(0.62- 2.02)
HU2	509	c	0	64	63	121	213	1.79	(1.18- 2.70)
HU2	510	c	0	123	101	121	213	2.14	(1.52- 3.03)
HU2	511	c	0	194	113	121	213	3.02	(2.19- 4.17)
Subtotal HU2								2.18	(1.79- 2.64)
JOLY	515	m	0	11	48	12	218	4.16	(1.73- 9.99)
JOLY	516	m	0	38	61	12	218	11.32	(5.57- 22.98)
JOLY	517	m	0	85	165	12	218	9.36	(4.95- 17.70)
JOLY	518	m	0	168	182	12	218	16.77	(9.04- 31.11)
JOLY	519	m	0	250	253	12	218	17.95	(9.78- 32.93)
JOLY	501	f	0	13	28	52	283	2.53	(1.23- 5.20)
JOLY	502	f	0	18	26	52	283	3.77	(1.93- 7.36)
JOLY	503	f	0	31	24	52	283	7.03	(3.82- 12.93)
JOLY	504	f	0	47	24	52	283	10.66	(6.00- 18.92)
JOLY	505	f	0	57	20	52	283	15.51	(8.61- 27.95)
Subtotal JOLY								9.03	(7.36- 11.08)
JUSSAW	510	m	0	16	20	149	624	3.35	(1.70- 6.62)
JUSSAW	511	m	0	34	19	149	624	7.49	(4.16- 13.51)
JUSSAW	512	m	0	38	23	149	624	6.92	(4.00- 11.97)
JUSSAW	513	m	0	27	9	149	624	12.56	(5.79- 27.28)
JUSSAW	514	m	0	11	6	149	624	7.68	(2.79- 21.09)
Subtotal JUSSAW								6.77	(5.01- 9.15)
KHUDER	501	m	0	16	61	23	309	3.52	(1.76- 7.06)
KHUDER	502	m	0	207	370	23	309	7.52	(4.76- 11.86)
KHUDER	503	m	0	236	354	23	309	8.96	(5.69- 14.11)
Subtotal KHUDER								7.07	(5.28- 9.47)
KREUZE	517	m	3	-	-	-	-	4.70	(3.10- 7.14)
KREUZE	518	m	3	-	-	-	-	29.23	(19.78- 43.20)
KREUZE	520	f	3	-	-	-	-	1.33	(0.80- 2.22)
KREUZE	521	f	3	-	-	-	-	7.14	(4.92- 10.35)
Subtotal KREUZE								7.26	(5.90- 8.93)
LETOUR	506	c	0	65	187	24	224	3.24	(1.95- 5.39)
LETOUR	507	c	0	264	160	24	224	15.40	(9.68- 24.51)
LETOUR	508	c	0	374	141	24	224	24.76	(15.58- 39.35)

Table 1I1 - 2

IESLC - Meta-analysis of Ever Smoking by Duration, Overview
All LC types, Any Product (or Cigarettes if Any not available)
Most adjusted

REF	NRR	SEX	AD	Number Exposed Case	Number Exposed Cont	Non-exposed Case	Non-exposed Cont	RR	95.00%CI
Subtotal LETOUR								11.50	(8.73- 15.14)
LEVIN	506	m	1	56	-	7	-	7.07	(3.07- 16.29)
LEVIN	507	m	1	63	-	7	-	8.96	(3.90- 20.61)
Subtotal LEVIN								7.96	(4.42- 14.35)
LIU3	510	m	2	30	-	4	-	1.07	(0.25- 4.59)
LIU3	511	m	2	22	-	4	-	1.71	(0.36- 8.12)
Subtotal LIU3								1.33	(0.46- 3.86)
LIU5	504	c	0	27	37	26	41	1.15	(0.57- 2.31)
LIU5	505	c	0	58	33	26	41	2.77	(1.45- 5.32)
Subtotal LIU5								1.84	(1.14- 2.96)
LUBIN	508	m	0	30	146	8	72	1.85	(0.81- 4.24)
LUBIN	509	m	0	124	294	8	72	3.80	(1.78- 8.12)
LUBIN	510	m	0	143	251	8	72	5.13	(2.40- 10.95)
LUBIN	511	m	0	59	86	8	72	6.17	(2.77- 13.77)
Subtotal LUBIN								3.94	(2.66- 5.83)
LUBIN2	531	m	0	953	2995	190	2616	4.38	(3.72- 5.16)
LUBIN2	532	m	0	2227	3470	190	2616	8.84	(7.56- 10.33)
LUBIN2	533	m	0	2079	2551	190	2616	11.22	(9.58- 13.14)
LUBIN2	534	m	0	1325	1484	190	2616	12.29	(10.42- 14.50)
LUBIN2	574	f	0	132	230	336	1188	2.03	(1.59- 2.59)
LUBIN2	575	f	0	187	186	336	1188	3.55	(2.81- 4.50)
LUBIN2	576	f	0	155	118	336	1188	4.64	(3.55- 6.07)
LUBIN2	577	f	0	81	32	336	1188	8.95	(5.84- 13.71)
Subtotal LUBIN2								6.83	(6.37- 7.32)
MATOS	536	m	2	20	-	11	-	2.20	(1.00- 4.90)
MATOS	537	m	2	82	-	11	-	7.20	(3.60- 14.50)
MATOS	538	m	2	86	-	11	-	12.70	(6.10- 26.10)
Subtotal MATOS								6.23	(4.07- 9.53)
MCCONN	501	c	0	3	4	9	23	1.92	(0.36- 10.32)
MCCONN	502	c	0	5	19	9	23	0.67	(0.19- 2.35)
MCCONN	503	c	0	46	57	9	23	2.06	(0.87- 4.89)
MCCONN	504	c	0	21	57	9	23	0.94	(0.38- 2.36)
MCCONN	505	c	0	16	40	9	23	1.02	(0.39- 2.68)
Subtotal MCCONN								1.22	(0.76- 1.94)
NOTAN2	513	c	0	7	15	107	201	0.88	(0.35- 2.22)
NOTAN2	514	c	0	15	15	107	201	1.88	(0.88- 3.99)
NOTAN2	515	c	0	17	16	107	201	2.00	(0.97- 4.11)
NOTAN2	516	c	0	12	7	107	201	3.22	(1.23- 8.42)
NOTAN2	517	c	0	5	5	107	201	1.88	(0.53- 6.63)
Subtotal NOTAN2								1.83	(1.24- 2.70)
OSANN2	504	f	1	23	-	33	-	1.60	(0.70- 3.50)
OSANN2	505	f	1	161	-	23	-	11.60	(5.80- 23.30)
Subtotal OSANN2								4.97	(2.94- 8.42)
PEZZOT	534	m	0	30	134	4	116	6.49	(2.22- 18.98)
PEZZOT	535	m	0	71	82	4	116	25.11	(8.82- 71.48)
PEZZOT	536	m	0	110	101	4	116	31.58	(11.25- 88.71)
Subtotal PEZZOT								17.64	(9.62- 32.34)
*QIAO2	516	m	1	7	-	10	-	0.40	(0.15- 1.05)
*QIAO2	517	m	1	54	-	10	-	1.46	(0.74- 2.87)
*QIAO2	518	m	1	170	-	10	-	2.05	(1.06- 3.94)
Subtotal QIAO2								1.32	(0.86- 2.01)
RACHTA	516	f	1	12	-	33	-	2.02	(0.87- 4.71)
RACHTA	517	f	1	49	-	33	-	7.55	(3.90- 14.63)
RACHTA	518	f	1	24	-	33	-	58.68	(7.56- 455.64)
Subtotal RACHTA								5.34	(3.22- 8.84)
TIZZAN	501	m	0	12	1	180	305	20.33	(2.62- 157.68)
TIZZAN	502	m	0	54	20	180	305	4.58	(2.65- 7.89)
TIZZAN	503	m	0	928	815	180	305	1.93	(1.57- 2.37)
TIZZAN	533	f	0	2	7	117	114	0.28	(0.06- 1.37)
TIZZAN	534	f	0	23	21	117	114	1.07	(0.56- 2.03)
Subtotal TIZZAN								2.01	(1.68- 2.42)
WANG2	501	c	0	4	17	11	43	0.92	(0.26- 3.29)
WANG2	503	c	0	8	18	11	43	1.74	(0.60- 5.03)
WANG2	504	c	0	26	38	11	43	2.67	(1.17- 6.13)
WANG2	505	c	0	22	26	11	43	3.31	(1.38- 7.91)
Subtotal WANG2								2.24	(1.38- 3.63)
WUWILL	516	f	3	137	-	417	-	1.35	(1.04- 1.73)
WUWILL	517	f	3	179	-	417	-	2.71	(2.05- 3.60)
WUWILL	518	f	3	223	-	417	-	3.49	(2.65- 4.59)
Subtotal WUWILL								2.27	(1.94- 2.65)
ZHENG	553	m	0	37	75	33	94	1.41	(0.80- 2.46)

Table 1I1 - 2

IESLC - Meta-analysis of Ever Smoking by Duration, Overview
All LC types, Any Product (or Cigarettes if Any not available)
Most adjusted

REF	NRR	SEX	AD	Number Exposed Case	Number Exposed Cont	Non-exposed Case	Non-exposed Cont	RR	95.00%CI
ZHENG	554	m	0	242	143	33	94	4.82	(3.08- 7.54)
ZHENG	558	f	0	17	17	152	184	1.21	(0.60- 2.45)
ZHENG	559	f	0	59	27	152	184	2.65	(1.60- 4.38)
Subtotal ZHENG								2.54	(1.94- 3.31)
ZHOU	501	c	0	170	12	507	68	1.90	(1.00- 3.60)
ZHOU	502	c	0	678	36	507	68	2.53	(1.66- 3.84)
Subtotal ZHOU								2.32	(1.63- 3.29)
Partial Totals				20841	23455	12003	34246		

*prospective study ~ With 0.5 adjustment for zero

REF	NRR	SEX	AD	Ys	Ws	Qs	Ps
AGUDO	510	f	3	0.25	2.80	4.95	0.6702
AGUDO	511	f	3	1.63	4.13	0.01	0.0009
Subtotal AGUDO				1.07	6.93	4.96	
ARMADA	506	m	1	0.96	4.31	1.71	0.0472
ARMADA	507	m	1	2.48	6.53	5.19	0.0000
ARMADA	508	m	1	3.29	4.86	14.10	0.0000
Subtotal ARMADA				2.31	15.71	21.00	
AUVINE	517	c	2	3.00	2.93	5.87	0.0000
AUVINE	518	c	2	3.50	5.39	19.80	0.0000
AUVINE	519	c	2	3.41	8.99	30.08	0.0000
Subtotal AUVINE				3.37	17.31	55.76	
AXELSS	519	m	6	0.45	5.97	7.69	0.2702
AXELSS	520	m	6	0.80	6.49	3.98	0.0411
AXELSS	521	m	6	2.03	9.33	1.85	0.0000
AXELSS	522	m	6	2.47	10.34	8.07	0.0000
AXELSS	523	m	6	3.30	8.71	25.58	0.0000
AXELSS	510	f	0	0.58	3.29	3.34	0.2943
AXELSS	511	f	0	1.26	5.56	0.57	0.0029
AXELSS	512	f	0	2.26	7.41	3.33	0.0000
AXELSS	513	f	0	2.94	7.42	13.52	0.0000
AXELSS	514	f	0	2.84	4.72	7.42	0.0000
Subtotal AXELSS				2.05	69.23	75.35	
BARBON	508	m	1	1.16	11.56	2.06	0.0001
BARBON	509	m	1	2.07	13.80	3.20	0.0000
BARBON	510	m	1	2.43	15.74	11.33	0.0000
BARBON	511	m	1	2.67	16.98	20.13	0.0000
Subtotal BARBON				2.16	58.09	36.72	
*BOUCOT	518	m	0	2.90	0.49	0.85	0.0421
*BOUCOT	519	m	0	3.99	0.50	2.87	0.0050
Subtotal BOUCOT				3.45	0.99	3.72	
BUFFLE	526	f	0	2.14	7.75	2.40	0.0000
BUFFLE	527	f	0	2.68	8.42	10.12	0.0000
BUFFLE	528	f	0	3.00	7.86	15.64	0.0000
Subtotal BUFFLE				2.61	24.04	28.16	
CHEN2	501	m	0	0.89	1.03	0.49	0.3653
CHEN2	502	m	0	1.59	1.38	0.00	0.0623
CHEN2	503	m	0	0.95	4.13	1.65	0.0523
CHEN2	504	m	0	1.59	4.85	0.00	0.0005
CHEN2	505	m	0	1.74	5.48	0.13	0.0000
CHEN2	510	f	0	-1.51	0.81	7.77	0.1734
CHEN2	511	f	0	0.28	0.93	1.60	0.7884
CHEN2	512	f	0	1.05	3.19	0.91	0.0607
CHEN2	513	f	0	0.61	5.42	5.11	0.1529
Subtotal CHEN2				1.10	27.21	17.65	
CHOI	501	m	0	0.93	6.32	2.75	0.0199
CHOI	502	m	0	1.07	9.21	2.48	0.0012
CHOI	503	m	0	1.54	9.66	0.02	0.0000
CHOI	504	m	0	1.92	8.35	0.96	0.0000
CHOI	505	m	0	1.99	5.33	0.87	0.0000
CHOI	510	f	0	-0.73	1.59	8.54	0.3546
CHOI	511	f	0	0.21	4.64	8.78	0.6519
CHOI	512	f	0	2.16	1.55	0.50	0.0072
CHOI	513	f	0	0.77	0.50	0.33	0.5883
Subtotal CHOI				1.29	47.14	25.23	
DAMBER	506	m	1	0.46	5.52	7.02	0.2825
DAMBER	507	m	1	1.30	12.09	1.00	0.0000
DAMBER	508	m	1	1.64	17.62	0.05	0.0000
DAMBER	509	m	1	2.16	21.28	7.14	0.0000
DAMBER	510	m	1	2.42	21.42	14.75	0.0000

Table 1I1 - 2

IESLC - Meta-analysis of Ever Smoking by Duration, Overview
All LC types, Any Product (or Cigarettes if Any not available)
Most adjusted

REF	NRR	SEX	AD	Ys	Ws	Qs	Ps
Subtotal DAMBER				1.86	77.93	29.96	
DESTEF	508	m	4	1.22	8.00	1.05	0.0005
DESTEF	509	m	4	1.65	12.22	0.05	0.0000
DESTEF	510	m	4	2.34	16.30	9.33	0.0000
DESTEF	511	m	4	2.38	15.97	10.07	0.0000
Subtotal DESTEF				2.02	52.48	20.49	
DOLL	515	m	0	1.94	3.23	0.41	0.0005
DOLL	516	m	0	1.52	4.90	0.02	0.0008
DOLL	517	m	0	2.19	6.17	2.28	0.0000
DOLL	518	m	0	2.29	6.13	3.07	0.0000
DOLL	522	f	0	0.14	5.92	12.41	0.7383
DOLL	523	f	0	0.79	4.00	2.50	0.1124
DOLL	524	f	0	0.98	8.35	3.10	0.0048
DOLL	525	f	0	1.08	1.85	0.47	0.1417
Subtotal DOLL				1.37	40.55	24.26	
DORGAN	570	m	2	1.69	10.46	0.12	0.0000
DORGAN	571	m	2	2.78	11.22	15.96	0.0000
DORGAN	562	f	3	1.45	47.84	0.92	0.0000
DORGAN	563	f	3	2.46	57.93	44.54	0.0000
Subtotal DORGAN				2.05	127.45	61.54	
DOSEME	501	m	2	0.00	14.17	35.61	1.0000
DOSEME	502	m	2	1.34	24.94	1.56	0.0000
DOSEME	503	m	2	1.59	31.98	0.00	0.0000
Subtotal DOSEME				1.18	71.09	37.17	
FAN	501	m	0	0.34	13.53	20.91	0.2079
FAN	502	m	0	0.86	15.89	8.35	0.0006
FAN	503	m	0	1.36	23.17	1.19	0.0000
FAN	506	f	0	0.91	4.78	2.21	0.0478
FAN	507	f	0	1.34	8.79	0.52	0.0001
FAN	508	f	0	1.46	18.96	0.28	0.0000
Subtotal FAN				1.10	85.12	33.45	
GAO	564	f	2	0.64	27.37	24.64	0.0009
GAO	565	f	2	1.52	37.80	0.15	0.0000
Subtotal GAO				1.15	65.17	24.79	
GARSHI	536	m	1	1.84	34.28	2.18	0.0000
GER	518	c	5	0.26	5.22	9.13	0.5490
GER	519	c	5	0.44	9.76	12.70	0.1647
GER	520	c	5	0.76	10.75	7.31	0.0126
Subtotal GER				0.54	25.73	29.15	
HAENSZ	542	f	0	0.58	8.51	8.53	0.0886
HAENSZ	543	f	0	0.79	21.36	13.65	0.0003
Subtotal HAENSZ				0.73	29.87	22.18	
HU	501	m	0	0.71	10.64	8.18	0.0209
HU	502	m	0	0.74	12.94	9.35	0.0082
HU	503	m	0	0.80	6.12	3.81	0.0488
HU	506	f	0	0.50	3.82	4.49	0.3277
HU	507	f	0	0.63	3.58	3.24	0.2303
HU	508	f	0	0.47	1.59	1.98	0.5535
Subtotal HU				0.69	38.69	31.05	
HU2	508	c	0	0.11	11.00	23.84	0.7065
HU2	509	c	0	0.58	22.49	22.68	0.0058
HU2	510	c	0	0.76	32.27	21.85	0.0000
HU2	511	c	0	1.11	37.09	8.52	0.0000
Subtotal HU2				0.78	102.85	76.88	
JOLY	515	m	0	1.43	5.01	0.13	0.0014
JOLY	516	m	0	2.43	7.66	5.41	0.0000
JOLY	517	m	0	2.24	9.46	4.01	0.0000
JOLY	518	m	0	2.82	10.06	15.33	0.0000
JOLY	519	m	0	2.89	10.43	17.69	0.0000
JOLY	501	f	0	0.93	7.39	3.20	0.0118
JOLY	502	f	0	1.33	8.56	0.57	0.0001
JOLY	503	f	0	1.95	10.34	1.38	0.0000
JOLY	504	f	0	2.37	11.67	7.12	0.0000
JOLY	505	f	0	2.74	11.07	14.80	0.0000
Subtotal JOLY				2.20	91.65	69.63	
JUSSAW	510	m	0	1.21	8.28	1.17	0.0005
JUSSAW	511	m	0	2.01	11.07	2.03	0.0000
JUSSAW	512	m	0	1.93	12.80	1.56	0.0000
JUSSAW	513	m	0	2.53	6.39	5.71	0.0000
JUSSAW	514	m	0	2.04	3.76	0.77	0.0001
Subtotal JUSSAW				1.91	42.30	11.25	

Table 1I1 - 2

IESLC - Meta-analysis of Ever Smoking by Duration, Overview
All LC types, Any Product (or Cigarettes if Any not available)
Most adjusted

REF	NRR	SEX	AD	Ys	Ws	Qs	Ps
KHUDER	501	m	0	1.26	7.96	0.85	0.0004
KHUDER	502	m	0	2.02	18.43	3.44	0.0000
KHUDER	503	m	0	2.19	18.60	6.85	0.0000
Subtotal KHUDER				1.96	44.99	11.13	
KREUZE	517	m	3	1.55	22.07	0.03	0.0000
KREUZE	518	m	3	3.38	25.18	80.67	0.0000
KREUZE	520	f	3	0.29	14.75	24.94	0.2734
KREUZE	521	f	3	1.97	27.78	4.02	0.0000
Subtotal KREUZE				1.98	89.79	109.65	
LETOUR	506	c	0	1.18	14.96	2.50	0.0000
LETOUR	507	c	0	2.73	17.80	23.50	0.0000
LETOUR	508	c	0	3.21	17.89	47.17	0.0000
Subtotal LETOUR				2.44	50.65	73.17	
LEVIN	506	m	1	1.96	5.52	0.76	0.0000
LEVIN	507	m	1	2.19	5.54	2.05	0.0000
Subtotal LEVIN				2.07	11.06	2.80	
LIU3	510	m	2	0.07	1.81	4.18	0.9274
LIU3	511	m	2	0.54	1.58	1.74	0.4997
Subtotal LIU3				0.29	3.40	5.92	
LIU5	504	c	0	0.14	7.88	16.45	0.6935
LIU5	505	c	0	1.02	9.06	2.90	0.0022
Subtotal LIU5				0.61	16.94	19.35	
LUBIN	508	m	0	0.61	5.58	5.26	0.1463
LUBIN	509	m	0	1.33	6.65	0.42	0.0006
LUBIN	510	m	0	1.63	6.67	0.02	0.0000
LUBIN	511	m	0	1.82	5.97	0.33	0.0000
Subtotal LUBIN				1.37	24.88	6.03	
LUBIN2	531	m	0	1.48	142.28	1.66	0.0000
LUBIN2	532	m	0	2.18	156.67	55.19	0.0000
LUBIN2	533	m	0	2.42	153.41	106.30	0.0000
LUBIN2	534	m	0	2.51	141.36	120.61	0.0000
LUBIN2	574	f	0	0.71	63.53	48.94	0.0000
LUBIN2	575	f	0	1.27	68.77	6.91	0.0000
LUBIN2	576	f	0	1.54	53.35	0.13	0.0000
LUBIN2	577	f	0	2.19	21.09	7.75	0.0000
Subtotal LUBIN2				1.92	800.46	347.50	
MATOS	536	m	2	0.79	6.08	3.86	0.0518
MATOS	537	m	2	1.97	7.92	1.20	0.0000
MATOS	538	m	2	2.54	7.27	6.65	0.0000
Subtotal MATOS				1.83	21.27	11.71	
MCCONN	501	c	0	0.65	1.36	1.18	0.4488
MCCONN	502	c	0	-0.40	2.46	9.65	0.5341
MCCONN	503	c	0	0.72	5.16	3.83	0.1002
MCCONN	504	c	0	-0.06	4.55	12.32	0.8977
MCCONN	505	c	0	0.02	4.13	10.10	0.9644
Subtotal MCCONN				0.20	17.65	37.08	
NOTAN2	513	c	0	-0.13	4.47	13.17	0.7808
NOTAN2	514	c	0	0.63	6.77	6.18	0.1008
NOTAN2	515	c	0	0.69	7.37	5.90	0.0606
NOTAN2	516	c	0	1.17	4.16	0.72	0.0171
NOTAN2	517	c	0	0.63	2.41	2.20	0.3273
Subtotal NOTAN2				0.60	25.18	28.16	
OSANN2	504	f	1	0.47	5.93	7.38	0.2523
OSANN2	505	f	1	2.45	7.95	5.95	0.0000
Subtotal OSANN2				1.60	13.88	13.33	
PEZZOT	534	m	0	1.87	3.34	0.27	0.0006
PEZZOT	535	m	0	3.22	3.51	9.42	0.0000
PEZZOT	536	m	0	3.45	3.60	12.56	0.0000
Subtotal PEZZOT				2.87	10.45	22.25	
*QIAO2	516	m	1	-0.92	4.06	25.40	0.0649
*QIAO2	517	m	1	0.38	8.36	12.18	0.2738
*QIAO2	518	m	1	0.72	8.91	6.71	0.0321
Subtotal QIAO2				0.27	21.34	44.29	
RACHTA	516	f	1	0.70	5.39	4.19	0.1027
RACHTA	517	f	1	2.02	8.79	1.67	0.0000
RACHTA	518	f	1	4.07	0.91	5.66	0.0001
Subtotal RACHTA				1.68	15.09	11.52	
TIZZAN	501	m	0	3.01	0.92	1.86	0.0039
TIZZAN	502	m	0	1.52	12.93	0.05	0.0000
TIZZAN	503	m	0	0.66	89.78	77.34	0.0000
TIZZAN	533	f	0	-1.28	1.51	12.43	0.1155

Table 1I1 - 2

IESLC - Meta-analysis of Ever Smoking by Duration, Overview
All LC types, Any Product (or Cigarettes if Any not available)
Most adjusted

REF	NRR	SEX	AD	Ys	Ws	Qs	Ps
TIZZAN	534	f	0	0.06	9.22	21.32	0.8435
Subtotal TIZZAN				0.70	114.36	113.01	
WANG2	501	c	0	-0.08	2.36	6.59	0.8977
WANG2	503	c	0	0.55	3.39	3.62	0.3089
WANG2	504	c	0	0.98	5.59	2.02	0.0200
WANG2	505	c	0	1.20	5.05	0.76	0.0072
Subtotal WANG2				0.81	16.39	12.99	
WUWILL	516	f	3	0.30	59.33	98.01	0.0208
WUWILL	517	f	3	1.00	48.46	16.78	0.0000
WUWILL	518	f	3	1.25	50.92	5.73	0.0000
Subtotal WUWILL				0.82	158.72	120.52	
ZHENG	553	m	0	0.34	12.30	19.07	0.2328
ZHENG	554	m	0	1.57	19.21	0.00	0.0000
ZHENG	558	f	0	0.19	7.71	14.99	0.5957
ZHENG	559	f	0	0.97	15.15	5.69	0.0002
Subtotal ZHENG				0.93	54.37	39.75	
ZHOU	501	c	0	0.64	9.44	8.41	0.0485
ZHOU	502	c	0	0.93	21.77	9.45	0.0000
Subtotal ZHOU				0.84	31.22	17.85	

N	178
NS	43

Table 1I1 - 3

IESLC - Meta-analysis of Ever Smoking by Duration, Overview
All LC types, Any Product (or Cigarettes if Any not available)
Most adjusted

	Sex combined	male	female	Total
N	31	90	57	178
NS	9	27	19	55

In this overview table, other than the “N” rows, entries in the “absent” and “Total” columns may be invalid and should be ignored

Duration of smoking (broad categories)

	absent	1-34k20	21-49k35	36+k50	Total
N	68	43	32	35	178
NS	31	34	26	28	119
Wt	989.14	633.79	552.54	518.43	2693.90
Het Chi	703.72	204.37	237.51	290.62	1790.54
Het df	67	42	31	34	177
Het P	***	***	***	***	***
Fixed RR	5.04	2.75	5.50	8.17	4.88
RRl	4.73	2.55	5.06	7.49	4.70
RRu	5.36	2.97	5.97	8.90	5.07
P	+++	+++	+++	+++	+++
Random RR	3.70	2.52	5.49	8.24	4.24
RRl	2.97	2.07	4.25	6.22	3.72
RRu	4.61	3.06	7.09	10.92	4.82
P	+++	+++	+++	+++	+++

Duration of smoking (narrow categories)

	absent	1-19k1	6-29k20	21-39k30	31-49k40	41-998k50	51+k999	Total
N	97	24	15	23	17	1	1	178
NS	42	17	11	17	13	1	1	101
Wt	1547.42	167.85	135.57	460.26	340.11	21.28	21.42	2693.90
Het Chi	1151.76	65.20	40.71	155.43	70.37	0.00	0.00	1790.54
Het df	96	23	14	22	16	0	0	177
Het P	***	***	***	***	***	N.S.	N.S.	***
Fixed RR	4.77	2.04	2.98	5.01	8.92	8.71	11.19	4.88
RRl	4.54	1.76	2.52	4.57	8.02	5.70	7.33	4.70
RRu	5.02	2.38	3.52	5.49	9.92	13.32	17.09	5.07
P	+++	+++	+++	+++	+++	+++	+++	+++
Random RR	4.70	1.87	2.92	4.38	8.31	8.71	11.19	4.24
RRl	3.90	1.42	2.15	3.31	6.34	5.70	7.33	3.72
RRu	5.66	2.46	3.96	5.80	10.89	13.32	17.09	4.82
P	+++	+++	+++	+++	+++	+++	+++	+++

MALES

Duration of smoking (broad categories)

	absent	1-34k20	21-49k35	36+k50	Total
N	34	21	16	19	90
NS	21	21	16	19	77
Wt	596.58	312.21	307.30	313.87	1529.97
Het Chi	385.88	83.05	66.04	79.16	806.99
Het df	33	20	15	18	89
Het P	***	***	***	***	***
Fixed RR	6.08	3.50	7.13	10.41	6.26
RRl	5.61	3.13	6.38	9.32	5.96
RRu	6.59	3.91	7.98	11.63	6.59
P	+++	+++	+++	+++	+++
Random RR	5.48	2.85	6.22	10.00	5.49
RRl	4.04	2.17	4.68	7.56	4.66
RRu	7.42	3.74	8.26	13.22	6.47
P	+++	+++	+++	+++	+++

Table 1I1 - 3

IESLC - Meta-analysis of Ever Smoking by Duration, Overview
All LC types, Any Product (or Cigarettes if Any not available)
Most adjusted

MALES

	Duration of smoking (narrow categories)							
	absent	1-19k1	6-29k20	21-39k30	31-49k40	41-998k50	51+k999	Total
N	49	10	7	12	10	1	1	90
NS	27	10	7	12	10	1	1	67
Wt	811.13	89.65	75.41	264.22	246.85	21.28	21.42	1529.97
Het Chi	534.93	30.95	20.87	44.71	22.45	0.00	0.00	806.99
Het df	48	9	6	11	9	0	0	89
Het P	***	***	**	***	**	N.S.	N.S.	***
Fixed RR	5.73	2.86	3.94	7.13	10.32	8.71	11.19	6.26
RRl	5.35	2.33	3.14	6.32	9.11	5.70	7.33	5.96
RRu	6.14	3.52	4.93	8.04	11.69	13.32	17.09	6.59
P	+++	+++	+++	+++	+++	+++	+++	+++
Random RR	5.60	2.84	4.02	5.70	9.45	8.71	11.19	5.49
RRl	4.35	1.88	2.55	4.18	7.26	5.70	7.33	4.66
RRu	7.22	4.29	6.33	7.77	12.30	13.32	17.09	6.47
P	+++	+++	+++	+++	+++	+++	+++	+++

FEMALES

	Duration of smoking (broad categories)				
	absent	1-34k20	21-49k35	36+k50	Total
N	24	14	9	10	57
NS	15	14	9	10	48
Wt	318.21	252.78	165.72	123.29	860.01
Het Chi	205.37	63.25	36.14	60.29	468.78
Het df	23	13	8	9	56
Het P	***	***	***	***	***
Fixed RR	4.45	2.20	4.04	5.69	3.68
RRl	3.99	1.94	3.47	4.77	3.44
RRu	4.97	2.48	4.70	6.79	3.93
P	+++	+++	+++	+++	+++
Random RR	2.91	2.24	5.43	7.33	3.53
RRl	2.02	1.62	3.67	4.24	2.85
RRu	4.18	3.08	8.01	12.69	4.36
P	+++	+++	+++	+++	+++

	Duration of smoking (narrow categories)							
	absent	1-19k1	6-29k20	21-39k30	31-49k40	41-998k50	51+k999	Total
N	32	9	4	7	5			57
NS	19	9	4	7	5			44
Wt	557.79	49.57	22.34	146.26	84.05			860.01
Het Chi	338.96	9.41	4.65	17.75	24.93			468.78
Het df	31	8	3	6	4			56
Het P	***	N.S.	N.S.	**	***			***
Fixed RR	3.73	1.45	2.63	3.61	6.49			3.68
RRl	3.43	1.09	1.74	3.07	5.24			3.44
RRu	4.05	1.91	3.99	4.25	8.04			3.93
P	+++	++	+++	+++	+++			+++
Random RR	3.86	1.43	2.53	4.32	8.52			3.53
RRl	2.86	1.04	1.49	3.01	4.41			2.85
RRu	5.22	1.95	4.28	6.21	16.47			4.36
P	+++	+	+++	+++	+++			+++

Table 1I1 - 4

IESLC - Meta-analysis of Ever Smoking by Duration, Overview
All LC types, Any Product (or Cigarettes if Any not available)
Least adjusted

REF	NRR	X	SEX	AGEL	AGEH	RACE	YF	LC TYPE	LOC	START	ST	NLC	R	VB	P	H	AD	PRODUCT	exL	exH	S1	S2	DENOM	De
AGUDO	507	x	f	0	0	all	-	all	Eu:wst	1989	CC	103	n	bl	n	n	0	cig only	1	16	0	1	nev cigs	st
AGUDO	508	x	f	0	0	all	-	all	Eu:wst	1989	CC	103	n	bl	n	n	0	cig only	17	999	0	0	nev cigs	st
ARMADA	501	x	m	0	0	all	-	all	Eu:wst	1986	CC	325	n	bl	n	y	0	cig+/-ot	1	24	1	0	nev cigs	st
ARMADA	502	x	m	0	0	all	-	all	Eu:wst	1986	CC	325	n	bl	n	y	0	cig+/-ot	25	49	2	0	nev cigs	st
ARMADA	503	x	m	0	0	all	-	all	Eu:wst	1986	CC	325	n	bl	n	y	0	cig+/-ot	50	999	3	0	nev cigs	st
AUVINE	501	x	c	0	0	all	-	all	Eu:Sca	1986	CC	517	n	bl	y	n	0	cig+/-ot	1	20	1	0	nev cigs	st
AUVINE	502	x	c	0	0	all	-	all	Eu:Sca	1986	CC	517	n	bl	y	n	0	cig+/-ot	21	40	2	0	nev cigs	st
AUVINE	503	x	c	0	0	all	-	all	Eu:Sca	1986	CC	517	n	bl	y	n	0	cig+/-ot	41	999	3	0	nev cigs	st
AXELSS	501	x	m	0	0	sca	-	all	Eu:Sca	1989	CC	436	n	bl	n	n	0	all/unsp	1	19	0	1	nev any	st
AXELSS	502	x	m	0	0	sca	-	all	Eu:Sca	1989	CC	436	n	bl	n	n	0	all/unsp	20	29	1	2	nev any	st
AXELSS	503	x	m	0	0	sca	-	all	Eu:Sca	1989	CC	436	n	bl	n	n	0	all/unsp	30	39	2	3	nev any	st
AXELSS	504	x	m	0	0	sca	-	all	Eu:Sca	1989	CC	436	n	bl	n	n	0	all/unsp	40	49	0	4	nev any	st
AXELSS	505	x	m	0	0	sca	-	all	Eu:Sca	1989	CC	436	n	bl	n	n	0	all/unsp	50	999	3	0	nev any	st
AXELSS	510		f	0	0	sca	-	all	Eu:Sca	1989	CC	436	n	bl	n	n	0	all/unsp	1	19	0	1	nev any	st
AXELSS	511		f	0	0	sca	-	all	Eu:Sca	1989	CC	436	n	bl	n	n	0	all/unsp	20	29	1	2	nev any	st
AXELSS	512		f	0	0	sca	-	all	Eu:Sca	1989	CC	436	n	bl	n	n	0	all/unsp	30	39	2	3	nev any	st
AXELSS	513		f	0	0	sca	-	all	Eu:Sca	1989	CC	436	n	bl	n	n	0	all/unsp	40	49	0	4	nev any	st
AXELSS	514		f	0	0	sca	-	all	Eu:Sca	1989	CC	436	n	bl	n	n	0	all/unsp	50	999	3	0	nev any	st
BARBON	501	x	m	0	0	all	-	all	Eu:wst	1979	CC	755	n	bl	y	y	0	all/unsp	1	29	1	0	nev any	st
BARBON	502	x	m	0	0	all	-	all	Eu:wst	1979	CC	755	n	bl	y	y	0	all/unsp	30	39	2	3	nev any	st
BARBON	503	x	m	0	0	all	-	all	Eu:wst	1979	CC	755	n	bl	y	y	0	all/unsp	40	49	0	4	nev any	st
BARBON	504	x	m	0	0	all	-	all	Eu:wst	1979	CC	755	n	bl	y	y	0	all/unsp	50	999	3	0	nev any	st
BOUCOT	518		m	0	0	all	9	all	NAmer	1951	pr	121	n	bl	n	n	0	cig+/-ot	1	39	0	0	nev any	ot
BOUCOT	519		m	0	0	all	9	all	NAmer	1951	pr	121	n	bl	n	n	0	cig+/-ot	40	999	3	0	nev any	ot
BUFFLE	526		f	0	0	w-hi	-	all	NAmer	1976	CC	943	n	bl	y	n	0	cig+/-ot	1	30	1	0	nev cigs	or
BUFFLE	527		f	0	0	w-hi	-	all	NAmer	1976	CC	943	n	bl	y	n	0	cig+/-ot	31	40	2	4	nev cigs	or
BUFFLE	528		f	0	0	w-hi	-	all	NAmer	1976	CC	943	n	bl	y	n	0	cig+/-ot	41	999	3	0	nev cigs	or
CHEN2	501		m	0	0	all	-	all	As:Chi	1983	CC	193	n	ot	y	n	0	all/unsp	1	9	0	1	nev any	st
CHEN2	502		m	0	0	all	-	all	As:Chi	1983	CC	193	n	ot	y	n	0	all/unsp	10	20	1	2	nev any	st
CHEN2	503		m	0	0	all	-	all	As:Chi	1983	CC	193	n	ot	y	n	0	all/unsp	21	30	0	3	nev any	st
CHEN2	504		m	0	0	all	-	all	As:Chi	1983	CC	193	n	ot	y	n	0	all/unsp	31	40	2	4	nev any	st
CHEN2	505		m	0	0	all	-	all	As:Chi	1983	CC	193	n	ot	y	n	0	all/unsp	41	999	3	0	nev any	st
CHEN2	510		f	0	0	all	-	all	As:Chi	1983	CC	193	n	ot	y	n	0	all/unsp	1	20	1	0	nev any	st
CHEN2	511		f	0	0	all	-	all	As:Chi	1983	CC	193	n	ot	y	n	0	all/unsp	21	30	0	3	nev any	st
CHEN2	512		f	0	0	all	-	all	As:Chi	1983	CC	193	n	ot	y	n	0	all/unsp	31	40	2	4	nev any	st
CHEN2	513		f	0	0	all	-	all	As:Chi	1983	CC	193	n	ot	y	n	0	all/unsp	41	999	3	0	nev any	st
CHOI	501		m	0	0	all	-	all	As:oth	1985	CC	375	n	bl	n	n	0	cig+/-ot	1	19	0	1	nev cigs	st
CHOI	502		m	0	0	all	-	all	As:oth	1985	CC	375	n	bl	n	n	0	cig+/-ot	20	29	1	2	nev cigs	st
CHOI	503		m	0	0	all	-	all	As:oth	1985	CC	375	n	bl	n	n	0	cig+/-ot	30	39	2	3	nev cigs	st
CHOI	504		m	0	0	all	-	all	As:oth	1985	CC	375	n	bl	n	n	0	cig+/-ot	40	49	0	4	nev cigs	st
CHOI	505		m	0	0	all	-	all	As:oth	1985	CC	375	n	bl	n	n	0	cig+/-ot	50	999	3	0	nev cigs	st
CHOI	510		f	0	0	all	-	all	As:oth	1985	CC	375	n	bl	n	n	0	cig+/-ot	1	19	0	1	nev cigs	st
CHOI	511		f	0	0	all	-	all	As:oth	1985	CC	375	n	bl	n	n	0	cig+/-ot	20	29	1	2	nev cigs	st
CHOI	512		f	0	0	all	-	all	As:oth	1985	CC	375	n	bl	n	n	0	cig+/-ot	30	39	2	3	nev cigs	st
CHOI	513		f	0	0	all	-	all	As:oth	1985	CC	375	n	bl	n	n	0	cig+/-ot	40	999	3	0	nev cigs	st
DAMBER	506		m	0	0	all	-	all	Eu:Sca	1972	CC	579	n	bl	y	n	1	all/unsp	1	20	1	0	nev any	ot
DAMBER	507		m	0	0	all	-	all	Eu:Sca	1972	CC	579	n	bl	y	n	1	all/unsp	21	30	0	3	nev any	ot
DAMBER	508		m	0	0	all	-	all	Eu:Sca	1972	CC	579	n	bl	y	n	1	all/unsp	31	40	2	4	nev any	ot
DAMBER	509		m	0	0	all	-	all	Eu:Sca	1972	CC	579	n	bl	y	n	1	all/unsp	41	50	3	5	nev any	ot
DAMBER	510		m	0	0	all	-	all	Eu:Sca	1972	CC	579	n	bl	y	n	1	all/unsp	51	999	0	6	nev any	ot
DESTEF	501	x	m	0	0	all	-	all	SCAmer	1988	CC	497	n	bl	n	y	0	all/unsp	1	29	1	0	nev any	st
DESTEF	502	x	m	0	0	all	-	all	SCAmer	1988	CC	497	n	bl	n	y	0	all/unsp	30	39	2	3	nev any	st
DESTEF	503	x	m	0	0	all	-	all	SCAmer	1988	CC	497	n	bl	n	y	0	all/unsp	40	49	0	4	nev any	st
DESTEF	504	x	m	0	0	all	-	all	SCAmer	1988	CC	497	n	bl	n	y	0	all/unsp	50	999	3	0	nev any	st
DOLL	515		m	0	0	all	-	all	Eu:UK	1948	CC	1465	n	V	n	n	0	all/unsp	1	9	0	1	nev any	st
DOLL	516		m	0	0	all	-	all	Eu:UK	1948	CC	1465	n	V	n	n	0	all/unsp	10	19	0	0	nev any	st
DOLL	517		m	0	0	all	-	all	Eu:UK	1948	CC	1465	n	V	n	n	0	all/unsp	20	39	0	0	nev any	st
DOLL	518		m	0	0	all	-	all	Eu:UK	1948	CC	1465	n	V	n	n	0	all/unsp	40	999	3	0	nev any	st
DOLL	522		f	0	0	all	-	all	Eu:UK	1948	CC	1465	n	V	n	n	0	all/unsp	1	9	0	1	nev any	st
DOLL	523		f	0	0	all	-	all	Eu:UK	1948	CC	1465	n	V	n	n	0	all/unsp	10	19	0	0	nev any	st
DOLL	524		f	0	0	all	-	all	Eu:UK	1948	CC	1465	n	V	n	n	0	all/unsp	20	39	0	0	nev any	st
DOLL	525		f	0	0	all	-	all	Eu:UK	1948	CC	1465	n	V	n	n	0	all/unsp	40	999	3	0	nev any	st
DORGAN	570		m	0	0	wh	-	all	NAmer	1980	CC	2026	n	bl	y	y	2	cig+/-ot	1	34	1	0	nev any	ot
DORGAN	571		m	0	0	wh	-	all	NAmer	1980	CC	2026	n	bl	y	y	2	cig+/-ot	35	999	0	0	nev any	ot
DORGAN	562		f	0	0	all	-	all	NAmer	1980	CC	2026	n	bl	y	y	3	cig+/-ot	1	34	1	0	nev any	ot
DORGAN	563		f	0	0	all	-	all	NAmer	1980	CC	2026	n	bl	y	y	3	cig+/-ot	35	999	0	0	nev any	ot
DOSEME	501		m	0	0	all	-	all	Eu:bal	1979	CC	1210	n	bl	n	n	2	cig+/-ot	1	10	0	1	nev cigs	or
DOSEME	502		m	0	0	all	-	all	Eu:bal	1979	CC	1210	n	bl	n	n	2	cig+/-ot	11	20	1	2	nev cigs	or
DOSEME	503		m	0	0	all	-	all	Eu:bal	1979	CC	1210	n	bl	n	n	2	cig+/-ot	21	999	0	0	nev cigs	or
FAN	501		m	0	0	all	-	all	As:Chi	1990	CC	403	n	ot	y	n	0	cig+/-ot	1	29	1	0	nev cigs	st
FAN	502		m	0	0	all	-	all	As:Chi	1990	CC	403	n	ot	y	n	0	cig+/-ot	30	39	2	3	nev cigs	st
FAN	503		m	0	0	all	-	all	As:Chi	1990	CC	403	n	ot	y	n	0	cig+/-ot	40	999	3	0	nev cigs	st

Table 1I1 - 4

IESLC - Meta-analysis of Ever Smoking by Duration, Overview
All LC types, Any Product (or Cigarettes if Any not available)
Least adjusted

REF	NRR	X	SEX	AGEL	AGEH	RACE	YF	LC TYPE	LOC	START	ST	NLC	R	VB	P	H	AD	PRODUCT	exL	exH	S1	S2	DENOM	De
FAN	506		f	0	0	all	-	all	As:Chi	1990	CC	403	n	ot	y	n	0	cig+/-ot	1	29	1	0	nev cigs	st
FAN	507		f	0	0	all	-	all	As:Chi	1990	CC	403	n	ot	y	n	0	cig+/-ot	30	39	2	3	nev cigs	st
FAN	508		f	0	0	all	-	all	As:Chi	1990	CC	403	n	ot	y	n	0	cig+/-ot	40	999	3	0	nev cigs	st
GAO	561	x	f	0	0	all	-	all	As:Chi	1984	CC	1405	n	ot	n	n	0	cig+/-ot	1	29	1	0	nev cigs	st
GAO	562	x	f	0	0	all	-	all	As:Chi	1984	CC	1405	n	ot	n	n	0	cig+/-ot	30	999	0	0	nev cigs	st
GARSHI	534	x	m	0	0	all	-	all	NAmer	1981	CC	1081	o	bl	y	n	0	all/unsp	20	999	0	0	nev any	st
GER	513	x	c	0	0	all	-	all	As:oth	1990	CC	141	n	ot	y	n	0	all/unsp	1	20	1	0	nev any	st
GER	514	x	c	0	0	all	-	all	As:oth	1990	CC	141	n	ot	y	n	0	all/unsp	21	40	2	0	nev any	st
GER	515	x	c	0	0	all	-	all	As:oth	1990	CC	141	n	ot	y	n	0	all/unsp	41	999	3	0	nev any	st
HAENSZ	542		f	0	0	all	-	not alv	NAmer	1955	CC	158	n	bl	n	y	0	cig+/-ot	1	14	0	1	nev any	st
HAENSZ	543		f	0	0	all	-	not alv	NAmer	1955	CC	158	n	bl	n	y	0	cig+/-ot	15	999	0	0	nev any	st
HU	501		m	0	0	all	-	all	As:Chi	1985	CC	227	n	ot	n	y	0	cig+/-ot	1	19	0	1	nev cigs	st
HU	502		m	0	0	all	-	all	As:Chi	1985	CC	227	n	ot	n	y	0	cig+/-ot	20	29	1	2	nev cigs	st
HU	503		m	0	0	all	-	all	As:Chi	1985	CC	227	n	ot	n	y	0	cig+/-ot	30	999	0	0	nev cigs	st
HU	506		f	0	0	all	-	all	As:Chi	1985	CC	227	n	ot	n	y	0	cig+/-ot	1	19	0	1	nev cigs	st
HU	507		f	0	0	all	-	all	As:Chi	1985	CC	227	n	ot	n	y	0	cig+/-ot	20	29	1	2	nev cigs	st
HU	508		f	0	0	all	-	all	As:Chi	1985	CC	227	n	ot	n	y	0	cig+/-ot	30	999	0	0	nev cigs	st
HU2	508		c	0	0	all	-	all	As:Chi	1977	CC	523	n	ot	y	n	0	cig+/-ot	1	19	0	1	nev cigs	ot
HU2	509		c	0	0	all	-	all	As:Chi	1977	CC	523	n	ot	y	n	0	cig+/-ot	20	29	1	2	nev cigs	or
HU2	510		c	0	0	all	-	all	As:Chi	1977	CC	523	n	ot	y	n	0	cig+/-ot	30	39	2	3	nev cigs	or
HU2	511		c	0	0	all	-	all	As:Chi	1977	CC	523	n	ot	y	n	0	cig+/-ot	40	999	3	0	nev cigs	st
JOLY	515		m	0	0	all	-	all	SCAmer	1978	CC	826	n	bl	n	n	0	cig+/-ot	1	19	0	1	nev any	st
JOLY	516		m	0	0	all	-	all	SCAmer	1978	CC	826	n	bl	n	n	0	cig+/-ot	20	29	1	2	nev any	st
JOLY	517		m	0	0	all	-	all	SCAmer	1978	CC	826	n	bl	n	n	0	cig+/-ot	30	39	2	3	nev any	st
JOLY	518		m	0	0	all	-	all	SCAmer	1978	CC	826	n	bl	n	n	0	cig+/-ot	40	49	0	4	nev any	st
JOLY	519		m	0	0	all	-	all	SCAmer	1978	CC	826	n	bl	n	n	0	cig+/-ot	50	999	3	0	nev any	st
JOLY	501		f	0	0	all	-	all	SCAmer	1978	CC	826	n	bl	n	n	0	cig+/-ot	1	19	0	1	nev any	st
JOLY	502		f	0	0	all	-	all	SCAmer	1978	CC	826	n	bl	n	n	0	cig+/-ot	20	29	1	2	nev any	st
JOLY	503		f	0	0	all	-	all	SCAmer	1978	CC	826	n	bl	n	n	0	cig+/-ot	30	39	2	3	nev any	st
JOLY	504		f	0	0	all	-	all	SCAmer	1978	CC	826	n	bl	n	n	0	cig+/-ot	40	49	0	4	nev any	st
JOLY	505		f	0	0	all	-	all	SCAmer	1978	CC	826	n	bl	n	n	0	cig+/-ot	50	999	3	0	nev any	st
JUSSAW	510		m	0	0	all	-	all	As:Ind	1964	CC	792	n	V	n	n	0	cig only	1	9	0	1	nev any	st
JUSSAW	511		m	0	0	all	-	all	As:Ind	1964	CC	792	n	V	n	n	0	cig only	10	19	0	0	nev any	st
JUSSAW	512		m	0	0	all	-	all	As:Ind	1964	CC	792	n	V	n	n	0	cig only	20	29	1	2	nev any	st
JUSSAW	513		m	0	0	all	-	all	As:Ind	1964	CC	792	n	V	n	n	0	cig only	30	39	2	3	nev any	st
JUSSAW	514		m	0	0	all	-	all	As:Ind	1964	CC	792	n	V	n	n	0	cig only	40	999	3	0	nev any	st
KHUDER	501		m	0	0	all	-	all	NAmer	1985	CC	482	n	bl	n	y	0	cig+/-ot	1	29	1	0	nev cigs	st
KHUDER	502		m	0	0	all	-	all	NAmer	1985	CC	482	n	bl	n	y	0	cig+/-ot	30	49	2	0	nev cigs	st
KHUDER	503		m	0	0	all	-	all	NAmer	1985	CC	482	n	bl	n	y	0	cig+/-ot	50	999	3	0	nev cigs	st
KREUZE	517		m	0	0	all	-	all	Eu:Ger	1990	CC	2260	n	bl	n	n	3	all/unsp	1	19	0	1	nev any	st
KREUZE	518		m	0	0	all	-	all	Eu:Ger	1990	CC	2260	n	bl	n	n	3	all/unsp	20	999	0	0	nev any	ot
KREUZE	520		f	0	0	all	-	all	Eu:Ger	1990	CC	2260	n	bl	n	n	3	all/unsp	1	19	0	1	nev any	ot
KREUZE	521		f	0	0	all	-	all	Eu:Ger	1990	CC	2260	n	bl	n	n	3	all/unsp	20	999	0	0	nev any	ot
LETOUR	506		c	0	0	all	-	all	NAmer	1983	CC	738	n	V	y	y	0	cig+/-ot	1	24	1	0	nev cigs	st
LETOUR	507		c	0	0	all	-	all	NAmer	1983	CC	738	n	V	y	y	0	cig+/-ot	25	40	2	0	nev cigs	st
LETOUR	508		c	0	0	all	-	all	NAmer	1983	CC	738	n	V	y	y	0	cig+/-ot	41	999	3	0	nev cigs	st
LEVIN	501	x	m	0	0	all	-	all	NAmer	1938	CC	475	n	bl	n	n	0	cig+/-ot	1	39	0	0	nev any	st
LEVIN	502	x	m	0	0	all	-	all	NAmer	1938	CC	475	n	bl	n	n	0	cig+/-ot	40	999	3	0	nev any	st
LIU3	507	x	m	0	0	all	-	all	As:Chi	1985	CC	110	n	ot	n	n	0	all/unsp	1	34	1	0	nev any	or
LIU3	508	x	m	0	0	all	-	all	As:Chi	1985	CC	110	n	ot	n	n	0	all/unsp	35	999	0	0	nev any	st
LIU5	504		c	0	0	all	-	all	As:Chi	1978	CC	111	n	ot	y	n	0	all/unsp	1	29	1	0	nev any	st
LIU5	505		c	0	0	all	-	all	As:Chi	1978	CC	111	n	ot	y	n	0	all/unsp	30	999	0	0	nev any	st
LUBIN	508		m	0	0	all	-	all	As:Chi	1984	CC	427	m	ot	y	n	0	cig+/-ot	1	29	1	0	nev any	st
LUBIN	509		m	0	0	all	-	all	As:Chi	1984	CC	427	m	ot	y	n	0	cig+/-ot	30	39	2	3	nev any	st
LUBIN	510		m	0	0	all	-	all	As:Chi	1984	CC	427	m	ot	y	n	0	cig+/-ot	40	49	0	4	nev any	st
LUBIN	511		m	0	0	all	-	all	As:Chi	1984	CC	427	m	ot	y	n	0	cig+/-ot	50	999	3	0	nev any	st
LUBIN2	531		m	0	0	all	-	all	Eu:mul	1976	CC	7804	n	bl	n	y	0	cig+/-ot	1	29	1	0	nev any	st
LUBIN2	532		m	0	0	all	-	all	Eu:mul	1976	CC	7804	n	bl	n	y	0	cig+/-ot	30	39	2	3	nev any	st
LUBIN2	533		m	0	0	all	-	all	Eu:mul	1976	CC	7804	n	bl	n	y	0	cig+/-ot	40	49	0	4	nev any	st
LUBIN2	534		m	0	0	all	-	all	Eu:mul	1976	CC	7804	n	bl	n	y	0	cig+/-ot	50	999	3	0	nev any	st
LUBIN2	574		f	0	0	all	-	all	Eu:mul	1976	CC	7804	n	bl	n	y	0	cig+/-ot	1	29	1	0	nev any	st
LUBIN2	575		f	0	0	all	-	all	Eu:mul	1976	CC	7804	n	bl	n	y	0	cig+/-ot	30	39	2	3	nev any	st
LUBIN2	576		f	0	0	all	-	all	Eu:mul	1976	CC	7804	n	bl	n	y	0	cig+/-ot	40	49	0	4	nev any	st
LUBIN2	577		f	0	0	all	-	all	Eu:mul	1976	CC	7804	n	bl	n	y	0	cig+/-ot	50	999	3	0	nev any	st
MATOS	516	x	m	0	0	all	-	all	SCAmer	1994	CC	200	n	bl	n	n	0	cig+/-ot	1	24	1	0	nev any	st
MATOS	517	x	m	0	0	all	-	all	SCAmer	1994	CC	200	n	bl	n	n	0	cig+/-ot	25	39	2	3	nev any	st
MATOS	518	x	m	0	0	all	-	all	SCAmer	1994	CC	200	n	bl	n	n	0	cig+/-ot	40	70	3	0	nev any	st
MCCONN	501		c	0	0	all	-	all	Eu:UK	1946	CC	100	n	V	n	y	0	all/unsp	1	9	0	1	nev any	st
MCCONN	502		c	0	0	all	-	all	Eu:UK	1946	CC	100	n	V	n	y	0	all/unsp	10	19	0	0	nev any	st
MCCONN	503		c	0	0	all	-	all	Eu:UK	1946	CC	100	n	V	n	y	0	all/unsp	20	29	1	2	nev any	st
MCCONN	504		c	0	0	all	-	all	Eu:UK	1946	CC	100	n	V	n	y	0	all/unsp	30	39	2	3	nev any	st
MCCONN	505		c	0	0	all	-	all	Eu:UK	1946	CC	100	n	V	n	y	0	all/unsp	40	999	3	0	nev any	st

Table 1I1 - 4

IESLC - Meta-analysis of Ever Smoking by Duration, Overview
All LC types, Any Product (or Cigarettes if Any not available)
Least adjusted

REF	NRR	X	SEX	AGEL	AGEH	RACE	YF	LC TYPE	LOC	START	ST	NLC	R	VB	P	H	AD	PRODUCT	exL	exH	S1	S2	DENOM	De
NOTAN2	513		c	0	0	all	-	all	As:Ind	1963	CC	683	n	V	n	n	0	cig only	1	10	0	1	nev any	st
NOTAN2	514		c	0	0	all	-	all	As:Ind	1963	CC	683	n	V	n	n	0	cig only	11	20	1	2	nev any	st
NOTAN2	515		c	0	0	all	-	all	As:Ind	1963	CC	683	n	V	n	n	0	cig only	21	30	0	3	nev any	st
NOTAN2	516		c	0	0	all	-	all	As:Ind	1963	CC	683	n	V	n	n	0	cig only	31	40	2	4	nev any	st
NOTAN2	517		c	0	0	all	-	all	As:Ind	1963	CC	683	n	V	n	n	0	cig only	41	999	3	0	nev any	st
OSANN2	501	x	f	0	0	all	-	all	NAmer	1964	ot	217	n	bl	n	y	0	cig+/-ot	1	20	1	0	nev cigs	st
OSANN2	502	x	f	0	0	all	-	all	NAmer	1964	ot	217	n	bl	n	y	0	cig+/-ot	21	999	0	0	nev cigs	st
PEZZOT	534		m	0	0	all	-	all	SCAmer	1987	CC	215	n	bl	n	y	0	cig only	1	30	1	0	nev cigs	st
PEZZOT	535		m	0	0	all	-	all	SCAmer	1987	CC	215	n	bl	n	y	0	cig only	31	40	2	4	nev cigs	st
PEZZOT	536		m	0	0	all	-	all	SCAmer	1987	CC	215	n	bl	n	y	0	cig only	41	999	3	0	nev cigs	st
QIAO2	511	x	m	0	0	all	0	all	As:Chi	1992	pr	241	m	ot	n	n	0	all/unsp	1	27	1	0	nev any	st
QIAO2	512	x	m	0	0	all	0	all	As:Chi	1992	pr	241	m	ot	n	n	0	all/unsp	28	41	2	0	nev any	st
QIAO2	513	x	m	0	0	all	0	all	As:Chi	1992	pr	241	m	ot	n	n	0	all/unsp	42	999	3	0	nev any	st
RACHTA	511	x	f	0	0	all	-	all	Eu:est	1991	CC	118	n	bl	n	y	0	cig+/-ot	1	20	1	0	nev cigs	st
RACHTA	512	x	f	0	0	all	-	all	Eu:est	1991	CC	118	n	bl	n	y	0	cig+/-ot	21	40	2	0	nev cigs	st
RACHTA	513	x	f	0	0	all	-	all	Eu:est	1991	CC	118	n	bl	n	y	0	cig+/-ot	41	999	3	0	nev cigs	st
TIZZAN	501		m	0	0	all	-	all	Eu:wst	1959	CC	1358	n	bl	n	n	0	cig only	1	4	0	0	nev any	st
TIZZAN	502		m	0	0	all	-	all	Eu:wst	1959	CC	1358	n	bl	n	n	0	cig only	5	10	0	1	nev any	st
TIZZAN	503		m	0	0	all	-	all	Eu:wst	1959	CC	1358	n	bl	n	n	0	cig only	11	999	0	0	nev any	st
TIZZAN	533		f	0	0	all	-	all	Eu:wst	1959	CC	1358	n	bl	n	n	0	all/unsp	1	10	0	1	nev any	st
TIZZAN	534		f	0	0	all	-	all	Eu:wst	1959	CC	1358	n	bl	n	n	0	all/unsp	11	999	0	0	nev any	st
WANG2	501		c	0	0	all	-	all	As:Chi	1980	CC	103	n	ot	n	n	0	cig+/-ot	1	19	0	1	nev cigs	st
WANG2	503		c	0	0	all	-	all	As:Chi	1980	CC	103	n	ot	n	n	0	cig+/-ot	20	29	1	2	nev cigs	st
WANG2	504		c	0	0	all	-	all	As:Chi	1980	CC	103	n	ot	n	n	0	cig+/-ot	30	39	2	3	nev cigs	st
WANG2	505		c	0	0	all	-	all	As:Chi	1980	CC	103	n	ot	n	n	0	cig+/-ot	40	49	0	4	nev cigs	st
WUWILL	501	x	f	0	0	all	-	all	As:Chi	1985	CC	965	n	ot	n	n	0	cig+/-ot	1	29	1	0	nev cigs	st
WUWILL	502	x	f	0	0	all	-	all	As:Chi	1985	CC	965	n	ot	n	n	0	cig+/-ot	30	39	2	3	nev cigs	st
WUWILL	503	x	f	0	0	all	-	all	As:Chi	1985	CC	965	n	ot	n	n	0	cig+/-ot	40	999	3	0	nev cigs	st
ZHENG	553		m	0	0	all	-	all	As:Chi	1982	CC	540	n	ot	*	y	0	cig+/-ot	1	29	1	0	nev cigs	st
ZHENG	554		m	0	0	all	-	all	As:Chi	1982	CC	540	n	ot	*	y	0	cig+/-ot	30	999	0	0	nev cigs	st
ZHENG	558		f	0	0	all	-	all	As:Chi	1982	CC	540	n	ot	*	y	0	cig+/-ot	1	29	1	0	nev cigs	st
ZHENG	559		f	0	0	all	-	all	As:Chi	1982	CC	540	n	ot	*	y	0	cig+/-ot	30	999	0	0	nev cigs	st
ZHOU	501		c	0	0	all	-	all	As:Chi	1978	CC	1360	n	ot	n	n	0	all/unsp	1	19	0	1	nev any	st
ZHOU	502		c	0	0	all	-	all	As:Chi	1978	CC	1360	n	ot	n	n	0	all/unsp	20	999	0	0	nev any	st

Cigarette type is all/unspec for all RRs

except for the following:

REF	NRR	CIGTYPE
JUSSAW	510	MC only
JUSSAW	511	MC only
JUSSAW	512	MC only
JUSSAW	513	MC only
JUSSAW	514	MC only
NOTAN2	513	MC only
NOTAN2	514	MC only
NOTAN2	515	MC only
NOTAN2	516	MC only
NOTAN2	517	MC only

In this overview table, subtotals and Qs values may be invalid and should be ignored

Table 1I1 - 5

IESLC - Meta-analysis of Ever Smoking by Duration, Overview
All LC types, Any Product (or Cigarettes if Any not available)
Least adjusted

REF	NRR	SEX	AD	Number Exposed Case	Cont	Non-exposed Case	Cont	RR	95.00%CI
AGUDO	507	f	0	5	12	80	183	0.95	(0.33- 2.79)
AGUDO	508	f	0	18	11	80	183	3.74	(1.69- 8.29)
Subtotal AGUDO								2.31	(1.22- 4.38)
ARMADA	501	m	0	21	55	8	71	3.39	(1.40- 8.23)
ARMADA	502	m	0	219	166	8	71	11.71	(5.49- 24.99)
ARMADA	503	m	0	77	33	8	71	20.71	(8.97- 47.82)
Subtotal ARMADA								9.86	(6.13- 15.85)
AUVINE	501	c	0	26	18	44	229	7.52	(3.80- 14.87)
AUVINE	502	c	0	10	5	44	229	10.41	(3.39- 31.93)
AUVINE	503	c	0	230	57	44	229	21.00	(13.61- 32.41)
Subtotal AUVINE								15.02	(10.61- 21.28)
AXELSS	501	m	0	13	84	16	160	1.55	(0.71- 3.37)
AXELSS	502	m	0	17	64	16	160	2.66	(1.27- 5.58)
AXELSS	503	m	0	57	71	16	160	8.03	(4.31- 14.94)
AXELSS	504	m	0	104	85	16	160	12.24	(6.79- 22.04)
AXELSS	505	m	0	101	40	16	160	25.25	(13.43- 47.46)
AXELSS	510	f	0	5	24	18	154	1.78	(0.61- 5.25)
AXELSS	511	f	0	12	29	18	154	3.54	(1.54- 8.13)
AXELSS	512	f	0	29	26	18	154	9.54	(4.64- 19.61)
AXELSS	513	f	0	44	20	18	154	18.82	(9.17- 38.65)
AXELSS	514	f	0	20	10	18	154	17.11	(6.94- 42.19)
Subtotal AXELSS								7.94	(6.31- 10.00)
BARBON	501	m	0	42	91	22	188	3.94	(2.22- 7.00)
BARBON	502	m	0	118	102	22	188	9.89	(5.91- 16.55)
BARBON	503	m	0	207	139	22	188	12.73	(7.79- 20.80)
BARBON	504	m	0	366	235	22	188	13.31	(8.31- 21.32)
Subtotal BARBON								9.63	(7.47- 12.42)
*BOUCOT	518	m	0	29	2621	0	805	18.13~	(1.11- 296.36)
*BOUCOT	519	m	0	52	1563	0	805	54.09~	(3.34- 875.17)
Subtotal BOUCOT								31.38	(4.37- 225.47)
BUFFLE	526	f	0	52	57	12	112	8.51	(4.21- 17.22)
BUFFLE	527	f	0	97	62	12	112	14.60	(7.43- 28.69)
BUFFLE	528	f	0	90	42	12	112	20.00	(9.94- 40.23)
Subtotal BUFFLE								13.60	(9.12- 20.29)
CHEN2	501	m	0	2	3	9	33	2.44	(0.35- 16.93)
CHEN2	502	m	0	4	3	9	33	4.89	(0.92- 25.93)
CHEN2	503	m	0	17	24	9	33	2.60	(0.99- 6.81)
CHEN2	504	m	0	36	27	9	33	4.89	(2.01- 11.91)
CHEN2	505	m	0	62	40	9	33	5.68	(2.46- 13.13)
CHEN2	510	f	0	1	6	25	33	0.22	(0.02- 1.95)
CHEN2	511	f	0	2	2	25	33	1.32	(0.17- 10.03)
CHEN2	512	f	0	13	6	25	33	2.86	(0.95- 8.58)
CHEN2	513	f	0	21	15	25	33	1.85	(0.80- 4.29)
Subtotal CHEN2								3.01	(2.07- 4.38)
CHOI	501	m	0	19	55	13	95	2.52	(1.16- 5.51)
CHOI	502	m	0	66	166	13	95	2.91	(1.52- 5.54)
CHOI	503	m	0	102	160	13	95	4.66	(2.48- 8.75)
CHOI	504	m	0	60	64	13	95	6.85	(3.48- 13.50)
CHOI	505	m	0	20	20	13	95	7.31	(3.13- 17.07)
CHOI	510	f	0	2	9	76	164	0.48	(0.10- 2.27)
CHOI	511	f	0	8	14	76	164	1.23	(0.50- 3.06)
CHOI	512	f	0	8	2	76	164	8.63	(1.79- 41.62)
CHOI	513	f	0	1	1	76	164	2.16	(0.13- 34.96)
Subtotal CHOI								3.63	(2.73- 4.83)
DAMBER	506	m	1	-	-	42	-	1.58	(0.69- 3.66)
DAMBER	507	m	1	-	-	42	-	3.66	(2.18- 6.73)
DAMBER	508	m	1	-	-	42	-	5.15	(3.27- 8.32)
DAMBER	509	m	1	-	-	42	-	8.71	(5.84- 13.66)
DAMBER	510	m	1	-	-	42	-	11.19	(7.43- 17.33)
Subtotal DAMBER								6.42	(5.14- 8.01)
DESTEF	501	m	0	43	55	27	163	4.72	(2.67- 8.35)
DESTEF	502	m	0	78	78	27	163	6.04	(3.61- 10.10)
DESTEF	503	m	0	171	93	27	163	11.10	(6.87- 17.92)
DESTEF	504	m	0	178	108	27	163	9.95	(6.20- 15.96)
Subtotal DESTEF								7.86	(6.10- 10.11)
DOLL	515	m	0	12	15	7	61	6.97	(2.34- 20.73)
DOLL	516	m	0	34	65	7	61	4.56	(1.88- 11.05)
DOLL	517	m	0	746	725	7	61	8.97	(4.07- 19.73)
DOLL	518	m	0	558	491	7	61	9.90	(4.49- 21.85)
DOLL	522	f	0	14	18	40	59	1.15	(0.51- 2.57)
DOLL	523	f	0	12	8	40	59	2.21	(0.83- 5.90)

Table 1I1 - 5

IESLC - Meta-analysis of Ever Smoking by Duration, Overview
All LC types, Any Product (or Cigarettes if Any not available)
Least adjusted

REF	NRR	SEX	AD	Number Exposed Case	Cont	Non-exposed Case	Cont	RR	95.00%CI
DOLL	524	f	0	36	20	40	59	2.66	(1.35- 5.23)
DOLL	525	f	0	6	3	40	59	2.95	(0.70- 12.49)
Subtotal DOLL								3.93	(2.89- 5.34)
DORGAN	570	m	2	-	-	-	-	5.44	(2.97- 9.98)
DORGAN	571	m	2	-	-	-	-	16.09	(8.96- 28.88)
DORGAN	562	f	3	-	-	-	-	4.25	(3.20- 5.64)
DORGAN	563	f	3	-	-	-	-	11.73	(9.07- 15.18)
Subtotal DORGAN								7.74	(6.50- 9.20)
DOSEME	501	m	2	32	-	142	-	1.00	(0.60- 1.70)
DOSEME	502	m	2	158	-	142	-	3.80	(2.60- 5.70)
DOSEME	503	m	2	466	-	142	-	4.90	(3.50- 7.00)
Subtotal DOSEME								3.27	(2.59- 4.12)
FAN	501	m	0	29	135	36	236	1.41	(0.83- 2.40)
FAN	502	m	0	44	122	36	236	2.36	(1.45- 3.87)
FAN	503	m	0	143	241	36	236	3.89	(2.59- 5.84)
FAN	506	f	0	8	15	69	320	2.47	(1.01- 6.06)
FAN	507	f	0	19	23	69	320	3.83	(1.98- 7.42)
FAN	508	f	0	55	59	69	320	4.32	(2.76- 6.78)
Subtotal FAN								3.01	(2.43- 3.72)
GAO	561	f	0	68	58	435	605	1.63	(1.12- 2.36)
GAO	562	f	0	168	72	435	605	3.25	(2.40- 4.39)
Subtotal GAO								2.47	(1.95- 3.12)
GARSHI	534	m	0	922	1314	41	363	6.21	(4.45- 8.67)
GER	513	c	0	10	40	51	246	1.21	(0.57- 2.57)
GER	514	c	0	31	123	51	246	1.22	(0.74- 2.00)
GER	515	c	0	49	155	51	246	1.52	(0.98- 2.37)
Subtotal GER								1.35	(1.00- 1.83)
HAENSZ	542	f	0	16	26	81	236	1.79	(0.92- 3.51)
HAENSZ	543	f	0	58	77	81	236	2.19	(1.44- 3.35)
Subtotal HAENSZ								2.07	(1.45- 2.97)
HU	501	m	0	41	33	41	67	2.03	(1.11- 3.70)
HU	502	m	0	60	47	41	67	2.09	(1.21- 3.60)
HU	503	m	0	19	14	41	67	2.22	(1.00- 4.90)
HU	506	f	0	11	8	40	48	1.65	(0.61- 4.50)
HU	507	f	0	11	7	40	48	1.89	(0.67- 5.32)
HU	508	f	0	4	3	40	48	1.60	(0.34- 7.57)
Subtotal HU								2.00	(1.46- 2.74)
HU2	508	c	0	21	33	121	213	1.12	(0.62- 2.02)
HU2	509	c	0	64	63	121	213	1.79	(1.18- 2.70)
HU2	510	c	0	123	101	121	213	2.14	(1.52- 3.03)
HU2	511	c	0	194	113	121	213	3.02	(2.19- 4.17)
Subtotal HU2								2.18	(1.79- 2.64)
JOLY	515	m	0	11	48	12	218	4.16	(1.73- 9.99)
JOLY	516	m	0	38	61	12	218	11.32	(5.57- 22.98)
JOLY	517	m	0	85	165	12	218	9.36	(4.95- 17.70)
JOLY	518	m	0	168	182	12	218	16.77	(9.04- 31.11)
JOLY	519	m	0	250	253	12	218	17.95	(9.78- 32.93)
JOLY	501	f	0	13	28	52	283	2.53	(1.23- 5.20)
JOLY	502	f	0	18	26	52	283	3.77	(1.93- 7.36)
JOLY	503	f	0	31	24	52	283	7.03	(3.82- 12.93)
JOLY	504	f	0	47	24	52	283	10.66	(6.00- 18.92)
JOLY	505	f	0	57	20	52	283	15.51	(8.61- 27.95)
Subtotal JOLY								9.03	(7.36- 11.08)
JUSSAW	510	m	0	16	20	149	624	3.35	(1.70- 6.62)
JUSSAW	511	m	0	34	19	149	624	7.49	(4.16- 13.51)
JUSSAW	512	m	0	38	23	149	624	6.92	(4.00- 11.97)
JUSSAW	513	m	0	27	9	149	624	12.56	(5.79- 27.28)
JUSSAW	514	m	0	11	6	149	624	7.68	(2.79- 21.09)
Subtotal JUSSAW								6.77	(5.01- 9.15)
KHUDER	501	m	0	16	61	23	309	3.52	(1.76- 7.06)
KHUDER	502	m	0	207	370	23	309	7.52	(4.76- 11.86)
KHUDER	503	m	0	236	354	23	309	8.96	(5.69- 14.11)
Subtotal KHUDER								7.07	(5.28- 9.47)
KREUZE	517	m	3	-	-	-	-	4.70	(3.10- 7.14)
KREUZE	518	m	3	-	-	-	-	29.23	(19.78- 43.20)
KREUZE	520	f	3	-	-	-	-	1.33	(0.80- 2.22)
KREUZE	521	f	3	-	-	-	-	7.14	(4.92- 10.35)
Subtotal KREUZE								7.26	(5.90- 8.93)
LETOUR	506	c	0	65	187	24	224	3.24	(1.95- 5.39)
LETOUR	507	c	0	264	160	24	224	15.40	(9.68- 24.51)
LETOUR	508	c	0	374	141	24	224	24.76	(15.58- 39.35)

Table 1I1 - 5

IESLC - Meta-analysis of Ever Smoking by Duration, Overview
All LC types, Any Product (or Cigarettes if Any not available)
Least adjusted

REF	NRR	SEX	AD	Number Exposed Case	Number Exposed Cont	Non-exposed Case	Non-exposed Cont	RR	95.00%CI
Subtotal LETOUR								11.50	(8.73- 15.14)
LEVIN	501	m	0	56	97	7	96	7.92	(3.44- 18.25)
LEVIN	502	m	0	63	91	7	96	9.49	(4.13- 21.81)
Subtotal LEVIN								8.67	(4.81- 15.63)
LIU3	507	m	0	30	146	4	19	0.98	(0.31- 3.07)
LIU3	508	m	0	22	59	4	19	1.77	(0.54- 5.79)
Subtotal LIU3								1.30	(0.57- 2.97)
LIU5	504	c	0	27	37	26	41	1.15	(0.57- 2.31)
LIU5	505	c	0	58	33	26	41	2.77	(1.45- 5.32)
Subtotal LIU5								1.84	(1.14- 2.96)
LUBIN	508	m	0	30	146	8	72	1.85	(0.81- 4.24)
LUBIN	509	m	0	124	294	8	72	3.80	(1.78- 8.12)
LUBIN	510	m	0	143	251	8	72	5.13	(2.40- 10.95)
LUBIN	511	m	0	59	86	8	72	6.17	(2.77- 13.77)
Subtotal LUBIN								3.94	(2.66- 5.83)
LUBIN2	531	m	0	953	2995	190	2616	4.38	(3.72- 5.16)
LUBIN2	532	m	0	2227	3470	190	2616	8.84	(7.56- 10.33)
LUBIN2	533	m	0	2079	2551	190	2616	11.22	(9.58- 13.14)
LUBIN2	534	m	0	1325	1484	190	2616	12.29	(10.42- 14.50)
LUBIN2	574	f	0	132	230	336	1188	2.03	(1.59- 2.59)
LUBIN2	575	f	0	187	186	336	1188	3.55	(2.81- 4.50)
LUBIN2	576	f	0	155	118	336	1188	4.64	(3.55- 6.07)
LUBIN2	577	f	0	81	32	336	1188	8.95	(5.84- 13.71)
Subtotal LUBIN2								6.83	(6.37- 7.32)
MATOS	516	m	0	20	84	11	110	2.38	(1.08- 5.24)
MATOS	517	m	0	82	110	11	110	7.45	(3.77- 14.75)
MATOS	518	m	0	86	89	11	110	9.66	(4.86- 19.21)
Subtotal MATOS								5.99	(3.96- 9.05)
MCCONN	501	c	0	3	4	9	23	1.92	(0.36- 10.32)
MCCONN	502	c	0	5	19	9	23	0.67	(0.19- 2.35)
MCCONN	503	c	0	46	57	9	23	2.06	(0.87- 4.89)
MCCONN	504	c	0	21	57	9	23	0.94	(0.38- 2.36)
MCCONN	505	c	0	16	40	9	23	1.02	(0.39- 2.68)
Subtotal MCCONN								1.22	(0.76- 1.94)
NOTAN2	513	c	0	7	15	107	201	0.88	(0.35- 2.22)
NOTAN2	514	c	0	15	15	107	201	1.88	(0.88- 3.99)
NOTAN2	515	c	0	17	16	107	201	2.00	(0.97- 4.11)
NOTAN2	516	c	0	12	7	107	201	3.22	(1.23- 8.42)
NOTAN2	517	c	0	5	5	107	201	1.88	(0.53- 6.63)
Subtotal NOTAN2								1.83	(1.24- 2.70)
OSANN2	501	f	0	23	47	33	109	1.62	(0.86- 3.04)
OSANN2	502	f	0	161	57	33	109	9.33	(5.70- 15.27)
Subtotal OSANN2								4.81	(3.26- 7.10)
PEZZOT	534	m	0	30	134	4	116	6.49	(2.22- 18.98)
PEZZOT	535	m	0	71	82	4	116	25.11	(8.82- 71.48)
PEZZOT	536	m	0	110	101	4	116	31.58	(11.25- 88.71)
Subtotal PEZZOT								17.64	(9.62- 32.34)
*QIAO2	511	m	0	7	2364	10	709	0.21	(0.08- 0.55)
*QIAO2	512	m	0	54	2257	10	709	1.70	(0.87- 3.31)
*QIAO2	513	m	0	170	2295	10	709	5.25	(2.79- 9.88)
Subtotal QIAO2								1.87	(1.24- 2.83)
RACHTA	511	f	0	12	19	33	98	1.88	(0.82- 4.27)
RACHTA	512	f	0	49	21	33	98	6.93	(3.63- 13.22)
RACHTA	513	f	0	24	1	33	98	71.27	(9.28- 547.53)
Subtotal RACHTA								4.97	(3.04- 8.14)
TIZZAN	501	m	0	12	1	180	305	20.33	(2.62- 157.68)
TIZZAN	502	m	0	54	20	180	305	4.58	(2.65- 7.89)
TIZZAN	503	m	0	928	815	180	305	1.93	(1.57- 2.37)
TIZZAN	533	f	0	2	7	117	114	0.28	(0.06- 1.37)
TIZZAN	534	f	0	23	21	117	114	1.07	(0.56- 2.03)
Subtotal TIZZAN								2.01	(1.68- 2.42)
WANG2	501	c	0	4	17	11	43	0.92	(0.26- 3.29)
WANG2	503	c	0	8	18	11	43	1.74	(0.60- 5.03)
WANG2	504	c	0	26	38	11	43	2.67	(1.17- 6.13)
WANG2	505	c	0	22	26	11	43	3.31	(1.38- 7.91)
Subtotal WANG2								2.24	(1.38- 3.63)
WUWILL	501	f	0	137	139	417	601	1.42	(1.09- 1.86)
WUWILL	502	f	0	179	98	417	601	2.63	(2.00- 3.47)
WUWILL	503	f	0	223	114	417	601	2.82	(2.18- 3.65)
Subtotal WUWILL								2.20	(1.88- 2.56)
ZHENG	553	m	0	37	75	33	94	1.41	(0.80- 2.46)

Table 1I1 - 5

IESLC - Meta-analysis of Ever Smoking by Duration, Overview
All LC types, Any Product (or Cigarettes if Any not available)
Least adjusted

REF	NRR	SEX	AD	Number Exposed Case	Cont	Non-exposed Case	Cont	RR	95.00%CI
ZHENG	554	m	0	242	143	33	94	4.82	(3.08- 7.54)
ZHENG	558	f	0	17	17	152	184	1.21	(0.60- 2.45)
ZHENG	559	f	0	59	27	152	184	2.65	(1.60- 4.38)
Subtotal ZHENG								2.54	(1.94- 3.31)
ZHOU	501	c	0	170	12	507	68	1.90	(1.00- 3.60)
ZHOU	502	c	0	678	36	507	68	2.53	(1.66- 3.84)
Subtotal ZHOU								2.32	(1.63- 3.29)
Partial Totals				20841	34907	12013	45029		

*prospective study ~ With 0.5 adjustment for zero

REF	NRR	SEX	AD	Ys	Ws	Qs	Ps
AGUDO	507	f	0	-0.05	3.32	8.72	0.9303
AGUDO	508	f	0	1.32	6.08	0.39	0.0011
Subtotal AGUDO				0.84	9.40	9.11	
ARMADA	501	m	0	1.22	4.88	0.61	0.0070
ARMADA	502	m	0	2.46	6.68	5.26	0.0000
ARMADA	503	m	0	3.03	5.48	11.65	0.0000
Subtotal ARMADA				2.29	17.05	17.52	
AUVINE	501	c	0	2.02	8.26	1.63	0.0000
AUVINE	502	c	0	2.34	3.06	1.81	0.0000
AUVINE	503	c	0	3.04	20.41	44.21	0.0000
Subtotal AUVINE				2.71	31.73	47.66	
AXELSS	501	m	0	0.44	6.35	8.19	0.2713
AXELSS	502	m	0	0.98	6.98	2.48	0.0098
AXELSS	503	m	0	2.08	9.96	2.59	0.0000
AXELSS	504	m	0	2.50	11.10	9.63	0.0000
AXELSS	505	m	0	3.23	9.65	26.46	0.0000
AXELSS	510	f	0	0.58	3.29	3.26	0.2943
AXELSS	511	f	0	1.26	5.56	0.53	0.0029
AXELSS	512	f	0	2.26	7.41	3.45	0.0000
AXELSS	513	f	0	2.94	7.42	13.77	0.0000
AXELSS	514	f	0	2.84	4.72	7.57	0.0000
Subtotal AXELSS				2.07	72.43	77.93	
BARBON	501	m	0	1.37	11.69	0.47	0.0000
BARBON	502	m	0	2.29	14.48	7.47	0.0000
BARBON	503	m	0	2.54	15.92	15.01	0.0000
BARBON	504	m	0	2.59	17.31	17.86	0.0000
Subtotal BARBON				2.26	59.40	40.80	
*BOUCOT	518	m	0	2.90	0.49	0.86	0.0421
*BOUCOT	519	m	0	3.99	0.50	2.90	0.0050
Subtotal BOUCOT				3.45	0.99	3.76	
BUFFLE	526	f	0	2.14	7.75	2.51	0.0000
BUFFLE	527	f	0	2.68	8.42	10.35	0.0000
BUFFLE	528	f	0	3.00	7.86	15.92	0.0000
Subtotal BUFFLE				2.61	24.04	28.78	
CHEN2	501	m	0	0.89	1.03	0.47	0.3653
CHEN2	502	m	0	1.59	1.38	0.00	0.0623
CHEN2	503	m	0	0.95	4.13	1.58	0.0523
CHEN2	504	m	0	1.59	4.85	0.00	0.0005
CHEN2	505	m	0	1.74	5.48	0.15	0.0000
CHEN2	510	f	0	-1.51	0.81	7.70	0.1734
CHEN2	511	f	0	0.28	0.93	1.57	0.7884
CHEN2	512	f	0	1.05	3.19	0.87	0.0607
CHEN2	513	f	0	0.61	5.42	4.98	0.1529
Subtotal CHEN2				1.10	27.21	17.32	
CHOI	501	m	0	0.93	6.32	2.64	0.0199
CHOI	502	m	0	1.07	9.21	2.36	0.0012
CHOI	503	m	0	1.54	9.66	0.01	0.0000
CHOI	504	m	0	1.92	8.35	1.03	0.0000
CHOI	505	m	0	1.99	5.33	0.92	0.0000
CHOI	510	f	0	-0.73	1.59	8.45	0.3546
CHOI	511	f	0	0.21	4.64	8.62	0.6519
CHOI	512	f	0	2.16	1.55	0.53	0.0072
CHOI	513	f	0	0.77	0.50	0.32	0.5883
Subtotal CHOI				1.29	47.14	24.88	
DAMBER	506	m	1	0.46	5.52	6.87	0.2825
DAMBER	507	m	1	1.30	12.09	0.92	0.0000
DAMBER	508	m	1	1.64	17.62	0.08	0.0000
DAMBER	509	m	1	2.16	21.28	7.45	0.0000
DAMBER	510	m	1	2.42	21.42	15.19	0.0000

Table 1I1 - 5

IESLC - Meta-analysis of Ever Smoking by Duration, Overview
All LC types, Any Product (or Cigarettes if Any not available)
Least adjusted

REF	NRR	SEX	AD	Ys	Ws	Qs	Ps
Subtotal DAMBER				1.86	77.93	30.50	
DESTEF	501	m	0	1.55	11.82	0.01	0.0000
DESTEF	502	m	0	1.80	14.53	0.74	0.0000
DESTEF	503	m	0	2.41	16.73	11.64	0.0000
DESTEF	504	m	0	2.30	17.23	9.05	0.0000
Subtotal DESTEF				2.06	60.31	21.43	
DOLL	515	m	0	1.94	3.23	0.44	0.0005
DOLL	516	m	0	1.52	4.90	0.02	0.0008
DOLL	517	m	0	2.19	6.17	2.38	0.0000
DOLL	518	m	0	2.29	6.13	3.18	0.0000
DOLL	522	f	0	0.14	5.92	12.20	0.7383
DOLL	523	f	0	0.79	4.00	2.42	0.1124
DOLL	524	f	0	0.98	8.35	2.97	0.0048
DOLL	525	f	0	1.08	1.85	0.44	0.1417
Subtotal DOLL				1.37	40.55	24.05	
DORGAN	570	m	2	1.69	10.46	0.15	0.0000
DORGAN	571	m	2	2.78	11.22	16.30	0.0000
DORGAN	562	f	3	1.45	47.84	0.76	0.0000
DORGAN	563	f	3	2.46	57.93	45.82	0.0000
Subtotal DORGAN				2.05	127.45	63.02	
DOSEME	501	m	2	0.00	14.17	35.05	1.0000
DOSEME	502	m	2	1.34	24.94	1.41	0.0000
DOSEME	503	m	2	1.59	31.98	0.01	0.0000
Subtotal DOSEME				1.18	71.09	36.47	
FAN	501	m	0	0.34	13.53	20.49	0.2079
FAN	502	m	0	0.86	15.89	8.06	0.0006
FAN	503	m	0	1.36	23.17	1.07	0.0000
FAN	506	f	0	0.91	4.78	2.13	0.0478
FAN	507	f	0	1.34	8.79	0.46	0.0001
FAN	508	f	0	1.46	18.96	0.22	0.0000
Subtotal FAN				1.10	85.12	32.43	
GAO	561	f	0	0.49	27.86	32.73	0.0099
GAO	562	f	0	1.18	42.03	6.58	0.0000
Subtotal GAO				0.90	69.89	39.31	
GARSHI	534	m	0	1.83	34.49	2.22	0.0000
GER	513	c	0	0.19	6.73	12.91	0.6273
GER	514	c	0	0.20	15.61	29.62	0.4403
GER	515	c	0	0.42	19.79	26.21	0.0605
Subtotal GER				0.30	42.13	68.75	
HAENSZ	542	f	0	0.58	8.51	8.32	0.0886
HAENSZ	543	f	0	0.79	21.36	13.23	0.0003
Subtotal HAENSZ				0.73	29.87	21.55	
HU	501	m	0	0.71	10.64	7.95	0.0209
HU	502	m	0	0.74	12.94	9.08	0.0082
HU	503	m	0	0.80	6.12	3.69	0.0488
HU	506	f	0	0.50	3.82	4.39	0.3277
HU	507	f	0	0.63	3.58	3.15	0.2303
HU	508	f	0	0.47	1.59	1.93	0.5535
Subtotal HU				0.69	38.69	30.20	
HU2	508	c	0	0.11	11.00	23.43	0.7065
HU2	509	c	0	0.58	22.49	22.12	0.0058
HU2	510	c	0	0.76	32.27	21.19	0.0000
HU2	511	c	0	1.11	37.09	8.08	0.0000
Subtotal HU2				0.78	102.85	74.82	
JOLY	515	m	0	1.43	5.01	0.11	0.0014
JOLY	516	m	0	2.43	7.66	5.58	0.0000
JOLY	517	m	0	2.24	9.46	4.16	0.0000
JOLY	518	m	0	2.82	10.06	15.64	0.0000
JOLY	519	m	0	2.89	10.43	18.03	0.0000
JOLY	501	f	0	0.93	7.39	3.08	0.0118
JOLY	502	f	0	1.33	8.56	0.52	0.0001
JOLY	503	f	0	1.95	10.34	1.47	0.0000
JOLY	504	f	0	2.37	11.67	7.35	0.0000
JOLY	505	f	0	2.74	11.07	15.12	0.0000
Subtotal JOLY				2.20	91.65	71.06	
JUSSAW	510	m	0	1.21	8.28	1.10	0.0005
JUSSAW	511	m	0	2.01	11.07	2.15	0.0000
JUSSAW	512	m	0	1.93	12.80	1.67	0.0000
JUSSAW	513	m	0	2.53	6.39	5.87	0.0000
JUSSAW	514	m	0	2.04	3.76	0.81	0.0001
Subtotal JUSSAW				1.91	42.30	11.60	

Table 1I1 - 5

IESLC - Meta-analysis of Ever Smoking by Duration, Overview
All LC types, Any Product (or Cigarettes if Any not available)
Least adjusted

REF	NRR	SEX	AD	Ys	Ws	Qs	Ps
KHUDER	501	m	0	1.26	7.96	0.78	0.0004
KHUDER	502	m	0	2.02	18.43	3.64	0.0000
KHUDER	503	m	0	2.19	18.60	7.14	0.0000
Subtotal KHUDER				1.96	44.99	11.56	
KREUZE	517	m	3	1.55	22.07	0.01	0.0000
KREUZE	518	m	3	3.38	25.18	81.80	0.0000
KREUZE	520	f	3	0.29	14.75	24.46	0.2734
KREUZE	521	f	3	1.97	27.78	4.29	0.0000
Subtotal KREUZE				1.98	89.79	110.56	
LETOUR	506	c	0	1.18	14.96	2.35	0.0000
LETOUR	507	c	0	2.73	17.80	24.02	0.0000
LETOUR	508	c	0	3.21	17.89	47.90	0.0000
Subtotal LETOUR				2.44	50.65	74.26	
LEVIN	501	m	0	2.07	5.51	1.36	0.0000
LEVIN	502	m	0	2.25	5.55	2.55	0.0000
Subtotal LEVIN				2.16	11.06	3.91	
LIU3	507	m	0	-0.02	2.92	7.44	0.9669
LIU3	508	m	0	0.57	2.74	2.75	0.3441
Subtotal LIU3				0.26	5.66	10.19	
LIU5	504	c	0	0.14	7.88	16.17	0.6935
LIU5	505	c	0	1.02	9.06	2.77	0.0022
Subtotal LIU5				0.61	16.94	18.94	
LUBIN	508	m	0	0.61	5.58	5.13	0.1463
LUBIN	509	m	0	1.33	6.65	0.38	0.0006
LUBIN	510	m	0	1.63	6.67	0.03	0.0000
LUBIN	511	m	0	1.82	5.97	0.37	0.0000
Subtotal LUBIN				1.37	24.88	5.90	
LUBIN2	531	m	0	1.48	142.28	1.30	0.0000
LUBIN2	532	m	0	2.18	156.67	57.54	0.0000
LUBIN2	533	m	0	2.42	153.41	109.52	0.0000
LUBIN2	534	m	0	2.51	141.36	123.90	0.0000
LUBIN2	574	f	0	0.71	63.53	47.56	0.0000
LUBIN2	575	f	0	1.27	68.77	6.38	0.0000
LUBIN2	576	f	0	1.54	53.35	0.07	0.0000
LUBIN2	577	f	0	2.19	21.09	8.08	0.0000
Subtotal LUBIN2				1.92	800.46	354.34	
MATOS	516	m	0	0.87	6.18	3.07	0.0311
MATOS	517	m	0	2.01	8.24	1.57	0.0000
MATOS	518	m	0	2.27	8.14	3.94	0.0000
Subtotal MATOS				1.79	22.56	8.58	
MCCONN	501	c	0	0.65	1.36	1.15	0.4488
MCCONN	502	c	0	-0.40	2.46	9.53	0.5341
MCCONN	503	c	0	0.72	5.16	3.72	0.1002
MCCONN	504	c	0	-0.06	4.55	12.14	0.8977
MCCONN	505	c	0	0.02	4.13	9.94	0.9644
Subtotal MCCONN				0.20	17.65	36.47	
NOTAN2	513	c	0	-0.13	4.47	12.98	0.7808
NOTAN2	514	c	0	0.63	6.77	6.01	0.1008
NOTAN2	515	c	0	0.69	7.37	5.73	0.0606
NOTAN2	516	c	0	1.17	4.16	0.68	0.0171
NOTAN2	517	c	0	0.63	2.41	2.14	0.3273
Subtotal NOTAN2				0.60	25.18	27.55	
OSANN2	501	f	0	0.48	9.59	11.45	0.1369
OSANN2	502	f	0	2.23	15.81	6.90	0.0000
Subtotal OSANN2				1.57	25.41	18.35	
PEZZOT	534	m	0	1.87	3.34	0.30	0.0006
PEZZOT	535	m	0	3.22	3.51	9.56	0.0000
PEZZOT	536	m	0	3.45	3.60	12.73	0.0000
Subtotal PEZZOT				2.87	10.45	22.59	
*QIAO2	511	m	0	-1.56	4.15	40.75	0.0015
*QIAO2	512	m	0	0.53	8.57	9.35	0.1218
*QIAO2	513	m	0	1.66	9.61	0.07	0.0000
Subtotal QIAO2				0.63	22.33	50.17	
RACHTA	511	f	0	0.63	5.67	5.05	0.1344
RACHTA	512	f	0	1.94	9.21	1.21	0.0000
RACHTA	513	f	0	4.27	0.92	6.70	0.0000
Subtotal RACHTA				1.60	15.80	12.97	
TIZZAN	501	m	0	3.01	0.92	1.90	0.0039
TIZZAN	502	m	0	1.52	12.93	0.04	0.0000
TIZZAN	503	m	0	0.66	89.78	75.27	0.0000
TIZZAN	533	f	0	-1.28	1.51	12.32	0.1155

Table 1I1 - 5

IESLC - Meta-analysis of Ever Smoking by Duration, Overview
All LC types, Any Product (or Cigarettes if Any not available)
Least adjusted

REF	NRR	SEX	AD	Ys	Ws	Qs	Ps
TIZZAN	534	f	0	0.06	9.22	20.97	0.8435
Subtotal TIZZAN				0.70	114.36	110.49	
WANG2	501	c	0	-0.08	2.36	6.49	0.8977
WANG2	503	c	0	0.55	3.39	3.53	0.3089
WANG2	504	c	0	0.98	5.59	1.94	0.0200
WANG2	505	c	0	1.20	5.05	0.72	0.0072
Subtotal WANG2				0.81	16.39	12.68	
WUWILL	501	f	0	0.35	53.89	80.46	0.0100
WUWILL	502	f	0	0.97	50.37	18.43	0.0000
WUWILL	503	f	0	1.04	57.74	16.61	0.0000
Subtotal WUWILL				0.79	162.01	115.50	
ZHENG	553	m	0	0.34	12.30	18.69	0.2328
ZHENG	554	m	0	1.57	19.21	0.00	0.0000
ZHENG	558	f	0	0.19	7.71	14.73	0.5957
ZHENG	559	f	0	0.97	15.15	5.46	0.0002
Subtotal ZHENG				0.93	54.37	38.87	
ZHOU	501	c	0	0.64	9.44	8.18	0.0485
ZHOU	502	c	0	0.93	21.77	9.09	0.0000
Subtotal ZHOU				0.84	31.22	17.28	

N	178
NS	43

Table 1I1 - 6

IESLC - Meta-analysis of Ever Smoking by Duration, Overview
All LC types, Any Product (or Cigarettes if Any not available)
Least adjusted

	Sex			
	combined	male	female	Total
N	31	90	57	178
NS	9	27	19	55

In this overview table, other than the "N" rows, entries in the "absent" and "Total" columns may be invalid and should be ignored

	Duration of smoking (broad categories)				
	absent	1-34k20	21-49k35	36+k50	Total
N	68	43	32	35	178
NS	31	34	26	28	119
Wt	1006.82	645.90	562.70	550.44	2765.86
Het Chi	724.86	219.19	244.63	337.22	1856.30
Het df	67	42	31	34	177
Het P	***	***	***	***	***
Fixed RR	4.97	2.78	5.35	7.80	4.82
RRl	4.68	2.57	4.93	7.17	4.64
RRu	5.29	3.00	5.82	8.48	5.00
P	+++	+++	+++	+++	+++
Random RR	3.65	2.50	5.32	8.09	4.16
RRl	2.93	2.05	4.12	6.04	3.65
RRu	4.55	3.04	6.88	10.84	4.73
P	+++	+++	+++	+++	+++

	Duration of smoking (narrow categories)							
	absent	1-19k1	6-29k20	21-39k30	31-49k40	41-998k50	51+k999	Total
N	97	24	15	23	17	1	1	178
NS	42	17	11	17	13	1	1	101
Wt	1610.75	168.75	136.06	466.12	341.48	21.28	21.42	2765.86
Het Chi	1197.39	66.60	40.25	163.10	72.11	0.00	0.00	1856.30
Het df	96	23	14	22	16	0	0	177
Het P	***	***	***	***	***	N.S.	N.S.	***
Fixed RR	4.66	2.03	3.00	5.06	9.01	8.71	11.19	4.82
RRl	4.43	1.74	2.54	4.62	8.11	5.70	7.33	4.64
RRu	4.89	2.36	3.55	5.54	10.02	13.32	17.09	5.00
P	+++	+++	+++	+++	+++	+++	+++	+++
Random RR	4.51	1.85	2.95	4.48	8.43	8.71	11.19	4.16
RRl	3.75	1.40	2.18	3.37	6.42	5.70	7.33	3.65
RRu	5.42	2.43	4.00	5.95	11.07	13.32	17.09	4.73
P	+++	+++	+++	+++	+++	+++	+++	+++

MALES

	Duration of smoking (broad categories)				
	absent	1-34k20	21-49k35	36+k50	Total
N	34	21	16	19	90
NS	21	21	16	19	77
Wt	599.69	318.51	311.61	318.59	1548.40
Het Chi	393.36	98.53	63.16	56.82	807.65
Het df	33	20	15	18	89
Het P	***	***	***	***	***
Fixed RR	6.11	3.54	7.28	10.51	6.33
RRl	5.64	3.18	6.52	9.41	6.02
RRu	6.61	3.96	8.14	11.73	6.65
P	+++	+++	+++	+++	+++
Random RR	5.50	2.88	6.46	10.09	5.57
RRl	4.05	2.16	4.91	7.96	4.73
RRu	7.46	3.84	8.50	12.79	6.55
P	+++	+++	+++	+++	+++

Table 1I1 - 6

IESLC - Meta-analysis of Ever Smoking by Duration, Overview
All LC types, Any Product (or Cigarettes if Any not available)
Least adjusted

MALES

		Duration of smoking (narrow categories)						
	absent	1-19k1	6-29k20	21-39k30	31-49k40	41-998k50	51+k999	Total
N	49	10	7	12	10	1	1	90
NS	27	10	7	12	10	1	1	67
Wt	823.37	90.02	75.91	268.17	248.23	21.28	21.42	1548.40
Het Chi	530.80	31.19	19.84	45.30	23.17	0.00	0.00	807.65
Het df	48	9	6	11	9	0	0	89
Het P	***	***	**	***	**	N.S.	N.S.	***
Fixed RR	5.78	2.85	3.99	7.28	10.46	8.71	11.19	6.33
RRl	5.39	2.32	3.18	6.46	9.24	5.70	7.33	6.02
RRu	6.18	3.51	4.99	8.21	11.85	13.32	17.09	6.65
P	+++	+++	+++	+++	+++	+++	+++	+++
Random RR	5.64	2.84	4.11	5.94	9.69	8.71	11.19	5.57
RRl	4.40	1.88	2.64	4.37	7.43	5.70	7.33	4.73
RRu	7.24	4.28	6.38	8.08	12.64	13.32	17.09	6.55
P	+++	+++	+++	+++	+++	+++	+++	+++

FEMALES

	Duration of smoking (broad categories)				
	absent	1-34k20	21-49k35	36+k50	Total
N	24	14	9	10	57
NS	15	14	9	10	48
Wt	332.78	251.76	168.06	130.13	882.73
Het Chi	214.05	61.70	36.85	75.69	476.41
Het df	23	13	8	9	56
Het P	***	***	***	***	***
Fixed RR	4.27	2.19	3.97	5.05	3.57
RRl	3.84	1.94	3.41	4.25	3.34
RRu	4.76	2.48	4.62	6.00	3.81
P	+++	+++	+++	+++	+++
Random RR	2.78	2.20	5.35	7.23	3.42
RRl	1.94	1.61	3.62	3.96	2.77
RRu	3.98	3.01	7.91	13.20	4.23
P	+++	+++	+++	+++	+++

		Duration of smoking (narrow categories)						
	absent	1-19k1	6-29k20	21-39k30	31-49k40	41-998k50	51+k999	Total
N	32	9	4	7	5			57
NS	19	9	4	7	5			44
Wt	578.08	50.10	22.34	148.17	84.05			882.73
Het Chi	343.04	9.92	4.65	18.76	24.93			476.41
Het df	31	8	3	6	4			56
Het P	***	N.S.	N.S.	**	***			***
Fixed RR	3.59	1.41	2.63	3.56	6.49			3.57
RRl	3.31	1.07	1.74	3.03	5.24			3.34
RRu	3.90	1.87	3.99	4.19	8.04			3.81
P	+++	+	+++	+++	+++			+++
Random RR	3.70	1.39	2.53	4.31	8.52			3.42
RRl	2.75	1.00	1.49	2.97	4.41			2.77
RRu	4.98	1.91	4.28	6.24	16.47			4.23
P	+++	+	+++	+++	+++			+++

Table 1I1 - 7

IESLC - Meta-analysis of Ever Smoking by Duration, Overview
All LC types, Any Product (or Cigarettes if Any not available)
Excluded studies (and stage at which they were excluded)

1	AKIBA AMANDU AMES BECHER BENSHL BEST BLOT1 BROSS BROWN3 CARPEN CEDERL CHYOU CPSI CPSII DARBY DEAN2 DEAN3 DOLL2 ENGELA GAO2 GARCIA GILLIS GRAHAM GURSEL HAMMO2 HIRAYA HOLE HUMBLE JAHN JAIN KAISE2 KATSOU KAUFMA LAUSSM LIAW MCDUFF MIGRAN MRFITR PEZZO2 PISANI PRESCO QIAO SEGI2 SPEIZE SVENSS TVERDA WAKAI WATSON WIGLE WU WYNDE3 WYNDE8
2	ALDERS BRESLO CHIAZZ DORN GUO HEGMAN KOO KOULUM LIU4 PERNU SOBUE SPITZ SUZUK2 VUTUC YUAN
3	GENG STASZE WU2 ZHANG
4	BOUCHA CHEN CORREA JEDRYC LUO WYNDE2 WYNDE6
5	HAMMON RESTRE SADOWS XU
7	BOFFET WYNDE7
14	BENHAM

Table 1I1 - 8

Potentially overlapping studies

REF	REFGP	PRINC	.	OVERLAP/LINK
LUBIN2	LUBIN2	1		Lubin-combined
OSANN2	KAISER	2		KAISER/OSANN2
LUBIN	XIANGZ	2		LUBIN/XIANGZ/QIAO

Table 1I1 - 9

Most adjusted - insufficient data for meta-analysis

REF	NRR	SEX	AGEL	AGEH	RACE	YF	LC TYPE	LOC	START	ST	NLC	R	VB	P	H	AD	PRODUCT	exL	exH	S1	S2	DENOM	De
BUFFLE	501	m	0	0	wh	-	all	NAmer	1976	CC	943	n	bl	y	n	0	cig+/-ot	1	33	1	0	nev cigs	or
BUFFLE	502	m	0	0	wh	-	all	NAmer	1976	CC	943	n	bl	y	n	0	cig+/-ot	34	43	2	4	nev cigs	or
BUFFLE	503	m	0	0	wh	-	all	NAmer	1976	CC	943	n	bl	y	n	0	cig+/-ot	44	49	0	0	nev cigs	or
BUFFLE	504	m	0	0	wh	-	all	NAmer	1976	CC	943	n	bl	y	n	0	cig+/-ot	50	999	3	0	nev cigs	or
GARSHI	535	m	0	0	all	-	all	NAmer	1981	CC	1081	o	bl	y	n	1	all/unsp	1	19	0	1	nev any	ot
HAMMON	513	m	0	0	wh	0	all	NAmer	1952	pr	448	n	bl	n	n	1	cig only	1	34	1	0	nev any	st
HAMMON	514	m	0	0	wh	0	all	NAmer	1952	pr	448	n	bl	n	n	1	cig only	35	999	0	0	nev any	st
SADOWS	522	m	0	0	wh	-	all	NAmer	1938	CC	477	n	bl	n	n	0	cig only	1	9	0	1	nev any	ot
SADOWS	523	m	0	0	wh	-	all	NAmer	1938	CC	477	n	bl	n	n	0	cig only	10	19	0	0	nev any	ot
SADOWS	524	m	0	0	wh	-	all	NAmer	1938	CC	477	n	bl	n	n	0	cig only	20	29	1	2	nev any	ot
SADOWS	525	m	0	0	wh	-	all	NAmer	1938	CC	477	n	bl	n	n	0	cig only	30	39	2	3	nev any	ot
SADOWS	526	m	0	0	wh	-	all	NAmer	1938	CC	477	n	bl	n	n	0	cig only	40	49	0	4	nev any	ot
SADOWS	527	m	0	0	wh	-	all	NAmer	1938	CC	477	n	bl	n	n	0	cig only	50	999	3	0	nev any	ot
XU	501	m	0	0	all	-	all	As:Chi	1985	CC	729	n	ot	n	n	2	all/unsp	1	29	1	0	nev any	or
XU	502	m	0	0	all	-	all	As:Chi	1985	CC	729	n	ot	n	n	2	all/unsp	30	39	2	3	nev any	or
XU	503	m	0	0	all	-	all	As:Chi	1985	CC	729	n	ot	n	n	2	all/unsp	40	999	3	0	nev any	or

REF	NRR	RR	SIG	RRDATA comment
BUFFLE	501	6.80		0
BUFFLE	502	11.10		0
BUFFLE	503	9.40		0
BUFFLE	504	14.50		0
GARSHI	535	*	gap	0
HAMMON	513	*		RR for <1/2 pack per day is 5.31, that for 1/2 to 1 pack per day is 6.56, that for 1 to 2 packs per day is 7.27 while that for 2+ packs per day 10.78
HAMMON	514	*		RR for <1/2 pack per day is 10.86, that for 1/2 to 1 pack per day is 8.20, that for 1 to 2 packs per day is 19.69 while that for 2+ packs per day 22.89
SADOWS	522	1.19		0
SADOWS	523	1.16		0
SADOWS	524	2.78		0
SADOWS	525	3.95		0
SADOWS	526	7.00		0
SADOWS	527	8.43		0

Table 1I1 - 9

IESLC - Meta-analysis of Ever Smoking by Duration, Overview
All LC types, Any Product (or Cigarettes if Any not available)
Most adjusted - insufficient data for meta-analysis

REF	NRR	RR	SIG	RRDATA comment
XU	501	*		RR for 1-19/day is 1.8(p<0.05), for 20-29/day is 1.5(p<0.05) and for >=30/ day is 5.3(p<0.05)
XU	502	*		RR for 1-19/day is 2.1(p<0.05), for 20-29/day is 2.7(p<0.05) and for >=30/ day is 4.9(p<0.05)
XU	503	*		RR for 1-19/day is 3.3(p<0.05), for 20-29/day is 6.0(p<0.05) and for >=30/ day is 17.1(p<0.05)

Least adjusted - insufficient data for meta-analysis: as for adjusted plus the following

REF	NRR	SEX	AGEL	AGEH	RACE	YF	LC TYPE	LOC	START	ST	NLC	R	VB	P	H	AD	PRODUCT	exL	exH	S1	S2	DENOM	De
GARSHI	533	m	0	0	all	-	all	NAmer	1981	CC	1081	o	bl	y	n	0	all/unsp	1	19	0	1	nev any	ot

REF	NRR	RR	SIG	RRDATA comment
GARSHI	533	*	gap	0

Table 1I2 -

IESLC - Meta-analysis of Ever Smoking, Duration, "Low"
All LC types, Any Product (or Cigarettes if Any not available)

This analysis is restricted to results for:
1) Ever smokers
2) Results by Duration
3) Categorical results by Duration
4) All LC types (or near equivalent)
5) Results complete enough for use in metaanalysis

Within each study, results are then selected (in the following order of preference, within each sex) for:
6) (not applicable)
7) PRODUCT: all/unspec, cigarettes regardless of other products, cigarettes only
8) CIGTYPE: all/unspecified, MC regardless of HR, MC only
9) (not applicable)
10) DENOM: never smoked anything, never smoked cigarettes, never any + low, never cigs + low
11) Followup period (YF, prospective studies): whole study (coded as 0) or longest available
12) LCtype: all or nearest available, at least Squamous and Adeno. (q = squamous, s = small,
l = large, a = adeno, mix = mixed, alv = alveolar)
13) Race: all or nearest available, otherwise by race (wh or w = white, bl or b = black, hi = hispanic
ch = chinese, jap = japanese, haw = hawaiian, w+o = white + oriental, sca = scandinavian, as = asian)
14) Duration "low" in key scheme 1 (key value 20, maximum range 1-34)
15) For overlapping studies: principal rather than subsidiary studies
Finally by Age: whole study (coded as 0) if available, otherwise by widest available age group
and then for single sex results (m, f) in preference to results for both sexes combined (c).

Results adjusted (AD) for the most potential confounders are then chosen in Sections -1 to -3
and results adjusted for the least confounders in Sections -4 to -6. (Those least adjusted results which
actually differ from the most adjusted are marked 'x' in column X in Section -4)

Section -7 shows excluded studies, together with the stage (as above) at which no qualifying
results were found.

Section -8 lists the potentially overlapping studies which have been included (1=principal, 2=subsidiary).

Section -9 lists any results which would have been included in preference except that they had data not complete
enough for use in meta-analysis, with their significance (yes/no), if known, and any further comment as entered
on the database. It also lists as "gap" any categories for which no data were presented by the original authors.

In addition to those mentioned above, the following fields, levels and abbreviations are used:
* or nk = not known, n = no, y = yes, ot = other
nev = never
all/unspec = all or unspecified, cig+/-ot = cigarettes irrespective of other products (cigar, pipe etc)
MC = manufactured cigarettes, HR = hand-rolled cigarettes
exL, exH = range of exposure (low and high) in the smoking group, in terms of Duration
REF: 6-character study reference
NRR: number of the RR on the database within the study
ST : study type (CC = case control, pr or prosp = prospective)
NLC: number of lung cancer cases in whole study
R : risky occupational population (n = no, m = mining, o = other risky)
VB : national cigarette type (V = at least 75% Virginia, bl = at least 75% blended, ot = other)
P : any proxy use
H : full histological confirmation
De : derivation of RR/CI (or = original, st = standard method, ot = other method of estimation)

Table 1I2 - 1

IESLC - Meta-analysis of Ever Smoking, Duration, "Low"
All LC types, Any Product (or Cigarettes if Any not available)
Most adjusted

REF	NRR	SEX	AGEL	AGEH	RACE	YF	LC TYPE	LOC	START	ST	NLC	R	VB	P	H	AD	PRODUCT	exL	exH	DENOM	De
ARMADA	506	m	0	0	all	-	all	Eu:wst	1986	CC	325	n	bl	n	y	1	cig+/-ot	1	24	nev cigs	or
AUVINE	517	c	0	0	all	-	all	Eu:Sca	1986	CC	517	n	bl	y	n	2	cig+/-ot	1	20	nev cigs	or
AXELSS	520	m	0	0	sca	-	all	Eu:Sca	1989	CC	436	n	bl	n	n	6	all/unsp	20	29	nev any	ot
AXELSS	511	f	0	0	sca	-	all	Eu:Sca	1989	CC	436	n	bl	n	n	0	all/unsp	20	29	nev any	st
BARBON	508	m	0	0	all	-	all	Eu:wst	1979	CC	755	n	bl	y	y	1	all/unsp	1	29	nev any	or
BUFFLE	526	f	0	0	w-hi	-	all	NAmer	1976	CC	943	n	bl	y	n	0	cig+/-ot	1	30	nev cigs	or
CHEN2	502	m	0	0	all	-	all	As:Chi	1983	CC	193	n	ot	y	n	0	all/unsp	10	20	nev any	st
CHEN2	510	f	0	0	all	-	all	As:Chi	1983	CC	193	n	ot	y	n	0	all/unsp	1	20	nev any	st
CHOI	502	m	0	0	all	-	all	As:oth	1985	CC	375	n	bl	n	n	0	cig+/-ot	20	29	nev cigs	st
CHOI	511	f	0	0	all	-	all	As:oth	1985	CC	375	n	bl	n	n	0	cig+/-ot	20	29	nev cigs	st
DAMBER	506	m	0	0	all	-	all	Eu:Sca	1972	CC	579	n	bl	y	n	1	all/unsp	1	20	nev any	ot
DESTEF	508	m	0	0	all	-	all	SCAmer	1988	CC	497	n	bl	n	y	4	all/unsp	1	29	nev any	or
DORGAN	570	m	0	0	wh	-	all	NAmer	1980	CC	2026	n	bl	y	y	2	cig+/-ot	1	34	nev any	ot
DORGAN	562	f	0	0	all	-	all	NAmer	1980	CC	2026	n	bl	y	y	3	cig+/-ot	1	34	nev any	ot
DOSEME	502	m	0	0	all	-	all	Eu:bal	1979	CC	1210	n	bl	n	n	2	cig+/-ot	11	20	nev cigs	or
FAN	501	m	0	0	all	-	all	As:Chi	1990	CC	403	n	ot	y	n	0	cig+/-ot	1	29	nev cigs	st
FAN	506	f	0	0	all	-	all	As:Chi	1990	CC	403	n	ot	y	n	0	cig+/-ot	1	29	nev cigs	st
GAO	564	f	0	0	all	-	all	As:Chi	1984	CC	1405	n	ot	n	n	2	cig+/-ot	1	29	nev cigs	ot
GER	518	c	0	0	all	-	all	As:oth	1990	CC	141	n	ot	y	n	5	all/unsp	1	20	nev any	ot
HU	502	m	0	0	all	-	all	As:Chi	1985	CC	227	n	ot	n	y	0	cig+/-ot	20	29	nev cigs	st
HU	507	f	0	0	all	-	all	As:Chi	1985	CC	227	n	ot	n	y	0	cig+/-ot	20	29	nev cigs	st
HU2	509	c	0	0	all	-	all	As:Chi	1977	CC	523	n	ot	y	n	0	cig+/-ot	20	29	nev cigs	or
JOLY	516	m	0	0	all	-	all	SCAmer	1978	CC	826	n	bl	n	n	0	cig+/-ot	20	29	nev any	st
JOLY	502	f	0	0	all	-	all	SCAmer	1978	CC	826	n	bl	n	n	0	cig+/-ot	20	29	nev any	st
JUSSAW	512	m	0	0	all	-	all	As:Ind	1964	CC	792	n	V	n	n	0	cig only	20	29	nev any	st
KHUDER	501	m	0	0	all	-	all	NAmer	1985	CC	482	n	bl	n	y	0	cig+/-ot	1	29	nev cigs	st
LETOUR	506	c	0	0	all	-	all	NAmer	1983	CC	738	n	V	y	y	0	cig+/-ot	1	24	nev cigs	st
LIU3	510	m	0	0	all	-	all	As:Chi	1985	CC	110	n	ot	n	n	2	all/unsp	1	34	nev any	or
LIU5	504	c	0	0	all	-	all	As:Chi	1978	CC	111	n	ot	y	n	0	all/unsp	1	29	nev any	st
LUBIN	508	m	0	0	all	-	all	As:Chi	1984	CC	427	m	ot	y	n	0	cig+/-ot	1	29	nev any	st
LUBIN2	531	m	0	0	all	-	all	Eu:mul	1976	CC	7804	n	bl	n	y	0	cig+/-ot	1	29	nev any	st
LUBIN2	574	f	0	0	all	-	all	Eu:mul	1976	CC	7804	n	bl	n	y	0	cig+/-ot	1	29	nev any	st
MATOS	536	m	0	0	all	-	all	SCAmer	1994	CC	200	n	bl	n	n	2	cig+/-ot	1	24	nev any	or
MCCONN	503	c	0	0	all	-	all	Eu:UK	1946	CC	100	n	V	n	y	0	all/unsp	20	29	nev any	st
NOTAN2	514	c	0	0	all	-	all	As:Ind	1963	CC	683	n	V	n	n	0	cig only	11	20	nev any	st
OSANN2	504	f	0	0	all	-	all	NAmer	1964	ot	217	n	bl	n	y	1	cig+/-ot	1	20	nev cigs	or
PEZZOT	534	m	0	0	all	-	all	SCAmer	1987	CC	215	n	bl	n	y	0	cig only	1	30	nev cigs	st
QIAO2	516	m	0	0	all	0	all	As:Chi	1992	pr	241	m	ot	n	n	1	all/unsp	1	27	nev any	or
RACHTA	516	f	0	0	all	-	all	Eu:est	1991	CC	118	n	bl	n	y	1	cig+/-ot	1	20	nev cigs	or
WANG2	503	c	0	0	all	-	all	As:Chi	1980	CC	103	n	ot	n	n	0	cig+/-ot	20	29	nev cigs	st
WUWILL	516	f	0	0	all	-	all	As:Chi	1985	CC	965	n	ot	n	n	3	cig+/-ot	1	29	nev cigs	ot
ZHENG	553	m	0	0	all	-	all	As:Chi	1982	CC	540	n	ot	*	y	0	cig+/-ot	1	29	nev cigs	st
ZHENG	558	f	0	0	all	-	all	As:Chi	1982	CC	540	n	ot	*	y	0	cig+/-ot	1	29	nev cigs	st

Cigarette type is all/unspec for all RRs

except for the following:

REF	NRR	CIGTYPE
JUSSAW	512	MC only
NOTAN2	514	MC only

Table 1I2 - 2

IESLC - Meta-analysis of Ever Smoking, Duration, "Low"
All LC types, Any Product (or Cigarettes if Any not available)
Most adjusted

REF	NRR	SEX	AD	Number Exposed Case	Cont	Non-exposed Case	Cont	RR	95.00%CI
ARMADA	506	m	1	21	-	8	-	2.60	(1.00- 6.60)
AUVINE	517	c	2	26	-	44	-	20.10	(6.69- 66.00)
AXELSS	520	m	6	17	-	16	-	2.23	(1.03- 4.80)
AXELSS	511	f	0	12	29	18	154	3.54	(1.54- 8.13)
Subtotal AXELSS								2.76	(1.57- 4.85)
BARBON	508	m	1	42	-	22	-	3.20	(1.80- 5.70)
BUFFLE	526	f	0	52	57	12	112	8.51	(4.21- 17.22)
CHEN2	502	m	0	4	3	9	33	4.89	(0.92- 25.93)
CHEN2	510	f	0	1	6	25	33	0.22	(0.02- 1.95)
Subtotal CHEN2								1.55	(0.41- 5.85)
CHOI	502	m	0	66	166	13	95	2.91	(1.52- 5.54)
CHOI	511	f	0	8	14	76	164	1.23	(0.50- 3.06)
Subtotal CHOI								2.18	(1.29- 3.69)
DAMBER	506	m	1	-	-	42	-	1.58	(0.69- 3.66)
DESTEF	508	m	4	43	-	27	-	3.40	(1.70- 6.80)
DORGAN	570	m	2	-	-	-	-	5.44	(2.97- 9.98)
DORGAN	562	f	3	-	-	-	-	4.25	(3.20- 5.64)
Subtotal DORGAN								4.44	(3.44- 5.74)
DOSEME	502	m	2	158	-	142	-	3.80	(2.60- 5.70)
FAN	501	m	0	29	135	36	236	1.41	(0.83- 2.40)
FAN	506	f	0	8	15	69	320	2.47	(1.01- 6.06)
Subtotal FAN								1.63	(1.03- 2.58)
GAO	564	f	2	68	-	435	-	1.89	(1.30- 2.75)
GER	518	c	5	10	-	51	-	1.30	(0.55- 3.06)
HU	502	m	0	60	47	41	67	2.09	(1.21- 3.60)
HU	507	f	0	11	7	40	48	1.89	(0.67- 5.32)
Subtotal HU								2.04	(1.26- 3.31)
HU2	509	c	0	64	63	121	213	1.79	(1.18- 2.70)
JOLY	516	m	0	38	61	12	218	11.32	(5.57- 22.98)
JOLY	502	f	0	18	26	52	283	3.77	(1.93- 7.36)
Subtotal JOLY								6.33	(3.89- 10.30)
JUSSAW	512	m	0	38	23	149	624	6.92	(4.00- 11.97)
KHUDER	501	m	0	16	61	23	309	3.52	(1.76- 7.06)
LETOUR	506	c	0	65	187	24	224	3.24	(1.95- 5.39)
LIU3	510	m	2	30	-	4	-	1.07	(0.25- 4.59)
LIU5	504	c	0	27	37	26	41	1.15	(0.57- 2.31)
LUBIN	508	m	0	30	146	8	72	1.85	(0.81- 4.24)
LUBIN2	531	m	0	953	2995	190	2616	4.38	(3.72- 5.16)
LUBIN2	574	f	0	132	230	336	1188	2.03	(1.59- 2.59)
Subtotal LUBIN2								3.45	(3.01- 3.96)
MATOS	536	m	2	20	-	11	-	2.20	(1.00- 4.90)
MCCONN	503	c	0	46	57	9	23	2.06	(0.87- 4.89)
NOTAN2	514	c	0	15	15	107	201	1.88	(0.88- 3.99)
OSANN2	504	f	1	23	-	33	-	1.60	(0.70- 3.50)
PEZZOT	534	m	0	30	134	4	116	6.49	(2.22- 18.98)
*QIAO2	516	m	1	7	-	10	-	0.40	(0.15- 1.05)
RACHTA	516	f	1	12	-	33	-	2.02	(0.87- 4.71)
WANG2	503	c	0	8	18	11	43	1.74	(0.60- 5.03)
WUWILL	516	f	3	137	-	417	-	1.35	(1.04- 1.73)
ZHENG	553	m	0	37	75	33	94	1.41	(0.80- 2.46)
ZHENG	558	f	0	17	17	152	184	1.21	(0.60- 2.45)
Subtotal ZHENG								1.33	(0.86- 2.06)
Partial Totals				2399	4624	2891	7711		

*prospective study

Table 1I2 - 2

IESLC - Meta-analysis of Ever Smoking, Duration, "Low"
All LC types, Any Product (or Cigarettes if Any not available)
Most adjusted

REF	NRR	SEX	AD	Ys	Ws	Qs	Ps
ARMADA	506	m	1	0.96	4.31	0.01	0.0472
AUVINE	517	c	2	3.00	2.93	11.60	0.0000
AXELSS	520	m	6	0.80	6.49	0.29	0.0411
AXELSS	511	f	0	1.26	5.56	0.35	0.0029
Subtotal AXELSS				1.02	12.05	0.64	
BARBON	508	m	1	1.16	11.56	0.26	0.0001
BUFFLE	526	f	0	2.14	7.75	9.89	0.0000
CHEN2	502	m	0	1.59	1.38	0.46	0.0623
CHEN2	510	f	0	-1.51	0.81	5.16	0.1734
Subtotal CHEN2				0.44	2.19	5.62	
CHOI	502	m	0	1.07	9.21	0.03	0.0012
CHOI	511	f	0	0.21	4.64	2.99	0.6519
Subtotal CHOI				0.78	13.84	3.01	
DAMBER	506	m	1	0.46	5.52	1.70	0.2825
DESTEF	508	m	4	1.22	8.00	0.36	0.0005
DORGAN	570	m	2	1.69	10.46	4.86	0.0000
DORGAN	562	f	3	1.45	47.84	9.04	0.0000
Subtotal DORGAN				1.49	58.30	13.90	
DOSEME	502	m	2	1.34	24.94	2.60	0.0000
FAN	501	m	0	0.34	13.53	6.07	0.2079
FAN	506	f	0	0.91	4.78	0.05	0.0478
Subtotal FAN				0.49	18.31	6.12	
GAO	564	f	2	0.64	27.37	3.86	0.0009
GER	518	c	5	0.26	5.22	2.93	0.5490
HU	502	m	0	0.74	12.94	0.99	0.0082
HU	507	f	0	0.63	3.58	0.51	0.2303
Subtotal HU				0.71	16.52	1.50	
HU2	509	c	0	0.58	22.49	4.18	0.0058
JOLY	516	m	0	2.43	7.66	15.31	0.0000
JOLY	502	f	0	1.33	8.56	0.85	0.0001
Subtotal JOLY				1.85	16.22	16.16	
JUSSAW	512	m	0	1.93	12.80	10.89	0.0000
KHUDER	501	m	0	1.26	7.96	0.49	0.0004
LETOUR	506	c	0	1.18	14.96	0.41	0.0000
LIU3	510	m	2	0.07	1.81	1.62	0.9274
LIU5	504	c	0	0.14	7.88	5.99	0.6935
LUBIN	508	m	0	0.61	5.58	0.88	0.1463
LUBIN2	531	m	0	1.48	142.28	30.78	0.0000
LUBIN2	574	f	0	0.71	63.53	5.89	0.0000
Subtotal LUBIN2				1.24	205.80	36.67	
MATOS	536	m	2	0.79	6.08	0.30	0.0518
MCCONN	503	c	0	0.72	5.16	0.43	0.1002
NOTAN2	514	c	0	0.63	6.77	0.99	0.1008
OSANN2	504	f	1	0.47	5.93	1.74	0.2523
PEZZOT	534	m	0	1.87	3.34	2.46	0.0006
*QIAO2	516	m	1	-0.92	4.06	15.09	0.0649
RACHTA	516	f	1	0.70	5.39	0.51	0.1027
WANG2	503	c	0	0.55	3.39	0.72	0.3089
WUWILL	516	f	3	0.30	59.33	30.08	0.0208
ZHENG	553	m	0	0.34	12.30	5.55	0.2328
ZHENG	558	f	0	0.19	7.71	5.20	0.5957
Subtotal ZHENG				0.28	20.01	10.75	

Table 1I2 - 2

IESLC - Meta-analysis of Ever Smoking, Duration, "Low"
All LC types, Any Product (or Cigarettes if Any not available)
Most adjusted

	N	43
	NS	34
	Wt	633.79
Het	Chi	204.37
Het	df	42
Het	P	***
Fixed	RR	2.75
	RRl	2.55
	RRu	2.97
	P	+++
Random	RR	2.52
	RRl	2.07
	RRu	3.06
	P	+++
Asymm	P	N.S.

Table 1I2 - 3

IESLC - Meta-analysis of Ever Smoking, Duration, "Low"
All LC types, Any Product (or Cigarettes if Any not available)
Most adjusted

	Sex			
	combined	male	female	Total
N	8	21	14	43
NS	8	21	14	43
Wt	68.80	312.21	252.78	633.79
Het Chi	22.62	83.05	63.25	204.37
Het df	7	20	13	42
Het P	**	***	***	***
Fixed RR	2.12	3.50	2.20	2.75
RRl	1.68	3.13	1.94	2.55
RRu	2.69	3.91	2.48	2.97
P	+++	+++	+++	+++
Random RR	2.25	2.85	2.24	2.52
RRl	1.42	2.17	1.62	2.07
RRu	3.56	3.74	3.08	3.06
P	+++	+++	+++	+++
Between Chi				35.44
Between df				2
Between P				***
Btwn(F) P				*
Btwn(R) P				N.S.

	Lung cancer type		
	all	other	Total
N	43		43
NS	34		34
Wt	633.79		633.79
Het Chi	204.37		204.37
Het df	42		42
Het P	***		***
Fixed RR	2.75		2.75
RRl	2.55		2.55
RRu	2.97		2.97
P	+++		+++
Random RR	2.52		2.52
RRl	2.07		2.07
RRu	3.06		3.06
P	+++		+++
Between Chi			
Between df			
Between P			N.S.
Btwn(F) P			N.S.
Btwn(R) P			N.S.

	Location								
	NAmer	UK	Scand	othEur	China	Japan	othAs	other	Total
N	6	1	4	6	16		5	5	43
NS	5	1	3	5	12		4	4	34
Wt	94.90	5.16	20.50	252.01	188.96		38.63	33.64	633.79
Het Chi	11.29	0.00	13.55	28.18	19.12		17.71	10.97	204.37
Het df	5	0	3	5	15		4	4	42
Het P	*	N.S.	**	***	N.S.		**	*	***
Fixed RR	4.10	2.06	3.16	3.42	1.51		2.90	4.52	2.75
RRl	3.35	0.87	2.05	3.02	1.31		2.12	3.23	2.55
RRu	5.02	4.89	4.86	3.87	1.75		3.98	6.34	2.97
P	+++	N.S.	+++	+++	+++		+++	+++	+++
Random RR	4.02	2.06	3.71	3.00	1.52		2.39	4.55	2.52
RRl	2.85	0.87	1.46	2.06	1.27		1.21	2.57	2.07
RRu	5.66	4.89	9.46	4.37	1.82		4.73	8.04	3.06
P	+++	N.S.	++	+++	+++		+	+++	+++
Between Chi									103.54
Between df									6
Between P									***
Btwn(F) P									***
Btwn(R) P									***

Table 1I2 - 3

IESLC - Meta-analysis of Ever Smoking, Duration, "Low"
All LC types, Any Product (or Cigarettes if Any not available)
Most adjusted

Detailed Country in "other Europe"

		multi	Germany	othWest	East	Balkans	Total
	N	2		2	1	1	6
	NS	1		2	1	1	5
	Wt	205.80		15.88	5.39	24.94	252.01
Het	Chi	26.02		0.14	0.00	0.00	28.18
Het	df	1		1	0	0	5
Het	P	***		N.S.	N.S.	N.S.	***
Fixed	RR	3.45		3.02	2.02	3.80	3.42
	RRl	3.01		1.85	0.87	2.57	3.02
	RRu	3.96		4.95	4.70	5.63	3.87
	P	+++		+++	N.S.	+++	+++
Random	RR	3.00		3.02	2.02	3.80	3.00
	RRl	1.41		1.85	0.87	2.57	2.06
	RRu	6.37		4.95	4.70	5.63	4.37
	P	++		+++	N.S.	+++	+++
Between	Chi						2.03
Between	df						3
Between	P						N.S.
Btwn(F)	P						N.S.
Btwn(R)	P						N.S.

Detailed Country in "other Asia"

		India	HongKong	other	Total
	N	2		3	5
	NS	2		2	4
	Wt	19.58		19.06	38.63
Het	Chi	7.53		3.28	17.71
Het	df	1		2	4
Het	P	**		N.S.	**
Fixed	RR	4.41		1.89	2.90
	RRl	2.83		1.21	2.12
	RRu	6.86		2.97	3.98
	P	+++		++	+++
Random	RR	3.70		1.79	2.39
	RRl	1.03		1.00	1.21
	RRu	13.27		3.23	4.73
	P	+		(+)	+
Between	Chi				6.90
Between	df				1
Between	P				**
Btwn(F)	P				N.S.
Btwn(R)	P				N.S.

Detailed other continent

		SCAmer	Total
	N	5	5
	NS	4	4
	Wt	33.64	33.64
Het	Chi	10.97	10.97
Het	df	4	4
Het	P	*	*
Fixed	RR	4.52	4.52
	RRl	3.23	3.23
	RRu	6.34	6.34
	P	+++	+++
Random	RR	4.55	4.55
	RRl	2.57	2.57
	RRu	8.04	8.04
	P	+++	+++
Between	Chi		
Between	df		
Between	P		N.S.
Btwn(F)	P		N.S.
Btwn(R)	P		N.S.

Table 1I2 - 3

IESLC - Meta-analysis of Ever Smoking, Duration, "Low"
All LC types, Any Product (or Cigarettes if Any not available)
Most adjusted

	Start year of study					
	<1960	1960-69	1970-79	1980-89	1990+	Total
N	1	3	10	23	6	43
NS	1	3	8	17	5	34
Wt	5.16	25.51	302.16	261.91	39.05	633.79
Het Chi	0.00	12.20	65.81	81.96	9.80	204.37
Het df	0	2	9	22	5	42
Het P	N.S.	**	***	***	(*)	***
Fixed RR	2.06	3.48	3.35	2.37	1.48	2.75
RRl	0.87	2.36	2.99	2.10	1.08	2.55
RRu	4.89	5.13	3.75	2.67	2.02	2.97
P	N.S.	+++	+++	+++	+	+++
Random RR	2.06	2.84	3.21	2.51	1.46	2.52
RRl	0.87	1.05	2.24	1.92	0.92	2.07
RRu	4.89	7.68	4.62	3.27	2.30	3.06
P	N.S.	+	+++	+++	N.S.	+++
Between Chi						34.58
Between df						4
Between P						***
Btwn(F) P						N.S.
Btwn(R) P						N.S.

	Study type (1)		
	CC	other	Total
N	41	2	43
NS	32	2	34
Wt	623.80	9.99	633.79
Het Chi	187.34	4.63	204.37
Het df	40	1	42
Het P	***	*	***
Fixed RR	2.80	0.91	2.75
RRl	2.59	0.49	2.55
RRu	3.03	1.69	2.97
P	+++	N.S.	+++
Random RR	2.64	0.82	2.52
RRl	2.17	0.21	2.07
RRu	3.20	3.20	3.06
P	+++	N.S.	+++
Between Chi			12.40
Between df			1
Between P			***
Btwn(F) P			N.S.
Btwn(R) P			(*)

	Study type (2)			
	CC	prosp	other	Total
N	41	1	1	43
NS	32	1	1	34
Wt	623.80	4.06	5.93	633.79
Het Chi	187.34	0.00	0.00	204.37
Het df	40	0	0	42
Het P	***	N.S.	N.S.	***
Fixed RR	2.80	0.40	1.60	2.75
RRl	2.59	0.15	0.72	2.55
RRu	3.03	1.06	3.58	2.97
P	+++	(-)	N.S.	+++
Random RR	2.64	0.40	1.60	2.52
RRl	2.17	0.15	0.72	2.07
RRu	3.20	1.06	3.58	3.06
P	+++	(-)	N.S.	+++
Between Chi				17.03
Between df				2
Between P				***
Btwn(F) P				N.S.
Btwn(R) P				***

Table 1I2 - 3

IESLC - Meta-analysis of Ever Smoking, Duration, "Low"
All LC types, Any Product (or Cigarettes if Any not available)
Most adjusted

	Study size (number of LC cases) 100-249	250-499	500-999	1000+	Total
N	14	10	13	6	43
NS	12	7	11	4	34
Wt	66.97	70.05	180.35	316.41	633.79
Het Chi	22.73	9.61	92.53	40.41	204.37
Het df	13	9	12	5	42
Het P	*	N.S.	***	***	***
Fixed RR	1.68	2.33	2.36	3.46	2.75
RRl	1.32	1.85	2.04	3.10	2.55
RRu	2.14	2.95	2.73	3.86	2.97
P	+++	+++	+++	+++	+++
Random RR	1.66	2.34	3.18	3.32	2.52
RRl	1.19	1.84	2.06	2.34	2.07
RRu	2.31	2.99	4.91	4.69	3.06
P	++	+++	+++	+++	+++
Between Chi					39.09
Between df					3
Between P					***
Btwn(F) P					*
Btwn(R) P					*

	Risky occupational population no	mining	othRisky	Total
N	41	2		43
NS	32	2		34
Wt	624.15	9.64		633.79
Het Chi	188.23	5.51		204.37
Het df	40	1		42
Het P	***	*		***
Fixed RR	2.80	0.97		2.75
RRl	2.59	0.52		2.55
RRu	3.02	1.83		2.97
P	+++	N.S.		+++
Random RR	2.63	0.88		2.52
RRl	2.17	0.20		2.07
RRu	3.19	3.94		3.06
P	+++	N.S.		+++
Between Chi				10.62
Between df				1
Between P				**
Btwn(F) P				N.S.
Btwn(R) P				N.S.

	National cigarette tobacco type Virginia	blended	other	Total
N	4	22	17	43
NS	4	17	13	34
Wt	39.69	399.93	194.17	633.79
Het Chi	10.09	73.97	19.24	204.37
Het df	3	21	16	42
Het P	*	***	N.S.	***
Fixed RR	3.56	3.59	1.51	2.75
RRl	2.61	3.26	1.31	2.55
RRu	4.86	3.96	1.74	2.97
P	+++	+++	+++	+++
Random RR	3.22	3.49	1.51	2.52
RRl	1.78	2.80	1.28	2.07
RRu	5.83	4.36	1.79	3.06
P	+++	+++	+++	+++
Between Chi				101.06
Between df				2
Between P				***
Btwn(F) P				***
Btwn(R) P				***

Table 1I2 - 3

IESLC - Meta-analysis of Ever Smoking, Duration, "Low"
All LC types, Any Product (or Cigarettes if Any not available)
Most adjusted

	Any proxy use No/nk	Yes	Total
N	28	15	43
NS	22	12	34
Wt	471.10	162.69	633.79
Het Chi	140.67	62.84	204.37
Het df	27	14	42
Het P	***	***	***
Fixed RR	2.69	2.93	2.75
RRl	2.46	2.51	2.55
RRu	2.95	3.42	2.97
P	+++	+++	+++
Random RR	2.41	2.73	2.52
RRl	1.90	1.90	2.07
RRu	3.06	3.92	3.06
P	+++	+++	+++
Between Chi			0.85
Between df			1
Between P			N.S.
Btwn(F) P			N.S.
Btwn(R) P			N.S.

	Full histological confirmation No	Yes	Total
N	26	17	43
NS	21	13	34
Wt	266.55	367.24	633.79
Het Chi	121.64	59.91	204.37
Het df	25	16	42
Het P	***	***	***
Fixed RR	2.20	3.23	2.75
RRl	1.95	2.92	2.55
RRu	2.48	3.58	2.97
P	+++	+++	+++
Random RR	2.37	2.75	2.52
RRl	1.77	2.16	2.07
RRu	3.16	3.49	3.06
P	+++	+++	+++
Between Chi			22.82
Between df			1
Between P			***
Btwn(F) P			*
Btwn(R) P			N.S.

	Number of adjustment variables (1) 0	1	2+/+nk	Total
N	26	6	11	43
NS	19	6	10	35
Wt	396.54	36.78	200.47	633.79
Het Chi	114.98	13.79	66.04	204.37
Het df	25	5	10	42
Het P	***	*	***	***
Fixed RR	2.98	1.87	2.52	2.75
RRl	2.70	1.35	2.20	2.55
RRu	3.29	2.58	2.90	2.97
P	+++	+++	+++	+++
Random RR	2.61	1.68	2.83	2.52
RRl	2.03	0.97	1.89	2.07
RRu	3.34	2.92	4.24	3.06
P	+++	(+)	+++	+++
Between Chi				9.56
Between df				2
Between P				**
Btwn(F) P				N.S.
Btwn(R) P				N.S.

Table 1I2 - 3

IESLC - Meta-analysis of Ever Smoking, Duration, "Low"
All LC types, Any Product (or Cigarettes if Any not available)
Most adjusted

	Number of adjustment variables (2) 0	1	2	3-5	6+/+nk	Total
N	26	6	6	4	1	43
NS	19	6	6	4	1	36
Wt	396.54	36.78	73.60	120.39	6.49	633.79
Het Chi	114.98	13.79	24.04	37.76	0.00	204.37
Het df	25	5	5	3	0	42
Het P	***	*	***	***	N.S.	***
Fixed RR	2.98	1.87	3.05	2.26	2.23	2.75
RRl	2.70	1.35	2.43	1.89	1.03	2.55
RRu	3.29	2.58	3.84	2.70	4.81	2.97
P	+++	+++	+++	+++	+	+++
Random RR	2.61	1.68	3.48	2.28	2.23	2.52
RRl	2.03	0.97	1.96	1.09	1.03	2.07
RRu	3.34	2.92	6.18	4.77	4.81	3.06
P	+++	(+)	+++	+	+	+++
Between Chi						13.79
Between df						4
Between P						**
Btwn(F) P						N.S.
Btwn(R) P						N.S.

	Product all/unsp	cig+/-ot	cig only	Total
N	12	28	3	43
NS	10	21	3	34
Wt	63.44	547.44	22.92	633.79
Het Chi	26.55	155.20	7.96	204.37
Het df	11	27	2	42
Het P	**	***	*	***
Fixed RR	1.93	2.81	4.66	2.75
RRl	1.51	2.58	3.10	2.55
RRu	2.46	3.05	7.02	2.97
P	+++	+++	+++	+++
Random RR	1.76	2.68	4.36	2.52
RRl	1.17	2.14	1.80	2.07
RRu	2.63	3.36	10.55	3.06
P	++	+++	++	+++
Between Chi				14.66
Between df				2
Between P				***
Btwn(F) P				N.S.
Btwn(R) P				(*)

	Denominator nev any	nev cigs	Total
N	22	21	43
NS	17	17	34
Wt	375.01	258.79	633.79
Het Chi	100.88	70.30	204.37
Het df	21	20	42
Het P	***	***	***
Fixed RR	3.33	2.09	2.75
RRl	3.01	1.85	2.55
RRu	3.68	2.36	2.97
P	+++	+++	+++
Random RR	2.65	2.38	2.52
RRl	2.02	1.85	2.07
RRu	3.47	3.05	3.06
P	+++	+++	+++
Between Chi			33.19
Between df			1
Between P			***
Btwn(F) P			**
Btwn(R) P			N.S.

Table 1I2 - 3

IESLC - Meta-analysis of Ever Smoking, Duration, "Low"
All LC types, Any Product (or Cigarettes if Any not available)
Most adjusted

	Derivation of RR/CI			
	Orig	StdCalc	Other	Total
N	12	24	7	43
NS	12	17	6	35
Wt	105.26	366.30	162.23	633.79
Het Chi	48.17	100.54	46.08	204.37
Het df	11	23	6	42
Het P	***	***	***	***
Fixed RR	2.76	3.01	2.25	2.75
RRl	2.28	2.71	1.93	2.55
RRu	3.34	3.33	2.62	2.97
P	+++	+++	+++	+++
Random RR	2.67	2.53	2.27	2.52
RRl	1.74	1.95	1.41	2.07
RRu	4.10	3.27	3.68	3.06
P	+++	+++	+++	+++
Between Chi				9.58
Between df				2
Between P				**
Btwn(F) P				N.S.
Btwn(R) P				N.S.

Table 1I2 - 4

IESLC - Meta-analysis of Ever Smoking, Duration, "Low"
All LC types, Any Product (or Cigarettes if Any not available)
Least adjusted

REF	NRR	X	SEX	AGEL	AGEH	RACE	YF	LC TYPE	LOC	START	ST	NLC	R	VB	P	H	AD	PRODUCT	exL	exH	DENOM	De
ARMADA	501	x	m	0	0	all	-	all	Eu:wst	1986	CC	325	n	bl	n	y	0	cig+/-ot	1	24	nev cigs	st
AUVINE	501	x	c	0	0	all	-	all	Eu:Sca	1986	CC	517	n	bl	y	n	0	cig+/-ot	1	20	nev cigs	st
AXELSS	502	x	m	0	0	sca	-	all	Eu:Sca	1989	CC	436	n	bl	n	n	0	all/unsp	20	29	nev any	st
AXELSS	511		f	0	0	sca	-	all	Eu:Sca	1989	CC	436	n	bl	n	n	0	all/unsp	20	29	nev any	st
BARBON	501	x	m	0	0	all	-	all	Eu:wst	1979	CC	755	n	bl	y	y	0	all/unsp	1	29	nev any	st
BUFFLE	526		f	0	0	w-hi	-	all	NAmer	1976	CC	943	n	bl	y	n	0	cig+/-ot	1	30	nev cigs	or
CHEN2	502		m	0	0	all	-	all	As:Chi	1983	CC	193	n	ot	y	n	0	all/unsp	10	20	nev any	st
CHEN2	510		f	0	0	all	-	all	As:Chi	1983	CC	193	n	ot	y	n	0	all/unsp	1	20	nev any	st
CHOI	502		m	0	0	all	-	all	As:oth	1985	CC	375	n	bl	n	n	0	cig+/-ot	20	29	nev cigs	st
CHOI	511		f	0	0	all	-	all	As:oth	1985	CC	375	n	bl	n	n	0	cig+/-ot	20	29	nev cigs	st
DAMBER	506		m	0	0	all	-	all	Eu:Sca	1972	CC	579	n	bl	y	n	1	all/unsp	1	20	nev any	ot
DESTEF	501	x	m	0	0	all	-	all	SCAmer	1988	CC	497	n	bl	n	y	0	all/unsp	1	29	nev any	st
DORGAN	570		m	0	0	wh	-	all	NAmer	1980	CC	2026	n	bl	y	y	2	cig+/-ot	1	34	nev any	ot
DORGAN	562		f	0	0	all	-	all	NAmer	1980	CC	2026	n	bl	y	y	3	cig+/-ot	1	34	nev any	ot
DOSEME	502		m	0	0	all	-	all	Eu:bal	1979	CC	1210	n	bl	n	n	2	cig+/-ot	11	20	nev cigs	or
FAN	501		m	0	0	all	-	all	As:Chi	1990	CC	403	n	ot	y	n	0	cig+/-ot	1	29	nev cigs	st
FAN	506		f	0	0	all	-	all	As:Chi	1990	CC	403	n	ot	y	n	0	cig+/-ot	1	29	nev cigs	st
GAO	561	x	f	0	0	all	-	all	As:Chi	1984	CC	1405	n	ot	n	n	0	cig+/-ot	1	29	nev cigs	st
GER	513	x	c	0	0	all	-	all	As:oth	1990	CC	141	n	ot	y	n	0	all/unsp	1	20	nev any	st
HU	502		m	0	0	all	-	all	As:Chi	1985	CC	227	n	ot	n	y	0	cig+/-ot	20	29	nev cigs	st
HU	507		f	0	0	all	-	all	As:Chi	1985	CC	227	n	ot	n	y	0	cig+/-ot	20	29	nev cigs	st
HU2	509		c	0	0	all	-	all	As:Chi	1977	CC	523	n	ot	y	n	0	cig+/-ot	20	29	nev cigs	or
JOLY	516		m	0	0	all	-	all	SCAmer	1978	CC	826	n	bl	n	n	0	cig+/-ot	20	29	nev any	st
JOLY	502		f	0	0	all	-	all	SCAmer	1978	CC	826	n	bl	n	n	0	cig+/-ot	20	29	nev any	st
JUSSAW	512		m	0	0	all	-	all	As:Ind	1964	CC	792	n	V	n	n	0	cig only	20	29	nev any	st
KHUDER	501		m	0	0	all	-	all	NAmer	1985	CC	482	n	bl	n	y	0	cig+/-ot	1	29	nev cigs	st
LETOUR	506		c	0	0	all	-	all	NAmer	1983	CC	738	n	V	y	y	0	cig+/-ot	1	24	nev cigs	st
LIU3	507	x	m	0	0	all	-	all	As:Chi	1985	CC	110	n	ot	n	n	0	all/unsp	1	34	nev any	or
LIU5	504		c	0	0	all	-	all	As:Chi	1978	CC	111	n	ot	y	n	0	all/unsp	1	29	nev any	st
LUBIN	508		m	0	0	all	-	all	As:Chi	1984	CC	427	m	ot	y	n	0	cig+/-ot	1	29	nev any	st
LUBIN2	531		m	0	0	all	-	all	Eu:mul	1976	CC	7804	n	bl	n	y	0	cig+/-ot	1	29	nev any	st
LUBIN2	574		f	0	0	all	-	all	Eu:mul	1976	CC	7804	n	bl	n	y	0	cig+/-ot	1	29	nev any	st
MATOS	516	x	m	0	0	all	-	all	SCAmer	1994	CC	200	n	bl	n	n	0	cig+/-ot	1	24	nev any	st
MCCONN	503		c	0	0	all	-	all	Eu:UK	1946	CC	100	n	V	n	y	0	all/unsp	20	29	nev any	st
NOTAN2	514		c	0	0	all	-	all	As:Ind	1963	CC	683	n	V	n	n	0	cig only	11	20	nev any	st
OSANN2	501	x	f	0	0	all	-	all	NAmer	1964	ot	217	n	bl	n	y	0	cig+/-ot	1	20	nev cigs	st
PEZZOT	534		m	0	0	all	-	all	SCAmer	1987	CC	215	n	bl	n	y	0	cig only	1	30	nev cigs	st
QIAO2	511	x	m	0	0	all	0	all	As:Chi	1992	pr	241	m	ot	n	n	0	all/unsp	1	27	nev any	st
RACHTA	511	x	f	0	0	all	-	all	Eu:est	1991	CC	118	n	bl	n	y	0	cig+/-ot	1	20	nev cigs	st
WANG2	503		c	0	0	all	-	all	As:Chi	1980	CC	103	n	ot	n	n	0	cig+/-ot	20	29	nev cigs	st
WUWILL	501	x	f	0	0	all	-	all	As:Chi	1985	CC	965	n	ot	n	n	0	cig+/-ot	1	29	nev cigs	st
ZHENG	553		m	0	0	all	-	all	As:Chi	1982	CC	540	n	ot	*	y	0	cig+/-ot	1	29	nev cigs	st
ZHENG	558		f	0	0	all	-	all	As:Chi	1982	CC	540	n	ot	*	y	0	cig+/-ot	1	29	nev cigs	st

Cigarette type is all/unspec for all RRs

except for the following:

REF	NRR	CIGTYPE
JUSSAW	512	MC only
NOTAN2	514	MC only

Table 1I2 - 5

IESLC - Meta-analysis of Ever Smoking, Duration, "Low"
All LC types, Any Product (or Cigarettes if Any not available)
Least adjusted

REF	NRR	SEX	AD	Number Exposed Case	Number Exposed Cont	Non-exposed Case	Non-exposed Cont	RR	95.00%CI
ARMADA	501	m	0	21	55	8	71	3.39	(1.40- 8.23)
AUVINE	501	c	0	26	18	44	229	7.52	(3.80- 14.87)
AXELSS	502	m	0	17	64	16	160	2.66	(1.27- 5.58)
AXELSS	511	f	0	12	29	18	154	3.54	(1.54- 8.13)
Subtotal AXELSS								3.02	(1.73- 5.25)
BARBON	501	m	0	42	91	22	188	3.94	(2.22- 7.00)
BUFFLE	526	f	0	52	57	12	112	8.51	(4.21- 17.22)
CHEN2	502	m	0	4	3	9	33	4.89	(0.92- 25.93)
CHEN2	510	f	0	1	6	25	33	0.22	(0.02- 1.95)
Subtotal CHEN2								1.55	(0.41- 5.85)
CHOI	502	m	0	66	166	13	95	2.91	(1.52- 5.54)
CHOI	511	f	0	8	14	76	164	1.23	(0.50- 3.06)
Subtotal CHOI								2.18	(1.29- 3.69)
DAMBER	506	m	1	-	-	42	-	1.58	(0.69- 3.66)
DESTEF	501	m	0	43	55	27	163	4.72	(2.67- 8.35)
DORGAN	570	m	2	-	-	-	-	5.44	(2.97- 9.98)
DORGAN	562	f	3	-	-	-	-	4.25	(3.20- 5.64)
Subtotal DORGAN								4.44	(3.44- 5.74)
DOSEME	502	m	2	158	-	142	-	3.80	(2.60- 5.70)
FAN	501	m	0	29	135	36	236	1.41	(0.83- 2.40)
FAN	506	f	0	8	15	69	320	2.47	(1.01- 6.06)
Subtotal FAN								1.63	(1.03- 2.58)
GAO	561	f	0	68	58	435	605	1.63	(1.12- 2.36)
GER	513	c	0	10	40	51	246	1.21	(0.57- 2.57)
HU	502	m	0	60	47	41	67	2.09	(1.21- 3.60)
HU	507	f	0	11	7	40	48	1.89	(0.67- 5.32)
Subtotal HU								2.04	(1.26- 3.31)
HU2	509	c	0	64	63	121	213	1.79	(1.18- 2.70)
JOLY	516	m	0	38	61	12	218	11.32	(5.57- 22.98)
JOLY	502	f	0	18	26	52	283	3.77	(1.93- 7.36)
Subtotal JOLY								6.33	(3.89- 10.30)
JUSSAW	512	m	0	38	23	149	624	6.92	(4.00- 11.97)
KHUDER	501	m	0	16	61	23	309	3.52	(1.76- 7.06)
LETOUR	506	c	0	65	187	24	224	3.24	(1.95- 5.39)
LIU3	507	m	0	30	146	4	19	0.98	(0.31- 3.07)
LIU5	504	c	0	27	37	26	41	1.15	(0.57- 2.31)
LUBIN	508	m	0	30	146	8	72	1.85	(0.81- 4.24)
LUBIN2	531	m	0	953	2995	190	2616	4.38	(3.72- 5.16)
LUBIN2	574	f	0	132	230	336	1188	2.03	(1.59- 2.59)
Subtotal LUBIN2								3.45	(3.01- 3.96)
MATOS	516	m	0	20	84	11	110	2.38	(1.08- 5.24)
MCCONN	503	c	0	46	57	9	23	2.06	(0.87- 4.89)
NOTAN2	514	c	0	15	15	107	201	1.88	(0.88- 3.99)
OSANN2	501	f	0	23	47	33	109	1.62	(0.86- 3.04)
PEZZOT	534	m	0	30	134	4	116	6.49	(2.22- 18.98)
*QIAO2	511	m	0	7	2364	10	709	0.21	(0.08- 0.55)
RACHTA	511	f	0	12	19	33	98	1.88	(0.82- 4.27)
WANG2	503	c	0	8	18	11	43	1.74	(0.60- 5.03)
WUWILL	501	f	0	137	139	417	601	1.42	(1.09- 1.86)
ZHENG	553	m	0	37	75	33	94	1.41	(0.80- 2.46)
ZHENG	558	f	0	17	17	152	184	1.21	(0.60- 2.45)
Subtotal ZHENG								1.33	(0.86- 2.06)
Partial Totals				2399	7804	2891	11019		

*prospective study

Table 1I2 - 5

IESLC - Meta-analysis of Ever Smoking, Duration, "Low"
All LC types, Any Product (or Cigarettes if Any not available)
Least adjusted

REF	NRR	SEX	AD	Ys	Ws	Qs	Ps
ARMADA	501	m	0	1.22	4.88	0.19	0.0070
AUVINE	501	c	0	2.02	8.26	8.17	0.0000
AXELSS	502	m	0	0.98	6.98	0.01	0.0098
AXELSS	511	f	0	1.26	5.56	0.33	0.0029
Subtotal AXELSS				1.10	12.54	0.34	
BARBON	501	m	0	1.37	11.69	1.43	0.0000
BUFFLE	526	f	0	2.14	7.75	9.71	0.0000
CHEN2	502	m	0	1.59	1.38	0.44	0.0623
CHEN2	510	f	0	-1.51	0.81	5.20	0.1734
Subtotal CHEN2				0.44	2.19	5.64	
CHOI	502	m	0	1.07	9.21	0.02	0.0012
CHOI	511	f	0	0.21	4.64	3.06	0.6519
Subtotal CHOI				0.78	13.84	3.08	
DAMBER	506	m	1	0.46	5.52	1.76	0.2825
DESTEF	501	m	0	1.55	11.82	3.31	0.0000
DORGAN	570	m	2	1.69	10.46	4.72	0.0000
DORGAN	562	f	3	1.45	47.84	8.62	0.0000
Subtotal DORGAN				1.49	58.30	13.34	
DOSEME	502	m	2	1.34	24.94	2.44	0.0000
FAN	501	m	0	0.34	13.53	6.26	0.2079
FAN	506	f	0	0.91	4.78	0.07	0.0478
Subtotal FAN				0.49	18.31	6.32	
GAO	561	f	0	0.49	27.86	7.93	0.0099
GER	513	c	0	0.19	6.73	4.69	0.6273
HU	502	m	0	0.74	12.94	1.07	0.0082
HU	507	f	0	0.63	3.58	0.54	0.2303
Subtotal HU				0.71	16.52	1.60	
HU2	509	c	0	0.58	22.49	4.38	0.0058
JOLY	516	m	0	2.43	7.66	15.09	0.0000
JOLY	502	f	0	1.33	8.56	0.79	0.0001
Subtotal JOLY				1.85	16.22	15.88	
JUSSAW	512	m	0	1.93	12.80	10.65	0.0000
KHUDER	501	m	0	1.26	7.96	0.45	0.0004
LETOUR	506	c	0	1.18	14.96	0.36	0.0000
LIU3	507	m	0	-0.02	2.92	3.20	0.9669
LIU5	504	c	0	0.14	7.88	6.13	0.6935
LUBIN	508	m	0	0.61	5.58	0.93	0.1463
LUBIN2	531	m	0	1.48	142.28	29.45	0.0000
LUBIN2	574	f	0	0.71	63.53	6.29	0.0000
Subtotal LUBIN2				1.24	205.80	35.74	
MATOS	516	m	0	0.87	6.18	0.15	0.0311
MCCONN	503	c	0	0.72	5.16	0.46	0.1002
NOTAN2	514	c	0	0.63	6.77	1.04	0.1008
OSANN2	501	f	0	0.48	9.59	2.82	0.1369
PEZZOT	534	m	0	1.87	3.34	2.40	0.0006
*QIAO2	511	m	0	-1.56	4.15	27.69	0.0015
RACHTA	511	f	0	0.63	5.67	0.88	0.1344
WANG2	503	c	0	0.55	3.39	0.75	0.3089
WUWILL	501	f	0	0.35	53.89	24.29	0.0100
ZHENG	553	m	0	0.34	12.30	5.72	0.2328
ZHENG	558	f	0	0.19	7.71	5.33	0.5957
Subtotal ZHENG				0.28	20.01	11.05	

Table 1I2 - 5

IESLC - Meta-analysis of Ever Smoking, Duration, "Low"
All LC types, Any Product (or Cigarettes if Any not available)
Least adjusted

	N	43
	NS	34
	Wt	645.90
Het	Chi	219.19
Het	df	42
Het	P	***
Fixed	RR	2.78
	RRl	2.57
	RRu	3.00
	P	+++
Random	RR	2.50
	RRl	2.05
	RRu	3.04
	P	+++
Asymm	P	N.S.

Table 1I2 - 6

IESLC - Meta-analysis of Ever Smoking, Duration, "Low"
All LC types, Any Product (or Cigarettes if Any not available)
Least adjusted

		combined	Sex<br>male	female	Total
	N	8	21	14	43
	NS	8	21	14	43
	Wt	75.64	318.51	251.76	645.90
Het	Chi	21.81	98.53	61.70	219.19
Het	df	7	20	13	42
Het	P	**	***	***	***
Fixed	RR	2.20	3.54	2.19	2.78
	RRl	1.75	3.18	1.94	2.57
	RRu	2.75	3.96	2.48	3.00
	P	+++	+++	+++	+++
Random	RR	2.16	2.88	2.20	2.50
	RRl	1.42	2.16	1.61	2.05
	RRu	3.29	3.84	3.01	3.04
	P	+++	+++	+++	+++
Between	Chi				37.15
Between	df				2
Between	P				***
Btwn(F)	P				*
Btwn(R)	P				N.S.

Table 1I2 - 7

IESLC - Meta-analysis of Ever Smoking, Duration, "Low"
All LC types, Any Product (or Cigarettes if Any not available)
Excluded studies (and stage at which they were excluded)

1	AKIBA AMANDU AMES BECHER BENSHL BEST BLOT1 BROSS BROWN3 CARPEN CEDERL CHYOU CPSI CPSII DARBY DEAN2 DEAN3 DOLL2 ENGELA GAO2 GARCIA GILLIS GRAHAM GURSEL HAMMO2 HIRAYA HOLE HUMBLE JAHN JAIN KAISE2 KATSOU KAUFMA LAUSSM LIAW MCDUFF MIGRAN MRFITR PEZZO2 PISANI PRESCO QIAO SEGI2 SPEIZE SVENSS TVERDA WAKAI WATSON WIGLE WU WYNDE3 WYNDE8
2	ALDERS BRESLO CHIAZZ DORN GUO HEGMAN KOO KOULUM LIU4 PERNU SOBUE SPITZ SUZUK2 VUTUC YUAN
3	GENG STASZE WU2 ZHANG
4	BOUCHA CHEN CORREA JEDRYC LUO WYNDE2 WYNDE6
5	HAMMON RESTRE SADOWS XU
7	BOFFET WYNDE7
14	AGUDO BOUCOT DOLL GARSHI HAENSZ KREUZE LEVIN TIZZAN ZHOU
15	BENHAM

Table 1I2 - 8
Potentially overlapping studies

REF	REFGP	PRINC	. OVERLAP/LINK
LUBIN2	LUBIN2	1	Lubin-combined
OSANN2	KAISER	2	KAISER/OSANN2
LUBIN	XIANGZ	2	LUBIN/XIANGZ/QIAO

Table 1I2 - 9
Most adjusted - insufficient data for meta-analysis

REF	NRR	SEX	AGEL	AGEH	RACE	YF	LC TYPE	LOC	START	ST	NLC	R	VB	P	H	AD	PRODUCT	exL	exH	DENOM	De
BUFFLE	501	m	0	0	wh	-	all	NAmer	1976	CC	943	n	bl	y	n	0	cig+/-ot	1	33	nev cigs	or
HAMMON	513	m	0	0	wh	0	all	NAmer	1952	pr	448	n	bl	n	n	1	cig only	1	34	nev any	st
SADOWS	524	m	0	0	wh	-	all	NAmer	1938	CC	477	n	bl	n	n	0	cig only	20	29	nev any	ot
XU	501	m	0	0	all	-	all	As:Chi	1985	CC	729	n	ot	n	n	2	all/unsp	1	29	nev any	or

REF	NRR	RR	SIG	RRDATA comment
BUFFLE	501	6.80		0
HAMMON	513	*		RR for <1/2 pack per day is 5.31, that for 1/2 to 1 pack per day is 6.56, that for 1 to 2 packs per day is 7.27 while that for 2+ packs per day 10.78
SADOWS	524	2.78		0
XU	501	*		RR for 1-19/day is 1.8(p<0.05), for 20-29/day is 1.5(p<0.05) and for >=30/ day is 5.3(p<0.05)

Table 1I3 -

IESLC - Meta-analysis of Ever Smoking, Duration, "Mid"
All LC types, Any Product (or Cigarettes if Any not available)

This analysis is restricted to results for:
1) Ever smokers
2) Results by Duration
3) Categorical results by Duration
4) All LC types (or near equivalent)
5) Results complete enough for use in metaanalysis

Within each study, results are then selected (in the following order of preference, within each sex) for:
6) (not applicable)
7) PRODUCT: all/unspec, cigarettes regardless of other products, cigarettes only
8) CIGTYPE: all/unspecified, MC regardless of HR, MC only
9) (not applicable)
10) DENOM: never smoked anything, never smoked cigarettes, never any + low, never cigs + low
11) Followup period (YF, prospective studies): whole study (coded as 0) or longest available
12) LCtype: all or nearest available, at least Squamous and Adeno. (q = squamous, s = small,
l = large, a = adeno, mix = mixed, alv = alveolar)
13) Race: all or nearest available, otherwise by race (wh or w = white, bl or b = black, hi = hispanic
ch = chinese, jap = japanese, haw = hawaiian, w+o = white + oriental, sca = scandinavian, as = asian)
14) Duration "mid" in key scheme 1 (key value 35, maximum range 21-49)
15) For overlapping studies: principal rather than subsidiary studies
Finally by Age: whole study (coded as 0) if available, otherwise by widest available age group
and then for single sex results (m, f) in preference to results for both sexes combined (c).

Results adjusted (AD) for the most potential confounders are then chosen in Sections -1 to -3
and results adjusted for the least confounders in Sections -4 to -6. (Those least adjusted results which
actually differ from the most adjusted are marked 'x' in column X in Section -4)

Section -7 shows excluded studies, together with the stage (as above) at which no qualifying
results were found.

Section -8 lists the potentially overlapping studies which have been included (1=principal, 2=subsidiary).

Section -9 lists any results which would have been included in preference except that they had data not complete
enough for use in meta-analysis, with their significance (yes/no), if known, and any further comment as entered
on the database. It also lists as "gap" any categories for which no data were presented by the original authors.

In addition to those mentioned above, the following fields, levels and abbreviations are used:
* or nk = not known, n = no, y = yes, ot = other
nev = never
all/unspec = all or unspecified, cig+/-ot = cigarettes irrespective of other products (cigar, pipe etc)
MC = manufactured cigarettes, HR = hand-rolled cigarettes
exL, exH = range of exposure (low and high) in the smoking group, in terms of Duration
REF: 6-character study reference
NRR: number of the RR on the database within the study
ST : study type (CC = case control, pr or prosp = prospective)
NLC: number of lung cancer cases in whole study
R : risky occupational population (n = no, m = mining, o = other risky)
VB : national cigarette type (V = at least 75% Virginia, bl = at least 75% blended, ot = other)
P : any proxy use
H : full histological confirmation
De : derivation of RR/CI (or = original, st = standard method, ot = other method of estimation)

Table 1I3 - 1

IESLC - Meta-analysis of Ever Smoking, Duration, "Mid"
All LC types, Any Product (or Cigarettes if Any not available)
Most adjusted

REF	NRR	SEX	AGEL	AGEH	RACE	YF	LC TYPE	LOC	START	ST	NLC	R	VB	P	H	AD	PRODUCT	exL	exH	DENOM	De
ARMADA	507	m	0	0	all	-	all	Eu:wst	1986	CC	325	n	bl	n	y	1	cig+/-ot	25	49	nev cigs	or
AUVINE	518	c	0	0	all	-	all	Eu:Sca	1986	CC	517	n	bl	y	n	2	cig+/-ot	21	40	nev cigs	or
AXELSS	521	m	0	0	sca	-	all	Eu:Sca	1989	CC	436	n	bl	n	n	6	all/unsp	30	39	nev any	ot
AXELSS	512	f	0	0	sca	-	all	Eu:Sca	1989	CC	436	n	bl	n	n	0	all/unsp	30	39	nev any	st
BARBON	509	m	0	0	all	-	all	Eu:wst	1979	CC	755	n	bl	y	y	1	all/unsp	30	39	nev any	or
BUFFLE	527	f	0	0	w-hi	-	all	NAmer	1976	CC	943	n	bl	y	n	0	cig+/-ot	31	40	nev cigs	or
CHEN2	504	m	0	0	all	-	all	As:Chi	1983	CC	193	n	ot	y	n	0	all/unsp	31	40	nev any	st
CHEN2	512	f	0	0	all	-	all	As:Chi	1983	CC	193	n	ot	y	n	0	all/unsp	31	40	nev any	st
CHOI	503	m	0	0	all	-	all	As:oth	1985	CC	375	n	bl	n	n	0	cig+/-ot	30	39	nev cigs	st
CHOI	512	f	0	0	all	-	all	As:oth	1985	CC	375	n	bl	n	n	0	cig+/-ot	30	39	nev cigs	st
DAMBER	508	m	0	0	all	-	all	Eu:Sca	1972	CC	579	n	bl	y	n	1	all/unsp	31	40	nev any	ot
DESTEF	509	m	0	0	all	-	all	SCAmer	1988	CC	497	n	bl	n	y	4	all/unsp	30	39	nev any	or
FAN	502	m	0	0	all	-	all	As:Chi	1990	CC	403	n	ot	y	n	0	cig+/-ot	30	39	nev cigs	st
FAN	507	f	0	0	all	-	all	As:Chi	1990	CC	403	n	ot	y	n	0	cig+/-ot	30	39	nev cigs	st
GER	519	c	0	0	all	-	all	As:oth	1990	CC	141	n	ot	y	n	5	all/unsp	21	40	nev any	ot
HU2	510	c	0	0	all	-	all	As:Chi	1977	CC	523	n	ot	y	n	0	cig+/-ot	30	39	nev cigs	or
JOLY	517	m	0	0	all	-	all	SCAmer	1978	CC	826	n	bl	n	n	0	cig+/-ot	30	39	nev any	st
JOLY	503	f	0	0	all	-	all	SCAmer	1978	CC	826	n	bl	n	n	0	cig+/-ot	30	39	nev any	st
JUSSAW	513	m	0	0	all	-	all	As:Ind	1964	CC	792	n	V	n	n	0	cig only	30	39	nev any	st
KHUDER	502	m	0	0	all	-	all	NAmer	1985	CC	482	n	bl	n	y	0	cig+/-ot	30	49	nev cigs	st
LETOUR	507	c	0	0	all	-	all	NAmer	1983	CC	738	n	V	y	y	0	cig+/-ot	25	40	nev cigs	st
LUBIN	509	m	0	0	all	-	all	As:Chi	1984	CC	427	m	ot	y	n	0	cig+/-ot	30	39	nev any	st
LUBIN2	532	m	0	0	all	-	all	Eu:mul	1976	CC	7804	n	bl	n	y	0	cig+/-ot	30	39	nev any	st
LUBIN2	575	f	0	0	all	-	all	Eu:mul	1976	CC	7804	n	bl	n	y	0	cig+/-ot	30	39	nev any	st
MATOS	537	m	0	0	all	-	all	SCAmer	1994	CC	200	n	bl	n	n	2	cig+/-ot	25	39	nev any	or
MCCONN	504	c	0	0	all	-	all	Eu:UK	1946	CC	100	n	V	n	y	0	all/unsp	30	39	nev any	st
NOTAN2	516	c	0	0	all	-	all	As:Ind	1963	CC	683	n	V	n	n	0	cig only	31	40	nev any	st
PEZZOT	535	m	0	0	all	-	all	SCAmer	1987	CC	215	n	bl	n	y	0	cig only	31	40	nev cigs	st
QIAO2	517	m	0	0	all	0	all	As:Chi	1992	pr	241	m	ot	n	n	1	all/unsp	28	41	nev any	or
RACHTA	517	f	0	0	all	-	all	Eu:est	1991	CC	118	n	bl	n	y	1	cig+/-ot	21	40	nev cigs	or
WANG2	504	c	0	0	all	-	all	As:Chi	1980	CC	103	n	ot	n	n	0	cig+/-ot	30	39	nev cigs	st
WUWILL	517	f	0	0	all	-	all	As:Chi	1985	CC	965	n	ot	n	n	3	cig+/-ot	30	39	nev cigs	ot

Cigarette type is all/unspec for all RRs

except for the following:

REF	NRR	CIGTYPE
JUSSAW	513	MC only
NOTAN2	516	MC only

Table 1I3 - 2

IESLC - Meta-analysis of Ever Smoking, Duration, "Mid"
All LC types, Any Product (or Cigarettes if Any not available)
Most adjusted

REF	NRR	SEX	AD	Number Exposed Case	Number Exposed Cont	Non-exposed Case	Non-exposed Cont	RR	95.00%CI
ARMADA	507	m	1	219	-	8	-	11.90	(5.50- 25.50)
AUVINE	518	c	2	10	-	44	-	33.20	(14.30- 77.40)
AXELSS	521	m	6	57	-	16	-	7.62	(4.01- 14.47)
AXELSS	512	f	0	29	26	18	154	9.54	(4.64- 19.61)
Subtotal AXELSS								8.42	(5.21- 13.59)
BARBON	509	m	1	118	-	22	-	7.90	(4.70- 13.50)
BUFFLE	527	f	0	97	62	12	112	14.60	(7.43- 28.69)
CHEN2	504	m	0	36	27	9	33	4.89	(2.01- 11.91)
CHEN2	512	f	0	13	6	25	33	2.86	(0.95- 8.58)
Subtotal CHEN2								3.95	(1.98- 7.89)
CHOI	503	m	0	102	160	13	95	4.66	(2.48- 8.75)
CHOI	512	f	0	8	2	76	164	8.63	(1.79- 41.62)
Subtotal CHOI								5.07	(2.83- 9.11)
DAMBER	508	m	1	-	-	42	-	5.15	(3.27- 8.32)
DESTEF	509	m	4	78	-	27	-	5.20	(2.90- 8.90)
FAN	502	m	0	44	122	36	236	2.36	(1.45- 3.87)
FAN	507	f	0	19	23	69	320	3.83	(1.98- 7.42)
Subtotal FAN								2.81	(1.89- 4.17)
GER	519	c	5	31	-	51	-	1.56	(0.83- 2.91)
HU2	510	c	0	123	101	121	213	2.14	(1.52- 3.03)
JOLY	517	m	0	85	165	12	218	9.36	(4.95- 17.70)
JOLY	503	f	0	31	24	52	283	7.03	(3.82- 12.93)
Subtotal JOLY								8.06	(5.19- 12.52)
JUSSAW	513	m	0	27	9	149	624	12.56	(5.79- 27.28)
KHUDER	502	m	0	207	370	23	309	7.52	(4.76- 11.86)
LETOUR	507	c	0	264	160	24	224	15.40	(9.68- 24.51)
LUBIN	509	m	0	124	294	8	72	3.80	(1.78- 8.12)
LUBIN2	532	m	0	2227	3470	190	2616	8.84	(7.56- 10.33)
LUBIN2	575	f	0	187	186	336	1188	3.55	(2.81- 4.50)
Subtotal LUBIN2								6.69	(5.87- 7.63)
MATOS	537	m	2	82	-	11	-	7.20	(3.60- 14.50)
MCCONN	504	c	0	21	57	9	23	0.94	(0.38- 2.36)
NOTAN2	516	c	0	12	7	107	201	3.22	(1.23- 8.42)
PEZZOT	535	m	0	71	82	4	116	25.11	(8.82- 71.48)
*QIAO2	517	m	1	54	-	10	-	1.46	(0.74- 2.87)
RACHTA	517	f	1	49	-	33	-	7.55	(3.90- 14.63)
WANG2	504	c	0	26	38	11	43	2.67	(1.17- 6.13)
WUWILL	517	f	3	179	-	417	-	2.71	(2.05- 3.60)
Partial Totals				4630	5391	1985	7277		

*prospective study

REF	NRR	SEX	AD	Ys	Ws	Qs	Ps
ARMADA	507	m	1	2.48	6.53	3.90	0.0000
AUVINE	518	c	2	3.50	5.39	17.43	0.0000
AXELSS	521	m	6	2.03	9.33	1.00	0.0000
AXELSS	512	f	0	2.26	7.41	2.25	0.0000
Subtotal AXELSS				2.13	16.74	3.25	
BARBON	509	m	1	2.07	13.80	1.82	0.0000
BUFFLE	527	f	0	2.68	8.42	8.04	0.0000
CHEN2	504	m	0	1.59	4.85	0.07	0.0005
CHEN2	512	f	0	1.05	3.19	1.36	0.0607
Subtotal CHEN2				1.37	8.03	1.43	
CHOI	503	m	0	1.54	9.66	0.26	0.0000
CHOI	512	f	0	2.16	1.55	0.32	0.0072
Subtotal CHOI				1.62	11.21	0.58	
DAMBER	508	m	1	1.64	17.62	0.07	0.0000
DESTEF	509	m	4	1.65	12.22	0.04	0.0000
FAN	502	m	0	0.86	15.89	11.31	0.0006
FAN	507	f	0	1.34	8.79	1.15	0.0001
Subtotal FAN				1.03	24.68	12.46	
GER	519	c	5	0.44	9.76	15.49	0.1647
HU2	510	c	0	0.76	32.27	28.61	0.0000
JOLY	517	m	0	2.24	9.46	2.68	0.0000
JOLY	503	f	0	1.95	10.34	0.63	0.0000
Subtotal JOLY				2.09	19.80	3.30	
JUSSAW	513	m	0	2.53	6.39	4.37	0.0000
KHUDER	502	m	0	2.02	18.43	1.80	0.0000
LETOUR	507	c	0	2.73	17.80	18.90	0.0000
LUBIN	509	m	0	1.33	6.65	0.91	0.0006
LUBIN2	532	m	0	2.18	156.67	35.31	0.0000

Table 1I3 - 2

IESLC - Meta-analysis of Ever Smoking, Duration, "Mid"
All LC types, Any Product (or Cigarettes if Any not available)
Most adjusted

REF	NRR	SEX	AD	Ys	Ws	Qs	Ps
LUBIN2	575	f	0	1.27	68.77	13.07	0.0000
Subtotal LUBIN2				1.90	225.44	48.37	
MATOS	537	m	2	1.97	7.92	0.58	0.0000
MCCONN	504	c	0	-0.06	4.55	14.17	0.8977
NOTAN2	516	c	0	1.17	4.16	1.19	0.0171
PEZZOT	535	m	0	3.22	3.51	8.10	0.0000
*QIAO2	517	m	1	0.38	8.36	14.70	0.2738
RACHTA	517	f	1	2.02	8.79	0.89	0.0000
WANG2	504	c	0	0.98	5.59	2.90	0.0200
WUWILL	517	f	3	1.00	48.46	24.24	0.0000

	N	32
	NS	26
	Wt	552.54
Het	Chi	237.51
Het	df	31
Het	P	***
Fixed	RR	5.50
	RRl	5.06
	RRu	5.97
	P	+++
Random	RR	5.49
	RRl	4.25
	RRu	7.09
	P	+++
Asymm	P	N.S.

Table 1I3 - 3

IESLC - Meta-analysis of Ever Smoking, Duration, "Mid"
All LC types, Any Product (or Cigarettes if Any not available)
Most adjusted

	Sex			
	combined	male	female	Total
N	7	16	9	32
NS	7	16	9	32
Wt	79.52	307.30	165.72	552.54
Het Chi	88.15	66.04	36.14	237.51
Het df	6	15	8	31
Het P	***	***	***	***
Fixed RR	3.82	7.13	4.04	5.50
RRl	3.07	6.38	3.47	5.06
RRu	4.76	7.98	4.70	5.97
P	+++	+++	+++	+++
Random RR	3.93	6.22	5.43	5.49
RRl	1.58	4.68	3.67	4.25
RRu	9.76	8.26	8.01	7.09
P	++	+++	+++	+++
Between Chi				47.18
Between df				2
Between P				***
Btwn(F) P				*
Btwn(R) P				N.S.

	Lung cancer type		
	all	other	Total
N	32		32
NS	26		26
Wt	552.54		552.54
Het Chi	237.51		237.51
Het df	31		31
Het P	***		***
Fixed RR	5.50		5.50
RRl	5.06		5.06
RRu	5.97		5.97
P	+++		+++
Random RR	5.49		5.49
RRl	4.25		4.25
RRu	7.09		7.09
P	+++		+++
Between Chi			
Between df			
Between P			N.S.
Btwn(F) P			N.S.
Btwn(R) P			N.S.

	Location								
	NAmer	UK	Scand	othEur	China	Japan	othAs	other	Total
N	3	1	4	5	9		5	5	32
NS	3	1	3	4	7		4	4	26
Wt	44.66	4.55	39.75	254.57	134.05		31.53	43.45	552.54
Het Chi	5.32	0.00	14.57	42.10	8.44		18.37	7.27	237.51
Het df	2	0	3	4	8		4	4	31
Het P	(*)	N.S.	**	***	N.S.		**	N.S.	***
Fixed RR	11.34	0.94	8.15	6.88	2.58		3.99	7.65	5.50
RRl	8.46	0.38	5.98	6.09	2.18		2.81	5.68	5.06
RRu	15.20	2.36	11.13	7.78	3.05		5.65	10.30	5.97
P	+++	N.S.	+++	+++	+++		+++	+++	+++
Random RR	11.66	0.94	9.98	7.13	2.58		4.52	8.08	5.49
RRl	7.12	0.38	4.87	4.28	2.16		2.04	5.36	4.25
RRu	19.08	2.36	20.46	11.87	3.09		10.00	12.18	7.09
P	+++	N.S.	+++	+++	+++		+++	+++	+++
Between Chi									141.44
Between df									6
Between P									***
Btwn(F) P									***
Btwn(R) P									***

Table 1I3 - 3

IESLC - Meta-analysis of Ever Smoking, Duration, "Mid"
All LC types, Any Product (or Cigarettes if Any not available)
Most adjusted

Detailed Country in "other Europe"

	multi	Germany	othWest	East	Balkans	Total
N	2		2	1		5
NS	1		2	1		4
Wt	225.44		20.33	8.79		254.57
Het Chi	39.63		0.74	0.00		42.10
Het df	1		1	0		4
Het P	***		N.S.	N.S.		***
Fixed RR	6.69		9.01	7.55		6.88
RRl	5.87		5.83	3.90		6.09
RRu	7.63		13.92	14.62		7.78
P	+++		+++	+++		+++
Random RR	5.63		9.01	7.55		7.13
RRl	2.31		5.83	3.90		4.28
RRu	13.74		13.92	14.62		11.87
P	+++		+++	+++		+++
Between Chi						1.73
Between df						2
Between P						N.S.
Btwn(F) P						N.S.
Btwn(R) P						N.S.

Detailed Country in "other Asia"

	India	HongKong	other	Total
N	2		3	5
NS	2		2	4
Wt	10.55		20.98	31.53
Het Chi	4.67		7.77	18.37
Het df	1		2	4
Het P	*		*	**
Fixed RR	7.35		2.93	3.99
RRl	4.02		1.91	2.81
RRu	13.43		4.50	5.65
P	+++		+++	+++
Random RR	6.56		3.43	4.52
RRl	1.73		1.33	2.04
RRu	24.87		8.84	10.00
P	++		+	+++
Between Chi				5.93
Between df				1
Between P				*
Btwn(F) P				N.S.
Btwn(R) P				N.S.

Detailed other continent

	SCAmer	Total
N	5	5
NS	4	4
Wt	43.45	43.45
Het Chi	7.27	7.27
Het df	4	4
Het P	N.S.	N.S.
Fixed RR	7.65	7.65
RRl	5.68	5.68
RRu	10.30	10.30
P	+++	+++
Random RR	8.08	8.08
RRl	5.36	5.36
RRu	12.18	12.18
P	+++	+++
Between Chi		
Between df		
Between P		N.S.
Btwn(F) P		N.S.
Btwn(R) P		N.S.

Table 1I3 - 3

IESLC - Meta-analysis of Ever Smoking, Duration, "Mid"
All LC types, Any Product (or Cigarettes if Any not available)
Most adjusted

	Start year of study					
	<1960	1960-69	1970-79	1980-89	1990+	Total
N	1	2	8	15	6	32
NS	1	2	6	12	5	26
Wt	4.55	10.55	317.35	160.57	59.52	552.54
Het Chi	0.00	4.67	86.64	83.22	23.48	237.51
Het df	0	1	7	14	5	31
Het P	N.S.	*	***	***	***	***
Fixed RR	0.94	7.35	6.11	5.72	3.05	5.50
RRl	0.38	4.02	5.48	4.90	2.37	5.06
RRu	2.36	13.43	6.82	6.67	3.93	5.97
P	N.S.	+++	+++	+++	+++	+++
Random RR	0.94	6.56	6.15	7.03	3.19	5.49
RRl	0.38	1.73	3.92	4.67	1.83	4.25
RRu	2.36	24.87	9.64	10.59	5.57	7.09
P	N.S.	++	+++	+++	+++	+++
Between Chi						39.51
Between df						4
Between P						***
Btwn(F) P						N.S.
Btwn(R) P						***

	Study type (1)		
	CC	other	Total
N	31	1	32
NS	25	1	26
Wt	544.18	8.36	552.54
Het Chi	222.59	0.00	237.51
Het df	30	0	31
Het P	***	N.S.	***
Fixed RR	5.61	1.46	5.50
RRl	5.16	0.74	5.06
RRu	6.10	2.88	5.97
P	+++	N.S.	+++
Random RR	5.73	1.46	5.49
RRl	4.44	0.74	4.25
RRu	7.39	2.88	7.09
P	+++	N.S.	+++
Between Chi			14.93
Between df			1
Between P			***
Btwn(F) P			N.S.
Btwn(R) P			***

	Study type (2)			
	CC	prosp	other	Total
N	31	1		32
NS	25	1		26
Wt	544.18	8.36		552.54
Het Chi	222.59	0.00		237.51
Het df	30	0		31
Het P	***	N.S.		***
Fixed RR	5.61	1.46		5.50
RRl	5.16	0.74		5.06
RRu	6.10	2.88		5.97
P	+++	N.S.		+++
Random RR	5.73	1.46		5.49
RRl	4.44	0.74		4.25
RRu	7.39	2.88		7.09
P	+++	N.S.		+++
Between Chi				14.93
Between df				1
Between P				***
Btwn(F) P				N.S.
Btwn(R) P				***

Table 1I3 - 3

IESLC - Meta-analysis of Ever Smoking, Duration, "Mid"
All LC types, Any Product (or Cigarettes if Any not available)
Most adjusted

	Study size (number of LC cases) 100-249	250-499	500-999	1000+	Total
N	9	10	11	2	32
NS	8	7	10	1	26
Wt	56.52	96.47	174.11	225.44	552.54
Het Chi	44.54	22.86	106.85	39.63	237.51
Het df	8	9	10	1	31
Het P	***	**	***	***	***
Fixed RR	3.36	5.35	5.08	6.69	5.50
RRl	2.59	4.38	4.37	5.87	5.06
RRu	4.36	6.53	5.89	7.63	5.97
P	+++	+++	+++	+++	+++
Random RR	3.56	5.57	7.37	5.63	5.49
RRl	1.91	4.00	4.41	2.31	4.25
RRu	6.67	7.75	12.31	13.74	7.09
P	+++	+++	+++	+++	+++
Between Chi					23.63
Between df					3
Between P					***
Btwn(F) P					N.S.
Btwn(R) P					N.S.

	Risky occupational population no	mining	othRisky	Total
N	30	2		32
NS	24	2		26
Wt	537.53	15.01		552.54
Het Chi	221.56	3.38		237.51
Het df	29	1		31
Het P	***	(*)		***
Fixed RR	5.64	2.23		5.50
RRl	5.18	1.34		5.06
RRu	6.13	3.70		5.97
P	+++	++		+++
Random RR	5.81	2.32		5.49
RRl	4.48	0.91		4.25
RRu	7.53	5.91		7.09
P	+++	(+)		+++
Between Chi				12.57
Between df				1
Between P				***
Btwn(F) P				N.S.
Btwn(R) P				(*)

	National cigarette tobacco type Virginia	blended	other	Total
N	4	18	10	32
NS	4	14	8	26
Wt	32.90	375.83	143.81	552.54
Het Chi	33.18	71.35	10.74	237.51
Het df	3	17	9	31
Het P	***	***	N.S.	***
Fixed RR	8.25	7.18	2.49	5.50
RRl	5.86	6.49	2.12	5.06
RRu	11.61	7.94	2.93	5.97
P	+++	+++	+++	+++
Random RR	5.12	8.04	2.50	5.49
RRl	1.48	6.27	2.06	4.25
RRu	17.77	10.31	3.02	7.09
P	+	+++	+++	+++
Between Chi				122.23
Between df				2
Between P				***
Btwn(F) P				***
Btwn(R) P				***

Table 1I3 - 3

IESLC - Meta-analysis of Ever Smoking, Duration, "Mid"
All LC types, Any Product (or Cigarettes if Any not available)
Most adjusted

	Any proxy use No/nk	Yes	Total
N	20	12	32
NS	16	10	26
Wt	408.11	144.44	552.54
Het Chi	130.93	101.07	237.51
Het df	19	11	31
Het P	***	***	***
Fixed RR	5.83	4.65	5.50
RRl	5.29	3.95	5.06
RRu	6.43	5.47	5.97
P	+++	+++	+++
Random RR	5.65	5.26	5.49
RRl	4.19	3.15	4.25
RRu	7.62	8.79	7.09
P	+++	+++	+++
Between Chi			5.51
Between df			1
Between P			*
Btwn(F) P			N.S.
Btwn(R) P			N.S.

	Full histological confirmation No	Yes	Total
N	22	10	32
NS	17	9	26
Wt	241.46	311.08	552.54
Het Chi	114.36	78.43	237.51
Het df	21	9	31
Het P	***	***	***
Fixed RR	3.98	7.06	5.50
RRl	3.51	6.32	5.06
RRu	4.51	7.89	5.97
P	+++	+++	+++
Random RR	4.85	7.07	5.49
RRl	3.55	4.74	4.25
RRu	6.63	10.54	7.09
P	+++	+++	+++
Between Chi			44.73
Between df			1
Between P			***
Btwn(F) P			*
Btwn(R) P			N.S.

	Number of adjustment variables (1) 0	1	2+/+nk	Total
N	21	5	6	32
NS	16	5	6	27
Wt	404.36	55.11	93.08	552.54
Het Chi	154.88	21.37	47.50	237.51
Het df	20	4	5	31
Het P	***	***	***	***
Fixed RR	5.95	5.56	3.88	5.50
RRl	5.39	4.27	3.17	5.06
RRu	6.55	7.24	4.76	5.97
P	+++	+++	+++	+++
Random RR	5.49	5.55	5.59	5.49
RRl	4.00	2.97	2.77	4.25
RRu	7.53	10.36	11.30	7.09
P	+++	+++	+++	+++
Between Chi				13.76
Between df				2
Between P				**
Btwn(F) P				N.S.
Btwn(R) P				N.S.

Table 1I3 - 3

IESLC - Meta-analysis of Ever Smoking, Duration, "Mid"
All LC types, Any Product (or Cigarettes if Any not available)
Most adjusted

	Number of adjustment variables (2) 0	1	2	3-5	6+/+nk	Total
N	21	5	2	3	1	32
NS	16	5	2	3	1	27
Wt	404.36	55.11	13.30	70.45	9.33	552.54
Het Chi	154.88	21.37	7.49	8.07	0.00	237.51
Het df	20	4	1	2	0	31
Het P	***	***	**	*	N.S.	***
Fixed RR	5.95	5.56	13.37	2.81	7.62	5.50
RRl	5.39	4.27	7.81	2.23	4.01	5.06
RRu	6.55	7.24	22.88	3.55	14.47	5.97
P	+++	+++	+++	+++	+++	+++
Random RR	5.49	5.55	15.16	2.83	7.62	5.49
RRl	4.00	2.97	3.39	1.62	4.01	4.25
RRu	7.53	10.36	67.78	4.95	14.47	7.09
P	+++	+++	+++	+++	+++	+++
Between Chi						45.70
Between df						4
Between P						***
Btwn(F) P						N.S.
Btwn(R) P						(*)

	Product all/unsp	cig+/-ot	cig only	Total
N	10	19	3	32
NS	8	15	3	26
Wt	91.09	447.39	14.06	552.54
Het Chi	44.77	172.37	8.65	237.51
Het df	9	18	2	31
Het P	***	***	*	***
Fixed RR	4.24	5.69	9.99	5.50
RRl	3.45	5.19	5.92	5.06
RRu	5.20	6.24	16.84	5.97
P	+++	+++	+++	+++
Random RR	3.81	6.10	9.99	5.49
RRl	2.38	4.41	3.30	4.25
RRu	6.11	8.43	30.26	7.09
P	+++	+++	+++	+++
Between Chi				11.73
Between df				2
Between P				**
Btwn(F) P				N.S.
Btwn(R) P				N.S.

	Denominator nev any	nev cigs	Total
N	18	14	32
NS	14	12	26
Wt	361.45	191.09	552.54
Het Chi	107.03	122.81	237.51
Het df	17	13	31
Het P	***	***	***
Fixed RR	5.99	4.67	5.50
RRl	5.40	4.06	5.06
RRu	6.64	5.39	5.97
P	+++	+++	+++
Random RR	4.83	6.68	5.49
RRl	3.55	4.19	4.25
RRu	6.56	10.65	7.09
P	+++	+++	+++
Between Chi			7.67
Between df			1
Between P			**
Btwn(F) P			N.S.
Btwn(R) P			N.S.

Table 1I3 - 3

IESLC - Meta-analysis of Ever Smoking, Duration, "Mid"
All LC types, Any Product (or Cigarettes if Any not available)
Most adjusted

		Derivation of RR/CI			
		Orig	StdCalc	Other	Total
	N	9	19	4	32
	NS	9	14	4	27
	Wt	103.70	363.66	85.17	552.54
Het	Chi	75.16	112.73	17.37	237.51
Het	df	8	18	3	31
Het	P	***	***	***	***
Fixed	RR	5.03	6.38	3.25	5.50
	RRl	4.15	5.75	2.63	5.06
	RRu	6.09	7.07	4.02	5.97
	P	+++	+++	+++	+++
Random	RR	6.80	5.54	3.56	5.49
	RRl	3.68	4.06	2.00	4.25
	RRu	12.56	7.56	6.33	7.09
	P	+++	+++	+++	+++
Between	Chi				32.25
Between	df				2
Between	P				***
Btwn(F)	P				N.S.
Btwn(R)	P				N.S.

Table 1I3 - 4

IESLC - Meta-analysis of Ever Smoking, Duration, "Mid"
All LC types, Any Product (or Cigarettes if Any not available)
Least adjusted

REF	NRR	X	SEX	AGEL	AGEH	RACE	YF	LC TYPE	LOC	START	ST	NLC	R	VB	P	H	AD	PRODUCT	exL	exH	DENOM	De
ARMADA	502	x	m	0	0	all	-	all	Eu:wst	1986	CC	325	n	bl	n	y	0	cig+/-ot	25	49	nev cigs	st
AUVINE	502	x	c	0	0	all	-	all	Eu:Sca	1986	CC	517	n	bl	y	n	0	cig+/-ot	21	40	nev cigs	st
AXELSS	503	x	m	0	0	sca	-	all	Eu:Sca	1989	CC	436	n	bl	n	n	0	all/unsp	30	39	nev any	st
AXELSS	512		f	0	0	sca	-	all	Eu:Sca	1989	CC	436	n	bl	n	n	0	all/unsp	30	39	nev any	st
BARBON	502	x	m	0	0	all	-	all	Eu:wst	1979	CC	755	n	bl	y	y	0	all/unsp	30	39	nev any	st
BUFFLE	527		f	0	0	w-hi	-	all	NAmer	1976	CC	943	n	bl	y	n	0	cig+/-ot	31	40	nev cigs	or
CHEN2	504		m	0	0	all	-	all	As:Chi	1983	CC	193	n	ot	y	n	0	all/unsp	31	40	nev any	st
CHEN2	512		f	0	0	all	-	all	As:Chi	1983	CC	193	n	ot	y	n	0	all/unsp	31	40	nev any	st
CHOI	503		m	0	0	all	-	all	As:oth	1985	CC	375	n	bl	n	n	0	cig+/-ot	30	39	nev cigs	st
CHOI	512		f	0	0	all	-	all	As:oth	1985	CC	375	n	bl	n	n	0	cig+/-ot	30	39	nev cigs	st
DAMBER	508		m	0	0	all	-	all	Eu:Sca	1972	CC	579	n	bl	y	n	1	all/unsp	31	40	nev any	ot
DESTEF	502	x	m	0	0	all	-	all	SCAmer	1988	CC	497	n	bl	n	y	0	all/unsp	30	39	nev any	st
FAN	502		m	0	0	all	-	all	As:Chi	1990	CC	403	n	ot	y	n	0	cig+/-ot	30	39	nev cigs	st
FAN	507		f	0	0	all	-	all	As:Chi	1990	CC	403	n	ot	y	n	0	cig+/-ot	30	39	nev cigs	st
GER	514	x	c	0	0	all	-	all	As:oth	1990	CC	141	n	ot	y	n	0	all/unsp	21	40	nev any	st
HU2	510		c	0	0	all	-	all	As:Chi	1977	CC	523	n	ot	y	n	0	cig+/-ot	30	39	nev cigs	or
JOLY	517		m	0	0	all	-	all	SCAmer	1978	CC	826	n	bl	n	n	0	cig+/-ot	30	39	nev any	st
JOLY	503		f	0	0	all	-	all	SCAmer	1978	CC	826	n	bl	n	n	0	cig+/-ot	30	39	nev any	st
JUSSAW	513		m	0	0	all	-	all	As:Ind	1964	CC	792	n	V	n	n	0	cig only	30	39	nev any	st
KHUDER	502		m	0	0	all	-	all	NAmer	1985	CC	482	n	bl	n	y	0	cig+/-ot	30	49	nev cigs	st
LETOUR	507		c	0	0	all	-	all	NAmer	1983	CC	738	n	V	y	y	0	cig+/-ot	25	40	nev cigs	st
LUBIN	509		m	0	0	all	-	all	As:Chi	1984	CC	427	m	ot	y	n	0	cig+/-ot	30	39	nev any	st
LUBIN2	532		m	0	0	all	-	all	Eu:mul	1976	CC	7804	n	bl	n	y	0	cig+/-ot	30	39	nev any	st
LUBIN2	575		f	0	0	all	-	all	Eu:mul	1976	CC	7804	n	bl	n	y	0	cig+/-ot	30	39	nev any	st
MATOS	517	x	m	0	0	all	-	all	SCAmer	1994	CC	200	n	bl	n	n	0	cig+/-ot	25	39	nev any	st
MCCONN	504		c	0	0	all	-	all	Eu:UK	1946	CC	100	n	V	n	y	0	all/unsp	30	39	nev any	st
NOTAN2	516		c	0	0	all	-	all	As:Ind	1963	CC	683	n	V	n	n	0	cig only	31	40	nev any	st
PEZZOT	535		m	0	0	all	-	all	SCAmer	1987	CC	215	n	bl	n	y	0	cig only	31	40	nev cigs	st
QIAO2	512	x	m	0	0	all	0	all	As:Chi	1992	pr	241	m	ot	n	n	0	all/unsp	28	41	nev any	st
RACHTA	512	x	f	0	0	all	-	all	Eu:est	1991	CC	118	n	bl	n	y	0	cig+/-ot	21	40	nev cigs	st
WANG2	504		c	0	0	all	-	all	As:Chi	1980	CC	103	n	ot	n	n	0	cig+/-ot	30	39	nev cigs	st
WUWILL	502	x	f	0	0	all	-	all	As:Chi	1985	CC	965	n	ot	n	n	0	cig+/-ot	30	39	nev cigs	st

Cigarette type is all/unspec for all RRs

except for the following:

REF	NRR	CIGTYPE
JUSSAW	513	MC only
NOTAN2	516	MC only

Table 1I3 - 5

IESLC - Meta-analysis of Ever Smoking, Duration, "Mid"
All LC types, Any Product (or Cigarettes if Any not available)
Least adjusted

REF	NRR	SEX	AD	Number Exposed Case	Cont	Non-exposed Case	Cont	RR	95.00%CI
ARMADA	502	m	0	219	166	8	71	11.71	(5.49- 24.99)
AUVINE	502	c	0	10	5	44	229	10.41	(3.39- 31.93)
AXELSS	503	m	0	57	71	16	160	8.03	(4.31- 14.94)
AXELSS	512	f	0	29	26	18	154	9.54	(4.64- 19.61)
Subtotal AXELSS								8.64	(5.40- 13.83)
BARBON	502	m	0	118	102	22	188	9.89	(5.91- 16.55)
BUFFLE	527	f	0	97	62	12	112	14.60	(7.43- 28.69)
CHEN2	504	m	0	36	27	9	33	4.89	(2.01- 11.91)
CHEN2	512	f	0	13	6	25	33	2.86	(0.95- 8.58)
Subtotal CHEN2								3.95	(1.98- 7.89)
CHOI	503	m	0	102	160	13	95	4.66	(2.48- 8.75)
CHOI	512	f	0	8	2	76	164	8.63	(1.79- 41.62)
Subtotal CHOI								5.07	(2.83- 9.11)
DAMBER	508	m	1	-	-	42	-	5.15	(3.27- 8.32)
DESTEF	502	m	0	78	78	27	163	6.04	(3.61- 10.10)
FAN	502	m	0	44	122	36	236	2.36	(1.45- 3.87)
FAN	507	f	0	19	23	69	320	3.83	(1.98- 7.42)
Subtotal FAN								2.81	(1.89- 4.17)
GER	514	c	0	31	123	51	246	1.22	(0.74- 2.00)
HU2	510	c	0	123	101	121	213	2.14	(1.52- 3.03)
JOLY	517	m	0	85	165	12	218	9.36	(4.95- 17.70)
JOLY	503	f	0	31	24	52	283	7.03	(3.82- 12.93)
Subtotal JOLY								8.06	(5.19- 12.52)
JUSSAW	513	m	0	27	9	149	624	12.56	(5.79- 27.28)
KHUDER	502	m	0	207	370	23	309	7.52	(4.76- 11.86)
LETOUR	507	c	0	264	160	24	224	15.40	(9.68- 24.51)
LUBIN	509	m	0	124	294	8	72	3.80	(1.78- 8.12)
LUBIN2	532	m	0	2227	3470	190	2616	8.84	(7.56- 10.33)
LUBIN2	575	f	0	187	186	336	1188	3.55	(2.81- 4.50)
Subtotal LUBIN2								6.69	(5.87- 7.63)
MATOS	517	m	0	82	110	11	110	7.45	(3.77- 14.75)
MCCONN	504	c	0	21	57	9	23	0.94	(0.38- 2.36)
NOTAN2	516	c	0	12	7	107	201	3.22	(1.23- 8.42)
PEZZOT	535	m	0	71	82	4	116	25.11	(8.82- 71.48)
*QIAO2	512	m	0	54	2257	10	709	1.70	(0.87- 3.31)
RACHTA	512	f	0	49	21	33	98	6.93	(3.63- 13.22)
WANG2	504	c	0	26	38	11	43	2.67	(1.17- 6.13)
WUWILL	502	f	0	179	98	417	601	2.63	(2.00- 3.47)
Partial Totals				4630	8422	1985	9852		

*prospective study

REF	NRR	SEX	AD	Ys	Ws	Qs	Ps
ARMADA	502	m	0	2.46	6.68	4.09	0.0000
AUVINE	502	c	0	2.34	3.06	1.35	0.0000
AXELSS	503	m	0	2.08	9.96	1.63	0.0000
AXELSS	512	f	0	2.26	7.41	2.47	0.0000
Subtotal AXELSS				2.16	17.37	4.11	
BARBON	502	m	0	2.29	14.48	5.45	0.0000
BUFFLE	527	f	0	2.68	8.42	8.48	0.0000
CHEN2	504	m	0	1.59	4.85	0.04	0.0005
CHEN2	512	f	0	1.05	3.19	1.25	0.0607
Subtotal CHEN2				1.37	8.03	1.29	
CHOI	503	m	0	1.54	9.66	0.19	0.0000
CHOI	512	f	0	2.16	1.55	0.35	0.0072
Subtotal CHOI				1.62	11.21	0.54	
DAMBER	508	m	1	1.64	17.62	0.03	0.0000
DESTEF	502	m	0	1.80	14.53	0.21	0.0000
FAN	502	m	0	0.86	15.89	10.61	0.0006
FAN	507	f	0	1.34	8.79	0.98	0.0001
Subtotal FAN				1.03	24.68	11.60	
GER	514	c	0	0.20	15.61	34.31	0.4403
HU2	510	c	0	0.76	32.27	27.03	0.0000
JOLY	517	m	0	2.24	9.46	2.95	0.0000
JOLY	503	f	0	1.95	10.34	0.77	0.0000
Subtotal JOLY				2.09	19.80	3.72	
JUSSAW	513	m	0	2.53	6.39	4.65	0.0000
KHUDER	502	m	0	2.02	18.43	2.12	0.0000
LETOUR	507	c	0	2.73	17.80	19.87	0.0000
LUBIN	509	m	0	1.33	6.65	0.79	0.0006
LUBIN2	532	m	0	2.18	156.67	39.33	0.0000

Table 1I3 - 5

IESLC - Meta-analysis of Ever Smoking, Duration, "Mid"
All LC types, Any Product (or Cigarettes if Any not available)
Least adjusted

REF	NRR	SEX	AD	Ys	Ws	Qs	Ps
LUBIN2	575	f	0	1.27	68.77	11.54	0.0000
Subtotal LUBIN2				1.90	225.44	50.87	
MATOS	517	m	0	2.01	8.24	0.90	0.0000
MCCONN	504	c	0	-0.06	4.55	13.75	0.8977
NOTAN2	516	c	0	1.17	4.16	1.07	0.0171
PEZZOT	535	m	0	3.22	3.51	8.38	0.0000
*QIAO2	512	m	0	0.53	8.57	11.32	0.1218
RACHTA	512	f	0	1.94	9.21	0.61	0.0000
WANG2	504	c	0	0.98	5.59	2.69	0.0200
WUWILL	502	f	0	0.97	50.37	25.39	0.0000

	N	32
	NS	26
	Wt	562.70
Het	Chi	244.63
Het	df	31
Het	P	***
Fixed	RR	5.35
	RRl	4.93
	RRu	5.82
	P	+++
Random	RR	5.32
	RRl	4.12
	RRu	6.88
	P	+++
Asymm	P	N.S.

Table 1I3 - 6

IESLC - Meta-analysis of Ever Smoking, Duration, "Mid"
All LC types, Any Product (or Cigarettes if Any not available)
Least adjusted

		combined	Sex male	female	Total
	N	7	16	9	32
	NS	7	16	9	32
	Wt	83.04	311.61	168.06	562.70
Het	Chi	74.89	63.16	36.85	244.63
Het	df	6	15	8	31
Het	P	***	***	***	***
Fixed	RR	3.09	7.28	3.97	5.35
	RRl	2.49	6.52	3.41	4.93
	RRu	3.83	8.14	4.62	5.82
	P	+++	+++	+++	+++
Random	RR	3.16	6.46	5.35	5.32
	RRl	1.38	4.91	3.62	4.12
	RRu	7.24	8.50	7.91	6.88
	P	++	+++	+++	+++
Between	Chi				69.72
Between	df				2
Between	P				***
Btwn(F)	P				**
Btwn(R)	P				N.S.

Table 1I3 - 7

IESLC - Meta-analysis of Ever Smoking, Duration, "Mid"
All LC types, Any Product (or Cigarettes if Any not available)
Excluded studies (and stage at which they were excluded)

1	AKIBA AMANDU AMES BECHER BENSHL BEST BLOT1 BROSS BROWN3 CARPEN CEDERL CHYOU CPSI CPSII DARBY DEAN2 DEAN3 DOLL2 ENGELA GAO2 GARCIA GILLIS GRAHAM GURSEL HAMMO2 HIRAYA HOLE HUMBLE JAHN JAIN KAISE2 KATSOU KAUFMA LAUSSM LIAW MCDUFF MIGRAN MRFITR PEZZO2 PISANI PRESCO QIAO SEGI2 SPEIZE SVENSS TVERDA WAKAI WATSON WIGLE WU WYNDE3 WYNDE8
2	ALDERS BRESLO CHIAZZ DORN GUO HEGMAN KOO KOULUM LIU4 PERNU SOBUE SPITZ SUZUK2 VUTUC YUAN
3	GENG STASZE WU2 ZHANG
4	BOUCHA CHEN CORREA JEDRYC LUO WYNDE2 WYNDE6
5	HAMMON RESTRE SADOWS XU
7	BOFFET WYNDE7
14	AGUDO BOUCOT DOLL DORGAN DOSEME GAO GARSHI HAENSZ HU KREUZE LEVIN LIU3 LIU5 OSANN2 TIZZAN ZHENG ZHOU
15	BENHAM

Table 1I3 - 8
Potentially overlapping studies

REF	REFGP	PRINC	. OVERLAP/LINK
LUBIN2	LUBIN2	1	Lubin-combined
LUBIN	XIANGZ	2	LUBIN/XIANGZ/QIAO

Table 1I3 - 9
Most adjusted - insufficient data for meta-analysis

REF	NRR	SEX	AGEL	AGEH	RACE	YF	LC TYPE	LOC	START	ST	NLC	R	VB	P	H	AD	PRODUCT	exL	exH	DENOM	De
BUFFLE	502	m	0	0	wh	-	all	NAmer	1976	CC	943	n	bl	y	n	0	cig+/-ot	34	43	nev cigs	or
SADOWS	525	m	0	0	wh	-	all	NAmer	1938	CC	477	n	bl	n	n	0	cig only	30	39	nev any	ot
XU	502	m	0	0	all	-	all	As:Chi	1985	CC	729	n	ot	n	n	2	all/unsp	30	39	nev any	or

REF	NRR	RR	SIG	RRDATA comment
BUFFLE	502	11.10		0
SADOWS	525	3.95		0
XU	502	*		RR for 1-19/day is 2.1(p<0.05), for 20-29/day is 2.7(p<0.05) and for >=30/ day is 4.9(p<0.05)

Table 1I4 -

IESLC - Meta-analysis of Ever Smoking, Duration, "High"
All LC types, Any Product (or Cigarettes if Any not available)

This analysis is restricted to results for:
1) Ever smokers
2) Results by Duration
3) Categorical results by Duration
4) All LC types (or near equivalent)
5) Results complete enough for use in metaanalysis

Within each study, results are then selected (in the following order of preference, within each sex) for:
6) PRODUCT: all/unspec, cigarettes regardless of other products, cigarettes only
7) CIGTYPE: all/unspecified, MC regardless of HR, MC only
8) (not applicable)
9) DENOM: never smoked anything, never smoked cigarettes, never any + low, never cigs + low
10) Followup period (YF, prospective studies): whole study (coded as 0) or longest available
11) LCtype: all or nearest available, at least Squamous and Adeno. (q = squamous, s = small,
l = large, a = adeno, mix = mixed, alv = alveolar)
12) Race: all or nearest available, otherwise by race (wh or w = white, bl or b = black, hi = hispanic
ch = chinese, jap = japanese, haw = hawaiian, w+o = white + oriental, sca = scandinavian, as = asian)
13) Duration "high" in key scheme 1 (key value 50, maximum range 36+)
14) For overlapping studies: principal rather than subsidiary studies
Finally by Age: whole study (coded as 0) if available, otherwise by widest available age group
and then for single sex results (m, f) in preference to results for both sexes combined (c).

Results adjusted (AD) for the most potential confounders are then chosen in Sections -1 to -3
and results adjusted for the least confounders in Sections -4 to -6. (Those least adjusted results which
actually differ from the most adjusted are marked 'x' in column X in Section -4)

Section -7 shows excluded studies, together with the stage (as above) at which no qualifying
results were found.

Section -8 lists the potentially overlapping studies which have been included (1=principal, 2=subsidiary).

Section -9 lists any results which would have been included in preference except that they had data not complete
enough for use in meta-analysis, with their significance (yes/no), if known, and any further comment as entered
on the database. It also lists as "gap" any categories for which no data were presented by the original authors.

In addition to those mentioned above, the following fields, levels and abbreviations are used:
* or nk = not known, n = no, y = yes, ot = other
nev = never
all/unspec = all or unspecified, cig+/-ot = cigarettes irrespective of other products (cigar, pipe etc)
MC = manufactured cigarettes, HR = hand-rolled cigarettes
exL, exH = range of exposure (low and high) in the smoking group, in terms of Duration
REF: 6-character study reference
NRR: number of the RR on the database within the study
ST : study type (CC = case control, pr or prosp = prospective)
NLC: number of lung cancer cases in whole study
R : risky occupational population (n = no, m = mining, o = other risky)
VB : national cigarette type (V = at least 75% Virginia, bl = at least 75% blended, ot = other)
P : any proxy use
H : full histological confirmation
De : derivation of RR/CI (or = original, st = standard method, ot = other method of estimation)

Table 1I4 - 1

IESLC - Meta-analysis of Ever Smoking, Duration, "High"
All LC types, Any Product (or Cigarettes if Any not available)
Most adjusted

REF	NRR	SEX	AGEL	AGEH	RACE	YF	LC TYPE	LOC	START	ST	NLC	R	VB	P	H	AD	PRODUCT	exL	exH	DENOM	De
ARMADA	508	m	0	0	all	-	all	Eu:wst	1986	CC	325	n	bl	n	y	1	cig+/-ot	50	999	nev cigs	or
AUVINE	519	c	0	0	all	-	all	Eu:Sca	1986	CC	517	n	bl	y	n	2	cig+/-ot	41	999	nev cigs	or
AXELSS	523	m	0	0	sca	-	all	Eu:Sca	1989	CC	436	n	bl	n	n	6	all/unsp	50	999	nev any	ot
AXELSS	514	f	0	0	sca	-	all	Eu:Sca	1989	CC	436	n	bl	n	n	0	all/unsp	50	999	nev any	st
BARBON	511	m	0	0	all	-	all	Eu:wst	1979	CC	755	n	bl	y	y	1	all/unsp	50	999	nev any	or
BOUCOT	519	m	0	0	all	9	all	NAmer	1951	pr	121	n	bl	n	n	0	cig+/-ot	40	999	nev any	ot
BUFFLE	528	f	0	0	w-hi	-	all	NAmer	1976	CC	943	n	bl	y	n	0	cig+/-ot	41	999	nev cigs	or
CHEN2	505	m	0	0	all	-	all	As:Chi	1983	CC	193	n	ot	y	n	0	all/unsp	41	999	nev any	st
CHEN2	513	f	0	0	all	-	all	As:Chi	1983	CC	193	n	ot	y	n	0	all/unsp	41	999	nev any	st
CHOI	505	m	0	0	all	-	all	As:oth	1985	CC	375	n	bl	n	n	0	cig+/-ot	50	999	nev cigs	st
CHOI	513	f	0	0	all	-	all	As:oth	1985	CC	375	n	bl	n	n	0	cig+/-ot	40	999	nev cigs	st
DAMBER	509	m	0	0	all	-	all	Eu:Sca	1972	CC	579	n	bl	y	n	1	all/unsp	41	50	nev any	ot
DESTEF	511	m	0	0	all	-	all	SCAmer	1988	CC	497	n	bl	n	y	4	all/unsp	50	999	nev any	or
DOLL	518	m	0	0	all	-	all	Eu:UK	1948	CC	1465	n	V	n	n	0	all/unsp	40	999	nev any	st
DOLL	525	f	0	0	all	-	all	Eu:UK	1948	CC	1465	n	V	n	n	0	all/unsp	40	999	nev any	st
FAN	503	m	0	0	all	-	all	As:Chi	1990	CC	403	n	ot	y	n	0	cig+/-ot	40	999	nev cigs	st
FAN	508	f	0	0	all	-	all	As:Chi	1990	CC	403	n	ot	y	n	0	cig+/-ot	40	999	nev cigs	st
GER	520	c	0	0	all	-	all	As:oth	1990	CC	141	n	ot	y	n	5	all/unsp	41	999	nev any	ot
HU2	511	c	0	0	all	-	all	As:Chi	1977	CC	523	n	ot	y	n	0	cig+/-ot	40	999	nev cigs	st
JOLY	519	m	0	0	all	-	all	SCAmer	1978	CC	826	n	bl	n	n	0	cig+/-ot	50	999	nev any	st
JOLY	505	f	0	0	all	-	all	SCAmer	1978	CC	826	n	bl	n	n	0	cig+/-ot	50	999	nev any	st
JUSSAW	514	m	0	0	all	-	all	As:Ind	1964	CC	792	n	V	n	n	0	cig only	40	999	nev any	st
KHUDER	503	m	0	0	all	-	all	NAmer	1985	CC	482	n	bl	n	y	0	cig+/-ot	50	999	nev cigs	st
KREUZE	507	m	55	69	all	-	all	Eu:Ger	1990	CC	2260	n	bl	n	n	3	all/unsp	40	999	nev any	or
KREUZE	510	f	55	69	all	-	all	Eu:Ger	1990	CC	2260	n	bl	n	n	3	all/unsp	40	999	nev any	or
LETOUR	508	c	0	0	all	-	all	NAmer	1983	CC	738	n	V	y	y	0	cig+/-ot	41	999	nev cigs	st
LEVIN	507	m	0	0	all	-	all	NAmer	1938	CC	475	n	bl	n	n	1	cig+/-ot	40	999	nev any	ot
LUBIN	511	m	0	0	all	-	all	As:Chi	1984	CC	427	m	ot	y	n	0	cig+/-ot	50	999	nev any	st
LUBIN2	534	m	0	0	all	-	all	Eu:mul	1976	CC	7804	n	bl	n	y	0	cig+/-ot	50	999	nev any	st
LUBIN2	577	f	0	0	all	-	all	Eu:mul	1976	CC	7804	n	bl	n	y	0	cig+/-ot	50	999	nev any	st
MATOS	538	m	0	0	all	-	all	SCAmer	1994	CC	200	n	bl	n	n	2	cig+/-ot	40	70	nev any	or
MCCONN	505	c	0	0	all	-	all	Eu:UK	1946	CC	100	n	V	n	y	0	all/unsp	40	999	nev any	st
NOTAN2	517	c	0	0	all	-	all	As:Ind	1963	CC	683	n	V	n	n	0	cig only	41	999	nev any	st
PEZZOT	536	m	0	0	all	-	all	SCAmer	1987	CC	215	n	bl	n	y	0	cig only	41	999	nev cigs	st
QIAO2	518	m	0	0	all	0	all	As:Chi	1992	pr	241	m	ot	n	n	1	all/unsp	42	999	nev any	or
RACHTA	518	f	0	0	all	-	all	Eu:est	1991	CC	118	n	bl	n	y	1	cig+/-ot	41	999	nev cigs	or
WUWILL	518	f	0	0	all	-	all	As:Chi	1985	CC	965	n	ot	n	n	3	cig+/-ot	40	999	nev cigs	ot

Cigarette type is all/unspec for all RRs

except for the following:

REF	NRR	CIGTYPE
JUSSAW	514	MC only
NOTAN2	517	MC only

Table 1I4 - 2

IESLC - Meta-analysis of Ever Smoking, Duration, "High"
All LC types, Any Product (or Cigarettes if Any not available)
Most adjusted

REF	NRR	SEX	AD	Number Exposed Case	Cont	Non-exposed Case	Cont	RR	95.00%CI
ARMADA	508	m	1	77	-	8	-	26.80	(11.00- 65.10)
AUVINE	519	c	2	230	-	44	-	30.40	(15.80- 58.40)
AXELSS	523	m	6	101	-	16	-	27.09	(13.94- 52.62)
AXELSS	514	f	0	20	10	18	154	17.11	(6.94- 42.19)
Subtotal AXELSS								23.05	(13.50- 39.36)
BARBON	511	m	1	366	-	22	-	14.50	(9.00- 23.30)
*BOUCOT	519	m	0	52	1563	0	805	54.09~	(3.34- 875.17)
BUFFLE	528	f	0	90	42	12	112	20.00	(9.94- 40.23)
CHEN2	505	m	0	62	40	9	33	5.68	(2.46- 13.13)
CHEN2	513	f	0	21	15	25	33	1.85	(0.80- 4.29)
Subtotal CHEN2								3.25	(1.80- 5.89)
CHOI	505	m	0	20	20	13	95	7.31	(3.13- 17.07)
CHOI	513	f	0	1	1	76	164	2.16	(0.13- 34.96)
Subtotal CHOI								6.59	(2.93- 14.84)
DAMBER	509	m	1	-	-	42	-	8.71	(5.84- 13.66)
DESTEF	511	m	4	178	-	27	-	10.80	(6.60- 17.60)
DOLL	518	m	0	558	491	7	61	9.90	(4.49- 21.85)
DOLL	525	f	0	6	3	40	59	2.95	(0.70- 12.49)
Subtotal DOLL								7.48	(3.74- 14.98)
FAN	503	m	0	143	241	36	236	3.89	(2.59- 5.84)
FAN	508	f	0	55	59	69	320	4.32	(2.76- 6.78)
Subtotal FAN								4.08	(3.02- 5.52)
GER	520	c	5	49	-	51	-	2.14	(1.18- 3.90)
HU2	511	c	0	194	113	121	213	3.02	(2.19- 4.17)
JOLY	519	m	0	250	253	12	218	17.95	(9.78- 32.93)
JOLY	505	f	0	57	20	52	283	15.51	(8.61- 27.95)
Subtotal JOLY								16.65	(10.91- 25.41)
JUSSAW	514	m	0	11	6	149	624	7.68	(2.79- 21.09)
KHUDER	503	m	0	236	354	23	309	8.96	(5.69- 14.11)
KREUZE	507	m	3	-	-	-	-	54.50	(34.90- 85.20)
KREUZE	510	f	3	-	-	-	-	8.30	(4.70- 14.50)
Subtotal KREUZE								26.38	(18.59- 37.42)
LETOUR	508	c	0	374	141	24	224	24.76	(15.58- 39.35)
LEVIN	507	m	1	63	-	7	-	8.96	(3.90- 20.61)
LUBIN	511	m	0	59	86	8	72	6.17	(2.77- 13.77)
LUBIN2	534	m	0	1325	1484	190	2616	12.29	(10.42- 14.50)
LUBIN2	577	f	0	81	32	336	1188	8.95	(5.84- 13.71)
Subtotal LUBIN2								11.80	(10.12- 13.76)
MATOS	538	m	2	86	-	11	-	12.70	(6.10- 26.10)
MCCONN	505	c	0	16	40	9	23	1.02	(0.39- 2.68)
NOTAN2	517	c	0	5	5	107	201	1.88	(0.53- 6.63)
PEZZOT	536	m	0	110	101	4	116	31.58	(11.25- 88.71)
*QIAO2	518	m	1	170	-	10	-	2.05	(1.06- 3.94)
RACHTA	518	f	1	24	-	33	-	58.68	(7.56- 455.64)
WUWILL	518	f	3	223	-	417	-	3.49	(2.65- 4.59)
Partial Totals				5313	5120	2028	8159		

*prospective study ~ With 0.5 adjustment for zero

Table 1I4 - 2

IESLC - Meta-analysis of Ever Smoking, Duration, "High"
All LC types, Any Product (or Cigarettes if Any not available)
Most adjusted

REF	NRR	SEX	AD	Ys	Ws	Qs	Ps
ARMADA	508	m	1	3.29	4.86	6.11	0.0000
AUVINE	519	c	2	3.41	8.99	13.99	0.0000
AXELSS	523	m	6	3.30	8.71	11.16	0.0000
AXELSS	514	f	0	2.84	4.72	2.13	0.0000
Subtotal AXELSS				3.14	13.42	13.30	
BARBON	511	m	1	2.67	16.98	4.37	0.0000
*BOUCOT	519	m	0	3.99	0.50	1.65	0.0050
BUFFLE	528	f	0	3.00	7.86	5.40	0.0000
CHEN2	505	m	0	1.74	5.48	1.01	0.0000
CHEN2	513	f	0	0.61	5.42	13.06	0.1529
Subtotal CHEN2				1.18	10.90	14.07	
CHOI	505	m	0	1.99	5.33	0.17	0.0000
CHOI	513	f	0	0.77	0.50	0.97	0.5883
Subtotal CHOI				1.89	5.83	1.14	
DAMBER	509	m	1	2.16	21.28	0.00	0.0000
DESTEF	511	m	4	2.38	15.97	0.72	0.0000
DOLL	518	m	0	2.29	6.13	0.10	0.0000
DOLL	525	f	0	1.08	1.85	2.17	0.1417
Subtotal DOLL				2.01	7.98	2.27	
FAN	503	m	0	1.36	23.17	15.15	0.0000
FAN	508	f	0	1.46	18.96	9.37	0.0000
Subtotal FAN				1.41	42.13	24.52	
GER	520	c	5	0.76	10.75	21.26	0.0126
HU2	511	c	0	1.11	37.09	41.75	0.0000
JOLY	519	m	0	2.89	10.43	5.42	0.0000
JOLY	505	f	0	2.74	11.07	3.66	0.0000
Subtotal JOLY				2.81	21.50	9.07	
JUSSAW	514	m	0	2.04	3.76	0.06	0.0001
KHUDER	503	m	0	2.19	18.60	0.01	0.0000
KREUZE	507	m	3	4.00	19.29	64.69	0.0000
KREUZE	510	f	3	2.12	12.11	0.03	0.0000
Subtotal KREUZE				3.27	31.40	64.72	
LETOUR	508	c	0	3.21	17.89	19.43	0.0000
LEVIN	507	m	1	2.19	5.54	0.00	0.0000
LUBIN	511	m	0	1.82	5.97	0.72	0.0000
LUBIN2	534	m	0	2.51	141.36	16.54	0.0000
LUBIN2	577	f	0	2.19	21.09	0.01	0.0000
Subtotal LUBIN2				2.47	162.45	16.56	
MATOS	538	m	2	2.54	7.27	1.02	0.0000
MCCONN	505	c	0	0.02	4.13	19.01	0.9644
NOTAN2	517	c	0	0.63	2.41	5.70	0.3273
PEZZOT	536	m	0	3.45	3.60	5.95	0.0000
*QIAO2	518	m	1	0.72	8.91	18.72	0.0321
RACHTA	518	f	1	4.07	0.91	3.32	0.0001
WUWILL	518	f	3	1.25	50.92	42.82	0.0000

	N	37
	NS	29
	Wt	549.82
Het	Chi	357.66
Het	df	36
Het	P	***
Fixed	RR	8.73
	RRl	8.03
	RRu	9.49
	P	+++
Random	RR	8.76
	RRl	6.55
	RRu	11.71
	P	+++
Asymm	P	N.S.

Table 1I4 - 3

IESLC - Meta-analysis of Ever Smoking, Duration, "High"
All LC types, Any Product (or Cigarettes if Any not available)
Most adjusted

	combined	Sex male	female	Total
N	6	20	11	37
NS	6	20	11	37
Wt	81.26	333.16	135.40	549.82
Het Chi	104.12	128.94	61.87	357.66
Het df	5	19	10	36
Het P	***	***	***	***
Fixed RR	5.53	11.46	5.88	8.73
RRl	4.45	10.29	4.97	8.03
RRu	6.87	12.76	6.96	9.49
P	+++	+++	+++	+++
Random RR	4.79	11.17	7.42	8.76
RRl	1.61	8.06	4.53	6.55
RRu	14.29	15.47	12.13	11.71
P	++	+++	+++	+++
Between Chi				62.73
Between df				2
Between P				***
Btwn(F) P				*
Btwn(R) P				N.S.

	Lung cancer type all	other	Total
N	37		37
NS	29		29
Wt	549.82		549.82
Het Chi	357.66		357.66
Het df	36		36
Het P	***		***
Fixed RR	8.73		8.73
RRl	8.03		8.03
RRu	9.49		9.49
P	+++		+++
Random RR	8.76		8.76
RRl	6.55		6.55
RRu	11.71		11.71
P	+++		+++
Between Chi			
Between df			
Between P			N.S.
Btwn(F) P			N.S.
Btwn(R) P			N.S.

	NAmer	UK	Scand	Location othEur	China	Japan	othAs	other	Total
N	5	3	4	7	8		5	5	37
NS	5	2	3	5	6		4	4	29
Wt	50.39	12.11	43.70	216.61	155.92		22.76	48.35	549.82
Het Chi	12.37	12.87	13.89	49.46	9.86		8.86	4.22	357.66
Het df	4	2	3	6	7		4	4	36
Het P	*	**	**	***	N.S.		(*)	N.S.	***
Fixed RR	14.83	3.79	15.19	13.82	3.47		3.48	14.53	8.73
RRl	11.25	2.16	11.29	12.09	2.97		2.31	10.96	8.03
RRu	19.54	6.66	20.43	15.78	4.06		5.24	19.26	9.49
P	+++	+++	+++	+++	+++		+++	+++	+++
Random RR	15.07	3.17	18.23	17.05	3.49		3.75	14.60	8.76
RRl	8.65	0.70	9.20	10.19	2.85		1.88	10.91	6.55
RRu	26.27	14.40	36.13	28.54	4.27		7.50	19.54	11.71
P	+++	N.S.	+++	+++	+++		+++	+++	+++
Between Chi									246.12
Between df									6
Between P									***
Btwn(F) P									***
Btwn(R) P									***

Table 1I4 - 3

IESLC - Meta-analysis of Ever Smoking, Duration, "High"
All LC types, Any Product (or Cigarettes if Any not available)
Most adjusted

Detailed Country in "other Europe"

	multi	Germany	othWest	East	Balkans	Total
N	2	2	2	1		7
NS	1	1	2	1		5
Wt	162.45	31.40	21.84	0.91		216.61
Het Chi	1.85	26.34	1.43	0.00		49.46
Het df	1	1	1	0		6
Het P	N.S.	***	N.S.	N.S.		***
Fixed RR	11.80	26.38	16.62	58.68		13.82
RRl	10.12	18.59	10.93	7.56		12.09
RRu	13.76	37.42	25.28	455.55		15.78
P	+++	+++	+++	+++		+++
Random RR	11.18	21.44	17.49	58.68		17.05
RRl	8.41	3.39	10.05	7.56		10.19
RRu	14.86	135.59	30.45	455.55		28.54
P	+++	++	+++	+++		+++
Between Chi						19.85
Between df						3
Between P						***
Btwn(F) P						N.S.
Btwn(R) P						N.S.

Detailed Country in "other Asia"

	India	HongKong	other	Total
N	2		3	5
NS	2		2	4
Wt	6.17		16.58	22.76
Het Chi	2.91		5.45	8.86
Het df	1		2	4
Het P	(*)		(*)	(*)
Fixed RR	4.43		3.18	3.48
RRl	2.01		1.96	2.31
RRu	9.75		5.14	5.24
P	+++		+++	+++
Random RR	4.00		3.55	3.75
RRl	1.01		1.31	1.88
RRu	15.85		9.59	7.50
P	+		+	+++
Between Chi				0.50
Between df				1
Between P				N.S.
Btwn(F) P				N.S.
Btwn(R) P				N.S.

Detailed other continent

	SCAmer	Total
N	5	5
NS	4	4
Wt	48.35	48.35
Het Chi	4.22	4.22
Het df	4	4
Het P	N.S.	N.S.
Fixed RR	14.53	14.53
RRl	10.96	10.96
RRu	19.26	19.26
P	+++	+++
Random RR	14.60	14.60
RRl	10.91	10.91
RRu	19.54	19.54
P	+++	+++
Between Chi		
Between df		
Between P		N.S.
Btwn(F) P		N.S.
Btwn(R) P		N.S.

Table 1I4 - 3

IESLC - Meta-analysis of Ever Smoking, Duration, "High"
All LC types, Any Product (or Cigarettes if Any not available)
Most adjusted

	Start year of study <1960	1960-69	1970-79	1980-89	1990+	Total
N	5	2	8	14	8	37
NS	4	2	6	11	6	29
Wt	18.15	6.17	267.17	156.95	101.38	549.82
Het Chi	18.42	2.91	71.60	118.15	129.24	357.66
Het df	4	1	7	13	7	36
Het P	**	(*)	***	***	***	***
Fixed RR	5.30	4.43	10.08	8.49	7.10	8.73
RRl	3.35	2.01	8.95	7.26	5.85	8.03
RRu	8.40	9.75	11.37	9.93	8.63	9.49
P	+++	+++	+++	+++	+++	+++
Random RR	5.41	4.00	10.77	10.71	7.62	8.76
RRl	1.83	1.01	6.89	6.38	3.17	6.55
RRu	15.98	15.85	16.83	17.97	18.29	11.71
P	++	+	+++	+++	+++	+++
Between Chi						17.33
Between df						4
Between P						**
Btwn(F) P						N.S.
Btwn(R) P						N.S.

	Study type (1) CC	other	Total
N	35	2	37
NS	27	2	29
Wt	540.41	9.41	549.82
Het Chi	337.03	5.03	357.66
Het df	34	1	36
Het P	***	*	***
Fixed RR	8.93	2.44	8.73
RRl	8.21	1.29	8.03
RRu	9.71	4.61	9.49
P	+++	++	+++
Random RR	9.01	7.87	8.76
RRl	6.73	0.34	6.55
RRu	12.06	184.85	11.71
P	+++	N.S.	+++
Between Chi			15.60
Between df			1
Between P			***
Btwn(F) P			N.S.
Btwn(R) P			N.S.

	Study type (2) CC	prosp	other	Total
N	35	2		37
NS	27	2		29
Wt	540.41	9.41		549.82
Het Chi	337.03	5.03		357.66
Het df	34	1		36
Het P	***	*		***
Fixed RR	8.93	2.44		8.73
RRl	8.21	1.29		8.03
RRu	9.71	4.61		9.49
P	+++	++		+++
Random RR	9.01	7.87		8.76
RRl	6.73	0.34		6.55
RRu	12.06	184.85		11.71
P	+++	N.S.		+++
Between Chi				15.60
Between df				1
Between P				***
Btwn(F) P				N.S.
Btwn(R) P				N.S.

Table 1I4 - 3

IESLC - Meta-analysis of Ever Smoking, Duration, "High"
All LC types, Any Product (or Cigarettes if Any not available)
Most adjusted

	Study size (number of LC cases)				
	100-249	250-499	500-999	1000+	Total
N	9	11	11	6	37
NS	8	8	10	3	29
Wt	46.98	112.33	188.69	201.83	549.82
Het Chi	54.81	44.95	137.36	49.94	357.66
Het df	8	10	10	5	36
Het P	***	***	***	***	***
Fixed RR	3.92	7.76	7.39	13.13	8.73
RRl	2.94	6.45	6.41	11.44	8.03
RRu	5.21	9.33	8.53	15.07	9.49
P	+++	+++	+++	+++	+++
Random RR	5.62	9.10	10.07	12.05	8.76
RRl	2.51	5.95	5.74	6.59	6.55
RRu	12.55	13.91	17.67	22.05	11.71
P	+++	+++	+++	+++	+++
Between Chi					70.60
Between df					3
Between P					***
Btwn(F) P					(*)
Btwn(R) P					N.S.

	Risky occupational population			
	no	mining	othRisky	Total
N	35	2		37
NS	27	2		29
Wt	534.94	14.89		549.82
Het Chi	337.81	4.35		357.66
Het df	34	1		36
Het P	***	*		***
Fixed RR	8.98	3.19		8.73
RRl	8.25	1.92		8.03
RRu	9.77	5.30		9.49
P	+++	+++		+++
Random RR	9.25	3.47		8.76
RRl	6.88	1.18		6.55
RRu	12.44	10.21		11.71
P	+++	+		+++
Between Chi				15.51
Between df				1
Between P				***
Btwn(F) P				N.S.
Btwn(R) P				(*)

	National cigarette tobacco type			
	Virginia	blended	other	Total
N	6	22	9	37
NS	5	17	7	29
Wt	36.17	346.98	166.67	549.82
Het Chi	45.94	78.44	12.22	357.66
Het df	5	21	8	36
Het P	***	***	N.S.	***
Fixed RR	9.85	13.63	3.36	8.73
RRl	7.11	12.27	2.89	8.03
RRu	13.65	15.15	3.92	9.49
P	+++	+++	+++	+++
Random RR	4.98	15.12	3.34	8.76
RRl	1.66	11.84	2.73	6.55
RRu	14.98	19.31	4.10	11.71
P	++	+++	+++	+++
Between Chi				221.07
Between df				2
Between P				***
Btwn(F) P				***
Btwn(R) P				***

Table 1I4 - 3

IESLC - Meta-analysis of Ever Smoking, Duration, "High"
All LC types, Any Product (or Cigarettes if Any not available)
Most adjusted

	Any proxy use No/nk	Yes	Total
N	25	12	37
NS	19	10	29
Wt	369.98	179.84	549.82
Het Chi	204.15	129.03	357.66
Het df	24	11	36
Het P	***	***	***
Fixed RR	10.12	6.45	8.73
RRl	9.14	5.57	8.03
RRu	11.20	7.47	9.49
P	+++	+++	+++
Random RR	9.91	7.02	8.76
RRl	6.98	4.19	6.55
RRu	14.09	11.76	11.71
P	+++	+++	+++
Between Chi			24.48
Between df			1
Between P			***
Btwn(F) P			N.S.
Btwn(R) P			N.S.

	Full histological confirmation No	Yes	Total
N	27	10	37
NS	20	9	29
Wt	304.42	245.40	549.82
Het Chi	259.57	47.43	357.66
Het df	26	9	36
Het P	***	***	***
Fixed RR	6.65	12.24	8.73
RRl	5.94	10.80	8.03
RRu	7.44	13.88	9.49
P	+++	+++	+++
Random RR	7.63	12.25	8.76
RRl	5.22	8.43	6.55
RRu	11.16	17.79	11.71
P	+++	+++	+++
Between Chi			50.67
Between df			1
Between P			***
Btwn(F) P			*
Btwn(R) P			(*)

	Number of adjustment variables (1) 0	1	2+/+nk	Total
N	23	6	8	37
NS	17	6	7	30
Wt	357.31	58.50	134.01	549.82
Het Chi	169.41	32.37	155.70	357.66
Het df	22	5	7	36
Het P	***	***	***	***
Fixed RR	8.66	9.19	8.74	8.73
RRl	7.80	7.11	7.38	8.03
RRu	9.60	11.87	10.35	9.49
P	+++	+++	+++	+++
Random RR	7.49	10.38	11.75	8.76
RRl	5.33	5.02	5.06	6.55
RRu	10.53	21.44	27.30	11.71
P	+++	+++	+++	+++
Between Chi				0.18
Between df				2
Between P				N.S.
Btwn(F) P				N.S.
Btwn(R) P				N.S.

Table 1I4 - 3

IESLC - Meta-analysis of Ever Smoking, Duration, "High"
All LC types, Any Product (or Cigarettes if Any not available)
Most adjusted

	Number of adjustment variables (2)					
	0	1	2	3-5	6+/+nk	Total
N	23	6	2	5	1	37
NS	17	6	2	4	1	30
Wt	357.31	58.50	16.26	109.04	8.71	549.82
Het Chi	169.41	32.37	3.06	124.37	0.00	357.66
Het df	22	5	1	4	0	36
Het P	***	***	(*)	***	N.S.	***
Fixed RR	8.66	9.19	20.58	7.03	27.09	8.73
RRl	7.80	7.11	12.66	5.82	13.94	8.03
RRu	9.60	11.87	33.45	8.48	52.63	9.49
P	+++	+++	+++	+++	+++	+++
Random RR	7.49	10.38	19.95	8.20	27.09	8.76
RRl	5.33	5.02	8.48	2.68	13.94	6.55
RRu	10.53	21.44	46.90	25.03	52.63	11.71
P	+++	+++	+++	+++	+++	+++
Between Chi						28.45
Between df						4
Between P						***
Btwn(F) P						N.S.
Btwn(R) P						**

	Product			
	all/unsp	cig+/-ot	cig only	Total
N	14	20	3	37
NS	10	16	3	29
Wt	141.73	398.32	9.78	549.82
Het Chi	157.61	187.20	11.69	357.66
Het df	13	19	2	36
Het P	***	***	**	***
Fixed RR	9.42	8.49	9.13	8.73
RRl	7.99	7.69	4.88	8.03
RRu	11.11	9.36	17.09	9.49
P	+++	+++	+++	+++
Random RR	7.01	10.12	7.96	8.76
RRl	3.88	7.07	1.72	6.55
RRu	12.65	14.48	36.79	11.71
P	+++	+++	++	+++
Between Chi				1.16
Between df				2
Between P				N.S.
Btwn(F) P				N.S.
Btwn(R) P				N.S.

	Denominator		
	nev any	nev cigs	Total
N	24	13	37
NS	18	11	29
Wt	351.14	198.68	549.82
Het Chi	178.83	139.02	357.66
Het df	23	12	36
Het P	***	***	***
Fixed RR	10.69	6.11	8.73
RRl	9.63	5.31	8.03
RRu	11.87	7.02	9.49
P	+++	+++	+++
Random RR	8.20	10.11	8.76
RRl	5.86	5.97	6.55
RRu	11.46	17.10	11.71
P	+++	+++	+++
Between Chi			39.81
Between df			1
Between P			***
Btwn(F) P			*
Btwn(R) P			N.S.

Table 1I4 - 3

IESLC - Meta-analysis of Ever Smoking, Duration, "High"
All LC types, Any Product (or Cigarettes if Any not available)
Most adjusted

	Derivation of RR/CI Orig	StdCalc	Other	Total
N	10	21	6	37
NS	9	15	6	30
Wt	103.17	348.96	97.70	549.82
Het Chi	81.77	162.07	50.32	357.66
Het df	9	20	5	36
Het P	***	***	***	***
Fixed RR	15.84	8.47	5.18	8.73
RRl	13.06	7.63	4.25	8.03
RRu	19.21	9.41	6.32	9.49
P	+++	+++	+++	+++
Random RR	15.66	6.95	7.57	8.76
RRl	8.53	4.89	3.55	6.55
RRu	28.76	9.87	16.17	11.71
P	+++	+++	+++	+++
Between Chi				63.50
Between df				2
Between P				***
Btwn(F) P				*
Btwn(R) P				(*)

Table 1I4 - 4

IESLC - Meta-analysis of Ever Smoking, Duration, "High"
All LC types, Any Product (or Cigarettes if Any not available)
Least adjusted

REF	NRR	X	SEX	AGEL	AGEH	RACE	YF	LC TYPE	LOC	START	ST	NLC	R	VB	P	H	AD	PRODUCT	exL	exH	DENOM	De
ARMADA	503	x	m	0	0	all	-	all	Eu:wst	1986	CC	325	n	bl	n	y	0	cig+/-ot	50	999	nev cigs	st
AUVINE	503	x	c	0	0	all	-	all	Eu:Sca	1986	CC	517	n	bl	y	n	0	cig+/-ot	41	999	nev cigs	st
AXELSS	505	x	m	0	0	sca	-	all	Eu:Sca	1989	CC	436	n	bl	n	n	0	all/unsp	50	999	nev any	st
AXELSS	514		f	0	0	sca	-	all	Eu:Sca	1989	CC	436	n	bl	n	n	0	all/unsp	50	999	nev any	st
BARBON	504	x	m	0	0	all	-	all	Eu:wst	1979	CC	755	n	bl	y	y	0	all/unsp	50	999	nev any	st
BOUCOT	519		m	0	0	all	9	all	NAmer	1951	pr	121	n	bl	n	n	0	cig+/-ot	40	999	nev any	ot
BUFFLE	528		f	0	0	w-hi	-	all	NAmer	1976	CC	943	n	bl	y	n	0	cig+/-ot	41	999	nev cigs	or
CHEN2	505		m	0	0	all	-	all	As:Chi	1983	CC	193	n	ot	y	n	0	all/unsp	41	999	nev any	st
CHEN2	513		f	0	0	all	-	all	As:Chi	1983	CC	193	n	ot	y	n	0	all/unsp	41	999	nev any	st
CHOI	505		m	0	0	all	-	all	As:oth	1985	CC	375	n	bl	n	n	0	cig+/-ot	50	999	nev cigs	st
CHOI	513		f	0	0	all	-	all	As:oth	1985	CC	375	n	bl	n	n	0	cig+/-ot	40	999	nev cigs	st
DAMBER	509		m	0	0	all	-	all	Eu:Sca	1972	CC	579	n	bl	y	n	1	all/unsp	41	50	nev any	ot
DESTEF	504	x	m	0	0	all	-	all	SCAmer	1988	CC	497	n	bl	n	y	0	all/unsp	50	999	nev any	st
DOLL	518		m	0	0	all	-	all	Eu:UK	1948	CC	1465	n	V	n	n	0	all/unsp	40	999	nev any	st
DOLL	525		f	0	0	all	-	all	Eu:UK	1948	CC	1465	n	V	n	n	0	all/unsp	40	999	nev any	st
FAN	503		m	0	0	all	-	all	As:Chi	1990	CC	403	n	ot	y	n	0	cig+/-ot	40	999	nev cigs	st
FAN	508		f	0	0	all	-	all	As:Chi	1990	CC	403	n	ot	y	n	0	cig+/-ot	40	999	nev cigs	st
GER	515	x	c	0	0	all	-	all	As:oth	1990	CC	141	n	ot	y	n	0	all/unsp	41	999	nev any	st
HU2	511		c	0	0	all	-	all	As:Chi	1977	CC	523	n	ot	y	n	0	cig+/-ot	40	999	nev cigs	st
JOLY	519		m	0	0	all	-	all	SCAmer	1978	CC	826	n	bl	n	n	0	cig+/-ot	50	999	nev any	st
JOLY	505		f	0	0	all	-	all	SCAmer	1978	CC	826	n	bl	n	n	0	cig+/-ot	50	999	nev any	st
JUSSAW	514		m	0	0	all	-	all	As:Ind	1964	CC	792	n	V	n	n	0	cig only	40	999	nev any	st
KHUDER	503		m	0	0	all	-	all	NAmer	1985	CC	482	n	bl	n	y	0	cig+/-ot	50	999	nev cigs	st
KREUZE	507		m	55	69	all	-	all	Eu:Ger	1990	CC	2260	n	bl	n	n	3	all/unsp	40	999	nev any	or
KREUZE	510		f	55	69	all	-	all	Eu:Ger	1990	CC	2260	n	bl	n	n	3	all/unsp	40	999	nev any	or
LETOUR	508		c	0	0	all	-	all	NAmer	1983	CC	738	n	V	y	y	0	cig+/-ot	41	999	nev cigs	st
LEVIN	502	x	m	0	0	all	-	all	NAmer	1938	CC	475	n	bl	n	n	0	cig+/-ot	40	999	nev any	st
LUBIN	511		m	0	0	all	-	all	As:Chi	1984	CC	427	m	ot	y	n	0	cig+/-ot	50	999	nev any	st
LUBIN2	534		m	0	0	all	-	all	Eu:mul	1976	CC	7804	n	bl	n	y	0	cig+/-ot	50	999	nev any	st
LUBIN2	577		f	0	0	all	-	all	Eu:mul	1976	CC	7804	n	bl	n	y	0	cig+/-ot	50	999	nev any	st
MATOS	518	x	m	0	0	all	-	all	SCAmer	1994	CC	200	n	bl	n	n	0	cig+/-ot	40	70	nev any	st
MCCONN	505		c	0	0	all	-	all	Eu:UK	1946	CC	100	n	V	n	y	0	all/unsp	40	999	nev any	st
NOTAN2	517		c	0	0	all	-	all	As:Ind	1963	CC	683	n	V	n	n	0	cig only	41	999	nev any	st
PEZZOT	536		m	0	0	all	-	all	SCAmer	1987	CC	215	n	bl	n	y	0	cig only	41	999	nev cigs	st
QIAO2	513	x	m	0	0	all	0	all	As:Chi	1992	pr	241	m	ot	n	n	0	all/unsp	42	999	nev any	st
RACHTA	513	x	f	0	0	all	-	all	Eu:est	1991	CC	118	n	bl	n	y	0	cig+/-ot	41	999	nev cigs	st
WUWILL	503	x	f	0	0	all	-	all	As:Chi	1985	CC	965	n	ot	n	n	0	cig+/-ot	40	999	nev cigs	st

Cigarette type is all/unspec for all RRs

except for the following:

REF	NRR	CIGTYPE
JUSSAW	514	MC only
NOTAN2	517	MC only

Table 1I4 - 5

IESLC - Meta-analysis of Ever Smoking, Duration, "High"
All LC types, Any Product (or Cigarettes if Any not available)
Least adjusted

REF	NRR	SEX	AD	Number Exposed Case	Number Exposed Cont	Non-exposed Case	Non-exposed Cont	RR	95.00%CI
ARMADA	503	m	0	77	33	8	71	20.71	(8.97- 47.82)
AUVINE	503	c	0	230	57	44	229	21.00	(13.61- 32.41)
AXELSS	505	m	0	101	40	16	160	25.25	(13.43- 47.46)
AXELSS	514	f	0	20	10	18	154	17.11	(6.94- 42.19)
Subtotal AXELSS								22.22	(13.25- 37.27)
BARBON	504	m	0	366	235	22	188	13.31	(8.31- 21.32)
*BOUCOT	519	m	0	52	1563	0	805	54.09~	(3.34- 875.17)
BUFFLE	528	f	0	90	42	12	112	20.00	(9.94- 40.23)
CHEN2	505	m	0	62	40	9	33	5.68	(2.46- 13.13)
CHEN2	513	f	0	21	15	25	33	1.85	(0.80- 4.29)
Subtotal CHEN2								3.25	(1.80- 5.89)
CHOI	505	m	0	20	20	13	95	7.31	(3.13- 17.07)
CHOI	513	f	0	1	1	76	164	2.16	(0.13- 34.96)
Subtotal CHOI								6.59	(2.93- 14.84)
DAMBER	509	m	1	-	-	42	-	8.71	(5.84- 13.66)
DESTEF	504	m	0	178	108	27	163	9.95	(6.20- 15.96)
DOLL	518	m	0	558	491	7	61	9.90	(4.49- 21.85)
DOLL	525	f	0	6	3	40	59	2.95	(0.70- 12.49)
Subtotal DOLL								7.48	(3.74- 14.98)
FAN	503	m	0	143	241	36	236	3.89	(2.59- 5.84)
FAN	508	f	0	55	59	69	320	4.32	(2.76- 6.78)
Subtotal FAN								4.08	(3.02- 5.52)
GER	515	c	0	49	155	51	246	1.52	(0.98- 2.37)
HU2	511	c	0	194	113	121	213	3.02	(2.19- 4.17)
JOLY	519	m	0	250	253	12	218	17.95	(9.78- 32.93)
JOLY	505	f	0	57	20	52	283	15.51	(8.61- 27.95)
Subtotal JOLY								16.65	(10.91- 25.41)
JUSSAW	514	m	0	11	6	149	624	7.68	(2.79- 21.09)
KHUDER	503	m	0	236	354	23	309	8.96	(5.69- 14.11)
KREUZE	507	m	3	-	-	-	-	54.50	(34.90- 85.20)
KREUZE	510	f	3	-	-	-	-	8.30	(4.70- 14.50)
Subtotal KREUZE								26.38	(18.59- 37.42)
LETOUR	508	c	0	374	141	24	224	24.76	(15.58- 39.35)
LEVIN	502	m	0	63	91	7	96	9.49	(4.13- 21.81)
LUBIN	511	m	0	59	86	8	72	6.17	(2.77- 13.77)
LUBIN2	534	m	0	1325	1484	190	2616	12.29	(10.42- 14.50)
LUBIN2	577	f	0	81	32	336	1188	8.95	(5.84- 13.71)
Subtotal LUBIN2								11.80	(10.12- 13.76)
MATOS	518	m	0	86	89	11	110	9.66	(4.86- 19.21)
MCCONN	505	c	0	16	40	9	23	1.02	(0.39- 2.68)
NOTAN2	517	c	0	5	5	107	201	1.88	(0.53- 6.63)
PEZZOT	536	m	0	110	101	4	116	31.58	(11.25- 88.71)
*QIAO2	513	m	0	170	2295	10	709	5.25	(2.79- 9.88)
RACHTA	513	f	0	24	1	33	98	71.27	(9.28- 547.53)
WUWILL	503	f	0	223	114	417	601	2.82	(2.18- 3.65)
Partial Totals				5313	8338	2028	10830		

*prospective study ~ With 0.5 adjustment for zero

Table 1I4 - 5

IESLC - Meta-analysis of Ever Smoking, Duration, "High"
All LC types, Any Product (or Cigarettes if Any not available)
Least adjusted

REF	NRR	SEX	AD	Ys	Ws	Qs	Ps
ARMADA	503	m	0	3.03	5.48	4.55	0.0000
AUVINE	503	c	0	3.04	20.41	17.46	0.0000
AXELSS	505	m	0	3.23	9.65	11.87	0.0000
AXELSS	514	f	0	2.84	4.72	2.44	0.0000
Subtotal AXELSS				3.10	14.36	14.31	
BARBON	504	m	0	2.59	17.31	3.80	0.0000
*BOUCOT	519	m	0	3.99	0.50	1.74	0.0050
BUFFLE	528	f	0	3.00	7.86	6.03	0.0000
CHEN2	505	m	0	1.74	5.48	0.80	0.0000
CHEN2	513	f	0	0.61	5.42	12.28	0.1529
Subtotal CHEN2				1.18	10.90	13.08	
CHOI	505	m	0	1.99	5.33	0.09	0.0000
CHOI	513	f	0	0.77	0.50	0.90	0.5883
Subtotal CHOI				1.89	5.83	0.99	
DAMBER	509	m	1	2.16	21.28	0.04	0.0000
DESTEF	504	m	0	2.30	17.23	0.54	0.0000
DOLL	518	m	0	2.29	6.13	0.18	0.0000
DOLL	525	f	0	1.08	1.85	1.99	0.1417
Subtotal DOLL				2.01	7.98	2.17	
FAN	503	m	0	1.36	23.17	13.43	0.0000
FAN	508	f	0	1.46	18.96	8.15	0.0000
Subtotal FAN				1.41	42.13	21.59	
GER	515	c	0	0.42	19.79	57.05	0.0605
HU2	511	c	0	1.11	37.09	38.12	0.0000
JOLY	519	m	0	2.89	10.43	6.15	0.0000
JOLY	505	f	0	2.74	11.07	4.28	0.0000
Subtotal JOLY				2.81	21.50	10.43	
JUSSAW	514	m	0	2.04	3.76	0.02	0.0001
KHUDER	503	m	0	2.19	18.60	0.10	0.0000
KREUZE	507	m	3	4.00	19.29	68.06	0.0000
KREUZE	510	f	3	2.12	12.11	0.00	0.0000
Subtotal KREUZE				3.27	31.40	68.06	
LETOUR	508	c	0	3.21	17.89	21.23	0.0000
LEVIN	502	m	0	2.25	5.55	0.10	0.0000
LUBIN	511	m	0	1.82	5.97	0.54	0.0000
LUBIN2	534	m	0	2.51	141.36	21.42	0.0000
LUBIN2	577	f	0	2.19	21.09	0.11	0.0000
Subtotal LUBIN2				2.47	162.45	21.53	
MATOS	518	m	0	2.27	8.14	0.18	0.0000
MCCONN	505	c	0	0.02	4.13	18.18	0.9644
NOTAN2	517	c	0	0.63	2.41	5.35	0.3273
PEZZOT	536	m	0	3.45	3.60	6.40	0.0000
*QIAO2	513	m	0	1.66	9.61	2.04	0.0000
RACHTA	513	f	0	4.27	0.92	4.26	0.0000
WUWILL	503	f	0	1.04	57.74	67.77	0.0000

	N	37
	NS	29
	Wt	581.83
Het	Chi	407.66
Het	df	36
Het	P	***
Fixed	RR	8.33
	RRl	7.68
	RRu	9.03
	P	+++
Random	RR	8.60
	RRl	6.37
	RRu	11.61
	P	+++
Asymm	P	N.S.

Table 1I4 - 6

IESLC - Meta-analysis of Ever Smoking, Duration, "High"
All LC types, Any Product (or Cigarettes if Any not available)
Least adjusted

		Sex			
		combined	male	female	Total
	N	6	20	11	37
	NS	6	20	11	37
	Wt	101.72	337.88	142.23	581.83
Het	Chi	137.40	106.11	78.42	407.66
Het	df	5	19	10	36
Het	P	***	***	***	***
Fixed	RR	5.35	11.54	5.27	8.33
	RRl	4.40	10.37	4.47	7.68
	RRu	6.49	12.84	6.21	9.03
	P	+++	+++	+++	+++
Random	RR	4.27	11.30	7.32	8.60
	RRl	1.44	8.42	4.25	6.37
	RRu	12.67	15.18	12.61	11.61
	P	++	+++	+++	+++
Between	Chi				85.73
Between	df				2
Between	P				***
Btwn(F)	P				*
Btwn(R)	P				N.S.

Table 1I4 - 7

IESLC - Meta-analysis of Ever Smoking, Duration, "High"
All LC types, Any Product (or Cigarettes if Any not available)
Excluded studies (and stage at which they were excluded)

1	AKIBA AMANDU AMES BECHER BENSHL BEST BLOT1 BROSS BROWN3 CARPEN CEDERL CHYOU CPSI CPSII DARBY DEAN2 DEAN3 DOLL2 ENGELA GAO2 GARCIA GILLIS GRAHAM GURSEL HAMMO2 HIRAYA HOLE HUMBLE JAHN JAIN KAISE2 KATSOU KAUFMA LAUSSM LIAW MCDUFF MIGRAN MRFITR PEZZO2 PISANI PRESCO QIAO SEGI2 SPEIZE SVENSS TVERDA WAKAI WATSON WIGLE WU WYNDE3 WYNDE8
2	ALDERS BRESLO CHIAZZ DORN GUO HEGMAN KOO KOULUM LIU4 PERNU SOBUE SPITZ SUZUK2 VUTUC YUAN
3	GENG STASZE WU2 ZHANG
4	BOUCHA CHEN CORREA JEDRYC LUO WYNDE2 WYNDE6
5	HAMMON RESTRE SADOWS XU
7	BOFFET WYNDE7
14	AGUDO DORGAN DOSEME GAO GARSHI HAENSZ HU LIU3 LIU5 OSANN2 TIZZAN WANG2 ZHENG ZHOU
15	BENHAM

Table 1I4 - 8
Potentially overlapping studies

REF	REFGP	PRINC	.	OVERLAP/LINK
LUBIN2	LUBIN2	1		Lubin-combined
LUBIN	XIANGZ	2		LUBIN/XIANGZ/QIAO

Table 1I4 - 9
Most adjusted - insufficient data for meta-analysis

REF	NRR	SEX	AGEL	AGEH	RACE	YF	LC TYPE	LOC	START	ST	NLC	R	VB	P	H	AD	PRODUCT	exL	exH	DENOM	De
BUFFLE	504	m	0	0	wh	-	all	NAmer	1976	CC	943	n	bl	y	n	0	cig+/-ot	50	999	nev cigs	or
SADOWS	527	m	0	0	wh	-	all	NAmer	1938	CC	477	n	bl	n	n	0	cig only	50	999	nev any	ot
XU	503	m	0	0	all	-	all	As:Chi	1985	CC	729	n	ot	n	n	2	all/unsp	40	999	nev any	or

REF	NRR	RR	SIG	RRDATA comment
BUFFLE	504	14.50		0
SADOWS	527	8.43		0
XU	503	*		RR for 1-19/day is 3.3(p<0.05), for 20-29/day is 6.0(p<0.05) and for >=30/ day is 17.1(p<0.05)

Table 1I5 -

IESLC - Meta-analysis of Ever Smoking, Duration, "Highest vs lowest"
All LC types, Any Product (or Cigarettes if Any not available)

This analysis is restricted to results for:
1) Ever smokers
2) Results by Duration
3) Categorical results by Duration
4) Denominator (unexposed) = "low"
5) All LC types (or near equivalent)
6) Results complete enough for use in metaanalysis

Within each study, results are then selected (in the following order of preference, within each sex) for:
7) (not applicable)
8) PRODUCT: all/unspec, cigarettes regardless of other products, cigarettes only
9) CIGTYPE: all/unspecified, MC regardless of HR, MC only
10) Results with least adjustment for other aspects of smoking (ADOS)
11) The highest vs lowest category
12) Followup period (YF, prospective studies): whole study (coded as 0) or longest available
13) LCtype: all or nearest available, at least Squamous and Adeno. (q = squamous, s = small,
l = large, a = adeno, mix = mixed, alv = alveolar)
14) Race: all or nearest available, otherwise by race (wh or w = white, bl or b = black, hi = hispanic
ch = chinese, jap = japanese, haw = hawaiian, w+o = white + oriental, sca = scandinavian, as = asian)
15) For overlapping studies: principal rather than subsidiary studies
Finally by Age: whole study (coded as 0) if available, otherwise by widest available age group
and then for single sex results (m, f) in preference to results for both sexes combined (c).

Results adjusted (AD) for the most potential confounders are then chosen in Sections -1 to -3
and results adjusted for the least confounders in Sections -4 to -6. (Those least adjusted results which
actually differ from the most adjusted are marked 'x' in column X in Section -4)

Section -7 shows excluded studies, together with the stage (as above) at which no qualifying
results were found.

Section -8 lists the potentially overlapping studies which have been included (1=principal, 2=subsidiary).

Section -9 lists any results which would have been included in preference except that they had data not complete
enough for use in meta-analysis, with their significance (yes/no), if known, and any further comment as entered
on the database. It also lists as "gap" any categories for which no data were presented by the original authors.

In addition to those mentioned above, the following fields, levels and abbreviations are used:
* or nk = not known, n = no, y = yes, ot = other
all/unspec = all or unspecified, cig+/-ot = cigarettes irrespective of other products (cigar, pipe etc)
MC = manufactured cigarettes, HR = hand-rolled cigarettes
exL, exH = range of exposure (low and high) in the "highest" group, in terms of Duration
unexL, unexH = range of exposure (low and high) in the "lowest" group, in terms of Duration
REF: 6-character study reference
NRR: number of the RR on the database within the study
ST : study type (CC = case control, pr or prosp = prospective)
NLC: number of lung cancer cases in whole study
R : risky occupational population (n = no, m = mining, o = other risky)
VB : national cigarette type (V = at least 75% Virginia, bl = at least 75% blended, ot = other)
P : any proxy use
H : full histological confirmation
De : derivation of RR/CI (or = original, st = standard method, ot = other method of estimation)

Table 1I5 - 1

IESLC - Meta-analysis of Ever Smoking, Duration, "Highest vs lowest"
All LC types, Any Product (or Cigarettes if Any not available)
Most adjusted

REF	NRR	SEX	AGEL	AGEH	RACE	YF	LC TYPE	LOC	START	ST	NLC	R	VB	P	H	AD	ADOS	PRODUCT	exL	exH	unexL	unexH	De
AGUDO	512	f	0	0	all	-	all	Eu:wst	1989	CC	103	n	bl	n	n	3	0	cig only	17	999	1	16	ot
ARMADA	510	m	0	0	all	-	all	Eu:wst	1986	CC	325	n	bl	n	y	1	0	cig+/-ot	50	999	1	24	ot
AUVINE	523	c	0	0	all	-	all	Eu:Sca	1986	CC	517	n	bl	y	n	2	0	cig+/-ot	41	999	1	20	ot
AXELSS	509	m	0	0	sca	-	all	Eu:Sca	1989	CC	436	n	bl	n	n	0	0	all/unsp	50	999	1	19	st
AXELSS	518	f	0	0	sca	-	all	Eu:Sca	1989	CC	436	n	bl	n	n	0	0	all/unsp	50	999	1	19	st
BARBON	514	m	0	0	all	-	all	Eu:wst	1979	CC	755	n	bl	y	y	1	0	all/unsp	50	999	1	29	ot
BOUCOT	520	m	0	0	all	9	all	NAmer	1951	pr	121	n	bl	n	n	0	0	cig+/-ot	40	999	1	39	st
BUFFLE	530	f	0	0	w-hi	-	all	NAmer	1976	CC	943	n	bl	y	n	0	0	cig+/-ot	41	999	1	30	st
CHEN2	509	m	0	0	all	-	all	As:Chi	1983	CC	193	n	ot	y	n	0	0	all/unsp	41	999	1	9	st
CHEN2	516	f	0	0	all	-	all	As:Chi	1983	CC	193	n	ot	y	n	0	0	all/unsp	41	999	1	20	st
CHOI	509	m	0	0	all	-	all	As:oth	1985	CC	375	n	bl	n	n	0	0	cig+/-ot	50	999	1	19	st
CHOI	516	f	0	0	all	-	all	As:oth	1985	CC	375	n	bl	n	n	0	0	cig+/-ot	40	999	1	19	st
DAMBER	514	m	0	0	all	-	all	Eu:Sca	1972	CC	579	n	bl	y	n	1	0	all/unsp	51	999	1	20	ot
DESTEF	514	m	0	0	all	-	all	SCAmer	1988	CC	497	n	bl	n	y	4	0	all/unsp	50	999	1	29	ot
DOLL	521	m	0	0	all	-	all	Eu:UK	1948	CC	1465	n	V	n	n	0	0	all/unsp	40	999	1	9	st
DOLL	528	f	0	0	all	-	all	Eu:UK	1948	CC	1465	n	V	n	n	0	0	all/unsp	40	999	1	9	st
DORGAN	526	m	0	0	wh	-	all	NAmer	1980	CC	2026	n	bl	y	y	2	0	cig+/-ot	35	999	1	34	ot
DORGAN	522	f	0	0	all	-	all	NAmer	1980	CC	2026	n	bl	y	y	3	0	cig+/-ot	35	999	1	34	ot
DOSEME	505	m	0	0	all	-	all	Eu:bal	1979	CC	1210	n	bl	n	n	2	0	cig+/-ot	21	999	1	10	ot
FAN	505	m	0	0	all	-	all	As:Chi	1990	CC	403	n	ot	y	n	0	0	cig+/-ot	40	999	1	29	st
FAN	510	f	0	0	all	-	all	As:Chi	1990	CC	403	n	ot	y	n	0	0	cig+/-ot	40	999	1	29	st
GAO	566	f	0	0	all	-	all	As:Chi	1984	CC	1405	n	ot	n	n	2	0	cig+/-ot	30	999	1	29	ot
GER	522	c	0	0	all	-	all	As:oth	1990	CC	141	n	ot	y	n	5	0	all/unsp	41	999	1	20	ot
HAENSZ	554	f	0	0	all	-	not alv	NAmer	1955	CC	158	n	bl	n	y	1	0	cig+/-ot	15	999	1	14	ot
HU	505	m	0	0	all	-	all	As:Chi	1985	CC	227	n	ot	n	y	0	0	cig+/-ot	30	999	1	19	st
HU	510	f	0	0	all	-	all	As:Chi	1985	CC	227	n	ot	n	y	0	0	cig+/-ot	30	999	1	19	st
HU2	514	c	0	0	all	-	all	As:Chi	1977	CC	523	n	ot	y	n	0	0	cig+/-ot	40	999	1	19	st
JOLY	523	m	0	0	all	-	all	SCAmer	1978	CC	826	n	bl	n	n	0	0	cig+/-ot	50	999	1	19	st
JOLY	509	f	0	0	all	-	all	SCAmer	1978	CC	826	n	bl	n	n	0	0	cig+/-ot	50	999	1	19	st
JUSSAW	518	m	0	0	all	-	all	As:Ind	1964	CC	792	n	V	n	n	0	0	cig only	40	999	1	9	st
KHUDER	505	m	0	0	all	-	all	NAmer	1985	CC	482	n	bl	n	y	0	0	cig+/-ot	50	999	1	29	st
KREUZE	514	m	55	69	all	-	all	Eu:Ger	1990	CC	2260	n	bl	n	n	3	0	all/unsp	40	999	1	19	ot
KREUZE	516	f	55	69	all	-	all	Eu:Ger	1990	CC	2260	n	bl	n	n	3	0	all/unsp	40	999	1	19	ot
LETOUR	510	c	0	0	all	-	all	NAmer	1983	CC	738	n	V	y	y	0	0	cig+/-ot	41	999	1	24	st
LEVIN	508	m	0	0	all	-	all	NAmer	1938	CC	475	n	bl	n	n	1	0	cig+/-ot	40	999	1	39	ot
LIU3	512	m	0	0	all	-	all	As:Chi	1985	CC	110	n	ot	n	n	2	0	all/unsp	35	999	1	34	ot
LIU5	506	c	0	0	all	-	all	As:Chi	1978	CC	111	n	ot	y	n	0	0	all/unsp	30	999	1	29	st
LUBIN	507	m	0	0	all	-	all	As:Chi	1984	CC	427	m	ot	y	n	0	0	cig+/-ot	50	999	1	29	st
LUBIN2	537	m	0	0	all	-	all	Eu:mul	1976	CC	7804	n	bl	n	y	0	0	cig+/-ot	50	999	1	29	st
LUBIN2	580	f	0	0	all	-	all	Eu:mul	1976	CC	7804	n	bl	n	y	0	0	cig+/-ot	50	999	1	29	st
MATOS	540	m	0	0	all	-	all	SCAmer	1994	CC	200	n	bl	n	n	2	0	cig+/-ot	40	70	1	24	ot
MCCONN	509	c	0	0	all	-	all	Eu:UK	1946	CC	100	n	V	n	y	0	0	all/unsp	40	999	1	9	st
NOTAN2	521	c	0	0	all	-	all	As:Ind	1963	CC	683	n	V	n	n	0	0	cig only	41	999	1	10	st
OSANN2	506	f	0	0	all	-	all	NAmer	1964	ot	217	n	bl	n	y	1	0	cig+/-ot	21	999	1	20	ot
PEZZOT	540	m	0	0	all	-	all	SCAmer	1987	CC	215	n	bl	n	y	2	0	cig only	41	999	1	30	ot
QIAO2	520	m	0	0	all	0	all	As:Chi	1992	pr	241	m	ot	n	n	1	0	all/unsp	42	999	1	27	ot
RACHTA	520	f	0	0	all	-	all	Eu:est	1991	CC	118	n	bl	n	y	1	0	cig+/-ot	41	999	1	20	ot
TIZZAN	505	m	0	0	all	-	all	Eu:wst	1959	CC	1358	n	bl	n	n	0	0	cig only	11	999	1	4	st
TIZZAN	535	f	0	0	all	-	all	Eu:wst	1959	CC	1358	n	bl	n	n	0	0	all/unsp	11	999	1	10	st
WANG2	509	c	0	0	all	-	all	As:Chi	1980	CC	103	n	ot	n	n	0	0	cig+/-ot	40	49	1	19	st
WUWILL	505	f	0	0	all	-	all	As:Chi	1985	CC	965	n	ot	n	n	0	0	cig+/-ot	40	999	1	29	st
ZHENG	556	m	0	0	all	-	all	As:Chi	1982	CC	540	n	ot	*	y	1	0	cig+/-ot	30	999	1	29	ot
ZHENG	561	f	0	0	all	-	all	As:Chi	1982	CC	540	n	ot	*	y	1	0	cig+/-ot	30	999	1	29	ot
ZHOU	503	c	0	0	all	-	all	As:Chi	1978	CC	1360	n	ot	n	n	0	0	all/unsp	20	999	1	19	st

Cigarette type is all/unspec for all RRs

except for the following:

REF	NRR	CIGTYPE
JUSSAW	518	MC only
NOTAN2	521	MC only

Table 1I5 - 2

IESLC - Meta-analysis of Ever Smoking, Duration, "Highest vs lowest"
All LC types, Any Product (or Cigarettes if Any not available)
Most adjusted

REF	NRR	SEX	AD	Number Exposed Case	Number Exposed Cont	Non-exposed Case	Non-exposed Cont	RR	95.00%CI
AGUDO	512	f	3	18	-	5	-	3.95	(0.93- 16.76)
ARMADA	510	m	1	77	-	21	-	10.31	(4.81- 22.07)
AUVINE	523	c	2	230	-	26	-	1.51	(0.51- 4.50)
AXELSS	509	m	0	101	40	13	84	16.32	(8.19- 32.51)
AXELSS	518	f	0	20	10	5	24	9.60	(2.82- 32.73)
Subtotal AXELSS								14.36	(7.88- 26.20)
BARBON	514	m	1	366	-	42	-	4.53	(3.02- 6.80)
*BOUCOT	520	m	0	52	1563	29	2621	3.01	(1.92- 4.72)
BUFFLE	530	f	0	90	42	52	57	2.35	(1.39- 3.97)
CHEN2	509	m	0	62	40	2	3	2.33	(0.37- 14.53)
CHEN2	516	f	0	21	15	1	6	8.40	(0.91- 77.21)
Subtotal CHEN2								3.92	(0.95- 16.08)
CHOI	509	m	0	20	20	19	55	2.89	(1.29- 6.51)
CHOI	516	f	0	1	1	2	9	4.50	(0.19- 106.82)
Subtotal CHOI								2.97	(1.36- 6.52)
DAMBER	514	m	1	-	-	-	-	7.08	(3.19- 15.74)
DESTEF	514	m	4	178	-	43	-	3.18	(1.74- 5.78)
DOLL	521	m	0	558	491	12	15	1.42	(0.66- 3.06)
DOLL	528	f	0	6	3	14	18	2.57	(0.54- 12.14)
Subtotal DOLL								1.60	(0.80- 3.18)
DORGAN	526	m	2	-	-	-	-	2.96	(2.31- 3.78)
DORGAN	522	f	3	-	-	-	-	2.76	(2.18- 3.50)
Subtotal DORGAN								2.85	(2.41- 3.39)
DOSEME	505	m	2	466	-	32	-	4.90	(2.91- 8.24)
FAN	505	m	0	143	241	29	135	2.76	(1.76- 4.34)
FAN	510	f	0	55	59	8	15	1.75	(0.69- 4.45)
Subtotal FAN								2.53	(1.69- 3.80)
GAO	566	f	2	168	-	68	-	2.43	(1.54- 3.83)
GER	522	c	5	49	-	10	-	1.65	(0.69- 3.91)
HAENSZ	554	f	1	58	-	16	-	1.21	(0.59- 2.47)
HU	505	m	0	19	14	41	33	1.09	(0.48- 2.50)
HU	510	f	0	4	3	11	8	0.97	(0.17- 5.59)
Subtotal HU								1.07	(0.51- 2.26)
HU2	514	c	0	194	113	21	33	2.70	(1.49- 4.89)
JOLY	523	m	0	250	253	11	48	4.31	(2.19- 8.49)
JOLY	509	f	0	57	20	13	28	6.14	(2.67- 14.11)
Subtotal JOLY								4.96	(2.94- 8.40)
JUSSAW	518	m	0	11	6	16	20	2.29	(0.70- 7.55)
KHUDER	505	m	0	236	354	16	61	2.54	(1.43- 4.52)
KREUZE	514	m	3	-	-	-	-	11.12	(8.68- 14.25)
KREUZE	516	f	3	-	-	-	-	9.22	(4.45- 19.09)
Subtotal KREUZE								10.91	(8.62- 13.79)
LETOUR	510	c	0	374	141	65	187	7.63	(5.42- 10.75)
LEVIN	508	m	1	63	-	56	-	1.27	(0.80- 2.00)
LIU3	512	m	2	22	-	30	-	1.60	(0.68- 3.77)
LIU5	506	c	0	58	33	27	37	2.41	(1.25- 4.64)
LUBIN	507	m	0	59	86	30	146	3.34	(2.00- 5.58)
LUBIN2	537	m	0	1325	1484	953	2995	2.81	(2.53- 3.11)
LUBIN2	580	f	0	81	32	132	230	4.41	(2.78- 7.00)
Subtotal LUBIN2								2.87	(2.59- 3.17)
MATOS	540	m	2	86	-	20	-	5.48	(2.97- 10.09)
MCCONN	509	c	0	16	40	3	4	0.53	(0.11- 2.66)
NOTAN2	521	c	0	5	5	7	15	2.14	(0.46- 9.90)
OSANN2	506	f	1	161	-	23	-	7.25	(3.41- 15.43)
PEZZOT	540	m	2	110	-	30	-	7.00	(4.33- 11.30)
*QIAO2	520	m	1	170	-	7	-	5.13	(2.36- 11.13)
RACHTA	520	f	1	24	-	12	-	29.05	(3.42- 246.78)
TIZZAN	505	m	0	928	815	12	1	0.09	(0.01- 0.73)
TIZZAN	535	f	0	23	21	2	7	3.83	(0.72- 20.55)
Subtotal TIZZAN								0.86	(0.24- 3.15)
WANG2	509	c	0	22	26	4	17	3.60	(1.05- 12.28)
WUWILL	505	f	0	223	114	137	139	1.98	(1.43- 2.75)
ZHENG	556	m	1	242	-	37	-	3.73	(2.39- 5.83)
ZHENG	561	f	1	59	-	17	-	3.16	(1.39- 7.16)
Subtotal ZHENG								3.59	(2.43- 5.31)
ZHOU	503	c	0	678	36	170	12	1.33	(0.68- 2.61)
Partial Totals				8239	6121	2352	7063		

*prospective study

Table 1I5 - 2

IESLC - Meta-analysis of Ever Smoking, Duration, "Highest vs lowest"
All LC types, Any Product (or Cigarettes if Any not available)
Most adjusted

REF	NRR	SEX	AD	Ys	Ws	Qs	Ps
AGUDO	512	f	3	1.37	1.84	0.05	0.0626
ARMADA	510	m	1	2.33	6.62	8.48	0.0000
AUVINE	523	c	2	0.41	3.24	2.02	0.4581
AXELSS	509	m	0	2.79	8.08	20.46	0.0000
AXELSS	518	f	0	2.26	2.55	2.87	0.0003
Subtotal AXELSS				2.66	10.64	23.33	
BARBON	514	m	1	1.51	23.32	2.23	0.0000
*BOUCOT	520	m	0	1.10	18.98	0.19	0.0000
BUFFLE	530	f	0	0.85	13.95	1.68	0.0014
CHEN2	509	m	0	0.84	1.14	0.15	0.3669
CHEN2	516	f	0	2.13	0.78	0.67	0.0601
Subtotal CHEN2				1.36	1.92	0.82	
CHOI	509	m	0	1.06	5.85	0.11	0.0101
CHOI	516	f	0	1.50	0.38	0.04	0.3520
Subtotal CHOI				1.09	6.24	0.15	
DAMBER	514	m	1	1.96	6.03	3.45	0.0000
DESTEF	514	m	4	1.16	10.66	0.02	0.0002
DOLL	521	m	0	0.35	6.50	4.70	0.3707
DOLL	528	f	0	0.94	1.59	0.11	0.2330
Subtotal DOLL				0.47	8.10	4.80	
DORGAN	526	m	2	1.09	63.36	0.85	0.0000
DORGAN	522	f	3	1.02	68.55	2.37	0.0000
Subtotal DORGAN				1.05	131.91	3.22	
DOSEME	505	m	2	1.59	14.18	2.14	0.0000
FAN	505	m	0	1.02	18.86	0.65	0.0000
FAN	510	f	0	0.56	4.41	1.82	0.2410
Subtotal FAN				0.93	23.27	2.47	
GAO	566	f	2	0.89	18.51	1.82	0.0001
GER	522	c	5	0.50	5.11	2.51	0.2578
HAENSZ	554	f	1	0.19	7.49	7.65	0.6018
HU	505	m	0	0.09	5.59	6.93	0.8345
HU	510	f	0	-0.03	1.25	1.90	0.9725
Subtotal HU				0.07	6.85	8.83	
HU2	514	c	0	0.99	10.88	0.47	0.0011
JOLY	523	m	0	1.46	8.35	0.57	0.0000
JOLY	509	f	0	1.81	5.55	2.09	0.0000
Subtotal JOLY				1.60	13.90	2.65	
JUSSAW	518	m	0	0.83	2.70	0.37	0.1728
KHUDER	505	m	0	0.93	11.63	0.84	0.0015
KREUZE	514	m	3	2.41	62.53	91.17	0.0000
KREUZE	516	f	3	2.22	7.25	7.54	0.0000
Subtotal KREUZE				2.39	69.77	98.71	
LETOUR	510	c	0	2.03	32.79	22.64	0.0000
LEVIN	508	m	1	0.24	18.30	16.94	0.3065
LIU3	512	m	2	0.47	5.24	2.80	0.2821
LIU5	506	c	0	0.88	8.96	0.93	0.0085
LUBIN	507	m	0	1.21	14.54	0.00	0.0000
LUBIN2	537	m	0	1.03	355.65	10.21	0.0000
LUBIN2	580	f	0	1.48	18.01	1.44	0.0000
Subtotal LUBIN2				1.05	373.66	11.65	
MATOS	540	m	2	1.70	10.27	2.57	0.0000
MCCONN	509	c	0	-0.63	1.49	4.99	0.4428
NOTAN2	521	c	0	0.76	1.64	0.32	0.3290
OSANN2	506	f	1	1.98	6.74	4.10	0.0000
PEZZOT	540	m	2	1.95	16.70	9.26	0.0000
*QIAO2	520	m	1	1.64	6.39	1.20	0.0000
RACHTA	520	f	1	3.37	0.84	3.94	0.0020
TIZZAN	505	m	0	-2.36	0.92	11.65	0.0238
TIZZAN	535	f	0	1.34	1.36	0.03	0.1168
Subtotal TIZZAN				-0.15	2.28	11.68	
WANG2	509	c	0	1.28	2.55	0.02	0.0411
WUWILL	505	f	0	0.69	36.04	9.59	0.0000
ZHENG	556	m	1	1.32	19.32	0.26	0.0000
ZHENG	561	f	1	1.15	5.72	0.01	0.0059
Subtotal ZHENG				1.28	25.04	0.27	
ZHOU	503	c	0	0.28	8.44	7.09	0.4081

Table 1I5 - 2

IESLC - Meta-analysis of Ever Smoking, Duration, "Highest vs lowest"
All LC types, Any Product (or Cigarettes if Any not available)
Most adjusted

	N	54
	NS	42
	Wt	999.65
Het	Chi	288.90
Het	df	53
Het	P	***
Fixed	RR	3.32
	RRl	3.12
	RRu	3.54
	P	+++
Random	RR	3.31
	RRl	2.77
	RRu	3.96
	P	+++
Asymm	P	N.S.

Table 1I5 - 3

IESLC - Meta-analysis of Ever Smoking, Duration, "Highest vs lowest"
All LC types, Any Product (or Cigarettes if Any not available)
Most adjusted

		Sex			
		combined	male	female	Total
	N	9	26	19	54
	NS	9	26	19	54
	Wt	75.09	721.74	202.82	999.65
Het	Chi	40.65	197.48	45.97	288.90
Het	df	8	25	18	53
Het	P	***	***	***	***
Fixed	RR	3.55	3.43	2.90	3.32
	RRl	2.83	3.19	2.53	3.12
	RRu	4.45	3.69	3.33	3.54
	P	+++	+++	+++	+++
Random	RR	2.26	3.55	3.34	3.31
	RRl	1.26	2.75	2.53	2.77
	RRu	4.07	4.58	4.40	3.96
	P	++	+++	+++	+++
Between	Chi				4.80
Between	df				2
Between	P				(*)
Btwn(F)	P				N.S.
Btwn(R)	P				N.S.

		Lung cancer type		
		all	other	Total
	N	53	1	54
	NS	41	1	42
	Wt	992.16	7.49	999.65
Het	Chi	281.19	0.00	288.90
Het	df	52	0	53
Het	P	***	N.S.	***
Fixed	RR	3.35	1.21	3.32
	RRl	3.15	0.59	3.12
	RRu	3.56	2.48	3.54
	P	+++	N.S.	+++
Random	RR	3.38	1.21	3.31
	RRl	2.83	0.59	2.77
	RRu	4.04	2.48	3.96
	P	+++	N.S.	+++
Between	Chi			7.71
Between	df			1
Between	P			**
Btwn(F)	P			N.S.
Btwn(R)	P			**

		Location								
		NAmer	UK	Scand	othEur	China	Japan	othAs	other	Total
	N	9	3	4	11	17		5	5	54
	NS	8	2	3	8	13		4	4	42
	Wt	241.80	9.59	19.91	492.52	168.62		15.69	51.54	999.65
Het	Chi	55.16	1.96	13.29	134.41	21.52		1.05	4.53	288.90
Het	df	8	2	3	10	16		4	4	53
Het	P	***	N.S.	**	***	N.S.		N.S.	N.S.	***
Fixed	RR	3.03	1.35	8.03	3.66	2.47		2.27	5.16	3.32
	RRl	2.67	0.71	5.18	3.35	2.13		1.38	3.93	3.12
	RRu	3.43	2.54	12.47	3.99	2.88		3.72	6.78	3.54
	P	+++	N.S.	+++	+++	+++		++	+++	+++
Random	RR	2.87	1.35	6.68	5.02	2.48		2.27	5.13	3.31
	RRl	2.01	0.71	2.54	2.98	2.05		1.38	3.83	2.77
	RRu	4.11	2.54	17.57	8.43	2.99		3.72	6.88	3.96
	P	+++	N.S.	+++	+++	+++		++	+++	+++
Between	Chi									56.98
Between	df									6
Between	P									***
Btwn(F)	P									(*)
Btwn(R)	P									***

Table 1I5 - 3

IESLC - Meta-analysis of Ever Smoking, Duration, "Highest vs lowest"
All LC types, Any Product (or Cigarettes if Any not available)
Most adjusted

Detailed Country in "other Europe"

	multi	Germany	othWest	East	Balkans	Total
N	2	2	5	1	1	11
NS	1	1	4	1	1	8
Wt	373.66	69.77	34.07	0.84	14.18	492.52
Het Chi	3.51	0.23	18.26	0.00	0.00	134.41
Het df	1	1	4	0	0	10
Het P	(*)	N.S.	**	N.S.	N.S.	***
Fixed RR	2.87	10.91	4.72	29.05	4.90	3.66
RRl	2.59	8.62	3.37	3.42	2.91	3.35
RRu	3.17	13.79	6.60	246.77	8.25	3.99
P	+++	+++	+++	++	+++	+++
Random RR	3.32	10.91	3.20	29.05	4.90	5.02
RRl	2.16	8.62	1.19	3.42	2.91	2.98
RRu	5.09	13.79	8.57	246.77	8.25	8.43
P	+++	+++	+	++	+++	+++
Between Chi						112.42
Between df						4
Between P						***
Btwn(F) P						*
Btwn(R) P						***

Detailed Country in "other Asia"

	India	HongKong	other	Total
N	2		3	5
NS	2		2	4
Wt	4.34		11.34	15.69
Het Chi	0.00		1.04	1.05
Het df	1		2	4
Het P	N.S.		N.S.	N.S.
Fixed RR	2.23		2.28	2.27
RRl	0.87		1.27	1.38
RRu	5.72		4.08	3.72
P	(+)		++	++
Random RR	2.23		2.28	2.27
RRl	0.87		1.27	1.38
RRu	5.72		4.08	3.72
P	(+)		++	++
Between Chi				0.00
Between df				1
Between P				N.S.
Btwn(F) P				N.S.
Btwn(R) P				N.S.

Detailed other continent

	SCAmer	Total
N	5	5
NS	4	4
Wt	51.54	51.54
Het Chi	4.53	4.53
Het df	4	4
Het P	N.S.	N.S.
Fixed RR	5.16	5.16
RRl	3.93	3.93
RRu	6.78	6.78
P	+++	+++
Random RR	5.13	5.13
RRl	3.83	3.83
RRu	6.88	6.88
P	+++	+++
Between Chi		
Between df		
Between P		N.S.
Btwn(F) P		N.S.
Btwn(R) P		N.S.

Table 1I5 - 3

IESLC - Meta-analysis of Ever Smoking, Duration, "Highest vs lowest"
All LC types, Any Product (or Cigarettes if Any not available)
Most adjusted

Start year of study

	<1960	1960-69	1970-79	1980-89	1990+	Total
N	8	3	11	24	8	54
NS	6	3	9	18	6	42
Wt	56.64	11.09	473.33	342.95	115.64	999.65
Het Chi	19.64	3.66	27.01	94.13	52.23	288.90
Het df	7	2	10	23	7	53
Het P	**	N.S.	**	***	***	***
Fixed RR	1.67	4.57	2.99	3.35	6.80	3.32
RRl	1.29	2.54	2.73	3.01	5.66	3.12
RRu	2.17	8.24	3.27	3.72	8.16	3.54
P	+++	+++	+++	+++	+++	+++
Random RR	1.43	3.87	3.43	3.50	4.86	3.31
RRl	0.85	1.62	2.70	2.72	2.65	2.77
RRu	2.41	9.26	4.35	4.51	8.91	3.96
P	N.S.	++	+++	+++	+++	+++
Between Chi						92.23
Between df						4
Between P						***
Btwn(F) P						***
Btwn(R) P						*

Study type (1)

	CC	other	Total
N	51	3	54
NS	39	3	42
Wt	967.55	32.11	999.65
Het Chi	283.37	4.32	288.90
Het df	50	2	53
Het P	***	N.S.	***
Fixed RR	3.30	4.02	3.32
RRl	3.10	2.85	3.12
RRu	3.52	5.69	3.54
P	+++	+++	+++
Random RR	3.24	4.48	3.31
RRl	2.69	2.59	2.77
RRu	3.90	7.77	3.96
P	+++	+++	+++
Between Chi			1.21
Between df			1
Between P			N.S.
Btwn(F) P			N.S.
Btwn(R) P			N.S.

Study type (2)

	CC	prosp	other	Total
N	51	2	1	54
NS	39	2	1	42
Wt	967.55	25.37	6.74	999.65
Het Chi	283.37	1.36	0.00	288.90
Het df	50	1	0	53
Het P	***	N.S.	N.S.	***
Fixed RR	3.30	3.44	7.25	3.32
RRl	3.10	2.33	3.41	3.12
RRu	3.52	5.08	15.42	3.54
P	+++	+++	+++	+++
Random RR	3.24	3.56	7.25	3.31
RRl	2.69	2.19	3.41	2.77
RRu	3.90	5.80	15.42	3.96
P	+++	+++	+++	+++
Between Chi				4.17
Between df				2
Between P				N.S.
Btwn(F) P				N.S.
Btwn(R) P				N.S.

Table 1I5 - 3

IESLC - Meta-analysis of Ever Smoking, Duration, "Highest vs lowest"
All LC types, Any Product (or Cigarettes if Any not available)
Most adjusted

	Study size (number of LC cases)				
	100-249	250-499	500-999	1000+	Total
N	17	11	13	13	54
NS	15	8	11	8	42
Wt	101.36	101.90	169.54	626.85	999.65
Het Chi	49.82	51.97	44.08	140.97	288.90
Het df	16	10	12	12	53
Het P	***	***	***	***	***
Fixed RR	3.26	3.16	3.67	3.27	3.32
RRl	2.68	2.60	3.15	3.03	3.12
RRu	3.96	3.84	4.26	3.54	3.54
P	+++	+++	+++	+++	+++
Random RR	2.94	3.73	3.55	3.19	3.31
RRl	2.01	2.32	2.58	2.22	2.77
RRu	4.31	6.00	4.88	4.58	3.96
P	+++	+++	+++	+++	+++
Between Chi					2.05
Between df					3
Between P					N.S.
Btwn(F) P					N.S.
Btwn(R) P					N.S.

	Risky occupational population			
	no	mining	othRisky	Total
N	52	2		54
NS	40	2		42
Wt	978.72	20.93		999.65
Het Chi	287.69	0.82		288.90
Het df	51	1		53
Het P	***	N.S.		***
Fixed RR	3.31	3.81		3.32
RRl	3.11	2.48		3.12
RRu	3.53	5.84		3.54
P	+++	+++		+++
Random RR	3.28	3.81		3.31
RRl	2.73	2.48		2.77
RRu	3.94	5.84		3.96
P	+++	+++		+++
Between Chi				0.39
Between df				1
Between P				N.S.
Btwn(F) P				N.S.
Btwn(R) P				N.S.

	National cigarette tobacco type			
	Virginia	blended	other	Total
N	6	30	18	54
NS	5	23	14	42
Wt	46.72	779.21	173.73	999.65
Het Chi	27.04	215.24	22.33	288.90
Het df	5	29	17	53
Het P	***	***	N.S.	***
Fixed RR	4.77	3.48	2.44	3.32
RRl	3.58	3.25	2.11	3.12
RRu	6.35	3.74	2.83	3.54
P	+++	+++	+++	+++
Random RR	2.25	4.12	2.44	3.31
RRl	0.89	3.23	2.03	2.77
RRu	5.68	5.25	2.93	3.96
P	(+)	+++	+++	+++
Between Chi				24.28
Between df				2
Between P				***
Btwn(F) P				N.S.
Btwn(R) P				**

Table 1I5 - 3

IESLC - Meta-analysis of Ever Smoking, Duration, "Highest vs lowest"
All LC types, Any Product (or Cigarettes if Any not available)
Most adjusted

	Any proxy use		
	No/nk	Yes	Total
N	39	15	54
NS	30	12	42
Wt	723.73	275.92	999.65
Het Chi	246.44	42.41	288.90
Het df	38	14	53
Het P	***	***	***
Fixed RR	3.34	3.29	3.32
RRl	3.10	2.92	3.12
RRu	3.59	3.70	3.54
P	+++	+++	+++
Random RR	3.37	3.18	3.31
RRl	2.65	2.50	2.77
RRu	4.29	4.04	3.96
P	+++	+++	+++
Between Chi			0.05
Between df			1
Between P			N.S.
Btwn(F) P			N.S.
Btwn(R) P			N.S.

	Full histological confirmation		
	No	Yes	Total
N	36	18	54
NS	28	14	42
Wt	343.90	655.75	999.65
Het Chi	197.49	86.43	288.90
Het df	35	17	53
Het P	***	***	***
Fixed RR	3.66	3.16	3.32
RRl	3.30	2.93	3.12
RRu	4.07	3.41	3.54
P	+++	+++	+++
Random RR	3.16	3.55	3.31
RRl	2.39	2.81	2.77
RRu	4.18	4.49	3.96
P	+++	+++	+++
Between Chi			4.99
Between df			1
Between P			*
Btwn(F) P			N.S.
Btwn(R) P			N.S.

	Number of adjustment variables (1)			
	0	1	2+/+nk	Total
N	31	10	13	54
NS	22	9	11	42
Wt	611.44	100.78	287.43	999.65
Het Chi	106.11	47.94	108.09	288.90
Het df	30	9	12	53
Het P	***	***	***	***
Fixed RR	2.94	3.52	4.24	3.32
RRl	2.71	2.90	3.78	3.12
RRu	3.18	4.28	4.76	3.54
P	+++	+++	+++	+++
Random RR	2.88	4.13	3.80	3.31
RRl	2.32	2.55	2.57	2.77
RRu	3.58	6.70	5.60	3.96
P	+++	+++	+++	+++
Between Chi				26.76
Between df				2
Between P				***
Btwn(F) P				(*)
Btwn(R) P				N.S.

Table 1I5 - 3

IESLC - Meta-analysis of Ever Smoking, Duration, "Highest vs lowest"
All LC types, Any Product (or Cigarettes if Any not available)
Most adjusted

	Number of adjustment variables (2) 0	1	2	3-5	6+/+nk	Total
N	31	10	7	6		54
NS	22	9	7	5		43
Wt	611.44	100.78	131.50	155.93		999.65
Het Chi	106.11	47.94	21.36	75.38		288.90
Het df	30	9	6	5		53
Het P	***	***	**	***		***
Fixed RR	2.94	3.52	3.41	5.09		3.32
RRl	2.71	2.90	2.88	4.35		3.12
RRu	3.18	4.28	4.05	5.95		3.54
P	+++	+++	+++	+++		+++
Random RR	2.88	4.13	3.46	4.36		3.31
RRl	2.32	2.55	2.38	2.09		2.77
RRu	3.58	6.70	5.02	9.09		3.96
P	+++	+++	+++	+++		+++
Between Chi						38.12
Between df						3
Between P						***
Btwn(F) P						(*)
Btwn(R) P						N.S.

	Product all/unsp	cig+/-ot	cig only	Total
N	18	31	5	54
NS	14	24	5	43
Wt	167.43	808.42	23.80	999.65
Het Chi	108.37	103.38	19.13	288.90
Het df	17	30	4	53
Het P	***	***	***	***
Fixed RR	5.57	2.96	4.60	3.32
RRl	4.79	2.76	3.08	3.12
RRu	6.48	3.17	6.88	3.54
P	+++	+++	+++	+++
Random RR	3.75	3.11	2.09	3.31
RRl	2.41	2.64	0.65	2.77
RRu	5.84	3.66	6.76	3.96
P	+++	+++	N.S.	+++
Between Chi				58.01
Between df				2
Between P				***
Btwn(F) P				**
Btwn(R) P				N.S.

	Derivation of RR/CI Orig	StdCalc	Other	Total
N		31	23	54
NS		22	20	42
Wt		611.44	388.22	999.65
Het Chi		106.11	158.59	288.90
Het df		30	22	53
Het P		***	***	***
Fixed RR		2.94	4.04	3.32
RRl		2.71	3.66	3.12
RRu		3.18	4.46	3.54
P		+++	+++	+++
Random RR		2.88	3.92	3.31
RRl		2.32	2.92	2.77
RRu		3.58	5.26	3.96
P		+++	+++	+++
Between Chi				24.21
Between df				1
Between P				***
Btwn(F) P				*
Btwn(R) P				(*)

Table 1I5 - 4

IESLC - Meta-analysis of Ever Smoking, Duration, "Highest vs lowest"
All LC types, Any Product (or Cigarettes if Any not available)
Least adjusted

REF	NRR	X	SEX	AGEL	AGEH	RACE	YF	LC TYPE	LOC	START	ST	NLC	R	VB	P	H	AD	ADOS	PRODUCT	exL	exH	unexL	unexH	De
AGUDO	509	x	f	0	0	all	-	all	Eu:wst	1989	CC	103	n	bl	n	n	0	0	cig only	17	999	1	16	st
ARMADA	505	x	m	0	0	all	-	all	Eu:wst	1986	CC	325	n	bl	n	y	0	0	cig+/-ot	50	999	1	24	st
AUVINE	505	x	c	0	0	all	-	all	Eu:Sca	1986	CC	517	n	bl	y	n	0	0	cig+/-ot	41	999	1	20	st
AXELSS	509		m	0	0	sca	-	all	Eu:Sca	1989	CC	436	n	bl	n	n	0	0	all/unsp	50	999	1	19	st
AXELSS	518		f	0	0	sca	-	all	Eu:Sca	1989	CC	436	n	bl	n	n	0	0	all/unsp	50	999	1	19	st
BARBON	507	x	m	0	0	all	-	all	Eu:wst	1979	CC	755	n	bl	y	y	0	0	all/unsp	50	999	1	29	st
BOUCOT	520		m	0	0	all	9	all	NAmer	1951	pr	121	n	bl	n	n	0	0	cig+/-ot	40	999	1	39	st
BUFFLE	530		f	0	0	w-hi	-	all	NAmer	1976	CC	943	n	bl	y	n	0	0	cig+/-ot	41	999	1	30	st
CHEN2	509		m	0	0	all	-	all	As:Chi	1983	CC	193	n	ot	y	n	0	0	all/unsp	41	999	1	9	st
CHEN2	516		f	0	0	all	-	all	As:Chi	1983	CC	193	n	ot	y	n	0	0	all/unsp	41	999	1	20	st
CHOI	509		m	0	0	all	-	all	As:oth	1985	CC	375	n	bl	n	n	0	0	cig+/-ot	50	999	1	19	st
CHOI	516		f	0	0	all	-	all	As:oth	1985	CC	375	n	bl	n	n	0	0	cig+/-ot	40	999	1	19	st
DAMBER	514		m	0	0	all	-	all	Eu:Sca	1972	CC	579	n	bl	y	n	1	0	all/unsp	51	999	1	20	ot
DESTEF	507	x	m	0	0	all	-	all	SCAmer	1988	CC	497	n	bl	n	y	0	0	all/unsp	50	999	1	29	st
DOLL	521		m	0	0	all	-	all	Eu:UK	1948	CC	1465	n	V	n	n	0	0	all/unsp	40	999	1	9	st
DOLL	528		f	0	0	all	-	all	Eu:UK	1948	CC	1465	n	V	n	n	0	0	all/unsp	40	999	1	9	st
DORGAN	526		m	0	0	wh	-	all	NAmer	1980	CC	2026	n	bl	y	y	2	0	cig+/-ot	35	999	1	34	ot
DORGAN	522		f	0	0	all	-	all	NAmer	1980	CC	2026	n	bl	y	y	3	0	cig+/-ot	35	999	1	34	ot
DOSEME	505		m	0	0	all	-	all	Eu:bal	1979	CC	1210	n	bl	n	n	2	0	cig+/-ot	21	999	1	10	ot
FAN	505		m	0	0	all	-	all	As:Chi	1990	CC	403	n	ot	y	n	0	0	cig+/-ot	40	999	1	29	st
FAN	510		f	0	0	all	-	all	As:Chi	1990	CC	403	n	ot	y	n	0	0	cig+/-ot	40	999	1	29	st
GAO	563	x	f	0	0	all	-	all	As:Chi	1984	CC	1405	n	ot	n	n	0	0	cig+/-ot	30	999	1	29	st
GER	517	x	c	0	0	all	-	all	As:oth	1990	CC	141	n	ot	y	n	0	0	all/unsp	41	999	1	20	st
HAENSZ	546	x	f	0	0	all	-	not alv	NAmer	1955	CC	158	n	bl	n	y	0	0	cig+/-ot	15	999	1	14	st
HU	505		m	0	0	all	-	all	As:Chi	1985	CC	227	n	ot	n	y	0	0	cig+/-ot	30	999	1	19	st
HU	510		f	0	0	all	-	all	As:Chi	1985	CC	227	n	ot	n	y	0	0	cig+/-ot	30	999	1	19	st
HU2	514		c	0	0	all	-	all	As:Chi	1977	CC	523	n	ot	y	n	0	0	cig+/-ot	40	999	1	19	st
JOLY	523		m	0	0	all	-	all	SCAmer	1978	CC	826	n	bl	n	n	0	0	cig+/-ot	50	999	1	19	st
JOLY	509		f	0	0	all	-	all	SCAmer	1978	CC	826	n	bl	n	n	0	0	cig+/-ot	50	999	1	19	st
JUSSAW	518		m	0	0	all	-	all	As:Ind	1964	CC	792	n	V	n	n	0	0	cig only	40	999	1	9	st
KHUDER	505		m	0	0	all	-	all	NAmer	1985	CC	482	n	bl	n	y	0	0	cig+/-ot	50	999	1	29	st
KREUZE	514		m	55	69	all	-	all	Eu:Ger	1990	CC	2260	n	bl	n	n	3	0	all/unsp	40	999	1	19	ot
KREUZE	516		f	55	69	all	-	all	Eu:Ger	1990	CC	2260	n	bl	n	n	3	0	all/unsp	40	999	1	19	ot
LETOUR	510		c	0	0	all	-	all	NAmer	1983	CC	738	n	V	y	y	0	0	cig+/-ot	41	999	1	24	st
LEVIN	503	x	m	0	0	all	-	all	NAmer	1938	CC	475	n	bl	n	n	0	0	cig+/-ot	40	999	1	39	st
LIU3	509	x	m	0	0	all	-	all	As:Chi	1985	CC	110	n	ot	n	n	0	0	all/unsp	35	999	1	34	st
LIU5	506		c	0	0	all	-	all	As:Chi	1978	CC	111	n	ot	y	n	0	0	all/unsp	30	999	1	29	st
LUBIN	507		m	0	0	all	-	all	As:Chi	1984	CC	427	m	ot	y	n	0	0	cig+/-ot	50	999	1	29	st
LUBIN2	537		m	0	0	all	-	all	Eu:mul	1976	CC	7804	n	bl	n	y	0	0	cig+/-ot	50	999	1	29	st
LUBIN2	580		f	0	0	all	-	all	Eu:mul	1976	CC	7804	n	bl	n	y	0	0	cig+/-ot	50	999	1	29	st
MATOS	520	x	m	0	0	all	-	all	SCAmer	1994	CC	200	n	bl	n	n	0	0	cig+/-ot	40	70	1	24	st
MCCONN	509		c	0	0	all	-	all	Eu:UK	1946	CC	100	n	V	n	y	0	0	all/unsp	40	999	1	9	st
NOTAN2	521		c	0	0	all	-	all	As:Ind	1963	CC	683	n	V	n	n	0	0	cig only	41	999	1	10	st
OSANN2	503	x	f	0	0	all	-	all	NAmer	1964	ot	217	n	bl	n	y	0	0	cig+/-ot	21	999	1	20	st
PEZZOT	538	x	m	0	0	all	-	all	SCAmer	1987	CC	215	n	bl	n	y	0	0	cig only	41	999	1	30	st
QIAO2	515	x	m	0	0	all	0	all	As:Chi	1992	pr	241	m	ot	n	n	0	0	all/unsp	42	999	1	27	st
RACHTA	515	x	f	0	0	all	-	all	Eu:est	1991	CC	118	n	bl	n	y	0	0	cig+/-ot	41	999	1	20	st
TIZZAN	505		m	0	0	all	-	all	Eu:wst	1959	CC	1358	n	bl	n	n	0	0	cig only	11	999	1	4	st
TIZZAN	535		f	0	0	all	-	all	Eu:wst	1959	CC	1358	n	bl	n	n	0	0	all/unsp	11	999	1	10	st
WANG2	509		c	0	0	all	-	all	As:Chi	1980	CC	103	n	ot	n	n	0	0	cig+/-ot	40	49	1	19	st
WUWILL	505		f	0	0	all	-	all	As:Chi	1985	CC	965	n	ot	n	n	0	0	cig+/-ot	40	999	1	29	st
ZHENG	555	x	m	0	0	all	-	all	As:Chi	1982	CC	540	n	ot	*	y	0	0	cig+/-ot	30	999	1	29	st
ZHENG	560	x	f	0	0	all	-	all	As:Chi	1982	CC	540	n	ot	*	y	0	0	cig+/-ot	30	999	1	29	st
ZHOU	503		c	0	0	all	-	all	As:Chi	1978	CC	1360	n	ot	n	n	0	0	all/unsp	20	999	1	19	st

Cigarette type is all/unspec for all RRs

except for the following:

REF	NRR	CIGTYPE
JUSSAW	518	MC only
NOTAN2	521	MC only

Table 1I5 - 5

IESLC - Meta-analysis of Ever Smoking, Duration, "Highest vs lowest"
All LC types, Any Product (or Cigarettes if Any not available)
Least adjusted

REF	NRR	SEX	AD	Number Exposed Case	Cont	Non-exposed Case	Cont	RR	95.00%CI
AGUDO	509	f	0	18	11	5	12	3.93	(1.09- 14.19)
ARMADA	505	m	0	77	33	21	55	6.11	(3.20- 11.68)
AUVINE	505	c	0	230	57	26	18	2.79	(1.43- 5.44)
AXELSS	509	m	0	101	40	13	84	16.32	(8.19- 32.51)
AXELSS	518	f	0	20	10	5	24	9.60	(2.82- 32.73)
Subtotal AXELSS								14.36	(7.88- 26.20)
BARBON	507	m	0	366	235	42	91	3.37	(2.26- 5.04)
*BOUCOT	520	m	0	52	1563	29	2621	3.01	(1.92- 4.72)
BUFFLE	530	f	0	90	42	52	57	2.35	(1.39- 3.97)
CHEN2	509	m	0	62	40	2	3	2.33	(0.37- 14.53)
CHEN2	516	f	0	21	15	1	6	8.40	(0.91- 77.21)
Subtotal CHEN2								3.92	(0.95- 16.08)
CHOI	509	m	0	20	20	19	55	2.89	(1.29- 6.51)
CHOI	516	f	0	1	1	2	9	4.50	(0.19- 106.82)
Subtotal CHOI								2.97	(1.36- 6.52)
DAMBER	514	m	1	-	-	-	-	7.08	(3.19- 15.74)
DESTEF	507	m	0	178	108	43	55	2.11	(1.32- 3.36)
DOLL	521	m	0	558	491	12	15	1.42	(0.66- 3.06)
DOLL	528	f	0	6	3	14	18	2.57	(0.54- 12.14)
Subtotal DOLL								1.60	(0.80- 3.18)
DORGAN	526	m	2	-	-	-	-	2.96	(2.31- 3.78)
DORGAN	522	f	3	-	-	-	-	2.76	(2.18- 3.50)
Subtotal DORGAN								2.85	(2.41- 3.39)
DOSEME	505	m	2	466	-	32	-	4.90	(2.91- 8.24)
FAN	505	m	0	143	241	29	135	2.76	(1.76- 4.34)
FAN	510	f	0	55	59	8	15	1.75	(0.69- 4.45)
Subtotal FAN								2.53	(1.69- 3.80)
GAO	563	f	0	168	72	68	58	1.99	(1.27- 3.11)
GER	517	c	0	49	155	10	40	1.26	(0.59- 2.71)
HAENSZ	546	f	0	58	77	16	26	1.22	(0.60- 2.49)
HU	505	m	0	19	14	41	33	1.09	(0.48- 2.50)
HU	510	f	0	4	3	11	8	0.97	(0.17- 5.59)
Subtotal HU								1.07	(0.51- 2.26)
HU2	514	c	0	194	113	21	33	2.70	(1.49- 4.89)
JOLY	523	m	0	250	253	11	48	4.31	(2.19- 8.49)
JOLY	509	f	0	57	20	13	28	6.14	(2.67- 14.11)
Subtotal JOLY								4.96	(2.94- 8.40)
JUSSAW	518	m	0	11	6	16	20	2.29	(0.70- 7.55)
KHUDER	505	m	0	236	354	16	61	2.54	(1.43- 4.52)
KREUZE	514	m	3	-	-	-	-	11.12	(8.68- 14.25)
KREUZE	516	f	3	-	-	-	-	9.22	(4.45- 19.09)
Subtotal KREUZE								10.91	(8.62- 13.79)
LETOUR	510	c	0	374	141	65	187	7.63	(5.42- 10.75)
LEVIN	503	m	0	63	91	56	97	1.20	(0.76- 1.90)
LIU3	509	m	0	22	59	30	146	1.81	(0.97- 3.40)
LIU5	506	c	0	58	33	27	37	2.41	(1.25- 4.64)
LUBIN	507	m	0	59	86	30	146	3.34	(2.00- 5.58)
LUBIN2	537	m	0	1325	1484	953	2995	2.81	(2.53- 3.11)
LUBIN2	580	f	0	81	32	132	230	4.41	(2.78- 7.00)
Subtotal LUBIN2								2.87	(2.59- 3.17)
MATOS	520	m	0	86	89	20	84	4.06	(2.29- 7.18)
MCCONN	509	c	0	16	40	3	4	0.53	(0.11- 2.66)
NOTAN2	521	c	0	5	5	7	15	2.14	(0.46- 9.90)
OSANN2	503	f	0	161	57	23	47	5.77	(3.22- 10.34)
PEZZOT	538	m	0	110	101	30	134	4.86	(3.01- 7.86)
*QIAO2	515	m	0	170	2295	7	2364	25.02	(11.77- 53.16)
RACHTA	515	f	0	24	1	12	19	38.00	(4.53- 318.78)
TIZZAN	505	m	0	928	815	12	1	0.09	(0.01- 0.73)
TIZZAN	535	f	0	23	21	2	7	3.83	(0.72- 20.55)
Subtotal TIZZAN								0.86	(0.24- 3.15)
WANG2	509	c	0	22	26	4	17	3.60	(1.05- 12.28)
WUWILL	505	f	0	223	114	137	139	1.98	(1.43- 2.75)
ZHENG	555	m	0	242	143	37	75	3.43	(2.20- 5.35)
ZHENG	560	f	0	59	27	17	17	2.19	(0.97- 4.92)
Subtotal ZHENG								3.09	(2.09- 4.57)
ZHOU	503	c	0	678	36	170	12	1.33	(0.68- 2.61)
Partial Totals				8239	9732	2352	10401		

*prospective study

Table 1I5 - 5

IESLC - Meta-analysis of Ever Smoking, Duration, "Highest vs lowest"
All LC types, Any Product (or Cigarettes if Any not available)
Least adjusted

REF	NRR	SEX	AD	Ys	Ws	Qs	Ps
AGUDO	509	f	0	1.37	2.33	0.09	0.0369
ARMADA	505	m	0	1.81	9.17	3.68	0.0000
AUVINE	505	c	0	1.03	8.63	0.19	0.0025
AXELSS	509	m	0	2.79	8.08	21.10	0.0000
AXELSS	518	f	0	2.26	2.55	3.01	0.0003
Subtotal AXELSS				2.66	10.64	24.11	
BARBON	507	m	0	1.22	23.93	0.04	0.0000
*BOUCOT	520	m	0	1.10	18.98	0.11	0.0000
BUFFLE	530	f	0	0.85	13.95	1.45	0.0014
CHEN2	509	m	0	0.84	1.14	0.13	0.3669
CHEN2	516	f	0	2.13	0.78	0.71	0.0601
Subtotal CHEN2				1.36	1.92	0.83	
CHOI	509	m	0	1.06	5.85	0.08	0.0101
CHOI	516	f	0	1.50	0.38	0.04	0.3520
Subtotal CHOI				1.09	6.24	0.12	
DAMBER	514	m	1	1.96	6.03	3.68	0.0000
DESTEF	507	m	0	0.75	17.76	3.29	0.0017
DOLL	521	m	0	0.35	6.50	4.43	0.3707
DOLL	528	f	0	0.94	1.59	0.09	0.2330
Subtotal DOLL				0.47	8.10	4.51	
DORGAN	526	m	2	1.09	63.36	0.53	0.0000
DORGAN	522	f	3	1.02	68.55	1.78	0.0000
Subtotal DORGAN				1.05	131.91	2.30	
DOSEME	505	m	2	1.59	14.18	2.42	0.0000
FAN	505	m	0	1.02	18.86	0.48	0.0000
FAN	510	f	0	0.56	4.41	1.68	0.2410
Subtotal FAN				0.93	23.27	2.17	
GAO	563	f	0	0.69	19.31	4.60	0.0025
GER	517	c	0	0.23	6.59	5.84	0.5470
HAENSZ	546	f	0	0.20	7.62	7.23	0.5768
HU	505	m	0	0.09	5.59	6.62	0.8345
HU	510	f	0	-0.03	1.25	1.82	0.9725
Subtotal HU				0.07	6.85	8.44	
HU2	514	c	0	0.99	10.88	0.37	0.0011
JOLY	523	m	0	1.46	8.35	0.68	0.0000
JOLY	509	f	0	1.81	5.55	2.26	0.0000
Subtotal JOLY				1.60	13.90	2.94	
JUSSAW	518	m	0	0.83	2.70	0.33	0.1728
KHUDER	505	m	0	0.93	11.63	0.69	0.0015
KREUZE	514	m	3	2.41	62.53	94.98	0.0000
KREUZE	516	f	3	2.22	7.25	7.91	0.0000
Subtotal KREUZE				2.39	69.77	102.89	
LETOUR	510	c	0	2.03	32.79	24.02	0.0000
LEVIN	503	m	0	0.18	18.17	17.98	0.4388
LIU3	509	m	0	0.60	9.75	3.28	0.0628
LIU5	506	c	0	0.88	8.96	0.79	0.0085
LUBIN	507	m	0	1.21	14.54	0.01	0.0000
LUBIN2	537	m	0	1.03	355.65	7.43	0.0000
LUBIN2	580	f	0	1.48	18.01	1.71	0.0000
Subtotal LUBIN2				1.05	373.66	9.13	
MATOS	520	m	0	1.40	11.80	0.59	0.0000
MCCONN	509	c	0	-0.63	1.49	4.86	0.4428
NOTAN2	521	c	0	0.76	1.64	0.28	0.3290
OSANN2	503	f	0	1.75	11.30	3.76	0.0000
PEZZOT	538	m	0	1.58	16.73	2.75	0.0000
*QIAO2	515	m	0	3.22	6.76	28.23	0.0000
RACHTA	515	f	0	3.64	0.85	5.14	0.0008
TIZZAN	505	m	0	-2.36	0.92	11.49	0.0238
TIZZAN	535	f	0	1.34	1.36	0.04	0.1168
Subtotal TIZZAN				-0.15	2.28	11.52	
WANG2	509	c	0	1.28	2.55	0.03	0.0411
WUWILL	505	f	0	0.69	36.04	8.68	0.0000
ZHENG	555	m	0	1.23	19.42	0.06	0.0000
ZHENG	560	f	0	0.78	5.83	0.91	0.0592
Subtotal ZHENG				1.13	25.25	0.97	
ZHOU	503	c	0	0.28	8.44	6.71	0.4081

Table 1I5 - 5

IESLC - Meta-analysis of Ever Smoking, Duration, "Highest vs lowest"
All LC types, Any Product (or Cigarettes if Any not available)
Least adjusted

	N	54
	NS	42
	Wt	1029.26
Het	Chi	311.07
Het	df	53
Het	P	***
Fixed	RR	3.24
	RRl	3.05
	RRu	3.45
	P	+++
Random	RR	3.23
	RRl	2.70
	RRu	3.86
	P	+++
Asymm	P	N.S.

Table 1I5 - 6

IESLC - Meta-analysis of Ever Smoking, Duration, "Highest vs lowest"
All LC types, Any Product (or Cigarettes if Any not available)
Least adjusted

	Sex<br>combined	male	female	Total
N	9	26	19	54
NS	9	26	19	54
Wt	81.96	738.39	208.91	1029.26
Het Chi	42.74	214.42	49.31	311.07
Het df	8	25	18	53
Het P	***	***	***	***
Fixed RR	3.46	3.34	2.84	3.24
RRl	2.79	3.11	2.48	3.05
RRu	4.29	3.59	3.26	3.45
P	+++	+++	+++	+++
Random RR	2.34	3.45	3.22	3.23
RRl	1.33	2.66	2.43	2.70
RRu	4.12	4.47	4.25	3.86
P	++	+++	+++	+++
Between Chi				4.59
Between df				2
Between P				N.S.
Btwn(F) P				N.S.
Btwn(R) P				N.S.

Table 1I5 - 7

IESLC - Meta-analysis of Ever Smoking, Duration, "Highest vs lowest"
All LC types, Any Product (or Cigarettes if Any not available)
Excluded studies (and stage at which they were excluded)

1	AKIBA AMANDU AMES BECHER BENSHL BEST BLOT1 BROSS BROWN3 CARPEN CEDERL CHYOU CPSI CPSII DARBY DEAN2 DEAN3 DOLL2 ENGELA GAO2 GARCIA GILLIS GRAHAM GURSEL HAMMO2 HIRAYA HOLE HUMBLE JAHN JAIN KAISE2 KATSOU KAUFMA LAUSSM LIAW MCDUFF MIGRAN MRFITR PEZZO2 PISANI PRESCO QIAO SEGI2 SPEIZE SVENSS TVERDA WAKAI WATSON WIGLE WU WYNDE3 WYNDE8
2	ALDERS BRESLO CHIAZZ DORN GUO HEGMAN KOO KOULUM LIU4 PERNU SOBUE SPITZ SUZUK2 VUTUC YUAN
3	GENG STASZE WU2 ZHANG
4	GARSHI
5	BOUCHA CHEN CORREA JEDRYC LUO WYNDE2 WYNDE6
6	HAMMON RESTRE SADOWS XU
8	BOFFET WYNDE7
15	BENHAM

Table 1I5 - 8
Potentially overlapping studies

REF	REFGP	PRINC	.	OVERLAP/LINK
LUBIN2	LUBIN2	1		Lubin-combined
OSANN2	KAISER	2		KAISER/OSANN2
LUBIN	XIANGZ	2		LUBIN/XIANGZ/QIAO

Table 1I5 - 9
Most adjusted - insufficient data for meta-analysis

REF	NRR	SEX	AGEL	AGEH	RACE	YF	LC TYPE	LOC	START	ST	NLC	R	VB	P	H	AD	ADOS	PRODUCT	exL	exH	unexL	unexH	De
BUFFLE	548	m	0	0	wh	-	all	NAmer	1976	CC	943	n	bl	y	n	0	0	cig+/-ot	50	999	1	33	st
HAMMON	512	m	0	0	wh	0	all	NAmer	1952	pr	448	n	bl	n	n	1	0	cig only	35	999	1	34	st
RESTRE	507	c	0	0	all	-	all	SCAmer	1978	CC	102	n	bl	n	n	3	1#	cig+/-ot	51	999	1	20	or
SADOWS	532	m	0	0	wh	-	all	NAmer	1938	CC	477	n	bl	n	n	0	0	cig only	50	999	1	9	ot
XU	505	m	0	0	all	-	all	As:Chi	1985	CC	729	n	ot	n	n	2	0	all/unsp	40	999	1	29	st

Comments on values in listings

RESTRE ADOS Number of cigarettes smoked per day

REF	NRR	RR	SIG	RRDATA comment
BUFFLE	548	2.13		0
HAMMON	512	*		RR for <1/2 pack per day is 2.04, that for 1/2 to 1 pack per day is 1.25, that for 1 to 2 packs per day is 2.71 while that for 2+ packs per day 2.12
RESTRE	507	24.01		Test for trend given as P = 0.006
SADOWS	532	7.09		0
XU	505	*		RR for 1-19/day is 1.8, for 20-29/day is 4.0 and for >=30/day is 3.2

Table 1I6 -

IESLC - Meta-analysis of Current Smoking by Duration, Overview
All LC types, Any Product (or Cigarettes if Any not available)

This analysis is restricted to results for:
1) Current smokers
2) Results by Duration
3) Categorical results by Duration
Results by Duration are grouped under 2 schemes (S1, S2). Each scheme has a set of "key values". An interval is allocated to the category whose key value it includes, and intervals which include none or more than one of the key values are excluded. (Open-ended intervals are coded as 999)

S1	key value	maximum range
1	20	1-34
2	35	21-49
3	50	36+

S2	key value	maximum range
1	5	1-19
2	20	6-29
3	30	21-39
4	40	31-49
5	50	41-998
6	999	51+

4) All LC types (or near equivalent)
5) Results complete enough for use in metaanalysis

Within each study, results are then selected (in the following order of preference, within each sex) for:
6) (not applicable)
7) PRODUCT: all/unspec, cigarettes regardless of other products, cigarettes only
8) CIGTYPE: all/unspecified, MC regardless of HR, MC only
9) (not applicable)
10) DENOM: never smoked anything, never smoked cigarettes, never any + low, never cigs + low
11) Followup period (YF, prospective studies): whole study (coded as 0) or longest available
12) LCtype: all or nearest available, at least Squamous and Adeno. (q = squamous, s = small, l = large, a = adeno, mix = mixed, alv = alveolar)
13) Race: all or nearest available, otherwise by race (wh or w = white, bl or b = black, hi = hispanic ch = chinese, jap = japanese, haw = hawaiian, w+o = white + oriental, sca = scandinavian, as = asian)
14) For overlapping studies: principal rather than subsidiary studies
Finally by Age: whole study (coded as 0) if available, otherwise by widest available age group and then for single sex results (m, f) in preference to results for both sexes combined (c).

Results adjusted (AD) for the most potential confounders are then chosen in Sections -1 to -3 and results adjusted for the least confounders in Sections -4 to -6. (Those least adjusted results which actually differ from the most adjusted are marked 'x' in column X in Section -4)

Section -7 shows excluded studies, together with the stage (as above) at which no qualifying results were found.

Section -8 lists the potentially overlapping studies which have been included (1=principal, 2=subsidiary).

Section -9 lists any results which would have been included in preference except that they had data not complete enough for use in meta-analysis, with their significance (yes/no), if known, and any further comment as entered on the database. It also lists as "gap" any categories for which no data were presented by the original authors.

In addition to those mentioned above, the following fields, levels and abbreviations are used:
* or nk = not known, n = no, y = yes, ot = other
nev = never
all/unspec = all or unspecified, cig+/-ot = cigarettes irrespective of other products (cigar, pipe etc)
MC = manufactured cigarettes, HR = hand-rolled cigarettes
exL, exH = range of exposure (low and high) in the smoking group, in terms of Duration
REF: 6-character study reference
NRR: number of the RR on the database within the study
ST : study type (CC = case control, pr or prosp = prospective)
NLC: number of lung cancer cases in whole study
R : risky occupational population (n = no, m = mining, o = other risky)
VB : national cigarette type (V = at least 75% Virginia, bl = at least 75% blended, ot = other)
P : any proxy use
H : full histological confirmation
De : derivation of RR/CI (or = original, st = standard method, ot = other method of estimation)

Table 1I6 - 1

IESLC - Meta-analysis of Current Smoking by Duration, Overview
All LC types, Any Product (or Cigarettes if Any not available)
Most adjusted

REF	NRR	SEX	AGEL	AGEH	RACE	YF	LC TYPE	LOC	START	ST	NLC	R	VB	P	H	AD	PRODUCT	exL	exH	S1	S2	DENOM	De
AMANDU	506	m	0	0	wh	0	all	NAmer	1959	pr	132	m	bl	n	n	2	cig+/-ot	0	24	1	0	nev cigs	ot
AMANDU	507	m	0	0	wh	0	all	NAmer	1959	pr	132	m	bl	n	n	2	cig+/-ot	25	999	0	0	nev cigs	ot
BEST	501	m	0	0	all	0	all	NAmer	1955	pr	381	n	V	n	n	1	cig only	1	4	0	0	nev any	ot
BEST	502	m	0	0	all	0	all	NAmer	1955	pr	381	n	V	n	n	1	cig only	5	9	0	1	nev any	ot
BEST	503	m	0	0	all	0	all	NAmer	1955	pr	381	n	V	n	n	1	cig only	10	14	0	0	nev any	ot
BEST	504	m	0	0	all	0	all	NAmer	1955	pr	381	n	V	n	n	1	cig only	15	19	0	0	nev any	ot
BEST	505	m	0	0	all	0	all	NAmer	1955	pr	381	n	V	n	n	1	cig only	20	29	1	2	nev any	ot
BEST	506	m	0	0	all	0	all	NAmer	1955	pr	381	n	V	n	n	1	cig only	30	39	2	3	nev any	ot
BEST	507	m	0	0	all	0	all	NAmer	1955	pr	381	n	V	n	n	1	cig only	40	999	3	0	nev any	ot
BOUCOT	521	m	0	0	all	0	all	NAmer	1951	pr	121	n	bl	n	n	2	cig only	1	39	0	0	nev any	ot
BOUCOT	522	m	0	0	all	0	all	NAmer	1951	pr	121	n	bl	n	n	2	cig only	40	999	3	0	nev any	ot
BUFFLE	531	f	0	0	w-hi	-	all	NAmer	1976	CC	943	n	bl	y	n	0	cig+/-ot	1	30	1	0	nev cigs	or
BUFFLE	532	f	0	0	w-hi	-	all	NAmer	1976	CC	943	n	bl	y	n	0	cig+/-ot	31	40	2	4	nev cigs	or
BUFFLE	533	f	0	0	w-hi	-	all	NAmer	1976	CC	943	n	bl	y	n	0	cig+/-ot	41	999	3	0	nev cigs	or
CEDERL	501	m	40	69	all	10	all	Eu:Sca	1963	pr	491	n	bl	n	n	1	cig only	1	29	1	0	nev any	ot
CEDERL	502	m	40	69	all	10	all	Eu:Sca	1963	pr	491	n	bl	n	n	1	cig only	30	999	0	0	nev any	ot
CEDERL	504	f	40	69	all	10	all	Eu:Sca	1963	pr	491	n	bl	n	n	1	cig only	1	29	1	0	nev any	ot
CEDERL	505	f	40	69	all	10	all	Eu:Sca	1963	pr	491	n	bl	n	n	1	cig only	30	999	0	0	nev any	ot
CPSI	580	m	40	84	wh	0	all	NAmer	1959	pr	5138	n	bl	n	n	0	cig only	1	29	1	0	nev cigs	st
CPSI	581	m	40	84	wh	0	all	NAmer	1959	pr	5138	n	bl	n	n	0	cig only	30	34	0	3	nev cigs	st
CPSI	582	m	40	84	wh	0	all	NAmer	1959	pr	5138	n	bl	n	n	0	cig only	35	39	2	0	nev cigs	st
CPSI	583	m	40	84	wh	0	all	NAmer	1959	pr	5138	n	bl	n	n	0	cig only	40	44	0	4	nev cigs	st
CPSI	584	m	40	84	wh	0	all	NAmer	1959	pr	5138	n	bl	n	n	0	cig only	45	49	0	0	nev cigs	st
CPSI	585	m	40	84	wh	0	all	NAmer	1959	pr	5138	n	bl	n	n	0	cig only	50	54	3	5	nev cigs	st
CPSI	586	m	40	84	wh	0	all	NAmer	1959	pr	5138	n	bl	n	n	0	cig only	55	59	0	0	nev cigs	st
CPSI	587	m	40	84	wh	0	all	NAmer	1959	pr	5138	n	bl	n	n	0	cig only	60	999	0	6	nev cigs	st
CPSI	676	f	40	84	wh	0	all	NAmer	1959	pr	5138	n	bl	n	n	0	cig only	1	29	1	0	nev cigs	st
CPSI	677	f	40	84	wh	0	all	NAmer	1959	pr	5138	n	bl	n	n	0	cig only	30	34	0	3	nev cigs	st
CPSI	678	f	40	84	wh	0	all	NAmer	1959	pr	5138	n	bl	n	n	0	cig only	35	39	2	0	nev cigs	st
CPSI	679	f	40	84	wh	0	all	NAmer	1959	pr	5138	n	bl	n	n	0	cig only	40	44	0	4	nev cigs	st
CPSI	680	f	40	84	wh	0	all	NAmer	1959	pr	5138	n	bl	n	n	0	cig only	45	49	0	0	nev cigs	st
CPSI	681	f	40	84	wh	0	all	NAmer	1959	pr	5138	n	bl	n	n	0	cig only	50	54	3	5	nev cigs	st
CPSI	682	f	40	84	wh	0	all	NAmer	1959	pr	5138	n	bl	n	n	0	cig only	55	999	0	6	nev cigs	st
CPSII	552	m	0	0	all	6	all	NAmer	1982	pr	3229	n	bl	n	n	0	cig only	1	29	1	0	nev any	st
CPSII	553	m	0	0	all	6	all	NAmer	1982	pr	3229	n	bl	n	n	0	cig only	30	34	0	3	nev any	st
CPSII	554	m	0	0	all	6	all	NAmer	1982	pr	3229	n	bl	n	n	0	cig only	35	39	2	0	nev any	st
CPSII	555	m	0	0	all	6	all	NAmer	1982	pr	3229	n	bl	n	n	0	cig only	40	44	0	4	nev any	st
CPSII	556	m	0	0	all	6	all	NAmer	1982	pr	3229	n	bl	n	n	0	cig only	45	49	0	0	nev any	st
CPSII	557	m	0	0	all	6	all	NAmer	1982	pr	3229	n	bl	n	n	0	cig only	50	54	3	5	nev any	st
CPSII	558	m	0	0	all	6	all	NAmer	1982	pr	3229	n	bl	n	n	0	cig only	55	59	0	0	nev any	st
CPSII	559	m	0	0	all	6	all	NAmer	1982	pr	3229	n	bl	n	n	0	cig only	60	999	0	6	nev any	st
CPSII	618	f	0	0	all	6	all	NAmer	1982	pr	3229	n	bl	n	n	0	cig+/-ot	1	29	1	0	nev cigs	st
CPSII	619	f	0	0	all	6	all	NAmer	1982	pr	3229	n	bl	n	n	0	cig+/-ot	30	34	0	3	nev cigs	st
CPSII	620	f	0	0	all	6	all	NAmer	1982	pr	3229	n	bl	n	n	0	cig+/-ot	35	39	2	0	nev cigs	st
CPSII	621	f	0	0	all	6	all	NAmer	1982	pr	3229	n	bl	n	n	0	cig+/-ot	40	44	0	4	nev cigs	st
CPSII	622	f	0	0	all	6	all	NAmer	1982	pr	3229	n	bl	n	n	0	cig+/-ot	45	49	0	0	nev cigs	st
CPSII	623	f	0	0	all	6	all	NAmer	1982	pr	3229	n	bl	n	n	0	cig+/-ot	50	54	3	5	nev cigs	st
CPSII	624	f	0	0	all	6	all	NAmer	1982	pr	3229	n	bl	n	n	0	cig+/-ot	55	59	0	0	nev cigs	st
CPSII	625	f	0	0	all	6	all	NAmer	1982	pr	3229	n	bl	n	n	0	cig+/-ot	60	999	0	6	nev cigs	st
DEAN2	501	m	0	0	all	-	all	Eu:UK	1960	CC	954	n	V	y	n	0	all/unsp	1	19	0	1	nev any	st
DEAN2	502	m	0	0	all	-	all	Eu:UK	1960	CC	954	n	V	y	n	0	all/unsp	20	999	0	0	nev any	st
DEAN2	504	f	0	0	all	-	all	Eu:UK	1960	CC	954	n	V	y	n	0	all/unsp	1	19	0	1	nev any	st
DEAN2	505	f	0	0	all	-	all	Eu:UK	1960	CC	954	n	V	y	n	0	all/unsp	20	999	0	0	nev any	st
HUMBLE	542	c	0	0	wh	-	not alv	NAmer	1980	CC	521	n	bl	y	n	3	cig+/-ot	1	29	1	0	nev cigs	ot
HUMBLE	543	c	0	0	wh	-	not alv	NAmer	1980	CC	521	n	bl	y	n	3	cig+/-ot	30	39	2	3	nev cigs	ot
HUMBLE	544	c	0	0	wh	-	not alv	NAmer	1980	CC	521	n	bl	y	n	3	cig+/-ot	40	49	0	4	nev cigs	ot
HUMBLE	545	c	0	0	wh	-	not alv	NAmer	1980	CC	521	n	bl	y	n	3	cig+/-ot	50	999	3	0	nev cigs	ot
KAISE2	596	m	0	0	all	9	all	NAmer	1979	pr	318	n	bl	n	n	1	cig only	1	39	0	0	nev any	st
KAISE2	597	m	0	0	all	9	all	NAmer	1979	pr	318	n	bl	n	n	1	cig only	40	999	3	0	nev any	st
KAISE2	516	f	0	0	all	9	all	NAmer	1979	pr	318	n	bl	n	n	1	cig only	1	39	0	0	nev any	st
KAISE2	517	f	0	0	all	9	all	NAmer	1979	pr	318	n	bl	n	n	1	cig only	40	999	3	0	nev any	st
KATSOU	512	f	0	0	all	-	all	Eu:bal	1987	CC	101	n	bl	n	n	1	all/unsp	1	29	1	0	nev any	or
KATSOU	513	f	0	0	all	-	all	Eu:bal	1987	CC	101	n	bl	n	n	1	all/unsp	30	999	0	0	nev any	or
LIAW	501	c	0	0	all	0	all	As:oth	1982	pr	127	n	ot	n	n	2	all/unsp	1	20	1	0	nev any	or
LIAW	502	c	0	0	all	0	all	As:oth	1982	pr	127	n	ot	n	n	2	all/unsp	21	30	0	3	nev any	or
LIAW	503	c	0	0	all	0	all	As:oth	1982	pr	127	n	ot	n	n	2	all/unsp	31	999	0	0	nev any	or
MATOS	521	m	0	0	all	-	all	SCAmer	1994	CC	200	n	bl	n	n	2	cig+/-ot	1	24	1	0	nev any	or
MATOS	522	m	0	0	all	-	all	SCAmer	1994	CC	200	n	bl	n	n	2	cig+/-ot	25	39	2	3	nev any	or
MATOS	523	m	0	0	all	-	all	SCAmer	1994	CC	200	n	bl	n	n	2	cig+/-ot	40	70	3	0	nev any	or
PEZZO2	507	m	0	0	all	-	all	SCAmer	1992	CC	367	n	bl	n	y	0	cig+/-ot	1	35	0	0	nev cigs	st
PEZZO2	508	m	0	0	all	-	all	SCAmer	1992	CC	367	n	bl	n	y	0	cig+/-ot	36	999	3	0	nev cigs	st
SOBUE	546	m	0	0	all	-	q+s+l+a	As:Jap	1986	CC	1376	n	bl	n	y	0	cig+/-ot	1	29	1	0	nev cigs	st

Table 1I6 - 1

IESLC - Meta-analysis of Current Smoking by Duration, Overview
All LC types, Any Product (or Cigarettes if Any not available)
Most adjusted

REF	NRR	SEX	AGEL	AGEH	RACE	YF	LC TYPE	LOC	START	ST	NLC	R	VB	P	H	AD	PRODUCT	exL	exH	S1	S2	DENOM	De
SOBUE	547	m	0	0	all	-	q+s+l+a	As:Jap	1986	CC	1376	n	bl	n	y	0	cig+/-ot	30	39	2	3	nev cigs	st
SOBUE	548	m	0	0	all	-	q+s+l+a	As:Jap	1986	CC	1376	n	bl	n	y	0	cig+/-ot	40	49	0	4	nev cigs	st
SOBUE	549	m	0	0	all	-	q+s+l+a	As:Jap	1986	CC	1376	n	bl	n	y	0	cig+/-ot	50	999	3	0	nev cigs	st

Cigarette type is all/unspec for all RRs

In this overview table, subtotals and Qs values may be invalid and should be ignored

Table 1I6 - 2

IESLC - Meta-analysis of Current Smoking by Duration, Overview
All LC types, Any Product (or Cigarettes if Any not available)
Most adjusted

REF	NRR	SEX	AD	Number Exposed Case	Number Exposed Cont	Non-exposed Case	Non-exposed Cont	RR	95.00%CI
*AMANDU	506	m	2	42	-	6	-	5.92	(2.13- 16.47)
*AMANDU	507	m	2	72	-	6	-	7.02	(2.67- 18.51)
Subtotal AMANDU								6.48	(3.21- 13.08)
*BEST	501	m	1	1	-	7	-	1.60	(0.20- 13.00)
*BEST	502	m	1	2	-	7	-	2.60	(0.54- 12.52)
*BEST	503	m	1	6	-	7	-	2.30	(0.77- 6.84)
*BEST	504	m	1	10	-	7	-	3.20	(1.22- 8.41)
*BEST	505	m	1	22	-	7	-	4.10	(1.75- 9.60)
*BEST	506	m	1	55	-	7	-	13.90	(6.33- 30.52)
*BEST	507	m	1	137	-	7	-	14.20	(6.64- 30.35)
Subtotal BEST								6.17	(4.26- 8.94)
*BOUCOT	521	m	2	32	-	0	-	42.40	(2.60- 692.28)
*BOUCOT	522	m	2	53	-	0	-	89.94	(5.55-1456.45)
Subtotal BOUCOT								61.82	(8.61- 444.17)
BUFFLE	531	f	0	36	24	12	112	14.00	(6.37- 30.79)
BUFFLE	532	f	0	74	47	12	112	14.70	(7.31- 29.55)
BUFFLE	533	f	0	70	36	12	112	18.15	(8.85- 37.22)
Subtotal BUFFLE								15.59	(10.22- 23.79)
*CEDERL	501	m	1	5	-	7	-	1.80	(0.57- 5.66)
*CEDERL	502	m	1	23	-	7	-	7.40	(3.18- 17.21)
*CEDERL	504	f	1	3	-	19	-	1.60	(0.47- 5.40)
*CEDERL	505	f	1	5	-	19	-	9.60	(3.60- 25.58)
Subtotal CEDERL								4.61	(2.78- 7.67)
*CPSI	580	m	0	95	266163	196	926068	1.69	(1.32- 2.15)
*CPSI	581	m	0	230	290031	196	926068	3.75	(3.10- 4.53)
*CPSI	582	m	0	470	367622	196	926068	6.04	(5.11- 7.14)
*CPSI	583	m	0	731	333292	196	926068	10.36	(8.85- 12.13)
*CPSI	584	m	0	764	221405	196	926068	16.30	(13.94- 19.07)
*CPSI	585	m	0	576	119633	196	926068	22.75	(19.35- 26.75)
*CPSI	586	m	0	356	53226	196	926068	31.60	(26.55- 37.61)
*CPSI	587	m	0	232	26906	196	926068	40.74	(33.70- 49.25)
*CPSI	676	f	0	105	694015	532	3877179	1.10	(0.89- 1.36)
*CPSI	677	f	0	141	383127	532	3877179	2.68	(2.23- 3.23)
*CPSI	678	f	0	154	315060	532	3877179	3.56	(2.98- 4.26)
*CPSI	679	f	0	120	163178	532	3877179	5.36	(4.40- 6.53)
*CPSI	680	f	0	54	53635	532	3877179	7.34	(5.55- 9.71)
*CPSI	681	f	0	16	14305	532	3877179	8.15	(4.96- 13.40)
*CPSI	682	f	0	10	5657	532	3877179	12.88	(6.90- 24.07)
Subtotal CPSI								8.01	(7.62- 8.43)
*CPSII	552	m	0	72	141932	124	742207	3.04	(2.27- 4.06)
*CPSII	553	m	0	145	113317	124	742207	7.66	(6.03- 9.73)
*CPSII	554	m	0	244	109788	124	742207	13.30	(10.72- 16.51)
*CPSII	555	m	0	413	103500	124	742207	23.88	(19.54- 29.19)
*CPSII	556	m	0	307	53805	124	742207	34.15	(27.73- 42.06)
*CPSII	557	m	0	332	39260	124	742207	50.62	(41.19- 62.20)
*CPSII	558	m	0	151	13598	124	742207	66.47	(52.45- 84.24)
*CPSII	559	m	0	117	8450	124	742207	82.88	(64.43- 106.60)
*CPSII	618	f	0	127	301244	310	2091302	2.84	(2.31- 3.50)
*CPSII	619	f	0	158	152833	310	2091302	6.97	(5.76- 8.45)
*CPSII	620	f	0	193	116270	310	2091302	11.20	(9.36- 13.40)
*CPSII	621	f	0	216	91501	310	2091302	15.93	(13.39- 18.94)
*CPSII	622	f	0	153	44769	310	2091302	23.06	(19.00- 27.98)
*CPSII	623	f	0	122	29119	310	2091302	28.26	(22.93- 34.84)
*CPSII	624	f	0	27	6262	310	2091302	29.09	(19.64- 43.07)
*CPSII	625	f	0	18	2224	310	2091302	54.60	(34.01- 87.65)
Subtotal CPSII								17.37	(16.44- 18.35)
DEAN2	501	m	0	34	36	33	112	3.21	(1.74- 5.89)
DEAN2	502	m	0	631	558	33	112	3.84	(2.56- 5.75)
DEAN2	504	f	0	10	14	88	121	0.98	(0.42- 2.31)
DEAN2	505	f	0	47	11	88	121	5.88	(2.88- 11.97)
Subtotal DEAN2								3.39	(2.55- 4.52)
HUMBLE	542	c	3	20	-	28	-	15.45	(6.19- 38.58)
HUMBLE	543	c	3	68	-	28	-	17.54	(8.46- 36.34)
HUMBLE	544	c	3	104	-	28	-	19.61	(11.20- 34.31)
HUMBLE	545	c	3	119	-	28	-	17.27	(10.38- 28.75)
Subtotal HUMBLE								17.79	(12.99- 24.36)
*KAISE2	596	m	1	17	-	14	-	4.86	(2.22- 10.61)
*KAISE2	597	m	1	34	-	14	-	15.64	(8.31- 29.40)
*KAISE2	516	f	1	24	-	11	-	9.09	(4.25- 19.43)
*KAISE2	517	f	1	26	-	11	-	30.41	(14.39- 64.25)
Subtotal KAISE2								12.59	(8.77- 18.07)

Table 1I6 - 2

IESLC - Meta-analysis of Current Smoking by Duration, Overview
All LC types, Any Product (or Cigarettes if Any not available)
Most adjusted

REF	NRR	SEX	AD	Number Exposed Case	Number Exposed Cont	Non-exposed Case	Non-exposed Cont	RR	95.00%CI
KATSOU	512	f	1	13	-	48	-	1.29	(0.54- 3.26)
KATSOU	513	f	1	32	-	48	-	7.43	(2.88- 19.13)
Subtotal KATSOU								2.96	(1.54- 5.68)
*LIAW	501	c	2	-	-	-	-	0.90	(0.30- 3.10)
*LIAW	502	c	2	-	-	-	-	2.60	(1.20- 5.90)
*LIAW	503	c	2	-	-	-	-	4.70	(2.70- 8.20)
Subtotal LIAW								3.19	(2.09- 4.88)
MATOS	521	m	2	10	-	11	-	5.20	(1.70- 16.40)
MATOS	522	m	2	47	-	11	-	7.40	(3.30- 16.60)
MATOS	523	m	2	55	-	11	-	10.20	(4.70- 22.10)
Subtotal MATOS								7.90	(4.79- 13.04)
PEZZO2	507	m	0	60	72	6	117	16.25	(6.68- 39.53)
PEZZO2	508	m	0	173	126	6	117	26.77	(11.42- 62.76)
Subtotal PEZZO2								21.08	(11.40- 39.00)
SOBUE	546	m	0	62	119	34	128	1.96	(1.21- 3.19)
SOBUE	547	m	0	159	200	34	128	2.99	(1.94- 4.61)
SOBUE	548	m	0	241	174	34	128	5.21	(3.41- 7.98)
SOBUE	549	m	0	147	73	34	128	7.58	(4.73- 12.14)
Subtotal SOBUE								3.96	(3.16- 4.96)
Partial Totals				9630	4636617	959457	218417		

*prospective study

REF	NRR	SEX	AD	Ys	Ws	Qs	Ps
*AMANDU	506	m	2	1.78	3.67	1.32	0.0007
*AMANDU	507	m	2	1.95	4.10	0.76	0.0001
Subtotal AMANDU				1.87	7.77	2.08	
*BEST	501	m	1	0.47	0.88	3.21	0.6590
*BEST	502	m	1	0.96	1.55	3.15	0.2335
*BEST	503	m	1	0.83	3.22	7.69	0.1350
*BEST	504	m	1	1.16	4.12	6.08	0.0182
*BEST	505	m	1	1.41	5.30	4.96	0.0012
*BEST	506	m	1	2.63	6.21	0.40	0.0000
*BEST	507	m	1	2.65	6.65	0.50	0.0000
Subtotal BEST				1.82	27.95	26.00	
*BOUCOT	521	m	2	3.75	0.49	0.92	0.0085
*BOUCOT	522	m	2	4.50	0.50	2.23	0.0015
Subtotal BOUCOT				4.12	0.99	3.15	
BUFFLE	531	f	0	2.64	6.18	0.42	0.0000
BUFFLE	532	f	0	2.69	7.87	0.75	0.0000
BUFFLE	533	f	0	2.90	7.44	2.02	0.0000
Subtotal BUFFLE				2.75	21.50	3.19	
*CEDERL	501	m	1	0.59	2.92	9.35	0.3155
*CEDERL	502	m	1	2.00	5.39	0.76	0.0000
*CEDERL	504	f	1	0.47	2.58	9.39	0.4505
*CEDERL	505	f	1	2.26	4.00	0.05	0.0000
Subtotal CEDERL				1.53	14.88	19.55	
*CPSI	580	m	0	0.52	64.01	220.35	0.0000
*CPSI	581	m	0	1.32	105.87	118.31	0.0000
*CPSI	582	m	0	1.80	138.39	46.48	0.0000
*CPSI	583	m	0	2.34	154.66	0.24	0.0000
*CPSI	584	m	0	2.79	156.12	26.68	0.0000
*CPSI	585	m	0	3.12	146.44	81.60	0.0000
*CPSI	586	m	0	3.45	126.72	146.50	0.0000
*CPSI	587	m	0	3.71	106.68	188.47	0.0000
*CPSI	676	f	0	0.10	87.71	456.06	0.3603
*CPSI	677	f	0	0.99	111.49	215.86	0.0000
*CPSI	678	f	0	1.27	119.48	146.58	0.0000
*CPSI	679	f	0	1.68	97.98	47.89	0.0000
*CPSI	680	f	0	1.99	49.07	7.27	0.0000
*CPSI	681	f	0	2.10	15.55	1.22	0.0000
*CPSI	682	f	0	2.56	9.83	0.31	0.0000
Subtotal CPSI				2.08	1489.99	1703.82	
*CPSII	552	m	0	1.11	45.57	73.19	0.0000
*CPSII	553	m	0	2.04	66.89	7.83	0.0000
*CPSII	554	m	0	2.59	82.29	3.63	0.0000
*CPSII	555	m	0	3.17	95.47	60.37	0.0000
*CPSII	556	m	0	3.53	88.48	117.59	0.0000
*CPSII	557	m	0	3.92	90.50	216.37	0.0000
*CPSII	558	m	0	4.20	68.44	226.36	0.0000
*CPSII	559	m	0	4.42	60.64	252.18	0.0000

Table 1I6 - 2

IESLC - Meta-analysis of Current Smoking by Duration, Overview
All LC types, Any Product (or Cigarettes if Any not available)
Most adjusted

REF	NRR	SEX	AD	Ys	Ws	Qs	Ps
*CPSII	618	f	0	1.05	90.12	160.09	0.0000
*CPSII	619	f	0	1.94	104.74	19.89	0.0000
*CPSII	620	f	0	2.42	119.07	0.17	0.0000
*CPSII	621	f	0	2.77	127.49	19.38	0.0000
*CPSII	622	f	0	3.14	102.68	59.29	0.0000
*CPSII	623	f	0	3.34	87.81	81.53	0.0000
*CPSII	624	f	0	3.37	24.94	24.55	0.0000
*CPSII	625	f	0	4.00	17.14	45.10	0.0000
Subtotal CPSII				2.85	1272.25	1367.51	
DEAN2	501	m	0	1.16	10.37	15.26	0.0002
DEAN2	502	m	0	1.34	23.47	25.05	0.0000
DEAN2	504	f	0	-0.02	5.23	30.05	0.9671
DEAN2	505	f	0	1.77	7.59	2.80	0.0000
Subtotal DEAN2				1.22	46.66	73.16	
HUMBLE	542	c	3	2.74	4.59	0.59	0.0000
HUMBLE	543	c	3	2.86	7.23	1.71	0.0000
HUMBLE	544	c	3	2.98	12.26	4.38	0.0000
HUMBLE	545	c	3	2.85	14.81	3.28	0.0000
Subtotal HUMBLE				2.88	38.89	9.97	
*KAISE2	596	m	1	1.58	6.28	3.99	0.0001
*KAISE2	597	m	1	2.75	9.62	1.33	0.0000
*KAISE2	516	f	1	2.21	6.65	0.19	0.0000
*KAISE2	517	f	1	3.41	6.86	7.38	0.0000
Subtotal KAISE2				2.53	29.42	12.89	
KATSOU	512	f	1	0.25	4.75	21.43	0.5788
KATSOU	513	f	1	2.01	4.29	0.59	0.0000
Subtotal KATSOU				1.08	9.04	22.03	
*LIAW	501	c	2	-0.11	2.82	17.38	0.8596
*LIAW	502	c	2	0.96	6.06	12.26	0.0187
*LIAW	503	c	2	1.55	12.45	8.59	0.0000
Subtotal LIAW				1.16	21.33	38.22	
MATOS	521	m	2	1.65	2.99	1.59	0.0044
MATOS	522	m	2	2.00	5.89	0.83	0.0000
MATOS	523	m	2	2.32	6.41	0.02	0.0000
Subtotal MATOS				2.07	15.29	2.45	
PEZZO2	507	m	0	2.79	4.86	0.82	0.0000
PEZZO2	508	m	0	3.29	5.29	4.38	0.0000
Subtotal PEZZO2				3.05	10.15	5.19	
SOBUE	546	m	0	0.67	16.19	47.04	0.0067
SOBUE	547	m	0	1.10	20.61	33.87	0.0000
SOBUE	548	m	0	1.65	21.22	11.20	0.0000
SOBUE	549	m	0	2.03	17.32	2.15	0.0000
Subtotal SOBUE				1.38	75.35	94.26	

N	75
NS	15

Table 1I6 - 3

IESLC - Meta-analysis of Current Smoking by Duration, Overview
All LC types, Any Product (or Cigarettes if Any not available)
Most adjusted

	Sex			
	combined	male	female	Total
N	7	42	26	75
NS	2	11	7	20

In this overview table, other than the “N” rows, entries in the “absent” and “Total” columns may be invalid and should be ignored

	Duration of smoking (broad categories)				
	absent	1-34k20	21-49k35	36+k50	Total
N	39	14	9	13	75
NS	13	11	7	10	41
Wt	1819.79	339.40	507.05	415.22	3081.46
Het Chi	1626.20	106.75	145.88	105.12	3383.47
Het df	38	13	8	12	74
Het P	***	***	***	***	***
Fixed RR	13.56	2.09	7.10	25.19	10.78
RRl	12.95	1.87	6.51	22.88	10.41
RRu	14.20	2.32	7.75	27.73	11.17
P	+++	+++	+++	+++	+++
Random RR	9.70	2.80	8.24	18.82	8.55
RRl	7.04	1.94	5.42	13.37	6.65
RRu	13.37	4.04	12.52	26.50	11.00
P	+++	+++	+++	+++	+++

	Duration of smoking (narrow categories)							
	absent	1-19k1	6-29k20	21-39k30	31-49k40	41-998k50	51+k999	Total
N	47	3	1	9	7	4	4	75
NS	15	2	1	7	5	2	2	33
Wt	1572.48	17.16	5.30	434.99	516.94	340.30	194.29	3081.46
Het Chi	1861.85	4.92	0.00	100.07	140.25	61.66	38.13	3383.47
Het df	46	2	0	8	6	3	3	74
Het P	***	(*)	N.S.	***	***	***	***	***
Fixed RR	9.08	2.19	4.10	4.63	11.77	28.40	49.23	10.78
RRl	8.65	1.37	1.75	4.21	10.80	25.54	42.77	10.41
RRu	9.54	3.52	9.60	5.09	12.83	31.58	56.66	11.17
P	+++	++	++	+++	+++	+++	+++	+++
Random RR	7.67	2.01	4.10	5.52	11.68	23.72	41.39	8.55
RRl	5.47	0.86	1.75	3.78	7.44	14.17	23.20	6.65
RRu	10.75	4.66	9.60	8.05	18.34	39.73	73.83	11.00
P	+++	N.S.	++	+++	+++	+++	+++	+++

MALES

	Duration of smoking (broad categories)				
	absent	1-34k20	21-49k35	36+k50	Total
N	22	7	5	8	42
NS	10	7	5	8	30
Wt	1115.92	140.65	253.39	282.74	1792.70
Het Chi	946.98	17.29	53.25	80.30	1835.19
Het df	21	6	4	7	41
Het P	***	**	***	***	***
Fixed RR	17.74	2.28	7.56	26.49	14.26
RRl	16.73	1.93	6.68	23.57	13.61
RRu	18.81	2.68	8.55	29.76	14.93
P	+++	+++	+++	+++	+++
Random RR	10.75	2.66	7.40	19.01	9.19
RRl	6.98	1.86	4.26	11.33	6.59
RRu	16.56	3.80	12.87	31.92	12.83
P	+++	+++	+++	+++	+++

Table 1I6 - 3

IESLC - Meta-analysis of Current Smoking by Duration, Overview
All LC types, Any Product (or Cigarettes if Any not available)
Most adjusted

MALES

Duration of smoking (narrow categories)

	absent	1-19k1	6-29k20	21-39k30	31-49k40	41-998k50	51+k999	Total
N	27	2	1	5	3	2	2	42
NS	11	2	1	5	3	2	2	25
Wt	894.40	11.93	5.30	205.47	271.35	236.94	167.31	1792.70
Het Chi	965.58	0.06	0.00	33.73	60.93	35.78	19.50	1835.19
Het df	26	1	0	4	2	1	1	41
Het P	***	N.S.	N.S.	***	***	***	***	***
Fixed RR	12.24	3.12	4.10	4.90	13.17	30.88	52.70	14.26
RRl	11.46	1.77	1.75	4.28	11.70	27.18	45.29	13.61
RRu	13.07	5.50	9.60	5.62	14.84	35.07	61.32	14.93
P	+++	+++	++	+++	+++	+++	+++	+++
Random RR	8.29	3.12	4.10	5.73	11.11	33.84	57.82	9.19
RRl	5.32	1.77	1.75	3.54	5.34	15.46	28.83	6.59
RRu	12.92	5.50	9.60	9.26	23.12	74.11	115.95	12.83
P	+++	+++	++	+++	+++	+++	+++	+++

FEMALES

Duration of smoking (broad categories)

	absent	1-34k20	21-49k35	36+k50	Total
N	14	5	3	4	26
NS	6	5	3	4	18
Wt	673.11	191.34	246.42	117.67	1228.55
Het Chi	449.47	66.08	83.68	21.38	1161.51
Het df	13	4	2	3	25
Het P	***	***	***	***	***
Fixed RR	8.94	1.89	6.48	23.42	7.21
RRl	8.29	1.64	5.72	19.55	6.82
RRu	9.64	2.17	7.34	28.06	7.63
P	+++	+++	+++	+++	+++
Random RR	9.16	2.45	8.11	18.82	7.91
RRl	5.68	1.17	3.22	9.71	5.30
RRu	14.77	5.11	20.46	36.46	11.82
P	+++	+	+++	+++	+++

Duration of smoking (narrow categories)

	absent	1-19k1	6-29k20	21-39k30	31-49k40	41-998k50	51+k999	Total
N	16	1		2	3	2	2	26
NS	7	1		2	3	2	2	17
Wt	643.41	5.23		216.23	233.33	103.36	26.98	1228.55
Het Chi	680.55	0.00		49.32	66.88	20.42	13.03	1161.51
Het df	15	0		1	2	1	1	25
Het P	***	N.S.		***	***	***	***	***
Fixed RR	6.03	0.98		4.26	10.05	23.44	32.25	7.21
RRl	5.58	0.42		3.73	8.84	19.33	22.12	6.82
RRu	6.51	2.31		4.87	11.43	28.43	47.04	7.63
P	+++	N.S.		+++	+++	+++	+++	+++
Random RR	7.14	0.98		4.32	10.58	15.51	26.92	7.91
RRl	4.07	0.42		1.69	4.49	4.59	6.54	5.30
RRu	12.52	2.31		11.03	24.94	52.40	110.82	11.82
P	+++	N.S.		++	+++	+++	+++	+++

Table 1I6 - 4

IESLC - Meta-analysis of Current Smoking by Duration, Overview
All LC types, Any Product (or Cigarettes if Any not available)
Least adjusted

REF	NRR	X	SEX	AGEL	AGEH	RACE	YF	LC TYPE	LOC	START	ST	NLC	R	VB	P	H	AD	PRODUCT	exL	exH	S1	S2	DENOM	De
AMANDU	501	x	m	0	0	wh	0	all	NAmer	1959	pr	132	m	bl	n	n	0	cig+/-ot	0	24	1	0	nev cigs	st
AMANDU	502	x	m	0	0	wh	0	all	NAmer	1959	pr	132	m	bl	n	n	0	cig+/-ot	25	999	0	0	nev cigs	st
BEST	501		m	0	0	all	0	all	NAmer	1955	pr	381	n	V	n	n	1	cig only	1	4	0	0	nev any	ot
BEST	502		m	0	0	all	0	all	NAmer	1955	pr	381	n	V	n	n	1	cig only	5	9	0	1	nev any	ot
BEST	503		m	0	0	all	0	all	NAmer	1955	pr	381	n	V	n	n	1	cig only	10	14	0	0	nev any	ot
BEST	504		m	0	0	all	0	all	NAmer	1955	pr	381	n	V	n	n	1	cig only	15	19	0	0	nev any	ot
BEST	505		m	0	0	all	0	all	NAmer	1955	pr	381	n	V	n	n	1	cig only	20	29	1	2	nev any	ot
BEST	506		m	0	0	all	0	all	NAmer	1955	pr	381	n	V	n	n	1	cig only	30	39	2	3	nev any	ot
BEST	507		m	0	0	all	0	all	NAmer	1955	pr	381	n	V	n	n	1	cig only	40	999	3	0	nev any	ot
BOUCOT	501	x	m	0	0	all	0	all	NAmer	1951	pr	121	n	bl	n	n	1	cig only	1	39	0	0	nev any	ot
BOUCOT	502	x	m	0	0	all	0	all	NAmer	1951	pr	121	n	bl	n	n	1	cig only	40	999	3	0	nev any	ot
BUFFLE	531		f	0	0	w-hi	-	all	NAmer	1976	CC	943	n	bl	y	n	0	cig+/-ot	1	30	1	0	nev cigs	or
BUFFLE	532		f	0	0	w-hi	-	all	NAmer	1976	CC	943	n	bl	y	n	0	cig+/-ot	31	40	2	4	nev cigs	or
BUFFLE	533		f	0	0	w-hi	-	all	NAmer	1976	CC	943	n	bl	y	n	0	cig+/-ot	41	999	3	0	nev cigs	or
CEDERL	501		m	40	69	all	10	all	Eu:Sca	1963	pr	491	n	bl	n	n	1	cig only	1	29	1	0	nev any	ot
CEDERL	502		m	40	69	all	10	all	Eu:Sca	1963	pr	491	n	bl	n	n	1	cig only	30	999	0	0	nev any	ot
CEDERL	504		f	40	69	all	10	all	Eu:Sca	1963	pr	491	n	bl	n	n	1	cig only	1	29	1	0	nev any	ot
CEDERL	505		f	40	69	all	10	all	Eu:Sca	1963	pr	491	n	bl	n	n	1	cig only	30	999	0	0	nev any	ot
CPSI	580		m	40	84	wh	0	all	NAmer	1959	pr	5138	n	bl	n	n	0	cig only	1	29	1	0	nev cigs	st
CPSI	581		m	40	84	wh	0	all	NAmer	1959	pr	5138	n	bl	n	n	0	cig only	30	34	0	3	nev cigs	st
CPSI	582		m	40	84	wh	0	all	NAmer	1959	pr	5138	n	bl	n	n	0	cig only	35	39	2	0	nev cigs	st
CPSI	583		m	40	84	wh	0	all	NAmer	1959	pr	5138	n	bl	n	n	0	cig only	40	44	0	4	nev cigs	st
CPSI	584		m	40	84	wh	0	all	NAmer	1959	pr	5138	n	bl	n	n	0	cig only	45	49	0	0	nev cigs	st
CPSI	585		m	40	84	wh	0	all	NAmer	1959	pr	5138	n	bl	n	n	0	cig only	50	54	3	5	nev cigs	st
CPSI	586		m	40	84	wh	0	all	NAmer	1959	pr	5138	n	bl	n	n	0	cig only	55	59	0	0	nev cigs	st
CPSI	587		m	40	84	wh	0	all	NAmer	1959	pr	5138	n	bl	n	n	0	cig only	60	999	0	6	nev cigs	st
CPSI	676		f	40	84	wh	0	all	NAmer	1959	pr	5138	n	bl	n	n	0	cig only	1	29	1	0	nev cigs	st
CPSI	677		f	40	84	wh	0	all	NAmer	1959	pr	5138	n	bl	n	n	0	cig only	30	34	0	3	nev cigs	st
CPSI	678		f	40	84	wh	0	all	NAmer	1959	pr	5138	n	bl	n	n	0	cig only	35	39	2	0	nev cigs	st
CPSI	679		f	40	84	wh	0	all	NAmer	1959	pr	5138	n	bl	n	n	0	cig only	40	44	0	4	nev cigs	st
CPSI	680		f	40	84	wh	0	all	NAmer	1959	pr	5138	n	bl	n	n	0	cig only	45	49	0	0	nev cigs	st
CPSI	681		f	40	84	wh	0	all	NAmer	1959	pr	5138	n	bl	n	n	0	cig only	50	54	3	5	nev cigs	st
CPSI	682		f	40	84	wh	0	all	NAmer	1959	pr	5138	n	bl	n	n	0	cig only	55	999	0	6	nev cigs	st
CPSII	552		m	0	0	all	6	all	NAmer	1982	pr	3229	n	bl	n	n	0	cig only	1	29	1	0	nev any	st
CPSII	553		m	0	0	all	6	all	NAmer	1982	pr	3229	n	bl	n	n	0	cig only	30	34	0	3	nev any	st
CPSII	554		m	0	0	all	6	all	NAmer	1982	pr	3229	n	bl	n	n	0	cig only	35	39	2	0	nev any	st
CPSII	555		m	0	0	all	6	all	NAmer	1982	pr	3229	n	bl	n	n	0	cig only	40	44	0	4	nev any	st
CPSII	556		m	0	0	all	6	all	NAmer	1982	pr	3229	n	bl	n	n	0	cig only	45	49	0	0	nev any	st
CPSII	557		m	0	0	all	6	all	NAmer	1982	pr	3229	n	bl	n	n	0	cig only	50	54	3	5	nev any	st
CPSII	558		m	0	0	all	6	all	NAmer	1982	pr	3229	n	bl	n	n	0	cig only	55	59	0	0	nev any	st
CPSII	559		m	0	0	all	6	all	NAmer	1982	pr	3229	n	bl	n	n	0	cig only	60	999	0	6	nev any	st
CPSII	618		f	0	0	all	6	all	NAmer	1982	pr	3229	n	bl	n	n	0	cig+/-ot	1	29	1	0	nev cigs	st
CPSII	619		f	0	0	all	6	all	NAmer	1982	pr	3229	n	bl	n	n	0	cig+/-ot	30	34	0	3	nev cigs	st
CPSII	620		f	0	0	all	6	all	NAmer	1982	pr	3229	n	bl	n	n	0	cig+/-ot	35	39	2	0	nev cigs	st
CPSII	621		f	0	0	all	6	all	NAmer	1982	pr	3229	n	bl	n	n	0	cig+/-ot	40	44	0	4	nev cigs	st
CPSII	622		f	0	0	all	6	all	NAmer	1982	pr	3229	n	bl	n	n	0	cig+/-ot	45	49	0	0	nev cigs	st
CPSII	623		f	0	0	all	6	all	NAmer	1982	pr	3229	n	bl	n	n	0	cig+/-ot	50	54	3	5	nev cigs	st
CPSII	624		f	0	0	all	6	all	NAmer	1982	pr	3229	n	bl	n	n	0	cig+/-ot	55	59	0	0	nev cigs	st
CPSII	625		f	0	0	all	6	all	NAmer	1982	pr	3229	n	bl	n	n	0	cig+/-ot	60	999	0	6	nev cigs	st
DEAN2	501		m	0	0	all	-	all	Eu:UK	1960	CC	954	n	V	y	n	0	all/unsp	1	19	0	1	nev any	st
DEAN2	502		m	0	0	all	-	all	Eu:UK	1960	CC	954	n	V	y	n	0	all/unsp	20	999	0	0	nev any	st
DEAN2	504		f	0	0	all	-	all	Eu:UK	1960	CC	954	n	V	y	n	0	all/unsp	1	19	0	1	nev any	st
DEAN2	505		f	0	0	all	-	all	Eu:UK	1960	CC	954	n	V	y	n	0	all/unsp	20	999	0	0	nev any	st
HUMBLE	517	x	c	0	0	wh	-	not alv	NAmer	1980	CC	521	n	bl	y	n	0	cig+/-ot	1	29	1	0	nev cigs	st
HUMBLE	518	x	c	0	0	wh	-	not alv	NAmer	1980	CC	521	n	bl	y	n	0	cig+/-ot	30	39	2	3	nev cigs	st
HUMBLE	519	x	c	0	0	wh	-	not alv	NAmer	1980	CC	521	n	bl	y	n	0	cig+/-ot	40	49	0	4	nev cigs	st
HUMBLE	520	x	c	0	0	wh	-	not alv	NAmer	1980	CC	521	n	bl	y	n	0	cig+/-ot	50	59	3	5	nev cigs	st
HUMBLE	521	x	c	0	0	wh	-	not alv	NAmer	1980	CC	521	n	bl	y	n	0	cig+/-ot	60	999	0	6	nev cigs	st
KAISE2	596		m	0	0	all	9	all	NAmer	1979	pr	318	n	bl	n	n	1	cig only	1	39	0	0	nev any	st
KAISE2	597		m	0	0	all	9	all	NAmer	1979	pr	318	n	bl	n	n	1	cig only	40	999	3	0	nev any	st
KAISE2	516		f	0	0	all	9	all	NAmer	1979	pr	318	n	bl	n	n	1	cig only	1	39	0	0	nev any	st
KAISE2	517		f	0	0	all	9	all	NAmer	1979	pr	318	n	bl	n	n	1	cig only	40	999	3	0	nev any	st
KATSOU	501	x	f	0	0	all	-	all	Eu:bal	1987	CC	101	n	bl	n	n	0	all/unsp	1	19	0	1	nev any	st
KATSOU	502	x	f	0	0	all	-	all	Eu:bal	1987	CC	101	n	bl	n	n	0	all/unsp	20	29	1	2	nev any	st
KATSOU	503	x	f	0	0	all	-	all	Eu:bal	1987	CC	101	n	bl	n	n	0	all/unsp	30	39	2	3	nev any	st
KATSOU	504	x	f	0	0	all	-	all	Eu:bal	1987	CC	101	n	bl	n	n	0	all/unsp	40	999	3	0	nev any	st
LIAW	501		c	0	0	all	0	all	As:oth	1982	pr	127	n	ot	n	n	2	all/unsp	1	20	1	0	nev any	or
LIAW	502		c	0	0	all	0	all	As:oth	1982	pr	127	n	ot	n	n	2	all/unsp	21	30	0	3	nev any	or
LIAW	503		c	0	0	all	0	all	As:oth	1982	pr	127	n	ot	n	n	2	all/unsp	31	999	0	0	nev any	or
MATOS	501	x	m	0	0	all	-	all	SCAmer	1994	CC	200	n	bl	n	n	0	cig+/-ot	1	24	1	0	nev any	st
MATOS	502	x	m	0	0	all	-	all	SCAmer	1994	CC	200	n	bl	n	n	0	cig+/-ot	25	39	2	3	nev any	st
MATOS	503	x	m	0	0	all	-	all	SCAmer	1994	CC	200	n	bl	n	n	0	cig+/-ot	40	70	3	0	nev any	st

Table 1I6 - 4

IESLC - Meta-analysis of Current Smoking by Duration, Overview
All LC types, Any Product (or Cigarettes if Any not available)
Least adjusted

REF	NRR	X	SEX	AGEL	AGEH	RACE	YF	LC TYPE	LOC	START	ST	NLC	R	VB	P	H	AD	PRODUCT	exL	exH	S1	S2	DENOM	De
PEZZO2	507		m	0	0	all	-	all	SCAmer	1992	CC	367	n	bl	n	y	0	cig+/-ot	1	35	0	0	nev cigs	st
PEZZO2	508		m	0	0	all	-	all	SCAmer	1992	CC	367	n	bl	n	y	0	cig+/-ot	36	999	3	0	nev cigs	st
SOBUE	546		m	0	0	all	-	q+s+l+a	As:Jap	1986	CC	1376	n	bl	n	y	0	cig+/-ot	1	29	1	0	nev cigs	st
SOBUE	547		m	0	0	all	-	q+s+l+a	As:Jap	1986	CC	1376	n	bl	n	y	0	cig+/-ot	30	39	2	3	nev cigs	st
SOBUE	548		m	0	0	all	-	q+s+l+a	As:Jap	1986	CC	1376	n	bl	n	y	0	cig+/-ot	40	49	0	4	nev cigs	st
SOBUE	549		m	0	0	all	-	q+s+l+a	As:Jap	1986	CC	1376	n	bl	n	y	0	cig+/-ot	50	999	3	0	nev cigs	st

Cigarette type is all/unspec for all RRs

In this overview table, subtotals and Qs values may be invalid and should be ignored

Table 1I6 - 5

IESLC - Meta-analysis of Current Smoking by Duration, Overview
All LC types, Any Product (or Cigarettes if Any not available)
Least adjusted

REF	NRR	SEX	AD	Number Exposed Case	Cont	Non-exposed Case	Cont	RR	95.00%CI
*AMANDU	501	m	0	42	68909	6	25350	2.58	(1.09- 6.06)
*AMANDU	502	m	0	72	27096	6	25350	11.23	(4.88- 25.81)
Subtotal AMANDU								5.48	(3.02- 9.96)
*BEST	501	m	1	1	-	7	-	1.60	(0.20- 13.00)
*BEST	502	m	1	2	-	7	-	2.60	(0.54- 12.52)
*BEST	503	m	1	6	-	7	-	2.30	(0.77- 6.84)
*BEST	504	m	1	10	-	7	-	3.20	(1.22- 8.41)
*BEST	505	m	1	22	-	7	-	4.10	(1.75- 9.60)
*BEST	506	m	1	55	-	7	-	13.90	(6.33- 30.52)
*BEST	507	m	1	137	-	7	-	14.20	(6.64- 30.35)
Subtotal BEST								6.17	(4.26- 8.94)
*BOUCOT	501	m	1	32	-	0	-	48.32	(2.96- 789.02)
*BOUCOT	502	m	1	53	-	0	-	91.60	(5.66-1483.27)
Subtotal BOUCOT								66.59	(9.27- 478.38)
BUFFLE	531	f	0	36	24	12	112	14.00	(6.37- 30.79)
BUFFLE	532	f	0	74	47	12	112	14.70	(7.31- 29.55)
BUFFLE	533	f	0	70	36	12	112	18.15	(8.85- 37.22)
Subtotal BUFFLE								15.59	(10.22- 23.79)
*CEDERL	501	m	1	5	-	7	-	1.80	(0.57- 5.66)
*CEDERL	502	m	1	23	-	7	-	7.40	(3.18- 17.21)
*CEDERL	504	f	1	3	-	19	-	1.60	(0.47- 5.40)
*CEDERL	505	f	1	5	-	19	-	9.60	(3.60- 25.58)
Subtotal CEDERL								4.61	(2.78- 7.67)
*CPSI	580	m	0	95	266163	196	926068	1.69	(1.32- 2.15)
*CPSI	581	m	0	230	290031	196	926068	3.75	(3.10- 4.53)
*CPSI	582	m	0	470	367622	196	926068	6.04	(5.11- 7.14)
*CPSI	583	m	0	731	333292	196	926068	10.36	(8.85- 12.13)
*CPSI	584	m	0	764	221405	196	926068	16.30	(13.94- 19.07)
*CPSI	585	m	0	576	119633	196	926068	22.75	(19.35- 26.75)
*CPSI	586	m	0	356	53226	196	926068	31.60	(26.55- 37.61)
*CPSI	587	m	0	232	26906	196	926068	40.74	(33.70- 49.25)
*CPSI	676	f	0	105	694015	532	3877179	1.10	(0.89- 1.36)
*CPSI	677	f	0	141	383127	532	3877179	2.68	(2.23- 3.23)
*CPSI	678	f	0	154	315060	532	3877179	3.56	(2.98- 4.26)
*CPSI	679	f	0	120	163178	532	3877179	5.36	(4.40- 6.53)
*CPSI	680	f	0	54	53635	532	3877179	7.34	(5.55- 9.71)
*CPSI	681	f	0	16	14305	532	3877179	8.15	(4.96- 13.40)
*CPSI	682	f	0	10	5657	532	3877179	12.88	(6.90- 24.07)
Subtotal CPSI								8.01	(7.62- 8.43)
*CPSII	552	m	0	72	141932	124	742207	3.04	(2.27- 4.06)
*CPSII	553	m	0	145	113317	124	742207	7.66	(6.03- 9.73)
*CPSII	554	m	0	244	109788	124	742207	13.30	(10.72- 16.51)
*CPSII	555	m	0	413	103500	124	742207	23.88	(19.54- 29.19)
*CPSII	556	m	0	307	53805	124	742207	34.15	(27.73- 42.06)
*CPSII	557	m	0	332	39260	124	742207	50.62	(41.19- 62.20)
*CPSII	558	m	0	151	13598	124	742207	66.47	(52.45- 84.24)
*CPSII	559	m	0	117	8450	124	742207	82.88	(64.43- 106.60)
*CPSII	618	f	0	127	301244	310	2091302	2.84	(2.31- 3.50)
*CPSII	619	f	0	158	152833	310	2091302	6.97	(5.76- 8.45)
*CPSII	620	f	0	193	116270	310	2091302	11.20	(9.36- 13.40)
*CPSII	621	f	0	216	91501	310	2091302	15.93	(13.39- 18.94)
*CPSII	622	f	0	153	44769	310	2091302	23.06	(19.00- 27.98)
*CPSII	623	f	0	122	29119	310	2091302	28.26	(22.93- 34.84)
*CPSII	624	f	0	27	6262	310	2091302	29.09	(19.64- 43.07)
*CPSII	625	f	0	18	2224	310	2091302	54.60	(34.01- 87.65)
Subtotal CPSII								17.37	(16.44- 18.35)
DEAN2	501	m	0	34	36	33	112	3.21	(1.74- 5.89)
DEAN2	502	m	0	631	558	33	112	3.84	(2.56- 5.75)
DEAN2	504	f	0	10	14	88	121	0.98	(0.42- 2.31)
DEAN2	505	f	0	47	11	88	121	5.88	(2.88- 11.97)
Subtotal DEAN2								3.39	(2.55- 4.52)
HUMBLE	517	c	0	20	33	28	285	6.17	(3.13- 12.15)
HUMBLE	518	c	0	68	58	28	285	11.93	(7.07- 20.13)
HUMBLE	519	c	0	104	59	28	285	17.94	(10.85- 29.66)
HUMBLE	520	c	0	90	55	28	285	16.66	(9.97- 27.82)
HUMBLE	521	c	0	29	22	28	285	13.42	(6.82- 26.39)
Subtotal HUMBLE								13.29	(10.33- 17.10)
*KAISE2	596	m	1	17	-	14	-	4.86	(2.22- 10.61)
*KAISE2	597	m	1	34	-	14	-	15.64	(8.31- 29.40)
*KAISE2	516	f	1	24	-	11	-	9.09	(4.25- 19.43)
*KAISE2	517	f	1	26	-	11	-	30.41	(14.39- 64.25)

Table 1I6 - 5

IESLC - Meta-analysis of Current Smoking by Duration, Overview
All LC types, Any Product (or Cigarettes if Any not available)
Least adjusted

REF	NRR	SEX	AD	Number Exposed Case	Number Exposed Cont	Non-exposed Case	Non-exposed Cont	RR	95.00%CI
Subtotal KAISE2								12.59	(8.77- 18.07)
KATSOU	501	f	0	5	5	48	67	1.40	(0.38- 5.09)
KATSOU	502	f	0	8	7	48	67	1.60	(0.54- 4.70)
KATSOU	503	f	0	15	2	48	67	10.47	(2.29- 47.93)
KATSOU	504	f	0	17	4	48	67	5.93	(1.88- 18.75)
Subtotal KATSOU								3.06	(1.66- 5.67)
*LIAW	501	c	2	-	-	-	-	0.90	(0.30- 3.10)
*LIAW	502	c	2	-	-	-	-	2.60	(1.20- 5.90)
*LIAW	503	c	2	-	-	-	-	4.70	(2.70- 8.20)
Subtotal LIAW								3.19	(2.09- 4.88)
MATOS	501	m	0	10	18	11	110	5.56	(2.06- 14.96)
MATOS	502	m	0	47	53	11	110	8.87	(4.26- 18.47)
MATOS	503	m	0	55	61	11	110	9.02	(4.39- 18.51)
Subtotal MATOS								8.09	(5.13- 12.76)
PEZZO2	507	m	0	60	72	6	117	16.25	(6.68- 39.53)
PEZZO2	508	m	0	173	126	6	117	26.77	(11.42- 62.76)
Subtotal PEZZO2								21.08	(11.40- 39.00)
SOBUE	546	m	0	62	119	34	128	1.96	(1.21- 3.19)
SOBUE	547	m	0	159	200	34	128	2.99	(1.94- 4.61)
SOBUE	548	m	0	241	174	34	128	5.21	(3.41- 7.98)
SOBUE	549	m	0	147	73	34	128	7.58	(4.73- 12.14)
Subtotal SOBUE								3.96	(3.16- 4.96)
Partial Totals				9630	4732999	971857271140			

*prospective study

REF	NRR	SEX	AD	Ys	Ws	Qs	Ps
*AMANDU	501	m	0	0.95	5.25	10.71	0.0302
*AMANDU	502	m	0	2.42	5.54	0.01	0.0000
Subtotal AMANDU				1.70	10.79	10.72	
*BEST	501	m	1	0.47	0.88	3.20	0.6590
*BEST	502	m	1	0.96	1.55	3.13	0.2335
*BEST	503	m	1	0.83	3.22	7.65	0.1350
*BEST	504	m	1	1.16	4.12	6.05	0.0182
*BEST	505	m	1	1.41	5.30	4.92	0.0012
*BEST	506	m	1	2.63	6.21	0.41	0.0000
*BEST	507	m	1	2.65	6.65	0.52	0.0000
Subtotal BEST				1.82	27.95	25.88	
*BOUCOT	501	m	1	3.88	0.49	1.11	0.0065
*BOUCOT	502	m	1	4.52	0.50	2.28	0.0015
Subtotal BOUCOT				4.20	0.99	3.39	
BUFFLE	531	f	0	2.64	6.18	0.43	0.0000
BUFFLE	532	f	0	2.69	7.87	0.77	0.0000
BUFFLE	533	f	0	2.90	7.44	2.05	0.0000
Subtotal BUFFLE				2.75	21.50	3.25	
*CEDERL	501	m	1	0.59	2.92	9.31	0.3155
*CEDERL	502	m	1	2.00	5.39	0.75	0.0000
*CEDERL	504	f	1	0.47	2.58	9.35	0.4505
*CEDERL	505	f	1	2.26	4.00	0.05	0.0000
Subtotal CEDERL				1.53	14.88	19.45	
*CPSI	580	m	0	0.52	64.01	219.45	0.0000
*CPSI	581	m	0	1.32	105.87	117.47	0.0000
*CPSI	582	m	0	1.80	138.39	45.88	0.0000
*CPSI	583	m	0	2.34	154.66	0.20	0.0000
*CPSI	584	m	0	2.79	156.12	27.17	0.0000
*CPSI	585	m	0	3.12	146.44	82.43	0.0000
*CPSI	586	m	0	3.45	126.72	147.52	0.0000
*CPSI	587	m	0	3.71	106.68	189.54	0.0000
*CPSI	676	f	0	0.10	87.71	454.56	0.3603
*CPSI	677	f	0	0.99	111.49	214.69	0.0000
*CPSI	678	f	0	1.27	119.48	145.58	0.0000
*CPSI	679	f	0	1.68	97.98	47.38	0.0000
*CPSI	680	f	0	1.99	49.07	7.13	0.0000
*CPSI	681	f	0	2.10	15.55	1.19	0.0000
*CPSI	682	f	0	2.56	9.83	0.32	0.0000
Subtotal CPSI				2.08	1489.99	1700.51	
*CPSII	552	m	0	1.11	45.57	72.76	0.0000
*CPSII	553	m	0	2.04	66.89	7.66	0.0000
*CPSII	554	m	0	2.59	82.29	3.76	0.0000
*CPSII	555	m	0	3.17	95.47	60.94	0.0000
*CPSII	556	m	0	3.53	88.48	118.36	0.0000

Table 1I6 - 5

IESLC - Meta-analysis of Current Smoking by Duration, Overview
All LC types, Any Product (or Cigarettes if Any not available)
Least adjusted

REF	NRR	SEX	AD	Ys	Ws	Qs	Ps
*CPSII	557	m	0	3.92	90.50	217.43	0.0000
*CPSII	558	m	0	4.20	68.44	227.30	0.0000
*CPSII	559	m	0	4.42	60.64	253.11	0.0000
*CPSII	618	f	0	1.05	90.12	159.18	0.0000
*CPSII	619	f	0	1.94	104.74	19.55	0.0000
*CPSII	620	f	0	2.42	119.07	0.20	0.0000
*CPSII	621	f	0	2.77	127.49	19.75	0.0000
*CPSII	622	f	0	3.14	102.68	59.88	0.0000
*CPSII	623	f	0	3.34	87.81	82.17	0.0000
*CPSII	624	f	0	3.37	24.94	24.74	0.0000
*CPSII	625	f	0	4.00	17.14	45.31	0.0000
Subtotal CPSII				2.85	1272.25	1372.10	
DEAN2	501	m	0	1.16	10.37	15.17	0.0002
DEAN2	502	m	0	1.34	23.47	24.87	0.0000
DEAN2	504	f	0	-0.02	5.23	29.95	0.9671
DEAN2	505	f	0	1.77	7.59	2.76	0.0000
Subtotal DEAN2				1.22	46.66	72.75	
HUMBLE	517	c	0	1.82	8.37	2.57	0.0000
HUMBLE	518	c	0	2.48	14.05	0.16	0.0000
HUMBLE	519	c	0	2.89	15.20	4.00	0.0000
HUMBLE	520	c	0	2.81	14.60	2.81	0.0000
HUMBLE	521	c	0	2.60	8.39	0.41	0.0000
Subtotal HUMBLE				2.59	60.60	9.95	
*KAISE2	596	m	1	1.58	6.28	3.95	0.0001
*KAISE2	597	m	1	2.75	9.62	1.36	0.0000
*KAISE2	516	f	1	2.21	6.65	0.19	0.0000
*KAISE2	517	f	1	3.41	6.86	7.43	0.0000
Subtotal KAISE2				2.53	29.42	12.93	
KATSOU	501	f	0	0.33	2.29	9.56	0.6134
KATSOU	502	f	0	0.47	3.29	11.98	0.3967
KATSOU	503	f	0	2.35	1.66	0.00	0.0025
KATSOU	504	f	0	1.78	2.90	1.02	0.0024
Subtotal KATSOU				1.12	10.15	22.56	
*LIAW	501	c	2	-0.11	2.82	17.32	0.8596
*LIAW	502	c	2	0.96	6.06	12.19	0.0187
*LIAW	503	c	2	1.55	12.45	8.51	0.0000
Subtotal LIAW				1.16	21.33	38.03	
MATOS	501	m	0	1.71	3.91	1.70	0.0007
MATOS	502	m	0	2.18	7.14	0.26	0.0000
MATOS	503	m	0	2.20	7.43	0.23	0.0000
Subtotal MATOS				2.09	18.48	2.19	
PEZZO2	507	m	0	2.79	4.86	0.83	0.0000
PEZZO2	508	m	0	3.29	5.29	4.41	0.0000
Subtotal PEZZO2				3.05	10.15	5.25	
SOBUE	546	m	0	0.67	16.19	46.83	0.0067
SOBUE	547	m	0	1.10	20.61	33.67	0.0000
SOBUE	548	m	0	1.65	21.22	11.09	0.0000
SOBUE	549	m	0	2.03	17.32	2.11	0.0000
Subtotal SOBUE				1.38	75.35	93.69	

N	78
NS	15

Table 1I6 - 6

IESLC - Meta-analysis of Current Smoking by Duration, Overview
All LC types, Any Product (or Cigarettes if Any not available)
Least adjusted

	Sex			
	combined	male	female	Total
N	8	42	28	78
NS	2	11	7	20

In this overview table, other than the “N” rows, entries in the “absent” and “Total” columns may be invalid and should be ignored

	Duration of smoking (broad categories)				
	absent	1-34k20	21-49k35	36+k50	Total
N	40	14	10	14	78
NS	13	11	8	11	43
Wt	1830.58	344.22	516.77	418.93	3110.50
Het Chi	1634.61	94.82	144.33	114.10	3392.65
Het df	39	13	9	13	77
Het P	***	***	***	***	***
Fixed RR	13.55	2.08	7.14	24.80	10.74
RRl	12.95	1.87	6.55	22.54	10.37
RRu	14.19	2.32	7.78	27.30	11.13
P	+++	+++	+++	+++	+++
Random RR	9.57	2.61	8.21	17.59	8.30
RRl	6.97	1.85	5.52	12.49	6.49
RRu	13.14	3.68	12.21	24.78	10.62
P	+++	+++	+++	+++	+++

	Duration of smoking (narrow categories)							
	absent	1-19k1	6-29k20	21-39k30	31-49k40	41-998k50	51+k999	Total
N	45	4	2	10	7	5	5	78
NS	15	3	2	8	5	3	3	39
Wt	1560.27	19.46	8.60	444.71	519.88	354.90	202.68	3110.50
Het Chi	1845.70	5.33	1.81	102.55	139.76	65.64	51.72	3392.65
Het df	44	3	1	9	6	4	4	77
Het P	***	N.S.	N.S.	***	***	***	***	***
Fixed RR	9.03	2.08	2.86	4.70	11.77	27.78	46.65	10.74
RRl	8.59	1.33	1.46	4.29	10.80	25.04	40.65	10.37
RRu	9.49	3.24	5.57	5.16	12.83	30.83	53.53	11.13
P	+++	++	++	+++	+++	+++	+++	+++
Random RR	7.56	1.89	2.72	5.64	11.57	22.31	33.76	8.30
RRl	5.35	0.98	1.09	3.92	7.40	14.06	18.94	6.49
RRu	10.67	3.66	6.80	8.11	18.11	35.42	60.18	10.62
P	+++	(+)	+	+++	+++	+++	+++	+++

MALES

	Duration of smoking (broad categories)				
	absent	1-34k20	21-49k35	36+k50	Total
N	22	7	5	8	42
NS	10	7	5	8	30
Wt	1117.36	143.15	254.64	283.76	1798.91
Het Chi	944.74	15.07	53.42	83.09	1846.51
Het df	21	6	4	7	41
Het P	***	*	***	***	***
Fixed RR	17.76	2.25	7.60	26.31	14.21
RRl	16.75	1.91	6.72	23.42	13.57
RRu	18.83	2.65	8.59	29.56	14.89
P	+++	+++	+++	+++	+++
Random RR	10.99	2.49	7.63	18.68	9.14
RRl	7.14	1.80	4.41	11.07	6.55
RRu	16.90	3.45	13.20	31.52	12.75
P	+++	+++	+++	+++	+++

Table 1I6 - 6

IESLC - Meta-analysis of Current Smoking by Duration, Overview
All LC types, Any Product (or Cigarettes if Any not available)
Least adjusted

MALES

Duration of smoking (narrow categories)

	absent	1-19k1	6-29k20	21-39k30	31-49k40	41-998k50	51+k999	Total
N	27	2	1	5	3	2	2	42
NS	11	2	1	5	3	2	2	25
Wt	899.36	11.93	5.30	206.72	271.35	236.94	167.31	1798.91
Het Chi	976.08	0.06	0.00	35.22	60.93	35.78	19.50	1846.51
Het df	26	1	0	4	2	1	1	41
Het P	***	N.S.	N.S.	***	***	***	***	***
Fixed RR	12.17	3.12	4.10	4.95	13.17	30.88	52.70	14.21
RRl	11.40	1.77	1.75	4.32	11.70	27.18	45.29	13.57
RRu	12.99	5.50	9.60	5.67	14.84	35.07	61.32	14.89
P	+++	+++	++	+++	+++	+++	+++	+++
Random RR	8.16	3.12	4.10	5.93	11.11	33.84	57.82	9.14
RRl	5.24	1.77	1.75	3.65	5.34	15.46	28.83	6.55
RRu	12.71	5.50	9.60	9.62	23.12	74.11	115.95	12.75
P	+++	+++	++	+++	+++	+++	+++	+++

FEMALES

Duration of smoking (broad categories)

	absent	1-34k20	21-49k35	36+k50	Total
N	14	5	4	5	28
NS	6	5	4	5	20
Wt	671.12	189.88	248.08	120.57	1229.66
Het Chi	457.21	65.48	84.06	26.72	1161.45
Het df	13	4	3	4	27
Het P	***	***	***	***	***
Fixed RR	8.89	1.90	6.50	22.66	7.21
RRl	8.24	1.65	5.74	18.95	6.82
RRu	9.59	2.19	7.37	27.08	7.62
P	+++	+++	+++	+++	+++
Random RR	8.42	2.57	8.43	15.98	7.63
RRl	5.19	1.22	3.63	8.38	5.16
RRu	13.69	5.41	19.55	30.49	11.28
P	+++	+	+++	+++	+++

Duration of smoking (narrow categories)

	absent	1-19k1	6-29k20	21-39k30	31-49k40	41-998k50	51+k999	Total
N	15	2	1	3	3	2	2	28
NS	7	2	1	3	3	2	2	19
Wt	637.27	7.53	3.29	217.89	233.33	103.36	26.98	1229.66
Het Chi	669.00	0.20	0.00	50.65	66.88	20.42	13.03	1161.45
Het df	14	1	0	2	2	1	1	27
Het P	***	N.S.	N.S.	***	***	***	***	***
Fixed RR	6.09	1.09	1.60	4.29	10.05	23.44	32.25	7.21
RRl	5.63	0.54	0.54	3.76	8.84	19.33	22.12	6.82
RRu	6.58	2.23	4.70	4.90	11.43	28.43	47.04	7.62
P	+++	N.S.	N.S.	+++	+++	+++	+++	+++
Random RR	7.84	1.09	1.60	5.06	10.58	15.51	26.92	7.63
RRl	4.39	0.54	0.54	2.18	4.49	4.59	6.54	5.16
RRu	13.98	2.23	4.70	11.75	24.94	52.40	110.82	11.28
P	+++	N.S.	N.S.	+++	+++	+++	+++	+++

Table 1I6 - 7

IESLC - Meta-analysis of Current Smoking by Duration, Overview
All LC types, Any Product (or Cigarettes if Any not available)
Excluded studies (and stage at which they were excluded)

1	AGUDO ALDERS ARMADA AUVINE AXELSS BARBON BECHER BENHAM BLOT1 BOFFET BOUCHA BRESLO BROWN3 CARPEN CHEN CHEN2 CHIAZZ CHOI CHYOU CORREA DAMBER DARBY DESTEF DOLL DOLL2 DORGAN DOSEME FAN GAO GARCIA GARSHI GENG GER GRAHAM GUO GURSEL HAENSZ HAMMO2 HAMMON HEGMAN HU HU2 JAHN JAIN JEDRYC JOLY JUSSAW KHUDER KOO KOULUM KREUZE LAUSSM LETOUR LEVIN LIU3 LIU4 LIU5 LUBIN LUBIN2 LUO MCCONN NOTAN2 OSANN2 PERNU PEZZOT PRESCO QIAO QIAO2 RACHTA RESTRE SADOWS STASZE SUZUK2 TIZZAN TVERDA VUTUC WANG2 WIGLE WU2 WUWILL WYNDE2 WYNDE3 XU YUAN ZHANG ZHENG ZHOU
2	BENSHL DEAN3 DORN ENGELA GAO2 GILLIS HIRAYA HOLE KAUFMA MIGRAN MRFITR SEGI2 SPEIZE SVENSS WAKAI WU
3	MCDUFF SPITZ WYNDE6
5	AKIBA PISANI
7	BROSS WYNDE7
10	AMES WATSON WYNDE8

Table 1I6 - 8
Potentially overlapping studies

REF	REFGP	PRINC	.	OVERLAP/LINK
CPSI	CPSI	1		CPSI overall

Table 1I7 -

IESLC - Meta-analysis of Current Smoking, Duration, "Low"
All LC types, Any Product (or Cigarettes if Any not available)

This analysis is restricted to results for:
1) Current smokers
2) Results by Duration
3) Categorical results by Duration
4) All LC types (or near equivalent)
5) Results complete enough for use in metaanalysis

Within each study, results are then selected (in the following order of preference, within each sex) for:
6) (not applicable)
7) PRODUCT: all/unspec, cigarettes regardless of other products, cigarettes only
8) CIGTYPE: all/unspecified, MC regardless of HR, MC only
9) (not applicable)
10) DENOM: never smoked anything, never smoked cigarettes, never any + low, never cigs + low
11) Followup period (YF, prospective studies): whole study (coded as 0) or longest available
12) LCtype: all or nearest available, at least Squamous and Adeno. (q = squamous, s = small,
l = large, a = adeno, mix = mixed, alv = alveolar)
13) Race: all or nearest available, otherwise by race (wh or w = white, bl or b = black, hi = hispanic
ch = chinese, jap = japanese, haw = hawaiian, w+o = white + oriental, sca = scandinavian, as = asian)
14) Duration "low" in key scheme 1 (key value 20, maximum range 1-34)
15) For overlapping studies: principal rather than subsidiary studies
Finally by Age: whole study (coded as 0) if available, otherwise by widest available age group
and then for single sex results (m, f) in preference to results for both sexes combined (c).

Results adjusted (AD) for the most potential confounders are then chosen in Sections -1 to -3
and results adjusted for the least confounders in Sections -4 to -6. (Those least adjusted results which
actually differ from the most adjusted are marked 'x' in column X in Section -4)

Section -7 shows excluded studies, together with the stage (as above) at which no qualifying
results were found.

Section -8 lists the potentially overlapping studies which have been included (1=principal, 2=subsidiary).

Section -9 lists any results which would have been included in preference except that they had data not complete
enough for use in meta-analysis, with their significance (yes/no), if known, and any further comment as entered
on the database. It also lists as "gap" any categories for which no data were presented by the original authors.

In addition to those mentioned above, the following fields, levels and abbreviations are used:
* or nk = not known, n = no, y = yes, ot = other
nev = never
all/unspec = all or unspecified, cig+/-ot = cigarettes irrespective of other products (cigar, pipe etc)
MC = manufactured cigarettes, HR = hand-rolled cigarettes
exL, exH = range of exposure (low and high) in the smoking group, in terms of Duration
REF: 6-character study reference
NRR: number of the RR on the database within the study
ST : study type (CC = case control, pr or prosp = prospective)
NLC: number of lung cancer cases in whole study
R : risky occupational population (n = no, m = mining, o = other risky)
VB : national cigarette type (V = at least 75% Virginia, bl = at least 75% blended, ot = other)
P : any proxy use
H : full histological confirmation
De : derivation of RR/CI (or = original, st = standard method, ot = other method of estimation)

Table 1I7 - 1

IESLC - Meta-analysis of Current Smoking, Duration, "Low"
All LC types, Any Product (or Cigarettes if Any not available)
Most adjusted

REF	NRR	SEX	AGEL	AGEH	RACE	YF	LC TYPE	LOC	START	ST	NLC	R	VB	P	H	AD	PRODUCT	exL	exH	DENOM	De
AMANDU	506	m	0	0	wh	0	all	NAmer	1959	pr	132	m	bl	n	n	2	cig+/-ot	0	24	nev cigs	ot
BEST	505	m	0	0	all	0	all	NAmer	1955	pr	381	n	V	n	n	1	cig only	20	29	nev any	ot
BUFFLE	531	f	0	0	w-hi	-	all	NAmer	1976	CC	943	n	bl	y	n	0	cig+/-ot	1	30	nev cigs	or
CEDERL	501	m	40	69	all	10	all	Eu:Sca	1963	pr	491	n	bl	n	n	1	cig only	1	29	nev any	ot
CEDERL	504	f	40	69	all	10	all	Eu:Sca	1963	pr	491	n	bl	n	n	1	cig only	1	29	nev any	ot
CPSI	580	m	40	84	wh	0	all	NAmer	1959	pr	5138	n	bl	n	n	0	cig only	1	29	nev cigs	st
CPSI	676	f	40	84	wh	0	all	NAmer	1959	pr	5138	n	bl	n	n	0	cig only	1	29	nev cigs	st
CPSII	552	m	0	0	all	6	all	NAmer	1982	pr	3229	n	bl	n	n	0	cig only	1	29	nev any	st
CPSII	618	f	0	0	all	6	all	NAmer	1982	pr	3229	n	bl	n	n	0	cig+/-ot	1	29	nev cigs	st
HUMBLE	542	c	0	0	wh	-	not alv	NAmer	1980	CC	521	n	bl	y	n	3	cig+/-ot	1	29	nev cigs	ot
KATSOU	512	f	0	0	all	-	all	Eu:bal	1987	CC	101	n	bl	n	n	1	all/unsp	1	29	nev any	or
LIAW	501	c	0	0	all	0	all	As:oth	1982	pr	127	n	ot	n	n	2	all/unsp	1	20	nev any	or
MATOS	521	m	0	0	all	-	all	SCAmer	1994	CC	200	n	bl	n	n	2	cig+/-ot	1	24	nev any	or
SOBUE	546	m	0	0	all	-	q+s+l+a	As:Jap	1986	CC	1376	n	bl	n	y	0	cig+/-ot	1	29	nev cigs	st

Cigarette type is all/unspec for all RRs

Table 1I7 - 2

IESLC - Meta-analysis of Current Smoking, Duration, "Low"
All LC types, Any Product (or Cigarettes if Any not available)
Most adjusted

REF	NRR	SEX	AD	Number Exposed Case	Number Exposed Cont	Non-exposed Case	Non-exposed Cont	RR	95.00%CI
*AMANDU	506	m	2	42	-	6	-	5.92	(2.13- 16.47)
*BEST	505	m	1	22	-	7	-	4.10	(1.75- 9.60)
BUFFLE	531	f	0	36	24	12	112	14.00	(6.37- 30.79)
*CEDERL	501	m	1	5	-	7	-	1.80	(0.57- 5.66)
*CEDERL	504	f	1	3	-	19	-	1.60	(0.47- 5.40)
Subtotal CEDERL								1.70	(0.74- 3.93)
*CPSI	580	m	0	95	266163	196	926068	1.69	(1.32- 2.15)
*CPSI	676	f	0	105	694015	532	3877179	1.10	(0.89- 1.36)
Subtotal CPSI								1.32	(1.13- 1.55)
*CPSII	552	m	0	72	141932	124	742207	3.04	(2.27- 4.06)
*CPSII	618	f	0	127	301244	310	2091302	2.84	(2.31- 3.50)
Subtotal CPSII								2.91	(2.46- 3.44)
HUMBLE	542	c	3	20	-	28	-	15.45	(6.19- 38.58)
KATSOU	512	f	1	13	-	48	-	1.29	(0.54- 3.26)
*LIAW	501	c	2	-	-	-	-	0.90	(0.30- 3.10)
MATOS	521	m	2	10	-	11	-	5.20	(1.70- 16.40)
SOBUE	546	m	0	62	119	34	128	1.96	(1.21- 3.19)
Partial Totals				612	1403497	1334	7636996		

*prospective study

REF	NRR	SEX	AD	Ys	Ws	Qs	Ps
*AMANDU	506	m	2	1.78	3.67	4.00	0.0007
*BEST	505	m	1	1.41	5.30	2.42	0.0012
BUFFLE	531	f	0	2.64	6.18	22.42	0.0000
*CEDERL	501	m	1	0.59	2.92	0.06	0.3155
*CEDERL	504	f	1	0.47	2.58	0.18	0.4505
Subtotal CEDERL				0.53	5.49	0.24	
*CPSI	580	m	0	0.52	64.01	2.88	0.0000
*CPSI	676	f	0	0.10	87.71	35.61	0.3603
Subtotal CPSI				0.28	151.71	38.49	
*CPSII	552	m	0	1.11	45.57	6.44	0.0000
*CPSII	618	f	0	1.05	90.12	8.68	0.0000
Subtotal CPSII				1.07	135.69	15.12	
HUMBLE	542	c	3	2.74	4.59	18.41	0.0000
KATSOU	512	f	1	0.25	4.75	1.10	0.5788
*LIAW	501	c	2	-0.11	2.82	1.99	0.8596
MATOS	521	m	2	1.65	2.99	2.50	0.0044
SOBUE	546	m	0	0.67	16.19	0.06	0.0067

	N	14
	NS	11
	Wt	339.40
Het	Chi	106.75
Het	df	13
Het	P	***
Fixed	RR	2.09
	RRl	1.87
	RRu	2.32
	P	+++
Random	RR	2.80
	RRl	1.94
	RRu	4.04
	P	+++
Asymm	P	N.S.

Table 1I7 - 3

IESLC - Meta-analysis of Current Smoking, Duration, "Low"
All LC types, Any Product (or Cigarettes if Any not available)
Most adjusted

	Sex			
	combined	male	female	Total
N	2	7	5	14
NS	2	7	5	14
Wt	7.41	140.65	191.34	339.40
Het Chi	14.11	17.29	66.08	106.75
Het df	1	6	4	13
Het P	***	**	***	***
Fixed RR	5.24	2.28	1.89	2.09
RRl	2.55	1.93	1.64	1.87
RRu	10.77	2.68	2.17	2.32
P	+++	+++	+++	+++
Random RR	3.82	2.66	2.45	2.80
RRl	0.24	1.86	1.17	1.94
RRu	61.92	3.80	5.11	4.04
P	N.S.	+++	+	+++
Between Chi				9.26
Between df				2
Between P				**
Btwn(F) P				N.S.
Btwn(R) P				N.S.

	Lung cancer type		
	all	other	Total
N	12	2	14
NS	9	2	11
Wt	318.62	20.78	339.40
Het Chi	88.07	15.23	106.75
Het df	11	1	13
Het P	***	***	***
Fixed RR	2.03	3.09	2.09
RRl	1.82	2.01	1.87
RRu	2.27	4.76	2.32
P	+++	+++	+++
Random RR	2.55	5.30	2.80
RRl	1.74	0.70	1.94
RRu	3.74	40.01	4.04
P	+++	N.S.	+++
Between Chi			3.45
Between df			1
Between P			(*)
Btwn(F) P			N.S.
Btwn(R) P			N.S.

				Location					
	NAmer	UK	Scand	othEur	China	Japan	othAs	other	Total
N	8		2	1		1	1	1	14
NS	6		1	1		1	1	1	11
Wt	307.15		5.49	4.75		16.19	2.82	2.99	339.40
Het Chi	100.81		0.02	0.00		0.00	0.00	0.00	106.75
Het df	7		1	0		0	0	0	13
Het P	***		N.S.	N.S.		N.S.	N.S.	N.S.	***
Fixed RR	2.11		1.70	1.29		1.96	0.90	5.20	2.09
RRl	1.89		0.74	0.53		1.21	0.28	1.67	1.87
RRu	2.36		3.93	3.17		3.19	2.89	16.15	2.32
P	+++		N.S.	N.S.		++	N.S.	++	+++
Random RR	3.61		1.70	1.29		1.96	0.90	5.20	2.80
RRl	2.22		0.74	0.53		1.21	0.28	1.67	1.94
RRu	5.87		3.93	3.17		3.19	2.89	16.15	4.04
P	+++		N.S.	N.S.		++	N.S.	++	+++
Between Chi									5.92
Between df									5
Between P									N.S.
Btwn(F) P									N.S.
Btwn(R) P									(*)

Table 1I7 - 3

IESLC - Meta-analysis of Current Smoking, Duration, "Low"
All LC types, Any Product (or Cigarettes if Any not available)
Most adjusted

Detailed Country in "other Europe"

	multi	Germany	othWest	East	Balkans	Total
N					1	1
NS					1	1
Wt					4.75	4.75
Het Chi					0.00	0.00
Het df					0	0
Het P					N.S.	N.S.
Fixed RR					1.29	1.29
RRl					0.53	0.53
RRu					3.17	3.17
P					N.S.	N.S.
Random RR					1.29	1.29
RRl					0.53	0.53
RRu					3.17	3.17
P					N.S.	N.S.
Between Chi						
Between df						
Between P						N.S.
Btwn(F) P						N.S.
Btwn(R) P						N.S.

Detailed Country in "other Asia"

	India	HongKong	other	Total
N			1	1
NS			1	1
Wt			2.82	2.82
Het Chi			0.00	0.00
Het df			0	0
Het P			N.S.	N.S.
Fixed RR			0.90	0.90
RRl			0.28	0.28
RRu			2.89	2.89
P			N.S.	N.S.
Random RR			0.90	0.90
RRl			0.28	0.28
RRu			2.89	2.89
P			N.S.	N.S.
Between Chi				
Between df				
Between P				N.S.
Btwn(F) P				N.S.
Btwn(R) P				N.S.

Detailed other continent

	SCAmer	Total
N	1	1
NS	1	1
Wt	2.99	2.99
Het Chi	0.00	0.00
Het df	0	0
Het P	N.S.	N.S.
Fixed RR	5.20	5.20
RRl	1.67	1.67
RRu	16.15	16.15
P	++	++
Random RR	5.20	5.20
RRl	1.67	1.67
RRu	16.15	16.15
P	++	++
Between Chi		
Between df		
Between P		N.S.
Btwn(F) P		N.S.
Btwn(R) P		N.S.

Table 1I7 - 3

IESLC - Meta-analysis of Current Smoking, Duration, "Low"
All LC types, Any Product (or Cigarettes if Any not available)
Most adjusted

	Start year of study					
	<1960	1960-69	1970-79	1980-89	1990+	Total
N	4	2	1	6	1	14
NS	3	1	1	5	1	11
Wt	160.69	5.49	6.18	164.04	2.99	339.40
Het Chi	20.95	0.02	0.00	22.24	0.00	106.75
Het df	3	1	0	5	0	13
Het P	***	N.S.	N.S.	***	N.S.	***
Fixed RR	1.42	1.70	14.00	2.81	5.20	2.09
RRl	1.21	0.74	6.37	2.41	1.67	1.87
RRu	1.65	3.93	30.79	3.27	16.15	2.32
P	+++	N.S.	+++	+++	++	+++
Random RR	2.09	1.70	14.00	2.70	5.20	2.80
RRl	1.23	0.74	6.37	1.77	1.67	1.94
RRu	3.54	3.93	30.79	4.10	16.15	4.04
P	++	N.S.	+++	+++	++	+++
Between Chi						63.53
Between df						4
Between P						***
Btwn(F) P						(*)
Btwn(R) P						***

	Study type (1)		
	CC	other	Total
N	5	9	14
NS	5	6	11
Wt	34.71	304.69	339.40
Het Chi	32.46	60.89	106.75
Het df	4	8	13
Het P	***	***	***
Fixed RR	3.76	1.95	2.09
RRl	2.69	1.74	1.87
RRu	5.24	2.18	2.32
P	+++	+++	+++
Random RR	4.83	2.15	2.80
RRl	1.74	1.47	1.94
RRu	13.37	3.16	4.04
P	++	+++	+++
Between Chi			13.39
Between df			1
Between P			***
Btwn(F) P			N.S.
Btwn(R) P			N.S.

	Study type (2)			
	CC	prosp	other	Total
N	5	9		14
NS	5	6		11
Wt	34.71	304.69		339.40
Het Chi	32.46	60.89		106.75
Het df	4	8		13
Het P	***	***		***
Fixed RR	3.76	1.95		2.09
RRl	2.69	1.74		1.87
RRu	5.24	2.18		2.32
P	+++	+++		+++
Random RR	4.83	2.15		2.80
RRl	1.74	1.47		1.94
RRu	13.37	3.16		4.04
P	++	+++		+++
Between Chi				13.39
Between df				1
Between P				***
Btwn(F) P				N.S.
Btwn(R) P				N.S.

Table 1I7 - 3

IESLC - Meta-analysis of Current Smoking, Duration, "Low"
All LC types, Any Product (or Cigarettes if Any not available)
Most adjusted

	100-249	250-499	500-999	1000+	Total
	Study size (number of LC cases)				
N	4	3	2	5	14
NS	4	2	2	3	11
Wt	14.23	10.80	10.77	303.59	339.40
Het Chi	9.32	2.10	0.03	51.55	106.75
Het df	3	2	1	4	13
Het P	*	N.S.	N.S.	***	***
Fixed RR	2.39	2.62	14.60	1.92	2.09
RRl	1.42	1.44	8.04	1.71	1.87
RRu	4.01	4.76	26.53	2.15	2.32
P	++	++	+++	+++	+++
Random RR	2.44	2.60	14.60	1.99	2.80
RRl	0.97	1.41	8.04	1.31	1.94
RRu	6.15	4.81	26.53	3.03	4.04
P	(+)	++	+++	++	+++
Between Chi					43.75
Between df					3
Between P					***
Btwn(F) P					N.S.
Btwn(R) P					***

	no	mining	othRisky	Total
	Risky occupational population			
N	13	1		14
NS	10	1		11
Wt	335.73	3.67		339.40
Het Chi	102.71	0.00		106.75
Het df	12	0		13
Het P	***	N.S.		***
Fixed RR	2.06	5.92		2.09
RRl	1.85	2.13		1.87
RRu	2.29	16.46		2.32
P	+++	+++		+++
Random RR	2.67	5.92		2.80
RRl	1.84	2.13		1.94
RRu	3.89	16.46		4.04
P	+++	+++		+++
Between Chi				4.04
Between df				1
Between P				*
Btwn(F) P				N.S.
Btwn(R) P				N.S.

	Virginia	blended	other	Total
	National cigarette tobacco type			
N	1	12	1	14
NS	1	9	1	11
Wt	5.30	331.28	2.82	339.40
Het Chi	0.00	102.33	0.00	106.75
Het df	0	11	0	13
Het P	N.S.	***	N.S.	***
Fixed RR	4.10	2.08	0.90	2.09
RRl	1.75	1.87	0.28	1.87
RRu	9.60	2.31	2.89	2.32
P	++	+++	N.S.	+++
Random RR	4.10	2.90	0.90	2.80
RRl	1.75	1.96	0.28	1.94
RRu	9.60	4.30	2.89	4.04
P	++	+++	N.S.	+++
Between Chi				4.42
Between df				2
Between P				N.S.
Btwn(F) P				N.S.
Btwn(R) P				N.S.

Table 1I7 - 3

IESLC - Meta-analysis of Current Smoking, Duration, "Low"
All LC types, Any Product (or Cigarettes if Any not available)
Most adjusted

	Any proxy use: No/nk	Yes	Total
N	12	2	14
NS	9	2	11
Wt	328.63	10.77	339.40
Het Chi	64.58	0.03	106.75
Het df	11	1	13
Het P	***	N.S.	***
Fixed RR	1.96	14.60	2.09
RRl	1.76	8.04	1.87
RRu	2.18	26.53	2.32
P	+++	+++	+++
Random RR	2.15	14.60	2.80
RRl	1.55	8.04	1.94
RRu	2.99	26.53	4.04
P	+++	+++	+++
Between Chi			42.14
Between df			1
Between P			***
Btwn(F) P			*
Btwn(R) P			***

	Full histological confirmation: No	Yes	Total
N	13	1	14
NS	10	1	11
Wt	323.21	16.19	339.40
Het Chi	106.69	0.00	106.75
Het df	12	0	13
Het P	***	N.S.	***
Fixed RR	2.09	1.96	2.09
RRl	1.88	1.21	1.87
RRu	2.33	3.19	2.32
P	+++	++	+++
Random RR	2.90	1.96	2.80
RRl	1.95	1.21	1.94
RRu	4.32	3.19	4.04
P	+++	++	+++
Between Chi			0.06
Between df			1
Between P			N.S.
Btwn(F) P			N.S.
Btwn(R) P			N.S.

	Number of adjustment variables (1): 0	1	2+/+nk	Total
N	6	4	4	14
NS	4	3	4	11
Wt	309.78	15.55	14.07	339.40
Het Chi	75.50	3.76	14.15	106.75
Het df	5	3	3	13
Het P	***	N.S.	**	***
Fixed RR	2.00	2.11	5.40	2.09
RRl	1.79	1.28	3.20	1.87
RRu	2.23	3.47	9.11	2.32
P	+++	++	+++	+++
Random RR	2.53	2.08	4.71	2.80
RRl	1.59	1.19	1.50	1.94
RRu	4.02	3.65	14.80	4.04
P	+++	+	++	+++
Between Chi				13.34
Between df				2
Between P				**
Btwn(F) P				N.S.
Btwn(R) P				N.S.

Table 1I7 - 3

IESLC - Meta-analysis of Current Smoking, Duration, "Low"
All LC types, Any Product (or Cigarettes if Any not available)
Most adjusted

	Number of adjustment variables (2) 0	1	2	3-5	6+/+nk	Total
N	6	4	3	1		14
NS	4	3	3	1		11
Wt	309.78	15.55	9.48	4.59		339.40
Het Chi	75.50	3.76	6.63	0.00		106.75
Het df	5	3	2	0		13
Het P	***	N.S.	*	N.S.		***
Fixed RR	2.00	2.11	3.25	15.45		2.09
RRl	1.79	1.28	1.72	6.19		1.87
RRu	2.23	3.47	6.14	38.57		2.32
P	+++	++	+++	+++		+++
Random RR	2.53	2.08	3.09	15.45		2.80
RRl	1.59	1.19	0.97	6.19		1.94
RRu	4.02	3.65	9.89	38.57		4.04
P	+++	+	(+)	+++		+++
Between Chi						20.87
Between df						3
Between P						***
Btwn(F) P						N.S.
Btwn(R) P						**

	Product all/unsp	cig+/-ot	cig only	Total
N	2	6	6	14
NS	2	6	4	12
Wt	7.57	123.75	208.08	339.40
Het Chi	0.23	32.06	35.64	106.75
Het df	1	5	5	13
Het P	N.S.	***	***	***
Fixed RR	1.13	3.24	1.64	2.09
RRl	0.55	2.72	1.43	1.87
RRu	2.30	3.86	1.88	2.32
P	N.S.	+++	+++	+++
Random RR	1.13	5.35	1.96	2.80
RRl	0.55	2.81	1.25	1.94
RRu	2.30	10.17	3.08	4.04
P	N.S.	+++	++	+++
Between Chi				38.82
Between df				2
Between P				***
Btwn(F) P				(*)
Btwn(R) P				**

	Denominator nev any	nev cigs	Total
N	7	7	14
NS	6	6	12
Wt	66.93	272.47	339.40
Het Chi	10.02	90.92	106.75
Het df	6	6	13
Het P	N.S.	***	***
Fixed RR	2.72	1.95	2.09
RRl	2.14	1.74	1.87
RRu	3.45	2.20	2.32
P	+++	+++	+++
Random RR	2.37	3.37	2.80
RRl	1.57	1.97	1.94
RRu	3.60	5.74	4.04
P	+++	+++	+++
Between Chi			5.82
Between df			1
Between P			*
Btwn(F) P			N.S.
Btwn(R) P			N.S.

Table 1I7 - 3

IESLC - Meta-analysis of Current Smoking, Duration, "Low"
All LC types, Any Product (or Cigarettes if Any not available)
Most adjusted

	Derivation of RR/CI Orig	StdCalc	Other	Total
N	4	5	5	14
NS	4	3	4	11
Wt	16.75	303.59	19.06	339.40
Het Chi	22.20	51.55	12.47	106.75
Het df	3	4	4	13
Het P	***	***	*	***
Fixed RR	3.76	1.92	4.70	2.09
RRl	2.33	1.71	3.00	1.87
RRu	6.07	2.15	7.37	2.32
P	+++	+++	+++	+++
Random RR	3.11	1.99	4.27	2.80
RRl	0.82	1.31	1.91	1.94
RRu	11.78	3.03	9.54	4.04
P	(+)	++	+++	+++
Between Chi				20.52
Between df				2
Between P				***
Btwn(F) P				N.S.
Btwn(R) P				N.S.

Table 1I7 - 4

IESLC - Meta-analysis of Current Smoking, Duration, "Low"
All LC types, Any Product (or Cigarettes if Any not available)
Least adjusted

REF	NRR	X	SEX	AGEL	AGEH	RACE	YF	LC TYPE	LOC	START	ST	NLC	R	VB	P	H	AD	PRODUCT	exL	exH	DENOM	De
AMANDU	501	x	m	0	0	wh	0	all	NAmer	1959	pr	132	m	bl	n	n	0	cig+/-ot	0	24	nev cigs	st
BEST	505		m	0	0	all	0	all	NAmer	1955	pr	381	n	V	n	n	1	cig only	20	29	nev any	ot
BUFFLE	531		f	0	0	w-hi	-	all	NAmer	1976	CC	943	n	bl	y	n	0	cig+/-ot	1	30	nev cigs	or
CEDERL	501		m	40	69	all	10	all	Eu:Sca	1963	pr	491	n	bl	n	n	1	cig only	1	29	nev any	ot
CEDERL	504		f	40	69	all	10	all	Eu:Sca	1963	pr	491	n	bl	n	n	1	cig only	1	29	nev any	ot
CPSI	580		m	40	84	wh	0	all	NAmer	1959	pr	5138	n	bl	n	n	0	cig only	1	29	nev cigs	st
CPSI	676		f	40	84	wh	0	all	NAmer	1959	pr	5138	n	bl	n	n	0	cig only	1	29	nev cigs	st
CPSII	552		m	0	0	all	6	all	NAmer	1982	pr	3229	n	bl	n	n	0	cig only	1	29	nev any	st
CPSII	618		f	0	0	all	6	all	NAmer	1982	pr	3229	n	bl	n	n	0	cig+/-ot	1	29	nev cigs	st
HUMBLE	517	x	c	0	0	wh	-	not alv	NAmer	1980	CC	521	n	bl	y	n	0	cig+/-ot	1	29	nev cigs	st
KATSOU	502	x	f	0	0	all	-	all	Eu:bal	1987	CC	101	n	bl	n	n	0	all/unsp	20	29	nev any	st
LIAW	501		c	0	0	all	0	all	As:oth	1982	pr	127	n	ot	n	n	2	all/unsp	1	20	nev any	or
MATOS	501	x	m	0	0	all	-	all	SCAmer	1994	CC	200	n	bl	n	n	0	cig+/-ot	1	24	nev any	st
SOBUE	546		m	0	0	all	-	q+s+l+a	As:Jap	1986	CC	1376	n	bl	n	y	0	cig+/-ot	1	29	nev cigs	st

Cigarette type is all/unspec for all RRs

Table 1I7 - 5

IESLC - Meta-analysis of Current Smoking, Duration, "Low"
All LC types, Any Product (or Cigarettes if Any not available)
Least adjusted

REF	NRR	SEX	AD	Number Exposed Case	Cont	Non-exposed Case	Cont	RR	95.00%CI
*AMANDU	501	m	0	42	68909	6	25350	2.58	(1.09- 6.06)
*BEST	505	m	1	22	-	7	-	4.10	(1.75- 9.60)
BUFFLE	531	f	0	36	24	12	112	14.00	(6.37- 30.79)
*CEDERL	501	m	1	5	-	7	-	1.80	(0.57- 5.66)
*CEDERL	504	f	1	3	-	19	-	1.60	(0.47- 5.40)
Subtotal CEDERL								1.70	(0.74- 3.93)
*CPSI	580	m	0	95	266163	196	926068	1.69	(1.32- 2.15)
*CPSI	676	f	0	105	694015	532	3877179	1.10	(0.89- 1.36)
Subtotal CPSI								1.32	(1.13- 1.55)
*CPSII	552	m	0	72	141932	124	742207	3.04	(2.27- 4.06)
*CPSII	618	f	0	127	301244	310	2091302	2.84	(2.31- 3.50)
Subtotal CPSII								2.91	(2.46- 3.44)
HUMBLE	517	c	0	20	33	28	285	6.17	(3.13- 12.15)
KATSOU	502	f	0	8	7	48	67	1.60	(0.54- 4.70)
*LIAW	501	c	2	-	-	-	-	0.90	(0.30- 3.10)
MATOS	501	m	0	10	18	11	110	5.56	(2.06- 14.96)
SOBUE	546	m	0	62	119	34	128	1.96	(1.21- 3.19)
Partial Totals				607	1472464	1334	7662808		

*prospective study

REF	NRR	SEX	AD	Ys	Ws	Qs	Ps
*AMANDU	501	m	0	0.95	5.25	0.24	0.0302
*BEST	505	m	1	1.41	5.30	2.43	0.0012
BUFFLE	531	f	0	2.64	6.18	22.45	0.0000
*CEDERL	501	m	1	0.59	2.92	0.06	0.3155
*CEDERL	504	f	1	0.47	2.58	0.18	0.4505
Subtotal CEDERL				0.53	5.49	0.24	
*CPSI	580	m	0	0.52	64.01	2.86	0.0000
*CPSI	676	f	0	0.10	87.71	35.50	0.3603
Subtotal CPSI				0.28	151.71	38.35	
*CPSII	552	m	0	1.11	45.57	6.47	0.0000
*CPSII	618	f	0	1.05	90.12	8.74	0.0000
Subtotal CPSII				1.07	135.69	15.21	
HUMBLE	517	c	0	1.82	8.37	9.86	0.0000
KATSOU	502	f	0	0.47	3.29	0.23	0.3967
*LIAW	501	c	2	-0.11	2.82	1.98	0.8596
MATOS	501	m	0	1.71	3.91	3.77	0.0007
SOBUE	546	m	0	0.67	16.19	0.06	0.0067

	N	14
	NS	11
	Wt	344.22
Het	Chi	94.82
Het	df	13
Het	P	***
Fixed	RR	2.08
	RRl	1.87
	RRu	2.32
	P	+++
Random	RR	2.61
	RRl	1.85
	RRu	3.68
	P	+++
Asymm	P	N.S.

Table 1I7 - 6

IESLC - Meta-analysis of Current Smoking, Duration, "Low"
All LC types, Any Product (or Cigarettes if Any not available)
Least adjusted

		Sex			
		combined	male	female	Total
	N	2	7	5	14
	NS	2	7	5	14
	Wt	11.18	143.15	189.88	344.22
Het	Chi	7.81	15.07	65.48	94.82
Het	df	1	6	4	13
Het	P	**	*	***	***
Fixed	RR	3.80	2.25	1.90	2.08
	RRl	2.11	1.91	1.65	1.87
	RRu	6.83	2.65	2.19	2.32
	P	+++	+++	+++	+++
Random	RR	2.50	2.49	2.57	2.61
	RRl	0.38	1.80	1.22	1.85
	RRu	16.46	3.45	5.41	3.68
	P	N.S.	+++	+	+++
Between	Chi				6.46
Between	df				2
Between	P				*
Btwn(F)	P				N.S.
Btwn(R)	P				N.S.

Table 1I7 - 7

IESLC - Meta-analysis of Current Smoking, Duration, "Low"
All LC types, Any Product (or Cigarettes if Any not available)
Excluded studies (and stage at which they were excluded)

1 AGUDO ALDERS ARMADA AUVINE AXELSS BARBON BECHER BENHAM BLOT1 BOFFET BOUCHA BRESLO BROWN3 CARPEN CHEN CHEN2
CHIAZZ CHOI CHYOU CORREA DAMBER DARBY DESTEF DOLL DOLL2 DORGAN DOSEME FAN GAO GARCIA GARSHI GENG
GER GRAHAM GUO GURSEL HAENSZ HAMMO2 HAMMON HEGMAN HU HU2 JAHN JAIN JEDRYC JOLY JUSSAW KHUDER
KOO KOULUM KREUZE LAUSSM LETOUR LEVIN LIU3 LIU4 LIU5 LUBIN LUBIN2 LUO MCCONN NOTAN2 OSANN2 PERNU
PEZZOT PRESCO QIAO QIAO2 RACHTA RESTRE SADOWS STASZE SUZUK2 TIZZAN TVERDA VUTUC WANG2 WIGLE WU2 WUWILL
WYNDE2 WYNDE3 XU YUAN ZHANG ZHENG ZHOU

2 BENSHL DEAN3 DORN ENGELA GAO2 GILLIS HIRAYA HOLE KAUFMA MIGRAN MRFITR SEGI2 SPEIZE SVENSS WAKAI WU

3 MCDUFF SPITZ WYNDE6

5 AKIBA PISANI

7 BROSS WYNDE7

10 AMES WATSON WYNDE8

14 BOUCOT DEAN2 KAISE2 PEZZO2

Table 1I7 - 8
Potentially overlapping studies

REF	REFGP	PRINC	.	OVERLAP/LINK
CPSI	CPSI	1		CPSI overall

Table 1I8 -

IESLC - Meta-analysis of Current Smoking, Duration, "Mid"
All LC types, Any Product (or Cigarettes if Any not available)

This analysis is restricted to results for:
1) Current smokers
2) Results by Duration
3) Categorical results by Duration
4) All LC types (or near equivalent)
5) Results complete enough for use in metaanalysis

Within each study, results are then selected (in the following order of preference, within each sex) for:
6) (not applicable)
7) PRODUCT: all/unspec, cigarettes regardless of other products, cigarettes only
8) CIGTYPE: all/unspecified, MC regardless of HR, MC only
9) (not applicable)
10) DENOM: never smoked anything, never smoked cigarettes, never any + low, never cigs + low
11) Followup period (YF, prospective studies): whole study (coded as 0) or longest available
12) LCtype: all or nearest available, at least Squamous and Adeno. (q = squamous, s = small, l = large, a = adeno, mix = mixed, alv = alveolar)
13) Race: all or nearest available, otherwise by race (wh or w = white, bl or b = black, hi = hispanic ch = chinese, jap = japanese, haw = hawaiian, w+o = white + oriental, sca = scandinavian, as = asian)
14) Duration "mid" in key scheme 1 (key value 35, maximum range 21-49)
15) For overlapping studies: principal rather than subsidiary studies
Finally by Age: whole study (coded as 0) if available, otherwise by widest available age group
and then for single sex results (m, f) in preference to results for both sexes combined (c).

Results adjusted (AD) for the most potential confounders are then chosen in Sections -1 to -3
and results adjusted for the least confounders in Sections -4 to -6. (Those least adjusted results which
actually differ from the most adjusted are marked 'x' in column X in Section -4)

Section -7 shows excluded studies, together with the stage (as above) at which no qualifying
results were found.

Section -8 lists the potentially overlapping studies which have been included (1=principal, 2=subsidiary).

Section -9 lists any results which would have been included in preference except that they had data not complete
enough for use in meta-analysis, with their significance (yes/no), if known, and any further comment as entered
on the database. It also lists as "gap" any categories for which no data were presented by the original authors.

In addition to those mentioned above, the following fields, levels and abbreviations are used:
* or nk = not known, n = no, y = yes, ot = other
nev = never
all/unspec = all or unspecified, cig+/-ot = cigarettes irrespective of other products (cigar, pipe etc)
MC = manufactured cigarettes, HR = hand-rolled cigarettes
exL, exH = range of exposure (low and high) in the smoking group, in terms of Duration
REF: 6-character study reference
NRR: number of the RR on the database within the study
ST : study type (CC = case control, pr or prosp = prospective)
NLC: number of lung cancer cases in whole study
R : risky occupational population (n = no, m = mining, o = other risky)
VB : national cigarette type (V = at least 75% Virginia, bl = at least 75% blended, ot = other)
P : any proxy use
H : full histological confirmation
De : derivation of RR/CI (or = original, st = standard method, ot = other method of estimation)

Table 1I8 - 1

IESLC - Meta-analysis of Current Smoking, Duration, "Mid"
All LC types, Any Product (or Cigarettes if Any not available)
Most adjusted

REF	NRR	SEX	AGEL	AGEH	RACE	YF	LC TYPE	LOC	START	ST	NLC	R	VB	P	H	AD	PRODUCT	exL	exH	DENOM	De
BEST	506	m	0	0	all	0	all	NAmer	1955	pr	381	n	V	n	n	1	cig only	30	39	nev any	ot
BUFFLE	532	f	0	0	w-hi	-	all	NAmer	1976	CC	943	n	bl	y	n	0	cig+/-ot	31	40	nev cigs	or
CPSI	582	m	40	84	wh	0	all	NAmer	1959	pr	5138	n	bl	n	n	0	cig only	35	39	nev cigs	st
CPSI	678	f	40	84	wh	0	all	NAmer	1959	pr	5138	n	bl	n	n	0	cig only	35	39	nev cigs	st
CPSII	554	m	0	0	all	6	all	NAmer	1982	pr	3229	n	bl	n	n	0	cig only	35	39	nev any	st
CPSII	620	f	0	0	all	6	all	NAmer	1982	pr	3229	n	bl	n	n	0	cig+/-ot	35	39	nev cigs	st
HUMBLE	543	c	0	0	wh	-	not alv	NAmer	1980	CC	521	n	bl	y	n	3	cig+/-ot	30	39	nev cigs	ot
KATSOU	503	f	0	0	all	-	all	Eu:bal	1987	CC	101	n	bl	n	n	0	all/unsp	30	39	nev any	st
MATOS	522	m	0	0	all	-	all	SCAmer	1994	CC	200	n	bl	n	n	2	cig+/-ot	25	39	nev any	or
SOBUE	547	m	0	0	all	-	q+s+l+a	As:Jap	1986	CC	1376	n	bl	n	y	0	cig+/-ot	30	39	nev cigs	st

Cigarette type is all/unspec for all RRs

Table 1I8 - 2

IESLC - Meta-analysis of Current Smoking, Duration, "Mid"
All LC types, Any Product (or Cigarettes if Any not available)
Most adjusted

REF	NRR	SEX	AD	Number Exposed Case	Cont	Non-exposed Case	Cont	RR	95.00%CI
*BEST	506	m	1	55	-	7	-	13.90	(6.33- 30.52)
BUFFLE	532	f	0	74	47	12	112	14.70	(7.31- 29.55)
*CPSI	582	m	0	470	367622	196	926068	6.04	(5.11- 7.14)
*CPSI	678	f	0	154	315060	532	3877179	3.56	(2.98- 4.26)
Subtotal CPSI								4.73	(4.19- 5.34)
*CPSII	554	m	0	244	109788	124	742207	13.30	(10.72- 16.51)
*CPSII	620	f	0	193	116270	310	2091302	11.20	(9.36- 13.40)
Subtotal CPSII								12.01	(10.46- 13.79)
HUMBLE	543	c	3	68	-	28	-	17.54	(8.46- 36.34)
KATSOU	503	f	0	15	2	48	67	10.47	(2.29- 47.93)
MATOS	522	m	2	47	-	11	-	7.40	(3.30- 16.60)
SOBUE	547	m	0	159	200	34	128	2.99	(1.94- 4.61)
Partial Totals				1479	908989	1302	7637063		

*prospective study

REF	NRR	SEX	AD	Ys	Ws	Qs	Ps
*BEST	506	m	1	2.63	6.21	2.79	0.0000
BUFFLE	532	f	0	2.69	7.87	4.15	0.0000
*CPSI	582	m	0	1.80	138.39	3.67	0.0000
*CPSI	678	f	0	1.27	119.48	57.05	0.0000
Subtotal CPSI				1.55	257.87	60.72	
*CPSII	554	m	0	2.59	82.29	32.30	0.0000
*CPSII	620	f	0	2.42	119.07	24.58	0.0000
Subtotal CPSII				2.49	201.36	56.88	
HUMBLE	543	c	3	2.86	7.23	5.90	0.0000
KATSOU	503	f	0	2.35	1.66	0.25	0.0025
MATOS	522	m	2	2.00	5.89	0.01	0.0000
SOBUE	547	m	0	1.10	20.61	15.43	0.0000

	N	10
	NS	8
	Wt	508.71
Het	Chi	146.13
Het	df	9
Het	P	***
Fixed	RR	7.11
	RRl	6.52
	RRu	7.75
	P	+++
Random	RR	8.33
	RRl	5.54
	RRu	12.51
	P	+++
Asymm	P	N.S.

Table 1I8 - 3

IESLC - Meta-analysis of Current Smoking, Duration, "Mid"
All LC types, Any Product (or Cigarettes if Any not available)
Most adjusted

	Sex			
	combined	male	female	Total
N	1	5	4	10
NS	1	5	4	10
Wt	7.23	253.39	248.08	508.71
Het Chi	0.00	53.25	84.06	146.13
Het df	0	4	3	9
Het P	N.S.	***	***	***
Fixed RR	17.54	7.56	6.50	7.11
RRl	8.46	6.68	5.74	6.52
RRu	36.35	8.55	7.37	7.75
P	+++	+++	+++	+++
Random RR	17.54	7.40	8.43	8.33
RRl	8.46	4.26	3.63	5.54
RRu	36.35	12.87	19.55	12.51
P	+++	+++	+++	+++
Between Chi				8.83
Between df				2
Between P				*
Btwn(F) P				N.S.
Btwn(R) P				N.S.

	Lung cancer type		
	all	other	Total
N	8	2	10
NS	6	2	8
Wt	480.86	27.85	508.71
Het Chi	124.54	16.74	146.13
Het df	7	1	9
Het P	***	***	***
Fixed RR	7.28	4.74	7.11
RRl	6.66	3.27	6.52
RRu	7.96	6.87	7.75
P	+++	+++	+++
Random RR	8.80	7.06	8.33
RRl	5.65	1.25	5.54
RRu	13.70	39.93	12.51
P	+++	+	+++
Between Chi			4.85
Between df			1
Between P			*
Btwn(F) P			N.S.
Btwn(R) P			N.S.

	Location								
	NAmer	UK	Scand	othEur	China	Japan	othAs	other	Total
N	7			1		1		1	10
NS	5			1		1		1	8
Wt	480.54			1.66		20.61		5.89	508.71
Het Chi	129.84			0.00		0.00		0.00	146.13
Het df	6			0		0		0	9
Het P	***			N.S.		N.S.		N.S.	***
Fixed RR	7.36			10.47		2.99		7.40	7.11
RRl	6.73			2.29		1.94		3.30	6.52
RRu	8.05			47.93		4.61		16.60	7.75
P	+++			++		+++		+++	+++
Random RR	9.65			10.47		2.99		7.40	8.33
RRl	6.07			2.29		1.94		3.30	5.54
RRu	15.36			47.93		4.61		16.60	12.51
P	+++			++		+++		+++	+++
Between Chi									16.29
Between df									3
Between P									***
Btwn(F) P									N.S.
Btwn(R) P									**

Table 1I8 - 3

IESLC - Meta-analysis of Current Smoking, Duration, "Mid"
All LC types, Any Product (or Cigarettes if Any not available)
Most adjusted

Detailed Country in "other Europe"

	multi	Germany	othWest	East	Balkans	Total
N					1	1
NS					1	1
Wt					1.66	1.66
Het Chi					0.00	0.00
Het df					0	0
Het P					N.S.	N.S.
Fixed RR					10.47	10.47
RRl					2.29	2.29
RRu					47.93	47.93
P					++	++
Random RR					10.47	10.47
RRl					2.29	2.29
RRu					47.93	47.93
P					++	++
Between Chi						
Between df						
Between P						N.S.
Btwn(F) P						N.S.
Btwn(R) P						N.S.

Detailed Country in "other Asia"

	India	HongKong	other	Total
N				
NS				
Wt				
Het Chi				
Het df				
Het P				
Fixed RR				
RRl				
RRu				
P				
Random RR				
RRl				
RRu				
P				
Between Chi				
Between df				
Between P				N.S.
Btwn(F) P				N.S.
Btwn(R) P				N.S.

Detailed other continent

	SCAmer	Total
N	1	1
NS	1	1
Wt	5.89	5.89
Het Chi	0.00	0.00
Het df	0	0
Het P	N.S.	N.S.
Fixed RR	7.40	7.40
RRl	3.30	3.30
RRu	16.60	16.60
P	+++	+++
Random RR	7.40	7.40
RRl	3.30	3.30
RRu	16.60	16.60
P	+++	+++
Between Chi		
Between df		
Between P		N.S.
Btwn(F) P		N.S.
Btwn(R) P		N.S.

Table 1I8 - 3

IESLC - Meta-analysis of Current Smoking, Duration, "Mid"
All LC types, Any Product (or Cigarettes if Any not available)
Most adjusted

	Start year of study					
	<1960	1960-69	1970-79	1980-89	1990+	Total
N	3		1	5	1	10
NS	2		1	4	1	8
Wt	264.08		7.87	230.87	5.89	508.71
Het Chi	24.93		0.00	39.37	0.00	146.13
Het df	2		0	4	0	9
Het P	***		N.S.	***	N.S.	***
Fixed RR	4.85		14.70	10.73	7.40	7.11
RRl	4.30		7.31	9.43	3.30	6.52
RRu	5.47		29.55	12.21	16.60	7.75
P	+++		+++	+++	+++	+++
Random RR	5.85		14.70	9.34	7.40	8.33
RRl	3.49		7.31	5.58	3.30	5.54
RRu	9.78		29.55	15.63	16.60	12.51
P	+++		+++	+++	+++	+++
Between Chi						81.83
Between df						3
Between P						***
Btwn(F) P						N.S.
Btwn(R) P						N.S.

	Study type (1)		
	CC	other	Total
N	5	5	10
NS	5	3	8
Wt	43.26	465.44	508.71
Het Chi	25.22	120.35	146.13
Het df	4	4	9
Het P	***	***	***
Fixed RR	6.38	7.18	7.11
RRl	4.73	6.56	6.52
RRu	8.59	7.86	7.75
P	+++	+++	+++
Random RR	8.70	8.23	8.33
RRl	3.80	4.84	5.54
RRu	19.93	13.97	12.51
P	+++	+++	+++
Between Chi			0.56
Between df			1
Between P			N.S.
Btwn(F) P			N.S.
Btwn(R) P			N.S.

	Study type (2)			
	CC	prosp	other	Total
N	5	5		10
NS	5	3		8
Wt	43.26	465.44		508.71
Het Chi	25.22	120.35		146.13
Het df	4	4		9
Het P	***	***		***
Fixed RR	6.38	7.18		7.11
RRl	4.73	6.56		6.52
RRu	8.59	7.86		7.75
P	+++	+++		+++
Random RR	8.70	8.23		8.33
RRl	3.80	4.84		5.54
RRu	19.93	13.97		12.51
P	+++	+++		+++
Between Chi				0.56
Between df				1
Between P				N.S.
Btwn(F) P				N.S.
Btwn(R) P				N.S.

Table 1I8 - 3

IESLC - Meta-analysis of Current Smoking, Duration, "Mid"
All LC types, Any Product (or Cigarettes if Any not available)
Most adjusted

	Study size (number of LC cases) 100-249	250-499	500-999	1000+	Total
N	2	1	2	5	10
NS	2	1	2	3	8
Wt	7.55	6.21	15.10	479.84	508.71
Het Chi	0.16	0.00	0.12	132.41	146.13
Het df	1	0	1	4	9
Het P	N.S.	N.S.	N.S.	***	***
Fixed RR	7.99	13.90	15.99	6.86	7.11
RRl	3.91	6.33	9.66	6.27	6.52
RRu	16.30	30.52	26.49	7.50	7.75
P	+++	+++	+++	+++	+++
Random RR	7.99	13.90	15.99	6.34	8.33
RRl	3.91	6.33	9.66	3.73	5.54
RRu	16.30	30.52	26.49	10.79	12.51
P	+++	+++	+++	+++	+++
Between Chi					13.45
Between df					3
Between P					**
Btwn(F) P					N.S.
Btwn(R) P					(*)

	Risky occupational population no	mining	othRisky	Total
N	10			10
NS	8			8
Wt	508.71			508.71
Het Chi	146.13			146.13
Het df	9			9
Het P	***			***
Fixed RR	7.11			7.11
RRl	6.52			6.52
RRu	7.75			7.75
P	+++			+++
Random RR	8.33			8.33
RRl	5.54			5.54
RRu	12.51			12.51
P	+++			+++
Between Chi				
Between df				
Between P				N.S.
Btwn(F) P				N.S.
Btwn(R) P				N.S.

	National cigarette tobacco type Virginia	blended	other	Total
N	1	9		10
NS	1	7		8
Wt	6.21	502.50		508.71
Het Chi	0.00	143.30		146.13
Het df	0	8		9
Het P	N.S.	***		***
Fixed RR	13.90	7.05		7.11
RRl	6.33	6.46		6.52
RRu	30.52	7.70		7.75
P	+++	+++		+++
Random RR	13.90	7.94		8.33
RRl	6.33	5.18		5.54
RRu	30.52	12.16		12.51
P	+++	+++		+++
Between Chi				2.83
Between df				1
Between P				(*)
Btwn(F) P				N.S.
Btwn(R) P				N.S.

Table 1I8 - 3

IESLC - Meta-analysis of Current Smoking, Duration, "Mid"
All LC types, Any Product (or Cigarettes if Any not available)
Most adjusted

	Any proxy use No/nk	Yes	Total
N	8	2	10
NS	6	2	8
Wt	493.60	15.10	508.71
Het Chi	135.78	0.12	146.13
Het df	7	1	9
Het P	***	N.S.	***
Fixed RR	6.94	15.99	7.11
RRl	6.35	9.66	6.52
RRu	7.57	26.49	7.75
P	+++	+++	+++
Random RR	7.19	15.99	8.33
RRl	4.60	9.66	5.54
RRu	11.25	26.49	12.51
P	+++	+++	+++
Between Chi			10.23
Between df			1
Between P			**
Btwn(F) P			N.S.
Btwn(R) P			*

	Full histological confirmation No	Yes	Total
N	9	1	10
NS	7	1	8
Wt	488.09	20.61	508.71
Het Chi	130.05	0.00	146.13
Het df	8	0	9
Het P	***	N.S.	***
Fixed RR	7.37	2.99	7.11
RRl	6.75	1.94	6.52
RRu	8.06	4.61	7.75
P	+++	+++	+++
Random RR	9.44	2.99	8.33
RRl	6.19	1.94	5.54
RRu	14.40	4.61	12.51
P	+++	+++	+++
Between Chi			16.08
Between df			1
Between P			***
Btwn(F) P			N.S.
Btwn(R) P			***

	Number of adjustment variables (1) 0	1	2+/+nk	Total
N	7	1	2	10
NS	5	1	2	8
Wt	489.38	6.21	13.12	508.71
Het Chi	137.19	0.00	2.42	146.13
Het df	6	0	1	9
Het P	***	N.S.	N.S.	***
Fixed RR	6.95	13.90	11.91	7.11
RRl	6.36	6.33	6.93	6.52
RRu	7.60	30.52	20.46	7.75
P	+++	+++	+++	+++
Random RR	7.27	13.90	11.60	8.33
RRl	4.51	6.33	4.98	5.54
RRu	11.71	30.52	27.01	12.51
P	+++	+++	+++	+++
Between Chi				6.53
Between df				2
Between P				*
Btwn(F) P				N.S.
Btwn(R) P				N.S.

Table 1I8 - 3

IESLC - Meta-analysis of Current Smoking, Duration, "Mid"
All LC types, Any Product (or Cigarettes if Any not available)
Most adjusted
Number of adjustment variables (2)

	0	1	2	3-5	6+/+nk	Total
N	7	1	1	1		10
NS	5	1	1	1		8
Wt	489.38	6.21	5.89	7.23		508.71
Het Chi	137.19	0.00	0.00	0.00		146.13
Het df	6	0	0	0		9
Het P	***	N.S.	N.S.	N.S.		***
Fixed RR	6.95	13.90	7.40	17.54		7.11
RRl	6.36	6.33	3.30	8.46		6.52
RRu	7.60	30.52	16.60	36.35		7.75
P	+++	+++	+++	+++		+++
Random RR	7.27	13.90	7.40	17.54		8.33
RRl	4.51	6.33	3.30	8.46		5.54
RRu	11.71	30.52	16.60	36.35		12.51
P	+++	+++	+++	+++		+++
Between Chi						8.94
Between df						3
Between P						*
Btwn(F) P						N.S.
Btwn(R) P						N.S.

Product

	all/unsp	cig+/-ot	cig only	Total
N	1	5	4	10
NS	1	5	3	9
Wt	1.66	160.68	346.37	508.71
Het Chi	0.00	35.28	88.78	146.13
Het df	0	4	3	9
Het P	N.S.	***	***	***
Fixed RR	10.47	9.63	6.16	7.11
RRl	2.29	8.25	5.55	6.52
RRu	47.93	11.24	6.85	7.75
P	++	+++	+++	+++
Random RR	10.47	8.94	7.59	8.33
RRl	2.29	4.68	4.05	5.54
RRu	47.93	17.08	14.24	12.51
P	++	+++	+++	+++
Between Chi				22.08
Between df				2
Between P				***
Btwn(F) P				N.S.
Btwn(R) P				N.S.

Denominator

	nev any	nev cigs	Total
N	4	6	10
NS	4	5	9
Wt	96.05	412.66	508.71
Het Chi	2.00	103.02	146.13
Het df	3	5	9
Het P	N.S.	***	***
Fixed RR	12.82	6.20	7.11
RRl	10.49	5.63	6.52
RRu	15.65	6.83	7.75
P	+++	+++	+++
Random RR	12.82	7.13	8.33
RRl	10.49	4.36	5.54
RRu	15.65	11.68	12.51
P	+++	+++	+++
Between Chi			41.11
Between df			1
Between P			***
Btwn(F) P			N.S.
Btwn(R) P			*

Table 1I8 - 3

IESLC - Meta-analysis of Current Smoking, Duration, "Mid"
All LC types, Any Product (or Cigarettes if Any not available)
Most adjusted

	Derivation of RR/CI Orig	StdCalc	Other	Total
N	2	6	2	10
NS	2	4	2	8
Wt	13.76	481.50	13.44	508.71
Het Chi	1.59	132.71	0.18	146.13
Het df	1	5	1	9
Het P	N.S.	***	N.S.	***
Fixed RR	10.96	6.87	15.75	7.11
RRl	6.46	6.28	9.23	6.52
RRu	18.58	7.51	26.89	7.75
P	+++	+++	+++	+++
Random RR	10.76	6.57	15.75	8.33
RRl	5.51	3.95	9.23	5.54
RRu	21.01	10.94	26.89	12.51
P	+++	+++	+++	+++
Between Chi				11.66
Between df				2
Between P				**
Btwn(F) P				N.S.
Btwn(R) P				(*)

Table 1I8 - 4

IESLC - Meta-analysis of Current Smoking, Duration, "Mid"
All LC types, Any Product (or Cigarettes if Any not available)
Least adjusted

REF	NRR	X	SEX	AGEL	AGEH	RACE	YF	LC TYPE	LOC	START	ST	NLC	R	VB	P	H	AD	PRODUCT	exL	exH	DENOM	De
BEST	506		m	0	0	all	0	all	NAmer	1955	pr	381	n	V	n	n	1	cig only	30	39	nev any	ot
BUFFLE	532		f	0	0	w-hi	-	all	NAmer	1976	CC	943	n	bl	y	n	0	cig+/-ot	31	40	nev cigs	or
CPSI	582		m	40	84	wh	0	all	NAmer	1959	pr	5138	n	bl	n	n	0	cig only	35	39	nev cigs	st
CPSI	678		f	40	84	wh	0	all	NAmer	1959	pr	5138	n	bl	n	n	0	cig only	35	39	nev cigs	st
CPSII	554		m	0	0	all	6	all	NAmer	1982	pr	3229	n	bl	n	n	0	cig only	35	39	nev any	st
CPSII	620		f	0	0	all	6	all	NAmer	1982	pr	3229	n	bl	n	n	0	cig+/-ot	35	39	nev cigs	st
HUMBLE	518	x	c	0	0	wh	-	not alv	NAmer	1980	CC	521	n	bl	y	n	0	cig+/-ot	30	39	nev cigs	st
KATSOU	503		f	0	0	all	-	all	Eu:bal	1987	CC	101	n	bl	n	n	0	all/unsp	30	39	nev any	st
MATOS	502	x	m	0	0	all	-	all	SCAmer	1994	CC	200	n	bl	n	n	0	cig+/-ot	25	39	nev any	st
SOBUE	547		m	0	0	all	-	q+s+l+a	As:Jap	1986	CC	1376	n	bl	n	y	0	cig+/-ot	30	39	nev cigs	st

Cigarette type is all/unspec for all RRs

Table 1I8 - 5

IESLC - Meta-analysis of Current Smoking, Duration, "Mid"
All LC types, Any Product (or Cigarettes if Any not available)
Least adjusted

REF	NRR	SEX	AD	Number Exposed Case	Number Exposed Cont	Non-exposed Case	Non-exposed Cont	RR	95.00%CI
*BEST	506	m	1	55	-	7	-	13.90	(6.33- 30.52)
BUFFLE	532	f	0	74	47	12	112	14.70	(7.31- 29.55)
*CPSI	582	m	0	470	367622	196	926068	6.04	(5.11- 7.14)
*CPSI	678	f	0	154	315060	532	3877179	3.56	(2.98- 4.26)
Subtotal CPSI								4.73	(4.19- 5.34)
*CPSII	554	m	0	244	109788	124	742207	13.30	(10.72- 16.51)
*CPSII	620	f	0	193	116270	310	2091302	11.20	(9.36- 13.40)
Subtotal CPSII								12.01	(10.46- 13.79)
HUMBLE	518	c	0	68	58	28	285	11.93	(7.07- 20.13)
KATSOU	503	f	0	15	2	48	67	10.47	(2.29- 47.93)
MATOS	502	m	0	47	53	11	110	8.87	(4.26- 18.47)
SOBUE	547	m	0	159	200	34	128	2.99	(1.94- 4.61)
Partial Totals				1479	909100	1302	7637458		

*prospective study

REF	NRR	SEX	AD	Ys	Ws	Qs	Ps
*BEST	506	m	1	2.63	6.21	2.76	0.0000
BUFFLE	532	f	0	2.69	7.87	4.10	0.0000
*CPSI	582	m	0	1.80	138.39	3.86	0.0000
*CPSI	678	f	0	1.27	119.48	57.72	0.0000
Subtotal CPSI				1.55	257.87	61.58	
*CPSII	554	m	0	2.59	82.29	31.89	0.0000
*CPSII	620	f	0	2.42	119.07	24.14	0.0000
Subtotal CPSII				2.49	201.36	56.03	
HUMBLE	518	c	0	2.48	14.05	3.71	0.0000
KATSOU	503	f	0	2.35	1.66	0.24	0.0025
MATOS	502	m	0	2.18	7.14	0.34	0.0000
SOBUE	547	m	0	1.10	20.61	15.57	0.0000

	N	10
	NS	8
	Wt	516.77
Het	Chi	144.33
Het	df	9
Het	P	***
Fixed	RR	7.14
	RRl	6.55
	RRu	7.78
	P	+++
Random	RR	8.21
	RRl	5.52
	RRu	12.21
	P	+++
Asymm	P	N.S.

Table 1I8 - 6

IESLC - Meta-analysis of Current Smoking, Duration, "Mid"
All LC types, Any Product (or Cigarettes if Any not available)
Least adjusted

	Sex combined	male	female	Total
N	1	5	4	10
NS	1	5	4	10
Wt	14.05	254.64	248.08	516.77
Het Chi	0.00	53.42	84.06	144.33
Het df	0	4	3	9
Het P	N.S.	***	***	***
Fixed RR	11.93	7.60	6.50	7.14
RRl	7.07	6.72	5.74	6.55
RRu	20.13	8.59	7.37	7.78
P	+++	+++	+++	+++
Random RR	11.93	7.63	8.43	8.21
RRl	7.07	4.41	3.63	5.52
RRu	20.13	13.20	19.55	12.21
P	+++	+++	+++	+++
Between Chi				6.85
Between df				2
Between P				*
Btwn(F) P				N.S.
Btwn(R) P				N.S.

Table 1I8 - 7

IESLC - Meta-analysis of Current Smoking, Duration, "Mid"
All LC types, Any Product (or Cigarettes if Any not available)
Excluded studies (and stage at which they were excluded)

1	AGUDO ALDERS ARMADA AUVINE AXELSS BARBON BECHER BENHAM BLOT1 BOFFET BOUCHA BRESLO BROWN3 CARPEN CHEN CHEN2 CHIAZZ CHOI CHYOU CORREA DAMBER DARBY DESTEF DOLL DOLL2 DORGAN DOSEME FAN GAO GARCIA GARSHI GENG GER GRAHAM GUO GURSEL HAENSZ HAMMO2 HAMMON HEGMAN HU HU2 JAHN JAIN JEDRYC JOLY JUSSAW KHUDER KOO KOULUM KREUZE LAUSSM LETOUR LEVIN LIU3 LIU4 LIU5 LUBIN LUBIN2 LUO MCCONN NOTAN2 OSANN2 PERNU PEZZOT PRESCO QIAO QIAO2 RACHTA RESTRE SADOWS STASZE SUZUK2 TIZZAN TVERDA VUTUC WANG2 WIGLE WU2 WUWILL WYNDE2 WYNDE3 XU YUAN ZHANG ZHENG ZHOU
2	BENSHL DEAN3 DORN ENGELA GAO2 GILLIS HIRAYA HOLE KAUFMA MIGRAN MRFITR SEGI2 SPEIZE SVENSS WAKAI WU
3	MCDUFF SPITZ WYNDE6
5	AKIBA PISANI
7	BROSS WYNDE7
10	AMES WATSON WYNDE8
14	AMANDU BOUCOT CEDERL DEAN2 KAISE2 LIAW PEZZO2

Table 1I8 - 8
Potentially overlapping studies

REF	REFGP	PRINC	.	OVERLAP/LINK
CPSI	CPSI	1		CPSI overall

Table 1I9 -

IESLC - Meta-analysis of Current Smoking, Duration, "High"
All LC types, Any Product (or Cigarettes if Any not available)

This analysis is restricted to results for:
1) Current smokers
2) Results by Duration
3) Categorical results by Duration
4) All LC types (or near equivalent)
5) Results complete enough for use in metaanalysis

Within each study, results are then selected (in the following order of preference, within each sex) for:
6) PRODUCT: all/unspec, cigarettes regardless of other products, cigarettes only
7) CIGTYPE: all/unspecified, MC regardless of HR, MC only
8) (not applicable)
9) DENOM: never smoked anything, never smoked cigarettes, never any + low, never cigs + low
10) Followup period (YF, prospective studies): whole study (coded as 0) or longest available
11) LCtype: all or nearest available, at least Squamous and Adeno. (q = squamous, s = small,
l = large, a = adeno, mix = mixed, alv = alveolar)
12) Race: all or nearest available, otherwise by race (wh or w = white, bl or b = black, hi = hispanic
ch = chinese, jap = japanese, haw = hawaiian, w+o = white + oriental, sca = scandinavian, as = asian)
13) Duration "high" in key scheme 1 (key value 50, maximum range 36+)
14) For overlapping studies: principal rather than subsidiary studies
Finally by Age: whole study (coded as 0) if available, otherwise by widest available age group
and then for single sex results (m, f) in preference to results for both sexes combined (c).

Results adjusted (AD) for the most potential confounders are then chosen in Sections -1 to -3
and results adjusted for the least confounders in Sections -4 to -6. (Those least adjusted results which
actually differ from the most adjusted are marked 'x' in column X in Section -4)

Section -7 shows excluded studies, together with the stage (as above) at which no qualifying
results were found.

Section -8 lists the potentially overlapping studies which have been included (1=principal, 2=subsidiary).

Section -9 lists any results which would have been included in preference except that they had data not complete
enough for use in meta-analysis, with their significance (yes/no), if known, and any further comment as entered
on the database. It also lists as "gap" any categories for which no data were presented by the original authors.

In addition to those mentioned above, the following fields, levels and abbreviations are used:
* or nk = not known, n = no, y = yes, ot = other
nev = never
all/unspec = all or unspecified, cig+/-ot = cigarettes irrespective of other products (cigar, pipe etc)
MC = manufactured cigarettes, HR = hand-rolled cigarettes
exL, exH = range of exposure (low and high) in the smoking group, in terms of Duration
REF: 6-character study reference
NRR: number of the RR on the database within the study
ST : study type (CC = case control, pr or prosp = prospective)
NLC: number of lung cancer cases in whole study
R : risky occupational population (n = no, m = mining, o = other risky)
VB : national cigarette type (V = at least 75% Virginia, bl = at least 75% blended, ot = other)
P : any proxy use
H : full histological confirmation
De : derivation of RR/CI (or = original, st = standard method, ot = other method of estimation)

Table 1I9 - 1

IESLC - Meta-analysis of Current Smoking, Duration, "High"
All LC types, Any Product (or Cigarettes if Any not available)
Most adjusted

REF	NRR	SEX	AGEL	AGEH	RACE	YF	LC TYPE	LOC	START	ST	NLC	R	VB	P	H	AD	PRODUCT	exL	exH	DENOM	De
BEST	507	m	0	0	all	0	all	NAmer	1955	pr	381	n	V	n	n	1	cig only	40	999	nev any	ot
BOUCOT	522	m	0	0	all	0	all	NAmer	1951	pr	121	n	bl	n	n	2	cig only	40	999	nev any	ot
BUFFLE	533	f	0	0	w-hi	-	all	NAmer	1976	CC	943	n	bl	y	n	0	cig+/-ot	41	999	nev cigs	or
CPSI	585	m	40	84	wh	0	all	NAmer	1959	pr	5138	n	bl	n	n	0	cig only	50	54	nev cigs	st
CPSI	681	f	40	84	wh	0	all	NAmer	1959	pr	5138	n	bl	n	n	0	cig only	50	54	nev cigs	st
CPSII	557	m	0	0	all	6	all	NAmer	1982	pr	3229	n	bl	n	n	0	cig only	50	54	nev any	st
CPSII	623	f	0	0	all	6	all	NAmer	1982	pr	3229	n	bl	n	n	0	cig+/-ot	50	54	nev cigs	st
HUMBLE	545	c	0	0	wh	-	not alv	NAmer	1980	CC	521	n	bl	y	n	3	cig+/-ot	50	999	nev cigs	ot
KAISE2	597	m	0	0	all	9	all	NAmer	1979	pr	318	n	bl	n	n	1	cig only	40	999	nev any	st
KAISE2	517	f	0	0	all	9	all	NAmer	1979	pr	318	n	bl	n	n	1	cig only	40	999	nev any	st
KATSOU	504	f	0	0	all	-	all	Eu:bal	1987	CC	101	n	bl	n	n	0	all/unsp	40	999	nev any	st
MATOS	523	m	0	0	all	-	all	SCAmer	1994	CC	200	n	bl	n	n	2	cig+/-ot	40	70	nev any	or
PEZZO2	508	m	0	0	all	-	all	SCAmer	1992	CC	367	n	bl	n	y	0	cig+/-ot	36	999	nev cigs	st
SOBUE	549	m	0	0	all	-	q+s+l+a	As:Jap	1986	CC	1376	n	bl	n	y	0	cig+/-ot	50	999	nev cigs	st

Cigarette type is all/unspec for all RRs

Table 1I9 - 2

IESLC - Meta-analysis of Current Smoking, Duration, "High"
All LC types, Any Product (or Cigarettes if Any not available)
Most adjusted

REF	NRR	SEX	AD	Number Exposed Case	Cont	Non-exposed Case	Cont	RR	95.00%CI
*BEST	507	m	1	137	-	7	-	14.20	(6.64- 30.35)
*BOUCOT	522	m	2	53	-	0	-	89.94	(5.55-1456.45)
BUFFLE	533	f	0	70	36	12	112	18.15	(8.85- 37.22)
*CPSI	585	m	0	576	119633	196	926068	22.75	(19.35- 26.75)
*CPSI	681	f	0	16	14305	532	3877179	8.15	(4.96- 13.40)
Subtotal CPSI								20.61	(17.67- 24.05)
*CPSII	557	m	0	332	39260	124	742207	50.62	(41.19- 62.20)
*CPSII	623	f	0	122	29119	310	2091302	28.26	(22.93- 34.84)
Subtotal CPSII								37.99	(32.80- 44.00)
HUMBLE	545	c	3	119	-	28	-	17.27	(10.38- 28.75)
*KAISE2	597	m	1	34	-	14	-	15.64	(8.31- 29.40)
*KAISE2	517	f	1	26	-	11	-	30.41	(14.39- 64.25)
Subtotal KAISE2								20.63	(12.73- 33.43)
KATSOU	504	f	0	17	4	48	67	5.93	(1.88- 18.75)
MATOS	523	m	2	55	-	11	-	10.20	(4.70- 22.10)
PEZZO2	508	m	0	173	126	6	117	26.77	(11.42- 62.76)
SOBUE	549	m	0	147	73	34	128	7.58	(4.73- 12.14)
Partial Totals				1877	202556	1333	7637180		

*prospective study

REF	NRR	SEX	AD	Ys	Ws	Qs	Ps
*BEST	507	m	1	2.65	6.65	2.11	0.0000
*BOUCOT	522	m	2	4.50	0.50	0.82	0.0015
BUFFLE	533	f	0	2.90	7.44	0.75	0.0000
*CPSI	585	m	0	3.12	146.44	1.24	0.0000
*CPSI	681	f	0	2.10	15.55	19.44	0.0000
Subtotal CPSI				3.03	161.99	20.68	
*CPSII	557	m	0	3.92	90.50	45.34	0.0000
*CPSII	623	f	0	3.34	87.81	1.38	0.0000
Subtotal CPSII				3.64	178.31	46.72	
HUMBLE	545	c	3	2.85	14.81	2.00	0.0000
*KAISE2	597	m	1	2.75	9.62	2.10	0.0000
*KAISE2	517	f	1	3.41	6.86	0.27	0.0000
Subtotal KAISE2				3.03	16.49	2.37	
KATSOU	504	f	0	1.78	2.90	5.98	0.0024
MATOS	523	m	2	2.32	6.41	5.13	0.0000
PEZZO2	508	m	0	3.29	5.29	0.03	0.0000
SOBUE	549	m	0	2.03	17.32	24.56	0.0000

	N	14
	NS	11
	Wt	418.12
Het	Chi	111.14
Het	df	13
Het	P	***
Fixed	RR	24.94
	RRl	22.66
	RRu	27.45
	P	+++
Random	RR	17.83
	RRl	12.70
	RRu	25.04
	P	+++
Asymm	P	(*)

Table 1I9 - 3

IESLC - Meta-analysis of Current Smoking, Duration, "High"
All LC types, Any Product (or Cigarettes if Any not available)
Most adjusted

	Sex: combined	male	female	Total
N	1	8	5	14
NS	1	8	5	14
Wt	14.81	282.74	120.57	418.12
Het Chi	0.00	80.30	26.72	111.14
Het df	0	7	4	13
Het P	N.S.	***	***	***
Fixed RR	17.27	26.49	22.66	24.94
RRl	10.38	23.57	18.95	22.66
RRu	28.74	29.76	27.08	27.45
P	+++	+++	+++	+++
Random RR	17.27	19.01	15.98	17.83
RRl	10.38	11.33	8.38	12.70
RRu	28.74	31.92	30.49	25.04
P	+++	+++	+++	+++
Between Chi				4.13
Between df				2
Between P				N.S.
Btwn(F) P				N.S.
Btwn(R) P				N.S.

	Lung cancer type: all	other	Total
N	12	2	14
NS	9	2	11
Wt	385.99	32.13	418.12
Het Chi	82.82	5.41	111.14
Het df	11	1	13
Het P	***	*	***
Fixed RR	26.68	11.08	24.94
RRl	24.15	7.84	22.66
RRu	29.48	15.66	27.45
P	+++	+++	+++
Random RR	19.63	11.37	17.83
RRl	13.89	5.08	12.70
RRu	27.75	25.49	25.04
P	+++	+++	+++
Between Chi			22.91
Between df			1
Between P			***
Btwn(F) P			N.S.
Btwn(R) P			N.S.

	Location: NAmer	UK	Scand	othEur	China	Japan	othAs	other	Total
N	10			1		1		2	14
NS	7			1		1		2	11
Wt	386.19			2.90		17.32		11.71	418.12
Het Chi	73.09			0.00		0.00		2.70	111.14
Het df	9			0		0		1	13
Het P	***			N.S.		N.S.		N.S.	***
Fixed RR	26.96			5.93		7.58		15.78	24.94
RRl	24.40			1.88		4.73		8.90	22.66
RRu	29.79			18.75		12.14		27.98	27.45
P	+++			++		+++		+++	+++
Random RR	21.47			5.93		7.58		16.25	17.83
RRl	15.15			1.88		4.73		6.31	12.70
RRu	30.44			18.75		12.14		41.80	25.04
P	+++			++		+++		+++	+++
Between Chi									35.35
Between df									3
Between P									***
Btwn(F) P									N.S.
Btwn(R) P									**

Table 1I9 - 3

IESLC - Meta-analysis of Current Smoking, Duration, "High"
All LC types, Any Product (or Cigarettes if Any not available)
Most adjusted

Detailed Country in "other Europe"

		multi	Germany	othWest	East	Balkans	Total
	N					1	1
	NS					1	1
	Wt					2.90	2.90
Het	Chi					0.00	0.00
Het	df					0	0
Het	P					N.S.	N.S.
Fixed	RR					5.93	5.93
	RRl					1.88	1.88
	RRu					18.75	18.75
	P					++	++
Random	RR					5.93	5.93
	RRl					1.88	1.88
	RRu					18.75	18.75
	P					++	++
Between	Chi						
Between	df						
Between	P						N.S.
Btwn(F)	P						N.S.
Btwn(R)	P						N.S.

Detailed Country in "other Asia"

		India	HongKong	other	Total
	N				
	NS				
	Wt				
Het	Chi				
Het	df				
Het	P				
Fixed	RR				
	RRl				
	RRu				
	P				
Random	RR				
	RRl				
	RRu				
	P				
Between	Chi				
Between	df				
Between	P				N.S.
Btwn(F)	P				N.S.
Btwn(R)	P				N.S.

Detailed other continent

		SCAmer	Total
	N	2	2
	NS	2	2
	Wt	11.71	11.71
Het	Chi	2.70	2.70
Het	df	1	1
Het	P	N.S.	N.S.
Fixed	RR	15.78	15.78
	RRl	8.90	8.90
	RRu	27.98	27.98
	P	+++	+++
Random	RR	16.25	16.25
	RRl	6.31	6.31
	RRu	41.80	41.80
	P	+++	+++
Between	Chi		
Between	df		
Between	P		N.S.
Btwn(F)	P		N.S.
Btwn(R)	P		N.S.

Table 1I9 - 3

IESLC - Meta-analysis of Current Smoking, Duration, "High"
All LC types, Any Product (or Cigarettes if Any not available)
Most adjusted

	Start year of study <1960	1960-69	1970-79	1980-89	1990+	Total
N	4		3	5	2	14
NS	3		2	4	2	11
Wt	169.14		23.93	213.34	11.71	418.12
Het Chi	16.79		1.86	69.85	2.70	111.14
Het df	3		2	4	1	13
Het P	***		N.S.	***	N.S.	***
Fixed RR	20.40		19.82	30.77	15.78	24.94
RRl	17.55		13.28	26.90	8.90	22.66
RRu	23.72		29.59	35.19	27.98	27.45
P	+++		+++	+++	+++	+++
Random RR	15.66		19.82	17.98	16.25	17.83
RRl	7.78		13.28	9.33	6.31	12.70
RRu	31.55		29.59	34.66	41.80	25.04
P	+++		+++	+++	+++	+++
Between Chi						19.95
Between df						3
Between P						***
Btwn(F) P						N.S.
Btwn(R) P						N.S.

	Study type (1) CC	other	Total
N	6	8	14
NS	6	5	11
Wt	54.18	363.94	418.12
Het Chi	11.86	68.73	111.14
Het df	5	7	13
Het P	*	***	***
Fixed RR	12.38	27.68	24.94
RRl	9.48	24.98	22.66
RRu	16.15	30.68	27.45
P	+++	+++	+++
Random RR	12.78	22.51	17.83
RRl	8.26	15.09	12.70
RRu	19.77	33.57	25.04
P	+++	+++	+++
Between Chi			30.55
Between df			1
Between P			***
Btwn(F) P			(*)
Btwn(R) P			(*)

	Study type (2) CC	prosp	other	Total
N	6	8		14
NS	6	5		11
Wt	54.18	363.94		418.12
Het Chi	11.86	68.73		111.14
Het df	5	7		13
Het P	*	***		***
Fixed RR	12.38	27.68		24.94
RRl	9.48	24.98		22.66
RRu	16.15	30.68		27.45
P	+++	+++		+++
Random RR	12.78	22.51		17.83
RRl	8.26	15.09		12.70
RRu	19.77	33.57		25.04
P	+++	+++		+++
Between Chi				30.55
Between df				1
Between P				***
Btwn(F) P				(*)
Btwn(R) P				(*)

Table 1I9 - 3

IESLC - Meta-analysis of Current Smoking, Duration, "High"
All LC types, Any Product (or Cigarettes if Any not available)
Most adjusted

	Study size (number of LC cases) 100-249	250-499	500-999	1000+	Total
N	3	4	2	5	14
NS	3	3	2	3	11
Wt	9.81	28.43	22.25	357.63	418.12
Het Chi	3.18	3.02	0.01	90.41	111.14
Het df	2	3	1	4	13
Het P	N.S.	N.S.	N.S.	***	***
Fixed RR	9.70	19.84	17.56	26.64	24.94
RRl	5.19	13.74	11.59	24.02	22.66
RRu	18.13	28.66	26.60	29.55	27.45
P	+++	+++	+++	+++	+++
Random RR	10.30	19.85	17.56	19.05	17.83
RRl	4.13	13.73	11.59	11.16	12.70
RRu	25.69	28.69	26.60	32.50	25.04
P	+++	+++	+++	+++	+++
Between Chi					14.53
Between df					3
Between P					**
Btwn(F) P					N.S.
Btwn(R) P					N.S.

	Risky occupational population no	mining	othRisky	Total
N	14			14
NS	11			11
Wt	418.12			418.12
Het Chi	111.14			111.14
Het df	13			13
Het P	***			***
Fixed RR	24.94			24.94
RRl	22.66			22.66
RRu	27.45			27.45
P	+++			+++
Random RR	17.83			17.83
RRl	12.70			12.70
RRu	25.04			25.04
P	+++			+++
Between Chi				
Between df				
Between P				N.S.
Btwn(F) P				N.S.
Btwn(R) P				N.S.

	National cigarette tobacco type Virginia	blended	other	Total
N	1	13		14
NS	1	10		11
Wt	6.65	411.47		418.12
Het Chi	0.00	109.00		111.14
Het df	0	12		13
Het P	N.S.	***		***
Fixed RR	14.20	25.17		24.94
RRl	6.64	22.85		22.66
RRu	30.36	27.72		27.45
P	+++	+++		+++
Random RR	14.20	18.12		17.83
RRl	6.64	12.73		12.70
RRu	30.36	25.79		25.04
P	+++	+++		+++
Between Chi				2.14
Between df				1
Between P				N.S.
Btwn(F) P				N.S.
Btwn(R) P				N.S.

Table 1I9 - 3

IESLC - Meta-analysis of Current Smoking, Duration, "High"
All LC types, Any Product (or Cigarettes if Any not available)
Most adjusted

	Any proxy use No/nk	Yes	Total
N	12	2	14
NS	9	2	11
Wt	395.87	22.25	418.12
Het Chi	108.24	0.01	111.14
Het df	11	1	13
Het P	***	N.S.	***
Fixed RR	25.44	17.56	24.94
RRl	23.05	11.59	22.66
RRu	28.07	26.60	27.45
P	+++	+++	+++
Random RR	17.82	17.56	17.83
RRl	12.19	11.59	12.70
RRu	26.05	26.60	25.04
P	+++	+++	+++
Between Chi			2.89
Between df			1
Between P			(*)
Btwn(F) P			N.S.
Btwn(R) P			N.S.

	Full histological confirmation No	Yes	Total
N	12	2	14
NS	9	2	11
Wt	395.51	22.62	418.12
Het Chi	85.52	6.45	111.14
Het df	11	1	13
Het P	***	*	***
Fixed RR	26.25	10.19	24.94
RRl	23.79	6.75	22.66
RRu	28.97	15.38	27.45
P	+++	+++	+++
Random RR	18.98	13.53	17.83
RRl	13.50	3.94	12.70
RRu	26.70	46.38	25.04
P	+++	+++	+++
Between Chi			19.17
Between df			1
Between P			***
Btwn(F) P			N.S.
Btwn(R) P			N.S.

	Number of adjustment variables (1) 0	1	2+/+nk	Total
N	8	3	3	14
NS	6	2	3	11
Wt	373.27	23.14	21.71	418.12
Het Chi	97.92	2.43	2.83	111.14
Het df	7	2	2	13
Het P	***	N.S.	N.S.	***
Fixed RR	26.13	18.53	15.35	24.94
RRl	23.61	12.33	10.08	22.66
RRu	28.92	27.85	23.38	27.45
P	+++	+++	+++	+++
Random RR	17.94	18.60	15.24	17.83
RRl	11.48	11.85	8.48	12.70
RRu	28.03	29.20	27.38	25.04
P	+++	+++	+++	+++
Between Chi				7.97
Between df				2
Between P				*
Btwn(F) P				N.S.
Btwn(R) P				N.S.

Table 1I9 - 3

IESLC - Meta-analysis of Current Smoking, Duration, "High"
All LC types, Any Product (or Cigarettes if Any not available)
Most adjusted

	Number of adjustment variables (2) 0	1	2	3-5	6+/+nk	Total
N	8	3	2	1		14
NS	6	2	2	1		11
Wt	373.27	23.14	6.91	14.81		418.12
Het Chi	97.92	2.43	2.18	0.00		111.14
Het df	7	2	1	0		13
Het P	***	N.S.	N.S.	N.S.		***
Fixed RR	26.13	18.53	11.92	17.27		24.94
RRl	23.61	12.33	5.66	10.38		22.66
RRu	28.92	27.85	25.13	28.74		27.45
P	+++	+++	+++	+++		+++
Random RR	17.94	18.60	19.75	17.27		17.83
RRl	11.48	11.85	2.78	10.38		12.70
RRu	28.03	29.20	140.40	28.74		25.04
P	+++	+++	++	+++		+++
Between Chi						8.62
Between df						3
Between P						*
Btwn(F) P						N.S.
Btwn(R) P						N.S.

	Product all/unsp	cig+/-ot	cig only	Total
N	1	6	7	14
NS	1	6	5	12
Wt	2.90	139.09	276.13	418.12
Het Chi	0.00	30.11	68.68	111.14
Het df	0	5	6	13
Het P	N.S.	***	***	***
Fixed RR	5.93	21.17	27.50	24.94
RRl	1.88	17.93	24.44	22.66
RRu	18.75	25.00	30.94	27.45
P	++	+++	+++	+++
Random RR	5.93	16.31	21.45	17.83
RRl	1.88	9.77	12.66	12.70
RRu	18.75	27.25	36.34	25.04
P	++	+++	+++	+++
Between Chi				12.36
Between df				2
Between P				**
Btwn(F) P				N.S.
Btwn(R) P				N.S.

	Denominator nev any	nev cigs	Total
N	7	7	14
NS	6	6	12
Wt	123.45	294.67	418.12
Het Chi	43.15	41.61	111.14
Het df	6	6	13
Het P	***	***	***
Fixed RR	36.77	21.20	24.94
RRl	30.82	18.91	22.66
RRu	43.86	23.76	27.45
P	+++	+++	+++
Random RR	19.21	16.59	17.83
RRl	9.50	11.47	12.70
RRu	38.85	23.99	25.04
P	+++	+++	+++
Between Chi			26.39
Between df			1
Between P			***
Btwn(F) P			(*)
Btwn(R) P			N.S.

Table 1I9 - 3

IESLC - Meta-analysis of Current Smoking, Duration, "High"
All LC types, Any Product (or Cigarettes if Any not available)
Most adjusted

	Derivation of RR/CI Orig	StdCalc	Other	Total
N	2	9	3	14
NS	2	6	3	11
Wt	13.86	382.31	21.95	418.12
Het Chi	1.14	99.62	1.59	111.14
Het df	1	8	2	13
Het P	N.S.	***	N.S.	***
Fixed RR	13.90	26.05	16.89	24.94
RRl	8.21	23.56	11.12	22.66
RRu	23.53	28.80	25.67	27.45
P	+++	+++	+++	+++
Random RR	13.86	18.61	16.89	17.83
RRl	7.89	12.26	11.12	12.70
RRu	24.35	28.24	25.67	25.04
P	+++	+++	+++	+++
Between Chi				8.79
Between df				2
Between P				*
Btwn(F) P				N.S.
Btwn(R) P				N.S.

Table 1I9 - 4

IESLC - Meta-analysis of Current Smoking, Duration, "High"
All LC types, Any Product (or Cigarettes if Any not available)
Least adjusted

REF	NRR	X	SEX	AGEL	AGEH	RACE	YF	LC TYPE	LOC	START	ST	NLC	R	VB	P	H	AD	PRODUCT	exL	exH	DENOM	De
BEST	507		m	0	0	all	0	all	NAmer	1955	pr	381	n	V	n	n	1	cig only	40	999	nev any	ot
BOUCOT	502	x	m	0	0	all	0	all	NAmer	1951	pr	121	n	bl	n	n	1	cig only	40	999	nev any	ot
BUFFLE	533		f	0	0	w-hi	-	all	NAmer	1976	CC	943	n	bl	y	n	0	cig+/-ot	41	999	nev cigs	or
CPSI	585		m	40	84	wh	0	all	NAmer	1959	pr	5138	n	bl	n	n	0	cig only	50	54	nev cigs	st
CPSI	681		f	40	84	wh	0	all	NAmer	1959	pr	5138	n	bl	n	n	0	cig only	50	54	nev cigs	st
CPSII	557		m	0	0	all	6	all	NAmer	1982	pr	3229	n	bl	n	n	0	cig only	50	54	nev any	st
CPSII	623		f	0	0	all	6	all	NAmer	1982	pr	3229	n	bl	n	n	0	cig+/-ot	50	54	nev cigs	st
HUMBLE	520	x	c	0	0	wh	-	not alv	NAmer	1980	CC	521	n	bl	y	n	0	cig+/-ot	50	59	nev cigs	st
KAISE2	597		m	0	0	all	9	all	NAmer	1979	pr	318	n	bl	n	n	1	cig only	40	999	nev any	st
KAISE2	517		f	0	0	all	9	all	NAmer	1979	pr	318	n	bl	n	n	1	cig only	40	999	nev any	st
KATSOU	504		f	0	0	all	-	all	Eu:bal	1987	CC	101	n	bl	n	n	0	all/unsp	40	999	nev any	st
MATOS	503	x	m	0	0	all	-	all	SCAmer	1994	CC	200	n	bl	n	n	0	cig+/-ot	40	70	nev any	st
PEZZO2	508		m	0	0	all	-	all	SCAmer	1992	CC	367	n	bl	n	y	0	cig+/-ot	36	999	nev cigs	st
SOBUE	549		m	0	0	all	-	q+s+l+a	As:Jap	1986	CC	1376	n	bl	n	y	0	cig+/-ot	50	999	nev cigs	st

Cigarette type is all/unspec for all RRs

Table 1I9 - 5

IESLC - Meta-analysis of Current Smoking, Duration, "High"
All LC types, Any Product (or Cigarettes if Any not available)
Least adjusted

REF	NRR	SEX	AD	Number Exposed Case	Exposed Cont	Non-exposed Case	Non-exposed Cont	RR	95.00%CI
*BEST	507	m	1	137	-	7	-	14.20	(6.64- 30.35)
*BOUCOT	502	m	1	53	-	0	-	91.60	(5.66-1483.27)
BUFFLE	533	f	0	70	36	12	112	18.15	(8.85- 37.22)
*CPSI	585	m	0	576	119633	196	926068	22.75	(19.35- 26.75)
*CPSI	681	f	0	16	14305	532	3877179	8.15	(4.96- 13.40)
Subtotal CPSI								20.61	(17.67- 24.05)
*CPSII	557	m	0	332	39260	124	742207	50.62	(41.19- 62.20)
*CPSII	623	f	0	122	29119	310	2091302	28.26	(22.93- 34.84)
Subtotal CPSII								37.99	(32.80- 44.00)
HUMBLE	520	c	0	90	55	28	285	16.66	(9.97- 27.82)
*KAISE2	597	m	1	34	-	14	-	15.64	(8.31- 29.40)
*KAISE2	517	f	1	26	-	11	-	30.41	(14.39- 64.25)
Subtotal KAISE2								20.63	(12.73- 33.43)
KATSOU	504	f	0	17	4	48	67	5.93	(1.88- 18.75)
MATOS	503	m	0	55	61	11	110	9.02	(4.39- 18.51)
PEZZO2	508	m	0	173	126	6	117	26.77	(11.42- 62.76)
SOBUE	549	m	0	147	73	34	128	7.58	(4.73- 12.14)
Partial Totals				1848	202672	1333	7637575		

*prospective study

REF	NRR	SEX	AD	Ys	Ws	Qs	Ps
*BEST	507	m	1	2.65	6.65	2.07	0.0000
*BOUCOT	502	m	1	4.52	0.50	0.85	0.0015
BUFFLE	533	f	0	2.90	7.44	0.73	0.0000
*CPSI	585	m	0	3.12	146.44	1.10	0.0000
*CPSI	681	f	0	2.10	15.55	19.26	0.0000
Subtotal CPSI				3.03	161.99	20.35	
*CPSII	557	m	0	3.92	90.50	46.04	0.0000
*CPSII	623	f	0	3.34	87.81	1.50	0.0000
Subtotal CPSII				3.64	178.31	47.54	
HUMBLE	520	c	0	2.81	14.60	2.31	0.0000
*KAISE2	597	m	1	2.75	9.62	2.05	0.0000
*KAISE2	517	f	1	3.41	6.86	0.28	0.0000
Subtotal KAISE2				3.03	16.49	2.33	
KATSOU	504	f	0	1.78	2.90	5.94	0.0024
MATOS	503	m	0	2.20	7.43	7.61	0.0000
PEZZO2	508	m	0	3.29	5.29	0.03	0.0000
SOBUE	549	m	0	2.03	17.32	24.34	0.0000

	N	14
	NS	11
	Wt	418.93
Het	Chi	114.10
Het	df	13
Het	P	***
Fixed	RR	24.80
	RRl	22.54
	RRu	27.30
	P	+++
Random	RR	17.59
	RRl	12.49
	RRu	24.78
	P	+++
Asymm	P	(*)

Table 1I9 - 6

IESLC - Meta-analysis of Current Smoking, Duration, "High"
All LC types, Any Product (or Cigarettes if Any not available)
Least adjusted

	Sex combined	male	female	Total
N	1	8	5	14
NS	1	8	5	14
Wt	14.60	283.76	120.57	418.93
Het Chi	0.00	83.09	26.72	114.10
Het df	0	7	4	13
Het P	N.S.	***	***	***
Fixed RR	16.66	26.31	22.66	24.80
RRl	9.97	23.42	18.95	22.54
RRu	27.82	29.56	27.08	27.30
P	+++	+++	+++	+++
Random RR	16.66	18.68	15.98	17.59
RRl	9.97	11.07	8.38	12.49
RRu	27.82	31.52	30.49	24.78
P	+++	+++	+++	+++
Between Chi				4.29
Between df				2
Between P				N.S.
Btwn(F) P				N.S.
Btwn(R) P				N.S.

Table 1I9 - 7

IESLC - Meta-analysis of Current Smoking, Duration, "High"
All LC types, Any Product (or Cigarettes if Any not available)
Excluded studies (and stage at which they were excluded)

1	AGUDO ALDERS ARMADA AUVINE AXELSS BARBON BECHER BENHAM BLOT1 BOFFET BOUCHA BRESLO BROWN3 CARPEN CHEN CHEN2 CHIAZZ CHOI CHYOU CORREA DAMBER DARBY DESTEF DOLL DOLL2 DORGAN DOSEME FAN GAO GARCIA GARSHI GENG GER GRAHAM GUO GURSEL HAENSZ HAMMO2 HAMMON HEGMAN HU HU2 JAHN JAIN JEDRYC JOLY JUSSAW KHUDER KOO KOULUM KREUZE LAUSSM LETOUR LEVIN LIU3 LIU4 LIU5 LUBIN LUBIN2 LUO MCCONN NOTAN2 OSANN2 PERNU PEZZOT PRESCO QIAO QIAO2 RACHTA RESTRE SADOWS STASZE SUZUK2 TIZZAN TVERDA VUTUC WANG2 WIGLE WU2 WUWILL WYNDE2 WYNDE3 XU YUAN ZHANG ZHENG ZHOU
2	BENSHL DEAN3 DORN ENGELA GAO2 GILLIS HIRAYA HOLE KAUFMA MIGRAN MRFITR SEGI2 SPEIZE SVENSS WAKAI WU
3	MCDUFF SPITZ WYNDE6
5	AKIBA PISANI
7	BROSS WYNDE7
10	AMES WATSON WYNDE8
14	AMANDU CEDERL DEAN2 LIAW

Table 1I9 - 8
Potentially overlapping studies

REF	REFGP	PRINC	.	OVERLAP/LINK
CPSI	CPSI	1		CPSI overall

Table 1I10 -

IESLC - Meta-analysis of Current Smoking, Duration, "Highest vs lowest"
All LC types, Any Product (or Cigarettes if Any not available)

This analysis is restricted to results for:
1) Current smokers
2) Results by Duration
3) Categorical results by Duration
4) Denominator (unexposed) = "low"
5) All LC types (or near equivalent)
6) Results complete enough for use in metaanalysis

Within each study, results are then selected (in the following order of preference, within each sex) for:
7) (not applicable)
8) PRODUCT: all/unspec, cigarettes regardless of other products, cigarettes only
9) CIGTYPE: all/unspecified, MC regardless of HR, MC only
10) Results with least adjustment for other aspects of smoking (ADOS)
11) The highest vs lowest category
12) Followup period (YF, prospective studies): whole study (coded as 0) or longest available
13) LCtype: all or nearest available, at least Squamous and Adeno. (q = squamous, s = small,
l = large, a = adeno, mix = mixed, alv = alveolar)
14) Race: all or nearest available, otherwise by race (wh or w = white, bl or b = black, hi = hispanic
ch = chinese, jap = japanese, haw = hawaiian, w+o = white + oriental, sca = scandinavian, as = asian)
15) For overlapping studies: principal rather than subsidiary studies
Finally by Age: whole study (coded as 0) if available, otherwise by widest available age group
and then for single sex results (m, f) in preference to results for both sexes combined (c).

Results adjusted (AD) for the most potential confounders are then chosen in Sections -1 to -3
and results adjusted for the least confounders in Sections -4 to -6. (Those least adjusted results which
actually differ from the most adjusted are marked 'x' in column X in Section -4)

Section -7 shows excluded studies, together with the stage (as above) at which no qualifying
results were found.

Section -8 lists the potentially overlapping studies which have been included (1=principal, 2=subsidiary).

Section -9 lists any results which would have been included in preference except that they had data not complete
enough for use in meta-analysis, with their significance (yes/no), if known, and any further comment as entered
on the database. It also lists as "gap" any categories for which no data were presented by the original authors.

In addition to those mentioned above, the following fields, levels and abbreviations are used:
* or nk = not known, n = no, y = yes, ot = other
all/unspec = all or unspecified, cig+/-ot = cigarettes irrespective of other products (cigar, pipe etc)
MC = manufactured cigarettes, HR = hand-rolled cigarettes
exL, exH = range of exposure (low and high) in the "highest" group, in terms of Duration
unexL, unexH = range of exposure (low and high) in the "lowest" group, in terms of Duration
REF: 6-character study reference
NRR: number of the RR on the database within the study
ST : study type (CC = case control, pr or prosp = prospective)
NLC: number of lung cancer cases in whole study
R : risky occupational population (n = no, m = mining, o = other risky)
VB : national cigarette type (V = at least 75% Virginia, bl = at least 75% blended, ot = other)
P : any proxy use
H : full histological confirmation
De : derivation of RR/CI (or = original, st = standard method, ot = other method of estimation)

Table 1I10 - 1

IESLC - Meta-analysis of Current Smoking, Duration, "Highest vs lowest"
All LC types, Any Product (or Cigarettes if Any not available)
Most adjusted

REF	NRR	SEX	AGEL	AGEH	RACE	YF	LC TYPE	LOC	START	ST	NLC	R	VB	P	H	AD	ADOS	PRODUCT	exL	exH	unexL	unexH	De
AMANDU	508	m	0	0	wh	0	all	NAmer	1959	pr	132	m	bl	n	n	2	0	cig+/-ot	25	999	1	24	ot
AMES	501	m	0	0	wh	-	all	NAmer	1959	ot	317	m	bl	n	n	2	0	all/unsp	30	999	1	29	or
BEST	513	m	0	0	all	0	all	NAmer	1955	pr	381	n	V	n	n	1	0	cig only	40	999	1	4	ot
BOUCOT	523	m	0	0	all	0	all	NAmer	1951	pr	121	n	bl	n	n	2	0	cig only	40	999	1	39	ot
BUFFLE	535	f	0	0	w-hi	-	all	NAmer	1976	CC	943	n	bl	y	n	0	0	cig+/-ot	41	999	1	30	st
CEDERL	503	m	40	69	all	10	all	Eu:Sca	1963	pr	491	n	bl	n	n	1	0	cig only	30	999	1	29	ot
CEDERL	506	f	40	69	all	10	all	Eu:Sca	1963	pr	491	n	bl	n	n	1	0	cig only	30	999	1	29	ot
CPSI	602	m	40	84	wh	0	all	NAmer	1959	pr	5138	n	bl	n	n	0	0	cig only	60	999	1	29	st
CPSI	695	f	40	84	wh	0	all	NAmer	1959	pr	5138	n	bl	n	n	0	0	cig only	55	999	1	29	st
CPSII	566	m	0	0	all	6	all	NAmer	1982	pr	3229	n	bl	n	n	0	0	cig only	60	999	1	29	st
CPSII	632	f	0	0	all	6	all	NAmer	1982	pr	3229	n	bl	n	n	0	0	cig+/-ot	60	999	1	29	st
DEAN2	503	m	0	0	all	-	all	Eu:UK	1960	CC	954	n	V	y	n	0	0	all/unsp	20	999	1	19	st
DEAN2	506	f	0	0	all	-	all	Eu:UK	1960	CC	954	n	V	y	n	0	0	all/unsp	20	999	1	19	st
HUMBLE	525	c	0	0	wh	-	not alv	NAmer	1980	CC	521	n	bl	y	n	0	0	cig+/-ot	60	999	1	29	st
KAISE2	600	m	0	0	all	9	all	NAmer	1979	pr	318	n	bl	n	n	1	0	cig only	40	999	1	39	st
KAISE2	520	f	0	0	all	9	all	NAmer	1979	pr	318	n	bl	n	n	1	0	cig only	40	999	1	39	st
KATSOU	507	f	0	0	all	-	all	Eu:bal	1987	CC	101	n	bl	n	n	0	0	all/unsp	40	999	1	19	st
LIAW	508	c	0	0	all	0	all	As:oth	1982	pr	127	n	ot	n	n	2	0	all/unsp	31	999	1	20	ot
MATOS	525	m	0	0	all	-	all	SCAmer	1994	CC	200	n	bl	n	n	2	0	cig+/-ot	40	70	1	24	ot
PEZZO2	509	m	0	0	all	-	all	SCAmer	1992	CC	367	n	bl	n	y	0	0	cig+/-ot	36	999	1	35	st
SOBUE	552	m	0	0	all	-	q+s+l+a	As:Jap	1986	CC	1376	n	bl	n	y	0	0	cig+/-ot	50	999	1	29	st
WATSON	503	m	0	0	all	-	all	NAmer	1950	CC	301	n	bl	n	y	0	0	all/unsp	20	999	1	19	st
WATSON	506	f	0	0	all	-	all	NAmer	1950	CC	301	n	bl	n	y	0	0	all/unsp	20	999	1	19	st
WYNDE8	502	m	0	0	all	-	all	NAmer	1985	CC	1044	n	bl	n	y	0	0	cig+/-ot	41	999	1	30	st
WYNDE8	504	f	0	0	all	-	all	NAmer	1985	CC	1044	n	bl	n	y	0	0	cig+/-ot	41	999	1	30	st

Cigarette type is all/unspec for all RRs

Table 1I10 - 2

IESLC - Meta-analysis of Current Smoking, Duration, "Highest vs lowest"
All LC types, Any Product (or Cigarettes if Any not available)
Most adjusted

REF	NRR	SEX	AD	Number Exposed Case	Cont	Non-exposed Case	Cont	RR	95.00%CI
*AMANDU	508	m	2	72	-	42	-	1.19	(0.76- 1.86)
AMES	501	m	2	-	-	-	-	2.28	(1.58- 3.29)
*BEST	513	m	1	137	-	1	-	8.88	(1.25- 62.98)
*BOUCOT	523	m	2	53	-	32	-	2.13	(1.38- 3.31)
BUFFLE	535	f	0	70	36	36	24	1.30	(0.67- 2.49)
*CEDERL	503	m	1	23	-	5	-	4.11	(1.48- 11.44)
*CEDERL	506	f	1	5	-	3	-	6.00	(1.44- 24.93)
Subtotal CEDERL								4.67	(2.04- 10.73)
*CPSI	602	m	0	232	26906	95	266163	24.16	(19.03- 30.66)
*CPSI	695	f	0	10	5657	105	694015	11.68	(6.11- 22.34)
Subtotal CPSI								22.15	(17.71- 27.71)
*CPSII	566	m	0	117	8450	72	141932	27.29	(20.37- 36.58)
*CPSII	632	f	0	18	2224	127	301244	19.20	(11.74- 31.40)
Subtotal CPSII								24.89	(19.36- 32.01)
DEAN2	503	m	0	631	558	34	36	1.20	(0.74- 1.94)
DEAN2	506	f	0	47	11	10	14	5.98	(2.11- 16.99)
Subtotal DEAN2								1.59	(1.03- 2.46)
HUMBLE	525	c	0	29	22	20	33	2.18	(0.99- 4.77)
*KAISE2	600	m	1	34	-	17	-	5.38	(2.95- 9.81)
*KAISE2	520	f	1	26	-	24	-	3.13	(1.63- 6.00)
Subtotal KAISE2								4.19	(2.70- 6.52)
KATSOU	507	f	0	17	4	5	5	4.25	(0.82- 22.13)
*LIAW	508	c	2	-	-	-	-	5.22	(1.75- 15.56)
MATOS	525	m	2	55	-	10	-	1.96	(0.73- 5.29)
PEZZO2	509	m	0	173	126	60	72	1.65	(1.09- 2.49)
SOBUE	552	m	0	147	73	62	119	3.87	(2.55- 5.86)
WATSON	503	m	0	252	231	8	20	2.73	(1.18- 6.31)
WATSON	506	f	0	10	13	5	20	3.08	(0.85- 11.07)
Subtotal WATSON								2.83	(1.40- 5.71)
WYNDE8	502	m	0	294	354	102	272	2.21	(1.68- 2.92)
WYNDE8	504	f	0	206	122	82	134	2.76	(1.94- 3.93)
Subtotal WYNDE8								2.41	(1.94- 2.99)
Partial Totals				2658	44787	957	1404103		

*prospective study

REF	NRR	SEX	AD	Ys	Ws	Qs	Ps
*AMANDU	508	m	2	0.17	19.18	39.88	0.4461
AMES	501	m	2	0.82	28.56	17.90	0.0000
*BEST	513	m	1	2.18	1.00	0.32	0.0290
*BOUCOT	523	m	2	0.76	20.03	14.74	0.0007
BUFFLE	535	f	0	0.26	8.97	16.50	0.4371
*CEDERL	503	m	1	1.41	3.67	0.15	0.0067
*CEDERL	506	f	1	1.79	1.89	0.06	0.0138
Subtotal CEDERL				1.54	5.56	0.21	
*CPSI	602	m	0	3.18	67.59	166.33	0.0000
*CPSI	695	f	0	2.46	9.15	6.49	0.0000
Subtotal CPSI				3.10	76.73	172.82	
*CPSII	566	m	0	3.31	44.82	128.14	0.0000
*CPSII	632	f	0	2.95	15.88	28.47	0.0000
Subtotal CPSII				3.21	60.70	156.61	
DEAN2	503	m	0	0.18	16.51	34.03	0.4643
DEAN2	506	f	0	1.79	3.53	0.11	0.0008
Subtotal DEAN2				0.46	20.04	34.14	
HUMBLE	525	c	0	0.78	6.24	4.39	0.0522
*KAISE2	600	m	1	1.68	10.64	0.05	0.0000
*KAISE2	520	f	1	1.14	9.05	2.04	0.0006
Subtotal KAISE2				1.43	19.69	2.09	
KATSOU	507	f	0	1.45	1.41	0.04	0.0857
*LIAW	508	c	2	1.65	3.22	0.00	0.0030
MATOS	525	m	2	0.67	3.92	3.48	0.1829
PEZZO2	509	m	0	0.50	22.59	28.16	0.0176
SOBUE	552	m	0	1.35	22.21	1.55	0.0000
WATSON	503	m	0	1.00	5.46	2.05	0.0191
WATSON	506	f	0	1.12	2.34	0.57	0.0854
Subtotal WATSON				1.04	7.80	2.61	
WYNDE8	502	m	0	0.80	50.74	34.18	0.0000
WYNDE8	504	f	0	1.01	30.57	11.04	0.0000
Subtotal WYNDE8				0.88	81.32	45.22	

Table 1I10 - 2

IESLC - Meta-analysis of Current Smoking, Duration, "Highest vs lowest"
All LC types, Any Product (or Cigarettes if Any not available)
Most adjusted

	N	25
	NS	18
	Wt	409.16
Het	Chi	540.67
Het	df	24
Het	P	***
Fixed	RR	5.03
	RRl	4.57
	RRu	5.54
	P	+++
Random	RR	3.92
	RRl	2.41
	RRu	6.38
	P	+++
Asymm	P	N.S.

Table 1I10 - 3

IESLC - Meta-analysis of Current Smoking, Duration, "Highest vs lowest"
All LC types, Any Product (or Cigarettes if Any not available)
Most adjusted

	Sex			
	combined	male	female	Total
N	2	14	9	25
NS	2	14	9	25
Wt	9.46	316.92	82.78	409.16
Het Chi	1.63	470.56	64.82	540.67
Het df	1	13	8	24
Het P	N.S.	***	***	***
Fixed RR	2.93	5.22	4.66	5.03
RRl	1.55	4.67	3.76	4.57
RRu	5.54	5.82	5.78	5.54
P	+++	+++	+++	+++
Random RR	3.09	3.57	4.77	3.92
RRl	1.33	1.78	2.41	2.41
RRu	7.17	7.18	9.44	6.38
P	++	+++	+++	+++
Between Chi				3.66
Between df				2
Between P				N.S.
Btwn(F) P				N.S.
Btwn(R) P				N.S.

	Lung cancer type		
	all	other	Total
N	23	2	25
NS	16	2	18
Wt	380.72	28.45	409.16
Het Chi	534.41	1.61	540.67
Het df	22	1	24
Het P	***	N.S.	***
Fixed RR	5.18	3.41	5.03
RRl	4.69	2.36	4.57
RRu	5.73	4.92	5.54
P	+++	+++	+++
Random RR	4.02	3.20	3.92
RRl	2.38	1.89	2.41
RRu	6.81	5.43	6.38
P	+++	+++	+++
Between Chi			4.65
Between df			1
Between P			*
Btwn(F) P			N.S.
Btwn(R) P			N.S.

				Location					
	NAmer	UK	Scand	othEur	China	Japan	othAs	other	Total
N	16	2	2	1		1	1	2	25
NS	11	1	1	1		1	1	2	18
Wt	330.22	20.04	5.56	1.41		22.21	3.22	26.50	409.16
Het Chi	462.76	7.52	0.18	0.00		0.00	0.00	0.10	540.67
Het df	15	1	1	0		0	0	1	24
Het P	***	**	N.S.	N.S.		N.S.	N.S.	N.S.	***
Fixed RR	6.01	1.59	4.67	4.25		3.87	5.22	1.69	5.03
RRl	5.39	1.03	2.04	0.82		2.55	1.75	1.16	4.57
RRu	6.69	2.46	10.73	22.13		5.86	15.57	2.47	5.54
P	+++	+	+++	(+)		+++	++	++	+++
Random RR	4.37	2.50	4.67	4.25		3.87	5.22	1.69	3.92
RRl	2.32	0.52	2.04	0.82		2.55	1.75	1.16	2.41
RRu	8.21	12.01	10.73	22.13		5.86	15.57	2.47	6.38
P	+++	N.S.	+++	(+)		+++	++	++	+++
Between Chi									70.11
Between df									6
Between P									***
Btwn(F) P									N.S.
Btwn(R) P									*

Table 1I10 - 3

IESLC - Meta-analysis of Current Smoking, Duration, "Highest vs lowest"
All LC types, Any Product (or Cigarettes if Any not available)
Most adjusted

Detailed Country in "other Europe"

	multi	Germany	othWest	East	Balkans	Total
N					1	1
NS					1	1
Wt					1.41	1.41
Het Chi					0.00	0.00
Het df					0	0
Het P					N.S.	N.S.
Fixed RR					4.25	4.25
RRl					0.82	0.82
RRu					22.13	22.13
P					(+)	(+)
Random RR					4.25	4.25
RRl					0.82	0.82
RRu					22.13	22.13
P					(+)	(+)
Between Chi						
Between df						
Between P						N.S.
Btwn(F) P						N.S.
Btwn(R) P						N.S.

Detailed Country in "other Asia"

	India	HongKong	other	Total
N			1	1
NS			1	1
Wt			3.22	3.22
Het Chi			0.00	0.00
Het df			0	0
Het P			N.S.	N.S.
Fixed RR			5.22	5.22
RRl			1.75	1.75
RRu			15.57	15.57
P			++	++
Random RR			5.22	5.22
RRl			1.75	1.75
RRu			15.57	15.57
P			++	++
Between Chi				
Between df				
Between P				N.S.
Btwn(F) P				N.S.
Btwn(R) P				N.S.

Detailed other continent

	SCAmer	Total
N	2	2
NS	2	2
Wt	26.50	26.50
Het Chi	0.10	0.10
Het df	1	1
Het P	N.S.	N.S.
Fixed RR	1.69	1.69
RRl	1.16	1.16
RRu	2.47	2.47
P	++	++
Random RR	1.69	1.69
RRl	1.16	1.16
RRu	2.47	2.47
P	++	++
Between Chi		
Between df		
Between P		N.S.
Btwn(F) P		N.S.
Btwn(R) P		N.S.

Table 1I10 - 3

IESLC - Meta-analysis of Current Smoking, Duration, "Highest vs lowest"
All LC types, Any Product (or Cigarettes if Any not available)
Most adjusted

	Start year of study					
	<1960	1960-69	1970-79	1980-89	1990+	Total
N	8	4	3	8	2	25
NS	6	2	2	6	2	18
Wt	153.31	25.60	28.66	175.09	26.50	409.16
Het Chi	236.58	12.76	9.93	204.01	0.10	540.67
Het df	7	3	2	7	1	24
Het P	***	**	**	***	N.S.	***
Fixed RR	6.63	2.01	2.90	5.83	1.69	5.03
RRl	5.66	1.36	2.01	5.03	1.16	4.57
RRu	7.77	2.96	4.19	6.76	2.47	5.54
P	+++	+++	+++	+++	++	+++
Random RR	4.24	3.29	2.82	5.36	1.69	3.92
RRl	1.50	1.26	1.24	2.25	1.16	2.41
RRu	11.96	8.62	6.38	12.74	2.47	6.38
P	++	+	+	+++	++	+++
Between Chi						77.29
Between df						4
Between P						***
Btwn(F) P						N.S.
Btwn(R) P						(*)

	Study type (1)		
	CC	other	Total
N	12	13	25
NS	9	9	18
Wt	174.48	234.68	409.16
Het Chi	23.73	321.04	540.67
Het df	11	12	24
Het P	*	***	***
Fixed RR	2.26	9.14	5.03
RRl	1.94	8.04	4.57
RRu	2.62	10.39	5.54
P	+++	+++	+++
Random RR	2.27	6.10	3.92
RRl	1.76	2.98	2.41
RRu	2.91	12.48	6.38
P	+++	+++	+++
Between Chi			195.90
Between df			1
Between P			***
Btwn(F) P			**
Btwn(R) P			*

	Study type (2)			
	CC	prosp	other	Total
N	12	12	1	25
NS	9	8	1	18
Wt	174.48	206.12	28.56	409.16
Het Chi	23.73	258.36	0.00	540.67
Het df	11	11	0	24
Het P	*	***	N.S.	***
Fixed RR	2.26	11.08	2.28	5.03
RRl	1.94	9.66	1.58	4.57
RRu	2.62	12.70	3.29	5.54
P	+++	+++	+++	+++
Random RR	2.27	6.67	2.28	3.92
RRl	1.76	3.22	1.58	2.41
RRu	2.91	13.83	3.29	6.38
P	+++	+++	+++	+++
Between Chi				258.58
Between df				2
Between P				***
Btwn(F) P				***
Btwn(R) P				*

Table 1I10 - 3

IESLC - Meta-analysis of Current Smoking, Duration, "Highest vs lowest"
All LC types, Any Product (or Cigarettes if Any not available)
Most adjusted

	Study size (number of LC cases)				
	100-249	250-499	500-999	1000+	Total
N	5	9	4	7	25
NS	5	6	3	4	18
Wt	47.76	85.20	35.25	240.96	409.16
Het Chi	8.58	14.81	8.50	289.06	540.67
Het df	4	8	3	6	24
Het P	(*)	(*)	*	***	***
Fixed RR	1.82	2.62	1.60	9.18	5.03
RRl	1.37	2.12	1.15	8.09	4.57
RRu	2.41	3.23	2.22	10.42	5.54
P	+++	+++	++	+++	+++
Random RR	2.08	2.98	1.88	8.59	3.92
RRl	1.27	2.13	1.04	3.48	2.41
RRu	3.41	4.15	3.41	21.21	6.38
P	++	+++	+	+++	+++
Between Chi					219.71
Between df					3
Between P					***
Btwn(F) P					*
Btwn(R) P					*

	Risky occupational population			
	no	mining	othRisky	Total
N	23	2		25
NS	16	2		18
Wt	361.42	47.74		409.16
Het Chi	475.89	4.85		540.67
Het df	22	1		24
Het P	***	*		***
Fixed RR	5.78	1.76		5.03
RRl	5.22	1.32		4.57
RRu	6.41	2.33		5.54
P	+++	+++		+++
Random RR	4.25	1.67		3.92
RRl	2.55	0.88		2.41
RRu	7.10	3.15		6.38
P	+++	N.S.		+++
Between Chi				59.92
Between df				1
Between P				***
Btwn(F) P				N.S.
Btwn(R) P				*

	National cigarette tobacco type			
	Virginia	blended	other	Total
N	3	21	1	25
NS	2	15	1	18
Wt	21.04	384.91	3.22	409.16
Het Chi	10.34	504.90	0.00	540.67
Het df	2	20	0	24
Het P	**	***	N.S.	***
Fixed RR	1.72	5.33	5.22	5.03
RRl	1.12	4.83	1.75	4.57
RRu	2.64	5.89	15.57	5.54
P	+	+++	++	+++
Random RR	3.35	3.93	5.22	3.92
RRl	0.85	2.32	1.75	2.41
RRu	13.27	6.67	15.57	6.38
P	(+)	+++	++	+++
Between Chi				25.43
Between df				2
Between P				***
Btwn(F) P				N.S.
Btwn(R) P				N.S.

Table 1I10 - 3

IESLC - Meta-analysis of Current Smoking, Duration, "Highest vs lowest"
All LC types, Any Product (or Cigarettes if Any not available)
Most adjusted

	Any proxy use No/nk	Yes	Total
N	21	4	25
NS	15	3	18
Wt	373.92	35.25	409.16
Het Chi	481.25	8.50	540.67
Het df	20	3	24
Het P	***	*	***
Fixed RR	5.61	1.60	5.03
RRl	5.07	1.15	4.57
RRu	6.21	2.22	5.54
P	+++	++	+++
Random RR	4.44	1.88	3.92
RRl	2.61	1.04	2.41
RRu	7.55	3.41	6.38
P	+++	+	+++
Between Chi			50.91
Between df			1
Between P			***
Btwn(F) P			N.S.
Btwn(R) P			*

	Full histological confirmation No	Yes	Total
N	19	6	25
NS	14	4	18
Wt	275.26	133.91	409.16
Het Chi	429.93	9.30	540.67
Het df	18	5	24
Het P	***	(*)	***
Fixed RR	7.12	2.46	5.03
RRl	6.33	2.08	4.57
RRu	8.01	2.92	5.54
P	+++	+++	+++
Random RR	4.49	2.52	3.92
RRl	2.42	1.95	2.41
RRu	8.34	3.25	6.38
P	+++	+++	+++
Between Chi			101.44
Between df			1
Between P			***
Btwn(F) P			*
Btwn(R) P			(*)

	Number of adjustment variables (1) 0	1	2+/+nk	Total
N	15	5	5	25
NS	10	3	5	18
Wt	308.00	26.25	74.91	409.16
Het Chi	444.04	2.17	8.66	540.67
Het df	14	4	4	24
Het P	***	N.S.	(*)	***
Fixed RR	6.41	4.42	1.95	5.03
RRl	5.73	3.01	1.55	4.57
RRu	7.17	6.47	2.45	5.54
P	+++	+++	+++	+++
Random RR	4.43	4.42	2.01	3.92
RRl	2.28	3.01	1.39	2.41
RRu	8.62	6.47	2.91	6.38
P	+++	+++	+++	+++
Between Chi				85.79
Between df				2
Between P				***
Btwn(F) P				N.S.
Btwn(R) P				**

Table 1I10 - 3

IESLC - Meta-analysis of Current Smoking, Duration, "Highest vs lowest"
All LC types, Any Product (or Cigarettes if Any not available)
Most adjusted

	Number of adjustment variables (2) 0	1	2	3-5	6+/+nk	Total
N	15	5	5			25
NS	10	3	5			18
Wt	308.00	26.25	74.91			409.16
Het Chi	444.04	2.17	8.66			540.67
Het df	14	4	4			24
Het P	***	N.S.	(*)			***
Fixed RR	6.41	4.42	1.95			5.03
RRl	5.73	3.01	1.55			4.57
RRu	7.17	6.47	2.45			5.54
P	+++	+++	+++			+++
Random RR	4.43	4.42	2.01			3.92
RRl	2.28	3.01	1.39			2.41
RRu	8.62	6.47	2.91			6.38
P	+++	+++	+++			+++
Between Chi						85.79
Between df						2
Between P						***
Btwn(F) P						N.S.
Btwn(R) P						**

	Product all/unsp	cig+/-ot	cig only	Total
N	7	9	9	25
NS	5	8	6	19
Wt	61.03	180.30	167.84	409.16
Het Chi	13.20	89.65	148.34	540.67
Het df	6	8	8	24
Het P	*	***	***	***
Fixed RR	2.21	2.61	13.77	5.03
RRl	1.72	2.25	11.83	4.57
RRu	2.84	3.02	16.01	5.54
P	+++	+++	+++	+++
Random RR	2.64	2.61	7.47	3.92
RRl	1.68	1.56	3.53	2.41
RRu	4.13	4.36	15.83	6.38
P	+++	+++	+++	+++
Between Chi				289.48
Between df				2
Between P				***
Btwn(F) P				***
Btwn(R) P				*

	Derivation of RR/CI Orig	StdCalc	Other	Total
N	1	17	7	25
NS	1	11	6	18
Wt	28.56	327.69	52.91	409.16
Het Chi	0.00	448.80	14.62	540.67
Het df	0	16	6	24
Het P	N.S.	***	*	***
Fixed RR	2.28	6.25	2.02	5.03
RRl	1.58	5.61	1.54	4.57
RRu	3.29	6.96	2.65	5.54
P	+++	+++	+++	+++
Random RR	2.28	4.39	2.64	3.92
RRl	1.58	2.40	1.60	2.41
RRu	3.29	8.04	4.37	6.38
P	+++	+++	+++	+++
Between Chi				77.24
Between df				2
Between P				***
Btwn(F) P				N.S.
Btwn(R) P				N.S.

Table 1I10 - 4

IESLC - Meta-analysis of Current Smoking, Duration, "Highest vs lowest"
All LC types, Any Product (or Cigarettes if Any not available)
Least adjusted

REF	NRR	X	SEX	AGEL	AGEH	RACE	YF	LC TYPE	LOC	START	ST	NLC	R	VB	P	H	AD	ADOS	PRODUCT	exL	exH	unexL	unexH	De
AMANDU	503	x	m	0	0	wh	0	all	NAmer	1959	pr	132	m	bl	n	n	0	0	cig+/-ot	25	999	1	24	st
AMES	501		m	0	0	wh	-	all	NAmer	1959	ot	317	m	bl	n	n	2	0	all/unsp	30	999	1	29	or
BEST	513		m	0	0	all	0	all	NAmer	1955	pr	381	n	V	n	n	1	0	cig only	40	999	1	4	ot
BOUCOT	503	x	m	0	0	all	0	all	NAmer	1951	pr	121	n	bl	n	n	1	0	cig only	40	999	1	39	ot
BUFFLE	535		f	0	0	w-hi	-	all	NAmer	1976	CC	943	n	bl	y	n	0	0	cig+/-ot	41	999	1	30	st
CEDERL	503		m	40	69	all	10	all	Eu:Sca	1963	pr	491	n	bl	n	n	1	0	cig only	30	999	1	29	ot
CEDERL	506		f	40	69	all	10	all	Eu:Sca	1963	pr	491	n	bl	n	n	1	0	cig only	30	999	1	29	ot
CPSI	602		m	40	84	wh	0	all	NAmer	1959	pr	5138	n	bl	n	n	0	0	cig only	60	999	1	29	st
CPSI	695		f	40	84	wh	0	all	NAmer	1959	pr	5138	n	bl	n	n	0	0	cig only	55	999	1	29	st
CPSII	566		m	0	0	all	6	all	NAmer	1982	pr	3229	n	bl	n	n	0	0	cig only	60	999	1	29	st
CPSII	632		f	0	0	all	6	all	NAmer	1982	pr	3229	n	bl	n	n	0	0	cig+/-ot	60	999	1	29	st
DEAN2	503		m	0	0	all	-	all	Eu:UK	1960	CC	954	n	V	y	n	0	0	all/unsp	20	999	1	19	st
DEAN2	506		f	0	0	all	-	all	Eu:UK	1960	CC	954	n	V	y	n	0	0	all/unsp	20	999	1	19	st
HUMBLE	525		c	0	0	wh	-	not alv	NAmer	1980	CC	521	n	bl	y	n	0	0	cig+/-ot	60	999	1	29	st
KAISE2	600		m	0	0	all	9	all	NAmer	1979	pr	318	n	bl	n	n	1	0	cig only	40	999	1	39	st
KAISE2	520		f	0	0	all	9	all	NAmer	1979	pr	318	n	bl	n	n	1	0	cig only	40	999	1	39	st
KATSOU	507		f	0	0	all	-	all	Eu:bal	1987	CC	101	n	bl	n	n	0	0	all/unsp	40	999	1	19	st
LIAW	508		c	0	0	all	0	all	As:oth	1982	pr	127	n	ot	n	n	2	0	all/unsp	31	999	1	20	ot
MATOS	505	x	m	0	0	all	-	all	SCAmer	1994	CC	200	n	bl	n	n	0	0	cig+/-ot	40	70	1	24	st
PEZZO2	509		m	0	0	all	-	all	SCAmer	1992	CC	367	n	bl	n	y	0	0	cig+/-ot	36	999	1	35	st
SOBUE	552		m	0	0	all	-	q+s+l+a	As:Jap	1986	CC	1376	n	bl	n	y	0	0	cig+/-ot	50	999	1	29	st
WATSON	503		m	0	0	all	-	all	NAmer	1950	CC	301	n	bl	n	y	0	0	all/unsp	20	999	1	19	st
WATSON	506		f	0	0	all	-	all	NAmer	1950	CC	301	n	bl	n	y	0	0	all/unsp	20	999	1	19	st
WYNDE8	502		m	0	0	all	-	all	NAmer	1985	CC	1044	n	bl	n	y	0	0	cig+/-ot	41	999	1	30	st
WYNDE8	504		f	0	0	all	-	all	NAmer	1985	CC	1044	n	bl	n	y	0	0	cig+/-ot	41	999	1	30	st

Cigarette type is all/unspec for all RRs

Table 1I10 - 5

IESLC - Meta-analysis of Current Smoking, Duration, "Highest vs lowest"
All LC types, Any Product (or Cigarettes if Any not available)
Least adjusted

REF	NRR	SEX	AD	Number Exposed Case	Number Exposed Cont	Non-exposed Case	Non-exposed Cont	RR	95.00%CI
*AMANDU	503	m	0	72	27096	42	68909	4.36	(2.98- 6.38)
AMES	501	m	2	-	-	-	-	2.28	(1.58- 3.29)
*BEST	513	m	1	137	-	1	-	8.88	(1.25- 62.98)
*BOUCOT	503	m	1	53	-	32	-	1.91	(1.23- 2.96)
BUFFLE	535	f	0	70	36	36	24	1.30	(0.67- 2.49)
*CEDERL	503	m	1	23	-	5	-	4.11	(1.48- 11.44)
*CEDERL	506	f	1	5	-	3	-	6.00	(1.44- 24.93)
Subtotal CEDERL								4.67	(2.04- 10.73)
*CPSI	602	m	0	232	26906	95	266163	24.16	(19.03- 30.66)
*CPSI	695	f	0	10	5657	105	694015	11.68	(6.11- 22.34)
Subtotal CPSI								22.15	(17.71- 27.71)
*CPSII	566	m	0	117	8450	72	141932	27.29	(20.37- 36.58)
*CPSII	632	f	0	18	2224	127	301244	19.20	(11.74- 31.40)
Subtotal CPSII								24.89	(19.36- 32.01)
DEAN2	503	m	0	631	558	34	36	1.20	(0.74- 1.94)
DEAN2	506	f	0	47	11	10	14	5.98	(2.11- 16.99)
Subtotal DEAN2								1.59	(1.03- 2.46)
HUMBLE	525	c	0	29	22	20	33	2.18	(0.99- 4.77)
*KAISE2	600	m	1	34	-	17	-	5.38	(2.95- 9.81)
*KAISE2	520	f	1	26	-	24	-	3.13	(1.63- 6.00)
Subtotal KAISE2								4.19	(2.70- 6.52)
KATSOU	507	f	0	17	4	5	5	4.25	(0.82- 22.13)
*LIAW	508	c	2	-	-	-	-	5.22	(1.75- 15.56)
MATOS	505	m	0	55	61	10	18	1.62	(0.69- 3.81)
PEZZO2	509	m	0	173	126	60	72	1.65	(1.09- 2.49)
SOBUE	552	m	0	147	73	62	119	3.87	(2.55- 5.86)
WATSON	503	m	0	252	231	8	20	2.73	(1.18- 6.31)
WATSON	506	f	0	10	13	5	20	3.08	(0.85- 11.07)
Subtotal WATSON								2.83	(1.40- 5.71)
WYNDE8	502	m	0	294	354	102	272	2.21	(1.68- 2.92)
WYNDE8	504	f	0	206	122	82	134	2.76	(1.94- 3.93)
Subtotal WYNDE8								2.41	(1.94- 2.99)
Partial Totals				2658	71944	957	1473030		

*prospective study

REF	NRR	SEX	AD	Ys	Ws	Qs	Ps
*AMANDU	503	m	0	1.47	26.56	0.96	0.0000
AMES	501	m	2	0.82	28.56	20.07	0.0000
*BEST	513	m	1	2.18	1.00	0.27	0.0290
*BOUCOT	503	m	1	0.65	19.92	20.54	0.0039
BUFFLE	535	f	0	0.26	8.97	17.65	0.4371
*CEDERL	503	m	1	1.41	3.67	0.23	0.0067
*CEDERL	506	f	1	1.79	1.89	0.03	0.0138
Subtotal CEDERL				1.54	5.56	0.26	
*CPSI	602	m	0	3.18	67.59	156.60	0.0000
*CPSI	695	f	0	2.46	9.15	5.79	0.0000
Subtotal CPSI				3.10	76.73	162.39	
*CPSII	566	m	0	3.31	44.82	121.18	0.0000
*CPSII	632	f	0	2.95	15.88	26.52	0.0000
Subtotal CPSII				3.21	60.70	147.70	
DEAN2	503	m	0	0.18	16.51	36.28	0.4643
DEAN2	506	f	0	1.79	3.53	0.06	0.0008
Subtotal DEAN2				0.46	20.04	36.34	
HUMBLE	525	c	0	0.78	6.24	4.89	0.0522
*KAISE2	600	m	1	1.68	10.64	0.00	0.0000
*KAISE2	520	f	1	1.14	9.05	2.46	0.0006
Subtotal KAISE2				1.43	19.69	2.46	
KATSOU	507	f	0	1.45	1.41	0.07	0.0857
*LIAW	508	c	2	1.65	3.22	0.00	0.0030
MATOS	505	m	0	0.48	5.26	7.30	0.2668
PEZZO2	509	m	0	0.50	22.59	30.56	0.0176
SOBUE	552	m	0	1.35	22.21	2.14	0.0000
WATSON	503	m	0	1.00	5.46	2.37	0.0191
WATSON	506	f	0	1.12	2.34	0.68	0.0854
Subtotal WATSON				1.04	7.80	3.05	
WYNDE8	502	m	0	0.80	50.74	38.17	0.0000
WYNDE8	504	f	0	1.01	30.57	12.82	0.0000
Subtotal WYNDE8				0.88	81.32	50.99	

Table 1I10 - 5

IESLC - Meta-analysis of Current Smoking, Duration, "Highest vs lowest"
All LC types, Any Product (or Cigarettes if Any not available)
Least adjusted

	N	25
	NS	18
	Wt	417.78
Het	Chi	507.64
Het	df	24
Het	P	***
Fixed	RR	5.27
	RRl	4.79
	RRu	5.80
	P	+++
Random	RR	4.09
	RRl	2.56
	RRu	6.53
	P	+++
Asymm	P	N.S.

Table 1I10 - 6

IESLC - Meta-analysis of Current Smoking, Duration, "Highest vs lowest"
All LC types, Any Product (or Cigarettes if Any not available)
Least adjusted

	Sex combined	male	female	Total
N	2	14	9	25
NS	2	14	9	25
Wt	9.46	325.54	82.78	417.78
Het Chi	1.63	435.92	64.82	507.64
Het df	1	13	8	24
Het P	N.S.	***	***	***
Fixed RR	2.93	5.53	4.66	5.27
RRl	1.55	4.96	3.76	4.79
RRu	5.54	6.17	5.78	5.80
P	+++	+++	+++	+++
Random RR	3.09	3.85	4.77	4.09
RRl	1.33	1.99	2.41	2.56
RRu	7.17	7.45	9.44	6.53
P	++	+++	+++	+++
Between Chi				5.28
Between df				2
Between P				(*)
Btwn(F) P				N.S.
Btwn(R) P				N.S.

Table 1I10 - 7

IESLC - Meta-analysis of Current Smoking, Duration, "Highest vs lowest"
All LC types, Any Product (or Cigarettes if Any not available)
Excluded studies (and stage at which they were excluded)

1	AGUDO ALDERS ARMADA AUVINE AXELSS BARBON BECHER BENHAM BLOT1 BOFFET BOUCHA BRESLO BROWN3 CARPEN CHEN CHEN2 CHIAZZ CHOI CHYOU CORREA DAMBER DARBY DESTEF DOLL DOLL2 DORGAN DOSEME FAN GAO GARCIA GARSHI GENG GER GRAHAM GUO GURSEL HAENSZ HAMMO2 HAMMON HEGMAN HU HU2 JAHN JAIN JEDRYC JOLY JUSSAW KHUDER KOO KOULUM KREUZE LAUSSM LETOUR LEVIN LIU3 LIU4 LIU5 LUBIN LUBIN2 LUO MCCONN NOTAN2 OSANN2 PERNU PEZZOT PRESCO QIAO QIAO2 RACHTA RESTRE SADOWS STASZE SUZUK2 TIZZAN TVERDA VUTUC WANG2 WIGLE WU2 WUWILL WYNDE2 WYNDE3 XU YUAN ZHANG ZHENG ZHOU
2	BENSHL DEAN3 DORN ENGELA GAO2 GILLIS HIRAYA HOLE KAUFMA MIGRAN MRFITR SEGI2 SPEIZE SVENSS WAKAI WU
3	MCDUFF SPITZ WYNDE6
4	AKIBA
6	PISANI
8	BROSS WYNDE7

Table 1I10 - 8
Potentially overlapping studies

REF	REFGP	PRINC	OVERLAP/LINK
WYNDE8	WYNDE6	2	WYNDE5/6/7/8
CPSI	CPSI	1	CPSI overall

Table 1I10 - 9
Most adjusted - insufficient data for meta-analysis

REF	NRR	SEX	AGEL	AGEH	RACE	YF	LC TYPE	LOC	START	ST	NLC	R	VB	P	H	AD	ADOS	PRODUCT	exL	exH	unexL	unexH	De
PISANI	504	c	0	0	all	-	all	Eu:wst	1980	CC	417	n	bl	n	n	10	1	#all/unsp	40	999	1	29	or

Comments on values in listings

PISANI ADOS Number of cigarettes per day

REF	NRR	RR	SIG	RRDATA comment
PISANI	504	1.20		0

Table 1I11 -

IESLC - Meta-analysis of Ever/current Smoking by Duration, Overview
All LC types, Any Product (or Cigarettes if Any not available)

This analysis is restricted to results for:
1) Ever/current smokers
2) Results by Duration
3) Categorical results by Duration
Results by Duration are grouped under 2 schemes (S1, S2). Each scheme has a set of "key values". An interval is allocated to the category whose key value it includes, and intervals which include none or more than one of the key values are excluded. (Open-ended intervals are coded as 999)

S1	key value	maximum range
1	20	1-34
2	35	21-49
3	50	36+

S2	key value	maximum range
1	5	1-19
2	20	6-29
3	30	21-39
4	40	31-49
5	50	41-998
6	999	51+

4) All LC types (or near equivalent)
5) Results complete enough for use in metaanalysis

Within each study, results are then selected (in the following order of preference, within each sex) for:
6) SMKSTA: ever, current
7) PRODUCT: all/unspec, cigarettes regardless of other products, cigarettes only
8) CIGTYPE: all/unspecified, MC regardless of HR, MC only
9) (not applicable)
10) DENOM: never smoked anything, never smoked cigarettes, never any + low, never cigs + low
11) Followup period (YF, prospective studies): whole study (coded as 0) or longest available
12) LCtype: all or nearest available, at least Squamous and Adeno. (q = squamous, s = small, l = large, a = adeno, mix = mixed, alv = alveolar)
13) Race: all or nearest available, otherwise by race (wh or w = white, bl or b = black, hi = hispanic ch = chinese, jap = japanese, haw = hawaiian, w+o = white + oriental, sca = scandinavian, as = asian)
14) For overlapping studies: principal rather than subsidiary studies
Finally by Age: whole study (coded as 0) if available, otherwise by widest available age group and then for single sex results (m, f) in preference to results for both sexes combined (c).

Results adjusted (AD) for the most potential confounders are then chosen in Sections -1 to -3 and results adjusted for the least confounders in Sections -4 to -6. (Those least adjusted results which actually differ from the most adjusted are marked 'x' in column X in Section -4)

Section -7 shows excluded studies, together with the stage (as above) at which no qualifying results were found.

Section -8 lists the potentially overlapping studies which have been included (1=principal, 2=subsidiary).

Section -9 lists any results which would have been included in preference except that they had data not complete enough for use in meta-analysis, with their significance (yes/no), if known, and any further comment as entered on the database. It also lists as "gap" any categories for which no data were presented by the original authors.

In addition to those mentioned above, the following fields, levels and abbreviations are used:
* or nk = not known, n = no, y = yes, ot = other
ev = ever, cu = current, nev = never
all/unspec = all or unspecified, cig+/-ot = cigarettes irrespective of other products (cigar, pipe etc)
MC = manufactured cigarettes, HR = hand-rolled cigarettes
exL, exH = range of exposure (low and high) in the smoking group, in terms of Duration
REF: 6-character study reference
NRR: number of the RR on the database within the study
ST : study type (CC = case control, pr or prosp = prospective)
NLC: number of lung cancer cases in whole study
R : risky occupational population (n = no, m = mining, o = other risky)
VB : national cigarette type (V = at least 75% Virginia, bl = at least 75% blended, ot = other)
P : any proxy use
H : full histological confirmation
De : derivation of RR/CI (or = original, st = standard method, ot = other method of estimation)

Table 1I11 - 1

IESLC - Meta-analysis of Ever/current Smoking by Duration, Overview
All LC types, Any Product (or Cigarettes if Any not available)
Most adjusted

REF	NRR	SEX	AGEL	AGEH	RACE	YF	LC TYPE	LOC	START	ST	NLC	R	VB	P	H	AD	SM	PRODUCT	exL	exH	S1	S2	DENOM	De
AGUDO	510	f	0	0	all	-	all	Eu:wst	1989	CC	103	n	bl	n	n	3	ev	cig only	1	16	0	1	nev cigs	or
AGUDO	511	f	0	0	all	-	all	Eu:wst	1989	CC	103	n	bl	n	n	3	ev	cig only	17	999	0	0	nev cigs	or
AMANDU	506	m	0	0	wh	0	all	NAmer	1959	pr	132	m	bl	n	n	2	cu	cig+/-ot	0	24	1	0	nev cigs	ot
AMANDU	507	m	0	0	wh	0	all	NAmer	1959	pr	132	m	bl	n	n	2	cu	cig+/-ot	25	999	0	0	nev cigs	ot
ARMADA	506	m	0	0	all	-	all	Eu:wst	1986	CC	325	n	bl	n	y	1	ev	cig+/-ot	1	24	1	0	nev cigs	or
ARMADA	507	m	0	0	all	-	all	Eu:wst	1986	CC	325	n	bl	n	y	1	ev	cig+/-ot	25	49	2	0	nev cigs	or
ARMADA	508	m	0	0	all	-	all	Eu:wst	1986	CC	325	n	bl	n	y	1	ev	cig+/-ot	50	999	3	0	nev cigs	or
AUVINE	517	c	0	0	all	-	all	Eu:Sca	1986	CC	517	n	bl	y	n	2	ev	cig+/-ot	1	20	1	0	nev cigs	or
AUVINE	518	c	0	0	all	-	all	Eu:Sca	1986	CC	517	n	bl	y	n	2	ev	cig+/-ot	21	40	2	0	nev cigs	or
AUVINE	519	c	0	0	all	-	all	Eu:Sca	1986	CC	517	n	bl	y	n	2	ev	cig+/-ot	41	999	3	0	nev cigs	or
AXELSS	519	m	0	0	sca	-	all	Eu:Sca	1989	CC	436	n	bl	n	n	6	ev	all/unsp	1	19	0	1	nev any	ot
AXELSS	520	m	0	0	sca	-	all	Eu:Sca	1989	CC	436	n	bl	n	n	6	ev	all/unsp	20	29	1	2	nev any	ot
AXELSS	521	m	0	0	sca	-	all	Eu:Sca	1989	CC	436	n	bl	n	n	6	ev	all/unsp	30	39	2	3	nev any	ot
AXELSS	522	m	0	0	sca	-	all	Eu:Sca	1989	CC	436	n	bl	n	n	6	ev	all/unsp	40	49	0	4	nev any	ot
AXELSS	523	m	0	0	sca	-	all	Eu:Sca	1989	CC	436	n	bl	n	n	6	ev	all/unsp	50	999	3	0	nev any	ot
AXELSS	510	f	0	0	sca	-	all	Eu:Sca	1989	CC	436	n	bl	n	n	0	ev	all/unsp	1	19	0	1	nev any	st
AXELSS	511	f	0	0	sca	-	all	Eu:Sca	1989	CC	436	n	bl	n	n	0	ev	all/unsp	20	29	1	2	nev any	st
AXELSS	512	f	0	0	sca	-	all	Eu:Sca	1989	CC	436	n	bl	n	n	0	ev	all/unsp	30	39	2	3	nev any	st
AXELSS	513	f	0	0	sca	-	all	Eu:Sca	1989	CC	436	n	bl	n	n	0	ev	all/unsp	40	49	0	4	nev any	st
AXELSS	514	f	0	0	sca	-	all	Eu:Sca	1989	CC	436	n	bl	n	n	0	ev	all/unsp	50	999	3	0	nev any	st
BARBON	508	m	0	0	all	-	all	Eu:wst	1979	CC	755	n	bl	y	y	1	ev	all/unsp	1	29	1	0	nev any	or
BARBON	509	m	0	0	all	-	all	Eu:wst	1979	CC	755	n	bl	y	y	1	ev	all/unsp	30	39	2	3	nev any	or
BARBON	510	m	0	0	all	-	all	Eu:wst	1979	CC	755	n	bl	y	y	1	ev	all/unsp	40	49	0	4	nev any	or
BARBON	511	m	0	0	all	-	all	Eu:wst	1979	CC	755	n	bl	y	y	1	ev	all/unsp	50	999	3	0	nev any	or
BEST	501	m	0	0	all	0	all	NAmer	1955	pr	381	n	V	n	n	1	cu	cig only	1	4	0	0	nev any	ot
BEST	502	m	0	0	all	0	all	NAmer	1955	pr	381	n	V	n	n	1	cu	cig only	5	9	0	1	nev any	ot
BEST	503	m	0	0	all	0	all	NAmer	1955	pr	381	n	V	n	n	1	cu	cig only	10	14	0	0	nev any	ot
BEST	504	m	0	0	all	0	all	NAmer	1955	pr	381	n	V	n	n	1	cu	cig only	15	19	0	0	nev any	ot
BEST	505	m	0	0	all	0	all	NAmer	1955	pr	381	n	V	n	n	1	cu	cig only	20	29	1	2	nev any	ot
BEST	506	m	0	0	all	0	all	NAmer	1955	pr	381	n	V	n	n	1	cu	cig only	30	39	2	3	nev any	ot
BEST	507	m	0	0	all	0	all	NAmer	1955	pr	381	n	V	n	n	1	cu	cig only	40	999	3	0	nev any	ot
BOUCOT	518	m	0	0	all	9	all	NAmer	1951	pr	121	n	bl	n	n	0	ev	cig+/-ot	1	39	0	0	nev any	ot
BOUCOT	519	m	0	0	all	9	all	NAmer	1951	pr	121	n	bl	n	n	0	ev	cig+/-ot	40	999	3	0	nev any	ot
BUFFLE	526	f	0	0	w-hi	-	all	NAmer	1976	CC	943	n	bl	y	n	0	ev	cig+/-ot	1	30	1	0	nev cigs	or
BUFFLE	527	f	0	0	w-hi	-	all	NAmer	1976	CC	943	n	bl	y	n	0	ev	cig+/-ot	31	40	2	4	nev cigs	or
BUFFLE	528	f	0	0	w-hi	-	all	NAmer	1976	CC	943	n	bl	y	n	0	ev	cig+/-ot	41	999	3	0	nev cigs	or
CEDERL	501	m	40	69	all	10	all	Eu:Sca	1963	pr	491	n	bl	n	n	1	cu	cig only	1	29	1	0	nev any	ot
CEDERL	502	m	40	69	all	10	all	Eu:Sca	1963	pr	491	n	bl	n	n	1	cu	cig only	30	999	0	0	nev any	ot
CEDERL	504	f	40	69	all	10	all	Eu:Sca	1963	pr	491	n	bl	n	n	1	cu	cig only	1	29	1	0	nev any	ot
CEDERL	505	f	40	69	all	10	all	Eu:Sca	1963	pr	491	n	bl	n	n	1	cu	cig only	30	999	0	0	nev any	ot
CHEN2	501	m	0	0	all	-	all	As:Chi	1983	CC	193	n	ot	y	n	0	ev	all/unsp	1	9	0	1	nev any	st
CHEN2	502	m	0	0	all	-	all	As:Chi	1983	CC	193	n	ot	y	n	0	ev	all/unsp	10	20	1	2	nev any	st
CHEN2	503	m	0	0	all	-	all	As:Chi	1983	CC	193	n	ot	y	n	0	ev	all/unsp	21	30	0	3	nev any	st
CHEN2	504	m	0	0	all	-	all	As:Chi	1983	CC	193	n	ot	y	n	0	ev	all/unsp	31	40	2	4	nev any	st
CHEN2	505	m	0	0	all	-	all	As:Chi	1983	CC	193	n	ot	y	n	0	ev	all/unsp	41	999	3	0	nev any	st
CHEN2	510	f	0	0	all	-	all	As:Chi	1983	CC	193	n	ot	y	n	0	ev	all/unsp	1	20	1	0	nev any	st
CHEN2	511	f	0	0	all	-	all	As:Chi	1983	CC	193	n	ot	y	n	0	ev	all/unsp	21	30	0	3	nev any	st
CHEN2	512	f	0	0	all	-	all	As:Chi	1983	CC	193	n	ot	y	n	0	ev	all/unsp	31	40	2	4	nev any	st
CHEN2	513	f	0	0	all	-	all	As:Chi	1983	CC	193	n	ot	y	n	0	ev	all/unsp	41	999	3	0	nev any	st
CHOI	501	m	0	0	all	-	all	As:oth	1985	CC	375	n	bl	n	n	0	ev	cig+/-ot	1	19	0	1	nev cigs	st
CHOI	502	m	0	0	all	-	all	As:oth	1985	CC	375	n	bl	n	n	0	ev	cig+/-ot	20	29	1	2	nev cigs	st
CHOI	503	m	0	0	all	-	all	As:oth	1985	CC	375	n	bl	n	n	0	ev	cig+/-ot	30	39	2	3	nev cigs	st
CHOI	504	m	0	0	all	-	all	As:oth	1985	CC	375	n	bl	n	n	0	ev	cig+/-ot	40	49	0	4	nev cigs	st
CHOI	505	m	0	0	all	-	all	As:oth	1985	CC	375	n	bl	n	n	0	ev	cig+/-ot	50	999	3	0	nev cigs	st
CHOI	510	f	0	0	all	-	all	As:oth	1985	CC	375	n	bl	n	n	0	ev	cig+/-ot	1	19	0	1	nev cigs	st
CHOI	511	f	0	0	all	-	all	As:oth	1985	CC	375	n	bl	n	n	0	ev	cig+/-ot	20	29	1	2	nev cigs	st
CHOI	512	f	0	0	all	-	all	As:oth	1985	CC	375	n	bl	n	n	0	ev	cig+/-ot	30	39	2	3	nev cigs	st
CHOI	513	f	0	0	all	-	all	As:oth	1985	CC	375	n	bl	n	n	0	ev	cig+/-ot	40	999	3	0	nev cigs	st
CPSI	580	m	40	84	wh	0	all	NAmer	1959	pr	5138	n	bl	n	n	0	cu	cig only	1	29	1	0	nev cigs	st
CPSI	581	m	40	84	wh	0	all	NAmer	1959	pr	5138	n	bl	n	n	0	cu	cig only	30	34	0	3	nev cigs	st
CPSI	582	m	40	84	wh	0	all	NAmer	1959	pr	5138	n	bl	n	n	0	cu	cig only	35	39	2	0	nev cigs	st
CPSI	583	m	40	84	wh	0	all	NAmer	1959	pr	5138	n	bl	n	n	0	cu	cig only	40	44	0	4	nev cigs	st
CPSI	584	m	40	84	wh	0	all	NAmer	1959	pr	5138	n	bl	n	n	0	cu	cig only	45	49	0	0	nev cigs	st
CPSI	585	m	40	84	wh	0	all	NAmer	1959	pr	5138	n	bl	n	n	0	cu	cig only	50	54	3	5	nev cigs	st
CPSI	586	m	40	84	wh	0	all	NAmer	1959	pr	5138	n	bl	n	n	0	cu	cig only	55	59	0	0	nev cigs	st
CPSI	587	m	40	84	wh	0	all	NAmer	1959	pr	5138	n	bl	n	n	0	cu	cig only	60	999	0	6	nev cigs	st
CPSI	676	f	40	84	wh	0	all	NAmer	1959	pr	5138	n	bl	n	n	0	cu	cig only	1	29	1	0	nev cigs	st
CPSI	677	f	40	84	wh	0	all	NAmer	1959	pr	5138	n	bl	n	n	0	cu	cig only	30	34	0	3	nev cigs	st
CPSI	678	f	40	84	wh	0	all	NAmer	1959	pr	5138	n	bl	n	n	0	cu	cig only	35	39	2	0	nev cigs	st
CPSI	679	f	40	84	wh	0	all	NAmer	1959	pr	5138	n	bl	n	n	0	cu	cig only	40	44	0	4	nev cigs	st
CPSI	680	f	40	84	wh	0	all	NAmer	1959	pr	5138	n	bl	n	n	0	cu	cig only	45	49	0	0	nev cigs	st
CPSI	681	f	40	84	wh	0	all	NAmer	1959	pr	5138	n	bl	n	n	0	cu	cig only	50	54	3	5	nev cigs	st

Table 1I11 - 1

IESLC - Meta-analysis of Ever/current Smoking by Duration, Overview
All LC types, Any Product (or Cigarettes if Any not available)
Most adjusted

REF	NRR	SEX	AGEL	AGEH	RACE	YF	LC TYPE	LOC	START	ST	NLC	R	VB	P	H	AD	SM	PRODUCT	exL	exH	S1	S2	DENOM	De
CPSI	682	f	40	84	wh	0	all	NAmer	1959	pr	5138	n	bl	n	n	0	cu	cig only	55	999	0	6	nev cigs	st
CPSII	552	m	0	0	all	6	all	NAmer	1982	pr	3229	n	bl	n	n	0	cu	cig only	1	29	1	0	nev any	st
CPSII	553	m	0	0	all	6	all	NAmer	1982	pr	3229	n	bl	n	n	0	cu	cig only	30	34	0	3	nev any	st
CPSII	554	m	0	0	all	6	all	NAmer	1982	pr	3229	n	bl	n	n	0	cu	cig only	35	39	2	0	nev any	st
CPSII	555	m	0	0	all	6	all	NAmer	1982	pr	3229	n	bl	n	n	0	cu	cig only	40	44	0	4	nev any	st
CPSII	556	m	0	0	all	6	all	NAmer	1982	pr	3229	n	bl	n	n	0	cu	cig only	45	49	0	0	nev any	st
CPSII	557	m	0	0	all	6	all	NAmer	1982	pr	3229	n	bl	n	n	0	cu	cig only	50	54	3	5	nev any	st
CPSII	558	m	0	0	all	6	all	NAmer	1982	pr	3229	n	bl	n	n	0	cu	cig only	55	59	0	0	nev any	st
CPSII	559	m	0	0	all	6	all	NAmer	1982	pr	3229	n	bl	n	n	0	cu	cig only	60	999	0	6	nev any	st
CPSII	618	f	0	0	all	6	all	NAmer	1982	pr	3229	n	bl	n	n	0	cu	cig+/-ot	1	29	1	0	nev cigs	st
CPSII	619	f	0	0	all	6	all	NAmer	1982	pr	3229	n	bl	n	n	0	cu	cig+/-ot	30	34	0	3	nev cigs	st
CPSII	620	f	0	0	all	6	all	NAmer	1982	pr	3229	n	bl	n	n	0	cu	cig+/-ot	35	39	2	0	nev cigs	st
CPSII	621	f	0	0	all	6	all	NAmer	1982	pr	3229	n	bl	n	n	0	cu	cig+/-ot	40	44	0	4	nev cigs	st
CPSII	622	f	0	0	all	6	all	NAmer	1982	pr	3229	n	bl	n	n	0	cu	cig+/-ot	45	49	0	0	nev cigs	st
CPSII	623	f	0	0	all	6	all	NAmer	1982	pr	3229	n	bl	n	n	0	cu	cig+/-ot	50	54	3	5	nev cigs	st
CPSII	624	f	0	0	all	6	all	NAmer	1982	pr	3229	n	bl	n	n	0	cu	cig+/-ot	55	59	0	0	nev cigs	st
CPSII	625	f	0	0	all	6	all	NAmer	1982	pr	3229	n	bl	n	n	0	cu	cig+/-ot	60	999	0	6	nev cigs	st
DAMBER	506	m	0	0	all	-	all	Eu:Sca	1972	CC	579	n	bl	y	n	1	ev	all/unsp	1	20	1	0	nev any	ot
DAMBER	507	m	0	0	all	-	all	Eu:Sca	1972	CC	579	n	bl	y	n	1	ev	all/unsp	21	30	0	3	nev any	ot
DAMBER	508	m	0	0	all	-	all	Eu:Sca	1972	CC	579	n	bl	y	n	1	ev	all/unsp	31	40	2	4	nev any	ot
DAMBER	509	m	0	0	all	-	all	Eu:Sca	1972	CC	579	n	bl	y	n	1	ev	all/unsp	41	50	3	5	nev any	ot
DAMBER	510	m	0	0	all	-	all	Eu:Sca	1972	CC	579	n	bl	y	n	1	ev	all/unsp	51	999	0	6	nev any	ot
DEAN2	501	m	0	0	all	-	all	Eu:UK	1960	CC	954	n	V	y	n	0	cu	all/unsp	1	19	0	1	nev any	st
DEAN2	502	m	0	0	all	-	all	Eu:UK	1960	CC	954	n	V	y	n	0	cu	all/unsp	20	999	0	0	nev any	st
DEAN2	504	f	0	0	all	-	all	Eu:UK	1960	CC	954	n	V	y	n	0	cu	all/unsp	1	19	0	1	nev any	st
DEAN2	505	f	0	0	all	-	all	Eu:UK	1960	CC	954	n	V	y	n	0	cu	all/unsp	20	999	0	0	nev any	st
DESTEF	508	m	0	0	all	-	all	SCAmer	1988	CC	497	n	bl	n	y	4	ev	all/unsp	1	29	1	0	nev any	or
DESTEF	509	m	0	0	all	-	all	SCAmer	1988	CC	497	n	bl	n	y	4	ev	all/unsp	30	39	2	3	nev any	or
DESTEF	510	m	0	0	all	-	all	SCAmer	1988	CC	497	n	bl	n	y	4	ev	all/unsp	40	49	0	4	nev any	or
DESTEF	511	m	0	0	all	-	all	SCAmer	1988	CC	497	n	bl	n	y	4	ev	all/unsp	50	999	3	0	nev any	or
DOLL	515	m	0	0	all	-	all	Eu:UK	1948	CC	1465	n	V	n	n	0	ev	all/unsp	1	9	0	1	nev any	st
DOLL	516	m	0	0	all	-	all	Eu:UK	1948	CC	1465	n	V	n	n	0	ev	all/unsp	10	19	0	0	nev any	st
DOLL	517	m	0	0	all	-	all	Eu:UK	1948	CC	1465	n	V	n	n	0	ev	all/unsp	20	39	0	0	nev any	st
DOLL	518	m	0	0	all	-	all	Eu:UK	1948	CC	1465	n	V	n	n	0	ev	all/unsp	40	999	3	0	nev any	st
DOLL	522	f	0	0	all	-	all	Eu:UK	1948	CC	1465	n	V	n	n	0	ev	all/unsp	1	9	0	1	nev any	st
DOLL	523	f	0	0	all	-	all	Eu:UK	1948	CC	1465	n	V	n	n	0	ev	all/unsp	10	19	0	0	nev any	st
DOLL	524	f	0	0	all	-	all	Eu:UK	1948	CC	1465	n	V	n	n	0	ev	all/unsp	20	39	0	0	nev any	st
DOLL	525	f	0	0	all	-	all	Eu:UK	1948	CC	1465	n	V	n	n	0	ev	all/unsp	40	999	3	0	nev any	st
DORGAN	570	m	0	0	wh	-	all	NAmer	1980	CC	2026	n	bl	y	y	2	ev	cig+/-ot	1	34	1	0	nev any	ot
DORGAN	571	m	0	0	wh	-	all	NAmer	1980	CC	2026	n	bl	y	y	2	ev	cig+/-ot	35	999	0	0	nev any	ot
DORGAN	562	f	0	0	all	-	all	NAmer	1980	CC	2026	n	bl	y	y	3	ev	cig+/-ot	1	34	1	0	nev any	ot
DORGAN	563	f	0	0	all	-	all	NAmer	1980	CC	2026	n	bl	y	y	3	ev	cig+/-ot	35	999	0	0	nev any	ot
DOSEME	501	m	0	0	all	-	all	Eu:bal	1979	CC	1210	n	bl	n	n	2	ev	cig+/-ot	1	10	0	1	nev cigs	or
DOSEME	502	m	0	0	all	-	all	Eu:bal	1979	CC	1210	n	bl	n	n	2	ev	cig+/-ot	11	20	1	2	nev cigs	or
DOSEME	503	m	0	0	all	-	all	Eu:bal	1979	CC	1210	n	bl	n	n	2	ev	cig+/-ot	21	999	0	0	nev cigs	or
FAN	501	m	0	0	all	-	all	As:Chi	1990	CC	403	n	ot	y	n	0	ev	cig+/-ot	1	29	1	0	nev cigs	st
FAN	502	m	0	0	all	-	all	As:Chi	1990	CC	403	n	ot	y	n	0	ev	cig+/-ot	30	39	2	3	nev cigs	st
FAN	503	m	0	0	all	-	all	As:Chi	1990	CC	403	n	ot	y	n	0	ev	cig+/-ot	40	999	3	0	nev cigs	st
FAN	506	f	0	0	all	-	all	As:Chi	1990	CC	403	n	ot	y	n	0	ev	cig+/-ot	1	29	1	0	nev cigs	st
FAN	507	f	0	0	all	-	all	As:Chi	1990	CC	403	n	ot	y	n	0	ev	cig+/-ot	30	39	2	3	nev cigs	st
FAN	508	f	0	0	all	-	all	As:Chi	1990	CC	403	n	ot	y	n	0	ev	cig+/-ot	40	999	3	0	nev cigs	st
GAO	564	f	0	0	all	-	all	As:Chi	1984	CC	1405	n	ot	n	n	2	ev	cig+/-ot	1	29	1	0	nev cigs	ot
GAO	565	f	0	0	all	-	all	As:Chi	1984	CC	1405	n	ot	n	n	2	ev	cig+/-ot	30	999	0	0	nev cigs	ot
GARSHI	536	m	0	0	all	-	all	NAmer	1981	CC	1081	o	bl	y	n	1	ev	all/unsp	20	999	0	0	nev any	st
GER	518	c	0	0	all	-	all	As:oth	1990	CC	141	n	ot	y	n	5	ev	all/unsp	1	20	1	0	nev any	ot
GER	519	c	0	0	all	-	all	As:oth	1990	CC	141	n	ot	y	n	5	ev	all/unsp	21	40	2	0	nev any	ot
GER	520	c	0	0	all	-	all	As:oth	1990	CC	141	n	ot	y	n	5	ev	all/unsp	41	999	3	0	nev any	ot
HAENSZ	542	f	0	0	all	-	not alv	NAmer	1955	CC	158	n	bl	n	y	0	ev	cig+/-ot	1	14	0	1	nev any	st
HAENSZ	543	f	0	0	all	-	not alv	NAmer	1955	CC	158	n	bl	n	y	0	ev	cig+/-ot	15	999	0	0	nev any	st
HU	501	m	0	0	all	-	all	As:Chi	1985	CC	227	n	ot	n	y	0	ev	cig+/-ot	1	19	0	1	nev cigs	st
HU	502	m	0	0	all	-	all	As:Chi	1985	CC	227	n	ot	n	y	0	ev	cig+/-ot	20	29	1	2	nev cigs	st
HU	503	m	0	0	all	-	all	As:Chi	1985	CC	227	n	ot	n	y	0	ev	cig+/-ot	30	999	0	0	nev cigs	st
HU	506	f	0	0	all	-	all	As:Chi	1985	CC	227	n	ot	n	y	0	ev	cig+/-ot	1	19	0	1	nev cigs	st
HU	507	f	0	0	all	-	all	As:Chi	1985	CC	227	n	ot	n	y	0	ev	cig+/-ot	20	29	1	2	nev cigs	st
HU	508	f	0	0	all	-	all	As:Chi	1985	CC	227	n	ot	n	y	0	ev	cig+/-ot	30	999	0	0	nev cigs	st
HU2	508	c	0	0	all	-	all	As:Chi	1977	CC	523	n	ot	y	n	0	ev	cig+/-ot	1	19	0	1	nev cigs	ot
HU2	509	c	0	0	all	-	all	As:Chi	1977	CC	523	n	ot	y	n	0	ev	cig+/-ot	20	29	1	2	nev cigs	or
HU2	510	c	0	0	all	-	all	As:Chi	1977	CC	523	n	ot	y	n	0	ev	cig+/-ot	30	39	2	3	nev cigs	or
HU2	511	c	0	0	all	-	all	As:Chi	1977	CC	523	n	ot	y	n	0	ev	cig+/-ot	40	999	3	0	nev cigs	st
HUMBLE	542	c	0	0	wh	-	not alv	NAmer	1980	CC	521	n	bl	y	n	3	cu	cig+/-ot	1	29	1	0	nev cigs	ot
HUMBLE	543	c	0	0	wh	-	not alv	NAmer	1980	CC	521	n	bl	y	n	3	cu	cig+/-ot	30	39	2	3	nev cigs	ot
HUMBLE	544	c	0	0	wh	-	not alv	NAmer	1980	CC	521	n	bl	y	n	3	cu	cig+/-ot	40	49	0	4	nev cigs	ot

Table 1I11 - 1

IESLC - Meta-analysis of Ever/current Smoking by Duration, Overview
All LC types, Any Product (or Cigarettes if Any not available)
Most adjusted

REF	NRR	SEX	AGEL	AGEH	RACE	YF	LC TYPE	LOC	START	ST	NLC	R	VB	P	H	AD	SM	PRODUCT	exL	exH	S1	S2	DENOM	De
HUMBLE	545	c	0	0	wh	-	not alv	NAmer	1980	CC	521	n	bl	y	n	3	cu	cig+/-ot	50	999	3	0	nev cigs	ot
JOLY	515	m	0	0	all	-	all	SCAmer	1978	CC	826	n	bl	n	n	0	ev	cig+/-ot	1	19	0	1	nev any	st
JOLY	516	m	0	0	all	-	all	SCAmer	1978	CC	826	n	bl	n	n	0	ev	cig+/-ot	20	29	1	2	nev any	st
JOLY	517	m	0	0	all	-	all	SCAmer	1978	CC	826	n	bl	n	n	0	ev	cig+/-ot	30	39	2	3	nev any	st
JOLY	518	m	0	0	all	-	all	SCAmer	1978	CC	826	n	bl	n	n	0	ev	cig+/-ot	40	49	0	4	nev any	st
JOLY	519	m	0	0	all	-	all	SCAmer	1978	CC	826	n	bl	n	n	0	ev	cig+/-ot	50	999	3	0	nev any	st
JOLY	501	f	0	0	all	-	all	SCAmer	1978	CC	826	n	bl	n	n	0	ev	cig+/-ot	1	19	0	1	nev any	st
JOLY	502	f	0	0	all	-	all	SCAmer	1978	CC	826	n	bl	n	n	0	ev	cig+/-ot	20	29	1	2	nev any	st
JOLY	503	f	0	0	all	-	all	SCAmer	1978	CC	826	n	bl	n	n	0	ev	cig+/-ot	30	39	2	3	nev any	st
JOLY	504	f	0	0	all	-	all	SCAmer	1978	CC	826	n	bl	n	n	0	ev	cig+/-ot	40	49	0	4	nev any	st
JOLY	505	f	0	0	all	-	all	SCAmer	1978	CC	826	n	bl	n	n	0	ev	cig+/-ot	50	999	3	0	nev any	st
JUSSAW	510	m	0	0	all	-	all	As:Ind	1964	CC	792	n	V	n	n	0	ev	cig only	1	9	0	1	nev any	st
JUSSAW	511	m	0	0	all	-	all	As:Ind	1964	CC	792	n	V	n	n	0	ev	cig only	10	19	0	0	nev any	st
JUSSAW	512	m	0	0	all	-	all	As:Ind	1964	CC	792	n	V	n	n	0	ev	cig only	20	29	1	2	nev any	st
JUSSAW	513	m	0	0	all	-	all	As:Ind	1964	CC	792	n	V	n	n	0	ev	cig only	30	39	2	3	nev any	st
JUSSAW	514	m	0	0	all	-	all	As:Ind	1964	CC	792	n	V	n	n	0	ev	cig only	40	999	3	0	nev any	st
KAISE2	596	m	0	0	all	9	all	NAmer	1979	pr	318	n	bl	n	n	1	cu	cig only	1	39	0	0	nev any	st
KAISE2	597	m	0	0	all	9	all	NAmer	1979	pr	318	n	bl	n	n	1	cu	cig only	40	999	3	0	nev any	st
KAISE2	516	f	0	0	all	9	all	NAmer	1979	pr	318	n	bl	n	n	1	cu	cig only	1	39	0	0	nev any	st
KAISE2	517	f	0	0	all	9	all	NAmer	1979	pr	318	n	bl	n	n	1	cu	cig only	40	999	3	0	nev any	st
KATSOU	512	f	0	0	all	-	all	Eu:bal	1987	CC	101	n	bl	n	n	1	cu	all/unsp	1	29	1	0	nev any	or
KATSOU	513	f	0	0	all	-	all	Eu:bal	1987	CC	101	n	bl	n	n	1	cu	all/unsp	30	999	0	0	nev any	or
KHUDER	501	m	0	0	all	-	all	NAmer	1985	CC	482	n	bl	n	y	0	ev	cig+/-ot	1	29	1	0	nev cigs	st
KHUDER	502	m	0	0	all	-	all	NAmer	1985	CC	482	n	bl	n	y	0	ev	cig+/-ot	30	49	2	0	nev cigs	st
KHUDER	503	m	0	0	all	-	all	NAmer	1985	CC	482	n	bl	n	y	0	ev	cig+/-ot	50	999	3	0	nev cigs	st
KREUZE	517	m	0	0	all	-	all	Eu:Ger	1990	CC	2260	n	bl	n	n	3	ev	all/unsp	1	19	0	1	nev any	st
KREUZE	518	m	0	0	all	-	all	Eu:Ger	1990	CC	2260	n	bl	n	n	3	ev	all/unsp	20	999	0	0	nev any	ot
KREUZE	520	f	0	0	all	-	all	Eu:Ger	1990	CC	2260	n	bl	n	n	3	ev	all/unsp	1	19	0	1	nev any	ot
KREUZE	521	f	0	0	all	-	all	Eu:Ger	1990	CC	2260	n	bl	n	n	3	ev	all/unsp	20	999	0	0	nev any	ot
LETOUR	506	c	0	0	all	-	all	NAmer	1983	CC	738	n	V	y	y	0	ev	cig+/-ot	1	24	1	0	nev cigs	st
LETOUR	507	c	0	0	all	-	all	NAmer	1983	CC	738	n	V	y	y	0	ev	cig+/-ot	25	40	2	0	nev cigs	st
LETOUR	508	c	0	0	all	-	all	NAmer	1983	CC	738	n	V	y	y	0	ev	cig+/-ot	41	999	3	0	nev cigs	st
LEVIN	506	m	0	0	all	-	all	NAmer	1938	CC	475	n	bl	n	n	1	ev	cig+/-ot	1	39	0	0	nev any	ot
LEVIN	507	m	0	0	all	-	all	NAmer	1938	CC	475	n	bl	n	n	1	ev	cig+/-ot	40	999	3	0	nev any	ot
LIAW	501	c	0	0	all	0	all	As:oth	1982	pr	127	n	ot	n	n	2	cu	all/unsp	1	20	1	0	nev any	or
LIAW	502	c	0	0	all	0	all	As:oth	1982	pr	127	n	ot	n	n	2	cu	all/unsp	21	30	0	3	nev any	or
LIAW	503	c	0	0	all	0	all	As:oth	1982	pr	127	n	ot	n	n	2	cu	all/unsp	31	999	0	0	nev any	or
LIU3	510	m	0	0	all	-	all	As:Chi	1985	CC	110	n	ot	n	n	2	ev	all/unsp	1	34	1	0	nev any	or
LIU3	511	m	0	0	all	-	all	As:Chi	1985	CC	110	n	ot	n	n	2	ev	all/unsp	35	999	0	0	nev any	or
LIU5	504	c	0	0	all	-	all	As:Chi	1978	CC	111	n	ot	y	n	0	ev	all/unsp	1	29	1	0	nev any	st
LIU5	505	c	0	0	all	-	all	As:Chi	1978	CC	111	n	ot	y	n	0	ev	all/unsp	30	999	0	0	nev any	st
LUBIN	508	m	0	0	all	-	all	As:Chi	1984	CC	427	m	ot	y	n	0	ev	cig+/-ot	1	29	1	0	nev any	st
LUBIN	509	m	0	0	all	-	all	As:Chi	1984	CC	427	m	ot	y	n	0	ev	cig+/-ot	30	39	2	3	nev any	st
LUBIN	510	m	0	0	all	-	all	As:Chi	1984	CC	427	m	ot	y	n	0	ev	cig+/-ot	40	49	0	4	nev any	st
LUBIN	511	m	0	0	all	-	all	As:Chi	1984	CC	427	m	ot	y	n	0	ev	cig+/-ot	50	999	3	0	nev any	st
LUBIN2	531	m	0	0	all	-	all	Eu:mul	1976	CC	7804	n	bl	n	y	0	ev	cig+/-ot	1	29	1	0	nev any	st
LUBIN2	532	m	0	0	all	-	all	Eu:mul	1976	CC	7804	n	bl	n	y	0	ev	cig+/-ot	30	39	2	3	nev any	st
LUBIN2	533	m	0	0	all	-	all	Eu:mul	1976	CC	7804	n	bl	n	y	0	ev	cig+/-ot	40	49	0	4	nev any	st
LUBIN2	534	m	0	0	all	-	all	Eu:mul	1976	CC	7804	n	bl	n	y	0	ev	cig+/-ot	50	999	3	0	nev any	st
LUBIN2	574	f	0	0	all	-	all	Eu:mul	1976	CC	7804	n	bl	n	y	0	ev	cig+/-ot	1	29	1	0	nev any	st
LUBIN2	575	f	0	0	all	-	all	Eu:mul	1976	CC	7804	n	bl	n	y	0	ev	cig+/-ot	30	39	2	3	nev any	st
LUBIN2	576	f	0	0	all	-	all	Eu:mul	1976	CC	7804	n	bl	n	y	0	ev	cig+/-ot	40	49	0	4	nev any	st
LUBIN2	577	f	0	0	all	-	all	Eu:mul	1976	CC	7804	n	bl	n	y	0	ev	cig+/-ot	50	999	3	0	nev any	st
MATOS	536	m	0	0	all	-	all	SCAmer	1994	CC	200	n	bl	n	n	2	ev	cig+/-ot	1	24	1	0	nev any	or
MATOS	537	m	0	0	all	-	all	SCAmer	1994	CC	200	n	bl	n	n	2	ev	cig+/-ot	25	39	2	3	nev any	or
MATOS	538	m	0	0	all	-	all	SCAmer	1994	CC	200	n	bl	n	n	2	ev	cig+/-ot	40	70	3	0	nev any	or
MCCONN	501	c	0	0	all	-	all	Eu:UK	1946	CC	100	n	V	n	y	0	ev	all/unsp	1	9	0	1	nev any	st
MCCONN	502	c	0	0	all	-	all	Eu:UK	1946	CC	100	n	V	n	y	0	ev	all/unsp	10	19	0	0	nev any	st
MCCONN	503	c	0	0	all	-	all	Eu:UK	1946	CC	100	n	V	n	y	0	ev	all/unsp	20	29	1	2	nev any	st
MCCONN	504	c	0	0	all	-	all	Eu:UK	1946	CC	100	n	V	n	y	0	ev	all/unsp	30	39	2	3	nev any	st
MCCONN	505	c	0	0	all	-	all	Eu:UK	1946	CC	100	n	V	n	y	0	ev	all/unsp	40	999	3	0	nev any	st
NOTAN2	513	c	0	0	all	-	all	As:Ind	1963	CC	683	n	V	n	n	0	ev	cig only	1	10	0	1	nev any	st
NOTAN2	514	c	0	0	all	-	all	As:Ind	1963	CC	683	n	V	n	n	0	ev	cig only	11	20	1	2	nev any	st
NOTAN2	515	c	0	0	all	-	all	As:Ind	1963	CC	683	n	V	n	n	0	ev	cig only	21	30	0	3	nev any	st
NOTAN2	516	c	0	0	all	-	all	As:Ind	1963	CC	683	n	V	n	n	0	ev	cig only	31	40	2	4	nev any	st
NOTAN2	517	c	0	0	all	-	all	As:Ind	1963	CC	683	n	V	n	n	0	ev	cig only	41	999	3	0	nev any	st
OSANN2	504	f	0	0	all	-	all	NAmer	1964	ot	217	n	bl	n	y	1	ev	cig+/-ot	1	20	1	0	nev cigs	or
OSANN2	505	f	0	0	all	-	all	NAmer	1964	ot	217	n	bl	n	y	1	ev	cig+/-ot	21	999	0	0	nev cigs	or
PEZZO2	507	m	0	0	all	-	all	SCAmer	1992	CC	367	n	bl	n	y	0	cu	cig+/-ot	1	35	0	0	nev cigs	st
PEZZO2	508	m	0	0	all	-	all	SCAmer	1992	CC	367	n	bl	n	y	0	cu	cig+/-ot	36	999	3	0	nev cigs	st
PEZZOT	534	m	0	0	all	-	all	SCAmer	1987	CC	215	n	bl	n	y	0	ev	cig only	1	30	1	0	nev cigs	st
PEZZOT	535	m	0	0	all	-	all	SCAmer	1987	CC	215	n	bl	n	y	0	ev	cig only	31	40	2	4	nev cigs	st

Table 1I11 - 1

IESLC - Meta-analysis of Ever/current Smoking by Duration, Overview
All LC types, Any Product (or Cigarettes if Any not available)
Most adjusted

REF	NRR	SEX	AGEL	AGEH	RACE	YF	LC TYPE	LOC	START	ST	NLC	R	VB	P	H	AD	SM	PRODUCT	exL	exH	S1	S2	DENOM	De
PEZZOT	536	m	0	0	all	-	all	SCAmer	1987	CC	215	n	bl	n	y	0	ev	cig only	41	999	3	0	nev cigs	st
QIAO2	516	m	0	0	all	0	all	As:Chi	1992	pr	241	m	ot	n	n	1	ev	all/unsp	1	27	1	0	nev any	or
QIAO2	517	m	0	0	all	0	all	As:Chi	1992	pr	241	m	ot	n	n	1	ev	all/unsp	28	41	2	0	nev any	or
QIAO2	518	m	0	0	all	0	all	As:Chi	1992	pr	241	m	ot	n	n	1	ev	all/unsp	42	999	3	0	nev any	or
RACHTA	516	f	0	0	all	-	all	Eu:est	1991	CC	118	n	bl	n	y	1	ev	cig+/-ot	1	20	1	0	nev cigs	or
RACHTA	517	f	0	0	all	-	all	Eu:est	1991	CC	118	n	bl	n	y	1	ev	cig+/-ot	21	40	2	0	nev cigs	or
RACHTA	518	f	0	0	all	-	all	Eu:est	1991	CC	118	n	bl	n	y	1	ev	cig+/-ot	41	999	3	0	nev cigs	or
SOBUE	546	m	0	0	all	-	q+s+l+a	As:Jap	1986	CC	1376	n	bl	n	y	0	cu	cig+/-ot	1	29	1	0	nev cigs	st
SOBUE	547	m	0	0	all	-	q+s+l+a	As:Jap	1986	CC	1376	n	bl	n	y	0	cu	cig+/-ot	30	39	2	3	nev cigs	st
SOBUE	548	m	0	0	all	-	q+s+l+a	As:Jap	1986	CC	1376	n	bl	n	y	0	cu	cig+/-ot	40	49	0	4	nev cigs	st
SOBUE	549	m	0	0	all	-	q+s+l+a	As:Jap	1986	CC	1376	n	bl	n	y	0	cu	cig+/-ot	50	999	3	0	nev cigs	st
TIZZAN	501	m	0	0	all	-	all	Eu:wst	1959	CC	1358	n	bl	n	n	0	ev	cig only	1	4	0	0	nev any	st
TIZZAN	502	m	0	0	all	-	all	Eu:wst	1959	CC	1358	n	bl	n	n	0	ev	cig only	5	10	0	1	nev any	st
TIZZAN	503	m	0	0	all	-	all	Eu:wst	1959	CC	1358	n	bl	n	n	0	ev	cig only	11	999	0	0	nev any	st
TIZZAN	533	f	0	0	all	-	all	Eu:wst	1959	CC	1358	n	bl	n	n	0	ev	all/unsp	1	10	0	1	nev any	st
TIZZAN	534	f	0	0	all	-	all	Eu:wst	1959	CC	1358	n	bl	n	n	0	ev	all/unsp	11	999	0	0	nev any	st
WANG2	501	c	0	0	all	-	all	As:Chi	1980	CC	103	n	ot	n	n	0	ev	cig+/-ot	1	19	0	1	nev cigs	st
WANG2	503	c	0	0	all	-	all	As:Chi	1980	CC	103	n	ot	n	n	0	ev	cig+/-ot	20	29	1	2	nev cigs	st
WANG2	504	c	0	0	all	-	all	As:Chi	1980	CC	103	n	ot	n	n	0	ev	cig+/-ot	30	39	2	3	nev cigs	st
WANG2	505	c	0	0	all	-	all	As:Chi	1980	CC	103	n	ot	n	n	0	ev	cig+/-ot	40	49	0	4	nev cigs	st
WUWILL	516	f	0	0	all	-	all	As:Chi	1985	CC	965	n	ot	n	n	3	ev	cig+/-ot	1	29	1	0	nev cigs	ot
WUWILL	517	f	0	0	all	-	all	As:Chi	1985	CC	965	n	ot	n	n	3	ev	cig+/-ot	30	39	2	3	nev cigs	ot
WUWILL	518	f	0	0	all	-	all	As:Chi	1985	CC	965	n	ot	n	n	3	ev	cig+/-ot	40	999	3	0	nev cigs	ot
ZHENG	553	m	0	0	all	-	all	As:Chi	1982	CC	540	n	ot	*	y	0	ev	cig+/-ot	1	29	1	0	nev cigs	st
ZHENG	554	m	0	0	all	-	all	As:Chi	1982	CC	540	n	ot	*	y	0	ev	cig+/-ot	30	999	0	0	nev cigs	st
ZHENG	558	f	0	0	all	-	all	As:Chi	1982	CC	540	n	ot	*	y	0	ev	cig+/-ot	1	29	1	0	nev cigs	st
ZHENG	559	f	0	0	all	-	all	As:Chi	1982	CC	540	n	ot	*	y	0	ev	cig+/-ot	30	999	0	0	nev cigs	st
ZHOU	501	c	0	0	all	-	all	As:Chi	1978	CC	1360	n	ot	n	n	0	ev	all/unsp	1	19	0	1	nev any	st
ZHOU	502	c	0	0	all	-	all	As:Chi	1978	CC	1360	n	ot	n	n	0	ev	all/unsp	20	999	0	0	nev any	st

Cigarette type is all/unspec for all RRs

except for the following:

REF	NRR	CIGTYPE
JUSSAW	510	MC only
JUSSAW	511	MC only
JUSSAW	512	MC only
JUSSAW	513	MC only
JUSSAW	514	MC only
NOTAN2	513	MC only
NOTAN2	514	MC only
NOTAN2	515	MC only
NOTAN2	516	MC only
NOTAN2	517	MC only

In this overview table, subtotals and Qs values may be invalid and should be ignored

Table 1I11 - 2

IESLC - Meta-analysis of Ever/current Smoking by Duration, Overview
All LC types, Any Product (or Cigarettes if Any not available)
Most adjusted

REF	NRR	SEX	AD	Number Exposed Case	Cont	Non-exposed Case	Cont	RR	95.00%CI
AGUDO	510	f	3	5	-	80	-	1.29	(0.40- 4.17)
AGUDO	511	f	3	18	-	80	-	5.09	(1.94- 13.35)
Subtotal AGUDO								2.92	(1.39- 6.16)
*AMANDU	506	m	2	42	-	6	-	5.92	(2.13- 16.47)
*AMANDU	507	m	2	72	-	6	-	7.02	(2.67- 18.51)
Subtotal AMANDU								6.48	(3.21- 13.08)
ARMADA	506	m	1	21	-	8	-	2.60	(1.00- 6.60)
ARMADA	507	m	1	219	-	8	-	11.90	(5.50- 25.50)
ARMADA	508	m	1	77	-	8	-	26.80	(11.00- 65.10)
Subtotal ARMADA								10.07	(6.14- 16.52)
AUVINE	517	c	2	26	-	44	-	20.10	(6.69- 66.00)
AUVINE	518	c	2	10	-	44	-	33.20	(14.30- 77.40)
AUVINE	519	c	2	230	-	44	-	30.40	(15.80- 58.40)
Subtotal AUVINE								29.13	(18.19- 46.66)
AXELSS	519	m	6	13	-	16	-	1.57	(0.70- 3.48)
AXELSS	520	m	6	17	-	16	-	2.23	(1.03- 4.80)
AXELSS	521	m	6	57	-	16	-	7.62	(4.01- 14.47)
AXELSS	522	m	6	104	-	16	-	11.81	(6.42- 21.73)
AXELSS	523	m	6	101	-	16	-	27.09	(13.94- 52.62)
AXELSS	510	f	0	5	24	18	154	1.78	(0.61- 5.25)
AXELSS	511	f	0	12	29	18	154	3.54	(1.54- 8.13)
AXELSS	512	f	0	29	26	18	154	9.54	(4.64- 19.61)
AXELSS	513	f	0	44	20	18	154	18.82	(9.17- 38.65)
AXELSS	514	f	0	20	10	18	154	17.11	(6.94- 42.19)
Subtotal AXELSS								7.77	(6.14- 9.83)
BARBON	508	m	1	42	-	22	-	3.20	(1.80- 5.70)
BARBON	509	m	1	118	-	22	-	7.90	(4.70- 13.50)
BARBON	510	m	1	207	-	22	-	11.40	(7.00- 18.80)
BARBON	511	m	1	366	-	22	-	14.50	(9.00- 23.30)
Subtotal BARBON								8.70	(6.73- 11.26)
*BEST	501	m	1	1	-	7	-	1.60	(0.20- 13.00)
*BEST	502	m	1	2	-	7	-	2.60	(0.54- 12.52)
*BEST	503	m	1	6	-	7	-	2.30	(0.77- 6.84)
*BEST	504	m	1	10	-	7	-	3.20	(1.22- 8.41)
*BEST	505	m	1	22	-	7	-	4.10	(1.75- 9.60)
*BEST	506	m	1	55	-	7	-	13.90	(6.33- 30.52)
*BEST	507	m	1	137	-	7	-	14.20	(6.64- 30.35)
Subtotal BEST								6.17	(4.26- 8.94)
*BOUCOT	518	m	0	29	2621	0	805	18.13~	(1.11- 296.36)
*BOUCOT	519	m	0	52	1563	0	805	54.09~	(3.34- 875.17)
Subtotal BOUCOT								31.38	(4.37- 225.47)
BUFFLE	526	f	0	52	57	12	112	8.51	(4.21- 17.22)
BUFFLE	527	f	0	97	62	12	112	14.60	(7.43- 28.69)
BUFFLE	528	f	0	90	42	12	112	20.00	(9.94- 40.23)
Subtotal BUFFLE								13.60	(9.12- 20.29)
*CEDERL	501	m	1	5	-	7	-	1.80	(0.57- 5.66)
*CEDERL	502	m	1	23	-	7	-	7.40	(3.18- 17.21)
*CEDERL	504	f	1	3	-	19	-	1.60	(0.47- 5.40)
*CEDERL	505	f	1	5	-	19	-	9.60	(3.60- 25.58)
Subtotal CEDERL								4.61	(2.78- 7.67)
CHEN2	501	m	0	2	3	9	33	2.44	(0.35- 16.93)
CHEN2	502	m	0	4	3	9	33	4.89	(0.92- 25.93)
CHEN2	503	m	0	17	24	9	33	2.60	(0.99- 6.81)
CHEN2	504	m	0	36	27	9	33	4.89	(2.01- 11.91)
CHEN2	505	m	0	62	40	9	33	5.68	(2.46- 13.13)
CHEN2	510	f	0	1	6	25	33	0.22	(0.02- 1.95)
CHEN2	511	f	0	2	2	25	33	1.32	(0.17- 10.03)
CHEN2	512	f	0	13	6	25	33	2.86	(0.95- 8.58)
CHEN2	513	f	0	21	15	25	33	1.85	(0.80- 4.29)
Subtotal CHEN2								3.01	(2.07- 4.38)
CHOI	501	m	0	19	55	13	95	2.52	(1.16- 5.51)
CHOI	502	m	0	66	166	13	95	2.91	(1.52- 5.54)
CHOI	503	m	0	102	160	13	95	4.66	(2.48- 8.75)
CHOI	504	m	0	60	64	13	95	6.85	(3.48- 13.50)
CHOI	505	m	0	20	20	13	95	7.31	(3.13- 17.07)
CHOI	510	f	0	2	9	76	164	0.48	(0.10- 2.27)
CHOI	511	f	0	8	14	76	164	1.23	(0.50- 3.06)
CHOI	512	f	0	8	2	76	164	8.63	(1.79- 41.62)
CHOI	513	f	0	1	1	76	164	2.16	(0.13- 34.96)
Subtotal CHOI								3.63	(2.73- 4.83)
*CPSI	580	m	0	95	266163	196	926068	1.69	(1.32- 2.15)

Table 1I11 - 2

IESLC - Meta-analysis of Ever/current Smoking by Duration, Overview
All LC types, Any Product (or Cigarettes if Any not available)
Most adjusted

REF	NRR	SEX	AD	Number Exposed Case	Number Exposed Cont	Non-exposed Case	Non-exposed Cont	RR	95.00%CI
*CPSI	581	m	0	230	290031	196	926068	3.75	(3.10- 4.53)
*CPSI	582	m	0	470	367622	196	926068	6.04	(5.11- 7.14)
*CPSI	583	m	0	731	333292	196	926068	10.36	(8.85- 12.13)
*CPSI	584	m	0	764	221405	196	926068	16.30	(13.94- 19.07)
*CPSI	585	m	0	576	119633	196	926068	22.75	(19.35- 26.75)
*CPSI	586	m	0	356	53226	196	926068	31.60	(26.55- 37.61)
*CPSI	587	m	0	232	26906	196	926068	40.74	(33.70- 49.25)
*CPSI	676	f	0	105	694015	532	3877179	1.10	(0.89- 1.36)
*CPSI	677	f	0	141	383127	532	3877179	2.68	(2.23- 3.23)
*CPSI	678	f	0	154	315060	532	3877179	3.56	(2.98- 4.26)
*CPSI	679	f	0	120	163178	532	3877179	5.36	(4.40- 6.53)
*CPSI	680	f	0	54	53635	532	3877179	7.34	(5.55- 9.71)
*CPSI	681	f	0	16	14305	532	3877179	8.15	(4.96- 13.40)
*CPSI	682	f	0	10	5657	532	3877179	12.88	(6.90- 24.07)
Subtotal CPSI								8.01	(7.62- 8.43)
*CPSII	552	m	0	72	141932	124	742207	3.04	(2.27- 4.06)
*CPSII	553	m	0	145	113317	124	742207	7.66	(6.03- 9.73)
*CPSII	554	m	0	244	109788	124	742207	13.30	(10.72- 16.51)
*CPSII	555	m	0	413	103500	124	742207	23.88	(19.54- 29.19)
*CPSII	556	m	0	307	53805	124	742207	34.15	(27.73- 42.06)
*CPSII	557	m	0	332	39260	124	742207	50.62	(41.19- 62.20)
*CPSII	558	m	0	151	13598	124	742207	66.47	(52.45- 84.24)
*CPSII	559	m	0	117	8450	124	742207	82.88	(64.43- 106.60)
*CPSII	618	f	0	127	301244	310	2091302	2.84	(2.31- 3.50)
*CPSII	619	f	0	158	152833	310	2091302	6.97	(5.76- 8.45)
*CPSII	620	f	0	193	116270	310	2091302	11.20	(9.36- 13.40)
*CPSII	621	f	0	216	91501	310	2091302	15.93	(13.39- 18.94)
*CPSII	622	f	0	153	44769	310	2091302	23.06	(19.00- 27.98)
*CPSII	623	f	0	122	29119	310	2091302	28.26	(22.93- 34.84)
*CPSII	624	f	0	27	6262	310	2091302	29.09	(19.64- 43.07)
*CPSII	625	f	0	18	2224	310	2091302	54.60	(34.01- 87.65)
Subtotal CPSII								17.37	(16.44- 18.35)
DAMBER	506	m	1	-	-	42	-	1.58	(0.69- 3.66)
DAMBER	507	m	1	-	-	42	-	3.66	(2.18- 6.73)
DAMBER	508	m	1	-	-	42	-	5.15	(3.27- 8.32)
DAMBER	509	m	1	-	-	42	-	8.71	(5.84- 13.66)
DAMBER	510	m	1	-	-	42	-	11.19	(7.43- 17.33)
Subtotal DAMBER								6.42	(5.14- 8.01)
DEAN2	501	m	0	34	36	33	112	3.21	(1.74- 5.89)
DEAN2	502	m	0	631	558	33	112	3.84	(2.56- 5.75)
DEAN2	504	f	0	10	14	88	121	0.98	(0.42- 2.31)
DEAN2	505	f	0	47	11	88	121	5.88	(2.88- 11.97)
Subtotal DEAN2								3.39	(2.55- 4.52)
DESTEF	508	m	4	43	-	27	-	3.40	(1.70- 6.80)
DESTEF	509	m	4	78	-	27	-	5.20	(2.90- 8.90)
DESTEF	510	m	4	171	-	27	-	10.40	(6.40- 16.90)
DESTEF	511	m	4	178	-	27	-	10.80	(6.60- 17.60)
Subtotal DESTEF								7.55	(5.76- 9.90)
DOLL	515	m	0	12	15	7	61	6.97	(2.34- 20.73)
DOLL	516	m	0	34	65	7	61	4.56	(1.88- 11.05)
DOLL	517	m	0	746	725	7	61	8.97	(4.07- 19.73)
DOLL	518	m	0	558	491	7	61	9.90	(4.49- 21.85)
DOLL	522	f	0	14	18	40	59	1.15	(0.51- 2.57)
DOLL	523	f	0	12	8	40	59	2.21	(0.83- 5.90)
DOLL	524	f	0	36	20	40	59	2.66	(1.35- 5.23)
DOLL	525	f	0	6	3	40	59	2.95	(0.70- 12.49)
Subtotal DOLL								3.93	(2.89- 5.34)
DORGAN	570	m	2	-	-	-	-	5.44	(2.97- 9.98)
DORGAN	571	m	2	-	-	-	-	16.09	(8.96- 28.88)
DORGAN	562	f	3	-	-	-	-	4.25	(3.20- 5.64)
DORGAN	563	f	3	-	-	-	-	11.73	(9.07- 15.18)
Subtotal DORGAN								7.74	(6.50- 9.20)
DOSEME	501	m	2	32	-	142	-	1.00	(0.60- 1.70)
DOSEME	502	m	2	158	-	142	-	3.80	(2.60- 5.70)
DOSEME	503	m	2	466	-	142	-	4.90	(3.50- 7.00)
Subtotal DOSEME								3.27	(2.59- 4.12)
FAN	501	m	0	29	135	36	236	1.41	(0.83- 2.40)
FAN	502	m	0	44	122	36	236	2.36	(1.45- 3.87)
FAN	503	m	0	143	241	36	236	3.89	(2.59- 5.84)
FAN	506	f	0	8	15	69	320	2.47	(1.01- 6.06)
FAN	507	f	0	19	23	69	320	3.83	(1.98- 7.42)

Table 1I11 - 2

IESLC - Meta-analysis of Ever/current Smoking by Duration, Overview
All LC types, Any Product (or Cigarettes if Any not available)
Most adjusted

REF	NRR	SEX	AD	Number Case	Exposed Cont	Non-exposed Case	Cont	RR	95.00%CI
FAN	508	f	0	55	59	69	320	4.32	(2.76- 6.78)
Subtotal FAN								3.01	(2.43- 3.72)
GAO	564	f	2	68	-	435	-	1.89	(1.30- 2.75)
GAO	565	f	2	168	-	435	-	4.58	(3.33- 6.30)
Subtotal GAO								3.16	(2.48- 4.03)
GARSHI	536	m	1	922	-	41	-	6.28	(4.49- 8.77)
GER	518	c	5	10	-	51	-	1.30	(0.55- 3.06)
GER	519	c	5	31	-	51	-	1.56	(0.83- 2.91)
GER	520	c	5	49	-	51	-	2.14	(1.18- 3.90)
Subtotal GER								1.72	(1.17- 2.52)
HAENSZ	542	f	0	16	26	81	236	1.79	(0.92- 3.51)
HAENSZ	543	f	0	58	77	81	236	2.19	(1.44- 3.35)
Subtotal HAENSZ								2.07	(1.45- 2.97)
HU	501	m	0	41	33	41	67	2.03	(1.11- 3.70)
HU	502	m	0	60	47	41	67	2.09	(1.21- 3.60)
HU	503	m	0	19	14	41	67	2.22	(1.00- 4.90)
HU	506	f	0	11	8	40	48	1.65	(0.61- 4.50)
HU	507	f	0	11	7	40	48	1.89	(0.67- 5.32)
HU	508	f	0	4	3	40	48	1.60	(0.34- 7.57)
Subtotal HU								2.00	(1.46- 2.74)
HU2	508	c	0	21	33	121	213	1.12	(0.62- 2.02)
HU2	509	c	0	64	63	121	213	1.79	(1.18- 2.70)
HU2	510	c	0	123	101	121	213	2.14	(1.52- 3.03)
HU2	511	c	0	194	113	121	213	3.02	(2.19- 4.17)
Subtotal HU2								2.18	(1.79- 2.64)
HUMBLE	542	c	3	20	-	28	-	15.45	(6.19- 38.58)
HUMBLE	543	c	3	68	-	28	-	17.54	(8.46- 36.34)
HUMBLE	544	c	3	104	-	28	-	19.61	(11.20- 34.31)
HUMBLE	545	c	3	119	-	28	-	17.27	(10.38- 28.75)
Subtotal HUMBLE								17.79	(12.99- 24.36)
JOLY	515	m	0	11	48	12	218	4.16	(1.73- 9.99)
JOLY	516	m	0	38	61	12	218	11.32	(5.57- 22.98)
JOLY	517	m	0	85	165	12	218	9.36	(4.95- 17.70)
JOLY	518	m	0	168	182	12	218	16.77	(9.04- 31.11)
JOLY	519	m	0	250	253	12	218	17.95	(9.78- 32.93)
JOLY	501	f	0	13	28	52	283	2.53	(1.23- 5.20)
JOLY	502	f	0	18	26	52	283	3.77	(1.93- 7.36)
JOLY	503	f	0	31	24	52	283	7.03	(3.82- 12.93)
JOLY	504	f	0	47	24	52	283	10.66	(6.00- 18.92)
JOLY	505	f	0	57	20	52	283	15.51	(8.61- 27.95)
Subtotal JOLY								9.03	(7.36- 11.08)
JUSSAW	510	m	0	16	20	149	624	3.35	(1.70- 6.62)
JUSSAW	511	m	0	34	19	149	624	7.49	(4.16- 13.51)
JUSSAW	512	m	0	38	23	149	624	6.92	(4.00- 11.97)
JUSSAW	513	m	0	27	9	149	624	12.56	(5.79- 27.28)
JUSSAW	514	m	0	11	6	149	624	7.68	(2.79- 21.09)
Subtotal JUSSAW								6.77	(5.01- 9.15)
*KAISE2	596	m	1	17	-	14	-	4.86	(2.22- 10.61)
*KAISE2	597	m	1	34	-	14	-	15.64	(8.31- 29.40)
*KAISE2	516	f	1	24	-	11	-	9.09	(4.25- 19.43)
*KAISE2	517	f	1	26	-	11	-	30.41	(14.39- 64.25)
Subtotal KAISE2								12.59	(8.77- 18.07)
KATSOU	512	f	1	13	-	48	-	1.29	(0.54- 3.26)
KATSOU	513	f	1	32	-	48	-	7.43	(2.88- 19.13)
Subtotal KATSOU								2.96	(1.54- 5.68)
KHUDER	501	m	0	16	61	23	309	3.52	(1.76- 7.06)
KHUDER	502	m	0	207	370	23	309	7.52	(4.76- 11.86)
KHUDER	503	m	0	236	354	23	309	8.96	(5.69- 14.11)
Subtotal KHUDER								7.07	(5.28- 9.47)
KREUZE	517	m	3	-	-	-	-	4.70	(3.10- 7.14)
KREUZE	518	m	3	-	-	-	-	29.23	(19.78- 43.20)
KREUZE	520	f	3	-	-	-	-	1.33	(0.80- 2.22)
KREUZE	521	f	3	-	-	-	-	7.14	(4.92- 10.35)
Subtotal KREUZE								7.26	(5.90- 8.93)
LETOUR	506	c	0	65	187	24	224	3.24	(1.95- 5.39)
LETOUR	507	c	0	264	160	24	224	15.40	(9.68- 24.51)
LETOUR	508	c	0	374	141	24	224	24.76	(15.58- 39.35)
Subtotal LETOUR								11.50	(8.73- 15.14)
LEVIN	506	m	1	56	-	7	-	7.07	(3.07- 16.29)
LEVIN	507	m	1	63	-	7	-	8.96	(3.90- 20.61)
Subtotal LEVIN								7.96	(4.42- 14.35)

Table 1I11 - 2

IESLC - Meta-analysis of Ever/current Smoking by Duration, Overview
All LC types, Any Product (or Cigarettes if Any not available)
Most adjusted

REF	NRR	SEX	AD	Number Exposed Case	Cont	Non-exposed Case	Cont	RR	95.00%CI
*LIAW	501	c	2	-	-	-	-	0.90	(0.30- 3.10)
*LIAW	502	c	2	-	-	-	-	2.60	(1.20- 5.90)
*LIAW	503	c	2	-	-	-	-	4.70	(2.70- 8.20)
Subtotal LIAW								3.19	(2.09- 4.88)
LIU3	510	m	2	30	-	4	-	1.07	(0.25- 4.59)
LIU3	511	m	2	22	-	4	-	1.71	(0.36- 8.12)
Subtotal LIU3								1.33	(0.46- 3.86)
LIU5	504	c	0	27	37	26	41	1.15	(0.57- 2.31)
LIU5	505	c	0	58	33	26	41	2.77	(1.45- 5.32)
Subtotal LIU5								1.84	(1.14- 2.96)
LUBIN	508	m	0	30	146	8	72	1.85	(0.81- 4.24)
LUBIN	509	m	0	124	294	8	72	3.80	(1.78- 8.12)
LUBIN	510	m	0	143	251	8	72	5.13	(2.40- 10.95)
LUBIN	511	m	0	59	86	8	72	6.17	(2.77- 13.77)
Subtotal LUBIN								3.94	(2.66- 5.83)
LUBIN2	531	m	0	953	2995	190	2616	4.38	(3.72- 5.16)
LUBIN2	532	m	0	2227	3470	190	2616	8.84	(7.56- 10.33)
LUBIN2	533	m	0	2079	2551	190	2616	11.22	(9.58- 13.14)
LUBIN2	534	m	0	1325	1484	190	2616	12.29	(10.42- 14.50)
LUBIN2	574	f	0	132	230	336	1188	2.03	(1.59- 2.59)
LUBIN2	575	f	0	187	186	336	1188	3.55	(2.81- 4.50)
LUBIN2	576	f	0	155	118	336	1188	4.64	(3.55- 6.07)
LUBIN2	577	f	0	81	32	336	1188	8.95	(5.84- 13.71)
Subtotal LUBIN2								6.83	(6.37- 7.32)
MATOS	536	m	2	20	-	11	-	2.20	(1.00- 4.90)
MATOS	537	m	2	82	-	11	-	7.20	(3.60- 14.50)
MATOS	538	m	2	86	-	11	-	12.70	(6.10- 26.10)
Subtotal MATOS								6.23	(4.07- 9.53)
MCCONN	501	c	0	3	4	9	23	1.92	(0.36- 10.32)
MCCONN	502	c	0	5	19	9	23	0.67	(0.19- 2.35)
MCCONN	503	c	0	46	57	9	23	2.06	(0.87- 4.89)
MCCONN	504	c	0	21	57	9	23	0.94	(0.38- 2.36)
MCCONN	505	c	0	16	40	9	23	1.02	(0.39- 2.68)
Subtotal MCCONN								1.22	(0.76- 1.94)
NOTAN2	513	c	0	7	15	107	201	0.88	(0.35- 2.22)
NOTAN2	514	c	0	15	15	107	201	1.88	(0.88- 3.99)
NOTAN2	515	c	0	17	16	107	201	2.00	(0.97- 4.11)
NOTAN2	516	c	0	12	7	107	201	3.22	(1.23- 8.42)
NOTAN2	517	c	0	5	5	107	201	1.88	(0.53- 6.63)
Subtotal NOTAN2								1.83	(1.24- 2.70)
OSANN2	504	f	1	23	-	33	-	1.60	(0.70- 3.50)
OSANN2	505	f	1	161	-	23	-	11.60	(5.80- 23.30)
Subtotal OSANN2								4.97	(2.94- 8.42)
PEZZO2	507	m	0	60	72	6	117	16.25	(6.68- 39.53)
PEZZO2	508	m	0	173	126	6	117	26.77	(11.42- 62.76)
Subtotal PEZZO2								21.08	(11.40- 39.00)
PEZZOT	534	m	0	30	134	4	116	6.49	(2.22- 18.98)
PEZZOT	535	m	0	71	82	4	116	25.11	(8.82- 71.48)
PEZZOT	536	m	0	110	101	4	116	31.58	(11.25- 88.71)
Subtotal PEZZOT								17.64	(9.62- 32.34)
*QIAO2	516	m	1	7	-	10	-	0.40	(0.15- 1.05)
*QIAO2	517	m	1	54	-	10	-	1.46	(0.74- 2.87)
*QIAO2	518	m	1	170	-	10	-	2.05	(1.06- 3.94)
Subtotal QIAO2								1.32	(0.86- 2.01)
RACHTA	516	f	1	12	-	33	-	2.02	(0.87- 4.71)
RACHTA	517	f	1	49	-	33	-	7.55	(3.90- 14.63)
RACHTA	518	f	1	24	-	33	-	58.68	(7.56- 455.64)
Subtotal RACHTA								5.34	(3.22- 8.84)
SOBUE	546	m	0	62	119	34	128	1.96	(1.21- 3.19)
SOBUE	547	m	0	159	200	34	128	2.99	(1.94- 4.61)
SOBUE	548	m	0	241	174	34	128	5.21	(3.41- 7.98)
SOBUE	549	m	0	147	73	34	128	7.58	(4.73- 12.14)
Subtotal SOBUE								3.96	(3.16- 4.96)
TIZZAN	501	m	0	12	1	180	305	20.33	(2.62- 157.68)
TIZZAN	502	m	0	54	20	180	305	4.58	(2.65- 7.89)
TIZZAN	503	m	0	928	815	180	305	1.93	(1.57- 2.37)
TIZZAN	533	f	0	2	7	117	114	0.28	(0.06- 1.37)
TIZZAN	534	f	0	23	21	117	114	1.07	(0.56- 2.03)
Subtotal TIZZAN								2.01	(1.68- 2.42)
WANG2	501	c	0	4	17	11	43	0.92	(0.26- 3.29)
WANG2	503	c	0	8	18	11	43	1.74	(0.60- 5.03)

Table 1I11 - 2

IESLC - Meta-analysis of Ever/current Smoking by Duration, Overview
All LC types, Any Product (or Cigarettes if Any not available)
Most adjusted

REF	NRR	SEX	AD	Number Exposed Case	Number Exposed Cont	Non-exposed Case	Non-exposed Cont	RR	95.00%CI
WANG2	504	c	0	26	38	11	43	2.67	(1.17- 6.13)
WANG2	505	c	0	22	26	11	43	3.31	(1.38- 7.91)
Subtotal WANG2								2.24	(1.38- 3.63)
WUWILL	516	f	3	137	-	417	-	1.35	(1.04- 1.73)
WUWILL	517	f	3	179	-	417	-	2.71	(2.05- 3.60)
WUWILL	518	f	3	223	-	417	-	3.49	(2.65- 4.59)
Subtotal WUWILL								2.27	(1.94- 2.65)
ZHENG	553	m	0	37	75	33	94	1.41	(0.80- 2.46)
ZHENG	554	m	0	242	143	33	94	4.82	(3.08- 7.54)
ZHENG	558	f	0	17	17	152	184	1.21	(0.60- 2.45)
ZHENG	559	f	0	59	27	152	184	2.65	(1.60- 4.38)
Subtotal ZHENG								2.54	(1.94- 3.31)
ZHOU	501	c	0	170	12	507	68	1.90	(1.00- 3.60)
ZHOU	502	c	0	678	36	507	68	2.53	(1.66- 3.84)
Subtotal ZHOU								2.32	(1.63- 3.29)
Partial Totals				30094	4659965	2152857252327			

*prospective study ~ With 0.5 adjustment for zero

REF	NRR	SEX	AD	Ys	Ws	Qs	Ps
AGUDO	510	f	3	0.25	2.80	8.57	0.6702
AGUDO	511	f	3	1.63	4.13	0.59	0.0009
Subtotal AGUDO				1.07	6.93	9.16	
*AMANDU	506	m	2	1.78	3.67	0.19	0.0007
*AMANDU	507	m	2	1.95	4.10	0.01	0.0001
Subtotal AMANDU				1.87	7.77	0.20	
ARMADA	506	m	1	0.96	4.31	4.75	0.0472
ARMADA	507	m	1	2.48	6.53	1.45	0.0000
ARMADA	508	m	1	3.29	4.86	8.01	0.0000
Subtotal ARMADA				2.31	15.71	14.21	
AUVINE	517	c	2	3.00	2.93	2.91	0.0000
AUVINE	518	c	2	3.50	5.39	12.08	0.0000
AUVINE	519	c	2	3.41	8.99	17.86	0.0000
Subtotal AUVINE				3.37	17.31	32.85	
AXELSS	519	m	6	0.45	5.97	14.43	0.2702
AXELSS	520	m	6	0.80	6.49	9.39	0.0411
AXELSS	521	m	6	2.03	9.33	0.01	0.0000
AXELSS	522	m	6	2.47	10.34	2.22	0.0000
AXELSS	523	m	6	3.30	8.71	14.59	0.0000
AXELSS	510	f	0	0.58	3.29	6.71	0.2943
AXELSS	511	f	0	1.26	5.56	3.05	0.0029
AXELSS	512	f	0	2.26	7.41	0.47	0.0000
AXELSS	513	f	0	2.94	7.42	6.42	0.0000
AXELSS	514	f	0	2.84	4.72	3.29	0.0000
Subtotal AXELSS				2.05	69.23	60.56	
BARBON	508	m	1	1.16	11.56	8.20	0.0001
BARBON	509	m	1	2.07	13.80	0.05	0.0000
BARBON	510	m	1	2.43	15.74	2.89	0.0000
BARBON	511	m	1	2.67	16.98	7.60	0.0000
Subtotal BARBON				2.16	58.09	18.74	
*BEST	501	m	1	0.47	0.88	2.08	0.6590
*BEST	502	m	1	0.96	1.55	1.71	0.2335
*BEST	503	m	1	0.83	3.22	4.43	0.1350
*BEST	504	m	1	1.16	4.12	2.92	0.0182
*BEST	505	m	1	1.41	5.30	1.87	0.0012
*BEST	506	m	1	2.63	6.21	2.44	0.0000
*BEST	507	m	1	2.65	6.65	2.80	0.0000
Subtotal BEST				1.82	27.95	18.24	
*BOUCOT	518	m	0	2.90	0.49	0.39	0.0421
*BOUCOT	519	m	0	3.99	0.50	1.95	0.0050
Subtotal BOUCOT				3.45	0.99	2.35	
BUFFLE	526	f	0	2.14	7.75	0.14	0.0000
BUFFLE	527	f	0	2.68	8.42	3.85	0.0000
BUFFLE	528	f	0	3.00	7.86	7.72	0.0000
Subtotal BUFFLE				2.61	24.04	11.71	
*CEDERL	501	m	1	0.59	2.92	5.86	0.3155
*CEDERL	502	m	1	2.00	5.39	0.00	0.0000
*CEDERL	504	f	1	0.47	2.58	6.07	0.4505
*CEDERL	505	f	1	2.26	4.00	0.26	0.0000
Subtotal CEDERL				1.53	14.88	12.19	
CHEN2	501	m	0	0.89	1.03	1.27	0.3653

Table 1I11 - 2

IESLC - Meta-analysis of Ever/current Smoking by Duration, Overview
All LC types, Any Product (or Cigarettes if Any not available)
Most adjusted

REF	NRR	SEX	AD	Ys	Ws	Qs	Ps
CHEN2	502	m	0	1.59	1.38	0.24	0.0623
CHEN2	503	m	0	0.95	4.13	4.56	0.0523
CHEN2	504	m	0	1.59	4.85	0.85	0.0005
CHEN2	505	m	0	1.74	5.48	0.39	0.0000
CHEN2	510	f	0	-1.51	0.81	10.01	0.1734
CHEN2	511	f	0	0.28	0.93	2.79	0.7884
CHEN2	512	f	0	1.05	3.19	2.90	0.0607
CHEN2	513	f	0	0.61	5.42	10.48	0.1529
Subtotal CHEN2				1.10	27.21	33.49	
CHOI	501	m	0	0.93	6.32	7.36	0.0199
CHOI	502	m	0	1.07	9.21	8.11	0.0012
CHOI	503	m	0	1.54	9.66	2.10	0.0000
CHOI	504	m	0	1.92	8.35	0.05	0.0000
CHOI	505	m	0	1.99	5.33	0.00	0.0000
CHOI	510	f	0	-0.73	1.59	11.91	0.3546
CHOI	511	f	0	0.21	4.64	14.95	0.6519
CHOI	512	f	0	2.16	1.55	0.04	0.0072
CHOI	513	f	0	0.77	0.50	0.76	0.5883
Subtotal CHOI				1.29	47.14	45.27	
*CPSI	580	m	0	0.52	64.01	140.65	0.0000
*CPSI	581	m	0	1.32	105.87	49.54	0.0000
*CPSI	582	m	0	1.80	138.39	5.90	0.0000
*CPSI	583	m	0	2.34	154.66	17.17	0.0000
*CPSI	584	m	0	2.79	156.12	96.55	0.0000
*CPSI	585	m	0	3.12	146.44	183.53	0.0000
*CPSI	586	m	0	3.45	126.72	265.78	0.0000
*CPSI	587	m	0	3.71	106.68	309.10	0.0000
*CPSI	676	f	0	0.10	87.71	319.06	0.3603
*CPSI	677	f	0	0.99	111.49	115.63	0.0000
*CPSI	678	f	0	1.27	119.48	64.47	0.0000
*CPSI	679	f	0	1.68	97.98	10.42	0.0000
*CPSI	680	f	0	1.99	49.07	0.01	0.0000
*CPSI	681	f	0	2.10	15.55	0.14	0.0000
*CPSI	682	f	0	2.56	9.83	2.98	0.0000
Subtotal CPSI				2.08	1489.99	1580.95	
*CPSII	552	m	0	1.11	45.57	36.45	0.0000
*CPSII	553	m	0	2.04	66.89	0.06	0.0000
*CPSII	554	m	0	2.59	82.29	27.96	0.0000
*CPSII	555	m	0	3.17	95.47	130.29	0.0000
*CPSII	556	m	0	3.53	88.48	205.99	0.0000
*CPSII	557	m	0	3.92	90.50	333.37	0.0000
*CPSII	558	m	0	4.20	68.44	328.74	0.0000
*CPSII	559	m	0	4.42	60.64	352.87	0.0000
*CPSII	618	f	0	1.05	90.12	83.02	0.0000
*CPSII	619	f	0	1.94	104.74	0.41	0.0000
*CPSII	620	f	0	2.42	119.07	20.09	0.0000
*CPSII	621	f	0	2.77	127.49	74.20	0.0000
*CPSII	622	f	0	3.14	102.68	131.78	0.0000
*CPSII	623	f	0	3.34	87.81	156.88	0.0000
*CPSII	624	f	0	3.37	24.94	46.48	0.0000
*CPSII	625	f	0	4.00	17.14	68.23	0.0000
Subtotal CPSII				2.85	1272.25	1996.82	
DAMBER	506	m	1	0.46	5.52	13.22	0.2825
DAMBER	507	m	1	1.30	12.09	6.05	0.0000
DAMBER	508	m	1	1.64	17.62	2.36	0.0000
DAMBER	509	m	1	2.16	21.28	0.54	0.0000
DAMBER	510	m	1	2.42	21.42	3.60	0.0000
Subtotal DAMBER				1.86	77.93	25.78	
DEAN2	501	m	0	1.16	10.37	7.32	0.0002
DEAN2	502	m	0	1.34	23.47	10.23	0.0000
DEAN2	504	f	0	-0.02	5.23	21.42	0.9671
DEAN2	505	f	0	1.77	7.59	0.42	0.0000
Subtotal DEAN2				1.22	46.66	39.38	
DESTEF	508	m	4	1.22	8.00	4.88	0.0005
DESTEF	509	m	4	1.65	12.22	1.55	0.0000
DESTEF	510	m	4	2.34	16.30	1.85	0.0000
DESTEF	511	m	4	2.38	15.97	2.24	0.0000
Subtotal DESTEF				2.02	52.48	10.52	
DOLL	515	m	0	1.94	3.23	0.01	0.0005
DOLL	516	m	0	1.52	4.90	1.17	0.0008
DOLL	517	m	0	2.19	6.17	0.22	0.0000

Table 1I11 - 2

IESLC - Meta-analysis of Ever/current Smoking by Duration, Overview
All LC types, Any Product (or Cigarettes if Any not available)
Most adjusted

REF	NRR	SEX	AD	Ys	Ws	Qs	Ps
DOLL	518	m	0	2.29	6.13	0.51	0.0000
DOLL	522	f	0	0.14	5.92	20.65	0.7383
DOLL	523	f	0	0.79	4.00	5.86	0.1124
DOLL	524	f	0	0.98	8.35	8.84	0.0048
DOLL	525	f	0	1.08	1.85	1.57	0.1417
Subtotal DOLL				1.37	40.55	38.82	
DORGAN	570	m	2	1.69	10.46	1.01	0.0000
DORGAN	571	m	2	2.78	11.22	6.71	0.0000
DORGAN	562	f	3	1.45	47.84	14.90	0.0000
DORGAN	563	f	3	2.46	57.93	12.11	0.0000
Subtotal DORGAN				2.05	127.45	34.73	
DOSEME	501	m	2	0.00	14.17	56.95	1.0000
DOSEME	502	m	2	1.34	24.94	11.19	0.0000
DOSEME	503	m	2	1.59	31.98	5.53	0.0000
Subtotal DOSEME				1.18	71.09	73.68	
FAN	501	m	0	0.34	13.53	37.41	0.2079
FAN	502	m	0	0.86	15.89	20.81	0.0006
FAN	503	m	0	1.36	23.17	9.69	0.0000
FAN	506	f	0	0.91	4.78	5.78	0.0478
FAN	507	f	0	1.34	8.79	3.85	0.0001
FAN	508	f	0	1.46	18.96	5.55	0.0000
Subtotal FAN				1.10	85.12	83.08	
GAO	564	f	2	0.64	27.37	51.26	0.0009
GAO	565	f	2	1.52	37.80	8.83	0.0000
Subtotal GAO				1.15	65.17	60.09	
GARSHI	536	m	1	1.84	34.28	0.96	0.0000
GER	518	c	5	0.26	5.22	15.84	0.5490
GER	519	c	5	0.44	9.76	23.77	0.1647
GER	520	c	5	0.76	10.75	16.64	0.0126
Subtotal GER				0.54	25.73	56.26	
HAENSZ	542	f	0	0.58	8.51	17.18	0.0886
HAENSZ	543	f	0	0.79	21.36	31.74	0.0003
Subtotal HAENSZ				0.73	29.87	48.92	
HU	501	m	0	0.71	10.64	17.89	0.0209
HU	502	m	0	0.74	12.94	20.87	0.0082
HU	503	m	0	0.80	6.12	8.94	0.0488
HU	506	f	0	0.50	3.82	8.64	0.3277
HU	507	f	0	0.63	3.58	6.72	0.2303
HU	508	f	0	0.47	1.59	3.75	0.5535
Subtotal HU				0.69	38.69	66.80	
HU2	508	c	0	0.11	11.00	39.37	0.7065
HU2	509	c	0	0.58	22.49	45.60	0.0058
HU2	510	c	0	0.76	32.27	49.81	0.0000
HU2	511	c	0	1.11	37.09	29.98	0.0000
Subtotal HU2				0.78	102.85	164.75	
HUMBLE	542	c	3	2.74	4.59	2.46	0.0000
HUMBLE	543	c	3	2.86	7.23	5.34	0.0000
HUMBLE	544	c	3	2.98	12.26	11.56	0.0000
HUMBLE	545	c	3	2.85	14.81	10.55	0.0000
Subtotal HUMBLE				2.88	38.89	29.91	
JOLY	515	m	0	1.43	5.01	1.68	0.0014
JOLY	516	m	0	2.43	7.66	1.36	0.0000
JOLY	517	m	0	2.24	9.46	0.51	0.0000
JOLY	518	m	0	2.82	10.06	6.68	0.0000
JOLY	519	m	0	2.89	10.43	8.13	0.0000
JOLY	501	f	0	0.93	7.39	8.58	0.0118
JOLY	502	f	0	1.33	8.56	3.94	0.0001
JOLY	503	f	0	1.95	10.34	0.03	0.0000
JOLY	504	f	0	2.37	11.67	1.52	0.0000
JOLY	505	f	0	2.74	11.07	6.01	0.0000
Subtotal JOLY				2.20	91.65	38.43	
JUSSAW	510	m	0	1.21	8.28	5.24	0.0005
JUSSAW	511	m	0	2.01	11.07	0.00	0.0000
JUSSAW	512	m	0	1.93	12.80	0.06	0.0000
JUSSAW	513	m	0	2.53	6.39	1.77	0.0000
JUSSAW	514	m	0	2.04	3.76	0.00	0.0001
Subtotal JUSSAW				1.91	42.30	7.08	
*KAISE2	596	m	1	1.58	6.28	1.13	0.0001
*KAISE2	597	m	1	2.75	9.62	5.34	0.0000
*KAISE2	516	f	1	2.21	6.65	0.27	0.0000
*KAISE2	517	f	1	3.41	6.86	13.64	0.0000

Table 1I11 - 2

IESLC - Meta-analysis of Ever/current Smoking by Duration, Overview
All LC types, Any Product (or Cigarettes if Any not available)
Most adjusted

REF	NRR	SEX	AD	Ys	Ws	Qs	Ps
Subtotal KAISE2				2.53	29.42	20.38	
KATSOU	512	f	1	0.25	4.75	14.56	0.5788
KATSOU	513	f	1	2.01	4.29	0.00	0.0000
Subtotal KATSOU				1.08	9.04	14.56	
KHUDER	501	m	0	1.26	7.96	4.42	0.0004
KHUDER	502	m	0	2.02	18.43	0.00	0.0000
KHUDER	503	m	0	2.19	18.60	0.65	0.0000
Subtotal KHUDER				1.96	44.99	5.08	
KREUZE	517	m	3	1.55	22.07	4.62	0.0000
KREUZE	518	m	3	3.38	25.18	47.27	0.0000
KREUZE	520	f	3	0.29	14.75	43.63	0.2734
KREUZE	521	f	3	1.97	27.78	0.04	0.0000
Subtotal KREUZE				1.98	89.79	95.57	
LETOUR	506	c	0	1.18	14.96	10.26	0.0000
LETOUR	507	c	0	2.73	17.80	9.47	0.0000
LETOUR	508	c	0	3.21	17.89	25.94	0.0000
Subtotal LETOUR				2.44	50.65	45.67	
LEVIN	506	m	1	1.96	5.52	0.01	0.0000
LEVIN	507	m	1	2.19	5.54	0.20	0.0000
Subtotal LEVIN				2.07	11.06	0.21	
*LIAW	501	c	2	-0.11	2.82	12.55	0.8596
*LIAW	502	c	2	0.96	6.06	6.67	0.0187
*LIAW	503	c	2	1.55	12.45	2.61	0.0000
Subtotal LIAW				1.16	21.33	21.83	
LIU3	510	m	2	0.07	1.81	6.81	0.9274
LIU3	511	m	2	0.54	1.58	3.41	0.4997
Subtotal LIU3				0.29	3.40	10.22	
LIU5	504	c	0	0.14	7.88	27.39	0.6935
LIU5	505	c	0	1.02	9.06	8.80	0.0022
Subtotal LIU5				0.61	16.94	36.19	
LUBIN	508	m	0	0.61	5.58	10.79	0.1463
LUBIN	509	m	0	1.33	6.65	3.00	0.0006
LUBIN	510	m	0	1.63	6.67	0.92	0.0000
LUBIN	511	m	0	1.82	5.97	0.20	0.0000
Subtotal LUBIN				1.37	24.88	14.91	
LUBIN2	531	m	0	1.48	142.28	39.62	0.0000
LUBIN2	532	m	0	2.18	156.67	4.74	0.0000
LUBIN2	533	m	0	2.42	153.41	26.14	0.0000
LUBIN2	534	m	0	2.51	141.36	35.91	0.0000
LUBIN2	574	f	0	0.71	63.53	106.92	0.0000
LUBIN2	575	f	0	1.27	68.77	37.32	0.0000
LUBIN2	576	f	0	1.54	53.35	11.75	0.0000
LUBIN2	577	f	0	2.19	21.09	0.73	0.0000
Subtotal LUBIN2				1.92	800.46	263.15	
MATOS	536	m	2	0.79	6.08	9.00	0.0518
MATOS	537	m	2	1.97	7.92	0.01	0.0000
MATOS	538	m	2	2.54	7.27	2.09	0.0000
Subtotal MATOS				1.83	21.27	11.11	
MCCONN	501	c	0	0.65	1.36	2.49	0.4488
MCCONN	502	c	0	-0.40	2.46	14.17	0.5341
MCCONN	503	c	0	0.72	5.16	8.47	0.1002
MCCONN	504	c	0	-0.06	4.55	19.41	0.8977
MCCONN	505	c	0	0.02	4.13	16.24	0.9644
Subtotal MCCONN				0.20	17.65	60.77	
NOTAN2	513	c	0	-0.13	4.47	20.40	0.7808
NOTAN2	514	c	0	0.63	6.77	12.80	0.1008
NOTAN2	515	c	0	0.69	7.37	12.73	0.0606
NOTAN2	516	c	0	1.17	4.16	2.90	0.0171
NOTAN2	517	c	0	0.63	2.41	4.56	0.3273
Subtotal NOTAN2				0.60	25.18	53.38	
OSANN2	504	f	1	0.47	5.93	13.98	0.2523
OSANN2	505	f	1	2.45	7.95	1.58	0.0000
Subtotal OSANN2				1.60	13.88	15.56	
PEZZO2	507	m	0	2.79	4.86	2.98	0.0000
PEZZO2	508	m	0	3.29	5.29	8.70	0.0000
Subtotal PEZZO2				3.05	10.15	11.68	
PEZZOT	534	m	0	1.87	3.34	0.06	0.0006
PEZZOT	535	m	0	3.22	3.51	5.21	0.0000
PEZZOT	536	m	0	3.45	3.60	7.55	0.0000
Subtotal PEZZOT				2.87	10.45	12.82	
*QIAO2	516	m	1	-0.92	4.06	34.63	0.0649

Table 1I11 - 2

IESLC - Meta-analysis of Ever/current Smoking by Duration, Overview
All LC types, Any Product (or Cigarettes if Any not available)
Most adjusted

REF	NRR	SEX	AD	Ys	Ws	Qs	Ps
*QIAO2	517	m	1	0.38	8.36	22.13	0.2738
*QIAO2	518	m	1	0.72	8.91	14.77	0.0321
Subtotal QIAO2				0.27	21.34	71.53	
RACHTA	516	f	1	0.70	5.39	9.13	0.1027
RACHTA	517	f	1	2.02	8.79	0.00	0.0000
RACHTA	518	f	1	4.07	0.91	3.91	0.0001
Subtotal RACHTA				1.68	15.09	13.04	
SOBUE	546	m	0	0.67	16.19	28.70	0.0067
SOBUE	547	m	0	1.10	20.61	17.02	0.0000
SOBUE	548	m	0	1.65	21.22	2.65	0.0000
SOBUE	549	m	0	2.03	17.32	0.01	0.0000
Subtotal SOBUE				1.38	75.35	48.38	
TIZZAN	501	m	0	3.01	0.92	0.93	0.0039
TIZZAN	502	m	0	1.52	12.93	3.03	0.0000
TIZZAN	503	m	0	0.66	89.78	163.09	0.0000
TIZZAN	533	f	0	-1.28	1.51	16.33	0.1155
TIZZAN	534	f	0	0.06	9.22	34.71	0.8435
Subtotal TIZZAN				0.70	114.36	218.10	
WANG2	501	c	0	-0.08	2.36	10.31	0.8977
WANG2	503	c	0	0.55	3.39	7.16	0.3089
WANG2	504	c	0	0.98	5.59	5.83	0.0200
WANG2	505	c	0	1.20	5.05	3.30	0.0072
Subtotal WANG2				0.81	16.39	26.60	
WUWILL	516	f	3	0.30	59.33	172.46	0.0208
WUWILL	517	f	3	1.00	48.46	49.25	0.0000
WUWILL	518	f	3	1.25	50.92	29.03	0.0000
Subtotal WUWILL				0.82	158.72	250.74	
ZHENG	553	m	0	0.34	12.30	34.09	0.2328
ZHENG	554	m	0	1.57	19.21	3.59	0.0000
ZHENG	558	f	0	0.19	7.71	25.38	0.5957
ZHENG	559	f	0	0.97	15.15	16.14	0.0002
Subtotal ZHENG				0.93	54.37	79.20	
ZHOU	501	c	0	0.64	9.44	17.55	0.0485
ZHOU	502	c	0	0.93	21.77	25.32	0.0000
Subtotal ZHOU				0.84	31.22	42.87	

N	245
NS	55

Table 1I11 - 3

IESLC - Meta-analysis of Ever/current Smoking by Duration, Overview
All LC types, Any Product (or Cigarettes if Any not available)
Most adjusted

	Sex			
	combined	male	female	Total
N	38	127	80	245
NS	11	36	25	72

In this overview table, other than the “N” rows, entries in the “absent” and “Total” columns may be invalid and should be ignored

	Duration of smoking (broad categories)				
	absent	1-34k20	21-49k35	36+k50	Total
N	106	55	39	45	245
NS	43	43	31	35	152
Wt	2808.44	964.02	1045.83	919.29	5737.58
Het Chi	2957.86	307.96	394.65	685.36	6059.48
Het df	105	54	38	44	244
Het P	***	***	***	***	***
Fixed RR	9.57	2.46	6.17	13.46	7.43
RRl	9.22	2.31	5.80	12.61	7.24
RRu	9.93	2.63	6.55	14.36	7.62
P	+++	+++	+++	+++	+++
Random RR	5.21	2.48	5.90	10.13	5.08
RRl	4.23	2.09	4.75	7.66	4.44
RRu	6.42	2.95	7.32	13.39	5.82
P	+++	+++	+++	+++	+++

	Duration of smoking (narrow categories)							
	absent	1-19k1	6-29k20	21-39k30	31-49k40	41-998k50	51+k999	Total
N	138	27	16	31	23	5	5	245
NS	54	19	12	23	17	3	3	131
Wt	3095.87	185.01	140.87	889.36	849.17	361.58	215.71	5737.58
Het Chi	3318.64	70.19	41.24	255.80	225.52	89.63	80.47	6059.48
Het df	137	26	15	30	22	4	4	244
Het P	***	***	***	***	***	***	***	***
Fixed RR	6.57	2.06	3.01	4.81	10.51	26.49	42.49	7.43
RRl	6.34	1.78	2.56	4.50	9.83	23.90	37.18	7.24
RRu	6.80	2.38	3.56	5.14	11.24	29.37	48.56	7.62
P	+++	+++	+++	+++	+++	+++	+++	+++
Random RR	5.33	1.89	2.97	4.66	9.14	19.48	31.39	5.08
RRl	4.44	1.46	2.22	3.75	7.15	11.51	16.07	4.44
RRu	6.41	2.43	3.98	5.79	11.68	32.98	61.30	5.82
P	+++	+++	+++	+++	+++	+++	+++	+++

MALES

	Duration of smoking (broad categories)				
	absent	1-34k20	21-49k35	36+k50	Total
N	55	27	20	25	127
NS	30	27	20	25	102
Wt	1712.01	449.87	554.80	589.70	3306.39
Het Chi	1777.53	117.41	119.76	286.24	3195.54
Het df	54	26	19	24	126
Het P	***	***	***	***	***
Fixed RR	12.21	3.05	7.32	16.27	9.77
RRl	11.65	2.78	6.74	15.00	9.44
RRu	12.81	3.34	7.96	17.63	10.11
P	+++	+++	+++	+++	+++
Random RR	7.02	2.80	6.47	12.06	6.37
RRl	5.24	2.22	5.07	8.78	5.31
RRu	9.39	3.52	8.25	16.56	7.64
P	+++	+++	+++	+++	+++

Table 1I11 - 3

IESLC - Meta-analysis of Ever/current Smoking by Duration, Overview
All LC types, Any Product (or Cigarettes if Any not available)
Most adjusted

MALES

	Duration of smoking (narrow categories)							
	absent	1-19k1	6-29k20	21-39k30	31-49k40	41-998k50	51+k999	Total
N	72	12	8	16	13	3	3	127
NS	36	12	8	16	13	3	3	91
Wt	1695.15	101.57	80.72	463.80	518.20	258.22	188.74	3306.39
Het Chi	1740.24	31.08	20.88	94.37	91.10	67.05	65.10	3195.54
Het df	71	11	7	15	12	2	2	126
Het P	***	**	**	***	***	***	***	***
Fixed RR	8.53	2.89	3.95	6.04	11.73	27.82	44.20	9.77
RRl	8.13	2.38	3.17	5.51	10.76	24.62	38.32	9.44
RRu	8.94	3.51	4.91	6.61	12.78	31.43	50.98	10.11
P	+++	+++	+++	+++	+++	+++	+++	+++
Random RR	6.26	2.86	4.03	5.62	9.93	22.05	34.17	6.37
RRl	4.86	2.01	2.68	4.31	7.48	10.07	14.06	5.31
RRu	8.06	4.07	6.04	7.34	13.17	48.29	83.07	7.64
P	+++	+++	+++	+++	+++	+++	+++	+++

FEMALES

	Duration of smoking (broad categories)				
	absent	1-34k20	21-49k35	36+k50	Total
N	38	18	11	13	80
NS	21	18	11	13	63
Wt	991.32	437.93	404.28	233.52	2067.05
Het Chi	759.91	108.76	133.88	200.52	1836.06
Het df	37	17	10	12	79
Het P	***	***	***	***	***
Fixed RR	7.14	2.00	5.25	11.19	5.40
RRl	6.71	1.82	4.77	9.84	5.18
RRu	7.60	2.20	5.79	12.72	5.64
P	+++	+++	+++	+++	+++
Random RR	4.54	2.08	5.70	9.50	4.36
RRl	3.34	1.57	3.75	5.17	3.49
RRu	6.17	2.75	8.65	17.45	5.45
P	+++	+++	+++	+++	+++

	Duration of smoking (narrow categories)							
	absent	1-19k1	6-29k20	21-39k30	31-49k40	41-998k50	51+k999	Total
N	46	10	4	9	7	2	2	80
NS	25	10	4	9	7	2	2	59
Wt	1187.57	54.81	22.34	362.49	309.51	103.36	26.98	2067.05
Het Chi	1067.90	10.11	4.65	69.45	101.63	20.42	13.03	1836.06
Het df	45	9	3	8	6	1	1	79
Het P	***	N.S.	N.S.	***	***	***	***	***
Fixed RR	4.76	1.39	2.63	3.99	8.84	23.44	32.25	5.40
RRl	4.49	1.07	1.74	3.60	7.91	19.33	22.12	5.18
RRu	5.04	1.82	3.99	4.42	9.88	28.43	47.04	5.64
P	+++	+	+++	+++	+++	+++	+++	+++
Random RR	4.52	1.38	2.53	4.40	8.76	15.51	26.92	4.36
RRl	3.36	1.03	1.49	3.04	5.12	4.59	6.54	3.49
RRu	6.10	1.84	4.28	6.38	14.98	52.40	110.82	5.45
P	+++	+	+++	+++	+++	+++	+++	+++

Table 1I11 - 4

IESLC - Meta-analysis of Ever/current Smoking by Duration, Overview
All LC types, Any Product (or Cigarettes if Any not available)
Least adjusted

REF	NRR	X	SEX	AGEL	AGEH	RACE	YF	LC TYPE	LOC	START	ST	NLC	R	VB	P	H	AD	SM	PRODUCT	exL	exH	S1	S2	DENOM	De
AGUDO	507	x	f	0	0	all	-	all	Eu:wst	1989	CC	103	n	bl	n	n	0	ev	cig only	1	16	0	1	nev cigs	st
AGUDO	508	x	f	0	0	all	-	all	Eu:wst	1989	CC	103	n	bl	n	n	0	ev	cig only	17	999	0	0	nev cigs	st
AMANDU	501	x	m	0	0	wh	0	all	NAmer	1959	pr	132	m	bl	n	n	0	cu	cig+/-ot	0	24	1	0	nev cigs	st
AMANDU	502	x	m	0	0	wh	0	all	NAmer	1959	pr	132	m	bl	n	n	0	cu	cig+/-ot	25	999	0	0	nev cigs	st
ARMADA	501	x	m	0	0	all	-	all	Eu:wst	1986	CC	325	n	bl	n	y	0	ev	cig+/-ot	1	24	1	0	nev cigs	st
ARMADA	502	x	m	0	0	all	-	all	Eu:wst	1986	CC	325	n	bl	n	y	0	ev	cig+/-ot	25	49	2	0	nev cigs	st
ARMADA	503	x	m	0	0	all	-	all	Eu:wst	1986	CC	325	n	bl	n	y	0	ev	cig+/-ot	50	999	3	0	nev cigs	st
AUVINE	501	x	c	0	0	all	-	all	Eu:Sca	1986	CC	517	n	bl	y	n	0	ev	cig+/-ot	1	20	1	0	nev cigs	st
AUVINE	502	x	c	0	0	all	-	all	Eu:Sca	1986	CC	517	n	bl	y	n	0	ev	cig+/-ot	21	40	2	0	nev cigs	st
AUVINE	503	x	c	0	0	all	-	all	Eu:Sca	1986	CC	517	n	bl	y	n	0	ev	cig+/-ot	41	999	3	0	nev cigs	st
AXELSS	501	x	m	0	0	sca	-	all	Eu:Sca	1989	CC	436	n	bl	n	n	0	ev	all/unsp	1	19	0	1	nev any	st
AXELSS	502	x	m	0	0	sca	-	all	Eu:Sca	1989	CC	436	n	bl	n	n	0	ev	all/unsp	20	29	1	2	nev any	st
AXELSS	503	x	m	0	0	sca	-	all	Eu:Sca	1989	CC	436	n	bl	n	n	0	ev	all/unsp	30	39	2	3	nev any	st
AXELSS	504	x	m	0	0	sca	-	all	Eu:Sca	1989	CC	436	n	bl	n	n	0	ev	all/unsp	40	49	0	4	nev any	st
AXELSS	505	x	m	0	0	sca	-	all	Eu:Sca	1989	CC	436	n	bl	n	n	0	ev	all/unsp	50	999	3	0	nev any	st
AXELSS	510		f	0	0	sca	-	all	Eu:Sca	1989	CC	436	n	bl	n	n	0	ev	all/unsp	1	19	0	1	nev any	st
AXELSS	511		f	0	0	sca	-	all	Eu:Sca	1989	CC	436	n	bl	n	n	0	ev	all/unsp	20	29	1	2	nev any	st
AXELSS	512		f	0	0	sca	-	all	Eu:Sca	1989	CC	436	n	bl	n	n	0	ev	all/unsp	30	39	2	3	nev any	st
AXELSS	513		f	0	0	sca	-	all	Eu:Sca	1989	CC	436	n	bl	n	n	0	ev	all/unsp	40	49	0	4	nev any	st
AXELSS	514		f	0	0	sca	-	all	Eu:Sca	1989	CC	436	n	bl	n	n	0	ev	all/unsp	50	999	3	0	nev any	st
BARBON	501	x	m	0	0	all	-	all	Eu:wst	1979	CC	755	n	bl	y	y	0	ev	all/unsp	1	29	1	0	nev any	st
BARBON	502	x	m	0	0	all	-	all	Eu:wst	1979	CC	755	n	bl	y	y	0	ev	all/unsp	30	39	2	3	nev any	st
BARBON	503	x	m	0	0	all	-	all	Eu:wst	1979	CC	755	n	bl	y	y	0	ev	all/unsp	40	49	0	4	nev any	st
BARBON	504	x	m	0	0	all	-	all	Eu:wst	1979	CC	755	n	bl	y	y	0	ev	all/unsp	50	999	3	0	nev any	st
BEST	501		m	0	0	all	0	all	NAmer	1955	pr	381	n	V	n	n	1	cu	cig only	1	4	0	0	nev any	ot
BEST	502		m	0	0	all	0	all	NAmer	1955	pr	381	n	V	n	n	1	cu	cig only	5	9	0	1	nev any	ot
BEST	503		m	0	0	all	0	all	NAmer	1955	pr	381	n	V	n	n	1	cu	cig only	10	14	0	0	nev any	ot
BEST	504		m	0	0	all	0	all	NAmer	1955	pr	381	n	V	n	n	1	cu	cig only	15	19	0	0	nev any	ot
BEST	505		m	0	0	all	0	all	NAmer	1955	pr	381	n	V	n	n	1	cu	cig only	20	29	1	2	nev any	ot
BEST	506		m	0	0	all	0	all	NAmer	1955	pr	381	n	V	n	n	1	cu	cig only	30	39	2	3	nev any	ot
BEST	507		m	0	0	all	0	all	NAmer	1955	pr	381	n	V	n	n	1	cu	cig only	40	999	3	0	nev any	ot
BOUCOT	518		m	0	0	all	9	all	NAmer	1951	pr	121	n	bl	n	n	0	ev	cig+/-ot	1	39	0	0	nev any	ot
BOUCOT	519		m	0	0	all	9	all	NAmer	1951	pr	121	n	bl	n	n	0	ev	cig+/-ot	40	999	3	0	nev any	ot
BUFFLE	526		f	0	0	w-hi	-	all	NAmer	1976	CC	943	n	bl	y	n	0	ev	cig+/-ot	1	30	1	0	nev cigs	or
BUFFLE	527		f	0	0	w-hi	-	all	NAmer	1976	CC	943	n	bl	y	n	0	ev	cig+/-ot	31	40	2	4	nev cigs	or
BUFFLE	528		f	0	0	w-hi	-	all	NAmer	1976	CC	943	n	bl	y	n	0	ev	cig+/-ot	41	999	3	0	nev cigs	or
CEDERL	501		m	40	69	all	10	all	Eu:Sca	1963	pr	491	n	bl	n	n	1	cu	cig only	1	29	1	0	nev any	ot
CEDERL	502		m	40	69	all	10	all	Eu:Sca	1963	pr	491	n	bl	n	n	1	cu	cig only	30	999	0	0	nev any	ot
CEDERL	504		f	40	69	all	10	all	Eu:Sca	1963	pr	491	n	bl	n	n	1	cu	cig only	1	29	1	0	nev any	ot
CEDERL	505		f	40	69	all	10	all	Eu:Sca	1963	pr	491	n	bl	n	n	1	cu	cig only	30	999	0	0	nev any	ot
CHEN2	501		m	0	0	all	-	all	As:Chi	1983	CC	193	n	ot	y	n	0	ev	all/unsp	1	9	0	1	nev any	st
CHEN2	502		m	0	0	all	-	all	As:Chi	1983	CC	193	n	ot	y	n	0	ev	all/unsp	10	20	1	2	nev any	st
CHEN2	503		m	0	0	all	-	all	As:Chi	1983	CC	193	n	ot	y	n	0	ev	all/unsp	21	30	0	3	nev any	st
CHEN2	504		m	0	0	all	-	all	As:Chi	1983	CC	193	n	ot	y	n	0	ev	all/unsp	31	40	2	4	nev any	st
CHEN2	505		m	0	0	all	-	all	As:Chi	1983	CC	193	n	ot	y	n	0	ev	all/unsp	41	999	3	0	nev any	st
CHEN2	510		f	0	0	all	-	all	As:Chi	1983	CC	193	n	ot	y	n	0	ev	all/unsp	1	20	1	0	nev any	st
CHEN2	511		f	0	0	all	-	all	As:Chi	1983	CC	193	n	ot	y	n	0	ev	all/unsp	21	30	0	3	nev any	st
CHEN2	512		f	0	0	all	-	all	As:Chi	1983	CC	193	n	ot	y	n	0	ev	all/unsp	31	40	2	4	nev any	st
CHEN2	513		f	0	0	all	-	all	As:Chi	1983	CC	193	n	ot	y	n	0	ev	all/unsp	41	999	3	0	nev any	st
CHOI	501		m	0	0	all	-	all	As:oth	1985	CC	375	n	bl	n	n	0	ev	cig+/-ot	1	19	0	1	nev cigs	st
CHOI	502		m	0	0	all	-	all	As:oth	1985	CC	375	n	bl	n	n	0	ev	cig+/-ot	20	29	1	2	nev cigs	st
CHOI	503		m	0	0	all	-	all	As:oth	1985	CC	375	n	bl	n	n	0	ev	cig+/-ot	30	39	2	3	nev cigs	st
CHOI	504		m	0	0	all	-	all	As:oth	1985	CC	375	n	bl	n	n	0	ev	cig+/-ot	40	49	0	4	nev cigs	st
CHOI	505		m	0	0	all	-	all	As:oth	1985	CC	375	n	bl	n	n	0	ev	cig+/-ot	50	999	3	0	nev cigs	st
CHOI	510		f	0	0	all	-	all	As:oth	1985	CC	375	n	bl	n	n	0	ev	cig+/-ot	1	19	0	1	nev cigs	st
CHOI	511		f	0	0	all	-	all	As:oth	1985	CC	375	n	bl	n	n	0	ev	cig+/-ot	20	29	1	2	nev cigs	st
CHOI	512		f	0	0	all	-	all	As:oth	1985	CC	375	n	bl	n	n	0	ev	cig+/-ot	30	39	2	3	nev cigs	st
CHOI	513		f	0	0	all	-	all	As:oth	1985	CC	375	n	bl	n	n	0	ev	cig+/-ot	40	999	3	0	nev cigs	st
CPSI	580		m	40	84	wh	0	all	NAmer	1959	pr	5138	n	bl	n	n	0	cu	cig only	1	29	1	0	nev cigs	st
CPSI	581		m	40	84	wh	0	all	NAmer	1959	pr	5138	n	bl	n	n	0	cu	cig only	30	34	0	3	nev cigs	st
CPSI	582		m	40	84	wh	0	all	NAmer	1959	pr	5138	n	bl	n	n	0	cu	cig only	35	39	2	0	nev cigs	st
CPSI	583		m	40	84	wh	0	all	NAmer	1959	pr	5138	n	bl	n	n	0	cu	cig only	40	44	0	4	nev cigs	st
CPSI	584		m	40	84	wh	0	all	NAmer	1959	pr	5138	n	bl	n	n	0	cu	cig only	45	49	0	0	nev cigs	st
CPSI	585		m	40	84	wh	0	all	NAmer	1959	pr	5138	n	bl	n	n	0	cu	cig only	50	54	3	5	nev cigs	st
CPSI	586		m	40	84	wh	0	all	NAmer	1959	pr	5138	n	bl	n	n	0	cu	cig only	55	59	0	0	nev cigs	st
CPSI	587		m	40	84	wh	0	all	NAmer	1959	pr	5138	n	bl	n	n	0	cu	cig only	60	999	0	6	nev cigs	st
CPSI	676		f	40	84	wh	0	all	NAmer	1959	pr	5138	n	bl	n	n	0	cu	cig only	1	29	1	0	nev cigs	st
CPSI	677		f	40	84	wh	0	all	NAmer	1959	pr	5138	n	bl	n	n	0	cu	cig only	30	34	0	3	nev cigs	st
CPSI	678		f	40	84	wh	0	all	NAmer	1959	pr	5138	n	bl	n	n	0	cu	cig only	35	39	2	0	nev cigs	st
CPSI	679		f	40	84	wh	0	all	NAmer	1959	pr	5138	n	bl	n	n	0	cu	cig only	40	44	0	4	nev cigs	st
CPSI	680		f	40	84	wh	0	all	NAmer	1959	pr	5138	n	bl	n	n	0	cu	cig only	45	49	0	0	nev cigs	st
CPSI	681		f	40	84	wh	0	all	NAmer	1959	pr	5138	n	bl	n	n	0	cu	cig only	50	54	3	5	nev cigs	st

Table 1I11 - 4

IESLC - Meta-analysis of Ever/current Smoking by Duration, Overview
All LC types, Any Product (or Cigarettes if Any not available)
Least adjusted

REF	NRR	X	SEX	AGEL	AGEH	RACE	YF	LC TYPE	LOC	START	ST	NLC	R	VB	P	H	AD	SM	PRODUCT	exL	exH	S1	S2	DENOM	De
CPSI	682		f	40	84	wh	0	all	NAmer	1959	pr	5138	n	bl	n	n	0	cu	cig only	55	999	0	6	nev cigs	st
CPSII	552		m	0	0	all	6	all	NAmer	1982	pr	3229	n	bl	n	n	0	cu	cig only	1	29	1	0	nev any	st
CPSII	553		m	0	0	all	6	all	NAmer	1982	pr	3229	n	bl	n	n	0	cu	cig only	30	34	0	3	nev any	st
CPSII	554		m	0	0	all	6	all	NAmer	1982	pr	3229	n	bl	n	n	0	cu	cig only	35	39	2	0	nev any	st
CPSII	555		m	0	0	all	6	all	NAmer	1982	pr	3229	n	bl	n	n	0	cu	cig only	40	44	0	4	nev any	st
CPSII	556		m	0	0	all	6	all	NAmer	1982	pr	3229	n	bl	n	n	0	cu	cig only	45	49	0	0	nev any	st
CPSII	557		m	0	0	all	6	all	NAmer	1982	pr	3229	n	bl	n	n	0	cu	cig only	50	54	3	5	nev any	st
CPSII	558		m	0	0	all	6	all	NAmer	1982	pr	3229	n	bl	n	n	0	cu	cig only	55	59	0	0	nev any	st
CPSII	559		m	0	0	all	6	all	NAmer	1982	pr	3229	n	bl	n	n	0	cu	cig only	60	999	0	6	nev any	st
CPSII	618		f	0	0	all	6	all	NAmer	1982	pr	3229	n	bl	n	n	0	cu	cig+/-ot	1	29	1	0	nev cigs	st
CPSII	619		f	0	0	all	6	all	NAmer	1982	pr	3229	n	bl	n	n	0	cu	cig+/-ot	30	34	0	3	nev cigs	st
CPSII	620		f	0	0	all	6	all	NAmer	1982	pr	3229	n	bl	n	n	0	cu	cig+/-ot	35	39	2	0	nev cigs	st
CPSII	621		f	0	0	all	6	all	NAmer	1982	pr	3229	n	bl	n	n	0	cu	cig+/-ot	40	44	0	4	nev cigs	st
CPSII	622		f	0	0	all	6	all	NAmer	1982	pr	3229	n	bl	n	n	0	cu	cig+/-ot	45	49	0	0	nev cigs	st
CPSII	623		f	0	0	all	6	all	NAmer	1982	pr	3229	n	bl	n	n	0	cu	cig+/-ot	50	54	3	5	nev cigs	st
CPSII	624		f	0	0	all	6	all	NAmer	1982	pr	3229	n	bl	n	n	0	cu	cig+/-ot	55	59	0	0	nev cigs	st
CPSII	625		f	0	0	all	6	all	NAmer	1982	pr	3229	n	bl	n	n	0	cu	cig+/-ot	60	999	0	6	nev cigs	st
DAMBER	506		m	0	0	all	-	all	Eu:Sca	1972	CC	579	n	bl	y	n	1	ev	all/unsp	1	20	1	0	nev any	ot
DAMBER	507		m	0	0	all	-	all	Eu:Sca	1972	CC	579	n	bl	y	n	1	ev	all/unsp	21	30	0	3	nev any	ot
DAMBER	508		m	0	0	all	-	all	Eu:Sca	1972	CC	579	n	bl	y	n	1	ev	all/unsp	31	40	2	4	nev any	ot
DAMBER	509		m	0	0	all	-	all	Eu:Sca	1972	CC	579	n	bl	y	n	1	ev	all/unsp	41	50	3	5	nev any	ot
DAMBER	510		m	0	0	all	-	all	Eu:Sca	1972	CC	579	n	bl	y	n	1	ev	all/unsp	51	999	0	6	nev any	ot
DEAN2	501		m	0	0	all	-	all	Eu:UK	1960	CC	954	n	V	y	n	0	cu	all/unsp	1	19	0	1	nev any	st
DEAN2	502		m	0	0	all	-	all	Eu:UK	1960	CC	954	n	V	y	n	0	cu	all/unsp	20	999	0	0	nev any	st
DEAN2	504		f	0	0	all	-	all	Eu:UK	1960	CC	954	n	V	y	n	0	cu	all/unsp	1	19	0	1	nev any	st
DEAN2	505		f	0	0	all	-	all	Eu:UK	1960	CC	954	n	V	y	n	0	cu	all/unsp	20	999	0	0	nev any	st
DESTEF	501	x	m	0	0	all	-	all	SCAmer	1988	CC	497	n	bl	n	y	0	ev	all/unsp	1	29	1	0	nev any	st
DESTEF	502	x	m	0	0	all	-	all	SCAmer	1988	CC	497	n	bl	n	y	0	ev	all/unsp	30	39	2	3	nev any	st
DESTEF	503	x	m	0	0	all	-	all	SCAmer	1988	CC	497	n	bl	n	y	0	ev	all/unsp	40	49	0	4	nev any	st
DESTEF	504	x	m	0	0	all	-	all	SCAmer	1988	CC	497	n	bl	n	y	0	ev	all/unsp	50	999	3	0	nev any	st
DOLL	515		m	0	0	all	-	all	Eu:UK	1948	CC	1465	n	V	n	n	0	ev	all/unsp	1	9	0	1	nev any	st
DOLL	516		m	0	0	all	-	all	Eu:UK	1948	CC	1465	n	V	n	n	0	ev	all/unsp	10	19	0	0	nev any	st
DOLL	517		m	0	0	all	-	all	Eu:UK	1948	CC	1465	n	V	n	n	0	ev	all/unsp	20	39	0	0	nev any	st
DOLL	518		m	0	0	all	-	all	Eu:UK	1948	CC	1465	n	V	n	n	0	ev	all/unsp	40	999	3	0	nev any	st
DOLL	522		f	0	0	all	-	all	Eu:UK	1948	CC	1465	n	V	n	n	0	ev	all/unsp	1	9	0	1	nev any	st
DOLL	523		f	0	0	all	-	all	Eu:UK	1948	CC	1465	n	V	n	n	0	ev	all/unsp	10	19	0	0	nev any	st
DOLL	524		f	0	0	all	-	all	Eu:UK	1948	CC	1465	n	V	n	n	0	ev	all/unsp	20	39	0	0	nev any	st
DOLL	525		f	0	0	all	-	all	Eu:UK	1948	CC	1465	n	V	n	n	0	ev	all/unsp	40	999	3	0	nev any	st
DORGAN	570		m	0	0	wh	-	all	NAmer	1980	CC	2026	n	bl	y	y	2	ev	cig+/-ot	1	34	1	0	nev any	ot
DORGAN	571		m	0	0	wh	-	all	NAmer	1980	CC	2026	n	bl	y	y	2	ev	cig+/-ot	35	999	0	0	nev any	ot
DORGAN	562		f	0	0	all	-	all	NAmer	1980	CC	2026	n	bl	y	y	3	ev	cig+/-ot	1	34	1	0	nev any	ot
DORGAN	563		f	0	0	all	-	all	NAmer	1980	CC	2026	n	bl	y	y	3	ev	cig+/-ot	35	999	0	0	nev any	ot
DOSEME	501		m	0	0	all	-	all	Eu:bal	1979	CC	1210	n	bl	n	n	2	ev	cig+/-ot	1	10	0	1	nev cigs	or
DOSEME	502		m	0	0	all	-	all	Eu:bal	1979	CC	1210	n	bl	n	n	2	ev	cig+/-ot	11	20	1	2	nev cigs	or
DOSEME	503		m	0	0	all	-	all	Eu:bal	1979	CC	1210	n	bl	n	n	2	ev	cig+/-ot	21	999	0	0	nev cigs	or
FAN	501		m	0	0	all	-	all	As:Chi	1990	CC	403	n	ot	y	n	0	ev	cig+/-ot	1	29	1	0	nev cigs	st
FAN	502		m	0	0	all	-	all	As:Chi	1990	CC	403	n	ot	y	n	0	ev	cig+/-ot	30	39	2	3	nev cigs	st
FAN	503		m	0	0	all	-	all	As:Chi	1990	CC	403	n	ot	y	n	0	ev	cig+/-ot	40	999	3	0	nev cigs	st
FAN	506		f	0	0	all	-	all	As:Chi	1990	CC	403	n	ot	y	n	0	ev	cig+/-ot	1	29	1	0	nev cigs	st
FAN	507		f	0	0	all	-	all	As:Chi	1990	CC	403	n	ot	y	n	0	ev	cig+/-ot	30	39	2	3	nev cigs	st
FAN	508		f	0	0	all	-	all	As:Chi	1990	CC	403	n	ot	y	n	0	ev	cig+/-ot	40	999	3	0	nev cigs	st
GAO	561	x	f	0	0	all	-	all	As:Chi	1984	CC	1405	n	ot	n	n	0	ev	cig+/-ot	1	29	1	0	nev cigs	st
GAO	562	x	f	0	0	all	-	all	As:Chi	1984	CC	1405	n	ot	n	n	0	ev	cig+/-ot	30	999	0	0	nev cigs	st
GARSHI	534	x	m	0	0	all	-	all	NAmer	1981	CC	1081	o	bl	y	n	0	ev	all/unsp	20	999	0	0	nev any	st
GER	513	x	c	0	0	all	-	all	As:oth	1990	CC	141	n	ot	y	n	0	ev	all/unsp	1	20	1	0	nev any	st
GER	514	x	c	0	0	all	-	all	As:oth	1990	CC	141	n	ot	y	n	0	ev	all/unsp	21	40	2	0	nev any	st
GER	515	x	c	0	0	all	-	all	As:oth	1990	CC	141	n	ot	y	n	0	ev	all/unsp	41	999	3	0	nev any	st
HAENSZ	542		f	0	0	all	-	not alv	NAmer	1955	CC	158	n	bl	n	y	0	ev	cig+/-ot	1	14	0	1	nev any	st
HAENSZ	543		f	0	0	all	-	not alv	NAmer	1955	CC	158	n	bl	n	y	0	ev	cig+/-ot	15	999	0	0	nev any	st
HU	501		m	0	0	all	-	all	As:Chi	1985	CC	227	n	ot	n	y	0	ev	cig+/-ot	1	19	0	1	nev cigs	st
HU	502		m	0	0	all	-	all	As:Chi	1985	CC	227	n	ot	n	y	0	ev	cig+/-ot	20	29	1	2	nev cigs	st
HU	503		m	0	0	all	-	all	As:Chi	1985	CC	227	n	ot	n	y	0	ev	cig+/-ot	30	999	0	0	nev cigs	st
HU	506		f	0	0	all	-	all	As:Chi	1985	CC	227	n	ot	n	y	0	ev	cig+/-ot	1	19	0	1	nev cigs	st
HU	507		f	0	0	all	-	all	As:Chi	1985	CC	227	n	ot	n	y	0	ev	cig+/-ot	20	29	1	2	nev cigs	st
HU	508		f	0	0	all	-	all	As:Chi	1985	CC	227	n	ot	n	y	0	ev	cig+/-ot	30	999	0	0	nev cigs	st
HU2	508		c	0	0	all	-	all	As:Chi	1977	CC	523	n	ot	y	n	0	ev	cig+/-ot	1	19	0	1	nev cigs	ot
HU2	509		c	0	0	all	-	all	As:Chi	1977	CC	523	n	ot	y	n	0	ev	cig+/-ot	20	29	1	2	nev cigs	or
HU2	510		c	0	0	all	-	all	As:Chi	1977	CC	523	n	ot	y	n	0	ev	cig+/-ot	30	39	2	3	nev cigs	or
HU2	511		c	0	0	all	-	all	As:Chi	1977	CC	523	n	ot	y	n	0	ev	cig+/-ot	40	999	3	0	nev cigs	st
HUMBLE	517	x	c	0	0	wh	-	not alv	NAmer	1980	CC	521	n	bl	y	n	0	cu	cig+/-ot	1	29	1	0	nev cigs	st
HUMBLE	518	x	c	0	0	wh	-	not alv	NAmer	1980	CC	521	n	bl	y	n	0	cu	cig+/-ot	30	39	2	3	nev cigs	st
HUMBLE	519	x	c	0	0	wh	-	not alv	NAmer	1980	CC	521	n	bl	y	n	0	cu	cig+/-ot	40	49	0	4	nev cigs	st

Table 1I11 - 4

IESLC - Meta-analysis of Ever/current Smoking by Duration, Overview
All LC types, Any Product (or Cigarettes if Any not available)
Least adjusted

REF	NRR	X	SEX	AGEL	AGEH	RACE	YF	LC TYPE	LOC	START	ST	NLC	R	VB	P	H	AD	SM	PRODUCT	exL	exH	S1	S2	DENOM	De
HUMBLE	520	x	c	0	0	wh	-	not alv	NAmer	1980	CC	521	n	bl	y	n	0	cu	cig+/-ot	50	59	3	5	nev cigs	st
HUMBLE	521	x	c	0	0	wh	-	not alv	NAmer	1980	CC	521	n	bl	y	n	0	cu	cig+/-ot	60	999	0	6	nev cigs	st
JOLY	515		m	0	0	all	-	all	SCAmer	1978	CC	826	n	bl	n	n	0	ev	cig+/-ot	1	19	0	1	nev any	st
JOLY	516		m	0	0	all	-	all	SCAmer	1978	CC	826	n	bl	n	n	0	ev	cig+/-ot	20	29	1	2	nev any	st
JOLY	517		m	0	0	all	-	all	SCAmer	1978	CC	826	n	bl	n	n	0	ev	cig+/-ot	30	39	2	3	nev any	st
JOLY	518		m	0	0	all	-	all	SCAmer	1978	CC	826	n	bl	n	n	0	ev	cig+/-ot	40	49	0	4	nev any	st
JOLY	519		m	0	0	all	-	all	SCAmer	1978	CC	826	n	bl	n	n	0	ev	cig+/-ot	50	999	3	0	nev any	st
JOLY	501		f	0	0	all	-	all	SCAmer	1978	CC	826	n	bl	n	n	0	ev	cig+/-ot	1	19	0	1	nev any	st
JOLY	502		f	0	0	all	-	all	SCAmer	1978	CC	826	n	bl	n	n	0	ev	cig+/-ot	20	29	1	2	nev any	st
JOLY	503		f	0	0	all	-	all	SCAmer	1978	CC	826	n	bl	n	n	0	ev	cig+/-ot	30	39	2	3	nev any	st
JOLY	504		f	0	0	all	-	all	SCAmer	1978	CC	826	n	bl	n	n	0	ev	cig+/-ot	40	49	0	4	nev any	st
JOLY	505		f	0	0	all	-	all	SCAmer	1978	CC	826	n	bl	n	n	0	ev	cig+/-ot	50	999	3	0	nev any	st
JUSSAW	510		m	0	0	all	-	all	As:Ind	1964	CC	792	n	V	n	n	0	ev	cig only	1	9	0	1	nev any	st
JUSSAW	511		m	0	0	all	-	all	As:Ind	1964	CC	792	n	V	n	n	0	ev	cig only	10	19	0	0	nev any	st
JUSSAW	512		m	0	0	all	-	all	As:Ind	1964	CC	792	n	V	n	n	0	ev	cig only	20	29	1	2	nev any	st
JUSSAW	513		m	0	0	all	-	all	As:Ind	1964	CC	792	n	V	n	n	0	ev	cig only	30	39	2	3	nev any	st
JUSSAW	514		m	0	0	all	-	all	As:Ind	1964	CC	792	n	V	n	n	0	ev	cig only	40	999	3	0	nev any	st
KAISE2	596		m	0	0	all	9	all	NAmer	1979	pr	318	n	bl	n	n	1	cu	cig only	1	39	0	0	nev any	st
KAISE2	597		m	0	0	all	9	all	NAmer	1979	pr	318	n	bl	n	n	1	cu	cig only	40	999	3	0	nev any	st
KAISE2	516		f	0	0	all	9	all	NAmer	1979	pr	318	n	bl	n	n	1	cu	cig only	1	39	0	0	nev any	st
KAISE2	517		f	0	0	all	9	all	NAmer	1979	pr	318	n	bl	n	n	1	cu	cig only	40	999	3	0	nev any	st
KATSOU	501	x	f	0	0	all	-	all	Eu:bal	1987	CC	101	n	bl	n	n	0	cu	all/unsp	1	19	0	1	nev any	st
KATSOU	502	x	f	0	0	all	-	all	Eu:bal	1987	CC	101	n	bl	n	n	0	cu	all/unsp	20	29	1	2	nev any	st
KATSOU	503	x	f	0	0	all	-	all	Eu:bal	1987	CC	101	n	bl	n	n	0	cu	all/unsp	30	39	2	3	nev any	st
KATSOU	504	x	f	0	0	all	-	all	Eu:bal	1987	CC	101	n	bl	n	n	0	cu	all/unsp	40	999	3	0	nev any	st
KHUDER	501		m	0	0	all	-	all	NAmer	1985	CC	482	n	bl	n	y	0	ev	cig+/-ot	1	29	1	0	nev cigs	st
KHUDER	502		m	0	0	all	-	all	NAmer	1985	CC	482	n	bl	n	y	0	ev	cig+/-ot	30	49	2	0	nev cigs	st
KHUDER	503		m	0	0	all	-	all	NAmer	1985	CC	482	n	bl	n	y	0	ev	cig+/-ot	50	999	3	0	nev cigs	st
KREUZE	517		m	0	0	all	-	all	Eu:Ger	1990	CC	2260	n	bl	n	n	3	ev	all/unsp	1	19	0	1	nev any	st
KREUZE	518		m	0	0	all	-	all	Eu:Ger	1990	CC	2260	n	bl	n	n	3	ev	all/unsp	20	999	0	0	nev any	ot
KREUZE	520		f	0	0	all	-	all	Eu:Ger	1990	CC	2260	n	bl	n	n	3	ev	all/unsp	1	19	0	1	nev any	ot
KREUZE	521		f	0	0	all	-	all	Eu:Ger	1990	CC	2260	n	bl	n	n	3	ev	all/unsp	20	999	0	0	nev any	ot
LETOUR	506		c	0	0	all	-	all	NAmer	1983	CC	738	n	V	y	y	0	ev	cig+/-ot	1	24	1	0	nev cigs	st
LETOUR	507		c	0	0	all	-	all	NAmer	1983	CC	738	n	V	y	y	0	ev	cig+/-ot	25	40	2	0	nev cigs	st
LETOUR	508		c	0	0	all	-	all	NAmer	1983	CC	738	n	V	y	y	0	ev	cig+/-ot	41	999	3	0	nev cigs	st
LEVIN	501	x	m	0	0	all	-	all	NAmer	1938	CC	475	n	bl	n	n	0	ev	cig+/-ot	1	39	0	0	nev any	st
LEVIN	502	x	m	0	0	all	-	all	NAmer	1938	CC	475	n	bl	n	n	0	ev	cig+/-ot	40	999	3	0	nev any	st
LIAW	501		c	0	0	all	0	all	As:oth	1982	pr	127	n	ot	n	n	2	cu	all/unsp	1	20	1	0	nev any	or
LIAW	502		c	0	0	all	0	all	As:oth	1982	pr	127	n	ot	n	n	2	cu	all/unsp	21	30	0	3	nev any	or
LIAW	503		c	0	0	all	0	all	As:oth	1982	pr	127	n	ot	n	n	2	cu	all/unsp	31	999	0	0	nev any	or
LIU3	507	x	m	0	0	all	-	all	As:Chi	1985	CC	110	n	ot	n	n	0	ev	all/unsp	1	34	1	0	nev any	or
LIU3	508	x	m	0	0	all	-	all	As:Chi	1985	CC	110	n	ot	n	n	0	ev	all/unsp	35	999	0	0	nev any	st
LIU5	504		c	0	0	all	-	all	As:Chi	1978	CC	111	n	ot	y	n	0	ev	all/unsp	1	29	1	0	nev any	st
LIU5	505		c	0	0	all	-	all	As:Chi	1978	CC	111	n	ot	y	n	0	ev	all/unsp	30	999	0	0	nev any	st
LUBIN	508		m	0	0	all	-	all	As:Chi	1984	CC	427	m	ot	y	n	0	ev	cig+/-ot	1	29	1	0	nev any	st
LUBIN	509		m	0	0	all	-	all	As:Chi	1984	CC	427	m	ot	y	n	0	ev	cig+/-ot	30	39	2	3	nev any	st
LUBIN	510		m	0	0	all	-	all	As:Chi	1984	CC	427	m	ot	y	n	0	ev	cig+/-ot	40	49	0	4	nev any	st
LUBIN	511		m	0	0	all	-	all	As:Chi	1984	CC	427	m	ot	y	n	0	ev	cig+/-ot	50	999	3	0	nev any	st
LUBIN2	531		m	0	0	all	-	all	Eu:mul	1976	CC	7804	n	bl	n	y	0	ev	cig+/-ot	1	29	1	0	nev any	st
LUBIN2	532		m	0	0	all	-	all	Eu:mul	1976	CC	7804	n	bl	n	y	0	ev	cig+/-ot	30	39	2	3	nev any	st
LUBIN2	533		m	0	0	all	-	all	Eu:mul	1976	CC	7804	n	bl	n	y	0	ev	cig+/-ot	40	49	0	4	nev any	st
LUBIN2	534		m	0	0	all	-	all	Eu:mul	1976	CC	7804	n	bl	n	y	0	ev	cig+/-ot	50	999	3	0	nev any	st
LUBIN2	574		f	0	0	all	-	all	Eu:mul	1976	CC	7804	n	bl	n	y	0	ev	cig+/-ot	1	29	1	0	nev any	st
LUBIN2	575		f	0	0	all	-	all	Eu:mul	1976	CC	7804	n	bl	n	y	0	ev	cig+/-ot	30	39	2	3	nev any	st
LUBIN2	576		f	0	0	all	-	all	Eu:mul	1976	CC	7804	n	bl	n	y	0	ev	cig+/-ot	40	49	0	4	nev any	st
LUBIN2	577		f	0	0	all	-	all	Eu:mul	1976	CC	7804	n	bl	n	y	0	ev	cig+/-ot	50	999	3	0	nev any	st
MATOS	516	x	m	0	0	all	-	all	SCAmer	1994	CC	200	n	bl	n	n	0	ev	cig+/-ot	1	24	1	0	nev any	st
MATOS	517	x	m	0	0	all	-	all	SCAmer	1994	CC	200	n	bl	n	n	0	ev	cig+/-ot	25	39	2	3	nev any	st
MATOS	518	x	m	0	0	all	-	all	SCAmer	1994	CC	200	n	bl	n	n	0	ev	cig+/-ot	40	70	3	0	nev any	st
MCCONN	501		c	0	0	all	-	all	Eu:UK	1946	CC	100	n	V	n	y	0	ev	all/unsp	1	9	0	1	nev any	st
MCCONN	502		c	0	0	all	-	all	Eu:UK	1946	CC	100	n	V	n	y	0	ev	all/unsp	10	19	0	0	nev any	st
MCCONN	503		c	0	0	all	-	all	Eu:UK	1946	CC	100	n	V	n	y	0	ev	all/unsp	20	29	1	2	nev any	st
MCCONN	504		c	0	0	all	-	all	Eu:UK	1946	CC	100	n	V	n	y	0	ev	all/unsp	30	39	2	3	nev any	st
MCCONN	505		c	0	0	all	-	all	Eu:UK	1946	CC	100	n	V	n	y	0	ev	all/unsp	40	999	3	0	nev any	st
NOTAN2	513		c	0	0	all	-	all	As:Ind	1963	CC	683	n	V	n	n	0	ev	cig only	1	10	0	1	nev any	st
NOTAN2	514		c	0	0	all	-	all	As:Ind	1963	CC	683	n	V	n	n	0	ev	cig only	11	20	1	2	nev any	st
NOTAN2	515		c	0	0	all	-	all	As:Ind	1963	CC	683	n	V	n	n	0	ev	cig only	21	30	0	3	nev any	st
NOTAN2	516		c	0	0	all	-	all	As:Ind	1963	CC	683	n	V	n	n	0	ev	cig only	31	40	2	4	nev any	st
NOTAN2	517		c	0	0	all	-	all	As:Ind	1963	CC	683	n	V	n	n	0	ev	cig only	41	999	3	0	nev any	st
OSANN2	501	x	f	0	0	all	-	all	NAmer	1964	ot	217	n	bl	n	y	0	ev	cig+/-ot	1	20	1	0	nev cigs	st
OSANN2	502	x	f	0	0	all	-	all	NAmer	1964	ot	217	n	bl	n	y	0	ev	cig+/-ot	21	999	0	0	nev cigs	st
PEZZO2	507		m	0	0	all	-	all	SCAmer	1992	CC	367	n	bl	n	y	0	cu	cig+/-ot	1	35	0	0	nev cigs	st

Table 1I11 - 4

IESLC - Meta-analysis of Ever/current Smoking by Duration, Overview
All LC types, Any Product (or Cigarettes if Any not available)
Least adjusted

REF	NRR	X	SEX	AGEL	AGEH	RACE	YF	LC TYPE	LOC	START	ST	NLC	R	VB	P	H	AD	SM	PRODUCT	exL	exH	S1	S2	DENOM	De
PEZZO2	508		m	0	0	all	-	all	SCAmer	1992	CC	367	n	bl	n	y	0	cu	cig+/-ot	36	999	3	0	nev cigs	st
PEZZOT	534		m	0	0	all	-	all	SCAmer	1987	CC	215	n	bl	n	y	0	ev	cig only	1	30	1	0	nev cigs	st
PEZZOT	535		m	0	0	all	-	all	SCAmer	1987	CC	215	n	bl	n	y	0	ev	cig only	31	40	2	4	nev cigs	st
PEZZOT	536		m	0	0	all	-	all	SCAmer	1987	CC	215	n	bl	n	y	0	ev	cig only	41	999	3	0	nev cigs	st
QIAO2	511	x	m	0	0	all	0	all	As:Chi	1992	pr	241	m	ot	n	n	0	ev	all/unsp	1	27	1	0	nev any	st
QIAO2	512	x	m	0	0	all	0	all	As:Chi	1992	pr	241	m	ot	n	n	0	ev	all/unsp	28	41	2	0	nev any	st
QIAO2	513	x	m	0	0	all	0	all	As:Chi	1992	pr	241	m	ot	n	n	0	ev	all/unsp	42	999	3	0	nev any	st
RACHTA	511	x	f	0	0	all	-	all	Eu:est	1991	CC	118	n	bl	n	y	0	ev	cig+/-ot	1	20	1	0	nev cigs	st
RACHTA	512	x	f	0	0	all	-	all	Eu:est	1991	CC	118	n	bl	n	y	0	ev	cig+/-ot	21	40	2	0	nev cigs	st
RACHTA	513	x	f	0	0	all	-	all	Eu:est	1991	CC	118	n	bl	n	y	0	ev	cig+/-ot	41	999	3	0	nev cigs	st
SOBUE	546		m	0	0	all	-	q+s+l+a	As:Jap	1986	CC	1376	n	bl	n	y	0	cu	cig+/-ot	1	29	1	0	nev cigs	st
SOBUE	547		m	0	0	all	-	q+s+l+a	As:Jap	1986	CC	1376	n	bl	n	y	0	cu	cig+/-ot	30	39	2	3	nev cigs	st
SOBUE	548		m	0	0	all	-	q+s+l+a	As:Jap	1986	CC	1376	n	bl	n	y	0	cu	cig+/-ot	40	49	0	4	nev cigs	st
SOBUE	549		m	0	0	all	-	q+s+l+a	As:Jap	1986	CC	1376	n	bl	n	y	0	cu	cig+/-ot	50	999	3	0	nev cigs	st
TIZZAN	501		m	0	0	all	-	all	Eu:wst	1959	CC	1358	n	bl	n	n	0	ev	cig only	1	4	0	0	nev any	st
TIZZAN	502		m	0	0	all	-	all	Eu:wst	1959	CC	1358	n	bl	n	n	0	ev	cig only	5	10	0	1	nev any	st
TIZZAN	503		m	0	0	all	-	all	Eu:wst	1959	CC	1358	n	bl	n	n	0	ev	cig only	11	999	0	0	nev any	st
TIZZAN	533		f	0	0	all	-	all	Eu:wst	1959	CC	1358	n	bl	n	n	0	ev	all/unsp	1	10	0	1	nev any	st
TIZZAN	534		f	0	0	all	-	all	Eu:wst	1959	CC	1358	n	bl	n	n	0	ev	all/unsp	11	999	0	0	nev any	st
WANG2	501		c	0	0	all	-	all	As:Chi	1980	CC	103	n	ot	n	n	0	ev	cig+/-ot	1	19	0	1	nev cigs	st
WANG2	503		c	0	0	all	-	all	As:Chi	1980	CC	103	n	ot	n	n	0	ev	cig+/-ot	20	29	1	2	nev cigs	st
WANG2	504		c	0	0	all	-	all	As:Chi	1980	CC	103	n	ot	n	n	0	ev	cig+/-ot	30	39	2	3	nev cigs	st
WANG2	505		c	0	0	all	-	all	As:Chi	1980	CC	103	n	ot	n	n	0	ev	cig+/-ot	40	49	0	4	nev cigs	st
WUWILL	501	x	f	0	0	all	-	all	As:Chi	1985	CC	965	n	ot	n	n	0	ev	cig+/-ot	1	29	1	0	nev cigs	st
WUWILL	502	x	f	0	0	all	-	all	As:Chi	1985	CC	965	n	ot	n	n	0	ev	cig+/-ot	30	39	2	3	nev cigs	st
WUWILL	503	x	f	0	0	all	-	all	As:Chi	1985	CC	965	n	ot	n	n	0	ev	cig+/-ot	40	999	3	0	nev cigs	st
ZHENG	553		m	0	0	all	-	all	As:Chi	1982	CC	540	n	ot	*	y	0	ev	cig+/-ot	1	29	1	0	nev cigs	st
ZHENG	554		m	0	0	all	-	all	As:Chi	1982	CC	540	n	ot	*	y	0	ev	cig+/-ot	30	999	0	0	nev cigs	st
ZHENG	558		f	0	0	all	-	all	As:Chi	1982	CC	540	n	ot	*	y	0	ev	cig+/-ot	1	29	1	0	nev cigs	st
ZHENG	559		f	0	0	all	-	all	As:Chi	1982	CC	540	n	ot	*	y	0	ev	cig+/-ot	30	999	0	0	nev cigs	st
ZHOU	501		c	0	0	all	-	all	As:Chi	1978	CC	1360	n	ot	n	n	0	ev	all/unsp	1	19	0	1	nev any	st
ZHOU	502		c	0	0	all	-	all	As:Chi	1978	CC	1360	n	ot	n	n	0	ev	all/unsp	20	999	0	0	nev any	st

Cigarette type is all/unspec for all RRs

except for the following:

REF	NRR	CIGTYPE
JUSSAW	510	MC only
JUSSAW	511	MC only
JUSSAW	512	MC only
JUSSAW	513	MC only
JUSSAW	514	MC only
NOTAN2	513	MC only
NOTAN2	514	MC only
NOTAN2	515	MC only
NOTAN2	516	MC only
NOTAN2	517	MC only

In this overview table, subtotals and Qs values may be invalid and should be ignored

Table 1I11 - 5

IESLC - Meta-analysis of Ever/current Smoking by Duration, Overview
All LC types, Any Product (or Cigarettes if Any not available)
Least adjusted

REF	NRR	SEX	AD	Number Exposed Case	Number Exposed Cont	Non-exposed Case	Non-exposed Cont	RR	95.00%CI
AGUDO	507	f	0	5	12	80	183	0.95	(0.33- 2.79)
AGUDO	508	f	0	18	11	80	183	3.74	(1.69- 8.29)
Subtotal AGUDO								2.31	(1.22- 4.38)
*AMANDU	501	m	0	42	68909	6	25350	2.58	(1.09- 6.06)
*AMANDU	502	m	0	72	27096	6	25350	11.23	(4.88- 25.81)
Subtotal AMANDU								5.48	(3.02- 9.96)
ARMADA	501	m	0	21	55	8	71	3.39	(1.40- 8.23)
ARMADA	502	m	0	219	166	8	71	11.71	(5.49- 24.99)
ARMADA	503	m	0	77	33	8	71	20.71	(8.97- 47.82)
Subtotal ARMADA								9.86	(6.13- 15.85)
AUVINE	501	c	0	26	18	44	229	7.52	(3.80- 14.87)
AUVINE	502	c	0	10	5	44	229	10.41	(3.39- 31.93)
AUVINE	503	c	0	230	57	44	229	21.00	(13.61- 32.41)
Subtotal AUVINE								15.02	(10.61- 21.28)
AXELSS	501	m	0	13	84	16	160	1.55	(0.71- 3.37)
AXELSS	502	m	0	17	64	16	160	2.66	(1.27- 5.58)
AXELSS	503	m	0	57	71	16	160	8.03	(4.31- 14.94)
AXELSS	504	m	0	104	85	16	160	12.24	(6.79- 22.04)
AXELSS	505	m	0	101	40	16	160	25.25	(13.43- 47.46)
AXELSS	510	f	0	5	24	18	154	1.78	(0.61- 5.25)
AXELSS	511	f	0	12	29	18	154	3.54	(1.54- 8.13)
AXELSS	512	f	0	29	26	18	154	9.54	(4.64- 19.61)
AXELSS	513	f	0	44	20	18	154	18.82	(9.17- 38.65)
AXELSS	514	f	0	20	10	18	154	17.11	(6.94- 42.19)
Subtotal AXELSS								7.94	(6.31- 10.00)
BARBON	501	m	0	42	91	22	188	3.94	(2.22- 7.00)
BARBON	502	m	0	118	102	22	188	9.89	(5.91- 16.55)
BARBON	503	m	0	207	139	22	188	12.73	(7.79- 20.80)
BARBON	504	m	0	366	235	22	188	13.31	(8.31- 21.32)
Subtotal BARBON								9.63	(7.47- 12.42)
*BEST	501	m	1	1	-	7	-	1.60	(0.20- 13.00)
*BEST	502	m	1	2	-	7	-	2.60	(0.54- 12.52)
*BEST	503	m	1	6	-	7	-	2.30	(0.77- 6.84)
*BEST	504	m	1	10	-	7	-	3.20	(1.22- 8.41)
*BEST	505	m	1	22	-	7	-	4.10	(1.75- 9.60)
*BEST	506	m	1	55	-	7	-	13.90	(6.33- 30.52)
*BEST	507	m	1	137	-	7	-	14.20	(6.64- 30.35)
Subtotal BEST								6.17	(4.26- 8.94)
*BOUCOT	518	m	0	29	2621	0	805	18.13~	(1.11- 296.36)
*BOUCOT	519	m	0	52	1563	0	805	54.09~	(3.34- 875.17)
Subtotal BOUCOT								31.38	(4.37- 225.47)
BUFFLE	526	f	0	52	57	12	112	8.51	(4.21- 17.22)
BUFFLE	527	f	0	97	62	12	112	14.60	(7.43- 28.69)
BUFFLE	528	f	0	90	42	12	112	20.00	(9.94- 40.23)
Subtotal BUFFLE								13.60	(9.12- 20.29)
*CEDERL	501	m	1	5	-	7	-	1.80	(0.57- 5.66)
*CEDERL	502	m	1	23	-	7	-	7.40	(3.18- 17.21)
*CEDERL	504	f	1	3	-	19	-	1.60	(0.47- 5.40)
*CEDERL	505	f	1	5	-	19	-	9.60	(3.60- 25.58)
Subtotal CEDERL								4.61	(2.78- 7.67)
CHEN2	501	m	0	2	3	9	33	2.44	(0.35- 16.93)
CHEN2	502	m	0	4	3	9	33	4.89	(0.92- 25.93)
CHEN2	503	m	0	17	24	9	33	2.60	(0.99- 6.81)
CHEN2	504	m	0	36	27	9	33	4.89	(2.01- 11.91)
CHEN2	505	m	0	62	40	9	33	5.68	(2.46- 13.13)
CHEN2	510	f	0	1	6	25	33	0.22	(0.02- 1.95)
CHEN2	511	f	0	2	2	25	33	1.32	(0.17- 10.03)
CHEN2	512	f	0	13	6	25	33	2.86	(0.95- 8.58)
CHEN2	513	f	0	21	15	25	33	1.85	(0.80- 4.29)
Subtotal CHEN2								3.01	(2.07- 4.38)
CHOI	501	m	0	19	55	13	95	2.52	(1.16- 5.51)
CHOI	502	m	0	66	166	13	95	2.91	(1.52- 5.54)
CHOI	503	m	0	102	160	13	95	4.66	(2.48- 8.75)
CHOI	504	m	0	60	64	13	95	6.85	(3.48- 13.50)
CHOI	505	m	0	20	20	13	95	7.31	(3.13- 17.07)
CHOI	510	f	0	2	9	76	164	0.48	(0.10- 2.27)
CHOI	511	f	0	8	14	76	164	1.23	(0.50- 3.06)
CHOI	512	f	0	8	2	76	164	8.63	(1.79- 41.62)
CHOI	513	f	0	1	1	76	164	2.16	(0.13- 34.96)
Subtotal CHOI								3.63	(2.73- 4.83)
*CPSI	580	m	0	95	266163	196	926068	1.69	(1.32- 2.15)

Table 1I11 - 5

IESLC - Meta-analysis of Ever/current Smoking by Duration, Overview
All LC types, Any Product (or Cigarettes if Any not available)
Least adjusted

REF	NRR	SEX	AD	Number Exposed Case	Number Exposed Cont	Non-exposed Case	Non-exposed Cont	RR	95.00%CI
*CPSI	581	m	0	230	290031	196	926068	3.75	(3.10- 4.53)
*CPSI	582	m	0	470	367622	196	926068	6.04	(5.11- 7.14)
*CPSI	583	m	0	731	333292	196	926068	10.36	(8.85- 12.13)
*CPSI	584	m	0	764	221405	196	926068	16.30	(13.94- 19.07)
*CPSI	585	m	0	576	119633	196	926068	22.75	(19.35- 26.75)
*CPSI	586	m	0	356	53226	196	926068	31.60	(26.55- 37.61)
*CPSI	587	m	0	232	26906	196	926068	40.74	(33.70- 49.25)
*CPSI	676	f	0	105	694015	532	3877179	1.10	(0.89- 1.36)
*CPSI	677	f	0	141	383127	532	3877179	2.68	(2.23- 3.23)
*CPSI	678	f	0	154	315060	532	3877179	3.56	(2.98- 4.26)
*CPSI	679	f	0	120	163178	532	3877179	5.36	(4.40- 6.53)
*CPSI	680	f	0	54	53635	532	3877179	7.34	(5.55- 9.71)
*CPSI	681	f	0	16	14305	532	3877179	8.15	(4.96- 13.40)
*CPSI	682	f	0	10	5657	532	3877179	12.88	(6.90- 24.07)
Subtotal CPSI								8.01	(7.62- 8.43)
*CPSII	552	m	0	72	141932	124	742207	3.04	(2.27- 4.06)
*CPSII	553	m	0	145	113317	124	742207	7.66	(6.03- 9.73)
*CPSII	554	m	0	244	109788	124	742207	13.30	(10.72- 16.51)
*CPSII	555	m	0	413	103500	124	742207	23.88	(19.54- 29.19)
*CPSII	556	m	0	307	53805	124	742207	34.15	(27.73- 42.06)
*CPSII	557	m	0	332	39260	124	742207	50.62	(41.19- 62.20)
*CPSII	558	m	0	151	13598	124	742207	66.47	(52.45- 84.24)
*CPSII	559	m	0	117	8450	124	742207	82.88	(64.43- 106.60)
*CPSII	618	f	0	127	301244	310	2091302	2.84	(2.31- 3.50)
*CPSII	619	f	0	158	152833	310	2091302	6.97	(5.76- 8.45)
*CPSII	620	f	0	193	116270	310	2091302	11.20	(9.36- 13.40)
*CPSII	621	f	0	216	91501	310	2091302	15.93	(13.39- 18.94)
*CPSII	622	f	0	153	44769	310	2091302	23.06	(19.00- 27.98)
*CPSII	623	f	0	122	29119	310	2091302	28.26	(22.93- 34.84)
*CPSII	624	f	0	27	6262	310	2091302	29.09	(19.64- 43.07)
*CPSII	625	f	0	18	2224	310	2091302	54.60	(34.01- 87.65)
Subtotal CPSII								17.37	(16.44- 18.35)
DAMBER	506	m	1	-	-	42	-	1.58	(0.69- 3.66)
DAMBER	507	m	1	-	-	42	-	3.66	(2.18- 6.73)
DAMBER	508	m	1	-	-	42	-	5.15	(3.27- 8.32)
DAMBER	509	m	1	-	-	42	-	8.71	(5.84- 13.66)
DAMBER	510	m	1	-	-	42	-	11.19	(7.43- 17.33)
Subtotal DAMBER								6.42	(5.14- 8.01)
DEAN2	501	m	0	34	36	33	112	3.21	(1.74- 5.89)
DEAN2	502	m	0	631	558	33	112	3.84	(2.56- 5.75)
DEAN2	504	f	0	10	14	88	121	0.98	(0.42- 2.31)
DEAN2	505	f	0	47	11	88	121	5.88	(2.88- 11.97)
Subtotal DEAN2								3.39	(2.55- 4.52)
DESTEF	501	m	0	43	55	27	163	4.72	(2.67- 8.35)
DESTEF	502	m	0	78	78	27	163	6.04	(3.61- 10.10)
DESTEF	503	m	0	171	93	27	163	11.10	(6.87- 17.92)
DESTEF	504	m	0	178	108	27	163	9.95	(6.20- 15.96)
Subtotal DESTEF								7.86	(6.10- 10.11)
DOLL	515	m	0	12	15	7	61	6.97	(2.34- 20.73)
DOLL	516	m	0	34	65	7	61	4.56	(1.88- 11.05)
DOLL	517	m	0	746	725	7	61	8.97	(4.07- 19.73)
DOLL	518	m	0	558	491	7	61	9.90	(4.49- 21.85)
DOLL	522	f	0	14	18	40	59	1.15	(0.51- 2.57)
DOLL	523	f	0	12	8	40	59	2.21	(0.83- 5.90)
DOLL	524	f	0	36	20	40	59	2.66	(1.35- 5.23)
DOLL	525	f	0	6	3	40	59	2.95	(0.70- 12.49)
Subtotal DOLL								3.93	(2.89- 5.34)
DORGAN	570	m	2	-	-	-	-	5.44	(2.97- 9.98)
DORGAN	571	m	2	-	-	-	-	16.09	(8.96- 28.88)
DORGAN	562	f	3	-	-	-	-	4.25	(3.20- 5.64)
DORGAN	563	f	3	-	-	-	-	11.73	(9.07- 15.18)
Subtotal DORGAN								7.74	(6.50- 9.20)
DOSEME	501	m	2	32	-	142	-	1.00	(0.60- 1.70)
DOSEME	502	m	2	158	-	142	-	3.80	(2.60- 5.70)
DOSEME	503	m	2	466	-	142	-	4.90	(3.50- 7.00)
Subtotal DOSEME								3.27	(2.59- 4.12)
FAN	501	m	0	29	135	36	236	1.41	(0.83- 2.40)
FAN	502	m	0	44	122	36	236	2.36	(1.45- 3.87)
FAN	503	m	0	143	241	36	236	3.89	(2.59- 5.84)
FAN	506	f	0	8	15	69	320	2.47	(1.01- 6.06)
FAN	507	f	0	19	23	69	320	3.83	(1.98- 7.42)

Table 1I11 - 5

IESLC - Meta-analysis of Ever/current Smoking by Duration, Overview
All LC types, Any Product (or Cigarettes if Any not available)
Least adjusted

REF	NRR	SEX	AD	Number Exposed Case	Cont	Non-exposed Case	Cont	RR	95.00%CI
FAN	508	f	0	55	59	69	320	4.32	(2.76- 6.78)
Subtotal FAN								3.01	(2.43- 3.72)
GAO	561	f	0	68	58	435	605	1.63	(1.12- 2.36)
GAO	562	f	0	168	72	435	605	3.25	(2.40- 4.39)
Subtotal GAO								2.47	(1.95- 3.12)
GARSHI	534	m	0	922	1314	41	363	6.21	(4.45- 8.67)
GER	513	c	0	10	40	51	246	1.21	(0.57- 2.57)
GER	514	c	0	31	123	51	246	1.22	(0.74- 2.00)
GER	515	c	0	49	155	51	246	1.52	(0.98- 2.37)
Subtotal GER								1.35	(1.00- 1.83)
HAENSZ	542	f	0	16	26	81	236	1.79	(0.92- 3.51)
HAENSZ	543	f	0	58	77	81	236	2.19	(1.44- 3.35)
Subtotal HAENSZ								2.07	(1.45- 2.97)
HU	501	m	0	41	33	41	67	2.03	(1.11- 3.70)
HU	502	m	0	60	47	41	67	2.09	(1.21- 3.60)
HU	503	m	0	19	14	41	67	2.22	(1.00- 4.90)
HU	506	f	0	11	8	40	48	1.65	(0.61- 4.50)
HU	507	f	0	11	7	40	48	1.89	(0.67- 5.32)
HU	508	f	0	4	3	40	48	1.60	(0.34- 7.57)
Subtotal HU								2.00	(1.46- 2.74)
HU2	508	c	0	21	33	121	213	1.12	(0.62- 2.02)
HU2	509	c	0	64	63	121	213	1.79	(1.18- 2.70)
HU2	510	c	0	123	101	121	213	2.14	(1.52- 3.03)
HU2	511	c	0	194	113	121	213	3.02	(2.19- 4.17)
Subtotal HU2								2.18	(1.79- 2.64)
HUMBLE	517	c	0	20	33	28	285	6.17	(3.13- 12.15)
HUMBLE	518	c	0	68	58	28	285	11.93	(7.07- 20.13)
HUMBLE	519	c	0	104	59	28	285	17.94	(10.85- 29.66)
HUMBLE	520	c	0	90	55	28	285	16.66	(9.97- 27.82)
HUMBLE	521	c	0	29	22	28	285	13.42	(6.82- 26.39)
Subtotal HUMBLE								13.29	(10.33- 17.10)
JOLY	515	m	0	11	48	12	218	4.16	(1.73- 9.99)
JOLY	516	m	0	38	61	12	218	11.32	(5.57- 22.98)
JOLY	517	m	0	85	165	12	218	9.36	(4.95- 17.70)
JOLY	518	m	0	168	182	12	218	16.77	(9.04- 31.11)
JOLY	519	m	0	250	253	12	218	17.95	(9.78- 32.93)
JOLY	501	f	0	13	28	52	283	2.53	(1.23- 5.20)
JOLY	502	f	0	18	26	52	283	3.77	(1.93- 7.36)
JOLY	503	f	0	31	24	52	283	7.03	(3.82- 12.93)
JOLY	504	f	0	47	24	52	283	10.66	(6.00- 18.92)
JOLY	505	f	0	57	20	52	283	15.51	(8.61- 27.95)
Subtotal JOLY								9.03	(7.36- 11.08)
JUSSAW	510	m	0	16	20	149	624	3.35	(1.70- 6.62)
JUSSAW	511	m	0	34	19	149	624	7.49	(4.16- 13.51)
JUSSAW	512	m	0	38	23	149	624	6.92	(4.00- 11.97)
JUSSAW	513	m	0	27	9	149	624	12.56	(5.79- 27.28)
JUSSAW	514	m	0	11	6	149	624	7.68	(2.79- 21.09)
Subtotal JUSSAW								6.77	(5.01- 9.15)
*KAISE2	596	m	1	17	-	14	-	4.86	(2.22- 10.61)
*KAISE2	597	m	1	34	-	14	-	15.64	(8.31- 29.40)
*KAISE2	516	f	1	24	-	11	-	9.09	(4.25- 19.43)
*KAISE2	517	f	1	26	-	11	-	30.41	(14.39- 64.25)
Subtotal KAISE2								12.59	(8.77- 18.07)
KATSOU	501	f	0	5	5	48	67	1.40	(0.38- 5.09)
KATSOU	502	f	0	8	7	48	67	1.60	(0.54- 4.70)
KATSOU	503	f	0	15	2	48	67	10.47	(2.29- 47.93)
KATSOU	504	f	0	17	4	48	67	5.93	(1.88- 18.75)
Subtotal KATSOU								3.06	(1.66- 5.67)
KHUDER	501	m	0	16	61	23	309	3.52	(1.76- 7.06)
KHUDER	502	m	0	207	370	23	309	7.52	(4.76- 11.86)
KHUDER	503	m	0	236	354	23	309	8.96	(5.69- 14.11)
Subtotal KHUDER								7.07	(5.28- 9.47)
KREUZE	517	m	3	-	-	-	-	4.70	(3.10- 7.14)
KREUZE	518	m	3	-	-	-	-	29.23	(19.78- 43.20)
KREUZE	520	f	3	-	-	-	-	1.33	(0.80- 2.22)
KREUZE	521	f	3	-	-	-	-	7.14	(4.92- 10.35)
Subtotal KREUZE								7.26	(5.90- 8.93)
LETOUR	506	c	0	65	187	24	224	3.24	(1.95- 5.39)
LETOUR	507	c	0	264	160	24	224	15.40	(9.68- 24.51)
LETOUR	508	c	0	374	141	24	224	24.76	(15.58- 39.35)
Subtotal LETOUR								11.50	(8.73- 15.14)

Table 1I11 - 5

IESLC - Meta-analysis of Ever/current Smoking by Duration, Overview
All LC types, Any Product (or Cigarettes if Any not available)
Least adjusted

REF	NRR	SEX	AD	Number Exposed Case	Number Exposed Cont	Non-exposed Case	Non-exposed Cont	RR	95.00%CI
LEVIN	501	m	0	56	97	7	96	7.92	(3.44- 18.25)
LEVIN	502	m	0	63	91	7	96	9.49	(4.13- 21.81)
Subtotal LEVIN								8.67	(4.81- 15.63)
*LIAW	501	c	2	-	-	-	-	0.90	(0.30- 3.10)
*LIAW	502	c	2	-	-	-	-	2.60	(1.20- 5.90)
*LIAW	503	c	2	-	-	-	-	4.70	(2.70- 8.20)
Subtotal LIAW								3.19	(2.09- 4.88)
LIU3	507	m	0	30	146	4	19	0.98	(0.31- 3.07)
LIU3	508	m	0	22	59	4	19	1.77	(0.54- 5.79)
Subtotal LIU3								1.30	(0.57- 2.97)
LIU5	504	c	0	27	37	26	41	1.15	(0.57- 2.31)
LIU5	505	c	0	58	33	26	41	2.77	(1.45- 5.32)
Subtotal LIU5								1.84	(1.14- 2.96)
LUBIN	508	m	0	30	146	8	72	1.85	(0.81- 4.24)
LUBIN	509	m	0	124	294	8	72	3.80	(1.78- 8.12)
LUBIN	510	m	0	143	251	8	72	5.13	(2.40- 10.95)
LUBIN	511	m	0	59	86	8	72	6.17	(2.77- 13.77)
Subtotal LUBIN								3.94	(2.66- 5.83)
LUBIN2	531	m	0	953	2995	190	2616	4.38	(3.72- 5.16)
LUBIN2	532	m	0	2227	3470	190	2616	8.84	(7.56- 10.33)
LUBIN2	533	m	0	2079	2551	190	2616	11.22	(9.58- 13.14)
LUBIN2	534	m	0	1325	1484	190	2616	12.29	(10.42- 14.50)
LUBIN2	574	f	0	132	230	336	1188	2.03	(1.59- 2.59)
LUBIN2	575	f	0	187	186	336	1188	3.55	(2.81- 4.50)
LUBIN2	576	f	0	155	118	336	1188	4.64	(3.55- 6.07)
LUBIN2	577	f	0	81	32	336	1188	8.95	(5.84- 13.71)
Subtotal LUBIN2								6.83	(6.37- 7.32)
MATOS	516	m	0	20	84	11	110	2.38	(1.08- 5.24)
MATOS	517	m	0	82	110	11	110	7.45	(3.77- 14.75)
MATOS	518	m	0	86	89	11	110	9.66	(4.86- 19.21)
Subtotal MATOS								5.99	(3.96- 9.05)
MCCONN	501	c	0	3	4	9	23	1.92	(0.36- 10.32)
MCCONN	502	c	0	5	19	9	23	0.67	(0.19- 2.35)
MCCONN	503	c	0	46	57	9	23	2.06	(0.87- 4.89)
MCCONN	504	c	0	21	57	9	23	0.94	(0.38- 2.36)
MCCONN	505	c	0	16	40	9	23	1.02	(0.39- 2.68)
Subtotal MCCONN								1.22	(0.76- 1.94)
NOTAN2	513	c	0	7	15	107	201	0.88	(0.35- 2.22)
NOTAN2	514	c	0	15	15	107	201	1.88	(0.88- 3.99)
NOTAN2	515	c	0	17	16	107	201	2.00	(0.97- 4.11)
NOTAN2	516	c	0	12	7	107	201	3.22	(1.23- 8.42)
NOTAN2	517	c	0	5	5	107	201	1.88	(0.53- 6.63)
Subtotal NOTAN2								1.83	(1.24- 2.70)
OSANN2	501	f	0	23	47	33	109	1.62	(0.86- 3.04)
OSANN2	502	f	0	161	57	33	109	9.33	(5.70- 15.27)
Subtotal OSANN2								4.81	(3.26- 7.10)
PEZZO2	507	m	0	60	72	6	117	16.25	(6.68- 39.53)
PEZZO2	508	m	0	173	126	6	117	26.77	(11.42- 62.76)
Subtotal PEZZO2								21.08	(11.40- 39.00)
PEZZOT	534	m	0	30	134	4	116	6.49	(2.22- 18.98)
PEZZOT	535	m	0	71	82	4	116	25.11	(8.82- 71.48)
PEZZOT	536	m	0	110	101	4	116	31.58	(11.25- 88.71)
Subtotal PEZZOT								17.64	(9.62- 32.34)
*QIAO2	511	m	0	7	2364	10	709	0.21	(0.08- 0.55)
*QIAO2	512	m	0	54	2257	10	709	1.70	(0.87- 3.31)
*QIAO2	513	m	0	170	2295	10	709	5.25	(2.79- 9.88)
Subtotal QIAO2								1.87	(1.24- 2.83)
RACHTA	511	f	0	12	19	33	98	1.88	(0.82- 4.27)
RACHTA	512	f	0	49	21	33	98	6.93	(3.63- 13.22)
RACHTA	513	f	0	24	1	33	98	71.27	(9.28- 547.53)
Subtotal RACHTA								4.97	(3.04- 8.14)
SOBUE	546	m	0	62	119	34	128	1.96	(1.21- 3.19)
SOBUE	547	m	0	159	200	34	128	2.99	(1.94- 4.61)
SOBUE	548	m	0	241	174	34	128	5.21	(3.41- 7.98)
SOBUE	549	m	0	147	73	34	128	7.58	(4.73- 12.14)
Subtotal SOBUE								3.96	(3.16- 4.96)
TIZZAN	501	m	0	12	1	180	305	20.33	(2.62- 157.68)
TIZZAN	502	m	0	54	20	180	305	4.58	(2.65- 7.89)
TIZZAN	503	m	0	928	815	180	305	1.93	(1.57- 2.37)
TIZZAN	533	f	0	2	7	117	114	0.28	(0.06- 1.37)
TIZZAN	534	f	0	23	21	117	114	1.07	(0.56- 2.03)

Table 1I11 - 5

IESLC - Meta-analysis of Ever/current Smoking by Duration, Overview
All LC types, Any Product (or Cigarettes if Any not available)
Least adjusted

REF	NRR	SEX	AD	Number Exposed Case	Cont	Non-exposed Case	Cont	RR	95.00%CI
Subtotal TIZZAN								2.01	(1.68- 2.42)
WANG2	501	c	0	4	17	11	43	0.92	(0.26- 3.29)
WANG2	503	c	0	8	18	11	43	1.74	(0.60- 5.03)
WANG2	504	c	0	26	38	11	43	2.67	(1.17- 6.13)
WANG2	505	c	0	22	26	11	43	3.31	(1.38- 7.91)
Subtotal WANG2								2.24	(1.38- 3.63)
WUWILL	501	f	0	137	139	417	601	1.42	(1.09- 1.86)
WUWILL	502	f	0	179	98	417	601	2.63	(2.00- 3.47)
WUWILL	503	f	0	223	114	417	601	2.82	(2.18- 3.65)
Subtotal WUWILL								2.20	(1.88- 2.56)
ZHENG	553	m	0	37	75	33	94	1.41	(0.80- 2.46)
ZHENG	554	m	0	242	143	33	94	4.82	(3.08- 7.54)
ZHENG	558	f	0	17	17	152	184	1.21	(0.60- 2.45)
ZHENG	559	f	0	59	27	152	184	2.65	(1.60- 4.38)
Subtotal ZHENG								2.54	(1.94- 3.31)
ZHOU	501	c	0	170	12	507	68	1.90	(1.00- 3.60)
ZHOU	502	c	0	678	36	507	68	2.53	(1.66- 3.84)
Subtotal ZHOU								2.32	(1.63- 3.29)
Partial Totals				30094	4767667	2166257315503			

*prospective study ~ With 0.5 adjustment for zero

REF	NRR	SEX	AD	Ys	Ws	Qs	Ps
AGUDO	507	f	0	-0.05	3.32	13.83	0.9303
AGUDO	508	f	0	1.32	6.08	2.76	0.0011
Subtotal AGUDO				0.84	9.40	16.59	
*AMANDU	501	m	0	0.95	5.25	5.76	0.0302
*AMANDU	502	m	0	2.42	5.54	1.00	0.0000
Subtotal AMANDU				1.70	10.79	6.76	
ARMADA	501	m	0	1.22	4.88	2.92	0.0070
ARMADA	502	m	0	2.46	6.68	1.46	0.0000
ARMADA	503	m	0	3.03	5.48	5.90	0.0000
Subtotal ARMADA				2.29	17.05	10.27	
AUVINE	501	c	0	2.02	8.26	0.00	0.0000
AUVINE	502	c	0	2.34	3.06	0.37	0.0000
AUVINE	503	c	0	3.04	20.41	22.55	0.0000
Subtotal AUVINE				2.71	31.73	22.92	
AXELSS	501	m	0	0.44	6.35	15.38	0.2713
AXELSS	502	m	0	0.98	6.98	7.22	0.0098
AXELSS	503	m	0	2.08	9.96	0.08	0.0000
AXELSS	504	m	0	2.50	11.10	2.89	0.0000
AXELSS	505	m	0	3.23	9.65	14.72	0.0000
AXELSS	510	f	0	0.58	3.29	6.60	0.2943
AXELSS	511	f	0	1.26	5.56	2.96	0.0029
AXELSS	512	f	0	2.26	7.41	0.51	0.0000
AXELSS	513	f	0	2.94	7.42	6.58	0.0000
AXELSS	514	f	0	2.84	4.72	3.38	0.0000
Subtotal AXELSS				2.07	72.43	60.31	
BARBON	501	m	0	1.37	11.69	4.51	0.0000
BARBON	502	m	0	2.29	14.48	1.28	0.0000
BARBON	503	m	0	2.54	15.92	4.82	0.0000
BARBON	504	m	0	2.59	17.31	6.13	0.0000
Subtotal BARBON				2.26	59.40	16.74	
*BEST	501	m	1	0.47	0.88	2.05	0.6590
*BEST	502	m	1	0.96	1.55	1.68	0.2335
*BEST	503	m	1	0.83	3.22	4.34	0.1350
*BEST	504	m	1	1.16	4.12	2.84	0.0182
*BEST	505	m	1	1.41	5.30	1.80	0.0012
*BEST	506	m	1	2.63	6.21	2.53	0.0000
*BEST	507	m	1	2.65	6.65	2.89	0.0000
Subtotal BEST				1.82	27.95	18.13	
*BOUCOT	518	m	0	2.90	0.49	0.40	0.0421
*BOUCOT	519	m	0	3.99	0.50	1.98	0.0050
Subtotal BOUCOT				3.45	0.99	2.38	
BUFFLE	526	f	0	2.14	7.75	0.17	0.0000
BUFFLE	527	f	0	2.68	8.42	3.98	0.0000
BUFFLE	528	f	0	3.00	7.86	7.90	0.0000
Subtotal BUFFLE				2.61	24.04	12.05	
*CEDERL	501	m	1	0.59	2.92	5.76	0.3155
*CEDERL	502	m	1	2.00	5.39	0.00	0.0000
*CEDERL	504	f	1	0.47	2.58	5.98	0.4505

Table 1I11 - 5

IESLC - Meta-analysis of Ever/current Smoking by Duration, Overview
All LC types, Any Product (or Cigarettes if Any not available)
Least adjusted

REF	NRR	SEX	AD	Ys	Ws	Qs	Ps
*CEDERL	505	f	1	2.26	4.00	0.29	0.0000
Subtotal CEDERL				1.53	14.88	12.04	
CHEN2	501	m	0	0.89	1.03	1.24	0.3653
CHEN2	502	m	0	1.59	1.38	0.23	0.0623
CHEN2	503	m	0	0.95	4.13	4.46	0.0523
CHEN2	504	m	0	1.59	4.85	0.80	0.0005
CHEN2	505	m	0	1.74	5.48	0.36	0.0000
CHEN2	510	f	0	-1.51	0.81	9.95	0.1734
CHEN2	511	f	0	0.28	0.93	2.75	0.7884
CHEN2	512	f	0	1.05	3.19	2.83	0.0607
CHEN2	513	f	0	0.61	5.42	10.31	0.1529
Subtotal CHEN2				1.10	27.21	32.93	
CHOI	501	m	0	0.93	6.32	7.20	0.0199
CHOI	502	m	0	1.07	9.21	7.91	0.0012
CHOI	503	m	0	1.54	9.66	2.00	0.0000
CHOI	504	m	0	1.92	8.35	0.04	0.0000
CHOI	505	m	0	1.99	5.33	0.00	0.0000
CHOI	510	f	0	-0.73	1.59	11.81	0.3546
CHOI	511	f	0	0.21	4.64	14.76	0.6519
CHOI	512	f	0	2.16	1.55	0.04	0.0072
CHOI	513	f	0	0.77	0.50	0.74	0.5883
Subtotal CHOI				1.29	47.14	44.50	
*CPSI	580	m	0	0.52	64.01	138.51	0.0000
*CPSI	581	m	0	1.32	105.87	47.91	0.0000
*CPSI	582	m	0	1.80	138.39	5.27	0.0000
*CPSI	583	m	0	2.34	154.66	18.36	0.0000
*CPSI	584	m	0	2.79	156.12	99.36	0.0000
*CPSI	585	m	0	3.12	146.44	187.28	0.0000
*CPSI	586	m	0	3.45	126.72	269.97	0.0000
*CPSI	587	m	0	3.71	106.68	313.24	0.0000
*CPSI	676	f	0	0.10	87.71	315.27	0.3603
*CPSI	677	f	0	0.99	111.49	113.07	0.0000
*CPSI	678	f	0	1.27	119.48	62.49	0.0000
*CPSI	679	f	0	1.68	97.98	9.71	0.0000
*CPSI	680	f	0	1.99	49.07	0.00	0.0000
*CPSI	681	f	0	2.10	15.55	0.17	0.0000
*CPSI	682	f	0	2.56	9.83	3.11	0.0000
Subtotal CPSI				2.08	1489.99	1583.72	
*CPSII	552	m	0	1.11	45.57	35.53	0.0000
*CPSII	553	m	0	2.04	66.89	0.12	0.0000
*CPSII	554	m	0	2.59	82.29	29.07	0.0000
*CPSII	555	m	0	3.17	95.47	132.84	0.0000
*CPSII	556	m	0	3.53	88.48	209.08	0.0000
*CPSII	557	m	0	3.92	90.50	337.33	0.0000
*CPSII	558	m	0	4.20	68.44	332.16	0.0000
*CPSII	559	m	0	4.42	60.64	356.20	0.0000
*CPSII	618	f	0	1.05	90.12	81.06	0.0000
*CPSII	619	f	0	1.94	104.74	0.28	0.0000
*CPSII	620	f	0	2.42	119.07	21.22	0.0000
*CPSII	621	f	0	2.77	127.49	76.43	0.0000
*CPSII	622	f	0	3.14	102.68	134.44	0.0000
*CPSII	623	f	0	3.34	87.81	159.56	0.0000
*CPSII	624	f	0	3.37	24.94	47.26	0.0000
*CPSII	625	f	0	4.00	17.14	69.01	0.0000
Subtotal CPSII				2.85	1272.25	2021.56	
DAMBER	506	m	1	0.46	5.52	13.03	0.2825
DAMBER	507	m	1	1.30	12.09	5.86	0.0000
DAMBER	508	m	1	1.64	17.62	2.22	0.0000
DAMBER	509	m	1	2.16	21.28	0.62	0.0000
DAMBER	510	m	1	2.42	21.42	3.80	0.0000
Subtotal DAMBER				1.86	77.93	25.53	
DEAN2	501	m	0	1.16	10.37	7.12	0.0002
DEAN2	502	m	0	1.34	23.47	9.88	0.0000
DEAN2	504	f	0	-0.02	5.23	21.18	0.9671
DEAN2	505	f	0	1.77	7.59	0.38	0.0000
Subtotal DEAN2				1.22	46.66	38.56	
DESTEF	501	m	0	1.55	11.82	2.31	0.0000
DESTEF	502	m	0	1.80	14.53	0.56	0.0000
DESTEF	503	m	0	2.41	16.73	2.86	0.0000
DESTEF	504	m	0	2.30	17.23	1.59	0.0000
Subtotal DESTEF				2.06	60.31	7.31	

Table 1I11 - 5

IESLC - Meta-analysis of Ever/current Smoking by Duration, Overview
All LC types, Any Product (or Cigarettes if Any not available)
Least adjusted

REF	NRR	SEX	AD	Ys	Ws	Qs	Ps
DOLL	515	m	0	1.94	3.23	0.01	0.0005
DOLL	516	m	0	1.52	4.90	1.11	0.0008
DOLL	517	m	0	2.19	6.17	0.25	0.0000
DOLL	518	m	0	2.29	6.13	0.55	0.0000
DOLL	522	f	0	0.14	5.92	20.40	0.7383
DOLL	523	f	0	0.79	4.00	5.75	0.1124
DOLL	524	f	0	0.98	8.35	8.64	0.0048
DOLL	525	f	0	1.08	1.85	1.53	0.1417
Subtotal DOLL				1.37	40.55	38.24	
DORGAN	570	m	2	1.69	10.46	0.94	0.0000
DORGAN	571	m	2	2.78	11.22	6.90	0.0000
DORGAN	562	f	3	1.45	47.84	14.30	0.0000
DORGAN	563	f	3	2.46	57.93	12.72	0.0000
Subtotal DORGAN				2.05	127.45	34.86	
DOSEME	501	m	2	0.00	14.17	56.31	1.0000
DOSEME	502	m	2	1.34	24.94	10.82	0.0000
DOSEME	503	m	2	1.59	31.98	5.23	0.0000
Subtotal DOSEME				1.18	71.09	72.36	
FAN	501	m	0	0.34	13.53	36.90	0.2079
FAN	502	m	0	0.86	15.89	20.40	0.0006
FAN	503	m	0	1.36	23.17	9.35	0.0000
FAN	506	f	0	0.91	4.78	5.66	0.0478
FAN	507	f	0	1.34	8.79	3.72	0.0001
FAN	508	f	0	1.46	18.96	5.32	0.0000
Subtotal FAN				1.10	85.12	81.34	
GAO	561	f	0	0.49	27.86	63.07	0.0099
GAO	562	f	0	1.18	42.03	28.02	0.0000
Subtotal GAO				0.90	69.89	91.08	
GARSHI	534	m	0	1.83	34.49	0.96	0.0000
GER	513	c	0	0.19	6.73	21.95	0.6273
GER	514	c	0	0.20	15.61	50.48	0.4403
GER	515	c	0	0.42	19.79	48.89	0.0605
Subtotal GER				0.30	42.13	121.32	
HAENSZ	542	f	0	0.58	8.51	16.91	0.0886
HAENSZ	543	f	0	0.79	21.36	31.15	0.0003
Subtotal HAENSZ				0.73	29.87	48.06	
HU	501	m	0	0.71	10.64	17.58	0.0209
HU	502	m	0	0.74	12.94	20.49	0.0082
HU	503	m	0	0.80	6.12	8.77	0.0488
HU	506	f	0	0.50	3.82	8.51	0.3277
HU	507	f	0	0.63	3.58	6.61	0.2303
HU	508	f	0	0.47	1.59	3.69	0.5535
Subtotal HU				0.69	38.69	65.66	
HU2	508	c	0	0.11	11.00	38.90	0.7065
HU2	509	c	0	0.58	22.49	44.87	0.0058
HU2	510	c	0	0.76	32.27	48.90	0.0000
HU2	511	c	0	1.11	37.09	29.22	0.0000
Subtotal HU2				0.78	102.85	161.89	
HUMBLE	517	c	0	1.82	8.37	0.25	0.0000
HUMBLE	518	c	0	2.48	14.05	3.31	0.0000
HUMBLE	519	c	0	2.89	15.20	12.14	0.0000
HUMBLE	520	c	0	2.81	14.60	9.79	0.0000
HUMBLE	521	c	0	2.60	8.39	3.05	0.0000
Subtotal HUMBLE				2.59	60.60	28.55	
JOLY	515	m	0	1.43	5.01	1.61	0.0014
JOLY	516	m	0	2.43	7.66	1.43	0.0000
JOLY	517	m	0	2.24	9.46	0.56	0.0000
JOLY	518	m	0	2.82	10.06	6.86	0.0000
JOLY	519	m	0	2.89	10.43	8.34	0.0000
JOLY	501	f	0	0.93	7.39	8.40	0.0118
JOLY	502	f	0	1.33	8.56	3.81	0.0001
JOLY	503	f	0	1.95	10.34	0.02	0.0000
JOLY	504	f	0	2.37	11.67	1.62	0.0000
JOLY	505	f	0	2.74	11.07	6.19	0.0000
Subtotal JOLY				2.20	91.65	38.85	
JUSSAW	510	m	0	1.21	8.28	5.10	0.0005
JUSSAW	511	m	0	2.01	11.07	0.00	0.0000
JUSSAW	512	m	0	1.93	12.80	0.05	0.0000
JUSSAW	513	m	0	2.53	6.39	1.84	0.0000
JUSSAW	514	m	0	2.04	3.76	0.01	0.0001
Subtotal JUSSAW				1.91	42.30	7.00	

Table 1I11 - 5

IESLC - Meta-analysis of Ever/current Smoking by Duration, Overview
All LC types, Any Product (or Cigarettes if Any not available)
Least adjusted

REF	NRR	SEX	AD	Ys	Ws	Qs	Ps
*KAISE2	596	m	1	1.58	6.28	1.07	0.0001
*KAISE2	597	m	1	2.75	9.62	5.50	0.0000
*KAISE2	516	f	1	2.21	6.65	0.30	0.0000
*KAISE2	517	f	1	3.41	6.86	13.86	0.0000
Subtotal KAISE2				2.53	29.42	20.74	
KATSOU	501	f	0	0.33	2.29	6.32	0.6134
KATSOU	502	f	0	0.47	3.29	7.68	0.3967
KATSOU	503	f	0	2.35	1.66	0.21	0.0025
KATSOU	504	f	0	1.78	2.90	0.13	0.0024
Subtotal KATSOU				1.12	10.15	14.34	
KHUDER	501	m	0	1.26	7.96	4.29	0.0004
KHUDER	502	m	0	2.02	18.43	0.01	0.0000
KHUDER	503	m	0	2.19	18.60	0.73	0.0000
Subtotal KHUDER				1.96	44.99	5.03	
KREUZE	517	m	3	1.55	22.07	4.39	0.0000
KREUZE	518	m	3	3.38	25.18	48.06	0.0000
KREUZE	520	f	3	0.29	14.75	43.05	0.2734
KREUZE	521	f	3	1.97	27.78	0.02	0.0000
Subtotal KREUZE				1.98	89.79	95.53	
LETOUR	506	c	0	1.18	14.96	9.98	0.0000
LETOUR	507	c	0	2.73	17.80	9.77	0.0000
LETOUR	508	c	0	3.21	17.89	26.43	0.0000
Subtotal LETOUR				2.44	50.65	46.18	
LEVIN	501	m	0	2.07	5.51	0.03	0.0000
LEVIN	502	m	0	2.25	5.55	0.37	0.0000
Subtotal LEVIN				2.16	11.06	0.40	
*LIAW	501	c	2	-0.11	2.82	12.41	0.8596
*LIAW	502	c	2	0.96	6.06	6.53	0.0187
*LIAW	503	c	2	1.55	12.45	2.48	0.0000
Subtotal LIAW				1.16	21.33	21.42	
LIU3	507	m	0	-0.02	2.92	11.88	0.9669
LIU3	508	m	0	0.57	2.74	5.54	0.3441
Subtotal LIU3				0.26	5.66	17.42	
LIU5	504	c	0	0.14	7.88	27.06	0.6935
LIU5	505	c	0	1.02	9.06	8.60	0.0022
Subtotal LIU5				0.61	16.94	35.66	
LUBIN	508	m	0	0.61	5.58	10.62	0.1463
LUBIN	509	m	0	1.33	6.65	2.89	0.0006
LUBIN	510	m	0	1.63	6.67	0.86	0.0000
LUBIN	511	m	0	1.82	5.97	0.18	0.0000
Subtotal LUBIN				1.37	24.88	14.55	
LUBIN2	531	m	0	1.48	142.28	37.93	0.0000
LUBIN2	532	m	0	2.18	156.67	5.38	0.0000
LUBIN2	533	m	0	2.42	153.41	27.60	0.0000
LUBIN2	534	m	0	2.51	141.36	37.55	0.0000
LUBIN2	574	f	0	0.71	63.53	105.06	0.0000
LUBIN2	575	f	0	1.27	68.77	36.18	0.0000
LUBIN2	576	f	0	1.54	53.35	11.19	0.0000
LUBIN2	577	f	0	2.19	21.09	0.83	0.0000
Subtotal LUBIN2				1.92	800.46	261.72	
MATOS	516	m	0	0.87	6.18	7.83	0.0311
MATOS	517	m	0	2.01	8.24	0.00	0.0000
MATOS	518	m	0	2.27	8.14	0.61	0.0000
Subtotal MATOS				1.79	22.56	8.45	
MCCONN	501	c	0	0.65	1.36	2.44	0.4488
MCCONN	502	c	0	-0.40	2.46	14.03	0.5341
MCCONN	503	c	0	0.72	5.16	8.32	0.1002
MCCONN	504	c	0	-0.06	4.55	19.20	0.8977
MCCONN	505	c	0	0.02	4.13	16.06	0.9644
Subtotal MCCONN				0.20	17.65	60.05	
NOTAN2	513	c	0	-0.13	4.47	20.18	0.7808
NOTAN2	514	c	0	0.63	6.77	12.58	0.1008
NOTAN2	515	c	0	0.69	7.37	12.51	0.0606
NOTAN2	516	c	0	1.17	4.16	2.82	0.0171
NOTAN2	517	c	0	0.63	2.41	4.48	0.3273
Subtotal NOTAN2				0.60	25.18	52.58	
OSANN2	501	f	0	0.48	9.59	21.98	0.1369
OSANN2	502	f	0	2.23	15.81	0.91	0.0000
Subtotal OSANN2				1.57	25.41	22.88	
PEZZO2	507	m	0	2.79	4.86	3.07	0.0000
PEZZO2	508	m	0	3.29	5.29	8.86	0.0000

Table 1I11 - 5

IESLC - Meta-analysis of Ever/current Smoking by Duration, Overview
All LC types, Any Product (or Cigarettes if Any not available)
Least adjusted

REF	NRR	SEX	AD	Ys	Ws	Qs	Ps
Subtotal PEZZO2				3.05	10.15	11.93	
PEZZOT	534	m	0	1.87	3.34	0.05	0.0006
PEZZOT	535	m	0	3.22	3.51	5.31	0.0000
PEZZOT	536	m	0	3.45	3.60	7.67	0.0000
Subtotal PEZZOT				2.87	10.45	13.03	
*QIAO2	511	m	0	-1.56	4.15	52.42	0.0015
*QIAO2	512	m	0	0.53	8.57	18.40	0.1218
*QIAO2	513	m	0	1.66	9.61	1.08	0.0000
Subtotal QIAO2				0.63	22.33	71.90	
RACHTA	511	f	0	0.63	5.67	10.55	0.1344
RACHTA	512	f	0	1.94	9.21	0.03	0.0000
RACHTA	513	f	0	4.27	0.92	4.77	0.0000
Subtotal RACHTA				1.60	15.80	15.36	
SOBUE	546	m	0	0.67	16.19	28.21	0.0067
SOBUE	547	m	0	1.10	20.61	16.60	0.0000
SOBUE	548	m	0	1.65	21.22	2.49	0.0000
SOBUE	549	m	0	2.03	17.32	0.02	0.0000
Subtotal SOBUE				1.38	75.35	47.31	
TIZZAN	501	m	0	3.01	0.92	0.95	0.0039
TIZZAN	502	m	0	1.52	12.93	2.89	0.0000
TIZZAN	503	m	0	0.66	89.78	160.35	0.0000
TIZZAN	533	f	0	-1.28	1.51	16.22	0.1155
TIZZAN	534	f	0	0.06	9.22	34.31	0.8435
Subtotal TIZZAN				0.70	114.36	214.72	
WANG2	501	c	0	-0.08	2.36	10.20	0.8977
WANG2	503	c	0	0.55	3.39	7.05	0.3089
WANG2	504	c	0	0.98	5.59	5.70	0.0200
WANG2	505	c	0	1.20	5.05	3.21	0.0072
Subtotal WANG2				0.81	16.39	26.16	
WUWILL	501	f	0	0.35	53.89	145.41	0.0100
WUWILL	502	f	0	0.97	50.37	52.99	0.0000
WUWILL	503	f	0	1.04	57.74	52.90	0.0000
Subtotal WUWILL				0.79	162.01	251.31	
ZHENG	553	m	0	0.34	12.30	33.63	0.2328
ZHENG	554	m	0	1.57	19.21	3.40	0.0000
ZHENG	558	f	0	0.19	7.71	25.06	0.5957
ZHENG	559	f	0	0.97	15.15	15.79	0.0002
Subtotal ZHENG				0.93	54.37	77.88	
ZHOU	501	c	0	0.64	9.44	17.26	0.0485
ZHOU	502	c	0	0.93	21.77	24.79	0.0000
Subtotal ZHOU				0.84	31.22	42.04	

N	248
NS	55

Table 1I11 - 6

IESLC - Meta-analysis of Ever/current Smoking by Duration, Overview
All LC types, Any Product (or Cigarettes if Any not available)
Least adjusted

	Sex combined	male	female	Total
N	39	127	82	248
NS	11	36	25	72

In this overview table, other than the “N” rows, entries in the “absent” and “Total” columns may be invalid and should be ignored

Duration of smoking (broad categories)

	absent	1-34k20	21-49k35	36+k50	Total
N	107	55	40	46	248
NS	43	43	32	36	154
Wt	2836.90	980.02	1064.47	954.00	5835.39
Het Chi	3010.77	311.82	404.21	766.01	6171.05
Het df	106	54	39	45	247
Het P	***	***	***	***	***
Fixed RR	9.49	2.48	6.09	12.85	7.34
RRl	9.15	2.33	5.73	12.06	7.16
RRu	9.85	2.64	6.47	13.69	7.53
P	+++	+++	+++	+++	+++
Random RR	5.16	2.43	5.75	9.88	4.99
RRl	4.19	2.05	4.64	7.43	4.36
RRu	6.35	2.88	7.14	13.13	5.71
P	+++	+++	+++	+++	+++

Duration of smoking (narrow categories)

	absent	1-19k1	6-29k20	21-39k30	31-49k40	41-998k50	51+k999	Total
N	136	28	17	32	23	6	6	248
NS	54	20	13	24	17	4	4	136
Wt	3145.06	188.20	144.66	903.70	853.49	376.18	224.10	5835.39
Het Chi	3372.29	71.94	42.08	264.29	225.72	92.66	91.21	6171.05
Het df	135	27	16	31	22	5	5	247
Het P	***	***	***	***	***	***	***	***
Fixed RR	6.41	2.03	2.99	4.86	10.56	26.02	40.70	7.34
RRl	6.19	1.76	2.54	4.55	9.87	23.52	35.70	7.16
RRu	6.64	2.34	3.52	5.19	11.29	28.78	46.39	7.53
P	+++	+++	+++	+++	+++	+++	+++	+++
Random RR	5.12	1.85	2.93	4.76	9.21	19.05	27.57	4.99
RRl	4.26	1.44	2.21	3.83	7.21	11.85	14.68	4.36
RRu	6.16	2.38	3.88	5.90	11.76	30.63	51.78	5.71
P	+++	+++	+++	+++	+++	+++	+++	+++

MALES

Duration of smoking (broad categories)

	absent	1-34k20	21-49k35	36+k50	Total
N	55	27	20	25	127
NS	30	27	20	25	102
Wt	1716.56	457.75	559.11	594.42	3327.84
Het Chi	1781.99	132.78	116.60	262.34	3194.50
Het df	54	26	19	24	126
Het P	***	***	***	***	***
Fixed RR	12.23	3.06	7.41	16.29	9.78
RRl	11.66	2.79	6.82	15.03	9.45
RRu	12.82	3.36	8.05	17.65	10.12
P	+++	+++	+++	+++	+++
Random RR	7.10	2.76	6.66	12.19	6.41
RRl	5.31	2.18	5.24	9.00	5.35
RRu	9.49	3.51	8.46	16.50	7.68
P	+++	+++	+++	+++	+++

Table 1I11 - 6

IESLC - Meta-analysis of Ever/current Smoking by Duration, Overview
All LC types, Any Product (or Cigarettes if Any not available)
Least adjusted

MALES

Duration of smoking (narrow categories)

	absent	1-19k1	6-29k20	21-39k30	31-49k40	41-998k50	51+k999	Total
N	72	12	8	16	13	3	3	127
NS	36	12	8	16	13	3	3	91
Wt	1710.40	101.94	81.21	467.75	519.57	258.22	188.74	3327.84
Het Chi	1740.56	31.33	19.85	96.96	91.01	67.05	65.10	3194.50
Het df	71	11	7	15	12	2	2	126
Het P	***	***	**	***	***	***	***	***
Fixed RR	8.52	2.88	3.99	6.12	11.80	27.82	44.20	9.78
RRl	8.12	2.37	3.21	5.59	10.83	24.62	38.32	9.45
RRu	8.93	3.50	4.96	6.70	12.86	31.43	50.98	10.12
P	+++	+++	+++	+++	+++	+++	+++	+++
Random RR	6.26	2.86	4.10	5.80	10.10	22.05	34.17	6.41
RRl	4.87	2.01	2.77	4.43	7.62	10.07	14.06	5.35
RRu	8.05	4.07	6.08	7.58	13.38	48.29	83.07	7.68
P	+++	+++	+++	+++	+++	+++	+++	+++

FEMALES

Duration of smoking (broad categories)

	absent	1-34k20	21-49k35	36+k50	Total
N	38	18	12	14	82
NS	21	18	12	14	65
Wt	1003.90	435.46	408.27	243.25	2090.88
Het Chi	790.73	106.39	137.02	240.96	1866.57
Het df	37	17	11	13	81
Het P	***	***	***	***	***
Fixed RR	6.97	2.01	5.23	10.22	5.32
RRl	6.55	1.83	4.74	9.01	5.09
RRu	7.42	2.20	5.76	11.59	5.55
P	+++	+++	+++	+++	+++
Random RR	4.24	2.08	5.79	9.11	4.26
RRl	3.10	1.57	3.85	4.87	3.41
RRu	5.78	2.74	8.71	17.03	5.31
P	+++	+++	+++	+++	+++

Duration of smoking (narrow categories)

	absent	1-19k1	6-29k20	21-39k30	31-49k40	41-998k50	51+k999	Total
N	45	11	5	10	7	2	2	82
NS	25	11	5	10	7	2	2	62
Wt	1201.72	57.62	25.63	366.06	309.51	103.36	26.98	2090.88
Het Chi	1075.62	10.55	5.37	72.45	101.63	20.42	13.03	1866.57
Het df	44	10	4	9	6	1	1	81
Het P	***	N.S.	N.S.	***	***	***	***	***
Fixed RR	4.67	1.37	2.47	3.98	8.84	23.44	32.25	5.32
RRl	4.41	1.06	1.68	3.59	7.91	19.33	22.12	5.09
RRu	4.94	1.77	3.64	4.41	9.88	28.43	47.04	5.55
P	+++	+	+++	+++	+++	+++	+++	+++
Random RR	4.49	1.36	2.38	4.55	8.76	15.51	26.92	4.26
RRl	3.32	1.04	1.51	3.16	5.12	4.59	6.54	3.41
RRu	6.06	1.78	3.76	6.56	14.98	52.40	110.82	5.31
P	+++	+	+++	+++	+++	+++	+++	+++

Table 1I11 - 7

IESLC - Meta-analysis of Ever/current Smoking by Duration, Overview
All LC types, Any Product (or Cigarettes if Any not available)
Excluded studies (and stage at which they were excluded)

1	BECHER BLOT1 BROWN3 CARPEN CHYOU DARBY DOLL2 GARCIA GRAHAM GURSEL HAMMO2 JAHN JAIN LAUSSM PRESCO QIAO TVERDA WIGLE WYNDE3
2	ALDERS BENSHL BRESLO CHIAZZ DEAN3 DORN ENGELA GAO2 GILLIS GUO HEGMAN HIRAYA HOLE KAUFMA KOO KOULUM LIU4 MIGRAN MRFITR PERNU SEGI2 SPEIZE SUZUK2 SVENSS VUTUC WAKAI WU YUAN
3	GENG MCDUFF SPITZ STASZE WU2 ZHANG
4	BOUCHA CHEN CORREA JEDRYC LUO WYNDE2 WYNDE6
5	AKIBA HAMMON PISANI RESTRE SADOWS XU
7	BOFFET BROSS WYNDE7
10	AMES WATSON WYNDE8
14	BENHAM

Table 1I11 - 8
Potentially overlapping studies

REF	REFGP	PRINC	.	OVERLAP/LINK
LUBIN2	LUBIN2	1		Lubin-combined
OSANN2	KAISER	2		KAISER/OSANN2
CPSI	CPSI	1		CPSI overall
LUBIN	XIANGZ	2		LUBIN/XIANGZ/QIAO

Table 1I11 - 9
Most adjusted - insufficient data for meta-analysis

REF	NRR	SEX	AGEL	AGEH	RACE	YF	LC TYPE	LOC	START	ST	NLC	R	VB	P	H	AD	SM	PRODUCT	exL	exH	S1	S2	DENOM	De
BUFFLE	501	m	0	0	wh	-	all	NAmer	1976	CC	943	n	bl	y	n	0	ev	cig+/-ot	1	33	1	0	nev cigs	or
BUFFLE	502	m	0	0	wh	-	all	NAmer	1976	CC	943	n	bl	y	n	0	ev	cig+/-ot	34	43	2	4	nev cigs	or
BUFFLE	503	m	0	0	wh	-	all	NAmer	1976	CC	943	n	bl	y	n	0	ev	cig+/-ot	44	49	0	0	nev cigs	or
BUFFLE	504	m	0	0	wh	-	all	NAmer	1976	CC	943	n	bl	y	n	0	ev	cig+/-ot	50	999	3	0	nev cigs	or
GARSHI	535	m	0	0	all	-	all	NAmer	1981	CC	1081	o	bl	y	n	1	ev	all/unsp	1	19	0	1	nev any	ot
HAMMON	513	m	0	0	wh	0	all	NAmer	1952	pr	448	n	bl	n	n	1	ev	cig only	1	34	1	0	nev any	st
HAMMON	514	m	0	0	wh	0	all	NAmer	1952	pr	448	n	bl	n	n	1	ev	cig only	35	999	0	0	nev any	st
SADOWS	522	m	0	0	wh	-	all	NAmer	1938	CC	477	n	bl	n	n	0	ev	cig only	1	9	0	1	nev any	ot
SADOWS	523	m	0	0	wh	-	all	NAmer	1938	CC	477	n	bl	n	n	0	ev	cig only	10	19	0	0	nev any	ot
SADOWS	524	m	0	0	wh	-	all	NAmer	1938	CC	477	n	bl	n	n	0	ev	cig only	20	29	1	2	nev any	ot
SADOWS	525	m	0	0	wh	-	all	NAmer	1938	CC	477	n	bl	n	n	0	ev	cig only	30	39	2	3	nev any	ot
SADOWS	526	m	0	0	wh	-	all	NAmer	1938	CC	477	n	bl	n	n	0	ev	cig only	40	49	0	4	nev any	ot
SADOWS	527	m	0	0	wh	-	all	NAmer	1938	CC	477	n	bl	n	n	0	ev	cig only	50	999	3	0	nev any	ot
XU	501	m	0	0	all	-	all	As:Chi	1985	CC	729	n	ot	n	n	2	ev	all/unsp	1	29	1	0	nev any	or
XU	502	m	0	0	all	-	all	As:Chi	1985	CC	729	n	ot	n	n	2	ev	all/unsp	30	39	2	3	nev any	or
XU	503	m	0	0	all	-	all	As:Chi	1985	CC	729	n	ot	n	n	2	ev	all/unsp	40	999	3	0	nev any	or

REF	NRR	RR	SIG	RRDATA comment
BUFFLE	501	6.80		0
BUFFLE	502	11.10		0
BUFFLE	503	9.40		0
BUFFLE	504	14.50		0
GARSHI	535	*	gap	0
HAMMON	513	*		RR for <1/2 pack per day is 5.31, that for 1/2 to 1 pack per day is 6.56, that for 1 to 2 packs per day is 7.27 while that for 2+ packs per day 10.78
HAMMON	514	*		RR for <1/2 pack per day is 10.86, that for 1/2 to 1 pack per day is 8.20, that for 1 to 2 packs per day is 19.69 while that for 2+ packs per day 22.89
SADOWS	522	1.19		0
SADOWS	523	1.16		0
SADOWS	524	2.78		0
SADOWS	525	3.95		0

Table 1I11 - 9

IESLC - Meta-analysis of Ever/current Smoking by Duration, Overview
All LC types, Any Product (or Cigarettes if Any not available)
Most adjusted - insufficient data for meta-analysis

REF	NRR	RR	SIG	RRDATA comment
SADOWS	526	7.00		0
SADOWS	527	8.43		0
XU	501		*	RR for 1-19/day is 1.8(p<0.05), for 20-29/day is 1.5(p<0.05) and for >=30/ day is 5.3(p<0.05)
XU	502		*	RR for 1-19/day is 2.1(p<0.05), for 20-29/day is 2.7(p<0.05) and for >=30/ day is 4.9(p<0.05)
XU	503		*	RR for 1-19/day is 3.3(p<0.05), for 20-29/day is 6.0(p<0.05) and for >=30/ day is 17.1(p<0.05)

Least adjusted - insufficient data for meta-analysis: as for adjusted plus the following

REF	NRR	SEX	AGEL	AGEH	RACE	YF	LC TYPE	LOC	START	ST	NLC	R	VB	P	H	AD	SM	PRODUCT	exL	exH	S1	S2	DENOM	De
GARSHI	533	m	0	0	all	-	all	NAmer	1981	CC	1081	o	bl	y	n	0	ev	all/unsp	1	19	0	1	nev any	ot

REF	NRR	RR	SIG	RRDATA comment
GARSHI	533		* gap	0

Table 1I12 -

IESLC - Meta-analysis of Ever/current Smoking, Duration, "Low"
All LC types, Any Product (or Cigarettes if Any not available)

This analysis is restricted to results for:
1) Ever/current smokers
2) Results by Duration
3) Categorical results by Duration
4) All LC types (or near equivalent)
5) Results complete enough for use in metaanalysis

Within each study, results are then selected (in the following order of preference, within each sex) for:
6) SMKSTA: ever, current
7) PRODUCT: all/unspec, cigarettes regardless of other products, cigarettes only
8) CIGTYPE: all/unspecified, MC regardless of HR, MC only
9) (not applicable)
10) DENOM: never smoked anything, never smoked cigarettes, never any + low, never cigs + low
11) Followup period (YF, prospective studies): whole study (coded as 0) or longest available
12) LCtype: all or nearest available, at least Squamous and Adeno. (q = squamous, s = small,
l = large, a = adeno, mix = mixed, alv = alveolar)
13) Race: all or nearest available, otherwise by race (wh or w = white, bl or b = black, hi = hispanic
ch = chinese, jap = japanese, haw = hawaiian, w+o = white + oriental, sca = scandinavian, as = asian)
14) Duration "low" in key scheme 1 (key value 20, maximum range 1-34)
15) For overlapping studies: principal rather than subsidiary studies
Finally by Age: whole study (coded as 0) if available, otherwise by widest available age group
and then for single sex results (m, f) in preference to results for both sexes combined (c).

Results adjusted (AD) for the most potential confounders are then chosen in Sections -1 to -3
and results adjusted for the least confounders in Sections -4 to -6. (Those least adjusted results which
actually differ from the most adjusted are marked 'x' in column X in Section -4)

Section -7 shows excluded studies, together with the stage (as above) at which no qualifying
results were found.

Section -8 lists the potentially overlapping studies which have been included (1=principal, 2=subsidiary).

Section -9 lists any results which would have been included in preference except that they had data not complete
enough for use in meta-analysis, with their significance (yes/no), if known, and any further comment as entered
on the database. It also lists as "gap" any categories for which no data were presented by the original authors.

In addition to those mentioned above, the following fields, levels and abbreviations are used:
* or nk = not known, n = no, y = yes, ot = other
ev = ever, cu = current, nev = never
all/unspec = all or unspecified, cig+/-ot = cigarettes irrespective of other products (cigar, pipe etc)
MC = manufactured cigarettes, HR = hand-rolled cigarettes
exL, exH = range of exposure (low and high) in the smoking group, in terms of Duration
REF: 6-character study reference
NRR: number of the RR on the database within the study
ST : study type (CC = case control, pr or prosp = prospective)
NLC: number of lung cancer cases in whole study
R : risky occupational population (n = no, m = mining, o = other risky)
VB : national cigarette type (V = at least 75% Virginia, bl = at least 75% blended, ot = other)
P : any proxy use
H : full histological confirmation
De : derivation of RR/CI (or = original, st = standard method, ot = other method of estimation)

Table 1I12 - 1

IESLC - Meta-analysis of Ever/current Smoking, Duration, "Low"
All LC types, Any Product (or Cigarettes if Any not available)
Most adjusted

REF	NRR	SEX	AGEL	AGEH	RACE	YF	LC TYPE	LOC	START	ST	NLC	R	VB	P	H	AD	SM	PRODUCT	exL	exH	DENOM	De
AMANDU	506	m	0	0	wh	0	all	NAmer	1959	pr	132	m	bl	n	n	2	cu	cig+/-ot	0	24	nev cigs	ot
ARMADA	506	m	0	0	all	-	all	Eu:wst	1986	CC	325	n	bl	n	y	1	ev	cig+/-ot	1	24	nev cigs	or
AUVINE	517	c	0	0	all	-	all	Eu:Sca	1986	CC	517	n	bl	y	n	2	ev	cig+/-ot	1	20	nev cigs	or
AXELSS	520	m	0	0	sca	-	all	Eu:Sca	1989	CC	436	n	bl	n	n	6	ev	all/unsp	20	29	nev any	ot
AXELSS	511	f	0	0	sca	-	all	Eu:Sca	1989	CC	436	n	bl	n	n	0	ev	all/unsp	20	29	nev any	st
BARBON	508	m	0	0	all	-	all	Eu:wst	1979	CC	755	n	bl	y	y	1	ev	all/unsp	1	29	nev any	or
BEST	505	m	0	0	all	0	all	NAmer	1955	pr	381	n	V	n	n	1	cu	cig only	20	29	nev any	ot
BUFFLE	526	f	0	0	w-hi	-	all	NAmer	1976	CC	943	n	bl	y	n	0	ev	cig+/-ot	1	30	nev cigs	or
CEDERL	501	m	40	69	all	10	all	Eu:Sca	1963	pr	491	n	bl	n	n	1	cu	cig only	1	29	nev any	ot
CEDERL	504	f	40	69	all	10	all	Eu:Sca	1963	pr	491	n	bl	n	n	1	cu	cig only	1	29	nev any	ot
CHEN2	502	m	0	0	all	-	all	As:Chi	1983	CC	193	n	ot	y	n	0	ev	all/unsp	10	20	nev any	st
CHEN2	510	f	0	0	all	-	all	As:Chi	1983	CC	193	n	ot	y	n	0	ev	all/unsp	1	20	nev any	st
CHOI	502	m	0	0	all	-	all	As:oth	1985	CC	375	n	bl	n	n	0	ev	cig+/-ot	20	29	nev cigs	st
CHOI	511	f	0	0	all	-	all	As:oth	1985	CC	375	n	bl	n	n	0	ev	cig+/-ot	20	29	nev cigs	st
CPSI	580	m	40	84	wh	0	all	NAmer	1959	pr	5138	n	bl	n	n	0	cu	cig only	1	29	nev cigs	st
CPSI	676	f	40	84	wh	0	all	NAmer	1959	pr	5138	n	bl	n	n	0	cu	cig only	1	29	nev cigs	st
CPSII	552	m	0	0	all	6	all	NAmer	1982	pr	3229	n	bl	n	n	0	cu	cig only	1	29	nev any	st
CPSII	618	f	0	0	all	6	all	NAmer	1982	pr	3229	n	bl	n	n	0	cu	cig+/-ot	1	29	nev cigs	st
DAMBER	506	m	0	0	all	-	all	Eu:Sca	1972	CC	579	n	bl	y	n	1	ev	all/unsp	1	20	nev any	ot
DESTEF	508	m	0	0	all	-	all	SCAmer	1988	CC	497	n	bl	n	y	4	ev	all/unsp	1	29	nev any	or
DORGAN	570	m	0	0	wh	-	all	NAmer	1980	CC	2026	n	bl	y	y	2	ev	cig+/-ot	1	34	nev any	ot
DORGAN	562	f	0	0	all	-	all	NAmer	1980	CC	2026	n	bl	y	y	3	ev	cig+/-ot	1	34	nev any	ot
DOSEME	502	m	0	0	all	-	all	Eu:bal	1979	CC	1210	n	bl	n	n	2	ev	cig+/-ot	11	20	nev cigs	or
FAN	501	m	0	0	all	-	all	As:Chi	1990	CC	403	n	ot	y	n	0	ev	cig+/-ot	1	29	nev cigs	st
FAN	506	f	0	0	all	-	all	As:Chi	1990	CC	403	n	ot	y	n	0	ev	cig+/-ot	1	29	nev cigs	st
GAO	564	f	0	0	all	-	all	As:Chi	1984	CC	1405	n	ot	n	n	2	ev	cig+/-ot	1	29	nev cigs	ot
GER	518	c	0	0	all	-	all	As:oth	1990	CC	141	n	ot	y	n	5	ev	all/unsp	1	20	nev any	ot
HU	502	m	0	0	all	-	all	As:Chi	1985	CC	227	n	ot	n	y	0	ev	cig+/-ot	20	29	nev cigs	st
HU	507	f	0	0	all	-	all	As:Chi	1985	CC	227	n	ot	n	y	0	ev	cig+/-ot	20	29	nev cigs	st
HU2	509	c	0	0	all	-	all	As:Chi	1977	CC	523	n	ot	y	n	0	ev	cig+/-ot	20	29	nev cigs	or
HUMBLE	542	c	0	0	wh	-	not alv	NAmer	1980	CC	521	n	bl	y	n	3	cu	cig+/-ot	1	29	nev cigs	ot
JOLY	516	m	0	0	all	-	all	SCAmer	1978	CC	826	n	bl	n	n	0	ev	cig+/-ot	20	29	nev any	st
JOLY	502	f	0	0	all	-	all	SCAmer	1978	CC	826	n	bl	n	n	0	ev	cig+/-ot	20	29	nev any	st
JUSSAW	512	m	0	0	all	-	all	As:Ind	1964	CC	792	n	V	n	n	0	ev	cig only	20	29	nev any	st
KATSOU	512	f	0	0	all	-	all	Eu:bal	1987	CC	101	n	bl	n	n	1	cu	all/unsp	1	29	nev any	or
KHUDER	501	m	0	0	all	-	all	NAmer	1985	CC	482	n	bl	n	y	0	ev	cig+/-ot	1	29	nev cigs	st
LETOUR	506	c	0	0	all	-	all	NAmer	1983	CC	738	n	V	y	y	0	ev	cig+/-ot	1	24	nev cigs	st
LIAW	501	c	0	0	all	0	all	As:oth	1982	pr	127	n	ot	n	n	2	cu	all/unsp	1	20	nev any	or
LIU3	510	m	0	0	all	-	all	As:Chi	1985	CC	110	n	ot	n	n	2	ev	all/unsp	1	34	nev any	or
LIU5	504	c	0	0	all	-	all	As:Chi	1978	CC	111	n	ot	y	n	0	ev	all/unsp	1	29	nev any	st
LUBIN	508	m	0	0	all	-	all	As:Chi	1984	CC	427	m	ot	y	n	0	ev	cig+/-ot	1	29	nev any	st
LUBIN2	531	m	0	0	all	-	all	Eu:mul	1976	CC	7804	n	bl	n	y	0	ev	cig+/-ot	1	29	nev any	st
LUBIN2	574	f	0	0	all	-	all	Eu:mul	1976	CC	7804	n	bl	n	y	0	ev	cig+/-ot	1	29	nev any	st
MATOS	536	m	0	0	all	-	all	SCAmer	1994	CC	200	n	bl	n	n	2	ev	cig+/-ot	1	24	nev any	or
MCCONN	503	c	0	0	all	-	all	Eu:UK	1946	CC	100	n	V	n	y	0	ev	all/unsp	20	29	nev any	st
NOTAN2	514	c	0	0	all	-	all	As:Ind	1963	CC	683	n	V	n	n	0	ev	cig only	11	20	nev any	st
OSANN2	504	f	0	0	all	-	all	NAmer	1964	ot	217	n	bl	n	y	1	ev	cig+/-ot	1	20	nev cigs	or
PEZZOT	534	m	0	0	all	-	all	SCAmer	1987	CC	215	n	bl	n	y	0	ev	cig only	1	30	nev cigs	st
QIAO2	516	m	0	0	all	0	all	As:Chi	1992	pr	241	m	ot	n	n	1	ev	all/unsp	1	27	nev any	or
RACHTA	516	f	0	0	all	-	all	Eu:est	1991	CC	118	n	bl	n	y	1	ev	cig+/-ot	1	20	nev cigs	or
SOBUE	546	m	0	0	all	-	q+s+l+a	As:Jap	1986	CC	1376	n	bl	n	y	0	cu	cig+/-ot	1	29	nev cigs	st
WANG2	503	c	0	0	all	-	all	As:Chi	1980	CC	103	n	ot	n	n	0	ev	cig+/-ot	20	29	nev cigs	st
WUWILL	516	f	0	0	all	-	all	As:Chi	1985	CC	965	n	ot	n	n	3	ev	cig+/-ot	1	29	nev cigs	ot
ZHENG	553	m	0	0	all	-	all	As:Chi	1982	CC	540	n	ot	*	y	0	ev	cig+/-ot	1	29	nev cigs	st
ZHENG	558	f	0	0	all	-	all	As:Chi	1982	CC	540	n	ot	*	y	0	ev	cig+/-ot	1	29	nev cigs	st

Cigarette type is all/unspec for all RRs

except for the following:

REF	NRR	CIGTYPE
JUSSAW	512	MC only
NOTAN2	514	MC only

Table 1I12 - 2

IESLC - Meta-analysis of Ever/current Smoking, Duration, "Low"
All LC types, Any Product (or Cigarettes if Any not available)
Most adjusted

REF	NRR	SEX	AD	Number Exposed Case	Number Exposed Cont	Non-exposed Case	Non-exposed Cont	RR	95.00%CI
*AMANDU	506	m	2	42	-	6	-	5.92	(2.13- 16.47)
ARMADA	506	m	1	21	-	8	-	2.60	(1.00- 6.60)
AUVINE	517	c	2	26	-	44	-	20.10	(6.69- 66.00)
AXELSS	520	m	6	17	-	16	-	2.23	(1.03- 4.80)
AXELSS	511	f	0	12	29	18	154	3.54	(1.54- 8.13)
Subtotal AXELSS								2.76	(1.57- 4.85)
BARBON	508	m	1	42	-	22	-	3.20	(1.80- 5.70)
*BEST	505	m	1	22	-	7	-	4.10	(1.75- 9.60)
BUFFLE	526	f	0	52	57	12	112	8.51	(4.21- 17.22)
*CEDERL	501	m	1	5	-	7	-	1.80	(0.57- 5.66)
*CEDERL	504	f	1	3	-	19	-	1.60	(0.47- 5.40)
Subtotal CEDERL								1.70	(0.74- 3.93)
CHEN2	502	m	0	4	3	9	33	4.89	(0.92- 25.93)
CHEN2	510	f	0	1	6	25	33	0.22	(0.02- 1.95)
Subtotal CHEN2								1.55	(0.41- 5.85)
CHOI	502	m	0	66	166	13	95	2.91	(1.52- 5.54)
CHOI	511	f	0	8	14	76	164	1.23	(0.50- 3.06)
Subtotal CHOI								2.18	(1.29- 3.69)
*CPSI	580	m	0	95	266163	196	926068	1.69	(1.32- 2.15)
*CPSI	676	f	0	105	694015	532	3877179	1.10	(0.89- 1.36)
Subtotal CPSI								1.32	(1.13- 1.55)
*CPSII	552	m	0	72	141932	124	742207	3.04	(2.27- 4.06)
*CPSII	618	f	0	127	301244	310	2091302	2.84	(2.31- 3.50)
Subtotal CPSII								2.91	(2.46- 3.44)
DAMBER	506	m	1	-	-	42	-	1.58	(0.69- 3.66)
DESTEF	508	m	4	43	-	27	-	3.40	(1.70- 6.80)
DORGAN	570	m	2	-	-	-	-	5.44	(2.97- 9.98)
DORGAN	562	f	3	-	-	-	-	4.25	(3.20- 5.64)
Subtotal DORGAN								4.44	(3.44- 5.74)
DOSEME	502	m	2	158	-	142	-	3.80	(2.60- 5.70)
FAN	501	m	0	29	135	36	236	1.41	(0.83- 2.40)
FAN	506	f	0	8	15	69	320	2.47	(1.01- 6.06)
Subtotal FAN								1.63	(1.03- 2.58)
GAO	564	f	2	68	-	435	-	1.89	(1.30- 2.75)
GER	518	c	5	10	-	51	-	1.30	(0.55- 3.06)
HU	502	m	0	60	47	41	67	2.09	(1.21- 3.60)
HU	507	f	0	11	7	40	48	1.89	(0.67- 5.32)
Subtotal HU								2.04	(1.26- 3.31)
HU2	509	c	0	64	63	121	213	1.79	(1.18- 2.70)
HUMBLE	542	c	3	20	-	28	-	15.45	(6.19- 38.58)
JOLY	516	m	0	38	61	12	218	11.32	(5.57- 22.98)
JOLY	502	f	0	18	26	52	283	3.77	(1.93- 7.36)
Subtotal JOLY								6.33	(3.89- 10.30)
JUSSAW	512	m	0	38	23	149	624	6.92	(4.00- 11.97)
KATSOU	512	f	1	13	-	48	-	1.29	(0.54- 3.26)
KHUDER	501	m	0	16	61	23	309	3.52	(1.76- 7.06)
LETOUR	506	c	0	65	187	24	224	3.24	(1.95- 5.39)
*LIAW	501	c	2	-	-	-	-	0.90	(0.30- 3.10)
LIU3	510	m	2	30	-	4	-	1.07	(0.25- 4.59)
LIU5	504	c	0	27	37	26	41	1.15	(0.57- 2.31)
LUBIN	508	m	0	30	146	8	72	1.85	(0.81- 4.24)
LUBIN2	531	m	0	953	2995	190	2616	4.38	(3.72- 5.16)
LUBIN2	574	f	0	132	230	336	1188	2.03	(1.59- 2.59)
Subtotal LUBIN2								3.45	(3.01- 3.96)
MATOS	536	m	2	20	-	11	-	2.20	(1.00- 4.90)
MCCONN	503	c	0	46	57	9	23	2.06	(0.87- 4.89)
NOTAN2	514	c	0	15	15	107	201	1.88	(0.88- 3.99)
OSANN2	504	f	1	23	-	33	-	1.60	(0.70- 3.50)
PEZZOT	534	m	0	30	134	4	116	6.49	(2.22- 18.98)
*QIAO2	516	m	1	7	-	10	-	0.40	(0.15- 1.05)
RACHTA	516	f	1	12	-	33	-	2.02	(0.87- 4.71)
SOBUE	546	m	0	62	119	34	128	1.96	(1.21- 3.19)
WANG2	503	c	0	8	18	11	43	1.74	(0.60- 5.03)
WUWILL	516	f	3	137	-	417	-	1.35	(1.04- 1.73)
ZHENG	553	m	0	37	75	33	94	1.41	(0.80- 2.46)
ZHENG	558	f	0	17	17	152	184	1.21	(0.60- 2.45)
Subtotal ZHENG								1.33	(0.86- 2.06)
Partial Totals				2965	1408097	4202	7644595		

*prospective study

Table 1I12 - 2

IESLC - Meta-analysis of Ever/current Smoking, Duration, "Low"
All LC types, Any Product (or Cigarettes if Any not available)
Most adjusted

REF	NRR	SEX	AD	Ys	Ws	Qs	Ps
*AMANDU	506	m	2	1.78	3.67	2.82	0.0007
ARMADA	506	m	1	0.96	4.31	0.01	0.0472
AUVINE	517	c	2	3.00	2.93	12.92	0.0000
AXELSS	520	m	6	0.80	6.49	0.06	0.0411
AXELSS	511	f	0	1.26	5.56	0.73	0.0029
Subtotal AXELSS				1.02	12.05	0.79	
BARBON	508	m	1	1.16	11.56	0.79	0.0001
*BEST	505	m	1	1.41	5.30	1.37	0.0012
BUFFLE	526	f	0	2.14	7.75	11.91	0.0000
*CEDERL	501	m	1	0.59	2.92	0.29	0.3155
*CEDERL	504	f	1	0.47	2.58	0.48	0.4505
Subtotal CEDERL				0.53	5.49	0.77	
CHEN2	502	m	0	1.59	1.38	0.65	0.0623
CHEN2	510	f	0	-1.51	0.81	4.72	0.1734
Subtotal CHEN2				0.44	2.19	5.37	
CHOI	502	m	0	1.07	9.21	0.25	0.0012
CHOI	511	f	0	0.21	4.64	2.22	0.6519
Subtotal CHOI				0.78	13.84	2.47	
*CPSI	580	m	0	0.52	64.01	9.22	0.0000
*CPSI	676	f	0	0.10	87.71	56.75	0.3603
Subtotal CPSI				0.28	151.71	65.97	
*CPSII	552	m	0	1.11	45.57	1.98	0.0000
*CPSII	618	f	0	1.05	90.12	1.85	0.0000
Subtotal CPSII				1.07	135.69	3.83	
DAMBER	506	m	1	0.46	5.52	1.09	0.2825
DESTEF	508	m	4	1.22	8.00	0.83	0.0005
DORGAN	570	m	2	1.69	10.46	6.56	0.0000
DORGAN	562	f	3	1.45	47.84	14.20	0.0000
Subtotal DORGAN				1.49	58.30	20.76	
DOSEME	502	m	2	1.34	24.94	4.67	0.0000
FAN	501	m	0	0.34	13.53	4.24	0.2079
FAN	506	f	0	0.91	4.78	0.00	0.0478
Subtotal FAN				0.49	18.31	4.24	
GAO	564	f	2	0.64	27.37	1.93	0.0009
GER	518	c	5	0.26	5.22	2.13	0.5490
HU	502	m	0	0.74	12.94	0.36	0.0082
HU	507	f	0	0.63	3.58	0.26	0.2303
Subtotal HU				0.71	16.52	0.62	
HU2	509	c	0	0.58	22.49	2.32	0.0058
HUMBLE	542	c	3	2.74	4.59	15.46	0.0000
JOLY	516	m	0	2.43	7.66	17.78	0.0000
JOLY	502	f	0	1.33	8.56	1.54	0.0001
Subtotal JOLY				1.85	16.22	19.33	
JUSSAW	512	m	0	1.93	12.80	13.64	0.0000
KATSOU	512	f	1	0.25	4.75	1.99	0.5788
KHUDER	501	m	0	1.26	7.96	1.02	0.0004
LETOUR	506	c	0	1.18	14.96	1.13	0.0000
*LIAW	501	c	2	-0.11	2.82	2.86	0.8596
LIU3	510	m	2	0.07	1.81	1.26	0.9274
LIU5	504	c	0	0.14	7.88	4.57	0.6935
LUBIN	508	m	0	0.61	5.58	0.46	0.1463
LUBIN2	531	m	0	1.48	142.28	47.07	0.0000
LUBIN2	574	f	0	0.71	63.53	2.40	0.0000
Subtotal LUBIN2				1.24	205.80	49.47	
MATOS	536	m	2	0.79	6.08	0.08	0.0518
MCCONN	503	c	0	0.72	5.16	0.16	0.1002
NOTAN2	514	c	0	0.63	6.77	0.50	0.1008
OSANN2	504	f	1	0.47	5.93	1.11	0.2523
PEZZOT	534	m	0	1.87	3.34	3.13	0.0006
*QIAO2	516	m	1	-0.92	4.06	13.42	0.0649
RACHTA	516	f	1	0.70	5.39	0.21	0.1027
SOBUE	546	m	0	0.67	16.19	0.84	0.0067
WANG2	503	c	0	0.55	3.39	0.41	0.3089
WUWILL	516	f	3	0.30	59.33	21.50	0.0208
ZHENG	553	m	0	0.34	12.30	3.88	0.2328
ZHENG	558	f	0	0.19	7.71	3.90	0.5957
Subtotal ZHENG				0.28	20.01	7.78	

Table 1I12 - 2

IESLC - Meta-analysis of Ever/current Smoking, Duration, "Low"
All LC types, Any Product (or Cigarettes if Any not available)
Most adjusted

	N	55
	NS	43
	Wt	964.02
Het	Chi	307.96
Het	df	54
Het	P	***
Fixed	RR	2.46
	RRl	2.31
	RRu	2.63
	P	+++
Random	RR	2.48
	RRl	2.09
	RRu	2.95
	P	+++
Asymm	P	N.S.

Table 1I12 - 3

IESLC - Meta-analysis of Ever/current Smoking, Duration, "Low"
All LC types, Any Product (or Cigarettes if Any not available)
Most adjusted

	Sex: combined	male	female	Total
N	10	27	18	55
NS	10	27	18	55
Wt	76.21	449.87	437.93	964.02
Het Chi	42.18	117.41	108.76	307.96
Het df	9	26	17	54
Het P	***	***	***	***
Fixed RR	2.32	3.05	2.00	2.46
RRl	1.85	2.78	1.82	2.31
RRu	2.90	3.34	2.20	2.63
P	+++	+++	+++	+++
Random RR	2.55	2.80	2.08	2.48
RRl	1.52	2.22	1.57	2.09
RRu	4.30	3.52	2.75	2.95
P	+++	+++	+++	+++
Between Chi				39.60
Between df				2
Between P				***
Btwn(F) P				*
Btwn(R) P				N.S.

	Lung cancer type: all	other	Total
N	53	2	55
NS	41	2	43
Wt	943.23	20.78	964.02
Het Chi	291.63	15.23	307.96
Het df	52	1	54
Het P	***	***	***
Fixed RR	2.45	3.09	2.46
RRl	2.30	2.01	2.31
RRu	2.61	4.76	2.63
P	+++	+++	+++
Random RR	2.42	5.30	2.48
RRl	2.04	0.70	2.09
RRu	2.88	40.01	2.95
P	+++	N.S.	+++
Between Chi			1.10
Between df			1
Between P			N.S.
Btwn(F) P			N.S.
Btwn(R) P			N.S.

	Location: NAmer	UK	Scand	othEur	China	Japan	othAs	other	Total
N	13	1	6	7	16	1	6	5	55
NS	10	1	4	6	12	1	5	4	43
Wt	395.87	5.16	25.99	256.76	188.96	16.19	41.45	33.64	964.02
Het Chi	125.13	0.00	15.22	32.61	19.12	0.00	21.31	10.97	307.96
Het df	12	0	5	6	15	0	5	4	54
Het P	***	N.S.	**	***	N.S.	N.S.	***	*	***
Fixed RR	2.40	2.06	2.77	3.36	1.51	1.96	2.68	4.52	2.46
RRl	2.18	0.87	1.89	2.97	1.31	1.21	1.98	3.23	2.31
RRu	2.65	4.89	4.07	3.79	1.75	3.19	3.64	6.34	2.63
P	+++	N.S.	+++	+++	+++	++	+++	+++	+++
Random RR	3.41	2.06	2.92	2.76	1.52	1.96	2.10	4.55	2.48
RRl	2.39	0.87	1.47	1.91	1.27	1.21	1.09	2.57	2.09
RRu	4.87	4.89	5.81	4.01	1.82	3.19	4.04	8.04	2.95
P	+++	N.S.	++	+++	+++	++	+	+++	+++
Between Chi									83.59
Between df									7
Between P									***
Btwn(F) P									*
Btwn(R) P									***

Table 1I12 - 3

IESLC - Meta-analysis of Ever/current Smoking, Duration, "Low"
All LC types, Any Product (or Cigarettes if Any not available)
Most adjusted

	Detailed Country in "other Europe" multi	Germany	othWest	East	Balkans	Total
N	2		2	1	2	7
NS	1		2	1	2	6
Wt	205.80		15.88	5.39	29.69	256.76
Het Chi	26.02		0.14	0.00	4.66	32.61
Het df	1		1	0	1	6
Het P	***		N.S.	N.S.	*	***
Fixed RR	3.45		3.02	2.02	3.20	3.36
RRl	3.01		1.85	0.87	2.23	2.97
RRu	3.96		4.95	4.70	4.58	3.79
P	+++		+++	N.S.	+++	+++
Random RR	3.00		3.02	2.02	2.40	2.76
RRl	1.41		1.85	0.87	0.84	1.91
RRu	6.37		4.95	4.70	6.83	4.01
P	++		+++	N.S.	N.S.	+++
Between Chi						1.80
Between df						3
Between P						N.S.
Btwn(F) P						N.S.
Btwn(R) P						N.S.

	Detailed Country in "other Asia" India	HongKong	other	Total
N	2		4	6
NS	2		3	5
Wt	19.58		21.88	41.45
Het Chi	7.53		4.63	21.31
Het df	1		3	5
Het P	**		N.S.	***
Fixed RR	4.41		1.72	2.68
RRl	2.83		1.13	1.98
RRu	6.86		2.62	3.64
P	+++		+	+++
Random RR	3.70		1.60	2.10
RRl	1.03		0.94	1.09
RRu	13.27		2.74	4.04
P	+		(+)	+
Between Chi				9.15
Between df				1
Between P				**
Btwn(F) P				N.S.
Btwn(R) P				N.S.

	Detailed other continent SCAmer	Total
N	5	5
NS	4	4
Wt	33.64	33.64
Het Chi	10.97	10.97
Het df	4	4
Het P	*	*
Fixed RR	4.52	4.52
RRl	3.23	3.23
RRu	6.34	6.34
P	+++	+++
Random RR	4.55	4.55
RRl	2.57	2.57
RRu	8.04	8.04
P	+++	+++
Between Chi		
Between df		
Between P		N.S.
Btwn(F) P		N.S.
Btwn(R) P		N.S.

Table 1I12 - 3

IESLC - Meta-analysis of Ever/current Smoking, Duration, "Low"
All LC types, Any Product (or Cigarettes if Any not available)
Most adjusted

	Start year of study					
	<1960	1960-69	1970-79	1980-89	1990+	Total
N	5	5	10	29	6	55
NS	4	4	8	22	5	43
Wt	165.85	31.00	302.16	425.95	39.05	964.02
Het Chi	21.66	14.53	65.81	107.12	9.80	307.96
Het df	4	4	9	28	5	54
Het P	***	**	***	***	(*)	***
Fixed RR	1.43	3.07	3.35	2.53	1.48	2.46
RRl	1.23	2.16	2.99	2.30	1.08	2.31
RRu	1.67	4.36	3.75	2.78	2.02	2.63
P	+++	+++	+++	+++	+	+++
Random RR	2.06	2.43	3.21	2.55	1.46	2.48
RRl	1.29	1.18	2.24	2.05	0.92	2.09
RRu	3.29	4.97	4.62	3.16	2.30	2.95
P	++	+	+++	+++	N.S.	+++
Between Chi						89.03
Between df						4
Between P						***
Btwn(F) P						**
Btwn(R) P						(*)

	Study type (1)		
	CC	other	Total
N	44	11	55
NS	35	8	43
Wt	649.34	314.68	964.02
Het Chi	205.63	71.13	307.96
Het df	43	10	54
Het P	***	***	***
Fixed RR	2.79	1.90	2.46
RRl	2.59	1.70	2.31
RRu	3.02	2.13	2.63
P	+++	+++	+++
Random RR	2.67	1.87	2.48
RRl	2.21	1.30	2.09
RRu	3.22	2.70	2.95
P	+++	+++	+++
Between Chi			31.21
Between df			1
Between P			***
Btwn(F) P			*
Btwn(R) P			(*)

	Study type (2)			
	CC	prosp	other	Total
N	44	10	1	55
NS	35	7	1	43
Wt	649.34	308.75	5.93	964.02
Het Chi	205.63	70.94	0.00	307.96
Het df	43	9	0	54
Het P	***	***	N.S.	***
Fixed RR	2.79	1.91	1.60	2.46
RRl	2.59	1.71	0.72	2.31
RRu	3.02	2.14	3.58	2.63
P	+++	+++	N.S.	+++
Random RR	2.67	1.89	1.60	2.48
RRl	2.21	1.28	0.72	2.09
RRu	3.22	2.79	3.58	2.95
P	+++	++	N.S.	+++
Between Chi				31.39
Between df				2
Between P				***
Btwn(F) P				(*)
Btwn(R) P				N.S.

Table 1I12 - 3

IESLC - Meta-analysis of Ever/current Smoking, Duration, "Low"
All LC types, Any Product (or Cigarettes if Any not available)
Most adjusted

	Study size (number of LC cases)				
	100-249	250-499	500-999	1000+	Total
N	17	13	14	11	55
NS	15	9	12	7	43
Wt	78.21	80.85	184.94	620.01	964.02
Het Chi	29.95	11.84	108.36	145.91	307.96
Het df	16	12	13	10	54
Het P	*	N.S.	***	***	***
Fixed RR	1.72	2.37	2.47	2.59	2.46
RRl	1.38	1.91	2.14	2.40	2.31
RRu	2.14	2.95	2.85	2.80	2.63
P	+++	+++	+++	+++	+++
Random RR	1.70	2.37	3.52	2.62	2.48
RRl	1.24	1.91	2.26	1.92	2.09
RRu	2.33	2.95	5.50	3.59	2.95
P	++	+++	+++	+++	+++
Between Chi					11.90
Between df					3
Between P					**
Btwn(F) P					N.S.
Btwn(R) P					(*)

	Risky occupational population			
	no	mining	othRisky	Total
N	52	3		55
NS	40	3		43
Wt	950.70	13.32		964.02
Het Chi	291.23	14.20		307.96
Het df	51	2		54
Het P	***	***		***
Fixed RR	2.48	1.60		2.46
RRl	2.33	0.93		2.31
RRu	2.64	2.74		2.63
P	+++	(+)		+++
Random RR	2.53	1.63		2.48
RRl	2.13	0.38		2.09
RRu	3.01	6.91		2.95
P	+++	N.S.		+++
Between Chi				2.53
Between df				1
Between P				N.S.
Btwn(F) P				N.S.
Btwn(R) P				N.S.

	National cigarette tobacco type			
	Virginia	blended	other	Total
N	5	32	18	55
NS	5	24	14	43
Wt	44.99	722.03	196.99	964.02
Het Chi	10.18	213.22	19.98	307.96
Het df	4	31	17	54
Het P	*	***	N.S.	***
Fixed RR	3.62	2.76	1.50	2.46
RRl	2.70	2.56	1.30	2.31
RRu	4.85	2.97	1.72	2.63
P	+++	+++	+++	+++
Random RR	3.38	3.10	1.50	2.48
RRl	2.08	2.49	1.27	2.09
RRu	5.50	3.86	1.77	2.95
P	+++	+++	+++	+++
Between Chi				64.58
Between df				2
Between P				***
Btwn(F) P				**
Btwn(R) P				***

Table 1I12 - 3

IESLC - Meta-analysis of Ever/current Smoking, Duration, "Low"
All LC types, Any Product (or Cigarettes if Any not available)
Most adjusted

	Any proxy use No/nk	Yes	Total
N	39	16	55
NS	30	13	43
Wt	796.73	167.28	964.02
Het Chi	223.15	75.19	307.96
Het df	38	15	54
Het P	***	***	***
Fixed RR	2.35	3.07	2.46
RRl	2.20	2.63	2.31
RRu	2.52	3.57	2.63
P	+++	+++	+++
Random RR	2.31	3.01	2.48
RRl	1.90	2.07	2.09
RRu	2.80	4.38	2.95
P	+++	+++	+++
Between Chi			9.63
Between df			1
Between P			**
Btwn(F) P			N.S.
Btwn(R) P			N.S.

	Full histological confirmation No	Yes	Total
N	37	18	55
NS	29	14	43
Wt	580.58	383.44	964.02
Het Chi	204.20	63.79	307.96
Het df	36	17	54
Het P	***	***	***
Fixed RR	2.09	3.17	2.46
RRl	1.93	2.87	2.31
RRu	2.27	3.50	2.63
P	+++	+++	+++
Random RR	2.39	2.69	2.48
RRl	1.91	2.13	2.09
RRu	2.98	3.39	2.95
P	+++	+++	+++
Between Chi			39.97
Between df			1
Between P			***
Btwn(F) P			**
Btwn(R) P			N.S.

	Number of adjustment variables (1) 0	1	2+/+nk	Total
N	31	10	14	55
NS	22	9	13	44
Wt	700.14	52.33	211.55	964.02
Het Chi	199.91	17.72	86.43	307.96
Het df	30	9	13	54
Het P	***	*	***	***
Fixed RR	2.46	1.94	2.63	2.46
RRl	2.29	1.48	2.30	2.31
RRu	2.65	2.54	3.01	2.63
P	+++	+++	+++	+++
Random RR	2.46	1.82	3.11	2.48
RRl	1.98	1.23	2.09	2.09
RRu	3.06	2.69	4.62	2.95
P	+++	++	+++	+++
Between Chi				3.91
Between df				2
Between P				N.S.
Btwn(F) P				N.S.
Btwn(R) P				N.S.

Table 1I12 - 3

IESLC - Meta-analysis of Ever/current Smoking, Duration, "Low"
All LC types, Any Product (or Cigarettes if Any not available)
Most adjusted

	Number of adjustment variables (2) 0	1	2	3-5	6+/+nk	Total
N	31	10	8	5	1	55
NS	22	9	8	5	1	45
Wt	700.14	52.33	80.09	124.97	6.49	964.02
Het Chi	199.91	17.72	29.84	54.09	0.00	307.96
Het df	30	9	7	4	0	54
Het P	***	*	***	***	N.S.	***
Fixed RR	2.46	1.94	3.01	2.43	2.23	2.46
RRl	2.29	1.48	2.42	2.04	1.03	2.31
RRu	2.65	2.54	3.75	2.89	4.81	2.63
P	+++	+++	+++	+++	+	+++
Random RR	2.46	1.82	3.24	3.18	2.23	2.48
RRl	1.98	1.23	1.92	1.49	1.03	2.09
RRu	3.06	2.69	5.45	6.79	4.81	2.95
P	+++	++	+++	++	+	+++
Between Chi						6.40
Between df						4
Between P						N.S.
Btwn(F) P						N.S.
Btwn(R) P						N.S.

	Smoking status ever	current	Total
N	43	12	55
NS	34	9	43
Wt	633.79	330.23	964.02
Het Chi	204.37	81.19	307.96
Het df	42	11	54
Het P	***	***	***
Fixed RR	2.75	2.00	2.46
RRl	2.55	1.79	2.31
RRu	2.97	2.22	2.63
P	+++	+++	+++
Random RR	2.52	2.36	2.48
RRl	2.07	1.65	2.09
RRu	3.06	3.37	2.95
P	+++	+++	+++
Between Chi			22.40
Between df			1
Between P			***
Btwn(F) P			*
Btwn(R) P			N.S.

	Product all/unsp	cig+/-ot	cig only	Total
N	14	32	9	55
NS	12	25	7	44
Wt	71.01	662.01	230.99	964.02
Het Chi	28.72	172.64	66.12	307.96
Het df	13	31	8	54
Het P	**	***	***	***
Fixed RR	1.82	2.83	1.82	2.46
RRl	1.44	2.62	1.60	2.31
RRu	2.30	3.05	2.07	2.63
P	+++	+++	+++	+++
Random RR	1.66	2.82	2.54	2.48
RRl	1.15	2.30	1.62	2.09
RRu	2.39	3.45	3.98	2.95
P	++	+++	+++	+++
Between Chi				40.49
Between df				2
Between P				***
Btwn(F) P				*
Btwn(R) P				*

Table 1I12 - 3

IESLC - Meta-analysis of Ever/current Smoking, Duration, "Low"
All LC types, Any Product (or Cigarettes if Any not available)
Most adjusted

		Denominator nev any	nev cigs	Total
	N	28	27	55
	NS	22	22	44
	Wt	438.94	525.07	964.02
Het	Chi	112.55	138.34	307.96
Het	df	27	26	54
Het	P	***	***	***
Fixed	RR	3.22	1.97	2.46
	RRl	2.93	1.81	2.31
	RRu	3.53	2.15	2.63
	P	+++	+++	+++
Random	RR	2.55	2.43	2.48
	RRl	2.02	1.94	2.09
	RRu	3.21	3.04	2.95
	P	+++	+++	+++
Between	Chi			57.07
Between	df			1
Between	P			***
Btwn(F)	P			**
Btwn(R)	P			N.S.

		Derivation of RR/CI Orig	StdCalc	Other	Total
	N	14	29	12	55
	NS	14	20	10	44
	Wt	112.84	669.89	181.29	964.02
Het	Chi	54.05	185.67	67.86	307.96
Het	df	13	28	11	54
Het	P	***	***	***	***
Fixed	RR	2.60	2.45	2.43	2.46
	RRl	2.16	2.27	2.10	2.31
	RRu	3.13	2.65	2.81	2.63
	P	+++	+++	+++	+++
Random	RR	2.39	2.39	2.83	2.48
	RRl	1.60	1.91	1.86	2.09
	RRu	3.58	3.00	4.30	2.95
	P	+++	+++	+++	+++
Between	Chi				0.38
Between	df				2
Between	P				N.S.
Btwn(F)	P				N.S.
Btwn(R)	P				N.S.

Table 1I12 - 4

IESLC - Meta-analysis of Ever/current Smoking, Duration, "Low"
All LC types, Any Product (or Cigarettes if Any not available)
Least adjusted

REF	NRR	X	SEX	AGEL	AGEH	RACE	YF	LC TYPE	LOC	START	ST	NLC	R	VB	P	H	AD	SM	PRODUCT	exL	exH	DENOM	De
AMANDU	501	x	m	0	0	wh	0	all	NAmer	1959	pr	132	m	bl	n	n	0	cu	cig+/-ot	0	24	nev cigs	st
ARMADA	501	x	m	0	0	all	-	all	Eu:wst	1986	CC	325	n	bl	n	y	0	ev	cig+/-ot	1	24	nev cigs	st
AUVINE	501	x	c	0	0	all	-	all	Eu:Sca	1986	CC	517	n	bl	y	n	0	ev	cig+/-ot	1	20	nev cigs	st
AXELSS	502	x	m	0	0	sca	-	all	Eu:Sca	1989	CC	436	n	bl	n	n	0	ev	all/unsp	20	29	nev any	st
AXELSS	511		f	0	0	sca	-	all	Eu:Sca	1989	CC	436	n	bl	n	n	0	ev	all/unsp	20	29	nev any	st
BARBON	501	x	m	0	0	all	-	all	Eu:wst	1979	CC	755	n	bl	y	y	0	ev	all/unsp	1	29	nev any	st
BEST	505		m	0	0	all	0	all	NAmer	1955	pr	381	n	V	n	n	1	cu	cig only	20	29	nev any	ot
BUFFLE	526		f	0	0	w-hi	-	all	NAmer	1976	CC	943	n	bl	y	n	0	ev	cig+/-ot	1	30	nev cigs	or
CEDERL	501		m	40	69	all	10	all	Eu:Sca	1963	pr	491	n	bl	n	n	1	cu	cig only	1	29	nev any	ot
CEDERL	504		f	40	69	all	10	all	Eu:Sca	1963	pr	491	n	bl	n	n	1	cu	cig only	1	29	nev any	ot
CHEN2	502		m	0	0	all	-	all	As:Chi	1983	CC	193	n	ot	y	n	0	ev	all/unsp	10	20	nev any	st
CHEN2	510		f	0	0	all	-	all	As:Chi	1983	CC	193	n	ot	y	n	0	ev	all/unsp	1	20	nev any	st
CHOI	502		m	0	0	all	-	all	As:oth	1985	CC	375	n	bl	n	n	0	ev	cig+/-ot	20	29	nev cigs	st
CHOI	511		f	0	0	all	-	all	As:oth	1985	CC	375	n	bl	n	n	0	ev	cig+/-ot	20	29	nev cigs	st
CPSI	580		m	40	84	wh	0	all	NAmer	1959	pr	5138	n	bl	n	n	0	cu	cig only	1	29	nev cigs	st
CPSI	676		f	40	84	wh	0	all	NAmer	1959	pr	5138	n	bl	n	n	0	cu	cig only	1	29	nev cigs	st
CPSII	552		m	0	0	all	6	all	NAmer	1982	pr	3229	n	bl	n	n	0	cu	cig only	1	29	nev any	st
CPSII	618		f	0	0	all	6	all	NAmer	1982	pr	3229	n	bl	n	n	0	cu	cig+/-ot	1	29	nev cigs	st
DAMBER	506		m	0	0	all	-	all	Eu:Sca	1972	CC	579	n	bl	y	n	1	ev	all/unsp	1	20	nev any	ot
DESTEF	501	x	m	0	0	all	-	all	SCAmer	1988	CC	497	n	bl	n	y	0	ev	all/unsp	1	29	nev any	st
DORGAN	570		m	0	0	wh	-	all	NAmer	1980	CC	2026	n	bl	y	y	2	ev	cig+/-ot	1	34	nev any	ot
DORGAN	562		f	0	0	all	-	all	NAmer	1980	CC	2026	n	bl	y	y	3	ev	cig+/-ot	1	34	nev any	ot
DOSEME	502		m	0	0	all	-	all	Eu:bal	1979	CC	1210	n	bl	n	n	2	ev	cig+/-ot	11	20	nev cigs	or
FAN	501		m	0	0	all	-	all	As:Chi	1990	CC	403	n	ot	y	n	0	ev	cig+/-ot	1	29	nev cigs	st
FAN	506		f	0	0	all	-	all	As:Chi	1990	CC	403	n	ot	y	n	0	ev	cig+/-ot	1	29	nev cigs	st
GAO	561	x	f	0	0	all	-	all	As:Chi	1984	CC	1405	n	ot	n	n	0	ev	cig+/-ot	1	29	nev cigs	st
GER	513	x	c	0	0	all	-	all	As:oth	1990	CC	141	n	ot	y	n	0	ev	all/unsp	1	20	nev any	st
HU	502		m	0	0	all	-	all	As:Chi	1985	CC	227	n	ot	n	y	0	ev	cig+/-ot	20	29	nev cigs	st
HU	507		f	0	0	all	-	all	As:Chi	1985	CC	227	n	ot	n	y	0	ev	cig+/-ot	20	29	nev cigs	st
HU2	509		c	0	0	all	-	all	As:Chi	1977	CC	523	n	ot	y	n	0	ev	cig+/-ot	20	29	nev cigs	or
HUMBLE	517	x	c	0	0	wh	-	not alv	NAmer	1980	CC	521	n	bl	y	n	0	cu	cig+/-ot	1	29	nev cigs	st
JOLY	516		m	0	0	all	-	all	SCAmer	1978	CC	826	n	bl	n	n	0	ev	cig+/-ot	20	29	nev any	st
JOLY	502		f	0	0	all	-	all	SCAmer	1978	CC	826	n	bl	n	n	0	ev	cig+/-ot	20	29	nev any	st
JUSSAW	512		m	0	0	all	-	all	As:Ind	1964	CC	792	n	V	n	n	0	ev	cig only	20	29	nev any	st
KATSOU	502	x	f	0	0	all	-	all	Eu:bal	1987	CC	101	n	bl	n	n	0	cu	all/unsp	20	29	nev any	st
KHUDER	501		m	0	0	all	-	all	NAmer	1985	CC	482	n	bl	n	y	0	ev	cig+/-ot	1	29	nev cigs	st
LETOUR	506		c	0	0	all	-	all	NAmer	1983	CC	738	n	V	y	y	0	ev	cig+/-ot	1	24	nev cigs	st
LIAW	501		c	0	0	all	0	all	As:oth	1982	pr	127	n	ot	n	n	2	cu	all/unsp	1	20	nev any	or
LIU3	507	x	m	0	0	all	-	all	As:Chi	1985	CC	110	n	ot	n	n	0	ev	all/unsp	1	34	nev any	or
LIU5	504		c	0	0	all	-	all	As:Chi	1978	CC	111	n	ot	y	n	0	ev	all/unsp	1	29	nev any	st
LUBIN	508		m	0	0	all	-	all	As:Chi	1984	CC	427	m	ot	y	n	0	ev	cig+/-ot	1	29	nev any	st
LUBIN2	531		m	0	0	all	-	all	Eu:mul	1976	CC	7804	n	bl	n	y	0	ev	cig+/-ot	1	29	nev any	st
LUBIN2	574		f	0	0	all	-	all	Eu:mul	1976	CC	7804	n	bl	n	y	0	ev	cig+/-ot	1	29	nev any	st
MATOS	516	x	m	0	0	all	-	all	SCAmer	1994	CC	200	n	bl	n	n	0	ev	cig+/-ot	1	24	nev any	st
MCCONN	503		c	0	0	all	-	all	Eu:UK	1946	CC	100	n	V	n	y	0	ev	all/unsp	20	29	nev any	st
NOTAN2	514		c	0	0	all	-	all	As:Ind	1963	CC	683	n	V	n	n	0	ev	cig only	11	20	nev any	st
OSANN2	501	x	f	0	0	all	-	all	NAmer	1964	ot	217	n	bl	n	y	0	ev	cig+/-ot	1	20	nev cigs	st
PEZZOT	534		m	0	0	all	-	all	SCAmer	1987	CC	215	n	bl	n	y	0	ev	cig only	1	30	nev cigs	st
QIAO2	511	x	m	0	0	all	0	all	As:Chi	1992	pr	241	m	ot	n	n	0	ev	all/unsp	1	27	nev any	st
RACHTA	511	x	f	0	0	all	-	all	Eu:est	1991	CC	118	n	bl	n	y	0	ev	cig+/-ot	1	20	nev cigs	st
SOBUE	546		m	0	0	all	-	q+s+l+a	As:Jap	1986	CC	1376	n	bl	n	y	0	cu	cig+/-ot	1	29	nev cigs	st
WANG2	503		c	0	0	all	-	all	As:Chi	1980	CC	103	n	ot	n	n	0	ev	cig+/-ot	20	29	nev cigs	st
WUWILL	501	x	f	0	0	all	-	all	As:Chi	1985	CC	965	n	ot	n	n	0	ev	cig+/-ot	1	29	nev cigs	st
ZHENG	553		m	0	0	all	-	all	As:Chi	1982	CC	540	n	ot	*	y	0	ev	cig+/-ot	1	29	nev cigs	st
ZHENG	558		f	0	0	all	-	all	As:Chi	1982	CC	540	n	ot	*	y	0	ev	cig+/-ot	1	29	nev cigs	st

Cigarette type is all/unspec for all RRs

except for the following:

REF	NRR	CIGTYPE
JUSSAW	512	MC only
NOTAN2	514	MC only

Table 1I12 - 5

IESLC - Meta-analysis of Ever/current Smoking, Duration, "Low"
All LC types, Any Product (or Cigarettes if Any not available)
Least adjusted

REF	NRR	SEX	AD	Number Exposed Case	Number Exposed Cont	Non-exposed Case	Non-exposed Cont	RR	95.00%CI
*AMANDU	501	m	0	42	68909	6	25350	2.58	(1.09- 6.06)
ARMADA	501	m	0	21	55	8	71	3.39	(1.40- 8.23)
AUVINE	501	c	0	26	18	44	229	7.52	(3.80- 14.87)
AXELSS	502	m	0	17	64	16	160	2.66	(1.27- 5.58)
AXELSS	511	f	0	12	29	18	154	3.54	(1.54- 8.13)
Subtotal AXELSS								3.02	(1.73- 5.25)
BARBON	501	m	0	42	91	22	188	3.94	(2.22- 7.00)
*BEST	505	m	1	22	-	7	-	4.10	(1.75- 9.60)
BUFFLE	526	f	0	52	57	12	112	8.51	(4.21- 17.22)
*CEDERL	501	m	1	5	-	7	-	1.80	(0.57- 5.66)
*CEDERL	504	f	1	3	-	19	-	1.60	(0.47- 5.40)
Subtotal CEDERL								1.70	(0.74- 3.93)
CHEN2	502	m	0	4	3	9	33	4.89	(0.92- 25.93)
CHEN2	510	f	0	1	6	25	33	0.22	(0.02- 1.95)
Subtotal CHEN2								1.55	(0.41- 5.85)
CHOI	502	m	0	66	166	13	95	2.91	(1.52- 5.54)
CHOI	511	f	0	8	14	76	164	1.23	(0.50- 3.06)
Subtotal CHOI								2.18	(1.29- 3.69)
*CPSI	580	m	0	95	266163	196	926068	1.69	(1.32- 2.15)
*CPSI	676	f	0	105	694015	532	3877179	1.10	(0.89- 1.36)
Subtotal CPSI								1.32	(1.13- 1.55)
*CPSII	552	m	0	72	141932	124	742207	3.04	(2.27- 4.06)
*CPSII	618	f	0	127	301244	310	2091302	2.84	(2.31- 3.50)
Subtotal CPSII								2.91	(2.46- 3.44)
DAMBER	506	m	1	-	-	42	-	1.58	(0.69- 3.66)
DESTEF	501	m	0	43	55	27	163	4.72	(2.67- 8.35)
DORGAN	570	m	2	-	-	-	-	5.44	(2.97- 9.98)
DORGAN	562	f	3	-	-	-	-	4.25	(3.20- 5.64)
Subtotal DORGAN								4.44	(3.44- 5.74)
DOSEME	502	m	2	158	-	142	-	3.80	(2.60- 5.70)
FAN	501	m	0	29	135	36	236	1.41	(0.83- 2.40)
FAN	506	f	0	8	15	69	320	2.47	(1.01- 6.06)
Subtotal FAN								1.63	(1.03- 2.58)
GAO	561	f	0	68	58	435	605	1.63	(1.12- 2.36)
GER	513	c	0	10	40	51	246	1.21	(0.57- 2.57)
HU	502	m	0	60	47	41	67	2.09	(1.21- 3.60)
HU	507	f	0	11	7	40	48	1.89	(0.67- 5.32)
Subtotal HU								2.04	(1.26- 3.31)
HU2	509	c	0	64	63	121	213	1.79	(1.18- 2.70)
HUMBLE	517	c	0	20	33	28	285	6.17	(3.13- 12.15)
JOLY	516	m	0	38	61	12	218	11.32	(5.57- 22.98)
JOLY	502	f	0	18	26	52	283	3.77	(1.93- 7.36)
Subtotal JOLY								6.33	(3.89- 10.30)
JUSSAW	512	m	0	38	23	149	624	6.92	(4.00- 11.97)
KATSOU	502	f	0	8	7	48	67	1.60	(0.54- 4.70)
KHUDER	501	m	0	16	61	23	309	3.52	(1.76- 7.06)
LETOUR	506	c	0	65	187	24	224	3.24	(1.95- 5.39)
*LIAW	501	c	2	-	-	-	-	0.90	(0.30- 3.10)
LIU3	507	m	0	30	146	4	19	0.98	(0.31- 3.07)
LIU5	504	c	0	27	37	26	41	1.15	(0.57- 2.31)
LUBIN	508	m	0	30	146	8	72	1.85	(0.81- 4.24)
LUBIN2	531	m	0	953	2995	190	2616	4.38	(3.72- 5.16)
LUBIN2	574	f	0	132	230	336	1188	2.03	(1.59- 2.59)
Subtotal LUBIN2								3.45	(3.01- 3.96)
MATOS	516	m	0	20	84	11	110	2.38	(1.08- 5.24)
MCCONN	503	c	0	46	57	9	23	2.06	(0.87- 4.89)
NOTAN2	514	c	0	15	15	107	201	1.88	(0.88- 3.99)
OSANN2	501	f	0	23	47	33	109	1.62	(0.86- 3.04)
PEZZOT	534	m	0	30	134	4	116	6.49	(2.22- 18.98)
*QIAO2	511	m	0	7	2364	10	709	0.21	(0.08- 0.55)
RACHTA	511	f	0	12	19	33	98	1.88	(0.82- 4.27)
SOBUE	546	m	0	62	119	34	128	1.96	(1.21- 3.19)
WANG2	503	c	0	8	18	11	43	1.74	(0.60- 5.03)
WUWILL	501	f	0	137	139	417	601	1.42	(1.09- 1.86)
ZHENG	553	m	0	37	75	33	94	1.41	(0.80- 2.46)
ZHENG	558	f	0	17	17	152	184	1.21	(0.60- 2.45)
Subtotal ZHENG								1.33	(0.86- 2.06)
Partial Totals				2960	1480226	4202	7673605		

*prospective study

Table 1I12 - 5

IESLC - Meta-analysis of Ever/current Smoking, Duration, "Low"
All LC types, Any Product (or Cigarettes if Any not available)
Least adjusted

REF	NRR	SEX	AD	Ys	Ws	Qs	Ps
*AMANDU	501	m	0	0.95	5.25	0.01	0.0302
ARMADA	501	m	0	1.22	4.88	0.48	0.0070
AUVINE	501	c	0	2.02	8.26	10.16	0.0000
AXELSS	502	m	0	0.98	6.98	0.03	0.0098
AXELSS	511	f	0	1.26	5.56	0.71	0.0029
Subtotal AXELSS				1.10	12.54	0.74	
BARBON	501	m	0	1.37	11.69	2.52	0.0000
*BEST	505	m	1	1.41	5.30	1.34	0.0012
BUFFLE	526	f	0	2.14	7.75	11.80	0.0000
*CEDERL	501	m	1	0.59	2.92	0.30	0.3155
*CEDERL	504	f	1	0.47	2.58	0.49	0.4505
Subtotal CEDERL				0.53	5.49	0.79	
CHEN2	502	m	0	1.59	1.38	0.64	0.0623
CHEN2	510	f	0	-1.51	0.81	4.74	0.1734
Subtotal CHEN2				0.44	2.19	5.38	
CHOI	502	m	0	1.07	9.21	0.23	0.0012
CHOI	511	f	0	0.21	4.64	2.26	0.6519
Subtotal CHOI				0.78	13.84	2.49	
*CPSI	580	m	0	0.52	64.01	9.51	0.0000
*CPSI	676	f	0	0.10	87.71	57.59	0.3603
Subtotal CPSI				0.28	151.71	67.10	
*CPSII	552	m	0	1.11	45.57	1.87	0.0000
*CPSII	618	f	0	1.05	90.12	1.70	0.0000
Subtotal CPSII				1.07	135.69	3.57	
DAMBER	506	m	1	0.46	5.52	1.12	0.2825
DESTEF	501	m	0	1.55	11.82	4.90	0.0000
DORGAN	570	m	2	1.69	10.46	6.46	0.0000
DORGAN	562	f	3	1.45	47.84	13.89	0.0000
Subtotal DORGAN				1.49	58.30	20.35	
DOSEME	502	m	2	1.34	24.94	4.55	0.0000
FAN	501	m	0	0.34	13.53	4.33	0.2079
FAN	506	f	0	0.91	4.78	0.00	0.0478
Subtotal FAN				0.49	18.31	4.33	
GAO	561	f	0	0.49	27.86	4.89	0.0099
GER	513	c	0	0.19	6.73	3.49	0.6273
HU	502	m	0	0.74	12.94	0.39	0.0082
HU	507	f	0	0.63	3.58	0.27	0.2303
Subtotal HU				0.71	16.52	0.65	
HU2	509	c	0	0.58	22.49	2.40	0.0058
HUMBLE	517	c	0	1.82	8.37	6.95	0.0000
JOLY	516	m	0	2.43	7.66	17.65	0.0000
JOLY	502	f	0	1.33	8.56	1.50	0.0001
Subtotal JOLY				1.85	16.22	19.15	
JUSSAW	512	m	0	1.93	12.80	13.48	0.0000
KATSOU	502	f	0	0.47	3.29	0.64	0.3967
KHUDER	501	m	0	1.26	7.96	0.98	0.0004
LETOUR	506	c	0	1.18	14.96	1.08	0.0000
*LIAW	501	c	2	-0.11	2.82	2.89	0.8596
LIU3	507	m	0	-0.02	2.92	2.54	0.9669
LIU5	504	c	0	0.14	7.88	4.64	0.6935
LUBIN	508	m	0	0.61	5.58	0.48	0.1463
LUBIN2	531	m	0	1.48	142.28	46.10	0.0000
LUBIN2	574	f	0	0.71	63.53	2.55	0.0000
Subtotal LUBIN2				1.24	205.80	48.65	
MATOS	516	m	0	0.87	6.18	0.01	0.0311
MCCONN	503	c	0	0.72	5.16	0.17	0.1002
NOTAN2	514	c	0	0.63	6.77	0.52	0.1008
OSANN2	501	f	0	0.48	9.59	1.76	0.1369
PEZZOT	534	m	0	1.87	3.34	3.09	0.0006
*QIAO2	511	m	0	-1.56	4.15	25.29	0.0015
RACHTA	511	f	0	0.63	5.67	0.44	0.1344
SOBUE	546	m	0	0.67	16.19	0.89	0.0067
WANG2	503	c	0	0.55	3.39	0.43	0.3089
WUWILL	501	f	0	0.35	53.89	16.72	0.0100
ZHENG	553	m	0	0.34	12.30	3.97	0.2328
ZHENG	558	f	0	0.19	7.71	3.96	0.5957
Subtotal ZHENG				0.28	20.01	7.93	

Table 1I12 - 5

IESLC - Meta-analysis of Ever/current Smoking, Duration, "Low"
All LC types, Any Product (or Cigarettes if Any not available)
Least adjusted

	N	55
	NS	43
	Wt	980.02
Het	Chi	311.82
Het	df	54
Het	P	***
Fixed	RR	2.48
	RRl	2.33
	RRu	2.64
	P	+++
Random	RR	2.43
	RRl	2.05
	RRu	2.88
	P	+++
Asymm	P	N.S.

Table 1I12 - 6

IESLC - Meta-analysis of Ever/current Smoking, Duration, "Low"
All LC types, Any Product (or Cigarettes if Any not available)
Least adjusted

	Sex combined	male	female	Total
N	10	27	18	55
NS	10	27	18	55
Wt	86.82	457.75	435.46	980.02
Het Chi	32.53	132.78	106.39	311.82
Het df	9	26	17	54
Het P	***	***	***	***
Fixed RR	2.36	3.06	2.01	2.48
RRl	1.91	2.79	1.83	2.33
RRu	2.91	3.36	2.20	2.64
P	+++	+++	+++	+++
Random RR	2.27	2.76	2.08	2.43
RRl	1.49	2.18	1.57	2.05
RRu	3.46	3.51	2.74	2.88
P	+++	+++	+++	+++
Between Chi				40.11
Between df				2
Between P				***
Btwn(F) P				*
Btwn(R) P				N.S.

Table 1I12 - 7

IESLC - Meta-analysis of Ever/current Smoking, Duration, "Low"
All LC types, Any Product (or Cigarettes if Any not available)
Excluded studies (and stage at which they were excluded)

1 BECHER BLOT1 BROWN3 CARPEN CHYOU DARBY DOLL2 GARCIA GRAHAM GURSEL HAMMO2 JAHN JAIN LAUSSM PRESCO QIAO TVERDA WIGLE WYNDE3

2 ALDERS BENSHL BRESLO CHIAZZ DEAN3 DORN ENGELA GAO2 GILLIS GUO HEGMAN HIRAYA HOLE KAUFMA KOO KOULUM LIU4 MIGRAN MRFITR PERNU SEGI2 SPEIZE SUZUK2 SVENSS VUTUC WAKAI WU YUAN

3 GENG MCDUFF SPITZ STASZE WU2 ZHANG

4 BOUCHA CHEN CORREA JEDRYC LUO WYNDE2 WYNDE6

5 AKIBA HAMMON PISANI RESTRE SADOWS XU

7 BOFFET BROSS WYNDE7

10 AMES WATSON WYNDE8

14 AGUDO BOUCOT DEAN2 DOLL GARSHI HAENSZ KAISE2 KREUZE LEVIN PEZZO2 TIZZAN ZHOU

15 BENHAM

Table 1I12 - 8
Potentially overlapping studies

REF	REFGP	PRINC	.	OVERLAP/LINK
LUBIN2	LUBIN2	1		Lubin-combined
OSANN2	KAISER	2		KAISER/OSANN2
CPSI	CPSI	1		CPSI overall
LUBIN	XIANGZ	2		LUBIN/XIANGZ/QIAO

Table 1I12 - 9
Most adjusted - insufficient data for meta-analysis

REF	NRR	SEX	AGEL	AGEH	RACE	YF	LC TYPE	LOC	START	ST	NLC	R	VB	P	H	AD	SM	PRODUCT	exL	exH	DENOM	De
BUFFLE	501	m	0	0	wh	-	all	NAmer	1976	CC	943	n	bl	y	n	0	ev	cig+/-ot	1	33	nev cigs	or
HAMMON	513	m	0	0	wh	0	all	NAmer	1952	pr	448	n	bl	n	n	1	ev	cig only	1	34	nev any	st
SADOWS	524	m	0	0	wh	-	all	NAmer	1938	CC	477	n	bl	n	n	0	ev	cig only	20	29	nev any	ot
XU	501	m	0	0	all	-	all	As:Chi	1985	CC	729	n	ot	n	n	2	ev	all/unsp	1	29	nev any	or

REF	NRR	RR	SIG	RRDATA comment
BUFFLE	501	6.80		0
HAMMON	513		*	RR for <1/2 pack per day is 5.31, that for 1/2 to 1 pack per day is 6.56, that for 1 to 2 packs per day is 7.27 while that for 2+ packs per day 10.78
SADOWS	524	2.78		0
XU	501		*	RR for 1-19/day is 1.8(p<0.05), for 20-29/day is 1.5(p<0.05) and for >=30/ day is 5.3(p<0.05)

Table 1I13 -

IESLC - Meta-analysis of Ever/current Smoking, Duration, "Mid"
All LC types, Any Product (or Cigarettes if Any not available)

This analysis is restricted to results for:
1) Ever/current smokers
2) Results by Duration
3) Categorical results by Duration
4) All LC types (or near equivalent)
5) Results complete enough for use in metaanalysis

Within each study, results are then selected (in the following order of preference, within each sex) for:
6) SMKSTA: ever, current
7) PRODUCT: all/unspec, cigarettes regardless of other products, cigarettes only
8) CIGTYPE: all/unspecified, MC regardless of HR, MC only
9) (not applicable)
10) DENOM: never smoked anything, never smoked cigarettes, never any + low, never cigs + low
11) Followup period (YF, prospective studies): whole study (coded as 0) or longest available
12) LCtype: all or nearest available, at least Squamous and Adeno. (q = squamous, s = small,
l = large, a = adeno, mix = mixed, alv = alveolar)
13) Race: all or nearest available, otherwise by race (wh or w = white, bl or b = black, hi = hispanic
ch = chinese, jap = japanese, haw = hawaiian, w+o = white + oriental, sca = scandinavian, as = asian)
14) Duration "mid" in key scheme 1 (key value 35, maximum range 21-49)
15) For overlapping studies: principal rather than subsidiary studies
Finally by Age: whole study (coded as 0) if available, otherwise by widest available age group
and then for single sex results (m, f) in preference to results for both sexes combined (c).

Results adjusted (AD) for the most potential confounders are then chosen in Sections -1 to -3
and results adjusted for the least confounders in Sections -4 to -6. (Those least adjusted results which
actually differ from the most adjusted are marked 'x' in column X in Section -4)

Section -7 shows excluded studies, together with the stage (as above) at which no qualifying
results were found.

Section -8 lists the potentially overlapping studies which have been included (1=principal, 2=subsidiary).

Section -9 lists any results which would have been included in preference except that they had data not complete
enough for use in meta-analysis, with their significance (yes/no), if known, and any further comment as entered
on the database. It also lists as "gap" any categories for which no data were presented by the original authors.

In addition to those mentioned above, the following fields, levels and abbreviations are used:
* or nk = not known, n = no, y = yes, ot = other
ev = ever, cu = current, nev = never
all/unspec = all or unspecified, cig+/-ot = cigarettes irrespective of other products (cigar, pipe etc)
MC = manufactured cigarettes, HR = hand-rolled cigarettes
exL, exH = range of exposure (low and high) in the smoking group, in terms of Duration
REF: 6-character study reference
NRR: number of the RR on the database within the study
ST : study type (CC = case control, pr or prosp = prospective)
NLC: number of lung cancer cases in whole study
R : risky occupational population (n = no, m = mining, o = other risky)
VB : national cigarette type (V = at least 75% Virginia, bl = at least 75% blended, ot = other)
P : any proxy use
H : full histological confirmation
De : derivation of RR/CI (or = original, st = standard method, ot = other method of estimation)

Table 1I13 - 1

IESLC - Meta-analysis of Ever/current Smoking, Duration, "Mid"
All LC types, Any Product (or Cigarettes if Any not available)
Most adjusted

REF	NRR	SEX	AGEL	AGEH	RACE	YF	LC TYPE	LOC	START	ST	NLC	R	VB	P	H	AD	SM	PRODUCT	exL	exH	DENOM	De
ARMADA	507	m	0	0	all	-	all	Eu:wst	1986	CC	325	n	bl	n	y	1	ev	cig+/-ot	25	49	nev cigs	or
AUVINE	518	c	0	0	all	-	all	Eu:Sca	1986	CC	517	n	bl	y	n	2	ev	cig+/-ot	21	40	nev cigs	or
AXELSS	521	m	0	0	sca	-	all	Eu:Sca	1989	CC	436	n	bl	n	n	6	ev	all/unsp	30	39	nev any	ot
AXELSS	512	f	0	0	sca	-	all	Eu:Sca	1989	CC	436	n	bl	n	n	0	ev	all/unsp	30	39	nev any	st
BARBON	509	m	0	0	all	-	all	Eu:wst	1979	CC	755	n	bl	y	y	1	ev	all/unsp	30	39	nev any	or
BEST	506	m	0	0	all	0	all	NAmer	1955	pr	381	n	V	n	n	1	cu	cig only	30	39	nev any	ot
BUFFLE	527	f	0	0	w-hi	-	all	NAmer	1976	CC	943	n	bl	y	n	0	ev	cig+/-ot	31	40	nev cigs	or
CHEN2	504	m	0	0	all	-	all	As:Chi	1983	CC	193	n	ot	y	n	0	ev	all/unsp	31	40	nev any	st
CHEN2	512	f	0	0	all	-	all	As:Chi	1983	CC	193	n	ot	y	n	0	ev	all/unsp	31	40	nev any	st
CHOI	503	m	0	0	all	-	all	As:oth	1985	CC	375	n	bl	n	n	0	ev	cig+/-ot	30	39	nev cigs	st
CHOI	512	f	0	0	all	-	all	As:oth	1985	CC	375	n	bl	n	n	0	ev	cig+/-ot	30	39	nev cigs	st
CPSI	582	m	40	84	wh	0	all	NAmer	1959	pr	5138	n	bl	n	n	0	cu	cig only	35	39	nev cigs	st
CPSI	678	f	40	84	wh	0	all	NAmer	1959	pr	5138	n	bl	n	n	0	cu	cig only	35	39	nev cigs	st
CPSII	554	m	0	0	all	6	all	NAmer	1982	pr	3229	n	bl	n	n	0	cu	cig only	35	39	nev any	st
CPSII	620	f	0	0	all	6	all	NAmer	1982	pr	3229	n	bl	n	n	0	cu	cig+/-ot	35	39	nev cigs	st
DAMBER	508	m	0	0	all	-	all	Eu:Sca	1972	CC	579	n	bl	y	n	1	ev	all/unsp	31	40	nev any	ot
DESTEF	509	m	0	0	all	-	all	SCAmer	1988	CC	497	n	bl	n	y	4	ev	all/unsp	30	39	nev any	or
FAN	502	m	0	0	all	-	all	As:Chi	1990	CC	403	n	ot	y	n	0	ev	cig+/-ot	30	39	nev cigs	st
FAN	507	f	0	0	all	-	all	As:Chi	1990	CC	403	n	ot	y	n	0	ev	cig+/-ot	30	39	nev cigs	st
GER	519	c	0	0	all	-	all	As:oth	1990	CC	141	n	ot	y	n	5	ev	all/unsp	21	40	nev any	ot
HU2	510	c	0	0	all	-	all	As:Chi	1977	CC	523	n	ot	y	n	0	ev	cig+/-ot	30	39	nev cigs	or
HUMBLE	543	c	0	0	wh	-	not alv	NAmer	1980	CC	521	n	bl	y	n	3	cu	cig+/-ot	30	39	nev cigs	ot
JOLY	517	m	0	0	all	-	all	SCAmer	1978	CC	826	n	bl	n	n	0	ev	cig+/-ot	30	39	nev any	st
JOLY	503	f	0	0	all	-	all	SCAmer	1978	CC	826	n	bl	n	n	0	ev	cig+/-ot	30	39	nev any	st
JUSSAW	513	m	0	0	all	-	all	As:Ind	1964	CC	792	n	V	n	n	0	ev	cig only	30	39	nev any	st
KATSOU	503	f	0	0	all	-	all	Eu:bal	1987	CC	101	n	bl	n	n	0	cu	all/unsp	30	39	nev any	st
KHUDER	502	m	0	0	all	-	all	NAmer	1985	CC	482	n	bl	n	y	0	ev	cig+/-ot	30	49	nev cigs	st
LETOUR	507	c	0	0	all	-	all	NAmer	1983	CC	738	n	V	y	y	0	ev	cig+/-ot	25	40	nev cigs	st
LUBIN	509	m	0	0	all	-	all	As:Chi	1984	CC	427	m	ot	y	n	0	ev	cig+/-ot	30	39	nev any	st
LUBIN2	532	m	0	0	all	-	all	Eu:mul	1976	CC	7804	n	bl	n	y	0	ev	cig+/-ot	30	39	nev any	st
LUBIN2	575	f	0	0	all	-	all	Eu:mul	1976	CC	7804	n	bl	n	y	0	ev	cig+/-ot	30	39	nev any	st
MATOS	537	m	0	0	all	-	all	SCAmer	1994	CC	200	n	bl	n	n	2	ev	cig+/-ot	25	39	nev any	or
MCCONN	504	c	0	0	all	-	all	Eu:UK	1946	CC	100	n	V	n	y	0	ev	all/unsp	30	39	nev any	st
NOTAN2	516	c	0	0	all	-	all	As:Ind	1963	CC	683	n	V	n	n	0	ev	cig only	31	40	nev any	st
PEZZOT	535	m	0	0	all	-	all	SCAmer	1987	CC	215	n	bl	n	y	0	ev	cig only	31	40	nev cigs	st
QIAO2	517	m	0	0	all	0	all	As:Chi	1992	pr	241	m	ot	n	n	1	ev	all/unsp	28	41	nev any	or
RACHTA	517	f	0	0	all	-	all	Eu:est	1991	CC	118	n	bl	n	y	1	ev	cig+/-ot	21	40	nev cigs	or
SOBUE	547	m	0	0	all	-	q+s+l+a	As:Jap	1986	CC	1376	n	bl	n	y	0	cu	cig+/-ot	30	39	nev cigs	st
WANG2	504	c	0	0	all	-	all	As:Chi	1980	CC	103	n	ot	n	n	0	ev	cig+/-ot	30	39	nev cigs	st
WUWILL	517	f	0	0	all	-	all	As:Chi	1985	CC	965	n	ot	n	n	3	ev	cig+/-ot	30	39	nev cigs	ot

Cigarette type is all/unspec for all RRs

except for the following:

REF	NRR	CIGTYPE
JUSSAW	513	MC only
NOTAN2	516	MC only

Table 1I13 - 2

IESLC - Meta-analysis of Ever/current Smoking, Duration, "Mid"
All LC types, Any Product (or Cigarettes if Any not available)
Most adjusted

REF	NRR	SEX	AD	Number Exposed Case	Cont	Non-exposed Case	Cont	RR	95.00%CI
ARMADA	507	m	1	219	-	8	-	11.90	(5.50- 25.50)
AUVINE	518	c	2	10	-	44	-	33.20	(14.30- 77.40)
AXELSS	521	m	6	57	-	16	-	7.62	(4.01- 14.47)
AXELSS	512	f	0	29	26	18	154	9.54	(4.64- 19.61)
Subtotal AXELSS								8.42	(5.21- 13.59)
BARBON	509	m	1	118	-	22	-	7.90	(4.70- 13.50)
*BEST	506	m	1	55	-	7	-	13.90	(6.33- 30.52)
BUFFLE	527	f	0	97	62	12	112	14.60	(7.43- 28.69)
CHEN2	504	m	0	36	27	9	33	4.89	(2.01- 11.91)
CHEN2	512	f	0	13	6	25	33	2.86	(0.95- 8.58)
Subtotal CHEN2								3.95	(1.98- 7.89)
CHOI	503	m	0	102	160	13	95	4.66	(2.48- 8.75)
CHOI	512	f	0	8	2	76	164	8.63	(1.79- 41.62)
Subtotal CHOI								5.07	(2.83- 9.11)
*CPSI	582	m	0	470	367622	196	926068	6.04	(5.11- 7.14)
*CPSI	678	f	0	154	315060	532	3877179	3.56	(2.98- 4.26)
Subtotal CPSI								4.73	(4.19- 5.34)
*CPSII	554	m	0	244	109788	124	742207	13.30	(10.72- 16.51)
*CPSII	620	f	0	193	116270	310	2091302	11.20	(9.36- 13.40)
Subtotal CPSII								12.01	(10.46- 13.79)
DAMBER	508	m	1	-	-	42	-	5.15	(3.27- 8.32)
DESTEF	509	m	4	78	-	27	-	5.20	(2.90- 8.90)
FAN	502	m	0	44	122	36	236	2.36	(1.45- 3.87)
FAN	507	f	0	19	23	69	320	3.83	(1.98- 7.42)
Subtotal FAN								2.81	(1.89- 4.17)
GER	519	c	5	31	-	51	-	1.56	(0.83- 2.91)
HU2	510	c	0	123	101	121	213	2.14	(1.52- 3.03)
HUMBLE	543	c	3	68	-	28	-	17.54	(8.46- 36.34)
JOLY	517	m	0	85	165	12	218	9.36	(4.95- 17.70)
JOLY	503	f	0	31	24	52	283	7.03	(3.82- 12.93)
Subtotal JOLY								8.06	(5.19- 12.52)
JUSSAW	513	m	0	27	9	149	624	12.56	(5.79- 27.28)
KATSOU	503	f	0	15	2	48	67	10.47	(2.29- 47.93)
KHUDER	502	m	0	207	370	23	309	7.52	(4.76- 11.86)
LETOUR	507	c	0	264	160	24	224	15.40	(9.68- 24.51)
LUBIN	509	m	0	124	294	8	72	3.80	(1.78- 8.12)
LUBIN2	532	m	0	2227	3470	190	2616	8.84	(7.56- 10.33)
LUBIN2	575	f	0	187	186	336	1188	3.55	(2.81- 4.50)
Subtotal LUBIN2								6.69	(5.87- 7.63)
MATOS	537	m	2	82	-	11	-	7.20	(3.60- 14.50)
MCCONN	504	c	0	21	57	9	23	0.94	(0.38- 2.36)
NOTAN2	516	c	0	12	7	107	201	3.22	(1.23- 8.42)
PEZZOT	535	m	0	71	82	4	116	25.11	(8.82- 71.48)
*QIAO2	517	m	1	54	-	10	-	1.46	(0.74- 2.87)
RACHTA	517	f	1	49	-	33	-	7.55	(3.90- 14.63)
SOBUE	547	m	0	159	200	34	128	2.99	(1.94- 4.61)
WANG2	504	c	0	26	38	11	43	2.67	(1.17- 6.13)
WUWILL	517	f	3	179	-	417	-	2.71	(2.05- 3.60)
Partial Totals				5988	914333	3264	7644228		

*prospective study

Table 1I13 - 2

IESLC - Meta-analysis of Ever/current Smoking, Duration, "Mid"
All LC types, Any Product (or Cigarettes if Any not available)
Most adjusted

REF	NRR	SEX	AD	Ys	Ws	Qs	Ps
ARMADA	507	m	1	2.48	6.53	2.81	0.0000
AUVINE	518	c	2	3.50	5.39	15.25	0.0000
AXELSS	521	m	6	2.03	9.33	0.41	0.0000
AXELSS	512	f	0	2.26	7.41	1.41	0.0000
Subtotal AXELSS				2.13	16.74	1.82	
BARBON	509	m	1	2.07	13.80	0.84	0.0000
*BEST	506	m	1	2.63	6.21	4.09	0.0000
BUFFLE	527	f	0	2.68	8.42	6.25	0.0000
CHEN2	504	m	0	1.59	4.85	0.26	0.0005
CHEN2	512	f	0	1.05	3.19	1.89	0.0607
Subtotal CHEN2				1.37	8.03	2.15	
CHOI	503	m	0	1.54	9.66	0.76	0.0000
CHOI	512	f	0	2.16	1.55	0.17	0.0072
Subtotal CHOI				1.62	11.21	0.94	
*CPSI	582	m	0	1.80	138.39	0.06	0.0000
*CPSI	678	f	0	1.27	119.48	36.09	0.0000
Subtotal CPSI				1.55	257.87	36.16	
*CPSII	554	m	0	2.59	82.29	48.53	0.0000
*CPSII	620	f	0	2.42	119.07	42.26	0.0000
Subtotal CPSII				2.49	201.36	90.78	
DAMBER	508	m	1	1.64	17.62	0.58	0.0000
DESTEF	509	m	4	1.65	12.22	0.36	0.0000
FAN	502	m	0	0.86	15.89	14.63	0.0006
FAN	507	f	0	1.34	8.79	2.00	0.0001
Subtotal FAN				1.03	24.68	16.63	
GER	519	c	5	0.44	9.76	18.47	0.1647
HU2	510	c	0	0.76	32.27	36.08	0.0000
HUMBLE	543	c	3	2.86	7.23	7.89	0.0000
JOLY	517	m	0	2.24	9.46	1.64	0.0000
JOLY	503	f	0	1.95	10.34	0.18	0.0000
Subtotal JOLY				2.09	19.80	1.81	
JUSSAW	513	m	0	2.53	6.39	3.23	0.0000
KATSOU	503	f	0	2.35	1.66	0.46	0.0025
KHUDER	502	m	0	2.02	18.43	0.72	0.0000
LETOUR	507	c	0	2.73	17.80	14.88	0.0000
LUBIN	509	m	0	1.33	6.65	1.57	0.0006
LUBIN2	532	m	0	2.18	156.67	20.17	0.0000
LUBIN2	575	f	0	1.27	68.77	20.94	0.0000
Subtotal LUBIN2				1.90	225.44	41.11	
MATOS	537	m	2	1.97	7.92	0.19	0.0000
MCCONN	504	c	0	-0.06	4.55	16.09	0.8977
NOTAN2	516	c	0	1.17	4.16	1.76	0.0171
PEZZOT	535	m	0	3.22	3.51	6.91	0.0000
*QIAO2	517	m	1	0.38	8.36	17.38	0.2738
RACHTA	517	f	1	2.02	8.79	0.36	0.0000
SOBUE	547	m	0	1.10	20.61	10.80	0.0000
WANG2	504	c	0	0.98	5.59	3.91	0.0200
WUWILL	517	f	3	1.00	48.46	32.83	0.0000

	N	40
	NS	32
	Wt	1047.49
Het	Chi	395.11
Het	df	39
Het	P	***
Fixed	RR	6.17
	RRl	5.81
	RRu	6.56
	P	+++
Random	RR	5.94
	RRl	4.79
	RRu	7.36
	P	+++
Asymm	P	N.S.

Table 1I13 - 3

IESLC - Meta-analysis of Ever/current Smoking, Duration, "Mid"
All LC types, Any Product (or Cigarettes if Any not available)
Most adjusted

	Sex: combined	male	female	Total
N	8	20	12	40
NS	8	20	12	40
Wt	86.75	554.80	405.94	1047.49
Het Chi	103.55	119.76	134.67	395.11
Het df	7	19	11	39
Het P	***	***	***	***
Fixed RR	4.34	7.32	5.27	6.17
RRl	3.51	6.74	4.78	5.81
RRu	5.35	7.96	5.81	6.56
P	+++	+++	+++	+++
Random RR	4.74	6.47	5.85	5.94
RRl	2.00	5.07	3.90	4.79
RRu	11.24	8.25	8.78	7.36
P	+++	+++	+++	+++
Between Chi				37.14
Between df				2
Between P				***
Btwn(F) P				N.S.
Btwn(R) P				N.S.

	Lung cancer type: all	other	Total
N	38	2	40
NS	30	2	32
Wt	1019.65	27.85	1047.49
Het Chi	376.37	16.74	395.11
Het df	37	1	39
Het P	***	***	***
Fixed RR	6.22	4.74	6.17
RRl	5.85	3.27	5.81
RRu	6.61	6.87	6.56
P	+++	+++	+++
Random RR	5.90	7.06	5.94
RRl	4.74	1.25	4.79
RRu	7.35	39.93	7.36
P	+++	+	+++
Between Chi			2.00
Between df			1
Between P			N.S.
Btwn(F) P			N.S.
Btwn(R) P			N.S.

	Location: NAmer	UK	Scand	othEur	China	Japan	othAs	other	Total
N	9	1	4	6	9	1	5	5	40
NS	7	1	3	5	7	1	4	4	32
Wt	517.34	4.55	39.75	256.23	134.05	20.61	31.53	43.45	1047.49
Het Chi	139.36	0.00	14.57	42.39	8.44	0.00	18.37	7.27	395.11
Het df	8	0	3	5	8	0	4	4	39
Het P	***	N.S.	**	***	N.S.	N.S.	**	N.S.	***
Fixed RR	7.56	0.94	8.15	6.90	2.58	2.99	3.99	7.65	6.17
RRl	6.94	0.38	5.98	6.11	2.18	1.94	2.81	5.68	5.81
RRu	8.25	2.36	11.13	7.80	3.05	4.61	5.65	10.30	6.56
P	+++	N.S.	+++	+++	+++	+++	+++	+++	+++
Random RR	9.87	0.94	9.98	7.32	2.58	2.99	4.52	8.08	5.94
RRl	6.62	0.38	4.87	4.51	2.16	1.94	2.04	5.36	4.79
RRu	14.72	2.36	20.46	11.88	3.09	4.61	10.00	12.18	7.36
P	+++	N.S.	+++	+++	+++	+++	+++	+++	+++
Between Chi									164.72
Between df									7
Between P									***
Btwn(F) P									**
Btwn(R) P									***

Table 1I13 - 3

IESLC - Meta-analysis of Ever/current Smoking, Duration, "Mid"
All LC types, Any Product (or Cigarettes if Any not available)
Most adjusted

	Detailed Country in "other Europe" multi	Germany	othWest	East	Balkans	Total
N	2		2	1	1	6
NS	1		2	1	1	5
Wt	225.44		20.33	8.79	1.66	256.23
Het Chi	39.63		0.74	0.00	0.00	42.39
Het df	1		1	0	0	5
Het P	***		N.S.	N.S.	N.S.	***
Fixed RR	6.69		9.01	7.55	10.47	6.90
RRl	5.87		5.83	3.90	2.29	6.11
RRu	7.63		13.92	14.62	47.93	7.80
P	+++		+++	+++	++	+++
Random RR	5.63		9.01	7.55	10.47	7.32
RRl	2.31		5.83	3.90	2.29	4.51
RRu	13.74		13.92	14.62	47.93	11.88
P	+++		+++	+++	++	+++
Between Chi						2.02
Between df						3
Between P						N.S.
Btwn(F) P						N.S.
Btwn(R) P						N.S.

	Detailed Country in "other Asia" India	HongKong	other	Total
N	2		3	5
NS	2		2	4
Wt	10.55		20.98	31.53
Het Chi	4.67		7.77	18.37
Het df	1		2	4
Het P	*		*	**
Fixed RR	7.35		2.93	3.99
RRl	4.02		1.91	2.81
RRu	13.43		4.50	5.65
P	+++		+++	+++
Random RR	6.56		3.43	4.52
RRl	1.73		1.33	2.04
RRu	24.87		8.84	10.00
P	++		+	+++
Between Chi				5.93
Between df				1
Between P				*
Btwn(F) P				N.S.
Btwn(R) P				N.S.

	Detailed other continent SCAmer	Total
N	5	5
NS	4	4
Wt	43.45	43.45
Het Chi	7.27	7.27
Het df	4	4
Het P	N.S.	N.S.
Fixed RR	7.65	7.65
RRl	5.68	5.68
RRu	10.30	10.30
P	+++	+++
Random RR	8.08	8.08
RRl	5.36	5.36
RRu	12.18	12.18
P	+++	+++
Between Chi		
Between df		
Between P		N.S.
Btwn(F) P		N.S.
Btwn(R) P		N.S.

Table 1I13 - 3

IESLC - Meta-analysis of Ever/current Smoking, Duration, "Mid"
All LC types, Any Product (or Cigarettes if Any not available)
Most adjusted

	Start year of study					
	<1960	1960-69	1970-79	1980-89	1990+	Total
N	4	2	8	20	6	40
NS	3	2	6	16	5	32
Wt	268.63	10.55	317.35	391.44	59.52	1047.49
Het Chi	36.95	4.67	86.64	160.10	23.48	395.11
Het df	3	1	7	19	5	39
Het P	***	*	***	***	***	***
Fixed RR	4.72	7.35	6.11	8.29	3.05	6.17
RRl	4.19	4.02	5.48	7.51	2.37	5.81
RRu	5.32	13.43	6.82	9.15	3.93	6.56
P	+++	+++	+++	+++	+++	+++
Random RR	4.38	6.56	6.15	7.58	3.19	5.94
RRl	2.54	1.73	3.92	5.44	1.83	4.79
RRu	7.56	24.87	9.64	10.56	5.57	7.36
P	+++	++	+++	+++	+++	+++
Between Chi						83.28
Between df						4
Between P						***
Btwn(F) P						(*)
Btwn(R) P						(*)

	Study type (1)		
	CC	other	Total
N	34	6	40
NS	28	4	32
Wt	573.69	473.81	1047.49
Het Chi	240.74	141.20	395.11
Het df	33	5	39
Het P	***	***	***
Fixed RR	5.57	6.98	6.17
RRl	5.14	6.38	5.81
RRu	6.05	7.64	6.56
P	+++	+++	+++
Random RR	5.84	6.46	5.94
RRl	4.57	3.83	4.79
RRu	7.47	10.87	7.36
P	+++	+++	+++
Between Chi			13.17
Between df			1
Between P			***
Btwn(F) P			N.S.
Btwn(R) P			N.S.

	Study type (2)			
	CC	prosp	other	Total
N	34	6		40
NS	28	4		32
Wt	573.69	473.81		1047.49
Het Chi	240.74	141.20		395.11
Het df	33	5		39
Het P	***	***		***
Fixed RR	5.57	6.98		6.17
RRl	5.14	6.38		5.81
RRu	6.05	7.64		6.56
P	+++	+++		+++
Random RR	5.84	6.46		5.94
RRl	4.57	3.83		4.79
RRu	7.47	10.87		7.36
P	+++	+++		+++
Between Chi				13.17
Between df				1
Between P				***
Btwn(F) P				N.S.
Btwn(R) P				N.S.

Table 1I13 - 3

IESLC - Meta-analysis of Ever/current Smoking, Duration, "Mid"
All LC types, Any Product (or Cigarettes if Any not available)
Most adjusted

	Study size (number of LC cases) 100-249	250-499	500-999	1000+	Total
N	10	11	12	7	40
NS	9	8	11	4	32
Wt	58.18	102.68	181.35	705.29	1047.49
Het Chi	46.62	28.19	117.53	172.13	395.11
Het df	9	10	11	6	39
Het P	***	**	***	***	***
Fixed RR	3.47	5.66	5.33	6.80	6.17
RRl	2.68	4.67	4.61	6.32	5.81
RRu	4.49	6.87	6.17	7.33	6.56
P	+++	+++	+++	+++	+++
Random RR	3.85	6.02	7.91	6.14	5.94
RRl	2.11	4.30	4.79	4.10	4.79
RRu	7.02	8.44	13.05	9.20	7.36
P	+++	+++	+++	+++	+++
Between Chi					30.65
Between df					3
Between P					***
Btwn(F) P					N.S.
Btwn(R) P					N.S.

	Risky occupational population no	mining	othRisky	Total
N	38	2		40
NS	30	2		32
Wt	1032.48	15.01		1047.49
Het Chi	375.93	3.38		395.11
Het df	37	1		39
Het P	***	(*)		***
Fixed RR	6.26	2.23		6.17
RRl	5.89	1.34		5.81
RRu	6.66	3.70		6.56
P	+++	++		+++
Random RR	6.22	2.32		5.94
RRl	5.00	0.91		4.79
RRu	7.73	5.91		7.36
P	+++	(+)		+++
Between Chi				15.80
Between df				1
Between P				***
Btwn(F) P				N.S.
Btwn(R) P				*

	National cigarette tobacco type Virginia	blended	other	Total
N	5	25	10	40
NS	5	19	8	32
Wt	39.11	864.57	143.81	1047.49
Het Chi	34.60	210.52	10.74	395.11
Het df	4	24	9	39
Het P	***	***	N.S.	***
Fixed RR	8.97	7.06	2.49	6.17
RRl	6.55	6.60	2.12	5.81
RRu	12.26	7.54	2.93	6.56
P	+++	+++	+++	+++
Random RR	6.32	7.88	2.50	5.94
RRl	2.36	6.27	2.06	4.79
RRu	16.87	9.89	3.02	7.36
P	+++	+++	+++	+++
Between Chi				139.25
Between df				2
Between P				***
Btwn(F) P				***
Btwn(R) P				***

Table 1I13 - 3

IESLC - Meta-analysis of Ever/current Smoking, Duration, "Mid"
All LC types, Any Product (or Cigarettes if Any not available)
Most adjusted

	Any proxy use No/nk	Yes	Total
N	27	13	40
NS	21	11	32
Wt	895.82	151.67	1047.49
Het Chi	273.27	113.22	395.11
Het df	26	12	39
Het P	***	***	***
Fixed RR	6.41	4.95	6.17
RRl	6.00	4.22	5.81
RRu	6.84	5.81	6.56
P	+++	+++	+++
Random RR	6.04	5.76	5.94
RRl	4.73	3.47	4.79
RRu	7.71	9.56	7.36
P	+++	+++	+++
Between Chi			8.62
Between df			1
Between P			**
Btwn(F) P			N.S.
Btwn(R) P			N.S.

	Full histological confirmation No	Yes	Total
N	29	11	40
NS	22	10	32
Wt	715.79	331.70	1047.49
Het Chi	299.22	92.68	395.11
Het df	28	10	39
Het P	***	***	***
Fixed RR	5.94	6.70	6.17
RRl	5.52	6.01	5.81
RRu	6.40	7.46	6.56
P	+++	+++	+++
Random RR	5.74	6.49	5.94
RRl	4.38	4.40	4.79
RRu	7.52	9.57	7.36
P	+++	+++	+++
Between Chi			3.22
Between df			1
Between P			(*)
Btwn(F) P			N.S.
Btwn(R) P			N.S.

	Number of adjustment variables (1) 0	1	2+/+nk	Total
N	27	6	7	40
NS	20	6	7	33
Wt	885.86	61.32	100.31	1047.49
Het Chi	292.15	26.06	62.77	395.11
Het df	26	5	6	39
Het P	***	***	***	***
Fixed RR	6.43	6.10	4.33	6.17
RRl	6.02	4.75	3.56	5.81
RRu	6.87	7.83	5.26	6.56
P	+++	+++	+++	+++
Random RR	5.74	6.38	6.58	5.94
RRl	4.46	3.55	3.27	4.79
RRu	7.39	11.46	13.25	7.36
P	+++	+++	+++	+++
Between Chi				14.14
Between df				2
Between P				***
Btwn(F) P				N.S.
Btwn(R) P				N.S.

Table 1I13 - 3

IESLC - Meta-analysis of Ever/current Smoking, Duration, "Mid"
All LC types, Any Product (or Cigarettes if Any not available)
Most adjusted

	Number of adjustment variables (2) 0	1	2	3-5	6+/+nk	Total
N	27	6	2	4	1	40
NS	20	6	2	4	1	33
Wt	885.86	61.32	13.30	77.68	9.33	1047.49
Het Chi	292.15	26.06	7.49	30.07	0.00	395.11
Het df	26	5	1	3	0	39
Het P	***	***	**	***	N.S.	***
Fixed RR	6.43	6.10	13.37	3.33	7.62	6.17
RRl	6.02	4.75	7.81	2.67	4.01	5.81
RRu	6.87	7.83	22.88	4.16	14.47	6.56
P	+++	+++	+++	+++	+++	+++
Random RR	5.74	6.38	15.16	4.30	7.62	5.94
RRl	4.46	3.55	3.39	1.89	4.01	4.79
RRu	7.39	11.46	67.78	9.78	14.47	7.36
P	+++	+++	+++	+++	+++	+++
Between Chi						39.35
Between df						4
Between P						***
Btwn(F) P						N.S.
Btwn(R) P						N.S.

	Smoking status ever	current	Total
N	32	8	40
NS	26	6	32
Wt	552.54	494.95	1047.49
Het Chi	237.51	141.90	395.11
Het df	31	7	39
Het P	***	***	***
Fixed RR	5.50	7.02	6.17
RRl	5.06	6.43	5.81
RRu	5.97	7.67	6.56
P	+++	+++	+++
Random RR	5.49	7.92	5.94
RRl	4.25	5.03	4.79
RRu	7.09	12.46	7.36
P	+++	+++	+++
Between Chi			15.70
Between df			1
Between P			***
Btwn(F) P			N.S.
Btwn(R) P			N.S.

	Product all/unsp	cig+/-ot	cig only	Total
N	11	22	7	40
NS	9	18	6	33
Wt	92.75	594.31	360.43	1047.49
Het Chi	46.10	235.05	100.56	395.11
Het df	10	21	6	39
Het P	***	***	***	***
Fixed RR	4.30	6.46	6.28	6.17
RRl	3.51	5.96	5.67	5.81
RRu	5.28	7.00	6.96	6.56
P	+++	+++	+++	+++
Random RR	4.02	6.36	8.34	5.94
RRl	2.54	4.71	4.99	4.79
RRu	6.35	8.58	13.95	7.36
P	+++	+++	+++	+++
Between Chi				13.39
Between df				2
Between P				**
Btwn(F) P				N.S.
Btwn(R) P				(*)

Table 1I13 - 3

IESLC - Meta-analysis of Ever/current Smoking, Duration, "Mid"
All LC types, Any Product (or Cigarettes if Any not available)
Most adjusted

	Denominator nev any	nev cigs	Total
N	21	19	40
NS	17	16	33
Wt	451.61	595.88	1047.49
Het Chi	152.94	228.99	395.11
Het df	20	18	39
Het P	***	***	***
Fixed RR	7.02	5.60	6.17
RRl	6.40	5.17	5.81
RRu	7.70	6.07	6.56
P	+++	+++	+++
Random RR	5.48	6.50	5.94
RRl	4.07	4.71	4.79
RRu	7.38	8.97	7.36
P	+++	+++	+++
Between Chi			13.18
Between df			1
Between P			***
Btwn(F) P			N.S.
Btwn(R) P			N.S.

	Derivation of RR/CI Orig	StdCalc	Other	Total
N	9	25	6	40
NS	9	18	6	33
Wt	103.70	845.17	98.62	1047.49
Het Chi	75.16	246.59	46.43	395.11
Het df	8	24	5	39
Het P	***	***	***	***
Fixed RR	5.03	6.65	4.03	6.17
RRl	4.15	6.22	3.31	5.81
RRu	6.09	7.12	4.91	6.56
P	+++	+++	+++	+++
Random RR	6.80	5.82	5.64	5.94
RRl	3.68	4.52	2.89	4.79
RRu	12.56	7.48	10.99	7.36
P	+++	+++	+++	+++
Between Chi				26.93
Between df				2
Between P				***
Btwn(F) P				N.S.
Btwn(R) P				N.S.

Table 1I13 - 4

IESLC - Meta-analysis of Ever/current Smoking, Duration, "Mid"
All LC types, Any Product (or Cigarettes if Any not available)
Least adjusted

REF	NRR	X	SEX	AGEL	AGEH	RACE	YF	LC TYPE	LOC	START	ST	NLC	R	VB	P	H	AD	SM	PRODUCT	exL	exH	DENOM	De
ARMADA	502	x	m	0	0	all	-	all	Eu:wst	1986	CC	325	n	bl	n	y	0	ev	cig+/-ot	25	49	nev cigs	st
AUVINE	502	x	c	0	0	all	-	all	Eu:Sca	1986	CC	517	n	bl	y	n	0	ev	cig+/-ot	21	40	nev cigs	st
AXELSS	503	x	m	0	0	sca	-	all	Eu:Sca	1989	CC	436	n	bl	n	n	0	ev	all/unsp	30	39	nev any	st
AXELSS	512		f	0	0	sca	-	all	Eu:Sca	1989	CC	436	n	bl	n	n	0	ev	all/unsp	30	39	nev any	st
BARBON	502	x	m	0	0	all	-	all	Eu:wst	1979	CC	755	n	bl	y	y	0	ev	all/unsp	30	39	nev any	st
BEST	506		m	0	0	all	0	all	NAmer	1955	pr	381	n	V	n	n	1	cu	cig only	30	39	nev any	ot
BUFFLE	527		f	0	0	w-hi	-	all	NAmer	1976	CC	943	n	bl	y	n	0	ev	cig+/-ot	31	40	nev cigs	or
CHEN2	504		m	0	0	all	-	all	As:Chi	1983	CC	193	n	ot	y	n	0	ev	all/unsp	31	40	nev any	st
CHEN2	512		f	0	0	all	-	all	As:Chi	1983	CC	193	n	ot	y	n	0	ev	all/unsp	31	40	nev any	st
CHOI	503		m	0	0	all	-	all	As:oth	1985	CC	375	n	bl	n	n	0	ev	cig+/-ot	30	39	nev cigs	st
CHOI	512		f	0	0	all	-	all	As:oth	1985	CC	375	n	bl	n	n	0	ev	cig+/-ot	30	39	nev cigs	st
CPSI	582		m	40	84	wh	0	all	NAmer	1959	pr	5138	n	bl	n	n	0	cu	cig only	35	39	nev cigs	st
CPSI	678		f	40	84	wh	0	all	NAmer	1959	pr	5138	n	bl	n	n	0	cu	cig only	35	39	nev cigs	st
CPSII	554		m	0	0	all	6	all	NAmer	1982	pr	3229	n	bl	n	n	0	cu	cig only	35	39	nev any	st
CPSII	620		f	0	0	all	6	all	NAmer	1982	pr	3229	n	bl	n	n	0	cu	cig+/-ot	35	39	nev cigs	st
DAMBER	508		m	0	0	all	-	all	Eu:Sca	1972	CC	579	n	bl	y	n	1	ev	all/unsp	31	40	nev any	ot
DESTEF	502	x	m	0	0	all	-	all	SCAmer	1988	CC	497	n	bl	n	y	0	ev	all/unsp	30	39	nev any	st
FAN	502		m	0	0	all	-	all	As:Chi	1990	CC	403	n	ot	y	n	0	ev	cig+/-ot	30	39	nev cigs	st
FAN	507		f	0	0	all	-	all	As:Chi	1990	CC	403	n	ot	y	n	0	ev	cig+/-ot	30	39	nev cigs	st
GER	514	x	c	0	0	all	-	all	As:oth	1990	CC	141	n	ot	y	n	0	ev	all/unsp	21	40	nev any	st
HU2	510		c	0	0	all	-	all	As:Chi	1977	CC	523	n	ot	y	n	0	ev	cig+/-ot	30	39	nev cigs	or
HUMBLE	518	x	c	0	0	wh	-	not alv	NAmer	1980	CC	521	n	bl	y	n	0	cu	cig+/-ot	30	39	nev cigs	st
JOLY	517		m	0	0	all	-	all	SCAmer	1978	CC	826	n	bl	n	n	0	ev	cig+/-ot	30	39	nev any	st
JOLY	503		f	0	0	all	-	all	SCAmer	1978	CC	826	n	bl	n	n	0	ev	cig+/-ot	30	39	nev any	st
JUSSAW	513		m	0	0	all	-	all	As:Ind	1964	CC	792	n	V	n	n	0	ev	cig only	30	39	nev any	st
KATSOU	503		f	0	0	all	-	all	Eu:bal	1987	CC	101	n	bl	n	n	0	cu	all/unsp	30	39	nev any	st
KHUDER	502		m	0	0	all	-	all	NAmer	1985	CC	482	n	bl	n	y	0	ev	cig+/-ot	30	49	nev cigs	st
LETOUR	507		c	0	0	all	-	all	NAmer	1983	CC	738	n	V	y	y	0	ev	cig+/-ot	25	40	nev cigs	st
LUBIN	509		m	0	0	all	-	all	As:Chi	1984	CC	427	m	ot	y	n	0	ev	cig+/-ot	30	39	nev any	st
LUBIN2	532		m	0	0	all	-	all	Eu:mul	1976	CC	7804	n	bl	n	y	0	ev	cig+/-ot	30	39	nev any	st
LUBIN2	575		f	0	0	all	-	all	Eu:mul	1976	CC	7804	n	bl	n	y	0	ev	cig+/-ot	30	39	nev any	st
MATOS	517	x	m	0	0	all	-	all	SCAmer	1994	CC	200	n	bl	n	n	0	ev	cig+/-ot	25	39	nev any	st
MCCONN	504		c	0	0	all	-	all	Eu:UK	1946	CC	100	n	V	n	y	0	ev	all/unsp	30	39	nev any	st
NOTAN2	516		c	0	0	all	-	all	As:Ind	1963	CC	683	n	V	n	n	0	ev	cig only	31	40	nev any	st
PEZZOT	535		m	0	0	all	-	all	SCAmer	1987	CC	215	n	bl	n	y	0	ev	cig only	31	40	nev cigs	st
QIAO2	512	x	m	0	0	all	0	all	As:Chi	1992	pr	241	m	ot	n	n	0	ev	all/unsp	28	41	nev any	st
RACHTA	512	x	f	0	0	all	-	all	Eu:est	1991	CC	118	n	bl	n	y	0	ev	cig+/-ot	21	40	nev cigs	st
SOBUE	547		m	0	0	all	-	q+s+l+a	As:Jap	1986	CC	1376	n	bl	n	y	0	cu	cig+/-ot	30	39	nev cigs	st
WANG2	504		c	0	0	all	-	all	As:Chi	1980	CC	103	n	ot	n	n	0	ev	cig+/-ot	30	39	nev cigs	st
WUWILL	502	x	f	0	0	all	-	all	As:Chi	1985	CC	965	n	ot	n	n	0	ev	cig+/-ot	30	39	nev cigs	st

Cigarette type is all/unspec for all RRs

except for the following:

REF	NRR	CIGTYPE
JUSSAW	513	MC only
NOTAN2	516	MC only

Table 1I13 - 5

IESLC - Meta-analysis of Ever/current Smoking, Duration, "Mid"
All LC types, Any Product (or Cigarettes if Any not available)
Least adjusted

REF	NRR	SEX	AD	Number Exposed Case	Number Exposed Cont	Non-exposed Case	Non-exposed Cont	RR	95.00%CI
ARMADA	502	m	0	219	166	8	71	11.71	(5.49- 24.99)
AUVINE	502	c	0	10	5	44	229	10.41	(3.39- 31.93)
AXELSS	503	m	0	57	71	16	160	8.03	(4.31- 14.94)
AXELSS	512	f	0	29	26	18	154	9.54	(4.64- 19.61)
Subtotal AXELSS								8.64	(5.40- 13.83)
BARBON	502	m	0	118	102	22	188	9.89	(5.91- 16.55)
*BEST	506	m	1	55	-	7	-	13.90	(6.33- 30.52)
BUFFLE	527	f	0	97	62	12	112	14.60	(7.43- 28.69)
CHEN2	504	m	0	36	27	9	33	4.89	(2.01- 11.91)
CHEN2	512	f	0	13	6	25	33	2.86	(0.95- 8.58)
Subtotal CHEN2								3.95	(1.98- 7.89)
CHOI	503	m	0	102	160	13	95	4.66	(2.48- 8.75)
CHOI	512	f	0	8	2	76	164	8.63	(1.79- 41.62)
Subtotal CHOI								5.07	(2.83- 9.11)
*CPSI	582	m	0	470	367622	196	926068	6.04	(5.11- 7.14)
*CPSI	678	f	0	154	315060	532	3877179	3.56	(2.98- 4.26)
Subtotal CPSI								4.73	(4.19- 5.34)
*CPSII	554	m	0	244	109788	124	742207	13.30	(10.72- 16.51)
*CPSII	620	f	0	193	116270	310	2091302	11.20	(9.36- 13.40)
Subtotal CPSII								12.01	(10.46- 13.79)
DAMBER	508	m	1	-	-	42	-	5.15	(3.27- 8.32)
DESTEF	502	m	0	78	78	27	163	6.04	(3.61- 10.10)
FAN	502	m	0	44	122	36	236	2.36	(1.45- 3.87)
FAN	507	f	0	19	23	69	320	3.83	(1.98- 7.42)
Subtotal FAN								2.81	(1.89- 4.17)
GER	514	c	0	31	123	51	246	1.22	(0.74- 2.00)
HU2	510	c	0	123	101	121	213	2.14	(1.52- 3.03)
HUMBLE	518	c	0	68	58	28	285	11.93	(7.07- 20.13)
JOLY	517	m	0	85	165	12	218	9.36	(4.95- 17.70)
JOLY	503	f	0	31	24	52	283	7.03	(3.82- 12.93)
Subtotal JOLY								8.06	(5.19- 12.52)
JUSSAW	513	m	0	27	9	149	624	12.56	(5.79- 27.28)
KATSOU	503	f	0	15	2	48	67	10.47	(2.29- 47.93)
KHUDER	502	m	0	207	370	23	309	7.52	(4.76- 11.86)
LETOUR	507	c	0	264	160	24	224	15.40	(9.68- 24.51)
LUBIN	509	m	0	124	294	8	72	3.80	(1.78- 8.12)
LUBIN2	532	m	0	2227	3470	190	2616	8.84	(7.56- 10.33)
LUBIN2	575	f	0	187	186	336	1188	3.55	(2.81- 4.50)
Subtotal LUBIN2								6.69	(5.87- 7.63)
MATOS	517	m	0	82	110	11	110	7.45	(3.77- 14.75)
MCCONN	504	c	0	21	57	9	23	0.94	(0.38- 2.36)
NOTAN2	516	c	0	12	7	107	201	3.22	(1.23- 8.42)
PEZZOT	535	m	0	71	82	4	116	25.11	(8.82- 71.48)
*QIAO2	512	m	0	54	2257	10	709	1.70	(0.87- 3.31)
RACHTA	512	f	0	49	21	33	98	6.93	(3.63- 13.22)
SOBUE	547	m	0	159	200	34	128	2.99	(1.94- 4.61)
WANG2	504	c	0	26	38	11	43	2.67	(1.17- 6.13)
WUWILL	502	f	0	179	98	417	601	2.63	(2.00- 3.47)
Partial Totals				5988	917422	3264	7647088		

*prospective study

Table 1I13 - 5

IESLC - Meta-analysis of Ever/current Smoking, Duration, "Mid"
All LC types, Any Product (or Cigarettes if Any not available)
Least adjusted

REF	NRR	SEX	AD	Ys	Ws	Qs	Ps
ARMADA	502	m	0	2.46	6.68	2.85	0.0000
AUVINE	502	c	0	2.34	3.06	0.88	0.0000
AXELSS	503	m	0	2.08	9.96	0.76	0.0000
AXELSS	512	f	0	2.26	7.41	1.49	0.0000
Subtotal AXELSS				2.16	17.37	2.26	
BARBON	502	m	0	2.29	14.48	3.40	0.0000
*BEST	506	m	1	2.63	6.21	4.23	0.0000
BUFFLE	527	f	0	2.68	8.42	6.44	0.0000
CHEN2	504	m	0	1.59	4.85	0.23	0.0005
CHEN2	512	f	0	1.05	3.19	1.82	0.0607
Subtotal CHEN2				1.37	8.03	2.05	
CHOI	503	m	0	1.54	9.66	0.69	0.0000
CHOI	512	f	0	2.16	1.55	0.19	0.0072
Subtotal CHOI				1.62	11.21	0.88	
*CPSI	582	m	0	1.80	138.39	0.01	0.0000
*CPSI	678	f	0	1.27	119.48	34.35	0.0000
Subtotal CPSI				1.55	257.87	34.36	
*CPSII	554	m	0	2.59	82.29	50.24	0.0000
*CPSII	620	f	0	2.42	119.07	44.18	0.0000
Subtotal CPSII				2.49	201.36	94.41	
DAMBER	508	m	1	1.64	17.62	0.50	0.0000
DESTEF	502	m	0	1.80	14.53	0.00	0.0000
FAN	502	m	0	0.86	15.89	14.22	0.0006
FAN	507	f	0	1.34	8.79	1.89	0.0001
Subtotal FAN				1.03	24.68	16.11	
GER	514	c	0	0.20	15.61	40.53	0.4403
HU2	510	c	0	0.76	32.27	35.17	0.0000
HUMBLE	518	c	0	2.48	14.05	6.36	0.0000
JOLY	517	m	0	2.24	9.46	1.75	0.0000
JOLY	503	f	0	1.95	10.34	0.21	0.0000
Subtotal JOLY				2.09	19.80	1.96	
JUSSAW	513	m	0	2.53	6.39	3.35	0.0000
KATSOU	503	f	0	2.35	1.66	0.49	0.0025
KHUDER	502	m	0	2.02	18.43	0.82	0.0000
LETOUR	507	c	0	2.73	17.80	15.32	0.0000
LUBIN	509	m	0	1.33	6.65	1.49	0.0006
LUBIN2	532	m	0	2.18	156.67	21.71	0.0000
LUBIN2	575	f	0	1.27	68.77	19.93	0.0000
Subtotal LUBIN2				1.90	225.44	41.64	
MATOS	517	m	0	2.01	8.24	0.34	0.0000
MCCONN	504	c	0	-0.06	4.55	15.86	0.8977
NOTAN2	516	c	0	1.17	4.16	1.69	0.0171
PEZZOT	535	m	0	3.22	3.51	7.04	0.0000
*QIAO2	512	m	0	0.53	8.57	14.00	0.1218
RACHTA	512	f	0	1.94	9.21	0.15	0.0000
SOBUE	547	m	0	1.10	20.61	10.40	0.0000
WANG2	504	c	0	0.98	5.59	3.78	0.0200
WUWILL	502	f	0	0.97	50.37	35.43	0.0000

	N	40
	NS	32
	Wt	1064.47
Het	Chi	404.21
Het	df	39
Het	P	***
Fixed	RR	6.09
	RRl	5.73
	RRu	6.47
	P	+++
Random	RR	5.75
	RRl	4.64
	RRu	7.14
	P	+++
Asymm	P	N.S.

Table 1I13 - 6

IESLC - Meta-analysis of Ever/current Smoking, Duration, "Mid"
All LC types, Any Product (or Cigarettes if Any not available)
Least adjusted

	Sex			
	combined	male	female	Total
N	8	20	12	40
NS	8	20	12	40
Wt	97.09	559.11	408.27	1064.47
Het Chi	96.86	116.60	137.02	404.21
Het df	7	19	11	39
Het P	***	***	***	***
Fixed RR	3.75	7.41	5.23	6.09
RRl	3.08	6.82	4.74	5.73
RRu	4.58	8.05	5.76	6.47
P	+++	+++	+++	+++
Random RR	3.77	6.66	5.79	5.75
RRl	1.71	5.24	3.85	4.64
RRu	8.30	8.46	8.71	7.14
P	+++	+++	+++	+++
Between Chi				53.72
Between df				2
Between P				***
Btwn(F) P				(*)
Btwn(R) P				N.S.

Table 1I13 - 7

IESLC - Meta-analysis of Ever/current Smoking, Duration, "Mid"
All LC types, Any Product (or Cigarettes if Any not available)
Excluded studies (and stage at which they were excluded)

1	BECHER BLOT1 BROWN3 CARPEN CHYOU DARBY DOLL2 GARCIA GRAHAM GURSEL HAMMO2 JAHN JAIN LAUSSM PRESCO QIAO TVERDA WIGLE WYNDE3
2	ALDERS BENSHL BRESLO CHIAZZ DEAN3 DORN ENGELA GAO2 GILLIS GUO HEGMAN HIRAYA HOLE KAUFMA KOO KOULUM LIU4 MIGRAN MRFITR PERNU SEGI2 SPEIZE SUZUK2 SVENSS VUTUC WAKAI WU YUAN
3	GENG MCDUFF SPITZ STASZE WU2 ZHANG
4	BOUCHA CHEN CORREA JEDRYC LUO WYNDE2 WYNDE6
5	AKIBA HAMMON PISANI RESTRE SADOWS XU
7	BOFFET BROSS WYNDE7
10	AMES WATSON WYNDE8
14	AGUDO AMANDU BOUCOT CEDERL DEAN2 DOLL DORGAN DOSEME GAO GARSHI HAENSZ HU KAISE2 KREUZE LEVIN LIAW LIU3 LIU5 OSANN2 PEZZO2 TIZZAN ZHENG ZHOU
15	BENHAM

Table 1I13 - 8
Potentially overlapping studies

REF	REFGP	PRINC	.	OVERLAP/LINK
LUBIN2	LUBIN2	1		Lubin-combined
CPSI	CPSI	1		CPSI overall
LUBIN	XIANGZ	2		LUBIN/XIANGZ/QIAO

Table 1I13 - 9
Most adjusted - insufficient data for meta-analysis

REF	NRR	SEX	AGEL	AGEH	RACE	YF	LC TYPE	LOC	START	ST	NLC	R	VB	P	H	AD	SM	PRODUCT	exL	exH	DENOM	De
BUFFLE	502	m	0	0	wh	-	all	NAmer	1976	CC	943	n	bl	y	n	0	ev	cig+/-ot	34	43	nev cigs	or
SADOWS	525	m	0	0	wh	-	all	NAmer	1938	CC	477	n	bl	n	n	0	ev	cig only	30	39	nev any	ot
XU	502	m	0	0	all	-	all	As:Chi	1985	CC	729	n	ot	n	n	2	ev	all/unsp	30	39	nev any	or

REF	NRR	RR	SIG	RRDATA comment
BUFFLE	502	11.10		0
SADOWS	525	3.95		0
XU	502	*		RR for 1-19/day is 2.1(p<0.05), for 20-29/day is 2.7(p<0.05) and for >=30/ day is 4.9(p<0.05)

Table 1I14 -

IESLC - Meta-analysis of Ever/current Smoking, Duration, "High"
All LC types, Any Product (or Cigarettes if Any not available)

This analysis is restricted to results for:
1) Ever/current smokers
2) Results by Duration
3) Categorical results by Duration
4) All LC types (or near equivalent)
5) Results complete enough for use in metaanalysis

Within each study, results are then selected (in the following order of preference, within each sex) for:
6) PRODUCT: all/unspec, cigarettes regardless of other products, cigarettes only
7) CIGTYPE: all/unspecified, MC regardless of HR, MC only
8) (not applicable)
9) DENOM: never smoked anything, never smoked cigarettes, never any + low, never cigs + low
10) Followup period (YF, prospective studies): whole study (coded as 0) or longest available
11) LCtype: all or nearest available, at least Squamous and Adeno. (q = squamous, s = small,
l = large, a = adeno, mix = mixed, alv = alveolar)
12) Race: all or nearest available, otherwise by race (wh or w = white, bl or b = black, hi = hispanic
ch = chinese, jap = japanese, haw = hawaiian, w+o = white + oriental, sca = scandinavian, as = asian)
13) Duration "high" in key scheme 1 (key value 50, maximum range 36+)
14) For overlapping studies: principal rather than subsidiary studies
Finally by Age: whole study (coded as 0) if available, otherwise by widest available age group
and then for single sex results (m, f) in preference to results for both sexes combined (c).

Results adjusted (AD) for the most potential confounders are then chosen in Sections -1 to -3
and results adjusted for the least confounders in Sections -4 to -6. (Those least adjusted results which
actually differ from the most adjusted are marked 'x' in column X in Section -4)

Section -7 shows excluded studies, together with the stage (as above) at which no qualifying
results were found.

Section -8 lists the potentially overlapping studies which have been included (1=principal, 2=subsidiary).

Section -9 lists any results which would have been included in preference except that they had data not complete
enough for use in meta-analysis, with their significance (yes/no), if known, and any further comment as entered
on the database. It also lists as "gap" any categories for which no data were presented by the original authors.

In addition to those mentioned above, the following fields, levels and abbreviations are used:
* or nk = not known, n = no, y = yes, ot = other
ev = ever, cu = current, nev = never
all/unspec = all or unspecified, cig+/-ot = cigarettes irrespective of other products (cigar, pipe etc)
MC = manufactured cigarettes, HR = hand-rolled cigarettes
exL, exH = range of exposure (low and high) in the smoking group, in terms of Duration
REF: 6-character study reference
NRR: number of the RR on the database within the study
ST : study type (CC = case control, pr or prosp = prospective)
NLC: number of lung cancer cases in whole study
R : risky occupational population (n = no, m = mining, o = other risky)
VB : national cigarette type (V = at least 75% Virginia, bl = at least 75% blended, ot = other)
P : any proxy use
H : full histological confirmation
De : derivation of RR/CI (or = original, st = standard method, ot = other method of estimation)

Table 1I14 - 1

IESLC - Meta-analysis of Ever/current Smoking, Duration, "High"
All LC types, Any Product (or Cigarettes if Any not available)
Most adjusted

REF	NRR	SEX	AGEL	AGEH	RACE	YF	LC TYPE	LOC	START	ST	NLC	R	VB	P	H	AD	SM	PRODUCT	exL	exH	DENOM	De
ARMADA	508	m	0	0	all	-	all	Eu:wst	1986	CC	325	n	bl	n	y	1	ev	cig+/-ot	50	999	nev cigs	or
AUVINE	519	c	0	0	all	-	all	Eu:Sca	1986	CC	517	n	bl	y	n	2	ev	cig+/-ot	41	999	nev cigs	or
AXELSS	523	m	0	0	sca	-	all	Eu:Sca	1989	CC	436	n	bl	n	n	6	ev	all/unsp	50	999	nev any	ot
AXELSS	514	f	0	0	sca	-	all	Eu:Sca	1989	CC	436	n	bl	n	n	0	ev	all/unsp	50	999	nev any	st
BARBON	511	m	0	0	all	-	all	Eu:wst	1979	CC	755	n	bl	y	y	1	ev	all/unsp	50	999	nev any	or
BEST	507	m	0	0	all	0	all	NAmer	1955	pr	381	n	V	n	n	1	cu	cig only	40	999	nev any	ot
BOUCOT	519	m	0	0	all	9	all	NAmer	1951	pr	121	n	bl	n	n	0	ev	cig+/-ot	40	999	nev any	ot
BUFFLE	528	f	0	0	w-hi	-	all	NAmer	1976	CC	943	n	bl	y	n	0	ev	cig+/-ot	41	999	nev cigs	or
CHEN2	505	m	0	0	all	-	all	As:Chi	1983	CC	193	n	ot	y	n	0	ev	all/unsp	41	999	nev any	st
CHEN2	513	f	0	0	all	-	all	As:Chi	1983	CC	193	n	ot	y	n	0	ev	all/unsp	41	999	nev any	st
CHOI	505	m	0	0	all	-	all	As:oth	1985	CC	375	n	bl	n	n	0	ev	cig+/-ot	50	999	nev cigs	st
CHOI	513	f	0	0	all	-	all	As:oth	1985	CC	375	n	bl	n	n	0	ev	cig+/-ot	40	999	nev cigs	st
CPSI	585	m	40	84	wh	0	all	NAmer	1959	pr	5138	n	bl	n	n	0	cu	cig only	50	54	nev cigs	st
CPSI	681	f	40	84	wh	0	all	NAmer	1959	pr	5138	n	bl	n	n	0	cu	cig only	50	54	nev cigs	st
CPSII	557	m	0	0	all	6	all	NAmer	1982	pr	3229	n	bl	n	n	0	cu	cig only	50	54	nev any	st
CPSII	623	f	0	0	all	6	all	NAmer	1982	pr	3229	n	bl	n	n	0	cu	cig+/-ot	50	54	nev cigs	st
DAMBER	509	m	0	0	all	-	all	Eu:Sca	1972	CC	579	n	bl	y	n	1	ev	all/unsp	41	50	nev any	ot
DESTEF	511	m	0	0	all	-	all	SCAmer	1988	CC	497	n	bl	n	y	4	ev	all/unsp	50	999	nev any	or
DOLL	518	m	0	0	all	-	all	Eu:UK	1948	CC	1465	n	V	n	n	0	ev	all/unsp	40	999	nev any	st
DOLL	525	f	0	0	all	-	all	Eu:UK	1948	CC	1465	n	V	n	n	0	ev	all/unsp	40	999	nev any	st
FAN	503	m	0	0	all	-	all	As:Chi	1990	CC	403	n	ot	y	n	0	ev	cig+/-ot	40	999	nev cigs	st
FAN	508	f	0	0	all	-	all	As:Chi	1990	CC	403	n	ot	y	n	0	ev	cig+/-ot	40	999	nev cigs	st
GER	520	c	0	0	all	-	all	As:oth	1990	CC	141	n	ot	y	n	5	ev	all/unsp	41	999	nev any	ot
HU2	511	c	0	0	all	-	all	As:Chi	1977	CC	523	n	ot	y	n	0	ev	cig+/-ot	40	999	nev cigs	st
HUMBLE	545	c	0	0	wh	-	not alv	NAmer	1980	CC	521	n	bl	y	n	3	cu	cig+/-ot	50	999	nev cigs	ot
JOLY	519	m	0	0	all	-	all	SCAmer	1978	CC	826	n	bl	n	n	0	ev	cig+/-ot	50	999	nev any	st
JOLY	505	f	0	0	all	-	all	SCAmer	1978	CC	826	n	bl	n	n	0	ev	cig+/-ot	50	999	nev any	st
JUSSAW	514	m	0	0	all	-	all	As:Ind	1964	CC	792	n	V	n	n	0	ev	cig only	40	999	nev any	st
KAISE2	597	m	0	0	all	9	all	NAmer	1979	pr	318	n	bl	n	n	1	cu	cig only	40	999	nev any	st
KAISE2	517	f	0	0	all	9	all	NAmer	1979	pr	318	n	bl	n	n	1	cu	cig only	40	999	nev any	st
KATSOU	504	f	0	0	all	-	all	Eu:bal	1987	CC	101	n	bl	n	n	0	cu	all/unsp	40	999	nev any	st
KHUDER	503	m	0	0	all	-	all	NAmer	1985	CC	482	n	bl	n	y	0	ev	cig+/-ot	50	999	nev cigs	st
KREUZE	507	m	55	69	all	-	all	Eu:Ger	1990	CC	2260	n	bl	n	n	3	ev	all/unsp	40	999	nev any	or
KREUZE	510	f	55	69	all	-	all	Eu:Ger	1990	CC	2260	n	bl	n	n	3	ev	all/unsp	40	999	nev any	or
LETOUR	508	c	0	0	all	-	all	NAmer	1983	CC	738	n	V	y	y	0	ev	cig+/-ot	41	999	nev cigs	st
LEVIN	507	m	0	0	all	-	all	NAmer	1938	CC	475	n	bl	n	n	1	ev	cig+/-ot	40	999	nev any	ot
LUBIN	511	m	0	0	all	-	all	As:Chi	1984	CC	427	m	ot	y	n	0	ev	cig+/-ot	50	999	nev any	st
LUBIN2	534	m	0	0	all	-	all	Eu:mul	1976	CC	7804	n	bl	n	y	0	ev	cig+/-ot	50	999	nev any	st
LUBIN2	577	f	0	0	all	-	all	Eu:mul	1976	CC	7804	n	bl	n	y	0	ev	cig+/-ot	50	999	nev any	st
MATOS	538	m	0	0	all	-	all	SCAmer	1994	CC	200	n	bl	n	n	2	ev	cig+/-ot	40	70	nev any	or
MCCONN	505	c	0	0	all	-	all	Eu:UK	1946	CC	100	n	V	n	y	0	ev	all/unsp	40	999	nev any	st
NOTAN2	517	c	0	0	all	-	all	As:Ind	1963	CC	683	n	V	n	n	0	ev	cig only	41	999	nev any	st
PEZZO2	508	m	0	0	all	-	all	SCAmer	1992	CC	367	n	bl	n	y	0	cu	cig+/-ot	36	999	nev cigs	st
PEZZOT	536	m	0	0	all	-	all	SCAmer	1987	CC	215	n	bl	n	y	0	ev	cig only	41	999	nev cigs	st
QIAO2	518	m	0	0	all	0	all	As:Chi	1992	pr	241	m	ot	n	n	1	ev	all/unsp	42	999	nev any	or
RACHTA	518	f	0	0	all	-	all	Eu:est	1991	CC	118	n	bl	n	y	1	ev	cig+/-ot	41	999	nev cigs	or
SOBUE	549	m	0	0	all	-	q+s+l+a	As:Jap	1986	CC	1376	n	bl	n	y	0	cu	cig+/-ot	50	999	nev cigs	st
WUWILL	518	f	0	0	all	-	all	As:Chi	1985	CC	965	n	ot	n	n	3	ev	cig+/-ot	40	999	nev cigs	ot

Cigarette type is all/unspec for all RRs

except for the following:

REF	NRR	CIGTYPE
JUSSAW	514	MC only
NOTAN2	517	MC only

Table 1I14 - 2

IESLC - Meta-analysis of Ever/current Smoking, Duration, "High"
All LC types, Any Product (or Cigarettes if Any not available)
Most adjusted

REF	NRR	SEX	AD	Number Exposed Case	Cont	Non-exposed Case	Cont	RR	95.00%CI
ARMADA	508	m	1	77	-	8	-	26.80	(11.00- 65.10)
AUVINE	519	c	2	230	-	44	-	30.40	(15.80- 58.40)
AXELSS	523	m	6	101	-	16	-	27.09	(13.94- 52.62)
AXELSS	514	f	0	20	10	18	154	17.11	(6.94- 42.19)
Subtotal AXELSS								23.05	(13.50- 39.36)
BARBON	511	m	1	366	-	22	-	14.50	(9.00- 23.30)
*BEST	507	m	1	137	-	7	-	14.20	(6.64- 30.35)
*BOUCOT	519	m	0	52	1563	0	805	54.09~	(3.34- 875.17)
BUFFLE	528	f	0	90	42	12	112	20.00	(9.94- 40.23)
CHEN2	505	m	0	62	40	9	33	5.68	(2.46- 13.13)
CHEN2	513	f	0	21	15	25	33	1.85	(0.80- 4.29)
Subtotal CHEN2								3.25	(1.80- 5.89)
CHOI	505	m	0	20	20	13	95	7.31	(3.13- 17.07)
CHOI	513	f	0	1	1	76	164	2.16	(0.13- 34.96)
Subtotal CHOI								6.59	(2.93- 14.84)
*CPSI	585	m	0	576	119633	196	926068	22.75	(19.35- 26.75)
*CPSI	681	f	0	16	14305	532	3877179	8.15	(4.96- 13.40)
Subtotal CPSI								20.61	(17.67- 24.05)
*CPSII	557	m	0	332	39260	124	742207	50.62	(41.19- 62.20)
*CPSII	623	f	0	122	29119	310	2091302	28.26	(22.93- 34.84)
Subtotal CPSII								37.99	(32.80- 44.00)
DAMBER	509	m	1	-	-	42	-	8.71	(5.84- 13.66)
DESTEF	511	m	4	178	-	27	-	10.80	(6.60- 17.60)
DOLL	518	m	0	558	491	7	61	9.90	(4.49- 21.85)
DOLL	525	f	0	6	3	40	59	2.95	(0.70- 12.49)
Subtotal DOLL								7.48	(3.74- 14.98)
FAN	503	m	0	143	241	36	236	3.89	(2.59- 5.84)
FAN	508	f	0	55	59	69	320	4.32	(2.76- 6.78)
Subtotal FAN								4.08	(3.02- 5.52)
GER	520	c	5	49	-	51	-	2.14	(1.18- 3.90)
HU2	511	c	0	194	113	121	213	3.02	(2.19- 4.17)
HUMBLE	545	c	3	119	-	28	-	17.27	(10.38- 28.75)
JOLY	519	m	0	250	253	12	218	17.95	(9.78- 32.93)
JOLY	505	f	0	57	20	52	283	15.51	(8.61- 27.95)
Subtotal JOLY								16.65	(10.91- 25.41)
JUSSAW	514	m	0	11	6	149	624	7.68	(2.79- 21.09)
*KAISE2	597	m	1	34	-	14	-	15.64	(8.31- 29.40)
*KAISE2	517	f	1	26	-	11	-	30.41	(14.39- 64.25)
Subtotal KAISE2								20.63	(12.73- 33.43)
KATSOU	504	f	0	17	4	48	67	5.93	(1.88- 18.75)
KHUDER	503	m	0	236	354	23	309	8.96	(5.69- 14.11)
KREUZE	507	m	3	-	-	-	-	54.50	(34.90- 85.20)
KREUZE	510	f	3	-	-	-	-	8.30	(4.70- 14.50)
Subtotal KREUZE								26.38	(18.59- 37.42)
LETOUR	508	c	0	374	141	24	224	24.76	(15.58- 39.35)
LEVIN	507	m	1	63	-	7	-	8.96	(3.90- 20.61)
LUBIN	511	m	0	59	86	8	72	6.17	(2.77- 13.77)
LUBIN2	534	m	0	1325	1484	190	2616	12.29	(10.42- 14.50)
LUBIN2	577	f	0	81	32	336	1188	8.95	(5.84- 13.71)
Subtotal LUBIN2								11.80	(10.12- 13.76)
MATOS	538	m	2	86	-	11	-	12.70	(6.10- 26.10)
MCCONN	505	c	0	16	40	9	23	1.02	(0.39- 2.68)
NOTAN2	517	c	0	5	5	107	201	1.88	(0.53- 6.63)
PEZZO2	508	m	0	173	126	6	117	26.77	(11.42- 62.76)
PEZZOT	536	m	0	110	101	4	116	31.58	(11.25- 88.71)
*QIAO2	518	m	1	170	-	10	-	2.05	(1.06- 3.94)
RACHTA	518	f	1	24	-	33	-	58.68	(7.56- 455.64)
SOBUE	549	m	0	147	73	34	128	7.58	(4.73- 12.14)
WUWILL	518	f	3	223	-	417	-	3.49	(2.65- 4.59)
Partial Totals				7012	207640	3338	7645227		

*prospective study

~ With 0.5 adjustment for zero

Table 1I14 - 2

IESLC - Meta-analysis of Ever/current Smoking, Duration, "High"
All LC types, Any Product (or Cigarettes if Any not available)
Most adjusted

REF	NRR	SEX	AD	Ys	Ws	Qs	Ps
ARMADA	508	m	1	3.29	4.86	2.18	0.0000
AUVINE	519	c	2	3.41	8.99	5.69	0.0000
AXELSS	523	m	6	3.30	8.71	4.03	0.0000
AXELSS	514	f	0	2.84	4.72	0.23	0.0000
Subtotal AXELSS				3.14	13.42	4.26	
BARBON	511	m	1	2.67	16.98	0.05	0.0000
*BEST	507	m	1	2.65	6.65	0.01	0.0000
*BOUCOT	519	m	0	3.99	0.50	0.93	0.0050
BUFFLE	528	f	0	3.00	7.86	1.12	0.0000
CHEN2	505	m	0	1.74	5.48	4.26	0.0000
CHEN2	513	f	0	0.61	5.42	21.78	0.1529
Subtotal CHEN2				1.18	10.90	26.04	
CHOI	505	m	0	1.99	5.33	2.12	0.0000
CHOI	513	f	0	0.77	0.50	1.69	0.5883
Subtotal CHOI				1.89	5.83	3.81	
*CPSI	585	m	0	3.12	146.44	37.40	0.0000
*CPSI	681	f	0	2.10	15.55	4.22	0.0000
Subtotal CPSI				3.03	161.99	41.62	
*CPSII	557	m	0	3.92	90.50	154.15	0.0000
*CPSII	623	f	0	3.34	87.81	45.83	0.0000
Subtotal CPSII				3.64	178.31	199.99	
DAMBER	509	m	1	2.16	21.28	4.40	0.0000
DESTEF	511	m	4	2.38	15.97	0.92	0.0000
DOLL	518	m	0	2.29	6.13	0.65	0.0000
DOLL	525	f	0	1.08	1.85	4.36	0.1417
Subtotal DOLL				2.01	7.98	5.01	
FAN	503	m	0	1.36	23.17	36.83	0.0000
FAN	508	f	0	1.46	18.96	25.30	0.0000
Subtotal FAN				1.41	42.13	62.13	
GER	520	c	5	0.76	10.75	37.13	0.0126
HU2	511	c	0	1.11	37.09	84.92	0.0000
HUMBLE	545	c	3	2.85	14.81	0.78	0.0000
JOLY	519	m	0	2.89	10.43	0.75	0.0000
JOLY	505	f	0	2.74	11.07	0.17	0.0000
Subtotal JOLY				2.81	21.50	0.92	
JUSSAW	514	m	0	2.04	3.76	1.27	0.0001
*KAISE2	597	m	1	2.75	9.62	0.16	0.0000
*KAISE2	517	f	1	3.41	6.86	4.34	0.0000
Subtotal KAISE2				3.03	16.49	4.51	
KATSOU	504	f	0	1.78	2.90	2.04	0.0024
KHUDER	503	m	0	2.19	18.60	3.39	0.0000
KREUZE	507	m	3	4.00	19.29	36.68	0.0000
KREUZE	510	f	3	2.12	12.11	3.06	0.0000
Subtotal KREUZE				3.27	31.40	39.75	
LETOUR	508	c	0	3.21	17.89	6.23	0.0000
LEVIN	507	m	1	2.19	5.54	1.01	0.0000
LUBIN	511	m	0	1.82	5.97	3.81	0.0000
LUBIN2	534	m	0	2.51	141.36	1.71	0.0000
LUBIN2	577	f	0	2.19	21.09	3.85	0.0000
Subtotal LUBIN2				2.47	162.45	5.57	
MATOS	538	m	2	2.54	7.27	0.04	0.0000
MCCONN	505	c	0	0.02	4.13	27.86	0.9644
NOTAN2	517	c	0	0.63	2.41	9.55	0.3273
PEZZO2	508	m	0	3.29	5.29	2.36	0.0000
PEZZOT	536	m	0	3.45	3.60	2.50	0.0000
*QIAO2	518	m	1	0.72	8.91	32.22	0.0321
RACHTA	518	f	1	4.07	0.91	1.93	0.0001
SOBUE	549	m	0	2.03	17.32	6.10	0.0000
WUWILL	518	f	3	1.25	50.92	95.47	0.0000

Table 1I14 - 2

IESLC - Meta-analysis of Ever/current Smoking, Duration, "High"
All LC types, Any Product (or Cigarettes if Any not available)
Most adjusted

	N	48
	NS	37
	Wt	953.59
Het	Chi	727.50
Het	df	47
Het	P	***
Fixed	RR	13.72
	RRl	12.88
	RRu	14.62
	P	+++
Random	RR	10.39
	RRl	7.92
	RRu	13.65
	P	+++
Asymm	P	*

Table 1I14 - 3

IESLC - Meta-analysis of Ever/current Smoking, Duration, "High"
All LC types, Any Product (or Cigarettes if Any not available)
Most adjusted

	Sex			
	combined	male	female	Total
N	7	26	15	48
NS	7	26	15	48
Wt	96.07	608.99	248.53	953.59
Het Chi	120.39	313.54	202.65	727.50
Het df	6	25	14	47
Het P	***	***	***	***
Fixed RR	6.59	16.90	10.94	13.72
RRl	5.39	15.61	9.66	12.88
RRu	8.04	18.30	12.39	14.62
P	+++	+++	+++	+++
Random RR	5.82	12.88	9.14	10.39
RRl	2.22	9.38	5.31	7.92
RRu	15.22	17.69	15.75	13.65
P	+++	+++	+++	+++
Between Chi				90.92
Between df				2
Between P				***
Btwn(F) P				*
Btwn(R) P				N.S.

	Lung cancer type		
	all	other	Total
N	46	2	48
NS	35	2	37
Wt	921.46	32.13	953.59
Het Chi	720.57	5.41	727.50
Het df	45	1	47
Het P	***	*	***
Fixed RR	13.83	11.08	13.72
RRl	12.96	7.84	12.88
RRu	14.75	15.66	14.62
P	+++	+++	+++
Random RR	10.34	11.37	10.39
RRl	7.80	5.08	7.92
RRu	13.72	25.49	13.65
P	+++	+++	+++
Between Chi			1.52
Between df			1
Between P			N.S.
Btwn(F) P			N.S.
Btwn(R) P			N.S.

	Location								
	NAmer	UK	Scand	othEur	China	Japan	othAs	other	Total
N	13	3	4	8	8	1	5	6	48
NS	10	2	3	6	6	1	4	5	37
Wt	428.64	12.11	43.70	219.51	155.92	17.32	22.76	53.64	953.59
Het Chi	99.79	12.87	13.89	51.51	9.86	0.00	8.86	6.00	727.50
Het df	12	2	3	7	7	0	4	5	47
Het P	***	**	**	***	N.S.	N.S.	(*)	N.S.	***
Fixed RR	25.27	3.79	15.19	13.66	3.47	7.58	3.48	15.44	13.72
RRl	22.99	2.16	11.29	11.97	2.97	4.73	2.31	11.81	12.88
RRu	27.78	6.66	20.43	15.59	4.06	12.14	5.24	20.17	14.62
P	+++	+++	+++	+++	+++	+++	+++	+++	+++
Random RR	19.05	3.17	18.23	15.53	3.49	7.58	3.75	15.77	10.39
RRl	13.78	0.70	9.20	9.51	2.85	4.73	1.88	11.70	7.92
RRu	26.34	14.40	36.13	25.33	4.27	12.14	7.50	21.26	13.65
P	+++	N.S.	+++	+++	+++	+++	+++	+++	+++
Between Chi									524.71
Between df									7
Between P									***
Btwn(F) P									***
Btwn(R) P									***

Table 1I14 - 3

IESLC - Meta-analysis of Ever/current Smoking, Duration, "High"
All LC types, Any Product (or Cigarettes if Any not available)
Most adjusted

Detailed Country in "other Europe"

	multi	Germany	othWest	East	Balkans	Total
N	2	2	2	1	1	8
NS	1	1	2	1	1	6
Wt	162.45	31.40	21.84	0.91	2.90	219.51
Het Chi	1.85	26.34	1.43	0.00	0.00	51.51
Het df	1	1	1	0	0	7
Het P	N.S.	***	N.S.	N.S.	N.S.	***
Fixed RR	11.80	26.38	16.62	58.68	5.93	13.66
RRl	10.12	18.59	10.93	7.56	1.88	11.97
RRu	13.76	37.42	25.28	455.55	18.75	15.59
P	+++	+++	+++	+++	++	+++
Random RR	11.18	21.44	17.49	58.68	5.93	15.53
RRl	8.41	3.39	10.05	7.56	1.88	9.51
RRu	14.86	135.59	30.45	455.55	18.75	25.33
P	+++	++	+++	+++	++	+++
Between Chi						21.89
Between df						4
Between P						***
Btwn(F) P						N.S.
Btwn(R) P						N.S.

Detailed Country in "other Asia"

	India	HongKong	other	Total
N	2		3	5
NS	2		2	4
Wt	6.17		16.58	22.76
Het Chi	2.91		5.45	8.86
Het df	1		2	4
Het P	(*)		(*)	(*)
Fixed RR	4.43		3.18	3.48
RRl	2.01		1.96	2.31
RRu	9.75		5.14	5.24
P	+++		+++	+++
Random RR	4.00		3.55	3.75
RRl	1.01		1.31	1.88
RRu	15.85		9.59	7.50
P	+		+	+++
Between Chi				0.50
Between df				1
Between P				N.S.
Btwn(F) P				N.S.
Btwn(R) P				N.S.

Detailed other continent

	SCAmer	Total
N	6	6
NS	5	5
Wt	53.64	53.64
Het Chi	6.00	6.00
Het df	5	5
Het P	N.S.	N.S.
Fixed RR	15.44	15.44
RRl	11.81	11.81
RRu	20.17	20.17
P	+++	+++
Random RR	15.77	15.77
RRl	11.70	11.70
RRu	21.26	21.26
P	+++	+++
Between Chi		
Between df		
Between P		N.S.
Btwn(F) P		N.S.
Btwn(R) P		N.S.

Table 1I14 - 3

IESLC - Meta-analysis of Ever/current Smoking, Duration, "High"
All LC types, Any Product (or Cigarettes if Any not available)
Most adjusted

	Start year of study					
	<1960	1960-69	1970-79	1980-89	1990+	Total
N	8	2	10	19	9	48
NS	6	2	7	15	7	37
Wt	186.79	6.17	283.66	370.30	106.67	953.59
Het Chi	63.65	2.91	81.33	337.82	138.09	727.50
Het df	7	1	9	18	8	47
Het P	***	(*)	***	***	***	***
Fixed RR	17.83	4.43	10.51	17.83	7.59	13.72
RRl	15.45	2.01	9.36	16.10	6.28	12.88
RRu	20.58	9.75	11.81	19.74	9.17	14.62
P	+++	+++	+++	+++	+++	+++
Random RR	8.23	4.00	12.18	12.19	8.74	10.39
RRl	4.02	1.01	8.12	7.49	3.83	7.92
RRu	16.85	15.85	18.27	19.85	19.95	13.65
P	+++	+	+++	+++	+++	+++
Between Chi						103.70
Between df						4
Between P						***
Btwn(F) P						N.S.
Btwn(R) P						N.S.

	Study type (1)		
	CC	other	Total
N	39	9	48
NS	31	6	37
Wt	580.74	372.86	953.59
Het Chi	350.57	127.19	727.50
Het df	38	8	47
Het P	***	***	***
Fixed RR	9.11	25.99	13.72
RRl	8.40	23.48	12.88
RRu	9.88	28.77	14.62
P	+++	+++	+++
Random RR	9.31	16.91	10.39
RRl	7.10	10.38	7.92
RRu	12.19	27.55	13.65
P	+++	+++	+++
Between Chi			249.74
Between df			1
Between P			***
Btwn(F) P			***
Btwn(R) P			*

	Study type (2)			
	CC	prosp	other	Total
N	39	9		48
NS	31	6		37
Wt	580.74	372.86		953.59
Het Chi	350.57	127.19		727.50
Het df	38	8		47
Het P	***	***		***
Fixed RR	9.11	25.99		13.72
RRl	8.40	23.48		12.88
RRu	9.88	28.77		14.62
P	+++	+++		+++
Random RR	9.31	16.91		10.39
RRl	7.10	10.38		7.92
RRu	12.19	27.55		13.65
P	+++	+++		+++
Between Chi				249.74
Between df				1
Between P				***
Btwn(F) P				***
Btwn(R) P				*

Table 1I14 - 3

IESLC - Meta-analysis of Ever/current Smoking, Duration, "High"
All LC types, Any Product (or Cigarettes if Any not available)
Most adjusted

	Study size (number of LC cases) 100-249	250-499	500-999	1000+	Total
N	10	15	12	11	48
NS	9	11	11	6	37
Wt	49.88	140.76	203.50	559.45	953.59
Het Chi	55.28	67.98	147.25	204.90	727.50
Het df	9	14	11	10	47
Het P	***	***	***	***	***
Fixed RR	4.01	9.38	7.86	20.64	13.72
RRl	3.04	7.95	6.85	19.00	12.88
RRu	5.30	11.06	9.02	22.42	14.62
P	+++	+++	+++	+++	+++
Random RR	5.59	11.36	10.56	15.09	10.39
RRl	2.68	7.72	6.21	9.94	7.92
RRu	11.67	16.71	17.95	22.90	13.65
P	+++	+++	+++	+++	+++
Between Chi					252.10
Between df					3
Between P					***
Btwn(F) P					***
Btwn(R) P					N.S.

	Risky occupational population no	mining	othRisky	Total
N	46	2		48
NS	35	2		37
Wt	938.71	14.89		953.59
Het Chi	690.97	4.35		727.50
Het df	45	1		47
Het P	***	*		***
Fixed RR	14.05	3.19		13.72
RRl	13.17	1.92		12.88
RRu	14.97	5.30		14.62
P	+++	+++		+++
Random RR	10.91	3.47		10.39
RRl	8.30	1.18		7.92
RRu	14.35	10.21		13.65
P	+++	+		+++
Between Chi				32.19
Between df				1
Between P				***
Btwn(F) P				N.S.
Btwn(R) P				*

	National cigarette tobacco type Virginia	blended	other	Total
N	7	32	9	48
NS	6	24	7	37
Wt	42.83	744.10	166.67	953.59
Het Chi	46.69	254.45	12.22	727.50
Het df	6	31	8	47
Het P	***	***	N.S.	***
Fixed RR	10.43	19.10	3.36	13.72
RRl	7.73	17.78	2.89	12.88
RRu	14.07	20.53	3.92	14.62
P	+++	+++	+++	+++
Random RR	5.92	16.31	3.34	10.39
RRl	2.39	12.87	2.73	7.92
RRu	14.66	20.67	4.10	13.65
P	+++	+++	+++	+++
Between Chi				414.14
Between df				2
Between P				***
Btwn(F) P				***
Btwn(R) P				***

Table 1I14 - 3

IESLC - Meta-analysis of Ever/current Smoking, Duration, "High"
All LC types, Any Product (or Cigarettes if Any not available)
Most adjusted

	Any proxy use No/nk	Yes	Total
N	35	13	48
NS	26	11	37
Wt	758.95	194.65	953.59
Het Chi	472.14	142.30	727.50
Het df	34	12	47
Het P	***	***	***
Fixed RR	16.34	6.95	13.72
RRl	15.22	6.04	12.88
RRu	17.54	8.00	14.62
P	+++	+++	+++
Random RR	11.91	7.53	10.39
RRl	8.82	4.58	7.92
RRu	16.09	12.39	13.65
P	+++	+++	+++
Between Chi			113.07
Between df			1
Between P			***
Btwn(F) P			**
Btwn(R) P			N.S.

	Full histological confirmation No	Yes	Total
N	36	12	48
NS	26	11	37
Wt	685.58	268.02	953.59
Het Chi	666.65	54.58	727.50
Het df	35	11	47
Het P	***	***	***
Fixed RR	14.44	12.06	13.72
RRl	13.40	10.70	12.88
RRu	15.56	13.59	14.62
P	+++	+++	+++
Random RR	9.65	12.32	10.39
RRl	6.76	8.81	7.92
RRu	13.77	17.22	13.65
P	+++	+++	+++
Between Chi			6.26
Between df			1
Between P			*
Btwn(F) P			N.S.
Btwn(R) P			N.S.

	Number of adjustment variables (1) 0	1	2+/+nk	Total
N	30	9	9	48
NS	22	8	8	38
Wt	723.14	81.64	148.82	953.59
Het Chi	489.89	42.96	161.89	727.50
Het df	29	8	8	47
Het P	***	***	***	***
Fixed RR	15.20	11.21	9.35	13.72
RRl	14.13	9.02	7.96	12.88
RRu	16.34	13.92	10.98	14.62
P	+++	+++	+++	+++
Random RR	9.25	12.66	12.26	10.39
RRl	6.59	7.40	5.76	7.92
RRu	12.97	21.66	26.10	13.65
P	+++	+++	+++	+++
Between Chi				32.76
Between df				2
Between P				***
Btwn(F) P				N.S.
Btwn(R) P				N.S.

Table 1I14 - 3

IESLC - Meta-analysis of Ever/current Smoking, Duration, "High"
All LC types, Any Product (or Cigarettes if Any not available)
Most adjusted

	Number of adjustment variables (2) 0	1	2	3-5	6+/+nk	Total
N	30	9	2	6	1	48
NS	22	8	2	5	1	38
Wt	723.14	81.64	16.26	123.85	8.71	953.59
Het Chi	489.89	42.96	3.06	134.91	0.00	727.50
Het df	29	8	1	5	0	47
Het P	***	***	(*)	***	N.S.	***
Fixed RR	15.20	11.21	20.58	7.82	27.09	13.72
RRl	14.13	9.02	12.66	6.56	13.94	12.88
RRu	16.34	13.92	33.45	9.33	52.63	14.62
P	+++	+++	+++	+++	+++	+++
Random RR	9.25	12.66	19.95	9.28	27.09	10.39
RRl	6.59	7.40	8.48	3.55	13.94	7.92
RRu	12.97	21.66	46.90	24.28	52.63	13.65
P	+++	+++	+++	+++	+++	+++
Between Chi						56.67
Between df						4
Between P						***
Btwn(F) P						N.S.
Btwn(R) P						*

	Smoking status ever	current	Total
N	37	11	48
NS	29	8	37
Wt	549.82	403.77	953.59
Het Chi	357.66	104.31	727.50
Het df	36	10	47
Het P	***	***	***
Fixed RR	8.73	25.40	13.72
RRl	8.03	23.04	12.88
RRu	9.49	28.01	14.62
P	+++	+++	+++
Random RR	8.76	18.13	10.39
RRl	6.55	12.52	7.92
RRu	11.71	26.24	13.65
P	+++	+++	+++
Between Chi			265.53
Between df			1
Between P			***
Btwn(F) P			***
Btwn(R) P			**

	Product all/unsp	cig+/-ot	cig only	Total
N	15	24	9	48
NS	11	20	7	38
Wt	144.63	523.56	285.41	953.59
Het Chi	158.22	301.30	91.11	727.50
Het df	14	23	8	47
Het P	***	***	***	***
Fixed RR	9.34	10.68	26.42	13.72
RRl	7.93	9.80	23.53	12.88
RRu	10.99	11.64	29.67	14.62
P	+++	+++	+++	+++
Random RR	6.95	11.28	16.21	10.39
RRl	3.94	7.96	9.78	7.92
RRu	12.24	16.00	26.85	13.65
P	+++	+++	+++	+++
Between Chi				176.87
Between df				2
Between P				***
Btwn(F) P				**
Btwn(R) P				(*)

Table 1I14 - 3

IESLC - Meta-analysis of Ever/current Smoking, Duration, "High"
All LC types, Any Product (or Cigarettes if Any not available)
Most adjusted

	Denominator nev any	nev cigs	Total
N	29	19	48
NS	22	16	38
Wt	467.68	485.91	953.59
Het Chi	358.84	363.55	727.50
Het df	28	18	47
Het P	***	***	***
Fixed RR	14.79	12.77	13.72
RRl	13.51	11.69	12.88
RRu	16.19	13.96	14.62
P	+++	+++	+++
Random RR	9.46	11.95	10.39
RRl	6.59	7.64	7.92
RRu	13.59	18.67	13.65
P	+++	+++	+++
Between Chi			5.12
Between df			1
Between P			*
Btwn(F) P			N.S.
Btwn(R) P			N.S.

	Derivation of RR/CI Orig	StdCalc	Other	Total
N	10	30	8	48
NS	9	21	8	38
Wt	103.17	731.27	119.16	953.59
Het Chi	81.77	491.80	73.48	727.50
Het df	9	29	7	47
Het P	***	***	***	***
Fixed RR	15.84	15.24	6.37	13.72
RRl	13.06	14.18	5.32	12.88
RRu	19.21	16.39	7.62	14.62
P	+++	+++	+++	+++
Random RR	15.66	9.38	9.25	10.39
RRl	8.53	6.72	4.77	7.92
RRu	28.76	13.08	17.95	13.65
P	+++	+++	+++	+++
Between Chi				80.45
Between df				2
Between P				***
Btwn(F) P				(*)
Btwn(R) P				N.S.

Table 1I14 - 4

IESLC - Meta-analysis of Ever/current Smoking, Duration, "High"
All LC types, Any Product (or Cigarettes if Any not available)
Least adjusted

REF	NRR	X	SEX	AGEL	AGEH	RACE	YF	LC TYPE	LOC	START	ST	NLC	R	VB	P	H	AD	SM	PRODUCT	exL	exH	DENOM	De
ARMADA	503	x	m	0	0	all	-	all	Eu:wst	1986	CC	325	n	bl	n	y	0	ev	cig+/-ot	50	999	nev cigs	st
AUVINE	503	x	c	0	0	all	-	all	Eu:Sca	1986	CC	517	n	bl	y	n	0	ev	cig+/-ot	41	999	nev cigs	st
AXELSS	505	x	m	0	0	sca	-	all	Eu:Sca	1989	CC	436	n	bl	n	n	0	ev	all/unsp	50	999	nev any	st
AXELSS	514		f	0	0	sca	-	all	Eu:Sca	1989	CC	436	n	bl	n	n	0	ev	all/unsp	50	999	nev any	st
BARBON	504	x	m	0	0	all	-	all	Eu:wst	1979	CC	755	n	bl	y	y	0	ev	all/unsp	50	999	nev any	st
BEST	507		m	0	0	all	0	all	NAmer	1955	pr	381	n	V	n	n	1	cu	cig only	40	999	nev any	ot
BOUCOT	519		m	0	0	all	9	all	NAmer	1951	pr	121	n	bl	n	n	0	ev	cig+/-ot	40	999	nev any	ot
BUFFLE	528		f	0	0	w-hi	-	all	NAmer	1976	CC	943	n	bl	y	n	0	ev	cig+/-ot	41	999	nev cigs	or
CHEN2	505		m	0	0	all	-	all	As:Chi	1983	CC	193	n	ot	y	n	0	ev	all/unsp	41	999	nev any	st
CHEN2	513		f	0	0	all	-	all	As:Chi	1983	CC	193	n	ot	y	n	0	ev	all/unsp	41	999	nev any	st
CHOI	505		m	0	0	all	-	all	As:oth	1985	CC	375	n	bl	n	n	0	ev	cig+/-ot	50	999	nev cigs	st
CHOI	513		f	0	0	all	-	all	As:oth	1985	CC	375	n	bl	n	n	0	ev	cig+/-ot	40	999	nev cigs	st
CPSI	585		m	40	84	wh	0	all	NAmer	1959	pr	5138	n	bl	n	n	0	cu	cig only	50	54	nev cigs	st
CPSI	681		f	40	84	wh	0	all	NAmer	1959	pr	5138	n	bl	n	n	0	cu	cig only	50	54	nev cigs	st
CPSII	557		m	0	0	all	6	all	NAmer	1982	pr	3229	n	bl	n	n	0	cu	cig only	50	54	nev any	st
CPSII	623		f	0	0	all	6	all	NAmer	1982	pr	3229	n	bl	n	n	0	cu	cig+/-ot	50	54	nev cigs	st
DAMBER	509		m	0	0	all	-	all	Eu:Sca	1972	CC	579	n	bl	y	n	1	ev	all/unsp	41	50	nev any	ot
DESTEF	504	x	m	0	0	all	-	all	SCAmer	1988	CC	497	n	bl	n	y	0	ev	all/unsp	50	999	nev any	st
DOLL	518		m	0	0	all	-	all	Eu:UK	1948	CC	1465	n	V	n	n	0	ev	all/unsp	40	999	nev any	st
DOLL	525		f	0	0	all	-	all	Eu:UK	1948	CC	1465	n	V	n	n	0	ev	all/unsp	40	999	nev any	st
FAN	503		m	0	0	all	-	all	As:Chi	1990	CC	403	n	ot	y	n	0	ev	cig+/-ot	40	999	nev cigs	st
FAN	508		f	0	0	all	-	all	As:Chi	1990	CC	403	n	ot	y	n	0	ev	cig+/-ot	40	999	nev cigs	st
GER	515	x	c	0	0	all	-	all	As:oth	1990	CC	141	n	ot	y	n	0	ev	all/unsp	41	999	nev any	st
HU2	511		c	0	0	all	-	all	As:Chi	1977	CC	523	n	ot	y	n	0	ev	cig+/-ot	40	999	nev cigs	st
HUMBLE	520	x	c	0	0	wh	-	not alv	NAmer	1980	CC	521	n	bl	y	n	0	cu	cig+/-ot	50	59	nev cigs	st
JOLY	519		m	0	0	all	-	all	SCAmer	1978	CC	826	n	bl	n	n	0	ev	cig+/-ot	50	999	nev any	st
JOLY	505		f	0	0	all	-	all	SCAmer	1978	CC	826	n	bl	n	n	0	ev	cig+/-ot	50	999	nev any	st
JUSSAW	514		m	0	0	all	-	all	As:Ind	1964	CC	792	n	V	n	n	0	ev	cig only	40	999	nev any	st
KAISE2	597		m	0	0	all	9	all	NAmer	1979	pr	318	n	bl	n	n	1	cu	cig only	40	999	nev any	st
KAISE2	517		f	0	0	all	9	all	NAmer	1979	pr	318	n	bl	n	n	1	cu	cig only	40	999	nev any	st
KATSOU	504		f	0	0	all	-	all	Eu:bal	1987	CC	101	n	bl	n	n	0	cu	all/unsp	40	999	nev any	st
KHUDER	503		m	0	0	all	-	all	NAmer	1985	CC	482	n	bl	n	y	0	ev	cig+/-ot	50	999	nev cigs	st
KREUZE	507		m	55	69	all	-	all	Eu:Ger	1990	CC	2260	n	bl	n	n	3	ev	all/unsp	40	999	nev any	or
KREUZE	510		f	55	69	all	-	all	Eu:Ger	1990	CC	2260	n	bl	n	n	3	ev	all/unsp	40	999	nev any	or
LETOUR	508		c	0	0	all	-	all	NAmer	1983	CC	738	n	V	y	y	0	ev	cig+/-ot	41	999	nev cigs	st
LEVIN	502	x	m	0	0	all	-	all	NAmer	1938	CC	475	n	bl	n	n	0	ev	cig+/-ot	40	999	nev any	st
LUBIN	511		m	0	0	all	-	all	As:Chi	1984	CC	427	m	ot	y	n	0	ev	cig+/-ot	50	999	nev any	st
LUBIN2	534		m	0	0	all	-	all	Eu:mul	1976	CC	7804	n	bl	n	y	0	ev	cig+/-ot	50	999	nev any	st
LUBIN2	577		f	0	0	all	-	all	Eu:mul	1976	CC	7804	n	bl	n	y	0	ev	cig+/-ot	50	999	nev any	st
MATOS	518	x	m	0	0	all	-	all	SCAmer	1994	CC	200	n	bl	n	n	0	ev	cig+/-ot	40	70	nev any	st
MCCONN	505		c	0	0	all	-	all	Eu:UK	1946	CC	100	n	V	n	y	0	ev	all/unsp	40	999	nev any	st
NOTAN2	517		c	0	0	all	-	all	As:Ind	1963	CC	683	n	V	n	n	0	ev	cig only	41	999	nev any	st
PEZZO2	508		m	0	0	all	-	all	SCAmer	1992	CC	367	n	bl	n	y	0	cu	cig+/-ot	36	999	nev cigs	st
PEZZOT	536		m	0	0	all	-	all	SCAmer	1987	CC	215	n	bl	n	y	0	ev	cig only	41	999	nev cigs	st
QIAO2	513	x	m	0	0	all	0	all	As:Chi	1992	pr	241	m	ot	n	n	0	ev	all/unsp	42	999	nev any	st
RACHTA	513	x	f	0	0	all	-	all	Eu:est	1991	CC	118	n	bl	n	y	0	ev	cig+/-ot	41	999	nev cigs	st
SOBUE	549		m	0	0	all	-	q+s+l+a	As:Jap	1986	CC	1376	n	bl	n	y	0	cu	cig+/-ot	50	999	nev cigs	st
WUWILL	503	x	f	0	0	all	-	all	As:Chi	1985	CC	965	n	ot	n	n	0	ev	cig+/-ot	40	999	nev cigs	st

Cigarette type is all/unspec for all RRs

except for the following:

REF	NRR	CIGTYPE
JUSSAW	514	MC only
NOTAN2	517	MC only

Table 1I14 - 5

IESLC - Meta-analysis of Ever/current Smoking, Duration, "High"
All LC types, Any Product (or Cigarettes if Any not available)
Least adjusted

REF	NRR	SEX	AD	Number Exposed Case	Number Exposed Cont	Non-exposed Case	Non-exposed Cont	RR	95.00%CI
ARMADA	503	m	0	77	33	8	71	20.71	(8.97- 47.82)
AUVINE	503	c	0	230	57	44	229	21.00	(13.61- 32.41)
AXELSS	505	m	0	101	40	16	160	25.25	(13.43- 47.46)
AXELSS	514	f	0	20	10	18	154	17.11	(6.94- 42.19)
Subtotal AXELSS								22.22	(13.25- 37.27)
BARBON	504	m	0	366	235	22	188	13.31	(8.31- 21.32)
*BEST	507	m	1	137	-	7	-	14.20	(6.64- 30.35)
*BOUCOT	519	m	0	52	1563	0	805	54.09~	(3.34- 875.17)
BUFFLE	528	f	0	90	42	12	112	20.00	(9.94- 40.23)
CHEN2	505	m	0	62	40	9	33	5.68	(2.46- 13.13)
CHEN2	513	f	0	21	15	25	33	1.85	(0.80- 4.29)
Subtotal CHEN2								3.25	(1.80- 5.89)
CHOI	505	m	0	20	20	13	95	7.31	(3.13- 17.07)
CHOI	513	f	0	1	1	76	164	2.16	(0.13- 34.96)
Subtotal CHOI								6.59	(2.93- 14.84)
*CPSI	585	m	0	576	119633	196	926068	22.75	(19.35- 26.75)
*CPSI	681	f	0	16	14305	532	3877179	8.15	(4.96- 13.40)
Subtotal CPSI								20.61	(17.67- 24.05)
*CPSII	557	m	0	332	39260	124	742207	50.62	(41.19- 62.20)
*CPSII	623	f	0	122	29119	310	2091302	28.26	(22.93- 34.84)
Subtotal CPSII								37.99	(32.80- 44.00)
DAMBER	509	m	1	-	-	42	-	8.71	(5.84- 13.66)
DESTEF	504	m	0	178	108	27	163	9.95	(6.20- 15.96)
DOLL	518	m	0	558	491	7	61	9.90	(4.49- 21.85)
DOLL	525	f	0	6	3	40	59	2.95	(0.70- 12.49)
Subtotal DOLL								7.48	(3.74- 14.98)
FAN	503	m	0	143	241	36	236	3.89	(2.59- 5.84)
FAN	508	f	0	55	59	69	320	4.32	(2.76- 6.78)
Subtotal FAN								4.08	(3.02- 5.52)
GER	515	c	0	49	155	51	246	1.52	(0.98- 2.37)
HU2	511	c	0	194	113	121	213	3.02	(2.19- 4.17)
HUMBLE	520	c	0	90	55	28	285	16.66	(9.97- 27.82)
JOLY	519	m	0	250	253	12	218	17.95	(9.78- 32.93)
JOLY	505	f	0	57	20	52	283	15.51	(8.61- 27.95)
Subtotal JOLY								16.65	(10.91- 25.41)
JUSSAW	514	m	0	11	6	149	624	7.68	(2.79- 21.09)
*KAISE2	597	m	1	34	-	14	-	15.64	(8.31- 29.40)
*KAISE2	517	f	1	26	-	11	-	30.41	(14.39- 64.25)
Subtotal KAISE2								20.63	(12.73- 33.43)
KATSOU	504	f	0	17	4	48	67	5.93	(1.88- 18.75)
KHUDER	503	m	0	236	354	23	309	8.96	(5.69- 14.11)
KREUZE	507	m	3	-	-	-	-	54.50	(34.90- 85.20)
KREUZE	510	f	3	-	-	-	-	8.30	(4.70- 14.50)
Subtotal KREUZE								26.38	(18.59- 37.42)
LETOUR	508	c	0	374	141	24	224	24.76	(15.58- 39.35)
LEVIN	502	m	0	63	91	7	96	9.49	(4.13- 21.81)
LUBIN	511	m	0	59	86	8	72	6.17	(2.77- 13.77)
LUBIN2	534	m	0	1325	1484	190	2616	12.29	(10.42- 14.50)
LUBIN2	577	f	0	81	32	336	1188	8.95	(5.84- 13.71)
Subtotal LUBIN2								11.80	(10.12- 13.76)
MATOS	518	m	0	86	89	11	110	9.66	(4.86- 19.21)
MCCONN	505	c	0	16	40	9	23	1.02	(0.39- 2.68)
NOTAN2	517	c	0	5	5	107	201	1.88	(0.53- 6.63)
PEZZO2	508	m	0	173	126	6	117	26.77	(11.42- 62.76)
PEZZOT	536	m	0	110	101	4	116	31.58	(11.25- 88.71)
*QIAO2	513	m	0	170	2295	10	709	5.25	(2.79- 9.88)
RACHTA	513	f	0	24	1	33	98	71.27	(9.28- 547.53)
SOBUE	549	m	0	147	73	34	128	7.58	(4.73- 12.14)
WUWILL	503	f	0	223	114	417	601	2.82	(2.18- 3.65)
Partial Totals				6983	210913	3338	7648183		

*prospective study ~ With 0.5 adjustment for zero

Table 1I14 - 5

IESLC - Meta-analysis of Ever/current Smoking, Duration, "High"
All LC types, Any Product (or Cigarettes if Any not available)
Least adjusted

REF	NRR	SEX	AD	Ys	Ws	Qs	Ps
ARMADA	503	m	0	3.03	5.48	1.13	0.0000
AUVINE	503	c	0	3.04	20.41	4.48	0.0000
AXELSS	505	m	0	3.23	9.65	4.11	0.0000
AXELSS	514	f	0	2.84	4.72	0.33	0.0000
Subtotal AXELSS				3.10	14.36	4.44	
BARBON	504	m	0	2.59	17.31	0.00	0.0000
*BEST	507	m	1	2.65	6.65	0.04	0.0000
*BOUCOT	519	m	0	3.99	0.50	0.99	0.0050
BUFFLE	528	f	0	3.00	7.86	1.38	0.0000
CHEN2	505	m	0	1.74	5.48	3.85	0.0000
CHEN2	513	f	0	0.61	5.42	20.85	0.1529
Subtotal CHEN2				1.18	10.90	24.70	
CHOI	505	m	0	1.99	5.33	1.84	0.0000
CHOI	513	f	0	0.77	0.50	1.62	0.5883
Subtotal CHOI				1.89	5.83	3.46	
*CPSI	585	m	0	3.12	146.44	44.05	0.0000
*CPSI	681	f	0	2.10	15.55	3.55	0.0000
Subtotal CPSI				3.03	161.99	47.60	
*CPSII	557	m	0	3.92	90.50	164.51	0.0000
*CPSII	623	f	0	3.34	87.81	51.47	0.0000
Subtotal CPSII				3.64	178.31	215.97	
DAMBER	509	m	1	2.16	21.28	3.60	0.0000
DESTEF	504	m	0	2.30	17.23	1.34	0.0000
DOLL	518	m	0	2.29	6.13	0.49	0.0000
DOLL	525	f	0	1.08	1.85	4.12	0.1417
Subtotal DOLL				2.01	7.98	4.61	
FAN	503	m	0	1.36	23.17	34.36	0.0000
FAN	508	f	0	1.46	18.96	23.44	0.0000
Subtotal FAN				1.41	42.13	57.80	
GER	515	c	0	0.42	19.79	91.83	0.0605
HU2	511	c	0	1.11	37.09	80.15	0.0000
HUMBLE	520	c	0	2.81	14.60	0.82	0.0000
JOLY	519	m	0	2.89	10.43	1.01	0.0000
JOLY	505	f	0	2.74	11.07	0.30	0.0000
Subtotal JOLY				2.81	21.50	1.32	
JUSSAW	514	m	0	2.04	3.76	1.09	0.0001
*KAISE2	597	m	1	2.75	9.62	0.29	0.0000
*KAISE2	517	f	1	3.41	6.86	4.83	0.0000
Subtotal KAISE2				3.03	16.49	5.12	
KATSOU	504	f	0	1.78	2.90	1.84	0.0024
KHUDER	503	m	0	2.19	18.60	2.74	0.0000
KREUZE	507	m	3	4.00	19.29	39.01	0.0000
KREUZE	510	f	3	2.12	12.11	2.56	0.0000
Subtotal KREUZE				3.27	31.40	41.57	
LETOUR	508	c	0	3.21	17.89	7.17	0.0000
LEVIN	502	m	0	2.25	5.55	0.59	0.0000
LUBIN	511	m	0	1.82	5.97	3.41	0.0000
LUBIN2	534	m	0	2.51	141.36	0.63	0.0000
LUBIN2	577	f	0	2.19	21.09	3.12	0.0000
Subtotal LUBIN2				2.47	162.45	3.75	
MATOS	518	m	0	2.27	8.14	0.77	0.0000
MCCONN	505	c	0	0.02	4.13	26.95	0.9644
NOTAN2	517	c	0	0.63	2.41	9.14	0.3273
PEZZO2	508	m	0	3.29	5.29	2.68	0.0000
PEZZOT	536	m	0	3.45	3.60	2.77	0.0000
*QIAO2	513	m	0	1.66	9.61	8.09	0.0000
RACHTA	513	f	0	4.27	0.92	2.64	0.0000
SOBUE	549	m	0	2.03	17.32	5.25	0.0000
WUWILL	503	f	0	1.04	57.74	136.86	0.0000

Table 1I14 - 5

IESLC - Meta-analysis of Ever/current Smoking, Duration, "High"
All LC types, Any Product (or Cigarettes if Any not available)
Least adjusted

	N	48
	NS	37
	Wt	985.39
Het	Chi	808.08
Het	df	47
Het	P	***
Fixed	RR	13.14
	RRl	12.35
	RRu	13.99
	P	+++
Random	RR	10.23
	RRl	7.73
	RRu	13.55
	P	+++
Asymm	P	(*)

Table 1I14 - 6

IESLC - Meta-analysis of Ever/current Smoking, Duration, "High"
All LC types, Any Product (or Cigarettes if Any not available)
Least adjusted

		Sex			
		combined	male	female	Total
	N	7	26	15	48
	NS	7	26	15	48
	Wt	116.32	613.71	255.36	985.39
Het	Chi	153.88	289.60	241.46	808.08
Het	df	6	25	14	47
Het	P	***	***	***	***
Fixed	RR	6.17	16.92	10.12	13.14
	RRl	5.14	15.63	8.95	12.35
	RRu	7.39	18.31	11.44	13.99
	P	+++	+++	+++	+++
Random	RR	5.23	13.01	9.05	10.23
	RRl	1.98	9.60	5.05	7.73
	RRu	13.79	17.64	16.21	13.55
	P	+++	+++	+++	+++
Between	Chi				123.15
Between	df				2
Between	P				***
Btwn(F)	P				*
Btwn(R)	P				N.S.

Table 1I14 - 7

IESLC - Meta-analysis of Ever/current Smoking, Duration, "High"
All LC types, Any Product (or Cigarettes if Any not available)
Excluded studies (and stage at which they were excluded)

1	BECHER BLOT1 BROWN3 CARPEN CHYOU DARBY DOLL2 GARCIA GRAHAM GURSEL HAMMO2 JAHN JAIN LAUSSM PRESCO QIAO TVERDA WIGLE WYNDE3
2	ALDERS BENSHL BRESLO CHIAZZ DEAN3 DORN ENGELA GAO2 GILLIS GUO HEGMAN HIRAYA HOLE KAUFMA KOO KOULUM LIU4 MIGRAN MRFITR PERNU SEGI2 SPEIZE SUZUK2 SVENSS VUTUC WAKAI WU YUAN
3	GENG MCDUFF SPITZ STASZE WU2 ZHANG
4	BOUCHA CHEN CORREA JEDRYC LUO WYNDE2 WYNDE6
5	AKIBA HAMMON PISANI RESTRE SADOWS XU
7	BOFFET BROSS WYNDE7
10	AMES WATSON WYNDE8
14	AGUDO AMANDU CEDERL DEAN2 DORGAN DOSEME GAO GARSHI HAENSZ HU LIAW LIU3 LIU5 OSANN2 TIZZAN WANG2 ZHENG ZHOU
15	BENHAM

Table 1I14 - 8

Potentially overlapping studies

REF	REFGP	PRINC	.	OVERLAP/LINK
LUBIN2	LUBIN2	1		Lubin-combined
CPSI	CPSI	1		CPSI overall
LUBIN	XIANGZ	2		LUBIN/XIANGZ/QIAO

Table 1I14 - 9

Most adjusted - insufficient data for meta-analysis

REF	NRR	SEX	AGEL	AGEH	RACE	YF	LC TYPE	LOC	START	ST	NLC	R	VB	P	H	AD	SM	PRODUCT	exL	exH	DENOM	De
BUFFLE	504	m	0	0	wh	-	all	NAmer	1976	CC	943	n	bl	y	n	0	ev	cig+/-ot	50	999	nev cigs	or
SADOWS	527	m	0	0	wh	-	all	NAmer	1938	CC	477	n	bl	n	n	0	ev	cig only	50	999	nev any	ot
XU	503	m	0	0	all	-	all	As:Chi	1985	CC	729	n	ot	n	n	2	ev	all/unsp	40	999	nev any	or

REF	NRR	RR	SIG	RRDATA comment
BUFFLE	504	14.50		0
SADOWS	527	8.43		0
XU	503	*		RR for 1-19/day is 3.3(p<0.05), for 20-29/day is 6.0(p<0.05) and for >=30/ day is 17.1(p<0.05)

Table 1I15 -

IESLC - Meta-analysis of Ever/current Smoking, Duration, "Highest vs lowest"
All LC types, Any Product (or Cigarettes if Any not available)

This analysis is restricted to results for:
1) Ever/current smokers
2) Results by Duration
3) Categorical results by Duration
4) Denominator (unexposed) = "low"
5) All LC types (or near equivalent)
6) Results complete enough for use in metaanalysis

Within each study, results are then selected (in the following order of preference, within each sex) for:
7) SMKSTA: ever, current
8) PRODUCT: all/unspec, cigarettes regardless of other products, cigarettes only
9) CIGTYPE: all/unspecified, MC regardless of HR, MC only
10) Results with least adjustment for other aspects of smoking (ADOS)
11) The highest vs lowest category
12) Followup period (YF, prospective studies): whole study (coded as 0) or longest available
13) LCtype: all or nearest available, at least Squamous and Adeno. (q = squamous, s = small,
l = large, a = adeno, mix = mixed, alv = alveolar)
14) Race: all or nearest available, otherwise by race (wh or w = white, bl or b = black, hi = hispanic
ch = chinese, jap = japanese, haw = hawaiian, w+o = white + oriental, sca = scandinavian, as = asian)
15) For overlapping studies: principal rather than subsidiary studies
Finally by Age: whole study (coded as 0) if available, otherwise by widest available age group
and then for single sex results (m, f) in preference to results for both sexes combined (c).

Results adjusted (AD) for the most potential confounders are then chosen in Sections -1 to -3
and results adjusted for the least confounders in Sections -4 to -6. (Those least adjusted results which
actually differ from the most adjusted are marked 'x' in column X in Section -4)

Section -7 shows excluded studies, together with the stage (as above) at which no qualifying
results were found.

Section -8 lists the potentially overlapping studies which have been included (1=principal, 2=subsidiary).

Section -9 lists any results which would have been included in preference except that they had data not complete
enough for use in meta-analysis, with their significance (yes/no), if known, and any further comment as entered
on the database. It also lists as "gap" any categories for which no data were presented by the original authors.

In addition to those mentioned above, the following fields, levels and abbreviations are used:
* or nk = not known, n = no, y = yes, ot = other
all/unspec = all or unspecified, cig+/-ot = cigarettes irrespective of other products (cigar, pipe etc)
MC = manufactured cigarettes, HR = hand-rolled cigarettes
exL, exH = range of exposure (low and high) in the "highest" group, in terms of Duration
unexL, unexH = range of exposure (low and high) in the "lowest" group, in terms of Duration
REF: 6-character study reference
NRR: number of the RR on the database within the study
ST : study type (CC = case control, pr or prosp = prospective)
NLC: number of lung cancer cases in whole study
R : risky occupational population (n = no, m = mining, o = other risky)
VB : national cigarette type (V = at least 75% Virginia, bl = at least 75% blended, ot = other)
P : any proxy use
H : full histological confirmation
De : derivation of RR/CI (or = original, st = standard method, ot = other method of estimation)

Table 1I15 - 1

IESLC - Meta-analysis of Ever/current Smoking, Duration, "Highest vs lowest"
All LC types, Any Product (or Cigarettes if Any not available)
Most adjusted

REF	NRR	SEX	AGEL	AGEH	RACE	YF	LC TYPE	LOC	START	ST	NLC	R	VB	P	H	AD	ADOS	SM	PRODUCT	exL	exH	unexL	unexH	De
AGUDO	512	f	0	0	all	-	all	Eu:wst	1989	CC	103	n	bl	n	n	3	0	ev	cig only	17	999	1	16	ot
AMANDU	508	m	0	0	wh	0	all	NAmer	1959	pr	132	m	bl	n	n	2	0	cu	cig+/-ot	25	999	1	24	ot
AMES	501	m	0	0	wh	-	all	NAmer	1959	ot	317	m	bl	n	n	2	0	cu	all/unsp	30	999	1	29	or
ARMADA	510	m	0	0	all	-	all	Eu:wst	1986	CC	325	n	bl	n	y	1	0	ev	cig+/-ot	50	999	1	24	ot
AUVINE	523	c	0	0	all	-	all	Eu:Sca	1986	CC	517	n	bl	y	n	2	0	ev	cig+/-ot	41	999	1	20	ot
AXELSS	509	m	0	0	sca	-	all	Eu:Sca	1989	CC	436	n	bl	n	n	0	0	ev	all/unsp	50	999	1	19	st
AXELSS	518	f	0	0	sca	-	all	Eu:Sca	1989	CC	436	n	bl	n	n	0	0	ev	all/unsp	50	999	1	19	st
BARBON	514	m	0	0	all	-	all	Eu:wst	1979	CC	755	n	bl	y	y	1	0	ev	all/unsp	50	999	1	29	ot
BEST	513	m	0	0	all	0	all	NAmer	1955	pr	381	n	V	n	n	1	0	cu	cig only	40	999	1	4	ot
BOUCOT	520	m	0	0	all	9	all	NAmer	1951	pr	121	n	bl	n	n	0	0	ev	cig+/-ot	40	999	1	39	st
BUFFLE	530	f	0	0	w-hi	-	all	NAmer	1976	CC	943	n	bl	y	n	0	0	ev	cig+/-ot	41	999	1	30	st
CEDERL	503	m	40	69	all	10	all	Eu:Sca	1963	pr	491	n	bl	n	n	1	0	cu	cig only	30	999	1	29	ot
CEDERL	506	f	40	69	all	10	all	Eu:Sca	1963	pr	491	n	bl	n	n	1	0	cu	cig only	30	999	1	29	ot
CHEN2	509	m	0	0	all	-	all	As:Chi	1983	CC	193	n	ot	y	n	0	0	ev	all/unsp	41	999	1	9	st
CHEN2	516	f	0	0	all	-	all	As:Chi	1983	CC	193	n	ot	y	n	0	0	ev	all/unsp	41	999	1	20	st
CHOI	509	m	0	0	all	-	all	As:oth	1985	CC	375	n	bl	n	n	0	0	ev	cig+/-ot	50	999	1	19	st
CHOI	516	f	0	0	all	-	all	As:oth	1985	CC	375	n	bl	n	n	0	0	ev	cig+/-ot	40	999	1	19	st
CPSI	602	m	40	84	wh	0	all	NAmer	1959	pr	5138	n	bl	n	n	0	0	cu	cig only	60	999	1	29	st
CPSI	695	f	40	84	wh	0	all	NAmer	1959	pr	5138	n	bl	n	n	0	0	cu	cig only	55	999	1	29	st
CPSII	566	m	0	0	all	6	all	NAmer	1982	pr	3229	n	bl	n	n	0	0	cu	cig only	60	999	1	29	st
CPSII	632	f	0	0	all	6	all	NAmer	1982	pr	3229	n	bl	n	n	0	0	cu	cig+/-ot	60	999	1	29	st
DAMBER	514	m	0	0	all	-	all	Eu:Sca	1972	CC	579	n	bl	y	n	1	0	ev	all/unsp	51	999	1	20	ot
DEAN2	503	m	0	0	all	-	all	Eu:UK	1960	CC	954	n	V	y	n	0	0	cu	all/unsp	20	999	1	19	st
DEAN2	506	f	0	0	all	-	all	Eu:UK	1960	CC	954	n	V	y	n	0	0	cu	all/unsp	20	999	1	19	st
DESTEF	514	m	0	0	all	-	all	SCAmer	1988	CC	497	n	bl	n	y	4	0	ev	all/unsp	50	999	1	29	ot
DOLL	521	m	0	0	all	-	all	Eu:UK	1948	CC	1465	n	V	n	n	0	0	ev	all/unsp	40	999	1	9	st
DOLL	528	f	0	0	all	-	all	Eu:UK	1948	CC	1465	n	V	n	n	0	0	ev	all/unsp	40	999	1	9	st
DORGAN	526	m	0	0	wh	-	all	NAmer	1980	CC	2026	n	bl	y	y	2	0	ev	cig+/-ot	35	999	1	34	ot
DORGAN	522	f	0	0	all	-	all	NAmer	1980	CC	2026	n	bl	y	y	3	0	ev	cig+/-ot	35	999	1	34	ot
DOSEME	505	m	0	0	all	-	all	Eu:bal	1979	CC	1210	n	bl	n	n	2	0	ev	cig+/-ot	21	999	1	10	ot
FAN	505	m	0	0	all	-	all	As:Chi	1990	CC	403	n	ot	y	n	0	0	ev	cig+/-ot	40	999	1	29	st
FAN	510	f	0	0	all	-	all	As:Chi	1990	CC	403	n	ot	y	n	0	0	ev	cig+/-ot	40	999	1	29	st
GAO	566	f	0	0	all	-	all	As:Chi	1984	CC	1405	n	ot	n	n	2	0	ev	cig+/-ot	30	999	1	29	ot
GER	522	c	0	0	all	-	all	As:oth	1990	CC	141	n	ot	y	n	5	0	ev	all/unsp	41	999	1	20	ot
HAENSZ	554	f	0	0	all	-	not alv	NAmer	1955	CC	158	n	bl	n	y	1	0	ev	cig+/-ot	15	999	1	14	ot
HU	505	m	0	0	all	-	all	As:Chi	1985	CC	227	n	ot	n	y	0	0	ev	cig+/-ot	30	999	1	19	st
HU	510	f	0	0	all	-	all	As:Chi	1985	CC	227	n	ot	n	y	0	0	ev	cig+/-ot	30	999	1	19	st
HU2	514	c	0	0	all	-	all	As:Chi	1977	CC	523	n	ot	y	n	0	0	ev	cig+/-ot	40	999	1	19	st
HUMBLE	525	c	0	0	wh	-	not alv	NAmer	1980	CC	521	n	bl	y	n	0	0	cu	cig+/-ot	60	999	1	29	st
JOLY	523	m	0	0	all	-	all	SCAmer	1978	CC	826	n	bl	n	n	0	0	ev	cig+/-ot	50	999	1	19	st
JOLY	509	f	0	0	all	-	all	SCAmer	1978	CC	826	n	bl	n	n	0	0	ev	cig+/-ot	50	999	1	19	st
JUSSAW	518	m	0	0	all	-	all	As:Ind	1964	CC	792	n	V	n	n	0	0	ev	cig only	40	999	1	9	st
KAISE2	600	m	0	0	all	9	all	NAmer	1979	pr	318	n	bl	n	n	1	0	cu	cig only	40	999	1	39	st
KAISE2	520	f	0	0	all	9	all	NAmer	1979	pr	318	n	bl	n	n	1	0	cu	cig only	40	999	1	39	st
KATSOU	507	f	0	0	all	-	all	Eu:bal	1987	CC	101	n	bl	n	n	0	0	cu	all/unsp	40	999	1	19	st
KHUDER	505	m	0	0	all	-	all	NAmer	1985	CC	482	n	bl	n	y	0	0	ev	cig+/-ot	50	999	1	29	st
KREUZE	514	m	55	69	all	-	all	Eu:Ger	1990	CC	2260	n	bl	n	n	3	0	ev	all/unsp	40	999	1	19	ot
KREUZE	516	f	55	69	all	-	all	Eu:Ger	1990	CC	2260	n	bl	n	n	3	0	ev	all/unsp	40	999	1	19	ot
LETOUR	510	c	0	0	all	-	all	NAmer	1983	CC	738	n	V	y	y	0	0	ev	cig+/-ot	41	999	1	24	st
LEVIN	508	m	0	0	all	-	all	NAmer	1938	CC	475	n	bl	n	n	1	0	ev	cig+/-ot	40	999	1	39	ot
LIAW	508	c	0	0	all	0	all	As:oth	1982	pr	127	n	ot	n	n	2	0	cu	all/unsp	31	999	1	20	ot
LIU3	512	m	0	0	all	-	all	As:Chi	1985	CC	110	n	ot	n	n	2	0	ev	all/unsp	35	999	1	34	ot
LIU5	506	c	0	0	all	-	all	As:Chi	1978	CC	111	n	ot	y	n	0	0	ev	all/unsp	30	999	1	29	st
LUBIN	507	m	0	0	all	-	all	As:Chi	1984	CC	427	m	ot	y	n	0	0	ev	cig+/-ot	50	999	1	29	st
LUBIN2	537	m	0	0	all	-	all	Eu:mul	1976	CC	7804	n	bl	n	y	0	0	ev	cig+/-ot	50	999	1	29	st
LUBIN2	580	f	0	0	all	-	all	Eu:mul	1976	CC	7804	n	bl	n	y	0	0	ev	cig+/-ot	50	999	1	29	st
MATOS	540	m	0	0	all	-	all	SCAmer	1994	CC	200	n	bl	n	n	2	0	ev	cig+/-ot	40	70	1	24	ot
MCCONN	509	c	0	0	all	-	all	Eu:UK	1946	CC	100	n	V	n	y	0	0	ev	all/unsp	40	999	1	9	st
NOTAN2	521	c	0	0	all	-	all	As:Ind	1963	CC	683	n	V	n	n	0	0	ev	cig only	41	999	1	10	st
OSANN2	506	f	0	0	all	-	all	NAmer	1964	ot	217	n	bl	n	y	1	0	ev	cig+/-ot	21	999	1	20	ot
PEZZO2	509	m	0	0	all	-	all	SCAmer	1992	CC	367	n	bl	n	y	0	0	cu	cig+/-ot	36	999	1	35	st
PEZZOT	540	m	0	0	all	-	all	SCAmer	1987	CC	215	n	bl	n	y	2	0	ev	cig only	41	999	1	30	ot
QIAO2	520	m	0	0	all	0	all	As:Chi	1992	pr	241	m	ot	n	n	1	0	ev	all/unsp	42	999	1	27	ot
RACHTA	520	f	0	0	all	-	all	Eu:est	1991	CC	118	n	bl	n	y	1	0	ev	cig+/-ot	41	999	1	20	ot
SOBUE	552	m	0	0	all	-	q+s+l+a	As:Jap	1986	CC	1376	n	bl	n	y	0	0	cu	cig+/-ot	50	999	1	29	st
TIZZAN	505	m	0	0	all	-	all	Eu:wst	1959	CC	1358	n	bl	n	n	0	0	ev	cig only	11	999	1	4	st
TIZZAN	535	f	0	0	all	-	all	Eu:wst	1959	CC	1358	n	bl	n	n	0	0	ev	all/unsp	11	999	1	10	st
WANG2	509	c	0	0	all	-	all	As:Chi	1980	CC	103	n	ot	n	n	0	0	ev	cig+/-ot	40	49	1	19	st
WATSON	503	m	0	0	all	-	all	NAmer	1950	CC	301	n	bl	n	y	0	0	cu	all/unsp	20	999	1	19	st
WATSON	506	f	0	0	all	-	all	NAmer	1950	CC	301	n	bl	n	y	0	0	cu	all/unsp	20	999	1	19	st
WUWILL	505	f	0	0	all	-	all	As:Chi	1985	CC	965	n	ot	n	n	0	0	ev	cig+/-ot	40	999	1	29	st
WYNDE8	502	m	0	0	all	-	all	NAmer	1985	CC	1044	n	bl	n	y	0	0	cu	cig+/-ot	41	999	1	30	st

Table 1I15 - 1

IESLC - Meta-analysis of Ever/current Smoking, Duration, "Highest vs lowest"
All LC types, Any Product (or Cigarettes if Any not available)
Most adjusted

REF	NRR	SEX	AGEL	AGEH	RACE	YF	LC TYPE	LOC	START	ST	NLC	R	VB	P	H	AD	ADOS	SM	PRODUCT	exL	exH	unexL	unexH	De
WYNDE8	504	f	0	0	all	-	all	NAmer	1985	CC	1044	n	bl	n	y	0	0	cu	cig+/-ot	41	999	1	30	st
ZHENG	556	m	0	0	all	-	all	As:Chi	1982	CC	540	n	ot	*	y	1	0	ev	cig+/-ot	30	999	1	29	ot
ZHENG	561	f	0	0	all	-	all	As:Chi	1982	CC	540	n	ot	*	y	1	0	ev	cig+/-ot	30	999	1	29	ot
ZHOU	503	c	0	0	all	-	all	As:Chi	1978	CC	1360	n	ot	n	n	0	0	ev	all/unsp	20	999	1	19	st

Cigarette type is all/unspec for all RRs

except for the following:

REF	NRR	CIGTYPE
JUSSAW	518	MC only
NOTAN2	521	MC only

Table 1I15 - 2

IESLC - Meta-analysis of Ever/current Smoking, Duration, "Highest vs lowest"
All LC types, Any Product (or Cigarettes if Any not available)
Most adjusted

REF	NRR	SEX	AD	Number Exposed Case	Cont	Non-exposed Case	Cont	RR	95.00%CI
AGUDO	512	f	3	18	-	5	-	3.95	(0.93- 16.76)
*AMANDU	508	m	2	72	-	42	-	1.19	(0.76- 1.86)
AMES	501	m	2	-	-	-	-	2.28	(1.58- 3.29)
ARMADA	510	m	1	77	-	21	-	10.31	(4.81- 22.07)
AUVINE	523	c	2	230	-	26	-	1.51	(0.51- 4.50)
AXELSS	509	m	0	101	40	13	84	16.32	(8.19- 32.51)
AXELSS	518	f	0	20	10	5	24	9.60	(2.82- 32.73)
Subtotal AXELSS								14.36	(7.88- 26.20)
BARBON	514	m	1	366	-	42	-	4.53	(3.02- 6.80)
*BEST	513	m	1	137	-	1	-	8.88	(1.25- 62.98)
*BOUCOT	520	m	0	52	1563	29	2621	3.01	(1.92- 4.72)
BUFFLE	530	f	0	90	42	52	57	2.35	(1.39- 3.97)
*CEDERL	503	m	1	23	-	5	-	4.11	(1.48- 11.44)
*CEDERL	506	f	1	5	-	3	-	6.00	(1.44- 24.93)
Subtotal CEDERL								4.67	(2.04- 10.73)
CHEN2	509	m	0	62	40	2	3	2.33	(0.37- 14.53)
CHEN2	516	f	0	21	15	1	6	8.40	(0.91- 77.21)
Subtotal CHEN2								3.92	(0.95- 16.08)
CHOI	509	m	0	20	20	19	55	2.89	(1.29- 6.51)
CHOI	516	f	0	1	1	2	9	4.50	(0.19- 106.82)
Subtotal CHOI								2.97	(1.36- 6.52)
*CPSI	602	m	0	232	26906	95	266163	24.16	(19.03- 30.66)
*CPSI	695	f	0	10	5657	105	694015	11.68	(6.11- 22.34)
Subtotal CPSI								22.15	(17.71- 27.71)
*CPSII	566	m	0	117	8450	72	141932	27.29	(20.37- 36.58)
*CPSII	632	f	0	18	2224	127	301244	19.20	(11.74- 31.40)
Subtotal CPSII								24.89	(19.36- 32.01)
DAMBER	514	m	1	-	-	-	-	7.08	(3.19- 15.74)
DEAN2	503	m	0	631	558	34	36	1.20	(0.74- 1.94)
DEAN2	506	f	0	47	11	10	14	5.98	(2.11- 16.99)
Subtotal DEAN2								1.59	(1.03- 2.46)
DESTEF	514	m	4	178	-	43	-	3.18	(1.74- 5.78)
DOLL	521	m	0	558	491	12	15	1.42	(0.66- 3.06)
DOLL	528	f	0	6	3	14	18	2.57	(0.54- 12.14)
Subtotal DOLL								1.60	(0.80- 3.18)
DORGAN	526	m	2	-	-	-	-	2.96	(2.31- 3.78)
DORGAN	522	f	3	-	-	-	-	2.76	(2.18- 3.50)
Subtotal DORGAN								2.85	(2.41- 3.39)
DOSEME	505	m	2	466	-	32	-	4.90	(2.91- 8.24)
FAN	505	m	0	143	241	29	135	2.76	(1.76- 4.34)
FAN	510	f	0	55	59	8	15	1.75	(0.69- 4.45)
Subtotal FAN								2.53	(1.69- 3.80)
GAO	566	f	2	168	-	68	-	2.43	(1.54- 3.83)
GER	522	c	5	49	-	10	-	1.65	(0.69- 3.91)
HAENSZ	554	f	1	58	-	16	-	1.21	(0.59- 2.47)
HU	505	m	0	19	14	41	33	1.09	(0.48- 2.50)
HU	510	f	0	4	3	11	8	0.97	(0.17- 5.59)
Subtotal HU								1.07	(0.51- 2.26)
HU2	514	c	0	194	113	21	33	2.70	(1.49- 4.89)
HUMBLE	525	c	0	29	22	20	33	2.18	(0.99- 4.77)
JOLY	523	m	0	250	253	11	48	4.31	(2.19- 8.49)
JOLY	509	f	0	57	20	13	28	6.14	(2.67- 14.11)
Subtotal JOLY								4.96	(2.94- 8.40)
JUSSAW	518	m	0	11	6	16	20	2.29	(0.70- 7.55)
*KAISE2	600	m	1	34	-	17	-	5.38	(2.95- 9.81)
*KAISE2	520	f	1	26	-	24	-	3.13	(1.63- 6.00)
Subtotal KAISE2								4.19	(2.70- 6.52)
KATSOU	507	f	0	17	4	5	5	4.25	(0.82- 22.13)
KHUDER	505	m	0	236	354	16	61	2.54	(1.43- 4.52)
KREUZE	514	m	3	-	-	-	-	11.12	(8.68- 14.25)
KREUZE	516	f	3	-	-	-	-	9.22	(4.45- 19.09)
Subtotal KREUZE								10.91	(8.62- 13.79)
LETOUR	510	c	0	374	141	65	187	7.63	(5.42- 10.75)
LEVIN	508	m	1	63	-	56	-	1.27	(0.80- 2.00)
*LIAW	508	c	2	-	-	-	-	5.22	(1.75- 15.56)
LIU3	512	m	2	22	-	30	-	1.60	(0.68- 3.77)
LIU5	506	c	0	58	33	27	37	2.41	(1.25- 4.64)
LUBIN	507	m	0	59	86	30	146	3.34	(2.00- 5.58)
LUBIN2	537	m	0	1325	1484	953	2995	2.81	(2.53- 3.11)
LUBIN2	580	f	0	81	32	132	230	4.41	(2.78- 7.00)
Subtotal LUBIN2								2.87	(2.59- 3.17)

Table 1I15 - 2

IESLC - Meta-analysis of Ever/current Smoking, Duration, "Highest vs lowest"
All LC types, Any Product (or Cigarettes if Any not available)
Most adjusted

REF	NRR	SEX	AD	Number Exposed Case	Number Exposed Cont	Non-exposed Case	Non-exposed Cont	RR	95.00%CI
MATOS	540	m	2	86	-	20	-	5.48	(2.97- 10.09)
MCCONN	509	c	0	16	40	3	4	0.53	(0.11- 2.66)
NOTAN2	521	c	0	5	5	7	15	2.14	(0.46- 9.90)
OSANN2	506	f	1	161	-	23	-	7.25	(3.41- 15.43)
PEZZO2	509	m	0	173	126	60	72	1.65	(1.09- 2.49)
PEZZOT	540	m	2	110	-	30	-	7.00	(4.33- 11.30)
*QIAO2	520	m	1	170	-	7	-	5.13	(2.36- 11.13)
RACHTA	520	f	1	24	-	12	-	29.05	(3.42- 246.78)
SOBUE	552	m	0	147	73	62	119	3.87	(2.55- 5.86)
TIZZAN	505	m	0	928	815	12	1	0.09	(0.01- 0.73)
TIZZAN	535	f	0	23	21	2	7	3.83	(0.72- 20.55)
Subtotal TIZZAN								0.86	(0.24- 3.15)
WANG2	509	c	0	22	26	4	17	3.60	(1.05- 12.28)
WATSON	503	m	0	252	231	8	20	2.73	(1.18- 6.31)
WATSON	506	f	0	10	13	5	20	3.08	(0.85- 11.07)
Subtotal WATSON								2.83	(1.40- 5.71)
WUWILL	505	f	0	223	114	137	139	1.98	(1.43- 2.75)
WYNDE8	502	m	0	294	354	102	272	2.21	(1.68- 2.92)
WYNDE8	504	f	0	206	122	82	134	2.76	(1.94- 3.93)
Subtotal WYNDE8								2.41	(1.94- 2.99)
ZHENG	556	m	1	242	-	37	-	3.73	(2.39- 5.83)
ZHENG	561	f	1	59	-	17	-	3.16	(1.39- 7.16)
Subtotal ZHENG								3.59	(2.43- 5.31)
ZHOU	503	c	0	678	36	170	12	1.33	(0.68- 2.61)
Partial Totals				10719	50872	3231	1411142		

*prospective study

REF	NRR	SEX	AD	Ys	Ws	Qs	Ps
AGUDO	512	f	3	1.37	1.84	0.00	0.0626
*AMANDU	508	m	2	0.17	19.18	26.02	0.4461
AMES	501	m	2	0.82	28.56	7.56	0.0000
ARMADA	510	m	1	2.33	6.62	6.55	0.0000
AUVINE	523	c	2	0.41	3.24	2.78	0.4581
AXELSS	509	m	0	2.79	8.08	17.07	0.0000
AXELSS	518	f	0	2.26	2.55	2.18	0.0003
Subtotal AXELSS				2.66	10.64	19.25	
BARBON	514	m	1	1.51	23.32	0.69	0.0000
*BEST	513	m	1	2.18	1.00	0.71	0.0290
*BOUCOT	520	m	0	1.10	18.98	1.07	0.0000
BUFFLE	530	f	0	0.85	13.95	3.28	0.0014
*CEDERL	503	m	1	1.41	3.67	0.02	0.0067
*CEDERL	506	f	1	1.79	1.89	0.39	0.0138
Subtotal CEDERL				1.54	5.56	0.41	
CHEN2	509	m	0	0.84	1.14	0.28	0.3669
CHEN2	516	f	0	2.13	0.78	0.49	0.0601
Subtotal CHEN2				1.36	1.92	0.77	
CHOI	509	m	0	1.06	5.85	0.45	0.0101
CHOI	516	f	0	1.50	0.38	0.01	0.3520
Subtotal CHOI				1.09	6.24	0.46	
*CPSI	602	m	0	3.18	67.59	230.32	0.0000
*CPSI	695	f	0	2.46	9.15	11.46	0.0000
Subtotal CPSI				3.10	76.73	241.78	
*CPSII	566	m	0	3.31	44.82	173.61	0.0000
*CPSII	632	f	0	2.95	15.88	41.48	0.0000
Subtotal CPSII				3.21	60.70	215.09	
DAMBER	514	m	1	1.96	6.03	2.31	0.0000
DEAN2	503	m	0	0.18	16.51	22.16	0.4643
DEAN2	506	f	0	1.79	3.53	0.71	0.0008
Subtotal DEAN2				0.46	20.04	22.87	
DESTEF	514	m	4	1.16	10.66	0.35	0.0002
DOLL	521	m	0	0.35	6.50	6.34	0.3707
DOLL	528	f	0	0.94	1.59	0.25	0.2330
Subtotal DOLL				0.47	8.10	6.59	
DORGAN	526	m	2	1.09	63.36	4.07	0.0000
DORGAN	522	f	3	1.02	68.55	7.17	0.0000
Subtotal DORGAN				1.05	131.91	11.24	
DOSEME	505	m	2	1.59	14.18	0.89	0.0000
FAN	505	m	0	1.02	18.86	1.96	0.0000
FAN	510	f	0	0.56	4.41	2.68	0.2410
Subtotal FAN				0.93	23.27	4.65	

Table 1I15 - 2

IESLC - Meta-analysis of Ever/current Smoking, Duration, "Highest vs lowest"
All LC types, Any Product (or Cigarettes if Any not available)
Most adjusted

REF	NRR	SEX	AD	Ys	Ws	Qs	Ps
GAO	566	f	2	0.89	18.51	3.76	0.0001
GER	522	c	5	0.50	5.11	3.58	0.2578
HAENSZ	554	f	1	0.19	7.49	9.88	0.6018
HU	505	m	0	0.09	5.59	8.75	0.8345
HU	510	f	0	-0.03	1.25	2.35	0.9725
Subtotal HU				0.07	6.85	11.09	
HU2	514	c	0	0.99	10.88	1.30	0.0011
HUMBLE	525	c	0	0.78	6.24	1.97	0.0522
JOLY	523	m	0	1.46	8.35	0.13	0.0000
JOLY	509	f	0	1.81	5.55	1.26	0.0000
Subtotal JOLY				1.60	13.90	1.38	
JUSSAW	518	m	0	0.83	2.70	0.70	0.1728
*KAISE2	600	m	1	1.68	10.64	1.26	0.0000
*KAISE2	520	f	1	1.14	9.05	0.35	0.0006
Subtotal KAISE2				1.43	19.69	1.61	
KATSOU	507	f	0	1.45	1.41	0.02	0.0857
KHUDER	505	m	0	0.93	11.63	1.92	0.0015
KREUZE	514	m	3	2.41	62.53	71.60	0.0000
KREUZE	516	f	3	2.22	7.25	5.65	0.0000
Subtotal KREUZE				2.39	69.77	77.25	
LETOUR	510	c	0	2.03	32.79	15.77	0.0000
LEVIN	508	m	1	0.24	18.30	22.13	0.3065
*LIAW	508	c	2	1.65	3.22	0.32	0.0030
LIU3	512	m	2	0.47	5.24	3.95	0.2821
LIU5	506	c	0	0.88	8.96	1.89	0.0085
LUBIN	507	m	0	1.21	14.54	0.26	0.0000
LUBIN2	537	m	0	1.03	355.65	33.49	0.0000
LUBIN2	580	f	0	1.48	18.01	0.38	0.0000
Subtotal LUBIN2				1.05	373.66	33.87	
MATOS	540	m	2	1.70	10.27	1.35	0.0000
MCCONN	509	c	0	-0.63	1.49	5.77	0.4428
NOTAN2	521	c	0	0.76	1.64	0.55	0.3290
OSANN2	506	f	1	1.98	6.74	2.78	0.0000
PEZZO2	509	m	0	0.50	22.59	15.91	0.0176
PEZZOT	540	m	2	1.95	16.70	6.16	0.0000
*QIAO2	520	m	1	1.64	6.39	0.56	0.0000
RACHTA	520	f	1	3.37	0.84	3.46	0.0020
SOBUE	552	m	0	1.35	22.21	0.00	0.0000
TIZZAN	505	m	0	-2.36	0.92	12.57	0.0238
TIZZAN	535	f	0	1.34	1.36	0.00	0.1168
Subtotal TIZZAN				-0.15	2.28	12.57	
WANG2	509	c	0	1.28	2.55	0.01	0.0411
WATSON	503	m	0	1.00	5.46	0.61	0.0191
WATSON	506	f	0	1.12	2.34	0.11	0.0854
Subtotal WATSON				1.04	7.80	0.72	
WUWILL	505	f	0	0.69	36.04	15.37	0.0000
WYNDE8	502	m	0	0.80	50.74	14.99	0.0000
WYNDE8	504	f	0	1.01	30.57	3.20	0.0000
Subtotal WYNDE8				0.88	81.32	18.19	
ZHENG	556	m	1	1.32	19.32	0.01	0.0000
ZHENG	561	f	1	1.15	5.72	0.20	0.0059
Subtotal ZHENG				1.28	25.04	0.21	
ZHOU	503	c	0	0.28	8.44	9.38	0.4081

Table 1I15 - 2

IESLC - Meta-analysis of Ever/current Smoking, Duration, "Highest vs lowest"
All LC types, Any Product (or Cigarettes if Any not available)
Most adjusted

	N	76
	NS	57
	Wt	1375.90
Het	Chi	860.96
Het	df	75
Het	P	***
Fixed	RR	3.81
	RRl	3.62
	RRu	4.02
	P	+++
Random	RR	3.56
	RRl	2.90
	RRu	4.35
	P	+++
Asymm	P	N.S.

Table 1I15 - 3

IESLC - Meta-analysis of Ever/current Smoking, Duration, "Highest vs lowest"
All LC types, Any Product (or Cigarettes if Any not available)
Most adjusted

	Sex combined	male	female	Total
N	11	38	27	76
NS	11	38	27	76
Wt	84.55	1014.71	276.64	1375.90
Het Chi	42.59	697.43	115.79	860.96
Het df	10	37	26	75
Het P	***	***	***	***
Fixed RR	3.48	3.96	3.43	3.81
RRl	2.81	3.72	3.05	3.62
RRu	4.30	4.21	3.86	4.02
P	+++	+++	+++	+++
Random RR	2.43	3.57	4.08	3.56
RRl	1.48	2.65	3.04	2.90
RRu	4.01	4.82	5.48	4.35
P	+++	+++	+++	+++
Between Chi				5.15
Between df				2
Between P				(*)
Btwn(F) P				N.S.
Btwn(R) P				N.S.

	Lung cancer type all	other	Total
N	73	3	76
NS	54	3	57
Wt	1339.96	35.94	1375.90
Het Chi	849.00	7.97	860.96
Het df	72	2	75
Het P	***	*	***
Fixed RR	3.85	2.75	3.81
RRl	3.65	1.98	3.62
RRu	4.06	3.81	4.02
P	+++	+++	+++
Random RR	3.63	2.27	3.56
RRl	2.95	1.10	2.90
RRu	4.47	4.69	4.35
P	+++	+	+++
Between Chi			3.99
Between df			1
Between P			*
Btwn(F) P			N.S.
Btwn(R) P			N.S.

	Location								
	NAmer	UK	Scand	othEur	China	Japan	othAs	other	Total
N	23	5	6	12	17	1	6	6	76
NS	17	3	4	9	13	1	5	5	57
Wt	543.02	29.62	25.47	493.93	168.62	22.21	18.91	74.13	1375.90
Het Chi	557.20	9.66	14.74	134.44	21.52	0.00	2.91	25.00	860.96
Het df	22	4	5	11	16	0	5	5	75
Het P	***	*	*	***	N.S.	N.S.	N.S.	***	***
Fixed RR	4.72	1.51	7.14	3.66	2.47	3.87	2.61	3.64	3.81
RRl	4.34	1.05	4.84	3.35	2.13	2.55	1.67	2.90	3.62
RRu	5.13	2.16	10.52	4.00	2.88	5.86	4.10	4.58	4.02
P	+++	+	+++	+++	+++	+++	+++	+++	+++
Random RR	4.01	1.71	6.15	4.98	2.48	3.87	2.61	4.08	3.56
RRl	2.57	0.88	3.06	3.01	2.05	2.55	1.67	2.41	2.90
RRu	6.24	3.32	12.35	8.22	2.99	5.86	4.10	6.90	4.35
P	+++	N.S.	+++	+++	+++	+++	+++	+++	+++
Between Chi									95.48
Between df									7
Between P									***
Btwn(F) P									N.S.
Btwn(R) P									**

Table 1I15 - 3

IESLC - Meta-analysis of Ever/current Smoking, Duration, "Highest vs lowest"
All LC types, Any Product (or Cigarettes if Any not available)
Most adjusted

Detailed Country in "other Europe"

	multi	Germany	othWest	East	Balkans	Total
N	2	2	5	1	2	12
NS	1	1	4	1	2	9
Wt	373.66	69.77	34.07	0.84	15.59	493.93
Het Chi	3.51	0.23	18.26	0.00	0.03	134.44
Het df	1	1	4	0	1	11
Het P	(*)	N.S.	**	N.S.	N.S.	***
Fixed RR	2.87	10.91	4.72	29.05	4.84	3.66
RRl	2.59	8.62	3.37	3.42	2.94	3.35
RRu	3.17	13.79	6.60	246.77	7.95	4.00
P	+++	+++	+++	++	+++	+++
Random RR	3.32	10.91	3.20	29.05	4.84	4.98
RRl	2.16	8.62	1.19	3.42	2.94	3.01
RRu	5.09	13.79	8.57	246.77	7.95	8.22
P	+++	+++	+	++	+++	+++
Between Chi						112.42
Between df						4
Between P						***
Btwn(F) P						**
Btwn(R) P						***

Detailed Country in "other Asia"

	India	HongKong	other	Total
N	2		4	6
NS	2		3	5
Wt	4.34		14.56	18.91
Het Chi	0.00		2.76	2.91
Het df	1		3	5
Het P	N.S.		N.S.	N.S.
Fixed RR	2.23		2.74	2.61
RRl	0.87		1.64	1.67
RRu	5.72		4.58	4.10
P	(+)		+++	+++
Random RR	2.23		2.74	2.61
RRl	0.87		1.64	1.67
RRu	5.72		4.58	4.10
P	(+)		+++	+++
Between Chi				0.14
Between df				1
Between P				N.S.
Btwn(F) P				N.S.
Btwn(R) P				N.S.

Detailed other continent

	SCAmer	Total
N	6	6
NS	5	5
Wt	74.13	74.13
Het Chi	25.00	25.00
Het df	5	5
Het P	***	***
Fixed RR	3.64	3.64
RRl	2.90	2.90
RRu	4.58	4.58
P	+++	+++
Random RR	4.08	4.08
RRl	2.41	2.41
RRu	6.90	6.90
P	+++	+++
Between Chi		
Between df		
Between P		N.S.
Btwn(F) P		N.S.
Btwn(R) P		N.S.

Table 1I15 - 3

IESLC - Meta-analysis of Ever/current Smoking, Duration, "Highest vs lowest"
All LC types, Any Product (or Cigarettes if Any not available)
Most adjusted

	Start year of study					
	<1960	1960-69	1970-79	1980-89	1990+	Total
N	15	7	13	32	9	76
NS	11	5	10	24	7	57
Wt	189.92	36.69	493.02	518.05	138.23	1375.90
Het Chi	321.71	21.66	30.61	333.76	90.18	860.96
Het df	14	6	12	31	8	75
Het P	***	**	**	***	***	***
Fixed RR	4.96	2.58	3.03	4.04	5.39	3.81
RRl	4.30	1.86	2.78	3.71	4.56	3.62
RRu	5.72	3.56	3.31	4.40	6.37	4.02
P	+++	+++	+++	+++	+++	+++
Random RR	2.38	3.40	3.52	3.93	4.25	3.56
RRl	1.11	1.70	2.83	2.87	2.23	2.90
RRu	5.13	6.78	4.37	5.38	8.11	4.35
P	+	+++	+++	+++	+++	+++
Between Chi						63.04
Between df						4
Between P						***
Btwn(F) P						N.S.
Btwn(R) P						N.S.

	Study type (1)		
	CC	other	Total
N	61	15	76
NS	46	11	57
Wt	1129.14	246.76	1375.90
Het Chi	320.79	304.58	860.96
Het df	60	14	75
Het P	***	***	***
Fixed RR	3.14	9.24	3.81
RRl	2.96	8.16	3.62
RRu	3.33	10.47	4.02
P	+++	+++	+++
Random RR	3.10	6.25	3.56
RRl	2.63	3.34	2.90
RRu	3.65	11.73	4.35
P	+++	+++	+++
Between Chi			235.59
Between df			1
Between P			***
Btwn(F) P			***
Btwn(R) P			*

	Study type (2)			
	CC	prosp	other	Total
N	61	13	2	76
NS	46	9	2	57
Wt	1129.14	211.45	35.31	1375.90
Het Chi	320.79	240.04	7.30	860.96
Het df	60	12	1	75
Het P	***	***	**	***
Fixed RR	3.14	11.25	2.84	3.81
RRl	2.96	9.83	2.04	3.62
RRu	3.33	12.88	3.95	4.02
P	+++	+++	+++	+++
Random RR	3.10	6.74	3.87	3.56
RRl	2.63	3.47	1.25	2.90
RRu	3.65	13.08	11.98	4.35
P	+++	+++	+	+++
Between Chi				292.83
Between df				2
Between P				***
Btwn(F) P				***
Btwn(R) P				(*)

Table 1I15 - 3

IESLC - Meta-analysis of Ever/current Smoking, Duration, "Highest vs lowest"
All LC types, Any Product (or Cigarettes if Any not available)
Most adjusted

	Study size (number of LC cases) 100-249	250-499	500-999	1000+	Total
N	20	20	16	20	76
NS	18	14	13	12	57
Wt	125.18	187.10	195.81	867.81	1375.90
Het Chi	67.68	68.45	65.25	615.06	860.96
Het df	19	19	15	19	75
Het P	***	***	***	***	***
Fixed RR	2.84	2.90	3.31	4.36	3.81
RRl	2.38	2.51	2.88	4.08	3.62
RRu	3.38	3.35	3.81	4.66	4.02
P	+++	+++	+++	+++	+++
Random RR	2.86	3.45	3.24	4.44	3.56
RRl	1.98	2.56	2.36	2.88	2.90
RRu	4.13	4.64	4.45	6.85	4.35
P	+++	+++	+++	+++	+++
Between Chi					44.52
Between df					3
Between P					***
Btwn(F) P					N.S.
Btwn(R) P					N.S.

	Risky occupational population no	mining	othRisky	Total
N	72	4		76
NS	53	4		57
Wt	1307.22	68.68		1375.90
Het Chi	825.51	14.38		860.96
Het df	71	3		75
Het P	***	**		***
Fixed RR	3.92	2.22		3.81
RRl	3.72	1.75		3.62
RRu	4.14	2.82		4.02
P	+++	+++		+++
Random RR	3.63	2.47		3.56
RRl	2.94	1.43		2.90
RRu	4.48	4.27		4.35
P	+++	++		+++
Between Chi				21.07
Between df				1
Between P				***
Btwn(F) P				N.S.
Btwn(R) P				N.S.

	National cigarette tobacco type Virginia	blended	other	Total
N	9	48	19	76
NS	7	35	15	57
Wt	67.75	1131.20	176.95	1375.90
Het Chi	52.39	744.86	24.16	860.96
Het df	8	47	18	75
Het P	***	***	N.S.	***
Fixed RR	3.48	4.10	2.48	3.81
RRl	2.74	3.87	2.14	3.62
RRu	4.41	4.35	2.87	4.02
P	+++	+++	+++	+++
Random RR	2.56	4.27	2.48	3.56
RRl	1.21	3.28	2.07	2.90
RRu	5.39	5.56	2.99	4.35
P	+	+++	+++	+++
Between Chi				39.56
Between df				2
Between P				***
Btwn(F) P				N.S.
Btwn(R) P				**

Table 1I15 - 3

IESLC - Meta-analysis of Ever/current Smoking, Duration, "Highest vs lowest"
All LC types, Any Product (or Cigarettes if Any not available)
Most adjusted

	Any proxy use No/nk	Yes	Total
N	58	18	76
NS	43	14	57
Wt	1073.70	302.20	1375.90
Het Chi	784.00	60.60	860.96
Het df	57	17	75
Het P	***	***	***
Fixed RR	4.04	3.11	3.81
RRl	3.81	2.77	3.62
RRu	4.29	3.48	4.02
P	+++	+++	+++
Random RR	3.77	2.96	3.56
RRl	2.92	2.31	2.90
RRu	4.86	3.78	4.35
P	+++	+++	+++
Between Chi			16.36
Between df			1
Between P			***
Btwn(F) P			N.S.
Btwn(R) P			N.S.

	Full histological confirmation No	Yes	Total
N	52	24	76
NS	39	18	57
Wt	586.24	789.66	1375.90
Het Chi	659.83	102.57	860.96
Het df	51	23	75
Het P	***	***	***
Fixed RR	5.20	3.03	3.81
RRl	4.80	2.82	3.62
RRu	5.64	3.25	4.02
P	+++	+++	+++
Random RR	3.71	3.25	3.56
RRl	2.71	2.70	2.90
RRu	5.08	3.93	4.35
P	+++	+++	+++
Between Chi			98.56
Between df			1
Between P			***
Btwn(F) P			**
Btwn(R) P			N.S.

	Number of adjustment variables (1) 0	1	2+/+nk	Total
N	45	15	16	76
NS	31	12	14	57
Wt	910.47	127.04	338.40	1375.90
Het Chi	664.33	51.17	145.12	860.96
Het df	44	14	15	75
Het P	***	***	***	***
Fixed RR	3.85	3.69	3.75	3.81
RRl	3.61	3.10	3.37	3.62
RRu	4.11	4.39	4.17	4.02
P	+++	+++	+++	+++
Random RR	3.34	4.22	3.42	3.56
RRl	2.48	2.94	2.37	2.90
RRu	4.50	6.06	4.92	4.35
P	+++	+++	+++	+++
Between Chi				0.33
Between df				2
Between P				N.S.
Btwn(F) P				N.S.
Btwn(R) P				N.S.

Table 1I15 - 3

IESLC - Meta-analysis of Ever/current Smoking, Duration, "Highest vs lowest"
All LC types, Any Product (or Cigarettes if Any not available)
Most adjusted

	Number of adjustment variables (2) 0	1	2	3-5	6+/+nk	Total
N	45	15	10	6		76
NS	31	12	10	5		58
Wt	910.47	127.04	182.47	155.93		1375.90
Het Chi	664.33	51.17	42.85	75.38		860.96
Het df	44	14	9	5		75
Het P	***	***	***	***		***
Fixed RR	3.85	3.69	2.89	5.09		3.81
RRl	3.61	3.10	2.50	4.35		3.62
RRu	4.11	4.39	3.34	5.95		4.02
P	+++	+++	+++	+++		+++
Random RR	3.34	4.22	2.96	4.36		3.56
RRl	2.48	2.94	2.08	2.09		2.90
RRu	4.50	6.06	4.20	9.09		4.35
P	+++	+++	+++	+++		+++
Between Chi						27.22
Between df						3
Between P						***
Btwn(F) P						N.S.
Btwn(R) P						N.S.

	Smoking status ever	current	Total
N	54	22	76
NS	42	15	57
Wt	999.65	376.25	1375.90
Het Chi	288.90	503.05	860.96
Het df	53	21	75
Het P	***	***	***
Fixed RR	3.32	5.49	3.81
RRl	3.12	4.97	3.62
RRu	3.54	6.08	4.02
P	+++	+++	+++
Random RR	3.31	4.38	3.56
RRl	2.77	2.59	2.90
RRu	3.96	7.43	4.35
P	+++	+++	+++
Between Chi			69.01
Between df			1
Between P			***
Btwn(F) P			*
Btwn(R) P			N.S.

	Product all/unsp	cig+/-ot	cig only	Total
N	25	38	13	76
NS	19	30	10	59
Wt	228.46	975.84	171.61	1375.90
Het Chi	159.90	188.96	125.68	860.96
Het df	24	37	12	75
Het P	***	***	***	***
Fixed RR	4.35	2.92	14.70	3.81
RRl	3.82	2.74	12.66	3.62
RRu	4.95	3.11	17.07	4.02
P	+++	+++	+++	+++
Random RR	3.50	3.07	5.65	3.56
RRl	2.40	2.59	3.14	2.90
RRu	5.10	3.64	10.15	4.35
P	+++	+++	+++	+++
Between Chi				386.42
Between df				2
Between P				***
Btwn(F) P				***
Btwn(R) P				N.S.

Table 1I15 - 3

IESLC - Meta-analysis of Ever/current Smoking, Duration, "Highest vs lowest"
All LC types, Any Product (or Cigarettes if Any not available)
Most adjusted

	Derivation of RR/CI			
	Orig	StdCalc	Other	Total
N	1	47	28	76
NS	1	32	24	57
Wt	28.56	930.16	417.18	1375.90
Het Chi	0.00	665.91	187.33	860.96
Het df	0	46	27	75
Het P	N.S.	***	***	***
Fixed RR	2.28	3.86	3.84	3.81
RRl	1.58	3.62	3.49	3.62
RRu	3.29	4.12	4.23	4.02
P	+++	+++	+++	+++
Random RR	2.28	3.37	3.84	3.56
RRl	1.58	2.53	2.90	2.90
RRu	3.29	4.50	5.09	4.35
P	+++	+++	+++	+++
Between Chi				7.73
Between df				2
Between P				*
Btwn(F) P				N.S.
Btwn(R) P				(*)

Table 1I15 - 4

IESLC - Meta-analysis of Ever/current Smoking, Duration, "Highest vs lowest"
All LC types, Any Product (or Cigarettes if Any not available)
Least adjusted

REF	NRR	X	SEX	AGEL	AGEH	RACE	YF	LC TYPE	LOC	START	ST	NLC	R	VB	P	H	AD	ADOS	SM	PRODUCT	exL	exH	unexL	unexH	De
AGUDO	509	x	f	0	0	all	-	all	Eu:wst	1989	CC	103	n	bl	n	n	0	0	ev	cig only	17	999	1	16	st
AMANDU	503	x	m	0	0	wh	0	all	NAmer	1959	pr	132	m	bl	n	n	0	0	cu	cig+/-ot	25	999	1	24	st
AMES	501		m	0	0	wh	-	all	NAmer	1959	ot	317	m	bl	n	n	2	0	cu	all/unsp	30	999	1	29	or
ARMADA	505	x	m	0	0	all	-	all	Eu:wst	1986	CC	325	n	bl	n	y	0	0	ev	cig+/-ot	50	999	1	24	st
AUVINE	505	x	c	0	0	all	-	all	Eu:Sca	1986	CC	517	n	bl	y	n	0	0	ev	cig+/-ot	41	999	1	20	st
AXELSS	509		m	0	0	sca	-	all	Eu:Sca	1989	CC	436	n	bl	n	n	0	0	ev	all/unsp	50	999	1	19	st
AXELSS	518		f	0	0	sca	-	all	Eu:Sca	1989	CC	436	n	bl	n	n	0	0	ev	all/unsp	50	999	1	19	st
BARBON	507	x	m	0	0	all	-	all	Eu:wst	1979	CC	755	n	bl	y	y	0	0	ev	all/unsp	50	999	1	29	st
BEST	513		m	0	0	all	0	all	NAmer	1955	pr	381	n	V	n	n	1	0	cu	cig only	40	999	1	4	ot
BOUCOT	520		m	0	0	all	9	all	NAmer	1951	pr	121	n	bl	n	n	0	0	ev	cig+/-ot	40	999	1	39	st
BUFFLE	530		f	0	0	w-hi	-	all	NAmer	1976	CC	943	n	bl	y	n	0	0	ev	cig+/-ot	41	999	1	30	st
CEDERL	503		m	40	69	all	10	all	Eu:Sca	1963	pr	491	n	bl	n	n	1	0	cu	cig only	30	999	1	29	ot
CEDERL	506		f	40	69	all	10	all	Eu:Sca	1963	pr	491	n	bl	n	n	1	0	cu	cig only	30	999	1	29	ot
CHEN2	509		m	0	0	all	-	all	As:Chi	1983	CC	193	n	ot	y	n	0	0	ev	all/unsp	41	999	1	9	st
CHEN2	516		f	0	0	all	-	all	As:Chi	1983	CC	193	n	ot	y	n	0	0	ev	all/unsp	41	999	1	20	st
CHOI	509		m	0	0	all	-	all	As:oth	1985	CC	375	n	bl	n	n	0	0	ev	cig+/-ot	50	999	1	19	st
CHOI	516		f	0	0	all	-	all	As:oth	1985	CC	375	n	bl	n	n	0	0	ev	cig+/-ot	40	999	1	19	st
CPSI	602		m	40	84	wh	0	all	NAmer	1959	pr	5138	n	bl	n	n	0	0	cu	cig only	60	999	1	29	st
CPSI	695		f	40	84	wh	0	all	NAmer	1959	pr	5138	n	bl	n	n	0	0	cu	cig only	55	999	1	29	st
CPSII	566		m	0	0	all	6	all	NAmer	1982	pr	3229	n	bl	n	n	0	0	cu	cig only	60	999	1	29	st
CPSII	632		f	0	0	all	6	all	NAmer	1982	pr	3229	n	bl	n	n	0	0	cu	cig+/-ot	60	999	1	29	st
DAMBER	514		m	0	0	all	-	all	Eu:Sca	1972	CC	579	n	bl	y	n	1	0	ev	all/unsp	51	999	1	20	ot
DEAN2	503		m	0	0	all	-	all	Eu:UK	1960	CC	954	n	V	y	n	0	0	cu	all/unsp	20	999	1	19	st
DEAN2	506		f	0	0	all	-	all	Eu:UK	1960	CC	954	n	V	y	n	0	0	cu	all/unsp	20	999	1	19	st
DESTEF	507	x	m	0	0	all	-	all	SCAmer	1988	CC	497	n	bl	n	y	0	0	ev	all/unsp	50	999	1	29	st
DOLL	521		m	0	0	all	-	all	Eu:UK	1948	CC	1465	n	V	n	n	0	0	ev	all/unsp	40	999	1	9	st
DOLL	528		f	0	0	all	-	all	Eu:UK	1948	CC	1465	n	V	n	n	0	0	ev	all/unsp	40	999	1	9	st
DORGAN	526		m	0	0	wh	-	all	NAmer	1980	CC	2026	n	bl	y	y	2	0	ev	cig+/-ot	35	999	1	34	ot
DORGAN	522		f	0	0	all	-	all	NAmer	1980	CC	2026	n	bl	y	y	3	0	ev	cig+/-ot	35	999	1	34	ot
DOSEME	505		m	0	0	all	-	all	Eu:bal	1979	CC	1210	n	bl	n	n	2	0	ev	cig+/-ot	21	999	1	10	ot
FAN	505		m	0	0	all	-	all	As:Chi	1990	CC	403	n	ot	y	n	0	0	ev	cig+/-ot	40	999	1	29	st
FAN	510		f	0	0	all	-	all	As:Chi	1990	CC	403	n	ot	y	n	0	0	ev	cig+/-ot	40	999	1	29	st
GAO	563	x	f	0	0	all	-	all	As:Chi	1984	CC	1405	n	ot	n	n	0	0	ev	cig+/-ot	30	999	1	29	st
GER	517	x	c	0	0	all	-	all	As:oth	1990	CC	141	n	ot	y	n	0	0	ev	all/unsp	41	999	1	20	st
HAENSZ	546	x	f	0	0	all	-	not alv	NAmer	1955	CC	158	n	bl	n	y	0	0	ev	cig+/-ot	15	999	1	14	st
HU	505		m	0	0	all	-	all	As:Chi	1985	CC	227	n	ot	n	y	0	0	ev	cig+/-ot	30	999	1	19	st
HU	510		f	0	0	all	-	all	As:Chi	1985	CC	227	n	ot	n	y	0	0	ev	cig+/-ot	30	999	1	19	st
HU2	514		c	0	0	all	-	all	As:Chi	1977	CC	523	n	ot	y	n	0	0	ev	cig+/-ot	40	999	1	19	st
HUMBLE	525		c	0	0	wh	-	not alv	NAmer	1980	CC	521	n	bl	y	n	0	0	cu	cig+/-ot	60	999	1	29	st
JOLY	523		m	0	0	all	-	all	SCAmer	1978	CC	826	n	bl	n	n	0	0	ev	cig+/-ot	50	999	1	19	st
JOLY	509		f	0	0	all	-	all	SCAmer	1978	CC	826	n	bl	n	n	0	0	ev	cig+/-ot	50	999	1	19	st
JUSSAW	518		m	0	0	all	-	all	As:Ind	1964	CC	792	n	V	n	n	0	0	ev	cig only	40	999	1	9	st
KAISE2	600		m	0	0	all	9	all	NAmer	1979	pr	318	n	bl	n	n	1	0	cu	cig only	40	999	1	39	st
KAISE2	520		f	0	0	all	9	all	NAmer	1979	pr	318	n	bl	n	n	1	0	cu	cig only	40	999	1	39	st
KATSOU	507		f	0	0	all	-	all	Eu:bal	1987	CC	101	n	bl	n	n	0	0	cu	all/unsp	40	999	1	19	st
KHUDER	505		m	0	0	all	-	all	NAmer	1985	CC	482	n	bl	n	y	0	0	ev	cig+/-ot	50	999	1	29	st
KREUZE	514		m	55	69	all	-	all	Eu:Ger	1990	CC	2260	n	bl	n	n	3	0	ev	all/unsp	40	999	1	19	ot
KREUZE	516		f	55	69	all	-	all	Eu:Ger	1990	CC	2260	n	bl	n	n	3	0	ev	all/unsp	40	999	1	19	ot
LETOUR	510		c	0	0	all	-	all	NAmer	1983	CC	738	n	V	y	y	0	0	ev	cig+/-ot	41	999	1	24	st
LEVIN	503	x	m	0	0	all	-	all	NAmer	1938	CC	475	n	bl	n	n	0	0	ev	cig+/-ot	40	999	1	39	st
LIAW	508		c	0	0	all	0	all	As:oth	1982	pr	127	n	ot	n	n	2	0	cu	all/unsp	31	999	1	20	ot
LIU3	509	x	m	0	0	all	-	all	As:Chi	1985	CC	110	n	ot	n	n	0	0	ev	all/unsp	35	999	1	34	st
LIU5	506		c	0	0	all	-	all	As:Chi	1978	CC	111	n	ot	y	n	0	0	ev	all/unsp	30	999	1	29	st
LUBIN	507		m	0	0	all	-	all	As:Chi	1984	CC	427	m	ot	y	n	0	0	ev	cig+/-ot	50	999	1	29	st
LUBIN2	537		m	0	0	all	-	all	Eu:mul	1976	CC	7804	n	bl	n	y	0	0	ev	cig+/-ot	50	999	1	29	st
LUBIN2	580		f	0	0	all	-	all	Eu:mul	1976	CC	7804	n	bl	n	y	0	0	ev	cig+/-ot	50	999	1	29	st
MATOS	520	x	m	0	0	all	-	all	SCAmer	1994	CC	200	n	bl	n	n	0	0	ev	cig+/-ot	40	70	1	24	st
MCCONN	509		c	0	0	all	-	all	Eu:UK	1946	CC	100	n	V	n	y	0	0	ev	all/unsp	40	999	1	9	st
NOTAN2	521		c	0	0	all	-	all	As:Ind	1963	CC	683	n	V	n	n	0	0	ev	cig only	41	999	1	10	st
OSANN2	503	x	f	0	0	all	-	all	NAmer	1964	ot	217	n	bl	n	y	0	0	ev	cig+/-ot	21	999	1	20	st
PEZZO2	509		m	0	0	all	-	all	SCAmer	1992	CC	367	n	bl	n	y	0	0	cu	cig+/-ot	36	999	1	35	st
PEZZOT	538	x	m	0	0	all	-	all	SCAmer	1987	CC	215	n	bl	n	y	0	0	ev	cig only	41	999	1	30	st
QIAO2	515	x	m	0	0	all	0	all	As:Chi	1992	pr	241	m	ot	n	n	0	0	ev	all/unsp	42	999	1	27	st
RACHTA	515	x	f	0	0	all	-	all	Eu:est	1991	CC	118	n	bl	n	y	0	0	ev	cig+/-ot	41	999	1	20	st
SOBUE	552		m	0	0	all	-	q+s+l+a	As:Jap	1986	CC	1376	n	bl	n	y	0	0	cu	cig+/-ot	50	999	1	29	st
TIZZAN	505		m	0	0	all	-	all	Eu:wst	1959	CC	1358	n	bl	n	n	0	0	ev	cig only	11	999	1	4	st
TIZZAN	535		f	0	0	all	-	all	Eu:wst	1959	CC	1358	n	bl	n	n	0	0	ev	all/unsp	11	999	1	10	st
WANG2	509		c	0	0	all	-	all	As:Chi	1980	CC	103	n	ot	n	n	0	0	ev	cig+/-ot	40	49	1	19	st
WATSON	503		m	0	0	all	-	all	NAmer	1950	CC	301	n	bl	n	y	0	0	cu	all/unsp	20	999	1	19	st
WATSON	506		f	0	0	all	-	all	NAmer	1950	CC	301	n	bl	n	y	0	0	cu	all/unsp	20	999	1	19	st
WUWILL	505		f	0	0	all	-	all	As:Chi	1985	CC	965	n	ot	n	n	0	0	ev	cig+/-ot	40	999	1	29	st
WYNDE8	502		m	0	0	all	-	all	NAmer	1985	CC	1044	n	bl	n	y	0	0	cu	cig+/-ot	41	999	1	30	st

Table 1I15 - 4

IESLC - Meta-analysis of Ever/current Smoking, Duration, "Highest vs lowest"
All LC types, Any Product (or Cigarettes if Any not available)
Least adjusted

REF	NRR	X	SEX	AGEL	AGEH	RACE	YF	LC TYPE	LOC	START	ST	NLC	R	VB	P	H	AD	ADOS	SM	PRODUCT	exL	exH	unexL	unexH	De
WYNDE8	504		f	0	0	all	-	all	NAmer	1985	CC	1044	n	bl	n	y	0	0	cu	cig+/-ot	41	999	1	30	st
ZHENG	555	x	m	0	0	all	-	all	As:Chi	1982	CC	540	n	ot	*	y	0	0	ev	cig+/-ot	30	999	1	29	st
ZHENG	560	x	f	0	0	all	-	all	As:Chi	1982	CC	540	n	ot	*	y	0	0	ev	cig+/-ot	30	999	1	29	st
ZHOU	503		c	0	0	all	-	all	As:Chi	1978	CC	1360	n	ot	n	n	0	0	ev	all/unsp	20	999	1	19	st

Cigarette type is all/unspec for all RRs

except for the following:

REF	NRR	CIGTYPE
JUSSAW	518	MC only
NOTAN2	521	MC only

Table 1I15 - 5

IESLC - Meta-analysis of Ever/current Smoking, Duration, "Highest vs lowest"
All LC types, Any Product (or Cigarettes if Any not available)
Least adjusted

REF	NRR	SEX	AD	Number Exposed Case	Number Exposed Cont	Non-exposed Case	Non-exposed Cont	RR	95.00%CI
AGUDO	509	f	0	18	11	5	12	3.93	(1.09- 14.19)
*AMANDU	503	m	0	72	27096	42	68909	4.36	(2.98- 6.38)
AMES	501	m	2	-	-	-	-	2.28	(1.58- 3.29)
ARMADA	505	m	0	77	33	21	55	6.11	(3.20- 11.68)
AUVINE	505	c	0	230	57	26	18	2.79	(1.43- 5.44)
AXELSS	509	m	0	101	40	13	84	16.32	(8.19- 32.51)
AXELSS	518	f	0	20	10	5	24	9.60	(2.82- 32.73)
Subtotal AXELSS								14.36	(7.88- 26.20)
BARBON	507	m	0	366	235	42	91	3.37	(2.26- 5.04)
*BEST	513	m	1	137	-	1	-	8.88	(1.25- 62.98)
*BOUCOT	520	m	0	52	1563	29	2621	3.01	(1.92- 4.72)
BUFFLE	530	f	0	90	42	52	57	2.35	(1.39- 3.97)
*CEDERL	503	m	1	23	-	5	-	4.11	(1.48- 11.44)
*CEDERL	506	f	1	5	-	3	-	6.00	(1.44- 24.93)
Subtotal CEDERL								4.67	(2.04- 10.73)
CHEN2	509	m	0	62	40	2	3	2.33	(0.37- 14.53)
CHEN2	516	f	0	21	15	1	6	8.40	(0.91- 77.21)
Subtotal CHEN2								3.92	(0.95- 16.08)
CHOI	509	m	0	20	20	19	55	2.89	(1.29- 6.51)
CHOI	516	f	0	1	1	2	9	4.50	(0.19- 106.82)
Subtotal CHOI								2.97	(1.36- 6.52)
*CPSI	602	m	0	232	26906	95	266163	24.16	(19.03- 30.66)
*CPSI	695	f	0	10	5657	105	694015	11.68	(6.11- 22.34)
Subtotal CPSI								22.15	(17.71- 27.71)
*CPSII	566	m	0	117	8450	72	141932	27.29	(20.37- 36.58)
*CPSII	632	f	0	18	2224	127	301244	19.20	(11.74- 31.40)
Subtotal CPSII								24.89	(19.36- 32.01)
DAMBER	514	m	1	-	-	-	-	7.08	(3.19- 15.74)
DEAN2	503	m	0	631	558	34	36	1.20	(0.74- 1.94)
DEAN2	506	f	0	47	11	10	14	5.98	(2.11- 16.99)
Subtotal DEAN2								1.59	(1.03- 2.46)
DESTEF	507	m	0	178	108	43	55	2.11	(1.32- 3.36)
DOLL	521	m	0	558	491	12	15	1.42	(0.66- 3.06)
DOLL	528	f	0	6	3	14	18	2.57	(0.54- 12.14)
Subtotal DOLL								1.60	(0.80- 3.18)
DORGAN	526	m	2	-	-	-	-	2.96	(2.31- 3.78)
DORGAN	522	f	3	-	-	-	-	2.76	(2.18- 3.50)
Subtotal DORGAN								2.85	(2.41- 3.39)
DOSEME	505	m	2	466	-	32	-	4.90	(2.91- 8.24)
FAN	505	m	0	143	241	29	135	2.76	(1.76- 4.34)
FAN	510	f	0	55	59	8	15	1.75	(0.69- 4.45)
Subtotal FAN								2.53	(1.69- 3.80)
GAO	563	f	0	168	72	68	58	1.99	(1.27- 3.11)
GER	517	c	0	49	155	10	40	1.26	(0.59- 2.71)
HAENSZ	546	f	0	58	77	16	26	1.22	(0.60- 2.49)
HU	505	m	0	19	14	41	33	1.09	(0.48- 2.50)
HU	510	f	0	4	3	11	8	0.97	(0.17- 5.59)
Subtotal HU								1.07	(0.51- 2.26)
HU2	514	c	0	194	113	21	33	2.70	(1.49- 4.89)
HUMBLE	525	c	0	29	22	20	33	2.18	(0.99- 4.77)
JOLY	523	m	0	250	253	11	48	4.31	(2.19- 8.49)
JOLY	509	f	0	57	20	13	28	6.14	(2.67- 14.11)
Subtotal JOLY								4.96	(2.94- 8.40)
JUSSAW	518	m	0	11	6	16	20	2.29	(0.70- 7.55)
*KAISE2	600	m	1	34	-	17	-	5.38	(2.95- 9.81)
*KAISE2	520	f	1	26	-	24	-	3.13	(1.63- 6.00)
Subtotal KAISE2								4.19	(2.70- 6.52)
KATSOU	507	f	0	17	4	5	5	4.25	(0.82- 22.13)
KHUDER	505	m	0	236	354	16	61	2.54	(1.43- 4.52)
KREUZE	514	m	3	-	-	-	-	11.12	(8.68- 14.25)
KREUZE	516	f	3	-	-	-	-	9.22	(4.45- 19.09)
Subtotal KREUZE								10.91	(8.62- 13.79)
LETOUR	510	c	0	374	141	65	187	7.63	(5.42- 10.75)
LEVIN	503	m	0	63	91	56	97	1.20	(0.76- 1.90)
*LIAW	508	c	2	-	-	-	-	5.22	(1.75- 15.56)
LIU3	509	m	0	22	59	30	146	1.81	(0.97- 3.40)
LIU5	506	c	0	58	33	27	37	2.41	(1.25- 4.64)
LUBIN	507	m	0	59	86	30	146	3.34	(2.00- 5.58)
LUBIN2	537	m	0	1325	1484	953	2995	2.81	(2.53- 3.11)
LUBIN2	580	f	0	81	32	132	230	4.41	(2.78- 7.00)
Subtotal LUBIN2								2.87	(2.59- 3.17)

Table 1I15 - 5

IESLC - Meta-analysis of Ever/current Smoking, Duration, "Highest vs lowest"
All LC types, Any Product (or Cigarettes if Any not available)
Least adjusted

REF	NRR	SEX	AD	Number Exposed Case	Cont	Non-exposed Case	Cont	RR	95.00%CI
MATOS	520	m	0	86	89	20	84	4.06	(2.29- 7.18)
MCCONN	509	c	0	16	40	3	4	0.53	(0.11- 2.66)
NOTAN2	521	c	0	5	5	7	15	2.14	(0.46- 9.90)
OSANN2	503	f	0	161	57	23	47	5.77	(3.22- 10.34)
PEZZO2	509	m	0	173	126	60	72	1.65	(1.09- 2.49)
PEZZOT	538	m	0	110	101	30	134	4.86	(3.01- 7.86)
*QIAO2	515	m	0	170	2295	7	2364	25.02	(11.77- 53.16)
RACHTA	515	f	0	24	1	12	19	38.00	(4.53- 318.78)
SOBUE	552	m	0	147	73	62	119	3.87	(2.55- 5.86)
TIZZAN	505	m	0	928	815	12	1	0.09	(0.01- 0.73)
TIZZAN	535	f	0	23	21	2	7	3.83	(0.72- 20.55)
Subtotal TIZZAN								0.86	(0.24- 3.15)
WANG2	509	c	0	22	26	4	17	3.60	(1.05- 12.28)
WATSON	503	m	0	252	231	8	20	2.73	(1.18- 6.31)
WATSON	506	f	0	10	13	5	20	3.08	(0.85- 11.07)
Subtotal WATSON								2.83	(1.40- 5.71)
WUWILL	505	f	0	223	114	137	139	1.98	(1.43- 2.75)
WYNDE8	502	m	0	294	354	102	272	2.21	(1.68- 2.92)
WYNDE8	504	f	0	206	122	82	134	2.76	(1.94- 3.93)
Subtotal WYNDE8								2.41	(1.94- 2.99)
ZHENG	555	m	0	242	143	37	75	3.43	(2.20- 5.35)
ZHENG	560	f	0	59	27	17	17	2.19	(0.97- 4.92)
Subtotal ZHENG								3.09	(2.09- 4.57)
ZHOU	503	c	0	678	36	170	12	1.33	(0.68- 2.61)
Partial Totals				10719	81579	3231	1483389		

*prospective study

REF	NRR	SEX	AD	Ys	Ws	Qs	Ps
AGUDO	509	f	0	1.37	2.33	0.00	0.0369
*AMANDU	503	m	0	1.47	26.56	0.50	0.0000
AMES	501	m	2	0.82	28.56	7.48	0.0000
ARMADA	505	m	0	1.81	9.17	2.06	0.0000
AUVINE	505	c	0	1.03	8.63	0.82	0.0025
AXELSS	509	m	0	2.79	8.08	17.14	0.0000
AXELSS	518	f	0	2.26	2.55	2.19	0.0003
Subtotal AXELSS				2.66	10.64	19.33	
BARBON	507	m	0	1.22	23.93	0.34	0.0000
*BEST	513	m	1	2.18	1.00	0.72	0.0290
*BOUCOT	520	m	0	1.10	18.98	1.05	0.0000
BUFFLE	530	f	0	0.85	13.95	3.24	0.0014
*CEDERL	503	m	1	1.41	3.67	0.02	0.0067
*CEDERL	506	f	1	1.79	1.89	0.39	0.0138
Subtotal CEDERL				1.54	5.56	0.41	
CHEN2	509	m	0	0.84	1.14	0.28	0.3669
CHEN2	516	f	0	2.13	0.78	0.49	0.0601
Subtotal CHEN2				1.36	1.92	0.77	
CHOI	509	m	0	1.06	5.85	0.44	0.0101
CHOI	516	f	0	1.50	0.38	0.01	0.3520
Subtotal CHOI				1.09	6.24	0.45	
*CPSI	602	m	0	3.18	67.59	231.00	0.0000
*CPSI	695	f	0	2.46	9.15	11.52	0.0000
Subtotal CPSI				3.10	76.73	242.52	
*CPSII	566	m	0	3.31	44.82	174.09	0.0000
*CPSII	632	f	0	2.95	15.88	41.62	0.0000
Subtotal CPSII				3.21	60.70	215.71	
DAMBER	514	m	1	1.96	6.03	2.33	0.0000
DEAN2	503	m	0	0.18	16.51	22.06	0.4643
DEAN2	506	f	0	1.79	3.53	0.72	0.0008
Subtotal DEAN2				0.46	20.04	22.78	
DESTEF	507	m	0	0.75	17.76	6.18	0.0017
DOLL	521	m	0	0.35	6.50	6.30	0.3707
DOLL	528	f	0	0.94	1.59	0.24	0.2330
Subtotal DOLL				0.47	8.10	6.55	
DORGAN	526	m	2	1.09	63.36	3.98	0.0000
DORGAN	522	f	3	1.02	68.55	7.05	0.0000
Subtotal DORGAN				1.05	131.91	11.03	
DOSEME	505	m	2	1.59	14.18	0.91	0.0000
FAN	505	m	0	1.02	18.86	1.93	0.0000
FAN	510	f	0	0.56	4.41	2.67	0.2410
Subtotal FAN				0.93	23.27	4.59	

Table 1I15 - 5

IESLC - Meta-analysis of Ever/current Smoking, Duration, "Highest vs lowest"
All LC types, Any Product (or Cigarettes if Any not available)
Least adjusted

REF	NRR	SEX	AD	Ys	Ws	Qs	Ps
GAO	563	f	0	0.69	19.31	8.10	0.0025
GER	517	c	0	0.23	6.59	7.99	0.5470
HAENSZ	546	f	0	0.20	7.62	9.80	0.5768
HU	505	m	0	0.09	5.59	8.71	0.8345
HU	510	f	0	-0.03	1.25	2.34	0.9725
Subtotal HU				0.07	6.85	11.04	
HU2	514	c	0	0.99	10.88	1.28	0.0011
HUMBLE	525	c	0	0.78	6.24	1.95	0.0522
JOLY	523	m	0	1.46	8.35	0.13	0.0000
JOLY	509	f	0	1.81	5.55	1.27	0.0000
Subtotal JOLY				1.60	13.90	1.40	
JUSSAW	518	m	0	0.83	2.70	0.69	0.1728
*KAISE2	600	m	1	1.68	10.64	1.28	0.0000
*KAISE2	520	f	1	1.14	9.05	0.34	0.0006
Subtotal KAISE2				1.43	19.69	1.62	
KATSOU	507	f	0	1.45	1.41	0.02	0.0857
KHUDER	505	m	0	0.93	11.63	1.89	0.0015
KREUZE	514	m	3	2.41	62.53	71.97	0.0000
KREUZE	516	f	3	2.22	7.25	5.68	0.0000
Subtotal KREUZE				2.39	69.77	77.65	
LETOUR	510	c	0	2.03	32.79	15.90	0.0000
LEVIN	503	m	0	0.18	18.17	24.21	0.4388
*LIAW	508	c	2	1.65	3.22	0.32	0.0030
LIU3	509	m	0	0.60	9.75	5.34	0.0628
LIU5	506	c	0	0.88	8.96	1.87	0.0085
LUBIN	507	m	0	1.21	14.54	0.25	0.0000
LUBIN2	537	m	0	1.03	355.65	32.89	0.0000
LUBIN2	580	f	0	1.48	18.01	0.40	0.0000
Subtotal LUBIN2				1.05	373.66	33.29	
MATOS	520	m	0	1.40	11.80	0.05	0.0000
MCCONN	509	c	0	-0.63	1.49	5.75	0.4428
NOTAN2	521	c	0	0.76	1.64	0.54	0.3290
OSANN2	503	f	0	1.75	11.30	1.97	0.0000
PEZZO2	509	m	0	0.50	22.59	15.81	0.0176
PEZZOT	538	m	0	1.58	16.73	1.01	0.0000
*QIAO2	515	m	0	3.22	6.76	23.99	0.0000
RACHTA	515	f	0	3.64	0.85	4.50	0.0008
SOBUE	552	m	0	1.35	22.21	0.01	0.0000
TIZZAN	505	m	0	-2.36	0.92	12.55	0.0238
TIZZAN	535	f	0	1.34	1.36	0.00	0.1168
Subtotal TIZZAN				-0.15	2.28	12.55	
WANG2	509	c	0	1.28	2.55	0.01	0.0411
WATSON	503	m	0	1.00	5.46	0.60	0.0191
WATSON	506	f	0	1.12	2.34	0.11	0.0854
Subtotal WATSON				1.04	7.80	0.71	
WUWILL	505	f	0	0.69	36.04	15.24	0.0000
WYNDE8	502	m	0	0.80	50.74	14.84	0.0000
WYNDE8	504	f	0	1.01	30.57	3.15	0.0000
Subtotal WYNDE8				0.88	81.32	17.99	
ZHENG	555	m	0	1.23	19.42	0.21	0.0000
ZHENG	560	f	0	0.78	5.83	1.79	0.0592
Subtotal ZHENG				1.13	25.25	2.00	
ZHOU	503	c	0	0.28	8.44	9.33	0.4081

Table 1I15 - 5

IESLC - Meta-analysis of Ever/current Smoking, Duration, "Highest vs lowest"
All LC types, Any Product (or Cigarettes if Any not available)
Least adjusted

	N	76
	NS	57
	Wt	1412.89
Het	Chi	865.83
Het	df	75
Het	P	***
Fixed	RR	3.80
	RRl	3.61
	RRu	4.01
	P	+++
Random	RR	3.58
	RRl	2.93
	RRu	4.37
	P	+++
Asymm	P	N.S.

Table 1I15 - 6

IESLC - Meta-analysis of Ever/current Smoking, Duration, "Highest vs lowest"
All LC types, Any Product (or Cigarettes if Any not available)
Least adjusted

		combined	Sex male	female	Total
	N	11	38	27	76
	NS	11	38	27	76
	Wt	91.42	1038.74	282.73	1412.89
Het	Chi	44.61	693.33	120.69	865.83
Het	df	10	37	26	75
Het	P	***	***	***	***
Fixed	RR	3.40	3.97	3.37	3.80
	RRl	2.77	3.74	3.00	3.61
	RRu	4.17	4.22	3.79	4.01
	P	+++	+++	+++	+++
Random	RR	2.50	3.66	3.97	3.58
	RRl	1.54	2.73	2.95	2.93
	RRu	4.04	4.91	5.33	4.37
	P	+++	+++	+++	+++
Between	Chi				7.20
Between	df				2
Between	P				*
Btwn(F)	P				N.S.
Btwn(R)	P				N.S.

Table 1I15 - 7

IESLC - Meta-analysis of Ever/current Smoking, Duration, "Highest vs lowest"
All LC types, Any Product (or Cigarettes if Any not available)
Excluded studies (and stage at which they were excluded)

1	BECHER BLOT1 BROWN3 CARPEN CHYOU DARBY DOLL2 GARCIA GRAHAM GURSEL HAMMO2 JAHN JAIN LAUSSM PRESCO QIAO TVERDA WIGLE WYNDE3
2	ALDERS BENSHL BRESLO CHIAZZ DEAN3 DORN ENGELA GAO2 GILLIS GUO HEGMAN HIRAYA HOLE KAUFMA KOO KOULUM LIU4 MIGRAN MRFITR PERNU SEGI2 SPEIZE SUZUK2 SVENSS VUTUC WAKAI WU YUAN
3	GENG MCDUFF SPITZ STASZE WU2 ZHANG
4	AKIBA GARSHI
5	BOUCHA CHEN CORREA JEDRYC LUO WYNDE2 WYNDE6
6	HAMMON PISANI RESTRE SADOWS XU
8	BOFFET BROSS WYNDE7
15	BENHAM

Table 1I15 - 8

Potentially overlapping studies

REF	REFGP	PRINC	. OVERLAP/LINK
LUBIN2	LUBIN2	1	Lubin-combined
OSANN2	KAISER	2	KAISER/OSANN2
WYNDE8	WYNDE6	2	WYNDE5/6/7/8
CPSI	CPSI	1	CPSI overall
LUBIN	XIANGZ	2	LUBIN/XIANGZ/QIAO

Table 1I15 - 9

Most adjusted - insufficient data for meta-analysis

REF	NRR	SEX	AGEL	AGEH	RACE	YF	LC TYPE	LOC	START	ST	NLC	R	VB	P	H	AD	ADOS	SM	PRODUCT	exL	exH	unexL	unexH	De
BUFFLE	548	m	0	0	wh	-	all	NAmer	1976	CC	943	n	bl	y	n	0	0	ev	cig+/-ot	50	999	1	33	st
HAMMON	512	m	0	0	wh	0	all	NAmer	1952	pr	448	n	bl	n	n	1	0	ev	cig only	35	999	1	34	st
RESTRE	507	c	0	0	all	-	all	SCAmer	1978	CC	102	n	bl	n	n	3	1#	ev	cig+/-ot	51	999	1	20	or
SADOWS	532	m	0	0	wh	-	all	NAmer	1938	CC	477	n	bl	n	n	0	0	ev	cig only	50	999	1	9	ot
XU	505	m	0	0	all	-	all	As:Chi	1985	CC	729	n	ot	n	n	2	0	ev	all/unsp	40	999	1	29	st

Comments on values in listings

RESTRE ADOS Number of cigarettes smoked per day

REF	NRR	RR	SIG	RRDATA comment
BUFFLE	548	2.13		0
HAMMON	512		*	RR for <1/2 pack per day is 2.04, that for 1/2 to 1 pack per day is 1.25, that for 1 to 2 packs per day is 2.71 while that for 2+ packs per day 2.12
RESTRE	507	24.01		Test for trend given as P = 0.006
SADOWS	532	7.09		0
XU	505		*	RR for 1-19/day is 1.8, for 20-29/day is 4.0 and for >=30/day is 3.2

Table 1I16 -

IESLC - Meta-analysis of Ever/current Smoking by Duration, Overview
All LC types, Cigarettes (or Any Product if Cigarettes not available)

This analysis is restricted to results for:
1) Ever/current smokers
2) Results by Duration
3) Categorical results by Duration
Results by Duration are grouped under 2 schemes (S1, S2). Each scheme has a set of "key values". An interval is allocated to the category whose key value it includes, and intervals which include none or more than one of the key values are excluded. (Open-ended intervals are coded as 999)

S1	key value	maximum range
1	20	1-34
2	35	21-49
3	50	36+

S2	key value	maximum range
1	5	1-19
2	20	6-29
3	30	21-39
4	40	31-49
5	50	41-998
6	999	51+

4) All LC types (or near equivalent)
5) Results complete enough for use in metaanalysis

Within each study, results are then selected (in the following order of preference, within each sex) for:
6) SMKSTA: ever, current
7) PRODUCT: cigarettes regardless of other products, cigarettes only, all/unspec
8) CIGTYPE: all/unspecified, MC regardless of HR, MC only
9) (not applicable)
10) DENOM: never smoked anything, never smoked cigarettes, never any + low, never cigs + low
11) Followup period (YF, prospective studies): whole study (coded as 0) or longest available
12) LCtype: all or nearest available, at least Squamous and Adeno. (q = squamous, s = small, l = large, a = adeno, mix = mixed, alv = alveolar)
13) Race: all or nearest available, otherwise by race (wh or w = white, bl or b = black, hi = hispanic ch = chinese, jap = japanese, haw = hawaiian, w+o = white + oriental, sca = scandinavian, as = asian)
14) For overlapping studies: principal rather than subsidiary studies
Finally by Age: whole study (coded as 0) if available, otherwise by widest available age group and then for single sex results (m, f) in preference to results for both sexes combined (c).

Results adjusted (AD) for the most potential confounders are then chosen in Sections -1 to -3 (and those which actually differ from the adjusted results in Table 1I11 - 1 are marked 'x' in Section -1) and results adjusted for the least confounders in Sections -4 to -6. (Those least adjusted results which actually differ from the most adjusted are marked 'x' in column X in Section -4)

Section -7 shows excluded studies, together with the stage (as above) at which no qualifying results were found.

Section -8 lists the potentially overlapping studies which have been included (1=principal, 2=subsidiary).

Section -9 lists any results which would have been included in preference except that they had data not complete enough for use in meta-analysis, with their significance (yes/no), if known, and any further comment as entered on the database. It also lists as "gap" any categories for which no data were presented by the original authors.

In addition to those mentioned above, the following fields, levels and abbreviations are used:
* or nk = not known, n = no, y = yes, ot = other
ev = ever, cu = current, nev = never
all/unspec = all or unspecified, cig+/-ot = cigarettes irrespective of other products (cigar, pipe etc)
MC = manufactured cigarettes, HR = hand-rolled cigarettes
exL, exH = range of exposure (low and high) in the smoking group, in terms of Duration
REF: 6-character study reference
NRR: number of the RR on the database within the study
ST : study type (CC = case control, pr or prosp = prospective)
NLC: number of lung cancer cases in whole study
R : risky occupational population (n = no, m = mining, o = other risky)
VB : national cigarette type (V = at least 75% Virginia, bl = at least 75% blended, ot = other)
P : any proxy use
H : full histological confirmation
De : derivation of RR/CI (or = original, st = standard method, ot = other method of estimation)

Table 1I16 - 1

IESLC - Meta-analysis of Ever/current Smoking by Duration, Overview
All LC types, Cigarettes (or Any Product if Cigarettes not available)
Most adjusted

REF	NRR	1I11	SEX	AGEL	AGEH	RACE	YF	LC TYPE	LOC	START	ST	NLC	R	VB	P	H	AD	SM	PRODUCT	exL	exH	S1	S2	DENOM	De
AGUDO	510		f	0	0	all	-	all	Eu:wst	1989	CC	103	n	bl	n	n	3	ev	cig only	1	16	0	1	nev cigs	or
AGUDO	511		f	0	0	all	-	all	Eu:wst	1989	CC	103	n	bl	n	n	3	ev	cig only	17	999	0	0	nev cigs	or
AMANDU	506		m	0	0	wh	0	all	NAmer	1959	pr	132	m	bl	n	n	2	cu	cig+/-ot	0	24	1	0	nev cigs	ot
AMANDU	507		m	0	0	wh	0	all	NAmer	1959	pr	132	m	bl	n	n	2	cu	cig+/-ot	25	999	0	0	nev cigs	ot
ARMADA	506		m	0	0	all	-	all	Eu:wst	1986	CC	325	n	bl	n	y	1	ev	cig+/-ot	1	24	1	0	nev cigs	or
ARMADA	507		m	0	0	all	-	all	Eu:wst	1986	CC	325	n	bl	n	y	1	ev	cig+/-ot	25	49	2	0	nev cigs	or
ARMADA	508		m	0	0	all	-	all	Eu:wst	1986	CC	325	n	bl	n	y	1	ev	cig+/-ot	50	999	3	0	nev cigs	or
AUVINE	517		c	0	0	all	-	all	Eu:Sca	1986	CC	517	n	bl	y	n	2	ev	cig+/-ot	1	20	1	0	nev cigs	or
AUVINE	518		c	0	0	all	-	all	Eu:Sca	1986	CC	517	n	bl	y	n	2	ev	cig+/-ot	21	40	2	0	nev cigs	or
AUVINE	519		c	0	0	all	-	all	Eu:Sca	1986	CC	517	n	bl	y	n	2	ev	cig+/-ot	41	999	3	0	nev cigs	or
AXELSS	519		m	0	0	sca	-	all	Eu:Sca	1989	CC	436	n	bl	n	n	6	ev	all/unsp	1	19	0	1	nev any	ot
AXELSS	520		m	0	0	sca	-	all	Eu:Sca	1989	CC	436	n	bl	n	n	6	ev	all/unsp	20	29	1	2	nev any	ot
AXELSS	521		m	0	0	sca	-	all	Eu:Sca	1989	CC	436	n	bl	n	n	6	ev	all/unsp	30	39	2	3	nev any	ot
AXELSS	522		m	0	0	sca	-	all	Eu:Sca	1989	CC	436	n	bl	n	n	6	ev	all/unsp	40	49	0	4	nev any	ot
AXELSS	523		m	0	0	sca	-	all	Eu:Sca	1989	CC	436	n	bl	n	n	6	ev	all/unsp	50	999	3	0	nev any	ot
AXELSS	510		f	0	0	sca	-	all	Eu:Sca	1989	CC	436	n	bl	n	n	0	ev	all/unsp	1	19	0	1	nev any	st
AXELSS	511		f	0	0	sca	-	all	Eu:Sca	1989	CC	436	n	bl	n	n	0	ev	all/unsp	20	29	1	2	nev any	st
AXELSS	512		f	0	0	sca	-	all	Eu:Sca	1989	CC	436	n	bl	n	n	0	ev	all/unsp	30	39	2	3	nev any	st
AXELSS	513		f	0	0	sca	-	all	Eu:Sca	1989	CC	436	n	bl	n	n	0	ev	all/unsp	40	49	0	4	nev any	st
AXELSS	514		f	0	0	sca	-	all	Eu:Sca	1989	CC	436	n	bl	n	n	0	ev	all/unsp	50	999	3	0	nev any	st
BARBON	508		m	0	0	all	-	all	Eu:wst	1979	CC	755	n	bl	y	y	1	ev	all/unsp	1	29	1	0	nev any	or
BARBON	509		m	0	0	all	-	all	Eu:wst	1979	CC	755	n	bl	y	y	1	ev	all/unsp	30	39	2	3	nev any	or
BARBON	510		m	0	0	all	-	all	Eu:wst	1979	CC	755	n	bl	y	y	1	ev	all/unsp	40	49	0	4	nev any	or
BARBON	511		m	0	0	all	-	all	Eu:wst	1979	CC	755	n	bl	y	y	1	ev	all/unsp	50	999	3	0	nev any	or
BEST	501		m	0	0	all	0	all	NAmer	1955	pr	381	n	V	n	n	1	cu	cig only	1	4	0	0	nev any	ot
BEST	502		m	0	0	all	0	all	NAmer	1955	pr	381	n	V	n	n	1	cu	cig only	5	9	0	1	nev any	ot
BEST	503		m	0	0	all	0	all	NAmer	1955	pr	381	n	V	n	n	1	cu	cig only	10	14	0	0	nev any	ot
BEST	504		m	0	0	all	0	all	NAmer	1955	pr	381	n	V	n	n	1	cu	cig only	15	19	0	0	nev any	ot
BEST	505		m	0	0	all	0	all	NAmer	1955	pr	381	n	V	n	n	1	cu	cig only	20	29	1	2	nev any	ot
BEST	506		m	0	0	all	0	all	NAmer	1955	pr	381	n	V	n	n	1	cu	cig only	30	39	2	3	nev any	ot
BEST	507		m	0	0	all	0	all	NAmer	1955	pr	381	n	V	n	n	1	cu	cig only	40	999	3	0	nev any	ot
BOUCOT	518		m	0	0	all	9	all	NAmer	1951	pr	121	n	bl	n	n	0	ev	cig+/-ot	1	39	0	0	nev any	ot
BOUCOT	519		m	0	0	all	9	all	NAmer	1951	pr	121	n	bl	n	n	0	ev	cig+/-ot	40	999	3	0	nev any	ot
BUFFLE	526		f	0	0	w-hi	-	all	NAmer	1976	CC	943	n	bl	y	n	0	ev	cig+/-ot	1	30	1	0	nev cigs	or
BUFFLE	527		f	0	0	w-hi	-	all	NAmer	1976	CC	943	n	bl	y	n	0	ev	cig+/-ot	31	40	2	4	nev cigs	or
BUFFLE	528		f	0	0	w-hi	-	all	NAmer	1976	CC	943	n	bl	y	n	0	ev	cig+/-ot	41	999	3	0	nev cigs	or
CEDERL	501		m	40	69	all	10	all	Eu:Sca	1963	pr	491	n	bl	n	n	1	cu	cig only	1	29	1	0	nev any	ot
CEDERL	502		m	40	69	all	10	all	Eu:Sca	1963	pr	491	n	bl	n	n	1	cu	cig only	30	999	0	0	nev any	ot
CEDERL	504		f	40	69	all	10	all	Eu:Sca	1963	pr	491	n	bl	n	n	1	cu	cig only	1	29	1	0	nev any	ot
CEDERL	505		f	40	69	all	10	all	Eu:Sca	1963	pr	491	n	bl	n	n	1	cu	cig only	30	999	0	0	nev any	ot
CHEN2	501		m	0	0	all	-	all	As:Chi	1983	CC	193	n	ot	y	n	0	ev	all/unsp	1	9	0	1	nev any	st
CHEN2	502		m	0	0	all	-	all	As:Chi	1983	CC	193	n	ot	y	n	0	ev	all/unsp	10	20	1	2	nev any	st
CHEN2	503		m	0	0	all	-	all	As:Chi	1983	CC	193	n	ot	y	n	0	ev	all/unsp	21	30	0	3	nev any	st
CHEN2	504		m	0	0	all	-	all	As:Chi	1983	CC	193	n	ot	y	n	0	ev	all/unsp	31	40	2	4	nev any	st
CHEN2	505		m	0	0	all	-	all	As:Chi	1983	CC	193	n	ot	y	n	0	ev	all/unsp	41	999	3	0	nev any	st
CHEN2	510		f	0	0	all	-	all	As:Chi	1983	CC	193	n	ot	y	n	0	ev	all/unsp	1	20	1	0	nev any	st
CHEN2	511		f	0	0	all	-	all	As:Chi	1983	CC	193	n	ot	y	n	0	ev	all/unsp	21	30	0	3	nev any	st
CHEN2	512		f	0	0	all	-	all	As:Chi	1983	CC	193	n	ot	y	n	0	ev	all/unsp	31	40	2	4	nev any	st
CHEN2	513		f	0	0	all	-	all	As:Chi	1983	CC	193	n	ot	y	n	0	ev	all/unsp	41	999	3	0	nev any	st
CHOI	501		m	0	0	all	-	all	As:oth	1985	CC	375	n	bl	n	n	0	ev	cig+/-ot	1	19	0	1	nev cigs	st
CHOI	502		m	0	0	all	-	all	As:oth	1985	CC	375	n	bl	n	n	0	ev	cig+/-ot	20	29	1	2	nev cigs	st
CHOI	503		m	0	0	all	-	all	As:oth	1985	CC	375	n	bl	n	n	0	ev	cig+/-ot	30	39	2	3	nev cigs	st
CHOI	504		m	0	0	all	-	all	As:oth	1985	CC	375	n	bl	n	n	0	ev	cig+/-ot	40	49	0	4	nev cigs	st
CHOI	505		m	0	0	all	-	all	As:oth	1985	CC	375	n	bl	n	n	0	ev	cig+/-ot	50	999	3	0	nev cigs	st
CHOI	510		f	0	0	all	-	all	As:oth	1985	CC	375	n	bl	n	n	0	ev	cig+/-ot	1	19	0	1	nev cigs	st
CHOI	511		f	0	0	all	-	all	As:oth	1985	CC	375	n	bl	n	n	0	ev	cig+/-ot	20	29	1	2	nev cigs	st
CHOI	512		f	0	0	all	-	all	As:oth	1985	CC	375	n	bl	n	n	0	ev	cig+/-ot	30	39	2	3	nev cigs	st
CHOI	513		f	0	0	all	-	all	As:oth	1985	CC	375	n	bl	n	n	0	ev	cig+/-ot	40	999	3	0	nev cigs	st
CPSI	580		m	40	84	wh	0	all	NAmer	1959	pr	5138	n	bl	n	n	0	cu	cig only	1	29	1	0	nev cigs	st
CPSI	581		m	40	84	wh	0	all	NAmer	1959	pr	5138	n	bl	n	n	0	cu	cig only	30	34	0	3	nev cigs	st
CPSI	582		m	40	84	wh	0	all	NAmer	1959	pr	5138	n	bl	n	n	0	cu	cig only	35	39	2	0	nev cigs	st
CPSI	583		m	40	84	wh	0	all	NAmer	1959	pr	5138	n	bl	n	n	0	cu	cig only	40	44	0	4	nev cigs	st
CPSI	584		m	40	84	wh	0	all	NAmer	1959	pr	5138	n	bl	n	n	0	cu	cig only	45	49	0	0	nev cigs	st
CPSI	585		m	40	84	wh	0	all	NAmer	1959	pr	5138	n	bl	n	n	0	cu	cig only	50	54	3	5	nev cigs	st
CPSI	586		m	40	84	wh	0	all	NAmer	1959	pr	5138	n	bl	n	n	0	cu	cig only	55	59	0	0	nev cigs	st
CPSI	587		m	40	84	wh	0	all	NAmer	1959	pr	5138	n	bl	n	n	0	cu	cig only	60	999	0	6	nev cigs	st
CPSI	676		f	40	84	wh	0	all	NAmer	1959	pr	5138	n	bl	n	n	0	cu	cig only	1	29	1	0	nev cigs	st
CPSI	677		f	40	84	wh	0	all	NAmer	1959	pr	5138	n	bl	n	n	0	cu	cig only	30	34	0	3	nev cigs	st
CPSI	678		f	40	84	wh	0	all	NAmer	1959	pr	5138	n	bl	n	n	0	cu	cig only	35	39	2	0	nev cigs	st
CPSI	679		f	40	84	wh	0	all	NAmer	1959	pr	5138	n	bl	n	n	0	cu	cig only	40	44	0	4	nev cigs	st
CPSI	680		f	40	84	wh	0	all	NAmer	1959	pr	5138	n	bl	n	n	0	cu	cig only	45	49	0	0	nev cigs	st
CPSI	681		f	40	84	wh	0	all	NAmer	1959	pr	5138	n	bl	n	n	0	cu	cig only	50	54	3	5	nev cigs	st

Table 1I16 - 1

IESLC - Meta-analysis of Ever/current Smoking by Duration, Overview
All LC types, Cigarettes (or Any Product if Cigarettes not available)
Most adjusted

REF	NRR	1I11	SEX	AGEL	AGEH	RACE	YF	LC TYPE	LOC	START	ST	NLC	R	VB	P	H	AD	SM	PRODUCT	exL	exH	S1	S2	DENOM	De
CPSI	682		f	40	84	wh	0	all	NAmer	1959	pr	5138	n	bl	n	n	0	cu	cig only	55	999	0	6	nev cigs	st
CPSII	552		m	0	0	all	6	all	NAmer	1982	pr	3229	n	bl	n	n	0	cu	cig only	1	29	1	0	nev any	st
CPSII	553		m	0	0	all	6	all	NAmer	1982	pr	3229	n	bl	n	n	0	cu	cig only	30	34	0	3	nev any	st
CPSII	554		m	0	0	all	6	all	NAmer	1982	pr	3229	n	bl	n	n	0	cu	cig only	35	39	2	0	nev any	st
CPSII	555		m	0	0	all	6	all	NAmer	1982	pr	3229	n	bl	n	n	0	cu	cig only	40	44	0	4	nev any	st
CPSII	556		m	0	0	all	6	all	NAmer	1982	pr	3229	n	bl	n	n	0	cu	cig only	45	49	0	0	nev any	st
CPSII	557		m	0	0	all	6	all	NAmer	1982	pr	3229	n	bl	n	n	0	cu	cig only	50	54	3	5	nev any	st
CPSII	558		m	0	0	all	6	all	NAmer	1982	pr	3229	n	bl	n	n	0	cu	cig only	55	59	0	0	nev any	st
CPSII	559		m	0	0	all	6	all	NAmer	1982	pr	3229	n	bl	n	n	0	cu	cig only	60	999	0	6	nev any	st
CPSII	618		f	0	0	all	6	all	NAmer	1982	pr	3229	n	bl	n	n	0	cu	cig+/-ot	1	29	1	0	nev cigs	st
CPSII	619		f	0	0	all	6	all	NAmer	1982	pr	3229	n	bl	n	n	0	cu	cig+/-ot	30	34	0	3	nev cigs	st
CPSII	620		f	0	0	all	6	all	NAmer	1982	pr	3229	n	bl	n	n	0	cu	cig+/-ot	35	39	2	0	nev cigs	st
CPSII	621		f	0	0	all	6	all	NAmer	1982	pr	3229	n	bl	n	n	0	cu	cig+/-ot	40	44	0	4	nev cigs	st
CPSII	622		f	0	0	all	6	all	NAmer	1982	pr	3229	n	bl	n	n	0	cu	cig+/-ot	45	49	0	0	nev cigs	st
CPSII	623		f	0	0	all	6	all	NAmer	1982	pr	3229	n	bl	n	n	0	cu	cig+/-ot	50	54	3	5	nev cigs	st
CPSII	624		f	0	0	all	6	all	NAmer	1982	pr	3229	n	bl	n	n	0	cu	cig+/-ot	55	59	0	0	nev cigs	st
CPSII	625		f	0	0	all	6	all	NAmer	1982	pr	3229	n	bl	n	n	0	cu	cig+/-ot	60	999	0	6	nev cigs	st
DAMBER	506		m	0	0	all	-	all	Eu:Sca	1972	CC	579	n	bl	y	n	1	ev	all/unsp	1	20	1	0	nev any	ot
DAMBER	507		m	0	0	all	-	all	Eu:Sca	1972	CC	579	n	bl	y	n	1	ev	all/unsp	21	30	0	3	nev any	ot
DAMBER	508		m	0	0	all	-	all	Eu:Sca	1972	CC	579	n	bl	y	n	1	ev	all/unsp	31	40	2	4	nev any	ot
DAMBER	509		m	0	0	all	-	all	Eu:Sca	1972	CC	579	n	bl	y	n	1	ev	all/unsp	41	50	3	5	nev any	ot
DAMBER	510		m	0	0	all	-	all	Eu:Sca	1972	CC	579	n	bl	y	n	1	ev	all/unsp	51	999	0	6	nev any	ot
DEAN2	501		m	0	0	all	-	all	Eu:UK	1960	CC	954	n	V	y	n	0	cu	all/unsp	1	19	0	1	nev any	st
DEAN2	502		m	0	0	all	-	all	Eu:UK	1960	CC	954	n	V	y	n	0	cu	all/unsp	20	999	0	0	nev any	st
DEAN2	504		f	0	0	all	-	all	Eu:UK	1960	CC	954	n	V	y	n	0	cu	all/unsp	1	19	0	1	nev any	st
DEAN2	505		f	0	0	all	-	all	Eu:UK	1960	CC	954	n	V	y	n	0	cu	all/unsp	20	999	0	0	nev any	st
DESTEF	508		m	0	0	all	-	all	SCAmer	1988	CC	497	n	bl	n	y	4	ev	all/unsp	1	29	1	0	nev any	or
DESTEF	509		m	0	0	all	-	all	SCAmer	1988	CC	497	n	bl	n	y	4	ev	all/unsp	30	39	2	3	nev any	or
DESTEF	510		m	0	0	all	-	all	SCAmer	1988	CC	497	n	bl	n	y	4	ev	all/unsp	40	49	0	4	nev any	or
DESTEF	511		m	0	0	all	-	all	SCAmer	1988	CC	497	n	bl	n	y	4	ev	all/unsp	50	999	3	0	nev any	or
DOLL	515		m	0	0	all	-	all	Eu:UK	1948	CC	1465	n	V	n	n	0	ev	all/unsp	1	9	0	1	nev any	st
DOLL	516		m	0	0	all	-	all	Eu:UK	1948	CC	1465	n	V	n	n	0	ev	all/unsp	10	19	0	0	nev any	st
DOLL	517		m	0	0	all	-	all	Eu:UK	1948	CC	1465	n	V	n	n	0	ev	all/unsp	20	39	0	0	nev any	st
DOLL	518		m	0	0	all	-	all	Eu:UK	1948	CC	1465	n	V	n	n	0	ev	all/unsp	40	999	3	0	nev any	st
DOLL	522		f	0	0	all	-	all	Eu:UK	1948	CC	1465	n	V	n	n	0	ev	all/unsp	1	9	0	1	nev any	st
DOLL	523		f	0	0	all	-	all	Eu:UK	1948	CC	1465	n	V	n	n	0	ev	all/unsp	10	19	0	0	nev any	st
DOLL	524		f	0	0	all	-	all	Eu:UK	1948	CC	1465	n	V	n	n	0	ev	all/unsp	20	39	0	0	nev any	st
DOLL	525		f	0	0	all	-	all	Eu:UK	1948	CC	1465	n	V	n	n	0	ev	all/unsp	40	999	3	0	nev any	st
DORGAN	570		m	0	0	wh	-	all	NAmer	1980	CC	2026	n	bl	y	y	2	ev	cig+/-ot	1	34	1	0	nev any	ot
DORGAN	571		m	0	0	wh	-	all	NAmer	1980	CC	2026	n	bl	y	y	2	ev	cig+/-ot	35	999	0	0	nev any	ot
DORGAN	562		f	0	0	all	-	all	NAmer	1980	CC	2026	n	bl	y	y	3	ev	cig+/-ot	1	34	1	0	nev any	ot
DORGAN	563		f	0	0	all	-	all	NAmer	1980	CC	2026	n	bl	y	y	3	ev	cig+/-ot	35	999	0	0	nev any	ot
DOSEME	501		m	0	0	all	-	all	Eu:bal	1979	CC	1210	n	bl	n	n	2	ev	cig+/-ot	1	10	0	1	nev cigs	or
DOSEME	502		m	0	0	all	-	all	Eu:bal	1979	CC	1210	n	bl	n	n	2	ev	cig+/-ot	11	20	1	2	nev cigs	or
DOSEME	503		m	0	0	all	-	all	Eu:bal	1979	CC	1210	n	bl	n	n	2	ev	cig+/-ot	21	999	0	0	nev cigs	or
FAN	501		m	0	0	all	-	all	As:Chi	1990	CC	403	n	ot	y	n	0	ev	cig+/-ot	1	29	1	0	nev cigs	st
FAN	502		m	0	0	all	-	all	As:Chi	1990	CC	403	n	ot	y	n	0	ev	cig+/-ot	30	39	2	3	nev cigs	st
FAN	503		m	0	0	all	-	all	As:Chi	1990	CC	403	n	ot	y	n	0	ev	cig+/-ot	40	999	3	0	nev cigs	st
FAN	506		f	0	0	all	-	all	As:Chi	1990	CC	403	n	ot	y	n	0	ev	cig+/-ot	1	29	1	0	nev cigs	st
FAN	507		f	0	0	all	-	all	As:Chi	1990	CC	403	n	ot	y	n	0	ev	cig+/-ot	30	39	2	3	nev cigs	st
FAN	508		f	0	0	all	-	all	As:Chi	1990	CC	403	n	ot	y	n	0	ev	cig+/-ot	40	999	3	0	nev cigs	st
GAO	564		f	0	0	all	-	all	As:Chi	1984	CC	1405	n	ot	n	n	2	ev	cig+/-ot	1	29	1	0	nev cigs	ot
GAO	565		f	0	0	all	-	all	As:Chi	1984	CC	1405	n	ot	n	n	2	ev	cig+/-ot	30	999	0	0	nev cigs	ot
GARSHI	536		m	0	0	all	-	all	NAmer	1981	CC	1081	o	bl	y	n	1	ev	all/unsp	20	999	0	0	nev any	st
GER	518		c	0	0	all	-	all	As:oth	1990	CC	141	n	ot	y	n	5	ev	all/unsp	1	20	1	0	nev any	ot
GER	519		c	0	0	all	-	all	As:oth	1990	CC	141	n	ot	y	n	5	ev	all/unsp	21	40	2	0	nev any	ot
GER	520		c	0	0	all	-	all	As:oth	1990	CC	141	n	ot	y	n	5	ev	all/unsp	41	999	3	0	nev any	ot
HAENSZ	542		f	0	0	all	-	not alv	NAmer	1955	CC	158	n	bl	n	y	0	ev	cig+/-ot	1	14	0	1	nev any	st
HAENSZ	543		f	0	0	all	-	not alv	NAmer	1955	CC	158	n	bl	n	y	0	ev	cig+/-ot	15	999	0	0	nev any	st
HU	501		m	0	0	all	-	all	As:Chi	1985	CC	227	n	ot	n	y	0	ev	cig+/-ot	1	19	0	1	nev cigs	st
HU	502		m	0	0	all	-	all	As:Chi	1985	CC	227	n	ot	n	y	0	ev	cig+/-ot	20	29	1	2	nev cigs	st
HU	503		m	0	0	all	-	all	As:Chi	1985	CC	227	n	ot	n	y	0	ev	cig+/-ot	30	999	0	0	nev cigs	st
HU	506		f	0	0	all	-	all	As:Chi	1985	CC	227	n	ot	n	y	0	ev	cig+/-ot	1	19	0	1	nev cigs	st
HU	507		f	0	0	all	-	all	As:Chi	1985	CC	227	n	ot	n	y	0	ev	cig+/-ot	20	29	1	2	nev cigs	st
HU	508		f	0	0	all	-	all	As:Chi	1985	CC	227	n	ot	n	y	0	ev	cig+/-ot	30	999	0	0	nev cigs	st
HU2	508		c	0	0	all	-	all	As:Chi	1977	CC	523	n	ot	y	n	0	ev	cig+/-ot	1	19	0	1	nev cigs	ot
HU2	509		c	0	0	all	-	all	As:Chi	1977	CC	523	n	ot	y	n	0	ev	cig+/-ot	20	29	1	2	nev cigs	or
HU2	510		c	0	0	all	-	all	As:Chi	1977	CC	523	n	ot	y	n	0	ev	cig+/-ot	30	39	2	3	nev cigs	or
HU2	511		c	0	0	all	-	all	As:Chi	1977	CC	523	n	ot	y	n	0	ev	cig+/-ot	40	999	3	0	nev cigs	st
HUMBLE	542		c	0	0	wh	-	not alv	NAmer	1980	CC	521	n	bl	y	n	3	cu	cig+/-ot	1	29	1	0	nev cigs	ot
HUMBLE	543		c	0	0	wh	-	not alv	NAmer	1980	CC	521	n	bl	y	n	3	cu	cig+/-ot	30	39	2	3	nev cigs	ot
HUMBLE	544		c	0	0	wh	-	not alv	NAmer	1980	CC	521	n	bl	y	n	3	cu	cig+/-ot	40	49	0	4	nev cigs	ot

Table 1I16 - 1

IESLC - Meta-analysis of Ever/current Smoking by Duration, Overview
All LC types, Cigarettes (or Any Product if Cigarettes not available)
Most adjusted

REF	NRR	1I11	SEX	AGEL	AGEH	RACE	YF	LC TYPE	LOC	START	ST	NLC	R	VB	P	H	AD	SM	PRODUCT	exL	exH	S1	S2	DENOM	De
HUMBLE	545		c	0	0	wh	-	not alv	NAmer	1980	CC	521	n	bl	y	n	3	cu	cig+/-ot	50	999	3	0	nev cigs	ot
JOLY	515		m	0	0	all	-	all	SCAmer	1978	CC	826	n	bl	n	n	0	ev	cig+/-ot	1	19	0	1	nev any	st
JOLY	516		m	0	0	all	-	all	SCAmer	1978	CC	826	n	bl	n	n	0	ev	cig+/-ot	20	29	1	2	nev any	st
JOLY	517		m	0	0	all	-	all	SCAmer	1978	CC	826	n	bl	n	n	0	ev	cig+/-ot	30	39	2	3	nev any	st
JOLY	518		m	0	0	all	-	all	SCAmer	1978	CC	826	n	bl	n	n	0	ev	cig+/-ot	40	49	0	4	nev any	st
JOLY	519		m	0	0	all	-	all	SCAmer	1978	CC	826	n	bl	n	n	0	ev	cig+/-ot	50	999	3	0	nev any	st
JOLY	501		f	0	0	all	-	all	SCAmer	1978	CC	826	n	bl	n	n	0	ev	cig+/-ot	1	19	0	1	nev any	st
JOLY	502		f	0	0	all	-	all	SCAmer	1978	CC	826	n	bl	n	n	0	ev	cig+/-ot	20	29	1	2	nev any	st
JOLY	503		f	0	0	all	-	all	SCAmer	1978	CC	826	n	bl	n	n	0	ev	cig+/-ot	30	39	2	3	nev any	st
JOLY	504		f	0	0	all	-	all	SCAmer	1978	CC	826	n	bl	n	n	0	ev	cig+/-ot	40	49	0	4	nev any	st
JOLY	505		f	0	0	all	-	all	SCAmer	1978	CC	826	n	bl	n	n	0	ev	cig+/-ot	50	999	3	0	nev any	st
JUSSAW	510		m	0	0	all	-	all	As:Ind	1964	CC	792	n	V	n	n	0	ev	cig only	1	9	0	1	nev any	st
JUSSAW	511		m	0	0	all	-	all	As:Ind	1964	CC	792	n	V	n	n	0	ev	cig only	10	19	0	0	nev any	st
JUSSAW	512		m	0	0	all	-	all	As:Ind	1964	CC	792	n	V	n	n	0	ev	cig only	20	29	1	2	nev any	st
JUSSAW	513		m	0	0	all	-	all	As:Ind	1964	CC	792	n	V	n	n	0	ev	cig only	30	39	2	3	nev any	st
JUSSAW	514		m	0	0	all	-	all	As:Ind	1964	CC	792	n	V	n	n	0	ev	cig only	40	999	3	0	nev any	st
KAISE2	596		m	0	0	all	9	all	NAmer	1979	pr	318	n	bl	n	n	1	cu	cig only	1	39	0	0	nev any	st
KAISE2	597		m	0	0	all	9	all	NAmer	1979	pr	318	n	bl	n	n	1	cu	cig only	40	999	3	0	nev any	st
KAISE2	516		f	0	0	all	9	all	NAmer	1979	pr	318	n	bl	n	n	1	cu	cig only	1	39	0	0	nev any	st
KAISE2	517		f	0	0	all	9	all	NAmer	1979	pr	318	n	bl	n	n	1	cu	cig only	40	999	3	0	nev any	st
KATSOU	512		f	0	0	all	-	all	Eu:bal	1987	CC	101	n	bl	n	n	1	cu	all/unsp	1	29	1	0	nev any	or
KATSOU	513		f	0	0	all	-	all	Eu:bal	1987	CC	101	n	bl	n	n	1	cu	all/unsp	30	999	0	0	nev any	or
KHUDER	501		m	0	0	all	-	all	NAmer	1985	CC	482	n	bl	n	y	0	ev	cig+/-ot	1	29	1	0	nev cigs	st
KHUDER	502		m	0	0	all	-	all	NAmer	1985	CC	482	n	bl	n	y	0	ev	cig+/-ot	30	49	2	0	nev cigs	st
KHUDER	503		m	0	0	all	-	all	NAmer	1985	CC	482	n	bl	n	y	0	ev	cig+/-ot	50	999	3	0	nev cigs	st
KREUZE	517		m	0	0	all	-	all	Eu:Ger	1990	CC	2260	n	bl	n	n	3	ev	all/unsp	1	19	0	1	nev any	st
KREUZE	518		m	0	0	all	-	all	Eu:Ger	1990	CC	2260	n	bl	n	n	3	ev	all/unsp	20	999	0	0	nev any	ot
KREUZE	520		f	0	0	all	-	all	Eu:Ger	1990	CC	2260	n	bl	n	n	3	ev	all/unsp	1	19	0	1	nev any	ot
KREUZE	521		f	0	0	all	-	all	Eu:Ger	1990	CC	2260	n	bl	n	n	3	ev	all/unsp	20	999	0	0	nev any	ot
LETOUR	506		c	0	0	all	-	all	NAmer	1983	CC	738	n	V	y	y	0	ev	cig+/-ot	1	24	1	0	nev cigs	st
LETOUR	507		c	0	0	all	-	all	NAmer	1983	CC	738	n	V	y	y	0	ev	cig+/-ot	25	40	2	0	nev cigs	st
LETOUR	508		c	0	0	all	-	all	NAmer	1983	CC	738	n	V	y	y	0	ev	cig+/-ot	41	999	3	0	nev cigs	st
LEVIN	506		m	0	0	all	-	all	NAmer	1938	CC	475	n	bl	n	n	1	ev	cig+/-ot	1	39	0	0	nev any	ot
LEVIN	507		m	0	0	all	-	all	NAmer	1938	CC	475	n	bl	n	n	1	ev	cig+/-ot	40	999	3	0	nev any	ot
LIAW	501		c	0	0	all	0	all	As:oth	1982	pr	127	n	ot	n	n	2	cu	all/unsp	1	20	1	0	nev any	or
LIAW	502		c	0	0	all	0	all	As:oth	1982	pr	127	n	ot	n	n	2	cu	all/unsp	21	30	0	3	nev any	or
LIAW	503		c	0	0	all	0	all	As:oth	1982	pr	127	n	ot	n	n	2	cu	all/unsp	31	999	0	0	nev any	or
LIU3	510		m	0	0	all	-	all	As:Chi	1985	CC	110	n	ot	n	n	2	ev	all/unsp	1	34	1	0	nev any	or
LIU3	511		m	0	0	all	-	all	As:Chi	1985	CC	110	n	ot	n	n	2	ev	all/unsp	35	999	0	0	nev any	or
LIU5	504		c	0	0	all	-	all	As:Chi	1978	CC	111	n	ot	y	n	0	ev	all/unsp	1	29	1	0	nev any	st
LIU5	505		c	0	0	all	-	all	As:Chi	1978	CC	111	n	ot	y	n	0	ev	all/unsp	30	999	0	0	nev any	st
LUBIN	508		m	0	0	all	-	all	As:Chi	1984	CC	427	m	ot	y	n	0	ev	cig+/-ot	1	29	1	0	nev any	st
LUBIN	509		m	0	0	all	-	all	As:Chi	1984	CC	427	m	ot	y	n	0	ev	cig+/-ot	30	39	2	3	nev any	st
LUBIN	510		m	0	0	all	-	all	As:Chi	1984	CC	427	m	ot	y	n	0	ev	cig+/-ot	40	49	0	4	nev any	st
LUBIN	511		m	0	0	all	-	all	As:Chi	1984	CC	427	m	ot	y	n	0	ev	cig+/-ot	50	999	3	0	nev any	st
LUBIN2	531		m	0	0	all	-	all	Eu:mul	1976	CC	7804	n	bl	n	y	0	ev	cig+/-ot	1	29	1	0	nev any	st
LUBIN2	532		m	0	0	all	-	all	Eu:mul	1976	CC	7804	n	bl	n	y	0	ev	cig+/-ot	30	39	2	3	nev any	st
LUBIN2	533		m	0	0	all	-	all	Eu:mul	1976	CC	7804	n	bl	n	y	0	ev	cig+/-ot	40	49	0	4	nev any	st
LUBIN2	534		m	0	0	all	-	all	Eu:mul	1976	CC	7804	n	bl	n	y	0	ev	cig+/-ot	50	999	3	0	nev any	st
LUBIN2	574		f	0	0	all	-	all	Eu:mul	1976	CC	7804	n	bl	n	y	0	ev	cig+/-ot	1	29	1	0	nev any	st
LUBIN2	575		f	0	0	all	-	all	Eu:mul	1976	CC	7804	n	bl	n	y	0	ev	cig+/-ot	30	39	2	3	nev any	st
LUBIN2	576		f	0	0	all	-	all	Eu:mul	1976	CC	7804	n	bl	n	y	0	ev	cig+/-ot	40	49	0	4	nev any	st
LUBIN2	577		f	0	0	all	-	all	Eu:mul	1976	CC	7804	n	bl	n	y	0	ev	cig+/-ot	50	999	3	0	nev any	st
MATOS	536		m	0	0	all	-	all	SCAmer	1994	CC	200	n	bl	n	n	2	ev	cig+/-ot	1	24	1	0	nev any	or
MATOS	537		m	0	0	all	-	all	SCAmer	1994	CC	200	n	bl	n	n	2	ev	cig+/-ot	25	39	2	3	nev any	or
MATOS	538		m	0	0	all	-	all	SCAmer	1994	CC	200	n	bl	n	n	2	ev	cig+/-ot	40	70	3	0	nev any	or
MCCONN	501		c	0	0	all	-	all	Eu:UK	1946	CC	100	n	V	n	y	0	ev	all/unsp	1	9	0	1	nev any	st
MCCONN	502		c	0	0	all	-	all	Eu:UK	1946	CC	100	n	V	n	y	0	ev	all/unsp	10	19	0	0	nev any	st
MCCONN	503		c	0	0	all	-	all	Eu:UK	1946	CC	100	n	V	n	y	0	ev	all/unsp	20	29	1	2	nev any	st
MCCONN	504		c	0	0	all	-	all	Eu:UK	1946	CC	100	n	V	n	y	0	ev	all/unsp	30	39	2	3	nev any	st
MCCONN	505		c	0	0	all	-	all	Eu:UK	1946	CC	100	n	V	n	y	0	ev	all/unsp	40	999	3	0	nev any	st
NOTAN2	513		c	0	0	all	-	all	As:Ind	1963	CC	683	n	V	n	n	0	ev	cig only	1	10	0	1	nev any	st
NOTAN2	514		c	0	0	all	-	all	As:Ind	1963	CC	683	n	V	n	n	0	ev	cig only	11	20	1	2	nev any	st
NOTAN2	515		c	0	0	all	-	all	As:Ind	1963	CC	683	n	V	n	n	0	ev	cig only	21	30	0	3	nev any	st
NOTAN2	516		c	0	0	all	-	all	As:Ind	1963	CC	683	n	V	n	n	0	ev	cig only	31	40	2	4	nev any	st
NOTAN2	517		c	0	0	all	-	all	As:Ind	1963	CC	683	n	V	n	n	0	ev	cig only	41	999	3	0	nev any	st
OSANN2	504		f	0	0	all	-	all	NAmer	1964	ot	217	n	bl	n	y	1	ev	cig+/-ot	1	20	1	0	nev cigs	or
OSANN2	505		f	0	0	all	-	all	NAmer	1964	ot	217	n	bl	n	y	1	ev	cig+/-ot	21	999	0	0	nev cigs	or
PEZZO2	507		m	0	0	all	-	all	SCAmer	1992	CC	367	n	bl	n	y	0	cu	cig+/-ot	1	35	0	0	nev cigs	st
PEZZO2	508		m	0	0	all	-	all	SCAmer	1992	CC	367	n	bl	n	y	0	cu	cig+/-ot	36	999	3	0	nev cigs	st
PEZZOT	534		m	0	0	all	-	all	SCAmer	1987	CC	215	n	bl	n	y	0	ev	cig only	1	30	1	0	nev cigs	st
PEZZOT	535		m	0	0	all	-	all	SCAmer	1987	CC	215	n	bl	n	y	0	ev	cig only	31	40	2	4	nev cigs	st

Table 1I16 - 1

IESLC - Meta-analysis of Ever/current Smoking by Duration, Overview
All LC types, Cigarettes (or Any Product if Cigarettes not available)
Most adjusted

REF	NRR	1I11	SEX	AGEL	AGEH	RACE	YF	LC TYPE	LOC	START	ST	NLC	R	VB	P	H	AD	SM	PRODUCT	exL	exH	S1	S2	DENOM	De
PEZZOT	536		m	0	0	all	-	all	SCAmer	1987	CC	215	n	bl	n	y	0	ev	cig only	41	999	3	0	nev cigs	st
QIAO2	516		m	0	0	all	0	all	As:Chi	1992	pr	241	m	ot	n	n	1	ev	all/unsp	1	27	1	0	nev any	or
QIAO2	517		m	0	0	all	0	all	As:Chi	1992	pr	241	m	ot	n	n	1	ev	all/unsp	28	41	2	0	nev any	or
QIAO2	518		m	0	0	all	0	all	As:Chi	1992	pr	241	m	ot	n	n	1	ev	all/unsp	42	999	3	0	nev any	or
RACHTA	516		f	0	0	all	-	all	Eu:est	1991	CC	118	n	bl	n	y	1	ev	cig+/-ot	1	20	1	0	nev cigs	or
RACHTA	517		f	0	0	all	-	all	Eu:est	1991	CC	118	n	bl	n	y	1	ev	cig+/-ot	21	40	2	0	nev cigs	or
RACHTA	518		f	0	0	all	-	all	Eu:est	1991	CC	118	n	bl	n	y	1	ev	cig+/-ot	41	999	3	0	nev cigs	or
SOBUE	546		m	0	0	all	-	q+s+l+a	As:Jap	1986	CC	1376	n	bl	n	y	0	cu	cig+/-ot	1	29	1	0	nev cigs	st
SOBUE	547		m	0	0	all	-	q+s+l+a	As:Jap	1986	CC	1376	n	bl	n	y	0	cu	cig+/-ot	30	39	2	3	nev cigs	st
SOBUE	548		m	0	0	all	-	q+s+l+a	As:Jap	1986	CC	1376	n	bl	n	y	0	cu	cig+/-ot	40	49	0	4	nev cigs	st
SOBUE	549		m	0	0	all	-	q+s+l+a	As:Jap	1986	CC	1376	n	bl	n	y	0	cu	cig+/-ot	50	999	3	0	nev cigs	st
TIZZAN	501		m	0	0	all	-	all	Eu:wst	1959	CC	1358	n	bl	n	n	0	ev	cig only	1	4	0	0	nev any	st
TIZZAN	502		m	0	0	all	-	all	Eu:wst	1959	CC	1358	n	bl	n	n	0	ev	cig only	5	10	0	1	nev any	st
TIZZAN	503		m	0	0	all	-	all	Eu:wst	1959	CC	1358	n	bl	n	n	0	ev	cig only	11	999	0	0	nev any	st
TIZZAN	530	x	f	0	0	all	-	all	Eu:wst	1959	CC	1358	n	bl	n	n	0	ev	cig only	1	10	0	1	nev any	st
TIZZAN	531	x	f	0	0	all	-	all	Eu:wst	1959	CC	1358	n	bl	n	n	0	ev	cig only	11	999	0	0	nev any	st
WANG2	501		c	0	0	all	-	all	As:Chi	1980	CC	103	n	ot	n	n	0	ev	cig+/-ot	1	19	0	1	nev cigs	st
WANG2	503		c	0	0	all	-	all	As:Chi	1980	CC	103	n	ot	n	n	0	ev	cig+/-ot	20	29	1	2	nev cigs	st
WANG2	504		c	0	0	all	-	all	As:Chi	1980	CC	103	n	ot	n	n	0	ev	cig+/-ot	30	39	2	3	nev cigs	st
WANG2	505		c	0	0	all	-	all	As:Chi	1980	CC	103	n	ot	n	n	0	ev	cig+/-ot	40	49	0	4	nev cigs	st
WUWILL	516		f	0	0	all	-	all	As:Chi	1985	CC	965	n	ot	n	n	3	ev	cig+/-ot	1	29	1	0	nev cigs	ot
WUWILL	517		f	0	0	all	-	all	As:Chi	1985	CC	965	n	ot	n	n	3	ev	cig+/-ot	30	39	2	3	nev cigs	ot
WUWILL	518		f	0	0	all	-	all	As:Chi	1985	CC	965	n	ot	n	n	3	ev	cig+/-ot	40	999	3	0	nev cigs	ot
ZHENG	553		m	0	0	all	-	all	As:Chi	1982	CC	540	n	ot	*	y	0	ev	cig+/-ot	1	29	1	0	nev cigs	st
ZHENG	554		m	0	0	all	-	all	As:Chi	1982	CC	540	n	ot	*	y	0	ev	cig+/-ot	30	999	0	0	nev cigs	st
ZHENG	558		f	0	0	all	-	all	As:Chi	1982	CC	540	n	ot	*	y	0	ev	cig+/-ot	1	29	1	0	nev cigs	st
ZHENG	559		f	0	0	all	-	all	As:Chi	1982	CC	540	n	ot	*	y	0	ev	cig+/-ot	30	999	0	0	nev cigs	st
ZHOU	501		c	0	0	all	-	all	As:Chi	1978	CC	1360	n	ot	n	n	0	ev	all/unsp	1	19	0	1	nev any	st
ZHOU	502		c	0	0	all	-	all	As:Chi	1978	CC	1360	n	ot	n	n	0	ev	all/unsp	20	999	0	0	nev any	st

Cigarette type is all/unspec for all RRs

except for the following:

REF	NRR	CIGTYPE
JUSSAW	510	MC only
JUSSAW	511	MC only
JUSSAW	512	MC only
JUSSAW	513	MC only
JUSSAW	514	MC only
NOTAN2	513	MC only
NOTAN2	514	MC only
NOTAN2	515	MC only
NOTAN2	516	MC only
NOTAN2	517	MC only

In this overview table, subtotals and Qs values may be invalid and should be ignored

Table 1I16 - 2

IESLC - Meta-analysis of Ever/current Smoking by Duration, Overview
All LC types, Cigarettes (or Any Product if Cigarettes not available)
Most adjusted

REF	NRR	SEX	AD	Number Exposed Case	Number Exposed Cont	Non-exposed Case	Non-exposed Cont	RR	95.00%CI
AGUDO	510	f	3	5	-	80	-	1.29	(0.40- 4.17)
AGUDO	511	f	3	18	-	80	-	5.09	(1.94- 13.35)
Subtotal AGUDO								2.92	(1.39- 6.16)
*AMANDU	506	m	2	42	-	6	-	5.92	(2.13- 16.47)
*AMANDU	507	m	2	72	-	6	-	7.02	(2.67- 18.51)
Subtotal AMANDU								6.48	(3.21- 13.08)
ARMADA	506	m	1	21	-	8	-	2.60	(1.00- 6.60)
ARMADA	507	m	1	219	-	8	-	11.90	(5.50- 25.50)
ARMADA	508	m	1	77	-	8	-	26.80	(11.00- 65.10)
Subtotal ARMADA								10.07	(6.14- 16.52)
AUVINE	517	c	2	26	-	44	-	20.10	(6.69- 66.00)
AUVINE	518	c	2	10	-	44	-	33.20	(14.30- 77.40)
AUVINE	519	c	2	230	-	44	-	30.40	(15.80- 58.40)
Subtotal AUVINE								29.13	(18.19- 46.66)
AXELSS	519	m	6	13	-	16	-	1.57	(0.70- 3.48)
AXELSS	520	m	6	17	-	16	-	2.23	(1.03- 4.80)
AXELSS	521	m	6	57	-	16	-	7.62	(4.01- 14.47)
AXELSS	522	m	6	104	-	16	-	11.81	(6.42- 21.73)
AXELSS	523	m	6	101	-	16	-	27.09	(13.94- 52.62)
AXELSS	510	f	0	5	24	18	154	1.78	(0.61- 5.25)
AXELSS	511	f	0	12	29	18	154	3.54	(1.54- 8.13)
AXELSS	512	f	0	29	26	18	154	9.54	(4.64- 19.61)
AXELSS	513	f	0	44	20	18	154	18.82	(9.17- 38.65)
AXELSS	514	f	0	20	10	18	154	17.11	(6.94- 42.19)
Subtotal AXELSS								7.77	(6.14- 9.83)
BARBON	508	m	1	42	-	22	-	3.20	(1.80- 5.70)
BARBON	509	m	1	118	-	22	-	7.90	(4.70- 13.50)
BARBON	510	m	1	207	-	22	-	11.40	(7.00- 18.80)
BARBON	511	m	1	366	-	22	-	14.50	(9.00- 23.30)
Subtotal BARBON								8.70	(6.73- 11.26)
*BEST	501	m	1	1	-	7	-	1.60	(0.20- 13.00)
*BEST	502	m	1	2	-	7	-	2.60	(0.54- 12.52)
*BEST	503	m	1	6	-	7	-	2.30	(0.77- 6.84)
*BEST	504	m	1	10	-	7	-	3.20	(1.22- 8.41)
*BEST	505	m	1	22	-	7	-	4.10	(1.75- 9.60)
*BEST	506	m	1	55	-	7	-	13.90	(6.33- 30.52)
*BEST	507	m	1	137	-	7	-	14.20	(6.64- 30.35)
Subtotal BEST								6.17	(4.26- 8.94)
*BOUCOT	518	m	0	29	2621	0	805	18.13~	(1.11- 296.36)
*BOUCOT	519	m	0	52	1563	0	805	54.09~	(3.34- 875.17)
Subtotal BOUCOT								31.38	(4.37- 225.47)
BUFFLE	526	f	0	52	57	12	112	8.51	(4.21- 17.22)
BUFFLE	527	f	0	97	62	12	112	14.60	(7.43- 28.69)
BUFFLE	528	f	0	90	42	12	112	20.00	(9.94- 40.23)
Subtotal BUFFLE								13.60	(9.12- 20.29)
*CEDERL	501	m	1	5	-	7	-	1.80	(0.57- 5.66)
*CEDERL	502	m	1	23	-	7	-	7.40	(3.18- 17.21)
*CEDERL	504	f	1	3	-	19	-	1.60	(0.47- 5.40)
*CEDERL	505	f	1	5	-	19	-	9.60	(3.60- 25.58)
Subtotal CEDERL								4.61	(2.78- 7.67)
CHEN2	501	m	0	2	3	9	33	2.44	(0.35- 16.93)
CHEN2	502	m	0	4	3	9	33	4.89	(0.92- 25.93)
CHEN2	503	m	0	17	24	9	33	2.60	(0.99- 6.81)
CHEN2	504	m	0	36	27	9	33	4.89	(2.01- 11.91)
CHEN2	505	m	0	62	40	9	33	5.68	(2.46- 13.13)
CHEN2	510	f	0	1	6	25	33	0.22	(0.02- 1.95)
CHEN2	511	f	0	2	2	25	33	1.32	(0.17- 10.03)
CHEN2	512	f	0	13	6	25	33	2.86	(0.95- 8.58)
CHEN2	513	f	0	21	15	25	33	1.85	(0.80- 4.29)
Subtotal CHEN2								3.01	(2.07- 4.38)
CHOI	501	m	0	19	55	13	95	2.52	(1.16- 5.51)
CHOI	502	m	0	66	166	13	95	2.91	(1.52- 5.54)
CHOI	503	m	0	102	160	13	95	4.66	(2.48- 8.75)
CHOI	504	m	0	60	64	13	95	6.85	(3.48- 13.50)
CHOI	505	m	0	20	20	13	95	7.31	(3.13- 17.07)
CHOI	510	f	0	2	9	76	164	0.48	(0.10- 2.27)
CHOI	511	f	0	8	14	76	164	1.23	(0.50- 3.06)
CHOI	512	f	0	8	2	76	164	8.63	(1.79- 41.62)
CHOI	513	f	0	1	1	76	164	2.16	(0.13- 34.96)
Subtotal CHOI								3.63	(2.73- 4.83)
*CPSI	580	m	0	95	266163	196	926068	1.69	(1.32- 2.15)

Table 1I16 - 2

IESLC - Meta-analysis of Ever/current Smoking by Duration, Overview
All LC types, Cigarettes (or Any Product if Cigarettes not available)
Most adjusted

REF	NRR	SEX	AD	Number Exposed Case	Number Exposed Cont	Non-exposed Case	Non-exposed Cont	RR	95.00%CI
*CPSI	581	m	0	230	290031	196	926068	3.75	(3.10- 4.53)
*CPSI	582	m	0	470	367622	196	926068	6.04	(5.11- 7.14)
*CPSI	583	m	0	731	333292	196	926068	10.36	(8.85- 12.13)
*CPSI	584	m	0	764	221405	196	926068	16.30	(13.94- 19.07)
*CPSI	585	m	0	576	119633	196	926068	22.75	(19.35- 26.75)
*CPSI	586	m	0	356	53226	196	926068	31.60	(26.55- 37.61)
*CPSI	587	m	0	232	26906	196	926068	40.74	(33.70- 49.25)
*CPSI	676	f	0	105	694015	532	3877179	1.10	(0.89- 1.36)
*CPSI	677	f	0	141	383127	532	3877179	2.68	(2.23- 3.23)
*CPSI	678	f	0	154	315060	532	3877179	3.56	(2.98- 4.26)
*CPSI	679	f	0	120	163178	532	3877179	5.36	(4.40- 6.53)
*CPSI	680	f	0	54	53635	532	3877179	7.34	(5.55- 9.71)
*CPSI	681	f	0	16	14305	532	3877179	8.15	(4.96- 13.40)
*CPSI	682	f	0	10	5657	532	3877179	12.88	(6.90- 24.07)
Subtotal CPSI								8.01	(7.62- 8.43)
*CPSII	552	m	0	72	141932	124	742207	3.04	(2.27- 4.06)
*CPSII	553	m	0	145	113317	124	742207	7.66	(6.03- 9.73)
*CPSII	554	m	0	244	109788	124	742207	13.30	(10.72- 16.51)
*CPSII	555	m	0	413	103500	124	742207	23.88	(19.54- 29.19)
*CPSII	556	m	0	307	53805	124	742207	34.15	(27.73- 42.06)
*CPSII	557	m	0	332	39260	124	742207	50.62	(41.19- 62.20)
*CPSII	558	m	0	151	13598	124	742207	66.47	(52.45- 84.24)
*CPSII	559	m	0	117	8450	124	742207	82.88	(64.43- 106.60)
*CPSII	618	f	0	127	301244	310	2091302	2.84	(2.31- 3.50)
*CPSII	619	f	0	158	152833	310	2091302	6.97	(5.76- 8.45)
*CPSII	620	f	0	193	116270	310	2091302	11.20	(9.36- 13.40)
*CPSII	621	f	0	216	91501	310	2091302	15.93	(13.39- 18.94)
*CPSII	622	f	0	153	44769	310	2091302	23.06	(19.00- 27.98)
*CPSII	623	f	0	122	29119	310	2091302	28.26	(22.93- 34.84)
*CPSII	624	f	0	27	6262	310	2091302	29.09	(19.64- 43.07)
*CPSII	625	f	0	18	2224	310	2091302	54.60	(34.01- 87.65)
Subtotal CPSII								17.37	(16.44- 18.35)
DAMBER	506	m	1	-	-	42	-	1.58	(0.69- 3.66)
DAMBER	507	m	1	-	-	42	-	3.66	(2.18- 6.73)
DAMBER	508	m	1	-	-	42	-	5.15	(3.27- 8.32)
DAMBER	509	m	1	-	-	42	-	8.71	(5.84- 13.66)
DAMBER	510	m	1	-	-	42	-	11.19	(7.43- 17.33)
Subtotal DAMBER								6.42	(5.14- 8.01)
DEAN2	501	m	0	34	36	33	112	3.21	(1.74- 5.89)
DEAN2	502	m	0	631	558	33	112	3.84	(2.56- 5.75)
DEAN2	504	f	0	10	14	88	121	0.98	(0.42- 2.31)
DEAN2	505	f	0	47	11	88	121	5.88	(2.88- 11.97)
Subtotal DEAN2								3.39	(2.55- 4.52)
DESTEF	508	m	4	43	-	27	-	3.40	(1.70- 6.80)
DESTEF	509	m	4	78	-	27	-	5.20	(2.90- 8.90)
DESTEF	510	m	4	171	-	27	-	10.40	(6.40- 16.90)
DESTEF	511	m	4	178	-	27	-	10.80	(6.60- 17.60)
Subtotal DESTEF								7.55	(5.76- 9.90)
DOLL	515	m	0	12	15	7	61	6.97	(2.34- 20.73)
DOLL	516	m	0	34	65	7	61	4.56	(1.88- 11.05)
DOLL	517	m	0	746	725	7	61	8.97	(4.07- 19.73)
DOLL	518	m	0	558	491	7	61	9.90	(4.49- 21.85)
DOLL	522	f	0	14	18	40	59	1.15	(0.51- 2.57)
DOLL	523	f	0	12	8	40	59	2.21	(0.83- 5.90)
DOLL	524	f	0	36	20	40	59	2.66	(1.35- 5.23)
DOLL	525	f	0	6	3	40	59	2.95	(0.70- 12.49)
Subtotal DOLL								3.93	(2.89- 5.34)
DORGAN	570	m	2	-	-	-	-	5.44	(2.97- 9.98)
DORGAN	571	m	2	-	-	-	-	16.09	(8.96- 28.88)
DORGAN	562	f	3	-	-	-	-	4.25	(3.20- 5.64)
DORGAN	563	f	3	-	-	-	-	11.73	(9.07- 15.18)
Subtotal DORGAN								7.74	(6.50- 9.20)
DOSEME	501	m	2	32	-	142	-	1.00	(0.60- 1.70)
DOSEME	502	m	2	158	-	142	-	3.80	(2.60- 5.70)
DOSEME	503	m	2	466	-	142	-	4.90	(3.50- 7.00)
Subtotal DOSEME								3.27	(2.59- 4.12)
FAN	501	m	0	29	135	36	236	1.41	(0.83- 2.40)
FAN	502	m	0	44	122	36	236	2.36	(1.45- 3.87)
FAN	503	m	0	143	241	36	236	3.89	(2.59- 5.84)
FAN	506	f	0	8	15	69	320	2.47	(1.01- 6.06)
FAN	507	f	0	19	23	69	320	3.83	(1.98- 7.42)

Table 1I16 - 2

IESLC - Meta-analysis of Ever/current Smoking by Duration, Overview
All LC types, Cigarettes (or Any Product if Cigarettes not available)
Most adjusted

REF	NRR	SEX	AD	Number Exposed Case	Number Exposed Cont	Non-exposed Case	Non-exposed Cont	RR	95.00%CI
FAN	508	f	0	55	59	69	320	4.32	(2.76- 6.78)
Subtotal FAN								3.01	(2.43- 3.72)
GAO	564	f	2	68	-	435	-	1.89	(1.30- 2.75)
GAO	565	f	2	168	-	435	-	4.58	(3.33- 6.30)
Subtotal GAO								3.16	(2.48- 4.03)
GARSHI	536	m	1	922	-	41	-	6.28	(4.49- 8.77)
GER	518	c	5	10	-	51	-	1.30	(0.55- 3.06)
GER	519	c	5	31	-	51	-	1.56	(0.83- 2.91)
GER	520	c	5	49	-	51	-	2.14	(1.18- 3.90)
Subtotal GER								1.72	(1.17- 2.52)
HAENSZ	542	f	0	16	26	81	236	1.79	(0.92- 3.51)
HAENSZ	543	f	0	58	77	81	236	2.19	(1.44- 3.35)
Subtotal HAENSZ								2.07	(1.45- 2.97)
HU	501	m	0	41	33	41	67	2.03	(1.11- 3.70)
HU	502	m	0	60	47	41	67	2.09	(1.21- 3.60)
HU	503	m	0	19	14	41	67	2.22	(1.00- 4.90)
HU	506	f	0	11	8	40	48	1.65	(0.61- 4.50)
HU	507	f	0	11	7	40	48	1.89	(0.67- 5.32)
HU	508	f	0	4	3	40	48	1.60	(0.34- 7.57)
Subtotal HU								2.00	(1.46- 2.74)
HU2	508	c	0	21	33	121	213	1.12	(0.62- 2.02)
HU2	509	c	0	64	63	121	213	1.79	(1.18- 2.70)
HU2	510	c	0	123	101	121	213	2.14	(1.52- 3.03)
HU2	511	c	0	194	113	121	213	3.02	(2.19- 4.17)
Subtotal HU2								2.18	(1.79- 2.64)
HUMBLE	542	c	3	20	-	28	-	15.45	(6.19- 38.58)
HUMBLE	543	c	3	68	-	28	-	17.54	(8.46- 36.34)
HUMBLE	544	c	3	104	-	28	-	19.61	(11.20- 34.31)
HUMBLE	545	c	3	119	-	28	-	17.27	(10.38- 28.75)
Subtotal HUMBLE								17.79	(12.99- 24.36)
JOLY	515	m	0	11	48	12	218	4.16	(1.73- 9.99)
JOLY	516	m	0	38	61	12	218	11.32	(5.57- 22.98)
JOLY	517	m	0	85	165	12	218	9.36	(4.95- 17.70)
JOLY	518	m	0	168	182	12	218	16.77	(9.04- 31.11)
JOLY	519	m	0	250	253	12	218	17.95	(9.78- 32.93)
JOLY	501	f	0	13	28	52	283	2.53	(1.23- 5.20)
JOLY	502	f	0	18	26	52	283	3.77	(1.93- 7.36)
JOLY	503	f	0	31	24	52	283	7.03	(3.82- 12.93)
JOLY	504	f	0	47	24	52	283	10.66	(6.00- 18.92)
JOLY	505	f	0	57	20	52	283	15.51	(8.61- 27.95)
Subtotal JOLY								9.03	(7.36- 11.08)
JUSSAW	510	m	0	16	20	149	624	3.35	(1.70- 6.62)
JUSSAW	511	m	0	34	19	149	624	7.49	(4.16- 13.51)
JUSSAW	512	m	0	38	23	149	624	6.92	(4.00- 11.97)
JUSSAW	513	m	0	27	9	149	624	12.56	(5.79- 27.28)
JUSSAW	514	m	0	11	6	149	624	7.68	(2.79- 21.09)
Subtotal JUSSAW								6.77	(5.01- 9.15)
*KAISE2	596	m	1	17	-	14	-	4.86	(2.22- 10.61)
*KAISE2	597	m	1	34	-	14	-	15.64	(8.31- 29.40)
*KAISE2	516	f	1	24	-	11	-	9.09	(4.25- 19.43)
*KAISE2	517	f	1	26	-	11	-	30.41	(14.39- 64.25)
Subtotal KAISE2								12.59	(8.77- 18.07)
KATSOU	512	f	1	13	-	48	-	1.29	(0.54- 3.26)
KATSOU	513	f	1	32	-	48	-	7.43	(2.88- 19.13)
Subtotal KATSOU								2.96	(1.54- 5.68)
KHUDER	501	m	0	16	61	23	309	3.52	(1.76- 7.06)
KHUDER	502	m	0	207	370	23	309	7.52	(4.76- 11.86)
KHUDER	503	m	0	236	354	23	309	8.96	(5.69- 14.11)
Subtotal KHUDER								7.07	(5.28- 9.47)
KREUZE	517	m	3	-	-	-	-	4.70	(3.10- 7.14)
KREUZE	518	m	3	-	-	-	-	29.23	(19.78- 43.20)
KREUZE	520	f	3	-	-	-	-	1.33	(0.80- 2.22)
KREUZE	521	f	3	-	-	-	-	7.14	(4.92- 10.35)
Subtotal KREUZE								7.26	(5.90- 8.93)
LETOUR	506	c	0	65	187	24	224	3.24	(1.95- 5.39)
LETOUR	507	c	0	264	160	24	224	15.40	(9.68- 24.51)
LETOUR	508	c	0	374	141	24	224	24.76	(15.58- 39.35)
Subtotal LETOUR								11.50	(8.73- 15.14)
LEVIN	506	m	1	56	-	7	-	7.07	(3.07- 16.29)
LEVIN	507	m	1	63	-	7	-	8.96	(3.90- 20.61)
Subtotal LEVIN								7.96	(4.42- 14.35)

Table 1I16 - 2

IESLC - Meta-analysis of Ever/current Smoking by Duration, Overview
All LC types, Cigarettes (or Any Product if Cigarettes not available)
Most adjusted

REF	NRR	SEX	AD	Number Exposed Case	Number Exposed Cont	Non-exposed Case	Non-exposed Cont	RR	95.00%CI
*LIAW	501	c	2	-	-	-	-	0.90	(0.30- 3.10)
*LIAW	502	c	2	-	-	-	-	2.60	(1.20- 5.90)
*LIAW	503	c	2	-	-	-	-	4.70	(2.70- 8.20)
Subtotal LIAW								3.19	(2.09- 4.88)
LIU3	510	m	2	30	-	4	-	1.07	(0.25- 4.59)
LIU3	511	m	2	22	-	4	-	1.71	(0.36- 8.12)
Subtotal LIU3								1.33	(0.46- 3.86)
LIU5	504	c	0	27	37	26	41	1.15	(0.57- 2.31)
LIU5	505	c	0	58	33	26	41	2.77	(1.45- 5.32)
Subtotal LIU5								1.84	(1.14- 2.96)
LUBIN	508	m	0	30	146	8	72	1.85	(0.81- 4.24)
LUBIN	509	m	0	124	294	8	72	3.80	(1.78- 8.12)
LUBIN	510	m	0	143	251	8	72	5.13	(2.40- 10.95)
LUBIN	511	m	0	59	86	8	72	6.17	(2.77- 13.77)
Subtotal LUBIN								3.94	(2.66- 5.83)
LUBIN2	531	m	0	953	2995	190	2616	4.38	(3.72- 5.16)
LUBIN2	532	m	0	2227	3470	190	2616	8.84	(7.56- 10.33)
LUBIN2	533	m	0	2079	2551	190	2616	11.22	(9.58- 13.14)
LUBIN2	534	m	0	1325	1484	190	2616	12.29	(10.42- 14.50)
LUBIN2	574	f	0	132	230	336	1188	2.03	(1.59- 2.59)
LUBIN2	575	f	0	187	186	336	1188	3.55	(2.81- 4.50)
LUBIN2	576	f	0	155	118	336	1188	4.64	(3.55- 6.07)
LUBIN2	577	f	0	81	32	336	1188	8.95	(5.84- 13.71)
Subtotal LUBIN2								6.83	(6.37- 7.32)
MATOS	536	m	2	20	-	11	-	2.20	(1.00- 4.90)
MATOS	537	m	2	82	-	11	-	7.20	(3.60- 14.50)
MATOS	538	m	2	86	-	11	-	12.70	(6.10- 26.10)
Subtotal MATOS								6.23	(4.07- 9.53)
MCCONN	501	c	0	3	4	9	23	1.92	(0.36- 10.32)
MCCONN	502	c	0	5	19	9	23	0.67	(0.19- 2.35)
MCCONN	503	c	0	46	57	9	23	2.06	(0.87- 4.89)
MCCONN	504	c	0	21	57	9	23	0.94	(0.38- 2.36)
MCCONN	505	c	0	16	40	9	23	1.02	(0.39- 2.68)
Subtotal MCCONN								1.22	(0.76- 1.94)
NOTAN2	513	c	0	7	15	107	201	0.88	(0.35- 2.22)
NOTAN2	514	c	0	15	15	107	201	1.88	(0.88- 3.99)
NOTAN2	515	c	0	17	16	107	201	2.00	(0.97- 4.11)
NOTAN2	516	c	0	12	7	107	201	3.22	(1.23- 8.42)
NOTAN2	517	c	0	5	5	107	201	1.88	(0.53- 6.63)
Subtotal NOTAN2								1.83	(1.24- 2.70)
OSANN2	504	f	1	23	-	33	-	1.60	(0.70- 3.50)
OSANN2	505	f	1	161	-	23	-	11.60	(5.80- 23.30)
Subtotal OSANN2								4.97	(2.94- 8.42)
PEZZO2	507	m	0	60	72	6	117	16.25	(6.68- 39.53)
PEZZO2	508	m	0	173	126	6	117	26.77	(11.42- 62.76)
Subtotal PEZZO2								21.08	(11.40- 39.00)
PEZZOT	534	m	0	30	134	4	116	6.49	(2.22- 18.98)
PEZZOT	535	m	0	71	82	4	116	25.11	(8.82- 71.48)
PEZZOT	536	m	0	110	101	4	116	31.58	(11.25- 88.71)
Subtotal PEZZOT								17.64	(9.62- 32.34)
*QIAO2	516	m	1	7	-	10	-	0.40	(0.15- 1.05)
*QIAO2	517	m	1	54	-	10	-	1.46	(0.74- 2.87)
*QIAO2	518	m	1	170	-	10	-	2.05	(1.06- 3.94)
Subtotal QIAO2								1.32	(0.86- 2.01)
RACHTA	516	f	1	12	-	33	-	2.02	(0.87- 4.71)
RACHTA	517	f	1	49	-	33	-	7.55	(3.90- 14.63)
RACHTA	518	f	1	24	-	33	-	58.68	(7.56- 455.64)
Subtotal RACHTA								5.34	(3.22- 8.84)
SOBUE	546	m	0	62	119	34	128	1.96	(1.21- 3.19)
SOBUE	547	m	0	159	200	34	128	2.99	(1.94- 4.61)
SOBUE	548	m	0	241	174	34	128	5.21	(3.41- 7.98)
SOBUE	549	m	0	147	73	34	128	7.58	(4.73- 12.14)
Subtotal SOBUE								3.96	(3.16- 4.96)
TIZZAN	501	m	0	12	1	180	305	20.33	(2.62- 157.68)
TIZZAN	502	m	0	54	20	180	305	4.58	(2.65- 7.89)
TIZZAN	503	m	0	928	815	180	305	1.93	(1.57- 2.37)
TIZZAN	530	f	0	2	7	117	114	0.28	(0.06- 1.37)
TIZZAN	531	f	0	23	21	117	114	1.07	(0.56- 2.03)
Subtotal TIZZAN								2.01	(1.68- 2.42)
WANG2	501	c	0	4	17	11	43	0.92	(0.26- 3.29)
WANG2	503	c	0	8	18	11	43	1.74	(0.60- 5.03)

Table 1I16 - 2

IESLC - Meta-analysis of Ever/current Smoking by Duration, Overview
All LC types, Cigarettes (or Any Product if Cigarettes not available)
Most adjusted

REF	NRR	SEX	AD	Number Exposed Case	Cont	Non-exposed Case	Cont	RR	95.00%CI
WANG2	504	c	0	26	38	11	43	2.67	(1.17- 6.13)
WANG2	505	c	0	22	26	11	43	3.31	(1.38- 7.91)
Subtotal WANG2								2.24	(1.38- 3.63)
WUWILL	516	f	3	137	-	417	-	1.35	(1.04- 1.73)
WUWILL	517	f	3	179	-	417	-	2.71	(2.05- 3.60)
WUWILL	518	f	3	223	-	417	-	3.49	(2.65- 4.59)
Subtotal WUWILL								2.27	(1.94- 2.65)
ZHENG	553	m	0	37	75	33	94	1.41	(0.80- 2.46)
ZHENG	554	m	0	242	143	33	94	4.82	(3.08- 7.54)
ZHENG	558	f	0	17	17	152	184	1.21	(0.60- 2.45)
ZHENG	559	f	0	59	27	152	184	2.65	(1.60- 4.38)
Subtotal ZHENG								2.54	(1.94- 3.31)
ZHOU	501	c	0	170	12	507	68	1.90	(1.00- 3.60)
ZHOU	502	c	0	678	36	507	68	2.53	(1.66- 3.84)
Subtotal ZHOU								2.32	(1.63- 3.29)
Partial Totals				30094	4659965	2152857252327			

*prospective study ~ With 0.5 adjustment for zero

REF	NRR	SEX	AD	Ys	Ws	Qs	Ps
AGUDO	510	f	3	0.25	2.80	8.57	0.6702
AGUDO	511	f	3	1.63	4.13	0.59	0.0009
Subtotal AGUDO				1.07	6.93	9.16	
*AMANDU	506	m	2	1.78	3.67	0.19	0.0007
*AMANDU	507	m	2	1.95	4.10	0.01	0.0001
Subtotal AMANDU				1.87	7.77	0.20	
ARMADA	506	m	1	0.96	4.31	4.75	0.0472
ARMADA	507	m	1	2.48	6.53	1.45	0.0000
ARMADA	508	m	1	3.29	4.86	8.01	0.0000
Subtotal ARMADA				2.31	15.71	14.21	
AUVINE	517	c	2	3.00	2.93	2.91	0.0000
AUVINE	518	c	2	3.50	5.39	12.08	0.0000
AUVINE	519	c	2	3.41	8.99	17.86	0.0000
Subtotal AUVINE				3.37	17.31	32.85	
AXELSS	519	m	6	0.45	5.97	14.43	0.2702
AXELSS	520	m	6	0.80	6.49	9.39	0.0411
AXELSS	521	m	6	2.03	9.33	0.01	0.0000
AXELSS	522	m	6	2.47	10.34	2.22	0.0000
AXELSS	523	m	6	3.30	8.71	14.59	0.0000
AXELSS	510	f	0	0.58	3.29	6.71	0.2943
AXELSS	511	f	0	1.26	5.56	3.05	0.0029
AXELSS	512	f	0	2.26	7.41	0.47	0.0000
AXELSS	513	f	0	2.94	7.42	6.42	0.0000
AXELSS	514	f	0	2.84	4.72	3.29	0.0000
Subtotal AXELSS				2.05	69.23	60.56	
BARBON	508	m	1	1.16	11.56	8.20	0.0001
BARBON	509	m	1	2.07	13.80	0.05	0.0000
BARBON	510	m	1	2.43	15.74	2.89	0.0000
BARBON	511	m	1	2.67	16.98	7.60	0.0000
Subtotal BARBON				2.16	58.09	18.74	
*BEST	501	m	1	0.47	0.88	2.08	0.6590
*BEST	502	m	1	0.96	1.55	1.71	0.2335
*BEST	503	m	1	0.83	3.22	4.43	0.1350
*BEST	504	m	1	1.16	4.12	2.92	0.0182
*BEST	505	m	1	1.41	5.30	1.87	0.0012
*BEST	506	m	1	2.63	6.21	2.44	0.0000
*BEST	507	m	1	2.65	6.65	2.80	0.0000
Subtotal BEST				1.82	27.95	18.24	
*BOUCOT	518	m	0	2.90	0.49	0.39	0.0421
*BOUCOT	519	m	0	3.99	0.50	1.95	0.0050
Subtotal BOUCOT				3.45	0.99	2.35	
BUFFLE	526	f	0	2.14	7.75	0.14	0.0000
BUFFLE	527	f	0	2.68	8.42	3.85	0.0000
BUFFLE	528	f	0	3.00	7.86	7.72	0.0000
Subtotal BUFFLE				2.61	24.04	11.71	
*CEDERL	501	m	1	0.59	2.92	5.86	0.3155
*CEDERL	502	m	1	2.00	5.39	0.00	0.0000
*CEDERL	504	f	1	0.47	2.58	6.07	0.4505
*CEDERL	505	f	1	2.26	4.00	0.26	0.0000
Subtotal CEDERL				1.53	14.88	12.19	
CHEN2	501	m	0	0.89	1.03	1.27	0.3653

Table 1I16 - 2

IESLC - Meta-analysis of Ever/current Smoking by Duration, Overview
All LC types, Cigarettes (or Any Product if Cigarettes not available)
Most adjusted

REF	NRR	SEX	AD	Ys	Ws	Qs	Ps
CHEN2	502	m	0	1.59	1.38	0.24	0.0623
CHEN2	503	m	0	0.95	4.13	4.56	0.0523
CHEN2	504	m	0	1.59	4.85	0.85	0.0005
CHEN2	505	m	0	1.74	5.48	0.39	0.0000
CHEN2	510	f	0	-1.51	0.81	10.01	0.1734
CHEN2	511	f	0	0.28	0.93	2.79	0.7884
CHEN2	512	f	0	1.05	3.19	2.90	0.0607
CHEN2	513	f	0	0.61	5.42	10.48	0.1529
Subtotal CHEN2				1.10	27.21	33.49	
CHOI	501	m	0	0.93	6.32	7.36	0.0199
CHOI	502	m	0	1.07	9.21	8.11	0.0012
CHOI	503	m	0	1.54	9.66	2.10	0.0000
CHOI	504	m	0	1.92	8.35	0.05	0.0000
CHOI	505	m	0	1.99	5.33	0.00	0.0000
CHOI	510	f	0	-0.73	1.59	11.91	0.3546
CHOI	511	f	0	0.21	4.64	14.95	0.6519
CHOI	512	f	0	2.16	1.55	0.04	0.0072
CHOI	513	f	0	0.77	0.50	0.76	0.5883
Subtotal CHOI				1.29	47.14	45.27	
*CPSI	580	m	0	0.52	64.01	140.65	0.0000
*CPSI	581	m	0	1.32	105.87	49.54	0.0000
*CPSI	582	m	0	1.80	138.39	5.90	0.0000
*CPSI	583	m	0	2.34	154.66	17.17	0.0000
*CPSI	584	m	0	2.79	156.12	96.55	0.0000
*CPSI	585	m	0	3.12	146.44	183.53	0.0000
*CPSI	586	m	0	3.45	126.72	265.78	0.0000
*CPSI	587	m	0	3.71	106.68	309.10	0.0000
*CPSI	676	f	0	0.10	87.71	319.06	0.3603
*CPSI	677	f	0	0.99	111.49	115.63	0.0000
*CPSI	678	f	0	1.27	119.48	64.47	0.0000
*CPSI	679	f	0	1.68	97.98	10.42	0.0000
*CPSI	680	f	0	1.99	49.07	0.01	0.0000
*CPSI	681	f	0	2.10	15.55	0.14	0.0000
*CPSI	682	f	0	2.56	9.83	2.98	0.0000
Subtotal CPSI				2.08	1489.99	1580.95	
*CPSII	552	m	0	1.11	45.57	36.45	0.0000
*CPSII	553	m	0	2.04	66.89	0.06	0.0000
*CPSII	554	m	0	2.59	82.29	27.96	0.0000
*CPSII	555	m	0	3.17	95.47	130.29	0.0000
*CPSII	556	m	0	3.53	88.48	205.99	0.0000
*CPSII	557	m	0	3.92	90.50	333.37	0.0000
*CPSII	558	m	0	4.20	68.44	328.74	0.0000
*CPSII	559	m	0	4.42	60.64	352.87	0.0000
*CPSII	618	f	0	1.05	90.12	83.02	0.0000
*CPSII	619	f	0	1.94	104.74	0.41	0.0000
*CPSII	620	f	0	2.42	119.07	20.09	0.0000
*CPSII	621	f	0	2.77	127.49	74.20	0.0000
*CPSII	622	f	0	3.14	102.68	131.78	0.0000
*CPSII	623	f	0	3.34	87.81	156.88	0.0000
*CPSII	624	f	0	3.37	24.94	46.48	0.0000
*CPSII	625	f	0	4.00	17.14	68.23	0.0000
Subtotal CPSII				2.85	1272.25	1996.82	
DAMBER	506	m	1	0.46	5.52	13.22	0.2825
DAMBER	507	m	1	1.30	12.09	6.05	0.0000
DAMBER	508	m	1	1.64	17.62	2.36	0.0000
DAMBER	509	m	1	2.16	21.28	0.54	0.0000
DAMBER	510	m	1	2.42	21.42	3.60	0.0000
Subtotal DAMBER				1.86	77.93	25.78	
DEAN2	501	m	0	1.16	10.37	7.32	0.0002
DEAN2	502	m	0	1.34	23.47	10.23	0.0000
DEAN2	504	f	0	-0.02	5.23	21.42	0.9671
DEAN2	505	f	0	1.77	7.59	0.42	0.0000
Subtotal DEAN2				1.22	46.66	39.38	
DESTEF	508	m	4	1.22	8.00	4.88	0.0005
DESTEF	509	m	4	1.65	12.22	1.55	0.0000
DESTEF	510	m	4	2.34	16.30	1.85	0.0000
DESTEF	511	m	4	2.38	15.97	2.24	0.0000
Subtotal DESTEF				2.02	52.48	10.52	
DOLL	515	m	0	1.94	3.23	0.01	0.0005
DOLL	516	m	0	1.52	4.90	1.17	0.0008
DOLL	517	m	0	2.19	6.17	0.22	0.0000

Table 1I16 - 2

IESLC - Meta-analysis of Ever/current Smoking by Duration, Overview
All LC types, Cigarettes (or Any Product if Cigarettes not available)
Most adjusted

REF	NRR	SEX	AD	Ys	Ws	Qs	Ps
DOLL	518	m	0	2.29	6.13	0.51	0.0000
DOLL	522	f	0	0.14	5.92	20.65	0.7383
DOLL	523	f	0	0.79	4.00	5.86	0.1124
DOLL	524	f	0	0.98	8.35	8.84	0.0048
DOLL	525	f	0	1.08	1.85	1.57	0.1417
Subtotal DOLL				1.37	40.55	38.82	
DORGAN	570	m	2	1.69	10.46	1.01	0.0000
DORGAN	571	m	2	2.78	11.22	6.71	0.0000
DORGAN	562	f	3	1.45	47.84	14.90	0.0000
DORGAN	563	f	3	2.46	57.93	12.11	0.0000
Subtotal DORGAN				2.05	127.45	34.73	
DOSEME	501	m	2	0.00	14.17	56.95	1.0000
DOSEME	502	m	2	1.34	24.94	11.19	0.0000
DOSEME	503	m	2	1.59	31.98	5.53	0.0000
Subtotal DOSEME				1.18	71.09	73.68	
FAN	501	m	0	0.34	13.53	37.41	0.2079
FAN	502	m	0	0.86	15.89	20.81	0.0006
FAN	503	m	0	1.36	23.17	9.69	0.0000
FAN	506	f	0	0.91	4.78	5.78	0.0478
FAN	507	f	0	1.34	8.79	3.85	0.0001
FAN	508	f	0	1.46	18.96	5.55	0.0000
Subtotal FAN				1.10	85.12	83.08	
GAO	564	f	2	0.64	27.37	51.26	0.0009
GAO	565	f	2	1.52	37.80	8.83	0.0000
Subtotal GAO				1.15	65.17	60.09	
GARSHI	536	m	1	1.84	34.28	0.96	0.0000
GER	518	c	5	0.26	5.22	15.84	0.5490
GER	519	c	5	0.44	9.76	23.77	0.1647
GER	520	c	5	0.76	10.75	16.64	0.0126
Subtotal GER				0.54	25.73	56.26	
HAENSZ	542	f	0	0.58	8.51	17.18	0.0886
HAENSZ	543	f	0	0.79	21.36	31.74	0.0003
Subtotal HAENSZ				0.73	29.87	48.92	
HU	501	m	0	0.71	10.64	17.89	0.0209
HU	502	m	0	0.74	12.94	20.87	0.0082
HU	503	m	0	0.80	6.12	8.94	0.0488
HU	506	f	0	0.50	3.82	8.64	0.3277
HU	507	f	0	0.63	3.58	6.72	0.2303
HU	508	f	0	0.47	1.59	3.75	0.5535
Subtotal HU				0.69	38.69	66.80	
HU2	508	c	0	0.11	11.00	39.37	0.7065
HU2	509	c	0	0.58	22.49	45.60	0.0058
HU2	510	c	0	0.76	32.27	49.81	0.0000
HU2	511	c	0	1.11	37.09	29.98	0.0000
Subtotal HU2				0.78	102.85	164.75	
HUMBLE	542	c	3	2.74	4.59	2.46	0.0000
HUMBLE	543	c	3	2.86	7.23	5.34	0.0000
HUMBLE	544	c	3	2.98	12.26	11.56	0.0000
HUMBLE	545	c	3	2.85	14.81	10.55	0.0000
Subtotal HUMBLE				2.88	38.89	29.91	
JOLY	515	m	0	1.43	5.01	1.68	0.0014
JOLY	516	m	0	2.43	7.66	1.36	0.0000
JOLY	517	m	0	2.24	9.46	0.51	0.0000
JOLY	518	m	0	2.82	10.06	6.68	0.0000
JOLY	519	m	0	2.89	10.43	8.13	0.0000
JOLY	501	f	0	0.93	7.39	8.58	0.0118
JOLY	502	f	0	1.33	8.56	3.94	0.0001
JOLY	503	f	0	1.95	10.34	0.03	0.0000
JOLY	504	f	0	2.37	11.67	1.52	0.0000
JOLY	505	f	0	2.74	11.07	6.01	0.0000
Subtotal JOLY				2.20	91.65	38.43	
JUSSAW	510	m	0	1.21	8.28	5.24	0.0005
JUSSAW	511	m	0	2.01	11.07	0.00	0.0000
JUSSAW	512	m	0	1.93	12.80	0.06	0.0000
JUSSAW	513	m	0	2.53	6.39	1.77	0.0000
JUSSAW	514	m	0	2.04	3.76	0.00	0.0001
Subtotal JUSSAW				1.91	42.30	7.08	
*KAISE2	596	m	1	1.58	6.28	1.13	0.0001
*KAISE2	597	m	1	2.75	9.62	5.34	0.0000
*KAISE2	516	f	1	2.21	6.65	0.27	0.0000
*KAISE2	517	f	1	3.41	6.86	13.64	0.0000

Table 1I16 - 2

IESLC - Meta-analysis of Ever/current Smoking by Duration, Overview
All LC types, Cigarettes (or Any Product if Cigarettes not available)
Most adjusted

REF	NRR	SEX	AD	Ys	Ws	Qs	Ps
Subtotal KAISE2				2.53	29.42	20.38	
KATSOU	512	f	1	0.25	4.75	14.56	0.5788
KATSOU	513	f	1	2.01	4.29	0.00	0.0000
Subtotal KATSOU				1.08	9.04	14.56	
KHUDER	501	m	0	1.26	7.96	4.42	0.0004
KHUDER	502	m	0	2.02	18.43	0.00	0.0000
KHUDER	503	m	0	2.19	18.60	0.65	0.0000
Subtotal KHUDER				1.96	44.99	5.08	
KREUZE	517	m	3	1.55	22.07	4.62	0.0000
KREUZE	518	m	3	3.38	25.18	47.27	0.0000
KREUZE	520	f	3	0.29	14.75	43.63	0.2734
KREUZE	521	f	3	1.97	27.78	0.04	0.0000
Subtotal KREUZE				1.98	89.79	95.57	
LETOUR	506	c	0	1.18	14.96	10.26	0.0000
LETOUR	507	c	0	2.73	17.80	9.47	0.0000
LETOUR	508	c	0	3.21	17.89	25.94	0.0000
Subtotal LETOUR				2.44	50.65	45.67	
LEVIN	506	m	1	1.96	5.52	0.01	0.0000
LEVIN	507	m	1	2.19	5.54	0.20	0.0000
Subtotal LEVIN				2.07	11.06	0.21	
*LIAW	501	c	2	-0.11	2.82	12.55	0.8596
*LIAW	502	c	2	0.96	6.06	6.67	0.0187
*LIAW	503	c	2	1.55	12.45	2.61	0.0000
Subtotal LIAW				1.16	21.33	21.83	
LIU3	510	m	2	0.07	1.81	6.81	0.9274
LIU3	511	m	2	0.54	1.58	3.41	0.4997
Subtotal LIU3				0.29	3.40	10.22	
LIU5	504	c	0	0.14	7.88	27.39	0.6935
LIU5	505	c	0	1.02	9.06	8.80	0.0022
Subtotal LIU5				0.61	16.94	36.19	
LUBIN	508	m	0	0.61	5.58	10.79	0.1463
LUBIN	509	m	0	1.33	6.65	3.00	0.0006
LUBIN	510	m	0	1.63	6.67	0.92	0.0000
LUBIN	511	m	0	1.82	5.97	0.20	0.0000
Subtotal LUBIN				1.37	24.88	14.91	
LUBIN2	531	m	0	1.48	142.28	39.62	0.0000
LUBIN2	532	m	0	2.18	156.67	4.74	0.0000
LUBIN2	533	m	0	2.42	153.41	26.14	0.0000
LUBIN2	534	m	0	2.51	141.36	35.91	0.0000
LUBIN2	574	f	0	0.71	63.53	106.92	0.0000
LUBIN2	575	f	0	1.27	68.77	37.32	0.0000
LUBIN2	576	f	0	1.54	53.35	11.75	0.0000
LUBIN2	577	f	0	2.19	21.09	0.73	0.0000
Subtotal LUBIN2				1.92	800.46	263.15	
MATOS	536	m	2	0.79	6.08	9.00	0.0518
MATOS	537	m	2	1.97	7.92	0.01	0.0000
MATOS	538	m	2	2.54	7.27	2.09	0.0000
Subtotal MATOS				1.83	21.27	11.11	
MCCONN	501	c	0	0.65	1.36	2.49	0.4488
MCCONN	502	c	0	-0.40	2.46	14.17	0.5341
MCCONN	503	c	0	0.72	5.16	8.47	0.1002
MCCONN	504	c	0	-0.06	4.55	19.41	0.8977
MCCONN	505	c	0	0.02	4.13	16.24	0.9644
Subtotal MCCONN				0.20	17.65	60.77	
NOTAN2	513	c	0	-0.13	4.47	20.40	0.7808
NOTAN2	514	c	0	0.63	6.77	12.80	0.1008
NOTAN2	515	c	0	0.69	7.37	12.73	0.0606
NOTAN2	516	c	0	1.17	4.16	2.90	0.0171
NOTAN2	517	c	0	0.63	2.41	4.56	0.3273
Subtotal NOTAN2				0.60	25.18	53.38	
OSANN2	504	f	1	0.47	5.93	13.98	0.2523
OSANN2	505	f	1	2.45	7.95	1.58	0.0000
Subtotal OSANN2				1.60	13.88	15.56	
PEZZO2	507	m	0	2.79	4.86	2.98	0.0000
PEZZO2	508	m	0	3.29	5.29	8.70	0.0000
Subtotal PEZZO2				3.05	10.15	11.68	
PEZZOT	534	m	0	1.87	3.34	0.06	0.0006
PEZZOT	535	m	0	3.22	3.51	5.21	0.0000
PEZZOT	536	m	0	3.45	3.60	7.55	0.0000
Subtotal PEZZOT				2.87	10.45	12.82	
*QIAO2	516	m	1	-0.92	4.06	34.63	0.0649

Table 1I16 - 2

IESLC - Meta-analysis of Ever/current Smoking by Duration, Overview
All LC types, Cigarettes (or Any Product if Cigarettes not available)
Most adjusted

REF	NRR	SEX	AD	Ys	Ws	Qs	Ps
*QIAO2	517	m	1	0.38	8.36	22.13	0.2738
*QIAO2	518	m	1	0.72	8.91	14.77	0.0321
Subtotal QIAO2				0.27	21.34	71.53	
RACHTA	516	f	1	0.70	5.39	9.13	0.1027
RACHTA	517	f	1	2.02	8.79	0.00	0.0000
RACHTA	518	f	1	4.07	0.91	3.91	0.0001
Subtotal RACHTA				1.68	15.09	13.04	
SOBUE	546	m	0	0.67	16.19	28.70	0.0067
SOBUE	547	m	0	1.10	20.61	17.02	0.0000
SOBUE	548	m	0	1.65	21.22	2.65	0.0000
SOBUE	549	m	0	2.03	17.32	0.01	0.0000
Subtotal SOBUE				1.38	75.35	48.38	
TIZZAN	501	m	0	3.01	0.92	0.93	0.0039
TIZZAN	502	m	0	1.52	12.93	3.03	0.0000
TIZZAN	503	m	0	0.66	89.78	163.09	0.0000
TIZZAN	530	f	0	-1.28	1.51	16.33	0.1155
TIZZAN	531	f	0	0.06	9.22	34.71	0.8435
Subtotal TIZZAN				0.70	114.36	218.10	
WANG2	501	c	0	-0.08	2.36	10.31	0.8977
WANG2	503	c	0	0.55	3.39	7.16	0.3089
WANG2	504	c	0	0.98	5.59	5.83	0.0200
WANG2	505	c	0	1.20	5.05	3.30	0.0072
Subtotal WANG2				0.81	16.39	26.60	
WUWILL	516	f	3	0.30	59.33	172.46	0.0208
WUWILL	517	f	3	1.00	48.46	49.25	0.0000
WUWILL	518	f	3	1.25	50.92	29.03	0.0000
Subtotal WUWILL				0.82	158.72	250.74	
ZHENG	553	m	0	0.34	12.30	34.09	0.2328
ZHENG	554	m	0	1.57	19.21	3.59	0.0000
ZHENG	558	f	0	0.19	7.71	25.38	0.5957
ZHENG	559	f	0	0.97	15.15	16.14	0.0002
Subtotal ZHENG				0.93	54.37	79.20	
ZHOU	501	c	0	0.64	9.44	17.55	0.0485
ZHOU	502	c	0	0.93	21.77	25.32	0.0000
Subtotal ZHOU				0.84	31.22	42.87	

N	245
NS	55

Table 1I16 - 3

IESLC - Meta-analysis of Ever/current Smoking by Duration, Overview
All LC types, Cigarettes (or Any Product if Cigarettes not available)
Most adjusted

	Sex combined	male	female	Total
N	38	127	80	245
NS	11	36	25	72

In this overview table, other than the “N” rows, entries in the “absent” and “Total” columns may be invalid and should be ignored

Duration of smoking (broad categories)

	absent	1-34k20	21-49k35	36+k50	Total
N	106	55	39	45	245
NS	43	43	31	35	152
Wt	2808.44	964.02	1045.83	919.29	5737.58
Het Chi	2957.86	307.96	394.65	685.36	6059.48
Het df	105	54	38	44	244
Het P	***	***	***	***	***
Fixed RR	9.57	2.46	6.17	13.46	7.43
RRl	9.22	2.31	5.80	12.61	7.24
RRu	9.93	2.63	6.55	14.36	7.62
P	+++	+++	+++	+++	+++
Random RR	5.21	2.48	5.90	10.13	5.08
RRl	4.23	2.09	4.75	7.66	4.44
RRu	6.42	2.95	7.32	13.39	5.82
P	+++	+++	+++	+++	+++

Duration of smoking (narrow categories)

	absent	1-19k1	6-29k20	21-39k30	31-49k40	41-998k50	51+k999	Total
N	138	27	16	31	23	5	5	245
NS	54	19	12	23	17	3	3	131
Wt	3095.87	185.01	140.87	889.36	849.17	361.58	215.71	5737.58
Het Chi	3318.64	70.19	41.24	255.80	225.52	89.63	80.47	6059.48
Het df	137	26	15	30	22	4	4	244
Het P	***	***	***	***	***	***	***	***
Fixed RR	6.57	2.06	3.01	4.81	10.51	26.49	42.49	7.43
RRl	6.34	1.78	2.56	4.50	9.83	23.90	37.18	7.24
RRu	6.80	2.38	3.56	5.14	11.24	29.37	48.56	7.62
P	+++	+++	+++	+++	+++	+++	+++	+++
Random RR	5.33	1.89	2.97	4.66	9.14	19.48	31.39	5.08
RRl	4.44	1.46	2.22	3.75	7.15	11.51	16.07	4.44
RRu	6.41	2.43	3.98	5.79	11.68	32.98	61.30	5.82
P	+++	+++	+++	+++	+++	+++	+++	+++

MALES

Duration of smoking (broad categories)

	absent	1-34k20	21-49k35	36+k50	Total
N	55	27	20	25	127
NS	30	27	20	25	102
Wt	1712.01	449.87	554.80	589.70	3306.39
Het Chi	1777.53	117.41	119.76	286.24	3195.54
Het df	54	26	19	24	126
Het P	***	***	***	***	***
Fixed RR	12.21	3.05	7.32	16.27	9.77
RRl	11.65	2.78	6.74	15.00	9.44
RRu	12.81	3.34	7.96	17.63	10.11
P	+++	+++	+++	+++	+++
Random RR	7.02	2.80	6.47	12.06	6.37
RRl	5.24	2.22	5.07	8.78	5.31
RRu	9.39	3.52	8.25	16.56	7.64
P	+++	+++	+++	+++	+++

Table 1I16 - 3

IESLC - Meta-analysis of Ever/current Smoking by Duration, Overview
All LC types, Cigarettes (or Any Product if Cigarettes not available)
Most adjusted

MALES

	Duration of smoking (narrow categories)							
	absent	1-19k1	6-29k20	21-39k30	31-49k40	41-998k50	51+k999	Total
N	72	12	8	16	13	3	3	127
NS	36	12	8	16	13	3	3	91
Wt	1695.15	101.57	80.72	463.80	518.20	258.22	188.74	3306.39
Het Chi	1740.24	31.08	20.88	94.37	91.10	67.05	65.10	3195.54
Het df	71	11	7	15	12	2	2	126
Het P	***	**	**	***	***	***	***	***
Fixed RR	8.53	2.89	3.95	6.04	11.73	27.82	44.20	9.77
RRl	8.13	2.38	3.17	5.51	10.76	24.62	38.32	9.44
RRu	8.94	3.51	4.91	6.61	12.78	31.43	50.98	10.11
P	+++	+++	+++	+++	+++	+++	+++	+++
Random RR	6.26	2.86	4.03	5.62	9.93	22.05	34.17	6.37
RRl	4.86	2.01	2.68	4.31	7.48	10.07	14.06	5.31
RRu	8.06	4.07	6.04	7.34	13.17	48.29	83.07	7.64
P	+++	+++	+++	+++	+++	+++	+++	+++

FEMALES

	Duration of smoking (broad categories)				
	absent	1-34k20	21-49k35	36+k50	Total
N	38	18	11	13	80
NS	21	18	11	13	63
Wt	991.32	437.93	404.28	233.52	2067.05
Het Chi	759.91	108.76	133.88	200.52	1836.06
Het df	37	17	10	12	79
Het P	***	***	***	***	***
Fixed RR	7.14	2.00	5.25	11.19	5.40
RRl	6.71	1.82	4.77	9.84	5.18
RRu	7.60	2.20	5.79	12.72	5.64
P	+++	+++	+++	+++	+++
Random RR	4.54	2.08	5.70	9.50	4.36
RRl	3.34	1.57	3.75	5.17	3.49
RRu	6.17	2.75	8.65	17.45	5.45
P	+++	+++	+++	+++	+++

	Duration of smoking (narrow categories)							
	absent	1-19k1	6-29k20	21-39k30	31-49k40	41-998k50	51+k999	Total
N	46	10	4	9	7	2	2	80
NS	25	10	4	9	7	2	2	59
Wt	1187.57	54.81	22.34	362.49	309.51	103.36	26.98	2067.05
Het Chi	1067.90	10.11	4.65	69.45	101.63	20.42	13.03	1836.06
Het df	45	9	3	8	6	1	1	79
Het P	***	N.S.	N.S.	***	***	***	***	***
Fixed RR	4.76	1.39	2.63	3.99	8.84	23.44	32.25	5.40
RRl	4.49	1.07	1.74	3.60	7.91	19.33	22.12	5.18
RRu	5.04	1.82	3.99	4.42	9.88	28.43	47.04	5.64
P	+++	+	+++	+++	+++	+++	+++	+++
Random RR	4.52	1.38	2.53	4.40	8.76	15.51	26.92	4.36
RRl	3.36	1.03	1.49	3.04	5.12	4.59	6.54	3.49
RRu	6.10	1.84	4.28	6.38	14.98	52.40	110.82	5.45
P	+++	+	+++	+++	+++	+++	+++	+++

Table 1I16 - 4

IESLC - Meta-analysis of Ever/current Smoking by Duration, Overview
All LC types, Cigarettes (or Any Product if Cigarettes not available)
Least adjusted

REF	NRR	X	SEX	AGEL	AGEH	RACE	YF	LC TYPE	LOC	START	ST	NLC	R	VB	P	H	AD	SM	PRODUCT	exL	exH	S1	S2	DENOM	De
AGUDO	507	x	f	0	0	all	-	all	Eu:wst	1989	CC	103	n	bl	n	n	0	ev	cig only	1	16	0	1	nev cigs	st
AGUDO	508	x	f	0	0	all	-	all	Eu:wst	1989	CC	103	n	bl	n	n	0	ev	cig only	17	999	0	0	nev cigs	st
AMANDU	501	x	m	0	0	wh	0	all	NAmer	1959	pr	132	m	bl	n	n	0	cu	cig+/-ot	0	24	1	0	nev cigs	st
AMANDU	502	x	m	0	0	wh	0	all	NAmer	1959	pr	132	m	bl	n	n	0	cu	cig+/-ot	25	999	0	0	nev cigs	st
ARMADA	501	x	m	0	0	all	-	all	Eu:wst	1986	CC	325	n	bl	n	y	0	ev	cig+/-ot	1	24	1	0	nev cigs	st
ARMADA	502	x	m	0	0	all	-	all	Eu:wst	1986	CC	325	n	bl	n	y	0	ev	cig+/-ot	25	49	2	0	nev cigs	st
ARMADA	503	x	m	0	0	all	-	all	Eu:wst	1986	CC	325	n	bl	n	y	0	ev	cig+/-ot	50	999	3	0	nev cigs	st
AUVINE	501	x	c	0	0	all	-	all	Eu:Sca	1986	CC	517	n	bl	y	n	0	ev	cig+/-ot	1	20	1	0	nev cigs	st
AUVINE	502	x	c	0	0	all	-	all	Eu:Sca	1986	CC	517	n	bl	y	n	0	ev	cig+/-ot	21	40	2	0	nev cigs	st
AUVINE	503	x	c	0	0	all	-	all	Eu:Sca	1986	CC	517	n	bl	y	n	0	ev	cig+/-ot	41	999	3	0	nev cigs	st
AXELSS	501	x	m	0	0	sca	-	all	Eu:Sca	1989	CC	436	n	bl	n	n	0	ev	all/unsp	1	19	0	1	nev any	st
AXELSS	502	x	m	0	0	sca	-	all	Eu:Sca	1989	CC	436	n	bl	n	n	0	ev	all/unsp	20	29	1	2	nev any	st
AXELSS	503	x	m	0	0	sca	-	all	Eu:Sca	1989	CC	436	n	bl	n	n	0	ev	all/unsp	30	39	2	3	nev any	st
AXELSS	504	x	m	0	0	sca	-	all	Eu:Sca	1989	CC	436	n	bl	n	n	0	ev	all/unsp	40	49	0	4	nev any	st
AXELSS	505	x	m	0	0	sca	-	all	Eu:Sca	1989	CC	436	n	bl	n	n	0	ev	all/unsp	50	999	3	0	nev any	st
AXELSS	510		f	0	0	sca	-	all	Eu:Sca	1989	CC	436	n	bl	n	n	0	ev	all/unsp	1	19	0	1	nev any	st
AXELSS	511		f	0	0	sca	-	all	Eu:Sca	1989	CC	436	n	bl	n	n	0	ev	all/unsp	20	29	1	2	nev any	st
AXELSS	512		f	0	0	sca	-	all	Eu:Sca	1989	CC	436	n	bl	n	n	0	ev	all/unsp	30	39	2	3	nev any	st
AXELSS	513		f	0	0	sca	-	all	Eu:Sca	1989	CC	436	n	bl	n	n	0	ev	all/unsp	40	49	0	4	nev any	st
AXELSS	514		f	0	0	sca	-	all	Eu:Sca	1989	CC	436	n	bl	n	n	0	ev	all/unsp	50	999	3	0	nev any	st
BARBON	501	x	m	0	0	all	-	all	Eu:wst	1979	CC	755	n	bl	y	y	0	ev	all/unsp	1	29	1	0	nev any	st
BARBON	502	x	m	0	0	all	-	all	Eu:wst	1979	CC	755	n	bl	y	y	0	ev	all/unsp	30	39	2	3	nev any	st
BARBON	503	x	m	0	0	all	-	all	Eu:wst	1979	CC	755	n	bl	y	y	0	ev	all/unsp	40	49	0	4	nev any	st
BARBON	504	x	m	0	0	all	-	all	Eu:wst	1979	CC	755	n	bl	y	y	0	ev	all/unsp	50	999	3	0	nev any	st
BEST	501		m	0	0	all	0	all	NAmer	1955	pr	381	n	V	n	n	1	cu	cig only	1	4	0	0	nev any	ot
BEST	502		m	0	0	all	0	all	NAmer	1955	pr	381	n	V	n	n	1	cu	cig only	5	9	0	1	nev any	ot
BEST	503		m	0	0	all	0	all	NAmer	1955	pr	381	n	V	n	n	1	cu	cig only	10	14	0	0	nev any	ot
BEST	504		m	0	0	all	0	all	NAmer	1955	pr	381	n	V	n	n	1	cu	cig only	15	19	0	0	nev any	ot
BEST	505		m	0	0	all	0	all	NAmer	1955	pr	381	n	V	n	n	1	cu	cig only	20	29	1	2	nev any	ot
BEST	506		m	0	0	all	0	all	NAmer	1955	pr	381	n	V	n	n	1	cu	cig only	30	39	2	3	nev any	ot
BEST	507		m	0	0	all	0	all	NAmer	1955	pr	381	n	V	n	n	1	cu	cig only	40	999	3	0	nev any	ot
BOUCOT	518		m	0	0	all	9	all	NAmer	1951	pr	121	n	bl	n	n	0	ev	cig+/-ot	1	39	0	0	nev any	ot
BOUCOT	519		m	0	0	all	9	all	NAmer	1951	pr	121	n	bl	n	n	0	ev	cig+/-ot	40	999	3	0	nev any	ot
BUFFLE	526		f	0	0	w-hi	-	all	NAmer	1976	CC	943	n	bl	y	n	0	ev	cig+/-ot	1	30	1	0	nev cigs	or
BUFFLE	527		f	0	0	w-hi	-	all	NAmer	1976	CC	943	n	bl	y	n	0	ev	cig+/-ot	31	40	2	4	nev cigs	or
BUFFLE	528		f	0	0	w-hi	-	all	NAmer	1976	CC	943	n	bl	y	n	0	ev	cig+/-ot	41	999	3	0	nev cigs	or
CEDERL	501		m	40	69	all	10	all	Eu:Sca	1963	pr	491	n	bl	n	n	1	cu	cig only	1	29	1	0	nev any	ot
CEDERL	502		m	40	69	all	10	all	Eu:Sca	1963	pr	491	n	bl	n	n	1	cu	cig only	30	999	0	0	nev any	ot
CEDERL	504		f	40	69	all	10	all	Eu:Sca	1963	pr	491	n	bl	n	n	1	cu	cig only	1	29	1	0	nev any	ot
CEDERL	505		f	40	69	all	10	all	Eu:Sca	1963	pr	491	n	bl	n	n	1	cu	cig only	30	999	0	0	nev any	ot
CHEN2	501		m	0	0	all	-	all	As:Chi	1983	CC	193	n	ot	y	n	0	ev	all/unsp	1	9	0	1	nev any	st
CHEN2	502		m	0	0	all	-	all	As:Chi	1983	CC	193	n	ot	y	n	0	ev	all/unsp	10	20	1	2	nev any	st
CHEN2	503		m	0	0	all	-	all	As:Chi	1983	CC	193	n	ot	y	n	0	ev	all/unsp	21	30	0	3	nev any	st
CHEN2	504		m	0	0	all	-	all	As:Chi	1983	CC	193	n	ot	y	n	0	ev	all/unsp	31	40	2	4	nev any	st
CHEN2	505		m	0	0	all	-	all	As:Chi	1983	CC	193	n	ot	y	n	0	ev	all/unsp	41	999	3	0	nev any	st
CHEN2	510		f	0	0	all	-	all	As:Chi	1983	CC	193	n	ot	y	n	0	ev	all/unsp	1	20	1	0	nev any	st
CHEN2	511		f	0	0	all	-	all	As:Chi	1983	CC	193	n	ot	y	n	0	ev	all/unsp	21	30	0	3	nev any	st
CHEN2	512		f	0	0	all	-	all	As:Chi	1983	CC	193	n	ot	y	n	0	ev	all/unsp	31	40	2	4	nev any	st
CHEN2	513		f	0	0	all	-	all	As:Chi	1983	CC	193	n	ot	y	n	0	ev	all/unsp	41	999	3	0	nev any	st
CHOI	501		m	0	0	all	-	all	As:oth	1985	CC	375	n	bl	n	n	0	ev	cig+/-ot	1	19	0	1	nev cigs	st
CHOI	502		m	0	0	all	-	all	As:oth	1985	CC	375	n	bl	n	n	0	ev	cig+/-ot	20	29	1	2	nev cigs	st
CHOI	503		m	0	0	all	-	all	As:oth	1985	CC	375	n	bl	n	n	0	ev	cig+/-ot	30	39	2	3	nev cigs	st
CHOI	504		m	0	0	all	-	all	As:oth	1985	CC	375	n	bl	n	n	0	ev	cig+/-ot	40	49	0	4	nev cigs	st
CHOI	505		m	0	0	all	-	all	As:oth	1985	CC	375	n	bl	n	n	0	ev	cig+/-ot	50	999	3	0	nev cigs	st
CHOI	510		f	0	0	all	-	all	As:oth	1985	CC	375	n	bl	n	n	0	ev	cig+/-ot	1	19	0	1	nev cigs	st
CHOI	511		f	0	0	all	-	all	As:oth	1985	CC	375	n	bl	n	n	0	ev	cig+/-ot	20	29	1	2	nev cigs	st
CHOI	512		f	0	0	all	-	all	As:oth	1985	CC	375	n	bl	n	n	0	ev	cig+/-ot	30	39	2	3	nev cigs	st
CHOI	513		f	0	0	all	-	all	As:oth	1985	CC	375	n	bl	n	n	0	ev	cig+/-ot	40	999	3	0	nev cigs	st
CPSI	580		m	40	84	wh	0	all	NAmer	1959	pr	5138	n	bl	n	n	0	cu	cig only	1	29	1	0	nev cigs	st
CPSI	581		m	40	84	wh	0	all	NAmer	1959	pr	5138	n	bl	n	n	0	cu	cig only	30	34	0	3	nev cigs	st
CPSI	582		m	40	84	wh	0	all	NAmer	1959	pr	5138	n	bl	n	n	0	cu	cig only	35	39	2	0	nev cigs	st
CPSI	583		m	40	84	wh	0	all	NAmer	1959	pr	5138	n	bl	n	n	0	cu	cig only	40	44	0	4	nev cigs	st
CPSI	584		m	40	84	wh	0	all	NAmer	1959	pr	5138	n	bl	n	n	0	cu	cig only	45	49	0	0	nev cigs	st
CPSI	585		m	40	84	wh	0	all	NAmer	1959	pr	5138	n	bl	n	n	0	cu	cig only	50	54	3	5	nev cigs	st
CPSI	586		m	40	84	wh	0	all	NAmer	1959	pr	5138	n	bl	n	n	0	cu	cig only	55	59	0	0	nev cigs	st
CPSI	587		m	40	84	wh	0	all	NAmer	1959	pr	5138	n	bl	n	n	0	cu	cig only	60	999	0	6	nev cigs	st
CPSI	676		f	40	84	wh	0	all	NAmer	1959	pr	5138	n	bl	n	n	0	cu	cig only	1	29	1	0	nev cigs	st
CPSI	677		f	40	84	wh	0	all	NAmer	1959	pr	5138	n	bl	n	n	0	cu	cig only	30	34	0	3	nev cigs	st
CPSI	678		f	40	84	wh	0	all	NAmer	1959	pr	5138	n	bl	n	n	0	cu	cig only	35	39	2	0	nev cigs	st
CPSI	679		f	40	84	wh	0	all	NAmer	1959	pr	5138	n	bl	n	n	0	cu	cig only	40	44	0	4	nev cigs	st
CPSI	680		f	40	84	wh	0	all	NAmer	1959	pr	5138	n	bl	n	n	0	cu	cig only	45	49	0	0	nev cigs	st
CPSI	681		f	40	84	wh	0	all	NAmer	1959	pr	5138	n	bl	n	n	0	cu	cig only	50	54	3	5	nev cigs	st

Table 1I16 - 4

IESLC - Meta-analysis of Ever/current Smoking by Duration, Overview
All LC types, Cigarettes (or Any Product if Cigarettes not available)
Least adjusted

REF	NRR	X	SEX	AGEL	AGEH	RACE	YF	LC TYPE	LOC	START	ST	NLC	R	VB	P	H	AD	SM	PRODUCT	exL	exH	S1	S2	DENOM	De
CPSI	682		f	40	84	wh	0	all	NAmer	1959	pr	5138	n	bl	n	n	0	cu	cig only	55	999	0	6	nev cigs	st
CPSII	552		m	0	0	all	6	all	NAmer	1982	pr	3229	n	bl	n	n	0	cu	cig only	1	29	1	0	nev any	st
CPSII	553		m	0	0	all	6	all	NAmer	1982	pr	3229	n	bl	n	n	0	cu	cig only	30	34	0	3	nev any	st
CPSII	554		m	0	0	all	6	all	NAmer	1982	pr	3229	n	bl	n	n	0	cu	cig only	35	39	2	0	nev any	st
CPSII	555		m	0	0	all	6	all	NAmer	1982	pr	3229	n	bl	n	n	0	cu	cig only	40	44	0	4	nev any	st
CPSII	556		m	0	0	all	6	all	NAmer	1982	pr	3229	n	bl	n	n	0	cu	cig only	45	49	0	0	nev any	st
CPSII	557		m	0	0	all	6	all	NAmer	1982	pr	3229	n	bl	n	n	0	cu	cig only	50	54	3	5	nev any	st
CPSII	558		m	0	0	all	6	all	NAmer	1982	pr	3229	n	bl	n	n	0	cu	cig only	55	59	0	0	nev any	st
CPSII	559		m	0	0	all	6	all	NAmer	1982	pr	3229	n	bl	n	n	0	cu	cig only	60	999	0	6	nev any	st
CPSII	618		f	0	0	all	6	all	NAmer	1982	pr	3229	n	bl	n	n	0	cu	cig+/-ot	1	29	1	0	nev cigs	st
CPSII	619		f	0	0	all	6	all	NAmer	1982	pr	3229	n	bl	n	n	0	cu	cig+/-ot	30	34	0	3	nev cigs	st
CPSII	620		f	0	0	all	6	all	NAmer	1982	pr	3229	n	bl	n	n	0	cu	cig+/-ot	35	39	2	0	nev cigs	st
CPSII	621		f	0	0	all	6	all	NAmer	1982	pr	3229	n	bl	n	n	0	cu	cig+/-ot	40	44	0	4	nev cigs	st
CPSII	622		f	0	0	all	6	all	NAmer	1982	pr	3229	n	bl	n	n	0	cu	cig+/-ot	45	49	0	0	nev cigs	st
CPSII	623		f	0	0	all	6	all	NAmer	1982	pr	3229	n	bl	n	n	0	cu	cig+/-ot	50	54	3	5	nev cigs	st
CPSII	624		f	0	0	all	6	all	NAmer	1982	pr	3229	n	bl	n	n	0	cu	cig+/-ot	55	59	0	0	nev cigs	st
CPSII	625		f	0	0	all	6	all	NAmer	1982	pr	3229	n	bl	n	n	0	cu	cig+/-ot	60	999	0	6	nev cigs	st
DAMBER	506		m	0	0	all	-	all	Eu:Sca	1972	CC	579	n	bl	y	n	1	ev	all/unsp	1	20	1	0	nev any	ot
DAMBER	507		m	0	0	all	-	all	Eu:Sca	1972	CC	579	n	bl	y	n	1	ev	all/unsp	21	30	0	3	nev any	ot
DAMBER	508		m	0	0	all	-	all	Eu:Sca	1972	CC	579	n	bl	y	n	1	ev	all/unsp	31	40	2	4	nev any	ot
DAMBER	509		m	0	0	all	-	all	Eu:Sca	1972	CC	579	n	bl	y	n	1	ev	all/unsp	41	50	3	5	nev any	ot
DAMBER	510		m	0	0	all	-	all	Eu:Sca	1972	CC	579	n	bl	y	n	1	ev	all/unsp	51	999	0	6	nev any	ot
DEAN2	501		m	0	0	all	-	all	Eu:UK	1960	CC	954	n	V	y	n	0	cu	all/unsp	1	19	0	1	nev any	st
DEAN2	502		m	0	0	all	-	all	Eu:UK	1960	CC	954	n	V	y	n	0	cu	all/unsp	20	999	0	0	nev any	st
DEAN2	504		f	0	0	all	-	all	Eu:UK	1960	CC	954	n	V	y	n	0	cu	all/unsp	1	19	0	1	nev any	st
DEAN2	505		f	0	0	all	-	all	Eu:UK	1960	CC	954	n	V	y	n	0	cu	all/unsp	20	999	0	0	nev any	st
DESTEF	501	x	m	0	0	all	-	all	SCAmer	1988	CC	497	n	bl	n	y	0	ev	all/unsp	1	29	1	0	nev any	st
DESTEF	502	x	m	0	0	all	-	all	SCAmer	1988	CC	497	n	bl	n	y	0	ev	all/unsp	30	39	2	3	nev any	st
DESTEF	503	x	m	0	0	all	-	all	SCAmer	1988	CC	497	n	bl	n	y	0	ev	all/unsp	40	49	0	4	nev any	st
DESTEF	504	x	m	0	0	all	-	all	SCAmer	1988	CC	497	n	bl	n	y	0	ev	all/unsp	50	999	3	0	nev any	st
DOLL	515		m	0	0	all	-	all	Eu:UK	1948	CC	1465	n	V	n	n	0	ev	all/unsp	1	9	0	1	nev any	st
DOLL	516		m	0	0	all	-	all	Eu:UK	1948	CC	1465	n	V	n	n	0	ev	all/unsp	10	19	0	0	nev any	st
DOLL	517		m	0	0	all	-	all	Eu:UK	1948	CC	1465	n	V	n	n	0	ev	all/unsp	20	39	0	0	nev any	st
DOLL	518		m	0	0	all	-	all	Eu:UK	1948	CC	1465	n	V	n	n	0	ev	all/unsp	40	999	3	0	nev any	st
DOLL	522		f	0	0	all	-	all	Eu:UK	1948	CC	1465	n	V	n	n	0	ev	all/unsp	1	9	0	1	nev any	st
DOLL	523		f	0	0	all	-	all	Eu:UK	1948	CC	1465	n	V	n	n	0	ev	all/unsp	10	19	0	0	nev any	st
DOLL	524		f	0	0	all	-	all	Eu:UK	1948	CC	1465	n	V	n	n	0	ev	all/unsp	20	39	0	0	nev any	st
DOLL	525		f	0	0	all	-	all	Eu:UK	1948	CC	1465	n	V	n	n	0	ev	all/unsp	40	999	3	0	nev any	st
DORGAN	570		m	0	0	wh	-	all	NAmer	1980	CC	2026	n	bl	y	y	2	ev	cig+/-ot	1	34	1	0	nev any	ot
DORGAN	571		m	0	0	wh	-	all	NAmer	1980	CC	2026	n	bl	y	y	2	ev	cig+/-ot	35	999	0	0	nev any	ot
DORGAN	562		f	0	0	all	-	all	NAmer	1980	CC	2026	n	bl	y	y	3	ev	cig+/-ot	1	34	1	0	nev any	ot
DORGAN	563		f	0	0	all	-	all	NAmer	1980	CC	2026	n	bl	y	y	3	ev	cig+/-ot	35	999	0	0	nev any	ot
DOSEME	501		m	0	0	all	-	all	Eu:bal	1979	CC	1210	n	bl	n	n	2	ev	cig+/-ot	1	10	0	1	nev cigs	or
DOSEME	502		m	0	0	all	-	all	Eu:bal	1979	CC	1210	n	bl	n	n	2	ev	cig+/-ot	11	20	1	2	nev cigs	or
DOSEME	503		m	0	0	all	-	all	Eu:bal	1979	CC	1210	n	bl	n	n	2	ev	cig+/-ot	21	999	0	0	nev cigs	or
FAN	501		m	0	0	all	-	all	As:Chi	1990	CC	403	n	ot	y	n	0	ev	cig+/-ot	1	29	1	0	nev cigs	st
FAN	502		m	0	0	all	-	all	As:Chi	1990	CC	403	n	ot	y	n	0	ev	cig+/-ot	30	39	2	3	nev cigs	st
FAN	503		m	0	0	all	-	all	As:Chi	1990	CC	403	n	ot	y	n	0	ev	cig+/-ot	40	999	3	0	nev cigs	st
FAN	506		f	0	0	all	-	all	As:Chi	1990	CC	403	n	ot	y	n	0	ev	cig+/-ot	1	29	1	0	nev cigs	st
FAN	507		f	0	0	all	-	all	As:Chi	1990	CC	403	n	ot	y	n	0	ev	cig+/-ot	30	39	2	3	nev cigs	st
FAN	508		f	0	0	all	-	all	As:Chi	1990	CC	403	n	ot	y	n	0	ev	cig+/-ot	40	999	3	0	nev cigs	st
GAO	561	x	f	0	0	all	-	all	As:Chi	1984	CC	1405	n	ot	n	n	0	ev	cig+/-ot	1	29	1	0	nev cigs	st
GAO	562	x	f	0	0	all	-	all	As:Chi	1984	CC	1405	n	ot	n	n	0	ev	cig+/-ot	30	999	0	0	nev cigs	st
GARSHI	534	x	m	0	0	all	-	all	NAmer	1981	CC	1081	o	bl	y	n	0	ev	all/unsp	20	999	0	0	nev any	st
GER	513	x	c	0	0	all	-	all	As:oth	1990	CC	141	n	ot	y	n	0	ev	all/unsp	1	20	1	0	nev any	st
GER	514	x	c	0	0	all	-	all	As:oth	1990	CC	141	n	ot	y	n	0	ev	all/unsp	21	40	2	0	nev any	st
GER	515	x	c	0	0	all	-	all	As:oth	1990	CC	141	n	ot	y	n	0	ev	all/unsp	41	999	3	0	nev any	st
HAENSZ	542		f	0	0	all	-	not alv	NAmer	1955	CC	158	n	bl	n	y	0	ev	cig+/-ot	1	14	0	1	nev any	st
HAENSZ	543		f	0	0	all	-	not alv	NAmer	1955	CC	158	n	bl	n	y	0	ev	cig+/-ot	15	999	0	0	nev any	st
HU	501		m	0	0	all	-	all	As:Chi	1985	CC	227	n	ot	n	y	0	ev	cig+/-ot	1	19	0	1	nev cigs	st
HU	502		m	0	0	all	-	all	As:Chi	1985	CC	227	n	ot	n	y	0	ev	cig+/-ot	20	29	1	2	nev cigs	st
HU	503		m	0	0	all	-	all	As:Chi	1985	CC	227	n	ot	n	y	0	ev	cig+/-ot	30	999	0	0	nev cigs	st
HU	506		f	0	0	all	-	all	As:Chi	1985	CC	227	n	ot	n	y	0	ev	cig+/-ot	1	19	0	1	nev cigs	st
HU	507		f	0	0	all	-	all	As:Chi	1985	CC	227	n	ot	n	y	0	ev	cig+/-ot	20	29	1	2	nev cigs	st
HU	508		f	0	0	all	-	all	As:Chi	1985	CC	227	n	ot	n	y	0	ev	cig+/-ot	30	999	0	0	nev cigs	st
HU2	508		c	0	0	all	-	all	As:Chi	1977	CC	523	n	ot	y	n	0	ev	cig+/-ot	1	19	0	1	nev cigs	ot
HU2	509		c	0	0	all	-	all	As:Chi	1977	CC	523	n	ot	y	n	0	ev	cig+/-ot	20	29	1	2	nev cigs	or
HU2	510		c	0	0	all	-	all	As:Chi	1977	CC	523	n	ot	y	n	0	ev	cig+/-ot	30	39	2	3	nev cigs	or
HU2	511		c	0	0	all	-	all	As:Chi	1977	CC	523	n	ot	y	n	0	ev	cig+/-ot	40	999	3	0	nev cigs	st
HUMBLE	517	x	c	0	0	wh	-	not alv	NAmer	1980	CC	521	n	bl	y	n	0	cu	cig+/-ot	1	29	1	0	nev cigs	st
HUMBLE	518	x	c	0	0	wh	-	not alv	NAmer	1980	CC	521	n	bl	y	n	0	cu	cig+/-ot	30	39	2	3	nev cigs	st
HUMBLE	519	x	c	0	0	wh	-	not alv	NAmer	1980	CC	521	n	bl	y	n	0	cu	cig+/-ot	40	49	0	4	nev cigs	st

Table 1I16 - 4

IESLC - Meta-analysis of Ever/current Smoking by Duration, Overview
All LC types, Cigarettes (or Any Product if Cigarettes not available)
Least adjusted

REF	NRR	X	SEX	AGEL	AGEH	RACE	YF	LC TYPE	LOC	START	ST	NLC	R	VB	P	H	AD	SM	PRODUCT	exL	exH	S1	S2	DENOM	De
HUMBLE	520	x	c	0	0	wh	-	not alv	NAmer	1980	CC	521	n	bl	y	n	0	cu	cig+/-ot	50	59	3	5	nev cigs	st
HUMBLE	521	x	c	0	0	wh	-	not alv	NAmer	1980	CC	521	n	bl	y	n	0	cu	cig+/-ot	60	999	0	6	nev cigs	st
JOLY	515		m	0	0	all	-	all	SCAmer	1978	CC	826	n	bl	n	n	0	ev	cig+/-ot	1	19	0	1	nev any	st
JOLY	516		m	0	0	all	-	all	SCAmer	1978	CC	826	n	bl	n	n	0	ev	cig+/-ot	20	29	1	2	nev any	st
JOLY	517		m	0	0	all	-	all	SCAmer	1978	CC	826	n	bl	n	n	0	ev	cig+/-ot	30	39	2	3	nev any	st
JOLY	518		m	0	0	all	-	all	SCAmer	1978	CC	826	n	bl	n	n	0	ev	cig+/-ot	40	49	0	4	nev any	st
JOLY	519		m	0	0	all	-	all	SCAmer	1978	CC	826	n	bl	n	n	0	ev	cig+/-ot	50	999	3	0	nev any	st
JOLY	501		f	0	0	all	-	all	SCAmer	1978	CC	826	n	bl	n	n	0	ev	cig+/-ot	1	19	0	1	nev any	st
JOLY	502		f	0	0	all	-	all	SCAmer	1978	CC	826	n	bl	n	n	0	ev	cig+/-ot	20	29	1	2	nev any	st
JOLY	503		f	0	0	all	-	all	SCAmer	1978	CC	826	n	bl	n	n	0	ev	cig+/-ot	30	39	2	3	nev any	st
JOLY	504		f	0	0	all	-	all	SCAmer	1978	CC	826	n	bl	n	n	0	ev	cig+/-ot	40	49	0	4	nev any	st
JOLY	505		f	0	0	all	-	all	SCAmer	1978	CC	826	n	bl	n	n	0	ev	cig+/-ot	50	999	3	0	nev any	st
JUSSAW	510		m	0	0	all	-	all	As:Ind	1964	CC	792	n	V	n	n	0	ev	cig only	1	9	0	1	nev any	st
JUSSAW	511		m	0	0	all	-	all	As:Ind	1964	CC	792	n	V	n	n	0	ev	cig only	10	19	0	0	nev any	st
JUSSAW	512		m	0	0	all	-	all	As:Ind	1964	CC	792	n	V	n	n	0	ev	cig only	20	29	1	2	nev any	st
JUSSAW	513		m	0	0	all	-	all	As:Ind	1964	CC	792	n	V	n	n	0	ev	cig only	30	39	2	3	nev any	st
JUSSAW	514		m	0	0	all	-	all	As:Ind	1964	CC	792	n	V	n	n	0	ev	cig only	40	999	3	0	nev any	st
KAISE2	596		m	0	0	all	9	all	NAmer	1979	pr	318	n	bl	n	n	1	cu	cig only	1	39	0	0	nev any	st
KAISE2	597		m	0	0	all	9	all	NAmer	1979	pr	318	n	bl	n	n	1	cu	cig only	40	999	3	0	nev any	st
KAISE2	516		f	0	0	all	9	all	NAmer	1979	pr	318	n	bl	n	n	1	cu	cig only	1	39	0	0	nev any	st
KAISE2	517		f	0	0	all	9	all	NAmer	1979	pr	318	n	bl	n	n	1	cu	cig only	40	999	3	0	nev any	st
KATSOU	501	x	f	0	0	all	-	all	Eu:bal	1987	CC	101	n	bl	n	n	0	cu	all/unsp	1	19	0	1	nev any	st
KATSOU	502	x	f	0	0	all	-	all	Eu:bal	1987	CC	101	n	bl	n	n	0	cu	all/unsp	20	29	1	2	nev any	st
KATSOU	503	x	f	0	0	all	-	all	Eu:bal	1987	CC	101	n	bl	n	n	0	cu	all/unsp	30	39	2	3	nev any	st
KATSOU	504	x	f	0	0	all	-	all	Eu:bal	1987	CC	101	n	bl	n	n	0	cu	all/unsp	40	999	3	0	nev any	st
KHUDER	501		m	0	0	all	-	all	NAmer	1985	CC	482	n	bl	n	y	0	ev	cig+/-ot	1	29	1	0	nev cigs	st
KHUDER	502		m	0	0	all	-	all	NAmer	1985	CC	482	n	bl	n	y	0	ev	cig+/-ot	30	49	2	0	nev cigs	st
KHUDER	503		m	0	0	all	-	all	NAmer	1985	CC	482	n	bl	n	y	0	ev	cig+/-ot	50	999	3	0	nev cigs	st
KREUZE	517		m	0	0	all	-	all	Eu:Ger	1990	CC	2260	n	bl	n	n	3	ev	all/unsp	1	19	0	1	nev any	st
KREUZE	518		m	0	0	all	-	all	Eu:Ger	1990	CC	2260	n	bl	n	n	3	ev	all/unsp	20	999	0	0	nev any	ot
KREUZE	520		f	0	0	all	-	all	Eu:Ger	1990	CC	2260	n	bl	n	n	3	ev	all/unsp	1	19	0	1	nev any	ot
KREUZE	521		f	0	0	all	-	all	Eu:Ger	1990	CC	2260	n	bl	n	n	3	ev	all/unsp	20	999	0	0	nev any	ot
LETOUR	506		c	0	0	all	-	all	NAmer	1983	CC	738	n	V	y	y	0	ev	cig+/-ot	1	24	1	0	nev cigs	st
LETOUR	507		c	0	0	all	-	all	NAmer	1983	CC	738	n	V	y	y	0	ev	cig+/-ot	25	40	2	0	nev cigs	st
LETOUR	508		c	0	0	all	-	all	NAmer	1983	CC	738	n	V	y	y	0	ev	cig+/-ot	41	999	3	0	nev cigs	st
LEVIN	501	x	m	0	0	all	-	all	NAmer	1938	CC	475	n	bl	n	n	0	ev	cig+/-ot	1	39	0	0	nev any	st
LEVIN	502	x	m	0	0	all	-	all	NAmer	1938	CC	475	n	bl	n	n	0	ev	cig+/-ot	40	999	3	0	nev any	st
LIAW	501		c	0	0	all	0	all	As:oth	1982	pr	127	n	ot	n	n	2	cu	all/unsp	1	20	1	0	nev any	or
LIAW	502		c	0	0	all	0	all	As:oth	1982	pr	127	n	ot	n	n	2	cu	all/unsp	21	30	0	3	nev any	or
LIAW	503		c	0	0	all	0	all	As:oth	1982	pr	127	n	ot	n	n	2	cu	all/unsp	31	999	0	0	nev any	or
LIU3	507	x	m	0	0	all	-	all	As:Chi	1985	CC	110	n	ot	n	n	0	ev	all/unsp	1	34	1	0	nev any	or
LIU3	508	x	m	0	0	all	-	all	As:Chi	1985	CC	110	n	ot	n	n	0	ev	all/unsp	35	999	0	0	nev any	st
LIU5	504		c	0	0	all	-	all	As:Chi	1978	CC	111	n	ot	y	n	0	ev	all/unsp	1	29	1	0	nev any	st
LIU5	505		c	0	0	all	-	all	As:Chi	1978	CC	111	n	ot	y	n	0	ev	all/unsp	30	999	0	0	nev any	st
LUBIN	508		m	0	0	all	-	all	As:Chi	1984	CC	427	m	ot	y	n	0	ev	cig+/-ot	1	29	1	0	nev any	st
LUBIN	509		m	0	0	all	-	all	As:Chi	1984	CC	427	m	ot	y	n	0	ev	cig+/-ot	30	39	2	3	nev any	st
LUBIN	510		m	0	0	all	-	all	As:Chi	1984	CC	427	m	ot	y	n	0	ev	cig+/-ot	40	49	0	4	nev any	st
LUBIN	511		m	0	0	all	-	all	As:Chi	1984	CC	427	m	ot	y	n	0	ev	cig+/-ot	50	999	3	0	nev any	st
LUBIN2	531		m	0	0	all	-	all	Eu:mul	1976	CC	7804	n	bl	n	y	0	ev	cig+/-ot	1	29	1	0	nev any	st
LUBIN2	532		m	0	0	all	-	all	Eu:mul	1976	CC	7804	n	bl	n	y	0	ev	cig+/-ot	30	39	2	3	nev any	st
LUBIN2	533		m	0	0	all	-	all	Eu:mul	1976	CC	7804	n	bl	n	y	0	ev	cig+/-ot	40	49	0	4	nev any	st
LUBIN2	534		m	0	0	all	-	all	Eu:mul	1976	CC	7804	n	bl	n	y	0	ev	cig+/-ot	50	999	3	0	nev any	st
LUBIN2	574		f	0	0	all	-	all	Eu:mul	1976	CC	7804	n	bl	n	y	0	ev	cig+/-ot	1	29	1	0	nev any	st
LUBIN2	575		f	0	0	all	-	all	Eu:mul	1976	CC	7804	n	bl	n	y	0	ev	cig+/-ot	30	39	2	3	nev any	st
LUBIN2	576		f	0	0	all	-	all	Eu:mul	1976	CC	7804	n	bl	n	y	0	ev	cig+/-ot	40	49	0	4	nev any	st
LUBIN2	577		f	0	0	all	-	all	Eu:mul	1976	CC	7804	n	bl	n	y	0	ev	cig+/-ot	50	999	3	0	nev any	st
MATOS	516	x	m	0	0	all	-	all	SCAmer	1994	CC	200	n	bl	n	n	0	ev	cig+/-ot	1	24	1	0	nev any	st
MATOS	517	x	m	0	0	all	-	all	SCAmer	1994	CC	200	n	bl	n	n	0	ev	cig+/-ot	25	39	2	3	nev any	st
MATOS	518	x	m	0	0	all	-	all	SCAmer	1994	CC	200	n	bl	n	n	0	ev	cig+/-ot	40	70	3	0	nev any	st
MCCONN	501		c	0	0	all	-	all	Eu:UK	1946	CC	100	n	V	n	y	0	ev	all/unsp	1	9	0	1	nev any	st
MCCONN	502		c	0	0	all	-	all	Eu:UK	1946	CC	100	n	V	n	y	0	ev	all/unsp	10	19	0	0	nev any	st
MCCONN	503		c	0	0	all	-	all	Eu:UK	1946	CC	100	n	V	n	y	0	ev	all/unsp	20	29	1	2	nev any	st
MCCONN	504		c	0	0	all	-	all	Eu:UK	1946	CC	100	n	V	n	y	0	ev	all/unsp	30	39	2	3	nev any	st
MCCONN	505		c	0	0	all	-	all	Eu:UK	1946	CC	100	n	V	n	y	0	ev	all/unsp	40	999	3	0	nev any	st
NOTAN2	513		c	0	0	all	-	all	As:Ind	1963	CC	683	n	V	n	n	0	ev	cig only	1	10	0	1	nev any	st
NOTAN2	514		c	0	0	all	-	all	As:Ind	1963	CC	683	n	V	n	n	0	ev	cig only	11	20	1	2	nev any	st
NOTAN2	515		c	0	0	all	-	all	As:Ind	1963	CC	683	n	V	n	n	0	ev	cig only	21	30	0	3	nev any	st
NOTAN2	516		c	0	0	all	-	all	As:Ind	1963	CC	683	n	V	n	n	0	ev	cig only	31	40	2	4	nev any	st
NOTAN2	517		c	0	0	all	-	all	As:Ind	1963	CC	683	n	V	n	n	0	ev	cig only	41	999	3	0	nev any	st
OSANN2	501	x	f	0	0	all	-	all	NAmer	1964	ot	217	n	bl	n	y	0	ev	cig+/-ot	1	20	1	0	nev cigs	st
OSANN2	502	x	f	0	0	all	-	all	NAmer	1964	ot	217	n	bl	n	y	0	ev	cig+/-ot	21	999	0	0	nev cigs	st
PEZZO2	507		m	0	0	all	-	all	SCAmer	1992	CC	367	n	bl	n	y	0	cu	cig+/-ot	1	35	0	0	nev cigs	st

Table 1I16 - 4

IESLC - Meta-analysis of Ever/current Smoking by Duration, Overview
All LC types, Cigarettes (or Any Product if Cigarettes not available)
Least adjusted

REF	NRR	X	SEX	AGEL	AGEH	RACE	YF	LC TYPE	LOC	START	ST	NLC	R	VB	P	H	AD	SM	PRODUCT	exL	exH	S1	S2	DENOM	De
PEZZO2	508		m	0	0	all	-	all	SCAmer	1992	CC	367	n	bl	n	y	0	cu	cig+/-ot	36	999	3	0	nev cigs	st
PEZZOT	534		m	0	0	all	-	all	SCAmer	1987	CC	215	n	bl	n	y	0	ev	cig only	1	30	1	0	nev cigs	st
PEZZOT	535		m	0	0	all	-	all	SCAmer	1987	CC	215	n	bl	n	y	0	ev	cig only	31	40	2	4	nev cigs	st
PEZZOT	536		m	0	0	all	-	all	SCAmer	1987	CC	215	n	bl	n	y	0	ev	cig only	41	999	3	0	nev cigs	st
QIAO2	511	x	m	0	0	all	0	all	As:Chi	1992	pr	241	m	ot	n	n	0	ev	all/unsp	1	27	1	0	nev any	st
QIAO2	512	x	m	0	0	all	0	all	As:Chi	1992	pr	241	m	ot	n	n	0	ev	all/unsp	28	41	2	0	nev any	st
QIAO2	513	x	m	0	0	all	0	all	As:Chi	1992	pr	241	m	ot	n	n	0	ev	all/unsp	42	999	3	0	nev any	st
RACHTA	511	x	f	0	0	all	-	all	Eu:est	1991	CC	118	n	bl	n	y	0	ev	cig+/-ot	1	20	1	0	nev cigs	st
RACHTA	512	x	f	0	0	all	-	all	Eu:est	1991	CC	118	n	bl	n	y	0	ev	cig+/-ot	21	40	2	0	nev cigs	st
RACHTA	513	x	f	0	0	all	-	all	Eu:est	1991	CC	118	n	bl	n	y	0	ev	cig+/-ot	41	999	3	0	nev cigs	st
SOBUE	546		m	0	0	all	-	q+s+l+a	As:Jap	1986	CC	1376	n	bl	n	y	0	cu	cig+/-ot	1	29	1	0	nev cigs	st
SOBUE	547		m	0	0	all	-	q+s+l+a	As:Jap	1986	CC	1376	n	bl	n	y	0	cu	cig+/-ot	30	39	2	3	nev cigs	st
SOBUE	548		m	0	0	all	-	q+s+l+a	As:Jap	1986	CC	1376	n	bl	n	y	0	cu	cig+/-ot	40	49	0	4	nev cigs	st
SOBUE	549		m	0	0	all	-	q+s+l+a	As:Jap	1986	CC	1376	n	bl	n	y	0	cu	cig+/-ot	50	999	3	0	nev cigs	st
TIZZAN	501		m	0	0	all	-	all	Eu:wst	1959	CC	1358	n	bl	n	n	0	ev	cig only	1	4	0	0	nev any	st
TIZZAN	502		m	0	0	all	-	all	Eu:wst	1959	CC	1358	n	bl	n	n	0	ev	cig only	5	10	0	1	nev any	st
TIZZAN	503		m	0	0	all	-	all	Eu:wst	1959	CC	1358	n	bl	n	n	0	ev	cig only	11	999	0	0	nev any	st
TIZZAN	530		f	0	0	all	-	all	Eu:wst	1959	CC	1358	n	bl	n	n	0	ev	cig only	1	10	0	1	nev any	st
TIZZAN	531		f	0	0	all	-	all	Eu:wst	1959	CC	1358	n	bl	n	n	0	ev	cig only	11	999	0	0	nev any	st
WANG2	501		c	0	0	all	-	all	As:Chi	1980	CC	103	n	ot	n	n	0	ev	cig+/-ot	1	19	0	1	nev cigs	st
WANG2	503		c	0	0	all	-	all	As:Chi	1980	CC	103	n	ot	n	n	0	ev	cig+/-ot	20	29	1	2	nev cigs	st
WANG2	504		c	0	0	all	-	all	As:Chi	1980	CC	103	n	ot	n	n	0	ev	cig+/-ot	30	39	2	3	nev cigs	st
WANG2	505		c	0	0	all	-	all	As:Chi	1980	CC	103	n	ot	n	n	0	ev	cig+/-ot	40	49	0	4	nev cigs	st
WUWILL	501	x	f	0	0	all	-	all	As:Chi	1985	CC	965	n	ot	n	n	0	ev	cig+/-ot	1	29	1	0	nev cigs	st
WUWILL	502	x	f	0	0	all	-	all	As:Chi	1985	CC	965	n	ot	n	n	0	ev	cig+/-ot	30	39	2	3	nev cigs	st
WUWILL	503	x	f	0	0	all	-	all	As:Chi	1985	CC	965	n	ot	n	n	0	ev	cig+/-ot	40	999	3	0	nev cigs	st
ZHENG	553		m	0	0	all	-	all	As:Chi	1982	CC	540	n	ot	*	y	0	ev	cig+/-ot	1	29	1	0	nev cigs	st
ZHENG	554		m	0	0	all	-	all	As:Chi	1982	CC	540	n	ot	*	y	0	ev	cig+/-ot	30	999	0	0	nev cigs	st
ZHENG	558		f	0	0	all	-	all	As:Chi	1982	CC	540	n	ot	*	y	0	ev	cig+/-ot	1	29	1	0	nev cigs	st
ZHENG	559		f	0	0	all	-	all	As:Chi	1982	CC	540	n	ot	*	y	0	ev	cig+/-ot	30	999	0	0	nev cigs	st
ZHOU	501		c	0	0	all	-	all	As:Chi	1978	CC	1360	n	ot	n	n	0	ev	all/unsp	1	19	0	1	nev any	st
ZHOU	502		c	0	0	all	-	all	As:Chi	1978	CC	1360	n	ot	n	n	0	ev	all/unsp	20	999	0	0	nev any	st

Cigarette type is all/unspec for all RRs

except for the following:

REF	NRR	CIGTYPE
JUSSAW	510	MC only
JUSSAW	511	MC only
JUSSAW	512	MC only
JUSSAW	513	MC only
JUSSAW	514	MC only
NOTAN2	513	MC only
NOTAN2	514	MC only
NOTAN2	515	MC only
NOTAN2	516	MC only
NOTAN2	517	MC only

In this overview table, subtotals and Qs values may be invalid and should be ignored

Table 1I16 - 5

IESLC - Meta-analysis of Ever/current Smoking by Duration, Overview
All LC types, Cigarettes (or Any Product if Cigarettes not available)
Least adjusted

				Number Exposed		Non-exposed			
REF	NRR	SEX	AD	Case	Cont	Case	Cont	RR	95.00%CI
AGUDO	507	f	0	5	12	80	183	0.95	(0.33- 2.79)
AGUDO	508	f	0	18	11	80	183	3.74	(1.69- 8.29)
Subtotal AGUDO								2.31	(1.22- 4.38)
*AMANDU	501	m	0	42	68909	6	25350	2.58	(1.09- 6.06)
*AMANDU	502	m	0	72	27096	6	25350	11.23	(4.88- 25.81)
Subtotal AMANDU								5.48	(3.02- 9.96)
ARMADA	501	m	0	21	55	8	71	3.39	(1.40- 8.23)
ARMADA	502	m	0	219	166	8	71	11.71	(5.49- 24.99)
ARMADA	503	m	0	77	33	8	71	20.71	(8.97- 47.82)
Subtotal ARMADA								9.86	(6.13- 15.85)
AUVINE	501	c	0	26	18	44	229	7.52	(3.80- 14.87)
AUVINE	502	c	0	10	5	44	229	10.41	(3.39- 31.93)
AUVINE	503	c	0	230	57	44	229	21.00	(13.61- 32.41)
Subtotal AUVINE								15.02	(10.61- 21.28)
AXELSS	501	m	0	13	84	16	160	1.55	(0.71- 3.37)
AXELSS	502	m	0	17	64	16	160	2.66	(1.27- 5.58)
AXELSS	503	m	0	57	71	16	160	8.03	(4.31- 14.94)
AXELSS	504	m	0	104	85	16	160	12.24	(6.79- 22.04)
AXELSS	505	m	0	101	40	16	160	25.25	(13.43- 47.46)
AXELSS	510	f	0	5	24	18	154	1.78	(0.61- 5.25)
AXELSS	511	f	0	12	29	18	154	3.54	(1.54- 8.13)
AXELSS	512	f	0	29	26	18	154	9.54	(4.64- 19.61)
AXELSS	513	f	0	44	20	18	154	18.82	(9.17- 38.65)
AXELSS	514	f	0	20	10	18	154	17.11	(6.94- 42.19)
Subtotal AXELSS								7.94	(6.31- 10.00)
BARBON	501	m	0	42	91	22	188	3.94	(2.22- 7.00)
BARBON	502	m	0	118	102	22	188	9.89	(5.91- 16.55)
BARBON	503	m	0	207	139	22	188	12.73	(7.79- 20.80)
BARBON	504	m	0	366	235	22	188	13.31	(8.31- 21.32)
Subtotal BARBON								9.63	(7.47- 12.42)
*BEST	501	m	1	1	-	7	-	1.60	(0.20- 13.00)
*BEST	502	m	1	2	-	7	-	2.60	(0.54- 12.52)
*BEST	503	m	1	6	-	7	-	2.30	(0.77- 6.84)
*BEST	504	m	1	10	-	7	-	3.20	(1.22- 8.41)
*BEST	505	m	1	22	-	7	-	4.10	(1.75- 9.60)
*BEST	506	m	1	55	-	7	-	13.90	(6.33- 30.52)
*BEST	507	m	1	137	-	7	-	14.20	(6.64- 30.35)
Subtotal BEST								6.17	(4.26- 8.94)
*BOUCOT	518	m	0	29	2621	0	805	18.13~	(1.11- 296.36)
*BOUCOT	519	m	0	52	1563	0	805	54.09~	(3.34- 875.17)
Subtotal BOUCOT								31.38	(4.37- 225.47)
BUFFLE	526	f	0	52	57	12	112	8.51	(4.21- 17.22)
BUFFLE	527	f	0	97	62	12	112	14.60	(7.43- 28.69)
BUFFLE	528	f	0	90	42	12	112	20.00	(9.94- 40.23)
Subtotal BUFFLE								13.60	(9.12- 20.29)
*CEDERL	501	m	1	5	-	7	-	1.80	(0.57- 5.66)
*CEDERL	502	m	1	23	-	7	-	7.40	(3.18- 17.21)
*CEDERL	504	f	1	3	-	19	-	1.60	(0.47- 5.40)
*CEDERL	505	f	1	5	-	19	-	9.60	(3.60- 25.58)
Subtotal CEDERL								4.61	(2.78- 7.67)
CHEN2	501	m	0	2	3	9	33	2.44	(0.35- 16.93)
CHEN2	502	m	0	4	3	9	33	4.89	(0.92- 25.93)
CHEN2	503	m	0	17	24	9	33	2.60	(0.99- 6.81)
CHEN2	504	m	0	36	27	9	33	4.89	(2.01- 11.91)
CHEN2	505	m	0	62	40	9	33	5.68	(2.46- 13.13)
CHEN2	510	f	0	1	6	25	33	0.22	(0.02- 1.95)
CHEN2	511	f	0	2	2	25	33	1.32	(0.17- 10.03)
CHEN2	512	f	0	13	6	25	33	2.86	(0.95- 8.58)
CHEN2	513	f	0	21	15	25	33	1.85	(0.80- 4.29)
Subtotal CHEN2								3.01	(2.07- 4.38)
CHOI	501	m	0	19	55	13	95	2.52	(1.16- 5.51)
CHOI	502	m	0	66	166	13	95	2.91	(1.52- 5.54)
CHOI	503	m	0	102	160	13	95	4.66	(2.48- 8.75)
CHOI	504	m	0	60	64	13	95	6.85	(3.48- 13.50)
CHOI	505	m	0	20	20	13	95	7.31	(3.13- 17.07)
CHOI	510	f	0	2	9	76	164	0.48	(0.10- 2.27)
CHOI	511	f	0	8	14	76	164	1.23	(0.50- 3.06)
CHOI	512	f	0	8	2	76	164	8.63	(1.79- 41.62)
CHOI	513	f	0	1	1	76	164	2.16	(0.13- 34.96)
Subtotal CHOI								3.63	(2.73- 4.83)
*CPSI	580	m	0	95	266163	196	926068	1.69	(1.32- 2.15)

Table 1I16 - 5

IESLC - Meta-analysis of Ever/current Smoking by Duration, Overview
All LC types, Cigarettes (or Any Product if Cigarettes not available)
Least adjusted

REF	NRR	SEX	AD	Number Exposed Case	Number Exposed Cont	Non-exposed Case	Non-exposed Cont	RR	95.00%CI
*CPSI	581	m	0	230	290031	196	926068	3.75	(3.10- 4.53)
*CPSI	582	m	0	470	367622	196	926068	6.04	(5.11- 7.14)
*CPSI	583	m	0	731	333292	196	926068	10.36	(8.85- 12.13)
*CPSI	584	m	0	764	221405	196	926068	16.30	(13.94- 19.07)
*CPSI	585	m	0	576	119633	196	926068	22.75	(19.35- 26.75)
*CPSI	586	m	0	356	53226	196	926068	31.60	(26.55- 37.61)
*CPSI	587	m	0	232	26906	196	926068	40.74	(33.70- 49.25)
*CPSI	676	f	0	105	694015	532	3877179	1.10	(0.89- 1.36)
*CPSI	677	f	0	141	383127	532	3877179	2.68	(2.23- 3.23)
*CPSI	678	f	0	154	315060	532	3877179	3.56	(2.98- 4.26)
*CPSI	679	f	0	120	163178	532	3877179	5.36	(4.40- 6.53)
*CPSI	680	f	0	54	53635	532	3877179	7.34	(5.55- 9.71)
*CPSI	681	f	0	16	14305	532	3877179	8.15	(4.96- 13.40)
*CPSI	682	f	0	10	5657	532	3877179	12.88	(6.90- 24.07)
Subtotal CPSI								8.01	(7.62- 8.43)
*CPSII	552	m	0	72	141932	124	742207	3.04	(2.27- 4.06)
*CPSII	553	m	0	145	113317	124	742207	7.66	(6.03- 9.73)
*CPSII	554	m	0	244	109788	124	742207	13.30	(10.72- 16.51)
*CPSII	555	m	0	413	103500	124	742207	23.88	(19.54- 29.19)
*CPSII	556	m	0	307	53805	124	742207	34.15	(27.73- 42.06)
*CPSII	557	m	0	332	39260	124	742207	50.62	(41.19- 62.20)
*CPSII	558	m	0	151	13598	124	742207	66.47	(52.45- 84.24)
*CPSII	559	m	0	117	8450	124	742207	82.88	(64.43- 106.60)
*CPSII	618	f	0	127	301244	310	2091302	2.84	(2.31- 3.50)
*CPSII	619	f	0	158	152833	310	2091302	6.97	(5.76- 8.45)
*CPSII	620	f	0	193	116270	310	2091302	11.20	(9.36- 13.40)
*CPSII	621	f	0	216	91501	310	2091302	15.93	(13.39- 18.94)
*CPSII	622	f	0	153	44769	310	2091302	23.06	(19.00- 27.98)
*CPSII	623	f	0	122	29119	310	2091302	28.26	(22.93- 34.84)
*CPSII	624	f	0	27	6262	310	2091302	29.09	(19.64- 43.07)
*CPSII	625	f	0	18	2224	310	2091302	54.60	(34.01- 87.65)
Subtotal CPSII								17.37	(16.44- 18.35)
DAMBER	506	m	1	-	-	42	-	1.58	(0.69- 3.66)
DAMBER	507	m	1	-	-	42	-	3.66	(2.18- 6.73)
DAMBER	508	m	1	-	-	42	-	5.15	(3.27- 8.32)
DAMBER	509	m	1	-	-	42	-	8.71	(5.84- 13.66)
DAMBER	510	m	1	-	-	42	-	11.19	(7.43- 17.33)
Subtotal DAMBER								6.42	(5.14- 8.01)
DEAN2	501	m	0	34	36	33	112	3.21	(1.74- 5.89)
DEAN2	502	m	0	631	558	33	112	3.84	(2.56- 5.75)
DEAN2	504	f	0	10	14	88	121	0.98	(0.42- 2.31)
DEAN2	505	f	0	47	11	88	121	5.88	(2.88- 11.97)
Subtotal DEAN2								3.39	(2.55- 4.52)
DESTEF	501	m	0	43	55	27	163	4.72	(2.67- 8.35)
DESTEF	502	m	0	78	78	27	163	6.04	(3.61- 10.10)
DESTEF	503	m	0	171	93	27	163	11.10	(6.87- 17.92)
DESTEF	504	m	0	178	108	27	163	9.95	(6.20- 15.96)
Subtotal DESTEF								7.86	(6.10- 10.11)
DOLL	515	m	0	12	15	7	61	6.97	(2.34- 20.73)
DOLL	516	m	0	34	65	7	61	4.56	(1.88- 11.05)
DOLL	517	m	0	746	725	7	61	8.97	(4.07- 19.73)
DOLL	518	m	0	558	491	7	61	9.90	(4.49- 21.85)
DOLL	522	f	0	14	18	40	59	1.15	(0.51- 2.57)
DOLL	523	f	0	12	8	40	59	2.21	(0.83- 5.90)
DOLL	524	f	0	36	20	40	59	2.66	(1.35- 5.23)
DOLL	525	f	0	6	3	40	59	2.95	(0.70- 12.49)
Subtotal DOLL								3.93	(2.89- 5.34)
DORGAN	570	m	2	-	-	-	-	5.44	(2.97- 9.98)
DORGAN	571	m	2	-	-	-	-	16.09	(8.96- 28.88)
DORGAN	562	f	3	-	-	-	-	4.25	(3.20- 5.64)
DORGAN	563	f	3	-	-	-	-	11.73	(9.07- 15.18)
Subtotal DORGAN								7.74	(6.50- 9.20)
DOSEME	501	m	2	32	-	142	-	1.00	(0.60- 1.70)
DOSEME	502	m	2	158	-	142	-	3.80	(2.60- 5.70)
DOSEME	503	m	2	466	-	142	-	4.90	(3.50- 7.00)
Subtotal DOSEME								3.27	(2.59- 4.12)
FAN	501	m	0	29	135	36	236	1.41	(0.83- 2.40)
FAN	502	m	0	44	122	36	236	2.36	(1.45- 3.87)
FAN	503	m	0	143	241	36	236	3.89	(2.59- 5.84)
FAN	506	f	0	8	15	69	320	2.47	(1.01- 6.06)
FAN	507	f	0	19	23	69	320	3.83	(1.98- 7.42)

Table 1I16 - 5

IESLC - Meta-analysis of Ever/current Smoking by Duration, Overview
All LC types, Cigarettes (or Any Product if Cigarettes not available)
Least adjusted

REF	NRR	SEX	AD	Number Exposed Case	Cont	Non-exposed Case	Cont	RR	95.00%CI
FAN	508	f	0	55	59	69	320	4.32	(2.76- 6.78)
Subtotal FAN								3.01	(2.43- 3.72)
GAO	561	f	0	68	58	435	605	1.63	(1.12- 2.36)
GAO	562	f	0	168	72	435	605	3.25	(2.40- 4.39)
Subtotal GAO								2.47	(1.95- 3.12)
GARSHI	534	m	0	922	1314	41	363	6.21	(4.45- 8.67)
GER	513	c	0	10	40	51	246	1.21	(0.57- 2.57)
GER	514	c	0	31	123	51	246	1.22	(0.74- 2.00)
GER	515	c	0	49	155	51	246	1.52	(0.98- 2.37)
Subtotal GER								1.35	(1.00- 1.83)
HAENSZ	542	f	0	16	26	81	236	1.79	(0.92- 3.51)
HAENSZ	543	f	0	58	77	81	236	2.19	(1.44- 3.35)
Subtotal HAENSZ								2.07	(1.45- 2.97)
HU	501	m	0	41	33	41	67	2.03	(1.11- 3.70)
HU	502	m	0	60	47	41	67	2.09	(1.21- 3.60)
HU	503	m	0	19	14	41	67	2.22	(1.00- 4.90)
HU	506	f	0	11	8	40	48	1.65	(0.61- 4.50)
HU	507	f	0	11	7	40	48	1.89	(0.67- 5.32)
HU	508	f	0	4	3	40	48	1.60	(0.34- 7.57)
Subtotal HU								2.00	(1.46- 2.74)
HU2	508	c	0	21	33	121	213	1.12	(0.62- 2.02)
HU2	509	c	0	64	63	121	213	1.79	(1.18- 2.70)
HU2	510	c	0	123	101	121	213	2.14	(1.52- 3.03)
HU2	511	c	0	194	113	121	213	3.02	(2.19- 4.17)
Subtotal HU2								2.18	(1.79- 2.64)
HUMBLE	517	c	0	20	33	28	285	6.17	(3.13- 12.15)
HUMBLE	518	c	0	68	58	28	285	11.93	(7.07- 20.13)
HUMBLE	519	c	0	104	59	28	285	17.94	(10.85- 29.66)
HUMBLE	520	c	0	90	55	28	285	16.66	(9.97- 27.82)
HUMBLE	521	c	0	29	22	28	285	13.42	(6.82- 26.39)
Subtotal HUMBLE								13.29	(10.33- 17.10)
JOLY	515	m	0	11	48	12	218	4.16	(1.73- 9.99)
JOLY	516	m	0	38	61	12	218	11.32	(5.57- 22.98)
JOLY	517	m	0	85	165	12	218	9.36	(4.95- 17.70)
JOLY	518	m	0	168	182	12	218	16.77	(9.04- 31.11)
JOLY	519	m	0	250	253	12	218	17.95	(9.78- 32.93)
JOLY	501	f	0	13	28	52	283	2.53	(1.23- 5.20)
JOLY	502	f	0	18	26	52	283	3.77	(1.93- 7.36)
JOLY	503	f	0	31	24	52	283	7.03	(3.82- 12.93)
JOLY	504	f	0	47	24	52	283	10.66	(6.00- 18.92)
JOLY	505	f	0	57	20	52	283	15.51	(8.61- 27.95)
Subtotal JOLY								9.03	(7.36- 11.08)
JUSSAW	510	m	0	16	20	149	624	3.35	(1.70- 6.62)
JUSSAW	511	m	0	34	19	149	624	7.49	(4.16- 13.51)
JUSSAW	512	m	0	38	23	149	624	6.92	(4.00- 11.97)
JUSSAW	513	m	0	27	9	149	624	12.56	(5.79- 27.28)
JUSSAW	514	m	0	11	6	149	624	7.68	(2.79- 21.09)
Subtotal JUSSAW								6.77	(5.01- 9.15)
*KAISE2	596	m	1	17	-	14	-	4.86	(2.22- 10.61)
*KAISE2	597	m	1	34	-	14	-	15.64	(8.31- 29.40)
*KAISE2	516	f	1	24	-	11	-	9.09	(4.25- 19.43)
*KAISE2	517	f	1	26	-	11	-	30.41	(14.39- 64.25)
Subtotal KAISE2								12.59	(8.77- 18.07)
KATSOU	501	f	0	5	5	48	67	1.40	(0.38- 5.09)
KATSOU	502	f	0	8	7	48	67	1.60	(0.54- 4.70)
KATSOU	503	f	0	15	2	48	67	10.47	(2.29- 47.93)
KATSOU	504	f	0	17	4	48	67	5.93	(1.88- 18.75)
Subtotal KATSOU								3.06	(1.66- 5.67)
KHUDER	501	m	0	16	61	23	309	3.52	(1.76- 7.06)
KHUDER	502	m	0	207	370	23	309	7.52	(4.76- 11.86)
KHUDER	503	m	0	236	354	23	309	8.96	(5.69- 14.11)
Subtotal KHUDER								7.07	(5.28- 9.47)
KREUZE	517	m	3	-	-	-	-	4.70	(3.10- 7.14)
KREUZE	518	m	3	-	-	-	-	29.23	(19.78- 43.20)
KREUZE	520	f	3	-	-	-	-	1.33	(0.80- 2.22)
KREUZE	521	f	3	-	-	-	-	7.14	(4.92- 10.35)
Subtotal KREUZE								7.26	(5.90- 8.93)
LETOUR	506	c	0	65	187	24	224	3.24	(1.95- 5.39)
LETOUR	507	c	0	264	160	24	224	15.40	(9.68- 24.51)
LETOUR	508	c	0	374	141	24	224	24.76	(15.58- 39.35)
Subtotal LETOUR								11.50	(8.73- 15.14)

Table 1I16 - 5

IESLC - Meta-analysis of Ever/current Smoking by Duration, Overview
All LC types, Cigarettes (or Any Product if Cigarettes not available)
Least adjusted

REF	NRR	SEX	AD	Number Exposed Case	Cont	Non-exposed Case	Cont	RR	95.00%CI
LEVIN	501	m	0	56	97	7	96	7.92	(3.44- 18.25)
LEVIN	502	m	0	63	91	7	96	9.49	(4.13- 21.81)
Subtotal LEVIN								8.67	(4.81- 15.63)
*LIAW	501	c	2	-	-	-	-	0.90	(0.30- 3.10)
*LIAW	502	c	2	-	-	-	-	2.60	(1.20- 5.90)
*LIAW	503	c	2	-	-	-	-	4.70	(2.70- 8.20)
Subtotal LIAW								3.19	(2.09- 4.88)
LIU3	507	m	0	30	146	4	19	0.98	(0.31- 3.07)
LIU3	508	m	0	22	59	4	19	1.77	(0.54- 5.79)
Subtotal LIU3								1.30	(0.57- 2.97)
LIU5	504	c	0	27	37	26	41	1.15	(0.57- 2.31)
LIU5	505	c	0	58	33	26	41	2.77	(1.45- 5.32)
Subtotal LIU5								1.84	(1.14- 2.96)
LUBIN	508	m	0	30	146	8	72	1.85	(0.81- 4.24)
LUBIN	509	m	0	124	294	8	72	3.80	(1.78- 8.12)
LUBIN	510	m	0	143	251	8	72	5.13	(2.40- 10.95)
LUBIN	511	m	0	59	86	8	72	6.17	(2.77- 13.77)
Subtotal LUBIN								3.94	(2.66- 5.83)
LUBIN2	531	m	0	953	2995	190	2616	4.38	(3.72- 5.16)
LUBIN2	532	m	0	2227	3470	190	2616	8.84	(7.56- 10.33)
LUBIN2	533	m	0	2079	2551	190	2616	11.22	(9.58- 13.14)
LUBIN2	534	m	0	1325	1484	190	2616	12.29	(10.42- 14.50)
LUBIN2	574	f	0	132	230	336	1188	2.03	(1.59- 2.59)
LUBIN2	575	f	0	187	186	336	1188	3.55	(2.81- 4.50)
LUBIN2	576	f	0	155	118	336	1188	4.64	(3.55- 6.07)
LUBIN2	577	f	0	81	32	336	1188	8.95	(5.84- 13.71)
Subtotal LUBIN2								6.83	(6.37- 7.32)
MATOS	516	m	0	20	84	11	110	2.38	(1.08- 5.24)
MATOS	517	m	0	82	110	11	110	7.45	(3.77- 14.75)
MATOS	518	m	0	86	89	11	110	9.66	(4.86- 19.21)
Subtotal MATOS								5.99	(3.96- 9.05)
MCCONN	501	c	0	3	4	9	23	1.92	(0.36- 10.32)
MCCONN	502	c	0	5	19	9	23	0.67	(0.19- 2.35)
MCCONN	503	c	0	46	57	9	23	2.06	(0.87- 4.89)
MCCONN	504	c	0	21	57	9	23	0.94	(0.38- 2.36)
MCCONN	505	c	0	16	40	9	23	1.02	(0.39- 2.68)
Subtotal MCCONN								1.22	(0.76- 1.94)
NOTAN2	513	c	0	7	15	107	201	0.88	(0.35- 2.22)
NOTAN2	514	c	0	15	15	107	201	1.88	(0.88- 3.99)
NOTAN2	515	c	0	17	16	107	201	2.00	(0.97- 4.11)
NOTAN2	516	c	0	12	7	107	201	3.22	(1.23- 8.42)
NOTAN2	517	c	0	5	5	107	201	1.88	(0.53- 6.63)
Subtotal NOTAN2								1.83	(1.24- 2.70)
OSANN2	501	f	0	23	47	33	109	1.62	(0.86- 3.04)
OSANN2	502	f	0	161	57	33	109	9.33	(5.70- 15.27)
Subtotal OSANN2								4.81	(3.26- 7.10)
PEZZO2	507	m	0	60	72	6	117	16.25	(6.68- 39.53)
PEZZO2	508	m	0	173	126	6	117	26.77	(11.42- 62.76)
Subtotal PEZZO2								21.08	(11.40- 39.00)
PEZZOT	534	m	0	30	134	4	116	6.49	(2.22- 18.98)
PEZZOT	535	m	0	71	82	4	116	25.11	(8.82- 71.48)
PEZZOT	536	m	0	110	101	4	116	31.58	(11.25- 88.71)
Subtotal PEZZOT								17.64	(9.62- 32.34)
*QIAO2	511	m	0	7	2364	10	709	0.21	(0.08- 0.55)
*QIAO2	512	m	0	54	2257	10	709	1.70	(0.87- 3.31)
*QIAO2	513	m	0	170	2295	10	709	5.25	(2.79- 9.88)
Subtotal QIAO2								1.87	(1.24- 2.83)
RACHTA	511	f	0	12	19	33	98	1.88	(0.82- 4.27)
RACHTA	512	f	0	49	21	33	98	6.93	(3.63- 13.22)
RACHTA	513	f	0	24	1	33	98	71.27	(9.28- 547.53)
Subtotal RACHTA								4.97	(3.04- 8.14)
SOBUE	546	m	0	62	119	34	128	1.96	(1.21- 3.19)
SOBUE	547	m	0	159	200	34	128	2.99	(1.94- 4.61)
SOBUE	548	m	0	241	174	34	128	5.21	(3.41- 7.98)
SOBUE	549	m	0	147	73	34	128	7.58	(4.73- 12.14)
Subtotal SOBUE								3.96	(3.16- 4.96)
TIZZAN	501	m	0	12	1	180	305	20.33	(2.62- 157.68)
TIZZAN	502	m	0	54	20	180	305	4.58	(2.65- 7.89)
TIZZAN	503	m	0	928	815	180	305	1.93	(1.57- 2.37)
TIZZAN	530	f	0	2	7	117	114	0.28	(0.06- 1.37)
TIZZAN	531	f	0	23	21	117	114	1.07	(0.56- 2.03)

Table 1I16 - 5

IESLC - Meta-analysis of Ever/current Smoking by Duration, Overview
All LC types, Cigarettes (or Any Product if Cigarettes not available)
Least adjusted

REF	NRR	SEX	AD	Number Exposed Case	Cont	Non-exposed Case	Cont	RR	95.00%CI
Subtotal TIZZAN								2.01	(1.68- 2.42)
WANG2	501	c	0	4	17	11	43	0.92	(0.26- 3.29)
WANG2	503	c	0	8	18	11	43	1.74	(0.60- 5.03)
WANG2	504	c	0	26	38	11	43	2.67	(1.17- 6.13)
WANG2	505	c	0	22	26	11	43	3.31	(1.38- 7.91)
Subtotal WANG2								2.24	(1.38- 3.63)
WUWILL	501	f	0	137	139	417	601	1.42	(1.09- 1.86)
WUWILL	502	f	0	179	98	417	601	2.63	(2.00- 3.47)
WUWILL	503	f	0	223	114	417	601	2.82	(2.18- 3.65)
Subtotal WUWILL								2.20	(1.88- 2.56)
ZHENG	553	m	0	37	75	33	94	1.41	(0.80- 2.46)
ZHENG	554	m	0	242	143	33	94	4.82	(3.08- 7.54)
ZHENG	558	f	0	17	17	152	184	1.21	(0.60- 2.45)
ZHENG	559	f	0	59	27	152	184	2.65	(1.60- 4.38)
Subtotal ZHENG								2.54	(1.94- 3.31)
ZHOU	501	c	0	170	12	507	68	1.90	(1.00- 3.60)
ZHOU	502	c	0	678	36	507	68	2.53	(1.66- 3.84)
Subtotal ZHOU								2.32	(1.63- 3.29)
Partial Totals				30094	4767667	21662573	15503		

*prospective study ~ With 0.5 adjustment for zero

REF	NRR	SEX	AD	Ys	Ws	Qs	Ps
AGUDO	507	f	0	-0.05	3.32	13.83	0.9303
AGUDO	508	f	0	1.32	6.08	2.76	0.0011
Subtotal AGUDO				0.84	9.40	16.59	
*AMANDU	501	m	0	0.95	5.25	5.76	0.0302
*AMANDU	502	m	0	2.42	5.54	1.00	0.0000
Subtotal AMANDU				1.70	10.79	6.76	
ARMADA	501	m	0	1.22	4.88	2.92	0.0070
ARMADA	502	m	0	2.46	6.68	1.46	0.0000
ARMADA	503	m	0	3.03	5.48	5.90	0.0000
Subtotal ARMADA				2.29	17.05	10.27	
AUVINE	501	c	0	2.02	8.26	0.00	0.0000
AUVINE	502	c	0	2.34	3.06	0.37	0.0000
AUVINE	503	c	0	3.04	20.41	22.55	0.0000
Subtotal AUVINE				2.71	31.73	22.92	
AXELSS	501	m	0	0.44	6.35	15.38	0.2713
AXELSS	502	m	0	0.98	6.98	7.22	0.0098
AXELSS	503	m	0	2.08	9.96	0.08	0.0000
AXELSS	504	m	0	2.50	11.10	2.89	0.0000
AXELSS	505	m	0	3.23	9.65	14.72	0.0000
AXELSS	510	f	0	0.58	3.29	6.60	0.2943
AXELSS	511	f	0	1.26	5.56	2.96	0.0029
AXELSS	512	f	0	2.26	7.41	0.51	0.0000
AXELSS	513	f	0	2.94	7.42	6.58	0.0000
AXELSS	514	f	0	2.84	4.72	3.38	0.0000
Subtotal AXELSS				2.07	72.43	60.31	
BARBON	501	m	0	1.37	11.69	4.51	0.0000
BARBON	502	m	0	2.29	14.48	1.28	0.0000
BARBON	503	m	0	2.54	15.92	4.82	0.0000
BARBON	504	m	0	2.59	17.31	6.13	0.0000
Subtotal BARBON				2.26	59.40	16.74	
*BEST	501	m	1	0.47	0.88	2.05	0.6590
*BEST	502	m	1	0.96	1.55	1.68	0.2335
*BEST	503	m	1	0.83	3.22	4.34	0.1350
*BEST	504	m	1	1.16	4.12	2.84	0.0182
*BEST	505	m	1	1.41	5.30	1.80	0.0012
*BEST	506	m	1	2.63	6.21	2.53	0.0000
*BEST	507	m	1	2.65	6.65	2.89	0.0000
Subtotal BEST				1.82	27.95	18.13	
*BOUCOT	518	m	0	2.90	0.49	0.40	0.0421
*BOUCOT	519	m	0	3.99	0.50	1.98	0.0050
Subtotal BOUCOT				3.45	0.99	2.38	
BUFFLE	526	f	0	2.14	7.75	0.17	0.0000
BUFFLE	527	f	0	2.68	8.42	3.98	0.0000
BUFFLE	528	f	0	3.00	7.86	7.90	0.0000
Subtotal BUFFLE				2.61	24.04	12.05	
*CEDERL	501	m	1	0.59	2.92	5.76	0.3155
*CEDERL	502	m	1	2.00	5.39	0.00	0.0000
*CEDERL	504	f	1	0.47	2.58	5.98	0.4505

Table 1I16 - 5

IESLC - Meta-analysis of Ever/current Smoking by Duration, Overview
All LC types, Cigarettes (or Any Product if Cigarettes not available)
Least adjusted

REF	NRR	SEX	AD	Ys	Ws	Qs	Ps
*CEDERL	505	f	1	2.26	4.00	0.29	0.0000
Subtotal CEDERL				1.53	14.88	12.04	
CHEN2	501	m	0	0.89	1.03	1.24	0.3653
CHEN2	502	m	0	1.59	1.38	0.23	0.0623
CHEN2	503	m	0	0.95	4.13	4.46	0.0523
CHEN2	504	m	0	1.59	4.85	0.80	0.0005
CHEN2	505	m	0	1.74	5.48	0.36	0.0000
CHEN2	510	f	0	-1.51	0.81	9.95	0.1734
CHEN2	511	f	0	0.28	0.93	2.75	0.7884
CHEN2	512	f	0	1.05	3.19	2.83	0.0607
CHEN2	513	f	0	0.61	5.42	10.31	0.1529
Subtotal CHEN2				1.10	27.21	32.93	
CHOI	501	m	0	0.93	6.32	7.20	0.0199
CHOI	502	m	0	1.07	9.21	7.91	0.0012
CHOI	503	m	0	1.54	9.66	2.00	0.0000
CHOI	504	m	0	1.92	8.35	0.04	0.0000
CHOI	505	m	0	1.99	5.33	0.00	0.0000
CHOI	510	f	0	-0.73	1.59	11.81	0.3546
CHOI	511	f	0	0.21	4.64	14.76	0.6519
CHOI	512	f	0	2.16	1.55	0.04	0.0072
CHOI	513	f	0	0.77	0.50	0.74	0.5883
Subtotal CHOI				1.29	47.14	44.50	
*CPSI	580	m	0	0.52	64.01	138.51	0.0000
*CPSI	581	m	0	1.32	105.87	47.91	0.0000
*CPSI	582	m	0	1.80	138.39	5.27	0.0000
*CPSI	583	m	0	2.34	154.66	18.36	0.0000
*CPSI	584	m	0	2.79	156.12	99.36	0.0000
*CPSI	585	m	0	3.12	146.44	187.28	0.0000
*CPSI	586	m	0	3.45	126.72	269.97	0.0000
*CPSI	587	m	0	3.71	106.68	313.24	0.0000
*CPSI	676	f	0	0.10	87.71	315.27	0.3603
*CPSI	677	f	0	0.99	111.49	113.07	0.0000
*CPSI	678	f	0	1.27	119.48	62.49	0.0000
*CPSI	679	f	0	1.68	97.98	9.71	0.0000
*CPSI	680	f	0	1.99	49.07	0.00	0.0000
*CPSI	681	f	0	2.10	15.55	0.17	0.0000
*CPSI	682	f	0	2.56	9.83	3.11	0.0000
Subtotal CPSI				2.08	1489.99	1583.72	
*CPSII	552	m	0	1.11	45.57	35.53	0.0000
*CPSII	553	m	0	2.04	66.89	0.12	0.0000
*CPSII	554	m	0	2.59	82.29	29.07	0.0000
*CPSII	555	m	0	3.17	95.47	132.84	0.0000
*CPSII	556	m	0	3.53	88.48	209.08	0.0000
*CPSII	557	m	0	3.92	90.50	337.33	0.0000
*CPSII	558	m	0	4.20	68.44	332.16	0.0000
*CPSII	559	m	0	4.42	60.64	356.20	0.0000
*CPSII	618	f	0	1.05	90.12	81.06	0.0000
*CPSII	619	f	0	1.94	104.74	0.28	0.0000
*CPSII	620	f	0	2.42	119.07	21.22	0.0000
*CPSII	621	f	0	2.77	127.49	76.43	0.0000
*CPSII	622	f	0	3.14	102.68	134.44	0.0000
*CPSII	623	f	0	3.34	87.81	159.56	0.0000
*CPSII	624	f	0	3.37	24.94	47.26	0.0000
*CPSII	625	f	0	4.00	17.14	69.01	0.0000
Subtotal CPSII				2.85	1272.25	2021.56	
DAMBER	506	m	1	0.46	5.52	13.03	0.2825
DAMBER	507	m	1	1.30	12.09	5.86	0.0000
DAMBER	508	m	1	1.64	17.62	2.22	0.0000
DAMBER	509	m	1	2.16	21.28	0.62	0.0000
DAMBER	510	m	1	2.42	21.42	3.80	0.0000
Subtotal DAMBER				1.86	77.93	25.53	
DEAN2	501	m	0	1.16	10.37	7.12	0.0002
DEAN2	502	m	0	1.34	23.47	9.88	0.0000
DEAN2	504	f	0	-0.02	5.23	21.18	0.9671
DEAN2	505	f	0	1.77	7.59	0.38	0.0000
Subtotal DEAN2				1.22	46.66	38.56	
DESTEF	501	m	0	1.55	11.82	2.31	0.0000
DESTEF	502	m	0	1.80	14.53	0.56	0.0000
DESTEF	503	m	0	2.41	16.73	2.86	0.0000
DESTEF	504	m	0	2.30	17.23	1.59	0.0000
Subtotal DESTEF				2.06	60.31	7.31	

Table 1I16 - 5

IESLC - Meta-analysis of Ever/current Smoking by Duration, Overview
All LC types, Cigarettes (or Any Product if Cigarettes not available)
Least adjusted

REF	NRR	SEX	AD	Ys	Ws	Qs	Ps
DOLL	515	m	0	1.94	3.23	0.01	0.0005
DOLL	516	m	0	1.52	4.90	1.11	0.0008
DOLL	517	m	0	2.19	6.17	0.25	0.0000
DOLL	518	m	0	2.29	6.13	0.55	0.0000
DOLL	522	f	0	0.14	5.92	20.40	0.7383
DOLL	523	f	0	0.79	4.00	5.75	0.1124
DOLL	524	f	0	0.98	8.35	8.64	0.0048
DOLL	525	f	0	1.08	1.85	1.53	0.1417
Subtotal DOLL				1.37	40.55	38.24	
DORGAN	570	m	2	1.69	10.46	0.94	0.0000
DORGAN	571	m	2	2.78	11.22	6.90	0.0000
DORGAN	562	f	3	1.45	47.84	14.30	0.0000
DORGAN	563	f	3	2.46	57.93	12.72	0.0000
Subtotal DORGAN				2.05	127.45	34.86	
DOSEME	501	m	2	0.00	14.17	56.31	1.0000
DOSEME	502	m	2	1.34	24.94	10.82	0.0000
DOSEME	503	m	2	1.59	31.98	5.23	0.0000
Subtotal DOSEME				1.18	71.09	72.36	
FAN	501	m	0	0.34	13.53	36.90	0.2079
FAN	502	m	0	0.86	15.89	20.40	0.0006
FAN	503	m	0	1.36	23.17	9.35	0.0000
FAN	506	f	0	0.91	4.78	5.66	0.0478
FAN	507	f	0	1.34	8.79	3.72	0.0001
FAN	508	f	0	1.46	18.96	5.32	0.0000
Subtotal FAN				1.10	85.12	81.34	
GAO	561	f	0	0.49	27.86	63.07	0.0099
GAO	562	f	0	1.18	42.03	28.02	0.0000
Subtotal GAO				0.90	69.89	91.08	
GARSHI	534	m	0	1.83	34.49	0.96	0.0000
GER	513	c	0	0.19	6.73	21.95	0.6273
GER	514	c	0	0.20	15.61	50.48	0.4403
GER	515	c	0	0.42	19.79	48.89	0.0605
Subtotal GER				0.30	42.13	121.32	
HAENSZ	542	f	0	0.58	8.51	16.91	0.0886
HAENSZ	543	f	0	0.79	21.36	31.15	0.0003
Subtotal HAENSZ				0.73	29.87	48.06	
HU	501	m	0	0.71	10.64	17.58	0.0209
HU	502	m	0	0.74	12.94	20.49	0.0082
HU	503	m	0	0.80	6.12	8.77	0.0488
HU	506	f	0	0.50	3.82	8.51	0.3277
HU	507	f	0	0.63	3.58	6.61	0.2303
HU	508	f	0	0.47	1.59	3.69	0.5535
Subtotal HU				0.69	38.69	65.66	
HU2	508	c	0	0.11	11.00	38.90	0.7065
HU2	509	c	0	0.58	22.49	44.87	0.0058
HU2	510	c	0	0.76	32.27	48.90	0.0000
HU2	511	c	0	1.11	37.09	29.22	0.0000
Subtotal HU2				0.78	102.85	161.89	
HUMBLE	517	c	0	1.82	8.37	0.25	0.0000
HUMBLE	518	c	0	2.48	14.05	3.31	0.0000
HUMBLE	519	c	0	2.89	15.20	12.14	0.0000
HUMBLE	520	c	0	2.81	14.60	9.79	0.0000
HUMBLE	521	c	0	2.60	8.39	3.05	0.0000
Subtotal HUMBLE				2.59	60.60	28.55	
JOLY	515	m	0	1.43	5.01	1.61	0.0014
JOLY	516	m	0	2.43	7.66	1.43	0.0000
JOLY	517	m	0	2.24	9.46	0.56	0.0000
JOLY	518	m	0	2.82	10.06	6.86	0.0000
JOLY	519	m	0	2.89	10.43	8.34	0.0000
JOLY	501	f	0	0.93	7.39	8.40	0.0118
JOLY	502	f	0	1.33	8.56	3.81	0.0001
JOLY	503	f	0	1.95	10.34	0.02	0.0000
JOLY	504	f	0	2.37	11.67	1.62	0.0000
JOLY	505	f	0	2.74	11.07	6.19	0.0000
Subtotal JOLY				2.20	91.65	38.85	
JUSSAW	510	m	0	1.21	8.28	5.10	0.0005
JUSSAW	511	m	0	2.01	11.07	0.00	0.0000
JUSSAW	512	m	0	1.93	12.80	0.05	0.0000
JUSSAW	513	m	0	2.53	6.39	1.84	0.0000
JUSSAW	514	m	0	2.04	3.76	0.01	0.0001
Subtotal JUSSAW				1.91	42.30	7.00	

Table 1I16 - 5

IESLC - Meta-analysis of Ever/current Smoking by Duration, Overview
All LC types, Cigarettes (or Any Product if Cigarettes not available)
Least adjusted

REF	NRR	SEX	AD	Ys	Ws	Qs	Ps
*KAISE2	596	m	1	1.58	6.28	1.07	0.0001
*KAISE2	597	m	1	2.75	9.62	5.50	0.0000
*KAISE2	516	f	1	2.21	6.65	0.30	0.0000
*KAISE2	517	f	1	3.41	6.86	13.86	0.0000
Subtotal KAISE2				2.53	29.42	20.74	
KATSOU	501	f	0	0.33	2.29	6.32	0.6134
KATSOU	502	f	0	0.47	3.29	7.68	0.3967
KATSOU	503	f	0	2.35	1.66	0.21	0.0025
KATSOU	504	f	0	1.78	2.90	0.13	0.0024
Subtotal KATSOU				1.12	10.15	14.34	
KHUDER	501	m	0	1.26	7.96	4.29	0.0004
KHUDER	502	m	0	2.02	18.43	0.01	0.0000
KHUDER	503	m	0	2.19	18.60	0.73	0.0000
Subtotal KHUDER				1.96	44.99	5.03	
KREUZE	517	m	3	1.55	22.07	4.39	0.0000
KREUZE	518	m	3	3.38	25.18	48.06	0.0000
KREUZE	520	f	3	0.29	14.75	43.05	0.2734
KREUZE	521	f	3	1.97	27.78	0.02	0.0000
Subtotal KREUZE				1.98	89.79	95.53	
LETOUR	506	c	0	1.18	14.96	9.98	0.0000
LETOUR	507	c	0	2.73	17.80	9.77	0.0000
LETOUR	508	c	0	3.21	17.89	26.43	0.0000
Subtotal LETOUR				2.44	50.65	46.18	
LEVIN	501	m	0	2.07	5.51	0.03	0.0000
LEVIN	502	m	0	2.25	5.55	0.37	0.0000
Subtotal LEVIN				2.16	11.06	0.40	
*LIAW	501	c	2	-0.11	2.82	12.41	0.8596
*LIAW	502	c	2	0.96	6.06	6.53	0.0187
*LIAW	503	c	2	1.55	12.45	2.48	0.0000
Subtotal LIAW				1.16	21.33	21.42	
LIU3	507	m	0	-0.02	2.92	11.88	0.9669
LIU3	508	m	0	0.57	2.74	5.54	0.3441
Subtotal LIU3				0.26	5.66	17.42	
LIU5	504	c	0	0.14	7.88	27.06	0.6935
LIU5	505	c	0	1.02	9.06	8.60	0.0022
Subtotal LIU5				0.61	16.94	35.66	
LUBIN	508	m	0	0.61	5.58	10.62	0.1463
LUBIN	509	m	0	1.33	6.65	2.89	0.0006
LUBIN	510	m	0	1.63	6.67	0.86	0.0000
LUBIN	511	m	0	1.82	5.97	0.18	0.0000
Subtotal LUBIN				1.37	24.88	14.55	
LUBIN2	531	m	0	1.48	142.28	37.93	0.0000
LUBIN2	532	m	0	2.18	156.67	5.38	0.0000
LUBIN2	533	m	0	2.42	153.41	27.60	0.0000
LUBIN2	534	m	0	2.51	141.36	37.55	0.0000
LUBIN2	574	f	0	0.71	63.53	105.06	0.0000
LUBIN2	575	f	0	1.27	68.77	36.18	0.0000
LUBIN2	576	f	0	1.54	53.35	11.19	0.0000
LUBIN2	577	f	0	2.19	21.09	0.83	0.0000
Subtotal LUBIN2				1.92	800.46	261.72	
MATOS	516	m	0	0.87	6.18	7.83	0.0311
MATOS	517	m	0	2.01	8.24	0.00	0.0000
MATOS	518	m	0	2.27	8.14	0.61	0.0000
Subtotal MATOS				1.79	22.56	8.45	
MCCONN	501	c	0	0.65	1.36	2.44	0.4488
MCCONN	502	c	0	-0.40	2.46	14.03	0.5341
MCCONN	503	c	0	0.72	5.16	8.32	0.1002
MCCONN	504	c	0	-0.06	4.55	19.20	0.8977
MCCONN	505	c	0	0.02	4.13	16.06	0.9644
Subtotal MCCONN				0.20	17.65	60.05	
NOTAN2	513	c	0	-0.13	4.47	20.18	0.7808
NOTAN2	514	c	0	0.63	6.77	12.58	0.1008
NOTAN2	515	c	0	0.69	7.37	12.51	0.0606
NOTAN2	516	c	0	1.17	4.16	2.82	0.0171
NOTAN2	517	c	0	0.63	2.41	4.48	0.3273
Subtotal NOTAN2				0.60	25.18	52.58	
OSANN2	501	f	0	0.48	9.59	21.98	0.1369
OSANN2	502	f	0	2.23	15.81	0.91	0.0000
Subtotal OSANN2				1.57	25.41	22.88	
PEZZO2	507	m	0	2.79	4.86	3.07	0.0000
PEZZO2	508	m	0	3.29	5.29	8.86	0.0000

Table 1I16 - 5

IESLC - Meta-analysis of Ever/current Smoking by Duration, Overview
All LC types, Cigarettes (or Any Product if Cigarettes not available)
Least adjusted

REF	NRR	SEX	AD	Ys	Ws	Qs	Ps
Subtotal PEZZO2				3.05	10.15	11.93	
PEZZOT	534	m	0	1.87	3.34	0.05	0.0006
PEZZOT	535	m	0	3.22	3.51	5.31	0.0000
PEZZOT	536	m	0	3.45	3.60	7.67	0.0000
Subtotal PEZZOT				2.87	10.45	13.03	
*QIAO2	511	m	0	-1.56	4.15	52.42	0.0015
*QIAO2	512	m	0	0.53	8.57	18.40	0.1218
*QIAO2	513	m	0	1.66	9.61	1.08	0.0000
Subtotal QIAO2				0.63	22.33	71.90	
RACHTA	511	f	0	0.63	5.67	10.55	0.1344
RACHTA	512	f	0	1.94	9.21	0.03	0.0000
RACHTA	513	f	0	4.27	0.92	4.77	0.0000
Subtotal RACHTA				1.60	15.80	15.36	
SOBUE	546	m	0	0.67	16.19	28.21	0.0067
SOBUE	547	m	0	1.10	20.61	16.60	0.0000
SOBUE	548	m	0	1.65	21.22	2.49	0.0000
SOBUE	549	m	0	2.03	17.32	0.02	0.0000
Subtotal SOBUE				1.38	75.35	47.31	
TIZZAN	501	m	0	3.01	0.92	0.95	0.0039
TIZZAN	502	m	0	1.52	12.93	2.89	0.0000
TIZZAN	503	m	0	0.66	89.78	160.35	0.0000
TIZZAN	530	f	0	-1.28	1.51	16.22	0.1155
TIZZAN	531	f	0	0.06	9.22	34.31	0.8435
Subtotal TIZZAN				0.70	114.36	214.72	
WANG2	501	c	0	-0.08	2.36	10.20	0.8977
WANG2	503	c	0	0.55	3.39	7.05	0.3089
WANG2	504	c	0	0.98	5.59	5.70	0.0200
WANG2	505	c	0	1.20	5.05	3.21	0.0072
Subtotal WANG2				0.81	16.39	26.16	
WUWILL	501	f	0	0.35	53.89	145.41	0.0100
WUWILL	502	f	0	0.97	50.37	52.99	0.0000
WUWILL	503	f	0	1.04	57.74	52.90	0.0000
Subtotal WUWILL				0.79	162.01	251.31	
ZHENG	553	m	0	0.34	12.30	33.63	0.2328
ZHENG	554	m	0	1.57	19.21	3.40	0.0000
ZHENG	558	f	0	0.19	7.71	25.06	0.5957
ZHENG	559	f	0	0.97	15.15	15.79	0.0002
Subtotal ZHENG				0.93	54.37	77.88	
ZHOU	501	c	0	0.64	9.44	17.26	0.0485
ZHOU	502	c	0	0.93	21.77	24.79	0.0000
Subtotal ZHOU				0.84	31.22	42.04	

N	248
NS	55

Table 1I16 - 6

IESLC - Meta-analysis of Ever/current Smoking by Duration, Overview
All LC types, Cigarettes (or Any Product if Cigarettes not available)
Least adjusted

	Sex combined	male	female	Total
N	39	127	82	248
NS	11	36	25	72

In this overview table, other than the “N” rows, entries in the “absent” and “Total” columns may be invalid and should be ignored

	Duration of smoking (broad categories) absent	1-34k20	21-49k35	36+k50	Total
N	107	55	40	46	248
NS	43	43	32	36	154
Wt	2836.90	980.02	1064.47	954.00	5835.39
Het Chi	3010.77	311.82	404.21	766.01	6171.05
Het df	106	54	39	45	247
Het P	***	***	***	***	***
Fixed RR	9.49	2.48	6.09	12.85	7.34
RRl	9.15	2.33	5.73	12.06	7.16
RRu	9.85	2.64	6.47	13.69	7.53
P	+++	+++	+++	+++	+++
Random RR	5.16	2.43	5.75	9.88	4.99
RRl	4.19	2.05	4.64	7.43	4.36
RRu	6.35	2.88	7.14	13.13	5.71
P	+++	+++	+++	+++	+++

Duration of smoking (narrow categories)

	absent	1-19k1	6-29k20	21-39k30	31-49k40	41-998k50	51+k999	Total
N	136	28	17	32	23	6	6	248
NS	54	20	13	24	17	4	4	136
Wt	3145.06	188.20	144.66	903.70	853.49	376.18	224.10	5835.39
Het Chi	3372.29	71.94	42.08	264.29	225.72	92.66	91.21	6171.05
Het df	135	27	16	31	22	5	5	247
Het P	***	***	***	***	***	***	***	***
Fixed RR	6.41	2.03	2.99	4.86	10.56	26.02	40.70	7.34
RRl	6.19	1.76	2.54	4.55	9.87	23.52	35.70	7.16
RRu	6.64	2.34	3.52	5.19	11.29	28.78	46.39	7.53
P	+++	+++	+++	+++	+++	+++	+++	+++
Random RR	5.12	1.85	2.93	4.76	9.21	19.05	27.57	4.99
RRl	4.26	1.44	2.21	3.83	7.21	11.85	14.68	4.36
RRu	6.16	2.38	3.88	5.90	11.76	30.63	51.78	5.71
P	+++	+++	+++	+++	+++	+++	+++	+++

MALES

	Duration of smoking (broad categories) absent	1-34k20	21-49k35	36+k50	Total
N	55	27	20	25	127
NS	30	27	20	25	102
Wt	1716.56	457.75	559.11	594.42	3327.84
Het Chi	1781.99	132.78	116.60	262.34	3194.50
Het df	54	26	19	24	126
Het P	***	***	***	***	***
Fixed RR	12.23	3.06	7.41	16.29	9.78
RRl	11.66	2.79	6.82	15.03	9.45
RRu	12.82	3.36	8.05	17.65	10.12
P	+++	+++	+++	+++	+++
Random RR	7.10	2.76	6.66	12.19	6.41
RRl	5.31	2.18	5.24	9.00	5.35
RRu	9.49	3.51	8.46	16.50	7.68
P	+++	+++	+++	+++	+++

Table 1I16 - 6

IESLC - Meta-analysis of Ever/current Smoking by Duration, Overview
All LC types, Cigarettes (or Any Product if Cigarettes not available)
Least adjusted

MALES

	Duration of smoking (narrow categories)							
	absent	1-19k1	6-29k20	21-39k30	31-49k40	41-998k50	51+k999	Total
N	72	12	8	16	13	3	3	127
NS	36	12	8	16	13	3	3	91
Wt	1710.40	101.94	81.21	467.75	519.57	258.22	188.74	3327.84
Het Chi	1740.56	31.33	19.85	96.96	91.01	67.05	65.10	3194.50
Het df	71	11	7	15	12	2	2	126
Het P	***	***	**	***	***	***	***	***
Fixed RR	8.52	2.88	3.99	6.12	11.80	27.82	44.20	9.78
RRl	8.12	2.37	3.21	5.59	10.83	24.62	38.32	9.45
RRu	8.93	3.50	4.96	6.70	12.86	31.43	50.98	10.12
P	+++	+++	+++	+++	+++	+++	+++	+++
Random RR	6.26	2.86	4.10	5.80	10.10	22.05	34.17	6.41
RRl	4.87	2.01	2.77	4.43	7.62	10.07	14.06	5.35
RRu	8.05	4.07	6.08	7.58	13.38	48.29	83.07	7.68
P	+++	+++	+++	+++	+++	+++	+++	+++

FEMALES

	Duration of smoking (broad categories)				
	absent	1-34k20	21-49k35	36+k50	Total
N	38	18	12	14	82
NS	21	18	12	14	65
Wt	1003.90	435.46	408.27	243.25	2090.88
Het Chi	790.73	106.39	137.02	240.96	1866.57
Het df	37	17	11	13	81
Het P	***	***	***	***	***
Fixed RR	6.97	2.01	5.23	10.22	5.32
RRl	6.55	1.83	4.74	9.01	5.09
RRu	7.42	2.20	5.76	11.59	5.55
P	+++	+++	+++	+++	+++
Random RR	4.24	2.08	5.79	9.11	4.26
RRl	3.10	1.57	3.85	4.87	3.41
RRu	5.78	2.74	8.71	17.03	5.31
P	+++	+++	+++	+++	+++

	Duration of smoking (narrow categories)							
	absent	1-19k1	6-29k20	21-39k30	31-49k40	41-998k50	51+k999	Total
N	45	11	5	10	7	2	2	82
NS	25	11	5	10	7	2	2	62
Wt	1201.72	57.62	25.63	366.06	309.51	103.36	26.98	2090.88
Het Chi	1075.62	10.55	5.37	72.45	101.63	20.42	13.03	1866.57
Het df	44	10	4	9	6	1	1	81
Het P	***	N.S.	N.S.	***	***	***	***	***
Fixed RR	4.67	1.37	2.47	3.98	8.84	23.44	32.25	5.32
RRl	4.41	1.06	1.68	3.59	7.91	19.33	22.12	5.09
RRu	4.94	1.77	3.64	4.41	9.88	28.43	47.04	5.55
P	+++	+	+++	+++	+++	+++	+++	+++
Random RR	4.49	1.36	2.38	4.55	8.76	15.51	26.92	4.26
RRl	3.32	1.04	1.51	3.16	5.12	4.59	6.54	3.41
RRu	6.06	1.78	3.76	6.56	14.98	52.40	110.82	5.31
P	+++	+	+++	+++	+++	+++	+++	+++

Table 1I16 - 7

IESLC - Meta-analysis of Ever/current Smoking by Duration, Overview
All LC types, Cigarettes (or Any Product if Cigarettes not available)
Excluded studies (and stage at which they were excluded)

1	BECHER BLOT1 BROWN3 CARPEN CHYOU DARBY DOLL2 GARCIA GRAHAM GURSEL HAMMO2 JAHN JAIN LAUSSM PRESCO QIAO TVERDA WIGLE WYNDE3
2	ALDERS BENSHL BRESLO CHIAZZ DEAN3 DORN ENGELA GAO2 GILLIS GUO HEGMAN HIRAYA HOLE KAUFMA KOO KOULUM LIU4 MIGRAN MRFITR PERNU SEGI2 SPEIZE SUZUK2 SVENSS VUTUC WAKAI WU YUAN
3	GENG MCDUFF SPITZ STASZE WU2 ZHANG
4	BOUCHA CHEN CORREA JEDRYC LUO WYNDE2 WYNDE6
5	AKIBA HAMMON PISANI RESTRE SADOWS XU
7	BOFFET BROSS WYNDE7
10	AMES WATSON WYNDE8
14	BENHAM

Table 1I16 - 8
Potentially overlapping studies

REF	REFGP	PRINC	. OVERLAP/LINK
LUBIN2	LUBIN2	1	Lubin-combined
OSANN2	KAISER	2	KAISER/OSANN2
CPSI	CPSI	1	CPSI overall
LUBIN	XIANGZ	2	LUBIN/XIANGZ/QIAO

Table 1I16 - 9
Most adjusted - insufficient data for meta-analysis

REF	NRR	SEX	AGEL	AGEH	RACE	YF	LC TYPE	LOC	START	ST	NLC	R	VB	P	H	AD	SM	PRODUCT	exL	exH	S1	S2	DENOM	De
BUFFLE	501	m	0	0	wh	-	all	NAmer	1976	CC	943	n	bl	y	n	0	ev	cig+/-ot	1	33	1	0	nev cigs	or
BUFFLE	502	m	0	0	wh	-	all	NAmer	1976	CC	943	n	bl	y	n	0	ev	cig+/-ot	34	43	2	4	nev cigs	or
BUFFLE	503	m	0	0	wh	-	all	NAmer	1976	CC	943	n	bl	y	n	0	ev	cig+/-ot	44	49	0	0	nev cigs	or
BUFFLE	504	m	0	0	wh	-	all	NAmer	1976	CC	943	n	bl	y	n	0	ev	cig+/-ot	50	999	3	0	nev cigs	or
GARSHI	535	m	0	0	all	-	all	NAmer	1981	CC	1081	o	bl	y	n	1	ev	all/unsp	1	19	0	1	nev any	ot
HAMMON	513	m	0	0	wh	0	all	NAmer	1952	pr	448	n	bl	n	n	1	ev	cig only	1	34	1	0	nev any	st
HAMMON	514	m	0	0	wh	0	all	NAmer	1952	pr	448	n	bl	n	n	1	ev	cig only	35	999	0	0	nev any	st
SADOWS	522	m	0	0	wh	-	all	NAmer	1938	CC	477	n	bl	n	n	0	ev	cig only	1	9	0	1	nev any	ot
SADOWS	523	m	0	0	wh	-	all	NAmer	1938	CC	477	n	bl	n	n	0	ev	cig only	10	19	0	0	nev any	ot
SADOWS	524	m	0	0	wh	-	all	NAmer	1938	CC	477	n	bl	n	n	0	ev	cig only	20	29	1	2	nev any	ot
SADOWS	525	m	0	0	wh	-	all	NAmer	1938	CC	477	n	bl	n	n	0	ev	cig only	30	39	2	3	nev any	ot
SADOWS	526	m	0	0	wh	-	all	NAmer	1938	CC	477	n	bl	n	n	0	ev	cig only	40	49	0	4	nev any	ot
SADOWS	527	m	0	0	wh	-	all	NAmer	1938	CC	477	n	bl	n	n	0	ev	cig only	50	999	3	0	nev any	ot
XU	501	m	0	0	all	-	all	As:Chi	1985	CC	729	n	ot	n	n	2	ev	all/unsp	1	29	1	0	nev any	or
XU	502	m	0	0	all	-	all	As:Chi	1985	CC	729	n	ot	n	n	2	ev	all/unsp	30	39	2	3	nev any	or
XU	503	m	0	0	all	-	all	As:Chi	1985	CC	729	n	ot	n	n	2	ev	all/unsp	40	999	3	0	nev any	or

REF	NRR	RR	SIG	RRDATA comment
BUFFLE	501	6.80		0
BUFFLE	502	11.10		0
BUFFLE	503	9.40		0
BUFFLE	504	14.50		0
GARSHI	535	*	gap	0
HAMMON	513	*		RR for <1/2 pack per day is 5.31, that for 1/2 to 1 pack per day is 6.56, that for 1 to 2 packs per day is 7.27 while that for 2+ packs per day 10.78
HAMMON	514	*		RR for <1/2 pack per day is 10.86, that for 1/2 to 1 pack per day is 8.20, that for 1 to 2 packs per day is 19.69 while that for 2+ packs per day 22.89
SADOWS	522	1.19		0
SADOWS	523	1.16		0
SADOWS	524	2.78		0
SADOWS	525	3.95		0

Table 1I16 - 9

IESLC - Meta-analysis of Ever/current Smoking by Duration, Overview
All LC types, Cigarettes (or Any Product if Cigarettes not available)
Most adjusted - insufficient data for meta-analysis

REF	NRR	RR	SIG	RRDATA	comment
SADOWS	526	7.00		0	
SADOWS	527	8.43		0	
XU	501		*	RR for 1-19/day is 1.8(p<0.05), for 20-29/day is 1.5(p<0.05) and for >=30/ day is 5.3(p<0.05)	
XU	502		*	RR for 1-19/day is 2.1(p<0.05), for 20-29/day is 2.7(p<0.05) and for >=30/ day is 4.9(p<0.05)	
XU	503		*	RR for 1-19/day is 3.3(p<0.05), for 20-29/day is 6.0(p<0.05) and for >=30/ day is 17.1(p<0.05)	

Least adjusted - insufficient data for meta-analysis: as for adjusted plus the following

REF	NRR	SEX	AGEL	AGEH	RACE	YF	LC TYPE	LOC	START	ST	NLC	R	VB	P	H	AD	SM	PRODUCT	exL	exH	S1	S2	DENOM	De
GARSHI	533	m	0	0	all	-	all	NAmer	1981	CC	1081	o	bl	y	n	0	ev	all/unsp	1	19	0	1	nev any	ot

REF	NRR	RR	SIG	RRDATA	comment
GARSHI	533		* gap	0	

Table 1I17 -

IESLC - Meta-analysis of Ever/current Smoking, Duration, "Low"
All LC types, Cigarettes (or Any Product if Cigarettes not available)

This analysis is restricted to results for:
1) Ever/current smokers
2) Results by Duration
3) Categorical results by Duration
4) All LC types (or near equivalent)
5) Results complete enough for use in metaanalysis

Within each study, results are then selected (in the following order of preference, within each sex) for:
6) SMKSTA: ever, current
7) PRODUCT: cigarettes regardless of other products, cigarettes only, all/unspec
8) CIGTYPE: all/unspecified, MC regardless of HR, MC only
9) (not applicable)
10) DENOM: never smoked anything, never smoked cigarettes, never any + low, never cigs + low
11) Followup period (YF, prospective studies): whole study (coded as 0) or longest available
12) LCtype: all or nearest available, at least Squamous and Adeno. (q = squamous, s = small,
l = large, a = adeno, mix = mixed, alv = alveolar)
13) Race: all or nearest available, otherwise by race (wh or w = white, bl or b = black, hi = hispanic
ch = chinese, jap = japanese, haw = hawaiian, w+o = white + oriental, sca = scandinavian, as = asian)
14) Duration "low" in key scheme 1 (key value 20, maximum range 1-34)
15) For overlapping studies: principal rather than subsidiary studies
Finally by Age: whole study (coded as 0) if available, otherwise by widest available age group
and then for single sex results (m, f) in preference to results for both sexes combined (c).

Results adjusted (AD) for the most potential confounders are then chosen in Sections -1 to -3
(and those which actually differ from the adjusted results in Table 1I12 - 1 are marked 'x' in Section -1)
and results adjusted for the least confounders in Sections -4 to -6. (Those least adjusted results which
actually differ from the most adjusted are marked 'x' in column X in Section -4)

Section -7 shows excluded studies, together with the stage (as above) at which no qualifying
results were found.

Section -8 lists the potentially overlapping studies which have been included (1=principal, 2=subsidiary).

Section -9 lists any results which would have been included in preference except that they had data not complete
enough for use in meta-analysis, with their significance (yes/no), if known, and any further comment as entered
on the database. It also lists as "gap" any categories for which no data were presented by the original authors.

In addition to those mentioned above, the following fields, levels and abbreviations are used:
* or nk = not known, n = no, y = yes, ot = other
ev = ever, cu = current, nev = never
all/unspec = all or unspecified, cig+/-ot = cigarettes irrespective of other products (cigar, pipe etc)
MC = manufactured cigarettes, HR = hand-rolled cigarettes
exL, exH = range of exposure (low and high) in the smoking group, in terms of Duration
REF: 6-character study reference
NRR: number of the RR on the database within the study
ST : study type (CC = case control, pr or prosp = prospective)
NLC: number of lung cancer cases in whole study
R : risky occupational population (n = no, m = mining, o = other risky)
VB : national cigarette type (V = at least 75% Virginia, bl = at least 75% blended, ot = other)
P : any proxy use
H : full histological confirmation
De : derivation of RR/CI (or = original, st = standard method, ot = other method of estimation)

Table 1I17 - 1

IESLC - Meta-analysis of Ever/current Smoking, Duration, "Low"
All LC types, Cigarettes (or Any Product if Cigarettes not available)
Most adjusted

REF	NRR	1I12	SEX	AGEL	AGEH	RACE	YF	LC TYPE	LOC	START	ST	NLC	R	VB	P	H	AD	SM	PRODUCT	exL	exH	DENOM	De
AMANDU	506		m	0	0	wh	0	all	NAmer	1959	pr	132	m	bl	n	n	2	cu	cig+/-ot	0	24	nev cigs	ot
ARMADA	506		m	0	0	all	-	all	Eu:wst	1986	CC	325	n	bl	n	y	1	ev	cig+/-ot	1	24	nev cigs	or
AUVINE	517		c	0	0	all	-	all	Eu:Sca	1986	CC	517	n	bl	y	n	2	ev	cig+/-ot	1	20	nev cigs	or
AXELSS	520		m	0	0	sca	-	all	Eu:Sca	1989	CC	436	n	bl	n	n	6	ev	all/unsp	20	29	nev any	ot
AXELSS	511		f	0	0	sca	-	all	Eu:Sca	1989	CC	436	n	bl	n	n	0	ev	all/unsp	20	29	nev any	st
BARBON	508		m	0	0	all	-	all	Eu:wst	1979	CC	755	n	bl	y	y	1	ev	all/unsp	1	29	nev any	or
BEST	505		m	0	0	all	0	all	NAmer	1955	pr	381	n	V	n	n	1	cu	cig only	20	29	nev any	ot
BUFFLE	526		f	0	0	w-hi	-	all	NAmer	1976	CC	943	n	bl	y	n	0	ev	cig+/-ot	1	30	nev cigs	or
CEDERL	501		m	40	69	all	10	all	Eu:Sca	1963	pr	491	n	bl	n	n	1	cu	cig only	1	29	nev any	ot
CEDERL	504		f	40	69	all	10	all	Eu:Sca	1963	pr	491	n	bl	n	n	1	cu	cig only	1	29	nev any	ot
CHEN2	502		m	0	0	all	-	all	As:Chi	1983	CC	193	n	ot	y	n	0	ev	all/unsp	10	20	nev any	st
CHEN2	510		f	0	0	all	-	all	As:Chi	1983	CC	193	n	ot	y	n	0	ev	all/unsp	1	20	nev any	st
CHOI	502		m	0	0	all	-	all	As:oth	1985	CC	375	n	bl	n	n	0	ev	cig+/-ot	20	29	nev cigs	st
CHOI	511		f	0	0	all	-	all	As:oth	1985	CC	375	n	bl	n	n	0	ev	cig+/-ot	20	29	nev cigs	st
CPSI	580		m	40	84	wh	0	all	NAmer	1959	pr	5138	n	bl	n	n	0	cu	cig only	1	29	nev cigs	st
CPSI	676		f	40	84	wh	0	all	NAmer	1959	pr	5138	n	bl	n	n	0	cu	cig only	1	29	nev cigs	st
CPSII	552		m	0	0	all	6	all	NAmer	1982	pr	3229	n	bl	n	n	0	cu	cig only	1	29	nev any	st
CPSII	618		f	0	0	all	6	all	NAmer	1982	pr	3229	n	bl	n	n	0	cu	cig+/-ot	1	29	nev cigs	st
DAMBER	506		m	0	0	all	-	all	Eu:Sca	1972	CC	579	n	bl	y	n	1	ev	all/unsp	1	20	nev any	ot
DESTEF	508		m	0	0	all	-	all	SCAmer	1988	CC	497	n	bl	n	y	4	ev	all/unsp	1	29	nev any	or
DORGAN	570		m	0	0	wh	-	all	NAmer	1980	CC	2026	n	bl	y	y	2	ev	cig+/-ot	1	34	nev any	ot
DORGAN	562		f	0	0	all	-	all	NAmer	1980	CC	2026	n	bl	y	y	3	ev	cig+/-ot	1	34	nev any	ot
DOSEME	502		m	0	0	all	-	all	Eu:bal	1979	CC	1210	n	bl	n	n	2	ev	cig+/-ot	11	20	nev cigs	or
FAN	501		m	0	0	all	-	all	As:Chi	1990	CC	403	n	ot	y	n	0	ev	cig+/-ot	1	29	nev cigs	st
FAN	506		f	0	0	all	-	all	As:Chi	1990	CC	403	n	ot	y	n	0	ev	cig+/-ot	1	29	nev cigs	st
GAO	564		f	0	0	all	-	all	As:Chi	1984	CC	1405	n	ot	n	n	2	ev	cig+/-ot	1	29	nev cigs	ot
GER	518		c	0	0	all	-	all	As:oth	1990	CC	141	n	ot	y	n	5	ev	all/unsp	1	20	nev any	ot
HU	502		m	0	0	all	-	all	As:Chi	1985	CC	227	n	ot	n	y	0	ev	cig+/-ot	20	29	nev cigs	st
HU	507		f	0	0	all	-	all	As:Chi	1985	CC	227	n	ot	n	y	0	ev	cig+/-ot	20	29	nev cigs	st
HU2	509		c	0	0	all	-	all	As:Chi	1977	CC	523	n	ot	y	n	0	ev	cig+/-ot	20	29	nev cigs	or
HUMBLE	542		c	0	0	wh	-	not alv	NAmer	1980	CC	521	n	bl	y	n	3	cu	cig+/-ot	1	29	nev cigs	ot
JOLY	516		m	0	0	all	-	all	SCAmer	1978	CC	826	n	bl	n	n	0	ev	cig+/-ot	20	29	nev any	st
JOLY	502		f	0	0	all	-	all	SCAmer	1978	CC	826	n	bl	n	n	0	ev	cig+/-ot	20	29	nev any	st
JUSSAW	512		m	0	0	all	-	all	As:Ind	1964	CC	792	n	V	n	n	0	ev	cig only	20	29	nev any	st
KATSOU	512		f	0	0	all	-	all	Eu:bal	1987	CC	101	n	bl	n	n	1	cu	all/unsp	1	29	nev any	or
KHUDER	501		m	0	0	all	-	all	NAmer	1985	CC	482	n	bl	n	y	0	ev	cig+/-ot	1	29	nev cigs	st
LETOUR	506		c	0	0	all	-	all	NAmer	1983	CC	738	n	V	y	y	0	ev	cig+/-ot	1	24	nev cigs	st
LIAW	501		c	0	0	all	0	all	As:oth	1982	pr	127	n	ot	n	n	2	cu	all/unsp	1	20	nev any	or
LIU3	510		m	0	0	all	-	all	As:Chi	1985	CC	110	n	ot	n	n	2	ev	all/unsp	1	34	nev any	or
LIU5	504		c	0	0	all	-	all	As:Chi	1978	CC	111	n	ot	y	n	0	ev	all/unsp	1	29	nev any	st
LUBIN	508		m	0	0	all	-	all	As:Chi	1984	CC	427	m	ot	y	n	0	ev	cig+/-ot	1	29	nev any	st
LUBIN2	531		m	0	0	all	-	all	Eu:mul	1976	CC	7804	n	bl	n	y	0	ev	cig+/-ot	1	29	nev any	st
LUBIN2	574		f	0	0	all	-	all	Eu:mul	1976	CC	7804	n	bl	n	y	0	ev	cig+/-ot	1	29	nev any	st
MATOS	536		m	0	0	all	-	all	SCAmer	1994	CC	200	n	bl	n	n	2	ev	cig+/-ot	1	24	nev any	or
MCCONN	503		c	0	0	all	-	all	Eu:UK	1946	CC	100	n	V	n	y	0	ev	all/unsp	20	29	nev any	st
NOTAN2	514		c	0	0	all	-	all	As:Ind	1963	CC	683	n	V	n	n	0	ev	cig only	11	20	nev any	st
OSANN2	504		f	0	0	all	-	all	NAmer	1964	ot	217	n	bl	n	y	1	ev	cig+/-ot	1	20	nev cigs	or
PEZZOT	534		m	0	0	all	-	all	SCAmer	1987	CC	215	n	bl	n	y	0	ev	cig only	1	30	nev cigs	st
QIAO2	516		m	0	0	all	0	all	As:Chi	1992	pr	241	m	ot	n	n	1	ev	all/unsp	1	27	nev any	or
RACHTA	516		f	0	0	all	-	all	Eu:est	1991	CC	118	n	bl	n	y	1	ev	cig+/-ot	1	20	nev cigs	or
SOBUE	546		m	0	0	all	-	q+s+l+a	As:Jap	1986	CC	1376	n	bl	n	y	0	cu	cig+/-ot	1	29	nev cigs	st
WANG2	503		c	0	0	all	-	all	As:Chi	1980	CC	103	n	ot	n	n	0	ev	cig+/-ot	20	29	nev cigs	st
WUWILL	516		f	0	0	all	-	all	As:Chi	1985	CC	965	n	ot	n	n	3	ev	cig+/-ot	1	29	nev cigs	ot
ZHENG	553		m	0	0	all	-	all	As:Chi	1982	CC	540	n	ot	*	y	0	ev	cig+/-ot	1	29	nev cigs	st
ZHENG	558		f	0	0	all	-	all	As:Chi	1982	CC	540	n	ot	*	y	0	ev	cig+/-ot	1	29	nev cigs	st

Cigarette type is all/unspec for all RRs

except for the following:

REF	NRR	CIGTYPE
JUSSAW	512	MC only
NOTAN2	514	MC only

Table 1I17 - 2

IESLC - Meta-analysis of Ever/current Smoking, Duration, "Low"
All LC types, Cigarettes (or Any Product if Cigarettes not available)
Most adjusted

REF	NRR	SEX	AD	Number Exposed Case	Cont	Non-exposed Case	Cont	RR	95.00%CI
*AMANDU	506	m	2	42	-	6	-	5.92	(2.13- 16.47)
ARMADA	506	m	1	21	-	8	-	2.60	(1.00- 6.60)
AUVINE	517	c	2	26	-	44	-	20.10	(6.69- 66.00)
AXELSS	520	m	6	17	-	16	-	2.23	(1.03- 4.80)
AXELSS	511	f	0	12	29	18	154	3.54	(1.54- 8.13)
Subtotal AXELSS								2.76	(1.57- 4.85)
BARBON	508	m	1	42	-	22	-	3.20	(1.80- 5.70)
*BEST	505	m	1	22	-	7	-	4.10	(1.75- 9.60)
BUFFLE	526	f	0	52	57	12	112	8.51	(4.21- 17.22)
*CEDERL	501	m	1	5	-	7	-	1.80	(0.57- 5.66)
*CEDERL	504	f	1	3	-	19	-	1.60	(0.47- 5.40)
Subtotal CEDERL								1.70	(0.74- 3.93)
CHEN2	502	m	0	4	3	9	33	4.89	(0.92- 25.93)
CHEN2	510	f	0	1	6	25	33	0.22	(0.02- 1.95)
Subtotal CHEN2								1.55	(0.41- 5.85)
CHOI	502	m	0	66	166	13	95	2.91	(1.52- 5.54)
CHOI	511	f	0	8	14	76	164	1.23	(0.50- 3.06)
Subtotal CHOI								2.18	(1.29- 3.69)
*CPSI	580	m	0	95	266163	196	926068	1.69	(1.32- 2.15)
*CPSI	676	f	0	105	694015	532	3877179	1.10	(0.89- 1.36)
Subtotal CPSI								1.32	(1.13- 1.55)
*CPSII	552	m	0	72	141932	124	742207	3.04	(2.27- 4.06)
*CPSII	618	f	0	127	301244	310	2091302	2.84	(2.31- 3.50)
Subtotal CPSII								2.91	(2.46- 3.44)
DAMBER	506	m	1	-	-	42	-	1.58	(0.69- 3.66)
DESTEF	508	m	4	43	-	27	-	3.40	(1.70- 6.80)
DORGAN	570	m	2	-	-	-	-	5.44	(2.97- 9.98)
DORGAN	562	f	3	-	-	-	-	4.25	(3.20- 5.64)
Subtotal DORGAN								4.44	(3.44- 5.74)
DOSEME	502	m	2	158	-	142	-	3.80	(2.60- 5.70)
FAN	501	m	0	29	135	36	236	1.41	(0.83- 2.40)
FAN	506	f	0	8	15	69	320	2.47	(1.01- 6.06)
Subtotal FAN								1.63	(1.03- 2.58)
GAO	564	f	2	68	-	435	-	1.89	(1.30- 2.75)
GER	518	c	5	10	-	51	-	1.30	(0.55- 3.06)
HU	502	m	0	60	47	41	67	2.09	(1.21- 3.60)
HU	507	f	0	11	7	40	48	1.89	(0.67- 5.32)
Subtotal HU								2.04	(1.26- 3.31)
HU2	509	c	0	64	63	121	213	1.79	(1.18- 2.70)
HUMBLE	542	c	3	20	-	28	-	15.45	(6.19- 38.58)
JOLY	516	m	0	38	61	12	218	11.32	(5.57- 22.98)
JOLY	502	f	0	18	26	52	283	3.77	(1.93- 7.36)
Subtotal JOLY								6.33	(3.89- 10.30)
JUSSAW	512	m	0	38	23	149	624	6.92	(4.00- 11.97)
KATSOU	512	f	1	13	-	48	-	1.29	(0.54- 3.26)
KHUDER	501	m	0	16	61	23	309	3.52	(1.76- 7.06)
LETOUR	506	c	0	65	187	24	224	3.24	(1.95- 5.39)
*LIAW	501	c	2	-	-	-	-	0.90	(0.30- 3.10)
LIU3	510	m	2	30	-	4	-	1.07	(0.25- 4.59)
LIU5	504	c	0	27	37	26	41	1.15	(0.57- 2.31)
LUBIN	508	m	0	30	146	8	72	1.85	(0.81- 4.24)
LUBIN2	531	m	0	953	2995	190	2616	4.38	(3.72- 5.16)
LUBIN2	574	f	0	132	230	336	1188	2.03	(1.59- 2.59)
Subtotal LUBIN2								3.45	(3.01- 3.96)
MATOS	536	m	2	20	-	11	-	2.20	(1.00- 4.90)
MCCONN	503	c	0	46	57	9	23	2.06	(0.87- 4.89)
NOTAN2	514	c	0	15	15	107	201	1.88	(0.88- 3.99)
OSANN2	504	f	1	23	-	33	-	1.60	(0.70- 3.50)
PEZZOT	534	m	0	30	134	4	116	6.49	(2.22- 18.98)
*QIAO2	516	m	1	7	-	10	-	0.40	(0.15- 1.05)
RACHTA	516	f	1	12	-	33	-	2.02	(0.87- 4.71)
SOBUE	546	m	0	62	119	34	128	1.96	(1.21- 3.19)
WANG2	503	c	0	8	18	11	43	1.74	(0.60- 5.03)
WUWILL	516	f	3	137	-	417	-	1.35	(1.04- 1.73)
ZHENG	553	m	0	37	75	33	94	1.41	(0.80- 2.46)
ZHENG	558	f	0	17	17	152	184	1.21	(0.60- 2.45)
Subtotal ZHENG								1.33	(0.86- 2.06)
Partial Totals				2965	1408097	4202	7644595		

*prospective study

Table 1I17 - 2

IESLC - Meta-analysis of Ever/current Smoking, Duration, "Low"
All LC types, Cigarettes (or Any Product if Cigarettes not available)
Most adjusted

REF	NRR	SEX	AD	Ys	Ws	Qs	Ps
*AMANDU	506	m	2	1.78	3.67	2.82	0.0007
ARMADA	506	m	1	0.96	4.31	0.01	0.0472
AUVINE	517	c	2	3.00	2.93	12.92	0.0000
AXELSS	520	m	6	0.80	6.49	0.06	0.0411
AXELSS	511	f	0	1.26	5.56	0.73	0.0029
Subtotal AXELSS				1.02	12.05	0.79	
BARBON	508	m	1	1.16	11.56	0.79	0.0001
*BEST	505	m	1	1.41	5.30	1.37	0.0012
BUFFLE	526	f	0	2.14	7.75	11.91	0.0000
*CEDERL	501	m	1	0.59	2.92	0.29	0.3155
*CEDERL	504	f	1	0.47	2.58	0.48	0.4505
Subtotal CEDERL				0.53	5.49	0.77	
CHEN2	502	m	0	1.59	1.38	0.65	0.0623
CHEN2	510	f	0	-1.51	0.81	4.72	0.1734
Subtotal CHEN2				0.44	2.19	5.37	
CHOI	502	m	0	1.07	9.21	0.25	0.0012
CHOI	511	f	0	0.21	4.64	2.22	0.6519
Subtotal CHOI				0.78	13.84	2.47	
*CPSI	580	m	0	0.52	64.01	9.22	0.0000
*CPSI	676	f	0	0.10	87.71	56.75	0.3603
Subtotal CPSI				0.28	151.71	65.97	
*CPSII	552	m	0	1.11	45.57	1.98	0.0000
*CPSII	618	f	0	1.05	90.12	1.85	0.0000
Subtotal CPSII				1.07	135.69	3.83	
DAMBER	506	m	1	0.46	5.52	1.09	0.2825
DESTEF	508	m	4	1.22	8.00	0.83	0.0005
DORGAN	570	m	2	1.69	10.46	6.56	0.0000
DORGAN	562	f	3	1.45	47.84	14.20	0.0000
Subtotal DORGAN				1.49	58.30	20.76	
DOSEME	502	m	2	1.34	24.94	4.67	0.0000
FAN	501	m	0	0.34	13.53	4.24	0.2079
FAN	506	f	0	0.91	4.78	0.00	0.0478
Subtotal FAN				0.49	18.31	4.24	
GAO	564	f	2	0.64	27.37	1.93	0.0009
GER	518	c	5	0.26	5.22	2.13	0.5490
HU	502	m	0	0.74	12.94	0.36	0.0082
HU	507	f	0	0.63	3.58	0.26	0.2303
Subtotal HU				0.71	16.52	0.62	
HU2	509	c	0	0.58	22.49	2.32	0.0058
HUMBLE	542	c	3	2.74	4.59	15.46	0.0000
JOLY	516	m	0	2.43	7.66	17.78	0.0000
JOLY	502	f	0	1.33	8.56	1.54	0.0001
Subtotal JOLY				1.85	16.22	19.33	
JUSSAW	512	m	0	1.93	12.80	13.64	0.0000
KATSOU	512	f	1	0.25	4.75	1.99	0.5788
KHUDER	501	m	0	1.26	7.96	1.02	0.0004
LETOUR	506	c	0	1.18	14.96	1.13	0.0000
*LIAW	501	c	2	-0.11	2.82	2.86	0.8596
LIU3	510	m	2	0.07	1.81	1.26	0.9274
LIU5	504	c	0	0.14	7.88	4.57	0.6935
LUBIN	508	m	0	0.61	5.58	0.46	0.1463
LUBIN2	531	m	0	1.48	142.28	47.07	0.0000
LUBIN2	574	f	0	0.71	63.53	2.40	0.0000
Subtotal LUBIN2				1.24	205.80	49.47	
MATOS	536	m	2	0.79	6.08	0.08	0.0518
MCCONN	503	c	0	0.72	5.16	0.16	0.1002
NOTAN2	514	c	0	0.63	6.77	0.50	0.1008
OSANN2	504	f	1	0.47	5.93	1.11	0.2523
PEZZOT	534	m	0	1.87	3.34	3.13	0.0006
*QIAO2	516	m	1	-0.92	4.06	13.42	0.0649
RACHTA	516	f	1	0.70	5.39	0.21	0.1027
SOBUE	546	m	0	0.67	16.19	0.84	0.0067
WANG2	503	c	0	0.55	3.39	0.41	0.3089
WUWILL	516	f	3	0.30	59.33	21.50	0.0208
ZHENG	553	m	0	0.34	12.30	3.88	0.2328
ZHENG	558	f	0	0.19	7.71	3.90	0.5957
Subtotal ZHENG				0.28	20.01	7.78	

Table 1I17 - 2

IESLC - Meta-analysis of Ever/current Smoking, Duration, "Low"
All LC types, Cigarettes (or Any Product if Cigarettes not available)
Most adjusted

	N	55
	NS	43
	Wt	964.02
Het	Chi	307.96
Het	df	54
Het	P	***
Fixed	RR	2.46
	RRl	2.31
	RRu	2.63
	P	+++
Random	RR	2.48
	RRl	2.09
	RRu	2.95
	P	+++
Asymm	P	N.S.

Table 1I17 - 3

IESLC - Meta-analysis of Ever/current Smoking, Duration, "Low"
All LC types, Cigarettes (or Any Product if Cigarettes not available)
Most adjusted

	Sex			
	combined	male	female	Total
N	10	27	18	55
NS	10	27	18	55
Wt	76.21	449.87	437.93	964.02
Het Chi	42.18	117.41	108.76	307.96
Het df	9	26	17	54
Het P	***	***	***	***
Fixed RR	2.32	3.05	2.00	2.46
RRl	1.85	2.78	1.82	2.31
RRu	2.90	3.34	2.20	2.63
P	+++	+++	+++	+++
Random RR	2.55	2.80	2.08	2.48
RRl	1.52	2.22	1.57	2.09
RRu	4.30	3.52	2.75	2.95
P	+++	+++	+++	+++
Between Chi				39.60
Between df				2
Between P				***
Btwn(F) P				*
Btwn(R) P				N.S.

	Lung cancer type		
	all	other	Total
N	53	2	55
NS	41	2	43
Wt	943.23	20.78	964.02
Het Chi	291.63	15.23	307.96
Het df	52	1	54
Het P	***	***	***
Fixed RR	2.45	3.09	2.46
RRl	2.30	2.01	2.31
RRu	2.61	4.76	2.63
P	+++	+++	+++
Random RR	2.42	5.30	2.48
RRl	2.04	0.70	2.09
RRu	2.88	40.01	2.95
P	+++	N.S.	+++
Between Chi			1.10
Between df			1
Between P			N.S.
Btwn(F) P			N.S.
Btwn(R) P			N.S.

	Location								
	NAmer	UK	Scand	othEur	China	Japan	othAs	other	Total
N	13	1	6	7	16	1	6	5	55
NS	10	1	4	6	12	1	5	4	43
Wt	395.87	5.16	25.99	256.76	188.96	16.19	41.45	33.64	964.02
Het Chi	125.13	0.00	15.22	32.61	19.12	0.00	21.31	10.97	307.96
Het df	12	0	5	6	15	0	5	4	54
Het P	***	N.S.	**	***	N.S.	N.S.	***	*	***
Fixed RR	2.40	2.06	2.77	3.36	1.51	1.96	2.68	4.52	2.46
RRl	2.18	0.87	1.89	2.97	1.31	1.21	1.98	3.23	2.31
RRu	2.65	4.89	4.07	3.79	1.75	3.19	3.64	6.34	2.63
P	+++	N.S.	+++	+++	+++	++	+++	+++	+++
Random RR	3.41	2.06	2.92	2.76	1.52	1.96	2.10	4.55	2.48
RRl	2.39	0.87	1.47	1.91	1.27	1.21	1.09	2.57	2.09
RRu	4.87	4.89	5.81	4.01	1.82	3.19	4.04	8.04	2.95
P	+++	N.S.	++	+++	+++	++	+	+++	+++
Between Chi									83.59
Between df									7
Between P									***
Btwn(F) P									*
Btwn(R) P									***

Table 1I17 - 3

IESLC - Meta-analysis of Ever/current Smoking, Duration, "Low"
All LC types, Cigarettes (or Any Product if Cigarettes not available)
Most adjusted

Detailed Country in "other Europe"

	multi	Germany	othWest	East	Balkans	Total
N	2		2	1	2	7
NS	1		2	1	2	6
Wt	205.80		15.88	5.39	29.69	256.76
Het Chi	26.02		0.14	0.00	4.66	32.61
Het df	1		1	0	1	6
Het P	***		N.S.	N.S.	*	***
Fixed RR	3.45		3.02	2.02	3.20	3.36
RRl	3.01		1.85	0.87	2.23	2.97
RRu	3.96		4.95	4.70	4.58	3.79
P	+++		+++	N.S.	+++	+++
Random RR	3.00		3.02	2.02	2.40	2.76
RRl	1.41		1.85	0.87	0.84	1.91
RRu	6.37		4.95	4.70	6.83	4.01
P	++		+++	N.S.	N.S.	+++
Between Chi						1.80
Between df						3
Between P						N.S.
Btwn(F) P						N.S.
Btwn(R) P						N.S.

Detailed Country in "other Asia"

	India	HongKong	other	Total
N	2		4	6
NS	2		3	5
Wt	19.58		21.88	41.45
Het Chi	7.53		4.63	21.31
Het df	1		3	5
Het P	**		N.S.	***
Fixed RR	4.41		1.72	2.68
RRl	2.83		1.13	1.98
RRu	6.86		2.62	3.64
P	+++		+	+++
Random RR	3.70		1.60	2.10
RRl	1.03		0.94	1.09
RRu	13.27		2.74	4.04
P	+		(+)	+
Between Chi				9.15
Between df				1
Between P				**
Btwn(F) P				N.S.
Btwn(R) P				N.S.

Detailed other continent

	SCAmer	Total
N	5	5
NS	4	4
Wt	33.64	33.64
Het Chi	10.97	10.97
Het df	4	4
Het P	*	*
Fixed RR	4.52	4.52
RRl	3.23	3.23
RRu	6.34	6.34
P	+++	+++
Random RR	4.55	4.55
RRl	2.57	2.57
RRu	8.04	8.04
P	+++	+++
Between Chi		
Between df		
Between P		N.S.
Btwn(F) P		N.S.
Btwn(R) P		N.S.

Table 1I17 - 3

IESLC - Meta-analysis of Ever/current Smoking, Duration, "Low"
All LC types, Cigarettes (or Any Product if Cigarettes not available)
Most adjusted

	Start year of study <1960	1960-69	1970-79	1980-89	1990+	Total
N	5	5	10	29	6	55
NS	4	4	8	22	5	43
Wt	165.85	31.00	302.16	425.95	39.05	964.02
Het Chi	21.66	14.53	65.81	107.12	9.80	307.96
Het df	4	4	9	28	5	54
Het P	***	**	***	***	(*)	***
Fixed RR	1.43	3.07	3.35	2.53	1.48	2.46
RRl	1.23	2.16	2.99	2.30	1.08	2.31
RRu	1.67	4.36	3.75	2.78	2.02	2.63
P	+++	+++	+++	+++	+	+++
Random RR	2.06	2.43	3.21	2.55	1.46	2.48
RRl	1.29	1.18	2.24	2.05	0.92	2.09
RRu	3.29	4.97	4.62	3.16	2.30	2.95
P	++	+	+++	+++	N.S.	+++
Between Chi						89.03
Between df						4
Between P						***
Btwn(F) P						**
Btwn(R) P						(*)

	Study type (1) CC	other	Total
N	44	11	55
NS	35	8	43
Wt	649.34	314.68	964.02
Het Chi	205.63	71.13	307.96
Het df	43	10	54
Het P	***	***	***
Fixed RR	2.79	1.90	2.46
RRl	2.59	1.70	2.31
RRu	3.02	2.13	2.63
P	+++	+++	+++
Random RR	2.67	1.87	2.48
RRl	2.21	1.30	2.09
RRu	3.22	2.70	2.95
P	+++	+++	+++
Between Chi			31.21
Between df			1
Between P			***
Btwn(F) P			*
Btwn(R) P			(*)

	Study type (2) CC	prosp	other	Total
N	44	10	1	55
NS	35	7	1	43
Wt	649.34	308.75	5.93	964.02
Het Chi	205.63	70.94	0.00	307.96
Het df	43	9	0	54
Het P	***	***	N.S.	***
Fixed RR	2.79	1.91	1.60	2.46
RRl	2.59	1.71	0.72	2.31
RRu	3.02	2.14	3.58	2.63
P	+++	+++	N.S.	+++
Random RR	2.67	1.89	1.60	2.48
RRl	2.21	1.28	0.72	2.09
RRu	3.22	2.79	3.58	2.95
P	+++	++	N.S.	+++
Between Chi				31.39
Between df				2
Between P				***
Btwn(F) P				(*)
Btwn(R) P				N.S.

Table 1I17 - 3

IESLC - Meta-analysis of Ever/current Smoking, Duration, "Low"
All LC types, Cigarettes (or Any Product if Cigarettes not available)
Most adjusted

	Study size (number of LC cases)				
	100-249	250-499	500-999	1000+	Total
N	17	13	14	11	55
NS	15	9	12	7	43
Wt	78.21	80.85	184.94	620.01	964.02
Het Chi	29.95	11.84	108.36	145.91	307.96
Het df	16	12	13	10	54
Het P	*	N.S.	***	***	***
Fixed RR	1.72	2.37	2.47	2.59	2.46
RRl	1.38	1.91	2.14	2.40	2.31
RRu	2.14	2.95	2.85	2.80	2.63
P	+++	+++	+++	+++	+++
Random RR	1.70	2.37	3.52	2.62	2.48
RRl	1.24	1.91	2.26	1.92	2.09
RRu	2.33	2.95	5.50	3.59	2.95
P	++	+++	+++	+++	+++
Between Chi					11.90
Between df					3
Between P					**
Btwn(F) P					N.S.
Btwn(R) P					(*)

	Risky occupational population			
	no	mining	othRisky	Total
N	52	3		55
NS	40	3		43
Wt	950.70	13.32		964.02
Het Chi	291.23	14.20		307.96
Het df	51	2		54
Het P	***	***		***
Fixed RR	2.48	1.60		2.46
RRl	2.33	0.93		2.31
RRu	2.64	2.74		2.63
P	+++	(+)		+++
Random RR	2.53	1.63		2.48
RRl	2.13	0.38		2.09
RRu	3.01	6.91		2.95
P	+++	N.S.		+++
Between Chi				2.53
Between df				1
Between P				N.S.
Btwn(F) P				N.S.
Btwn(R) P				N.S.

	National cigarette tobacco type			
	Virginia	blended	other	Total
N	5	32	18	55
NS	5	24	14	43
Wt	44.99	722.03	196.99	964.02
Het Chi	10.18	213.22	19.98	307.96
Het df	4	31	17	54
Het P	*	***	N.S.	***
Fixed RR	3.62	2.76	1.50	2.46
RRl	2.70	2.56	1.30	2.31
RRu	4.85	2.97	1.72	2.63
P	+++	+++	+++	+++
Random RR	3.38	3.10	1.50	2.48
RRl	2.08	2.49	1.27	2.09
RRu	5.50	3.86	1.77	2.95
P	+++	+++	+++	+++
Between Chi				64.58
Between df				2
Between P				***
Btwn(F) P				**
Btwn(R) P				***

Table 1I17 - 3

IESLC - Meta-analysis of Ever/current Smoking, Duration, "Low"
All LC types, Cigarettes (or Any Product if Cigarettes not available)
Most adjusted

	Any proxy use No/nk	Yes	Total
N	39	16	55
NS	30	13	43
Wt	796.73	167.28	964.02
Het Chi	223.15	75.19	307.96
Het df	38	15	54
Het P	***	***	***
Fixed RR	2.35	3.07	2.46
RRl	2.20	2.63	2.31
RRu	2.52	3.57	2.63
P	+++	+++	+++
Random RR	2.31	3.01	2.48
RRl	1.90	2.07	2.09
RRu	2.80	4.38	2.95
P	+++	+++	+++
Between Chi			9.63
Between df			1
Between P			**
Btwn(F) P			N.S.
Btwn(R) P			N.S.

	Full histological confirmation No	Yes	Total
N	37	18	55
NS	29	14	43
Wt	580.58	383.44	964.02
Het Chi	204.20	63.79	307.96
Het df	36	17	54
Het P	***	***	***
Fixed RR	2.09	3.17	2.46
RRl	1.93	2.87	2.31
RRu	2.27	3.50	2.63
P	+++	+++	+++
Random RR	2.39	2.69	2.48
RRl	1.91	2.13	2.09
RRu	2.98	3.39	2.95
P	+++	+++	+++
Between Chi			39.97
Between df			1
Between P			***
Btwn(F) P			**
Btwn(R) P			N.S.

	Number of adjustment variables (1) 0	1	2+/+nk	Total
N	31	10	14	55
NS	22	9	13	44
Wt	700.14	52.33	211.55	964.02
Het Chi	199.91	17.72	86.43	307.96
Het df	30	9	13	54
Het P	***	*	***	***
Fixed RR	2.46	1.94	2.63	2.46
RRl	2.29	1.48	2.30	2.31
RRu	2.65	2.54	3.01	2.63
P	+++	+++	+++	+++
Random RR	2.46	1.82	3.11	2.48
RRl	1.98	1.23	2.09	2.09
RRu	3.06	2.69	4.62	2.95
P	+++	++	+++	+++
Between Chi				3.91
Between df				2
Between P				N.S.
Btwn(F) P				N.S.
Btwn(R) P				N.S.

Table 1I17 - 3

IESLC - Meta-analysis of Ever/current Smoking, Duration, "Low"
All LC types, Cigarettes (or Any Product if Cigarettes not available)
Most adjusted

	Number of adjustment variables (2) 0	1	2	3-5	6+/+nk	Total
N	31	10	8	5	1	55
NS	22	9	8	5	1	45
Wt	700.14	52.33	80.09	124.97	6.49	964.02
Het Chi	199.91	17.72	29.84	54.09	0.00	307.96
Het df	30	9	7	4	0	54
Het P	***	*	***	***	N.S.	***
Fixed RR	2.46	1.94	3.01	2.43	2.23	2.46
RRl	2.29	1.48	2.42	2.04	1.03	2.31
RRu	2.65	2.54	3.75	2.89	4.81	2.63
P	+++	+++	+++	+++	+	+++
Random RR	2.46	1.82	3.24	3.18	2.23	2.48
RRl	1.98	1.23	1.92	1.49	1.03	2.09
RRu	3.06	2.69	5.45	6.79	4.81	2.95
P	+++	++	+++	++	+	+++
Between Chi						6.40
Between df						4
Between P						N.S.
Btwn(F) P						N.S.
Btwn(R) P						N.S.

	Smoking status ever	current	Total
N	43	12	55
NS	34	9	43
Wt	633.79	330.23	964.02
Het Chi	204.37	81.19	307.96
Het df	42	11	54
Het P	***	***	***
Fixed RR	2.75	2.00	2.46
RRl	2.55	1.79	2.31
RRu	2.97	2.22	2.63
P	+++	+++	+++
Random RR	2.52	2.36	2.48
RRl	2.07	1.65	2.09
RRu	3.06	3.37	2.95
P	+++	+++	+++
Between Chi			22.40
Between df			1
Between P			***
Btwn(F) P			*
Btwn(R) P			N.S.

	Product all/unsp	cig+/-ot	cig only	Total
N	14	32	9	55
NS	12	25	7	44
Wt	71.01	662.01	230.99	964.02
Het Chi	28.72	172.64	66.12	307.96
Het df	13	31	8	54
Het P	**	***	***	***
Fixed RR	1.82	2.83	1.82	2.46
RRl	1.44	2.62	1.60	2.31
RRu	2.30	3.05	2.07	2.63
P	+++	+++	+++	+++
Random RR	1.66	2.82	2.54	2.48
RRl	1.15	2.30	1.62	2.09
RRu	2.39	3.45	3.98	2.95
P	++	+++	+++	+++
Between Chi				40.49
Between df				2
Between P				***
Btwn(F) P				*
Btwn(R) P				*

Table 1I17 - 3

IESLC - Meta-analysis of Ever/current Smoking, Duration, "Low"
All LC types, Cigarettes (or Any Product if Cigarettes not available)
Most adjusted

		Denominator nev any	nev cigs	Total
	N	28	27	55
	NS	22	22	44
	Wt	438.94	525.07	964.02
Het	Chi	112.55	138.34	307.96
Het	df	27	26	54
Het	P	***	***	***
Fixed	RR	3.22	1.97	2.46
	RRl	2.93	1.81	2.31
	RRu	3.53	2.15	2.63
	P	+++	+++	+++
Random	RR	2.55	2.43	2.48
	RRl	2.02	1.94	2.09
	RRu	3.21	3.04	2.95
	P	+++	+++	+++
Between	Chi			57.07
Between	df			1
Between	P			***
Btwn(F)	P			**
Btwn(R)	P			N.S.

		Derivation of RR/CI Orig	StdCalc	Other	Total
	N	14	29	12	55
	NS	14	20	10	44
	Wt	112.84	669.89	181.29	964.02
Het	Chi	54.05	185.67	67.86	307.96
Het	df	13	28	11	54
Het	P	***	***	***	***
Fixed	RR	2.60	2.45	2.43	2.46
	RRl	2.16	2.27	2.10	2.31
	RRu	3.13	2.65	2.81	2.63
	P	+++	+++	+++	+++
Random	RR	2.39	2.39	2.83	2.48
	RRl	1.60	1.91	1.86	2.09
	RRu	3.58	3.00	4.30	2.95
	P	+++	+++	+++	+++
Between	Chi				0.38
Between	df				2
Between	P				N.S.
Btwn(F)	P				N.S.
Btwn(R)	P				N.S.

Table 1I17 - 4

IESLC - Meta-analysis of Ever/current Smoking, Duration, "Low"
All LC types, Cigarettes (or Any Product if Cigarettes not available)
Least adjusted

REF	NRR	X	SEX	AGEL	AGEH	RACE	YF	LC TYPE	LOC	START	ST	NLC	R	VB	P	H	AD	SM	PRODUCT	exL	exH	DENOM	De
AMANDU	501	x	m	0	0	wh	0	all	NAmer	1959	pr	132	m	bl	n	n	0	cu	cig+/-ot	0	24	nev cigs	st
ARMADA	501	x	m	0	0	all	-	all	Eu:wst	1986	CC	325	n	bl	n	y	0	ev	cig+/-ot	1	24	nev cigs	st
AUVINE	501	x	c	0	0	all	-	all	Eu:Sca	1986	CC	517	n	bl	y	n	0	ev	cig+/-ot	1	20	nev cigs	st
AXELSS	502	x	m	0	0	sca	-	all	Eu:Sca	1989	CC	436	n	bl	n	n	0	ev	all/unsp	20	29	nev any	st
AXELSS	511		f	0	0	sca	-	all	Eu:Sca	1989	CC	436	n	bl	n	n	0	ev	all/unsp	20	29	nev any	st
BARBON	501	x	m	0	0	all	-	all	Eu:wst	1979	CC	755	n	bl	y	y	0	ev	all/unsp	1	29	nev any	st
BEST	505		m	0	0	all	0	all	NAmer	1955	pr	381	n	V	n	n	1	cu	cig only	20	29	nev any	ot
BUFFLE	526		f	0	0	w-hi	-	all	NAmer	1976	CC	943	n	bl	y	n	0	ev	cig+/-ot	1	30	nev cigs	or
CEDERL	501		m	40	69	all	10	all	Eu:Sca	1963	pr	491	n	bl	n	n	1	cu	cig only	1	29	nev any	ot
CEDERL	504		f	40	69	all	10	all	Eu:Sca	1963	pr	491	n	bl	n	n	1	cu	cig only	1	29	nev any	ot
CHEN2	502		m	0	0	all	-	all	As:Chi	1983	CC	193	n	ot	y	n	0	ev	all/unsp	10	20	nev any	st
CHEN2	510		f	0	0	all	-	all	As:Chi	1983	CC	193	n	ot	y	n	0	ev	all/unsp	1	20	nev any	st
CHOI	502		m	0	0	all	-	all	As:oth	1985	CC	375	n	bl	n	n	0	ev	cig+/-ot	20	29	nev cigs	st
CHOI	511		f	0	0	all	-	all	As:oth	1985	CC	375	n	bl	n	n	0	ev	cig+/-ot	20	29	nev cigs	st
CPSI	580		m	40	84	wh	0	all	NAmer	1959	pr	5138	n	bl	n	n	0	cu	cig only	1	29	nev cigs	st
CPSI	676		f	40	84	wh	0	all	NAmer	1959	pr	5138	n	bl	n	n	0	cu	cig only	1	29	nev cigs	st
CPSII	552		m	0	0	all	6	all	NAmer	1982	pr	3229	n	bl	n	n	0	cu	cig only	1	29	nev any	st
CPSII	618		f	0	0	all	6	all	NAmer	1982	pr	3229	n	bl	n	n	0	cu	cig+/-ot	1	29	nev cigs	st
DAMBER	506		m	0	0	all	-	all	Eu:Sca	1972	CC	579	n	bl	y	n	1	ev	all/unsp	1	20	nev any	ot
DESTEF	501	x	m	0	0	all	-	all	SCAmer	1988	CC	497	n	bl	n	y	0	ev	all/unsp	1	29	nev any	st
DORGAN	570		m	0	0	wh	-	all	NAmer	1980	CC	2026	n	bl	y	y	2	ev	cig+/-ot	1	34	nev any	ot
DORGAN	562		f	0	0	all	-	all	NAmer	1980	CC	2026	n	bl	y	y	3	ev	cig+/-ot	1	34	nev any	ot
DOSEME	502		m	0	0	all	-	all	Eu:bal	1979	CC	1210	n	bl	n	n	2	ev	cig+/-ot	11	20	nev cigs	or
FAN	501		m	0	0	all	-	all	As:Chi	1990	CC	403	n	ot	y	n	0	ev	cig+/-ot	1	29	nev cigs	st
FAN	506		f	0	0	all	-	all	As:Chi	1990	CC	403	n	ot	y	n	0	ev	cig+/-ot	1	29	nev cigs	st
GAO	561	x	f	0	0	all	-	all	As:Chi	1984	CC	1405	n	ot	n	n	0	ev	cig+/-ot	1	29	nev cigs	st
GER	513	x	c	0	0	all	-	all	As:oth	1990	CC	141	n	ot	y	n	0	ev	all/unsp	1	20	nev any	st
HU	502		m	0	0	all	-	all	As:Chi	1985	CC	227	n	ot	n	y	0	ev	cig+/-ot	20	29	nev cigs	st
HU	507		f	0	0	all	-	all	As:Chi	1985	CC	227	n	ot	n	y	0	ev	cig+/-ot	20	29	nev cigs	st
HU2	509		c	0	0	all	-	all	As:Chi	1977	CC	523	n	ot	y	n	0	ev	cig+/-ot	20	29	nev cigs	or
HUMBLE	517	x	c	0	0	wh	-	not alv	NAmer	1980	CC	521	n	bl	y	n	0	cu	cig+/-ot	1	29	nev cigs	st
JOLY	516		m	0	0	all	-	all	SCAmer	1978	CC	826	n	bl	n	n	0	ev	cig+/-ot	20	29	nev any	st
JOLY	502		f	0	0	all	-	all	SCAmer	1978	CC	826	n	bl	n	n	0	ev	cig+/-ot	20	29	nev any	st
JUSSAW	512		m	0	0	all	-	all	As:Ind	1964	CC	792	n	V	n	n	0	ev	cig only	20	29	nev any	st
KATSOU	502	x	f	0	0	all	-	all	Eu:bal	1987	CC	101	n	bl	n	n	0	cu	all/unsp	20	29	nev any	st
KHUDER	501		m	0	0	all	-	all	NAmer	1985	CC	482	n	bl	n	y	0	ev	cig+/-ot	1	29	nev cigs	st
LETOUR	506		c	0	0	all	-	all	NAmer	1983	CC	738	n	V	y	y	0	ev	cig+/-ot	1	24	nev cigs	st
LIAW	501		c	0	0	all	0	all	As:oth	1982	pr	127	n	ot	n	n	2	cu	all/unsp	1	20	nev any	or
LIU3	507	x	m	0	0	all	-	all	As:Chi	1985	CC	110	n	ot	n	n	0	ev	all/unsp	1	34	nev any	or
LIU5	504		c	0	0	all	-	all	As:Chi	1978	CC	111	n	ot	y	n	0	ev	all/unsp	1	29	nev any	st
LUBIN	508		m	0	0	all	-	all	As:Chi	1984	CC	427	m	ot	y	n	0	ev	cig+/-ot	1	29	nev any	st
LUBIN2	531		m	0	0	all	-	all	Eu:mul	1976	CC	7804	n	bl	n	y	0	ev	cig+/-ot	1	29	nev any	st
LUBIN2	574		f	0	0	all	-	all	Eu:mul	1976	CC	7804	n	bl	n	y	0	ev	cig+/-ot	1	29	nev any	st
MATOS	516	x	m	0	0	all	-	all	SCAmer	1994	CC	200	n	bl	n	n	0	ev	cig+/-ot	1	24	nev any	st
MCCONN	503		c	0	0	all	-	all	Eu:UK	1946	CC	100	n	V	n	y	0	ev	all/unsp	20	29	nev any	st
NOTAN2	514		c	0	0	all	-	all	As:Ind	1963	CC	683	n	V	n	n	0	ev	cig only	11	20	nev any	st
OSANN2	501	x	f	0	0	all	-	all	NAmer	1964	ot	217	n	bl	n	y	0	ev	cig+/-ot	1	20	nev cigs	st
PEZZOT	534		m	0	0	all	-	all	SCAmer	1987	CC	215	n	bl	n	y	0	ev	cig only	1	30	nev cigs	st
QIAO2	511	x	m	0	0	all	0	all	As:Chi	1992	pr	241	m	ot	n	n	0	ev	all/unsp	1	27	nev any	st
RACHTA	511	x	f	0	0	all	-	all	Eu:est	1991	CC	118	n	bl	n	y	0	ev	cig+/-ot	1	20	nev cigs	st
SOBUE	546		m	0	0	all	-	q+s+l+a	As:Jap	1986	CC	1376	n	bl	n	y	0	cu	cig+/-ot	1	29	nev cigs	st
WANG2	503		c	0	0	all	-	all	As:Chi	1980	CC	103	n	ot	n	n	0	ev	cig+/-ot	20	29	nev cigs	st
WUWILL	501	x	f	0	0	all	-	all	As:Chi	1985	CC	965	n	ot	n	n	0	ev	cig+/-ot	1	29	nev cigs	st
ZHENG	553		m	0	0	all	-	all	As:Chi	1982	CC	540	n	ot	*	y	0	ev	cig+/-ot	1	29	nev cigs	st
ZHENG	558		f	0	0	all	-	all	As:Chi	1982	CC	540	n	ot	*	y	0	ev	cig+/-ot	1	29	nev cigs	st

Cigarette type is all/unspec for all RRs

except for the following:

REF	NRR	CIGTYPE
JUSSAW	512	MC only
NOTAN2	514	MC only

Table 1I17 - 5

IESLC - Meta-analysis of Ever/current Smoking, Duration, "Low"
All LC types, Cigarettes (or Any Product if Cigarettes not available)
Least adjusted

REF	NRR	SEX	AD	Number Exposed Case	Number Exposed Cont	Non-exposed Case	Non-exposed Cont	RR	95.00%CI
*AMANDU	501	m	0	42	68909	6	25350	2.58	(1.09- 6.06)
ARMADA	501	m	0	21	55	8	71	3.39	(1.40- 8.23)
AUVINE	501	c	0	26	18	44	229	7.52	(3.80- 14.87)
AXELSS	502	m	0	17	64	16	160	2.66	(1.27- 5.58)
AXELSS	511	f	0	12	29	18	154	3.54	(1.54- 8.13)
Subtotal AXELSS								3.02	(1.73- 5.25)
BARBON	501	m	0	42	91	22	188	3.94	(2.22- 7.00)
*BEST	505	m	1	22	-	7	-	4.10	(1.75- 9.60)
BUFFLE	526	f	0	52	57	12	112	8.51	(4.21- 17.22)
*CEDERL	501	m	1	5	-	7	-	1.80	(0.57- 5.66)
*CEDERL	504	f	1	3	-	19	-	1.60	(0.47- 5.40)
Subtotal CEDERL								1.70	(0.74- 3.93)
CHEN2	502	m	0	4	3	9	33	4.89	(0.92- 25.93)
CHEN2	510	f	0	1	6	25	33	0.22	(0.02- 1.95)
Subtotal CHEN2								1.55	(0.41- 5.85)
CHOI	502	m	0	66	166	13	95	2.91	(1.52- 5.54)
CHOI	511	f	0	8	14	76	164	1.23	(0.50- 3.06)
Subtotal CHOI								2.18	(1.29- 3.69)
*CPSI	580	m	0	95	266163	196	926068	1.69	(1.32- 2.15)
*CPSI	676	f	0	105	694015	532	3877179	1.10	(0.89- 1.36)
Subtotal CPSI								1.32	(1.13- 1.55)
*CPSII	552	m	0	72	141932	124	742207	3.04	(2.27- 4.06)
*CPSII	618	f	0	127	301244	310	2091302	2.84	(2.31- 3.50)
Subtotal CPSII								2.91	(2.46- 3.44)
DAMBER	506	m	1	-	-	42	-	1.58	(0.69- 3.66)
DESTEF	501	m	0	43	55	27	163	4.72	(2.67- 8.35)
DORGAN	570	m	2	-	-	-	-	5.44	(2.97- 9.98)
DORGAN	562	f	3	-	-	-	-	4.25	(3.20- 5.64)
Subtotal DORGAN								4.44	(3.44- 5.74)
DOSEME	502	m	2	158	-	142	-	3.80	(2.60- 5.70)
FAN	501	m	0	29	135	36	236	1.41	(0.83- 2.40)
FAN	506	f	0	8	15	69	320	2.47	(1.01- 6.06)
Subtotal FAN								1.63	(1.03- 2.58)
GAO	561	f	0	68	58	435	605	1.63	(1.12- 2.36)
GER	513	c	0	10	40	51	246	1.21	(0.57- 2.57)
HU	502	m	0	60	47	41	67	2.09	(1.21- 3.60)
HU	507	f	0	11	7	40	48	1.89	(0.67- 5.32)
Subtotal HU								2.04	(1.26- 3.31)
HU2	509	c	0	64	63	121	213	1.79	(1.18- 2.70)
HUMBLE	517	c	0	20	33	28	285	6.17	(3.13- 12.15)
JOLY	516	m	0	38	61	12	218	11.32	(5.57- 22.98)
JOLY	502	f	0	18	26	52	283	3.77	(1.93- 7.36)
Subtotal JOLY								6.33	(3.89- 10.30)
JUSSAW	512	m	0	38	23	149	624	6.92	(4.00- 11.97)
KATSOU	502	f	0	8	7	48	67	1.60	(0.54- 4.70)
KHUDER	501	m	0	16	61	23	309	3.52	(1.76- 7.06)
LETOUR	506	c	0	65	187	24	224	3.24	(1.95- 5.39)
*LIAW	501	c	2	-	-	-	-	0.90	(0.30- 3.10)
LIU3	507	m	0	30	146	4	19	0.98	(0.31- 3.07)
LIU5	504	c	0	27	37	26	41	1.15	(0.57- 2.31)
LUBIN	508	m	0	30	146	8	72	1.85	(0.81- 4.24)
LUBIN2	531	m	0	953	2995	190	2616	4.38	(3.72- 5.16)
LUBIN2	574	f	0	132	230	336	1188	2.03	(1.59- 2.59)
Subtotal LUBIN2								3.45	(3.01- 3.96)
MATOS	516	m	0	20	84	11	110	2.38	(1.08- 5.24)
MCCONN	503	c	0	46	57	9	23	2.06	(0.87- 4.89)
NOTAN2	514	c	0	15	15	107	201	1.88	(0.88- 3.99)
OSANN2	501	f	0	23	47	33	109	1.62	(0.86- 3.04)
PEZZOT	534	m	0	30	134	4	116	6.49	(2.22- 18.98)
*QIAO2	511	m	0	7	2364	10	709	0.21	(0.08- 0.55)
RACHTA	511	f	0	12	19	33	98	1.88	(0.82- 4.27)
SOBUE	546	m	0	62	119	34	128	1.96	(1.21- 3.19)
WANG2	503	c	0	8	18	11	43	1.74	(0.60- 5.03)
WUWILL	501	f	0	137	139	417	601	1.42	(1.09- 1.86)
ZHENG	553	m	0	37	75	33	94	1.41	(0.80- 2.46)
ZHENG	558	f	0	17	17	152	184	1.21	(0.60- 2.45)
Subtotal ZHENG								1.33	(0.86- 2.06)
Partial Totals				2960	1480226	4202	7673605		

*prospective study

Table 1I17 - 5

IESLC - Meta-analysis of Ever/current Smoking, Duration, "Low"
All LC types, Cigarettes (or Any Product if Cigarettes not available)
Least adjusted

REF	NRR	SEX	AD	Ys	Ws	Qs	Ps
*AMANDU	501	m	0	0.95	5.25	0.01	0.0302
ARMADA	501	m	0	1.22	4.88	0.48	0.0070
AUVINE	501	c	0	2.02	8.26	10.16	0.0000
AXELSS	502	m	0	0.98	6.98	0.03	0.0098
AXELSS	511	f	0	1.26	5.56	0.71	0.0029
Subtotal AXELSS				1.10	12.54	0.74	
BARBON	501	m	0	1.37	11.69	2.52	0.0000
*BEST	505	m	1	1.41	5.30	1.34	0.0012
BUFFLE	526	f	0	2.14	7.75	11.80	0.0000
*CEDERL	501	m	1	0.59	2.92	0.30	0.3155
*CEDERL	504	f	1	0.47	2.58	0.49	0.4505
Subtotal CEDERL				0.53	5.49	0.79	
CHEN2	502	m	0	1.59	1.38	0.64	0.0623
CHEN2	510	f	0	-1.51	0.81	4.74	0.1734
Subtotal CHEN2				0.44	2.19	5.38	
CHOI	502	m	0	1.07	9.21	0.23	0.0012
CHOI	511	f	0	0.21	4.64	2.26	0.6519
Subtotal CHOI				0.78	13.84	2.49	
*CPSI	580	m	0	0.52	64.01	9.51	0.0000
*CPSI	676	f	0	0.10	87.71	57.59	0.3603
Subtotal CPSI				0.28	151.71	67.10	
*CPSII	552	m	0	1.11	45.57	1.87	0.0000
*CPSII	618	f	0	1.05	90.12	1.70	0.0000
Subtotal CPSII				1.07	135.69	3.57	
DAMBER	506	m	1	0.46	5.52	1.12	0.2825
DESTEF	501	m	0	1.55	11.82	4.90	0.0000
DORGAN	570	m	2	1.69	10.46	6.46	0.0000
DORGAN	562	f	3	1.45	47.84	13.89	0.0000
Subtotal DORGAN				1.49	58.30	20.35	
DOSEME	502	m	2	1.34	24.94	4.55	0.0000
FAN	501	m	0	0.34	13.53	4.33	0.2079
FAN	506	f	0	0.91	4.78	0.00	0.0478
Subtotal FAN				0.49	18.31	4.33	
GAO	561	f	0	0.49	27.86	4.89	0.0099
GER	513	c	0	0.19	6.73	3.49	0.6273
HU	502	m	0	0.74	12.94	0.39	0.0082
HU	507	f	0	0.63	3.58	0.27	0.2303
Subtotal HU				0.71	16.52	0.65	
HU2	509	c	0	0.58	22.49	2.40	0.0058
HUMBLE	517	c	0	1.82	8.37	6.95	0.0000
JOLY	516	m	0	2.43	7.66	17.65	0.0000
JOLY	502	f	0	1.33	8.56	1.50	0.0001
Subtotal JOLY				1.85	16.22	19.15	
JUSSAW	512	m	0	1.93	12.80	13.48	0.0000
KATSOU	502	f	0	0.47	3.29	0.64	0.3967
KHUDER	501	m	0	1.26	7.96	0.98	0.0004
LETOUR	506	c	0	1.18	14.96	1.08	0.0000
*LIAW	501	c	2	-0.11	2.82	2.89	0.8596
LIU3	507	m	0	-0.02	2.92	2.54	0.9669
LIU5	504	c	0	0.14	7.88	4.64	0.6935
LUBIN	508	m	0	0.61	5.58	0.48	0.1463
LUBIN2	531	m	0	1.48	142.28	46.10	0.0000
LUBIN2	574	f	0	0.71	63.53	2.55	0.0000
Subtotal LUBIN2				1.24	205.80	48.65	
MATOS	516	m	0	0.87	6.18	0.01	0.0311
MCCONN	503	c	0	0.72	5.16	0.17	0.1002
NOTAN2	514	c	0	0.63	6.77	0.52	0.1008
OSANN2	501	f	0	0.48	9.59	1.76	0.1369
PEZZOT	534	m	0	1.87	3.34	3.09	0.0006
*QIAO2	511	m	0	-1.56	4.15	25.29	0.0015
RACHTA	511	f	0	0.63	5.67	0.44	0.1344
SOBUE	546	m	0	0.67	16.19	0.89	0.0067
WANG2	503	c	0	0.55	3.39	0.43	0.3089
WUWILL	501	f	0	0.35	53.89	16.72	0.0100
ZHENG	553	m	0	0.34	12.30	3.97	0.2328
ZHENG	558	f	0	0.19	7.71	3.96	0.5957
Subtotal ZHENG				0.28	20.01	7.93	

Table 1I17 - 5

IESLC - Meta-analysis of Ever/current Smoking, Duration, "Low"
All LC types, Cigarettes (or Any Product if Cigarettes not available)
Least adjusted

	N	55
	NS	43
	Wt	980.02
Het	Chi	311.82
Het	df	54
Het	P	***
Fixed	RR	2.48
	RRl	2.33
	RRu	2.64
	P	+++
Random	RR	2.43
	RRl	2.05
	RRu	2.88
	P	+++
Asymm	P	N.S.

Table 1I17 - 6

IESLC - Meta-analysis of Ever/current Smoking, Duration, "Low"
All LC types, Cigarettes (or Any Product if Cigarettes not available)
Least adjusted

		Sex		
	combined	male	female	Total
N	10	27	18	55
NS	10	27	18	55
Wt	86.82	457.75	435.46	980.02
Het Chi	32.53	132.78	106.39	311.82
Het df	9	26	17	54
Het P	***	***	***	***
Fixed RR	2.36	3.06	2.01	2.48
RRl	1.91	2.79	1.83	2.33
RRu	2.91	3.36	2.20	2.64
P	+++	+++	+++	+++
Random RR	2.27	2.76	2.08	2.43
RRl	1.49	2.18	1.57	2.05
RRu	3.46	3.51	2.74	2.88
P	+++	+++	+++	+++
Between Chi				40.11
Between df				2
Between P				***
Btwn(F) P				*
Btwn(R) P				N.S.

Table 1I17 - 7

IESLC - Meta-analysis of Ever/current Smoking, Duration, "Low"
All LC types, Cigarettes (or Any Product if Cigarettes not available)
Excluded studies (and stage at which they were excluded)

1	BECHER BLOT1 BROWN3 CARPEN CHYOU DARBY DOLL2 GARCIA GRAHAM GURSEL HAMMO2 JAHN JAIN LAUSSM PRESCO QIAO TVERDA WIGLE WYNDE3
2	ALDERS BENSHL BRESLO CHIAZZ DEAN3 DORN ENGELA GAO2 GILLIS GUO HEGMAN HIRAYA HOLE KAUFMA KOO KOULUM LIU4 MIGRAN MRFITR PERNU SEGI2 SPEIZE SUZUK2 SVENSS VUTUC WAKAI WU YUAN
3	GENG MCDUFF SPITZ STASZE WU2 ZHANG
4	BOUCHA CHEN CORREA JEDRYC LUO WYNDE2 WYNDE6
5	AKIBA HAMMON PISANI RESTRE SADOWS XU
7	BOFFET BROSS WYNDE7
10	AMES WATSON WYNDE8
14	AGUDO BOUCOT DEAN2 DOLL GARSHI HAENSZ KAISE2 KREUZE LEVIN PEZZO2 TIZZAN ZHOU
15	BENHAM

Table 1I17 - 8
Potentially overlapping studies

REF	REFGP	PRINC	. OVERLAP/LINK
LUBIN2	LUBIN2	1	Lubin-combined
OSANN2	KAISER	2	KAISER/OSANN2
CPSI	CPSI	1	CPSI overall
LUBIN	XIANGZ	2	LUBIN/XIANGZ/QIAO

Table 1I17 - 9
Most adjusted - insufficient data for meta-analysis

REF	NRR	SEX	AGEL	AGEH	RACE	YF	LC TYPE	LOC	START	ST	NLC	R	VB	P	H	AD	SM	PRODUCT	exL	exH	DENOM	De
BUFFLE	501	m	0	0	wh	-	all	NAmer	1976	CC	943	n	bl	y	n	0	ev	cig+/-ot	1	33	nev cigs	or
HAMMON	513	m	0	0	wh	0	all	NAmer	1952	pr	448	n	bl	n	n	1	ev	cig only	1	34	nev any	st
SADOWS	524	m	0	0	wh	-	all	NAmer	1938	CC	477	n	bl	n	n	0	ev	cig only	20	29	nev any	ot
XU	501	m	0	0	all	-	all	As:Chi	1985	CC	729	n	ot	n	n	2	ev	all/unsp	1	29	nev any	or

REF	NRR	RR	SIG	RRDATA comment
BUFFLE	501	6.80		0
HAMMON	513	*		RR for <1/2 pack per day is 5.31, that for 1/2 to 1 pack per day is 6.56, that for 1 to 2 packs per day is 7.27 while that for 2+ packs per day 10.78
SADOWS	524	2.78		0
XU	501	*		RR for 1-19/day is 1.8(p<0.05), for 20-29/day is 1.5(p<0.05) and for >=30/ day is 5.3(p<0.05)

Table 1I18 -

IESLC - Meta-analysis of Ever/current Smoking, Duration, "Mid"
All LC types, Cigarettes (or Any Product if Cigarettes not available)

This analysis is restricted to results for:
1) Ever/current smokers
2) Results by Duration
3) Categorical results by Duration
4) All LC types (or near equivalent)
5) Results complete enough for use in metaanalysis

Within each study, results are then selected (in the following order of preference, within each sex) for:
6) SMKSTA: ever, current
7) PRODUCT: cigarettes regardless of other products, cigarettes only, all/unspec
8) CIGTYPE: all/unspecified, MC regardless of HR, MC only
9) (not applicable)
10) DENOM: never smoked anything, never smoked cigarettes, never any + low, never cigs + low
11) Followup period (YF, prospective studies): whole study (coded as 0) or longest available
12) LCtype: all or nearest available, at least Squamous and Adeno. (q = squamous, s = small,
l = large, a = adeno, mix = mixed, alv = alveolar)
13) Race: all or nearest available, otherwise by race (wh or w = white, bl or b = black, hi = hispanic
ch = chinese, jap = japanese, haw = hawaiian, w+o = white + oriental, sca = scandinavian, as = asian)
14) Duration "mid" in key scheme 1 (key value 35, maximum range 21-49)
15) For overlapping studies: principal rather than subsidiary studies
Finally by Age: whole study (coded as 0) if available, otherwise by widest available age group
and then for single sex results (m, f) in preference to results for both sexes combined (c).

Results adjusted (AD) for the most potential confounders are then chosen in Sections -1 to -3
(and those which actually differ from the adjusted results in Table 1I13 - 1 are marked 'x' in Section -1)
and results adjusted for the least confounders in Sections -4 to -6. (Those least adjusted results which
actually differ from the most adjusted are marked 'x' in column X in Section -4)

Section -7 shows excluded studies, together with the stage (as above) at which no qualifying
results were found.

Section -8 lists the potentially overlapping studies which have been included (1=principal, 2=subsidiary).

Section -9 lists any results which would have been included in preference except that they had data not complete
enough for use in meta-analysis, with their significance (yes/no), if known, and any further comment as entered
on the database. It also lists as "gap" any categories for which no data were presented by the original authors.

In addition to those mentioned above, the following fields, levels and abbreviations are used:
* or nk = not known, n = no, y = yes, ot = other
ev = ever, cu = current, nev = never
all/unspec = all or unspecified, cig+/-ot = cigarettes irrespective of other products (cigar, pipe etc)
MC = manufactured cigarettes, HR = hand-rolled cigarettes
exL, exH = range of exposure (low and high) in the smoking group, in terms of Duration
REF: 6-character study reference
NRR: number of the RR on the database within the study
ST : study type (CC = case control, pr or prosp = prospective)
NLC: number of lung cancer cases in whole study
R : risky occupational population (n = no, m = mining, o = other risky)
VB : national cigarette type (V = at least 75% Virginia, bl = at least 75% blended, ot = other)
P : any proxy use
H : full histological confirmation
De : derivation of RR/CI (or = original, st = standard method, ot = other method of estimation)

Table 1I18 - 1

IESLC - Meta-analysis of Ever/current Smoking, Duration, "Mid"
All LC types, Cigarettes (or Any Product if Cigarettes not available)
Most adjusted

REF	NRR	1I13	SEX	AGEL	AGEH	RACE	YF	LC TYPE	LOC	START	ST	NLC	R	VB	P	H	AD	SM	PRODUCT	exL	exH	DENOM	De
ARMADA	507		m	0	0	all	-	all	Eu:wst	1986	CC	325	n	bl	n	y	1	ev	cig+/-ot	25	49	nev cigs	or
AUVINE	518		c	0	0	all	-	all	Eu:Sca	1986	CC	517	n	bl	y	n	2	ev	cig+/-ot	21	40	nev cigs	or
AXELSS	521		m	0	0	sca	-	all	Eu:Sca	1989	CC	436	n	bl	n	n	6	ev	all/unsp	30	39	nev any	ot
AXELSS	512		f	0	0	sca	-	all	Eu:Sca	1989	CC	436	n	bl	n	n	0	ev	all/unsp	30	39	nev any	st
BARBON	509		m	0	0	all	-	all	Eu:wst	1979	CC	755	n	bl	y	y	1	ev	all/unsp	30	39	nev any	or
BEST	506		m	0	0	all	0	all	NAmer	1955	pr	381	n	V	n	n	1	cu	cig only	30	39	nev any	ot
BUFFLE	527		f	0	0	w-hi	-	all	NAmer	1976	CC	943	n	bl	y	n	0	ev	cig+/-ot	31	40	nev cigs	or
CHEN2	504		m	0	0	all	-	all	As:Chi	1983	CC	193	n	ot	y	n	0	ev	all/unsp	31	40	nev any	st
CHEN2	512		f	0	0	all	-	all	As:Chi	1983	CC	193	n	ot	y	n	0	ev	all/unsp	31	40	nev any	st
CHOI	503		m	0	0	all	-	all	As:oth	1985	CC	375	n	bl	n	n	0	ev	cig+/-ot	30	39	nev cigs	st
CHOI	512		f	0	0	all	-	all	As:oth	1985	CC	375	n	bl	n	n	0	ev	cig+/-ot	30	39	nev cigs	st
CPSI	582		m	40	84	wh	0	all	NAmer	1959	pr	5138	n	bl	n	n	0	cu	cig only	35	39	nev cigs	st
CPSI	678		f	40	84	wh	0	all	NAmer	1959	pr	5138	n	bl	n	n	0	cu	cig only	35	39	nev cigs	st
CPSII	554		m	0	0	all	6	all	NAmer	1982	pr	3229	n	bl	n	n	0	cu	cig only	35	39	nev any	st
CPSII	620		f	0	0	all	6	all	NAmer	1982	pr	3229	n	bl	n	n	0	cu	cig+/-ot	35	39	nev cigs	st
DAMBER	508		m	0	0	all	-	all	Eu:Sca	1972	CC	579	n	bl	y	n	1	ev	all/unsp	31	40	nev any	ot
DESTEF	509		m	0	0	all	-	all	SCAmer	1988	CC	497	n	bl	n	y	4	ev	all/unsp	30	39	nev any	or
FAN	502		m	0	0	all	-	all	As:Chi	1990	CC	403	n	ot	y	n	0	ev	cig+/-ot	30	39	nev cigs	st
FAN	507		f	0	0	all	-	all	As:Chi	1990	CC	403	n	ot	y	n	0	ev	cig+/-ot	30	39	nev cigs	st
GER	519		c	0	0	all	-	all	As:oth	1990	CC	141	n	ot	y	n	5	ev	all/unsp	21	40	nev any	ot
HU2	510		c	0	0	all	-	all	As:Chi	1977	CC	523	n	ot	y	n	0	ev	cig+/-ot	30	39	nev cigs	or
HUMBLE	543		c	0	0	wh	-	not alv	NAmer	1980	CC	521	n	bl	y	n	3	cu	cig+/-ot	30	39	nev cigs	ot
JOLY	517		m	0	0	all	-	all	SCAmer	1978	CC	826	n	bl	n	n	0	ev	cig+/-ot	30	39	nev any	st
JOLY	503		f	0	0	all	-	all	SCAmer	1978	CC	826	n	bl	n	n	0	ev	cig+/-ot	30	39	nev any	st
JUSSAW	513		m	0	0	all	-	all	As:Ind	1964	CC	792	n	V	n	n	0	ev	cig only	30	39	nev any	st
KATSOU	503		f	0	0	all	-	all	Eu:bal	1987	CC	101	n	bl	n	n	0	cu	all/unsp	30	39	nev any	st
KHUDER	502		m	0	0	all	-	all	NAmer	1985	CC	482	n	bl	n	y	0	ev	cig+/-ot	30	49	nev cigs	st
LETOUR	507		c	0	0	all	-	all	NAmer	1983	CC	738	n	V	y	y	0	ev	cig+/-ot	25	40	nev cigs	st
LUBIN	509		m	0	0	all	-	all	As:Chi	1984	CC	427	m	ot	y	n	0	ev	cig+/-ot	30	39	nev any	st
LUBIN2	532		m	0	0	all	-	all	Eu:mul	1976	CC	7804	n	bl	n	y	0	ev	cig+/-ot	30	39	nev any	st
LUBIN2	575		f	0	0	all	-	all	Eu:mul	1976	CC	7804	n	bl	n	y	0	ev	cig+/-ot	30	39	nev any	st
MATOS	537		m	0	0	all	-	all	SCAmer	1994	CC	200	n	bl	n	n	2	ev	cig+/-ot	25	39	nev any	or
MCCONN	504		c	0	0	all	-	all	Eu:UK	1946	CC	100	n	V	n	y	0	ev	all/unsp	30	39	nev any	st
NOTAN2	516		c	0	0	all	-	all	As:Ind	1963	CC	683	n	V	n	n	0	ev	cig only	31	40	nev any	st
PEZZOT	535		m	0	0	all	-	all	SCAmer	1987	CC	215	n	bl	n	y	0	ev	cig only	31	40	nev cigs	st
QIAO2	517		m	0	0	all	0	all	As:Chi	1992	pr	241	m	ot	n	n	1	ev	all/unsp	28	41	nev any	or
RACHTA	517		f	0	0	all	-	all	Eu:est	1991	CC	118	n	bl	n	y	1	ev	cig+/-ot	21	40	nev cigs	or
SOBUE	547		m	0	0	all	-	q+s+l+a	As:Jap	1986	CC	1376	n	bl	n	y	0	cu	cig+/-ot	30	39	nev cigs	st
WANG2	504		c	0	0	all	-	all	As:Chi	1980	CC	103	n	ot	n	n	0	ev	cig+/-ot	30	39	nev cigs	st
WUWILL	517		f	0	0	all	-	all	As:Chi	1985	CC	965	n	ot	n	n	3	ev	cig+/-ot	30	39	nev cigs	ot

Cigarette type is all/unspec for all RRs

except for the following:

REF	NRR	CIGTYPE
JUSSAW	513	MC only
NOTAN2	516	MC only

Table 1I18 - 2

IESLC - Meta-analysis of Ever/current Smoking, Duration, "Mid"
All LC types, Cigarettes (or Any Product if Cigarettes not available)
Most adjusted

REF	NRR	SEX	AD	Number Exposed Case	Cont	Non-exposed Case	Cont	RR	95.00%CI
ARMADA	507	m	1	219	-	8	-	11.90	(5.50- 25.50)
AUVINE	518	c	2	10	-	44	-	33.20	(14.30- 77.40)
AXELSS	521	m	6	57	-	16	-	7.62	(4.01- 14.47)
AXELSS	512	f	0	29	26	18	154	9.54	(4.64- 19.61)
Subtotal AXELSS								8.42	(5.21- 13.59)
BARBON	509	m	1	118	-	22	-	7.90	(4.70- 13.50)
*BEST	506	m	1	55	-	7	-	13.90	(6.33- 30.52)
BUFFLE	527	f	0	97	62	12	112	14.60	(7.43- 28.69)
CHEN2	504	m	0	36	27	9	33	4.89	(2.01- 11.91)
CHEN2	512	f	0	13	6	25	33	2.86	(0.95- 8.58)
Subtotal CHEN2								3.95	(1.98- 7.89)
CHOI	503	m	0	102	160	13	95	4.66	(2.48- 8.75)
CHOI	512	f	0	8	2	76	164	8.63	(1.79- 41.62)
Subtotal CHOI								5.07	(2.83- 9.11)
*CPSI	582	m	0	470	367622	196	926068	6.04	(5.11- 7.14)
*CPSI	678	f	0	154	315060	532	3877179	3.56	(2.98- 4.26)
Subtotal CPSI								4.73	(4.19- 5.34)
*CPSII	554	m	0	244	109788	124	742207	13.30	(10.72- 16.51)
*CPSII	620	f	0	193	116270	310	2091302	11.20	(9.36- 13.40)
Subtotal CPSII								12.01	(10.46- 13.79)
DAMBER	508	m	1	-	-	42	-	5.15	(3.27- 8.32)
DESTEF	509	m	4	78	-	27	-	5.20	(2.90- 8.90)
FAN	502	m	0	44	122	36	236	2.36	(1.45- 3.87)
FAN	507	f	0	19	23	69	320	3.83	(1.98- 7.42)
Subtotal FAN								2.81	(1.89- 4.17)
GER	519	c	5	31	-	51	-	1.56	(0.83- 2.91)
HU2	510	c	0	123	101	121	213	2.14	(1.52- 3.03)
HUMBLE	543	c	3	68	-	28	-	17.54	(8.46- 36.34)
JOLY	517	m	0	85	165	12	218	9.36	(4.95- 17.70)
JOLY	503	f	0	31	24	52	283	7.03	(3.82- 12.93)
Subtotal JOLY								8.06	(5.19- 12.52)
JUSSAW	513	m	0	27	9	149	624	12.56	(5.79- 27.28)
KATSOU	503	f	0	15	2	48	67	10.47	(2.29- 47.93)
KHUDER	502	m	0	207	370	23	309	7.52	(4.76- 11.86)
LETOUR	507	c	0	264	160	24	224	15.40	(9.68- 24.51)
LUBIN	509	m	0	124	294	8	72	3.80	(1.78- 8.12)
LUBIN2	532	m	0	2227	3470	190	2616	8.84	(7.56- 10.33)
LUBIN2	575	f	0	187	186	336	1188	3.55	(2.81- 4.50)
Subtotal LUBIN2								6.69	(5.87- 7.63)
MATOS	537	m	2	82	-	11	-	7.20	(3.60- 14.50)
MCCONN	504	c	0	21	57	9	23	0.94	(0.38- 2.36)
NOTAN2	516	c	0	12	7	107	201	3.22	(1.23- 8.42)
PEZZOT	535	m	0	71	82	4	116	25.11	(8.82- 71.48)
*QIAO2	517	m	1	54	-	10	-	1.46	(0.74- 2.87)
RACHTA	517	f	1	49	-	33	-	7.55	(3.90- 14.63)
SOBUE	547	m	0	159	200	34	128	2.99	(1.94- 4.61)
WANG2	504	c	0	26	38	11	43	2.67	(1.17- 6.13)
WUWILL	517	f	3	179	-	417	-	2.71	(2.05- 3.60)
Partial Totals				5988	914333	3264	7644228		

*prospective study

Table 1I18 - 2

IESLC - Meta-analysis of Ever/current Smoking, Duration, "Mid"
All LC types, Cigarettes (or Any Product if Cigarettes not available)
Most adjusted

REF	NRR	SEX	AD	Ys	Ws	Qs	Ps
ARMADA	507	m	1	2.48	6.53	2.81	0.0000
AUVINE	518	c	2	3.50	5.39	15.25	0.0000
AXELSS	521	m	6	2.03	9.33	0.41	0.0000
AXELSS	512	f	0	2.26	7.41	1.41	0.0000
Subtotal AXELSS				2.13	16.74	1.82	
BARBON	509	m	1	2.07	13.80	0.84	0.0000
*BEST	506	m	1	2.63	6.21	4.09	0.0000
BUFFLE	527	f	0	2.68	8.42	6.25	0.0000
CHEN2	504	m	0	1.59	4.85	0.26	0.0005
CHEN2	512	f	0	1.05	3.19	1.89	0.0607
Subtotal CHEN2				1.37	8.03	2.15	
CHOI	503	m	0	1.54	9.66	0.76	0.0000
CHOI	512	f	0	2.16	1.55	0.17	0.0072
Subtotal CHOI				1.62	11.21	0.94	
*CPSI	582	m	0	1.80	138.39	0.06	0.0000
*CPSI	678	f	0	1.27	119.48	36.09	0.0000
Subtotal CPSI				1.55	257.87	36.16	
*CPSII	554	m	0	2.59	82.29	48.53	0.0000
*CPSII	620	f	0	2.42	119.07	42.26	0.0000
Subtotal CPSII				2.49	201.36	90.78	
DAMBER	508	m	1	1.64	17.62	0.58	0.0000
DESTEF	509	m	4	1.65	12.22	0.36	0.0000
FAN	502	m	0	0.86	15.89	14.63	0.0006
FAN	507	f	0	1.34	8.79	2.00	0.0001
Subtotal FAN				1.03	24.68	16.63	
GER	519	c	5	0.44	9.76	18.47	0.1647
HU2	510	c	0	0.76	32.27	36.08	0.0000
HUMBLE	543	c	3	2.86	7.23	7.89	0.0000
JOLY	517	m	0	2.24	9.46	1.64	0.0000
JOLY	503	f	0	1.95	10.34	0.18	0.0000
Subtotal JOLY				2.09	19.80	1.81	
JUSSAW	513	m	0	2.53	6.39	3.23	0.0000
KATSOU	503	f	0	2.35	1.66	0.46	0.0025
KHUDER	502	m	0	2.02	18.43	0.72	0.0000
LETOUR	507	c	0	2.73	17.80	14.88	0.0000
LUBIN	509	m	0	1.33	6.65	1.57	0.0006
LUBIN2	532	m	0	2.18	156.67	20.17	0.0000
LUBIN2	575	f	0	1.27	68.77	20.94	0.0000
Subtotal LUBIN2				1.90	225.44	41.11	
MATOS	537	m	2	1.97	7.92	0.19	0.0000
MCCONN	504	c	0	-0.06	4.55	16.09	0.8977
NOTAN2	516	c	0	1.17	4.16	1.76	0.0171
PEZZOT	535	m	0	3.22	3.51	6.91	0.0000
*QIAO2	517	m	1	0.38	8.36	17.38	0.2738
RACHTA	517	f	1	2.02	8.79	0.36	0.0000
SOBUE	547	m	0	1.10	20.61	10.80	0.0000
WANG2	504	c	0	0.98	5.59	3.91	0.0200
WUWILL	517	f	3	1.00	48.46	32.83	0.0000

	N	40
	NS	32
	Wt	1047.49
Het	Chi	395.11
Het	df	39
Het	P	***
Fixed	RR	6.17
	RRl	5.81
	RRu	6.56
	P	+++
Random	RR	5.94
	RRl	4.79
	RRu	7.36
	P	+++
Asymm	P	N.S.

Table 1I18 - 3

IESLC - Meta-analysis of Ever/current Smoking, Duration, "Mid"
All LC types, Cigarettes (or Any Product if Cigarettes not available)
Most adjusted

	Sex			
	combined	male	female	Total
N	8	20	12	40
NS	8	20	12	40
Wt	86.75	554.80	405.94	1047.49
Het Chi	103.55	119.76	134.67	395.11
Het df	7	19	11	39
Het P	***	***	***	***
Fixed RR	4.34	7.32	5.27	6.17
RRl	3.51	6.74	4.78	5.81
RRu	5.35	7.96	5.81	6.56
P	+++	+++	+++	+++
Random RR	4.74	6.47	5.85	5.94
RRl	2.00	5.07	3.90	4.79
RRu	11.24	8.25	8.78	7.36
P	+++	+++	+++	+++
Between Chi				37.14
Between df				2
Between P				***
Btwn(F) P				N.S.
Btwn(R) P				N.S.

	Lung cancer type		
	all	other	Total
N	38	2	40
NS	30	2	32
Wt	1019.65	27.85	1047.49
Het Chi	376.37	16.74	395.11
Het df	37	1	39
Het P	***	***	***
Fixed RR	6.22	4.74	6.17
RRl	5.85	3.27	5.81
RRu	6.61	6.87	6.56
P	+++	+++	+++
Random RR	5.90	7.06	5.94
RRl	4.74	1.25	4.79
RRu	7.35	39.93	7.36
P	+++	+	+++
Between Chi			2.00
Between df			1
Between P			N.S.
Btwn(F) P			N.S.
Btwn(R) P			N.S.

				Location					
	NAmer	UK	Scand	othEur	China	Japan	othAs	other	Total
N	9	1	4	6	9	1	5	5	40
NS	7	1	3	5	7	1	4	4	32
Wt	517.34	4.55	39.75	256.23	134.05	20.61	31.53	43.45	1047.49
Het Chi	139.36	0.00	14.57	42.39	8.44	0.00	18.37	7.27	395.11
Het df	8	0	3	5	8	0	4	4	39
Het P	***	N.S.	**	***	N.S.	N.S.	**	N.S.	***
Fixed RR	7.56	0.94	8.15	6.90	2.58	2.99	3.99	7.65	6.17
RRl	6.94	0.38	5.98	6.11	2.18	1.94	2.81	5.68	5.81
RRu	8.25	2.36	11.13	7.80	3.05	4.61	5.65	10.30	6.56
P	+++	N.S.	+++	+++	+++	+++	+++	+++	+++
Random RR	9.87	0.94	9.98	7.32	2.58	2.99	4.52	8.08	5.94
RRl	6.62	0.38	4.87	4.51	2.16	1.94	2.04	5.36	4.79
RRu	14.72	2.36	20.46	11.88	3.09	4.61	10.00	12.18	7.36
P	+++	N.S.	+++	+++	+++	+++	+++	+++	+++
Between Chi									164.72
Between df									7
Between P									***
Btwn(F) P									**
Btwn(R) P									***

Table 1I18 - 3

IESLC - Meta-analysis of Ever/current Smoking, Duration, "Mid"
All LC types, Cigarettes (or Any Product if Cigarettes not available)
Most adjusted

Detailed Country in "other Europe"

	multi	Germany	othWest	East	Balkans	Total
N	2		2	1	1	6
NS	1		2	1	1	5
Wt	225.44		20.33	8.79	1.66	256.23
Het Chi	39.63		0.74	0.00	0.00	42.39
Het df	1		1	0	0	5
Het P	***		N.S.	N.S.	N.S.	***
Fixed RR	6.69		9.01	7.55	10.47	6.90
RRl	5.87		5.83	3.90	2.29	6.11
RRu	7.63		13.92	14.62	47.93	7.80
P	+++		+++	+++	++	+++
Random RR	5.63		9.01	7.55	10.47	7.32
RRl	2.31		5.83	3.90	2.29	4.51
RRu	13.74		13.92	14.62	47.93	11.88
P	+++		+++	+++	++	+++
Between Chi						2.02
Between df						3
Between P						N.S.
Btwn(F) P						N.S.
Btwn(R) P						N.S.

Detailed Country in "other Asia"

	India	HongKong	other	Total
N	2		3	5
NS	2		2	4
Wt	10.55		20.98	31.53
Het Chi	4.67		7.77	18.37
Het df	1		2	4
Het P	*		*	**
Fixed RR	7.35		2.93	3.99
RRl	4.02		1.91	2.81
RRu	13.43		4.50	5.65
P	+++		+++	+++
Random RR	6.56		3.43	4.52
RRl	1.73		1.33	2.04
RRu	24.87		8.84	10.00
P	++		+	+++
Between Chi				5.93
Between df				1
Between P				*
Btwn(F) P				N.S.
Btwn(R) P				N.S.

Detailed other continent

	SCAmer	Total
N	5	5
NS	4	4
Wt	43.45	43.45
Het Chi	7.27	7.27
Het df	4	4
Het P	N.S.	N.S.
Fixed RR	7.65	7.65
RRl	5.68	5.68
RRu	10.30	10.30
P	+++	+++
Random RR	8.08	8.08
RRl	5.36	5.36
RRu	12.18	12.18
P	+++	+++
Between Chi		
Between df		
Between P		N.S.
Btwn(F) P		N.S.
Btwn(R) P		N.S.

Table 1I18 - 3

IESLC - Meta-analysis of Ever/current Smoking, Duration, "Mid"
All LC types, Cigarettes (or Any Product if Cigarettes not available)
Most adjusted

	Start year of study <1960	1960-69	1970-79	1980-89	1990+	Total
N	4	2	8	20	6	40
NS	3	2	6	16	5	32
Wt	268.63	10.55	317.35	391.44	59.52	1047.49
Het Chi	36.95	4.67	86.64	160.10	23.48	395.11
Het df	3	1	7	19	5	39
Het P	***	*	***	***	***	***
Fixed RR	4.72	7.35	6.11	8.29	3.05	6.17
RRl	4.19	4.02	5.48	7.51	2.37	5.81
RRu	5.32	13.43	6.82	9.15	3.93	6.56
P	+++	+++	+++	+++	+++	+++
Random RR	4.38	6.56	6.15	7.58	3.19	5.94
RRl	2.54	1.73	3.92	5.44	1.83	4.79
RRu	7.56	24.87	9.64	10.56	5.57	7.36
P	+++	++	+++	+++	+++	+++
Between Chi						83.28
Between df						4
Between P						***
Btwn(F) P						(*)
Btwn(R) P						(*)

	Study type (1) CC	other	Total
N	34	6	40
NS	28	4	32
Wt	573.69	473.81	1047.49
Het Chi	240.74	141.20	395.11
Het df	33	5	39
Het P	***	***	***
Fixed RR	5.57	6.98	6.17
RRl	5.14	6.38	5.81
RRu	6.05	7.64	6.56
P	+++	+++	+++
Random RR	5.84	6.46	5.94
RRl	4.57	3.83	4.79
RRu	7.47	10.87	7.36
P	+++	+++	+++
Between Chi			13.17
Between df			1
Between P			***
Btwn(F) P			N.S.
Btwn(R) P			N.S.

	Study type (2) CC	prosp	other	Total
N	34	6		40
NS	28	4		32
Wt	573.69	473.81		1047.49
Het Chi	240.74	141.20		395.11
Het df	33	5		39
Het P	***	***		***
Fixed RR	5.57	6.98		6.17
RRl	5.14	6.38		5.81
RRu	6.05	7.64		6.56
P	+++	+++		+++
Random RR	5.84	6.46		5.94
RRl	4.57	3.83		4.79
RRu	7.47	10.87		7.36
P	+++	+++		+++
Between Chi				13.17
Between df				1
Between P				***
Btwn(F) P				N.S.
Btwn(R) P				N.S.

Table 1I18 - 3

IESLC - Meta-analysis of Ever/current Smoking, Duration, "Mid"
All LC types, Cigarettes (or Any Product if Cigarettes not available)
Most adjusted

	Study size (number of LC cases) 100-249	250-499	500-999	1000+	Total
N	10	11	12	7	40
NS	9	8	11	4	32
Wt	58.18	102.68	181.35	705.29	1047.49
Het Chi	46.62	28.19	117.53	172.13	395.11
Het df	9	10	11	6	39
Het P	***	**	***	***	***
Fixed RR	3.47	5.66	5.33	6.80	6.17
RRl	2.68	4.67	4.61	6.32	5.81
RRu	4.49	6.87	6.17	7.33	6.56
P	+++	+++	+++	+++	+++
Random RR	3.85	6.02	7.91	6.14	5.94
RRl	2.11	4.30	4.79	4.10	4.79
RRu	7.02	8.44	13.05	9.20	7.36
P	+++	+++	+++	+++	+++
Between Chi					30.65
Between df					3
Between P					***
Btwn(F) P					N.S.
Btwn(R) P					N.S.

	Risky occupational population no	mining	othRisky	Total
N	38	2		40
NS	30	2		32
Wt	1032.48	15.01		1047.49
Het Chi	375.93	3.38		395.11
Het df	37	1		39
Het P	***	(*)		***
Fixed RR	6.26	2.23		6.17
RRl	5.89	1.34		5.81
RRu	6.66	3.70		6.56
P	+++	++		+++
Random RR	6.22	2.32		5.94
RRl	5.00	0.91		4.79
RRu	7.73	5.91		7.36
P	+++	(+)		+++
Between Chi				15.80
Between df				1
Between P				***
Btwn(F) P				N.S.
Btwn(R) P				*

	National cigarette tobacco type Virginia	blended	other	Total
N	5	25	10	40
NS	5	19	8	32
Wt	39.11	864.57	143.81	1047.49
Het Chi	34.60	210.52	10.74	395.11
Het df	4	24	9	39
Het P	***	***	N.S.	***
Fixed RR	8.97	7.06	2.49	6.17
RRl	6.55	6.60	2.12	5.81
RRu	12.26	7.54	2.93	6.56
P	+++	+++	+++	+++
Random RR	6.32	7.88	2.50	5.94
RRl	2.36	6.27	2.06	4.79
RRu	16.87	9.89	3.02	7.36
P	+++	+++	+++	+++
Between Chi				139.25
Between df				2
Between P				***
Btwn(F) P				***
Btwn(R) P				***

Table 1I18 - 3

IESLC - Meta-analysis of Ever/current Smoking, Duration, "Mid"
All LC types, Cigarettes (or Any Product if Cigarettes not available)
Most adjusted

	Any proxy use No/nk	Yes	Total
N	27	13	40
NS	21	11	32
Wt	895.82	151.67	1047.49
Het Chi	273.27	113.22	395.11
Het df	26	12	39
Het P	***	***	***
Fixed RR	6.41	4.95	6.17
RRl	6.00	4.22	5.81
RRu	6.84	5.81	6.56
P	+++	+++	+++
Random RR	6.04	5.76	5.94
RRl	4.73	3.47	4.79
RRu	7.71	9.56	7.36
P	+++	+++	+++
Between Chi			8.62
Between df			1
Between P			**
Btwn(F) P			N.S.
Btwn(R) P			N.S.

	Full histological confirmation No	Yes	Total
N	29	11	40
NS	22	10	32
Wt	715.79	331.70	1047.49
Het Chi	299.22	92.68	395.11
Het df	28	10	39
Het P	***	***	***
Fixed RR	5.94	6.70	6.17
RRl	5.52	6.01	5.81
RRu	6.40	7.46	6.56
P	+++	+++	+++
Random RR	5.74	6.49	5.94
RRl	4.38	4.40	4.79
RRu	7.52	9.57	7.36
P	+++	+++	+++
Between Chi			3.22
Between df			1
Between P			(*)
Btwn(F) P			N.S.
Btwn(R) P			N.S.

	Number of adjustment variables (1) 0	1	2+/+nk	Total
N	27	6	7	40
NS	20	6	7	33
Wt	885.86	61.32	100.31	1047.49
Het Chi	292.15	26.06	62.77	395.11
Het df	26	5	6	39
Het P	***	***	***	***
Fixed RR	6.43	6.10	4.33	6.17
RRl	6.02	4.75	3.56	5.81
RRu	6.87	7.83	5.26	6.56
P	+++	+++	+++	+++
Random RR	5.74	6.38	6.58	5.94
RRl	4.46	3.55	3.27	4.79
RRu	7.39	11.46	13.25	7.36
P	+++	+++	+++	+++
Between Chi				14.14
Between df				2
Between P				***
Btwn(F) P				N.S.
Btwn(R) P				N.S.

Table 1I18 - 3

IESLC - Meta-analysis of Ever/current Smoking, Duration, "Mid"
All LC types, Cigarettes (or Any Product if Cigarettes not available)
Most adjusted

	Number of adjustment variables (2) 0	1	2	3-5	6+/+nk	Total
N	27	6	2	4	1	40
NS	20	6	2	4	1	33
Wt	885.86	61.32	13.30	77.68	9.33	1047.49
Het Chi	292.15	26.06	7.49	30.07	0.00	395.11
Het df	26	5	1	3	0	39
Het P	***	***	**	***	N.S.	***
Fixed RR	6.43	6.10	13.37	3.33	7.62	6.17
RRl	6.02	4.75	7.81	2.67	4.01	5.81
RRu	6.87	7.83	22.88	4.16	14.47	6.56
P	+++	+++	+++	+++	+++	+++
Random RR	5.74	6.38	15.16	4.30	7.62	5.94
RRl	4.46	3.55	3.39	1.89	4.01	4.79
RRu	7.39	11.46	67.78	9.78	14.47	7.36
P	+++	+++	+++	+++	+++	+++
Between Chi						39.35
Between df						4
Between P						***
Btwn(F) P						N.S.
Btwn(R) P						N.S.

	Smoking status ever	current	Total
N	32	8	40
NS	26	6	32
Wt	552.54	494.95	1047.49
Het Chi	237.51	141.90	395.11
Het df	31	7	39
Het P	***	***	***
Fixed RR	5.50	7.02	6.17
RRl	5.06	6.43	5.81
RRu	5.97	7.67	6.56
P	+++	+++	+++
Random RR	5.49	7.92	5.94
RRl	4.25	5.03	4.79
RRu	7.09	12.46	7.36
P	+++	+++	+++
Between Chi			15.70
Between df			1
Between P			***
Btwn(F) P			N.S.
Btwn(R) P			N.S.

	Product all/unsp	cig+/-ot	cig only	Total
N	11	22	7	40
NS	9	18	6	33
Wt	92.75	594.31	360.43	1047.49
Het Chi	46.10	235.05	100.56	395.11
Het df	10	21	6	39
Het P	***	***	***	***
Fixed RR	4.30	6.46	6.28	6.17
RRl	3.51	5.96	5.67	5.81
RRu	5.28	7.00	6.96	6.56
P	+++	+++	+++	+++
Random RR	4.02	6.36	8.34	5.94
RRl	2.54	4.71	4.99	4.79
RRu	6.35	8.58	13.95	7.36
P	+++	+++	+++	+++
Between Chi				13.39
Between df				2
Between P				**
Btwn(F) P				N.S.
Btwn(R) P				(*)

Table 1I18 - 3

IESLC - Meta-analysis of Ever/current Smoking, Duration, "Mid"
All LC types, Cigarettes (or Any Product if Cigarettes not available)
Most adjusted

		Denominator nev any	nev cigs	Total
	N	21	19	40
	NS	17	16	33
	Wt	451.61	595.88	1047.49
Het	Chi	152.94	228.99	395.11
Het	df	20	18	39
Het	P	***	***	***
Fixed	RR	7.02	5.60	6.17
	RRl	6.40	5.17	5.81
	RRu	7.70	6.07	6.56
	P	+++	+++	+++
Random	RR	5.48	6.50	5.94
	RRl	4.07	4.71	4.79
	RRu	7.38	8.97	7.36
	P	+++	+++	+++
Between	Chi			13.18
Between	df			1
Between	P			***
Btwn(F)	P			N.S.
Btwn(R)	P			N.S.

Derivation of RR/CI

		Orig	StdCalc	Other	Total
	N	9	25	6	40
	NS	9	18	6	33
	Wt	103.70	845.17	98.62	1047.49
Het	Chi	75.16	246.59	46.43	395.11
Het	df	8	24	5	39
Het	P	***	***	***	***
Fixed	RR	5.03	6.65	4.03	6.17
	RRl	4.15	6.22	3.31	5.81
	RRu	6.09	7.12	4.91	6.56
	P	+++	+++	+++	+++
Random	RR	6.80	5.82	5.64	5.94
	RRl	3.68	4.52	2.89	4.79
	RRu	12.56	7.48	10.99	7.36
	P	+++	+++	+++	+++
Between	Chi				26.93
Between	df				2
Between	P				***
Btwn(F)	P				N.S.
Btwn(R)	P				N.S.

Table 1I18 - 4

IESLC - Meta-analysis of Ever/current Smoking, Duration, "Mid"
All LC types, Cigarettes (or Any Product if Cigarettes not available)
Least adjusted

REF	NRR	X	SEX	AGEL	AGEH	RACE	YF	LC TYPE	LOC	START	ST	NLC	R	VB	P	H	AD	SM	PRODUCT	exL	exH	DENOM	De
ARMADA	502	x	m	0	0	all	-	all	Eu:wst	1986	CC	325	n	bl	n	y	0	ev	cig+/-ot	25	49	nev cigs	st
AUVINE	502	x	c	0	0	all	-	all	Eu:Sca	1986	CC	517	n	bl	y	n	0	ev	cig+/-ot	21	40	nev cigs	st
AXELSS	503	x	m	0	0	sca	-	all	Eu:Sca	1989	CC	436	n	bl	n	n	0	ev	all/unsp	30	39	nev any	st
AXELSS	512		f	0	0	sca	-	all	Eu:Sca	1989	CC	436	n	bl	n	n	0	ev	all/unsp	30	39	nev any	st
BARBON	502	x	m	0	0	all	-	all	Eu:wst	1979	CC	755	n	bl	y	y	0	ev	all/unsp	30	39	nev any	st
BEST	506		m	0	0	all	0	all	NAmer	1955	pr	381	n	V	n	n	1	cu	cig only	30	39	nev any	ot
BUFFLE	527		f	0	0	w-hi	-	all	NAmer	1976	CC	943	n	bl	y	n	0	ev	cig+/-ot	31	40	nev cigs	or
CHEN2	504		m	0	0	all	-	all	As:Chi	1983	CC	193	n	ot	y	n	0	ev	all/unsp	31	40	nev any	st
CHEN2	512		f	0	0	all	-	all	As:Chi	1983	CC	193	n	ot	y	n	0	ev	all/unsp	31	40	nev any	st
CHOI	503		m	0	0	all	-	all	As:oth	1985	CC	375	n	bl	n	n	0	ev	cig+/-ot	30	39	nev cigs	st
CHOI	512		f	0	0	all	-	all	As:oth	1985	CC	375	n	bl	n	n	0	ev	cig+/-ot	30	39	nev cigs	st
CPSI	582		m	40	84	wh	0	all	NAmer	1959	pr	5138	n	bl	n	n	0	cu	cig only	35	39	nev cigs	st
CPSI	678		f	40	84	wh	0	all	NAmer	1959	pr	5138	n	bl	n	n	0	cu	cig only	35	39	nev cigs	st
CPSII	554		m	0	0	all	6	all	NAmer	1982	pr	3229	n	bl	n	n	0	cu	cig only	35	39	nev any	st
CPSII	620		f	0	0	all	6	all	NAmer	1982	pr	3229	n	bl	n	n	0	cu	cig+/-ot	35	39	nev cigs	st
DAMBER	508		m	0	0	all	-	all	Eu:Sca	1972	CC	579	n	bl	y	n	1	ev	all/unsp	31	40	nev any	ot
DESTEF	502	x	m	0	0	all	-	all	SCAmer	1988	CC	497	n	bl	n	y	0	ev	all/unsp	30	39	nev any	st
FAN	502		m	0	0	all	-	all	As:Chi	1990	CC	403	n	ot	y	n	0	ev	cig+/-ot	30	39	nev cigs	st
FAN	507		f	0	0	all	-	all	As:Chi	1990	CC	403	n	ot	y	n	0	ev	cig+/-ot	30	39	nev cigs	st
GER	514	x	c	0	0	all	-	all	As:oth	1990	CC	141	n	ot	y	n	0	ev	all/unsp	21	40	nev any	st
HU2	510		c	0	0	all	-	all	As:Chi	1977	CC	523	n	ot	y	n	0	ev	cig+/-ot	30	39	nev cigs	or
HUMBLE	518	x	c	0	0	wh	-	not alv	NAmer	1980	CC	521	n	bl	y	n	0	cu	cig+/-ot	30	39	nev cigs	st
JOLY	517		m	0	0	all	-	all	SCAmer	1978	CC	826	n	bl	n	n	0	ev	cig+/-ot	30	39	nev any	st
JOLY	503		f	0	0	all	-	all	SCAmer	1978	CC	826	n	bl	n	n	0	ev	cig+/-ot	30	39	nev any	st
JUSSAW	513		m	0	0	all	-	all	As:Ind	1964	CC	792	n	V	n	n	0	ev	cig only	30	39	nev any	st
KATSOU	503		f	0	0	all	-	all	Eu:bal	1987	CC	101	n	bl	n	n	0	cu	all/unsp	30	39	nev any	st
KHUDER	502		m	0	0	all	-	all	NAmer	1985	CC	482	n	bl	n	y	0	ev	cig+/-ot	30	49	nev cigs	st
LETOUR	507		c	0	0	all	-	all	NAmer	1983	CC	738	n	V	y	y	0	ev	cig+/-ot	25	40	nev cigs	st
LUBIN	509		m	0	0	all	-	all	As:Chi	1984	CC	427	m	ot	y	n	0	ev	cig+/-ot	30	39	nev any	st
LUBIN2	532		m	0	0	all	-	all	Eu:mul	1976	CC	7804	n	bl	n	y	0	ev	cig+/-ot	30	39	nev any	st
LUBIN2	575		f	0	0	all	-	all	Eu:mul	1976	CC	7804	n	bl	n	y	0	ev	cig+/-ot	30	39	nev any	st
MATOS	517	x	m	0	0	all	-	all	SCAmer	1994	CC	200	n	bl	n	n	0	ev	cig+/-ot	25	39	nev any	st
MCCONN	504		c	0	0	all	-	all	Eu:UK	1946	CC	100	n	V	n	y	0	ev	all/unsp	30	39	nev any	st
NOTAN2	516		c	0	0	all	-	all	As:Ind	1963	CC	683	n	V	n	n	0	ev	cig only	31	40	nev any	st
PEZZOT	535		m	0	0	all	-	all	SCAmer	1987	CC	215	n	bl	n	y	0	ev	cig only	31	40	nev cigs	st
QIAO2	512	x	m	0	0	all	0	all	As:Chi	1992	pr	241	m	ot	n	n	0	ev	all/unsp	28	41	nev any	st
RACHTA	512	x	f	0	0	all	-	all	Eu:est	1991	CC	118	n	bl	n	y	0	ev	cig+/-ot	21	40	nev cigs	st
SOBUE	547		m	0	0	all	-	q+s+l+a	As:Jap	1986	CC	1376	n	bl	n	y	0	cu	cig+/-ot	30	39	nev cigs	st
WANG2	504		c	0	0	all	-	all	As:Chi	1980	CC	103	n	ot	n	n	0	ev	cig+/-ot	30	39	nev cigs	st
WUWILL	502	x	f	0	0	all	-	all	As:Chi	1985	CC	965	n	ot	n	n	0	ev	cig+/-ot	30	39	nev cigs	st

Cigarette type is all/unspec for all RRs

except for the following:

REF	NRR	CIGTYPE
JUSSAW	513	MC only
NOTAN2	516	MC only

Table 1I18 - 5

IESLC - Meta-analysis of Ever/current Smoking, Duration, "Mid"
All LC types, Cigarettes (or Any Product if Cigarettes not available)
Least adjusted

REF	NRR	SEX	AD	Number Exposed Case	Number Exposed Cont	Non-exposed Case	Non-exposed Cont	RR	95.00%CI
ARMADA	502	m	0	219	166	8	71	11.71	(5.49- 24.99)
AUVINE	502	c	0	10	5	44	229	10.41	(3.39- 31.93)
AXELSS	503	m	0	57	71	16	160	8.03	(4.31- 14.94)
AXELSS	512	f	0	29	26	18	154	9.54	(4.64- 19.61)
Subtotal AXELSS								8.64	(5.40- 13.83)
BARBON	502	m	0	118	102	22	188	9.89	(5.91- 16.55)
*BEST	506	m	1	55	-	7	-	13.90	(6.33- 30.52)
BUFFLE	527	f	0	97	62	12	112	14.60	(7.43- 28.69)
CHEN2	504	m	0	36	27	9	33	4.89	(2.01- 11.91)
CHEN2	512	f	0	13	6	25	33	2.86	(0.95- 8.58)
Subtotal CHEN2								3.95	(1.98- 7.89)
CHOI	503	m	0	102	160	13	95	4.66	(2.48- 8.75)
CHOI	512	f	0	8	2	76	164	8.63	(1.79- 41.62)
Subtotal CHOI								5.07	(2.83- 9.11)
*CPSI	582	m	0	470	367622	196	926068	6.04	(5.11- 7.14)
*CPSI	678	f	0	154	315060	532	3877179	3.56	(2.98- 4.26)
Subtotal CPSI								4.73	(4.19- 5.34)
*CPSII	554	m	0	244	109788	124	742207	13.30	(10.72- 16.51)
*CPSII	620	f	0	193	116270	310	2091302	11.20	(9.36- 13.40)
Subtotal CPSII								12.01	(10.46- 13.79)
DAMBER	508	m	1	-	-	42	-	5.15	(3.27- 8.32)
DESTEF	502	m	0	78	78	27	163	6.04	(3.61- 10.10)
FAN	502	m	0	44	122	36	236	2.36	(1.45- 3.87)
FAN	507	f	0	19	23	69	320	3.83	(1.98- 7.42)
Subtotal FAN								2.81	(1.89- 4.17)
GER	514	c	0	31	123	51	246	1.22	(0.74- 2.00)
HU2	510	c	0	123	101	121	213	2.14	(1.52- 3.03)
HUMBLE	518	c	0	68	58	28	285	11.93	(7.07- 20.13)
JOLY	517	m	0	85	165	12	218	9.36	(4.95- 17.70)
JOLY	503	f	0	31	24	52	283	7.03	(3.82- 12.93)
Subtotal JOLY								8.06	(5.19- 12.52)
JUSSAW	513	m	0	27	9	149	624	12.56	(5.79- 27.28)
KATSOU	503	f	0	15	2	48	67	10.47	(2.29- 47.93)
KHUDER	502	m	0	207	370	23	309	7.52	(4.76- 11.86)
LETOUR	507	c	0	264	160	24	224	15.40	(9.68- 24.51)
LUBIN	509	m	0	124	294	8	72	3.80	(1.78- 8.12)
LUBIN2	532	m	0	2227	3470	190	2616	8.84	(7.56- 10.33)
LUBIN2	575	f	0	187	186	336	1188	3.55	(2.81- 4.50)
Subtotal LUBIN2								6.69	(5.87- 7.63)
MATOS	517	m	0	82	110	11	110	7.45	(3.77- 14.75)
MCCONN	504	c	0	21	57	9	23	0.94	(0.38- 2.36)
NOTAN2	516	c	0	12	7	107	201	3.22	(1.23- 8.42)
PEZZOT	535	m	0	71	82	4	116	25.11	(8.82- 71.48)
*QIAO2	512	m	0	54	2257	10	709	1.70	(0.87- 3.31)
RACHTA	512	f	0	49	21	33	98	6.93	(3.63- 13.22)
SOBUE	547	m	0	159	200	34	128	2.99	(1.94- 4.61)
WANG2	504	c	0	26	38	11	43	2.67	(1.17- 6.13)
WUWILL	502	f	0	179	98	417	601	2.63	(2.00- 3.47)
Partial Totals				5988	917422	3264	7647088		

*prospective study

Table 1I18 - 5

IESLC - Meta-analysis of Ever/current Smoking, Duration, "Mid"
All LC types, Cigarettes (or Any Product if Cigarettes not available)
Least adjusted

REF	NRR	SEX	AD	Ys	Ws	Qs	Ps
ARMADA	502	m	0	2.46	6.68	2.85	0.0000
AUVINE	502	c	0	2.34	3.06	0.88	0.0000
AXELSS	503	m	0	2.08	9.96	0.76	0.0000
AXELSS	512	f	0	2.26	7.41	1.49	0.0000
Subtotal AXELSS				2.16	17.37	2.26	
BARBON	502	m	0	2.29	14.48	3.40	0.0000
*BEST	506	m	1	2.63	6.21	4.23	0.0000
BUFFLE	527	f	0	2.68	8.42	6.44	0.0000
CHEN2	504	m	0	1.59	4.85	0.23	0.0005
CHEN2	512	f	0	1.05	3.19	1.82	0.0607
Subtotal CHEN2				1.37	8.03	2.05	
CHOI	503	m	0	1.54	9.66	0.69	0.0000
CHOI	512	f	0	2.16	1.55	0.19	0.0072
Subtotal CHOI				1.62	11.21	0.88	
*CPSI	582	m	0	1.80	138.39	0.01	0.0000
*CPSI	678	f	0	1.27	119.48	34.35	0.0000
Subtotal CPSI				1.55	257.87	34.36	
*CPSII	554	m	0	2.59	82.29	50.24	0.0000
*CPSII	620	f	0	2.42	119.07	44.18	0.0000
Subtotal CPSII				2.49	201.36	94.41	
DAMBER	508	m	1	1.64	17.62	0.50	0.0000
DESTEF	502	m	0	1.80	14.53	0.00	0.0000
FAN	502	m	0	0.86	15.89	14.22	0.0006
FAN	507	f	0	1.34	8.79	1.89	0.0001
Subtotal FAN				1.03	24.68	16.11	
GER	514	c	0	0.20	15.61	40.53	0.4403
HU2	510	c	0	0.76	32.27	35.17	0.0000
HUMBLE	518	c	0	2.48	14.05	6.36	0.0000
JOLY	517	m	0	2.24	9.46	1.75	0.0000
JOLY	503	f	0	1.95	10.34	0.21	0.0000
Subtotal JOLY				2.09	19.80	1.96	
JUSSAW	513	m	0	2.53	6.39	3.35	0.0000
KATSOU	503	f	0	2.35	1.66	0.49	0.0025
KHUDER	502	m	0	2.02	18.43	0.82	0.0000
LETOUR	507	c	0	2.73	17.80	15.32	0.0000
LUBIN	509	m	0	1.33	6.65	1.49	0.0006
LUBIN2	532	m	0	2.18	156.67	21.71	0.0000
LUBIN2	575	f	0	1.27	68.77	19.93	0.0000
Subtotal LUBIN2				1.90	225.44	41.64	
MATOS	517	m	0	2.01	8.24	0.34	0.0000
MCCONN	504	c	0	-0.06	4.55	15.86	0.8977
NOTAN2	516	c	0	1.17	4.16	1.69	0.0171
PEZZOT	535	m	0	3.22	3.51	7.04	0.0000
*QIAO2	512	m	0	0.53	8.57	14.00	0.1218
RACHTA	512	f	0	1.94	9.21	0.15	0.0000
SOBUE	547	m	0	1.10	20.61	10.40	0.0000
WANG2	504	c	0	0.98	5.59	3.78	0.0200
WUWILL	502	f	0	0.97	50.37	35.43	0.0000

	N	40
	NS	32
	Wt	1064.47
Het	Chi	404.21
Het	df	39
Het	P	***
Fixed	RR	6.09
	RRl	5.73
	RRu	6.47
	P	+++
Random	RR	5.75
	RRl	4.64
	RRu	7.14
	P	+++
Asymm	P	N.S.

Table 1I18 - 6

IESLC - Meta-analysis of Ever/current Smoking, Duration, "Mid"
All LC types, Cigarettes (or Any Product if Cigarettes not available)
Least adjusted

		Sex			
		combined	male	female	Total
	N	8	20	12	40
	NS	8	20	12	40
	Wt	97.09	559.11	408.27	1064.47
Het	Chi	96.86	116.60	137.02	404.21
Het	df	7	19	11	39
Het	P	***	***	***	***
Fixed	RR	3.75	7.41	5.23	6.09
	RRl	3.08	6.82	4.74	5.73
	RRu	4.58	8.05	5.76	6.47
	P	+++	+++	+++	+++
Random	RR	3.77	6.66	5.79	5.75
	RRl	1.71	5.24	3.85	4.64
	RRu	8.30	8.46	8.71	7.14
	P	+++	+++	+++	+++
Between	Chi				53.72
Between	df				2
Between	P				***
Btwn(F)	P				(*)
Btwn(R)	P				N.S.

Table 1I18 - 7

IESLC - Meta-analysis of Ever/current Smoking, Duration, "Mid"
All LC types, Cigarettes (or Any Product if Cigarettes not available)
Excluded studies (and stage at which they were excluded)

1	BECHER BLOT1 BROWN3 CARPEN CHYOU DARBY DOLL2 GARCIA GRAHAM GURSEL HAMMO2 JAHN JAIN LAUSSM PRESCO QIAO TVERDA WIGLE WYNDE3
2	ALDERS BENSHL BRESLO CHIAZZ DEAN3 DORN ENGELA GAO2 GILLIS GUO HEGMAN HIRAYA HOLE KAUFMA KOO KOULUM LIU4 MIGRAN MRFITR PERNU SEGI2 SPEIZE SUZUK2 SVENSS VUTUC WAKAI WU YUAN
3	GENG MCDUFF SPITZ STASZE WU2 ZHANG
4	BOUCHA CHEN CORREA JEDRYC LUO WYNDE2 WYNDE6
5	AKIBA HAMMON PISANI RESTRE SADOWS XU
7	BOFFET BROSS WYNDE7
10	AMES WATSON WYNDE8
14	AGUDO AMANDU BOUCOT CEDERL DEAN2 DOLL DORGAN DOSEME GAO GARSHI HAENSZ HU KAISE2 KREUZE LEVIN LIAW LIU3 LIU5 OSANN2 PEZZO2 TIZZAN ZHENG ZHOU
15	BENHAM

Table 1I18 - 8
Potentially overlapping studies

REF	REFGP	PRINC	.	OVERLAP/LINK
LUBIN2	LUBIN2	1		Lubin-combined
CPSI	CPSI	1		CPSI overall
LUBIN	XIANGZ	2		LUBIN/XIANGZ/QIAO

Table 1I18 - 9
Most adjusted - insufficient data for meta-analysis

REF	NRR	SEX	AGEL	AGEH	RACE	YF	LC TYPE	LOC	START	ST	NLC	R	VB	P	H	AD	SM	PRODUCT	exL	exH	DENOM	De
BUFFLE	502	m	0	0	wh	-	all	NAmer	1976	CC	943	n	bl	y	n	0	ev	cig+/-ot	34	43	nev cigs	or
SADOWS	525	m	0	0	wh	-	all	NAmer	1938	CC	477	n	bl	n	n	0	ev	cig only	30	39	nev any	ot
XU	502	m	0	0	all	-	all	As:Chi	1985	CC	729	n	ot	n	n	2	ev	all/unsp	30	39	nev any	or

REF	NRR	RR	SIG	RRDATA comment
BUFFLE	502	11.10		0
SADOWS	525	3.95		0
XU	502	*		RR for 1-19/day is 2.1(p<0.05), for 20-29/day is 2.7(p<0.05) and for >=30/ day is 4.9(p<0.05)

Table 1I19 -

IESLC - Meta-analysis of Ever/current Smoking, Duration, "High"
All LC types, Cigarettes (or Any Product if Cigarettes not available)

This analysis is restricted to results for:
1) Ever/current smokers
2) Results by Duration
3) Categorical results by Duration
4) All LC types (or near equivalent)
5) Results complete enough for use in metaanalysis

Within each study, results are then selected (in the following order of preference, within each sex) for:
6) PRODUCT: cigarettes regardless of other products, cigarettes only, all/unspec
7) CIGTYPE: all/unspecified, MC regardless of HR, MC only
8) (not applicable)
9) DENOM: never smoked anything, never smoked cigarettes, never any + low, never cigs + low
10) Followup period (YF, prospective studies): whole study (coded as 0) or longest available
11) LCtype: all or nearest available, at least Squamous and Adeno. (q = squamous, s = small,
l = large, a = adeno, mix = mixed, alv = alveolar)
12) Race: all or nearest available, otherwise by race (wh or w = white, bl or b = black, hi = hispanic
ch = chinese, jap = japanese, haw = hawaiian, w+o = white + oriental, sca = scandinavian, as = asian)
13) Duration "high" in key scheme 1 (key value 50, maximum range 36+)
14) For overlapping studies: principal rather than subsidiary studies
Finally by Age: whole study (coded as 0) if available, otherwise by widest available age group
and then for single sex results (m, f) in preference to results for both sexes combined (c).

Results adjusted (AD) for the most potential confounders are then chosen in Sections -1 to -3
(and those which actually differ from the adjusted results in Table 1I14 - 1 are marked 'x' in Section -1)
and results adjusted for the least confounders in Sections -4 to -6. (Those least adjusted results which
actually differ from the most adjusted are marked 'x' in column X in Section -4)

Section -7 shows excluded studies, together with the stage (as above) at which no qualifying
results were found.

Section -8 lists the potentially overlapping studies which have been included (1=principal, 2=subsidiary).

Section -9 lists any results which would have been included in preference except that they had data not complete
enough for use in meta-analysis, with their significance (yes/no), if known, and any further comment as entered
on the database. It also lists as "gap" any categories for which no data were presented by the original authors.

In addition to those mentioned above, the following fields, levels and abbreviations are used:
* or nk = not known, n = no, y = yes, ot = other
ev = ever, cu = current, nev = never
all/unspec = all or unspecified, cig+/-ot = cigarettes irrespective of other products (cigar, pipe etc)
MC = manufactured cigarettes, HR = hand-rolled cigarettes
exL, exH = range of exposure (low and high) in the smoking group, in terms of Duration
REF: 6-character study reference
NRR: number of the RR on the database within the study
ST : study type (CC = case control, pr or prosp = prospective)
NLC: number of lung cancer cases in whole study
R : risky occupational population (n = no, m = mining, o = other risky)
VB : national cigarette type (V = at least 75% Virginia, bl = at least 75% blended, ot = other)
P : any proxy use
H : full histological confirmation
De : derivation of RR/CI (or = original, st = standard method, ot = other method of estimation)

Table 1I19 - 1

IESLC - Meta-analysis of Ever/current Smoking, Duration, "High"
All LC types, Cigarettes (or Any Product if Cigarettes not available)
Most adjusted

REF	NRR	1I14	SEX	AGEL	AGEH	RACE	YF	LC TYPE	LOC	START	ST	NLC	R	VB	P	H	AD	SM	PRODUCT	exL	exH	DENOM	De
ARMADA	508		m	0	0	all	-	all	Eu:wst	1986	CC	325	n	bl	n	y	1	ev	cig+/-ot	50	999	nev cigs	or
AUVINE	519		c	0	0	all	-	all	Eu:Sca	1986	CC	517	n	bl	y	n	2	ev	cig+/-ot	41	999	nev cigs	or
AXELSS	523		m	0	0	sca	-	all	Eu:Sca	1989	CC	436	n	bl	n	n	6	ev	all/unsp	50	999	nev any	ot
AXELSS	514		f	0	0	sca	-	all	Eu:Sca	1989	CC	436	n	bl	n	n	0	ev	all/unsp	50	999	nev any	st
BARBON	511		m	0	0	all	-	all	Eu:wst	1979	CC	755	n	bl	y	y	1	ev	all/unsp	50	999	nev any	or
BEST	507		m	0	0	all	0	all	NAmer	1955	pr	381	n	V	n	n	1	cu	cig only	40	999	nev any	ot
BOUCOT	519		m	0	0	all	9	all	NAmer	1951	pr	121	n	bl	n	n	0	ev	cig+/-ot	40	999	nev any	ot
BUFFLE	528		f	0	0	w-hi	-	all	NAmer	1976	CC	943	n	bl	y	n	0	ev	cig+/-ot	41	999	nev cigs	or
CHEN2	505		m	0	0	all	-	all	As:Chi	1983	CC	193	n	ot	y	n	0	ev	all/unsp	41	999	nev any	st
CHEN2	513		f	0	0	all	-	all	As:Chi	1983	CC	193	n	ot	y	n	0	ev	all/unsp	41	999	nev any	st
CHOI	505		m	0	0	all	-	all	As:oth	1985	CC	375	n	bl	n	n	0	ev	cig+/-ot	50	999	nev cigs	st
CHOI	513		f	0	0	all	-	all	As:oth	1985	CC	375	n	bl	n	n	0	ev	cig+/-ot	40	999	nev cigs	st
CPSI	585		m	40	84	wh	0	all	NAmer	1959	pr	5138	n	bl	n	n	0	cu	cig only	50	54	nev cigs	st
CPSI	681		f	40	84	wh	0	all	NAmer	1959	pr	5138	n	bl	n	n	0	cu	cig only	50	54	nev cigs	st
CPSII	557		m	0	0	all	6	all	NAmer	1982	pr	3229	n	bl	n	n	0	cu	cig only	50	54	nev any	st
CPSII	623		f	0	0	all	6	all	NAmer	1982	pr	3229	n	bl	n	n	0	cu	cig+/-ot	50	54	nev cigs	st
DAMBER	509		m	0	0	all	-	all	Eu:Sca	1972	CC	579	n	bl	y	n	1	ev	all/unsp	41	50	nev any	ot
DESTEF	511		m	0	0	all	-	all	SCAmer	1988	CC	497	n	bl	n	y	4	ev	all/unsp	50	999	nev any	or
DOLL	518		m	0	0	all	-	all	Eu:UK	1948	CC	1465	n	V	n	n	0	ev	all/unsp	40	999	nev any	st
DOLL	525		f	0	0	all	-	all	Eu:UK	1948	CC	1465	n	V	n	n	0	ev	all/unsp	40	999	nev any	st
FAN	503		m	0	0	all	-	all	As:Chi	1990	CC	403	n	ot	y	n	0	ev	cig+/-ot	40	999	nev cigs	st
FAN	508		f	0	0	all	-	all	As:Chi	1990	CC	403	n	ot	y	n	0	ev	cig+/-ot	40	999	nev cigs	st
GER	520		c	0	0	all	-	all	As:oth	1990	CC	141	n	ot	y	n	5	ev	all/unsp	41	999	nev any	ot
HU2	511		c	0	0	all	-	all	As:Chi	1977	CC	523	n	ot	y	n	0	ev	cig+/-ot	40	999	nev cigs	st
HUMBLE	545		c	0	0	wh	-	not alv	NAmer	1980	CC	521	n	bl	y	n	3	cu	cig+/-ot	50	999	nev cigs	ot
JOLY	519		m	0	0	all	-	all	SCAmer	1978	CC	826	n	bl	n	n	0	ev	cig+/-ot	50	999	nev any	st
JOLY	505		f	0	0	all	-	all	SCAmer	1978	CC	826	n	bl	n	n	0	ev	cig+/-ot	50	999	nev any	st
JUSSAW	514		m	0	0	all	-	all	As:Ind	1964	CC	792	n	V	n	n	0	ev	cig only	40	999	nev any	st
KAISE2	597		m	0	0	all	9	all	NAmer	1979	pr	318	n	bl	n	n	1	cu	cig only	40	999	nev any	st
KAISE2	517		f	0	0	all	9	all	NAmer	1979	pr	318	n	bl	n	n	1	cu	cig only	40	999	nev any	st
KATSOU	504		f	0	0	all	-	all	Eu:bal	1987	CC	101	n	bl	n	n	0	cu	all/unsp	40	999	nev any	st
KHUDER	503		m	0	0	all	-	all	NAmer	1985	CC	482	n	bl	n	y	0	ev	cig+/-ot	50	999	nev cigs	st
KREUZE	507		m	55	69	all	-	all	Eu:Ger	1990	CC	2260	n	bl	n	n	3	ev	all/unsp	40	999	nev any	or
KREUZE	510		f	55	69	all	-	all	Eu:Ger	1990	CC	2260	n	bl	n	n	3	ev	all/unsp	40	999	nev any	or
LETOUR	508		c	0	0	all	-	all	NAmer	1983	CC	738	n	V	y	y	0	ev	cig+/-ot	41	999	nev cigs	st
LEVIN	507		m	0	0	all	-	all	NAmer	1938	CC	475	n	bl	n	n	1	ev	cig+/-ot	40	999	nev any	ot
LUBIN	511		m	0	0	all	-	all	As:Chi	1984	CC	427	m	ot	y	n	0	ev	cig+/-ot	50	999	nev any	st
LUBIN2	534		m	0	0	all	-	all	Eu:mul	1976	CC	7804	n	bl	n	y	0	ev	cig+/-ot	50	999	nev any	st
LUBIN2	577		f	0	0	all	-	all	Eu:mul	1976	CC	7804	n	bl	n	y	0	ev	cig+/-ot	50	999	nev any	st
MATOS	538		m	0	0	all	-	all	SCAmer	1994	CC	200	n	bl	n	n	2	ev	cig+/-ot	40	70	nev any	or
MCCONN	505		c	0	0	all	-	all	Eu:UK	1946	CC	100	n	V	n	y	0	ev	all/unsp	40	999	nev any	st
NOTAN2	517		c	0	0	all	-	all	As:Ind	1963	CC	683	n	V	n	n	0	ev	cig only	41	999	nev any	st
PEZZO2	508		m	0	0	all	-	all	SCAmer	1992	CC	367	n	bl	n	y	0	cu	cig+/-ot	36	999	nev cigs	st
PEZZOT	536		m	0	0	all	-	all	SCAmer	1987	CC	215	n	bl	n	y	0	ev	cig only	41	999	nev cigs	st
QIAO2	518		m	0	0	all	0	all	As:Chi	1992	pr	241	m	ot	n	n	1	ev	all/unsp	42	999	nev any	or
RACHTA	518		f	0	0	all	-	all	Eu:est	1991	CC	118	n	bl	n	y	1	ev	cig+/-ot	41	999	nev cigs	or
SOBUE	549		m	0	0	all	-	q+s+l+a	As:Jap	1986	CC	1376	n	bl	n	y	0	cu	cig+/-ot	50	999	nev cigs	st
WUWILL	518		f	0	0	all	-	all	As:Chi	1985	CC	965	n	ot	n	n	3	ev	cig+/-ot	40	999	nev cigs	ot

Cigarette type is all/unspec for all RRs

except for the following:

REF	NRR	CIGTYPE
JUSSAW	514	MC only
NOTAN2	517	MC only

Table 1I19 - 2

IESLC - Meta-analysis of Ever/current Smoking, Duration, "High"
All LC types, Cigarettes (or Any Product if Cigarettes not available)
Most adjusted

REF	NRR	SEX	AD	Number Exposed Case	Cont	Non-exposed Case	Cont	RR	95.00%CI
ARMADA	508	m	1	77	-	8	-	26.80	(11.00- 65.10)
AUVINE	519	c	2	230	-	44	-	30.40	(15.80- 58.40)
AXELSS	523	m	6	101	-	16	-	27.09	(13.94- 52.62)
AXELSS	514	f	0	20	10	18	154	17.11	(6.94- 42.19)
Subtotal AXELSS								23.05	(13.50- 39.36)
BARBON	511	m	1	366	-	22	-	14.50	(9.00- 23.30)
*BEST	507	m	1	137	-	7	-	14.20	(6.64- 30.35)
*BOUCOT	519	m	0	52	1563	0	805	54.09~	(3.34- 875.17)
BUFFLE	528	f	0	90	42	12	112	20.00	(9.94- 40.23)
CHEN2	505	m	0	62	40	9	33	5.68	(2.46- 13.13)
CHEN2	513	f	0	21	15	25	33	1.85	(0.80- 4.29)
Subtotal CHEN2								3.25	(1.80- 5.89)
CHOI	505	m	0	20	20	13	95	7.31	(3.13- 17.07)
CHOI	513	f	0	1	1	76	164	2.16	(0.13- 34.96)
Subtotal CHOI								6.59	(2.93- 14.84)
*CPSI	585	m	0	576	119633	196	926068	22.75	(19.35- 26.75)
*CPSI	681	f	0	16	14305	532	3877179	8.15	(4.96- 13.40)
Subtotal CPSI								20.61	(17.67- 24.05)
*CPSII	557	m	0	332	39260	124	742207	50.62	(41.19- 62.20)
*CPSII	623	f	0	122	29119	310	2091302	28.26	(22.93- 34.84)
Subtotal CPSII								37.99	(32.80- 44.00)
DAMBER	509	m	1	-	-	42	-	8.71	(5.84- 13.66)
DESTEF	511	m	4	178	-	27	-	10.80	(6.60- 17.60)
DOLL	518	m	0	558	491	7	61	9.90	(4.49- 21.85)
DOLL	525	f	0	6	3	40	59	2.95	(0.70- 12.49)
Subtotal DOLL								7.48	(3.74- 14.98)
FAN	503	m	0	143	241	36	236	3.89	(2.59- 5.84)
FAN	508	f	0	55	59	69	320	4.32	(2.76- 6.78)
Subtotal FAN								4.08	(3.02- 5.52)
GER	520	c	5	49	-	51	-	2.14	(1.18- 3.90)
HU2	511	c	0	194	113	121	213	3.02	(2.19- 4.17)
HUMBLE	545	c	3	119	-	28	-	17.27	(10.38- 28.75)
JOLY	519	m	0	250	253	12	218	17.95	(9.78- 32.93)
JOLY	505	f	0	57	20	52	283	15.51	(8.61- 27.95)
Subtotal JOLY								16.65	(10.91- 25.41)
JUSSAW	514	m	0	11	6	149	624	7.68	(2.79- 21.09)
*KAISE2	597	m	1	34	-	14	-	15.64	(8.31- 29.40)
*KAISE2	517	f	1	26	-	11	-	30.41	(14.39- 64.25)
Subtotal KAISE2								20.63	(12.73- 33.43)
KATSOU	504	f	0	17	4	48	67	5.93	(1.88- 18.75)
KHUDER	503	m	0	236	354	23	309	8.96	(5.69- 14.11)
KREUZE	507	m	3	-	-	-	-	54.50	(34.90- 85.20)
KREUZE	510	f	3	-	-	-	-	8.30	(4.70- 14.50)
Subtotal KREUZE								26.38	(18.59- 37.42)
LETOUR	508	c	0	374	141	24	224	24.76	(15.58- 39.35)
LEVIN	507	m	1	63	-	7	-	8.96	(3.90- 20.61)
LUBIN	511	m	0	59	86	8	72	6.17	(2.77- 13.77)
LUBIN2	534	m	0	1325	1484	190	2616	12.29	(10.42- 14.50)
LUBIN2	577	f	0	81	32	336	1188	8.95	(5.84- 13.71)
Subtotal LUBIN2								11.80	(10.12- 13.76)
MATOS	538	m	2	86	-	11	-	12.70	(6.10- 26.10)
MCCONN	505	c	0	16	40	9	23	1.02	(0.39- 2.68)
NOTAN2	517	c	0	5	5	107	201	1.88	(0.53- 6.63)
PEZZO2	508	m	0	173	126	6	117	26.77	(11.42- 62.76)
PEZZOT	536	m	0	110	101	4	116	31.58	(11.25- 88.71)
*QIAO2	518	m	1	170	-	10	-	2.05	(1.06- 3.94)
RACHTA	518	f	1	24	-	33	-	58.68	(7.56- 455.64)
SOBUE	549	m	0	147	73	34	128	7.58	(4.73- 12.14)
WUWILL	518	f	3	223	-	417	-	3.49	(2.65- 4.59)
Partial Totals				7012	207640	3338	7645227		

*prospective study ~ With 0.5 adjustment for zero

Table 1I19 - 2

IESLC - Meta-analysis of Ever/current Smoking, Duration, "High"
All LC types, Cigarettes (or Any Product if Cigarettes not available)
Most adjusted

REF	NRR	SEX	AD	Ys	Ws	Qs	Ps
ARMADA	508	m	1	3.29	4.86	2.18	0.0000
AUVINE	519	c	2	3.41	8.99	5.69	0.0000
AXELSS	523	m	6	3.30	8.71	4.03	0.0000
AXELSS	514	f	0	2.84	4.72	0.23	0.0000
Subtotal AXELSS				3.14	13.42	4.26	
BARBON	511	m	1	2.67	16.98	0.05	0.0000
*BEST	507	m	1	2.65	6.65	0.01	0.0000
*BOUCOT	519	m	0	3.99	0.50	0.93	0.0050
BUFFLE	528	f	0	3.00	7.86	1.12	0.0000
CHEN2	505	m	0	1.74	5.48	4.26	0.0000
CHEN2	513	f	0	0.61	5.42	21.78	0.1529
Subtotal CHEN2				1.18	10.90	26.04	
CHOI	505	m	0	1.99	5.33	2.12	0.0000
CHOI	513	f	0	0.77	0.50	1.69	0.5883
Subtotal CHOI				1.89	5.83	3.81	
*CPSI	585	m	0	3.12	146.44	37.40	0.0000
*CPSI	681	f	0	2.10	15.55	4.22	0.0000
Subtotal CPSI				3.03	161.99	41.62	
*CPSII	557	m	0	3.92	90.50	154.15	0.0000
*CPSII	623	f	0	3.34	87.81	45.83	0.0000
Subtotal CPSII				3.64	178.31	199.99	
DAMBER	509	m	1	2.16	21.28	4.40	0.0000
DESTEF	511	m	4	2.38	15.97	0.92	0.0000
DOLL	518	m	0	2.29	6.13	0.65	0.0000
DOLL	525	f	0	1.08	1.85	4.36	0.1417
Subtotal DOLL				2.01	7.98	5.01	
FAN	503	m	0	1.36	23.17	36.83	0.0000
FAN	508	f	0	1.46	18.96	25.30	0.0000
Subtotal FAN				1.41	42.13	62.13	
GER	520	c	5	0.76	10.75	37.13	0.0126
HU2	511	c	0	1.11	37.09	84.92	0.0000
HUMBLE	545	c	3	2.85	14.81	0.78	0.0000
JOLY	519	m	0	2.89	10.43	0.75	0.0000
JOLY	505	f	0	2.74	11.07	0.17	0.0000
Subtotal JOLY				2.81	21.50	0.92	
JUSSAW	514	m	0	2.04	3.76	1.27	0.0001
*KAISE2	597	m	1	2.75	9.62	0.16	0.0000
*KAISE2	517	f	1	3.41	6.86	4.34	0.0000
Subtotal KAISE2				3.03	16.49	4.51	
KATSOU	504	f	0	1.78	2.90	2.04	0.0024
KHUDER	503	m	0	2.19	18.60	3.39	0.0000
KREUZE	507	m	3	4.00	19.29	36.68	0.0000
KREUZE	510	f	3	2.12	12.11	3.06	0.0000
Subtotal KREUZE				3.27	31.40	39.75	
LETOUR	508	c	0	3.21	17.89	6.23	0.0000
LEVIN	507	m	1	2.19	5.54	1.01	0.0000
LUBIN	511	m	0	1.82	5.97	3.81	0.0000
LUBIN2	534	m	0	2.51	141.36	1.71	0.0000
LUBIN2	577	f	0	2.19	21.09	3.85	0.0000
Subtotal LUBIN2				2.47	162.45	5.57	
MATOS	538	m	2	2.54	7.27	0.04	0.0000
MCCONN	505	c	0	0.02	4.13	27.86	0.9644
NOTAN2	517	c	0	0.63	2.41	9.55	0.3273
PEZZO2	508	m	0	3.29	5.29	2.36	0.0000
PEZZOT	536	m	0	3.45	3.60	2.50	0.0000
*QIAO2	518	m	1	0.72	8.91	32.22	0.0321
RACHTA	518	f	1	4.07	0.91	1.93	0.0001
SOBUE	549	m	0	2.03	17.32	6.10	0.0000
WUWILL	518	f	3	1.25	50.92	95.47	0.0000

Table 1I19 - 2

IESLC - Meta-analysis of Ever/current Smoking, Duration, "High"
All LC types, Cigarettes (or Any Product if Cigarettes not available)
Most adjusted

	N	48
	NS	37
	Wt	953.59
Het	Chi	727.50
Het	df	47
Het	P	***
Fixed	RR	13.72
	RRl	12.88
	RRu	14.62
	P	+++
Random	RR	10.39
	RRl	7.92
	RRu	13.65
	P	+++
Asymm	P	*

Table 1I19 - 3

IESLC - Meta-analysis of Ever/current Smoking, Duration, "High"
All LC types, Cigarettes (or Any Product if Cigarettes not available)
Most adjusted

	Sex			
	combined	male	female	Total
N	7	26	15	48
NS	7	26	15	48
Wt	96.07	608.99	248.53	953.59
Het Chi	120.39	313.54	202.65	727.50
Het df	6	25	14	47
Het P	***	***	***	***
Fixed RR	6.59	16.90	10.94	13.72
RRl	5.39	15.61	9.66	12.88
RRu	8.04	18.30	12.39	14.62
P	+++	+++	+++	+++
Random RR	5.82	12.88	9.14	10.39
RRl	2.22	9.38	5.31	7.92
RRu	15.22	17.69	15.75	13.65
P	+++	+++	+++	+++
Between Chi				90.92
Between df				2
Between P				***
Btwn(F) P				*
Btwn(R) P				N.S.

	Lung cancer type		
	all	other	Total
N	46	2	48
NS	35	2	37
Wt	921.46	32.13	953.59
Het Chi	720.57	5.41	727.50
Het df	45	1	47
Het P	***	*	***
Fixed RR	13.83	11.08	13.72
RRl	12.96	7.84	12.88
RRu	14.75	15.66	14.62
P	+++	+++	+++
Random RR	10.34	11.37	10.39
RRl	7.80	5.08	7.92
RRu	13.72	25.49	13.65
P	+++	+++	+++
Between Chi			1.52
Between df			1
Between P			N.S.
Btwn(F) P			N.S.
Btwn(R) P			N.S.

	Location								
	NAmer	UK	Scand	othEur	China	Japan	othAs	other	Total
N	13	3	4	8	8	1	5	6	48
NS	10	2	3	6	6	1	4	5	37
Wt	428.64	12.11	43.70	219.51	155.92	17.32	22.76	53.64	953.59
Het Chi	99.79	12.87	13.89	51.51	9.86	0.00	8.86	6.00	727.50
Het df	12	2	3	7	7	0	4	5	47
Het P	***	**	**	***	N.S.	N.S.	(*)	N.S.	***
Fixed RR	25.27	3.79	15.19	13.66	3.47	7.58	3.48	15.44	13.72
RRl	22.99	2.16	11.29	11.97	2.97	4.73	2.31	11.81	12.88
RRu	27.78	6.66	20.43	15.59	4.06	12.14	5.24	20.17	14.62
P	+++	+++	+++	+++	+++	+++	+++	+++	+++
Random RR	19.05	3.17	18.23	15.53	3.49	7.58	3.75	15.77	10.39
RRl	13.78	0.70	9.20	9.51	2.85	4.73	1.88	11.70	7.92
RRu	26.34	14.40	36.13	25.33	4.27	12.14	7.50	21.26	13.65
P	+++	N.S.	+++	+++	+++	+++	+++	+++	+++
Between Chi									524.71
Between df									7
Between P									***
Btwn(F) P									***
Btwn(R) P									***

Table 1I19 - 3

IESLC - Meta-analysis of Ever/current Smoking, Duration, "High"
All LC types, Cigarettes (or Any Product if Cigarettes not available)
Most adjusted

Detailed Country in "other Europe"

	multi	Germany	othWest	East	Balkans	Total
N	2	2	2	1	1	8
NS	1	1	2	1	1	6
Wt	162.45	31.40	21.84	0.91	2.90	219.51
Het Chi	1.85	26.34	1.43	0.00	0.00	51.51
Het df	1	1	1	0	0	7
Het P	N.S.	***	N.S.	N.S.	N.S.	***
Fixed RR	11.80	26.38	16.62	58.68	5.93	13.66
RRl	10.12	18.59	10.93	7.56	1.88	11.97
RRu	13.76	37.42	25.28	455.55	18.75	15.59
P	+++	+++	+++	+++	++	+++
Random RR	11.18	21.44	17.49	58.68	5.93	15.53
RRl	8.41	3.39	10.05	7.56	1.88	9.51
RRu	14.86	135.59	30.45	455.55	18.75	25.33
P	+++	++	+++	+++	++	+++
Between Chi						21.89
Between df						4
Between P						***
Btwn(F) P						N.S.
Btwn(R) P						N.S.

Detailed Country in "other Asia"

	India	HongKong	other	Total
N	2		3	5
NS	2		2	4
Wt	6.17		16.58	22.76
Het Chi	2.91		5.45	8.86
Het df	1		2	4
Het P	(*)		(*)	(*)
Fixed RR	4.43		3.18	3.48
RRl	2.01		1.96	2.31
RRu	9.75		5.14	5.24
P	+++		+++	+++
Random RR	4.00		3.55	3.75
RRl	1.01		1.31	1.88
RRu	15.85		9.59	7.50
P	+		+	+++
Between Chi				0.50
Between df				1
Between P				N.S.
Btwn(F) P				N.S.
Btwn(R) P				N.S.

Detailed other continent

	SCAmer	Total
N	6	6
NS	5	5
Wt	53.64	53.64
Het Chi	6.00	6.00
Het df	5	5
Het P	N.S.	N.S.
Fixed RR	15.44	15.44
RRl	11.81	11.81
RRu	20.17	20.17
P	+++	+++
Random RR	15.77	15.77
RRl	11.70	11.70
RRu	21.26	21.26
P	+++	+++
Between Chi		
Between df		
Between P		N.S.
Btwn(F) P		N.S.
Btwn(R) P		N.S.

Table 1I19 - 3

IESLC - Meta-analysis of Ever/current Smoking, Duration, "High"
All LC types, Cigarettes (or Any Product if Cigarettes not available)
Most adjusted

	Start year of study					
	<1960	1960-69	1970-79	1980-89	1990+	Total
N	8	2	10	19	9	48
NS	6	2	7	15	7	37
Wt	186.79	6.17	283.66	370.30	106.67	953.59
Het Chi	63.65	2.91	81.33	337.82	138.09	727.50
Het df	7	1	9	18	8	47
Het P	***	(*)	***	***	***	***
Fixed RR	17.83	4.43	10.51	17.83	7.59	13.72
RRl	15.45	2.01	9.36	16.10	6.28	12.88
RRu	20.58	9.75	11.81	19.74	9.17	14.62
P	+++	+++	+++	+++	+++	+++
Random RR	8.23	4.00	12.18	12.19	8.74	10.39
RRl	4.02	1.01	8.12	7.49	3.83	7.92
RRu	16.85	15.85	18.27	19.85	19.95	13.65
P	+++	+	+++	+++	+++	+++
Between Chi						103.70
Between df						4
Between P						***
Btwn(F) P						N.S.
Btwn(R) P						N.S.

	Study type (1)		
	CC	other	Total
N	39	9	48
NS	31	6	37
Wt	580.74	372.86	953.59
Het Chi	350.57	127.19	727.50
Het df	38	8	47
Het P	***	***	***
Fixed RR	9.11	25.99	13.72
RRl	8.40	23.48	12.88
RRu	9.88	28.77	14.62
P	+++	+++	+++
Random RR	9.31	16.91	10.39
RRl	7.10	10.38	7.92
RRu	12.19	27.55	13.65
P	+++	+++	+++
Between Chi			249.74
Between df			1
Between P			***
Btwn(F) P			***
Btwn(R) P			*

	Study type (2)			
	CC	prosp	other	Total
N	39	9		48
NS	31	6		37
Wt	580.74	372.86		953.59
Het Chi	350.57	127.19		727.50
Het df	38	8		47
Het P	***	***		***
Fixed RR	9.11	25.99		13.72
RRl	8.40	23.48		12.88
RRu	9.88	28.77		14.62
P	+++	+++		+++
Random RR	9.31	16.91		10.39
RRl	7.10	10.38		7.92
RRu	12.19	27.55		13.65
P	+++	+++		+++
Between Chi				249.74
Between df				1
Between P				***
Btwn(F) P				***
Btwn(R) P				*

Table 1I19 - 3

IESLC - Meta-analysis of Ever/current Smoking, Duration, "High"
All LC types, Cigarettes (or Any Product if Cigarettes not available)
Most adjusted

	Study size (number of LC cases) 100-249	250-499	500-999	1000+	Total
N	10	15	12	11	48
NS	9	11	11	6	37
Wt	49.88	140.76	203.50	559.45	953.59
Het Chi	55.28	67.98	147.25	204.90	727.50
Het df	9	14	11	10	47
Het P	***	***	***	***	***
Fixed RR	4.01	9.38	7.86	20.64	13.72
RRl	3.04	7.95	6.85	19.00	12.88
RRu	5.30	11.06	9.02	22.42	14.62
P	+++	+++	+++	+++	+++
Random RR	5.59	11.36	10.56	15.09	10.39
RRl	2.68	7.72	6.21	9.94	7.92
RRu	11.67	16.71	17.95	22.90	13.65
P	+++	+++	+++	+++	+++
Between Chi					252.10
Between df					3
Between P					***
Btwn(F) P					***
Btwn(R) P					N.S.

	Risky occupational population no	mining	othRisky	Total
N	46	2		48
NS	35	2		37
Wt	938.71	14.89		953.59
Het Chi	690.97	4.35		727.50
Het df	45	1		47
Het P	***	*		***
Fixed RR	14.05	3.19		13.72
RRl	13.17	1.92		12.88
RRu	14.97	5.30		14.62
P	+++	+++		+++
Random RR	10.91	3.47		10.39
RRl	8.30	1.18		7.92
RRu	14.35	10.21		13.65
P	+++	+		+++
Between Chi				32.19
Between df				1
Between P				***
Btwn(F) P				N.S.
Btwn(R) P				*

	National cigarette tobacco type Virginia	blended	other	Total
N	7	32	9	48
NS	6	24	7	37
Wt	42.83	744.10	166.67	953.59
Het Chi	46.69	254.45	12.22	727.50
Het df	6	31	8	47
Het P	***	***	N.S.	***
Fixed RR	10.43	19.10	3.36	13.72
RRl	7.73	17.78	2.89	12.88
RRu	14.07	20.53	3.92	14.62
P	+++	+++	+++	+++
Random RR	5.92	16.31	3.34	10.39
RRl	2.39	12.87	2.73	7.92
RRu	14.66	20.67	4.10	13.65
P	+++	+++	+++	+++
Between Chi				414.14
Between df				2
Between P				***
Btwn(F) P				***
Btwn(R) P				***

Table 1I19 - 3

IESLC - Meta-analysis of Ever/current Smoking, Duration, "High"
All LC types, Cigarettes (or Any Product if Cigarettes not available)
Most adjusted

	Any proxy use No/nk	Yes	Total
N	35	13	48
NS	26	11	37
Wt	758.95	194.65	953.59
Het Chi	472.14	142.30	727.50
Het df	34	12	47
Het P	***	***	***
Fixed RR	16.34	6.95	13.72
RRl	15.22	6.04	12.88
RRu	17.54	8.00	14.62
P	+++	+++	+++
Random RR	11.91	7.53	10.39
RRl	8.82	4.58	7.92
RRu	16.09	12.39	13.65
P	+++	+++	+++
Between Chi			113.07
Between df			1
Between P			***
Btwn(F) P			**
Btwn(R) P			N.S.

	Full histological confirmation No	Yes	Total
N	36	12	48
NS	26	11	37
Wt	685.58	268.02	953.59
Het Chi	666.65	54.58	727.50
Het df	35	11	47
Het P	***	***	***
Fixed RR	14.44	12.06	13.72
RRl	13.40	10.70	12.88
RRu	15.56	13.59	14.62
P	+++	+++	+++
Random RR	9.65	12.32	10.39
RRl	6.76	8.81	7.92
RRu	13.77	17.22	13.65
P	+++	+++	+++
Between Chi			6.26
Between df			1
Between P			*
Btwn(F) P			N.S.
Btwn(R) P			N.S.

	Number of adjustment variables (1) 0	1	2+/+nk	Total
N	30	9	9	48
NS	22	8	8	38
Wt	723.14	81.64	148.82	953.59
Het Chi	489.89	42.96	161.89	727.50
Het df	29	8	8	47
Het P	***	***	***	***
Fixed RR	15.20	11.21	9.35	13.72
RRl	14.13	9.02	7.96	12.88
RRu	16.34	13.92	10.98	14.62
P	+++	+++	+++	+++
Random RR	9.25	12.66	12.26	10.39
RRl	6.59	7.40	5.76	7.92
RRu	12.97	21.66	26.10	13.65
P	+++	+++	+++	+++
Between Chi				32.76
Between df				2
Between P				***
Btwn(F) P				N.S.
Btwn(R) P				N.S.

Table 1I19 - 3

IESLC - Meta-analysis of Ever/current Smoking, Duration, "High"
All LC types, Cigarettes (or Any Product if Cigarettes not available)
Most adjusted

	Number of adjustment variables (2) 0	1	2	3-5	6+/+nk	Total
N	30	9	2	6	1	48
NS	22	8	2	5	1	38
Wt	723.14	81.64	16.26	123.85	8.71	953.59
Het Chi	489.89	42.96	3.06	134.91	0.00	727.50
Het df	29	8	1	5	0	47
Het P	***	***	(*)	***	N.S.	***
Fixed RR	15.20	11.21	20.58	7.82	27.09	13.72
RRl	14.13	9.02	12.66	6.56	13.94	12.88
RRu	16.34	13.92	33.45	9.33	52.63	14.62
P	+++	+++	+++	+++	+++	+++
Random RR	9.25	12.66	19.95	9.28	27.09	10.39
RRl	6.59	7.40	8.48	3.55	13.94	7.92
RRu	12.97	21.66	46.90	24.28	52.63	13.65
P	+++	+++	+++	+++	+++	+++
Between Chi						56.67
Between df						4
Between P						***
Btwn(F) P						N.S.
Btwn(R) P						*

	Smoking status ever	current	Total
N	37	11	48
NS	29	8	37
Wt	549.82	403.77	953.59
Het Chi	357.66	104.31	727.50
Het df	36	10	47
Het P	***	***	***
Fixed RR	8.73	25.40	13.72
RRl	8.03	23.04	12.88
RRu	9.49	28.01	14.62
P	+++	+++	+++
Random RR	8.76	18.13	10.39
RRl	6.55	12.52	7.92
RRu	11.71	26.24	13.65
P	+++	+++	+++
Between Chi			265.53
Between df			1
Between P			***
Btwn(F) P			***
Btwn(R) P			**

	Product all/unsp	cig+/-ot	cig only	Total
N	15	24	9	48
NS	11	20	7	38
Wt	144.63	523.56	285.41	953.59
Het Chi	158.22	301.30	91.11	727.50
Het df	14	23	8	47
Het P	***	***	***	***
Fixed RR	9.34	10.68	26.42	13.72
RRl	7.93	9.80	23.53	12.88
RRu	10.99	11.64	29.67	14.62
P	+++	+++	+++	+++
Random RR	6.95	11.28	16.21	10.39
RRl	3.94	7.96	9.78	7.92
RRu	12.24	16.00	26.85	13.65
P	+++	+++	+++	+++
Between Chi				176.87
Between df				2
Between P				***
Btwn(F) P				**
Btwn(R) P				(*)

Table 1I19 - 3

IESLC - Meta-analysis of Ever/current Smoking, Duration, "High"
All LC types, Cigarettes (or Any Product if Cigarettes not available)
Most adjusted

	Denominator nev any	nev cigs	Total
N	29	19	48
NS	22	16	38
Wt	467.68	485.91	953.59
Het Chi	358.84	363.55	727.50
Het df	28	18	47
Het P	***	***	***
Fixed RR	14.79	12.77	13.72
RRl	13.51	11.69	12.88
RRu	16.19	13.96	14.62
P	+++	+++	+++
Random RR	9.46	11.95	10.39
RRl	6.59	7.64	7.92
RRu	13.59	18.67	13.65
P	+++	+++	+++
Between Chi			5.12
Between df			1
Between P			*
Btwn(F) P			N.S.
Btwn(R) P			N.S.

	Derivation of RR/CI Orig	StdCalc	Other	Total
N	10	30	8	48
NS	9	21	8	38
Wt	103.17	731.27	119.16	953.59
Het Chi	81.77	491.80	73.48	727.50
Het df	9	29	7	47
Het P	***	***	***	***
Fixed RR	15.84	15.24	6.37	13.72
RRl	13.06	14.18	5.32	12.88
RRu	19.21	16.39	7.62	14.62
P	+++	+++	+++	+++
Random RR	15.66	9.38	9.25	10.39
RRl	8.53	6.72	4.77	7.92
RRu	28.76	13.08	17.95	13.65
P	+++	+++	+++	+++
Between Chi				80.45
Between df				2
Between P				***
Btwn(F) P				(*)
Btwn(R) P				N.S.

Table 1I19 - 4

IESLC - Meta-analysis of Ever/current Smoking, Duration, "High"
All LC types, Cigarettes (or Any Product if Cigarettes not available)
Least adjusted

REF	NRR	X	SEX	AGEL	AGEH	RACE	YF	LC TYPE	LOC	START	ST	NLC	R	VB	P	H	AD	SM	PRODUCT	exL	exH	DENOM	De
ARMADA	503	x	m	0	0	all	-	all	Eu:wst	1986	CC	325	n	bl	n	y	0	ev	cig+/-ot	50	999	nev cigs	st
AUVINE	503	x	c	0	0	all	-	all	Eu:Sca	1986	CC	517	n	bl	y	n	0	ev	cig+/-ot	41	999	nev cigs	st
AXELSS	505	x	m	0	0	sca	-	all	Eu:Sca	1989	CC	436	n	bl	n	n	0	ev	all/unsp	50	999	nev any	st
AXELSS	514		f	0	0	sca	-	all	Eu:Sca	1989	CC	436	n	bl	n	n	0	ev	all/unsp	50	999	nev any	st
BARBON	504	x	m	0	0	all	-	all	Eu:wst	1979	CC	755	n	bl	y	y	0	ev	all/unsp	50	999	nev any	st
BEST	507		m	0	0	all	0	all	NAmer	1955	pr	381	n	V	n	n	1	cu	cig only	40	999	nev any	ot
BOUCOT	519		m	0	0	all	9	all	NAmer	1951	pr	121	n	bl	n	n	0	ev	cig+/-ot	40	999	nev any	ot
BUFFLE	528		f	0	0	w-hi	-	all	NAmer	1976	CC	943	n	bl	y	n	0	ev	cig+/-ot	41	999	nev cigs	or
CHEN2	505		m	0	0	all	-	all	As:Chi	1983	CC	193	n	ot	y	n	0	ev	all/unsp	41	999	nev any	st
CHEN2	513		f	0	0	all	-	all	As:Chi	1983	CC	193	n	ot	y	n	0	ev	all/unsp	41	999	nev any	st
CHOI	505		m	0	0	all	-	all	As:oth	1985	CC	375	n	bl	n	n	0	ev	cig+/-ot	50	999	nev cigs	st
CHOI	513		f	0	0	all	-	all	As:oth	1985	CC	375	n	bl	n	n	0	ev	cig+/-ot	40	999	nev cigs	st
CPSI	585		m	40	84	wh	0	all	NAmer	1959	pr	5138	n	bl	n	n	0	cu	cig only	50	54	nev cigs	st
CPSI	681		f	40	84	wh	0	all	NAmer	1959	pr	5138	n	bl	n	n	0	cu	cig only	50	54	nev cigs	st
CPSII	557		m	0	0	all	6	all	NAmer	1982	pr	3229	n	bl	n	n	0	cu	cig only	50	54	nev any	st
CPSII	623		f	0	0	all	6	all	NAmer	1982	pr	3229	n	bl	n	n	0	cu	cig+/-ot	50	54	nev cigs	st
DAMBER	509		m	0	0	all	-	all	Eu:Sca	1972	CC	579	n	bl	y	n	1	ev	all/unsp	41	50	nev any	ot
DESTEF	504	x	m	0	0	all	-	all	SCAmer	1988	CC	497	n	bl	n	y	0	ev	all/unsp	50	999	nev any	st
DOLL	518		m	0	0	all	-	all	Eu:UK	1948	CC	1465	n	V	n	n	0	ev	all/unsp	40	999	nev any	st
DOLL	525		f	0	0	all	-	all	Eu:UK	1948	CC	1465	n	V	n	n	0	ev	all/unsp	40	999	nev any	st
FAN	503		m	0	0	all	-	all	As:Chi	1990	CC	403	n	ot	y	n	0	ev	cig+/-ot	40	999	nev cigs	st
FAN	508		f	0	0	all	-	all	As:Chi	1990	CC	403	n	ot	y	n	0	ev	cig+/-ot	40	999	nev cigs	st
GER	515	x	c	0	0	all	-	all	As:oth	1990	CC	141	n	ot	y	n	0	ev	all/unsp	41	999	nev any	st
HU2	511		c	0	0	all	-	all	As:Chi	1977	CC	523	n	ot	y	n	0	ev	cig+/-ot	40	999	nev cigs	st
HUMBLE	520	x	c	0	0	wh	-	not alv	NAmer	1980	CC	521	n	bl	y	n	0	cu	cig+/-ot	50	59	nev cigs	st
JOLY	519		m	0	0	all	-	all	SCAmer	1978	CC	826	n	bl	n	n	0	ev	cig+/-ot	50	999	nev any	st
JOLY	505		f	0	0	all	-	all	SCAmer	1978	CC	826	n	bl	n	n	0	ev	cig+/-ot	50	999	nev any	st
JUSSAW	514		m	0	0	all	-	all	As:Ind	1964	CC	792	n	V	n	n	0	ev	cig only	40	999	nev any	st
KAISE2	597		m	0	0	all	9	all	NAmer	1979	pr	318	n	bl	n	n	1	cu	cig only	40	999	nev any	st
KAISE2	517		f	0	0	all	9	all	NAmer	1979	pr	318	n	bl	n	n	1	cu	cig only	40	999	nev any	st
KATSOU	504		f	0	0	all	-	all	Eu:bal	1987	CC	101	n	bl	n	n	0	cu	all/unsp	40	999	nev any	st
KHUDER	503		m	0	0	all	-	all	NAmer	1985	CC	482	n	bl	n	y	0	ev	cig+/-ot	50	999	nev cigs	st
KREUZE	507		m	55	69	all	-	all	Eu:Ger	1990	CC	2260	n	bl	n	n	3	ev	all/unsp	40	999	nev any	or
KREUZE	510		f	55	69	all	-	all	Eu:Ger	1990	CC	2260	n	bl	n	n	3	ev	all/unsp	40	999	nev any	or
LETOUR	508		c	0	0	all	-	all	NAmer	1983	CC	738	n	V	y	y	0	ev	cig+/-ot	41	999	nev cigs	st
LEVIN	502	x	m	0	0	all	-	all	NAmer	1938	CC	475	n	bl	n	n	0	ev	cig+/-ot	40	999	nev any	st
LUBIN	511		m	0	0	all	-	all	As:Chi	1984	CC	427	m	ot	y	n	0	ev	cig+/-ot	50	999	nev any	st
LUBIN2	534		m	0	0	all	-	all	Eu:mul	1976	CC	7804	n	bl	n	y	0	ev	cig+/-ot	50	999	nev any	st
LUBIN2	577		f	0	0	all	-	all	Eu:mul	1976	CC	7804	n	bl	n	y	0	ev	cig+/-ot	50	999	nev any	st
MATOS	518	x	m	0	0	all	-	all	SCAmer	1994	CC	200	n	bl	n	n	0	ev	cig+/-ot	40	70	nev any	st
MCCONN	505		c	0	0	all	-	all	Eu:UK	1946	CC	100	n	V	n	y	0	ev	all/unsp	40	999	nev any	st
NOTAN2	517		c	0	0	all	-	all	As:Ind	1963	CC	683	n	V	n	n	0	ev	cig only	41	999	nev any	st
PEZZO2	508		m	0	0	all	-	all	SCAmer	1992	CC	367	n	bl	n	y	0	cu	cig+/-ot	36	999	nev cigs	st
PEZZOT	536		m	0	0	all	-	all	SCAmer	1987	CC	215	n	bl	n	y	0	ev	cig only	41	999	nev cigs	st
QIAO2	513	x	m	0	0	all	0	all	As:Chi	1992	pr	241	m	ot	n	n	0	ev	all/unsp	42	999	nev any	st
RACHTA	513	x	f	0	0	all	-	all	Eu:est	1991	CC	118	n	bl	n	y	0	ev	cig+/-ot	41	999	nev cigs	st
SOBUE	549		m	0	0	all	-	q+s+l+a	As:Jap	1986	CC	1376	n	bl	n	y	0	cu	cig+/-ot	50	999	nev cigs	st
WUWILL	503	x	f	0	0	all	-	all	As:Chi	1985	CC	965	n	ot	n	n	0	ev	cig+/-ot	40	999	nev cigs	st

Cigarette type is all/unspec for all RRs

except for the following:

REF	NRR	CIGTYPE
JUSSAW	514	MC only
NOTAN2	517	MC only

Table 1I19 - 5

IESLC - Meta-analysis of Ever/current Smoking, Duration, "High"
All LC types, Cigarettes (or Any Product if Cigarettes not available)
Least adjusted

REF	NRR	SEX	AD	Number Exposed Case	Cont	Non-exposed Case	Cont	RR	95.00%CI
ARMADA	503	m	0	77	33	8	71	20.71	(8.97- 47.82)
AUVINE	503	c	0	230	57	44	229	21.00	(13.61- 32.41)
AXELSS	505	m	0	101	40	16	160	25.25	(13.43- 47.46)
AXELSS	514	f	0	20	10	18	154	17.11	(6.94- 42.19)
Subtotal AXELSS								22.22	(13.25- 37.27)
BARBON	504	m	0	366	235	22	188	13.31	(8.31- 21.32)
*BEST	507	m	1	137	-	7	-	14.20	(6.64- 30.35)
*BOUCOT	519	m	0	52	1563	0	805	54.09~	(3.34- 875.17)
BUFFLE	528	f	0	90	42	12	112	20.00	(9.94- 40.23)
CHEN2	505	m	0	62	40	9	33	5.68	(2.46- 13.13)
CHEN2	513	f	0	21	15	25	33	1.85	(0.80- 4.29)
Subtotal CHEN2								3.25	(1.80- 5.89)
CHOI	505	m	0	20	20	13	95	7.31	(3.13- 17.07)
CHOI	513	f	0	1	1	76	164	2.16	(0.13- 34.96)
Subtotal CHOI								6.59	(2.93- 14.84)
*CPSI	585	m	0	576	119633	196	926068	22.75	(19.35- 26.75)
*CPSI	681	f	0	16	14305	532	3877179	8.15	(4.96- 13.40)
Subtotal CPSI								20.61	(17.67- 24.05)
*CPSII	557	m	0	332	39260	124	742207	50.62	(41.19- 62.20)
*CPSII	623	f	0	122	29119	310	2091302	28.26	(22.93- 34.84)
Subtotal CPSII								37.99	(32.80- 44.00)
DAMBER	509	m	1	-	-	42	-	8.71	(5.84- 13.66)
DESTEF	504	m	0	178	108	27	163	9.95	(6.20- 15.96)
DOLL	518	m	0	558	491	7	61	9.90	(4.49- 21.85)
DOLL	525	f	0	6	3	40	59	2.95	(0.70- 12.49)
Subtotal DOLL								7.48	(3.74- 14.98)
FAN	503	m	0	143	241	36	236	3.89	(2.59- 5.84)
FAN	508	f	0	55	59	69	320	4.32	(2.76- 6.78)
Subtotal FAN								4.08	(3.02- 5.52)
GER	515	c	0	49	155	51	246	1.52	(0.98- 2.37)
HU2	511	c	0	194	113	121	213	3.02	(2.19- 4.17)
HUMBLE	520	c	0	90	55	28	285	16.66	(9.97- 27.82)
JOLY	519	m	0	250	253	12	218	17.95	(9.78- 32.93)
JOLY	505	f	0	57	20	52	283	15.51	(8.61- 27.95)
Subtotal JOLY								16.65	(10.91- 25.41)
JUSSAW	514	m	0	11	6	149	624	7.68	(2.79- 21.09)
*KAISE2	597	m	1	34	-	14	-	15.64	(8.31- 29.40)
*KAISE2	517	f	1	26	-	11	-	30.41	(14.39- 64.25)
Subtotal KAISE2								20.63	(12.73- 33.43)
KATSOU	504	f	0	17	4	48	67	5.93	(1.88- 18.75)
KHUDER	503	m	0	236	354	23	309	8.96	(5.69- 14.11)
KREUZE	507	m	3	-	-	-	-	54.50	(34.90- 85.20)
KREUZE	510	f	3	-	-	-	-	8.30	(4.70- 14.50)
Subtotal KREUZE								26.38	(18.59- 37.42)
LETOUR	508	c	0	374	141	24	224	24.76	(15.58- 39.35)
LEVIN	502	m	0	63	91	7	96	9.49	(4.13- 21.81)
LUBIN	511	m	0	59	86	8	72	6.17	(2.77- 13.77)
LUBIN2	534	m	0	1325	1484	190	2616	12.29	(10.42- 14.50)
LUBIN2	577	f	0	81	32	336	1188	8.95	(5.84- 13.71)
Subtotal LUBIN2								11.80	(10.12- 13.76)
MATOS	518	m	0	86	89	11	110	9.66	(4.86- 19.21)
MCCONN	505	c	0	16	40	9	23	1.02	(0.39- 2.68)
NOTAN2	517	c	0	5	5	107	201	1.88	(0.53- 6.63)
PEZZO2	508	m	0	173	126	6	117	26.77	(11.42- 62.76)
PEZZOT	536	m	0	110	101	4	116	31.58	(11.25- 88.71)
*QIAO2	513	m	0	170	2295	10	709	5.25	(2.79- 9.88)
RACHTA	513	f	0	24	1	33	98	71.27	(9.28- 547.53)
SOBUE	549	m	0	147	73	34	128	7.58	(4.73- 12.14)
WUWILL	503	f	0	223	114	417	601	2.82	(2.18- 3.65)
Partial Totals				6983	210913	3338	7648183		

*prospective study ~ With 0.5 adjustment for zero

Table 1I19 - 5

IESLC - Meta-analysis of Ever/current Smoking, Duration, "High"
All LC types, Cigarettes (or Any Product if Cigarettes not available)
Least adjusted

REF	NRR	SEX	AD	Ys	Ws	Qs	Ps
ARMADA	503	m	0	3.03	5.48	1.13	0.0000
AUVINE	503	c	0	3.04	20.41	4.48	0.0000
AXELSS	505	m	0	3.23	9.65	4.11	0.0000
AXELSS	514	f	0	2.84	4.72	0.33	0.0000
Subtotal AXELSS				3.10	14.36	4.44	
BARBON	504	m	0	2.59	17.31	0.00	0.0000
*BEST	507	m	1	2.65	6.65	0.04	0.0000
*BOUCOT	519	m	0	3.99	0.50	0.99	0.0050
BUFFLE	528	f	0	3.00	7.86	1.38	0.0000
CHEN2	505	m	0	1.74	5.48	3.85	0.0000
CHEN2	513	f	0	0.61	5.42	20.85	0.1529
Subtotal CHEN2				1.18	10.90	24.70	
CHOI	505	m	0	1.99	5.33	1.84	0.0000
CHOI	513	f	0	0.77	0.50	1.62	0.5883
Subtotal CHOI				1.89	5.83	3.46	
*CPSI	585	m	0	3.12	146.44	44.05	0.0000
*CPSI	681	f	0	2.10	15.55	3.55	0.0000
Subtotal CPSI				3.03	161.99	47.60	
*CPSII	557	m	0	3.92	90.50	164.51	0.0000
*CPSII	623	f	0	3.34	87.81	51.47	0.0000
Subtotal CPSII				3.64	178.31	215.97	
DAMBER	509	m	1	2.16	21.28	3.60	0.0000
DESTEF	504	m	0	2.30	17.23	1.34	0.0000
DOLL	518	m	0	2.29	6.13	0.49	0.0000
DOLL	525	f	0	1.08	1.85	4.12	0.1417
Subtotal DOLL				2.01	7.98	4.61	
FAN	503	m	0	1.36	23.17	34.36	0.0000
FAN	508	f	0	1.46	18.96	23.44	0.0000
Subtotal FAN				1.41	42.13	57.80	
GER	515	c	0	0.42	19.79	91.83	0.0605
HU2	511	c	0	1.11	37.09	80.15	0.0000
HUMBLE	520	c	0	2.81	14.60	0.82	0.0000
JOLY	519	m	0	2.89	10.43	1.01	0.0000
JOLY	505	f	0	2.74	11.07	0.30	0.0000
Subtotal JOLY				2.81	21.50	1.32	
JUSSAW	514	m	0	2.04	3.76	1.09	0.0001
*KAISE2	597	m	1	2.75	9.62	0.29	0.0000
*KAISE2	517	f	1	3.41	6.86	4.83	0.0000
Subtotal KAISE2				3.03	16.49	5.12	
KATSOU	504	f	0	1.78	2.90	1.84	0.0024
KHUDER	503	m	0	2.19	18.60	2.74	0.0000
KREUZE	507	m	3	4.00	19.29	39.01	0.0000
KREUZE	510	f	3	2.12	12.11	2.56	0.0000
Subtotal KREUZE				3.27	31.40	41.57	
LETOUR	508	c	0	3.21	17.89	7.17	0.0000
LEVIN	502	m	0	2.25	5.55	0.59	0.0000
LUBIN	511	m	0	1.82	5.97	3.41	0.0000
LUBIN2	534	m	0	2.51	141.36	0.63	0.0000
LUBIN2	577	f	0	2.19	21.09	3.12	0.0000
Subtotal LUBIN2				2.47	162.45	3.75	
MATOS	518	m	0	2.27	8.14	0.77	0.0000
MCCONN	505	c	0	0.02	4.13	26.95	0.9644
NOTAN2	517	c	0	0.63	2.41	9.14	0.3273
PEZZO2	508	m	0	3.29	5.29	2.68	0.0000
PEZZOT	536	m	0	3.45	3.60	2.77	0.0000
*QIAO2	513	m	0	1.66	9.61	8.09	0.0000
RACHTA	513	f	0	4.27	0.92	2.64	0.0000
SOBUE	549	m	0	2.03	17.32	5.25	0.0000
WUWILL	503	f	0	1.04	57.74	136.86	0.0000

Table 1I19 - 5

IESLC - Meta-analysis of Ever/current Smoking, Duration, "High"
All LC types, Cigarettes (or Any Product if Cigarettes not available)
Least adjusted

	N	48
	NS	37
	Wt	985.39
Het	Chi	808.08
Het	df	47
Het	P	***
Fixed	RR	13.14
	RRl	12.35
	RRu	13.99
	P	+++
Random	RR	10.23
	RRl	7.73
	RRu	13.55
	P	+++
Asymm	P	(*)

Table 1I19 - 6

IESLC - Meta-analysis of Ever/current Smoking, Duration, "High"
All LC types, Cigarettes (or Any Product if Cigarettes not available)
Least adjusted

		Sex		
	combined	male	female	Total
N	7	26	15	48
NS	7	26	15	48
Wt	116.32	613.71	255.36	985.39
Het Chi	153.88	289.60	241.46	808.08
Het df	6	25	14	47
Het P	***	***	***	***
Fixed RR	6.17	16.92	10.12	13.14
RRl	5.14	15.63	8.95	12.35
RRu	7.39	18.31	11.44	13.99
P	+++	+++	+++	+++
Random RR	5.23	13.01	9.05	10.23
RRl	1.98	9.60	5.05	7.73
RRu	13.79	17.64	16.21	13.55
P	+++	+++	+++	+++
Between Chi				123.15
Between df				2
Between P				***
Btwn(F) P				*
Btwn(R) P				N.S.

Table 1I19 - 7

IESLC - Meta-analysis of Ever/current Smoking, Duration, "High"
All LC types, Cigarettes (or Any Product if Cigarettes not available)
Excluded studies (and stage at which they were excluded)

1	BECHER BLOT1 BROWN3 CARPEN CHYOU DARBY DOLL2 GARCIA GRAHAM GURSEL HAMMO2 JAHN JAIN LAUSSM PRESCO QIAO TVERDA WIGLE WYNDE3
2	ALDERS BENSHL BRESLO CHIAZZ DEAN3 DORN ENGELA GAO2 GILLIS GUO HEGMAN HIRAYA HOLE KAUFMA KOO KOULUM LIU4 MIGRAN MRFITR PERNU SEGI2 SPEIZE SUZUK2 SVENSS VUTUC WAKAI WU YUAN
3	GENG MCDUFF SPITZ STASZE WU2 ZHANG
4	BOUCHA CHEN CORREA JEDRYC LUO WYNDE2 WYNDE6
5	AKIBA HAMMON PISANI RESTRE SADOWS XU
7	BOFFET BROSS WYNDE7
10	AMES WATSON WYNDE8
14	AGUDO AMANDU CEDERL DEAN2 DORGAN DOSEME GAO GARSHI HAENSZ HU LIAW LIU3 LIU5 OSANN2 TIZZAN WANG2 ZHENG ZHOU
15	BENHAM

Table 1I19 - 8

Potentially overlapping studies

REF	REFGP	PRINC	.	OVERLAP/LINK
LUBIN2	LUBIN2	1		Lubin-combined
CPSI	CPSI	1		CPSI overall
LUBIN	XIANGZ	2		LUBIN/XIANGZ/QIAO

Table 1I19 - 9

Most adjusted - insufficient data for meta-analysis

REF	NRR	SEX	AGEL	AGEH	RACE	YF	LC TYPE	LOC	START	ST	NLC	R	VB	P	H	AD	SM	PRODUCT	exL	exH	DENOM	De
BUFFLE	504	m	0	0	wh	-	all	NAmer	1976	CC	943	n	bl	y	n	0	ev	cig+/-ot	50	999	nev cigs	or
SADOWS	527	m	0	0	wh	-	all	NAmer	1938	CC	477	n	bl	n	n	0	ev	cig only	50	999	nev any	ot
XU	503	m	0	0	all	-	all	As:Chi	1985	CC	729	n	ot	n	n	2	ev	all/unsp	40	999	nev any	or

REF	NRR	RR	SIG	RRDATA comment
BUFFLE	504	14.50		0
SADOWS	527	8.43		0
XU	503	*		RR for 1-19/day is 3.3(p<0.05), for 20-29/day is 6.0(p<0.05) and for >=30/ day is 17.1(p<0.05)

Table 1I20 -

IESLC - Meta-analysis of Ever/current Smoking, Duration, "Highest vs lowest"
All LC types, Cigarettes (or Any Product if Cigarettes not available)

This analysis is restricted to results for:
1) Ever/current smokers
2) Results by Duration
3) Categorical results by Duration
4) Denominator (unexposed) = "low"
5) All LC types (or near equivalent)
6) Results complete enough for use in metaanalysis

Within each study, results are then selected (in the following order of preference, within each sex) for:
7) SMKSTA: ever, current
8) PRODUCT: cigarettes regardless of other products, cigarettes only, all/unspec
9) CIGTYPE: all/unspecified, MC regardless of HR, MC only
10) Results with least adjustment for other aspects of smoking (ADOS)
11) The highest vs lowest category
12) Followup period (YF, prospective studies): whole study (coded as 0) or longest available
13) LCtype: all or nearest available, at least Squamous and Adeno. (q = squamous, s = small, l = large, a = adeno, mix = mixed, alv = alveolar)
14) Race: all or nearest available, otherwise by race (wh or w = white, bl or b = black, hi = hispanic ch = chinese, jap = japanese, haw = hawaiian, w+o = white + oriental, sca = scandinavian, as = asian)
15) For overlapping studies: principal rather than subsidiary studies
Finally by Age: whole study (coded as 0) if available, otherwise by widest available age group and then for single sex results (m, f) in preference to results for both sexes combined (c).

Results adjusted (AD) for the most potential confounders are then chosen in Sections -1 to -3 (and those which actually differ from the adjusted results in Table 1I15 - 1 are marked 'x' in Section -1) and results adjusted for the least confounders in Sections -4 to -6. (Those least adjusted results which actually differ from the most adjusted are marked 'x' in column X in Section -4)

Section -7 shows excluded studies, together with the stage (as above) at which no qualifying results were found.

Section -8 lists the potentially overlapping studies which have been included (1=principal, 2=subsidiary).

Section -9 lists any results which would have been included in preference except that they had data not complete enough for use in meta-analysis, with their significance (yes/no), if known, and any further comment as entered on the database. It also lists as "gap" any categories for which no data were presented by the original authors.

In addition to those mentioned above, the following fields, levels and abbreviations are used:
* or nk = not known, n = no, y = yes, ot = other
all/unspec = all or unspecified, cig+/-ot = cigarettes irrespective of other products (cigar, pipe etc)
MC = manufactured cigarettes, HR = hand-rolled cigarettes
exL, exH = range of exposure (low and high) in the "highest" group, in terms of Duration
unexL, unexH = range of exposure (low and high) in the "lowest" group, in terms of Duration
REF: 6-character study reference
NRR: number of the RR on the database within the study
ST : study type (CC = case control, pr or prosp = prospective)
NLC: number of lung cancer cases in whole study
R : risky occupational population (n = no, m = mining, o = other risky)
VB : national cigarette type (V = at least 75% Virginia, bl = at least 75% blended, ot = other)
P : any proxy use
H : full histological confirmation
De : derivation of RR/CI (or = original, st = standard method, ot = other method of estimation)

Table 1I20 - 1

IESLC - Meta-analysis of Ever/current Smoking, Duration, "Highest vs lowest"
All LC types, Cigarettes (or Any Product if Cigarettes not available)
Most adjusted

REF	NRR	1I15	SEX	AGEL	AGEH	RACE	YF	LC TYPE	LOC	START	ST	NLC	R	VB	P	H	AD	ADOS	SM	PRODUCT	exL	exH	unexL	unexH	De
AGUDO	512		f	0	0	all	-	all	Eu:wst	1989	CC	103	n	bl	n	n	3	0	ev	cig only	17	999	1	16	ot
AMANDU	508		m	0	0	wh	0	all	NAmer	1959	pr	132	m	bl	n	n	2	0	cu	cig+/-ot	25	999	1	24	ot
AMES	501		m	0	0	wh	-	all	NAmer	1959	ot	317	m	bl	n	n	2	0	cu	all/unsp	30	999	1	29	or
ARMADA	510		m	0	0	all	-	all	Eu:wst	1986	CC	325	n	bl	n	y	1	0	ev	cig+/-ot	50	999	1	24	ot
AUVINE	523		c	0	0	all	-	all	Eu:Sca	1986	CC	517	n	bl	y	n	2	0	ev	cig+/-ot	41	999	1	20	ot
AXELSS	509		m	0	0	sca	-	all	Eu:Sca	1989	CC	436	n	bl	n	n	0	0	ev	all/unsp	50	999	1	19	st
AXELSS	518		f	0	0	sca	-	all	Eu:Sca	1989	CC	436	n	bl	n	n	0	0	ev	all/unsp	50	999	1	19	st
BARBON	514		m	0	0	all	-	all	Eu:wst	1979	CC	755	n	bl	y	y	1	0	ev	all/unsp	50	999	1	29	ot
BEST	513		m	0	0	all	0	all	NAmer	1955	pr	381	n	V	n	n	1	0	cu	cig only	40	999	1	4	ot
BOUCOT	520		m	0	0	all	9	all	NAmer	1951	pr	121	n	bl	n	n	0	0	ev	cig+/-ot	40	999	1	39	st
BUFFLE	530		f	0	0	w-hi	-	all	NAmer	1976	CC	943	n	bl	y	n	0	0	ev	cig+/-ot	41	999	1	30	st
CEDERL	503		m	40	69	all	10	all	Eu:Sca	1963	pr	491	n	bl	n	n	1	0	cu	cig only	30	999	1	29	ot
CEDERL	506		f	40	69	all	10	all	Eu:Sca	1963	pr	491	n	bl	n	n	1	0	cu	cig only	30	999	1	29	ot
CHEN2	509		m	0	0	all	-	all	As:Chi	1983	CC	193	n	ot	y	n	0	0	ev	all/unsp	41	999	1	9	st
CHEN2	516		f	0	0	all	-	all	As:Chi	1983	CC	193	n	ot	y	n	0	0	ev	all/unsp	41	999	1	20	st
CHOI	509		m	0	0	all	-	all	As:oth	1985	CC	375	n	bl	n	n	0	0	ev	cig+/-ot	50	999	1	19	st
CHOI	516		f	0	0	all	-	all	As:oth	1985	CC	375	n	bl	n	n	0	0	ev	cig+/-ot	40	999	1	19	st
CPSI	602		m	40	84	wh	0	all	NAmer	1959	pr	5138	n	bl	n	n	0	0	cu	cig only	60	999	1	29	st
CPSI	695		f	40	84	wh	0	all	NAmer	1959	pr	5138	n	bl	n	n	0	0	cu	cig only	55	999	1	29	st
CPSII	566		m	0	0	all	6	all	NAmer	1982	pr	3229	n	bl	n	n	0	0	cu	cig only	60	999	1	29	st
CPSII	632		f	0	0	all	6	all	NAmer	1982	pr	3229	n	bl	n	n	0	0	cu	cig+/-ot	60	999	1	29	st
DAMBER	514		m	0	0	all	-	all	Eu:Sca	1972	CC	579	n	bl	y	n	1	0	ev	all/unsp	51	999	1	20	ot
DEAN2	503		m	0	0	all	-	all	Eu:UK	1960	CC	954	n	V	y	n	0	0	cu	all/unsp	20	999	1	19	st
DEAN2	506		f	0	0	all	-	all	Eu:UK	1960	CC	954	n	V	y	n	0	0	cu	all/unsp	20	999	1	19	st
DESTEF	537	x	m	0	0	all	-	all	SCAmer	1988	CC	497	n	bl	n	y	0	0	ev	cig+/-ot	53	999	1	31	st
DOLL	521		m	0	0	all	-	all	Eu:UK	1948	CC	1465	n	V	n	n	0	0	ev	all/unsp	40	999	1	9	st
DOLL	528		f	0	0	all	-	all	Eu:UK	1948	CC	1465	n	V	n	n	0	0	ev	all/unsp	40	999	1	9	st
DORGAN	526		m	0	0	wh	-	all	NAmer	1980	CC	2026	n	bl	y	y	2	0	ev	cig+/-ot	35	999	1	34	ot
DORGAN	522		f	0	0	all	-	all	NAmer	1980	CC	2026	n	bl	y	y	3	0	ev	cig+/-ot	35	999	1	34	ot
DOSEME	505		m	0	0	all	-	all	Eu:bal	1979	CC	1210	n	bl	n	n	2	0	ev	cig+/-ot	21	999	1	10	ot
FAN	505		m	0	0	all	-	all	As:Chi	1990	CC	403	n	ot	y	n	0	0	ev	cig+/-ot	40	999	1	29	st
FAN	510		f	0	0	all	-	all	As:Chi	1990	CC	403	n	ot	y	n	0	0	ev	cig+/-ot	40	999	1	29	st
GAO	566		f	0	0	all	-	all	As:Chi	1984	CC	1405	n	ot	n	n	2	0	ev	cig+/-ot	30	999	1	29	ot
GER	522		c	0	0	all	-	all	As:oth	1990	CC	141	n	ot	y	n	5	0	ev	all/unsp	41	999	1	20	ot
HAENSZ	554		f	0	0	all	-	not alv	NAmer	1955	CC	158	n	bl	n	y	1	0	ev	cig+/-ot	15	999	1	14	ot
HU	505		m	0	0	all	-	all	As:Chi	1985	CC	227	n	ot	n	y	0	0	ev	cig+/-ot	30	999	1	19	st
HU	510		f	0	0	all	-	all	As:Chi	1985	CC	227	n	ot	n	y	0	0	ev	cig+/-ot	30	999	1	19	st
HU2	514		c	0	0	all	-	all	As:Chi	1977	CC	523	n	ot	y	n	0	0	ev	cig+/-ot	40	999	1	19	st
HUMBLE	525		c	0	0	wh	-	not alv	NAmer	1980	CC	521	n	bl	y	n	0	0	cu	cig+/-ot	60	999	1	29	st
JOLY	523		m	0	0	all	-	all	SCAmer	1978	CC	826	n	bl	n	n	0	0	ev	cig+/-ot	50	999	1	19	st
JOLY	509		f	0	0	all	-	all	SCAmer	1978	CC	826	n	bl	n	n	0	0	ev	cig+/-ot	50	999	1	19	st
JUSSAW	518		m	0	0	all	-	all	As:Ind	1964	CC	792	n	V	n	n	0	0	ev	cig only	40	999	1	9	st
KAISE2	600		m	0	0	all	9	all	NAmer	1979	pr	318	n	bl	n	n	1	0	cu	cig only	40	999	1	39	st
KAISE2	520		f	0	0	all	9	all	NAmer	1979	pr	318	n	bl	n	n	1	0	cu	cig only	40	999	1	39	st
KATSOU	507		f	0	0	all	-	all	Eu:bal	1987	CC	101	n	bl	n	n	0	0	cu	all/unsp	40	999	1	19	st
KHUDER	505		m	0	0	all	-	all	NAmer	1985	CC	482	n	bl	n	y	0	0	ev	cig+/-ot	50	999	1	29	st
KREUZE	514		m	55	69	all	-	all	Eu:Ger	1990	CC	2260	n	bl	n	n	3	0	ev	all/unsp	40	999	1	19	ot
KREUZE	516		f	55	69	all	-	all	Eu:Ger	1990	CC	2260	n	bl	n	n	3	0	ev	all/unsp	40	999	1	19	ot
LETOUR	510		c	0	0	all	-	all	NAmer	1983	CC	738	n	V	y	y	0	0	ev	cig+/-ot	41	999	1	24	st
LEVIN	508		m	0	0	all	-	all	NAmer	1938	CC	475	n	bl	n	n	1	0	ev	cig+/-ot	40	999	1	39	ot
LIAW	508		c	0	0	all	0	all	As:oth	1982	pr	127	n	ot	n	n	2	0	cu	all/unsp	31	999	1	20	ot
LIU3	512		m	0	0	all	-	all	As:Chi	1985	CC	110	n	ot	n	n	2	0	ev	all/unsp	35	999	1	34	ot
LIU5	506		c	0	0	all	-	all	As:Chi	1978	CC	111	n	ot	y	n	0	0	ev	all/unsp	30	999	1	29	st
LUBIN	507		m	0	0	all	-	all	As:Chi	1984	CC	427	m	ot	y	n	0	0	ev	cig+/-ot	50	999	1	29	st
LUBIN2	537		m	0	0	all	-	all	Eu:mul	1976	CC	7804	n	bl	n	y	0	0	ev	cig+/-ot	50	999	1	29	st
LUBIN2	580		f	0	0	all	-	all	Eu:mul	1976	CC	7804	n	bl	n	y	0	0	ev	cig+/-ot	50	999	1	29	st
MATOS	540		m	0	0	all	-	all	SCAmer	1994	CC	200	n	bl	n	n	2	0	ev	cig+/-ot	40	70	1	24	ot
MCCONN	509		c	0	0	all	-	all	Eu:UK	1946	CC	100	n	V	n	y	0	0	ev	all/unsp	40	999	1	9	st
NOTAN2	521		c	0	0	all	-	all	As:Ind	1963	CC	683	n	V	n	n	0	0	ev	cig only	41	999	1	10	st
OSANN2	506		f	0	0	all	-	all	NAmer	1964	ot	217	n	bl	n	y	1	0	ev	cig+/-ot	21	999	1	20	ot
PEZZO2	509		m	0	0	all	-	all	SCAmer	1992	CC	367	n	bl	n	y	0	0	cu	cig+/-ot	36	999	1	35	st
PEZZOT	540		m	0	0	all	-	all	SCAmer	1987	CC	215	n	bl	n	y	2	0	ev	cig only	41	999	1	30	ot
QIAO2	520		m	0	0	all	0	all	As:Chi	1992	pr	241	m	ot	n	n	1	0	ev	all/unsp	42	999	1	27	ot
RACHTA	520		f	0	0	all	-	all	Eu:est	1991	CC	118	n	bl	n	y	1	0	ev	cig+/-ot	41	999	1	20	ot
SOBUE	552		m	0	0	all	-	q+s+l+a	As:Jap	1986	CC	1376	n	bl	n	y	0	0	cu	cig+/-ot	50	999	1	29	st
TIZZAN	505		m	0	0	all	-	all	Eu:wst	1959	CC	1358	n	bl	n	n	0	0	ev	cig only	11	999	1	4	st
TIZZAN	532	x	f	0	0	all	-	all	Eu:wst	1959	CC	1358	n	bl	n	n	0	0	ev	cig only	11	999	1	10	st
WANG2	509		c	0	0	all	-	all	As:Chi	1980	CC	103	n	ot	n	n	0	0	ev	cig+/-ot	40	49	1	19	st
WATSON	503		m	0	0	all	-	all	NAmer	1950	CC	301	n	bl	n	y	0	0	cu	all/unsp	20	999	1	19	st
WATSON	506		f	0	0	all	-	all	NAmer	1950	CC	301	n	bl	n	y	0	0	cu	all/unsp	20	999	1	19	st
WUWILL	505		f	0	0	all	-	all	As:Chi	1985	CC	965	n	ot	n	n	0	0	ev	cig+/-ot	40	999	1	29	st
WYNDE8	502		m	0	0	all	-	all	NAmer	1985	CC	1044	n	bl	n	y	0	0	cu	cig+/-ot	41	999	1	30	st

Table 1I20 - 1

IESLC - Meta-analysis of Ever/current Smoking, Duration, "Highest vs lowest"
All LC types, Cigarettes (or Any Product if Cigarettes not available)
Most adjusted

REF	NRR	1I15	SEX	AGEL	AGEH	RACE	YF	LC TYPE	LOC	START	ST	NLC	R	VB	P	H	AD	ADOS	SM	PRODUCT	exL	exH	unexL	unexH	De
WYNDE8	504		f	0	0	all	-	all	NAmer	1985	CC	1044	n	bl	n	y	0	0	cu	cig+/-ot	41	999	1	30	st
ZHENG	556		m	0	0	all	-	all	As:Chi	1982	CC	540	n	ot	*	y	1	0	ev	cig+/-ot	30	999	1	29	ot
ZHENG	561		f	0	0	all	-	all	As:Chi	1982	CC	540	n	ot	*	y	1	0	ev	cig+/-ot	30	999	1	29	ot
ZHOU	503		c	0	0	all	-	all	As:Chi	1978	CC	1360	n	ot	n	n	0	0	ev	all/unsp	20	999	1	19	st

Cigarette type is all/unspec for all RRs

except for the following:

REF	NRR	CIGTYPE
DESTEF	537	MC only
JUSSAW	518	MC only
NOTAN2	521	MC only

Table 1I20 - 2

IESLC - Meta-analysis of Ever/current Smoking, Duration, "Highest vs lowest"
All LC types, Cigarettes (or Any Product if Cigarettes not available)
Most adjusted

REF	NRR	SEX	AD	Number Exposed Case	Cont	Non-exposed Case	Cont	RR	95.00%CI
AGUDO	512	f	3	18	-	5	-	3.95	(0.93- 16.76)
*AMANDU	508	m	2	72	-	42	-	1.19	(0.76- 1.86)
AMES	501	m	2	-	-	-	-	2.28	(1.58- 3.29)
ARMADA	510	m	1	77	-	21	-	10.31	(4.81- 22.07)
AUVINE	523	c	2	230	-	26	-	1.51	(0.51- 4.50)
AXELSS	509	m	0	101	40	13	84	16.32	(8.19- 32.51)
AXELSS	518	f	0	20	10	5	24	9.60	(2.82- 32.73)
Subtotal AXELSS								14.36	(7.88- 26.20)
BARBON	514	m	1	366	-	42	-	4.53	(3.02- 6.80)
*BEST	513	m	1	137	-	1	-	8.88	(1.25- 62.98)
*BOUCOT	520	m	0	52	1563	29	2621	3.01	(1.92- 4.72)
BUFFLE	530	f	0	90	42	52	57	2.35	(1.39- 3.97)
*CEDERL	503	m	1	23	-	5	-	4.11	(1.48- 11.44)
*CEDERL	506	f	1	5	-	3	-	6.00	(1.44- 24.93)
Subtotal CEDERL								4.67	(2.04- 10.73)
CHEN2	509	m	0	62	40	2	3	2.33	(0.37- 14.53)
CHEN2	516	f	0	21	15	1	6	8.40	(0.91- 77.21)
Subtotal CHEN2								3.92	(0.95- 16.08)
CHOI	509	m	0	20	20	19	55	2.89	(1.29- 6.51)
CHOI	516	f	0	1	1	2	9	4.50	(0.19- 106.82)
Subtotal CHOI								2.97	(1.36- 6.52)
*CPSI	602	m	0	232	26906	95	266163	24.16	(19.03- 30.66)
*CPSI	695	f	0	10	5657	105	694015	11.68	(6.11- 22.34)
Subtotal CPSI								22.15	(17.71- 27.71)
*CPSII	566	m	0	117	8450	72	141932	27.29	(20.37- 36.58)
*CPSII	632	f	0	18	2224	127	301244	19.20	(11.74- 31.40)
Subtotal CPSII								24.89	(19.36- 32.01)
DAMBER	514	m	1	-	-	-	-	7.08	(3.19- 15.74)
DEAN2	503	m	0	631	558	34	36	1.20	(0.74- 1.94)
DEAN2	506	f	0	47	11	10	14	5.98	(2.11- 16.99)
Subtotal DEAN2								1.59	(1.03- 2.46)
DESTEF	537	m	0	25	18	25	96	5.33	(2.52- 11.28)
DOLL	521	m	0	558	491	12	15	1.42	(0.66- 3.06)
DOLL	528	f	0	6	3	14	18	2.57	(0.54- 12.14)
Subtotal DOLL								1.60	(0.80- 3.18)
DORGAN	526	m	2	-	-	-	-	2.96	(2.31- 3.78)
DORGAN	522	f	3	-	-	-	-	2.76	(2.18- 3.50)
Subtotal DORGAN								2.85	(2.41- 3.39)
DOSEME	505	m	2	466	-	32	-	4.90	(2.91- 8.24)
FAN	505	m	0	143	241	29	135	2.76	(1.76- 4.34)
FAN	510	f	0	55	59	8	15	1.75	(0.69- 4.45)
Subtotal FAN								2.53	(1.69- 3.80)
GAO	566	f	2	168	-	68	-	2.43	(1.54- 3.83)
GER	522	c	5	49	-	10	-	1.65	(0.69- 3.91)
HAENSZ	554	f	1	58	-	16	-	1.21	(0.59- 2.47)
HU	505	m	0	19	14	41	33	1.09	(0.48- 2.50)
HU	510	f	0	4	3	11	8	0.97	(0.17- 5.59)
Subtotal HU								1.07	(0.51- 2.26)
HU2	514	c	0	194	113	21	33	2.70	(1.49- 4.89)
HUMBLE	525	c	0	29	22	20	33	2.18	(0.99- 4.77)
JOLY	523	m	0	250	253	11	48	4.31	(2.19- 8.49)
JOLY	509	f	0	57	20	13	28	6.14	(2.67- 14.11)
Subtotal JOLY								4.96	(2.94- 8.40)
JUSSAW	518	m	0	11	6	16	20	2.29	(0.70- 7.55)
*KAISE2	600	m	1	34	-	17	-	5.38	(2.95- 9.81)
*KAISE2	520	f	1	26	-	24	-	3.13	(1.63- 6.00)
Subtotal KAISE2								4.19	(2.70- 6.52)
KATSOU	507	f	0	17	4	5	5	4.25	(0.82- 22.13)
KHUDER	505	m	0	236	354	16	61	2.54	(1.43- 4.52)
KREUZE	514	m	3	-	-	-	-	11.12	(8.68- 14.25)
KREUZE	516	f	3	-	-	-	-	9.22	(4.45- 19.09)
Subtotal KREUZE								10.91	(8.62- 13.79)
LETOUR	510	c	0	374	141	65	187	7.63	(5.42- 10.75)
LEVIN	508	m	1	63	-	56	-	1.27	(0.80- 2.00)
*LIAW	508	c	2	-	-	-	-	5.22	(1.75- 15.56)
LIU3	512	m	2	22	-	30	-	1.60	(0.68- 3.77)
LIU5	506	c	0	58	33	27	37	2.41	(1.25- 4.64)
LUBIN	507	m	0	59	86	30	146	3.34	(2.00- 5.58)
LUBIN2	537	m	0	1325	1484	953	2995	2.81	(2.53- 3.11)
LUBIN2	580	f	0	81	32	132	230	4.41	(2.78- 7.00)
Subtotal LUBIN2								2.87	(2.59- 3.17)

Table 1I20 - 2

IESLC - Meta-analysis of Ever/current Smoking, Duration, "Highest vs lowest"
All LC types, Cigarettes (or Any Product if Cigarettes not available)
Most adjusted

REF	NRR	SEX	AD	Number Case	Exposed Cont	Non-exposed Case	Non-exposed Cont	RR	95.00%CI
MATOS	540	m	2	86	-	20	-	5.48	(2.97- 10.09)
MCCONN	509	c	0	16	40	3	4	0.53	(0.11- 2.66)
NOTAN2	521	c	0	5	5	7	15	2.14	(0.46- 9.90)
OSANN2	506	f	1	161	-	23	-	7.25	(3.41- 15.43)
PEZZO2	509	m	0	173	126	60	72	1.65	(1.09- 2.49)
PEZZOT	540	m	2	110	-	30	-	7.00	(4.33- 11.30)
*QIAO2	520	m	1	170	-	7	-	5.13	(2.36- 11.13)
RACHTA	520	f	1	24	-	12	-	29.05	(3.42- 246.78)
SOBUE	552	m	0	147	73	62	119	3.87	(2.55- 5.86)
TIZZAN	505	m	0	928	815	12	1	0.09	(0.01- 0.73)
TIZZAN	532	f	0	23	21	2	7	3.83	(0.72- 20.55)
Subtotal TIZZAN								0.86	(0.24- 3.15)
WANG2	509	c	0	22	26	4	17	3.60	(1.05- 12.28)
WATSON	503	m	0	252	231	8	20	2.73	(1.18- 6.31)
WATSON	506	f	0	10	13	5	20	3.08	(0.85- 11.07)
Subtotal WATSON								2.83	(1.40- 5.71)
WUWILL	505	f	0	223	114	137	139	1.98	(1.43- 2.75)
WYNDE8	502	m	0	294	354	102	272	2.21	(1.68- 2.92)
WYNDE8	504	f	0	206	122	82	134	2.76	(1.94- 3.93)
Subtotal WYNDE8								2.41	(1.94- 2.99)
ZHENG	556	m	1	242	-	37	-	3.73	(2.39- 5.83)
ZHENG	561	f	1	59	-	17	-	3.16	(1.39- 7.16)
Subtotal ZHENG								3.59	(2.43- 5.31)
ZHOU	503	c	0	678	36	170	12	1.33	(0.68- 2.61)
Partial Totals				10566	50890	3213	1411238		

*prospective study

REF	NRR	SEX	AD	Ys	Ws	Qs	Ps
AGUDO	512	f	3	1.37	1.84	0.00	0.0626
*AMANDU	508	m	2	0.17	19.18	26.16	0.4461
AMES	501	m	2	0.82	28.56	7.65	0.0000
ARMADA	510	m	1	2.33	6.62	6.51	0.0000
AUVINE	523	c	2	0.41	3.24	2.80	0.4581
AXELSS	509	m	0	2.79	8.08	17.00	0.0000
AXELSS	518	f	0	2.26	2.55	2.16	0.0003
Subtotal AXELSS				2.66	10.64	19.16	
BARBON	514	m	1	1.51	23.32	0.67	0.0000
*BEST	513	m	1	2.18	1.00	0.71	0.0290
*BOUCOT	520	m	0	1.10	18.98	1.10	0.0000
BUFFLE	530	f	0	0.85	13.95	3.32	0.0014
*CEDERL	503	m	1	1.41	3.67	0.02	0.0067
*CEDERL	506	f	1	1.79	1.89	0.38	0.0138
Subtotal CEDERL				1.54	5.56	0.40	
CHEN2	509	m	0	0.84	1.14	0.28	0.3669
CHEN2	516	f	0	2.13	0.78	0.48	0.0601
Subtotal CHEN2				1.36	1.92	0.77	
CHOI	509	m	0	1.06	5.85	0.46	0.0101
CHOI	516	f	0	1.50	0.38	0.01	0.3520
Subtotal CHOI				1.09	6.24	0.47	
*CPSI	602	m	0	3.18	67.59	229.55	0.0000
*CPSI	695	f	0	2.46	9.15	11.40	0.0000
Subtotal CPSI				3.10	76.73	240.95	
*CPSII	566	m	0	3.31	44.82	173.07	0.0000
*CPSII	632	f	0	2.95	15.88	41.32	0.0000
Subtotal CPSII				3.21	60.70	214.38	
DAMBER	514	m	1	1.96	6.03	2.29	0.0000
DEAN2	503	m	0	0.18	16.51	22.28	0.4643
DEAN2	506	f	0	1.79	3.53	0.70	0.0008
Subtotal DEAN2				0.46	20.04	22.98	
DESTEF	537	m	0	1.67	6.85	0.76	0.0000
DOLL	521	m	0	0.35	6.50	6.38	0.3707
DOLL	528	f	0	0.94	1.59	0.25	0.2330
Subtotal DOLL				0.47	8.10	6.63	
DORGAN	526	m	2	1.09	63.36	4.17	0.0000
DORGAN	522	f	3	1.02	68.55	7.31	0.0000
Subtotal DORGAN				1.05	131.91	11.48	
DOSEME	505	m	2	1.59	14.18	0.87	0.0000
FAN	505	m	0	1.02	18.86	2.00	0.0000
FAN	510	f	0	0.56	4.41	2.71	0.2410
Subtotal FAN				0.93	23.27	4.71	

Table 1I20 - 2

IESLC - Meta-analysis of Ever/current Smoking, Duration, "Highest vs lowest"
All LC types, Cigarettes (or Any Product if Cigarettes not available)
Most adjusted

REF	NRR	SEX	AD	Ys	Ws	Qs	Ps
GAO	566	f	2	0.89	18.51	3.81	0.0001
GER	522	c	5	0.50	5.11	3.61	0.2578
HAENSZ	554	f	1	0.19	7.49	9.93	0.6018
HU	505	m	0	0.09	5.59	8.79	0.8345
HU	510	f	0	-0.03	1.25	2.36	0.9725
Subtotal HU				0.07	6.85	11.15	
HU2	514	c	0	0.99	10.88	1.33	0.0011
HUMBLE	525	c	0	0.78	6.24	1.99	0.0522
JOLY	523	m	0	1.46	8.35	0.12	0.0000
JOLY	509	f	0	1.81	5.55	1.24	0.0000
Subtotal JOLY				1.60	13.90	1.36	
JUSSAW	518	m	0	0.83	2.70	0.71	0.1728
*KAISE2	600	m	1	1.68	10.64	1.24	0.0000
*KAISE2	520	f	1	1.14	9.05	0.36	0.0006
Subtotal KAISE2				1.43	19.69	1.60	
KATSOU	507	f	0	1.45	1.41	0.02	0.0857
KHUDER	505	m	0	0.93	11.63	1.95	0.0015
KREUZE	514	m	3	2.41	62.53	71.19	0.0000
KREUZE	516	f	3	2.22	7.25	5.61	0.0000
Subtotal KREUZE				2.39	69.77	76.80	
LETOUR	510	c	0	2.03	32.79	15.63	0.0000
LEVIN	508	m	1	0.24	18.30	22.25	0.3065
*LIAW	508	c	2	1.65	3.22	0.31	0.0030
LIU3	512	m	2	0.47	5.24	3.98	0.2821
LIU5	506	c	0	0.88	8.96	1.92	0.0085
LUBIN	507	m	0	1.21	14.54	0.27	0.0000
LUBIN2	537	m	0	1.03	355.65	34.17	0.0000
LUBIN2	580	f	0	1.48	18.01	0.36	0.0000
Subtotal LUBIN2				1.05	373.66	34.53	
MATOS	540	m	2	1.70	10.27	1.33	0.0000
MCCONN	509	c	0	-0.63	1.49	5.79	0.4428
NOTAN2	521	c	0	0.76	1.64	0.55	0.3290
OSANN2	506	f	1	1.98	6.74	2.76	0.0000
PEZZO2	509	m	0	0.50	22.59	16.03	0.0176
PEZZOT	540	m	2	1.95	16.70	6.10	0.0000
*QIAO2	520	m	1	1.64	6.39	0.55	0.0000
RACHTA	520	f	1	3.37	0.84	3.45	0.0020
SOBUE	552	m	0	1.35	22.21	0.00	0.0000
TIZZAN	505	m	0	-2.36	0.92	12.59	0.0238
TIZZAN	532	f	0	1.34	1.36	0.00	0.1168
Subtotal TIZZAN				-0.15	2.28	12.59	
WANG2	509	c	0	1.28	2.55	0.01	0.0411
WATSON	503	m	0	1.00	5.46	0.62	0.0191
WATSON	506	f	0	1.12	2.34	0.11	0.0854
Subtotal WATSON				1.04	7.80	0.74	
WUWILL	505	f	0	0.69	36.04	15.52	0.0000
WYNDE8	502	m	0	0.80	50.74	15.16	0.0000
WYNDE8	504	f	0	1.01	30.57	3.26	0.0000
Subtotal WYNDE8				0.88	81.32	18.42	
ZHENG	556	m	1	1.32	19.32	0.01	0.0000
ZHENG	561	f	1	1.15	5.72	0.21	0.0059
Subtotal ZHENG				1.28	25.04	0.22	
ZHOU	503	c	0	0.28	8.44	9.43	0.4081

Table 1I20 - 2

IESLC - Meta-analysis of Ever/current Smoking, Duration, "Highest vs lowest"
All LC types, Cigarettes (or Any Product if Cigarettes not available)
Most adjusted

	N	76
	NS	57
	Wt	1372.09
Het	Chi	861.36
Het	df	75
Het	P	***
Fixed	RR	3.83
	RRl	3.63
	RRu	4.03
	P	+++
Random	RR	3.58
	RRl	2.92
	RRu	4.39
	P	+++
Asymm	P	N.S.

Table 1I20 - 3

IESLC - Meta-analysis of Ever/current Smoking, Duration, "Highest vs lowest"
All LC types, Cigarettes (or Any Product if Cigarettes not available)
Most adjusted

	Sex			
	combined	male	female	Total
N	11	38	27	76
NS	11	38	27	76
Wt	84.55	1010.90	276.64	1372.09
Het Chi	42.59	697.51	115.79	861.36
Het df	10	37	26	75
Het P	***	***	***	***
Fixed RR	3.48	3.97	3.43	3.83
RRl	2.81	3.74	3.05	3.63
RRu	4.30	4.23	3.86	4.03
P	+++	+++	+++	+++
Random RR	2.43	3.62	4.08	3.58
RRl	1.48	2.68	3.04	2.92
RRu	4.01	4.89	5.48	4.39
P	+++	+++	+++	+++
Between Chi				5.47
Between df				2
Between P				(*)
Btwn(F) P				N.S.
Btwn(R) P				N.S.

	Lung cancer type		
	all	other	Total
N	73	3	76
NS	54	3	57
Wt	1336.15	35.94	1372.09
Het Chi	849.33	7.97	861.36
Het df	72	2	75
Het P	***	*	***
Fixed RR	3.86	2.75	3.83
RRl	3.66	1.98	3.63
RRu	4.07	3.81	4.03
P	+++	+++	+++
Random RR	3.66	2.27	3.58
RRl	2.97	1.10	2.92
RRu	4.51	4.69	4.39
P	+++	+	+++
Between Chi			4.06
Between df			1
Between P			*
Btwn(F) P			N.S.
Btwn(R) P			N.S.

	Location								
	NAmer	UK	Scand	othEur	China	Japan	othAs	other	Total
N	23	5	6	12	17	1	6	6	76
NS	17	3	4	9	13	1	5	5	57
Wt	543.02	29.62	25.47	493.93	168.62	22.21	18.91	70.32	1372.09
Het Chi	557.20	9.66	14.74	134.44	21.52	0.00	2.91	25.56	861.36
Het df	22	4	5	11	16	0	5	5	75
Het P	***	*	*	***	N.S.	N.S.	N.S.	***	***
Fixed RR	4.72	1.51	7.14	3.66	2.47	3.87	2.61	3.86	3.83
RRl	4.34	1.05	4.84	3.35	2.13	2.55	1.67	3.06	3.63
RRu	5.13	2.16	10.52	4.00	2.88	5.86	4.10	4.88	4.03
P	+++	+	+++	+++	+++	+++	+++	+++	+++
Random RR	4.01	1.71	6.15	4.98	2.48	3.87	2.61	4.44	3.58
RRl	2.57	0.88	3.06	3.01	2.05	2.55	1.67	2.56	2.92
RRu	6.24	3.32	12.35	8.22	2.99	5.86	4.10	7.70	4.39
P	+++	N.S.	+++	+++	+++	+++	+++	+++	+++
Between Chi									95.32
Between df									7
Between P									***
Btwn(F) P									N.S.
Btwn(R) P									**

Table 1I20 - 3

IESLC - Meta-analysis of Ever/current Smoking, Duration, "Highest vs lowest"
All LC types, Cigarettes (or Any Product if Cigarettes not available)
Most adjusted

Detailed Country in "other Europe"

	multi	Germany	othWest	East	Balkans	Total
N	2	2	5	1	2	12
NS	1	1	4	1	2	9
Wt	373.66	69.77	34.07	0.84	15.59	493.93
Het Chi	3.51	0.23	18.26	0.00	0.03	134.44
Het df	1	1	4	0	1	11
Het P	(*)	N.S.	**	N.S.	N.S.	***
Fixed RR	2.87	10.91	4.72	29.05	4.84	3.66
RRl	2.59	8.62	3.37	3.42	2.94	3.35
RRu	3.17	13.79	6.60	246.77	7.95	4.00
P	+++	+++	+++	++	+++	+++
Random RR	3.32	10.91	3.20	29.05	4.84	4.98
RRl	2.16	8.62	1.19	3.42	2.94	3.01
RRu	5.09	13.79	8.57	246.77	7.95	8.22
P	+++	+++	+	++	+++	+++
Between Chi						112.42
Between df						4
Between P						***
Btwn(F) P						**
Btwn(R) P						***

Detailed Country in "other Asia"

	India	HongKong	other	Total
N	2		4	6
NS	2		3	5
Wt	4.34		14.56	18.91
Het Chi	0.00		2.76	2.91
Het df	1		3	5
Het P	N.S.		N.S.	N.S.
Fixed RR	2.23		2.74	2.61
RRl	0.87		1.64	1.67
RRu	5.72		4.58	4.10
P	(+)		+++	+++
Random RR	2.23		2.74	2.61
RRl	0.87		1.64	1.67
RRu	5.72		4.58	4.10
P	(+)		+++	+++
Between Chi				0.14
Between df				1
Between P				N.S.
Btwn(F) P				N.S.
Btwn(R) P				N.S.

Detailed other continent

	SCAmer	Total
N	6	6
NS	5	5
Wt	70.32	70.32
Het Chi	25.56	25.56
Het df	5	5
Het P	***	***
Fixed RR	3.86	3.86
RRl	3.06	3.06
RRu	4.88	4.88
P	+++	+++
Random RR	4.44	4.44
RRl	2.56	2.56
RRu	7.70	7.70
P	+++	+++
Between Chi		
Between df		
Between P		N.S.
Btwn(F) P		N.S.
Btwn(R) P		N.S.

Table 1I20 - 3

IESLC - Meta-analysis of Ever/current Smoking, Duration, "Highest vs lowest"
All LC types, Cigarettes (or Any Product if Cigarettes not available)
Most adjusted

	Start year of study					
	<1960	1960-69	1970-79	1980-89	1990+	Total
N	15	7	13	32	9	76
NS	11	5	10	24	7	57
Wt	189.92	36.69	493.02	514.23	138.23	1372.09
Het Chi	321.71	21.66	30.61	333.64	90.18	861.36
Het df	14	6	12	31	8	75
Het P	***	**	**	***	***	***
Fixed RR	4.96	2.58	3.03	4.08	5.39	3.83
RRl	4.30	1.86	2.78	3.74	4.56	3.63
RRu	5.72	3.56	3.31	4.44	6.37	4.03
P	+++	+++	+++	+++	+++	+++
Random RR	2.38	3.40	3.52	4.00	4.25	3.58
RRl	1.11	1.70	2.83	2.92	2.23	2.92
RRu	5.13	6.78	4.37	5.48	8.11	4.39
P	+	+++	+++	+++	+++	+++
Between Chi						63.57
Between df						4
Between P						***
Btwn(F) P						N.S.
Btwn(R) P						N.S.

	Study type (1)		
	CC	other	Total
N	61	15	76
NS	46	11	57
Wt	1125.33	246.76	1372.09
Het Chi	322.69	304.58	861.36
Het df	60	14	75
Het P	***	***	***
Fixed RR	3.15	9.24	3.83
RRl	2.97	8.16	3.63
RRu	3.34	10.47	4.03
P	+++	+++	+++
Random RR	3.12	6.25	3.58
RRl	2.65	3.34	2.92
RRu	3.68	11.73	4.39
P	+++	+++	+++
Between Chi			234.09
Between df			1
Between P			***
Btwn(F) P			***
Btwn(R) P			*

	Study type (2)			
	CC	prosp	other	Total
N	61	13	2	76
NS	46	9	2	57
Wt	1125.33	211.45	35.31	1372.09
Het Chi	322.69	240.04	7.30	861.36
Het df	60	12	1	75
Het P	***	***	**	***
Fixed RR	3.15	11.25	2.84	3.83
RRl	2.97	9.83	2.04	3.63
RRu	3.34	12.88	3.95	4.03
P	+++	+++	+++	+++
Random RR	3.12	6.74	3.87	3.58
RRl	2.65	3.47	1.25	2.92
RRu	3.68	13.08	11.98	4.39
P	+++	+++	+	+++
Between Chi				291.33
Between df				2
Between P				***
Btwn(F) P				***
Btwn(R) P				(*)

Table 1I20 - 3

IESLC - Meta-analysis of Ever/current Smoking, Duration, "Highest vs lowest"
All LC types, Cigarettes (or Any Product if Cigarettes not available)
Most adjusted

	Study size (number of LC cases)				
	100-249	250-499	500-999	1000+	Total
N	20	20	16	20	76
NS	18	14	13	12	57
Wt	125.18	183.29	195.81	867.81	1372.09
Het Chi	67.68	70.85	65.25	615.06	861.36
Het df	19	19	15	19	75
Het P	***	***	***	***	***
Fixed RR	2.84	2.95	3.31	4.36	3.83
RRl	2.38	2.55	2.88	4.08	3.63
RRu	3.38	3.41	3.81	4.66	4.03
P	+++	+++	+++	+++	+++
Random RR	2.86	3.56	3.24	4.44	3.58
RRl	1.98	2.62	2.36	2.88	2.92
RRu	4.13	4.83	4.45	6.85	4.39
P	+++	+++	+++	+++	+++
Between Chi					42.53
Between df					3
Between P					***
Btwn(F) P					N.S.
Btwn(R) P					N.S.

	Risky occupational population			
	no	mining	othRisky	Total
N	72	4		76
NS	53	4		57
Wt	1303.41	68.68		1372.09
Het Chi	825.67	14.38		861.36
Het df	71	3		75
Het P	***	**		***
Fixed RR	3.94	2.22		3.83
RRl	3.73	1.75		3.63
RRu	4.16	2.82		4.03
P	+++	+++		+++
Random RR	3.66	2.47		3.58
RRl	2.96	1.43		2.92
RRu	4.52	4.27		4.39
P	+++	++		+++
Between Chi				21.31
Between df				1
Between P				***
Btwn(F) P				N.S.
Btwn(R) P				N.S.

	National cigarette tobacco type			
	Virginia	blended	other	Total
N	9	48	19	76
NS	7	35	15	57
Wt	67.75	1127.39	176.95	1372.09
Het Chi	52.39	744.62	24.16	861.36
Het df	8	47	18	75
Het P	***	***	N.S.	***
Fixed RR	3.48	4.12	2.48	3.83
RRl	2.74	3.89	2.14	3.63
RRu	4.41	4.37	2.87	4.03
P	+++	+++	+++	+++
Random RR	2.56	4.32	2.48	3.58
RRl	1.21	3.32	2.07	2.92
RRu	5.39	5.62	2.99	4.39
P	+	+++	+++	+++
Between Chi				40.20
Between df				2
Between P				***
Btwn(F) P				N.S.
Btwn(R) P				**

Table 1I20 - 3

IESLC - Meta-analysis of Ever/current Smoking, Duration, "Highest vs lowest"
All LC types, Cigarettes (or Any Product if Cigarettes not available)
Most adjusted

	Any proxy use No/nk	Yes	Total
N	58	18	76
NS	43	14	57
Wt	1069.89	302.20	1372.09
Het Chi	783.90	60.60	861.36
Het df	57	17	75
Het P	***	***	***
Fixed RR	4.06	3.11	3.83
RRl	3.82	2.77	3.63
RRu	4.31	3.48	4.03
P	+++	+++	+++
Random RR	3.80	2.96	3.58
RRl	2.94	2.31	2.92
RRu	4.91	3.78	4.39
P	+++	+++	+++
Between Chi			16.87
Between df			1
Between P			***
Btwn(F) P			N.S.
Btwn(R) P			N.S.

	Full histological confirmation No	Yes	Total
N	52	24	76
NS	39	18	57
Wt	586.24	785.85	1372.09
Het Chi	659.83	104.73	861.36
Het df	51	23	75
Het P	***	***	***
Fixed RR	5.20	3.04	3.83
RRl	4.80	2.84	3.63
RRu	5.64	3.26	4.03
P	+++	+++	+++
Random RR	3.71	3.31	3.58
RRl	2.71	2.73	2.92
RRu	5.08	4.01	4.39
P	+++	+++	+++
Between Chi			96.81
Between df			1
Between P			***
Btwn(F) P			**
Btwn(R) P			N.S.

	Number of adjustment variables (1) 0	1	2+/+nk	Total
N	46	15	15	76
NS	32	12	13	57
Wt	917.32	127.04	327.73	1372.09
Het Chi	665.05	51.17	144.82	861.36
Het df	45	14	14	75
Het P	***	***	***	***
Fixed RR	3.86	3.69	3.77	3.83
RRl	3.62	3.10	3.38	3.63
RRu	4.12	4.39	4.20	4.03
P	+++	+++	+++	+++
Random RR	3.38	4.22	3.43	3.58
RRl	2.52	2.94	2.34	2.92
RRu	4.53	6.06	5.04	4.39
P	+++	+++	+++	+++
Between Chi				0.32
Between df				2
Between P				N.S.
Btwn(F) P				N.S.
Btwn(R) P				N.S.

Table 1I20 - 3

IESLC - Meta-analysis of Ever/current Smoking, Duration, "Highest vs lowest"
All LC types, Cigarettes (or Any Product if Cigarettes not available)
Most adjusted

	Number of adjustment variables (2)					
	0	1	2	3-5	6+/+nk	Total
N	46	15	10	5		76
NS	32	12	10	4		58
Wt	917.32	127.04	182.47	145.27		1372.09
Het Chi	665.05	51.17	42.85	72.85		861.36
Het df	45	14	9	4		75
Het P	***	***	***	***		***
Fixed RR	3.86	3.69	2.89	5.27		3.83
RRl	3.62	3.10	2.50	4.48		3.63
RRu	4.12	4.39	3.34	6.20		4.03
P	+++	+++	+++	+++		+++
Random RR	3.38	4.22	2.96	4.65		3.58
RRl	2.52	2.94	2.08	1.97		2.92
RRu	4.53	6.06	4.20	10.94		4.39
P	+++	+++	+++	+++		+++
Between Chi						29.44
Between df						3
Between P						***
Btwn(F) P						N.S.
Btwn(R) P						N.S.

	Smoking status		
	ever	current	Total
N	54	22	76
NS	42	15	57
Wt	995.84	376.25	1372.09
Het Chi	290.40	503.05	861.36
Het df	53	21	75
Het P	***	***	***
Fixed RR	3.34	5.49	3.83
RRl	3.14	4.97	3.63
RRu	3.55	6.08	4.03
P	+++	+++	+++
Random RR	3.35	4.38	3.58
RRl	2.80	2.59	2.92
RRu	4.00	7.43	4.39
P	+++	+++	+++
Between Chi			67.92
Between df			1
Between P			***
Btwn(F) P			*
Btwn(R) P			N.S.

	Product			
	all/unsp	cig+/-ot	cig only	Total
N	23	39	14	76
NS	17	31	10	58
Wt	216.43	982.69	172.97	1372.09
Het Chi	158.78	191.43	128.12	861.36
Het df	22	38	13	75
Het P	***	***	***	***
Fixed RR	4.42	2.93	14.55	3.83
RRl	3.87	2.75	12.53	3.63
RRu	5.05	3.12	16.88	4.03
P	+++	+++	+++	+++
Random RR	3.50	3.11	5.53	3.58
RRl	2.34	2.63	3.13	2.92
RRu	5.24	3.68	9.79	4.39
P	+++	+++	+++	+++
Between Chi				383.03
Between df				2
Between P				***
Btwn(F) P				***
Btwn(R) P				N.S.

Table 1I20 - 3

IESLC - Meta-analysis of Ever/current Smoking, Duration, "Highest vs lowest"
All LC types, Cigarettes (or Any Product if Cigarettes not available)
Most adjusted

	Derivation of RR/CI			
	Orig	StdCalc	Other	Total
N	1	48	27	76
NS	1	33	23	57
Wt	28.56	937.01	406.52	1372.09
Het Chi	0.00	666.62	186.93	861.36
Het df	0	47	26	75
Het P	N.S.	***	***	***
Fixed RR	2.28	3.87	3.86	3.83
RRl	1.58	3.63	3.50	3.63
RRu	3.29	4.13	4.26	4.03
P	+++	+++	+++	+++
Random RR	2.28	3.41	3.87	3.58
RRl	1.58	2.57	2.89	2.92
RRu	3.29	4.53	5.18	4.39
P	+++	+++	+++	+++
Between Chi				7.81
Between df				2
Between P				*
Btwn(F) P				N.S.
Btwn(R) P				(*)

Table 1I20 - 4

IESLC - Meta-analysis of Ever/current Smoking, Duration, "Highest vs lowest"
All LC types, Cigarettes (or Any Product if Cigarettes not available)
Least adjusted

REF	NRR	X	SEX	AGEL	AGEH	RACE	YF	LC TYPE	LOC	START	ST	NLC	R	VB	P	H	AD	ADOS	SM	PRODUCT	exL	exH	unexL	unexH	De
AGUDO	509	x	f	0	0	all	-	all	Eu:wst	1989	CC	103	n	bl	n	n	0	0	ev	cig only	17	999	1	16	st
AMANDU	503	x	m	0	0	wh	0	all	NAmer	1959	pr	132	m	bl	n	n	0	0	cu	cig+/-ot	25	999	1	24	st
AMES	501		m	0	0	wh	-	all	NAmer	1959	ot	317	m	bl	n	n	2	0	cu	all/unsp	30	999	1	29	or
ARMADA	505	x	m	0	0	all	-	all	Eu:wst	1986	CC	325	n	bl	n	y	0	0	ev	cig+/-ot	50	999	1	24	st
AUVINE	505	x	c	0	0	all	-	all	Eu:Sca	1986	CC	517	n	bl	y	n	0	0	ev	cig+/-ot	41	999	1	20	st
AXELSS	509		m	0	0	sca	-	all	Eu:Sca	1989	CC	436	n	bl	n	n	0	0	ev	all/unsp	50	999	1	19	st
AXELSS	518		f	0	0	sca	-	all	Eu:Sca	1989	CC	436	n	bl	n	n	0	0	ev	all/unsp	50	999	1	19	st
BARBON	507	x	m	0	0	all	-	all	Eu:wst	1979	CC	755	n	bl	y	y	0	0	ev	all/unsp	50	999	1	29	st
BEST	513		m	0	0	all	0	all	NAmer	1955	pr	381	n	V	n	n	1	0	cu	cig only	40	999	1	4	ot
BOUCOT	520		m	0	0	all	9	all	NAmer	1951	pr	121	n	bl	n	n	0	0	ev	cig+/-ot	40	999	1	39	st
BUFFLE	530		f	0	0	w-hi	-	all	NAmer	1976	CC	943	n	bl	y	n	0	0	ev	cig+/-ot	41	999	1	30	st
CEDERL	503		m	40	69	all	10	all	Eu:Sca	1963	pr	491	n	bl	n	n	1	0	cu	cig only	30	999	1	29	ot
CEDERL	506		f	40	69	all	10	all	Eu:Sca	1963	pr	491	n	bl	n	n	1	0	cu	cig only	30	999	1	29	ot
CHEN2	509		m	0	0	all	-	all	As:Chi	1983	CC	193	n	ot	y	n	0	0	ev	all/unsp	41	999	1	9	st
CHEN2	516		f	0	0	all	-	all	As:Chi	1983	CC	193	n	ot	y	n	0	0	ev	all/unsp	41	999	1	20	st
CHOI	509		m	0	0	all	-	all	As:oth	1985	CC	375	n	bl	n	n	0	0	ev	cig+/-ot	50	999	1	19	st
CHOI	516		f	0	0	all	-	all	As:oth	1985	CC	375	n	bl	n	n	0	0	ev	cig+/-ot	40	999	1	19	st
CPSI	602		m	40	84	wh	0	all	NAmer	1959	pr	5138	n	bl	n	n	0	0	cu	cig only	60	999	1	29	st
CPSI	695		f	40	84	wh	0	all	NAmer	1959	pr	5138	n	bl	n	n	0	0	cu	cig only	55	999	1	29	st
CPSII	566		m	0	0	all	6	all	NAmer	1982	pr	3229	n	bl	n	n	0	0	cu	cig only	60	999	1	29	st
CPSII	632		f	0	0	all	6	all	NAmer	1982	pr	3229	n	bl	n	n	0	0	cu	cig+/-ot	60	999	1	29	st
DAMBER	514		m	0	0	all	-	all	Eu:Sca	1972	CC	579	n	bl	y	n	1	0	ev	all/unsp	51	999	1	20	ot
DEAN2	503		m	0	0	all	-	all	Eu:UK	1960	CC	954	n	V	y	n	0	0	cu	all/unsp	20	999	1	19	st
DEAN2	506		f	0	0	all	-	all	Eu:UK	1960	CC	954	n	V	y	n	0	0	cu	all/unsp	20	999	1	19	st
DESTEF	537		m	0	0	all	-	all	SCAmer	1988	CC	497	n	bl	n	y	0	0	ev	cig+/-ot	53	999	1	31	st
DOLL	521		m	0	0	all	-	all	Eu:UK	1948	CC	1465	n	V	n	n	0	0	ev	all/unsp	40	999	1	9	st
DOLL	528		f	0	0	all	-	all	Eu:UK	1948	CC	1465	n	V	n	n	0	0	ev	all/unsp	40	999	1	9	st
DORGAN	526		m	0	0	wh	-	all	NAmer	1980	CC	2026	n	bl	y	y	2	0	ev	cig+/-ot	35	999	1	34	ot
DORGAN	522		f	0	0	all	-	all	NAmer	1980	CC	2026	n	bl	y	y	3	0	ev	cig+/-ot	35	999	1	34	ot
DOSEME	505		m	0	0	all	-	all	Eu:bal	1979	CC	1210	n	bl	n	n	2	0	ev	cig+/-ot	21	999	1	10	ot
FAN	505		m	0	0	all	-	all	As:Chi	1990	CC	403	n	ot	y	n	0	0	ev	cig+/-ot	40	999	1	29	st
FAN	510		f	0	0	all	-	all	As:Chi	1990	CC	403	n	ot	y	n	0	0	ev	cig+/-ot	40	999	1	29	st
GAO	563	x	f	0	0	all	-	all	As:Chi	1984	CC	1405	n	ot	n	n	0	0	ev	cig+/-ot	30	999	1	29	st
GER	517	x	c	0	0	all	-	all	As:oth	1990	CC	141	n	ot	y	n	0	0	ev	all/unsp	41	999	1	20	st
HAENSZ	546	x	f	0	0	all	-	not alv	NAmer	1955	CC	158	n	bl	n	y	0	0	ev	cig+/-ot	15	999	1	14	st
HU	505		m	0	0	all	-	all	As:Chi	1985	CC	227	n	ot	n	y	0	0	ev	cig+/-ot	30	999	1	19	st
HU	510		f	0	0	all	-	all	As:Chi	1985	CC	227	n	ot	n	y	0	0	ev	cig+/-ot	30	999	1	19	st
HU2	514		c	0	0	all	-	all	As:Chi	1977	CC	523	n	ot	y	n	0	0	ev	cig+/-ot	40	999	1	19	st
HUMBLE	525		c	0	0	wh	-	not alv	NAmer	1980	CC	521	n	bl	y	n	0	0	cu	cig+/-ot	60	999	1	29	st
JOLY	523		m	0	0	all	-	all	SCAmer	1978	CC	826	n	bl	n	n	0	0	ev	cig+/-ot	50	999	1	19	st
JOLY	509		f	0	0	all	-	all	SCAmer	1978	CC	826	n	bl	n	n	0	0	ev	cig+/-ot	50	999	1	19	st
JUSSAW	518		m	0	0	all	-	all	As:Ind	1964	CC	792	n	V	n	n	0	0	ev	cig only	40	999	1	9	st
KAISE2	600		m	0	0	all	9	all	NAmer	1979	pr	318	n	bl	n	n	1	0	cu	cig only	40	999	1	39	st
KAISE2	520		f	0	0	all	9	all	NAmer	1979	pr	318	n	bl	n	n	1	0	cu	cig only	40	999	1	39	st
KATSOU	507		f	0	0	all	-	all	Eu:bal	1987	CC	101	n	bl	n	n	0	0	cu	all/unsp	40	999	1	19	st
KHUDER	505		m	0	0	all	-	all	NAmer	1985	CC	482	n	bl	n	y	0	0	ev	cig+/-ot	50	999	1	29	st
KREUZE	514		m	55	69	all	-	all	Eu:Ger	1990	CC	2260	n	bl	n	n	3	0	ev	all/unsp	40	999	1	19	ot
KREUZE	516		f	55	69	all	-	all	Eu:Ger	1990	CC	2260	n	bl	n	n	3	0	ev	all/unsp	40	999	1	19	ot
LETOUR	510		c	0	0	all	-	all	NAmer	1983	CC	738	n	V	y	y	0	0	ev	cig+/-ot	41	999	1	24	st
LEVIN	503	x	m	0	0	all	-	all	NAmer	1938	CC	475	n	bl	n	n	0	0	ev	cig+/-ot	40	999	1	39	st
LIAW	508		c	0	0	all	0	all	As:oth	1982	pr	127	n	ot	n	n	2	0	cu	all/unsp	31	999	1	20	ot
LIU3	509	x	m	0	0	all	-	all	As:Chi	1985	CC	110	n	ot	n	n	0	0	ev	all/unsp	35	999	1	34	st
LIU5	506		c	0	0	all	-	all	As:Chi	1978	CC	111	n	ot	y	n	0	0	ev	all/unsp	30	999	1	29	st
LUBIN	507		m	0	0	all	-	all	As:Chi	1984	CC	427	m	ot	y	n	0	0	ev	cig+/-ot	50	999	1	29	st
LUBIN2	537		m	0	0	all	-	all	Eu:mul	1976	CC	7804	n	bl	n	y	0	0	ev	cig+/-ot	50	999	1	29	st
LUBIN2	580		f	0	0	all	-	all	Eu:mul	1976	CC	7804	n	bl	n	y	0	0	ev	cig+/-ot	50	999	1	29	st
MATOS	520	x	m	0	0	all	-	all	SCAmer	1994	CC	200	n	bl	n	n	0	0	ev	cig+/-ot	40	70	1	24	st
MCCONN	509		c	0	0	all	-	all	Eu:UK	1946	CC	100	n	V	n	y	0	0	ev	all/unsp	40	999	1	9	st
NOTAN2	521		c	0	0	all	-	all	As:Ind	1963	CC	683	n	V	n	n	0	0	ev	cig only	41	999	1	10	st
OSANN2	503	x	f	0	0	all	-	all	NAmer	1964	ot	217	n	bl	n	y	0	0	ev	cig+/-ot	21	999	1	20	st
PEZZO2	509		m	0	0	all	-	all	SCAmer	1992	CC	367	n	bl	n	y	0	0	cu	cig+/-ot	36	999	1	35	st
PEZZOT	538	x	m	0	0	all	-	all	SCAmer	1987	CC	215	n	bl	n	y	0	0	ev	cig only	41	999	1	30	st
QIAO2	515	x	m	0	0	all	0	all	As:Chi	1992	pr	241	m	ot	n	n	0	0	ev	all/unsp	42	999	1	27	st
RACHTA	515	x	f	0	0	all	-	all	Eu:est	1991	CC	118	n	bl	n	y	0	0	ev	cig+/-ot	41	999	1	20	st
SOBUE	552		m	0	0	all	-	q+s+l+a	As:Jap	1986	CC	1376	n	bl	n	y	0	0	cu	cig+/-ot	50	999	1	29	st
TIZZAN	505		m	0	0	all	-	all	Eu:wst	1959	CC	1358	n	bl	n	n	0	0	ev	cig only	11	999	1	4	st
TIZZAN	532		f	0	0	all	-	all	Eu:wst	1959	CC	1358	n	bl	n	n	0	0	ev	cig only	11	999	1	10	st
WANG2	509		c	0	0	all	-	all	As:Chi	1980	CC	103	n	ot	n	n	0	0	ev	cig+/-ot	40	49	1	19	st
WATSON	503		m	0	0	all	-	all	NAmer	1950	CC	301	n	bl	n	y	0	0	cu	all/unsp	20	999	1	19	st
WATSON	506		f	0	0	all	-	all	NAmer	1950	CC	301	n	bl	n	y	0	0	cu	all/unsp	20	999	1	19	st
WUWILL	505		f	0	0	all	-	all	As:Chi	1985	CC	965	n	ot	n	n	0	0	ev	cig+/-ot	40	999	1	29	st
WYNDE8	502		m	0	0	all	-	all	NAmer	1985	CC	1044	n	bl	n	y	0	0	cu	cig+/-ot	41	999	1	30	st

Table 1I20 - 4

IESLC - Meta-analysis of Ever/current Smoking, Duration, "Highest vs lowest"
All LC types, Cigarettes (or Any Product if Cigarettes not available)
Least adjusted

REF	NRR	X	SEX	AGEL	AGEH	RACE	YF	LC TYPE	LOC	START	ST	NLC	R	VB	P	H	AD	ADOS	SM	PRODUCT	exL	exH	unexL	unexH	De
WYNDE8	504		f	0	0	all	-	all	NAmer	1985	CC	1044	n	bl	n	y	0	0	cu	cig+/-ot	41	999	1	30	st
ZHENG	555	x	m	0	0	all	-	all	As:Chi	1982	CC	540	n	ot	*	y	0	0	ev	cig+/-ot	30	999	1	29	st
ZHENG	560	x	f	0	0	all	-	all	As:Chi	1982	CC	540	n	ot	*	y	0	0	ev	cig+/-ot	30	999	1	29	st
ZHOU	503		c	0	0	all	-	all	As:Chi	1978	CC	1360	n	ot	n	n	0	0	ev	all/unsp	20	999	1	19	st

Cigarette type is all/unspec for all RRs

except for the following:

REF	NRR	CIGTYPE
DESTEF	537	MC only
JUSSAW	518	MC only
NOTAN2	521	MC only

Table 1I20 - 5

IESLC - Meta-analysis of Ever/current Smoking, Duration, "Highest vs lowest"
All LC types, Cigarettes (or Any Product if Cigarettes not available)
Least adjusted

				Number Exposed		Non-exposed			
REF	NRR	SEX	AD	Case	Cont	Case	Cont	RR	95.00%CI
AGUDO	509	f	0	18	11	5	12	3.93	(1.09- 14.19)
*AMANDU	503	m	0	72	27096	42	68909	4.36	(2.98- 6.38)
AMES	501	m	2	-	-	-	-	2.28	(1.58- 3.29)
ARMADA	505	m	0	77	33	21	55	6.11	(3.20- 11.68)
AUVINE	505	c	0	230	57	26	18	2.79	(1.43- 5.44)
AXELSS	509	m	0	101	40	13	84	16.32	(8.19- 32.51)
AXELSS	518	f	0	20	10	5	24	9.60	(2.82- 32.73)
Subtotal AXELSS								14.36	(7.88- 26.20)
BARBON	507	m	0	366	235	42	91	3.37	(2.26- 5.04)
*BEST	513	m	1	137	-	1	-	8.88	(1.25- 62.98)
*BOUCOT	520	m	0	52	1563	29	2621	3.01	(1.92- 4.72)
BUFFLE	530	f	0	90	42	52	57	2.35	(1.39- 3.97)
*CEDERL	503	m	1	23	-	5	-	4.11	(1.48- 11.44)
*CEDERL	506	f	1	5	-	3	-	6.00	(1.44- 24.93)
Subtotal CEDERL								4.67	(2.04- 10.73)
CHEN2	509	m	0	62	40	2	3	2.33	(0.37- 14.53)
CHEN2	516	f	0	21	15	1	6	8.40	(0.91- 77.21)
Subtotal CHEN2								3.92	(0.95- 16.08)
CHOI	509	m	0	20	20	19	55	2.89	(1.29- 6.51)
CHOI	516	f	0	1	1	2	9	4.50	(0.19- 106.82)
Subtotal CHOI								2.97	(1.36- 6.52)
*CPSI	602	m	0	232	26906	95	266163	24.16	(19.03- 30.66)
*CPSI	695	f	0	10	5657	105	694015	11.68	(6.11- 22.34)
Subtotal CPSI								22.15	(17.71- 27.71)
*CPSII	566	m	0	117	8450	72	141932	27.29	(20.37- 36.58)
*CPSII	632	f	0	18	2224	127	301244	19.20	(11.74- 31.40)
Subtotal CPSII								24.89	(19.36- 32.01)
DAMBER	514	m	1	-	-	-	-	7.08	(3.19- 15.74)
DEAN2	503	m	0	631	558	34	36	1.20	(0.74- 1.94)
DEAN2	506	f	0	47	11	10	14	5.98	(2.11- 16.99)
Subtotal DEAN2								1.59	(1.03- 2.46)
DESTEF	537	m	0	25	18	25	96	5.33	(2.52- 11.28)
DOLL	521	m	0	558	491	12	15	1.42	(0.66- 3.06)
DOLL	528	f	0	6	3	14	18	2.57	(0.54- 12.14)
Subtotal DOLL								1.60	(0.80- 3.18)
DORGAN	526	m	2	-	-	-	-	2.96	(2.31- 3.78)
DORGAN	522	f	3	-	-	-	-	2.76	(2.18- 3.50)
Subtotal DORGAN								2.85	(2.41- 3.39)
DOSEME	505	m	2	466	-	32	-	4.90	(2.91- 8.24)
FAN	505	m	0	143	241	29	135	2.76	(1.76- 4.34)
FAN	510	f	0	55	59	8	15	1.75	(0.69- 4.45)
Subtotal FAN								2.53	(1.69- 3.80)
GAO	563	f	0	168	72	68	58	1.99	(1.27- 3.11)
GER	517	c	0	49	155	10	40	1.26	(0.59- 2.71)
HAENSZ	546	f	0	58	77	16	26	1.22	(0.60- 2.49)
HU	505	m	0	19	14	41	33	1.09	(0.48- 2.50)
HU	510	f	0	4	3	11	8	0.97	(0.17- 5.59)
Subtotal HU								1.07	(0.51- 2.26)
HU2	514	c	0	194	113	21	33	2.70	(1.49- 4.89)
HUMBLE	525	c	0	29	22	20	33	2.18	(0.99- 4.77)
JOLY	523	m	0	250	253	11	48	4.31	(2.19- 8.49)
JOLY	509	f	0	57	20	13	28	6.14	(2.67- 14.11)
Subtotal JOLY								4.96	(2.94- 8.40)
JUSSAW	518	m	0	11	6	16	20	2.29	(0.70- 7.55)
*KAISE2	600	m	1	34	-	17	-	5.38	(2.95- 9.81)
*KAISE2	520	f	1	26	-	24	-	3.13	(1.63- 6.00)
Subtotal KAISE2								4.19	(2.70- 6.52)
KATSOU	507	f	0	17	4	5	5	4.25	(0.82- 22.13)
KHUDER	505	m	0	236	354	16	61	2.54	(1.43- 4.52)
KREUZE	514	m	3	-	-	-	-	11.12	(8.68- 14.25)
KREUZE	516	f	3	-	-	-	-	9.22	(4.45- 19.09)
Subtotal KREUZE								10.91	(8.62- 13.79)
LETOUR	510	c	0	374	141	65	187	7.63	(5.42- 10.75)
LEVIN	503	m	0	63	91	56	97	1.20	(0.76- 1.90)
*LIAW	508	c	2	-	-	-	-	5.22	(1.75- 15.56)
LIU3	509	m	0	22	59	30	146	1.81	(0.97- 3.40)
LIU5	506	c	0	58	33	27	37	2.41	(1.25- 4.64)
LUBIN	507	m	0	59	86	30	146	3.34	(2.00- 5.58)
LUBIN2	537	m	0	1325	1484	953	2995	2.81	(2.53- 3.11)
LUBIN2	580	f	0	81	32	132	230	4.41	(2.78- 7.00)
Subtotal LUBIN2								2.87	(2.59- 3.17)

Table 1I20 - 5

IESLC - Meta-analysis of Ever/current Smoking, Duration, "Highest vs lowest"
All LC types, Cigarettes (or Any Product if Cigarettes not available)
Least adjusted

REF	NRR	SEX	AD	Number Exposed Case	Number Exposed Cont	Non-exposed Case	Non-exposed Cont	RR	95.00%CI
MATOS	520	m	0	86	89	20	84	4.06	(2.29- 7.18)
MCCONN	509	c	0	16	40	3	4	0.53	(0.11- 2.66)
NOTAN2	521	c	0	5	5	7	15	2.14	(0.46- 9.90)
OSANN2	503	f	0	161	57	23	47	5.77	(3.22- 10.34)
PEZZO2	509	m	0	173	126	60	72	1.65	(1.09- 2.49)
PEZZOT	538	m	0	110	101	30	134	4.86	(3.01- 7.86)
*QIAO2	515	m	0	170	2295	7	2364	25.02	(11.77- 53.16)
RACHTA	515	f	0	24	1	12	19	38.00	(4.53- 318.78)
SOBUE	552	m	0	147	73	62	119	3.87	(2.55- 5.86)
TIZZAN	505	m	0	928	815	12	1	0.09	(0.01- 0.73)
TIZZAN	532	f	0	23	21	2	7	3.83	(0.72- 20.55)
Subtotal TIZZAN								0.86	(0.24- 3.15)
WANG2	509	c	0	22	26	4	17	3.60	(1.05- 12.28)
WATSON	503	m	0	252	231	8	20	2.73	(1.18- 6.31)
WATSON	506	f	0	10	13	5	20	3.08	(0.85- 11.07)
Subtotal WATSON								2.83	(1.40- 5.71)
WUWILL	505	f	0	223	114	137	139	1.98	(1.43- 2.75)
WYNDE8	502	m	0	294	354	102	272	2.21	(1.68- 2.92)
WYNDE8	504	f	0	206	122	82	134	2.76	(1.94- 3.93)
Subtotal WYNDE8								2.41	(1.94- 2.99)
ZHENG	555	m	0	242	143	37	75	3.43	(2.20- 5.35)
ZHENG	560	f	0	59	27	17	17	2.19	(0.97- 4.92)
Subtotal ZHENG								3.09	(2.09- 4.57)
ZHOU	503	c	0	678	36	170	12	1.33	(0.68- 2.61)
Partial Totals				10566	81489	3213	1483430		

*prospective study

REF	NRR	SEX	AD	Ys	Ws	Qs	Ps
AGUDO	509	f	0	1.37	2.33	0.00	0.0369
*AMANDU	503	m	0	1.47	26.56	0.43	0.0000
AMES	501	m	2	0.82	28.56	7.75	0.0000
ARMADA	505	m	0	1.81	9.17	1.98	0.0000
AUVINE	505	c	0	1.03	8.63	0.87	0.0025
AXELSS	509	m	0	2.79	8.08	16.93	0.0000
AXELSS	518	f	0	2.26	2.55	2.15	0.0003
Subtotal AXELSS				2.66	10.64	19.07	
BARBON	507	m	0	1.22	23.93	0.40	0.0000
*BEST	513	m	1	2.18	1.00	0.70	0.0290
*BOUCOT	520	m	0	1.10	18.98	1.13	0.0000
BUFFLE	530	f	0	0.85	13.95	3.36	0.0014
*CEDERL	503	m	1	1.41	3.67	0.02	0.0067
*CEDERL	506	f	1	1.79	1.89	0.38	0.0138
Subtotal CEDERL				1.54	5.56	0.39	
CHEN2	509	m	0	0.84	1.14	0.29	0.3669
CHEN2	516	f	0	2.13	0.78	0.48	0.0601
Subtotal CHEN2				1.36	1.92	0.77	
CHOI	509	m	0	1.06	5.85	0.47	0.0101
CHOI	516	f	0	1.50	0.38	0.01	0.3520
Subtotal CHOI				1.09	6.24	0.48	
*CPSI	602	m	0	3.18	67.59	228.73	0.0000
*CPSI	695	f	0	2.46	9.15	11.33	0.0000
Subtotal CPSI				3.10	76.73	240.06	
*CPSII	566	m	0	3.31	44.82	172.48	0.0000
*CPSII	632	f	0	2.95	15.88	41.15	0.0000
Subtotal CPSII				3.21	60.70	213.63	
DAMBER	514	m	1	1.96	6.03	2.26	0.0000
DEAN2	503	m	0	0.18	16.51	22.40	0.4643
DEAN2	506	f	0	1.79	3.53	0.69	0.0008
Subtotal DEAN2				0.46	20.04	23.10	
DESTEF	537	m	0	1.67	6.85	0.74	0.0000
DOLL	521	m	0	0.35	6.50	6.42	0.3707
DOLL	528	f	0	0.94	1.59	0.26	0.2330
Subtotal DOLL				0.47	8.10	6.68	
DORGAN	526	m	2	1.09	63.36	4.28	0.0000
DORGAN	522	f	3	1.02	68.55	7.46	0.0000
Subtotal DORGAN				1.05	131.91	11.73	
DOSEME	505	m	2	1.59	14.18	0.85	0.0000
FAN	505	m	0	1.02	18.86	2.04	0.0000
FAN	510	f	0	0.56	4.41	2.73	0.2410
Subtotal FAN				0.93	23.27	4.77	

Table 1I20 - 5

IESLC - Meta-analysis of Ever/current Smoking, Duration, "Highest vs lowest"
All LC types, Cigarettes (or Any Product if Cigarettes not available)
Least adjusted

REF	NRR	SEX	AD	Ys	Ws	Qs	Ps
GAO	563	f	0	0.69	19.31	8.33	0.0025
GER	517	c	0	0.23	6.59	8.12	0.5470
HAENSZ	546	f	0	0.20	7.62	9.96	0.5768
HU	505	m	0	0.09	5.59	8.83	0.8345
HU	510	f	0	-0.03	1.25	2.37	0.9725
Subtotal HU				0.07	6.85	11.20	
HU2	514	c	0	0.99	10.88	1.35	0.0011
HUMBLE	525	c	0	0.78	6.24	2.01	0.0522
JOLY	523	m	0	1.46	8.35	0.11	0.0000
JOLY	509	f	0	1.81	5.55	1.22	0.0000
Subtotal JOLY				1.60	13.90	1.34	
JUSSAW	518	m	0	0.83	2.70	0.72	0.1728
*KAISE2	600	m	1	1.68	10.64	1.21	0.0000
*KAISE2	520	f	1	1.14	9.05	0.38	0.0006
Subtotal KAISE2				1.43	19.69	1.59	
KATSOU	507	f	0	1.45	1.41	0.01	0.0857
KHUDER	505	m	0	0.93	11.63	1.98	0.0015
KREUZE	514	m	3	2.41	62.53	70.75	0.0000
KREUZE	516	f	3	2.22	7.25	5.56	0.0000
Subtotal KREUZE				2.39	69.77	76.32	
LETOUR	510	c	0	2.03	32.79	15.48	0.0000
LEVIN	503	m	0	0.18	18.17	24.60	0.4388
*LIAW	508	c	2	1.65	3.22	0.30	0.0030
LIU3	509	m	0	0.60	9.75	5.47	0.0628
LIU5	506	c	0	0.88	8.96	1.95	0.0085
LUBIN	507	m	0	1.21	14.54	0.28	0.0000
LUBIN2	537	m	0	1.03	355.65	34.90	0.0000
LUBIN2	580	f	0	1.48	18.01	0.35	0.0000
Subtotal LUBIN2				1.05	373.66	35.25	
MATOS	520	m	0	1.40	11.80	0.04	0.0000
MCCONN	509	c	0	-0.63	1.49	5.81	0.4428
NOTAN2	521	c	0	0.76	1.64	0.56	0.3290
OSANN2	503	f	0	1.75	11.30	1.88	0.0000
PEZZO2	509	m	0	0.50	22.59	16.15	0.0176
PEZZOT	538	m	0	1.58	16.73	0.94	0.0000
*QIAO2	515	m	0	3.22	6.76	23.76	0.0000
RACHTA	515	f	0	3.64	0.85	4.46	0.0008
SOBUE	552	m	0	1.35	22.21	0.00	0.0000
TIZZAN	505	m	0	-2.36	0.92	12.61	0.0238
TIZZAN	532	f	0	1.34	1.36	0.00	0.1168
Subtotal TIZZAN				-0.15	2.28	12.61	
WANG2	509	c	0	1.28	2.55	0.01	0.0411
WATSON	503	m	0	1.00	5.46	0.64	0.0191
WATSON	506	f	0	1.12	2.34	0.11	0.0854
Subtotal WATSON				1.04	7.80	0.75	
WUWILL	505	f	0	0.69	36.04	15.68	0.0000
WYNDE8	502	m	0	0.80	50.74	15.34	0.0000
WYNDE8	504	f	0	1.01	30.57	3.33	0.0000
Subtotal WYNDE8				0.88	81.32	18.67	
ZHENG	555	m	0	1.23	19.42	0.25	0.0000
ZHENG	560	f	0	0.78	5.83	1.85	0.0592
Subtotal ZHENG				1.13	25.25	2.09	
ZHOU	503	c	0	0.28	8.44	9.49	0.4081

Table 1I20 - 5

IESLC - Meta-analysis of Ever/current Smoking, Duration, "Highest vs lowest"
All LC types, Cigarettes (or Any Product if Cigarettes not available)
Least adjusted

	N	76
	NS	57
	Wt	1401.98
Het	Chi	860.31
Het	df	75
Het	P	***
Fixed	RR	3.84
	RRl	3.64
	RRu	4.04
	P	+++
Random	RR	3.63
	RRl	2.97
	RRu	4.43
	P	+++
Asymm	P	N.S.

Table 1I20 - 6

IESLC - Meta-analysis of Ever/current Smoking, Duration, "Highest vs lowest"
All LC types, Cigarettes (or Any Product if Cigarettes not available)
Least adjusted

		combined	Sex male	female	Total
	N	11	38	27	76
	NS	11	38	27	76
	Wt	91.42	1027.84	282.73	1401.98
Het	Chi	44.61	686.64	120.69	860.31
Het	df	10	37	26	75
Het	P	***	***	***	***
Fixed	RR	3.40	4.02	3.37	3.84
	RRl	2.77	3.78	3.00	3.64
	RRu	4.17	4.27	3.79	4.04
	P	+++	+++	+++	+++
Random	RR	2.50	3.75	3.97	3.63
	RRl	1.54	2.80	2.95	2.97
	RRu	4.04	5.04	5.33	4.43
	P	+++	+++	+++	+++
Between	Chi				8.37
Between	df				2
Between	P				*
Btwn(F)	P				N.S.
Btwn(R)	P				N.S.

Table 1I20 - 7

IESLC - Meta-analysis of Ever/current Smoking, Duration, "Highest vs lowest"
All LC types, Cigarettes (or Any Product if Cigarettes not available)
Excluded studies (and stage at which they were excluded)

1	BECHER BLOT1 BROWN3 CARPEN CHYOU DARBY DOLL2 GARCIA GRAHAM GURSEL HAMMO2 JAHN JAIN LAUSSM PRESCO QIAO TVERDA WIGLE WYNDE3
2	ALDERS BENSHL BRESLO CHIAZZ DEAN3 DORN ENGELA GAO2 GILLIS GUO HEGMAN HIRAYA HOLE KAUFMA KOO KOULUM LIU4 MIGRAN MRFITR PERNU SEGI2 SPEIZE SUZUK2 SVENSS VUTUC WAKAI WU YUAN
3	GENG MCDUFF SPITZ STASZE WU2 ZHANG
4	AKIBA GARSHI
5	BOUCHA CHEN CORREA JEDRYC LUO WYNDE2 WYNDE6
6	HAMMON PISANI RESTRE SADOWS XU
8	BOFFET BROSS WYNDE7
15	BENHAM

Table 1I20 - 8
Potentially overlapping studies

REF	REFGP	PRINC	.	OVERLAP/LINK
LUBIN2	LUBIN2	1		Lubin-combined
OSANN2	KAISER	2		KAISER/OSANN2
WYNDE8	WYNDE6	2		WYNDE5/6/7/8
CPSI	CPSI	1		CPSI overall
LUBIN	XIANGZ	2		LUBIN/XIANGZ/QIAO

Table 1I20 - 9
Most adjusted - insufficient data for meta-analysis

REF	NRR	SEX	AGEL	AGEH	RACE	YF	LC TYPE	LOC	START	ST	NLC	R	VB	P	H	AD	ADOS	SM	PRODUCT	exL	exH	unexL	unexH	De
BUFFLE	548	m	0	0	wh	-	all	NAmer	1976	CC	943	n	bl	y	n	0	0	ev	cig+/-ot	50	999	1	33	st
HAMMON	512	m	0	0	wh	0	all	NAmer	1952	pr	448	n	bl	n	n	1	0	ev	cig only	35	999	1	34	st
RESTRE	507	c	0	0	all	-	all	SCAmer	1978	CC	102	n	bl	n	n	3	1#	ev	cig+/-ot	51	999	1	20	or
SADOWS	532	m	0	0	wh	-	all	NAmer	1938	CC	477	n	bl	n	n	0	0	ev	cig only	50	999	1	9	ot
XU	505	m	0	0	all	-	all	As:Chi	1985	CC	729	n	ot	n	n	2	0	ev	all/unsp	40	999	1	29	st

Comments on values in listings

RESTRE ADOS Number of cigarettes smoked per day

REF	NRR	RR	SIG	RRDATA comment
BUFFLE	548	2.13		0
HAMMON	512	*		RR for <1/2 pack per day is 2.04, that for 1/2 to 1 pack per day is 1.25, that for 1 to 2 packs per day is 2.71 while that for 2+ packs per day 2.12
RESTRE	507	24.01		Test for trend given as P = 0.006
SADOWS	532	7.09		0
XU	505	*		RR for 1-19/day is 1.8, for 20-29/day is 4.0 and for >=30/day is 3.2

Table 1I21 -

IESLC - Meta-analysis of Ever/current Smoking by Duration, Overview
All LC types, Cigarettes only

This analysis is restricted to results for:
1) Ever/current smokers
2) Results by Duration
3) Categorical results by Duration
Results by Duration are grouped under 2 schemes (S1, S2). Each scheme has a set of "key values". An interval is allocated to the category whose key value it includes, and intervals which include none or more than one of the key values are excluded. (Open-ended intervals are coded as 999)

S1	key value	maximum range
1	20	1-34
2	35	21-49
3	50	36+

S2	key value	maximum range
1	5	1-19
2	20	6-29
3	30	21-39
4	40	31-49
5	50	41-998
6	999	51+

4) All LC types (or near equivalent)
5) Results complete enough for use in metaanalysis

Within each study, results are then selected (in the following order of preference, within each sex) for:
6) SMKSTA: ever, current
7) PRODUCT: cigarettes only
8) CIGTYPE: all/unspecified, MC regardless of HR, MC only
9) (not applicable)
10) DENOM: never smoked anything, never smoked cigarettes, never any + low, never cigs + low
11) Followup period (YF, prospective studies): whole study (coded as 0) or longest available
12) LCtype: all or nearest available, at least Squamous and Adeno. (q = squamous, s = small, l = large, a = adeno, mix = mixed, alv = alveolar)
13) Race: all or nearest available, otherwise by race (wh or w = white, bl or b = black, hi = hispanic ch = chinese, jap = japanese, haw = hawaiian, w+o = white + oriental, sca = scandinavian, as = asian)
14) For overlapping studies: principal rather than subsidiary studies
Finally by Age: whole study (coded as 0) if available, otherwise by widest available age group and then for single sex results (m, f) in preference to results for both sexes combined (c).

Results adjusted (AD) for the most potential confounders are then chosen in Sections -1 to -3 (and those which actually differ from the adjusted results in Table 1I11 - 1 are marked 'x' in Section -1) and results adjusted for the least confounders in Sections -4 to -6. (Those least adjusted results which actually differ from the most adjusted are marked 'x' in column X in Section -4)

Section -7 shows excluded studies, together with the stage (as above) at which no qualifying results were found.

Section -8 lists the potentially overlapping studies which have been included (1=principal, 2=subsidiary).

Section -9 lists any results which would have been included in preference except that they had data not complete enough for use in meta-analysis, with their significance (yes/no), if known, and any further comment as entered on the database. It also lists as "gap" any categories for which no data were presented by the original authors.

In addition to those mentioned above, the following fields, levels and abbreviations are used:
* or nk = not known, n = no, y = yes, ot = other
ev = ever, cu = current, nev = never
all/unspec = all or unspecified, MC = manufactured cigarettes, HR = hand-rolled cigarettes
exL, exH = range of exposure (low and high) in the smoking group, in terms of Duration
REF: 6-character study reference
NRR: number of the RR on the database within the study
ST : study type (CC = case control, pr or prosp = prospective)
NLC: number of lung cancer cases in whole study
R : risky occupational population (n = no, m = mining, o = other risky)
VB : national cigarette type (V = at least 75% Virginia, bl = at least 75% blended, ot = other)
P : any proxy use
H : full histological confirmation
De : derivation of RR/CI (or = original, st = standard method, ot = other method of estimation)

Table 1I21 - 1

IESLC - Meta-analysis of Ever/current Smoking by Duration, Overview
All LC types, Cigarettes only
Most adjusted

REF	NRR	1I11	SEX	AGEL	AGEH	RACE	YF	LC TYPE	LOC	START	ST	NLC	R	VB	P	H	AD	SM	PRODUCT	exL	exH	S1	S2	DENOM	De
AGUDO	510		f	0	0	all	-	all	Eu:wst	1989	CC	103	n	bl	n	n	3	ev	cig only	1	16	0	1	nev cigs	or
AGUDO	511		f	0	0	all	-	all	Eu:wst	1989	CC	103	n	bl	n	n	3	ev	cig only	17	999	0	0	nev cigs	or
BENHAM	511	x	m	0	0	all	-	not mix	Eu:wst	1976	CC	1625	n	bl	n	y	0	ev	cig only	1	35	0	0	nev any	st
BENHAM	512	x	m	0	0	all	-	not mix	Eu:wst	1976	CC	1625	n	bl	n	y	0	ev	cig only	36	999	3	0	nev any	st
BEST	501		m	0	0	all	0	all	NAmer	1955	pr	381	n	V	n	n	1	cu	cig only	1	4	0	0	nev any	ot
BEST	502		m	0	0	all	0	all	NAmer	1955	pr	381	n	V	n	n	1	cu	cig only	5	9	0	1	nev any	ot
BEST	503		m	0	0	all	0	all	NAmer	1955	pr	381	n	V	n	n	1	cu	cig only	10	14	0	0	nev any	ot
BEST	504		m	0	0	all	0	all	NAmer	1955	pr	381	n	V	n	n	1	cu	cig only	15	19	0	0	nev any	ot
BEST	505		m	0	0	all	0	all	NAmer	1955	pr	381	n	V	n	n	1	cu	cig only	20	29	1	2	nev any	ot
BEST	506		m	0	0	all	0	all	NAmer	1955	pr	381	n	V	n	n	1	cu	cig only	30	39	2	3	nev any	ot
BEST	507		m	0	0	all	0	all	NAmer	1955	pr	381	n	V	n	n	1	cu	cig only	40	999	3	0	nev any	ot
BOUCOT	512	x	m	0	0	all	9	all	NAmer	1951	pr	121	n	bl	n	n	0	ev	cig only	1	39	0	0	nev any	ot
BOUCOT	513	x	m	0	0	all	9	all	NAmer	1951	pr	121	n	bl	n	n	0	ev	cig only	40	999	3	0	nev any	ot
CEDERL	501		m	40	69	all	10	all	Eu:Sca	1963	pr	491	n	bl	n	n	1	cu	cig only	1	29	1	0	nev any	ot
CEDERL	502		m	40	69	all	10	all	Eu:Sca	1963	pr	491	n	bl	n	n	1	cu	cig only	30	999	0	0	nev any	ot
CEDERL	504		f	40	69	all	10	all	Eu:Sca	1963	pr	491	n	bl	n	n	1	cu	cig only	1	29	1	0	nev any	ot
CEDERL	505		f	40	69	all	10	all	Eu:Sca	1963	pr	491	n	bl	n	n	1	cu	cig only	30	999	0	0	nev any	ot
CPSI	580		m	40	84	wh	0	all	NAmer	1959	pr	5138	n	bl	n	n	0	cu	cig only	1	29	1	0	nev cigs	st
CPSI	581		m	40	84	wh	0	all	NAmer	1959	pr	5138	n	bl	n	n	0	cu	cig only	30	34	0	3	nev cigs	st
CPSI	582		m	40	84	wh	0	all	NAmer	1959	pr	5138	n	bl	n	n	0	cu	cig only	35	39	2	0	nev cigs	st
CPSI	583		m	40	84	wh	0	all	NAmer	1959	pr	5138	n	bl	n	n	0	cu	cig only	40	44	0	4	nev cigs	st
CPSI	584		m	40	84	wh	0	all	NAmer	1959	pr	5138	n	bl	n	n	0	cu	cig only	45	49	0	0	nev cigs	st
CPSI	585		m	40	84	wh	0	all	NAmer	1959	pr	5138	n	bl	n	n	0	cu	cig only	50	54	3	5	nev cigs	st
CPSI	586		m	40	84	wh	0	all	NAmer	1959	pr	5138	n	bl	n	n	0	cu	cig only	55	59	0	0	nev cigs	st
CPSI	587		m	40	84	wh	0	all	NAmer	1959	pr	5138	n	bl	n	n	0	cu	cig only	60	999	0	6	nev cigs	st
CPSI	676		f	40	84	wh	0	all	NAmer	1959	pr	5138	n	bl	n	n	0	cu	cig only	1	29	1	0	nev cigs	st
CPSI	677		f	40	84	wh	0	all	NAmer	1959	pr	5138	n	bl	n	n	0	cu	cig only	30	34	0	3	nev cigs	st
CPSI	678		f	40	84	wh	0	all	NAmer	1959	pr	5138	n	bl	n	n	0	cu	cig only	35	39	2	0	nev cigs	st
CPSI	679		f	40	84	wh	0	all	NAmer	1959	pr	5138	n	bl	n	n	0	cu	cig only	40	44	0	4	nev cigs	st
CPSI	680		f	40	84	wh	0	all	NAmer	1959	pr	5138	n	bl	n	n	0	cu	cig only	45	49	0	0	nev cigs	st
CPSI	681		f	40	84	wh	0	all	NAmer	1959	pr	5138	n	bl	n	n	0	cu	cig only	50	54	3	5	nev cigs	st
CPSI	682		f	40	84	wh	0	all	NAmer	1959	pr	5138	n	bl	n	n	0	cu	cig only	55	999	0	6	nev cigs	st
CPSII	552		m	0	0	all	6	all	NAmer	1982	pr	3229	n	bl	n	n	0	cu	cig only	1	29	1	0	nev any	st
CPSII	553		m	0	0	all	6	all	NAmer	1982	pr	3229	n	bl	n	n	0	cu	cig only	30	34	0	3	nev any	st
CPSII	554		m	0	0	all	6	all	NAmer	1982	pr	3229	n	bl	n	n	0	cu	cig only	35	39	2	0	nev any	st
CPSII	555		m	0	0	all	6	all	NAmer	1982	pr	3229	n	bl	n	n	0	cu	cig only	40	44	0	4	nev any	st
CPSII	556		m	0	0	all	6	all	NAmer	1982	pr	3229	n	bl	n	n	0	cu	cig only	45	49	0	0	nev any	st
CPSII	557		m	0	0	all	6	all	NAmer	1982	pr	3229	n	bl	n	n	0	cu	cig only	50	54	3	5	nev any	st
CPSII	558		m	0	0	all	6	all	NAmer	1982	pr	3229	n	bl	n	n	0	cu	cig only	55	59	0	0	nev any	st
CPSII	559		m	0	0	all	6	all	NAmer	1982	pr	3229	n	bl	n	n	0	cu	cig only	60	999	0	6	nev any	st
JUSSAW	510		m	0	0	all	-	all	As:Ind	1964	CC	792	n	V	n	n	0	ev	cig only	1	9	0	1	nev any	st
JUSSAW	511		m	0	0	all	-	all	As:Ind	1964	CC	792	n	V	n	n	0	ev	cig only	10	19	0	0	nev any	st
JUSSAW	512		m	0	0	all	-	all	As:Ind	1964	CC	792	n	V	n	n	0	ev	cig only	20	29	1	2	nev any	st
JUSSAW	513		m	0	0	all	-	all	As:Ind	1964	CC	792	n	V	n	n	0	ev	cig only	30	39	2	3	nev any	st
JUSSAW	514		m	0	0	all	-	all	As:Ind	1964	CC	792	n	V	n	n	0	ev	cig only	40	999	3	0	nev any	st
KAISE2	596		m	0	0	all	9	all	NAmer	1979	pr	318	n	bl	n	n	1	cu	cig only	1	39	0	0	nev any	st
KAISE2	597		m	0	0	all	9	all	NAmer	1979	pr	318	n	bl	n	n	1	cu	cig only	40	999	3	0	nev any	st
KAISE2	516		f	0	0	all	9	all	NAmer	1979	pr	318	n	bl	n	n	1	cu	cig only	1	39	0	0	nev any	st
KAISE2	517		f	0	0	all	9	all	NAmer	1979	pr	318	n	bl	n	n	1	cu	cig only	40	999	3	0	nev any	st
LUBIN	526	x	m	0	0	all	-	all	As:Chi	1984	CC	427	m	ot	y	n	4	ev	cig only	1	29	1	0	nev any	ot
LUBIN	527	x	m	0	0	all	-	all	As:Chi	1984	CC	427	m	ot	y	n	4	ev	cig only	30	39	2	3	nev any	ot
LUBIN	528	x	m	0	0	all	-	all	As:Chi	1984	CC	427	m	ot	y	n	4	ev	cig only	40	49	0	4	nev any	ot
LUBIN	529	x	m	0	0	all	-	all	As:Chi	1984	CC	427	m	ot	y	n	4	ev	cig only	50	999	3	0	nev any	ot
NOTAN2	513		c	0	0	all	-	all	As:Ind	1963	CC	683	n	V	n	n	0	ev	cig only	1	10	0	1	nev any	st
NOTAN2	514		c	0	0	all	-	all	As:Ind	1963	CC	683	n	V	n	n	0	ev	cig only	11	20	1	2	nev any	st
NOTAN2	515		c	0	0	all	-	all	As:Ind	1963	CC	683	n	V	n	n	0	ev	cig only	21	30	0	3	nev any	st
NOTAN2	516		c	0	0	all	-	all	As:Ind	1963	CC	683	n	V	n	n	0	ev	cig only	31	40	2	4	nev any	st
NOTAN2	517		c	0	0	all	-	all	As:Ind	1963	CC	683	n	V	n	n	0	ev	cig only	41	999	3	0	nev any	st
PEZZOT	534		m	0	0	all	-	all	SCAmer	1987	CC	215	n	bl	n	y	0	ev	cig only	1	30	1	0	nev cigs	st
PEZZOT	535		m	0	0	all	-	all	SCAmer	1987	CC	215	n	bl	n	y	0	ev	cig only	31	40	2	4	nev cigs	st
PEZZOT	536		m	0	0	all	-	all	SCAmer	1987	CC	215	n	bl	n	y	0	ev	cig only	41	999	3	0	nev cigs	st
TIZZAN	501		m	0	0	all	-	all	Eu:wst	1959	CC	1358	n	bl	n	n	0	ev	cig only	1	4	0	0	nev any	st
TIZZAN	502		m	0	0	all	-	all	Eu:wst	1959	CC	1358	n	bl	n	n	0	ev	cig only	5	10	0	1	nev any	st
TIZZAN	503		m	0	0	all	-	all	Eu:wst	1959	CC	1358	n	bl	n	n	0	ev	cig only	11	999	0	0	nev any	st
TIZZAN	530	x	f	0	0	all	-	all	Eu:wst	1959	CC	1358	n	bl	n	n	0	ev	cig only	1	10	0	1	nev any	st
TIZZAN	531	x	f	0	0	all	-	all	Eu:wst	1959	CC	1358	n	bl	n	n	0	ev	cig only	11	999	0	0	nev any	st

Cigarette type is all/unspec for all RRs

except for the following:

Table 1I21 - 1

IESLC - Meta-analysis of Ever/current Smoking by Duration, Overview
All LC types, Cigarettes only
Most adjusted

REF	NRR	CIGTYPE
JUSSAW	510	MC only
JUSSAW	511	MC only
JUSSAW	512	MC only
JUSSAW	513	MC only
JUSSAW	514	MC only
NOTAN2	513	MC only
NOTAN2	514	MC only
NOTAN2	515	MC only
NOTAN2	516	MC only
NOTAN2	517	MC only

In this overview table, subtotals and Qs values may be invalid and should be ignored

Table 1I21 - 2

IESLC - Meta-analysis of Ever/current Smoking by Duration, Overview
All LC types, Cigarettes only
Most adjusted

REF	NRR	SEX	AD	Number Exposed Case	Cont	Non-exposed Case	Cont	RR	95.00%CI
AGUDO	510	f	3	5	-	80	-	1.29	(0.40- 4.17)
AGUDO	511	f	3	18	-	80	-	5.09	(1.94- 13.35)
Subtotal AGUDO								2.92	(1.39- 6.16)
BENHAM	511	m	0	490	772	33	523	10.06	(6.95- 14.56)
BENHAM	512	m	0	694	620	33	523	17.74	(12.28- 25.63)
Subtotal BENHAM								13.37	(10.30- 17.36)
*BEST	501	m	1	1	-	7	-	1.60	(0.20- 13.00)
*BEST	502	m	1	2	-	7	-	2.60	(0.54- 12.52)
*BEST	503	m	1	6	-	7	-	2.30	(0.77- 6.84)
*BEST	504	m	1	10	-	7	-	3.20	(1.22- 8.41)
*BEST	505	m	1	22	-	7	-	4.10	(1.75- 9.60)
*BEST	506	m	1	55	-	7	-	13.90	(6.33- 30.52)
*BEST	507	m	1	137	-	7	-	14.20	(6.64- 30.35)
Subtotal BEST								6.17	(4.26- 8.94)
*BOUCOT	512	m	0	19	2088	0	805	15.04~	(0.91- 248.83)
*BOUCOT	513	m	0	44	1177	0	805	60.88~	(3.75- 987.17)
Subtotal BOUCOT								30.41	(4.21- 219.61)
*CEDERL	501	m	1	5	-	7	-	1.80	(0.57- 5.66)
*CEDERL	502	m	1	23	-	7	-	7.40	(3.18- 17.21)
*CEDERL	504	f	1	3	-	19	-	1.60	(0.47- 5.40)
*CEDERL	505	f	1	5	-	19	-	9.60	(3.60- 25.58)
Subtotal CEDERL								4.61	(2.78- 7.67)
*CPSI	580	m	0	95	266163	196	926068	1.69	(1.32- 2.15)
*CPSI	581	m	0	230	290031	196	926068	3.75	(3.10- 4.53)
*CPSI	582	m	0	470	367622	196	926068	6.04	(5.11- 7.14)
*CPSI	583	m	0	731	333292	196	926068	10.36	(8.85- 12.13)
*CPSI	584	m	0	764	221405	196	926068	16.30	(13.94- 19.07)
*CPSI	585	m	0	576	119633	196	926068	22.75	(19.35- 26.75)
*CPSI	586	m	0	356	53226	196	926068	31.60	(26.55- 37.61)
*CPSI	587	m	0	232	26906	196	926068	40.74	(33.70- 49.25)
*CPSI	676	f	0	105	694015	532	3877179	1.10	(0.89- 1.36)
*CPSI	677	f	0	141	383127	532	3877179	2.68	(2.23- 3.23)
*CPSI	678	f	0	154	315060	532	3877179	3.56	(2.98- 4.26)
*CPSI	679	f	0	120	163178	532	3877179	5.36	(4.40- 6.53)
*CPSI	680	f	0	54	53635	532	3877179	7.34	(5.55- 9.71)
*CPSI	681	f	0	16	14305	532	3877179	8.15	(4.96- 13.40)
*CPSI	682	f	0	10	5657	532	3877179	12.88	(6.90- 24.07)
Subtotal CPSI								8.01	(7.62- 8.43)
*CPSII	552	m	0	72	141932	124	742207	3.04	(2.27- 4.06)
*CPSII	553	m	0	145	113317	124	742207	7.66	(6.03- 9.73)
*CPSII	554	m	0	244	109788	124	742207	13.30	(10.72- 16.51)
*CPSII	555	m	0	413	103500	124	742207	23.88	(19.54- 29.19)
*CPSII	556	m	0	307	53805	124	742207	34.15	(27.73- 42.06)
*CPSII	557	m	0	332	39260	124	742207	50.62	(41.19- 62.20)
*CPSII	558	m	0	151	13598	124	742207	66.47	(52.45- 84.24)
*CPSII	559	m	0	117	8450	124	742207	82.88	(64.43- 106.60)
Subtotal CPSII								24.98	(23.06- 27.07)
JUSSAW	510	m	0	16	20	149	624	3.35	(1.70- 6.62)
JUSSAW	511	m	0	34	19	149	624	7.49	(4.16- 13.51)
JUSSAW	512	m	0	38	23	149	624	6.92	(4.00- 11.97)
JUSSAW	513	m	0	27	9	149	624	12.56	(5.79- 27.28)
JUSSAW	514	m	0	11	6	149	624	7.68	(2.79- 21.09)
Subtotal JUSSAW								6.77	(5.01- 9.15)
*KAISE2	596	m	1	17	-	14	-	4.86	(2.22- 10.61)
*KAISE2	597	m	1	34	-	14	-	15.64	(8.31- 29.40)
*KAISE2	516	f	1	24	-	11	-	9.09	(4.25- 19.43)
*KAISE2	517	f	1	26	-	11	-	30.41	(14.39- 64.25)
Subtotal KAISE2								12.59	(8.77- 18.07)
LUBIN	526	m	4	7	-	8	-	1.35	(0.46- 3.96)
LUBIN	527	m	4	16	-	8	-	2.27	(0.91- 5.65)
LUBIN	528	m	4	21	-	8	-	4.39	(1.81- 10.64)
LUBIN	529	m	4	8	-	8	-	9.56	(3.12- 29.29)
Subtotal LUBIN								3.29	(2.01- 5.39)
NOTAN2	513	c	0	7	15	107	201	0.88	(0.35- 2.22)
NOTAN2	514	c	0	15	15	107	201	1.88	(0.88- 3.99)
NOTAN2	515	c	0	17	16	107	201	2.00	(0.97- 4.11)
NOTAN2	516	c	0	12	7	107	201	3.22	(1.23- 8.42)
NOTAN2	517	c	0	5	5	107	201	1.88	(0.53- 6.63)
Subtotal NOTAN2								1.83	(1.24- 2.70)
PEZZOT	534	m	0	30	134	4	116	6.49	(2.22- 18.98)
PEZZOT	535	m	0	71	82	4	116	25.11	(8.82- 71.48)

Table 1I21 - 2

IESLC - Meta-analysis of Ever/current Smoking by Duration, Overview
All LC types, Cigarettes only
Most adjusted

REF	NRR	SEX	AD	Number Exposed Case	Number Exposed Cont	Non-exposed Case	Non-exposed Cont	RR	95.00%CI
PEZZOT	536	m	0	110	101	4	116	31.58	(11.25- 88.71)
Subtotal PEZZOT								17.64	(9.62- 32.34)
TIZZAN	501	m	0	12	1	180	305	20.33	(2.62- 157.68)
TIZZAN	502	m	0	54	20	180	305	4.58	(2.65- 7.89)
TIZZAN	503	m	0	928	815	180	305	1.93	(1.57- 2.37)
TIZZAN	530	f	0	2	7	117	114	0.28	(0.06- 1.37)
TIZZAN	531	f	0	23	21	117	114	1.07	(0.56- 2.03)
Subtotal TIZZAN								2.01	(1.68- 2.42)
Partial Totals				8939	3896878	87594	0494725		

*prospective study ~ With 0.5 adjustment for zero

REF	NRR	SEX	AD	Ys	Ws	Qs	Ps
AGUDO	510	f	3	0.25	2.80	11.51	0.6702
AGUDO	511	f	3	1.63	4.13	1.78	0.0009
Subtotal AGUDO				1.07	6.93	13.29	
BENHAM	511	m	0	2.31	28.13	0.02	0.0000
BENHAM	512	m	0	2.88	28.35	9.94	0.0000
Subtotal BENHAM				2.59	56.48	9.96	
*BEST	501	m	1	0.47	0.88	2.90	0.6590
*BEST	502	m	1	0.96	1.55	2.74	0.2335
*BEST	503	m	1	0.83	3.22	6.78	0.1350
*BEST	504	m	1	1.16	4.12	5.18	0.0182
*BEST	505	m	1	1.41	5.30	4.04	0.0012
*BEST	506	m	1	2.63	6.21	0.75	0.0000
*BEST	507	m	1	2.65	6.65	0.91	0.0000
Subtotal BEST				1.82	27.95	23.30	
*BOUCOT	512	m	0	2.71	0.49	0.09	0.0583
*BOUCOT	513	m	0	4.11	0.49	1.65	0.0038
Subtotal BOUCOT				3.41	0.98	1.74	
*CEDERL	501	m	1	0.59	2.92	8.39	0.3155
*CEDERL	502	m	1	2.00	5.39	0.43	0.0000
*CEDERL	504	f	1	0.47	2.58	8.48	0.4505
*CEDERL	505	f	1	2.26	4.00	0.00	0.0000
Subtotal CEDERL				1.53	14.88	17.30	
*CPSI	580	m	0	0.52	64.01	198.51	0.0000
*CPSI	581	m	0	1.32	105.87	98.14	0.0000
*CPSI	582	m	0	1.80	138.39	32.58	0.0000
*CPSI	583	m	0	2.34	154.66	0.46	0.0000
*CPSI	584	m	0	2.79	156.12	40.24	0.0000
*CPSI	585	m	0	3.12	146.44	103.53	0.0000
*CPSI	586	m	0	3.45	126.72	173.33	0.0000
*CPSI	587	m	0	3.71	106.68	216.17	0.0000
*CPSI	676	f	0	0.10	87.71	419.11	0.3603
*CPSI	677	f	0	0.99	111.49	187.58	0.0000
*CPSI	678	f	0	1.27	119.48	122.67	0.0000
*CPSI	679	f	0	1.68	97.98	35.84	0.0000
*CPSI	680	f	0	1.99	49.07	4.15	0.0000
*CPSI	681	f	0	2.10	15.55	0.54	0.0000
*CPSI	682	f	0	2.56	9.83	0.73	0.0000
Subtotal CPSI				2.08	1489.99	1633.58	
*CPSII	552	m	0	1.11	45.57	62.70	0.0000
*CPSII	553	m	0	2.04	66.89	4.11	0.0000
*CPSII	554	m	0	2.59	82.29	7.62	0.0000
*CPSII	555	m	0	3.17	95.47	75.54	0.0000
*CPSII	556	m	0	3.53	88.48	137.62	0.0000
*CPSII	557	m	0	3.92	90.50	243.58	0.0000
*CPSII	558	m	0	4.20	68.44	250.45	0.0000
*CPSII	559	m	0	4.42	60.64	276.04	0.0000
Subtotal CPSII				3.22	598.26	1057.65	
JUSSAW	510	m	0	1.21	8.28	9.56	0.0005
JUSSAW	511	m	0	2.01	11.07	0.80	0.0000
JUSSAW	512	m	0	1.93	12.80	1.56	0.0000
JUSSAW	513	m	0	2.53	6.39	0.39	0.0000
JUSSAW	514	m	0	2.04	3.76	0.23	0.0001
Subtotal JUSSAW				1.91	42.30	12.54	
*KAISE2	596	m	1	1.58	6.28	3.10	0.0001
*KAISE2	597	m	1	2.75	9.62	2.09	0.0000
*KAISE2	516	f	1	2.21	6.65	0.04	0.0000
*KAISE2	517	f	1	3.41	6.86	8.78	0.0000
Subtotal KAISE2				2.53	29.42	14.01	

Table 1I21 - 2

IESLC - Meta-analysis of Ever/current Smoking by Duration, Overview
All LC types, Cigarettes only
Most adjusted

REF	NRR	SEX	AD	Ys	Ws	Qs	Ps
LUBIN	526	m	4	0.30	3.32	13.05	0.5848
LUBIN	527	m	4	0.82	4.61	9.88	0.0784
LUBIN	528	m	4	1.48	4.90	3.17	0.0011
LUBIN	529	m	4	2.26	3.06	0.00	0.0001
Subtotal LUBIN				1.19	15.89	26.09	
NOTAN2	513	c	0	-0.13	4.47	26.06	0.7808
NOTAN2	514	c	0	0.63	6.77	18.51	0.1008
NOTAN2	515	c	0	0.69	7.37	18.70	0.0606
NOTAN2	516	c	0	1.17	4.16	5.16	0.0171
NOTAN2	517	c	0	0.63	2.41	6.60	0.3273
Subtotal NOTAN2				0.60	25.18	75.03	
PEZZOT	534	m	0	1.87	3.34	0.57	0.0006
PEZZOT	535	m	0	3.22	3.51	3.10	0.0000
PEZZOT	536	m	0	3.45	3.60	4.92	0.0000
Subtotal PEZZOT				2.87	10.45	8.59	
TIZZAN	501	m	0	3.01	0.92	0.49	0.0039
TIZZAN	502	m	0	1.52	12.93	7.53	0.0000
TIZZAN	503	m	0	0.66	89.78	237.50	0.0000
TIZZAN	530	f	0	-1.28	1.51	19.22	0.1155
TIZZAN	531	f	0	0.06	9.22	45.41	0.8435
Subtotal TIZZAN				0.70	114.36	310.15	

N	66
NS	13

Table 1I21 - 3

IESLC - Meta-analysis of Ever/current Smoking by Duration, Overview
All LC types, Cigarettes only
Most adjusted

	Sex			
	combined	male	female	Total
N	5	46	15	66
NS	1	11	5	17

In this overview table, other than the “N” rows, entries in the “absent” and “Total” columns may be invalid and should be ignored

	Duration of smoking (broad categories)				
	absent	1-34k20	21-49k35	36+k50	Total
N	36	10	8	12	66
NS	12	8	7	10	37
Wt	1516.41	234.31	365.03	317.32	2433.07
Het Chi	1857.80	66.41	105.28	98.50	3203.24
Het df	35	9	7	11	65
Het P	***	***	***	***	***
Fixed RR	11.67	1.81	6.20	25.28	9.81
RRl	11.10	1.59	5.60	22.65	9.43
RRu	12.27	2.06	6.87	28.22	10.21
P	+++	+++	+++	+++	+++
Random RR	6.61	2.42	7.29	16.38	6.70
RRl	4.45	1.58	4.47	10.68	4.98
RRu	9.81	3.70	11.90	25.10	9.02
P	+++	+++	+++	+++	+++

	Duration of smoking (narrow categories)							
	absent	1-19k1	6-29k20	21-39k30	31-49k40	41-998k50	51+k999	Total
N	38	6	3	7	6	3	3	66
NS	13	5	3	6	5	2	2	36
Wt	1277.52	31.54	24.88	308.83	360.66	252.49	177.15	2433.07
Het Chi	1773.79	18.86	7.55	69.53	120.97	61.66	37.92	3203.24
Het df	37	5	2	6	5	2	2	65
Het P	***	**	*	***	***	***	***	***
Fixed RR	8.13	2.54	4.34	3.99	10.63	28.44	48.73	9.81
RRl	7.69	1.79	2.93	3.57	9.59	25.14	42.06	9.43
RRu	8.59	3.59	6.43	4.46	11.78	32.18	56.47	10.21
P	+++	+++	+++	+++	+++	+++	+++	+++
Random RR	6.84	1.76	3.87	4.73	9.08	21.83	37.41	6.70
RRl	4.52	0.82	1.75	3.00	4.93	10.05	17.80	4.98
RRu	10.33	3.74	8.60	7.45	16.71	47.46	78.63	9.02
P	+++	N.S.	+++	+++	+++	+++	+++	+++

MALES

	Duration of smoking (broad categories)				
	absent	1-34k20	21-49k35	36+k50	Total
N	24	7	6	9	46
NS	10	7	6	9	32
Wt	1207.88	137.25	241.40	292.49	1879.03
Het Chi	1314.71	30.82	47.07	60.28	2067.41
Het df	23	6	5	8	45
Het P	***	***	***	***	***
Fixed RR	15.29	2.49	8.25	27.32	13.54
RRl	14.45	2.11	7.27	24.36	12.94
RRu	16.18	2.94	9.36	30.63	14.17
P	+++	+++	+++	+++	+++
Random RR	9.65	3.04	9.46	20.16	9.23
RRl	6.07	1.89	5.60	13.12	6.63
RRu	15.35	4.90	16.00	30.97	12.85
P	+++	+++	+++	+++	+++

Table 1I21 - 3

IESLC - Meta-analysis of Ever/current Smoking by Duration, Overview
All LC types, Cigarettes only
Most adjusted

MALES

Duration of smoking (narrow categories)

	absent	1-19k1	6-29k20	21-39k30	31-49k40	41-998k50	51+k999	Total
N	28	3	2	5	4	2	2	46
NS	11	3	2	5	4	2	2	29
Wt	985.41	22.76	18.11	189.97	258.53	236.94	167.31	1879.03
Het Chi	1189.54	0.77	1.03	35.54	49.02	35.78	19.50	2067.41
Het df	27	2	1	4	3	1	1	45
Het P	***	N.S.	N.S.	***	***	***	***	***
Fixed RR	10.98	3.93	5.94	5.18	14.05	30.88	52.70	13.54
RRl	10.32	2.61	3.74	4.49	12.43	27.18	45.29	12.94
RRu	11.69	5.93	9.41	5.97	15.87	35.07	61.32	14.17
P	+++	+++	+++	+++	+++	+++	+++	+++
Random RR	8.29	3.93	5.92	6.40	13.19	33.84	57.82	9.23
RRl	5.23	2.61	3.70	3.70	6.84	15.46	28.83	6.63
RRu	13.13	5.93	9.46	11.04	25.43	74.11	115.95	12.85
P	+++	+++	+++	+++	+++	+++	+++	+++

FEMALES

Duration of smoking (broad categories)

	absent	1-34k20	21-49k35	36+k50	Total
N	10	2	1	2	15
NS	5	2	1	2	8
Wt	296.68	90.28	119.48	22.41	528.86
Het Chi	95.08	0.35	0.00	8.25	266.72
Het df	9	1	0	1	14
Het P	***	N.S.	N.S.	**	***
Fixed RR	4.22	1.11	3.56	12.20	3.38
RRl	3.77	0.91	2.98	8.06	3.11
RRu	4.73	1.37	4.26	18.46	3.69
P	+++	N.S.	+++	+++	+++
Random RR	4.02	1.11	3.56	15.27	4.06
RRl	2.54	0.91	2.98	4.21	2.65
RRu	6.36	1.37	4.26	55.39	6.22
P	+++	N.S.	+++	+++	+++

Duration of smoking (narrow categories)

	absent	1-19k1	6-29k20	21-39k30	31-49k40	41-998k50	51+k999	Total
N	9	2		1	1	1	1	15
NS	5	2		1	1	1	1	8
Wt	289.70	4.31		111.49	97.98	15.55	9.83	528.86
Het Chi	193.01	2.31		0.00	0.00	0.00	0.00	266.72
Het df	8	1		0	0	0	0	14
Het P	***	N.S.		N.S.	N.S.	N.S.	N.S.	***
Fixed RR	2.95	0.75		2.68	5.36	8.15	12.88	3.38
RRl	2.63	0.29		2.23	4.40	4.96	6.90	3.11
RRu	3.32	1.93		3.23	6.53	13.40	24.07	3.69
P	+++	N.S.		+++	+++	+++	+++	+++
Random RR	4.38	0.66		2.68	5.36	8.15	12.88	4.06
RRl	2.25	0.15		2.23	4.40	4.96	6.90	2.65
RRu	8.52	2.94		3.23	6.53	13.40	24.07	6.22
P	+++	N.S.		+++	+++	+++	+++	+++

Table 1I21 - 4

IESLC - Meta-analysis of Ever/current Smoking by Duration, Overview
All LC types, Cigarettes only
Least adjusted

REF	NRR	X	SEX	AGEL	AGEH	RACE	YF	LC TYPE	LOC	START	ST	NLC	R	VB	P	H	AD	SM	PRODUCT	exL	exH	S1	S2	DENOM	De
AGUDO	507	x	f	0	0	all	-	all	Eu:wst	1989	CC	103	n	bl	n	n	0	ev	cig only	1	16	0	1	nev cigs	st
AGUDO	508	x	f	0	0	all	-	all	Eu:wst	1989	CC	103	n	bl	n	n	0	ev	cig only	17	999	0	0	nev cigs	st
BENHAM	511		m	0	0	all	-	not mix	Eu:wst	1976	CC	1625	n	bl	n	y	0	ev	cig only	1	35	0	0	nev any	st
BENHAM	512		m	0	0	all	-	not mix	Eu:wst	1976	CC	1625	n	bl	n	y	0	ev	cig only	36	999	3	0	nev any	st
BEST	501		m	0	0	all	0	all	NAmer	1955	pr	381	n	V	n	n	1	cu	cig only	1	4	0	0	nev any	ot
BEST	502		m	0	0	all	0	all	NAmer	1955	pr	381	n	V	n	n	1	cu	cig only	5	9	0	1	nev any	ot
BEST	503		m	0	0	all	0	all	NAmer	1955	pr	381	n	V	n	n	1	cu	cig only	10	14	0	0	nev any	ot
BEST	504		m	0	0	all	0	all	NAmer	1955	pr	381	n	V	n	n	1	cu	cig only	15	19	0	0	nev any	ot
BEST	505		m	0	0	all	0	all	NAmer	1955	pr	381	n	V	n	n	1	cu	cig only	20	29	1	2	nev any	ot
BEST	506		m	0	0	all	0	all	NAmer	1955	pr	381	n	V	n	n	1	cu	cig only	30	39	2	3	nev any	ot
BEST	507		m	0	0	all	0	all	NAmer	1955	pr	381	n	V	n	n	1	cu	cig only	40	999	3	0	nev any	ot
BOUCOT	512		m	0	0	all	9	all	NAmer	1951	pr	121	n	bl	n	n	0	ev	cig only	1	39	0	0	nev any	ot
BOUCOT	513		m	0	0	all	9	all	NAmer	1951	pr	121	n	bl	n	n	0	ev	cig only	40	999	3	0	nev any	ot
CEDERL	501		m	40	69	all	10	all	Eu:Sca	1963	pr	491	n	bl	n	n	1	cu	cig only	1	29	1	0	nev any	ot
CEDERL	502		m	40	69	all	10	all	Eu:Sca	1963	pr	491	n	bl	n	n	1	cu	cig only	30	999	0	0	nev any	ot
CEDERL	504		f	40	69	all	10	all	Eu:Sca	1963	pr	491	n	bl	n	n	1	cu	cig only	1	29	1	0	nev any	ot
CEDERL	505		f	40	69	all	10	all	Eu:Sca	1963	pr	491	n	bl	n	n	1	cu	cig only	30	999	0	0	nev any	ot
CPSI	580		m	40	84	wh	0	all	NAmer	1959	pr	5138	n	bl	n	n	0	cu	cig only	1	29	1	0	nev cigs	st
CPSI	581		m	40	84	wh	0	all	NAmer	1959	pr	5138	n	bl	n	n	0	cu	cig only	30	34	0	3	nev cigs	st
CPSI	582		m	40	84	wh	0	all	NAmer	1959	pr	5138	n	bl	n	n	0	cu	cig only	35	39	2	0	nev cigs	st
CPSI	583		m	40	84	wh	0	all	NAmer	1959	pr	5138	n	bl	n	n	0	cu	cig only	40	44	0	4	nev cigs	st
CPSI	584		m	40	84	wh	0	all	NAmer	1959	pr	5138	n	bl	n	n	0	cu	cig only	45	49	0	0	nev cigs	st
CPSI	585		m	40	84	wh	0	all	NAmer	1959	pr	5138	n	bl	n	n	0	cu	cig only	50	54	3	5	nev cigs	st
CPSI	586		m	40	84	wh	0	all	NAmer	1959	pr	5138	n	bl	n	n	0	cu	cig only	55	59	0	0	nev cigs	st
CPSI	587		m	40	84	wh	0	all	NAmer	1959	pr	5138	n	bl	n	n	0	cu	cig only	60	999	0	6	nev cigs	st
CPSI	676		f	40	84	wh	0	all	NAmer	1959	pr	5138	n	bl	n	n	0	cu	cig only	1	29	1	0	nev cigs	st
CPSI	677		f	40	84	wh	0	all	NAmer	1959	pr	5138	n	bl	n	n	0	cu	cig only	30	34	0	3	nev cigs	st
CPSI	678		f	40	84	wh	0	all	NAmer	1959	pr	5138	n	bl	n	n	0	cu	cig only	35	39	2	0	nev cigs	st
CPSI	679		f	40	84	wh	0	all	NAmer	1959	pr	5138	n	bl	n	n	0	cu	cig only	40	44	0	4	nev cigs	st
CPSI	680		f	40	84	wh	0	all	NAmer	1959	pr	5138	n	bl	n	n	0	cu	cig only	45	49	0	0	nev cigs	st
CPSI	681		f	40	84	wh	0	all	NAmer	1959	pr	5138	n	bl	n	n	0	cu	cig only	50	54	3	5	nev cigs	st
CPSI	682		f	40	84	wh	0	all	NAmer	1959	pr	5138	n	bl	n	n	0	cu	cig only	55	999	0	6	nev cigs	st
CPSII	552		m	0	0	all	6	all	NAmer	1982	pr	3229	n	bl	n	n	0	cu	cig only	1	29	1	0	nev any	st
CPSII	553		m	0	0	all	6	all	NAmer	1982	pr	3229	n	bl	n	n	0	cu	cig only	30	34	0	3	nev any	st
CPSII	554		m	0	0	all	6	all	NAmer	1982	pr	3229	n	bl	n	n	0	cu	cig only	35	39	2	0	nev any	st
CPSII	555		m	0	0	all	6	all	NAmer	1982	pr	3229	n	bl	n	n	0	cu	cig only	40	44	0	4	nev any	st
CPSII	556		m	0	0	all	6	all	NAmer	1982	pr	3229	n	bl	n	n	0	cu	cig only	45	49	0	0	nev any	st
CPSII	557		m	0	0	all	6	all	NAmer	1982	pr	3229	n	bl	n	n	0	cu	cig only	50	54	3	5	nev any	st
CPSII	558		m	0	0	all	6	all	NAmer	1982	pr	3229	n	bl	n	n	0	cu	cig only	55	59	0	0	nev any	st
CPSII	559		m	0	0	all	6	all	NAmer	1982	pr	3229	n	bl	n	n	0	cu	cig only	60	999	0	6	nev any	st
JUSSAW	510		m	0	0	all	-	all	As:Ind	1964	CC	792	n	V	n	n	0	ev	cig only	1	9	0	1	nev any	st
JUSSAW	511		m	0	0	all	-	all	As:Ind	1964	CC	792	n	V	n	n	0	ev	cig only	10	19	0	0	nev any	st
JUSSAW	512		m	0	0	all	-	all	As:Ind	1964	CC	792	n	V	n	n	0	ev	cig only	20	29	1	2	nev any	st
JUSSAW	513		m	0	0	all	-	all	As:Ind	1964	CC	792	n	V	n	n	0	ev	cig only	30	39	2	3	nev any	st
JUSSAW	514		m	0	0	all	-	all	As:Ind	1964	CC	792	n	V	n	n	0	ev	cig only	40	999	3	0	nev any	st
KAISE2	596		m	0	0	all	9	all	NAmer	1979	pr	318	n	bl	n	n	1	cu	cig only	1	39	0	0	nev any	st
KAISE2	597		m	0	0	all	9	all	NAmer	1979	pr	318	n	bl	n	n	1	cu	cig only	40	999	3	0	nev any	st
KAISE2	516		f	0	0	all	9	all	NAmer	1979	pr	318	n	bl	n	n	1	cu	cig only	1	39	0	0	nev any	st
KAISE2	517		f	0	0	all	9	all	NAmer	1979	pr	318	n	bl	n	n	1	cu	cig only	40	999	3	0	nev any	st
LUBIN	519	x	m	0	0	all	-	all	As:Chi	1984	CC	427	m	ot	y	n	0	ev	cig only	1	29	1	0	nev any	st
LUBIN	520	x	m	0	0	all	-	all	As:Chi	1984	CC	427	m	ot	y	n	0	ev	cig only	30	39	2	3	nev any	st
LUBIN	521	x	m	0	0	all	-	all	As:Chi	1984	CC	427	m	ot	y	n	0	ev	cig only	40	49	0	4	nev any	st
LUBIN	522	x	m	0	0	all	-	all	As:Chi	1984	CC	427	m	ot	y	n	0	ev	cig only	50	999	3	0	nev any	st
NOTAN2	513		c	0	0	all	-	all	As:Ind	1963	CC	683	n	V	n	n	0	ev	cig only	1	10	0	1	nev any	st
NOTAN2	514		c	0	0	all	-	all	As:Ind	1963	CC	683	n	V	n	n	0	ev	cig only	11	20	1	2	nev any	st
NOTAN2	515		c	0	0	all	-	all	As:Ind	1963	CC	683	n	V	n	n	0	ev	cig only	21	30	0	3	nev any	st
NOTAN2	516		c	0	0	all	-	all	As:Ind	1963	CC	683	n	V	n	n	0	ev	cig only	31	40	2	4	nev any	st
NOTAN2	517		c	0	0	all	-	all	As:Ind	1963	CC	683	n	V	n	n	0	ev	cig only	41	999	3	0	nev any	st
PEZZOT	534		m	0	0	all	-	all	SCAmer	1987	CC	215	n	bl	n	y	0	ev	cig only	1	30	1	0	nev cigs	st
PEZZOT	535		m	0	0	all	-	all	SCAmer	1987	CC	215	n	bl	n	y	0	ev	cig only	31	40	2	4	nev cigs	st
PEZZOT	536		m	0	0	all	-	all	SCAmer	1987	CC	215	n	bl	n	y	0	ev	cig only	41	999	3	0	nev cigs	st
TIZZAN	501		m	0	0	all	-	all	Eu:wst	1959	CC	1358	n	bl	n	n	0	ev	cig only	1	4	0	0	nev any	st
TIZZAN	502		m	0	0	all	-	all	Eu:wst	1959	CC	1358	n	bl	n	n	0	ev	cig only	5	10	0	1	nev any	st
TIZZAN	503		m	0	0	all	-	all	Eu:wst	1959	CC	1358	n	bl	n	n	0	ev	cig only	11	999	0	0	nev any	st
TIZZAN	530		f	0	0	all	-	all	Eu:wst	1959	CC	1358	n	bl	n	n	0	ev	cig only	1	10	0	1	nev any	st
TIZZAN	531		f	0	0	all	-	all	Eu:wst	1959	CC	1358	n	bl	n	n	0	ev	cig only	11	999	0	0	nev any	st

Cigarette type is all/unspec for all RRs

except for the following:

Table 1I21 - 4

IESLC - Meta-analysis of Ever/current Smoking by Duration, Overview
All LC types, Cigarettes only
Least adjusted

REF	NRR	CIGTYPE
JUSSAW	510	MC only
JUSSAW	511	MC only
JUSSAW	512	MC only
JUSSAW	513	MC only
JUSSAW	514	MC only
NOTAN2	513	MC only
NOTAN2	514	MC only
NOTAN2	515	MC only
NOTAN2	516	MC only
NOTAN2	517	MC only

In this overview table, subtotals and Qs values may be invalid and should be ignored

Table 1I21 - 5

IESLC - Meta-analysis of Ever/current Smoking by Duration, Overview
All LC types, Cigarettes only
Least adjusted

REF	NRR	SEX	AD	Number Exposed Case	Cont	Non-exposed Case	Cont	RR	95.00%CI
AGUDO	507	f	0	5	12	80	183	0.95	(0.33- 2.79)
AGUDO	508	f	0	18	11	80	183	3.74	(1.69- 8.29)
Subtotal AGUDO								2.31	(1.22- 4.38)
BENHAM	511	m	0	490	772	33	523	10.06	(6.95- 14.56)
BENHAM	512	m	0	694	620	33	523	17.74	(12.28- 25.63)
Subtotal BENHAM								13.37	(10.30- 17.36)
*BEST	501	m	1	1	-	7	-	1.60	(0.20- 13.00)
*BEST	502	m	1	2	-	7	-	2.60	(0.54- 12.52)
*BEST	503	m	1	6	-	7	-	2.30	(0.77- 6.84)
*BEST	504	m	1	10	-	7	-	3.20	(1.22- 8.41)
*BEST	505	m	1	22	-	7	-	4.10	(1.75- 9.60)
*BEST	506	m	1	55	-	7	-	13.90	(6.33- 30.52)
*BEST	507	m	1	137	-	7	-	14.20	(6.64- 30.35)
Subtotal BEST								6.17	(4.26- 8.94)
*BOUCOT	512	m	0	19	2088	0	805	15.04~	(0.91- 248.83)
*BOUCOT	513	m	0	44	1177	0	805	60.88~	(3.75- 987.17)
Subtotal BOUCOT								30.41	(4.21- 219.61)
*CEDERL	501	m	1	5	-	7	-	1.80	(0.57- 5.66)
*CEDERL	502	m	1	23	-	7	-	7.40	(3.18- 17.21)
*CEDERL	504	f	1	3	-	19	-	1.60	(0.47- 5.40)
*CEDERL	505	f	1	5	-	19	-	9.60	(3.60- 25.58)
Subtotal CEDERL								4.61	(2.78- 7.67)
*CPSI	580	m	0	95	266163	196	926068	1.69	(1.32- 2.15)
*CPSI	581	m	0	230	290031	196	926068	3.75	(3.10- 4.53)
*CPSI	582	m	0	470	367622	196	926068	6.04	(5.11- 7.14)
*CPSI	583	m	0	731	333292	196	926068	10.36	(8.85- 12.13)
*CPSI	584	m	0	764	221405	196	926068	16.30	(13.94- 19.07)
*CPSI	585	m	0	576	119633	196	926068	22.75	(19.35- 26.75)
*CPSI	586	m	0	356	53226	196	926068	31.60	(26.55- 37.61)
*CPSI	587	m	0	232	26906	196	926068	40.74	(33.70- 49.25)
*CPSI	676	f	0	105	694015	532	3877179	1.10	(0.89- 1.36)
*CPSI	677	f	0	141	383127	532	3877179	2.68	(2.23- 3.23)
*CPSI	678	f	0	154	315060	532	3877179	3.56	(2.98- 4.26)
*CPSI	679	f	0	120	163178	532	3877179	5.36	(4.40- 6.53)
*CPSI	680	f	0	54	53635	532	3877179	7.34	(5.55- 9.71)
*CPSI	681	f	0	16	14305	532	3877179	8.15	(4.96- 13.40)
*CPSI	682	f	0	10	5657	532	3877179	12.88	(6.90- 24.07)
Subtotal CPSI								8.01	(7.62- 8.43)
*CPSII	552	m	0	72	141932	124	742207	3.04	(2.27- 4.06)
*CPSII	553	m	0	145	113317	124	742207	7.66	(6.03- 9.73)
*CPSII	554	m	0	244	109788	124	742207	13.30	(10.72- 16.51)
*CPSII	555	m	0	413	103500	124	742207	23.88	(19.54- 29.19)
*CPSII	556	m	0	307	53805	124	742207	34.15	(27.73- 42.06)
*CPSII	557	m	0	332	39260	124	742207	50.62	(41.19- 62.20)
*CPSII	558	m	0	151	13598	124	742207	66.47	(52.45- 84.24)
*CPSII	559	m	0	117	8450	124	742207	82.88	(64.43- 106.60)
Subtotal CPSII								24.98	(23.06- 27.07)
JUSSAW	510	m	0	16	20	149	624	3.35	(1.70- 6.62)
JUSSAW	511	m	0	34	19	149	624	7.49	(4.16- 13.51)
JUSSAW	512	m	0	38	23	149	624	6.92	(4.00- 11.97)
JUSSAW	513	m	0	27	9	149	624	12.56	(5.79- 27.28)
JUSSAW	514	m	0	11	6	149	624	7.68	(2.79- 21.09)
Subtotal JUSSAW								6.77	(5.01- 9.15)
*KAISE2	596	m	1	17	-	14	-	4.86	(2.22- 10.61)
*KAISE2	597	m	1	34	-	14	-	15.64	(8.31- 29.40)
*KAISE2	516	f	1	24	-	11	-	9.09	(4.25- 19.43)
*KAISE2	517	f	1	26	-	11	-	30.41	(14.39- 64.25)
Subtotal KAISE2								12.59	(8.77- 18.07)
LUBIN	519	m	0	7	51	8	72	1.24	(0.42- 3.62)
LUBIN	520	m	0	16	67	8	72	2.15	(0.86- 5.35)
LUBIN	521	m	0	21	57	8	72	3.32	(1.37- 8.04)
LUBIN	522	m	0	8	16	8	72	4.50	(1.47- 13.79)
Subtotal LUBIN								2.52	(1.54- 4.12)
NOTAN2	513	c	0	7	15	107	201	0.88	(0.35- 2.22)
NOTAN2	514	c	0	15	15	107	201	1.88	(0.88- 3.99)
NOTAN2	515	c	0	17	16	107	201	2.00	(0.97- 4.11)
NOTAN2	516	c	0	12	7	107	201	3.22	(1.23- 8.42)
NOTAN2	517	c	0	5	5	107	201	1.88	(0.53- 6.63)
Subtotal NOTAN2								1.83	(1.24- 2.70)
PEZZOT	534	m	0	30	134	4	116	6.49	(2.22- 18.98)
PEZZOT	535	m	0	71	82	4	116	25.11	(8.82- 71.48)

Table 1I21 - 5

IESLC - Meta-analysis of Ever/current Smoking by Duration, Overview
All LC types, Cigarettes only
Least adjusted

REF	NRR	SEX	AD	Number Exposed Case	Cont	Non-exposed Case	Cont	RR	95.00%CI
PEZZOT	536	m	0	110	101	4	116	31.58	(11.25- 88.71)
Subtotal PEZZOT								17.64	(9.62- 32.34)
TIZZAN	501	m	0	12	1	180	305	20.33	(2.62- 157.68)
TIZZAN	502	m	0	54	20	180	305	4.58	(2.65- 7.89)
TIZZAN	503	m	0	928	815	180	305	1.93	(1.57- 2.37)
TIZZAN	530	f	0	2	7	117	114	0.28	(0.06- 1.37)
TIZZAN	531	f	0	23	21	117	114	1.07	(0.56- 2.03)
Subtotal TIZZAN								2.01	(1.68- 2.42)
Partial Totals				8939	3897092	875940495379			

*prospective study ~ With 0.5 adjustment for zero

REF	NRR	SEX	AD	Ys	Ws	Qs	Ps
AGUDO	507	f	0	-0.05	3.32	17.98	0.9303
AGUDO	508	f	0	1.32	6.08	5.60	0.0011
Subtotal AGUDO				0.84	9.40	23.59	
BENHAM	511	m	0	2.31	28.13	0.02	0.0000
BENHAM	512	m	0	2.88	28.35	10.07	0.0000
Subtotal BENHAM				2.59	56.48	10.10	
*BEST	501	m	1	0.47	0.88	2.89	0.6590
*BEST	502	m	1	0.96	1.55	2.73	0.2335
*BEST	503	m	1	0.83	3.22	6.74	0.1350
*BEST	504	m	1	1.16	4.12	5.14	0.0182
*BEST	505	m	1	1.41	5.30	4.00	0.0012
*BEST	506	m	1	2.63	6.21	0.77	0.0000
*BEST	507	m	1	2.65	6.65	0.93	0.0000
Subtotal BEST				1.82	27.95	23.20	
*BOUCOT	512	m	0	2.71	0.49	0.09	0.0583
*BOUCOT	513	m	0	4.11	0.49	1.66	0.0038
Subtotal BOUCOT				3.41	0.98	1.75	
*CEDERL	501	m	1	0.59	2.92	8.35	0.3155
*CEDERL	502	m	1	2.00	5.39	0.42	0.0000
*CEDERL	504	f	1	0.47	2.58	8.44	0.4505
*CEDERL	505	f	1	2.26	4.00	0.00	0.0000
Subtotal CEDERL				1.53	14.88	17.21	
*CPSI	580	m	0	0.52	64.01	197.64	0.0000
*CPSI	581	m	0	1.32	105.87	97.35	0.0000
*CPSI	582	m	0	1.80	138.39	32.06	0.0000
*CPSI	583	m	0	2.34	154.66	0.53	0.0000
*CPSI	584	m	0	2.79	156.12	40.86	0.0000
*CPSI	585	m	0	3.12	146.44	104.49	0.0000
*CPSI	586	m	0	3.45	126.72	174.48	0.0000
*CPSI	587	m	0	3.71	106.68	217.35	0.0000
*CPSI	676	f	0	0.10	87.71	417.62	0.3603
*CPSI	677	f	0	0.99	111.49	186.46	0.0000
*CPSI	678	f	0	1.27	119.48	121.73	0.0000
*CPSI	679	f	0	1.68	97.98	35.38	0.0000
*CPSI	680	f	0	1.99	49.07	4.04	0.0000
*CPSI	681	f	0	2.10	15.55	0.51	0.0000
*CPSI	682	f	0	2.56	9.83	0.75	0.0000
Subtotal CPSI				2.08	1489.99	1631.25	
*CPSII	552	m	0	1.11	45.57	62.29	0.0000
*CPSII	553	m	0	2.04	66.89	3.98	0.0000
*CPSII	554	m	0	2.59	82.29	7.81	0.0000
*CPSII	555	m	0	3.17	95.47	76.20	0.0000
*CPSII	556	m	0	3.53	88.48	138.48	0.0000
*CPSII	557	m	0	3.92	90.50	244.74	0.0000
*CPSII	558	m	0	4.20	68.44	251.47	0.0000
*CPSII	559	m	0	4.42	60.64	277.05	0.0000
Subtotal CPSII				3.22	598.26	1062.01	
JUSSAW	510	m	0	1.21	8.28	9.49	0.0005
JUSSAW	511	m	0	2.01	11.07	0.78	0.0000
JUSSAW	512	m	0	1.93	12.80	1.53	0.0000
JUSSAW	513	m	0	2.53	6.39	0.40	0.0000
JUSSAW	514	m	0	2.04	3.76	0.22	0.0001
Subtotal JUSSAW				1.91	42.30	12.42	
*KAISE2	596	m	1	1.58	6.28	3.07	0.0001
*KAISE2	597	m	1	2.75	9.62	2.13	0.0000
*KAISE2	516	f	1	2.21	6.65	0.04	0.0000
*KAISE2	517	f	1	3.41	6.86	8.84	0.0000
Subtotal KAISE2				2.53	29.42	14.07	

Table 1I21 - 5

IESLC - Meta-analysis of Ever/current Smoking by Duration, Overview
All LC types, Cigarettes only
Least adjusted

REF	NRR	SEX	AD	Ys	Ws	Qs	Ps
LUBIN	519	m	0	0.21	3.32	14.20	0.7003
LUBIN	520	m	0	0.77	4.62	10.61	0.1000
LUBIN	521	m	0	1.20	4.90	5.73	0.0080
LUBIN	522	m	0	1.50	3.06	1.84	0.0085
Subtotal LUBIN				0.93	15.91	32.38	
NOTAN2	513	c	0	-0.13	4.47	25.98	0.7808
NOTAN2	514	c	0	0.63	6.77	18.42	0.1008
NOTAN2	515	c	0	0.69	7.37	18.61	0.0606
NOTAN2	516	c	0	1.17	4.16	5.13	0.0171
NOTAN2	517	c	0	0.63	2.41	6.57	0.3273
Subtotal NOTAN2				0.60	25.18	74.70	
PEZZOT	534	m	0	1.87	3.34	0.56	0.0006
PEZZOT	535	m	0	3.22	3.51	3.12	0.0000
PEZZOT	536	m	0	3.45	3.60	4.95	0.0000
Subtotal PEZZOT				2.87	10.45	8.64	
TIZZAN	501	m	0	3.01	0.92	0.49	0.0039
TIZZAN	502	m	0	1.52	12.93	7.45	0.0000
TIZZAN	503	m	0	0.66	89.78	236.37	0.0000
TIZZAN	530	f	0	-1.28	1.51	19.18	0.1155
TIZZAN	531	f	0	0.06	9.22	45.25	0.8435
Subtotal TIZZAN				0.70	114.36	308.74	

N	66
NS	13

Table 1I21 - 6

IESLC - Meta-analysis of Ever/current Smoking by Duration, Overview
All LC types, Cigarettes only
Least adjusted

	Sex			
	combined	male	female	Total
N	5	46	15	66
NS	1	11	5	17

In this overview table, other than the “N” rows, entries in the “absent” and “Total” columns may be invalid and should be ignored

		Duration of smoking (broad categories)				
		absent	1-34k20	21-49k35	36+k50	Total
	N	36	10	8	12	66
	NS	12	8	7	10	37
	Wt	1518.88	234.31	365.05	317.32	2435.56
Het	Chi	1873.13	66.61	105.81	104.72	3220.05
Het	df	35	9	7	11	65
Het	P	***	***	***	***	***
Fixed	RR	11.62	1.81	6.20	25.10	9.77
	RRl	11.05	1.59	5.59	22.49	9.39
	RRu	12.22	2.06	6.87	28.02	10.17
	P	+++	+++	+++	+++	+++
Random	RR	6.44	2.40	7.25	15.52	6.52
	RRl	4.34	1.57	4.44	10.01	4.84
	RRu	9.57	3.68	11.85	24.06	8.79
	P	+++	+++	+++	+++	+++

			Duration of smoking (narrow categories)					
		absent	1-19k1	6-29k20	21-39k30	31-49k40	41-998k50	51+k999	Total
	N	38	6	3	7	6	3	3	66
	NS	13	5	3	6	5	2	2	36
	Wt	1279.47	32.06	24.88	308.85	360.67	252.49	177.15	2435.56
Het	Chi	1778.60	20.70	7.55	69.84	123.78	61.66	37.92	3220.05
Het	df	37	5	2	6	5	2	2	65
Het	P	***	***	*	***	***	***	***	***
Fixed	RR	8.09	2.43	4.34	3.99	10.59	28.44	48.73	9.77
	RRl	7.66	1.72	2.93	3.57	9.55	25.14	42.06	9.39
	RRu	8.55	3.44	6.43	4.46	11.74	32.18	56.47	10.17
	P	+++	+++	+++	+++	+++	+++	+++	+++
Random	RR	6.64	1.65	3.87	4.71	8.72	21.83	37.41	6.52
	RRl	4.39	0.76	1.75	2.99	4.71	10.05	17.80	4.84
	RRu	10.03	3.60	8.60	7.42	16.15	47.46	78.63	8.79
	P	+++	N.S.	+++	+++	+++	+++	+++	+++

MALES

		Duration of smoking (broad categories)				
		absent	1-34k20	21-49k35	36+k50	Total
	N	24	7	6	9	46
	NS	10	7	6	9	32
	Wt	1207.88	137.25	241.41	292.49	1879.05
Het	Chi	1318.53	31.21	47.76	66.85	2076.60
Het	df	23	6	5	8	45
Het	P	***	***	***	***	***
Fixed	RR	15.27	2.49	8.24	27.10	13.51
	RRl	14.44	2.10	7.27	24.17	12.91
	RRu	16.16	2.94	9.35	30.39	14.14
	P	+++	+++	+++	+++	+++
Random	RR	9.54	3.01	9.39	18.84	9.01
	RRl	5.99	1.87	5.54	12.02	6.47
	RRu	15.18	4.86	15.93	29.52	12.56
	P	+++	+++	+++	+++	+++

Table 1I21 - 6

IESLC - Meta-analysis of Ever/current Smoking by Duration, Overview
All LC types, Cigarettes only
Least adjusted

MALES

Duration of smoking (narrow categories)

	absent	1-19k1	6-29k20	21-39k30	31-49k40	41-998k50	51+k999	Total
N	28	3	2	5	4	2	2	46
NS	11	3	2	5	4	2	2	29
Wt	985.41	22.76	18.11	189.98	258.53	236.94	167.31	1879.05
Het Chi	1193.19	0.77	1.03	35.98	52.60	35.78	19.50	2076.60
Het df	27	2	1	4	3	1	1	45
Het P	***	N.S.	N.S.	***	***	***	***	***
Fixed RR	10.96	3.93	5.94	5.17	13.97	30.88	52.70	13.51
RRl	10.29	2.61	3.74	4.48	12.37	27.18	45.29	12.91
RRu	11.66	5.93	9.41	5.96	15.78	35.07	61.32	14.14
P	+++	+++	+++	+++	+++	+++	+++	+++
Random RR	8.06	3.93	5.92	6.34	12.43	33.84	57.82	9.01
RRl	5.08	2.61	3.70	3.66	6.31	15.46	28.83	6.47
RRu	12.77	5.93	9.46	10.99	24.46	74.11	115.95	12.56
P	+++	+++	+++	+++	+++	+++	+++	+++

FEMALES

Duration of smoking (broad categories)

	absent	1-34k20	21-49k35	36+k50	Total
N	10	2	1	2	15
NS	5	2	1	2	8
Wt	299.16	90.28	119.48	22.41	531.33
Het Chi	98.41	0.35	0.00	8.25	268.81
Het df	9	1	0	1	14
Het P	***	N.S.	N.S.	**	***
Fixed RR	4.18	1.11	3.56	12.20	3.37
RRl	3.73	0.91	2.98	8.06	3.09
RRu	4.68	1.37	4.26	18.46	3.67
P	+++	N.S.	+++	+++	+++
Random RR	3.80	1.11	3.56	15.27	3.91
RRl	2.40	0.91	2.98	4.21	2.56
RRu	6.01	1.37	4.26	55.39	5.98
P	+++	N.S.	+++	+++	+++

Duration of smoking (narrow categories)

	absent	1-19k1	6-29k20	21-39k30	31-49k40	41-998k50	51+k999	Total
N	9	2		1	1	1	1	15
NS	5	2		1	1	1	1	8
Wt	291.65	4.83		111.49	97.98	15.55	9.83	531.33
Het Chi	192.12	1.58		0.00	0.00	0.00	0.00	268.81
Het df	8	1		0	0	0	0	14
Het P	***	N.S.		N.S.	N.S.	N.S.	N.S.	***
Fixed RR	2.95	0.65		2.68	5.36	8.15	12.88	3.37
RRl	2.63	0.27		2.23	4.40	4.96	6.90	3.09
RRu	3.30	1.58		3.23	6.53	13.40	24.07	3.67
P	+++	N.S.		+++	+++	+++	+++	+++
Random RR	4.24	0.60		2.68	5.36	8.15	12.88	3.91
RRl	2.20	0.18		2.23	4.40	4.96	6.90	2.56
RRu	8.19	1.92		3.23	6.53	13.40	24.07	5.98
P	+++	N.S.		+++	+++	+++	+++	+++

Table 1I21 - 7

IESLC - Meta-analysis of Ever/current Smoking by Duration, Overview
All LC types, Cigarettes only
Excluded studies (and stage at which they were excluded)

1	BECHER BLOT1 BROWN3 CARPEN CHYOU DARBY DOLL2 GARCIA GRAHAM GURSEL HAMMO2 JAHN JAIN LAUSSM PRESCO QIAO TVERDA WIGLE WYNDE3
2	ALDERS BENSHL BRESLO CHIAZZ DEAN3 DORN ENGELA GAO2 GILLIS GUO HEGMAN HIRAYA HOLE KAUFMA KOO KOULUM LIU4 MIGRAN MRFITR PERNU SEGI2 SPEIZE SUZUK2 SVENSS VUTUC WAKAI WU YUAN
3	GENG MCDUFF SPITZ STASZE WU2 ZHANG
4	BOUCHA CHEN CORREA JEDRYC LUO WYNDE2 WYNDE6
5	AKIBA HAMMON PISANI RESTRE SADOWS XU
7	AMANDU AMES ARMADA AUVINE AXELSS BARBON BOFFET BROSS BUFFLE CHEN2 CHOI DAMBER DEAN2 DESTEF DOLL DORGAN DOSEME FAN GAO GARSHI GER HAENSZ HU HU2 HUMBLE JOLY KATSOU KHUDER KREUZE LETOUR LEVIN LIAW LIU3 LIU5 LUBIN2 MATOS MCCONN OSANN2 PEZZO2 QIAO2 RACHTA SOBUE WANG2 WATSON WUWILL WYNDE7 WYNDE8 ZHENG ZHOU

Table 1I21 - 8
Potentially overlapping studies

REF	REFGP	PRINC	. OVERLAP/LINK
BENHAM	LUBIN2	2	Subset of Lubin2
CPSI	CPSI	1	CPSI overall
LUBIN	XIANGZ	2	LUBIN/XIANGZ/QIAO

Table 1I21 - 9
Most adjusted - insufficient data for meta-analysis

REF	NRR	SEX	AGEL	AGEH	RACE	YF	LC TYPE	LOC	START	ST	NLC	R	VB	P	H	AD	SM	PRODUCT	exL	exH	S1	S2	DENOM	De
HAMMON	513	m	0	0	wh	0	all	NAmer	1952	pr	448	n	bl	n	n	1	ev	cig only	1	34	1	0	nev any	st
HAMMON	514	m	0	0	wh	0	all	NAmer	1952	pr	448	n	bl	n	n	1	ev	cig only	35	999	0	0	nev any	st
LUBIN2	1059	m	0	0	all	-	all	Eu:mul	1976	CC	7804	n	bl	n	y	1	ev	cig only	1	39	0	0	nev any	or
LUBIN2	1060	m	0	0	all	-	all	Eu:mul	1976	CC	7804	n	bl	n	y	1	ev	cig only	40	999	3	0	nev any	or
SADOWS	522	m	0	0	wh	-	all	NAmer	1938	CC	477	n	bl	n	n	0	ev	cig only	1	9	0	1	nev any	ot
SADOWS	523	m	0	0	wh	-	all	NAmer	1938	CC	477	n	bl	n	n	0	ev	cig only	10	19	0	0	nev any	ot
SADOWS	524	m	0	0	wh	-	all	NAmer	1938	CC	477	n	bl	n	n	0	ev	cig only	20	29	1	2	nev any	ot
SADOWS	525	m	0	0	wh	-	all	NAmer	1938	CC	477	n	bl	n	n	0	ev	cig only	30	39	2	3	nev any	ot
SADOWS	526	m	0	0	wh	-	all	NAmer	1938	CC	477	n	bl	n	n	0	ev	cig only	40	49	0	4	nev any	ot
SADOWS	527	m	0	0	wh	-	all	NAmer	1938	CC	477	n	bl	n	n	0	ev	cig only	50	999	3	0	nev any	ot

REF	NRR	RR	SIG	RRDATA comment
HAMMON	513		*	RR for <1/2 pack per day is 5.31, that for 1/2 to 1 pack per day is 6.56, that for 1 to 2 packs per day is 7.27 while that for 2+ packs per day 10.78
HAMMON	514		*	RR for <1/2 pack per day is 10.86, that for 1/2 to 1 pack per day is 8.20, that for 1 to 2 packs per day is 19.69 while that for 2+ packs per day 22.89
LUBIN2	1059	7.00		0
LUBIN2	1060	12.36		0
SADOWS	522	1.19		0
SADOWS	523	1.16		0
SADOWS	524	2.78		0
SADOWS	525	3.95		0
SADOWS	526	7.00		0
SADOWS	527	8.43		0

Table 1I22 -

IESLC - Meta-analysis of Ever/current Smoking, Duration, "Low"
All LC types, Cigarettes only

This analysis is restricted to results for:
1) Ever/current smokers
2) Results by Duration
3) Categorical results by Duration
4) All LC types (or near equivalent)
5) Results complete enough for use in metaanalysis

Within each study, results are then selected (in the following order of preference, within each sex) for:
6) SMKSTA: ever, current
7) PRODUCT: cigarettes only
8) CIGTYPE: all/unspecified, MC regardless of HR, MC only
9) (not applicable)
10) DENOM: never smoked anything, never smoked cigarettes, never any + low, never cigs + low
11) Followup period (YF, prospective studies): whole study (coded as 0) or longest available
12) LCtype: all or nearest available, at least Squamous and Adeno. (q = squamous, s = small,
l = large, a = adeno, mix = mixed, alv = alveolar)
13) Race: all or nearest available, otherwise by race (wh or w = white, bl or b = black, hi = hispanic
ch = chinese, jap = japanese, haw = hawaiian, w+o = white + oriental, sca = scandinavian, as = asian)
14) Duration "low" in key scheme 1 (key value 20, maximum range 1-34)
15) For overlapping studies: principal rather than subsidiary studies
Finally by Age: whole study (coded as 0) if available, otherwise by widest available age group
and then for single sex results (m, f) in preference to results for both sexes combined (c).

Results adjusted (AD) for the most potential confounders are then chosen in Sections -1 to -3
(and those which actually differ from the adjusted results in Table 1I12 - 1 are marked 'x' in Section -1)
and results adjusted for the least confounders in Sections -4 to -6. (Those least adjusted results which
actually differ from the most adjusted are marked 'x' in column X in Section -4)

Section -7 shows excluded studies, together with the stage (as above) at which no qualifying
results were found.

Section -8 lists the potentially overlapping studies which have been included (1=principal, 2=subsidiary).

Section -9 lists any results which would have been included in preference except that they had data not complete
enough for use in meta-analysis, with their significance (yes/no), if known, and any further comment as entered
on the database. It also lists as "gap" any categories for which no data were presented by the original authors.

In addition to those mentioned above, the following fields, levels and abbreviations are used:
* or nk = not known, n = no, y = yes, ot = other
ev = ever, cu = current, nev = never
all/unspec = all or unspecified, MC = manufactured cigarettes, HR = hand-rolled cigarettes
exL, exH = range of exposure (low and high) in the smoking group, in terms of Duration
REF: 6-character study reference
NRR: number of the RR on the database within the study
ST : study type (CC = case control, pr or prosp = prospective)
NLC: number of lung cancer cases in whole study
R : risky occupational population (n = no, m = mining, o = other risky)
VB : national cigarette type (V = at least 75% Virginia, bl = at least 75% blended, ot = other)
P : any proxy use
H : full histological confirmation
De : derivation of RR/CI (or = original, st = standard method, ot = other method of estimation)

Table 1I22 - 1

IESLC - Meta-analysis of Ever/current Smoking, Duration, "Low"
All LC types, Cigarettes only
Most adjusted

REF	NRR	1I12	SEX	AGEL	AGEH	RACE	YF	LC TYPE	LOC	START	ST	NLC	R	VB	P	H	AD	SM	PRODUCT	exL	exH	DENOM	De
BEST	505		m	0	0	all	0	all	NAmer	1955	pr	381	n	V	n	n	1	cu	cig only	20	29	nev any	ot
CEDERL	501		m	40	69	all	10	all	Eu:Sca	1963	pr	491	n	bl	n	n	1	cu	cig only	1	29	nev any	ot
CEDERL	504		f	40	69	all	10	all	Eu:Sca	1963	pr	491	n	bl	n	n	1	cu	cig only	1	29	nev any	ot
CPSI	580		m	40	84	wh	0	all	NAmer	1959	pr	5138	n	bl	n	n	0	cu	cig only	1	29	nev cigs	st
CPSI	676		f	40	84	wh	0	all	NAmer	1959	pr	5138	n	bl	n	n	0	cu	cig only	1	29	nev cigs	st
CPSII	552		m	0	0	all	6	all	NAmer	1982	pr	3229	n	bl	n	n	0	cu	cig only	1	29	nev any	st
JUSSAW	512		m	0	0	all	-	all	As:Ind	1964	CC	792	n	V	n	n	0	ev	cig only	20	29	nev any	st
LUBIN	526	x	m	0	0	all	-	all	As:Chi	1984	CC	427	m	ot	y	n	4	ev	cig only	1	29	nev any	ot
NOTAN2	514		c	0	0	all	-	all	As:Ind	1963	CC	683	n	V	n	n	0	ev	cig only	11	20	nev any	st
PEZZOT	534		m	0	0	all	-	all	SCAmer	1987	CC	215	n	bl	n	y	0	ev	cig only	1	30	nev cigs	st

Cigarette type is all/unspec for all RRs

except for the following:

REF	NRR	CIGTYPE
JUSSAW	512	MC only
NOTAN2	514	MC only

Table 1I22 - 2

IESLC - Meta-analysis of Ever/current Smoking, Duration, "Low"
All LC types, Cigarettes only
Most adjusted

REF	NRR	SEX	AD	Number Exposed Case	Cont	Non-exposed Case	Cont	RR	95.00%CI
*BEST	505	m	1	22	-	7	-	4.10	(1.75- 9.60)
*CEDERL	501	m	1	5	-	7	-	1.80	(0.57- 5.66)
*CEDERL	504	f	1	3	-	19	-	1.60	(0.47- 5.40)
Subtotal CEDERL								1.70	(0.74- 3.93)
*CPSI	580	m	0	95	266163	196	926068	1.69	(1.32- 2.15)
*CPSI	676	f	0	105	694015	532	3877179	1.10	(0.89- 1.36)
Subtotal CPSI								1.32	(1.13- 1.55)
*CPSII	552	m	0	72	141932	124	742207	3.04	(2.27- 4.06)
JUSSAW	512	m	0	38	23	149	624	6.92	(4.00- 11.97)
LUBIN	526	m	4	7	-	8	-	1.35	(0.46- 3.96)
NOTAN2	514	c	0	15	15	107	201	1.88	(0.88- 3.99)
PEZZOT	534	m	0	30	134	4	116	6.49	(2.22- 18.98)
Partial Totals				392	1102282	1153	5546395		

*prospective study

REF	NRR	SEX	AD	Ys	Ws	Qs	Ps
*BEST	505	m	1	1.41	5.30	3.53	0.0012
*CEDERL	501	m	1	0.59	2.92	0.00	0.3155
*CEDERL	504	f	1	0.47	2.58	0.04	0.4505
Subtotal CEDERL				0.53	5.49	0.04	
*CPSI	580	m	0	0.52	64.01	0.33	0.0000
*CPSI	676	f	0	0.10	87.71	21.65	0.3603
Subtotal CPSI				0.28	151.71	21.99	
*CPSII	552	m	0	1.11	45.57	12.14	0.0000
JUSSAW	512	m	0	1.93	12.80	22.98	0.0000
LUBIN	526	m	4	0.30	3.32	0.29	0.5848
NOTAN2	514	c	0	0.63	6.77	0.01	0.1008
PEZZOT	534	m	0	1.87	3.34	5.44	0.0006

	N	10
	NS	8
	Wt	234.31
Het	Chi	66.41
Het	df	9
Het	P	***
Fixed	RR	1.81
	RRl	1.59
	RRu	2.06
	P	+++
Random	RR	2.42
	RRl	1.58
	RRu	3.70
	P	+++
Asymm	P	N.S.

Table 1I22 - 3

IESLC - Meta-analysis of Ever/current Smoking, Duration, "Low"
All LC types, Cigarettes only
Most adjusted

	Sex			
	combined	male	female	Total
N	1	7	2	10
NS	1	7	2	10
Wt	6.77	137.25	90.28	234.31
Het Chi	0.00	30.82	0.35	66.41
Het df	0	6	1	9
Het P	N.S.	***	N.S.	***
Fixed RR	1.88	2.49	1.11	1.81
RRl	0.88	2.11	0.91	1.59
RRu	3.99	2.94	1.37	2.06
P	N.S.	+++	N.S.	+++
Random RR	1.88	3.04	1.11	2.42
RRl	0.88	1.89	0.91	1.58
RRu	3.99	4.90	1.37	3.70
P	N.S.	+++	N.S.	+++
Between Chi				35.24
Between df				2
Between P				***
Btwn(F) P				(*)
Btwn(R) P				***

	Lung cancer type		
	all	other	Total
N	10		10
NS	8		8
Wt	234.31		234.31
Het Chi	66.41		66.41
Het df	9		9
Het P	***		***
Fixed RR	1.81		1.81
RRl	1.59		1.59
RRu	2.06		2.06
P	+++		+++
Random RR	2.42		2.42
RRl	1.58		1.58
RRu	3.70		3.70
P	+++		+++
Between Chi			
Between df			
Between P			N.S.
Btwn(F) P			N.S.
Btwn(R) P			N.S.

	Location								
	NAmer	UK	Scand	othEur	China	Japan	othAs	other	Total
N	4		2		1		2	1	10
NS	3		1		1		2	1	8
Wt	202.58		5.49		3.32		19.58	3.34	234.31
Het Chi	35.62		0.02		0.00		7.53	0.00	66.41
Het df	3		1		0		1	0	9
Het P	***		N.S.		N.S.		**	N.S.	***
Fixed RR	1.64		1.70		1.35		4.41	6.49	1.81
RRl	1.43		0.74		0.46		2.83	2.22	1.59
RRu	1.88		3.93		3.96		6.86	18.98	2.06
P	+++		N.S.		N.S.		+++	+++	+++
Random RR	2.03		1.70		1.35		3.70	6.49	2.42
RRl	1.20		0.74		0.46		1.03	2.22	1.58
RRu	3.44		3.93		3.96		13.27	18.98	3.70
P	++		N.S.		N.S.		+	+++	+++
Between Chi									23.25
Between df									4
Between P									***
Btwn(F) P									N.S.
Btwn(R) P									N.S.

Table 1I22 - 3

IESLC - Meta-analysis of Ever/current Smoking, Duration, "Low"
All LC types, Cigarettes only
Most adjusted

Detailed Country in "other Europe"

		multi	Germany	othWest	East	Balkans	Total
	N						
	NS						
	Wt						
Het	Chi						
Het	df						
Het	P						
Fixed	RR						
	RRl						
	RRu						
	P						
Random	RR						
	RRl						
	RRu						
	P						
Between	Chi						
Between	df						
Between	P						N.S.
Btwn(F)	P						N.S.
Btwn(R)	P						N.S.

Detailed Country in "other Asia"

		India	HongKong	other	Total
	N	2			2
	NS	2			2
	Wt	19.58			19.58
Het	Chi	7.53			7.53
Het	df	1			1
Het	P	**			**
Fixed	RR	4.41			4.41
	RRl	2.83			2.83
	RRu	6.86			6.86
	P	+++			+++
Random	RR	3.70			3.70
	RRl	1.03			1.03
	RRu	13.27			13.27
	P	+			+
Between	Chi				
Between	df				
Between	P				N.S.
Btwn(F)	P				N.S.
Btwn(R)	P				N.S.

Detailed other continent

		SCAmer	Total
	N	1	1
	NS	1	1
	Wt	3.34	3.34
Het	Chi	0.00	0.00
Het	df	0	0
Het	P	N.S.	N.S.
Fixed	RR	6.49	6.49
	RRl	2.22	2.22
	RRu	18.98	18.98
	P	+++	+++
Random	RR	6.49	6.49
	RRl	2.22	2.22
	RRu	18.98	18.98
	P	+++	+++
Between	Chi		
Between	df		
Between	P		N.S.
Btwn(F)	P		N.S.
Btwn(R)	P		N.S.

Table 1I22 - 3

IESLC - Meta-analysis of Ever/current Smoking, Duration, "Low"
All LC types, Cigarettes only
Most adjusted

	Start year of study					
	<1960	1960-69	1970-79	1980-89	1990+	Total
N	3	4		3		10
NS	2	3		3		8
Wt	157.01	25.07		52.22		234.31
Het Chi	13.27	11.43		4.11		66.41
Het df	2	3		2		9
Het P	**	**		N.S.		***
Fixed RR	1.37	3.58		3.03		1.81
RRl	1.17	2.42		2.31		1.59
RRu	1.60	5.29		3.97		2.06
P	+++	+++		+++		+++
Random RR	1.67	2.69		3.00		2.42
RRl	1.03	1.16		1.58		1.58
RRu	2.72	6.25		5.72		3.70
P	+	+		+++		+++
Between Chi						37.61
Between df						2
Between P						***
Btwn(F) P						(*)
Btwn(R) P						N.S.

	Study type (1)		
	CC	other	Total
N	4	6	10
NS	4	4	8
Wt	26.23	208.08	234.31
Het Chi	12.41	35.64	66.41
Het df	3	5	9
Het P	**	***	***
Fixed RR	3.99	1.64	1.81
RRl	2.72	1.43	1.59
RRu	5.85	1.88	2.06
P	+++	+++	+++
Random RR	3.37	1.96	2.42
RRl	1.46	1.25	1.58
RRu	7.82	3.08	3.70
P	++	++	+++
Between Chi			18.36
Between df			1
Between P			***
Btwn(F) P			N.S.
Btwn(R) P			N.S.

	Study type (2)			
	CC	prosp	other	Total
N	4	6		10
NS	4	4		8
Wt	26.23	208.08		234.31
Het Chi	12.41	35.64		66.41
Het df	3	5		9
Het P	**	***		***
Fixed RR	3.99	1.64		1.81
RRl	2.72	1.43		1.59
RRu	5.85	1.88		2.06
P	+++	+++		+++
Random RR	3.37	1.96		2.42
RRl	1.46	1.25		1.58
RRu	7.82	3.08		3.70
P	++	++		+++
Between Chi				18.36
Between df				1
Between P				***
Btwn(F) P				N.S.
Btwn(R) P				N.S.

Table 1I22 - 3

IESLC - Meta-analysis of Ever/current Smoking, Duration, "Low"
All LC types, Cigarettes only
Most adjusted

	Study size (number of LC cases)				
	100-249	250-499	500-999	1000+	Total
N	1	4	2	3	10
NS	1	3	2	2	8
Wt	3.34	14.11	19.58	197.28	234.31
Het Chi	0.00	3.22	7.53	31.04	66.41
Het df	0	3	1	2	9
Het P	N.S.	N.S.	**	***	***
Fixed RR	6.49	2.24	4.41	1.60	1.81
RRl	2.22	1.33	2.83	1.39	1.59
RRu	18.98	3.78	6.86	1.84	2.06
P	+++	++	+++	+++	+++
Random RR	6.49	2.22	3.70	1.77	2.42
RRl	2.22	1.29	1.03	1.01	1.58
RRu	18.98	3.82	13.27	3.09	3.70
P	+++	++	+	+	+++
Between Chi					24.62
Between df					3
Between P					***
Btwn(F) P					N.S.
Btwn(R) P					N.S.

	Risky occupational population			
	no	mining	othRisky	Total
N	9	1		10
NS	7	1		8
Wt	230.99	3.32		234.31
Het Chi	66.12	0.00		66.41
Het df	8	0		9
Het P	***	N.S.		***
Fixed RR	1.82	1.35		1.81
RRl	1.60	0.46		1.59
RRu	2.07	3.96		2.06
P	+++	N.S.		+++
Random RR	2.54	1.35		2.42
RRl	1.62	0.46		1.58
RRu	3.98	3.96		3.70
P	+++	N.S.		+++
Between Chi				0.29
Between df				1
Between P				N.S.
Btwn(F) P				N.S.
Btwn(R) P				N.S.

	National cigarette tobacco type			
	Virginia	blended	other	Total
N	3	6	1	10
NS	3	4	1	8
Wt	24.88	206.11	3.32	234.31
Het Chi	7.55	37.51	0.00	66.41
Het df	2	5	0	9
Het P	*	***	N.S.	***
Fixed RR	4.34	1.64	1.35	1.81
RRl	2.93	1.43	0.46	1.59
RRu	6.43	1.88	3.96	2.06
P	+++	+++	N.S.	+++
Random RR	3.87	2.02	1.35	2.42
RRl	1.75	1.26	0.46	1.58
RRu	8.60	3.24	3.96	3.70
P	+++	++	N.S.	+++
Between Chi				21.35
Between df				2
Between P				***
Btwn(F) P				N.S.
Btwn(R) P				N.S.

Table 1I22 - 3

IESLC - Meta-analysis of Ever/current Smoking, Duration, "Low"
All LC types, Cigarettes only
Most adjusted

	Any proxy use No/nk	Yes	Total
N	9	1	10
NS	7	1	8
Wt	230.99	3.32	234.31
Het Chi	66.12	0.00	66.41
Het df	8	0	9
Het P	***	N.S.	***
Fixed RR	1.82	1.35	1.81
RRl	1.60	0.46	1.59
RRu	2.07	3.96	2.06
P	+++	N.S.	+++
Random RR	2.54	1.35	2.42
RRl	1.62	0.46	1.58
RRu	3.98	3.96	3.70
P	+++	N.S.	+++
Between Chi			0.29
Between df			1
Between P			N.S.
Btwn(F) P			N.S.
Btwn(R) P			N.S.

	Full histological confirmation No	Yes	Total
N	9	1	10
NS	7	1	8
Wt	230.97	3.34	234.31
Het Chi	60.89	0.00	66.41
Het df	8	0	9
Het P	***	N.S.	***
Fixed RR	1.78	6.49	1.81
RRl	1.56	2.22	1.59
RRu	2.02	18.98	2.06
P	+++	+++	+++
Random RR	2.23	6.49	2.42
RRl	1.45	2.22	1.58
RRu	3.44	18.98	3.70
P	+++	+++	+++
Between Chi			5.52
Between df			1
Between P			*
Btwn(F) P			N.S.
Btwn(R) P			(*)

	Number of adjustment variables (1) 0	1	2+/+nk	Total
N	6	3	1	10
NS	5	2	1	8
Wt	220.20	10.80	3.32	234.31
Het Chi	62.51	2.10	0.00	66.41
Het df	5	2	0	9
Het P	***	N.S.	N.S.	***
Fixed RR	1.79	2.62	1.35	1.81
RRl	1.57	1.44	0.46	1.59
RRu	2.04	4.76	3.96	2.06
P	+++	++	N.S.	+++
Random RR	2.59	2.60	1.35	2.42
RRl	1.52	1.41	0.46	1.58
RRu	4.42	4.81	3.96	3.70
P	+++	++	N.S.	+++
Between Chi				1.80
Between df				2
Between P				N.S.
Btwn(F) P				N.S.
Btwn(R) P				N.S.

Table 1I22 - 3

IESLC - Meta-analysis of Ever/current Smoking, Duration, "Low"
All LC types, Cigarettes only
Most adjusted

Number of adjustment variables (2)

	0	1	2	3-5	6+/+nk	Total
N	6	3		1		10
NS	5	2		1		8
Wt	220.20	10.80		3.32		234.31
Het Chi	62.51	2.10		0.00		66.41
Het df	5	2		0		9
Het P	***	N.S.		N.S.		***
Fixed RR	1.79	2.62		1.35		1.81
RRl	1.57	1.44		0.46		1.59
RRu	2.04	4.76		3.96		2.06
P	+++	++		N.S.		+++
Random RR	2.59	2.60		1.35		2.42
RRl	1.52	1.41		0.46		1.58
RRu	4.42	4.81		3.96		3.70
P	+++	++		N.S.		+++
Between Chi						1.80
Between df						2
Between P						N.S.
Btwn(F) P						N.S.
Btwn(R) P						N.S.

Smoking status

	ever	current	Total
N	4	6	10
NS	4	4	8
Wt	26.23	208.08	234.31
Het Chi	12.41	35.64	66.41
Het df	3	5	9
Het P	**	***	***
Fixed RR	3.99	1.64	1.81
RRl	2.72	1.43	1.59
RRu	5.85	1.88	2.06
P	+++	+++	+++
Random RR	3.37	1.96	2.42
RRl	1.46	1.25	1.58
RRu	7.82	3.08	3.70
P	++	++	+++
Between Chi			18.36
Between df			1
Between P			***
Btwn(F) P			N.S.
Btwn(R) P			N.S.

Denominator

	nev any	nev cigs	Total
N	7	3	10
NS	6	2	8
Wt	79.26	155.05	234.31
Het Chi	14.64	14.98	66.41
Het df	6	2	9
Het P	*	***	***
Fixed RR	3.15	1.37	1.81
RRl	2.53	1.17	1.59
RRu	3.93	1.60	2.06
P	+++	+++	+++
Random RR	2.86	1.75	2.42
RRl	1.86	1.02	1.58
RRu	4.39	2.99	3.70
P	+++	+	+++
Between Chi			36.79
Between df			1
Between P			***
Btwn(F) P			*
Btwn(R) P			N.S.

Table 1I22 - 3

IESLC - Meta-analysis of Ever/current Smoking, Duration, "Low"
All LC types, Cigarettes only
Most adjusted

	Derivation of RR/CI Orig	StdCalc	Other	Total
N		6	4	10
NS		5	3	8
Wt		220.20	14.11	234.31
Het Chi		62.51	3.22	66.41
Het df		5	3	9
Het P		***	N.S.	***
Fixed RR		1.79	2.24	1.81
RRl		1.57	1.33	1.59
RRu		2.04	3.78	2.06
P		+++	++	+++
Random RR		2.59	2.22	2.42
RRl		1.52	1.29	1.58
RRu		4.42	3.82	3.70
P		+++	++	+++
Between Chi				0.68
Between df				1
Between P				N.S.
Btwn(F) P				N.S.
Btwn(R) P				N.S.

Table 1I22 - 4

IESLC - Meta-analysis of Ever/current Smoking, Duration, "Low"
All LC types, Cigarettes only
Least adjusted

REF	NRR	X	SEX	AGEL	AGEH	RACE	YF	LC TYPE	LOC	START	ST	NLC	R	VB	P	H	AD	SM	PRODUCT	exL	exH	DENOM	De
BEST	505		m	0	0	all	0	all	NAmer	1955	pr	381	n	V	n	n	1	cu	cig only	20	29	nev any	ot
CEDERL	501		m	40	69	all	10	all	Eu:Sca	1963	pr	491	n	bl	n	n	1	cu	cig only	1	29	nev any	ot
CEDERL	504		f	40	69	all	10	all	Eu:Sca	1963	pr	491	n	bl	n	n	1	cu	cig only	1	29	nev any	ot
CPSI	580		m	40	84	wh	0	all	NAmer	1959	pr	5138	n	bl	n	n	0	cu	cig only	1	29	nev cigs	st
CPSI	676		f	40	84	wh	0	all	NAmer	1959	pr	5138	n	bl	n	n	0	cu	cig only	1	29	nev cigs	st
CPSII	552		m	0	0	all	6	all	NAmer	1982	pr	3229	n	bl	n	n	0	cu	cig only	1	29	nev any	st
JUSSAW	512		m	0	0	all	-	all	As:Ind	1964	CC	792	n	V	n	n	0	ev	cig only	20	29	nev any	st
LUBIN	519	x	m	0	0	all	-	all	As:Chi	1984	CC	427	m	ot	y	n	0	ev	cig only	1	29	nev any	st
NOTAN2	514		c	0	0	all	-	all	As:Ind	1963	CC	683	n	V	n	n	0	ev	cig only	11	20	nev any	st
PEZZOT	534		m	0	0	all	-	all	SCAmer	1987	CC	215	n	bl	n	y	0	ev	cig only	1	30	nev cigs	st

Cigarette type is all/unspec for all RRs

except for the following:

REF	NRR	CIGTYPE
JUSSAW	512	MC only
NOTAN2	514	MC only

Table 1I22 - 5

IESLC - Meta-analysis of Ever/current Smoking, Duration, "Low"
All LC types, Cigarettes only
Least adjusted

REF	NRR	SEX	AD	Number Exposed Case	Cont	Non-exposed Case	Cont	RR	95.00%CI
*BEST	505	m	1	22	-	7	-	4.10	(1.75- 9.60)
*CEDERL	501	m	1	5	-	7	-	1.80	(0.57- 5.66)
*CEDERL	504	f	1	3	-	19	-	1.60	(0.47- 5.40)
Subtotal CEDERL								1.70	(0.74- 3.93)
*CPSI	580	m	0	95	266163	196	926068	1.69	(1.32- 2.15)
*CPSI	676	f	0	105	694015	532	3877179	1.10	(0.89- 1.36)
Subtotal CPSI								1.32	(1.13- 1.55)
*CPSII	552	m	0	72	141932	124	742207	3.04	(2.27- 4.06)
JUSSAW	512	m	0	38	23	149	624	6.92	(4.00- 11.97)
LUBIN	519	m	0	7	51	8	72	1.24	(0.42- 3.62)
NOTAN2	514	c	0	15	15	107	201	1.88	(0.88- 3.99)
PEZZOT	534	m	0	30	134	4	116	6.49	(2.22- 18.98)
Partial Totals				392	1102333	1153	5546467		

*prospective study

REF	NRR	SEX	AD	Ys	Ws	Qs	Ps
*BEST	505	m	1	1.41	5.30	3.55	0.0012
*CEDERL	501	m	1	0.59	2.92	0.00	0.3155
*CEDERL	504	f	1	0.47	2.58	0.04	0.4505
Subtotal CEDERL				0.53	5.49	0.04	
*CPSI	580	m	0	0.52	64.01	0.32	0.0000
*CPSI	676	f	0	0.10	87.71	21.54	0.3603
Subtotal CPSI				0.28	151.71	21.86	
*CPSII	552	m	0	1.11	45.57	12.20	0.0000
JUSSAW	512	m	0	1.93	12.80	23.02	0.0000
LUBIN	519	m	0	0.21	3.32	0.48	0.7003
NOTAN2	514	c	0	0.63	6.77	0.01	0.1008
PEZZOT	534	m	0	1.87	3.34	5.45	0.0006

	N	10
	NS	8
	Wt	234.31
Het	Chi	66.61
Het	df	9
Het	P	***
Fixed	RR	1.81
	RRl	1.59
	RRu	2.06
	P	+++
Random	RR	2.40
	RRl	1.57
	RRu	3.68
	P	+++
Asymm	P	N.S.

Table 1I22 - 6

IESLC - Meta-analysis of Ever/current Smoking, Duration, "Low"
All LC types, Cigarettes only
Least adjusted

	Sex combined	male	female	Total
N	1	7	2	10
NS	1	7	2	10
Wt	6.77	137.25	90.28	234.31
Het Chi	0.00	31.21	0.35	66.61
Het df	0	6	1	9
Het P	N.S.	***	N.S.	***
Fixed RR	1.88	2.49	1.11	1.81
RRl	0.88	2.10	0.91	1.59
RRu	3.99	2.94	1.37	2.06
P	N.S.	+++	N.S.	+++
Random RR	1.88	3.01	1.11	2.40
RRl	0.88	1.87	0.91	1.57
RRu	3.99	4.86	1.37	3.68
P	N.S.	+++	N.S.	+++
Between Chi				35.06
Between df				2
Between P				***
Btwn(F) P				(*)
Btwn(R) P				***

Table 1I22 - 7

IESLC - Meta-analysis of Ever/current Smoking, Duration, "Low"
All LC types, Cigarettes only
Excluded studies (and stage at which they were excluded)

1	BECHER BLOT1 BROWN3 CARPEN CHYOU DARBY DOLL2 GARCIA GRAHAM GURSEL HAMMO2 JAHN JAIN LAUSSM PRESCO QIAO TVERDA WIGLE WYNDE3
2	ALDERS BENSHL BRESLO CHIAZZ DEAN3 DORN ENGELA GAO2 GILLIS GUO HEGMAN HIRAYA HOLE KAUFMA KOO KOULUM LIU4 MIGRAN MRFITR PERNU SEGI2 SPEIZE SUZUK2 SVENSS VUTUC WAKAI WU YUAN
3	GENG MCDUFF SPITZ STASZE WU2 ZHANG
4	BOUCHA CHEN CORREA JEDRYC LUO WYNDE2 WYNDE6
5	AKIBA HAMMON PISANI RESTRE SADOWS XU
7	AMANDU AMES ARMADA AUVINE AXELSS BARBON BOFFET BROSS BUFFLE CHEN2 CHOI DAMBER DEAN2 DESTEF DOLL DORGAN DOSEME FAN GAO GARSHI GER HAENSZ HU HU2 HUMBLE JOLY KATSOU KHUDER KREUZE LETOUR LEVIN LIAW LIU3 LIU5 LUBIN2 MATOS MCCONN OSANN2 PEZZO2 QIAO2 RACHTA SOBUE WANG2 WATSON WUWILL WYNDE7 WYNDE8 ZHENG ZHOU
14	AGUDO BENHAM BOUCOT KAISE2 TIZZAN

Table 1I22 - 8
Potentially overlapping studies

REF	REFGP	PRINC	.	OVERLAP/LINK
CPSI	CPSI	1		CPSI overall
LUBIN	XIANGZ	2		LUBIN/XIANGZ/QIAO

Table 1I22 - 9
Most adjusted - insufficient data for meta-analysis

REF	NRR	SEX	AGEL	AGEH	RACE	YF	LC TYPE	LOC	START	ST	NLC	R	VB	P	H	AD	SM	PRODUCT	exL	exH	DENOM	De
HAMMON	513	m	0	0	wh	0	all	NAmer	1952	pr	448	n	bl	n	n	1	ev	cig only	1	34	nev any	st
SADOWS	524	m	0	0	wh	-	all	NAmer	1938	CC	477	n	bl	n	n	0	ev	cig only	20	29	nev any	ot

REF	NRR	RR	SIG	RRDATA comment
HAMMON	513		*	RR for <1/2 pack per day is 5.31, that for 1/2 to 1 pack per day is 6.56, that for 1 to 2 packs per day is 7.27 while that for 2+ packs per day 10.78
SADOWS	524	2.78		0

Table 1I23 -

IESLC - Meta-analysis of Ever/current Smoking, Duration, "Mid"
All LC types, Cigarettes only

This analysis is restricted to results for:
1) Ever/current smokers
2) Results by Duration
3) Categorical results by Duration
4) All LC types (or near equivalent)
5) Results complete enough for use in metaanalysis

Within each study, results are then selected (in the following order of preference, within each sex) for:
6) SMKSTA: ever, current
7) PRODUCT: cigarettes only
8) CIGTYPE: all/unspecified, MC regardless of HR, MC only
9) (not applicable)
10) DENOM: never smoked anything, never smoked cigarettes, never any + low, never cigs + low
11) Followup period (YF, prospective studies): whole study (coded as 0) or longest available
12) LCtype: all or nearest available, at least Squamous and Adeno. (q = squamous, s = small, l = large, a = adeno, mix = mixed, alv = alveolar)
13) Race: all or nearest available, otherwise by race (wh or w = white, bl or b = black, hi = hispanic ch = chinese, jap = japanese, haw = hawaiian, w+o = white + oriental, sca = scandinavian, as = asian)
14) Duration "mid" in key scheme 1 (key value 35, maximum range 21-49)
15) For overlapping studies: principal rather than subsidiary studies
Finally by Age: whole study (coded as 0) if available, otherwise by widest available age group and then for single sex results (m, f) in preference to results for both sexes combined (c).

Results adjusted (AD) for the most potential confounders are then chosen in Sections -1 to -3 (and those which actually differ from the adjusted results in Table 1I13 - 1 are marked 'x' in Section -1) and results adjusted for the least confounders in Sections -4 to -6. (Those least adjusted results which actually differ from the most adjusted are marked 'x' in column X in Section -4)

Section -7 shows excluded studies, together with the stage (as above) at which no qualifying results were found.

Section -8 lists the potentially overlapping studies which have been included (1=principal, 2=subsidiary).

Section -9 lists any results which would have been included in preference except that they had data not complete enough for use in meta-analysis, with their significance (yes/no), if known, and any further comment as entered on the database. It also lists as "gap" any categories for which no data were presented by the original authors.

In addition to those mentioned above, the following fields, levels and abbreviations are used:
* or nk = not known, n = no, y = yes, ot = other
ev = ever, cu = current, nev = never
all/unspec = all or unspecified, MC = manufactured cigarettes, HR = hand-rolled cigarettes
exL, exH = range of exposure (low and high) in the smoking group, in terms of Duration
REF: 6-character study reference
NRR: number of the RR on the database within the study
ST : study type (CC = case control, pr or prosp = prospective)
NLC: number of lung cancer cases in whole study
R : risky occupational population (n = no, m = mining, o = other risky)
VB : national cigarette type (V = at least 75% Virginia, bl = at least 75% blended, ot = other)
P : any proxy use
H : full histological confirmation
De : derivation of RR/CI (or = original, st = standard method, ot = other method of estimation)

Table 1I23 - 1

IESLC - Meta-analysis of Ever/current Smoking, Duration, "Mid"
All LC types, Cigarettes only
Most adjusted

REF	NRR	1I13	SEX	AGEL	AGEH	RACE	YF	LC TYPE	LOC	START	ST	NLC	R	VB	P	H	AD	SM	PRODUCT	exL	exH	DENOM	De
BEST	506		m	0	0	all	0	all	NAmer	1955	pr	381	n	V	n	n	1	cu	cig only	30	39	nev any	ot
CPSI	582		m	40	84	wh	0	all	NAmer	1959	pr	5138	n	bl	n	n	0	cu	cig only	35	39	nev cigs	st
CPSI	678		f	40	84	wh	0	all	NAmer	1959	pr	5138	n	bl	n	n	0	cu	cig only	35	39	nev cigs	st
CPSII	554		m	0	0	all	6	all	NAmer	1982	pr	3229	n	bl	n	n	0	cu	cig only	35	39	nev any	st
JUSSAW	513		m	0	0	all	-	all	As:Ind	1964	CC	792	n	V	n	n	0	ev	cig only	30	39	nev any	st
LUBIN	527	x	m	0	0	all	-	all	As:Chi	1984	CC	427	m	ot	y	n	4	ev	cig only	30	39	nev any	ot
NOTAN2	516		c	0	0	all	-	all	As:Ind	1963	CC	683	n	V	n	n	0	ev	cig only	31	40	nev any	st
PEZZOT	535		m	0	0	all	-	all	SCAmer	1987	CC	215	n	bl	n	y	0	ev	cig only	31	40	nev cigs	st

Cigarette type is all/unspec for all RRs

except for the following:

REF	NRR	CIGTYPE
JUSSAW	513	MC only
NOTAN2	516	MC only

Table 1I23 - 2

IESLC - Meta-analysis of Ever/current Smoking, Duration, "Mid"
All LC types, Cigarettes only
Most adjusted

REF	NRR	SEX	AD	Number Exposed Case	Number Exposed Cont	Non-exposed Case	Non-exposed Cont	RR	95.00%CI
*BEST	506	m	1	55	-	7	-	13.90	(6.33- 30.52)
*CPSI	582	m	0	470	367622	196	926068	6.04	(5.11- 7.14)
*CPSI	678	f	0	154	315060	532	3877179	3.56	(2.98- 4.26)
Subtotal CPSI								4.73	(4.19- 5.34)
*CPSII	554	m	0	244	109788	124	742207	13.30	(10.72- 16.51)
JUSSAW	513	m	0	27	9	149	624	12.56	(5.79- 27.28)
LUBIN	527	m	4	16	-	8	-	2.27	(0.91- 5.65)
NOTAN2	516	c	0	12	7	107	201	3.22	(1.23- 8.42)
PEZZOT	535	m	0	71	82	4	116	25.11	(8.82- 71.48)
Partial Totals				1049	792568	1127	5546395		

*prospective study

REF	NRR	SEX	AD	Ys	Ws	Qs	Ps
*BEST	506	m	1	2.63	6.21	4.04	0.0000
*CPSI	582	m	0	1.80	138.39	0.10	0.0000
*CPSI	678	f	0	1.27	119.48	36.72	0.0000
Subtotal CPSI				1.55	257.87	36.82	
*CPSII	554	m	0	2.59	82.29	47.93	0.0000
JUSSAW	513	m	0	2.53	6.39	3.19	0.0000
LUBIN	527	m	4	0.82	4.61	4.65	0.0784
NOTAN2	516	c	0	1.17	4.16	1.79	0.0171
PEZZOT	535	m	0	3.22	3.51	6.86	0.0000

	N	8
	NS	7
	Wt	365.03
Het	Chi	105.28
Het	df	7
Het	P	***
Fixed	RR	6.20
	RRl	5.60
	RRu	6.87
	P	+++
Random	RR	7.29
	RRl	4.47
	RRu	11.90
	P	+++
Asymm	P	N.S.

Table 1I23 - 3

IESLC - Meta-analysis of Ever/current Smoking, Duration, "Mid"
All LC types, Cigarettes only
Most adjusted

		Sex		
	combined	male	female	Total
N	1	6	1	8
NS	1	6	1	8
Wt	4.16	241.40	119.48	365.03
Het Chi	0.00	47.07	0.00	105.28
Het df	0	5	0	7
Het P	N.S.	***	N.S.	***
Fixed RR	3.22	8.25	3.56	6.20
RRl	1.23	7.27	2.98	5.60
RRu	8.42	9.36	4.26	6.87
P	+	+++	+++	+++
Random RR	3.22	9.46	3.56	7.29
RRl	1.23	5.60	2.98	4.47
RRu	8.42	16.00	4.26	11.90
P	+	+++	+++	+++
Between Chi				58.21
Between df				2
Between P				***
Btwn(F) P				N.S.
Btwn(R) P				**

Too few RRs for analysis by factor

Table 1I23 - 4

IESLC - Meta-analysis of Ever/current Smoking, Duration, "Mid"
All LC types, Cigarettes only
Least adjusted

REF	NRR	X	SEX	AGEL	AGEH	RACE	YF	LC TYPE	LOC	START	ST	NLC	R	VB	P	H	AD	SM	PRODUCT	exL	exH	DENOM	De
BEST	506		m	0	0	all	0	all	NAmer	1955	pr	381	n	V	n	n	1	cu	cig only	30	39	nev any	ot
CPSI	582		m	40	84	wh	0	all	NAmer	1959	pr	5138	n	bl	n	n	0	cu	cig only	35	39	nev cigs	st
CPSI	678		f	40	84	wh	0	all	NAmer	1959	pr	5138	n	bl	n	n	0	cu	cig only	35	39	nev cigs	st
CPSII	554		m	0	0	all	6	all	NAmer	1982	pr	3229	n	bl	n	n	0	cu	cig only	35	39	nev any	st
JUSSAW	513		m	0	0	all	-	all	As:Ind	1964	CC	792	n	V	n	n	0	ev	cig only	30	39	nev any	st
LUBIN	520	x	m	0	0	all	-	all	As:Chi	1984	CC	427	m	ot	y	n	0	ev	cig only	30	39	nev any	st
NOTAN2	516		c	0	0	all	-	all	As:Ind	1963	CC	683	n	V	n	n	0	ev	cig only	31	40	nev any	st
PEZZOT	535		m	0	0	all	-	all	SCAmer	1987	CC	215	n	bl	n	y	0	ev	cig only	31	40	nev cigs	st

Cigarette type is all/unspec for all RRs

except for the following:

REF	NRR	CIGTYPE
JUSSAW	513	MC only
NOTAN2	516	MC only

Table 1I23 - 5

IESLC - Meta-analysis of Ever/current Smoking, Duration, "Mid"
All LC types, Cigarettes only
Least adjusted

REF	NRR	SEX	AD	Number Exposed Case	Cont	Non-exposed Case	Cont	RR	95.00%CI
*BEST	506	m	1	55	-	7	-	13.90	(6.33- 30.52)
*CPSI	582	m	0	470	367622	196	926068	6.04	(5.11- 7.14)
*CPSI	678	f	0	154	315060	532	3877179	3.56	(2.98- 4.26)
Subtotal CPSI								4.73	(4.19- 5.34)
*CPSII	554	m	0	244	109788	124	742207	13.30	(10.72- 16.51)
JUSSAW	513	m	0	27	9	149	624	12.56	(5.79- 27.28)
LUBIN	520	m	0	16	67	8	72	2.15	(0.86- 5.35)
NOTAN2	516	c	0	12	7	107	201	3.22	(1.23- 8.42)
PEZZOT	535	m	0	71	82	4	116	25.11	(8.82- 71.48)
Partial Totals				1049	792635	1127	5546467		

*prospective study

REF	NRR	SEX	AD	Ys	Ws	Qs	Ps
*BEST	506	m	1	2.63	6.21	4.05	0.0000
*CPSI	582	m	0	1.80	138.39	0.09	0.0000
*CPSI	678	f	0	1.27	119.48	36.62	0.0000
Subtotal CPSI				1.55	257.87	36.71	
*CPSII	554	m	0	2.59	82.29	48.02	0.0000
JUSSAW	513	m	0	2.53	6.39	3.19	0.0000
LUBIN	520	m	0	0.77	4.62	5.18	0.1000
NOTAN2	516	c	0	1.17	4.16	1.78	0.0171
PEZZOT	535	m	0	3.22	3.51	6.87	0.0000

	N	8
	NS	7
	Wt	365.05
Het	Chi	105.81
Het	df	7
Het	P	***
Fixed	RR	6.20
	RRl	5.59
	RRu	6.87
	P	+++
Random	RR	7.25
	RRl	4.44
	RRu	11.85
	P	+++
Asymm	P	N.S.

Table 1I23 - 6

IESLC - Meta-analysis of Ever/current Smoking, Duration, "Mid"
All LC types, Cigarettes only
Least adjusted

	Sex			
	combined	male	female	Total
N	1	6	1	8
NS	1	6	1	8
Wt	4.16	241.41	119.48	365.05
Het Chi	0.00	47.76	0.00	105.81
Het df	0	5	0	7
Het P	N.S.	***	N.S.	***
Fixed RR	3.22	8.24	3.56	6.20
RRl	1.23	7.27	2.98	5.59
RRu	8.42	9.35	4.26	6.87
P	+	+++	+++	+++
Random RR	3.22	9.39	3.56	7.25
RRl	1.23	5.54	2.98	4.44
RRu	8.42	15.93	4.26	11.85
P	+	+++	+++	+++
Between Chi				58.05
Between df				2
Between P				***
Btwn(F) P				N.S.
Btwn(R) P				**

Table 1I23 - 7

IESLC - Meta-analysis of Ever/current Smoking, Duration, "Mid"
All LC types, Cigarettes only
Excluded studies (and stage at which they were excluded)

1	BECHER BLOT1 BROWN3 CARPEN CHYOU DARBY DOLL2 GARCIA GRAHAM GURSEL HAMMO2 JAHN JAIN LAUSSM PRESCO QIAO TVERDA WIGLE WYNDE3
2	ALDERS BENSHL BRESLO CHIAZZ DEAN3 DORN ENGELA GAO2 GILLIS GUO HEGMAN HIRAYA HOLE KAUFMA KOO KOULUM LIU4 MIGRAN MRFITR PERNU SEGI2 SPEIZE SUZUK2 SVENSS VUTUC WAKAI WU YUAN
3	GENG MCDUFF SPITZ STASZE WU2 ZHANG
4	BOUCHA CHEN CORREA JEDRYC LUO WYNDE2 WYNDE6
5	AKIBA HAMMON PISANI RESTRE SADOWS XU
7	AMANDU AMES ARMADA AUVINE AXELSS BARBON BOFFET BROSS BUFFLE CHEN2 CHOI DAMBER DEAN2 DESTEF DOLL DORGAN DOSEME FAN GAO GARSHI GER HAENSZ HU HU2 HUMBLE JOLY KATSOU KHUDER KREUZE LETOUR LEVIN LIAW LIU3 LIU5 LUBIN2 MATOS MCCONN OSANN2 PEZZO2 QIAO2 RACHTA SOBUE WANG2 WATSON WUWILL WYNDE7 WYNDE8 ZHENG ZHOU
14	AGUDO BENHAM BOUCOT CEDERL KAISE2 TIZZAN

Table 1I23 - 8
Potentially overlapping studies

REF	REFGP	PRINC	.	OVERLAP/LINK
CPSI	CPSI	1	CPSI overall
LUBIN	XIANGZ	2	LUBIN/XIANGZ/QIAO

Table 1I23 - 9
Most adjusted - insufficient data for meta-analysis

REF	NRR	SEX	AGEL	AGEH	RACE	YF	LC TYPE	LOC	START	ST	NLC	R	VB	P	H	AD	SM	PRODUCT	exL	exH	DENOM	De
SADOWS	525	m	0	0	wh	-	all	NAmer	1938	CC	477	n	bl	n	n	0	ev	cig only	30	39	nev	any ot

REF	NRR	RR	SIG	RRDATA comment
SADOWS	525	3.95		0

Table 1I24 -

IESLC - Meta-analysis of Ever/current Smoking, Duration, "High"
All LC types, Cigarettes only

This analysis is restricted to results for:
1) Ever/current smokers
2) Results by Duration
3) Categorical results by Duration
4) All LC types (or near equivalent)
5) Results complete enough for use in metaanalysis

Within each study, results are then selected (in the following order of preference, within each sex) for:
6) PRODUCT: cigarettes only
7) CIGTYPE: all/unspecified, MC regardless of HR, MC only
8) (not applicable)
9) DENOM: never smoked anything, never smoked cigarettes, never any + low, never cigs + low
10) Followup period (YF, prospective studies): whole study (coded as 0) or longest available
11) LCtype: all or nearest available, at least Squamous and Adeno. (q = squamous, s = small,
l = large, a = adeno, mix = mixed, alv = alveolar)
12) Race: all or nearest available, otherwise by race (wh or w = white, bl or b = black, hi = hispanic
ch = chinese, jap = japanese, haw = hawaiian, w+o = white + oriental, sca = scandinavian, as = asian)
13) Duration "high" in key scheme 1 (key value 50, maximum range 36+)
14) For overlapping studies: principal rather than subsidiary studies
Finally by Age: whole study (coded as 0) if available, otherwise by widest available age group
and then for single sex results (m, f) in preference to results for both sexes combined (c).

Results adjusted (AD) for the most potential confounders are then chosen in Sections -1 to -3
(and those which actually differ from the adjusted results in Table 1I14 - 1 are marked 'x' in Section -1)
and results adjusted for the least confounders in Sections -4 to -6. (Those least adjusted results which
actually differ from the most adjusted are marked 'x' in column X in Section -4)

Section -7 shows excluded studies, together with the stage (as above) at which no qualifying
results were found.

Section -8 lists the potentially overlapping studies which have been included (1=principal, 2=subsidiary).

Section -9 lists any results which would have been included in preference except that they had data not complete
enough for use in meta-analysis, with their significance (yes/no), if known, and any further comment as entered
on the database. It also lists as "gap" any categories for which no data were presented by the original authors.

In addition to those mentioned above, the following fields, levels and abbreviations are used:
* or nk = not known, n = no, y = yes, ot = other
ev = ever, cu = current, nev = never
all/unspec = all or unspecified, MC = manufactured cigarettes, HR = hand-rolled cigarettes
exL, exH = range of exposure (low and high) in the smoking group, in terms of Duration
REF: 6-character study reference
NRR: number of the RR on the database within the study
ST : study type (CC = case control, pr or prosp = prospective)
NLC: number of lung cancer cases in whole study
R : risky occupational population (n = no, m = mining, o = other risky)
VB : national cigarette type (V = at least 75% Virginia, bl = at least 75% blended, ot = other)
P : any proxy use
H : full histological confirmation
De : derivation of RR/CI (or = original, st = standard method, ot = other method of estimation)

Table 1I24 - 1

IESLC - Meta-analysis of Ever/current Smoking, Duration, "High"
All LC types, Cigarettes only
Most adjusted

REF	NRR	1I14	SEX	AGEL	AGEH	RACE	YF	LC TYPE	LOC	START	ST	NLC	R	VB	P	H	AD	SM	PRODUCT	exL	exH	DENOM	De
BENHAM	512	x	m	0	0	all	-	not mix	Eu:wst	1976	CC	1625	n	bl	n	y	0	ev	cig only	36	999	nev any	st
BEST	507		m	0	0	all	0	all	NAmer	1955	pr	381	n	V	n	n	1	cu	cig only	40	999	nev any	ot
BOUCOT	513	x	m	0	0	all	9	all	NAmer	1951	pr	121	n	bl	n	n	0	ev	cig only	40	999	nev any	ot
CPSI	585		m	40	84	wh	0	all	NAmer	1959	pr	5138	n	bl	n	n	0	cu	cig only	50	54	nev cigs	st
CPSI	681		f	40	84	wh	0	all	NAmer	1959	pr	5138	n	bl	n	n	0	cu	cig only	50	54	nev cigs	st
CPSII	557		m	0	0	all	6	all	NAmer	1982	pr	3229	n	bl	n	n	0	cu	cig only	50	54	nev any	st
JUSSAW	514		m	0	0	all	-	all	As:Ind	1964	CC	792	n	V	n	n	0	ev	cig only	40	999	nev any	st
KAISE2	597		m	0	0	all	9	all	NAmer	1979	pr	318	n	bl	n	n	1	cu	cig only	40	999	nev any	st
KAISE2	517		f	0	0	all	9	all	NAmer	1979	pr	318	n	bl	n	n	1	cu	cig only	40	999	nev any	st
LUBIN	529	x	m	0	0	all	-	all	As:Chi	1984	CC	427	m	ot	y	n	4	ev	cig only	50	999	nev any	ot
NOTAN2	517		c	0	0	all	-	all	As:Ind	1963	CC	683	n	V	n	n	0	ev	cig only	41	999	nev any	st
PEZZOT	536		m	0	0	all	-	all	SCAmer	1987	CC	215	n	bl	n	y	0	ev	cig only	41	999	nev cigs	st

Cigarette type is all/unspec for all RRs

except for the following:

REF	NRR	CIGTYPE
JUSSAW	514	MC only
NOTAN2	517	MC only

Table 1I24 - 2

IESLC - Meta-analysis of Ever/current Smoking, Duration, "High"
All LC types, Cigarettes only
Most adjusted

REF	NRR	SEX	AD	Number Exposed Case	Number Exposed Cont	Non-exposed Case	Non-exposed Cont	RR	95.00%CI
BENHAM	512	m	0	694	620	33	523	17.74	(12.28- 25.63)
*BEST	507	m	1	137	-	7	-	14.20	(6.64- 30.35)
*BOUCOT	513	m	0	44	1177	0	805	60.88~	(3.75- 987.17)
*CPSI	585	m	0	576	119633	196	926068	22.75	(19.35- 26.75)
*CPSI	681	f	0	16	14305	532	3877179	8.15	(4.96- 13.40)
Subtotal CPSI								20.61	(17.67- 24.05)
*CPSII	557	m	0	332	39260	124	742207	50.62	(41.19- 62.20)
JUSSAW	514	m	0	11	6	149	624	7.68	(2.79- 21.09)
*KAISE2	597	m	1	34	-	14	-	15.64	(8.31- 29.40)
*KAISE2	517	f	1	26	-	11	-	30.41	(14.39- 64.25)
Subtotal KAISE2								20.63	(12.73- 33.43)
LUBIN	529	m	4	8	-	8	-	9.56	(3.12- 29.29)
NOTAN2	517	c	0	5	5	107	201	1.88	(0.53- 6.63)
PEZZOT	536	m	0	110	101	4	116	31.58	(11.25- 88.71)
Partial Totals				1993	175107	1185	5547723		

*prospective study ~ With 0.5 adjustment for zero

REF	NRR	SEX	AD	Ys	Ws	Qs	Ps
BENHAM	512	m	0	2.88	28.35	3.56	0.0000
*BEST	507	m	1	2.65	6.65	2.21	0.0000
*BOUCOT	513	m	0	4.11	0.49	0.38	0.0038
*CPSI	585	m	0	3.12	146.44	1.63	0.0000
*CPSI	681	f	0	2.10	15.55	19.92	0.0000
Subtotal CPSI				3.03	161.99	21.56	
*CPSII	557	m	0	3.92	90.50	43.60	0.0000
JUSSAW	514	m	0	2.04	3.76	5.34	0.0001
*KAISE2	597	m	1	2.75	9.62	2.22	0.0000
*KAISE2	517	f	1	3.41	6.86	0.23	0.0000
Subtotal KAISE2				3.03	16.49	2.45	
LUBIN	529	m	4	2.26	3.06	2.90	0.0001
NOTAN2	517	c	0	0.63	2.41	16.31	0.3273
PEZZOT	536	m	0	3.45	3.60	0.18	0.0000

	N	12
	NS	10
	Wt	317.32
Het	Chi	98.50
Het	df	11
Het	P	***
Fixed	RR	25.28
	RRl	22.65
	RRu	28.22
	P	+++
Random	RR	16.38
	RRl	10.68
	RRu	25.10
	P	+++
Asymm	P	N.S.

Table 1I24 - 3

IESLC - Meta-analysis of Ever/current Smoking, Duration, "High"
All LC types, Cigarettes only
Most adjusted

	Sex combined	male	female	Total
N	1	9	2	12
NS	1	9	2	12
Wt	2.41	292.49	22.41	317.32
Het Chi	0.00	60.28	8.25	98.50
Het df	0	8	1	11
Het P	N.S.	***	**	***
Fixed RR	1.88	27.32	12.20	25.28
RRl	0.53	24.36	8.06	22.65
RRu	6.63	30.63	18.46	28.22
P	N.S.	+++	+++	+++
Random RR	1.88	20.16	15.27	16.38
RRl	0.53	13.12	4.21	10.68
RRu	6.63	30.97	55.39	25.10
P	N.S.	+++	+++	+++
Between Chi				29.96
Between df				2
Between P				***
Btwn(F) P				N.S.
Btwn(R) P				**

	Lung cancer type all	other	Total
N	11	1	12
NS	9	1	10
Wt	288.97	28.35	317.32
Het Chi	94.59	0.00	98.50
Het df	10	0	11
Het P	***	N.S.	***
Fixed RR	26.18	17.74	25.28
RRl	23.33	12.28	22.65
RRu	29.38	25.63	28.22
P	+++	+++	+++
Random RR	16.08	17.74	16.38
RRl	9.98	12.28	10.68
RRu	25.92	25.63	25.10
P	+++	+++	+++
Between Chi			3.91
Between df			1
Between P			*
Btwn(F) P			N.S.
Btwn(R) P			N.S.

	Location								
	NAmer	UK	Scand	othEur	China	Japan	othAs	other	Total
N	7			1	1		2	1	12
NS	5			1	1		2	1	10
Wt	276.13			28.35	3.06		6.17	3.60	317.32
Het Chi	68.30			0.00	0.00		2.91	0.00	98.50
Het df	6			0	0		1	0	11
Het P	***			N.S.	N.S.		(*)	N.S.	***
Fixed RR	27.48			17.74	9.56		4.43	31.58	25.28
RRl	24.42			12.28	3.12		2.01	11.25	22.65
RRu	30.92			25.63	29.29		9.75	88.71	28.22
P	+++			+++	+++		+++	+++	+++
Random RR	21.20			17.74	9.56		4.00	31.58	16.38
RRl	12.53			12.28	3.12		1.01	11.25	10.68
RRu	35.86			25.63	29.29		15.85	88.71	25.10
P	+++			+++	+++		+	+++	+++
Between Chi									27.29
Between df									4
Between P									***
Btwn(F) P									N.S.
Btwn(R) P									N.S.

Table 1I24 - 3

IESLC - Meta-analysis of Ever/current Smoking, Duration, "High"
All LC types, Cigarettes only
Most adjusted

Detailed Country in "other Europe"

	multi	Germany	othWest	East	Balkans	Total
N			1			1
NS			1			1
Wt			28.35			28.35
Het Chi			0.00			0.00
Het df			0			0
Het P			N.S.			N.S.
Fixed RR			17.74			17.74
RRl			12.28			12.28
RRu			25.63			25.63
P			+++			+++
Random RR			17.74			17.74
RRl			12.28			12.28
RRu			25.63			25.63
P			+++			+++
Between Chi						
Between df						
Between P						N.S.
Btwn(F) P						N.S.
Btwn(R) P						N.S.

Detailed Country in "other Asia"

	India	HongKong	other	Total
N	2			2
NS	2			2
Wt	6.17			6.17
Het Chi	2.91			2.91
Het df	1			1
Het P	(*)			(*)
Fixed RR	4.43			4.43
RRl	2.01			2.01
RRu	9.75			9.75
P	+++			+++
Random RR	4.00			4.00
RRl	1.01			1.01
RRu	15.85			15.85
P	+			+
Between Chi				
Between df				
Between P				N.S.
Btwn(F) P				N.S.
Btwn(R) P				N.S.

Detailed other continent

	SCAmer	Total
N	1	1
NS	1	1
Wt	3.60	3.60
Het Chi	0.00	0.00
Het df	0	0
Het P	N.S.	N.S.
Fixed RR	31.58	31.58
RRl	11.25	11.25
RRu	88.71	88.71
P	+++	+++
Random RR	31.58	31.58
RRl	11.25	11.25
RRu	88.71	88.71
P	+++	+++
Between Chi		
Between df		
Between P		N.S.
Btwn(F) P		N.S.
Btwn(R) P		N.S.

Table 1I24 - 3

IESLC - Meta-analysis of Ever/current Smoking, Duration, "High"
All LC types, Cigarettes only
Most adjusted

	Start year of study					
	<1960	1960-69	1970-79	1980-89	1990+	Total
N	4	2	3	3		12
NS	3	2	2	3		10
Wt	169.14	6.17	44.84	97.17		317.32
Het Chi	16.29	2.91	2.01	8.84		98.50
Het df	3	1	2	2		11
Het P	***	(*)	N.S.	*		***
Fixed RR	20.38	4.43	18.75	47.19		25.28
RRl	17.53	2.01	13.99	38.68		22.65
RRu	23.69	9.75	25.13	57.57		28.22
P	+++	+++	+++	+++		+++
Random RR	15.32	4.00	18.76	27.87		16.38
RRl	7.69	1.01	13.98	10.70		10.68
RRu	30.54	15.85	25.16	72.63		25.10
P	+++	+	+++	+++		+++
Between Chi						68.46
Between df						3
Between P						***
Btwn(F) P						*
Btwn(R) P						N.S.

	Study type (1)		
	CC	other	Total
N	5	7	12
NS	5	5	10
Wt	41.19	276.13	317.32
Het Chi	15.47	68.30	98.50
Het df	4	6	11
Het P	**	***	***
Fixed RR	14.47	27.48	25.28
RRl	10.66	24.42	22.65
RRu	19.64	30.92	28.22
P	+++	+++	+++
Random RR	10.41	21.20	16.38
RRl	4.76	12.53	10.68
RRu	22.77	35.86	25.10
P	+++	+++	+++
Between Chi			14.73
Between df			1
Between P			***
Btwn(F) P			N.S.
Btwn(R) P			N.S.

	Study type (2)			
	CC	prosp	other	Total
N	5	7		12
NS	5	5		10
Wt	41.19	276.13		317.32
Het Chi	15.47	68.30		98.50
Het df	4	6		11
Het P	**	***		***
Fixed RR	14.47	27.48		25.28
RRl	10.66	24.42		22.65
RRu	19.64	30.92		28.22
P	+++	+++		+++
Random RR	10.41	21.20		16.38
RRl	4.76	12.53		10.68
RRu	22.77	35.86		25.10
P	+++	+++		+++
Between Chi				14.73
Between df				1
Between P				***
Btwn(F) P				N.S.
Btwn(R) P				N.S.

Table 1I24 - 3

IESLC - Meta-analysis of Ever/current Smoking, Duration, "High"
All LC types, Cigarettes only
Most adjusted

	Study size (number of LC cases) 100-249	250-499	500-999	1000+	Total
N	2	4	2	4	12
NS	2	3	2	3	10
Wt	4.10	26.21	6.17	280.84	317.32
Het Chi	0.19	3.62	2.91	67.34	98.50
Het df	1	3	1	3	11
Het P	N.S.	N.S.	(*)	***	***
Fixed RR	34.19	17.15	4.43	27.12	25.28
RRl	12.98	11.69	2.01	24.13	22.65
RRu	90.04	25.15	9.75	30.49	28.22
P	+++	+++	+++	+++	+++
Random RR	34.19	17.05	4.00	20.84	16.38
RRl	12.98	11.14	1.01	11.19	10.68
RRu	90.04	26.11	15.85	38.82	25.10
P	+++	+++	+	+++	+++
Between Chi					24.45
Between df					3
Between P					***
Btwn(F) P					N.S.
Btwn(R) P					(*)

	Risky occupational population no	mining	othRisky	Total
N	11	1		12
NS	9	1		10
Wt	314.26	3.06		317.32
Het Chi	95.58	0.00		98.50
Het df	10	0		11
Het P	***	N.S.		***
Fixed RR	25.52	9.56		25.28
RRl	22.85	3.12		22.65
RRu	28.51	29.29		28.22
P	+++	+++		+++
Random RR	17.01	9.56		16.38
RRl	10.94	3.12		10.68
RRu	26.44	29.29		25.10
P	+++	+++		+++
Between Chi				2.93
Between df				1
Between P				(*)
Btwn(F) P				N.S.
Btwn(R) P				N.S.

	National cigarette tobacco type Virginia	blended	other	Total
N	3	8	1	12
NS	3	6	1	10
Wt	12.83	301.43	3.06	317.32
Het Chi	7.26	70.71	0.00	98.50
Het df	2	7	0	11
Het P	*	***	N.S.	***
Fixed RR	8.10	26.80	9.56	25.28
RRl	4.69	23.94	3.12	22.65
RRu	14.01	30.01	29.29	28.22
P	+++	+++	+++	+++
Random RR	6.47	22.44	9.56	16.38
RRl	2.16	14.16	3.12	10.68
RRu	19.35	35.57	29.29	25.10
P	+++	+++	+++	+++
Between Chi				20.53
Between df				2
Between P				***
Btwn(F) P				N.S.
Btwn(R) P				(*)

Table 1I24 - 3

IESLC - Meta-analysis of Ever/current Smoking, Duration, "High"
All LC types, Cigarettes only
Most adjusted

	Any proxy use No/nk	Yes	Total
N	11	1	12
NS	9	1	10
Wt	314.26	3.06	317.32
Het Chi	95.58	0.00	98.50
Het df	10	0	11
Het P	***	N.S.	***
Fixed RR	25.52	9.56	25.28
RRl	22.85	3.12	22.65
RRu	28.51	29.29	28.22
P	+++	+++	+++
Random RR	17.01	9.56	16.38
RRl	10.94	3.12	10.68
RRu	26.44	29.29	25.10
P	+++	+++	+++
Between Chi			2.93
Between df			1
Between P			(*)
Btwn(F) P			N.S.
Btwn(R) P			N.S.

	Full histological confirmation No	Yes	Total
N	10	2	12
NS	8	2	10
Wt	285.37	31.96	317.32
Het Chi	94.47	1.06	98.50
Het df	9	1	11
Het P	***	N.S.	***
Fixed RR	26.12	18.93	25.28
RRl	23.26	13.38	22.65
RRu	29.33	26.78	28.22
P	+++	+++	+++
Random RR	15.12	19.19	16.38
RRl	9.12	13.02	10.68
RRu	25.06	28.26	25.10
P	+++	+++	+++
Between Chi			2.97
Between df			1
Between P			(*)
Btwn(F) P			N.S.
Btwn(R) P			N.S.

	Number of adjustment variables (1) 0	1	2+/+nk	Total
N	8	3	1	12
NS	7	2	1	10
Wt	291.12	23.14	3.06	317.32
Het Chi	90.58	2.43	0.00	98.50
Het df	7	2	0	11
Het P	***	N.S.	N.S.	***
Fixed RR	26.18	18.53	9.56	25.28
RRl	23.34	12.33	3.12	22.65
RRu	29.37	27.85	29.29	28.22
P	+++	+++	+++	+++
Random RR	16.18	18.60	9.56	16.38
RRl	9.33	11.85	3.12	10.68
RRu	28.05	29.20	29.29	25.10
P	+++	+++	+++	+++
Between Chi				5.49
Between df				2
Between P				(*)
Btwn(F) P				N.S.
Btwn(R) P				N.S.

Table 1I24 - 3

IESLC - Meta-analysis of Ever/current Smoking, Duration, "High"
All LC types, Cigarettes only
Most adjusted

	Number of adjustment variables (2) 0	1	2	3-5	6+/+nk	Total
N	8	3		1		12
NS	7	2		1		10
Wt	291.12	23.14		3.06		317.32
Het Chi	90.58	2.43		0.00		98.50
Het df	7	2		0		11
Het P	***	N.S.		N.S.		***
Fixed RR	26.18	18.53		9.56		25.28
RRl	23.34	12.33		3.12		22.65
RRu	29.37	27.85		29.29		28.22
P	+++	+++		+++		+++
Random RR	16.18	18.60		9.56		16.38
RRl	9.33	11.85		3.12		10.68
RRu	28.05	29.20		29.29		25.10
P	+++	+++		+++		+++
Between Chi						5.49
Between df						2
Between P						(*)
Btwn(F) P						N.S.
Btwn(R) P						N.S.

	Smoking status ever	current	Total
N	6	6	12
NS	6	4	10
Wt	41.69	275.63	317.32
Het Chi	16.48	67.99	98.50
Het df	5	5	11
Het P	**	***	***
Fixed RR	14.72	27.44	25.28
RRl	10.87	24.38	22.65
RRu	19.94	30.88	28.22
P	+++	+++	+++
Random RR	11.55	20.51	16.38
RRl	5.45	11.98	10.68
RRu	24.45	35.10	25.10
P	+++	+++	+++
Between Chi			14.04
Between df			1
Between P			***
Btwn(F) P			N.S.
Btwn(R) P			N.S.

	Denominator nev any	nev cigs	Total
N	9	3	12
NS	8	2	10
Wt	151.73	165.59	317.32
Het Chi	69.90	15.45	98.50
Het df	8	2	11
Het P	***	***	***
Fixed RR	31.28	20.81	25.28
RRl	26.68	17.87	22.65
RRu	36.67	24.23	28.22
P	+++	+++	+++
Random RR	15.47	17.12	16.38
RRl	8.29	7.81	10.68
RRu	28.86	37.54	25.10
P	+++	+++	+++
Between Chi			13.15
Between df			1
Between P			***
Btwn(F) P			N.S.
Btwn(R) P			N.S.

Table 1I24 - 3

IESLC - Meta-analysis of Ever/current Smoking, Duration, "High"
All LC types, Cigarettes only
Most adjusted

	Orig	StdCalc	Other	Total
	Derivation of RR/CI			
N		9	3	12
NS		7	3	10
Wt		307.11	10.21	317.32
Het Chi		92.88	1.50	98.50
Het df		8	2	11
Het P		***	N.S.	***
Fixed RR		25.81	13.53	25.28
RRl		23.08	7.33	22.65
RRu		28.87	24.99	28.22
P		+++	+++	+++
Random RR		16.77	13.53	16.38
RRl		10.43	7.33	10.68
RRu		26.98	24.99	25.10
P		+++	+++	+++
Between Chi				4.12
Between df				1
Between P				*
Btwn(F) P				N.S.
Btwn(R) P				N.S.

Table 1I24 - 4

IESLC - Meta-analysis of Ever/current Smoking, Duration, "High"
All LC types, Cigarettes only
Least adjusted

REF	NRR	X	SEX	AGEL	AGEH	RACE	YF	LC TYPE	LOC	START	ST	NLC	R	VB	P	H	AD	SM	PRODUCT	exL	exH	DENOM	De
BENHAM	512		m	0	0	all	-	not mix	Eu:wst	1976	CC	1625	n	bl	n	y	0	ev	cig only	36	999	nev any	st
BEST	507		m	0	0	all	0	all	NAmer	1955	pr	381	n	V	n	n	1	cu	cig only	40	999	nev any	ot
BOUCOT	513		m	0	0	all	9	all	NAmer	1951	pr	121	n	bl	n	n	0	ev	cig only	40	999	nev any	ot
CPSI	585		m	40	84	wh	0	all	NAmer	1959	pr	5138	n	bl	n	n	0	cu	cig only	50	54	nev cigs	st
CPSI	681		f	40	84	wh	0	all	NAmer	1959	pr	5138	n	bl	n	n	0	cu	cig only	50	54	nev cigs	st
CPSII	557		m	0	0	all	6	all	NAmer	1982	pr	3229	n	bl	n	n	0	cu	cig only	50	54	nev any	st
JUSSAW	514		m	0	0	all	-	all	As:Ind	1964	CC	792	n	V	n	n	0	ev	cig only	40	999	nev any	st
KAISE2	597		m	0	0	all	9	all	NAmer	1979	pr	318	n	bl	n	n	1	cu	cig only	40	999	nev any	st
KAISE2	517		f	0	0	all	9	all	NAmer	1979	pr	318	n	bl	n	n	1	cu	cig only	40	999	nev any	st
LUBIN	522	x	m	0	0	all	-	all	As:Chi	1984	CC	427	m	ot	y	n	0	ev	cig only	50	999	nev any	st
NOTAN2	517		c	0	0	all	-	all	As:Ind	1963	CC	683	n	V	n	n	0	ev	cig only	41	999	nev any	st
PEZZOT	536		m	0	0	all	-	all	SCAmer	1987	CC	215	n	bl	n	y	0	ev	cig only	41	999	nev cigs	st

Cigarette type is all/unspec for all RRs

except for the following:

REF	NRR	CIGTYPE
JUSSAW	514	MC only
NOTAN2	517	MC only

Table 1I24 - 5

IESLC - Meta-analysis of Ever/current Smoking, Duration, "High"
All LC types, Cigarettes only
Least adjusted

REF	NRR	SEX	AD	Number Exposed Case	Cont	Non-exposed Case	Cont	RR	95.00%CI
BENHAM	512	m	0	694	620	33	523	17.74	(12.28- 25.63)
*BEST	507	m	1	137	-	7	-	14.20	(6.64- 30.35)
*BOUCOT	513	m	0	44	1177	0	805	60.88~	(3.75- 987.17)
*CPSI	585	m	0	576	119633	196	926068	22.75	(19.35- 26.75)
*CPSI	681	f	0	16	14305	532	3877179	8.15	(4.96- 13.40)
Subtotal CPSI								20.61	(17.67- 24.05)
*CPSII	557	m	0	332	39260	124	742207	50.62	(41.19- 62.20)
JUSSAW	514	m	0	11	6	149	624	7.68	(2.79- 21.09)
*KAISE2	597	m	1	34	-	14	-	15.64	(8.31- 29.40)
*KAISE2	517	f	1	26	-	11	-	30.41	(14.39- 64.25)
Subtotal KAISE2								20.63	(12.73- 33.43)
LUBIN	522	m	0	8	16	8	72	4.50	(1.47- 13.79)
NOTAN2	517	c	0	5	5	107	201	1.88	(0.53- 6.63)
PEZZOT	536	m	0	110	101	4	116	31.58	(11.25- 88.71)
Partial Totals				1993	175123	1185	5547795		

*prospective study ~ With 0.5 adjustment for zero

REF	NRR	SEX	AD	Ys	Ws	Qs	Ps
BENHAM	512	m	0	2.88	28.35	3.42	0.0000
*BEST	507	m	1	2.65	6.65	2.16	0.0000
*BOUCOT	513	m	0	4.11	0.49	0.39	0.0038
*CPSI	585	m	0	3.12	146.44	1.42	0.0000
*CPSI	681	f	0	2.10	15.55	19.67	0.0000
Subtotal CPSI				3.03	161.99	21.09	
*CPSII	557	m	0	3.92	90.50	44.52	0.0000
JUSSAW	514	m	0	2.04	3.76	5.28	0.0001
*KAISE2	597	m	1	2.75	9.62	2.15	0.0000
*KAISE2	517	f	1	3.41	6.86	0.25	0.0000
Subtotal KAISE2				3.03	16.49	2.41	
LUBIN	522	m	0	1.50	3.06	9.05	0.0085
NOTAN2	517	c	0	0.63	2.41	16.22	0.3273
PEZZOT	536	m	0	3.45	3.60	0.19	0.0000

	N	12
	NS	10
	Wt	317.32
Het	Chi	104.72
Het	df	11
Het	P	***
Fixed	RR	25.10
	RRl	22.49
	RRu	28.02
	P	+++
Random	RR	15.52
	RRl	10.01
	RRu	24.06
	P	+++
Asymm	P	(*)

Table 1I24 - 6

IESLC - Meta-analysis of Ever/current Smoking, Duration, "High"
All LC types, Cigarettes only
Least adjusted

	combined	Sex male	female	Total
N	1	9	2	12
NS	1	9	2	12
Wt	2.41	292.49	22.41	317.32
Het Chi	0.00	66.85	8.25	104.72
Het df	0	8	1	11
Het P	N.S.	***	**	***
Fixed RR	1.88	27.10	12.20	25.10
RRl	0.53	24.17	8.06	22.49
RRu	6.63	30.39	18.46	28.02
P	N.S.	+++	+++	+++
Random RR	1.88	18.84	15.27	15.52
RRl	0.53	12.02	4.21	10.01
RRu	6.63	29.52	55.39	24.06
P	N.S.	+++	+++	+++
Between Chi				29.61
Between df				2
Between P				***
Btwn(F) P				N.S.
Btwn(R) P				**

Table 1I24 - 7

IESLC - Meta-analysis of Ever/current Smoking, Duration, "High"
All LC types, Cigarettes only
Excluded studies (and stage at which they were excluded)

Stage	Studies
1	BECHER BLOT1 BROWN3 CARPEN CHYOU DARBY DOLL2 GARCIA GRAHAM GURSEL HAMMO2 JAHN JAIN LAUSSM PRESCO QIAO TVERDA WIGLE WYNDE3
2	ALDERS BENSHL BRESLO CHIAZZ DEAN3 DORN ENGELA GAO2 GILLIS GUO HEGMAN HIRAYA HOLE KAUFMA KOO KOULUM LIU4 MIGRAN MRFITR PERNU SEGI2 SPEIZE SUZUK2 SVENSS VUTUC WAKAI WU YUAN
3	GENG MCDUFF SPITZ STASZE WU2 ZHANG
4	BOUCHA CHEN CORREA JEDRYC LUO WYNDE2 WYNDE6
5	AKIBA HAMMON PISANI RESTRE SADOWS XU
7	AMANDU AMES ARMADA AUVINE AXELSS BARBON BOFFET BROSS BUFFLE CHEN2 CHOI DAMBER DEAN2 DESTEF DOLL DORGAN DOSEME FAN GAO GARSHI GER HAENSZ HU HU2 HUMBLE JOLY KATSOU KHUDER KREUZE LETOUR LEVIN LIAW LIU3 LIU5 LUBIN2 MATOS MCCONN OSANN2 PEZZO2 QIAO2 RACHTA SOBUE WANG2 WATSON WUWILL WYNDE7 WYNDE8 ZHENG ZHOU
14	AGUDO CEDERL TIZZAN

Table 1I24 - 8
Potentially overlapping studies

REF	REFGP	PRINC	. OVERLAP/LINK
BENHAM	LUBIN2	2	Subset of Lubin2
CPSI	CPSI	1	CPSI overall
LUBIN	XIANGZ	2	LUBIN/XIANGZ/QIAO

Table 1I24 - 9
Most adjusted - insufficient data for meta-analysis

REF	NRR	SEX	AGEL	AGEH	RACE	YF	LC TYPE	LOC	START	ST	NLC	R	VB	P	H	AD	SM	PRODUCT	exL	exH	DENOM	De
LUBIN2	1060	m	0	0	all	-	all	Eu:mul	1976	CC	7804	n	bl	n	y	1	ev	cig only	40	999	nev any	or
SADOWS	527	m	0	0	wh	-	all	NAmer	1938	CC	477	n	bl	n	n	0	ev	cig only	50	999	nev any	ot

REF	NRR	RR	SIG	RRDATA comment
LUBIN2	1060	12.36		0
SADOWS	527	8.43		0

Table 1I25 -

IESLC - Meta-analysis of Ever/current Smoking, Duration, "Highest vs lowest"
All LC types, Cigarettes only

This analysis is restricted to results for:
1) Ever/current smokers
2) Results by Duration
3) Categorical results by Duration
4) Denominator (unexposed) = "low"
5) All LC types (or near equivalent)
6) Results complete enough for use in metaanalysis

Within each study, results are then selected (in the following order of preference, within each sex) for:
7) SMKSTA: ever, current
8) PRODUCT: cigarettes only
9) CIGTYPE: all/unspecified, MC regardless of HR, MC only
10) Results with least adjustment for other aspects of smoking (ADOS)
11) The highest vs lowest category
12) Followup period (YF, prospective studies): whole study (coded as 0) or longest available
13) LCtype: all or nearest available, at least Squamous and Adeno. (q = squamous, s = small,
l = large, a = adeno, mix = mixed, alv = alveolar)
14) Race: all or nearest available, otherwise by race (wh or w = white, bl or b = black, hi = hispanic
ch = chinese, jap = japanese, haw = hawaiian, w+o = white + oriental, sca = scandinavian, as = asian)
15) For overlapping studies: principal rather than subsidiary studies
Finally by Age: whole study (coded as 0) if available, otherwise by widest available age group
and then for single sex results (m, f) in preference to results for both sexes combined (c).

Results adjusted (AD) for the most potential confounders are then chosen in Sections -1 to -3
(and those which actually differ from the adjusted results in Table 1I15 - 1 are marked 'x' in Section -1)
and results adjusted for the least confounders in Sections -4 to -6. (Those least adjusted results which
actually differ from the most adjusted are marked 'x' in column X in Section -4)

Section -7 shows excluded studies, together with the stage (as above) at which no qualifying
results were found.

Section -8 lists the potentially overlapping studies which have been included (1=principal, 2=subsidiary).

Section -9 lists any results which would have been included in preference except that they had data not complete
enough for use in meta-analysis, with their significance (yes/no), if known, and any further comment as entered
on the database. It also lists as "gap" any categories for which no data were presented by the original authors.

In addition to those mentioned above, the following fields, levels and abbreviations are used:
* or nk = not known, n = no, y = yes, ot = other
all/unspec = all or unspecified, MC = manufactured cigarettes, HR = hand-rolled cigarettes
exL, exH = range of exposure (low and high) in the "highest" group, in terms of Duration
unexL, unexH = range of exposure (low and high) in the "lowest" group, in terms of Duration
REF: 6-character study reference
NRR: number of the RR on the database within the study
ST : study type (CC = case control, pr or prosp = prospective)
NLC: number of lung cancer cases in whole study
R : risky occupational population (n = no, m = mining, o = other risky)
VB : national cigarette type (V = at least 75% Virginia, bl = at least 75% blended, ot = other)
P : any proxy use
H : full histological confirmation
De : derivation of RR/CI (or = original, st = standard method, ot = other method of estimation)

Table 1I25 - 1

IESLC - Meta-analysis of Ever/current Smoking, Duration, "Highest vs lowest"
All LC types, Cigarettes only
Most adjusted

REF	NRR	1I15	SEX	AGEL	AGEH	RACE	YF	LC TYPE	LOC	START	ST	NLC	R	VB	P	H	AD	ADOS	SM	PRODUCT	exL	exH	unexL	unexH	De
AGUDO	512		f	0	0	all	-	all	Eu:wst	1989	CC	103	n	bl	n	n	3	0	ev	cig only	17	999	1	16	ot
BENHAM	613	x	m	0	0	all	-	all	Eu:wst	1976	CC	1625	n	bl	n	y	0	0	ev	cig only	46	999	1	25	st
BEST	513		m	0	0	all	0	all	NAmer	1955	pr	381	n	V	n	n	1	0	cu	cig only	40	999	1	4	ot
BOUCOT	514	x	m	0	0	all	9	all	NAmer	1951	pr	121	n	bl	n	n	0	0	ev	cig only	40	999	1	39	st
CEDERL	503		m	40	69	all	10	all	Eu:Sca	1963	pr	491	n	bl	n	n	1	0	cu	cig only	30	999	1	29	ot
CEDERL	506		f	40	69	all	10	all	Eu:Sca	1963	pr	491	n	bl	n	n	1	0	cu	cig only	30	999	1	29	ot
CPSI	602		m	40	84	wh	0	all	NAmer	1959	pr	5138	n	bl	n	n	0	0	cu	cig only	60	999	1	29	st
CPSI	695		f	40	84	wh	0	all	NAmer	1959	pr	5138	n	bl	n	n	0	0	cu	cig only	55	999	1	29	st
CPSII	566		m	0	0	all	6	all	NAmer	1982	pr	3229	n	bl	n	n	0	0	cu	cig only	60	999	1	29	st
JUSSAW	518		m	0	0	all	-	all	As:Ind	1964	CC	792	n	V	n	n	0	0	ev	cig only	40	999	1	9	st
KAISE2	600		m	0	0	all	9	all	NAmer	1979	pr	318	n	bl	n	n	1	0	cu	cig only	40	999	1	39	st
KAISE2	520		f	0	0	all	9	all	NAmer	1979	pr	318	n	bl	n	n	1	0	cu	cig only	40	999	1	39	st
LUBIN	532	x	m	0	0	all	-	all	As:Chi	1984	CC	427	m	ot	y	n	4	0	ev	cig only	50	999	1	29	ot
NOTAN2	521		c	0	0	all	-	all	As:Ind	1963	CC	683	n	V	n	n	0	0	ev	cig only	41	999	1	10	st
PEZZOT	540		m	0	0	all	-	all	SCAmer	1987	CC	215	n	bl	n	y	2	0	ev	cig only	41	999	1	30	ot
TIZZAN	505		m	0	0	all	-	all	Eu:wst	1959	CC	1358	n	bl	n	n	0	0	ev	cig only	11	999	1	4	st
TIZZAN	532	x	f	0	0	all	-	all	Eu:wst	1959	CC	1358	n	bl	n	n	0	0	ev	cig only	11	999	1	10	st

Cigarette type is all/unspec for all RRs

except for the following:

REF	NRR	CIGTYPE
JUSSAW	518	MC only
NOTAN2	521	MC only

Table 1I25 - 2

IESLC - Meta-analysis of Ever/current Smoking, Duration, "Highest vs lowest"
All LC types, Cigarettes only
Most adjusted

REF	NRR	SEX	AD	Number Exposed Case	Cont	Non-exposed Case	Cont	RR	95.00%CI
AGUDO	512	f	3	18	-	5	-	3.95	(0.93- 16.76)
BENHAM	613	m	0	278	245	104	358	3.91	(2.96- 5.16)
*BEST	513	m	1	137	-	1	-	8.88	(1.25- 62.98)
*BOUCOT	514	m	0	44	1177	19	2088	4.11	(2.41- 7.00)
*CEDERL	503	m	1	23	-	5	-	4.11	(1.48- 11.44)
*CEDERL	506	f	1	5	-	3	-	6.00	(1.44- 24.93)
Subtotal CEDERL								4.67	(2.04- 10.73)
*CPSI	602	m	0	232	26906	95	266163	24.16	(19.03- 30.66)
*CPSI	695	f	0	10	5657	105	694015	11.68	(6.11- 22.34)
Subtotal CPSI								22.15	(17.71- 27.71)
*CPSII	566	m	0	117	8450	72	141932	27.29	(20.37- 36.58)
JUSSAW	518	m	0	11	6	16	20	2.29	(0.70- 7.55)
*KAISE2	600	m	1	34	-	17	-	5.38	(2.95- 9.81)
*KAISE2	520	f	1	26	-	24	-	3.13	(1.63- 6.00)
Subtotal KAISE2								4.19	(2.70- 6.52)
LUBIN	532	m	4	8	-	7	-	7.08	(2.28- 21.98)
NOTAN2	521	c	0	5	5	7	15	2.14	(0.46- 9.90)
PEZZOT	540	m	2	110	-	30	-	7.00	(4.33- 11.30)
TIZZAN	505	m	0	928	815	12	1	0.09	(0.01- 0.73)
TIZZAN	532	f	0	23	21	2	7	3.83	(0.72- 20.55)
Subtotal TIZZAN								0.86	(0.24- 3.15)
Partial Totals				2009	43282	524	1104599		

*prospective study

REF	NRR	SEX	AD	Ys	Ws	Qs	Ps
AGUDO	512	f	3	1.37	1.84	1.66	0.0626
BENHAM	613	m	0	1.36	49.78	45.96	0.0000
*BEST	513	m	1	2.18	1.00	0.02	0.0290
*BOUCOT	514	m	0	1.41	13.51	11.19	0.0000
*CEDERL	503	m	1	1.41	3.67	3.04	0.0067
*CEDERL	506	f	1	1.79	1.89	0.53	0.0138
Subtotal CEDERL				1.54	5.56	3.58	
*CPSI	602	m	0	3.18	67.59	50.14	0.0000
*CPSI	695	f	0	2.46	9.15	0.17	0.0000
Subtotal CPSI				3.10	76.73	50.30	
*CPSII	566	m	0	3.31	44.82	43.34	0.0000
JUSSAW	518	m	0	0.83	2.70	6.03	0.1728
*KAISE2	600	m	1	1.68	10.64	4.37	0.0000
*KAISE2	520	f	1	1.14	9.05	12.65	0.0006
Subtotal KAISE2				1.43	19.69	17.02	
LUBIN	532	m	4	1.96	2.99	0.40	0.0007
NOTAN2	521	c	0	0.76	1.64	4.00	0.3290
PEZZOT	540	m	2	1.95	16.70	2.38	0.0000
TIZZAN	505	m	0	-2.36	0.92	20.16	0.0238
TIZZAN	532	f	0	1.34	1.36	1.31	0.1168
Subtotal TIZZAN				-0.15	2.28	21.47	

	N	17
	NS	13
	Wt	239.26
Het	Chi	207.35
Het	df	16
Het	P	***
Fixed	RR	10.21
	RRl	8.99
	RRu	11.59
	P	+++
Random	RR	5.30
	RRl	3.12
	RRu	9.00
	P	+++
Asymm	P	*

Table 1I25 - 3

IESLC - Meta-analysis of Ever/current Smoking, Duration, "Highest vs lowest"
All LC types, Cigarettes only
Most adjusted

		combined	Sex male	female	Total
	N	1	11	5	17
	NS	1	11	5	17
	Wt	1.64	214.33	23.28	239.26
Het	Chi	0.00	185.82	8.43	207.35
Het	df	0	10	4	16
Het	P	N.S.	***	(*)	***
Fixed	RR	2.14	11.01	5.71	10.21
	RRl	0.46	9.63	3.80	8.99
	RRu	9.90	12.58	8.56	11.59
	P	N.S.	+++	+++	+++
Random	RR	2.14	5.62	5.40	5.30
	RRl	0.46	2.89	2.76	3.12
	RRu	9.90	10.93	10.55	9.00
	P	N.S.	+++	+++	+++
Between	Chi				13.09
Between	df				2
Between	P				**
Btwn(F)	P				N.S.
Btwn(R)	P				N.S.

		Lung cancer type all	other	Total
	N	17		17
	NS	13		13
	Wt	239.26		239.26
Het	Chi	207.35		207.35
Het	df	16		16
Het	P	***		***
Fixed	RR	10.21		10.21
	RRl	8.99		8.99
	RRu	11.59		11.59
	P	+++		+++
Random	RR	5.30		5.30
	RRl	3.12		3.12
	RRu	9.00		9.00
	P	+++		+++
Between	Chi			
Between	df			
Between	P			N.S.
Btwn(F)	P			N.S.
Btwn(R)	P			N.S.

		NAmer	UK	Scand	Location othEur	China	Japan	othAs	other	Total
	N	7		2	4	1		2	1	17
	NS	5		1	3	1		2	1	13
	Wt	155.75		5.56	53.90	2.99		4.34	16.70	239.26
Het	Chi	87.12		0.18	12.51	0.00		0.00	0.00	207.35
Het	df	6		1	3	0		1	0	16
Het	P	***		N.S.	**	N.S.		N.S.	N.S.	***
Fixed	RR	16.37		4.67	3.67	7.08		2.23	7.00	10.21
	RRl	13.99		2.04	2.81	2.28		0.87	4.33	8.99
	RRu	19.16		10.73	4.79	21.98		5.72	11.31	11.59
	P	+++		+++	+++	+++		(+)	+++	+++
Random	RR	9.28		4.67	1.95	7.08		2.23	7.00	5.30
	RRl	4.65		2.04	0.54	2.28		0.87	4.33	3.12
	RRu	18.51		10.73	7.04	21.98		5.72	11.31	9.00
	P	+++		+++	N.S.	+++		(+)	+++	+++
Between	Chi									107.52
Between	df									5
Between	P									***
Btwn(F)	P									N.S.
Btwn(R)	P									(*)

Table 1I25 - 3

IESLC - Meta-analysis of Ever/current Smoking, Duration, "Highest vs lowest"
All LC types, Cigarettes only
Most adjusted

Detailed Country in "other Europe"

	multi	Germany	othWest	East	Balkans	Total
N			4			4
NS			3			3
Wt			53.90			53.90
Het Chi			12.51			12.51
Het df			3			3
Het P			**			**
Fixed RR			3.67			3.67
RRl			2.81			2.81
RRu			4.79			4.79
P			+++			+++
Random RR			1.95			1.95
RRl			0.54			0.54
RRu			7.04			7.04
P			N.S.			N.S.
Between Chi						
Between df						
Between P						N.S.
Btwn(F) P						N.S.
Btwn(R) P						N.S.

Detailed Country in "other Asia"

	India	HongKong	other	Total
N	2			2
NS	2			2
Wt	4.34			4.34
Het Chi	0.00			0.00
Het df	1			1
Het P	N.S.			N.S.
Fixed RR	2.23			2.23
RRl	0.87			0.87
RRu	5.72			5.72
P	(+)			(+)
Random RR	2.23			2.23
RRl	0.87			0.87
RRu	5.72			5.72
P	(+)			(+)
Between Chi				
Between df				
Between P				N.S.
Btwn(F) P				N.S.
Btwn(R) P				N.S.

Detailed other continent

	SCAmer	Total
N	1	1
NS	1	1
Wt	16.70	16.70
Het Chi	0.00	0.00
Het df	0	0
Het P	N.S.	N.S.
Fixed RR	7.00	7.00
RRl	4.33	4.33
RRu	11.31	11.31
P	+++	+++
Random RR	7.00	7.00
RRl	4.33	4.33
RRu	11.31	11.31
P	+++	+++
Between Chi		
Between df		
Between P		N.S.
Btwn(F) P		N.S.
Btwn(R) P		N.S.

Table 1I25 - 3

IESLC - Meta-analysis of Ever/current Smoking, Duration, "Highest vs lowest"
All LC types, Cigarettes only
Most adjusted

	Start year of study <1960	1960-69	1970-79	1980-89	1990+	Total
N	6	4	3	4		17
NS	4	3	2	4		13
Wt	93.52	9.91	69.47	66.35		239.26
Het Chi	64.69	1.51	1.51	29.39		207.35
Het df	5	3	2	3		16
Het P	***	N.S.	N.S.	***		***
Fixed RR	15.89	3.38	3.99	17.28		10.21
RRl	12.97	1.81	3.15	13.59		8.99
RRu	19.46	6.30	5.04	21.99		11.59
P	+++	+++	+++	+++		+++
Random RR	5.08	3.38	3.99	9.51		5.30
RRl	1.72	1.81	3.15	3.51		3.12
RRu	14.98	6.30	5.04	25.74		9.00
P	++	+++	+++	+++		+++
Between Chi						110.25
Between df						3
Between P						***
Btwn(F) P						*
Btwn(R) P						N.S.

	Study type (1) CC	other	Total
N	8	9	17
NS	7	6	13
Wt	77.94	161.32	239.26
Het Chi	20.42	95.75	207.35
Het df	7	8	16
Het P	**	***	***
Fixed RR	4.20	15.68	10.21
RRl	3.36	13.44	8.99
RRu	5.24	18.30	11.59
P	+++	+++	+++
Random RR	3.43	8.24	5.30
RRl	1.97	4.39	3.12
RRu	5.97	15.47	9.00
P	+++	+++	+++
Between Chi			91.18
Between df			1
Between P			***
Btwn(F) P			**
Btwn(R) P			*

	Study type (2) CC	prosp	other	Total
N	8	9		17
NS	7	6		13
Wt	77.94	161.32		239.26
Het Chi	20.42	95.75		207.35
Het df	7	8		16
Het P	**	***		***
Fixed RR	4.20	15.68		10.21
RRl	3.36	13.44		8.99
RRu	5.24	18.30		11.59
P	+++	+++		+++
Random RR	3.43	8.24		5.30
RRl	1.97	4.39		3.12
RRu	5.97	15.47		9.00
P	+++	+++		+++
Between Chi				91.18
Between df				1
Between P				***
Btwn(F) P				**
Btwn(R) P				*

Table 1I25 - 3

IESLC - Meta-analysis of Ever/current Smoking, Duration, "Highest vs lowest"
All LC types, Cigarettes only
Most adjusted

	Study size (number of LC cases) 100-249	250-499	500-999	1000+	Total
N	3	6	2	6	17
NS	3	4	2	4	13
Wt	32.05	29.25	4.34	173.62	239.26
Het Chi	2.31	2.77	0.00	146.65	207.35
Het df	2	5	1	5	16
Het P	N.S.	N.S.	N.S.	***	***
Fixed RR	5.41	4.63	2.23	13.62	10.21
RRl	3.83	3.23	0.87	11.74	8.99
RRu	7.65	6.66	5.72	15.80	11.59
P	+++	+++	(+)	+++	+++
Random RR	5.36	4.63	2.23	6.80	5.30
RRl	3.64	3.23	0.87	2.63	3.12
RRu	7.90	6.66	5.72	17.54	9.00
P	+++	+++	(+)	+++	+++
Between Chi					55.61
Between df					3
Between P					***
Btwn(F) P					N.S.
Btwn(R) P					N.S.

	Risky occupational population no	mining	othRisky	Total
N	16	1		17
NS	12	1		13
Wt	236.26	2.99		239.26
Het Chi	206.94	0.00		207.35
Het df	15	0		16
Het P	***	N.S.		***
Fixed RR	10.26	7.08		10.21
RRl	9.03	2.28		8.99
RRu	11.65	21.98		11.59
P	+++	+++		+++
Random RR	5.20	7.08		5.30
RRl	3.00	2.28		3.12
RRu	9.01	21.98		9.00
P	+++	+++		+++
Between Chi				0.41
Between df				1
Between P				N.S.
Btwn(F) P				N.S.
Btwn(R) P				N.S.

	National cigarette tobacco type Virginia	blended	other	Total
N	3	13	1	17
NS	3	9	1	13
Wt	5.34	230.92	2.99	239.26
Het Chi	1.55	196.63	0.00	207.35
Het df	2	12	0	16
Het P	N.S.	***	N.S.	***
Fixed RR	2.89	10.56	7.08	10.21
RRl	1.24	9.28	2.28	8.99
RRu	6.75	12.02	21.98	11.59
P	+	+++	+++	+++
Random RR	2.89	5.65	7.08	5.30
RRl	1.24	3.12	2.28	3.12
RRu	6.75	10.23	21.98	9.00
P	+	+++	+++	+++
Between Chi				9.16
Between df				2
Between P				*
Btwn(F) P				N.S.
Btwn(R) P				N.S.

Table 1I25 - 3

IESLC - Meta-analysis of Ever/current Smoking, Duration, "Highest vs lowest"
All LC types, Cigarettes only
Most adjusted

	Any proxy use No/nk	Yes	Total
N	16	1	17
NS	12	1	13
Wt	236.26	2.99	239.26
Het Chi	206.94	0.00	207.35
Het df	15	0	16
Het P	***	N.S.	***
Fixed RR	10.26	7.08	10.21
RRl	9.03	2.28	8.99
RRu	11.65	21.98	11.59
P	+++	+++	+++
Random RR	5.20	7.08	5.30
RRl	3.00	2.28	3.12
RRu	9.01	21.98	9.00
P	+++	+++	+++
Between Chi			0.41
Between df			1
Between P			N.S.
Btwn(F) P			N.S.
Btwn(R) P			N.S.

	Full histological confirmation No	Yes	Total
N	15	2	17
NS	11	2	13
Wt	172.77	66.48	239.26
Het Chi	142.04	4.26	207.35
Het df	14	1	16
Het P	***	*	***
Fixed RR	13.97	4.52	10.21
RRl	12.03	3.56	8.99
RRu	16.21	5.75	11.59
P	+++	+++	+++
Random RR	5.31	5.05	5.30
RRl	2.99	2.86	3.12
RRu	9.44	8.92	9.00
P	+++	+++	+++
Between Chi			61.05
Between df			1
Between P			***
Btwn(F) P			*
Btwn(R) P			N.S.

	Number of adjustment variables (1) 0	1	2+/+nk	Total
N	9	5	3	17
NS	7	3	3	13
Wt	191.47	26.25	21.53	239.26
Het Chi	177.23	2.17	0.55	207.35
Het df	8	4	2	16
Het P	***	N.S.	N.S.	***
Fixed RR	12.01	4.42	6.68	10.21
RRl	10.43	3.01	4.38	8.99
RRu	13.84	6.47	10.19	11.59
P	+++	+++	+++	+++
Random RR	5.13	4.42	6.68	5.30
RRl	2.34	3.01	4.38	3.12
RRu	11.25	6.47	10.19	9.00
P	+++	+++	+++	+++
Between Chi				27.39
Between df				2
Between P				***
Btwn(F) P				N.S.
Btwn(R) P				N.S.

Table 1I25 - 3

IESLC - Meta-analysis of Ever/current Smoking, Duration, "Highest vs lowest"
All LC types, Cigarettes only
Most adjusted

	Number of adjustment variables (2) 0	1	2	3-5	6+/+nk	Total
N	9	5	1	2		17
NS	7	3	1	2		13
Wt	191.47	26.25	16.70	4.83		239.26
Het Chi	177.23	2.17	0.00	0.39		207.35
Het df	8	4	0	1		16
Het P	***	N.S.	N.S.	N.S.		***
Fixed RR	12.01	4.42	7.00	5.67		10.21
RRl	10.43	3.01	4.33	2.32		8.99
RRu	13.84	6.47	11.31	13.83		11.59
P	+++	+++	+++	+++		+++
Random RR	5.13	4.42	7.00	5.67		5.30
RRl	2.34	3.01	4.33	2.32		3.12
RRu	11.25	6.47	11.31	13.83		9.00
P	+++	+++	+++	+++		+++
Between Chi						27.56
Between df						3
Between P						***
Btwn(F) P						N.S.
Btwn(R) P						N.S.

	Smoking status ever	current	Total
N	9	8	17
NS	8	5	13
Wt	91.45	147.81	239.26
Het Chi	20.43	69.30	207.35
Het df	8	7	16
Het P	**	***	***
Fixed RR	4.19	17.72	10.21
RRl	3.41	15.08	8.99
RRu	5.14	20.82	11.59
P	+++	+++	+++
Random RR	3.69	9.22	5.30
RRl	2.37	4.95	3.12
RRu	5.76	17.17	9.00
P	+++	+++	+++
Between Chi			117.62
Between df			1
Between P			***
Btwn(F) P			***
Btwn(R) P			*

	Derivation of RR/CI Orig	StdCalc	Other	Total
N		11	6	17
NS		8	5	13
Wt		211.16	28.09	239.26
Het Chi		198.43	1.42	207.35
Het df		10	5	16
Het P		***	N.S.	***
Fixed RR		10.89	6.28	10.21
RRl		9.52	4.34	8.99
RRu		12.46	9.10	11.59
P		+++	+++	+++
Random RR		4.94	6.28	5.30
RRl		2.48	4.34	3.12
RRu		9.84	9.10	9.00
P		+++	+++	+++
Between Chi				7.49
Between df				1
Between P				**
Btwn(F) P				N.S.
Btwn(R) P				N.S.

Table 1I25 - 4

IESLC - Meta-analysis of Ever/current Smoking, Duration, "Highest vs lowest"
All LC types, Cigarettes only
Least adjusted

REF	NRR	X	SEX	AGEL	AGEH	RACE	YF	LC TYPE	LOC	START	ST	NLC	R	VB	P	H	AD	ADOS	SM	PRODUCT	exL	exH	unexL	unexH	De
AGUDO	509	x	f	0	0	all	-	all	Eu:wst	1989	CC	103	n	bl	n	n	0	0	ev	cig only	17	999	1	16	st
BENHAM	613		m	0	0	all	-	all	Eu:wst	1976	CC	1625	n	bl	n	y	0	0	ev	cig only	46	999	1	25	st
BEST	513		m	0	0	all	0	all	NAmer	1955	pr	381	n	V	n	n	1	0	cu	cig only	40	999	1	4	ot
BOUCOT	514		m	0	0	all	9	all	NAmer	1951	pr	121	n	bl	n	n	0	0	ev	cig only	40	999	1	39	st
CEDERL	503		m	40	69	all	10	all	Eu:Sca	1963	pr	491	n	bl	n	n	1	0	cu	cig only	30	999	1	29	ot
CEDERL	506		f	40	69	all	10	all	Eu:Sca	1963	pr	491	n	bl	n	n	1	0	cu	cig only	30	999	1	29	ot
CPSI	602		m	40	84	wh	0	all	NAmer	1959	pr	5138	n	bl	n	n	0	0	cu	cig only	60	999	1	29	st
CPSI	695		f	40	84	wh	0	all	NAmer	1959	pr	5138	n	bl	n	n	0	0	cu	cig only	55	999	1	29	st
CPSII	566		m	0	0	all	6	all	NAmer	1982	pr	3229	n	bl	n	n	0	0	cu	cig only	60	999	1	29	st
JUSSAW	518		m	0	0	all	-	all	As:Ind	1964	CC	792	n	V	n	n	0	0	ev	cig only	40	999	1	9	st
KAISE2	600		m	0	0	all	9	all	NAmer	1979	pr	318	n	bl	n	n	1	0	cu	cig only	40	999	1	39	st
KAISE2	520		f	0	0	all	9	all	NAmer	1979	pr	318	n	bl	n	n	1	0	cu	cig only	40	999	1	39	st
LUBIN	525	x	m	0	0	all	-	all	As:Chi	1984	CC	427	m	ot	y	n	0	0	ev	cig only	50	999	1	29	st
NOTAN2	521		c	0	0	all	-	all	As:Ind	1963	CC	683	n	V	n	n	0	0	ev	cig only	41	999	1	10	st
PEZZOT	538	x	m	0	0	all	-	all	SCAmer	1987	CC	215	n	bl	n	y	0	0	ev	cig only	41	999	1	30	st
TIZZAN	505		m	0	0	all	-	all	Eu:wst	1959	CC	1358	n	bl	n	n	0	0	ev	cig only	11	999	1	4	st
TIZZAN	532		f	0	0	all	-	all	Eu:wst	1959	CC	1358	n	bl	n	n	0	0	ev	cig only	11	999	1	10	st

Cigarette type is all/unspec for all RRs

except for the following:

REF	NRR	CIGTYPE
JUSSAW	518	MC only
NOTAN2	521	MC only

Table 1I25 - 5

IESLC - Meta-analysis of Ever/current Smoking, Duration, "Highest vs lowest"
All LC types, Cigarettes only
Least adjusted

REF	NRR	SEX	AD	Number Exposed Case	Cont	Non-exposed Case	Cont	RR	95.00%CI
AGUDO	509	f	0	18	11	5	12	3.93	(1.09- 14.19)
BENHAM	613	m	0	278	245	104	358	3.91	(2.96- 5.16)
*BEST	513	m	1	137	-	1	-	8.88	(1.25- 62.98)
*BOUCOT	514	m	0	44	1177	19	2088	4.11	(2.41- 7.00)
*CEDERL	503	m	1	23	-	5	-	4.11	(1.48- 11.44)
*CEDERL	506	f	1	5	-	3	-	6.00	(1.44- 24.93)
Subtotal CEDERL								4.67	(2.04- 10.73)
*CPSI	602	m	0	232	26906	95	266163	24.16	(19.03- 30.66)
*CPSI	695	f	0	10	5657	105	694015	11.68	(6.11- 22.34)
Subtotal CPSI								22.15	(17.71- 27.71)
*CPSII	566	m	0	117	8450	72	141932	27.29	(20.37- 36.58)
JUSSAW	518	m	0	11	6	16	20	2.29	(0.70- 7.55)
*KAISE2	600	m	1	34	-	17	-	5.38	(2.95- 9.81)
*KAISE2	520	f	1	26	-	24	-	3.13	(1.63- 6.00)
Subtotal KAISE2								4.19	(2.70- 6.52)
LUBIN	525	m	0	8	16	7	51	3.64	(1.14- 11.61)
NOTAN2	521	c	0	5	5	7	15	2.14	(0.46- 9.90)
PEZZOT	538	m	0	110	101	30	134	4.86	(3.01- 7.86)
TIZZAN	505	m	0	928	815	12	1	0.09	(0.01- 0.73)
TIZZAN	532	f	0	23	21	2	7	3.83	(0.72- 20.55)
Subtotal TIZZAN								0.86	(0.24- 3.15)
Partial Totals				2009	43410	524	1104796		

*prospective study

REF	NRR	SEX	AD	Ys	Ws	Qs	Ps
AGUDO	509	f	0	1.37	2.33	1.97	0.0369
BENHAM	613	m	0	1.36	49.78	42.66	0.0000
*BEST	513	m	1	2.18	1.00	0.01	0.0290
*BOUCOT	514	m	0	1.41	13.51	10.35	0.0000
*CEDERL	503	m	1	1.41	3.67	2.81	0.0067
*CEDERL	506	f	1	1.79	1.89	0.47	0.0138
Subtotal CEDERL				1.54	5.56	3.28	
*CPSI	602	m	0	3.18	67.59	54.31	0.0000
*CPSI	695	f	0	2.46	9.15	0.26	0.0000
Subtotal CPSI				3.10	76.73	54.58	
*CPSII	566	m	0	3.31	44.82	46.49	0.0000
JUSSAW	518	m	0	0.83	2.70	5.75	0.1728
*KAISE2	600	m	1	1.68	10.64	3.90	0.0000
*KAISE2	520	f	1	1.14	9.05	11.91	0.0006
Subtotal KAISE2				1.43	19.69	15.81	
LUBIN	525	m	0	1.29	2.86	2.83	0.0289
NOTAN2	521	c	0	0.76	1.64	3.82	0.3290
PEZZOT	538	m	0	1.58	16.73	8.34	0.0000
TIZZAN	505	m	0	-2.36	0.92	19.86	0.0238
TIZZAN	532	f	0	1.34	1.36	1.22	0.1168
Subtotal TIZZAN				-0.15	2.28	21.07	

	N	17
	NS	13
	Wt	239.64
Het	Chi	216.96
Het	df	16
Het	P	***
Fixed	RR	9.86
	RRl	8.68
	RRu	11.19
	P	+++
Random	RR	4.95
	RRl	2.88
	RRu	8.48
	P	+++
Asymm	P	*

Table 1I25 - 6

IESLC - Meta-analysis of Ever/current Smoking, Duration, "Highest vs lowest"
All LC types, Cigarettes only
Least adjusted

		Sex		
	combined	male	female	Total
N	1	11	5	17
NS	1	11	5	17
Wt	1.64	214.22	23.77	239.64
Het Chi	0.00	196.17	8.50	216.96
Het df	0	10	4	16
Het P	N.S.	***	(*)	***
Fixed RR	2.14	10.61	5.66	9.86
RRl	0.46	9.28	3.79	8.68
RRu	9.90	12.13	8.46	11.19
P	N.S.	+++	+++	+++
Random RR	2.14	5.09	5.36	4.95
RRl	0.46	2.57	2.77	2.88
RRu	9.90	10.06	10.36	8.48
P	N.S.	+++	+++	+++
Between Chi				12.29
Between df				2
Between P				**
Btwn(F) P				N.S.
Btwn(R) P				N.S.

Table 1I25 - 7

IESLC - Meta-analysis of Ever/current Smoking, Duration, "Highest vs lowest"
All LC types, Cigarettes only
Excluded studies (and stage at which they were excluded)

1	BECHER BLOT1 BROWN3 CARPEN CHYOU DARBY DOLL2 GARCIA GRAHAM GURSEL HAMMO2 JAHN JAIN LAUSSM PRESCO QIAO TVERDA WIGLE WYNDE3
2	ALDERS BENSHL BRESLO CHIAZZ DEAN3 DORN ENGELA GAO2 GILLIS GUO HEGMAN HIRAYA HOLE KAUFMA KOO KOULUM LIU4 MIGRAN MRFITR PERNU SEGI2 SPEIZE SUZUK2 SVENSS VUTUC WAKAI WU YUAN
3	GENG MCDUFF SPITZ STASZE WU2 ZHANG
4	AKIBA GARSHI
5	BOUCHA CHEN CORREA JEDRYC LUO WYNDE2 WYNDE6
6	HAMMON PISANI RESTRE SADOWS XU
8	AMANDU AMES ARMADA AUVINE AXELSS BARBON BOFFET BROSS BUFFLE CHEN2 CHOI DAMBER DEAN2 DESTEF DOLL DORGAN DOSEME FAN GAO GER HAENSZ HU HU2 HUMBLE JOLY KATSOU KHUDER KREUZE LETOUR LEVIN LIAW LIU3 LIU5 LUBIN2 MATOS MCCONN OSANN2 PEZZO2 QIAO2 RACHTA SOBUE WANG2 WATSON WUWILL WYNDE7 WYNDE8 ZHENG ZHOU

Table 1I25 - 8
Potentially overlapping studies

REF	REFGP	PRINC	.	OVERLAP/LINK
BENHAM	LUBIN2	2		Subset of Lubin2
CPSI	CPSI	1		CPSI overall
LUBIN	XIANGZ	2		LUBIN/XIANGZ/QIAO

Table 1I25 - 9
Most adjusted - insufficient data for meta-analysis

REF	NRR	SEX	AGEL	AGEH	RACE	YF	LC TYPE	LOC	START	ST	NLC	R	VB	P	H	AD	ADOS	SM	PRODUCT	exL	exH	unexL	unexH	De
HAMMON	512	m	0	0	wh	0	all	NAmer	1952	pr	448	n	bl	n	n	1	0	ev	cig only	35	999	1	34	st
SADOWS	532	m	0	0	wh	-	all	NAmer	1938	CC	477	n	bl	n	n	0	0	ev	cig only	50	999	1	9	ot

REF	NRR	RR	SIG	RRDATA comment
HAMMON	512		*	RR for <1/2 pack per day is 2.04, that for 1/2 to 1 pack per day is 1.25, that for 1 to 2 packs per day is 2.71 while that for 2+ packs per day 2.12
SADOWS	532	7.09		0

Table 1I26 -

IESLC - Meta-analysis of Ever Smoking by Duration, Overview
All LC types, Pipes and/or cigars (not cigs)

This analysis is restricted to results for:
1) Ever smokers
2) Results by Duration
3) Categorical results by Duration
Results by Duration are grouped under 2 schemes (S1, S2). Each scheme has a set of "key values". An interval is allocated to the category whose key value it includes, and intervals which include none or more than one of the key values are excluded. (Open-ended intervals are coded as 999)

S1	key value	maximum range
1	20	1-34
2	35	21-49
3	50	36+

S2	key value	maximum range
1	5	1-19
2	20	6-29
3	30	21-39
4	40	31-49
5	50	41-998
6	999	51+

4) All LC types (or near equivalent)
5) Results complete enough for use in metaanalysis
6) pipes and/or cigars (not cigs)

Within each study, results are then selected (in the following order of preference, within each sex) for:
7) (not applicable)
8) DENOM: never smoked anything, never any + low
9) Followup period (YF, prospective studies): whole study (coded as 0) or longest available
10) LCtype: all or nearest available, at least Squamous and Adeno. (q = squamous, s = small, l = large, a = adeno, mix = mixed, alv = alveolar)
11) Race: all or nearest available, otherwise by race (wh or w = white, bl or b = black, hi = hispanic ch = chinese, jap = japanese, haw = hawaiian, w+o = white + oriental, sca = scandinavian, as = asian)
12) For overlapping studies: principal rather than subsidiary studies
Finally by Age: whole study (coded as 0) if available, otherwise by widest available age group and then for single sex results (m, f) in preference to results for both sexes combined (c).

Results adjusted (AD) for the most potential confounders are then chosen in Sections -1 to -3 and results adjusted for the least confounders in Sections -4 to -6. (Those least adjusted results which actually differ from the most adjusted are marked 'x' in column X in Section -4)

Section -7 shows excluded studies, together with the stage (as above) at which no qualifying results were found.

Section -8 lists the potentially overlapping studies which have been included (1=principal, 2=subsidiary).

Section -9 lists any results which would have been included in preference except that they had data not complete enough for use in meta-analysis, with their significance (yes/no), if known, and any further comment as entered on the database. It also lists as "gap" any categories for which no data were presented by the original authors.

In addition to those mentioned above, the following fields, levels and abbreviations are used:
* or nk = not known, n = no, y = yes, ot = other
nev = never
all/unspec = all or unspecified, cig+/-ot = cigarettes irrespective of other products (cigar, pipe etc)
MC = manufactured cigarettes, HR = hand-rolled cigarettes
exL, exH = range of exposure (low and high) in the smoking group, in terms of Duration
REF: 6-character study reference
NRR: number of the RR on the database within the study
ST : study type (CC = case control, pr or prosp = prospective)
NLC: number of lung cancer cases in whole study
R : risky occupational population (n = no, m = mining, o = other risky)
VB : national cigarette type (V = at least 75% Virginia, bl = at least 75% blended, ot = other)
P : any proxy use
H : full histological confirmation
De : derivation of RR/CI (or = original, st = standard method, ot = other method of estimation)

Table 1I26 - 1

IESLC - Meta-analysis of Ever Smoking by Duration, Overview
All LC types, Pipes and/or cigars (not cigs)
Most adjusted

REF	NRR	SEX	AGEL	AGEH	RACE	YF	LC TYPE	LOC	START	ST	NLC	R	VB	P	H	AD	exL	exH	S1	S2	DENOM	De
WYNDE7	521	m	0	0	all	-	all	NAmer	1977	CC	2085	n	bl	n	y	0	1	9	0	1	nev any	st
WYNDE7	522	m	0	0	all	-	all	NAmer	1977	CC	2085	n	bl	n	y	0	10	19	0	0	nev any	st
WYNDE7	523	m	0	0	all	-	all	NAmer	1977	CC	2085	n	bl	n	y	0	20	29	1	2	nev any	st
WYNDE7	524	m	0	0	all	-	all	NAmer	1977	CC	2085	n	bl	n	y	0	30	39	2	3	nev any	st
WYNDE7	525	m	0	0	all	-	all	NAmer	1977	CC	2085	n	bl	n	y	0	40	999	3	0	nev any	st

In this overview table, subtotals and Qs values may be invalid and should be ignored

Table 1I26 - 2

IESLC - Meta-analysis of Ever Smoking by Duration, Overview
All LC types, Pipes and/or cigars (not cigs)
Most adjusted

REF	NRR	SEX	AD	Number Exposed Case	Cont	Non-exposed Case	Cont	RR	95.00%CI
WYNDE7	521	m	0	3	34	64	918	1.27	(0.38- 4.23)
WYNDE7	522	m	0	5	41	64	918	1.75	(0.67- 4.58)
WYNDE7	523	m	0	6	66	64	918	1.30	(0.54- 3.12)
WYNDE7	524	m	0	16	81	64	918	2.83	(1.57- 5.13)
WYNDE7	525	m	0	19	84	64	918	3.24	(1.86- 5.67)
Subtotal WYNDE7								2.36	(1.70- 3.29)
Totals				49	306	320	4590		

*prospective study

REF	NRR	SEX	AD	Ys	Ws	Qs	Ps
WYNDE7	521	m	0	0.24	2.64	1.03	0.7022
WYNDE7	522	m	0	0.56	4.15	0.37	0.2548
WYNDE7	523	m	0	0.27	5.04	1.78	0.5514
WYNDE7	524	m	0	1.04	10.92	0.36	0.0006
WYNDE7	525	m	0	1.18	12.31	1.24	0.0000
Subtotal WYNDE7				0.86	35.05	4.78	

N	5
NS	1

Table 1I26 - 3

IESLC - Meta-analysis of Ever Smoking by Duration, Overview
All LC types, Pipes and/or cigars (not cigs)
Most adjusted

	Sex combined	male	female	Total
N		5		5
NS		1		1

In this overview table, other than the "N" rows, entries in the "absent" and "Total" columns may be invalid and should be ignored

	Duration of smoking (broad categories) absent	1-34k20	21-49k35	36+k50	Total
N	2	1	1	1	5
NS	1	1	1	1	1
Wt	6.78	5.04	10.92	12.31	35.05
Het Chi	0.17	0.00	0.00	0.00	4.78
Het df	1	0	0	0	4
Het P	N.S.	N.S.	N.S.	N.S.	N.S.
Fixed RR	1.54	1.30	2.83	3.24	2.36
RRl	0.73	0.54	1.57	1.86	1.70
RRu	3.27	3.12	5.13	5.67	3.29
P	N.S.	N.S.	+++	+++	+++
Random RR	1.54	1.30	2.83	3.24	2.29
RRl	0.73	0.54	1.57	1.86	1.58
RRu	3.27	3.12	5.13	5.67	3.32
P	N.S.	N.S.	+++	+++	+++

	absent	Duration of smoking (narrow categories) 1-19k1	6-29k20	21-39k30	31-49k40	41-998k50	51+k999	Total
N	2	1	1	1				5
NS	1	1	1	1				1
Wt	16.46	2.64	5.04	10.92				35.05
Het Chi	1.18	0.00	0.00	0.00				4.78
Het df	1	0	0	0				4
Het P	N.S.	N.S.	N.S.	N.S.				N.S.
Fixed RR	2.78	1.27	1.30	2.83				2.36
RRl	1.71	0.38	0.54	1.57				1.70
RRu	4.50	4.23	3.12	5.13				3.29
P	+++	N.S.	N.S.	+++				+++
Random RR	2.71	1.27	1.30	2.83				2.29
RRl	1.56	0.38	0.54	1.57				1.58
RRu	4.70	4.23	3.12	5.13				3.32
P	+++	N.S.	N.S.	+++				+++

Table 1I26 - 4

IESLC - Meta-analysis of Ever Smoking by Duration, Overview
All LC types, Pipes and/or cigars (not cigs)
Least adjusted

REF	NRR	X	SEX	AGEL	AGEH	RACE	YF	LC TYPE	LOC	START	ST	NLC	R	VB	P	H	AD	exL	exH	S1	S2	DENOM	De
WYNDE7	521		m	0	0	all	-	all	NAmer	1977	CC	2085	n	bl	n	y	0	1	9	0	1	nev any	st
WYNDE7	522		m	0	0	all	-	all	NAmer	1977	CC	2085	n	bl	n	y	0	10	19	0	0	nev any	st
WYNDE7	523		m	0	0	all	-	all	NAmer	1977	CC	2085	n	bl	n	y	0	20	29	1	2	nev any	st
WYNDE7	524		m	0	0	all	-	all	NAmer	1977	CC	2085	n	bl	n	y	0	30	39	2	3	nev any	st
WYNDE7	525		m	0	0	all	-	all	NAmer	1977	CC	2085	n	bl	n	y	0	40	999	3	0	nev any	st

In this overview table, subtotals and Qs values may be invalid and should be ignored

Table 1I26 - 5

IESLC - Meta-analysis of Ever Smoking by Duration, Overview
All LC types, Pipes and/or cigars (not cigs)
Least adjusted

REF	NRR	SEX	AD	Number Exposed Case	Cont	Non-exposed Case	Cont	RR	95.00%CI
WYNDE7	521	m	0	3	34	64	918	1.27	(0.38- 4.23)
WYNDE7	522	m	0	5	41	64	918	1.75	(0.67- 4.58)
WYNDE7	523	m	0	6	66	64	918	1.30	(0.54- 3.12)
WYNDE7	524	m	0	16	81	64	918	2.83	(1.57- 5.13)
WYNDE7	525	m	0	19	84	64	918	3.24	(1.86- 5.67)
Subtotal WYNDE7								2.36	(1.70- 3.29)
Totals				49	306	320	4590		

*prospective study

REF	NRR	SEX	AD	Ys	Ws	Qs	Ps
WYNDE7	521	m	0	0.24	2.64	1.03	0.7022
WYNDE7	522	m	0	0.56	4.15	0.37	0.2548
WYNDE7	523	m	0	0.27	5.04	1.78	0.5514
WYNDE7	524	m	0	1.04	10.92	0.36	0.0006
WYNDE7	525	m	0	1.18	12.31	1.24	0.0000
Subtotal WYNDE7				0.86	35.05	4.78	

N	5
NS	1

Table 1I26 - 6

IESLC - Meta-analysis of Ever Smoking by Duration, Overview
All LC types, Pipes and/or cigars (not cigs)
Least adjusted

	Sex			
	combined	male	female	Total
N		5		5
NS		1		1

In this overview table, other than the "N" rows, entries in the "absent" and "Total" columns may be invalid and should be ignored

	Duration of smoking (broad categories)				
	absent	1-34k20	21-49k35	36+k50	Total
N	2	1	1	1	5
NS	1	1	1	1	1
Wt	6.78	5.04	10.92	12.31	35.05
Het Chi	0.17	0.00	0.00	0.00	4.78
Het df	1	0	0	0	4
Het P	N.S.	N.S.	N.S.	N.S.	N.S.
Fixed RR	1.54	1.30	2.83	3.24	2.36
RRl	0.73	0.54	1.57	1.86	1.70
RRu	3.27	3.12	5.13	5.67	3.29
P	N.S.	N.S.	+++	+++	+++
Random RR	1.54	1.30	2.83	3.24	2.29
RRl	0.73	0.54	1.57	1.86	1.58
RRu	3.27	3.12	5.13	5.67	3.32
P	N.S.	N.S.	+++	+++	+++

	Duration of smoking (narrow categories)							
	absent	1-19k1	6-29k20	21-39k30	31-49k40	41-998k50	51+k999	Total
N	2	1	1	1				5
NS	1	1	1	1				1
Wt	16.46	2.64	5.04	10.92				35.05
Het Chi	1.18	0.00	0.00	0.00				4.78
Het df	1	0	0	0				4
Het P	N.S.	N.S.	N.S.	N.S.				N.S.
Fixed RR	2.78	1.27	1.30	2.83				2.36
RRl	1.71	0.38	0.54	1.57				1.70
RRu	4.50	4.23	3.12	5.13				3.29
P	+++	N.S.	N.S.	+++				+++
Random RR	2.71	1.27	1.30	2.83				2.29
RRl	1.56	0.38	0.54	1.57				1.58
RRu	4.70	4.23	3.12	5.13				3.32
P	+++	N.S.	N.S.	+++				+++

Table 1I26 - 7

IESLC - Meta-analysis of Ever Smoking by Duration, Overview
All LC types, Pipes and/or cigars (not cigs)
Excluded studies (and stage at which they were excluded)

1	AKIBA AMANDU AMES BECHER BENSHL BEST BLOT1 BROSS BROWN3 CARPEN CEDERL CHYOU CPSI CPSII DARBY DEAN2 DEAN3 DOLL2 ENGELA GAO2 GARCIA GILLIS GRAHAM GURSEL HAMMO2 HIRAYA HOLE HUMBLE JAHN JAIN KAISE2 KATSOU KAUFMA LAUSSM LIAW MCDUFF MIGRAN MRFITR PEZZO2 PISANI PRESCO QIAO SEGI2 SPEIZE SVENSS TVERDA WAKAI WATSON WIGLE WU WYNDE3 WYNDE8
2	ALDERS BRESLO CHIAZZ DORN GUO HEGMAN KOO KOULUM LIU4 PERNU SOBUE SPITZ SUZUK2 VUTUC YUAN
3	GENG STASZE WU2 ZHANG
4	BOUCHA CHEN CORREA JEDRYC LUO WYNDE2 WYNDE6
5	HAMMON RESTRE SADOWS XU
7	AGUDO ARMADA AUVINE AXELSS BARBON BENHAM BOFFET BOUCOT BUFFLE CHEN2 CHOI DAMBER DESTEF DOLL DORGAN DOSEME FAN GAO GARSHI GER HAENSZ HU HU2 JOLY JUSSAW KHUDER KREUZE LETOUR LEVIN LIU3 LIU5 LUBIN LUBIN2 MATOS MCCONN NOTAN2 OSANN2 PEZZOT QIAO2 RACHTA TIZZAN WANG2 WUWILL ZHENG ZHOU

Table 1I26 - 8
Potentially overlapping studies

REF	REFGP	PRINC	.	OVERLAP/LINK
WYNDE7	WYNDE6	2		WYNDE5/6/7/8

Table 1I26 - 9
Most adjusted - insufficient data for meta-analysis

REF	NRR	SEX	AGEL	AGEH	RACE	YF	LC TYPE	LOC	START	ST	NLC	R	VB	P	H	AD	exL	exH	S1	S2	DENOM	De
SADOWS	582	m	0	0	wh	-	all	NAmer	1938	CC	477	n	bl	n	n	0	1	19	0	1	nev any	ot
SADOWS	583	m	0	0	wh	-	all	NAmer	1938	CC	477	n	bl	n	n	0	20	29	1	2	nev any	ot
SADOWS	584	m	0	0	wh	-	all	NAmer	1938	CC	477	n	bl	n	n	0	30	39	2	3	nev any	ot
SADOWS	585	m	0	0	wh	-	all	NAmer	1938	CC	477	n	bl	n	n	0	40	999	3	0	nev any	ot

REF	NRR	RR	SIG	RRDATA comment
SADOWS	582	0.81		0
SADOWS	583	1.62		0
SADOWS	584	2.97		0
SADOWS	585	2.22		0

Table 1I27 -

IESLC - Meta-analysis of Ever Smoking, Duration, "Highest vs lowest"
All LC types, Pipes and/or cigars (not cigs)

This analysis is restricted to results for:
1) Ever smokers
2) Results by Duration
3) Categorical results by Duration
4) Denominator (unexposed) = "low"
5) All LC types (or near equivalent)
6) Results complete enough for use in metaanalysis
7) (not applicable)
8) PRODUCT: pipes and/or cigars (not cigs)

Within each study, results are then selected (in the following order of preference, within each sex) for:
9) Results with least adjustment for other aspects of smoking (ADOS)
10) The highest vs lowest category
11) Followup period (YF, prospective studies): whole study (coded as 0) or longest available
12) LCtype: all or nearest available, at least Squamous and Adeno. (q = squamous, s = small,
l = large, a = adeno, mix = mixed, alv = alveolar)
13) Race: all or nearest available, otherwise by race (wh or w = white, bl or b = black, hi = hispanic
ch = chinese, jap = japanese, haw = hawaiian, w+o = white + oriental, sca = scandinavian, as = asian)
14) For overlapping studies: principal rather than subsidiary studies
Finally by Age: whole study (coded as 0) if available, otherwise by widest available age group
and then for single sex results (m, f) in preference to results for both sexes combined (c).

Results adjusted (AD) for the most potential confounders are then chosen in Sections -1 to -3
and results adjusted for the least confounders in Sections -4 to -6. (Those least adjusted results which
actually differ from the most adjusted are marked 'x' in column X in Section -4)

Section -7 shows excluded studies, together with the stage (as above) at which no qualifying
results were found.

Section -8 lists the potentially overlapping studies which have been included (1=principal, 2=subsidiary).

Section -9 lists any results which would have been included in preference except that they had data not complete
enough for use in meta-analysis, with their significance (yes/no), if known, and any further comment as entered
on the database. It also lists as "gap" any categories for which no data were presented by the original authors.

In addition to those mentioned above, the following fields, levels and abbreviations are used:
* or nk = not known, n = no, y = yes, ot = other
all/unspec = all or unspecified, cig+/-ot = cigarettes irrespective of other products (cigar, pipe etc)
MC = manufactured cigarettes, HR = hand-rolled cigarettes
exL, exH = range of exposure (low and high) in the "highest" group, in terms of Duration
unexL, unexH = range of exposure (low and high) in the "lowest" group, in terms of Duration
REF: 6-character study reference
NRR: number of the RR on the database within the study
ST : study type (CC = case control, pr or prosp = prospective)
NLC: number of lung cancer cases in whole study
R : risky occupational population (n = no, m = mining, o = other risky)
VB : national cigarette type (V = at least 75% Virginia, bl = at least 75% blended, ot = other)
P : any proxy use
H : full histological confirmation
De : derivation of RR/CI (or = original, st = standard method, ot = other method of estimation)

Table 1I27 - 1

IESLC - Meta-analysis of Ever Smoking, Duration, "Highest vs lowest"
All LC types, Pipes and/or cigars (not cigs)
Most adjusted

REF	NRR	SEX	AGEL	AGEH	RACE	YF	LC TYPE	LOC	START	ST	NLC	R	VB	P	H	AD	ADOS	exL	exH	unexL	unexH	De
WYNDE7	529	m	0	0	all	-	all	NAmer	1977	CC	2085	n	bl	n	y	0	0	40	999	1	9	st

Table 1I27 - 2

IESLC - Meta-analysis of Ever Smoking, Duration, "Highest vs lowest"
All LC types, Pipes and/or cigars (not cigs)
Most adjusted

REF	NRR	SEX	AD	Number Exposed Case	Exposed Cont	Non-exposed Case	Non-exposed Cont	RR	95.00%CI
WYNDE7	529	m	0	19	84	3	34	2.56	(0.71- 9.23)
Totals				19	84	3	34		

*prospective study

REF	NRR	SEX	AD	Ys	Ws	Qs	Ps
WYNDE7	529	m	0	0.94	2.34	0.00	0.1498

N	1
NS	1
Wt	2.34
Het Chi	0.00
Het df	0
Het P	N.S.
Fixed RR	2.56
RRl	0.71
RRu	9.23
P	N.S.
Random RR	2.56
RRl	0.71
RRu	9.23
P	N.S.
Asymm P	

Table 1I27 - 3

IESLC - Meta-analysis of Ever Smoking, Duration, "Highest vs lowest"
All LC types, Pipes and/or cigars (not cigs)
Most adjusted

	Sex combined	male	female	Total
N		1		1
NS		1		1
Wt		2.34		2.34
Het Chi		0.00		0.00
Het df		0		0
Het P		N.S.		N.S.
Fixed RR		2.56		2.56
RRl		0.71		0.71
RRu		9.23		9.23
P		N.S.		N.S.
Random RR		2.56		2.56
RRl		0.71		0.71
RRu		9.23		9.23
P		N.S.		N.S.
Between Chi				
Between df				
Between P				N.S.
Btwn(F) P				N.S.
Btwn(R) P				N.S.

MALES

Too few RRs for analysis by factor

Table 1I27 - 4

IESLC - Meta-analysis of Ever Smoking, Duration, "Highest vs lowest"
All LC types, Pipes and/or cigars (not cigs)
Least adjusted

REF	NRR	X	SEX	AGEL	AGEH	RACE	YF	LC TYPE	LOC	START	ST	NLC	R	VB	P	H	AD	ADOS	exL	exH	unexL	unexH	De
WYNDE7	529		m	0	0	all	-	all	NAmer	1977	CC	2085	n	bl	n	y	0	0	40	999	1	9	st

Table 1I27 - 5

IESLC - Meta-analysis of Ever Smoking, Duration, "Highest vs lowest"
All LC types, Pipes and/or cigars (not cigs)
Least adjusted

REF	NRR	SEX	AD	Number Exposed Case	Number Exposed Cont	Non-exposed Case	Non-exposed Cont	RR	95.00%CI
WYNDE7	529	m	0	19	84	3	34	2.56	(0.71- 9.23)
Totals				19	84	3	34		

*prospective study

REF	NRR	SEX	AD	Ys	Ws	Qs	Ps
WYNDE7	529	m	0	0.94	2.34	0.00	0.1498

	N	1
	NS	1
	Wt	2.34
Het	Chi	0.00
Het	df	0
Het	P	N.S.
Fixed	RR	2.56
	RRl	0.71
	RRu	9.23
	P	N.S.
Random	RR	2.56
	RRl	0.71
	RRu	9.23
	P	N.S.
Asymm	P	

Table 1I27 - 6

IESLC - Meta-analysis of Ever Smoking, Duration, "Highest vs lowest"
All LC types, Pipes and/or cigars (not cigs)
Least adjusted

	Sex combined	male	female	Total
N		1		1
NS		1		1
Wt		2.34		2.34
Het Chi		0.00		0.00
Het df		0		0
Het P		N.S.		N.S.
Fixed RR		2.56		2.56
RRl		0.71		0.71
RRu		9.23		9.23
P		N.S.		N.S.
Random RR		2.56		2.56
RRl		0.71		0.71
RRu		9.23		9.23
P		N.S.		N.S.
Between Chi				
Between df				
Between P				N.S.
Btwn(F) P				N.S.
Btwn(R) P				N.S.

Table 1I27 - 7

IESLC - Meta-analysis of Ever Smoking, Duration, "Highest vs lowest"
All LC types, Pipes and/or cigars (not cigs)
Excluded studies (and stage at which they were excluded)

1	AKIBA AMANDU AMES BECHER BENSHL BEST BLOT1 BROSS BROWN3 CARPEN CEDERL CHYOU CPSI CPSII DARBY DEAN2 DEAN3 DOLL2 ENGELA GAO2 GARCIA GILLIS GRAHAM GURSEL HAMMO2 HIRAYA HOLE HUMBLE JAHN JAIN KAISE2 KATSOU KAUFMA LAUSSM LIAW MCDUFF MIGRAN MRFITR PEZZO2 PISANI PRESCO QIAO SEGI2 SPEIZE SVENSS TVERDA WAKAI WATSON WIGLE WU WYNDE3 WYNDE8
2	ALDERS BRESLO CHIAZZ DORN GUO HEGMAN KOO KOULUM LIU4 PERNU SOBUE SPITZ SUZUK2 VUTUC YUAN
3	GENG STASZE WU2 ZHANG
4	GARSHI
5	BOUCHA CHEN CORREA JEDRYC LUO WYNDE2 WYNDE6
6	HAMMON RESTRE SADOWS XU
8	AGUDO ARMADA AUVINE AXELSS BARBON BENHAM BOFFET BOUCOT BUFFLE CHEN2 CHOI DAMBER DESTEF DOLL DORGAN DOSEME FAN GAO GER HAENSZ HU HU2 JOLY JUSSAW KHUDER KREUZE LETOUR LEVIN LIU3 LIU5 LUBIN LUBIN2 MATOS MCCONN NOTAN2 OSANN2 PEZZOT QIAO2 RACHTA TIZZAN WANG2 WUWILL ZHENG ZHOU

Table 1I27 - 8

Potentially overlapping studies

REF	REFGP	PRINC	.	OVERLAP/LINK
WYNDE7	WYNDE6	2		WYNDE5/6/7/8

Table 1I27 - 9

Most adjusted - insufficient data for meta-analysis

REF	NRR	SEX	AGEL	AGEH	RACE	YF	LC TYPE	LOC	START	ST	NLC	R	VB	P	H	AD	ADOS	exL	exH	unexL	unexH	De
SADOWS	588	m	0	0	wh	-	all	NAmer	1938	CC	477	n	bl	n	n	0	0	40	999	1	19	ot

REF	NRR	RR	SIG	RRDATA comment
SADOWS	588	2.73		0

Table 1I28 -

IESLC - Meta-analysis of Current Smoking by Duration, Overview
All LC types, Pipes and/or cigars (not cigs)

This analysis is restricted to results for:
1) Current smokers
2) Results by Duration
3) Categorical results by Duration
Results by Duration are grouped under 2 schemes (S1, S2). Each scheme has a set of "key values". An interval is allocated to the category whose key value it includes, and intervals which include none or more than one of the key values are excluded. (Open-ended intervals are coded as 999)

S1	key value	maximum range
1	20	1-34
2	35	21-49
3	50	36+

S2	key value	maximum range
1	5	1-19
2	20	6-29
3	30	21-39
4	40	31-49
5	50	41-998
6	999	51+

4) All LC types (or near equivalent)
5) Results complete enough for use in metaanalysis
6) pipes and/or cigars (not cigs)

Within each study, results are then selected (in the following order of preference, within each sex) for:
7) (not applicable)
8) DENOM: never smoked anything, never any + low
9) Followup period (YF, prospective studies): whole study (coded as 0) or longest available
10) LCtype: all or nearest available, at least Squamous and Adeno. (q = squamous, s = small, l = large, a = adeno, mix = mixed, alv = alveolar)
11) Race: all or nearest available, otherwise by race (wh or w = white, bl or b = black, hi = hispanic ch = chinese, jap = japanese, haw = hawaiian, w+o = white + oriental, sca = scandinavian, as = asian)
12) For overlapping studies: principal rather than subsidiary studies
Finally by Age: whole study (coded as 0) if available, otherwise by widest available age group and then for single sex results (m, f) in preference to results for both sexes combined (c).

Results adjusted (AD) for the most potential confounders are then chosen in Sections -1 to -3 and results adjusted for the least confounders in Sections -4 to -6. (Those least adjusted results which actually differ from the most adjusted are marked 'x' in column X in Section -4)

Section -7 shows excluded studies, together with the stage (as above) at which no qualifying results were found.

Section -8 lists the potentially overlapping studies which have been included (1=principal, 2=subsidiary).

Section -9 lists any results which would have been included in preference except that they had data not complete enough for use in meta-analysis, with their significance (yes/no), if known, and any further comment as entered on the database. It also lists as "gap" any categories for which no data were presented by the original authors.

In addition to those mentioned above, the following fields, levels and abbreviations are used:
* or nk = not known, n = no, y = yes, ot = other
nev = never
all/unspec = all or unspecified, cig+/-ot = cigarettes irrespective of other products (cigar, pipe etc)
MC = manufactured cigarettes, HR = hand-rolled cigarettes
exL, exH = range of exposure (low and high) in the smoking group, in terms of Duration
REF: 6-character study reference
NRR: number of the RR on the database within the study
ST : study type (CC = case control, pr or prosp = prospective)
NLC: number of lung cancer cases in whole study
R : risky occupational population (n = no, m = mining, o = other risky)
VB : national cigarette type (V = at least 75% Virginia, bl = at least 75% blended, ot = other)
P : any proxy use
H : full histological confirmation
De : derivation of RR/CI (or = original, st = standard method, ot = other method of estimation)

Table 1I28 - 1

IESLC - Meta-analysis of Current Smoking by Duration, Overview
All LC types, Pipes and/or cigars (not cigs)
Most adjusted

REF	NRR	SEX	AGEL	AGEH	RACE	YF	LC TYPE	LOC	START	ST	NLC	R	VB	P	H	AD	exL	exH	S1	S2	DENOM	De
WYNDE7	501	m	0	0	all	-	all	NAmer	1977	CC	2085	n	bl	n	y	0	1	19	0	1	nev any	st
WYNDE7	502	m	0	0	all	-	all	NAmer	1977	CC	2085	n	bl	n	y	0	20	29	1	2	nev any	st
WYNDE7	503	m	0	0	all	-	all	NAmer	1977	CC	2085	n	bl	n	y	0	30	39	2	3	nev any	st
WYNDE7	504	m	0	0	all	-	all	NAmer	1977	CC	2085	n	bl	n	y	0	40	999	3	0	nev any	st

In this overview table, subtotals and Qs values may be invalid and should be ignored

Table 1I28 - 2

IESLC - Meta-analysis of Current Smoking by Duration, Overview
All LC types, Pipes and/or cigars (not cigs)
Most adjusted

REF	NRR	SEX	AD	Number Exposed Case	Exposed Cont	Non-exposed Case	Non-exposed Cont	RR	95.00%CI
WYNDE7	501	m	0	3	26	64	918	1.66	(0.49- 5.62)
WYNDE7	502	m	0	4	36	64	918	1.59	(0.55- 4.62)
WYNDE7	503	m	0	9	48	64	918	2.69	(1.26- 5.73)
WYNDE7	504	m	0	15	60	64	918	3.59	(1.93- 6.67)
Subtotal WYNDE7								2.67	(1.77- 4.03)
Totals				31	170	256	3672		

*prospective study

REF	NRR	SEX	AD	Ys	Ws	Qs	Ps
WYNDE7	501	m	0	0.50	2.57	0.59	0.4189
WYNDE7	502	m	0	0.47	3.40	0.91	0.3904
WYNDE7	503	m	0	0.99	6.73	0.00	0.0103
WYNDE7	504	m	0	1.28	10.00	0.87	0.0001
Subtotal WYNDE7				0.98	22.69	2.36	

N	4
NS	1

Table 1I28 - 3

IESLC - Meta-analysis of Current Smoking by Duration, Overview
All LC types, Pipes and/or cigars (not cigs)
Most adjusted

	combined	Sex male	female	Total
N		4		4
NS		1		1

In this overview table, other than the "N" rows, entries in the "absent" and "Total" columns may be invalid and should be ignored

MALES

Duration of smoking (broad categories)

	absent	1-34k20	21-49k35	36+k50	Total
N	1	1	1	1	4
NS	1	1	1	1	1
Wt	2.57	3.40	6.73	10.00	22.69
Het Chi	0.00	0.00	0.00	0.00	2.36
Het df	0	0	0	0	3
Het P	N.S.	N.S.	N.S.	N.S.	N.S.
Fixed RR	1.66	1.59	2.69	3.59	2.67
RRl	0.49	0.55	1.26	1.93	1.77
RRu	5.62	4.62	5.73	6.67	4.03
P	N.S.	N.S.	+	+++	+++
Random RR	1.66	1.59	2.69	3.59	2.67
RRl	0.49	0.55	1.26	1.93	1.77
RRu	5.62	4.62	5.73	6.67	4.03
P	N.S.	N.S.	+	+++	+++

Duration of smoking (narrow categories)

	absent	1-19k1	6-29k20	21-39k30	31-49k40	41-998k50	51+k999	Total
N	1	1	1	1				4
NS	1	1	1	1				1
Wt	10.00	2.57	3.40	6.73				22.69
Het Chi	0.00	0.00	0.00	0.00				2.36
Het df	0	0	0	0				3
Het P	N.S.	N.S.	N.S.	N.S.				N.S.
Fixed RR	3.59	1.66	1.59	2.69				2.67
RRl	1.93	0.49	0.55	1.26				1.77
RRu	6.67	5.62	4.62	5.73				4.03
P	+++	N.S.	N.S.	+				+++
Random RR	3.59	1.66	1.59	2.69				2.67
RRl	1.93	0.49	0.55	1.26				1.77
RRu	6.67	5.62	4.62	5.73				4.03
P	+++	N.S.	N.S.	+				+++

Table 1I28 - 4

IESLC - Meta-analysis of Current Smoking by Duration, Overview
All LC types, Pipes and/or cigars (not cigs)
Least adjusted

REF	NRR	X	SEX	AGEL	AGEH	RACE	YF	LC TYPE	LOC	START	ST	NLC	R	VB	P	H	AD	exL	exH	S1	S2	DENOM	De
WYNDE7	501		m	0	0	all	-	all	NAmer	1977	CC	2085	n	bl	n	y	0	1	19	0	1	nev any	st
WYNDE7	502		m	0	0	all	-	all	NAmer	1977	CC	2085	n	bl	n	y	0	20	29	1	2	nev any	st
WYNDE7	503		m	0	0	all	-	all	NAmer	1977	CC	2085	n	bl	n	y	0	30	39	2	3	nev any	st
WYNDE7	504		m	0	0	all	-	all	NAmer	1977	CC	2085	n	bl	n	y	0	40	999	3	0	nev any	st

In this overview table, subtotals and Qs values may be invalid and should be ignored

Table 1I28 - 5

IESLC - Meta-analysis of Current Smoking by Duration, Overview
All LC types, Pipes and/or cigars (not cigs)
Least adjusted

REF	NRR	SEX	AD	Number Exposed Case	Number Exposed Cont	Non-exposed Case	Non-exposed Cont	RR	95.00%CI
WYNDE7	501	m	0	3	26	64	918	1.66	(0.49- 5.62)
WYNDE7	502	m	0	4	36	64	918	1.59	(0.55- 4.62)
WYNDE7	503	m	0	9	48	64	918	2.69	(1.26- 5.73)
WYNDE7	504	m	0	15	60	64	918	3.59	(1.93- 6.67)
Subtotal WYNDE7								2.67	(1.77- 4.03)
Totals				31	170	256	3672		

*prospective study

REF	NRR	SEX	AD	Ys	Ws	Qs	Ps
WYNDE7	501	m	0	0.50	2.57	0.59	0.4189
WYNDE7	502	m	0	0.47	3.40	0.91	0.3904
WYNDE7	503	m	0	0.99	6.73	0.00	0.0103
WYNDE7	504	m	0	1.28	10.00	0.87	0.0001
Subtotal WYNDE7				0.98	22.69	2.36	

N	4
NS	1

Table 1I28 - 6

IESLC - Meta-analysis of Current Smoking by Duration, Overview
All LC types, Pipes and/or cigars (not cigs)
Least adjusted

	Sex		
combined	male	female	Total
N	4		4
NS	1		1

In this overview table, other than the "N" rows, entries in the "absent" and "Total" columns may be invalid and should be ignored

MALES

	Duration of smoking (broad categories)				
	absent	1-34k20	21-49k35	36+k50	Total
N	1	1	1	1	4
NS	1	1	1	1	1
Wt	2.57	3.40	6.73	10.00	22.69
Het Chi	0.00	0.00	0.00	0.00	2.36
Het df	0	0	0	0	3
Het P	N.S.	N.S.	N.S.	N.S.	N.S.
Fixed RR	1.66	1.59	2.69	3.59	2.67
RRl	0.49	0.55	1.26	1.93	1.77
RRu	5.62	4.62	5.73	6.67	4.03
P	N.S.	N.S.	+	+++	+++
Random RR	1.66	1.59	2.69	3.59	2.67
RRl	0.49	0.55	1.26	1.93	1.77
RRu	5.62	4.62	5.73	6.67	4.03
P	N.S.	N.S.	+	+++	+++

	Duration of smoking (narrow categories)							
	absent	1-19k1	6-29k20	21-39k30	31-49k40	41-998k50	51+k999	Total
N	1	1	1	1				4
NS	1	1	1	1				1
Wt	10.00	2.57	3.40	6.73				22.69
Het Chi	0.00	0.00	0.00	0.00				2.36
Het df	0	0	0	0				3
Het P	N.S.	N.S.	N.S.	N.S.				N.S.
Fixed RR	3.59	1.66	1.59	2.69				2.67
RRl	1.93	0.49	0.55	1.26				1.77
RRu	6.67	5.62	4.62	5.73				4.03
P	+++	N.S.	N.S.	+				+++
Random RR	3.59	1.66	1.59	2.69				2.67
RRl	1.93	0.49	0.55	1.26				1.77
RRu	6.67	5.62	4.62	5.73				4.03
P	+++	N.S.	N.S.	+				+++

Table 1I28 - 7

IESLC - Meta-analysis of Current Smoking by Duration, Overview
All LC types, Pipes and/or cigars (not cigs)
Excluded studies (and stage at which they were excluded)

1	AGUDO ALDERS ARMADA AUVINE AXELSS BARBON BECHER BENHAM BLOT1 BOFFET BOUCHA BRESLO BROWN3 CARPEN CHEN CHEN2 CHIAZZ CHOI CHYOU CORREA DAMBER DARBY DESTEF DOLL DOLL2 DORGAN DOSEME FAN GAO GARCIA GARSHI GENG GER GRAHAM GUO GURSEL HAENSZ HAMMO2 HAMMON HEGMAN HU HU2 JAHN JAIN JEDRYC JOLY JUSSAW KHUDER KOO KOULUM KREUZE LAUSSM LETOUR LEVIN LIU3 LIU4 LIU5 LUBIN LUBIN2 LUO MCCONN NOTAN2 OSANN2 PERNU PEZZOT PRESCO QIAO QIAO2 RACHTA RESTRE SADOWS STASZE SUZUK2 TIZZAN TVERDA VUTUC WANG2 WIGLE WU2 WUWILL WYNDE2 WYNDE3 XU YUAN ZHANG ZHENG ZHOU
2	BENSHL DEAN3 DORN ENGELA GAO2 GILLIS HIRAYA HOLE KAUFMA MIGRAN MRFITR SEGI2 SPEIZE SVENSS WAKAI WU
3	MCDUFF SPITZ WYNDE6
5	AKIBA PISANI
7	AMANDU AMES BEST BOUCOT BROSS BUFFLE CEDERL CPSI CPSII DEAN2 HUMBLE KAISE2 KATSOU LIAW MATOS PEZZO2 SOBUE WATSON WYNDE8

Table 1I28 - 8
Potentially overlapping studies

REF	REFGP	PRINC	.	OVERLAP/LINK
WYNDE7	WYNDE6	2		WYNDE5/6/7/8

Table 1I29 -

IESLC - Meta-analysis of Current Smoking, Duration, "Highest vs lowest"
All LC types, Pipes and/or cigars (not cigs)

This analysis is restricted to results for:
1) Current smokers
2) Results by Duration
3) Categorical results by Duration
4) Denominator (unexposed) = "low"
5) All LC types (or near equivalent)
6) Results complete enough for use in metaanalysis
7) (not applicable)
8) PRODUCT: pipes and/or cigars (not cigs)

Within each study, results are then selected (in the following order of preference, within each sex) for:
9) Results with least adjustment for other aspects of smoking (ADOS)
10) The highest vs lowest category
11) Followup period (YF, prospective studies): whole study (coded as 0) or longest available
12) LCtype: all or nearest available, at least Squamous and Adeno. (q = squamous, s = small,
l = large, a = adeno, mix = mixed, alv = alveolar)
13) Race: all or nearest available, otherwise by race (wh or w = white, bl or b = black, hi = hispanic
ch = chinese, jap = japanese, haw = hawaiian, w+o = white + oriental, sca = scandinavian, as = asian)
14) For overlapping studies: principal rather than subsidiary studies
Finally by Age: whole study (coded as 0) if available, otherwise by widest available age group
and then for single sex results (m, f) in preference to results for both sexes combined (c).

Results adjusted (AD) for the most potential confounders are then chosen in Sections -1 to -3
and results adjusted for the least confounders in Sections -4 to -6. (Those least adjusted results which
actually differ from the most adjusted are marked 'x' in column X in Section -4)

Section -7 shows excluded studies, together with the stage (as above) at which no qualifying
results were found.

Section -8 lists the potentially overlapping studies which have been included (1=principal, 2=subsidiary).

Section -9 lists any results which would have been included in preference except that they had data not complete
enough for use in meta-analysis, with their significance (yes/no), if known, and any further comment as entered
on the database. It also lists as "gap" any categories for which no data were presented by the original authors.

In addition to those mentioned above, the following fields, levels and abbreviations are used:
* or nk = not known, n = no, y = yes, ot = other
all/unspec = all or unspecified, cig+/-ot = cigarettes irrespective of other products (cigar, pipe etc)
MC = manufactured cigarettes, HR = hand-rolled cigarettes
exL, exH = range of exposure (low and high) in the "highest" group, in terms of Duration
unexL, unexH = range of exposure (low and high) in the "lowest" group, in terms of Duration
REF: 6-character study reference
NRR: number of the RR on the database within the study
ST : study type (CC = case control, pr or prosp = prospective)
NLC: number of lung cancer cases in whole study
R : risky occupational population (n = no, m = mining, o = other risky)
VB : national cigarette type (V = at least 75% Virginia, bl = at least 75% blended, ot = other)
P : any proxy use
H : full histological confirmation
De : derivation of RR/CI (or = original, st = standard method, ot = other method of estimation)

Table 1I29 - 1

IESLC - Meta-analysis of Current Smoking, Duration, "Highest vs lowest"
All LC types, Pipes and/or cigars (not cigs)
Most adjusted

REF	NRR	SEX	AGEL	AGEH	RACE	YF	LC TYPE	LOC	START	ST	NLC	R	VB	P	H	AD	ADOS	exL	exH	unexL	unexH	De
WYNDE7	511	m	0	0	all	-	all	NAmer	1977	CC	2085	n	bl	n	y	0	0	40	999	1	19	st

Table 1I29 - 2

IESLC - Meta-analysis of Current Smoking, Duration, "Highest vs lowest"
All LC types, Pipes and/or cigars (not cigs)
Most adjusted

REF	NRR	SEX	AD	Number Case	Exposed Cont	Non-exposed Case	Cont	RR	95.00%CI
WYNDE7	511	m	0	15	60	3	26	2.17	(0.58- 8.13)
Totals				15	60	3	26		

*prospective study

REF	NRR	SEX	AD	Ys	Ws	Qs	Ps
WYNDE7	511	m	0	0.77	2.20	0.00	0.2518

	N	1
	NS	1
	Wt	2.20
Het	Chi	0.00
Het	df	0
Het	P	N.S.
Fixed	RR	2.17
	RRl	0.58
	RRu	8.13
	P	N.S.
Random	RR	2.17
	RRl	0.58
	RRu	8.13
	P	N.S.
Asymm	P	

Table 1I29 - 3

IESLC - Meta-analysis of Current Smoking, Duration, "Highest vs lowest"
All LC types, Pipes and/or cigars (not cigs)
Most adjusted

	Sex combined	male	female	Total
N		1		1
NS		1		1
Wt		2.20		2.20
Het Chi		0.00		0.00
Het df		0		0
Het P		N.S.		N.S.
Fixed RR		2.17		2.17
RRl		0.58		0.58
RRu		8.13		8.13
P		N.S.		N.S.
Random RR		2.17		2.17
RRl		0.58		0.58
RRu		8.13		8.13
P		N.S.		N.S.
Between Chi				
Between df				
Between P				N.S.
Btwn(F) P				N.S.
Btwn(R) P				N.S.

MALES

Too few RRs for analysis by factor

Table 1I29 - 4

IESLC - Meta-analysis of Current Smoking, Duration, "Highest vs lowest"
All LC types, Pipes and/or cigars (not cigs)
Least adjusted

REF	NRR	X	SEX	AGEL	AGEH	RACE	YF	LC TYPE	LOC	START	ST	NLC	R	VB	P	H	AD	ADOS	exL	exH	unexL	unexH	De
WYNDE7	511		m	0	0	all	-	all	NAmer	1977	CC	2085	n	bl	n	y	0	0	40	999	1	19	st

Table 1I29 - 5

IESLC - Meta-analysis of Current Smoking, Duration, "Highest vs lowest"
All LC types, Pipes and/or cigars (not cigs)
Least adjusted

REF	NRR	SEX	AD	Number Exposed Case	Number Exposed Cont	Non-exposed Case	Non-exposed Cont	RR	95.00%CI
WYNDE7	511	m	0	15	60	3	26	2.17	(0.58- 8.13)
Totals				15	60	3	26		

*prospective study

REF	NRR	SEX	AD	Ys	Ws	Qs	Ps
WYNDE7	511	m	0	0.77	2.20	0.00	0.2518

	N	1
	NS	1
	Wt	2.20
Het	Chi	0.00
Het	df	0
Het	P	N.S.
Fixed	RR	2.17
	RRl	0.58
	RRu	8.13
	P	N.S.
Random	RR	2.17
	RRl	0.58
	RRu	8.13
	P	N.S.
Asymm	P	

Table 1I29 - 6

IESLC - Meta-analysis of Current Smoking, Duration, "Highest vs lowest"
All LC types, Pipes and/or cigars (not cigs)
Least adjusted

	Sex: combined	male	female	Total
N		1		1
NS		1		1
Wt		2.20		2.20
Het Chi		0.00		0.00
Het df		0		0
Het P		N.S.		N.S.
Fixed RR		2.17		2.17
RRl		0.58		0.58
RRu		8.13		8.13
P		N.S.		N.S.
Random RR		2.17		2.17
RRl		0.58		0.58
RRu		8.13		8.13
P		N.S.		N.S.
Between Chi				
Between df				
Between P				N.S.
Btwn(F) P				N.S.
Btwn(R) P				N.S.

Table 1I29 - 7

IESLC - Meta-analysis of Current Smoking, Duration, "Highest vs lowest"
All LC types, Pipes and/or cigars (not cigs)
Excluded studies (and stage at which they were excluded)

1	AGUDO ALDERS ARMADA AUVINE AXELSS BARBON BECHER BENHAM BLOT1 BOFFET BOUCHA BRESLO BROWN3 CARPEN CHEN CHEN2 CHIAZZ CHOI CHYOU CORREA DAMBER DARBY DESTEF DOLL DOLL2 DORGAN DOSEME FAN GAO GARCIA GARSHI GENG GER GRAHAM GUO GURSEL HAENSZ HAMMO2 HAMMON HEGMAN HU HU2 JAHN JAIN JEDRYC JOLY JUSSAW KHUDER KOO KOULUM KREUZE LAUSSM LETOUR LEVIN LIU3 LIU4 LIU5 LUBIN LUBIN2 LUO MCCONN NOTAN2 OSANN2 PERNU PEZZOT PRESCO QIAO QIAO2 RACHTA RESTRE SADOWS STASZE SUZUK2 TIZZAN TVERDA VUTUC WANG2 WIGLE WU2 WUWILL WYNDE2 WYNDE3 XU YUAN ZHANG ZHENG ZHOU
2	BENSHL DEAN3 DORN ENGELA GAO2 GILLIS HIRAYA HOLE KAUFMA MIGRAN MRFITR SEGI2 SPEIZE SVENSS WAKAI WU
3	MCDUFF SPITZ WYNDE6
4	AKIBA
6	PISANI
8	AMANDU AMES BEST BOUCOT BROSS BUFFLE CEDERL CPSI CPSII DEAN2 HUMBLE KAISE2 KATSOU LIAW MATOS PEZZO2 SOBUE WATSON WYNDE8

Table 1I29 - 8
Potentially overlapping studies

REF	REFGP	PRINC	.	OVERLAP/LINK
WYNDE7	WYNDE6	2		WYNDE5/6/7/8

Table 1I30 -

IESLC - Meta-analysis of Ever/current Smoking by Duration, Overview
All LC types, Pipes and/or cigars (not cigs)

This analysis is restricted to results for:
1) Ever/current smokers
2) Results by Duration
3) Categorical results by Duration
Results by Duration are grouped under 2 schemes (S1, S2). Each scheme has a set of "key values". An interval is allocated to the category whose key value it includes, and intervals which include none or more than one of the key values are excluded. (Open-ended intervals are coded as 999)

S1	key value	maximum range
1	20	1-34
2	35	21-49
3	50	36+

S2	key value	maximum range
1	5	1-19
2	20	6-29
3	30	21-39
4	40	31-49
5	50	41-998
6	999	51+

4) All LC types (or near equivalent)
5) Results complete enough for use in metaanalysis
6) pipes and/or cigars (not cigs)

Within each study, results are then selected (in the following order of preference, within each sex) for:
7) (not applicable)
8) DENOM: never smoked anything, never any + low
9) Followup period (YF, prospective studies): whole study (coded as 0) or longest available
10) LCtype: all or nearest available, at least Squamous and Adeno. (q = squamous, s = small, l = large, a = adeno, mix = mixed, alv = alveolar)
11) Race: all or nearest available, otherwise by race (wh or w = white, bl or b = black, hi = hispanic ch = chinese, jap = japanese, haw = hawaiian, w+o = white + oriental, sca = scandinavian, as = asian)
12) For overlapping studies: principal rather than subsidiary studies
Finally by Age: whole study (coded as 0) if available, otherwise by widest available age group and then for single sex results (m, f) in preference to results for both sexes combined (c).

Results adjusted (AD) for the most potential confounders are then chosen in Sections -1 to -3 and results adjusted for the least confounders in Sections -4 to -6. (Those least adjusted results which actually differ from the most adjusted are marked 'x' in column X in Section -4)

Section -7 shows excluded studies, together with the stage (as above) at which no qualifying results were found.

Section -8 lists the potentially overlapping studies which have been included (1=principal, 2=subsidiary).

Section -9 lists any results which would have been included in preference except that they had data not complete enough for use in meta-analysis, with their significance (yes/no), if known, and any further comment as entered on the database. It also lists as "gap" any categories for which no data were presented by the original authors.

In addition to those mentioned above, the following fields, levels and abbreviations are used:
* or nk = not known, n = no, y = yes, ot = other
ev = ever, cu = current, nev = never
all/unspec = all or unspecified, cig+/-ot = cigarettes irrespective of other products (cigar, pipe etc)
MC = manufactured cigarettes, HR = hand-rolled cigarettes
exL, exH = range of exposure (low and high) in the smoking group, in terms of Duration
REF: 6-character study reference
NRR: number of the RR on the database within the study
ST : study type (CC = case control, pr or prosp = prospective)
NLC: number of lung cancer cases in whole study
R : risky occupational population (n = no, m = mining, o = other risky)
VB : national cigarette type (V = at least 75% Virginia, bl = at least 75% blended, ot = other)
P : any proxy use
H : full histological confirmation
De : derivation of RR/CI (or = original, st = standard method, ot = other method of estimation)

Table 1I30 - 1

IESLC - Meta-analysis of Ever/current Smoking by Duration, Overview
All LC types, Pipes and/or cigars (not cigs)
Most adjusted

REF	NRR	SEX	AGEL	AGEH	RACE	YF	LC TYPE	LOC	START	ST	NLC	R	VB	P	H	AD	SM	exL	exH	S1	S2	DENOM	De
WYNDE7	521	m	0	0	all	-	all	NAmer	1977	CC	2085	n	bl	n	y	0	ev	1	9	0	1	nev any	st
WYNDE7	522	m	0	0	all	-	all	NAmer	1977	CC	2085	n	bl	n	y	0	ev	10	19	0	0	nev any	st
WYNDE7	523	m	0	0	all	-	all	NAmer	1977	CC	2085	n	bl	n	y	0	ev	20	29	1	2	nev any	st
WYNDE7	524	m	0	0	all	-	all	NAmer	1977	CC	2085	n	bl	n	y	0	ev	30	39	2	3	nev any	st
WYNDE7	525	m	0	0	all	-	all	NAmer	1977	CC	2085	n	bl	n	y	0	ev	40	999	3	0	nev any	st

In this overview table, subtotals and Qs values may be invalid and should be ignored

Table 1I30 - 2

IESLC - Meta-analysis of Ever/current Smoking by Duration, Overview
All LC types, Pipes and/or cigars (not cigs)
Most adjusted

REF	NRR	SEX	AD	Number Exposed Case	Exposed Cont	Non-exposed Case	Non-exposed Cont	RR	95.00%CI
WYNDE7	521	m	0	3	34	64	918	1.27	(0.38- 4.23)
WYNDE7	522	m	0	5	41	64	918	1.75	(0.67- 4.58)
WYNDE7	523	m	0	6	66	64	918	1.30	(0.54- 3.12)
WYNDE7	524	m	0	16	81	64	918	2.83	(1.57- 5.13)
WYNDE7	525	m	0	19	84	64	918	3.24	(1.86- 5.67)
Subtotal WYNDE7								2.36	(1.70- 3.29)
Totals				49	306	320	4590		

*prospective study

REF	NRR	SEX	AD	Ys	Ws	Qs	Ps
WYNDE7	521	m	0	0.24	2.64	1.03	0.7022
WYNDE7	522	m	0	0.56	4.15	0.37	0.2548
WYNDE7	523	m	0	0.27	5.04	1.78	0.5514
WYNDE7	524	m	0	1.04	10.92	0.36	0.0006
WYNDE7	525	m	0	1.18	12.31	1.24	0.0000
Subtotal WYNDE7				0.86	35.05	4.78	

N	5
NS	1

Table 1I30 - 3

IESLC - Meta-analysis of Ever/current Smoking by Duration, Overview
All LC types, Pipes and/or cigars (not cigs)
Most adjusted

	Sex			
	combined	male	female	Total
N		5		5
NS		1		1

In this overview table, other than the "N" rows, entries in the "absent" and "Total" columns may be invalid and should be ignored

MALES

	Duration of smoking (broad categories)				
	absent	1-34k20	21-49k35	36+k50	Total
N	2	1	1	1	5
NS	1	1	1	1	1
Wt	6.78	5.04	10.92	12.31	35.05
Het Chi	0.17	0.00	0.00	0.00	4.78
Het df	1	0	0	0	4
Het P	N.S.	N.S.	N.S.	N.S.	N.S.
Fixed RR	1.54	1.30	2.83	3.24	2.36
RRl	0.73	0.54	1.57	1.86	1.70
RRu	3.27	3.12	5.13	5.67	3.29
P	N.S.	N.S.	+++	+++	+++
Random RR	1.54	1.30	2.83	3.24	2.29
RRl	0.73	0.54	1.57	1.86	1.58
RRu	3.27	3.12	5.13	5.67	3.32
P	N.S.	N.S.	+++	+++	+++

	Duration of smoking (narrow categories)							
	absent	1-19k1	6-29k20	21-39k30	31-49k40	41-998k50	51+k999	Total
N	2	1	1	1				5
NS	1	1	1	1				1
Wt	16.46	2.64	5.04	10.92				35.05
Het Chi	1.18	0.00	0.00	0.00				4.78
Het df	1	0	0	0				4
Het P	N.S.	N.S.	N.S.	N.S.				N.S.
Fixed RR	2.78	1.27	1.30	2.83				2.36
RRl	1.71	0.38	0.54	1.57				1.70
RRu	4.50	4.23	3.12	5.13				3.29
P	+++	N.S.	N.S.	+++				+++
Random RR	2.71	1.27	1.30	2.83				2.29
RRl	1.56	0.38	0.54	1.57				1.58
RRu	4.70	4.23	3.12	5.13				3.32
P	+++	N.S.	N.S.	+++				+++

Table 1I30 - 4

IESLC - Meta-analysis of Ever/current Smoking by Duration, Overview
All LC types, Pipes and/or cigars (not cigs)
Least adjusted

REF	NRR	X	SEX	AGEL	AGEH	RACE	YF	LC TYPE	LOC	START	ST	NLC	R	VB	P	H	AD	SM	exL	exH	S1	S2	DENOM	De
WYNDE7	521		m	0	0	all	-	all	NAmer	1977	CC	2085	n	bl	n	y	0	ev	1	9	0	1	nev any	st
WYNDE7	522		m	0	0	all	-	all	NAmer	1977	CC	2085	n	bl	n	y	0	ev	10	19	0	0	nev any	st
WYNDE7	523		m	0	0	all	-	all	NAmer	1977	CC	2085	n	bl	n	y	0	ev	20	29	1	2	nev any	st
WYNDE7	524		m	0	0	all	-	all	NAmer	1977	CC	2085	n	bl	n	y	0	ev	30	39	2	3	nev any	st
WYNDE7	525		m	0	0	all	-	all	NAmer	1977	CC	2085	n	bl	n	y	0	ev	40	999	3	0	nev any	st

In this overview table, subtotals and Qs values may be invalid and should be ignored

Table 1I30 - 5

IESLC - Meta-analysis of Ever/current Smoking by Duration, Overview
All LC types, Pipes and/or cigars (not cigs)
Least adjusted

REF	NRR	SEX	AD	Number Exposed Case	Cont	Non-exposed Case	Cont	RR	95.00%CI
WYNDE7	521	m	0	3	34	64	918	1.27	(0.38- 4.23)
WYNDE7	522	m	0	5	41	64	918	1.75	(0.67- 4.58)
WYNDE7	523	m	0	6	66	64	918	1.30	(0.54- 3.12)
WYNDE7	524	m	0	16	81	64	918	2.83	(1.57- 5.13)
WYNDE7	525	m	0	19	84	64	918	3.24	(1.86- 5.67)
Subtotal WYNDE7								2.36	(1.70- 3.29)
Totals				49	306	320	4590		

*prospective study

REF	NRR	SEX	AD	Ys	Ws	Qs	Ps
WYNDE7	521	m	0	0.24	2.64	1.03	0.7022
WYNDE7	522	m	0	0.56	4.15	0.37	0.2548
WYNDE7	523	m	0	0.27	5.04	1.78	0.5514
WYNDE7	524	m	0	1.04	10.92	0.36	0.0006
WYNDE7	525	m	0	1.18	12.31	1.24	0.0000
Subtotal WYNDE7				0.86	35.05	4.78	

N	5
NS	1

Table 1I30 - 6

IESLC - Meta-analysis of Ever/current Smoking by Duration, Overview
All LC types, Pipes and/or cigars (not cigs)
Least adjusted

	Sex: combined	male	female	Total
N		5		5
NS		1		1

In this overview table, other than the "N" rows, entries in the "absent" and "Total" columns may be invalid and should be ignored

MALES

Duration of smoking (broad categories)

	absent	1-34k20	21-49k35	36+k50	Total
N	2	1	1	1	5
NS	1	1	1	1	1
Wt	6.78	5.04	10.92	12.31	35.05
Het Chi	0.17	0.00	0.00	0.00	4.78
Het df	1	0	0	0	4
Het P	N.S.	N.S.	N.S.	N.S.	N.S.
Fixed RR	1.54	1.30	2.83	3.24	2.36
RRl	0.73	0.54	1.57	1.86	1.70
RRu	3.27	3.12	5.13	5.67	3.29
P	N.S.	N.S.	+++	+++	+++
Random RR	1.54	1.30	2.83	3.24	2.29
RRl	0.73	0.54	1.57	1.86	1.58
RRu	3.27	3.12	5.13	5.67	3.32
P	N.S.	N.S.	+++	+++	+++

Duration of smoking (narrow categories)

	absent	1-19k1	6-29k20	21-39k30	31-49k40	41-998k50	51+k999	Total
N	2	1	1	1				5
NS	1	1	1	1				1
Wt	16.46	2.64	5.04	10.92				35.05
Het Chi	1.18	0.00	0.00	0.00				4.78
Het df	1	0	0	0				4
Het P	N.S.	N.S.	N.S.	N.S.				N.S.
Fixed RR	2.78	1.27	1.30	2.83				2.36
RRl	1.71	0.38	0.54	1.57				1.70
RRu	4.50	4.23	3.12	5.13				3.29
P	+++	N.S.	N.S.	+++				+++
Random RR	2.71	1.27	1.30	2.83				2.29
RRl	1.56	0.38	0.54	1.57				1.58
RRu	4.70	4.23	3.12	5.13				3.32
P	+++	N.S.	N.S.	+++				+++

Table 1I30 - 7

IESLC - Meta-analysis of Ever/current Smoking by Duration, Overview
All LC types, Pipes and/or cigars (not cigs)
Excluded studies (and stage at which they were excluded)

1	BECHER BLOT1 BROWN3 CARPEN CHYOU DARBY DOLL2 GARCIA GRAHAM GURSEL HAMMO2 JAHN JAIN LAUSSM PRESCO QIAO TVERDA WIGLE WYNDE3
2	ALDERS BENSHL BRESLO CHIAZZ DEAN3 DORN ENGELA GAO2 GILLIS GUO HEGMAN HIRAYA HOLE KAUFMA KOO KOULUM LIU4 MIGRAN MRFITR PERNU SEGI2 SPEIZE SUZUK2 SVENSS VUTUC WAKAI WU YUAN
3	GENG MCDUFF SPITZ STASZE WU2 ZHANG
4	BOUCHA CHEN CORREA JEDRYC LUO WYNDE2 WYNDE6
5	AKIBA HAMMON PISANI RESTRE SADOWS XU
7	AGUDO AMANDU AMES ARMADA AUVINE AXELSS BARBON BENHAM BEST BOFFET BOUCOT BROSS BUFFLE CEDERL CHEN2 CHOI CPSI CPSII DAMBER DEAN2 DESTEF DOLL DORGAN DOSEME FAN GAO GARSHI GER HAENSZ HU HU2 HUMBLE JOLY JUSSAW KAISE2 KATSOU KHUDER KREUZE LETOUR LEVIN LIAW LIU3 LIU5 LUBIN LUBIN2 MATOS MCCONN NOTAN2 OSANN2 PEZZO2 PEZZOT QIAO2 RACHTA SOBUE TIZZAN WANG2 WATSON WUWILL WYNDE8 ZHENG ZHOU

Table 1I30 - 8
Potentially overlapping studies

REF	REFGP	PRINC	.	OVERLAP/LINK
WYNDE7	WYNDE6	2		WYNDE5/6/7/8

Table 1I30 - 9
Most adjusted - insufficient data for meta-analysis

REF	NRR	SEX	AGEL	AGEH	RACE	YF	LC TYPE	LOC	START	ST	NLC	R	VB	P	H	AD	SM	exL	exH	S1	S2	DENOM	De
SADOWS	582	m	0	0	wh	-	all	NAmer	1938	CC	477	n	bl	n	n	0	ev	1	19	0	1	nev any	ot
SADOWS	583	m	0	0	wh	-	all	NAmer	1938	CC	477	n	bl	n	n	0	ev	20	29	1	2	nev any	ot
SADOWS	584	m	0	0	wh	-	all	NAmer	1938	CC	477	n	bl	n	n	0	ev	30	39	2	3	nev any	ot
SADOWS	585	m	0	0	wh	-	all	NAmer	1938	CC	477	n	bl	n	n	0	ev	40	999	3	0	nev any	ot

REF	NRR	RR	SIG	RRDATA comment
SADOWS	582	0.81		0
SADOWS	583	1.62		0
SADOWS	584	2.97		0
SADOWS	585	2.22		0

Table 1I31 -

IESLC - Meta-analysis of Ever/current Smoking, Duration, "Highest vs lowest"
All LC types, Pipes and/or cigars (not cigs)

This analysis is restricted to results for:
1) Ever/current smokers
2) Results by Duration
3) Categorical results by Duration
4) Denominator (unexposed) = "low"
5) All LC types (or near equivalent)
6) Results complete enough for use in metaanalysis
7) SMKSTA: ever, current
8) PRODUCT: pipes and/or cigars (not cigs)

Within each study, results are then selected (in the following order of preference, within each sex) for:
9) Results with least adjustment for other aspects of smoking (ADOS)
10) The highest vs lowest category
11) Followup period (YF, prospective studies): whole study (coded as 0) or longest available
12) LCtype: all or nearest available, at least Squamous and Adeno. (q = squamous, s = small,
l = large, a = adeno, mix = mixed, alv = alveolar)
13) Race: all or nearest available, otherwise by race (wh or w = white, bl or b = black, hi = hispanic
ch = chinese, jap = japanese, haw = hawaiian, w+o = white + oriental, sca = scandinavian, as = asian)
14) For overlapping studies: principal rather than subsidiary studies
Finally by Age: whole study (coded as 0) if available, otherwise by widest available age group
and then for single sex results (m, f) in preference to results for both sexes combined (c).

Results adjusted (AD) for the most potential confounders are then chosen in Sections -1 to -3
and results adjusted for the least confounders in Sections -4 to -6. (Those least adjusted results which
actually differ from the most adjusted are marked 'x' in column X in Section -4)

Section -7 shows excluded studies, together with the stage (as above) at which no qualifying
results were found.

Section -8 lists the potentially overlapping studies which have been included (1=principal, 2=subsidiary).

Section -9 lists any results which would have been included in preference except that they had data not complete
enough for use in meta-analysis, with their significance (yes/no), if known, and any further comment as entered
on the database. It also lists as "gap" any categories for which no data were presented by the original authors.

In addition to those mentioned above, the following fields, levels and abbreviations are used:
* or nk = not known, n = no, y = yes, ot = other
all/unspec = all or unspecified, cig+/-ot = cigarettes irrespective of other products (cigar, pipe etc)
MC = manufactured cigarettes, HR = hand-rolled cigarettes
exL, exH = range of exposure (low and high) in the "highest" group, in terms of Duration
unexL, unexH = range of exposure (low and high) in the "lowest" group, in terms of Duration
REF: 6-character study reference
NRR: number of the RR on the database within the study
ST : study type (CC = case control, pr or prosp = prospective)
NLC: number of lung cancer cases in whole study
R : risky occupational population (n = no, m = mining, o = other risky)
VB : national cigarette type (V = at least 75% Virginia, bl = at least 75% blended, ot = other)
P : any proxy use
H : full histological confirmation
De : derivation of RR/CI (or = original, st = standard method, ot = other method of estimation)

Table 1I31 - 1

IESLC - Meta-analysis of Ever/current Smoking, Duration, "Highest vs lowest"
All LC types, Pipes and/or cigars (not cigs)
Most adjusted

REF	NRR	SEX	AGEL	AGEH	RACE	YF	LC TYPE	LOC	START	ST	NLC	R	VB	P	H	AD	ADOS	SM	exL	exH	unexL	unexH	De
WYNDE7	529	m	0	0	all	-	all	NAmer	1977	CC	2085	n	bl	n	y	0	0	ev	40	999	1	9	st

Table 1I31 - 2

IESLC - Meta-analysis of Ever/current Smoking, Duration, "Highest vs lowest"
All LC types, Pipes and/or cigars (not cigs)
Most adjusted

REF	NRR	SEX	AD	Number Exposed Case	Cont	Non-exposed Case	Cont	RR	95.00%CI
WYNDE7	529	m	0	19	84	3	34	2.56	(0.71- 9.23)
Totals				19	84	3	34		

*prospective study

REF	NRR	SEX	AD	Ys	Ws	Qs	Ps
WYNDE7	529	m	0	0.94	2.34	0.00	0.1498

N	1
NS	1
Wt	2.34
Het Chi	0.00
Het df	0
Het P	N.S.
Fixed RR	2.56
RRl	0.71
RRu	9.23
P	N.S.
Random RR	2.56
RRl	0.71
RRu	9.23
P	N.S.
Asymm P	

Table 1I31 - 3

IESLC - Meta-analysis of Ever/current Smoking, Duration, "Highest vs lowest"
All LC types, Pipes and/or cigars (not cigs)
Most adjusted

	Sex combined	male	female	Total
N		1		1
NS		1		1
Wt		2.34		2.34
Het Chi		0.00		0.00
Het df		0		0
Het P		N.S.		N.S.
Fixed RR		2.56		2.56
RRl		0.71		0.71
RRu		9.23		9.23
P		N.S.		N.S.
Random RR		2.56		2.56
RRl		0.71		0.71
RRu		9.23		9.23
P		N.S.		N.S.
Between Chi				
Between df				
Between P				N.S.
Btwn(F) P				N.S.
Btwn(R) P				N.S.

MALES

Too few RRs for analysis by factor

Table 1I31 - 4

IESLC - Meta-analysis of Ever/current Smoking, Duration, "Highest vs lowest"
All LC types, Pipes and/or cigars (not cigs)
Least adjusted

REF	NRR	X	SEX	AGEL	AGEH	RACE	YF	LC TYPE	LOC	START	ST	NLC	R	VB	P	H	AD	ADOS	SM	exL	exH	unexL	unexH	De
WYNDE7	529		m	0	0	all	-	all	NAmer	1977	CC	2085	n	bl	n	y	0	0	ev	40	999	1	9	st

Table 1I31 - 5

IESLC - Meta-analysis of Ever/current Smoking, Duration, "Highest vs lowest"
All LC types, Pipes and/or cigars (not cigs)
Least adjusted

REF	NRR	SEX	AD	Number Exposed Case	Number Exposed Cont	Non-exposed Case	Non-exposed Cont	RR	95.00%CI
WYNDE7	529	m	0	19	84	3	34	2.56	(0.71- 9.23)
Totals				19	84	3	34		

*prospective study

REF	NRR	SEX	AD	Ys	Ws	Qs	Ps
WYNDE7	529	m	0	0.94	2.34	0.00	0.1498

	N	1
	NS	1
	Wt	2.34
Het	Chi	0.00
Het	df	0
Het	P	N.S.
Fixed	RR	2.56
	RRl	0.71
	RRu	9.23
	P	N.S.
Random	RR	2.56
	RRl	0.71
	RRu	9.23
	P	N.S.
Asymm	P	

Table 1I31 - 6

IESLC - Meta-analysis of Ever/current Smoking, Duration, "Highest vs lowest"
All LC types, Pipes and/or cigars (not cigs)
Least adjusted

	Sex combined	male	female	Total
N		1		1
NS		1		1
Wt		2.34		2.34
Het Chi		0.00		0.00
Het df		0		0
Het P		N.S.		N.S.
Fixed RR		2.56		2.56
RRl		0.71		0.71
RRu		9.23		9.23
P		N.S.		N.S.
Random RR		2.56		2.56
RRl		0.71		0.71
RRu		9.23		9.23
P		N.S.		N.S.
Between Chi				
Between df				
Between P				N.S.
Btwn(F) P				N.S.
Btwn(R) P				N.S.

Table 1I31 - 7

IESLC - Meta-analysis of Ever/current Smoking, Duration, "Highest vs lowest"
All LC types, Pipes and/or cigars (not cigs)
Excluded studies (and stage at which they were excluded)

1	BECHER BLOT1 BROWN3 CARPEN CHYOU DARBY DOLL2 GARCIA GRAHAM GURSEL HAMMO2 JAHN JAIN LAUSSM PRESCO QIAO TVERDA WIGLE WYNDE3
2	ALDERS BENSHL BRESLO CHIAZZ DEAN3 DORN ENGELA GAO2 GILLIS GUO HEGMAN HIRAYA HOLE KAUFMA KOO KOULUM LIU4 MIGRAN MRFITR PERNU SEGI2 SPEIZE SUZUK2 SVENSS VUTUC WAKAI WU YUAN
3	GENG MCDUFF SPITZ STASZE WU2 ZHANG
4	AKIBA GARSHI
5	BOUCHA CHEN CORREA JEDRYC LUO WYNDE2 WYNDE6
6	HAMMON PISANI RESTRE SADOWS XU
8	AGUDO AMANDU AMES ARMADA AUVINE AXELSS BARBON BENHAM BEST BOFFET BOUCOT BROSS BUFFLE CEDERL CHEN2 CHOI CPSI CPSII DAMBER DEAN2 DESTEF DOLL DORGAN DOSEME FAN GAO GER HAENSZ HU HU2 HUMBLE JOLY JUSSAW KAISE2 KATSOU KHUDER KREUZE LETOUR LEVIN LIAW LIU3 LIU5 LUBIN LUBIN2 MATOS MCCONN NOTAN2 OSANN2 PEZZO2 PEZZOT QIAO2 RACHTA SOBUE TIZZAN WANG2 WATSON WUWILL WYNDE8 ZHENG ZHOU

Table 1I31 - 8
Potentially overlapping studies

REF	REFGP	PRINC .	OVERLAP/LINK
WYNDE7	WYNDE6	2	WYNDE5/6/7/8

Table 1I31 - 9
Most adjusted - insufficient data for meta-analysis

REF	NRR	SEX	AGEL	AGEH	RACE	YF	LC TYPE	LOC	START	ST	NLC	R	VB	P	H	AD	ADOS	SM	exL	exH	unexL	unexH	De
SADOWS	588	m	0	0	wh	-	all	NAmer	1938	CC	477	n	bl	n	n	0	0	ev	40	999	1	19	ot

REF	NRR	RR	SIG	RRDATA comment
SADOWS	588	2.73		0

Table 1I32 -

IESLC - Meta-analysis of Ever/current Smoking by Duration, Overview
All LC types, Pipes only

This analysis is restricted to results for:
1) Ever/current smokers
2) Results by Duration
3) Categorical results by Duration
Results by Duration are grouped under 2 schemes (S1, S2). Each scheme has a set of "key values". An interval is allocated to the category whose key value it includes, and intervals which include none or more than one of the key values are excluded. (Open-ended intervals are coded as 999)

S1	key value	maximum range
1	20	1-34
2	35	21-49
3	50	36+

S2	key value	maximum range
1	5	1-19
2	20	6-29
3	30	21-39
4	40	31-49
5	50	41-998
6	999	51+

4) All LC types (or near equivalent)
5) Results complete enough for use in metaanalysis
6) pipes only

Within each study, results are then selected (in the following order of preference, within each sex) for:
7) (not applicable)
8) DENOM: never smoked anything, never any + low
9) Followup period (YF, prospective studies): whole study (coded as 0) or longest available
10) LCtype: all or nearest available, at least Squamous and Adeno. (q = squamous, s = small, l = large, a = adeno, mix = mixed, alv = alveolar)
11) Race: all or nearest available, otherwise by race (wh or w = white, bl or b = black, hi = hispanic ch = chinese, jap = japanese, haw = hawaiian, w+o = white + oriental, sca = scandinavian, as = asian)
12) For overlapping studies: principal rather than subsidiary studies
Finally by Age: whole study (coded as 0) if available, otherwise by widest available age group and then for single sex results (m, f) in preference to results for both sexes combined (c).

Results adjusted (AD) for the most potential confounders are then chosen in Sections -1 to -3 and results adjusted for the least confounders in Sections -4 to -6. (Those least adjusted results which actually differ from the most adjusted are marked 'x' in column X in Section -4)

Section -7 shows excluded studies, together with the stage (as above) at which no qualifying results were found.

Section -8 lists the potentially overlapping studies which have been included (1=principal, 2=subsidiary).

Section -9 lists any results which would have been included in preference except that they had data not complete enough for use in meta-analysis, with their significance (yes/no), if known, and any further comment as entered on the database. It also lists as "gap" any categories for which no data were presented by the original authors.

In addition to those mentioned above, the following fields, levels and abbreviations are used:
* or nk = not known, n = no, y = yes, ot = other
ev = ever, cu = current, nev = never
all/unspec = all or unspecified, cig+/-ot = cigarettes irrespective of other products (cigar, pipe etc)
MC = manufactured cigarettes, HR = hand-rolled cigarettes
exL, exH = range of exposure (low and high) in the smoking group, in terms of Duration
REF: 6-character study reference
NRR: number of the RR on the database within the study
ST : study type (CC = case control, pr or prosp = prospective)
NLC: number of lung cancer cases in whole study
R : risky occupational population (n = no, m = mining, o = other risky)
VB : national cigarette type (V = at least 75% Virginia, bl = at least 75% blended, ot = other)
P : any proxy use
H : full histological confirmation
De : derivation of RR/CI (or = original, st = standard method, ot = other method of estimation)

Table 1I32 - 1

IESLC - Meta-analysis of Ever/current Smoking by Duration, Overview
All LC types, Pipes only
Most adjusted

REF	NRR	SEX	AGEL	AGEH	RACE	YF	LC TYPE	LOC	START	ST	NLC	R	VB	P	H	AD	SM	exL	exH	S1	S2	DENOM	De
BOFFET	532	m	0	0	all	-	all	Eu:mul	1988	CC	5621	n	bl	y	n	2	ev	0	20	1	0	nev any	or
BOFFET	533	m	0	0	all	-	all	Eu:mul	1988	CC	5621	n	bl	y	n	2	ev	20	32	0	3	nev any	or
BOFFET	534	m	0	0	all	-	all	Eu:mul	1988	CC	5621	n	bl	y	n	2	ev	32	44	2	4	nev any	or
BOFFET	535	m	0	0	all	-	all	Eu:mul	1988	CC	5621	n	bl	y	n	2	ev	44	999	3	0	nev any	or
CEDERL	507	m	40	69	all	10	all	Eu:Sca	1963	pr	491	n	bl	n	n	1	cu	1	29	1	0	nev any	ot
CEDERL	508	m	40	69	all	10	all	Eu:Sca	1963	pr	491	n	bl	n	n	1	cu	30	999	0	0	nev any	ot
DAMBER	515	m	0	0	all	-	all	Eu:Sca	1972	CC	579	n	bl	y	n	1	ev	1	30	1	0	nev any	ot
DAMBER	516	m	0	0	all	-	all	Eu:Sca	1972	CC	579	n	bl	y	n	1	ev	31	50	0	0	nev any	ot
DAMBER	517	m	0	0	all	-	all	Eu:Sca	1972	CC	579	n	bl	y	n	1	ev	51	999	0	6	nev any	ot
LUBIN	558	m	0	0	all	-	all	As:Chi	1984	CC	427	m	ot	y	n	4	ev	1	29	1	0	nev any	ot
LUBIN	559	m	0	0	all	-	all	As:Chi	1984	CC	427	m	ot	y	n	4	ev	30	39	2	3	nev any	ot
LUBIN	560	m	0	0	all	-	all	As:Chi	1984	CC	427	m	ot	y	n	4	ev	40	49	0	4	nev any	ot
LUBIN	561	m	0	0	all	-	all	As:Chi	1984	CC	427	m	ot	y	n	4	ev	50	999	3	0	nev any	ot
LUBIN2	1165	m	0	0	all	-	all	Eu:mul	1976	CC	7804	n	bl	n	y	1	ev	1	19	0	1	nev any	ot
LUBIN2	1166	m	0	0	all	-	all	Eu:mul	1976	CC	7804	n	bl	n	y	1	ev	20	29	1	2	nev any	ot
LUBIN2	1167	m	0	0	all	-	all	Eu:mul	1976	CC	7804	n	bl	n	y	1	ev	30	39	2	3	nev any	ot
LUBIN2	1168	m	0	0	all	-	all	Eu:mul	1976	CC	7804	n	bl	n	y	1	ev	40	999	3	0	nev any	ot

In this overview table, subtotals and Qs values may be invalid and should be ignored

Table 1I32 - 2

IESLC - Meta-analysis of Ever/current Smoking by Duration, Overview
All LC types, Pipes only
Most adjusted

REF	NRR	SEX	AD	Number Exposed Case	Number Exposed Cont	Non-exposed Case	Non-exposed Cont	RR	95.00%CI
BOFFET	532	m	2	3	-	117	-	1.30	(0.40- 4.50)
BOFFET	533	m	2	7	-	117	-	3.40	(1.40- 8.00)
BOFFET	534	m	2	21	-	117	-	13.30	(7.20- 24.90)
BOFFET	535	m	2	30	-	117	-	19.10	(10.40- 35.10)
Subtotal BOFFET								9.57	(6.61- 13.85)
*CEDERL	507	m	1	1	-	7	-	0.40	(0.05- 3.25)
*CEDERL	508	m	1	26	-	7	-	10.00	(4.35- 22.99)
Subtotal CEDERL								6.43	(2.97- 13.93)
DAMBER	515	m	1	-	-	42	-	4.13	(1.90- 9.21)
DAMBER	516	m	1	-	-	42	-	5.56	(3.49- 9.21)
DAMBER	517	m	1	-	-	42	-	9.84	(6.35- 16.35)
Subtotal DAMBER								6.80	(4.98- 9.28)
LUBIN	558	m	4	7	-	8	-	2.09	(0.68- 6.44)
LUBIN	559	m	4	13	-	8	-	2.71	(1.02- 7.19)
LUBIN	560	m	4	24	-	8	-	2.53	(1.07- 6.01)
LUBIN	561	m	4	10	-	8	-	2.09	(0.75- 5.81)
Subtotal LUBIN								2.38	(1.45- 3.88)
LUBIN2	1165	m	1	3	-	190	-	1.10	(0.34- 3.60)
LUBIN2	1166	m	1	3	-	190	-	1.02	(0.31- 3.32)
LUBIN2	1167	m	1	11	-	190	-	2.51	(1.29- 4.88)
LUBIN2	1168	m	1	22	-	190	-	4.43	(2.67- 7.35)
Subtotal LUBIN2								2.86	(1.99- 4.11)
Partial Totals				181	0	1400	0		

*prospective study

REF	NRR	SEX	AD	Ys	Ws	Qs	Ps
BOFFET	532	m	2	0.26	2.62	4.99	0.6709
BOFFET	533	m	2	1.22	5.06	0.88	0.0059
BOFFET	534	m	2	2.59	9.98	8.93	0.0000
BOFFET	535	m	2	2.95	10.38	17.77	0.0000
Subtotal BOFFET				2.26	28.05	32.57	
*CEDERL	507	m	1	-0.92	0.88	5.77	0.3895
*CEDERL	508	m	1	2.30	5.54	2.42	0.0000
Subtotal CEDERL				1.86	6.43	8.19	
DAMBER	515	m	1	1.42	6.17	0.31	0.0004
DAMBER	516	m	1	1.72	16.32	0.09	0.0000
DAMBER	517	m	1	2.29	17.18	7.14	0.0000
Subtotal DAMBER				1.92	39.66	7.54	
LUBIN	558	m	4	0.74	3.04	2.49	0.1987
LUBIN	559	m	4	1.00	4.03	1.67	0.0454
LUBIN	560	m	4	0.93	5.16	2.63	0.0350
LUBIN	561	m	4	0.74	3.67	3.00	0.1581
Subtotal LUBIN				0.87	15.89	9.79	
LUBIN2	1165	m	1	0.10	2.76	6.60	0.8742
LUBIN2	1166	m	1	0.02	2.73	7.19	0.9739
LUBIN2	1167	m	1	0.92	8.68	4.52	0.0067
LUBIN2	1168	m	1	1.49	14.99	0.35	0.0000
Subtotal LUBIN2				1.05	29.16	18.66	

N	17
NS	5

Table 1I32 - 3

IESLC - Meta-analysis of Ever/current Smoking by Duration, Overview
All LC types, Pipes only
Most adjusted

	Sex			
	combined	male	female	Total
N		17		17
NS		5		5

In this overview table, other than the "N" rows, entries in the "absent" and "Total" columns may be invalid and should be ignored

MALES

	Duration of smoking (broad categories)				
	absent	1-34k20	21-49k35	36+k50	Total
N	6	5	3	3	17
NS	5	5	3	3	16
Wt	52.02	15.45	22.69	29.04	119.19
Het Chi	19.09	7.26	15.11	18.93	76.75
Het df	5	4	2	2	16
Het P	**	N.S.	***	***	***
Fixed RR	5.78	2.03	5.30	6.79	5.16
RRl	4.41	1.23	3.51	4.72	4.32
RRu	7.59	3.34	8.00	9.78	6.18
P	+++	++	+++	+++	+++
Random RR	4.64	1.73	4.60	5.87	3.77
RRl	2.62	0.85	1.43	1.77	2.49
RRu	8.24	3.51	14.84	19.53	5.72
P	+++	N.S.	+	++	+++

	Duration of smoking (narrow categories)							
	absent	1-19k1	6-29k20	21-39k30	31-49k40	41-998k50	51+k999	Total
N	9	1	1	3	2		1	17
NS	5	1	1	3	2		1	11
Wt	63.61	2.76	2.73	17.77	15.14		17.18	119.19
Het Chi	37.06	0.00	0.00	0.30	9.37		0.00	76.75
Het df	8	0	0	2	1		0	16
Het P	***	N.S.	N.S.	N.S.	**		N.S.	***
Fixed RR	5.40	1.10	1.02	2.78	7.56		9.84	5.16
RRl	4.22	0.34	0.31	1.75	4.57		6.13	4.32
RRu	6.90	3.58	3.34	4.43	12.50		15.79	6.18
P	+++	N.S.	N.S.	+++	+++		+++	+++
Random RR	4.11	1.10	1.02	2.78	5.97		9.84	3.77
RRl	2.31	0.34	0.31	1.75	1.17		6.13	2.49
RRu	7.31	3.58	3.34	4.43	30.31		15.79	5.72
P	+++	N.S.	N.S.	+++	+		+++	+++

Table 1I32 - 4

IESLC - Meta-analysis of Ever/current Smoking by Duration, Overview
All LC types, Pipes only
Least adjusted

REF	NRR	X	SEX	AGEL	AGEH	RACE	YF	LC TYPE	LOC	START	ST	NLC	R	VB	P	H	AD	SM	exL	exH	S1	S2	DENOM	De
BOFFET	525	x	m	0	0	all	-	all	Eu:mul	1988	CC	5621	n	bl	y	n	0	ev	0	20	1	0	nev any	st
BOFFET	526	x	m	0	0	all	-	all	Eu:mul	1988	CC	5621	n	bl	y	n	0	ev	20	32	0	3	nev any	st
BOFFET	527	x	m	0	0	all	-	all	Eu:mul	1988	CC	5621	n	bl	y	n	0	ev	32	44	2	4	nev any	st
BOFFET	528	x	m	0	0	all	-	all	Eu:mul	1988	CC	5621	n	bl	y	n	0	ev	44	999	3	0	nev any	st
CEDERL	507		m	40	69	all	10	all	Eu:Sca	1963	pr	491	n	bl	n	n	1	cu	1	29	1	0	nev any	ot
CEDERL	508		m	40	69	all	10	all	Eu:Sca	1963	pr	491	n	bl	n	n	1	cu	30	999	0	0	nev any	ot
DAMBER	515		m	0	0	all	-	all	Eu:Sca	1972	CC	579	n	bl	y	n	1	ev	1	30	1	0	nev any	ot
DAMBER	516		m	0	0	all	-	all	Eu:Sca	1972	CC	579	n	bl	y	n	1	ev	31	50	0	0	nev any	ot
DAMBER	517		m	0	0	all	-	all	Eu:Sca	1972	CC	579	n	bl	y	n	1	ev	51	999	0	6	nev any	ot
LUBIN	551	x	m	0	0	all	-	all	As:Chi	1984	CC	427	m	ot	y	n	0	ev	1	29	1	0	nev any	st
LUBIN	552	x	m	0	0	all	-	all	As:Chi	1984	CC	427	m	ot	y	n	0	ev	30	39	2	3	nev any	st
LUBIN	553	x	m	0	0	all	-	all	As:Chi	1984	CC	427	m	ot	y	n	0	ev	40	49	0	4	nev any	st
LUBIN	554	x	m	0	0	all	-	all	As:Chi	1984	CC	427	m	ot	y	n	0	ev	50	999	3	0	nev any	st
LUBIN2	1045	x	m	0	0	all	-	all	Eu:mul	1976	CC	7804	n	bl	n	y	0	ev	1	19	0	1	nev any	st
LUBIN2	1046	x	m	0	0	all	-	all	Eu:mul	1976	CC	7804	n	bl	n	y	0	ev	20	29	1	2	nev any	st
LUBIN2	1047	x	m	0	0	all	-	all	Eu:mul	1976	CC	7804	n	bl	n	y	0	ev	30	39	2	3	nev any	st
LUBIN2	1048	x	m	0	0	all	-	all	Eu:mul	1976	CC	7804	n	bl	n	y	0	ev	40	999	3	0	nev any	st

In this overview table, subtotals and Qs values may be invalid and should be ignored

Table 1I32 - 5

IESLC - Meta-analysis of Ever/current Smoking by Duration, Overview
All LC types, Pipes only
Least adjusted

REF	NRR	SEX	AD	Number Exposed Case	Number Exposed Cont	Non-exposed Case	Non-exposed Cont	RR	95.00%CI
BOFFET	525	m	0	3	33	117	1750	1.36	(0.41- 4.50)
BOFFET	526	m	0	7	33	117	1750	3.17	(1.37- 7.33)
BOFFET	527	m	0	21	33	117	1750	9.52	(5.34- 16.97)
BOFFET	528	m	0	30	30	117	1750	14.96	(8.72- 25.66)
Subtotal BOFFET								8.10	(5.76- 11.41)
*CEDERL	507	m	1	1	-	7	-	0.40	(0.05- 3.25)
*CEDERL	508	m	1	26	-	7	-	10.00	(4.35- 22.99)
Subtotal CEDERL								6.43	(2.97- 13.93)
DAMBER	515	m	1	-	-	42	-	4.13	(1.90- 9.21)
DAMBER	516	m	1	-	-	42	-	5.56	(3.49- 9.21)
DAMBER	517	m	1	-	-	42	-	9.84	(6.35- 16.35)
Subtotal DAMBER								6.80	(4.98- 9.28)
LUBIN	551	m	0	7	21	8	72	3.00	(0.97- 9.24)
LUBIN	552	m	0	13	31	8	72	3.77	(1.42- 10.02)
LUBIN	553	m	0	24	70	8	72	3.09	(1.30- 7.33)
LUBIN	554	m	0	10	30	8	72	3.00	(1.08- 8.34)
Subtotal LUBIN								3.21	(1.96- 5.25)
LUBIN2	1045	m	0	3	36	190	2616	1.15	(0.35- 3.76)
LUBIN2	1046	m	0	3	43	190	2616	0.96	(0.30- 3.12)
LUBIN2	1047	m	0	11	54	190	2616	2.80	(1.44- 5.45)
LUBIN2	1048	m	0	22	64	190	2616	4.73	(2.85- 7.85)
Subtotal LUBIN2								3.05	(2.12- 4.38)
Partial Totals				181	478	1400	17752		

*prospective study

REF	NRR	SEX	AD	Ys	Ws	Qs	Ps
BOFFET	525	m	0	0.31	2.68	5.02	0.6147
BOFFET	526	m	0	1.15	5.49	1.49	0.0068
BOFFET	527	m	0	2.25	11.49	3.83	0.0000
BOFFET	528	m	0	2.71	13.20	13.99	0.0000
Subtotal BOFFET				2.09	32.85	24.33	
*CEDERL	507	m	1	-0.92	0.88	5.92	0.3895
*CEDERL	508	m	1	2.30	5.54	2.18	0.0000
Subtotal CEDERL				1.86	6.43	8.10	
DAMBER	515	m	1	1.42	6.17	0.41	0.0004
DAMBER	516	m	1	1.72	16.32	0.03	0.0000
DAMBER	517	m	1	2.29	17.18	6.41	0.0000
Subtotal DAMBER				1.92	39.66	6.84	
LUBIN	551	m	0	1.10	3.04	1.01	0.0556
LUBIN	552	m	0	1.33	4.03	0.49	0.0077
LUBIN	553	m	0	1.13	5.13	1.55	0.0107
LUBIN	554	m	0	1.10	3.67	1.22	0.0352
Subtotal LUBIN				1.17	15.87	4.27	
LUBIN2	1045	m	0	0.14	2.73	6.45	0.8204
LUBIN2	1046	m	0	-0.04	2.76	8.13	0.9467
LUBIN2	1047	m	0	1.03	8.69	3.61	0.0024
LUBIN2	1048	m	0	1.55	14.99	0.22	0.0000
Subtotal LUBIN2				1.12	29.16	18.41	

N	17
NS	5

Table 1I32 - 6

IESLC - Meta-analysis of Ever/current Smoking by Duration, Overview
All LC types, Pipes only
Least adjusted

	Sex combined	male	female	Total
N		17		17
NS		5		5

In this overview table, other than the "N" rows, entries in the "absent" and "Total" columns may be invalid and should be ignored

MALES

	Duration of smoking (broad categories) absent	1-34k20	21-49k35	36+k50	Total
N	6	5	3	3	17
NS	5	5	3	3	16
Wt	52.38	15.53	24.21	31.86	123.98
Het Chi	17.67	7.81	7.92	12.51	61.95
Het df	5	4	2	2	16
Het P	**	(*)	*	**	***
Fixed RR	5.85	2.16	5.26	7.23	5.34
RRl	4.46	1.32	3.53	5.11	4.48
RRu	7.67	3.56	7.84	10.24	6.37
P	+++	++	+++	+++	+++
Random RR	4.80	1.83	4.79	6.32	4.05
RRl	2.77	0.88	2.09	2.48	2.80
RRu	8.31	3.83	10.99	16.09	5.86
P	+++	N.S.	+++	+++	+++

	Duration of smoking (narrow categories) absent	1-19k1	6-29k20	21-39k30	31-49k40	41-998k50	51+k999	Total
N	9	1	1	3	2		1	17
NS	5	1	1	3	2		1	11
Wt	66.49	2.73	2.76	18.21	16.62		17.18	123.98
Het Chi	29.70	0.00	0.00	0.25	4.50		0.00	61.95
Het df	8	0	0	2	1		0	16
Het P	***	N.S.	N.S.	N.S.	*		N.S.	***
Fixed RR	5.71	1.15	0.96	3.11	6.72		9.84	5.34
RRl	4.49	0.35	0.30	1.96	4.16		6.13	4.48
RRu	7.27	3.76	3.12	4.92	10.87		15.79	6.37
P	+++	N.S.	N.S.	+++	+++		+++	+++
Random RR	4.52	1.15	0.96	3.11	5.69		9.84	4.05
RRl	2.72	0.35	0.30	1.96	1.89		6.13	2.80
RRu	7.51	3.76	3.12	4.92	17.08		15.79	5.86
P	+++	N.S.	N.S.	+++	++		+++	+++

Table 1I32 - 7

IESLC - Meta-analysis of Ever/current Smoking by Duration, Overview
All LC types, Pipes only
Excluded studies (and stage at which they were excluded)

1 BECHER BLOT1 BROWN3 CARPEN CHYOU DARBY DOLL2 GARCIA GRAHAM GURSEL HAMMO2 JAHN JAIN LAUSSM PRESCO QIAO TVERDA WIGLE WYNDE3

2 ALDERS BENSHL BRESLO CHIAZZ DEAN3 DORN ENGELA GAO2 GILLIS GUO HEGMAN HIRAYA HOLE KAUFMA KOO KOULUM LIU4 MIGRAN MRFITR PERNU SEGI2 SPEIZE SUZUK2 SVENSS VUTUC WAKAI WU YUAN

3 GENG MCDUFF SPITZ STASZE WU2 ZHANG

4 BOUCHA CHEN CORREA JEDRYC LUO WYNDE2 WYNDE6

5 AKIBA HAMMON PISANI RESTRE SADOWS XU

7 AGUDO AMANDU AMES ARMADA AUVINE AXELSS BARBON BEST BOUCOT BROSS BUFFLE CHEN2 CHOI CPSI CPSII DEAN2 DESTEF DOLL DORGAN DOSEME FAN GAO GARSHI GER HAENSZ HU HU2 HUMBLE JOLY JUSSAW KAISE2 KATSOU KHUDER KREUZE LETOUR LEVIN LIAW LIU3 LIU5 MATOS MCCONN NOTAN2 OSANN2 PEZZO2 PEZZOT QIAO2 RACHTA SOBUE TIZZAN WANG2 WATSON WUWILL WYNDE7 WYNDE8 ZHENG ZHOU

13 BENHAM

Table 1I32 - 8
Potentially overlapping studies

REF	REFGP	PRINC	. OVERLAP/LINK
LUBIN2	LUBIN2	1	Lubin-combined
BOFFET	BOFFET	1	BOFFET-combined
LUBIN	XIANGZ	2	LUBIN/XIANGZ/QIAO

Table 1I32 - 9
Most adjusted - insufficient data for meta-analysis

REF	NRR	SEX	AGEL	AGEH	RACE	YF	LC TYPE	LOC	START	ST	NLC	R	VB	P	H	AD	SM	exL	exH	S1	S2	DENOM	De
SADOWS	600	m	0	0	wh	-	all	NAmer	1938	CC	477	n	bl	n	n	0	ev	1	9	0	1	nev any	ot
SADOWS	601	m	0	0	wh	-	all	NAmer	1938	CC	477	n	bl	n	n	0	ev	10	19	0	0	nev any	ot
SADOWS	602	m	0	0	wh	-	all	NAmer	1938	CC	477	n	bl	n	n	0	ev	20	29	1	2	nev any	ot
SADOWS	603	m	0	0	wh	-	all	NAmer	1938	CC	477	n	bl	n	n	0	ev	30	39	2	3	nev any	ot
SADOWS	604	m	0	0	wh	-	all	NAmer	1938	CC	477	n	bl	n	n	0	ev	40	49	0	4	nev any	ot
SADOWS	605	m	0	0	wh	-	all	NAmer	1938	CC	477	n	bl	n	n	0	ev	50	999	3	0	nev any	ot

REF	NRR	RR	SIG	RRDATA comment
SADOWS	600	2.95		0
SADOWS	601	0.00		0
SADOWS	602	0.38		0
SADOWS	603	3.46		0
SADOWS	604	2.22		0
SADOWS	605	0.00		0

Table 1I33 -

IESLC - Meta-analysis of Ever/current Smoking, Duration, "Highest vs lowest"
All LC types, Pipes only

This analysis is restricted to results for:
1) Ever/current smokers
2) Results by Duration
3) Categorical results by Duration
4) Denominator (unexposed) = "low"
5) All LC types (or near equivalent)
6) Results complete enough for use in metaanalysis
7) SMKSTA: ever, current
8) PRODUCT: pipes only

Within each study, results are then selected (in the following order of preference, within each sex) for:
9) Results with least adjustment for other aspects of smoking (ADOS)
10) The highest vs lowest category
11) Followup period (YF, prospective studies): whole study (coded as 0) or longest available
12) LCtype: all or nearest available, at least Squamous and Adeno. (q = squamous, s = small, l = large, a = adeno, mix = mixed, alv = alveolar)
13) Race: all or nearest available, otherwise by race (wh or w = white, bl or b = black, hi = hispanic ch = chinese, jap = japanese, haw = hawaiian, w+o = white + oriental, sca = scandinavian, as = asian)
14) For overlapping studies: principal rather than subsidiary studies
Finally by Age: whole study (coded as 0) if available, otherwise by widest available age group and then for single sex results (m, f) in preference to results for both sexes combined (c).

Results adjusted (AD) for the most potential confounders are then chosen in Sections -1 to -3 and results adjusted for the least confounders in Sections -4 to -6. (Those least adjusted results which actually differ from the most adjusted are marked 'x' in column X in Section -4)

Section -7 shows excluded studies, together with the stage (as above) at which no qualifying results were found.

Section -8 lists the potentially overlapping studies which have been included (1=principal, 2=subsidiary).

Section -9 lists any results which would have been included in preference except that they had data not complete enough for use in meta-analysis, with their significance (yes/no), if known, and any further comment as entered on the database. It also lists as "gap" any categories for which no data were presented by the original authors.

In addition to those mentioned above, the following fields, levels and abbreviations are used:
* or nk = not known, n = no, y = yes, ot = other
all/unspec = all or unspecified, cig+/-ot = cigarettes irrespective of other products (cigar, pipe etc)
MC = manufactured cigarettes, HR = hand-rolled cigarettes
exL, exH = range of exposure (low and high) in the "highest" group, in terms of Duration
unexL, unexH = range of exposure (low and high) in the "lowest" group, in terms of Duration
REF: 6-character study reference
NRR: number of the RR on the database within the study
ST : study type (CC = case control, pr or prosp = prospective)
NLC: number of lung cancer cases in whole study
R : risky occupational population (n = no, m = mining, o = other risky)
VB : national cigarette type (V = at least 75% Virginia, bl = at least 75% blended, ot = other)
P : any proxy use
H : full histological confirmation
De : derivation of RR/CI (or = original, st = standard method, ot = other method of estimation)

Table 1I33 - 1

IESLC - Meta-analysis of Ever/current Smoking, Duration, "Highest vs lowest"
All LC types, Pipes only
Most adjusted

REF	NRR	SEX	AGEL	AGEH	RACE	YF	LC TYPE	LOC	START	ST	NLC	R	VB	P	H	AD	ADOS	SM	exL	exH	unexL	unexH	De
BOFFET	538	m	0	0	all	-	all	Eu:mul	1988	CC	5621	n	bl	y	n	2	0	ev	44	999	0	20	ot
CEDERL	509	m	40	69	all	10	all	Eu:Sca	1963	pr	491	n	bl	n	n	1	0	cu	30	999	1	29	ot
DAMBER	519	m	0	0	all	-	all	Eu:Sca	1972	CC	579	n	bl	y	n	1	0	ev	51	999	1	30	ot
LUBIN	564	m	0	0	all	-	all	As:Chi	1984	CC	427	m	ot	y	n	4	0	ev	50	999	1	29	ot
LUBIN2	1171	m	0	0	all	-	all	Eu:mul	1976	CC	7804	n	bl	n	y	1	0	ev	40	999	1	19	ot

Table 1I33 - 2

IESLC - Meta-analysis of Ever/current Smoking, Duration, "Highest vs lowest"
All LC types, Pipes only
Most adjusted

REF	NRR	SEX	AD	Number Exposed Case	Cont	Non-exposed Case	Cont	RR	95.00%CI
BOFFET	538	m	2	30	-	3	-	14.69	(3.91- 55.15)
*CEDERL	509	m	1	26	-	1	-	25.00	(3.25- 192.28)
DAMBER	519	m	1	-	-	-	-	2.38	(1.09- 5.20)
LUBIN	564	m	4	10	-	7	-	1.00	(0.31- 3.19)
LUBIN2	1171	m	1	22	-	3	-	4.03	(1.13- 14.39)
Partial Totals				88	0	14	0		

*prospective study

REF	NRR	SEX	AD	Ys	Ws	Qs	Ps
BOFFET	538	m	2	2.69	2.19	4.81	0.0001
*CEDERL	509	m	1	3.22	0.92	3.74	0.0020
DAMBER	519	m	1	0.87	6.29	0.73	0.0296
LUBIN	564	m	4	0.00	2.83	4.12	1.0000
LUBIN2	1171	m	1	1.39	2.37	0.08	0.0318

	N	5
	NS	5
	Wt	14.61
Het	Chi	13.47
Het	df	4
Het	P	**
Fixed	RR	3.34
	RRl	2.00
	RRu	5.58
	P	+++
Random	RR	4.32
	RRl	1.57
	RRu	11.89
	P	++
Asymm	P	N.S.

Table 1I33 - 3

IESLC - Meta-analysis of Ever/current Smoking, Duration, "Highest vs lowest"
All LC types, Pipes only
Most adjusted

	Sex combined	male	female	Total
N		5		5
NS		5		5
Wt		14.61		14.61
Het Chi		13.47		13.47
Het df		4		4
Het P		**		**
Fixed RR		3.34		3.34
RRl		2.00		2.00
RRu		5.58		5.58
P		+++		+++
Random RR		4.32		4.32
RRl		1.57		1.57
RRu		11.89		11.89
P		++		++
Between Chi				
Between df				
Between P				N.S.
Btwn(F) P				N.S.
Btwn(R) P				N.S.

MALES

Too few RRs for analysis by factor

Table 1I33 - 4

IESLC - Meta-analysis of Ever/current Smoking, Duration, "Highest vs lowest"
All LC types, Pipes only
Least adjusted

REF	NRR	X	SEX	AGEL	AGEH	RACE	YF	LC TYPE	LOC	START	ST	NLC	R	VB	P	H	AD	ADOS	SM	exL	exH	unexL	unexH	De
BOFFET	531	x	m	0	0	all	-	all	Eu:mul	1988	CC	5621	n	bl	y	n	0	0	ev	44	999	0	20	st
CEDERL	509		m	40	69	all	10	all	Eu:Sca	1963	pr	491	n	bl	n	n	1	0	cu	30	999	1	29	ot
DAMBER	519		m	0	0	all	-	all	Eu:Sca	1972	CC	579	n	bl	y	n	1	0	ev	51	999	1	30	ot
LUBIN	557	x	m	0	0	all	-	all	As:Chi	1984	CC	427	m	ot	y	n	0	0	ev	50	999	1	29	st
LUBIN2	1051	x	m	0	0	all	-	all	Eu:mul	1976	CC	7804	n	bl	n	y	0	0	ev	40	999	1	19	st

Table 1I33 - 5

IESLC - Meta-analysis of Ever/current Smoking, Duration, "Highest vs lowest"
All LC types, Pipes only
Least adjusted

REF	NRR	SEX	AD	Number Exposed Case	Cont	Non-exposed Case	Cont	RR	95.00%CI
BOFFET	531	m	0	30	30	3	33	11.00	(3.04- 39.79)
*CEDERL	509	m	1	26	-	1	-	25.00	(3.25- 192.28)
DAMBER	519	m	1	-	-	-	-	2.38	(1.09- 5.20)
LUBIN	557	m	0	10	30	7	21	1.00	(0.33- 3.05)
LUBIN2	1051	m	0	22	64	3	36	4.13	(1.15- 14.74)
Partial Totals				88	124	14	90		

*prospective study

REF	NRR	SEX	AD	Ys	Ws	Qs	Ps
BOFFET	531	m	0	2.40	2.32	3.58	0.0003
*CEDERL	509	m	1	3.22	0.92	3.92	0.0020
DAMBER	519	m	1	0.87	6.29	0.53	0.0296
LUBIN	557	m	0	0.00	3.09	4.14	1.0000
LUBIN2	1051	m	0	1.42	2.37	0.16	0.0292

	N	5
	NS	5
	Wt	15.00
Het	Chi	12.33
Het	df	4
Het	P	*
Fixed	RR	3.18
	RRl	1.92
	RRu	5.28
	P	+++
Random	RR	4.03
	RRl	1.55
	RRu	10.44
	P	++
Asymm	P	N.S.

Table 1I33 - 6

IESLC - Meta-analysis of Ever/current Smoking, Duration, "Highest vs lowest"
All LC types, Pipes only
Least adjusted

	Sex combined male	female	Total
N	5		5
NS	5		5
Wt	15.00		15.00
Het Chi	12.33		12.33
Het df	4		4
Het P	*		*
Fixed RR	3.18		3.18
RRl	1.92		1.92
RRu	5.28		5.28
P	+++		+++
Random RR	4.03		4.03
RRl	1.55		1.55
RRu	10.44		10.44
P	++		++
Between Chi			
Between df			
Between P			N.S.
Btwn(F) P			N.S.
Btwn(R) P			N.S.

Table 1I33 - 7

IESLC - Meta-analysis of Ever/current Smoking, Duration, "Highest vs lowest"
All LC types, Pipes only
Excluded studies (and stage at which they were excluded)

1	BECHER BLOT1 BROWN3 CARPEN CHYOU DARBY DOLL2 GARCIA GRAHAM GURSEL HAMMO2 JAHN JAIN LAUSSM PRESCO QIAO TVERDA WIGLE WYNDE3
2	ALDERS BENSHL BRESLO CHIAZZ DEAN3 DORN ENGELA GAO2 GILLIS GUO HEGMAN HIRAYA HOLE KAUFMA KOO KOULUM LIU4 MIGRAN MRFITR PERNU SEGI2 SPEIZE SUZUK2 SVENSS VUTUC WAKAI WU YUAN
3	GENG MCDUFF SPITZ STASZE WU2 ZHANG
4	AKIBA GARSHI
5	BOUCHA CHEN CORREA JEDRYC LUO WYNDE2 WYNDE6
6	HAMMON PISANI RESTRE SADOWS XU
8	AGUDO AMANDU AMES ARMADA AUVINE AXELSS BARBON BEST BOUCOT BROSS BUFFLE CHEN2 CHOI CPSI CPSII DEAN2 DESTEF DOLL DORGAN DOSEME FAN GAO GER HAENSZ HU HU2 HUMBLE JOLY JUSSAW KAISE2 KATSOU KHUDER KREUZE LETOUR LEVIN LIAW LIU3 LIU5 MATOS MCCONN NOTAN2 OSANN2 PEZZO2 PEZZOT QIAO2 RACHTA SOBUE TIZZAN WANG2 WATSON WUWILL WYNDE7 WYNDE8 ZHENG ZHOU
14	BENHAM

Table 1I33 - 8
Potentially overlapping studies

REF	REFGP	PRINC	.	OVERLAP/LINK
LUBIN2	LUBIN2	1		Lubin-combined
BOFFET	BOFFET	1		BOFFET-combined
LUBIN	XIANGZ	2		LUBIN/XIANGZ/QIAO

Table 1I33 - 9
Most adjusted - insufficient data for meta-analysis

REF	NRR	SEX	AGEL	AGEH	RACE	YF	LC TYPE	LOC	START	ST	NLC	R	VB	P	H	AD	ADOS	SM	exL	exH	unexL	unexH	De
SADOWS	610	m	0	0	wh	-	all	NAmer	1938	CC	477	n	bl	n	n	0	0	ev	50	999	1	9	ot

REF	NRR	RR	SIG	RRDATA comment
SADOWS	610	0.00		0

Table 1I34 -

IESLC - Meta-analysis of Ever/current Smoking by Duration, Overview
All LC types, Cigars only

This analysis is restricted to results for:
1) Ever/current smokers
2) Results by Duration
3) Categorical results by Duration
   Results by Duration are grouped under 2 schemes (S1, S2). Each scheme has a set of "key values". An interval is allocated to the category whose key value it includes, and intervals which include none or more than one of the key values are excluded. (Open-ended intervals are coded as 999)

S1	key value	maximum range
1	20	1-34
2	35	21-49
3	50	36+

S2	key value	maximum range
1	5	1-19
2	20	6-29
3	30	21-39
4	40	31-49
5	50	41-998
6	999	51+

4) All LC types (or near equivalent)
5) Results complete enough for use in metaanalysis
6) cigars only

Within each study, results are then selected (in the following order of preference, within each sex) for:
7) (not applicable)
8) DENOM: never smoked anything, never any + low
9) Followup period (YF, prospective studies): whole study (coded as 0) or longest available
10) LCtype: all or nearest available, at least Squamous and Adeno. (q = squamous, s = small,
          l = large, a = adeno, mix = mixed, alv = alveolar)
11) Race: all or nearest available, otherwise by race (wh or w = white, bl or b = black, hi = hispanic
          ch = chinese, jap = japanese, haw = hawaiian, w+o = white + oriental, sca = scandinavian, as = asian)
12) For overlapping studies: principal rather than subsidiary studies
Finally by Age: whole study (coded as 0) if available, otherwise by widest available age group
and then for single sex results (m, f) in preference to results for both sexes combined (c).

Results adjusted (AD) for the most potential confounders are then chosen in Sections -1 to -3
and results adjusted for the least confounders in Sections -4 to -6. (Those least adjusted results which
actually differ from the most adjusted are marked 'x' in column X in Section -4)

Section -7 shows excluded studies, together with the stage (as above) at which no qualifying
results were found.

Section -8 lists the potentially overlapping studies which have been included (1=principal, 2=subsidiary).

Section -9 lists any results which would have been included in preference except that they had data not complete
enough for use in meta-analysis, with their significance (yes/no), if known, and any further comment as entered
on the database. It also lists as "gap" any categories for which no data were presented by the original authors.

In addition to those mentioned above, the following fields, levels and abbreviations are used:
* or nk = not known, n = no, y = yes, ot = other
ev = ever, cu = current, nev = never
all/unspec = all or unspecified, MC = manufactured cigarettes, HR = hand-rolled cigarettes
exL, exH = range of exposure (low and high) in the smoking group, in terms of Duration
REF: 6-character study reference
NRR: number of the RR on the database within the study
ST : study type (CC = case control, pr or prosp = prospective)
NLC: number of lung cancer cases in whole study
R : risky occupational population (n = no, m = mining, o = other risky)
VB : national cigarette type (V = at least 75% Virginia, bl = at least 75% blended, ot = other)
P : any proxy use
H : full histological confirmation
De : derivation of RR/CI (or = original, st = standard method, ot = other method of estimation)

Table 1I34 - 1

IESLC - Meta-analysis of Ever/current Smoking by Duration, Overview
All LC types, Cigars only
Most adjusted

REF	NRR	SEX	AGEL	AGEH	RACE	YF	LC TYPE	LOC	START	ST	NLC	R	VB	P	H	AD	SM	exL	exH	S1	S2	DENOM	De
BOFFET	508	m	0	0	all	-	all	Eu:mul	1988	CC	5621	n	bl	y	n	2	ev	0	13	0	1	nev any	or
BOFFET	509	m	0	0	all	-	all	Eu:mul	1988	CC	5621	n	bl	y	n	2	ev	13	26	1	2	nev any	or
BOFFET	510	m	0	0	all	-	all	Eu:mul	1988	CC	5621	n	bl	y	n	2	ev	26	39	2	3	nev any	or
BOFFET	511	m	0	0	all	-	all	Eu:mul	1988	CC	5621	n	bl	y	n	2	ev	39	999	3	0	nev any	or
JOLY	667	m	0	0	all	-	all	SCAmer	1978	CC	826	n	bl	n	n	0	ev	1	39	0	0	nev any	st
JOLY	668	m	0	0	all	-	all	SCAmer	1978	CC	826	n	bl	n	n	0	ev	40	49	0	4	nev any	st
JOLY	669	m	0	0	all	-	all	SCAmer	1978	CC	826	n	bl	n	n	0	ev	50	999	3	0	nev any	st
LUBIN2	1172	m	0	0	all	-	all	Eu:mul	1976	CC	7804	n	bl	n	y	1	ev	1	19	0	1	nev any	ot
LUBIN2	1173	m	0	0	all	-	all	Eu:mul	1976	CC	7804	n	bl	n	y	1	ev	20	29	1	2	nev any	ot
LUBIN2	1174	m	0	0	all	-	all	Eu:mul	1976	CC	7804	n	bl	n	y	1	ev	30	39	2	3	nev any	ot
LUBIN2	1175	m	0	0	all	-	all	Eu:mul	1976	CC	7804	n	bl	n	y	1	ev	40	999	3	0	nev any	ot

In this overview table, subtotals and Qs values may be invalid and should be ignored

Table 1I34 - 2

IESLC - Meta-analysis of Ever/current Smoking by Duration, Overview
All LC types, Cigars only
Most adjusted

REF	NRR	SEX	AD	Number Case	Exposed Cont	Non-exposed Case	Cont	RR	95.00%CI
BOFFET	508	m	2	4	-	117	-	3.10	(1.00- 9.40)
BOFFET	509	m	2	5	-	117	-	4.30	(1.60- 11.90)
BOFFET	510	m	2	12	-	117	-	10.30	(4.70- 22.70)
BOFFET	511	m	2	22	-	117	-	20.70	(10.50- 41.10)
Subtotal BOFFET								9.71	(6.35- 14.85)
JOLY	667	m	0	8	49	12	218	2.97	(1.15- 7.64)
JOLY	668	m	0	9	54	12	218	3.03	(1.21- 7.55)
JOLY	669	m	0	26	76	12	218	6.21	(2.99- 12.92)
Subtotal JOLY								4.15	(2.55- 6.77)
LUBIN2	1172	m	1	5	-	190	-	2.44	(0.94- 6.36)
LUBIN2	1173	m	1	10	-	190	-	4.23	(2.03- 8.81)
LUBIN2	1174	m	1	8	-	190	-	2.37	(1.09- 5.17)
LUBIN2	1175	m	1	14	-	190	-	3.01	(1.63- 5.54)
Subtotal LUBIN2								3.01	(2.08- 4.37)
Partial Totals				123	179	1264	654		

*prospective study

REF	NRR	SEX	AD	Ys	Ws	Qs	Ps
BOFFET	508	m	2	1.13	3.06	0.57	0.0478
BOFFET	509	m	2	1.46	3.82	0.04	0.0044
BOFFET	510	m	2	2.33	6.20	3.66	0.0000
BOFFET	511	m	2	3.03	8.25	17.74	0.0000
Subtotal BOFFET				2.27	21.32	22.01	
JOLY	667	m	0	1.09	4.29	0.97	0.0244
JOLY	668	m	0	1.11	4.60	0.96	0.0175
JOLY	669	m	0	1.83	7.17	0.50	0.0000
Subtotal JOLY				1.42	16.05	2.43	
LUBIN2	1172	m	1	0.89	4.20	1.90	0.0674
LUBIN2	1173	m	1	1.44	7.13	0.11	0.0001
LUBIN2	1174	m	1	0.86	6.34	3.12	0.0298
LUBIN2	1175	m	1	1.10	10.27	2.19	0.0004
Subtotal LUBIN2				1.10	27.94	7.31	

N	11
NS	3

Table 1I34 - 3

IESLC - Meta-analysis of Ever/current Smoking by Duration, Overview
All LC types, Cigars only
Most adjusted

	Sex combined	male	female	Total
N		11		11
NS		3		3

In this overview table, other than the "N" rows, entries in the "absent" and "Total" columns may be invalid and should be ignored

MALES

	Duration of smoking (broad categories) absent	1-34k20	21-49k35	36+k50	Total
N	4	2	2	3	11
NS	3	2	2	3	10
Wt	16.15	10.95	12.54	25.68	65.32
Het Chi	0.15	0.00	6.76	17.10	31.75
Het df	3	1	1	2	10
Het P	N.S.	N.S.	**	***	***
Fixed RR	2.86	4.25	4.90	6.85	4.78
RRl	1.76	2.35	2.82	4.65	3.75
RRu	4.66	7.69	8.52	10.08	6.09
P	+++	+++	+++	+++	+++
Random RR	2.86	4.25	4.93	7.24	4.53
RRl	1.76	2.35	1.17	2.32	2.92
RRu	4.66	7.69	20.82	22.60	7.03
P	+++	+++	+	+++	+++

	Duration of smoking (narrow categories) absent	1-19k1	6-29k20	21-39k30	31-49k40	41-998k50	51+k999	Total
N	4	2	2	2	1			11
NS	3	2	2	2	1			10
Wt	29.97	7.26	10.95	12.54	4.60			65.32
Het Chi	19.67	0.10	0.00	6.76	0.00			31.75
Het df	3	1	1	1	0			10
Het P	***	N.S.	N.S.	**	N.S.			***
Fixed RR	6.07	2.70	4.25	4.90	3.03			4.78
RRl	4.25	1.30	2.35	2.82	1.21			3.75
RRu	8.69	5.58	7.69	8.52	7.55			6.09
P	+++	++	+++	+++	+			+++
Random RR	5.91	2.70	4.25	4.93	3.03			4.53
RRl	2.32	1.30	2.35	1.17	1.21			2.92
RRu	15.03	5.58	7.69	20.82	7.55			7.03
P	+++	++	+++	+	+			+++

Table 1I34 - 4

IESLC - Meta-analysis of Ever/current Smoking by Duration, Overview
All LC types, Cigars only
Least adjusted

REF	NRR	X	SEX	AGEL	AGEH	RACE	YF	LC TYPE	LOC	START	ST	NLC	R	VB	P	H	AD	SM	exL	exH	S1	S2	DENOM	De
BOFFET	501	x	m	0	0	all	-	all	Eu:mul	1988	CC	5621	n	bl	y	n	0	ev	0	13	0	1	nev any	st
BOFFET	502	x	m	0	0	all	-	all	Eu:mul	1988	CC	5621	n	bl	y	n	0	ev	13	26	1	2	nev any	st
BOFFET	503	x	m	0	0	all	-	all	Eu:mul	1988	CC	5621	n	bl	y	n	0	ev	26	39	2	3	nev any	st
BOFFET	504	x	m	0	0	all	-	all	Eu:mul	1988	CC	5621	n	bl	y	n	0	ev	39	999	3	0	nev any	st
JOLY	667		m	0	0	all	-	all	SCAmer	1978	CC	826	n	bl	n	n	0	ev	1	39	0	0	nev any	st
JOLY	668		m	0	0	all	-	all	SCAmer	1978	CC	826	n	bl	n	n	0	ev	40	49	0	4	nev any	st
JOLY	669		m	0	0	all	-	all	SCAmer	1978	CC	826	n	bl	n	n	0	ev	50	999	3	0	nev any	st
LUBIN2	1052	x	m	0	0	all	-	all	Eu:mul	1976	CC	7804	n	bl	n	y	0	ev	1	19	0	1	nev any	st
LUBIN2	1053	x	m	0	0	all	-	all	Eu:mul	1976	CC	7804	n	bl	n	y	0	ev	20	29	1	2	nev any	st
LUBIN2	1054	x	m	0	0	all	-	all	Eu:mul	1976	CC	7804	n	bl	n	y	0	ev	30	39	2	3	nev any	st
LUBIN2	1055	x	m	0	0	all	-	all	Eu:mul	1976	CC	7804	n	bl	n	y	0	ev	40	999	3	0	nev any	st

In this overview table, subtotals and Qs values may be invalid and should be ignored

Table 1I34 - 5

IESLC - Meta-analysis of Ever/current Smoking by Duration, Overview
All LC types, Cigars only
Least adjusted

REF	NRR	SEX	AD	Number Exposed Case	Exposed Cont	Non-exposed Case	Non-exposed Cont	RR	95.00%CI
BOFFET	501	m	0	4	21	117	1750	2.85	(0.96- 8.44)
BOFFET	502	m	0	5	20	117	1750	3.74	(1.38- 10.14)
BOFFET	503	m	0	12	17	117	1750	10.56	(4.93- 22.63)
BOFFET	504	m	0	22	19	117	1750	17.32	(9.12- 32.90)
Subtotal BOFFET								9.01	(5.99- 13.55)
JOLY	667	m	0	8	49	12	218	2.97	(1.15- 7.64)
JOLY	668	m	0	9	54	12	218	3.03	(1.21- 7.55)
JOLY	669	m	0	26	76	12	218	6.21	(2.99- 12.92)
Subtotal JOLY								4.15	(2.55- 6.77)
LUBIN2	1052	m	0	5	30	190	2616	2.29	(0.88- 5.98)
LUBIN2	1053	m	0	10	29	190	2616	4.75	(2.28- 9.89)
LUBIN2	1054	m	0	8	36	190	2616	3.06	(1.40- 6.68)
LUBIN2	1055	m	0	14	50	190	2616	3.86	(2.09- 7.10)
Subtotal LUBIN2								3.57	(2.46- 5.17)
Totals				123	401	1264	18118		

*prospective study

REF	NRR	SEX	AD	Ys	Ws	Qs	Ps
BOFFET	501	m	0	1.05	3.26	1.10	0.0587
BOFFET	502	m	0	1.32	3.86	0.37	0.0096
BOFFET	503	m	0	2.36	6.61	3.52	0.0000
BOFFET	504	m	0	2.85	9.33	13.99	0.0000
Subtotal BOFFET				2.20	23.06	18.98	
JOLY	667	m	0	1.09	4.29	1.25	0.0244
JOLY	668	m	0	1.11	4.60	1.24	0.0175
JOLY	669	m	0	1.83	7.17	0.29	0.0000
Subtotal JOLY				1.42	16.05	2.77	
LUBIN2	1052	m	0	0.83	4.18	2.65	0.0893
LUBIN2	1053	m	0	1.56	7.14	0.03	0.0000
LUBIN2	1054	m	0	1.12	6.31	1.63	0.0050
LUBIN2	1055	m	0	1.35	10.30	0.79	0.0000
Subtotal LUBIN2				1.27	27.93	5.12	

N	11
NS	3

Table 1I34 - 6

IESLC - Meta-analysis of Ever/current Smoking by Duration, Overview
All LC types, Cigars only
Least adjusted

	Sex			
	combined	male	female	Total
N		11		11
NS		3		3

In this overview table, other than the "N" rows, entries in the "absent" and "Total" columns may be invalid and should be ignored

MALES

	Duration of smoking (broad categories)				
	absent	1-34k20	21-49k35	36+k50	Total
N	4	2	2	3	11
NS	3	2	2	3	10
Wt	16.33	11.00	12.92	26.80	67.04
Het Chi	0.21	0.14	4.95	11.34	26.87
Het df	3	1	1	2	10
Het P	N.S.	N.S.	*	**	**
Fixed RR	2.77	4.37	5.77	7.39	5.09
RRl	1.71	2.42	3.34	5.06	4.01
RRu	4.50	7.88	9.95	10.79	6.47
P	+++	+++	+++	+++	+++
Random RR	2.77	4.37	5.70	7.45	4.70
RRl	1.71	2.42	1.69	3.01	3.15
RRu	4.50	7.88	19.19	18.47	7.01
P	+++	+++	++	+++	+++

	Duration of smoking (narrow categories)							
	absent	1-19k1	6-29k20	21-39k30	31-49k40	41-998k50	51+k999	Total
N	4	2	2	2	1			11
NS	3	2	2	2	1			10
Wt	31.08	7.44	11.00	12.92	4.60			67.04
Het Chi	14.42	0.09	0.14	4.95	0.00			26.87
Het df	3	1	1	1	0			10
Het P	**	N.S.	N.S.	*	N.S.			**
Fixed RR	6.52	2.52	4.37	5.77	3.03			5.09
RRl	4.58	1.23	2.42	3.34	1.21			4.01
RRu	9.26	5.17	7.88	9.95	7.55			6.47
P	+++	+	+++	+++	+			+++
Random RR	6.09	2.52	4.37	5.70	3.03			4.70
RRl	2.77	1.23	2.42	1.69	1.21			3.15
RRu	13.39	5.17	7.88	19.19	7.55			7.01
P	+++	+	+++	++	+			+++

Table 1I34 - 7

IESLC - Meta-analysis of Ever/current Smoking by Duration, Overview
All LC types, Cigars only
Excluded studies (and stage at which they were excluded)

1 BECHER BLOT1 BROWN3 CARPEN CHYOU DARBY DOLL2 GARCIA GRAHAM GURSEL HAMMO2 JAHN JAIN LAUSSM PRESCO QIAO TVERDA WIGLE WYNDE3

2 ALDERS BENSHL BRESLO CHIAZZ DEAN3 DORN ENGELA GAO2 GILLIS GUO HEGMAN HIRAYA HOLE KAUFMA KOO KOULUM LIU4 MIGRAN MRFITR PERNU SEGI2 SPEIZE SUZUK2 SVENSS VUTUC WAKAI WU YUAN

3 GENG MCDUFF SPITZ STASZE WU2 ZHANG

4 BOUCHA CHEN CORREA JEDRYC LUO WYNDE2 WYNDE6

5 AKIBA HAMMON PISANI RESTRE SADOWS XU

7 AGUDO AMANDU AMES ARMADA AUVINE AXELSS BARBON BEST BOUCOT BROSS BUFFLE CEDERL CHEN2 CHOI CPSI CPSII DAMBER DEAN2 DESTEF DOLL DORGAN DOSEME FAN GAO GARSHI GER HAENSZ HU HU2 HUMBLE JUSSAW KAISE2 KATSOU KHUDER KREUZE LETOUR LEVIN LIAW LIU3 LIU5 LUBIN MATOS MCCONN NOTAN2 OSANN2 PEZZO2 PEZZOT QIAO2 RACHTA SOBUE TIZZAN WANG2 WATSON WUWILL WYNDE7 WYNDE8 ZHENG ZHOU

13 BENHAM

Table 1I34 - 8
Potentially overlapping studies

REF	REFGP	PRINC	.	OVERLAP/LINK
LUBIN2	LUBIN2	1		Lubin-combined
BOFFET	BOFFET	1		BOFFET-combined

Table 1I34 - 9
Most adjusted - insufficient data for meta-analysis

REF	NRR	SEX	AGEL	AGEH	RACE	YF	LC TYPE	LOC	START	ST	NLC	R	VB	P	H	AD	SM	exL	exH	S1	S2	DENOM	De
SADOWS	589	m	0	0	wh	-	all	NAmer	1938	CC	477	n	bl	n	n	0	ev	1	9	0	1	nev any	ot
SADOWS	590	m	0	0	wh	-	all	NAmer	1938	CC	477	n	bl	n	n	0	ev	10	19	0	0	nev any	ot
SADOWS	591	m	0	0	wh	-	all	NAmer	1938	CC	477	n	bl	n	n	0	ev	20	29	1	2	nev any	ot
SADOWS	592	m	0	0	wh	-	all	NAmer	1938	CC	477	n	bl	n	n	0	ev	30	39	2	3	nev any	ot
SADOWS	593	m	0	0	wh	-	all	NAmer	1938	CC	477	n	bl	n	n	0	ev	40	49	0	4	nev any	ot
SADOWS	594	m	0	0	wh	-	all	NAmer	1938	CC	477	n	bl	n	n	0	ev	50	999	3	0	nev any	ot

REF	NRR	RR	SIG	RRDATA comment
SADOWS	589	1.49		0
SADOWS	590	0.65		0
SADOWS	591	4.46		0
SADOWS	592	3.35		0
SADOWS	593	0.00		0
SADOWS	594	4.38		0

Table 1I35 -

IESLC - Meta-analysis of Ever/current Smoking, Duration, "Highest vs lowest"
All LC types, Cigars only

This analysis is restricted to results for:
1) Ever/current smokers
2) Results by Duration
3) Categorical results by Duration
4) Denominator (unexposed) = "low"
5) All LC types (or near equivalent)
6) Results complete enough for use in metaanalysis
7) SMKSTA: ever, current
8) PRODUCT: cigars only

Within each study, results are then selected (in the following order of preference, within each sex) for:
9) Results with least adjustment for other aspects of smoking (ADOS)
10) The highest vs lowest category
11) Followup period (YF, prospective studies): whole study (coded as 0) or longest available
12) LCtype: all or nearest available, at least Squamous and Adeno. (q = squamous, s = small, l = large, a = adeno, mix = mixed, alv = alveolar)
13) Race: all or nearest available, otherwise by race (wh or w = white, bl or b = black, hi = hispanic ch = chinese, jap = japanese, haw = hawaiian, w+o = white + oriental, sca = scandinavian, as = asian)
14) For overlapping studies: principal rather than subsidiary studies
Finally by Age: whole study (coded as 0) if available, otherwise by widest available age group and then for single sex results (m, f) in preference to results for both sexes combined (c).

Results adjusted (AD) for the most potential confounders are then chosen in Sections -1 to -3 and results adjusted for the least confounders in Sections -4 to -6. (Those least adjusted results which actually differ from the most adjusted are marked 'x' in column X in Section -4)

Section -7 shows excluded studies, together with the stage (as above) at which no qualifying results were found.

Section -8 lists the potentially overlapping studies which have been included (1=principal, 2=subsidiary).

Section -9 lists any results which would have been included in preference except that they had data not complete enough for use in meta-analysis, with their significance (yes/no), if known, and any further comment as entered on the database. It also lists as "gap" any categories for which no data were presented by the original authors.

In addition to those mentioned above, the following fields, levels and abbreviations are used:
* or nk = not known, n = no, y = yes, ot = other
all/unspec = all or unspecified, MC = manufactured cigarettes, HR = hand-rolled cigarettes
exL, exH = range of exposure (low and high) in the "highest" group, in terms of Duration
unexL, unexH = range of exposure (low and high) in the "lowest" group, in terms of Duration
REF: 6-character study reference
NRR: number of the RR on the database within the study
ST : study type (CC = case control, pr or prosp = prospective)
NLC: number of lung cancer cases in whole study
R : risky occupational population (n = no, m = mining, o = other risky)
VB : national cigarette type (V = at least 75% Virginia, bl = at least 75% blended, ot = other)
P : any proxy use
H : full histological confirmation
De : derivation of RR/CI (or = original, st = standard method, ot = other method of estimation)

Table 1I35 - 1

IESLC - Meta-analysis of Ever/current Smoking, Duration, "Highest vs lowest"
All LC types, Cigars only
Most adjusted

REF	NRR	SEX	AGEL	AGEH	RACE	YF	LC TYPE	LOC	START	ST	NLC	R	VB	P	H	AD	ADOS	SM	exL	exH	unexL	unexH	De
BOFFET	514	m	0	0	all	-	all	Eu:mul	1988	CC	5621	n	bl	y	n	2	0	ev	39	999	0	13	ot
JOLY	671	m	0	0	all	-	all	SCAmer	1978	CC	826	n	bl	n	n	0	0	ev	50	999	1	39	st
LUBIN2	1178	m	0	0	all	-	all	Eu:mul	1976	CC	7804	n	bl	n	y	1	0	ev	40	999	1	19	ot

Table 1I35 - 2

IESLC - Meta-analysis of Ever/current Smoking, Duration, "Highest vs lowest"
All LC types, Cigars only
Most adjusted

REF	NRR	SEX	AD	Number Exposed Case	Exposed Cont	Non-exposed Case	Non-exposed Cont	RR	95.00%CI
BOFFET	514	m	2	22	-	4	-	6.68	(1.85- 24.14)
JOLY	671	m	0	26	76	8	49	2.10	(0.88- 5.00)
LUBIN2	1178	m	1	14	-	5	-	1.23	(0.40- 3.77)
Partial Totals				62	76	17	49		

*prospective study

REF	NRR	SEX	AD	Ys	Ws	Qs	Ps
BOFFET	514	m	2	1.90	2.33	2.60	0.0038
JOLY	671	m	0	0.74	5.08	0.05	0.0956
LUBIN2	1178	m	1	0.21	3.05	1.23	0.7176

	N	3
	NS	3
	Wt	10.46
Het	Chi	3.89
Het	df	2
Het	P	N.S.
Fixed	RR	2.32
	RRl	1.27
	RRu	4.26
	P	++
Random	RR	2.43
	RRl	1.02
	RRu	5.79
	P	+
Asymm	P	N.S.

Table 1I35 - 3

IESLC - Meta-analysis of Ever/current Smoking, Duration, "Highest vs lowest"
All LC types, Cigars only
Most adjusted

	Sex combined	male	female	Total
N		3		3
NS		3		3
Wt		10.46		10.46
Het Chi		3.89		3.89
Het df		2		2
Het P		N.S.		N.S.
Fixed RR		2.32		2.32
RRl		1.27		1.27
RRu		4.26		4.26
P		++		++
Random RR		2.43		2.43
RRl		1.02		1.02
RRu		5.79		5.79
P		+		+
Between Chi				
Between df				
Between P				N.S.
Btwn(F) P				N.S.
Btwn(R) P				N.S.

MALES

Too few RRs for analysis by factor

Table 1I35 - 4

IESLC - Meta-analysis of Ever/current Smoking, Duration, "Highest vs lowest"
All LC types, Cigars only
Least adjusted

REF	NRR	X	SEX	AGEL	AGEH	RACE	YF	LC TYPE	LOC	START	ST	NLC	R	VB	P	H	AD	ADOS	SM	exL	exH	unexL	unexH	De
BOFFET	507	x	m	0	0	all	-	all	Eu:mul	1988	CC	5621	n	bl	y	n	0	0	ev	39	999	0	13	st
JOLY	671		m	0	0	all	-	all	SCAmer	1978	CC	826	n	bl	n	n	0	0	ev	50	999	1	39	st
LUBIN2	1058	x	m	0	0	all	-	all	Eu:mul	1976	CC	7804	n	bl	n	y	0	0	ev	40	999	1	19	st

Table 1I35 - 5

IESLC - Meta-analysis of Ever/current Smoking, Duration, "Highest vs lowest"
All LC types, Cigars only
Least adjusted

REF	NRR	SEX	AD	Number Case	Exposed Cont	Non-exposed Case	Cont	RR	95.00%CI
BOFFET	507	m	0	22	19	4	21	6.08	(1.77- 20.86)
JOLY	671	m	0	26	76	8	49	2.10	(0.88- 5.00)
LUBIN2	1058	m	0	14	50	5	30	1.68	(0.55- 5.13)
Totals				62	145	17	100		

*prospective study

REF	NRR	SEX	AD	Ys	Ws	Qs	Ps
BOFFET	507	m	0	1.80	2.53	1.94	0.0041
JOLY	671	m	0	0.74	5.08	0.18	0.0956
LUBIN2	1058	m	0	0.52	3.08	0.52	0.3626

	N	3
	NS	3
	Wt	10.68
Het	Chi	2.64
Het	df	2
Het	P	N.S.
Fixed	RR	2.53
	RRl	1.39
	RRu	4.61
	P	++
Random	RR	2.58
	RRl	1.28
	RRu	5.20
	P	++
Asymm	P	N.S.

Table 1I35 - 6

IESLC - Meta-analysis of Ever/current Smoking, Duration, "Highest vs lowest"
All LC types, Cigars only
Least adjusted

	Sex combined	male	female	Total
N		3		3
NS		3		3
Wt		10.68		10.68
Het Chi		2.64		2.64
Het df		2		2
Het P		N.S.		N.S.
Fixed RR		2.53		2.53
RRl		1.39		1.39
RRu		4.61		4.61
P		++		++
Random RR		2.58		2.58
RRl		1.28		1.28
RRu		5.20		5.20
P		++		++
Between Chi				
Between df				
Between P				N.S.
Btwn(F) P				N.S.
Btwn(R) P				N.S.

Table 1I35 - 7

IESLC - Meta-analysis of Ever/current Smoking, Duration, "Highest vs lowest"
All LC types, Cigars only
Excluded studies (and stage at which they were excluded)

1	BECHER BLOT1 BROWN3 CARPEN CHYOU DARBY DOLL2 GARCIA GRAHAM GURSEL HAMMO2 JAHN JAIN LAUSSM PRESCO QIAO TVERDA WIGLE WYNDE3
2	ALDERS BENSHL BRESLO CHIAZZ DEAN3 DORN ENGELA GAO2 GILLIS GUO HEGMAN HIRAYA HOLE KAUFMA KOO KOULUM LIU4 MIGRAN MRFITR PERNU SEGI2 SPEIZE SUZUK2 SVENSS VUTUC WAKAI WU YUAN
3	GENG MCDUFF SPITZ STASZE WU2 ZHANG
4	AKIBA GARSHI
5	BOUCHA CHEN CORREA JEDRYC LUO WYNDE2 WYNDE6
6	HAMMON PISANI RESTRE SADOWS XU
8	AGUDO AMANDU AMES ARMADA AUVINE AXELSS BARBON BEST BOUCOT BROSS BUFFLE CEDERL CHEN2 CHOI CPSI CPSII DAMBER DEAN2 DESTEF DOLL DORGAN DOSEME FAN GAO GER HAENSZ HU HU2 HUMBLE JUSSAW KAISE2 KATSOU KHUDER KREUZE LETOUR LEVIN LIAW LIU3 LIU5 LUBIN MATOS MCCONN NOTAN2 OSANN2 PEZZO2 PEZZOT QIAO2 RACHTA SOBUE TIZZAN WANG2 WATSON WUWILL WYNDE7 WYNDE8 ZHENG ZHOU
14	BENHAM

Table 1I35 - 8
Potentially overlapping studies

REF	REFGP	PRINC	.	OVERLAP/LINK
LUBIN2	LUBIN2	1		Lubin-combined
BOFFET	BOFFET	1		BOFFET-combined

Table 1I35 - 9
Most adjusted - insufficient data for meta-analysis

REF	NRR	SEX	AGEL	AGEH	RACE	YF	LC TYPE	LOC	START	ST	NLC	R	VB	P	H	AD	ADOS	SM	exL	exH	unexL	unexH	De
SADOWS	599	m	0	0	wh	-	all	NAmer	1938	CC	477	n	bl	n	n	0	0	ev	50	999	1	9	ot

REF	NRR	RR	SIG	RRDATA comment
SADOWS	599	2.95		0

Table 1I36 -

IESLC - Meta-analysis of Ever/current Smoking by Duration, Overview
All LC types, Mixed smokers

This analysis is restricted to results for:
1) Ever/current smokers
2) Results by Duration
3) Categorical results by Duration
Results by Duration are grouped under 2 schemes (S1, S2). Each scheme has a set of "key values". An interval is allocated to the category whose key value it includes, and intervals which include none or more than one of the key values are excluded. (Open-ended intervals are coded as 999)

S1	key value	maximum range
1	20	1-34
2	35	21-49
3	50	36+

S2	key value	maximum range
1	5	1-19
2	20	6-29
3	30	21-39
4	40	31-49
5	50	41-998
6	999	51+

4) All LC types (or near equivalent)
5) Results complete enough for use in metaanalysis
6) mixed smokers (cigarettes and pipe/cigar)

Within each study, results are then selected (in the following order of preference, within each sex) for:
7) (not applicable)
8) DENOM: never smoked anything, never any + low
9) Followup period (YF, prospective studies): whole study (coded as 0) or longest available
10) LCtype: all or nearest available, at least Squamous and Adeno. (q = squamous, s = small, l = large, a = adeno, mix = mixed, alv = alveolar)
11) Race: all or nearest available, otherwise by race (wh or w = white, bl or b = black, hi = hispanic ch = chinese, jap = japanese, haw = hawaiian, w+o = white + oriental, sca = scandinavian, as = asian)
12) For overlapping studies: principal rather than subsidiary studies
Finally by Age: whole study (coded as 0) if available, otherwise by widest available age group and then for single sex results (m, f) in preference to results for both sexes combined (c).

Results adjusted (AD) for the most potential confounders are then chosen in Sections -1 to -3 and results adjusted for the least confounders in Sections -4 to -6. (Those least adjusted results which actually differ from the most adjusted are marked 'x' in column X in Section -4)

Section -7 shows excluded studies, together with the stage (as above) at which no qualifying results were found.

Section -8 lists the potentially overlapping studies which have been included (1=principal, 2=subsidiary).

Section -9 lists any results which would have been included in preference except that they had data not complete enough for use in meta-analysis, with their significance (yes/no), if known, and any further comment as entered on the database. It also lists as "gap" any categories for which no data were presented by the original authors.

In addition to those mentioned above, the following fields, levels and abbreviations are used:
* or nk = not known, n = no, y = yes, ot = other
ev = ever, cu = current, nev = never
exL, exH = range of exposure (low and high) in the smoking group, in terms of Duration
REF: 6-character study reference
NRR: number of the RR on the database within the study
ST : study type (CC = case control, pr or prosp = prospective)
NLC: number of lung cancer cases in whole study
R : risky occupational population (n = no, m = mining, o = other risky)
VB : national cigarette type (V = at least 75% Virginia, bl = at least 75% blended, ot = other)
P : any proxy use
H : full histological confirmation
De : derivation of RR/CI (or = original, st = standard method, ot = other method of estimation)

Table 1I36 - 1

IESLC - Meta-analysis of Ever/current Smoking by Duration, Overview
All LC types, Mixed smokers
Most adjusted

REF	NRR	SEX	AGEL	AGEH	RACE	YF	LC TYPE	LOC	START	ST	NLC	R	VB	P	H	AD	SM	exL	exH	S1	S2	DENOM		De
BOUCOT	515	m	0	0	all	9	all	NAmer	1951	pr	121	n	bl	n	n	0	ev	1	39	0	0	nev	any	ot
BOUCOT	516	m	0	0	all	9	all	NAmer	1951	pr	121	n	bl	n	n	0	ev	40	999	3	0	nev	any	ot
BROSS	501	m	0	0	wh	-	all	NAmer	1960	CC	974	n	bl	n	n	0	cu	1	29	1	0	nev	any	st
BROSS	508	m	0	0	wh	-	all	NAmer	1960	CC	974	n	bl	n	n	0	cu	1	29	1	0	nev	any	st
BROSS	502	m	0	0	wh	-	all	NAmer	1960	CC	974	n	bl	n	n	0	cu	30	39	2	3	nev	any	st
BROSS	509	m	0	0	wh	-	all	NAmer	1960	CC	974	n	bl	n	n	0	cu	30	39	2	3	nev	any	st
BROSS	510	m	0	0	wh	-	all	NAmer	1960	CC	974	n	bl	n	n	0	cu	40	49	0	4	nev	any	st
BROSS	503	m	0	0	wh	-	all	NAmer	1960	CC	974	n	bl	n	n	0	cu	40	49	0	4	nev	any	st
BROSS	511	m	0	0	wh	-	all	NAmer	1960	CC	974	n	bl	n	n	0	cu	50	999	3	0	nev	any	st
BROSS	504	m	0	0	wh	-	all	NAmer	1960	CC	974	n	bl	n	n	0	cu	50	999	3	0	nev	any	st
JOLY	672	m	0	0	all	-	all	SCAmer	1978	CC	826	n	bl	n	n	0	ev	1	39	0	0	nev	any	st
JOLY	673	m	0	0	all	-	all	SCAmer	1978	CC	826	n	bl	n	n	0	ev	40	49	0	4	nev	any	st
JOLY	674	m	0	0	all	-	all	SCAmer	1978	CC	826	n	bl	n	n	0	ev	50	999	3	0	nev	any	st

In this overview table, subtotals and Qs values may be invalid and should be ignored

Table 1I36 - 2

IESLC - Meta-analysis of Ever/current Smoking by Duration, Overview
All LC types, Mixed smokers
Most adjusted

REF	NRR	SEX	AD	Number Exposed Case	Cont	Non-exposed Case	Cont	RR	95.00%CI
*BOUCOT	515	m	0	10	533	0	805	31.71~	(1.86- 539.94)
*BOUCOT	516	m	0	8	386	0	805	35.43~	(2.05- 612.24)
Subtotal BOUCOT								33.51	(4.49- 250.01)
BROSS	501	m	0	21	32	38	170	2.94	(1.53- 5.64)
BROSS	508	m	0	5	16	38	170	1.40	(0.48- 4.05)
BROSS	502	m	0	58	31	38	170	8.37	(4.78- 14.66)
BROSS	509	m	0	20	27	38	170	3.31	(1.68- 6.52)
BROSS	503	m	0	70	52	38	170	6.02	(3.64- 9.95)
BROSS	510	m	0	27	24	38	170	5.03	(2.62- 9.67)
BROSS	504	m	0	51	23	38	170	9.92	(5.42- 18.17)
BROSS	511	m	0	13	9	38	170	6.46	(2.58- 16.21)
Subtotal BROSS								5.36	(4.25- 6.75)
JOLY	672	m	0	78	112	12	218	12.65	(6.61- 24.21)
JOLY	673	m	0	42	47	12	218	16.23	(7.94- 33.18)
JOLY	674	m	0	53	51	12	218	18.88	(9.41- 37.90)
Subtotal JOLY								15.54	(10.46- 23.07)
Totals				456	1343	340	3624		

*prospective study ~ With 0.5 adjustment for zero

REF	NRR	SEX	AD	Ys	Ws	Qs	Ps
*BOUCOT	515	m	0	3.46	0.48	1.07	0.0169
*BOUCOT	516	m	0	3.57	0.47	1.22	0.0141
Subtotal BOUCOT				3.51	0.95	2.28	
BROSS	501	m	0	1.08	9.00	7.08	0.0012
BROSS	508	m	0	0.34	3.39	9.00	0.5371
BROSS	502	m	0	2.12	12.24	0.32	0.0000
BROSS	509	m	0	1.20	8.39	4.91	0.0005
BROSS	503	m	0	1.80	15.22	0.43	0.0000
BROSS	510	m	0	1.62	9.02	1.09	0.0000
BROSS	504	m	0	2.29	10.49	1.15	0.0000
BROSS	511	m	0	1.87	4.54	0.04	0.0001
Subtotal BROSS				1.68	72.29	24.02	
JOLY	672	m	0	2.54	9.12	3.01	0.0000
JOLY	673	m	0	2.79	7.52	5.10	0.0000
JOLY	674	m	0	2.94	7.91	7.51	0.0000
Subtotal JOLY				2.74	24.55	15.62	

N	13
NS	3

Table 1I36 - 3

IESLC - Meta-analysis of Ever/current Smoking by Duration, Overview
All LC types, Mixed smokers
Most adjusted

	Sex combined	male	female	Total
N		13		13
NS		3		3

In this overview table, other than the "N" rows, entries in the "absent" and "Total" columns may be invalid and should be ignored

MALES

	Duration of smoking (broad categories) absent	1-34k20	21-49k35	36+k50	Total
N	5	2	2	4	13
NS	3	1	1	3	7
Wt	41.35	12.40	20.63	23.42	97.79
Het Chi	9.68	1.36	4.27	4.28	41.92
Het df	4	1	1	3	12
Het P	*	N.S.	*	N.S.	***
Fixed RR	8.33	2.40	5.74	11.64	7.12
RRl	6.14	1.37	3.73	7.76	5.84
RRu	11.29	4.18	8.84	17.45	8.69
P	+++	++	+++	+++	+++
Random RR	9.00	2.29	5.37	11.62	7.11
RRl	5.36	1.16	2.17	6.89	4.78
RRu	15.13	4.55	13.31	19.60	10.58
P	+++	+	+++	+++	+++

	absent	1-19k1	6-29k20	Duration of smoking (narrow categories) 21-39k30	31-49k40	41-998k50	51+k999	Total
N	8			2	3			13
NS	3			1	2			5
Wt	45.41			20.63	31.75			97.79
Het Chi	29.73			4.27	6.61			41.92
Het df	7			1	2			12
Het P	***			*	*			***
Fixed RR	7.77			5.74	7.24			7.12
RRl	5.81			3.73	5.11			5.84
RRu	10.39			8.84	10.25			8.69
P	+++			+++	+++			+++
Random RR	7.63			5.37	7.68			7.11
RRl	3.91			2.17	4.01			4.78
RRu	14.88			13.31	14.71			10.58
P	+++			+++	+++			+++

Table 1I36 - 4

IESLC - Meta-analysis of Ever/current Smoking by Duration, Overview
All LC types, Mixed smokers
Least adjusted

REF	NRR	X	SEX	AGEL	AGEH	RACE	YF	LC TYPE	LOC	START	ST	NLC	R	VB	P	H	AD	SM	exL	exH	S1	S2	DENOM	De
BOUCOT	515		m	0	0	all	9	all	NAmer	1951	pr	121	n	bl	n	n	0	ev	1	39	0	0	nev any	ot
BOUCOT	516		m	0	0	all	9	all	NAmer	1951	pr	121	n	bl	n	n	0	ev	40	999	3	0	nev any	ot
BROSS	501		m	0	0	wh	-	all	NAmer	1960	CC	974	n	bl	n	n	0	cu	1	29	1	0	nev any	st
BROSS	508		m	0	0	wh	-	all	NAmer	1960	CC	974	n	bl	n	n	0	cu	1	29	1	0	nev any	st
BROSS	502		m	0	0	wh	-	all	NAmer	1960	CC	974	n	bl	n	n	0	cu	30	39	2	3	nev any	st
BROSS	509		m	0	0	wh	-	all	NAmer	1960	CC	974	n	bl	n	n	0	cu	30	39	2	3	nev any	st
BROSS	510		m	0	0	wh	-	all	NAmer	1960	CC	974	n	bl	n	n	0	cu	40	49	0	4	nev any	st
BROSS	503		m	0	0	wh	-	all	NAmer	1960	CC	974	n	bl	n	n	0	cu	40	49	0	4	nev any	st
BROSS	511		m	0	0	wh	-	all	NAmer	1960	CC	974	n	bl	n	n	0	cu	50	999	3	0	nev any	st
BROSS	504		m	0	0	wh	-	all	NAmer	1960	CC	974	n	bl	n	n	0	cu	50	999	3	0	nev any	st
JOLY	672		m	0	0	all	-	all	SCAmer	1978	CC	826	n	bl	n	n	0	ev	1	39	0	0	nev any	st
JOLY	673		m	0	0	all	-	all	SCAmer	1978	CC	826	n	bl	n	n	0	ev	40	49	0	4	nev any	st
JOLY	674		m	0	0	all	-	all	SCAmer	1978	CC	826	n	bl	n	n	0	ev	50	999	3	0	nev any	st

In this overview table, subtotals and Qs values may be invalid and should be ignored

Table 1I36 - 5

IESLC - Meta-analysis of Ever/current Smoking by Duration, Overview
All LC types, Mixed smokers
Least adjusted

REF	NRR	SEX	AD	Number Exposed Case	Cont	Non-exposed Case	Cont	RR	95.00%CI
*BOUCOT	515	m	0	10	533	0	805	31.71~	(1.86- 539.94)
*BOUCOT	516	m	0	8	386	0	805	35.43~	(2.05- 612.24)
Subtotal BOUCOT								33.51	(4.49- 250.01)
BROSS	501	m	0	21	32	38	170	2.94	(1.53- 5.64)
BROSS	508	m	0	5	16	38	170	1.40	(0.48- 4.05)
BROSS	502	m	0	58	31	38	170	8.37	(4.78- 14.66)
BROSS	509	m	0	20	27	38	170	3.31	(1.68- 6.52)
BROSS	503	m	0	70	52	38	170	6.02	(3.64- 9.95)
BROSS	510	m	0	27	24	38	170	5.03	(2.62- 9.67)
BROSS	504	m	0	51	23	38	170	9.92	(5.42- 18.17)
BROSS	511	m	0	13	9	38	170	6.46	(2.58- 16.21)
Subtotal BROSS								5.36	(4.25- 6.75)
JOLY	672	m	0	78	112	12	218	12.65	(6.61- 24.21)
JOLY	673	m	0	42	47	12	218	16.23	(7.94- 33.18)
JOLY	674	m	0	53	51	12	218	18.88	(9.41- 37.90)
Subtotal JOLY								15.54	(10.46- 23.07)
Totals				456	1343	340	3624		

*prospective study ~ With 0.5 adjustment for zero

REF	NRR	SEX	AD	Ys	Ws	Qs	Ps
*BOUCOT	515	m	0	3.46	0.48	1.07	0.0169
*BOUCOT	516	m	0	3.57	0.47	1.22	0.0141
Subtotal BOUCOT				3.51	0.95	2.28	
BROSS	501	m	0	1.08	9.00	7.08	0.0012
BROSS	508	m	0	0.34	3.39	9.00	0.5371
BROSS	502	m	0	2.12	12.24	0.32	0.0000
BROSS	509	m	0	1.20	8.39	4.91	0.0005
BROSS	503	m	0	1.80	15.22	0.43	0.0000
BROSS	510	m	0	1.62	9.02	1.09	0.0000
BROSS	504	m	0	2.29	10.49	1.15	0.0000
BROSS	511	m	0	1.87	4.54	0.04	0.0001
Subtotal BROSS				1.68	72.29	24.02	
JOLY	672	m	0	2.54	9.12	3.01	0.0000
JOLY	673	m	0	2.79	7.52	5.10	0.0000
JOLY	674	m	0	2.94	7.91	7.51	0.0000
Subtotal JOLY				2.74	24.55	15.62	

N	13
NS	3

Table 1I36 - 6

IESLC - Meta-analysis of Ever/current Smoking by Duration, Overview
All LC types, Mixed smokers
Least adjusted

	Sex combined	male	female	Total
N		13		13
NS		3		3

In this overview table, other than the "N" rows, entries in the "absent" and "Total" columns may be invalid and should be ignored

MALES

	Duration of smoking (broad categories) absent	1-34k20	21-49k35	36+k50	Total
N	5	2	2	4	13
NS	3	1	1	3	7
Wt	41.35	12.40	20.63	23.42	97.79
Het Chi	9.68	1.36	4.27	4.28	41.92
Het df	4	1	1	3	12
Het P	*	N.S.	*	N.S.	***
Fixed RR	8.33	2.40	5.74	11.64	7.12
RRl	6.14	1.37	3.73	7.76	5.84
RRu	11.29	4.18	8.84	17.45	8.69
P	+++	++	+++	+++	+++
Random RR	9.00	2.29	5.37	11.62	7.11
RRl	5.36	1.16	2.17	6.89	4.78
RRu	15.13	4.55	13.31	19.60	10.58
P	+++	+	+++	+++	+++

	absent	1-19k1	6-29k20	Duration of smoking (narrow categories) 21-39k30	31-49k40	41-998k50	51+k999	Total
N	8			2	3			13
NS	3			1	2			5
Wt	45.41			20.63	31.75			97.79
Het Chi	29.73			4.27	6.61			41.92
Het df	7			1	2			12
Het P	***			*	*			***
Fixed RR	7.77			5.74	7.24			7.12
RRl	5.81			3.73	5.11			5.84
RRu	10.39			8.84	10.25			8.69
P	+++			+++	+++			+++
Random RR	7.63			5.37	7.68			7.11
RRl	3.91			2.17	4.01			4.78
RRu	14.88			13.31	14.71			10.58
P	+++			+++	+++			+++

Table 1I36 - 7

IESLC - Meta-analysis of Ever/current Smoking by Duration, Overview
All LC types, Mixed smokers
Excluded studies (and stage at which they were excluded)

1 BECHER BLOT1 BROWN3 CARPEN CHYOU DARBY DOLL2 GARCIA GRAHAM GURSEL HAMMO2 JAHN JAIN LAUSSM PRESCO QIAO TVERDA WIGLE WYNDE3

2 ALDERS BENSHL BRESLO CHIAZZ DEAN3 DORN ENGELA GAO2 GILLIS GUO HEGMAN HIRAYA HOLE KAUFMA KOO KOULUM LIU4 MIGRAN MRFITR PERNU SEGI2 SPEIZE SUZUK2 SVENSS VUTUC WAKAI WU YUAN

3 GENG MCDUFF SPITZ STASZE WU2 ZHANG

4 BOUCHA CHEN CORREA JEDRYC LUO WYNDE2 WYNDE6

5 AKIBA HAMMON PISANI RESTRE SADOWS XU

7 AGUDO AMANDU AMES ARMADA AUVINE AXELSS BARBON BENHAM BEST BOFFET BUFFLE CEDERL CHEN2 CHOI CPSI CPSII DAMBER DEAN2 DESTEF DOLL DORGAN DOSEME FAN GAO GARSHI GER HAENSZ HU HU2 HUMBLE JUSSAW KAISE2 KATSOU KHUDER KREUZE LETOUR LEVIN LIAW LIU3 LIU5 LUBIN LUBIN2 MATOS MCCONN NOTAN2 OSANN2 PEZZO2 PEZZOT QIAO2 RACHTA SOBUE TIZZAN WANG2 WATSON WUWILL WYNDE7 WYNDE8 ZHENG ZHOU

Table 1I36 - 8
Potentially overlapping studies

REF	REFGP	PRINC	. OVERLAP/LINK
BROSS	BYERS1	1	GRAHAM/BROSS/BYERS1

Table 1I36 - 9
Most adjusted - insufficient data for meta-analysis

REF	NRR	SEX	AGEL	AGEH	RACE	YF	LC TYPE	LOC	START	ST	NLC	R	VB	P	H	AD	SM	exL	exH	S1	S2	DENOM	De
DAMBER	520	m	0	0	all	-	all	Eu:Sca	1972	CC	579	n	bl	y	n	1	ev	1	30	1	0	nev any	or
DAMBER	521	m	0	0	all	-	all	Eu:Sca	1972	CC	579	n	bl	y	n	1	ev	31	50	0	0	nev any	ot
DAMBER	522	m	0	0	all	-	all	Eu:Sca	1972	CC	579	n	bl	y	n	1	ev	51	999	0	6	nev any	or
SADOWS	554	m	0	0	wh	-	all	NAmer	1938	CC	477	n	bl	n	n	0	ev	1	19	0	1	nev any	ot
SADOWS	555	m	0	0	wh	-	all	NAmer	1938	CC	477	n	bl	n	n	0	ev	20	29	1	2	nev any	ot
SADOWS	556	m	0	0	wh	-	all	NAmer	1938	CC	477	n	bl	n	n	0	ev	30	39	2	3	nev any	ot
SADOWS	557	m	0	0	wh	-	all	NAmer	1938	CC	477	n	bl	n	n	0	ev	40	999	3	0	nev any	ot

REF	NRR	RR	SIG	RRDATA comment
DAMBER	520	2.30		0
DAMBER	521	*	gap	0
DAMBER	522	16.20		0
SADOWS	554	2.95		0
SADOWS	555	3.68		0
SADOWS	556	8.19		0
SADOWS	557	5.86		0

Table 1I37 -

IESLC - Meta-analysis of Ever/current Smoking, Duration, "Highest vs lowest"
All LC types, Mixed smokers

This analysis is restricted to results for:
1) Ever/current smokers
2) Results by Duration
3) Categorical results by Duration
4) Denominator (unexposed) = "low"
5) All LC types (or near equivalent)
6) Results complete enough for use in metaanalysis
7) SMKSTA: ever, current
8) PRODUCT: mixed smokers (cigarettes and pipe/cigar)

Within each study, results are then selected (in the following order of preference, within each sex) for:
9) Results with least adjustment for other aspects of smoking (ADOS)
10) The highest vs lowest category
11) Followup period (YF, prospective studies): whole study (coded as 0) or longest available
12) LCtype: all or nearest available, at least Squamous and Adeno. (q = squamous, s = small,
l = large, a = adeno, mix = mixed, alv = alveolar)
13) Race: all or nearest available, otherwise by race (wh or w = white, bl or b = black, hi = hispanic
ch = chinese, jap = japanese, haw = hawaiian, w+o = white + oriental, sca = scandinavian, as = asian)
14) For overlapping studies: principal rather than subsidiary studies
Finally by Age: whole study (coded as 0) if available, otherwise by widest available age group
and then for single sex results (m, f) in preference to results for both sexes combined (c).

Results adjusted (AD) for the most potential confounders are then chosen in Sections -1 to -3
and results adjusted for the least confounders in Sections -4 to -6. (Those least adjusted results which
actually differ from the most adjusted are marked 'x' in column X in Section -4)

Section -7 shows excluded studies, together with the stage (as above) at which no qualifying
results were found.

Section -8 lists the potentially overlapping studies which have been included (1=principal, 2=subsidiary).

Section -9 lists any results which would have been included in preference except that they had data not complete
enough for use in meta-analysis, with their significance (yes/no), if known, and any further comment as entered
on the database. It also lists as "gap" any categories for which no data were presented by the original authors.

In addition to those mentioned above, the following fields, levels and abbreviations are used:
* or nk = not known, n = no, y = yes, ot = other
exL, exH = range of exposure (low and high) in the "highest" group, in terms of Duration
unexL, unexH = range of exposure (low and high) in the "lowest" group, in terms of Duration
REF: 6-character study reference
NRR: number of the RR on the database within the study
ST : study type (CC = case control, pr or prosp = prospective)
NLC: number of lung cancer cases in whole study
R : risky occupational population (n = no, m = mining, o = other risky)
VB : national cigarette type (V = at least 75% Virginia, bl = at least 75% blended, ot = other)
P : any proxy use
H : full histological confirmation
De : derivation of RR/CI (or = original, st = standard method, ot = other method of estimation)

Table 1I37 - 1

IESLC - Meta-analysis of Ever/current Smoking, Duration, "Highest vs lowest"
All LC types, Mixed smokers
Most adjusted

REF	NRR	SEX	AGEL	AGEH	RACE	YF	LC TYPE	LOC	START	ST	NLC	R	VB	P	H	AD	ADOS	SM	exL	exH	unexL	unexH	De
BOUCOT	517	m	0	0	all	9	all	NAmer	1951	pr	121	n	bl	n	n	0	0	ev	40	999	1	39	st
BROSS	507	m	0	0	wh	-	all	NAmer	1960	CC	974	n	bl	n	n	0	0	cu	50	999	1	29	st
BROSS	514	m	0	0	wh	-	all	NAmer	1960	CC	974	n	bl	n	n	0	0	cu	50	999	1	29	st
JOLY	676	m	0	0	all	-	all	SCAmer	1978	CC	826	n	bl	n	n	0	0	ev	50	999	1	39	st

Table 1I37 - 2

IESLC - Meta-analysis of Ever/current Smoking, Duration, "Highest vs lowest"
All LC types, Mixed smokers
Most adjusted

REF	NRR	SEX	AD	Number Exposed Case	Exposed Cont	Non-exposed Case	Non-exposed Cont	RR	95.00%CI
*BOUCOT	517	m	0	8	386	10	533	1.10	(0.44- 2.77)
BROSS	507	m	0	51	23	21	32	3.38	(1.61- 7.07)
BROSS	514	m	0	13	9	5	16	4.62	(1.24- 17.23)
Subtotal BROSS								3.64	(1.91- 6.93)
JOLY	676	m	0	53	51	78	112	1.49	(0.92- 2.41)
Totals				125	469	114	693		

*prospective study

REF	NRR	SEX	AD	Ys	Ws	Qs	Ps
*BOUCOT	517	m	0	0.10	4.53	1.26	0.8321
BROSS	507	m	0	1.22	7.04	2.45	0.0012
BROSS	514	m	0	1.53	2.22	1.81	0.0226
Subtotal BROSS				1.29	9.26	4.27	
JOLY	676	m	0	0.40	16.60	0.86	0.1029

	N	4
	NS	3
	Wt	30.40
Het	Chi	6.39
Het	df	3
Het	P	(*)
Fixed	RR	1.87
	RRl	1.31
	RRu	2.67
	P	+++
Random	RR	2.05
	RRl	1.14
	RRu	3.66
	P	+
Asymm	P	N.S.

Table 1I37 - 3

IESLC - Meta-analysis of Ever/current Smoking, Duration, "Highest vs lowest"
All LC types, Mixed smokers
Most adjusted

	Sex combined	male	female	Total
N		4		4
NS		3		3
Wt		30.40		30.40
Het Chi		6.39		6.39
Het df		3		3
Het P		(*)		(*)
Fixed RR		1.87		1.87
RRl		1.31		1.31
RRu		2.67		2.67
P		+++		+++
Random RR		2.05		2.05
RRl		1.14		1.14
RRu		3.66		3.66
P		+		+
Between Chi				
Between df				
Between P				N.S.
Btwn(F) P				N.S.
Btwn(R) P				N.S.

MALES

Too few RRs for analysis by factor

Table 1I37 - 4

IESLC - Meta-analysis of Ever/current Smoking, Duration, "Highest vs lowest"
All LC types, Mixed smokers
Least adjusted

REF	NRR	X	SEX	AGEL	AGEH	RACE	YF	LC TYPE	LOC	START	ST	NLC	R	VB	P	H	AD	ADOS	SM	exL	exH	unexL	unexH	De
BOUCOT	517		m	0	0	all	9	all	NAmer	1951	pr	121	n	bl	n	n	0	0	ev	40	999	1	39	st
BROSS	507		m	0	0	wh	-	all	NAmer	1960	CC	974	n	bl	n	n	0	0	cu	50	999	1	29	st
BROSS	514		m	0	0	wh	-	all	NAmer	1960	CC	974	n	bl	n	n	0	0	cu	50	999	1	29	st
JOLY	676		m	0	0	all	-	all	SCAmer	1978	CC	826	n	bl	n	n	0	0	ev	50	999	1	39	st

Table 1I37 - 5

IESLC - Meta-analysis of Ever/current Smoking, Duration, "Highest vs lowest"
All LC types, Mixed smokers
Least adjusted

REF	NRR	SEX	AD	Number Exposed Case	Number Exposed Cont	Non-exposed Case	Non-exposed Cont	RR	95.00%CI
*BOUCOT	517	m	0	8	386	10	533	1.10	(0.44- 2.77)
BROSS	507	m	0	51	23	21	32	3.38	(1.61- 7.07)
BROSS	514	m	0	13	9	5	16	4.62	(1.24- 17.23)
Subtotal BROSS								3.64	(1.91- 6.93)
JOLY	676	m	0	53	51	78	112	1.49	(0.92- 2.41)
Totals				125	469	114	693		

*prospective study

REF	NRR	SEX	AD	Ys	Ws	Qs	Ps
*BOUCOT	517	m	0	0.10	4.53	1.26	0.8321
BROSS	507	m	0	1.22	7.04	2.45	0.0012
BROSS	514	m	0	1.53	2.22	1.81	0.0226
Subtotal BROSS				1.29	9.26	4.27	
JOLY	676	m	0	0.40	16.60	0.86	0.1029

	N	4
	NS	3
	Wt	30.40
Het	Chi	6.39
Het	df	3
Het	P	(*)
Fixed	RR	1.87
	RRl	1.31
	RRu	2.67
	P	+++
Random	RR	2.05
	RRl	1.14
	RRu	3.66
	P	+
Asymm	P	N.S.

Table 1I37 - 6

IESLC - Meta-analysis of Ever/current Smoking, Duration, "Highest vs lowest"
All LC types, Mixed smokers
Least adjusted

	Sex combined	male	female	Total
N		4		4
NS		3		3
Wt		30.40		30.40
Het Chi		6.39		6.39
Het df		3		3
Het P		(*)		(*)
Fixed RR		1.87		1.87
RRl		1.31		1.31
RRu		2.67		2.67
P		+++		+++
Random RR		2.05		2.05
RRl		1.14		1.14
RRu		3.66		3.66
P		+		+
Between Chi				
Between df				
Between P				N.S.
Btwn(F) P				N.S.
Btwn(R) P				N.S.

Table 1I37 - 7

IESLC - Meta-analysis of Ever/current Smoking, Duration, "Highest vs lowest"
All LC types, Mixed smokers
Excluded studies (and stage at which they were excluded)

1	BECHER BLOT1 BROWN3 CARPEN CHYOU DARBY DOLL2 GARCIA GRAHAM GURSEL HAMMO2 JAHN JAIN LAUSSM PRESCO QIAO TVERDA WIGLE WYNDE3
2	ALDERS BENSHL BRESLO CHIAZZ DEAN3 DORN ENGELA GAO2 GILLIS GUO HEGMAN HIRAYA HOLE KAUFMA KOO KOULUM LIU4 MIGRAN MRFITR PERNU SEGI2 SPEIZE SUZUK2 SVENSS VUTUC WAKAI WU YUAN
3	GENG MCDUFF SPITZ STASZE WU2 ZHANG
4	AKIBA GARSHI
5	BOUCHA CHEN CORREA JEDRYC LUO WYNDE2 WYNDE6
6	HAMMON PISANI RESTRE SADOWS XU
8	AGUDO AMANDU AMES ARMADA AUVINE AXELSS BARBON BENHAM BEST BOFFET BUFFLE CEDERL CHEN2 CHOI CPSI CPSII DAMBER DEAN2 DESTEF DOLL DORGAN DOSEME FAN GAO GER HAENSZ HU HU2 HUMBLE JUSSAW KAISE2 KATSOU KHUDER KREUZE LETOUR LEVIN LIAW LIU3 LIU5 LUBIN LUBIN2 MATOS MCCONN NOTAN2 OSANN2 PEZZO2 PEZZOT QIAO2 RACHTA SOBUE TIZZAN WANG2 WATSON WUWILL WYNDE7 WYNDE8 ZHENG ZHOU

Table 1I37 - 8
Potentially overlapping studies

REF	REFGP	PRINC	.	OVERLAP/LINK
BROSS	BYERS1	1		GRAHAM/BROSS/BYERS1

Table 1I37 - 9
Most adjusted - insufficient data for meta-analysis

REF	NRR	SEX	AGEL	AGEH	RACE	YF	LC TYPE	LOC	START	ST	NLC	R	VB	P	H	AD	ADOS	SM	exL	exH	unexL	unexH	De
SADOWS	560	m	0	0	wh	-	all	NAmer	1938	CC	477	n	bl	n	n	0	0	ev	40	999	1	19	ot

REF	NRR	RR	SIG	RRDATA comment
SADOWS	560	1.99		0
