# Supplementary material for: Systematic review with meta-analysis of the epidemiological evidence in the 1900s relating smoking to lung cancer
Source: BMC Cancer. 2012 Sep 3;12:385. doi: 10.1186/1471-2407-12-385 (PMC3505152; doi:10.1186/1471-2407-12-385)
Supplement: Additional file 5 — Detailed Analysis Tables (Individual file names as described in Additional file 1: Methods, Table1). [file 1471-2407-12-385-S5.zip › PDF/5ABCD.pdf]

Table 5A1 -

IESLC - Meta-analysis of Ever Smoking, Any product (or Cigarettes if Any not available)
Small

This analysis is restricted to results for:
1) Non-dose-response data
2) Ever smokers
3) Results complete enough for use in metaanalysis

Within each study, results are then selected (in the following order of preference, within each sex) for:
4) PRODUCT: all/unspec, cigarettes regardless of other products, cigarettes only
5) CIGTYPE: all/unspecified, MC regardless of HR, MC only
6) DENOM: never smoked anything, never smoked cigarettes, (never +1 = +long term ex, +2 = +amount unknown,
+3 = never cigs+long term ex)
7) Followup period (YF, prospective studies): whole study (coded as 0) or longest available
8) LCtype: small (specifically)

9) Race: all or nearest available, otherwise by race (wh or w = white, bl or b = black, hi = hispanic
ch = chinese, jap = japanese, haw = hawaiian, w+o = white + oriental, sca = scandinavian, as = asian)
10) For overlapping studies: principal rather than subsidiary studies
Finally by Age: whole study (coded as 0) if available, otherwise by widest available age group
and then for single sex results (m, f) in preference to combined sex results (c).

Results adjusted (AD) for the most potential confounders are then chosen in Sections -1 to -3
and results adjusted for the least confounders in Sections -4 to -6. (Those least adjusted results which
actually differ from the most adjusted as marked 'x' in column X in Section -4)
(Results adjusted for an unknown number of confounder(s) are coded as 20.)

Section -7 shows excluded studies, together with the stage (as above) at which no qualifying
results were found.

Section -8 lists the potentially overlapping studies which have been included (1=principal, 2=subsidiary).

Section -9 lists any results which would have been included in preference except that they had data not complete
enough for use in meta-analysis, with their significance (yes/no), if known, and any further comment as entered
on the database.

In addition to those mentioned above, the following fields, levels and abbreviations are used:
* or nk = not known, n = no, y = yes, ot = other
nev = never
all/unspec = all or unspecified, cig+/-ot = cigarettes irrespective of other products (cigar, pipe etc)
MC = manufactured cigarettes, HR = hand-rolled cigarettes
REF: 6-character study reference
NRR: number of the RR on the database within the study
ST : study type (CC = case control, pr or prosp = prospective)
NLC: number of lung cancer cases in whole study
R : risky occupational population (n = no, m = mining, o = other risky)
VB : national cigarette type (V = at least 75% Virginia, bl = at least 75% blended, ot = other)
P : any proxy use
H : full histological confirmation
De : derivation of RR/CI (or = original, st = standard method, ot = other method of estimation)

Table 5A1 - 1

IESLC - Meta-analysis of Ever Smoking, Any product (or Cigarettes if Any not available)
Small
Most adjusted

REF	NRR	SEX	AGEL	AGEH	RACE	YF	LC TYPE	LOC	START	ST	NLC	R	VB	P	H	AD	PRODUCT	DENOM	De
ABRAHA	3	m	0	0	all	0	small	Eu:est	1975	pr	571	n	bl	n	n	0	all/unsp	nev any	ot
ABRAHA	6	f	0	0	all	0	small	Eu:est	1975	pr	571	n	bl	n	n	0	all/unsp	nev any	ot
ALDERS	53	m	0	0	all	-	small	Eu:UK	1977	CC	1448	n	V	n	n	2	all/unsp	nev any	or
ALDERS	56	f	0	0	all	-	small	Eu:UK	1977	CC	1448	n	V	n	n	2	all/unsp	nev any	or
ANDERS	9	f	0	0	all	0	small	NAmer	1986	pr	343	n	bl	n	n	0	cig+/-ot	nev cigs	st
BAND	3	m	0	0	all	-	small	NAmer	1983	CC	2831	n	V	y	y	2	cig only	nev any	ot
BARBON	128	m	0	0	all	-	small	Eu:wst	1979	CC	755	n	bl	y	y	3	all/unsp	nev any	ot
BROWN2	8	m	0	0	wh	-	small	NAmer	1984	CC	14596	n	bl	n	y	2	cig+/-ot	nev cigs	or
BROWN2	7	f	0	0	wh	-	small	NAmer	1984	CC	14596	n	bl	n	y	2	cig+/-ot	nev cigs	or
BUFFLE	51	m	0	0	wh	-	small	NAmer	1976	CC	943	n	bl	y	n	0	cig+/-ot	nev cigs	ot
BUFFLE	66	f	0	0	w-hi	-	small	NAmer	1976	CC	943	n	bl	y	n	0	cig+/-ot	nev cigs	st
BYERS1	2	m	0	0	wh	-	small	NAmer	1957	CC	1002	n	bl	n	n	0	cig+/-ot	nev cigs	st
COMSTO	65	m	0	0	all	-	small	NAmer	1975	ot	258	n	bl	n	n	0	cig+/-ot	nev cigs	st
COMSTO	77	f	0	0	all	-	small	NAmer	1975	ot	258	n	bl	n	n	0	cig+/-ot	nev cigs	st
DAMBER	31	m	0	0	all	-	small	Eu:Sca	1972	CC	579	n	bl	y	n	1	all/unsp	nev any	or
DORGAN	119	m	0	0	wh	-	small	NAmer	1980	CC	2026	n	bl	y	y	2	cig+/-ot	nev any	or
DORGAN	101	f	0	0	all	-	small	NAmer	1980	CC	2026	n	bl	y	y	3	cig+/-ot	nev any	or
DOSEME	2	m	0	0	all	-	small	Eu:bal	1979	CC	1210	n	bl	n	n	2	cig+/-ot	nev cigs	or
ENGELA	90	m	0	0	all	0	small	Eu:Sca	1964	pr	435	n	bl	n	n	7	cig+/-ot	nev cigs	ot
FAN	5	c	0	0	all	-	small	As:Chi	1990	CC	403	n	ot	y	n	0	cig+/-ot	nev cigs	ot
GAO	4	m	0	0	all	-	small	As:Chi	1984	CC	1405	n	ot	n	n	2	cig+/-ot	nev cigs	or
GAO	14	f	0	0	all	-	small	As:Chi	1984	CC	1405	n	ot	n	n	2	cig+/-ot	nev cigs	or
HEGMAN	3	c	0	0	all	-	small	NAmer	1989	CC	282	n	bl	y	y	0	all/unsp	nev any	st
ISHIMA	7	c	0	0	all	-	small	As:Jap	1961	CC	180	n	bl	y	y	5	all/unsp	nev any	st
JAHN	45	m	0	0	all	-	small	Eu:Ger	1988	CC	1004	n	bl	n	n	0	all/unsp	nev any	st
JAIN	49	m	0	0	all	-	small	NAmer	1981	CC	845	n	V	y	n	2	cig+/-ot	nev cigs	or
JAIN	44	f	0	0	all	-	small	NAmer	1981	CC	845	n	V	y	n	2	cig+/-ot	nev cigs	or
JEDRYC	55	m	0	0	all	-	small	Eu:est	1980	CC	1630	n	bl	y	n	3	cig+/-ot	nev any	ot
KHUDER	25	m	0	0	all	-	small	NAmer	1985	CC	482	n	bl	n	y	0	cig+/-ot	nev cigs	ot
KIHARA	27	c	0	0	jap	-	small	As:Jap	1991	CC	440	n	bl	n	n	0	all/unsp	nev any	st
LAMTH	2	f	0	0	ch	-	small	As:HK	1983	CC	445	n	bl	n	n	0	all/unsp	nev any	or
LAMWK	3	f	0	0	ch	-	small	As:HK	1981	CC	163	n	bl	n	n	0	all/unsp	nev any	st
LAMWK2	2	m	0	0	all	-	small	As:HK	1976	CC	480	n	bl	n	n	0	all/unsp	nev any	st
LAMWK2	6	f	0	0	all	-	small	As:HK	1976	CC	480	n	bl	n	n	0	all/unsp	nev any	st
LUBIN2	146	m	0	0	all	-	small	Eu:mul	1976	CC	7804	n	bl	n	y	0	cig+/-ot	nev any	st
LUBIN2	166	f	0	0	all	-	small	Eu:mul	1976	CC	7804	n	bl	n	y	0	cig+/-ot	nev any	st
NOU	2	m	0	0	all	-	small	Eu:Sca	1971	CC	273	n	bl	y	n	0	all/unsp	nev any	st
NOU	7	f	0	0	all	-	small	Eu:Sca	1971	CC	273	n	bl	y	n	0	all/unsp	nev any	st
ORMOS	12	m	0	0	all	-	small	Eu:est	1947	CC	119	n	bl	y	y	0	cig+/-ot	nev any	st
OSANN	45	m	0	0	all	-	small	NAmer	1984	CC	1986	n	bl	n	n	2	cig+/-ot	nev cigs	or
OSANN	46	f	0	0	all	-	small	NAmer	1984	CC	1986	n	bl	n	n	2	cig+/-ot	nev cigs	or
PEZZOT	8	m	0	0	all	-	small	SCAmer	1987	CC	215	n	bl	n	y	0	cig only	nev cigs	ot
SEOW	4	f	0	0	ch	-	small	As:oth	1997	CC	153	n	bl	n	y	0	cig+/-ot	nev cigs	st
SIEMIA	6	m	0	0	all	-	small	NAmer	1979	CC	857	n	V	y	y	7	cig+/-ot	nev cigs	or
SOBUE	101	m	0	0	all	-	small	As:Jap	1986	CC	1376	n	bl	n	y	1	cig+/-ot	nev cigs	ot
SOBUE	111	f	0	0	all	-	small	As:Jap	1986	CC	1376	n	bl	n	y	1	cig+/-ot	nev cigs	ot
STASZE	29	m	0	0	all	-	small	Eu:est	1954	CC	281	n	bl	n	y	0	all/unsp	nev any	st
STASZE	39	f	0	0	all	-	small	Eu:est	1954	CC	281	n	bl	n	y	0	all/unsp	nev any	st
STAYNE	2	m	0	0	all	-	small	NAmer	1969	CC	420	n	bl	n	n	0	all/unsp	nev any	st
SVENSS	73	f	0	0	all	-	small	Eu:Sca	1983	CC	210	n	bl	n	n	1	all/unsp	nev any	ot
TIZZAN	20	c	0	0	all	-	small	Eu:wst	1959	CC	1358	n	bl	n	n	0	all/unsp	nev any	st
WUWILL	10	f	0	0	all	-	small	As:Chi	1985	CC	965	n	ot	n	n	3	cig+/-ot	nev cigs	or
ZHOU	14	m	0	0	all	-	small	As:Chi	1978	CC	1360	n	ot	n	n	0	all/unsp	nev any	st
ZHOU	15	f	0	0	all	-	small	As:Chi	1978	CC	1360	n	ot	n	n	0	all/unsp	nev any	st

Cigarette type is all/unspec for all RRs

Table 5A1 - 2

IESLC - Meta-analysis of Ever Smoking, Any product (or Cigarettes if Any not available)
Small
Most adjusted

REF	NRR	SEX	AD	Number Exposed Case	Number Exposed Cont	Non-exposed Case	Non-exposed Cont	RR	95.00%CI
*ABRAHA	3	m	0	68	10351	2	3365	11.05	(2.71- 45.07)
*ABRAHA	6	f	0	26	5256	5	11589	11.47	(4.41- 29.84)
Subtotal ABRAHA								11.33	(5.14- 24.99)
ALDERS	53	m	2	-	-	-	-	6.99	(1.50- 32.57)
ALDERS	56	f	2	-	-	-	-	7.47	(3.17- 17.63)
Subtotal ALDERS								7.35	(3.48- 15.56)
*ANDERS	9	f	0	76	96164	4	195158	38.56	(14.11- 105.38)
BAND	3	m	2	-	-	-	-	45.79	(14.41- 145.53)
BARBON	128	m	3	-	-	-	-	11.01	(4.81- 25.22)
BROWN2	8	m	2	-	-	-	-	11.40	(9.10- 14.20)
BROWN2	7	f	2	-	-	-	-	37.60	(28.50- 49.30)
Subtotal BROWN2								18.32	(15.41- 21.77)
BUFFLE	51	m	0	-	-	-	-	14.38	(2.81- 73.55)
BUFFLE	66	f	0	61	166	1	112	41.16	(5.62- 301.22)
Subtotal BUFFLE								21.95	(6.21- 77.55)
BYERS1	2	m	0	85	695	4	424	12.96	(4.72- 35.59)
COMSTO	65	m	0	25	229	2	84	4.59	(1.06- 19.78)
COMSTO	77	f	0	27	87	2	115	17.84	(4.13- 77.08)
Subtotal COMSTO								9.04	(3.21- 25.43)
DAMBER	31	m	1	-	-	-	-	13.80	(5.20- 45.60)
DORGAN	119	m	2	-	-	-	-	22.90	(3.20- 166.00)
DORGAN	101	f	3	-	-	-	-	62.60	(23.00- 171.00)
Subtotal DORGAN								50.93	(20.83- 124.56)
DOSEME	2	m	2	-	-	-	-	5.40	(2.90- 10.00)
*ENGELA	90	m	7	-	-	-	-	4.35	(1.19- 15.88)
FAN	5	c	0	40	595	15	556	2.49	(1.36- 4.56)
GAO	4	m	2	-	-	-	-	7.40	(2.30- 24.10)
GAO	14	f	2	-	-	-	-	7.90	(3.60- 17.00)
Subtotal GAO								7.74	(4.05- 14.80)
HEGMAN	3	c	0	50	1202	1	2080	86.52	(11.94- 627.12)
ISHIMA	7	c	5	-	-	-	-	3.00	(0.75- 17.23)
JAHN	45	m	0	166	701	1	138	32.68	(4.54- 235.34)
JAIN	49	m	2	-	-	-	-	6.33	(2.16- 27.00)
JAIN	44	f	2	-	-	-	-	48.00	(10.50- 849.00)
Subtotal JAIN								10.47	(3.50- 31.29)
JEDRYC	55	m	3	-	-	-	-	10.81	(3.36- 34.80)
KHUDER	25	m	0	74	-	1	-	27.02	(3.66- 199.46)
KIHARA	27	c	0	56	232	9	237	6.36	(3.07- 13.15)
LAMTH	2	f	0	42	14	9	36	12.00	(4.65- 30.98)
LAMWK	3	f	0	29	41	3	144	33.95	(9.84- 117.12)
LAMWK2	2	m	0	39	161	1	43	10.42	(1.39- 77.99)
LAMWK2	6	f	0	12	50	4	139	8.34	(2.57- 27.06)
Subtotal LAMWK2								8.83	(3.20- 24.38)
LUBIN2	146	m	0	1129	10433	34	2616	8.33	(5.90- 11.74)
LUBIN2	166	f	0	145	567	55	1180	5.49	(3.96- 7.60)
Subtotal LUBIN2								6.68	(5.28- 8.47)
NOU	2	m	0	42	247	1	122	20.74	(2.82- 152.52)
NOU	7	f	0	5	92	2	261	7.09	(1.35- 37.19)
Subtotal NOU								10.99	(3.07- 39.32)
ORMOS	12	m	0	41	1034	2	777	15.40	(3.71- 63.88)
OSANN	45	m	2	-	-	-	-	37.50	(13.90- 102.00)
OSANN	46	f	2	-	-	-	-	86.00	(31.60- 234.00)
Subtotal OSANN								56.68	(27.97- 114.86)
PEZZOT	8	m	0	36	317	0	116	26.79~	(1.63- 439.95)
SEOW	4	f	0	13	15	2	125	54.17	(11.13- 263.53)
SIEMIA	6	m	7	-	-	-	-	15.50	(3.70- 64.50)
SOBUE	101	m	1	-	-	-	-	21.15	(5.22- 85.73)
SOBUE	111	f	1	-	-	-	-	10.77	(5.63- 20.57)
Subtotal SOBUE								12.13	(6.74- 21.84)
STASZE	29	m	0	29	754	3	158	2.03	(0.61- 6.73)
STASZE	39	f	0	1	153	4	1660	2.71	(0.30- 24.42)
Subtotal STASZE								2.17	(0.76- 6.21)
STAYNE	2	m	0	41	567	4	333	6.02	(2.14- 16.96)
SVENSS	73	f	1	-	-	-	-	34.32	(6.13- 192.17)
TIZZAN	20	c	0	101	939	18	419	2.50	(1.50- 4.19)
WUWILL	10	f	3	-	-	-	-	2.20	(1.40- 3.20)
ZHOU	14	m	0	74	41	17	36	3.82	(1.91- 7.63)
ZHOU	15	f	0	9	7	28	32	1.47	(0.48- 4.46)
Subtotal ZHOU								2.93	(1.63- 5.26)
Partial Totals				2542	131110	234	222055		

*prospective study ~ With 0.5 adjustment for zero

Table 5A1 - 2

IESLC - Meta-analysis of Ever Smoking, Any product (or Cigarettes if Any not available)
Small
Most adjusted

REF	NRR	SEX	AD	Ys	Ws	Qs	Ps
*ABRAHA	3	m	0	2.40	1.94	0.02	0.0008
*ABRAHA	6	f	0	2.44	4.20	0.09	0.0000
Subtotal ABRAHA				2.43	6.14	0.12	
ALDERS	53	m	2	1.94	1.62	0.19	0.0133
ALDERS	56	f	2	2.01	5.22	0.41	0.0000
Subtotal ALDERS				2.00	6.84	0.60	
*ANDERS	9	f	0	3.65	3.80	7.05	0.0000
BAND	3	m	2	3.82	2.87	6.76	0.0000
BARBON	128	m	3	2.40	5.60	0.07	0.0000
BROWN2	8	m	2	2.43	77.61	1.60	0.0000
BROWN2	7	f	2	3.63	51.16	91.43	0.0000
Subtotal BROWN2				2.91	128.77	93.02	
BUFFLE	51	m	0	2.67	1.44	0.20	0.0014
BUFFLE	66	f	0	3.72	0.97	1.97	0.0003
Subtotal BUFFLE				3.09	2.41	2.18	
BYERS1	2	m	0	2.56	3.77	0.28	0.0000
COMSTO	65	m	0	1.52	1.80	1.06	0.0412
COMSTO	77	f	0	2.88	1.79	0.63	0.0001
Subtotal COMSTO				2.20	3.59	1.69	
DAMBER	31	m	1	2.62	3.26	0.36	0.0000
DORGAN	119	m	2	3.13	0.99	0.70	0.0019
DORGAN	101	f	3	4.14	3.82	13.02	0.0000
Subtotal DORGAN				3.93	4.80	13.71	
DOSEME	2	m	2	1.69	10.03	3.66	0.0000
*ENGELA	90	m	7	1.47	2.29	1.54	0.0261
FAN	5	c	0	0.91	10.51	19.93	0.0031
GAO	4	m	2	2.00	2.78	0.23	0.0008
GAO	14	f	2	2.07	6.38	0.32	0.0000
Subtotal GAO				2.05	9.16	0.55	
HEGMAN	3	c	0	4.46	0.98	4.61	0.0000
ISHIMA	7	c	5	1.10	1.56	2.22	0.1695
JAHN	45	m	0	3.49	0.99	1.41	0.0005
JAIN	49	m	2	1.85	2.41	0.48	0.0042
JAIN	44	f	2	3.87	0.80	1.99	0.0006
Subtotal JAIN				2.35	3.21	2.47	
JEDRYC	55	m	3	2.38	2.81	0.02	0.0001
KHUDER	25	m	0	3.30	0.96	0.97	0.0012
KIHARA	27	c	0	1.85	7.27	1.41	0.0000
LAMTH	2	f	0	2.48	4.27	0.16	0.0000
LAMWK	3	f	0	3.52	2.51	3.82	0.0000
LAMWK2	2	m	0	2.34	0.95	0.00	0.0225
LAMWK2	6	f	0	2.12	2.77	0.08	0.0004
Subtotal LAMWK2				2.18	3.72	0.08	
LUBIN2	146	m	0	2.12	32.49	0.95	0.0000
LUBIN2	166	f	0	1.70	36.11	12.48	0.0000
Subtotal LUBIN2				1.90	68.61	13.43	
NOU	2	m	0	3.03	0.97	0.53	0.0029
NOU	7	f	0	1.96	1.40	0.15	0.0205
Subtotal NOU				2.40	2.36	0.68	
ORMOS	12	m	0	2.73	1.90	0.38	0.0002
OSANN	45	m	2	3.62	3.87	6.88	0.0000
OSANN	46	f	2	4.45	3.83	17.95	0.0000
Subtotal OSANN				4.04	7.70	24.84	
PEZZOT	8	m	0	3.29	0.49	0.49	0.0213
SEOW	4	f	0	3.99	1.53	4.44	0.0000
SIEMIA	6	m	7	2.74	1.88	0.38	0.0002
SOBUE	101	m	1	3.05	1.96	1.14	0.0000
SOBUE	111	f	1	2.38	9.15	0.07	0.0000
Subtotal SOBUE				2.50	11.11	1.21	
STASZE	29	m	0	0.71	2.66	6.69	0.2493
STASZE	39	f	0	1.00	0.80	1.33	0.3735
Subtotal STASZE				0.77	3.46	8.01	
STAYNE	2	m	0	1.80	3.58	0.88	0.0007
SVENSS	73	f	1	3.54	1.29	2.01	0.0001
TIZZAN	20	c	0	0.92	14.51	27.33	0.0005
WUWILL	10	f	3	0.79	22.48	50.71	0.0002
ZHOU	14	m	0	1.34	8.03	7.24	0.0001
ZHOU	15	f	0	0.38	3.12	11.31	0.4969
Subtotal ZHOU				1.07	11.15	18.55	

Table 5A1 - 2

IESLC - Meta-analysis of Ever Smoking, Any product (or Cigarettes if Any not available)
Small
Most adjusted

	N	54
	NS	39
	Wt	384.19
Het	Chi	322.05
Het	df	53
Het	P	***
Fixed	RR	9.88
	RRl	8.94
	RRu	10.92
	P	+++
Random	RR	10.98
	RRl	8.25
	RRu	14.61
	P	+++
Asymm	P	N.S.

Table 5A1 - 3

IESLC - Meta-analysis of Ever Smoking, Any product (or Cigarettes if Any not available)
Small
Most adjusted

	Sex			
	combined	male	female	Total
N	5	28	21	54
NS	5	28	21	54
Wt	34.84	181.94	167.41	384.19
Het Chi	15.50	45.10	213.51	322.05
Het df	4	27	20	53
Het P	**	*	***	***
Fixed RR	3.38	9.93	12.28	9.88
RRl	2.43	8.59	10.55	8.94
RRu	4.71	11.48	14.28	10.92
P	+++	+++	+++	+++
Random RR	4.55	9.94	14.31	10.98
RRl	2.14	7.74	8.11	8.25
RRu	9.67	12.75	25.23	14.61
P	+++	+++	+++	+++
Between Chi				47.94
Between df				2
Between P				***
Btwn(F) P				*

	Lung cancer type	
	small	Total
N	54	54
NS	39	39
Wt	384.19	384.19
Het Chi	322.05	322.05
Het df	53	53
Het P	***	***
Fixed RR	9.88	9.88
RRl	8.94	8.94
RRu	10.92	10.92
P	+++	+++
Random RR	10.98	10.98
RRl	8.25	8.25
RRu	14.61	14.61
P	+++	+++
Between Chi		
Between df		
Between P		N.S.
Btwn(F) P		N.S.

	Location								
	NAmer	UK	Scand	othEur	China	Japan	othAs	other	Total
N	19	2	5	12	6	4	5	1	54
NS	13	1	4	9	4	3	4	1	39
Wt	168.33	6.84	9.21	114.04	53.30	19.95	12.03	0.49	384.19
Het Chi	79.69	0.01	4.47	28.60	12.83	4.45	5.40	0.00	322.05
Het df	18	1	4	11	5	3	4	0	53
Het P	***	N.S.	N.S.	**	*	N.S.	N.S.	N.S.	***
Fixed RR	19.61	7.35	11.10	6.14	2.97	8.59	16.42	26.79	9.88
RRl	16.86	3.48	5.82	5.11	2.27	5.54	9.33	1.63	8.94
RRu	22.81	15.56	21.19	7.37	3.89	13.32	28.90	439.95	10.92
P	+++	+++	+++	+++	+++	+++	+++	+	+++
Random RR	23.13	7.35	11.19	6.58	3.33	8.49	17.00	26.79	10.98
RRl	14.94	3.48	5.60	4.57	2.08	4.75	8.65	1.63	8.25
RRu	35.80	15.56	22.34	9.48	5.35	15.17	33.44	439.95	14.61
P	+++	+++	+++	+++	+++	+++	+++	+	+++
Between Chi									186.61
Between df									7
Between P									***
Btwn(F) P									***

Table 5A1 - 3

IESLC - Meta-analysis of Ever Smoking, Any product (or Cigarettes if Any not available)
Small
Most adjusted

	Detailed Country in "other Europe"					
	multi	Germany	othWest	East	Balkans	Total
N	2	1	2	6	1	12
NS	1	1	2	4	1	9
Wt	68.61	0.99	20.11	14.31	10.03	114.04
Het Chi	2.98	0.00	8.86	7.77	0.00	28.60
Het df	1	0	1	5	0	11
Het P	(*)	N.S.	**	N.S.	N.S.	**
Fixed RR	6.68	32.68	3.78	7.84	5.40	6.14
RRl	5.28	4.54	2.44	4.67	2.91	5.11
RRu	8.47	235.34	5.85	13.16	10.03	7.37
P	+++	+++	+++	+++	+++	+++
Random RR	6.73	32.68	5.06	7.63	5.40	6.58
RRl	4.47	4.54	1.19	3.92	2.91	4.57
RRu	10.13	235.34	21.56	14.84	10.03	9.48
P	+++	+++	+	+++	+++	+++
Between Chi						9.00
Between df						4
Between P						(*)
Btwn(F) P						N.S.

	Detailed Country in "other Asia"			
	India	HongKong	other	Total
N		4	1	5
NS		3	1	4
Wt		10.50	1.53	12.03
Het Chi		2.89	0.00	5.40
Het df		3	0	4
Het P		N.S.	N.S.	N.S.
Fixed RR		13.79	54.17	16.42
RRl		7.53	11.13	9.33
RRu		25.26	263.53	28.90
P		+++	+++	+++
Random RR		13.79	54.17	17.00
RRl		7.53	11.13	8.65
RRu		25.26	263.53	33.44
P		+++	+++	+++
Between Chi				2.51
Between df				1
Between P				N.S.
Btwn(F) P				N.S.

	Detailed other continent			
	SCAmer	Auslia	Africa	Total
N	1			1
NS	1			1
Wt	0.49			0.49
Het Chi	0.00			0.00
Het df	0			0
Het P	N.S.			N.S.
Fixed RR	26.79			26.79
RRl	1.63			1.63
RRu	439.95			439.95
P	+			+
Random RR	26.79			26.79
RRl	1.63			1.63
RRu	439.95			439.95
P	+			+
Between Chi				
Between df				
Between P				N.S.
Btwn(F) P				N.S.

Table 5A1 - 3

IESLC - Meta-analysis of Ever Smoking, Any product (or Cigarettes if Any not available)
Small
Most adjusted

	Start year of study <1960	1960-69	1970-79	1980-89	1990+	Total
N	5	3	20	23	3	54
NS	4	3	12	17	3	39
Wt	23.64	7.43	125.59	208.21	19.32	384.19
Het Chi	13.04	0.55	26.68	177.07	14.04	322.05
Het df	4	2	19	22	2	53
Het P	*	N.S.	N.S.	***	***	***
Fixed RR	3.69	4.70	6.98	15.03	4.53	9.88
RRl	2.46	2.29	5.86	13.12	2.90	8.94
RRu	5.52	9.65	8.31	17.22	7.07	10.92
P	+++	+++	+++	+++	+++	+++
Random RR	4.83	4.70	7.39	20.26	7.77	10.98
RRl	1.97	2.29	5.76	12.58	2.06	8.25
RRu	11.86	9.65	9.48	32.63	29.30	14.61
P	+++	+++	+++	+++	++	+++
Between Chi						90.68
Between df						4
Between P						***
Btwn(F) P						**

	Study type (1) CC	other	Total
N	48	6	54
NS	35	4	39
Wt	368.37	15.82	384.19
Het Chi	311.62	9.47	322.05
Het df	47	5	53
Het P	***	(*)	***
Fixed RR	9.78	12.58	9.88
RRl	8.83	7.69	8.94
RRu	10.83	20.59	10.92
P	+++	+++	+++
Random RR	10.95	11.73	10.98
RRl	8.06	5.86	8.25
RRu	14.87	23.47	14.61
P	+++	+++	+++
Between Chi			0.96
Between df			1
Between P			N.S.
Btwn(F) P			N.S.

	Study type (2) CC	prosp	other	Total
N	48	4	2	54
NS	35	3	1	39
Wt	368.37	12.23	3.59	384.19
Het Chi	311.62	7.30	1.66	322.05
Het df	47	3	1	53
Het P	***	(*)	N.S.	***
Fixed RR	9.78	13.86	9.04	9.88
RRl	8.83	7.91	3.21	8.94
RRu	10.83	24.27	25.43	10.92
P	+++	+++	+++	+++
Random RR	10.95	12.81	9.04	10.98
RRl	8.06	5.24	2.39	8.25
RRu	14.87	31.33	34.25	14.61
P	+++	+++	++	+++
Between Chi				1.47
Between df				2
Between P				N.S.
Btwn(F) P				N.S.

Table 5A1 - 3

IESLC - Meta-analysis of Ever Smoking, Any product (or Cigarettes if Any not available)
Small
Most adjusted

	Study size (number of LC cases)				
	100-249	250-499	500-999	1000+	Total
N	6	16	10	22	54
NS	6	12	7	14	39
Wt	9.29	46.80	44.98	283.12	384.19
Het Chi	8.40	40.13	38.53	199.88	322.05
Het df	5	15	9	21	53
Het P	N.S.	***	***	***	***
Fixed RR	20.51	6.73	5.27	11.35	9.88
RRl	10.78	5.05	3.93	10.11	8.94
RRu	39.02	8.96	7.06	12.76	10.92
P	+++	+++	+++	+++	+++
Random RR	20.30	8.30	10.85	11.57	10.98
RRl	8.62	4.98	5.26	7.66	8.25
RRu	47.82	13.82	22.36	17.47	14.61
P	+++	+++	+++	+++	+++
Between Chi					35.11
Between df					3
Between P					***
Btwn(F) P					N.S.

	Risky occupational population			
	no	mining	othRisky	Total
N	54			54
NS	39			39
Wt	384.19			384.19
Het Chi	322.05			322.05
Het df	53			53
Het P	***			***
Fixed RR	9.88			9.88
RRl	8.94			8.94
RRu	10.92			10.92
P	+++			+++
Random RR	10.98			10.98
RRl	8.25			8.25
RRu	14.61			14.61
P	+++			+++
Between Chi				
Between df				
Between P				N.S.
Btwn(F) P				N.S.

	National cigarette tobacco type			
	Virginia	blended	other	Total
N	6	42	6	54
NS	4	31	4	39
Wt	14.80	316.09	53.30	384.19
Het Chi	9.42	210.47	12.83	322.05
Het df	5	41	5	53
Het P	(*)	***	*	***
Fixed RR	12.45	11.96	2.97	9.88
RRl	7.48	10.72	2.27	8.94
RRu	20.72	13.36	3.89	10.92
P	+++	+++	+++	+++
Random RR	13.42	13.00	3.33	10.98
RRl	6.42	9.61	2.08	8.25
RRu	28.05	17.58	5.35	14.61
P	+++	+++	+++	+++
Between Chi				89.33
Between df				2
Between P				***
Btwn(F) P				***

Table 5A1 - 3

IESLC - Meta-analysis of Ever Smoking, Any product (or Cigarettes if Any not available)
Small
Most adjusted

	Any proxy use No/nk	Yes	Total
N	37	17	54
NS	26	13	39
Wt	340.04	44.16	384.19
Het Chi	268.22	53.41	322.05
Het df	36	16	53
Het P	***	***	***
Fixed RR	9.76	10.83	9.88
RRl	8.78	8.07	8.94
RRu	10.85	14.55	10.92
P	+++	+++	+++
Random RR	9.80	14.94	10.98
RRl	7.00	8.36	8.25
RRu	13.71	26.68	14.61
P	+++	+++	+++
Between Chi			0.43
Between df			1
Between P			N.S.
Btwn(F) P			N.S.

	Full histological confirmation No	Yes	Total
N	35	19	54
NS	25	14	39
Wt	149.66	234.53	384.19
Het Chi	140.81	129.59	322.05
Het df	34	18	53
Het P	***	***	***
Fixed RR	6.24	13.24	9.88
RRl	5.32	11.65	8.94
RRu	7.33	15.05	10.92
P	+++	+++	+++
Random RR	9.39	14.62	10.98
RRl	6.60	9.38	8.25
RRu	13.35	22.80	14.61
P	+++	+++	+++
Between Chi			51.65
Between df			1
Between P			***
Btwn(F) P			**

	Number of adjustment variables (1) 0	1	2+/+nk	Total
N	30	4	20	54
NS	22	3	14	39
Wt	158.52	15.67	210.01	384.19
Het Chi	91.81	1.99	183.78	322.05
Het df	29	3	19	53
Het P	***	N.S.	***	***
Fixed RR	6.58	13.58	13.10	9.88
RRl	5.63	8.28	11.45	8.94
RRu	7.69	22.28	15.00	10.92
P	+++	+++	+++	+++
Random RR	8.79	13.58	12.81	10.98
RRl	6.31	8.28	7.75	8.25
RRu	12.23	22.28	21.16	14.61
P	+++	+++	+++	+++
Between Chi				44.47
Between df				2
Between P				***
Btwn(F) P				*

Table 5A1 - 3

IESLC - Meta-analysis of Ever Smoking, Any product (or Cigarettes if Any not available)
Small
Most adjusted

	Number of adjustment variables (2) 0	1	2	3-5	6+/+nk	Total
N	30	4	13	5	2	54
NS	22	3	8	5	2	40
Wt	158.52	15.67	169.57	36.28	4.17	384.19
Het Chi	91.81	1.99	86.82	44.86	1.67	322.05
Het df	29	3	12	4	1	53
Het P	***	N.S.	***	***	N.S.	***
Fixed RR	6.58	13.58	16.61	4.60	7.72	9.88
RRl	5.63	8.28	14.29	3.32	2.95	8.94
RRu	7.69	22.28	19.30	6.37	20.15	10.92
P	+++	+++	+++	+++	+++	+++
Random RR	8.79	13.58	15.85	8.69	7.91	10.98
RRl	6.31	8.28	9.39	2.34	2.28	8.25
RRu	12.23	22.28	26.77	32.30	27.42	14.61
P	+++	+++	+++	++	++	+++
Between Chi						94.91
Between df						4
Between P						***
Btwn(F) P						**

	Product all/unsp	cig+/-ot	cig only	Total
N	23	29	2	54
NS	17	20	2	39
Wt	79.50	301.33	3.36	384.19
Het Chi	55.70	239.17	0.12	322.05
Het df	22	28	1	53
Het P	***	***	N.S.	***
Fixed RR	6.25	10.96	42.35	9.88
RRl	5.02	9.79	14.55	8.94
RRu	7.79	12.28	123.29	10.92
P	+++	+++	+++	+++
Random RR	7.71	13.10	42.35	10.98
RRl	5.27	8.89	14.55	8.25
RRu	11.28	19.31	123.29	14.61
P	+++	+++	+++	+++
Between Chi				27.06
Between df				2
Between P				***
Btwn(F) P				N.S.

	Denominator nev any	nev cigs	Total
N	30	24	54
NS	22	17	39
Wt	160.49	223.70	384.19
Het Chi	91.21	204.33	322.05
Het df	29	23	53
Het P	***	***	***
Fixed RR	7.24	12.34	9.88
RRl	6.21	10.82	8.94
RRu	8.46	14.07	10.92
P	+++	+++	+++
Random RR	9.18	13.35	10.98
RRl	6.66	8.34	8.25
RRu	12.65	21.37	14.61
P	+++	+++	+++
Between Chi			26.51
Between df			1
Between P			***
Btwn(F) P			*

Table 5A1 - 3

IESLC - Meta-analysis of Ever Smoking, Any product (or Cigarettes if Any not available)

Small
Most adjusted

		Derivation of RR/CI			
		Orig	StdCalc	Other	Total
	N	17	24	13	54
	NS	11	18	11	40
	Wt	202.41	136.26	45.53	384.19
Het	Chi	172.83	75.74	32.91	322.05
Het	df	16	23	12	53
Het	P	***	***	**	***
Fixed	RR	13.27	6.58	8.98	9.88
	RRl	11.56	5.56	6.72	8.94
	RRu	15.23	7.78	12.01	10.92
	P	+++	+++	+++	+++
Random	RR	13.77	8.38	11.78	10.98
	RRl	8.01	5.79	6.94	8.25
	RRu	23.67	12.13	20.00	14.61
	P	+++	+++	+++	+++
Between	Chi				40.57
Between	df				2
Between	P				***
Btwn(F)	P				*

Table 5A1 - 4

IESLC - Meta-analysis of Ever Smoking, Any product (or Cigarettes if Any not available)
Small
Least adjusted

REF	NRR	X	SEX	AGEL	AGEH	RACE	YF	LC TYPE	LOC	START	ST	NLC	R	VB	P	H	AD	PRODUCT	DENOM	De
ABRAHA	3		m	0	0	all	0	small	Eu:est	1975	pr	571	n	bl	n	n	0	all/unsp	nev any	ot
ABRAHA	6		f	0	0	all	0	small	Eu:est	1975	pr	571	n	bl	n	n	0	all/unsp	nev any	ot
ALDERS	53		m	0	0	all	-	small	Eu:UK	1977	CC	1448	n	V	n	n	2	all/unsp	nev any	or
ALDERS	56		f	0	0	all	-	small	Eu:UK	1977	CC	1448	n	V	n	n	2	all/unsp	nev any	or
ANDERS	9		f	0	0	all	0	small	NAmer	1986	pr	343	n	bl	n	n	0	cig+/-ot	nev cigs	st
BAND	3		m	0	0	all	-	small	NAmer	1983	CC	2831	n	V	y	y	2	cig only	nev any	ot
BARBON	114	x	m	0	0	all	-	small	Eu:wst	1979	CC	755	n	bl	y	y	0	all/unsp	nev any	st
BROWN2	8		m	0	0	wh	-	small	NAmer	1984	CC	14596	n	bl	n	y	2	cig+/-ot	nev cigs	or
BROWN2	7		f	0	0	wh	-	small	NAmer	1984	CC	14596	n	bl	n	y	2	cig+/-ot	nev cigs	or
BUFFLE	51		m	0	0	wh	-	small	NAmer	1976	CC	943	n	bl	y	n	0	cig+/-ot	nev cigs	ot
BUFFLE	66		f	0	0	w-hi	-	small	NAmer	1976	CC	943	n	bl	y	n	0	cig+/-ot	nev cigs	st
BYERS1	2		m	0	0	wh	-	small	NAmer	1957	CC	1002	n	bl	n	n	0	cig+/-ot	nev cigs	st
COMSTO	65		m	0	0	all	-	small	NAmer	1975	ot	258	n	bl	n	n	0	cig+/-ot	nev cigs	st
COMSTO	77		f	0	0	all	-	small	NAmer	1975	ot	258	n	bl	n	n	0	cig+/-ot	nev cigs	st
DAMBER	10	x	m	0	0	all	-	small	Eu:Sca	1972	CC	579	n	bl	y	n	0	all/unsp	nev any	st
DORGAN	119		m	0	0	wh	-	small	NAmer	1980	CC	2026	n	bl	y	y	2	cig+/-ot	nev any	or
DORGAN	101		f	0	0	all	-	small	NAmer	1980	CC	2026	n	bl	y	y	3	cig+/-ot	nev any	or
DOSEME	18	x	m	0	0	all	-	small	Eu:bal	1979	CC	1210	n	bl	n	n	0	cig+/-ot	nev cigs	st
ENGELA	90		m	0	0	all	0	small	Eu:Sca	1964	pr	435	n	bl	n	n	7	cig+/-ot	nev cigs	ot
FAN	5		c	0	0	all	-	small	As:Chi	1990	CC	403	n	ot	y	n	0	cig+/-ot	nev cigs	ot
GAO	9	x	m	0	0	all	-	small	As:Chi	1984	CC	1405	n	ot	n	n	0	cig+/-ot	nev cigs	st
GAO	19	x	f	0	0	all	-	small	As:Chi	1984	CC	1405	n	ot	n	n	0	cig+/-ot	nev cigs	st
HEGMAN	3		c	0	0	all	-	small	NAmer	1989	CC	282	n	bl	y	y	0	all/unsp	nev any	st
ISHIMA	2	x	c	0	0	all	-	small	As:Jap	1961	CC	180	n	bl	y	y	0	all/unsp	nev any	st
JAHN	45		m	0	0	all	-	small	Eu:Ger	1988	CC	1004	n	bl	n	n	0	all/unsp	nev any	st
JAIN	9	x	m	0	0	all	-	small	NAmer	1981	CC	845	n	V	y	n	0	cig+/-ot	nev cigs	st
JAIN	4	x	f	0	0	all	-	small	NAmer	1981	CC	845	n	V	y	n	0	cig+/-ot	nev cigs	st
JEDRYC	14	x	m	0	0	all	-	small	Eu:est	1980	CC	1630	n	bl	y	n	0	cig+/-ot	nev any	st
KHUDER	25		m	0	0	all	-	small	NAmer	1985	CC	482	n	bl	n	y	0	cig+/-ot	nev cigs	ot
KIHARA	27		c	0	0	jap	-	small	As:Jap	1991	CC	440	n	bl	n	n	0	all/unsp	nev any	st
LAMTH	2		f	0	0	ch	-	small	As:HK	1983	CC	445	n	bl	n	n	0	all/unsp	nev any	or
LAMWK	3		f	0	0	ch	-	small	As:HK	1981	CC	163	n	bl	n	n	0	all/unsp	nev any	st
LAMWK2	2		m	0	0	all	-	small	As:HK	1976	CC	480	n	bl	n	n	0	all/unsp	nev any	st
LAMWK2	6		f	0	0	all	-	small	As:HK	1976	CC	480	n	bl	n	n	0	all/unsp	nev any	st
LUBIN2	146		m	0	0	all	-	small	Eu:mul	1976	CC	7804	n	bl	n	y	0	cig+/-ot	nev any	st
LUBIN2	166		f	0	0	all	-	small	Eu:mul	1976	CC	7804	n	bl	n	y	0	cig+/-ot	nev any	st
NOU	2		m	0	0	all	-	small	Eu:Sca	1971	CC	273	n	bl	y	n	0	all/unsp	nev any	st
NOU	7		f	0	0	all	-	small	Eu:Sca	1971	CC	273	n	bl	y	n	0	all/unsp	nev any	st
ORMOS	12		m	0	0	all	-	small	Eu:est	1947	CC	119	n	bl	y	y	0	cig+/-ot	nev any	st
OSANN	20	x	m	0	0	all	-	small	NAmer	1984	CC	1986	n	bl	n	n	0	cig+/-ot	nev cigs	st
OSANN	24	x	f	0	0	all	-	small	NAmer	1984	CC	1986	n	bl	n	n	0	cig+/-ot	nev cigs	st
PEZZOT	8		m	0	0	all	-	small	SCAmer	1987	CC	215	n	bl	n	y	0	cig only	nev cigs	ot
SEOW	4		f	0	0	ch	-	small	As:oth	1997	CC	153	n	bl	n	y	0	cig+/-ot	nev cigs	st
SIEMIA	10	x	m	0	0	all	-	small	NAmer	1979	CC	857	n	V	y	y	0	cig+/-ot	nev cigs	st
SOBUE	11	x	m	0	0	all	-	small	As:Jap	1986	CC	1376	n	bl	n	y	0	cig+/-ot	nev cigs	st
SOBUE	27	x	f	0	0	all	-	small	As:Jap	1986	CC	1376	n	bl	n	y	0	cig+/-ot	nev cigs	st
STASZE	29		m	0	0	all	-	small	Eu:est	1954	CC	281	n	bl	n	y	0	all/unsp	nev any	st
STASZE	39		f	0	0	all	-	small	Eu:est	1954	CC	281	n	bl	n	y	0	all/unsp	nev any	st
STAYNE	2		m	0	0	all	-	small	NAmer	1969	CC	420	n	bl	n	n	0	all/unsp	nev any	st
SVENSS	58	x	f	0	0	all	-	small	Eu:Sca	1983	CC	210	n	bl	n	n	0	all/unsp	nev any	st
TIZZAN	20		c	0	0	all	-	small	Eu:wst	1959	CC	1358	n	bl	n	n	0	all/unsp	nev any	st
WUWILL	10		f	0	0	all	-	small	As:Chi	1985	CC	965	n	ot	n	n	3	cig+/-ot	nev cigs	or
ZHOU	14		m	0	0	all	-	small	As:Chi	1978	CC	1360	n	ot	n	n	0	all/unsp	nev any	st
ZHOU	15		f	0	0	all	-	small	As:Chi	1978	CC	1360	n	ot	n	n	0	all/unsp	nev any	st

Cigarette type is all/unspec for all RRs

Table 5A1 - 5

IESLC - Meta-analysis of Ever Smoking, Any product (or Cigarettes if Any not available)
Small
Least adjusted

REF	NRR	SEX	AD	Number Exposed Case	Number Exposed Cont	Non-exposed Case	Non-exposed Cont	RR	95.00%CI
*ABRAHA	3	m	0	68	10351	2	3365	11.05	(2.71- 45.07)
*ABRAHA	6	f	0	26	5256	5	11589	11.47	(4.41- 29.84)
Subtotal ABRAHA								11.33	(5.14- 24.99)
ALDERS	53	m	2	-	-	-	-	6.99	(1.50- 32.57)
ALDERS	56	f	2	-	-	-	-	7.47	(3.17- 17.63)
Subtotal ALDERS								7.35	(3.48- 15.56)
*ANDERS	9	f	0	76	96164	4	195158	38.56	(14.11- 105.38)
BAND	3	m	2	-	-	-	-	45.79	(14.41- 145.53)
BARBON	114	m	0	212	567	6	188	11.72	(5.12- 26.81)
BROWN2	8	m	2	-	-	-	-	11.40	(9.10- 14.20)
BROWN2	7	f	2	-	-	-	-	37.60	(28.50- 49.30)
Subtotal BROWN2								18.32	(15.41- 21.77)
BUFFLE	51	m	0	-	-	-	-	14.38	(2.81- 73.55)
BUFFLE	66	f	0	61	166	1	112	41.16	(5.62- 301.22)
Subtotal BUFFLE								21.95	(6.21- 77.55)
BYERS1	2	m	0	85	695	4	424	12.96	(4.72- 35.59)
COMSTO	65	m	0	25	229	2	84	4.59	(1.06- 19.78)
COMSTO	77	f	0	27	87	2	115	17.84	(4.13- 77.08)
Subtotal COMSTO								9.04	(3.21- 25.43)
DAMBER	10	m	0	145	99	5	47	13.77	(5.29- 35.84)
DORGAN	119	m	2	-	-	-	-	22.90	(3.20- 166.00)
DORGAN	101	f	3	-	-	-	-	62.60	(23.00- 171.00)
Subtotal DORGAN								50.93	(20.83- 124.56)
DOSEME	18	m	0	143	536	13	293	6.01	(3.35- 10.80)
*ENGELA	90	m	7	-	-	-	-	4.35	(1.19- 15.88)
FAN	5	c	0	40	595	15	556	2.49	(1.36- 4.56)
GAO	9	m	0	60	558	3	202	7.24	(2.25- 23.34)
GAO	19	f	0	17	130	17	605	4.65	(2.31- 9.36)
Subtotal GAO								5.23	(2.87- 9.52)
HEGMAN	3	c	0	50	1202	1	2080	86.52	(11.94- 627.12)
ISHIMA	2	c	0	31	25	4	10	3.10	(0.87- 11.08)
JAHN	45	m	0	166	701	1	138	32.68	(4.54- 235.34)
JAIN	9	m	0	80	277	3	85	8.18	(2.52- 26.58)
JAIN	4	f	0	103	196	2	214	56.23	(13.69- 230.92)
Subtotal JAIN								18.04	(7.30- 44.58)
JEDRYC	14	m	0	148	1054	3	289	13.53	(4.28- 42.74)
KHUDER	25	m	0	74	-	1	-	27.02	(3.66- 199.46)
KIHARA	27	c	0	56	232	9	237	6.36	(3.07- 13.15)
LAMTH	2	f	0	42	14	9	36	12.00	(4.65- 30.98)
LAMWK	3	f	0	29	41	3	144	33.95	(9.84- 117.12)
LAMWK2	2	m	0	39	161	1	43	10.42	(1.39- 77.99)
LAMWK2	6	f	0	12	50	4	139	8.34	(2.57- 27.06)
Subtotal LAMWK2								8.83	(3.20- 24.38)
LUBIN2	146	m	0	1129	10433	34	2616	8.33	(5.90- 11.74)
LUBIN2	166	f	0	145	567	55	1180	5.49	(3.96- 7.60)
Subtotal LUBIN2								6.68	(5.28- 8.47)
NOU	2	m	0	42	247	1	122	20.74	(2.82- 152.52)
NOU	7	f	0	5	92	2	261	7.09	(1.35- 37.19)
Subtotal NOU								10.99	(3.07- 39.32)
ORMOS	12	m	0	41	1034	2	777	15.40	(3.71- 63.88)
OSANN	20	m	0	191	1018	4	833	39.07	(14.45- 105.62)
OSANN	24	f	0	165	563	4	1093	80.08	(29.55- 217.03)
Subtotal OSANN								55.89	(27.64- 113.00)
PEZZOT	8	m	0	36	317	0	116	26.79~	(1.63- 439.95)
SEOW	4	f	0	13	15	2	125	54.17	(11.13- 263.53)
SIEMIA	10	m	0	157	428	2	105	19.26	(4.70- 78.95)
SOBUE	11	m	0	130	1013	1	128	16.43	(2.28- 118.50)
SOBUE	27	f	0	26	232	9	857	10.67	(4.93- 23.09)
Subtotal SOBUE								11.30	(5.51- 23.19)
STASZE	29	m	0	29	754	3	158	2.03	(0.61- 6.73)
STASZE	39	f	0	1	153	4	1660	2.71	(0.30- 24.42)
Subtotal STASZE								2.17	(0.76- 6.21)
STAYNE	2	m	0	41	567	4	333	6.02	(2.14- 16.96)
SVENSS	58	f	0	43	89	2	120	28.99	(6.84- 122.85)
TIZZAN	20	c	0	101	939	18	419	2.50	(1.50- 4.19)
WUWILL	10	f	3	-	-	-	-	2.20	(1.40- 3.20)
ZHOU	14	m	0	74	41	17	36	3.82	(1.91- 7.63)
ZHOU	15	f	0	9	7	28	32	1.47	(0.48- 4.46)
Subtotal ZHOU								2.93	(1.63- 5.26)
Partial Totals				4193	137895	312	227124		

*prospective study ~ With 0.5 adjustment for zero

Table 5A1 - 5

IESLC - Meta-analysis of Ever Smoking, Any product (or Cigarettes if Any not available)
Small
Least adjusted

REF	NRR	SEX	AD	Ys	Ws	Qs	Ps
*ABRAHA	3	m	0	2.40	1.94	0.03	0.0008
*ABRAHA	6	f	0	2.44	4.20	0.10	0.0000
Subtotal ABRAHA				2.43	6.14	0.12	
ALDERS	53	m	2	1.94	1.62	0.19	0.0133
ALDERS	56	f	2	2.01	5.22	0.40	0.0000
Subtotal ALDERS				2.00	6.84	0.59	
*ANDERS	9	f	0	3.65	3.80	7.09	0.0000
BAND	3	m	2	3.82	2.87	6.79	0.0000
BARBON	114	m	0	2.46	5.60	0.17	0.0000
BROWN2	8	m	2	2.43	77.61	1.68	0.0000
BROWN2	7	f	2	3.63	51.16	91.96	0.0000
Subtotal BROWN2				2.91	128.77	93.64	
BUFFLE	51	m	0	2.67	1.44	0.21	0.0014
BUFFLE	66	f	0	3.72	0.97	1.99	0.0003
Subtotal BUFFLE				3.09	2.41	2.19	
BYERS1	2	m	0	2.56	3.77	0.29	0.0000
COMSTO	65	m	0	1.52	1.80	1.05	0.0412
COMSTO	77	f	0	2.88	1.79	0.64	0.0001
Subtotal COMSTO				2.20	3.59	1.68	
DAMBER	10	m	0	2.62	4.20	0.47	0.0000
DORGAN	119	m	2	3.13	0.99	0.70	0.0019
DORGAN	101	f	3	4.14	3.82	13.07	0.0000
Subtotal DORGAN				3.93	4.80	13.78	
DOSEME	18	m	0	1.79	11.21	2.72	0.0000
*ENGELA	90	m	7	1.47	2.29	1.52	0.0261
FAN	5	c	0	0.91	10.51	19.82	0.0031
GAO	9	m	0	1.98	2.80	0.26	0.0009
GAO	19	f	0	1.54	7.87	4.41	0.0000
Subtotal GAO				1.65	10.68	4.68	
HEGMAN	3	c	0	4.46	0.98	4.63	0.0000
ISHIMA	2	c	0	1.13	2.37	3.16	0.0817
JAHN	45	m	0	3.49	0.99	1.42	0.0005
JAIN	9	m	0	2.10	2.77	0.09	0.0005
JAIN	4	f	0	4.03	1.92	5.85	0.0000
Subtotal JAIN				2.89	4.69	5.94	
JEDRYC	14	m	0	2.60	2.90	0.29	0.0000
KHUDER	25	m	0	3.30	0.96	0.98	0.0012
KIHARA	27	c	0	1.85	7.27	1.39	0.0000
LAMTH	2	f	0	2.48	4.27	0.17	0.0000
LAMWK	3	f	0	3.52	2.51	3.84	0.0000
LAMWK2	2	m	0	2.34	0.95	0.00	0.0225
LAMWK2	6	f	0	2.12	2.77	0.08	0.0004
Subtotal LAMWK2				2.18	3.72	0.08	
LUBIN2	146	m	0	2.12	32.49	0.91	0.0000
LUBIN2	166	f	0	1.70	36.11	12.32	0.0000
Subtotal LUBIN2				1.90	68.61	13.22	
NOU	2	m	0	3.03	0.97	0.54	0.0029
NOU	7	f	0	1.96	1.40	0.15	0.0205
Subtotal NOU				2.40	2.36	0.69	
ORMOS	12	m	0	2.73	1.90	0.38	0.0002
OSANN	20	m	0	3.67	3.88	7.39	0.0000
OSANN	24	f	0	4.38	3.86	16.99	0.0000
Subtotal OSANN				4.02	7.75	24.38	
PEZZOT	8	m	0	3.29	0.49	0.49	0.0213
SEOW	4	f	0	3.99	1.53	4.47	0.0000
SIEMIA	10	m	0	2.96	1.93	0.87	0.0000
SOBUE	11	m	0	2.80	0.98	0.26	0.0055
SOBUE	27	f	0	2.37	6.45	0.04	0.0000
Subtotal SOBUE				2.42	7.43	0.30	
STASZE	29	m	0	0.71	2.66	6.65	0.2493
STASZE	39	f	0	1.00	0.80	1.32	0.3735
Subtotal STASZE				0.77	3.46	7.97	
STAYNE	2	m	0	1.80	3.58	0.86	0.0007
SVENSS	58	f	0	3.37	1.84	2.15	0.0000
TIZZAN	20	c	0	0.92	14.51	27.18	0.0005
WUWILL	10	f	3	0.79	22.48	50.45	0.0002
ZHOU	14	m	0	1.34	8.03	7.18	0.0001
ZHOU	15	f	0	0.38	3.12	11.27	0.4969
Subtotal ZHOU				1.07	11.15	18.45	

Table 5A1 - 5

IESLC - Meta-analysis of Ever Smoking, Any product (or Cigarettes if Any not available)
Small
Least adjusted

	N	54
	NS	39
	Wt	387.19
Het	Chi	329.33
Het	df	53
Het	P	***
Fixed	RR	9.84
	RRl	8.91
	RRu	10.87
	P	+++
Random	RR	11.04
	RRl	8.30
	RRu	14.70
	P	+++
Asymm	P	N.S.

Table 5A1 - 6

IESLC - Meta-analysis of Ever Smoking, Any product (or Cigarettes if Any not available)
Small
Least adjusted

	Sex			
	combined	male	female	Total
N	5	28	21	54
NS	5	28	21	54
Wt	35.64	183.63	167.91	387.19
Het Chi	15.50	44.34	221.64	329.33
Het df	4	27	20	53
Het P	**	*	***	***
Fixed RR	3.38	10.03	12.08	9.84
RRl	2.43	8.68	10.39	8.91
RRu	4.69	11.59	14.06	10.87
P	+++	+++	+++	+++
Random RR	4.49	10.13	14.11	11.04
RRl	2.16	7.92	7.97	8.30
RRu	9.33	12.95	24.99	14.70
P	+++	+++	+++	+++
Between Chi				47.84
Between df				2
Between P				***
Btwn(F) P				*

	Lung cancer type	
	small	Total
N	54	54
NS	39	39
Wt	387.19	387.19
Het Chi	329.33	329.33
Het df	53	53
Het P	***	***
Fixed RR	9.84	9.84
RRl	8.91	8.91
RRu	10.87	10.87
P	+++	+++
Random RR	11.04	11.04
RRl	8.30	8.30
RRu	14.70	14.70
P	+++	+++
Between Chi		
Between df		
Between P		N.S.
Btwn(F) P		N.S.

	Location								
	NAmer	UK	Scand	othEur	China	Japan	othAs	other	Total
N	19	2	5	12	6	4	5	1	54
NS	13	1	4	9	4	3	4	1	39
Wt	169.91	6.84	10.69	115.32	54.82	17.07	12.03	0.49	387.19
Het Chi	79.59	0.01	4.54	29.74	8.08	3.45	5.40	0.00	329.33
Het df	18	1	4	11	5	3	4	0	53
Het P	***	N.S.	N.S.	**	N.S.	N.S.	N.S.	N.S.	***
Fixed RR	19.84	7.35	11.64	6.25	2.83	7.39	16.42	26.79	9.84
RRl	17.07	3.48	6.39	5.21	2.17	4.60	9.33	1.63	8.91
RRu	23.06	15.56	21.19	7.50	3.68	11.88	28.90	439.95	10.87
P	+++	+++	+++	+++	+++	+++	+++	+	+++
Random RR	24.05	7.35	11.62	6.82	2.99	7.34	17.00	26.79	11.04
RRl	15.66	3.48	6.06	4.72	2.08	4.34	8.65	1.63	8.30
RRu	36.95	15.56	22.26	9.86	4.31	12.43	33.44	439.95	14.70
P	+++	+++	+++	+++	+++	+++	+++	+	+++
Between Chi									198.52
Between df									7
Between P									***
Btwn(F) P									***

Table 5A1 - 6

IESLC - Meta-analysis of Ever Smoking, Any product (or Cigarettes if Any not available)
Small
Least adjusted

	Detailed Country in "other Europe"					
	multi	Germany	othWest	East	Balkans	Total
N	2	1	2	6	1	12
NS	1	1	2	4	1	9
Wt	68.61	0.99	20.12	14.40	11.21	115.32
Het Chi	2.98	0.00	9.63	8.31	0.00	29.74
Het df	1	0	1	5	0	11
Het P	(*)	N.S.	**	N.S.	N.S.	**
Fixed RR	6.68	32.68	3.85	8.22	6.01	6.25
RRl	5.28	4.54	2.49	4.90	3.35	5.21
RRu	8.47	235.34	5.96	13.78	10.80	7.50
P	+++	+++	+++	+++	+++	+++
Random RR	6.73	32.68	5.23	7.95	6.01	6.82
RRl	4.47	4.54	1.15	4.00	3.35	4.72
RRu	10.13	235.34	23.68	15.82	10.80	9.86
P	+++	+++	+	+++	+++	+++
Between Chi						8.83
Between df						4
Between P						(*)
Btwn(F) P						N.S.

	Detailed Country in "other Asia"			
	India	HongKong	other	Total
N		4	1	5
NS		3	1	4
Wt		10.50	1.53	12.03
Het Chi		2.89	0.00	5.40
Het df		3	0	4
Het P		N.S.	N.S.	N.S.
Fixed RR		13.79	54.17	16.42
RRl		7.53	11.13	9.33
RRu		25.26	263.53	28.90
P		+++	+++	+++
Random RR		13.79	54.17	17.00
RRl		7.53	11.13	8.65
RRu		25.26	263.53	33.44
P		+++	+++	+++
Between Chi				2.51
Between df				1
Between P				N.S.
Btwn(F) P				N.S.

	Detailed other continent			
	SCAmer	Auslia	Africa	Total
N	1			1
NS	1			1
Wt	0.49			0.49
Het Chi	0.00			0.00
Het df	0			0
Het P	N.S.			N.S.
Fixed RR	26.79			26.79
RRl	1.63			1.63
RRu	439.95			439.95
P	+			+
Random RR	26.79			26.79
RRl	1.63			1.63
RRu	439.95			439.95
P	+			+
Between Chi				
Between df				
Between P				N.S.
Btwn(F) P				N.S.

Table 5A1 - 6

IESLC - Meta-analysis of Ever Smoking, Any product (or Cigarettes if Any not available)
Small
Least adjusted

	Start year of study <1960	1960-69	1970-79	1980-89	1990+	Total
N	5	3	20	23	3	54
NS	4	3	12	17	3	39
Wt	23.64	8.24	127.77	208.22	19.32	387.19
Het Chi	13.04	0.63	27.77	185.45	14.04	329.33
Het df	4	2	19	22	2	53
Het P	*	N.S.	(*)	***	***	***
Fixed RR	3.69	4.55	7.11	14.88	4.53	9.84
RRl	2.46	2.30	5.98	12.99	2.90	8.91
RRu	5.52	9.00	8.46	17.04	7.07	10.87
P	+++	+++	+++	+++	+++	+++
Random RR	4.83	4.55	7.61	20.19	7.77	11.04
RRl	1.97	2.30	5.91	12.45	2.06	8.30
RRu	11.86	9.00	9.79	32.75	29.30	14.70
P	+++	+++	+++	+++	++	+++
Between Chi						88.39
Between df						4
Between P						***
Btwn(F) P						**

	Study type (1) CC	other	Total
N	48	6	54
NS	35	4	39
Wt	371.36	15.82	387.19
Het Chi	318.87	9.47	329.33
Het df	47	5	53
Het P	***	(*)	***
Fixed RR	9.74	12.58	9.84
RRl	8.79	7.69	8.91
RRu	10.78	20.59	10.87
P	+++	+++	+++
Random RR	11.02	11.73	11.04
RRl	8.11	5.86	8.30
RRu	14.98	23.47	14.70
P	+++	+++	+++
Between Chi			1.00
Between df			1
Between P			N.S.
Btwn(F) P			N.S.

	Study type (2) CC	prosp	other	Total
N	48	4	2	54
NS	35	3	1	39
Wt	371.36	12.23	3.59	387.19
Het Chi	318.87	7.30	1.66	329.33
Het df	47	3	1	53
Het P	***	(*)	N.S.	***
Fixed RR	9.74	13.86	9.04	9.84
RRl	8.79	7.91	3.21	8.91
RRu	10.78	24.27	25.43	10.87
P	+++	+++	+++	+++
Random RR	11.02	12.81	9.04	11.04
RRl	8.11	5.24	2.39	8.30
RRu	14.98	31.33	34.25	14.70
P	+++	+++	++	+++
Between Chi				1.50
Between df				2
Between P				N.S.
Btwn(F) P				N.S.

Table 5A1 - 6

IESLC - Meta-analysis of Ever Smoking, Any product (or Cigarettes if Any not available)
Small
Least adjusted

	Study size (number of LC cases)				
	100-249	250-499	500-999	1000+	Total
N	6	16	10	22	54
NS	6	12	7	14	39
Wt	10.64	46.80	47.46	282.28	387.19
Het Chi	10.74	40.13	47.73	203.18	329.33
Het df	5	15	9	21	53
Het P	(*)	***	***	***	***
Fixed RR	17.82	6.73	5.89	11.17	9.84
RRl	9.77	5.05	4.43	9.94	8.91
RRu	32.50	8.96	7.83	12.55	10.87
P	+++	+++	+++	+++	+++
Random RR	18.90	8.30	12.33	11.25	11.04
RRl	7.62	4.98	5.78	7.41	8.30
RRu	46.88	13.82	26.33	17.10	14.70
P	+++	+++	+++	+++	+++
Between Chi					27.54
Between df					3
Between P					***
Btwn(F) P					N.S.

	Risky occupational population			
	no	mining	othRisky	Total
N	54			54
NS	39			39
Wt	387.19			387.19
Het Chi	329.33			329.33
Het df	53			53
Het P	***			***
Fixed RR	9.84			9.84
RRl	8.91			8.91
RRu	10.87			10.87
P	+++			+++
Random RR	11.04			11.04
RRl	8.30			8.30
RRu	14.70			14.70
P	+++			+++
Between Chi				
Between df				
Between P				N.S.
Btwn(F) P				N.S.

	National cigarette tobacco type			
	Virginia	blended	other	Total
N	6	42	6	54
NS	4	31	4	39
Wt	16.34	316.03	54.82	387.19
Het Chi	11.55	209.65	8.08	329.33
Het df	5	41	5	53
Het P	*	***	N.S.	***
Fixed RR	14.71	11.96	2.83	9.84
RRl	9.06	10.72	2.17	8.91
RRu	23.88	13.36	3.68	10.87
P	+++	+++	+++	+++
Random RR	16.08	12.97	2.99	11.04
RRl	7.51	9.59	2.08	8.30
RRu	34.41	17.53	4.31	14.70
P	+++	+++	+++	+++
Between Chi				100.04
Between df				2
Between P				***
Btwn(F) P				***

Table 5A1 - 6

IESLC - Meta-analysis of Ever Smoking, Any product (or Cigarettes if Any not available)
Small
Least adjusted

	Any proxy use No/nk	Yes	Total
N	37	17	54
NS	26	13	39
Wt	339.65	47.53	387.19
Het Chi	269.96	57.92	329.33
Het df	36	16	53
Het P	***	***	***
Fixed RR	9.62	11.59	9.84
RRl	8.65	8.72	8.91
RRu	10.70	15.40	10.87
P	+++	+++	+++
Random RR	9.56	15.98	11.04
RRl	6.82	8.98	8.30
RRu	13.40	28.42	14.70
P	+++	+++	+++
Between Chi			1.45
Between df			1
Between P			N.S.
Btwn(F) P			N.S.

	Full histological confirmation No	Yes	Total
N	35	19	54
NS	25	14	39
Wt	155.47	231.71	387.19
Het Chi	148.54	130.78	329.33
Het df	34	18	53
Het P	***	***	***
Fixed RR	6.34	13.21	9.84
RRl	5.42	11.61	8.91
RRu	7.42	15.02	10.87
P	+++	+++	+++
Random RR	9.56	14.48	11.04
RRl	6.72	9.22	8.30
RRu	13.60	22.72	14.70
P	+++	+++	+++
Between Chi			50.01
Between df			1
Between P			***
Btwn(F) P			**

	Number of adjustment variables (1) 0	1	2+/+nk	Total
N	45		9	54
NS	33		6	39
Wt	219.12		168.06	387.19
Het Chi	148.85		148.90	329.33
Het df	44		8	53
Het P	***		***	***
Fixed RR	7.66		13.63	9.84
RRl	6.71		11.72	8.91
RRu	8.75		15.86	10.87
P	+++		+++	+++
Random RR	10.32		13.16	11.04
RRl	7.83		5.88	8.30
RRu	13.59		29.43	14.70
P	+++		+++	+++
Between Chi				31.57
Between df				1
Between P				***
Btwn(F) P				*

Table 5A1 - 6

IESLC - Meta-analysis of Ever Smoking, Any product (or Cigarettes if Any not available)
Small
Least adjusted

	Number of adjustment variables (2) 0	1	2	3-5	6+/+nk	Total
N	45		6	2	1	54
NS	33		4	2	1	39
Wt	219.12		139.47	26.30	2.29	387.19
Het Chi	148.85		52.00	36.59	0.00	329.33
Het df	44		5	1	0	53
Het P	***		***	***	N.S.	***
Fixed RR	7.66		17.88	3.58	4.35	9.84
RRl	6.71		15.14	2.44	1.19	8.91
RRu	8.75		21.10	5.24	15.89	10.87
P	+++		+++	+++	+	+++
Random RR	10.32		17.21	11.36	4.35	11.04
RRl	7.83		8.18	0.43	1.19	8.30
RRu	13.59		36.25	302.11	15.89	14.70
P	+++		+++	N.S.	+	+++
Between Chi						91.89
Between df						3
Between P						***
Btwn(F) P						***

	Product all/unsp	cig+/-ot	cig only	Total
N	23	29	2	54
NS	17	20	2	39
Wt	81.79	302.03	3.36	387.19
Het Chi	57.58	245.52	0.12	329.33
Het df	22	28	1	53
Het P	***	***	N.S.	***
Fixed RR	6.34	10.90	42.35	9.84
RRl	5.11	9.74	14.55	8.91
RRu	7.88	12.20	123.29	10.87
P	+++	+++	+++	+++
Random RR	7.75	13.26	42.35	11.04
RRl	5.31	8.97	14.55	8.30
RRu	11.33	19.60	123.29	14.70
P	+++	+++	+++	+++
Between Chi				26.11
Between df				2
Between P				***
Btwn(F) P				N.S.

	Denominator nev any	nev cigs	Total
N	30	24	54
NS	22	17	39
Wt	162.88	224.30	387.19
Het Chi	93.47	211.10	329.33
Het df	29	23	53
Het P	***	***	***
Fixed RR	7.31	12.20	9.84
RRl	6.27	10.71	8.91
RRu	8.53	13.91	10.87
P	+++	+++	+++
Random RR	9.26	13.42	11.04
RRl	6.73	8.34	8.30
RRu	12.75	21.59	14.70
P	+++	+++	+++
Between Chi			24.76
Between df			1
Between P			***
Btwn(F) P			*

Table 5A1 - 6

IESLC - Meta-analysis of Ever Smoking, Any product (or Cigarettes if Any not available)

Small

Least adjusted

	Derivation of RR/CI			
	Orig	StdCalc	Other	Total
N	8	38	8	54
NS	5	28	7	40
Wt	167.17	195.31	24.71	387.19
Het Chi	141.76	130.81	26.66	329.33
Het df	7	37	7	53
Het P	***	***	***	***
Fixed RR	13.52	7.85	6.83	9.84
RRl	11.62	6.82	4.61	8.91
RRu	15.73	9.03	10.14	10.87
P	+++	+++	+++	+++
Random RR	12.70	10.49	11.02	11.04
RRl	5.52	7.77	4.60	8.30
RRu	29.20	14.15	26.36	14.70
P	+++	+++	+++	+++
Between Chi				30.10
Between df				2
Between P				***
Btwn(F) P				(*)

Table 5A1 - 7

IESLC - Meta-analysis of Ever Smoking, Any product (or Cigarettes if Any not available)
Small
Excluded studies (and stage at which they were excluded)

1 BOUCHA BUELL LAURIL MZILEN

2 BYERS2 ENSTRO HIRAY2 KANELL LIAW LICKIN MRFIT MURATA NOTANI RESTRE RIMING SAARIK SEGI2 SHIMIZ SITAS SOBUE2
TANG2 WALD WARSIN WATSON WU2 WYNDE5 WYNDE8 WYNDER

3 BERRIN CHEN TAO ULMER

6 BLOT1 BLOT2 BLOT3 HIRAOK JONES MOLLO PERSHA SCHWA2 VANDER

8 ABELIN AGUDO AKIBA AMANDU AMES ARCHER ARMADA AUSTIN AUVINE AXELSO AXELSS BECHER BENHAM BENSHL BEST BLOHMK
BLOT4 BOFFET BOUCOT BRESLO BRETT BROCKM BROSS BROWN1 CARPEN CASCO2 CASCOR CEDERL CHAN CHANG CHATZI CHEN2
CHEN3 CHIAZZ CHOI CHOW CHYOU COOKSO CORREA CPSI CPSII DARBY DAVEYS DEAN DEAN2 DEAN3 DEKLER DESTE2
DESTEF DOCKER DOLL DOLL2 DORANT DORN DROSTE DU DUNN EBELIN ESAKI GAO2 GARCIA GARDIN GARSHI GENG
GER GILLIS GODLEY GOLLED GOODMA GRAHAM GREGOR GSELL HAENSZ HAMMO2 HAMMON HANSEN HEIN HENNEK HINDS HIRAYA
HITOSU HOLE HOROWI HORWIT HU HU2 HUANG HUMBLE JARUP JARVHO JIANG JOLY JUSSAW KAISE2 KAISER KATSOU
KAUFMA KELLER KINLEN KJUUS KNEKT KO KOHLME KOO KOULUM KREUZE KREYBE KUBIK LANGE LAUSSM LEI LEMARC
LETOUR LEVIN LIDDEL LIU LIU2 LIU3 LIU4 LIU5 LOMBA2 LOMBAR LUBIN LUO MACLEN MAGNUS MARSH MARSH2
MARTIS MASTRA MATOS MATSUD MCCONN MCDUFF MCLAUG MIGRAN MILLER MILLS MRFITR NAM NOTAN2 ODRISC OSANN2 PARKIN
PASTOR PAWLEG PERNU PERSH2 PETO PEZZO2 PIKE PISANI POFFIJ POLEDN PRESCO QIAO QIAO2 RACHTA RADZIK RANDIG
REN RONCO ROOTS ROTHSC SADOWS SANKAR SCHWAR SEGI SHAW SIMARA SIMONA SPEIZE SPITZ STOCKS STOCKW STUCKE
SUN SUZUK2 SUZUKI TANG TENKAN TOKARS TOUSEY TSUGAN TULINI TVERDA VEIERO VUTUC WAKAI WANG WANG2 WANG3
WANG4 WICKLU WIGLE WILKIN WU WUNSCH WYNDE2 WYNDE3 WYNDE4 WYNDE6 WYNDE7 XIANGZ XU XU2 XU3 XU4
YAMAGU YONG YUAN ZHANG ZHENG

Table 5A1 - 8
Potentially overlapping studies

REF	REFGP	PRINC	.	OVERLAP/LINK
LUBIN2	LUBIN2	1		Lubin-combined
LAMWK2	LAMWK2	1		Chan/LamWK2
LAMTH	LAMTH	1		KOO/LAMTH/LAMWK
LAMWK	LAMWK	1		KOO/LAMTH/LAMWK
ISHIMA	AKIBA	2		AKIBA/ISHIMA
BYERS1	BYERS1	2		GRAHAM/BROSS/BYERS1
JAHN	BOFFET	2		Subset of BOFFET

Table 5A1 - 9
Most adjusted - insufficient data for metaanalysis

REF	NRR	SEX	AGEL	AGEH	RACE	YF	LC TYPE	LOC	START	ST	NLC	R	VB	P	H	AD	PRODUCT	DENOM	De
BUFFLE	48	f	0	0	wh	-	small	NAmer	1976	CC	943	n	bl	y	n	0	cig+/-ot	nev cigs	or
CHEN	6	c	0	0	all	-	small	As:oth	1987	CC	323	n	ot	n	y	2	cig+/-ot	nev cigs	ot
KOO	3	f	0	0	all	-	small	As:HK	1981	CC	200	n	bl	n	n	0	all/unsp	nev any	or

REF	NRR	RR	SIG	RRdata comment
BUFFLE	48	52.30		0
CHEN	6	3.59	y	P < 0.001
KOO	3	5.14		0

Table 5B1 -

IESLC - Meta-analysis of Current Smoking (vs never smoking), Any product (or Cigarettes if Any not available)
Small

This analysis is restricted to results for:
1) Non-dose-response data
2) Current smokers
3) Results complete enough for use in metaanalysis

Within each study, results are then selected (in the following order of preference, within each sex) for:
4) PRODUCT: all/unspec, cigarettes regardless of other products, cigarettes only
5) CIGTYPE: all/unspecified, MC regardless of HR, MC only
6) DENOM: never smoked anything, never smoked cigarettes, (never +1 = +long term ex, +2 = +amount unknown,
+3 = never cigs+long term ex)
7) Followup period (YF, prospective studies): whole study (coded as 0) or longest available
8) LCtype: small (specifically)

9) Race: all or nearest available, otherwise by race (wh or w = white, bl or b = black, hi = hispanic
ch = chinese, jap = japanese, haw = hawaiian, w+o = white + oriental, sca = scandinavian, as = asian)
10) For overlapping studies: principal rather than subsidiary studies
Finally by Age: whole study (coded as 0) if available, otherwise by widest available age group
and then for single sex results (m, f) in preference to combined sex results (c).

Results adjusted (AD) for the most potential confounders are then chosen in Sections -1 to -3
and results adjusted for the least confounders in Sections -4 to -6. (Those least adjusted results which
actually differ from the most adjusted as marked 'x' in column X in Section -4)
(Results adjusted for an unknown number of confounder(s) are coded as 20.)

Section -7 shows excluded studies, together with the stage (as above) at which no qualifying
results were found.

Section -8 lists the potentially overlapping studies which have been included (1=principal, 2=subsidiary).

Section -9 lists any results which would have been included in preference except that they had data not complete
enough for use in meta-analysis, with their significance (yes/no), if known, and any further comment as entered
on the database.

In addition to those mentioned above, the following fields, levels and abbreviations are used:
* or nk = not known, n = no, y = yes, ot = other
nev = never
all/unspec = all or unspecified, cig+/-ot = cigarettes irrespective of other products (cigar, pipe etc)
MC = manufactured cigarettes, HR = hand-rolled cigarettes
REF: 6-character study reference
NRR: number of the RR on the database within the study
ST : study type (CC = case control, pr or prosp = prospective)
NLC: number of lung cancer cases in whole study
R : risky occupational population (n = no, m = mining, o = other risky)
VB : national cigarette type (V = at least 75% Virginia, bl = at least 75% blended, ot = other)
P : any proxy use
H : full histological confirmation
De : derivation of RR/CI (or = original, st = standard method, ot = other method of estimation)

Table 5B1 - 1

IESLC - Meta-analysis of Current Smoking (vs never smoking), Any product (or Cigarettes if Any not available)
Small
Most adjusted

REF	NRR	SEX	AGEL	AGEH	RACE	YF	LC TYPE	LOC	START	ST	NLC	R	VB	P	H	AD	PRODUCT	DENOM	De
BARBON	86	m	0	0	all	-	small	Eu:wst	1979	CC	755	n	bl	y	y	1	all/unsp	nev any	or
BOUCOT	144	m	0	0	all	0	small	NAmer	1951	pr	121	n	bl	n	n	2	cig only	nev any	ot
BROWN2	18	m	0	0	wh	-	small	NAmer	1984	CC	14596	n	bl	n	y	2	cig+/-ot	nev cigs	or
BROWN2	17	f	0	0	wh	-	small	NAmer	1984	CC	14596	n	bl	n	y	2	cig+/-ot	nev cigs	or
BUFFLE	67	f	0	0	w-hi	-	small	NAmer	1976	CC	943	n	bl	y	n	0	cig+/-ot	nev cigs	st
COMSTO	22	m	0	0	all	-	small	NAmer	1975	ot	258	n	bl	n	n	0	cig+/-ot	nev cigs	st
COMSTO	29	f	0	0	all	-	small	NAmer	1975	ot	258	n	bl	n	n	0	cig+/-ot	nev cigs	st
CPSI	407	f	0	0	all	2	small	NAmer	1959	pr	5138	n	bl	n	n	1	cig only	nev any	ot
CPSII	116	m	0	0	all	2	small	NAmer	1982	pr	3229	n	bl	n	n	1	cig only	nev any	ot
CPSII	119	f	0	0	all	2	small	NAmer	1982	pr	3229	n	bl	n	n	1	cig+/-ot	nev cigs	ot
DORN	339	m	0	0	wh	8	small	NAmer	1954	pr	5097	n	bl	n	n	1	cig only	nev any	ot
ENGELA	84	m	0	0	all	0	small	Eu:Sca	1964	pr	435	n	bl	n	n	7	cig+/-ot	nev cigs	ot
JAHN	6	m	0	0	all	-	small	Eu:Ger	1988	CC	1004	n	bl	n	n	0	cig+/-ot	nev any	st
JAIN	19	m	0	0	all	-	small	NAmer	1981	CC	845	n	V	y	n	0	cig+/-ot	nev cigs	st
JAIN	14	f	0	0	all	-	small	NAmer	1981	CC	845	n	V	y	n	0	cig+/-ot	nev cigs	st
JEDRYC	24	m	0	0	all	-	small	Eu:est	1980	CC	1630	n	bl	y	n	0	cig+/-ot	nev any	st
KHUDER	15	m	0	0	all	-	small	NAmer	1985	CC	482	n	bl	n	y	0	cig+/-ot	nev cigs	or
KIHARA	3	c	0	0	jap	-	small	As:Jap	1991	CC	440	n	bl	n	n	0	all/unsp	nev any	st
LUBIN2	250	m	0	0	all	-	small	Eu:mul	1976	CC	7804	n	bl	n	y	0	cig+/-ot	nev any	st
LUBIN2	262	f	0	0	all	-	small	Eu:mul	1976	CC	7804	n	bl	n	y	0	cig+/-ot	nev any	st
OSANN	37	m	0	0	all	-	small	NAmer	1984	CC	1986	n	bl	n	n	2	cig+/-ot	nev cigs	or
OSANN	38	f	0	0	all	-	small	NAmer	1984	CC	1986	n	bl	n	n	2	cig+/-ot	nev cigs	or
SOBUE	38	m	0	0	all	-	small	As:Jap	1986	CC	1376	n	bl	n	y	1	cig+/-ot	nev cigs	or
SOBUE	48	f	0	0	all	-	small	As:Jap	1986	CC	1376	n	bl	n	y	1	cig+/-ot	nev cigs	or
SOBUE2	3	m	0	0	all	-	small	As:Jap	1965	CC	2083	n	bl	n	n	2	cig+/-ot	nev any	or
SOBUE2	7	f	0	0	all	-	small	As:Jap	1965	CC	2083	n	bl	n	n	2	cig+/-ot	nev any	or
SVENSS	98	f	0	0	all	-	small	Eu:Sca	1983	CC	210	n	bl	n	n	1	all/unsp	nev any	ot

Cigarette type is all/unspec for all RRs

Table 5B1 - 2

IESLC - Meta-analysis of Current Smoking (vs never smoking), Any product (or Cigarettes if Any not available)
Small
Most adjusted

REF	NRR	SEX	AD	Number Exposed Case	Number Exposed Cont	Non-exposed Case	Non-exposed Cont	RR	95.00%CI
BARBON	86	m	1	-	-	-	-	14.30	(6.20- 33.00)
*BOUCOT	144	m	2	-	-	-	-	6.42	(0.37- 111.22)
BROWN2	18	m	2	-	-	-	-	15.10	(12.00- 19.00)
BROWN2	17	f	2	-	-	-	-	42.50	(32.10- 56.60)
Subtotal BROWN2								22.76	(19.04- 27.20)
BUFFLE	67	f	0	49	110	1	112	49.89	(6.77- 367.64)
COMSTO	22	m	0	20	100	2	84	8.40	(1.91- 36.98)
COMSTO	29	f	0	25	52	2	115	27.64	(6.31- 121.08)
Subtotal COMSTO								15.27	(5.36- 43.47)
*CPSI	407	f	1	-	-	-	-	2.12	(0.39- 11.60)
*CPSII	116	m	1	-	-	-	-	72.00	(5.22- 992.66)
*CPSII	119	f	1	-	-	-	-	20.91	(6.10- 71.69)
Subtotal CPSII								26.14	(8.57- 79.74)
*DORN	339	m	1	-	-	-	-	41.49	(14.52- 118.57)
*ENGELA	84	m	7	-	-	-	-	9.64	(2.59- 35.94)
JAHN	6	m	0	86	269	1	138	44.12	(6.08- 320.18)
JAIN	19	m	0	50	118	3	85	12.01	(3.62- 39.78)
JAIN	14	f	0	83	99	2	214	89.71	(21.63- 372.09)
Subtotal JAIN								27.66	(11.06- 69.14)
JEDRYC	24	m	0	105	516	3	289	19.60	(6.17- 62.32)
KHUDER	15	m	0	38	-	1	-	23.10	(3.10- 174.20)
KIHARA	3	c	0	50	162	9	237	8.13	(3.89- 16.99)
LUBIN2	250	m	0	826	6209	34	2616	10.24	(7.24- 14.47)
LUBIN2	262	f	0	119	410	55	1180	6.23	(4.44- 8.74)
Subtotal LUBIN2								7.94	(6.23- 10.12)
OSANN	37	m	2	-	-	-	-	62.00	(22.80- 169.00)
OSANN	38	f	2	-	-	-	-	119.10	(43.40- 327.00)
Subtotal OSANN								85.70	(42.09- 174.51)
SOBUE	38	m	1	-	-	-	-	21.40	(5.30- 87.10)
SOBUE	48	f	1	-	-	-	-	12.10	(6.30- 23.40)
Subtotal SOBUE								13.41	(7.40- 24.29)
SOBUE2	3	m	2	-	-	-	-	6.90	(4.60- 10.30)
SOBUE2	7	f	2	-	-	-	-	14.40	(9.30- 22.20)
Subtotal SOBUE2								9.69	(7.21- 13.03)
SVENSS	98	f	1	-	-	-	-	55.62	(9.76- 316.85)
Partial Totals				1451	8045	113	5070		

*prospective study

REF	NRR	SEX	AD	Ys	Ws	Qs	Ps
BARBON	86	m	1	2.66	5.50	0.03	0.0000
*BOUCOT	144	m	2	1.86	0.47	0.36	0.2014
BROWN2	18	m	2	2.71	72.77	0.01	0.0000
BROWN2	17	f	2	3.75	47.77	49.77	0.0000
Subtotal BROWN2				3.12	120.54	49.78	
BUFFLE	67	f	0	3.91	0.96	1.34	0.0001
COMSTO	22	m	0	2.13	1.75	0.63	0.0049
COMSTO	29	f	0	3.32	1.76	0.61	0.0000
Subtotal COMSTO				2.73	3.51	1.24	
*CPSI	407	f	1	0.75	1.34	5.22	0.3853
*CPSII	116	m	1	4.28	0.56	1.34	0.0014
*CPSII	119	f	1	3.04	2.53	0.25	0.0000
Subtotal CPSII				3.26	3.09	1.58	
*DORN	339	m	1	3.73	3.48	3.46	0.0000
*ENGELA	84	m	7	2.27	2.22	0.48	0.0007
JAHN	6	m	0	3.79	0.98	1.09	0.0002
JAIN	19	m	0	2.49	2.68	0.16	0.0000
JAIN	14	f	0	4.50	1.90	5.93	0.0000
Subtotal JAIN				3.32	4.58	6.09	
JEDRYC	24	m	0	2.98	2.87	0.17	0.0000
KHUDER	15	m	0	3.14	0.95	0.16	0.0023
KIHARA	3	c	0	2.10	7.07	2.84	0.0000
LUBIN2	250	m	0	2.33	32.09	5.21	0.0000
LUBIN2	262	f	0	1.83	33.48	27.11	0.0000
Subtotal LUBIN2				2.07	65.56	32.32	
OSANN	37	m	2	4.13	3.83	7.49	0.0000
OSANN	38	f	2	4.78	3.77	15.85	0.0000
Subtotal OSANN				4.45	7.60	23.34	
SOBUE	38	m	1	3.06	1.96	0.22	0.0000
SOBUE	48	f	1	2.49	8.92	0.50	0.0000
Subtotal SOBUE				2.60	10.88	0.71	

Table 5B1 - 2

IESLC - Meta-analysis of Current Smoking (vs never smoking), Any product (or Cigarettes if Any not available)
Small
Most adjusted

REF	NRR	SEX	AD	Ys	Ws	Qs	Ps
SOBUE2	3	m	2	1.93	23.65	15.03	0.0000
SOBUE2	7	f	2	2.67	20.30	0.08	0.0000
Subtotal SOBUE2				2.27	43.95	15.11	
SVENSS	98	f	1	4.02	1.27	2.11	0.0000

	N	27
	NS	19
	Wt	286.80
Het	Chi	147.44
Het	df	26
Het	P	***
Fixed	RR	15.31
	RRl	13.64
	RRu	17.19
	P	+++
Random	RR	18.17
	RRl	12.92
	RRu	25.56
	P	+++
Asymm	P	N.S.

Table 5B1 - 3

IESLC - Meta-analysis of Current Smoking (vs never smoking), Any product (or Cigarettes if Any not available)

Small

Most adjusted

	Sex combined	Sex male	Sex female	Total
N	1	15	11	27
NS	1	15	11	27
Wt	7.07	155.74	123.99	286.80
Het Chi	0.00	32.19	102.30	147.44
Het df	0	14	10	26
Het P	N.S.	**	***	***
Fixed RR	8.13	13.14	19.25	15.31
RRl	3.89	11.23	16.14	13.64
RRu	16.99	15.38	22.95	17.19
P	+++	+++	+++	+++
Random RR	8.13	15.26	23.17	18.17
RRl	3.89	10.90	11.70	12.92
RRu	16.99	21.36	45.88	25.56
P	+++	+++	+++	+++
Between Chi				12.96
Between df				2
Between P				**
Btwn(F) P				N.S.

	Lung cancer type small	Total
N	27	27
NS	19	19
Wt	286.80	286.80
Het Chi	147.44	147.44
Het df	26	26
Het P	***	***
Fixed RR	15.31	15.31
RRl	13.64	13.64
RRu	17.19	17.19
P	+++	+++
Random RR	18.17	18.17
RRl	12.92	12.92
RRu	25.56	25.56
P	+++	+++
Between Chi		
Between df		
Between P		N.S.
Btwn(F) P		N.S.

	Location NAmer	UK	Scand	othEur	China	Japan	othAs	other	Total
N	15		2	5		5			27
NS	10		2	4		3			19
Wt	146.51		3.49	74.91		61.90			286.80
Het Chi	61.84		2.48	10.42		7.72			147.44
Het df	14		1	4		4			26
Het P	***		N.S.	*		N.S.			***
Fixed RR	24.21		18.23	8.78		10.06			15.31
RRl	20.59		6.38	7.00		7.84			13.64
RRu	28.47		52.05	11.01		12.90			17.19
P	+++		+++	+++		+++			+++
Random RR	27.59		21.03	10.65		10.43			18.17
RRl	16.80		3.81	6.69		7.13			12.92
RRu	45.29		115.92	16.97		15.26			25.56
P	+++		+++	+++		+++			+++
Between Chi									64.99
Between df									3
Between P									***
Btwn(F) P									**

Table 5B1 - 3

IESLC - Meta-analysis of Current Smoking (vs never smoking), Any product (or Cigarettes if Any not available)
Small
Most adjusted

	Detailed Country in "other Europe"					
	multi	Germany	othWest	East	Balkans	Total
N	2	1	1	1		5
NS	1	1	1	1		4
Wt	65.56	0.98	5.50	2.87		74.91
Het Chi	4.05	0.00	0.00	0.00		10.42
Het df	1	0	0	0		4
Het P	*	N.S.	N.S.	N.S.		*
Fixed RR	7.94	44.12	14.30	19.60		8.78
RRl	6.23	6.08	6.20	6.17		7.00
RRu	10.12	320.18	32.99	62.32		11.01
P	+++	+++	+++	+++		+++
Random RR	7.97	44.12	14.30	19.60		10.65
RRl	4.90	6.08	6.20	6.17		6.69
RRu	12.98	320.18	32.99	62.32		16.97
P	+++	+++	+++	+++		+++
Between Chi						6.37
Between df						3
Between P						(*)
Btwn(F) P						N.S.

	Detailed Country in "other Asia"			
	India	HongKong	other	Total
N				
NS				
Wt				
Het Chi				
Het df				
Het P				N.S.
Fixed RR				
RRl				
RRu				
P				+++
Random RR				
RRl				
RRu				
P				+++
Between Chi				
Between df				
Between P				N.S.
Btwn(F) P				N.S.

	Detailed other continent			
	SCAmer	Auslia	Africa	Total
N				
NS				
Wt				
Het Chi				
Het df				
Het P				N.S.
Fixed RR				
RRl				
RRu				
P				+++
Random RR				
RRl				
RRu				
P				+++
Between Chi				
Between df				
Between P				N.S.
Btwn(F) P				N.S.

Table 5B1 - 3

IESLC - Meta-analysis of Current Smoking (vs never smoking), Any product (or Cigarettes if Any not available)
Small
Most adjusted

		Start year of study				
	<1960	1960-69	1970-79	1980-89	1990+	Total
N	3	3	6	14	1	27
NS	3	2	4	9	1	19
Wt	5.29	46.17	75.53	152.75	7.07	286.80
Het Chi	9.00	5.91	11.24	55.23	0.00	147.44
Het df	2	2	5	13	0	26
Het P	*	(*)	*	***	N.S.	***
Fixed RR	16.59	9.69	8.75	23.82	8.13	15.31
RRl	7.08	7.26	6.98	20.33	3.89	13.64
RRu	38.89	12.93	10.96	27.92	16.99	17.19
P	+++	+++	+++	+++	+++	+++
Random RR	9.25	9.87	10.52	30.34	8.13	18.17
RRl	1.09	5.54	6.71	19.15	3.89	12.92
RRu	78.71	17.57	16.48	48.07	16.99	25.56
P	+	+++	+++	+++	+++	+++
Between Chi						66.06
Between df						4
Between P						***
Btwn(F) P						**

	Study type (1)		
	CC	other	Total
N	19	8	27
NS	13	6	19
Wt	272.69	14.11	286.80
Het Chi	135.10	12.26	147.44
Het df	18	7	26
Het P	***	(*)	***
Fixed RR	15.26	16.48	15.31
RRl	13.55	9.78	13.64
RRu	17.18	27.77	17.19
P	+++	+++	+++
Random RR	19.17	15.12	18.17
RRl	13.00	7.33	12.92
RRu	28.27	31.19	25.56
P	+++	+++	+++
Between Chi			0.08
Between df			1
Between P			N.S.
Btwn(F) P			N.S.

	Study type (2)			
	CC	prosp	other	Total
N	19	6	2	27
NS	13	5	1	19
Wt	272.69	10.60	3.51	286.80
Het Chi	135.10	10.99	1.24	147.44
Het df	18	5	1	26
Het P	***	(*)	N.S.	***
Fixed RR	15.26	16.90	15.27	15.31
RRl	13.55	9.26	5.36	13.64
RRu	17.18	30.86	43.47	17.19
P	+++	+++	+++	+++
Random RR	19.17	14.72	15.26	18.17
RRl	13.00	5.61	4.75	12.92
RRu	28.27	38.60	49.05	25.56
P	+++	+++	+++	+++
Between Chi				0.11
Between df				2
Between P				N.S.
Btwn(F) P				N.S.

Table 5B1 - 3

IESLC - Meta-analysis of Current Smoking (vs never smoking), Any product (or Cigarettes if Any not available)

Small
Most adjusted

	Study size (number of LC cases) 100-249	250-499	500-999	1000+	Total
N	2	5	4	16	27
NS	2	4	3	10	19
Wt	1.74	13.74	11.03	260.28	286.80
Het Chi	1.60	2.81	6.37	132.80	147.44
Het df	1	4	3	15	26
Het P	N.S.	N.S.	(*)	***	***
Fixed RR	30.97	10.55	20.96	15.34	15.31
RRl	7.01	6.22	11.62	13.59	13.64
RRu	136.82	17.89	37.82	17.32	17.19
P	+++	+++	+++	+++	+++
Random RR	25.72	10.55	25.06	18.49	18.17
RRl	3.38	6.22	9.85	12.03	12.92
RRu	195.39	17.89	63.74	28.42	25.56
P	++	+++	+++	+++	+++
Between Chi					3.86
Between df					3
Between P					N.S.
Btwn(F) P					N.S.

	Risky occupational population no	mining	othRisky	Total
N	27			27
NS	19			19
Wt	286.80			286.80
Het Chi	147.44			147.44
Het df	26			26
Het P	***			***
Fixed RR	15.31			15.31
RRl	13.64			13.64
RRu	17.19			17.19
P	+++			+++
Random RR	18.17			18.17
RRl	12.92			12.92
RRu	25.56			25.56
P	+++			+++
Between Chi				
Between df				
Between P				N.S.
Btwn(F) P				N.S.

	National cigarette tobacco type Virginia	blended	other	Total
N	2	25		27
NS	1	18		19
Wt	4.58	282.23		286.80
Het Chi	4.49	141.33		147.44
Het df	1	24		26
Het P	*	***		***
Fixed RR	27.66	15.17		15.31
RRl	11.06	13.50		13.64
RRu	69.14	17.05		17.19
P	+++	+++		+++
Random RR	31.59	17.53		18.17
RRl	4.41	12.34		12.92
RRu	226.41	24.90		25.56
P	+++	+++		+++
Between Chi				1.62
Between df				1
Between P				N.S.
Btwn(F) P				N.S.

Table 5B1 - 3

IESLC - Meta-analysis of Current Smoking (vs never smoking), Any product (or Cigarettes if Any not available)
Small
Most adjusted

	Any proxy use		
	No/nk	Yes	Total
N	22	5	27
NS	15	4	19
Wt	272.90	13.91	286.80
Het Chi	139.74	6.38	147.44
Het df	21	4	26
Het P	***	N.S.	***
Fixed RR	15.08	20.67	15.31
RRl	13.39	12.22	13.64
RRu	16.98	34.97	17.19
P	+++	+++	+++
Random RR	17.25	22.72	18.17
RRl	11.81	11.32	12.92
RRu	25.21	45.63	25.56
P	+++	+++	+++
Between Chi			1.32
Between df			1
Between P			N.S.
Btwn(F) P			N.S.

	Full histological confirmation		
	No	Yes	Total
N	19	8	27
NS	14	5	19
Wt	83.38	203.43	286.80
Het Chi	64.31	82.95	147.44
Het df	18	7	26
Het P	***	***	***
Fixed RR	14.73	15.56	15.31
RRl	11.89	13.56	13.64
RRu	18.26	17.85	17.19
P	+++	+++	+++
Random RR	20.91	14.80	18.17
RRl	12.99	8.52	12.92
RRu	33.68	25.72	25.56
P	+++	+++	+++
Between Chi			0.18
Between df			1
Between P			N.S.
Btwn(F) P			N.S.

	Number of adjustment variables (1)			
	0	1	2+/+nk	Total
N	11	8	8	27
NS	8	6	5	19
Wt	86.47	25.56	174.77	286.80
Het Chi	25.28	12.91	80.24	147.44
Het df	10	7	7	26
Het P	**	(*)	***	***
Fixed RR	9.47	16.76	19.17	15.31
RRl	7.67	11.37	16.53	13.64
RRu	11.69	24.69	22.24	17.19
P	+++	+++	+++	+++
Random RR	13.86	18.10	21.78	18.17
RRl	8.84	10.12	11.80	12.92
RRu	21.75	32.36	40.22	25.56
P	+++	+++	+++	+++
Between Chi				29.01
Between df				2
Between P				***
Btwn(F) P				(*)

Table 5B1 - 3

IESLC - Meta-analysis of Current Smoking (vs never smoking), Any product (or Cigarettes if Any not available)

Small

Most adjusted

	Number of adjustment variables (2)					
	0	1	2	3-5	6+/+nk	Total
N	11	8	7		1	27
NS	8	6	4		1	19
Wt	86.47	25.56	172.55		2.22	286.80
Het Chi	25.28	12.91	79.17		0.00	147.44
Het df	10	7	6		0	26
Het P	**	(*)	***		N.S.	***
Fixed RR	9.47	16.76	19.34		9.64	15.31
RRl	7.67	11.37	16.66		2.59	13.64
RRu	11.69	24.69	22.46		35.91	17.19
P	+++	+++	+++		+++	+++
Random RR	13.86	18.10	23.74		9.64	18.17
RRl	8.84	10.12	12.39		2.59	12.92
RRu	21.75	32.36	45.51		35.91	25.56
P	+++	+++	+++		+++	+++
Between Chi						30.08
Between df						3
Between P						***
Btwn(F) P						N.S.

	Product			
	all/unsp	cig+/-ot	cig only	Total
N	3	20	4	27
NS	3	13	4	20
Wt	13.83	267.12	5.85	286.80
Het Chi	4.22	132.08	10.09	147.44
Het df	2	19	3	26
Het P	N.S.	***	*	***
Fixed RR	12.14	15.43	19.08	15.31
RRl	7.16	13.68	8.48	13.64
RRu	20.56	17.39	42.91	17.19
P	+++	+++	+++	+++
Random RR	14.12	19.07	14.32	18.17
RRl	6.11	12.97	2.51	12.92
RRu	32.67	28.04	81.85	25.56
P	+++	+++	++	+++
Between Chi				1.05
Between df				2
Between P				N.S.
Btwn(F) P				N.S.

	Denominator		
	nev any	nev cigs	Total
N	13	14	27
NS	11	9	20
Wt	133.04	153.76	286.80
Het Chi	33.88	57.30	147.44
Het df	12	13	26
Het P	***	***	***
Fixed RR	9.51	23.12	15.31
RRl	8.03	19.74	13.64
RRu	11.27	27.08	17.19
P	+++	+++	+++
Random RR	11.65	25.90	18.17
RRl	8.14	16.39	12.92
RRu	16.66	40.95	25.56
P	+++	+++	+++
Between Chi			56.26
Between df			1
Between P			***
Btwn(F) P			***

Table 5B1 - 3

IESLC - Meta-analysis of Current Smoking (vs never smoking), Any product (or Cigarettes if Any not available)

Small
Most adjusted

	Derivation of RR/CI			
	Orig	StdCalc	Other	Total
N	10	10	7	27
NS	6	7	6	19
Wt	189.41	85.53	11.87	286.80
Het Chi	80.99	24.52	12.60	147.44
Het df	9	9	6	26
Het P	***	**	*	***
Fixed RR	18.84	9.38	19.20	15.31
RRl	16.34	7.59	10.87	13.64
RRu	21.73	11.59	33.91	17.19
P	+++	+++	+++	+++
Random RR	21.40	13.64	17.69	18.17
RRl	12.87	8.57	7.33	12.92
RRu	35.59	21.71	42.68	25.56
P	+++	+++	+++	+++
Between Chi				29.33
Between df				2
Between P				***
Btwn(F) P				(*)

Table 5B1 - 4

IESLC - Meta-analysis of Current Smoking (vs never smoking), Any product (or Cigarettes if Any not available)
Small
Least adjusted

REF	NRR	X	SEX	AGEL	AGEH	RACE	YF	LC TYPE	LOC	START	ST	NLC	R	VB	P	H	AD	PRODUCT	DENOM	De
BARBON	30	x	m	0	0	all	-	small	Eu:wst	1979	CC	755	n	bl	y	y	0	all/unsp	nev any	st
BOUCOT	71	x	m	0	0	all	0	small	NAmer	1951	pr	121	n	bl	n	n	0	cig only	nev any	ot
BROWN2	18		m	0	0	wh	-	small	NAmer	1984	CC	14596	n	bl	n	y	2	cig+/-ot	nev cigs	or
BROWN2	17		f	0	0	wh	-	small	NAmer	1984	CC	14596	n	bl	n	y	2	cig+/-ot	nev cigs	or
BUFFLE	67		f	0	0	w-hi	-	small	NAmer	1976	CC	943	n	bl	y	n	0	cig+/-ot	nev cigs	st
COMSTO	22		m	0	0	all	-	small	NAmer	1975	ot	258	n	bl	n	n	0	cig+/-ot	nev cigs	st
COMSTO	29		f	0	0	all	-	small	NAmer	1975	ot	258	n	bl	n	n	0	cig+/-ot	nev cigs	st
CPSI	407		f	0	0	all	2	small	NAmer	1959	pr	5138	n	bl	n	n	1	cig only	nev any	ot
CPSII	116		m	0	0	all	2	small	NAmer	1982	pr	3229	n	bl	n	n	1	cig only	nev any	ot
CPSII	119		f	0	0	all	2	small	NAmer	1982	pr	3229	n	bl	n	n	1	cig+/-ot	nev cigs	ot
DORN	339		m	0	0	wh	8	small	NAmer	1954	pr	5097	n	bl	n	n	1	cig only	nev any	ot
ENGELA	84		m	0	0	all	0	small	Eu:Sca	1964	pr	435	n	bl	n	n	7	cig+/-ot	nev cigs	ot
JAHN	6		m	0	0	all	-	small	Eu:Ger	1988	CC	1004	n	bl	n	n	0	cig+/-ot	nev any	st
JAIN	19		m	0	0	all	-	small	NAmer	1981	CC	845	n	V	y	n	0	cig+/-ot	nev cigs	st
JAIN	14		f	0	0	all	-	small	NAmer	1981	CC	845	n	V	y	n	0	cig+/-ot	nev cigs	st
JEDRYC	24		m	0	0	all	-	small	Eu:est	1980	CC	1630	n	bl	y	n	0	cig+/-ot	nev any	st
KHUDER	15		m	0	0	all	-	small	NAmer	1985	CC	482	n	bl	n	y	0	cig+/-ot	nev cigs	or
KIHARA	3		c	0	0	jap	-	small	As:Jap	1991	CC	440	n	bl	n	n	0	all/unsp	nev any	st
LUBIN2	250		m	0	0	all	-	small	Eu:mul	1976	CC	7804	n	bl	n	y	0	cig+/-ot	nev any	st
LUBIN2	262		f	0	0	all	-	small	Eu:mul	1976	CC	7804	n	bl	n	y	0	cig+/-ot	nev any	st
OSANN	12	x	m	0	0	all	-	small	NAmer	1984	CC	1986	n	bl	n	n	0	cig+/-ot	nev cigs	st
OSANN	16	x	f	0	0	all	-	small	NAmer	1984	CC	1986	n	bl	n	n	0	cig+/-ot	nev cigs	st
SOBUE	10	x	m	0	0	all	-	small	As:Jap	1986	CC	1376	n	bl	n	y	0	cig+/-ot	nev cigs	st
SOBUE	26	x	f	0	0	all	-	small	As:Jap	1986	CC	1376	n	bl	n	y	0	cig+/-ot	nev cigs	st
SOBUE2	3		m	0	0	all	-	small	As:Jap	1965	CC	2083	n	bl	n	n	2	cig+/-ot	nev any	or
SOBUE2	7		f	0	0	all	-	small	As:Jap	1965	CC	2083	n	bl	n	n	2	cig+/-ot	nev any	or
SVENSS	63	x	f	0	0	all	-	small	Eu:Sca	1983	CC	210	n	bl	n	n	0	all/unsp	nev any	st

Cigarette type is all/unspec for all RRs

Table 5B1 - 5

IESLC - Meta-analysis of Current Smoking (vs never smoking), Any product (or Cigarettes if Any not available)
Small
Least adjusted

REF	NRR	SEX	AD	Number Exposed Case	Number Exposed Cont	Non-exposed Case	Non-exposed Cont	RR	95.00%CI
BARBON	30	m	0	170	362	6	188	14.71	(6.40- 33.85)
*BOUCOT	71	m	0	8	22177	0	7551	5.79~	(0.33- 100.28)
BROWN2	18	m	2	-	-	-	-	15.10	(12.00- 19.00)
BROWN2	17	f	2	-	-	-	-	42.50	(32.10- 56.60)
Subtotal BROWN2								22.76	(19.04- 27.20)
BUFFLE	67	f	0	49	110	1	112	49.89	(6.77- 367.64)
COMSTO	22	m	0	20	100	2	84	8.40	(1.91- 36.98)
COMSTO	29	f	0	25	52	2	115	27.64	(6.31- 121.08)
Subtotal COMSTO								15.27	(5.36- 43.47)
*CPSI	407	f	1	-	-	-	-	2.12	(0.39- 11.60)
*CPSII	116	m	1	-	-	-	-	72.00	(5.22- 992.66)
*CPSII	119	f	1	-	-	-	-	20.91	(6.10- 71.69)
Subtotal CPSII								26.14	(8.57- 79.74)
*DORN	339	m	1	-	-	-	-	41.49	(14.52- 118.57)
*ENGELA	84	m	7	-	-	-	-	9.64	(2.59- 35.94)
JAHN	6	m	0	86	269	1	138	44.12	(6.08- 320.18)
JAIN	19	m	0	50	118	3	85	12.01	(3.62- 39.78)
JAIN	14	f	0	83	99	2	214	89.71	(21.63- 372.09)
Subtotal JAIN								27.66	(11.06- 69.14)
JEDRYC	24	m	0	105	516	3	289	19.60	(6.17- 62.32)
KHUDER	15	m	0	38	-	1	-	23.10	(3.10- 174.20)
KIHARA	3	c	0	50	162	9	237	8.13	(3.89- 16.99)
LUBIN2	250	m	0	826	6209	34	2616	10.24	(7.24- 14.47)
LUBIN2	262	f	0	119	410	55	1180	6.23	(4.44- 8.74)
Subtotal LUBIN2								7.94	(6.23- 10.12)
OSANN	12	m	0	157	541	4	833	60.43	(22.27- 164.00)
OSANN	16	f	0	134	367	4	1093	99.77	(36.65- 271.61)
Subtotal OSANN								77.59	(38.26- 157.34)
SOBUE	10	m	0	102	650	1	128	20.09	(2.78- 145.28)
SOBUE	26	f	0	23	168	9	857	13.04	(5.93- 28.67)
Subtotal SOBUE								13.83	(6.65- 28.76)
SOBUE2	3	m	2	-	-	-	-	6.90	(4.60- 10.30)
SOBUE2	7	f	2	-	-	-	-	14.40	(9.30- 22.20)
Subtotal SOBUE2								9.69	(7.21- 13.03)
SVENSS	63	f	0	38	53	2	120	43.02	(10.01- 184.90)
Partial Totals				2083	32363	139	15840		

*prospective study ~ With 0.5 adjustment for zero

REF	NRR	SEX	AD	Ys	Ws	Qs	Ps
BARBON	30	m	0	2.69	5.54	0.01	0.0000
*BOUCOT	71	m	0	1.76	0.47	0.45	0.2276
BROWN2	18	m	2	2.71	72.77	0.02	0.0000
BROWN2	17	f	2	3.75	47.77	49.62	0.0000
Subtotal BROWN2				3.12	120.54	49.63	
BUFFLE	67	f	0	3.91	0.96	1.34	0.0001
COMSTO	22	m	0	2.13	1.75	0.63	0.0049
COMSTO	29	f	0	3.32	1.76	0.61	0.0000
Subtotal COMSTO				2.73	3.51	1.24	
*CPSI	407	f	1	0.75	1.34	5.23	0.3853
*CPSII	116	m	1	4.28	0.56	1.33	0.0014
*CPSII	119	f	1	3.04	2.53	0.24	0.0000
Subtotal CPSII				3.26	3.09	1.58	
*DORN	339	m	1	3.73	3.48	3.45	0.0000
*ENGELA	84	m	7	2.27	2.22	0.48	0.0007
JAHN	6	m	0	3.79	0.98	1.09	0.0002
JAIN	19	m	0	2.49	2.68	0.16	0.0000
JAIN	14	f	0	4.50	1.90	5.92	0.0000
Subtotal JAIN				3.32	4.58	6.08	
JEDRYC	24	m	0	2.98	2.87	0.17	0.0000
KHUDER	15	m	0	3.14	0.95	0.16	0.0023
KIHARA	3	c	0	2.10	7.07	2.85	0.0000
LUBIN2	250	m	0	2.33	32.09	5.25	0.0000
LUBIN2	262	f	0	1.83	33.48	27.20	0.0000
Subtotal LUBIN2				2.07	65.56	32.45	
OSANN	12	m	0	4.10	3.85	7.25	0.0000
OSANN	16	f	0	4.60	3.83	13.43	0.0000
Subtotal OSANN				4.35	7.68	20.68	
SOBUE	10	m	0	3.00	0.98	0.07	0.0030
SOBUE	26	f	0	2.57	6.18	0.16	0.0000
Subtotal SOBUE				2.63	7.17	0.23	

Table 5B1 - 5

IESLC - Meta-analysis of Current Smoking (vs never smoking), Any product (or Cigarettes if Any not available)
Small
Least adjusted

REF	NRR	SEX	AD	Ys	Ws	Qs	Ps
SOBUE2	3	m	2	1.93	23.65	15.09	0.0000
SOBUE2	7	f	2	2.67	20.30	0.08	0.0000
Subtotal SOBUE2				2.27	43.95	15.17	
SVENSS	63	f	0	3.76	1.81	1.92	0.0000

	N	27
	NS	19
	Wt	283.75
Het	Chi	144.22
Het	df	26
Het	P	***
Fixed	RR	15.34
	RRl	13.65
	RRu	17.23
	P	+++
Random	RR	18.03
	RRl	12.83
	RRu	25.36
	P	+++
Asymm	P	N.S.

Table 5B1 - 6

IESLC - Meta-analysis of Current Smoking (vs never smoking), Any product (or Cigarettes if Any not available)
Small
Least adjusted

	Sex: combined	male	female	Total
N	1	15	11	27
NS	1	15	11	27
Wt	7.07	154.83	121.85	283.75
Het Chi	0.00	31.76	98.89	144.22
Het df	0	14	10	26
Het P	N.S.	**	***	***
Fixed RR	8.13	13.10	19.45	15.34
RRl	3.89	11.19	16.28	13.65
RRu	16.99	15.33	23.23	17.23
P	+++	+++	+++	+++
Random RR	8.13	15.12	22.78	18.03
RRl	3.89	10.78	11.56	12.83
RRu	16.99	21.20	44.88	25.36
P	+++	+++	+++	+++
Between Chi				13.57
Between df				2
Between P				**
Btwn(F) P				N.S.

	Lung cancer type: small	Total
N	27	27
NS	19	19
Wt	283.75	283.75
Het Chi	144.22	144.22
Het df	26	26
Het P	***	***
Fixed RR	15.34	15.34
RRl	13.65	13.65
RRu	17.23	17.23
P	+++	+++
Random RR	18.03	18.03
RRl	12.83	12.83
RRu	25.36	25.36
P	+++	+++
Between Chi		
Between df		
Between P		N.S.
Btwn(F) P		N.S.

	Location: NAmer	UK	Scand	othEur	China	Japan	othAs	other	Total
N	15		2	5		5			27
NS	10		2	4		3			19
Wt	146.59		4.03	74.95		58.18			283.75
Het Chi	59.93		2.23	10.58		7.17			144.22
Het df	14		1	4		4			26
Het P	***		N.S.	*		N.S.			***
Fixed RR	24.10		18.86	8.80		9.91			15.34
RRl	20.50		7.10	7.02		7.67			13.65
RRu	28.33		50.07	11.03		12.82			17.23
P	+++		+++	+++		+++			+++
Random RR	27.12		19.67	10.74		10.23			18.03
RRl	16.63		4.55	6.71		6.94			12.83
RRu	44.22		85.07	17.17		15.07			25.36
P	+++		+++	+++		+++			+++
Between Chi									64.32
Between df									3
Between P									***
Btwn(F) P									**

Table 5B1 - 6

IESLC - Meta-analysis of Current Smoking (vs never smoking), Any product (or Cigarettes if Any not available)
Small
Least adjusted

	Detailed Country in "other Europe"					
	multi	Germany	othWest	East	Balkans	Total
N	2	1	1	1		5
NS	1	1	1	1		4
Wt	65.56	0.98	5.54	2.87		74.95
Het Chi	4.05	0.00	0.00	0.00		10.58
Het df	1	0	0	0		4
Het P	*	N.S.	N.S.	N.S.		*
Fixed RR	7.94	44.12	14.71	19.60		8.80
RRl	6.23	6.08	6.40	6.17		7.02
RRu	10.12	320.18	33.85	62.32		11.03
P	+++	+++	+++	+++		+++
Random RR	7.97	44.12	14.71	19.60		10.74
RRl	4.90	6.08	6.40	6.17		6.71
RRu	12.98	320.18	33.85	62.32		17.17
P	+++	+++	+++	+++		+++
Between Chi						6.54
Between df						3
Between P						(*)
Btwn(F) P						N.S.

	Detailed Country in "other Asia"			
	India	HongKong	other	Total
N				
NS				
Wt				
Het Chi				
Het df				
Het P				N.S.
Fixed RR				
RRl				
RRu				
P				+++
Random RR				
RRl				
RRu				
P				+++
Between Chi				
Between df				
Between P				N.S.
Btwn(F) P				N.S.

	Detailed other continent			
	SCAmer	Auslia	Africa	Total
N				
NS				
Wt				
Het Chi				
Het df				
Het P				N.S.
Fixed RR				
RRl				
RRu				
P				+++
Random RR				
RRl				
RRu				
P				+++
Between Chi				
Between df				
Between P				N.S.
Btwn(F) P				N.S.

Table 5B1 - 6

IESLC - Meta-analysis of Current Smoking (vs never smoking), Any product (or Cigarettes if Any not available)
Small
Least adjusted

	Start year of study <1960	1960-69	1970-79	1980-89	1990+	Total
N	3	3	6	14	1	27
NS	3	2	4	9	1	19
Wt	5.29	46.17	75.57	149.65	7.07	283.75
Het Chi	9.10	5.91	11.41	51.02	0.00	144.22
Het df	2	2	5	13	0	26
Het P	*	(*)	*	***	N.S.	***
Fixed RR	16.43	9.69	8.77	24.10	8.13	15.34
RRl	7.01	7.26	7.00	20.53	3.89	13.65
RRu	38.53	12.93	10.99	28.28	16.99	17.23
P	+++	+++	+++	+++	+++	+++
Random RR	9.00	9.87	10.60	30.17	8.13	18.03
RRl	1.05	5.54	6.74	19.14	3.89	12.83
RRu	77.47	17.57	16.67	47.55	16.99	25.36
P	+	+++	+++	+++	+++	+++
Between Chi						66.78
Between df						4
Between P						***
Btwn(F) P						**

	Study type (1) CC	other	Total
N	19	8	27
NS	13	6	19
Wt	269.64	14.11	283.75
Het Chi	131.79	12.36	144.22
Het df	18	7	26
Het P	***	(*)	***
Fixed RR	15.28	16.42	15.34
RRl	13.56	9.75	13.65
RRu	17.22	27.67	17.23
P	+++	+++	+++
Random RR	19.01	15.02	18.03
RRl	12.89	7.26	12.83
RRu	28.03	31.09	25.36
P	+++	+++	+++
Between Chi			0.07
Between df			1
Between P			N.S.
Btwn(F) P			N.S.

	Study type (2) CC	prosp	other	Total
N	19	6	2	27
NS	13	5	1	19
Wt	269.64	10.60	3.51	283.75
Het Chi	131.79	11.09	1.24	144.22
Het df	18	5	1	26
Het P	***	*	N.S.	***
Fixed RR	15.28	16.82	15.27	15.34
RRl	13.56	9.22	5.36	13.65
RRu	17.22	30.72	43.47	17.23
P	+++	+++	+++	+++
Random RR	19.01	14.58	15.26	18.03
RRl	12.89	5.53	4.75	12.83
RRu	28.03	38.41	49.05	25.36
P	+++	+++	+++	+++
Between Chi				0.09
Between df				2
Between P				N.S.
Btwn(F) P				N.S.

Table 5B1 - 6

IESLC - Meta-analysis of Current Smoking (vs never smoking), Any product (or Cigarettes if Any not available)

Small

Least adjusted

	Study size (number of LC cases)				
	100-249	250-499	500-999	1000+	Total
N	2	5	4	16	27
NS	2	4	3	10	19
Wt	2.28	13.74	11.07	256.65	283.75
Het Chi	1.51	2.81	6.26	129.69	144.22
Het df	1	4	3	15	26
Het P	N.S.	N.S.	(*)	***	***
Fixed RR	28.39	10.55	21.24	15.35	15.34
RRl	7.75	6.22	11.78	13.58	13.65
RRu	103.99	17.89	38.27	17.34	17.23
P	+++	+++	+++	+++	+++
Random RR	23.30	10.55	25.23	18.33	18.03
RRl	3.81	6.22	10.01	11.88	12.83
RRu	142.54	17.89	63.56	28.26	25.36
P	+++	+++	+++	+++	+++
Between Chi					3.96
Between df					3
Between P					N.S.
Btwn(F) P					N.S.

	Risky occupational population			
	no	mining	othRisky	Total
N	27			27
NS	19			19
Wt	283.75			283.75
Het Chi	144.22			144.22
Het df	26			26
Het P	***			***
Fixed RR	15.34			15.34
RRl	13.65			13.65
RRu	17.23			17.23
P	+++			+++
Random RR	18.03			18.03
RRl	12.83			12.83
RRu	25.36			25.36
P	+++			+++
Between Chi				
Between df				
Between P				N.S.
Btwn(F) P				N.S.

	National cigarette tobacco type			
	Virginia	blended	other	Total
N	2	25		27
NS	1	18		19
Wt	4.58	279.17		283.75
Het Chi	4.49	138.12		144.22
Het df	1	24		26
Het P	*	***		***
Fixed RR	27.66	15.19		15.34
RRl	11.06	13.51		13.65
RRu	69.14	17.08		17.23
P	+++	+++		+++
Random RR	31.59	17.38		18.03
RRl	4.41	12.24		12.83
RRu	226.41	24.69		25.36
P	+++	+++		+++
Between Chi				1.62
Between df				1
Between P				N.S.
Btwn(F) P				N.S.

Table 5B1 - 6

IESLC - Meta-analysis of Current Smoking (vs never smoking), Any product (or Cigarettes if Any not available)

Small

Least adjusted

	Any proxy use No/nk	Yes	Total
N	22	5	27
NS	15	4	19
Wt	269.80	13.95	283.75
Het Chi	136.55	6.27	144.22
Het df	21	4	26
Het P	***	N.S.	***
Fixed RR	15.10	20.89	15.34
RRl	13.40	12.36	13.65
RRu	17.01	35.31	17.23
P	+++	+++	+++
Random RR	17.06	22.85	18.03
RRl	11.68	11.46	12.83
RRu	24.93	45.56	25.36
P	+++	+++	+++
Between Chi			1.40
Between df			1
Between P			N.S.
Btwn(F) P			N.S.

	Full histological confirmation No	Yes	Total
N	19	8	27
NS	14	5	19
Wt	84.00	199.75	283.75
Het Chi	61.56	82.42	144.22
Het df	18	7	26
Het P	***	***	***
Fixed RR	14.66	15.63	15.34
RRl	11.84	13.61	13.65
RRu	18.15	17.96	17.23
P	+++	+++	+++
Random RR	20.46	14.83	18.03
RRl	12.87	8.40	12.83
RRu	32.53	26.17	25.36
P	+++	+++	+++
Between Chi			0.24
Between df			1
Between P			N.S.
Btwn(F) P			N.S.

	Number of adjustment variables (1) 0	1	2+/+nk	Total
N	18	4	5	27
NS	13	3	3	19
Wt	109.14	7.91	166.70	283.75
Het Chi	61.08	9.48	61.12	144.22
Het df	17	3	4	26
Het P	***	*	***	***
Fixed RR	11.78	20.97	17.96	15.34
RRl	9.76	10.44	15.43	13.65
RRu	14.21	42.10	20.91	17.23
P	+++	+++	+++	+++
Random RR	19.43	18.18	15.08	18.03
RRl	12.53	4.73	7.75	12.83
RRu	30.12	69.88	29.34	25.36
P	+++	+++	+++	+++
Between Chi				12.54
Between df				2
Between P				**
Btwn(F) P				N.S.

Table 5B1 - 6

IESLC - Meta-analysis of Current Smoking (vs never smoking), Any product (or Cigarettes if Any not available)
Small
Least adjusted

	Number of adjustment variables (2) 0	1	2	3-5	6+/+nk	Total
N	18	4	4		1	27
NS	13	3	2		1	19
Wt	109.14	7.91	164.48		2.22	283.75
Het Chi	61.08	9.48	60.25		0.00	144.22
Het df	17	3	3		0	26
Het P	***	*	***		N.S.	***
Fixed RR	11.78	20.97	18.12		9.64	15.34
RRl	9.76	10.44	15.55		2.59	13.65
RRu	14.21	42.10	21.11		35.91	17.23
P	+++	+++	+++		+++	+++
Random RR	19.43	18.18	16.04		9.64	18.03
RRl	12.53	4.73	7.81		2.59	12.83
RRu	30.12	69.88	32.96		35.91	25.36
P	+++	+++	+++		+++	+++
Between Chi						13.41
Between df						3
Between P						**
Btwn(F) P						N.S.

	Product all/unsp	cig+/-ot	cig only	Total
N	3	20	4	27
NS	3	13	4	20
Wt	14.41	263.49	5.85	283.75
Het Chi	4.22	128.97	10.20	144.22
Het df	2	19	3	26
Het P	N.S.	***	*	***
Fixed RR	12.58	15.43	18.92	15.34
RRl	7.51	13.68	8.41	13.65
RRu	21.08	17.41	42.55	17.23
P	+++	+++	+++	+++
Random RR	14.32	18.94	14.04	18.03
RRl	6.44	12.86	2.43	12.83
RRu	31.84	27.90	81.00	25.36
P	+++	+++	++	+++
Between Chi				0.83
Between df				2
Between P				N.S.
Btwn(F) P				N.S.

	Denominator nev any	nev cigs	Total
N	13	14	27
NS	11	9	20
Wt	133.62	150.13	283.75
Het Chi	34.22	53.47	144.22
Het df	12	13	26
Het P	***	***	***
Fixed RR	9.56	23.37	15.34
RRl	8.07	19.92	13.65
RRu	11.32	27.42	17.23
P	+++	+++	+++
Random RR	11.73	25.90	18.03
RRl	8.20	16.38	12.83
RRu	16.76	40.94	25.36
P	+++	+++	+++
Between Chi			56.53
Between df			1
Between P			***
Btwn(F) P			***

Table 5B1 - 6

IESLC - Meta-analysis of Current Smoking (vs never smoking), Any product (or Cigarettes if Any not available)

Small
Least adjusted

	Derivation of RR/CI			
	Orig	StdCalc	Other	Total
N	5	16	6	27
NS	3	11	5	19
Wt	165.43	107.72	10.60	283.75
Het Chi	60.31	60.41	11.09	144.22
Het df	4	15	5	26
Het P	***	***	*	***
Fixed RR	18.14	11.75	16.82	15.34
RRl	15.58	9.73	9.22	13.65
RRu	21.13	14.19	30.72	17.23
P	+++	+++	+++	+++
Random RR	16.51	19.89	14.58	18.03
RRl	8.34	12.59	5.53	12.83
RRu	32.66	31.42	38.41	25.36
P	+++	+++	+++	+++
Between Chi				12.42
Between df				2
Between P				**
Btwn(F) P				N.S.

Table 5B1 - 7

IESLC - Meta-analysis of Current Smoking (vs never smoking), Any product (or Cigarettes if Any not available)
Small
Excluded studies (and stage at which they were excluded)

1 BOUCHA BUELL LAURIL MZILEN

2 ABELIN ABRAHA AUVINE AXELSO BAND BERRIN BLOT1 BLOT2 BLOT3 BLOT4 BRESLO BROCKM BROWN1 BYERS1 CASCO2 CASCOR
CHAN CHATZI CHEN CHEN2 CHEN3 CHIAZZ COOKSO DAVEYS DEAN DOSEME DU DUNN EBELIN ESAKI FAN GENG
GER GODLEY GOLLED GSELL HANSEN HEGMAN HINDS HIRAOK HOROWI HORWIT HU HU2 HUANG ISHIMA JARUP JIANG
JONES JUSSAW KO KOHLME KOULUM KREYBE LAMTH LAMWK LAMWK2 LAUSSM LEI LETOUR LEVIN LIU LIU2 LIU3
LIU4 LIU5 LOMBA2 LUO MAGNUS MARSH MARSH2 MARTIS MASTRA MATSUD MCCONN MCDUFF MCLAUG MILLER MILLS MOLLO
NOTAN2 NOU ORMOS PASTOR PAWLEG PERNU PERSHA PIKE POFFIJ POLEDN PRESCO QIAO RADZIK RANDIG REN RONCO
ROOTS ROTHSC SADOWS SANKAR SCHWA2 SEGI SEOW SIEMIA SIMARA STASZE STOCKS SUN TAO ULMER VANDER WANG
WANG3 WANG4 WICKLU WILKIN WUWILL WYNDE2 WYNDE4 XIANGZ XU XU2 XU3 XU4 YUAN ZHANG ZHENG ZHOU

3 RESTRE

4 BOFFET

5 RIMING TANG2 WYNDE5 WYNDE8

6 BYERS2 HIRAY2 LICKIN MRFIT MURATA NOTANI SAARIK SHIMIZ SITAS STAYNE WARSIN WATSON WYNDER

8 AGUDO AKIBA ALDERS AMANDU AMES ANDERS ARCHER ARMADA AUSTIN AXELSS BECHER BENHAM BENSHL BEST BLOHMK BRETT
BROSS CARPEN CEDERL CHANG CHOI CHOW CHYOU CORREA DAMBER DARBY DEAN2 DEAN3 DEKLER DESTE2 DESTEF DOCKER
DOLL DOLL2 DORANT DORGAN DROSTE ENSTRO GAO GAO2 GARCIA GARDIN GARSHI GILLIS GOODMA GRAHAM GREGOR HAENSZ
HAMMO2 HAMMON HEIN HENNEK HIRAYA HITOSU HOLE HUMBLE JARVHO JOLY KAISE2 KAISER KANELL KATSOU KAUFMA KELLER
KINLEN KJUUS KNEKT KOO KREUZE KUBIK LANGE LEMARC LIAW LIDDEL LOMBAR LUBIN MACLEN MATOS MIGRAN MRFITR
NAM ODRISC OSANN2 PARKIN PERSH2 PETO PEZZO2 PEZZOT PISANI QIAO2 RACHTA SCHWAR SEGI2 SHAW SIMONA SPEIZE
SPITZ STOCKW STUCKE SUZUK2 SUZUKI TANG TENKAN TIZZAN TOKARS TOUSEY TSUGAN TULINI TVERDA VEIERO VUTUC WAKAI
WALD WANG2 WIGLE WU WU2 WUNSCH WYNDE3 WYNDE6 WYNDE7 YAMAGU YONG

Table 5B1 - 8

Potentially overlapping studies

REF	REFGP	PRINC	. OVERLAP/LINK
LUBIN2	LUBIN2	1	Lubin-combined
CPSI	CPSI	1	CPSI overall
SOBUE2	SOBUE2	1	SOBUE2/MATSUD
JAHN	BOFFET	2	Subset of BOFFET

Table 5C1 -

IESLC - Meta-anal of Ever Smoking (or Current if Ever not available), Any prod (or Cigs if Any not avail)
Small

This analysis is restricted to results for:
1) Non-dose-response data
2) Results complete enough for use in metaanalysis

Within each study, results are then selected (in the following order of preference, within each sex) for:
3) SMKSTA: ever smokers, current smokers
4) PRODUCT: all/unspec, cigarettes regardless of other products, cigarettes only
5) CIGTYPE: all/unspecified, MC regardless of HR, MC only
6) DENOM: never smoked anything, never smoked cigarettes, (never +1 = +long term ex, +2 = +amount unknown, +3 = never cigs+long term ex)
7) Followup period (YF, prospective studies): whole study (coded as 0) or longest available
8) LCtype: small (specifically)

9) Race: all or nearest available, otherwise by race (wh or w = white, bl or b = black, hi = hispanic ch = chinese, jap = japanese, haw = hawaiian, w+o = white + oriental, sca = scandinavian, as = asian)
10) For overlapping studies: principal rather than subsidiary studies
Finally by Age: whole study (coded as 0) if available, otherwise by widest available age group
and then for single sex results (m, f) in preference to combined sex results (c).

Results adjusted (AD) for the most potential confounders are then chosen in Sections -1 to -3 and results adjusted for the least confounders in Sections -4 to -6. (Those least adjusted results which actually differ from the most adjusted as marked 'x' in column X in Section -4)
(Results adjusted for an unknown number of confounder(s) are coded as 20.)

Section -7 shows excluded studies, together with the stage (as above) at which no qualifying results were found.

Section -8 lists the potentially overlapping studies which have been included (1=principal, 2=subsidiary).

Section -9 lists any results which would have been included in preference except that they had data not complete enough for use in meta-analysis, with their significance (yes/no), if known, and any further comment as entered on the database.

In addition to those mentioned above, the following fields, levels and abbreviations are used:
* or nk = not known, n = no, y = yes, ot = other
ev = ever, cu = current, nev = never
all/unspec = all or unspecified, cig+/-ot = cigarettes irrespective of other products (cigar, pipe etc)
MC = manufactured cigarettes, HR = hand-rolled cigarettes
REF: 6-character study reference
NRR: number of the RR on the database within the study
ST : study type (CC = case control, pr or prosp = prospective)
NLC: number of lung cancer cases in whole study
R : risky occupational population (n = no, m = mining, o = other risky)
VB : national cigarette type (V = at least 75% Virginia, bl = at least 75% blended, ot = other)
P : any proxy use
H : full histological confirmation
De : derivation of RR/CI (or = original, st = standard method, ot = other method of estimation)

Table 5C1 - 1

IESLC - Meta-anal of Ever Smoking (or Current if Ever not available), Any prod (or Cigs if Any not avail)
Small
Most adjusted

REF	NRR	SEX	AGEL	AGEH	RACE	YF	LC TYPE	LOC	START	ST	NLC	R	VB	P	H	AD	SM	PRODUCT	DENOM	De
ABRAHA	3	m	0	0	all	0	small	Eu:est	1975	pr	571	n	bl	n	n	0	ev	all/unsp	nev any	ot
ABRAHA	6	f	0	0	all	0	small	Eu:est	1975	pr	571	n	bl	n	n	0	ev	all/unsp	nev any	ot
ALDERS	53	m	0	0	all	-	small	Eu:UK	1977	CC	1448	n	V	n	n	2	ev	all/unsp	nev any	or
ALDERS	56	f	0	0	all	-	small	Eu:UK	1977	CC	1448	n	V	n	n	2	ev	all/unsp	nev any	or
ANDERS	9	f	0	0	all	0	small	NAmer	1986	pr	343	n	bl	n	n	0	ev	cig+/-ot	nev cigs	st
BAND	3	m	0	0	all	-	small	NAmer	1983	CC	2831	n	V	y	y	2	ev	cig only	nev any	ot
BARBON	128	m	0	0	all	-	small	Eu:wst	1979	CC	755	n	bl	y	y	3	ev	all/unsp	nev any	ot
BOUCOT	144	m	0	0	all	0	small	NAmer	1951	pr	121	n	bl	n	n	2	cu	cig only	nev any	ot
BROWN2	8	m	0	0	wh	-	small	NAmer	1984	CC	14596	n	bl	n	y	2	ev	cig+/-ot	nev cigs	or
BROWN2	7	f	0	0	wh	-	small	NAmer	1984	CC	14596	n	bl	n	y	2	ev	cig+/-ot	nev cigs	or
BUFFLE	51	m	0	0	wh	-	small	NAmer	1976	CC	943	n	bl	y	n	0	ev	cig+/-ot	nev cigs	ot
BUFFLE	66	f	0	0	w-hi	-	small	NAmer	1976	CC	943	n	bl	y	n	0	ev	cig+/-ot	nev cigs	st
BYERS1	2	m	0	0	wh	-	small	NAmer	1957	CC	1002	n	bl	n	n	0	ev	cig+/-ot	nev cigs	st
COMSTO	65	m	0	0	all	-	small	NAmer	1975	ot	258	n	bl	n	n	0	ev	cig+/-ot	nev cigs	st
COMSTO	77	f	0	0	all	-	small	NAmer	1975	ot	258	n	bl	n	n	0	ev	cig+/-ot	nev cigs	st
CPSI	407	f	0	0	all	2	small	NAmer	1959	pr	5138	n	bl	n	n	1	cu	cig only	nev any	ot
CPSII	116	m	0	0	all	2	small	NAmer	1982	pr	3229	n	bl	n	n	1	cu	cig only	nev any	ot
CPSII	119	f	0	0	all	2	small	NAmer	1982	pr	3229	n	bl	n	n	1	cu	cig+/-ot	nev cigs	ot
DAMBER	31	m	0	0	all	-	small	Eu:Sca	1972	CC	579	n	bl	y	n	1	ev	all/unsp	nev any	or
DORGAN	119	m	0	0	wh	-	small	NAmer	1980	CC	2026	n	bl	y	y	2	ev	cig+/-ot	nev any	or
DORGAN	101	f	0	0	all	-	small	NAmer	1980	CC	2026	n	bl	y	y	3	ev	cig+/-ot	nev any	or
DORN	339	m	0	0	wh	8	small	NAmer	1954	pr	5097	n	bl	n	n	1	cu	cig only	nev any	ot
DOSEME	2	m	0	0	all	-	small	Eu:bal	1979	CC	1210	n	bl	n	n	2	ev	cig+/-ot	nev cigs	or
ENGELA	90	m	0	0	all	0	small	Eu:Sca	1964	pr	435	n	bl	n	n	7	ev	cig+/-ot	nev cigs	ot
FAN	5	c	0	0	all	-	small	As:Chi	1990	CC	403	n	ot	y	n	0	ev	cig+/-ot	nev cigs	ot
GAO	4	m	0	0	all	-	small	As:Chi	1984	CC	1405	n	ot	n	n	2	ev	cig+/-ot	nev cigs	or
GAO	14	f	0	0	all	-	small	As:Chi	1984	CC	1405	n	ot	n	n	2	ev	cig+/-ot	nev cigs	or
HEGMAN	3	c	0	0	all	-	small	NAmer	1989	CC	282	n	bl	y	y	0	ev	all/unsp	nev any	st
ISHIMA	7	c	0	0	all	-	small	As:Jap	1961	CC	180	n	bl	y	y	5	ev	all/unsp	nev any	st
JAHN	45	m	0	0	all	-	small	Eu:Ger	1988	CC	1004	n	bl	n	n	0	ev	all/unsp	nev any	st
JAIN	49	m	0	0	all	-	small	NAmer	1981	CC	845	n	V	y	n	2	ev	cig+/-ot	nev cigs	or
JAIN	44	f	0	0	all	-	small	NAmer	1981	CC	845	n	V	y	n	2	ev	cig+/-ot	nev cigs	or
JEDRYC	55	m	0	0	all	-	small	Eu:est	1980	CC	1630	n	bl	y	n	3	ev	cig+/-ot	nev any	ot
KHUDER	25	m	0	0	all	-	small	NAmer	1985	CC	482	n	bl	n	y	0	ev	cig+/-ot	nev cigs	ot
KIHARA	27	c	0	0	jap	-	small	As:Jap	1991	CC	440	n	bl	n	n	0	ev	all/unsp	nev any	st
LAMTH	2	f	0	0	ch	-	small	As:HK	1983	CC	445	n	bl	n	n	0	ev	all/unsp	nev any	or
LAMWK	3	f	0	0	ch	-	small	As:HK	1981	CC	163	n	bl	n	n	0	ev	all/unsp	nev any	st
LAMWK2	2	m	0	0	all	-	small	As:HK	1976	CC	480	n	bl	n	n	0	ev	all/unsp	nev any	st
LAMWK2	6	f	0	0	all	-	small	As:HK	1976	CC	480	n	bl	n	n	0	ev	all/unsp	nev any	st
LUBIN2	146	m	0	0	all	-	small	Eu:mul	1976	CC	7804	n	bl	n	y	0	ev	cig+/-ot	nev any	st
LUBIN2	166	f	0	0	all	-	small	Eu:mul	1976	CC	7804	n	bl	n	y	0	ev	cig+/-ot	nev any	st
NOU	2	m	0	0	all	-	small	Eu:Sca	1971	CC	273	n	bl	y	n	0	ev	all/unsp	nev any	st
NOU	7	f	0	0	all	-	small	Eu:Sca	1971	CC	273	n	bl	y	n	0	ev	all/unsp	nev any	st
ORMOS	12	m	0	0	all	-	small	Eu:est	1947	CC	119	n	bl	y	y	0	ev	cig+/-ot	nev any	st
OSANN	45	m	0	0	all	-	small	NAmer	1984	CC	1986	n	bl	n	n	2	ev	cig+/-ot	nev cigs	or
OSANN	46	f	0	0	all	-	small	NAmer	1984	CC	1986	n	bl	n	n	2	ev	cig+/-ot	nev cigs	or
PEZZOT	8	m	0	0	all	-	small	SCAmer	1987	CC	215	n	bl	n	y	0	ev	cig only	nev cigs	ot
SEOW	4	f	0	0	ch	-	small	As:oth	1997	CC	153	n	bl	n	y	0	ev	cig+/-ot	nev cigs	st
SIEMIA	6	m	0	0	all	-	small	NAmer	1979	CC	857	n	V	y	y	7	ev	cig+/-ot	nev cigs	or
SOBUE	101	m	0	0	all	-	small	As:Jap	1986	CC	1376	n	bl	n	y	1	ev	cig+/-ot	nev cigs	ot
SOBUE	111	f	0	0	all	-	small	As:Jap	1986	CC	1376	n	bl	n	y	1	ev	cig+/-ot	nev cigs	ot
SOBUE2	3	m	0	0	all	-	small	As:Jap	1965	CC	2083	n	bl	n	n	2	cu	cig+/-ot	nev any	or
SOBUE2	7	f	0	0	all	-	small	As:Jap	1965	CC	2083	n	bl	n	n	2	cu	cig+/-ot	nev any	or
STASZE	29	m	0	0	all	-	small	Eu:est	1954	CC	281	n	bl	n	y	0	ev	all/unsp	nev any	st
STASZE	39	f	0	0	all	-	small	Eu:est	1954	CC	281	n	bl	n	y	0	ev	all/unsp	nev any	st
STAYNE	2	m	0	0	all	-	small	NAmer	1969	CC	420	n	bl	n	n	0	ev	all/unsp	nev any	st
SVENSS	73	f	0	0	all	-	small	Eu:Sca	1983	CC	210	n	bl	n	n	1	ev	all/unsp	nev any	ot
TIZZAN	20	c	0	0	all	-	small	Eu:wst	1959	CC	1358	n	bl	n	n	0	ev	all/unsp	nev any	st
WUWILL	10	f	0	0	all	-	small	As:Chi	1985	CC	965	n	ot	n	n	3	ev	cig+/-ot	nev cigs	or
ZHOU	14	m	0	0	all	-	small	As:Chi	1978	CC	1360	n	ot	n	n	0	ev	all/unsp	nev any	st
ZHOU	15	f	0	0	all	-	small	As:Chi	1978	CC	1360	n	ot	n	n	0	ev	all/unsp	nev any	st

Cigarette type is all/unspec for all RRs

Table 5C1 - 2

IESLC - Meta-anal of Ever Smoking (or Current if Ever not available), Any prod (or Cigs if Any not avail)
Small
Most adjusted

REF	NRR	SEX	AD	Number Exposed Case	Number Exposed Cont	Non-exposed Case	Non-exposed Cont	RR	95.00%CI
*ABRAHA	3	m	0	68	10351	2	3365	11.05	(2.71- 45.07)
*ABRAHA	6	f	0	26	5256	5	11589	11.47	(4.41- 29.84)
Subtotal ABRAHA								11.33	(5.14- 24.99)
ALDERS	53	m	2	-	-	-	-	6.99	(1.50- 32.57)
ALDERS	56	f	2	-	-	-	-	7.47	(3.17- 17.63)
Subtotal ALDERS								7.35	(3.48- 15.56)
*ANDERS	9	f	0	76	96164	4	195158	38.56	(14.11- 105.38)
BAND	3	m	2	-	-	-	-	45.79	(14.41- 145.53)
BARBON	128	m	3	-	-	-	-	11.01	(4.81- 25.22)
*BOUCOT	144	m	2	-	-	-	-	6.42	(0.37- 111.22)
BROWN2	8	m	2	-	-	-	-	11.40	(9.10- 14.20)
BROWN2	7	f	2	-	-	-	-	37.60	(28.50- 49.30)
Subtotal BROWN2								18.32	(15.41- 21.77)
BUFFLE	51	m	0	-	-	-	-	14.38	(2.81- 73.55)
BUFFLE	66	f	0	61	166	1	112	41.16	(5.62- 301.22)
Subtotal BUFFLE								21.95	(6.21- 77.55)
BYERS1	2	m	0	85	695	4	424	12.96	(4.72- 35.59)
COMSTO	65	m	0	25	229	2	84	4.59	(1.06- 19.78)
COMSTO	77	f	0	27	87	2	115	17.84	(4.13- 77.08)
Subtotal COMSTO								9.04	(3.21- 25.43)
*CPSI	407	f	1	-	-	-	-	2.12	(0.39- 11.60)
*CPSII	116	m	1	-	-	-	-	72.00	(5.22- 992.66)
*CPSII	119	f	1	-	-	-	-	20.91	(6.10- 71.69)
Subtotal CPSII								26.14	(8.57- 79.74)
DAMBER	31	m	1	-	-	-	-	13.80	(5.20- 45.60)
DORGAN	119	m	2	-	-	-	-	22.90	(3.20- 166.00)
DORGAN	101	f	3	-	-	-	-	62.60	(23.00- 171.00)
Subtotal DORGAN								50.93	(20.83- 124.56)
*DORN	339	m	1	-	-	-	-	41.49	(14.52- 118.57)
DOSEME	2	m	2	-	-	-	-	5.40	(2.90- 10.00)
*ENGELA	90	m	7	-	-	-	-	4.35	(1.19- 15.88)
FAN	5	c	0	40	595	15	556	2.49	(1.36- 4.56)
GAO	4	m	2	-	-	-	-	7.40	(2.30- 24.10)
GAO	14	f	2	-	-	-	-	7.90	(3.60- 17.00)
Subtotal GAO								7.74	(4.05- 14.80)
HEGMAN	3	c	0	50	1202	1	2080	86.52	(11.94- 627.12)
ISHIMA	7	c	5	-	-	-	-	3.00	(0.75- 17.23)
JAHN	45	m	0	166	701	1	138	32.68	(4.54- 235.34)
JAIN	49	m	2	-	-	-	-	6.33	(2.16- 27.00)
JAIN	44	f	2	-	-	-	-	48.00	(10.50- 849.00)
Subtotal JAIN								10.47	(3.50- 31.29)
JEDRYC	55	m	3	-	-	-	-	10.81	(3.36- 34.80)
KHUDER	25	m	0	74	-	1	-	27.02	(3.66- 199.46)
KIHARA	27	c	0	56	232	9	237	6.36	(3.07- 13.15)
LAMTH	2	f	0	42	14	9	36	12.00	(4.65- 30.98)
LAMWK	3	f	0	29	41	3	144	33.95	(9.84- 117.12)
LAMWK2	2	m	0	39	161	1	43	10.42	(1.39- 77.99)
LAMWK2	6	f	0	12	50	4	139	8.34	(2.57- 27.06)
Subtotal LAMWK2								8.83	(3.20- 24.38)
LUBIN2	146	m	0	1129	10433	34	2616	8.33	(5.90- 11.74)
LUBIN2	166	f	0	145	567	55	1180	5.49	(3.96- 7.60)
Subtotal LUBIN2								6.68	(5.28- 8.47)
NOU	2	m	0	42	247	1	122	20.74	(2.82- 152.52)
NOU	7	f	0	5	92	2	261	7.09	(1.35- 37.19)
Subtotal NOU								10.99	(3.07- 39.32)
ORMOS	12	m	0	41	1034	2	777	15.40	(3.71- 63.88)
OSANN	45	m	2	-	-	-	-	37.50	(13.90- 102.00)
OSANN	46	f	2	-	-	-	-	86.00	(31.60- 234.00)
Subtotal OSANN								56.68	(27.97- 114.86)
PEZZOT	8	m	0	36	317	0	116	26.79~	(1.63- 439.95)
SEOW	4	f	0	13	15	2	125	54.17	(11.13- 263.53)
SIEMIA	6	m	7	-	-	-	-	15.50	(3.70- 64.50)
SOBUE	101	m	1	-	-	-	-	21.15	(5.22- 85.73)
SOBUE	111	f	1	-	-	-	-	10.77	(5.63- 20.57)
Subtotal SOBUE								12.13	(6.74- 21.84)
SOBUE2	3	m	2	-	-	-	-	6.90	(4.60- 10.30)
SOBUE2	7	f	2	-	-	-	-	14.40	(9.30- 22.20)
Subtotal SOBUE2								9.69	(7.21- 13.03)
STASZE	29	m	0	29	754	3	158	2.03	(0.61- 6.73)
STASZE	39	f	0	1	153	4	1660	2.71	(0.30- 24.42)
Subtotal STASZE								2.17	(0.76- 6.21)

Table 5C1 - 2

IESLC - Meta-anal of Ever Smoking (or Current if Ever not available), Any prod (or Cigs if Any not avail)
Small
Most adjusted

REF	NRR	SEX	AD	Number Exposed Case	Cont	Non-exposed Case	Cont	RR	95.00%CI
STAYNE	2	m	0	41	567	4	333	6.02	(2.14- 16.96)
SVENSS	73	f	1	-	-	-	-	34.32	(6.13- 192.17)
TIZZAN	20	c	0	101	939	18	419	2.50	(1.50- 4.19)
WUWILL	10	f	3	-	-	-	-	2.20	(1.40- 3.20)
ZHOU	14	m	0	74	41	17	36	3.82	(1.91- 7.63)
ZHOU	15	f	0	9	7	28	32	1.47	(0.48- 4.46)
Subtotal ZHOU								2.93	(1.63- 5.26)
Partial Totals				2542	131110	234	222055		

*prospective study ~ With 0.5 adjustment for zero

REF	NRR	SEX	AD	Ys	Ws	Qs	Ps
*ABRAHA	3	m	0	2.40	1.94	0.02	0.0008
*ABRAHA	6	f	0	2.44	4.20	0.08	0.0000
Subtotal ABRAHA				2.43	6.14	0.10	
ALDERS	53	m	2	1.94	1.62	0.21	0.0133
ALDERS	56	f	2	2.01	5.22	0.44	0.0000
Subtotal ALDERS				2.00	6.84	0.65	
*ANDERS	9	f	0	3.65	3.80	6.93	0.0000
BAND	3	m	2	3.82	2.87	6.66	0.0000
BARBON	128	m	3	2.40	5.60	0.05	0.0000
*BOUCOT	144	m	2	1.86	0.47	0.09	0.2014
BROWN2	8	m	2	2.43	77.61	1.35	0.0000
BROWN2	7	f	2	3.63	51.16	89.89	0.0000
Subtotal BROWN2				2.91	128.77	91.25	
BUFFLE	51	m	0	2.67	1.44	0.19	0.0014
BUFFLE	66	f	0	3.72	0.97	1.94	0.0003
Subtotal BUFFLE				3.09	2.41	2.14	
BYERS1	2	m	0	2.56	3.77	0.26	0.0000
COMSTO	65	m	0	1.52	1.80	1.09	0.0412
COMSTO	77	f	0	2.88	1.79	0.60	0.0001
Subtotal COMSTO				2.20	3.59	1.69	
*CPSI	407	f	1	0.75	1.34	3.21	0.3853
*CPSII	116	m	1	4.28	0.56	2.18	0.0014
*CPSII	119	f	1	3.04	2.53	1.38	0.0000
Subtotal CPSII				3.26	3.09	3.56	
DAMBER	31	m	1	2.62	3.26	0.34	0.0000
DORGAN	119	m	2	3.13	0.99	0.68	0.0019
DORGAN	101	f	3	4.14	3.82	12.86	0.0000
Subtotal DORGAN				3.93	4.80	13.54	
*DORN	339	m	1	3.73	3.48	7.07	0.0000
DOSEME	2	m	2	1.69	10.03	3.79	0.0000
*ENGELA	90	m	7	1.47	2.29	1.58	0.0261
FAN	5	c	0	0.91	10.51	20.26	0.0031
GAO	4	m	2	2.00	2.78	0.25	0.0008
GAO	14	f	2	2.07	6.38	0.35	0.0000
Subtotal GAO				2.05	9.16	0.60	
HEGMAN	3	c	0	4.46	0.98	4.56	0.0000
ISHIMA	7	c	5	1.10	1.56	2.26	0.1695
JAHN	45	m	0	3.49	0.99	1.38	0.0005
JAIN	49	m	2	1.85	2.41	0.50	0.0042
JAIN	44	f	2	3.87	0.80	1.96	0.0006
Subtotal JAIN				2.35	3.21	2.46	
JEDRYC	55	m	3	2.38	2.81	0.02	0.0001
KHUDER	25	m	0	3.30	0.96	0.95	0.0012
KIHARA	27	c	0	1.85	7.27	1.49	0.0000
LAMTH	2	f	0	2.48	4.27	0.14	0.0000
LAMWK	3	f	0	3.52	2.51	3.75	0.0000
LAMWK2	2	m	0	2.34	0.95	0.00	0.0225
LAMWK2	6	f	0	2.12	2.77	0.09	0.0004
Subtotal LAMWK2				2.18	3.72	0.09	
LUBIN2	146	m	0	2.12	32.49	1.08	0.0000
LUBIN2	166	f	0	1.70	36.11	12.97	0.0000
Subtotal LUBIN2				1.90	68.61	14.04	
NOU	2	m	0	3.03	0.97	0.52	0.0029
NOU	7	f	0	1.96	1.40	0.16	0.0205
Subtotal NOU				2.40	2.36	0.68	
ORMOS	12	m	0	2.73	1.90	0.36	0.0002
OSANN	45	m	2	3.62	3.87	6.77	0.0000
OSANN	46	f	2	4.45	3.83	17.77	0.0000
Subtotal OSANN				4.04	7.70	24.53	

Table 5C1 - 2

IESLC - Meta-anal of Ever Smoking (or Current if Ever not available), Any prod (or Cigs if Any not avail)
Small
Most adjusted

REF	NRR	SEX	AD	Ys	Ws	Qs	Ps
PEZZOT	8	m	0	3.29	0.49	0.48	0.0213
SEOW	4	f	0	3.99	1.53	4.39	0.0000
SIEMIA	6	m	7	2.74	1.88	0.36	0.0002
SOBUE	101	m	1	3.05	1.96	1.10	0.0000
SOBUE	111	f	1	2.38	9.15	0.05	0.0000
Subtotal SOBUE				2.50	11.11	1.16	
SOBUE2	3	m	2	1.93	23.65	3.24	0.0000
SOBUE2	7	f	2	2.67	20.30	2.71	0.0000
Subtotal SOBUE2				2.27	43.95	5.95	
STASZE	29	m	0	0.71	2.66	6.78	0.2493
STASZE	39	f	0	1.00	0.80	1.35	0.3735
Subtotal STASZE				0.77	3.46	8.13	
STAYNE	2	m	0	1.80	3.58	0.92	0.0007
SVENSS	73	f	1	3.54	1.29	1.97	0.0001
TIZZAN	20	c	0	0.92	14.51	27.79	0.0005
WUWILL	10	f	3	0.79	22.48	51.47	0.0002
ZHOU	14	m	0	1.34	8.03	7.41	0.0001
ZHOU	15	f	0	0.38	3.12	11.45	0.4969
Subtotal ZHOU				1.07	11.15	18.86	

	N	61
	NS	44

	Wt	436.52
Het	Chi	341.97
Het	df	60
Het	P	***
Fixed	RR	9.99
	RRl	9.09
	RRu	10.97
	P	+++
Random	RR	11.14
	RRl	8.59
	RRu	14.46
	P	+++
Asymm	P	N.S.

Table 5C1 - 3

IESLC - Meta-anal of Ever Smoking (or Current if Ever not available), Any prod (or Cigs if Any not avail)
Small
Most adjusted

	Sex			
	combined	male	female	Total
N	5	32	24	61
NS	5	32	24	61
Wt	34.84	210.11	191.57	436.52
Het Chi	15.50	57.60	218.84	341.97
Het df	4	31	23	60
Het P	**	**	***	***
Fixed RR	3.38	9.80	12.42	9.99
RRl	2.43	8.56	10.78	9.09
RRu	4.71	11.22	14.31	10.97
P	+++	+++	+++	+++
Random RR	4.55	10.34	13.58	11.14
RRl	2.14	8.11	8.25	8.59
RRu	9.67	13.18	22.34	14.46
P	+++	+++	+++	+++
Between Chi				50.03
Between df				2
Between P				***
Btwn(F) P				*

	Lung cancer type	
	small	Total
N	61	61
NS	44	44
Wt	436.52	436.52
Het Chi	341.97	341.97
Het df	60	60
Het P	***	***
Fixed RR	9.99	9.99
RRl	9.09	9.09
RRu	10.97	10.97
P	+++	+++
Random RR	11.14	11.14
RRl	8.59	8.59
RRu	14.46	14.46
P	+++	+++
Between Chi		
Between df		
Between P		N.S.
Btwn(F) P		N.S.

	Location								
	NAmer	UK	Scand	othEur	China	Japan	othAs	other	Total
N	24	2	5	12	6	6	5	1	61
NS	17	1	4	9	4	4	4	1	44
Wt	176.71	6.84	9.21	114.04	53.30	63.90	12.03	0.49	436.52
Het Chi	89.80	0.01	4.47	28.60	12.83	10.56	5.40	0.00	341.97
Het df	23	1	4	11	5	5	4	0	60
Het P	***	N.S.	N.S.	**	*	(*)	N.S.	N.S.	***
Fixed RR	19.61	7.35	11.10	6.14	2.97	9.33	16.42	26.79	9.99
RRl	16.92	3.48	5.82	5.11	2.27	7.30	9.33	1.63	9.09
RRu	22.72	15.56	21.19	7.37	3.89	11.93	28.90	439.95	10.97
P	+++	+++	+++	+++	+++	+++	+++	+	+++
Random RR	21.95	7.35	11.19	6.58	3.33	9.19	17.00	26.79	11.14
RRl	14.73	3.48	5.60	4.57	2.08	6.12	8.65	1.63	8.59
RRu	32.71	15.56	22.34	9.48	5.35	13.80	33.44	439.95	14.46
P	+++	+++	+++	+++	+++	+++	+++	+	+++
Between Chi									190.31
Between df									7
Between P									***
Btwn(F) P									***

Table 5C1 - 3

IESLC - Meta-anal of Ever Smoking (or Current if Ever not available), Any prod (or Cigs if Any not avail)
Small
Most adjusted

	Detailed Country in "other Europe"					
	multi	Germany	othWest	East	Balkans	Total
N	2	1	2	6	1	12
NS	1	1	2	4	1	9
Wt	68.61	0.99	20.11	14.31	10.03	114.04
Het Chi	2.98	0.00	8.86	7.77	0.00	28.60
Het df	1	0	1	5	0	11
Het P	(*)	N.S.	**	N.S.	N.S.	**
Fixed RR	6.68	32.68	3.78	7.84	5.40	6.14
RRl	5.28	4.54	2.44	4.67	2.91	5.11
RRu	8.47	235.34	5.85	13.16	10.03	7.37
P	+++	+++	+++	+++	+++	+++
Random RR	6.73	32.68	5.06	7.63	5.40	6.58
RRl	4.47	4.54	1.19	3.92	2.91	4.57
RRu	10.13	235.34	21.56	14.84	10.03	9.48
P	+++	+++	+	+++	+++	+++
Between Chi						9.00
Between df						4
Between P						(*)
Btwn(F) P						N.S.

	Detailed Country in "other Asia"			
	India	HongKong	other	Total
N		4	1	5
NS		3	1	4
Wt		10.50	1.53	12.03
Het Chi		2.89	0.00	5.40
Het df		3	0	4
Het P		N.S.	N.S.	N.S.
Fixed RR		13.79	54.17	16.42
RRl		7.53	11.13	9.33
RRu		25.26	263.53	28.90
P		+++	+++	+++
Random RR		13.79	54.17	17.00
RRl		7.53	11.13	8.65
RRu		25.26	263.53	33.44
P		+++	+++	+++
Between Chi				2.51
Between df				1
Between P				N.S.
Btwn(F) P				N.S.

	Detailed other continent			
	SCAmer	Auslia	Africa	Total
N	1			1
NS	1			1
Wt	0.49			0.49
Het Chi	0.00			0.00
Het df	0			0
Het P	N.S.			N.S.
Fixed RR	26.79			26.79
RRl	1.63			1.63
RRu	439.95			439.95
P	+			+
Random RR	26.79			26.79
RRl	1.63			1.63
RRu	439.95			439.95
P	+			+
Between Chi				
Between df				
Between P				N.S.
Btwn(F) P				N.S.

Table 5C1 - 3

IESLC - Meta-anal of Ever Smoking (or Current if Ever not available), Any prod (or Cigs if Any not avail)
Small
Most adjusted

	Start year of study <1960	1960-69	1970-79	1980-89	1990+	Total
N	8	5	20	25	3	61
NS	7	4	12	18	3	44
Wt	28.93	51.38	125.59	211.30	19.32	436.52
Het Chi	31.82	9.78	26.68	178.70	14.04	341.97
Het df	7	4	19	24	2	60
Het P	***	*	N.S.	***	***	***
Fixed RR	4.85	8.73	6.98	15.16	4.53	9.99
RRl	3.37	6.64	5.86	13.24	2.90	9.09
RRu	6.99	11.47	8.31	17.34	7.07	10.97
P	+++	+++	+++	+++	+++	+++
Random RR	6.21	7.49	7.39	20.78	7.77	11.14
RRl	2.47	4.43	5.76	13.15	2.06	8.59
RRu	15.62	12.68	9.48	32.83	29.30	14.46
P	+++	+++	+++	+++	++	+++
Between Chi						80.95
Between df						4
Between P						***
Btwn(F) P						**

	Study type (1) CC	other	Total
N	50	11	61
NS	36	8	44
Wt	412.32	24.20	436.52
Het Chi	317.53	20.66	341.97
Het df	49	10	60
Het P	***	*	***
Fixed RR	9.77	14.67	9.99
RRl	8.87	9.85	9.09
RRu	10.76	21.85	10.97
P	+++	+++	+++
Random RR	10.82	13.34	11.14
RRl	8.13	7.31	8.59
RRu	14.39	24.33	14.46
P	+++	+++	+++
Between Chi			3.79
Between df			1
Between P			(*)
Btwn(F) P			N.S.

	Study type (2) CC	prosp	other	Total
N	50	9	2	61
NS	36	7	1	44
Wt	412.32	20.61	3.59	436.52
Het Chi	317.53	18.01	1.66	341.97
Het df	49	8	1	60
Het P	***	*	N.S.	***
Fixed RR	9.77	15.96	9.04	9.99
RRl	8.87	10.37	3.21	9.09
RRu	10.76	24.58	25.43	10.97
P	+++	+++	+++	+++
Random RR	10.82	14.43	9.04	11.14
RRl	8.13	7.26	2.39	8.59
RRu	14.39	28.71	34.25	14.46
P	+++	+++	++	+++
Between Chi				4.77
Between df				2
Between P				(*)
Btwn(F) P				N.S.

Table 5C1 - 3

IESLC - Meta-anal of Ever Smoking (or Current if Ever not available), Any prod (or Cigs if Any not avail)
Small
Most adjusted

	Study size (number of LC cases)				
	100-249	250-499	500-999	1000+	Total
N	7	16	10	28	61
NS	7	12	7	18	44
Wt	9.76	46.80	44.98	334.98	436.52
Het Chi	9.00	40.13	38.53	219.34	341.97
Het df	6	15	9	27	60
Het P	N.S.	***	***	***	***
Fixed RR	19.39	6.73	5.27	11.28	9.99
RRl	10.35	5.05	3.93	10.14	9.09
RRu	36.31	8.96	7.06	12.56	10.97
P	+++	+++	+++	+++	+++
Random RR	18.81	8.30	10.85	11.91	11.14
RRl	8.48	4.98	5.26	8.38	8.59
RRu	41.73	13.82	22.36	16.92	14.46
P	+++	+++	+++	+++	+++
Between Chi					34.97
Between df					3
Between P					***
Btwn(F) P					N.S.

	Risky occupational population			
	no	mining	othRisky	Total
N	61			61
NS	44			44
Wt	436.52			436.52
Het Chi	341.97			341.97
Het df	60			60
Het P	***			***
Fixed RR	9.99			9.99
RRl	9.09			9.09
RRu	10.97			10.97
P	+++			+++
Random RR	11.14			11.14
RRl	8.59			8.59
RRu	14.46			14.46
P	+++			+++
Between Chi				
Between df				
Between P				N.S.
Btwn(F) P				N.S.

	National cigarette tobacco type			
	Virginia	blended	other	Total
N	6	49	6	61
NS	4	36	4	44
Wt	14.80	368.42	53.30	436.52
Het Chi	9.42	230.42	12.83	341.97
Het df	5	48	5	60
Het P	(*)	***	*	***
Fixed RR	12.45	11.80	2.97	9.99
RRl	7.48	10.65	2.27	9.09
RRu	20.72	13.07	3.89	10.97
P	+++	+++	+++	+++
Random RR	13.42	12.96	3.33	11.14
RRl	6.42	9.89	2.08	8.59
RRu	28.05	17.00	5.35	14.46
P	+++	+++	+++	+++
Between Chi				89.31
Between df				2
Between P				***
Btwn(F) P				***

Table 5C1 - 3

IESLC - Meta-anal of Ever Smoking (or Current if Ever not available), Any prod (or Cigs if Any not avail)
Small
Most adjusted

	Any proxy use No/nk	Yes	Total
N	44	17	61
NS	31	13	44
Wt	392.36	44.16	436.52
Het Chi	288.24	53.41	341.97
Het df	43	16	60
Het P	***	***	***
Fixed RR	9.90	10.83	9.99
RRl	8.97	8.07	9.09
RRu	10.93	14.55	10.97
P	+++	+++	+++
Random RR	10.18	14.94	11.14
RRl	7.55	8.36	8.59
RRu	13.73	26.68	14.46
P	+++	+++	+++
Between Chi			0.32
Between df			1
Between P			N.S.
Btwn(F) P			N.S.

	Full histological confirmation No	Yes	Total
N	42	19	61
NS	30	14	44
Wt	201.99	234.53	436.52
Het Chi	172.18	129.59	341.97
Het df	41	18	60
Het P	***	***	***
Fixed RR	7.20	13.24	9.99
RRl	6.28	11.65	9.09
RRu	8.27	15.05	10.97
P	+++	+++	+++
Random RR	9.84	14.62	11.14
RRl	7.19	9.38	8.59
RRu	13.45	22.80	14.46
P	+++	+++	+++
Between Chi			40.20
Between df			1
Between P			***
Btwn(F) P			**

	Number of adjustment variables (1) 0	1	2+/+nk	Total
N	30	8	23	61
NS	22	6	16	44
Wt	158.52	23.58	254.43	436.52
Het Chi	91.81	12.46	193.20	341.97
Het df	29	7	22	60
Het P	***	(*)	***	***
Fixed RR	6.58	15.71	12.42	9.99
RRl	5.63	10.49	10.99	9.09
RRu	7.69	23.52	14.05	10.97
P	+++	+++	+++	+++
Random RR	8.79	16.98	12.27	11.14
RRl	6.31	9.38	7.98	8.59
RRu	12.23	30.74	18.86	14.46
P	+++	+++	+++	+++
Between Chi				44.50
Between df				2
Between P				***
Btwn(F) P				*

Table 5C1 - 3

IESLC - Meta-anal of Ever Smoking (or Current if Ever not available), Any prod (or Cigs if Any not avail)

Small

Most adjusted

	Number of adjustment variables (2) 0	1	2	3-5	6+/+nk	Total
N	30	8	16	5	2	61
NS	22	6	10	5	2	45
Wt	158.52	23.58	213.98	36.28	4.17	436.52
Het Chi	91.81	12.46	103.18	44.86	1.67	341.97
Het df	29	7	15	4	1	60
Het P	***	(*)	***	***	N.S.	***
Fixed RR	6.58	15.71	14.84	4.60	7.72	9.99
RRl	5.63	10.49	12.98	3.32	2.95	9.09
RRu	7.69	23.52	16.96	6.37	20.15	10.97
P	+++	+++	+++	+++	+++	+++
Random RR	8.79	16.98	14.33	8.69	7.91	11.14
RRl	6.31	9.38	9.24	2.34	2.28	8.59
RRu	12.23	30.74	22.20	32.30	27.42	14.46
P	+++	+++	+++	++	++	+++
Between Chi						88.00
Between df						4
Between P						***
Btwn(F) P						**

	Product all/unsp	cig+/-ot	cig only	Total
N	23	32	6	61
NS	17	22	6	45
Wt	79.50	347.81	9.21	436.52
Het Chi	55.70	246.77	11.57	341.97
Het df	22	31	5	60
Het P	***	***	*	***
Fixed RR	6.25	10.85	25.53	9.99
RRl	5.02	9.76	13.38	9.09
RRu	7.79	12.05	48.69	10.97
P	+++	+++	+++	+++
Random RR	7.71	12.86	20.61	11.14
RRl	5.27	9.10	6.82	8.59
RRu	11.28	18.17	62.29	14.46
P	+++	+++	+++	+++
Between Chi				27.94
Between df				2
Between P				***
Btwn(F) P				(*)

	Denominator nev any	nev cigs	Total
N	36	25	61
NS	27	18	45
Wt	210.29	226.23	436.52
Het Chi	114.81	205.03	341.97
Het df	35	24	60
Het P	***	***	***
Fixed RR	7.91	12.41	9.99
RRl	6.91	10.90	9.09
RRu	9.05	14.14	10.97
P	+++	+++	+++
Random RR	9.57	13.57	11.14
RRl	7.17	8.58	8.59
RRu	12.78	21.46	14.46
P	+++	+++	+++
Between Chi			22.14
Between df			1
Between P			***
Btwn(F) P			*

Table 5C1 - 3

IESLC - Meta-anal of Ever Smoking (or Current if Ever not available), Any prod (or Cigs if Any not avail)

Small
Most adjusted

	Derivation of RR/CI			
	Orig	StdCalc	Other	Total
N	19	24	18	61
NS	12	18	15	45
Wt	246.35	136.26	53.91	436.52
Het Chi	182.31	75.74	47.33	341.97
Het df	18	23	17	60
Het P	***	***	***	***
Fixed RR	12.55	6.58	10.14	9.99
RRl	11.07	5.56	7.76	9.09
RRu	14.21	7.78	13.24	10.97
P	+++	+++	+++	+++
Random RR	13.11	8.38	12.85	11.14
RRl	8.25	5.79	7.86	8.59
RRu	20.81	12.13	21.00	14.46
P	+++	+++	+++	+++
Between Chi				36.60
Between df				2
Between P				***
Btwn(F) P				*

	Smoking status		
	ever	current	Total
N	54	7	61
NS	39	5	44
Wt	384.19	52.33	436.52
Het Chi	322.05	19.52	341.97
Het df	53	6	60
Het P	***	**	***
Fixed RR	9.88	10.85	9.99
RRl	8.94	8.28	9.09
RRu	10.92	14.23	10.97
P	+++	+++	+++
Random RR	10.98	13.01	11.14
RRl	8.25	6.73	8.59
RRu	14.61	25.15	14.46
P	+++	+++	+++
Between Chi			0.41
Between df			1
Between P			N.S.
Btwn(F) P			N.S.

Table 5C1 - 4

IESLC - Meta-anal of Ever Smoking (or Current if Ever not available), Any prod (or Cigs if Any not avail)
Small
Least adjusted

REF	NRR	X	SEX	AGEL	AGEH	RACE	YF	LC TYPE	LOC	START	ST	NLC	R	VB	P	H	AD	SM	PRODUCT	DENOM	De
ABRAHA	3		m	0	0	all	0	small	Eu:est	1975	pr	571	n	bl	n	n	0	ev	all/unsp	nev any	ot
ABRAHA	6		f	0	0	all	0	small	Eu:est	1975	pr	571	n	bl	n	n	0	ev	all/unsp	nev any	ot
ALDERS	53		m	0	0	all	-	small	Eu:UK	1977	CC	1448	n	V	n	n	2	ev	all/unsp	nev any	or
ALDERS	56		f	0	0	all	-	small	Eu:UK	1977	CC	1448	n	V	n	n	2	ev	all/unsp	nev any	or
ANDERS	9		f	0	0	all	0	small	NAmer	1986	pr	343	n	bl	n	n	0	ev	cig+/-ot	nev cigs	st
BAND	3		m	0	0	all	-	small	NAmer	1983	CC	2831	n	V	y	y	2	ev	cig only	nev any	ot
BARBON	114	x	m	0	0	all	-	small	Eu:wst	1979	CC	755	n	bl	y	y	0	ev	all/unsp	nev any	st
BOUCOT	71	x	m	0	0	all	0	small	NAmer	1951	pr	121	n	bl	n	n	0	cu	cig only	nev any	ot
BROWN2	8		m	0	0	wh	-	small	NAmer	1984	CC	14596	n	bl	n	y	2	ev	cig+/-ot	nev cigs	or
BROWN2	7		f	0	0	wh	-	small	NAmer	1984	CC	14596	n	bl	n	y	2	ev	cig+/-ot	nev cigs	or
BUFFLE	51		m	0	0	wh	-	small	NAmer	1976	CC	943	n	bl	y	n	0	ev	cig+/-ot	nev cigs	ot
BUFFLE	66		f	0	0	w-hi	-	small	NAmer	1976	CC	943	n	bl	y	n	0	ev	cig+/-ot	nev cigs	st
BYERS1	2		m	0	0	wh	-	small	NAmer	1957	CC	1002	n	bl	n	n	0	ev	cig+/-ot	nev cigs	st
COMSTO	65		m	0	0	all	-	small	NAmer	1975	ot	258	n	bl	n	n	0	ev	cig+/-ot	nev cigs	st
COMSTO	77		f	0	0	all	-	small	NAmer	1975	ot	258	n	bl	n	n	0	ev	cig+/-ot	nev cigs	st
CPSI	407		f	0	0	all	2	small	NAmer	1959	pr	5138	n	bl	n	n	1	cu	cig only	nev any	ot
CPSII	116		m	0	0	all	2	small	NAmer	1982	pr	3229	n	bl	n	n	1	cu	cig only	nev any	ot
CPSII	119		f	0	0	all	2	small	NAmer	1982	pr	3229	n	bl	n	n	1	cu	cig+/-ot	nev cigs	ot
DAMBER	10	x	m	0	0	all	-	small	Eu:Sca	1972	CC	579	n	bl	y	n	0	ev	all/unsp	nev any	st
DORGAN	119		m	0	0	wh	-	small	NAmer	1980	CC	2026	n	bl	y	y	2	ev	cig+/-ot	nev any	or
DORGAN	101		f	0	0	all	-	small	NAmer	1980	CC	2026	n	bl	y	y	3	ev	cig+/-ot	nev any	or
DORN	339		m	0	0	wh	8	small	NAmer	1954	pr	5097	n	bl	n	n	1	cu	cig only	nev any	ot
DOSEME	18	x	m	0	0	all	-	small	Eu:bal	1979	CC	1210	n	bl	n	n	0	ev	cig+/-ot	nev cigs	st
ENGELA	90		m	0	0	all	0	small	Eu:Sca	1964	pr	435	n	bl	n	n	7	ev	cig+/-ot	nev cigs	ot
FAN	5		c	0	0	all	-	small	As:Chi	1990	CC	403	n	ot	y	n	0	ev	cig+/-ot	nev cigs	ot
GAO	9	x	m	0	0	all	-	small	As:Chi	1984	CC	1405	n	ot	n	n	0	ev	cig+/-ot	nev cigs	st
GAO	19	x	f	0	0	all	-	small	As:Chi	1984	CC	1405	n	ot	n	n	0	ev	cig+/-ot	nev cigs	st
HEGMAN	3		c	0	0	all	-	small	NAmer	1989	CC	282	n	bl	y	y	0	ev	all/unsp	nev any	st
ISHIMA	2	x	c	0	0	all	-	small	As:Jap	1961	CC	180	n	bl	y	y	0	ev	all/unsp	nev any	st
JAHN	45		m	0	0	all	-	small	Eu:Ger	1988	CC	1004	n	bl	n	n	0	ev	all/unsp	nev any	st
JAIN	9	x	m	0	0	all	-	small	NAmer	1981	CC	845	n	V	y	n	0	ev	cig+/-ot	nev cigs	st
JAIN	4	x	f	0	0	all	-	small	NAmer	1981	CC	845	n	V	y	n	0	ev	cig+/-ot	nev cigs	st
JEDRYC	14	x	m	0	0	all	-	small	Eu:est	1980	CC	1630	n	bl	y	n	0	ev	cig+/-ot	nev any	st
KHUDER	25		m	0	0	all	-	small	NAmer	1985	CC	482	n	bl	n	y	0	ev	cig+/-ot	nev cigs	ot
KIHARA	27		c	0	0	jap	-	small	As:Jap	1991	CC	440	n	bl	n	n	0	ev	all/unsp	nev any	st
LAMTH	2		f	0	0	ch	-	small	As:HK	1983	CC	445	n	bl	n	n	0	ev	all/unsp	nev any	or
LAMWK	3		f	0	0	ch	-	small	As:HK	1981	CC	163	n	bl	n	n	0	ev	all/unsp	nev any	st
LAMWK2	2		m	0	0	all	-	small	As:HK	1976	CC	480	n	bl	n	n	0	ev	all/unsp	nev any	st
LAMWK2	6		f	0	0	all	-	small	As:HK	1976	CC	480	n	bl	n	n	0	ev	all/unsp	nev any	st
LUBIN2	146		m	0	0	all	-	small	Eu:mul	1976	CC	7804	n	bl	n	y	0	ev	cig+/-ot	nev any	st
LUBIN2	166		f	0	0	all	-	small	Eu:mul	1976	CC	7804	n	bl	n	y	0	ev	cig+/-ot	nev any	st
NOU	2		m	0	0	all	-	small	Eu:Sca	1971	CC	273	n	bl	y	n	0	ev	all/unsp	nev any	st
NOU	7		f	0	0	all	-	small	Eu:Sca	1971	CC	273	n	bl	y	n	0	ev	all/unsp	nev any	st
ORMOS	12		m	0	0	all	-	small	Eu:est	1947	CC	119	n	bl	y	y	0	ev	cig+/-ot	nev any	st
OSANN	20	x	m	0	0	all	-	small	NAmer	1984	CC	1986	n	bl	n	n	0	ev	cig+/-ot	nev cigs	st
OSANN	24	x	f	0	0	all	-	small	NAmer	1984	CC	1986	n	bl	n	n	0	ev	cig+/-ot	nev cigs	st
PEZZOT	8		m	0	0	all	-	small	SCAmer	1987	CC	215	n	bl	n	y	0	ev	cig only	nev cigs	ot
SEOW	4		f	0	0	ch	-	small	As:oth	1997	CC	153	n	bl	n	y	0	ev	cig+/-ot	nev cigs	st
SIEMIA	10	x	m	0	0	all	-	small	NAmer	1979	CC	857	n	V	y	y	0	ev	cig+/-ot	nev cigs	st
SOBUE	11	x	m	0	0	all	-	small	As:Jap	1986	CC	1376	n	bl	n	y	0	ev	cig+/-ot	nev cigs	st
SOBUE	27	x	f	0	0	all	-	small	As:Jap	1986	CC	1376	n	bl	n	y	0	ev	cig+/-ot	nev cigs	st
SOBUE2	3		m	0	0	all	-	small	As:Jap	1965	CC	2083	n	bl	n	n	2	cu	cig+/-ot	nev any	or
SOBUE2	7		f	0	0	all	-	small	As:Jap	1965	CC	2083	n	bl	n	n	2	cu	cig+/-ot	nev any	or
STASZE	29		m	0	0	all	-	small	Eu:est	1954	CC	281	n	bl	n	y	0	ev	all/unsp	nev any	st
STASZE	39		f	0	0	all	-	small	Eu:est	1954	CC	281	n	bl	n	y	0	ev	all/unsp	nev any	st
STAYNE	2		m	0	0	all	-	small	NAmer	1969	CC	420	n	bl	n	n	0	ev	all/unsp	nev any	st
SVENSS	58	x	f	0	0	all	-	small	Eu:Sca	1983	CC	210	n	bl	n	n	0	ev	all/unsp	nev any	st
TIZZAN	20		c	0	0	all	-	small	Eu:wst	1959	CC	1358	n	bl	n	n	0	ev	all/unsp	nev any	st
WUWILL	10		f	0	0	all	-	small	As:Chi	1985	CC	965	n	ot	n	n	3	ev	cig+/-ot	nev cigs	or
ZHOU	14		m	0	0	all	-	small	As:Chi	1978	CC	1360	n	ot	n	n	0	ev	all/unsp	nev any	st
ZHOU	15		f	0	0	all	-	small	As:Chi	1978	CC	1360	n	ot	n	n	0	ev	all/unsp	nev any	st

Cigarette type is all/unspec for all RRs

Table 5C1 - 5

IESLC - Meta-anal of Ever Smoking (or Current if Ever not available), Any prod (or Cigs if Any not avail)
Small
Least adjusted

REF	NRR	SEX	AD	Number Exposed Case	Number Exposed Cont	Non-exposed Case	Non-exposed Cont	RR	95.00%CI
*ABRAHA	3	m	0	68	10351	2	3365	11.05	(2.71- 45.07)
*ABRAHA	6	f	0	26	5256	5	11589	11.47	(4.41- 29.84)
Subtotal ABRAHA								11.33	(5.14- 24.99)
ALDERS	53	m	2	-	-	-	-	6.99	(1.50- 32.57)
ALDERS	56	f	2	-	-	-	-	7.47	(3.17- 17.63)
Subtotal ALDERS								7.35	(3.48- 15.56)
*ANDERS	9	f	0	76	96164	4	195158	38.56	(14.11- 105.38)
BAND	3	m	2	-	-	-	-	45.79	(14.41- 145.53)
BARBON	114	m	0	212	567	6	188	11.72	(5.12- 26.81)
*BOUCOT	71	m	0	8	22177	0	7551	5.79~	(0.33- 100.28)
BROWN2	8	m	2	-	-	-	-	11.40	(9.10- 14.20)
BROWN2	7	f	2	-	-	-	-	37.60	(28.50- 49.30)
Subtotal BROWN2								18.32	(15.41- 21.77)
BUFFLE	51	m	0	-	-	-	-	14.38	(2.81- 73.55)
BUFFLE	66	f	0	61	166	1	112	41.16	(5.62- 301.22)
Subtotal BUFFLE								21.95	(6.21- 77.55)
BYERS1	2	m	0	85	695	4	424	12.96	(4.72- 35.59)
COMSTO	65	m	0	25	229	2	84	4.59	(1.06- 19.78)
COMSTO	77	f	0	27	87	2	115	17.84	(4.13- 77.08)
Subtotal COMSTO								9.04	(3.21- 25.43)
*CPSI	407	f	1	-	-	-	-	2.12	(0.39- 11.60)
*CPSII	116	m	1	-	-	-	-	72.00	(5.22- 992.66)
*CPSII	119	f	1	-	-	-	-	20.91	(6.10- 71.69)
Subtotal CPSII								26.14	(8.57- 79.74)
DAMBER	10	m	0	145	99	5	47	13.77	(5.29- 35.84)
DORGAN	119	m	2	-	-	-	-	22.90	(3.20- 166.00)
DORGAN	101	f	3	-	-	-	-	62.60	(23.00- 171.00)
Subtotal DORGAN								50.93	(20.83- 124.56)
*DORN	339	m	1	-	-	-	-	41.49	(14.52- 118.57)
DOSEME	18	m	0	143	536	13	293	6.01	(3.35- 10.80)
*ENGELA	90	m	7	-	-	-	-	4.35	(1.19- 15.88)
FAN	5	c	0	40	595	15	556	2.49	(1.36- 4.56)
GAO	9	m	0	60	558	3	202	7.24	(2.25- 23.34)
GAO	19	f	0	17	130	17	605	4.65	(2.31- 9.36)
Subtotal GAO								5.23	(2.87- 9.52)
HEGMAN	3	c	0	50	1202	1	2080	86.52	(11.94- 627.12)
ISHIMA	2	c	0	31	25	4	10	3.10	(0.87- 11.08)
JAHN	45	m	0	166	701	1	138	32.68	(4.54- 235.34)
JAIN	9	m	0	80	277	3	85	8.18	(2.52- 26.58)
JAIN	4	f	0	103	196	2	214	56.23	(13.69- 230.92)
Subtotal JAIN								18.04	(7.30- 44.58)
JEDRYC	14	m	0	148	1054	3	289	13.53	(4.28- 42.74)
KHUDER	25	m	0	74	-	1	-	27.02	(3.66- 199.46)
KIHARA	27	c	0	56	232	9	237	6.36	(3.07- 13.15)
LAMTH	2	f	0	42	14	9	36	12.00	(4.65- 30.98)
LAMWK	3	f	0	29	41	3	144	33.95	(9.84- 117.12)
LAMWK2	2	m	0	39	161	1	43	10.42	(1.39- 77.99)
LAMWK2	6	f	0	12	50	4	139	8.34	(2.57- 27.06)
Subtotal LAMWK2								8.83	(3.20- 24.38)
LUBIN2	146	m	0	1129	10433	34	2616	8.33	(5.90- 11.74)
LUBIN2	166	f	0	145	567	55	1180	5.49	(3.96- 7.60)
Subtotal LUBIN2								6.68	(5.28- 8.47)
NOU	2	m	0	42	247	1	122	20.74	(2.82- 152.52)
NOU	7	f	0	5	92	2	261	7.09	(1.35- 37.19)
Subtotal NOU								10.99	(3.07- 39.32)
ORMOS	12	m	0	41	1034	2	777	15.40	(3.71- 63.88)
OSANN	20	m	0	191	1018	4	833	39.07	(14.45- 105.62)
OSANN	24	f	0	165	563	4	1093	80.08	(29.55- 217.03)
Subtotal OSANN								55.89	(27.64- 113.00)
PEZZOT	8	m	0	36	317	0	116	26.79~	(1.63- 439.95)
SEOW	4	f	0	13	15	2	125	54.17	(11.13- 263.53)
SIEMIA	10	m	0	157	428	2	105	19.26	(4.70- 78.95)
SOBUE	11	m	0	130	1013	1	128	16.43	(2.28- 118.50)
SOBUE	27	f	0	26	232	9	857	10.67	(4.93- 23.09)
Subtotal SOBUE								11.30	(5.51- 23.19)
SOBUE2	3	m	2	-	-	-	-	6.90	(4.60- 10.30)
SOBUE2	7	f	2	-	-	-	-	14.40	(9.30- 22.20)
Subtotal SOBUE2								9.69	(7.21- 13.03)
STASZE	29	m	0	29	754	3	158	2.03	(0.61- 6.73)
STASZE	39	f	0	1	153	4	1660	2.71	(0.30- 24.42)
Subtotal STASZE								2.17	(0.76- 6.21)

Table 5C1 - 5

IESLC - Meta-anal of Ever Smoking (or Current if Ever not available), Any prod (or Cigs if Any not avail)
Small
Least adjusted

REF	NRR	SEX	AD	Number Exposed Case	Number Exposed Cont	Non-exposed Case	Non-exposed Cont	RR	95.00%CI
STAYNE	2	m	0	41	567	4	333	6.02	(2.14- 16.96)
SVENSS	58	f	0	43	89	2	120	28.99	(6.84- 122.85)
TIZZAN	20	c	0	101	939	18	419	2.50	(1.50- 4.19)
WUWILL	10	f	3	-	-	-	-	2.20	(1.40- 3.20)
ZHOU	14	m	0	74	41	17	36	3.82	(1.91- 7.63)
ZHOU	15	f	0	9	7	28	32	1.47	(0.48- 4.46)
Subtotal ZHOU								2.93	(1.63- 5.26)
Partial Totals				4201	160072	312	234675		

*prospective study ~ With 0.5 adjustment for zero

REF	NRR	SEX	AD	Ys	Ws	Qs	Ps
*ABRAHA	3	m	0	2.40	1.94	0.02	0.0008
*ABRAHA	6	f	0	2.44	4.20	0.08	0.0000
Subtotal ABRAHA				2.43	6.14	0.11	
ALDERS	53	m	2	1.94	1.62	0.20	0.0133
ALDERS	56	f	2	2.01	5.22	0.43	0.0000
Subtotal ALDERS				2.00	6.84	0.63	
*ANDERS	9	f	0	3.65	3.80	6.97	0.0000
BAND	3	m	2	3.82	2.87	6.69	0.0000
BARBON	114	m	0	2.46	5.60	0.15	0.0000
*BOUCOT	71	m	0	1.76	0.47	0.14	0.2276
BROWN2	8	m	2	2.43	77.61	1.43	0.0000
BROWN2	7	f	2	3.63	51.16	90.38	0.0000
Subtotal BROWN2				2.91	128.77	91.81	
BUFFLE	51	m	0	2.67	1.44	0.20	0.0014
BUFFLE	66	f	0	3.72	0.97	1.95	0.0003
Subtotal BUFFLE				3.09	2.41	2.15	
BYERS1	2	m	0	2.56	3.77	0.26	0.0000
COMSTO	65	m	0	1.52	1.80	1.08	0.0412
COMSTO	77	f	0	2.88	1.79	0.61	0.0001
Subtotal COMSTO				2.20	3.59	1.69	
*CPSI	407	f	1	0.75	1.34	3.19	0.3853
*CPSII	116	m	1	4.28	0.56	2.18	0.0014
*CPSII	119	f	1	3.04	2.53	1.39	0.0000
Subtotal CPSII				3.26	3.09	3.58	
DAMBER	10	m	0	2.62	4.20	0.44	0.0000
DORGAN	119	m	2	3.13	0.99	0.68	0.0019
DORGAN	101	f	3	4.14	3.82	12.91	0.0000
Subtotal DORGAN				3.93	4.80	13.59	
*DORN	339	m	1	3.73	3.48	7.10	0.0000
DOSEME	18	m	0	1.79	11.21	2.85	0.0000
*ENGELA	90	m	7	1.47	2.29	1.57	0.0261
FAN	5	c	0	0.91	10.51	20.16	0.0031
GAO	9	m	0	1.98	2.80	0.28	0.0009
GAO	19	f	0	1.54	7.87	4.55	0.0000
Subtotal GAO				1.65	10.68	4.83	
HEGMAN	3	c	0	4.46	0.98	4.58	0.0000
ISHIMA	2	c	0	1.13	2.37	3.22	0.0817
JAHN	45	m	0	3.49	0.99	1.39	0.0005
JAIN	9	m	0	2.10	2.77	0.11	0.0005
JAIN	4	f	0	4.03	1.92	5.77	0.0000
Subtotal JAIN				2.89	4.69	5.88	
JEDRYC	14	m	0	2.60	2.90	0.27	0.0000
KHUDER	25	m	0	3.30	0.96	0.96	0.0012
KIHARA	27	c	0	1.85	7.27	1.46	0.0000
LAMTH	2	f	0	2.48	4.27	0.15	0.0000
LAMWK	3	f	0	3.52	2.51	3.77	0.0000
LAMWK2	2	m	0	2.34	0.95	0.00	0.0225
LAMWK2	6	f	0	2.12	2.77	0.09	0.0004
Subtotal LAMWK2				2.18	3.72	0.09	
LUBIN2	146	m	0	2.12	32.49	1.04	0.0000
LUBIN2	166	f	0	1.70	36.11	12.81	0.0000
Subtotal LUBIN2				1.90	68.61	13.85	
NOU	2	m	0	3.03	0.97	0.52	0.0029
NOU	7	f	0	1.96	1.40	0.16	0.0205
Subtotal NOU				2.40	2.36	0.68	
ORMOS	12	m	0	2.73	1.90	0.36	0.0002
OSANN	20	m	0	3.67	3.88	7.27	0.0000
OSANN	24	f	0	4.38	3.86	16.80	0.0000
Subtotal OSANN				4.02	7.75	24.07	

Table 5C1 - 5

IESLC - Meta-anal of Ever Smoking (or Current if Ever not available), Any prod (or Cigs if Any not avail)
Small
Least adjusted

REF	NRR	SEX	AD	Ys	Ws	Qs	Ps
PEZZOT	8	m	0	3.29	0.49	0.48	0.0213
SEOW	4	f	0	3.99	1.53	4.40	0.0000
SIEMIA	10	m	0	2.96	1.93	0.84	0.0000
SOBUE	11	m	0	2.80	0.98	0.25	0.0055
SOBUE	27	f	0	2.37	6.45	0.03	0.0000
Subtotal SOBUE				2.42	7.43	0.28	
SOBUE2	3	m	2	1.93	23.65	3.17	0.0000
SOBUE2	7	f	2	2.67	20.30	2.77	0.0000
Subtotal SOBUE2				2.27	43.95	5.94	
STASZE	29	m	0	0.71	2.66	6.75	0.2493
STASZE	39	f	0	1.00	0.80	1.34	0.3735
Subtotal STASZE				0.77	3.46	8.09	
STAYNE	2	m	0	1.80	3.58	0.91	0.0007
SVENSS	58	f	0	3.37	1.84	2.11	0.0000
TIZZAN	20	c	0	0.92	14.51	27.64	0.0005
WUWILL	10	f	3	0.79	22.48	51.23	0.0002
ZHOU	14	m	0	1.34	8.03	7.36	0.0001
ZHOU	15	f	0	0.38	3.12	11.40	0.4969
Subtotal ZHOU				1.07	11.15	18.76	

	N	61
	NS	44

	Wt	439.51
Het	Chi	349.34
Het	df	60
Het	P	***
Fixed	RR	9.95
	RRl	9.06
	RRu	10.93
	P	+++
Random	RR	11.19
	RRl	8.62
	RRu	14.54
	P	+++
Asymm	P	N.S.

Table 5C1 - 6

IESLC - Meta-anal of Ever Smoking (or Current if Ever not available), Any prod (or Cigs if Any not avail)
Small
Least adjusted

	Sex			
	combined	male	female	Total
N	5	32	24	61
NS	5	32	24	61
Wt	35.64	211.79	192.08	439.51
Het Chi	15.50	56.95	227.04	349.34
Het df	4	31	23	60
Het P	**	**	***	***
Fixed RR	3.38	9.89	12.25	9.95
RRl	2.43	8.64	10.63	9.06
RRu	4.69	11.31	14.11	10.93
P	+++	+++	+++	+++
Random RR	4.49	10.50	13.43	11.19
RRl	2.16	8.26	8.13	8.62
RRu	9.33	13.35	22.18	14.54
P	+++	+++	+++	+++
Between Chi				49.86
Between df				2
Between P				***
Btwn(F) P				*

	Lung cancer type	
	small	Total
N	61	61
NS	44	44
Wt	439.51	439.51
Het Chi	349.34	349.34
Het df	60	60
Het P	***	***
Fixed RR	9.95	9.95
RRl	9.06	9.06
RRu	10.93	10.93
P	+++	+++
Random RR	11.19	11.19
RRl	8.62	8.62
RRu	14.54	14.54
P	+++	+++
Between Chi		
Between df		
Between P		N.S.
Btwn(F) P		N.S.

	Location								
	NAmer	UK	Scand	othEur	China	Japan	othAs	other	Total
N	24	2	5	12	6	6	5	1	61
NS	17	1	4	9	4	4	4	1	44
Wt	178.29	6.84	10.69	115.32	54.82	61.02	12.03	0.49	439.51
Het Chi	89.82	0.01	4.54	29.74	8.08	10.27	5.40	0.00	349.34
Het df	23	1	4	11	5	5	4	0	60
Het P	***	N.S.	N.S.	**	N.S.	(*)	N.S.	N.S.	***
Fixed RR	19.82	7.35	11.64	6.25	2.83	8.98	16.42	26.79	9.95
RRl	17.11	3.48	6.39	5.21	2.17	6.99	9.33	1.63	9.06
RRu	22.95	15.56	21.19	7.50	3.68	11.55	28.90	439.95	10.93
P	+++	+++	+++	+++	+++	+++	+++	+	+++
Random RR	22.68	7.35	11.62	6.82	2.99	8.59	17.00	26.79	11.19
RRl	15.31	3.48	6.06	4.72	2.08	5.67	8.65	1.63	8.62
RRu	33.61	15.56	22.26	9.86	4.31	13.03	33.44	439.95	14.54
P	+++	+++	+++	+++	+++	+++	+++	+	+++
Between Chi									201.49
Between df									7
Between P									***
Btwn(F) P									***

Table 5C1 - 6

IESLC - Meta-anal of Ever Smoking (or Current if Ever not available), Any prod (or Cigs if Any not avail)
Small
Least adjusted

	Detailed Country in "other Europe"					
	multi	Germany	othWest	East	Balkans	Total
N	2	1	2	6	1	12
NS	1	1	2	4	1	9
Wt	68.61	0.99	20.12	14.40	11.21	115.32
Het Chi	2.98	0.00	9.63	8.31	0.00	29.74
Het df	1	0	1	5	0	11
Het P	(*)	N.S.	**	N.S.	N.S.	**
Fixed RR	6.68	32.68	3.85	8.22	6.01	6.25
RRl	5.28	4.54	2.49	4.90	3.35	5.21
RRu	8.47	235.34	5.96	13.78	10.80	7.50
P	+++	+++	+++	+++	+++	+++
Random RR	6.73	32.68	5.23	7.95	6.01	6.82
RRl	4.47	4.54	1.15	4.00	3.35	4.72
RRu	10.13	235.34	23.68	15.82	10.80	9.86
P	+++	+++	+	+++	+++	+++
Between Chi						8.83
Between df						4
Between P						(*)
Btwn(F) P						N.S.

	Detailed Country in "other Asia"			
	India	HongKong	other	Total
N		4	1	5
NS		3	1	4
Wt		10.50	1.53	12.03
Het Chi		2.89	0.00	5.40
Het df		3	0	4
Het P		N.S.	N.S.	N.S.
Fixed RR		13.79	54.17	16.42
RRl		7.53	11.13	9.33
RRu		25.26	263.53	28.90
P		+++	+++	+++
Random RR		13.79	54.17	17.00
RRl		7.53	11.13	8.65
RRu		25.26	263.53	33.44
P		+++	+++	+++
Between Chi				2.51
Between df				1
Between P				N.S.
Btwn(F) P				N.S.

	Detailed other continent			
	SCAmer	Auslia	Africa	Total
N	1			1
NS	1			1
Wt	0.49			0.49
Het Chi	0.00			0.00
Het df	0			0
Het P	N.S.			N.S.
Fixed RR	26.79			26.79
RRl	1.63			1.63
RRu	439.95			439.95
P	+			+
Random RR	26.79			26.79
RRl	1.63			1.63
RRu	439.95			439.95
P	+			+
Between Chi				
Between df				
Between P				N.S.
Btwn(F) P				N.S.

Table 5C1 - 6

IESLC - Meta-anal of Ever Smoking (or Current if Ever not available), Any prod (or Cigs if Any not avail)
Small
Least adjusted

	Start year of study					
	<1960	1960-69	1970-79	1980-89	1990+	Total
N	8	5	20	25	3	61
NS	7	4	12	18	3	44
Wt	28.93	52.18	127.77	211.31	19.32	439.51
Het Chi	31.80	10.52	27.77	187.12	14.04	349.34
Het df	7	4	19	24	2	60
Het P	***	*	(*)	***	***	***
Fixed RR	4.85	8.60	7.11	15.00	4.53	9.95
RRl	3.37	6.56	5.98	13.11	2.90	9.06
RRu	6.98	11.28	8.46	17.16	7.07	10.93
P	+++	+++	+++	+++	+++	+++
Random RR	6.17	7.26	7.61	20.72	7.77	11.19
RRl	2.45	4.26	5.91	13.03	2.06	8.62
RRu	15.51	12.36	9.79	32.97	29.30	14.54
P	+++	+++	+++	+++	++	+++
Between Chi						78.08
Between df						4
Between P						***
Btwn(F) P						**

	Study type (1)		
	CC	other	Total
N	50	11	61
NS	36	8	44
Wt	415.31	24.20	439.51
Het Chi	324.78	20.74	349.34
Het df	49	10	60
Het P	***	*	***
Fixed RR	9.73	14.64	9.95
RRl	8.84	9.83	9.06
RRu	10.71	21.81	10.93
P	+++	+++	+++
Random RR	10.88	13.28	11.19
RRl	8.18	7.27	8.62
RRu	14.49	24.26	14.54
P	+++	+++	+++
Between Chi			3.81
Between df			1
Between P			(*)
Btwn(F) P			N.S.

	Study type (2)			
	CC	prosp	other	Total
N	50	9	2	61
NS	36	7	1	44
Wt	415.31	20.61	3.59	439.51
Het Chi	324.78	18.10	1.66	349.34
Het df	49	8	1	60
Het P	***	*	N.S.	***
Fixed RR	9.73	15.92	9.04	9.95
RRl	8.84	10.34	3.21	9.06
RRu	10.71	24.52	25.43	10.93
P	+++	+++	+++	+++
Random RR	10.88	14.36	9.04	11.19
RRl	8.18	7.20	2.39	8.62
RRu	14.49	28.61	34.25	14.54
P	+++	+++	++	+++
Between Chi				4.80
Between df				2
Between P				(*)
Btwn(F) P				N.S.

Table 5C1 - 6

IESLC - Meta-anal of Ever Smoking (or Current if Ever not available), Any prod (or Cigs if Any not avail)
Small
Least adjusted

	Study size (number of LC cases) 100-249	250-499	500-999	1000+	Total
N	7	16	10	28	61
NS	7	12	7	18	44
Wt	11.11	46.80	47.46	334.13	439.51
Het Chi	11.31	40.13	47.73	222.59	349.34
Het df	6	15	9	27	60
Het P	(*)	***	***	***	***
Fixed RR	16.99	6.73	5.89	11.13	9.95
RRl	9.44	5.05	4.43	10.00	9.06
RRu	30.58	8.96	7.83	12.39	10.93
P	+++	+++	+++	+++	+++
Random RR	17.35	8.30	12.33	11.65	11.19
RRl	7.45	4.98	5.78	8.16	8.62
RRu	40.40	13.82	26.33	16.62	14.54
P	+++	+++	+++	+++	+++
Between Chi					27.56
Between df					3
Between P					***
Btwn(F) P					N.S.

	Risky occupational population no	mining	othRisky	Total
N	61			61
NS	44			44
Wt	439.51			439.51
Het Chi	349.34			349.34
Het df	60			60
Het P	***			***
Fixed RR	9.95			9.95
RRl	9.06			9.06
RRu	10.93			10.93
P	+++			+++
Random RR	11.19			11.19
RRl	8.62			8.62
RRu	14.54			14.54
P	+++			+++
Between Chi				
Between df				
Between P				N.S.
Btwn(F) P				N.S.

	National cigarette tobacco type Virginia	blended	other	Total
N	6	49	6	61
NS	4	36	4	44
Wt	16.34	368.35	54.82	439.51
Het Chi	11.55	229.66	8.08	349.34
Het df	5	48	5	60
Het P	*	***	N.S.	***
Fixed RR	14.71	11.80	2.83	9.95
RRl	9.06	10.65	2.17	9.06
RRu	23.88	13.07	3.68	10.93
P	+++	+++	+++	+++
Random RR	16.08	12.93	2.99	11.19
RRl	7.51	9.87	2.08	8.62
RRu	34.41	16.94	4.31	14.54
P	+++	+++	+++	+++
Between Chi				100.04
Between df				2
Between P				***
Btwn(F) P				***

Table 5C1 - 6

IESLC - Meta-anal of Ever Smoking (or Current if Ever not available), Any prod (or Cigs if Any not avail)
Small
Least adjusted

	Any proxy use		
	No/nk	Yes	Total
N	44	17	61
NS	31	13	44
Wt	391.98	47.53	439.51
Het Chi	290.18	57.92	349.34
Het df	43	16	60
Het P	***	***	***
Fixed RR	9.77	11.59	9.95
RRl	8.85	8.72	9.06
RRu	10.79	15.40	10.93
P	+++	+++	+++
Random RR	9.96	15.98	11.19
RRl	7.38	8.98	8.62
RRu	13.45	28.42	14.54
P	+++	+++	+++
Between Chi			1.23
Between df			1
Between P			N.S.
Btwn(F) P			N.S.

	Full histological confirmation		
	No	Yes	Total
N	42	19	61
NS	30	14	44
Wt	207.80	231.71	439.51
Het Chi	179.35	130.78	349.34
Het df	41	18	60
Het P	***	***	***
Fixed RR	7.26	13.21	9.95
RRl	6.34	11.61	9.06
RRu	8.32	15.02	10.93
P	+++	+++	+++
Random RR	9.97	14.48	11.19
RRl	7.30	9.22	8.62
RRu	13.64	22.72	14.54
P	+++	+++	+++
Between Chi			39.21
Between df			1
Between P			***
Btwn(F) P			**

	Number of adjustment variables (1)			
	0	1	2+/+nk	Total
N	46	4	11	61
NS	34	3	7	44
Wt	219.60	7.91	212.01	439.51
Het Chi	148.89	9.48	158.87	349.34
Het df	45	3	10	60
Het P	***	*	***	***
Fixed RR	7.66	20.97	12.70	9.95
RRl	6.71	10.44	11.10	9.06
RRu	8.74	42.10	14.53	10.93
P	+++	+++	+++	+++
Random RR	10.26	18.18	12.34	11.19
RRl	7.81	4.73	6.60	8.62
RRu	13.50	69.88	23.09	14.54
P	+++	+++	+++	+++
Between Chi				32.09
Between df				2
Between P				***
Btwn(F) P				(*)

Table 5C1 - 6

IESLC - Meta-anal of Ever Smoking (or Current if Ever not available), Any prod (or Cigs if Any not avail)
Small
Least adjusted

	Number of adjustment variables (2) 0	1	2	3-5	6+/+nk	Total
N	46	4	8	2	1	61
NS	34	3	5	2	1	45
Wt	219.60	7.91	183.42	26.30	2.29	439.51
Het Chi	148.89	9.48	70.43	36.59	0.00	349.34
Het df	45	3	7	1	0	60
Het P	***	*	***	***	N.S.	***
Fixed RR	7.66	20.97	15.44	3.58	4.35	9.95
RRl	6.71	10.44	13.36	2.44	1.19	9.06
RRu	8.74	42.10	17.84	5.24	15.89	10.93
P	+++	+++	+++	+++	+	+++
Random RR	10.26	18.18	14.53	11.36	4.35	11.19
RRl	7.81	4.73	8.26	0.43	1.19	8.62
RRu	13.50	69.88	25.58	302.11	15.89	14.54
P	+++	+++	+++	N.S.	+	+++
Between Chi						83.94
Between df						4
Between P						***
Btwn(F) P						**

	Product all/unsp	cig+/-ot	cig only	Total
N	23	32	6	61
NS	17	22	6	45
Wt	81.79	348.50	9.21	439.51
Het Chi	57.58	253.08	11.71	349.34
Het df	22	31	5	60
Het P	***	***	*	***
Fixed RR	6.34	10.79	25.39	9.95
RRl	5.11	9.72	13.31	9.06
RRu	7.88	11.99	48.43	10.93
P	+++	+++	+++	+++
Random RR	7.75	13.00	20.36	11.19
RRl	5.31	9.18	6.69	8.62
RRu	11.33	18.41	61.97	14.54
P	+++	+++	+++	+++
Between Chi				26.97
Between df				2
Between P				***
Btwn(F) P				(*)

	Denominator nev any	nev cigs	Total
N	36	25	61
NS	27	18	45
Wt	212.68	226.84	439.51
Het Chi	116.85	211.83	349.34
Het df	35	24	60
Het P	***	***	***
Fixed RR	7.96	12.28	9.95
RRl	6.96	10.78	9.06
RRu	9.10	13.98	10.93
P	+++	+++	+++
Random RR	9.62	13.64	11.19
RRl	7.21	8.59	8.62
RRu	12.84	21.67	14.54
P	+++	+++	+++
Between Chi			20.66
Between df			1
Between P			***
Btwn(F) P			(*)

Table 5C1 - 6

IESLC - Meta-anal of Ever Smoking (or Current if Ever not available), Any prod (or Cigs if Any not avail)

Small
Least adjusted

	Derivation of RR/CI			
	Orig	StdCalc	Other	Total
N	10	38	13	61
NS	6	28	11	45
Wt	211.12	195.31	33.09	439.51
Het Chi	151.52	130.81	43.77	349.34
Het df	9	37	12	60
Het P	***	***	***	***
Fixed RR	12.61	7.85	8.91	9.95
RRl	11.02	6.82	6.34	9.06
RRu	14.43	9.03	12.53	10.93
P	+++	+++	+++	+++
Random RR	11.94	10.49	12.55	11.19
RRl	6.31	7.77	6.08	8.62
RRu	22.62	14.15	25.90	14.54
P	+++	+++	+++	+++
Between Chi				23.24
Between df				2
Between P				***
Btwn(F) P				N.S.

	Smoking status		
	ever	current	Total
N	54	7	61
NS	39	5	44
Wt	387.19	52.33	439.51
Het Chi	329.33	19.57	349.34
Het df	53	6	60
Het P	***	**	***
Fixed RR	9.84	10.84	9.95
RRl	8.91	8.27	9.06
RRu	10.87	14.21	10.93
P	+++	+++	+++
Random RR	11.04	12.95	11.19
RRl	8.30	6.70	8.62
RRu	14.70	25.06	14.54
P	+++	+++	+++
Between Chi			0.43
Between df			1
Between P			N.S.
Btwn(F) P			N.S.

Table 5C1 - 7

IESLC - Meta-anal of Ever Smoking (or Current if Ever not available), Any prod (or Cigs if Any not avail)
Small
Excluded studies (and stage at which they were excluded)

1	BOUCHA BUELL LAURIL MZILEN
2	BERRIN CHEN RESTRE TAO ULMER
5	RIMING TANG2 WYNDE5 WYNDE8
6	BLOT1 BLOT2 BLOT3 BYERS2 HIRAOK HIRAY2 JONES LICKIN MOLLO MRFIT MURATA NOTANI PERSHA SAARIK SCHWA2 SHIMIZ SITAS VANDER WARSIN WATSON WYNDER
8	ABELIN AGUDO AKIBA AMANDU AMES ARCHER ARMADA AUSTIN AUVINE AXELSO AXELSS BECHER BENHAM BENSHL BEST BLOHMK BLOT4 BOFFET BRESLO BRETT BROCKM BROSS BROWN1 CARPEN CASCO2 CASCOR CEDERL CHAN CHANG CHATZI CHEN2 CHEN3 CHIAZZ CHOI CHOW CHYOU COOKSO CORREA DARBY DAVEYS DEAN DEAN2 DEAN3 DEKLER DESTE2 DESTEF DOCKER DOLL DOLL2 DORANT DROSTE DU DUNN EBELIN ENSTRO ESAKI GAO2 GARCIA GARDIN GARSHI GENG GER GILLIS GODLEY GOLLED GOODMA GRAHAM GREGOR GSELL HAENSZ HAMMO2 HAMMON HANSEN HEIN HENNEK HINDS HIRAYA HITOSU HOLE HOROWI HORWIT HU HU2 HUANG HUMBLE JARUP JARVHO JIANG JOLY JUSSAW KAISE2 KAISER KANELL KATSOU KAUFMA KELLER KINLEN KJUUS KNEKT KO KOHLME KOO KOULUM KREUZE KREYBE KUBIK LANGE LAUSSM LEI LEMARC LETOUR LEVIN LIAW LIDDEL LIU LIU2 LIU3 LIU4 LIU5 LOMBA2 LOMBAR LUBIN LUO MACLEN MAGNUS MARSH MARSH2 MARTIS MASTRA MATOS MATSUD MCCONN MCDUFF MCLAUG MIGRAN MILLER MILLS MRFITR NAM NOTAN2 ODRISC OSANN2 PARKIN PASTOR PAWLEG PERNU PERSH2 PETO PEZZO2 PIKE PISANI POFFIJ POLEDN PRESCO QIAO QIAO2 RACHTA RADZIK RANDIG REN RONCO ROOTS ROTHSC SADOWS SANKAR SCHWAR SEGI SEGI2 SHAW SIMARA SIMONA SPEIZE SPITZ STOCKS STOCKW STUCKE SUN SUZUK2 SUZUKI TANG TENKAN TOKARS TOUSEY TSUGAN TULINI TVERDA VEIERO VUTUC WAKAI WALD WANG WANG2 WANG3 WANG4 WICKLU WIGLE WILKIN WU WU2 WUNSCH WYNDE2 WYNDE3 WYNDE4 WYNDE6 WYNDE7 XIANGZ XU XU2 XU3 XU4 YAMAGU YONG YUAN ZHANG ZHENG

Table 5C1 - 8
Potentially overlapping studies

REF	REFGP	PRINC	. OVERLAP/LINK
LUBIN2	LUBIN2	1	Lubin-combined
LAMWK2	LAMWK2	1	Chan/LamWK2
LAMTH	LAMTH	1	KOO/LAMTH/LAMWK
LAMWK	LAMWK	1	KOO/LAMTH/LAMWK
ISHIMA	AKIBA	2	AKIBA/ISHIMA
BYERS1	BYERS1	2	GRAHAM/BROSS/BYERS1
CPSI	CPSI	1	CPSI overall
SOBUE2	SOBUE2	1	SOBUE2/MATSUD
JAHN	BOFFET	2	Subset of BOFFET

Table 5C1 - 9
Most adjusted - insufficient data for metaanalysis

REF	NRR	SEX	AGEL	AGEH	RACE	YF	LC TYPE	LOC	START	ST	NLC	R	VB	P	H	AD	SM	PRODUCT	DENOM	De
BUFFLE	48	f	0	0	wh	-	small	NAmer	1976	CC	943	n	bl	y	n	0	ev	cig+/-ot	nev cigs	or
CHEN	6	c	0	0	all	-	small	As:oth	1987	CC	323	n	ot	n	y	2	ev	cig+/-ot	nev cigs	ot
KOO	3	f	0	0	all	-	small	As:HK	1981	CC	200	n	bl	n	n	0	ev	all/unsp	nev any	or

REF	NRR	RR	SIG	RRdata comment
BUFFLE	48	52.30		0
CHEN	6	3.59	y	P < 0.001
KOO	3	5.14		0

Table 5D1 -

IESLC - Meta-analysis of Ex Smoking, Any product (or Cigarettes if Any not available)
Small

This analysis is restricted to results for:
1) Non-dose-response data
2) Ex smokers
3) Results complete enough for use in metaanalysis

Within each study, results are then selected (in the following order of preference, within each sex) for:
4) PRODUCT: all/unspec, cigarettes regardless of other products, cigarettes only
5) CIGTYPE: all/unspecified, MC regardless of HR, MC only
6) DENOM: never smoked anything, never smoked cigarettes, (never +1 = +long term ex, +2 = +amount unknown, +3 = never cigs+long term ex)
7) Followup period (YF, prospective studies): whole study (coded as 0) or longest available
8) LCtype: small (specifically)

9) Race: all or nearest available, otherwise by race (wh or w = white, bl or b = black, hi = hispanic ch = chinese, jap = japanese, haw = hawaiian, w+o = white + oriental, sca = scandinavian, as = asian)
10) For overlapping studies: principal rather than subsidiary studies
Finally by Age: whole study (coded as 0) if available, otherwise by widest available age group
and then for single sex results (m, f) in preference to combined sex results (c).

Results adjusted (AD) for the most potential confounders are then chosen in Sections -1 to -3 and results adjusted for the least confounders in Sections -4 to -6. (Those least adjusted results which actually differ from the most adjusted as marked 'x' in column X in Section -4)
(Results adjusted for an unknown number of confounder(s) are coded as 20.)

Section -7 shows excluded studies, together with the stage (as above) at which no qualifying results were found.

Section -8 lists the potentially overlapping studies which have been included (1=principal, 2=subsidiary).

Section -9 lists any results which would have been included in preference except that they had data not complete enough for use in meta-analysis, with their significance (yes/no), if known, and any further comment as entered on the database.

In addition to those mentioned above, the following fields, levels and abbreviations are used:
* or nk = not known, n = no, y = yes, ot = other
nev = never
all/unspec = all or unspecified, cig+/-ot = cigarettes irrespective of other products (cigar, pipe etc)
MC = manufactured cigarettes, HR = hand-rolled cigarettes
REF: 6-character study reference
NRR: number of the RR on the database within the study
ST : study type (CC = case control, pr or prosp = prospective)
NLC: number of lung cancer cases in whole study
R : risky occupational population (n = no, m = mining, o = other risky)
VB : national cigarette type (V = at least 75% Virginia, bl = at least 75% blended, ot = other)
P : any proxy use
H : full histological confirmation
De : derivation of RR/CI (or = original, st = standard method, ot = other method of estimation)

Table 5D1 - 1

IESLC - Meta-analysis of Ex Smoking, Any product (or Cigarettes if Any not available)
Small
Most adjusted

REF	NRR	SEX	AGEL	AGEH	RACE	YF	LC TYPE	LOC	START	ST	NLC	R	VB	P	H	AD	PRODUCT	DENOM	De
BARBON	85	m	0	0	all	-	small	Eu:wst	1979	CC	755	n	bl	y	y	1	all/unsp	nev any	or
BROWN2	28	m	0	0	wh	-	small	NAmer	1984	CC	14596	n	bl	n	y	2	cig+/-ot	nev cigs	or
BROWN2	27	f	0	0	wh	-	small	NAmer	1984	CC	14596	n	bl	n	y	2	cig+/-ot	nev cigs	or
BUFFLE	68	f	0	0	w-hi	-	small	NAmer	1976	CC	943	n	bl	y	n	0	cig+/-ot	nev cigs	st
COMSTO	18	m	0	0	all	-	small	NAmer	1975	ot	258	n	bl	n	n	0	cig+/-ot	nev cigs	st
COMSTO	26	f	0	0	all	-	small	NAmer	1975	ot	258	n	bl	n	n	0	cig+/-ot	nev cigs	st
ENGELA	83	m	0	0	all	0	small	Eu:Sca	1964	pr	435	n	bl	n	n	7	cig+/-ot	nev cigs	or
JAHN	11	m	0	0	all	-	small	Eu:Ger	1988	CC	1004	n	bl	n	n	0	cig+/-ot	nev any	st
JAIN	29	m	0	0	all	-	small	NAmer	1981	CC	845	n	V	y	n	0	cig+/-ot	nev cigs	st
JAIN	24	f	0	0	all	-	small	NAmer	1981	CC	845	n	V	y	n	0	cig+/-ot	nev cigs	st
JEDRYC	25	m	0	0	all	-	small	Eu:est	1980	CC	1630	n	bl	y	n	0	cig+/-ot	nev any	st
KHUDER	9	m	0	0	all	-	small	NAmer	1985	CC	482	n	bl	n	y	0	cig+/-ot	nev cigs	or
KIHARA	11	c	0	0	jap	-	small	As:Jap	1991	CC	440	n	bl	n	n	0	all/unsp	nev any	st
LUBIN2	258	m	0	0	all	-	small	Eu:mul	1976	CC	7804	n	bl	n	y	0	cig+/-ot	nev any	st
LUBIN2	270	f	0	0	all	-	small	Eu:mul	1976	CC	7804	n	bl	n	y	0	cig+/-ot	nev any	st
OSANN	29	m	0	0	all	-	small	NAmer	1984	CC	1986	n	bl	n	n	2	cig+/-ot	nev cigs	or
OSANN	30	f	0	0	all	-	small	NAmer	1984	CC	1986	n	bl	n	n	2	cig+/-ot	nev cigs	or
SOBUE	37	m	0	0	all	-	small	As:Jap	1986	CC	1376	n	bl	n	y	1	cig+/-ot	nev cigs	or
SOBUE	47	f	0	0	all	-	small	As:Jap	1986	CC	1376	n	bl	n	y	1	cig+/-ot	nev cigs	or
SVENSS	3	f	0	0	all	-	small	Eu:Sca	1983	CC	210	n	bl	n	n	1	all/unsp	nev any	or

Cigarette type is all/unspec for all RRs

Table 5D1 - 2

IESLC - Meta-analysis of Ex Smoking, Any product (or Cigarettes if Any not available)
Small
Most adjusted

REF	NRR	SEX	AD	Number Exposed Case	Number Exposed Cont	Non-exposed Case	Non-exposed Cont	RR	95.00%CI
BARBON	85	m	1	-	-	-	-	6.50	(2.70- 15.60)
BROWN2	28	m	2	-	-	-	-	7.90	(6.20- 10.00)
BROWN2	27	f	2	-	-	-	-	29.80	(22.00- 40.30)
Subtotal BROWN2								13.16	(10.91- 15.87)
BUFFLE	68	f	0	12	56	1	112	24.00	(3.04- 189.26)
COMSTO	18	m	0	5	129	2	84	1.63	(0.31- 8.58)
COMSTO	26	f	0	2	35	2	115	3.29	(0.45- 24.19)
Subtotal COMSTO								2.17	(0.60- 7.79)
*ENGELA	83	m	7	-	-	-	-	2.00	(0.50- 8.10)
JAHN	11	m	0	79	402	1	138	27.12	(3.74- 196.77)
JAIN	29	m	0	30	159	3	85	5.35	(1.59- 18.03)
JAIN	24	f	0	20	97	2	214	22.06	(5.06- 96.26)
Subtotal JAIN								9.49	(3.72- 24.25)
JEDRYC	25	m	0	28	312	3	289	8.65	(2.60- 28.74)
KHUDER	9	m	0	36	-	1	-	34.40	(4.50- 262.00)
KIHARA	11	c	0	6	70	9	237	2.26	(0.78- 6.56)
LUBIN2	258	m	0	297	4228	34	2616	5.40	(3.78- 7.73)
LUBIN2	270	f	0	25	157	55	1180	3.42	(2.07- 5.64)
Subtotal LUBIN2								4.63	(3.46- 6.19)
OSANN	29	m	2	-	-	-	-	14.00	(5.00- 39.80)
OSANN	30	f	2	-	-	-	-	43.30	(15.10- 124.00)
Subtotal OSANN								24.41	(11.66- 51.11)
SOBUE	37	m	1	-	-	-	-	9.20	(1.50- 56.80)
SOBUE	47	f	1	-	-	-	-	4.70	(1.30- 17.40)
Subtotal SOBUE								5.90	(2.05- 16.94)
SVENSS	3	f	1	-	-	-	-	9.10	(1.40- 69.70)
Partial Totals				540	5645	113	5070		

*prospective study

REF	NRR	SEX	AD	Ys	Ws	Qs	Ps
BARBON	85	m	1	1.87	4.99	0.67	0.0000
BROWN2	28	m	2	2.07	67.24	1.97	0.0000
BROWN2	27	f	2	3.39	41.94	56.11	0.0000
Subtotal BROWN2				2.58	109.18	58.07	
BUFFLE	68	f	0	3.18	0.90	0.80	0.0026
COMSTO	18	m	0	0.49	1.39	4.26	0.5657
COMSTO	26	f	0	1.19	0.96	1.06	0.2428
Subtotal COMSTO				0.77	2.35	5.32	
*ENGELA	83	m	7	0.69	1.98	4.73	0.3293
JAHN	11	m	0	3.30	0.98	1.10	0.0011
JAIN	29	m	0	1.68	2.60	0.82	0.0069
JAIN	24	f	0	3.09	1.77	1.30	0.0000
Subtotal JAIN				2.25	4.37	2.12	
JEDRYC	25	m	0	2.16	2.66	0.02	0.0004
KHUDER	9	m	0	3.54	0.93	1.57	0.0006
KIHARA	11	c	0	0.81	3.38	6.84	0.1347
LUBIN2	258	m	0	1.69	29.94	9.08	0.0000
LUBIN2	270	f	0	1.23	15.29	15.58	0.0000
Subtotal LUBIN2				1.53	45.23	24.65	
OSANN	29	m	2	2.64	3.57	0.57	0.0000
OSANN	30	f	2	3.77	3.47	8.12	0.0000
Subtotal OSANN				3.20	7.04	8.69	
SOBUE	37	m	1	2.22	1.16	0.00	0.0167
SOBUE	47	f	1	1.55	2.28	1.09	0.0194
Subtotal SOBUE				1.77	3.45	1.09	
SVENSS	3	f	1	2.21	1.01	0.00	0.0267

Table 5D1 - 2

IESLC - Meta-analysis of Ex Smoking, Any product (or Cigarettes if Any not available)
Small
Most adjusted

	N	20
	NS	14
	Wt	188.45
Het	Chi	115.67
Het	df	19
Het	P	***
Fixed	RR	9.37
	RRl	8.13
	RRu	10.81
	P	+++
Random	RR	8.21
	RRl	5.24
	RRu	12.86
	P	+++
Asymm	P	N.S.

Table 5D1 - 3

IESLC - Meta-analysis of Ex Smoking, Any product (or Cigarettes if Any not available)

Small

Most adjusted

	Sex			
	combined	male	female	Total
N	1	11	8	20
NS	1	11	8	20
Wt	3.38	117.45	67.62	188.45
Het Chi	0.00	15.33	62.55	115.67
Het df	0	10	7	19
Het P	N.S.	N.S.	***	***
Fixed RR	2.26	7.06	16.47	9.37
RRl	0.78	5.89	12.98	8.13
RRu	6.56	8.46	20.91	10.81
P	N.S.	+++	+++	+++
Random RR	2.26	6.90	11.91	8.21
RRl	0.78	4.99	4.52	5.24
RRu	6.56	9.52	31.35	12.86
P	N.S.	+++	+++	+++
Between Chi				37.79
Between df				2
Between P				***
Btwn(F) P				*

	Lung cancer type	
	small	Total
N	20	20
NS	14	14
Wt	188.45	188.45
Het Chi	115.67	115.67
Het df	19	19
Het P	***	***
Fixed RR	9.37	9.37
RRl	8.13	8.13
RRu	10.81	10.81
P	+++	+++
Random RR	8.21	8.21
RRl	5.24	5.24
RRu	12.86	12.86
P	+++	+++
Between Chi		
Between df		
Between P		N.S.
Btwn(F) P		N.S.

	Location								
	NAmer	UK	Scand	othEur	China	Japan	othAs	other	Total
N	10		2	5		3			20
NS	6		2	4		2			14
Wt	124.77		2.99	53.87		6.82			188.45
Het Chi	62.15		1.53	6.32		1.92			115.67
Het df	9		1	4		2			19
Het P	***		N.S.	N.S.		N.S.			***
Fixed RR	13.17		3.33	5.09		3.67			9.37
RRl	11.05		1.07	3.89		1.73			8.13
RRu	15.69		10.36	6.64		7.77			10.81
P	+++		+	+++		+++			+++
Random RR	12.95		3.63	5.37		3.67			8.21
RRl	6.68		0.85	3.59		1.73			5.24
RRu	25.09		15.49	8.04		7.77			12.86
P	+++		(+)	+++		+++			+++
Between Chi									43.75
Between df									3
Between P									***
Btwn(F) P									*

Table 5D1 - 3

IESLC - Meta-analysis of Ex Smoking, Any product (or Cigarettes if Any not available)
Small
Most adjusted

	Detailed Country in "other Europe"					
	multi	Germany	othWest	East	Balkans	Total
N	2	1	1	1		5
NS	1	1	1	1		4
Wt	45.23	0.98	4.99	2.66		53.87
Het Chi	2.13	0.00	0.00	0.00		6.32
Het df	1	0	0	0		4
Het P	N.S.	N.S.	N.S.	N.S.		N.S.
Fixed RR	4.63	27.12	6.50	8.65		5.09
RRl	3.46	3.74	2.70	2.60		3.89
RRu	6.19	196.77	15.62	28.74		6.64
P	+++	++	+++	+++		+++
Random RR	4.45	27.12	6.50	8.65		5.37
RRl	2.85	3.74	2.70	2.60		3.59
RRu	6.94	196.77	15.62	28.74		8.04
P	+++	++	+++	+++		+++
Between Chi						4.19
Between df						3
Between P						N.S.
Btwn(F) P						N.S.

	Detailed Country in "other Asia"			
	India	HongKong	other	Total
N				
NS				
Wt				
Het Chi				
Het df				
Het P				N.S.
Fixed RR				
RRl				
RRu				
P				+++
Random RR				
RRl				
RRu				
P				+++
Between Chi				
Between df				
Between P				N.S.
Btwn(F) P				N.S.

	Detailed other continent			
	SCAmer	Auslia	Africa	Total
N				
NS				
Wt				
Het Chi				
Het df				
Het P				N.S.
Fixed RR				
RRl				
RRu				
P				+++
Random RR				
RRl				
RRu				
P				+++
Between Chi				
Between df				
Between P				N.S.
Btwn(F) P				N.S.

Table 5D1 - 3

IESLC - Meta-analysis of Ex Smoking, Any product (or Cigarettes if Any not available)

Small

Most adjusted

	Start year of study					
	<1960	1960-69	1970-79	1980-89	1990+	Total
N		1	6	12	1	20
NS		1	4	8	1	14
Wt		1.98	53.48	129.61	3.38	188.45
Het Chi		0.00	6.74	57.58	0.00	115.67
Het df		0	5	11	0	19
Het P		N.S.	N.S.	***	N.S.	***
Fixed RR		2.00	4.75	13.18	2.26	9.37
RRl		0.50	3.63	11.10	0.78	8.13
RRu		8.05	6.21	15.66	6.56	10.81
P		N.S.	+++	+++	N.S.	+++
Random RR		2.00	4.68	13.77	2.26	8.21
RRl		0.50	3.20	7.86	0.78	5.24
RRu		8.05	6.85	24.15	6.56	12.86
P		N.S.	+++	+++	N.S.	+++
Between Chi						51.34
Between df						3
Between P						***
Btwn(F) P						*

	Study type (1)		
	CC	other	Total
N	17	3	20
NS	12	2	14
Wt	184.11	4.33	188.45
Het Chi	105.39	0.29	115.67
Het df	16	2	19
Het P	***	N.S.	***
Fixed RR	9.71	2.09	9.37
RRl	8.40	0.82	8.13
RRu	11.22	5.36	10.81
P	+++	N.S.	+++
Random RR	9.76	2.09	8.21
RRl	6.10	0.82	5.24
RRu	15.60	5.36	12.86
P	+++	N.S.	+++
Between Chi			9.99
Between df			1
Between P			**
Btwn(F) P			N.S.

	Study type (2)			
	CC	prosp	other	Total
N	17	1	2	20
NS	12	1	1	14
Wt	184.11	1.98	2.35	188.45
Het Chi	105.39	0.00	0.28	115.67
Het df	16	0	1	19
Het P	***	N.S.	N.S.	***
Fixed RR	9.71	2.00	2.17	9.37
RRl	8.40	0.50	0.60	8.13
RRu	11.22	8.05	7.79	10.81
P	+++	N.S.	N.S.	+++
Random RR	9.76	2.00	2.17	8.21
RRl	6.10	0.50	0.60	5.24
RRu	15.60	8.05	7.79	12.86
P	+++	N.S.	N.S.	+++
Between Chi				9.99
Between df				2
Between P				**
Btwn(F) P				N.S.

Table 5D1 - 3

IESLC - Meta-analysis of Ex Smoking, Any product (or Cigarettes if Any not available)
Small
Most adjusted

	Study size (number of LC cases)				
	100-249	250-499	500-999	1000+	Total
N	1	5	4	10	20
NS	1	4	3	6	14
Wt	1.01	8.64	10.26	168.53	188.45
Het Chi	0.00	6.65	3.50	92.90	115.67
Het df	0	4	3	9	19
Het P	N.S.	N.S.	N.S.	***	***
Fixed RR	9.10	2.91	8.56	10.01	9.37
RRl	1.29	1.50	4.65	8.61	8.13
RRu	64.21	5.67	15.79	11.64	10.81
P	+	++	+++	+++	+++
Random RR	9.10	3.21	8.90	10.46	8.21
RRl	1.29	1.31	4.50	5.77	5.24
RRu	64.21	7.89	17.61	18.99	12.86
P	+	+	+++	+++	+++
Between Chi					12.61
Between df					3
Between P					**
Btwn(F) P					N.S.

	Risky occupational population			
	no	mining	othRisky	Total
N	20			20
NS	14			14
Wt	188.45			188.45
Het Chi	115.67			115.67
Het df	19			19
Het P	***			***
Fixed RR	9.37			9.37
RRl	8.13			8.13
RRu	10.81			10.81
P	+++			+++
Random RR	8.21			8.21
RRl	5.24			5.24
RRu	12.86			12.86
P	+++			+++
Between Chi				
Between df				
Between P				N.S.
Btwn(F) P				N.S.

	National cigarette tobacco type			
	Virginia	blended	other	Total
N	2	18		20
NS	1	13		14
Wt	4.37	184.08		188.45
Het Chi	2.12	113.55		115.67
Het df	1	17		19
Het P	N.S.	***		***
Fixed RR	9.49	9.37		9.37
RRl	3.72	8.11		8.13
RRu	24.25	10.83		10.81
P	+++	+++		+++
Random RR	10.19	8.03		8.21
RRl	2.55	4.98		5.24
RRu	40.65	12.93		12.86
P	++	+++		+++
Between Chi				0.00
Between df				1
Between P				N.S.
Btwn(F) P				N.S.

Table 5D1 - 3

IESLC - Meta-analysis of Ex Smoking, Any product (or Cigarettes if Any not available)
Small
Most adjusted

	Any proxy use No/nk	Yes	Total
N	15	5	20
NS	10	4	14
Wt	175.52	12.93	188.45
Het Chi	112.06	3.50	115.67
Het df	14	4	19
Het P	***	N.S.	***
Fixed RR	9.43	8.58	9.37
RRl	8.14	4.98	8.13
RRu	10.94	14.80	10.81
P	+++	+++	+++
Random RR	7.77	8.58	8.21
RRl	4.54	4.98	5.24
RRu	13.29	14.80	12.86
P	+++	+++	+++
Between Chi			0.11
Between df			1
Between P			N.S.
Btwn(F) P			N.S.

	Full histological confirmation No	Yes	Total
N	12	8	20
NS	9	5	14
Wt	24.66	163.78	188.45
Het Chi	29.28	86.00	115.67
Het df	11	7	19
Het P	**	***	***
Fixed RR	8.34	9.54	9.37
RRl	5.62	8.18	8.13
RRu	12.38	11.12	10.81
P	+++	+++	+++
Random RR	8.10	8.34	8.21
RRl	4.15	4.34	5.24
RRu	15.79	16.03	12.86
P	+++	+++	+++
Between Chi			0.39
Between df			1
Between P			N.S.
Btwn(F) P			N.S.

	Number of adjustment variables (1) 0	1	2+/+nk	Total
N	11	4	5	20
NS	8	3	3	14
Wt	60.80	9.45	118.20	188.45
Het Chi	19.62	0.49	57.49	115.67
Het df	10	3	4	19
Het P	*	N.S.	***	***
Fixed RR	5.08	6.50	13.22	9.37
RRl	3.95	3.44	11.04	8.13
RRu	6.53	12.30	15.84	10.81
P	+++	+++	+++	+++
Random RR	5.88	6.50	13.33	8.21
RRl	3.69	3.44	5.51	5.24
RRu	9.36	12.30	32.25	12.86
P	+++	+++	+++	+++
Between Chi				38.07
Between df				2
Between P				***
Btwn(F) P				*

Table 5D1 - 3

IESLC - Meta-analysis of Ex Smoking, Any product (or Cigarettes if Any not available)
Small
Most adjusted

		Number of adjustment variables (2)					
		0	1	2	3-5	6+/+nk	Total
	N	11	4	4		1	20
	NS	8	3	2		1	14
	Wt	60.80	9.45	116.22		1.98	188.45
Het	Chi	19.62	0.49	50.30		0.00	115.67
Het	df	10	3	3		0	19
Het	P	*	N.S.	***		N.S.	***
Fixed	RR	5.08	6.50	13.66		2.00	9.37
	RRl	3.95	3.44	11.39		0.50	8.13
	RRu	6.53	12.30	16.38		8.05	10.81
	P	+++	+++	+++		N.S.	+++
Random	RR	5.88	6.50	18.72		2.00	8.21
	RRl	3.69	3.44	7.41		0.50	5.24
	RRu	9.36	12.30	47.34		8.05	12.86
	P	+++	+++	+++		N.S.	+++
Between	Chi						45.26
Between	df						3
Between	P						***
Btwn(F)	P						*

		Product			
		all/unsp	cig+/-ot	cig only	Total
	N	3	17		20
	NS	3	11		14
	Wt	9.38	179.07		188.45
Het	Chi	2.78	107.91		115.67
Het	df	2	16		19
Het	P	N.S.	***		***
Fixed	RR	4.61	9.73		9.37
	RRl	2.43	8.40		8.13
	RRu	8.73	11.26		10.81
	P	+++	+++		+++
Random	RR	4.59	9.05		8.21
	RRl	2.07	5.54		5.24
	RRu	10.18	14.78		12.86
	P	+++	+++		+++
Between	Chi				4.98
Between	df				1
Between	P				*
Btwn(F)	P				N.S.

		Denominator		
		nev any	nev cigs	Total
	N	7	13	20
	NS	6	8	14
	Wt	58.25	130.20	188.45
Het	Chi	8.81	71.43	115.67
Het	df	6	12	19
Het	P	N.S.	***	***
Fixed	RR	4.90	12.53	9.37
	RRl	3.79	10.55	8.13
	RRu	6.34	14.87	10.81
	P	+++	+++	+++
Random	RR	5.02	10.21	8.21
	RRl	3.44	5.65	5.24
	RRu	7.33	18.45	12.86
	P	+++	+++	+++
Between	Chi			35.42
Between	df			1
Between	P			***
Btwn(F)	P			*

Table 5D1 - 3

IESLC - Meta-analysis of Ex Smoking, Any product (or Cigarettes if Any not available)

Small

Most adjusted

	Derivation of RR/CI			
	Orig	StdCalc	Other	Total
N	10	10		20
NS	7	7		14
Wt	128.57	59.87		188.45
Het Chi	63.33	16.17		115.67
Het df	9	9		19
Het P	***	(*)		***
Fixed RR	12.64	4.93		9.37
RRl	10.63	3.83		8.13
RRu	15.02	6.35		10.81
P	+++	+++		+++
Random RR	11.26	5.33		8.21
RRl	5.98	3.42		5.24
RRu	21.22	8.32		12.86
P	+++	+++		+++
Between Chi				36.17
Between df				1
Between P				***
Btwn(F) P				*

Table 5D1 - 4

IESLC - Meta-analysis of Ex Smoking, Any product (or Cigarettes if Any not available)
Small
Least adjusted

REF	NRR	X	SEX	AGEL	AGEH	RACE	YF	LC TYPE	LOC	START	ST	NLC	R	VB	P	H	AD	PRODUCT	DENOM	De
BARBON	29	x	m	0	0	all	-	small	Eu:wst	1979	CC	755	n	bl	y	y	0	all/unsp	nev any	st
BROWN2	28		m	0	0	wh	-	small	NAmer	1984	CC	14596	n	bl	n	y	2	cig+/-ot	nev cigs	or
BROWN2	27		f	0	0	wh	-	small	NAmer	1984	CC	14596	n	bl	n	y	2	cig+/-ot	nev cigs	or
BUFFLE	68		f	0	0	w-hi	-	small	NAmer	1976	CC	943	n	bl	y	n	0	cig+/-ot	nev cigs	st
COMSTO	18		m	0	0	all	-	small	NAmer	1975	ot	258	n	bl	n	n	0	cig+/-ot	nev cigs	st
COMSTO	26		f	0	0	all	-	small	NAmer	1975	ot	258	n	bl	n	n	0	cig+/-ot	nev cigs	st
ENGELA	83		m	0	0	all	0	small	Eu:Sca	1964	pr	435	n	bl	n	n	7	cig+/-ot	nev cigs	or
JAHN	11		m	0	0	all	-	small	Eu:Ger	1988	CC	1004	n	bl	n	n	0	cig+/-ot	nev any	st
JAIN	29		m	0	0	all	-	small	NAmer	1981	CC	845	n	V	y	n	0	cig+/-ot	nev cigs	st
JAIN	24		f	0	0	all	-	small	NAmer	1981	CC	845	n	V	y	n	0	cig+/-ot	nev cigs	st
JEDRYC	25		m	0	0	all	-	small	Eu:est	1980	CC	1630	n	bl	y	n	0	cig+/-ot	nev any	st
KHUDER	9		m	0	0	all	-	small	NAmer	1985	CC	482	n	bl	n	y	0	cig+/-ot	nev cigs	or
KIHARA	11		c	0	0	jap	-	small	As:Jap	1991	CC	440	n	bl	n	n	0	all/unsp	nev any	st
LUBIN2	258		m	0	0	all	-	small	Eu:mul	1976	CC	7804	n	bl	n	y	0	cig+/-ot	nev any	st
LUBIN2	270		f	0	0	all	-	small	Eu:mul	1976	CC	7804	n	bl	n	y	0	cig+/-ot	nev any	st
OSANN	4	x	m	0	0	all	-	small	NAmer	1984	CC	1986	n	bl	n	n	0	cig+/-ot	nev cigs	st
OSANN	8	x	f	0	0	all	-	small	NAmer	1984	CC	1986	n	bl	n	n	0	cig+/-ot	nev cigs	st
SOBUE	9	x	m	0	0	all	-	small	As:Jap	1986	CC	1376	n	bl	n	y	0	cig+/-ot	nev cigs	st
SOBUE	25	x	f	0	0	all	-	small	As:Jap	1986	CC	1376	n	bl	n	y	0	cig+/-ot	nev cigs	st
SVENSS	23	x	f	0	0	all	-	small	Eu:Sca	1983	CC	210	n	bl	n	n	0	all/unsp	nev any	st

Cigarette type is all/unspec for all RRs

Table 5D1 - 5

IESLC - Meta-analysis of Ex Smoking, Any product (or Cigarettes if Any not available)
Small
Least adjusted

REF	NRR	SEX	AD	Number Exposed Case	Cont	Non-exposed Case	Cont	RR	95.00%CI
BARBON	29	m	0	42	205	6	188	6.42	(2.67- 15.45)
BROWN2	28	m	2	-	-	-	-	7.90	(6.20- 10.00)
BROWN2	27	f	2	-	-	-	-	29.80	(22.00- 40.30)
Subtotal BROWN2								13.16	(10.91- 15.87)
BUFFLE	68	f	0	12	56	1	112	24.00	(3.04- 189.26)
COMSTO	18	m	0	5	129	2	84	1.63	(0.31- 8.58)
COMSTO	26	f	0	2	35	2	115	3.29	(0.45- 24.19)
Subtotal COMSTO								2.17	(0.60- 7.79)
*ENGELA	83	m	7	-	-	-	-	2.00	(0.50- 8.10)
JAHN	11	m	0	79	402	1	138	27.12	(3.74- 196.77)
JAIN	29	m	0	30	159	3	85	5.35	(1.59- 18.03)
JAIN	24	f	0	20	97	2	214	22.06	(5.06- 96.26)
Subtotal JAIN								9.49	(3.72- 24.25)
JEDRYC	25	m	0	28	312	3	289	8.65	(2.60- 28.74)
KHUDER	9	m	0	36	-	1	-	34.40	(4.50- 262.00)
KIHARA	11	c	0	6	70	9	237	2.26	(0.78- 6.56)
LUBIN2	258	m	0	297	4228	34	2616	5.40	(3.78- 7.73)
LUBIN2	270	f	0	25	157	55	1180	3.42	(2.07- 5.64)
Subtotal LUBIN2								4.63	(3.46- 6.19)
OSANN	4	m	0	34	477	4	833	14.84	(5.24- 42.09)
OSANN	8	f	0	31	196	4	1093	43.22	(15.09- 123.79)
Subtotal OSANN								25.20	(12.02- 52.84)
SOBUE	9	m	0	28	363	1	128	9.87	(1.33- 73.31)
SOBUE	25	f	0	3	64	9	857	4.46	(1.18- 16.90)
Subtotal SOBUE								5.69	(1.88- 17.25)
SVENSS	23	f	0	5	36	2	120	8.33	(1.55- 44.78)
Partial Totals				683	6986	139	8289		

*prospective study

REF	NRR	SEX	AD	Ys	Ws	Qs	Ps
BARBON	29	m	0	1.86	4.98	0.71	0.0000
BROWN2	28	m	2	2.07	67.24	1.97	0.0000
BROWN2	27	f	2	3.39	41.94	56.08	0.0000
Subtotal BROWN2				2.58	109.18	58.06	
BUFFLE	68	f	0	3.18	0.90	0.80	0.0026
COMSTO	18	m	0	0.49	1.39	4.26	0.5657
COMSTO	26	f	0	1.19	0.96	1.06	0.2428
Subtotal COMSTO				0.77	2.35	5.32	
*ENGELA	83	m	7	0.69	1.98	4.73	0.3293
JAHN	11	m	0	3.30	0.98	1.10	0.0011
JAIN	29	m	0	1.68	2.60	0.82	0.0069
JAIN	24	f	0	3.09	1.77	1.30	0.0000
Subtotal JAIN				2.25	4.37	2.12	
JEDRYC	25	m	0	2.16	2.66	0.02	0.0004
KHUDER	9	m	0	3.54	0.93	1.57	0.0006
KIHARA	11	c	0	0.81	3.38	6.84	0.1347
LUBIN2	258	m	0	1.69	29.94	9.08	0.0000
LUBIN2	270	f	0	1.23	15.29	15.58	0.0000
Subtotal LUBIN2				1.53	45.23	24.67	
OSANN	4	m	0	2.70	3.54	0.75	0.0000
OSANN	8	f	0	3.77	3.47	8.10	0.0000
Subtotal OSANN				3.23	7.01	8.85	
SOBUE	9	m	0	2.29	0.96	0.00	0.0252
SOBUE	25	f	0	1.50	2.17	1.19	0.0276
Subtotal SOBUE				1.74	3.12	1.20	
SVENSS	23	f	0	2.12	1.36	0.02	0.0135

Table 5D1 - 5

IESLC - Meta-analysis of Ex Smoking, Any product (or Cigarettes if Any not available)
Small
Least adjusted

	N	20
	NS	14
	Wt	188.43
Het	Chi	116.00
Het	df	19
Het	P	***
Fixed	RR	9.38
	RRl	8.13
	RRu	10.81
	P	+++
Random	RR	8.21
	RRl	5.24
	RRu	12.86
	P	+++
Asymm	P	N.S.

Table 5D1 - 6

IESLC - Meta-analysis of Ex Smoking, Any product (or Cigarettes if Any not available)
Small
Least adjusted

	Sex			
	combined	male	female	Total
N	1	11	8	20
NS	1	11	8	20
Wt	3.38	117.20	67.86	188.43
Het Chi	0.00	15.65	62.92	116.00
Het df	0	10	7	19
Het P	N.S.	N.S.	***	***
Fixed RR	2.26	7.07	16.40	9.38
RRl	0.78	5.90	12.93	8.13
RRu	6.56	8.47	20.80	10.81
P	N.S.	+++	+++	+++
Random RR	2.26	6.92	11.70	8.21
RRl	0.78	4.98	4.47	5.24
RRu	6.56	9.61	30.62	12.86
P	N.S.	+++	+++	+++
Between Chi				37.43
Between df				2
Between P				***
Btwn(F) P				*

	Lung cancer type	
	small	Total
N	20	20
NS	14	14
Wt	188.43	188.43
Het Chi	116.00	116.00
Het df	19	19
Het P	***	***
Fixed RR	9.38	9.38
RRl	8.13	8.13
RRu	10.81	10.81
P	+++	+++
Random RR	8.21	8.21
RRl	5.24	5.24
RRu	12.86	12.86
P	+++	+++
Between Chi		
Between df		
Between P		N.S.
Btwn(F) P		N.S.

	Location								
	NAmer	UK	Scand	othEur	China	Japan	othAs	other	Total
N	10		2	5		3			20
NS	6		2	4		2			14
Wt	124.74		3.34	53.86		6.50			188.43
Het Chi	62.17		1.64	6.29		1.81			116.00
Het df	9		1	4		2			19
Het P	***		N.S.	N.S.		N.S.			***
Fixed RR	13.19		3.57	5.08		3.52			9.38
RRl	11.07		1.22	3.89		1.63			8.13
RRu	15.72		10.45	6.64		7.59			10.81
P	+++		+	+++		++			+++
Random RR	13.03		3.76	5.36		3.52			8.21
RRl	6.72		0.94	3.58		1.63			5.24
RRu	25.26		15.11	8.01		7.59			12.86
P	+++		(+)	+++		++			+++
Between Chi									44.09
Between df									3
Between P									***
Btwn(F) P									*

Table 5D1 - 6

IESLC - Meta-analysis of Ex Smoking, Any product (or Cigarettes if Any not available)
Small
Least adjusted

Detailed Country in "other Europe"

	multi	Germany	othWest	East	Balkans	Total
N	2	1	1	1		5
NS	1	1	1	1		4
Wt	45.23	0.98	4.98	2.66		53.86
Het Chi	2.13	0.00	0.00	0.00		6.29
Het df	1	0	0	0		4
Het P	N.S.	N.S.	N.S.	N.S.		N.S.
Fixed RR	4.63	27.12	6.42	8.65		5.08
RRl	3.46	3.74	2.67	2.60		3.89
RRu	6.19	196.77	15.45	28.74		6.64
P	+++	++	+++	+++		+++
Random RR	4.45	27.12	6.42	8.65		5.36
RRl	2.85	3.74	2.67	2.60		3.58
RRu	6.94	196.77	15.45	28.74		8.01
P	+++	++	+++	+++		+++
Between Chi						4.16
Between df						3
Between P						N.S.
Btwn(F) P						N.S.

Detailed Country in "other Asia"

	India	HongKong	other	Total
N				
NS				
Wt				
Het Chi				
Het df				
Het P				N.S.
Fixed RR				
RRl				
RRu				
P				+++
Random RR				
RRl				
RRu				
P				+++
Between Chi				
Between df				
Between P				N.S.
Btwn(F) P				N.S.

Detailed other continent

	SCAmer	Auslia	Africa	Total
N				
NS				
Wt				
Het Chi				
Het df				
Het P				N.S.
Fixed RR				
RRl				
RRu				
P				+++
Random RR				
RRl				
RRu				
P				+++
Between Chi				
Between df				
Between P				N.S.
Btwn(F) P				N.S.

Table 5D1 - 6

IESLC - Meta-analysis of Ex Smoking, Any product (or Cigarettes if Any not available)
Small
Least adjusted

	Start year of study					
	<1960	1960-69	1970-79	1980-89	1990+	Total
N		1	6	12	1	20
NS		1	4	8	1	14
Wt		1.98	53.47	129.61	3.38	188.43
Het Chi		0.00	6.70	57.80	0.00	116.00
Het df		0	5	11	0	19
Het P		N.S.	N.S.	***	N.S.	***
Fixed RR		2.00	4.75	13.19	2.26	9.38
RRl		0.50	3.63	11.11	0.78	8.13
RRu		8.05	6.20	15.67	6.56	10.81
P		N.S.	+++	+++	N.S.	+++
Random RR		2.00	4.67	13.77	2.26	8.21
RRl		0.50	3.20	7.85	0.78	5.24
RRu		8.05	6.82	24.15	6.56	12.86
P		N.S.	+++	+++	N.S.	+++
Between Chi						51.50
Between df						3
Between P						***
Btwn(F) P						*

	Study type (1)		
	CC	other	Total
N	17	3	20
NS	12	2	14
Wt	184.10	4.33	188.43
Het Chi	105.72	0.29	116.00
Het df	16	2	19
Het P	***	N.S.	***
Fixed RR	9.71	2.09	9.38
RRl	8.41	0.82	8.13
RRu	11.22	5.36	10.81
P	+++	N.S.	+++
Random RR	9.75	2.09	8.21
RRl	6.10	0.82	5.24
RRu	15.60	5.36	12.86
P	+++	N.S.	+++
Between Chi			9.99
Between df			1
Between P			**
Btwn(F) P			N.S.

	Study type (2)			
	CC	prosp	other	Total
N	17	1	2	20
NS	12	1	1	14
Wt	184.10	1.98	2.35	188.43
Het Chi	105.72	0.00	0.28	116.00
Het df	16	0	1	19
Het P	***	N.S.	N.S.	***
Fixed RR	9.71	2.00	2.17	9.38
RRl	8.41	0.50	0.60	8.13
RRu	11.22	8.05	7.79	10.81
P	+++	N.S.	N.S.	+++
Random RR	9.75	2.00	2.17	8.21
RRl	6.10	0.50	0.60	5.24
RRu	15.60	8.05	7.79	12.86
P	+++	N.S.	N.S.	+++
Between Chi				10.00
Between df				2
Between P				**
Btwn(F) P				N.S.

Table 5D1 - 6

IESLC - Meta-analysis of Ex Smoking, Any product (or Cigarettes if Any not available)
Small
Least adjusted

	Study size (number of LC cases) 100-249	250-499	500-999	1000+	Total
N	1	5	4	10	20
NS	1	4	3	6	14
Wt	1.36	8.64	10.25	168.18	188.43
Het Chi	0.00	6.65	3.53	93.14	116.00
Het df	0	4	3	9	19
Het P	N.S.	N.S.	N.S.	***	***
Fixed RR	8.33	2.91	8.52	10.02	9.38
RRl	1.55	1.50	4.62	8.62	8.13
RRu	44.78	5.67	15.71	11.66	10.81
P	+	++	+++	+++	+++
Random RR	8.33	3.21	8.87	10.54	8.21
RRl	1.55	1.31	4.46	5.79	5.24
RRu	44.78	7.89	17.64	19.21	12.86
P	+	+	+++	+++	+++
Between Chi					12.68
Between df					3
Between P					**
Btwn(F) P					N.S.

	Risky occupational population no	mining	othRisky	Total
N	20			20
NS	14			14
Wt	188.43			188.43
Het Chi	116.00			116.00
Het df	19			19
Het P	***			***
Fixed RR	9.38			9.38
RRl	8.13			8.13
RRu	10.81			10.81
P	+++			+++
Random RR	8.21			8.21
RRl	5.24			5.24
RRu	12.86			12.86
P	+++			+++
Between Chi				
Between df				
Between P				N.S.
Btwn(F) P				N.S.

	National cigarette tobacco type Virginia	blended	other	Total
N	2	18		20
NS	1	13		14
Wt	4.37	184.06		188.43
Het Chi	2.12	113.88		116.00
Het df	1	17		19
Het P	N.S.	***		***
Fixed RR	9.49	9.37		9.38
RRl	3.72	8.11		8.13
RRu	24.25	10.83		10.81
P	+++	+++		+++
Random RR	10.19	8.02		8.21
RRl	2.55	4.98		5.24
RRu	40.65	12.93		12.86
P	++	+++		+++
Between Chi				0.00
Between df				1
Between P				N.S.
Btwn(F) P				N.S.

Table 5D1 - 6

IESLC - Meta-analysis of Ex Smoking, Any product (or Cigarettes if Any not available)
Small
Least adjusted

	Any proxy use No/nk	Yes	Total
N	15	5	20
NS	10	4	14
Wt	175.52	12.91	188.43
Het Chi	112.34	3.53	116.00
Het df	14	4	19
Het P	***	N.S.	***
Fixed RR	9.44	8.54	9.38
RRl	8.14	4.95	8.13
RRu	10.94	14.74	10.81
P	+++	+++	+++
Random RR	7.77	8.54	8.21
RRl	4.54	4.95	5.24
RRu	13.30	14.74	12.86
P	+++	+++	+++
Between Chi			0.12
Between df			1
Between P			N.S.
Btwn(F) P			N.S.

	Full histological confirmation No	Yes	Total
N	12	8	20
NS	9	5	14
Wt	24.98	163.45	188.43
Het Chi	29.47	86.16	116.00
Het df	11	7	19
Het P	**	***	***
Fixed RR	8.37	9.54	9.38
RRl	5.66	8.18	8.13
RRu	12.40	11.12	10.81
P	+++	+++	+++
Random RR	8.10	8.32	8.21
RRl	4.17	4.31	5.24
RRu	15.73	16.08	12.86
P	+++	+++	+++
Between Chi			0.37
Between df			1
Between P			N.S.
Btwn(F) P			N.S.

	Number of adjustment variables (1) 0	1	2+/+nk	Total
N	17		3	20
NS	12		2	14
Wt	77.27		111.16	188.43
Het Chi	38.32		52.43	116.00
Het df	16		2	19
Het P	**		***	***
Fixed RR	6.04		12.72	9.38
RRl	4.84		10.56	8.13
RRu	7.55		15.32	10.81
P	+++		+++	+++
Random RR	7.61		9.23	8.21
RRl	4.99		2.88	5.24
RRu	11.61		29.65	12.86
P	+++		+++	+++
Between Chi				25.25
Between df				1
Between P				***
Btwn(F) P				*

Table 5D1 - 6

IESLC - Meta-analysis of Ex Smoking, Any product (or Cigarettes if Any not available)
Small
Least adjusted

	Number of adjustment variables (2) 0	1	2	3-5	6+/+nk	Total
N	17		2		1	20
NS	12		1		1	14
Wt	77.27		109.18		1.98	188.43
Het Chi	38.32		45.53		0.00	116.00
Het df	16		1		0	19
Het P	**		***		N.S.	***
Fixed RR	6.04		13.16		2.00	9.38
RRl	4.84		10.91		0.50	8.13
RRu	7.55		15.87		8.05	10.81
P	+++		+++		N.S.	+++
Random RR	7.61		15.29		2.00	8.21
RRl	4.99		4.16		0.50	5.24
RRu	11.61		56.17		8.05	12.86
P	+++		+++		N.S.	+++
Between Chi						32.15
Between df						2
Between P						***
Btwn(F) P						(*)

	Product all/unsp	cig+/-ot	cig only	Total
N	3	17		20
NS	3	11		14
Wt	9.72	178.72		188.43
Het Chi	2.74	108.16		116.00
Het df	2	16		19
Het P	N.S.	***		***
Fixed RR	4.63	9.74		9.38
RRl	2.47	8.41		8.13
RRu	8.68	11.28		10.81
P	+++	+++		+++
Random RR	4.62	9.09		8.21
RRl	2.15	5.55		5.24
RRu	9.93	14.88		12.86
P	+++	+++		+++
Between Chi				5.10
Between df				1
Between P				*
Btwn(F) P				N.S.

	Denominator nev any	nev cigs	Total
N	7	13	20
NS	6	8	14
Wt	58.59	129.84	188.43
Het Chi	8.77	71.54	116.00
Het df	6	12	19
Het P	N.S.	***	***
Fixed RR	4.90	12.56	9.38
RRl	3.80	10.57	8.13
RRu	6.34	14.92	10.81
P	+++	+++	+++
Random RR	5.02	10.28	8.21
RRl	3.46	5.67	5.24
RRu	7.30	18.64	12.86
P	+++	+++	+++
Between Chi			35.69
Between df			1
Between P			***
Btwn(F) P			*

Table 5D1 - 6

IESLC - Meta-analysis of Ex Smoking, Any product (or Cigarettes if Any not available)

Small

Least adjusted

	Derivation of RR/CI			
	Orig	StdCalc	Other	Total
N	4	16		20
NS	3	11		14
Wt	112.09	76.34		188.43
Het Chi	53.34	35.47		116.00
Het df	3	15		19
Het P	***	**		***
Fixed RR	12.83	5.92		9.38
RRl	10.66	4.73		8.13
RRu	15.44	7.41		10.81
P	+++	+++		+++
Random RR	11.27	7.22		8.21
RRl	3.89	4.74		5.24
RRu	32.62	10.99		12.86
P	+++	+++		+++
Between Chi				27.18
Between df				1
Between P				***
Btwn(F) P				*

Table 5D1 - 7

IESLC - Meta-analysis of Ex Smoking, Any product (or Cigarettes if Any not available)
Small
Excluded studies (and stage at which they were excluded)

1 BOUCHA BUELL LAURIL MZILEN

2 ABELIN ABRAHA ALDERS AUVINE AXELSO BAND BERRIN BLOT1 BLOT2 BLOT3 BLOT4 BRESLO BROCKM BROWN1 BYERS1 BYERS2
CASCO2 CASCOR CHAN CHATZI CHEN CHEN2 CHEN3 CHIAZZ COOKSO DAVEYS DEAN DOSEME DU DUNN EBELIN ENSTRO
ESAKI FAN GENG GER GODLEY GOLLED GSELL HANSEN HEGMAN HINDS HIRAOK HIRAY2 HOROWI HORWIT HU HU2
HUANG ISHIMA JARUP JIANG JONES JUSSAW KANELL KO KOHLME KOULUM KREYBE LAMTH LAMWK LAMWK2 LAUSSM LEI
LETOUR LEVIN LIAW LICKIN LIU LIU2 LIU3 LIU4 LIU5 LOMBA2 LUO MAGNUS MARSH MARSH2 MARTIS MASTRA
MATSUD MCCONN MCDUFF MCLAUG MILLER MILLS MOLLO MRFIT MURATA NOTAN2 NOTANI NOU ORMOS PASTOR PAWLEG PERNU
PERSHA PIKE POFFIJ POLEDN QIAO RADZIK RANDIG REN RIMING RONCO ROOTS ROTHSC SAARIK SADOWS SANKAR SCHWA2
SEGI SEGI2 SEOW SHIMIZ SIEMIA SIMARA SITAS SOBUE2 STASZE STAYNE STOCKS SUN TANG2 TAO ULMER VANDER
WALD WANG WANG3 WANG4 WARSIN WATSON WICKLU WILKIN WU2 WUWILL WYNDE2 WYNDE4 WYNDE5 WYNDE8 WYNDER XIANGZ
XU XU2 XU3 XU4 YUAN ZHANG ZHENG ZHOU

3 RESTRE

4 BOFFET

8 AGUDO AKIBA AMANDU AMES ANDERS ARCHER ARMADA AUSTIN AXELSS BECHER BENHAM BENSHL BEST BLOHMK BOUCOT BRETT
BROSS CARPEN CEDERL CHANG CHOI CHOW CHYOU CORREA CPSI CPSII DAMBER DARBY DEAN2 DEAN3 DEKLER DESTE2
DESTEF DOCKER DOLL DOLL2 DORANT DORGAN DORN DROSTE GAO GAO2 GARCIA GARDIN GARSHI GILLIS GOODMA GRAHAM
GREGOR HAENSZ HAMMO2 HAMMON HEIN HENNEK HIRAYA HITOSU HOLE HUMBLE JARVHO JOLY KAISE2 KAISER KATSOU KAUFMA
KELLER KINLEN KJUUS KNEKT KOO KREUZE KUBIK LANGE LEMARC LIDDEL LOMBAR LUBIN MACLEN MATOS MIGRAN MRFITR
NAM ODRISC OSANN2 PARKIN PERSH2 PETO PEZZO2 PEZZOT PISANI PRESCO QIAO2 RACHTA SCHWAR SHAW SIMONA SPEIZE
SPITZ STOCKW STUCKE SUZUK2 SUZUKI TANG TENKAN TIZZAN TOKARS TOUSEY TSUGAN TULINI TVERDA VEIERO VUTUC WAKAI
WANG2 WIGLE WU WUNSCH WYNDE3 WYNDE6 WYNDE7 YAMAGU YONG

Table 5D1 - 8

Potentially overlapping studies

REF	REFGP	PRINC	.	OVERLAP/LINK
LUBIN2	LUBIN2	1		Lubin-combined
JAHN	BOFFET	2		Subset of BOFFET
