# Supplementary material for: Systematic review with meta-analysis of the epidemiological evidence in the 1900s relating smoking to lung cancer
Source: BMC Cancer. 2012 Sep 3;12:385. doi: 10.1186/1471-2407-12-385 (PMC3505152; doi:10.1186/1471-2407-12-385)
Supplement: Additional file 5 — Detailed Analysis Tables (Individual file names as described in Additional file 1: Methods, Table1). [file 1471-2407-12-385-S5.zip › PDF/2H.pdf]

Table 2H1 -

IESLC - Meta-analysis of Ever Smoking by Age started, Overview
Squamous, Any Product (or Cigarettes if Any not available)

This analysis is restricted to results for:
1) Ever smokers
2) Results by Age started
3) Categorical results by Age started
Results by Age started are grouped under 2 schemes (S1, S2). Each scheme has a set of "key values". An interval is allocated to the category whose key value it includes, and intervals which include none or more than one of the key values are excluded. (Open-ended intervals are coded as 999)

S1	key value	maximum range
1	26	19+
2	18	15-25
3	14	1-17

S2	key value	maximum range
1	30	27+
2	26	23-29
3	22	19-25
4	18	15-21
5	14	11-17
6	10	1-13

4) Squamous (or near equivalent)
5) Results complete enough for use in metaanalysis

Within each study, results are then selected (in the following order of preference, within each sex) for:
6) (not applicable)
7) PRODUCT: all/unspec, cigarettes regardless of other products, cigarettes only
8) CIGTYPE: all/unspecified, MC regardless of HR, MC only
9) (not applicable)
10) DENOM: never smoked anything, never smoked cigarettes, never any + low, never cigs + low
11) Followup period (YF, prospective studies): whole study (coded as 0) or longest available
12) LCtype: squamous or nearest available, but not adeno. (q = squamous, s = small,
a = adeno, KI = Kreyberg I, u = undifferentiated)
13) Race: all or nearest available, otherwise by race (wh or w = white, bl or b = black, hi = hispanic
ch = chinese, jap = japanese, haw = hawaiian, w+o = white + oriental, sca = scandinavian, as = asian)
14) For overlapping studies: principal rather than subsidiary studies
Finally by Age: whole study (coded as 0) if available, otherwise by widest available age group
and then for single sex results (m, f) in preference to results for both sexes combined (c).

Results adjusted (AD) for the most potential confounders are then chosen in Sections -1 to -3 and results adjusted for the least confounders in Sections -4 to -6. (Those least adjusted results which actually differ from the most adjusted are marked 'x' in column X in Section -4)

Section -7 shows excluded studies, together with the stage (as above) at which no qualifying results were found.

Section -8 lists the potentially overlapping studies which have been included (1=principal, 2=subsidiary).

Section -9 lists any results which would have been included in preference except that they had data not complete enough for use in meta-analysis, with their significance (yes/no), if known, and any further comment as entered on the database. It also lists as "gap" any categories for which no data were presented by the original authors.

In addition to those mentioned above, the following fields, levels and abbreviations are used:
* or nk = not known, n = no, y = yes, ot = other
nev = never
all/unspec = all or unspecified, cig+/-ot = cigarettes irrespective of other products (cigar, pipe etc)
MC = manufactured cigarettes, HR = hand-rolled cigarettes
exL, exH = range of exposure (low and high) in the smoking group, in terms of Age started
REF: 6-character study reference
NRR: number of the RR on the database within the study
ST : study type (CC = case control, pr or prosp = prospective)
NLC: number of lung cancer cases in whole study
R : risky occupational population (n = no, m = mining, o = other risky)
VB : national cigarette type (V = at least 75% Virginia, bl = at least 75% blended, ot = other)
P : any proxy use
H : full histological confirmation
De : derivation of RR/CI (or = original, st = standard method, ot = other method of estimation)

Table 2H1 - 1

IESLC - Meta-analysis of Ever Smoking by Age started, Overview
Squamous, Any Product (or Cigarettes if Any not available)
Most adjusted

REF	NRR	SEX	AGEL	AGEH	RACE	YF	LC TYPE	LOC	START	ST	NLC	R	VB	P	H	AD	PRODUCT	exL	exH	S1	S2	DENOM	De
BARBON	574	m	0	0	all	-	q	Eu:wst	1979	CC	755	n	bl	y	y	1	all/unsp	20	999	1	0	nev any	or
BARBON	575	m	0	0	all	-	q	Eu:wst	1979	CC	755	n	bl	y	y	1	all/unsp	15	19	2	4	nev any	or
BARBON	576	m	0	0	all	-	q	Eu:wst	1979	CC	755	n	bl	y	y	1	all/unsp	1	14	3	0	nev any	or
BENHAM	514	m	0	0	all	-	KI	Eu:wst	1976	CC	1625	n	bl	n	y	0	cig only	25	999	1	0	nev any	st
BENHAM	515	m	0	0	all	-	KI	Eu:wst	1976	CC	1625	n	bl	n	y	0	cig only	20	24	0	3	nev any	st
BENHAM	516	m	0	0	all	-	KI	Eu:wst	1976	CC	1625	n	bl	n	y	0	cig only	17	19	2	4	nev any	st
BENHAM	517	m	0	0	all	-	KI	Eu:wst	1976	CC	1625	n	bl	n	y	0	cig only	1	16	3	0	nev any	st
HAENSZ	501	f	0	0	all	-	q+u	NAmer	1955	CC	158	n	bl	n	y	0	cig+/-ot	25	999	1	0	nev any	st
HAENSZ	502	f	0	0	all	-	q+u	NAmer	1955	CC	158	n	bl	n	y	0	cig+/-ot	1	24	0	0	nev any	st
JEDRYC	528	m	0	0	all	-	q	Eu:est	1980	CC	1630	n	bl	y	n	0	cig+/-ot	19	999	1	0	nev any	st
JEDRYC	529	m	0	0	all	-	q	Eu:est	1980	CC	1630	n	bl	y	n	0	cig+/-ot	17	18	2	4	nev any	st
JEDRYC	530	m	0	0	all	-	q	Eu:est	1980	CC	1630	n	bl	y	n	0	cig+/-ot	1	16	3	0	nev any	st
LUO	512	c	0	0	all	-	q	As:Chi	1990	CC	102	n	ot	n	y	20	cig+/-ot	40	999	0	0	nev cigs	or
LUO	513	c	0	0	all	-	q	As:Chi	1990	CC	102	n	ot	n	y	20	cig+/-ot	20	39	1	0	nev cigs	or
LUO	514	c	0	0	all	-	q	As:Chi	1990	CC	102	n	ot	n	y	20	cig+/-ot	0	19	0	0	nev cigs	or
MATOS	616	m	0	0	all	-	q	SCAmer	1994	CC	200	n	bl	n	n	2	cig+/-ot	20	999	1	0	nev any	or
MATOS	617	m	0	0	all	-	q	SCAmer	1994	CC	200	n	bl	n	n	2	cig+/-ot	15	19	2	4	nev any	or
MATOS	618	m	0	0	all	-	q	SCAmer	1994	CC	200	n	bl	n	n	2	cig+/-ot	1	14	3	0	nev any	or
PEZZOT	543	m	0	0	all	-	q	SCAmer	1987	CC	215	n	bl	n	y	0	cig only	19	999	1	0	nev cigs	ot
PEZZOT	544	m	0	0	all	-	q	SCAmer	1987	CC	215	n	bl	n	y	0	cig only	14	18	0	0	nev cigs	ot
PEZZOT	545	m	0	0	all	-	q	SCAmer	1987	CC	215	n	bl	n	y	0	cig only	1	13	0	6	nev cigs	ot
WYNDE6	608	m	0	0	wh	-	q	NAmer	1969	CC	4423	n	bl	n	y	0	cig+/-ot	21	999	1	0	nev cigs	st
WYNDE6	609	m	0	0	wh	-	q	NAmer	1969	CC	4423	n	bl	n	y	0	cig+/-ot	18	20	2	4	nev cigs	st
WYNDE6	610	m	0	0	wh	-	q	NAmer	1969	CC	4423	n	bl	n	y	0	cig+/-ot	1	17	3	0	nev cigs	st
WYNDE6	632	f	0	0	wh	-	q	NAmer	1969	CC	4423	n	bl	n	y	0	cig+/-ot	21	999	1	0	nev cigs	st
WYNDE6	633	f	0	0	wh	-	q	NAmer	1969	CC	4423	n	bl	n	y	0	cig+/-ot	18	20	2	4	nev cigs	st
WYNDE6	634	f	0	0	wh	-	q	NAmer	1969	CC	4423	n	bl	n	y	0	cig+/-ot	1	17	3	0	nev cigs	st
ZHENG	520	m	0	0	all	-	q	As:Chi	1982	CC	540	n	ot	*	y	0	cig+/-ot	30	999	0	1	nev cigs	st
ZHENG	521	m	0	0	all	-	q	As:Chi	1982	CC	540	n	ot	*	y	0	cig+/-ot	20	29	1	0	nev cigs	st
ZHENG	522	m	0	0	all	-	q	As:Chi	1982	CC	540	n	ot	*	y	0	cig+/-ot	1	19	0	0	nev cigs	st
ZHENG	527	f	0	0	all	-	q	As:Chi	1982	CC	540	n	ot	*	y	0	cig+/-ot	30	999	0	1	nev cigs	st
ZHENG	528	f	0	0	all	-	q	As:Chi	1982	CC	540	n	ot	*	y	0	cig+/-ot	1	29	0	0	nev cigs	st

Cigarette type is all/unspec for all RRs

In this overview table, subtotals and Qs values may be invalid and should be ignored

Table 2H1 - 2

IESLC - Meta-analysis of Ever Smoking by Age started, Overview
Squamous, Any Product (or Cigarettes if Any not available)
Most adjusted

REF	NRR	SEX	AD	Number Exposed Case	Cont	Non-exposed Case	Cont	RR	95.00%CI
BARBON	574	m	1	64	-	6	-	9.40	(4.20- 23.40)
BARBON	575	m	1	145	-	6	-	13.70	(5.90- 31.70)
BARBON	576	m	1	52	-	6	-	71.30	(27.60- 184.00)
Subtotal BARBON								19.26	(11.59- 32.00)
BENHAM	514	m	0	98	137	24	481	14.34	(8.83- 23.29)
BENHAM	515	m	0	323	398	24	481	16.26	(10.52- 25.14)
BENHAM	516	m	0	311	373	24	481	16.71	(10.80- 25.86)
BENHAM	517	m	0	342	341	24	481	20.10	(12.99- 31.10)
Subtotal BENHAM								16.86	(13.48- 21.08)
HAENSZ	501	f	0	32	66	44	236	2.60	(1.53- 4.42)
HAENSZ	502	f	0	24	37	44	236	3.48	(1.90- 6.38)
Subtotal HAENSZ								2.95	(1.98- 4.40)
JEDRYC	528	m	0	134	502	6	289	12.86	(5.60- 29.50)
JEDRYC	529	m	0	96	221	6	289	20.92	(9.00- 48.62)
JEDRYC	530	m	0	53	111	6	289	23.00	(9.61- 55.01)
Subtotal JEDRYC								18.20	(11.15- 29.70)
LUO	512	c	20	1	-	5	-	3.10	(0.30- 40.20)
LUO	513	c	20	19	-	5	-	10.50	(2.30- 48.60)
LUO	514	c	20	14	-	5	-	12.40	(2.70- 57.70)
Subtotal LUO								9.22	(3.43- 24.79)
MATOS	616	m	2	6	-	3	-	3.30	(0.80- 13.90)
MATOS	617	m	2	25	-	3	-	7.90	(2.30- 27.10)
MATOS	618	m	2	16	-	3	-	7.20	(2.00- 25.90)
Subtotal MATOS								6.00	(2.82- 12.75)
PEZZOT	543	m	0	10	105	0	116	23.19~	(1.34- 400.59)
PEZZOT	544	m	0	45	145	0	116	72.86~	(4.44-1195.36)
PEZZOT	545	m	0	30	67	0	116	105.28~	(6.34-1749.54)
Subtotal PEZZOT								56.74	(11.15- 288.86)
WYNDE6	608	m	0	44	92	9	589	31.30	(14.79- 66.26)
WYNDE6	609	m	0	81	139	9	589	38.14	(18.69- 77.81)
WYNDE6	610	m	0	262	301	9	589	56.96	(28.89- 112.31)
WYNDE6	632	f	0	32	90	12	673	19.94	(9.91- 40.11)
WYNDE6	633	f	0	49	94	12	673	29.23	(15.00- 56.97)
WYNDE6	634	f	0	72	91	12	673	44.37	(23.18- 84.93)
Subtotal WYNDE6								35.10	(26.47- 46.53)
ZHENG	520	m	0	13	66	4	94	4.63	(1.45- 14.82)
ZHENG	521	m	0	81	109	4	94	17.46	(6.17- 49.46)
ZHENG	522	m	0	62	43	4	94	33.88	(11.58- 99.14)
ZHENG	527	f	0	9	16	33	184	3.14	(1.28- 7.69)
ZHENG	528	f	0	34	28	33	184	6.77	(3.63- 12.62)
Subtotal ZHENG								7.97	(5.36- 11.85)
Partial Totals				2579	3572	385	8047		

*prospective study ~ With 0.5 adjustment for zero

REF	NRR	SEX	AD	Ys	Ws	Qs	Ps
BARBON	574	m	1	2.24	5.21	1.05	0.0000
BARBON	575	m	1	2.62	5.44	0.03	0.0000
BARBON	576	m	1	4.27	4.27	10.61	0.0000
Subtotal BARBON				2.96	14.91	11.70	
BENHAM	514	m	0	2.66	16.33	0.01	0.0000
BENHAM	515	m	0	2.79	20.26	0.20	0.0000
BENHAM	516	m	0	2.82	20.14	0.32	0.0000
BENHAM	517	m	0	3.00	20.16	1.95	0.0000
Subtotal BENHAM				2.82	76.89	2.47	
HAENSZ	501	f	0	0.96	13.63	41.00	0.0004
HAENSZ	502	f	0	1.25	10.45	21.78	0.0001
Subtotal HAENSZ				1.08	24.08	62.78	
JEDRYC	528	m	0	2.55	5.57	0.10	0.0000
JEDRYC	529	m	0	3.04	5.40	0.66	0.0000
JEDRYC	530	m	0	3.14	5.05	1.00	0.0000
Subtotal JEDRYC				2.90	16.02	1.77	
LUO	512	c	20	1.13	0.64	1.56	0.3652
LUO	513	c	20	2.35	1.65	0.19	0.0025
LUO	514	c	20	2.52	1.64	0.05	0.0013
Subtotal LUO				2.22	3.93	1.79	
MATOS	616	m	2	1.19	1.89	4.22	0.1012
MATOS	617	m	2	2.07	2.53	0.98	0.0010
MATOS	618	m	2	1.97	2.34	1.20	0.0025
Subtotal MATOS				1.79	6.75	6.40	
PEZZOT	543	m	0	3.14	0.47	0.10	0.0306

Table 2H1 - 2

IESLC - Meta-analysis of Ever Smoking by Age started, Overview
Squamous, Any Product (or Cigarettes if Any not available)
Most adjusted

REF	NRR	SEX	AD	Ys	Ws	Qs	Ps
PEZZOT	544	m	0	4.29	0.49	1.25	0.0027
PEZZOT	545	m	0	4.66	0.49	1.88	0.0012
Subtotal PEZZOT				4.04	1.45	3.23	
WYNDE6	608	m	0	3.44	6.83	3.88	0.0000
WYNDE6	609	m	0	3.64	7.56	6.83	0.0000
WYNDE6	610	m	0	4.04	8.34	15.25	0.0000
WYNDE6	632	f	0	2.99	7.86	0.72	0.0000
WYNDE6	633	f	0	3.38	8.63	4.05	0.0000
WYNDE6	634	f	0	3.79	9.12	11.08	0.0000
Subtotal WYNDE6				3.56	48.33	41.81	
ZHENG	520	m	0	1.53	2.84	3.80	0.0099
ZHENG	521	m	0	2.86	3.54	0.10	0.0000
ZHENG	522	m	0	3.52	3.33	2.31	0.0000
ZHENG	527	f	0	1.14	4.78	11.43	0.0125
ZHENG	528	f	0	1.91	9.91	5.99	0.0000
Subtotal ZHENG				2.08	24.40	23.64	

N	32
NS	9

Table 2H1 - 3

IESLC - Meta-analysis of Ever Smoking by Age started, Overview
Squamous, Any Product (or Cigarettes if Any not available)
Most adjusted

	Sex			
	combined	male	female	Total
N	3	22	7	32
NS	1	7	3	11

In this overview table, other than the "N" rows, entries in the "absent" and "Total" columns may be invalid and should be ignored

	Age started (broad categories)				
	absent	19+k26	15-25k18	1-17k14	Total
N	10	10	6	6	32
NS	5	9	5	5	24
Wt	54.83	62.98	49.70	49.28	216.78
Het Chi	35.72	43.54	8.01	16.38	155.60
Het df	9	9	5	5	31
Het P	***	***	N.S.	**	***
Fixed RR	8.80	10.35	20.15	29.91	14.73
RRl	6.76	8.09	15.26	22.62	12.90
RRu	11.47	13.25	26.61	39.54	16.83
P	+++	+++	+++	+++	+++
Random RR	9.12	11.17	20.28	31.07	14.70
RRl	4.84	6.15	13.92	17.93	10.63
RRu	17.20	20.31	29.53	53.85	20.32
P	+++	+++	+++	+++	+++

	Age started (narrow categories)							
	absent	27+k30	23-29k26	19-25k22	15-21k18	11-17k14	1-13k10	Total
N	22	2		1	6		1	32
NS	9	1		1	5		1	16
Wt	138.73	7.61		20.26	49.70		0.49	216.78
Het Chi	124.96	0.27		0.00	8.01		0.00	155.60
Het df	21	1		0	5		0	31
Het P	***	N.S.		N.S.	N.S.		N.S.	***
Fixed RR	13.92	3.63		16.26	20.15		105.28	14.73
RRl	11.79	1.78		10.52	15.26		6.34	12.90
RRu	16.44	7.38		25.14	26.61		1749.54	16.83
P	+++	+++		+++	+++		++	+++
Random RR	14.70	3.63		16.26	20.28		105.28	14.70
RRl	9.46	1.78		10.52	13.92		6.34	10.63
RRu	22.85	7.38		25.14	29.53		1749.54	20.32
P	+++	+++		+++	+++		++	+++

Table 2H1 - 3

IESLC - Meta-analysis of Ever Smoking by Age started, Overview
Squamous, Any Product (or Cigarettes if Any not available)
Most adjusted

MALES

	Age started (broad categories) absent	19+k26	15-25k18	1-17k14	Total
N	5	7	5	5	22
NS	3	7	5	5	20
Wt	27.41	39.84	41.06	40.16	148.47
Het Chi	9.06	9.46	6.56	14.64	48.36
Het df	4	6	4	4	21
Het P	(*)	N.S.	N.S.	**	***
Fixed RR	16.58	14.59	18.64	27.35	18.94
RRl	11.40	10.69	13.72	20.07	16.13
RRu	24.11	19.90	25.30	37.26	22.25
P	+++	+++	+++	+++	+++
Random RR	18.66	14.12	18.61	28.44	18.83
RRl	8.10	9.17	12.12	14.64	14.34
RRu	42.97	21.72	28.56	55.25	24.73
P	+++	+++	+++	+++	+++

		Age started (narrow categories)						
	absent	27+k30	23-29k26	19-25k22	15-21k18	11-17k14	1-13k10	Total
N	14	1		1	5		1	22
NS	7	1		1	5		1	14
Wt	83.82	2.84		20.26	41.06		0.49	148.47
Het Chi	33.69	0.00		0.00	6.56		0.00	48.36
Het df	13	0		0	4		0	21
Het P	**	N.S.		N.S.	N.S.		N.S.	***
Fixed RR	20.58	4.63		16.26	18.64		105.28	18.94
RRl	16.61	1.45		10.52	13.72		6.34	16.13
RRu	25.49	14.82		25.14	25.30		1749.54	22.25
P	+++	++		+++	+++		++	+++
Random RR	20.40	4.63		16.26	18.61		105.28	18.83
RRl	13.88	1.45		10.52	12.12		6.34	14.34
RRu	29.99	14.82		25.14	28.56		1749.54	24.73
P	+++	++		+++	+++		++	+++

FEMALES

	Age started (broad categories) absent	19+k26	15-25k18	1-17k14	Total
N	3	2	1	1	7
NS	2	2	1	1	4
Wt	25.14	21.49	8.63	9.12	64.38
Het Chi	2.96	20.69	0.00	0.00	76.50
Het df	2	1	0	0	6
Het P	N.S.	***	N.S.	N.S.	***
Fixed RR	4.44	5.48	29.23	44.37	8.49
RRl	3.00	3.59	15.00	23.18	6.65
RRu	6.56	8.36	56.97	84.93	10.84
P	+++	+++	+++	+++	+++
Random RR	4.38	7.11	29.23	44.37	9.06
RRl	2.69	0.97	15.00	23.18	3.76
RRu	7.11	52.31	56.97	84.93	21.85
P	+++	(+)	+++	+++	+++

Table 2H1 - 3

IESLC - Meta-analysis of Ever Smoking by Age started, Overview
Squamous, Any Product (or Cigarettes if Any not available)
Most adjusted

FEMALES

	absent	Age started (narrow categories) 27+k30	23-29k26	19-25k22	15-21k18	11-17k14	1-13k10	Total
N	5	1			1			7
NS	3	1			1			4
Wt	50.98	4.78			8.63			64.38
Het Chi	57.89	0.00			0.00			76.50
Het df	4	0			0			6
Het P	***	N.S.			N.S.			***
Fixed RR	7.56	3.14			29.23			8.49
RRl	5.75	1.28			15.00			6.65
RRu	9.95	7.69			56.97			10.84
P	+++	+			+++			+++
Random RR	8.76	3.14			29.23			9.06
RRl	3.06	1.28			15.00			3.76
RRu	25.02	7.69			56.97			21.85
P	+++	+			+++			+++

Table 2H1 - 4

IESLC - Meta-analysis of Ever Smoking by Age started, Overview
Squamous, Any Product (or Cigarettes if Any not available)
Least adjusted

REF	NRR	X	SEX	AGEL	AGEH	RACE	YF	LC TYPE	LOC	START	ST	NLC	R	VB	P	H	AD	PRODUCT	exL	exH	S1	S2	DENOM	De
BARBON	569	x	m	0	0	all	-	q	Eu:wst	1979	CC	755	n	bl	y	y	0	all/unsp	20	999	1	0	nev any	st
BARBON	570	x	m	0	0	all	-	q	Eu:wst	1979	CC	755	n	bl	y	y	0	all/unsp	15	19	2	4	nev any	st
BARBON	571	x	m	0	0	all	-	q	Eu:wst	1979	CC	755	n	bl	y	y	0	all/unsp	1	14	3	0	nev any	st
BENHAM	514		m	0	0	all	-	KI	Eu:wst	1976	CC	1625	n	bl	n	y	0	cig only	25	999	1	0	nev any	st
BENHAM	515		m	0	0	all	-	KI	Eu:wst	1976	CC	1625	n	bl	n	y	0	cig only	20	24	0	3	nev any	st
BENHAM	516		m	0	0	all	-	KI	Eu:wst	1976	CC	1625	n	bl	n	y	0	cig only	17	19	2	4	nev any	st
BENHAM	517		m	0	0	all	-	KI	Eu:wst	1976	CC	1625	n	bl	n	y	0	cig only	1	16	3	0	nev any	st
HAENSZ	501		f	0	0	all	-	q+u	NAmer	1955	CC	158	n	bl	n	y	0	cig+/-ot	25	999	1	0	nev any	st
HAENSZ	502		f	0	0	all	-	q+u	NAmer	1955	CC	158	n	bl	n	y	0	cig+/-ot	1	24	0	0	nev any	st
JEDRYC	528		m	0	0	all	-	q	Eu:est	1980	CC	1630	n	bl	y	n	0	cig+/-ot	19	999	1	0	nev any	st
JEDRYC	529		m	0	0	all	-	q	Eu:est	1980	CC	1630	n	bl	y	n	0	cig+/-ot	17	18	2	4	nev any	st
JEDRYC	530		m	0	0	all	-	q	Eu:est	1980	CC	1630	n	bl	y	n	0	cig+/-ot	1	16	3	0	nev any	st
LUO	507	x	c	0	0	all	-	q	As:Chi	1990	CC	102	n	ot	n	y	0	cig+/-ot	40	999	0	0	nev cigs	st
LUO	508	x	c	0	0	all	-	q	As:Chi	1990	CC	102	n	ot	n	y	0	cig+/-ot	20	39	1	0	nev cigs	st
LUO	509	x	c	0	0	all	-	q	As:Chi	1990	CC	102	n	ot	n	y	0	cig+/-ot	0	19	0	0	nev cigs	st
MATOS	611	x	m	0	0	all	-	q	SCAmer	1994	CC	200	n	bl	n	n	0	cig+/-ot	20	999	1	0	nev any	st
MATOS	612	x	m	0	0	all	-	q	SCAmer	1994	CC	200	n	bl	n	n	0	cig+/-ot	15	19	2	4	nev any	st
MATOS	613	x	m	0	0	all	-	q	SCAmer	1994	CC	200	n	bl	n	n	0	cig+/-ot	1	14	3	0	nev any	st
PEZZOT	543		m	0	0	all	-	q	SCAmer	1987	CC	215	n	bl	n	y	0	cig only	19	999	1	0	nev cigs	ot
PEZZOT	544		m	0	0	all	-	q	SCAmer	1987	CC	215	n	bl	n	y	0	cig only	14	18	0	0	nev cigs	ot
PEZZOT	545		m	0	0	all	-	q	SCAmer	1987	CC	215	n	bl	n	y	0	cig only	1	13	0	6	nev cigs	ot
WYNDE6	608		m	0	0	wh	-	q	NAmer	1969	CC	4423	n	bl	n	y	0	cig+/-ot	21	999	1	0	nev cigs	st
WYNDE6	609		m	0	0	wh	-	q	NAmer	1969	CC	4423	n	bl	n	y	0	cig+/-ot	18	20	2	4	nev cigs	st
WYNDE6	610		m	0	0	wh	-	q	NAmer	1969	CC	4423	n	bl	n	y	0	cig+/-ot	1	17	3	0	nev cigs	st
WYNDE6	632		f	0	0	wh	-	q	NAmer	1969	CC	4423	n	bl	n	y	0	cig+/-ot	21	999	1	0	nev cigs	st
WYNDE6	633		f	0	0	wh	-	q	NAmer	1969	CC	4423	n	bl	n	y	0	cig+/-ot	18	20	2	4	nev cigs	st
WYNDE6	634		f	0	0	wh	-	q	NAmer	1969	CC	4423	n	bl	n	y	0	cig+/-ot	1	17	3	0	nev cigs	st
ZHENG	520		m	0	0	all	-	q	As:Chi	1982	CC	540	n	ot	*	y	0	cig+/-ot	30	999	0	1	nev cigs	st
ZHENG	521		m	0	0	all	-	q	As:Chi	1982	CC	540	n	ot	*	y	0	cig+/-ot	20	29	1	0	nev cigs	st
ZHENG	522		m	0	0	all	-	q	As:Chi	1982	CC	540	n	ot	*	y	0	cig+/-ot	1	19	0	0	nev cigs	st
ZHENG	527		f	0	0	all	-	q	As:Chi	1982	CC	540	n	ot	*	y	0	cig+/-ot	30	999	0	1	nev cigs	st
ZHENG	528		f	0	0	all	-	q	As:Chi	1982	CC	540	n	ot	*	y	0	cig+/-ot	1	29	0	0	nev cigs	st

Cigarette type is all/unspec for all RRs

In this overview table, subtotals and Qs values may be invalid and should be ignored

Table 2H1 - 5

IESLC - Meta-analysis of Ever Smoking by Age started, Overview
Squamous, Any Product (or Cigarettes if Any not available)
Least adjusted

REF	NRR	SEX	AD	Number Exposed Case	Cont	Non-exposed Case	Cont	RR	95.00%CI
BARBON	569	m	0	64	207	6	188	9.69	(4.10- 22.89)
BARBON	570	m	0	145	337	6	188	13.48	(5.84- 31.10)
BARBON	571	m	0	52	23	6	188	70.84	(27.41- 183.08)
Subtotal BARBON								19.28	(11.62- 32.01)
BENHAM	514	m	0	98	137	24	481	14.34	(8.83- 23.29)
BENHAM	515	m	0	323	398	24	481	16.26	(10.52- 25.14)
BENHAM	516	m	0	311	373	24	481	16.71	(10.80- 25.86)
BENHAM	517	m	0	342	341	24	481	20.10	(12.99- 31.10)
Subtotal BENHAM								16.86	(13.48- 21.08)
HAENSZ	501	f	0	32	66	44	236	2.60	(1.53- 4.42)
HAENSZ	502	f	0	24	37	44	236	3.48	(1.90- 6.38)
Subtotal HAENSZ								2.95	(1.98- 4.40)
JEDRYC	528	m	0	134	502	6	289	12.86	(5.60- 29.50)
JEDRYC	529	m	0	96	221	6	289	20.92	(9.00- 48.62)
JEDRYC	530	m	0	53	111	6	289	23.00	(9.61- 55.01)
Subtotal JEDRYC								18.20	(11.15- 29.70)
LUO	507	c	0	1	8	5	51	1.28	(0.13- 12.37)
LUO	508	c	0	19	38	5	51	5.10	(1.75- 14.88)
LUO	509	c	0	14	20	5	51	7.14	(2.27- 22.43)
Subtotal LUO								5.07	(2.42- 10.62)
MATOS	611	m	0	6	73	3	110	3.01	(0.73- 12.43)
MATOS	612	m	0	25	120	3	110	7.64	(2.24- 26.01)
MATOS	613	m	0	16	90	3	110	6.52	(1.84- 23.08)
Subtotal MATOS								5.58	(2.64- 11.78)
PEZZOT	543	m	0	10	105	0	116	23.19~	(1.34- 400.59)
PEZZOT	544	m	0	45	145	0	116	72.86~	(4.44-1195.36)
PEZZOT	545	m	0	30	67	0	116	105.28~	(6.34-1749.54)
Subtotal PEZZOT								56.74	(11.15- 288.86)
WYNDE6	608	m	0	44	92	9	589	31.30	(14.79- 66.26)
WYNDE6	609	m	0	81	139	9	589	38.14	(18.69- 77.81)
WYNDE6	610	m	0	262	301	9	589	56.96	(28.89- 112.31)
WYNDE6	632	f	0	32	90	12	673	19.94	(9.91- 40.11)
WYNDE6	633	f	0	49	94	12	673	29.23	(15.00- 56.97)
WYNDE6	634	f	0	72	91	12	673	44.37	(23.18- 84.93)
Subtotal WYNDE6								35.10	(26.47- 46.53)
ZHENG	520	m	0	13	66	4	94	4.63	(1.45- 14.82)
ZHENG	521	m	0	81	109	4	94	17.46	(6.17- 49.46)
ZHENG	522	m	0	62	43	4	94	33.88	(11.58- 99.14)
ZHENG	527	f	0	9	16	33	184	3.14	(1.28- 7.69)
ZHENG	528	f	0	34	28	33	184	6.77	(3.63- 12.62)
Subtotal ZHENG								7.97	(5.36- 11.85)
Totals				2579	4488	385	9094		

*prospective study ~ With 0.5 adjustment for zero

REF	NRR	SEX	AD	Ys	Ws	Qs	Ps
BARBON	569	m	0	2.27	5.20	0.79	0.0000
BARBON	570	m	0	2.60	5.50	0.02	0.0000
BARBON	571	m	0	4.26	4.26	10.89	0.0000
Subtotal BARBON				2.96	14.96	11.70	
BENHAM	514	m	0	2.66	16.33	0.00	0.0000
BENHAM	515	m	0	2.79	20.26	0.33	0.0000
BENHAM	516	m	0	2.82	20.14	0.48	0.0000
BENHAM	517	m	0	3.00	20.16	2.32	0.0000
Subtotal BENHAM				2.82	76.89	3.12	
HAENSZ	501	f	0	0.96	13.63	39.67	0.0004
HAENSZ	502	f	0	1.25	10.45	20.93	0.0001
Subtotal HAENSZ				1.08	24.08	60.60	
JEDRYC	528	m	0	2.55	5.57	0.06	0.0000
JEDRYC	529	m	0	3.04	5.40	0.78	0.0000
JEDRYC	530	m	0	3.14	5.05	1.13	0.0000
Subtotal JEDRYC				2.90	16.02	1.97	
LUO	507	c	0	0.24	0.74	4.35	0.8340
LUO	508	c	0	1.63	3.35	3.57	0.0029
LUO	509	c	0	1.97	2.93	1.42	0.0008
Subtotal LUO				1.62	7.03	9.34	
MATOS	611	m	0	1.10	1.91	4.65	0.1271
MATOS	612	m	0	2.03	2.56	1.01	0.0011
MATOS	613	m	0	1.87	2.40	1.49	0.0037
Subtotal MATOS				1.72	6.88	7.15	
PEZZOT	543	m	0	3.14	0.47	0.11	0.0306

Table 2H1 - 5

IESLC - Meta-analysis of Ever Smoking by Age started, Overview
Squamous, Any Product (or Cigarettes if Any not available)
Least adjusted

REF	NRR	SEX	AD	Ys	Ws	Qs	Ps
PEZZOT	544	m	0	4.29	0.49	1.30	0.0027
PEZZOT	545	m	0	4.66	0.49	1.94	0.0012
Subtotal PEZZOT				4.04	1.45	3.34	
WYNDE6	608	m	0	3.44	6.83	4.17	0.0000
WYNDE6	609	m	0	3.64	7.56	7.25	0.0000
WYNDE6	610	m	0	4.04	8.34	15.89	0.0000
WYNDE6	632	f	0	2.99	7.86	0.86	0.0000
WYNDE6	633	f	0	3.38	8.63	4.39	0.0000
WYNDE6	634	f	0	3.79	9.12	11.66	0.0000
Subtotal WYNDE6				3.56	48.33	44.23	
ZHENG	520	m	0	1.53	2.84	3.62	0.0099
ZHENG	521	m	0	2.86	3.54	0.14	0.0000
ZHENG	522	m	0	3.52	3.33	2.47	0.0000
ZHENG	527	f	0	1.14	4.78	11.02	0.0125
ZHENG	528	f	0	1.91	9.91	5.57	0.0000
Subtotal ZHENG				2.08	24.40	22.81	

N	32
NS	9

Table 2H1 - 6

IESLC - Meta-analysis of Ever Smoking by Age started, Overview
Squamous, Any Product (or Cigarettes if Any not available)
Least adjusted

		Sex		
	combined	male	female	Total
N	3	22	7	32
NS	1	7	3	11

In this overview table, other than the "N" rows, entries in the "absent" and "Total" columns may be invalid and should be ignored

	Age started (broad categories)				
	absent	19+k26	15-25k18	1-17k14	Total
N	10	10	6	6	32
NS	5	9	5	5	24
Wt	56.23	64.69	49.79	49.33	220.04
Het Chi	37.67	45.55	8.28	17.16	164.28
Het df	9	9	5	5	31
Het P	***	***	N.S.	**	***
Fixed RR	8.51	9.97	20.06	29.69	14.32
RRl	6.55	7.81	15.19	22.46	12.55
RRu	11.05	12.72	26.48	39.25	16.34
P	+++	+++	+++	+++	+++
Random RR	8.25	10.37	20.14	30.58	13.82
RRl	4.38	5.72	13.74	17.44	9.96
RRu	15.53	18.81	29.52	53.64	19.18
P	+++	+++	+++	+++	+++

				Age started (narrow categories)				
	absent	27+k30	23-29k26	19-25k22	15-21k18	11-17k14	1-13k10	Total
N	22	2		1	6		1	32
NS	9	1		1	5		1	16
Wt	141.89	7.61		20.26	49.79		0.49	220.04
Het Chi	132.77	0.27		0.00	8.28		0.00	164.28
Het df	21	1		0	5		0	31
Het P	***	N.S.		N.S.	N.S.		N.S.	***
Fixed RR	13.36	3.63		16.26	20.06		105.28	14.32
RRl	11.33	1.78		10.52	15.19		6.34	12.55
RRu	15.75	7.38		25.14	26.48		1749.54	16.34
P	+++	+++		+++	+++		++	+++
Random RR	13.41	3.63		16.26	20.14		105.28	13.82
RRl	8.60	1.78		10.52	13.74		6.34	9.96
RRu	20.92	7.38		25.14	29.52		1749.54	19.18
P	+++	+++		+++	+++		++	+++

Table 2H1 - 6

IESLC - Meta-analysis of Ever Smoking by Age started, Overview
Squamous, Any Product (or Cigarettes if Any not available)
Least adjusted

MALES

		Age started (broad categories)				
		absent	19+k26	15-25k18	1-17k14	Total
	N	5	7	5	5	22
	NS	3	7	5	5	20
	Wt	27.41	39.85	41.16	40.21	148.63
Het	Chi	9.06	9.92	6.80	15.35	49.55
Het	df	4	6	4	4	21
Het	P	(*)	N.S.	N.S.	**	***
Fixed	RR	16.58	14.57	18.53	27.11	18.87
	RRl	11.40	10.68	13.66	19.90	16.06
	RRu	24.11	19.87	25.16	36.92	22.16
	P	+++	+++	+++	+++	+++
Random	RR	18.66	14.02	18.45	27.89	18.67
	RRl	8.10	9.00	11.93	14.15	14.17
	RRu	42.97	21.83	28.54	54.95	24.60
	P	+++	+++	+++	+++	+++

				Age started (narrow categories)					
		absent	27+k30	23-29k26	19-25k22	15-21k18	11-17k14	1-13k10	Total
	N	14	1		1	5		1	22
	NS	7	1		1	5		1	14
	Wt	83.89	2.84		20.26	41.16		0.49	148.63
Het	Chi	34.69	0.00		0.00	6.80		0.00	49.55
Het	df	13	0		0	4		0	21
Het	P	***	N.S.		N.S.	N.S.		N.S.	***
Fixed	RR	20.48	4.63		16.26	18.53		105.28	18.87
	RRl	16.53	1.45		10.52	13.66		6.34	16.06
	RRu	25.37	14.82		25.14	25.16		1749.54	22.16
	P	+++	++		+++	+++		++	+++
Random	RR	20.19	4.63		16.26	18.45		105.28	18.67
	RRl	13.67	1.45		10.52	11.93		6.34	14.17
	RRu	29.83	14.82		25.14	28.54		1749.54	24.60
	P	+++	++		+++	+++		++	+++

FEMALES

		Age started (broad categories)				
		absent	19+k26	15-25k18	1-17k14	Total
	N	3	2	1	1	7
	NS	2	2	1	1	4
	Wt	25.14	21.49	8.63	9.12	64.38
Het	Chi	2.96	20.69	0.00	0.00	76.50
Het	df	2	1	0	0	6
Het	P	N.S.	***	N.S.	N.S.	***
Fixed	RR	4.44	5.48	29.23	44.37	8.49
	RRl	3.00	3.59	15.00	23.18	6.65
	RRu	6.56	8.36	56.97	84.93	10.84
	P	+++	+++	+++	+++	+++
Random	RR	4.38	7.11	29.23	44.37	9.06
	RRl	2.69	0.97	15.00	23.18	3.76
	RRu	7.11	52.31	56.97	84.93	21.85
	P	+++	(+)	+++	+++	+++

Table 2H1 - 6

IESLC - Meta-analysis of Ever Smoking by Age started, Overview
Squamous, Any Product (or Cigarettes if Any not available)
Least adjusted

FEMALES

		absent	27+k30	23-29k26	19-25k22	15-21k18	11-17k14	1-13k10	Total
	N	5	1			1			7
	NS	3	1			1			4
	Wt	50.98	4.78			8.63			64.38
Het	Chi	57.89	0.00			0.00			76.50
Het	df	4	0			0			6
Het	P	***	N.S.			N.S.			***
Fixed	RR	7.56	3.14			29.23			8.49
	RRl	5.75	1.28			15.00			6.65
	RRu	9.95	7.69			56.97			10.84
	P	+++	+			+++			+++
Random	RR	8.76	3.14			29.23			9.06
	RRl	3.06	1.28			15.00			3.76
	RRu	25.02	7.69			56.97			21.85
	P	+++	+			+++			+++

(Column group heading: Age started (narrow categories))

Table 2H1 - 7

IESLC - Meta-analysis of Ever Smoking by Age started, Overview
Squamous, Any Product (or Cigarettes if Any not available)
Excluded studies (and stage at which they were excluded)

1	AKIBA AMANDU AMES BECHER BENSHL BEST BLOT1 BROSS BROWN3 CARPEN CEDERL CHYOU CPSI CPSII DARBY DEAN2 DEAN3 DOLL2 ENGELA GAO2 GARCIA GILLIS GRAHAM GURSEL HAMMO2 HIRAYA HOLE HUMBLE JAHN JAIN KAISE2 KATSOU KAUFMA LAUSSM LIAW MCDUFF MIGRAN MRFITR PEZZO2 PISANI PRESCO QIAO SEGI2 SPEIZE SVENSS TVERDA WAKAI WATSON WIGLE WU WYNDE3 WYNDE8
2	AXELSS BOUCHA BOUCOT CHEN DESTEF DORGAN DOSEME FAN GARSHI GER HAMMON JUSSAW KOO KREUZE LEVIN MCCONN NOTAN2 OSANN2 RESTRE SADOWS VUTUC WANG2 WU2 WUWILL WYNDE2 XU ZHOU
3	GUO SPITZ STASZE ZHANG
4	AGUDO ARMADA AUVINE BOFFET BRESLO BUFFLE CHEN2 CHIAZZ CHOI CORREA DAMBER DOLL DORN GAO GENG HU HU2 JOLY KOULUM LETOUR LIU3 LIU4 LIU5 LUBIN LUBIN2 PERNU QIAO2 RACHTA SOBUE SUZUK2 TIZZAN WYNDE7 YUAN
5	ALDERS
10	HEGMAN KHUDER

Table 2H1 - 8
Potentially overlapping studies

REF	REFGP	PRINC	.	OVERLAP/LINK
BENHAM	LUBIN2	2		Subset of Lubin2
WYNDE6	WYNDE6	1		WYNDE5/6/7/8

Table 2H2 -

IESLC - Meta-analysis of Ever Smoking, Age started, "Low"
Squamous, Any Product (or Cigarettes if Any not available)

This analysis is restricted to results for:
1) Ever smokers
2) Results by Age started
3) Categorical results by Age started
4) Squamous (or near equivalent)
5) Results complete enough for use in metaanalysis

Within each study, results are then selected (in the following order of preference, within each sex) for:
6) (not applicable)
7) PRODUCT: all/unspec, cigarettes regardless of other products, cigarettes only
8) CIGTYPE: all/unspecified, MC regardless of HR, MC only
9) (not applicable)
10) DENOM: never smoked anything, never smoked cigarettes, never any + low, never cigs + low
11) Followup period (YF, prospective studies): whole study (coded as 0) or longest available
12) LCtype: squamous or nearest available, but not adeno. (q = squamous, s = small,
a = adeno, KI = Kreyberg I, u = undifferentiated)
13) Race: all or nearest available, otherwise by race (wh or w = white, bl or b = black, hi = hispanic
ch = chinese, jap = japanese, haw = hawaiian, w+o = white + oriental, sca = scandinavian, as = asian)
14) Age started "low" in key scheme 1 (key value 26, maximum range 19+)
15) For overlapping studies: principal rather than subsidiary studies
Finally by Age: whole study (coded as 0) if available, otherwise by widest available age group
and then for single sex results (m, f) in preference to results for both sexes combined (c).

Results adjusted (AD) for the most potential confounders are then chosen in Sections -1 to -3
and results adjusted for the least confounders in Sections -4 to -6. (Those least adjusted results which
actually differ from the most adjusted are marked 'x' in column X in Section -4)

Section -7 shows excluded studies, together with the stage (as above) at which no qualifying
results were found.

Section -8 lists the potentially overlapping studies which have been included (1=principal, 2=subsidiary).

Section -9 lists any results which would have been included in preference except that they had data not complete
enough for use in meta-analysis, with their significance (yes/no), if known, and any further comment as entered
on the database. It also lists as "gap" any categories for which no data were presented by the original authors.

In addition to those mentioned above, the following fields, levels and abbreviations are used:
* or nk = not known, n = no, y = yes, ot = other
nev = never
all/unspec = all or unspecified, cig+/-ot = cigarettes irrespective of other products (cigar, pipe etc)
MC = manufactured cigarettes, HR = hand-rolled cigarettes
exL, exH = range of exposure (low and high) in the smoking group, in terms of Age started
REF: 6-character study reference
NRR: number of the RR on the database within the study
ST : study type (CC = case control, pr or prosp = prospective)
NLC: number of lung cancer cases in whole study
R : risky occupational population (n = no, m = mining, o = other risky)
VB : national cigarette type (V = at least 75% Virginia, bl = at least 75% blended, ot = other)
P : any proxy use
H : full histological confirmation
De : derivation of RR/CI (or = original, st = standard method, ot = other method of estimation)

Table 2H2 - 1

IESLC - Meta-analysis of Ever Smoking, Age started, "Low"
Squamous, Any Product (or Cigarettes if Any not available)
Most adjusted

REF	NRR	SEX	AGEL	AGEH	RACE	YF	LC TYPE	LOC	START	ST	NLC	R	VB	P	H	AD	PRODUCT	exL	exH	DENOM	De
BARBON	574	m	0	0	all	-	q	Eu:wst	1979	CC	755	n	bl	y	y	1	all/unsp	20	999	nev any	or
BENHAM	514	m	0	0	all	-	KI	Eu:wst	1976	CC	1625	n	bl	n	y	0	cig only	25	999	nev any	st
HAENSZ	501	f	0	0	all	-	q+u	NAmer	1955	CC	158	n	bl	n	y	0	cig+/-ot	25	999	nev any	st
JEDRYC	528	m	0	0	all	-	q	Eu:est	1980	CC	1630	n	bl	y	n	0	cig+/-ot	19	999	nev any	st
LUO	513	c	0	0	all	-	q	As:Chi	1990	CC	102	n	ot	n	y	20	cig+/-ot	20	39	nev cigs	or
MATOS	616	m	0	0	all	-	q	SCAmer	1994	CC	200	n	bl	n	n	2	cig+/-ot	20	999	nev any	or
PEZZOT	543	m	0	0	all	-	q	SCAmer	1987	CC	215	n	bl	n	y	0	cig only	19	999	nev cigs	ot
WYNDE6	608	m	0	0	wh	-	q	NAmer	1969	CC	4423	n	bl	n	y	0	cig+/-ot	21	999	nev cigs	st
WYNDE6	632	f	0	0	wh	-	q	NAmer	1969	CC	4423	n	bl	n	y	0	cig+/-ot	21	999	nev cigs	st
ZHENG	521	m	0	0	all	-	q	As:Chi	1982	CC	540	n	ot	*	y	0	cig+/-ot	20	29	nev cigs	st

Cigarette type is all/unspec for all RRs

Table 2H2 - 2

IESLC - Meta-analysis of Ever Smoking, Age started, "Low"
Squamous, Any Product (or Cigarettes if Any not available)
Most adjusted

REF	NRR	SEX	AD	Number Exposed Case	Number Exposed Cont	Non-exposed Case	Non-exposed Cont	RR	95.00%CI
BARBON	574	m	1	64	-	6	-	9.40	(4.20- 23.40)
BENHAM	514	m	0	98	137	24	481	14.34	(8.83- 23.29)
HAENSZ	501	f	0	32	66	44	236	2.60	(1.53- 4.42)
JEDRYC	528	m	0	134	502	6	289	12.86	(5.60- 29.50)
LUO	513	c	20	19	-	5	-	10.50	(2.30- 48.60)
MATOS	616	m	2	6	-	3	-	3.30	(0.80- 13.90)
PEZZOT	543	m	0	10	105	0	116	23.19~	(1.34- 400.59)
WYNDE6	608	m	0	44	92	9	589	31.30	(14.79- 66.26)
WYNDE6	632	f	0	32	90	12	673	19.94	(9.91- 40.11)
Subtotal WYNDE6								24.59	(14.75- 41.00)
ZHENG	521	m	0	81	109	4	94	17.46	(6.17- 49.46)
Partial Totals				520	1101	113	2478		

*prospective study ~ With 0.5 adjustment for zero

REF	NRR	SEX	AD	Ys	Ws	Qs	Ps
BARBON	574	m	1	2.24	5.21	0.05	0.0000
BENHAM	514	m	0	2.66	16.33	1.73	0.0000
HAENSZ	501	f	0	0.96	13.63	26.02	0.0004
JEDRYC	528	m	0	2.55	5.57	0.26	0.0000
LUO	513	c	20	2.35	1.65	0.00	0.0025
MATOS	616	m	2	1.19	1.89	2.46	0.1012
PEZZOT	543	m	0	3.14	0.47	0.31	0.0306
WYNDE6	608	m	0	3.44	6.83	8.36	0.0000
WYNDE6	632	f	0	2.99	7.86	3.38	0.0000
Subtotal WYNDE6				3.20	14.69	11.74	
ZHENG	521	m	0	2.86	3.54	0.97	0.0000

	N	10
	NS	9
	Wt	62.98
Het	Chi	43.54
Het	df	9
Het	P	***
Fixed	RR	10.35
	RRl	8.09
	RRu	13.25
	P	+++
Random	RR	11.17
	RRl	6.15
	RRu	20.31
	P	+++
Asymm	P	N.S.

Table 2H2 - 3

IESLC - Meta-analysis of Ever Smoking, Age started, "Low"
Squamous, Any Product (or Cigarettes if Any not available)
Most adjusted

	Sex<br>combined	male	female	Total
N	1	7	2	10
NS	1	7	2	10
Wt	1.65	39.84	21.49	62.98
Het Chi	0.00	9.46	20.69	43.54
Het df	0	6	1	9
Het P	N.S.	N.S.	***	***
Fixed RR	10.50	14.59	5.48	10.35
RRl	2.28	10.69	3.59	8.09
RRu	48.27	19.90	8.36	13.25
P	++	+++	+++	+++
Random RR	10.50	14.12	7.11	11.17
RRl	2.28	9.17	0.97	6.15
RRu	48.27	21.72	52.31	20.31
P	++	+++	(+)	+++
Between Chi				13.38
Between df				2
Between P				**
Btwn(F) P				N.S.
Btwn(R) P				N.S.

Table 2H2 - 4

IESLC - Meta-analysis of Ever Smoking, Age started, "Low"
Squamous, Any Product (or Cigarettes if Any not available)
Least adjusted

REF	NRR	X	SEX	AGEL	AGEH	RACE	YF	LC TYPE	LOC	START	ST	NLC	R	VB	P	H	AD	PRODUCT	exL	exH	DENOM	De
BARBON	569	x	m	0	0	all	-	q	Eu:wst	1979	CC	755	n	bl	y	y	0	all/unsp	20	999	nev any	st
BENHAM	514		m	0	0	all	-	KI	Eu:wst	1976	CC	1625	n	bl	n	y	0	cig only	25	999	nev any	st
HAENSZ	501		f	0	0	all	-	q+u	NAmer	1955	CC	158	n	bl	n	y	0	cig+/-ot	25	999	nev any	st
JEDRYC	528		m	0	0	all	-	q	Eu:est	1980	CC	1630	n	bl	y	n	0	cig+/-ot	19	999	nev any	st
LUO	508	x	c	0	0	all	-	q	As:Chi	1990	CC	102	n	ot	n	y	0	cig+/-ot	20	39	nev cigs	st
MATOS	611	x	m	0	0	all	-	q	SCAmer	1994	CC	200	n	bl	n	n	0	cig+/-ot	20	999	nev any	st
PEZZOT	543		m	0	0	all	-	q	SCAmer	1987	CC	215	n	bl	n	y	0	cig only	19	999	nev cigs	ot
WYNDE6	608		m	0	0	wh	-	q	NAmer	1969	CC	4423	n	bl	n	y	0	cig+/-ot	21	999	nev cigs	st
WYNDE6	632		f	0	0	wh	-	q	NAmer	1969	CC	4423	n	bl	n	y	0	cig+/-ot	21	999	nev cigs	st
ZHENG	521		m	0	0	all	-	q	As:Chi	1982	CC	540	n	ot	*	y	0	cig+/-ot	20	29	nev cigs	st

Cigarette type is all/unspec for all RRs

Table 2H2 - 5

IESLC - Meta-analysis of Ever Smoking, Age started, "Low"
Squamous, Any Product (or Cigarettes if Any not available)
Least adjusted

REF	NRR	SEX	AD	Number Exposed Case	Cont	Non-exposed Case	Cont	RR	95.00%CI
BARBON	569	m	0	64	207	6	188	9.69	(4.10- 22.89)
BENHAM	514	m	0	98	137	24	481	14.34	(8.83- 23.29)
HAENSZ	501	f	0	32	66	44	236	2.60	(1.53- 4.42)
JEDRYC	528	m	0	134	502	6	289	12.86	(5.60- 29.50)
LUO	508	c	0	19	38	5	51	5.10	(1.75- 14.88)
MATOS	611	m	0	6	73	3	110	3.01	(0.73- 12.43)
PEZZOT	543	m	0	10	105	0	116	23.19~	(1.34- 400.59)
WYNDE6	608	m	0	44	92	9	589	31.30	(14.79- 66.26)
WYNDE6	632	f	0	32	90	12	673	19.94	(9.91- 40.11)
Subtotal WYNDE6								24.59	(14.75- 41.00)
ZHENG	521	m	0	81	109	4	94	17.46	(6.17- 49.46)
Totals				520	1419	113	2827		

*prospective study ~ With 0.5 adjustment for zero

REF	NRR	SEX	AD	Ys	Ws	Qs	Ps
BARBON	569	m	0	2.27	5.20	0.00	0.0000
BENHAM	514	m	0	2.66	16.33	2.15	0.0000
HAENSZ	501	f	0	0.96	13.63	24.61	0.0004
JEDRYC	528	m	0	2.55	5.57	0.36	0.0000
LUO	508	c	0	1.63	3.35	1.50	0.0029
MATOS	611	m	0	1.10	1.91	2.74	0.1271
PEZZOT	543	m	0	3.14	0.47	0.34	0.0306
WYNDE6	608	m	0	3.44	6.83	8.94	0.0000
WYNDE6	632	f	0	2.99	7.86	3.78	0.0000
Subtotal WYNDE6				3.20	14.69	12.72	
ZHENG	521	m	0	2.86	3.54	1.11	0.0000

	N	10
	NS	9
	Wt	64.69
Het	Chi	45.55
Het	df	9
Het	P	***
Fixed	RR	9.97
	RRl	7.81
	RRu	12.72
	P	+++
Random	RR	10.37
	RRl	5.72
	RRu	18.81
	P	+++
Asymm	P	N.S.

Table 2H2 - 6

IESLC - Meta-analysis of Ever Smoking, Age started, "Low"
Squamous, Any Product (or Cigarettes if Any not available)
Least adjusted

	Sex combined	male	female	Total
N	1	7	2	10
NS	1	7	2	10
Wt	3.35	39.85	21.49	64.69
Het Chi	0.00	9.92	20.69	45.55
Het df	0	6	1	9
Het P	N.S.	N.S.	***	***
Fixed RR	5.10	14.57	5.48	9.97
RRl	1.75	10.68	3.59	7.81
RRu	14.88	19.87	8.36	12.72
P	++	+++	+++	+++
Random RR	5.10	14.02	7.11	10.37
RRl	1.75	9.00	0.97	5.72
RRu	14.88	21.83	52.31	18.81
P	++	+++	(+)	+++
Between Chi				14.94
Between df				2
Between P				***
Btwn(F) P				N.S.
Btwn(R) P				N.S.

Table 2H2 - 7

IESLC - Meta-analysis of Ever Smoking, Age started, "Low"
Squamous, Any Product (or Cigarettes if Any not available)
Excluded studies (and stage at which they were excluded)

1	AKIBA AMANDU AMES BECHER BENSHL BEST BLOT1 BROSS BROWN3 CARPEN CEDERL CHYOU CPSI CPSII DARBY DEAN2 DEAN3 DOLL2 ENGELA GAO2 GARCIA GILLIS GRAHAM GURSEL HAMMO2 HIRAYA HOLE HUMBLE JAHN JAIN KAISE2 KATSOU KAUFMA LAUSSM LIAW MCDUFF MIGRAN MRFITR PEZZO2 PISANI PRESCO QIAO SEGI2 SPEIZE SVENSS TVERDA WAKAI WATSON WIGLE WU WYNDE3 WYNDE8
2	AXELSS BOUCHA BOUCOT CHEN DESTEF DORGAN DOSEME FAN GARSHI GER HAMMON JUSSAW KOO KREUZE LEVIN MCCONN NOTAN2 OSANN2 RESTRE SADOWS VUTUC WANG2 WU2 WUWILL WYNDE2 XU ZHOU
3	GUO SPITZ STASZE ZHANG
4	AGUDO ARMADA AUVINE BOFFET BRESLO BUFFLE CHEN2 CHIAZZ CHOI CORREA DAMBER DOLL DORN GAO GENG HU HU2 JOLY KOULUM LETOUR LIU3 LIU4 LIU5 LUBIN LUBIN2 PERNU QIAO2 RACHTA SOBUE SUZUK2 TIZZAN WYNDE7 YUAN
5	ALDERS
10	HEGMAN KHUDER

Table 2H2 - 8

Potentially overlapping studies

REF	REFGP	PRINC	. OVERLAP/LINK
BENHAM	LUBIN2	2	Subset of Lubin2
WYNDE6	WYNDE6	1	WYNDE5/6/7/8

Table 2H3 -

IESLC - Meta-analysis of Ever Smoking, Age started, "Mid"
Squamous, Any Product (or Cigarettes if Any not available)

This analysis is restricted to results for:
1) Ever smokers
2) Results by Age started
3) Categorical results by Age started
4) Squamous (or near equivalent)
5) Results complete enough for use in metaanalysis

Within each study, results are then selected (in the following order of preference, within each sex) for:
6) (not applicable)
7) PRODUCT: all/unspec, cigarettes regardless of other products, cigarettes only
8) CIGTYPE: all/unspecified, MC regardless of HR, MC only
9) (not applicable)
10) DENOM: never smoked anything, never smoked cigarettes, never any + low, never cigs + low
11) Followup period (YF, prospective studies): whole study (coded as 0) or longest available
12) LCtype: squamous or nearest available, but not adeno. (q = squamous, s = small,
a = adeno, KI = Kreyberg I, u = undifferentiated)
13) Race: all or nearest available, otherwise by race (wh or w = white, bl or b = black, hi = hispanic
ch = chinese, jap = japanese, haw = hawaiian, w+o = white + oriental, sca = scandinavian, as = asian)
14) Age started "mid" in key scheme 1 (key value 18, maximum range 15-25)
15) For overlapping studies: principal rather than subsidiary studies
Finally by Age: whole study (coded as 0) if available, otherwise by widest available age group
and then for single sex results (m, f) in preference to results for both sexes combined (c).

Results adjusted (AD) for the most potential confounders are then chosen in Sections -1 to -3
and results adjusted for the least confounders in Sections -4 to -6. (Those least adjusted results which
actually differ from the most adjusted are marked 'x' in column X in Section -4)

Section -7 shows excluded studies, together with the stage (as above) at which no qualifying
results were found.

Section -8 lists the potentially overlapping studies which have been included (1=principal, 2=subsidiary).

Section -9 lists any results which would have been included in preference except that they had data not complete
enough for use in meta-analysis, with their significance (yes/no), if known, and any further comment as entered
on the database. It also lists as "gap" any categories for which no data were presented by the original authors.

In addition to those mentioned above, the following fields, levels and abbreviations are used:
* or nk = not known, n = no, y = yes, ot = other
nev = never
all/unspec = all or unspecified, cig+/-ot = cigarettes irrespective of other products (cigar, pipe etc)
MC = manufactured cigarettes, HR = hand-rolled cigarettes
exL, exH = range of exposure (low and high) in the smoking group, in terms of Age started
REF: 6-character study reference
NRR: number of the RR on the database within the study
ST : study type (CC = case control, pr or prosp = prospective)
NLC: number of lung cancer cases in whole study
R : risky occupational population (n = no, m = mining, o = other risky)
VB : national cigarette type (V = at least 75% Virginia, bl = at least 75% blended, ot = other)
P : any proxy use
H : full histological confirmation
De : derivation of RR/CI (or = original, st = standard method, ot = other method of estimation)

Table 2H3 - 1

IESLC - Meta-analysis of Ever Smoking, Age started, "Mid"
Squamous, Any Product (or Cigarettes if Any not available)
Most adjusted

REF	NRR	SEX	AGEL	AGEH	RACE	YF	LC TYPE	LOC	START	ST	NLC	R	VB	P	H	AD	PRODUCT	exL	exH	DENOM	De
BARBON	575	m	0	0	all	-	q	Eu:wst	1979	CC	755	n	bl	y	y	1	all/unsp	15	19	nev any	or
BENHAM	516	m	0	0	all	-	KI	Eu:wst	1976	CC	1625	n	bl	n	y	0	cig only	17	19	nev any	st
JEDRYC	529	m	0	0	all	-	q	Eu:est	1980	CC	1630	n	bl	y	n	0	cig+/-ot	17	18	nev any	st
MATOS	617	m	0	0	all	-	q	SCAmer	1994	CC	200	n	bl	n	n	2	cig+/-ot	15	19	nev any	or
WYNDE6	609	m	0	0	wh	-	q	NAmer	1969	CC	4423	n	bl	n	y	0	cig+/-ot	18	20	nev cigs	st
WYNDE6	633	f	0	0	wh	-	q	NAmer	1969	CC	4423	n	bl	n	y	0	cig+/-ot	18	20	nev cigs	st

Cigarette type is all/unspec for all RRs

Table 2H3 - 2

IESLC - Meta-analysis of Ever Smoking, Age started, "Mid"
Squamous, Any Product (or Cigarettes if Any not available)
Most adjusted

REF	NRR	SEX	AD	Number Case	Exposed Cont	Non-exposed Case	Cont	RR	95.00%CI
BARBON	575	m	1	145	-	6	-	13.70	(5.90- 31.70)
BENHAM	516	m	0	311	373	24	481	16.71	(10.80- 25.86)
JEDRYC	529	m	0	96	221	6	289	20.92	(9.00- 48.62)
MATOS	617	m	2	25	-	3	-	7.90	(2.30- 27.10)
WYNDE6	609	m	0	81	139	9	589	38.14	(18.69- 77.81)
WYNDE6	633	f	0	49	94	12	673	29.23	(15.00- 56.97)
Subtotal WYNDE6								33.10	(20.33- 53.87)
Partial Totals				707	827	60	2032		

*prospective study

REF	NRR	SEX	AD	Ys	Ws	Qs	Ps
BARBON	575	m	1	2.62	5.44	0.81	0.0000
BENHAM	516	m	0	2.82	20.14	0.71	0.0000
JEDRYC	529	m	0	3.04	5.40	0.01	0.0000
MATOS	617	m	2	2.07	2.53	2.21	0.0010
WYNDE6	609	m	0	3.64	7.56	3.07	0.0000
WYNDE6	633	f	0	3.38	8.63	1.19	0.0000
Subtotal WYNDE6				3.50	16.19	4.27	

	N	6
	NS	5
	Wt	49.70
Het	Chi	8.01
Het	df	5
Het	P	N.S.
Fixed	RR	20.15
	RRl	15.26
	RRu	26.61
	P	+++
Random	RR	20.28
	RRl	13.92
	RRu	29.53
	P	+++
Asymm	P	N.S.

Table 2H3 - 3

IESLC - Meta-analysis of Ever Smoking, Age started, "Mid"
Squamous, Any Product (or Cigarettes if Any not available)
Most adjusted

	Sex combined	male	female	Total
N		5	1	6
NS		5	1	5
Wt		41.06	8.63	49.70
Het Chi		6.56	0.00	8.01
Het df		4	0	5
Het P		N.S.	N.S.	N.S.
Fixed RR		18.64	29.23	20.15
RRl		13.72	15.00	15.26
RRu		25.30	56.97	26.61
P		+++	+++	+++
Random RR		18.61	29.23	20.28
RRl		12.12	15.00	13.92
RRu		28.56	56.97	29.53
P		+++	+++	+++
Between Chi				1.45
Between df				1
Between P				N.S.
Btwn(F) P				N.S.
Btwn(R) P				N.S.

Too few RRs for analysis by factor

Table 2H3 - 4

IESLC - Meta-analysis of Ever Smoking, Age started, "Mid"
Squamous, Any Product (or Cigarettes if Any not available)
Least adjusted

REF	NRR	X	SEX	AGEL	AGEH	RACE	YF	LC TYPE	LOC	START	ST	NLC	R	VB	P	H	AD	PRODUCT	exL	exH	DENOM	De
BARBON	570	x	m	0	0	all	-	q	Eu:wst	1979	CC	755	n	bl	y	y	0	all/unsp	15	19	nev any	st
BENHAM	516		m	0	0	all	-	KI	Eu:wst	1976	CC	1625	n	bl	n	y	0	cig only	17	19	nev any	st
JEDRYC	529		m	0	0	all	-	q	Eu:est	1980	CC	1630	n	bl	y	n	0	cig+/-ot	17	18	nev any	st
MATOS	612	x	m	0	0	all	-	q	SCAmer	1994	CC	200	n	bl	n	n	0	cig+/-ot	15	19	nev any	st
WYNDE6	609		m	0	0	wh	-	q	NAmer	1969	CC	4423	n	bl	n	y	0	cig+/-ot	18	20	nev cigs	st
WYNDE6	633		f	0	0	wh	-	q	NAmer	1969	CC	4423	n	bl	n	y	0	cig+/-ot	18	20	nev cigs	st

Cigarette type is all/unspec for all RRs

Table 2H3 - 5

IESLC - Meta-analysis of Ever Smoking, Age started, "Mid"
Squamous, Any Product (or Cigarettes if Any not available)
Least adjusted

REF	NRR	SEX	AD	Number Exposed Case	Number Exposed Cont	Non-exposed Case	Non-exposed Cont	RR	95.00%CI
BARBON	570	m	0	145	337	6	188	13.48	(5.84- 31.10)
BENHAM	516	m	0	311	373	24	481	16.71	(10.80- 25.86)
JEDRYC	529	m	0	96	221	6	289	20.92	(9.00- 48.62)
MATOS	612	m	0	25	120	3	110	7.64	(2.24- 26.01)
WYNDE6	609	m	0	81	139	9	589	38.14	(18.69- 77.81)
WYNDE6	633	f	0	49	94	12	673	29.23	(15.00- 56.97)
Subtotal WYNDE6								33.10	(20.33- 53.87)
Totals				707	1284	60	2330		

*prospective study

REF	NRR	SEX	AD	Ys	Ws	Qs	Ps
BARBON	570	m	0	2.60	5.50	0.87	0.0000
BENHAM	516	m	0	2.82	20.14	0.67	0.0000
JEDRYC	529	m	0	3.04	5.40	0.01	0.0000
MATOS	612	m	0	2.03	2.56	2.39	0.0011
WYNDE6	609	m	0	3.64	7.56	3.12	0.0000
WYNDE6	633	f	0	3.38	8.63	1.22	0.0000
Subtotal WYNDE6				3.50	16.19	4.34	

	N	6
	NS	5
	Wt	49.79
Het	Chi	8.28
Het	df	5
Het	P	N.S.
Fixed	RR	20.06
	RRl	15.19
	RRu	26.48
	P	+++
Random	RR	20.14
	RRl	13.74
	RRu	29.52
	P	+++
Asymm	P	N.S.

Table 2H3 - 6

IESLC - Meta-analysis of Ever Smoking, Age started, "Mid"
Squamous, Any Product (or Cigarettes if Any not available)
Least adjusted

	Sex combined	male	female	Total
N		5	1	6
NS		5	1	5
Wt		41.16	8.63	49.79
Het Chi		6.80	0.00	8.28
Het df		4	0	5
Het P		N.S.	N.S.	N.S.
Fixed RR		18.53	29.23	20.06
RRl		13.66	15.00	15.19
RRu		25.16	56.97	26.48
P		+++	+++	+++
Random RR		18.45	29.23	20.14
RRl		11.93	15.00	13.74
RRu		28.54	56.97	29.52
P		+++	+++	+++
Between Chi				1.48
Between df				1
Between P				N.S.
Btwn(F) P				N.S.
Btwn(R) P				N.S.

Table 2H3 - 7

IESLC - Meta-analysis of Ever Smoking, Age started, "Mid"
Squamous, Any Product (or Cigarettes if Any not available)
Excluded studies (and stage at which they were excluded)

1	AKIBA AMANDU AMES BECHER BENSHL BEST BLOT1 BROSS BROWN3 CARPEN CEDERL CHYOU CPSI CPSII DARBY DEAN2 DEAN3 DOLL2 ENGELA GAO2 GARCIA GILLIS GRAHAM GURSEL HAMMO2 HIRAYA HOLE HUMBLE JAHN JAIN KAISE2 KATSOU KAUFMA LAUSSM LIAW MCDUFF MIGRAN MRFITR PEZZO2 PISANI PRESCO QIAO SEGI2 SPEIZE SVENSS TVERDA WAKAI WATSON WIGLE WU WYNDE3 WYNDE8
2	AXELSS BOUCHA BOUCOT CHEN DESTEF DORGAN DOSEME FAN GARSHI GER HAMMON JUSSAW KOO KREUZE LEVIN MCCONN NOTAN2 OSANN2 RESTRE SADOWS VUTUC WANG2 WU2 WUWILL WYNDE2 XU ZHOU
3	GUO SPITZ STASZE ZHANG
4	AGUDO ARMADA AUVINE BOFFET BRESLO BUFFLE CHEN2 CHIAZZ CHOI CORREA DAMBER DOLL DORN GAO GENG HU HU2 JOLY KOULUM LETOUR LIU3 LIU4 LIU5 LUBIN LUBIN2 PERNU QIAO2 RACHTA SOBUE SUZUK2 TIZZAN WYNDE7 YUAN
5	ALDERS
10	HEGMAN KHUDER
14	HAENSZ LUO PEZZOT ZHENG

Table 2H3 - 8
Potentially overlapping studies

REF	REFGP	PRINC	. OVERLAP/LINK
BENHAM	LUBIN2	2	Subset of Lubin2
WYNDE6	WYNDE6	1	WYNDE5/6/7/8

Table 2H4 -

IESLC - Meta-analysis of Ever Smoking, Age started, "High"
Squamous, Any Product (or Cigarettes if Any not available)

This analysis is restricted to results for:
1) Ever smokers
2) Results by Age started
3) Categorical results by Age started
4) Squamous (or near equivalent)
5) Results complete enough for use in metaanalysis

Within each study, results are then selected (in the following order of preference, within each sex) for:
6) PRODUCT: all/unspec, cigarettes regardless of other products, cigarettes only
7) CIGTYPE: all/unspecified, MC regardless of HR, MC only
8) (not applicable)
9) DENOM: never smoked anything, never smoked cigarettes, never any + low, never cigs + low
10) Followup period (YF, prospective studies): whole study (coded as 0) or longest available
11) LCtype: squamous or nearest available, but not adeno. (q = squamous, s = small,
a = adeno, KI = Kreyberg I, u = undifferentiated)
12) Race: all or nearest available, otherwise by race (wh or w = white, bl or b = black, hi = hispanic
ch = chinese, jap = japanese, haw = hawaiian, w+o = white + oriental, sca = scandinavian, as = asian)
13) Age started "high" in key scheme 1 (key value 14, maximum range 1-17)
14) For overlapping studies: principal rather than subsidiary studies
Finally by Age: whole study (coded as 0) if available, otherwise by widest available age group
and then for single sex results (m, f) in preference to results for both sexes combined (c).

Results adjusted (AD) for the most potential confounders are then chosen in Sections -1 to -3
and results adjusted for the least confounders in Sections -4 to -6. (Those least adjusted results which
actually differ from the most adjusted are marked 'x' in column X in Section -4)

Section -7 shows excluded studies, together with the stage (as above) at which no qualifying
results were found.

Section -8 lists the potentially overlapping studies which have been included (1=principal, 2=subsidiary).

Section -9 lists any results which would have been included in preference except that they had data not complete
enough for use in meta-analysis, with their significance (yes/no), if known, and any further comment as entered
on the database. It also lists as "gap" any categories for which no data were presented by the original authors.

In addition to those mentioned above, the following fields, levels and abbreviations are used:
* or nk = not known, n = no, y = yes, ot = other
nev = never
all/unspec = all or unspecified, cig+/-ot = cigarettes irrespective of other products (cigar, pipe etc)
MC = manufactured cigarettes, HR = hand-rolled cigarettes
exL, exH = range of exposure (low and high) in the smoking group, in terms of Age started
REF: 6-character study reference
NRR: number of the RR on the database within the study
ST : study type (CC = case control, pr or prosp = prospective)
NLC: number of lung cancer cases in whole study
R : risky occupational population (n = no, m = mining, o = other risky)
VB : national cigarette type (V = at least 75% Virginia, bl = at least 75% blended, ot = other)
P : any proxy use
H : full histological confirmation
De : derivation of RR/CI (or = original, st = standard method, ot = other method of estimation)

Table 2H4 - 1

IESLC - Meta-analysis of Ever Smoking, Age started, "High"
Squamous, Any Product (or Cigarettes if Any not available)
Most adjusted

REF	NRR	SEX	AGEL	AGEH	RACE	YF	LC TYPE	LOC	START	ST	NLC	R	VB	P	H	AD	PRODUCT	exL	exH	DENOM	De
BARBON	576	m	0	0	all	-	q	Eu:wst	1979	CC	755	n	bl	y	y	1	all/unsp	1	14	nev any	or
BENHAM	517	m	0	0	all	-	KI	Eu:wst	1976	CC	1625	n	bl	n	y	0	cig only	1	16	nev any	st
JEDRYC	530	m	0	0	all	-	q	Eu:est	1980	CC	1630	n	bl	y	n	0	cig+/-ot	1	16	nev any	st
MATOS	618	m	0	0	all	-	q	SCAmer	1994	CC	200	n	bl	n	n	2	cig+/-ot	1	14	nev any	or
WYNDE6	610	m	0	0	wh	-	q	NAmer	1969	CC	4423	n	bl	n	y	0	cig+/-ot	1	17	nev cigs	st
WYNDE6	634	f	0	0	wh	-	q	NAmer	1969	CC	4423	n	bl	n	y	0	cig+/-ot	1	17	nev cigs	st

Cigarette type is all/unspec for all RRs

Table 2H4 - 2

IESLC - Meta-analysis of Ever Smoking, Age started, "High"
Squamous, Any Product (or Cigarettes if Any not available)
Most adjusted

REF	NRR	SEX	AD	Number Exposed Case	Number Exposed Cont	Non-exposed Case	Non-exposed Cont	RR	95.00%CI
BARBON	576	m	1	52	-	6	-	71.30	(27.60- 184.00)
BENHAM	517	m	0	342	341	24	481	20.10	(12.99- 31.10)
JEDRYC	530	m	0	53	111	6	289	23.00	(9.61- 55.01)
MATOS	618	m	2	16	-	3	-	7.20	(2.00- 25.90)
WYNDE6	610	m	0	262	301	9	589	56.96	(28.89- 112.31)
WYNDE6	634	f	0	72	91	12	673	44.37	(23.18- 84.93)
Subtotal WYNDE6								50.00	(31.27- 79.93)
Partial Totals				797	844	60	2032		

*prospective study

REF	NRR	SEX	AD	Ys	Ws	Qs	Ps
BARBON	576	m	1	4.27	4.27	3.22	0.0000
BENHAM	517	m	0	3.00	20.16	3.18	0.0000
JEDRYC	530	m	0	3.14	5.05	0.35	0.0000
MATOS	618	m	2	1.97	2.34	4.75	0.0025
WYNDE6	610	m	0	4.04	8.34	3.46	0.0000
WYNDE6	634	f	0	3.79	9.12	1.42	0.0000
Subtotal WYNDE6				3.91	17.45	4.88	

	N	6
	NS	5
	Wt	49.28
Het	Chi	16.38
Het	df	5
Het	P	**
Fixed	RR	29.91
	RRl	22.62
	RRu	39.54
	P	+++
Random	RR	31.07
	RRl	17.93
	RRu	53.85
	P	+++
Asymm	P	N.S.

Table 2H4 - 3

IESLC - Meta-analysis of Ever Smoking, Age started, "High"
Squamous, Any Product (or Cigarettes if Any not available)
Most adjusted

	Sex			
	combined	male	female	Total
N		5	1	6
NS		5	1	5
Wt		40.16	9.12	49.28
Het Chi		14.64	0.00	16.38
Het df		4	0	5
Het P		**	N.S.	**
Fixed RR		27.35	44.37	29.91
RRl		20.07	23.18	22.62
RRu		37.26	84.93	39.54
P		+++	+++	+++
Random RR		28.44	44.37	31.07
RRl		14.64	23.18	17.93
RRu		55.25	84.93	53.85
P		+++	+++	+++
Between Chi				1.74
Between df				1
Between P				N.S.
Btwn(F) P				N.S.
Btwn(R) P				N.S.

Too few RRs for analysis by factor

Table 2H4 - 4

IESLC - Meta-analysis of Ever Smoking, Age started, "High"
Squamous, Any Product (or Cigarettes if Any not available)
Least adjusted

REF	NRR	X	SEX	AGEL	AGEH	RACE	YF	LC TYPE	LOC	START	ST	NLC	R	VB	P	H	AD	PRODUCT	exL	exH	DENOM	De
BARBON	571	x	m	0	0	all	-	q	Eu:wst	1979	CC	755	n	bl	y	y	0	all/unsp	1	14	nev any	st
BENHAM	517		m	0	0	all	-	KI	Eu:wst	1976	CC	1625	n	bl	n	y	0	cig only	1	16	nev any	st
JEDRYC	530		m	0	0	all	-	q	Eu:est	1980	CC	1630	n	bl	y	n	0	cig+/-ot	1	16	nev any	st
MATOS	613	x	m	0	0	all	-	q	SCAmer	1994	CC	200	n	bl	n	n	0	cig+/-ot	1	14	nev any	st
WYNDE6	610		m	0	0	wh	-	q	NAmer	1969	CC	4423	n	bl	n	y	0	cig+/-ot	1	17	nev cigs	st
WYNDE6	634		f	0	0	wh	-	q	NAmer	1969	CC	4423	n	bl	n	y	0	cig+/-ot	1	17	nev cigs	st

Cigarette type is all/unspec for all RRs

Table 2H4 - 5

IESLC - Meta-analysis of Ever Smoking, Age started, "High"
Squamous, Any Product (or Cigarettes if Any not available)
Least adjusted

REF	NRR	SEX	AD	Number Exposed Case	Cont	Non-exposed Case	Cont	RR	95.00%CI
BARBON	571	m	0	52	23	6	188	70.84	(27.41- 183.08)
BENHAM	517	m	0	342	341	24	481	20.10	(12.99- 31.10)
JEDRYC	530	m	0	53	111	6	289	23.00	(9.61- 55.01)
MATOS	613	m	0	16	90	3	110	6.52	(1.84- 23.08)
WYNDE6	610	m	0	262	301	9	589	56.96	(28.89- 112.31)
WYNDE6	634	f	0	72	91	12	673	44.37	(23.18- 84.93)
Subtotal WYNDE6								50.00	(31.27- 79.93)
Totals				797	957	60	2330		

*prospective study

REF	NRR	SEX	AD	Ys	Ws	Qs	Ps
BARBON	571	m	0	4.26	4.26	3.22	0.0000
BENHAM	517	m	0	3.00	20.16	3.07	0.0000
JEDRYC	530	m	0	3.14	5.05	0.33	0.0000
MATOS	613	m	0	1.87	2.40	5.53	0.0037
WYNDE6	610	m	0	4.04	8.34	3.54	0.0000
WYNDE6	634	f	0	3.79	9.12	1.47	0.0000
Subtotal WYNDE6				3.91	17.45	5.01	

	N	6
	NS	5
	Wt	49.33
Het	Chi	17.16
Het	df	5
Het	P	**
Fixed	RR	29.69
	RRl	22.46
	RRu	39.25
	P	+++
Random	RR	30.58
	RRl	17.44
	RRu	53.64
	P	+++
Asymm	P	N.S.

Table 2H4 - 6

IESLC - Meta-analysis of Ever Smoking, Age started, "High"
Squamous, Any Product (or Cigarettes if Any not available)
Least adjusted

	Sex		
combined	male	female	Total
N	5	1	6
NS	5	1	5
Wt	40.21	9.12	49.33
Het Chi	15.35	0.00	17.16
Het df	4	0	5
Het P	**	N.S.	**
Fixed RR	27.11	44.37	29.69
RRl	19.90	23.18	22.46
RRu	36.92	84.93	39.25
P	+++	+++	+++
Random RR	27.89	44.37	30.58
RRl	14.15	23.18	17.44
RRu	54.95	84.93	53.64
P	+++	+++	+++
Between Chi			1.81
Between df			1
Between P			N.S.
Btwn(F) P			N.S.
Btwn(R) P			N.S.

Table 2H4 - 7

IESLC - Meta-analysis of Ever Smoking, Age started, "High"
Squamous, Any Product (or Cigarettes if Any not available)
Excluded studies (and stage at which they were excluded)

1	AKIBA AMANDU AMES BECHER BENSHL BEST BLOT1 BROSS BROWN3 CARPEN CEDERL CHYOU CPSI CPSII DARBY DEAN2 DEAN3 DOLL2 ENGELA GAO2 GARCIA GILLIS GRAHAM GURSEL HAMMO2 HIRAYA HOLE HUMBLE JAHN JAIN KAISE2 KATSOU KAUFMA LAUSSM LIAW MCDUFF MIGRAN MRFITR PEZZO2 PISANI PRESCO QIAO SEGI2 SPEIZE SVENSS TVERDA WAKAI WATSON WIGLE WU WYNDE3 WYNDE8
2	AXELSS BOUCHA BOUCOT CHEN DESTEF DORGAN DOSEME FAN GARSHI GER HAMMON JUSSAW KOO KREUZE LEVIN MCCONN NOTAN2 OSANN2 RESTRE SADOWS VUTUC WANG2 WU2 WUWILL WYNDE2 XU ZHOU
3	GUO SPITZ STASZE ZHANG
4	AGUDO ARMADA AUVINE BOFFET BRESLO BUFFLE CHEN2 CHIAZZ CHOI CORREA DAMBER DOLL DORN GAO GENG HU HU2 JOLY KOULUM LETOUR LIU3 LIU4 LIU5 LUBIN LUBIN2 PERNU QIAO2 RACHTA SOBUE SUZUK2 TIZZAN WYNDE7 YUAN
5	ALDERS
10	HEGMAN KHUDER
14	HAENSZ LUO PEZZOT ZHENG

Table 2H4 - 8

Potentially overlapping studies

REF	REFGP	PRINC	OVERLAP/LINK
BENHAM	LUBIN2	2	Subset of Lubin2
WYNDE6	WYNDE6	1	WYNDE5/6/7/8

Table 2H5 -

IESLC - Meta-analysis of Ever Smoking, Age started, "Highest vs lowest"
Squamous, Any Product (or Cigarettes if Any not available)

This analysis is restricted to results for:
1) Ever smokers
2) Results by Age started
3) Categorical results by Age started
4) Denominator (unexposed) = "low"
5) Squamous (or near equivalent)
6) Results complete enough for use in metaanalysis

Within each study, results are then selected (in the following order of preference, within each sex) for:
7) (not applicable)
8) PRODUCT: all/unspec, cigarettes regardless of other products, cigarettes only
9) CIGTYPE: all/unspecified, MC regardless of HR, MC only
10) Results with least adjustment for other aspects of smoking (ADOS)
11) The highest vs lowest category
12) Followup period (YF, prospective studies): whole study (coded as 0) or longest available
13) LCtype: squamous or nearest available, but not adeno. (q = squamous, s = small,
a = adeno, KI = Kreyberg I, u = undifferentiated)
14) Race: all or nearest available, otherwise by race (wh or w = white, bl or b = black, hi = hispanic
ch = chinese, jap = japanese, haw = hawaiian, w+o = white + oriental, sca = scandinavian, as = asian)
15) For overlapping studies: principal rather than subsidiary studies
Finally by Age: whole study (coded as 0) if available, otherwise by widest available age group
and then for single sex results (m, f) in preference to results for both sexes combined (c).

Results adjusted (AD) for the most potential confounders are then chosen in Sections -1 to -3
and results adjusted for the least confounders in Sections -4 to -6. (Those least adjusted results which
actually differ from the most adjusted are marked 'x' in column X in Section -4)

Section -7 shows excluded studies, together with the stage (as above) at which no qualifying
results were found.

Section -8 lists the potentially overlapping studies which have been included (1=principal, 2=subsidiary).

Section -9 lists any results which would have been included in preference except that they had data not complete
enough for use in meta-analysis, with their significance (yes/no), if known, and any further comment as entered
on the database. It also lists as "gap" any categories for which no data were presented by the original authors.

In addition to those mentioned above, the following fields, levels and abbreviations are used:
* or nk = not known, n = no, y = yes, ot = other
all/unspec = all or unspecified, cig+/-ot = cigarettes irrespective of other products (cigar, pipe etc)
MC = manufactured cigarettes, HR = hand-rolled cigarettes
exL, exH = range of exposure (low and high) in the "highest" group, in terms of Age started
unexL, unexH = range of exposure (low and high) in the "lowest" group, in terms of Age started
REF: 6-character study reference
NRR: number of the RR on the database within the study
ST : study type (CC = case control, pr or prosp = prospective)
NLC: number of lung cancer cases in whole study
R : risky occupational population (n = no, m = mining, o = other risky)
VB : national cigarette type (V = at least 75% Virginia, bl = at least 75% blended, ot = other)
P : any proxy use
H : full histological confirmation
De : derivation of RR/CI (or = original, st = standard method, ot = other method of estimation)

Table 2H5 - 1

IESLC - Meta-analysis of Ever Smoking, Age started, "Highest vs lowest"
Squamous, Any Product (or Cigarettes if Any not available)
Most adjusted

REF	NRR	SEX	AGEL	AGEH	RACE	YF	LC TYPE	LOC	START	ST	NLC	R	VB	P	H	AD	ADOS	PRODUCT	exL	exH	unexL	unexH	De
BARBON	578	m	0	0	all	-	q	Eu:wst	1979	CC	755	n	bl	y	y	1	0	all/unsp	1	14	20	999	ot
BENHAM	520	m	0	0	all	-	KI	Eu:wst	1976	CC	1625	n	bl	n	y	0	0	cig only	1	16	25	999	st
HAENSZ	533	f	0	0	all	-	q+u	NAmer	1955	CC	158	n	bl	n	y	2	0	cig+/-ot	1	24	25	999	ot
HEGMAN	503	m	0	0	all	-	q	NAmer	1989	CC	282	n	bl	y	y	0	0	all/unsp	1	19	20	999	st
HEGMAN	508	f	0	0	all	-	q	NAmer	1989	CC	282	n	bl	y	y	0	0	all/unsp	1	25	26	999	ot
JEDRYC	532	m	0	0	all	-	q	Eu:est	1980	CC	1630	n	bl	y	n	0	0	cig+/-ot	1	16	19	999	st
KHUDER	524	m	0	0	all	-	q	NAmer	1985	CC	482	n	bl	n	y	5	3#	cig+/-ot	1	15	20	999	or
LUO	516	c	0	0	all	-	q	As:Chi	1990	CC	102	n	ot	n	y	20	0	cig+/-ot	0	19	40	999	ot
MATOS	620	m	0	0	all	-	q	SCAmer	1994	CC	200	n	bl	n	n	2	0	cig+/-ot	1	14	20	999	ot
PEZZOT	549	m	0	0	all	-	q	SCAmer	1987	CC	215	n	bl	n	y	2	0	cig only	1	13	19	999	ot
WYNDE6	612	m	0	0	wh	-	q	NAmer	1969	CC	4423	n	bl	n	y	0	0	cig+/-ot	1	17	21	999	st
WYNDE6	636	f	0	0	wh	-	q	NAmer	1969	CC	4423	n	bl	n	y	0	0	cig+/-ot	1	17	21	999	st
ZHENG	526	m	0	0	all	-	q	As:Chi	1982	CC	540	n	ot	*	y	1	0	cig+/-ot	1	19	30	999	ot
ZHENG	530	f	0	0	all	-	q	As:Chi	1982	CC	540	n	ot	*	y	1	0	cig+/-ot	1	29	30	999	ot

Comments on values in listings

KHUDER ADOS Duration of smoking (years), number of cigarettes per day and Quitted smoking

Cigarette type is all/unspec for all RRs

Table 2H5 - 2

IESLC - Meta-analysis of Ever Smoking, Age started, "Highest vs lowest"
Squamous, Any Product (or Cigarettes if Any not available)
Most adjusted

REF	NRR	SEX	AD	Number Exposed Case	Cont	Non-exposed Case	Cont	RR	95.00%CI
BARBON	578	m	1	52	-	64	-	7.59	(4.36- 13.19)
BENHAM	520	m	0	342	341	98	137	1.40	(1.04- 1.89)
HAENSZ	533	f	2	24	-	32	-	1.38	(0.71- 2.67)
HEGMAN	503	m	0	57	716	15	289	1.53	(0.85- 2.75)
HEGMAN	508	f	0	17	169	0	28	5.88~	(0.34- 100.62)
Subtotal HEGMAN								1.62	(0.91- 2.87)
JEDRYC	532	m	0	53	111	134	502	1.79	(1.22- 2.61)
KHUDER	524	m	5	-	-	-	-	1.00	(0.50- 1.80)
LUO	516	c	20	14	-	1	-	4.00	(0.47- 33.90)
MATOS	620	m	2	16	-	6	-	2.18	(0.81- 5.85)
PEZZOT	549	m	2	30	-	10	-	3.33	(1.43- 7.79)
WYNDE6	612	m	0	262	301	44	92	1.82	(1.23- 2.70)
WYNDE6	636	f	0	72	91	32	90	2.23	(1.34- 3.70)
Subtotal WYNDE6								1.96	(1.44- 2.68)
ZHENG	526	m	1	62	-	13	-	7.08	(3.48- 14.41)
ZHENG	530	f	1	34	-	9	-	1.63	(0.63- 4.25)
Subtotal ZHENG								4.19	(2.37- 7.42)
Partial Totals				1035	1729	458	1138		

*prospective study ~ With 0.5 adjustment for zero

REF	NRR	SEX	AD	Ys	Ws	Qs	Ps
BARBON	578	m	1	2.03	12.54	22.69	0.0000
BENHAM	520	m	0	0.34	42.81	5.06	0.0270
HAENSZ	533	f	2	0.32	8.76	1.13	0.3405
HEGMAN	503	m	0	0.43	11.23	0.72	0.1518
HEGMAN	508	f	0	1.77	0.48	0.57	0.2211
Subtotal HEGMAN				0.48	11.70	1.29	
JEDRYC	532	m	0	0.58	26.79	0.27	0.0026
KHUDER	524	m	5	0.00	9.36	4.35	1.0000
LUO	516	c	20	1.39	0.84	0.42	0.2040
MATOS	620	m	2	0.78	3.93	0.04	0.1223
PEZZOT	549	m	2	1.20	5.35	1.45	0.0054
WYNDE6	612	m	0	0.60	24.55	0.17	0.0030
WYNDE6	636	f	0	0.80	14.87	0.21	0.0020
Subtotal WYNDE6				0.67	39.42	0.38	
ZHENG	526	m	1	1.96	7.61	12.39	0.0000
ZHENG	530	f	1	0.49	4.22	0.16	0.3157
Subtotal ZHENG				1.43	11.83	12.54	

	N	14
	NS	11
	Wt	173.33
Het	Chi	49.62
Het	df	13
Het	P	***
Fixed	RR	1.98
	RRl	1.70
	RRu	2.29
	P	+++
Random	RR	2.23
	RRl	1.61
	RRu	3.08
	P	+++
Asymm	P	N.S.

Table 2H5 - 3

IESLC - Meta-analysis of Ever Smoking, Age started, "Highest vs lowest"
Squamous, Any Product (or Cigarettes if Any not available)
Most adjusted

		Sex			
	combined	male	female	Total
N	1	9	4	14
NS	1	9	4	14
Wt	0.84	144.16	28.32	173.33
Het Chi	0.00	47.13	1.96	49.62
Het df	0	8	3	13
Het P	N.S.	***	N.S.	***
Fixed RR	4.00	1.99	1.86	1.98
RRl	0.47	1.69	1.29	1.70
RRu	33.97	2.35	2.69	2.29
P	N.S.	+++	+++	+++
Random RR	4.00	2.34	1.86	2.23
RRl	0.47	1.53	1.29	1.61
RRu	33.97	3.58	2.69	3.08
P	N.S.	+++	+++	+++
Between Chi				0.53
Between df				2
Between P				N.S.
Btwn(F) P				N.S.
Btwn(R) P				N.S.

	Lung cancer type					
	q	q+s	q+u	KI	not a	Total
N	12		1	1		14
NS	9		1	1		11
Wt	121.76		8.76	42.81		173.33
Het Chi	40.81		0.00	0.00		49.62
Het df	11		0	0		13
Het P	***		N.S.	N.S.		***
Fixed RR	2.29		1.38	1.40		1.98
RRl	1.92		0.71	1.04		1.70
RRu	2.73		2.68	1.89		2.29
P	+++		N.S.	+		+++
Random RR	2.48		1.38	1.40		2.23
RRl	1.69		0.71	1.04		1.61
RRu	3.63		2.68	1.89		3.08
P	+++		N.S.	+		+++
Between Chi						8.81
Between df						2
Between P						*
Btwn(F) P						N.S.
Btwn(R) P						(*)

	Location								
	NAmer	UK	Scand	othEur	China	Japan	othAs	other	Total
N	6			3	3			2	14
NS	4			3	2			2	11
Wt	69.25			82.13	12.67			9.28	173.33
Het Chi	5.02			28.01	5.85			0.41	49.62
Het df	5			2	2			1	13
Het P	N.S.			***	(*)			N.S.	***
Fixed RR	1.66			1.96	4.18			2.78	1.98
RRl	1.31			1.58	2.41			1.46	1.70
RRu	2.10			2.44	7.25			5.30	2.29
P	+++			+++	+++			++	+++
Random RR	1.66			2.59	3.65			2.78	2.23
RRl	1.31			1.10	1.22			1.46	1.61
RRu	2.10			6.11	10.92			5.30	3.08
P	+++			+	+			++	+++
Between Chi									10.32
Between df									3
Between P									*
Btwn(F) P									N.S.
Btwn(R) P									N.S.

Table 2H5 - 3

IESLC - Meta-analysis of Ever Smoking, Age started, "Highest vs lowest"
Squamous, Any Product (or Cigarettes if Any not available)
Most adjusted

Detailed Country in "other Europe"

	multi	Germany	othWest	East	Balkans	Total
N			2	1		3
NS			2	1		3
Wt			55.35	26.79		82.13
Het Chi			27.66	0.00		28.01
Het df			1	0		2
Het P			***	N.S.		***
Fixed RR			2.06	1.79		1.96
RRl			1.58	1.22		1.58
RRu			2.68	2.61		2.44
P			+++	++		+++
Random RR			3.21	1.79		2.59
RRl			0.61	1.22		1.10
RRu			16.78	2.61		6.11
P			N.S.	++		+
Between Chi						0.35
Between df						1
Between P						N.S.
Btwn(F) P						N.S.
Btwn(R) P						N.S.

Detailed Country in "other Asia"

	India	HongKong	other	Total
N				
NS				
Wt				
Het Chi				
Het df				
Het P				N.S.
Fixed RR				
RRl				
RRu				
P				++
Random RR				
RRl				
RRu				
P				++
Between Chi				
Between df				
Between P				N.S.
Btwn(F) P				N.S.
Btwn(R) P				N.S.

Detailed other continent

	SCAmer	Total
N	2	2
NS	2	2
Wt	9.28	9.28
Het Chi	0.41	0.41
Het df	1	1
Het P	N.S.	N.S.
Fixed RR	2.78	2.78
RRl	1.46	1.46
RRu	5.30	5.30
P	++	++
Random RR	2.78	2.78
RRl	1.46	1.46
RRu	5.30	5.30
P	++	++
Between Chi		
Between df		
Between P		N.S.
Btwn(F) P		N.S.
Btwn(R) P		N.S.

Table 2H5 - 3

IESLC - Meta-analysis of Ever Smoking, Age started, "Highest vs lowest"
Squamous, Any Product (or Cigarettes if Any not available)
Most adjusted

	<1960	Start year of study 1960-69	1970-79	1980-89	1990+	Total
N	1	2	2	7	2	14
NS	1	1	2	5	2	11
Wt	8.76	39.42	55.35	65.03	4.77	173.33
Het Chi	0.00	0.37	27.66	19.90	0.25	49.62
Het df	0	1	1	6	1	13
Het P	N.S.	N.S.	***	**	N.S.	***
Fixed RR	1.38	1.96	2.06	1.99	2.43	1.98
RRl	0.71	1.44	1.58	1.56	0.99	1.70
RRu	2.68	2.68	2.68	2.53	5.95	2.29
P	N.S.	+++	+++	+++	(+)	+++
Random RR	1.38	1.96	3.21	2.19	2.43	2.23
RRl	0.71	1.44	0.61	1.32	0.99	1.61
RRu	2.68	2.68	16.78	3.61	5.95	3.08
P	N.S.	+++	N.S.	++	(+)	+++
Between Chi						1.42
Between df						4
Between P						N.S.
Btwn(F) P						N.S.
Btwn(R) P						N.S.

	Study type (1) CC	other	Total
N	14		14
NS	11		11
Wt	173.33		173.33
Het Chi	49.62		49.62
Het df	13		13
Het P	***		***
Fixed RR	1.98		1.98
RRl	1.70		1.70
RRu	2.29		2.29
P	+++		+++
Random RR	2.23		2.23
RRl	1.61		1.61
RRu	3.08		3.08
P	+++		+++
Between Chi			
Between df			
Between P			N.S.
Btwn(F) P			N.S.
Btwn(R) P			N.S.

	Study type (2) CC	prosp	other	Total
N	14			14
NS	11			11
Wt	173.33			173.33
Het Chi	49.62			49.62
Het df	13			13
Het P	***			***
Fixed RR	1.98			1.98
RRl	1.70			1.70
RRu	2.29			2.29
P	+++			+++
Random RR	2.23			2.23
RRl	1.61			1.61
RRu	3.08			3.08
P	+++			+++
Between Chi				
Between df				
Between P				N.S.
Btwn(F) P				N.S.
Btwn(R) P				N.S.

Table 2H5 - 3

IESLC - Meta-analysis of Ever Smoking, Age started, "Highest vs lowest"
Squamous, Any Product (or Cigarettes if Any not available)
Most adjusted

	Study size (number of LC cases) 100-249	250-499	500-999	1000+	Total
N	4	3	3	4	14
NS	4	2	2	3	11
Wt	18.88	21.07	24.37	109.02	173.33
Het Chi	3.02	2.04	7.99	2.84	49.62
Het df	3	2	2	3	13
Het P	N.S.	N.S.	*	N.S.	***
Fixed RR	2.04	1.31	5.69	1.68	1.98
RRl	1.30	0.85	3.83	1.39	1.70
RRu	3.21	2.00	8.47	2.03	2.29
P	++	N.S.	+++	+++	+++
Random RR	2.05	1.31	4.79	1.68	2.23
RRl	1.30	0.85	2.07	1.39	1.61
RRu	3.22	2.02	11.06	2.03	3.08
P	++	N.S.	+++	+++	+++
Between Chi					33.73
Between df					3
Between P					***
Btwn(F) P					**
Btwn(R) P					*

	Risky occupational population no	mining	othRisky	Total
N	14			14
NS	11			11
Wt	173.33			173.33
Het Chi	49.62			49.62
Het df	13			13
Het P	***			***
Fixed RR	1.98			1.98
RRl	1.70			1.70
RRu	2.29			2.29
P	+++			+++
Random RR	2.23			2.23
RRl	1.61			1.61
RRu	3.08			3.08
P	+++			+++
Between Chi				
Between df				
Between P				N.S.
Btwn(F) P				N.S.
Btwn(R) P				N.S.

	National cigarette tobacco type Virginia	blended	other	Total
N		11	3	14
NS		9	2	11
Wt		160.66	12.67	173.33
Het Chi		36.10	5.85	49.62
Het df		10	2	13
Het P		***	(*)	***
Fixed RR		1.86	4.18	1.98
RRl		1.60	2.41	1.70
RRu		2.18	7.25	2.29
P		+++	+++	+++
Random RR		2.02	3.65	2.23
RRl		1.46	1.22	1.61
RRu		2.79	10.92	3.08
P		+++	+	+++
Between Chi				7.67
Between df				1
Between P				**
Btwn(F) P				N.S.
Btwn(R) P				N.S.

Table 2H5 - 3

IESLC - Meta-analysis of Ever Smoking, Age started, "Highest vs lowest"
Squamous, Any Product (or Cigarettes if Any not available)
Most adjusted

	Any proxy use No/nk	Yes	Total
N	10	4	14
NS	8	3	11
Wt	122.30	51.03	173.33
Het Chi	24.22	21.50	49.62
Het df	9	3	13
Het P	**	***	***
Fixed RR	1.79	2.49	1.98
RRl	1.50	1.90	1.70
RRu	2.14	3.28	2.29
P	+++	+++	+++
Random RR	2.01	2.89	2.23
RRl	1.44	1.20	1.61
RRu	2.79	6.99	3.08
P	+++	+	+++
Between Chi			3.90
Between df			1
Between P			*
Btwn(F) P			N.S.
Btwn(R) P			N.S.

	Full histological confirmation No	Yes	Total
N	2	12	14
NS	2	9	11
Wt	30.72	142.61	173.33
Het Chi	0.13	49.27	49.62
Het df	1	11	13
Het P	N.S.	***	***
Fixed RR	1.83	2.01	1.98
RRl	1.29	1.71	1.70
RRu	2.61	2.37	2.29
P	+++	+++	+++
Random RR	1.83	2.30	2.23
RRl	1.29	1.56	1.61
RRu	2.61	3.41	3.08
P	+++	+++	+++
Between Chi			0.21
Between df			1
Between P			N.S.
Btwn(F) P			N.S.
Btwn(R) P			N.S.

	Number of adjustment variables (1) 0	1	2+/+nk	Total
N	6	3	5	14
NS	4	2	5	11
Wt	120.72	24.37	28.24	173.33
Het Chi	3.68	7.99	6.21	49.62
Het df	5	2	4	13
Het P	N.S.	*	N.S.	***
Fixed RR	1.68	5.69	1.61	1.98
RRl	1.40	3.83	1.11	1.70
RRu	2.00	8.47	2.33	2.29
P	+++	+++	+	+++
Random RR	1.68	4.79	1.72	2.23
RRl	1.40	2.07	1.06	1.61
RRu	2.00	11.06	2.80	3.08
P	+++	+++	+	+++
Between Chi				31.73
Between df				2
Between P				***
Btwn(F) P				**
Btwn(R) P				(*)

Table 2H5 - 3

IESLC - Meta-analysis of Ever Smoking, Age started, "Highest vs lowest"
Squamous, Any Product (or Cigarettes if Any not available)
Most adjusted

	Number of adjustment variables (2) 0	1	2	3-5	6+/+nk	Total
N	6	3	3	1	1	14
NS	4	2	3	1	1	11
Wt	120.72	24.37	18.04	9.36	0.84	173.33
Het Chi	3.68	7.99	2.62	0.00	0.00	49.62
Het df	5	2	2	0	0	13
Het P	N.S.	*	N.S.	N.S.	N.S.	***
Fixed RR	1.68	5.69	1.98	1.00	4.00	1.98
RRl	1.40	3.83	1.25	0.53	0.47	1.70
RRu	2.00	8.47	3.14	1.90	33.97	2.29
P	+++	+++	++	N.S.	N.S.	+++
Random RR	1.68	4.79	2.03	1.00	4.00	2.23
RRl	1.40	2.07	1.18	0.53	0.47	1.61
RRu	2.00	11.06	3.47	1.90	33.97	3.08
P	+++	+++	++	N.S.	N.S.	+++
Between Chi						35.32
Between df						4
Between P						***
Btwn(F) P						*
Btwn(R) P						*

	Product all/unsp	cig+/-ot	cig only	Total
N	3	9	2	14
NS	2	7	2	11
Wt	24.24	100.93	48.16	173.33
Het Chi	15.26	19.06	3.56	49.62
Het df	2	8	1	13
Het P	***	*	(*)	***
Fixed RR	3.60	1.93	1.54	1.98
RRl	2.42	1.59	1.16	1.70
RRu	5.36	2.34	2.05	2.29
P	+++	+++	++	+++
Random RR	3.72	2.00	1.97	2.23
RRl	0.95	1.43	0.86	1.61
RRu	14.53	2.78	4.50	3.08
P	(+)	+++	N.S.	+++
Between Chi				11.74
Between df				2
Between P				**
Btwn(F) P				N.S.
Btwn(R) P				N.S.

	Derivation of RR/CI Orig	StdCalc	Other	Total
N	1	5	8	14
NS	1	4	7	12
Wt	9.36	120.24	43.72	173.33
Het Chi	0.00	2.92	22.29	49.62
Het df	0	4	7	13
Het P	N.S.	N.S.	**	***
Fixed RR	1.00	1.67	3.66	1.98
RRl	0.53	1.39	2.72	1.70
RRu	1.90	1.99	4.92	2.29
P	N.S.	+++	+++	+++
Random RR	1.00	1.67	3.33	2.23
RRl	0.53	1.39	1.85	1.61
RRu	1.90	1.99	5.98	3.08
P	N.S.	+++	+++	+++
Between Chi				24.40
Between df				2
Between P				***
Btwn(F) P				*
Btwn(R) P				*

Table 2H5 - 4

IESLC - Meta-analysis of Ever Smoking, Age started, "Highest vs lowest"
Squamous, Any Product (or Cigarettes if Any not available)
Least adjusted

REF	NRR	X	SEX	AGEL	AGEH	RACE	YF	LC TYPE	LOC	START	ST	NLC	R	VB	P	H	AD	ADOS	PRODUCT	exL	exH	unexL	unexH	De
BARBON	573	x	m	0	0	all	-	q	Eu:wst	1979	CC	755	n	bl	y	y	0	0	all/unsp	1	14	20	999	st
BENHAM	520		m	0	0	all	-	KI	Eu:wst	1976	CC	1625	n	bl	n	y	0	0	cig only	1	16	25	999	st
HAENSZ	505	x	f	0	0	all	-	q+u	NAmer	1955	CC	158	n	bl	n	y	0	0	cig+/-ot	1	24	25	999	st
HEGMAN	503		m	0	0	all	-	q	NAmer	1989	CC	282	n	bl	y	y	0	0	all/unsp	1	19	20	999	st
HEGMAN	508		f	0	0	all	-	q	NAmer	1989	CC	282	n	bl	y	y	0	0	all/unsp	1	25	26	999	ot
JEDRYC	532		m	0	0	all	-	q	Eu:est	1980	CC	1630	n	bl	y	n	0	0	cig+/-ot	1	16	19	999	st
KHUDER	524		m	0	0	all	-	q	NAmer	1985	CC	482	n	bl	n	y	5	3#	cig+/-ot	1	15	20	999	or
LUO	511	x	c	0	0	all	-	q	As:Chi	1990	CC	102	n	ot	n	y	0	0	cig+/-ot	0	19	40	999	st
MATOS	615	x	m	0	0	all	-	q	SCAmer	1994	CC	200	n	bl	n	n	0	0	cig+/-ot	1	14	20	999	st
PEZZOT	547	x	m	0	0	all	-	q	SCAmer	1987	CC	215	n	bl	n	y	0	0	cig only	1	13	19	999	st
WYNDE6	612		m	0	0	wh	-	q	NAmer	1969	CC	4423	n	bl	n	y	0	0	cig+/-ot	1	17	21	999	st
WYNDE6	636		f	0	0	wh	-	q	NAmer	1969	CC	4423	n	bl	n	y	0	0	cig+/-ot	1	17	21	999	st
ZHENG	524	x	m	0	0	all	-	q	As:Chi	1982	CC	540	n	ot	*	y	0	0	cig+/-ot	1	19	30	999	st
ZHENG	529	x	f	0	0	all	-	q	As:Chi	1982	CC	540	n	ot	*	y	0	0	cig+/-ot	1	29	30	999	st

Comments on values in listings

KHUDER ADOS Duration of smoking (years), number of cigarettes per day and Quitted smoking

Cigarette type is all/unspec for all RRs

Table 2H5 - 5

IESLC - Meta-analysis of Ever Smoking, Age started, "Highest vs lowest"
Squamous, Any Product (or Cigarettes if Any not available)
Least adjusted

REF	NRR	SEX	AD	Number Exposed Case	Exposed Cont	Non-exposed Case	Non-exposed Cont	RR	95.00%CI
BARBON	573	m	0	52	23	64	207	7.31	(4.16- 12.87)
BENHAM	520	m	0	342	341	98	137	1.40	(1.04- 1.89)
HAENSZ	505	f	0	24	37	32	66	1.34	(0.69- 2.60)
HEGMAN	503	m	0	57	716	15	289	1.53	(0.85- 2.75)
HEGMAN	508	f	0	17	169	0	28	5.88~	(0.34- 100.62)
Subtotal HEGMAN								1.62	(0.91- 2.87)
JEDRYC	532	m	0	53	111	134	502	1.79	(1.22- 2.61)
KHUDER	524	m	5	-	-	-	-	1.00	(0.50- 1.80)
LUO	511	c	0	14	20	1	8	5.60	(0.63- 49.95)
MATOS	615	m	0	16	90	6	73	2.16	(0.81- 5.81)
PEZZOT	547	m	0	30	34	10	32	2.82	(1.19- 6.69)
WYNDE6	612	m	0	262	301	44	92	1.82	(1.23- 2.70)
WYNDE6	636	f	0	72	91	32	90	2.23	(1.34- 3.70)
Subtotal WYNDE6								1.96	(1.44- 2.68)
ZHENG	524	m	0	62	43	13	66	7.32	(3.60- 14.90)
ZHENG	529	f	0	34	28	9	16	2.16	(0.83- 5.62)
Subtotal ZHENG								4.74	(2.68- 8.40)
Partial Totals				1035	2004	458	1606		

*prospective study ~ With 0.5 adjustment for zero

REF	NRR	SEX	AD	Ys	Ws	Qs	Ps
BARBON	573	m	0	1.99	12.02	20.70	0.0000
BENHAM	520	m	0	0.34	42.81	4.94	0.0270
HAENSZ	505	f	0	0.29	8.69	1.30	0.3909
HEGMAN	503	m	0	0.43	11.23	0.70	0.1518
HEGMAN	508	f	0	1.77	0.48	0.57	0.2211
Subtotal HEGMAN				0.48	11.70	1.27	
JEDRYC	532	m	0	0.58	26.79	0.25	0.0026
KHUDER	524	m	5	0.00	9.36	4.30	1.0000
LUO	511	c	0	1.72	0.80	0.88	0.1228
MATOS	615	m	0	0.77	3.94	0.03	0.1258
PEZZOT	547	m	0	1.04	5.15	0.67	0.0184
WYNDE6	612	m	0	0.60	24.55	0.15	0.0030
WYNDE6	636	f	0	0.80	14.87	0.22	0.0020
Subtotal WYNDE6				0.67	39.42	0.37	
ZHENG	524	m	0	1.99	7.61	13.12	0.0000
ZHENG	529	f	0	0.77	4.19	0.04	0.1153
Subtotal ZHENG				1.56	11.80	13.15	

	N	14
	NS	11
	Wt	172.49
Het	Chi	47.86
Het	df	13
Het	P	***
Fixed	RR	1.97
	RRl	1.70
	RRu	2.29
	P	+++
Random	RR	2.24
	RRl	1.62
	RRu	3.09
	P	+++
Asymm	P	N.S.

Table 2H5 - 6

IESLC - Meta-analysis of Ever Smoking, Age started, "Highest vs lowest"
Squamous, Any Product (or Cigarettes if Any not available)
Least adjusted

		Sex		
	combined	male	female	Total
N	1	9	4	14
NS	1	9	4	14
Wt	0.80	143.46	28.23	172.49
Het Chi	0.00	44.86	2.11	47.86
Het df	0	8	3	13
Het P	N.S.	***	N.S.	***
Fixed RR	5.60	1.97	1.93	1.97
RRl	0.63	1.67	1.33	1.70
RRu	49.95	2.32	2.78	2.29
P	N.S.	+++	+++	+++
Random RR	5.60	2.30	1.93	2.24
RRl	0.63	1.51	1.33	1.62
RRu	49.95	3.49	2.78	3.09
P	N.S.	+++	+++	+++
Between Chi				0.89
Between df				2
Between P				N.S.
Btwn(F) P				N.S.
Btwn(R) P				N.S.

Table 2H5 - 7

IESLC - Meta-analysis of Ever Smoking, Age started, "Highest vs lowest"
Squamous, Any Product (or Cigarettes if Any not available)
Excluded studies (and stage at which they were excluded)

1	AKIBA AMANDU AMES BECHER BENSHL BEST BLOT1 BROSS BROWN3 CARPEN CEDERL CHYOU CPSI CPSII DARBY DEAN2 DEAN3 DOLL2 ENGELA GAO2 GARCIA GILLIS GRAHAM GURSEL HAMMO2 HIRAYA HOLE HUMBLE JAHN JAIN KAISE2 KATSOU KAUFMA LAUSSM LIAW MCDUFF MIGRAN MRFITR PEZZO2 PISANI PRESCO QIAO SEGI2 SPEIZE SVENSS TVERDA WAKAI WATSON WIGLE WU WYNDE3 WYNDE8
2	AXELSS BOUCHA BOUCOT CHEN DESTEF DORGAN DOSEME FAN GARSHI GER HAMMON JUSSAW KOO KREUZE LEVIN MCCONN NOTAN2 OSANN2 RESTRE SADOWS VUTUC WANG2 WU2 WUWILL WYNDE2 XU ZHOU
3	GUO SPITZ STASZE ZHANG
5	AGUDO ARMADA AUVINE BOFFET BRESLO BUFFLE CHEN2 CHIAZZ CHOI CORREA DAMBER DOLL DORN GAO GENG HU HU2 JOLY KOULUM LETOUR LIU3 LIU4 LIU5 LUBIN LUBIN2 PERNU QIAO2 RACHTA SOBUE SUZUK2 TIZZAN WYNDE7 YUAN
6	ALDERS

Table 2H5 - 8
Potentially overlapping studies

REF	REFGP	PRINC	.	OVERLAP/LINK
BENHAM	LUBIN2	2		Subset of Lubin2
WYNDE6	WYNDE6	1		WYNDE5/6/7/8

Table 2H5 - 9
Most adjusted - insufficient data for meta-analysis

REF	NRR	SEX	AGEL	AGEH	RACE	YF	LC TYPE	LOC	START	ST	NLC	R	VB	P	H	AD	ADOS	PRODUCT	exL	exH	unexL	unexH	De
ALDERS	533	m	0	0	all	-	q+s	Eu:UK	1977	CC	1448	n	V	n	n	2	1#cig	only	1	14	25	999	ot
ALDERS	536	f	0	0	all	-	q+s	Eu:UK	1977	CC	1448	n	V	n	n	2	1#cig	only	1	14	25	999	ot

Comments on values in listings

ALDERS	ADOS	Number of cigs/day
ALDERS	ADOS	Number of cigs/day

REF	NRR	RR	SIG	RRDATA comment
ALDERS	533	5.56	n	0
ALDERS	536	1.56	n	0

Table 2H6 -

IESLC - Meta-analysis of Current Smoking by Age started, Overview
Squamous, Any Product (or Cigarettes if Any not available)

This analysis is restricted to results for:
1) Current smokers
2) Results by Age started
3) Categorical results by Age started
Results by Age started are grouped under 2 schemes (S1, S2). Each scheme has a set of "key values". An interval is allocated to the category whose key value it includes, and intervals which include none or more than one of the key values are excluded. (Open-ended intervals are coded as 999)

S1	key value	maximum range
1	26	19+
2	18	15-25
3	14	1-17

S2	key value	maximum range
1	30	27+
2	26	23-29
3	22	19-25
4	18	15-21
5	14	11-17
6	10	1-13

4) Squamous (or near equivalent)
5) Results complete enough for use in metaanalysis

Within each study, results are then selected (in the following order of preference, within each sex) for:
6) (not applicable)
7) PRODUCT: all/unspec, cigarettes regardless of other products, cigarettes only
8) CIGTYPE: all/unspecified, MC regardless of HR, MC only
9) (not applicable)
10) DENOM: never smoked anything, never smoked cigarettes, never any + low, never cigs + low
11) Followup period (YF, prospective studies): whole study (coded as 0) or longest available
12) LCtype: squamous or nearest available, but not adeno. (q = squamous, s = small,
a = adeno, KI = Kreyberg I, u = undifferentiated)
13) Race: all or nearest available, otherwise by race (wh or w = white, bl or b = black, hi = hispanic
ch = chinese, jap = japanese, haw = hawaiian, w+o = white + oriental, sca = scandinavian, as = asian)
14) For overlapping studies: principal rather than subsidiary studies
Finally by Age: whole study (coded as 0) if available, otherwise by widest available age group
and then for single sex results (m, f) in preference to results for both sexes combined (c).

Results adjusted (AD) for the most potential confounders are then chosen in Sections -1 to -3
and results adjusted for the least confounders in Sections -4 to -6. (Those least adjusted results which actually differ from the most adjusted are marked 'x' in column X in Section -4)

Section -7 shows excluded studies, together with the stage (as above) at which no qualifying results were found.

Section -8 lists the potentially overlapping studies which have been included (1=principal, 2=subsidiary).

Section -9 lists any results which would have been included in preference except that they had data not complete enough for use in meta-analysis, with their significance (yes/no), if known, and any further comment as entered on the database. It also lists as "gap" any categories for which no data were presented by the original authors.

In addition to those mentioned above, the following fields, levels and abbreviations are used:
* or nk = not known, n = no, y = yes, ot = other
nev = never
all/unspec = all or unspecified, cig+/-ot = cigarettes irrespective of other products (cigar, pipe etc)
MC = manufactured cigarettes, HR = hand-rolled cigarettes
exL, exH = range of exposure (low and high) in the smoking group, in terms of Age started
REF: 6-character study reference
NRR: number of the RR on the database within the study
ST : study type (CC = case control, pr or prosp = prospective)
NLC: number of lung cancer cases in whole study
R : risky occupational population (n = no, m = mining, o = other risky)
VB : national cigarette type (V = at least 75% Virginia, bl = at least 75% blended, ot = other)
P : any proxy use
H : full histological confirmation
De : derivation of RR/CI (or = original, st = standard method, ot = other method of estimation)

Table 2H6 - 1

IESLC - Meta-analysis of Current Smoking by Age started, Overview
Squamous, Any Product (or Cigarettes if Any not available)
Most adjusted

REF	NRR	SEX	AGEL	AGEH	RACE	YF	LC TYPE	LOC	START	ST	NLC	R	VB	P	H	AD	PRODUCT	exL	exH	S1	S2	DENOM	De
ENGELA	517	m	0	0	all	0	q	Eu:Sca	1964	pr	435	n	bl	n	n	0	cig+/-ot	30	999	0	1	nev cigs	st
ENGELA	518	m	0	0	all	0	q	Eu:Sca	1964	pr	435	n	bl	n	n	0	cig+/-ot	20	29	1	0	nev cigs	st
ENGELA	519	m	0	0	all	0	q	Eu:Sca	1964	pr	435	n	bl	n	n	0	cig+/-ot	1	19	0	0	nev cigs	st
SVENSS	509	f	0	0	all	-	q	Eu:Sca	1983	CC	210	n	bl	n	n	0	all/unsp	26	999	1	0	nev any	st
SVENSS	510	f	0	0	all	-	q	Eu:Sca	1983	CC	210	n	bl	n	n	0	all/unsp	19	25	0	3	nev any	st
SVENSS	511	f	0	0	all	-	q	Eu:Sca	1983	CC	210	n	bl	n	n	0	all/unsp	0	18	0	0	nev any	st
WAKAI	506	m	0	0	all	-	q	As:Jap	1988	CC	333	n	bl	n	y	0	cig+/-ot	30	999	0	1	nev any	st
WAKAI	507	m	0	0	all	-	q	As:Jap	1988	CC	333	n	bl	n	y	0	cig+/-ot	20	29	1	0	nev any	st
WAKAI	508	m	0	0	all	-	q	As:Jap	1988	CC	333	n	bl	n	y	0	cig+/-ot	1	19	0	0	nev any	st
WU	533	f	0	0	wh	-	q	NAmer	1981	CC	220	n	bl	n	y	2	all/unsp	25	999	1	0	nev any	or
WU	534	f	0	0	wh	-	q	NAmer	1981	CC	220	n	bl	n	y	2	all/unsp	19	24	0	3	nev any	or
WU	535	f	0	0	wh	-	q	NAmer	1981	CC	220	n	bl	n	y	2	all/unsp	0	18	0	0	nev any	or

Cigarette type is all/unspec for all RRs

In this overview table, subtotals and Qs values may be invalid and should be ignored

Table 2H6 - 2

IESLC - Meta-analysis of Current Smoking by Age started, Overview
Squamous, Any Product (or Cigarettes if Any not available)
Most adjusted

REF	NRR	SEX	AD	Number Exposed Case	Cont	Non-exposed Case	Cont	RR	95.00%CI
*ENGELA	517	m	0	6	9762	3	58716	12.03	(3.01- 48.09)
*ENGELA	518	m	0	17	30195	3	58716	11.02	(3.23- 37.60)
*ENGELA	519	m	0	64	50732	3	58716	24.69	(7.76- 78.58)
Subtotal ENGELA								15.41	(7.50- 31.65)
SVENSS	509	f	0	9	18	5	120	12.00	(3.61- 39.85)
SVENSS	510	f	0	18	14	5	120	30.86	(9.92- 96.00)
SVENSS	511	f	0	15	21	5	120	17.14	(5.63- 52.19)
Subtotal SVENSS								18.79	(9.69- 36.45)
WAKAI	506	m	0	4	25	2	65	5.20	(0.90- 30.19)
WAKAI	507	m	0	61	183	2	65	10.83	(2.58- 45.57)
WAKAI	508	m	0	21	74	2	65	9.22	(2.08- 40.85)
Subtotal WAKAI								8.47	(3.47- 20.65)
WU	533	f	2	6	-	2	-	7.80	(0.80- 73.70)
WU	534	f	2	18	-	2	-	47.10	(4.40- 498.50)
WU	535	f	2	37	-	2	-	115.70	(9.80-1371.20)
Subtotal WU								32.22	(8.24- 125.94)
Partial Totals				276	91024	36	176703		

*prospective study

REF	NRR	SEX	AD	Ys	Ws	Qs	Ps
*ENGELA	517	m	0	2.49	2.00	0.14	0.0004
*ENGELA	518	m	0	2.40	2.55	0.31	0.0001
*ENGELA	519	m	0	3.21	2.87	0.59	0.0000
Subtotal ENGELA				2.74	7.42	1.05	
SVENSS	509	f	0	2.48	2.67	0.19	0.0000
SVENSS	510	f	0	3.43	2.98	1.37	0.0000
SVENSS	511	f	0	2.84	3.10	0.03	0.0000
Subtotal SVENSS				2.93	8.75	1.59	
WAKAI	506	m	0	1.65	1.24	1.51	0.0662
WAKAI	507	m	0	2.38	1.86	0.25	0.0012
WAKAI	508	m	0	2.22	1.73	0.49	0.0034
Subtotal WAKAI				2.14	4.84	2.25	
WU	533	f	2	2.05	0.75	0.36	0.0750
WU	534	f	2	3.85	0.69	0.83	0.0014
WU	535	f	2	4.75	0.63	2.52	0.0002
Subtotal WU				3.47	2.07	3.72	

N	12
NS	4

Table 2H6 - 3

IESLC - Meta-analysis of Current Smoking by Age started, Overview
Squamous, Any Product (or Cigarettes if Any not available)
Most adjusted

	Sex			
	combined	male	female	Total
N		6	6	12
NS		2	2	4

In this overview table, other than the "N" rows, entries in the "absent" and "Total" columns may be invalid and should be ignored

	Age started (broad categories)				
	absent	19+k26	15-25k18	1-17k14	Total
N	8	4			12
NS	4	4			8
Wt	15.24	7.83			23.07
Het Chi	6.96	0.11			8.60
Het df	7	3			11
Het P	N.S.	N.S.			N.S.
Fixed RR	18.83	10.93			15.66
RRl	11.40	5.43			10.41
RRu	31.11	22.02			23.54
P	+++	+++			+++
Random RR	18.83	10.93			15.66
RRl	11.40	5.43			10.41
RRu	31.11	22.02			23.54
P	+++	+++			+++

		Age started (narrow categories)						
	absent	27+k30	23-29k26	19-25k22	15-21k18	11-17k14	1-13k10	Total
N	8	2		2				12
NS	4	2		2				8
Wt	16.16	3.24		3.67				23.07
Het Chi	4.70	0.54		0.10				8.60
Het df	7	1		1				11
Het P	N.S.	N.S.		N.S.				N.S.
Fixed RR	14.82	8.72		33.40				15.66
RRl	9.10	2.94		12.00				10.41
RRu	24.13	25.91		92.92				23.54
P	+++	+++		+++				+++
Random RR	14.82	8.72		33.40				15.66
RRl	9.10	2.94		12.00				10.41
RRu	24.13	25.91		92.92				23.54
P	+++	+++		+++				+++

Table 2H6 - 3

IESLC - Meta-analysis of Current Smoking by Age started, Overview
Squamous, Any Product (or Cigarettes if Any not available)
Most adjusted

MALES

	Age started (broad categories) absent	19+k26	15-25k18	1-17k14	Total
N	4	2			6
NS	2	2			4
Wt	7.84	4.41			12.25
Het Chi	2.44	0.00			2.52
Het df	3	1			5
Het P	N.S.	N.S.			N.S.
Fixed RR	12.92	10.94			12.17
RRl	6.42	4.30			6.95
RRu	26.01	27.82			21.30
P	+++	+++			+++
Random RR	12.92	10.94			12.17
RRl	6.42	4.30			6.95
RRu	26.01	27.82			21.30
P	+++	+++			+++

			Age started (narrow categories)					
	absent	27+k30	23-29k26	19-25k22	15-21k18	11-17k14	1-13k10	Total
N	4	2						6
NS	2	2						4
Wt	9.01	3.24						12.25
Het Chi	1.49	0.54						2.52
Het df	3	1						5
Het P	N.S.	N.S.						N.S.
Fixed RR	13.71	8.72						12.17
RRl	7.14	2.94						6.95
RRu	26.35	25.91						21.30
P	+++	+++						+++
Random RR	13.71	8.72						12.17
RRl	7.14	2.94						6.95
RRu	26.35	25.91						21.30
P	+++	+++						+++

FEMALES

	Age started (broad categories) absent	19+k26	15-25k18	1-17k14	Total
N	4	2			6
NS	2	2			4
Wt	7.40	3.42			10.82
Het Chi	2.23	0.11			4.42
Het df	3	1			5
Het P	N.S.	N.S.			N.S.
Fixed RR	28.07	10.92			20.83
RRl	13.66	3.78			11.48
RRu	57.71	31.51			37.80
P	+++	+++			+++
Random RR	28.07	10.92			20.83
RRl	13.66	3.78			11.48
RRu	57.71	31.51			37.80
P	+++	+++			+++

Table 2H6 - 3

IESLC - Meta-analysis of Current Smoking by Age started, Overview
Squamous, Any Product (or Cigarettes if Any not available)
Most adjusted

FEMALES

		absent	27+k30	23-29k26	19-25k22	15-21k18	11-17k14	1-13k10	Total
			Age started (narrow categories)						
	N	4			2				6
	NS	2			2				4
	Wt	7.15			3.67				10.82
Het	Chi	3.08			0.10				4.42
Het	df	3			1				5
Het	P	N.S.			N.S.				N.S.
Fixed	RR	16.34			33.40				20.83
	RRl	7.85			12.00				11.48
	RRu	34.02			92.92				37.80
	P	+++			+++				+++
Random	RR	16.40			33.40				20.83
	RRl	7.75			12.00				11.48
	RRu	34.69			92.92				37.80
	P	+++			+++				+++

Table 2H6 - 4

IESLC - Meta-analysis of Current Smoking by Age started, Overview
Squamous, Any Product (or Cigarettes if Any not available)
Least adjusted

REF	NRR	X	SEX	AGEL	AGEH	RACE	YF	LC TYPE	LOC	START	ST	NLC	R	VB	P	H	AD	PRODUCT	exL	exH	S1	S2	DENOM	De
ENGELA	517		m	0	0	all	0	q	Eu:Sca	1964	pr	435	n	bl	n	n	0	cig+/-ot	30	999	0	1	nev cigs	st
ENGELA	518		m	0	0	all	0	q	Eu:Sca	1964	pr	435	n	bl	n	n	0	cig+/-ot	20	29	1	0	nev cigs	st
ENGELA	519		m	0	0	all	0	q	Eu:Sca	1964	pr	435	n	bl	n	n	0	cig+/-ot	1	19	0	0	nev cigs	st
SVENSS	509		f	0	0	all	-	q	Eu:Sca	1983	CC	210	n	bl	n	n	0	all/unsp	26	999	1	0	nev any	st
SVENSS	510		f	0	0	all	-	q	Eu:Sca	1983	CC	210	n	bl	n	n	0	all/unsp	19	25	0	3	nev any	st
SVENSS	511		f	0	0	all	-	q	Eu:Sca	1983	CC	210	n	bl	n	n	0	all/unsp	0	18	0	0	nev any	st
WAKAI	506		m	0	0	all	-	q	As:Jap	1988	CC	333	n	bl	n	y	0	cig+/-ot	30	999	0	1	nev any	st
WAKAI	507		m	0	0	all	-	q	As:Jap	1988	CC	333	n	bl	n	y	0	cig+/-ot	20	29	1	0	nev any	st
WAKAI	508		m	0	0	all	-	q	As:Jap	1988	CC	333	n	bl	n	y	0	cig+/-ot	1	19	0	0	nev any	st
WU	509	x	f	0	0	wh	-	q	NAmer	1981	CC	220	n	bl	n	y	0	all/unsp	25	999	1	0	nev any	st
WU	510	x	f	0	0	wh	-	q	NAmer	1981	CC	220	n	bl	n	y	0	all/unsp	19	24	0	3	nev any	st
WU	511	x	f	0	0	wh	-	q	NAmer	1981	CC	220	n	bl	n	y	0	all/unsp	0	18	0	0	nev any	st

Cigarette type is all/unspec for all RRs

In this overview table, subtotals and Qs values may be invalid and should be ignored

Table 2H6 - 5

IESLC - Meta-analysis of Current Smoking by Age started, Overview
Squamous, Any Product (or Cigarettes if Any not available)
Least adjusted

REF	NRR	SEX	AD	Number Exposed Case	Cont	Non-exposed Case	Cont	RR	95.00%CI
*ENGELA	517	m	0	6	9762	3	58716	12.03	(3.01- 48.09)
*ENGELA	518	m	0	17	30195	3	58716	11.02	(3.23- 37.60)
*ENGELA	519	m	0	64	50732	3	58716	24.69	(7.76- 78.58)
Subtotal ENGELA								15.41	(7.50- 31.65)
SVENSS	509	f	0	9	18	5	120	12.00	(3.61- 39.85)
SVENSS	510	f	0	18	14	5	120	30.86	(9.92- 96.00)
SVENSS	511	f	0	15	21	5	120	17.14	(5.63- 52.19)
Subtotal SVENSS								18.79	(9.69- 36.45)
WAKAI	506	m	0	4	25	2	65	5.20	(0.90- 30.19)
WAKAI	507	m	0	61	183	2	65	10.83	(2.58- 45.57)
WAKAI	508	m	0	21	74	2	65	9.22	(2.08- 40.85)
Subtotal WAKAI								8.47	(3.47- 20.65)
WU	509	f	0	6	5	2	30	18.00	(2.80- 115.56)
WU	510	f	0	18	7	2	30	38.57	(7.21- 206.25)
WU	511	f	0	37	11	2	30	50.45	(10.37- 245.38)
Subtotal WU								34.61	(13.01- 92.08)
Totals				276	91047	36	176793		

*prospective study

REF	NRR	SEX	AD	Ys	Ws	Qs	Ps
*ENGELA	517	m	0	2.49	2.00	0.22	0.0004
*ENGELA	518	m	0	2.40	2.55	0.45	0.0001
*ENGELA	519	m	0	3.21	2.87	0.43	0.0000
Subtotal ENGELA				2.74	7.42	1.10	
SVENSS	509	f	0	2.48	2.67	0.30	0.0000
SVENSS	510	f	0	3.43	2.98	1.11	0.0000
SVENSS	511	f	0	2.84	3.10	0.00	0.0000
Subtotal SVENSS				2.93	8.75	1.41	
WAKAI	506	m	0	1.65	1.24	1.70	0.0662
WAKAI	507	m	0	2.38	1.86	0.35	0.0012
WAKAI	508	m	0	2.22	1.73	0.62	0.0034
Subtotal WAKAI				2.14	4.84	2.67	
WU	509	f	0	2.89	1.11	0.01	0.0023
WU	510	f	0	3.65	1.37	0.95	0.0000
WU	511	f	0	3.92	1.54	1.87	0.0000
Subtotal WU				3.54	4.01	2.82	

N	12
NS	4

Table 2H6 - 6

IESLC - Meta-analysis of Current Smoking by Age started, Overview
Squamous, Any Product (or Cigarettes if Any not available)
Least adjusted

	Sex			
	combined	male	female	Total
N		6	6	12
NS		2	2	4

In this overview table, other than the "N" rows, entries in the "absent" and "Total" columns may be invalid and should be ignored

	Age started (broad categories)				
	absent	19+k26	15-25k18	1-17k14	Total
N	8	4			12
NS	4	4			8
Wt	16.83	8.19			25.02
Het Chi	6.47	0.22			8.00
Het df	7	3			11
Het P	N.S.	N.S.			N.S.
Fixed RR	19.65	12.06			16.75
RRl	12.19	6.08			11.32
RRu	31.69	23.93			24.79
P	+++	+++			+++
Random RR	19.65	12.06			16.75
RRl	12.19	6.08			11.32
RRu	31.69	23.93			24.79
P	+++	+++			+++

			Age started (narrow categories)					
	absent	27+k30	23-29k26	19-25k22	15-21k18	11-17k14	1-13k10	Total
N	8	2		2				12
NS	4	2		2				8
Wt	17.43	3.24		4.35				25.02
Het Chi	3.98	0.54		0.05				8.00
Het df	7	1		1				11
Het P	N.S.	N.S.		N.S.				N.S.
Fixed RR	15.96	8.72		33.10				16.75
RRl	9.98	2.94		12.93				11.32
RRu	25.52	25.91		84.72				24.79
P	+++	+++		+++				+++
Random RR	15.96	8.72		33.10				16.75
RRl	9.98	2.94		12.93				11.32
RRu	25.52	25.91		84.72				24.79
P	+++	+++		+++				+++

Table 2H6 - 6

IESLC - Meta-analysis of Current Smoking by Age started, Overview
Squamous, Any Product (or Cigarettes if Any not available)
Least adjusted

MALES

	Age started (broad categories) absent	19+k26	15-25k18	1-17k14	Total
N	4	2			6
NS	2	2			4
Wt	7.84	4.41			12.25
Het Chi	2.44	0.00			2.52
Het df	3	1			5
Het P	N.S.	N.S.			N.S.
Fixed RR	12.92	10.94			12.17
RRl	6.42	4.30			6.95
RRu	26.01	27.82			21.30
P	+++	+++			+++
Random RR	12.92	10.94			12.17
RRl	6.42	4.30			6.95
RRu	26.01	27.82			21.30
P	+++	+++			+++

	absent	27+k30	23-29k26	19-25k22 Age started (narrow categories)	15-21k18	11-17k14	1-13k10	Total
N	4	2						6
NS	2	2						4
Wt	9.01	3.24						12.25
Het Chi	1.49	0.54						2.52
Het df	3	1						5
Het P	N.S.	N.S.						N.S.
Fixed RR	13.71	8.72						12.17
RRl	7.14	2.94						6.95
RRu	26.35	25.91						21.30
P	+++	+++						+++
Random RR	13.71	8.72						12.17
RRl	7.14	2.94						6.95
RRu	26.35	25.91						21.30
P	+++	+++						+++

FEMALES

	Age started (broad categories) absent	19+k26	15-25k18	1-17k14	Total
N	4	2			6
NS	2	2			4
Wt	8.98	3.78			12.76
Het Chi	1.45	0.13			3.03
Het df	3	1			5
Het P	N.S.	N.S.			N.S.
Fixed RR	28.35	13.52			22.77
RRl	14.74	4.93			13.15
RRu	54.51	37.06			39.41
P	+++	+++			+++
Random RR	28.35	13.52			22.77
RRl	14.74	4.93			13.15
RRu	54.51	37.06			39.41
P	+++	+++			+++

Table 2H6 - 6

IESLC - Meta-analysis of Current Smoking by Age started, Overview
Squamous, Any Product (or Cigarettes if Any not available)
Least adjusted

FEMALES

		Age started (narrow categories)						
	absent	27+k30	23-29k26	19-25k22	15-21k18	11-17k14	1-13k10	Total
N	4			2				6
NS	2			2				4
Wt	8.41			4.35				12.76
Het Chi	2.06			0.05				3.03
Het df	3			1				5
Het P	N.S.			N.S.				N.S.
Fixed RR	18.76			33.10				22.77
RRl	9.55			12.93				13.15
RRu	36.88			84.72				39.41
P	+++			+++				+++
Random RR	18.76			33.10				22.77
RRl	9.55			12.93				13.15
RRu	36.88			84.72				39.41
P	+++			+++				+++

Table 2H6 - 7

IESLC - Meta-analysis of Current Smoking by Age started, Overview
Squamous, Any Product (or Cigarettes if Any not available)
Excluded studies (and stage at which they were excluded)

1	AGUDO	ALDERS	ARMADA	AUVINE	AXELSS	BARBON	BECHER	BENHAM	BLOT1	BOFFET	BOUCHA	BRESLO	BROWN3	CARPEN	CHEN	CHEN2
	CHIAZZ	CHOI	CHYOU	CORREA	DAMBER	DARBY	DESTEF	DOLL	DOLL2	DORGAN	DOSEME	FAN	GAO	GARCIA	GARSHI	GENG
	GER	GRAHAM	GUO	GURSEL	HAENSZ	HAMMO2	HAMMON	HEGMAN	HU	HU2	JAHN	JAIN	JEDRYC	JOLY	JUSSAW	KHUDER
	KOO	KOULUM	KREUZE	LAUSSM	LETOUR	LEVIN	LIU3	LIU4	LIU5	LUBIN	LUBIN2	LUO	MCCONN	NOTAN2	OSANN2	PERNU
	PEZZOT	PRESCO	QIAO	QIAO2	RACHTA	RESTRE	SADOWS	STASZE	SUZUK2	TIZZAN	TVERDA	VUTUC	WANG2	WIGLE	WU2	WUWILL
	WYNDE2	WYNDE3	XU	YUAN	ZHANG	ZHENG	ZHOU									
2	AKIBA	AMANDU	AMES	BENSHL	BEST	BOUCOT	BROSS	BUFFLE	CPSII	DEAN2	GILLIS	HUMBLE	KAISE2	KATSOU	KAUFMA	PEZZO2
	PISANI	SPITZ	WATSON	WYNDE8												
3	MCDUFF	WYNDE6														
4	CEDERL	CPSI	DEAN3	DORN	GAO2	HIRAYA	HOLE	LIAW	MATOS	MIGRAN	MRFITR	SEGI2	SOBUE	SPEIZE	WYNDE7	

Table 2H7 -

IESLC - Meta-analysis of Current Smoking, Age started, "Low"
Squamous, Any Product (or Cigarettes if Any not available)

This analysis is restricted to results for:
1) Current smokers
2) Results by Age started
3) Categorical results by Age started
4) Squamous (or near equivalent)
5) Results complete enough for use in metaanalysis

Within each study, results are then selected (in the following order of preference, within each sex) for:
6) (not applicable)
7) PRODUCT: all/unspec, cigarettes regardless of other products, cigarettes only
8) CIGTYPE: all/unspecified, MC regardless of HR, MC only
9) (not applicable)
10) DENOM: never smoked anything, never smoked cigarettes, never any + low, never cigs + low
11) Followup period (YF, prospective studies): whole study (coded as 0) or longest available
12) LCtype: squamous or nearest available, but not adeno. (q = squamous, s = small,
a = adeno, KI = Kreyberg I, u = undifferentiated)
13) Race: all or nearest available, otherwise by race (wh or w = white, bl or b = black, hi = hispanic
ch = chinese, jap = japanese, haw = hawaiian, w+o = white + oriental, sca = scandinavian, as = asian)
14) Age started "low" in key scheme 1 (key value 26, maximum range 19+)
15) For overlapping studies: principal rather than subsidiary studies
Finally by Age: whole study (coded as 0) if available, otherwise by widest available age group
and then for single sex results (m, f) in preference to results for both sexes combined (c).

Results adjusted (AD) for the most potential confounders are then chosen in Sections -1 to -3
and results adjusted for the least confounders in Sections -4 to -6. (Those least adjusted results which
actually differ from the most adjusted are marked 'x' in column X in Section -4)

Section -7 shows excluded studies, together with the stage (as above) at which no qualifying
results were found.

Section -8 lists the potentially overlapping studies which have been included (1=principal, 2=subsidiary).

Section -9 lists any results which would have been included in preference except that they had data not complete
enough for use in meta-analysis, with their significance (yes/no), if known, and any further comment as entered
on the database. It also lists as "gap" any categories for which no data were presented by the original authors.

In addition to those mentioned above, the following fields, levels and abbreviations are used:
* or nk = not known, n = no, y = yes, ot = other
nev = never
all/unspec = all or unspecified, cig+/-ot = cigarettes irrespective of other products (cigar, pipe etc)
MC = manufactured cigarettes, HR = hand-rolled cigarettes
exL, exH = range of exposure (low and high) in the smoking group, in terms of Age started
REF: 6-character study reference
NRR: number of the RR on the database within the study
ST : study type (CC = case control, pr or prosp = prospective)
NLC: number of lung cancer cases in whole study
R : risky occupational population (n = no, m = mining, o = other risky)
VB : national cigarette type (V = at least 75% Virginia, bl = at least 75% blended, ot = other)
P : any proxy use
H : full histological confirmation
De : derivation of RR/CI (or = original, st = standard method, ot = other method of estimation)

Table 2H7 - 1

IESLC - Meta-analysis of Current Smoking, Age started, "Low"
Squamous, Any Product (or Cigarettes if Any not available)
Most adjusted

REF	NRR	SEX	AGEL	AGEH	RACE	YF	LC TYPE	LOC	START	ST	NLC	R	VB	P	H	AD	PRODUCT	exL	exH	DENOM	De
ENGELA	518	m	0	0	all	0	q	Eu:Sca	1964	pr	435	n	bl	n	n	0	cig+/-ot	20	29	nev cigs	st
SVENSS	509	f	0	0	all	-	q	Eu:Sca	1983	CC	210	n	bl	n	n	0	all/unsp	26	999	nev any	st
WAKAI	507	m	0	0	all	-	q	As:Jap	1988	CC	333	n	bl	n	y	0	cig+/-ot	20	29	nev any	st
WU	533	f	0	0	wh	-	q	NAmer	1981	CC	220	n	bl	n	y	2	all/unsp	25	999	nev any	or

Cigarette type is all/unspec for all RRs

Table 2H7 - 2

IESLC - Meta-analysis of Current Smoking, Age started, "Low"
Squamous, Any Product (or Cigarettes if Any not available)
Most adjusted

REF	NRR	SEX	AD	Number Case	Exposed Cont	Non-exposed Case	Cont	RR	95.00%CI
*ENGELA	518	m	0	17	30195	3	58716	11.02	(3.23- 37.60)
SVENSS	509	f	0	9	18	5	120	12.00	(3.61- 39.85)
WAKAI	507	m	0	61	183	2	65	10.83	(2.58- 45.57)
WU	533	f	2	6	-	2	-	7.80	(0.80- 73.70)
Partial Totals				93	30396	12	58901		

*prospective study

REF	NRR	SEX	AD	Ys	Ws	Qs	Ps
*ENGELA	518	m	0	2.40	2.55	0.00	0.0001
SVENSS	509	f	0	2.48	2.67	0.02	0.0000
WAKAI	507	m	0	2.38	1.86	0.00	0.0012
WU	533	f	2	2.05	0.75	0.09	0.0750

	N	4
	NS	4
	Wt	7.83
Het	Chi	0.11
Het	df	3
Het	P	N.S.
Fixed	RR	10.93
	RRl	5.43
	RRu	22.02
	P	+++
Random	RR	10.93
	RRl	5.43
	RRu	22.02
	P	+++
Asymm	P	*

Table 2H7 - 3

IESLC - Meta-analysis of Current Smoking, Age started, "Low"
Squamous, Any Product (or Cigarettes if Any not available)
Most adjusted

	combined	Sex: male	female	Total
N		2	2	4
NS		2	2	4
Wt		4.41	3.42	7.83
Het Chi		0.00	0.11	0.11
Het df		1	1	3
Het P		N.S.	N.S.	N.S.
Fixed RR		10.94	10.92	10.93
RRl		4.30	3.78	5.43
RRu		27.82	31.51	22.02
P		+++	+++	+++
Random RR		10.94	10.92	10.93
RRl		4.30	3.78	5.43
RRu		27.82	31.51	22.02
P		+++	+++	+++
Between Chi				0.00
Between df				1
Between P				N.S.
Btwn(F) P				N.S.
Btwn(R) P				N.S.

Too few RRs for analysis by factor

Table 2H7 - 4

IESLC - Meta-analysis of Current Smoking, Age started, "Low"
Squamous, Any Product (or Cigarettes if Any not available)
Least adjusted

REF	NRR	X	SEX	AGEL	AGEH	RACE	YF	LC TYPE	LOC	START	ST	NLC	R	VB	P	H	AD	PRODUCT	exL	exH	DENOM	De
ENGELA	518		m	0	0	all	0	q	Eu:Sca	1964	pr	435	n	bl	n	n	0	cig+/-ot	20	29	nev cigs	st
SVENSS	509		f	0	0	all	-	q	Eu:Sca	1983	CC	210	n	bl	n	n	0	all/unsp	26	999	nev any	st
WAKAI	507		m	0	0	all	-	q	As:Jap	1988	CC	333	n	bl	n	y	0	cig+/-ot	20	29	nev any	st
WU	509	x	f	0	0	wh	-	q	NAmer	1981	CC	220	n	bl	n	y	0	all/unsp	25	999	nev any	st

Cigarette type is all/unspec for all RRs

Table 2H7 - 5

IESLC - Meta-analysis of Current Smoking, Age started, "Low"
Squamous, Any Product (or Cigarettes if Any not available)
Least adjusted

REF	NRR	SEX	AD	Number Exposed Case	Number Exposed Cont	Non-exposed Case	Non-exposed Cont	RR	95.00%CI
*ENGELA	518	m	0	17	30195	3	58716	11.02	(3.23- 37.60)
SVENSS	509	f	0	9	18	5	120	12.00	(3.61- 39.85)
WAKAI	507	m	0	61	183	2	65	10.83	(2.58- 45.57)
WU	509	f	0	6	5	2	30	18.00	(2.80- 115.56)
Totals				93	30401	12	58931		

*prospective study

REF	NRR	SEX	AD	Ys	Ws	Qs	Ps
*ENGELA	518	m	0	2.40	2.55	0.02	0.0001
SVENSS	509	f	0	2.48	2.67	0.00	0.0000
WAKAI	507	m	0	2.38	1.86	0.02	0.0012
WU	509	f	0	2.89	1.11	0.18	0.0023

	N	4
	NS	4
	Wt	8.19
Het	Chi	0.22
Het	df	3
Het	P	N.S.
Fixed	RR	12.06
	RRl	6.08
	RRu	23.93
	P	+++
Random	RR	12.06
	RRl	6.08
	RRu	23.93
	P	+++
Asymm	P	N.S.

Table 2H7 - 6

IESLC - Meta-analysis of Current Smoking, Age started, "Low"
Squamous, Any Product (or Cigarettes if Any not available)
Least adjusted

	Sex combined	male	female	Total
N		2	2	4
NS		2	2	4
Wt		4.41	3.78	8.19
Het Chi		0.00	0.13	0.22
Het df		1	1	3
Het P		N.S.	N.S.	N.S.
Fixed RR		10.94	13.52	12.06
RRl		4.30	4.93	6.08
RRu		27.82	37.06	23.93
P		+++	+++	+++
Random RR		10.94	13.52	12.06
RRl		4.30	4.93	6.08
RRu		27.82	37.06	23.93
P		+++	+++	+++
Between Chi				0.09
Between df				1
Between P				N.S.
Btwn(F) P				N.S.
Btwn(R) P				N.S.

Table 2H7 - 7

IESLC - Meta-analysis of Current Smoking, Age started, "Low"
Squamous, Any Product (or Cigarettes if Any not available)
Excluded studies (and stage at which they were excluded)

1	AGUDO ALDERS ARMADA AUVINE AXELSS BARBON BECHER BENHAM BLOT1 BOFFET BOUCHA BRESLO BROWN3 CARPEN CHEN CHEN2 CHIAZZ CHOI CHYOU CORREA DAMBER DARBY DESTEF DOLL DOLL2 DORGAN DOSEME FAN GAO GARCIA GARSHI GENG GER GRAHAM GUO GURSEL HAENSZ HAMMO2 HAMMON HEGMAN HU HU2 JAHN JAIN JEDRYC JOLY JUSSAW KHUDER KOO KOULUM KREUZE LAUSSM LETOUR LEVIN LIU3 LIU4 LIU5 LUBIN LUBIN2 LUO MCCONN NOTAN2 OSANN2 PERNU PEZZOT PRESCO QIAO QIAO2 RACHTA RESTRE SADOWS STASZE SUZUK2 TIZZAN TVERDA VUTUC WANG2 WIGLE WU2 WUWILL WYNDE2 WYNDE3 XU YUAN ZHANG ZHENG ZHOU
2	AKIBA AMANDU AMES BENSHL BEST BOUCOT BROSS BUFFLE CPSII DEAN2 GILLIS HUMBLE KAISE2 KATSOU KAUFMA PEZZO2 PISANI SPITZ WATSON WYNDE8
3	MCDUFF WYNDE6
4	CEDERL CPSI DEAN3 DORN GAO2 HIRAYA HOLE LIAW MATOS MIGRAN MRFITR SEGI2 SOBUE SPEIZE WYNDE7

Table 2H8 -

IESLC - Meta-analysis of Current Smoking, Age started, "Mid"
Squamous, Any Product (or Cigarettes if Any not available)

This analysis is restricted to results for:
1) Current smokers
2) Results by Age started
3) Categorical results by Age started
4) Squamous (or near equivalent)
5) Results complete enough for use in metaanalysis

Within each study, results are then selected (in the following order of preference, within each sex) for:
6) (not applicable)
7) PRODUCT: all/unspec, cigarettes regardless of other products, cigarettes only
8) CIGTYPE: all/unspecified, MC regardless of HR, MC only
9) (not applicable)
10) DENOM: never smoked anything, never smoked cigarettes, never any + low, never cigs + low
11) Followup period (YF, prospective studies): whole study (coded as 0) or longest available
12) LCtype: squamous or nearest available, but not adeno. (q = squamous, s = small,
a = adeno, KI = Kreyberg I, u = undifferentiated)
13) Race: all or nearest available, otherwise by race (wh or w = white, bl or b = black, hi = hispanic
ch = chinese, jap = japanese, haw = hawaiian, w+o = white + oriental, sca = scandinavian, as = asian)
14) Age started "mid" in key scheme 1 (key value 18, maximum range 15-25)
15) For overlapping studies: principal rather than subsidiary studies
Finally by Age: whole study (coded as 0) if available, otherwise by widest available age group
and then for single sex results (m, f) in preference to results for both sexes combined (c).

Results adjusted (AD) for the most potential confounders are then chosen in Sections -1 to -3
and results adjusted for the least confounders in Sections -4 to -6. (Those least adjusted results which
actually differ from the most adjusted are marked 'x' in column X in Section -4)

Section -7 shows excluded studies, together with the stage (as above) at which no qualifying
results were found.

Section -8 lists the potentially overlapping studies which have been included (1=principal, 2=subsidiary).

Section -9 lists any results which would have been included in preference except that they had data not complete
enough for use in meta-analysis, with their significance (yes/no), if known, and any further comment as entered
on the database. It also lists as "gap" any categories for which no data were presented by the original authors.

In addition to those mentioned above, the following fields, levels and abbreviations are used:
* or nk = not known, n = no, y = yes, ot = other
nev = never
all/unspec = all or unspecified, cig+/-ot = cigarettes irrespective of other products (cigar, pipe etc)
MC = manufactured cigarettes, HR = hand-rolled cigarettes
exL, exH = range of exposure (low and high) in the smoking group, in terms of Age started
REF: 6-character study reference
NRR: number of the RR on the database within the study
ST : study type (CC = case control, pr or prosp = prospective)
NLC: number of lung cancer cases in whole study
R : risky occupational population (n = no, m = mining, o = other risky)
VB : national cigarette type (V = at least 75% Virginia, bl = at least 75% blended, ot = other)
P : any proxy use
H : full histological confirmation
De : derivation of RR/CI (or = original, st = standard method, ot = other method of estimation)

Table 2H8 - 0

No RRs selected for this analysis

Table 2H8 - 7

IESLC - Meta-analysis of Current Smoking, Age started, "Mid"
Squamous, Any Product (or Cigarettes if Any not available)
Excluded studies (and stage at which they were excluded)

1 AGUDO ALDERS ARMADA AUVINE AXELSS BARBON BECHER BENHAM BLOT1 BOFFET BOUCHA BRESLO BROWN3 CARPEN CHEN CHEN2 CHIAZZ CHOI CHYOU CORREA DAMBER DARBY DESTEF DOLL DOLL2 DORGAN DOSEME FAN GAO GARCIA GARSHI GENG GER GRAHAM GUO GURSEL HAENSZ HAMMO2 HAMMON HEGMAN HU HU2 JAHN JAIN JEDRYC JOLY JUSSAW KHUDER KOO KOULUM KREUZE LAUSSM LETOUR LEVIN LIU3 LIU4 LIU5 LUBIN LUBIN2 LUO MCCONN NOTAN2 OSANN2 PERNU PEZZOT PRESCO QIAO QIAO2 RACHTA RESTRE SADOWS STASZE SUZUK2 TIZZAN TVERDA VUTUC WANG2 WIGLE WU2 WUWILL WYNDE2 WYNDE3 XU YUAN ZHANG ZHENG ZHOU

2 AKIBA AMANDU AMES BENSHL BEST BOUCOT BROSS BUFFLE CPSII DEAN2 GILLIS HUMBLE KAISE2 KATSOU KAUFMA PEZZO2 PISANI SPITZ WATSON WYNDE8

3 MCDUFF WYNDE6

4 CEDERL CPSI DEAN3 DORN GAO2 HIRAYA HOLE LIAW MATOS MIGRAN MRFITR SEGI2 SOBUE SPEIZE WYNDE7

14 ENGELA SVENSS WAKAI WU

Table 2H9 -

IESLC - Meta-analysis of Current Smoking, Age started, "High"
Squamous, Any Product (or Cigarettes if Any not available)

This analysis is restricted to results for:
1) Current smokers
2) Results by Age started
3) Categorical results by Age started
4) Squamous (or near equivalent)
5) Results complete enough for use in metaanalysis

Within each study, results are then selected (in the following order of preference, within each sex) for:
6) PRODUCT: all/unspec, cigarettes regardless of other products, cigarettes only
7) CIGTYPE: all/unspecified, MC regardless of HR, MC only
8) (not applicable)
9) DENOM: never smoked anything, never smoked cigarettes, never any + low, never cigs + low
10) Followup period (YF, prospective studies): whole study (coded as 0) or longest available
11) LCtype: squamous or nearest available, but not adeno. (q = squamous, s = small,
a = adeno, KI = Kreyberg I, u = undifferentiated)
12) Race: all or nearest available, otherwise by race (wh or w = white, bl or b = black, hi = hispanic
ch = chinese, jap = japanese, haw = hawaiian, w+o = white + oriental, sca = scandinavian, as = asian)
13) Age started "high" in key scheme 1 (key value 14, maximum range 1-17)
14) For overlapping studies: principal rather than subsidiary studies
Finally by Age: whole study (coded as 0) if available, otherwise by widest available age group
and then for single sex results (m, f) in preference to results for both sexes combined (c).

Results adjusted (AD) for the most potential confounders are then chosen in Sections -1 to -3
and results adjusted for the least confounders in Sections -4 to -6. (Those least adjusted results which
actually differ from the most adjusted are marked 'x' in column X in Section -4)

Section -7 shows excluded studies, together with the stage (as above) at which no qualifying
results were found.

Section -8 lists the potentially overlapping studies which have been included (1=principal, 2=subsidiary).

Section -9 lists any results which would have been included in preference except that they had data not complete
enough for use in meta-analysis, with their significance (yes/no), if known, and any further comment as entered
on the database. It also lists as "gap" any categories for which no data were presented by the original authors.

In addition to those mentioned above, the following fields, levels and abbreviations are used:
* or nk = not known, n = no, y = yes, ot = other
nev = never
all/unspec = all or unspecified, cig+/-ot = cigarettes irrespective of other products (cigar, pipe etc)
MC = manufactured cigarettes, HR = hand-rolled cigarettes
exL, exH = range of exposure (low and high) in the smoking group, in terms of Age started
REF: 6-character study reference
NRR: number of the RR on the database within the study
ST : study type (CC = case control, pr or prosp = prospective)
NLC: number of lung cancer cases in whole study
R : risky occupational population (n = no, m = mining, o = other risky)
VB : national cigarette type (V = at least 75% Virginia, bl = at least 75% blended, ot = other)
P : any proxy use
H : full histological confirmation
De : derivation of RR/CI (or = original, st = standard method, ot = other method of estimation)

Table 2H9 - 0

No RRs selected for this analysis

Table 2H9 - 7

IESLC - Meta-analysis of Current Smoking, Age started, "High"
Squamous, Any Product (or Cigarettes if Any not available)
Excluded studies (and stage at which they were excluded)

1 AGUDO ALDERS ARMADA AUVINE AXELSS BARBON BECHER BENHAM BLOT1 BOFFET BOUCHA BRESLO BROWN3 CARPEN CHEN CHEN2 CHIAZZ CHOI CHYOU CORREA DAMBER DARBY DESTEF DOLL DOLL2 DORGAN DOSEME FAN GAO GARCIA GARSHI GENG GER GRAHAM GUO GURSEL HAENSZ HAMMO2 HAMMON HEGMAN HU HU2 JAHN JAIN JEDRYC JOLY JUSSAW KHUDER KOO KOULUM KREUZE LAUSSM LETOUR LEVIN LIU3 LIU4 LIU5 LUBIN LUBIN2 LUO MCCONN NOTAN2 OSANN2 PERNU PEZZOT PRESCO QIAO QIAO2 RACHTA RESTRE SADOWS STASZE SUZUK2 TIZZAN TVERDA VUTUC WANG2 WIGLE WU2 WUWILL WYNDE2 WYNDE3 XU YUAN ZHANG ZHENG ZHOU

2 AKIBA AMANDU AMES BENSHL BEST BOUCOT BROSS BUFFLE CPSII DEAN2 GILLIS HUMBLE KAISE2 KATSOU KAUFMA PEZZO2 PISANI SPITZ WATSON WYNDE8

3 MCDUFF WYNDE6

4 CEDERL CPSI DEAN3 DORN GAO2 HIRAYA HOLE LIAW MATOS MIGRAN MRFITR SEGI2 SOBUE SPEIZE WYNDE7

14 ENGELA SVENSS WAKAI WU

Table 2H10 -

IESLC - Meta-analysis of Current Smoking, Age started, "Highest vs lowest"
Squamous, Any Product (or Cigarettes if Any not available)

This analysis is restricted to results for:
1) Current smokers
2) Results by Age started
3) Categorical results by Age started
4) Denominator (unexposed) = "low"
5) Squamous (or near equivalent)
6) Results complete enough for use in metaanalysis

Within each study, results are then selected (in the following order of preference, within each sex) for:
7) (not applicable)
8) PRODUCT: all/unspec, cigarettes regardless of other products, cigarettes only
9) CIGTYPE: all/unspecified, MC regardless of HR, MC only
10) Results with least adjustment for other aspects of smoking (ADOS)
11) The highest vs lowest category
12) Followup period (YF, prospective studies): whole study (coded as 0) or longest available
13) LCtype: squamous or nearest available, but not adeno. (q = squamous, s = small,
a = adeno, KI = Kreyberg I, u = undifferentiated)
14) Race: all or nearest available, otherwise by race (wh or w = white, bl or b = black, hi = hispanic
ch = chinese, jap = japanese, haw = hawaiian, w+o = white + oriental, sca = scandinavian, as = asian)
15) For overlapping studies: principal rather than subsidiary studies
Finally by Age: whole study (coded as 0) if available, otherwise by widest available age group
and then for single sex results (m, f) in preference to results for both sexes combined (c).

Results adjusted (AD) for the most potential confounders are then chosen in Sections -1 to -3
and results adjusted for the least confounders in Sections -4 to -6. (Those least adjusted results which
actually differ from the most adjusted are marked 'x' in column X in Section -4)

Section -7 shows excluded studies, together with the stage (as above) at which no qualifying
results were found.

Section -8 lists the potentially overlapping studies which have been included (1=principal, 2=subsidiary).

Section -9 lists any results which would have been included in preference except that they had data not complete
enough for use in meta-analysis, with their significance (yes/no), if known, and any further comment as entered
on the database. It also lists as "gap" any categories for which no data were presented by the original authors.

In addition to those mentioned above, the following fields, levels and abbreviations are used:
* or nk = not known, n = no, y = yes, ot = other
all/unspec = all or unspecified, cig+/-ot = cigarettes irrespective of other products (cigar, pipe etc)
MC = manufactured cigarettes, HR = hand-rolled cigarettes
exL, exH = range of exposure (low and high) in the "highest" group, in terms of Age started
unexL, unexH = range of exposure (low and high) in the "lowest" group, in terms of Age started
REF: 6-character study reference
NRR: number of the RR on the database within the study
ST : study type (CC = case control, pr or prosp = prospective)
NLC: number of lung cancer cases in whole study
R : risky occupational population (n = no, m = mining, o = other risky)
VB : national cigarette type (V = at least 75% Virginia, bl = at least 75% blended, ot = other)
P : any proxy use
H : full histological confirmation
De : derivation of RR/CI (or = original, st = standard method, ot = other method of estimation)

Table 2H10 - 1

IESLC - Meta-analysis of Current Smoking, Age started, "Highest vs lowest"
Squamous, Any Product (or Cigarettes if Any not available)
Most adjusted

REF	NRR	SEX	AGEL	AGEH	RACE	YF	LC TYPE	LOC	START	ST	NLC	R	VB	P	H	AD	ADOS	PRODUCT	exL	exH	unexL	unexH	De
ENGELA	521	m	0	0	all	0	q	Eu:Sca	1964	pr	435	n	bl	n	n	0	0	cig+/-ot	1	19	30	999	st
SVENSS	513	f	0	0	all	-	q	Eu:Sca	1983	CC	210	n	bl	n	n	0	0	all/unsp	0	18	26	999	st
WAKAI	510	m	0	0	all	-	q	As:Jap	1988	CC	333	n	bl	n	y	0	0	cig+/-ot	1	19	30	999	st
WU	537	f	0	0	wh	-	q	NAmer	1981	CC	220	n	bl	n	y	2	0	all/unsp	0	18	25	999	ot

Cigarette type is all/unspec for all RRs

Table 2H10 - 2

IESLC - Meta-analysis of Current Smoking, Age started, "Highest vs lowest"
Squamous, Any Product (or Cigarettes if Any not available)
Most adjusted

REF	NRR	SEX	AD	Number Case	Exposed Cont	Non-exposed Case	Cont	RR	95.00%CI
*ENGELA	521	m	0	64	50732	6	9762	2.05	(0.89- 4.74)
SVENSS	513	f	0	15	21	9	18	1.43	(0.51- 4.04)
WAKAI	510	m	0	21	74	4	25	1.77	(0.56- 5.67)
WU	537	f	2	37	-	6	-	14.83	(2.14- 102.93)
Partial Totals				137	50827	25	9805		

*prospective study

REF	NRR	SEX	AD	Ys	Ws	Qs	Ps
*ENGELA	521	m	0	0.72	5.49	0.00	0.0920
SVENSS	513	f	0	0.36	3.56	0.53	0.5010
WAKAI	510	m	0	0.57	2.85	0.08	0.3335
WU	537	f	2	2.70	1.02	3.91	0.0063

	N	4
	NS	4
	Wt	12.92
Het	Chi	4.53
Het	df	3
Het	P	N.S.
Fixed	RR	2.10
	RRl	1.22
	RRu	3.63
	P	++
Random	RR	2.23
	RRl	1.11
	RRu	4.49
	P	+
Asymm	P	N.S.

Table 2H10 - 3

IESLC - Meta-analysis of Current Smoking, Age started, "Highest vs lowest"
Squamous, Any Product (or Cigarettes if Any not available)
Most adjusted

	Sex		
combined	male	female	Total
N	2	2	4
NS	2	2	4
Wt	8.34	4.58	12.92
Het Chi	0.04	4.36	4.53
Het df	1	1	3
Het P	N.S.	*	N.S.
Fixed RR	1.95	2.41	2.10
RRl	0.99	0.96	1.22
RRu	3.85	6.02	3.63
P	(+)	(+)	++
Random RR	1.95	3.97	2.23
RRl	0.99	0.41	1.11
RRu	3.85	38.58	4.49
P	(+)	N.S.	+
Between Chi			0.13
Between df			1
Between P			N.S.
Btwn(F) P			N.S.
Btwn(R) P			N.S.

Too few RRs for analysis by factor

Table 2H10 - 4

IESLC - Meta-analysis of Current Smoking, Age started, "Highest vs lowest"
Squamous, Any Product (or Cigarettes if Any not available)
Least adjusted

REF	NRR	X	SEX	AGEL	AGEH	RACE	YF	LC TYPE	LOC	START	ST	NLC	R	VB	P	H	AD	ADOS	PRODUCT	exL	exH	unexL	unexH	De
ENGELA	521		m	0	0	all	0	q	Eu:Sca	1964	pr	435	n	bl	n	n	0	0	cig+/-ot	1	19	30	999	st
SVENSS	513		f	0	0	all	-	q	Eu:Sca	1983	CC	210	n	bl	n	n	0	0	all/unsp	0	18	26	999	st
WAKAI	510		m	0	0	all	-	q	As:Jap	1988	CC	333	n	bl	n	y	0	0	cig+/-ot	1	19	30	999	st
WU	513	x	f	0	0	wh	-	q	NAmer	1981	CC	220	n	bl	n	y	0	0	all/unsp	0	18	25	999	st

Cigarette type is all/unspec for all RRs

Table 2H10 - 5

IESLC - Meta-analysis of Current Smoking, Age started, "Highest vs lowest"
Squamous, Any Product (or Cigarettes if Any not available)
Least adjusted

REF	NRR	SEX	AD	Number Case	Exposed Cont	Non-exposed Case	Cont	RR	95.00%CI
*ENGELA	521	m	0	64	50732	6	9762	2.05	(0.89- 4.74)
SVENSS	513	f	0	15	21	9	18	1.43	(0.51- 4.04)
WAKAI	510	m	0	21	74	4	25	1.77	(0.56- 5.67)
WU	513	f	0	37	11	6	5	2.80	(0.72- 10.97)
Totals				137	50838	25	9810		

*prospective study

REF	NRR	SEX	AD	Ys	Ws	Qs	Ps
*ENGELA	521	m	0	0.72	5.49	0.03	0.0920
SVENSS	513	f	0	0.36	3.56	0.29	0.5010
WAKAI	510	m	0	0.57	2.85	0.01	0.3335
WU	513	f	0	1.03	2.06	0.31	0.1387

	N	4
	NS	4
	Wt	13.96
Het	Chi	0.65
Het	df	3
Het	P	N.S.
Fixed	RR	1.90
	RRl	1.13
	RRu	3.21
	P	+
Random	RR	1.90
	RRl	1.13
	RRu	3.21
	P	+
Asymm	P	N.S.

Table 2H10 - 6

IESLC - Meta-analysis of Current Smoking, Age started, "Highest vs lowest"
Squamous, Any Product (or Cigarettes if Any not available)
Least adjusted

	Sex combined	male	female	Total
N		2	2	4
NS		2	2	4
Wt		8.34	5.62	13.96
Het Chi		0.04	0.59	0.65
Het df		1	1	3
Het P		N.S.	N.S.	N.S.
Fixed RR		1.95	1.83	1.90
RRl		0.99	0.80	1.13
RRu		3.85	4.18	3.21
P		(+)	N.S.	+
Random RR		1.95	1.83	1.90
RRl		0.99	0.80	1.13
RRu		3.85	4.18	3.21
P		(+)	N.S.	+
Between Chi				0.01
Between df				1
Between P				N.S.
Btwn(F) P				N.S.
Btwn(R) P				N.S.

Table 2H10 - 7

IESLC - Meta-analysis of Current Smoking, Age started, "Highest vs lowest"
Squamous, Any Product (or Cigarettes if Any not available)
Excluded studies (and stage at which they were excluded)

1	AGUDO ALDERS ARMADA AUVINE AXELSS BARBON BECHER BENHAM BLOT1 BOFFET BOUCHA BRESLO BROWN3 CARPEN CHEN CHEN2 CHIAZZ CHOI CHYOU CORREA DAMBER DARBY DESTEF DOLL DOLL2 DORGAN DOSEME FAN GAO GARCIA GARSHI GENG GER GRAHAM GUO GURSEL HAENSZ HAMMO2 HAMMON HEGMAN HU HU2 JAHN JAIN JEDRYC JOLY JUSSAW KHUDER KOO KOULUM KREUZE LAUSSM LETOUR LEVIN LIU3 LIU4 LIU5 LUBIN LUBIN2 LUO MCCONN NOTAN2 OSANN2 PERNU PEZZOT PRESCO QIAO QIAO2 RACHTA RESTRE SADOWS STASZE SUZUK2 TIZZAN TVERDA VUTUC WANG2 WIGLE WU2 WUWILL WYNDE2 WYNDE3 XU YUAN ZHANG ZHENG ZHOU
2	AKIBA AMANDU AMES BENSHL BEST BOUCOT BROSS BUFFLE CPSII DEAN2 GILLIS HUMBLE KAISE2 KATSOU KAUFMA PEZZO2 PISANI SPITZ WATSON WYNDE8
3	MCDUFF WYNDE6
5	CEDERL CPSI DEAN3 DORN GAO2 HIRAYA HOLE LIAW MATOS MIGRAN MRFITR SEGI2 SOBUE SPEIZE WYNDE7

Table 2H11 -

IESLC - Meta-analysis of Ever/current Smoking by Age started, Overview
Squamous, Any Product (or Cigarettes if Any not available)

This analysis is restricted to results for:
1) Ever/current smokers
2) Results by Age started
3) Categorical results by Age started
   Results by Age started are grouped under 2 schemes (S1, S2). Each scheme has a set of "key values". An interval is allocated to the category whose key value it includes, and intervals which include none or more than one of the key values are excluded. (Open-ended intervals are coded as 999)

S1	key value	maximum range
1	26	19+
2	18	15-25
3	14	1-17

S2	key value	maximum range
1	30	27+
2	26	23-29
3	22	19-25
4	18	15-21
5	14	11-17
6	10	1-13

4) Squamous (or near equivalent)
5) Results complete enough for use in metaanalysis

Within each study, results are then selected (in the following order of preference, within each sex) for:
6) SMKSTA: ever, current
7) PRODUCT: all/unspec, cigarettes regardless of other products, cigarettes only
8) CIGTYPE: all/unspecified, MC regardless of HR, MC only
9) (not applicable)
10) DENOM: never smoked anything, never smoked cigarettes, never any + low, never cigs + low
11) Followup period (YF, prospective studies): whole study (coded as 0) or longest available
12) LCtype: squamous or nearest available, but not adeno. (q = squamous, s = small,
          a = adeno, KI = Kreyberg I, u = undifferentiated)
13) Race: all or nearest available, otherwise by race (wh or w = white, bl or b = black, hi = hispanic
          ch = chinese, jap = japanese, haw = hawaiian, w+o = white + oriental, sca = scandinavian, as = asian)
14) For overlapping studies: principal rather than subsidiary studies
Finally by Age: whole study (coded as 0) if available, otherwise by widest available age group
and then for single sex results (m, f) in preference to results for both sexes combined (c).

Results adjusted (AD) for the most potential confounders are then chosen in Sections -1 to -3 and results adjusted for the least confounders in Sections -4 to -6. (Those least adjusted results which actually differ from the most adjusted are marked 'x' in column X in Section -4)

Section -7 shows excluded studies, together with the stage (as above) at which no qualifying results were found.

Section -8 lists the potentially overlapping studies which have been included (1=principal, 2=subsidiary).

Section -9 lists any results which would have been included in preference except that they had data not complete enough for use in meta-analysis, with their significance (yes/no), if known, and any further comment as entered on the database. It also lists as "gap" any categories for which no data were presented by the original authors.

In addition to those mentioned above, the following fields, levels and abbreviations are used:
* or nk = not known, n = no, y = yes, ot = other
ev = ever, cu = current, nev = never
all/unspec = all or unspecified, cig+/-ot = cigarettes irrespective of other products (cigar, pipe etc)
MC = manufactured cigarettes, HR = hand-rolled cigarettes
exL, exH = range of exposure (low and high) in the smoking group, in terms of Age started
REF: 6-character study reference
NRR: number of the RR on the database within the study
ST : study type (CC = case control, pr or prosp = prospective)
NLC: number of lung cancer cases in whole study
R : risky occupational population (n = no, m = mining, o = other risky)
VB : national cigarette type (V = at least 75% Virginia, bl = at least 75% blended, ot = other)
P : any proxy use
H : full histological confirmation
De : derivation of RR/CI (or = original, st = standard method, ot = other method of estimation)

Table 2H11 - 1

IESLC - Meta-analysis of Ever/current Smoking by Age started, Overview
Squamous, Any Product (or Cigarettes if Any not available)
Most adjusted

REF	NRR	SEX	AGEL	AGEH	RACE	YF	LC TYPE	LOC	START	ST	NLC	R	VB	P	H	AD	SM	PRODUCT	exL	exH	S1	S2	DENOM	De
BARBON	574	m	0	0	all	-	q	Eu:wst	1979	CC	755	n	bl	y	y	1	ev	all/unsp	20	999	1	0	nev any	or
BARBON	575	m	0	0	all	-	q	Eu:wst	1979	CC	755	n	bl	y	y	1	ev	all/unsp	15	19	2	4	nev any	or
BARBON	576	m	0	0	all	-	q	Eu:wst	1979	CC	755	n	bl	y	y	1	ev	all/unsp	1	14	3	0	nev any	or
BENHAM	514	m	0	0	all	-	KI	Eu:wst	1976	CC	1625	n	bl	n	y	0	ev	cig only	25	999	1	0	nev any	st
BENHAM	515	m	0	0	all	-	KI	Eu:wst	1976	CC	1625	n	bl	n	y	0	ev	cig only	20	24	0	3	nev any	st
BENHAM	516	m	0	0	all	-	KI	Eu:wst	1976	CC	1625	n	bl	n	y	0	ev	cig only	17	19	2	4	nev any	st
BENHAM	517	m	0	0	all	-	KI	Eu:wst	1976	CC	1625	n	bl	n	y	0	ev	cig only	1	16	3	0	nev any	st
ENGELA	517	m	0	0	all	0	q	Eu:Sca	1964	pr	435	n	bl	n	n	0	cu	cig+/-ot	30	999	0	1	nev cigs	st
ENGELA	518	m	0	0	all	0	q	Eu:Sca	1964	pr	435	n	bl	n	n	0	cu	cig+/-ot	20	29	1	0	nev cigs	st
ENGELA	519	m	0	0	all	0	q	Eu:Sca	1964	pr	435	n	bl	n	n	0	cu	cig+/-ot	1	19	0	0	nev cigs	st
HAENSZ	501	f	0	0	all	-	q+u	NAmer	1955	CC	158	n	bl	n	y	0	ev	cig+/-ot	25	999	1	0	nev any	st
HAENSZ	502	f	0	0	all	-	q+u	NAmer	1955	CC	158	n	bl	n	y	0	ev	cig+/-ot	1	24	0	0	nev any	st
JEDRYC	528	m	0	0	all	-	q	Eu:est	1980	CC	1630	n	bl	y	n	0	ev	cig+/-ot	19	999	1	0	nev any	st
JEDRYC	529	m	0	0	all	-	q	Eu:est	1980	CC	1630	n	bl	y	n	0	ev	cig+/-ot	17	18	2	4	nev any	st
JEDRYC	530	m	0	0	all	-	q	Eu:est	1980	CC	1630	n	bl	y	n	0	ev	cig+/-ot	1	16	3	0	nev any	st
LUO	512	c	0	0	all	-	q	As:Chi	1990	CC	102	n	ot	n	y	20	ev	cig+/-ot	40	999	0	0	nev cigs	or
LUO	513	c	0	0	all	-	q	As:Chi	1990	CC	102	n	ot	n	y	20	ev	cig+/-ot	20	39	1	0	nev cigs	or
LUO	514	c	0	0	all	-	q	As:Chi	1990	CC	102	n	ot	n	y	20	ev	cig+/-ot	0	19	0	0	nev cigs	or
MATOS	616	m	0	0	all	-	q	SCAmer	1994	CC	200	n	bl	n	n	2	ev	cig+/-ot	20	999	1	0	nev any	or
MATOS	617	m	0	0	all	-	q	SCAmer	1994	CC	200	n	bl	n	n	2	ev	cig+/-ot	15	19	2	4	nev any	or
MATOS	618	m	0	0	all	-	q	SCAmer	1994	CC	200	n	bl	n	n	2	ev	cig+/-ot	1	14	3	0	nev any	or
PEZZOT	543	m	0	0	all	-	q	SCAmer	1987	CC	215	n	bl	n	y	0	ev	cig only	19	999	1	0	nev cigs	ot
PEZZOT	544	m	0	0	all	-	q	SCAmer	1987	CC	215	n	bl	n	y	0	ev	cig only	14	18	0	0	nev cigs	ot
PEZZOT	545	m	0	0	all	-	q	SCAmer	1987	CC	215	n	bl	n	y	0	ev	cig only	1	13	0	6	nev cigs	ot
SVENSS	509	f	0	0	all	-	q	Eu:Sca	1983	CC	210	n	bl	n	n	0	cu	all/unsp	26	999	1	0	nev any	st
SVENSS	510	f	0	0	all	-	q	Eu:Sca	1983	CC	210	n	bl	n	n	0	cu	all/unsp	19	25	0	3	nev any	st
SVENSS	511	f	0	0	all	-	q	Eu:Sca	1983	CC	210	n	bl	n	n	0	cu	all/unsp	0	18	0	0	nev any	st
WAKAI	506	m	0	0	all	-	q	As:Jap	1988	CC	333	n	bl	n	y	0	cu	cig+/-ot	30	999	0	1	nev any	st
WAKAI	507	m	0	0	all	-	q	As:Jap	1988	CC	333	n	bl	n	y	0	cu	cig+/-ot	20	29	1	0	nev any	st
WAKAI	508	m	0	0	all	-	q	As:Jap	1988	CC	333	n	bl	n	y	0	cu	cig+/-ot	1	19	0	0	nev any	st
WU	533	f	0	0	wh	-	q	NAmer	1981	CC	220	n	bl	n	y	2	cu	all/unsp	25	999	1	0	nev any	or
WU	534	f	0	0	wh	-	q	NAmer	1981	CC	220	n	bl	n	y	2	cu	all/unsp	19	24	0	3	nev any	or
WU	535	f	0	0	wh	-	q	NAmer	1981	CC	220	n	bl	n	y	2	cu	all/unsp	0	18	0	0	nev any	or
WYNDE6	608	m	0	0	wh	-	q	NAmer	1969	CC	4423	n	bl	n	y	0	ev	cig+/-ot	21	999	1	0	nev cigs	st
WYNDE6	609	m	0	0	wh	-	q	NAmer	1969	CC	4423	n	bl	n	y	0	ev	cig+/-ot	18	20	2	4	nev cigs	st
WYNDE6	610	m	0	0	wh	-	q	NAmer	1969	CC	4423	n	bl	n	y	0	ev	cig+/-ot	1	17	3	0	nev cigs	st
WYNDE6	632	f	0	0	wh	-	q	NAmer	1969	CC	4423	n	bl	n	y	0	ev	cig+/-ot	21	999	1	0	nev cigs	st
WYNDE6	633	f	0	0	wh	-	q	NAmer	1969	CC	4423	n	bl	n	y	0	ev	cig+/-ot	18	20	2	4	nev cigs	st
WYNDE6	634	f	0	0	wh	-	q	NAmer	1969	CC	4423	n	bl	n	y	0	ev	cig+/-ot	1	17	3	0	nev cigs	st
ZHENG	520	m	0	0	all	-	q	As:Chi	1982	CC	540	n	ot	*	y	0	ev	cig+/-ot	30	999	0	1	nev cigs	st
ZHENG	521	m	0	0	all	-	q	As:Chi	1982	CC	540	n	ot	*	y	0	ev	cig+/-ot	20	29	1	0	nev cigs	st
ZHENG	522	m	0	0	all	-	q	As:Chi	1982	CC	540	n	ot	*	y	0	ev	cig+/-ot	1	19	0	0	nev cigs	st
ZHENG	527	f	0	0	all	-	q	As:Chi	1982	CC	540	n	ot	*	y	0	ev	cig+/-ot	30	999	0	1	nev cigs	st
ZHENG	528	f	0	0	all	-	q	As:Chi	1982	CC	540	n	ot	*	y	0	ev	cig+/-ot	1	29	0	0	nev cigs	st

Cigarette type is all/unspec for all RRs

In this overview table, subtotals and Qs values may be invalid and should be ignored

Table 2H11 - 2

IESLC - Meta-analysis of Ever/current Smoking by Age started, Overview
Squamous, Any Product (or Cigarettes if Any not available)
Most adjusted

REF	NRR	SEX	AD	Number Exposed Case	Number Exposed Cont	Non-exposed Case	Non-exposed Cont	RR	95.00%CI
BARBON	574	m	1	64	-	6	-	9.40	(4.20- 23.40)
BARBON	575	m	1	145	-	6	-	13.70	(5.90- 31.70)
BARBON	576	m	1	52	-	6	-	71.30	(27.60- 184.00)
Subtotal BARBON								19.26	(11.59- 32.00)
BENHAM	514	m	0	98	137	24	481	14.34	(8.83- 23.29)
BENHAM	515	m	0	323	398	24	481	16.26	(10.52- 25.14)
BENHAM	516	m	0	311	373	24	481	16.71	(10.80- 25.86)
BENHAM	517	m	0	342	341	24	481	20.10	(12.99- 31.10)
Subtotal BENHAM								16.86	(13.48- 21.08)
*ENGELA	517	m	0	6	9762	3	58716	12.03	(3.01- 48.09)
*ENGELA	518	m	0	17	30195	3	58716	11.02	(3.23- 37.60)
*ENGELA	519	m	0	64	50732	3	58716	24.69	(7.76- 78.58)
Subtotal ENGELA								15.41	(7.50- 31.65)
HAENSZ	501	f	0	32	66	44	236	2.60	(1.53- 4.42)
HAENSZ	502	f	0	24	37	44	236	3.48	(1.90- 6.38)
Subtotal HAENSZ								2.95	(1.98- 4.40)
JEDRYC	528	m	0	134	502	6	289	12.86	(5.60- 29.50)
JEDRYC	529	m	0	96	221	6	289	20.92	(9.00- 48.62)
JEDRYC	530	m	0	53	111	6	289	23.00	(9.61- 55.01)
Subtotal JEDRYC								18.20	(11.15- 29.70)
LUO	512	c	20	1	-	5	-	3.10	(0.30- 40.20)
LUO	513	c	20	19	-	5	-	10.50	(2.30- 48.60)
LUO	514	c	20	14	-	5	-	12.40	(2.70- 57.70)
Subtotal LUO								9.22	(3.43- 24.79)
MATOS	616	m	2	6	-	3	-	3.30	(0.80- 13.90)
MATOS	617	m	2	25	-	3	-	7.90	(2.30- 27.10)
MATOS	618	m	2	16	-	3	-	7.20	(2.00- 25.90)
Subtotal MATOS								6.00	(2.82- 12.75)
PEZZOT	543	m	0	10	105	0	116	23.19~	(1.34- 400.59)
PEZZOT	544	m	0	45	145	0	116	72.86~	(4.44-1195.36)
PEZZOT	545	m	0	30	67	0	116	105.28~	(6.34-1749.54)
Subtotal PEZZOT								56.74	(11.15- 288.86)
SVENSS	509	f	0	9	18	5	120	12.00	(3.61- 39.85)
SVENSS	510	f	0	18	14	5	120	30.86	(9.92- 96.00)
SVENSS	511	f	0	15	21	5	120	17.14	(5.63- 52.19)
Subtotal SVENSS								18.79	(9.69- 36.45)
WAKAI	506	m	0	4	25	2	65	5.20	(0.90- 30.19)
WAKAI	507	m	0	61	183	2	65	10.83	(2.58- 45.57)
WAKAI	508	m	0	21	74	2	65	9.22	(2.08- 40.85)
Subtotal WAKAI								8.47	(3.47- 20.65)
WU	533	f	2	6	-	2	-	7.80	(0.80- 73.70)
WU	534	f	2	18	-	2	-	47.10	(4.40- 498.50)
WU	535	f	2	37	-	2	-	115.70	(9.80-1371.20)
Subtotal WU								32.22	(8.24- 125.94)
WYNDE6	608	m	0	44	92	9	589	31.30	(14.79- 66.26)
WYNDE6	609	m	0	81	139	9	589	38.14	(18.69- 77.81)
WYNDE6	610	m	0	262	301	9	589	56.96	(28.89- 112.31)
WYNDE6	632	f	0	32	90	12	673	19.94	(9.91- 40.11)
WYNDE6	633	f	0	49	94	12	673	29.23	(15.00- 56.97)
WYNDE6	634	f	0	72	91	12	673	44.37	(23.18- 84.93)
Subtotal WYNDE6								35.10	(26.47- 46.53)
ZHENG	520	m	0	13	66	4	94	4.63	(1.45- 14.82)
ZHENG	521	m	0	81	109	4	94	17.46	(6.17- 49.46)
ZHENG	522	m	0	62	43	4	94	33.88	(11.58- 99.14)
ZHENG	527	f	0	9	16	33	184	3.14	(1.28- 7.69)
ZHENG	528	f	0	34	28	33	184	6.77	(3.63- 12.62)
Subtotal ZHENG								7.97	(5.36- 11.85)
Partial Totals				2855	94596	421	184750		

*prospective study

~ With 0.5 adjustment for zero

Table 2H11 - 2

IESLC - Meta-analysis of Ever/current Smoking by Age started, Overview
Squamous, Any Product (or Cigarettes if Any not available)
Most adjusted

REF	NRR	SEX	AD	Ys	Ws	Qs	Ps
BARBON	574	m	1	2.24	5.21	1.08	0.0000
BARBON	575	m	1	2.62	5.44	0.03	0.0000
BARBON	576	m	1	4.27	4.27	10.54	0.0000
Subtotal BARBON				2.96	14.91	11.65	
BENHAM	514	m	0	2.66	16.33	0.02	0.0000
BENHAM	515	m	0	2.79	20.26	0.18	0.0000
BENHAM	516	m	0	2.82	20.14	0.29	0.0000
BENHAM	517	m	0	3.00	20.16	1.87	0.0000
Subtotal BENHAM				2.82	76.89	2.36	
*ENGELA	517	m	0	2.49	2.00	0.09	0.0004
*ENGELA	518	m	0	2.40	2.55	0.22	0.0001
*ENGELA	519	m	0	3.21	2.87	0.75	0.0000
Subtotal ENGELA				2.74	7.42	1.06	
HAENSZ	501	f	0	0.96	13.63	41.28	0.0004
HAENSZ	502	f	0	1.25	10.45	21.95	0.0001
Subtotal HAENSZ				1.08	24.08	63.23	
JEDRYC	528	m	0	2.55	5.57	0.11	0.0000
JEDRYC	529	m	0	3.04	5.40	0.64	0.0000
JEDRYC	530	m	0	3.14	5.05	0.98	0.0000
Subtotal JEDRYC				2.90	16.02	1.73	
LUO	512	c	20	1.13	0.64	1.57	0.3652
LUO	513	c	20	2.35	1.65	0.20	0.0025
LUO	514	c	20	2.52	1.64	0.05	0.0013
Subtotal LUO				2.22	3.93	1.82	
MATOS	616	m	2	1.19	1.89	4.25	0.1012
MATOS	617	m	2	2.07	2.53	1.00	0.0010
MATOS	618	m	2	1.97	2.34	1.22	0.0025
Subtotal MATOS				1.79	6.75	6.47	
PEZZOT	543	m	0	3.14	0.47	0.09	0.0306
PEZZOT	544	m	0	4.29	0.49	1.24	0.0027
PEZZOT	545	m	0	4.66	0.49	1.87	0.0012
Subtotal PEZZOT				4.04	1.45	3.21	
SVENSS	509	f	0	2.48	2.67	0.12	0.0000
SVENSS	510	f	0	3.43	2.98	1.60	0.0000
SVENSS	511	f	0	2.84	3.10	0.07	0.0000
Subtotal SVENSS				2.93	8.75	1.79	
WAKAI	506	m	0	1.65	1.24	1.36	0.0662
WAKAI	507	m	0	2.38	1.86	0.18	0.0012
WAKAI	508	m	0	2.22	1.73	0.39	0.0034
Subtotal WAKAI				2.14	4.84	1.93	
WU	533	f	2	2.05	0.75	0.31	0.0750
WU	534	f	2	3.85	0.69	0.92	0.0014
WU	535	f	2	4.75	0.63	2.66	0.0002
Subtotal WU				3.47	2.07	3.89	
WYNDE6	608	m	0	3.44	6.83	3.82	0.0000
WYNDE6	609	m	0	3.64	7.56	6.75	0.0000
WYNDE6	610	m	0	4.04	8.34	15.11	0.0000
WYNDE6	632	f	0	2.99	7.86	0.69	0.0000
WYNDE6	633	f	0	3.38	8.63	3.98	0.0000
WYNDE6	634	f	0	3.79	9.12	10.96	0.0000
Subtotal WYNDE6				3.56	48.33	41.32	
ZHENG	520	m	0	1.53	2.84	3.84	0.0099
ZHENG	521	m	0	2.86	3.54	0.10	0.0000
ZHENG	522	m	0	3.52	3.33	2.28	0.0000
ZHENG	527	f	0	1.14	4.78	11.52	0.0125
ZHENG	528	f	0	1.91	9.91	6.08	0.0000
Subtotal ZHENG				2.08	24.40	23.82	

N	44
NS	13

Table 2H11 - 3

IESLC - Meta-analysis of Ever/current Smoking by Age started, Overview
Squamous, Any Product (or Cigarettes if Any not available)
Most adjusted

	Sex			
	combined	male	female	Total
N	3	28	13	44
NS	1	9	5	15

In this overview table, other than the "N" rows, entries in the "absent" and "Total" columns may be invalid and should be ignored

	Age started (broad categories)				
	absent	19+k26	15-25k18	1-17k14	Total
N	18	14	6	6	44
NS	9	13	5	5	32
Wt	70.07	70.81	49.70	49.28	239.85
Het Chi	49.57	43.67	8.01	16.38	164.28
Het df	17	13	5	5	43
Het P	***	***	N.S.	**	***
Fixed RR	10.39	10.42	20.15	29.91	14.82
RRl	8.22	8.25	15.26	22.62	13.06
RRu	13.13	13.15	26.61	39.54	16.82
P	+++	+++	+++	+++	+++
Random RR	12.12	11.06	20.28	31.07	14.89
RRl	7.59	6.87	13.92	17.93	11.32
RRu	19.35	17.81	29.53	53.85	19.57
P	+++	+++	+++	+++	+++

	Age started (narrow categories)							
	absent	27+k30	23-29k26	19-25k22	15-21k18	11-17k14	1-13k10	Total
N	30	4		3	6		1	44
NS	13	3		3	5		1	25
Wt	154.89	10.85		23.93	49.70		0.49	239.85
Het Chi	129.72	2.56		1.71	8.01		0.00	164.28
Het df	29	3		2	5		0	43
Het P	***	N.S.		N.S.	N.S.		N.S.	***
Fixed RR	14.01	4.71		18.16	20.15		105.28	14.82
RRl	11.97	2.60		12.17	15.26		6.34	13.06
RRu	16.40	8.54		27.11	26.61		1749.54	16.82
P	+++	+++		+++	+++		++	+++
Random RR	14.79	4.71		18.16	20.28		105.28	14.89
RRl	10.26	2.60		12.17	13.92		6.34	11.32
RRu	21.34	8.54		27.11	29.53		1749.54	19.57
P	+++	+++		+++	+++		++	+++

Table 2H11 - 3

IESLC - Meta-analysis of Ever/current Smoking by Age started, Overview
Squamous, Any Product (or Cigarettes if Any not available)
Most adjusted

MALES

	Age started (broad categories) absent	19+k26	15-25k18	1-17k14	Total
N	9	9	5	5	28
NS	5	9	5	5	24
Wt	35.25	44.25	41.06	40.16	160.72
Het Chi	11.88	9.79	6.56	14.64	53.10
Het df	8	8	4	4	27
Het P	N.S.	N.S.	N.S.	**	**
Fixed RR	15.69	14.17	18.64	27.35	18.32
RRl	11.28	10.56	13.72	20.07	15.69
RRu	21.82	19.03	25.30	37.26	21.38
P	+++	+++	+++	+++	+++
Random RR	15.33	13.87	18.61	28.44	17.70
RRl	9.28	9.79	12.12	14.64	13.87
RRu	25.35	19.66	28.56	55.25	22.58
P	+++	+++	+++	+++	+++

	absent	Age started (narrow categories) 27+k30	23-29k26	19-25k22	15-21k18	11-17k14	1-13k10	Total
N	18	3		1	5		1	28
NS	9	3		1	5		1	19
Wt	92.83	6.08		20.26	41.06		0.49	160.72
Het Chi	36.52	1.15		0.00	6.56		0.00	53.10
Het df	17	2		0	4		0	27
Het P	**	N.S.		N.S.	N.S.		N.S.	**
Fixed RR	19.78	6.49		16.26	18.64		105.28	18.32
RRl	16.14	2.93		10.52	13.72		6.34	15.69
RRu	24.24	14.38		25.14	25.30		1749.54	21.38
P	+++	+++		+++	+++		++	+++
Random RR	19.06	6.49		16.26	18.61		105.28	17.70
RRl	13.67	2.93		10.52	12.12		6.34	13.87
RRu	26.58	14.38		25.14	28.56		1749.54	22.58
P	+++	+++		+++	+++		++	+++

FEMALES

	Age started (broad categories) absent	19+k26	15-25k18	1-17k14	Total
N	7	4	1	1	13
NS	4	4	1	1	8
Wt	32.54	24.91	8.63	9.12	75.20
Het Chi	24.65	22.20	0.00	0.00	88.38
Het df	6	3	0	0	12
Het P	***	***	N.S.	N.S.	***
Fixed RR	6.75	6.02	29.23	44.37	9.66
RRl	4.79	4.07	15.00	23.18	7.71
RRu	9.51	8.92	56.97	84.93	12.11
P	+++	+++	+++	+++	+++
Random RR	10.63	8.18	29.23	44.37	12.72
RRl	4.82	2.30	15.00	23.18	6.54
RRu	23.45	29.06	56.97	84.93	24.75
P	+++	++	+++	+++	+++

Table 2H11 - 3

IESLC - Meta-analysis of Ever/current Smoking by Age started, Overview
Squamous, Any Product (or Cigarettes if Any not available)
Most adjusted

FEMALES

	absent	Age started (narrow categories) 27+k30	23-29k26	19-25k22	15-21k18	11-17k14	1-13k10	Total
N	9	1		2	1			13
NS	5	1		2	1			8
Wt	58.12	4.78		3.67	8.63			75.20
Het Chi	64.70	0.00		0.10	0.00			88.38
Het df	8	0		1	0			12
Het P	***	N.S.		N.S.	N.S.			***
Fixed RR	8.31	3.14		33.40	29.23			9.66
RRl	6.43	1.28		12.00	15.00			7.71
RRu	10.75	7.69		92.92	56.97			12.11
P	+++	+		+++	+++			+++
Random RR	11.31	3.14		33.40	29.23			12.72
RRl	5.09	1.28		12.00	15.00			6.54
RRu	25.13	7.69		92.92	56.97			24.75
P	+++	+		+++	+++			+++

Table 2H11 - 4

IESLC - Meta-analysis of Ever/current Smoking by Age started, Overview
Squamous, Any Product (or Cigarettes if Any not available)
Least adjusted

REF	NRR	X	SEX	AGEL	AGEH	RACE	YF	LC TYPE	LOC	START	ST	NLC	R	VB	P	H	AD	SM	PRODUCT	exL	exH	S1	S2	DENOM	De
BARBON	569	x	m	0	0	all	-	q	Eu:wst	1979	CC	755	n	bl	y	y	0	ev	all/unsp	20	999	1	0	nev any	st
BARBON	570	x	m	0	0	all	-	q	Eu:wst	1979	CC	755	n	bl	y	y	0	ev	all/unsp	15	19	2	4	nev any	st
BARBON	571	x	m	0	0	all	-	q	Eu:wst	1979	CC	755	n	bl	y	y	0	ev	all/unsp	1	14	3	0	nev any	st
BENHAM	514		m	0	0	all	-	KI	Eu:wst	1976	CC	1625	n	bl	n	y	0	ev	cig only	25	999	1	0	nev any	st
BENHAM	515		m	0	0	all	-	KI	Eu:wst	1976	CC	1625	n	bl	n	y	0	ev	cig only	20	24	0	3	nev any	st
BENHAM	516		m	0	0	all	-	KI	Eu:wst	1976	CC	1625	n	bl	n	y	0	ev	cig only	17	19	2	4	nev any	st
BENHAM	517		m	0	0	all	-	KI	Eu:wst	1976	CC	1625	n	bl	n	y	0	ev	cig only	1	16	3	0	nev any	st
ENGELA	517		m	0	0	all	0	q	Eu:Sca	1964	pr	435	n	bl	n	n	0	cu	cig+/-ot	30	999	0	1	nev cigs	st
ENGELA	518		m	0	0	all	0	q	Eu:Sca	1964	pr	435	n	bl	n	n	0	cu	cig+/-ot	20	29	1	0	nev cigs	st
ENGELA	519		m	0	0	all	0	q	Eu:Sca	1964	pr	435	n	bl	n	n	0	cu	cig+/-ot	1	19	0	0	nev cigs	st
HAENSZ	501		f	0	0	all	-	q+u	NAmer	1955	CC	158	n	bl	n	y	0	ev	cig+/-ot	25	999	1	0	nev any	st
HAENSZ	502		f	0	0	all	-	q+u	NAmer	1955	CC	158	n	bl	n	y	0	ev	cig+/-ot	1	24	0	0	nev any	st
JEDRYC	528		m	0	0	all	-	q	Eu:est	1980	CC	1630	n	bl	y	n	0	ev	cig+/-ot	19	999	1	0	nev any	st
JEDRYC	529		m	0	0	all	-	q	Eu:est	1980	CC	1630	n	bl	y	n	0	ev	cig+/-ot	17	18	2	4	nev any	st
JEDRYC	530		m	0	0	all	-	q	Eu:est	1980	CC	1630	n	bl	y	n	0	ev	cig+/-ot	1	16	3	0	nev any	st
LUO	507	x	c	0	0	all	-	q	As:Chi	1990	CC	102	n	ot	n	y	0	ev	cig+/-ot	40	999	0	0	nev cigs	st
LUO	508	x	c	0	0	all	-	q	As:Chi	1990	CC	102	n	ot	n	y	0	ev	cig+/-ot	20	39	1	0	nev cigs	st
LUO	509	x	c	0	0	all	-	q	As:Chi	1990	CC	102	n	ot	n	y	0	ev	cig+/-ot	0	19	0	0	nev cigs	st
MATOS	611	x	m	0	0	all	-	q	SCAmer	1994	CC	200	n	bl	n	n	0	ev	cig+/-ot	20	999	1	0	nev any	st
MATOS	612	x	m	0	0	all	-	q	SCAmer	1994	CC	200	n	bl	n	n	0	ev	cig+/-ot	15	19	2	4	nev any	st
MATOS	613	x	m	0	0	all	-	q	SCAmer	1994	CC	200	n	bl	n	n	0	ev	cig+/-ot	1	14	3	0	nev any	st
PEZZOT	543		m	0	0	all	-	q	SCAmer	1987	CC	215	n	bl	n	y	0	ev	cig only	19	999	1	0	nev cigs	ot
PEZZOT	544		m	0	0	all	-	q	SCAmer	1987	CC	215	n	bl	n	y	0	ev	cig only	14	18	0	0	nev cigs	ot
PEZZOT	545		m	0	0	all	-	q	SCAmer	1987	CC	215	n	bl	n	y	0	ev	cig only	1	13	0	6	nev cigs	ot
SVENSS	509		f	0	0	all	-	q	Eu:Sca	1983	CC	210	n	bl	n	n	0	cu	all/unsp	26	999	1	0	nev any	st
SVENSS	510		f	0	0	all	-	q	Eu:Sca	1983	CC	210	n	bl	n	n	0	cu	all/unsp	19	25	0	3	nev any	st
SVENSS	511		f	0	0	all	-	q	Eu:Sca	1983	CC	210	n	bl	n	n	0	cu	all/unsp	0	18	0	0	nev any	st
WAKAI	506		m	0	0	all	-	q	As:Jap	1988	CC	333	n	bl	n	y	0	cu	cig+/-ot	30	999	0	1	nev any	st
WAKAI	507		m	0	0	all	-	q	As:Jap	1988	CC	333	n	bl	n	y	0	cu	cig+/-ot	20	29	1	0	nev any	st
WAKAI	508		m	0	0	all	-	q	As:Jap	1988	CC	333	n	bl	n	y	0	cu	cig+/-ot	1	19	0	0	nev any	st
WU	509	x	f	0	0	wh	-	q	NAmer	1981	CC	220	n	bl	n	y	0	cu	all/unsp	25	999	1	0	nev any	st
WU	510	x	f	0	0	wh	-	q	NAmer	1981	CC	220	n	bl	n	y	0	cu	all/unsp	19	24	0	3	nev any	st
WU	511	x	f	0	0	wh	-	q	NAmer	1981	CC	220	n	bl	n	y	0	cu	all/unsp	0	18	0	0	nev any	st
WYNDE6	608		m	0	0	wh	-	q	NAmer	1969	CC	4423	n	bl	n	y	0	ev	cig+/-ot	21	999	1	0	nev cigs	st
WYNDE6	609		m	0	0	wh	-	q	NAmer	1969	CC	4423	n	bl	n	y	0	ev	cig+/-ot	18	20	2	4	nev cigs	st
WYNDE6	610		m	0	0	wh	-	q	NAmer	1969	CC	4423	n	bl	n	y	0	ev	cig+/-ot	1	17	3	0	nev cigs	st
WYNDE6	632		f	0	0	wh	-	q	NAmer	1969	CC	4423	n	bl	n	y	0	ev	cig+/-ot	21	999	1	0	nev cigs	st
WYNDE6	633		f	0	0	wh	-	q	NAmer	1969	CC	4423	n	bl	n	y	0	ev	cig+/-ot	18	20	2	4	nev cigs	st
WYNDE6	634		f	0	0	wh	-	q	NAmer	1969	CC	4423	n	bl	n	y	0	ev	cig+/-ot	1	17	3	0	nev cigs	st
ZHENG	520		m	0	0	all	-	q	As:Chi	1982	CC	540	n	ot	*	y	0	ev	cig+/-ot	30	999	0	1	nev cigs	st
ZHENG	521		m	0	0	all	-	q	As:Chi	1982	CC	540	n	ot	*	y	0	ev	cig+/-ot	20	29	1	0	nev cigs	st
ZHENG	522		m	0	0	all	-	q	As:Chi	1982	CC	540	n	ot	*	y	0	ev	cig+/-ot	1	19	0	0	nev cigs	st
ZHENG	527		f	0	0	all	-	q	As:Chi	1982	CC	540	n	ot	*	y	0	ev	cig+/-ot	30	999	0	1	nev cigs	st
ZHENG	528		f	0	0	all	-	q	As:Chi	1982	CC	540	n	ot	*	y	0	ev	cig+/-ot	1	29	0	0	nev cigs	st

Cigarette type is all/unspec for all RRs

In this overview table, subtotals and Qs values may be invalid and should be ignored

Table 2H11 - 5

IESLC - Meta-analysis of Ever/current Smoking by Age started, Overview
Squamous, Any Product (or Cigarettes if Any not available)
Least adjusted

REF	NRR	SEX	AD	Number Exposed Case	Number Exposed Cont	Non-exposed Case	Non-exposed Cont	RR	95.00%CI
BARBON	569	m	0	64	207	6	188	9.69	(4.10- 22.89)
BARBON	570	m	0	145	337	6	188	13.48	(5.84- 31.10)
BARBON	571	m	0	52	23	6	188	70.84	(27.41- 183.08)
Subtotal BARBON								19.28	(11.62- 32.01)
BENHAM	514	m	0	98	137	24	481	14.34	(8.83- 23.29)
BENHAM	515	m	0	323	398	24	481	16.26	(10.52- 25.14)
BENHAM	516	m	0	311	373	24	481	16.71	(10.80- 25.86)
BENHAM	517	m	0	342	341	24	481	20.10	(12.99- 31.10)
Subtotal BENHAM								16.86	(13.48- 21.08)
*ENGELA	517	m	0	6	9762	3	58716	12.03	(3.01- 48.09)
*ENGELA	518	m	0	17	30195	3	58716	11.02	(3.23- 37.60)
*ENGELA	519	m	0	64	50732	3	58716	24.69	(7.76- 78.58)
Subtotal ENGELA								15.41	(7.50- 31.65)
HAENSZ	501	f	0	32	66	44	236	2.60	(1.53- 4.42)
HAENSZ	502	f	0	24	37	44	236	3.48	(1.90- 6.38)
Subtotal HAENSZ								2.95	(1.98- 4.40)
JEDRYC	528	m	0	134	502	6	289	12.86	(5.60- 29.50)
JEDRYC	529	m	0	96	221	6	289	20.92	(9.00- 48.62)
JEDRYC	530	m	0	53	111	6	289	23.00	(9.61- 55.01)
Subtotal JEDRYC								18.20	(11.15- 29.70)
LUO	507	c	0	1	8	5	51	1.28	(0.13- 12.37)
LUO	508	c	0	19	38	5	51	5.10	(1.75- 14.88)
LUO	509	c	0	14	20	5	51	7.14	(2.27- 22.43)
Subtotal LUO								5.07	(2.42- 10.62)
MATOS	611	m	0	6	73	3	110	3.01	(0.73- 12.43)
MATOS	612	m	0	25	120	3	110	7.64	(2.24- 26.01)
MATOS	613	m	0	16	90	3	110	6.52	(1.84- 23.08)
Subtotal MATOS								5.58	(2.64- 11.78)
PEZZOT	543	m	0	10	105	0	116	23.19~	(1.34- 400.59)
PEZZOT	544	m	0	45	145	0	116	72.86~	(4.44-1195.36)
PEZZOT	545	m	0	30	67	0	116	105.28~	(6.34-1749.54)
Subtotal PEZZOT								56.74	(11.15- 288.86)
SVENSS	509	f	0	9	18	5	120	12.00	(3.61- 39.85)
SVENSS	510	f	0	18	14	5	120	30.86	(9.92- 96.00)
SVENSS	511	f	0	15	21	5	120	17.14	(5.63- 52.19)
Subtotal SVENSS								18.79	(9.69- 36.45)
WAKAI	506	m	0	4	25	2	65	5.20	(0.90- 30.19)
WAKAI	507	m	0	61	183	2	65	10.83	(2.58- 45.57)
WAKAI	508	m	0	21	74	2	65	9.22	(2.08- 40.85)
Subtotal WAKAI								8.47	(3.47- 20.65)
WU	509	f	0	6	5	2	30	18.00	(2.80- 115.56)
WU	510	f	0	18	7	2	30	38.57	(7.21- 206.25)
WU	511	f	0	37	11	2	30	50.45	(10.37- 245.38)
Subtotal WU								34.61	(13.01- 92.08)
WYNDE6	608	m	0	44	92	9	589	31.30	(14.79- 66.26)
WYNDE6	609	m	0	81	139	9	589	38.14	(18.69- 77.81)
WYNDE6	610	m	0	262	301	9	589	56.96	(28.89- 112.31)
WYNDE6	632	f	0	32	90	12	673	19.94	(9.91- 40.11)
WYNDE6	633	f	0	49	94	12	673	29.23	(15.00- 56.97)
WYNDE6	634	f	0	72	91	12	673	44.37	(23.18- 84.93)
Subtotal WYNDE6								35.10	(26.47- 46.53)
ZHENG	520	m	0	13	66	4	94	4.63	(1.45- 14.82)
ZHENG	521	m	0	81	109	4	94	17.46	(6.17- 49.46)
ZHENG	522	m	0	62	43	4	94	33.88	(11.58- 99.14)
ZHENG	527	f	0	9	16	33	184	3.14	(1.28- 7.69)
ZHENG	528	f	0	34	28	33	184	6.77	(3.63- 12.62)
Subtotal ZHENG								7.97	(5.36- 11.85)
Totals				2855	95535	421	185887		

*prospective study ~ With 0.5 adjustment for zero

Table 2H11 - 5

IESLC - Meta-analysis of Ever/current Smoking by Age started, Overview
Squamous, Any Product (or Cigarettes if Any not available)
Least adjusted

REF	NRR	SEX	AD	Ys	Ws	Qs	Ps
BARBON	569	m	0	2.27	5.20	0.86	0.0000
BARBON	570	m	0	2.60	5.50	0.03	0.0000
BARBON	571	m	0	4.26	4.26	10.67	0.0000
Subtotal BARBON				2.96	14.96	11.57	
BENHAM	514	m	0	2.66	16.33	0.00	0.0000
BENHAM	515	m	0	2.79	20.26	0.25	0.0000
BENHAM	516	m	0	2.82	20.14	0.38	0.0000
BENHAM	517	m	0	3.00	20.16	2.10	0.0000
Subtotal BENHAM				2.82	76.89	2.74	
*ENGELA	517	m	0	2.49	2.00	0.07	0.0004
*ENGELA	518	m	0	2.40	2.55	0.20	0.0001
*ENGELA	519	m	0	3.21	2.87	0.80	0.0000
Subtotal ENGELA				2.74	7.42	1.07	
HAENSZ	501	f	0	0.96	13.63	40.42	0.0004
HAENSZ	502	f	0	1.25	10.45	21.41	0.0001
Subtotal HAENSZ				1.08	24.08	61.83	
JEDRYC	528	m	0	2.55	5.57	0.09	0.0000
JEDRYC	529	m	0	3.04	5.40	0.71	0.0000
JEDRYC	530	m	0	3.14	5.05	1.06	0.0000
Subtotal JEDRYC				2.90	16.02	1.86	
LUO	507	c	0	0.24	0.74	4.41	0.8340
LUO	508	c	0	1.63	3.35	3.68	0.0029
LUO	509	c	0	1.97	2.93	1.49	0.0008
Subtotal LUO				1.62	7.03	9.58	
MATOS	611	m	0	1.10	1.91	4.74	0.1271
MATOS	612	m	0	2.03	2.56	1.06	0.0011
MATOS	613	m	0	1.87	2.40	1.55	0.0037
Subtotal MATOS				1.72	6.88	7.36	
PEZZOT	543	m	0	3.14	0.47	0.10	0.0306
PEZZOT	544	m	0	4.29	0.49	1.27	0.0027
PEZZOT	545	m	0	4.66	0.49	1.90	0.0012
Subtotal PEZZOT				4.04	1.45	3.28	
SVENSS	509	f	0	2.48	2.67	0.10	0.0000
SVENSS	510	f	0	3.43	2.98	1.68	0.0000
SVENSS	511	f	0	2.84	3.10	0.08	0.0000
Subtotal SVENSS				2.93	8.75	1.87	
WAKAI	506	m	0	1.65	1.24	1.32	0.0662
WAKAI	507	m	0	2.38	1.86	0.16	0.0012
WAKAI	508	m	0	2.22	1.73	0.36	0.0034
Subtotal WAKAI				2.14	4.84	1.84	
WU	509	f	0	2.89	1.11	0.05	0.0023
WU	510	f	0	3.65	1.37	1.30	0.0000
WU	511	f	0	3.92	1.54	2.37	0.0000
Subtotal WU				3.54	4.01	3.72	
WYNDE6	608	m	0	3.44	6.83	4.01	0.0000
WYNDE6	609	m	0	3.64	7.56	7.01	0.0000
WYNDE6	610	m	0	4.04	8.34	15.53	0.0000
WYNDE6	632	f	0	2.99	7.86	0.78	0.0000
WYNDE6	633	f	0	3.38	8.63	4.20	0.0000
WYNDE6	634	f	0	3.79	9.12	11.33	0.0000
Subtotal WYNDE6				3.56	48.33	42.85	
ZHENG	520	m	0	1.53	2.84	3.72	0.0099
ZHENG	521	m	0	2.86	3.54	0.12	0.0000
ZHENG	522	m	0	3.52	3.33	2.38	0.0000
ZHENG	527	f	0	1.14	4.78	11.25	0.0125
ZHENG	528	f	0	1.91	9.91	5.81	0.0000
Subtotal ZHENG				2.08	24.40	23.27	

N	44
NS	13

Table 2H11 - 6

IESLC - Meta-analysis of Ever/current Smoking by Age started, Overview
Squamous, Any Product (or Cigarettes if Any not available)
Least adjusted

	Sex			
	combined	male	female	Total
N	3	28	13	44
NS	1	9	5	15

In this overview table, other than the "N" rows, entries in the "absent" and "Total" columns may be invalid and should be ignored

	Age started (broad categories)				
	absent	19+k26	15-25k18	1-17k14	Total
N	18	14	6	6	44
NS	9	13	5	5	32
Wt	73.05	72.88	49.79	49.33	245.06
Het Chi	53.22	46.03	8.28	17.16	172.83
Het df	17	13	5	5	43
Het P	***	***	N.S.	**	***
Fixed RR	10.31	10.19	20.06	29.69	14.55
RRl	8.20	8.10	15.19	22.46	12.84
RRu	12.97	12.81	26.48	39.25	16.49
P	+++	+++	+++	+++	+++
Random RR	11.65	10.79	20.14	30.58	14.40
RRl	7.33	6.71	13.74	17.44	10.95
RRu	18.51	17.35	29.52	53.64	18.94
P	+++	+++	+++	+++	+++

	Age started (narrow categories)							
	absent	27+k30	23-29k26	19-25k22	15-21k18	11-17k14	1-13k10	Total
N	30	4		3	6		1	44
NS	13	3		3	5		1	25
Wt	159.32	10.85		24.61	49.79		0.49	245.06
Het Chi	137.24	2.56		1.85	8.28		0.00	172.83
Het df	29	3		2	5		0	43
Het P	***	N.S.		N.S.	N.S.		N.S.	***
Fixed RR	13.62	4.71		18.44	20.06		105.28	14.55
RRl	11.66	2.60		12.42	15.19		6.34	12.84
RRu	15.91	8.54		27.38	26.48		1749.54	16.49
P	+++	+++		+++	+++		++	+++
Random RR	14.02	4.71		18.44	20.14		105.28	14.40
RRl	9.72	2.60		12.42	13.74		6.34	10.95
RRu	20.21	8.54		27.38	29.52		1749.54	18.94
P	+++	+++		+++	+++		++	+++

Table 2H11 - 6

IESLC - Meta-analysis of Ever/current Smoking by Age started, Overview
Squamous, Any Product (or Cigarettes if Any not available)
Least adjusted

MALES

		Age started (broad categories)				
		absent	19+k26	15-25k18	1-17k14	Total
	N	9	9	5	5	28
	NS	5	9	5	5	24
	Wt	35.25	44.26	41.16	40.21	160.89
Het	Chi	11.88	10.24	6.80	15.35	54.24
Het	df	8	8	4	4	27
Het	P	N.S.	N.S.	N.S.	**	**
Fixed	RR	15.69	14.16	18.53	27.11	18.25
	RRl	11.28	10.54	13.66	19.90	15.63
	RRu	21.82	19.01	25.16	36.92	21.29
	P	+++	+++	+++	+++	+++
Random	RR	15.33	13.78	18.45	27.89	17.56
	RRl	9.28	9.62	11.93	14.15	13.73
	RRu	25.35	19.75	28.54	54.95	22.46
	P	+++	+++	+++	+++	+++

			Age started (narrow categories)						
		absent	27+k30	23-29k26	19-25k22	15-21k18	11-17k14	1-13k10	Total
	N	18	3		1	5		1	28
	NS	9	3		1	5		1	19
	Wt	92.90	6.08		20.26	41.16		0.49	160.89
Het	Chi	37.49	1.15		0.00	6.80		0.00	54.24
Het	df	17	2		0	4		0	27
Het	P	**	N.S.		N.S.	N.S.		N.S.	**
Fixed	RR	19.70	6.49		16.26	18.53		105.28	18.25
	RRl	16.07	2.93		10.52	13.66		6.34	15.63
	RRu	24.14	14.38		25.14	25.16		1749.54	21.29
	P	+++	+++		+++	+++		++	+++
Random	RR	18.89	6.49		16.26	18.45		105.28	17.56
	RRl	13.49	2.93		10.52	11.93		6.34	13.73
	RRu	26.45	14.38		25.14	28.54		1749.54	22.46
	P	+++	+++		+++	+++		++	+++

FEMALES

		Age started (broad categories)				
		absent	19+k26	15-25k18	1-17k14	Total
	N	7	4	1	1	13
	NS	4	4	1	1	8
	Wt	34.13	25.27	8.63	9.12	77.15
Het	Chi	27.18	23.44	0.00	0.00	89.90
Het	df	6	3	0	0	12
Het	P	***	***	N.S.	N.S.	***
Fixed	RR	7.23	6.27	29.23	44.37	10.00
	RRl	5.17	4.25	15.00	23.18	8.00
	RRu	10.11	9.26	56.97	84.93	12.49
	P	+++	+++	+++	+++	+++
Random	RR	11.17	9.54	29.23	44.37	13.30
	RRl	5.14	2.70	15.00	23.18	6.97
	RRu	24.29	33.79	56.97	84.93	25.40
	P	+++	+++	+++	+++	+++

Table 2H11 - 6

IESLC - Meta-analysis of Ever/current Smoking by Age started, Overview
Squamous, Any Product (or Cigarettes if Any not available)
Least adjusted

FEMALES

		Age started (narrow categories)						
	absent	27+k30	23-29k26	19-25k22	15-21k18	11-17k14	1-13k10	Total
N	9	1		2	1			13
NS	5	1		2	1			8
Wt	59.39	4.78		4.35	8.63			77.15
Het Chi	65.92	0.00		0.05	0.00			89.90
Het df	8	0		1	0			12
Het P	***	N.S.		N.S.	N.S.			***
Fixed RR	8.60	3.14		33.10	29.23			10.00
RRl	6.67	1.28		12.93	15.00			8.00
RRu	11.09	7.69		84.72	56.97			12.49
P	+++	+		+++	+++			+++
Random RR	11.95	3.14		33.10	29.23			13.30
RRl	5.49	1.28		12.93	15.00			6.97
RRu	26.04	7.69		84.72	56.97			25.40
P	+++	+		+++	+++			+++

Table 2H11 - 7

IESLC - Meta-analysis of Ever/current Smoking by Age started, Overview
Squamous, Any Product (or Cigarettes if Any not available)
Excluded studies (and stage at which they were excluded)

1	BECHER BLOT1 BROWN3 CARPEN CHYOU DARBY DOLL2 GARCIA GRAHAM GURSEL HAMMO2 JAHN JAIN LAUSSM PRESCO QIAO TVERDA WIGLE WYNDE3
2	AKIBA AMANDU AMES AXELSS BENSHL BEST BOUCHA BOUCOT BROSS CHEN CPSII DEAN2 DESTEF DORGAN DOSEME FAN GARSHI GER GILLIS HAMMON HUMBLE JUSSAW KAISE2 KATSOU KAUFMA KOO KREUZE LEVIN MCCONN NOTAN2 OSANN2 PEZZO2 PISANI RESTRE SADOWS VUTUC WANG2 WATSON WU2 WUWILL WYNDE2 WYNDE8 XU ZHOU
3	GUO MCDUFF SPITZ STASZE ZHANG
4	AGUDO ARMADA AUVINE BOFFET BRESLO BUFFLE CEDERL CHEN2 CHIAZZ CHOI CORREA CPSI DAMBER DEAN3 DOLL DORN GAO GAO2 GENG HIRAYA HOLE HU HU2 JOLY KOULUM LETOUR LIAW LIU3 LIU4 LIU5 LUBIN LUBIN2 MIGRAN MRFITR PERNU QIAO2 RACHTA SEGI2 SOBUE SPEIZE SUZUK2 TIZZAN WYNDE7 YUAN
5	ALDERS
10	HEGMAN KHUDER

Table 2H11 - 8
Potentially overlapping studies

REF	REFGP	PRINC	OVERLAP/LINK
BENHAM	LUBIN2	2	Subset of Lubin2
WYNDE6	WYNDE6	1	WYNDE5/6/7/8

Table 2H12 -

IESLC - Meta-analysis of Ever/current Smoking, Age started, "Low"
Squamous, Any Product (or Cigarettes if Any not available)

This analysis is restricted to results for:
1) Ever/current smokers
2) Results by Age started
3) Categorical results by Age started
4) Squamous (or near equivalent)
5) Results complete enough for use in metaanalysis

Within each study, results are then selected (in the following order of preference, within each sex) for:
6) SMKSTA: ever, current
7) PRODUCT: all/unspec, cigarettes regardless of other products, cigarettes only
8) CIGTYPE: all/unspecified, MC regardless of HR, MC only
9) (not applicable)
10) DENOM: never smoked anything, never smoked cigarettes, never any + low, never cigs + low
11) Followup period (YF, prospective studies): whole study (coded as 0) or longest available
12) LCtype: squamous or nearest available, but not adeno. (q = squamous, s = small,
a = adeno, KI = Kreyberg I, u = undifferentiated)
13) Race: all or nearest available, otherwise by race (wh or w = white, bl or b = black, hi = hispanic
ch = chinese, jap = japanese, haw = hawaiian, w+o = white + oriental, sca = scandinavian, as = asian)
14) Age started "low" in key scheme 1 (key value 26, maximum range 19+)
15) For overlapping studies: principal rather than subsidiary studies
Finally by Age: whole study (coded as 0) if available, otherwise by widest available age group
and then for single sex results (m, f) in preference to results for both sexes combined (c).

Results adjusted (AD) for the most potential confounders are then chosen in Sections -1 to -3
and results adjusted for the least confounders in Sections -4 to -6. (Those least adjusted results which
actually differ from the most adjusted are marked 'x' in column X in Section -4)

Section -7 shows excluded studies, together with the stage (as above) at which no qualifying
results were found.

Section -8 lists the potentially overlapping studies which have been included (1=principal, 2=subsidiary).

Section -9 lists any results which would have been included in preference except that they had data not complete
enough for use in meta-analysis, with their significance (yes/no), if known, and any further comment as entered
on the database. It also lists as "gap" any categories for which no data were presented by the original authors.

In addition to those mentioned above, the following fields, levels and abbreviations are used:
* or nk = not known, n = no, y = yes, ot = other
ev = ever, cu = current, nev = never
all/unspec = all or unspecified, cig+/-ot = cigarettes irrespective of other products (cigar, pipe etc)
MC = manufactured cigarettes, HR = hand-rolled cigarettes
exL, exH = range of exposure (low and high) in the smoking group, in terms of Age started
REF: 6-character study reference
NRR: number of the RR on the database within the study
ST : study type (CC = case control, pr or prosp = prospective)
NLC: number of lung cancer cases in whole study
R : risky occupational population (n = no, m = mining, o = other risky)
VB : national cigarette type (V = at least 75% Virginia, bl = at least 75% blended, ot = other)
P : any proxy use
H : full histological confirmation
De : derivation of RR/CI (or = original, st = standard method, ot = other method of estimation)

Table 2H12 - 1

IESLC - Meta-analysis of Ever/current Smoking, Age started, "Low"
Squamous, Any Product (or Cigarettes if Any not available)
Most adjusted

REF	NRR	SEX	AGEL	AGEH	RACE	YF	LC TYPE	LOC	START	ST	NLC	R	VB	P	H	AD	SM	PRODUCT	exL	exH	DENOM	De
BARBON	574	m	0	0	all	-	q	Eu:wst	1979	CC	755	n	bl	y	y	1	ev	all/unsp	20	999	nev any	or
BENHAM	514	m	0	0	all	-	KI	Eu:wst	1976	CC	1625	n	bl	n	y	0	ev	cig only	25	999	nev any	st
ENGELA	518	m	0	0	all	0	q	Eu:Sca	1964	pr	435	n	bl	n	n	0	cu	cig+/-ot	20	29	nev cigs	st
HAENSZ	501	f	0	0	all	-	q+u	NAmer	1955	CC	158	n	bl	n	y	0	ev	cig+/-ot	25	999	nev any	st
JEDRYC	528	m	0	0	all	-	q	Eu:est	1980	CC	1630	n	bl	y	n	0	ev	cig+/-ot	19	999	nev any	st
LUO	513	c	0	0	all	-	q	As:Chi	1990	CC	102	n	ot	n	y	20	ev	cig+/-ot	20	39	nev cigs	or
MATOS	616	m	0	0	all	-	q	SCAmer	1994	CC	200	n	bl	n	n	2	ev	cig+/-ot	20	999	nev any	or
PEZZOT	543	m	0	0	all	-	q	SCAmer	1987	CC	215	n	bl	n	y	0	ev	cig only	19	999	nev cigs	ot
SVENSS	509	f	0	0	all	-	q	Eu:Sca	1983	CC	210	n	bl	n	n	0	cu	all/unsp	26	999	nev any	st
WAKAI	507	m	0	0	all	-	q	As:Jap	1988	CC	333	n	bl	n	y	0	cu	cig+/-ot	20	29	nev any	st
WU	533	f	0	0	wh	-	q	NAmer	1981	CC	220	n	bl	n	y	2	cu	all/unsp	25	999	nev any	or
WYNDE6	608	m	0	0	wh	-	q	NAmer	1969	CC	4423	n	bl	n	y	0	ev	cig+/-ot	21	999	nev cigs	st
WYNDE6	632	f	0	0	wh	-	q	NAmer	1969	CC	4423	n	bl	n	y	0	ev	cig+/-ot	21	999	nev cigs	st
ZHENG	521	m	0	0	all	-	q	As:Chi	1982	CC	540	n	ot	*	y	0	ev	cig+/-ot	20	29	nev cigs	st

Cigarette type is all/unspec for all RRs

Table 2H12 - 2

IESLC - Meta-analysis of Ever/current Smoking, Age started, "Low"
Squamous, Any Product (or Cigarettes if Any not available)
Most adjusted

REF	NRR	SEX	AD	Number Exposed Case	Cont	Non-exposed Case	Cont	RR	95.00%CI
BARBON	574	m	1	64	-	6	-	9.40	(4.20- 23.40)
BENHAM	514	m	0	98	137	24	481	14.34	(8.83- 23.29)
*ENGELA	518	m	0	17	30195	3	58716	11.02	(3.23- 37.60)
HAENSZ	501	f	0	32	66	44	236	2.60	(1.53- 4.42)
JEDRYC	528	m	0	134	502	6	289	12.86	(5.60- 29.50)
LUO	513	c	20	19	-	5	-	10.50	(2.30- 48.60)
MATOS	616	m	2	6	-	3	-	3.30	(0.80- 13.90)
PEZZOT	543	m	0	10	105	0	116	23.19~	(1.34- 400.59)
SVENSS	509	f	0	9	18	5	120	12.00	(3.61- 39.85)
WAKAI	507	m	0	61	183	2	65	10.83	(2.58- 45.57)
WU	533	f	2	6	-	2	-	7.80	(0.80- 73.70)
WYNDE6	608	m	0	44	92	9	589	31.30	(14.79- 66.26)
WYNDE6	632	f	0	32	90	12	673	19.94	(9.91- 40.11)
Subtotal WYNDE6								24.59	(14.75- 41.00)
ZHENG	521	m	0	81	109	4	94	17.46	(6.17- 49.46)
Partial Totals				613	31497	125	61379		

*prospective study ~ With 0.5 adjustment for zero

REF	NRR	SEX	AD	Ys	Ws	Qs	Ps
BARBON	574	m	1	2.24	5.21	0.05	0.0000
BENHAM	514	m	0	2.66	16.33	1.67	0.0000
*ENGELA	518	m	0	2.40	2.55	0.01	0.0001
HAENSZ	501	f	0	0.96	13.63	26.24	0.0004
JEDRYC	528	m	0	2.55	5.57	0.25	0.0000
LUO	513	c	20	2.35	1.65	0.00	0.0025
MATOS	616	m	2	1.19	1.89	2.49	0.1012
PEZZOT	543	m	0	3.14	0.47	0.30	0.0306
SVENSS	509	f	0	2.48	2.67	0.05	0.0000
WAKAI	507	m	0	2.38	1.86	0.00	0.0012
WU	533	f	2	2.05	0.75	0.06	0.0750
WYNDE6	608	m	0	3.44	6.83	8.27	0.0000
WYNDE6	632	f	0	2.99	7.86	3.32	0.0000
Subtotal WYNDE6				3.20	14.69	11.59	
ZHENG	521	m	0	2.86	3.54	0.95	0.0000

	N	14
	NS	13
	Wt	70.81
Het	Chi	43.67
Het	df	13
Het	P	***
Fixed	RR	10.42
	RRl	8.25
	RRu	13.15
	P	+++
Random	RR	11.06
	RRl	6.87
	RRu	17.81
	P	+++
Asymm	P	N.S.

Table 2H12 - 3

IESLC - Meta-analysis of Ever/current Smoking, Age started, "Low"
Squamous, Any Product (or Cigarettes if Any not available)
Most adjusted

	Sex			
	combined	male	female	Total
N	1	9	4	14
NS	1	9	4	14
Wt	1.65	44.25	24.91	70.81
Het Chi	0.00	9.79	22.20	43.67
Het df	0	8	3	13
Het P	N.S.	N.S.	***	***
Fixed RR	10.50	14.17	6.02	10.42
RRl	2.28	10.56	4.07	8.25
RRu	48.27	19.03	8.92	13.15
P	++	+++	+++	+++
Random RR	10.50	13.87	8.18	11.06
RRl	2.28	9.79	2.30	6.87
RRu	48.27	19.66	29.06	17.81
P	++	+++	++	+++
Between Chi				11.67
Between df				2
Between P				**
Btwn(F) P				N.S.
Btwn(R) P				N.S.

	Lung cancer type					
	q	q+s	q+u	KI	not a	Total
N	12		1	1		14
NS	11		1	1		13
Wt	40.85		13.63	16.33		70.81
Het Chi	11.16		0.00	0.00		43.67
Het df	11		0	0		13
Het P	N.S.		N.S.	N.S.		***
Fixed RR	14.56		2.60	14.34		10.42
RRl	10.72		1.53	8.83		8.25
RRu	19.79		4.42	23.29		13.15
P	+++		+++	+++		+++
Random RR	14.51		2.60	14.34		11.06
RRl	10.64		1.53	8.83		6.87
RRu	19.79		4.42	23.29		17.81
P	+++		+++	+++		+++
Between Chi						32.50
Between df						2
Between P						***
Btwn(F) P						***
Btwn(R) P						***

	Location								
	NAmer	UK	Scand	othEur	China	Japan	othAs	other	Total
N	4		2	3	2	1		2	14
NS	3		2	3	2	1		2	13
Wt	29.07		5.22	27.10	5.20	1.86		2.36	70.81
Het Chi	36.44		0.01	0.70	0.29	0.00		1.44	43.67
Het df	3		1	2	1	0		1	13
Het P	***		N.S.	N.S.	N.S.	N.S.		N.S.	***
Fixed RR	8.33		11.51	12.93	14.86	10.83		4.88	10.42
RRl	5.79		4.88	8.87	6.29	2.58		1.36	8.25
RRu	11.98		27.15	18.84	35.10	45.57		17.49	13.15
P	+++		+++	+++	+++	++		+	+++
Random RR	10.81		11.51	12.93	14.86	10.83		5.83	11.06
RRl	2.62		4.88	8.87	6.29	2.58		1.03	6.87
RRu	44.50		27.15	18.84	35.10	45.57		33.12	17.81
P	+++		+++	+++	+++	++		+	+++
Between Chi									4.79
Between df									5
Between P									N.S.
Btwn(F) P									N.S.
Btwn(R) P									N.S.

Table 2H12 - 3

IESLC - Meta-analysis of Ever/current Smoking, Age started, "Low"
Squamous, Any Product (or Cigarettes if Any not available)
Most adjusted

Detailed Country in "other Europe"

	multi	Germany	othWest	East	Balkans	Total
N			2	1		3
NS			2	1		3
Wt			21.53	5.57		27.10
Het Chi			0.70	0.00		0.70
Het df			1	0		2
Het P			N.S.	N.S.		N.S.
Fixed RR			12.95	12.86		12.93
RRl			8.49	5.60		8.87
RRu			19.75	29.50		18.84
P			+++	+++		+++
Random RR			12.95	12.86		12.93
RRl			8.49	5.60		8.87
RRu			19.75	29.50		18.84
P			+++	+++		+++
Between Chi						0.00
Between df						1
Between P						N.S.
Btwn(F) P						N.S.
Btwn(R) P						N.S.

Detailed Country in "other Asia"

	India	HongKong	other	Total
N				
NS				
Wt				
Het Chi				
Het df				
Het P				N.S.
Fixed RR				
RRl				
RRu				
P				+++
Random RR				
RRl				
RRu				
P				+++
Between Chi				
Between df				
Between P				N.S.
Btwn(F) P				N.S.
Btwn(R) P				N.S.

Detailed other continent

	SCAmer	Total
N	2	2
NS	2	2
Wt	2.36	2.36
Het Chi	1.44	1.44
Het df	1	1
Het P	N.S.	N.S.
Fixed RR	4.88	4.88
RRl	1.36	1.36
RRu	17.49	17.49
P	+	+
Random RR	5.83	5.83
RRl	1.03	1.03
RRu	33.12	33.12
P	+	+
Between Chi		
Between df		
Between P		N.S.
Btwn(F) P		N.S.
Btwn(R) P		N.S.

Table 2H12 - 3

IESLC - Meta-analysis of Ever/current Smoking, Age started, "Low"
Squamous, Any Product (or Cigarettes if Any not available)
Most adjusted

	Start year of study					
	<1960	1960-69	1970-79	1980-89	1990+	Total
N	1	3	2	6	2	14
NS	1	2	2	6	2	13
Wt	13.63	17.24	21.53	14.86	3.54	70.81
Het Chi	0.00	2.14	0.70	0.74	1.18	43.67
Het df	0	2	1	5	1	13
Het P	N.S.	N.S.	N.S.	N.S.	N.S.	***
Fixed RR	2.60	21.84	12.95	13.28	5.67	10.42
RRl	1.53	13.62	8.49	7.99	2.00	8.25
RRu	4.42	35.01	19.75	22.09	16.06	13.15
P	+++	+++	+++	+++	++	+++
Random RR	2.60	21.73	12.95	13.28	5.70	11.06
RRl	1.53	13.27	8.49	7.99	1.84	6.87
RRu	4.42	35.56	19.75	22.09	17.68	17.81
P	+++	+++	+++	+++	++	+++
Between Chi						38.90
Between df						4
Between P						***
Btwn(F) P						***
Btwn(R) P						***

	Study type (1)		
	CC	other	Total
N	13	1	14
NS	12	1	13
Wt	68.26	2.55	70.81
Het Chi	43.66	0.00	43.67
Het df	12	0	13
Het P	***	N.S.	***
Fixed RR	10.39	11.02	10.42
RRl	8.20	3.23	8.25
RRu	13.18	37.60	13.15
P	+++	+++	+++
Random RR	11.06	11.02	11.06
RRl	6.67	3.23	6.87
RRu	18.35	37.60	17.81
P	+++	+++	+++
Between Chi			0.01
Between df			1
Between P			N.S.
Btwn(F) P			N.S.
Btwn(R) P			N.S.

	Study type (2)			
	CC	prosp	other	Total
N	13	1		14
NS	12	1		13
Wt	68.26	2.55		70.81
Het Chi	43.66	0.00		43.67
Het df	12	0		13
Het P	***	N.S.		***
Fixed RR	10.39	11.02		10.42
RRl	8.20	3.23		8.25
RRu	13.18	37.60		13.15
P	+++	+++		+++
Random RR	11.06	11.02		11.06
RRl	6.67	3.23		6.87
RRu	18.35	37.60		17.81
P	+++	+++		+++
Between Chi				0.01
Between df				1
Between P				N.S.
Btwn(F) P				N.S.
Btwn(R) P				N.S.

Table 2H12 - 3

IESLC - Meta-analysis of Ever/current Smoking, Age started, "Low"
Squamous, Any Product (or Cigarettes if Any not available)
Most adjusted

	Study size (number of LC cases) 100-249	250-499	500-999	1000+	Total
N	6	2	2	4	14
NS	6	2	2	3	13
Wt	21.06	4.41	8.75	36.59	70.81
Het Chi	9.14	0.00	0.81	3.62	43.67
Het df	5	1	1	3	13
Het P	N.S.	N.S.	N.S.	N.S.	***
Fixed RR	3.93	10.94	12.08	17.51	10.42
RRl	2.56	4.30	6.23	12.67	8.25
RRu	6.02	27.82	23.43	24.21	13.15
P	+++	+++	+++	+++	+++
Random RR	5.67	10.94	12.08	17.76	11.06
RRl	2.68	4.30	6.23	12.34	6.87
RRu	12.01	27.82	23.43	25.55	17.81
P	+++	+++	+++	+++	+++
Between Chi					30.09
Between df					3
Between P					***
Btwn(F) P					**
Btwn(R) P					(*)

	Risky occupational population no	mining	othRisky	Total
N	14			14
NS	13			13
Wt	70.81			70.81
Het Chi	43.67			43.67
Het df	13			13
Het P	***			***
Fixed RR	10.42			10.42
RRl	8.25			8.25
RRu	13.15			13.15
P	+++			+++
Random RR	11.06			11.06
RRl	6.87			6.87
RRu	17.81			17.81
P	+++			+++
Between Chi				
Between df				
Between P				N.S.
Btwn(F) P				N.S.
Btwn(R) P				N.S.

	National cigarette tobacco type Virginia	blended	other	Total
N		12	2	14
NS		11	2	13
Wt		65.61	5.20	70.81
Het Chi		42.67	0.29	43.67
Het df		11	1	13
Het P		***	N.S.	***
Fixed RR		10.13	14.86	10.42
RRl		7.95	6.29	8.25
RRu		12.90	35.10	13.15
P		+++	+++	+++
Random RR		10.66	14.86	11.06
RRl		6.25	6.29	6.87
RRu		18.18	35.10	17.81
P		+++	+++	+++
Between Chi				0.71
Between df				1
Between P				N.S.
Btwn(F) P				N.S.
Btwn(R) P				N.S.

Table 2H12 - 3

IESLC - Meta-analysis of Ever/current Smoking, Age started, "Low"
Squamous, Any Product (or Cigarettes if Any not available)
Most adjusted

	Any proxy use No/nk	Yes	Total
N	12	2	14
NS	11	2	13
Wt	60.03	10.78	70.81
Het Chi	43.36	0.26	43.67
Het df	11	1	13
Het P	***	N.S.	***
Fixed RR	10.31	11.05	10.42
RRl	8.00	6.08	8.25
RRu	13.27	20.08	13.15
P	+++	+++	+++
Random RR	11.06	11.05	11.06
RRl	6.24	6.08	6.87
RRu	19.60	20.08	17.81
P	+++	+++	+++
Between Chi			0.04
Between df			1
Between P			N.S.
Btwn(F) P			N.S.
Btwn(R) P			N.S.

	Full histological confirmation No	Yes	Total
N	4	10	14
NS	4	9	13
Wt	12.67	58.14	70.81
Het Chi	2.78	40.86	43.67
Het df	3	9	13
Het P	N.S.	***	***
Fixed RR	10.03	10.50	10.42
RRl	5.79	8.12	8.25
RRu	17.40	13.58	13.15
P	+++	+++	+++
Random RR	10.03	11.93	11.06
RRl	5.79	6.40	6.87
RRu	17.40	22.24	17.81
P	+++	+++	+++
Between Chi			0.02
Between df			1
Between P			N.S.
Btwn(F) P			N.S.
Btwn(R) P			N.S.

	Number of adjustment variables (1) 0	1	2+/+nk	Total
N	10	1	3	14
NS	9	1	3	13
Wt	61.31	5.21	4.29	70.81
Het Chi	40.92	0.00	1.24	43.67
Het df	9	0	2	13
Het P	***	N.S.	N.S.	***
Fixed RR	10.92	9.40	5.99	10.42
RRl	8.50	3.98	2.33	8.25
RRu	14.03	22.19	15.44	13.15
P	+++	+++	+++	+++
Random RR	12.62	9.40	5.99	11.06
RRl	7.00	3.98	2.33	6.87
RRu	22.73	22.19	15.44	17.81
P	+++	+++	+++	+++
Between Chi				1.50
Between df				2
Between P				N.S.
Btwn(F) P				N.S.
Btwn(R) P				N.S.

Table 2H12 - 3

IESLC - Meta-analysis of Ever/current Smoking, Age started, "Low"
Squamous, Any Product (or Cigarettes if Any not available)
Most adjusted

	Number of adjustment variables (2) 0	1	2	3-5	6+/+nk	Total
N	10	1	2		1	14
NS	9	1	2		1	13
Wt	61.31	5.21	2.64		1.65	70.81
Het Chi	40.92	0.00	0.40		0.00	43.67
Het df	9	0	1		0	13
Het P	***	N.S.	N.S.		N.S.	***
Fixed RR	10.92	9.40	4.22		10.50	10.42
RRl	8.50	3.98	1.26		2.28	8.25
RRu	14.03	22.19	14.10		48.27	13.15
P	+++	+++	+		++	+++
Random RR	12.62	9.40	4.22		10.50	11.06
RRl	7.00	3.98	1.26		2.28	6.87
RRu	22.73	22.19	14.10		48.27	17.81
P	+++	+++	+		++	+++
Between Chi						2.35
Between df						3
Between P						N.S.
Btwn(F) P						N.S.
Btwn(R) P						N.S.

	Smoking status ever	current	Total
N	10	4	14
NS	9	4	13
Wt	62.98	7.83	70.81
Het Chi	43.54	0.11	43.67
Het df	9	3	13
Het P	***	N.S.	***
Fixed RR	10.35	10.93	10.42
RRl	8.09	5.43	8.25
RRu	13.25	22.02	13.15
P	+++	+++	+++
Random RR	11.17	10.93	11.06
RRl	6.15	5.43	6.87
RRu	20.31	22.02	17.81
P	+++	+++	+++
Between Chi			0.02
Between df			1
Between P			N.S.
Btwn(F) P			N.S.
Btwn(R) P			N.S.

	Product all/unsp	cig+/-ot	cig only	Total
N	3	9	2	14
NS	3	8	2	13
Wt	8.63	45.38	16.80	70.81
Het Chi	0.15	40.92	0.11	43.67
Het df	2	8	1	13
Het P	N.S.	***	N.S.	***
Fixed RR	9.97	9.28	14.53	10.42
RRl	5.12	6.94	9.01	8.25
RRu	19.44	12.42	23.44	13.15
P	+++	+++	+++	+++
Random RR	9.97	10.65	14.53	11.06
RRl	5.12	5.26	9.01	6.87
RRu	19.44	21.55	23.44	17.81
P	+++	+++	+++	+++
Between Chi				2.48
Between df				2
Between P				N.S.
Btwn(F) P				N.S.
Btwn(R) P				N.S.

Table 2H12 - 3

IESLC - Meta-analysis of Ever/current Smoking, Age started, "Low"
Squamous, Any Product (or Cigarettes if Any not available)
Most adjusted

		Denominator nev any	nev cigs	Total
	N	8	6	14
	NS	8	5	13
	Wt	47.90	22.91	70.81
Het	Chi	26.13	3.04	43.67
Het	df	7	5	13
Het	P	***	N.S.	***
Fixed	RR	7.62	20.04	10.42
	RRl	5.74	13.30	8.25
	RRu	10.11	30.17	13.15
	P	+++	+++	+++
Random	RR	7.92	20.04	11.06
	RRl	4.24	13.30	6.87
	RRu	14.80	30.17	17.81
	P	+++	+++	+++
Between	Chi			14.50
Between	df			1
Between	P			***
Btwn(F)	P			*
Btwn(R)	P			*

		Derivation of RR/CI Orig	StdCalc	Other	Total
	N	4	9	1	14
	NS	4	8	1	13
	Wt	9.50	60.84	0.47	70.81
Het	Chi	1.72	40.65	0.00	43.67
Het	df	3	8	0	13
Het	P	N.S.	***	N.S.	***
Fixed	RR	7.67	10.86	23.19	10.42
	RRl	4.06	8.44	1.34	8.25
	RRu	14.49	13.96	400.59	13.15
	P	+++	+++	+	+++
Random	RR	7.67	12.36	23.19	11.06
	RRl	4.06	6.74	1.34	6.87
	RRu	14.49	22.67	400.59	17.81
	P	+++	+++	+	+++
Between	Chi				1.30
Between	df				2
Between	P				N.S.
Btwn(F)	P				N.S.
Btwn(R)	P				N.S.

Table 2H12 - 4

IESLC - Meta-analysis of Ever/current Smoking, Age started, "Low"
Squamous, Any Product (or Cigarettes if Any not available)
Least adjusted

REF	NRR	X	SEX	AGEL	AGEH	RACE	YF	LC TYPE	LOC	START	ST	NLC	R	VB	P	H	AD	SM	PRODUCT	exL	exH	DENOM	De
BARBON	569	x	m	0	0	all	-	q	Eu:wst	1979	CC	755	n	bl	y	y	0	ev	all/unsp	20	999	nev any	st
BENHAM	514		m	0	0	all	-	KI	Eu:wst	1976	CC	1625	n	bl	n	y	0	ev	cig only	25	999	nev any	st
ENGELA	518		m	0	0	all	0	q	Eu:Sca	1964	pr	435	n	bl	n	n	0	cu	cig+/-ot	20	29	nev cigs	st
HAENSZ	501		f	0	0	all	-	q+u	NAmer	1955	CC	158	n	bl	n	y	0	ev	cig+/-ot	25	999	nev any	st
JEDRYC	528		m	0	0	all	-	q	Eu:est	1980	CC	1630	n	bl	y	n	0	ev	cig+/-ot	19	999	nev any	st
LUO	508	x	c	0	0	all	-	q	As:Chi	1990	CC	102	n	ot	n	y	0	ev	cig+/-ot	20	39	nev cigs	st
MATOS	611	x	m	0	0	all	-	q	SCAmer	1994	CC	200	n	bl	n	n	0	ev	cig+/-ot	20	999	nev any	st
PEZZOT	543		m	0	0	all	-	q	SCAmer	1987	CC	215	n	bl	n	y	0	ev	cig only	19	999	nev cigs	ot
SVENSS	509		f	0	0	all	-	q	Eu:Sca	1983	CC	210	n	bl	n	n	0	cu	all/unsp	26	999	nev any	st
WAKAI	507		m	0	0	all	-	q	As:Jap	1988	CC	333	n	bl	n	y	0	cu	cig+/-ot	20	29	nev any	st
WU	509	x	f	0	0	wh	-	q	NAmer	1981	CC	220	n	bl	n	y	0	cu	all/unsp	25	999	nev any	st
WYNDE6	608		m	0	0	wh	-	q	NAmer	1969	CC	4423	n	bl	n	y	0	ev	cig+/-ot	21	999	nev cigs	st
WYNDE6	632		f	0	0	wh	-	q	NAmer	1969	CC	4423	n	bl	n	y	0	ev	cig+/-ot	21	999	nev cigs	st
ZHENG	521		m	0	0	all	-	q	As:Chi	1982	CC	540	n	ot	*	y	0	ev	cig+/-ot	20	29	nev cigs	st

Cigarette type is all/unspec for all RRs

Table 2H12 - 5

IESLC - Meta-analysis of Ever/current Smoking, Age started, "Low"
Squamous, Any Product (or Cigarettes if Any not available)
Least adjusted

REF	NRR	SEX	AD	Number Exposed Case	Exposed Cont	Non-exposed Case	Non-exposed Cont	RR	95.00%CI
BARBON	569	m	0	64	207	6	188	9.69	(4.10- 22.89)
BENHAM	514	m	0	98	137	24	481	14.34	(8.83- 23.29)
*ENGELA	518	m	0	17	30195	3	58716	11.02	(3.23- 37.60)
HAENSZ	501	f	0	32	66	44	236	2.60	(1.53- 4.42)
JEDRYC	528	m	0	134	502	6	289	12.86	(5.60- 29.50)
LUO	508	c	0	19	38	5	51	5.10	(1.75- 14.88)
MATOS	611	m	0	6	73	3	110	3.01	(0.73- 12.43)
PEZZOT	543	m	0	10	105	0	116	23.19~	(1.34- 400.59)
SVENSS	509	f	0	9	18	5	120	12.00	(3.61- 39.85)
WAKAI	507	m	0	61	183	2	65	10.83	(2.58- 45.57)
WU	509	f	0	6	5	2	30	18.00	(2.80- 115.56)
WYNDE6	608	m	0	44	92	9	589	31.30	(14.79- 66.26)
WYNDE6	632	f	0	32	90	12	673	19.94	(9.91- 40.11)
Subtotal WYNDE6								24.59	(14.75- 41.00)
ZHENG	521	m	0	81	109	4	94	17.46	(6.17- 49.46)
Totals				613	31820	125	61758		

*prospective study ~ With 0.5 adjustment for zero

REF	NRR	SEX	AD	Ys	Ws	Qs	Ps
BARBON	569	m	0	2.27	5.20	0.01	0.0000
BENHAM	514	m	0	2.66	16.33	1.91	0.0000
*ENGELA	518	m	0	2.40	2.55	0.02	0.0001
HAENSZ	501	f	0	0.96	13.63	25.40	0.0004
JEDRYC	528	m	0	2.55	5.57	0.30	0.0000
LUO	508	c	0	1.63	3.35	1.60	0.0029
MATOS	611	m	0	1.10	1.91	2.84	0.1271
PEZZOT	543	m	0	3.14	0.47	0.32	0.0306
SVENSS	509	f	0	2.48	2.67	0.07	0.0000
WAKAI	507	m	0	2.38	1.86	0.01	0.0012
WU	509	f	0	2.89	1.11	0.36	0.0023
WYNDE6	608	m	0	3.44	6.83	8.61	0.0000
WYNDE6	632	f	0	2.99	7.86	3.55	0.0000
Subtotal WYNDE6				3.20	14.69	12.16	
ZHENG	521	m	0	2.86	3.54	1.03	0.0000

	N	14
	NS	13
	Wt	72.88
Het	Chi	46.03
Het	df	13
Het	P	***
Fixed	RR	10.19
	RRl	8.10
	RRu	12.81
	P	+++
Random	RR	10.79
	RRl	6.71
	RRu	17.35
	P	+++
Asymm	P	N.S.

Table 2H12 - 6

IESLC - Meta-analysis of Ever/current Smoking, Age started, "Low"
Squamous, Any Product (or Cigarettes if Any not available)
Least adjusted

		Sex			
		combined	male	female	Total
	N	1	9	4	14
	NS	1	9	4	14
	Wt	3.35	44.26	25.27	72.88
Het	Chi	0.00	10.24	23.44	46.03
Het	df	0	8	3	13
Het	P	N.S.	N.S.	***	***
Fixed	RR	5.10	14.16	6.27	10.19
	RRl	1.75	10.54	4.25	8.10
	RRu	14.88	19.01	9.26	12.81
	P	++	+++	+++	+++
Random	RR	5.10	13.78	9.54	10.79
	RRl	1.75	9.62	2.70	6.71
	RRu	14.88	19.75	33.79	17.35
	P	++	+++	+++	+++
Between	Chi				12.34
Between	df				2
Between	P				**
Btwn(F)	P				N.S.
Btwn(R)	P				N.S.

Table 2H12 - 7

IESLC - Meta-analysis of Ever/current Smoking, Age started, "Low"
Squamous, Any Product (or Cigarettes if Any not available)
Excluded studies (and stage at which they were excluded)

1	BECHER BLOT1 BROWN3 CARPEN CHYOU DARBY DOLL2 GARCIA GRAHAM GURSEL HAMMO2 JAHN JAIN LAUSSM PRESCO QIAO TVERDA WIGLE WYNDE3
2	AKIBA AMANDU AMES AXELSS BENSHL BEST BOUCHA BOUCOT BROSS CHEN CPSII DEAN2 DESTEF DORGAN DOSEME FAN GARSHI GER GILLIS HAMMON HUMBLE JUSSAW KAISE2 KATSOU KAUFMA KOO KREUZE LEVIN MCCONN NOTAN2 OSANN2 PEZZO2 PISANI RESTRE SADOWS VUTUC WANG2 WATSON WU2 WUWILL WYNDE2 WYNDE8 XU ZHOU
3	GUO MCDUFF SPITZ STASZE ZHANG
4	AGUDO ARMADA AUVINE BOFFET BRESLO BUFFLE CEDERL CHEN2 CHIAZZ CHOI CORREA CPSI DAMBER DEAN3 DOLL DORN GAO GAO2 GENG HIRAYA HOLE HU HU2 JOLY KOULUM LETOUR LIAW LIU3 LIU4 LIU5 LUBIN LUBIN2 MIGRAN MRFITR PERNU QIAO2 RACHTA SEGI2 SOBUE SPEIZE SUZUK2 TIZZAN WYNDE7 YUAN
5	ALDERS
10	HEGMAN KHUDER

Table 2H12 - 8
Potentially overlapping studies

REF	REFGP	PRINC	OVERLAP/LINK
BENHAM	LUBIN2	2	Subset of Lubin2
WYNDE6	WYNDE6	1	WYNDE5/6/7/8

Table 2H13 -

IESLC - Meta-analysis of Ever/current Smoking, Age started, "Mid"
Squamous, Any Product (or Cigarettes if Any not available)

This analysis is restricted to results for:
1) Ever/current smokers
2) Results by Age started
3) Categorical results by Age started
4) Squamous (or near equivalent)
5) Results complete enough for use in metaanalysis

Within each study, results are then selected (in the following order of preference, within each sex) for:
6) SMKSTA: ever, current
7) PRODUCT: all/unspec, cigarettes regardless of other products, cigarettes only
8) CIGTYPE: all/unspecified, MC regardless of HR, MC only
9) (not applicable)
10) DENOM: never smoked anything, never smoked cigarettes, never any + low, never cigs + low
11) Followup period (YF, prospective studies): whole study (coded as 0) or longest available
12) LCtype: squamous or nearest available, but not adeno. (q = squamous, s = small,
a = adeno, KI = Kreyberg I, u = undifferentiated)
13) Race: all or nearest available, otherwise by race (wh or w = white, bl or b = black, hi = hispanic
ch = chinese, jap = japanese, haw = hawaiian, w+o = white + oriental, sca = scandinavian, as = asian)
14) Age started "mid" in key scheme 1 (key value 18, maximum range 15-25)
15) For overlapping studies: principal rather than subsidiary studies
Finally by Age: whole study (coded as 0) if available, otherwise by widest available age group
and then for single sex results (m, f) in preference to results for both sexes combined (c).

Results adjusted (AD) for the most potential confounders are then chosen in Sections -1 to -3
and results adjusted for the least confounders in Sections -4 to -6. (Those least adjusted results which
actually differ from the most adjusted are marked 'x' in column X in Section -4)

Section -7 shows excluded studies, together with the stage (as above) at which no qualifying
results were found.

Section -8 lists the potentially overlapping studies which have been included (1=principal, 2=subsidiary).

Section -9 lists any results which would have been included in preference except that they had data not complete
enough for use in meta-analysis, with their significance (yes/no), if known, and any further comment as entered
on the database. It also lists as "gap" any categories for which no data were presented by the original authors.

In addition to those mentioned above, the following fields, levels and abbreviations are used:
* or nk = not known, n = no, y = yes, ot = other
ev = ever, cu = current, nev = never
all/unspec = all or unspecified, cig+/-ot = cigarettes irrespective of other products (cigar, pipe etc)
MC = manufactured cigarettes, HR = hand-rolled cigarettes
exL, exH = range of exposure (low and high) in the smoking group, in terms of Age started
REF: 6-character study reference
NRR: number of the RR on the database within the study
ST : study type (CC = case control, pr or prosp = prospective)
NLC: number of lung cancer cases in whole study
R : risky occupational population (n = no, m = mining, o = other risky)
VB : national cigarette type (V = at least 75% Virginia, bl = at least 75% blended, ot = other)
P : any proxy use
H : full histological confirmation
De : derivation of RR/CI (or = original, st = standard method, ot = other method of estimation)

Table 2H13 - 1

IESLC - Meta-analysis of Ever/current Smoking, Age started, "Mid"
Squamous, Any Product (or Cigarettes if Any not available)
Most adjusted

REF	NRR	SEX	AGEL	AGEH	RACE	YF	LC TYPE	LOC	START	ST	NLC	R	VB	P	H	AD	SM	PRODUCT	exL	exH	DENOM	De
BARBON	575	m	0	0	all	-	q	Eu:wst	1979	CC	755	n	bl	y	y	1	ev	all/unsp	15	19	nev any	or
BENHAM	516	m	0	0	all	-	KI	Eu:wst	1976	CC	1625	n	bl	n	y	0	ev	cig only	17	19	nev any	st
JEDRYC	529	m	0	0	all	-	q	Eu:est	1980	CC	1630	n	bl	y	n	0	ev	cig+/-ot	17	18	nev any	st
MATOS	617	m	0	0	all	-	q	SCAmer	1994	CC	200	n	bl	n	n	2	ev	cig+/-ot	15	19	nev any	or
WYNDE6	609	m	0	0	wh	-	q	NAmer	1969	CC	4423	n	bl	n	y	0	ev	cig+/-ot	18	20	nev cigs	st
WYNDE6	633	f	0	0	wh	-	q	NAmer	1969	CC	4423	n	bl	n	y	0	ev	cig+/-ot	18	20	nev cigs	st

Cigarette type is all/unspec for all RRs

Table 2H13 - 2

IESLC - Meta-analysis of Ever/current Smoking, Age started, "Mid"
Squamous, Any Product (or Cigarettes if Any not available)
Most adjusted

REF	NRR	SEX	AD	Number Exposed Case	Cont	Non-exposed Case	Cont	RR	95.00%CI
BARBON	575	m	1	145	-	6	-	13.70	(5.90- 31.70)
BENHAM	516	m	0	311	373	24	481	16.71	(10.80- 25.86)
JEDRYC	529	m	0	96	221	6	289	20.92	(9.00- 48.62)
MATOS	617	m	2	25	-	3	-	7.90	(2.30- 27.10)
WYNDE6	609	m	0	81	139	9	589	38.14	(18.69- 77.81)
WYNDE6	633	f	0	49	94	12	673	29.23	(15.00- 56.97)
Subtotal WYNDE6								33.10	(20.33- 53.87)
Partial Totals				707	827	60	2032		

*prospective study

REF	NRR	SEX	AD	Ys	Ws	Qs	Ps
BARBON	575	m	1	2.62	5.44	0.81	0.0000
BENHAM	516	m	0	2.82	20.14	0.71	0.0000
JEDRYC	529	m	0	3.04	5.40	0.01	0.0000
MATOS	617	m	2	2.07	2.53	2.21	0.0010
WYNDE6	609	m	0	3.64	7.56	3.07	0.0000
WYNDE6	633	f	0	3.38	8.63	1.19	0.0000
Subtotal WYNDE6				3.50	16.19	4.27	

	N	6
	NS	5
	Wt	49.70
Het	Chi	8.01
Het	df	5
Het	P	N.S.
Fixed	RR	20.15
	RRl	15.26
	RRu	26.61
	P	+++
Random	RR	20.28
	RRl	13.92
	RRu	29.53
	P	+++
Asymm	P	N.S.

Table 2H13 - 3

IESLC - Meta-analysis of Ever/current Smoking, Age started, "Mid"
Squamous, Any Product (or Cigarettes if Any not available)
Most adjusted

	Sex			
	combined	male	female	Total
N		5	1	6
NS		5	1	5
Wt		41.06	8.63	49.70
Het Chi		6.56	0.00	8.01
Het df		4	0	5
Het P		N.S.	N.S.	N.S.
Fixed RR		18.64	29.23	20.15
RRl		13.72	15.00	15.26
RRu		25.30	56.97	26.61
P		+++	+++	+++
Random RR		18.61	29.23	20.28
RRl		12.12	15.00	13.92
RRu		28.56	56.97	29.53
P		+++	+++	+++
Between Chi				1.45
Between df				1
Between P				N.S.
Btwn(F) P				N.S.
Btwn(R) P				N.S.

Too few RRs for analysis by factor

Table 2H13 - 4

IESLC - Meta-analysis of Ever/current Smoking, Age started, "Mid"
Squamous, Any Product (or Cigarettes if Any not available)
Least adjusted

REF	NRR	X	SEX	AGEL	AGEH	RACE	YF	LC TYPE	LOC	START	ST	NLC	R	VB	P	H	AD	SM	PRODUCT	exL	exH	DENOM	De
BARBON	570	x	m	0	0	all	-	q	Eu:wst	1979	CC	755	n	bl	y	y	0	ev	all/unsp	15	19	nev any	st
BENHAM	516		m	0	0	all	-	KI	Eu:wst	1976	CC	1625	n	bl	n	y	0	ev	cig only	17	19	nev any	st
JEDRYC	529		m	0	0	all	-	q	Eu:est	1980	CC	1630	n	bl	y	n	0	ev	cig+/-ot	17	18	nev any	st
MATOS	612	x	m	0	0	all	-	q	SCAmer	1994	CC	200	n	bl	n	n	0	ev	cig+/-ot	15	19	nev any	st
WYNDE6	609		m	0	0	wh	-	q	NAmer	1969	CC	4423	n	bl	n	y	0	ev	cig+/-ot	18	20	nev cigs	st
WYNDE6	633		f	0	0	wh	-	q	NAmer	1969	CC	4423	n	bl	n	y	0	ev	cig+/-ot	18	20	nev cigs	st

Cigarette type is all/unspec for all RRs

Table 2H13 - 5

IESLC - Meta-analysis of Ever/current Smoking, Age started, "Mid"
Squamous, Any Product (or Cigarettes if Any not available)
Least adjusted

REF	NRR	SEX	AD	Number Exposed Case	Cont	Non-exposed Case	Cont	RR	95.00%CI
BARBON	570	m	0	145	337	6	188	13.48	(5.84- 31.10)
BENHAM	516	m	0	311	373	24	481	16.71	(10.80- 25.86)
JEDRYC	529	m	0	96	221	6	289	20.92	(9.00- 48.62)
MATOS	612	m	0	25	120	3	110	7.64	(2.24- 26.01)
WYNDE6	609	m	0	81	139	9	589	38.14	(18.69- 77.81)
WYNDE6	633	f	0	49	94	12	673	29.23	(15.00- 56.97)
Subtotal WYNDE6								33.10	(20.33- 53.87)
Totals				707	1284	60	2330		

*prospective study

REF	NRR	SEX	AD	Ys	Ws	Qs	Ps
BARBON	570	m	0	2.60	5.50	0.87	0.0000
BENHAM	516	m	0	2.82	20.14	0.67	0.0000
JEDRYC	529	m	0	3.04	5.40	0.01	0.0000
MATOS	612	m	0	2.03	2.56	2.39	0.0011
WYNDE6	609	m	0	3.64	7.56	3.12	0.0000
WYNDE6	633	f	0	3.38	8.63	1.22	0.0000
Subtotal WYNDE6				3.50	16.19	4.34	

	N	6
	NS	5
	Wt	49.79
Het	Chi	8.28
Het	df	5
Het	P	N.S.
Fixed	RR	20.06
	RRl	15.19
	RRu	26.48
	P	+++
Random	RR	20.14
	RRl	13.74
	RRu	29.52
	P	+++
Asymm	P	N.S.

Table 2H13 - 6

IESLC - Meta-analysis of Ever/current Smoking, Age started, "Mid"
Squamous, Any Product (or Cigarettes if Any not available)
Least adjusted

	Sex combined	male	female	Total
N		5	1	6
NS		5	1	5
Wt		41.16	8.63	49.79
Het Chi		6.80	0.00	8.28
Het df		4	0	5
Het P		N.S.	N.S.	N.S.
Fixed RR		18.53	29.23	20.06
RRl		13.66	15.00	15.19
RRu		25.16	56.97	26.48
P		+++	+++	+++
Random RR		18.45	29.23	20.14
RRl		11.93	15.00	13.74
RRu		28.54	56.97	29.52
P		+++	+++	+++
Between Chi				1.48
Between df				1
Between P				N.S.
Btwn(F) P				N.S.
Btwn(R) P				N.S.

Table 2H13 - 7

IESLC - Meta-analysis of Ever/current Smoking, Age started, "Mid"
Squamous, Any Product (or Cigarettes if Any not available)
Excluded studies (and stage at which they were excluded)

Stage	Studies
1	BECHER BLOT1 BROWN3 CARPEN CHYOU DARBY DOLL2 GARCIA GRAHAM GURSEL HAMMO2 JAHN JAIN LAUSSM PRESCO QIAO TVERDA WIGLE WYNDE3
2	AKIBA AMANDU AMES AXELSS BENSHL BEST BOUCHA BOUCOT BROSS CHEN CPSII DEAN2 DESTEF DORGAN DOSEME FAN GARSHI GER GILLIS HAMMON HUMBLE JUSSAW KAISE2 KATSOU KAUFMA KOO KREUZE LEVIN MCCONN NOTAN2 OSANN2 PEZZO2 PISANI RESTRE SADOWS VUTUC WANG2 WATSON WU2 WUWILL WYNDE2 WYNDE8 XU ZHOU
3	GUO MCDUFF SPITZ STASZE ZHANG
4	AGUDO ARMADA AUVINE BOFFET BRESLO BUFFLE CEDERL CHEN2 CHIAZZ CHOI CORREA CPSI DAMBER DEAN3 DOLL DORN GAO GAO2 GENG HIRAYA HOLE HU HU2 JOLY KOULUM LETOUR LIAW LIU3 LIU4 LIU5 LUBIN LUBIN2 MIGRAN MRFITR PERNU QIAO2 RACHTA SEGI2 SOBUE SPEIZE SUZUK2 TIZZAN WYNDE7 YUAN
5	ALDERS
10	HEGMAN KHUDER
14	ENGELA HAENSZ LUO PEZZOT SVENSS WAKAI WU ZHENG

Table 2H13 - 8
Potentially overlapping studies

REF	REFGP	PRINC	OVERLAP/LINK
BENHAM	LUBIN2	2	Subset of Lubin2
WYNDE6	WYNDE6	1	WYNDE5/6/7/8

Table 2H14 -

IESLC - Meta-analysis of Ever/current Smoking, Age started, "High"
Squamous, Any Product (or Cigarettes if Any not available)

This analysis is restricted to results for:
1) Ever/current smokers
2) Results by Age started
3) Categorical results by Age started
4) Squamous (or near equivalent)
5) Results complete enough for use in metaanalysis

Within each study, results are then selected (in the following order of preference, within each sex) for:
6) PRODUCT: all/unspec, cigarettes regardless of other products, cigarettes only
7) CIGTYPE: all/unspecified, MC regardless of HR, MC only
8) (not applicable)
9) DENOM: never smoked anything, never smoked cigarettes, never any + low, never cigs + low
10) Followup period (YF, prospective studies): whole study (coded as 0) or longest available
11) LCtype: squamous or nearest available, but not adeno. (q = squamous, s = small,
a = adeno, KI = Kreyberg I, u = undifferentiated)
12) Race: all or nearest available, otherwise by race (wh or w = white, bl or b = black, hi = hispanic
ch = chinese, jap = japanese, haw = hawaiian, w+o = white + oriental, sca = scandinavian, as = asian)
13) Age started "high" in key scheme 1 (key value 14, maximum range 1-17)
14) For overlapping studies: principal rather than subsidiary studies
Finally by Age: whole study (coded as 0) if available, otherwise by widest available age group
and then for single sex results (m, f) in preference to results for both sexes combined (c).

Results adjusted (AD) for the most potential confounders are then chosen in Sections -1 to -3
and results adjusted for the least confounders in Sections -4 to -6. (Those least adjusted results which
actually differ from the most adjusted are marked 'x' in column X in Section -4)

Section -7 shows excluded studies, together with the stage (as above) at which no qualifying
results were found.

Section -8 lists the potentially overlapping studies which have been included (1=principal, 2=subsidiary).

Section -9 lists any results which would have been included in preference except that they had data not complete
enough for use in meta-analysis, with their significance (yes/no), if known, and any further comment as entered
on the database. It also lists as "gap" any categories for which no data were presented by the original authors.

In addition to those mentioned above, the following fields, levels and abbreviations are used:
* or nk = not known, n = no, y = yes, ot = other
ev = ever, cu = current, nev = never
all/unspec = all or unspecified, cig+/-ot = cigarettes irrespective of other products (cigar, pipe etc)
MC = manufactured cigarettes, HR = hand-rolled cigarettes
exL, exH = range of exposure (low and high) in the smoking group, in terms of Age started
REF: 6-character study reference
NRR: number of the RR on the database within the study
ST : study type (CC = case control, pr or prosp = prospective)
NLC: number of lung cancer cases in whole study
R : risky occupational population (n = no, m = mining, o = other risky)
VB : national cigarette type (V = at least 75% Virginia, bl = at least 75% blended, ot = other)
P : any proxy use
H : full histological confirmation
De : derivation of RR/CI (or = original, st = standard method, ot = other method of estimation)

Table 2H14 - 1

IESLC - Meta-analysis of Ever/current Smoking, Age started, "High"
Squamous, Any Product (or Cigarettes if Any not available)
Most adjusted

REF	NRR	SEX	AGEL	AGEH	RACE	YF	LC TYPE	LOC	START	ST	NLC	R	VB	P	H	AD	SM	PRODUCT	exL	exH	DENOM	De
BARBON	576	m	0	0	all	-	q	Eu:wst	1979	CC	755	n	bl	y	y	1	ev	all/unsp	1	14	nev any	or
BENHAM	517	m	0	0	all	-	KI	Eu:wst	1976	CC	1625	n	bl	n	y	0	ev	cig only	1	16	nev any	st
JEDRYC	530	m	0	0	all	-	q	Eu:est	1980	CC	1630	n	bl	y	n	0	ev	cig+/-ot	1	16	nev any	st
MATOS	618	m	0	0	all	-	q	SCAmer	1994	CC	200	n	bl	n	n	2	ev	cig+/-ot	1	14	nev any	or
WYNDE6	610	m	0	0	wh	-	q	NAmer	1969	CC	4423	n	bl	n	y	0	ev	cig+/-ot	1	17	nev cigs	st
WYNDE6	634	f	0	0	wh	-	q	NAmer	1969	CC	4423	n	bl	n	y	0	ev	cig+/-ot	1	17	nev cigs	st

Cigarette type is all/unspec for all RRs

Table 2H14 - 2

IESLC - Meta-analysis of Ever/current Smoking, Age started, "High"
Squamous, Any Product (or Cigarettes if Any not available)
Most adjusted

REF	NRR	SEX	AD	Number Case	Exposed Cont	Non-exposed Case	Cont	RR	95.00%CI
BARBON	576	m	1	52	-	6	-	71.30	(27.60- 184.00)
BENHAM	517	m	0	342	341	24	481	20.10	(12.99- 31.10)
JEDRYC	530	m	0	53	111	6	289	23.00	(9.61- 55.01)
MATOS	618	m	2	16	-	3	-	7.20	(2.00- 25.90)
WYNDE6	610	m	0	262	301	9	589	56.96	(28.89- 112.31)
WYNDE6	634	f	0	72	91	12	673	44.37	(23.18- 84.93)
Subtotal WYNDE6								50.00	(31.27- 79.93)
Partial Totals				797	844	60	2032		

*prospective study

REF	NRR	SEX	AD	Ys	Ws	Qs	Ps
BARBON	576	m	1	4.27	4.27	3.22	0.0000
BENHAM	517	m	0	3.00	20.16	3.18	0.0000
JEDRYC	530	m	0	3.14	5.05	0.35	0.0000
MATOS	618	m	2	1.97	2.34	4.75	0.0025
WYNDE6	610	m	0	4.04	8.34	3.46	0.0000
WYNDE6	634	f	0	3.79	9.12	1.42	0.0000
Subtotal WYNDE6				3.91	17.45	4.88	

	N	6
	NS	5
	Wt	49.28
Het	Chi	16.38
Het	df	5
Het	P	**
Fixed	RR	29.91
	RRl	22.62
	RRu	39.54
	P	+++
Random	RR	31.07
	RRl	17.93
	RRu	53.85
	P	+++
Asymm	P	N.S.

Table 2H14 - 3

IESLC - Meta-analysis of Ever/current Smoking, Age started, "High"
Squamous, Any Product (or Cigarettes if Any not available)
Most adjusted

	Sex combined	male	female	Total
N		5	1	6
NS		5	1	5
Wt		40.16	9.12	49.28
Het Chi		14.64	0.00	16.38
Het df		4	0	5
Het P		**	N.S.	**
Fixed RR		27.35	44.37	29.91
RRl		20.07	23.18	22.62
RRu		37.26	84.93	39.54
P		+++	+++	+++
Random RR		28.44	44.37	31.07
RRl		14.64	23.18	17.93
RRu		55.25	84.93	53.85
P		+++	+++	+++
Between Chi				1.74
Between df				1
Between P				N.S.
Btwn(F) P				N.S.
Btwn(R) P				N.S.

Too few RRs for analysis by factor

Table 2H14 - 4

IESLC - Meta-analysis of Ever/current Smoking, Age started, "High"
Squamous, Any Product (or Cigarettes if Any not available)
Least adjusted

REF	NRR	X	SEX	AGEL	AGEH	RACE	YF	LC TYPE	LOC	START	ST	NLC	R	VB	P	H	AD	SM	PRODUCT	exL	exH	DENOM	De
BARBON	571	x	m	0	0	all	-	q	Eu:wst	1979	CC	755	n	bl	y	y	0	ev	all/unsp	1	14	nev any	st
BENHAM	517		m	0	0	all	-	KI	Eu:wst	1976	CC	1625	n	bl	n	y	0	ev	cig only	1	16	nev any	st
JEDRYC	530		m	0	0	all	-	q	Eu:est	1980	CC	1630	n	bl	y	n	0	ev	cig+/-ot	1	16	nev any	st
MATOS	613	x	m	0	0	all	-	q	SCAmer	1994	CC	200	n	bl	n	n	0	ev	cig+/-ot	1	14	nev any	st
WYNDE6	610		m	0	0	wh	-	q	NAmer	1969	CC	4423	n	bl	n	y	0	ev	cig+/-ot	1	17	nev cigs	st
WYNDE6	634		f	0	0	wh	-	q	NAmer	1969	CC	4423	n	bl	n	y	0	ev	cig+/-ot	1	17	nev cigs	st

Cigarette type is all/unspec for all RRs

Table 2H14 - 5

IESLC - Meta-analysis of Ever/current Smoking, Age started, "High"
Squamous, Any Product (or Cigarettes if Any not available)
Least adjusted

REF	NRR	SEX	AD	Number Exposed Case	Cont	Non-exposed Case	Cont	RR	95.00%CI
BARBON	571	m	0	52	23	6	188	70.84	(27.41- 183.08)
BENHAM	517	m	0	342	341	24	481	20.10	(12.99- 31.10)
JEDRYC	530	m	0	53	111	6	289	23.00	(9.61- 55.01)
MATOS	613	m	0	16	90	3	110	6.52	(1.84- 23.08)
WYNDE6	610	m	0	262	301	9	589	56.96	(28.89- 112.31)
WYNDE6	634	f	0	72	91	12	673	44.37	(23.18- 84.93)
Subtotal WYNDE6								50.00	(31.27- 79.93)
Totals				797	957	60	2330		

*prospective study

REF	NRR	SEX	AD	Ys	Ws	Qs	Ps
BARBON	571	m	0	4.26	4.26	3.22	0.0000
BENHAM	517	m	0	3.00	20.16	3.07	0.0000
JEDRYC	530	m	0	3.14	5.05	0.33	0.0000
MATOS	613	m	0	1.87	2.40	5.53	0.0037
WYNDE6	610	m	0	4.04	8.34	3.54	0.0000
WYNDE6	634	f	0	3.79	9.12	1.47	0.0000
Subtotal WYNDE6				3.91	17.45	5.01	

	N	6
	NS	5
	Wt	49.33
Het	Chi	17.16
Het	df	5
Het	P	**
Fixed	RR	29.69
	RRl	22.46
	RRu	39.25
	P	+++
Random	RR	30.58
	RRl	17.44
	RRu	53.64
	P	+++
Asymm	P	N.S.

Table 2H14 - 6

IESLC - Meta-analysis of Ever/current Smoking, Age started, "High"
Squamous, Any Product (or Cigarettes if Any not available)
Least adjusted

	Sex combined	male	female	Total
N		5	1	6
NS		5	1	5
Wt		40.21	9.12	49.33
Het Chi		15.35	0.00	17.16
Het df		4	0	5
Het P		**	N.S.	**
Fixed RR		27.11	44.37	29.69
RRl		19.90	23.18	22.46
RRu		36.92	84.93	39.25
P		+++	+++	+++
Random RR		27.89	44.37	30.58
RRl		14.15	23.18	17.44
RRu		54.95	84.93	53.64
P		+++	+++	+++
Between Chi				1.81
Between df				1
Between P				N.S.
Btwn(F) P				N.S.
Btwn(R) P				N.S.

Table 2H14 - 7

IESLC - Meta-analysis of Ever/current Smoking, Age started, "High"
Squamous, Any Product (or Cigarettes if Any not available)
Excluded studies (and stage at which they were excluded)

1	BECHER BLOT1 BROWN3 CARPEN CHYOU DARBY DOLL2 GARCIA GRAHAM GURSEL HAMMO2 JAHN JAIN LAUSSM PRESCO QIAO TVERDA WIGLE WYNDE3
2	AKIBA AMANDU AMES AXELSS BENSHL BEST BOUCHA BOUCOT BROSS CHEN CPSII DEAN2 DESTEF DORGAN DOSEME FAN GARSHI GER GILLIS HAMMON HUMBLE JUSSAW KAISE2 KATSOU KAUFMA KOO KREUZE LEVIN MCCONN NOTAN2 OSANN2 PEZZO2 PISANI RESTRE SADOWS VUTUC WANG2 WATSON WU2 WUWILL WYNDE2 WYNDE8 XU ZHOU
3	GUO MCDUFF SPITZ STASZE ZHANG
4	AGUDO ARMADA AUVINE BOFFET BRESLO BUFFLE CEDERL CHEN2 CHIAZZ CHOI CORREA CPSI DAMBER DEAN3 DOLL DORN GAO GAO2 GENG HIRAYA HOLE HU HU2 JOLY KOULUM LETOUR LIAW LIU3 LIU4 LIU5 LUBIN LUBIN2 MIGRAN MRFITR PERNU QIAO2 RACHTA SEGI2 SOBUE SPEIZE SUZUK2 TIZZAN WYNDE7 YUAN
5	ALDERS
10	HEGMAN KHUDER
14	ENGELA HAENSZ LUO PEZZOT SVENSS WAKAI WU ZHENG

Table 2H14 - 8
Potentially overlapping studies

REF	REFGP	PRINC	. OVERLAP/LINK
BENHAM	LUBIN2	2	Subset of Lubin2
WYNDE6	WYNDE6	1	WYNDE5/6/7/8

Table 2H15 -

IESLC - Meta-analysis of Ever/current Smoking, Age started, "Highest vs lowest"
Squamous, Any Product (or Cigarettes if Any not available)

This analysis is restricted to results for:
1) Ever/current smokers
2) Results by Age started
3) Categorical results by Age started
4) Denominator (unexposed) = "low"
5) Squamous (or near equivalent)
6) Results complete enough for use in metaanalysis

Within each study, results are then selected (in the following order of preference, within each sex) for:
7) SMKSTA: ever, current
8) PRODUCT: all/unspec, cigarettes regardless of other products, cigarettes only
9) CIGTYPE: all/unspecified, MC regardless of HR, MC only
10) Results with least adjustment for other aspects of smoking (ADOS)
11) The highest vs lowest category
12) Followup period (YF, prospective studies): whole study (coded as 0) or longest available
13) LCtype: squamous or nearest available, but not adeno. (q = squamous, s = small,
a = adeno, KI = Kreyberg I, u = undifferentiated)
14) Race: all or nearest available, otherwise by race (wh or w = white, bl or b = black, hi = hispanic
ch = chinese, jap = japanese, haw = hawaiian, w+o = white + oriental, sca = scandinavian, as = asian)
15) For overlapping studies: principal rather than subsidiary studies
Finally by Age: whole study (coded as 0) if available, otherwise by widest available age group
and then for single sex results (m, f) in preference to results for both sexes combined (c).

Results adjusted (AD) for the most potential confounders are then chosen in Sections -1 to -3
and results adjusted for the least confounders in Sections -4 to -6. (Those least adjusted results which
actually differ from the most adjusted are marked 'x' in column X in Section -4)

Section -7 shows excluded studies, together with the stage (as above) at which no qualifying
results were found.

Section -8 lists the potentially overlapping studies which have been included (1=principal, 2=subsidiary).

Section -9 lists any results which would have been included in preference except that they had data not complete
enough for use in meta-analysis, with their significance (yes/no), if known, and any further comment as entered
on the database. It also lists as "gap" any categories for which no data were presented by the original authors.

In addition to those mentioned above, the following fields, levels and abbreviations are used:
* or nk = not known, n = no, y = yes, ot = other
all/unspec = all or unspecified, cig+/-ot = cigarettes irrespective of other products (cigar, pipe etc)
MC = manufactured cigarettes, HR = hand-rolled cigarettes
exL, exH = range of exposure (low and high) in the "highest" group, in terms of Age started
unexL, unexH = range of exposure (low and high) in the "lowest" group, in terms of Age started
REF: 6-character study reference
NRR: number of the RR on the database within the study
ST : study type (CC = case control, pr or prosp = prospective)
NLC: number of lung cancer cases in whole study
R : risky occupational population (n = no, m = mining, o = other risky)
VB : national cigarette type (V = at least 75% Virginia, bl = at least 75% blended, ot = other)
P : any proxy use
H : full histological confirmation
De : derivation of RR/CI (or = original, st = standard method, ot = other method of estimation)

Table 2H15 - 1

IESLC - Meta-analysis of Ever/current Smoking, Age started, "Highest vs lowest"
Squamous, Any Product (or Cigarettes if Any not available)
Most adjusted

REF	NRR	SEX	AGEL	AGEH	RACE	YF	LC TYPE	LOC	START	ST	NLC	R	VB	P	H	AD	ADOS	SM	PRODUCT	exL	exH	unexL	unexH	De
BARBON	578	m	0	0	all	-	q	Eu:wst	1979	CC	755	n	bl	y	y	1	0	ev	all/unsp	1	14	20	999	ot
BENHAM	520	m	0	0	all	-	KI	Eu:wst	1976	CC	1625	n	bl	n	y	0	0	ev	cig only	1	16	25	999	st
ENGELA	521	m	0	0	all	0	q	Eu:Sca	1964	pr	435	n	bl	n	n	0	0	cu	cig+/-ot	1	19	30	999	st
HAENSZ	533	f	0	0	all	-	q+u	NAmer	1955	CC	158	n	bl	n	y	2	0	ev	cig+/-ot	1	24	25	999	ot
HEGMAN	503	m	0	0	all	-	q	NAmer	1989	CC	282	n	bl	y	y	0	0	ev	all/unsp	1	19	20	999	st
HEGMAN	508	f	0	0	all	-	q	NAmer	1989	CC	282	n	bl	y	y	0	0	ev	all/unsp	1	25	26	999	ot
JEDRYC	532	m	0	0	all	-	q	Eu:est	1980	CC	1630	n	bl	y	n	0	0	ev	cig+/-ot	1	16	19	999	st
KHUDER	524	m	0	0	all	-	q	NAmer	1985	CC	482	n	bl	n	y	5	3#	ev	cig+/-ot	1	15	20	999	or
LUO	516	c	0	0	all	-	q	As:Chi	1990	CC	102	n	ot	n	y	20	0	ev	cig+/-ot	0	19	40	999	ot
MATOS	620	m	0	0	all	-	q	SCAmer	1994	CC	200	n	bl	n	n	2	0	ev	cig+/-ot	1	14	20	999	ot
PEZZOT	549	m	0	0	all	-	q	SCAmer	1987	CC	215	n	bl	n	y	2	0	ev	cig only	1	13	19	999	ot
SVENSS	513	f	0	0	all	-	q	Eu:Sca	1983	CC	210	n	bl	n	n	0	0	cu	all/unsp	0	18	26	999	st
WAKAI	510	m	0	0	all	-	q	As:Jap	1988	CC	333	n	bl	n	y	0	0	cu	cig+/-ot	1	19	30	999	st
WU	537	f	0	0	wh	-	q	NAmer	1981	CC	220	n	bl	n	y	2	0	cu	all/unsp	0	18	25	999	ot
WYNDE6	612	m	0	0	wh	-	q	NAmer	1969	CC	4423	n	bl	n	y	0	0	ev	cig+/-ot	1	17	21	999	st
WYNDE6	636	f	0	0	wh	-	q	NAmer	1969	CC	4423	n	bl	n	y	0	0	ev	cig+/-ot	1	17	21	999	st
ZHENG	526	m	0	0	all	-	q	As:Chi	1982	CC	540	n	ot	*	y	1	0	ev	cig+/-ot	1	19	30	999	ot
ZHENG	530	f	0	0	all	-	q	As:Chi	1982	CC	540	n	ot	*	y	1	0	ev	cig+/-ot	1	29	30	999	ot

Comments on values in listings

KHUDER ADOS Duration of smoking (years), number of cigarettes per day and Quitted smoking

Cigarette type is all/unspec for all RRs

Table 2H15 - 2

IESLC - Meta-analysis of Ever/current Smoking, Age started, "Highest vs lowest"
Squamous, Any Product (or Cigarettes if Any not available)
Most adjusted

REF	NRR	SEX	AD	Number Exposed Case	Number Exposed Cont	Non-exposed Case	Non-exposed Cont	RR	95.00%CI
BARBON	578	m	1	52	-	64	-	7.59	(4.36- 13.19)
BENHAM	520	m	0	342	341	98	137	1.40	(1.04- 1.89)
*ENGELA	521	m	0	64	50732	6	9762	2.05	(0.89- 4.74)
HAENSZ	533	f	2	24	-	32	-	1.38	(0.71- 2.67)
HEGMAN	503	m	0	57	716	15	289	1.53	(0.85- 2.75)
HEGMAN	508	f	0	17	169	0	28	5.88~	(0.34- 100.62)
Subtotal HEGMAN								1.62	(0.91- 2.87)
JEDRYC	532	m	0	53	111	134	502	1.79	(1.22- 2.61)
KHUDER	524	m	5	-	-	-	-	1.00	(0.50- 1.80)
LUO	516	c	20	14	-	1	-	4.00	(0.47- 33.90)
MATOS	620	m	2	16	-	6	-	2.18	(0.81- 5.85)
PEZZOT	549	m	2	30	-	10	-	3.33	(1.43- 7.79)
SVENSS	513	f	0	15	21	9	18	1.43	(0.51- 4.04)
WAKAI	510	m	0	21	74	4	25	1.77	(0.56- 5.67)
WU	537	f	2	37	-	6	-	14.83	(2.14- 102.93)
WYNDE6	612	m	0	262	301	44	92	1.82	(1.23- 2.70)
WYNDE6	636	f	0	72	91	32	90	2.23	(1.34- 3.70)
Subtotal WYNDE6								1.96	(1.44- 2.68)
ZHENG	526	m	1	62	-	13	-	7.08	(3.48- 14.41)
ZHENG	530	f	1	34	-	9	-	1.63	(0.63- 4.25)
Subtotal ZHENG								4.19	(2.37- 7.42)
Partial Totals				1172	52556	483	10943		

*prospective study ~ With 0.5 adjustment for zero

REF	NRR	SEX	AD	Ys	Ws	Qs	Ps
BARBON	578	m	1	2.03	12.54	22.55	0.0000
BENHAM	520	m	0	0.34	42.81	5.18	0.0270
*ENGELA	521	m	0	0.72	5.49	0.01	0.0920
HAENSZ	533	f	2	0.32	8.76	1.16	0.3405
HEGMAN	503	m	0	0.43	11.23	0.75	0.1518
HEGMAN	508	f	0	1.77	0.48	0.56	0.2211
Subtotal HEGMAN				0.48	11.70	1.31	
JEDRYC	532	m	0	0.58	26.79	0.29	0.0026
KHUDER	524	m	5	0.00	9.36	4.41	1.0000
LUO	516	c	20	1.39	0.84	0.41	0.2040
MATOS	620	m	2	0.78	3.93	0.03	0.1223
PEZZOT	549	m	2	1.20	5.35	1.43	0.0054
SVENSS	513	f	0	0.36	3.56	0.39	0.5010
WAKAI	510	m	0	0.57	2.85	0.04	0.3335
WU	537	f	2	2.70	1.02	4.14	0.0063
WYNDE6	612	m	0	0.60	24.55	0.19	0.0030
WYNDE6	636	f	0	0.80	14.87	0.19	0.0020
Subtotal WYNDE6				0.67	39.42	0.38	
ZHENG	526	m	1	1.96	7.61	12.30	0.0000
ZHENG	530	f	1	0.49	4.22	0.16	0.3157
Subtotal ZHENG				1.43	11.83	12.47	

	N	18
	NS	15
	Wt	186.25
Het	Chi	54.19
Het	df	17
Het	P	***
Fixed	RR	1.99
	RRl	1.72
	RRu	2.29
	P	+++
Random	RR	2.23
	RRl	1.66
	RRu	2.98
	P	+++
Asymm	P	N.S.

Table 2H15 - 3

IESLC - Meta-analysis of Ever/current Smoking, Age started, "Highest vs lowest"
Squamous, Any Product (or Cigarettes if Any not available)
Most adjusted

	Sex			
	combined	male	female	Total
N	1	11	6	18
NS	1	11	6	18
Wt	0.84	152.50	32.91	186.25
Het Chi	0.00	47.17	6.58	54.19
Het df	0	10	5	17
Het P	N.S.	***	N.S.	***
Fixed RR	4.00	1.99	1.93	1.99
RRl	0.47	1.70	1.37	1.72
RRu	33.97	2.33	2.72	2.29
P	N.S.	+++	+++	+++
Random RR	4.00	2.28	1.96	2.23
RRl	0.47	1.56	1.27	1.66
RRu	33.97	3.31	3.02	2.98
P	N.S.	+++	++	+++
Between Chi				0.44
Between df				2
Between P				N.S.
Btwn(F) P				N.S.
Btwn(R) P				N.S.

	Lung cancer type					
	q	q+s	q+u	KI	not a	Total
N	16		1	1		18
NS	13		1	1		15
Wt	134.68		8.76	42.81		186.25
Het Chi	45.42		0.00	0.00		54.19
Het df	15		0	0		17
Het P	***		N.S.	N.S.		***
Fixed RR	2.27		1.38	1.40		1.99
RRl	1.92		0.71	1.04		1.72
RRu	2.69		2.68	1.89		2.29
P	+++		N.S.	+		+++
Random RR	2.43		1.38	1.40		2.23
RRl	1.75		0.71	1.04		1.66
RRu	3.39		2.68	1.89		2.98
P	+++		N.S.	+		+++
Between Chi						8.77
Between df						2
Between P						*
Btwn(F) P						N.S.
Btwn(R) P						*

	Location								
	NAmer	UK	Scand	othEur	China	Japan	othAs	other	Total
N	7		2	3	3	1		2	18
NS	5		2	3	2	1		2	15
Wt	70.27		9.05	82.13	12.67	2.85		9.28	186.25
Het Chi	9.87		0.28	28.01	5.85	0.00		0.41	54.19
Het df	6		1	2	2	0		1	17
Het P	N.S.		N.S.	***	(*)	N.S.		N.S.	***
Fixed RR	1.71		1.78	1.96	4.18	1.77		2.78	1.99
RRl	1.36		0.93	1.58	2.41	0.56		1.46	1.72
RRu	2.16		3.41	2.44	7.25	5.67		5.30	2.29
P	+++		(+)	+++	+++	N.S.		++	+++
Random RR	1.72		1.78	2.59	3.65	1.77		2.78	2.23
RRl	1.24		0.93	1.10	1.22	0.56		1.46	1.66
RRu	2.40		3.41	6.11	10.92	5.67		5.30	2.98
P	++		(+)	+	+	N.S.		++	+++
Between Chi									9.77
Between df									5
Between P									(*)
Btwn(F) P									N.S.
Btwn(R) P									N.S.

Table 2H15 - 3

IESLC - Meta-analysis of Ever/current Smoking, Age started, "Highest vs lowest"
Squamous, Any Product (or Cigarettes if Any not available)
Most adjusted

Detailed Country in "other Europe"

	multi	Germany	othWest	East	Balkans	Total
N			2	1		3
NS			2	1		3
Wt			55.35	26.79		82.13
Het Chi			27.66	0.00		28.01
Het df			1	0		2
Het P			***	N.S.		***
Fixed RR			2.06	1.79		1.96
RRl			1.58	1.22		1.58
RRu			2.68	2.61		2.44
P			+++	++		+++
Random RR			3.21	1.79		2.59
RRl			0.61	1.22		1.10
RRu			16.78	2.61		6.11
P			N.S.	++		+
Between Chi						0.35
Between df						1
Between P						N.S.
Btwn(F) P						N.S.
Btwn(R) P						N.S.

Detailed Country in "other Asia"

	India	HongKong	other	Total
N				
NS				
Wt				
Het Chi				
Het df				
Het P				N.S.
Fixed RR				
RRl				
RRu				
P				++
Random RR				
RRl				
RRu				
P				++
Between Chi				
Between df				
Between P				N.S.
Btwn(F) P				N.S.
Btwn(R) P				N.S.

Detailed other continent

	SCAmer	Total
N	2	2
NS	2	2
Wt	9.28	9.28
Het Chi	0.41	0.41
Het df	1	1
Het P	N.S.	N.S.
Fixed RR	2.78	2.78
RRl	1.46	1.46
RRu	5.30	5.30
P	++	++
Random RR	2.78	2.78
RRl	1.46	1.46
RRu	5.30	5.30
P	++	++
Between Chi		
Between df		
Between P		N.S.
Btwn(F) P		N.S.
Btwn(R) P		N.S.

Table 2H15 - 3

IESLC - Meta-analysis of Ever/current Smoking, Age started, "Highest vs lowest"
Squamous, Any Product (or Cigarettes if Any not available)
Most adjusted

	Start year of study					
	<1960	1960-69	1970-79	1980-89	1990+	Total
N	1	3	2	10	2	18
NS	1	2	2	8	2	15
Wt	8.76	44.91	55.35	72.46	4.77	186.25
Het Chi	0.00	0.38	27.66	24.46	0.25	54.19
Het df	0	2	1	9	1	17
Het P	N.S.	N.S.	***	**	N.S.	***
Fixed RR	1.38	1.97	2.06	2.00	2.43	1.99
RRl	0.71	1.47	1.58	1.59	0.99	1.72
RRu	2.68	2.64	2.68	2.52	5.95	2.29
P	N.S.	+++	+++	+++	(+)	+++
Random RR	1.38	1.97	3.21	2.23	2.43	2.23
RRl	0.71	1.47	0.61	1.44	0.99	1.66
RRu	2.68	2.64	16.78	3.45	5.95	2.98
P	N.S.	+++	N.S.	+++	(+)	+++
Between Chi						1.42
Between df						4
Between P						N.S.
Btwn(F) P						N.S.
Btwn(R) P						N.S.

	Study type (1)		
	CC	other	Total
N	17	1	18
NS	14	1	15
Wt	180.76	5.49	186.25
Het Chi	54.18	0.00	54.19
Het df	16	0	17
Het P	***	N.S.	***
Fixed RR	1.98	2.05	1.99
RRl	1.71	0.89	1.72
RRu	2.29	4.74	2.29
P	+++	(+)	+++
Random RR	2.24	2.05	2.23
RRl	1.65	0.89	1.66
RRu	3.05	4.74	2.98
P	+++	(+)	+++
Between Chi			0.01
Between df			1
Between P			N.S.
Btwn(F) P			N.S.
Btwn(R) P			N.S.

	Study type (2)			
	CC	prosp	other	Total
N	17	1		18
NS	14	1		15
Wt	180.76	5.49		186.25
Het Chi	54.18	0.00		54.19
Het df	16	0		17
Het P	***	N.S.		***
Fixed RR	1.98	2.05		1.99
RRl	1.71	0.89		1.72
RRu	2.29	4.74		2.29
P	+++	(+)		+++
Random RR	2.24	2.05		2.23
RRl	1.65	0.89		1.66
RRu	3.05	4.74		2.98
P	+++	(+)		+++
Between Chi				0.01
Between df				1
Between P				N.S.
Btwn(F) P				N.S.
Btwn(R) P				N.S.

Table 2H15 - 3

IESLC - Meta-analysis of Ever/current Smoking, Age started, "Highest vs lowest"
Squamous, Any Product (or Cigarettes if Any not available)
Most adjusted

	Study size (number of LC cases)				
	100-249	250-499	500-999	1000+	Total
N	6	5	3	4	18
NS	6	4	2	3	15
Wt	23.46	29.41	24.37	109.02	186.25
Het Chi	7.48	3.04	7.99	2.84	54.19
Het df	5	4	2	3	17
Het P	N.S.	N.S.	*	N.S.	***
Fixed RR	2.11	1.46	5.69	1.68	1.99
RRl	1.41	1.02	3.83	1.39	1.72
RRu	3.16	2.10	8.47	2.03	2.29
P	+++	+	+++	+++	+++
Random RR	2.29	1.46	4.79	1.68	2.23
RRl	1.34	1.02	2.07	1.39	1.66
RRu	3.90	2.10	11.06	2.03	2.98
P	++	+	+++	+++	+++
Between Chi					32.84
Between df					3
Between P					***
Btwn(F) P					**
Btwn(R) P					(*)

	Risky occupational population			
	no	mining	othRisky	Total
N	18			18
NS	15			15
Wt	186.25			186.25
Het Chi	54.19			54.19
Het df	17			17
Het P	***			***
Fixed RR	1.99			1.99
RRl	1.72			1.72
RRu	2.29			2.29
P	+++			+++
Random RR	2.23			2.23
RRl	1.66			1.66
RRu	2.98			2.98
P	+++			+++
Between Chi				
Between df				
Between P				N.S.
Btwn(F) P				N.S.
Btwn(R) P				N.S.

	National cigarette tobacco type			
	Virginia	blended	other	Total
N		15	3	18
NS		13	2	15
Wt		173.58	12.67	186.25
Het Chi		40.80	5.85	54.19
Het df		14	2	17
Het P		***	(*)	***
Fixed RR		1.88	4.18	1.99
RRl		1.62	2.41	1.72
RRu		2.18	7.25	2.29
P		+++	+++	+++
Random RR		2.05	3.65	2.23
RRl		1.54	1.22	1.66
RRu		2.73	10.92	2.98
P		+++	+	+++
Between Chi				7.54
Between df				1
Between P				**
Btwn(F) P				N.S.
Btwn(R) P				N.S.

Table 2H15 - 3

IESLC - Meta-analysis of Ever/current Smoking, Age started, "Highest vs lowest"
Squamous, Any Product (or Cigarettes if Any not available)
Most adjusted

	Any proxy use No/nk	Yes	Total
N	14	4	18
NS	12	3	15
Wt	135.22	51.03	186.25
Het Chi	29.04	21.50	54.19
Het df	13	3	17
Het P	**	***	***
Fixed RR	1.82	2.49	1.99
RRl	1.54	1.90	1.72
RRu	2.16	3.28	2.29
P	+++	+++	+++
Random RR	2.04	2.89	2.23
RRl	1.52	1.20	1.66
RRu	2.73	6.99	2.98
P	+++	+	+++
Between Chi			3.66
Between df			1
Between P			(*)
Btwn(F) P			N.S.
Btwn(R) P			N.S.

	Full histological confirmation No	Yes	Total
N	4	14	18
NS	4	11	15
Wt	39.77	146.48	186.25
Het Chi	0.42	53.39	54.19
Het df	3	13	17
Het P	N.S.	***	***
Fixed RR	1.82	2.03	1.99
RRl	1.34	1.73	1.72
RRu	2.49	2.39	2.29
P	+++	+++	+++
Random RR	1.82	2.40	2.23
RRl	1.34	1.64	1.66
RRu	2.49	3.50	2.98
P	+++	+++	+++
Between Chi			0.37
Between df			1
Between P			N.S.
Btwn(F) P			N.S.
Btwn(R) P			N.S.

	Number of adjustment variables (1) 0	1	2+/+nk	Total
N	9	3	6	18
NS	7	2	6	15
Wt	132.62	24.37	29.26	186.25
Het Chi	4.00	7.99	11.08	54.19
Het df	8	2	5	17
Het P	N.S.	*	*	***
Fixed RR	1.68	5.69	1.74	1.99
RRl	1.42	3.83	1.21	1.72
RRu	2.00	8.47	2.50	2.29
P	+++	+++	++	+++
Random RR	1.68	4.79	2.11	2.23
RRl	1.42	2.07	1.16	1.66
RRu	2.00	11.06	3.84	2.98
P	+++	+++	+	+++
Between Chi				31.11
Between df				2
Between P				***
Btwn(F) P				**
Btwn(R) P				*

Table 2H15 - 3

IESLC - Meta-analysis of Ever/current Smoking, Age started, "Highest vs lowest"
Squamous, Any Product (or Cigarettes if Any not available)
Most adjusted

	Number of adjustment variables (2) 0	1	2	3-5	6+/+nk	Total
N	9	3	4	1	1	18
NS	7	2	4	1	1	15
Wt	132.62	24.37	19.06	9.36	0.84	186.25
Het Chi	4.00	7.99	6.55	0.00	0.00	54.19
Het df	8	2	3	0	0	17
Het P	N.S.	*	(*)	N.S.	N.S.	***
Fixed RR	1.68	5.69	2.21	1.00	4.00	1.99
RRl	1.42	3.83	1.41	0.53	0.47	1.72
RRu	2.00	8.47	3.46	1.90	33.97	2.29
P	+++	+++	+++	N.S.	N.S.	+++
Random RR	1.68	4.79	2.59	1.00	4.00	2.23
RRl	1.42	2.07	1.26	0.53	0.47	1.66
RRu	2.00	11.06	5.35	1.90	33.97	2.98
P	+++	+++	++	N.S.	N.S.	+++
Between Chi						35.64
Between df						4
Between P						***
Btwn(F) P						**
Btwn(R) P						*

	Smoking status ever	current	Total
N	14	4	18
NS	11	4	15
Wt	173.33	12.92	186.25
Het Chi	49.62	4.53	54.19
Het df	13	3	17
Het P	***	N.S.	***
Fixed RR	1.98	2.10	1.99
RRl	1.70	1.22	1.72
RRu	2.29	3.63	2.29
P	+++	++	+++
Random RR	2.23	2.23	2.23
RRl	1.61	1.11	1.66
RRu	3.08	4.49	2.98
P	+++	+	+++
Between Chi			0.05
Between df			1
Between P			N.S.
Btwn(F) P			N.S.
Btwn(R) P			N.S.

	Product all/unsp	cig+/-ot	cig only	Total
N	5	11	2	18
NS	4	9	2	15
Wt	28.83	109.27	48.16	186.25
Het Chi	20.24	19.10	3.56	54.19
Het df	4	10	1	17
Het P	***	*	(*)	***
Fixed RR	3.38	1.93	1.54	1.99
RRl	2.35	1.60	1.16	1.72
RRu	4.87	2.33	2.05	2.29
P	+++	+++	++	+++
Random RR	3.63	1.98	1.97	2.23
RRl	1.33	1.49	0.86	1.66
RRu	9.89	2.64	4.50	2.98
P	+	+++	N.S.	+++
Between Chi				11.29
Between df				2
Between P				**
Btwn(F) P				N.S.
Btwn(R) P				N.S.

Table 2H15 - 3

IESLC - Meta-analysis of Ever/current Smoking, Age started, "Highest vs lowest"
Squamous, Any Product (or Cigarettes if Any not available)
Most adjusted

	Derivation of RR/CI			
	Orig	StdCalc	Other	Total
N	1	8	9	18
NS	1	7	8	16
Wt	9.36	132.14	44.74	186.25
Het Chi	0.00	3.25	24.25	54.19
Het df	0	7	8	17
Het P	N.S.	N.S.	**	***
Fixed RR	1.00	1.68	3.78	1.99
RRl	0.53	1.41	2.82	1.72
RRu	1.90	1.99	5.06	2.29
P	N.S.	+++	+++	+++
Random RR	1.00	1.68	3.64	2.23
RRl	0.53	1.41	2.05	1.66
RRu	1.90	1.99	6.46	2.98
P	N.S.	+++	+++	+++
Between Chi				26.68
Between df				2
Between P				***
Btwn(F) P				**
Btwn(R) P				**

Table 2H15 - 4

IESLC - Meta-analysis of Ever/current Smoking, Age started, "Highest vs lowest"
Squamous, Any Product (or Cigarettes if Any not available)
Least adjusted

REF	NRR	X	SEX	AGEL	AGEH	RACE	YF	LC TYPE	LOC	START	ST	NLC	R	VB	P	H	AD	ADOS	SM	PRODUCT	exL	exH	unexL	unexH	De
BARBON	573	x	m	0	0	all	-	q	Eu:wst	1979	CC	755	n	bl	y	y	0	0	ev	all/unsp	1	14	20	999	st
BENHAM	520		m	0	0	all	-	KI	Eu:wst	1976	CC	1625	n	bl	n	y	0	0	ev	cig only	1	16	25	999	st
ENGELA	521		m	0	0	all	0	q	Eu:Sca	1964	pr	435	n	bl	n	n	0	0	cu	cig+/-ot	1	19	30	999	st
HAENSZ	505	x	f	0	0	all	-	q+u	NAmer	1955	CC	158	n	bl	n	y	0	0	ev	cig+/-ot	1	24	25	999	st
HEGMAN	503		m	0	0	all	-	q	NAmer	1989	CC	282	n	bl	y	y	0	0	ev	all/unsp	1	19	20	999	st
HEGMAN	508		f	0	0	all	-	q	NAmer	1989	CC	282	n	bl	y	y	0	0	ev	all/unsp	1	25	26	999	ot
JEDRYC	532		m	0	0	all	-	q	Eu:est	1980	CC	1630	n	bl	y	n	0	0	ev	cig+/-ot	1	16	19	999	st
KHUDER	524		m	0	0	all	-	q	NAmer	1985	CC	482	n	bl	n	y	5	3#	ev	cig+/-ot	1	15	20	999	or
LUO	511	x	c	0	0	all	-	q	As:Chi	1990	CC	102	n	ot	n	y	0	0	ev	cig+/-ot	0	19	40	999	st
MATOS	615	x	m	0	0	all	-	q	SCAmer	1994	CC	200	n	bl	n	n	0	0	ev	cig+/-ot	1	14	20	999	st
PEZZOT	547	x	m	0	0	all	-	q	SCAmer	1987	CC	215	n	bl	n	y	0	0	ev	cig only	1	13	19	999	st
SVENSS	513		f	0	0	all	-	q	Eu:Sca	1983	CC	210	n	bl	n	n	0	0	cu	all/unsp	0	18	26	999	st
WAKAI	510		m	0	0	all	-	q	As:Jap	1988	CC	333	n	bl	n	y	0	0	cu	cig+/-ot	1	19	30	999	st
WU	513	x	f	0	0	wh	-	q	NAmer	1981	CC	220	n	bl	n	y	0	0	cu	all/unsp	0	18	25	999	st
WYNDE6	612		m	0	0	wh	-	q	NAmer	1969	CC	4423	n	bl	n	y	0	0	ev	cig+/-ot	1	17	21	999	st
WYNDE6	636		f	0	0	wh	-	q	NAmer	1969	CC	4423	n	bl	n	y	0	0	ev	cig+/-ot	1	17	21	999	st
ZHENG	524	x	m	0	0	all	-	q	As:Chi	1982	CC	540	n	ot	*	y	0	0	ev	cig+/-ot	1	19	30	999	st
ZHENG	529	x	f	0	0	all	-	q	As:Chi	1982	CC	540	n	ot	*	y	0	0	ev	cig+/-ot	1	29	30	999	st

Comments on values in listings

KHUDER ADOS Duration of smoking (years), number of cigarettes per day and Quitted smoking

Cigarette type is all/unspec for all RRs

Table 2H15 - 5

IESLC - Meta-analysis of Ever/current Smoking, Age started, "Highest vs lowest"
Squamous, Any Product (or Cigarettes if Any not available)
Least adjusted

REF	NRR	SEX	AD	Number Exposed Case	Cont	Non-exposed Case	Cont	RR	95.00%CI
BARBON	573	m	0	52	23	64	207	7.31	(4.16- 12.87)
BENHAM	520	m	0	342	341	98	137	1.40	(1.04- 1.89)
*ENGELA	521	m	0	64	50732	6	9762	2.05	(0.89- 4.74)
HAENSZ	505	f	0	24	37	32	66	1.34	(0.69- 2.60)
HEGMAN	503	m	0	57	716	15	289	1.53	(0.85- 2.75)
HEGMAN	508	f	0	17	169	0	28	5.88~	(0.34- 100.62)
Subtotal HEGMAN								1.62	(0.91- 2.87)
JEDRYC	532	m	0	53	111	134	502	1.79	(1.22- 2.61)
KHUDER	524	m	5	-	-	-	-	1.00	(0.50- 1.80)
LUO	511	c	0	14	20	1	8	5.60	(0.63- 49.95)
MATOS	615	m	0	16	90	6	73	2.16	(0.81- 5.81)
PEZZOT	547	m	0	30	34	10	32	2.82	(1.19- 6.69)
SVENSS	513	f	0	15	21	9	18	1.43	(0.51- 4.04)
WAKAI	510	m	0	21	74	4	25	1.77	(0.56- 5.67)
WU	513	f	0	37	11	6	5	2.80	(0.72- 10.97)
WYNDE6	612	m	0	262	301	44	92	1.82	(1.23- 2.70)
WYNDE6	636	f	0	72	91	32	90	2.23	(1.34- 3.70)
Subtotal WYNDE6								1.96	(1.44- 2.68)
ZHENG	524	m	0	62	43	13	66	7.32	(3.60- 14.90)
ZHENG	529	f	0	34	28	9	16	2.16	(0.83- 5.62)
Subtotal ZHENG								4.74	(2.68- 8.40)
Partial Totals				1172	52842	483	11416		

*prospective study

~ With 0.5 adjustment for zero

REF	NRR	SEX	AD	Ys	Ws	Qs	Ps
BARBON	573	m	0	1.99	12.02	20.78	0.0000
BENHAM	520	m	0	0.34	42.81	4.86	0.0270
*ENGELA	521	m	0	0.72	5.49	0.01	0.0920
HAENSZ	505	f	0	0.29	8.69	1.28	0.3909
HEGMAN	503	m	0	0.43	11.23	0.69	0.1518
HEGMAN	508	f	0	1.77	0.48	0.57	0.2211
Subtotal HEGMAN				0.48	11.70	1.26	
JEDRYC	532	m	0	0.58	26.79	0.23	0.0026
KHUDER	524	m	5	0.00	9.36	4.27	1.0000
LUO	511	c	0	1.72	0.80	0.88	0.1228
MATOS	615	m	0	0.77	3.94	0.04	0.1258
PEZZOT	547	m	0	1.04	5.15	0.68	0.0184
SVENSS	513	f	0	0.36	3.56	0.36	0.5010
WAKAI	510	m	0	0.57	2.85	0.03	0.3335
WU	513	f	0	1.03	2.06	0.26	0.1387
WYNDE6	612	m	0	0.60	24.55	0.14	0.0030
WYNDE6	636	f	0	0.80	14.87	0.23	0.0020
Subtotal WYNDE6				0.67	39.42	0.37	
ZHENG	524	m	0	1.99	7.61	13.17	0.0000
ZHENG	529	f	0	0.77	4.19	0.04	0.1153
Subtotal ZHENG				1.56	11.80	13.20	

	N	18
	NS	15
	Wt	186.45
Het	Chi	48.52
Het	df	17
Het	P	***
Fixed	RR	1.96
	RRl	1.70
	RRu	2.27
	P	+++
Random	RR	2.17
	RRl	1.65
	RRu	2.86
	P	+++
Asymm	P	N.S.

Table 2H15 - 6

IESLC - Meta-analysis of Ever/current Smoking, Age started, "Highest vs lowest"
Squamous, Any Product (or Cigarettes if Any not available)
Least adjusted

		Sex			
		combined	male	female	Total
	N	1	11	6	18
	NS	1	11	6	18
	Wt	0.80	151.80	33.85	186.45
Het	Chi	0.00	44.90	2.72	48.52
Het	df	0	10	5	17
Het	P	N.S.	***	N.S.	***
Fixed	RR	5.60	1.97	1.91	1.96
	RRl	0.63	1.68	1.36	1.70
	RRu	49.95	2.30	2.67	2.27
	P	N.S.	+++	+++	+++
Random	RR	5.60	2.24	1.91	2.17
	RRl	0.63	1.55	1.36	1.65
	RRu	49.95	3.24	2.67	2.86
	P	N.S.	+++	+++	+++
Between	Chi				0.91
Between	df				2
Between	P				N.S.
Btwn(F)	P				N.S.
Btwn(R)	P				N.S.

Table 2H15 - 7

IESLC - Meta-analysis of Ever/current Smoking, Age started, "Highest vs lowest"
Squamous, Any Product (or Cigarettes if Any not available)
Excluded studies (and stage at which they were excluded)

1 BECHER BLOT1 BROWN3 CARPEN CHYOU DARBY DOLL2 GARCIA GRAHAM GURSEL HAMMO2 JAHN JAIN LAUSSM PRESCO QIAO
TVERDA WIGLE WYNDE3

2 AKIBA AMANDU AMES AXELSS BENSHL BEST BOUCHA BOUCOT BROSS CHEN CPSII DEAN2 DESTEF DORGAN DOSEME FAN
GARSHI GER GILLIS HAMMON HUMBLE JUSSAW KAISE2 KATSOU KAUFMA KOO KREUZE LEVIN MCCONN NOTAN2 OSANN2 PEZZO2
PISANI RESTRE SADOWS VUTUC WANG2 WATSON WU2 WUWILL WYNDE2 WYNDE8 XU ZHOU

3 GUO MCDUFF SPITZ STASZE ZHANG

5 AGUDO ARMADA AUVINE BOFFET BRESLO BUFFLE CEDERL CHEN2 CHIAZZ CHOI CORREA CPSI DAMBER DEAN3 DOLL DORN
GAO GAO2 GENG HIRAYA HOLE HU HU2 JOLY KOULUM LETOUR LIAW LIU3 LIU4 LIU5 LUBIN LUBIN2
MIGRAN MRFITR PERNU QIAO2 RACHTA SEGI2 SOBUE SPEIZE SUZUK2 TIZZAN WYNDE7 YUAN

6 ALDERS

Table 2H15 - 8
Potentially overlapping studies

REF	REFGP	PRINC	. OVERLAP/LINK
BENHAM	LUBIN2	2	Subset of Lubin2
WYNDE6	WYNDE6	1	WYNDE5/6/7/8

Table 2H15 - 9
Most adjusted - insufficient data for meta-analysis

REF	NRR	SEX	AGEL	AGEH	RACE	YF	LC TYPE	LOC	START	ST	NLC	R	VB	P	H	AD	ADOS	SM	PRODUCT	exL	exH	unexL	unexH	De
ALDERS	533	m	0	0	all	-	q+s	Eu:UK	1977	CC	1448	n	V	n	n	2	1#	ev	cig only	1	14	25	999	ot
ALDERS	536	f	0	0	all	-	q+s	Eu:UK	1977	CC	1448	n	V	n	n	2	1#	ev	cig only	1	14	25	999	ot

Comments on values in listings

ALDERS ADOS Number of cigs/day
ALDERS ADOS Number of cigs/day

REF	NRR	RR	SIG	RRDATA comment
ALDERS	533	5.56	n	0
ALDERS	536	1.56	n	0

Table 2H16 -

IESLC - Meta-analysis of Ever/current Smoking by Age started, Overview
Squamous, Cigarettes (or Any Product if Cigarettes not available)

This analysis is restricted to results for:
1) Ever/current smokers
2) Results by Age started
3) Categorical results by Age started
   Results by Age started are grouped under 2 schemes (S1, S2). Each scheme has a set of "key values". An interval is allocated to the category whose key value it includes, and intervals which include none or more than one of the key values are excluded. (Open-ended intervals are coded as 999)

S1	key value	maximum range
1	26	19+
2	18	15-25
3	14	1-17

S2	key value	maximum range
1	30	27+
2	26	23-29
3	22	19-25
4	18	15-21
5	14	11-17
6	10	1-13

4) Squamous (or near equivalent)
5) Results complete enough for use in metaanalysis

Within each study, results are then selected (in the following order of preference, within each sex) for:
6) SMKSTA: ever, current
7) PRODUCT: cigarettes regardless of other products, cigarettes only, all/unspec
8) CIGTYPE: all/unspecified, MC regardless of HR, MC only
9) (not applicable)
10) DENOM: never smoked anything, never smoked cigarettes, never any + low, never cigs + low
11) Followup period (YF, prospective studies): whole study (coded as 0) or longest available
12) LCtype: squamous or nearest available, but not adeno. (q = squamous, s = small,
        a = adeno, KI = Kreyberg I, u = undifferentiated)
13) Race: all or nearest available, otherwise by race (wh or w = white, bl or b = black, hi = hispanic
        ch = chinese, jap = japanese, haw = hawaiian, w+o = white + oriental, sca = scandinavian, as = asian)
14) For overlapping studies: principal rather than subsidiary studies
Finally by Age: whole study (coded as 0) if available, otherwise by widest available age group and then for single sex results (m, f) in preference to results for both sexes combined (c).

Results adjusted (AD) for the most potential confounders are then chosen in Sections -1 to -3 (and those which actually differ from the adjusted results in Table 2H11 - 1 are marked 'x' in Section -1) and results adjusted for the least confounders in Sections -4 to -6. (Those least adjusted results which actually differ from the most adjusted are marked 'x' in column X in Section -4)

Section -7 shows excluded studies, together with the stage (as above) at which no qualifying results were found.

Section -8 lists the potentially overlapping studies which have been included (1=principal, 2=subsidiary).

Section -9 lists any results which would have been included in preference except that they had data not complete enough for use in meta-analysis, with their significance (yes/no), if known, and any further comment as entered on the database. It also lists as "gap" any categories for which no data were presented by the original authors.

In addition to those mentioned above, the following fields, levels and abbreviations are used:
* or nk = not known, n = no, y = yes, ot = other
ev = ever, cu = current, nev = never
all/unspec = all or unspecified, cig+/-ot = cigarettes irrespective of other products (cigar, pipe etc)
MC = manufactured cigarettes, HR = hand-rolled cigarettes
exL, exH = range of exposure (low and high) in the smoking group, in terms of Age started
REF: 6-character study reference
NRR: number of the RR on the database within the study
ST : study type (CC = case control, pr or prosp = prospective)
NLC: number of lung cancer cases in whole study
R : risky occupational population (n = no, m = mining, o = other risky)
VB : national cigarette type (V = at least 75% Virginia, bl = at least 75% blended, ot = other)
P : any proxy use
H : full histological confirmation
De : derivation of RR/CI (or = original, st = standard method, ot = other method of estimation)

Table 2H16 - 1

IESLC - Meta-analysis of Ever/current Smoking by Age started, Overview
Squamous, Cigarettes (or Any Product if Cigarettes not available)
Most adjusted

REF	NRR	2H11	SEX	AGEL	AGEH	RACE	YF	LC TYPE	LOC	START	ST	NLC	R	VB	P	H	AD	SM	PRODUCT	exL	exH	S1	S2	DENOM	De
BARBON	574		m	0	0	all	-	q	Eu:wst	1979	CC	755	n	bl	y	y	1	ev	all/unsp	20	999	1	0	nev any	or
BARBON	575		m	0	0	all	-	q	Eu:wst	1979	CC	755	n	bl	y	y	1	ev	all/unsp	15	19	2	4	nev any	or
BARBON	576		m	0	0	all	-	q	Eu:wst	1979	CC	755	n	bl	y	y	1	ev	all/unsp	1	14	3	0	nev any	or
BENHAM	514		m	0	0	all	-	KI	Eu:wst	1976	CC	1625	n	bl	n	y	0	ev	cig only	25	999	1	0	nev any	st
BENHAM	515		m	0	0	all	-	KI	Eu:wst	1976	CC	1625	n	bl	n	y	0	ev	cig only	20	24	0	3	nev any	st
BENHAM	516		m	0	0	all	-	KI	Eu:wst	1976	CC	1625	n	bl	n	y	0	ev	cig only	17	19	2	4	nev any	st
BENHAM	517		m	0	0	all	-	KI	Eu:wst	1976	CC	1625	n	bl	n	y	0	ev	cig only	1	16	3	0	nev any	st
ENGELA	517		m	0	0	all	0	q	Eu:Sca	1964	pr	435	n	bl	n	n	0	cu	cig+/-ot	30	999	0	1	nev cigs	st
ENGELA	518		m	0	0	all	0	q	Eu:Sca	1964	pr	435	n	bl	n	n	0	cu	cig+/-ot	20	29	1	0	nev cigs	st
ENGELA	519		m	0	0	all	0	q	Eu:Sca	1964	pr	435	n	bl	n	n	0	cu	cig+/-ot	1	19	0	0	nev cigs	st
HAENSZ	501		f	0	0	all	-	q+u	NAmer	1955	CC	158	n	bl	n	y	0	ev	cig+/-ot	25	999	1	0	nev any	st
HAENSZ	502		f	0	0	all	-	q+u	NAmer	1955	CC	158	n	bl	n	y	0	ev	cig+/-ot	1	24	0	0	nev any	st
JEDRYC	528		m	0	0	all	-	q	Eu:est	1980	CC	1630	n	bl	y	n	0	ev	cig+/-ot	19	999	1	0	nev any	st
JEDRYC	529		m	0	0	all	-	q	Eu:est	1980	CC	1630	n	bl	y	n	0	ev	cig+/-ot	17	18	2	4	nev any	st
JEDRYC	530		m	0	0	all	-	q	Eu:est	1980	CC	1630	n	bl	y	n	0	ev	cig+/-ot	1	16	3	0	nev any	st
LUO	512		c	0	0	all	-	q	As:Chi	1990	CC	102	n	ot	n	y	20	ev	cig+/-ot	40	999	0	0	nev cigs	or
LUO	513		c	0	0	all	-	q	As:Chi	1990	CC	102	n	ot	n	y	20	ev	cig+/-ot	20	39	1	0	nev cigs	or
LUO	514		c	0	0	all	-	q	As:Chi	1990	CC	102	n	ot	n	y	20	ev	cig+/-ot	0	19	0	0	nev cigs	or
MATOS	616		m	0	0	all	-	q	SCAmer	1994	CC	200	n	bl	n	n	2	ev	cig+/-ot	20	999	1	0	nev any	or
MATOS	617		m	0	0	all	-	q	SCAmer	1994	CC	200	n	bl	n	n	2	ev	cig+/-ot	15	19	2	4	nev any	or
MATOS	618		m	0	0	all	-	q	SCAmer	1994	CC	200	n	bl	n	n	2	ev	cig+/-ot	1	14	3	0	nev any	or
PEZZOT	543		m	0	0	all	-	q	SCAmer	1987	CC	215	n	bl	n	y	0	ev	cig only	19	999	1	0	nev cigs	ot
PEZZOT	544		m	0	0	all	-	q	SCAmer	1987	CC	215	n	bl	n	y	0	ev	cig only	14	18	0	0	nev cigs	ot
PEZZOT	545		m	0	0	all	-	q	SCAmer	1987	CC	215	n	bl	n	y	0	ev	cig only	1	13	0	6	nev cigs	ot
SVENSS	509		f	0	0	all	-	q	Eu:Sca	1983	CC	210	n	bl	n	n	0	cu	all/unsp	26	999	1	0	nev any	st
SVENSS	510		f	0	0	all	-	q	Eu:Sca	1983	CC	210	n	bl	n	n	0	cu	all/unsp	19	25	0	3	nev any	st
SVENSS	511		f	0	0	all	-	q	Eu:Sca	1983	CC	210	n	bl	n	n	0	cu	all/unsp	0	18	0	0	nev any	st
WAKAI	506		m	0	0	all	-	q	As:Jap	1988	CC	333	n	bl	n	y	0	cu	cig+/-ot	30	999	0	1	nev any	st
WAKAI	507		m	0	0	all	-	q	As:Jap	1988	CC	333	n	bl	n	y	0	cu	cig+/-ot	20	29	1	0	nev any	st
WAKAI	508		m	0	0	all	-	q	As:Jap	1988	CC	333	n	bl	n	y	0	cu	cig+/-ot	1	19	0	0	nev any	st
WU	533		f	0	0	wh	-	q	NAmer	1981	CC	220	n	bl	n	y	2	cu	all/unsp	25	999	1	0	nev any	or
WU	534		f	0	0	wh	-	q	NAmer	1981	CC	220	n	bl	n	y	2	cu	all/unsp	19	24	0	3	nev any	or
WU	535		f	0	0	wh	-	q	NAmer	1981	CC	220	n	bl	n	y	2	cu	all/unsp	0	18	0	0	nev any	or
WYNDE6	608		m	0	0	wh	-	q	NAmer	1969	CC	4423	n	bl	n	y	0	ev	cig+/-ot	21	999	1	0	nev cigs	st
WYNDE6	609		m	0	0	wh	-	q	NAmer	1969	CC	4423	n	bl	n	y	0	ev	cig+/-ot	18	20	2	4	nev cigs	st
WYNDE6	610		m	0	0	wh	-	q	NAmer	1969	CC	4423	n	bl	n	y	0	ev	cig+/-ot	1	17	3	0	nev cigs	st
WYNDE6	632		f	0	0	wh	-	q	NAmer	1969	CC	4423	n	bl	n	y	0	ev	cig+/-ot	21	999	1	0	nev cigs	st
WYNDE6	633		f	0	0	wh	-	q	NAmer	1969	CC	4423	n	bl	n	y	0	ev	cig+/-ot	18	20	2	4	nev cigs	st
WYNDE6	634		f	0	0	wh	-	q	NAmer	1969	CC	4423	n	bl	n	y	0	ev	cig+/-ot	1	17	3	0	nev cigs	st
ZHENG	520		m	0	0	all	-	q	As:Chi	1982	CC	540	n	ot	*	y	0	ev	cig+/-ot	30	999	0	1	nev cigs	st
ZHENG	521		m	0	0	all	-	q	As:Chi	1982	CC	540	n	ot	*	y	0	ev	cig+/-ot	20	29	1	0	nev cigs	st
ZHENG	522		m	0	0	all	-	q	As:Chi	1982	CC	540	n	ot	*	y	0	ev	cig+/-ot	1	19	0	0	nev cigs	st
ZHENG	527		f	0	0	all	-	q	As:Chi	1982	CC	540	n	ot	*	y	0	ev	cig+/-ot	30	999	0	1	nev cigs	st
ZHENG	528		f	0	0	all	-	q	As:Chi	1982	CC	540	n	ot	*	y	0	ev	cig+/-ot	1	29	0	0	nev cigs	st

Cigarette type is all/unspec for all RRs

In this overview table, subtotals and Qs values may be invalid and should be ignored

Table 2H16 - 2

IESLC - Meta-analysis of Ever/current Smoking by Age started, Overview
Squamous, Cigarettes (or Any Product if Cigarettes not available)
Most adjusted

REF	NRR	SEX	AD	Number Exposed Case	Cont	Non-exposed Case	Cont	RR	95.00%CI
BARBON	574	m	1	64	-	6	-	9.40	(4.20- 23.40)
BARBON	575	m	1	145	-	6	-	13.70	(5.90- 31.70)
BARBON	576	m	1	52	-	6	-	71.30	(27.60- 184.00)
Subtotal BARBON								19.26	(11.59- 32.00)
BENHAM	514	m	0	98	137	24	481	14.34	(8.83- 23.29)
BENHAM	515	m	0	323	398	24	481	16.26	(10.52- 25.14)
BENHAM	516	m	0	311	373	24	481	16.71	(10.80- 25.86)
BENHAM	517	m	0	342	341	24	481	20.10	(12.99- 31.10)
Subtotal BENHAM								16.86	(13.48- 21.08)
*ENGELA	517	m	0	6	9762	3	58716	12.03	(3.01- 48.09)
*ENGELA	518	m	0	17	30195	3	58716	11.02	(3.23- 37.60)
*ENGELA	519	m	0	64	50732	3	58716	24.69	(7.76- 78.58)
Subtotal ENGELA								15.41	(7.50- 31.65)
HAENSZ	501	f	0	32	66	44	236	2.60	(1.53- 4.42)
HAENSZ	502	f	0	24	37	44	236	3.48	(1.90- 6.38)
Subtotal HAENSZ								2.95	(1.98- 4.40)
JEDRYC	528	m	0	134	502	6	289	12.86	(5.60- 29.50)
JEDRYC	529	m	0	96	221	6	289	20.92	(9.00- 48.62)
JEDRYC	530	m	0	53	111	6	289	23.00	(9.61- 55.01)
Subtotal JEDRYC								18.20	(11.15- 29.70)
LUO	512	c	20	1	-	5	-	3.10	(0.30- 40.20)
LUO	513	c	20	19	-	5	-	10.50	(2.30- 48.60)
LUO	514	c	20	14	-	5	-	12.40	(2.70- 57.70)
Subtotal LUO								9.22	(3.43- 24.79)
MATOS	616	m	2	6	-	3	-	3.30	(0.80- 13.90)
MATOS	617	m	2	25	-	3	-	7.90	(2.30- 27.10)
MATOS	618	m	2	16	-	3	-	7.20	(2.00- 25.90)
Subtotal MATOS								6.00	(2.82- 12.75)
PEZZOT	543	m	0	10	105	0	116	23.19~	(1.34- 400.59)
PEZZOT	544	m	0	45	145	0	116	72.86~	(4.44-1195.36)
PEZZOT	545	m	0	30	67	0	116	105.28~	(6.34-1749.54)
Subtotal PEZZOT								56.74	(11.15- 288.86)
SVENSS	509	f	0	9	18	5	120	12.00	(3.61- 39.85)
SVENSS	510	f	0	18	14	5	120	30.86	(9.92- 96.00)
SVENSS	511	f	0	15	21	5	120	17.14	(5.63- 52.19)
Subtotal SVENSS								18.79	(9.69- 36.45)
WAKAI	506	m	0	4	25	2	65	5.20	(0.90- 30.19)
WAKAI	507	m	0	61	183	2	65	10.83	(2.58- 45.57)
WAKAI	508	m	0	21	74	2	65	9.22	(2.08- 40.85)
Subtotal WAKAI								8.47	(3.47- 20.65)
WU	533	f	2	6	-	2	-	7.80	(0.80- 73.70)
WU	534	f	2	18	-	2	-	47.10	(4.40- 498.50)
WU	535	f	2	37	-	2	-	115.70	(9.80-1371.20)
Subtotal WU								32.22	(8.24- 125.94)
WYNDE6	608	m	0	44	92	9	589	31.30	(14.79- 66.26)
WYNDE6	609	m	0	81	139	9	589	38.14	(18.69- 77.81)
WYNDE6	610	m	0	262	301	9	589	56.96	(28.89- 112.31)
WYNDE6	632	f	0	32	90	12	673	19.94	(9.91- 40.11)
WYNDE6	633	f	0	49	94	12	673	29.23	(15.00- 56.97)
WYNDE6	634	f	0	72	91	12	673	44.37	(23.18- 84.93)
Subtotal WYNDE6								35.10	(26.47- 46.53)
ZHENG	520	m	0	13	66	4	94	4.63	(1.45- 14.82)
ZHENG	521	m	0	81	109	4	94	17.46	(6.17- 49.46)
ZHENG	522	m	0	62	43	4	94	33.88	(11.58- 99.14)
ZHENG	527	f	0	9	16	33	184	3.14	(1.28- 7.69)
ZHENG	528	f	0	34	28	33	184	6.77	(3.63- 12.62)
Subtotal ZHENG								7.97	(5.36- 11.85)
Partial Totals				2855	94596	421	184750		

*prospective study ~ With 0.5 adjustment for zero

Table 2H16 - 2

IESLC - Meta-analysis of Ever/current Smoking by Age started, Overview
Squamous, Cigarettes (or Any Product if Cigarettes not available)
Most adjusted

REF	NRR	SEX	AD	Ys	Ws	Qs	Ps
BARBON	574	m	1	2.24	5.21	1.08	0.0000
BARBON	575	m	1	2.62	5.44	0.03	0.0000
BARBON	576	m	1	4.27	4.27	10.54	0.0000
Subtotal BARBON				2.96	14.91	11.65	
BENHAM	514	m	0	2.66	16.33	0.02	0.0000
BENHAM	515	m	0	2.79	20.26	0.18	0.0000
BENHAM	516	m	0	2.82	20.14	0.29	0.0000
BENHAM	517	m	0	3.00	20.16	1.87	0.0000
Subtotal BENHAM				2.82	76.89	2.36	
*ENGELA	517	m	0	2.49	2.00	0.09	0.0004
*ENGELA	518	m	0	2.40	2.55	0.22	0.0001
*ENGELA	519	m	0	3.21	2.87	0.75	0.0000
Subtotal ENGELA				2.74	7.42	1.06	
HAENSZ	501	f	0	0.96	13.63	41.28	0.0004
HAENSZ	502	f	0	1.25	10.45	21.95	0.0001
Subtotal HAENSZ				1.08	24.08	63.23	
JEDRYC	528	m	0	2.55	5.57	0.11	0.0000
JEDRYC	529	m	0	3.04	5.40	0.64	0.0000
JEDRYC	530	m	0	3.14	5.05	0.98	0.0000
Subtotal JEDRYC				2.90	16.02	1.73	
LUO	512	c	20	1.13	0.64	1.57	0.3652
LUO	513	c	20	2.35	1.65	0.20	0.0025
LUO	514	c	20	2.52	1.64	0.05	0.0013
Subtotal LUO				2.22	3.93	1.82	
MATOS	616	m	2	1.19	1.89	4.25	0.1012
MATOS	617	m	2	2.07	2.53	1.00	0.0010
MATOS	618	m	2	1.97	2.34	1.22	0.0025
Subtotal MATOS				1.79	6.75	6.47	
PEZZOT	543	m	0	3.14	0.47	0.09	0.0306
PEZZOT	544	m	0	4.29	0.49	1.24	0.0027
PEZZOT	545	m	0	4.66	0.49	1.87	0.0012
Subtotal PEZZOT				4.04	1.45	3.21	
SVENSS	509	f	0	2.48	2.67	0.12	0.0000
SVENSS	510	f	0	3.43	2.98	1.60	0.0000
SVENSS	511	f	0	2.84	3.10	0.07	0.0000
Subtotal SVENSS				2.93	8.75	1.79	
WAKAI	506	m	0	1.65	1.24	1.36	0.0662
WAKAI	507	m	0	2.38	1.86	0.18	0.0012
WAKAI	508	m	0	2.22	1.73	0.39	0.0034
Subtotal WAKAI				2.14	4.84	1.93	
WU	533	f	2	2.05	0.75	0.31	0.0750
WU	534	f	2	3.85	0.69	0.92	0.0014
WU	535	f	2	4.75	0.63	2.66	0.0002
Subtotal WU				3.47	2.07	3.89	
WYNDE6	608	m	0	3.44	6.83	3.82	0.0000
WYNDE6	609	m	0	3.64	7.56	6.75	0.0000
WYNDE6	610	m	0	4.04	8.34	15.11	0.0000
WYNDE6	632	f	0	2.99	7.86	0.69	0.0000
WYNDE6	633	f	0	3.38	8.63	3.98	0.0000
WYNDE6	634	f	0	3.79	9.12	10.96	0.0000
Subtotal WYNDE6				3.56	48.33	41.32	
ZHENG	520	m	0	1.53	2.84	3.84	0.0099
ZHENG	521	m	0	2.86	3.54	0.10	0.0000
ZHENG	522	m	0	3.52	3.33	2.28	0.0000
ZHENG	527	f	0	1.14	4.78	11.52	0.0125
ZHENG	528	f	0	1.91	9.91	6.08	0.0000
Subtotal ZHENG				2.08	24.40	23.82	

N	44
NS	13

Table 2H16 - 3

IESLC - Meta-analysis of Ever/current Smoking by Age started, Overview
Squamous, Cigarettes (or Any Product if Cigarettes not available)
Most adjusted

	Sex			
	combined	male	female	Total
N	3	28	13	44
NS	1	9	5	15

In this overview table, other than the "N" rows, entries in the "absent" and "Total" columns may be invalid and should be ignored

	Age started (broad categories)				
	absent	19+k26	15-25k18	1-17k14	Total
N	18	14	6	6	44
NS	9	13	5	5	32
Wt	70.07	70.81	49.70	49.28	239.85
Het Chi	49.57	43.67	8.01	16.38	164.28
Het df	17	13	5	5	43
Het P	***	***	N.S.	**	***
Fixed RR	10.39	10.42	20.15	29.91	14.82
RRl	8.22	8.25	15.26	22.62	13.06
RRu	13.13	13.15	26.61	39.54	16.82
P	+++	+++	+++	+++	+++
Random RR	12.12	11.06	20.28	31.07	14.89
RRl	7.59	6.87	13.92	17.93	11.32
RRu	19.35	17.81	29.53	53.85	19.57
P	+++	+++	+++	+++	+++

		Age started (narrow categories)						
	absent	27+k30	23-29k26	19-25k22	15-21k18	11-17k14	1-13k10	Total
N	30	4		3	6		1	44
NS	13	3		3	5		1	25
Wt	154.89	10.85		23.93	49.70		0.49	239.85
Het Chi	129.72	2.56		1.71	8.01		0.00	164.28
Het df	29	3		2	5		0	43
Het P	***	N.S.		N.S.	N.S.		N.S.	***
Fixed RR	14.01	4.71		18.16	20.15		105.28	14.82
RRl	11.97	2.60		12.17	15.26		6.34	13.06
RRu	16.40	8.54		27.11	26.61		1749.54	16.82
P	+++	+++		+++	+++		++	+++
Random RR	14.79	4.71		18.16	20.28		105.28	14.89
RRl	10.26	2.60		12.17	13.92		6.34	11.32
RRu	21.34	8.54		27.11	29.53		1749.54	19.57
P	+++	+++		+++	+++		++	+++

Table 2H16 - 3

IESLC - Meta-analysis of Ever/current Smoking by Age started, Overview
Squamous, Cigarettes (or Any Product if Cigarettes not available)
Most adjusted

MALES

		Age started (broad categories)				
		absent	19+k26	15-25k18	1-17k14	Total
	N	9	9	5	5	28
	NS	5	9	5	5	24
	Wt	35.25	44.25	41.06	40.16	160.72
Het	Chi	11.88	9.79	6.56	14.64	53.10
Het	df	8	8	4	4	27
Het	P	N.S.	N.S.	N.S.	**	**
Fixed	RR	15.69	14.17	18.64	27.35	18.32
	RRl	11.28	10.56	13.72	20.07	15.69
	RRu	21.82	19.03	25.30	37.26	21.38
	P	+++	+++	+++	+++	+++
Random	RR	15.33	13.87	18.61	28.44	17.70
	RRl	9.28	9.79	12.12	14.64	13.87
	RRu	25.35	19.66	28.56	55.25	22.58
	P	+++	+++	+++	+++	+++

				Age started (narrow categories)					
		absent	27+k30	23-29k26	19-25k22	15-21k18	11-17k14	1-13k10	Total
	N	18	3		1	5		1	28
	NS	9	3		1	5		1	19
	Wt	92.83	6.08		20.26	41.06		0.49	160.72
Het	Chi	36.52	1.15		0.00	6.56		0.00	53.10
Het	df	17	2		0	4		0	27
Het	P	**	N.S.		N.S.	N.S.		N.S.	**
Fixed	RR	19.78	6.49		16.26	18.64		105.28	18.32
	RRl	16.14	2.93		10.52	13.72		6.34	15.69
	RRu	24.24	14.38		25.14	25.30		1749.54	21.38
	P	+++	+++		+++	+++		++	+++
Random	RR	19.06	6.49		16.26	18.61		105.28	17.70
	RRl	13.67	2.93		10.52	12.12		6.34	13.87
	RRu	26.58	14.38		25.14	28.56		1749.54	22.58
	P	+++	+++		+++	+++		++	+++

FEMALES

		Age started (broad categories)				
		absent	19+k26	15-25k18	1-17k14	Total
	N	7	4	1	1	13
	NS	4	4	1	1	8
	Wt	32.54	24.91	8.63	9.12	75.20
Het	Chi	24.65	22.20	0.00	0.00	88.38
Het	df	6	3	0	0	12
Het	P	***	***	N.S.	N.S.	***
Fixed	RR	6.75	6.02	29.23	44.37	9.66
	RRl	4.79	4.07	15.00	23.18	7.71
	RRu	9.51	8.92	56.97	84.93	12.11
	P	+++	+++	+++	+++	+++
Random	RR	10.63	8.18	29.23	44.37	12.72
	RRl	4.82	2.30	15.00	23.18	6.54
	RRu	23.45	29.06	56.97	84.93	24.75
	P	+++	++	+++	+++	+++

Table 2H16 - 3

IESLC - Meta-analysis of Ever/current Smoking by Age started, Overview
Squamous, Cigarettes (or Any Product if Cigarettes not available)
Most adjusted

FEMALES

	absent	Age started (narrow categories) 27+k30	23-29k26	19-25k22	15-21k18	11-17k14	1-13k10	Total
N	9	1		2	1			13
NS	5	1		2	1			8
Wt	58.12	4.78		3.67	8.63			75.20
Het Chi	64.70	0.00		0.10	0.00			88.38
Het df	8	0		1	0			12
Het P	***	N.S.		N.S.	N.S.			***
Fixed RR	8.31	3.14		33.40	29.23			9.66
RRl	6.43	1.28		12.00	15.00			7.71
RRu	10.75	7.69		92.92	56.97			12.11
P	+++	+		+++	+++			+++
Random RR	11.31	3.14		33.40	29.23			12.72
RRl	5.09	1.28		12.00	15.00			6.54
RRu	25.13	7.69		92.92	56.97			24.75
P	+++	+		+++	+++			+++

Table 2H16 - 4

IESLC - Meta-analysis of Ever/current Smoking by Age started, Overview
Squamous, Cigarettes (or Any Product if Cigarettes not available)
Least adjusted

REF	NRR	X	SEX	AGEL	AGEH	RACE	YF	LC TYPE	LOC	START	ST	NLC	R	VB	P	H	AD	SM	PRODUCT	exL	exH	S1	S2	DENOM	De
BARBON	569	x	m	0	0	all	-	q	Eu:wst	1979	CC	755	n	bl	y	y	0	ev	all/unsp	20	999	1	0	nev any	st
BARBON	570	x	m	0	0	all	-	q	Eu:wst	1979	CC	755	n	bl	y	y	0	ev	all/unsp	15	19	2	4	nev any	st
BARBON	571	x	m	0	0	all	-	q	Eu:wst	1979	CC	755	n	bl	y	y	0	ev	all/unsp	1	14	3	0	nev any	st
BENHAM	514		m	0	0	all	-	KI	Eu:wst	1976	CC	1625	n	bl	n	y	0	ev	cig only	25	999	1	0	nev any	st
BENHAM	515		m	0	0	all	-	KI	Eu:wst	1976	CC	1625	n	bl	n	y	0	ev	cig only	20	24	0	3	nev any	st
BENHAM	516		m	0	0	all	-	KI	Eu:wst	1976	CC	1625	n	bl	n	y	0	ev	cig only	17	19	2	4	nev any	st
BENHAM	517		m	0	0	all	-	KI	Eu:wst	1976	CC	1625	n	bl	n	y	0	ev	cig only	1	16	3	0	nev any	st
ENGELA	517		m	0	0	all	0	q	Eu:Sca	1964	pr	435	n	bl	n	n	0	cu	cig+/-ot	30	999	0	1	nev cigs	st
ENGELA	518		m	0	0	all	0	q	Eu:Sca	1964	pr	435	n	bl	n	n	0	cu	cig+/-ot	20	29	1	0	nev cigs	st
ENGELA	519		m	0	0	all	0	q	Eu:Sca	1964	pr	435	n	bl	n	n	0	cu	cig+/-ot	1	19	0	0	nev cigs	st
HAENSZ	501		f	0	0	all	-	q+u	NAmer	1955	CC	158	n	bl	n	y	0	ev	cig+/-ot	25	999	1	0	nev any	st
HAENSZ	502		f	0	0	all	-	q+u	NAmer	1955	CC	158	n	bl	n	y	0	ev	cig+/-ot	1	24	0	0	nev any	st
JEDRYC	528		m	0	0	all	-	q	Eu:est	1980	CC	1630	n	bl	y	n	0	ev	cig+/-ot	19	999	1	0	nev any	st
JEDRYC	529		m	0	0	all	-	q	Eu:est	1980	CC	1630	n	bl	y	n	0	ev	cig+/-ot	17	18	2	4	nev any	st
JEDRYC	530		m	0	0	all	-	q	Eu:est	1980	CC	1630	n	bl	y	n	0	ev	cig+/-ot	1	16	3	0	nev any	st
LUO	507	x	c	0	0	all	-	q	As:Chi	1990	CC	102	n	ot	n	y	0	ev	cig+/-ot	40	999	0	0	nev cigs	st
LUO	508	x	c	0	0	all	-	q	As:Chi	1990	CC	102	n	ot	n	y	0	ev	cig+/-ot	20	39	1	0	nev cigs	st
LUO	509	x	c	0	0	all	-	q	As:Chi	1990	CC	102	n	ot	n	y	0	ev	cig+/-ot	0	19	0	0	nev cigs	st
MATOS	611	x	m	0	0	all	-	q	SCAmer	1994	CC	200	n	bl	n	n	0	ev	cig+/-ot	20	999	1	0	nev any	st
MATOS	612	x	m	0	0	all	-	q	SCAmer	1994	CC	200	n	bl	n	n	0	ev	cig+/-ot	15	19	2	4	nev any	st
MATOS	613	x	m	0	0	all	-	q	SCAmer	1994	CC	200	n	bl	n	n	0	ev	cig+/-ot	1	14	3	0	nev any	st
PEZZOT	543		m	0	0	all	-	q	SCAmer	1987	CC	215	n	bl	n	y	0	ev	cig only	19	999	1	0	nev cigs	ot
PEZZOT	544		m	0	0	all	-	q	SCAmer	1987	CC	215	n	bl	n	y	0	ev	cig only	14	18	0	0	nev cigs	ot
PEZZOT	545		m	0	0	all	-	q	SCAmer	1987	CC	215	n	bl	n	y	0	ev	cig only	1	13	0	6	nev cigs	ot
SVENSS	509		f	0	0	all	-	q	Eu:Sca	1983	CC	210	n	bl	n	n	0	cu	all/unsp	26	999	1	0	nev any	st
SVENSS	510		f	0	0	all	-	q	Eu:Sca	1983	CC	210	n	bl	n	n	0	cu	all/unsp	19	25	0	3	nev any	st
SVENSS	511		f	0	0	all	-	q	Eu:Sca	1983	CC	210	n	bl	n	n	0	cu	all/unsp	0	18	0	0	nev any	st
WAKAI	506		m	0	0	all	-	q	As:Jap	1988	CC	333	n	bl	n	y	0	cu	cig+/-ot	30	999	0	1	nev any	st
WAKAI	507		m	0	0	all	-	q	As:Jap	1988	CC	333	n	bl	n	y	0	cu	cig+/-ot	20	29	1	0	nev any	st
WAKAI	508		m	0	0	all	-	q	As:Jap	1988	CC	333	n	bl	n	y	0	cu	cig+/-ot	1	19	0	0	nev any	st
WU	509	x	f	0	0	wh	-	q	NAmer	1981	CC	220	n	bl	n	y	0	cu	all/unsp	25	999	1	0	nev any	st
WU	510	x	f	0	0	wh	-	q	NAmer	1981	CC	220	n	bl	n	y	0	cu	all/unsp	19	24	0	3	nev any	st
WU	511	x	f	0	0	wh	-	q	NAmer	1981	CC	220	n	bl	n	y	0	cu	all/unsp	0	18	0	0	nev any	st
WYNDE6	608		m	0	0	wh	-	q	NAmer	1969	CC	4423	n	bl	n	y	0	ev	cig+/-ot	21	999	1	0	nev cigs	st
WYNDE6	609		m	0	0	wh	-	q	NAmer	1969	CC	4423	n	bl	n	y	0	ev	cig+/-ot	18	20	2	4	nev cigs	st
WYNDE6	610		m	0	0	wh	-	q	NAmer	1969	CC	4423	n	bl	n	y	0	ev	cig+/-ot	1	17	3	0	nev cigs	st
WYNDE6	632		f	0	0	wh	-	q	NAmer	1969	CC	4423	n	bl	n	y	0	ev	cig+/-ot	21	999	1	0	nev cigs	st
WYNDE6	633		f	0	0	wh	-	q	NAmer	1969	CC	4423	n	bl	n	y	0	ev	cig+/-ot	18	20	2	4	nev cigs	st
WYNDE6	634		f	0	0	wh	-	q	NAmer	1969	CC	4423	n	bl	n	y	0	ev	cig+/-ot	1	17	3	0	nev cigs	st
ZHENG	520		m	0	0	all	-	q	As:Chi	1982	CC	540	n	ot	*	y	0	ev	cig+/-ot	30	999	0	1	nev cigs	st
ZHENG	521		m	0	0	all	-	q	As:Chi	1982	CC	540	n	ot	*	y	0	ev	cig+/-ot	20	29	1	0	nev cigs	st
ZHENG	522		m	0	0	all	-	q	As:Chi	1982	CC	540	n	ot	*	y	0	ev	cig+/-ot	1	19	0	0	nev cigs	st
ZHENG	527		f	0	0	all	-	q	As:Chi	1982	CC	540	n	ot	*	y	0	ev	cig+/-ot	30	999	0	1	nev cigs	st
ZHENG	528		f	0	0	all	-	q	As:Chi	1982	CC	540	n	ot	*	y	0	ev	cig+/-ot	1	29	0	0	nev cigs	st

Cigarette type is all/unspec for all RRs

In this overview table, subtotals and Qs values may be invalid and should be ignored

Table 2H16 - 5

IESLC - Meta-analysis of Ever/current Smoking by Age started, Overview
Squamous, Cigarettes (or Any Product if Cigarettes not available)
Least adjusted

REF	NRR	SEX	AD	Number Exposed Case	Number Exposed Cont	Non-exposed Case	Non-exposed Cont	RR	95.00%CI
BARBON	569	m	0	64	207	6	188	9.69	(4.10- 22.89)
BARBON	570	m	0	145	337	6	188	13.48	(5.84- 31.10)
BARBON	571	m	0	52	23	6	188	70.84	(27.41- 183.08)
Subtotal BARBON								19.28	(11.62- 32.01)
BENHAM	514	m	0	98	137	24	481	14.34	(8.83- 23.29)
BENHAM	515	m	0	323	398	24	481	16.26	(10.52- 25.14)
BENHAM	516	m	0	311	373	24	481	16.71	(10.80- 25.86)
BENHAM	517	m	0	342	341	24	481	20.10	(12.99- 31.10)
Subtotal BENHAM								16.86	(13.48- 21.08)
*ENGELA	517	m	0	6	9762	3	58716	12.03	(3.01- 48.09)
*ENGELA	518	m	0	17	30195	3	58716	11.02	(3.23- 37.60)
*ENGELA	519	m	0	64	50732	3	58716	24.69	(7.76- 78.58)
Subtotal ENGELA								15.41	(7.50- 31.65)
HAENSZ	501	f	0	32	66	44	236	2.60	(1.53- 4.42)
HAENSZ	502	f	0	24	37	44	236	3.48	(1.90- 6.38)
Subtotal HAENSZ								2.95	(1.98- 4.40)
JEDRYC	528	m	0	134	502	6	289	12.86	(5.60- 29.50)
JEDRYC	529	m	0	96	221	6	289	20.92	(9.00- 48.62)
JEDRYC	530	m	0	53	111	6	289	23.00	(9.61- 55.01)
Subtotal JEDRYC								18.20	(11.15- 29.70)
LUO	507	c	0	1	8	5	51	1.28	(0.13- 12.37)
LUO	508	c	0	19	38	5	51	5.10	(1.75- 14.88)
LUO	509	c	0	14	20	5	51	7.14	(2.27- 22.43)
Subtotal LUO								5.07	(2.42- 10.62)
MATOS	611	m	0	6	73	3	110	3.01	(0.73- 12.43)
MATOS	612	m	0	25	120	3	110	7.64	(2.24- 26.01)
MATOS	613	m	0	16	90	3	110	6.52	(1.84- 23.08)
Subtotal MATOS								5.58	(2.64- 11.78)
PEZZOT	543	m	0	10	105	0	116	23.19~	(1.34- 400.59)
PEZZOT	544	m	0	45	145	0	116	72.86~	(4.44-1195.36)
PEZZOT	545	m	0	30	67	0	116	105.28~	(6.34-1749.54)
Subtotal PEZZOT								56.74	(11.15- 288.86)
SVENSS	509	f	0	9	18	5	120	12.00	(3.61- 39.85)
SVENSS	510	f	0	18	14	5	120	30.86	(9.92- 96.00)
SVENSS	511	f	0	15	21	5	120	17.14	(5.63- 52.19)
Subtotal SVENSS								18.79	(9.69- 36.45)
WAKAI	506	m	0	4	25	2	65	5.20	(0.90- 30.19)
WAKAI	507	m	0	61	183	2	65	10.83	(2.58- 45.57)
WAKAI	508	m	0	21	74	2	65	9.22	(2.08- 40.85)
Subtotal WAKAI								8.47	(3.47- 20.65)
WU	509	f	0	6	5	2	30	18.00	(2.80- 115.56)
WU	510	f	0	18	7	2	30	38.57	(7.21- 206.25)
WU	511	f	0	37	11	2	30	50.45	(10.37- 245.38)
Subtotal WU								34.61	(13.01- 92.08)
WYNDE6	608	m	0	44	92	9	589	31.30	(14.79- 66.26)
WYNDE6	609	m	0	81	139	9	589	38.14	(18.69- 77.81)
WYNDE6	610	m	0	262	301	9	589	56.96	(28.89- 112.31)
WYNDE6	632	f	0	32	90	12	673	19.94	(9.91- 40.11)
WYNDE6	633	f	0	49	94	12	673	29.23	(15.00- 56.97)
WYNDE6	634	f	0	72	91	12	673	44.37	(23.18- 84.93)
Subtotal WYNDE6								35.10	(26.47- 46.53)
ZHENG	520	m	0	13	66	4	94	4.63	(1.45- 14.82)
ZHENG	521	m	0	81	109	4	94	17.46	(6.17- 49.46)
ZHENG	522	m	0	62	43	4	94	33.88	(11.58- 99.14)
ZHENG	527	f	0	9	16	33	184	3.14	(1.28- 7.69)
ZHENG	528	f	0	34	28	33	184	6.77	(3.63- 12.62)
Subtotal ZHENG								7.97	(5.36- 11.85)
Totals				2855	95535	421	185887		

*prospective study ~ With 0.5 adjustment for zero

Table 2H16 - 5

IESLC - Meta-analysis of Ever/current Smoking by Age started, Overview
Squamous, Cigarettes (or Any Product if Cigarettes not available)
Least adjusted

REF	NRR	SEX	AD	Ys	Ws	Qs	Ps
BARBON	569	m	0	2.27	5.20	0.86	0.0000
BARBON	570	m	0	2.60	5.50	0.03	0.0000
BARBON	571	m	0	4.26	4.26	10.67	0.0000
Subtotal BARBON				2.96	14.96	11.57	
BENHAM	514	m	0	2.66	16.33	0.00	0.0000
BENHAM	515	m	0	2.79	20.26	0.25	0.0000
BENHAM	516	m	0	2.82	20.14	0.38	0.0000
BENHAM	517	m	0	3.00	20.16	2.10	0.0000
Subtotal BENHAM				2.82	76.89	2.74	
*ENGELA	517	m	0	2.49	2.00	0.07	0.0004
*ENGELA	518	m	0	2.40	2.55	0.20	0.0001
*ENGELA	519	m	0	3.21	2.87	0.80	0.0000
Subtotal ENGELA				2.74	7.42	1.07	
HAENSZ	501	f	0	0.96	13.63	40.42	0.0004
HAENSZ	502	f	0	1.25	10.45	21.41	0.0001
Subtotal HAENSZ				1.08	24.08	61.83	
JEDRYC	528	m	0	2.55	5.57	0.09	0.0000
JEDRYC	529	m	0	3.04	5.40	0.71	0.0000
JEDRYC	530	m	0	3.14	5.05	1.06	0.0000
Subtotal JEDRYC				2.90	16.02	1.86	
LUO	507	c	0	0.24	0.74	4.41	0.8340
LUO	508	c	0	1.63	3.35	3.68	0.0029
LUO	509	c	0	1.97	2.93	1.49	0.0008
Subtotal LUO				1.62	7.03	9.58	
MATOS	611	m	0	1.10	1.91	4.74	0.1271
MATOS	612	m	0	2.03	2.56	1.06	0.0011
MATOS	613	m	0	1.87	2.40	1.55	0.0037
Subtotal MATOS				1.72	6.88	7.36	
PEZZOT	543	m	0	3.14	0.47	0.10	0.0306
PEZZOT	544	m	0	4.29	0.49	1.27	0.0027
PEZZOT	545	m	0	4.66	0.49	1.90	0.0012
Subtotal PEZZOT				4.04	1.45	3.28	
SVENSS	509	f	0	2.48	2.67	0.10	0.0000
SVENSS	510	f	0	3.43	2.98	1.68	0.0000
SVENSS	511	f	0	2.84	3.10	0.08	0.0000
Subtotal SVENSS				2.93	8.75	1.87	
WAKAI	506	m	0	1.65	1.24	1.32	0.0662
WAKAI	507	m	0	2.38	1.86	0.16	0.0012
WAKAI	508	m	0	2.22	1.73	0.36	0.0034
Subtotal WAKAI				2.14	4.84	1.84	
WU	509	f	0	2.89	1.11	0.05	0.0023
WU	510	f	0	3.65	1.37	1.30	0.0000
WU	511	f	0	3.92	1.54	2.37	0.0000
Subtotal WU				3.54	4.01	3.72	
WYNDE6	608	m	0	3.44	6.83	4.01	0.0000
WYNDE6	609	m	0	3.64	7.56	7.01	0.0000
WYNDE6	610	m	0	4.04	8.34	15.53	0.0000
WYNDE6	632	f	0	2.99	7.86	0.78	0.0000
WYNDE6	633	f	0	3.38	8.63	4.20	0.0000
WYNDE6	634	f	0	3.79	9.12	11.33	0.0000
Subtotal WYNDE6				3.56	48.33	42.85	
ZHENG	520	m	0	1.53	2.84	3.72	0.0099
ZHENG	521	m	0	2.86	3.54	0.12	0.0000
ZHENG	522	m	0	3.52	3.33	2.38	0.0000
ZHENG	527	f	0	1.14	4.78	11.25	0.0125
ZHENG	528	f	0	1.91	9.91	5.81	0.0000
Subtotal ZHENG				2.08	24.40	23.27	

N	44
NS	13

Table 2H16 - 6

IESLC - Meta-analysis of Ever/current Smoking by Age started, Overview
Squamous, Cigarettes (or Any Product if Cigarettes not available)
Least adjusted

	Sex			
	combined	male	female	Total
N	3	28	13	44
NS	1	9	5	15

In this overview table, other than the "N" rows, entries in the "absent" and "Total" columns may be invalid and should be ignored

	Age started (broad categories)				
	absent	19+k26	15-25k18	1-17k14	Total
N	18	14	6	6	44
NS	9	13	5	5	32
Wt	73.05	72.88	49.79	49.33	245.06
Het Chi	53.22	46.03	8.28	17.16	172.83
Het df	17	13	5	5	43
Het P	***	***	N.S.	**	***
Fixed RR	10.31	10.19	20.06	29.69	14.55
RRl	8.20	8.10	15.19	22.46	12.84
RRu	12.97	12.81	26.48	39.25	16.49
P	+++	+++	+++	+++	+++
Random RR	11.65	10.79	20.14	30.58	14.40
RRl	7.33	6.71	13.74	17.44	10.95
RRu	18.51	17.35	29.52	53.64	18.94
P	+++	+++	+++	+++	+++

			Age started (narrow categories)					
	absent	27+k30	23-29k26	19-25k22	15-21k18	11-17k14	1-13k10	Total
N	30	4		3	6		1	44
NS	13	3		3	5		1	25
Wt	159.32	10.85		24.61	49.79		0.49	245.06
Het Chi	137.24	2.56		1.85	8.28		0.00	172.83
Het df	29	3		2	5		0	43
Het P	***	N.S.		N.S.	N.S.		N.S.	***
Fixed RR	13.62	4.71		18.44	20.06		105.28	14.55
RRl	11.66	2.60		12.42	15.19		6.34	12.84
RRu	15.91	8.54		27.38	26.48		1749.54	16.49
P	+++	+++		+++	+++		++	+++
Random RR	14.02	4.71		18.44	20.14		105.28	14.40
RRl	9.72	2.60		12.42	13.74		6.34	10.95
RRu	20.21	8.54		27.38	29.52		1749.54	18.94
P	+++	+++		+++	+++		++	+++

Table 2H16 - 6

IESLC - Meta-analysis of Ever/current Smoking by Age started, Overview
Squamous, Cigarettes (or Any Product if Cigarettes not available)
Least adjusted

MALES

		Age started (broad categories)				
		absent	19+k26	15-25k18	1-17k14	Total
	N	9	9	5	5	28
	NS	5	9	5	5	24
	Wt	35.25	44.26	41.16	40.21	160.89
Het	Chi	11.88	10.24	6.80	15.35	54.24
Het	df	8	8	4	4	27
Het	P	N.S.	N.S.	N.S.	**	**
Fixed	RR	15.69	14.16	18.53	27.11	18.25
	RRl	11.28	10.54	13.66	19.90	15.63
	RRu	21.82	19.01	25.16	36.92	21.29
	P	+++	+++	+++	+++	+++
Random	RR	15.33	13.78	18.45	27.89	17.56
	RRl	9.28	9.62	11.93	14.15	13.73
	RRu	25.35	19.75	28.54	54.95	22.46
	P	+++	+++	+++	+++	+++

			Age started (narrow categories)						
		absent	27+k30	23-29k26	19-25k22	15-21k18	11-17k14	1-13k10	Total
	N	18	3		1	5		1	28
	NS	9	3		1	5		1	19
	Wt	92.90	6.08		20.26	41.16		0.49	160.89
Het	Chi	37.49	1.15		0.00	6.80		0.00	54.24
Het	df	17	2		0	4		0	27
Het	P	**	N.S.		N.S.	N.S.		N.S.	**
Fixed	RR	19.70	6.49		16.26	18.53		105.28	18.25
	RRl	16.07	2.93		10.52	13.66		6.34	15.63
	RRu	24.14	14.38		25.14	25.16		1749.54	21.29
	P	+++	+++		+++	+++		++	+++
Random	RR	18.89	6.49		16.26	18.45		105.28	17.56
	RRl	13.49	2.93		10.52	11.93		6.34	13.73
	RRu	26.45	14.38		25.14	28.54		1749.54	22.46
	P	+++	+++		+++	+++		++	+++

FEMALES

		Age started (broad categories)				
		absent	19+k26	15-25k18	1-17k14	Total
	N	7	4	1	1	13
	NS	4	4	1	1	8
	Wt	34.13	25.27	8.63	9.12	77.15
Het	Chi	27.18	23.44	0.00	0.00	89.90
Het	df	6	3	0	0	12
Het	P	***	***	N.S.	N.S.	***
Fixed	RR	7.23	6.27	29.23	44.37	10.00
	RRl	5.17	4.25	15.00	23.18	8.00
	RRu	10.11	9.26	56.97	84.93	12.49
	P	+++	+++	+++	+++	+++
Random	RR	11.17	9.54	29.23	44.37	13.30
	RRl	5.14	2.70	15.00	23.18	6.97
	RRu	24.29	33.79	56.97	84.93	25.40
	P	+++	+++	+++	+++	+++

Table 2H16 - 6

IESLC - Meta-analysis of Ever/current Smoking by Age started, Overview
Squamous, Cigarettes (or Any Product if Cigarettes not available)
Least adjusted

FEMALES

	absent	Age started (narrow categories) 27+k30	23-29k26	19-25k22	15-21k18	11-17k14	1-13k10	Total
N	9	1		2	1			13
NS	5	1		2	1			8
Wt	59.39	4.78		4.35	8.63			77.15
Het Chi	65.92	0.00		0.05	0.00			89.90
Het df	8	0		1	0			12
Het P	***	N.S.		N.S.	N.S.			***
Fixed RR	8.60	3.14		33.10	29.23			10.00
RRl	6.67	1.28		12.93	15.00			8.00
RRu	11.09	7.69		84.72	56.97			12.49
P	+++	+		+++	+++			+++
Random RR	11.95	3.14		33.10	29.23			13.30
RRl	5.49	1.28		12.93	15.00			6.97
RRu	26.04	7.69		84.72	56.97			25.40
P	+++	+		+++	+++			+++

Table 2H16 - 7

IESLC - Meta-analysis of Ever/current Smoking by Age started, Overview
Squamous, Cigarettes (or Any Product if Cigarettes not available)
Excluded studies (and stage at which they were excluded)

1	BECHER BLOT1 BROWN3 CARPEN CHYOU DARBY DOLL2 GARCIA GRAHAM GURSEL HAMMO2 JAHN JAIN LAUSSM PRESCO QIAO TVERDA WIGLE WYNDE3
2	AKIBA AMANDU AMES AXELSS BENSHL BEST BOUCHA BOUCOT BROSS CHEN CPSII DEAN2 DESTEF DORGAN DOSEME FAN GARSHI GER GILLIS HAMMON HUMBLE JUSSAW KAISE2 KATSOU KAUFMA KOO KREUZE LEVIN MCCONN NOTAN2 OSANN2 PEZZO2 PISANI RESTRE SADOWS VUTUC WANG2 WATSON WU2 WUWILL WYNDE2 WYNDE8 XU ZHOU
3	GUO MCDUFF SPITZ STASZE ZHANG
4	AGUDO ARMADA AUVINE BOFFET BRESLO BUFFLE CEDERL CHEN2 CHIAZZ CHOI CORREA CPSI DAMBER DEAN3 DOLL DORN GAO GAO2 GENG HIRAYA HOLE HU HU2 JOLY KOULUM LETOUR LIAW LIU3 LIU4 LIU5 LUBIN LUBIN2 MIGRAN MRFITR PERNU QIAO2 RACHTA SEGI2 SOBUE SPEIZE SUZUK2 TIZZAN WYNDE7 YUAN
5	ALDERS
10	HEGMAN KHUDER

Table 2H16 - 8
Potentially overlapping studies

REF	REFGP	PRINC	. OVERLAP/LINK
BENHAM	LUBIN2	2	Subset of Lubin2
WYNDE6	WYNDE6	1	WYNDE5/6/7/8

Table 2H17 -

IESLC - Meta-analysis of Ever/current Smoking, Age started, "Low"
Squamous, Cigarettes (or Any Product if Cigarettes not available)

This analysis is restricted to results for:
1) Ever/current smokers
2) Results by Age started
3) Categorical results by Age started
4) Squamous (or near equivalent)
5) Results complete enough for use in metaanalysis

Within each study, results are then selected (in the following order of preference, within each sex) for:
6) SMKSTA: ever, current
7) PRODUCT: cigarettes regardless of other products, cigarettes only, all/unspec
8) CIGTYPE: all/unspecified, MC regardless of HR, MC only
9) (not applicable)
10) DENOM: never smoked anything, never smoked cigarettes, never any + low, never cigs + low
11) Followup period (YF, prospective studies): whole study (coded as 0) or longest available
12) LCtype: squamous or nearest available, but not adeno. (q = squamous, s = small,
a = adeno, KI = Kreyberg I, u = undifferentiated)
13) Race: all or nearest available, otherwise by race (wh or w = white, bl or b = black, hi = hispanic
ch = chinese, jap = japanese, haw = hawaiian, w+o = white + oriental, sca = scandinavian, as = asian)
14) Age started "low" in key scheme 1 (key value 26, maximum range 19+)
15) For overlapping studies: principal rather than subsidiary studies
Finally by Age: whole study (coded as 0) if available, otherwise by widest available age group
and then for single sex results (m, f) in preference to results for both sexes combined (c).

Results adjusted (AD) for the most potential confounders are then chosen in Sections -1 to -3
(and those which actually differ from the adjusted results in Table 2H12 - 1 are marked 'x' in Section -1)
and results adjusted for the least confounders in Sections -4 to -6. (Those least adjusted results which
actually differ from the most adjusted are marked 'x' in column X in Section -4)

Section -7 shows excluded studies, together with the stage (as above) at which no qualifying
results were found.

Section -8 lists the potentially overlapping studies which have been included (1=principal, 2=subsidiary).

Section -9 lists any results which would have been included in preference except that they had data not complete
enough for use in meta-analysis, with their significance (yes/no), if known, and any further comment as entered
on the database. It also lists as "gap" any categories for which no data were presented by the original authors.

In addition to those mentioned above, the following fields, levels and abbreviations are used:
* or nk = not known, n = no, y = yes, ot = other
ev = ever, cu = current, nev = never
all/unspec = all or unspecified, cig+/-ot = cigarettes irrespective of other products (cigar, pipe etc)
MC = manufactured cigarettes, HR = hand-rolled cigarettes
exL, exH = range of exposure (low and high) in the smoking group, in terms of Age started
REF: 6-character study reference
NRR: number of the RR on the database within the study
ST : study type (CC = case control, pr or prosp = prospective)
NLC: number of lung cancer cases in whole study
R : risky occupational population (n = no, m = mining, o = other risky)
VB : national cigarette type (V = at least 75% Virginia, bl = at least 75% blended, ot = other)
P : any proxy use
H : full histological confirmation
De : derivation of RR/CI (or = original, st = standard method, ot = other method of estimation)

Table 2H17 - 1

IESLC - Meta-analysis of Ever/current Smoking, Age started, "Low"
Squamous, Cigarettes (or Any Product if Cigarettes not available)
Most adjusted

REF	NRR	2H12	SEX	AGEL	AGEH	RACE	YF	LC TYPE	LOC	START	ST	NLC	R	VB	P	H	AD	SM	PRODUCT	exL	exH	DENOM	De
BARBON	574		m	0	0	all	-	q	Eu:wst	1979	CC	755	n	bl	y	y	1	ev	all/unsp	20	999	nev any	or
BENHAM	514		m	0	0	all	-	KI	Eu:wst	1976	CC	1625	n	bl	n	y	0	ev	cig only	25	999	nev any	st
ENGELA	518		m	0	0	all	0	q	Eu:Sca	1964	pr	435	n	bl	n	n	0	cu	cig+/-ot	20	29	nev cigs	st
HAENSZ	501		f	0	0	all	-	q+u	NAmer	1955	CC	158	n	bl	n	y	0	ev	cig+/-ot	25	999	nev any	st
JEDRYC	528		m	0	0	all	-	q	Eu:est	1980	CC	1630	n	bl	y	n	0	ev	cig+/-ot	19	999	nev any	st
LUO	513		c	0	0	all	-	q	As:Chi	1990	CC	102	n	ot	n	y	20	ev	cig+/-ot	20	39	nev cigs	or
MATOS	616		m	0	0	all	-	q	SCAmer	1994	CC	200	n	bl	n	n	2	ev	cig+/-ot	20	999	nev any	or
PEZZOT	543		m	0	0	all	-	q	SCAmer	1987	CC	215	n	bl	n	y	0	ev	cig only	19	999	nev cigs	ot
SVENSS	509		f	0	0	all	-	q	Eu:Sca	1983	CC	210	n	bl	n	n	0	cu	all/unsp	26	999	nev any	st
WAKAI	507		m	0	0	all	-	q	As:Jap	1988	CC	333	n	bl	n	y	0	cu	cig+/-ot	20	29	nev any	st
WU	533		f	0	0	wh	-	q	NAmer	1981	CC	220	n	bl	n	y	2	cu	all/unsp	25	999	nev any	or
WYNDE6	608		m	0	0	wh	-	q	NAmer	1969	CC	4423	n	bl	n	y	0	ev	cig+/-ot	21	999	nev cigs	st
WYNDE6	632		f	0	0	wh	-	q	NAmer	1969	CC	4423	n	bl	n	y	0	ev	cig+/-ot	21	999	nev cigs	st
ZHENG	521		m	0	0	all	-	q	As:Chi	1982	CC	540	n	ot	*	y	0	ev	cig+/-ot	20	29	nev cigs	st

Cigarette type is all/unspec for all RRs

Table 2H17 - 2

IESLC - Meta-analysis of Ever/current Smoking, Age started, "Low"
Squamous, Cigarettes (or Any Product if Cigarettes not available)
Most adjusted

REF	NRR	SEX	AD	Number Case	Exposed Cont	Non-exposed Case	Cont	RR	95.00%CI
BARBON	574	m	1	64	-	6	-	9.40	(4.20- 23.40)
BENHAM	514	m	0	98	137	24	481	14.34	(8.83- 23.29)
*ENGELA	518	m	0	17	30195	3	58716	11.02	(3.23- 37.60)
HAENSZ	501	f	0	32	66	44	236	2.60	(1.53- 4.42)
JEDRYC	528	m	0	134	502	6	289	12.86	(5.60- 29.50)
LUO	513	c	20	19	-	5	-	10.50	(2.30- 48.60)
MATOS	616	m	2	6	-	3	-	3.30	(0.80- 13.90)
PEZZOT	543	m	0	10	105	0	116	23.19~	(1.34- 400.59)
SVENSS	509	f	0	9	18	5	120	12.00	(3.61- 39.85)
WAKAI	507	m	0	61	183	2	65	10.83	(2.58- 45.57)
WU	533	f	2	6	-	2	-	7.80	(0.80- 73.70)
WYNDE6	608	m	0	44	92	9	589	31.30	(14.79- 66.26)
WYNDE6	632	f	0	32	90	12	673	19.94	(9.91- 40.11)
Subtotal WYNDE6								24.59	(14.75- 41.00)
ZHENG	521	m	0	81	109	4	94	17.46	(6.17- 49.46)
Partial Totals				613	31497	125	61379		

*prospective study ~ With 0.5 adjustment for zero

REF	NRR	SEX	AD	Ys	Ws	Qs	Ps
BARBON	574	m	1	2.24	5.21	0.05	0.0000
BENHAM	514	m	0	2.66	16.33	1.67	0.0000
*ENGELA	518	m	0	2.40	2.55	0.01	0.0001
HAENSZ	501	f	0	0.96	13.63	26.24	0.0004
JEDRYC	528	m	0	2.55	5.57	0.25	0.0000
LUO	513	c	20	2.35	1.65	0.00	0.0025
MATOS	616	m	2	1.19	1.89	2.49	0.1012
PEZZOT	543	m	0	3.14	0.47	0.30	0.0306
SVENSS	509	f	0	2.48	2.67	0.05	0.0000
WAKAI	507	m	0	2.38	1.86	0.00	0.0012
WU	533	f	2	2.05	0.75	0.06	0.0750
WYNDE6	608	m	0	3.44	6.83	8.27	0.0000
WYNDE6	632	f	0	2.99	7.86	3.32	0.0000
Subtotal WYNDE6				3.20	14.69	11.59	
ZHENG	521	m	0	2.86	3.54	0.95	0.0000

	N	14
	NS	13
	Wt	70.81
Het	Chi	43.67
Het	df	13
Het	P	***
Fixed	RR	10.42
	RRl	8.25
	RRu	13.15
	P	+++
Random	RR	11.06
	RRl	6.87
	RRu	17.81
	P	+++
Asymm	P	N.S.

Table 2H17 - 3

IESLC - Meta-analysis of Ever/current Smoking, Age started, "Low"
Squamous, Cigarettes (or Any Product if Cigarettes not available)
Most adjusted

	Sex: combined	male	female	Total
N	1	9	4	14
NS	1	9	4	14
Wt	1.65	44.25	24.91	70.81
Het Chi	0.00	9.79	22.20	43.67
Het df	0	8	3	13
Het P	N.S.	N.S.	***	***
Fixed RR	10.50	14.17	6.02	10.42
RRl	2.28	10.56	4.07	8.25
RRu	48.27	19.03	8.92	13.15
P	++	+++	+++	+++
Random RR	10.50	13.87	8.18	11.06
RRl	2.28	9.79	2.30	6.87
RRu	48.27	19.66	29.06	17.81
P	++	+++	++	+++
Between Chi				11.67
Between df				2
Between P				**
Btwn(F) P				N.S.
Btwn(R) P				N.S.

Table 2H17 - 4

IESLC - Meta-analysis of Ever/current Smoking, Age started, "Low"
Squamous, Cigarettes (or Any Product if Cigarettes not available)
Least adjusted

REF	NRR	X	SEX	AGEL	AGEH	RACE	YF	LC TYPE	LOC	START	ST	NLC	R	VB	P	H	AD	SM	PRODUCT	exL	exH	DENOM	De
BARBON	569	x	m	0	0	all	-	q	Eu:wst	1979	CC	755	n	bl	y	y	0	ev	all/unsp	20	999	nev any	st
BENHAM	514		m	0	0	all	-	KI	Eu:wst	1976	CC	1625	n	bl	n	y	0	ev	cig only	25	999	nev any	st
ENGELA	518		m	0	0	all	0	q	Eu:Sca	1964	pr	435	n	bl	n	n	0	cu	cig+/-ot	20	29	nev cigs	st
HAENSZ	501		f	0	0	all	-	q+u	NAmer	1955	CC	158	n	bl	n	y	0	ev	cig+/-ot	25	999	nev any	st
JEDRYC	528		m	0	0	all	-	q	Eu:est	1980	CC	1630	n	bl	y	n	0	ev	cig+/-ot	19	999	nev any	st
LUO	508	x	c	0	0	all	-	q	As:Chi	1990	CC	102	n	ot	n	y	0	ev	cig+/-ot	20	39	nev cigs	st
MATOS	611	x	m	0	0	all	-	q	SCAmer	1994	CC	200	n	bl	n	n	0	ev	cig+/-ot	20	999	nev any	st
PEZZOT	543		m	0	0	all	-	q	SCAmer	1987	CC	215	n	bl	n	y	0	ev	cig only	19	999	nev cigs	ot
SVENSS	509		f	0	0	all	-	q	Eu:Sca	1983	CC	210	n	bl	n	n	0	cu	all/unsp	26	999	nev any	st
WAKAI	507		m	0	0	all	-	q	As:Jap	1988	CC	333	n	bl	n	y	0	cu	cig+/-ot	20	29	nev any	st
WU	509	x	f	0	0	wh	-	q	NAmer	1981	CC	220	n	bl	n	y	0	cu	all/unsp	25	999	nev any	st
WYNDE6	608		m	0	0	wh	-	q	NAmer	1969	CC	4423	n	bl	n	y	0	ev	cig+/-ot	21	999	nev cigs	st
WYNDE6	632		f	0	0	wh	-	q	NAmer	1969	CC	4423	n	bl	n	y	0	ev	cig+/-ot	21	999	nev cigs	st
ZHENG	521		m	0	0	all	-	q	As:Chi	1982	CC	540	n	ot	*	y	0	ev	cig+/-ot	20	29	nev cigs	st

Cigarette type is all/unspec for all RRs

Table 2H17 - 5

IESLC - Meta-analysis of Ever/current Smoking, Age started, "Low"
Squamous, Cigarettes (or Any Product if Cigarettes not available)
Least adjusted

REF	NRR	SEX	AD	Number Exposed Case	Cont	Non-exposed Case	Cont	RR	95.00%CI
BARBON	569	m	0	64	207	6	188	9.69	(4.10- 22.89)
BENHAM	514	m	0	98	137	24	481	14.34	(8.83- 23.29)
*ENGELA	518	m	0	17	30195	3	58716	11.02	(3.23- 37.60)
HAENSZ	501	f	0	32	66	44	236	2.60	(1.53- 4.42)
JEDRYC	528	m	0	134	502	6	289	12.86	(5.60- 29.50)
LUO	508	c	0	19	38	5	51	5.10	(1.75- 14.88)
MATOS	611	m	0	6	73	3	110	3.01	(0.73- 12.43)
PEZZOT	543	m	0	10	105	0	116	23.19~	(1.34- 400.59)
SVENSS	509	f	0	9	18	5	120	12.00	(3.61- 39.85)
WAKAI	507	m	0	61	183	2	65	10.83	(2.58- 45.57)
WU	509	f	0	6	5	2	30	18.00	(2.80- 115.56)
WYNDE6	608	m	0	44	92	9	589	31.30	(14.79- 66.26)
WYNDE6	632	f	0	32	90	12	673	19.94	(9.91- 40.11)
Subtotal WYNDE6								24.59	(14.75- 41.00)
ZHENG	521	m	0	81	109	4	94	17.46	(6.17- 49.46)
Totals				613	31820	125	61758		

*prospective study ~ With 0.5 adjustment for zero

REF	NRR	SEX	AD	Ys	Ws	Qs	Ps
BARBON	569	m	0	2.27	5.20	0.01	0.0000
BENHAM	514	m	0	2.66	16.33	1.91	0.0000
*ENGELA	518	m	0	2.40	2.55	0.02	0.0001
HAENSZ	501	f	0	0.96	13.63	25.40	0.0004
JEDRYC	528	m	0	2.55	5.57	0.30	0.0000
LUO	508	c	0	1.63	3.35	1.60	0.0029
MATOS	611	m	0	1.10	1.91	2.84	0.1271
PEZZOT	543	m	0	3.14	0.47	0.32	0.0306
SVENSS	509	f	0	2.48	2.67	0.07	0.0000
WAKAI	507	m	0	2.38	1.86	0.01	0.0012
WU	509	f	0	2.89	1.11	0.36	0.0023
WYNDE6	608	m	0	3.44	6.83	8.61	0.0000
WYNDE6	632	f	0	2.99	7.86	3.55	0.0000
Subtotal WYNDE6				3.20	14.69	12.16	
ZHENG	521	m	0	2.86	3.54	1.03	0.0000

	N	14
	NS	13
	Wt	72.88
Het	Chi	46.03
Het	df	13
Het	P	***
Fixed	RR	10.19
	RRl	8.10
	RRu	12.81
	P	+++
Random	RR	10.79
	RRl	6.71
	RRu	17.35
	P	+++
Asymm	P	N.S.

Table 2H17 - 6

IESLC - Meta-analysis of Ever/current Smoking, Age started, "Low"
Squamous, Cigarettes (or Any Product if Cigarettes not available)
Least adjusted

		Sex		
	combined	male	female	Total
N	1	9	4	14
NS	1	9	4	14
Wt	3.35	44.26	25.27	72.88
Het Chi	0.00	10.24	23.44	46.03
Het df	0	8	3	13
Het P	N.S.	N.S.	***	***
Fixed RR	5.10	14.16	6.27	10.19
RRl	1.75	10.54	4.25	8.10
RRu	14.88	19.01	9.26	12.81
P	++	+++	+++	+++
Random RR	5.10	13.78	9.54	10.79
RRl	1.75	9.62	2.70	6.71
RRu	14.88	19.75	33.79	17.35
P	++	+++	+++	+++
Between Chi				12.34
Between df				2
Between P				**
Btwn(F) P				N.S.
Btwn(R) P				N.S.

Table 2H17 - 7

IESLC - Meta-analysis of Ever/current Smoking, Age started, "Low"
Squamous, Cigarettes (or Any Product if Cigarettes not available)
Excluded studies (and stage at which they were excluded)

1	BECHER BLOT1 BROWN3 CARPEN CHYOU DARBY DOLL2 GARCIA GRAHAM GURSEL HAMMO2 JAHN JAIN LAUSSM PRESCO QIAO TVERDA WIGLE WYNDE3
2	AKIBA AMANDU AMES AXELSS BENSHL BEST BOUCHA BOUCOT BROSS CHEN CPSII DEAN2 DESTEF DORGAN DOSEME FAN GARSHI GER GILLIS HAMMON HUMBLE JUSSAW KAISE2 KATSOU KAUFMA KOO KREUZE LEVIN MCCONN NOTAN2 OSANN2 PEZZO2 PISANI RESTRE SADOWS VUTUC WANG2 WATSON WU2 WUWILL WYNDE2 WYNDE8 XU ZHOU
3	GUO MCDUFF SPITZ STASZE ZHANG
4	AGUDO ARMADA AUVINE BOFFET BRESLO BUFFLE CEDERL CHEN2 CHIAZZ CHOI CORREA CPSI DAMBER DEAN3 DOLL DORN GAO GAO2 GENG HIRAYA HOLE HU HU2 JOLY KOULUM LETOUR LIAW LIU3 LIU4 LIU5 LUBIN LUBIN2 MIGRAN MRFITR PERNU QIAO2 RACHTA SEGI2 SOBUE SPEIZE SUZUK2 TIZZAN WYNDE7 YUAN
5	ALDERS
10	HEGMAN KHUDER

Table 2H17 - 8
Potentially overlapping studies

REF	REFGP	PRINC	. OVERLAP/LINK
BENHAM	LUBIN2	2	Subset of Lubin2
WYNDE6	WYNDE6	1	WYNDE5/6/7/8

Table 2H18 -

IESLC - Meta-analysis of Ever/current Smoking, Age started, "Mid"
Squamous, Cigarettes (or Any Product if Cigarettes not available)

This analysis is restricted to results for:
1) Ever/current smokers
2) Results by Age started
3) Categorical results by Age started
4) Squamous (or near equivalent)
5) Results complete enough for use in metaanalysis

Within each study, results are then selected (in the following order of preference, within each sex) for:
6) SMKSTA: ever, current
7) PRODUCT: cigarettes regardless of other products, cigarettes only, all/unspec
8) CIGTYPE: all/unspecified, MC regardless of HR, MC only
9) (not applicable)
10) DENOM: never smoked anything, never smoked cigarettes, never any + low, never cigs + low
11) Followup period (YF, prospective studies): whole study (coded as 0) or longest available
12) LCtype: squamous or nearest available, but not adeno. (q = squamous, s = small,
a = adeno, KI = Kreyberg I, u = undifferentiated)
13) Race: all or nearest available, otherwise by race (wh or w = white, bl or b = black, hi = hispanic
ch = chinese, jap = japanese, haw = hawaiian, w+o = white + oriental, sca = scandinavian, as = asian)
14) Age started "mid" in key scheme 1 (key value 18, maximum range 15-25)
15) For overlapping studies: principal rather than subsidiary studies
Finally by Age: whole study (coded as 0) if available, otherwise by widest available age group
and then for single sex results (m, f) in preference to results for both sexes combined (c).

Results adjusted (AD) for the most potential confounders are then chosen in Sections -1 to -3
(and those which actually differ from the adjusted results in Table 2H13 - 1 are marked 'x' in Section -1)
and results adjusted for the least confounders in Sections -4 to -6. (Those least adjusted results which
actually differ from the most adjusted are marked 'x' in column X in Section -4)

Section -7 shows excluded studies, together with the stage (as above) at which no qualifying
results were found.

Section -8 lists the potentially overlapping studies which have been included (1=principal, 2=subsidiary).

Section -9 lists any results which would have been included in preference except that they had data not complete
enough for use in meta-analysis, with their significance (yes/no), if known, and any further comment as entered
on the database. It also lists as "gap" any categories for which no data were presented by the original authors.

In addition to those mentioned above, the following fields, levels and abbreviations are used:
* or nk = not known, n = no, y = yes, ot = other
ev = ever, cu = current, nev = never
all/unspec = all or unspecified, cig+/-ot = cigarettes irrespective of other products (cigar, pipe etc)
MC = manufactured cigarettes, HR = hand-rolled cigarettes
exL, exH = range of exposure (low and high) in the smoking group, in terms of Age started
REF: 6-character study reference
NRR: number of the RR on the database within the study
ST : study type (CC = case control, pr or prosp = prospective)
NLC: number of lung cancer cases in whole study
R : risky occupational population (n = no, m = mining, o = other risky)
VB : national cigarette type (V = at least 75% Virginia, bl = at least 75% blended, ot = other)
P : any proxy use
H : full histological confirmation
De : derivation of RR/CI (or = original, st = standard method, ot = other method of estimation)

Table 2H18 - 1

IESLC - Meta-analysis of Ever/current Smoking, Age started, "Mid"
Squamous, Cigarettes (or Any Product if Cigarettes not available)
Most adjusted

REF	NRR	2H13	SEX	AGEL	AGEH	RACE	YF	LC TYPE	LOC	START	ST	NLC	R	VB	P	H	AD	SM	PRODUCT	exL	exH	DENOM	De
BARBON	575		m	0	0	all	-	q	Eu:wst	1979	CC	755	n	bl	y	y	1	ev	all/unsp	15	19	nev any	or
BENHAM	516		m	0	0	all	-	KI	Eu:wst	1976	CC	1625	n	bl	n	y	0	ev	cig only	17	19	nev any	st
JEDRYC	529		m	0	0	all	-	q	Eu:est	1980	CC	1630	n	bl	y	n	0	ev	cig+/-ot	17	18	nev any	st
MATOS	617		m	0	0	all	-	q	SCAmer	1994	CC	200	n	bl	n	n	2	ev	cig+/-ot	15	19	nev any	or
WYNDE6	609		m	0	0	wh	-	q	NAmer	1969	CC	4423	n	bl	n	y	0	ev	cig+/-ot	18	20	nev cigs	st
WYNDE6	633		f	0	0	wh	-	q	NAmer	1969	CC	4423	n	bl	n	y	0	ev	cig+/-ot	18	20	nev cigs	st

Cigarette type is all/unspec for all RRs

Table 2H18 - 2

IESLC - Meta-analysis of Ever/current Smoking, Age started, "Mid"
Squamous, Cigarettes (or Any Product if Cigarettes not available)
Most adjusted

REF	NRR	SEX	AD	Number Case	Exposed Cont	Non-exposed Case	Cont	RR	95.00%CI
BARBON	575	m	1	145	-	6	-	13.70	(5.90- 31.70)
BENHAM	516	m	0	311	373	24	481	16.71	(10.80- 25.86)
JEDRYC	529	m	0	96	221	6	289	20.92	(9.00- 48.62)
MATOS	617	m	2	25	-	3	-	7.90	(2.30- 27.10)
WYNDE6	609	m	0	81	139	9	589	38.14	(18.69- 77.81)
WYNDE6	633	f	0	49	94	12	673	29.23	(15.00- 56.97)
Subtotal WYNDE6								33.10	(20.33- 53.87)
Partial Totals				707	827	60	2032		

*prospective study

REF	NRR	SEX	AD	Ys	Ws	Qs	Ps
BARBON	575	m	1	2.62	5.44	0.81	0.0000
BENHAM	516	m	0	2.82	20.14	0.71	0.0000
JEDRYC	529	m	0	3.04	5.40	0.01	0.0000
MATOS	617	m	2	2.07	2.53	2.21	0.0010
WYNDE6	609	m	0	3.64	7.56	3.07	0.0000
WYNDE6	633	f	0	3.38	8.63	1.19	0.0000
Subtotal WYNDE6				3.50	16.19	4.27	

	N	6
	NS	5
	Wt	49.70
Het	Chi	8.01
Het	df	5
Het	P	N.S.
Fixed	RR	20.15
	RRl	15.26
	RRu	26.61
	P	+++
Random	RR	20.28
	RRl	13.92
	RRu	29.53
	P	+++
Asymm	P	N.S.

Table 2H18 - 3

IESLC - Meta-analysis of Ever/current Smoking, Age started, "Mid"
Squamous, Cigarettes (or Any Product if Cigarettes not available)
Most adjusted

	combined	Sex male	female	Total
N		5	1	6
NS		5	1	5
Wt		41.06	8.63	49.70
Het Chi		6.56	0.00	8.01
Het df		4	0	5
Het P		N.S.	N.S.	N.S.
Fixed RR		18.64	29.23	20.15
RRl		13.72	15.00	15.26
RRu		25.30	56.97	26.61
P		+++	+++	+++
Random RR		18.61	29.23	20.28
RRl		12.12	15.00	13.92
RRu		28.56	56.97	29.53
P		+++	+++	+++
Between Chi				1.45
Between df				1
Between P				N.S.
Btwn(F) P				N.S.
Btwn(R) P				N.S.

Too few RRs for analysis by factor

Table 2H18 - 4

IESLC - Meta-analysis of Ever/current Smoking, Age started, "Mid"
Squamous, Cigarettes (or Any Product if Cigarettes not available)
Least adjusted

REF	NRR	X	SEX	AGEL	AGEH	RACE	YF	LC TYPE	LOC	START	ST	NLC	R	VB	P	H	AD	SM	PRODUCT	exL	exH	DENOM	De
BARBON	570	x	m	0	0	all	-	q	Eu:wst	1979	CC	755	n	bl	y	y	0	ev	all/unsp	15	19	nev any	st
BENHAM	516		m	0	0	all	-	KI	Eu:wst	1976	CC	1625	n	bl	n	y	0	ev	cig only	17	19	nev any	st
JEDRYC	529		m	0	0	all	-	q	Eu:est	1980	CC	1630	n	bl	y	n	0	ev	cig+/-ot	17	18	nev any	st
MATOS	612	x	m	0	0	all	-	q	SCAmer	1994	CC	200	n	bl	n	n	0	ev	cig+/-ot	15	19	nev any	st
WYNDE6	609		m	0	0	wh	-	q	NAmer	1969	CC	4423	n	bl	n	y	0	ev	cig+/-ot	18	20	nev cigs	st
WYNDE6	633		f	0	0	wh	-	q	NAmer	1969	CC	4423	n	bl	n	y	0	ev	cig+/-ot	18	20	nev cigs	st

Cigarette type is all/unspec for all RRs

Table 2H18 - 5

IESLC - Meta-analysis of Ever/current Smoking, Age started, "Mid"
Squamous, Cigarettes (or Any Product if Cigarettes not available)
Least adjusted

REF	NRR	SEX	AD	Number Exposed Case	Number Exposed Cont	Non-exposed Case	Non-exposed Cont	RR	95.00%CI
BARBON	570	m	0	145	337	6	188	13.48	(5.84- 31.10)
BENHAM	516	m	0	311	373	24	481	16.71	(10.80- 25.86)
JEDRYC	529	m	0	96	221	6	289	20.92	(9.00- 48.62)
MATOS	612	m	0	25	120	3	110	7.64	(2.24- 26.01)
WYNDE6	609	m	0	81	139	9	589	38.14	(18.69- 77.81)
WYNDE6	633	f	0	49	94	12	673	29.23	(15.00- 56.97)
Subtotal WYNDE6								33.10	(20.33- 53.87)
Totals				707	1284	60	2330		

*prospective study

REF	NRR	SEX	AD	Ys	Ws	Qs	Ps
BARBON	570	m	0	2.60	5.50	0.87	0.0000
BENHAM	516	m	0	2.82	20.14	0.67	0.0000
JEDRYC	529	m	0	3.04	5.40	0.01	0.0000
MATOS	612	m	0	2.03	2.56	2.39	0.0011
WYNDE6	609	m	0	3.64	7.56	3.12	0.0000
WYNDE6	633	f	0	3.38	8.63	1.22	0.0000
Subtotal WYNDE6				3.50	16.19	4.34	

	N	6
	NS	5
	Wt	49.79
Het	Chi	8.28
Het	df	5
Het	P	N.S.
Fixed	RR	20.06
	RRl	15.19
	RRu	26.48
	P	+++
Random	RR	20.14
	RRl	13.74
	RRu	29.52
	P	+++
Asymm	P	N.S.

Table 2H18 - 6

IESLC - Meta-analysis of Ever/current Smoking, Age started, "Mid"
Squamous, Cigarettes (or Any Product if Cigarettes not available)
Least adjusted

	Sex combined	male	female	Total
N		5	1	6
NS		5	1	5
Wt		41.16	8.63	49.79
Het Chi		6.80	0.00	8.28
Het df		4	0	5
Het P		N.S.	N.S.	N.S.
Fixed RR		18.53	29.23	20.06
RRl		13.66	15.00	15.19
RRu		25.16	56.97	26.48
P		+++	+++	+++
Random RR		18.45	29.23	20.14
RRl		11.93	15.00	13.74
RRu		28.54	56.97	29.52
P		+++	+++	+++
Between Chi				1.48
Between df				1
Between P				N.S.
Btwn(F) P				N.S.
Btwn(R) P				N.S.

Table 2H18 - 7

IESLC - Meta-analysis of Ever/current Smoking, Age started, "Mid"
Squamous, Cigarettes (or Any Product if Cigarettes not available)
Excluded studies (and stage at which they were excluded)

Stage	Excluded studies
1	BECHER BLOT1 BROWN3 CARPEN CHYOU DARBY DOLL2 GARCIA GRAHAM GURSEL HAMMO2 JAHN JAIN LAUSSM PRESCO QIAO TVERDA WIGLE WYNDE3
2	AKIBA AMANDU AMES AXELSS BENSHL BEST BOUCHA BOUCOT BROSS CHEN CPSII DEAN2 DESTEF DORGAN DOSEME FAN GARSHI GER GILLIS HAMMON HUMBLE JUSSAW KAISE2 KATSOU KAUFMA KOO KREUZE LEVIN MCCONN NOTAN2 OSANN2 PEZZO2 PISANI RESTRE SADOWS VUTUC WANG2 WATSON WU2 WUWILL WYNDE2 WYNDE8 XU ZHOU
3	GUO MCDUFF SPITZ STASZE ZHANG
4	AGUDO ARMADA AUVINE BOFFET BRESLO BUFFLE CEDERL CHEN2 CHIAZZ CHOI CORREA CPSI DAMBER DEAN3 DOLL DORN GAO GAO2 GENG HIRAYA HOLE HU HU2 JOLY KOULUM LETOUR LIAW LIU3 LIU4 LIU5 LUBIN LUBIN2 MIGRAN MRFITR PERNU QIAO2 RACHTA SEGI2 SOBUE SPEIZE SUZUK2 TIZZAN WYNDE7 YUAN
5	ALDERS
10	HEGMAN KHUDER
14	ENGELA HAENSZ LUO PEZZOT SVENSS WAKAI WU ZHENG

Table 2H18 - 8
Potentially overlapping studies

REF	REFGP	PRINC	. OVERLAP/LINK
BENHAM	LUBIN2	2	Subset of Lubin2
WYNDE6	WYNDE6	1	WYNDE5/6/7/8

Table 2H19 -

IESLC - Meta-analysis of Ever/current Smoking, Age started, "High"
Squamous, Cigarettes (or Any Product if Cigarettes not available)

This analysis is restricted to results for:
1) Ever/current smokers
2) Results by Age started
3) Categorical results by Age started
4) Squamous (or near equivalent)
5) Results complete enough for use in metaanalysis

Within each study, results are then selected (in the following order of preference, within each sex) for:
6) PRODUCT: cigarettes regardless of other products, cigarettes only, all/unspec
7) CIGTYPE: all/unspecified, MC regardless of HR, MC only
8) (not applicable)
9) DENOM: never smoked anything, never smoked cigarettes, never any + low, never cigs + low
10) Followup period (YF, prospective studies): whole study (coded as 0) or longest available
11) LCtype: squamous or nearest available, but not adeno. (q = squamous, s = small,
a = adeno, KI = Kreyberg I, u = undifferentiated)
12) Race: all or nearest available, otherwise by race (wh or w = white, bl or b = black, hi = hispanic
ch = chinese, jap = japanese, haw = hawaiian, w+o = white + oriental, sca = scandinavian, as = asian)
13) Age started "high" in key scheme 1 (key value 14, maximum range 1-17)
14) For overlapping studies: principal rather than subsidiary studies
Finally by Age: whole study (coded as 0) if available, otherwise by widest available age group
and then for single sex results (m, f) in preference to results for both sexes combined (c).

Results adjusted (AD) for the most potential confounders are then chosen in Sections -1 to -3
(and those which actually differ from the adjusted results in Table 2H14 - 1 are marked 'x' in Section -1)
and results adjusted for the least confounders in Sections -4 to -6. (Those least adjusted results which
actually differ from the most adjusted are marked 'x' in column X in Section -4)

Section -7 shows excluded studies, together with the stage (as above) at which no qualifying
results were found.

Section -8 lists the potentially overlapping studies which have been included (1=principal, 2=subsidiary).

Section -9 lists any results which would have been included in preference except that they had data not complete
enough for use in meta-analysis, with their significance (yes/no), if known, and any further comment as entered
on the database. It also lists as "gap" any categories for which no data were presented by the original authors.

In addition to those mentioned above, the following fields, levels and abbreviations are used:
* or nk = not known, n = no, y = yes, ot = other
ev = ever, cu = current, nev = never
all/unspec = all or unspecified, cig+/-ot = cigarettes irrespective of other products (cigar, pipe etc)
MC = manufactured cigarettes, HR = hand-rolled cigarettes
exL, exH = range of exposure (low and high) in the smoking group, in terms of Age started
REF: 6-character study reference
NRR: number of the RR on the database within the study
ST : study type (CC = case control, pr or prosp = prospective)
NLC: number of lung cancer cases in whole study
R : risky occupational population (n = no, m = mining, o = other risky)
VB : national cigarette type (V = at least 75% Virginia, bl = at least 75% blended, ot = other)
P : any proxy use
H : full histological confirmation
De : derivation of RR/CI (or = original, st = standard method, ot = other method of estimation)

Table 2H19 - 1

IESLC - Meta-analysis of Ever/current Smoking, Age started, "High"
Squamous, Cigarettes (or Any Product if Cigarettes not available)
Most adjusted

REF	NRR	2H14	SEX	AGEL	AGEH	RACE	YF	LC TYPE	LOC	START	ST	NLC	R	VB	P	H	AD	SM	PRODUCT	exL	exH	DENOM	De
BARBON	576		m	0	0	all	-	q	Eu:wst	1979	CC	755	n	bl	y	y	1	ev	all/unsp	1	14	nev any	or
BENHAM	517		m	0	0	all	-	KI	Eu:wst	1976	CC	1625	n	bl	n	y	0	ev	cig only	1	16	nev any	st
JEDRYC	530		m	0	0	all	-	q	Eu:est	1980	CC	1630	n	bl	y	n	0	ev	cig+/-ot	1	16	nev any	st
MATOS	618		m	0	0	all	-	q	SCAmer	1994	CC	200	n	bl	n	n	2	ev	cig+/-ot	1	14	nev any	or
WYNDE6	610		m	0	0	wh	-	q	NAmer	1969	CC	4423	n	bl	n	y	0	ev	cig+/-ot	1	17	nev cigs	st
WYNDE6	634		f	0	0	wh	-	q	NAmer	1969	CC	4423	n	bl	n	y	0	ev	cig+/-ot	1	17	nev cigs	st

Cigarette type is all/unspec for all RRs

Table 2H19 - 2

IESLC - Meta-analysis of Ever/current Smoking, Age started, "High"
Squamous, Cigarettes (or Any Product if Cigarettes not available)
Most adjusted

REF	NRR	SEX	AD	Number Exposed Case	Number Exposed Cont	Non-exposed Case	Non-exposed Cont	RR	95.00%CI
BARBON	576	m	1	52	-	6	-	71.30	(27.60- 184.00)
BENHAM	517	m	0	342	341	24	481	20.10	(12.99- 31.10)
JEDRYC	530	m	0	53	111	6	289	23.00	(9.61- 55.01)
MATOS	618	m	2	16	-	3	-	7.20	(2.00- 25.90)
WYNDE6	610	m	0	262	301	9	589	56.96	(28.89- 112.31)
WYNDE6	634	f	0	72	91	12	673	44.37	(23.18- 84.93)
Subtotal WYNDE6								50.00	(31.27- 79.93)
Partial Totals				797	844	60	2032		

*prospective study

REF	NRR	SEX	AD	Ys	Ws	Qs	Ps
BARBON	576	m	1	4.27	4.27	3.22	0.0000
BENHAM	517	m	0	3.00	20.16	3.18	0.0000
JEDRYC	530	m	0	3.14	5.05	0.35	0.0000
MATOS	618	m	2	1.97	2.34	4.75	0.0025
WYNDE6	610	m	0	4.04	8.34	3.46	0.0000
WYNDE6	634	f	0	3.79	9.12	1.42	0.0000
Subtotal WYNDE6				3.91	17.45	4.88	

	N	6
	NS	5
	Wt	49.28
Het	Chi	16.38
Het	df	5
Het	P	**
Fixed	RR	29.91
	RRl	22.62
	RRu	39.54
	P	+++
Random	RR	31.07
	RRl	17.93
	RRu	53.85
	P	+++
Asymm	P	N.S.

Table 2H19 - 3

IESLC - Meta-analysis of Ever/current Smoking, Age started, "High"
Squamous, Cigarettes (or Any Product if Cigarettes not available)
Most adjusted

	Sex combined	male	female	Total
N		5	1	6
NS		5	1	5
Wt		40.16	9.12	49.28
Het Chi		14.64	0.00	16.38
Het df		4	0	5
Het P		**	N.S.	**
Fixed RR		27.35	44.37	29.91
RRl		20.07	23.18	22.62
RRu		37.26	84.93	39.54
P		+++	+++	+++
Random RR		28.44	44.37	31.07
RRl		14.64	23.18	17.93
RRu		55.25	84.93	53.85
P		+++	+++	+++
Between Chi				1.74
Between df				1
Between P				N.S.
Btwn(F) P				N.S.
Btwn(R) P				N.S.

Too few RRs for analysis by factor

Table 2H19 - 4

IESLC - Meta-analysis of Ever/current Smoking, Age started, "High"
Squamous, Cigarettes (or Any Product if Cigarettes not available)
Least adjusted

REF	NRR	X	SEX	AGEL	AGEH	RACE	YF	LC TYPE	LOC	START	ST	NLC	R	VB	P	H	AD	SM	PRODUCT	exL	exH	DENOM	De
BARBON	571	x	m	0	0	all	-	q	Eu:wst	1979	CC	755	n	bl	y	y	0	ev	all/unsp	1	14	nev any	st
BENHAM	517		m	0	0	all	-	KI	Eu:wst	1976	CC	1625	n	bl	n	y	0	ev	cig only	1	16	nev any	st
JEDRYC	530		m	0	0	all	-	q	Eu:est	1980	CC	1630	n	bl	y	n	0	ev	cig+/-ot	1	16	nev any	st
MATOS	613	x	m	0	0	all	-	q	SCAmer	1994	CC	200	n	bl	n	n	0	ev	cig+/-ot	1	14	nev any	st
WYNDE6	610		m	0	0	wh	-	q	NAmer	1969	CC	4423	n	bl	n	y	0	ev	cig+/-ot	1	17	nev cigs	st
WYNDE6	634		f	0	0	wh	-	q	NAmer	1969	CC	4423	n	bl	n	y	0	ev	cig+/-ot	1	17	nev cigs	st

Cigarette type is all/unspec for all RRs

Table 2H19 - 5

IESLC - Meta-analysis of Ever/current Smoking, Age started, "High"
Squamous, Cigarettes (or Any Product if Cigarettes not available)
Least adjusted

REF	NRR	SEX	AD	Number Exposed Case	Exposed Cont	Non-exposed Case	Non-exposed Cont	RR	95.00%CI
BARBON	571	m	0	52	23	6	188	70.84	(27.41- 183.08)
BENHAM	517	m	0	342	341	24	481	20.10	(12.99- 31.10)
JEDRYC	530	m	0	53	111	6	289	23.00	(9.61- 55.01)
MATOS	613	m	0	16	90	3	110	6.52	(1.84- 23.08)
WYNDE6	610	m	0	262	301	9	589	56.96	(28.89- 112.31)
WYNDE6	634	f	0	72	91	12	673	44.37	(23.18- 84.93)
Subtotal WYNDE6								50.00	(31.27- 79.93)
Totals				797	957	60	2330		

*prospective study

REF	NRR	SEX	AD	Ys	Ws	Qs	Ps
BARBON	571	m	0	4.26	4.26	3.22	0.0000
BENHAM	517	m	0	3.00	20.16	3.07	0.0000
JEDRYC	530	m	0	3.14	5.05	0.33	0.0000
MATOS	613	m	0	1.87	2.40	5.53	0.0037
WYNDE6	610	m	0	4.04	8.34	3.54	0.0000
WYNDE6	634	f	0	3.79	9.12	1.47	0.0000
Subtotal WYNDE6				3.91	17.45	5.01	

	N	6
	NS	5
	Wt	49.33
Het	Chi	17.16
Het	df	5
Het	P	**
Fixed	RR	29.69
	RRl	22.46
	RRu	39.25
	P	+++
Random	RR	30.58
	RRl	17.44
	RRu	53.64
	P	+++
Asymm	P	N.S.

Table 2H19 - 6

IESLC - Meta-analysis of Ever/current Smoking, Age started, "High"
Squamous, Cigarettes (or Any Product if Cigarettes not available)
Least adjusted

	Sex combined	male	female	Total
N		5	1	6
NS		5	1	5
Wt		40.21	9.12	49.33
Het Chi		15.35	0.00	17.16
Het df		4	0	5
Het P		**	N.S.	**
Fixed RR		27.11	44.37	29.69
RRl		19.90	23.18	22.46
RRu		36.92	84.93	39.25
P		+++	+++	+++
Random RR		27.89	44.37	30.58
RRl		14.15	23.18	17.44
RRu		54.95	84.93	53.64
P		+++	+++	+++
Between Chi				1.81
Between df				1
Between P				N.S.
Btwn(F) P				N.S.
Btwn(R) P				N.S.

Table 2H19 - 7

IESLC - Meta-analysis of Ever/current Smoking, Age started, "High"
Squamous, Cigarettes (or Any Product if Cigarettes not available)
Excluded studies (and stage at which they were excluded)

1	BECHER BLOT1 BROWN3 CARPEN CHYOU DARBY DOLL2 GARCIA GRAHAM GURSEL HAMMO2 JAHN JAIN LAUSSM PRESCO QIAO TVERDA WIGLE WYNDE3
2	AKIBA AMANDU AMES AXELSS BENSHL BEST BOUCHA BOUCOT BROSS CHEN CPSII DEAN2 DESTEF DORGAN DOSEME FAN GARSHI GER GILLIS HAMMON HUMBLE JUSSAW KAISE2 KATSOU KAUFMA KOO KREUZE LEVIN MCCONN NOTAN2 OSANN2 PEZZO2 PISANI RESTRE SADOWS VUTUC WANG2 WATSON WU2 WUWILL WYNDE2 WYNDE8 XU ZHOU
3	GUO MCDUFF SPITZ STASZE ZHANG
4	AGUDO ARMADA AUVINE BOFFET BRESLO BUFFLE CEDERL CHEN2 CHIAZZ CHOI CORREA CPSI DAMBER DEAN3 DOLL DORN GAO GAO2 GENG HIRAYA HOLE HU HU2 JOLY KOULUM LETOUR LIAW LIU3 LIU4 LIU5 LUBIN LUBIN2 MIGRAN MRFITR PERNU QIAO2 RACHTA SEGI2 SOBUE SPEIZE SUZUK2 TIZZAN WYNDE7 YUAN
5	ALDERS
10	HEGMAN KHUDER
14	ENGELA HAENSZ LUO PEZZOT SVENSS WAKAI WU ZHENG

Table 2H19 - 8

Potentially overlapping studies

REF	REFGP	PRINC	OVERLAP/LINK
BENHAM	LUBIN2	2	Subset of Lubin2
WYNDE6	WYNDE6	1	WYNDE5/6/7/8

Table 2H20 -

IESLC - Meta-analysis of Ever/current Smoking, Age started, "Highest vs lowest"
Squamous, Cigarettes (or Any Product if Cigarettes not available)

This analysis is restricted to results for:
1) Ever/current smokers
2) Results by Age started
3) Categorical results by Age started
4) Denominator (unexposed) = "low"
5) Squamous (or near equivalent)
6) Results complete enough for use in metaanalysis

Within each study, results are then selected (in the following order of preference, within each sex) for:
7) SMKSTA: ever, current
8) PRODUCT: cigarettes regardless of other products, cigarettes only, all/unspec
9) CIGTYPE: all/unspecified, MC regardless of HR, MC only
10) Results with least adjustment for other aspects of smoking (ADOS)
11) The highest vs lowest category
12) Followup period (YF, prospective studies): whole study (coded as 0) or longest available
13) LCtype: squamous or nearest available, but not adeno. (q = squamous, s = small,
a = adeno, KI = Kreyberg I, u = undifferentiated)
14) Race: all or nearest available, otherwise by race (wh or w = white, bl or b = black, hi = hispanic
ch = chinese, jap = japanese, haw = hawaiian, w+o = white + oriental, sca = scandinavian, as = asian)
15) For overlapping studies: principal rather than subsidiary studies
Finally by Age: whole study (coded as 0) if available, otherwise by widest available age group
and then for single sex results (m, f) in preference to results for both sexes combined (c).

Results adjusted (AD) for the most potential confounders are then chosen in Sections -1 to -3
(and those which actually differ from the adjusted results in Table 2H15 - 1 are marked 'x' in Section -1)
and results adjusted for the least confounders in Sections -4 to -6. (Those least adjusted results which
actually differ from the most adjusted are marked 'x' in column X in Section -4)

Section -7 shows excluded studies, together with the stage (as above) at which no qualifying
results were found.

Section -8 lists the potentially overlapping studies which have been included (1=principal, 2=subsidiary).

Section -9 lists any results which would have been included in preference except that they had data not complete
enough for use in meta-analysis, with their significance (yes/no), if known, and any further comment as entered
on the database. It also lists as "gap" any categories for which no data were presented by the original authors.

In addition to those mentioned above, the following fields, levels and abbreviations are used:
* or nk = not known, n = no, y = yes, ot = other
all/unspec = all or unspecified, cig+/-ot = cigarettes irrespective of other products (cigar, pipe etc)
MC = manufactured cigarettes, HR = hand-rolled cigarettes
exL, exH = range of exposure (low and high) in the "highest" group, in terms of Age started
unexL, unexH = range of exposure (low and high) in the "lowest" group, in terms of Age started
REF: 6-character study reference
NRR: number of the RR on the database within the study
ST : study type (CC = case control, pr or prosp = prospective)
NLC: number of lung cancer cases in whole study
R : risky occupational population (n = no, m = mining, o = other risky)
VB : national cigarette type (V = at least 75% Virginia, bl = at least 75% blended, ot = other)
P : any proxy use
H : full histological confirmation
De : derivation of RR/CI (or = original, st = standard method, ot = other method of estimation)

Table 2H20 - 1

IESLC - Meta-analysis of Ever/current Smoking, Age started, "Highest vs lowest"
Squamous, Cigarettes (or Any Product if Cigarettes not available)
Most adjusted

REF	NRR	2H15	SEX	AGEL	AGEH	RACE	YF	LC TYPE	LOC	START	ST	NLC	R	VB	P	H	AD	ADOS	SM	PRODUCT	exL	exH	unexL	unexH	De
BARBON	578		m	0	0	all	-	q	Eu:wst	1979	CC	755	n	bl	y	y	1	0	ev	all/unsp	1	14	20	999	ot
BENHAM	520		m	0	0	all	-	KI	Eu:wst	1976	CC	1625	n	bl	n	y	0	0	ev	cig only	1	16	25	999	st
ENGELA	521		m	0	0	all	0	q	Eu:Sca	1964	pr	435	n	bl	n	n	0	0	cu	cig+/-ot	1	19	30	999	st
HAENSZ	533		f	0	0	all	-	q+u	NAmer	1955	CC	158	n	bl	n	y	2	0	ev	cig+/-ot	1	24	25	999	ot
HEGMAN	503		m	0	0	all	-	q	NAmer	1989	CC	282	n	bl	y	y	0	0	ev	all/unsp	1	19	20	999	st
HEGMAN	508		f	0	0	all	-	q	NAmer	1989	CC	282	n	bl	y	y	0	0	ev	all/unsp	1	25	26	999	ot
JEDRYC	532		m	0	0	all	-	q	Eu:est	1980	CC	1630	n	bl	y	n	0	0	ev	cig+/-ot	1	16	19	999	st
KHUDER	524		m	0	0	all	-	q	NAmer	1985	CC	482	n	bl	n	y	5	3#	ev	cig+/-ot	1	15	20	999	or
LUO	516		c	0	0	all	-	q	As:Chi	1990	CC	102	n	ot	n	y	20	0	ev	cig+/-ot	0	19	40	999	ot
MATOS	620		m	0	0	all	-	q	SCAmer	1994	CC	200	n	bl	n	n	2	0	ev	cig+/-ot	1	14	20	999	ot
PEZZOT	549		m	0	0	all	-	q	SCAmer	1987	CC	215	n	bl	n	y	2	0	ev	cig only	1	13	19	999	ot
SVENSS	513		f	0	0	all	-	q	Eu:Sca	1983	CC	210	n	bl	n	n	0	0	cu	all/unsp	0	18	26	999	st
WAKAI	510		m	0	0	all	-	q	As:Jap	1988	CC	333	n	bl	n	y	0	0	cu	cig+/-ot	1	19	30	999	st
WU	537		f	0	0	wh	-	q	NAmer	1981	CC	220	n	bl	n	y	2	0	cu	all/unsp	0	18	25	999	ot
WYNDE6	612		m	0	0	wh	-	q	NAmer	1969	CC	4423	n	bl	n	y	0	0	ev	cig+/-ot	1	17	21	999	st
WYNDE6	636		f	0	0	wh	-	q	NAmer	1969	CC	4423	n	bl	n	y	0	0	ev	cig+/-ot	1	17	21	999	st
ZHENG	526		m	0	0	all	-	q	As:Chi	1982	CC	540	n	ot	*	y	1	0	ev	cig+/-ot	1	19	30	999	ot
ZHENG	530		f	0	0	all	-	q	As:Chi	1982	CC	540	n	ot	*	y	1	0	ev	cig+/-ot	1	29	30	999	ot

Comments on values in listings

KHUDER ADOS Duration of smoking (years), number of cigarettes per day and Quitted smoking

Cigarette type is all/unspec for all RRs

Table 2H20 - 2

IESLC - Meta-analysis of Ever/current Smoking, Age started, "Highest vs lowest"
Squamous, Cigarettes (or Any Product if Cigarettes not available)
Most adjusted

REF	NRR	SEX	AD	Number Case	Exposed Cont	Non-exposed Case	Cont	RR	95.00%CI
BARBON	578	m	1	52	-	64	-	7.59	(4.36- 13.19)
BENHAM	520	m	0	342	341	98	137	1.40	(1.04- 1.89)
*ENGELA	521	m	0	64	50732	6	9762	2.05	(0.89- 4.74)
HAENSZ	533	f	2	24	-	32	-	1.38	(0.71- 2.67)
HEGMAN	503	m	0	57	716	15	289	1.53	(0.85- 2.75)
HEGMAN	508	f	0	17	169	0	28	5.88~	(0.34- 100.62)
Subtotal HEGMAN								1.62	(0.91- 2.87)
JEDRYC	532	m	0	53	111	134	502	1.79	(1.22- 2.61)
KHUDER	524	m	5	-	-	-	-	1.00	(0.50- 1.80)
LUO	516	c	20	14	-	1	-	4.00	(0.47- 33.90)
MATOS	620	m	2	16	-	6	-	2.18	(0.81- 5.85)
PEZZOT	549	m	2	30	-	10	-	3.33	(1.43- 7.79)
SVENSS	513	f	0	15	21	9	18	1.43	(0.51- 4.04)
WAKAI	510	m	0	21	74	4	25	1.77	(0.56- 5.67)
WU	537	f	2	37	-	6	-	14.83	(2.14- 102.93)
WYNDE6	612	m	0	262	301	44	92	1.82	(1.23- 2.70)
WYNDE6	636	f	0	72	91	32	90	2.23	(1.34- 3.70)
Subtotal WYNDE6								1.96	(1.44- 2.68)
ZHENG	526	m	1	62	-	13	-	7.08	(3.48- 14.41)
ZHENG	530	f	1	34	-	9	-	1.63	(0.63- 4.25)
Subtotal ZHENG								4.19	(2.37- 7.42)
Partial Totals				1172	52556	483	10943		

*prospective study ~ With 0.5 adjustment for zero

REF	NRR	SEX	AD	Ys	Ws	Qs	Ps
BARBON	578	m	1	2.03	12.54	22.55	0.0000
BENHAM	520	m	0	0.34	42.81	5.18	0.0270
*ENGELA	521	m	0	0.72	5.49	0.01	0.0920
HAENSZ	533	f	2	0.32	8.76	1.16	0.3405
HEGMAN	503	m	0	0.43	11.23	0.75	0.1518
HEGMAN	508	f	0	1.77	0.48	0.56	0.2211
Subtotal HEGMAN				0.48	11.70	1.31	
JEDRYC	532	m	0	0.58	26.79	0.29	0.0026
KHUDER	524	m	5	0.00	9.36	4.41	1.0000
LUO	516	c	20	1.39	0.84	0.41	0.2040
MATOS	620	m	2	0.78	3.93	0.03	0.1223
PEZZOT	549	m	2	1.20	5.35	1.43	0.0054
SVENSS	513	f	0	0.36	3.56	0.39	0.5010
WAKAI	510	m	0	0.57	2.85	0.04	0.3335
WU	537	f	2	2.70	1.02	4.14	0.0063
WYNDE6	612	m	0	0.60	24.55	0.19	0.0030
WYNDE6	636	f	0	0.80	14.87	0.19	0.0020
Subtotal WYNDE6				0.67	39.42	0.38	
ZHENG	526	m	1	1.96	7.61	12.30	0.0000
ZHENG	530	f	1	0.49	4.22	0.16	0.3157
Subtotal ZHENG				1.43	11.83	12.47	

	N	18
	NS	15
	Wt	186.25
Het	Chi	54.19
Het	df	17
Het	P	***
Fixed	RR	1.99
	RRl	1.72
	RRu	2.29
	P	+++
Random	RR	2.23
	RRl	1.66
	RRu	2.98
	P	+++
Asymm	P	N.S.

Table 2H20 - 3

IESLC - Meta-analysis of Ever/current Smoking, Age started, "Highest vs lowest"
Squamous, Cigarettes (or Any Product if Cigarettes not available)
Most adjusted

		Sex			
	combined	male	female	Total
N	1	11	6	18
NS	1	11	6	18
Wt	0.84	152.50	32.91	186.25
Het Chi	0.00	47.17	6.58	54.19
Het df	0	10	5	17
Het P	N.S.	***	N.S.	***
Fixed RR	4.00	1.99	1.93	1.99
RRl	0.47	1.70	1.37	1.72
RRu	33.97	2.33	2.72	2.29
P	N.S.	+++	+++	+++
Random RR	4.00	2.28	1.96	2.23
RRl	0.47	1.56	1.27	1.66
RRu	33.97	3.31	3.02	2.98
P	N.S.	+++	++	+++
Between Chi				0.44
Between df				2
Between P				N.S.
Btwn(F) P				N.S.
Btwn(R) P				N.S.

	Lung cancer type					
	q	q+s	q+u	KI	not a	Total
N	16		1	1		18
NS	13		1	1		15
Wt	134.68		8.76	42.81		186.25
Het Chi	45.42		0.00	0.00		54.19
Het df	15		0	0		17
Het P	***		N.S.	N.S.		***
Fixed RR	2.27		1.38	1.40		1.99
RRl	1.92		0.71	1.04		1.72
RRu	2.69		2.68	1.89		2.29
P	+++		N.S.	+		+++
Random RR	2.43		1.38	1.40		2.23
RRl	1.75		0.71	1.04		1.66
RRu	3.39		2.68	1.89		2.98
P	+++		N.S.	+		+++
Between Chi						8.77
Between df						2
Between P						*
Btwn(F) P						N.S.
Btwn(R) P						*

	Location								
	NAmer	UK	Scand	othEur	China	Japan	othAs	other	Total
N	7		2	3	3	1		2	18
NS	5		2	3	2	1		2	15
Wt	70.27		9.05	82.13	12.67	2.85		9.28	186.25
Het Chi	9.87		0.28	28.01	5.85	0.00		0.41	54.19
Het df	6		1	2	2	0		1	17
Het P	N.S.		N.S.	***	(*)	N.S.		N.S.	***
Fixed RR	1.71		1.78	1.96	4.18	1.77		2.78	1.99
RRl	1.36		0.93	1.58	2.41	0.56		1.46	1.72
RRu	2.16		3.41	2.44	7.25	5.67		5.30	2.29
P	+++		(+)	+++	+++	N.S.		++	+++
Random RR	1.72		1.78	2.59	3.65	1.77		2.78	2.23
RRl	1.24		0.93	1.10	1.22	0.56		1.46	1.66
RRu	2.40		3.41	6.11	10.92	5.67		5.30	2.98
P	++		(+)	+	+	N.S.		++	+++
Between Chi									9.77
Between df									5
Between P									(*)
Btwn(F) P									N.S.
Btwn(R) P									N.S.

Table 2H20 - 3

IESLC - Meta-analysis of Ever/current Smoking, Age started, "Highest vs lowest"
Squamous, Cigarettes (or Any Product if Cigarettes not available)
Most adjusted

Detailed Country in "other Europe"

	multi	Germany	othWest	East	Balkans	Total
N			2	1		3
NS			2	1		3
Wt			55.35	26.79		82.13
Het Chi			27.66	0.00		28.01
Het df			1	0		2
Het P			***	N.S.		***
Fixed RR			2.06	1.79		1.96
RRl			1.58	1.22		1.58
RRu			2.68	2.61		2.44
P			+++	++		+++
Random RR			3.21	1.79		2.59
RRl			0.61	1.22		1.10
RRu			16.78	2.61		6.11
P			N.S.	++		+
Between Chi						0.35
Between df						1
Between P						N.S.
Btwn(F) P						N.S.
Btwn(R) P						N.S.

Detailed Country in "other Asia"

	India	HongKong	other	Total
N				
NS				
Wt				
Het Chi				
Het df				
Het P				N.S.
Fixed RR				
RRl				
RRu				
P				++
Random RR				
RRl				
RRu				
P				++
Between Chi				
Between df				
Between P				N.S.
Btwn(F) P				N.S.
Btwn(R) P				N.S.

Detailed other continent

	SCAmer	Total
N	2	2
NS	2	2
Wt	9.28	9.28
Het Chi	0.41	0.41
Het df	1	1
Het P	N.S.	N.S.
Fixed RR	2.78	2.78
RRl	1.46	1.46
RRu	5.30	5.30
P	++	++
Random RR	2.78	2.78
RRl	1.46	1.46
RRu	5.30	5.30
P	++	++
Between Chi		
Between df		
Between P		N.S.
Btwn(F) P		N.S.
Btwn(R) P		N.S.

Table 2H20 - 3

IESLC - Meta-analysis of Ever/current Smoking, Age started, "Highest vs lowest"
Squamous, Cigarettes (or Any Product if Cigarettes not available)
Most adjusted

	Start year of study					
	<1960	1960-69	1970-79	1980-89	1990+	Total
N	1	3	2	10	2	18
NS	1	2	2	8	2	15
Wt	8.76	44.91	55.35	72.46	4.77	186.25
Het Chi	0.00	0.38	27.66	24.46	0.25	54.19
Het df	0	2	1	9	1	17
Het P	N.S.	N.S.	***	**	N.S.	***
Fixed RR	1.38	1.97	2.06	2.00	2.43	1.99
RRl	0.71	1.47	1.58	1.59	0.99	1.72
RRu	2.68	2.64	2.68	2.52	5.95	2.29
P	N.S.	+++	+++	+++	(+)	+++
Random RR	1.38	1.97	3.21	2.23	2.43	2.23
RRl	0.71	1.47	0.61	1.44	0.99	1.66
RRu	2.68	2.64	16.78	3.45	5.95	2.98
P	N.S.	+++	N.S.	+++	(+)	+++
Between Chi						1.42
Between df						4
Between P						N.S.
Btwn(F) P						N.S.
Btwn(R) P						N.S.

	Study type (1)		
	CC	other	Total
N	17	1	18
NS	14	1	15
Wt	180.76	5.49	186.25
Het Chi	54.18	0.00	54.19
Het df	16	0	17
Het P	***	N.S.	***
Fixed RR	1.98	2.05	1.99
RRl	1.71	0.89	1.72
RRu	2.29	4.74	2.29
P	+++	(+)	+++
Random RR	2.24	2.05	2.23
RRl	1.65	0.89	1.66
RRu	3.05	4.74	2.98
P	+++	(+)	+++
Between Chi			0.01
Between df			1
Between P			N.S.
Btwn(F) P			N.S.
Btwn(R) P			N.S.

	Study type (2)			
	CC	prosp	other	Total
N	17	1		18
NS	14	1		15
Wt	180.76	5.49		186.25
Het Chi	54.18	0.00		54.19
Het df	16	0		17
Het P	***	N.S.		***
Fixed RR	1.98	2.05		1.99
RRl	1.71	0.89		1.72
RRu	2.29	4.74		2.29
P	+++	(+)		+++
Random RR	2.24	2.05		2.23
RRl	1.65	0.89		1.66
RRu	3.05	4.74		2.98
P	+++	(+)		+++
Between Chi				0.01
Between df				1
Between P				N.S.
Btwn(F) P				N.S.
Btwn(R) P				N.S.

Table 2H20 - 3

IESLC - Meta-analysis of Ever/current Smoking, Age started, "Highest vs lowest"
Squamous, Cigarettes (or Any Product if Cigarettes not available)
Most adjusted

	Study size (number of LC cases)				
	100-249	250-499	500-999	1000+	Total
N	6	5	3	4	18
NS	6	4	2	3	15
Wt	23.46	29.41	24.37	109.02	186.25
Het Chi	7.48	3.04	7.99	2.84	54.19
Het df	5	4	2	3	17
Het P	N.S.	N.S.	*	N.S.	***
Fixed RR	2.11	1.46	5.69	1.68	1.99
RRl	1.41	1.02	3.83	1.39	1.72
RRu	3.16	2.10	8.47	2.03	2.29
P	+++	+	+++	+++	+++
Random RR	2.29	1.46	4.79	1.68	2.23
RRl	1.34	1.02	2.07	1.39	1.66
RRu	3.90	2.10	11.06	2.03	2.98
P	++	+	+++	+++	+++
Between Chi					32.84
Between df					3
Between P					***
Btwn(F) P					**
Btwn(R) P					(*)

	Risky occupational population			
	no	mining	othRisky	Total
N	18			18
NS	15			15
Wt	186.25			186.25
Het Chi	54.19			54.19
Het df	17			17
Het P	***			***
Fixed RR	1.99			1.99
RRl	1.72			1.72
RRu	2.29			2.29
P	+++			+++
Random RR	2.23			2.23
RRl	1.66			1.66
RRu	2.98			2.98
P	+++			+++
Between Chi				
Between df				
Between P				N.S.
Btwn(F) P				N.S.
Btwn(R) P				N.S.

	National cigarette tobacco type			
	Virginia	blended	other	Total
N		15	3	18
NS		13	2	15
Wt		173.58	12.67	186.25
Het Chi		40.80	5.85	54.19
Het df		14	2	17
Het P		***	(*)	***
Fixed RR		1.88	4.18	1.99
RRl		1.62	2.41	1.72
RRu		2.18	7.25	2.29
P		+++	+++	+++
Random RR		2.05	3.65	2.23
RRl		1.54	1.22	1.66
RRu		2.73	10.92	2.98
P		+++	+	+++
Between Chi				7.54
Between df				1
Between P				**
Btwn(F) P				N.S.
Btwn(R) P				N.S.

Table 2H20 - 3

IESLC - Meta-analysis of Ever/current Smoking, Age started, "Highest vs lowest"
Squamous, Cigarettes (or Any Product if Cigarettes not available)
Most adjusted

	Any proxy use No/nk	Yes	Total
N	14	4	18
NS	12	3	15
Wt	135.22	51.03	186.25
Het Chi	29.04	21.50	54.19
Het df	13	3	17
Het P	**	***	***
Fixed RR	1.82	2.49	1.99
RRl	1.54	1.90	1.72
RRu	2.16	3.28	2.29
P	+++	+++	+++
Random RR	2.04	2.89	2.23
RRl	1.52	1.20	1.66
RRu	2.73	6.99	2.98
P	+++	+	+++
Between Chi			3.66
Between df			1
Between P			(*)
Btwn(F) P			N.S.
Btwn(R) P			N.S.

	Full histological confirmation No	Yes	Total
N	4	14	18
NS	4	11	15
Wt	39.77	146.48	186.25
Het Chi	0.42	53.39	54.19
Het df	3	13	17
Het P	N.S.	***	***
Fixed RR	1.82	2.03	1.99
RRl	1.34	1.73	1.72
RRu	2.49	2.39	2.29
P	+++	+++	+++
Random RR	1.82	2.40	2.23
RRl	1.34	1.64	1.66
RRu	2.49	3.50	2.98
P	+++	+++	+++
Between Chi			0.37
Between df			1
Between P			N.S.
Btwn(F) P			N.S.
Btwn(R) P			N.S.

	Number of adjustment variables (1) 0	1	2+/+nk	Total
N	9	3	6	18
NS	7	2	6	15
Wt	132.62	24.37	29.26	186.25
Het Chi	4.00	7.99	11.08	54.19
Het df	8	2	5	17
Het P	N.S.	*	*	***
Fixed RR	1.68	5.69	1.74	1.99
RRl	1.42	3.83	1.21	1.72
RRu	2.00	8.47	2.50	2.29
P	+++	+++	++	+++
Random RR	1.68	4.79	2.11	2.23
RRl	1.42	2.07	1.16	1.66
RRu	2.00	11.06	3.84	2.98
P	+++	+++	+	+++
Between Chi				31.11
Between df				2
Between P				***
Btwn(F) P				**
Btwn(R) P				*

Table 2H20 - 3

IESLC - Meta-analysis of Ever/current Smoking, Age started, "Highest vs lowest"
Squamous, Cigarettes (or Any Product if Cigarettes not available)
Most adjusted

	Number of adjustment variables (2) 0	1	2	3-5	6+/+nk	Total
N	9	3	4	1	1	18
NS	7	2	4	1	1	15
Wt	132.62	24.37	19.06	9.36	0.84	186.25
Het Chi	4.00	7.99	6.55	0.00	0.00	54.19
Het df	8	2	3	0	0	17
Het P	N.S.	*	(*)	N.S.	N.S.	***
Fixed RR	1.68	5.69	2.21	1.00	4.00	1.99
RRl	1.42	3.83	1.41	0.53	0.47	1.72
RRu	2.00	8.47	3.46	1.90	33.97	2.29
P	+++	+++	+++	N.S.	N.S.	+++
Random RR	1.68	4.79	2.59	1.00	4.00	2.23
RRl	1.42	2.07	1.26	0.53	0.47	1.66
RRu	2.00	11.06	5.35	1.90	33.97	2.98
P	+++	+++	++	N.S.	N.S.	+++
Between Chi						35.64
Between df						4
Between P						***
Btwn(F) P						**
Btwn(R) P						*

	Smoking status ever	current	Total
N	14	4	18
NS	11	4	15
Wt	173.33	12.92	186.25
Het Chi	49.62	4.53	54.19
Het df	13	3	17
Het P	***	N.S.	***
Fixed RR	1.98	2.10	1.99
RRl	1.70	1.22	1.72
RRu	2.29	3.63	2.29
P	+++	++	+++
Random RR	2.23	2.23	2.23
RRl	1.61	1.11	1.66
RRu	3.08	4.49	2.98
P	+++	+	+++
Between Chi			0.05
Between df			1
Between P			N.S.
Btwn(F) P			N.S.
Btwn(R) P			N.S.

	Product all/unsp	cig+/-ot	cig only	Total
N	5	11	2	18
NS	4	9	2	15
Wt	28.83	109.27	48.16	186.25
Het Chi	20.24	19.10	3.56	54.19
Het df	4	10	1	17
Het P	***	*	(*)	***
Fixed RR	3.38	1.93	1.54	1.99
RRl	2.35	1.60	1.16	1.72
RRu	4.87	2.33	2.05	2.29
P	+++	+++	++	+++
Random RR	3.63	1.98	1.97	2.23
RRl	1.33	1.49	0.86	1.66
RRu	9.89	2.64	4.50	2.98
P	+	+++	N.S.	+++
Between Chi				11.29
Between df				2
Between P				**
Btwn(F) P				N.S.
Btwn(R) P				N.S.

Table 2H20 - 3

IESLC - Meta-analysis of Ever/current Smoking, Age started, "Highest vs lowest"
Squamous, Cigarettes (or Any Product if Cigarettes not available)
Most adjusted

	Derivation of RR/CI			
	Orig	StdCalc	Other	Total
N	1	8	9	18
NS	1	7	8	16
Wt	9.36	132.14	44.74	186.25
Het Chi	0.00	3.25	24.25	54.19
Het df	0	7	8	17
Het P	N.S.	N.S.	**	***
Fixed RR	1.00	1.68	3.78	1.99
RRl	0.53	1.41	2.82	1.72
RRu	1.90	1.99	5.06	2.29
P	N.S.	+++	+++	+++
Random RR	1.00	1.68	3.64	2.23
RRl	0.53	1.41	2.05	1.66
RRu	1.90	1.99	6.46	2.98
P	N.S.	+++	+++	+++
Between Chi				26.68
Between df				2
Between P				***
Btwn(F) P				**
Btwn(R) P				**

Table 2H20 - 4

IESLC - Meta-analysis of Ever/current Smoking, Age started, "Highest vs lowest"
Squamous, Cigarettes (or Any Product if Cigarettes not available)
Least adjusted

REF	NRR	X	SEX	AGEL	AGEH	RACE	YF	LC TYPE	LOC	START	ST	NLC	R	VB	P	H	AD	ADOS	SM	PRODUCT	exL	exH	unexL	unexH	De
BARBON	573	x	m	0	0	all	-	q	Eu:wst	1979	CC	755	n	bl	y	y	0	0	ev	all/unsp	1	14	20	999	st
BENHAM	520		m	0	0	all	-	KI	Eu:wst	1976	CC	1625	n	bl	n	y	0	0	ev	cig only	1	16	25	999	st
ENGELA	521		m	0	0	all	0	q	Eu:Sca	1964	pr	435	n	bl	n	n	0	0	cu	cig+/-ot	1	19	30	999	st
HAENSZ	505	x	f	0	0	all	-	q+u	NAmer	1955	CC	158	n	bl	n	y	0	0	ev	cig+/-ot	1	24	25	999	st
HEGMAN	503		m	0	0	all	-	q	NAmer	1989	CC	282	n	bl	y	y	0	0	ev	all/unsp	1	19	20	999	st
HEGMAN	508		f	0	0	all	-	q	NAmer	1989	CC	282	n	bl	y	y	0	0	ev	all/unsp	1	25	26	999	ot
JEDRYC	532		m	0	0	all	-	q	Eu:est	1980	CC	1630	n	bl	y	n	0	0	ev	cig+/-ot	1	16	19	999	st
KHUDER	524		m	0	0	all	-	q	NAmer	1985	CC	482	n	bl	n	y	5	3#	ev	cig+/-ot	1	15	20	999	or
LUO	511	x	c	0	0	all	-	q	As:Chi	1990	CC	102	n	ot	n	y	0	0	ev	cig+/-ot	0	19	40	999	st
MATOS	615	x	m	0	0	all	-	q	SCAmer	1994	CC	200	n	bl	n	n	0	0	ev	cig+/-ot	1	14	20	999	st
PEZZOT	547	x	m	0	0	all	-	q	SCAmer	1987	CC	215	n	bl	n	y	0	0	ev	cig only	1	13	19	999	st
SVENSS	513		f	0	0	all	-	q	Eu:Sca	1983	CC	210	n	bl	n	n	0	0	cu	all/unsp	0	18	26	999	st
WAKAI	510		m	0	0	all	-	q	As:Jap	1988	CC	333	n	bl	n	y	0	0	cu	cig+/-ot	1	19	30	999	st
WU	513	x	f	0	0	wh	-	q	NAmer	1981	CC	220	n	bl	n	y	0	0	cu	all/unsp	0	18	25	999	st
WYNDE6	612		m	0	0	wh	-	q	NAmer	1969	CC	4423	n	bl	n	y	0	0	ev	cig+/-ot	1	17	21	999	st
WYNDE6	636		f	0	0	wh	-	q	NAmer	1969	CC	4423	n	bl	n	y	0	0	ev	cig+/-ot	1	17	21	999	st
ZHENG	524	x	m	0	0	all	-	q	As:Chi	1982	CC	540	n	ot	*	y	0	0	ev	cig+/-ot	1	19	30	999	st
ZHENG	529	x	f	0	0	all	-	q	As:Chi	1982	CC	540	n	ot	*	y	0	0	ev	cig+/-ot	1	29	30	999	st

Comments on values in listings

KHUDER ADOS Duration of smoking (years), number of cigarettes per day and Quitted smoking

Cigarette type is all/unspec for all RRs

Table 2H20 - 5

IESLC - Meta-analysis of Ever/current Smoking, Age started, "Highest vs lowest"
Squamous, Cigarettes (or Any Product if Cigarettes not available)
Least adjusted

REF	NRR	SEX	AD	Number Exposed Case	Number Exposed Cont	Non-exposed Case	Non-exposed Cont	RR	95.00%CI
BARBON	573	m	0	52	23	64	207	7.31	(4.16- 12.87)
BENHAM	520	m	0	342	341	98	137	1.40	(1.04- 1.89)
*ENGELA	521	m	0	64	50732	6	9762	2.05	(0.89- 4.74)
HAENSZ	505	f	0	24	37	32	66	1.34	(0.69- 2.60)
HEGMAN	503	m	0	57	716	15	289	1.53	(0.85- 2.75)
HEGMAN	508	f	0	17	169	0	28	5.88~	(0.34- 100.62)
Subtotal HEGMAN								1.62	(0.91- 2.87)
JEDRYC	532	m	0	53	111	134	502	1.79	(1.22- 2.61)
KHUDER	524	m	5	-	-	-	-	1.00	(0.50- 1.80)
LUO	511	c	0	14	20	1	8	5.60	(0.63- 49.95)
MATOS	615	m	0	16	90	6	73	2.16	(0.81- 5.81)
PEZZOT	547	m	0	30	34	10	32	2.82	(1.19- 6.69)
SVENSS	513	f	0	15	21	9	18	1.43	(0.51- 4.04)
WAKAI	510	m	0	21	74	4	25	1.77	(0.56- 5.67)
WU	513	f	0	37	11	6	5	2.80	(0.72- 10.97)
WYNDE6	612	m	0	262	301	44	92	1.82	(1.23- 2.70)
WYNDE6	636	f	0	72	91	32	90	2.23	(1.34- 3.70)
Subtotal WYNDE6								1.96	(1.44- 2.68)
ZHENG	524	m	0	62	43	13	66	7.32	(3.60- 14.90)
ZHENG	529	f	0	34	28	9	16	2.16	(0.83- 5.62)
Subtotal ZHENG								4.74	(2.68- 8.40)
Partial Totals				1172	52842	483	11416		

*prospective study ~ With 0.5 adjustment for zero

REF	NRR	SEX	AD	Ys	Ws	Qs	Ps
BARBON	573	m	0	1.99	12.02	20.78	0.0000
BENHAM	520	m	0	0.34	42.81	4.86	0.0270
*ENGELA	521	m	0	0.72	5.49	0.01	0.0920
HAENSZ	505	f	0	0.29	8.69	1.28	0.3909
HEGMAN	503	m	0	0.43	11.23	0.69	0.1518
HEGMAN	508	f	0	1.77	0.48	0.57	0.2211
Subtotal HEGMAN				0.48	11.70	1.26	
JEDRYC	532	m	0	0.58	26.79	0.23	0.0026
KHUDER	524	m	5	0.00	9.36	4.27	1.0000
LUO	511	c	0	1.72	0.80	0.88	0.1228
MATOS	615	m	0	0.77	3.94	0.04	0.1258
PEZZOT	547	m	0	1.04	5.15	0.68	0.0184
SVENSS	513	f	0	0.36	3.56	0.36	0.5010
WAKAI	510	m	0	0.57	2.85	0.03	0.3335
WU	513	f	0	1.03	2.06	0.26	0.1387
WYNDE6	612	m	0	0.60	24.55	0.14	0.0030
WYNDE6	636	f	0	0.80	14.87	0.23	0.0020
Subtotal WYNDE6				0.67	39.42	0.37	
ZHENG	524	m	0	1.99	7.61	13.17	0.0000
ZHENG	529	f	0	0.77	4.19	0.04	0.1153
Subtotal ZHENG				1.56	11.80	13.20	

	N	18
	NS	15
	Wt	186.45
Het	Chi	48.52
Het	df	17
Het	P	***
Fixed	RR	1.96
	RRl	1.70
	RRu	2.27
	P	+++
Random	RR	2.17
	RRl	1.65
	RRu	2.86
	P	+++
Asymm	P	N.S.

Table 2H20 - 6

IESLC - Meta-analysis of Ever/current Smoking, Age started, "Highest vs lowest"
Squamous, Cigarettes (or Any Product if Cigarettes not available)
Least adjusted

		Sex			
		combined	male	female	Total
	N	1	11	6	18
	NS	1	11	6	18
	Wt	0.80	151.80	33.85	186.45
Het	Chi	0.00	44.90	2.72	48.52
Het	df	0	10	5	17
Het	P	N.S.	***	N.S.	***
Fixed	RR	5.60	1.97	1.91	1.96
	RRl	0.63	1.68	1.36	1.70
	RRu	49.95	2.30	2.67	2.27
	P	N.S.	+++	+++	+++
Random	RR	5.60	2.24	1.91	2.17
	RRl	0.63	1.55	1.36	1.65
	RRu	49.95	3.24	2.67	2.86
	P	N.S.	+++	+++	+++
Between	Chi				0.91
Between	df				2
Between	P				N.S.
Btwn(F)	P				N.S.
Btwn(R)	P				N.S.

Table 2H20 - 7

IESLC - Meta-analysis of Ever/current Smoking, Age started, "Highest vs lowest"
Squamous, Cigarettes (or Any Product if Cigarettes not available)
Excluded studies (and stage at which they were excluded)

1	BECHER BLOT1 BROWN3 CARPEN CHYOU DARBY DOLL2 GARCIA GRAHAM GURSEL HAMMO2 JAHN JAIN LAUSSM PRESCO QIAO TVERDA WIGLE WYNDE3
2	AKIBA AMANDU AMES AXELSS BENSHL BEST BOUCHA BOUCOT BROSS CHEN CPSII DEAN2 DESTEF DORGAN DOSEME FAN GARSHI GER GILLIS HAMMON HUMBLE JUSSAW KAISE2 KATSOU KAUFMA KOO KREUZE LEVIN MCCONN NOTAN2 OSANN2 PEZZO2 PISANI RESTRE SADOWS VUTUC WANG2 WATSON WU2 WUWILL WYNDE2 WYNDE8 XU ZHOU
3	GUO MCDUFF SPITZ STASZE ZHANG
5	AGUDO ARMADA AUVINE BOFFET BRESLO BUFFLE CEDERL CHEN2 CHIAZZ CHOI CORREA CPSI DAMBER DEAN3 DOLL DORN GAO GAO2 GENG HIRAYA HOLE HU HU2 JOLY KOULUM LETOUR LIAW LIU3 LIU4 LIU5 LUBIN LUBIN2 MIGRAN MRFITR PERNU QIAO2 RACHTA SEGI2 SOBUE SPEIZE SUZUK2 TIZZAN WYNDE7 YUAN
6	ALDERS

Table 2H20 - 8
Potentially overlapping studies

REF	REFGP	PRINC	. OVERLAP/LINK
BENHAM	LUBIN2	2	Subset of Lubin2
WYNDE6	WYNDE6	1	WYNDE5/6/7/8

Table 2H20 - 9
Most adjusted - insufficient data for meta-analysis

REF	NRR	SEX	AGEL	AGEH	RACE	YF	LC TYPE	LOC	START	ST	NLC	R	VB	P	H	AD	ADOS	SM	PRODUCT	exL	exH	unexL	unexH	De
ALDERS	533	m	0	0	all	-	q+s	Eu:UK	1977	CC	1448	n	V	n	n	2		1#ev	cig only	1	14	25	999	ot
ALDERS	536	f	0	0	all	-	q+s	Eu:UK	1977	CC	1448	n	V	n	n	2		1#ev	cig only	1	14	25	999	ot

Comments on values in listings

ALDERS ADOS Number of cigs/day
ALDERS ADOS Number of cigs/day

REF	NRR	RR	SIG	RRDATA comment
ALDERS	533	5.56	n	0
ALDERS	536	1.56	n	0

Table 2H21 -

IESLC - Meta-analysis of Ever/current Smoking by Age started, Overview
Squamous, Cigarettes only

This analysis is restricted to results for:
1) Ever/current smokers
2) Results by Age started
3) Categorical results by Age started
Results by Age started are grouped under 2 schemes (S1, S2). Each scheme has a set of "key values". An interval is allocated to the category whose key value it includes, and intervals which include none or more than one of the key values are excluded. (Open-ended intervals are coded as 999)

S1	key value	maximum range
1	26	19+
2	18	15-25
3	14	1-17

S2	key value	maximum range
1	30	27+
2	26	23-29
3	22	19-25
4	18	15-21
5	14	11-17
6	10	1-13

4) Squamous (or near equivalent)
5) Results complete enough for use in metaanalysis

Within each study, results are then selected (in the following order of preference, within each sex) for:
6) SMKSTA: ever, current
7) PRODUCT: cigarettes only
8) CIGTYPE: all/unspecified, MC regardless of HR, MC only
9) (not applicable)
10) DENOM: never smoked anything, never smoked cigarettes, never any + low, never cigs + low
11) Followup period (YF, prospective studies): whole study (coded as 0) or longest available
12) LCtype: squamous or nearest available, but not adeno. (q = squamous, s = small,
a = adeno, KI = Kreyberg I, u = undifferentiated)
13) Race: all or nearest available, otherwise by race (wh or w = white, bl or b = black, hi = hispanic
ch = chinese, jap = japanese, haw = hawaiian, w+o = white + oriental, sca = scandinavian, as = asian)
14) For overlapping studies: principal rather than subsidiary studies
Finally by Age: whole study (coded as 0) if available, otherwise by widest available age group
and then for single sex results (m, f) in preference to results for both sexes combined (c).

Results adjusted (AD) for the most potential confounders are then chosen in Sections -1 to -3
(and those which actually differ from the adjusted results in Table 2H11 - 1 are marked 'x' in Section -1)
and results adjusted for the least confounders in Sections -4 to -6. (Those least adjusted results which
actually differ from the most adjusted are marked 'x' in column X in Section -4)

Section -7 shows excluded studies, together with the stage (as above) at which no qualifying
results were found.

Section -8 lists the potentially overlapping studies which have been included (1=principal, 2=subsidiary).

Section -9 lists any results which would have been included in preference except that they had data not complete
enough for use in meta-analysis, with their significance (yes/no), if known, and any further comment as entered
on the database. It also lists as "gap" any categories for which no data were presented by the original authors.

In addition to those mentioned above, the following fields, levels and abbreviations are used:
* or nk = not known, n = no, y = yes, ot = other
ev = ever, cu = current, nev = never
all/unspec = all or unspecified, MC = manufactured cigarettes, HR = hand-rolled cigarettes
exL, exH = range of exposure (low and high) in the smoking group, in terms of Age started
REF: 6-character study reference
NRR: number of the RR on the database within the study
ST : study type (CC = case control, pr or prosp = prospective)
NLC: number of lung cancer cases in whole study
R : risky occupational population (n = no, m = mining, o = other risky)
VB : national cigarette type (V = at least 75% Virginia, bl = at least 75% blended, ot = other)
P : any proxy use
H : full histological confirmation
De : derivation of RR/CI (or = original, st = standard method, ot = other method of estimation)

Table 2H21 - 1

IESLC - Meta-analysis of Ever/current Smoking by Age started, Overview
Squamous, Cigarettes only
Most adjusted

REF	NRR	2H11	SEX	AGEL	AGEH	RACE	YF	LC TYPE	LOC	START	ST	NLC	R	VB	P	H	AD	SM	PRODUCT	exL	exH	S1	S2	DENOM	De
BENHAM	514		m	0	0	all	-	KI	Eu:wst	1976	CC	1625	n	bl	n	y	0	ev	cig only	25	999	1	0	nev any	st
BENHAM	515		m	0	0	all	-	KI	Eu:wst	1976	CC	1625	n	bl	n	y	0	ev	cig only	20	24	0	3	nev any	st
BENHAM	516		m	0	0	all	-	KI	Eu:wst	1976	CC	1625	n	bl	n	y	0	ev	cig only	17	19	2	4	nev any	st
BENHAM	517		m	0	0	all	-	KI	Eu:wst	1976	CC	1625	n	bl	n	y	0	ev	cig only	1	16	3	0	nev any	st
PEZZOT	543		m	0	0	all	-	q	SCAmer	1987	CC	215	n	bl	n	y	0	ev	cig only	19	999	1	0	nev cigs	ot
PEZZOT	544		m	0	0	all	-	q	SCAmer	1987	CC	215	n	bl	n	y	0	ev	cig only	14	18	0	0	nev cigs	ot
PEZZOT	545		m	0	0	all	-	q	SCAmer	1987	CC	215	n	bl	n	y	0	ev	cig only	1	13	0	6	nev cigs	ot

Cigarette type is all/unspec for all RRs

In this overview table, subtotals and Qs values may be invalid and should be ignored

Table 2H21 - 2

IESLC - Meta-analysis of Ever/current Smoking by Age started, Overview
Squamous, Cigarettes only
Most adjusted

REF	NRR	SEX	AD	Number Exposed Case	Cont	Non-exposed Case	Cont	RR	95.00%CI
BENHAM	514	m	0	98	137	24	481	14.34	(8.83- 23.29)
BENHAM	515	m	0	323	398	24	481	16.26	(10.52- 25.14)
BENHAM	516	m	0	311	373	24	481	16.71	(10.80- 25.86)
BENHAM	517	m	0	342	341	24	481	20.10	(12.99- 31.10)
Subtotal BENHAM								16.86	(13.48- 21.08)
PEZZOT	543	m	0	10	105	0	116	23.19~	(1.34- 400.59)
PEZZOT	544	m	0	45	145	0	116	72.86~	(4.44-1195.36)
PEZZOT	545	m	0	30	67	0	116	105.28~	(6.34-1749.54)
Subtotal PEZZOT								56.74	(11.15- 288.86)
Totals				1159	1566	96	2272		

*prospective study ~ With 0.5 adjustment for zero

REF	NRR	SEX	AD	Ys	Ws	Qs	Ps
BENHAM	514	m	0	2.66	16.33	0.56	0.0000
BENHAM	515	m	0	2.79	20.26	0.07	0.0000
BENHAM	516	m	0	2.82	20.14	0.02	0.0000
BENHAM	517	m	0	3.00	20.16	0.47	0.0000
Subtotal BENHAM				2.82	76.89	1.12	
PEZZOT	543	m	0	3.14	0.47	0.04	0.0306
PEZZOT	544	m	0	4.29	0.49	1.02	0.0027
PEZZOT	545	m	0	4.66	0.49	1.59	0.0012
Subtotal PEZZOT				4.04	1.45	2.65	

N	7
NS	2

Table 2H21 - 3

IESLC - Meta-analysis of Ever/current Smoking by Age started, Overview
Squamous, Cigarettes only
Most adjusted

	Sex combined	male	female	Total
N		7		7
NS		2		2

In this overview table, other than the "N" rows, entries in the "absent" and "Total" columns may be invalid and should be ignored

	Age started (broad categories) absent	19+k26	15-25k18	1-17k14	Total
N	3	2	1	1	7
NS	2	2	1	1	5
Wt	21.24	16.80	20.14	20.16	78.34
Het Chi	2.67	0.11	0.00	0.00	3.77
Het df	2	1	0	0	6
Het P	N.S.	N.S.	N.S.	N.S.	N.S.
Fixed RR	17.57	14.53	16.71	20.10	17.24
RRl	11.49	9.01	10.80	12.99	13.82
RRu	26.89	23.44	25.86	31.10	21.51
P	+++	+++	+++	+++	+++
Random RR	24.88	14.53	16.71	20.10	17.24
RRl	8.45	9.01	10.80	12.99	13.82
RRu	73.22	23.44	25.86	31.10	21.51
P	+++	+++	+++	+++	+++

	absent	27+k30	23-29k26	Age started (narrow categories) 19-25k22	15-21k18	11-17k14	1-13k10	Total
N	4			1	1		1	7
NS	2			1	1		1	4
Wt	37.45			20.26	20.14		0.49	78.34
Het Chi	2.07			0.00	0.00		0.00	3.77
Het df	3			0	0		0	6
Het P	N.S.			N.S.	N.S.		N.S.	N.S.
Fixed RR	17.67			16.26	16.71		105.28	17.24
RRl	12.83			10.52	10.80		6.34	13.82
RRu	24.35			25.14	25.86		1749.54	21.51
P	+++			+++	+++		++	+++
Random RR	17.67			16.26	16.71		105.28	17.24
RRl	12.83			10.52	10.80		6.34	13.82
RRu	24.35			25.14	25.86		1749.54	21.51
P	+++			+++	+++		++	+++

Table 2H21 - 3

IESLC - Meta-analysis of Ever/current Smoking by Age started, Overview
Squamous, Cigarettes only
Most adjusted

MALES

		Age started (broad categories)				
		absent	19+k26	15-25k18	1-17k14	Total
	N	3	2	1	1	7
	NS	2	2	1	1	5
	Wt	21.24	16.80	20.14	20.16	78.34
Het	Chi	2.67	0.11	0.00	0.00	3.77
Het	df	2	1	0	0	6
Het	P	N.S.	N.S.	N.S.	N.S.	N.S.
Fixed	RR	17.57	14.53	16.71	20.10	17.24
	RRl	11.49	9.01	10.80	12.99	13.82
	RRu	26.89	23.44	25.86	31.10	21.51
	P	+++	+++	+++	+++	+++
Random	RR	24.88	14.53	16.71	20.10	17.24
	RRl	8.45	9.01	10.80	12.99	13.82
	RRu	73.22	23.44	25.86	31.10	21.51
	P	+++	+++	+++	+++	+++

				Age started (narrow categories)				
		absent	27+k30	23-29k26	19-25k22	15-21k18	11-17k14	1-13k10	Total
	N	4			1	1		1	7
	NS	2			1	1		1	4
	Wt	37.45			20.26	20.14		0.49	78.34
Het	Chi	2.07			0.00	0.00		0.00	3.77
Het	df	3			0	0		0	6
Het	P	N.S.			N.S.	N.S.		N.S.	N.S.
Fixed	RR	17.67			16.26	16.71		105.28	17.24
	RRl	12.83			10.52	10.80		6.34	13.82
	RRu	24.35			25.14	25.86		1749.54	21.51
	P	+++			+++	+++		++	+++
Random	RR	17.67			16.26	16.71		105.28	17.24
	RRl	12.83			10.52	10.80		6.34	13.82
	RRu	24.35			25.14	25.86		1749.54	21.51
	P	+++			+++	+++		++	+++

Table 2H21 - 4

IESLC - Meta-analysis of Ever/current Smoking by Age started, Overview
Squamous, Cigarettes only
Least adjusted

REF	NRR	X	SEX	AGEL	AGEH	RACE	YF	LC TYPE	LOC	START	ST	NLC	R	VB	P	H	AD	SM	PRODUCT	exL	exH	S1	S2	DENOM	De
BENHAM	514		m	0	0	all	-	KI	Eu:wst	1976	CC	1625	n	bl	n	y	0	ev	cig only	25	999	1	0	nev any	st
BENHAM	515		m	0	0	all	-	KI	Eu:wst	1976	CC	1625	n	bl	n	y	0	ev	cig only	20	24	0	3	nev any	st
BENHAM	516		m	0	0	all	-	KI	Eu:wst	1976	CC	1625	n	bl	n	y	0	ev	cig only	17	19	2	4	nev any	st
BENHAM	517		m	0	0	all	-	KI	Eu:wst	1976	CC	1625	n	bl	n	y	0	ev	cig only	1	16	3	0	nev any	st
PEZZOT	543		m	0	0	all	-	q	SCAmer	1987	CC	215	n	bl	n	y	0	ev	cig only	19	999	1	0	nev cigs	ot
PEZZOT	544		m	0	0	all	-	q	SCAmer	1987	CC	215	n	bl	n	y	0	ev	cig only	14	18	0	0	nev cigs	ot
PEZZOT	545		m	0	0	all	-	q	SCAmer	1987	CC	215	n	bl	n	y	0	ev	cig only	1	13	0	6	nev cigs	ot

Cigarette type is all/unspec for all RRs

In this overview table, subtotals and Qs values may be invalid and should be ignored

Table 2H21 - 5

IESLC - Meta-analysis of Ever/current Smoking by Age started, Overview
Squamous, Cigarettes only
Least adjusted

REF	NRR	SEX	AD	Number Exposed Case	Cont	Non-exposed Case	Cont	RR	95.00%CI
BENHAM	514	m	0	98	137	24	481	14.34	(8.83- 23.29)
BENHAM	515	m	0	323	398	24	481	16.26	(10.52- 25.14)
BENHAM	516	m	0	311	373	24	481	16.71	(10.80- 25.86)
BENHAM	517	m	0	342	341	24	481	20.10	(12.99- 31.10)
Subtotal BENHAM								16.86	(13.48- 21.08)
PEZZOT	543	m	0	10	105	0	116	23.19~	(1.34- 400.59)
PEZZOT	544	m	0	45	145	0	116	72.86~	(4.44-1195.36)
PEZZOT	545	m	0	30	67	0	116	105.28~	(6.34-1749.54)
Subtotal PEZZOT								56.74	(11.15- 288.86)
Totals				1159	1566	96	2272		

*prospective study ~ With 0.5 adjustment for zero

REF	NRR	SEX	AD	Ys	Ws	Qs	Ps
BENHAM	514	m	0	2.66	16.33	0.56	0.0000
BENHAM	515	m	0	2.79	20.26	0.07	0.0000
BENHAM	516	m	0	2.82	20.14	0.02	0.0000
BENHAM	517	m	0	3.00	20.16	0.47	0.0000
Subtotal BENHAM				2.82	76.89	1.12	
PEZZOT	543	m	0	3.14	0.47	0.04	0.0306
PEZZOT	544	m	0	4.29	0.49	1.02	0.0027
PEZZOT	545	m	0	4.66	0.49	1.59	0.0012
Subtotal PEZZOT				4.04	1.45	2.65	

N	7
NS	2

Table 2H21 - 6

IESLC - Meta-analysis of Ever/current Smoking by Age started, Overview
Squamous, Cigarettes only
Least adjusted

	Sex: combined	male	female	Total
N		7		7
NS		2		2

In this overview table, other than the "N" rows, entries in the "absent" and "Total" columns may be invalid and should be ignored

	Age started (broad categories): absent	19+k26	15-25k18	1-17k14	Total
N	3	2	1	1	7
NS	2	2	1	1	5
Wt	21.24	16.80	20.14	20.16	78.34
Het Chi	2.67	0.11	0.00	0.00	3.77
Het df	2	1	0	0	6
Het P	N.S.	N.S.	N.S.	N.S.	N.S.
Fixed RR	17.57	14.53	16.71	20.10	17.24
RRl	11.49	9.01	10.80	12.99	13.82
RRu	26.89	23.44	25.86	31.10	21.51
P	+++	+++	+++	+++	+++
Random RR	24.88	14.53	16.71	20.10	17.24
RRl	8.45	9.01	10.80	12.99	13.82
RRu	73.22	23.44	25.86	31.10	21.51
P	+++	+++	+++	+++	+++

	absent	Age started (narrow categories): 27+k30	23-29k26	19-25k22	15-21k18	11-17k14	1-13k10	Total
N	4			1	1		1	7
NS	2			1	1		1	4
Wt	37.45			20.26	20.14		0.49	78.34
Het Chi	2.07			0.00	0.00		0.00	3.77
Het df	3			0	0		0	6
Het P	N.S.			N.S.	N.S.		N.S.	N.S.
Fixed RR	17.67			16.26	16.71		105.28	17.24
RRl	12.83			10.52	10.80		6.34	13.82
RRu	24.35			25.14	25.86		1749.54	21.51
P	+++			+++	+++		++	+++
Random RR	17.67			16.26	16.71		105.28	17.24
RRl	12.83			10.52	10.80		6.34	13.82
RRu	24.35			25.14	25.86		1749.54	21.51
P	+++			+++	+++		++	+++

Table 2H21 - 6

IESLC - Meta-analysis of Ever/current Smoking by Age started, Overview
Squamous, Cigarettes only
Least adjusted

MALES

	Age started (broad categories) absent	19+k26	15-25k18	1-17k14	Total
N	3	2	1	1	7
NS	2	2	1	1	5
Wt	21.24	16.80	20.14	20.16	78.34
Het Chi	2.67	0.11	0.00	0.00	3.77
Het df	2	1	0	0	6
Het P	N.S.	N.S.	N.S.	N.S.	N.S.
Fixed RR	17.57	14.53	16.71	20.10	17.24
RRl	11.49	9.01	10.80	12.99	13.82
RRu	26.89	23.44	25.86	31.10	21.51
P	+++	+++	+++	+++	+++
Random RR	24.88	14.53	16.71	20.10	17.24
RRl	8.45	9.01	10.80	12.99	13.82
RRu	73.22	23.44	25.86	31.10	21.51
P	+++	+++	+++	+++	+++

		Age started (narrow categories)					
	absent	27+k30	23-29k26	19-25k22	15-21k18	11-17k14	1-13k10	Total
N	4			1	1		1	7
NS	2			1	1		1	4
Wt	37.45			20.26	20.14		0.49	78.34
Het Chi	2.07			0.00	0.00		0.00	3.77
Het df	3			0	0		0	6
Het P	N.S.			N.S.	N.S.		N.S.	N.S.
Fixed RR	17.67			16.26	16.71		105.28	17.24
RRl	12.83			10.52	10.80		6.34	13.82
RRu	24.35			25.14	25.86		1749.54	21.51
P	+++			+++	+++		++	+++
Random RR	17.67			16.26	16.71		105.28	17.24
RRl	12.83			10.52	10.80		6.34	13.82
RRu	24.35			25.14	25.86		1749.54	21.51
P	+++			+++	+++		++	+++

Table 2H21 - 7

IESLC - Meta-analysis of Ever/current Smoking by Age started, Overview
Squamous, Cigarettes only
Excluded studies (and stage at which they were excluded)

1	BECHER BLOT1 BROWN3 CARPEN CHYOU DARBY DOLL2 GARCIA GRAHAM GURSEL HAMMO2 JAHN JAIN LAUSSM PRESCO QIAO TVERDA WIGLE WYNDE3
2	AKIBA AMANDU AMES AXELSS BENSHL BEST BOUCHA BOUCOT BROSS CHEN CPSII DEAN2 DESTEF DORGAN DOSEME FAN GARSHI GER GILLIS HAMMON HUMBLE JUSSAW KAISE2 KATSOU KAUFMA KOO KREUZE LEVIN MCCONN NOTAN2 OSANN2 PEZZO2 PISANI RESTRE SADOWS VUTUC WANG2 WATSON WU2 WUWILL WYNDE2 WYNDE8 XU ZHOU
3	GUO MCDUFF SPITZ STASZE ZHANG
4	AGUDO ARMADA AUVINE BOFFET BRESLO BUFFLE CEDERL CHEN2 CHIAZZ CHOI CORREA CPSI DAMBER DEAN3 DOLL DORN GAO GAO2 GENG HIRAYA HOLE HU HU2 JOLY KOULUM LETOUR LIAW LIU3 LIU4 LIU5 LUBIN LUBIN2 MIGRAN MRFITR PERNU QIAO2 RACHTA SEGI2 SOBUE SPEIZE SUZUK2 TIZZAN WYNDE7 YUAN
5	ALDERS
7	BARBON ENGELA HAENSZ HEGMAN JEDRYC KHUDER LUO MATOS SVENSS WAKAI WU WYNDE6 ZHENG

Table 2H21 - 8
Potentially overlapping studies

REF	REFGP	PRINC	.	OVERLAP/LINK
BENHAM	LUBIN2	2		Subset of Lubin2

Table 2H22 -

IESLC - Meta-analysis of Ever/current Smoking, Age started, "Low"
Squamous, Cigarettes only

This analysis is restricted to results for:
1) Ever/current smokers
2) Results by Age started
3) Categorical results by Age started
4) Squamous (or near equivalent)
5) Results complete enough for use in metaanalysis

Within each study, results are then selected (in the following order of preference, within each sex) for:
6) SMKSTA: ever, current
7) PRODUCT: cigarettes only
8) CIGTYPE: all/unspecified, MC regardless of HR, MC only
9) (not applicable)
10) DENOM: never smoked anything, never smoked cigarettes, never any + low, never cigs + low
11) Followup period (YF, prospective studies): whole study (coded as 0) or longest available
12) LCtype: squamous or nearest available, but not adeno. (q = squamous, s = small,
a = adeno, KI = Kreyberg I, u = undifferentiated)
13) Race: all or nearest available, otherwise by race (wh or w = white, bl or b = black, hi = hispanic
ch = chinese, jap = japanese, haw = hawaiian, w+o = white + oriental, sca = scandinavian, as = asian)
14) Age started "low" in key scheme 1 (key value 26, maximum range 19+)
15) For overlapping studies: principal rather than subsidiary studies
Finally by Age: whole study (coded as 0) if available, otherwise by widest available age group
and then for single sex results (m, f) in preference to results for both sexes combined (c).

Results adjusted (AD) for the most potential confounders are then chosen in Sections -1 to -3
(and those which actually differ from the adjusted results in Table 2H12 - 1 are marked 'x' in Section -1)
and results adjusted for the least confounders in Sections -4 to -6. (Those least adjusted results which
actually differ from the most adjusted are marked 'x' in column X in Section -4)

Section -7 shows excluded studies, together with the stage (as above) at which no qualifying
results were found.

Section -8 lists the potentially overlapping studies which have been included (1=principal, 2=subsidiary).

Section -9 lists any results which would have been included in preference except that they had data not complete
enough for use in meta-analysis, with their significance (yes/no), if known, and any further comment as entered
on the database. It also lists as "gap" any categories for which no data were presented by the original authors.

In addition to those mentioned above, the following fields, levels and abbreviations are used:
* or nk = not known, n = no, y = yes, ot = other
ev = ever, cu = current, nev = never
all/unspec = all or unspecified, MC = manufactured cigarettes, HR = hand-rolled cigarettes
exL, exH = range of exposure (low and high) in the smoking group, in terms of Age started
REF: 6-character study reference
NRR: number of the RR on the database within the study
ST : study type (CC = case control, pr or prosp = prospective)
NLC: number of lung cancer cases in whole study
R : risky occupational population (n = no, m = mining, o = other risky)
VB : national cigarette type (V = at least 75% Virginia, bl = at least 75% blended, ot = other)
P : any proxy use
H : full histological confirmation
De : derivation of RR/CI (or = original, st = standard method, ot = other method of estimation)

Table 2H22 - 1

IESLC - Meta-analysis of Ever/current Smoking, Age started, "Low"
Squamous, Cigarettes only
Most adjusted

REF	NRR	2H12	SEX	AGEL	AGEH	RACE	YF	LC TYPE	LOC	START	ST	NLC	R	VB	P	H	AD	SM	PRODUCT	exL	exH	DENOM	De
BENHAM	514		m	0	0	all	-	KI	Eu:wst	1976	CC	1625	n	bl	n	y	0	ev	cig only	25	999	nev any	st
PEZZOT	543		m	0	0	all	-	q	SCAmer	1987	CC	215	n	bl	n	y	0	ev	cig only	19	999	nev cigs	ot

Cigarette type is all/unspec for all RRs

Table 2H22 - 2

IESLC - Meta-analysis of Ever/current Smoking, Age started, "Low"
Squamous, Cigarettes only
Most adjusted

REF	NRR	SEX	AD	Number Exposed Case	Exposed Cont	Non-exposed Case	Non-exposed Cont	RR	95.00%CI
BENHAM	514	m	0	98	137	24	481	14.34	(8.83- 23.29)
PEZZOT	543	m	0	10	105	0	116	23.19~	(1.34- 400.59)
Totals				108	242	24	597		

*prospective study ~ With 0.5 adjustment for zero

REF	NRR	SEX	AD	Ys	Ws	Qs	Ps
BENHAM	514	m	0	2.66	16.33	0.00	0.0000
PEZZOT	543	m	0	3.14	0.47	0.10	0.0306

	N	2
	NS	2
	Wt	16.80
Het	Chi	0.11
Het	df	1
Het	P	N.S.
Fixed	RR	14.53
	RRl	9.01
	RRu	23.44
	P	+++
Random	RR	14.53
	RRl	9.01
	RRu	23.44
	P	+++
Asymm	P	

Table 2H22 - 3

IESLC - Meta-analysis of Ever/current Smoking, Age started, "Low"
Squamous, Cigarettes only
Most adjusted

		Sex		
	combined	male	female	Total
N		2		2
NS		2		2
Wt		16.80		16.80
Het Chi		0.11		0.11
Het df		1		1
Het P		N.S.		N.S.
Fixed RR		14.53		14.53
RRl		9.01		9.01
RRu		23.44		23.44
P		+++		+++
Random RR		14.53		14.53
RRl		9.01		9.01
RRu		23.44		23.44
P		+++		+++
Between Chi				
Between df				
Between P				N.S.
Btwn(F) P				N.S.
Btwn(R) P				N.S.

Too few RRs for analysis by factor

Table 2H22 - 4

IESLC - Meta-analysis of Ever/current Smoking, Age started, "Low"
Squamous, Cigarettes only
Least adjusted

REF	NRR	X	SEX	AGEL	AGEH	RACE	YF	LC TYPE	LOC	START	ST	NLC	R	VB	P	H	AD	SM	PRODUCT	exL	exH	DENOM	De
BENHAM	514		m	0	0	all	-	KI	Eu:wst	1976	CC	1625	n	bl	n	y	0	ev	cig only	25	999	nev any	st
PEZZOT	543		m	0	0	all	-	q	SCAmer	1987	CC	215	n	bl	n	y	0	ev	cig only	19	999	nev cigs	ot

Cigarette type is all/unspec for all RRs

Table 2H22 - 5

IESLC - Meta-analysis of Ever/current Smoking, Age started, "Low"
Squamous, Cigarettes only
Least adjusted

REF	NRR	SEX	AD	Number Case	Exposed Cont	Non-exposed Case	Cont	RR	95.00%CI
BENHAM	514	m	0	98	137	24	481	14.34	(8.83- 23.29)
PEZZOT	543	m	0	10	105	0	116	23.19~	(1.34- 400.59)
Totals				108	242	24	597		

*prospective study ~ With 0.5 adjustment for zero

REF	NRR	SEX	AD	Ys	Ws	Qs	Ps
BENHAM	514	m	0	2.66	16.33	0.00	0.0000
PEZZOT	543	m	0	3.14	0.47	0.10	0.0306

	N	2
	NS	2
	Wt	16.80
Het	Chi	0.11
Het	df	1
Het	P	N.S.
Fixed	RR	14.53
	RRl	9.01
	RRu	23.44
	P	+++
Random	RR	14.53
	RRl	9.01
	RRu	23.44
	P	+++
Asymm	P	

Table 2H22 - 6

IESLC - Meta-analysis of Ever/current Smoking, Age started, "Low"
Squamous, Cigarettes only
Least adjusted

	Sex combined	male	female	Total
N		2		2
NS		2		2
Wt		16.80		16.80
Het Chi		0.11		0.11
Het df		1		1
Het P		N.S.		N.S.
Fixed RR		14.53		14.53
RRl		9.01		9.01
RRu		23.44		23.44
P		+++		+++
Random RR		14.53		14.53
RRl		9.01		9.01
RRu		23.44		23.44
P		+++		+++
Between Chi				
Between df				
Between P				N.S.
Btwn(F) P				N.S.
Btwn(R) P				N.S.

Table 2H22 - 7

IESLC - Meta-analysis of Ever/current Smoking, Age started, "Low"
Squamous, Cigarettes only
Excluded studies (and stage at which they were excluded)

1	BECHER BLOT1 BROWN3 CARPEN CHYOU DARBY DOLL2 GARCIA GRAHAM GURSEL HAMMO2 JAHN JAIN LAUSSM PRESCO QIAO TVERDA WIGLE WYNDE3
2	AKIBA AMANDU AMES AXELSS BENSHL BEST BOUCHA BOUCOT BROSS CHEN CPSII DEAN2 DESTEF DORGAN DOSEME FAN GARSHI GER GILLIS HAMMON HUMBLE JUSSAW KAISE2 KATSOU KAUFMA KOO KREUZE LEVIN MCCONN NOTAN2 OSANN2 PEZZO2 PISANI RESTRE SADOWS VUTUC WANG2 WATSON WU2 WUWILL WYNDE2 WYNDE8 XU ZHOU
3	GUO MCDUFF SPITZ STASZE ZHANG
4	AGUDO ARMADA AUVINE BOFFET BRESLO BUFFLE CEDERL CHEN2 CHIAZZ CHOI CORREA CPSI DAMBER DEAN3 DOLL DORN GAO GAO2 GENG HIRAYA HOLE HU HU2 JOLY KOULUM LETOUR LIAW LIU3 LIU4 LIU5 LUBIN LUBIN2 MIGRAN MRFITR PERNU QIAO2 RACHTA SEGI2 SOBUE SPEIZE SUZUK2 TIZZAN WYNDE7 YUAN
5	ALDERS
7	BARBON ENGELA HAENSZ HEGMAN JEDRYC KHUDER LUO MATOS SVENSS WAKAI WU WYNDE6 ZHENG

Table 2H22 - 8
Potentially overlapping studies

REF	REFGP	PRINC	.	OVERLAP/LINK
BENHAM	LUBIN2	2		Subset of Lubin2

Table 2H23 -

IESLC - Meta-analysis of Ever/current Smoking, Age started, "Mid"
Squamous, Cigarettes only

This analysis is restricted to results for:
1) Ever/current smokers
2) Results by Age started
3) Categorical results by Age started
4) Squamous (or near equivalent)
5) Results complete enough for use in metaanalysis

Within each study, results are then selected (in the following order of preference, within each sex) for:
6) SMKSTA: ever, current
7) PRODUCT: cigarettes only
8) CIGTYPE: all/unspecified, MC regardless of HR, MC only
9) (not applicable)
10) DENOM: never smoked anything, never smoked cigarettes, never any + low, never cigs + low
11) Followup period (YF, prospective studies): whole study (coded as 0) or longest available
12) LCtype: squamous or nearest available, but not adeno. (q = squamous, s = small,
a = adeno, KI = Kreyberg I, u = undifferentiated)
13) Race: all or nearest available, otherwise by race (wh or w = white, bl or b = black, hi = hispanic
ch = chinese, jap = japanese, haw = hawaiian, w+o = white + oriental, sca = scandinavian, as = asian)
14) Age started "mid" in key scheme 1 (key value 18, maximum range 15-25)
15) For overlapping studies: principal rather than subsidiary studies
Finally by Age: whole study (coded as 0) if available, otherwise by widest available age group
and then for single sex results (m, f) in preference to results for both sexes combined (c).

Results adjusted (AD) for the most potential confounders are then chosen in Sections -1 to -3
(and those which actually differ from the adjusted results in Table 2H13 - 1 are marked 'x' in Section -1)
and results adjusted for the least confounders in Sections -4 to -6. (Those least adjusted results which
actually differ from the most adjusted are marked 'x' in column X in Section -4)

Section -7 shows excluded studies, together with the stage (as above) at which no qualifying
results were found.

Section -8 lists the potentially overlapping studies which have been included (1=principal, 2=subsidiary).

Section -9 lists any results which would have been included in preference except that they had data not complete
enough for use in meta-analysis, with their significance (yes/no), if known, and any further comment as entered
on the database. It also lists as "gap" any categories for which no data were presented by the original authors.

In addition to those mentioned above, the following fields, levels and abbreviations are used:
* or nk = not known, n = no, y = yes, ot = other
ev = ever, cu = current, nev = never
all/unspec = all or unspecified, MC = manufactured cigarettes, HR = hand-rolled cigarettes
exL, exH = range of exposure (low and high) in the smoking group, in terms of Age started
REF: 6-character study reference
NRR: number of the RR on the database within the study
ST : study type (CC = case control, pr or prosp = prospective)
NLC: number of lung cancer cases in whole study
R : risky occupational population (n = no, m = mining, o = other risky)
VB : national cigarette type (V = at least 75% Virginia, bl = at least 75% blended, ot = other)
P : any proxy use
H : full histological confirmation
De : derivation of RR/CI (or = original, st = standard method, ot = other method of estimation)

Table 2H23 - 1

IESLC - Meta-analysis of Ever/current Smoking, Age started, "Mid"
Squamous, Cigarettes only
Most adjusted

REF	NRR	2H13	SEX	AGEL	AGEH	RACE	YF	LC TYPE	LOC	START	ST	NLC	R	VB	P	H	AD	SM	PRODUCT	exL	exH	DENOM	De
BENHAM	516		m	0	0	all	-	KI	Eu:wst	1976	CC	1625	n	bl	n	y	0	ev	cig only	17	19	nev any	st

Cigarette type is all/unspec for all RRs

Table 2H23 - 2

IESLC - Meta-analysis of Ever/current Smoking, Age started, "Mid"
Squamous, Cigarettes only
Most adjusted

REF	NRR	SEX	AD	Number Exposed Case	Cont	Non-exposed Case	Cont	RR	95.00%CI
BENHAM	516	m	0	311	373	24	481	16.71	(10.80- 25.86)
Totals				311	373	24	481		

*prospective study

REF	NRR	SEX	AD	Ys	Ws	Qs	Ps
BENHAM	516	m	0	2.82	20.14	0.00	0.0000

	N	1
	NS	1
	Wt	20.14
Het	Chi	0.00
Het	df	0
Het	P	N.S.
Fixed	RR	16.71
	RRl	10.80
	RRu	25.86
	P	+++
Random	RR	16.71
	RRl	10.80
	RRu	25.86
	P	+++
Asymm	P	

Table 2H23 - 3

IESLC - Meta-analysis of Ever/current Smoking, Age started, "Mid"
Squamous, Cigarettes only
Most adjusted

	Sex combined	male	female	Total
N		1		1
NS		1		1
Wt		20.14		20.14
Het Chi		0.00		0.00
Het df		0		0
Het P		N.S.		N.S.
Fixed RR		16.71		16.71
RRl		10.80		10.80
RRu		25.86		25.86
P		+++		+++
Random RR		16.71		16.71
RRl		10.80		10.80
RRu		25.86		25.86
P		+++		+++
Between Chi				
Between df				
Between P				N.S.
Btwn(F) P				N.S.
Btwn(R) P				N.S.

Too few RRs for analysis by factor

Table 2H23 - 4

IESLC - Meta-analysis of Ever/current Smoking, Age started, "Mid"
Squamous, Cigarettes only
Least adjusted

REF	NRR	X	SEX	AGEL	AGEH	RACE	YF	LC TYPE	LOC	START	ST	NLC	R	VB	P	H	AD	SM	PRODUCT	exL	exH	DENOM	De
BENHAM	516		m	0	0	all	-	KI	Eu:wst	1976	CC	1625	n	bl	n	y	0	ev	cig only	17	19	nev any	st

Cigarette type is all/unspec for all RRs

Table 2H23 - 5

IESLC - Meta-analysis of Ever/current Smoking, Age started, "Mid"
Squamous, Cigarettes only
Least adjusted

REF	NRR	SEX	AD	Number Exposed Case	Cont	Non-exposed Case	Cont	RR	95.00%CI
BENHAM	516	m	0	311	373	24	481	16.71	(10.80- 25.86)
Totals				311	373	24	481		

*prospective study

REF	NRR	SEX	AD	Ys	Ws	Qs	Ps
BENHAM	516	m	0	2.82	20.14	0.00	0.0000

	N	1
	NS	1
	Wt	20.14
Het	Chi	0.00
Het	df	0
Het	P	N.S.
Fixed	RR	16.71
	RRl	10.80
	RRu	25.86
	P	+++
Random	RR	16.71
	RRl	10.80
	RRu	25.86
	P	+++
Asymm	P	

Table 2H23 - 6

IESLC - Meta-analysis of Ever/current Smoking, Age started, "Mid"
Squamous, Cigarettes only
Least adjusted

	Sex combined	male	female	Total
N		1		1
NS		1		1
Wt		20.14		20.14
Het Chi		0.00		0.00
Het df		0		0
Het P		N.S.		N.S.
Fixed RR		16.71		16.71
RRl		10.80		10.80
RRu		25.86		25.86
P		+++		+++
Random RR		16.71		16.71
RRl		10.80		10.80
RRu		25.86		25.86
P		+++		+++
Between Chi				
Between df				
Between P				N.S.
Btwn(F) P				N.S.
Btwn(R) P				N.S.

Table 2H23 - 7

IESLC - Meta-analysis of Ever/current Smoking, Age started, "Mid"
Squamous, Cigarettes only
Excluded studies (and stage at which they were excluded)

1	BECHER BLOT1 BROWN3 CARPEN CHYOU DARBY DOLL2 GARCIA GRAHAM GURSEL HAMMO2 JAHN JAIN LAUSSM PRESCO QIAO TVERDA WIGLE WYNDE3
2	AKIBA AMANDU AMES AXELSS BENSHL BEST BOUCHA BOUCOT BROSS CHEN CPSII DEAN2 DESTEF DORGAN DOSEME FAN GARSHI GER GILLIS HAMMON HUMBLE JUSSAW KAISE2 KATSOU KAUFMA KOO KREUZE LEVIN MCCONN NOTAN2 OSANN2 PEZZO2 PISANI RESTRE SADOWS VUTUC WANG2 WATSON WU2 WUWILL WYNDE2 WYNDE8 XU ZHOU
3	GUO MCDUFF SPITZ STASZE ZHANG
4	AGUDO ARMADA AUVINE BOFFET BRESLO BUFFLE CEDERL CHEN2 CHIAZZ CHOI CORREA CPSI DAMBER DEAN3 DOLL DORN GAO GAO2 GENG HIRAYA HOLE HU HU2 JOLY KOULUM LETOUR LIAW LIU3 LIU4 LIU5 LUBIN LUBIN2 MIGRAN MRFITR PERNU QIAO2 RACHTA SEGI2 SOBUE SPEIZE SUZUK2 TIZZAN WYNDE7 YUAN
5	ALDERS
7	BARBON ENGELA HAENSZ HEGMAN JEDRYC KHUDER LUO MATOS SVENSS WAKAI WU WYNDE6 ZHENG
14	PEZZOT

Table 2H23 - 8
Potentially overlapping studies

REF	REFGP	PRINC	.	OVERLAP/LINK
BENHAM	LUBIN2	2		Subset of Lubin2

Table 2H24 -

IESLC - Meta-analysis of Ever/current Smoking, Age started, "High"
Squamous, Cigarettes only

This analysis is restricted to results for:
1) Ever/current smokers
2) Results by Age started
3) Categorical results by Age started
4) Squamous (or near equivalent)
5) Results complete enough for use in metaanalysis

Within each study, results are then selected (in the following order of preference, within each sex) for:
6) PRODUCT: cigarettes only
7) CIGTYPE: all/unspecified, MC regardless of HR, MC only
8) (not applicable)
9) DENOM: never smoked anything, never smoked cigarettes, never any + low, never cigs + low
10) Followup period (YF, prospective studies): whole study (coded as 0) or longest available
11) LCtype: squamous or nearest available, but not adeno. (q = squamous, s = small,
a = adeno, KI = Kreyberg I, u = undifferentiated)
12) Race: all or nearest available, otherwise by race (wh or w = white, bl or b = black, hi = hispanic
ch = chinese, jap = japanese, haw = hawaiian, w+o = white + oriental, sca = scandinavian, as = asian)
13) Age started "high" in key scheme 1 (key value 14, maximum range 1-17)
14) For overlapping studies: principal rather than subsidiary studies
Finally by Age: whole study (coded as 0) if available, otherwise by widest available age group
and then for single sex results (m, f) in preference to results for both sexes combined (c).

Results adjusted (AD) for the most potential confounders are then chosen in Sections -1 to -3
(and those which actually differ from the adjusted results in Table 2H14 - 1 are marked 'x' in Section -1)
and results adjusted for the least confounders in Sections -4 to -6. (Those least adjusted results which
actually differ from the most adjusted are marked 'x' in column X in Section -4)

Section -7 shows excluded studies, together with the stage (as above) at which no qualifying
results were found.

Section -8 lists the potentially overlapping studies which have been included (1=principal, 2=subsidiary).

Section -9 lists any results which would have been included in preference except that they had data not complete
enough for use in meta-analysis, with their significance (yes/no), if known, and any further comment as entered
on the database. It also lists as "gap" any categories for which no data were presented by the original authors.

In addition to those mentioned above, the following fields, levels and abbreviations are used:
* or nk = not known, n = no, y = yes, ot = other
ev = ever, cu = current, nev = never
all/unspec = all or unspecified, MC = manufactured cigarettes, HR = hand-rolled cigarettes
exL, exH = range of exposure (low and high) in the smoking group, in terms of Age started
REF: 6-character study reference
NRR: number of the RR on the database within the study
ST : study type (CC = case control, pr or prosp = prospective)
NLC: number of lung cancer cases in whole study
R : risky occupational population (n = no, m = mining, o = other risky)
VB : national cigarette type (V = at least 75% Virginia, bl = at least 75% blended, ot = other)
P : any proxy use
H : full histological confirmation
De : derivation of RR/CI (or = original, st = standard method, ot = other method of estimation)

Table 2H24 - 1

IESLC - Meta-analysis of Ever/current Smoking, Age started, "High"
Squamous, Cigarettes only
Most adjusted

REF	NRR	2H14	SEX	AGEL	AGEH	RACE	YF	LC TYPE	LOC	START	ST	NLC	R	VB	P	H	AD	SM	PRODUCT	exL	exH	DENOM	De
BENHAM	517		m	0	0	all	-	KI	Eu:wst	1976	CC	1625	n	bl	n	y	0	ev	cig only	1	16	nev any	st

Cigarette type is all/unspec for all RRs

Table 2H24 - 2

IESLC - Meta-analysis of Ever/current Smoking, Age started, "High"
Squamous, Cigarettes only
Most adjusted

REF	NRR	SEX	AD	Number Exposed Case	Number Exposed Cont	Non-exposed Case	Non-exposed Cont	RR	95.00%CI
BENHAM	517	m	0	342	341	24	481	20.10	(12.99- 31.10)
Totals				342	341	24	481		

*prospective study

REF	NRR	SEX	AD	Ys	Ws	Qs	Ps
BENHAM	517	m	0	3.00	20.16	0.00	0.0000

	N	1
	NS	1
	Wt	20.16
Het	Chi	0.00
Het	df	0
Het	P	N.S.
Fixed	RR	20.10
	RRl	12.99
	RRu	31.10
	P	+++
Random	RR	20.10
	RRl	12.99
	RRu	31.10
	P	+++
Asymm	P	

Table 2H24 - 3

IESLC - Meta-analysis of Ever/current Smoking, Age started, "High"
Squamous, Cigarettes only
Most adjusted

	Sex: combined	male	female	Total
N		1		1
NS		1		1
Wt		20.16		20.16
Het Chi		0.00		0.00
Het df		0		0
Het P		N.S.		N.S.
Fixed RR		20.10		20.10
RRl		12.99		12.99
RRu		31.10		31.10
P		+++		+++
Random RR		20.10		20.10
RRl		12.99		12.99
RRu		31.10		31.10
P		+++		+++
Between Chi				
Between df				
Between P				N.S.
Btwn(F) P				N.S.
Btwn(R) P				N.S.

Too few RRs for analysis by factor

Table 2H24 - 4

IESLC - Meta-analysis of Ever/current Smoking, Age started, "High"
Squamous, Cigarettes only
Least adjusted

REF	NRR	X	SEX	AGEL	AGEH	RACE	YF	LC TYPE	LOC	START	ST	NLC	R	VB	P	H	AD	SM	PRODUCT	exL	exH	DENOM	De
BENHAM	517		m	0	0	all	-	KI	Eu:wst	1976	CC	1625	n	bl	n	y	0	ev	cig only	1	16	nev any	st

Cigarette type is all/unspec for all RRs

Table 2H24 - 5

IESLC - Meta-analysis of Ever/current Smoking, Age started, "High"
Squamous, Cigarettes only
Least adjusted

REF	NRR	SEX	AD	Number Exposed Case	Number Exposed Cont	Non-exposed Case	Non-exposed Cont	RR	95.00%CI
BENHAM	517	m	0	342	341	24	481	20.10	(12.99- 31.10)
Totals				342	341	24	481		

*prospective study

REF	NRR	SEX	AD	Ys	Ws	Qs	Ps
BENHAM	517	m	0	3.00	20.16	0.00	0.0000

	N	1
	NS	1
	Wt	20.16
Het	Chi	0.00
Het	df	0
Het	P	N.S.
Fixed	RR	20.10
	RRl	12.99
	RRu	31.10
	P	+++
Random	RR	20.10
	RRl	12.99
	RRu	31.10
	P	+++
Asymm	P	

Table 2H24 - 6

IESLC - Meta-analysis of Ever/current Smoking, Age started, "High"
Squamous, Cigarettes only
Least adjusted

	Sex combined	male	female	Total
N		1		1
NS		1		1
Wt		20.16		20.16
Het Chi		0.00		0.00
Het df		0		0
Het P		N.S.		N.S.
Fixed RR		20.10		20.10
RRl		12.99		12.99
RRu		31.10		31.10
P		+++		+++
Random RR		20.10		20.10
RRl		12.99		12.99
RRu		31.10		31.10
P		+++		+++
Between Chi				
Between df				
Between P				N.S.
Btwn(F) P				N.S.
Btwn(R) P				N.S.

Table 2H24 - 7

IESLC - Meta-analysis of Ever/current Smoking, Age started, "High"
Squamous, Cigarettes only
Excluded studies (and stage at which they were excluded)

1	BECHER BLOT1 BROWN3 CARPEN CHYOU DARBY DOLL2 GARCIA GRAHAM GURSEL HAMMO2 JAHN JAIN LAUSSM PRESCO QIAO TVERDA WIGLE WYNDE3
2	AKIBA AMANDU AMES AXELSS BENSHL BEST BOUCHA BOUCOT BROSS CHEN CPSII DEAN2 DESTEF DORGAN DOSEME FAN GARSHI GER GILLIS HAMMON HUMBLE JUSSAW KAISE2 KATSOU KAUFMA KOO KREUZE LEVIN MCCONN NOTAN2 OSANN2 PEZZO2 PISANI RESTRE SADOWS VUTUC WANG2 WATSON WU2 WUWILL WYNDE2 WYNDE8 XU ZHOU
3	GUO MCDUFF SPITZ STASZE ZHANG
4	AGUDO ARMADA AUVINE BOFFET BRESLO BUFFLE CEDERL CHEN2 CHIAZZ CHOI CORREA CPSI DAMBER DEAN3 DOLL DORN GAO GAO2 GENG HIRAYA HOLE HU HU2 JOLY KOULUM LETOUR LIAW LIU3 LIU4 LIU5 LUBIN LUBIN2 MIGRAN MRFITR PERNU QIAO2 RACHTA SEGI2 SOBUE SPEIZE SUZUK2 TIZZAN WYNDE7 YUAN
5	ALDERS
7	BARBON ENGELA HAENSZ HEGMAN JEDRYC KHUDER LUO MATOS SVENSS WAKAI WU WYNDE6 ZHENG
14	PEZZOT

Table 2H24 - 8
Potentially overlapping studies

REF	REFGP	PRINC.	OVERLAP/LINK
BENHAM	LUBIN2	2	Subset of Lubin2

Table 2H25 -

IESLC - Meta-analysis of Ever/current Smoking, Age started, "Highest vs lowest"
Squamous, Cigarettes only

This analysis is restricted to results for:
1) Ever/current smokers
2) Results by Age started
3) Categorical results by Age started
4) Denominator (unexposed) = "low"
5) Squamous (or near equivalent)
6) Results complete enough for use in metaanalysis

Within each study, results are then selected (in the following order of preference, within each sex) for:
7) SMKSTA: ever, current
8) PRODUCT: cigarettes only
9) CIGTYPE: all/unspecified, MC regardless of HR, MC only
10) Results with least adjustment for other aspects of smoking (ADOS)
11) The highest vs lowest category
12) Followup period (YF, prospective studies): whole study (coded as 0) or longest available
13) LCtype: squamous or nearest available, but not adeno. (q = squamous, s = small,
a = adeno, KI = Kreyberg I, u = undifferentiated)
14) Race: all or nearest available, otherwise by race (wh or w = white, bl or b = black, hi = hispanic
ch = chinese, jap = japanese, haw = hawaiian, w+o = white + oriental, sca = scandinavian, as = asian)
15) For overlapping studies: principal rather than subsidiary studies
Finally by Age: whole study (coded as 0) if available, otherwise by widest available age group
and then for single sex results (m, f) in preference to results for both sexes combined (c).

Results adjusted (AD) for the most potential confounders are then chosen in Sections -1 to -3
(and those which actually differ from the adjusted results in Table 2H15 - 1 are marked 'x' in Section -1)
and results adjusted for the least confounders in Sections -4 to -6. (Those least adjusted results which
actually differ from the most adjusted are marked 'x' in column X in Section -4)

Section -7 shows excluded studies, together with the stage (as above) at which no qualifying
results were found.

Section -8 lists the potentially overlapping studies which have been included (1=principal, 2=subsidiary).

Section -9 lists any results which would have been included in preference except that they had data not complete
enough for use in meta-analysis, with their significance (yes/no), if known, and any further comment as entered
on the database. It also lists as "gap" any categories for which no data were presented by the original authors.

In addition to those mentioned above, the following fields, levels and abbreviations are used:
* or nk = not known, n = no, y = yes, ot = other
all/unspec = all or unspecified, MC = manufactured cigarettes, HR = hand-rolled cigarettes
exL, exH = range of exposure (low and high) in the "highest" group, in terms of Age started
unexL, unexH = range of exposure (low and high) in the "lowest" group, in terms of Age started
REF: 6-character study reference
NRR: number of the RR on the database within the study
ST : study type (CC = case control, pr or prosp = prospective)
NLC: number of lung cancer cases in whole study
R : risky occupational population (n = no, m = mining, o = other risky)
VB : national cigarette type (V = at least 75% Virginia, bl = at least 75% blended, ot = other)
P : any proxy use
H : full histological confirmation
De : derivation of RR/CI (or = original, st = standard method, ot = other method of estimation)

Table 2H25 - 1

IESLC - Meta-analysis of Ever/current Smoking, Age started, "Highest vs lowest"
Squamous, Cigarettes only
Most adjusted

REF	NRR	2H15	SEX	AGEL	AGEH	RACE	YF	LC TYPE	LOC	START	ST	NLC	R	VB	P	H	AD	ADOS	SM	PRODUCT	exL	exH	unexL	unexH	De
BENHAM	520		m	0	0	all	-	KI	Eu:wst	1976	CC	1625	n	bl	n	y	0	0	ev	cig only	1	16	25	999	st
PEZZOT	549		m	0	0	all	-	q	SCAmer	1987	CC	215	n	bl	n	y	2	0	ev	cig only	1	13	19	999	ot

Cigarette type is all/unspec for all RRs

Table 2H25 - 2

IESLC - Meta-analysis of Ever/current Smoking, Age started, "Highest vs lowest"
Squamous, Cigarettes only
Most adjusted

REF	NRR	SEX	AD	Number Exposed Case	Cont	Non-exposed Case	Cont	RR	95.00%CI
BENHAM	520	m	0	342	341	98	137	1.40	(1.04- 1.89)
PEZZOT	549	m	2	30	-	10	-	3.33	(1.43- 7.79)
Partial Totals				372	341	108	137		

*prospective study

REF	NRR	SEX	AD	Ys	Ws	Qs	Ps
BENHAM	520	m	0	0.34	42.81	0.39	0.0270
PEZZOT	549	m	2	1.20	5.35	3.16	0.0054

	N	2
	NS	2
	Wt	48.16
Het	Chi	3.56
Het	df	1
Het	P	(*)
Fixed	RR	1.54
	RRl	1.16
	RRu	2.05
	P	++
Random	RR	1.97
	RRl	0.86
	RRu	4.50
	P	N.S.
Asymm	P	

Table 2H25 - 3

IESLC - Meta-analysis of Ever/current Smoking, Age started, "Highest vs lowest"
Squamous, Cigarettes only
Most adjusted

	Sex combined	male	female	Total
N		2		2
NS		2		2
Wt		48.16		48.16
Het Chi		3.56		3.56
Het df		1		1
Het P		(*)		(*)
Fixed RR		1.54		1.54
RRl		1.16		1.16
RRu		2.05		2.05
P		++		++
Random RR		1.97		1.97
RRl		0.86		0.86
RRu		4.50		4.50
P		N.S.		N.S.
Between Chi				
Between df				
Between P				N.S.
Btwn(F) P				N.S.
Btwn(R) P				N.S.

Too few RRs for analysis by factor

Table 2H25 - 4

IESLC - Meta-analysis of Ever/current Smoking, Age started, "Highest vs lowest"
Squamous, Cigarettes only
Least adjusted

REF	NRR	X	SEX	AGEL	AGEH	RACE	YF	LC TYPE	LOC	START	ST	NLC	R	VB	P	H	AD	ADOS	SM	PRODUCT	exL	exH	unexL	unexH	De
BENHAM	520		m	0	0	all	-	KI	Eu:wst	1976	CC	1625	n	bl	n	y	0	0	ev	cig only	1	16	25	999	st
PEZZOT	547	x	m	0	0	all	-	q	SCAmer	1987	CC	215	n	bl	n	y	0	0	ev	cig only	1	13	19	999	st

Cigarette type is all/unspec for all RRs

Table 2H25 - 5

IESLC - Meta-analysis of Ever/current Smoking, Age started, "Highest vs lowest"
Squamous, Cigarettes only
Least adjusted

REF	NRR	SEX	AD	Number Exposed Case	Cont	Non-exposed Case	Cont	RR	95.00%CI
BENHAM	520	m	0	342	341	98	137	1.40	(1.04- 1.89)
PEZZOT	547	m	0	30	34	10	32	2.82	(1.19- 6.69)
Totals				372	375	108	169		

*prospective study

REF	NRR	SEX	AD	Ys	Ws	Qs	Ps
BENHAM	520	m	0	0.34	42.81	0.24	0.0270
PEZZOT	547	m	0	1.04	5.15	2.01	0.0184

	N	2
	NS	2
	Wt	47.96
Het	Chi	2.25
Het	df	1
Het	P	N.S.
Fixed	RR	1.51
	RRl	1.14
	RRu	2.01
	P	++
Random	RR	1.76
	RRl	0.93
	RRu	3.35
	P	(+)
Asymm	P	

Table 2H25 - 6

IESLC - Meta-analysis of Ever/current Smoking, Age started, "Highest vs lowest"
Squamous, Cigarettes only
Least adjusted

	Sex			
	combined	male	female	Total
N		2		2
NS		2		2
Wt		47.96		47.96
Het Chi		2.25		2.25
Het df		1		1
Het P		N.S.		N.S.
Fixed RR		1.51		1.51
RRl		1.14		1.14
RRu		2.01		2.01
P		++		++
Random RR		1.76		1.76
RRl		0.93		0.93
RRu		3.35		3.35
P		(+)		(+)
Between Chi				
Between df				
Between P				N.S.
Btwn(F) P				N.S.
Btwn(R) P				N.S.

Table 2H25 - 7

IESLC - Meta-analysis of Ever/current Smoking, Age started, "Highest vs lowest"
Squamous, Cigarettes only
Excluded studies (and stage at which they were excluded)

1	BECHER BLOT1 BROWN3 CARPEN CHYOU DARBY DOLL2 GARCIA GRAHAM GURSEL HAMMO2 JAHN JAIN LAUSSM PRESCO QIAO TVERDA WIGLE WYNDE3
2	AKIBA AMANDU AMES AXELSS BENSHL BEST BOUCHA BOUCOT BROSS CHEN CPSII DEAN2 DESTEF DORGAN DOSEME FAN GARSHI GER GILLIS HAMMON HUMBLE JUSSAW KAISE2 KATSOU KAUFMA KOO KREUZE LEVIN MCCONN NOTAN2 OSANN2 PEZZO2 PISANI RESTRE SADOWS VUTUC WANG2 WATSON WU2 WUWILL WYNDE2 WYNDE8 XU ZHOU
3	GUO MCDUFF SPITZ STASZE ZHANG
5	AGUDO ARMADA AUVINE BOFFET BRESLO BUFFLE CEDERL CHEN2 CHIAZZ CHOI CORREA CPSI DAMBER DEAN3 DOLL DORN GAO GAO2 GENG HIRAYA HOLE HU HU2 JOLY KOULUM LETOUR LIAW LIU3 LIU4 LIU5 LUBIN LUBIN2 MIGRAN MRFITR PERNU QIAO2 RACHTA SEGI2 SOBUE SPEIZE SUZUK2 TIZZAN WYNDE7 YUAN
6	ALDERS
8	BARBON ENGELA HAENSZ HEGMAN JEDRYC KHUDER LUO MATOS SVENSS WAKAI WU WYNDE6 ZHENG

Table 2H25 - 8
Potentially overlapping studies

REF	REFGP	PRINC	.	OVERLAP/LINK
BENHAM	LUBIN2	2		Subset of Lubin2

Table 2H25 - 9
Most adjusted - insufficient data for meta-analysis

REF	NRR	SEX	AGEL	AGEH	RACE	YF	LC TYPE	LOC	START	ST	NLC	R	VB	P	H	AD	ADOS	SM	PRODUCT	exL	exH	unexL	unexH	De
ALDERS	533	m	0	0	all	-	q+s	Eu:UK	1977	CC	1448	n		V	n	n	2	1#ev	cig only	1	14	25	999	ot
ALDERS	536	f	0	0	all	-	q+s	Eu:UK	1977	CC	1448	n		V	n	n	2	1#ev	cig only	1	14	25	999	ot

Comments on values in listings

ALDERS ADOS Number of cigs/day
ALDERS ADOS Number of cigs/day

REF	NRR	RR	SIG	RRDATA comment
ALDERS	533	5.56	n	0
ALDERS	536	1.56	n	0
