# Supplementary material for: Systematic review with meta-analysis of the epidemiological evidence in the 1900s relating smoking to lung cancer
Source: BMC Cancer. 2012 Sep 3;12:385. doi: 10.1186/1471-2407-12-385 (PMC3505152; doi:10.1186/1471-2407-12-385)
Supplement: Additional file 5 — Detailed Analysis Tables (Individual file names as described in Additional file 1: Methods, Table1). [file 1471-2407-12-385-S5.zip › PDF/3J.pdf]

Table 3J1 -

IESLC - Meta-analysis of Ex Smoking by Years quit (vs never), Overview
Adenocarcinoma, Any Product (or Cigarettes if Any not available)

This analysis is restricted to results for:
1) Ex smokers
2) Results by Years quit (vs never)
3) Categorical results by Years quit (vs never)
   Results by Years quit (vs never) are grouped under 2 schemes (S1, S2). Each scheme has a set of "key values". An interv
   is allocated to the category whose key value it includes, and intervals which include none or more than
   one of the key values are excluded. (Open-ended intervals are coded as 999)

S1	key value	maximum range
1	12	8+
2	7	4-11
3	3	1-6

S2	key value	maximum range
1	20	13+
2	12	4-19
3	3	1-11

4) Adenocarcinoma (or near equivalent)
5) Results complete enough for use in metaanalysis

Within each study, results are then selected (in the following order of preference, within each sex) for:
6) (not applicable)
7) PRODUCT: all/unspec, cigarettes regardless of other products, cigarettes only
8) CIGTYPE: all/unspecified, MC regardless of HR, MC only
9) (not applicable)
10) DENOM: never smoked anything, never smoked cigarettes, never any + low, never cigs + low
11) Followup period (YF, prospective studies): whole study (coded as 0) or longest available
12) LCtype: adeno or nearest available, but not squamous. (q = squamous, s = small,
       a = adeno, l = large, KII = Kreyberg II, al = alveolar, br = bronchiolar, u = undifferentiated)
13) Race: all or nearest available, otherwise by race (wh or w = white, bl or b = black, hi = hispanic
       ch = chinese, jap = japanese, haw = hawaiian, w+o = white + oriental, sca = scandinavian, as = asian)
14) For overlapping studies: principal rather than subsidiary studies
Finally by Age: whole study (coded as 0) if available, otherwise by widest available age group
and then for single sex results (m, f) in preference to results for both sexes combined (c).

Results adjusted (AD) for the most potential confounders are then chosen in Sections -1 to -3
and results adjusted for the least confounders in Sections -4 to -6. (Those least adjusted results which
actually differ from the most adjusted are marked 'x' in column X in Section -4)

Section -7 shows excluded studies, together with the stage (as above) at which no qualifying
results were found.

Section -8 lists the potentially overlapping studies which have been included (1=principal, 2=subsidiary).

Section -9 lists any results which would have been included in preference except that they had data not complete
enough for use in meta-analysis, with their significance (yes/no), if known, and any further comment as entered
on the database. It also lists as "gap" any categories for which no data were presented by the original authors.
This is commonly due to recent quitters having been combined with current smokers

In addition to those mentioned above, the following fields, levels and abbreviations are used:
* or nk = not known, n = no, y = yes, ot = other
nev = never
all/unspec = all or unspecified, cig+/-ot = cigarettes irrespective of other products (cigar, pipe etc)
MC = manufactured cigarettes, HR = hand-rolled cigarettes
exL, exH = range of exposure (low and high) in the smoking group, in terms of Years quit (vs never)
REF: 6-character study reference
NRR: number of the RR on the database within the study
ST : study type (CC = case control, pr or prosp = prospective)
NLC: number of lung cancer cases in whole study
R : risky occupational population (n = no, m = mining, o = other risky)
VB : national cigarette type (V = at least 75% Virginia, bl = at least 75% blended, ot = other)
P : any proxy use
H : full histological confirmation
De : derivation of RR/CI (or = original, st = standard method, ot = other method of estimation)

Table 3J1 - 1

IESLC - Meta-analysis of Ex Smoking by Years quit (vs never), Overview
Adenocarcinoma, Any Product (or Cigarettes if Any not available)
Most adjusted

REF	NRR	SEX	AGEL	AGEH	RACE	YF	LC TYPE	LOC	START	ST	NLC	R	VB	P	H	AD	PRODUCT	exL	exH	S1	S2	DENOM	De
BARBON	756	m	0	0	all	-	a	Eu:wst	1979	CC	755	n	bl	y	y	1	all/unsp	25	999	0	0	nev any	or
BARBON	757	m	0	0	all	-	a	Eu:wst	1979	CC	755	n	bl	y	y	1	all/unsp	15	24	0	1	nev any	or
BARBON	758	m	0	0	all	-	a	Eu:wst	1979	CC	755	n	bl	y	y	1	all/unsp	5	14	0	2	nev any	or
BARBON	759	m	0	0	all	-	a	Eu:wst	1979	CC	755	n	bl	y	y	1	all/unsp	0.1	4	3	3	nev any	or
BROWN3	505	f	0	0	wh	-	a	NAmer		CC	618		bl	y	n	0	all/unsp	15	999	0	1	nev any	st
JAHN	639	m	0	0	all	-	a	Eu:Ger	1988	CC	1004	n	bl	n	n	0	cig+/-ot	21	999	0	0	nev any	st
JAHN	640	m	0	0	all	-	a	Eu:Ger	1988	CC	1004	n	bl	n	n	0	cig+/-ot	11	20	1	0	nev any	st
JAHN	641	m	0	0	all	-	a	Eu:Ger	1988	CC	1004	n	bl	n	n	0	cig+/-ot	6	10	2	0	nev any	st
JAHN	642	m	0	0	all	-	a	Eu:Ger	1988	CC	1004	n	bl	n	n	0	cig+/-ot	2	5	3	3	nev any	st
JAHN	643	m	0	0	all	-	a	Eu:Ger	1988	CC	1004	n	bl	n	n	0	cig+/-ot	1.0	1.9	0	0	nev any	st
JAHN	644	m	0	0	all	-	a	Eu:Ger	1988	CC	1004	n	bl	n	n	0	cig+/-ot	0.1	0.9	0	0	nev any	st
JAIN	537	m	0	0	all	-	a	NAmer	1981	CC	845	n	V	y	n	0	cig+/-ot	10	999	1	0	nev cigs	st
JAIN	538	m	0	0	all	-	a	NAmer	1981	CC	845	n	V	y	n	0	cig+/-ot	2	9	0	3	nev cigs	st
JAIN	501	f	0	0	all	-	a	NAmer	1981	CC	845	n	V	y	n	0	cig+/-ot	10	999	1	0	nev cigs	st
JAIN	502	f	0	0	all	-	a	NAmer	1981	CC	845	n	V	y	n	0	cig+/-ot	2	9	0	3	nev cigs	st
JEDRYC	559	m	0	0	all	-	a	Eu:est	1980	CC	1630	n	bl	y	n	0	cig+/-ot	10	999	1	0	nev any	st
JEDRYC	560	m	0	0	all	-	a	Eu:est	1980	CC	1630	n	bl	y	n	0	cig+/-ot	5	9	2	0	nev any	st
LUBIN2	861	m	0	0	all	-	a	Eu:mul	1976	CC	7804	n	bl	n	y	0	cig+/-ot	20	999	0	1	nev any	st
LUBIN2	862	m	0	0	all	-	a	Eu:mul	1976	CC	7804	n	bl	n	y	0	cig+/-ot	15	19	0	0	nev any	st
LUBIN2	863	m	0	0	all	-	a	Eu:mul	1976	CC	7804	n	bl	n	y	0	cig+/-ot	10	14	1	2	nev any	st
LUBIN2	864	m	0	0	all	-	a	Eu:mul	1976	CC	7804	n	bl	n	y	0	cig+/-ot	5	9	2	0	nev any	st
LUBIN2	865	m	0	0	all	-	a	Eu:mul	1976	CC	7804	n	bl	n	y	0	cig+/-ot	0.1	4	3	3	nev any	st
LUBIN2	965	f	0	0	all	-	a	Eu:mul	1976	CC	7804	n	bl	n	y	0	cig+/-ot	20	999	0	1	nev any	st
LUBIN2	966	f	0	0	all	-	a	Eu:mul	1976	CC	7804	n	bl	n	y	0	cig+/-ot	10	19	1	2	nev any	st
LUBIN2	967	f	0	0	all	-	a	Eu:mul	1976	CC	7804	n	bl	n	y	0	cig+/-ot	0.1	9	0	3	nev any	st
MATOS	671	m	0	0	all	-	a	SCAmer	1994	CC	200	n	bl	n	n	2	cig+/-ot	11	999	1	0	nev any	ot
MATOS	672	m	0	0	all	-	a	SCAmer	1994	CC	200	n	bl	n	n	2	cig+/-ot	6	10	2	0	nev any	ot
MATOS	673	m	0	0	all	-	a	SCAmer	1994	CC	200	n	bl	n	n	2	cig+/-ot	1.0	5	3	3	nev any	ot
PEZZOT	585	m	0	0	all	-	a	SCAmer	1987	CC	215	n	bl	n	y	0	cig only	11	999	1	0	nev cigs	st
PEZZOT	586	m	0	0	all	-	a	SCAmer	1987	CC	215	n	bl	n	y	0	cig only	1.0	10	0	3	nev cigs	st
SVENSS	569	f	0	0	all	-	a	Eu:Sca	1983	CC	210	n	bl	n	n	0	all/unsp	11	999	1	0	nev any	st
SVENSS	570	f	0	0	all	-	a	Eu:Sca	1983	CC	210	n	bl	n	n	0	all/unsp	3	10	0	3	nev any	st
WAKAI	562	m	0	0	all	-	a	As:Jap	1988	CC	333	n	bl	n	y	1	cig+/-ot	20	999	0	1	nev any	or
WAKAI	563	m	0	0	all	-	a	As:Jap	1988	CC	333	n	bl	n	y	1	cig+/-ot	10	19	1	2	nev any	or
WAKAI	564	m	0	0	all	-	a	As:Jap	1988	CC	333	n	bl	n	y	1	cig+/-ot	5	9	2	0	nev any	or
WU2	502	f	0	0	all	-	a	NAmer	1983	CC	336	n	bl	n	y	2	all/unsp	1.0	9	0	3	nev any	or
WYNDE3	518	m	0	0	all	-	KII	NAmer	1966	CC	350	n	bl	n	y	0	all/unsp	13	999	0	1	nev any	st
WYNDE3	519	m	0	0	all	-	KII	NAmer	1966	CC	350	n	bl	n	y	0	all/unsp	7	12	0	2	nev any	st
WYNDE3	520	m	0	0	all	-	KII	NAmer	1966	CC	350	n	bl	n	y	0	all/unsp	4	6	0	0	nev any	st
WYNDE3	521	m	0	0	all	-	KII	NAmer	1966	CC	350	n	bl	n	y	0	all/unsp	1.0	3	3	3	nev any	st
WYNDE3	580	f	0	0	all	-	KII	NAmer	1966	CC	350	n	bl	n	y	0	cig+/-ot	10	999	1	0	nev any	ot
WYNDE6	817	m	0	0	all	-	KII	NAmer	1969	CC	4423	n	bl	n	y	2	cig+/-ot	16	999	0	1	nev any	ot
WYNDE6	818	m	0	0	all	-	KII	NAmer	1969	CC	4423	n	bl	n	y	2	cig+/-ot	11	15	1	2	nev any	ot
WYNDE6	819	m	0	0	all	-	KII	NAmer	1969	CC	4423	n	bl	n	y	2	cig+/-ot	7	10	2	0	nev any	ot
WYNDE6	820	m	0	0	all	-	KII	NAmer	1969	CC	4423	n	bl	n	y	2	cig+/-ot	4	6	0	0	nev any	ot
WYNDE6	821	m	0	0	all	-	KII	NAmer	1969	CC	4423	n	bl	n	y	2	cig+/-ot	1.0	3	3	3	nev any	ot

Cigarette type is all/unspec for all RRs

In this overview table, subtotals and Qs values may be invalid and should be ignored

Table 3J1 - 2

IESLC - Meta-analysis of Ex Smoking by Years quit (vs never), Overview
Adenocarcinoma, Any Product (or Cigarettes if Any not available)
Most adjusted

REF	NRR	SEX	AD	Number Exposed Case	Number Exposed Cont	Non-exposed Case	Non-exposed Cont	RR	95.00%CI
BARBON	756	m	1	4	-	7	-	1.80	(0.50- 6.40)
BARBON	757	m	1	7	-	7	-	4.60	(1.50- 13.80)
BARBON	758	m	1	23	-	7	-	7.30	(3.00- 17.60)
BARBON	759	m	1	7	-	7	-	9.40	(3.00- 29.70)
Subtotal BARBON								5.40	(3.16- 9.24)
BROWN3	505	f	0	73	219	432	1168	0.90	(0.68- 1.20)
JAHN	639	m	0	15	146	8	138	1.77	(0.73- 4.31)
JAHN	640	m	0	22	130	8	138	2.92	(1.26- 6.79)
JAHN	641	m	0	13	63	8	138	3.56	(1.40- 9.02)
JAHN	642	m	0	19	46	8	138	7.13	(2.92- 17.37)
JAHN	643	m	0	18	9	8	138	34.50	(11.82- 100.74)
JAHN	644	m	0	40	8	8	138	86.25	(30.45- 244.33)
Subtotal JAHN								6.98	(4.77- 10.22)
JAIN	537	m	0	14	113	4	85	2.63	(0.84- 8.28)
JAIN	538	m	0	16	46	4	85	7.39	(2.33- 23.41)
JAIN	501	f	0	3	61	24	214	0.44	(0.13- 1.51)
JAIN	502	f	0	14	36	24	214	3.47	(1.64- 7.32)
Subtotal JAIN								2.69	(1.63- 4.45)
JEDRYC	559	m	0	12	230	7	289	2.15	(0.83- 5.56)
JEDRYC	560	m	0	9	82	7	289	4.53	(1.64- 12.54)
Subtotal JEDRYC								3.04	(1.52- 6.09)
LUBIN2	861	m	0	35	1128	57	2616	1.42	(0.93- 2.18)
LUBIN2	862	m	0	21	478	57	2616	2.02	(1.21- 3.36)
LUBIN2	863	m	0	30	693	57	2616	1.99	(1.27- 3.12)
LUBIN2	864	m	0	50	882	57	2616	2.60	(1.77- 3.83)
LUBIN2	865	m	0	77	1047	57	2616	3.38	(2.38- 4.79)
LUBIN2	965	f	0	1	29	138	1180	0.29	(0.04- 2.18)
LUBIN2	966	f	0	3	33	138	1180	0.78	(0.24- 2.57)
LUBIN2	967	f	0	13	95	138	1180	1.17	(0.64- 2.14)
Subtotal LUBIN2								2.10	(1.76- 2.50)
MATOS	671	m	2	12	-	5	-	3.00	(1.09- 8.23)
MATOS	672	m	2	9	-	5	-	10.00	(3.08- 32.51)
MATOS	673	m	2	12	-	5	-	13.00	(4.21- 40.17)
Subtotal MATOS								6.76	(3.59- 12.75)
PEZZOT	585	m	0	7	106	3	116	2.55	(0.64- 10.13)
PEZZOT	586	m	0	11	82	3	116	5.19	(1.40- 19.18)
Subtotal PEZZOT								3.71	(1.44- 9.57)
SVENSS	569	f	0	7	24	22	120	1.59	(0.61- 4.14)
SVENSS	570	f	0	5	13	22	120	2.10	(0.68- 6.48)
Subtotal SVENSS								1.79	(0.86- 3.70)
WAKAI	562	m	1	3	-	8	-	0.54	(0.14- 2.16)
WAKAI	563	m	1	13	-	8	-	2.49	(0.95- 6.53)
WAKAI	564	m	1	7	-	8	-	1.23	(0.42- 3.64)
Subtotal WAKAI								1.40	(0.74- 2.65)
WU2	502	f	2	-	-	-	-	2.30	(1.20- 4.40)
WYNDE3	518	m	0	3	55	6	88	0.80	(0.19- 3.33)
WYNDE3	519	m	0	3	31	6	88	1.42	(0.33- 6.02)
WYNDE3	520	m	0	3	17	6	88	2.59	(0.59- 11.37)
WYNDE3	521	m	0	3	22	6	88	2.00	(0.46- 8.63)
WYNDE3	580	f	0	0	3	15	76	0.71~	(0.03- 14.35)
Subtotal WYNDE3								1.47	(0.73- 2.99)
WYNDE6	817	m	2	6	-	11	-	1.20	(0.44- 3.26)
WYNDE6	818	m	2	11	-	11	-	5.40	(2.32- 12.58)
WYNDE6	819	m	2	18	-	11	-	6.60	(3.09- 14.10)
WYNDE6	820	m	2	14	-	11	-	5.90	(2.65- 13.11)
WYNDE6	821	m	2	29	-	11	-	14.20	(7.02- 28.73)
Subtotal WYNDE6								6.10	(4.25- 8.74)
Partial Totals				715	5927	1460	20692		

*prospective study ~ With 0.5 adjustment for zero

Table 3J1 - 2

IESLC - Meta-analysis of Ex Smoking by Years quit (vs never), Overview
Adenocarcinoma, Any Product (or Cigarettes if Any not available)
Most adjusted

REF	NRR	SEX	AD	Ys	Ws	Qs	Ps
BARBON	756	m	1	0.59	2.36	0.23	0.3661
BARBON	757	m	1	1.53	3.12	1.22	0.0070
BARBON	758	m	1	1.99	4.91	5.80	0.0000
BARBON	759	m	1	2.24	2.92	5.25	0.0001
Subtotal BARBON				1.69	13.32	12.50	
BROWN3	505	f	0	-0.10	46.65	47.12	0.4775
JAHN	639	m	0	0.57	4.86	0.53	0.2071
JAHN	640	m	0	1.07	5.39	0.16	0.0128
JAHN	641	m	0	1.27	4.44	0.60	0.0074
JAHN	642	m	0	1.96	4.84	5.47	0.0000
JAHN	643	m	0	3.54	3.35	23.32	0.0000
JAHN	644	m	0	4.46	3.54	44.81	0.0000
Subtotal JAHN				1.94	26.43	74.88	
JAIN	537	m	0	0.97	2.92	0.01	0.0979
JAIN	538	m	0	2.00	2.89	3.49	0.0007
JAIN	501	f	0	-0.82	2.52	7.52	0.1902
JAIN	502	f	0	1.24	6.87	0.81	0.0011
Subtotal JAIN				0.99	15.21	11.83	
JEDRYC	559	m	0	0.77	4.27	0.08	0.1127
JEDRYC	560	m	0	1.51	3.71	1.38	0.0036
Subtotal JEDRYC				1.11	7.98	1.46	
LUBIN2	861	m	0	0.35	21.10	6.33	0.1044
LUBIN2	862	m	0	0.70	14.78	0.59	0.0070
LUBIN2	863	m	0	0.69	18.97	0.87	0.0028
LUBIN2	864	m	0	0.96	25.60	0.08	0.0000
LUBIN2	865	m	0	1.22	31.38	3.12	0.0000
LUBIN2	965	f	0	-1.22	0.96	4.32	0.2317
LUBIN2	966	f	0	-0.25	2.69	3.58	0.6795
LUBIN2	967	f	0	0.16	10.47	5.79	0.6113
Subtotal LUBIN2				0.74	125.96	24.68	
MATOS	671	m	2	1.10	3.76	0.15	0.0332
MATOS	672	m	2	2.30	2.77	5.44	0.0001
MATOS	673	m	2	2.56	3.02	8.36	0.0000
Subtotal MATOS				1.91	9.55	13.94	
PEZZOT	585	m	0	0.94	2.02	0.00	0.1824
PEZZOT	586	m	0	1.65	2.25	1.25	0.0136
Subtotal PEZZOT				1.31	4.27	1.25	
SVENSS	569	f	0	0.46	4.20	0.80	0.3415
SVENSS	570	f	0	0.74	3.02	0.08	0.1976
Subtotal SVENSS				0.58	7.22	0.88	
WAKAI	562	m	1	-0.62	2.05	4.72	0.3774
WAKAI	563	m	1	0.91	4.14	0.00	0.0636
WAKAI	564	m	1	0.21	3.30	1.59	0.7071
Subtotal WAKAI				0.34	9.48	6.31	
WU2	502	f	2	0.83	9.10	0.04	0.0120
WYNDE3	518	m	0	-0.22	1.89	2.39	0.7591
WYNDE3	519	m	0	0.35	1.84	0.56	0.6348
WYNDE3	520	m	0	0.95	1.75	0.00	0.2079
WYNDE3	521	m	0	0.69	1.80	0.08	0.3529
WYNDE3	580	f	0	-0.35	0.42	0.66	0.8202
Subtotal WYNDE3				0.39	7.70	3.69	
WYNDE6	817	m	2	0.18	3.83	1.98	0.7212
WYNDE6	818	m	2	1.69	5.38	3.32	0.0001
WYNDE6	819	m	2	1.89	6.67	6.48	0.0000
WYNDE6	820	m	2	1.77	6.01	4.59	0.0000
WYNDE6	821	m	2	2.65	7.74	23.76	0.0000
Subtotal WYNDE6				1.81	29.62	40.13	

N	46
NS	13

Table 3J1 - 3

IESLC - Meta-analysis of Ex Smoking by Years quit (vs never), Overview
Adenocarcinoma, Any Product (or Cigarettes if Any not available)
Most adjusted

	Sex			
	combined	male	female	Total
N		36	10	46
NS		10	6	16

In this overview table, other than the "N" rows, entries in the "absent" and "Total" columns may be invalid and should be ignored

	Years quit vs never (lower focus)				
	absent	8+k12	4-11k7	1-6k3	Total
N	22	12	6	6	46
NS	11	10	6	6	33
Wt	157.62	56.69	46.48	51.70	312.49
Het Chi	148.55	15.94	11.66	19.31	238.69
Het df	21	11	5	5	45
Het P	***	N.S.	*	**	***
Fixed RR	1.88	2.13	3.29	5.05	2.46
RRl	1.61	1.64	2.47	3.85	2.20
RRu	2.20	2.76	4.39	6.64	2.75
P	+++	+++	+++	+++	+++
Random RR	2.74	2.10	3.74	6.73	2.99
RRl	1.73	1.49	2.23	3.46	2.27
RRu	4.34	2.94	6.25	13.12	3.95
P	+++	+++	+++	+++	+++

	Years quit vs never (higher focus)				
	absent	13+k20	4-19k12	1-11k3	Total
N	21	7	6	12	46
NS	11	6	5	10	32
Wt	108.66	79.61	37.92	86.30	312.49
Het Chi	90.94	12.50	14.07	42.90	238.69
Het df	20	6	5	11	45
Het P	***	(*)	*	***	***
Fixed RR	3.23	1.07	2.56	3.71	2.46
RRl	2.68	0.86	1.86	3.01	2.20
RRu	3.90	1.33	3.51	4.59	2.75
P	+++	N.S.	+++	+++	+++
Random RR	3.46	1.14	2.64	4.32	2.99
RRl	2.25	0.74	1.44	2.71	2.27
RRu	5.32	1.77	4.83	6.88	3.95
P	+++	N.S.	++	+++	+++

Table 3J1 - 3

IESLC - Meta-analysis of Ex Smoking by Years quit (vs never), Overview
Adenocarcinoma, Any Product (or Cigarettes if Any not available)
Most adjusted

MALES

Years quit vs never (lower focus)

	absent	8+k12	4-11k7	1-6k3	Total
N	16	8	6	6	36
NS	8	8	6	6	28
Wt	80.54	46.86	46.48	51.70	225.58
Het Chi	99.68	4.53	11.66	19.31	149.10
Het df	15	7	5	5	35
Het P	***	N.S.	*	**	***
Fixed RR	2.90	2.55	3.29	5.05	3.29
RRl	2.33	1.91	2.47	3.85	2.89
RRu	3.60	3.39	4.39	6.64	3.75
P	+++	+++	+++	+++	+++
Random RR	3.52	2.55	3.74	6.73	3.78
RRl	1.93	1.91	2.23	3.46	2.83
RRu	6.43	3.39	6.25	13.12	5.06
P	+++	+++	+++	+++	+++

Years quit vs never (higher focus)

	absent	13+k20	4-19k12	1-11k3	Total
N	18	5	5	8	36
NS	10	5	5	8	28
Wt	101.52	32.00	35.23	56.83	225.58
Het Chi	77.03	6.96	9.97	19.70	149.10
Het df	17	4	4	7	35
Het P	***	N.S.	*	**	***
Fixed RR	3.52	1.42	2.80	5.16	3.29
RRl	2.90	1.00	2.01	3.98	2.89
RRu	4.28	2.01	3.89	6.69	3.75
P	+++	+	+++	+++	+++
Random RR	4.10	1.39	3.18	6.61	3.78
RRl	2.64	0.78	1.75	3.88	2.83
RRu	6.39	2.45	5.76	11.28	5.06
P	+++	N.S.	+++	+++	+++

FEMALES

Years quit vs never (lower focus)

	absent	8+k12	4-11k7	1-6k3	Total
N	6	4			10
NS	5	4			9
Wt	77.07	9.83			86.91
Het Chi	18.25	2.75			21.68
Het df	5	3			9
Het P	**	N.S.			**
Fixed RR	1.20	0.91			1.16
RRl	0.96	0.49			0.94
RRu	1.50	1.69			1.43
P	N.S.	N.S.			N.S.
Random RR	1.50	0.91			1.29
RRl	0.87	0.49			0.85
RRu	2.59	1.69			1.96
P	N.S.	N.S.			N.S.

Table 3J1 - 3

IESLC - Meta-analysis of Ex Smoking by Years quit (vs never), Overview
Adenocarcinoma, Any Product (or Cigarettes if Any not available)
Most adjusted

FEMALES

		Years quit vs never (higher focus) absent	13+k20	4-19k12	1-11k3	Total
	N	3	2	1	4	10
	NS	3	2	1	4	9
	Wt	7.14	47.61	2.69	29.46	86.91
Het	Chi	2.66	1.17	0.00	5.27	21.68
Het	df	2	1	0	3	9
Het	P	N.S.	N.S.	N.S.	N.S.	**
Fixed	RR	0.96	0.88	0.78	1.97	1.16
	RRl	0.46	0.66	0.24	1.37	0.94
	RRu	2.00	1.17	2.57	2.83	1.43
	P	N.S.	N.S.	N.S.	+++	N.S.
Random	RR	0.90	0.81	0.78	2.04	1.29
	RRl	0.36	0.43	0.24	1.24	0.85
	RRu	2.26	1.53	2.57	3.34	1.96
	P	N.S.	N.S.	N.S.	++	N.S.

Table 3J1 - 4

IESLC - Meta-analysis of Ex Smoking by Years quit (vs never), Overview
Adenocarcinoma, Any Product (or Cigarettes if Any not available)
Least adjusted

REF	NRR	X	SEX	AGEL	AGEH	RACE	YF	LC TYPE	LOC	START	ST	NLC	R	VB	P	H	AD	PRODUCT	exL	exH	S1	S2	DENOM	De
BARBON	741	x	m	0	0	all	-	a	Eu:wst	1979	CC	755	n	bl	y	y	0	all/unsp	25	999	0	0	nev any	st
BARBON	742	x	m	0	0	all	-	a	Eu:wst	1979	CC	755	n	bl	y	y	0	all/unsp	15	24	0	1	nev any	st
BARBON	743	x	m	0	0	all	-	a	Eu:wst	1979	CC	755	n	bl	y	y	0	all/unsp	5	14	0	2	nev any	st
BARBON	744	x	m	0	0	all	-	a	Eu:wst	1979	CC	755	n	bl	y	y	0	all/unsp	0.1	4	3	3	nev any	st
BROWN3	505		f	0	0	wh	-	a	NAmer		CC	618		bl	y	n	0	all/unsp	15	999	0	1	nev any	st
JAHN	639		m	0	0	all	-	a	Eu:Ger	1988	CC	1004	n	bl	n	n	0	cig+/-ot	21	999	0	0	nev any	st
JAHN	640		m	0	0	all	-	a	Eu:Ger	1988	CC	1004	n	bl	n	n	0	cig+/-ot	11	20	1	0	nev any	st
JAHN	641		m	0	0	all	-	a	Eu:Ger	1988	CC	1004	n	bl	n	n	0	cig+/-ot	6	10	2	0	nev any	st
JAHN	642		m	0	0	all	-	a	Eu:Ger	1988	CC	1004	n	bl	n	n	0	cig+/-ot	2	5	3	3	nev any	st
JAHN	643		m	0	0	all	-	a	Eu:Ger	1988	CC	1004	n	bl	n	n	0	cig+/-ot	1.0	1.9	0	0	nev any	st
JAHN	644		m	0	0	all	-	a	Eu:Ger	1988	CC	1004	n	bl	n	n	0	cig+/-ot	0.1	0.9	0	0	nev any	st
JAIN	537		m	0	0	all	-	a	NAmer	1981	CC	845	n	V	y	n	0	cig+/-ot	10	999	1	0	nev cigs	st
JAIN	538		m	0	0	all	-	a	NAmer	1981	CC	845	n	V	y	n	0	cig+/-ot	2	9	0	3	nev cigs	st
JAIN	501		f	0	0	all	-	a	NAmer	1981	CC	845	n	V	y	n	0	cig+/-ot	10	999	1	0	nev cigs	st
JAIN	502		f	0	0	all	-	a	NAmer	1981	CC	845	n	V	y	n	0	cig+/-ot	2	9	0	3	nev cigs	st
JEDRYC	559		m	0	0	all	-	a	Eu:est	1980	CC	1630	n	bl	y	n	0	cig+/-ot	10	999	1	0	nev any	st
JEDRYC	560		m	0	0	all	-	a	Eu:est	1980	CC	1630	n	bl	y	n	0	cig+/-ot	5	9	2	0	nev any	st
LUBIN2	861		m	0	0	all	-	a	Eu:mul	1976	CC	7804	n	bl	n	y	0	cig+/-ot	20	999	0	1	nev any	st
LUBIN2	862		m	0	0	all	-	a	Eu:mul	1976	CC	7804	n	bl	n	y	0	cig+/-ot	15	19	0	0	nev any	st
LUBIN2	863		m	0	0	all	-	a	Eu:mul	1976	CC	7804	n	bl	n	y	0	cig+/-ot	10	14	1	2	nev any	st
LUBIN2	864		m	0	0	all	-	a	Eu:mul	1976	CC	7804	n	bl	n	y	0	cig+/-ot	5	9	2	0	nev any	st
LUBIN2	865		m	0	0	all	-	a	Eu:mul	1976	CC	7804	n	bl	n	y	0	cig+/-ot	0.1	4	3	3	nev any	st
LUBIN2	965		f	0	0	all	-	a	Eu:mul	1976	CC	7804	n	bl	n	y	0	cig+/-ot	20	999	0	1	nev any	st
LUBIN2	966		f	0	0	all	-	a	Eu:mul	1976	CC	7804	n	bl	n	y	0	cig+/-ot	10	19	1	2	nev any	st
LUBIN2	967		f	0	0	all	-	a	Eu:mul	1976	CC	7804	n	bl	n	y	0	cig+/-ot	0.1	9	0	3	nev any	st
MATOS	661	x	m	0	0	all	-	a	SCAmer	1994	CC	200	n	bl	n	n	0	cig+/-ot	11	999	1	0	nev any	st
MATOS	662	x	m	0	0	all	-	a	SCAmer	1994	CC	200	n	bl	n	n	0	cig+/-ot	6	10	2	0	nev any	st
MATOS	663	x	m	0	0	all	-	a	SCAmer	1994	CC	200	n	bl	n	n	0	cig+/-ot	1.0	5	3	3	nev any	st
PEZZOT	585		m	0	0	all	-	a	SCAmer	1987	CC	215	n	bl	n	y	0	cig only	11	999	1	0	nev cigs	st
PEZZOT	586		m	0	0	all	-	a	SCAmer	1987	CC	215	n	bl	n	y	0	cig only	1.0	10	0	3	nev cigs	st
SVENSS	569		f	0	0	all	-	a	Eu:Sca	1983	CC	210	n	bl	n	n	0	all/unsp	11	999	1	0	nev any	st
SVENSS	570		f	0	0	all	-	a	Eu:Sca	1983	CC	210	n	bl	n	n	0	all/unsp	3	10	0	3	nev any	st
WAKAI	554	x	m	0	0	all	-	a	As:Jap	1988	CC	333	n	bl	n	y	0	cig+/-ot	20	999	0	1	nev any	st
WAKAI	555	x	m	0	0	all	-	a	As:Jap	1988	CC	333	n	bl	n	y	0	cig+/-ot	10	19	1	2	nev any	st
WAKAI	556	x	m	0	0	all	-	a	As:Jap	1988	CC	333	n	bl	n	y	0	cig+/-ot	5	9	2	0	nev any	st
WU2	502		f	0	0	all	-	a	NAmer	1983	CC	336	n	bl	n	y	2	all/unsp	1.0	9	0	3	nev any	or
WYNDE3	518		m	0	0	all	-	KII	NAmer	1966	CC	350	n	bl	n	y	0	all/unsp	13	999	0	1	nev any	st
WYNDE3	519		m	0	0	all	-	KII	NAmer	1966	CC	350	n	bl	n	y	0	all/unsp	7	12	0	2	nev any	st
WYNDE3	520		m	0	0	all	-	KII	NAmer	1966	CC	350	n	bl	n	y	0	all/unsp	4	6	0	0	nev any	st
WYNDE3	521		m	0	0	all	-	KII	NAmer	1966	CC	350	n	bl	n	y	0	all/unsp	1.0	3	3	3	nev any	st
WYNDE3	580		f	0	0	all	-	KII	NAmer	1966	CC	350	n	bl	n	y	0	cig+/-ot	10	999	1	0	nev any	ot
WYNDE6	802	x	m	0	0	all	-	KII	NAmer	1969	CC	4423	n	bl	n	y	0	cig+/-ot	16	999	0	1	nev any	st
WYNDE6	803	x	m	0	0	all	-	KII	NAmer	1969	CC	4423	n	bl	n	y	0	cig+/-ot	11	15	1	2	nev any	st
WYNDE6	804	x	m	0	0	all	-	KII	NAmer	1969	CC	4423	n	bl	n	y	0	cig+/-ot	7	10	2	0	nev any	st
WYNDE6	805	x	m	0	0	all	-	KII	NAmer	1969	CC	4423	n	bl	n	y	0	cig+/-ot	4	6	0	0	nev any	st
WYNDE6	806	x	m	0	0	all	-	KII	NAmer	1969	CC	4423	n	bl	n	y	0	cig+/-ot	1.0	3	3	3	nev any	st

Cigarette type is all/unspec for all RRs

In this overview table, subtotals and Qs values may be invalid and should be ignored

Table 3J1 - 5

IESLC - Meta-analysis of Ex Smoking by Years quit (vs never), Overview
Adenocarcinoma, Any Product (or Cigarettes if Any not available)
Least adjusted

REF	NRR	SEX	AD	Number Exposed Case	Cont	Non-exposed Case	Cont	RR	95.00%CI
BARBON	741	m	0	4	59	7	188	1.82	(0.52- 6.44)
BARBON	742	m	0	7	41	7	188	4.59	(1.53- 13.79)
BARBON	743	m	0	23	85	7	188	7.27	(3.00- 17.59)
BARBON	744	m	0	7	20	7	188	9.40	(2.99- 29.53)
Subtotal BARBON								5.38	(3.15- 9.18)
BROWN3	505	f	0	73	219	432	1168	0.90	(0.68- 1.20)
JAHN	639	m	0	15	146	8	138	1.77	(0.73- 4.31)
JAHN	640	m	0	22	130	8	138	2.92	(1.26- 6.79)
JAHN	641	m	0	13	63	8	138	3.56	(1.40- 9.02)
JAHN	642	m	0	19	46	8	138	7.13	(2.92- 17.37)
JAHN	643	m	0	18	9	8	138	34.50	(11.82- 100.74)
JAHN	644	m	0	40	8	8	138	86.25	(30.45- 244.33)
Subtotal JAHN								6.98	(4.77- 10.22)
JAIN	537	m	0	14	113	4	85	2.63	(0.84- 8.28)
JAIN	538	m	0	16	46	4	85	7.39	(2.33- 23.41)
JAIN	501	f	0	3	61	24	214	0.44	(0.13- 1.51)
JAIN	502	f	0	14	36	24	214	3.47	(1.64- 7.32)
Subtotal JAIN								2.69	(1.63- 4.45)
JEDRYC	559	m	0	12	230	7	289	2.15	(0.83- 5.56)
JEDRYC	560	m	0	9	82	7	289	4.53	(1.64- 12.54)
Subtotal JEDRYC								3.04	(1.52- 6.09)
LUBIN2	861	m	0	35	1128	57	2616	1.42	(0.93- 2.18)
LUBIN2	862	m	0	21	478	57	2616	2.02	(1.21- 3.36)
LUBIN2	863	m	0	30	693	57	2616	1.99	(1.27- 3.12)
LUBIN2	864	m	0	50	882	57	2616	2.60	(1.77- 3.83)
LUBIN2	865	m	0	77	1047	57	2616	3.38	(2.38- 4.79)
LUBIN2	965	f	0	1	29	138	1180	0.29	(0.04- 2.18)
LUBIN2	966	f	0	3	33	138	1180	0.78	(0.24- 2.57)
LUBIN2	967	f	0	13	95	138	1180	1.17	(0.64- 2.14)
Subtotal LUBIN2								2.10	(1.76- 2.50)
MATOS	661	m	0	12	101	5	110	2.61	(0.89- 7.68)
MATOS	662	m	0	9	27	5	110	7.33	(2.27- 23.66)
MATOS	663	m	0	12	23	5	110	11.48	(3.69- 35.74)
Subtotal MATOS								5.83	(3.04- 11.18)
PEZZOT	585	m	0	7	106	3	116	2.55	(0.64- 10.13)
PEZZOT	586	m	0	11	82	3	116	5.19	(1.40- 19.18)
Subtotal PEZZOT								3.71	(1.44- 9.57)
SVENSS	569	f	0	7	24	22	120	1.59	(0.61- 4.14)
SVENSS	570	f	0	5	13	22	120	2.10	(0.68- 6.48)
Subtotal SVENSS								1.79	(0.86- 3.70)
WAKAI	554	m	0	3	47	8	65	0.52	(0.13- 2.06)
WAKAI	555	m	0	13	44	8	65	2.40	(0.92- 6.27)
WAKAI	556	m	0	7	48	8	65	1.18	(0.40- 3.49)
Subtotal WAKAI								1.36	(0.72- 2.56)
WU2	502	f	2	-	-	-	-	2.30	(1.20- 4.40)
WYNDE3	518	m	0	3	55	6	88	0.80	(0.19- 3.33)
WYNDE3	519	m	0	3	31	6	88	1.42	(0.33- 6.02)
WYNDE3	520	m	0	3	17	6	88	2.59	(0.59- 11.37)
WYNDE3	521	m	0	3	22	6	88	2.00	(0.46- 8.63)
WYNDE3	580	f	0	0	3	15	76	0.71~	(0.03- 14.35)
Subtotal WYNDE3								1.47	(0.73- 2.99)
WYNDE6	802	m	0	6	530	11	1667	1.72	(0.63- 4.66)
WYNDE6	803	m	0	11	259	11	1667	6.44	(2.76- 15.00)
WYNDE6	804	m	0	18	340	11	1667	8.02	(3.76- 17.14)
WYNDE6	805	m	0	14	321	11	1667	6.61	(2.97- 14.69)
WYNDE6	806	m	0	29	307	11	1667	14.32	(7.08- 28.96)
Subtotal WYNDE6								7.06	(4.92- 10.12)
Partial Totals				715	8179	1460	30304		

*prospective study ~ With 0.5 adjustment for zero

Table 3J1 - 5

IESLC - Meta-analysis of Ex Smoking by Years quit (vs never), Overview
Adenocarcinoma, Any Product (or Cigarettes if Any not available)
Least adjusted

REF	NRR	SEX	AD	Ys	Ws	Qs	Ps
BARBON	741	m	0	0.60	2.41	0.23	0.3523
BARBON	742	m	0	1.52	3.17	1.20	0.0067
BARBON	743	m	0	1.98	4.92	5.68	0.0000
BARBON	744	m	0	2.24	2.93	5.21	0.0001
Subtotal BARBON				1.68	13.43	12.32	
BROWN3	505	f	0	-0.10	46.65	47.80	0.4775
JAHN	639	m	0	0.57	4.86	0.55	0.2071
JAHN	640	m	0	1.07	5.39	0.14	0.0128
JAHN	641	m	0	1.27	4.44	0.58	0.0074
JAHN	642	m	0	1.96	4.84	5.39	0.0000
JAHN	643	m	0	3.54	3.35	23.19	0.0000
JAHN	644	m	0	4.46	3.54	44.63	0.0000
Subtotal JAHN				1.94	26.43	74.48	
JAIN	537	m	0	0.97	2.92	0.01	0.0979
JAIN	538	m	0	2.00	2.89	3.45	0.0007
JAIN	501	f	0	-0.82	2.52	7.58	0.1902
JAIN	502	f	0	1.24	6.87	0.77	0.0011
Subtotal JAIN				0.99	15.21	11.81	
JEDRYC	559	m	0	0.77	4.27	0.08	0.1127
JEDRYC	560	m	0	1.51	3.71	1.35	0.0036
Subtotal JEDRYC				1.11	7.98	1.43	
LUBIN2	861	m	0	0.35	21.10	6.50	0.1044
LUBIN2	862	m	0	0.70	14.78	0.63	0.0070
LUBIN2	863	m	0	0.69	18.97	0.93	0.0028
LUBIN2	864	m	0	0.96	25.60	0.06	0.0000
LUBIN2	865	m	0	1.22	31.38	2.98	0.0000
LUBIN2	965	f	0	-1.22	0.96	4.35	0.2317
LUBIN2	966	f	0	-0.25	2.69	3.62	0.6795
LUBIN2	967	f	0	0.16	10.47	5.91	0.6113
Subtotal LUBIN2				0.74	125.96	24.98	
MATOS	661	m	0	0.96	3.31	0.01	0.0806
MATOS	662	m	0	1.99	2.80	3.29	0.0009
MATOS	663	m	0	2.44	2.98	6.99	0.0000
Subtotal MATOS				1.76	9.08	10.29	
PEZZOT	585	m	0	0.94	2.02	0.00	0.1824
PEZZOT	586	m	0	1.65	2.25	1.22	0.0136
Subtotal PEZZOT				1.31	4.27	1.23	
SVENSS	569	f	0	0.46	4.20	0.83	0.3415
SVENSS	570	f	0	0.74	3.02	0.08	0.1976
Subtotal SVENSS				0.58	7.22	0.91	
WAKAI	554	m	0	-0.66	2.02	4.95	0.3507
WAKAI	555	m	0	0.88	4.17	0.00	0.0739
WAKAI	556	m	0	0.17	3.29	1.79	0.7583
Subtotal WAKAI				0.30	9.47	6.75	
WU2	502	f	2	0.83	9.10	0.05	0.0120
WYNDE3	518	m	0	-0.22	1.89	2.42	0.7591
WYNDE3	519	m	0	0.35	1.84	0.57	0.6348
WYNDE3	520	m	0	0.95	1.75	0.00	0.2079
WYNDE3	521	m	0	0.69	1.80	0.08	0.3529
WYNDE3	580	f	0	-0.35	0.42	0.67	0.8202
Subtotal WYNDE3				0.39	7.70	3.75	
WYNDE6	802	m	0	0.54	3.85	0.52	0.2899
WYNDE6	803	m	0	1.86	5.37	4.88	0.0000
WYNDE6	804	m	0	2.08	6.67	9.19	0.0000
WYNDE6	805	m	0	1.89	6.02	5.79	0.0000
WYNDE6	806	m	0	2.66	7.74	23.78	0.0000
Subtotal WYNDE6				1.95	29.64	44.16	

N	46
NS	13

Table 3J1 - 6

IESLC - Meta-analysis of Ex Smoking by Years quit (vs never), Overview
Adenocarcinoma, Any Product (or Cigarettes if Any not available)
Least adjusted

	Sex			
	combined	male	female	Total
N		36	10	46
NS		10	6	16

In this overview table, other than the "N" rows, entries in the "absent" and "Total" columns may be invalid and should be ignored

	Years quit vs never (lower focus)				
	absent	8+k12	4-11k7	1-6k3	Total
N	22	12	6	6	46
NS	11	10	6	6	33
Wt	157.71	56.27	46.51	51.66	312.15
Het Chi	149.57	17.50	12.34	18.75	239.94
Het df	21	11	5	5	45
Het P	***	(*)	*	**	***
Fixed RR	1.91	2.13	3.32	5.02	2.48
RRl	1.63	1.64	2.49	3.82	2.22
RRu	2.23	2.77	4.42	6.59	2.77
P	+++	+++	+++	+++	+++
Random RR	2.80	2.09	3.72	6.62	3.01
RRl	1.76	1.46	2.19	3.43	2.28
RRu	4.44	2.99	6.32	12.79	3.98
P	+++	+++	+++	+++	+++

	Years quit vs never (higher focus)				
	absent	13+k20	4-19k12	1-11k3	Total
N	21	7	6	12	46
NS	11	6	5	10	32
Wt	108.29	79.64	37.95	86.26	312.15
Het Chi	92.65	13.46	15.60	42.12	239.94
Het df	20	6	5	11	45
Het P	***	*	**	***	***
Fixed RR	3.25	1.09	2.61	3.70	2.48
RRl	2.69	0.87	1.90	3.00	2.22
RRu	3.93	1.35	3.59	4.57	2.77
P	+++	N.S.	+++	+++	+++
Random RR	3.44	1.19	2.69	4.28	3.01
RRl	2.23	0.76	1.42	2.70	2.28
RRu	5.32	1.88	5.09	6.79	3.98
P	+++	N.S.	++	+++	+++

Table 3J1 - 6

IESLC - Meta-analysis of Ex Smoking by Years quit (vs never), Overview
Adenocarcinoma, Any Product (or Cigarettes if Any not available)
Least adjusted

MALES

	Years quit vs never (lower focus) absent	8+k12	4-11k7	1-6k3	Total
N	16	8	6	6	36
NS	8	8	6	6	28
Wt	80.64	46.43	46.51	51.66	225.24
Het Chi	98.90	6.02	12.34	18.75	148.99
Het df	15	7	5	5	35
Het P	***	N.S.	*	**	***
Fixed RR	2.97	2.56	3.32	5.02	3.32
RRl	2.39	1.92	2.49	3.82	2.92
RRu	3.69	3.41	4.42	6.59	3.79
P	+++	+++	+++	+++	+++
Random RR	3.63	2.56	3.72	6.62	3.81
RRl	1.99	1.92	2.19	3.43	2.85
RRu	6.61	3.41	6.32	12.79	5.10
P	+++	+++	+++	+++	+++

	Years quit vs never (higher focus) absent	13+k20	4-19k12	1-11k3	Total
N	18	5	5	8	36
NS	10	5	5	8	28
Wt	101.15	32.03	35.26	56.80	225.24
Het Chi	78.63	7.11	11.36	19.16	148.99
Het df	17	4	4	7	35
Het P	***	N.S.	*	**	***
Fixed RR	3.54	1.48	2.86	5.13	3.32
RRl	2.92	1.05	2.06	3.95	2.92
RRu	4.31	2.10	3.98	6.65	3.79
P	+++	+	+++	+++	+++
Random RR	4.09	1.48	3.27	6.52	3.81
RRl	2.61	0.83	1.73	3.85	2.85
RRu	6.40	2.64	6.18	11.05	5.10
P	+++	N.S.	+++	+++	+++

FEMALES

	Years quit vs never (lower focus) absent	8+k12	4-11k7	1-6k3	Total
N	6	4			10
NS	5	4			9
Wt	77.07	9.83			86.91
Het Chi	18.25	2.75			21.68
Het df	5	3			9
Het P	**	N.S.			**
Fixed RR	1.20	0.91			1.16
RRl	0.96	0.49			0.94
RRu	1.50	1.69			1.43
P	N.S.	N.S.			N.S.
Random RR	1.50	0.91			1.29
RRl	0.87	0.49			0.85
RRu	2.59	1.69			1.96
P	N.S.	N.S.			N.S.

Table 3J1 - 6

IESLC - Meta-analysis of Ex Smoking by Years quit (vs never), Overview
Adenocarcinoma, Any Product (or Cigarettes if Any not available)
Least adjusted

FEMALES

	Years quit vs never (higher focus)				
	absent	13+k20	4-19k12	1-11k3	Total
N	3	2	1	4	10
NS	3	2	1	4	9
Wt	7.14	47.61	2.69	29.46	86.91
Het Chi	2.66	1.17	0.00	5.27	21.68
Het df	2	1	0	3	9
Het P	N.S.	N.S.	N.S.	N.S.	**
Fixed RR	0.96	0.88	0.78	1.97	1.16
RRl	0.46	0.66	0.24	1.37	0.94
RRu	2.00	1.17	2.57	2.83	1.43
P	N.S.	N.S.	N.S.	+++	N.S.
Random RR	0.90	0.81	0.78	2.04	1.29
RRl	0.36	0.43	0.24	1.24	0.85
RRu	2.26	1.53	2.57	3.34	1.96
P	N.S.	N.S.	N.S.	++	N.S.

Table 3J1 - 7

IESLC - Meta-analysis of Ex Smoking by Years quit (vs never), Overview
Adenocarcinoma, Any Product (or Cigarettes if Any not available)
Excluded studies (and stage at which they were excluded)

Stage	Studies
1	AGUDO AKIBA AMANDU AMES AXELSS BEST BOUCHA BOUCOT BRESLO CHEN CHEN2 CHIAZZ DEAN2 DOSEME ENGELA FAN GENG GER GUO HAENSZ HEGMAN HOLE HU HU2 JUSSAW KATSOU KAUFMA KOO KOULUM KREUZE LETOUR LEVIN LIAW LIU3 LIU4 LIU5 MCCONN MIGRAN MRFITR NOTAN2 OSANN2 PERNU QIAO2 RACHTA RESTRE SADOWS SEGI2 STASZE TIZZAN VUTUC WATSON WU WUWILL WYNDE2 WYNDE8 XU YUAN ZHANG ZHENG ZHOU
2	BUFFLE HUMBLE PISANI PRESCO WYNDE7
3	MCDUFF SPITZ
4	ARMADA AUVINE BECHER BENSHL BLOT1 BOFFET BROSS CARPEN CEDERL CHOI CHYOU CORREA CPSI CPSII DAMBER DARBY DEAN3 DESTEF DOLL DOLL2 DORGAN DORN GAO GAO2 GARCIA GARSHI GILLIS GRAHAM GURSEL HAMMO2 HAMMON HIRAYA JOLY KAISE2 KHUDER LAUSSM LUBIN LUO PEZZO2 QIAO SPEIZE SUZUK2 TVERDA WANG2 WIGLE
5	ALDERS
10	SOBUE
14	BENHAM

Table 3J1 - 8

Potentially overlapping studies

REF	REFGP	PRINC	OVERLAP/LINK
LUBIN2	LUBIN2	1	Lubin-combined
WYNDE6	WYNDE6	1	WYNDE5/6/7/8
JAHN	BOFFET	2	Subset of BOFFET

Table 3J1 - 9

Most adjusted - insufficient data for meta-analysis

REF	NRR	SEX	AGEL	AGEH	RACE	YF	LC TYPE	LOC	START	ST	NLC	R	VB	P	H	AD	PRODUCT	exL	exH	S1	S2	DENOM	De
ALDERS	567	m	0	0	all	-	not q+s	Eu:UK	1977	CC	1448	n	V	n	n	1	cig only	10	999	1	0	nev any	st
ALDERS	568	m	0	0	all	-	not q+s	Eu:UK	1977	CC	1448	n	V	n	n	1	cig only	3	9	0	3	nev any	st
ALDERS	569	m	0	0	all	-	not q+s	Eu:UK	1977	CC	1448	n	V	n	n	1	cig only	0.1	2	0	0	nev any	st
ALDERS	578	f	0	0	all	-	not q+s	Eu:UK	1977	CC	1448	n	V	n	n	1	cig only	10	999	1	0	nev any	st
ALDERS	579	f	0	0	all	-	not q+s	Eu:UK	1977	CC	1448	n	V	n	n	1	cig only	3	9	0	3	nev any	st
ALDERS	580	f	0	0	all	-	not q+s	Eu:UK	1977	CC	1448	n	V	n	n	1	cig only	0.1	2	0	0	nev any	st
BROWN3	506	f	0	0	wh	-	a	NAmer		CC	618		bl	y	n	0	all/unsp	1.0	14	0	0	nev any	ot
JAIN	586	m	0	0	all	-	a	NAmer	1981	CC	845	n	V	y	n	0	cig+/-ot	0.1	1.9	0	0	nev cigs	ot
JAIN	574	f	0	0	all	-	a	NAmer	1981	CC	845	n	V	y	n	0	cig+/-ot	0.1	1.9	0	0	nev cigs	ot
JEDRYC	561	m	0	0	all	-	a	Eu:est	1980	CC	1630	n	bl	y	n	0	cig+/-ot	1.0	4	3	3	nev any	ot
MATOS	711	m	0	0	all	-	a	SCAmer	1994	CC	200	n	bl	n	n	2	cig+/-ot	0.1	0.9	0	0	nev any	ot
PEZZOT	601	m	0	0	all	-	a	SCAmer	1987	CC	215	n	bl	n	y	0	cig only	0.1	0.9	0	0	nev cigs	ot
SVENSS	597	f	0	0	all	-	a	Eu:Sca	1983	CC	210	n	bl	n	n	0	all/unsp	1.0	2	0	0	nev any	ot
WAKAI	619	m	0	0	all	-	a	As:Jap	1988	CC	333	n	bl	n	y	1	cig+/-ot	1.0	4	3	3	nev any	ot
WU2	501	f	0	0	all	-	a	NAmer	1983	CC	336	n	bl	n	y	2	all/unsp	10	999	1	0	nev any	ot
WYNDE3	522	m	0	0	all	-	KII	NAmer	1966	CC	350	n	bl	n	y	0	all/unsp	0.1	0.9	0	0	nev any	ot
WYNDE3	581	f	0	0	all	-	KII	NAmer	1966	CC	350	n	bl	n	y	0	cig+/-ot	1.0	9	0	3	nev any	ot
WYNDE6	822	m	0	0	all	-	KII	NAmer	1969	CC	4423	n	bl	n	y	2	cig+/-ot	0.1	0.9	0	0	nev any	ot

REF	NRR	RR	SIG	RRDATA comment
ALDERS	567	2.22		0
ALDERS	568	3.80		0
ALDERS	569	5.05		0
ALDERS	578	1.64		0
ALDERS	579	0.73		0
ALDERS	580	6.27		0
BROWN3	506	*	gap	0
JAIN	586	*	gap	0
JAIN	574	*	gap	0
JEDRYC	561	*	gap	0
MATOS	711	*	gap	0
PEZZOT	601	*	gap	0
SVENSS	597	*	gap	0
WAKAI	619	*	gap	0
WU2	501	*	gap	0
WYNDE3	522	*	gap	0
WYNDE3	581	*	gap	0
WYNDE6	822	*	gap	0

Table 3J1 - 9

IESLC - Meta-analysis of Ex Smoking by Years quit (vs never), Overview
Adenocarcinoma, Any Product (or Cigarettes if Any not available)

Least adjusted - insufficient data for meta-analysis: as for adjusted plus the following

REF	NRR	SEX	AGEL	AGEH	RACE	YF	LC TYPE	LOC	START	ST	NLC	R	VB	P	H	AD	PRODUCT	exL	exH	S1	S2	DENOM	De
MATOS	709	m	0	0	all	-	a	SCAmer	1994	CC	200	n	bl	n	n	0	cig+/-ot	0.1	0.9	0	0	nev any	ot
WAKAI	617	m	0	0	all	-	a	As:Jap	1988	CC	333	n	bl	n	y	0	cig+/-ot	1.0	4	3	3	nev any	ot
WYNDE6	807	m	0	0	all	-	KII	NAmer	1969	CC	4423	n	bl	n	y	0	cig+/-ot	0.1	0.9	0	0	nev any	ot

REF	NRR	RR	SIG	RRDATA comment
MATOS	709	*	gap	0
WAKAI	617	*	gap	0
WYNDE6	807	*	gap	0

Table 3J2 -

IESLC - Meta-analysis of Ex Smoking, Years quit (vs never), "Low"
Adenocarcinoma, Any Product (or Cigarettes if Any not available)

This analysis is restricted to results for:
1) Ex smokers
2) Results by Years quit (vs never)
3) Categorical results by Years quit (vs never)
4) Adenocarcinoma (or near equivalent)
5) Results complete enough for use in metaanalysis

Within each study, results are then selected (in the following order of preference, within each sex) for:
6) (not applicable)
7) PRODUCT: all/unspec, cigarettes regardless of other products, cigarettes only
8) CIGTYPE: all/unspecified, MC regardless of HR, MC only
9) (not applicable)
10) DENOM: never smoked anything, never smoked cigarettes, never any + low, never cigs + low
11) Followup period (YF, prospective studies): whole study (coded as 0) or longest available
12) LCtype: adeno or nearest available, but not squamous. (q = squamous, s = small,
a = adeno, l = large, KII = Kreyberg II, al = alveolar, br = bronchiolar, u = undifferentiated)
13) Race: all or nearest available, otherwise by race (wh or w = white, bl or b = black, hi = hispanic
ch = chinese, jap = japanese, haw = hawaiian, w+o = white + oriental, sca = scandinavian, as = asian)
14) Years quit (vs never) "low" in key scheme 1 (key value 12, maximum range 8+)
15) For overlapping studies: principal rather than subsidiary studies
Finally by Age: whole study (coded as 0) if available, otherwise by widest available age group
and then for single sex results (m, f) in preference to results for both sexes combined (c).

Results adjusted (AD) for the most potential confounders are then chosen in Sections -1 to -3
and results adjusted for the least confounders in Sections -4 to -6. (Those least adjusted results which
actually differ from the most adjusted are marked 'x' in column X in Section -4)

Section -7 shows excluded studies, together with the stage (as above) at which no qualifying
results were found.

Section -8 lists the potentially overlapping studies which have been included (1=principal, 2=subsidiary).

Section -9 lists any results which would have been included in preference except that they had data not complete
enough for use in meta-analysis, with their significance (yes/no), if known, and any further comment as entered
on the database. It also lists as "gap" any categories for which no data were presented by the original authors.
This is commonly due to recent quitters having been combined with current smokers

In addition to those mentioned above, the following fields, levels and abbreviations are used:
* or nk = not known, n = no, y = yes, ot = other
nev = never
all/unspec = all or unspecified, cig+/-ot = cigarettes irrespective of other products (cigar, pipe etc)
MC = manufactured cigarettes, HR = hand-rolled cigarettes
exL, exH = range of exposure (low and high) in the smoking group, in terms of Years quit (vs never)
REF: 6-character study reference
NRR: number of the RR on the database within the study
ST : study type (CC = case control, pr or prosp = prospective)
NLC: number of lung cancer cases in whole study
R : risky occupational population (n = no, m = mining, o = other risky)
VB : national cigarette type (V = at least 75% Virginia, bl = at least 75% blended, ot = other)
P : any proxy use
H : full histological confirmation
De : derivation of RR/CI (or = original, st = standard method, ot = other method of estimation)

Table 3J2 - 1

IESLC - Meta-analysis of Ex Smoking, Years quit (vs never), "Low"
Adenocarcinoma, Any Product (or Cigarettes if Any not available)
Most adjusted

REF	NRR	SEX	AGEL	AGEH	RACE	YF	LC TYPE	LOC	START	ST	NLC	R	VB	P	H	AD	PRODUCT	exL	exH	DENOM	De
JAHN	640	m	0	0	all	-	a	Eu:Ger	1988	CC	1004	n	bl	n	n	0	cig+/-ot	11	20	nev any	st
JAIN	537	m	0	0	all	-	a	NAmer	1981	CC	845	n	V	y	n	0	cig+/-ot	10	999	nev cigs	st
JAIN	501	f	0	0	all	-	a	NAmer	1981	CC	845	n	V	y	n	0	cig+/-ot	10	999	nev cigs	st
JEDRYC	559	m	0	0	all	-	a	Eu:est	1980	CC	1630	n	bl	y	n	0	cig+/-ot	10	999	nev any	st
LUBIN2	863	m	0	0	all	-	a	Eu:mul	1976	CC	7804	n	bl	n	y	0	cig+/-ot	10	14	nev any	st
LUBIN2	966	f	0	0	all	-	a	Eu:mul	1976	CC	7804	n	bl	n	y	0	cig+/-ot	10	19	nev any	st
MATOS	671	m	0	0	all	-	a	SCAmer	1994	CC	200	n	bl	n	n	2	cig+/-ot	11	999	nev any	ot
PEZZOT	585	m	0	0	all	-	a	SCAmer	1987	CC	215	n	bl	n	y	0	cig only	11	999	nev cigs	st
SVENSS	569	f	0	0	all	-	a	Eu:Sca	1983	CC	210	n	bl	n	n	0	all/unsp	11	999	nev any	st
WAKAI	563	m	0	0	all	-	a	As:Jap	1988	CC	333	n	bl	n	y	1	cig+/-ot	10	19	nev any	or
WYNDE3	559	m	0	0	all	-	KII	NAmer	1966	CC	350	n	bl	n	y	0	cig+/-ot	10	999	nev any	st
WYNDE3	580	f	0	0	all	-	KII	NAmer	1966	CC	350	n	bl	n	y	0	cig+/-ot	10	999	nev any	ot
WYNDE6	818	m	0	0	all	-	KII	NAmer	1969	CC	4423	n	bl	n	y	2	cig+/-ot	11	15	nev any	ot

Cigarette type is all/unspec for all RRs

Table 3J2 - 2

IESLC - Meta-analysis of Ex Smoking, Years quit (vs never), "Low"
Adenocarcinoma, Any Product (or Cigarettes if Any not available)
Most adjusted

REF	NRR	SEX	AD	Number Exposed Case	Number Exposed Cont	Non-exposed Case	Non-exposed Cont	RR	95.00%CI
JAHN	640	m	0	22	130	8	138	2.92	(1.26- 6.79)
JAIN	537	m	0	14	113	4	85	2.63	(0.84- 8.28)
JAIN	501	f	0	3	61	24	214	0.44	(0.13- 1.51)
Subtotal JAIN								1.15	(0.50- 2.66)
JEDRYC	559	m	0	12	230	7	289	2.15	(0.83- 5.56)
LUBIN2	863	m	0	30	693	57	2616	1.99	(1.27- 3.12)
LUBIN2	966	f	0	3	33	138	1180	0.78	(0.24- 2.57)
Subtotal LUBIN2								1.77	(1.16- 2.69)
MATOS	671	m	2	12	-	5	-	3.00	(1.09- 8.23)
PEZZOT	585	m	0	7	106	3	116	2.55	(0.64- 10.13)
SVENSS	569	f	0	7	24	22	120	1.59	(0.61- 4.14)
WAKAI	563	m	1	13	-	8	-	2.49	(0.95- 6.53)
WYNDE3	559	m	0	3	65	6	88	0.68	(0.16- 2.81)
WYNDE3	580	f	0	0	3	15	76	0.71~	(0.03- 14.35)
Subtotal WYNDE3								0.68	(0.19- 2.47)
WYNDE6	818	m	2	11	-	11	-	5.40	(2.32- 12.58)
Partial Totals				137	1458	308	4922		

*prospective study ~ With 0.5 adjustment for zero

REF	NRR	SEX	AD	Ys	Ws	Qs	Ps
JAHN	640	m	0	1.07	5.39	0.67	0.0128
JAIN	537	m	0	0.97	2.92	0.18	0.0979
JAIN	501	f	0	-0.82	2.52	6.01	0.1902
Subtotal JAIN				0.14	5.45	6.19	
JEDRYC	559	m	0	0.77	4.27	0.01	0.1127
LUBIN2	863	m	0	0.69	18.97	0.02	0.0028
LUBIN2	966	f	0	-0.25	2.69	2.53	0.6795
Subtotal LUBIN2				0.57	21.66	2.55	
MATOS	671	m	2	1.10	3.76	0.54	0.0332
PEZZOT	585	m	0	0.94	2.02	0.10	0.1824
SVENSS	569	f	0	0.46	4.20	0.27	0.3415
WAKAI	563	m	1	0.91	4.14	0.16	0.0636
WYNDE3	559	m	0	-0.39	1.90	2.33	0.5908
WYNDE3	580	f	0	-0.35	0.42	0.48	0.8202
Subtotal WYNDE3				-0.38	2.32	2.81	
WYNDE6	818	m	2	1.69	5.38	5.04	0.0001

	N	13
	NS	10
	Wt	58.59
Het	Chi	18.35
Het	df	12
Het	P	N.S.
Fixed	RR	2.05
	RRl	1.59
	RRu	2.65
	P	+++
Random	RR	1.98
	RRl	1.40
	RRu	2.79
	P	+++
Asymm	P	N.S.

Table 3J2 - 3

IESLC - Meta-analysis of Ex Smoking, Years quit (vs never), "Low"
Adenocarcinoma, Any Product (or Cigarettes if Any not available)
Most adjusted

		Sex		
	combined	male	female	Total
N		9	4	13
NS		9	4	13
Wt		48.76	9.83	58.59
Het Chi		7.73	2.75	18.35
Het df		8	3	12
Het P		N.S.	N.S.	N.S.
Fixed RR		2.42	0.91	2.05
RRl		1.83	0.49	1.59
RRu		3.20	1.69	2.65
P		+++	N.S.	+++
Random RR		2.42	0.91	1.98
RRl		1.83	0.49	1.40
RRu		3.20	1.69	2.79
P		+++	N.S.	+++
Between Chi				7.86
Between df				1
Between P				**
Btwn(F) P				*
Btwn(R) P				**

		Lung cancer type					
	a	a+l	a+al+br	KII	not q+u	not q+s	Total
N	10			3			13
NS	8			2			10
Wt	50.89			7.70			58.59
Het Chi	10.35			6.94			18.35
Het df	9			2			12
Het P	N.S.			*			N.S.
Fixed RR	1.95			2.89			2.05
RRl	1.48			1.43			1.59
RRu	2.56			5.86			2.65
P	+++			++			+++
Random RR	1.93			1.72			1.98
RRl	1.42			0.33			1.40
RRu	2.63			9.03			2.79
P	+++			N.S.			+++
Between Chi							1.05
Between df							1
Between P							N.S.
Btwn(F) P							N.S.
Btwn(R) P							N.S.

				Location					
	NAmer	UK	Scand	othEur	China	Japan	othAs	other	Total
N	5		1	4		1		2	13
NS	3		1	3		1		2	10
Wt	13.15		4.20	31.33		4.14		5.78	58.59
Het Chi	14.02		0.00	3.20		0.00		0.03	18.35
Het df	4		0	3		0		1	12
Het P	**		N.S.	N.S.		N.S.		N.S.	N.S.
Fixed RR	1.97		1.59	1.98		2.49		2.84	2.05
RRl	1.15		0.61	1.40		0.95		1.26	1.59
RRu	3.39		4.14	2.81		6.53		6.41	2.65
P	+		N.S.	+++		(+)		+	+++
Random RR	1.44		1.59	1.98		2.49		2.84	1.98
RRl	0.48		0.61	1.36		0.95		1.26	1.40
RRu	4.34		4.14	2.87		6.53		6.41	2.79
P	N.S.		N.S.	+++		(+)		+	+++
Between Chi									1.09
Between df									4
Between P									N.S.
Btwn(F) P									N.S.
Btwn(R) P									N.S.

Table 3J2 - 3

IESLC - Meta-analysis of Ex Smoking, Years quit (vs never), "Low"
Adenocarcinoma, Any Product (or Cigarettes if Any not available)
Most adjusted

Detailed Country in "other Europe"

	multi	Germany	othWest	East	Balkans	Total
N	2	1		1		4
NS	1	1		1		3
Wt	21.66	5.39		4.27		31.33
Het Chi	2.07	0.00		0.00		3.20
Het df	1	0		0		3
Het P	N.S.	N.S.		N.S.		N.S.
Fixed RR	1.77	2.92		2.15		1.98
RRl	1.16	1.26		0.83		1.40
RRu	2.69	6.79		5.56		2.81
P	++	+		N.S.		+++
Random RR	1.47	2.92		2.15		1.98
RRl	0.63	1.26		0.83		1.36
RRu	3.47	6.79		5.56		2.87
P	N.S.	+		N.S.		+++
Between Chi						1.12
Between df						2
Between P						N.S.
Btwn(F) P						N.S.
Btwn(R) P						N.S.

Detailed Country in "other Asia"

	India	HongKong	other	Total
N				
NS				
Wt				
Het Chi				
Het df				
Het P				N.S.
Fixed RR				
RRl				
RRu				
P				N.S.
Random RR				
RRl				
RRu				
P				N.S.
Between Chi				
Between df				
Between P				N.S.
Btwn(F) P				N.S.
Btwn(R) P				N.S.

Detailed other continent

	SCAmer	Total
N	2	2
NS	2	2
Wt	5.78	5.78
Het Chi	0.03	0.03
Het df	1	1
Het P	N.S.	N.S.
Fixed RR	2.84	2.84
RRl	1.26	1.26
RRu	6.41	6.41
P	+	+
Random RR	2.84	2.84
RRl	1.26	1.26
RRu	6.41	6.41
P	+	+
Between Chi		
Between df		
Between P		N.S.
Btwn(F) P		N.S.
Btwn(R) P		N.S.

Table 3J2 - 3

IESLC - Meta-analysis of Ex Smoking, Years quit (vs never), "Low"
Adenocarcinoma, Any Product (or Cigarettes if Any not available)
Most adjusted

	Start year of study					
	<1960	1960-69	1970-79	1980-89	1990+	Total
N		3	2	7	1	13
NS		2	1	6	1	10
Wt		7.70	21.66	25.47	3.76	58.59
Het Chi		6.94	2.07	7.37	0.00	18.35
Het df		2	1	6	0	12
Het P		*	N.S.	N.S.	N.S.	N.S.
Fixed RR		2.89	1.77	1.98	3.00	2.05
RRl		1.43	1.16	1.34	1.09	1.59
RRu		5.86	2.69	2.92	8.24	2.65
P		++	++	+++	+	+++
Random RR		1.72	1.47	1.96	3.00	1.98
RRl		0.33	0.63	1.27	1.09	1.40
RRu		9.03	3.47	3.02	8.24	2.79
P		N.S.	N.S.	++	+	+++
Between Chi						1.96
Between df						3
Between P						N.S.
Btwn(F) P						N.S.
Btwn(R) P						N.S.

	Study type (1)		
	CC	other	Total
N	13		13
NS	10		10
Wt	58.59		58.59
Het Chi	18.35		18.35
Het df	12		12
Het P	N.S.		N.S.
Fixed RR	2.05		2.05
RRl	1.59		1.59
RRu	2.65		2.65
P	+++		+++
Random RR	1.98		1.98
RRl	1.40		1.40
RRu	2.79		2.79
P	+++		+++
Between Chi			
Between df			
Between P			N.S.
Btwn(F) P			N.S.
Btwn(R) P			N.S.

	Study type (2)			
	CC	prosp	other	Total
N	13			13
NS	10			10
Wt	58.59			58.59
Het Chi	18.35			18.35
Het df	12			12
Het P	N.S.			N.S.
Fixed RR	2.05			2.05
RRl	1.59			1.59
RRu	2.65			2.65
P	+++			+++
Random RR	1.98			1.98
RRl	1.40			1.40
RRu	2.79			2.79
P	+++			+++
Between Chi				
Between df				
Between P				N.S.
Btwn(F) P				N.S.
Btwn(R) P				N.S.

Table 3J2 - 3

IESLC - Meta-analysis of Ex Smoking, Years quit (vs never), "Low"
Adenocarcinoma, Any Product (or Cigarettes if Any not available)
Most adjusted

	Study size (number of LC cases) 100-249	250-499	500-999	1000+	Total
N	3	3	2	5	13
NS	3	2	1	4	10
Wt	9.98	6.46	5.45	36.71	58.59
Het Chi	0.85	2.49	4.35	7.81	18.35
Het df	2	2	1	4	12
Het P	N.S.	N.S.	*	(*)	N.S.
Fixed RR	2.22	1.56	1.15	2.29	2.05
RRl	1.20	0.72	0.50	1.66	1.59
RRu	4.14	3.38	2.66	3.17	2.65
P	+	N.S.	N.S.	+++	+++
Random RR	2.22	1.44	1.09	2.34	1.98
RRl	1.20	0.56	0.19	1.42	1.40
RRu	4.14	3.66	6.32	3.87	2.79
P	+	N.S.	N.S.	+++	+++
Between Chi					2.84
Between df					3
Between P					N.S.
Btwn(F) P					N.S.
Btwn(R) P					N.S.

	Risky occupational population no	mining	othRisky	Total
N	13			13
NS	10			10
Wt	58.59			58.59
Het Chi	18.35			18.35
Het df	12			12
Het P	N.S.			N.S.
Fixed RR	2.05			2.05
RRl	1.59			1.59
RRu	2.65			2.65
P	+++			+++
Random RR	1.98			1.98
RRl	1.40			1.40
RRu	2.79			2.79
P	+++			+++
Between Chi				
Between df				
Between P				N.S.
Btwn(F) P				N.S.
Btwn(R) P				N.S.

	National cigarette tobacco type Virginia	blended	other	Total
N	2	11		13
NS	1	9		10
Wt	5.45	53.14		58.59
Het Chi	4.35	11.97		18.35
Het df	1	10		12
Het P	*	N.S.		N.S.
Fixed RR	1.15	2.18		2.05
RRl	0.50	1.66		1.59
RRu	2.66	2.85		2.65
P	N.S.	+++		+++
Random RR	1.09	2.17		1.98
RRl	0.19	1.59		1.40
RRu	6.32	2.97		2.79
P	N.S.	+++		+++
Between Chi				2.03
Between df				1
Between P				N.S.
Btwn(F) P				N.S.
Btwn(R) P				N.S.

Table 3J2 - 3

IESLC - Meta-analysis of Ex Smoking, Years quit (vs never), "Low"
Adenocarcinoma, Any Product (or Cigarettes if Any not available)
Most adjusted

	Any proxy use No/nk	Yes	Total
N	10	3	13
NS	8	2	10
Wt	48.87	9.72	58.59
Het Chi	11.97	5.30	18.35
Het df	9	2	12
Het P	N.S.	(*)	N.S.
Fixed RR	2.18	1.51	2.05
RRl	1.65	0.81	1.59
RRu	2.88	2.84	2.65
P	+++	N.S.	+++
Random RR	2.16	1.41	1.98
RRl	1.52	0.50	1.40
RRu	3.08	3.99	2.79
P	+++	N.S.	+++
Between Chi			1.08
Between df			1
Between P			N.S.
Btwn(F) P			N.S.
Btwn(R) P			N.S.

	Full histological confirmation No	Yes	Total
N	6	7	13
NS	5	5	10
Wt	23.07	35.52	58.59
Het Chi	7.67	10.65	18.35
Het df	5	6	12
Het P	N.S.	(*)	N.S.
Fixed RR	1.99	2.09	2.05
RRl	1.32	1.50	1.59
RRu	2.99	2.90	2.65
P	+++	+++	+++
Random RR	1.93	1.98	1.98
RRl	1.16	1.17	1.40
RRu	3.22	3.35	2.79
P	+	+	+++
Between Chi			0.03
Between df			1
Between P			N.S.
Btwn(F) P			N.S.
Btwn(R) P			N.S.

	Number of adjustment variables (1) 0	1	2+/+nk	Total
N	10	1	2	13
NS	7	1	2	10
Wt	45.32	4.14	9.14	58.59
Het Chi	11.39	0.00	0.76	18.35
Het df	9	0	1	12
Het P	N.S.	N.S.	N.S.	N.S.
Fixed RR	1.74	2.49	4.24	2.05
RRl	1.30	0.95	2.22	1.59
RRu	2.33	6.53	8.11	2.65
P	+++	(+)	+++	+++
Random RR	1.65	2.49	4.24	1.98
RRl	1.15	0.95	2.22	1.40
RRu	2.37	6.53	8.11	2.79
P	++	(+)	+++	+++
Between Chi				6.20
Between df				2
Between P				*
Btwn(F) P				N.S.
Btwn(R) P				*

Table 3J2 - 3

IESLC - Meta-analysis of Ex Smoking, Years quit (vs never), "Low"
Adenocarcinoma, Any Product (or Cigarettes if Any not available)
Most adjusted

	Number of adjustment variables (2) 0	1	2	3-5	6+/+nk	Total
N	10	1	2			13
NS	7	1	2			10
Wt	45.32	4.14	9.14			58.59
Het Chi	11.39	0.00	0.76			18.35
Het df	9	0	1			12
Het P	N.S.	N.S.	N.S.			N.S.
Fixed RR	1.74	2.49	4.24			2.05
RRl	1.30	0.95	2.22			1.59
RRu	2.33	6.53	8.11			2.65
P	+++	(+)	+++			+++
Random RR	1.65	2.49	4.24			1.98
RRl	1.15	0.95	2.22			1.40
RRu	2.37	6.53	8.11			2.79
P	++	(+)	+++			+++
Between Chi						6.20
Between df						2
Between P						*
Btwn(F) P						N.S.
Btwn(R) P						*

	Product all/unsp	cig+/-ot	cig only	Total
N	1	11	1	13
NS	1	8	1	10
Wt	4.20	52.37	2.02	58.59
Het Chi	0.00	17.97	0.00	18.35
Het df	0	10	0	12
Het P	N.S.	(*)	N.S.	N.S.
Fixed RR	1.59	2.08	2.55	2.05
RRl	0.61	1.58	0.64	1.59
RRu	4.14	2.72	10.13	2.65
P	N.S.	+++	N.S.	+++
Random RR	1.59	1.96	2.55	1.98
RRl	0.61	1.31	0.64	1.40
RRu	4.14	2.93	10.13	2.79
P	N.S.	++	N.S.	+++
Between Chi				0.38
Between df				2
Between P				N.S.
Btwn(F) P				N.S.
Btwn(R) P				N.S.

	Denominator nev any	nev cigs	Total
N	10	3	13
NS	8	2	10
Wt	51.12	7.47	58.59
Het Chi	11.91	5.30	18.35
Het df	9	2	12
Het P	N.S.	(*)	N.S.
Fixed RR	2.16	1.42	2.05
RRl	1.64	0.70	1.59
RRu	2.84	2.92	2.65
P	+++	N.S.	+++
Random RR	2.15	1.43	1.98
RRl	1.53	0.44	1.40
RRu	3.02	4.61	2.79
P	+++	N.S.	+++
Between Chi			1.14
Between df			1
Between P			N.S.
Btwn(F) P			N.S.
Btwn(R) P			N.S.

Table 3J2 - 3

IESLC - Meta-analysis of Ex Smoking, Years quit (vs never), "Low"
Adenocarcinoma, Any Product (or Cigarettes if Any not available)
Most adjusted

	Derivation of RR/CI			
	Orig	StdCalc	Other	Total
N	1	9	3	13
NS	1	7	3	11
Wt	4.14	44.90	9.56	58.59
Het Chi	0.00	11.04	2.07	18.35
Het df	0	8	2	12
Het P	N.S.	N.S.	N.S.	N.S.
Fixed RR	2.49	1.76	3.92	2.05
RRl	0.95	1.31	2.08	1.59
RRu	6.53	2.35	7.38	2.65
P	(+)	+++	+++	+++
Random RR	2.49	1.66	3.89	1.98
RRl	0.95	1.14	2.03	1.40
RRu	6.53	2.42	7.47	2.79
P	(+)	++	+++	+++
Between Chi				5.24
Between df				2
Between P				(*)
Btwn(F) P				N.S.
Btwn(R) P				(*)

Table 3J2 - 4

IESLC - Meta-analysis of Ex Smoking, Years quit (vs never), "Low"
Adenocarcinoma, Any Product (or Cigarettes if Any not available)
Least adjusted

REF	NRR	X	SEX	AGEL	AGEH	RACE	YF	LC TYPE	LOC	START	ST	NLC	R	VB	P	H	AD	PRODUCT	exL	exH	DENOM	De
JAHN	640		m	0	0	all	-	a	Eu:Ger	1988	CC	1004	n	bl	n	n	0	cig+/-ot	11	20	nev any	st
JAIN	537		m	0	0	all	-	a	NAmer	1981	CC	845	n	V	y	n	0	cig+/-ot	10	999	nev cigs	st
JAIN	501		f	0	0	all	-	a	NAmer	1981	CC	845	n	V	y	n	0	cig+/-ot	10	999	nev cigs	st
JEDRYC	559		m	0	0	all	-	a	Eu:est	1980	CC	1630	n	bl	y	n	0	cig+/-ot	10	999	nev any	st
LUBIN2	863		m	0	0	all	-	a	Eu:mul	1976	CC	7804	n	bl	n	y	0	cig+/-ot	10	14	nev any	st
LUBIN2	966		f	0	0	all	-	a	Eu:mul	1976	CC	7804	n	bl	n	y	0	cig+/-ot	10	19	nev any	st
MATOS	661	x	m	0	0	all	-	a	SCAmer	1994	CC	200	n	bl	n	n	0	cig+/-ot	11	999	nev any	st
PEZZOT	585		m	0	0	all	-	a	SCAmer	1987	CC	215	n	bl	n	y	0	cig only	11	999	nev cigs	st
SVENSS	569		f	0	0	all	-	a	Eu:Sca	1983	CC	210	n	bl	n	n	0	all/unsp	11	999	nev any	st
WAKAI	555	x	m	0	0	all	-	a	As:Jap	1988	CC	333	n	bl	n	y	0	cig+/-ot	10	19	nev any	st
WYNDE3	559		m	0	0	all	-	KII	NAmer	1966	CC	350	n	bl	n	y	0	cig+/-ot	10	999	nev any	st
WYNDE3	580		f	0	0	all	-	KII	NAmer	1966	CC	350	n	bl	n	y	0	cig+/-ot	10	999	nev any	ot
WYNDE6	803	x	m	0	0	all	-	KII	NAmer	1969	CC	4423	n	bl	n	y	0	cig+/-ot	11	15	nev any	st

Cigarette type is all/unspec for all RRs

Table 3J2 - 5

IESLC - Meta-analysis of Ex Smoking, Years quit (vs never), "Low"
Adenocarcinoma, Any Product (or Cigarettes if Any not available)
Least adjusted

REF	NRR	SEX	AD	Number Exposed Case	Cont	Non-exposed Case	Cont	RR	95.00%CI
JAHN	640	m	0	22	130	8	138	2.92	(1.26- 6.79)
JAIN	537	m	0	14	113	4	85	2.63	(0.84- 8.28)
JAIN	501	f	0	3	61	24	214	0.44	(0.13- 1.51)
Subtotal JAIN								1.15	(0.50- 2.66)
JEDRYC	559	m	0	12	230	7	289	2.15	(0.83- 5.56)
LUBIN2	863	m	0	30	693	57	2616	1.99	(1.27- 3.12)
LUBIN2	966	f	0	3	33	138	1180	0.78	(0.24- 2.57)
Subtotal LUBIN2								1.77	(1.16- 2.69)
MATOS	661	m	0	12	101	5	110	2.61	(0.89- 7.68)
PEZZOT	585	m	0	7	106	3	116	2.55	(0.64- 10.13)
SVENSS	569	f	0	7	24	22	120	1.59	(0.61- 4.14)
WAKAI	555	m	0	13	44	8	65	2.40	(0.92- 6.27)
WYNDE3	559	m	0	3	65	6	88	0.68	(0.16- 2.81)
WYNDE3	580	f	0	0	3	15	76	0.71~	(0.03- 14.35)
Subtotal WYNDE3								0.68	(0.19- 2.47)
WYNDE6	803	m	0	11	259	11	1667	6.44	(2.76- 15.00)
Totals				137	1862	308	6764		

*prospective study ~ With 0.5 adjustment for zero

REF	NRR	SEX	AD	Ys	Ws	Qs	Ps
JAHN	640	m	0	1.07	5.39	0.66	0.0128
JAIN	537	m	0	0.97	2.92	0.18	0.0979
JAIN	501	f	0	-0.82	2.52	6.03	0.1902
Subtotal JAIN				0.14	5.45	6.21	
JEDRYC	559	m	0	0.77	4.27	0.01	0.1127
LUBIN2	863	m	0	0.69	18.97	0.02	0.0028
LUBIN2	966	f	0	-0.25	2.69	2.55	0.6795
Subtotal LUBIN2				0.57	21.66	2.57	
MATOS	661	m	0	0.96	3.31	0.19	0.0806
PEZZOT	585	m	0	0.94	2.02	0.09	0.1824
SVENSS	569	f	0	0.46	4.20	0.28	0.3415
WAKAI	555	m	0	0.88	4.17	0.10	0.0739
WYNDE3	559	m	0	-0.39	1.90	2.34	0.5908
WYNDE3	580	f	0	-0.35	0.42	0.48	0.8202
Subtotal WYNDE3				-0.38	2.32	2.83	
WYNDE6	803	m	0	1.86	5.37	6.99	0.0000

	N	13
	NS	10
	Wt	58.16
Het	Chi	19.93
Het	df	12
Het	P	(*)
Fixed	RR	2.06
	RRl	1.59
	RRu	2.66
	P	+++
Random	RR	1.97
	RRl	1.37
	RRu	2.83
	P	+++
Asymm	P	N.S.

Table 3J2 - 6

IESLC - Meta-analysis of Ex Smoking, Years quit (vs never), "Low"
Adenocarcinoma, Any Product (or Cigarettes if Any not available)
Least adjusted

	Sex		
combined	male	female	Total
N	9	4	13
NS	9	4	13
Wt	48.33	9.83	58.16
Het Chi	9.25	2.75	19.93
Het df	8	3	12
Het P	N.S.	N.S.	(*)
Fixed RR	2.43	0.91	2.06
RRl	1.83	0.49	1.59
RRu	3.22	1.69	2.66
P	+++	N.S.	+++
Random RR	2.47	0.91	1.97
RRl	1.80	0.49	1.37
RRu	3.40	1.69	2.83
P	+++	N.S.	+++
Between Chi			7.93
Between df			1
Between P			**
Btwn(F) P			*
Btwn(R) P			**

Table 3J2 - 7

IESLC - Meta-analysis of Ex Smoking, Years quit (vs never), "Low"
Adenocarcinoma, Any Product (or Cigarettes if Any not available)
Excluded studies (and stage at which they were excluded)

1	AGUDO AKIBA AMANDU AMES AXELSS BEST BOUCHA BOUCOT BRESLO CHEN CHEN2 CHIAZZ DEAN2 DOSEME ENGELA FAN GENG GER GUO HAENSZ HEGMAN HOLE HU HU2 JUSSAW KATSOU KAUFMA KOO KOULUM KREUZE LETOUR LEVIN LIAW LIU3 LIU4 LIU5 MCCONN MIGRAN MRFITR NOTAN2 OSANN2 PERNU QIAO2 RACHTA RESTRE SADOWS SEGI2 STASZE TIZZAN VUTUC WATSON WU WUWILL WYNDE2 WYNDE8 XU YUAN ZHANG ZHENG ZHOU
2	BUFFLE HUMBLE PISANI PRESCO WYNDE7
3	MCDUFF SPITZ
4	ARMADA AUVINE BECHER BENSHL BLOT1 BOFFET BROSS CARPEN CEDERL CHOI CHYOU CORREA CPSI CPSII DAMBER DARBY DEAN3 DESTEF DOLL DOLL2 DORGAN DORN GAO GAO2 GARCIA GARSHI GILLIS GRAHAM GURSEL HAMMO2 HAMMON HIRAYA JOLY KAISE2 KHUDER LAUSSM LUBIN LUO PEZZO2 QIAO SPEIZE SUZUK2 TVERDA WANG2 WIGLE
5	ALDERS
10	SOBUE
14	BARBON BROWN3 WU2
15	BENHAM

Table 3J2 - 8
Potentially overlapping studies

REF	REFGP	PRINC	. OVERLAP/LINK
LUBIN2	LUBIN2	1	Lubin-combined
WYNDE6	WYNDE6	1	WYNDE5/6/7/8
JAHN	BOFFET	2	Subset of BOFFET

Table 3J2 - 9
Most adjusted - insufficient data for meta-analysis

REF	NRR	SEX	AGEL	AGEH	RACE	YF	LC TYPE	LOC	START	ST	NLC	R	VB	P	H	AD	PRODUCT	exL	exH	DENOM	De
ALDERS	567	m	0	0	all	-	not q+s	Eu:UK	1977	CC	1448	n	V	n	n	1	cig only	10	999	nev any	st
ALDERS	578	f	0	0	all	-	not q+s	Eu:UK	1977	CC	1448	n	V	n	n	1	cig only	10	999	nev any	st
WU2	501	f	0	0	all	-	a	NAmer	1983	CC	336	n	bl	n	y	2	all/unsp	10	999	nev any	ot

REF	NRR	RR	SIG	RRDATA comment
ALDERS	567	2.22		0
ALDERS	578	1.64		0
WU2	501	*	gap	0

Table 3J3 -

IESLC - Meta-analysis of Ex Smoking, Years quit (vs never), "Mid"
Adenocarcinoma, Any Product (or Cigarettes if Any not available)

This analysis is restricted to results for:
1) Ex smokers
2) Results by Years quit (vs never)
3) Categorical results by Years quit (vs never)
4) Adenocarcinoma (or near equivalent)
5) Results complete enough for use in metaanalysis

Within each study, results are then selected (in the following order of preference, within each sex) for:
6) (not applicable)
7) PRODUCT: all/unspec, cigarettes regardless of other products, cigarettes only
8) CIGTYPE: all/unspecified, MC regardless of HR, MC only
9) (not applicable)
10) DENOM: never smoked anything, never smoked cigarettes, never any + low, never cigs + low
11) Followup period (YF, prospective studies): whole study (coded as 0) or longest available
12) LCtype: adeno or nearest available, but not squamous. (q = squamous, s = small,
a = adeno, l = large, KII = Kreyberg II, al = alveolar, br = bronchiolar, u = undifferentiated)
13) Race: all or nearest available, otherwise by race (wh or w = white, bl or b = black, hi = hispanic
ch = chinese, jap = japanese, haw = hawaiian, w+o = white + oriental, sca = scandinavian, as = asian)
14) Years quit (vs never) "mid" in key scheme 1 (key value 7, maximum range 4-11)
15) For overlapping studies: principal rather than subsidiary studies
Finally by Age: whole study (coded as 0) if available, otherwise by widest available age group
and then for single sex results (m, f) in preference to results for both sexes combined (c).

Results adjusted (AD) for the most potential confounders are then chosen in Sections -1 to -3
and results adjusted for the least confounders in Sections -4 to -6. (Those least adjusted results which
actually differ from the most adjusted are marked 'x' in column X in Section -4)

Section -7 shows excluded studies, together with the stage (as above) at which no qualifying
results were found.

Section -8 lists the potentially overlapping studies which have been included (1=principal, 2=subsidiary).

Section -9 lists any results which would have been included in preference except that they had data not complete
enough for use in meta-analysis, with their significance (yes/no), if known, and any further comment as entered
on the database. It also lists as "gap" any categories for which no data were presented by the original authors.
This is commonly due to recent quitters having been combined with current smokers

In addition to those mentioned above, the following fields, levels and abbreviations are used:
* or nk = not known, n = no, y = yes, ot = other
nev = never
all/unspec = all or unspecified, cig+/-ot = cigarettes irrespective of other products (cigar, pipe etc)
MC = manufactured cigarettes, HR = hand-rolled cigarettes
exL, exH = range of exposure (low and high) in the smoking group, in terms of Years quit (vs never)
REF: 6-character study reference
NRR: number of the RR on the database within the study
ST : study type (CC = case control, pr or prosp = prospective)
NLC: number of lung cancer cases in whole study
R : risky occupational population (n = no, m = mining, o = other risky)
VB : national cigarette type (V = at least 75% Virginia, bl = at least 75% blended, ot = other)
P : any proxy use
H : full histological confirmation
De : derivation of RR/CI (or = original, st = standard method, ot = other method of estimation)

Table 3J3 - 1

IESLC - Meta-analysis of Ex Smoking, Years quit (vs never), "Mid"
Adenocarcinoma, Any Product (or Cigarettes if Any not available)
Most adjusted

REF	NRR	SEX	AGEL	AGEH	RACE	YF	LC TYPE	LOC	START	ST	NLC	R	VB	P	H	AD	PRODUCT	exL	exH	DENOM	De
JAHN	641	m	0	0	all	-	a	Eu:Ger	1988	CC	1004	n	bl	n	n	0	cig+/-ot	6	10	nev any	st
JEDRYC	560	m	0	0	all	-	a	Eu:est	1980	CC	1630	n	bl	y	n	0	cig+/-ot	5	9	nev any	st
LUBIN2	864	m	0	0	all	-	a	Eu:mul	1976	CC	7804	n	bl	n	y	0	cig+/-ot	5	9	nev any	st
MATOS	672	m	0	0	all	-	a	SCAmer	1994	CC	200	n	bl	n	n	2	cig+/-ot	6	10	nev any	ot
WAKAI	564	m	0	0	all	-	a	As:Jap	1988	CC	333	n	bl	n	y	1	cig+/-ot	5	9	nev any	or
WYNDE6	819	m	0	0	all	-	KII	NAmer	1969	CC	4423	n	bl	n	y	2	cig+/-ot	7	10	nev any	ot

Cigarette type is all/unspec for all RRs

Table 3J3 - 2

IESLC - Meta-analysis of Ex Smoking, Years quit (vs never), "Mid"
Adenocarcinoma, Any Product (or Cigarettes if Any not available)
Most adjusted

REF	NRR	SEX	AD	Number Exposed Case	Cont	Non-exposed Case	Cont	RR	95.00%CI
JAHN	641	m	0	13	63	8	138	3.56	(1.40- 9.02)
JEDRYC	560	m	0	9	82	7	289	4.53	(1.64- 12.54)
LUBIN2	864	m	0	50	882	57	2616	2.60	(1.77- 3.83)
MATOS	672	m	2	9	-	5	-	10.00	(3.08- 32.51)
WAKAI	564	m	1	7	-	8	-	1.23	(0.42- 3.64)
WYNDE6	819	m	2	18	-	11	-	6.60	(3.09- 14.10)
Partial Totals				106	1027	96	3043		

*prospective study

REF	NRR	SEX	AD	Ys	Ws	Qs	Ps
JAHN	641	m	0	1.27	4.44	0.03	0.0074
JEDRYC	560	m	0	1.51	3.71	0.38	0.0036
LUBIN2	864	m	0	0.96	25.60	1.41	0.0000
MATOS	672	m	2	2.30	2.77	3.42	0.0001
WAKAI	564	m	1	0.21	3.30	3.19	0.7071
WYNDE6	819	m	2	1.89	6.67	3.23	0.0000

	N	6
	NS	6
	Wt	46.48
Het	Chi	11.66
Het	df	5
Het	P	*
Fixed	RR	3.29
	RRl	2.47
	RRu	4.39
	P	+++
Random	RR	3.74
	RRl	2.23
	RRu	6.25
	P	+++
Asymm	P	N.S.

Table 3J3 - 3

IESLC - Meta-analysis of Ex Smoking, Years quit (vs never), "Mid"
Adenocarcinoma, Any Product (or Cigarettes if Any not available)
Most adjusted

	Sex: combined male	female	Total
N	6		6
NS	6		6
Wt	46.48		46.48
Het Chi	11.66		11.66
Het df	5		5
Het P	*		*
Fixed RR	3.29		3.29
RRl	2.47		2.47
RRu	4.39		4.39
P	+++		+++
Random RR	3.74		3.74
RRl	2.23		2.23
RRu	6.25		6.25
P	+++		+++
Between Chi			
Between df			
Between P			N.S.
Btwn(F) P			N.S.
Btwn(R) P			N.S.

Too few RRs for analysis by factor

Table 3J3 - 4

IESLC - Meta-analysis of Ex Smoking, Years quit (vs never), "Mid"
Adenocarcinoma, Any Product (or Cigarettes if Any not available)
Least adjusted

REF	NRR	X	SEX	AGEL	AGEH	RACE	YF	LC TYPE	LOC	START	ST	NLC	R	VB	P	H	AD	PRODUCT	exL	exH	DENOM	De
JAHN	641		m	0	0	all	-	a	Eu:Ger	1988	CC	1004	n	bl	n	n	0	cig+/-ot	6	10	nev any	st
JEDRYC	560		m	0	0	all	-	a	Eu:est	1980	CC	1630	n	bl	y	n	0	cig+/-ot	5	9	nev any	st
LUBIN2	864		m	0	0	all	-	a	Eu:mul	1976	CC	7804	n	bl	n	y	0	cig+/-ot	5	9	nev any	st
MATOS	662	x	m	0	0	all	-	a	SCAmer	1994	CC	200	n	bl	n	n	0	cig+/-ot	6	10	nev any	st
WAKAI	556	x	m	0	0	all	-	a	As:Jap	1988	CC	333	n	bl	n	y	0	cig+/-ot	5	9	nev any	st
WYNDE6	804	x	m	0	0	all	-	KII	NAmer	1969	CC	4423	n	bl	n	y	0	cig+/-ot	7	10	nev any	st

Cigarette type is all/unspec for all RRs

Table 3J3 - 5

IESLC - Meta-analysis of Ex Smoking, Years quit (vs never), "Mid"
Adenocarcinoma, Any Product (or Cigarettes if Any not available)
Least adjusted

REF	NRR	SEX	AD	Number Exposed Case	Cont	Non-exposed Case	Cont	RR	95.00%CI
JAHN	641	m	0	13	63	8	138	3.56	(1.40- 9.02)
JEDRYC	560	m	0	9	82	7	289	4.53	(1.64- 12.54)
LUBIN2	864	m	0	50	882	57	2616	2.60	(1.77- 3.83)
MATOS	662	m	0	9	27	5	110	7.33	(2.27- 23.66)
WAKAI	556	m	0	7	48	8	65	1.18	(0.40- 3.49)
WYNDE6	804	m	0	18	340	11	1667	8.02	(3.76- 17.14)
Totals				106	1442	96	4885		

*prospective study

REF	NRR	SEX	AD	Ys	Ws	Qs	Ps
JAHN	641	m	0	1.27	4.44	0.02	0.0074
JEDRYC	560	m	0	1.51	3.71	0.36	0.0036
LUBIN2	864	m	0	0.96	25.60	1.50	0.0000
MATOS	662	m	0	1.99	2.80	1.76	0.0009
WAKAI	556	m	0	0.17	3.29	3.48	0.7583
WYNDE6	804	m	0	2.08	6.67	5.21	0.0000

	N	6
	NS	6
	Wt	46.51
Het	Chi	12.34
Het	df	5
Het	P	*
Fixed	RR	3.32
	RRl	2.49
	RRu	4.42
	P	+++
Random	RR	3.72
	RRl	2.19
	RRu	6.32
	P	+++
Asymm	P	N.S.

Table 3J3 - 6

IESLC - Meta-analysis of Ex Smoking, Years quit (vs never), "Mid"
Adenocarcinoma, Any Product (or Cigarettes if Any not available)
Least adjusted

	Sex combined	male	female	Total
N		6		6
NS		6		6
Wt		46.51		46.51
Het Chi		12.34		12.34
Het df		5		5
Het P		*		*
Fixed RR		3.32		3.32
RRl		2.49		2.49
RRu		4.42		4.42
P		+++		+++
Random RR		3.72		3.72
RRl		2.19		2.19
RRu		6.32		6.32
P		+++		+++
Between Chi				
Between df				
Between P				N.S.
Btwn(F) P				N.S.
Btwn(R) P				N.S.

Table 3J3 - 7

IESLC - Meta-analysis of Ex Smoking, Years quit (vs never), "Mid"
Adenocarcinoma, Any Product (or Cigarettes if Any not available)
Excluded studies (and stage at which they were excluded)

1	AGUDO AKIBA AMANDU AMES AXELSS BEST BOUCHA BOUCOT BRESLO CHEN CHEN2 CHIAZZ DEAN2 DOSEME ENGELA FAN GENG GER GUO HAENSZ HEGMAN HOLE HU HU2 JUSSAW KATSOU KAUFMA KOO KOULUM KREUZE LETOUR LEVIN LIAW LIU3 LIU4 LIU5 MCCONN MIGRAN MRFITR NOTAN2 OSANN2 PERNU QIAO2 RACHTA RESTRE SADOWS SEGI2 STASZE TIZZAN VUTUC WATSON WU WUWILL WYNDE2 WYNDE8 XU YUAN ZHANG ZHENG ZHOU
2	BUFFLE HUMBLE PISANI PRESCO WYNDE7
3	MCDUFF SPITZ
4	ARMADA AUVINE BECHER BENSHL BLOT1 BOFFET BROSS CARPEN CEDERL CHOI CHYOU CORREA CPSI CPSII DAMBER DARBY DEAN3 DESTEF DOLL DOLL2 DORGAN DORN GAO GAO2 GARCIA GARSHI GILLIS GRAHAM GURSEL HAMMO2 HAMMON HIRAYA JOLY KAISE2 KHUDER LAUSSM LUBIN LUO PEZZO2 QIAO SPEIZE SUZUK2 TVERDA WANG2 WIGLE
5	ALDERS
10	SOBUE
14	BARBON BROWN3 JAIN PEZZOT SVENSS WU2 WYNDE3
15	BENHAM

Table 3J3 - 8
Potentially overlapping studies

REF	REFGP	PRINC	OVERLAP/LINK
LUBIN2	LUBIN2	1	Lubin-combined
WYNDE6	WYNDE6	1	WYNDE5/6/7/8
JAHN	BOFFET	2	Subset of BOFFET

Table 3J4 -

IESLC - Meta-analysis of Ex Smoking, Years quit (vs never), "High"
Adenocarcinoma, Any Product (or Cigarettes if Any not available)

This analysis is restricted to results for:
1) Ex smokers
2) Results by Years quit (vs never)
3) Categorical results by Years quit (vs never)
4) Adenocarcinoma (or near equivalent)
5) Results complete enough for use in metaanalysis

Within each study, results are then selected (in the following order of preference, within each sex) for:
6) PRODUCT: all/unspec, cigarettes regardless of other products, cigarettes only
7) CIGTYPE: all/unspecified, MC regardless of HR, MC only
8) (not applicable)
9) DENOM: never smoked anything, never smoked cigarettes, never any + low, never cigs + low
10) Followup period (YF, prospective studies): whole study (coded as 0) or longest available
11) LCtype: adeno or nearest available, but not squamous. (q = squamous, s = small,
a = adeno, l = large, KII = Kreyberg II, al = alveolar, br = bronchiolar, u = undifferentiated)
12) Race: all or nearest available, otherwise by race (wh or w = white, bl or b = black, hi = hispanic
ch = chinese, jap = japanese, haw = hawaiian, w+o = white + oriental, sca = scandinavian, as = asian)
13) Years quit (vs never) "high" in key scheme 1 (key value 3, maximum range 1-6)
14) For overlapping studies: principal rather than subsidiary studies
Finally by Age: whole study (coded as 0) if available, otherwise by widest available age group
and then for single sex results (m, f) in preference to results for both sexes combined (c).

Results adjusted (AD) for the most potential confounders are then chosen in Sections -1 to -3
and results adjusted for the least confounders in Sections -4 to -6. (Those least adjusted results which
actually differ from the most adjusted are marked 'x' in column X in Section -4)

Section -7 shows excluded studies, together with the stage (as above) at which no qualifying
results were found.

Section -8 lists the potentially overlapping studies which have been included (1=principal, 2=subsidiary).

Section -9 lists any results which would have been included in preference except that they had data not complete
enough for use in meta-analysis, with their significance (yes/no), if known, and any further comment as entered
on the database. It also lists as "gap" any categories for which no data were presented by the original authors.
This is commonly due to recent quitters having been combined with current smokers

In addition to those mentioned above, the following fields, levels and abbreviations are used:
* or nk = not known, n = no, y = yes, ot = other
nev = never
all/unspec = all or unspecified, cig+/-ot = cigarettes irrespective of other products (cigar, pipe etc)
MC = manufactured cigarettes, HR = hand-rolled cigarettes
exL, exH = range of exposure (low and high) in the smoking group, in terms of Years quit (vs never)
REF: 6-character study reference
NRR: number of the RR on the database within the study
ST : study type (CC = case control, pr or prosp = prospective)
NLC: number of lung cancer cases in whole study
R : risky occupational population (n = no, m = mining, o = other risky)
VB : national cigarette type (V = at least 75% Virginia, bl = at least 75% blended, ot = other)
P : any proxy use
H : full histological confirmation
De : derivation of RR/CI (or = original, st = standard method, ot = other method of estimation)

Table 3J4 - 1

IESLC - Meta-analysis of Ex Smoking, Years quit (vs never), "High"
Adenocarcinoma, Any Product (or Cigarettes if Any not available)
Most adjusted

REF	NRR	SEX	AGEL	AGEH	RACE	YF	LC TYPE	LOC	START	ST	NLC	R	VB	P	H	AD	PRODUCT	exL	exH	DENOM	De
BARBON	759	m	0	0	all	-	a	Eu:wst	1979	CC	755	n	bl	y	y	1	all/unsp	0.1	4	nev any	or
JAHN	642	m	0	0	all	-	a	Eu:Ger	1988	CC	1004	n	bl	n	n	0	cig+/-ot	2	5	nev any	st
LUBIN2	865	m	0	0	all	-	a	Eu:mul	1976	CC	7804	n	bl	n	y	0	cig+/-ot	0.1	4	nev any	st
MATOS	673	m	0	0	all	-	a	SCAmer	1994	CC	200	n	bl	n	n	2	cig+/-ot	1.0	5	nev any	ot
WYNDE3	521	m	0	0	all	-	KII	NAmer	1966	CC	350	n	bl	n	y	0	all/unsp	1.0	3	nev any	st
WYNDE6	821	m	0	0	all	-	KII	NAmer	1969	CC	4423	n	bl	n	y	2	cig+/-ot	1.0	3	nev any	ot

Cigarette type is all/unspec for all RRs

Table 3J4 - 2

IESLC - Meta-analysis of Ex Smoking, Years quit (vs never), "High"
Adenocarcinoma, Any Product (or Cigarettes if Any not available)
Most adjusted

REF	NRR	SEX	AD	Number Exposed Case	Exposed Cont	Non-exposed Case	Non-exposed Cont	RR	95.00%CI
BARBON	759	m	1	7	-	7	-	9.40	(3.00- 29.70)
JAHN	642	m	0	19	46	8	138	7.13	(2.92- 17.37)
LUBIN2	865	m	0	77	1047	57	2616	3.38	(2.38- 4.79)
MATOS	673	m	2	12	-	5	-	13.00	(4.21- 40.17)
WYNDE3	521	m	0	3	22	6	88	2.00	(0.46- 8.63)
WYNDE6	821	m	2	29	-	11	-	14.20	(7.02- 28.73)
Partial Totals				147	1115	94	2842		

*prospective study

REF	NRR	SEX	AD	Ys	Ws	Qs	Ps
BARBON	759	m	1	2.24	2.92	1.13	0.0001
JAHN	642	m	0	1.96	4.84	0.57	0.0000
LUBIN2	865	m	0	1.22	31.38	5.11	0.0000
MATOS	673	m	2	2.56	3.02	2.70	0.0000
WYNDE3	521	m	0	0.69	1.80	1.54	0.3529
WYNDE6	821	m	2	2.65	7.74	8.26	0.0000

	N	6
	NS	6
	Wt	51.70
Het	Chi	19.31
Het	df	5
Het	P	**
Fixed	RR	5.05
	RRl	3.85
	RRu	6.64
	P	+++
Random	RR	6.73
	RRl	3.46
	RRu	13.12
	P	+++
Asymm	P	N.S.

Table 3J4 - 3

IESLC - Meta-analysis of Ex Smoking, Years quit (vs never), "High"
Adenocarcinoma, Any Product (or Cigarettes if Any not available)
Most adjusted

	Sex			
	combined	male	female	Total
N		6		6
NS		6		6
Wt		51.70		51.70
Het Chi		19.31		19.31
Het df		5		5
Het P		**		**
Fixed RR		5.05		5.05
RRl		3.85		3.85
RRu		6.64		6.64
P		+++		+++
Random RR		6.73		6.73
RRl		3.46		3.46
RRu		13.12		13.12
P		+++		+++
Between Chi				
Between df				
Between P				N.S.
Btwn(F) P				N.S.
Btwn(R) P				N.S.

Too few RRs for analysis by factor

Table 3J4 - 4

IESLC - Meta-analysis of Ex Smoking, Years quit (vs never), "High"
Adenocarcinoma, Any Product (or Cigarettes if Any not available)
Least adjusted

REF	NRR	X	SEX	AGEL	AGEH	RACE	YF	LC TYPE	LOC	START	ST	NLC	R	VB	P	H	AD	PRODUCT	exL	exH	DENOM	De
BARBON	744	x	m	0	0	all	-	a	Eu:wst	1979	CC	755	n	bl	y	y	0	all/unsp	0.1	4	nev any	st
JAHN	642		m	0	0	all	-	a	Eu:Ger	1988	CC	1004	n	bl	n	n	0	cig+/-ot	2	5	nev any	st
LUBIN2	865		m	0	0	all	-	a	Eu:mul	1976	CC	7804	n	bl	n	y	0	cig+/-ot	0.1	4	nev any	st
MATOS	663	x	m	0	0	all	-	a	SCAmer	1994	CC	200	n	bl	n	n	0	cig+/-ot	1.0	5	nev any	st
WYNDE3	521		m	0	0	all	-	KII	NAmer	1966	CC	350	n	bl	n	y	0	all/unsp	1.0	3	nev any	st
WYNDE6	806	x	m	0	0	all	-	KII	NAmer	1969	CC	4423	n	bl	n	y	0	cig+/-ot	1.0	3	nev any	st

Cigarette type is all/unspec for all RRs

Table 3J4 - 5

IESLC - Meta-analysis of Ex Smoking, Years quit (vs never), "High"
Adenocarcinoma, Any Product (or Cigarettes if Any not available)
Least adjusted

REF	NRR	SEX	AD	Number Exposed Case	Cont	Non-exposed Case	Cont	RR	95.00%CI
BARBON	744	m	0	7	20	7	188	9.40	(2.99- 29.53)
JAHN	642	m	0	19	46	8	138	7.13	(2.92- 17.37)
LUBIN2	865	m	0	77	1047	57	2616	3.38	(2.38- 4.79)
MATOS	663	m	0	12	23	5	110	11.48	(3.69- 35.74)
WYNDE3	521	m	0	3	22	6	88	2.00	(0.46- 8.63)
WYNDE6	806	m	0	29	307	11	1667	14.32	(7.08- 28.96)
Totals				147	1465	94	4807		

*prospective study

REF	NRR	SEX	AD	Ys	Ws	Qs	Ps
BARBON	744	m	0	2.24	2.93	1.15	0.0001
JAHN	642	m	0	1.96	4.84	0.59	0.0000
LUBIN2	865	m	0	1.22	31.38	4.94	0.0000
MATOS	663	m	0	2.44	2.98	2.04	0.0000
WYNDE3	521	m	0	0.69	1.80	1.52	0.3529
WYNDE6	806	m	0	2.66	7.74	8.50	0.0000

	N	6
	NS	6
	Wt	51.66
Het	Chi	18.75
Het	df	5
Het	P	**
Fixed	RR	5.02
	RRl	3.82
	RRu	6.59
	P	+++
Random	RR	6.62
	RRl	3.43
	RRu	12.79
	P	+++
Asymm	P	N.S.

Table 3J4 - 6

IESLC - Meta-analysis of Ex Smoking, Years quit (vs never), "High"
Adenocarcinoma, Any Product (or Cigarettes if Any not available)
Least adjusted

	Sex combined	male	female	Total
N		6		6
NS		6		6
Wt		51.66		51.66
Het Chi		18.75		18.75
Het df		5		5
Het P		**		**
Fixed RR		5.02		5.02
RRl		3.82		3.82
RRu		6.59		6.59
P		+++		+++
Random RR		6.62		6.62
RRl		3.43		3.43
RRu		12.79		12.79
P		+++		+++
Between Chi				
Between df				
Between P				N.S.
Btwn(F) P				N.S.
Btwn(R) P				N.S.

Table 3J4 - 7

IESLC - Meta-analysis of Ex Smoking, Years quit (vs never), "High"
Adenocarcinoma, Any Product (or Cigarettes if Any not available)
Excluded studies (and stage at which they were excluded)

1	AGUDO AKIBA AMANDU AMES AXELSS BEST BOUCHA BOUCOT BRESLO CHEN CHEN2 CHIAZZ DEAN2 DOSEME ENGELA FAN GENG GER GUO HAENSZ HEGMAN HOLE HU HU2 JUSSAW KATSOU KAUFMA KOO KOULUM KREUZE LETOUR LEVIN LIAW LIU3 LIU4 LIU5 MCCONN MIGRAN MRFITR NOTAN2 OSANN2 PERNU QIAO2 RACHTA RESTRE SADOWS SEGI2 STASZE TIZZAN VUTUC WATSON WU WUWILL WYNDE2 WYNDE8 XU YUAN ZHANG ZHENG ZHOU
2	BUFFLE HUMBLE PISANI PRESCO WYNDE7
3	MCDUFF SPITZ
4	ARMADA AUVINE BECHER BENSHL BLOT1 BOFFET BROSS CARPEN CEDERL CHOI CHYOU CORREA CPSI CPSII DAMBER DARBY DEAN3 DESTEF DOLL DOLL2 DORGAN DORN GAO GAO2 GARCIA GARSHI GILLIS GRAHAM GURSEL HAMMO2 HAMMON HIRAYA JOLY KAISE2 KHUDER LAUSSM LUBIN LUO PEZZO2 QIAO SPEIZE SUZUK2 TVERDA WANG2 WIGLE
5	ALDERS
10	SOBUE
14	BROWN3 JAIN JEDRYC PEZZOT SVENSS WAKAI WU2
15	BENHAM

Table 3J4 - 8
Potentially overlapping studies

REF	REFGP	PRINC	OVERLAP/LINK
LUBIN2	LUBIN2	1	Lubin-combined
WYNDE6	WYNDE6	1	WYNDE5/6/7/8
JAHN	BOFFET	2	Subset of BOFFET

Table 3J4 - 9
Most adjusted - insufficient data for meta-analysis

REF	NRR	SEX	AGEL	AGEH	RACE	YF	LC TYPE	LOC	START	ST	NLC	R	VB	P	H	AD	PRODUCT	exL	exH	DENOM	De
JEDRYC	561	m	0	0	all	-	a	Eu:est	1980	CC	1630	n	bl	y	n	0	cig+/-ot	1.0	4	nev any	ot
WAKAI	619	m	0	0	all	-	a	As:Jap	1988	CC	333	n	bl	n	y	1	cig+/-ot	1.0	4	nev any	ot

REF	NRR	RR	SIG	RRDATA comment
JEDRYC	561	*	gap	0
WAKAI	619	*	gap	0

Least adjusted - insufficient data for meta-analysis: as for adjusted plus the following

REF	NRR	SEX	AGEL	AGEH	RACE	YF	LC TYPE	LOC	START	ST	NLC	R	VB	P	H	AD	PRODUCT	exL	exH	DENOM	De
WAKAI	617	m	0	0	all	-	a	As:Jap	1988	CC	333	n	bl	n	y	0	cig+/-ot	1.0	4	nev any	ot

REF	NRR	RR	SIG	RRDATA comment
WAKAI	617	*	gap	0

Table 3J5 -

IESLC - Meta-analysis of Ex Smoking, Years quit (vs never), "Highest vs lowest"
Adenocarcinoma, Any Product (or Cigarettes if Any not available)

This analysis is restricted to results for:
1) Ex smokers
2) Results by Years quit (vs never)
3) Categorical results by Years quit (vs never)
4) Denominator (unexposed) = "low"
5) Adenocarcinoma (or near equivalent)
6) Results complete enough for use in metaanalysis

Within each study, results are then selected (in the following order of preference, within each sex) for:
7) (not applicable)
8) PRODUCT: all/unspec, cigarettes regardless of other products, cigarettes only
9) CIGTYPE: all/unspecified, MC regardless of HR, MC only
10) Results with least adjustment for other aspects of smoking (ADOS)
11) The highest vs lowest category
12) Followup period (YF, prospective studies): whole study (coded as 0) or longest available
13) LCtype: adeno or nearest available, but not squamous. (q = squamous, s = small,
a = adeno, l = large, KII = Kreyberg II, al = alveolar, br = bronchiolar, u = undifferentiated)
14) Race: all or nearest available, otherwise by race (wh or w = white, bl or b = black, hi = hispanic
ch = chinese, jap = japanese, haw = hawaiian, w+o = white + oriental, sca = scandinavian, as = asian)
15) For overlapping studies: principal rather than subsidiary studies
Finally by Age: whole study (coded as 0) if available, otherwise by widest available age group
and then for single sex results (m, f) in preference to results for both sexes combined (c).

Results adjusted (AD) for the most potential confounders are then chosen in Sections -1 to -3
and results adjusted for the least confounders in Sections -4 to -6. (Those least adjusted results which
actually differ from the most adjusted are marked 'x' in column X in Section -4)

Section -7 shows excluded studies, together with the stage (as above) at which no qualifying
results were found.

Section -8 lists the potentially overlapping studies which have been included (1=principal, 2=subsidiary).

Section -9 lists any results which would have been included in preference except that they had data not complete
enough for use in meta-analysis, with their significance (yes/no), if known, and any further comment as entered
on the database. It also lists as "gap" any categories for which no data were presented by the original authors.
This is commonly due to recent quitters having been combined with current smokers

In addition to those mentioned above, the following fields, levels and abbreviations are used:
* or nk = not known, n = no, y = yes, ot = other
all/unspec = all or unspecified, cig+/-ot = cigarettes irrespective of other products (cigar, pipe etc)
MC = manufactured cigarettes, HR = hand-rolled cigarettes
exL, exH = range of exposure (low and high) in the "highest" group, in terms of Years quit (vs never)
unexL, unexH = range of exposure (low and high) in the "lowest" group, in terms of Years quit (vs never)
REF: 6-character study reference
NRR: number of the RR on the database within the study
ST : study type (CC = case control, pr or prosp = prospective)
NLC: number of lung cancer cases in whole study
R : risky occupational population (n = no, m = mining, o = other risky)
VB : national cigarette type (V = at least 75% Virginia, bl = at least 75% blended, ot = other)
P : any proxy use
H : full histological confirmation
De : derivation of RR/CI (or = original, st = standard method, ot = other method of estimation)

Table 3J5 - 1

IESLC - Meta-analysis of Ex Smoking, Years quit (vs never), "Highest vs lowest"
Adenocarcinoma, Any Product (or Cigarettes if Any not available)
Most adjusted

REF	NRR	SEX	AGEL	AGEH	RACE	YF	LC TYPE	LOC	START	ST	NLC	R	VB	P	H	AD	ADOS	PRODUCT	exL	exH	unexL	unexH	De
BARBON	763	m	0	0	all	-	a	Eu:wst	1979	CC	755	n	bl	y	y	1	0	all/unsp	0.1	4	25	999	ot
JAHN	650	m	0	0	all	-	a	Eu:Ger	1988	CC	1004	n	bl	n	n	0	0	cig+/-ot	0.1	0.9	21	999	st
JAIN	539	m	0	0	all	-	a	NAmer	1981	CC	845	n	V	y	n	0	0	cig+/-ot	2	9	10	999	st
JAIN	503	f	0	0	all	-	a	NAmer	1981	CC	845	n	V	y	n	0	0	cig+/-ot	2	9	10	999	st
JEDRYC	563	m	0	0	all	-	a	Eu:est	1980	CC	1630	n	bl	y	n	0	0	cig+/-ot	5	9	10	999	st
LUBIN2	870	m	0	0	all	-	a	Eu:mul	1976	CC	7804	n	bl	n	y	0	0	cig+/-ot	0.1	4	20	999	st
LUBIN2	970	f	0	0	all	-	a	Eu:mul	1976	CC	7804	n	bl	n	y	0	0	cig+/-ot	0.1	9	20	999	st
MATOS	675	m	0	0	all	-	a	SCAmer	1994	CC	200	n	bl	n	n	2	0	cig+/-ot	1.0	5	11	999	ot
PEZZOT	587	m	0	0	all	-	a	SCAmer	1987	CC	215	n	bl	n	y	0	0	cig only	1.0	10	11	999	st
SOBUE	785	m	0	0	all	-	a	As:Jap	1986	CC	1376	n	bl	n	y	0	0	cig+/-ot	1.0	4	10	999	st
SVENSS	571	f	0	0	all	-	a	Eu:Sca	1983	CC	210	n	bl	n	n	0	0	all/unsp	3	10	11	999	st
WAKAI	566	m	0	0	all	-	a	As:Jap	1988	CC	333	n	bl	n	y	1	0	cig+/-ot	5	9	20	999	ot
WYNDE3	526	m	0	0	all	-	KII	NAmer	1966	CC	350	n	bl	n	y	0	0	all/unsp	1.0	3	13	999	st
WYNDE6	826	m	0	0	all	-	KII	NAmer	1969	CC	4423	n	bl	n	y	2	0	cig+/-ot	1.0	3	16	999	ot

Cigarette type is all/unspec for all RRs

Table 3J5 - 2

IESLC - Meta-analysis of Ex Smoking, Years quit (vs never), "Highest vs lowest"
Adenocarcinoma, Any Product (or Cigarettes if Any not available)
Most adjusted

REF	NRR	SEX	AD	Number Exposed Case	Cont	Non-exposed Case	Cont	RR	95.00%CI
BARBON	763	m	1	7	-	4	-	5.22	(1.37- 19.83)
JAHN	650	m	0	40	8	15	146	48.67	(19.27- 122.93)
JAIN	539	m	0	16	46	14	113	2.81	(1.27- 6.22)
JAIN	503	f	0	14	36	3	61	7.91	(2.13- 29.40)
Subtotal JAIN								3.71	(1.88- 7.32)
JEDRYC	563	m	0	9	82	12	230	2.10	(0.86- 5.18)
LUBIN2	870	m	0	77	1047	35	1128	2.37	(1.58- 3.57)
LUBIN2	970	f	0	13	95	1	29	3.97	(0.50- 31.64)
Subtotal LUBIN2								2.42	(1.62- 3.61)
MATOS	675	m	2	12	-	12	-	4.33	(1.71- 10.97)
PEZZOT	587	m	0	11	21	7	31	2.32	(0.77- 6.95)
SOBUE	785	m	0	44	116	49	144	1.11	(0.69- 1.79)
SVENSS	571	f	0	5	13	7	24	1.32	(0.35- 4.99)
WAKAI	566	m	1	7	-	3	-	2.28	(0.56- 9.20)
WYNDE3	526	m	0	3	22	3	55	2.50	(0.47- 13.35)
WYNDE6	826	m	2	29	-	6	-	2.84	(1.17- 6.92)
Partial Totals				287	1486	171	1961		

*prospective study

REF	NRR	SEX	AD	Ys	Ws	Qs	Ps
BARBON	763	m	1	1.65	2.15	1.01	0.0154
JAHN	650	m	0	3.88	4.47	38.09	0.0000
JAIN	539	m	0	1.03	6.08	0.03	0.0109
JAIN	503	f	0	2.07	2.23	2.70	0.0020
Subtotal JAIN				1.31	8.31	2.72	
JEDRYC	563	m	0	0.74	4.74	0.24	0.1054
LUBIN2	870	m	0	0.86	23.04	0.25	0.0000
LUBIN2	970	f	0	1.38	0.89	0.15	0.1932
Subtotal LUBIN2				0.88	23.93	0.40	
MATOS	675	m	2	1.47	4.45	1.10	0.0020
PEZZOT	587	m	0	0.84	3.19	0.05	0.1330
SOBUE	785	m	0	0.11	17.04	12.56	0.6540
SVENSS	571	f	0	0.28	2.17	1.03	0.6838
WAKAI	566	m	1	0.82	1.96	0.04	0.2484
WYNDE3	526	m	0	0.92	1.37	0.00	0.2836
WYNDE6	826	m	2	1.04	4.86	0.03	0.0213

	N	14
	NS	12
	Wt	78.64
Het	Chi	57.28
Het	df	13
Het	P	***
Fixed	RR	2.63
	RRl	2.11
	RRu	3.28
	P	+++
Random	RR	3.32
	RRl	1.98
	RRu	5.58
	P	+++
Asymm	P	N.S.

Table 3J5 - 3

IESLC - Meta-analysis of Ex Smoking, Years quit (vs never), "Highest vs lowest"
Adenocarcinoma, Any Product (or Cigarettes if Any not available)
Most adjusted

	Sex combined	Sex male	Sex female	Total
N		11	3	14
NS		11	3	14
Wt		73.35	5.29	78.64
Het Chi		53.37	3.55	57.28
Het df		10	2	13
Het P		***	N.S.	***
Fixed RR		2.58	3.38	2.63
RRl		2.06	1.44	2.11
RRu		3.25	7.92	3.28
P		+++	++	+++
Random RR		3.31	3.40	3.32
RRl		1.85	1.05	1.98
RRu		5.93	10.98	5.58
P		+++	+	+++
Between Chi				0.35
Between df				1
Between P				N.S.
Btwn(F) P				N.S.
Btwn(R) P				N.S.

	Lung cancer type a	a+l	a+al+br	KII	not q+u	not q+s	Total
N	12			2			14
NS	10			2			12
Wt	72.40			6.23			78.64
Het Chi	57.25			0.02			57.28
Het df	11			1			13
Het P	***			N.S.			***
Fixed RR	2.62			2.76			2.63
RRl	2.08			1.26			2.11
RRu	3.30			6.05			3.28
P	+++			+			+++
Random RR	3.44			2.76			3.32
RRl	1.91			1.26			1.98
RRu	6.19			6.05			5.58
P	+++			+			+++
Between Chi							0.02
Between df							1
Between P							N.S.
Btwn(F) P							N.S.
Btwn(R) P							N.S.

	Location NAmer	UK	Scand	othEur	China	Japan	othAs	other	Total
N	4		1	5		2		2	14
NS	3		1	4		2		2	12
Wt	14.54		2.17	35.30		19.00		7.64	78.64
Het Chi	2.07		0.00	36.03		0.90		0.72	57.28
Het df	3		0	4		1		1	13
Het P	N.S.		N.S.	***		N.S.		N.S.	***
Fixed RR	3.27		1.32	3.64		1.20		3.34	2.63
RRl	1.95		0.35	2.61		0.77		1.64	2.11
RRu	5.46		4.99	5.06		1.88		6.78	3.28
P	+++		N.S.	+++		N.S.		+++	+++
Random RR	3.27		1.32	5.52		1.20		3.34	3.32
RRl	1.95		0.35	1.57		0.77		1.64	1.98
RRu	5.46		4.99	19.35		1.88		6.78	5.58
P	+++		N.S.	++		N.S.		+++	+++
Between Chi									17.55
Between df									4
Between P									**
Btwn(F) P									N.S.
Btwn(R) P									*

Table 3J5 - 3

IESLC - Meta-analysis of Ex Smoking, Years quit (vs never), "Highest vs lowest"
Adenocarcinoma, Any Product (or Cigarettes if Any not available)
Most adjusted

Detailed Country in "other Europe"

	multi	Germany	othWest	East	Balkans	Total
N	2	1	1	1		5
NS	1	1	1	1		4
Wt	23.93	4.47	2.15	4.74		35.30
Het Chi	0.23	0.00	0.00	0.00		36.03
Het df	1	0	0	0		4
Het P	N.S.	N.S.	N.S.	N.S.		***
Fixed RR	2.42	48.67	5.22	2.10		3.64
RRl	1.62	19.27	1.37	0.86		2.61
RRu	3.61	122.93	19.86	5.18		5.06
P	+++	+++	+	N.S.		+++
Random RR	2.42	48.67	5.22	2.10		5.52
RRl	1.62	19.27	1.37	0.86		1.57
RRu	3.61	122.93	19.86	5.18		19.35
P	+++	+++	+	N.S.		++
Between Chi						35.81
Between df						3
Between P						***
Btwn(F) P						N.S.
Btwn(R) P						***

Detailed Country in "other Asia"

	India	HongKong	other	Total
N				
NS				
Wt				
Het Chi				
Het df				
Het P				N.S.
Fixed RR				
RRl				
RRu				
P				N.S.
Random RR				
RRl				
RRu				
P				N.S.
Between Chi				
Between df				
Between P				N.S.
Btwn(F) P				N.S.
Btwn(R) P				N.S.

Detailed other continent

	SCAmer	Total
N	2	2
NS	2	2
Wt	7.64	7.64
Het Chi	0.72	0.72
Het df	1	1
Het P	N.S.	N.S.
Fixed RR	3.34	3.34
RRl	1.64	1.64
RRu	6.78	6.78
P	+++	+++
Random RR	3.34	3.34
RRl	1.64	1.64
RRu	6.78	6.78
P	+++	+++
Between Chi		
Between df		
Between P		N.S.
Btwn(F) P		N.S.
Btwn(R) P		N.S.

Table 3J5 - 3

IESLC - Meta-analysis of Ex Smoking, Years quit (vs never), "Highest vs lowest"
Adenocarcinoma, Any Product (or Cigarettes if Any not available)
Most adjusted

	<1960	Start year of study 1960-69	1970-79	1980-89	1990+	Total
N		2	3	8	1	14
NS		2	2	7	1	12
Wt		6.23	26.08	41.87	4.45	78.64
Het Chi		0.02	1.40	54.64	0.00	57.28
Het df		1	2	7	0	13
Het P		N.S.	N.S.	***	N.S.	***
Fixed RR		2.76	2.57	2.51	4.33	2.63
RRl		1.26	1.75	1.85	1.71	2.11
RRu		6.05	3.78	3.40	10.97	3.28
P		+	+++	+++	++	+++
Random RR		2.76	2.57	3.38	4.33	3.32
RRl		1.26	1.75	1.35	1.71	1.98
RRu		6.05	3.78	8.46	10.97	5.58
P		+	+++	++	++	+++
Between Chi						1.22
Between df						3
Between P						N.S.
Btwn(F) P						N.S.
Btwn(R) P						N.S.

	Study type (1) CC	other	Total
N	14		14
NS	12		12
Wt	78.64		78.64
Het Chi	57.28		57.28
Het df	13		13
Het P	***		***
Fixed RR	2.63		2.63
RRl	2.11		2.11
RRu	3.28		3.28
P	+++		+++
Random RR	3.32		3.32
RRl	1.98		1.98
RRu	5.58		5.58
P	+++		+++
Between Chi			
Between df			
Between P			N.S.
Btwn(F) P			N.S.
Btwn(R) P			N.S.

	Study type (2) CC	prosp	other	Total
N	14			14
NS	12			12
Wt	78.64			78.64
Het Chi	57.28			57.28
Het df	13			13
Het P	***			***
Fixed RR	2.63			2.63
RRl	2.11			2.11
RRu	3.28			3.28
P	+++			+++
Random RR	3.32			3.32
RRl	1.98			1.98
RRu	5.58			5.58
P	+++			+++
Between Chi				
Between df				
Between P				N.S.
Btwn(F) P				N.S.
Btwn(R) P				N.S.

Table 3J5 - 3

IESLC - Meta-analysis of Ex Smoking, Years quit (vs never), "Highest vs lowest"
Adenocarcinoma, Any Product (or Cigarettes if Any not available)
Most adjusted

	Study size (number of LC cases)				
	100-249	250-499	500-999	1000+	Total
N	3	2	3	6	14
NS	3	2	2	5	12
Wt	9.80	3.33	10.46	55.05	78.64
Het Chi	2.18	0.01	1.95	50.98	57.28
Het df	2	1	2	5	13
Het P	N.S.	N.S.	N.S.	***	***
Fixed RR	2.72	2.37	3.98	2.43	2.63
RRl	1.45	0.81	2.17	1.87	2.11
RRu	5.08	6.93	7.29	3.17	3.28
P	++	N.S.	+++	+++	+++
Random RR	2.69	2.37	3.98	3.65	3.32
RRl	1.39	0.81	2.17	1.40	1.98
RRu	5.18	6.93	7.29	9.48	5.58
P	++	N.S.	+++	++	+++
Between Chi					2.17
Between df					3
Between P					N.S.
Btwn(F) P					N.S.
Btwn(R) P					N.S.

	Risky occupational population			
	no	mining	othRisky	Total
N	14			14
NS	12			12
Wt	78.64			78.64
Het Chi	57.28			57.28
Het df	13			13
Het P	***			***
Fixed RR	2.63			2.63
RRl	2.11			2.11
RRu	3.28			3.28
P	+++			+++
Random RR	3.32			3.32
RRl	1.98			1.98
RRu	5.58			5.58
P	+++			+++
Between Chi				
Between df				
Between P				N.S.
Btwn(F) P				N.S.
Btwn(R) P				N.S.

	National cigarette tobacco type			
	Virginia	blended	other	Total
N	2	12		14
NS	1	11		12
Wt	8.31	70.33		78.64
Het Chi	1.75	54.44		57.28
Het df	1	11		13
Het P	N.S.	***		***
Fixed RR	3.71	2.53		2.63
RRl	1.88	2.00		2.11
RRu	7.32	3.19		3.28
P	+++	+++		+++
Random RR	4.11	3.18		3.32
RRl	1.54	1.76		1.98
RRu	10.93	5.72		5.58
P	++	+++		+++
Between Chi				1.09
Between df				1
Between P				N.S.
Btwn(F) P				N.S.
Btwn(R) P				N.S.

Table 3J5 - 3

IESLC - Meta-analysis of Ex Smoking, Years quit (vs never), "Highest vs lowest"
Adenocarcinoma, Any Product (or Cigarettes if Any not available)
Most adjusted

	Any proxy use No/nk	Yes	Total
N	10	4	14
NS	9	3	12
Wt	63.44	15.20	78.64
Het Chi	53.14	3.27	57.28
Het df	9	3	13
Het P	***	N.S.	***
Fixed RR	2.50	3.26	2.63
RRl	1.95	1.97	2.11
RRu	3.20	5.39	3.28
P	+++	+++	+++
Random RR	3.19	3.31	3.32
RRl	1.61	1.94	1.98
RRu	6.32	5.62	5.58
P	+++	+++	+++
Between Chi			0.87
Between df			1
Between P			N.S.
Btwn(F) P			N.S.
Btwn(R) P			N.S.

	Full histological confirmation No	Yes	Total
N	6	8	14
NS	5	7	12
Wt	24.13	54.50	78.64
Het Chi	33.15	9.65	57.28
Het df	5	7	13
Het P	***	N.S.	***
Fixed RR	5.01	1.98	2.63
RRl	3.36	1.52	2.11
RRu	7.47	2.58	3.28
P	+++	+++	+++
Random RR	4.91	2.10	3.32
RRl	1.72	1.46	1.98
RRu	14.01	3.03	5.58
P	++	+++	+++
Between Chi			14.48
Between df			1
Between P			***
Btwn(F) P			(*)
Btwn(R) P			N.S.

	Number of adjustment variables (1) 0	1	2+/+nk	Total
N	10	2	2	14
NS	8	2	2	12
Wt	65.21	4.11	9.31	78.64
Het Chi	54.88	0.70	0.41	57.28
Het df	9	1	1	13
Het P	***	N.S.	N.S.	***
Fixed RR	2.48	3.52	3.47	2.63
RRl	1.95	1.34	1.83	2.11
RRu	3.16	9.24	6.60	3.28
P	+++	+	+++	+++
Random RR	3.28	3.52	3.47	3.32
RRl	1.66	1.34	1.83	1.98
RRu	6.50	9.24	6.60	5.58
P	+++	+	+++	+++
Between Chi				1.29
Between df				2
Between P				N.S.
Btwn(F) P				N.S.
Btwn(R) P				N.S.

Table 3J5 - 3

IESLC - Meta-analysis of Ex Smoking, Years quit (vs never), "Highest vs lowest"
Adenocarcinoma, Any Product (or Cigarettes if Any not available)
Most adjusted

	Number of adjustment variables (2) 0	1	2	3-5	6+/+nk	Total
N	10	2	2			14
NS	8	2	2			12
Wt	65.21	4.11	9.31			78.64
Het Chi	54.88	0.70	0.41			57.28
Het df	9	1	1			13
Het P	***	N.S.	N.S.			***
Fixed RR	2.48	3.52	3.47			2.63
RRl	1.95	1.34	1.83			2.11
RRu	3.16	9.24	6.60			3.28
P	+++	+	+++			+++
Random RR	3.28	3.52	3.47			3.32
RRl	1.66	1.34	1.83			1.98
RRu	6.50	9.24	6.60			5.58
P	+++	+	+++			+++
Between Chi						1.29
Between df						2
Between P						N.S.
Btwn(F) P						N.S.
Btwn(R) P						N.S.

	Product all/unsp	cig+/-ot	cig only	Total
N	3	10	1	14
NS	3	8	1	12
Wt	5.69	69.76	3.19	78.64
Het Chi	2.05	55.18	0.00	57.28
Het df	2	9	0	13
Het P	N.S.	***	N.S.	***
Fixed RR	2.59	2.65	2.32	2.63
RRl	1.14	2.09	0.77	2.11
RRu	5.89	3.35	6.95	3.28
P	+	+++	N.S.	+++
Random RR	2.59	3.67	2.32	3.32
RRl	1.13	1.92	0.77	1.98
RRu	5.95	7.00	6.95	5.58
P	+	+++	N.S.	+++
Between Chi				0.06
Between df				2
Between P				N.S.
Btwn(F) P				N.S.
Btwn(R) P				N.S.

	Derivation of RR/CI Orig	StdCalc	Other	Total
N		10	4	14
NS		8	4	12
Wt		65.21	13.42	78.64
Het Chi		54.88	1.12	57.28
Het df		9	3	13
Het P		***	N.S.	***
Fixed RR		2.48	3.49	2.63
RRl		1.95	2.04	2.11
RRu		3.16	5.95	3.28
P		+++	+++	+++
Random RR		3.28	3.49	3.32
RRl		1.66	2.04	1.98
RRu		6.50	5.95	5.58
P		+++	+++	+++
Between Chi				1.29
Between df				1
Between P				N.S.
Btwn(F) P				N.S.
Btwn(R) P				N.S.

Table 3J5 - 4

IESLC - Meta-analysis of Ex Smoking, Years quit (vs never), "Highest vs lowest"
Adenocarcinoma, Any Product (or Cigarettes if Any not available)
Least adjusted

REF	NRR	X	SEX	AGEL	AGEH	RACE	YF	LC TYPE	LOC	START	ST	NLC	R	VB	P	H	AD	ADOS	PRODUCT	exL	exH	unexL	unexH	De
BARBON	748	x	m	0	0	all	-	a	Eu:wst	1979	CC	755	n	bl	y	y	0	0	all/unsp	0.1	4	25	999	st
JAHN	650		m	0	0	all	-	a	Eu:Ger	1988	CC	1004	n	bl	n	n	0	0	cig+/-ot	0.1	0.9	21	999	st
JAIN	539		m	0	0	all	-	a	NAmer	1981	CC	845	n	V	y	n	0	0	cig+/-ot	2	9	10	999	st
JAIN	503		f	0	0	all	-	a	NAmer	1981	CC	845	n	V	y	n	0	0	cig+/-ot	2	9	10	999	st
JEDRYC	563		m	0	0	all	-	a	Eu:est	1980	CC	1630	n	bl	y	n	0	0	cig+/-ot	5	9	10	999	st
LUBIN2	870		m	0	0	all	-	a	Eu:mul	1976	CC	7804	n	bl	n	y	0	0	cig+/-ot	0.1	4	20	999	st
LUBIN2	970		f	0	0	all	-	a	Eu:mul	1976	CC	7804	n	bl	n	y	0	0	cig+/-ot	0.1	9	20	999	st
MATOS	665	x	m	0	0	all	-	a	SCAmer	1994	CC	200	n	bl	n	n	0	0	cig+/-ot	1.0	5	11	999	st
PEZZOT	587		m	0	0	all	-	a	SCAmer	1987	CC	215	n	bl	n	y	0	0	cig only	1.0	10	11	999	st
SOBUE	785		m	0	0	all	-	a	As:Jap	1986	CC	1376	n	bl	n	y	0	0	cig+/-ot	1.0	4	10	999	st
SVENSS	571		f	0	0	all	-	a	Eu:Sca	1983	CC	210	n	bl	n	n	0	0	all/unsp	3	10	11	999	st
WAKAI	558	x	m	0	0	all	-	a	As:Jap	1988	CC	333	n	bl	n	y	0	0	cig+/-ot	5	9	20	999	st
WYNDE3	526		m	0	0	all	-	KII	NAmer	1966	CC	350	n	bl	n	y	0	0	all/unsp	1.0	3	13	999	st
WYNDE6	811	x	m	0	0	all	-	KII	NAmer	1969	CC	4423	n	bl	n	y	0	0	cig+/-ot	1.0	3	16	999	st

Cigarette type is all/unspec for all RRs

Table 3J5 - 5

IESLC - Meta-analysis of Ex Smoking, Years quit (vs never), "Highest vs lowest"
Adenocarcinoma, Any Product (or Cigarettes if Any not available)
Least adjusted

REF	NRR	SEX	AD	Number Exposed Case	Cont	Non-exposed Case	Cont	RR	95.00%CI
BARBON	748	m	0	7	20	4	59	5.16	(1.37- 19.50)
JAHN	650	m	0	40	8	15	146	48.67	(19.27- 122.93)
JAIN	539	m	0	16	46	14	113	2.81	(1.27- 6.22)
JAIN	503	f	0	14	36	3	61	7.91	(2.13- 29.40)
Subtotal JAIN								3.71	(1.88- 7.32)
JEDRYC	563	m	0	9	82	12	230	2.10	(0.86- 5.18)
LUBIN2	870	m	0	77	1047	35	1128	2.37	(1.58- 3.57)
LUBIN2	970	f	0	13	95	1	29	3.97	(0.50- 31.64)
Subtotal LUBIN2								2.42	(1.62- 3.61)
MATOS	665	m	0	12	23	12	101	4.39	(1.75- 11.01)
PEZZOT	587	m	0	11	21	7	31	2.32	(0.77- 6.95)
SOBUE	785	m	0	44	116	49	144	1.11	(0.69- 1.79)
SVENSS	571	f	0	5	13	7	24	1.32	(0.35- 4.99)
WAKAI	558	m	0	7	48	3	47	2.28	(0.56- 9.37)
WYNDE3	526	m	0	3	22	3	55	2.50	(0.47- 13.35)
WYNDE6	811	m	0	29	307	6	530	8.34	(3.43- 20.32)
Totals				287	1884	171	2698		

*prospective study

REF	NRR	SEX	AD	Ys	Ws	Qs	Ps
BARBON	748	m	0	1.64	2.17	0.80	0.0155
JAHN	650	m	0	3.88	4.47	36.34	0.0000
JAIN	539	m	0	1.03	6.08	0.00	0.0109
JAIN	503	f	0	2.07	2.23	2.38	0.0020
Subtotal JAIN				1.31	8.31	2.38	
JEDRYC	563	m	0	0.74	4.74	0.40	0.1054
LUBIN2	870	m	0	0.86	23.04	0.68	0.0000
LUBIN2	970	f	0	1.38	0.89	0.11	0.1932
Subtotal LUBIN2				0.88	23.93	0.79	
MATOS	665	m	0	1.48	4.54	0.90	0.0016
PEZZOT	587	m	0	0.84	3.19	0.12	0.1330
SOBUE	785	m	0	0.11	17.04	14.62	0.6540
SVENSS	571	f	0	0.28	2.17	1.25	0.6838
WAKAI	558	m	0	0.83	1.93	0.08	0.2511
WYNDE3	526	m	0	0.92	1.37	0.02	0.2836
WYNDE6	811	m	0	2.12	4.85	5.72	0.0000

	N	14
	NS	12
	Wt	78.71
Het	Chi	63.42
Het	df	13
Het	P	***
Fixed	RR	2.81
	RRl	2.26
	RRu	3.51
	P	+++
Random	RR	3.63
	RRl	2.11
	RRu	6.25
	P	+++
Asymm	P	N.S.

Table 3J5 - 6

IESLC - Meta-analysis of Ex Smoking, Years quit (vs never), "Highest vs lowest"
Adenocarcinoma, Any Product (or Cigarettes if Any not available)
Least adjusted

combined	Sex male	female	Total
N	11	3	14
NS	11	3	14
Wt	73.42	5.29	78.71
Het Chi	59.68	3.55	63.42
Het df	10	2	13
Het P	***	N.S.	***
Fixed RR	2.78	3.38	2.81
RRl	2.21	1.44	2.26
RRu	3.49	7.92	3.51
P	+++	++	+++
Random RR	3.68	3.40	3.63
RRl	1.99	1.05	2.11
RRu	6.81	10.98	6.25
P	+++	+	+++
Between Chi			0.19
Between df			1
Between P			N.S.
Btwn(F) P			N.S.
Btwn(R) P			N.S.

Table 3J5 - 7

IESLC - Meta-analysis of Ex Smoking, Years quit (vs never), "Highest vs lowest"
Adenocarcinoma, Any Product (or Cigarettes if Any not available)
Excluded studies (and stage at which they were excluded)

1	AGUDO AKIBA AMANDU AMES AXELSS BEST BOUCHA BOUCOT BRESLO CHEN CHEN2 CHIAZZ DEAN2 DOSEME ENGELA FAN GENG GER GUO HAENSZ HEGMAN HOLE HU HU2 JUSSAW KATSOU KAUFMA KOO KOULUM KREUZE LETOUR LEVIN LIAW LIU3 LIU4 LIU5 MCCONN MIGRAN MRFITR NOTAN2 OSANN2 PERNU QIAO2 RACHTA RESTRE SADOWS SEGI2 STASZE TIZZAN VUTUC WATSON WU WUWILL WYNDE2 WYNDE8 XU YUAN ZHANG ZHENG ZHOU
2	BUFFLE HUMBLE PISANI PRESCO WYNDE7
3	MCDUFF SPITZ
4	AUVINE BLOT1 BROWN3 GURSEL LAUSSM LUO WU2
5	ARMADA BECHER BENSHL BOFFET BROSS CARPEN CEDERL CHOI CHYOU CORREA CPSI CPSII DAMBER DARBY DEAN3 DESTEF DOLL DOLL2 DORGAN DORN GAO GAO2 GARCIA GARSHI GILLIS GRAHAM HAMMO2 HAMMON HIRAYA JOLY KAISE2 KHUDER LUBIN PEZZO2 QIAO SPEIZE SUZUK2 TVERDA WANG2 WIGLE
6	ALDERS
15	BENHAM

Table 3J5 - 8
Potentially overlapping studies

REF	REFGP	PRINC	. OVERLAP/LINK
LUBIN2	LUBIN2	1	Lubin-combined
WYNDE6	WYNDE6	1	WYNDE5/6/7/8
JAHN	BOFFET	2	Subset of BOFFET

Table 3J5 - 9
Most adjusted - insufficient data for meta-analysis

REF	NRR	SEX	AGEL	AGEH	RACE	YF	LC TYPE	LOC	START	ST	NLC	R	VB	P	H	AD	ADOS	PRODUCT	exL	exH	unexL	unexH	De
ALDERS	572	m	0	0	all	-	not q+s	Eu:UK	1977	CC	1448	n	V	n	n	1	0	cig only	0.1	2	10	999	st
ALDERS	583	f	0	0	all	-	not q+s	Eu:UK	1977	CC	1448	n	V	n	n	1	0	cig only	0.1	2	10	999	st
JAIN	587	m	0	0	all	-	a	NAmer	1981	CC	845	n	V	y	n	0	0	cig+/-ot	0.1	1.9	10	999	ot
JAIN	575	f	0	0	all	-	a	NAmer	1981	CC	845	n	V	y	n	0	0	cig+/-ot	0.1	1.9	10	999	ot
JEDRYC	564	m	0	0	all	-	a	Eu:est	1980	CC	1630	n	bl	y	n	0	0	cig+/-ot	0.1	4	10	999	ot
MATOS	712	m	0	0	all	-	a	SCAmer	1994	CC	200	n	bl	n	n	2	0	cig+/-ot	0.1	0.9	11	999	ot
PEZZOT	602	m	0	0	all	-	a	SCAmer	1987	CC	215	n	bl	n	y	0	0	cig only	0.1	0.9	11	999	ot
SOBUE	786	m	0	0	all	-	a	As:Jap	1986	CC	1376	n	bl	n	y	0	0	cig+/-ot	0.1	0.9	10	999	ot
SVENSS	598	f	0	0	all	-	a	Eu:Sca	1983	CC	210	n	bl	n	n	0	0	all/unsp	0.1	2	11	999	ot
WAKAI	620	m	0	0	all	-	a	As:Jap	1988	CC	333	n	bl	n	y	1	0	cig+/-ot	0.1	4	20	999	ot
WYNDE3	527	m	0	0	all	-	KII	NAmer	1966	CC	350	n	bl	n	y	0	0	all/unsp	0.1	0.9	13	999	ot
WYNDE6	827	m	0	0	all	-	KII	NAmer	1969	CC	4423	n	bl	n	y	2	0	cig+/-ot	0.1	0.9	16	999	ot

REF	NRR	RR	SIG	RRDATA comment
ALDERS	572	2.27		0
ALDERS	583	3.83		0
JAIN	587	*	gap	0
JAIN	575	*	gap	0
JEDRYC	564	*	gap	0
MATOS	712	*	gap	0
PEZZOT	602	*	gap	0
SOBUE	786	*	gap	0
SVENSS	598	*	gap	0
WAKAI	620	*	gap	0
WYNDE3	527	*	gap	0
WYNDE6	827	*	gap	0

Least adjusted - insufficient data for meta-analysis: as for adjusted plus the following

REF	NRR	SEX	AGEL	AGEH	RACE	YF	LC TYPE	LOC	START	ST	NLC	R	VB	P	H	AD	ADOS	PRODUCT	exL	exH	unexL	unexH	De
MATOS	710	m	0	0	all	-	a	SCAmer	1994	CC	200	n	bl	n	n	0	0	cig+/-ot	0.1	0.9	11	999	ot
WAKAI	618	m	0	0	all	-	a	As:Jap	1988	CC	333	n	bl	n	y	0	0	cig+/-ot	0.1	4	20	999	ot
WYNDE6	812	m	0	0	all	-	KII	NAmer	1969	CC	4423	n	bl	n	y	0	0	cig+/-ot	0.1	0.9	16	999	ot

REF	NRR	RR	SIG	RRDATA comment
MATOS	710	*	gap	0

Table 3J5 - 9

IESLC - Meta-analysis of Ex Smoking, Years quit (vs never), "Highest vs lowest"
Adenocarcinoma, Any Product (or Cigarettes if Any not available)
Least adjusted - insufficient data for meta-analysis: as for adjusted plus the following

REF	NRR	RR	SIG	RRDATA comment
WAKAI	618	*	gap	0
WYNDE6	812	*	gap	0

Table 3J6 -

IESLC - Meta-analysis of Ex Smoking by Years quit (vs never), Overview
Adenocarcinoma, Cigarettes (or Any Product if Cigarettes not available)

This analysis is restricted to results for:
1) Ex smokers
2) Results by Years quit (vs never)
3) Categorical results by Years quit (vs never)
   Results by Years quit (vs never) are grouped under 2 schemes (S1, S2). Each scheme has a set of "key values". An interv
   is allocated to the category whose key value it includes, and intervals which include none or more than
   one of the key values are excluded. (Open-ended intervals are coded as 999)

S1	key value	maximum range
1	12	8+
2	7	4-11
3	3	1-6

S2	key value	maximum range
1	20	13+
2	12	4-19
3	3	1-11

4) Adenocarcinoma (or near equivalent)
5) Results complete enough for use in metaanalysis

Within each study, results are then selected (in the following order of preference, within each sex) for:
6) (not applicable)
7) PRODUCT: cigarettes regardless of other products, cigarettes only, all/unspec
8) CIGTYPE: all/unspecified, MC regardless of HR, MC only
9) (not applicable)
10) DENOM: never smoked anything, never smoked cigarettes, never any + low, never cigs + low
11) Followup period (YF, prospective studies): whole study (coded as 0) or longest available
12) LCtype: adeno or nearest available, but not squamous. (q = squamous, s = small,
 a = adeno, l = large, KII = Kreyberg II, al = alveolar, br = bronchiolar, u = undifferentiated)
13) Race: all or nearest available, otherwise by race (wh or w = white, bl or b = black, hi = hispanic
 ch = chinese, jap = japanese, haw = hawaiian, w+o = white + oriental, sca = scandinavian, as = asian)
14) For overlapping studies: principal rather than subsidiary studies
Finally by Age: whole study (coded as 0) if available, otherwise by widest available age group
and then for single sex results (m, f) in preference to results for both sexes combined (c).

Results adjusted (AD) for the most potential confounders are then chosen in Sections -1 to -3
(and those which actually differ from the adjusted results in Table 3J1 - 1 are marked 'x' in Section -1)
and results adjusted for the least confounders in Sections -4 to -6. (Those least adjusted results which
actually differ from the most adjusted are marked 'x' in column X in Section -4)

Section -7 shows excluded studies, together with the stage (as above) at which no qualifying
results were found.

Section -8 lists the potentially overlapping studies which have been included (1=principal, 2=subsidiary).

Section -9 lists any results which would have been included in preference except that they had data not complete
enough for use in meta-analysis, with their significance (yes/no), if known, and any further comment as entered
on the database. It also lists as "gap" any categories for which no data were presented by the original authors.
This is commonly due to recent quitters having been combined with current smokers

In addition to those mentioned above, the following fields, levels and abbreviations are used:
* or nk = not known, n = no, y = yes, ot = other
nev = never
all/unspec = all or unspecified, cig+/-ot = cigarettes irrespective of other products (cigar, pipe etc)
MC = manufactured cigarettes, HR = hand-rolled cigarettes
exL, exH = range of exposure (low and high) in the smoking group, in terms of Years quit (vs never)
REF: 6-character study reference
NRR: number of the RR on the database within the study
ST : study type (CC = case control, pr or prosp = prospective)
NLC: number of lung cancer cases in whole study
R : risky occupational population (n = no, m = mining, o = other risky)
VB : national cigarette type (V = at least 75% Virginia, bl = at least 75% blended, ot = other)
P : any proxy use
H : full histological confirmation
De : derivation of RR/CI (or = original, st = standard method, ot = other method of estimation)

Table 3J6 - 1

IESLC - Meta-analysis of Ex Smoking by Years quit (vs never), Overview
Adenocarcinoma, Cigarettes (or Any Product if Cigarettes not available)
Most adjusted

REF	NRR	3J1	SEX	AGEL	AGEH	RACE	YF	LC TYPE	LOC	START	ST	NLC	R	VB	P	H	AD	PRODUCT	exL	exH	S1	S2	DENOM	De
BARBON	756		m	0	0	all	-	a	Eu:wst	1979	CC	755	n	bl	y	y	1	all/unsp	25	999	0	0	nev any	or
BARBON	757		m	0	0	all	-	a	Eu:wst	1979	CC	755	n	bl	y	y	1	all/unsp	15	24	0	1	nev any	or
BARBON	758		m	0	0	all	-	a	Eu:wst	1979	CC	755	n	bl	y	y	1	all/unsp	5	14	0	2	nev any	or
BARBON	759		m	0	0	all	-	a	Eu:wst	1979	CC	755	n	bl	y	y	1	all/unsp	0.1	4	3	3	nev any	or
BROWN3	505		f	0	0	wh	-	a	NAmer		CC	618		bl	y	n	0	all/unsp	15	999	0	1	nev any	st
JAHN	639		m	0	0	all	-	a	Eu:Ger	1988	CC	1004	n	bl	n	n	0	cig+/-ot	21	999	0	0	nev any	st
JAHN	640		m	0	0	all	-	a	Eu:Ger	1988	CC	1004	n	bl	n	n	0	cig+/-ot	11	20	1	0	nev any	st
JAHN	641		m	0	0	all	-	a	Eu:Ger	1988	CC	1004	n	bl	n	n	0	cig+/-ot	6	10	2	0	nev any	st
JAHN	642		m	0	0	all	-	a	Eu:Ger	1988	CC	1004	n	bl	n	n	0	cig+/-ot	2	5	3	3	nev any	st
JAHN	643		m	0	0	all	-	a	Eu:Ger	1988	CC	1004	n	bl	n	n	0	cig+/-ot	1.0	1.9	0	0	nev any	st
JAHN	644		m	0	0	all	-	a	Eu:Ger	1988	CC	1004	n	bl	n	n	0	cig+/-ot	0.1	0.9	0	0	nev any	st
JAIN	537		m	0	0	all	-	a	NAmer	1981	CC	845	n	V	y	n	0	cig+/-ot	10	999	1	0	nev cigs	st
JAIN	538		m	0	0	all	-	a	NAmer	1981	CC	845	n	V	y	n	0	cig+/-ot	2	9	0	3	nev cigs	st
JAIN	501		f	0	0	all	-	a	NAmer	1981	CC	845	n	V	y	n	0	cig+/-ot	10	999	1	0	nev cigs	st
JAIN	502		f	0	0	all	-	a	NAmer	1981	CC	845	n	V	y	n	0	cig+/-ot	2	9	0	3	nev cigs	st
JEDRYC	559		m	0	0	all	-	a	Eu:est	1980	CC	1630	n	bl	y	n	0	cig+/-ot	10	999	1	0	nev any	st
JEDRYC	560		m	0	0	all	-	a	Eu:est	1980	CC	1630	n	bl	y	n	0	cig+/-ot	5	9	2	0	nev any	st
LUBIN2	861		m	0	0	all	-	a	Eu:mul	1976	CC	7804	n	bl	n	y	0	cig+/-ot	20	999	0	1	nev any	st
LUBIN2	862		m	0	0	all	-	a	Eu:mul	1976	CC	7804	n	bl	n	y	0	cig+/-ot	15	19	0	0	nev any	st
LUBIN2	863		m	0	0	all	-	a	Eu:mul	1976	CC	7804	n	bl	n	y	0	cig+/-ot	10	14	1	2	nev any	st
LUBIN2	864		m	0	0	all	-	a	Eu:mul	1976	CC	7804	n	bl	n	y	0	cig+/-ot	5	9	2	0	nev any	st
LUBIN2	865		m	0	0	all	-	a	Eu:mul	1976	CC	7804	n	bl	n	y	0	cig+/-ot	0.1	4	3	3	nev any	st
LUBIN2	965		f	0	0	all	-	a	Eu:mul	1976	CC	7804	n	bl	n	y	0	cig+/-ot	20	999	0	1	nev any	st
LUBIN2	966		f	0	0	all	-	a	Eu:mul	1976	CC	7804	n	bl	n	y	0	cig+/-ot	10	19	1	2	nev any	st
LUBIN2	967		f	0	0	all	-	a	Eu:mul	1976	CC	7804	n	bl	n	y	0	cig+/-ot	0.1	9	0	3	nev any	st
MATOS	671		m	0	0	all	-	a	SCAmer	1994	CC	200	n	bl	n	n	2	cig+/-ot	11	999	1	0	nev any	ot
MATOS	672		m	0	0	all	-	a	SCAmer	1994	CC	200	n	bl	n	n	2	cig+/-ot	6	10	2	0	nev any	ot
MATOS	673		m	0	0	all	-	a	SCAmer	1994	CC	200	n	bl	n	n	2	cig+/-ot	1.0	5	3	3	nev any	ot
PEZZOT	585		m	0	0	all	-	a	SCAmer	1987	CC	215	n	bl	n	y	0	cig only	11	999	1	0	nev cigs	st
PEZZOT	586		m	0	0	all	-	a	SCAmer	1987	CC	215	n	bl	n	y	0	cig only	1.0	10	0	3	nev cigs	st
SVENSS	569		f	0	0	all	-	a	Eu:Sca	1983	CC	210	n	bl	n	n	0	all/unsp	11	999	1	0	nev any	st
SVENSS	570		f	0	0	all	-	a	Eu:Sca	1983	CC	210	n	bl	n	n	0	all/unsp	3	10	0	3	nev any	st
WAKAI	562		m	0	0	all	-	a	As:Jap	1988	CC	333	n	bl	n	y	1	cig+/-ot	20	999	0	1	nev any	or
WAKAI	563		m	0	0	all	-	a	As:Jap	1988	CC	333	n	bl	n	y	1	cig+/-ot	10	19	1	2	nev any	or
WAKAI	564		m	0	0	all	-	a	As:Jap	1988	CC	333	n	bl	n	y	1	cig+/-ot	5	9	2	0	nev any	or
WU2	502		f	0	0	all	-	a	NAmer	1983	CC	336	n	bl	n	y	2	all/unsp	1.0	9	0	3	nev any	or
WYNDE3	559	x	m	0	0	all	-	KII	NAmer	1966	CC	350	n	bl	n	y	0	cig+/-ot	10	999	1	0	nev any	st
WYNDE3	580		f	0	0	all	-	KII	NAmer	1966	CC	350	n	bl	n	y	0	cig+/-ot	10	999	1	0	nev any	ot
WYNDE6	817		m	0	0	all	-	KII	NAmer	1969	CC	4423	n	bl	n	y	2	cig+/-ot	16	999	0	1	nev any	ot
WYNDE6	818		m	0	0	all	-	KII	NAmer	1969	CC	4423	n	bl	n	y	2	cig+/-ot	11	15	1	2	nev any	ot
WYNDE6	819		m	0	0	all	-	KII	NAmer	1969	CC	4423	n	bl	n	y	2	cig+/-ot	7	10	2	0	nev any	ot
WYNDE6	820		m	0	0	all	-	KII	NAmer	1969	CC	4423	n	bl	n	y	2	cig+/-ot	4	6	0	0	nev any	ot
WYNDE6	821		m	0	0	all	-	KII	NAmer	1969	CC	4423	n	bl	n	y	2	cig+/-ot	1.0	3	3	3	nev any	ot

Cigarette type is all/unspec for all RRs

In this overview table, subtotals and Qs values may be invalid and should be ignored

Table 3J6 - 2

IESLC - Meta-analysis of Ex Smoking by Years quit (vs never), Overview
Adenocarcinoma, Cigarettes (or Any Product if Cigarettes not available)
Most adjusted

REF	NRR	SEX	AD	Number Exposed Case	Number Exposed Cont	Non-exposed Case	Non-exposed Cont	RR	95.00%CI
BARBON	756	m	1	4	-	7	-	1.80	(0.50- 6.40)
BARBON	757	m	1	7	-	7	-	4.60	(1.50- 13.80)
BARBON	758	m	1	23	-	7	-	7.30	(3.00- 17.60)
BARBON	759	m	1	7	-	7	-	9.40	(3.00- 29.70)
Subtotal BARBON								5.40	(3.16- 9.24)
BROWN3	505	f	0	73	219	432	1168	0.90	(0.68- 1.20)
JAHN	639	m	0	15	146	8	138	1.77	(0.73- 4.31)
JAHN	640	m	0	22	130	8	138	2.92	(1.26- 6.79)
JAHN	641	m	0	13	63	8	138	3.56	(1.40- 9.02)
JAHN	642	m	0	19	46	8	138	7.13	(2.92- 17.37)
JAHN	643	m	0	18	9	8	138	34.50	(11.82- 100.74)
JAHN	644	m	0	40	8	8	138	86.25	(30.45- 244.33)
Subtotal JAHN								6.98	(4.77- 10.22)
JAIN	537	m	0	14	113	4	85	2.63	(0.84- 8.28)
JAIN	538	m	0	16	46	4	85	7.39	(2.33- 23.41)
JAIN	501	f	0	3	61	24	214	0.44	(0.13- 1.51)
JAIN	502	f	0	14	36	24	214	3.47	(1.64- 7.32)
Subtotal JAIN								2.69	(1.63- 4.45)
JEDRYC	559	m	0	12	230	7	289	2.15	(0.83- 5.56)
JEDRYC	560	m	0	9	82	7	289	4.53	(1.64- 12.54)
Subtotal JEDRYC								3.04	(1.52- 6.09)
LUBIN2	861	m	0	35	1128	57	2616	1.42	(0.93- 2.18)
LUBIN2	862	m	0	21	478	57	2616	2.02	(1.21- 3.36)
LUBIN2	863	m	0	30	693	57	2616	1.99	(1.27- 3.12)
LUBIN2	864	m	0	50	882	57	2616	2.60	(1.77- 3.83)
LUBIN2	865	m	0	77	1047	57	2616	3.38	(2.38- 4.79)
LUBIN2	965	f	0	1	29	138	1180	0.29	(0.04- 2.18)
LUBIN2	966	f	0	3	33	138	1180	0.78	(0.24- 2.57)
LUBIN2	967	f	0	13	95	138	1180	1.17	(0.64- 2.14)
Subtotal LUBIN2								2.10	(1.76- 2.50)
MATOS	671	m	2	12	-	5	-	3.00	(1.09- 8.23)
MATOS	672	m	2	9	-	5	-	10.00	(3.08- 32.51)
MATOS	673	m	2	12	-	5	-	13.00	(4.21- 40.17)
Subtotal MATOS								6.76	(3.59- 12.75)
PEZZOT	585	m	0	7	106	3	116	2.55	(0.64- 10.13)
PEZZOT	586	m	0	11	82	3	116	5.19	(1.40- 19.18)
Subtotal PEZZOT								3.71	(1.44- 9.57)
SVENSS	569	f	0	7	24	22	120	1.59	(0.61- 4.14)
SVENSS	570	f	0	5	13	22	120	2.10	(0.68- 6.48)
Subtotal SVENSS								1.79	(0.86- 3.70)
WAKAI	562	m	1	3	-	8	-	0.54	(0.14- 2.16)
WAKAI	563	m	1	13	-	8	-	2.49	(0.95- 6.53)
WAKAI	564	m	1	7	-	8	-	1.23	(0.42- 3.64)
Subtotal WAKAI								1.40	(0.74- 2.65)
WU2	502	f	2	-	-	-	-	2.30	(1.20- 4.40)
WYNDE3	559	m	0	3	65	6	88	0.68	(0.16- 2.81)
WYNDE3	580	f	0	0	3	15	76	0.71~	(0.03- 14.35)
Subtotal WYNDE3								0.68	(0.19- 2.47)
WYNDE6	817	m	2	6	-	11	-	1.20	(0.44- 3.26)
WYNDE6	818	m	2	11	-	11	-	5.40	(2.32- 12.58)
WYNDE6	819	m	2	18	-	11	-	6.60	(3.09- 14.10)
WYNDE6	820	m	2	14	-	11	-	5.90	(2.65- 13.11)
WYNDE6	821	m	2	29	-	11	-	14.20	(7.02- 28.73)
Subtotal WYNDE6								6.10	(4.25- 8.74)
Partial Totals				706	5867	1442	20428		

*prospective study

~ With 0.5 adjustment for zero

Table 3J6 - 2

IESLC - Meta-analysis of Ex Smoking by Years quit (vs never), Overview
Adenocarcinoma, Cigarettes (or Any Product if Cigarettes not available)
Most adjusted

REF	NRR	SEX	AD	Ys	Ws	Qs	Ps
BARBON	756	m	1	0.59	2.36	0.24	0.3661
BARBON	757	m	1	1.53	3.12	1.21	0.0070
BARBON	758	m	1	1.99	4.91	5.77	0.0000
BARBON	759	m	1	2.24	2.92	5.22	0.0001
Subtotal BARBON				1.69	13.32	12.43	
BROWN3	505	f	0	-0.10	46.65	47.41	0.4775
JAHN	639	m	0	0.57	4.86	0.54	0.2071
JAHN	640	m	0	1.07	5.39	0.15	0.0128
JAHN	641	m	0	1.27	4.44	0.59	0.0074
JAHN	642	m	0	1.96	4.84	5.43	0.0000
JAHN	643	m	0	3.54	3.35	23.26	0.0000
JAHN	644	m	0	4.46	3.54	44.73	0.0000
Subtotal JAHN				1.94	26.43	74.70	
JAIN	537	m	0	0.97	2.92	0.01	0.0979
JAIN	538	m	0	2.00	2.89	3.47	0.0007
JAIN	501	f	0	-0.82	2.52	7.54	0.1902
JAIN	502	f	0	1.24	6.87	0.79	0.0011
Subtotal JAIN				0.99	15.21	11.82	
JEDRYC	559	m	0	0.77	4.27	0.08	0.1127
JEDRYC	560	m	0	1.51	3.71	1.37	0.0036
Subtotal JEDRYC				1.11	7.98	1.45	
LUBIN2	861	m	0	0.35	21.10	6.40	0.1044
LUBIN2	862	m	0	0.70	14.78	0.61	0.0070
LUBIN2	863	m	0	0.69	18.97	0.90	0.0028
LUBIN2	864	m	0	0.96	25.60	0.07	0.0000
LUBIN2	865	m	0	1.22	31.38	3.06	0.0000
LUBIN2	965	f	0	-1.22	0.96	4.33	0.2317
LUBIN2	966	f	0	-0.25	2.69	3.60	0.6795
LUBIN2	967	f	0	0.16	10.47	5.84	0.6113
Subtotal LUBIN2				0.74	125.96	24.81	
MATOS	671	m	2	1.10	3.76	0.14	0.0332
MATOS	672	m	2	2.30	2.77	5.41	0.0001
MATOS	673	m	2	2.56	3.02	8.33	0.0000
Subtotal MATOS				1.91	9.55	13.88	
PEZZOT	585	m	0	0.94	2.02	0.00	0.1824
PEZZOT	586	m	0	1.65	2.25	1.24	0.0136
Subtotal PEZZOT				1.31	4.27	1.24	
SVENSS	569	f	0	0.46	4.20	0.81	0.3415
SVENSS	570	f	0	0.74	3.02	0.08	0.1976
Subtotal SVENSS				0.58	7.22	0.89	
WAKAI	562	m	1	-0.62	2.05	4.74	0.3774
WAKAI	563	m	1	0.91	4.14	0.00	0.0636
WAKAI	564	m	1	0.21	3.30	1.60	0.7071
Subtotal WAKAI				0.34	9.48	6.35	
WU2	502	f	2	0.83	9.10	0.05	0.0120
WYNDE3	559	m	0	-0.39	1.90	3.18	0.5908
WYNDE3	580	f	0	-0.35	0.42	0.66	0.8202
Subtotal WYNDE3				-0.38	2.32	3.85	
WYNDE6	817	m	2	0.18	3.83	2.00	0.7212
WYNDE6	818	m	2	1.69	5.38	3.29	0.0001
WYNDE6	819	m	2	1.89	6.67	6.44	0.0000
WYNDE6	820	m	2	1.77	6.01	4.56	0.0000
WYNDE6	821	m	2	2.65	7.74	23.67	0.0000
Subtotal WYNDE6				1.81	29.62	39.96	

N	43
NS	13

Table 3J6 - 3

IESLC - Meta-analysis of Ex Smoking by Years quit (vs never), Overview
Adenocarcinoma, Cigarettes (or Any Product if Cigarettes not available)
Most adjusted

	Sex			
	combined	male	female	Total
N		33	10	43
NS		10	6	16

In this overview table, other than the "N" rows, entries in the "absent" and "Total" columns may be invalid and should be ignored

	Years quit vs never (lower focus)				
	absent	8+k12	4-11k7	1-6k3	Total
N	19	13	6	5	43
NS	10	10	6	5	31
Wt	152.13	58.59	46.48	49.90	307.11
Het Chi	146.83	18.35	11.66	17.71	238.83
Het df	18	12	5	4	42
Het P	***	N.S.	*	**	***
Fixed RR	1.90	2.05	3.29	5.22	2.47
RRl	1.62	1.59	2.47	3.96	2.21
RRu	2.23	2.65	4.39	6.90	2.76
P	+++	+++	+++	+++	+++
Random RR	2.98	1.98	3.74	7.88	3.05
RRl	1.81	1.40	2.23	3.84	2.28
RRu	4.90	2.79	6.25	16.18	4.07
P	+++	+++	+++	+++	+++

	Years quit vs never (higher focus)				
	absent	13+k20	4-19k12	1-11k3	Total
N	21	6	5	11	43
NS	11	5	4	9	29
Wt	108.81	77.72	36.08	84.50	307.11
Het Chi	95.44	12.34	13.40	42.20	238.83
Het df	20	5	4	10	42
Het P	***	*	**	***	***
Fixed RR	3.16	1.08	2.63	3.76	2.47
RRl	2.62	0.86	1.90	3.04	2.21
RRu	3.81	1.34	3.65	4.66	2.76
P	+++	N.S.	+++	+++	+++
Random RR	3.28	1.18	2.84	4.53	3.05
RRl	2.11	0.73	1.45	2.79	2.28
RRu	5.09	1.90	5.55	7.36	4.07
P	+++	N.S.	++	+++	+++

Table 3J6 - 3

IESLC - Meta-analysis of Ex Smoking by Years quit (vs never), Overview
Adenocarcinoma, Cigarettes (or Any Product if Cigarettes not available)
Most adjusted

MALES

Years quit vs never (lower focus)

	absent	8+k12	4-11k7	1-6k3	Total
N	13	9	6	5	33
NS	7	9	6	5	27
Wt	75.06	48.76	46.48	49.90	220.21
Het Chi	95.39	7.73	11.66	17.71	148.20
Het df	12	8	5	4	32
Het P	***	N.S.	*	**	***
Fixed RR	3.05	2.42	3.29	5.22	3.33
RRl	2.43	1.83	2.47	3.96	2.91
RRu	3.83	3.20	4.39	6.90	3.80
P	+++	+++	+++	+++	+++
Random RR	4.20	2.42	3.74	7.88	3.93
RRl	2.13	1.83	2.23	3.84	2.90
RRu	8.28	3.20	6.25	16.18	5.34
P	+++	+++	+++	+++	+++

Years quit vs never (higher focus)

	absent	13+k20	4-19k12	1-11k3	Total
N	18	4	4	7	33
NS	10	4	4	7	25
Wt	101.67	30.11	33.39	55.04	220.21
Het Chi	81.96	6.30	9.07	18.04	148.20
Het df	17	3	3	6	32
Het P	***	(*)	*	**	***
Fixed RR	3.43	1.47	2.91	5.32	3.33
RRl	2.83	1.03	2.07	4.08	2.91
RRu	4.17	2.11	4.08	6.93	3.80
P	+++	+	+++	+++	+++
Random RR	3.87	1.49	3.55	7.37	3.93
RRl	2.46	0.77	1.83	4.21	2.90
RRu	6.10	2.89	6.86	12.90	5.34
P	+++	N.S.	+++	+++	+++

FEMALES

Years quit vs never (lower focus)

	absent	8+k12	4-11k7	1-6k3	Total
N	6	4			10
NS	5	4			9
Wt	77.07	9.83			86.91
Het Chi	18.25	2.75			21.68
Het df	5	3			9
Het P	**	N.S.			**
Fixed RR	1.20	0.91			1.16
RRl	0.96	0.49			0.94
RRu	1.50	1.69			1.43
P	N.S.	N.S.			N.S.
Random RR	1.50	0.91			1.29
RRl	0.87	0.49			0.85
RRu	2.59	1.69			1.96
P	N.S.	N.S.			N.S.

Table 3J6 - 3

IESLC - Meta-analysis of Ex Smoking by Years quit (vs never), Overview
Adenocarcinoma, Cigarettes (or Any Product if Cigarettes not available)
Most adjusted

FEMALES

	Years quit vs never (higher focus)				
	absent	13+k20	4-19k12	1-11k3	Total
N	3	2	1	4	10
NS	3	2	1	4	9
Wt	7.14	47.61	2.69	29.46	86.91
Het Chi	2.66	1.17	0.00	5.27	21.68
Het df	2	1	0	3	9
Het P	N.S.	N.S.	N.S.	N.S.	**
Fixed RR	0.96	0.88	0.78	1.97	1.16
RRl	0.46	0.66	0.24	1.37	0.94
RRu	2.00	1.17	2.57	2.83	1.43
P	N.S.	N.S.	N.S.	+++	N.S.
Random RR	0.90	0.81	0.78	2.04	1.29
RRl	0.36	0.43	0.24	1.24	0.85
RRu	2.26	1.53	2.57	3.34	1.96
P	N.S.	N.S.	N.S.	++	N.S.

Table 3J6 - 4

IESLC - Meta-analysis of Ex Smoking by Years quit (vs never), Overview
Adenocarcinoma, Cigarettes (or Any Product if Cigarettes not available)
Least adjusted

REF	NRR	X	SEX	AGEL	AGEH	RACE	YF	LC TYPE	LOC	START	ST	NLC	R	VB	P	H	AD	PRODUCT	exL	exH	S1	S2	DENOM	De
BARBON	741	x	m	0	0	all	-	a	Eu:wst	1979	CC	755	n	bl	y	y	0	all/unsp	25	999	0	0	nev any	st
BARBON	742	x	m	0	0	all	-	a	Eu:wst	1979	CC	755	n	bl	y	y	0	all/unsp	15	24	0	1	nev any	st
BARBON	743	x	m	0	0	all	-	a	Eu:wst	1979	CC	755	n	bl	y	y	0	all/unsp	5	14	0	2	nev any	st
BARBON	744	x	m	0	0	all	-	a	Eu:wst	1979	CC	755	n	bl	y	y	0	all/unsp	0.1	4	3	3	nev any	st
BROWN3	505		f	0	0	wh	-	a	NAmer		CC	618		bl	y	n	0	all/unsp	15	999	0	1	nev any	st
JAHN	639		m	0	0	all	-	a	Eu:Ger	1988	CC	1004	n	bl	n	n	0	cig+/-ot	21	999	0	0	nev any	st
JAHN	640		m	0	0	all	-	a	Eu:Ger	1988	CC	1004	n	bl	n	n	0	cig+/-ot	11	20	1	0	nev any	st
JAHN	641		m	0	0	all	-	a	Eu:Ger	1988	CC	1004	n	bl	n	n	0	cig+/-ot	6	10	2	0	nev any	st
JAHN	642		m	0	0	all	-	a	Eu:Ger	1988	CC	1004	n	bl	n	n	0	cig+/-ot	2	5	3	3	nev any	st
JAHN	643		m	0	0	all	-	a	Eu:Ger	1988	CC	1004	n	bl	n	n	0	cig+/-ot	1.0	1.9	0	0	nev any	st
JAHN	644		m	0	0	all	-	a	Eu:Ger	1988	CC	1004	n	bl	n	n	0	cig+/-ot	0.1	0.9	0	0	nev any	st
JAIN	537		m	0	0	all	-	a	NAmer	1981	CC	845	n	V	y	n	0	cig+/-ot	10	999	1	0	nev cigs	st
JAIN	538		m	0	0	all	-	a	NAmer	1981	CC	845	n	V	y	n	0	cig+/-ot	2	9	0	3	nev cigs	st
JAIN	501		f	0	0	all	-	a	NAmer	1981	CC	845	n	V	y	n	0	cig+/-ot	10	999	1	0	nev cigs	st
JAIN	502		f	0	0	all	-	a	NAmer	1981	CC	845	n	V	y	n	0	cig+/-ot	2	9	0	3	nev cigs	st
JEDRYC	559		m	0	0	all	-	a	Eu:est	1980	CC	1630	n	bl	y	n	0	cig+/-ot	10	999	1	0	nev any	st
JEDRYC	560		m	0	0	all	-	a	Eu:est	1980	CC	1630	n	bl	y	n	0	cig+/-ot	5	9	2	0	nev any	st
LUBIN2	861		m	0	0	all	-	a	Eu:mul	1976	CC	7804	n	bl	n	y	0	cig+/-ot	20	999	0	1	nev any	st
LUBIN2	862		m	0	0	all	-	a	Eu:mul	1976	CC	7804	n	bl	n	y	0	cig+/-ot	15	19	0	0	nev any	st
LUBIN2	863		m	0	0	all	-	a	Eu:mul	1976	CC	7804	n	bl	n	y	0	cig+/-ot	10	14	1	2	nev any	st
LUBIN2	864		m	0	0	all	-	a	Eu:mul	1976	CC	7804	n	bl	n	y	0	cig+/-ot	5	9	2	0	nev any	st
LUBIN2	865		m	0	0	all	-	a	Eu:mul	1976	CC	7804	n	bl	n	y	0	cig+/-ot	0.1	4	3	3	nev any	st
LUBIN2	965		f	0	0	all	-	a	Eu:mul	1976	CC	7804	n	bl	n	y	0	cig+/-ot	20	999	0	1	nev any	st
LUBIN2	966		f	0	0	all	-	a	Eu:mul	1976	CC	7804	n	bl	n	y	0	cig+/-ot	10	19	1	2	nev any	st
LUBIN2	967		f	0	0	all	-	a	Eu:mul	1976	CC	7804	n	bl	n	y	0	cig+/-ot	0.1	9	0	3	nev any	st
MATOS	661	x	m	0	0	all	-	a	SCAmer	1994	CC	200	n	bl	n	n	0	cig+/-ot	11	999	1	0	nev any	st
MATOS	662	x	m	0	0	all	-	a	SCAmer	1994	CC	200	n	bl	n	n	0	cig+/-ot	6	10	2	0	nev any	st
MATOS	663	x	m	0	0	all	-	a	SCAmer	1994	CC	200	n	bl	n	n	0	cig+/-ot	1.0	5	3	3	nev any	st
PEZZOT	585		m	0	0	all	-	a	SCAmer	1987	CC	215	n	bl	n	y	0	cig only	11	999	1	0	nev cigs	st
PEZZOT	586		m	0	0	all	-	a	SCAmer	1987	CC	215	n	bl	n	y	0	cig only	1.0	10	0	3	nev cigs	st
SVENSS	569		f	0	0	all	-	a	Eu:Sca	1983	CC	210	n	bl	n	n	0	all/unsp	11	999	1	0	nev any	st
SVENSS	570		f	0	0	all	-	a	Eu:Sca	1983	CC	210	n	bl	n	n	0	all/unsp	3	10	0	3	nev any	st
WAKAI	554	x	m	0	0	all	-	a	As:Jap	1988	CC	333	n	bl	n	y	0	cig+/-ot	20	999	0	1	nev any	st
WAKAI	555	x	m	0	0	all	-	a	As:Jap	1988	CC	333	n	bl	n	y	0	cig+/-ot	10	19	1	2	nev any	st
WAKAI	556	x	m	0	0	all	-	a	As:Jap	1988	CC	333	n	bl	n	y	0	cig+/-ot	5	9	2	0	nev any	st
WU2	502		f	0	0	all	-	a	NAmer	1983	CC	336	n	bl	n	y	2	all/unsp	1.0	9	0	3	nev any	or
WYNDE3	559		m	0	0	all	-	KII	NAmer	1966	CC	350	n	bl	n	y	0	cig+/-ot	10	999	1	0	nev any	st
WYNDE3	580		f	0	0	all	-	KII	NAmer	1966	CC	350	n	bl	n	y	0	cig+/-ot	10	999	1	0	nev any	ot
WYNDE6	802	x	m	0	0	all	-	KII	NAmer	1969	CC	4423	n	bl	n	y	0	cig+/-ot	16	999	0	1	nev any	st
WYNDE6	803	x	m	0	0	all	-	KII	NAmer	1969	CC	4423	n	bl	n	y	0	cig+/-ot	11	15	1	2	nev any	st
WYNDE6	804	x	m	0	0	all	-	KII	NAmer	1969	CC	4423	n	bl	n	y	0	cig+/-ot	7	10	2	0	nev any	st
WYNDE6	805	x	m	0	0	all	-	KII	NAmer	1969	CC	4423	n	bl	n	y	0	cig+/-ot	4	6	0	0	nev any	st
WYNDE6	806	x	m	0	0	all	-	KII	NAmer	1969	CC	4423	n	bl	n	y	0	cig+/-ot	1.0	3	3	3	nev any	st

Cigarette type is all/unspec for all RRs

In this overview table, subtotals and Qs values may be invalid and should be ignored

Table 3J6 - 5

IESLC - Meta-analysis of Ex Smoking by Years quit (vs never), Overview
Adenocarcinoma, Cigarettes (or Any Product if Cigarettes not available)
Least adjusted

REF	NRR	SEX	AD	Number Exposed Case	Cont	Non-exposed Case	Cont	RR	95.00%CI
BARBON	741	m	0	4	59	7	188	1.82	(0.52- 6.44)
BARBON	742	m	0	7	41	7	188	4.59	(1.53- 13.79)
BARBON	743	m	0	23	85	7	188	7.27	(3.00- 17.59)
BARBON	744	m	0	7	20	7	188	9.40	(2.99- 29.53)
Subtotal BARBON								5.38	(3.15- 9.18)
BROWN3	505	f	0	73	219	432	1168	0.90	(0.68- 1.20)
JAHN	639	m	0	15	146	8	138	1.77	(0.73- 4.31)
JAHN	640	m	0	22	130	8	138	2.92	(1.26- 6.79)
JAHN	641	m	0	13	63	8	138	3.56	(1.40- 9.02)
JAHN	642	m	0	19	46	8	138	7.13	(2.92- 17.37)
JAHN	643	m	0	18	9	8	138	34.50	(11.82- 100.74)
JAHN	644	m	0	40	8	8	138	86.25	(30.45- 244.33)
Subtotal JAHN								6.98	(4.77- 10.22)
JAIN	537	m	0	14	113	4	85	2.63	(0.84- 8.28)
JAIN	538	m	0	16	46	4	85	7.39	(2.33- 23.41)
JAIN	501	f	0	3	61	24	214	0.44	(0.13- 1.51)
JAIN	502	f	0	14	36	24	214	3.47	(1.64- 7.32)
Subtotal JAIN								2.69	(1.63- 4.45)
JEDRYC	559	m	0	12	230	7	289	2.15	(0.83- 5.56)
JEDRYC	560	m	0	9	82	7	289	4.53	(1.64- 12.54)
Subtotal JEDRYC								3.04	(1.52- 6.09)
LUBIN2	861	m	0	35	1128	57	2616	1.42	(0.93- 2.18)
LUBIN2	862	m	0	21	478	57	2616	2.02	(1.21- 3.36)
LUBIN2	863	m	0	30	693	57	2616	1.99	(1.27- 3.12)
LUBIN2	864	m	0	50	882	57	2616	2.60	(1.77- 3.83)
LUBIN2	865	m	0	77	1047	57	2616	3.38	(2.38- 4.79)
LUBIN2	965	f	0	1	29	138	1180	0.29	(0.04- 2.18)
LUBIN2	966	f	0	3	33	138	1180	0.78	(0.24- 2.57)
LUBIN2	967	f	0	13	95	138	1180	1.17	(0.64- 2.14)
Subtotal LUBIN2								2.10	(1.76- 2.50)
MATOS	661	m	0	12	101	5	110	2.61	(0.89- 7.68)
MATOS	662	m	0	9	27	5	110	7.33	(2.27- 23.66)
MATOS	663	m	0	12	23	5	110	11.48	(3.69- 35.74)
Subtotal MATOS								5.83	(3.04- 11.18)
PEZZOT	585	m	0	7	106	3	116	2.55	(0.64- 10.13)
PEZZOT	586	m	0	11	82	3	116	5.19	(1.40- 19.18)
Subtotal PEZZOT								3.71	(1.44- 9.57)
SVENSS	569	f	0	7	24	22	120	1.59	(0.61- 4.14)
SVENSS	570	f	0	5	13	22	120	2.10	(0.68- 6.48)
Subtotal SVENSS								1.79	(0.86- 3.70)
WAKAI	554	m	0	3	47	8	65	0.52	(0.13- 2.06)
WAKAI	555	m	0	13	44	8	65	2.40	(0.92- 6.27)
WAKAI	556	m	0	7	48	8	65	1.18	(0.40- 3.49)
Subtotal WAKAI								1.36	(0.72- 2.56)
WU2	502	f	2	-	-	-	-	2.30	(1.20- 4.40)
WYNDE3	559	m	0	3	65	6	88	0.68	(0.16- 2.81)
WYNDE3	580	f	0	0	3	15	76	0.71~	(0.03- 14.35)
Subtotal WYNDE3								0.68	(0.19- 2.47)
WYNDE6	802	m	0	6	530	11	1667	1.72	(0.63- 4.66)
WYNDE6	803	m	0	11	259	11	1667	6.44	(2.76- 15.00)
WYNDE6	804	m	0	18	340	11	1667	8.02	(3.76- 17.14)
WYNDE6	805	m	0	14	321	11	1667	6.61	(2.97- 14.69)
WYNDE6	806	m	0	29	307	11	1667	14.32	(7.08- 28.96)
Subtotal WYNDE6								7.06	(4.92- 10.12)
Partial Totals				706	8119	1442	30040		

*prospective study ~ With 0.5 adjustment for zero

Table 3J6 - 5

IESLC - Meta-analysis of Ex Smoking by Years quit (vs never), Overview
Adenocarcinoma, Cigarettes (or Any Product if Cigarettes not available)
Least adjusted

REF	NRR	SEX	AD	Ys	Ws	Qs	Ps
BARBON	741	m	0	0.60	2.41	0.23	0.3523
BARBON	742	m	0	1.52	3.17	1.18	0.0067
BARBON	743	m	0	1.98	4.92	5.65	0.0000
BARBON	744	m	0	2.24	2.93	5.18	0.0001
Subtotal BARBON				1.68	13.43	12.25	
BROWN3	505	f	0	-0.10	46.65	48.11	0.4775
JAHN	639	m	0	0.57	4.86	0.56	0.2071
JAHN	640	m	0	1.07	5.39	0.14	0.0128
JAHN	641	m	0	1.27	4.44	0.57	0.0074
JAHN	642	m	0	1.96	4.84	5.36	0.0000
JAHN	643	m	0	3.54	3.35	23.13	0.0000
JAHN	644	m	0	4.46	3.54	44.54	0.0000
Subtotal JAHN				1.94	26.43	74.30	
JAIN	537	m	0	0.97	2.92	0.01	0.0979
JAIN	538	m	0	2.00	2.89	3.43	0.0007
JAIN	501	f	0	-0.82	2.52	7.61	0.1902
JAIN	502	f	0	1.24	6.87	0.76	0.0011
Subtotal JAIN				0.99	15.21	11.80	
JEDRYC	559	m	0	0.77	4.27	0.09	0.1127
JEDRYC	560	m	0	1.51	3.71	1.33	0.0036
Subtotal JEDRYC				1.11	7.98	1.42	
LUBIN2	861	m	0	0.35	21.10	6.57	0.1044
LUBIN2	862	m	0	0.70	14.78	0.65	0.0070
LUBIN2	863	m	0	0.69	18.97	0.96	0.0028
LUBIN2	864	m	0	0.96	25.60	0.05	0.0000
LUBIN2	865	m	0	1.22	31.38	2.92	0.0000
LUBIN2	965	f	0	-1.22	0.96	4.36	0.2317
LUBIN2	966	f	0	-0.25	2.69	3.64	0.6795
LUBIN2	967	f	0	0.16	10.47	5.96	0.6113
Subtotal LUBIN2				0.74	125.96	25.12	
MATOS	661	m	0	0.96	3.31	0.01	0.0806
MATOS	662	m	0	1.99	2.80	3.27	0.0009
MATOS	663	m	0	2.44	2.98	6.96	0.0000
Subtotal MATOS				1.76	9.08	10.24	
PEZZOT	585	m	0	0.94	2.02	0.00	0.1824
PEZZOT	586	m	0	1.65	2.25	1.21	0.0136
Subtotal PEZZOT				1.31	4.27	1.21	
SVENSS	569	f	0	0.46	4.20	0.84	0.3415
SVENSS	570	f	0	0.74	3.02	0.09	0.1976
Subtotal SVENSS				0.58	7.22	0.93	
WAKAI	554	m	0	-0.66	2.02	4.97	0.3507
WAKAI	555	m	0	0.88	4.17	0.01	0.0739
WAKAI	556	m	0	0.17	3.29	1.81	0.7583
Subtotal WAKAI				0.30	9.47	6.78	
WU2	502	f	2	0.83	9.10	0.06	0.0120
WYNDE3	559	m	0	-0.39	1.90	3.22	0.5908
WYNDE3	580	f	0	-0.35	0.42	0.67	0.8202
Subtotal WYNDE3				-0.38	2.32	3.89	
WYNDE6	802	m	0	0.54	3.85	0.53	0.2899
WYNDE6	803	m	0	1.86	5.37	4.85	0.0000
WYNDE6	804	m	0	2.08	6.67	9.14	0.0000
WYNDE6	805	m	0	1.89	6.02	5.75	0.0000
WYNDE6	806	m	0	2.66	7.74	23.69	0.0000
Subtotal WYNDE6				1.95	29.64	43.95	

N	43
NS	13

Table 3J6 - 6

IESLC - Meta-analysis of Ex Smoking by Years quit (vs never), Overview
Adenocarcinoma, Cigarettes (or Any Product if Cigarettes not available)
Least adjusted

	Sex			
	combined	male	female	Total
N		33	10	43
NS		10	6	16

In this overview table, other than the "N" rows, entries in the "absent" and "Total" columns may be invalid and should be ignored

	Years quit vs never (lower focus)				
	absent	8+k12	4-11k7	1-6k3	Total
N	19	13	6	5	43
NS	10	10	6	5	31
Wt	152.23	58.16	46.51	49.87	306.77
Het Chi	147.80	19.93	12.34	17.17	240.06
Het df	18	12	5	4	42
Het P	***	(*)	*	**	***
Fixed RR	1.93	2.06	3.32	5.19	2.49
RRl	1.64	1.59	2.49	3.93	2.22
RRu	2.26	2.66	4.42	6.85	2.78
P	+++	+++	+++	+++	+++
Random RR	3.05	1.97	3.72	7.72	3.07
RRl	1.85	1.37	2.19	3.80	2.30
RRu	5.03	2.83	6.32	15.69	4.10
P	+++	+++	+++	+++	+++

	Years quit vs never (higher focus)				
	absent	13+k20	4-19k12	1-11k3	Total
N	21	6	5	11	43
NS	11	5	4	9	29
Wt	108.44	77.75	36.11	84.47	306.77
Het Chi	97.17	13.28	14.88	41.43	240.06
Het df	20	5	4	10	42
Het P	***	*	**	***	***
Fixed RR	3.18	1.09	2.69	3.75	2.49
RRl	2.63	0.88	1.94	3.03	2.22
RRu	3.83	1.37	3.73	4.64	2.78
P	+++	N.S.	+++	+++	+++
Random RR	3.26	1.24	2.91	4.48	3.07
RRl	2.09	0.75	1.44	2.77	2.30
RRu	5.09	2.04	5.90	7.25	4.10
P	+++	N.S.	++	+++	+++

Table 3J6 - 6

IESLC - Meta-analysis of Ex Smoking by Years quit (vs never), Overview
Adenocarcinoma, Cigarettes (or Any Product if Cigarettes not available)
Least adjusted

MALES

Years quit vs never (lower focus)

		absent	8+k12	4-11k7	1-6k3	Total
	N	13	9	6	5	33
	NS	7	9	6	5	27
	Wt	75.16	48.33	46.51	49.87	219.86
Het	Chi	94.40	9.25	12.34	17.17	148.03
Het	df	12	8	5	4	32
Het	P	***	N.S.	*	**	***
Fixed	RR	3.13	2.43	3.32	5.19	3.36
	RRl	2.50	1.83	2.49	3.93	2.95
	RRu	3.93	3.22	4.42	6.85	3.84
	P	+++	+++	+++	+++	+++
Random	RR	4.34	2.47	3.72	7.72	3.97
	RRl	2.21	1.80	2.19	3.80	2.93
	RRu	8.54	3.40	6.32	15.69	5.39
	P	+++	+++	+++	+++	+++

Years quit vs never (higher focus)

		absent	13+k20	4-19k12	1-11k3	Total
	N	18	4	4	7	33
	NS	10	4	4	7	25
	Wt	101.29	30.14	33.42	55.00	219.86
Het	Chi	83.60	6.34	10.40	17.51	148.03
Het	df	17	3	3	6	32
Het	P	***	(*)	*	**	***
Fixed	RR	3.45	1.54	2.97	5.29	3.36
	RRl	2.84	1.08	2.12	4.06	2.95
	RRu	4.20	2.20	4.17	6.88	3.84
	P	+++	+	+++	+++	+++
Random	RR	3.85	1.62	3.69	7.25	3.97
	RRl	2.43	0.84	1.82	4.18	2.93
	RRu	6.11	3.13	7.49	12.60	5.39
	P	+++	N.S.	+++	+++	+++

FEMALES

Years quit vs never (lower focus)

		absent	8+k12	4-11k7	1-6k3	Total
	N	6	4			10
	NS	5	4			9
	Wt	77.07	9.83			86.91
Het	Chi	18.25	2.75			21.68
Het	df	5	3			9
Het	P	**	N.S.			**
Fixed	RR	1.20	0.91			1.16
	RRl	0.96	0.49			0.94
	RRu	1.50	1.69			1.43
	P	N.S.	N.S.			N.S.
Random	RR	1.50	0.91			1.29
	RRl	0.87	0.49			0.85
	RRu	2.59	1.69			1.96
	P	N.S.	N.S.			N.S.

Table 3J6 - 6

IESLC - Meta-analysis of Ex Smoking by Years quit (vs never), Overview
Adenocarcinoma, Cigarettes (or Any Product if Cigarettes not available)
Least adjusted

FEMALES

		Years quit vs never (higher focus)				
		absent	13+k20	4-19k12	1-11k3	Total
	N	3	2	1	4	10
	NS	3	2	1	4	9
	Wt	7.14	47.61	2.69	29.46	86.91
Het	Chi	2.66	1.17	0.00	5.27	21.68
Het	df	2	1	0	3	9
Het	P	N.S.	N.S.	N.S.	N.S.	**
Fixed	RR	0.96	0.88	0.78	1.97	1.16
	RRl	0.46	0.66	0.24	1.37	0.94
	RRu	2.00	1.17	2.57	2.83	1.43
	P	N.S.	N.S.	N.S.	+++	N.S.
Random	RR	0.90	0.81	0.78	2.04	1.29
	RRl	0.36	0.43	0.24	1.24	0.85
	RRu	2.26	1.53	2.57	3.34	1.96
	P	N.S.	N.S.	N.S.	++	N.S.

Table 3J6 - 7

IESLC - Meta-analysis of Ex Smoking by Years quit (vs never), Overview
Adenocarcinoma, Cigarettes (or Any Product if Cigarettes not available)
Excluded studies (and stage at which they were excluded)

Stage	Studies
1	AGUDO AKIBA AMANDU AMES AXELSS BEST BOUCHA BOUCOT BRESLO CHEN CHEN2 CHIAZZ DEAN2 DOSEME ENGELA FAN GENG GER GUO HAENSZ HEGMAN HOLE HU HU2 JUSSAW KATSOU KAUFMA KOO KOULUM KREUZE LETOUR LEVIN LIAW LIU3 LIU4 LIU5 MCCONN MIGRAN MRFITR NOTAN2 OSANN2 PERNU QIAO2 RACHTA RESTRE SADOWS SEGI2 STASZE TIZZAN VUTUC WATSON WU WUWILL WYNDE2 WYNDE8 XU YUAN ZHANG ZHENG ZHOU
2	BUFFLE HUMBLE PISANI PRESCO WYNDE7
3	MCDUFF SPITZ
4	ARMADA AUVINE BECHER BENSHL BLOT1 BOFFET BROSS CARPEN CEDERL CHOI CHYOU CORREA CPSI CPSII DAMBER DARBY DEAN3 DESTEF DOLL DOLL2 DORGAN DORN GAO GAO2 GARCIA GARSHI GILLIS GRAHAM GURSEL HAMMO2 HAMMON HIRAYA JOLY KAISE2 KHUDER LAUSSM LUBIN LUO PEZZO2 QIAO SPEIZE SUZUK2 TVERDA WANG2 WIGLE
5	ALDERS
10	SOBUE
14	BENHAM

Table 3J6 - 8

Potentially overlapping studies

REF	REFGP	PRINC	OVERLAP/LINK
LUBIN2	LUBIN2	1	Lubin-combined
WYNDE6	WYNDE6	1	WYNDE5/6/7/8
JAHN	BOFFET	2	Subset of BOFFET

Table 3J6 - 9

Most adjusted - insufficient data for meta-analysis

REF	NRR	SEX	AGEL	AGEH	RACE	YF	LC TYPE	LOC	START	ST	NLC	R	VB	P	H	AD	PRODUCT	exL	exH	S1	S2	DENOM	De
ALDERS	567	m	0	0	all	-	not q+s	Eu:UK	1977	CC	1448	n	V	n	n	1	cig only	10	999	1	0	nev any	st
ALDERS	568	m	0	0	all	-	not q+s	Eu:UK	1977	CC	1448	n	V	n	n	1	cig only	3	9	0	3	nev any	st
ALDERS	569	m	0	0	all	-	not q+s	Eu:UK	1977	CC	1448	n	V	n	n	1	cig only	0.1	2	0	0	nev any	st
ALDERS	578	f	0	0	all	-	not q+s	Eu:UK	1977	CC	1448	n	V	n	n	1	cig only	10	999	1	0	nev any	st
ALDERS	579	f	0	0	all	-	not q+s	Eu:UK	1977	CC	1448	n	V	n	n	1	cig only	3	9	0	3	nev any	st
ALDERS	580	f	0	0	all	-	not q+s	Eu:UK	1977	CC	1448	n	V	n	n	1	cig only	0.1	2	0	0	nev any	st
BROWN3	506	f	0	0	wh	-	a	NAmer		CC	618		bl	y	n	0	all/unsp	1.0	14	0	0	nev any	ot
JAIN	586	m	0	0	all	-	a	NAmer	1981	CC	845	n	V	y	n	0	cig+/-ot	0.1	1.9	0	0	nev cigs	ot
JAIN	574	f	0	0	all	-	a	NAmer	1981	CC	845	n	V	y	n	0	cig+/-ot	0.1	1.9	0	0	nev cigs	ot
JEDRYC	561	m	0	0	all	-	a	Eu:est	1980	CC	1630	n	bl	y	n	0	cig+/-ot	1.0	4	3	3	nev any	ot
MATOS	711	m	0	0	all	-	a	SCAmer	1994	CC	200	n	bl	n	n	2	cig+/-ot	0.1	0.9	0	0	nev any	ot
PEZZOT	601	m	0	0	all	-	a	SCAmer	1987	CC	215	n	bl	n	y	0	cig only	0.1	0.9	0	0	nev cigs	ot
SVENSS	597	f	0	0	all	-	a	Eu:Sca	1983	CC	210	n	bl	n	n	0	all/unsp	1.0	2	0	0	nev any	ot
WAKAI	619	m	0	0	all	-	a	As:Jap	1988	CC	333	n	bl	n	y	1	cig+/-ot	1.0	4	3	3	nev any	ot
WU2	501	f	0	0	all	-	a	NAmer	1983	CC	336	n	bl	n	y	2	all/unsp	10	999	1	0	nev any	ot
WYNDE3	560	m	0	0	all	-	KII	NAmer	1966	CC	350	n	bl	n	y	0	cig+/-ot	1.0	9	0	3	nev any	ot
WYNDE3	581	f	0	0	all	-	KII	NAmer	1966	CC	350	n	bl	n	y	0	cig+/-ot	1.0	9	0	3	nev any	ot
WYNDE6	822	m	0	0	all	-	KII	NAmer	1969	CC	4423	n	bl	n	y	2	cig+/-ot	0.1	0.9	0	0	nev any	ot

REF	NRR	RR	SIG	RRDATA comment
ALDERS	567	2.22		0
ALDERS	568	3.80		0
ALDERS	569	5.05		0
ALDERS	578	1.64		0
ALDERS	579	0.73		0
ALDERS	580	6.27		0
BROWN3	506	*	gap	0
JAIN	586	*	gap	0
JAIN	574	*	gap	0
JEDRYC	561	*	gap	0
MATOS	711	*	gap	0
PEZZOT	601	*	gap	0
SVENSS	597	*	gap	0
WAKAI	619	*	gap	0
WU2	501	*	gap	0
WYNDE3	560	*	gap	0
WYNDE3	581	*	gap	0
WYNDE6	822	*	gap	0

Table 3J6 - 9

IESLC - Meta-analysis of Ex Smoking by Years quit (vs never), Overview
Adenocarcinoma, Cigarettes (or Any Product if Cigarettes not available)

Least adjusted - insufficient data for meta-analysis: as for adjusted plus the following

REF	NRR	SEX	AGEL	AGEH	RACE	YF	LC TYPE	LOC	START	ST	NLC	R	VB	P	H	AD	PRODUCT	exL	exH	S1	S2	DENOM	De
MATOS	709	m	0	0	all	-	a	SCAmer	1994	CC	200	n	bl	n	n	0	cig+/-ot	0.1	0.9	0	0	nev any	ot
WAKAI	617	m	0	0	all	-	a	As:Jap	1988	CC	333	n	bl	n	y	0	cig+/-ot	1.0	4	3	3	nev any	ot
WYNDE6	807	m	0	0	all	-	KII	NAmer	1969	CC	4423	n	bl	n	y	0	cig+/-ot	0.1	0.9	0	0	nev any	ot

REF	NRR	RR	SIG	RRDATA comment
MATOS	709	*	gap	0
WAKAI	617	*	gap	0
WYNDE6	807	*	gap	0

Table 3J7 -

IESLC - Meta-analysis of Ex Smoking, Years quit (vs never), "Low"
Adenocarcinoma, Cigarettes (or Any Product if Cigarettes not available)

This analysis is restricted to results for:
1) Ex smokers
2) Results by Years quit (vs never)
3) Categorical results by Years quit (vs never)
4) Adenocarcinoma (or near equivalent)
5) Results complete enough for use in metaanalysis

Within each study, results are then selected (in the following order of preference, within each sex) for:
6) (not applicable)
7) PRODUCT: cigarettes regardless of other products, cigarettes only, all/unspec
8) CIGTYPE: all/unspecified, MC regardless of HR, MC only
9) (not applicable)
10) DENOM: never smoked anything, never smoked cigarettes, never any + low, never cigs + low
11) Followup period (YF, prospective studies): whole study (coded as 0) or longest available
12) LCtype: adeno or nearest available, but not squamous. (q = squamous, s = small,
a = adeno, l = large, KII = Kreyberg II, al = alveolar, br = bronchiolar, u = undifferentiated)
13) Race: all or nearest available, otherwise by race (wh or w = white, bl or b = black, hi = hispanic
ch = chinese, jap = japanese, haw = hawaiian, w+o = white + oriental, sca = scandinavian, as = asian)
14) Years quit (vs never) "low" in key scheme 1 (key value 12, maximum range 8+)
15) For overlapping studies: principal rather than subsidiary studies
Finally by Age: whole study (coded as 0) if available, otherwise by widest available age group
and then for single sex results (m, f) in preference to results for both sexes combined (c).

Results adjusted (AD) for the most potential confounders are then chosen in Sections -1 to -3
(and those which actually differ from the adjusted results in Table 3J2 - 1 are marked 'x' in Section -1)
and results adjusted for the least confounders in Sections -4 to -6. (Those least adjusted results which
actually differ from the most adjusted are marked 'x' in column X in Section -4)

Section -7 shows excluded studies, together with the stage (as above) at which no qualifying
results were found.

Section -8 lists the potentially overlapping studies which have been included (1=principal, 2=subsidiary).

Section -9 lists any results which would have been included in preference except that they had data not complete
enough for use in meta-analysis, with their significance (yes/no), if known, and any further comment as entered
on the database. It also lists as "gap" any categories for which no data were presented by the original authors.
This is commonly due to recent quitters having been combined with current smokers

In addition to those mentioned above, the following fields, levels and abbreviations are used:
* or nk = not known, n = no, y = yes, ot = other
nev = never
all/unspec = all or unspecified, cig+/-ot = cigarettes irrespective of other products (cigar, pipe etc)
MC = manufactured cigarettes, HR = hand-rolled cigarettes
exL, exH = range of exposure (low and high) in the smoking group, in terms of Years quit (vs never)
REF: 6-character study reference
NRR: number of the RR on the database within the study
ST : study type (CC = case control, pr or prosp = prospective)
NLC: number of lung cancer cases in whole study
R : risky occupational population (n = no, m = mining, o = other risky)
VB : national cigarette type (V = at least 75% Virginia, bl = at least 75% blended, ot = other)
P : any proxy use
H : full histological confirmation
De : derivation of RR/CI (or = original, st = standard method, ot = other method of estimation)

Table 3J7 - 1

IESLC - Meta-analysis of Ex Smoking, Years quit (vs never), "Low"
Adenocarcinoma, Cigarettes (or Any Product if Cigarettes not available)
Most adjusted

REF	NRR	3J2	SEX	AGEL	AGEH	RACE	YF	LC TYPE	LOC	START	ST	NLC	R	VB	P	H	AD	PRODUCT	exL	exH	DENOM	De
JAHN	640		m	0	0	all	-	a	Eu:Ger	1988	CC	1004	n	bl	n	n	0	cig+/-ot	11	20	nev any	st
JAIN	537		m	0	0	all	-	a	NAmer	1981	CC	845	n	V	y	n	0	cig+/-ot	10	999	nev cigs	st
JAIN	501		f	0	0	all	-	a	NAmer	1981	CC	845	n	V	y	n	0	cig+/-ot	10	999	nev cigs	st
JEDRYC	559		m	0	0	all	-	a	Eu:est	1980	CC	1630	n	bl	y	n	0	cig+/-ot	10	999	nev any	st
LUBIN2	863		m	0	0	all	-	a	Eu:mul	1976	CC	7804	n	bl	n	y	0	cig+/-ot	10	14	nev any	st
LUBIN2	966		f	0	0	all	-	a	Eu:mul	1976	CC	7804	n	bl	n	y	0	cig+/-ot	10	19	nev any	st
MATOS	671		m	0	0	all	-	a	SCAmer	1994	CC	200	n	bl	n	n	2	cig+/-ot	11	999	nev any	ot
PEZZOT	585		m	0	0	all	-	a	SCAmer	1987	CC	215	n	bl	n	y	0	cig only	11	999	nev cigs	st
SVENSS	569		f	0	0	all	-	a	Eu:Sca	1983	CC	210	n	bl	n	n	0	all/unsp	11	999	nev any	st
WAKAI	563		m	0	0	all	-	a	As:Jap	1988	CC	333	n	bl	n	y	1	cig+/-ot	10	19	nev any	or
WYNDE3	559		m	0	0	all	-	KII	NAmer	1966	CC	350	n	bl	n	y	0	cig+/-ot	10	999	nev any	st
WYNDE3	580		f	0	0	all	-	KII	NAmer	1966	CC	350	n	bl	n	y	0	cig+/-ot	10	999	nev any	ot
WYNDE6	818		m	0	0	all	-	KII	NAmer	1969	CC	4423	n	bl	n	y	2	cig+/-ot	11	15	nev any	ot

Cigarette type is all/unspec for all RRs

Table 3J7 - 2

IESLC - Meta-analysis of Ex Smoking, Years quit (vs never), "Low"
Adenocarcinoma, Cigarettes (or Any Product if Cigarettes not available)
Most adjusted

REF	NRR	SEX	AD	Number Exposed Case	Number Exposed Cont	Non-exposed Case	Non-exposed Cont	RR	95.00%CI
JAHN	640	m	0	22	130	8	138	2.92	(1.26- 6.79)
JAIN	537	m	0	14	113	4	85	2.63	(0.84- 8.28)
JAIN	501	f	0	3	61	24	214	0.44	(0.13- 1.51)
Subtotal JAIN								1.15	(0.50- 2.66)
JEDRYC	559	m	0	12	230	7	289	2.15	(0.83- 5.56)
LUBIN2	863	m	0	30	693	57	2616	1.99	(1.27- 3.12)
LUBIN2	966	f	0	3	33	138	1180	0.78	(0.24- 2.57)
Subtotal LUBIN2								1.77	(1.16- 2.69)
MATOS	671	m	2	12	-	5	-	3.00	(1.09- 8.23)
PEZZOT	585	m	0	7	106	3	116	2.55	(0.64- 10.13)
SVENSS	569	f	0	7	24	22	120	1.59	(0.61- 4.14)
WAKAI	563	m	1	13	-	8	-	2.49	(0.95- 6.53)
WYNDE3	559	m	0	3	65	6	88	0.68	(0.16- 2.81)
WYNDE3	580	f	0	0	3	15	76	0.71~	(0.03- 14.35)
Subtotal WYNDE3								0.68	(0.19- 2.47)
WYNDE6	818	m	2	11	-	11	-	5.40	(2.32- 12.58)
Partial Totals				137	1458	308	4922		

*prospective study

~ With 0.5 adjustment for zero

REF	NRR	SEX	AD	Ys	Ws	Qs	Ps
JAHN	640	m	0	1.07	5.39	0.67	0.0128
JAIN	537	m	0	0.97	2.92	0.18	0.0979
JAIN	501	f	0	-0.82	2.52	6.01	0.1902
Subtotal JAIN				0.14	5.45	6.19	
JEDRYC	559	m	0	0.77	4.27	0.01	0.1127
LUBIN2	863	m	0	0.69	18.97	0.02	0.0028
LUBIN2	966	f	0	-0.25	2.69	2.53	0.6795
Subtotal LUBIN2				0.57	21.66	2.55	
MATOS	671	m	2	1.10	3.76	0.54	0.0332
PEZZOT	585	m	0	0.94	2.02	0.10	0.1824
SVENSS	569	f	0	0.46	4.20	0.27	0.3415
WAKAI	563	m	1	0.91	4.14	0.16	0.0636
WYNDE3	559	m	0	-0.39	1.90	2.33	0.5908
WYNDE3	580	f	0	-0.35	0.42	0.48	0.8202
Subtotal WYNDE3				-0.38	2.32	2.81	
WYNDE6	818	m	2	1.69	5.38	5.04	0.0001

	N	13
	NS	10
	Wt	58.59
Het	Chi	18.35
Het	df	12
Het	P	N.S.
Fixed	RR	2.05
	RRl	1.59
	RRu	2.65
	P	+++
Random	RR	1.98
	RRl	1.40
	RRu	2.79
	P	+++
Asymm	P	N.S.

Table 3J7 - 3

IESLC - Meta-analysis of Ex Smoking, Years quit (vs never), "Low"
Adenocarcinoma, Cigarettes (or Any Product if Cigarettes not available)
Most adjusted

	Sex			
	combined	male	female	Total
N		9	4	13
NS		9	4	13
Wt		48.76	9.83	58.59
Het Chi		7.73	2.75	18.35
Het df		8	3	12
Het P		N.S.	N.S.	N.S.
Fixed RR		2.42	0.91	2.05
RRl		1.83	0.49	1.59
RRu		3.20	1.69	2.65
P		+++	N.S.	+++
Random RR		2.42	0.91	1.98
RRl		1.83	0.49	1.40
RRu		3.20	1.69	2.79
P		+++	N.S.	+++
Between Chi				7.86
Between df				1
Between P				**
Btwn(F) P				*
Btwn(R) P				**

	Lung cancer type						
	a	a+l	a+al+br	KII	not q+u	not q+s	Total
N	10			3			13
NS	8			2			10
Wt	50.89			7.70			58.59
Het Chi	10.35			6.94			18.35
Het df	9			2			12
Het P	N.S.			*			N.S.
Fixed RR	1.95			2.89			2.05
RRl	1.48			1.43			1.59
RRu	2.56			5.86			2.65
P	+++			++			+++
Random RR	1.93			1.72			1.98
RRl	1.42			0.33			1.40
RRu	2.63			9.03			2.79
P	+++			N.S.			+++
Between Chi							1.05
Between df							1
Between P							N.S.
Btwn(F) P							N.S.
Btwn(R) P							N.S.

	Location								
	NAmer	UK	Scand	othEur	China	Japan	othAs	other	Total
N	5		1	4		1		2	13
NS	3		1	3		1		2	10
Wt	13.15		4.20	31.33		4.14		5.78	58.59
Het Chi	14.02		0.00	3.20		0.00		0.03	18.35
Het df	4		0	3		0		1	12
Het P	**		N.S.	N.S.		N.S.		N.S.	N.S.
Fixed RR	1.97		1.59	1.98		2.49		2.84	2.05
RRl	1.15		0.61	1.40		0.95		1.26	1.59
RRu	3.39		4.14	2.81		6.53		6.41	2.65
P	+		N.S.	+++		(+)		+	+++
Random RR	1.44		1.59	1.98		2.49		2.84	1.98
RRl	0.48		0.61	1.36		0.95		1.26	1.40
RRu	4.34		4.14	2.87		6.53		6.41	2.79
P	N.S.		N.S.	+++		(+)		+	+++
Between Chi									1.09
Between df									4
Between P									N.S.
Btwn(F) P									N.S.
Btwn(R) P									N.S.

Table 3J7 - 3

IESLC - Meta-analysis of Ex Smoking, Years quit (vs never), "Low"
Adenocarcinoma, Cigarettes (or Any Product if Cigarettes not available)
Most adjusted

Detailed Country in "other Europe"

	multi	Germany	othWest	East	Balkans	Total
N	2	1		1		4
NS	1	1		1		3
Wt	21.66	5.39		4.27		31.33
Het Chi	2.07	0.00		0.00		3.20
Het df	1	0		0		3
Het P	N.S.	N.S.		N.S.		N.S.
Fixed RR	1.77	2.92		2.15		1.98
RRl	1.16	1.26		0.83		1.40
RRu	2.69	6.79		5.56		2.81
P	++	+		N.S.		+++
Random RR	1.47	2.92		2.15		1.98
RRl	0.63	1.26		0.83		1.36
RRu	3.47	6.79		5.56		2.87
P	N.S.	+		N.S.		+++
Between Chi						1.12
Between df						2
Between P						N.S.
Btwn(F) P						N.S.
Btwn(R) P						N.S.

Detailed Country in "other Asia"

	India	HongKong	other	Total
N				
NS				
Wt				
Het Chi				
Het df				
Het P				N.S.
Fixed RR				
RRl				
RRu				
P				N.S.
Random RR				
RRl				
RRu				
P				N.S.
Between Chi				
Between df				
Between P				N.S.
Btwn(F) P				N.S.
Btwn(R) P				N.S.

Detailed other continent

	SCAmer	Total
N	2	2
NS	2	2
Wt	5.78	5.78
Het Chi	0.03	0.03
Het df	1	1
Het P	N.S.	N.S.
Fixed RR	2.84	2.84
RRl	1.26	1.26
RRu	6.41	6.41
P	+	+
Random RR	2.84	2.84
RRl	1.26	1.26
RRu	6.41	6.41
P	+	+
Between Chi		
Between df		
Between P		N.S.
Btwn(F) P		N.S.
Btwn(R) P		N.S.

Table 3J7 - 3

IESLC - Meta-analysis of Ex Smoking, Years quit (vs never), "Low"
Adenocarcinoma, Cigarettes (or Any Product if Cigarettes not available)
Most adjusted

	<1960	1960-69	1970-79	1980-89	1990+	Total
	Start year of study					
N		3	2	7	1	13
NS		2	1	6	1	10
Wt		7.70	21.66	25.47	3.76	58.59
Het Chi		6.94	2.07	7.37	0.00	18.35
Het df		2	1	6	0	12
Het P		*	N.S.	N.S.	N.S.	N.S.
Fixed RR		2.89	1.77	1.98	3.00	2.05
RRl		1.43	1.16	1.34	1.09	1.59
RRu		5.86	2.69	2.92	8.24	2.65
P		++	++	+++	+	+++
Random RR		1.72	1.47	1.96	3.00	1.98
RRl		0.33	0.63	1.27	1.09	1.40
RRu		9.03	3.47	3.02	8.24	2.79
P		N.S.	N.S.	++	+	+++
Between Chi						1.96
Between df						3
Between P						N.S.
Btwn(F) P						N.S.
Btwn(R) P						N.S.

	CC	other	Total
	Study type (1)		
N	13		13
NS	10		10
Wt	58.59		58.59
Het Chi	18.35		18.35
Het df	12		12
Het P	N.S.		N.S.
Fixed RR	2.05		2.05
RRl	1.59		1.59
RRu	2.65		2.65
P	+++		+++
Random RR	1.98		1.98
RRl	1.40		1.40
RRu	2.79		2.79
P	+++		+++
Between Chi			
Between df			
Between P			N.S.
Btwn(F) P			N.S.
Btwn(R) P			N.S.

	CC	prosp	other	Total
	Study type (2)			
N	13			13
NS	10			10
Wt	58.59			58.59
Het Chi	18.35			18.35
Het df	12			12
Het P	N.S.			N.S.
Fixed RR	2.05			2.05
RRl	1.59			1.59
RRu	2.65			2.65
P	+++			+++
Random RR	1.98			1.98
RRl	1.40			1.40
RRu	2.79			2.79
P	+++			+++
Between Chi				
Between df				
Between P				N.S.
Btwn(F) P				N.S.
Btwn(R) P				N.S.

Table 3J7 - 3

IESLC - Meta-analysis of Ex Smoking, Years quit (vs never), "Low"
Adenocarcinoma, Cigarettes (or Any Product if Cigarettes not available)
Most adjusted

	Study size (number of LC cases) 100-249	250-499	500-999	1000+	Total
N	3	3	2	5	13
NS	3	2	1	4	10
Wt	9.98	6.46	5.45	36.71	58.59
Het Chi	0.85	2.49	4.35	7.81	18.35
Het df	2	2	1	4	12
Het P	N.S.	N.S.	*	(*)	N.S.
Fixed RR	2.22	1.56	1.15	2.29	2.05
RRl	1.20	0.72	0.50	1.66	1.59
RRu	4.14	3.38	2.66	3.17	2.65
P	+	N.S.	N.S.	+++	+++
Random RR	2.22	1.44	1.09	2.34	1.98
RRl	1.20	0.56	0.19	1.42	1.40
RRu	4.14	3.66	6.32	3.87	2.79
P	+	N.S.	N.S.	+++	+++
Between Chi					2.84
Between df					3
Between P					N.S.
Btwn(F) P					N.S.
Btwn(R) P					N.S.

	Risky occupational population no	mining	othRisky	Total
N	13			13
NS	10			10
Wt	58.59			58.59
Het Chi	18.35			18.35
Het df	12			12
Het P	N.S.			N.S.
Fixed RR	2.05			2.05
RRl	1.59			1.59
RRu	2.65			2.65
P	+++			+++
Random RR	1.98			1.98
RRl	1.40			1.40
RRu	2.79			2.79
P	+++			+++
Between Chi				
Between df				
Between P				N.S.
Btwn(F) P				N.S.
Btwn(R) P				N.S.

	National cigarette tobacco type Virginia	blended	other	Total
N	2	11		13
NS	1	9		10
Wt	5.45	53.14		58.59
Het Chi	4.35	11.97		18.35
Het df	1	10		12
Het P	*	N.S.		N.S.
Fixed RR	1.15	2.18		2.05
RRl	0.50	1.66		1.59
RRu	2.66	2.85		2.65
P	N.S.	+++		+++
Random RR	1.09	2.17		1.98
RRl	0.19	1.59		1.40
RRu	6.32	2.97		2.79
P	N.S.	+++		+++
Between Chi				2.03
Between df				1
Between P				N.S.
Btwn(F) P				N.S.
Btwn(R) P				N.S.

Table 3J7 - 3

IESLC - Meta-analysis of Ex Smoking, Years quit (vs never), "Low"
Adenocarcinoma, Cigarettes (or Any Product if Cigarettes not available)
Most adjusted

	Any proxy use		
	No/nk	Yes	Total
N	10	3	13
NS	8	2	10
Wt	48.87	9.72	58.59
Het Chi	11.97	5.30	18.35
Het df	9	2	12
Het P	N.S.	(*)	N.S.
Fixed RR	2.18	1.51	2.05
RRl	1.65	0.81	1.59
RRu	2.88	2.84	2.65
P	+++	N.S.	+++
Random RR	2.16	1.41	1.98
RRl	1.52	0.50	1.40
RRu	3.08	3.99	2.79
P	+++	N.S.	+++
Between Chi			1.08
Between df			1
Between P			N.S.
Btwn(F) P			N.S.
Btwn(R) P			N.S.

	Full histological confirmation		
	No	Yes	Total
N	6	7	13
NS	5	5	10
Wt	23.07	35.52	58.59
Het Chi	7.67	10.65	18.35
Het df	5	6	12
Het P	N.S.	(*)	N.S.
Fixed RR	1.99	2.09	2.05
RRl	1.32	1.50	1.59
RRu	2.99	2.90	2.65
P	+++	+++	+++
Random RR	1.93	1.98	1.98
RRl	1.16	1.17	1.40
RRu	3.22	3.35	2.79
P	+	+	+++
Between Chi			0.03
Between df			1
Between P			N.S.
Btwn(F) P			N.S.
Btwn(R) P			N.S.

	Number of adjustment variables (1)			
	0	1	2+/+nk	Total
N	10	1	2	13
NS	7	1	2	10
Wt	45.32	4.14	9.14	58.59
Het Chi	11.39	0.00	0.76	18.35
Het df	9	0	1	12
Het P	N.S.	N.S.	N.S.	N.S.
Fixed RR	1.74	2.49	4.24	2.05
RRl	1.30	0.95	2.22	1.59
RRu	2.33	6.53	8.11	2.65
P	+++	(+)	+++	+++
Random RR	1.65	2.49	4.24	1.98
RRl	1.15	0.95	2.22	1.40
RRu	2.37	6.53	8.11	2.79
P	++	(+)	+++	+++
Between Chi				6.20
Between df				2
Between P				*
Btwn(F) P				N.S.
Btwn(R) P				*

Table 3J7 - 3

IESLC - Meta-analysis of Ex Smoking, Years quit (vs never), "Low"
Adenocarcinoma, Cigarettes (or Any Product if Cigarettes not available)
Most adjusted

	Number of adjustment variables (2) 0	1	2	3-5	6+/+nk	Total
N	10	1	2			13
NS	7	1	2			10
Wt	45.32	4.14	9.14			58.59
Het Chi	11.39	0.00	0.76			18.35
Het df	9	0	1			12
Het P	N.S.	N.S.	N.S.			N.S.
Fixed RR	1.74	2.49	4.24			2.05
RRl	1.30	0.95	2.22			1.59
RRu	2.33	6.53	8.11			2.65
P	+++	(+)	+++			+++
Random RR	1.65	2.49	4.24			1.98
RRl	1.15	0.95	2.22			1.40
RRu	2.37	6.53	8.11			2.79
P	++	(+)	+++			+++
Between Chi						6.20
Between df						2
Between P						*
Btwn(F) P						N.S.
Btwn(R) P						*

	Product all/unsp	cig+/-ot	cig only	Total
N	1	11	1	13
NS	1	8	1	10
Wt	4.20	52.37	2.02	58.59
Het Chi	0.00	17.97	0.00	18.35
Het df	0	10	0	12
Het P	N.S.	(*)	N.S.	N.S.
Fixed RR	1.59	2.08	2.55	2.05
RRl	0.61	1.58	0.64	1.59
RRu	4.14	2.72	10.13	2.65
P	N.S.	+++	N.S.	+++
Random RR	1.59	1.96	2.55	1.98
RRl	0.61	1.31	0.64	1.40
RRu	4.14	2.93	10.13	2.79
P	N.S.	++	N.S.	+++
Between Chi				0.38
Between df				2
Between P				N.S.
Btwn(F) P				N.S.
Btwn(R) P				N.S.

	Denominator nev any	nev cigs	Total
N	10	3	13
NS	8	2	10
Wt	51.12	7.47	58.59
Het Chi	11.91	5.30	18.35
Het df	9	2	12
Het P	N.S.	(*)	N.S.
Fixed RR	2.16	1.42	2.05
RRl	1.64	0.70	1.59
RRu	2.84	2.92	2.65
P	+++	N.S.	+++
Random RR	2.15	1.43	1.98
RRl	1.53	0.44	1.40
RRu	3.02	4.61	2.79
P	+++	N.S.	+++
Between Chi			1.14
Between df			1
Between P			N.S.
Btwn(F) P			N.S.
Btwn(R) P			N.S.

Table 3J7 - 3

IESLC - Meta-analysis of Ex Smoking, Years quit (vs never), "Low"
Adenocarcinoma, Cigarettes (or Any Product if Cigarettes not available)
Most adjusted

	Derivation of RR/CI			
	Orig	StdCalc	Other	Total
N	1	9	3	13
NS	1	7	3	11
Wt	4.14	44.90	9.56	58.59
Het Chi	0.00	11.04	2.07	18.35
Het df	0	8	2	12
Het P	N.S.	N.S.	N.S.	N.S.
Fixed RR	2.49	1.76	3.92	2.05
RRl	0.95	1.31	2.08	1.59
RRu	6.53	2.35	7.38	2.65
P	(+)	+++	+++	+++
Random RR	2.49	1.66	3.89	1.98
RRl	0.95	1.14	2.03	1.40
RRu	6.53	2.42	7.47	2.79
P	(+)	++	+++	+++
Between Chi				5.24
Between df				2
Between P				(*)
Btwn(F) P				N.S.
Btwn(R) P				(*)

Table 3J7 - 4

IESLC - Meta-analysis of Ex Smoking, Years quit (vs never), "Low"
Adenocarcinoma, Cigarettes (or Any Product if Cigarettes not available)
Least adjusted

REF	NRR	X	SEX	AGEL	AGEH	RACE	YF	LC TYPE	LOC	START	ST	NLC	R	VB	P	H	AD	PRODUCT	exL	exH	DENOM	De
JAHN	640		m	0	0	all	-	a	Eu:Ger	1988	CC	1004	n	bl	n	n	0	cig+/-ot	11	20	nev any	st
JAIN	537		m	0	0	all	-	a	NAmer	1981	CC	845	n	V	y	n	0	cig+/-ot	10	999	nev cigs	st
JAIN	501		f	0	0	all	-	a	NAmer	1981	CC	845	n	V	y	n	0	cig+/-ot	10	999	nev cigs	st
JEDRYC	559		m	0	0	all	-	a	Eu:est	1980	CC	1630	n	bl	y	n	0	cig+/-ot	10	999	nev any	st
LUBIN2	863		m	0	0	all	-	a	Eu:mul	1976	CC	7804	n	bl	n	y	0	cig+/-ot	10	14	nev any	st
LUBIN2	966		f	0	0	all	-	a	Eu:mul	1976	CC	7804	n	bl	n	y	0	cig+/-ot	10	19	nev any	st
MATOS	661	x	m	0	0	all	-	a	SCAmer	1994	CC	200	n	bl	n	n	0	cig+/-ot	11	999	nev any	st
PEZZOT	585		m	0	0	all	-	a	SCAmer	1987	CC	215	n	bl	n	y	0	cig only	11	999	nev cigs	st
SVENSS	569		f	0	0	all	-	a	Eu:Sca	1983	CC	210	n	bl	n	n	0	all/unsp	11	999	nev any	st
WAKAI	555	x	m	0	0	all	-	a	As:Jap	1988	CC	333	n	bl	n	y	0	cig+/-ot	10	19	nev any	st
WYNDE3	559		m	0	0	all	-	KII	NAmer	1966	CC	350	n	bl	n	y	0	cig+/-ot	10	999	nev any	st
WYNDE3	580		f	0	0	all	-	KII	NAmer	1966	CC	350	n	bl	n	y	0	cig+/-ot	10	999	nev any	ot
WYNDE6	803	x	m	0	0	all	-	KII	NAmer	1969	CC	4423	n	bl	n	y	0	cig+/-ot	11	15	nev any	st

Cigarette type is all/unspec for all RRs

Table 3J7 - 5

IESLC - Meta-analysis of Ex Smoking, Years quit (vs never), "Low"
Adenocarcinoma, Cigarettes (or Any Product if Cigarettes not available)
Least adjusted

REF	NRR	SEX	AD	Number Exposed Case	Cont	Non-exposed Case	Cont	RR	95.00%CI
JAHN	640	m	0	22	130	8	138	2.92	(1.26- 6.79)
JAIN	537	m	0	14	113	4	85	2.63	(0.84- 8.28)
JAIN	501	f	0	3	61	24	214	0.44	(0.13- 1.51)
Subtotal JAIN								1.15	(0.50- 2.66)
JEDRYC	559	m	0	12	230	7	289	2.15	(0.83- 5.56)
LUBIN2	863	m	0	30	693	57	2616	1.99	(1.27- 3.12)
LUBIN2	966	f	0	3	33	138	1180	0.78	(0.24- 2.57)
Subtotal LUBIN2								1.77	(1.16- 2.69)
MATOS	661	m	0	12	101	5	110	2.61	(0.89- 7.68)
PEZZOT	585	m	0	7	106	3	116	2.55	(0.64- 10.13)
SVENSS	569	f	0	7	24	22	120	1.59	(0.61- 4.14)
WAKAI	555	m	0	13	44	8	65	2.40	(0.92- 6.27)
WYNDE3	559	m	0	3	65	6	88	0.68	(0.16- 2.81)
WYNDE3	580	f	0	0	3	15	76	0.71~	(0.03- 14.35)
Subtotal WYNDE3								0.68	(0.19- 2.47)
WYNDE6	803	m	0	11	259	11	1667	6.44	(2.76- 15.00)
Totals				137	1862	308	6764		

*prospective study ~ With 0.5 adjustment for zero

REF	NRR	SEX	AD	Ys	Ws	Qs	Ps
JAHN	640	m	0	1.07	5.39	0.66	0.0128
JAIN	537	m	0	0.97	2.92	0.18	0.0979
JAIN	501	f	0	-0.82	2.52	6.03	0.1902
Subtotal JAIN				0.14	5.45	6.21	
JEDRYC	559	m	0	0.77	4.27	0.01	0.1127
LUBIN2	863	m	0	0.69	18.97	0.02	0.0028
LUBIN2	966	f	0	-0.25	2.69	2.55	0.6795
Subtotal LUBIN2				0.57	21.66	2.57	
MATOS	661	m	0	0.96	3.31	0.19	0.0806
PEZZOT	585	m	0	0.94	2.02	0.09	0.1824
SVENSS	569	f	0	0.46	4.20	0.28	0.3415
WAKAI	555	m	0	0.88	4.17	0.10	0.0739
WYNDE3	559	m	0	-0.39	1.90	2.34	0.5908
WYNDE3	580	f	0	-0.35	0.42	0.48	0.8202
Subtotal WYNDE3				-0.38	2.32	2.83	
WYNDE6	803	m	0	1.86	5.37	6.99	0.0000

	N	13
	NS	10
	Wt	58.16
Het	Chi	19.93
Het	df	12
Het	P	(*)
Fixed	RR	2.06
	RRl	1.59
	RRu	2.66
	P	+++
Random	RR	1.97
	RRl	1.37
	RRu	2.83
	P	+++
Asymm	P	N.S.

Table 3J7 - 6

IESLC - Meta-analysis of Ex Smoking, Years quit (vs never), "Low"
Adenocarcinoma, Cigarettes (or Any Product if Cigarettes not available)
Least adjusted

combined	Sex male	female	Total
N	9	4	13
NS	9	4	13
Wt	48.33	9.83	58.16
Het Chi	9.25	2.75	19.93
Het df	8	3	12
Het P	N.S.	N.S.	(*)
Fixed RR	2.43	0.91	2.06
RRl	1.83	0.49	1.59
RRu	3.22	1.69	2.66
P	+++	N.S.	+++
Random RR	2.47	0.91	1.97
RRl	1.80	0.49	1.37
RRu	3.40	1.69	2.83
P	+++	N.S.	+++
Between Chi			7.93
Between df			1
Between P			**
Btwn(F) P			*
Btwn(R) P			**

Table 3J7 - 7

IESLC - Meta-analysis of Ex Smoking, Years quit (vs never), "Low"
Adenocarcinoma, Cigarettes (or Any Product if Cigarettes not available)
Excluded studies (and stage at which they were excluded)

1	AGUDO AKIBA AMANDU AMES AXELSS BEST BOUCHA BOUCOT BRESLO CHEN CHEN2 CHIAZZ DEAN2 DOSEME ENGELA FAN GENG GER GUO HAENSZ HEGMAN HOLE HU HU2 JUSSAW KATSOU KAUFMA KOO KOULUM KREUZE LETOUR LEVIN LIAW LIU3 LIU4 LIU5 MCCONN MIGRAN MRFITR NOTAN2 OSANN2 PERNU QIAO2 RACHTA RESTRE SADOWS SEGI2 STASZE TIZZAN VUTUC WATSON WU WUWILL WYNDE2 WYNDE8 XU YUAN ZHANG ZHENG ZHOU
2	BUFFLE HUMBLE PISANI PRESCO WYNDE7
3	MCDUFF SPITZ
4	ARMADA AUVINE BECHER BENSHL BLOT1 BOFFET BROSS CARPEN CEDERL CHOI CHYOU CORREA CPSI CPSII DAMBER DARBY DEAN3 DESTEF DOLL DOLL2 DORGAN DORN GAO GAO2 GARCIA GARSHI GILLIS GRAHAM GURSEL HAMMO2 HAMMON HIRAYA JOLY KAISE2 KHUDER LAUSSM LUBIN LUO PEZZO2 QIAO SPEIZE SUZUK2 TVERDA WANG2 WIGLE
5	ALDERS
10	SOBUE
14	BARBON BROWN3 WU2
15	BENHAM

Table 3J7 - 8
Potentially overlapping studies

REF	REFGP	PRINC	OVERLAP/LINK
LUBIN2	LUBIN2	1	Lubin-combined
WYNDE6	WYNDE6	1	WYNDE5/6/7/8
JAHN	BOFFET	2	Subset of BOFFET

Table 3J7 - 9
Most adjusted - insufficient data for meta-analysis

REF	NRR	SEX	AGEL	AGEH	RACE	YF	LC TYPE	LOC	START	ST	NLC	R	VB	P	H	AD	PRODUCT	exL	exH	DENOM	De
ALDERS	567	m	0	0	all	-	not q+s	Eu:UK	1977	CC	1448	n	V	n	n	1	cig only	10	999	nev any	st
ALDERS	578	f	0	0	all	-	not q+s	Eu:UK	1977	CC	1448	n	V	n	n	1	cig only	10	999	nev any	st
WU2	501	f	0	0	all	-	a	NAmer	1983	CC	336	n	bl	n	y	2	all/unsp	10	999	nev any	ot

REF	NRR	RR	SIG	RRDATA comment
ALDERS	567	2.22		0
ALDERS	578	1.64		0
WU2	501	*	gap	0

Table 3J8 -

IESLC - Meta-analysis of Ex Smoking, Years quit (vs never), "Mid"
Adenocarcinoma, Cigarettes (or Any Product if Cigarettes not available)

This analysis is restricted to results for:
1) Ex smokers
2) Results by Years quit (vs never)
3) Categorical results by Years quit (vs never)
4) Adenocarcinoma (or near equivalent)
5) Results complete enough for use in metaanalysis

Within each study, results are then selected (in the following order of preference, within each sex) for:
6) (not applicable)
7) PRODUCT: cigarettes regardless of other products, cigarettes only, all/unspec
8) CIGTYPE: all/unspecified, MC regardless of HR, MC only
9) (not applicable)
10) DENOM: never smoked anything, never smoked cigarettes, never any + low, never cigs + low
11) Followup period (YF, prospective studies): whole study (coded as 0) or longest available
12) LCtype: adeno or nearest available, but not squamous. (q = squamous, s = small,
a = adeno, l = large, KII = Kreyberg II, al = alveolar, br = bronchiolar, u = undifferentiated)
13) Race: all or nearest available, otherwise by race (wh or w = white, bl or b = black, hi = hispanic
ch = chinese, jap = japanese, haw = hawaiian, w+o = white + oriental, sca = scandinavian, as = asian)
14) Years quit (vs never) "mid" in key scheme 1 (key value 7, maximum range 4-11)
15) For overlapping studies: principal rather than subsidiary studies
Finally by Age: whole study (coded as 0) if available, otherwise by widest available age group
and then for single sex results (m, f) in preference to results for both sexes combined (c).

Results adjusted (AD) for the most potential confounders are then chosen in Sections -1 to -3
(and those which actually differ from the adjusted results in Table 3J3 - 1 are marked 'x' in Section -1)
and results adjusted for the least confounders in Sections -4 to -6. (Those least adjusted results which
actually differ from the most adjusted are marked 'x' in column X in Section -4)

Section -7 shows excluded studies, together with the stage (as above) at which no qualifying
results were found.

Section -8 lists the potentially overlapping studies which have been included (1=principal, 2=subsidiary).

Section -9 lists any results which would have been included in preference except that they had data not complete
enough for use in meta-analysis, with their significance (yes/no), if known, and any further comment as entered
on the database. It also lists as "gap" any categories for which no data were presented by the original authors.
This is commonly due to recent quitters having been combined with current smokers

In addition to those mentioned above, the following fields, levels and abbreviations are used:
* or nk = not known, n = no, y = yes, ot = other
nev = never
all/unspec = all or unspecified, cig+/-ot = cigarettes irrespective of other products (cigar, pipe etc)
MC = manufactured cigarettes, HR = hand-rolled cigarettes
exL, exH = range of exposure (low and high) in the smoking group, in terms of Years quit (vs never)
REF: 6-character study reference
NRR: number of the RR on the database within the study
ST : study type (CC = case control, pr or prosp = prospective)
NLC: number of lung cancer cases in whole study
R : risky occupational population (n = no, m = mining, o = other risky)
VB : national cigarette type (V = at least 75% Virginia, bl = at least 75% blended, ot = other)
P : any proxy use
H : full histological confirmation
De : derivation of RR/CI (or = original, st = standard method, ot = other method of estimation)

Table 3J8 - 1

IESLC - Meta-analysis of Ex Smoking, Years quit (vs never), "Mid"
Adenocarcinoma, Cigarettes (or Any Product if Cigarettes not available)
Most adjusted

REF	NRR	3J3	SEX	AGEL	AGEH	RACE	YF	LC TYPE	LOC	START	ST	NLC	R	VB	P	H	AD	PRODUCT	exL	exH	DENOM	De
JAHN	641		m	0	0	all	-	a	Eu:Ger	1988	CC	1004	n	bl	n	n	0	cig+/-ot	6	10	nev any	st
JEDRYC	560		m	0	0	all	-	a	Eu:est	1980	CC	1630	n	bl	y	n	0	cig+/-ot	5	9	nev any	st
LUBIN2	864		m	0	0	all	-	a	Eu:mul	1976	CC	7804	n	bl	n	y	0	cig+/-ot	5	9	nev any	st
MATOS	672		m	0	0	all	-	a	SCAmer	1994	CC	200	n	bl	n	n	2	cig+/-ot	6	10	nev any	ot
WAKAI	564		m	0	0	all	-	a	As:Jap	1988	CC	333	n	bl	n	y	1	cig+/-ot	5	9	nev any	or
WYNDE6	819		m	0	0	all	-	KII	NAmer	1969	CC	4423	n	bl	n	y	2	cig+/-ot	7	10	nev any	ot

Cigarette type is all/unspec for all RRs

Table 3J8 - 2

IESLC - Meta-analysis of Ex Smoking, Years quit (vs never), "Mid"
Adenocarcinoma, Cigarettes (or Any Product if Cigarettes not available)
Most adjusted

REF	NRR	SEX	AD	Number Exposed Case	Cont	Non-exposed Case	Cont	RR	95.00%CI
JAHN	641	m	0	13	63	8	138	3.56	(1.40- 9.02)
JEDRYC	560	m	0	9	82	7	289	4.53	(1.64- 12.54)
LUBIN2	864	m	0	50	882	57	2616	2.60	(1.77- 3.83)
MATOS	672	m	2	9	-	5	-	10.00	(3.08- 32.51)
WAKAI	564	m	1	7	-	8	-	1.23	(0.42- 3.64)
WYNDE6	819	m	2	18	-	11	-	6.60	(3.09- 14.10)
Partial Totals				106	1027	96	3043		

*prospective study

REF	NRR	SEX	AD	Ys	Ws	Qs	Ps
JAHN	641	m	0	1.27	4.44	0.03	0.0074
JEDRYC	560	m	0	1.51	3.71	0.38	0.0036
LUBIN2	864	m	0	0.96	25.60	1.41	0.0000
MATOS	672	m	2	2.30	2.77	3.42	0.0001
WAKAI	564	m	1	0.21	3.30	3.19	0.7071
WYNDE6	819	m	2	1.89	6.67	3.23	0.0000

	N	6
	NS	6
	Wt	46.48
Het	Chi	11.66
Het	df	5
Het	P	*
Fixed	RR	3.29
	RRl	2.47
	RRu	4.39
	P	+++
Random	RR	3.74
	RRl	2.23
	RRu	6.25
	P	+++
Asymm	P	N.S.

Table 3J8 - 3

IESLC - Meta-analysis of Ex Smoking, Years quit (vs never), "Mid"
Adenocarcinoma, Cigarettes (or Any Product if Cigarettes not available)
Most adjusted

	Sex combined	male	female	Total
N		6		6
NS		6		6
Wt		46.48		46.48
Het Chi		11.66		11.66
Het df		5		5
Het P		*		*
Fixed RR		3.29		3.29
RRl		2.47		2.47
RRu		4.39		4.39
P		+++		+++
Random RR		3.74		3.74
RRl		2.23		2.23
RRu		6.25		6.25
P		+++		+++
Between Chi				
Between df				
Between P				N.S.
Btwn(F) P				N.S.
Btwn(R) P				N.S.

Too few RRs for analysis by factor

Table 3J8 - 4

IESLC - Meta-analysis of Ex Smoking, Years quit (vs never), "Mid"
Adenocarcinoma, Cigarettes (or Any Product if Cigarettes not available)
Least adjusted

REF	NRR	X	SEX	AGEL	AGEH	RACE	YF	LC TYPE	LOC	START	ST	NLC	R	VB	P	H	AD	PRODUCT	exL	exH	DENOM	De
JAHN	641		m	0	0	all	-	a	Eu:Ger	1988	CC	1004	n	bl	n	n	0	cig+/-ot	6	10	nev any	st
JEDRYC	560		m	0	0	all	-	a	Eu:est	1980	CC	1630	n	bl	y	n	0	cig+/-ot	5	9	nev any	st
LUBIN2	864		m	0	0	all	-	a	Eu:mul	1976	CC	7804	n	bl	n	y	0	cig+/-ot	5	9	nev any	st
MATOS	662	x	m	0	0	all	-	a	SCAmer	1994	CC	200	n	bl	n	n	0	cig+/-ot	6	10	nev any	st
WAKAI	556	x	m	0	0	all	-	a	As:Jap	1988	CC	333	n	bl	n	y	0	cig+/-ot	5	9	nev any	st
WYNDE6	804	x	m	0	0	all	-	KII	NAmer	1969	CC	4423	n	bl	n	y	0	cig+/-ot	7	10	nev any	st

Cigarette type is all/unspec for all RRs

Table 3J8 - 5

IESLC - Meta-analysis of Ex Smoking, Years quit (vs never), "Mid"
Adenocarcinoma, Cigarettes (or Any Product if Cigarettes not available)
Least adjusted

REF	NRR	SEX	AD	Number Exposed Case	Exposed Cont	Non-exposed Case	Non-exposed Cont	RR	95.00%CI
JAHN	641	m	0	13	63	8	138	3.56	(1.40- 9.02)
JEDRYC	560	m	0	9	82	7	289	4.53	(1.64- 12.54)
LUBIN2	864	m	0	50	882	57	2616	2.60	(1.77- 3.83)
MATOS	662	m	0	9	27	5	110	7.33	(2.27- 23.66)
WAKAI	556	m	0	7	48	8	65	1.18	(0.40- 3.49)
WYNDE6	804	m	0	18	340	11	1667	8.02	(3.76- 17.14)
Totals				106	1442	96	4885		

*prospective study

REF	NRR	SEX	AD	Ys	Ws	Qs	Ps
JAHN	641	m	0	1.27	4.44	0.02	0.0074
JEDRYC	560	m	0	1.51	3.71	0.36	0.0036
LUBIN2	864	m	0	0.96	25.60	1.50	0.0000
MATOS	662	m	0	1.99	2.80	1.76	0.0009
WAKAI	556	m	0	0.17	3.29	3.48	0.7583
WYNDE6	804	m	0	2.08	6.67	5.21	0.0000

	N	6
	NS	6
	Wt	46.51
Het	Chi	12.34
Het	df	5
Het	P	*
Fixed	RR	3.32
	RRl	2.49
	RRu	4.42
	P	+++
Random	RR	3.72
	RRl	2.19
	RRu	6.32
	P	+++
Asymm	P	N.S.

Table 3J8 - 6

IESLC - Meta-analysis of Ex Smoking, Years quit (vs never), "Mid"
Adenocarcinoma, Cigarettes (or Any Product if Cigarettes not available)
Least adjusted

	Sex combined	male	female	Total
N		6		6
NS		6		6
Wt		46.51		46.51
Het Chi		12.34		12.34
Het df		5		5
Het P		*		*
Fixed RR		3.32		3.32
RRl		2.49		2.49
RRu		4.42		4.42
P		+++		+++
Random RR		3.72		3.72
RRl		2.19		2.19
RRu		6.32		6.32
P		+++		+++
Between Chi				
Between df				
Between P				N.S.
Btwn(F) P				N.S.
Btwn(R) P				N.S.

Table 3J8 - 7

IESLC - Meta-analysis of Ex Smoking, Years quit (vs never), "Mid"
Adenocarcinoma, Cigarettes (or Any Product if Cigarettes not available)
Excluded studies (and stage at which they were excluded)

1	AGUDO AKIBA AMANDU AMES AXELSS BEST BOUCHA BOUCOT BRESLO CHEN CHEN2 CHIAZZ DEAN2 DOSEME ENGELA FAN GENG GER GUO HAENSZ HEGMAN HOLE HU HU2 JUSSAW KATSOU KAUFMA KOO KOULUM KREUZE LETOUR LEVIN LIAW LIU3 LIU4 LIU5 MCCONN MIGRAN MRFITR NOTAN2 OSANN2 PERNU QIAO2 RACHTA RESTRE SADOWS SEGI2 STASZE TIZZAN VUTUC WATSON WU WUWILL WYNDE2 WYNDE8 XU YUAN ZHANG ZHENG ZHOU
2	BUFFLE HUMBLE PISANI PRESCO WYNDE7
3	MCDUFF SPITZ
4	ARMADA AUVINE BECHER BENSHL BLOT1 BOFFET BROSS CARPEN CEDERL CHOI CHYOU CORREA CPSI CPSII DAMBER DARBY DEAN3 DESTEF DOLL DOLL2 DORGAN DORN GAO GAO2 GARCIA GARSHI GILLIS GRAHAM GURSEL HAMMO2 HAMMON HIRAYA JOLY KAISE2 KHUDER LAUSSM LUBIN LUO PEZZO2 QIAO SPEIZE SUZUK2 TVERDA WANG2 WIGLE
5	ALDERS
10	SOBUE
14	BARBON BROWN3 JAIN PEZZOT SVENSS WU2 WYNDE3
15	BENHAM

Table 3J8 - 8
Potentially overlapping studies

REF	REFGP	PRINC	OVERLAP/LINK
LUBIN2	LUBIN2	1	Lubin-combined
WYNDE6	WYNDE6	1	WYNDE5/6/7/8
JAHN	BOFFET	2	Subset of BOFFET

Table 3J9 -

IESLC - Meta-analysis of Ex Smoking, Years quit (vs never), "High"
Adenocarcinoma, Cigarettes (or Any Product if Cigarettes not available)

This analysis is restricted to results for:
1) Ex smokers
2) Results by Years quit (vs never)
3) Categorical results by Years quit (vs never)
4) Adenocarcinoma (or near equivalent)
5) Results complete enough for use in metaanalysis

Within each study, results are then selected (in the following order of preference, within each sex) for:
6) PRODUCT: cigarettes regardless of other products, cigarettes only, all/unspec
7) CIGTYPE: all/unspecified, MC regardless of HR, MC only
8) (not applicable)
9) DENOM: never smoked anything, never smoked cigarettes, never any + low, never cigs + low
10) Followup period (YF, prospective studies): whole study (coded as 0) or longest available
11) LCtype: adeno or nearest available, but not squamous. (q = squamous, s = small,
a = adeno, l = large, KII = Kreyberg II, al = alveolar, br = bronchiolar, u = undifferentiated)
12) Race: all or nearest available, otherwise by race (wh or w = white, bl or b = black, hi = hispanic
ch = chinese, jap = japanese, haw = hawaiian, w+o = white + oriental, sca = scandinavian, as = asian)
13) Years quit (vs never) "high" in key scheme 1 (key value 3, maximum range 1-6)
14) For overlapping studies: principal rather than subsidiary studies
Finally by Age: whole study (coded as 0) if available, otherwise by widest available age group
and then for single sex results (m, f) in preference to results for both sexes combined (c).

Results adjusted (AD) for the most potential confounders are then chosen in Sections -1 to -3
(and those which actually differ from the adjusted results in Table 3J4 - 1 are marked 'x' in Section -1)
and results adjusted for the least confounders in Sections -4 to -6. (Those least adjusted results which
actually differ from the most adjusted are marked 'x' in column X in Section -4)

Section -7 shows excluded studies, together with the stage (as above) at which no qualifying
results were found.

Section -8 lists the potentially overlapping studies which have been included (1=principal, 2=subsidiary).

Section -9 lists any results which would have been included in preference except that they had data not complete
enough for use in meta-analysis, with their significance (yes/no), if known, and any further comment as entered
on the database. It also lists as "gap" any categories for which no data were presented by the original authors.
This is commonly due to recent quitters having been combined with current smokers

In addition to those mentioned above, the following fields, levels and abbreviations are used:
* or nk = not known, n = no, y = yes, ot = other
nev = never
all/unspec = all or unspecified, cig+/-ot = cigarettes irrespective of other products (cigar, pipe etc)
MC = manufactured cigarettes, HR = hand-rolled cigarettes
exL, exH = range of exposure (low and high) in the smoking group, in terms of Years quit (vs never)
REF: 6-character study reference
NRR: number of the RR on the database within the study
ST : study type (CC = case control, pr or prosp = prospective)
NLC: number of lung cancer cases in whole study
R : risky occupational population (n = no, m = mining, o = other risky)
VB : national cigarette type (V = at least 75% Virginia, bl = at least 75% blended, ot = other)
P : any proxy use
H : full histological confirmation
De : derivation of RR/CI (or = original, st = standard method, ot = other method of estimation)

Table 3J9 - 1

IESLC - Meta-analysis of Ex Smoking, Years quit (vs never), "High"
Adenocarcinoma, Cigarettes (or Any Product if Cigarettes not available)
Most adjusted

REF	NRR	3J4	SEX	AGEL	AGEH	RACE	YF	LC TYPE	LOC	START	ST	NLC	R	VB	P	H	AD	PRODUCT	exL	exH	DENOM	De
BARBON	759		m	0	0	all	-	a	Eu:wst	1979	CC	755	n	bl	y	y	1	all/unsp	0.1	4	nev any	or
JAHN	642		m	0	0	all	-	a	Eu:Ger	1988	CC	1004	n	bl	n	n	0	cig+/-ot	2	5	nev any	st
LUBIN2	865		m	0	0	all	-	a	Eu:mul	1976	CC	7804	n	bl	n	y	0	cig+/-ot	0.1	4	nev any	st
MATOS	673		m	0	0	all	-	a	SCAmer	1994	CC	200	n	bl	n	n	2	cig+/-ot	1.0	5	nev any	ot
WYNDE3	521		m	0	0	all	-	KII	NAmer	1966	CC	350	n	bl	n	y	0	all/unsp	1.0	3	nev any	st
WYNDE6	821		m	0	0	all	-	KII	NAmer	1969	CC	4423	n	bl	n	y	2	cig+/-ot	1.0	3	nev any	ot

Cigarette type is all/unspec for all RRs

Table 3J9 - 2

IESLC - Meta-analysis of Ex Smoking, Years quit (vs never), "High"
Adenocarcinoma, Cigarettes (or Any Product if Cigarettes not available)
Most adjusted

REF	NRR	SEX	AD	Number Exposed Case	Cont	Non-exposed Case	Cont	RR	95.00%CI
BARBON	759	m	1	7	-	7	-	9.40	(3.00- 29.70)
JAHN	642	m	0	19	46	8	138	7.13	(2.92- 17.37)
LUBIN2	865	m	0	77	1047	57	2616	3.38	(2.38- 4.79)
MATOS	673	m	2	12	-	5	-	13.00	(4.21- 40.17)
WYNDE3	521	m	0	3	22	6	88	2.00	(0.46- 8.63)
WYNDE6	821	m	2	29	-	11	-	14.20	(7.02- 28.73)
Partial Totals				147	1115	94	2842		

*prospective study

REF	NRR	SEX	AD	Ys	Ws	Qs	Ps
BARBON	759	m	1	2.24	2.92	1.13	0.0001
JAHN	642	m	0	1.96	4.84	0.57	0.0000
LUBIN2	865	m	0	1.22	31.38	5.11	0.0000
MATOS	673	m	2	2.56	3.02	2.70	0.0000
WYNDE3	521	m	0	0.69	1.80	1.54	0.3529
WYNDE6	821	m	2	2.65	7.74	8.26	0.0000

	N	6
	NS	6
	Wt	51.70
Het	Chi	19.31
Het	df	5
Het	P	**
Fixed	RR	5.05
	RRl	3.85
	RRu	6.64
	P	+++
Random	RR	6.73
	RRl	3.46
	RRu	13.12
	P	+++
Asymm	P	N.S.

Table 3J9 - 3

IESLC - Meta-analysis of Ex Smoking, Years quit (vs never), "High"
Adenocarcinoma, Cigarettes (or Any Product if Cigarettes not available)
Most adjusted

	Sex combined	male	female	Total
N		6		6
NS		6		6
Wt		51.70		51.70
Het Chi		19.31		19.31
Het df		5		5
Het P		**		**
Fixed RR		5.05		5.05
RRl		3.85		3.85
RRu		6.64		6.64
P		+++		+++
Random RR		6.73		6.73
RRl		3.46		3.46
RRu		13.12		13.12
P		+++		+++
Between Chi				
Between df				
Between P				N.S.
Btwn(F) P				N.S.
Btwn(R) P				N.S.

Too few RRs for analysis by factor

Table 3J9 - 4

IESLC - Meta-analysis of Ex Smoking, Years quit (vs never), "High"
Adenocarcinoma, Cigarettes (or Any Product if Cigarettes not available)
Least adjusted

REF	NRR	X	SEX	AGEL	AGEH	RACE	YF	LC TYPE	LOC	START	ST	NLC	R	VB	P	H	AD	PRODUCT	exL	exH	DENOM	De
BARBON	744	x	m	0	0	all	-	a	Eu:wst	1979	CC	755	n	bl	y	y	0	all/unsp	0.1	4	nev any	st
JAHN	642		m	0	0	all	-	a	Eu:Ger	1988	CC	1004	n	bl	n	n	0	cig+/-ot	2	5	nev any	st
LUBIN2	865		m	0	0	all	-	a	Eu:mul	1976	CC	7804	n	bl	n	y	0	cig+/-ot	0.1	4	nev any	st
MATOS	663	x	m	0	0	all	-	a	SCAmer	1994	CC	200	n	bl	n	n	0	cig+/-ot	1.0	5	nev any	st
WYNDE3	521		m	0	0	all	-	KII	NAmer	1966	CC	350	n	bl	n	y	0	all/unsp	1.0	3	nev any	st
WYNDE6	806	x	m	0	0	all	-	KII	NAmer	1969	CC	4423	n	bl	n	y	0	cig+/-ot	1.0	3	nev any	st

Cigarette type is all/unspec for all RRs

Table 3J9 - 5

IESLC - Meta-analysis of Ex Smoking, Years quit (vs never), "High"
Adenocarcinoma, Cigarettes (or Any Product if Cigarettes not available)
Least adjusted

REF	NRR	SEX	AD	Number Exposed Case	Exposed Cont	Non-exposed Case	Non-exposed Cont	RR	95.00%CI
BARBON	744	m	0	7	20	7	188	9.40	(2.99- 29.53)
JAHN	642	m	0	19	46	8	138	7.13	(2.92- 17.37)
LUBIN2	865	m	0	77	1047	57	2616	3.38	(2.38- 4.79)
MATOS	663	m	0	12	23	5	110	11.48	(3.69- 35.74)
WYNDE3	521	m	0	3	22	6	88	2.00	(0.46- 8.63)
WYNDE6	806	m	0	29	307	11	1667	14.32	(7.08- 28.96)
Totals				147	1465	94	4807		

*prospective study

REF	NRR	SEX	AD	Ys	Ws	Qs	Ps
BARBON	744	m	0	2.24	2.93	1.15	0.0001
JAHN	642	m	0	1.96	4.84	0.59	0.0000
LUBIN2	865	m	0	1.22	31.38	4.94	0.0000
MATOS	663	m	0	2.44	2.98	2.04	0.0000
WYNDE3	521	m	0	0.69	1.80	1.52	0.3529
WYNDE6	806	m	0	2.66	7.74	8.50	0.0000

	N	6
	NS	6
	Wt	51.66
Het	Chi	18.75
Het	df	5
Het	P	**
Fixed	RR	5.02
	RRl	3.82
	RRu	6.59
	P	+++
Random	RR	6.62
	RRl	3.43
	RRu	12.79
	P	+++
Asymm	P	N.S.

Table 3J9 - 6

IESLC - Meta-analysis of Ex Smoking, Years quit (vs never), "High"
Adenocarcinoma, Cigarettes (or Any Product if Cigarettes not available)
Least adjusted

	Sex combined	male	female	Total
N		6		6
NS		6		6
Wt		51.66		51.66
Het Chi		18.75		18.75
Het df		5		5
Het P		**		**
Fixed RR		5.02		5.02
RRl		3.82		3.82
RRu		6.59		6.59
P		+++		+++
Random RR		6.62		6.62
RRl		3.43		3.43
RRu		12.79		12.79
P		+++		+++
Between Chi				
Between df				
Between P				N.S.
Btwn(F) P				N.S.
Btwn(R) P				N.S.

Table 3J9 - 7

IESLC - Meta-analysis of Ex Smoking, Years quit (vs never), "High"
Adenocarcinoma, Cigarettes (or Any Product if Cigarettes not available)
Excluded studies (and stage at which they were excluded)

1	AGUDO AKIBA AMANDU AMES AXELSS BEST BOUCHA BOUCOT BRESLO CHEN CHEN2 CHIAZZ DEAN2 DOSEME ENGELA FAN GENG GER GUO HAENSZ HEGMAN HOLE HU HU2 JUSSAW KATSOU KAUFMA KOO KOULUM KREUZE LETOUR LEVIN LIAW LIU3 LIU4 LIU5 MCCONN MIGRAN MRFITR NOTAN2 OSANN2 PERNU QIAO2 RACHTA RESTRE SADOWS SEGI2 STASZE TIZZAN VUTUC WATSON WU WUWILL WYNDE2 WYNDE8 XU YUAN ZHANG ZHENG ZHOU
2	BUFFLE HUMBLE PISANI PRESCO WYNDE7
3	MCDUFF SPITZ
4	ARMADA AUVINE BECHER BENSHL BLOT1 BOFFET BROSS CARPEN CEDERL CHOI CHYOU CORREA CPSI CPSII DAMBER DARBY DEAN3 DESTEF DOLL DOLL2 DORGAN DORN GAO GAO2 GARCIA GARSHI GILLIS GRAHAM GURSEL HAMMO2 HAMMON HIRAYA JOLY KAISE2 KHUDER LAUSSM LUBIN LUO PEZZO2 QIAO SPEIZE SUZUK2 TVERDA WANG2 WIGLE
5	ALDERS
10	SOBUE
14	BROWN3 JAIN JEDRYC PEZZOT SVENSS WAKAI WU2
15	BENHAM

Table 3J9 - 8
Potentially overlapping studies

REF	REFGP	PRINC	.	OVERLAP/LINK
LUBIN2	LUBIN2	1		Lubin-combined
WYNDE6	WYNDE6	1		WYNDE5/6/7/8
JAHN	BOFFET	2		Subset of BOFFET

Table 3J9 - 9
Most adjusted - insufficient data for meta-analysis

REF	NRR	SEX	AGEL	AGEH	RACE	YF	LC TYPE	LOC	START	ST	NLC	R	VB	P	H	AD	PRODUCT	exL	exH	DENOM	De
JEDRYC	561	m	0	0	all	-	a	Eu:est	1980	CC	1630	n	bl	y	n	0	cig+/-ot	1.0	4	nev any	ot
WAKAI	619	m	0	0	all	-	a	As:Jap	1988	CC	333	n	bl	n	y	1	cig+/-ot	1.0	4	nev any	ot

REF	NRR	RR	SIG	RRDATA comment
JEDRYC	561	*	gap	0
WAKAI	619	*	gap	0

Least adjusted - insufficient data for meta-analysis: as for adjusted plus the following

REF	NRR	SEX	AGEL	AGEH	RACE	YF	LC TYPE	LOC	START	ST	NLC	R	VB	P	H	AD	PRODUCT	exL	exH	DENOM	De
WAKAI	617	m	0	0	all	-	a	As:Jap	1988	CC	333	n	bl	n	y	0	cig+/-ot	1.0	4	nev any	ot

REF	NRR	RR	SIG	RRDATA comment
WAKAI	617	*	gap	0

Table 3J10 -

IESLC - Meta-analysis of Ex Smoking, Years quit (vs never), "Highest vs lowest"
Adenocarcinoma, Cigarettes (or Any Product if Cigarettes not available)

This analysis is restricted to results for:
1) Ex smokers
2) Results by Years quit (vs never)
3) Categorical results by Years quit (vs never)
4) Denominator (unexposed) = "low"
5) Adenocarcinoma (or near equivalent)
6) Results complete enough for use in metaanalysis

Within each study, results are then selected (in the following order of preference, within each sex) for:
7) (not applicable)
8) PRODUCT: cigarettes regardless of other products, cigarettes only, all/unspec
9) CIGTYPE: all/unspecified, MC regardless of HR, MC only
10) Results with least adjustment for other aspects of smoking (ADOS)
11) The highest vs lowest category
12) Followup period (YF, prospective studies): whole study (coded as 0) or longest available
13) LCtype: adeno or nearest available, but not squamous. (q = squamous, s = small,
a = adeno, l = large, KII = Kreyberg II, al = alveolar, br = bronchiolar, u = undifferentiated)
14) Race: all or nearest available, otherwise by race (wh or w = white, bl or b = black, hi = hispanic
ch = chinese, jap = japanese, haw = hawaiian, w+o = white + oriental, sca = scandinavian, as = asian)
15) For overlapping studies: principal rather than subsidiary studies
Finally by Age: whole study (coded as 0) if available, otherwise by widest available age group
and then for single sex results (m, f) in preference to results for both sexes combined (c).

Results adjusted (AD) for the most potential confounders are then chosen in Sections -1 to -3
(and those which actually differ from the adjusted results in Table 3J5 - 1 are marked 'x' in Section -1)
and results adjusted for the least confounders in Sections -4 to -6. (Those least adjusted results which
actually differ from the most adjusted are marked 'x' in column X in Section -4)

Section -7 shows excluded studies, together with the stage (as above) at which no qualifying
results were found.

Section -8 lists the potentially overlapping studies which have been included (1=principal, 2=subsidiary).

Section -9 lists any results which would have been included in preference except that they had data not complete
enough for use in meta-analysis, with their significance (yes/no), if known, and any further comment as entered
on the database. It also lists as "gap" any categories for which no data were presented by the original authors.
This is commonly due to recent quitters having been combined with current smokers

In addition to those mentioned above, the following fields, levels and abbreviations are used:
* or nk = not known, n = no, y = yes, ot = other
all/unspec = all or unspecified, cig+/-ot = cigarettes irrespective of other products (cigar, pipe etc)
MC = manufactured cigarettes, HR = hand-rolled cigarettes
exL, exH = range of exposure (low and high) in the "highest" group, in terms of Years quit (vs never)
unexL, unexH = range of exposure (low and high) in the "lowest" group, in terms of Years quit (vs never)
REF: 6-character study reference
NRR: number of the RR on the database within the study
ST : study type (CC = case control, pr or prosp = prospective)
NLC: number of lung cancer cases in whole study
R : risky occupational population (n = no, m = mining, o = other risky)
VB : national cigarette type (V = at least 75% Virginia, bl = at least 75% blended, ot = other)
P : any proxy use
H : full histological confirmation
De : derivation of RR/CI (or = original, st = standard method, ot = other method of estimation)

Table 3J10 - 1

IESLC - Meta-analysis of Ex Smoking, Years quit (vs never), "Highest vs lowest"
Adenocarcinoma, Cigarettes (or Any Product if Cigarettes not available)
Most adjusted

REF	NRR	3J5	SEX	AGEL	AGEH	RACE	YF	LC TYPE	LOC	START	ST	NLC	R	VB	P	H	AD	ADOS	PRODUCT	exL	exH	unexL	unexH	De
BARBON	763		m	0	0	all	-	a	Eu:wst	1979	CC	755	n	bl	y	y	1	0	all/unsp	0.1	4	25	999	ot
JAHN	650		m	0	0	all	-	a	Eu:Ger	1988	CC	1004	n	bl	n	n	0	0	cig+/-ot	0.1	0.9	21	999	st
JAIN	539		m	0	0	all	-	a	NAmer	1981	CC	845	n	V	y	n	0	0	cig+/-ot	2	9	10	999	st
JAIN	503		f	0	0	all	-	a	NAmer	1981	CC	845	n	V	y	n	0	0	cig+/-ot	2	9	10	999	st
JEDRYC	563		m	0	0	all	-	a	Eu:est	1980	CC	1630	n	bl	y	n	0	0	cig+/-ot	5	9	10	999	st
LUBIN2	870		m	0	0	all	-	a	Eu:mul	1976	CC	7804	n	bl	n	y	0	0	cig+/-ot	0.1	4	20	999	st
LUBIN2	970		f	0	0	all	-	a	Eu:mul	1976	CC	7804	n	bl	n	y	0	0	cig+/-ot	0.1	9	20	999	st
MATOS	675		m	0	0	all	-	a	SCAmer	1994	CC	200	n	bl	n	n	2	0	cig+/-ot	1.0	5	11	999	ot
PEZZOT	587		m	0	0	all	-	a	SCAmer	1987	CC	215	n	bl	n	y	0	0	cig only	1.0	10	11	999	st
SOBUE	785		m	0	0	all	-	a	As:Jap	1986	CC	1376	n	bl	n	y	0	0	cig+/-ot	1.0	4	10	999	st
SVENSS	571		f	0	0	all	-	a	Eu:Sca	1983	CC	210	n	bl	n	n	0	0	all/unsp	3	10	11	999	st
WAKAI	566		m	0	0	all	-	a	As:Jap	1988	CC	333	n	bl	n	y	1	0	cig+/-ot	5	9	20	999	ot
WYNDE3	526		m	0	0	all	-	KII	NAmer	1966	CC	350	n	bl	n	y	0	0	all/unsp	1.0	3	13	999	st
WYNDE6	826		m	0	0	all	-	KII	NAmer	1969	CC	4423	n	bl	n	y	2	0	cig+/-ot	1.0	3	16	999	ot

Cigarette type is all/unspec for all RRs

Table 3J10 - 2

IESLC - Meta-analysis of Ex Smoking, Years quit (vs never), "Highest vs lowest"
Adenocarcinoma, Cigarettes (or Any Product if Cigarettes not available)
Most adjusted

REF	NRR	SEX	AD	Number Exposed Case	Cont	Non-exposed Case	Cont	RR	95.00%CI
BARBON	763	m	1	7	-	4	-	5.22	(1.37- 19.83)
JAHN	650	m	0	40	8	15	146	48.67	(19.27- 122.93)
JAIN	539	m	0	16	46	14	113	2.81	(1.27- 6.22)
JAIN	503	f	0	14	36	3	61	7.91	(2.13- 29.40)
Subtotal JAIN								3.71	(1.88- 7.32)
JEDRYC	563	m	0	9	82	12	230	2.10	(0.86- 5.18)
LUBIN2	870	m	0	77	1047	35	1128	2.37	(1.58- 3.57)
LUBIN2	970	f	0	13	95	1	29	3.97	(0.50- 31.64)
Subtotal LUBIN2								2.42	(1.62- 3.61)
MATOS	675	m	2	12	-	12	-	4.33	(1.71- 10.97)
PEZZOT	587	m	0	11	21	7	31	2.32	(0.77- 6.95)
SOBUE	785	m	0	44	116	49	144	1.11	(0.69- 1.79)
SVENSS	571	f	0	5	13	7	24	1.32	(0.35- 4.99)
WAKAI	566	m	1	7	-	3	-	2.28	(0.56- 9.20)
WYNDE3	526	m	0	3	22	3	55	2.50	(0.47- 13.35)
WYNDE6	826	m	2	29	-	6	-	2.84	(1.17- 6.92)
Partial Totals				287	1486	171	1961		

*prospective study

REF	NRR	SEX	AD	Ys	Ws	Qs	Ps
BARBON	763	m	1	1.65	2.15	1.01	0.0154
JAHN	650	m	0	3.88	4.47	38.09	0.0000
JAIN	539	m	0	1.03	6.08	0.03	0.0109
JAIN	503	f	0	2.07	2.23	2.70	0.0020
Subtotal JAIN				1.31	8.31	2.72	
JEDRYC	563	m	0	0.74	4.74	0.24	0.1054
LUBIN2	870	m	0	0.86	23.04	0.25	0.0000
LUBIN2	970	f	0	1.38	0.89	0.15	0.1932
Subtotal LUBIN2				0.88	23.93	0.40	
MATOS	675	m	2	1.47	4.45	1.10	0.0020
PEZZOT	587	m	0	0.84	3.19	0.05	0.1330
SOBUE	785	m	0	0.11	17.04	12.56	0.6540
SVENSS	571	f	0	0.28	2.17	1.03	0.6838
WAKAI	566	m	1	0.82	1.96	0.04	0.2484
WYNDE3	526	m	0	0.92	1.37	0.00	0.2836
WYNDE6	826	m	2	1.04	4.86	0.03	0.0213

	N	14
	NS	12
	Wt	78.64
Het	Chi	57.28
Het	df	13
Het	P	***
Fixed	RR	2.63
	RRl	2.11
	RRu	3.28
	P	+++
Random	RR	3.32
	RRl	1.98
	RRu	5.58
	P	+++
Asymm	P	N.S.

Table 3J10 - 3

IESLC - Meta-analysis of Ex Smoking, Years quit (vs never), "Highest vs lowest"
Adenocarcinoma, Cigarettes (or Any Product if Cigarettes not available)
Most adjusted

	combined	Sex male	female	Total
N		11	3	14
NS		11	3	14
Wt		73.35	5.29	78.64
Het Chi		53.37	3.55	57.28
Het df		10	2	13
Het P		***	N.S.	***
Fixed RR		2.58	3.38	2.63
RRl		2.06	1.44	2.11
RRu		3.25	7.92	3.28
P		+++	++	+++
Random RR		3.31	3.40	3.32
RRl		1.85	1.05	1.98
RRu		5.93	10.98	5.58
P		+++	+	+++
Between Chi				0.35
Between df				1
Between P				N.S.
Btwn(F) P				N.S.
Btwn(R) P				N.S.

	Lung cancer type a	a+l	a+al+br	KII	not q+u	not q+s	Total
N	12			2			14
NS	10			2			12
Wt	72.40			6.23			78.64
Het Chi	57.25			0.02			57.28
Het df	11			1			13
Het P	***			N.S.			***
Fixed RR	2.62			2.76			2.63
RRl	2.08			1.26			2.11
RRu	3.30			6.05			3.28
P	+++			+			+++
Random RR	3.44			2.76			3.32
RRl	1.91			1.26			1.98
RRu	6.19			6.05			5.58
P	+++			+			+++
Between Chi							0.02
Between df							1
Between P							N.S.
Btwn(F) P							N.S.
Btwn(R) P							N.S.

	Location NAmer	UK	Scand	othEur	China	Japan	othAs	other	Total
N	4		1	5		2		2	14
NS	3		1	4		2		2	12
Wt	14.54		2.17	35.30		19.00		7.64	78.64
Het Chi	2.07		0.00	36.03		0.90		0.72	57.28
Het df	3		0	4		1		1	13
Het P	N.S.		N.S.	***		N.S.		N.S.	***
Fixed RR	3.27		1.32	3.64		1.20		3.34	2.63
RRl	1.95		0.35	2.61		0.77		1.64	2.11
RRu	5.46		4.99	5.06		1.88		6.78	3.28
P	+++		N.S.	+++		N.S.		+++	+++
Random RR	3.27		1.32	5.52		1.20		3.34	3.32
RRl	1.95		0.35	1.57		0.77		1.64	1.98
RRu	5.46		4.99	19.35		1.88		6.78	5.58
P	+++		N.S.	++		N.S.		+++	+++
Between Chi									17.55
Between df									4
Between P									**
Btwn(F) P									N.S.
Btwn(R) P									*

Table 3J10 - 3

IESLC - Meta-analysis of Ex Smoking, Years quit (vs never), "Highest vs lowest"
Adenocarcinoma, Cigarettes (or Any Product if Cigarettes not available)
Most adjusted

Detailed Country in "other Europe"

	multi	Germany	othWest	East	Balkans	Total
N	2	1	1	1		5
NS	1	1	1	1		4
Wt	23.93	4.47	2.15	4.74		35.30
Het Chi	0.23	0.00	0.00	0.00		36.03
Het df	1	0	0	0		4
Het P	N.S.	N.S.	N.S.	N.S.		***
Fixed RR	2.42	48.67	5.22	2.10		3.64
RRl	1.62	19.27	1.37	0.86		2.61
RRu	3.61	122.93	19.86	5.18		5.06
P	+++	+++	+	N.S.		+++
Random RR	2.42	48.67	5.22	2.10		5.52
RRl	1.62	19.27	1.37	0.86		1.57
RRu	3.61	122.93	19.86	5.18		19.35
P	+++	+++	+	N.S.		++
Between Chi						35.81
Between df						3
Between P						***
Btwn(F) P						N.S.
Btwn(R) P						***

Detailed Country in "other Asia"

	India	HongKong	other	Total
N				
NS				
Wt				
Het Chi				
Het df				
Het P				N.S.
Fixed RR				
RRl				
RRu				
P				N.S.
Random RR				
RRl				
RRu				
P				N.S.
Between Chi				
Between df				
Between P				N.S.
Btwn(F) P				N.S.
Btwn(R) P				N.S.

Detailed other continent

	SCAmer	Total
N	2	2
NS	2	2
Wt	7.64	7.64
Het Chi	0.72	0.72
Het df	1	1
Het P	N.S.	N.S.
Fixed RR	3.34	3.34
RRl	1.64	1.64
RRu	6.78	6.78
P	+++	+++
Random RR	3.34	3.34
RRl	1.64	1.64
RRu	6.78	6.78
P	+++	+++
Between Chi		
Between df		
Between P		N.S.
Btwn(F) P		N.S.
Btwn(R) P		N.S.

Table 3J10 - 3

IESLC - Meta-analysis of Ex Smoking, Years quit (vs never), "Highest vs lowest"
Adenocarcinoma, Cigarettes (or Any Product if Cigarettes not available)
Most adjusted

	Start year of study					
	<1960	1960-69	1970-79	1980-89	1990+	Total
N		2	3	8	1	14
NS		2	2	7	1	12
Wt		6.23	26.08	41.87	4.45	78.64
Het Chi		0.02	1.40	54.64	0.00	57.28
Het df		1	2	7	0	13
Het P		N.S.	N.S.	***	N.S.	***
Fixed RR		2.76	2.57	2.51	4.33	2.63
RRl		1.26	1.75	1.85	1.71	2.11
RRu		6.05	3.78	3.40	10.97	3.28
P		+	+++	+++	++	+++
Random RR		2.76	2.57	3.38	4.33	3.32
RRl		1.26	1.75	1.35	1.71	1.98
RRu		6.05	3.78	8.46	10.97	5.58
P		+	+++	++	++	+++
Between Chi						1.22
Between df						3
Between P						N.S.
Btwn(F) P						N.S.
Btwn(R) P						N.S.

	Study type (1)		
	CC	other	Total
N	14		14
NS	12		12
Wt	78.64		78.64
Het Chi	57.28		57.28
Het df	13		13
Het P	***		***
Fixed RR	2.63		2.63
RRl	2.11		2.11
RRu	3.28		3.28
P	+++		+++
Random RR	3.32		3.32
RRl	1.98		1.98
RRu	5.58		5.58
P	+++		+++
Between Chi			
Between df			
Between P			N.S.
Btwn(F) P			N.S.
Btwn(R) P			N.S.

	Study type (2)			
	CC	prosp	other	Total
N	14			14
NS	12			12
Wt	78.64			78.64
Het Chi	57.28			57.28
Het df	13			13
Het P	***			***
Fixed RR	2.63			2.63
RRl	2.11			2.11
RRu	3.28			3.28
P	+++			+++
Random RR	3.32			3.32
RRl	1.98			1.98
RRu	5.58			5.58
P	+++			+++
Between Chi				
Between df				
Between P				N.S.
Btwn(F) P				N.S.
Btwn(R) P				N.S.

Table 3J10 - 3

IESLC - Meta-analysis of Ex Smoking, Years quit (vs never), "Highest vs lowest"
Adenocarcinoma, Cigarettes (or Any Product if Cigarettes not available)
Most adjusted

	Study size (number of LC cases)				
	100-249	250-499	500-999	1000+	Total
N	3	2	3	6	14
NS	3	2	2	5	12
Wt	9.80	3.33	10.46	55.05	78.64
Het Chi	2.18	0.01	1.95	50.98	57.28
Het df	2	1	2	5	13
Het P	N.S.	N.S.	N.S.	***	***
Fixed RR	2.72	2.37	3.98	2.43	2.63
RRl	1.45	0.81	2.17	1.87	2.11
RRu	5.08	6.93	7.29	3.17	3.28
P	++	N.S.	+++	+++	+++
Random RR	2.69	2.37	3.98	3.65	3.32
RRl	1.39	0.81	2.17	1.40	1.98
RRu	5.18	6.93	7.29	9.48	5.58
P	++	N.S.	+++	++	+++
Between Chi					2.17
Between df					3
Between P					N.S.
Btwn(F) P					N.S.
Btwn(R) P					N.S.

	Risky occupational population			
	no	mining	othRisky	Total
N	14			14
NS	12			12
Wt	78.64			78.64
Het Chi	57.28			57.28
Het df	13			13
Het P	***			***
Fixed RR	2.63			2.63
RRl	2.11			2.11
RRu	3.28			3.28
P	+++			+++
Random RR	3.32			3.32
RRl	1.98			1.98
RRu	5.58			5.58
P	+++			+++
Between Chi				
Between df				
Between P				N.S.
Btwn(F) P				N.S.
Btwn(R) P				N.S.

	National cigarette tobacco type			
	Virginia	blended	other	Total
N	2	12		14
NS	1	11		12
Wt	8.31	70.33		78.64
Het Chi	1.75	54.44		57.28
Het df	1	11		13
Het P	N.S.	***		***
Fixed RR	3.71	2.53		2.63
RRl	1.88	2.00		2.11
RRu	7.32	3.19		3.28
P	+++	+++		+++
Random RR	4.11	3.18		3.32
RRl	1.54	1.76		1.98
RRu	10.93	5.72		5.58
P	++	+++		+++
Between Chi				1.09
Between df				1
Between P				N.S.
Btwn(F) P				N.S.
Btwn(R) P				N.S.

Table 3J10 - 3

IESLC - Meta-analysis of Ex Smoking, Years quit (vs never), "Highest vs lowest"
Adenocarcinoma, Cigarettes (or Any Product if Cigarettes not available)
Most adjusted

	Any proxy use No/nk	Yes	Total
N	10	4	14
NS	9	3	12
Wt	63.44	15.20	78.64
Het Chi	53.14	3.27	57.28
Het df	9	3	13
Het P	***	N.S.	***
Fixed RR	2.50	3.26	2.63
RRl	1.95	1.97	2.11
RRu	3.20	5.39	3.28
P	+++	+++	+++
Random RR	3.19	3.31	3.32
RRl	1.61	1.94	1.98
RRu	6.32	5.62	5.58
P	+++	+++	+++
Between Chi			0.87
Between df			1
Between P			N.S.
Btwn(F) P			N.S.
Btwn(R) P			N.S.

	Full histological confirmation No	Yes	Total
N	6	8	14
NS	5	7	12
Wt	24.13	54.50	78.64
Het Chi	33.15	9.65	57.28
Het df	5	7	13
Het P	***	N.S.	***
Fixed RR	5.01	1.98	2.63
RRl	3.36	1.52	2.11
RRu	7.47	2.58	3.28
P	+++	+++	+++
Random RR	4.91	2.10	3.32
RRl	1.72	1.46	1.98
RRu	14.01	3.03	5.58
P	++	+++	+++
Between Chi			14.48
Between df			1
Between P			***
Btwn(F) P			(*)
Btwn(R) P			N.S.

	Number of adjustment variables (1) 0	1	2+/+nk	Total
N	10	2	2	14
NS	8	2	2	12
Wt	65.21	4.11	9.31	78.64
Het Chi	54.88	0.70	0.41	57.28
Het df	9	1	1	13
Het P	***	N.S.	N.S.	***
Fixed RR	2.48	3.52	3.47	2.63
RRl	1.95	1.34	1.83	2.11
RRu	3.16	9.24	6.60	3.28
P	+++	+	+++	+++
Random RR	3.28	3.52	3.47	3.32
RRl	1.66	1.34	1.83	1.98
RRu	6.50	9.24	6.60	5.58
P	+++	+	+++	+++
Between Chi				1.29
Between df				2
Between P				N.S.
Btwn(F) P				N.S.
Btwn(R) P				N.S.

Table 3J10 - 3

IESLC - Meta-analysis of Ex Smoking, Years quit (vs never), "Highest vs lowest"
Adenocarcinoma, Cigarettes (or Any Product if Cigarettes not available)
Most adjusted

	Number of adjustment variables (2) 0	1	2	3-5	6+/+nk	Total
N	10	2	2			14
NS	8	2	2			12
Wt	65.21	4.11	9.31			78.64
Het Chi	54.88	0.70	0.41			57.28
Het df	9	1	1			13
Het P	***	N.S.	N.S.			***
Fixed RR	2.48	3.52	3.47			2.63
RRl	1.95	1.34	1.83			2.11
RRu	3.16	9.24	6.60			3.28
P	+++	+	+++			+++
Random RR	3.28	3.52	3.47			3.32
RRl	1.66	1.34	1.83			1.98
RRu	6.50	9.24	6.60			5.58
P	+++	+	+++			+++
Between Chi						1.29
Between df						2
Between P						N.S.
Btwn(F) P						N.S.
Btwn(R) P						N.S.

	Product all/unsp	cig+/-ot	cig only	Total
N	3	10	1	14
NS	3	8	1	12
Wt	5.69	69.76	3.19	78.64
Het Chi	2.05	55.18	0.00	57.28
Het df	2	9	0	13
Het P	N.S.	***	N.S.	***
Fixed RR	2.59	2.65	2.32	2.63
RRl	1.14	2.09	0.77	2.11
RRu	5.89	3.35	6.95	3.28
P	+	+++	N.S.	+++
Random RR	2.59	3.67	2.32	3.32
RRl	1.13	1.92	0.77	1.98
RRu	5.95	7.00	6.95	5.58
P	+	+++	N.S.	+++
Between Chi				0.06
Between df				2
Between P				N.S.
Btwn(F) P				N.S.
Btwn(R) P				N.S.

	Derivation of RR/CI Orig	StdCalc	Other	Total
N		10	4	14
NS		8	4	12
Wt		65.21	13.42	78.64
Het Chi		54.88	1.12	57.28
Het df		9	3	13
Het P		***	N.S.	***
Fixed RR		2.48	3.49	2.63
RRl		1.95	2.04	2.11
RRu		3.16	5.95	3.28
P		+++	+++	+++
Random RR		3.28	3.49	3.32
RRl		1.66	2.04	1.98
RRu		6.50	5.95	5.58
P		+++	+++	+++
Between Chi				1.29
Between df				1
Between P				N.S.
Btwn(F) P				N.S.
Btwn(R) P				N.S.

Table 3J10 - 4

IESLC - Meta-analysis of Ex Smoking, Years quit (vs never), "Highest vs lowest"
Adenocarcinoma, Cigarettes (or Any Product if Cigarettes not available)
Least adjusted

REF	NRR	X	SEX	AGEL	AGEH	RACE	YF	LC TYPE	LOC	START	ST	NLC	R	VB	P	H	AD	ADOS	PRODUCT	exL	exH	unexL	unexH	De
BARBON	748	x	m	0	0	all	-	a	Eu:wst	1979	CC	755	n	bl	y	y	0	0	all/unsp	0.1	4	25	999	st
JAHN	650		m	0	0	all	-	a	Eu:Ger	1988	CC	1004	n	bl	n	n	0	0	cig+/-ot	0.1	0.9	21	999	st
JAIN	539		m	0	0	all	-	a	NAmer	1981	CC	845	n	V	y	n	0	0	cig+/-ot	2	9	10	999	st
JAIN	503		f	0	0	all	-	a	NAmer	1981	CC	845	n	V	y	n	0	0	cig+/-ot	2	9	10	999	st
JEDRYC	563		m	0	0	all	-	a	Eu:est	1980	CC	1630	n	bl	y	n	0	0	cig+/-ot	5	9	10	999	st
LUBIN2	870		m	0	0	all	-	a	Eu:mul	1976	CC	7804	n	bl	n	y	0	0	cig+/-ot	0.1	4	20	999	st
LUBIN2	970		f	0	0	all	-	a	Eu:mul	1976	CC	7804	n	bl	n	y	0	0	cig+/-ot	0.1	9	20	999	st
MATOS	665	x	m	0	0	all	-	a	SCAmer	1994	CC	200	n	bl	n	n	0	0	cig+/-ot	1.0	5	11	999	st
PEZZOT	587		m	0	0	all	-	a	SCAmer	1987	CC	215	n	bl	n	y	0	0	cig only	1.0	10	11	999	st
SOBUE	785		m	0	0	all	-	a	As:Jap	1986	CC	1376	n	bl	n	y	0	0	cig+/-ot	1.0	4	10	999	st
SVENSS	571		f	0	0	all	-	a	Eu:Sca	1983	CC	210	n	bl	n	n	0	0	all/unsp	3	10	11	999	st
WAKAI	558	x	m	0	0	all	-	a	As:Jap	1988	CC	333	n	bl	n	y	0	0	cig+/-ot	5	9	20	999	st
WYNDE3	526		m	0	0	all	-	KII	NAmer	1966	CC	350	n	bl	n	y	0	0	all/unsp	1.0	3	13	999	st
WYNDE6	811	x	m	0	0	all	-	KII	NAmer	1969	CC	4423	n	bl	n	y	0	0	cig+/-ot	1.0	3	16	999	st

Cigarette type is all/unspec for all RRs

Table 3J10 - 5

IESLC - Meta-analysis of Ex Smoking, Years quit (vs never), "Highest vs lowest"
Adenocarcinoma, Cigarettes (or Any Product if Cigarettes not available)
Least adjusted

REF	NRR	SEX	AD	Number Exposed Case	Cont	Non-exposed Case	Cont	RR	95.00%CI
BARBON	748	m	0	7	20	4	59	5.16	(1.37- 19.50)
JAHN	650	m	0	40	8	15	146	48.67	(19.27- 122.93)
JAIN	539	m	0	16	46	14	113	2.81	(1.27- 6.22)
JAIN	503	f	0	14	36	3	61	7.91	(2.13- 29.40)
Subtotal JAIN								3.71	(1.88- 7.32)
JEDRYC	563	m	0	9	82	12	230	2.10	(0.86- 5.18)
LUBIN2	870	m	0	77	1047	35	1128	2.37	(1.58- 3.57)
LUBIN2	970	f	0	13	95	1	29	3.97	(0.50- 31.64)
Subtotal LUBIN2								2.42	(1.62- 3.61)
MATOS	665	m	0	12	23	12	101	4.39	(1.75- 11.01)
PEZZOT	587	m	0	11	21	7	31	2.32	(0.77- 6.95)
SOBUE	785	m	0	44	116	49	144	1.11	(0.69- 1.79)
SVENSS	571	f	0	5	13	7	24	1.32	(0.35- 4.99)
WAKAI	558	m	0	7	48	3	47	2.28	(0.56- 9.37)
WYNDE3	526	m	0	3	22	3	55	2.50	(0.47- 13.35)
WYNDE6	811	m	0	29	307	6	530	8.34	(3.43- 20.32)
Totals				287	1884	171	2698		

*prospective study

REF	NRR	SEX	AD	Ys	Ws	Qs	Ps
BARBON	748	m	0	1.64	2.17	0.80	0.0155
JAHN	650	m	0	3.88	4.47	36.34	0.0000
JAIN	539	m	0	1.03	6.08	0.00	0.0109
JAIN	503	f	0	2.07	2.23	2.38	0.0020
Subtotal JAIN				1.31	8.31	2.38	
JEDRYC	563	m	0	0.74	4.74	0.40	0.1054
LUBIN2	870	m	0	0.86	23.04	0.68	0.0000
LUBIN2	970	f	0	1.38	0.89	0.11	0.1932
Subtotal LUBIN2				0.88	23.93	0.79	
MATOS	665	m	0	1.48	4.54	0.90	0.0016
PEZZOT	587	m	0	0.84	3.19	0.12	0.1330
SOBUE	785	m	0	0.11	17.04	14.62	0.6540
SVENSS	571	f	0	0.28	2.17	1.25	0.6838
WAKAI	558	m	0	0.83	1.93	0.08	0.2511
WYNDE3	526	m	0	0.92	1.37	0.02	0.2836
WYNDE6	811	m	0	2.12	4.85	5.72	0.0000

	N	14
	NS	12
	Wt	78.71
Het	Chi	63.42
Het	df	13
Het	P	***
Fixed	RR	2.81
	RRl	2.26
	RRu	3.51
	P	+++
Random	RR	3.63
	RRl	2.11
	RRu	6.25
	P	+++
Asymm	P	N.S.

Table 3J10 - 6

IESLC - Meta-analysis of Ex Smoking, Years quit (vs never), "Highest vs lowest"
Adenocarcinoma, Cigarettes (or Any Product if Cigarettes not available)
Least adjusted

	combined	Sex male	female	Total
N		11	3	14
NS		11	3	14
Wt		73.42	5.29	78.71
Het Chi		59.68	3.55	63.42
Het df		10	2	13
Het P		***	N.S.	***
Fixed RR		2.78	3.38	2.81
RRl		2.21	1.44	2.26
RRu		3.49	7.92	3.51
P		+++	++	+++
Random RR		3.68	3.40	3.63
RRl		1.99	1.05	2.11
RRu		6.81	10.98	6.25
P		+++	+	+++
Between Chi				0.19
Between df				1
Between P				N.S.
Btwn(F) P				N.S.
Btwn(R) P				N.S.

Table 3J10 - 7

IESLC - Meta-analysis of Ex Smoking, Years quit (vs never), "Highest vs lowest"
Adenocarcinoma, Cigarettes (or Any Product if Cigarettes not available)
Excluded studies (and stage at which they were excluded)

1	AGUDO AKIBA AMANDU AMES AXELSS BEST BOUCHA BOUCOT BRESLO CHEN CHEN2 CHIAZZ DEAN2 DOSEME ENGELA FAN GENG GER GUO HAENSZ HEGMAN HOLE HU HU2 JUSSAW KATSOU KAUFMA KOO KOULUM KREUZE LETOUR LEVIN LIAW LIU3 LIU4 LIU5 MCCONN MIGRAN MRFITR NOTAN2 OSANN2 PERNU QIAO2 RACHTA RESTRE SADOWS SEGI2 STASZE TIZZAN VUTUC WATSON WU WUWILL WYNDE2 WYNDE8 XU YUAN ZHANG ZHENG ZHOU
2	BUFFLE HUMBLE PISANI PRESCO WYNDE7
3	MCDUFF SPITZ
4	AUVINE BLOT1 BROWN3 GURSEL LAUSSM LUO WU2
5	ARMADA BECHER BENSHL BOFFET BROSS CARPEN CEDERL CHOI CHYOU CORREA CPSI CPSII DAMBER DARBY DEAN3 DESTEF DOLL DOLL2 DORGAN DORN GAO GAO2 GARCIA GARSHI GILLIS GRAHAM HAMMO2 HAMMON HIRAYA JOLY KAISE2 KHUDER LUBIN PEZZO2 QIAO SPEIZE SUZUK2 TVERDA WANG2 WIGLE
6	ALDERS
15	BENHAM

Table 3J10 - 8

Potentially overlapping studies

REF	REFGP	PRINC	OVERLAP/LINK
LUBIN2	LUBIN2	1	Lubin-combined
WYNDE6	WYNDE6	1	WYNDE5/6/7/8
JAHN	BOFFET	2	Subset of BOFFET

Table 3J10 - 9

Most adjusted - insufficient data for meta-analysis

REF	NRR	SEX	AGEL	AGEH	RACE	YF	LC TYPE	LOC	START	ST	NLC	R	VB	P	H	AD	ADOS	PRODUCT	exL	exH	unexL	unexH	De
ALDERS	572	m	0	0	all	-	not q+s	Eu:UK	1977	CC	1448	n	V	n	n	1	0	cig only	0.1	2	10	999	st
ALDERS	583	f	0	0	all	-	not q+s	Eu:UK	1977	CC	1448	n	V	n	n	1	0	cig only	0.1	2	10	999	st
JAIN	587	m	0	0	all	-	a	NAmer	1981	CC	845	n	V	y	n	0	0	cig+/-ot	0.1	1.9	10	999	ot
JAIN	575	f	0	0	all	-	a	NAmer	1981	CC	845	n	V	y	n	0	0	cig+/-ot	0.1	1.9	10	999	ot
JEDRYC	564	m	0	0	all	-	a	Eu:est	1980	CC	1630	n	bl	y	n	0	0	cig+/-ot	0.1	4	10	999	ot
MATOS	712	m	0	0	all	-	a	SCAmer	1994	CC	200	n	bl	n	n	2	0	cig+/-ot	0.1	0.9	11	999	ot
PEZZOT	602	m	0	0	all	-	a	SCAmer	1987	CC	215	n	bl	n	y	0	0	cig only	0.1	0.9	11	999	ot
SOBUE	786	m	0	0	all	-	a	As:Jap	1986	CC	1376	n	bl	n	y	0	0	cig+/-ot	0.1	0.9	10	999	ot
SVENSS	598	f	0	0	all	-	a	Eu:Sca	1983	CC	210	n	bl	n	n	0	0	all/unsp	0.1	2	11	999	ot
WAKAI	620	m	0	0	all	-	a	As:Jap	1988	CC	333	n	bl	n	y	1	0	cig+/-ot	0.1	4	20	999	ot
WYNDE3	527	m	0	0	all	-	KII	NAmer	1966	CC	350	n	bl	n	y	0	0	all/unsp	0.1	0.9	13	999	ot
WYNDE6	827	m	0	0	all	-	KII	NAmer	1969	CC	4423	n	bl	n	y	2	0	cig+/-ot	0.1	0.9	16	999	ot

REF	NRR	RR	SIG	RRDATA comment
ALDERS	572	2.27		0
ALDERS	583	3.83		0
JAIN	587	*	gap	0
JAIN	575	*	gap	0
JEDRYC	564	*	gap	0
MATOS	712	*	gap	0
PEZZOT	602	*	gap	0
SOBUE	786	*	gap	0
SVENSS	598	*	gap	0
WAKAI	620	*	gap	0
WYNDE3	527	*	gap	0
WYNDE6	827	*	gap	0

Least adjusted - insufficient data for meta-analysis: as for adjusted plus the following

REF	NRR	SEX	AGEL	AGEH	RACE	YF	LC TYPE	LOC	START	ST	NLC	R	VB	P	H	AD	ADOS	PRODUCT	exL	exH	unexL	unexH	De
MATOS	710	m	0	0	all	-	a	SCAmer	1994	CC	200	n	bl	n	n	0	0	cig+/-ot	0.1	0.9	11	999	ot
WAKAI	618	m	0	0	all	-	a	As:Jap	1988	CC	333	n	bl	n	y	0	0	cig+/-ot	0.1	4	20	999	ot
WYNDE6	812	m	0	0	all	-	KII	NAmer	1969	CC	4423	n	bl	n	y	0	0	cig+/-ot	0.1	0.9	16	999	ot

REF	NRR	RR	SIG	RRDATA comment
MATOS	710	*	gap	0

Table 3J10 - 9

IESLC - Meta-analysis of Ex Smoking, Years quit (vs never), "Highest vs lowest"
Adenocarcinoma, Cigarettes (or Any Product if Cigarettes not available)
Least adjusted - insufficient data for meta-analysis: as for adjusted plus the following

REF	NRR	RR	SIG	RRDATA comment
WAKAI	618	*	gap	0
WYNDE6	812	*	gap	0

Table 3J11 -

IESLC - Meta-analysis of Ex Smoking by Years quit (vs never), Overview
Adenocarcinoma, Cigarettes only

This analysis is restricted to results for:
1) Ex smokers
2) Results by Years quit (vs never)
3) Categorical results by Years quit (vs never)
Results by Years quit (vs never) are grouped under 2 schemes (S1, S2). Each scheme has a set of "key values". An interv is allocated to the category whose key value it includes, and intervals which include none or more than one of the key values are excluded. (Open-ended intervals are coded as 999)

S1	key value	maximum range
1	12	8+
2	7	4-11
3	3	1-6

S2	key value	maximum range
1	20	13+
2	12	4-19
3	3	1-11

4) Adenocarcinoma (or near equivalent)
5) Results complete enough for use in metaanalysis

Within each study, results are then selected (in the following order of preference, within each sex) for:
6) (not applicable)
7) PRODUCT: cigarettes only
8) CIGTYPE: all/unspecified, MC regardless of HR, MC only
9) (not applicable)
10) DENOM: never smoked anything, never smoked cigarettes, never any + low, never cigs + low
11) Followup period (YF, prospective studies): whole study (coded as 0) or longest available
12) LCtype: adeno or nearest available, but not squamous. (q = squamous, s = small, a = adeno, l = large, KII = Kreyberg II, al = alveolar, br = bronchiolar, u = undifferentiated)
13) Race: all or nearest available, otherwise by race (wh or w = white, bl or b = black, hi = hispanic ch = chinese, jap = japanese, haw = hawaiian, w+o = white + oriental, sca = scandinavian, as = asian)
14) For overlapping studies: principal rather than subsidiary studies
Finally by Age: whole study (coded as 0) if available, otherwise by widest available age group and then for single sex results (m, f) in preference to results for both sexes combined (c).

Results adjusted (AD) for the most potential confounders are then chosen in Sections -1 to -3 (and those which actually differ from the adjusted results in Table 3J1 - 1 are marked 'x' in Section -1) and results adjusted for the least confounders in Sections -4 to -6. (Those least adjusted results which actually differ from the most adjusted are marked 'x' in column X in Section -4)

Section -7 shows excluded studies, together with the stage (as above) at which no qualifying results were found.

Section -8 lists the potentially overlapping studies which have been included (1=principal, 2=subsidiary).

Section -9 lists any results which would have been included in preference except that they had data not complete enough for use in meta-analysis, with their significance (yes/no), if known, and any further comment as entered on the database. It also lists as "gap" any categories for which no data were presented by the original authors. This is commonly due to recent quitters having been combined with current smokers

In addition to those mentioned above, the following fields, levels and abbreviations are used:
* or nk = not known, n = no, y = yes, ot = other
nev = never
all/unspec = all or unspecified, MC = manufactured cigarettes, HR = hand-rolled cigarettes
exL, exH = range of exposure (low and high) in the smoking group, in terms of Years quit (vs never)
REF: 6-character study reference
NRR: number of the RR on the database within the study
ST : study type (CC = case control, pr or prosp = prospective)
NLC: number of lung cancer cases in whole study
R : risky occupational population (n = no, m = mining, o = other risky)
VB : national cigarette type (V = at least 75% Virginia, bl = at least 75% blended, ot = other)
P : any proxy use
H : full histological confirmation
De : derivation of RR/CI (or = original, st = standard method, ot = other method of estimation)

Table 3J11 - 1

IESLC - Meta-analysis of Ex Smoking by Years quit (vs never), Overview
Adenocarcinoma, Cigarettes only
Most adjusted

REF	NRR	3J1	SEX	AGEL	AGEH	RACE	YF	LC TYPE	LOC	START	ST	NLC	R	VB	P	H	AD	PRODUCT	exL	exH	S1	S2	DENOM	De
BENHAM	548	x	m	0	0	all	-	KII	Eu:wst	1976	CC	1625	n	bl	n	y	0	cig only	11	999	1	0	nev any	st
BENHAM	549	x	m	0	0	all	-	KII	Eu:wst	1976	CC	1625	n	bl	n	y	0	cig only	4	10	2	0	nev any	st
BENHAM	550	x	m	0	0	all	-	KII	Eu:wst	1976	CC	1625	n	bl	n	y	0	cig only	1.0	3	3	3	nev any	st
PEZZOT	585		m	0	0	all	-	a	SCAmer	1987	CC	215	n	bl	n	y	0	cig only	11	999	1	0	nev cigs	st
PEZZOT	586		m	0	0	all	-	a	SCAmer	1987	CC	215	n	bl	n	y	0	cig only	1.0	10	0	3	nev cigs	st

Cigarette type is all/unspec for all RRs

In this overview table, subtotals and Qs values may be invalid and should be ignored

Table 3J11 - 2

IESLC - Meta-analysis of Ex Smoking by Years quit (vs never), Overview
Adenocarcinoma, Cigarettes only
Most adjusted

REF	NRR	SEX	AD	Number Exposed Case	Number Exposed Cont	Non-exposed Case	Non-exposed Cont	RR	95.00%CI
BENHAM	548	m	0	4	21	9	42	0.89	(0.24- 3.23)
BENHAM	549	m	0	8	18	9	42	2.07	(0.69- 6.24)
BENHAM	550	m	0	13	9	9	42	6.74	(2.21- 20.53)
Subtotal BENHAM								2.53	(1.29- 4.93)
PEZZOT	585	m	0	7	106	3	116	2.55	(0.64- 10.13)
PEZZOT	586	m	0	11	82	3	116	5.19	(1.40- 19.18)
Subtotal PEZZOT								3.71	(1.44- 9.57)
Totals				43	236	33	358		

*prospective study

REF	NRR	SEX	AD	Ys	Ws	Qs	Ps
BENHAM	548	m	0	-0.12	2.31	3.18	0.8579
BENHAM	549	m	0	0.73	3.17	0.33	0.1940
BENHAM	550	m	0	1.91	3.10	2.26	0.0008
Subtotal BENHAM				0.93	8.58	5.77	
PEZZOT	585	m	0	0.94	2.02	0.03	0.1824
PEZZOT	586	m	0	1.65	2.25	0.79	0.0136
Subtotal PEZZOT				1.31	4.27	0.82	

N	5
NS	2

Table 3J11 - 3

IESLC - Meta-analysis of Ex Smoking by Years quit (vs never), Overview
Adenocarcinoma, Cigarettes only
Most adjusted

	Sex combined	male	female	Total
N		5		5
NS		2		2

In this overview table, other than the "N" rows, entries in the "absent" and "Total" columns may be invalid and should be ignored

Years quit vs never (lower focus)

	absent	8+k12	4-11k7	1-6k3	Total
N	1	2	1	1	5
NS	1	2	1	1	4
Wt	2.25	4.34	3.17	3.10	12.85
Het Chi	0.00	1.20	0.00	0.00	6.58
Het df	0	1	0	0	4
Het P	N.S.	N.S.	N.S.	N.S.	N.S.
Fixed RR	5.19	1.45	2.07	6.74	2.87
RRl	1.40	0.57	0.69	2.21	1.66
RRu	19.18	3.73	6.24	20.53	4.96
P	+	N.S.	N.S.	+++	+++
Random RR	5.19	1.46	2.07	6.74	2.83
RRl	1.40	0.52	0.69	2.21	1.40
RRu	19.18	4.11	6.24	20.53	5.73
P	+	N.S.	N.S.	+++	++

Years quit vs never (higher focus)

	absent	13+k20	4-19k12	1-11k3	Total
N	3			2	5
NS	2			2	4
Wt	7.51			5.34	12.85
Het Chi	1.43			0.09	6.58
Het df	2			1	4
Het P	N.S.			N.S.	N.S.
Fixed RR	1.69			6.04	2.87
RRl	0.83			2.59	1.66
RRu	3.46			14.10	4.96
P	N.S.			+++	+++
Random RR	1.69			6.04	2.83
RRl	0.83			2.59	1.40
RRu	3.46			14.10	5.73
P	N.S.			+++	++

Table 3J11 - 3

IESLC - Meta-analysis of Ex Smoking by Years quit (vs never), Overview
Adenocarcinoma, Cigarettes only
Most adjusted

MALES

Years quit vs never (lower focus)

	absent	8+k12	4-11k7	1-6k3	Total
N	1	2	1	1	5
NS	1	2	1	1	4
Wt	2.25	4.34	3.17	3.10	12.85
Het Chi	0.00	1.20	0.00	0.00	6.58
Het df	0	1	0	0	4
Het P	N.S.	N.S.	N.S.	N.S.	N.S.
Fixed RR	5.19	1.45	2.07	6.74	2.87
RRl	1.40	0.57	0.69	2.21	1.66
RRu	19.18	3.73	6.24	20.53	4.96
P	+	N.S.	N.S.	+++	+++
Random RR	5.19	1.46	2.07	6.74	2.83
RRl	1.40	0.52	0.69	2.21	1.40
RRu	19.18	4.11	6.24	20.53	5.73
P	+	N.S.	N.S.	+++	++

Years quit vs never (higher focus)

	absent	13+k20	4-19k12	1-11k3	Total
N	3			2	5
NS	2			2	4
Wt	7.51			5.34	12.85
Het Chi	1.43			0.09	6.58
Het df	2			1	4
Het P	N.S.			N.S.	N.S.
Fixed RR	1.69			6.04	2.87
RRl	0.83			2.59	1.66
RRu	3.46			14.10	4.96
P	N.S.			+++	+++
Random RR	1.69			6.04	2.83
RRl	0.83			2.59	1.40
RRu	3.46			14.10	5.73
P	N.S.			+++	++

Table 3J11 - 4

IESLC - Meta-analysis of Ex Smoking by Years quit (vs never), Overview
Adenocarcinoma, Cigarettes only
Least adjusted

REF	NRR	X	SEX	AGEL	AGEH	RACE	YF	LC TYPE	LOC	START	ST	NLC	R	VB	P	H	AD	PRODUCT	exL	exH	S1	S2	DENOM	De
BENHAM	548		m	0	0	all	-	KII	Eu:wst	1976	CC	1625	n	bl	n	y	0	cig only	11	999	1	0	nev any	st
BENHAM	549		m	0	0	all	-	KII	Eu:wst	1976	CC	1625	n	bl	n	y	0	cig only	4	10	2	0	nev any	st
BENHAM	550		m	0	0	all	-	KII	Eu:wst	1976	CC	1625	n	bl	n	y	0	cig only	1.0	3	3	3	nev any	st
PEZZOT	585		m	0	0	all	-	a	SCAmer	1987	CC	215	n	bl	n	y	0	cig only	11	999	1	0	nev cigs	st
PEZZOT	586		m	0	0	all	-	a	SCAmer	1987	CC	215	n	bl	n	y	0	cig only	1.0	10	0	3	nev cigs	st

Cigarette type is all/unspec for all RRs

In this overview table, subtotals and Qs values may be invalid and should be ignored

Table 3J11 - 5

IESLC - Meta-analysis of Ex Smoking by Years quit (vs never), Overview
Adenocarcinoma, Cigarettes only
Least adjusted

REF	NRR	SEX	AD	Number Exposed Case	Number Exposed Cont	Non-exposed Case	Non-exposed Cont	RR	95.00%CI
BENHAM	548	m	0	4	21	9	42	0.89	(0.24- 3.23)
BENHAM	549	m	0	8	18	9	42	2.07	(0.69- 6.24)
BENHAM	550	m	0	13	9	9	42	6.74	(2.21- 20.53)
Subtotal BENHAM								2.53	(1.29- 4.93)
PEZZOT	585	m	0	7	106	3	116	2.55	(0.64- 10.13)
PEZZOT	586	m	0	11	82	3	116	5.19	(1.40- 19.18)
Subtotal PEZZOT								3.71	(1.44- 9.57)
Totals				43	236	33	358		

*prospective study

REF	NRR	SEX	AD	Ys	Ws	Qs	Ps
BENHAM	548	m	0	-0.12	2.31	3.18	0.8579
BENHAM	549	m	0	0.73	3.17	0.33	0.1940
BENHAM	550	m	0	1.91	3.10	2.26	0.0008
Subtotal BENHAM				0.93	8.58	5.77	
PEZZOT	585	m	0	0.94	2.02	0.03	0.1824
PEZZOT	586	m	0	1.65	2.25	0.79	0.0136
Subtotal PEZZOT				1.31	4.27	0.82	

N	5
NS	2

Table 3J11 - 6

IESLC - Meta-analysis of Ex Smoking by Years quit (vs never), Overview
Adenocarcinoma, Cigarettes only
Least adjusted

	combined	Sex male	female	Total
N		5		5
NS		2		2

In this overview table, other than the "N" rows, entries in the "absent" and "Total" columns may be invalid and should be ignored

Years quit vs never (lower focus)	absent	8+k12	4-11k7	1-6k3	Total
N	1	2	1	1	5
NS	1	2	1	1	4
Wt	2.25	4.34	3.17	3.10	12.85
Het Chi	0.00	1.20	0.00	0.00	6.58
Het df	0	1	0	0	4
Het P	N.S.	N.S.	N.S.	N.S.	N.S.
Fixed RR	5.19	1.45	2.07	6.74	2.87
RRl	1.40	0.57	0.69	2.21	1.66
RRu	19.18	3.73	6.24	20.53	4.96
P	+	N.S.	N.S.	+++	+++
Random RR	5.19	1.46	2.07	6.74	2.83
RRl	1.40	0.52	0.69	2.21	1.40
RRu	19.18	4.11	6.24	20.53	5.73
P	+	N.S.	N.S.	+++	++

Years quit vs never (higher focus)	absent	13+k20	4-19k12	1-11k3	Total
N	3			2	5
NS	2			2	4
Wt	7.51			5.34	12.85
Het Chi	1.43			0.09	6.58
Het df	2			1	4
Het P	N.S.			N.S.	N.S.
Fixed RR	1.69			6.04	2.87
RRl	0.83			2.59	1.66
RRu	3.46			14.10	4.96
P	N.S.			+++	+++
Random RR	1.69			6.04	2.83
RRl	0.83			2.59	1.40
RRu	3.46			14.10	5.73
P	N.S.			+++	++

Table 3J11 - 6

IESLC - Meta-analysis of Ex Smoking by Years quit (vs never), Overview
Adenocarcinoma, Cigarettes only
Least adjusted

MALES

	Years quit vs never (lower focus) absent	8+k12	4-11k7	1-6k3	Total
N	1	2	1	1	5
NS	1	2	1	1	4
Wt	2.25	4.34	3.17	3.10	12.85
Het Chi	0.00	1.20	0.00	0.00	6.58
Het df	0	1	0	0	4
Het P	N.S.	N.S.	N.S.	N.S.	N.S.
Fixed RR	5.19	1.45	2.07	6.74	2.87
RRl	1.40	0.57	0.69	2.21	1.66
RRu	19.18	3.73	6.24	20.53	4.96
P	+	N.S.	N.S.	+++	+++
Random RR	5.19	1.46	2.07	6.74	2.83
RRl	1.40	0.52	0.69	2.21	1.40
RRu	19.18	4.11	6.24	20.53	5.73
P	+	N.S.	N.S.	+++	++

	Years quit vs never (higher focus) absent	13+k20	4-19k12	1-11k3	Total
N	3			2	5
NS	2			2	4
Wt	7.51			5.34	12.85
Het Chi	1.43			0.09	6.58
Het df	2			1	4
Het P	N.S.			N.S.	N.S.
Fixed RR	1.69			6.04	2.87
RRl	0.83			2.59	1.66
RRu	3.46			14.10	4.96
P	N.S.			+++	+++
Random RR	1.69			6.04	2.83
RRl	0.83			2.59	1.40
RRu	3.46			14.10	5.73
P	N.S.			+++	++

Table 3J11 - 7

IESLC - Meta-analysis of Ex Smoking by Years quit (vs never), Overview
Adenocarcinoma, Cigarettes only
Excluded studies (and stage at which they were excluded)

1 AGUDO AKIBA AMANDU AMES AXELSS BEST BOUCHA BOUCOT BRESLO CHEN CHEN2 CHIAZZ DEAN2 DOSEME ENGELA FAN GENG GER GUO HAENSZ HEGMAN HOLE HU HU2 JUSSAW KATSOU KAUFMA KOO KOULUM KREUZE LETOUR LEVIN LIAW LIU3 LIU4 LIU5 MCCONN MIGRAN MRFITR NOTAN2 OSANN2 PERNU QIAO2 RACHTA RESTRE SADOWS SEGI2 STASZE TIZZAN VUTUC WATSON WU WUWILL WYNDE2 WYNDE8 XU YUAN ZHANG ZHENG ZHOU

2 BUFFLE HUMBLE PISANI PRESCO WYNDE7

3 MCDUFF SPITZ

4 ARMADA AUVINE BECHER BENSHL BLOT1 BOFFET BROSS CARPEN CEDERL CHOI CHYOU CORREA CPSI CPSII DAMBER DARBY DEAN3 DESTEF DOLL DOLL2 DORGAN DORN GAO GAO2 GARCIA GARSHI GILLIS GRAHAM GURSEL HAMMO2 HAMMON HIRAYA JOLY KAISE2 KHUDER LAUSSM LUBIN LUO PEZZO2 QIAO SPEIZE SUZUK2 TVERDA WANG2 WIGLE

5 ALDERS

7 BARBON BROWN3 JAHN JAIN JEDRYC LUBIN2 MATOS SOBUE SVENSS WAKAI WU2 WYNDE3 WYNDE6

Table 3J11 - 8

Potentially overlapping studies

REF	REFGP	PRINC .	OVERLAP/LINK
BENHAM	LUBIN2	2	Subset of Lubin2

Table 3J11 - 9

Most adjusted - insufficient data for meta-analysis

REF	NRR	SEX	AGEL	AGEH	RACE	YF	LC TYPE	LOC	START	ST	NLC	R	VB	P	H	AD	PRODUCT	exL	exH	S1	S2	DENOM	De
ALDERS	567	m	0	0	all	-	not q+s	Eu:UK	1977	CC	1448	n	V	n	n	1	cig only	10	999	1	0	nev any	st
ALDERS	568	m	0	0	all	-	not q+s	Eu:UK	1977	CC	1448	n	V	n	n	1	cig only	3	9	0	3	nev any	st
ALDERS	569	m	0	0	all	-	not q+s	Eu:UK	1977	CC	1448	n	V	n	n	1	cig only	0.1	2	0	0	nev any	st
ALDERS	578	f	0	0	all	-	not q+s	Eu:UK	1977	CC	1448	n	V	n	n	1	cig only	10	999	1	0	nev any	st
ALDERS	579	f	0	0	all	-	not q+s	Eu:UK	1977	CC	1448	n	V	n	n	1	cig only	3	9	0	3	nev any	st
ALDERS	580	f	0	0	all	-	not q+s	Eu:UK	1977	CC	1448	n	V	n	n	1	cig only	0.1	2	0	0	nev any	st
BENHAM	551	m	0	0	all	-	KII	Eu:wst	1976	CC	1625	n	bl	n	y	0	cig only	0.1	0.9	0	0	nev any	ot
PEZZOT	601	m	0	0	all	-	a	SCAmer	1987	CC	215	n	bl	n	y	0	cig only	0.1	0.9	0	0	nev cigs	ot

REF	NRR	RR	SIG	RRDATA comment
ALDERS	567	2.22		0
ALDERS	568	3.80		0
ALDERS	569	5.05		0
ALDERS	578	1.64		0
ALDERS	579	0.73		0
ALDERS	580	6.27		0
BENHAM	551	*	gap	0
PEZZOT	601	*	gap	0

Table 3J12 -

IESLC - Meta-analysis of Ex Smoking, Years quit (vs never), "Low"
Adenocarcinoma, Cigarettes only

This analysis is restricted to results for:
1) Ex smokers
2) Results by Years quit (vs never)
3) Categorical results by Years quit (vs never)
4) Adenocarcinoma (or near equivalent)
5) Results complete enough for use in metaanalysis

Within each study, results are then selected (in the following order of preference, within each sex) for:
6) (not applicable)
7) PRODUCT: cigarettes only
8) CIGTYPE: all/unspecified, MC regardless of HR, MC only
9) (not applicable)
10) DENOM: never smoked anything, never smoked cigarettes, never any + low, never cigs + low
11) Followup period (YF, prospective studies): whole study (coded as 0) or longest available
12) LCtype: adeno or nearest available, but not squamous. (q = squamous, s = small,
a = adeno, l = large, KII = Kreyberg II, al = alveolar, br = bronchiolar, u = undifferentiated)
13) Race: all or nearest available, otherwise by race (wh or w = white, bl or b = black, hi = hispanic
ch = chinese, jap = japanese, haw = hawaiian, w+o = white + oriental, sca = scandinavian, as = asian)
14) Years quit (vs never) "low" in key scheme 1 (key value 12, maximum range 8+)
15) For overlapping studies: principal rather than subsidiary studies
Finally by Age: whole study (coded as 0) if available, otherwise by widest available age group
and then for single sex results (m, f) in preference to results for both sexes combined (c).

Results adjusted (AD) for the most potential confounders are then chosen in Sections -1 to -3
(and those which actually differ from the adjusted results in Table 3J2 - 1 are marked 'x' in Section -1)
and results adjusted for the least confounders in Sections -4 to -6. (Those least adjusted results which
actually differ from the most adjusted are marked 'x' in column X in Section -4)

Section -7 shows excluded studies, together with the stage (as above) at which no qualifying
results were found.

Section -8 lists the potentially overlapping studies which have been included (1=principal, 2=subsidiary).

Section -9 lists any results which would have been included in preference except that they had data not complete
enough for use in meta-analysis, with their significance (yes/no), if known, and any further comment as entered
on the database. It also lists as "gap" any categories for which no data were presented by the original authors.
This is commonly due to recent quitters having been combined with current smokers

In addition to those mentioned above, the following fields, levels and abbreviations are used:
* or nk = not known, n = no, y = yes, ot = other
nev = never
all/unspec = all or unspecified, MC = manufactured cigarettes, HR = hand-rolled cigarettes
exL, exH = range of exposure (low and high) in the smoking group, in terms of Years quit (vs never)
REF: 6-character study reference
NRR: number of the RR on the database within the study
ST : study type (CC = case control, pr or prosp = prospective)
NLC: number of lung cancer cases in whole study
R : risky occupational population (n = no, m = mining, o = other risky)
VB : national cigarette type (V = at least 75% Virginia, bl = at least 75% blended, ot = other)
P : any proxy use
H : full histological confirmation
De : derivation of RR/CI (or = original, st = standard method, ot = other method of estimation)

Table 3J12 - 1

IESLC - Meta-analysis of Ex Smoking, Years quit (vs never), "Low"
Adenocarcinoma, Cigarettes only
Most adjusted

REF	NRR	3J2	SEX	AGEL	AGEH	RACE	YF	LC TYPE	LOC	START	ST	NLC	R	VB	P	H	AD	PRODUCT	exL	exH	DENOM	De
BENHAM	548	x	m	0	0	all	-	KII	Eu:wst	1976	CC	1625	n	bl	n	y	0	cig only	11	999	nev any	st
PEZZOT	585		m	0	0	all	-	a	SCAmer	1987	CC	215	n	bl	n	y	0	cig only	11	999	nev cigs	st

Cigarette type is all/unspec for all RRs

Table 3J12 - 2

IESLC - Meta-analysis of Ex Smoking, Years quit (vs never), "Low"
Adenocarcinoma, Cigarettes only
Most adjusted

REF	NRR	SEX	AD	Number Exposed Case	Cont	Non-exposed Case	Cont	RR	95.00%CI
BENHAM	548	m	0	4	21	9	42	0.89	(0.24- 3.23)
PEZZOT	585	m	0	7	106	3	116	2.55	(0.64- 10.13)
Totals				11	127	12	158		

*prospective study

REF	NRR	SEX	AD	Ys	Ws	Qs	Ps
BENHAM	548	m	0	-0.12	2.31	0.56	0.8579
PEZZOT	585	m	0	0.94	2.02	0.64	0.1824

	N	2
	NS	2
	Wt	4.34
Het	Chi	1.20
Het	df	1
Het	P	N.S.
Fixed	RR	1.45
	RRl	0.57
	RRu	3.73
	P	N.S.
Random	RR	1.46
	RRl	0.52
	RRu	4.11
	P	N.S.
Asymm	P	

Table 3J12 - 3

IESLC - Meta-analysis of Ex Smoking, Years quit (vs never), "Low"
Adenocarcinoma, Cigarettes only
Most adjusted

	Sex combined	male	female	Total
N		2		2
NS		2		2
Wt		4.34		4.34
Het Chi		1.20		1.20
Het df		1		1
Het P		N.S.		N.S.
Fixed RR		1.45		1.45
RRl		0.57		0.57
RRu		3.73		3.73
P		N.S.		N.S.
Random RR		1.46		1.46
RRl		0.52		0.52
RRu		4.11		4.11
P		N.S.		N.S.
Between Chi				
Between df				
Between P				N.S.
Btwn(F) P				N.S.
Btwn(R) P				N.S.

Too few RRs for analysis by factor

Table 3J12 - 4

IESLC - Meta-analysis of Ex Smoking, Years quit (vs never), "Low"
Adenocarcinoma, Cigarettes only
Least adjusted

REF	NRR	X	SEX	AGEL	AGEH	RACE	YF	LC TYPE	LOC	START	ST	NLC	R	VB	P	H	AD	PRODUCT	exL	exH	DENOM	De
BENHAM	548		m	0	0	all	-	KII	Eu:wst	1976	CC	1625	n	bl	n	y	0	cig only	11	999	nev any	st
PEZZOT	585		m	0	0	all	-	a	SCAmer	1987	CC	215	n	bl	n	y	0	cig only	11	999	nev cigs	st

Cigarette type is all/unspec for all RRs

Table 3J12 - 5

IESLC - Meta-analysis of Ex Smoking, Years quit (vs never), "Low"
Adenocarcinoma, Cigarettes only
Least adjusted

REF	NRR	SEX	AD	Number Exposed Case	Number Exposed Cont	Non-exposed Case	Non-exposed Cont	RR	95.00%CI
BENHAM	548	m	0	4	21	9	42	0.89	(0.24- 3.23)
PEZZOT	585	m	0	7	106	3	116	2.55	(0.64- 10.13)
Totals				11	127	12	158		

*prospective study

REF	NRR	SEX	AD	Ys	Ws	Qs	Ps
BENHAM	548	m	0	-0.12	2.31	0.56	0.8579
PEZZOT	585	m	0	0.94	2.02	0.64	0.1824

	N	2
	NS	2
	Wt	4.34
Het	Chi	1.20
Het	df	1
Het	P	N.S.
Fixed	RR	1.45
	RRl	0.57
	RRu	3.73
	P	N.S.
Random	RR	1.46
	RRl	0.52
	RRu	4.11
	P	N.S.
Asymm	P	

Table 3J12 - 6

IESLC - Meta-analysis of Ex Smoking, Years quit (vs never), "Low"
Adenocarcinoma, Cigarettes only
Least adjusted

	Sex combined	male	female	Total
N		2		2
NS		2		2
Wt		4.34		4.34
Het Chi		1.20		1.20
Het df		1		1
Het P		N.S.		N.S.
Fixed RR		1.45		1.45
RRl		0.57		0.57
RRu		3.73		3.73
P		N.S.		N.S.
Random RR		1.46		1.46
RRl		0.52		0.52
RRu		4.11		4.11
P		N.S.		N.S.
Between Chi				
Between df				
Between P				N.S.
Btwn(F) P				N.S.
Btwn(R) P				N.S.

Table 3J12 - 7

IESLC - Meta-analysis of Ex Smoking, Years quit (vs never), "Low"
Adenocarcinoma, Cigarettes only
Excluded studies (and stage at which they were excluded)

```
1        AGUDO  AKIBA AMANDU   AMES AXELSS   BEST BOUCHA BOUCOT BRESLO   CHEN  CHEN2 CHIAZZ  DEAN2 DOSEME ENGELA    FAN
          GENG    GER    GUO HAENSZ HEGMAN   HOLE     HU    HU2 JUSSAW KATSOU KAUFMA    KOO KOULUM KREUZE LETOUR  LEVIN
          LIAW   LIU3   LIU4   LIU5 MCCONN MIGRAN MRFITR NOTAN2 OSANN2  PERNU  QIAO2 RACHTA RESTRE SADOWS  SEGI2 STASZE
       TIZZAN  VUTUC WATSON     WU WUWILL WYNDE2 WYNDE8     XU   YUAN  ZHANG  ZHENG   ZHOU

2      BUFFLE HUMBLE PISANI PRESCO WYNDE7

3      MCDUFF  SPITZ

4      ARMADA AUVINE BECHER BENSHL  BLOT1 BOFFET  BROSS CARPEN CEDERL   CHOI  CHYOU CORREA   CPSI  CPSII DAMBER  DARBY
        DEAN3 DESTEF   DOLL  DOLL2 DORGAN   DORN    GAO   GAO2 GARCIA GARSHI GILLIS GRAHAM GURSEL HAMMO2 HAMMON HIRAYA
         JOLY KAISE2 KHUDER LAUSSM  LUBIN    LUO PEZZO2   QIAO SPEIZE SUZUK2 TVERDA  WANG2  WIGLE

5      ALDERS

7      BARBON BROWN3   JAHN   JAIN JEDRYC LUBIN2  MATOS  SOBUE SVENSS  WAKAI    WU2 WYNDE3 WYNDE6
```

Table 3J12 - 8
Potentially overlapping studies

REF	REFGP	PRINC .	OVERLAP/LINK
BENHAM	LUBIN2	2	Subset of Lubin2

Table 3J12 - 9
Most adjusted - insufficient data for meta-analysis

REF	NRR	SEX	AGEL	AGEH	RACE	YF	LC TYPE	LOC	START	ST	NLC	R	VB	P	H	AD	PRODUCT	exL	exH	DENOM	De
ALDERS	567	m	0	0	all	-	not q+s	Eu:UK	1977	CC	1448	n	V	n	n	1	cig only	10	999	nev	any st
ALDERS	578	f	0	0	all	-	not q+s	Eu:UK	1977	CC	1448	n	V	n	n	1	cig only	10	999	nev	any st

REF	NRR	RR	SIG	RRDATA comment
ALDERS	567	2.22		0
ALDERS	578	1.64		0

Table 3J13 -

IESLC - Meta-analysis of Ex Smoking, Years quit (vs never), "Mid"
Adenocarcinoma, Cigarettes only

This analysis is restricted to results for:
1) Ex smokers
2) Results by Years quit (vs never)
3) Categorical results by Years quit (vs never)
4) Adenocarcinoma (or near equivalent)
5) Results complete enough for use in metaanalysis

Within each study, results are then selected (in the following order of preference, within each sex) for:
6) (not applicable)
7) PRODUCT: cigarettes only
8) CIGTYPE: all/unspecified, MC regardless of HR, MC only
9) (not applicable)
10) DENOM: never smoked anything, never smoked cigarettes, never any + low, never cigs + low
11) Followup period (YF, prospective studies): whole study (coded as 0) or longest available
12) LCtype: adeno or nearest available, but not squamous. (q = squamous, s = small,
a = adeno, l = large, KII = Kreyberg II, al = alveolar, br = bronchiolar, u = undifferentiated)
13) Race: all or nearest available, otherwise by race (wh or w = white, bl or b = black, hi = hispanic
ch = chinese, jap = japanese, haw = hawaiian, w+o = white + oriental, sca = scandinavian, as = asian)
14) Years quit (vs never) "mid" in key scheme 1 (key value 7, maximum range 4-11)
15) For overlapping studies: principal rather than subsidiary studies
Finally by Age: whole study (coded as 0) if available, otherwise by widest available age group
and then for single sex results (m, f) in preference to results for both sexes combined (c).

Results adjusted (AD) for the most potential confounders are then chosen in Sections -1 to -3
(and those which actually differ from the adjusted results in Table 3J3 - 1 are marked 'x' in Section -1)
and results adjusted for the least confounders in Sections -4 to -6. (Those least adjusted results which
actually differ from the most adjusted are marked 'x' in column X in Section -4)

Section -7 shows excluded studies, together with the stage (as above) at which no qualifying
results were found.

Section -8 lists the potentially overlapping studies which have been included (1=principal, 2=subsidiary).

Section -9 lists any results which would have been included in preference except that they had data not complete
enough for use in meta-analysis, with their significance (yes/no), if known, and any further comment as entered
on the database. It also lists as "gap" any categories for which no data were presented by the original authors.
This is commonly due to recent quitters having been combined with current smokers

In addition to those mentioned above, the following fields, levels and abbreviations are used:
* or nk = not known, n = no, y = yes, ot = other
nev = never
all/unspec = all or unspecified, MC = manufactured cigarettes, HR = hand-rolled cigarettes
exL, exH = range of exposure (low and high) in the smoking group, in terms of Years quit (vs never)
REF: 6-character study reference
NRR: number of the RR on the database within the study
ST : study type (CC = case control, pr or prosp = prospective)
NLC: number of lung cancer cases in whole study
R : risky occupational population (n = no, m = mining, o = other risky)
VB : national cigarette type (V = at least 75% Virginia, bl = at least 75% blended, ot = other)
P : any proxy use
H : full histological confirmation
De : derivation of RR/CI (or = original, st = standard method, ot = other method of estimation)

Table 3J13 - 1

IESLC - Meta-analysis of Ex Smoking, Years quit (vs never), "Mid"
Adenocarcinoma, Cigarettes only
Most adjusted

REF	NRR	3J3	SEX	AGEL	AGEH	RACE	YF	LC TYPE	LOC	START	ST	NLC	R	VB	P	H	AD	PRODUCT	exL	exH	DENOM	De
BENHAM	549	x	m	0	0	all	-	KII	Eu:wst	1976	CC	1625	n	bl	n	y	0	cig only	4	10	nev any	st

Cigarette type is all/unspec for all RRs

Table 3J13 - 2

IESLC - Meta-analysis of Ex Smoking, Years quit (vs never), "Mid"
Adenocarcinoma, Cigarettes only
Most adjusted

REF	NRR	SEX	AD	Number Exposed Case	Number Exposed Cont	Non-exposed Case	Non-exposed Cont	RR	95.00%CI
BENHAM	549	m	0	8	18	9	42	2.07	(0.69- 6.24)
Totals				8	18	9	42		

*prospective study

REF	NRR	SEX	AD	Ys	Ws	Qs	Ps
BENHAM	549	m	0	0.73	3.17	0.00	0.1940

	N	1
	NS	1
	Wt	3.17
Het	Chi	0.00
Het	df	0
Het	P	N.S.
Fixed	RR	2.07
	RRl	0.69
	RRu	6.24
	P	N.S.
Random	RR	2.07
	RRl	0.69
	RRu	6.24
	P	N.S.
Asymm	P	

Table 3J13 - 3

IESLC - Meta-analysis of Ex Smoking, Years quit (vs never), "Mid"
Adenocarcinoma, Cigarettes only
Most adjusted

	Sex: combined	male	female	Total
N		1		1
NS		1		1
Wt		3.17		3.17
Het Chi		0.00		0.00
Het df		0		0
Het P		N.S.		N.S.
Fixed RR		2.07		2.07
RRl		0.69		0.69
RRu		6.24		6.24
P		N.S.		N.S.
Random RR		2.07		2.07
RRl		0.69		0.69
RRu		6.24		6.24
P		N.S.		N.S.
Between Chi				
Between df				
Between P				N.S.
Btwn(F) P				N.S.
Btwn(R) P				N.S.

Too few RRs for analysis by factor

Table 3J13 - 4

IESLC - Meta-analysis of Ex Smoking, Years quit (vs never), "Mid"
Adenocarcinoma, Cigarettes only
Least adjusted

REF	NRR	X	SEX	AGEL	AGEH	RACE	YF	LC TYPE	LOC	START	ST	NLC	R	VB	P	H	AD	PRODUCT	exL	exH	DENOM	De
BENHAM	549		m	0	0	all	-	KII	Eu:wst	1976	CC	1625	n	bl	n	y	0	cig only	4	10	nev any	st

Cigarette type is all/unspec for all RRs

Table 3J13 - 5

IESLC - Meta-analysis of Ex Smoking, Years quit (vs never), "Mid"
Adenocarcinoma, Cigarettes only
Least adjusted

REF	NRR	SEX	AD	Number Exposed Case	Exposed Cont	Non-exposed Case	Non-exposed Cont	RR	95.00%CI
BENHAM	549	m	0	8	18	9	42	2.07	(0.69- 6.24)
Totals				8	18	9	42		

*prospective study

REF	NRR	SEX	AD	Ys	Ws	Qs	Ps
BENHAM	549	m	0	0.73	3.17	0.00	0.1940

	N	1
	NS	1
	Wt	3.17
Het	Chi	0.00
Het	df	0
Het	P	N.S.
Fixed	RR	2.07
	RRl	0.69
	RRu	6.24
	P	N.S.
Random	RR	2.07
	RRl	0.69
	RRu	6.24
	P	N.S.
Asymm	P	

Table 3J13 - 6

IESLC - Meta-analysis of Ex Smoking, Years quit (vs never), "Mid"
Adenocarcinoma, Cigarettes only
Least adjusted

	Sex			
	combined	male	female	Total
N		1		1
NS		1		1
Wt		3.17		3.17
Het Chi		0.00		0.00
Het df		0		0
Het P		N.S.		N.S.
Fixed RR		2.07		2.07
RRl		0.69		0.69
RRu		6.24		6.24
P		N.S.		N.S.
Random RR		2.07		2.07
RRl		0.69		0.69
RRu		6.24		6.24
P		N.S.		N.S.
Between Chi				
Between df				
Between P				N.S.
Btwn(F) P				N.S.
Btwn(R) P				N.S.

Table 3J13 - 7

IESLC - Meta-analysis of Ex Smoking, Years quit (vs never), "Mid"
Adenocarcinoma, Cigarettes only
Excluded studies (and stage at which they were excluded)

1	AGUDO AKIBA AMANDU AMES AXELSS BEST BOUCHA BOUCOT BRESLO CHEN CHEN2 CHIAZZ DEAN2 DOSEME ENGELA FAN GENG GER GUO HAENSZ HEGMAN HOLE HU HU2 JUSSAW KATSOU KAUFMA KOO KOULUM KREUZE LETOUR LEVIN LIAW LIU3 LIU4 LIU5 MCCONN MIGRAN MRFITR NOTAN2 OSANN2 PERNU QIAO2 RACHTA RESTRE SADOWS SEGI2 STASZE TIZZAN VUTUC WATSON WU WUWILL WYNDE2 WYNDE8 XU YUAN ZHANG ZHENG ZHOU
2	BUFFLE HUMBLE PISANI PRESCO WYNDE7
3	MCDUFF SPITZ
4	ARMADA AUVINE BECHER BENSHL BLOT1 BOFFET BROSS CARPEN CEDERL CHOI CHYOU CORREA CPSI CPSII DAMBER DARBY DEAN3 DESTEF DOLL DOLL2 DORGAN DORN GAO GAO2 GARCIA GARSHI GILLIS GRAHAM GURSEL HAMMO2 HAMMON HIRAYA JOLY KAISE2 KHUDER LAUSSM LUBIN LUO PEZZO2 QIAO SPEIZE SUZUK2 TVERDA WANG2 WIGLE
5	ALDERS
7	BARBON BROWN3 JAHN JAIN JEDRYC LUBIN2 MATOS SOBUE SVENSS WAKAI WU2 WYNDE3 WYNDE6
14	PEZZOT

Table 3J13 - 8
Potentially overlapping studies

REF	REFGP	PRINC	.	OVERLAP/LINK
BENHAM	LUBIN2	2		Subset of Lubin2

Table 3J14 -

IESLC - Meta-analysis of Ex Smoking, Years quit (vs never), "High"
Adenocarcinoma, Cigarettes only

This analysis is restricted to results for:
1) Ex smokers
2) Results by Years quit (vs never)
3) Categorical results by Years quit (vs never)
4) Adenocarcinoma (or near equivalent)
5) Results complete enough for use in metaanalysis

Within each study, results are then selected (in the following order of preference, within each sex) for:
6) PRODUCT: cigarettes only
7) CIGTYPE: all/unspecified, MC regardless of HR, MC only
8) (not applicable)
9) DENOM: never smoked anything, never smoked cigarettes, never any + low, never cigs + low
10) Followup period (YF, prospective studies): whole study (coded as 0) or longest available
11) LCtype: adeno or nearest available, but not squamous. (q = squamous, s = small,
a = adeno, l = large, KII = Kreyberg II, al = alveolar, br = bronchiolar, u = undifferentiated)
12) Race: all or nearest available, otherwise by race (wh or w = white, bl or b = black, hi = hispanic
ch = chinese, jap = japanese, haw = hawaiian, w+o = white + oriental, sca = scandinavian, as = asian)
13) Years quit (vs never) "high" in key scheme 1 (key value 3, maximum range 1-6)
14) For overlapping studies: principal rather than subsidiary studies
Finally by Age: whole study (coded as 0) if available, otherwise by widest available age group
and then for single sex results (m, f) in preference to results for both sexes combined (c).

Results adjusted (AD) for the most potential confounders are then chosen in Sections -1 to -3
(and those which actually differ from the adjusted results in Table 3J4 - 1 are marked 'x' in Section -1)
and results adjusted for the least confounders in Sections -4 to -6. (Those least adjusted results which
actually differ from the most adjusted are marked 'x' in column X in Section -4)

Section -7 shows excluded studies, together with the stage (as above) at which no qualifying
results were found.

Section -8 lists the potentially overlapping studies which have been included (1=principal, 2=subsidiary).

Section -9 lists any results which would have been included in preference except that they had data not complete
enough for use in meta-analysis, with their significance (yes/no), if known, and any further comment as entered
on the database. It also lists as "gap" any categories for which no data were presented by the original authors.
This is commonly due to recent quitters having been combined with current smokers

In addition to those mentioned above, the following fields, levels and abbreviations are used:
* or nk = not known, n = no, y = yes, ot = other
nev = never
all/unspec = all or unspecified, MC = manufactured cigarettes, HR = hand-rolled cigarettes
exL, exH = range of exposure (low and high) in the smoking group, in terms of Years quit (vs never)
REF: 6-character study reference
NRR: number of the RR on the database within the study
ST : study type (CC = case control, pr or prosp = prospective)
NLC: number of lung cancer cases in whole study
R : risky occupational population (n = no, m = mining, o = other risky)
VB : national cigarette type (V = at least 75% Virginia, bl = at least 75% blended, ot = other)
P : any proxy use
H : full histological confirmation
De : derivation of RR/CI (or = original, st = standard method, ot = other method of estimation)

Table 3J14 - 1

IESLC - Meta-analysis of Ex Smoking, Years quit (vs never), "High"
Adenocarcinoma, Cigarettes only
Most adjusted

REF	NRR	3J4	SEX	AGEL	AGEH	RACE	YF	LC TYPE	LOC	START	ST	NLC	R	VB	P	H	AD	PRODUCT	exL	exH	DENOM	De
BENHAM	550	x	m	0	0	all	-	KII	Eu:wst	1976	CC	1625	n	bl	n	y	0	cig only	1.0	3	nev any	st

Cigarette type is all/unspec for all RRs

Table 3J14 - 2

IESLC - Meta-analysis of Ex Smoking, Years quit (vs never), "High"
Adenocarcinoma, Cigarettes only
Most adjusted

REF	NRR	SEX	AD	Number Exposed Case	Number Exposed Cont	Non-exposed Case	Non-exposed Cont	RR	95.00%CI
BENHAM	550	m	0	13	9	9	42	6.74	(2.21- 20.53)
Totals				13	9	9	42		

*prospective study

REF	NRR	SEX	AD	Ys	Ws	Qs	Ps
BENHAM	550	m	0	1.91	3.10	0.00	0.0008

	N	1
	NS	1
	Wt	3.10
Het	Chi	0.00
Het	df	0
Het	P	N.S.
Fixed	RR	6.74
	RRl	2.21
	RRu	20.53
	P	+++
Random	RR	6.74
	RRl	2.21
	RRu	20.53
	P	+++
Asymm	P	

Table 3J14 - 3

IESLC - Meta-analysis of Ex Smoking, Years quit (vs never), "High"
Adenocarcinoma, Cigarettes only
Most adjusted

	Sex combined	male	female	Total
N		1		1
NS		1		1
Wt		3.10		3.10
Het Chi		0.00		0.00
Het df		0		0
Het P		N.S.		N.S.
Fixed RR		6.74		6.74
RRl		2.21		2.21
RRu		20.53		20.53
P		+++		+++
Random RR		6.74		6.74
RRl		2.21		2.21
RRu		20.53		20.53
P		+++		+++
Between Chi				
Between df				
Between P				N.S.
Btwn(F) P				N.S.
Btwn(R) P				N.S.

Too few RRs for analysis by factor

Table 3J14 - 4

IESLC - Meta-analysis of Ex Smoking, Years quit (vs never), "High"
Adenocarcinoma, Cigarettes only
Least adjusted

REF	NRR	X	SEX	AGEL	AGEH	RACE	YF	LC TYPE	LOC	START	ST	NLC	R	VB	P	H	AD	PRODUCT	exL	exH	DENOM	De
BENHAM	550		m	0	0	all	-	KII	Eu:wst	1976	CC	1625	n	bl	n	y	0	cig only	1.0	3	nev any	st

Cigarette type is all/unspec for all RRs

Table 3J14 - 5

IESLC - Meta-analysis of Ex Smoking, Years quit (vs never), "High"
Adenocarcinoma, Cigarettes only
Least adjusted

REF	NRR	SEX	AD	Number Case	Exposed Cont	Non-exposed Case	Cont	RR	95.00%CI
BENHAM	550	m	0	13	9	9	42	6.74	(2.21- 20.53)
Totals				13	9	9	42		

*prospective study

REF	NRR	SEX	AD	Ys	Ws	Qs	Ps
BENHAM	550	m	0	1.91	3.10	0.00	0.0008

N	1
NS	1
Wt	3.10
Het Chi	0.00
Het df	0
Het P	N.S.
Fixed RR	6.74
RRl	2.21
RRu	20.53
P	+++
Random RR	6.74
RRl	2.21
RRu	20.53
P	+++
Asymm P	

Table 3J14 - 6

IESLC - Meta-analysis of Ex Smoking, Years quit (vs never), "High"
Adenocarcinoma, Cigarettes only
Least adjusted

	Sex combined	male	female	Total
N		1		1
NS		1		1
Wt		3.10		3.10
Het Chi		0.00		0.00
Het df		0		0
Het P		N.S.		N.S.
Fixed RR		6.74		6.74
RRl		2.21		2.21
RRu		20.53		20.53
P		+++		+++
Random RR		6.74		6.74
RRl		2.21		2.21
RRu		20.53		20.53
P		+++		+++
Between Chi				
Between df				
Between P				N.S.
Btwn(F) P				N.S.
Btwn(R) P				N.S.

Table 3J14 - 7

IESLC - Meta-analysis of Ex Smoking, Years quit (vs never), "High"
Adenocarcinoma, Cigarettes only
Excluded studies (and stage at which they were excluded)

1	AGUDO AKIBA AMANDU AMES AXELSS BEST BOUCHA BOUCOT BRESLO CHEN CHEN2 CHIAZZ DEAN2 DOSEME ENGELA FAN GENG GER GUO HAENSZ HEGMAN HOLE HU HU2 JUSSAW KATSOU KAUFMA KOO KOULUM KREUZE LETOUR LEVIN LIAW LIU3 LIU4 LIU5 MCCONN MIGRAN MRFITR NOTAN2 OSANN2 PERNU QIAO2 RACHTA RESTRE SADOWS SEGI2 STASZE TIZZAN VUTUC WATSON WU WUWILL WYNDE2 WYNDE8 XU YUAN ZHANG ZHENG ZHOU
2	BUFFLE HUMBLE PISANI PRESCO WYNDE7
3	MCDUFF SPITZ
4	ARMADA AUVINE BECHER BENSHL BLOT1 BOFFET BROSS CARPEN CEDERL CHOI CHYOU CORREA CPSI CPSII DAMBER DARBY DEAN3 DESTEF DOLL DOLL2 DORGAN DORN GAO GAO2 GARCIA GARSHI GILLIS GRAHAM GURSEL HAMMO2 HAMMON HIRAYA JOLY KAISE2 KHUDER LAUSSM LUBIN LUO PEZZO2 QIAO SPEIZE SUZUK2 TVERDA WANG2 WIGLE
5	ALDERS
7	BARBON BROWN3 JAHN JAIN JEDRYC LUBIN2 MATOS SOBUE SVENSS WAKAI WU2 WYNDE3 WYNDE6
14	PEZZOT

Table 3J14 - 8
Potentially overlapping studies

REF	REFGP	PRINC	.	OVERLAP/LINK
BENHAM	LUBIN2	2		Subset of Lubin2

Table 3J15 -

IESLC - Meta-analysis of Ex Smoking, Years quit (vs never), "Highest vs lowest"
Adenocarcinoma, Cigarettes only

This analysis is restricted to results for:
1) Ex smokers
2) Results by Years quit (vs never)
3) Categorical results by Years quit (vs never)
4) Denominator (unexposed) = "low"
5) Adenocarcinoma (or near equivalent)
6) Results complete enough for use in metaanalysis

Within each study, results are then selected (in the following order of preference, within each sex) for:
7) (not applicable)
8) PRODUCT: cigarettes only
9) CIGTYPE: all/unspecified, MC regardless of HR, MC only
10) Results with least adjustment for other aspects of smoking (ADOS)
11) The highest vs lowest category
12) Followup period (YF, prospective studies): whole study (coded as 0) or longest available
13) LCtype: adeno or nearest available, but not squamous. (q = squamous, s = small,
a = adeno, l = large, KII = Kreyberg II, al = alveolar, br = bronchiolar, u = undifferentiated)
14) Race: all or nearest available, otherwise by race (wh or w = white, bl or b = black, hi = hispanic
ch = chinese, jap = japanese, haw = hawaiian, w+o = white + oriental, sca = scandinavian, as = asian)
15) For overlapping studies: principal rather than subsidiary studies
Finally by Age: whole study (coded as 0) if available, otherwise by widest available age group
and then for single sex results (m, f) in preference to results for both sexes combined (c).

Results adjusted (AD) for the most potential confounders are then chosen in Sections -1 to -3
(and those which actually differ from the adjusted results in Table 3J5 - 1 are marked 'x' in Section -1)
and results adjusted for the least confounders in Sections -4 to -6. (Those least adjusted results which
actually differ from the most adjusted are marked 'x' in column X in Section -4)

Section -7 shows excluded studies, together with the stage (as above) at which no qualifying
results were found.

Section -8 lists the potentially overlapping studies which have been included (1=principal, 2=subsidiary).

Section -9 lists any results which would have been included in preference except that they had data not complete
enough for use in meta-analysis, with their significance (yes/no), if known, and any further comment as entered
on the database. It also lists as "gap" any categories for which no data were presented by the original authors.
This is commonly due to recent quitters having been combined with current smokers

In addition to those mentioned above, the following fields, levels and abbreviations are used:
* or nk = not known, n = no, y = yes, ot = other
all/unspec = all or unspecified, MC = manufactured cigarettes, HR = hand-rolled cigarettes
exL, exH = range of exposure (low and high) in the "highest" group, in terms of Years quit (vs never)
unexL, unexH = range of exposure (low and high) in the "lowest" group, in terms of Years quit (vs never)
REF: 6-character study reference
NRR: number of the RR on the database within the study
ST : study type (CC = case control, pr or prosp = prospective)
NLC: number of lung cancer cases in whole study
R : risky occupational population (n = no, m = mining, o = other risky)
VB : national cigarette type (V = at least 75% Virginia, bl = at least 75% blended, ot = other)
P : any proxy use
H : full histological confirmation
De : derivation of RR/CI (or = original, st = standard method, ot = other method of estimation)

Table 3J15 - 1

IESLC - Meta-analysis of Ex Smoking, Years quit (vs never), "Highest vs lowest"
Adenocarcinoma, Cigarettes only
Most adjusted

REF	NRR	3J5	SEX	AGEL	AGEH	RACE	YF	LC TYPE	LOC	START	ST	NLC	R	VB	P	H	AD	ADOS	PRODUCT	exL	exH	unexL	unexH	De
BENHAM	554	x	m	0	0	all	-	KII	Eu:wst	1976	CC	1625	n	bl	n	y	0	0	cig only	1.0	3	11	999	st
PEZZOT	587		m	0	0	all	-	a	SCAmer	1987	CC	215	n	bl	n	y	0	0	cig only	1.0	10	11	999	st

Cigarette type is all/unspec for all RRs

Table 3J15 - 2

IESLC - Meta-analysis of Ex Smoking, Years quit (vs never), "Highest vs lowest"
Adenocarcinoma, Cigarettes only
Most adjusted

REF	NRR	SEX	AD	Number Case	Exposed Cont	Non-exposed Case	Cont	RR	95.00%CI
BENHAM	554	m	0	13	9	4	21	7.58	(1.93- 29.72)
PEZZOT	587	m	0	11	21	7	31	2.32	(0.77- 6.95)
Totals				24	30	11	52		

*prospective study

REF	NRR	SEX	AD	Ys	Ws	Qs	Ps
BENHAM	554	m	0	2.03	2.06	1.07	0.0036
PEZZOT	587	m	0	0.84	3.19	0.69	0.1330

	N	2
	NS	2
	Wt	5.25
Het	Chi	1.76
Het	df	1
Het	P	N.S.
Fixed	RR	3.69
	RRl	1.57
	RRu	8.69
	P	++
Random	RR	3.90
	RRl	1.23
	RRu	12.34
	P	+
Asymm	P	

Table 3J15 - 3

IESLC - Meta-analysis of Ex Smoking, Years quit (vs never), "Highest vs lowest"
Adenocarcinoma, Cigarettes only
Most adjusted

	Sex combined	male	female	Total
N		2		2
NS		2		2
Wt		5.25		5.25
Het Chi		1.76		1.76
Het df		1		1
Het P		N.S.		N.S.
Fixed RR		3.69		3.69
RRl		1.57		1.57
RRu		8.69		8.69
P		++		++
Random RR		3.90		3.90
RRl		1.23		1.23
RRu		12.34		12.34
P		+		+
Between Chi				
Between df				
Between P				N.S.
Btwn(F) P				N.S.
Btwn(R) P				N.S.

Too few RRs for analysis by factor

Table 3J15 - 4

IESLC - Meta-analysis of Ex Smoking, Years quit (vs never), "Highest vs lowest"
Adenocarcinoma, Cigarettes only
Least adjusted

REF	NRR	X	SEX	AGEL	AGEH	RACE	YF	LC TYPE	LOC	START	ST	NLC	R	VB	P	H	AD	ADOS	PRODUCT	exL	exH	unexL	unexH	De
BENHAM	554		m	0	0	all	-	KII	Eu:wst	1976	CC	1625	n	bl	n	y	0	0	cig only	1.0	3	11	999	st
PEZZOT	587		m	0	0	all	-	a	SCAmer	1987	CC	215	n	bl	n	y	0	0	cig only	1.0	10	11	999	st

Cigarette type is all/unspec for all RRs

Table 3J15 - 5

IESLC - Meta-analysis of Ex Smoking, Years quit (vs never), "Highest vs lowest"
Adenocarcinoma, Cigarettes only
Least adjusted

REF	NRR	SEX	AD	Number Case	Exposed Cont	Non-exposed Case	Cont	RR	95.00%CI
BENHAM	554	m	0	13	9	4	21	7.58	(1.93- 29.72)
PEZZOT	587	m	0	11	21	7	31	2.32	(0.77- 6.95)
Totals				24	30	11	52		

*prospective study

REF	NRR	SEX	AD	Ys	Ws	Qs	Ps
BENHAM	554	m	0	2.03	2.06	1.07	0.0036
PEZZOT	587	m	0	0.84	3.19	0.69	0.1330

	N	2
	NS	2
	Wt	5.25
Het	Chi	1.76
Het	df	1
Het	P	N.S.
Fixed	RR	3.69
	RRl	1.57
	RRu	8.69
	P	++
Random	RR	3.90
	RRl	1.23
	RRu	12.34
	P	+
Asymm	P	

Table 3J15 - 6

IESLC - Meta-analysis of Ex Smoking, Years quit (vs never), "Highest vs lowest"
Adenocarcinoma, Cigarettes only
Least adjusted

		Sex		
	combined	male	female	Total
N		2		2
NS		2		2
Wt		5.25		5.25
Het Chi		1.76		1.76
Het df		1		1
Het P		N.S.		N.S.
Fixed RR		3.69		3.69
RRl		1.57		1.57
RRu		8.69		8.69
P		++		++
Random RR		3.90		3.90
RRl		1.23		1.23
RRu		12.34		12.34
P		+		+
Between Chi				
Between df				
Between P				N.S.
Btwn(F) P				N.S.
Btwn(R) P				N.S.

Table 3J15 - 7

IESLC - Meta-analysis of Ex Smoking, Years quit (vs never), "Highest vs lowest"
Adenocarcinoma, Cigarettes only
Excluded studies (and stage at which they were excluded)

Stage	Studies
1	AGUDO AKIBA AMANDU AMES AXELSS BEST BOUCHA BOUCOT BRESLO CHEN CHEN2 CHIAZZ DEAN2 DOSEME ENGELA FAN GENG GER GUO HAENSZ HEGMAN HOLE HU HU2 JUSSAW KATSOU KAUFMA KOO KOULUM KREUZE LETOUR LEVIN LIAW LIU3 LIU4 LIU5 MCCONN MIGRAN MRFITR NOTAN2 OSANN2 PERNU QIAO2 RACHTA RESTRE SADOWS SEGI2 STASZE TIZZAN VUTUC WATSON WU WUWILL WYNDE2 WYNDE8 XU YUAN ZHANG ZHENG ZHOU
2	BUFFLE HUMBLE PISANI PRESCO WYNDE7
3	MCDUFF SPITZ
4	AUVINE BLOT1 BROWN3 GURSEL LAUSSM LUO WU2
5	ARMADA BECHER BENSHL BOFFET BROSS CARPEN CEDERL CHOI CHYOU CORREA CPSI CPSII DAMBER DARBY DEAN3 DESTEF DOLL DOLL2 DORGAN DORN GAO GAO2 GARCIA GARSHI GILLIS GRAHAM HAMMO2 HAMMON HIRAYA JOLY KAISE2 KHUDER LUBIN PEZZO2 QIAO SPEIZE SUZUK2 TVERDA WANG2 WIGLE
6	ALDERS
8	BARBON JAHN JAIN JEDRYC LUBIN2 MATOS SOBUE SVENSS WAKAI WYNDE3 WYNDE6

Table 3J15 - 8

Potentially overlapping studies

REF	REFGP	PRINC	.	OVERLAP/LINK
BENHAM	LUBIN2	2		Subset of Lubin2

Table 3J15 - 9

Most adjusted - insufficient data for meta-analysis

REF	NRR	SEX	AGEL	AGEH	RACE	YF	LC TYPE	LOC	START	ST	NLC	R	VB	P	H	AD	ADOS	PRODUCT	exL	exH	unexL	unexH	De
ALDERS	572	m	0	0	all	-	not q+s	Eu:UK	1977	CC	1448	n	V	n	n	1	0	cig only	0.1	2	10	999	st
ALDERS	583	f	0	0	all	-	not q+s	Eu:UK	1977	CC	1448	n	V	n	n	1	0	cig only	0.1	2	10	999	st
BENHAM	555	m	0	0	all	-	KII	Eu:wst	1976	CC	1625	n	bl	n	y	0	0	cig only	0.1	0.9	11	999	ot
PEZZOT	602	m	0	0	all	-	a	SCAmer	1987	CC	215	n	bl	n	y	0	0	cig only	0.1	0.9	11	999	ot

REF	NRR	RR	SIG	RRDATA comment
ALDERS	572	2.27		0
ALDERS	583	3.83		0
BENHAM	555	*	gap	0
PEZZOT	602	*	gap	0
