# Supplementary material for: Systematic review with meta-analysis of the epidemiological evidence in the 1900s relating smoking to lung cancer
Source: BMC Cancer. 2012 Sep 3;12:385. doi: 10.1186/1471-2407-12-385 (PMC3505152; doi:10.1186/1471-2407-12-385)
Supplement: Additional file 5 — Detailed Analysis Tables (Individual file names as described in Additional file 1: Methods, Table1). [file 1471-2407-12-385-S5.zip › PDF/2E.pdf]

Table 2E1 -

IESLC - Meta-analysis of Ever Smoking, Pipe and/or Cigars (not cigs)
Squamous

This analysis is restricted to results for:
1) Non-dose-response data
2) Smokers of pipe and/or cigars (but not cigarettes)
3) Ever smokers
4) Results complete enough for use in metaanalysis

Within each study, results are then selected (in the following order of preference, within each sex) for:
5) DENOM: never smoked anything, (never +1 = +long term ex)
6) Followup period (prospective studies): whole study (coded as 0) or longest available
7) LCtype: all or nearest available, at least Squamous and Adeno. (q = squamous, s = small,
l = large, a = adeno, mix = mixed, alv = alveolar)
8) Race: all or nearest available, otherwise by race (wh or w = white, bl or b = black, hi = hispanic
ch = chinese, jap = japanese, haw = hawaiian, w+o = white + oriental, sca = scandinavian, as = asian)
9) For overlapping studies: principal rather than subsidiary studies
Finally by Age: whole study (coded as 0) if available, otherwise by widest available age group
and then for single sex results (m, f) in preference to combined sex results (c).

Results adjusted (AD) for the most potential confounders are then chosen in Sections -1 to -3
and results adjusted for the least confounders in Sections -4 to -6. (Those least adjusted results which
actually differ from the most adjusted as marked 'x' in column X in Section -4)
(Results adjusted for an unknown number of confounder(s) are coded as 20.)

Section -7 shows excluded studies, together with the stage (as above) at which no qualifying
results were found.

Section -8 lists the potentially overlapping studies which have been included (1=principal, 2=subsidiary).

Section -9 lists any results which would have been included in preference except that they had data not complete
enough for use in meta-analysis, with their significance (yes/no), if known, and any further comment as entered
on the database.

In addition to those mentioned above, the following fields, levels and abbreviations are used:
* or nk = not known, n = no, y = yes, ot = other
nev = never
REF: 6-character study reference
NRR: number of the RR on the database within the study
ST : study type (CC = case control, pr or prosp = prospective)
NLC: number of lung cancer cases in whole study
R : risky occupational population (n = no, m = mining, o = other risky)
VB : national cigarette type (V = at least 75% Virginia, bl = at least 75% blended, ot = other)
P : any proxy use
H : full histological confirmation
De : derivation of RR/CI (or = original, st = standard method, ot = other method of estimation)

Table 2E1 - 1

IESLC - Meta-analysis of Ever Smoking, Pipe and/or Cigars (not cigs)
Squamous
Most adjusted

REF	NRR	SEX	AGEL	AGEH	RACE	YF	LC TYPE	LOC	START	ST	NLC	R	VB	P	H	AD	DENOM	De
ALDERS	28	m	0	0	all	-	q+s	Eu:UK	1977	CC	1448	n	V	n	n	1	nev any	ot
BRESLO	32	c	0	0	all	-	not a	NAmer	1949	CC	518	n	bl	n	y	0	nev+1	st
HAMMON	61	m	0	0	wh	0	not a	NAmer	1952	pr	448	n	bl	n	n	1	nev any	ot
JAHN	17	m	0	0	all	-	q	Eu:Ger	1988	CC	1004	n	bl	n	n	0	nev any	st
STASZE	14	m	0	0	all	-	q	Eu:est	1954	CC	281	n	bl	n	y	0	nev any	ot
WYNDE2	1	m	0	0	all	-	KI	NAmer	1962	CC	404	n	bl	n	y	0	nev any	st
WYNDE3	150	m	0	0	all	-	KI	NAmer	1966	CC	350	n	bl	n	y	0	nev any	st
WYNDE6	57	m	0	0	all	-	KI	NAmer	1969	CC	4423	n	bl	n	y	0	nev any	st

Table 2E1 - 2

IESLC - Meta-analysis of Ever Smoking, Pipe and/or Cigars (not cigs)
Squamous
Most adjusted

REF	NRR	SEX	AD	Number Exposed Case	Cont	Non-exposed Case	Cont	RR	95.00%CI
ALDERS	28	m	1	-	-	-	-	5.76	(1.60- 20.79)
BRESLO	32	c	0	13	68	15	56	0.71	(0.31- 1.62)
*HAMMON	61	m	1	-	-	-	-	4.46	(1.53- 13.00)
JAHN	17	m	0	8	30	3	138	12.27	(3.07- 48.97)
STASZE	14	m	0	2	101	0	158	7.81~	(0.37- 164.30)
WYNDE2	1	m	0	11	104	3	105	3.70	(1.00- 13.65)
WYNDE3	150	m	0	10	68	3	88	4.31	(1.14- 16.29)
WYNDE6	57	m	0	38	199	29	617	4.06	(2.44- 6.76)
Partial Totals				82	570	53	1162		

*prospective study ~ With 0.5 adjustment for zero

REF	NRR	SEX	AD	Ys	Ws	Qs	Ps
ALDERS	28	m	1	1.75	2.34	0.68	0.0074
BRESLO	32	c	0	-0.34	5.68	13.59	0.4217
*HAMMON	61	m	1	1.50	3.36	0.27	0.0062
JAHN	17	m	0	2.51	2.00	3.37	0.0004
STASZE	14	m	0	2.06	0.41	0.30	0.1861
WYNDE2	1	m	0	1.31	2.26	0.02	0.0493
WYNDE3	150	m	0	1.46	2.18	0.14	0.0310
WYNDE6	57	m	0	1.40	14.83	0.55	0.0000

	N	8
	NS	8
	Wt	33.05
Het	Chi	18.92
Het	df	7
Het	P	**
Fixed	RR	3.35
	RRl	2.38
	RRu	4.72
	P	+++
Random	RR	3.72
	RRl	1.95
	RRu	7.10
	P	+++
Asymm	P	N.S.

Table 2E1 - 3

IESLC - Meta-analysis of Ever Smoking, Pipe and/or Cigars (not cigs)
Squamous
Most adjusted

	Sex			
	combined	male	female	Total
N	1	7		8
NS	1	7		8
Wt	5.68	27.37		33.05
Het Chi	0.00	2.51		18.92
Het df	0	6		7
Het P	N.S.	N.S.		**
Fixed RR	0.71	4.62		3.35
RRl	0.31	3.18		2.38
RRu	1.62	6.72		4.72
P	N.S.	+++		+++
Random RR	0.71	4.62		3.72
RRl	0.31	3.18		1.95
RRu	1.62	6.72		7.10
P	N.S.	+++		+++
Between Chi				16.41
Between df				1
Between P				***
Btwn(F) P				***
Btwn(R) P				***

Too few RRs for analysis by factor

Table 2E1 - 4

IESLC - Meta-analysis of Ever Smoking, Pipe and/or Cigars (not cigs)
Squamous
Least adjusted

REF	NRR	X	SEX	AGEL	AGEH	RACE	YF	LC TYPE	LOC	START	ST	NLC	R	VB	P	H	AD	DENOM	De
ALDERS	79	x	m	0	0	all	-	q+s	Eu:UK	1977	CC	1448	n	V	n	n	0	nev any	st
BRESLO	32		c	0	0	all	-	not a	NAmer	1949	CC	518	n	bl	n	y	0	nev+1	st
HAMMON	74	x	m	0	0	wh	0	not a	NAmer	1952	pr	448	n	bl	n	n	0	nev any	st
JAHN	17		m	0	0	all	-	q	Eu:Ger	1988	CC	1004	n	bl	n	n	0	nev any	st
STASZE	14		m	0	0	all	-	q	Eu:est	1954	CC	281	n	bl	n	y	0	nev any	ot
WYNDE2	1		m	0	0	all	-	KI	NAmer	1962	CC	404	n	bl	n	y	0	nev any	st
WYNDE3	150		m	0	0	all	-	KI	NAmer	1966	CC	350	n	bl	n	y	0	nev any	st
WYNDE6	57		m	0	0	all	-	KI	NAmer	1969	CC	4423	n	bl	n	y	0	nev any	st

Table 2E1 - 5

IESLC - Meta-analysis of Ever Smoking, Pipe and/or Cigars (not cigs)
Squamous
Least adjusted

REF	NRR	SEX	AD	Number Exposed Case	Cont	Non-exposed Case	Cont	RR	95.00%CI
ALDERS	79	m	0	7	35	4	133	6.65	(1.84- 24.01)
BRESLO	32	c	0	13	68	15	56	0.71	(0.31- 1.62)
*HAMMON	74	m	0	21	127770	4	115884	4.76	(1.63- 13.87)
JAHN	17	m	0	8	30	3	138	12.27	(3.07- 48.97)
STASZE	14	m	0	2	101	0	158	7.81~	(0.37- 164.30)
WYNDE2	1	m	0	11	104	3	105	3.70	(1.00- 13.65)
WYNDE3	150	m	0	10	68	3	88	4.31	(1.14- 16.29)
WYNDE6	57	m	0	38	199	29	617	4.06	(2.44- 6.76)
Totals				110	128375	61	117179		

*prospective study ~ With 0.5 adjustment for zero

REF	NRR	SEX	AD	Ys	Ws	Qs	Ps
ALDERS	79	m	0	1.89	2.33	1.04	0.0038
BRESLO	32	c	0	-0.34	5.68	13.89	0.4217
*HAMMON	74	m	0	1.56	3.36	0.37	0.0042
JAHN	17	m	0	2.51	2.00	3.28	0.0004
STASZE	14	m	0	2.06	0.41	0.28	0.1861
WYNDE2	1	m	0	1.31	2.26	0.02	0.0493
WYNDE3	150	m	0	1.46	2.18	0.12	0.0310
WYNDE6	57	m	0	1.40	14.83	0.45	0.0000

	N	8
	NS	8
	Wt	33.05
Het	Chi	19.46
Het	df	7
Het	P	**
Fixed	RR	3.41
	RRl	2.43
	RRu	4.80
	P	+++
Random	RR	3.83
	RRl	1.99
	RRu	7.38
	P	+++
Asymm	P	N.S.

Table 2E1 - 6

IESLC - Meta-analysis of Ever Smoking, Pipe and/or Cigars (not cigs)
Squamous
Least adjusted

	Sex: combined	male	female	Total
N	1	7		8
NS	1	7		8
Wt	5.68	27.37		33.05
Het Chi	0.00	2.69		19.46
Het df	0	6		7
Het P	N.S.	N.S.		**
Fixed RR	0.71	4.72		3.41
RRl	0.31	3.24		2.43
RRu	1.62	6.86		4.80
P	N.S.	+++		+++
Random RR	0.71	4.72		3.83
RRl	0.31	3.24		1.99
RRu	1.62	6.86		7.38
P	N.S.	+++		+++
Between Chi				16.77
Between df				1
Between P				***
Btwn(F) P				***
Btwn(R) P				***

Table 2E1 - 7

IESLC - Meta-analysis of Ever Smoking, Pipe and/or Cigars (not cigs)
Squamous
Excluded studies (and stage at which they were excluded)

1	BOUCHA BUELL LAURIL MZILEN
2	ABRAHA AGUDO AKIBA AMANDU AMES ANDERS ARCHER AUSTIN AUVINE AXELSO AXELSS BAND BARBON BERRIN BLOHMK BLOT1 BLOT2 BLOT3 BLOT4 BRETT BROCKM BROWN1 BROWN2 BUFFLE BYERS1 BYERS2 CARPEN CASCO2 CASCOR CEDERL CHAN CHANG CHATZI CHEN CHEN2 CHEN3 CHIAZZ CHOI CHYOU COOKSO CORREA CPSII DAVEYS DEAN3 DESTE2 DESTEF DOCKER DOLL DORANT DOSEME DROSTE DU DUNN EBELIN ENSTRO ESAKI FAN GAO GAO2 GARCIA GARDIN GARSHI GENG GER GILLIS GODLEY GOODMA GREGOR GSELL HAENSZ HANSEN HEGMAN HEIN HENNEK HINDS HIRAOK HIRAY2 HIRAYA HITOSU HOROWI HORWIT HU HU2 HUANG HUMBLE ISHIMA JAIN JARUP JARVHO JEDRYC JIANG JOLY JONES JUSSAW KAISE2 KAISER KANELL KATSOU KAUFMA KELLER KHUDER KIHARA KO KOHLME KOO KOULUM KREYBE KUBIK LAMTH LAMWK LAMWK2 LANGE LAUSSM LEI LEMARC LETOUR LIAW LICKIN LIDDEL LIU LIU2 LIU3 LIU4 LIU5 LOMBA2 LUBIN LUO MACLEN MAGNUS MARTIS MASTRA MATSUD MCCONN MCDUFF MCLAUG MILLER MILLS MOLLO MRFIT MRFITR MURATA NAM NOTAN2 NOTANI NOU ODRISC ORMOS OSANN OSANN2 PARKIN PASTOR PAWLEG PERNU PERSH2 PERSHA PETO PEZZO2 PEZZOT PIKE PISANI POFFIJ POLEDN PRESCO QIAO QIAO2 RACHTA RADZIK RANDIG REN RESTRE RIMING RONCO ROOTS ROTHSC SAARIK SANKAR SCHWA2 SCHWAR SEGI SEGI2 SEOW SHAW SHIMIZ SIEMIA SIMARA SIMONA SITAS SOBUE SOBUE2 SPEIZE SPITZ STAYNE STOCKS STUCKE SUN SUZUK2 SUZUKI SVENSS TANG TANG2 TAO TENKAN TIZZAN TOKARS TSUGAN TVERDA ULMER VANDER VEIERO VUTUC WAKAI WANG WANG2 WANG3 WANG4 WARSIN WATSON WICKLU WILKIN WU WU2 WUNSCH WUWILL WYNDE4 WYNDE5 WYNDE8 WYNDER XIANGZ XU XU2 XU3 XU4 YAMAGU YONG YUAN ZHANG ZHENG ZHOU
3	BENSHL BROSS DARBY DEKLER ENGELA HOLE KINLEN KNEKT KREUZE MATOS MIGRAN TULINI WALD WYNDE7
7	ABELIN ARMADA BECHER BENHAM BEST BOFFET BOUCOT CHOW COMSTO CPSI DAMBER DEAN DEAN2 DOLL2 DORGAN DORN GOLLED GRAHAM HAMMO2 KJUUS LEVIN LOMBAR LUBIN2 MARSH MARSH2 SADOWS STOCKW TOUSEY WIGLE

Table 2E1 - 8
Potentially overlapping studies

REF	REFGP	PRINC	. OVERLAP/LINK
WYNDE6	WYNDE6	1	WYNDE5/6/7/8
JAHN	BOFFET	2	Subset of BOFFET

Table 2E2 -

IESLC - Meta-analysis of Current Smoking, Pipe and/or Cigars (not cigs)
Squamous

This analysis is restricted to results for:
1) Non-dose-response data
2) Smokers of pipe and/or cigars (but not cigarettes)
3) Current smokers
4) Results complete enough for use in metaanalysis

Within each study, results are then selected (in the following order of preference, within each sex) for:
5) DENOM: never smoked anything, (never +1 = +long term ex)
6) Followup period (prospective studies): whole study (coded as 0) or longest available
7) LCtype: all or nearest available, at least Squamous and Adeno. (q = squamous, s = small,
l = large, a = adeno, mix = mixed, alv = alveolar)
8) Race: all or nearest available, otherwise by race (wh or w = white, bl or b = black, hi = hispanic
ch = chinese, jap = japanese, haw = hawaiian, w+o = white + oriental, sca = scandinavian, as = asian)
9) For overlapping studies: principal rather than subsidiary studies
Finally by Age: whole study (coded as 0) if available, otherwise by widest available age group
and then for single sex results (m, f) in preference to combined sex results (c).

Results adjusted (AD) for the most potential confounders are then chosen in Sections -1 to -3
and results adjusted for the least confounders in Sections -4 to -6. (Those least adjusted results which
actually differ from the most adjusted as marked 'x' in column X in Section -4)
(Results adjusted for an unknown number of confounder(s) are coded as 20.)

Section -7 shows excluded studies, together with the stage (as above) at which no qualifying
results were found.

Section -8 lists the potentially overlapping studies which have been included (1=principal, 2=subsidiary).

Section -9 lists any results which would have been included in preference except that they had data not complete
enough for use in meta-analysis, with their significance (yes/no), if known, and any further comment as entered
on the database.

In addition to those mentioned above, the following fields, levels and abbreviations are used:
* or nk = not known, n = no, y = yes, ot = other
nev = never
REF: 6-character study reference
NRR: number of the RR on the database within the study
ST : study type (CC = case control, pr or prosp = prospective)
NLC: number of lung cancer cases in whole study
R : risky occupational population (n = no, m = mining, o = other risky)
VB : national cigarette type (V = at least 75% Virginia, bl = at least 75% blended, ot = other)
P : any proxy use
H : full histological confirmation
De : derivation of RR/CI (or = original, st = standard method, ot = other method of estimation)

Table 2E2 - 1

IESLC - Meta-analysis of Current Smoking, Pipe and/or Cigars (not cigs)
Squamous
Most adjusted

REF	NRR	SEX	AGEL	AGEH	RACE	YF	LC TYPE	LOC	START	ST	NLC	R	VB	P	H	AD	DENOM	De
WYNDE7	67	m	0	0	all	-	KI	NAmer	1977	CC	2085	n	bl	n	y	0	nev any	st

Table 2E2 - 2

IESLC - Meta-analysis of Current Smoking, Pipe and/or Cigars (not cigs)
Squamous
Most adjusted

REF	NRR	SEX	AD	Number Case	Exposed Cont	Non-exposed Case	Cont	RR	95.00%CI
WYNDE7	67	m	0	24	171	22	918	5.86	(3.21- 10.68)
Totals				24	171	22	918		

*prospective study

REF	NRR	SEX	AD	Ys	Ws	Qs	Ps
WYNDE7	67	m	0	1.77	10.63	0.00	0.0000

N	1
NS	1
Wt	10.63
Het Chi	0.00
Het df	0
Het P	N.S.
Fixed RR	5.86
RRl	3.21
RRu	10.68
P	+++
Random RR	5.86
RRl	3.21
RRu	10.68
P	+++
Asymm P	

Table 2E2 - 3

IESLC - Meta-analysis of Current Smoking, Pipe and/or Cigars (not cigs)
Squamous
Most adjusted

	Sex			
	combined	male	female	Total
N		1		1
NS		1		1
Wt		10.63		10.63
Het Chi		0.00		0.00
Het df		0		0
Het P		N.S.		N.S.
Fixed RR		5.86		5.86
RRl		3.21		3.21
RRu		10.68		10.68
P		+++		+++
Random RR		5.86		5.86
RRl		3.21		3.21
RRu		10.68		10.68
P		+++		+++
Between Chi				
Between df				
Between P				N.S.
Btwn(F) P				N.S.
Btwn(R) P				N.S.

Too few RRs for analysis by factor

Table 2E2 - 4

IESLC - Meta-analysis of Current Smoking, Pipe and/or Cigars (not cigs)
Squamous
Least adjusted

REF	NRR	X	SEX	AGEL	AGEH	RACE	YF	LC TYPE	LOC	START	ST	NLC	R	VB	P	H	AD	DENOM	De
WYNDE7	67		m	0	0	all	-	KI	NAmer	1977	CC	2085	n	bl	n	y	0	nev any	st

Table 2E2 - 5

IESLC - Meta-analysis of Current Smoking, Pipe and/or Cigars (not cigs)
Squamous
Least adjusted

REF	NRR	SEX	AD	Number Case	Exposed Cont	Non-exposed Case	Cont	RR	95.00%CI
WYNDE7	67	m	0	24	171	22	918	5.86	(3.21- 10.68)
Totals				24	171	22	918		

*prospective study

REF	NRR	SEX	AD	Ys	Ws	Qs	Ps
WYNDE7	67	m	0	1.77	10.63	0.00	0.0000

	N	1
	NS	1
	Wt	10.63
Het	Chi	0.00
Het	df	0
Het	P	N.S.
Fixed	RR	5.86
	RRl	3.21
	RRu	10.68
	P	+++
Random	RR	5.86
	RRl	3.21
	RRu	10.68
	P	+++
Asymm	P	

Table 2E2 - 6

IESLC - Meta-analysis of Current Smoking, Pipe and/or Cigars (not cigs)
Squamous
Least adjusted

	Sex combined	male	female	Total
N		1		1
NS		1		1
Wt		10.63		10.63
Het Chi		0.00		0.00
Het df		0		0
Het P		N.S.		N.S.
Fixed RR		5.86		5.86
RRl		3.21		3.21
RRu		10.68		10.68
P		+++		+++
Random RR		5.86		5.86
RRl		3.21		3.21
RRu		10.68		10.68
P		+++		+++
Between Chi				
Between df				
Between P				N.S.
Btwn(F) P				N.S.
Btwn(R) P				N.S.

Table 2E2 - 7

IESLC - Meta-analysis of Current Smoking, Pipe and/or Cigars (not cigs)
Squamous
Excluded studies (and stage at which they were excluded)

1	BOUCHA BUELL LAURIL MZILEN
2	ABRAHA AGUDO AKIBA AMANDU AMES ANDERS ARCHER AUSTIN AUVINE AXELSO AXELSS BAND BARBON BERRIN BLOHMK BLOT1 BLOT2 BLOT3 BLOT4 BRETT BROCKM BROWN1 BROWN2 BUFFLE BYERS1 BYERS2 CARPEN CASCO2 CASCOR CEDERL CHAN CHANG CHATZI CHEN CHEN2 CHEN3 CHIAZZ CHOI CHYOU COOKSO CORREA CPSII DAVEYS DEAN3 DESTE2 DESTEF DOCKER DOLL DORANT DOSEME DROSTE DU DUNN EBELIN ENSTRO ESAKI FAN GAO GAO2 GARCIA GARDIN GARSHI GENG GER GILLIS GODLEY GOODMA GREGOR GSELL HAENSZ HANSEN HEGMAN HEIN HENNEK HINDS HIRAOK HIRAY2 HIRAYA HITOSU HOROWI HORWIT HU HU2 HUANG HUMBLE ISHIMA JAIN JARUP JARVHO JEDRYC JIANG JOLY JONES JUSSAW KAISE2 KAISER KANELL KATSOU KAUFMA KELLER KHUDER KIHARA KO KOHLME KOO KOULUM KREYBE KUBIK LAMTH LAMWK LAMWK2 LANGE LAUSSM LEI LEMARC LETOUR LIAW LICKIN LIDDEL LIU LIU2 LIU3 LIU4 LIU5 LOMBA2 LUBIN LUO MACLEN MAGNUS MARTIS MASTRA MATSUD MCCONN MCDUFF MCLAUG MILLER MILLS MOLLO MRFIT MRFITR MURATA NAM NOTAN2 NOTANI NOU ODRISC ORMOS OSANN OSANN2 PARKIN PASTOR PAWLEG PERNU PERSH2 PERSHA PETO PEZZO2 PEZZOT PIKE PISANI POFFIJ POLEDN PRESCO QIAO QIAO2 RACHTA RADZIK RANDIG REN RESTRE RIMING RONCO ROOTS ROTHSC SAARIK SANKAR SCHWA2 SCHWAR SEGI SEGI2 SEOW SHAW SHIMIZ SIEMIA SIMARA SIMONA SITAS SOBUE SOBUE2 SPEIZE SPITZ STAYNE STOCKS STUCKE SUN SUZUK2 SUZUKI SVENSS TANG TANG2 TAO TENKAN TIZZAN TOKARS TSUGAN TVERDA ULMER VANDER VEIERO VUTUC WAKAI WANG WANG2 WANG3 WANG4 WARSIN WATSON WICKLU WILKIN WU WU2 WUNSCH WUWILL WYNDE4 WYNDE5 WYNDE8 WYNDER XIANGZ XU XU2 XU3 XU4 YAMAGU YONG YUAN ZHANG ZHENG ZHOU
3	ABELIN ALDERS ARMADA BECHER BENHAM BEST BOFFET BOUCOT BRESLO COMSTO CPSI DAMBER DEAN DEAN2 DORGAN GOLLED HAMMO2 HAMMON JAHN KJUUS LEVIN LOMBAR MARSH MARSH2 SADOWS STASZE STOCKW TOUSEY WYNDE2 WYNDE3 WYNDE6
7	BENSHL BROSS CHOW DARBY DEKLER DOLL2 DORN ENGELA GRAHAM HOLE KINLEN KNEKT KREUZE LUBIN2 MATOS MIGRAN TULINI WALD WIGLE

Table 2E2 - 8
Potentially overlapping studies

REF	REFGP	PRINC	.	OVERLAP/LINK
WYNDE7	WYNDE6	2		WYNDE5/6/7/8

Table 2E3 -

IESLC - Meta-analysis of Ever Smoking (or Current if Ever not available), Pipe and/or Cigars (not cigs)
Squamous

This analysis is restricted to results for:
1) Non-dose-response data
2) Smokers of pipe and/or cigars (but not cigarettes)
3) Results complete enough for use in metaanalysis

Within each study, results are then selected (in the following order of preference, within each sex) for:
4) SMKSTA: ever smokers, current smokers
5) DENOM: never smoked anything, (never +1 = +long term ex)
6) Followup period (prospective studies): whole study (coded as 0) or longest available
7) LCtype: all or nearest available, at least Squamous and Adeno. (q = squamous, s = small,
l = large, a = adeno, mix = mixed, alv = alveolar)
8) Race: all or nearest available, otherwise by race (wh or w = white, bl or b = black, hi = hispanic
ch = chinese, jap = japanese, haw = hawaiian, w+o = white + oriental, sca = scandinavian, as = asian)
9) For overlapping studies: principal rather than subsidiary studies
Finally by Age: whole study (coded as 0) if available, otherwise by widest available age group
and then for single sex results (m, f) in preference to combined sex results (c).

Results adjusted (AD) for the most potential confounders are then chosen in Sections -1 to -3
and results adjusted for the least confounders in Sections -4 to -6. (Those least adjusted results which
actually differ from the most adjusted as marked 'x' in column X in Section -4)
(Results adjusted for an unknown number of confounder(s) are coded as 20.)

Section -7 shows excluded studies, together with the stage (as above) at which no qualifying
results were found.

Section -8 lists the potentially overlapping studies which have been included (1=principal, 2=subsidiary).

Section -9 lists any results which would have been included in preference except that they had data not complete
enough for use in meta-analysis, with their significance (yes/no), if known, and any further comment as entered
on the database.

In addition to those mentioned above, the following fields, levels and abbreviations are used:
* or nk = not known, n = no, y = yes, ot = other
ev = ever, cu = current, nev = never
REF: 6-character study reference
NRR: number of the RR on the database within the study
ST : study type (CC = case control, pr or prosp = prospective)
NLC: number of lung cancer cases in whole study
R : risky occupational population (n = no, m = mining, o = other risky)
VB : national cigarette type (V = at least 75% Virginia, bl = at least 75% blended, ot = other)
P : any proxy use
H : full histological confirmation
De : derivation of RR/CI (or = original, st = standard method, ot = other method of estimation)

Table 2E3 - 1

IESLC - Meta-analysis of Ever Smoking (or Current if Ever not available), Pipe and/or Cigars (not cigs)
Squamous
Most adjusted

REF	NRR	SEX	AGEL	AGEH	RACE	YF	LC TYPE	LOC	START	ST	NLC	R	VB	P	H	AD	SM	DENOM	De
ALDERS	28	m	0	0	all	-	q+s	Eu:UK	1977	CC	1448	n	V	n	n	1	ev	nev any	ot
BRESLO	32	c	0	0	all	-	not a	NAmer	1949	CC	518	n	bl	n	y	0	ev	nev+1	st
HAMMON	61	m	0	0	wh	0	not a	NAmer	1952	pr	448	n	bl	n	n	1	ev	nev any	ot
JAHN	17	m	0	0	all	-	q	Eu:Ger	1988	CC	1004	n	bl	n	n	0	ev	nev any	st
STASZE	14	m	0	0	all	-	q	Eu:est	1954	CC	281	n	bl	n	y	0	ev	nev any	ot
WYNDE2	1	m	0	0	all	-	KI	NAmer	1962	CC	404	n	bl	n	y	0	ev	nev any	st
WYNDE3	150	m	0	0	all	-	KI	NAmer	1966	CC	350	n	bl	n	y	0	ev	nev any	st
WYNDE6	57	m	0	0	all	-	KI	NAmer	1969	CC	4423	n	bl	n	y	0	ev	nev any	st

Table 2E3 - 2

IESLC - Meta-analysis of Ever Smoking (or Current if Ever not available), Pipe and/or Cigars (not cigs)
Squamous
Most adjusted

REF	NRR	SEX	AD	Number Exposed Case	Number Exposed Cont	Non-exposed Case	Non-exposed Cont	RR	95.00%CI
ALDERS	28	m	1	-	-	-	-	5.76	(1.60- 20.79)
BRESLO	32	c	0	13	68	15	56	0.71	(0.31- 1.62)
*HAMMON	61	m	1	-	-	-	-	4.46	(1.53- 13.00)
JAHN	17	m	0	8	30	3	138	12.27	(3.07- 48.97)
STASZE	14	m	0	2	101	0	158	7.81~	(0.37- 164.30)
WYNDE2	1	m	0	11	104	3	105	3.70	(1.00- 13.65)
WYNDE3	150	m	0	10	68	3	88	4.31	(1.14- 16.29)
WYNDE6	57	m	0	38	199	29	617	4.06	(2.44- 6.76)
Partial Totals				82	570	53	1162		

*prospective study ~ With 0.5 adjustment for zero

REF	NRR	SEX	AD	Ys	Ws	Qs	Ps
ALDERS	28	m	1	1.75	2.34	0.68	0.0074
BRESLO	32	c	0	-0.34	5.68	13.59	0.4217
*HAMMON	61	m	1	1.50	3.36	0.27	0.0062
JAHN	17	m	0	2.51	2.00	3.37	0.0004
STASZE	14	m	0	2.06	0.41	0.30	0.1861
WYNDE2	1	m	0	1.31	2.26	0.02	0.0493
WYNDE3	150	m	0	1.46	2.18	0.14	0.0310
WYNDE6	57	m	0	1.40	14.83	0.55	0.0000

	N	8
	NS	8
	Wt	33.05
Het	Chi	18.92
Het	df	7
Het	P	**
Fixed	RR	3.35
	RRl	2.38
	RRu	4.72
	P	+++
Random	RR	3.72
	RRl	1.95
	RRu	7.10
	P	+++
Asymm	P	N.S.

Table 2E3 - 3

IESLC - Meta-analysis of Ever Smoking (or Current if Ever not available), Pipe and/or Cigars (not cigs)
Squamous
Most adjusted

	combined	Sex: male	female	Total
N	1	7		8
NS	1	7		8
Wt	5.68	27.37		33.05
Het Chi	0.00	2.51		18.92
Het df	0	6		7
Het P	N.S.	N.S.		**
Fixed RR	0.71	4.62		3.35
RRl	0.31	3.18		2.38
RRu	1.62	6.72		4.72
P	N.S.	+++		+++
Random RR	0.71	4.62		3.72
RRl	0.31	3.18		1.95
RRu	1.62	6.72		7.10
P	N.S.	+++		+++
Between Chi				16.41
Between df				1
Between P				***
Btwn(F) P				***
Btwn(R) P				***

Too few RRs for analysis by factor

Table 2E3 - 4

IESLC - Meta-analysis of Ever Smoking (or Current if Ever not available), Pipe and/or Cigars (not cigs)
Squamous
Least adjusted

REF	NRR	X	SEX	AGEL	AGEH	RACE	YF	LC TYPE	LOC	START	ST	NLC	R	VB	P	H	AD	SM	DENOM	De
ALDERS	79	x	m	0	0	all	-	q+s	Eu:UK	1977	CC	1448	n	V	n	n	0	ev	nev any	st
BRESLO	32		c	0	0	all	-	not a	NAmer	1949	CC	518	n	bl	n	y	0	ev	nev+1	st
HAMMON	74	x	m	0	0	wh	0	not a	NAmer	1952	pr	448	n	bl	n	n	0	ev	nev any	st
JAHN	17		m	0	0	all	-	q	Eu:Ger	1988	CC	1004	n	bl	n	n	0	ev	nev any	st
STASZE	14		m	0	0	all	-	q	Eu:est	1954	CC	281	n	bl	n	y	0	ev	nev any	ot
WYNDE2	1		m	0	0	all	-	KI	NAmer	1962	CC	404	n	bl	n	y	0	ev	nev any	st
WYNDE3	150		m	0	0	all	-	KI	NAmer	1966	CC	350	n	bl	n	y	0	ev	nev any	st
WYNDE6	57		m	0	0	all	-	KI	NAmer	1969	CC	4423	n	bl	n	y	0	ev	nev any	st

Table 2E3 - 5

IESLC - Meta-analysis of Ever Smoking (or Current if Ever not available), Pipe and/or Cigars (not cigs)
Squamous
Least adjusted

				Number Exposed		Non-exposed			
REF	NRR	SEX	AD	Case	Cont	Case	Cont	RR	95.00%CI
ALDERS	79	m	0	7	35	4	133	6.65	(1.84- 24.01)
BRESLO	32	c	0	13	68	15	56	0.71	(0.31- 1.62)
*HAMMON	74	m	0	21	127770	4	115884	4.76	(1.63- 13.87)
JAHN	17	m	0	8	30	3	138	12.27	(3.07- 48.97)
STASZE	14	m	0	2	101	0	158	7.81~	(0.37- 164.30)
WYNDE2	1	m	0	11	104	3	105	3.70	(1.00- 13.65)
WYNDE3	150	m	0	10	68	3	88	4.31	(1.14- 16.29)
WYNDE6	57	m	0	38	199	29	617	4.06	(2.44- 6.76)
Totals				110	128375	61	117179		

*prospective study

~ With 0.5 adjustment for zero

REF	NRR	SEX	AD	Ys	Ws	Qs	Ps
ALDERS	79	m	0	1.89	2.33	1.04	0.0038
BRESLO	32	c	0	-0.34	5.68	13.89	0.4217
*HAMMON	74	m	0	1.56	3.36	0.37	0.0042
JAHN	17	m	0	2.51	2.00	3.28	0.0004
STASZE	14	m	0	2.06	0.41	0.28	0.1861
WYNDE2	1	m	0	1.31	2.26	0.02	0.0493
WYNDE3	150	m	0	1.46	2.18	0.12	0.0310
WYNDE6	57	m	0	1.40	14.83	0.45	0.0000

	N	8
	NS	8
	Wt	33.05
Het	Chi	19.46
Het	df	7
Het	P	**
Fixed	RR	3.41
	RRl	2.43
	RRu	4.80
	P	+++
Random	RR	3.83
	RRl	1.99
	RRu	7.38
	P	+++
Asymm	P	N.S.

Table 2E3 - 6

IESLC - Meta-analysis of Ever Smoking (or Current if Ever not available), Pipe and/or Cigars (not cigs)
Squamous
Least adjusted

	Sex			
	combined	male	female	Total
N	1	7		8
NS	1	7		8
Wt	5.68	27.37		33.05
Het Chi	0.00	2.69		19.46
Het df	0	6		7
Het P	N.S.	N.S.		**
Fixed RR	0.71	4.72		3.41
RRl	0.31	3.24		2.43
RRu	1.62	6.86		4.80
P	N.S.	+++		+++
Random RR	0.71	4.72		3.83
RRl	0.31	3.24		1.99
RRu	1.62	6.86		7.38
P	N.S.	+++		+++
Between Chi				16.77
Between df				1
Between P				***
Btwn(F) P				***
Btwn(R) P				***

Table 2E3 - 7

IESLC - Meta-analysis of Ever Smoking (or Current if Ever not available), Pipe and/or Cigars (not cigs)
Squamous
Excluded studies (and stage at which they were excluded)

```
1      BOUCHA  BUELL LAURIL MZILEN

2      ABRAHA  AGUDO  AKIBA AMANDU   AMES ANDERS ARCHER AUSTIN AUVINE AXELSO AXELSS   BAND BARBON BERRIN BLOHMK  BLOT1
        BLOT2  BLOT3  BLOT4  BRETT BROCKM BROWN1 BROWN2 BUFFLE BYERS1 BYERS2 CARPEN CASCO2 CASCOR CEDERL   CHAN  CHANG
       CHATZI   CHEN  CHEN2  CHEN3 CHIAZZ   CHOI  CHYOU COOKSO CORREA  CPSII DAVEYS  DEAN3 DESTE2 DESTEF DOCKER   DOLL
       DORANT DOSEME DROSTE     DU   DUNN EBELIN ENSTRO  ESAKI    FAN    GAO   GAO2 GARCIA GARDIN GARSHI   GENG    GER
       GILLIS GODLEY GOODMA GREGOR  GSELL HAENSZ HANSEN HEGMAN   HEIN HENNEK  HINDS HIRAOK HIRAY2 HIRAYA HITOSU HOROWI
       HORWIT     HU    HU2  HUANG HUMBLE ISHIMA   JAIN  JARUP JARVHO JEDRYC  JIANG   JOLY  JONES JUSSAW KAISE2 KAISER
       KANELL KATSOU KAUFMA KELLER KHUDER KIHARA     KO KOHLME    KOO KOULUM KREYBE  KUBIK  LAMTH  LAMWK LAMWK2  LANGE
       LAUSSM    LEI LEMARC LETOUR   LIAW LICKIN LIDDEL    LIU   LIU2   LIU3   LIU4   LIU5 LOMBA2  LUBIN    LUO MACLEN
       MAGNUS MARTIS MASTRA MATSUD MCCONN MCDUFF MCLAUG MILLER  MILLS  MOLLO  MRFIT MRFITR MURATA    NAM NOTAN2 NOTANI
          NOU ODRISC  ORMOS  OSANN OSANN2 PARKIN PASTOR PAWLEG  PERNU PERSH2 PERSHA   PETO PEZZO2 PEZZOT   PIKE PISANI
       POFFIJ POLEDN PRESCO   QIAO  QIAO2 RACHTA RADZIK RANDIG    REN RESTRE RIMING  RONCO  ROOTS ROTHSC SAARIK SANKAR
       SCHWA2 SCHWAR   SEGI  SEGI2   SEOW   SHAW SHIMIZ SIEMIA SIMARA SIMONA  SITAS  SOBUE SOBUE2 SPEIZE  SPITZ STAYNE
       STOCKS STUCKE    SUN SUZUK2 SUZUKI SVENSS   TANG  TANG2    TAO TENKAN TIZZAN TOKARS TSUGAN TVERDA  ULMER VANDER
       VEIERO  VUTUC  WAKAI   WANG  WANG2  WANG3  WANG4 WARSIN WATSON WICKLU WILKIN     WU    WU2 WUNSCH WUWILL WYNDE4
       WYNDE5 WYNDE8 WYNDER XIANGZ     XU    XU2    XU3    XU4 YAMAGU   YONG   YUAN  ZHANG  ZHENG   ZHOU

7      ABELIN ARMADA BECHER BENHAM BENSHL   BEST BOFFET BOUCOT  BROSS   CHOW COMSTO   CPSI DAMBER  DARBY   DEAN  DEAN2
       DEKLER  DOLL2 DORGAN   DORN ENGELA GOLLED GRAHAM HAMMO2   HOLE KINLEN  KJUUS  KNEKT KREUZE  LEVIN LOMBAR LUBIN2
        MARSH MARSH2  MATOS MIGRAN SADOWS STOCKW TOUSEY TULINI   WALD  WIGLE

9      WYNDE7
```

Table 2E3 - 8

Potentially overlapping studies

REF	REFGP	PRINC	.	OVERLAP/LINK
WYNDE6	WYNDE6	1		WYNDE5/6/7/8
JAHN	BOFFET	2		Subset of BOFFET

Table 2E4 -

IESLC - Meta-analysis of Current Smoking (or Ever if Current not available), Pipe and/or Cigars (not cigs)
Squamous

This analysis is restricted to results for:
1) Non-dose-response data
2) Smokers of pipe and/or cigars (but not cigarettes)
3) Results complete enough for use in metaanalysis

Within each study, results are then selected (in the following order of preference, within each sex) for:
4) SMKSTA: current smokers, ever smokers
5) DENOM: never smoked anything, (never +1 = +long term ex)
6) Followup period (prospective studies): whole study (coded as 0) or longest available
7) LCtype: all or nearest available, at least Squamous and Adeno. (q = squamous, s = small,
l = large, a = adeno, mix = mixed, alv = alveolar)
8) Race: all or nearest available, otherwise by race (wh or w = white, bl or b = black, hi = hispanic
ch = chinese, jap = japanese, haw = hawaiian, w+o = white + oriental, sca = scandinavian, as = asian)
9) For overlapping studies: principal rather than subsidiary studies
Finally by Age: whole study (coded as 0) if available, otherwise by widest available age group
and then for single sex results (m, f) in preference to combined sex results (c).

Results adjusted (AD) for the most potential confounders are then chosen in Sections -1 to -3
(and those which actually differ from the adjusted results in Table 2E3 - 1 are marked 'x' in Section -1)
and results adjusted for the least confounders in Sections -4 to -6. (Those least adjusted results which
actually differ from the most adjusted as marked 'x' in column X in Section -4)
(Results adjusted for an unknown number of confounder(s) are coded as 20.)

Section -7 shows excluded studies, together with the stage (as above) at which no qualifying
results were found.

Section -8 lists the potentially overlapping studies which have been included (1=principal, 2=subsidiary).

Section -9 lists any results which would have been included in preference except that they had data not complete
enough for use in meta-analysis, with their significance (yes/no), if known, and any further comment as entered
on the database.

In addition to those mentioned above, the following fields, levels and abbreviations are used:
* or nk = not known, n = no, y = yes, ot = other
ev = ever, cu = current, nev = never
REF: 6-character study reference
NRR: number of the RR on the database within the study
ST : study type (CC = case control, pr or prosp = prospective)
NLC: number of lung cancer cases in whole study
R : risky occupational population (n = no, m = mining, o = other risky)
VB : national cigarette type (V = at least 75% Virginia, bl = at least 75% blended, ot = other)
P : any proxy use
H : full histological confirmation
De : derivation of RR/CI (or = original, st = standard method, ot = other method of estimation)

Table 2E4 - 1

IESLC - Meta-analysis of Current Smoking (or Ever if Current not available), Pipe and/or Cigars (not cigs)
Squamous
Most adjusted

REF	NRR	2E3	SEX	AGEL	AGEH	RACE	YF	LC TYPE	LOC	START	ST	NLC	R	VB	P	H	AD	SM	DENOM	De
ALDERS	28		m	0	0	all	-	q+s	Eu:UK	1977	CC	1448	n	V	n	n	1	ev	nev any	ot
BRESLO	32		c	0	0	all	-	not a	NAmer	1949	CC	518	n	bl	n	y	0	ev	nev+1	st
HAMMON	61		m	0	0	wh	0	not a	NAmer	1952	pr	448	n	bl	n	n	1	ev	nev any	ot
JAHN	17		m	0	0	all	-	q	Eu:Ger	1988	CC	1004	n	bl	n	n	0	ev	nev any	st
STASZE	14		m	0	0	all	-	q	Eu:est	1954	CC	281	n	bl	n	y	0	ev	nev any	ot
WYNDE2	1		m	0	0	all	-	KI	NAmer	1962	CC	404	n	bl	n	y	0	ev	nev any	st
WYNDE3	150		m	0	0	all	-	KI	NAmer	1966	CC	350	n	bl	n	y	0	ev	nev any	st
WYNDE6	57		m	0	0	all	-	KI	NAmer	1969	CC	4423	n	bl	n	y	0	ev	nev any	st

Table 2E4 - 2

IESLC - Meta-analysis of Current Smoking (or Ever if Current not available), Pipe and/or Cigars (not cigs)
Squamous
Most adjusted

REF	NRR	SEX	AD	Number Exposed Case	Number Exposed Cont	Non-exposed Case	Non-exposed Cont	RR	95.00%CI
ALDERS	28	m	1	-	-	-	-	5.76	(1.60- 20.79)
BRESLO	32	c	0	13	68	15	56	0.71	(0.31- 1.62)
*HAMMON	61	m	1	-	-	-	-	4.46	(1.53- 13.00)
JAHN	17	m	0	8	30	3	138	12.27	(3.07- 48.97)
STASZE	14	m	0	2	101	0	158	7.81~	(0.37- 164.30)
WYNDE2	1	m	0	11	104	3	105	3.70	(1.00- 13.65)
WYNDE3	150	m	0	10	68	3	88	4.31	(1.14- 16.29)
WYNDE6	57	m	0	38	199	29	617	4.06	(2.44- 6.76)
Partial Totals				82	570	53	1162		

*prospective study

~ With 0.5 adjustment for zero

REF	NRR	SEX	AD	Ys	Ws	Qs	Ps
ALDERS	28	m	1	1.75	2.34	0.68	0.0074
BRESLO	32	c	0	-0.34	5.68	13.59	0.4217
*HAMMON	61	m	1	1.50	3.36	0.27	0.0062
JAHN	17	m	0	2.51	2.00	3.37	0.0004
STASZE	14	m	0	2.06	0.41	0.30	0.1861
WYNDE2	1	m	0	1.31	2.26	0.02	0.0493
WYNDE3	150	m	0	1.46	2.18	0.14	0.0310
WYNDE6	57	m	0	1.40	14.83	0.55	0.0000

	N	8
	NS	8
	Wt	33.05
Het	Chi	18.92
Het	df	7
Het	P	**
Fixed	RR	3.35
	RRl	2.38
	RRu	4.72
	P	+++
Random	RR	3.72
	RRl	1.95
	RRu	7.10
	P	+++
Asymm	P	N.S.

Table 2E4 - 3

IESLC - Meta-analysis of Current Smoking (or Ever if Current not available), Pipe and/or Cigars (not cigs)
Squamous
Most adjusted

	combined	Sex<br>male	female	Total
N	1	7		8
NS	1	7		8
Wt	5.68	27.37		33.05
Het Chi	0.00	2.51		18.92
Het df	0	6		7
Het P	N.S.	N.S.		**
Fixed RR	0.71	4.62		3.35
RRl	0.31	3.18		2.38
RRu	1.62	6.72		4.72
P	N.S.	+++		+++
Random RR	0.71	4.62		3.72
RRl	0.31	3.18		1.95
RRu	1.62	6.72		7.10
P	N.S.	+++		+++
Between Chi				16.41
Between df				1
Between P				***
Btwn(F) P				***
Btwn(R) P				***

Too few RRs for analysis by factor

Table 2E4 - 4

IESLC - Meta-analysis of Current Smoking (or Ever if Current not available), Pipe and/or Cigars (not cigs)
Squamous
Least adjusted

REF	NRR	X	SEX	AGEL	AGEH	RACE	YF	LC TYPE	LOC	START	ST	NLC	R	VB	P	H	AD	SM	DENOM	De
ALDERS	79	x	m	0	0	all	-	q+s	Eu:UK	1977	CC	1448	n	V	n	n	0	ev	nev any	st
BRESLO	32		c	0	0	all	-	not a	NAmer	1949	CC	518	n	bl	n	y	0	ev	nev+1	st
HAMMON	74	x	m	0	0	wh	0	not a	NAmer	1952	pr	448	n	bl	n	n	0	ev	nev any	st
JAHN	17		m	0	0	all	-	q	Eu:Ger	1988	CC	1004	n	bl	n	n	0	ev	nev any	st
STASZE	14		m	0	0	all	-	q	Eu:est	1954	CC	281	n	bl	n	y	0	ev	nev any	ot
WYNDE2	1		m	0	0	all	-	KI	NAmer	1962	CC	404	n	bl	n	y	0	ev	nev any	st
WYNDE3	150		m	0	0	all	-	KI	NAmer	1966	CC	350	n	bl	n	y	0	ev	nev any	st
WYNDE6	57		m	0	0	all	-	KI	NAmer	1969	CC	4423	n	bl	n	y	0	ev	nev any	st

Table 2E4 - 5

IESLC - Meta-analysis of Current Smoking (or Ever if Current not available), Pipe and/or Cigars (not cigs)
Squamous
Least adjusted

REF	NRR	SEX	AD	Number Exposed Case	Cont	Non-exposed Case	Cont	RR	95.00%CI
ALDERS	79	m	0	7	35	4	133	6.65	(1.84- 24.01)
BRESLO	32	c	0	13	68	15	56	0.71	(0.31- 1.62)
*HAMMON	74	m	0	21	127770	4	115884	4.76	(1.63- 13.87)
JAHN	17	m	0	8	30	3	138	12.27	(3.07- 48.97)
STASZE	14	m	0	2	101	0	158	7.81~	(0.37- 164.30)
WYNDE2	1	m	0	11	104	3	105	3.70	(1.00- 13.65)
WYNDE3	150	m	0	10	68	3	88	4.31	(1.14- 16.29)
WYNDE6	57	m	0	38	199	29	617	4.06	(2.44- 6.76)
Totals				110	128375	61	117179		

*prospective study ~ With 0.5 adjustment for zero

REF	NRR	SEX	AD	Ys	Ws	Qs	Ps
ALDERS	79	m	0	1.89	2.33	1.04	0.0038
BRESLO	32	c	0	-0.34	5.68	13.89	0.4217
*HAMMON	74	m	0	1.56	3.36	0.37	0.0042
JAHN	17	m	0	2.51	2.00	3.28	0.0004
STASZE	14	m	0	2.06	0.41	0.28	0.1861
WYNDE2	1	m	0	1.31	2.26	0.02	0.0493
WYNDE3	150	m	0	1.46	2.18	0.12	0.0310
WYNDE6	57	m	0	1.40	14.83	0.45	0.0000

	N	8
	NS	8
	Wt	33.05
Het	Chi	19.46
Het	df	7
Het	P	**
Fixed	RR	3.41
	RRl	2.43
	RRu	4.80
	P	+++
Random	RR	3.83
	RRl	1.99
	RRu	7.38
	P	+++
Asymm	P	N.S.

Table 2E4 - 6

IESLC - Meta-analysis of Current Smoking (or Ever if Current not available), Pipe and/or Cigars (not cigs)
Squamous
Least adjusted

		Sex		
	combined	male	female	Total
N	1	7		8
NS	1	7		8
Wt	5.68	27.37		33.05
Het Chi	0.00	2.69		19.46
Het df	0	6		7
Het P	N.S.	N.S.		**
Fixed RR	0.71	4.72		3.41
RRl	0.31	3.24		2.43
RRu	1.62	6.86		4.80
P	N.S.	+++		+++
Random RR	0.71	4.72		3.83
RRl	0.31	3.24		1.99
RRu	1.62	6.86		7.38
P	N.S.	+++		+++
Between Chi				16.77
Between df				1
Between P				***
Btwn(F) P				***
Btwn(R) P				***

Table 2E4 - 7

IESLC - Meta-analysis of Current Smoking (or Ever if Current not available), Pipe and/or Cigars (not cigs)
Squamous
Excluded studies (and stage at which they were excluded)

1	BOUCHA BUELL LAURIL MZILEN
2	ABRAHA AGUDO AKIBA AMANDU AMES ANDERS ARCHER AUSTIN AUVINE AXELSO AXELSS BAND BARBON BERRIN BLOHMK BLOT1 BLOT2 BLOT3 BLOT4 BRETT BROCKM BROWN1 BROWN2 BUFFLE BYERS1 BYERS2 CARPEN CASCO2 CASCOR CEDERL CHAN CHANG CHATZI CHEN CHEN2 CHEN3 CHIAZZ CHOI CHYOU COOKSO CORREA CPSII DAVEYS DEAN3 DESTE2 DESTEF DOCKER DOLL DORANT DOSEME DROSTE DU DUNN EBELIN ENSTRO ESAKI FAN GAO GAO2 GARCIA GARDIN GARSHI GENG GER GILLIS GODLEY GOODMA GREGOR GSELL HAENSZ HANSEN HEGMAN HEIN HENNEK HINDS HIRAOK HIRAY2 HIRAYA HITOSU HOROWI HORWIT HU HU2 HUANG HUMBLE ISHIMA JAIN JARUP JARVHO JEDRYC JIANG JOLY JONES JUSSAW KAISE2 KAISER KANELL KATSOU KAUFMA KELLER KHUDER KIHARA KO KOHLME KOO KOULUM KREYBE KUBIK LAMTH LAMWK LAMWK2 LANGE LAUSSM LEI LEMARC LETOUR LIAW LICKIN LIDDEL LIU LIU2 LIU3 LIU4 LIU5 LOMBA2 LUBIN LUO MACLEN MAGNUS MARTIS MASTRA MATSUD MCCONN MCDUFF MCLAUG MILLER MILLS MOLLO MRFIT MRFITR MURATA NAM NOTAN2 NOTANI NOU ODRISC ORMOS OSANN OSANN2 PARKIN PASTOR PAWLEG PERNU PERSH2 PERSHA PETO PEZZO2 PEZZOT PIKE PISANI POFFIJ POLEDN PRESCO QIAO QIAO2 RACHTA RADZIK RANDIG REN RESTRE RIMING RONCO ROOTS ROTHSC SAARIK SANKAR SCHWA2 SCHWAR SEGI SEGI2 SEOW SHAW SHIMIZ SIEMIA SIMARA SIMONA SITAS SOBUE SOBUE2 SPEIZE SPITZ STAYNE STOCKS STUCKE SUN SUZUK2 SUZUKI SVENSS TANG TANG2 TAO TENKAN TIZZAN TOKARS TSUGAN TVERDA ULMER VANDER VEIERO VUTUC WAKAI WANG WANG2 WANG3 WANG4 WARSIN WATSON WICKLU WILKIN WU WU2 WUNSCH WUWILL WYNDE4 WYNDE5 WYNDE8 WYNDER XIANGZ XU XU2 XU3 XU4 YAMAGU YONG YUAN ZHANG ZHENG ZHOU
7	ABELIN ARMADA BECHER BENHAM BENSHL BEST BOFFET BOUCOT BROSS CHOW COMSTO CPSI DAMBER DARBY DEAN DEAN2 DEKLER DOLL2 DORGAN DORN ENGELA GOLLED GRAHAM HAMMO2 HOLE KINLEN KJUUS KNEKT KREUZE LEVIN LOMBAR LUBIN2 MARSH MARSH2 MATOS MIGRAN SADOWS STOCKW TOUSEY TULINI WALD WIGLE
9	WYNDE7

Table 2E4 - 8
Potentially overlapping studies

REF	REFGP	PRINC	. OVERLAP/LINK
WYNDE6	WYNDE6	1	WYNDE5/6/7/8
JAHN	BOFFET	2	Subset of BOFFET

Table 2E5 -

IESLC - Meta-analysis of Ex Smoking, Pipe and/or Cigars (not cigs)
Squamous

This analysis is restricted to results for:
1) Non-dose-response data
2) Smokers of pipe and/or cigars (but not cigarettes)
3) Ex smokers
4) Results complete enough for use in metaanalysis

Within each study, results are then selected (in the following order of preference, within each sex) for:
5) DENOM: never smoked anything, (never +1 = +long term ex)
6) Followup period (prospective studies): whole study (coded as 0) or longest available
7) LCtype: all or nearest available, at least Squamous and Adeno. (q = squamous, s = small,
l = large, a = adeno, mix = mixed, alv = alveolar)
8) Race: all or nearest available, otherwise by race (wh or w = white, bl or b = black, hi = hispanic
ch = chinese, jap = japanese, haw = hawaiian, w+o = white + oriental, sca = scandinavian, as = asian)
9) For overlapping studies: principal rather than subsidiary studies
Finally by Age: whole study (coded as 0) if available, otherwise by widest available age group
and then for single sex results (m, f) in preference to combined sex results (c).

Results adjusted (AD) for the most potential confounders are then chosen in Sections -1 to -3
and results adjusted for the least confounders in Sections -4 to -6. (Those least adjusted results which
actually differ from the most adjusted as marked 'x' in column X in Section -4)
(Results adjusted for an unknown number of confounder(s) are coded as 20.)

Section -7 shows excluded studies, together with the stage (as above) at which no qualifying
results were found.

Section -8 lists the potentially overlapping studies which have been included (1=principal, 2=subsidiary).

Section -9 lists any results which would have been included in preference except that they had data not complete
enough for use in meta-analysis, with their significance (yes/no), if known, and any further comment as entered
on the database.

In addition to those mentioned above, the following fields, levels and abbreviations are used:
* or nk = not known, n = no, y = yes, ot = other
nev = never
REF: 6-character study reference
NRR: number of the RR on the database within the study
ST : study type (CC = case control, pr or prosp = prospective)
NLC: number of lung cancer cases in whole study
R : risky occupational population (n = no, m = mining, o = other risky)
VB : national cigarette type (V = at least 75% Virginia, bl = at least 75% blended, ot = other)
P : any proxy use
H : full histological confirmation
De : derivation of RR/CI (or = original, st = standard method, ot = other method of estimation)

Table 2E5 - 1

IESLC - Meta-analysis of Ex Smoking, Pipe and/or Cigars (not cigs)
Squamous
Most adjusted

REF	NRR	SEX	AGEL	AGEH	RACE	YF	LC TYPE	LOC	START	ST	NLC	R	VB	P	H	AD	DENOM	De
WYNDE7	71	m	0	0	all	-	KI	NAmer	1977	CC	2085	n	bl	n	y	0	nev any	st

Table 2E5 - 2

IESLC - Meta-analysis of Ex Smoking, Pipe and/or Cigars (not cigs)
Squamous
Most adjusted

REF	NRR	SEX	AD	Number Exposed Case	Cont	Non-exposed Case	Cont	RR	95.00%CI
WYNDE7	71	m	0	9	137	22	918	2.74	(1.24- 6.08)
Totals				9	137	22	918		

*prospective study

REF	NRR	SEX	AD	Ys	Ws	Qs	Ps
WYNDE7	71	m	0	1.01	6.06	0.00	0.0130

	N	1
	NS	1
	Wt	6.06
Het	Chi	0.00
Het	df	0
Het	P	N.S.
Fixed	RR	2.74
	RRl	1.24
	RRu	6.08
	P	+
Random	RR	2.74
	RRl	1.24
	RRu	6.08
	P	+
Asymm	P	

Table 2E5 - 3

IESLC - Meta-analysis of Ex Smoking, Pipe and/or Cigars (not cigs)
Squamous
Most adjusted

	Sex			
	combined	male	female	Total
N		1		1
NS		1		1
Wt		6.06		6.06
Het Chi		0.00		0.00
Het df		0		0
Het P		N.S.		N.S.
Fixed RR		2.74		2.74
RRl		1.24		1.24
RRu		6.08		6.08
P		+		+
Random RR		2.74		2.74
RRl		1.24		1.24
RRu		6.08		6.08
P		+		+
Between Chi				
Between df				
Between P				N.S.
Btwn(F) P				N.S.
Btwn(R) P				N.S.

Too few RRs for analysis by factor

Table 2E5 - 4

IESLC - Meta-analysis of Ex Smoking, Pipe and/or Cigars (not cigs)
Squamous
Least adjusted

REF	NRR	X	SEX	AGEL	AGEH	RACE	YF	LC TYPE	LOC	START	ST	NLC	R	VB	P	H	AD	DENOM	De
WYNDE7	71		m	0	0	all	-	KI	NAmer	1977	CC	2085	n	bl	n	y	0	nev any	st

Table 2E5 - 5

IESLC - Meta-analysis of Ex Smoking, Pipe and/or Cigars (not cigs)
Squamous
Least adjusted

REF	NRR	SEX	AD	Number Case	Exposed Cont	Non-exposed Case	Cont	RR	95.00%CI
WYNDE7	71	m	0	9	137	22	918	2.74	(1.24- 6.08)
Totals				9	137	22	918		

*prospective study

REF	NRR	SEX	AD	Ys	Ws	Qs	Ps
WYNDE7	71	m	0	1.01	6.06	0.00	0.0130

N	1
NS	1
Wt	6.06
Het Chi	0.00
Het df	0
Het P	N.S.
Fixed RR	2.74
RRl	1.24
RRu	6.08
P	+
Random RR	2.74
RRl	1.24
RRu	6.08
P	+
Asymm P	

Table 2E5 - 6

IESLC - Meta-analysis of Ex Smoking, Pipe and/or Cigars (not cigs)
Squamous
Least adjusted

	Sex combined	male	female	Total
N		1		1
NS		1		1
Wt		6.06		6.06
Het Chi		0.00		0.00
Het df		0		0
Het P		N.S.		N.S.
Fixed RR		2.74		2.74
RRl		1.24		1.24
RRu		6.08		6.08
P		+		+
Random RR		2.74		2.74
RRl		1.24		1.24
RRu		6.08		6.08
P		+		+
Between Chi				
Between df				
Between P				N.S.
Btwn(F) P				N.S.
Btwn(R) P				N.S.

Table 2E5 - 7

IESLC - Meta-analysis of Ex Smoking, Pipe and/or Cigars (not cigs)
Squamous
Excluded studies (and stage at which they were excluded)

1	BOUCHA	BUELL	LAURIL	MZILEN												
2	ABRAHA	AGUDO	AKIBA	AMANDU	AMES	ANDERS	ARCHER	AUSTIN	AUVINE	AXELSO	AXELSS	BAND	BARBON	BERRIN	BLOHMK	BLOT1
	BLOT2	BLOT3	BLOT4	BRETT	BROCKM	BROWN1	BROWN2	BUFFLE	BYERS1	BYERS2	CARPEN	CASCO2	CASCOR	CEDERL	CHAN	CHANG
	CHATZI	CHEN	CHEN2	CHEN3	CHIAZZ	CHOI	CHYOU	COOKSO	CORREA	CPSII	DAVEYS	DEAN3	DESTE2	DESTEF	DOCKER	DOLL
	DORANT	DOSEME	DROSTE	DU	DUNN	EBELIN	ENSTRO	ESAKI	FAN	GAO	GAO2	GARCIA	GARDIN	GARSHI	GENG	GER
	GILLIS	GODLEY	GOODMA	GREGOR	GSELL	HAENSZ	HANSEN	HEGMAN	HEIN	HENNEK	HINDS	HIRAOK	HIRAY2	HIRAYA	HITOSU	HOROWI
	HORWIT	HU	HU2	HUANG	HUMBLE	ISHIMA	JAIN	JARUP	JARVHO	JEDRYC	JIANG	JOLY	JONES	JUSSAW	KAISE2	KAISER
	KANELL	KATSOU	KAUFMA	KELLER	KHUDER	KIHARA	KO	KOHLME	KOO	KOULUM	KREYBE	KUBIK	LAMTH	LAMWK	LAMWK2	LANGE
	LAUSSM	LEI	LEMARC	LETOUR	LIAW	LICKIN	LIDDEL	LIU	LIU2	LIU3	LIU4	LIU5	LOMBA2	LUBIN	LUO	MACLEN
	MAGNUS	MARTIS	MASTRA	MATSUD	MCCONN	MCDUFF	MCLAUG	MILLER	MILLS	MOLLO	MRFIT	MRFITR	MURATA	NAM	NOTAN2	NOTANI
	NOU	ODRISC	ORMOS	OSANN	OSANN2	PARKIN	PASTOR	PAWLEG	PERNU	PERSH2	PERSHA	PETO	PEZZO2	PEZZOT	PIKE	PISANI
	POFFIJ	POLEDN	PRESCO	QIAO	QIAO2	RACHTA	RADZIK	RANDIG	REN	RESTRE	RIMING	RONCO	ROOTS	ROTHSC	SAARIK	SANKAR
	SCHWA2	SCHWAR	SEGI	SEGI2	SEOW	SHAW	SHIMIZ	SIEMIA	SIMARA	SIMONA	SITAS	SOBUE	SOBUE2	SPEIZE	SPITZ	STAYNE
	STOCKS	STUCKE	SUN	SUZUK2	SUZUKI	SVENSS	TANG	TANG2	TAO	TENKAN	TIZZAN	TOKARS	TSUGAN	TVERDA	ULMER	VANDER
	VEIERO	VUTUC	WAKAI	WANG	WANG2	WANG3	WANG4	WARSIN	WATSON	WICKLU	WILKIN	WU	WU2	WUNSCH	WUWILL	WYNDE4
	WYNDE5	WYNDE8	WYNDER	XIANGZ	XU	XU2	XU3	XU4	YAMAGU	YONG	YUAN	ZHANG	ZHENG	ZHOU		
3	ABELIN	ALDERS	ARMADA	BECHER	BENHAM	BENSHL	BEST	BOFFET	BOUCOT	BRESLO	BROSS	COMSTO	CPSI	DAMBER	DARBY	DEAN
	DEAN2	DEKLER	DORGAN	ENGELA	GOLLED	GRAHAM	HAMMO2	HAMMON	HOLE	JAHN	KINLEN	KJUUS	KNEKT	KREUZE	LEVIN	LOMBAR
	MARSH	MARSH2	MATOS	SADOWS	STASZE	STOCKW	TOUSEY	TULINI	WALD	WYNDE2	WYNDE3	WYNDE6				
7	CHOW	DOLL2	DORN	LUBIN2	MIGRAN	WIGLE										

Table 2E5 - 8
Potentially overlapping studies

REF	REFGP	PRINC	.	OVERLAP/LINK
WYNDE7	WYNDE6	2		WYNDE5/6/7/8

Table 2E6 -

IESLC - Meta-analysis of Ever Smoking, Pipe only
Squamous

This analysis is restricted to results for:
1) Non-dose-response data
2) Smokers of pipe only
3) Ever smokers
4) Results complete enough for use in metaanalysis

Within each study, results are then selected (in the following order of preference, within each sex) for:
5) DENOM: never smoked anything, (never +1 = +long term ex)
6) Followup period (prospective studies): whole study (coded as 0) or longest available
7) LCtype: all or nearest available, at least Squamous and Adeno. (q = squamous, s = small,
l = large, a = adeno, mix = mixed, alv = alveolar)
8) Race: all or nearest available, otherwise by race (wh or w = white, bl or b = black, hi = hispanic
ch = chinese, jap = japanese, haw = hawaiian, w+o = white + oriental, sca = scandinavian, as = asian)
9) For overlapping studies: principal rather than subsidiary studies
Finally by Age: whole study (coded as 0) if available, otherwise by widest available age group
and then for single sex results (m, f) in preference to combined sex results (c).

Results adjusted (AD) for the most potential confounders are then chosen in Sections -1 to -3
and results adjusted for the least confounders in Sections -4 to -6. (Those least adjusted results which
actually differ from the most adjusted as marked 'x' in column X in Section -4)
(Results adjusted for an unknown number of confounder(s) are coded as 20.)

Section -7 shows excluded studies, together with the stage (as above) at which no qualifying
results were found.

Section -8 lists the potentially overlapping studies which have been included (1=principal, 2=subsidiary).

Section -9 lists any results which would have been included in preference except that they had data not complete
enough for use in meta-analysis, with their significance (yes/no), if known, and any further comment as entered
on the database.

In addition to those mentioned above, the following fields, levels and abbreviations are used:
* or nk = not known, n = no, y = yes, ot = other
nev = never
REF: 6-character study reference
NRR: number of the RR on the database within the study
ST : study type (CC = case control, pr or prosp = prospective)
NLC: number of lung cancer cases in whole study
R : risky occupational population (n = no, m = mining, o = other risky)
VB : national cigarette type (V = at least 75% Virginia, bl = at least 75% blended, ot = other)
P : any proxy use
H : full histological confirmation
De : derivation of RR/CI (or = original, st = standard method, ot = other method of estimation)

Table 2E6 - 1

IESLC - Meta-analysis of Ever Smoking, Pipe only
Squamous
Most adjusted

REF	NRR	SEX	AGEL	AGEH	RACE	YF	LC TYPE	LOC	START	ST	NLC	R	VB	P	H	AD	DENOM	De
HAMMON	55	m	0	0	wh	0	not a	NAmer	1952	pr	448	n	bl	n	n	1	nev any	ot
LUBIN	16	m	0	0	all	-	KI	As:Chi	1984	CC	427	m	ot	y	n	0	nev any	st
LUBIN2	137	m	0	0	all	-	q	Eu:mul	1976	CC	7804	n	bl	n	y	0	nev any	st
WYNDE7	57	m	0	0	all	-	KI	NAmer	1977	CC	2085	n	bl	n	y	0	nev any	st

Table 2E6 - 2

IESLC - Meta-analysis of Ever Smoking, Pipe only
Squamous
Most adjusted

REF	NRR	SEX	AD	Number Exposed Case	Cont	Non-exposed Case	Cont	RR	95.00%CI
*HAMMON	55	m	1	-	-	-	-	8.50	(2.77- 26.07)
LUBIN	16	m	0	39	151	4	72	4.65	(1.60- 13.51)
LUBIN2	137	m	0	19	197	127	2616	1.99	(1.20- 3.29)
WYNDE7	57	m	0	7	97	22	918	3.01	(1.25- 7.23)
Partial Totals				65	445	153	3606		

*prospective study

REF	NRR	SEX	AD	Ys	Ws	Qs	Ps
*HAMMON	55	m	1	2.14	3.06	3.70	0.0002
LUBIN	16	m	0	1.54	3.38	0.83	0.0047
LUBIN2	137	m	0	0.69	15.16	1.89	0.0075
WYNDE7	57	m	0	1.10	5.01	0.02	0.0136

	N	4
	NS	4
	Wt	26.60
Het	Chi	6.45
Het	df	3
Het	P	(*)
Fixed	RR	2.83
	RRl	1.93
	RRu	4.14
	P	+++
Random	RR	3.43
	RRl	1.84
	RRu	6.41
	P	+++
Asymm	P	(*)

Table 2E6 - 3

IESLC - Meta-analysis of Ever Smoking, Pipe only
Squamous
Most adjusted

	Sex combined	male	female	Total
N		4		4
NS		4		4
Wt		26.60		26.60
Het Chi		6.45		6.45
Het df		3		3
Het P		(*)		(*)
Fixed RR		2.83		2.83
RRl		1.93		1.93
RRu		4.14		4.14
P		+++		+++
Random RR		3.43		3.43
RRl		1.84		1.84
RRu		6.41		6.41
P		+++		+++
Between Chi				
Between df				
Between P				N.S.
Btwn(F) P				N.S.
Btwn(R) P				N.S.

Too few RRs for analysis by factor

Table 2E6 - 4

IESLC - Meta-analysis of Ever Smoking, Pipe only
Squamous
Least adjusted

REF	NRR	X	SEX	AGEL	AGEH	RACE	YF	LC TYPE	LOC	START	ST	NLC	R	VB	P	H	AD	DENOM	De
HAMMON	68	x	m	0	0	wh	0	not a	NAmer	1952	pr	448	n	bl	n	n	0	nev any	st
LUBIN	16		m	0	0	all	-	KI	As:Chi	1984	CC	427	m	ot	y	n	0	nev any	st
LUBIN2	137		m	0	0	all	-	q	Eu:mul	1976	CC	7804	n	bl	n	y	0	nev any	st
WYNDE7	57		m	0	0	all	-	KI	NAmer	1977	CC	2085	n	bl	n	y	0	nev any	st

Table 2E6 - 5

IESLC - Meta-analysis of Ever Smoking, Pipe only
Squamous
Least adjusted

REF	NRR	SEX	AD	Number Exposed Case	Number Exposed Cont	Non-exposed Case	Non-exposed Cont	RR	95.00%CI
*HAMMON	68	m	0	13	43041	4	115884	8.75	(2.85- 26.83)
LUBIN	16	m	0	39	151	4	72	4.65	(1.60- 13.51)
LUBIN2	137	m	0	19	197	127	2616	1.99	(1.20- 3.29)
WYNDE7	57	m	0	7	97	22	918	3.01	(1.25- 7.23)
Totals				78	43486	157	119490		

*prospective study

REF	NRR	SEX	AD	Ys	Ws	Qs	Ps
*HAMMON	68	m	0	2.17	3.06	3.88	0.0001
LUBIN	16	m	0	1.54	3.38	0.82	0.0047
LUBIN2	137	m	0	0.69	15.16	1.93	0.0075
WYNDE7	57	m	0	1.10	5.01	0.02	0.0136

	N	4
	NS	4
	Wt	26.60
Het	Chi	6.65
Het	df	3
Het	P	(*)
Fixed	RR	2.84
	RRl	1.94
	RRu	4.15
	P	+++
Random	RR	3.46
	RRl	1.84
	RRu	6.54
	P	+++
Asymm	P	(*)

Table 2E6 - 6

IESLC - Meta-analysis of Ever Smoking, Pipe only
Squamous
Least adjusted

	Sex combined	male	female	Total
N		4		4
NS		4		4
Wt		26.60		26.60
Het Chi		6.65		6.65
Het df		3		3
Het P		(*)		(*)
Fixed RR		2.84		2.84
RRl		1.94		1.94
RRu		4.15		4.15
P		+++		+++
Random RR		3.46		3.46
RRl		1.84		1.84
RRu		6.54		6.54
P		+++		+++
Between Chi				
Between df				
Between P				N.S.
Btwn(F) P				N.S.
Btwn(R) P				N.S.

Table 2E6 - 7

IESLC - Meta-analysis of Ever Smoking, Pipe only
Squamous
Excluded studies (and stage at which they were excluded)

1	BOUCHA BUELL LAURIL MZILEN
2	ABRAHA AGUDO AKIBA ALDERS AMANDU AMES ANDERS ARCHER AUSTIN AUVINE AXELSO AXELSS BAND BARBON BECHER BERRIN BLOHMK BLOT1 BLOT2 BLOT3 BLOT4 BRESLO BRETT BROCKM BROSS BROWN1 BROWN2 BUFFLE BYERS1 BYERS2 CARPEN CASCO2 CASCOR CHAN CHANG CHATZI CHEN CHEN2 CHEN3 CHIAZZ CHOI CHOW CHYOU COMSTO CORREA CPSII DARBY DAVEYS DEAN2 DEKLER DESTE2 DESTEF DOCKER DORANT DORGAN DOSEME DROSTE DU DUNN EBELIN ENGELA ENSTRO ESAKI FAN GAO GAO2 GARCIA GARSHI GENG GER GILLIS GODLEY GOLLED GOODMA GRAHAM GREGOR GSELL HAENSZ HAMMO2 HANSEN HEGMAN HENNEK HINDS HIRAOK HIRAYA HITOSU HOLE HOROWI HORWIT HU HU2 HUANG HUMBLE ISHIMA JAHN JAIN JARUP JARVHO JEDRYC JIANG JOLY JONES JUSSAW KAISE2 KAISER KANELL KATSOU KAUFMA KELLER KHUDER KIHARA KINLEN KJUUS KNEKT KO KOHLME KOO KREUZE KREYBE KUBIK LAMTH LAMWK LAMWK2 LAUSSM LEI LEMARC LETOUR LIAW LICKIN LIDDEL LIU LIU2 LIU3 LIU4 LIU5 LOMBA2 LOMBAR LUO MACLEN MAGNUS MARSH MARSH2 MARTIS MASTRA MATOS MATSUD MCDUFF MCLAUG MIGRAN MILLER MILLS MOLLO MRFIT MRFITR MURATA NAM NOTAN2 NOTANI NOU ODRISC ORMOS OSANN OSANN2 PARKIN PASTOR PAWLEG PERSH2 PERSHA PETO PEZZO2 PEZZOT PIKE PISANI POFFIJ POLEDN PRESCO QIAO2 RACHTA RADZIK RANDIG REN RESTRE RIMING RONCO ROOTS ROTHSC SAARIK SANKAR SCHWAR SEGI SEGI2 SEOW SHAW SHIMIZ SIEMIA SIMARA SIMONA SITAS SOBUE SOBUE2 SPEIZE SPITZ STASZE STAYNE STOCKW STUCKE SUN SUZUK2 SUZUKI SVENSS TANG TANG2 TAO TENKAN TOKARS TOUSEY TSUGAN ULMER VANDER VEIERO VUTUC WAKAI WALD WANG WANG2 WANG3 WANG4 WARSIN WATSON WICKLU WIGLE WILKIN WU WU2 WUNSCH WUWILL WYNDE2 WYNDE3 WYNDE4 WYNDE5 WYNDE6 WYNDE8 WYNDER XU XU2 XU3 XU4 YAMAGU YONG YUAN ZHANG ZHENG ZHOU
3	BENSHL CEDERL DEAN3 GARDIN HEIN HIRAY2 LANGE TULINI TVERDA
4	DOLL2
5	SCHWA2
7	ABELIN ARMADA BENHAM BEST BOFFET BOUCOT COOKSO CPSI DAMBER DEAN DOLL DORN KOULUM LEVIN MCCONN PERNU QIAO SADOWS STOCKS TIZZAN XIANGZ

Table 2E6 - 8
Potentially overlapping studies

REF	REFGP	PRINC	. OVERLAP/LINK
LUBIN2	LUBIN2	1	Lubin-combined
WYNDE7	WYNDE6	2	WYNDE5/6/7/8
LUBIN	XIANGZ	2	LUBIN/XIANGZ/QIAO

Table 2E7 -

IESLC - Meta-analysis of Current Smoking, Pipe only
Squamous

This analysis is restricted to results for:
1) Non-dose-response data
2) Smokers of pipe only
3) Current smokers
4) Results complete enough for use in metaanalysis

Within each study, results are then selected (in the following order of preference, within each sex) for:
5) DENOM: never smoked anything, (never +1 = +long term ex)
6) Followup period (prospective studies): whole study (coded as 0) or longest available
7) LCtype: all or nearest available, at least Squamous and Adeno. (q = squamous, s = small,
l = large, a = adeno, mix = mixed, alv = alveolar)
8) Race: all or nearest available, otherwise by race (wh or w = white, bl or b = black, hi = hispanic
ch = chinese, jap = japanese, haw = hawaiian, w+o = white + oriental, sca = scandinavian, as = asian)
9) For overlapping studies: principal rather than subsidiary studies
Finally by Age: whole study (coded as 0) if available, otherwise by widest available age group
and then for single sex results (m, f) in preference to combined sex results (c).

Results adjusted (AD) for the most potential confounders are then chosen in Sections -1 to -3
and results adjusted for the least confounders in Sections -4 to -6. (Those least adjusted results which
actually differ from the most adjusted as marked 'x' in column X in Section -4)
(Results adjusted for an unknown number of confounder(s) are coded as 20.)

Section -7 shows excluded studies, together with the stage (as above) at which no qualifying
results were found.

Section -8 lists the potentially overlapping studies which have been included (1=principal, 2=subsidiary).

Section -9 lists any results which would have been included in preference except that they had data not complete
enough for use in meta-analysis, with their significance (yes/no), if known, and any further comment as entered
on the database.

In addition to those mentioned above, the following fields, levels and abbreviations are used:
* or nk = not known, n = no, y = yes, ot = other
nev = never
REF: 6-character study reference
NRR: number of the RR on the database within the study
ST : study type (CC = case control, pr or prosp = prospective)
NLC: number of lung cancer cases in whole study
R : risky occupational population (n = no, m = mining, o = other risky)
VB : national cigarette type (V = at least 75% Virginia, bl = at least 75% blended, ot = other)
P : any proxy use
H : full histological confirmation
De : derivation of RR/CI (or = original, st = standard method, ot = other method of estimation)

Table 2E7 - 1

IESLC - Meta-analysis of Current Smoking, Pipe only
Squamous
Most adjusted

REF	NRR	SEX	AGEL	AGEH	RACE	YF	LC TYPE	LOC	START	ST	NLC	R	VB	P	H	AD	DENOM	De
WYNDE7	20	m	0	0	all	-	KI	NAmer	1977	CC	2085	n	bl	n	y	0	nev any	st

Table 2E7 - 2

IESLC - Meta-analysis of Current Smoking, Pipe only
Squamous
Most adjusted

REF	NRR	SEX	AD	Number Exposed Case	Number Exposed Cont	Non-exposed Case	Non-exposed Cont	RR	95.00%CI
WYNDE7	20	m	0	6	54	22	918	4.64	(1.80- 11.91)
Totals				6	54	22	918		

*prospective study

REF	NRR	SEX	AD	Ys	Ws	Qs	Ps
WYNDE7	20	m	0	1.53	4.32	0.00	0.0014

	N	1
	NS	1
	Wt	4.32
Het	Chi	0.00
Het	df	0
Het	P	N.S.
Fixed	RR	4.64
	RRl	1.80
	RRu	11.91
	P	++
Random	RR	4.64
	RRl	1.80
	RRu	11.91
	P	++
Asymm	P	

Table 2E7 - 3

IESLC - Meta-analysis of Current Smoking, Pipe only
Squamous
Most adjusted

	combined	Sex male	female	Total
N		1		1
NS		1		1
Wt		4.32		4.32
Het Chi		0.00		0.00
Het df		0		0
Het P		N.S.		N.S.
Fixed RR		4.64		4.64
RRl		1.80		1.80
RRu		11.91		11.91
P		++		++
Random RR		4.64		4.64
RRl		1.80		1.80
RRu		11.91		11.91
P		++		++
Between Chi				
Between df				
Between P				N.S.
Btwn(F) P				N.S.
Btwn(R) P				N.S.

Too few RRs for analysis by factor

Table 2E7 - 4

IESLC - Meta-analysis of Current Smoking, Pipe only
Squamous
Least adjusted

REF	NRR	X	SEX	AGEL	AGEH	RACE	YF	LC TYPE	LOC	START	ST	NLC	R	VB	P	H	AD	DENOM	De
WYNDE7	20		m	0	0	all	-	KI	NAmer	1977	CC	2085	n	bl	n	y	0	nev any	st

Table 2E7 - 5

IESLC - Meta-analysis of Current Smoking, Pipe only
Squamous
Least adjusted

REF	NRR	SEX	AD	Number Case	Exposed Cont	Non-exposed Case	Cont	RR	95.00%CI
WYNDE7	20	m	0	6	54	22	918	4.64	(1.80- 11.91)
Totals				6	54	22	918		

*prospective study

REF	NRR	SEX	AD	Ys	Ws	Qs	Ps
WYNDE7	20	m	0	1.53	4.32	0.00	0.0014

	N	1
	NS	1
	Wt	4.32
Het	Chi	0.00
Het	df	0
Het	P	N.S.
Fixed	RR	4.64
	RRl	1.80
	RRu	11.91
	P	++
Random	RR	4.64
	RRl	1.80
	RRu	11.91
	P	++
Asymm	P	

Table 2E7 - 6

IESLC - Meta-analysis of Current Smoking, Pipe only
Squamous
Least adjusted

	Sex		
combined	male	female	Total
N	1		1
NS	1		1
Wt	4.32		4.32
Het Chi	0.00		0.00
Het df	0		0
Het P	N.S.		N.S.
Fixed RR	4.64		4.64
RRl	1.80		1.80
RRu	11.91		11.91
P	++		++
Random RR	4.64		4.64
RRl	1.80		1.80
RRu	11.91		11.91
P	++		++
Between Chi			
Between df			
Between P			N.S.
Btwn(F) P			N.S.
Btwn(R) P			N.S.

Table 2E7 - 7

IESLC - Meta-analysis of Current Smoking, Pipe only
Squamous
Excluded studies (and stage at which they were excluded)

1 BOUCHA BUELL LAURIL MZILEN

2 ABRAHA AGUDO AKIBA ALDERS AMANDU AMES ANDERS ARCHER AUSTIN AUVINE AXELSO AXELSS BAND BARBON BECHER BERRIN
BLOHMK BLOT1 BLOT2 BLOT3 BLOT4 BRESLO BRETT BROCKM BROSS BROWN1 BROWN2 BUFFLE BYERS1 BYERS2 CARPEN CASCO2
CASCOR CHAN CHANG CHATZI CHEN CHEN2 CHEN3 CHIAZZ CHOI CHOW CHYOU COMSTO CORREA CPSII DARBY DAVEYS
DEAN2 DEKLER DESTE2 DESTEF DOCKER DORANT DORGAN DOSEME DROSTE DU DUNN EBELIN ENGELA ENSTRO ESAKI FAN
GAO GAO2 GARCIA GARSHI GENG GER GILLIS GODLEY GOLLED GOODMA GRAHAM GREGOR GSELL HAENSZ HAMMO2 HANSEN
HEGMAN HENNEK HINDS HIRAOK HIRAYA HITOSU HOLE HOROWI HORWIT HU HU2 HUANG HUMBLE ISHIMA JAHN JAIN
JARUP JARVHO JEDRYC JIANG JOLY JONES JUSSAW KAISE2 KAISER KANELL KATSOU KAUFMA KELLER KHUDER KIHARA KINLEN
KJUUS KNEKT KO KOHLME KOO KREUZE KREYBE KUBIK LAMTH LAMWK LAMWK2 LAUSSM LEI LEMARC LETOUR LIAW
LICKIN LIDDEL LIU LIU2 LIU3 LIU4 LIU5 LOMBA2 LOMBAR LUO MACLEN MAGNUS MARSH MARSH2 MARTIS MASTRA
MATOS MATSUD MCDUFF MCLAUG MIGRAN MILLER MILLS MOLLO MRFIT MRFITR MURATA NAM NOTAN2 NOTANI NOU ODRISC
ORMOS OSANN OSANN2 PARKIN PASTOR PAWLEG PERSH2 PERSHA PETO PEZZO2 PEZZOT PIKE PISANI POFFIJ POLEDN PRESCO
QIAO2 RACHTA RADZIK RANDIG REN RESTRE RIMING RONCO ROOTS ROTHSC SAARIK SANKAR SCHWAR SEGI SEGI2 SEOW
SHAW SHIMIZ SIEMIA SIMARA SIMONA SITAS SOBUE SOBUE2 SPEIZE SPITZ STASZE STAYNE STOCKW STUCKE SUN SUZUK2
SUZUKI SVENSS TANG TANG2 TAO TENKAN TOKARS TOUSEY TSUGAN ULMER VANDER VEIERO VUTUC WAKAI WALD WANG
WANG2 WANG3 WANG4 WARSIN WATSON WICKLU WIGLE WILKIN WU WU2 WUNSCH WUWILL WYNDE2 WYNDE3 WYNDE4 WYNDE5
WYNDE6 WYNDE8 WYNDER XU XU2 XU3 XU4 YAMAGU YONG YUAN ZHANG ZHENG ZHOU

3 ABELIN ARMADA BENHAM BEST BOUCOT COOKSO CPSI DEAN DOLL DOLL2 HAMMON KOULUM LEVIN LUBIN MCCONN PERNU
QIAO SADOWS SCHWA2 STOCKS TIZZAN XIANGZ

5 HIRAY2 TVERDA

7 BENSHL BOFFET CEDERL DAMBER DEAN3 DORN GARDIN HEIN LANGE LUBIN2 TULINI

Table 2E7 - 8

Potentially overlapping studies

REF	REFGP	PRINC	.	OVERLAP/LINK
WYNDE7	WYNDE6	2		WYNDE5/6/7/8

Table 2E8 -

IESLC - Meta-analysis of Ever Smoking (or Current if Ever not available), Pipe only
Squamous

This analysis is restricted to results for:
1) Non-dose-response data
2) Smokers of pipe only
3) Results complete enough for use in metaanalysis

Within each study, results are then selected (in the following order of preference, within each sex) for:
4) SMKSTA: ever smokers, current smokers
5) DENOM: never smoked anything, (never +1 = +long term ex)
6) Followup period (prospective studies): whole study (coded as 0) or longest available
7) LCtype: all or nearest available, at least Squamous and Adeno. (q = squamous, s = small,
l = large, a = adeno, mix = mixed, alv = alveolar)
8) Race: all or nearest available, otherwise by race (wh or w = white, bl or b = black, hi = hispanic
ch = chinese, jap = japanese, haw = hawaiian, w+o = white + oriental, sca = scandinavian, as = asian)
9) For overlapping studies: principal rather than subsidiary studies
Finally by Age: whole study (coded as 0) if available, otherwise by widest available age group
and then for single sex results (m, f) in preference to combined sex results (c).

Results adjusted (AD) for the most potential confounders are then chosen in Sections -1 to -3
and results adjusted for the least confounders in Sections -4 to -6. (Those least adjusted results which
actually differ from the most adjusted as marked 'x' in column X in Section -4)
(Results adjusted for an unknown number of confounder(s) are coded as 20.)

Section -7 shows excluded studies, together with the stage (as above) at which no qualifying
results were found.

Section -8 lists the potentially overlapping studies which have been included (1=principal, 2=subsidiary).

Section -9 lists any results which would have been included in preference except that they had data not complete
enough for use in meta-analysis, with their significance (yes/no), if known, and any further comment as entered
on the database.

In addition to those mentioned above, the following fields, levels and abbreviations are used:
* or nk = not known, n = no, y = yes, ot = other
ev = ever, cu = current, nev = never
REF: 6-character study reference
NRR: number of the RR on the database within the study
ST : study type (CC = case control, pr or prosp = prospective)
NLC: number of lung cancer cases in whole study
R : risky occupational population (n = no, m = mining, o = other risky)
VB : national cigarette type (V = at least 75% Virginia, bl = at least 75% blended, ot = other)
P : any proxy use
H : full histological confirmation
De : derivation of RR/CI (or = original, st = standard method, ot = other method of estimation)

Table 2E8 - 1

IESLC - Meta-analysis of Ever Smoking (or Current if Ever not available), Pipe only
Squamous
Most adjusted

REF	NRR	SEX	AGEL	AGEH	RACE	YF	LC TYPE	LOC	START	ST	NLC	R	VB	P	H	AD	SM	DENOM	De
HAMMON	55	m	0	0	wh	0	not a	NAmer	1952	pr	448	n	bl	n	n	1	ev	nev any	ot
LUBIN	16	m	0	0	all	-	KI	As:Chi	1984	CC	427	m	ot	y	n	0	ev	nev any	st
LUBIN2	137	m	0	0	all	-	q	Eu:mul	1976	CC	7804	n	bl	n	y	0	ev	nev any	st
WYNDE7	57	m	0	0	all	-	KI	NAmer	1977	CC	2085	n	bl	n	y	0	ev	nev any	st

Table 2E8 - 2

IESLC - Meta-analysis of Ever Smoking (or Current if Ever not available), Pipe only
Squamous
Most adjusted

REF	NRR	SEX	AD	Number Exposed Case	Number Exposed Cont	Non-exposed Case	Non-exposed Cont	RR	95.00%CI
*HAMMON	55	m	1	-	-	-	-	8.50	(2.77- 26.07)
LUBIN	16	m	0	39	151	4	72	4.65	(1.60- 13.51)
LUBIN2	137	m	0	19	197	127	2616	1.99	(1.20- 3.29)
WYNDE7	57	m	0	7	97	22	918	3.01	(1.25- 7.23)
Partial Totals				65	445	153	3606		

*prospective study

REF	NRR	SEX	AD	Ys	Ws	Qs	Ps
*HAMMON	55	m	1	2.14	3.06	3.70	0.0002
LUBIN	16	m	0	1.54	3.38	0.83	0.0047
LUBIN2	137	m	0	0.69	15.16	1.89	0.0075
WYNDE7	57	m	0	1.10	5.01	0.02	0.0136

	N	4
	NS	4
	Wt	26.60
Het	Chi	6.45
Het	df	3
Het	P	(*)
Fixed	RR	2.83
	RRl	1.93
	RRu	4.14
	P	+++
Random	RR	3.43
	RRl	1.84
	RRu	6.41
	P	+++
Asymm	P	(*)

Table 2E8 - 3

IESLC - Meta-analysis of Ever Smoking (or Current if Ever not available), Pipe only
Squamous
Most adjusted

	Sex combined	male	female	Total
N		4		4
NS		4		4
Wt		26.60		26.60
Het Chi		6.45		6.45
Het df		3		3
Het P		(*)		(*)
Fixed RR		2.83		2.83
RRl		1.93		1.93
RRu		4.14		4.14
P		+++		+++
Random RR		3.43		3.43
RRl		1.84		1.84
RRu		6.41		6.41
P		+++		+++
Between Chi				
Between df				
Between P				N.S.
Btwn(F) P				N.S.
Btwn(R) P				N.S.

Too few RRs for analysis by factor

Table 2E8 - 4

IESLC - Meta-analysis of Ever Smoking (or Current if Ever not available), Pipe only
Squamous
Least adjusted

REF	NRR	X	SEX	AGEL	AGEH	RACE	YF	LC TYPE	LOC	START	ST	NLC	R	VB	P	H	AD	SM	DENOM	De
HAMMON	68	x	m	0	0	wh	0	not a	NAmer	1952	pr	448	n	bl	n	n	0	ev	nev any	st
LUBIN	16		m	0	0	all	-	KI	As:Chi	1984	CC	427	m	ot	y	n	0	ev	nev any	st
LUBIN2	137		m	0	0	all	-	q	Eu:mul	1976	CC	7804	n	bl	n	y	0	ev	nev any	st
WYNDE7	57		m	0	0	all	-	KI	NAmer	1977	CC	2085	n	bl	n	y	0	ev	nev any	st

Table 2E8 - 5

IESLC - Meta-analysis of Ever Smoking (or Current if Ever not available), Pipe only
Squamous
Least adjusted

REF	NRR	SEX	AD	Number Exposed Case	Cont	Non-exposed Case	Cont	RR	95.00%CI
*HAMMON	68	m	0	13	43041	4	115884	8.75	(2.85- 26.83)
LUBIN	16	m	0	39	151	4	72	4.65	(1.60- 13.51)
LUBIN2	137	m	0	19	197	127	2616	1.99	(1.20- 3.29)
WYNDE7	57	m	0	7	97	22	918	3.01	(1.25- 7.23)
Totals				78	43486	157	119490		

*prospective study

REF	NRR	SEX	AD	Ys	Ws	Qs	Ps
*HAMMON	68	m	0	2.17	3.06	3.88	0.0001
LUBIN	16	m	0	1.54	3.38	0.82	0.0047
LUBIN2	137	m	0	0.69	15.16	1.93	0.0075
WYNDE7	57	m	0	1.10	5.01	0.02	0.0136

	N	4
	NS	4
	Wt	26.60
Het	Chi	6.65
Het	df	3
Het	P	(*)
Fixed	RR	2.84
	RRl	1.94
	RRu	4.15
	P	+++
Random	RR	3.46
	RRl	1.84
	RRu	6.54
	P	+++
Asymm	P	(*)

Table 2E8 - 6

IESLC - Meta-analysis of Ever Smoking (or Current if Ever not available), Pipe only
Squamous
Least adjusted

	Sex combined	male	female	Total
N		4		4
NS		4		4
Wt		26.60		26.60
Het Chi		6.65		6.65
Het df		3		3
Het P		(*)		(*)
Fixed RR		2.84		2.84
RRl		1.94		1.94
RRu		4.15		4.15
P		+++		+++
Random RR		3.46		3.46
RRl		1.84		1.84
RRu		6.54		6.54
P		+++		+++
Between Chi				
Between df				
Between P				N.S.
Btwn(F) P				N.S.
Btwn(R) P				N.S.

Table 2E8 - 7

IESLC - Meta-analysis of Ever Smoking (or Current if Ever not available), Pipe only
Squamous
Excluded studies (and stage at which they were excluded)

1	BOUCHA BUELL LAURIL MZILEN
2	ABRAHA AGUDO AKIBA ALDERS AMANDU AMES ANDERS ARCHER AUSTIN AUVINE AXELSO AXELSS BAND BARBON BECHER BERRIN BLOHMK BLOT1 BLOT2 BLOT3 BLOT4 BRESLO BRETT BROCKM BROSS BROWN1 BROWN2 BUFFLE BYERS1 BYERS2 CARPEN CASCO2 CASCOR CHAN CHANG CHATZI CHEN CHEN2 CHEN3 CHIAZZ CHOI CHOW CHYOU COMSTO CORREA CPSII DARBY DAVEYS DEAN2 DEKLER DESTE2 DESTEF DOCKER DORANT DORGAN DOSEME DROSTE DU DUNN EBELIN ENGELA ENSTRO ESAKI FAN GAO GAO2 GARCIA GARSHI GENG GER GILLIS GODLEY GOLLED GOODMA GRAHAM GREGOR GSELL HAENSZ HAMMO2 HANSEN HEGMAN HENNEK HINDS HIRAOK HIRAYA HITOSU HOLE HOROWI HORWIT HU HU2 HUANG HUMBLE ISHIMA JAHN JAIN JARUP JARVHO JEDRYC JIANG JOLY JONES JUSSAW KAISE2 KAISER KANELL KATSOU KAUFMA KELLER KHUDER KIHARA KINLEN KJUUS KNEKT KO KOHLME KOO KREUZE KREYBE KUBIK LAMTH LAMWK LAMWK2 LAUSSM LEI LEMARC LETOUR LIAW LICKIN LIDDEL LIU LIU2 LIU3 LIU4 LIU5 LOMBA2 LOMBAR LUO MACLEN MAGNUS MARSH MARSH2 MARTIS MASTRA MATOS MATSUD MCDUFF MCLAUG MIGRAN MILLER MILLS MOLLO MRFIT MRFITR MURATA NAM NOTAN2 NOTANI NOU ODRISC ORMOS OSANN OSANN2 PARKIN PASTOR PAWLEG PERSH2 PERSHA PETO PEZZO2 PEZZOT PIKE PISANI POFFIJ POLEDN PRESCO QIAO2 RACHTA RADZIK RANDIG REN RESTRE RIMING RONCO ROOTS ROTHSC SAARIK SANKAR SCHWAR SEGI SEGI2 SEOW SHAW SHIMIZ SIEMIA SIMARA SIMONA SITAS SOBUE SOBUE2 SPEIZE SPITZ STASZE STAYNE STOCKW STUCKE SUN SUZUK2 SUZUKI SVENSS TANG TANG2 TAO TENKAN TOKARS TOUSEY TSUGAN ULMER VANDER VEIERO VUTUC WAKAI WALD WANG WANG2 WANG3 WANG4 WARSIN WATSON WICKLU WIGLE WILKIN WU WU2 WUNSCH WUWILL WYNDE2 WYNDE3 WYNDE4 WYNDE5 WYNDE6 WYNDE8 WYNDER XU XU2 XU3 XU4 YAMAGU YONG YUAN ZHANG ZHENG ZHOU
3	DOLL2
5	HIRAY2 SCHWA2 TVERDA
7	ABELIN ARMADA BENHAM BENSHL BEST BOFFET BOUCOT CEDERL COOKSO CPSI DAMBER DEAN DEAN3 DOLL DORN GARDIN HEIN KOULUM LANGE LEVIN MCCONN PERNU QIAO SADOWS STOCKS TIZZAN TULINI XIANGZ

Table 2E8 - 8
Potentially overlapping studies

REF	REFGP	PRINC	OVERLAP/LINK
LUBIN2	LUBIN2	1	Lubin-combined
WYNDE7	WYNDE6	2	WYNDE5/6/7/8
LUBIN	XIANGZ	2	LUBIN/XIANGZ/QIAO

Table 2E9 -

IESLC - Meta-analysis of Current Smoking (or Ever if Current not available), Pipe only
Squamous

This analysis is restricted to results for:
1) Non-dose-response data
2) Smokers of pipe only
3) Results complete enough for use in metaanalysis

Within each study, results are then selected (in the following order of preference, within each sex) for:
4) SMKSTA: current smokers, ever smokers
5) DENOM: never smoked anything, (never +1 = +long term ex)
6) Followup period (prospective studies): whole study (coded as 0) or longest available
7) LCtype: all or nearest available, at least Squamous and Adeno. (q = squamous, s = small,
l = large, a = adeno, mix = mixed, alv = alveolar)
8) Race: all or nearest available, otherwise by race (wh or w = white, bl or b = black, hi = hispanic
ch = chinese, jap = japanese, haw = hawaiian, w+o = white + oriental, sca = scandinavian, as = asian)
9) For overlapping studies: principal rather than subsidiary studies
Finally by Age: whole study (coded as 0) if available, otherwise by widest available age group
and then for single sex results (m, f) in preference to combined sex results (c).

Results adjusted (AD) for the most potential confounders are then chosen in Sections -1 to -3
(and those which actually differ from the adjusted results in Table 2E8 - 1 are marked 'x' in Section -1)
and results adjusted for the least confounders in Sections -4 to -6. (Those least adjusted results which
actually differ from the most adjusted as marked 'x' in column X in Section -4)
(Results adjusted for an unknown number of confounder(s) are coded as 20.)

Section -7 shows excluded studies, together with the stage (as above) at which no qualifying
results were found.

Section -8 lists the potentially overlapping studies which have been included (1=principal, 2=subsidiary).

Section -9 lists any results which would have been included in preference except that they had data not complete
enough for use in meta-analysis, with their significance (yes/no), if known, and any further comment as entered
on the database.

In addition to those mentioned above, the following fields, levels and abbreviations are used:
* or nk = not known, n = no, y = yes, ot = other
ev = ever, cu = current, nev = never
REF: 6-character study reference
NRR: number of the RR on the database within the study
ST : study type (CC = case control, pr or prosp = prospective)
NLC: number of lung cancer cases in whole study
R : risky occupational population (n = no, m = mining, o = other risky)
VB : national cigarette type (V = at least 75% Virginia, bl = at least 75% blended, ot = other)
P : any proxy use
H : full histological confirmation
De : derivation of RR/CI (or = original, st = standard method, ot = other method of estimation)

Table 2E9 - 1

IESLC - Meta-analysis of Current Smoking (or Ever if Current not available), Pipe only
Squamous
Most adjusted

REF	NRR	2E8	SEX	AGEL	AGEH	RACE	YF	LC TYPE	LOC	START	ST	NLC	R	VB	P	H	AD	SM	DENOM	De
HAMMON	55		m	0	0	wh	0	not a	NAmer	1952	pr	448	n	bl	n	n	1	ev	nev any	ot
LUBIN	16		m	0	0	all	-	KI	As:Chi	1984	CC	427	m	ot	y	n	0	ev	nev any	st
LUBIN2	137		m	0	0	all	-	q	Eu:mul	1976	CC	7804	n	bl	n	y	0	ev	nev any	st
WYNDE7	20	x	m	0	0	all	-	KI	NAmer	1977	CC	2085	n	bl	n	y	0	cu	nev any	st

Table 2E9 - 2

IESLC - Meta-analysis of Current Smoking (or Ever if Current not available), Pipe only
Squamous
Most adjusted

REF	NRR	SEX	AD	Number Exposed Case	Number Exposed Cont	Non-exposed Case	Non-exposed Cont	RR	95.00%CI
*HAMMON	55	m	1	-	-	-	-	8.50	(2.77- 26.07)
LUBIN	16	m	0	39	151	4	72	4.65	(1.60- 13.51)
LUBIN2	137	m	0	19	197	127	2616	1.99	(1.20- 3.29)
WYNDE7	20	m	0	6	54	22	918	4.64	(1.80- 11.91)
Partial Totals				64	402	153	3606		

*prospective study

REF	NRR	SEX	AD	Ys	Ws	Qs	Ps
*HAMMON	55	m	1	2.14	3.06	3.24	0.0002
LUBIN	16	m	0	1.54	3.38	0.61	0.0047
LUBIN2	137	m	0	0.69	15.16	2.72	0.0075
WYNDE7	20	m	0	1.53	4.32	0.78	0.0014

	N	4
	NS	4
	Wt	25.91
Het	Chi	7.35
Het	df	3
Het	P	(*)
Fixed	RR	3.03
	RRl	2.06
	RRu	4.46
	P	+++
Random	RR	3.89
	RRl	1.96
	RRu	7.71
	P	+++
Asymm	P	*

Table 2E9 - 3

IESLC - Meta-analysis of Current Smoking (or Ever if Current not available), Pipe only
Squamous
Most adjusted

	Sex combined	male	female	Total
N		4		4
NS		4		4
Wt		25.91		25.91
Het Chi		7.35		7.35
Het df		3		3
Het P		(*)		(*)
Fixed RR		3.03		3.03
RRl		2.06		2.06
RRu		4.46		4.46
P		+++		+++
Random RR		3.89		3.89
RRl		1.96		1.96
RRu		7.71		7.71
P		+++		+++
Between Chi				
Between df				
Between P				N.S.
Btwn(F) P				N.S.
Btwn(R) P				N.S.

Too few RRs for analysis by factor

Table 2E9 - 4

IESLC - Meta-analysis of Current Smoking (or Ever if Current not available), Pipe only
Squamous
Least adjusted

REF	NRR	X	SEX	AGEL	AGEH	RACE	YF	LC TYPE	LOC	START	ST	NLC	R	VB	P	H	AD	SM	DENOM	De
HAMMON	68	x	m	0	0	wh	0	not a	NAmer	1952	pr	448	n	bl	n	n	0	ev	nev any	st
LUBIN	16		m	0	0	all	-	KI	As:Chi	1984	CC	427	m	ot	y	n	0	ev	nev any	st
LUBIN2	137		m	0	0	all	-	q	Eu:mul	1976	CC	7804	n	bl	n	y	0	ev	nev any	st
WYNDE7	20		m	0	0	all	-	KI	NAmer	1977	CC	2085	n	bl	n	y	0	cu	nev any	st

Table 2E9 - 5

IESLC - Meta-analysis of Current Smoking (or Ever if Current not available), Pipe only
Squamous
Least adjusted

REF	NRR	SEX	AD	Number Exposed Case	Cont	Non-exposed Case	Cont	RR	95.00%CI
*HAMMON	68	m	0	13	43041	4	115884	8.75	(2.85- 26.83)
LUBIN	16	m	0	39	151	4	72	4.65	(1.60- 13.51)
LUBIN2	137	m	0	19	197	127	2616	1.99	(1.20- 3.29)
WYNDE7	20	m	0	6	54	22	918	4.64	(1.80- 11.91)
Totals				77	43443	157	119490		

*prospective study

REF	NRR	SEX	AD	Ys	Ws	Qs	Ps
*HAMMON	68	m	0	2.17	3.06	3.41	0.0001
LUBIN	16	m	0	1.54	3.38	0.60	0.0047
LUBIN2	137	m	0	0.69	15.16	2.76	0.0075
WYNDE7	20	m	0	1.53	4.32	0.76	0.0014

	N	4
	NS	4
	Wt	25.91
Het	Chi	7.54
Het	df	3
Het	P	(*)
Fixed	RR	3.04
	RRl	2.07
	RRu	4.47
	P	+++
Random	RR	3.93
	RRl	1.96
	RRu	7.85
	P	+++
Asymm	P	*

Table 2E9 - 6

IESLC - Meta-analysis of Current Smoking (or Ever if Current not available), Pipe only
Squamous
Least adjusted

	Sex combined	male	female	Total
N		4		4
NS		4		4
Wt		25.91		25.91
Het Chi		7.54		7.54
Het df		3		3
Het P		(*)		(*)
Fixed RR		3.04		3.04
RRl		2.07		2.07
RRu		4.47		4.47
P		+++		+++
Random RR		3.93		3.93
RRl		1.96		1.96
RRu		7.85		7.85
P		+++		+++
Between Chi				
Between df				
Between P				N.S.
Btwn(F) P				N.S.
Btwn(R) P				N.S.

Table 2E9 - 7

IESLC - Meta-analysis of Current Smoking (or Ever if Current not available), Pipe only
Squamous
Excluded studies (and stage at which they were excluded)

1 BOUCHA BUELL LAURIL MZILEN

2 ABRAHA AGUDO AKIBA ALDERS AMANDU AMES ANDERS ARCHER AUSTIN AUVINE AXELSO AXELSS BAND BARBON BECHER BERRIN
BLOHMK BLOT1 BLOT2 BLOT3 BLOT4 BRESLO BRETT BROCKM BROSS BROWN1 BROWN2 BUFFLE BYERS1 BYERS2 CARPEN CASCO2
CASCOR CHAN CHANG CHATZI CHEN CHEN2 CHEN3 CHIAZZ CHOI CHOW CHYOU COMSTO CORREA CPSII DARBY DAVEYS
DEAN2 DEKLER DESTE2 DESTEF DOCKER DORANT DORGAN DOSEME DROSTE DU DUNN EBELIN ENGELA ENSTRO ESAKI FAN
GAO GAO2 GARCIA GARSHI GENG GER GILLIS GODLEY GOLLED GOODMA GRAHAM GREGOR GSELL HAENSZ HAMMO2 HANSEN
HEGMAN HENNEK HINDS HIRAOK HIRAYA HITOSU HOLE HOROWI HORWIT HU HU2 HUANG HUMBLE ISHIMA JAHN JAIN
JARUP JARVHO JEDRYC JIANG JOLY JONES JUSSAW KAISE2 KAISER KANELL KATSOU KAUFMA KELLER KHUDER KIHARA KINLEN
KJUUS KNEKT KO KOHLME KOO KREUZE KREYBE KUBIK LAMTH LAMWK LAMWK2 LAUSSM LEI LEMARC LETOUR LIAW
LICKIN LIDDEL LIU LIU2 LIU3 LIU4 LIU5 LOMBA2 LOMBAR LUO MACLEN MAGNUS MARSH MARSH2 MARTIS MASTRA
MATOS MATSUD MCDUFF MCLAUG MIGRAN MILLER MILLS MOLLO MRFIT MRFITR MURATA NAM NOTAN2 NOTANI NOU ODRISC
ORMOS OSANN OSANN2 PARKIN PASTOR PAWLEG PERSH2 PERSHA PETO PEZZO2 PEZZOT PIKE PISANI POFFIJ POLEDN PRESCO
QIAO2 RACHTA RADZIK RANDIG REN RESTRE RIMING RONCO ROOTS ROTHSC SAARIK SANKAR SCHWAR SEGI SEGI2 SEOW
SHAW SHIMIZ SIEMIA SIMARA SIMONA SITAS SOBUE SOBUE2 SPEIZE SPITZ STASZE STAYNE STOCKW STUCKE SUN SUZUK2
SUZUKI SVENSS TANG TANG2 TAO TENKAN TOKARS TOUSEY TSUGAN ULMER VANDER VEIERO VUTUC WAKAI WALD WANG
WANG2 WANG3 WANG4 WARSIN WATSON WICKLU WIGLE WILKIN WU WU2 WUNSCH WUWILL WYNDE2 WYNDE3 WYNDE4 WYNDE5
WYNDE6 WYNDE8 WYNDER XU XU2 XU3 XU4 YAMAGU YONG YUAN ZHANG ZHENG ZHOU

3 DOLL2

5 HIRAY2 SCHWA2 TVERDA

7 ABELIN ARMADA BENHAM BENSHL BEST BOFFET BOUCOT CEDERL COOKSO CPSI DAMBER DEAN DEAN3 DOLL DORN GARDIN
HEIN KOULUM LANGE LEVIN MCCONN PERNU QIAO SADOWS STOCKS TIZZAN TULINI XIANGZ

Table 2E9 - 8

Potentially overlapping studies

REF	REFGP	PRINC	. OVERLAP/LINK
LUBIN2	LUBIN2	1	Lubin-combined
WYNDE7	WYNDE6	2	WYNDE5/6/7/8
LUBIN	XIANGZ	2	LUBIN/XIANGZ/QIAO

Table 2E10 -

IESLC - Meta-analysis of Ex Smoking, Pipe only
Squamous

This analysis is restricted to results for:
1) Non-dose-response data
2) Smokers of pipe only
3) Ex smokers
4) Results complete enough for use in metaanalysis

Within each study, results are then selected (in the following order of preference, within each sex) for:
5) DENOM: never smoked anything, (never +1 = +long term ex)
6) Followup period (prospective studies): whole study (coded as 0) or longest available
7) LCtype: all or nearest available, at least Squamous and Adeno. (q = squamous, s = small,
l = large, a = adeno, mix = mixed, alv = alveolar)
8) Race: all or nearest available, otherwise by race (wh or w = white, bl or b = black, hi = hispanic
ch = chinese, jap = japanese, haw = hawaiian, w+o = white + oriental, sca = scandinavian, as = asian)
9) For overlapping studies: principal rather than subsidiary studies
Finally by Age: whole study (coded as 0) if available, otherwise by widest available age group
and then for single sex results (m, f) in preference to combined sex results (c).

Results adjusted (AD) for the most potential confounders are then chosen in Sections -1 to -3
and results adjusted for the least confounders in Sections -4 to -6. (Those least adjusted results which
actually differ from the most adjusted as marked 'x' in column X in Section -4)
(Results adjusted for an unknown number of confounder(s) are coded as 20.)

Section -7 shows excluded studies, together with the stage (as above) at which no qualifying
results were found.

Section -8 lists the potentially overlapping studies which have been included (1=principal, 2=subsidiary).

Section -9 lists any results which would have been included in preference except that they had data not complete
enough for use in meta-analysis, with their significance (yes/no), if known, and any further comment as entered
on the database.

In addition to those mentioned above, the following fields, levels and abbreviations are used:
* or nk = not known, n = no, y = yes, ot = other
nev = never
REF: 6-character study reference
NRR: number of the RR on the database within the study
ST : study type (CC = case control, pr or prosp = prospective)
NLC: number of lung cancer cases in whole study
R : risky occupational population (n = no, m = mining, o = other risky)
VB : national cigarette type (V = at least 75% Virginia, bl = at least 75% blended, ot = other)
P : any proxy use
H : full histological confirmation
De : derivation of RR/CI (or = original, st = standard method, ot = other method of estimation)

Table 2E10 - 1

IESLC - Meta-analysis of Ex Smoking, Pipe only
Squamous
Most adjusted

REF	NRR	SEX	AGEL	AGEH	RACE	YF	LC TYPE	LOC	START	ST	NLC	R	VB	P	H	AD	DENOM	De
WYNDE7	25	m	0	0	all	-	KI	NAmer	1977	CC	2085	n	bl	n	y	0	nev	any st

Table 2E10 - 2

IESLC - Meta-analysis of Ex Smoking, Pipe only
Squamous
Most adjusted

REF	NRR	SEX	AD	Number Exposed Case	Cont	Non-exposed Case	Cont	RR	95.00%CI
WYNDE7	25	m	0	1	43	22	918	0.97	(0.13- 7.37)
Totals				1	43	22	918		

*prospective study

REF	NRR	SEX	AD	Ys	Ws	Qs	Ps
WYNDE7	25	m	0	-0.03	0.93	0.00	0.9768

	N	1
	NS	1
	Wt	0.93
Het	Chi	0.00
Het	df	0
Het	P	N.S.
Fixed	RR	0.97
	RRl	0.13
	RRu	7.37
	P	N.S.
Random	RR	0.97
	RRl	0.13
	RRu	7.37
	P	N.S.
Asymm	P	

Table 2E10 - 3

IESLC - Meta-analysis of Ex Smoking, Pipe only
Squamous
Most adjusted

	Sex combined	male	female	Total
N		1		1
NS		1		1
Wt		0.93		0.93
Het Chi		0.00		0.00
Het df		0		0
Het P		N.S.		N.S.
Fixed RR		0.97		0.97
RRl		0.13		0.13
RRu		7.37		7.37
P		N.S.		N.S.
Random RR		0.97		0.97
RRl		0.13		0.13
RRu		7.37		7.37
P		N.S.		N.S.
Between Chi				
Between df				
Between P				N.S.
Btwn(F) P				N.S.
Btwn(R) P				N.S.

Too few RRs for analysis by factor

Table 2E10 - 4

IESLC - Meta-analysis of Ex Smoking, Pipe only
Squamous
Least adjusted

REF	NRR	X	SEX	AGEL	AGEH	RACE	YF	LC TYPE	LOC	START	ST	NLC	R	VB	P	H	AD	DENOM	De
WYNDE7	25		m	0	0	all	-	KI	NAmer	1977	CC	2085	n	bl	n	y	0	nev any	st

Table 2E10 - 5

IESLC - Meta-analysis of Ex Smoking, Pipe only
Squamous
Least adjusted

REF	NRR	SEX	AD	Number Exposed Case	Number Exposed Cont	Non-exposed Case	Non-exposed Cont	RR	95.00%CI
WYNDE7	25	m	0	1	43	22	918	0.97	(0.13- 7.37)
Totals				1	43	22	918		

*prospective study

REF	NRR	SEX	AD	Ys	Ws	Qs	Ps
WYNDE7	25	m	0	-0.03	0.93	0.00	0.9768

	N	1
	NS	1
	Wt	0.93
Het	Chi	0.00
Het	df	0
Het	P	N.S.
Fixed	RR	0.97
	RRl	0.13
	RRu	7.37
	P	N.S.
Random	RR	0.97
	RRl	0.13
	RRu	7.37
	P	N.S.
Asymm	P	

Table 2E10 - 6

IESLC - Meta-analysis of Ex Smoking, Pipe only
Squamous
Least adjusted

	Sex combined	male	female	Total
N		1		1
NS		1		1
Wt		0.93		0.93
Het Chi		0.00		0.00
Het df		0		0
Het P		N.S.		N.S.
Fixed RR		0.97		0.97
RRl		0.13		0.13
RRu		7.37		7.37
P		N.S.		N.S.
Random RR		0.97		0.97
RRl		0.13		0.13
RRu		7.37		7.37
P		N.S.		N.S.
Between Chi				
Between df				
Between P				N.S.
Btwn(F) P				N.S.
Btwn(R) P				N.S.

Table 2E10 - 7

IESLC - Meta-analysis of Ex Smoking, Pipe only
Squamous
Excluded studies (and stage at which they were excluded)

1	BOUCHA BUELL LAURIL MZILEN
2	ABRAHA AGUDO AKIBA ALDERS AMANDU AMES ANDERS ARCHER AUSTIN AUVINE AXELSO AXELSS BAND BARBON BECHER BERRIN BLOHMK BLOT1 BLOT2 BLOT3 BLOT4 BRESLO BRETT BROCKM BROSS BROWN1 BROWN2 BUFFLE BYERS1 BYERS2 CARPEN CASCO2 CASCOR CHAN CHANG CHATZI CHEN CHEN2 CHEN3 CHIAZZ CHOI CHOW CHYOU COMSTO CORREA CPSII DARBY DAVEYS DEAN2 DEKLER DESTE2 DESTEF DOCKER DORANT DORGAN DOSEME DROSTE DU DUNN EBELIN ENGELA ENSTRO ESAKI FAN GAO GAO2 GARCIA GARSHI GENG GER GILLIS GODLEY GOLLED GOODMA GRAHAM GREGOR GSELL HAENSZ HAMMO2 HANSEN HEGMAN HENNEK HINDS HIRAOK HIRAYA HITOSU HOLE HOROWI HORWIT HU HU2 HUANG HUMBLE ISHIMA JAHN JAIN JARUP JARVHO JEDRYC JIANG JOLY JONES JUSSAW KAISE2 KAISER KANELL KATSOU KAUFMA KELLER KHUDER KIHARA KINLEN KJUUS KNEKT KO KOHLME KOO KREUZE KREYBE KUBIK LAMTH LAMWK LAMWK2 LAUSSM LEI LEMARC LETOUR LIAW LICKIN LIDDEL LIU LIU2 LIU3 LIU4 LIU5 LOMBA2 LOMBAR LUO MACLEN MAGNUS MARSH MARSH2 MARTIS MASTRA MATOS MATSUD MCDUFF MCLAUG MIGRAN MILLER MILLS MOLLO MRFIT MRFITR MURATA NAM NOTAN2 NOTANI NOU ODRISC ORMOS OSANN OSANN2 PARKIN PASTOR PAWLEG PERSH2 PERSHA PETO PEZZO2 PEZZOT PIKE PISANI POFFIJ POLEDN PRESCO QIAO2 RACHTA RADZIK RANDIG REN RESTRE RIMING RONCO ROOTS ROTHSC SAARIK SANKAR SCHWAR SEGI SEGI2 SEOW SHAW SHIMIZ SIEMIA SIMARA SIMONA SITAS SOBUE SOBUE2 SPEIZE SPITZ STASZE STAYNE STOCKW STUCKE SUN SUZUK2 SUZUKI SVENSS TANG TANG2 TAO TENKAN TOKARS TOUSEY TSUGAN ULMER VANDER VEIERO VUTUC WAKAI WALD WANG WANG2 WANG3 WANG4 WARSIN WATSON WICKLU WIGLE WILKIN WU WU2 WUNSCH WUWILL WYNDE2 WYNDE3 WYNDE4 WYNDE5 WYNDE6 WYNDE8 WYNDER XU XU2 XU3 XU4 YAMAGU YONG YUAN ZHANG ZHENG ZHOU
3	ABELIN ARMADA BENHAM BENSHL BEST BOUCOT CEDERL COOKSO CPSI DEAN DEAN3 DOLL DOLL2 GARDIN HAMMON HEIN HIRAY2 KOULUM LANGE LEVIN LUBIN MCCONN PERNU QIAO SADOWS SCHWA2 STOCKS TIZZAN TULINI TVERDA XIANGZ
7	BOFFET DAMBER DORN LUBIN2

Table 2E10 - 8
Potentially overlapping studies

REF	REFGP	PRINC	.	OVERLAP/LINK
WYNDE7	WYNDE6	2		WYNDE5/6/7/8

Table 2E11 -

IESLC - Meta-analysis of Ever Smoking, Cigars only
Squamous

This analysis is restricted to results for:
1) Non-dose-response data
2) Smokers of cigars only
3) Ever smokers
4) Results complete enough for use in metaanalysis

Within each study, results are then selected (in the following order of preference, within each sex) for:
5) DENOM: never smoked anything, (never +1 = +long term ex)
6) Followup period (prospective studies): whole study (coded as 0) or longest available
7) LCtype: all or nearest available, at least Squamous and Adeno. (q = squamous, s = small,
l = large, a = adeno, mix = mixed, alv = alveolar)
8) Race: all or nearest available, otherwise by race (wh or w = white, bl or b = black, hi = hispanic
ch = chinese, jap = japanese, haw = hawaiian, w+o = white + oriental, sca = scandinavian, as = asian)
9) For overlapping studies: principal rather than subsidiary studies
Finally by Age: whole study (coded as 0) if available, otherwise by widest available age group
and then for single sex results (m, f) in preference to combined sex results (c).

Results adjusted (AD) for the most potential confounders are then chosen in Sections -1 to -3
and results adjusted for the least confounders in Sections -4 to -6. (Those least adjusted results which
actually differ from the most adjusted as marked 'x' in column X in Section -4)
(Results adjusted for an unknown number of confounder(s) are coded as 20.)

Section -7 shows excluded studies, together with the stage (as above) at which no qualifying
results were found.

Section -8 lists the potentially overlapping studies which have been included (1=principal, 2=subsidiary).

Section -9 lists any results which would have been included in preference except that they had data not complete
enough for use in meta-analysis, with their significance (yes/no), if known, and any further comment as entered
on the database.

In addition to those mentioned above, the following fields, levels and abbreviations are used:
* or nk = not known, n = no, y = yes, ot = other
nev = never
REF: 6-character study reference
NRR: number of the RR on the database within the study
ST : study type (CC = case control, pr or prosp = prospective)
NLC: number of lung cancer cases in whole study
R : risky occupational population (n = no, m = mining, o = other risky)
VB : national cigarette type (V = at least 75% Virginia, bl = at least 75% blended, ot = other)
P : any proxy use
H : full histological confirmation
De : derivation of RR/CI (or = original, st = standard method, ot = other method of estimation)

Table 2E11 - 1

IESLC - Meta-analysis of Ever Smoking, Cigars only
Squamous
Most adjusted

REF	NRR	SEX	AGEL	AGEH	RACE	YF	LC TYPE	LOC	START	ST	NLC	R	VB	P	H	AD	DENOM	De
HAMMON	54	m	0	0	wh	0	not a	NAmer	1952	pr	448	n	bl	n	n	1	nev any	ot
LUBIN2	133	m	0	0	all	-	q	Eu:mul	1976	CC	7804	n	bl	n	y	0	nev any	st
WYNDE7	56	m	0	0	all	-	KI	NAmer	1977	CC	2085	n	bl	n	y	0	nev any	st

Table 2E11 - 2

IESLC - Meta-analysis of Ever Smoking, Cigars only
Squamous
Most adjusted

REF	NRR	SEX	AD	Number Exposed Case	Number Exposed Cont	Non-exposed Case	Non-exposed Cont	RR	95.00%CI
*HAMMON	54	m	1	-	-	-	-	3.35	(0.95- 11.88)
LUBIN2	133	m	0	21	145	127	2616	2.98	(1.83- 4.87)
WYNDE7	56	m	0	21	152	22	918	5.76	(3.09- 10.74)
Partial Totals				42	297	149	3534		

*prospective study

REF	NRR	SEX	AD	Ys	Ws	Qs	Ps
*HAMMON	54	m	1	1.21	2.41	0.04	0.0607
LUBIN2	133	m	0	1.09	15.93	0.93	0.0000
WYNDE7	56	m	0	1.75	9.93	1.73	0.0000

	N	3
	NS	3
	Wt	28.26
Het	Chi	2.70
Het	df	2
Het	P	N.S.
Fixed	RR	3.80
	RRl	2.63
	RRu	5.49
	P	+++
Random	RR	3.87
	RRl	2.45
	RRu	6.12
	P	+++
Asymm	P	N.S.

Table 2E11 - 3

IESLC - Meta-analysis of Ever Smoking, Cigars only
Squamous
Most adjusted

	combined	Sex male	female	Total
N		3		3
NS		3		3
Wt		28.26		28.26
Het Chi		2.70		2.70
Het df		2		2
Het P		N.S.		N.S.
Fixed RR		3.80		3.80
RRl		2.63		2.63
RRu		5.49		5.49
P		+++		+++
Random RR		3.87		3.87
RRl		2.45		2.45
RRu		6.12		6.12
P		+++		+++
Between Chi				
Between df				
Between P				N.S.
Btwn(F) P				N.S.
Btwn(R) P				N.S.

Too few RRs for analysis by factor

Table 2E11 - 4

IESLC - Meta-analysis of Ever Smoking, Cigars only
Squamous
Least adjusted

REF	NRR	X	SEX	AGEL	AGEH	RACE	YF	LC TYPE	LOC	START	ST	NLC	R	VB	P	H	AD	DENOM	De
HAMMON	67	x	m	0	0	wh	0	not a	NAmer	1952	pr	448	n	bl	n	n	0	nev any	st
LUBIN2	133		m	0	0	all	-	q	Eu:mul	1976	CC	7804	n	bl	n	y	0	nev any	st
WYNDE7	56		m	0	0	all	-	KI	NAmer	1977	CC	2085	n	bl	n	y	0	nev any	st

Table 2E11 - 5

IESLC - Meta-analysis of Ever Smoking, Cigars only
Squamous
Least adjusted

REF	NRR	SEX	AD	Number Exposed Case	Cont	Non-exposed Case	Cont	RR	95.00%CI
*HAMMON	67	m	0	6	51480	4	115884	3.38	(0.95- 11.96)
LUBIN2	133	m	0	21	145	127	2616	2.98	(1.83- 4.87)
WYNDE7	56	m	0	21	152	22	918	5.76	(3.09- 10.74)
Totals				48	51777	153	119418		

*prospective study

REF	NRR	SEX	AD	Ys	Ws	Qs	Ps
*HAMMON	67	m	0	1.22	2.40	0.03	0.0594
LUBIN2	133	m	0	1.09	15.93	0.93	0.0000
WYNDE7	56	m	0	1.75	9.93	1.72	0.0000

	N	3
	NS	3
	Wt	28.26
Het	Chi	2.69
Het	df	2
Het	P	N.S.
Fixed	RR	3.80
	RRl	2.63
	RRu	5.49
	P	+++
Random	RR	3.87
	RRl	2.45
	RRu	6.12
	P	+++
Asymm	P	N.S.

Table 2E11 - 6

IESLC - Meta-analysis of Ever Smoking, Cigars only
Squamous
Least adjusted

	Sex combined	male	female	Total
N		3		3
NS		3		3
Wt		28.26		28.26
Het Chi		2.69		2.69
Het df		2		2
Het P		N.S.		N.S.
Fixed RR		3.80		3.80
RRl		2.63		2.63
RRu		5.49		5.49
P		+++		+++
Random RR		3.87		3.87
RRl		2.45		2.45
RRu		6.12		6.12
P		+++		+++
Between Chi				
Between df				
Between P				N.S.
Btwn(F) P				N.S.
Btwn(R) P				N.S.

Table 2E11 - 7

IESLC - Meta-analysis of Ever Smoking, Cigars only
Squamous
Excluded studies (and stage at which they were excluded)

1	BOUCHA BUELL LAURIL MZILEN
2	ABRAHA AGUDO AKIBA ALDERS AMANDU AMES ANDERS ARCHER AUSTIN AUVINE AXELSO AXELSS BAND BARBON BECHER BERRIN BLOHMK BLOT1 BLOT2 BLOT3 BLOT4 BRESLO BRETT BROCKM BROSS BROWN1 BROWN2 BUFFLE BYERS1 BYERS2 CARPEN CASCO2 CASCOR CHAN CHANG CHATZI CHEN CHEN2 CHEN3 CHIAZZ CHOI CHOW CHYOU COMSTO COOKSO CORREA DARBY DAVEYS DEAN DEAN2 DEKLER DESTE2 DESTEF DOCKER DOLL DOLL2 DORANT DORGAN DOSEME DROSTE DU DUNN EBELIN ENGELA ENSTRO ESAKI FAN GAO GAO2 GARCIA GARSHI GENG GER GILLIS GODLEY GOLLED GOODMA GRAHAM GREGOR GSELL HAENSZ HAMMO2 HANSEN HEGMAN HENNEK HINDS HIRAOK HIRAY2 HIRAYA HITOSU HOLE HOROWI HORWIT HU HU2 HUANG HUMBLE ISHIMA JAHN JAIN JARUP JARVHO JEDRYC JIANG JONES JUSSAW KAISE2 KAISER KANELL KATSOU KAUFMA KELLER KHUDER KIHARA KINLEN KJUUS KNEKT KO KOHLME KOO KOULUM KREUZE KREYBE KUBIK LAMTH LAMWK LAMWK2 LAUSSM LEI LEMARC LETOUR LIAW LICKIN LIDDEL LIU LIU2 LIU3 LIU4 LIU5 LOMBA2 LOMBAR LUBIN LUO MACLEN MAGNUS MARSH MARSH2 MARTIS MASTRA MATOS MATSUD MCCONN MCDUFF MCLAUG MIGRAN MILLER MILLS MOLLO MRFIT MRFITR MURATA NAM NOTAN2 NOTANI NOU ODRISC ORMOS OSANN OSANN2 PARKIN PASTOR PAWLEG PERNU PERSH2 PERSHA PETO PEZZO2 PEZZOT PIKE PISANI POFFIJ POLEDN PRESCO QIAO QIAO2 RACHTA RADZIK RANDIG REN RESTRE RIMING RONCO ROOTS ROTHSC SAARIK SANKAR SCHWA2 SCHWAR SEGI SEGI2 SEOW SHAW SHIMIZ SIEMIA SIMARA SIMONA SITAS SOBUE SOBUE2 SPEIZE SPITZ STASZE STAYNE STOCKS STOCKW STUCKE SUN SUZUK2 SUZUKI SVENSS TANG TANG2 TAO TENKAN TOKARS TOUSEY TSUGAN TVERDA ULMER VANDER VEIERO VUTUC WAKAI WALD WANG WANG2 WANG3 WANG4 WARSIN WATSON WICKLU WIGLE WILKIN WU WU2 WUNSCH WUWILL WYNDE2 WYNDE3 WYNDE4 WYNDE5 WYNDE6 WYNDE8 XIANGZ XU XU2 XU3 XU4 YAMAGU YONG YUAN ZHANG ZHENG ZHOU
3	BENSHL CEDERL CPSII DEAN3 GARDIN HEIN LANGE TULINI WYNDER
7	ABELIN ARMADA BENHAM BEST BOFFET BOUCOT CPSI DAMBER DORN JOLY LEVIN SADOWS TIZZAN

Table 2E11 - 8

Potentially overlapping studies

REF	REFGP	PRINC	.	OVERLAP/LINK
LUBIN2	LUBIN2	1		Lubin-combined
WYNDE7	WYNDE6	2		WYNDE5/6/7/8

Table 2E12 -

IESLC - Meta-analysis of Current Smoking, Cigars only
Squamous

This analysis is restricted to results for:
1) Non-dose-response data
2) Smokers of cigars only
3) Current smokers
4) Results complete enough for use in metaanalysis

Within each study, results are then selected (in the following order of preference, within each sex) for:
5) DENOM: never smoked anything, (never +1 = +long term ex)
6) Followup period (prospective studies): whole study (coded as 0) or longest available
7) LCtype: all or nearest available, at least Squamous and Adeno. (q = squamous, s = small,
l = large, a = adeno, mix = mixed, alv = alveolar)
8) Race: all or nearest available, otherwise by race (wh or w = white, bl or b = black, hi = hispanic
ch = chinese, jap = japanese, haw = hawaiian, w+o = white + oriental, sca = scandinavian, as = asian)
9) For overlapping studies: principal rather than subsidiary studies
Finally by Age: whole study (coded as 0) if available, otherwise by widest available age group
and then for single sex results (m, f) in preference to combined sex results (c).

Results adjusted (AD) for the most potential confounders are then chosen in Sections -1 to -3
and results adjusted for the least confounders in Sections -4 to -6. (Those least adjusted results which
actually differ from the most adjusted as marked 'x' in column X in Section -4)
(Results adjusted for an unknown number of confounder(s) are coded as 20.)

Section -7 shows excluded studies, together with the stage (as above) at which no qualifying
results were found.

Section -8 lists the potentially overlapping studies which have been included (1=principal, 2=subsidiary).

Section -9 lists any results which would have been included in preference except that they had data not complete
enough for use in meta-analysis, with their significance (yes/no), if known, and any further comment as entered
on the database.

In addition to those mentioned above, the following fields, levels and abbreviations are used:
* or nk = not known, n = no, y = yes, ot = other
nev = never
REF: 6-character study reference
NRR: number of the RR on the database within the study
ST : study type (CC = case control, pr or prosp = prospective)
NLC: number of lung cancer cases in whole study
R : risky occupational population (n = no, m = mining, o = other risky)
VB : national cigarette type (V = at least 75% Virginia, bl = at least 75% blended, ot = other)
P : any proxy use
H : full histological confirmation
De : derivation of RR/CI (or = original, st = standard method, ot = other method of estimation)

Table 2E12 - 1

IESLC - Meta-analysis of Current Smoking, Cigars only
Squamous
Most adjusted

REF	NRR	SEX	AGEL	AGEH	RACE	YF	LC TYPE	LOC	START	ST	NLC	R	VB	P	H	AD	DENOM	De
WYNDE7	19	m	0	0	all	-	KI	NAmer	1977	CC	2085	n	bl	n	y	0	nev any	st

Table 2E12 - 2

IESLC - Meta-analysis of Current Smoking, Cigars only
Squamous
Most adjusted

REF	NRR	SEX	AD	Number Case	Exposed Cont	Non-exposed Case	Cont	RR	95.00%CI
WYNDE7	19	m	0	15	82	22	918	7.63	(3.81- 15.28)
Totals				15	82	22	918		

*prospective study

REF	NRR	SEX	AD	Ys	Ws	Qs	Ps
WYNDE7	19	m	0	2.03	7.97	0.00	0.0000

	N	1
	NS	1
	Wt	7.97
Het	Chi	0.00
Het	df	0
Het	P	N.S.
Fixed	RR	7.63
	RRl	3.81
	RRu	15.28
	P	+++
Random	RR	7.63
	RRl	3.81
	RRu	15.28
	P	+++
Asymm	P	

Table 2E12 - 3

IESLC - Meta-analysis of Current Smoking, Cigars only
Squamous
Most adjusted

	Sex combined	male	female	Total
N		1		1
NS		1		1
Wt		7.97		7.97
Het Chi		0.00		0.00
Het df		0		0
Het P		N.S.		N.S.
Fixed RR		7.63		7.63
RRl		3.81		3.81
RRu		15.28		15.28
P		+++		+++
Random RR		7.63		7.63
RRl		3.81		3.81
RRu		15.28		15.28
P		+++		+++
Between Chi				
Between df				
Between P				N.S.
Btwn(F) P				N.S.
Btwn(R) P				N.S.

Too few RRs for analysis by factor

Table 2E12 - 4

IESLC - Meta-analysis of Current Smoking, Cigars only
Squamous
Least adjusted

REF	NRR	X	SEX	AGEL	AGEH	RACE	YF	LC TYPE	LOC	START	ST	NLC	R	VB	P	H	AD	DENOM	De
WYNDE7	19		m	0	0	all	-	KI	NAmer	1977	CC	2085	n	bl	n	y	0	nev any	st

Table 2E12 - 5

IESLC - Meta-analysis of Current Smoking, Cigars only
Squamous
Least adjusted

REF	NRR	SEX	AD	Number Exposed Case	Number Exposed Cont	Non-exposed Case	Non-exposed Cont	RR	95.00%CI
WYNDE7	19	m	0	15	82	22	918	7.63	(3.81- 15.28)
Totals				15	82	22	918		

*prospective study

REF	NRR	SEX	AD	Ys	Ws	Qs	Ps
WYNDE7	19	m	0	2.03	7.97	0.00	0.0000

	N	1
	NS	1
	Wt	7.97
Het	Chi	0.00
Het	df	0
Het	P	N.S.
Fixed	RR	7.63
	RRl	3.81
	RRu	15.28
	P	+++
Random	RR	7.63
	RRl	3.81
	RRu	15.28
	P	+++
Asymm	P	

Table 2E12 - 6

IESLC - Meta-analysis of Current Smoking, Cigars only
Squamous
Least adjusted

	Sex			
	combined	male	female	Total
N		1		1
NS		1		1
Wt		7.97		7.97
Het Chi		0.00		0.00
Het df		0		0
Het P		N.S.		N.S.
Fixed RR		7.63		7.63
RRl		3.81		3.81
RRu		15.28		15.28
P		+++		+++
Random RR		7.63		7.63
RRl		3.81		3.81
RRu		15.28		15.28
P		+++		+++
Between Chi				
Between df				
Between P				N.S.
Btwn(F) P				N.S.
Btwn(R) P				N.S.

Table 2E12 - 7

IESLC - Meta-analysis of Current Smoking, Cigars only
Squamous
Excluded studies (and stage at which they were excluded)

1	BOUCHA BUELL LAURIL MZILEN
2	ABRAHA AGUDO AKIBA ALDERS AMANDU AMES ANDERS ARCHER AUSTIN AUVINE AXELSO AXELSS BAND BARBON BECHER BERRIN BLOHMK BLOT1 BLOT2 BLOT3 BLOT4 BRESLO BRETT BROCKM BROSS BROWN1 BROWN2 BUFFLE BYERS1 BYERS2 CARPEN CASCO2 CASCOR CHAN CHANG CHATZI CHEN CHEN2 CHEN3 CHIAZZ CHOI CHOW CHYOU COMSTO COOKSO CORREA DARBY DAVEYS DEAN DEAN2 DEKLER DESTE2 DESTEF DOCKER DOLL DOLL2 DORANT DORGAN DOSEME DROSTE DU DUNN EBELIN ENGELA ENSTRO ESAKI FAN GAO GAO2 GARCIA GARSHI GENG GER GILLIS GODLEY GOLLED GOODMA GRAHAM GREGOR GSELL HAENSZ HAMMO2 HANSEN HEGMAN HENNEK HINDS HIRAOK HIRAY2 HIRAYA HITOSU HOLE HOROWI HORWIT HU HU2 HUANG HUMBLE ISHIMA JAHN JAIN JARUP JARVHO JEDRYC JIANG JONES JUSSAW KAISE2 KAISER KANELL KATSOU KAUFMA KELLER KHUDER KIHARA KINLEN KJUUS KNEKT KO KOHLME KOO KOULUM KREUZE KREYBE KUBIK LAMTH LAMWK LAMWK2 LAUSSM LEI LEMARC LETOUR LIAW LICKIN LIDDEL LIU LIU2 LIU3 LIU4 LIU5 LOMBA2 LOMBAR LUBIN LUO MACLEN MAGNUS MARSH MARSH2 MARTIS MASTRA MATOS MATSUD MCCONN MCDUFF MCLAUG MIGRAN MILLER MILLS MOLLO MRFIT MRFITR MURATA NAM NOTAN2 NOTANI NOU ODRISC ORMOS OSANN OSANN2 PARKIN PASTOR PAWLEG PERNU PERSH2 PERSHA PETO PEZZO2 PEZZOT PIKE PISANI POFFIJ POLEDN PRESCO QIAO QIAO2 RACHTA RADZIK RANDIG REN RESTRE RIMING RONCO ROOTS ROTHSC SAARIK SANKAR SCHWA2 SCHWAR SEGI SEGI2 SEOW SHAW SHIMIZ SIEMIA SIMARA SIMONA SITAS SOBUE SOBUE2 SPEIZE SPITZ STASZE STAYNE STOCKS STOCKW STUCKE SUN SUZUK2 SUZUKI SVENSS TANG TANG2 TAO TENKAN TOKARS TOUSEY TSUGAN TVERDA ULMER VANDER VEIERO VUTUC WAKAI WALD WANG WANG2 WANG3 WANG4 WARSIN WATSON WICKLU WIGLE WILKIN WU WU2 WUNSCH WUWILL WYNDE2 WYNDE3 WYNDE4 WYNDE5 WYNDE6 WYNDE8 XIANGZ XU XU2 XU3 XU4 YAMAGU YONG YUAN ZHANG ZHENG ZHOU
3	ABELIN ARMADA BENHAM BEST BOUCOT DAMBER HAMMON LEVIN SADOWS TIZZAN
5	WYNDER
7	BENSHL BOFFET CEDERL CPSI CPSII DEAN3 DORN GARDIN HEIN JOLY LANGE LUBIN2 TULINI

Table 2E12 - 8
Potentially overlapping studies

REF	REFGP	PRINC	.	OVERLAP/LINK
WYNDE7	WYNDE6	2		WYNDE5/6/7/8

Table 2E13 -

IESLC - Meta-analysis of Ever Smoking (or Current if Ever not available), Cigars only
Squamous

This analysis is restricted to results for:
1) Non-dose-response data
2) Smokers of cigars only
3) Results complete enough for use in metaanalysis

Within each study, results are then selected (in the following order of preference, within each sex) for:
4) SMKSTA: ever smokers, current smokers
5) DENOM: never smoked anything, (never +1 = +long term ex)
6) Followup period (prospective studies): whole study (coded as 0) or longest available
7) LCtype: all or nearest available, at least Squamous and Adeno. (q = squamous, s = small,
l = large, a = adeno, mix = mixed, alv = alveolar)
8) Race: all or nearest available, otherwise by race (wh or w = white, bl or b = black, hi = hispanic
ch = chinese, jap = japanese, haw = hawaiian, w+o = white + oriental, sca = scandinavian, as = asian)
9) For overlapping studies: principal rather than subsidiary studies
Finally by Age: whole study (coded as 0) if available, otherwise by widest available age group
and then for single sex results (m, f) in preference to combined sex results (c).

Results adjusted (AD) for the most potential confounders are then chosen in Sections -1 to -3
and results adjusted for the least confounders in Sections -4 to -6. (Those least adjusted results which
actually differ from the most adjusted as marked 'x' in column X in Section -4)
(Results adjusted for an unknown number of confounder(s) are coded as 20.)

Section -7 shows excluded studies, together with the stage (as above) at which no qualifying
results were found.

Section -8 lists the potentially overlapping studies which have been included (1=principal, 2=subsidiary).

Section -9 lists any results which would have been included in preference except that they had data not complete
enough for use in meta-analysis, with their significance (yes/no), if known, and any further comment as entered
on the database.

In addition to those mentioned above, the following fields, levels and abbreviations are used:
* or nk = not known, n = no, y = yes, ot = other
ev = ever, cu = current, nev = never
REF: 6-character study reference
NRR: number of the RR on the database within the study
ST : study type (CC = case control, pr or prosp = prospective)
NLC: number of lung cancer cases in whole study
R : risky occupational population (n = no, m = mining, o = other risky)
VB : national cigarette type (V = at least 75% Virginia, bl = at least 75% blended, ot = other)
P : any proxy use
H : full histological confirmation
De : derivation of RR/CI (or = original, st = standard method, ot = other method of estimation)

Table 2E13 - 1

IESLC - Meta-analysis of Ever Smoking (or Current if Ever not available), Cigars only
Squamous
Most adjusted

REF	NRR	SEX	AGEL	AGEH	RACE	YF	LC TYPE	LOC	START	ST	NLC	R	VB	P	H	AD	SM	DENOM	De
HAMMON	54	m	0	0	wh	0	not a	NAmer	1952	pr	448	n	bl	n	n	1	ev	nev any	ot
LUBIN2	133	m	0	0	all	-	q	Eu:mul	1976	CC	7804	n	bl	n	y	0	ev	nev any	st
WYNDE7	56	m	0	0	all	-	KI	NAmer	1977	CC	2085	n	bl	n	y	0	ev	nev any	st

Table 2E13 - 2

IESLC - Meta-analysis of Ever Smoking (or Current if Ever not available), Cigars only
Squamous
Most adjusted

REF	NRR	SEX	AD	Number Exposed Case	Cont	Non-exposed Case	Cont	RR	95.00%CI
*HAMMON	54	m	1	-	-	-	-	3.35	(0.95- 11.88)
LUBIN2	133	m	0	21	145	127	2616	2.98	(1.83- 4.87)
WYNDE7	56	m	0	21	152	22	918	5.76	(3.09- 10.74)
Partial Totals				42	297	149	3534		

*prospective study

REF	NRR	SEX	AD	Ys	Ws	Qs	Ps
*HAMMON	54	m	1	1.21	2.41	0.04	0.0607
LUBIN2	133	m	0	1.09	15.93	0.93	0.0000
WYNDE7	56	m	0	1.75	9.93	1.73	0.0000

	N	3
	NS	3
	Wt	28.26
Het	Chi	2.70
Het	df	2
Het	P	N.S.
Fixed	RR	3.80
	RRl	2.63
	RRu	5.49
	P	+++
Random	RR	3.87
	RRl	2.45
	RRu	6.12
	P	+++
Asymm	P	N.S.

Table 2E13 - 3

IESLC - Meta-analysis of Ever Smoking (or Current if Ever not available), Cigars only
Squamous
Most adjusted

	combined	Sex: male	female	Total
N		3		3
NS		3		3
Wt		28.26		28.26
Het Chi		2.70		2.70
Het df		2		2
Het P		N.S.		N.S.
Fixed RR		3.80		3.80
RRl		2.63		2.63
RRu		5.49		5.49
P		+++		+++
Random RR		3.87		3.87
RRl		2.45		2.45
RRu		6.12		6.12
P		+++		+++
Between Chi				
Between df				
Between P				N.S.
Btwn(F) P				N.S.
Btwn(R) P				N.S.

Too few RRs for analysis by factor

Table 2E13 - 4

IESLC - Meta-analysis of Ever Smoking (or Current if Ever not available), Cigars only
Squamous
Least adjusted

REF	NRR	X	SEX	AGEL	AGEH	RACE	YF	LC TYPE	LOC	START	ST	NLC	R	VB	P	H	AD	SM	DENOM	De
HAMMON	67	x	m	0	0	wh	0	not a	NAmer	1952	pr	448	n	bl	n	n	0	ev	nev any	st
LUBIN2	133		m	0	0	all	-	q	Eu:mul	1976	CC	7804	n	bl	n	y	0	ev	nev any	st
WYNDE7	56		m	0	0	all	-	KI	NAmer	1977	CC	2085	n	bl	n	y	0	ev	nev any	st

Table 2E13 - 5

IESLC - Meta-analysis of Ever Smoking (or Current if Ever not available), Cigars only
Squamous
Least adjusted

REF	NRR	SEX	AD	Number Exposed Case	Number Exposed Cont	Non-exposed Case	Non-exposed Cont	RR	95.00%CI
*HAMMON	67	m	0	6	51480	4	115884	3.38	(0.95- 11.96)
LUBIN2	133	m	0	21	145	127	2616	2.98	(1.83- 4.87)
WYNDE7	56	m	0	21	152	22	918	5.76	(3.09- 10.74)
Totals				48	51777	153	119418		

*prospective study

REF	NRR	SEX	AD	Ys	Ws	Qs	Ps
*HAMMON	67	m	0	1.22	2.40	0.03	0.0594
LUBIN2	133	m	0	1.09	15.93	0.93	0.0000
WYNDE7	56	m	0	1.75	9.93	1.72	0.0000

	N	3
	NS	3
	Wt	28.26
Het	Chi	2.69
Het	df	2
Het	P	N.S.
Fixed	RR	3.80
	RRl	2.63
	RRu	5.49
	P	+++
Random	RR	3.87
	RRl	2.45
	RRu	6.12
	P	+++
Asymm	P	N.S.

Table 2E13 - 6

IESLC - Meta-analysis of Ever Smoking (or Current if Ever not available), Cigars only
Squamous
Least adjusted

	Sex combined	male	female	Total
N		3		3
NS		3		3
Wt		28.26		28.26
Het Chi		2.69		2.69
Het df		2		2
Het P		N.S.		N.S.
Fixed RR		3.80		3.80
RRl		2.63		2.63
RRu		5.49		5.49
P		+++		+++
Random RR		3.87		3.87
RRl		2.45		2.45
RRu		6.12		6.12
P		+++		+++
Between Chi				
Between df				
Between P				N.S.
Btwn(F) P				N.S.
Btwn(R) P				N.S.

Table 2E13 - 7

IESLC - Meta-analysis of Ever Smoking (or Current if Ever not available), Cigars only
Squamous
Excluded studies (and stage at which they were excluded)

Stage	Studies
1	BOUCHA BUELL LAURIL MZILEN
2	ABRAHA AGUDO AKIBA ALDERS AMANDU AMES ANDERS ARCHER AUSTIN AUVINE AXELSO AXELSS BAND BARBON BECHER BERRIN BLOHMK BLOT1 BLOT2 BLOT3 BLOT4 BRESLO BRETT BROCKM BROSS BROWN1 BROWN2 BUFFLE BYERS1 BYERS2 CARPEN CASCO2 CASCOR CHAN CHANG CHATZI CHEN CHEN2 CHEN3 CHIAZZ CHOI CHOW CHYOU COMSTO COOKSO CORREA DARBY DAVEYS DEAN DEAN2 DEKLER DESTE2 DESTEF DOCKER DOLL DOLL2 DORANT DORGAN DOSEME DROSTE DU DUNN EBELIN ENGELA ENSTRO ESAKI FAN GAO GAO2 GARCIA GARSHI GENG GER GILLIS GODLEY GOLLED GOODMA GRAHAM GREGOR GSELL HAENSZ HAMMO2 HANSEN HEGMAN HENNEK HINDS HIRAOK HIRAY2 HIRAYA HITOSU HOLE HOROWI HORWIT HU HU2 HUANG HUMBLE ISHIMA JAHN JAIN JARUP JARVHO JEDRYC JIANG JONES JUSSAW KAISE2 KAISER KANELL KATSOU KAUFMA KELLER KHUDER KIHARA KINLEN KJUUS KNEKT KO KOHLME KOO KOULUM KREUZE KREYBE KUBIK LAMTH LAMWK LAMWK2 LAUSSM LEI LEMARC LETOUR LIAW LICKIN LIDDEL LIU LIU2 LIU3 LIU4 LIU5 LOMBA2 LOMBAR LUBIN LUO MACLEN MAGNUS MARSH MARSH2 MARTIS MASTRA MATOS MATSUD MCCONN MCDUFF MCLAUG MIGRAN MILLER MILLS MOLLO MRFIT MRFITR MURATA NAM NOTAN2 NOTANI NOU ODRISC ORMOS OSANN OSANN2 PARKIN PASTOR PAWLEG PERNU PERSH2 PERSHA PETO PEZZO2 PEZZOT PIKE PISANI POFFIJ POLEDN PRESCO QIAO QIAO2 RACHTA RADZIK RANDIG REN RESTRE RIMING RONCO ROOTS ROTHSC SAARIK SANKAR SCHWA2 SCHWAR SEGI SEGI2 SEOW SHAW SHIMIZ SIEMIA SIMARA SIMONA SITAS SOBUE SOBUE2 SPEIZE SPITZ STASZE STAYNE STOCKS STOCKW STUCKE SUN SUZUK2 SUZUKI SVENSS TANG TANG2 TAO TENKAN TOKARS TOUSEY TSUGAN TVERDA ULMER VANDER VEIERO VUTUC WAKAI WALD WANG WANG2 WANG3 WANG4 WARSIN WATSON WICKLU WIGLE WILKIN WU WU2 WUNSCH WUWILL WYNDE2 WYNDE3 WYNDE4 WYNDE5 WYNDE6 WYNDE8 XIANGZ XU XU2 XU3 XU4 YAMAGU YONG YUAN ZHANG ZHENG ZHOU
5	WYNDER
7	ABELIN ARMADA BENHAM BENSHL BEST BOFFET BOUCOT CEDERL CPSI CPSII DAMBER DEAN3 DORN GARDIN HEIN JOLY LANGE LEVIN SADOWS TIZZAN TULINI

Table 2E13 - 8

Potentially overlapping studies

REF	REFGP	PRINC	.	OVERLAP/LINK
LUBIN2	LUBIN2	1		Lubin-combined
WYNDE7	WYNDE6	2		WYNDE5/6/7/8

Table 2E14 -

IESLC - Meta-analysis of Current Smoking (or Ever if Current not available), Cigars only
Squamous

This analysis is restricted to results for:
1) Non-dose-response data
2) Smokers of cigars only
3) Results complete enough for use in metaanalysis

Within each study, results are then selected (in the following order of preference, within each sex) for:
4) SMKSTA: current smokers, ever smokers
5) DENOM: never smoked anything, (never +1 = +long term ex)
6) Followup period (prospective studies): whole study (coded as 0) or longest available
7) LCtype: all or nearest available, at least Squamous and Adeno. (q = squamous, s = small,
l = large, a = adeno, mix = mixed, alv = alveolar)
8) Race: all or nearest available, otherwise by race (wh or w = white, bl or b = black, hi = hispanic
ch = chinese, jap = japanese, haw = hawaiian, w+o = white + oriental, sca = scandinavian, as = asian)
9) For overlapping studies: principal rather than subsidiary studies
Finally by Age: whole study (coded as 0) if available, otherwise by widest available age group
and then for single sex results (m, f) in preference to combined sex results (c).

Results adjusted (AD) for the most potential confounders are then chosen in Sections -1 to -3
(and those which actually differ from the adjusted results in Table 2E13 - 1 are marked 'x' in Section -1)
and results adjusted for the least confounders in Sections -4 to -6. (Those least adjusted results which
actually differ from the most adjusted as marked 'x' in column X in Section -4)
(Results adjusted for an unknown number of confounder(s) are coded as 20.)

Section -7 shows excluded studies, together with the stage (as above) at which no qualifying
results were found.

Section -8 lists the potentially overlapping studies which have been included (1=principal, 2=subsidiary).

Section -9 lists any results which would have been included in preference except that they had data not complete
enough for use in meta-analysis, with their significance (yes/no), if known, and any further comment as entered
on the database.

In addition to those mentioned above, the following fields, levels and abbreviations are used:
* or nk = not known, n = no, y = yes, ot = other
ev = ever, cu = current, nev = never
REF: 6-character study reference
NRR: number of the RR on the database within the study
ST : study type (CC = case control, pr or prosp = prospective)
NLC: number of lung cancer cases in whole study
R : risky occupational population (n = no, m = mining, o = other risky)
VB : national cigarette type (V = at least 75% Virginia, bl = at least 75% blended, ot = other)
P : any proxy use
H : full histological confirmation
De : derivation of RR/CI (or = original, st = standard method, ot = other method of estimation)

Table 2E14 - 1

IESLC - Meta-analysis of Current Smoking (or Ever if Current not available), Cigars only
Squamous
Most adjusted

REF	NRR	2E13	SEX	AGEL	AGEH	RACE	YF	LC TYPE	LOC	START	ST	NLC	R	VB	P	H	AD	SM	DENOM	De
HAMMON	54		m	0	0	wh	0	not a	NAmer	1952	pr	448	n	bl	n	n	1	ev	nev any	ot
LUBIN2	133		m	0	0	all	-	q	Eu:mul	1976	CC	7804	n	bl	n	y	0	ev	nev any	st
WYNDE7	19	x	m	0	0	all	-	KI	NAmer	1977	CC	2085	n	bl	n	y	0	cu	nev any	st

Table 2E14 - 2

IESLC - Meta-analysis of Current Smoking (or Ever if Current not available), Cigars only
Squamous
Most adjusted

REF	NRR	SEX	AD	Number Exposed Case	Number Exposed Cont	Non-exposed Case	Non-exposed Cont	RR	95.00%CI
*HAMMON	54	m	1	-	-	-	-	3.35	(0.95- 11.88)
LUBIN2	133	m	0	21	145	127	2616	2.98	(1.83- 4.87)
WYNDE7	19	m	0	15	82	22	918	7.63	(3.81- 15.28)
Partial Totals				36	227	149	3534		

*prospective study

REF	NRR	SEX	AD	Ys	Ws	Qs	Ps
*HAMMON	54	m	1	1.21	2.41	0.08	0.0607
LUBIN2	133	m	0	1.09	15.93	1.39	0.0000
WYNDE7	19	m	0	2.03	7.97	3.31	0.0000

	N	3
	NS	3
	Wt	26.31
Het	Chi	4.78
Het	df	2
Het	P	(*)
Fixed	RR	4.01
	RRl	2.74
	RRu	5.87
	P	+++
Random	RR	4.28
	RRl	2.19
	RRu	8.37
	P	+++
Asymm	P	N.S.

Table 2E14 - 3

IESLC - Meta-analysis of Current Smoking (or Ever if Current not available), Cigars only
Squamous
Most adjusted

	Sex: combined	male	female	Total
N		3		3
NS		3		3
Wt		26.31		26.31
Het Chi		4.78		4.78
Het df		2		2
Het P		(*)		(*)
Fixed RR		4.01		4.01
RRl		2.74		2.74
RRu		5.87		5.87
P		+++		+++
Random RR		4.28		4.28
RRl		2.19		2.19
RRu		8.37		8.37
P		+++		+++
Between Chi				
Between df				
Between P				N.S.
Btwn(F) P				N.S.
Btwn(R) P				N.S.

Too few RRs for analysis by factor

Table 2E14 - 4

IESLC - Meta-analysis of Current Smoking (or Ever if Current not available), Cigars only
Squamous
Least adjusted

REF	NRR	X	SEX	AGEL	AGEH	RACE	YF	LC TYPE	LOC	START	ST	NLC	R	VB	P	H	AD	SM	DENOM	De
HAMMON	67	x	m	0	0	wh	0	not a	NAmer	1952	pr	448	n	bl	n	n	0	ev	nev any	st
LUBIN2	133		m	0	0	all	-	q	Eu:mul	1976	CC	7804	n	bl	n	y	0	ev	nev any	st
WYNDE7	19		m	0	0	all	-	KI	NAmer	1977	CC	2085	n	bl	n	y	0	cu	nev any	st

Table 2E14 - 5

IESLC - Meta-analysis of Current Smoking (or Ever if Current not available), Cigars only
Squamous
Least adjusted

REF	NRR	SEX	AD	Number Exposed Case	Cont	Non-exposed Case	Cont	RR	95.00%CI
*HAMMON	67	m	0	6	51480	4	115884	3.38	(0.95- 11.96)
LUBIN2	133	m	0	21	145	127	2616	2.98	(1.83- 4.87)
WYNDE7	19	m	0	15	82	22	918	7.63	(3.81- 15.28)
Totals				42	51707	153	119418		

*prospective study

REF	NRR	SEX	AD	Ys	Ws	Qs	Ps
*HAMMON	67	m	0	1.22	2.40	0.07	0.0594
LUBIN2	133	m	0	1.09	15.93	1.40	0.0000
WYNDE7	19	m	0	2.03	7.97	3.30	0.0000

	N	3
	NS	3
	Wt	26.30
Het	Chi	4.77
Het	df	2
Het	P	(*)
Fixed	RR	4.01
	RRl	2.74
	RRu	5.88
	P	+++
Random	RR	4.29
	RRl	2.20
	RRu	8.38
	P	+++
Asymm	P	N.S.

Table 2E14 - 6

IESLC - Meta-analysis of Current Smoking (or Ever if Current not available), Cigars only
Squamous
Least adjusted

	Sex			
	combined	male	female	Total
N		3		3
NS		3		3
Wt		26.30		26.30
Het Chi		4.77		4.77
Het df		2		2
Het P		(*)		(*)
Fixed RR		4.01		4.01
RRl		2.74		2.74
RRu		5.88		5.88
P		+++		+++
Random RR		4.29		4.29
RRl		2.20		2.20
RRu		8.38		8.38
P		+++		+++
Between Chi				
Between df				
Between P				N.S.
Btwn(F) P				N.S.
Btwn(R) P				N.S.

Table 2E14 - 7

IESLC - Meta-analysis of Current Smoking (or Ever if Current not available), Cigars only
Squamous
Excluded studies (and stage at which they were excluded)

1 BOUCHA BUELL LAURIL MZILEN

2 ABRAHA AGUDO AKIBA ALDERS AMANDU AMES ANDERS ARCHER AUSTIN AUVINE AXELSO AXELSS BAND BARBON BECHER BERRIN
BLOHMK BLOT1 BLOT2 BLOT3 BLOT4 BRESLO BRETT BROCKM BROSS BROWN1 BROWN2 BUFFLE BYERS1 BYERS2 CARPEN CASCO2
CASCOR CHAN CHANG CHATZI CHEN CHEN2 CHEN3 CHIAZZ CHOI CHOW CHYOU COMSTO COOKSO CORREA DARBY DAVEYS
DEAN DEAN2 DEKLER DESTE2 DESTEF DOCKER DOLL DOLL2 DORANT DORGAN DOSEME DROSTE DU DUNN EBELIN ENGELA
ENSTRO ESAKI FAN GAO GAO2 GARCIA GARSHI GENG GER GILLIS GODLEY GOLLED GOODMA GRAHAM GREGOR GSELL
HAENSZ HAMMO2 HANSEN HEGMAN HENNEK HINDS HIRAOK HIRAY2 HIRAYA HITOSU HOLE HOROWI HORWIT HU HU2 HUANG
HUMBLE ISHIMA JAHN JAIN JARUP JARVHO JEDRYC JIANG JONES JUSSAW KAISE2 KAISER KANELL KATSOU KAUFMA KELLER
KHUDER KIHARA KINLEN KJUUS KNEKT KO KOHLME KOO KOULUM KREUZE KREYBE KUBIK LAMTH LAMWK LAMWK2 LAUSSM
LEI LEMARC LETOUR LIAW LICKIN LIDDEL LIU LIU2 LIU3 LIU4 LIU5 LOMBA2 LOMBAR LUBIN LUO MACLEN
MAGNUS MARSH MARSH2 MARTIS MASTRA MATOS MATSUD MCCONN MCDUFF MCLAUG MIGRAN MILLER MILLS MOLLO MRFIT MRFITR
MURATA NAM NOTAN2 NOTANI NOU ODRISC ORMOS OSANN OSANN2 PARKIN PASTOR PAWLEG PERNU PERSH2 PERSHA PETO
PEZZO2 PEZZOT PIKE PISANI POFFIJ POLEDN PRESCO QIAO QIAO2 RACHTA RADZIK RANDIG REN RESTRE RIMING RONCO
ROOTS ROTHSC SAARIK SANKAR SCHWA2 SCHWAR SEGI SEGI2 SEOW SHAW SHIMIZ SIEMIA SIMARA SIMONA SITAS SOBUE
SOBUE2 SPEIZE SPITZ STASZE STAYNE STOCKS STOCKW STUCKE SUN SUZUK2 SUZUKI SVENSS TANG TANG2 TAO TENKAN
TOKARS TOUSEY TSUGAN TVERDA ULMER VANDER VEIERO VUTUC WAKAI WALD WANG WANG2 WANG3 WANG4 WARSIN WATSON
WICKLU WIGLE WILKIN WU WU2 WUNSCH WUWILL WYNDE2 WYNDE3 WYNDE4 WYNDE5 WYNDE6 WYNDE8 XIANGZ XU XU2
XU3 XU4 YAMAGU YONG YUAN ZHANG ZHENG ZHOU

5 WYNDER

7 ABELIN ARMADA BENHAM BENSHL BEST BOFFET BOUCOT CEDERL CPSI CPSII DAMBER DEAN3 DORN GARDIN HEIN JOLY
LANGE LEVIN SADOWS TIZZAN TULINI

Table 2E14 - 8

Potentially overlapping studies

REF	REFGP	PRINC	. OVERLAP/LINK
LUBIN2	LUBIN2	1	Lubin-combined
WYNDE7	WYNDE6	2	WYNDE5/6/7/8

Table 2E15 -

IESLC - Meta-analysis of Ex Smoking, Cigars only
Squamous

This analysis is restricted to results for:
1) Non-dose-response data
2) Smokers of cigars only
3) Ex smokers
4) Results complete enough for use in metaanalysis

Within each study, results are then selected (in the following order of preference, within each sex) for:
5) DENOM: never smoked anything, (never +1 = +long term ex)
6) Followup period (prospective studies): whole study (coded as 0) or longest available
7) LCtype: all or nearest available, at least Squamous and Adeno. (q = squamous, s = small,
l = large, a = adeno, mix = mixed, alv = alveolar)
8) Race: all or nearest available, otherwise by race (wh or w = white, bl or b = black, hi = hispanic
ch = chinese, jap = japanese, haw = hawaiian, w+o = white + oriental, sca = scandinavian, as = asian)
9) For overlapping studies: principal rather than subsidiary studies
Finally by Age: whole study (coded as 0) if available, otherwise by widest available age group
and then for single sex results (m, f) in preference to combined sex results (c).

Results adjusted (AD) for the most potential confounders are then chosen in Sections -1 to -3
and results adjusted for the least confounders in Sections -4 to -6. (Those least adjusted results which
actually differ from the most adjusted as marked 'x' in column X in Section -4)
(Results adjusted for an unknown number of confounder(s) are coded as 20.)

Section -7 shows excluded studies, together with the stage (as above) at which no qualifying
results were found.

Section -8 lists the potentially overlapping studies which have been included (1=principal, 2=subsidiary).

Section -9 lists any results which would have been included in preference except that they had data not complete
enough for use in meta-analysis, with their significance (yes/no), if known, and any further comment as entered
on the database.

In addition to those mentioned above, the following fields, levels and abbreviations are used:
* or nk = not known, n = no, y = yes, ot = other
nev = never
REF: 6-character study reference
NRR: number of the RR on the database within the study
ST : study type (CC = case control, pr or prosp = prospective)
NLC: number of lung cancer cases in whole study
R : risky occupational population (n = no, m = mining, o = other risky)
VB : national cigarette type (V = at least 75% Virginia, bl = at least 75% blended, ot = other)
P : any proxy use
H : full histological confirmation
De : derivation of RR/CI (or = original, st = standard method, ot = other method of estimation)

Table 2E15 - 1

IESLC - Meta-analysis of Ex Smoking, Cigars only
Squamous
Most adjusted

REF	NRR	SEX	AGEL	AGEH	RACE	YF	LC TYPE	LOC	START	ST	NLC	R	VB	P	H	AD	DENOM	De
WYNDE7	24	m	0	0	all	-	KI	NAmer	1977	CC	2085	n	bl	n	y	0	nev any	st

Table 2E15 - 2

IESLC - Meta-analysis of Ex Smoking, Cigars only
Squamous
Most adjusted

REF	NRR	SEX	AD	Number Exposed Case	Number Exposed Cont	Non-exposed Case	Non-exposed Cont	RR	95.00%CI
WYNDE7	24	m	0	6	70	22	918	3.58	(1.40- 9.11)
Totals				6	70	22	918		

*prospective study

REF	NRR	SEX	AD	Ys	Ws	Qs	Ps
WYNDE7	24	m	0	1.27	4.40	0.00	0.0075

	N	1
	NS	1
	Wt	4.40
Het	Chi	0.00
Het	df	0
Het	P	N.S.
Fixed	RR	3.58
	RRl	1.40
	RRu	9.11
	P	++
Random	RR	3.58
	RRl	1.40
	RRu	9.11
	P	++
Asymm	P	

Table 2E15 - 3

IESLC - Meta-analysis of Ex Smoking, Cigars only
Squamous
Most adjusted

	Sex combined	male	female	Total
N		1		1
NS		1		1
Wt		4.40		4.40
Het Chi		0.00		0.00
Het df		0		0
Het P		N.S.		N.S.
Fixed RR		3.58		3.58
RRl		1.40		1.40
RRu		9.11		9.11
P		++		++
Random RR		3.58		3.58
RRl		1.40		1.40
RRu		9.11		9.11
P		++		++
Between Chi				
Between df				
Between P				N.S.
Btwn(F) P				N.S.
Btwn(R) P				N.S.

Too few RRs for analysis by factor

Table 2E15 - 4

IESLC - Meta-analysis of Ex Smoking, Cigars only
Squamous
Least adjusted

REF	NRR	X	SEX	AGEL	AGEH	RACE	YF	LC TYPE	LOC	START	ST	NLC	R	VB	P	H	AD	DENOM	De
WYNDE7	24		m	0	0	all	-	KI	NAmer	1977	CC	2085	n	bl	n	y	0	nev any	st

Table 2E15 - 5

IESLC - Meta-analysis of Ex Smoking, Cigars only
Squamous
Least adjusted

REF	NRR	SEX	AD	Number Case	Exposed Cont	Non-exposed Case	Cont	RR	95.00%CI
WYNDE7	24	m	0	6	70	22	918	3.58	(1.40- 9.11)
Totals				6	70	22	918		

*prospective study

REF	NRR	SEX	AD	Ys	Ws	Qs	Ps
WYNDE7	24	m	0	1.27	4.40	0.00	0.0075

	N	1
	NS	1
	Wt	4.40
Het	Chi	0.00
Het	df	0
Het	P	N.S.
Fixed	RR	3.58
	RRl	1.40
	RRu	9.11
	P	++
Random	RR	3.58
	RRl	1.40
	RRu	9.11
	P	++
Asymm	P	

Table 2E15 - 6

IESLC - Meta-analysis of Ex Smoking, Cigars only
Squamous
Least adjusted

	Sex combined	male	female	Total
N		1		1
NS		1		1
Wt		4.40		4.40
Het Chi		0.00		0.00
Het df		0		0
Het P		N.S.		N.S.
Fixed RR		3.58		3.58
RRl		1.40		1.40
RRu		9.11		9.11
P		++		++
Random RR		3.58		3.58
RRl		1.40		1.40
RRu		9.11		9.11
P		++		++
Between Chi				
Between df				
Between P				N.S.
Btwn(F) P				N.S.
Btwn(R) P				N.S.

Table 2E15 - 7

IESLC - Meta-analysis of Ex Smoking, Cigars only
Squamous
Excluded studies (and stage at which they were excluded)

1	BOUCHA BUELL LAURIL MZILEN
2	ABRAHA AGUDO AKIBA ALDERS AMANDU AMES ANDERS ARCHER AUSTIN AUVINE AXELSO AXELSS BAND BARBON BECHER BERRIN BLOHMK BLOT1 BLOT2 BLOT3 BLOT4 BRESLO BRETT BROCKM BROSS BROWN1 BROWN2 BUFFLE BYERS1 BYERS2 CARPEN CASCO2 CASCOR CHAN CHANG CHATZI CHEN CHEN2 CHEN3 CHIAZZ CHOI CHOW CHYOU COMSTO COOKSO CORREA DARBY DAVEYS DEAN DEAN2 DEKLER DESTE2 DESTEF DOCKER DOLL DOLL2 DORANT DORGAN DOSEME DROSTE DU DUNN EBELIN ENGELA ENSTRO ESAKI FAN GAO GAO2 GARCIA GARSHI GENG GER GILLIS GODLEY GOLLED GOODMA GRAHAM GREGOR GSELL HAENSZ HAMMO2 HANSEN HEGMAN HENNEK HINDS HIRAOK HIRAY2 HIRAYA HITOSU HOLE HOROWI HORWIT HU HU2 HUANG HUMBLE ISHIMA JAHN JAIN JARUP JARVHO JEDRYC JIANG JONES JUSSAW KAISE2 KAISER KANELL KATSOU KAUFMA KELLER KHUDER KIHARA KINLEN KJUUS KNEKT KO KOHLME KOO KOULUM KREUZE KREYBE KUBIK LAMTH LAMWK LAMWK2 LAUSSM LEI LEMARC LETOUR LIAW LICKIN LIDDEL LIU LIU2 LIU3 LIU4 LIU5 LOMBA2 LOMBAR LUBIN LUO MACLEN MAGNUS MARSH MARSH2 MARTIS MASTRA MATOS MATSUD MCCONN MCDUFF MCLAUG MIGRAN MILLER MILLS MOLLO MRFIT MRFITR MURATA NAM NOTAN2 NOTANI NOU ODRISC ORMOS OSANN OSANN2 PARKIN PASTOR PAWLEG PERNU PERSH2 PERSHA PETO PEZZO2 PEZZOT PIKE PISANI POFFIJ POLEDN PRESCO QIAO QIAO2 RACHTA RADZIK RANDIG REN RESTRE RIMING RONCO ROOTS ROTHSC SAARIK SANKAR SCHWA2 SCHWAR SEGI SEGI2 SEOW SHAW SHIMIZ SIEMIA SIMARA SIMONA SITAS SOBUE SOBUE2 SPEIZE SPITZ STASZE STAYNE STOCKS STOCKW STUCKE SUN SUZUK2 SUZUKI SVENSS TANG TANG2 TAO TENKAN TOKARS TOUSEY TSUGAN TVERDA ULMER VANDER VEIERO VUTUC WAKAI WALD WANG WANG2 WANG3 WANG4 WARSIN WATSON WICKLU WIGLE WILKIN WU WU2 WUNSCH WUWILL WYNDE2 WYNDE3 WYNDE4 WYNDE5 WYNDE6 WYNDE8 XIANGZ XU XU2 XU3 XU4 YAMAGU YONG YUAN ZHANG ZHENG ZHOU
3	ABELIN ARMADA BENHAM BENSHL BEST BOUCOT CEDERL CPSI CPSII DAMBER DEAN3 GARDIN HAMMON HEIN LANGE LEVIN SADOWS TIZZAN TULINI WYNDER
7	BOFFET DORN JOLY LUBIN2

Table 2E15 - 8
Potentially overlapping studies

REF	REFGP	PRINC	.	OVERLAP/LINK
WYNDE7	WYNDE6	2		WYNDE5/6/7/8

Table 2E16 -

IESLC - Meta-analysis of Ever Smoking, Mixed smokers
Squamous

This analysis is restricted to results for:
1) Non-dose-response data
2) Mixed smokers (cigarettes and pipe/cigar)
3) Ever smokers
4) Results complete enough for use in metaanalysis

Within each study, results are then selected (in the following order of preference, within each sex) for:
5) DENOM: never smoked anything, (never +1 = +long term ex)
6) Followup period (prospective studies): whole study (coded as 0) or longest available
7) LCtype: all or nearest available, at least Squamous and Adeno. (q = squamous, s = small,
l = large, a = adeno, mix = mixed, alv = alveolar)
8) Race: all or nearest available, otherwise by race (wh or w = white, bl or b = black, hi = hispanic
ch = chinese, jap = japanese, haw = hawaiian, w+o = white + oriental, sca = scandinavian, as = asian)
9) For overlapping studies: principal rather than subsidiary studies
Finally by Age: whole study (coded as 0) if available, otherwise by widest available age group
and then for single sex results (m, f) in preference to combined sex results (c).

Results adjusted (AD) for the most potential confounders are then chosen in Sections -1 to -3
and results adjusted for the least confounders in Sections -4 to -6. (Those least adjusted results which
actually differ from the most adjusted as marked 'x' in column X in Section -4)
(Results adjusted for an unknown number of confounder(s) are coded as 20.)

Section -7 shows excluded studies, together with the stage (as above) at which no qualifying
results were found.

Section -8 lists the potentially overlapping studies which have been included (1=principal, 2=subsidiary).

Section -9 lists any results which would have been included in preference except that they had data not complete
enough for use in meta-analysis, with their significance (yes/no), if known, and any further comment as entered
on the database.

In addition to those mentioned above, the following fields, levels and abbreviations are used:
* or nk = not known, n = no, y = yes, ot = other
nev = never
REF: 6-character study reference
NRR: number of the RR on the database within the study
ST : study type (CC = case control, pr or prosp = prospective)
NLC: number of lung cancer cases in whole study
R : risky occupational population (n = no, m = mining, o = other risky)
VB : national cigarette type (V = at least 75% Virginia, bl = at least 75% blended, ot = other)
P : any proxy use
H : full histological confirmation
De : derivation of RR/CI (or = original, st = standard method, ot = other method of estimation)

Table 2E16 - 1

IESLC - Meta-analysis of Ever Smoking, Mixed smokers
Squamous
Most adjusted

REF	NRR	SEX	AGEL	AGEH	RACE	YF	LC TYPE	LOC	START	ST	NLC	R	VB	P	H	AD	DENOM	De
ALDERS	29	m	0	0	all	-	q+s	Eu:UK	1977	CC	1448	n	V	n	n	1	nev any	ot
BRESLO	9	c	0	0	all	-	not a	NAmer	1949	CC	518	n	bl	n	y	0	nev+1	st
HAMMON	57	m	0	0	wh	0	not a	NAmer	1952	pr	448	n	bl	n	n	1	nev any	ot
LUBIN	17	m	0	0	all	-	KI	As:Chi	1984	CC	427	m	ot	y	n	0	nev any	st
STASZE	15	m	0	0	all	-	q	Eu:est	1954	CC	281	n	bl	n	y	0	nev any	ot
WYNDE7	60	m	0	0	all	-	KI	NAmer	1977	CC	2085	n	bl	n	y	0	nev any	st

Table 2E16 - 2

IESLC - Meta-analysis of Ever Smoking, Mixed smokers
Squamous
Most adjusted

REF	NRR	SEX	AD	Number Exposed Case	Cont	Non-exposed Case	Cont	RR	95.00%CI
ALDERS	29	m	1	-	-	-	-	7.58	(2.70- 21.28)
BRESLO	9	c	0	146	154	15	56	3.54	(1.92- 6.53)
*HAMMON	57	m	1	-	-	-	-	19.71	(7.26- 53.50)
LUBIN	17	m	0	249	597	4	72	7.51	(2.71- 20.77)
STASZE	15	m	0	18	101	0	158	57.78~	(3.44- 969.38)
WYNDE7	60	m	0	223	614	22	918	15.16	(9.67- 23.76)
Partial Totals				636	1466	41	1204		

*prospective study

~ With 0.5 adjustment for zero

REF	NRR	SEX	AD	Ys	Ws	Qs	Ps
ALDERS	29	m	1	2.03	3.61	0.22	0.0001
BRESLO	9	c	0	1.26	10.22	10.35	0.0001
*HAMMON	57	m	1	2.98	3.85	1.95	0.0000
LUBIN	17	m	0	2.02	3.71	0.24	0.0001
STASZE	15	m	0	4.06	0.48	1.54	0.0048
WYNDE7	60	m	0	2.72	18.99	3.81	0.0000

	N	6
	NS	6
	Wt	40.86
Het	Chi	18.10
Het	df	5
Het	P	**
Fixed	RR	9.68
	RRl	7.13
	RRu	13.16
	P	+++
Random	RR	9.78
	RRl	4.94
	RRu	19.35
	P	+++
Asymm	P	N.S.

Table 2E16 - 3

IESLC - Meta-analysis of Ever Smoking, Mixed smokers
Squamous
Most adjusted

	Sex			
	combined	male	female	Total
N	1	5		6
NS	1	5		6
Wt	10.22	30.64		40.86
Het Chi	0.00	4.30		18.10
Het df	0	4		5
Het P	N.S.	N.S.		**
Fixed RR	3.54	13.54		9.68
RRl	1.92	9.51		7.13
RRu	6.53	19.30		13.16
P	+++	+++		+++
Random RR	3.54	13.35		9.78
RRl	1.92	9.03		4.94
RRu	6.53	19.71		19.35
P	+++	+++		+++
Between Chi				13.80
Between df				1
Between P				***
Btwn(F) P				*
Btwn(R) P				***

Too few RRs for analysis by factor

Table 2E16 - 4

IESLC - Meta-analysis of Ever Smoking, Mixed smokers
Squamous
Least adjusted

REF	NRR	X	SEX	AGEL	AGEH	RACE	YF	LC TYPE	LOC	START	ST	NLC	R	VB	P	H	AD	DENOM	De
ALDERS	80	x	m	0	0	all	-	q+s	Eu:UK	1977	CC	1448	n	V	n	n	0	nev any	st
BRESLO	9		c	0	0	all	-	not a	NAmer	1949	CC	518	n	bl	n	y	0	nev+1	st
HAMMON	70	x	m	0	0	wh	0	not a	NAmer	1952	pr	448	n	bl	n	n	0	nev any	st
LUBIN	17		m	0	0	all	-	KI	As:Chi	1984	CC	427	m	ot	y	n	0	nev any	st
STASZE	15		m	0	0	all	-	q	Eu:est	1954	CC	281	n	bl	n	y	0	nev any	ot
WYNDE7	60		m	0	0	all	-	KI	NAmer	1977	CC	2085	n	bl	n	y	0	nev any	st

Table 2E16 - 5

IESLC - Meta-analysis of Ever Smoking, Mixed smokers
Squamous
Least adjusted

REF	NRR	SEX	AD	Number Exposed Case	Cont	Non-exposed Case	Cont	RR	95.00%CI
ALDERS	80	m	0	70	179	4	133	13.00	(4.63- 36.50)
BRESLO	9	c	0	146	154	15	56	3.54	(1.92- 6.53)
*HAMMON	70	m	0	103	156773	4	115884	19.03	(7.01- 51.68)
LUBIN	17	m	0	249	597	4	72	7.51	(2.71- 20.77)
STASZE	15	m	0	18	101	0	158	57.78~	(3.44- 969.38)
WYNDE7	60	m	0	223	614	22	918	15.16	(9.67- 23.76)
Totals				809	158418	49	117221		

*prospective study ~ With 0.5 adjustment for zero

REF	NRR	SEX	AD	Ys	Ws	Qs	Ps
ALDERS	80	m	0	2.57	3.61	0.23	0.0000
BRESLO	9	c	0	1.26	10.22	11.28	0.0001
*HAMMON	70	m	0	2.95	3.85	1.54	0.0000
LUBIN	17	m	0	2.02	3.71	0.33	0.0001
STASZE	15	m	0	4.06	0.48	1.47	0.0048
WYNDE7	60	m	0	2.72	18.99	3.10	0.0000

	N	6
	NS	6
	Wt	40.86
Het	Chi	17.93
Het	df	5
Het	P	**
Fixed	RR	10.12
	RRl	7.45
	RRu	13.75
	P	+++
Random	RR	10.62
	RRl	5.38
	RRu	20.94
	P	+++
Asymm	P	N.S.

Table 2E16 - 6

IESLC - Meta-analysis of Ever Smoking, Mixed smokers
Squamous
Least adjusted

		combined	Sex male	female	Total
	N	1	5		6
	NS	1	5		6
	Wt	10.22	30.64		40.86
Het	Chi	0.00	2.89		17.93
Het	df	0	4		5
Het	P	N.S.	N.S.		**
Fixed	RR	3.54	14.37		10.12
	RRl	1.92	10.08		7.45
	RRu	6.53	20.47		13.75
	P	+++	+++		+++
Random	RR	3.54	14.37		10.62
	RRl	1.92	10.08		5.38
	RRu	6.53	20.47		20.94
	P	+++	+++		+++
Between	Chi				15.04
Between	df				1
Between	P				***
Btwn(F)	P				*
Btwn(R)	P				***

Table 2E16 - 7

IESLC - Meta-analysis of Ever Smoking, Mixed smokers
Squamous
Excluded studies (and stage at which they were excluded)

1 BOUCHA BUELL LAURIL MZILEN

2 ABRAHA AGUDO AKIBA AMANDU AMES ANDERS ARCHER AUSTIN AUVINE AXELSO AXELSS BAND BARBON BECHER BENSHL BERRIN
BLOHMK BLOT1 BLOT2 BLOT3 BLOT4 BRETT BROCKM BROSS BROWN1 BROWN2 BUFFLE BYERS1 BYERS2 CARPEN CASCO2 CASCOR
CHAN CHANG CHATZI CHEN CHEN2 CHEN3 CHIAZZ CHOI CHYOU COMSTO COOKSO CORREA CPSII DARBY DAVEYS DEAN3
DEKLER DESTE2 DESTEF DOCKER DOLL DORANT DORGAN DOSEME DROSTE DU DUNN EBELIN ENGELA ENSTRO ESAKI FAN
GAO GAO2 GARCIA GARDIN GARSHI GENG GER GILLIS GODLEY GOODMA GREGOR GSELL HAENSZ HAMMO2 HANSEN HEGMAN
HEIN HENNEK HINDS HIRAOK HIRAYA HITOSU HOLE HOROWI HORWIT HU HU2 HUANG HUMBLE ISHIMA JAHN JAIN
JARUP JARVHO JEDRYC JIANG JONES JUSSAW KAISE2 KAISER KANELL KATSOU KAUFMA KELLER KHUDER KIHARA KINLEN KJUUS
KNEKT KO KOHLME KOO KOULUM KREUZE KREYBE KUBIK LAMTH LAMWK LAMWK2 LANGE LAUSSM LEI LEMARC LETOUR
LEVIN LIAW LICKIN LIDDEL LIU LIU2 LIU3 LIU4 LIU5 LOMBA2 LUO MACLEN MAGNUS MARSH MARSH2 MARTIS
MASTRA MATOS MATSUD MCDUFF MCLAUG MILLER MILLS MOLLO MRFIT MRFITR MURATA NAM NOTAN2 NOTANI NOU ODRISC
ORMOS OSANN OSANN2 PARKIN PASTOR PAWLEG PERNU PERSH2 PERSHA PETO PEZZO2 PEZZOT PIKE PISANI POFFIJ POLEDN
PRESCO QIAO2 RACHTA RADZIK RANDIG REN RESTRE RIMING RONCO ROOTS ROTHSC SAARIK SANKAR SCHWAR SEGI SEGI2
SEOW SHAW SHIMIZ SIEMIA SIMARA SIMONA SITAS SOBUE SOBUE2 SPEIZE SPITZ STAYNE STOCKS STOCKW STUCKE SUN
SUZUK2 SUZUKI SVENSS TANG TANG2 TAO TENKAN TIZZAN TOKARS TOUSEY TSUGAN TULINI ULMER VANDER VEIERO VUTUC
WAKAI WALD WANG WANG2 WANG3 WANG4 WARSIN WATSON WICKLU WIGLE WILKIN WU WU2 WUNSCH WUWILL WYNDE2
WYNDE3 WYNDE4 WYNDE5 WYNDE6 WYNDE8 XU XU2 XU3 XU4 YAMAGU YONG YUAN ZHANG ZHENG ZHOU

3 CEDERL HIRAY2 MIGRAN TVERDA WYNDER

5 SCHWA2

7 ABELIN ARMADA BENHAM BEST BOFFET BOUCOT CHOW CPSI DAMBER DEAN DEAN2 DOLL2 DORN GOLLED GRAHAM JOLY
LOMBAR LUBIN2 MCCONN QIAO SADOWS XIANGZ

Table 2E16 - 8

Potentially overlapping studies

REF	REFGP	PRINC	.	OVERLAP/LINK
WYNDE7	WYNDE6	2		WYNDE5/6/7/8
LUBIN	XIANGZ	2		LUBIN/XIANGZ/QIAO

Table 2E17 -

IESLC - Meta-analysis of Current Smoking, Mixed smokers
Squamous

This analysis is restricted to results for:
1) Non-dose-response data
2) Mixed smokers (cigarettes and pipe/cigar)
3) Current smokers
4) Results complete enough for use in metaanalysis

Within each study, results are then selected (in the following order of preference, within each sex) for:
5) DENOM: never smoked anything, (never +1 = +long term ex)
6) Followup period (prospective studies): whole study (coded as 0) or longest available
7) LCtype: all or nearest available, at least Squamous and Adeno. (q = squamous, s = small,
l = large, a = adeno, mix = mixed, alv = alveolar)
8) Race: all or nearest available, otherwise by race (wh or w = white, bl or b = black, hi = hispanic
ch = chinese, jap = japanese, haw = hawaiian, w+o = white + oriental, sca = scandinavian, as = asian)
9) For overlapping studies: principal rather than subsidiary studies
Finally by Age: whole study (coded as 0) if available, otherwise by widest available age group
and then for single sex results (m, f) in preference to combined sex results (c).

Results adjusted (AD) for the most potential confounders are then chosen in Sections -1 to -3
and results adjusted for the least confounders in Sections -4 to -6. (Those least adjusted results which
actually differ from the most adjusted as marked 'x' in column X in Section -4)
(Results adjusted for an unknown number of confounder(s) are coded as 20.)

Section -7 shows excluded studies, together with the stage (as above) at which no qualifying
results were found.

Section -8 lists the potentially overlapping studies which have been included (1=principal, 2=subsidiary).

Section -9 lists any results which would have been included in preference except that they had data not complete
enough for use in meta-analysis, with their significance (yes/no), if known, and any further comment as entered
on the database.

In addition to those mentioned above, the following fields, levels and abbreviations are used:
* or nk = not known, n = no, y = yes, ot = other
nev = never
REF: 6-character study reference
NRR: number of the RR on the database within the study
ST : study type (CC = case control, pr or prosp = prospective)
NLC: number of lung cancer cases in whole study
R : risky occupational population (n = no, m = mining, o = other risky)
VB : national cigarette type (V = at least 75% Virginia, bl = at least 75% blended, ot = other)
P : any proxy use
H : full histological confirmation
De : derivation of RR/CI (or = original, st = standard method, ot = other method of estimation)

Table 2E17 - 1

IESLC - Meta-analysis of Current Smoking, Mixed smokers
Squamous
Most adjusted

REF	NRR	SEX	AGEL	AGEH	RACE	YF	LC TYPE	LOC	START	ST	NLC	R	VB	P	H	AD	DENOM	De
WYNDE7	23	m	0	0	all	-	KI	NAmer	1977	CC	2085	n	bl	n	y	0	nev any	st

Table 2E17 - 2

IESLC - Meta-analysis of Current Smoking, Mixed smokers
Squamous
Most adjusted

REF	NRR	SEX	AD	Number Exposed Case	Cont	Non-exposed Case	Cont	RR	95.00%CI
WYNDE7	23	m	0	152	287	22	918	22.10	(13.86- 35.23)
Totals				152	287	22	918		

*prospective study

REF	NRR	SEX	AD	Ys	Ws	Qs	Ps
WYNDE7	23	m	0	3.10	17.67	0.00	0.0000

	N	1
	NS	1
	Wt	17.67
Het	Chi	0.00
Het	df	0
Het	P	N.S.
Fixed	RR	22.10
	RRl	13.86
	RRu	35.23
	P	+++
Random	RR	22.10
	RRl	13.86
	RRu	35.23
	P	+++
Asymm	P	

Table 2E17 - 3

IESLC - Meta-analysis of Current Smoking, Mixed smokers
Squamous
Most adjusted

	Sex			
	combined	male	female	Total
N		1		1
NS		1		1
Wt		17.67		17.67
Het Chi		0.00		0.00
Het df		0		0
Het P		N.S.		N.S.
Fixed RR		22.10		22.10
RRl		13.86		13.86
RRu		35.23		35.23
P		+++		+++
Random RR		22.10		22.10
RRl		13.86		13.86
RRu		35.23		35.23
P		+++		+++
Between Chi				
Between df				
Between P				N.S.
Btwn(F) P				N.S.
Btwn(R) P				N.S.

Too few RRs for analysis by factor

Table 2E17 - 4

IESLC - Meta-analysis of Current Smoking, Mixed smokers
Squamous
Least adjusted

REF	NRR	X	SEX	AGEL	AGEH	RACE	YF	LC TYPE	LOC	START	ST	NLC	R	VB	P	H	AD	DENOM	De
WYNDE7	23		m	0	0	all	-	KI	NAmer	1977	CC	2085	n	bl	n	y	0	nev any	st

Table 2E17 - 5

IESLC - Meta-analysis of Current Smoking, Mixed smokers
Squamous
Least adjusted

REF	NRR	SEX	AD	Number Exposed Case	Cont	Non-exposed Case	Cont	RR	95.00%CI
WYNDE7	23	m	0	152	287	22	918	22.10	(13.86- 35.23)
Totals				152	287	22	918		

*prospective study

REF	NRR	SEX	AD	Ys	Ws	Qs	Ps
WYNDE7	23	m	0	3.10	17.67	0.00	0.0000

	N	1
	NS	1
	Wt	17.67
Het	Chi	0.00
Het	df	0
Het	P	N.S.
Fixed	RR	22.10
	RRl	13.86
	RRu	35.23
	P	+++
Random	RR	22.10
	RRl	13.86
	RRu	35.23
	P	+++
Asymm	P	

Table 2E17 - 6

IESLC - Meta-analysis of Current Smoking, Mixed smokers
Squamous
Least adjusted

	Sex combined	male	female	Total
N		1		1
NS		1		1
Wt		17.67		17.67
Het Chi		0.00		0.00
Het df		0		0
Het P		N.S.		N.S.
Fixed RR		22.10		22.10
RRl		13.86		13.86
RRu		35.23		35.23
P		+++		+++
Random RR		22.10		22.10
RRl		13.86		13.86
RRu		35.23		35.23
P		+++		+++
Between Chi				
Between df				
Between P				N.S.
Btwn(F) P				N.S.
Btwn(R) P				N.S.

Table 2E17 - 7

IESLC - Meta-analysis of Current Smoking, Mixed smokers
Squamous
Excluded studies (and stage at which they were excluded)

```
1      BOUCHA  BUELL LAURIL MZILEN

2      ABRAHA  AGUDO  AKIBA AMANDU   AMES ANDERS ARCHER AUSTIN AUVINE AXELSO AXELSS   BAND BARBON BECHER BENSHL BERRIN
       BLOHMK  BLOT1  BLOT2  BLOT3  BLOT4  BRETT BROCKM  BROSS BROWN1 BROWN2 BUFFLE BYERS1 BYERS2 CARPEN CASCO2 CASCOR
         CHAN  CHANG CHATZI   CHEN  CHEN2  CHEN3 CHIAZZ   CHOI  CHYOU COMSTO COOKSO CORREA  CPSII  DARBY DAVEYS  DEAN3
       DEKLER DESTE2 DESTEF DOCKER   DOLL DORANT DORGAN DOSEME DROSTE     DU   DUNN EBELIN ENGELA ENSTRO  ESAKI    FAN
          GAO   GAO2 GARCIA GARDIN GARSHI   GENG    GER GILLIS GODLEY GOODMA GREGOR  GSELL HAENSZ HAMMO2 HANSEN HEGMAN
         HEIN HENNEK  HINDS HIRAOK HIRAYA HITOSU   HOLE HOROWI HORWIT     HU    HU2  HUANG HUMBLE ISHIMA   JAHN   JAIN
        JARUP JARVHO JEDRYC  JIANG  JONES JUSSAW KAISE2 KAISER KANELL KATSOU KAUFMA KELLER KHUDER KIHARA KINLEN  KJUUS
        KNEKT     KO KOHLME    KOO KOULUM KREUZE KREYBE  KUBIK  LAMTH  LAMWK LAMWK2  LANGE LAUSSM    LEI LEMARC LETOUR
        LEVIN   LIAW LICKIN LIDDEL    LIU   LIU2   LIU3   LIU4   LIU5 LOMBA2    LUO MACLEN MAGNUS  MARSH MARSH2 MARTIS
       MASTRA  MATOS MATSUD MCDUFF MCLAUG MILLER  MILLS  MOLLO  MRFIT MRFITR MURATA    NAM NOTAN2 NOTANI    NOU ODRISC
        ORMOS  OSANN OSANN2 PARKIN PASTOR PAWLEG  PERNU PERSH2 PERSHA   PETO PEZZO2 PEZZOT   PIKE PISANI POFFIJ POLEDN
       PRESCO  QIAO2 RACHTA RADZIK RANDIG    REN RESTRE RIMING  RONCO  ROOTS ROTHSC SAARIK SANKAR SCHWAR   SEGI  SEGI2
         SEOW   SHAW SHIMIZ SIEMIA SIMARA SIMONA  SITAS  SOBUE SOBUE2 SPEIZE  SPITZ STAYNE STOCKS STOCKW STUCKE    SUN
       SUZUK2 SUZUKI SVENSS   TANG  TANG2    TAO TENKAN TIZZAN TOKARS TOUSEY TSUGAN TULINI  ULMER VANDER VEIERO  VUTUC
        WAKAI   WALD   WANG  WANG2  WANG3  WANG4 WARSIN WATSON WICKLU  WIGLE WILKIN     WU    WU2 WUNSCH WUWILL WYNDE2
       WYNDE3 WYNDE4 WYNDE5 WYNDE6 WYNDE8     XU    XU2    XU3    XU4 YAMAGU   YONG   YUAN  ZHANG  ZHENG   ZHOU

3      ABELIN ALDERS ARMADA BENHAM   BEST BOFFET BOUCOT BRESLO   CPSI DAMBER   DEAN  DEAN2 GOLLED HAMMON   JOLY  LUBIN
       MCCONN   QIAO SADOWS SCHWA2 STASZE XIANGZ

5      HIRAY2 TVERDA WYNDER

7      CEDERL   CHOW  DOLL2   DORN GRAHAM LOMBAR LUBIN2 MIGRAN
```

Table 2E17 - 8

Potentially overlapping studies

REF	REFGP	PRINC	.	OVERLAP/LINK
WYNDE7	WYNDE6	2		WYNDE5/6/7/8

Table 2E18 -

IESLC - Meta-analysis of Ever Smoking (or Current if Ever not available), Mixed smokers
Squamous

This analysis is restricted to results for:
1) Non-dose-response data
2) Mixed smokers (cigarettes and pipe/cigar)
3) Results complete enough for use in metaanalysis

Within each study, results are then selected (in the following order of preference, within each sex) for:
4) SMKSTA: ever smokers, current smokers
5) DENOM: never smoked anything, (never +1 = +long term ex)
6) Followup period (prospective studies): whole study (coded as 0) or longest available
7) LCtype: all or nearest available, at least Squamous and Adeno. (q = squamous, s = small,
l = large, a = adeno, mix = mixed, alv = alveolar)
8) Race: all or nearest available, otherwise by race (wh or w = white, bl or b = black, hi = hispanic
ch = chinese, jap = japanese, haw = hawaiian, w+o = white + oriental, sca = scandinavian, as = asian)
9) For overlapping studies: principal rather than subsidiary studies
Finally by Age: whole study (coded as 0) if available, otherwise by widest available age group
and then for single sex results (m, f) in preference to combined sex results (c).

Results adjusted (AD) for the most potential confounders are then chosen in Sections -1 to -3
and results adjusted for the least confounders in Sections -4 to -6. (Those least adjusted results which
actually differ from the most adjusted as marked 'x' in column X in Section -4)
(Results adjusted for an unknown number of confounder(s) are coded as 20.)

Section -7 shows excluded studies, together with the stage (as above) at which no qualifying
results were found.

Section -8 lists the potentially overlapping studies which have been included (1=principal, 2=subsidiary).

Section -9 lists any results which would have been included in preference except that they had data not complete
enough for use in meta-analysis, with their significance (yes/no), if known, and any further comment as entered
on the database.

In addition to those mentioned above, the following fields, levels and abbreviations are used:
* or nk = not known, n = no, y = yes, ot = other
ev = ever, cu = current, nev = never
REF: 6-character study reference
NRR: number of the RR on the database within the study
ST : study type (CC = case control, pr or prosp = prospective)
NLC: number of lung cancer cases in whole study
R : risky occupational population (n = no, m = mining, o = other risky)
VB : national cigarette type (V = at least 75% Virginia, bl = at least 75% blended, ot = other)
P : any proxy use
H : full histological confirmation
De : derivation of RR/CI (or = original, st = standard method, ot = other method of estimation)

Table 2E18 - 1

IESLC - Meta-analysis of Ever Smoking (or Current if Ever not available), Mixed smokers
Squamous
Most adjusted

REF	NRR	SEX	AGEL	AGEH	RACE	YF	LC TYPE	LOC	START	ST	NLC	R	VB	P	H	AD	SM	DENOM	De
ALDERS	29	m	0	0	all	-	q+s	Eu:UK	1977	CC	1448	n	V	n	n	1	ev	nev any	ot
BRESLO	9	c	0	0	all	-	not a	NAmer	1949	CC	518	n	bl	n	y	0	ev	nev+1	st
HAMMON	57	m	0	0	wh	0	not a	NAmer	1952	pr	448	n	bl	n	n	1	ev	nev any	ot
LUBIN	17	m	0	0	all	-	KI	As:Chi	1984	CC	427	m	ot	y	n	0	ev	nev any	st
STASZE	15	m	0	0	all	-	q	Eu:est	1954	CC	281	n	bl	n	y	0	ev	nev any	ot
WYNDE7	60	m	0	0	all	-	KI	NAmer	1977	CC	2085	n	bl	n	y	0	ev	nev any	st

Table 2E18 - 2

IESLC - Meta-analysis of Ever Smoking (or Current if Ever not available), Mixed smokers
Squamous
Most adjusted

REF	NRR	SEX	AD	Number Exposed Case	Number Exposed Cont	Non-exposed Case	Non-exposed Cont	RR	95.00%CI
ALDERS	29	m	1	-	-	-	-	7.58	(2.70- 21.28)
BRESLO	9	c	0	146	154	15	56	3.54	(1.92- 6.53)
*HAMMON	57	m	1	-	-	-	-	19.71	(7.26- 53.50)
LUBIN	17	m	0	249	597	4	72	7.51	(2.71- 20.77)
STASZE	15	m	0	18	101	0	158	57.78~	(3.44- 969.38)
WYNDE7	60	m	0	223	614	22	918	15.16	(9.67- 23.76)
Partial Totals				636	1466	41	1204		

*prospective study ~ With 0.5 adjustment for zero

REF	NRR	SEX	AD	Ys	Ws	Qs	Ps
ALDERS	29	m	1	2.03	3.61	0.22	0.0001
BRESLO	9	c	0	1.26	10.22	10.35	0.0001
*HAMMON	57	m	1	2.98	3.85	1.95	0.0000
LUBIN	17	m	0	2.02	3.71	0.24	0.0001
STASZE	15	m	0	4.06	0.48	1.54	0.0048
WYNDE7	60	m	0	2.72	18.99	3.81	0.0000

	N	6
	NS	6
	Wt	40.86
Het	Chi	18.10
Het	df	5
Het	P	**
Fixed	RR	9.68
	RRl	7.13
	RRu	13.16
	P	+++
Random	RR	9.78
	RRl	4.94
	RRu	19.35
	P	+++
Asymm	P	N.S.

Table 2E18 - 3

IESLC - Meta-analysis of Ever Smoking (or Current if Ever not available), Mixed smokers
Squamous
Most adjusted

	Sex			
	combined	male	female	Total
N	1	5		6
NS	1	5		6
Wt	10.22	30.64		40.86
Het Chi	0.00	4.30		18.10
Het df	0	4		5
Het P	N.S.	N.S.		**
Fixed RR	3.54	13.54		9.68
RRl	1.92	9.51		7.13
RRu	6.53	19.30		13.16
P	+++	+++		+++
Random RR	3.54	13.35		9.78
RRl	1.92	9.03		4.94
RRu	6.53	19.71		19.35
P	+++	+++		+++
Between Chi				13.80
Between df				1
Between P				***
Btwn(F) P				*
Btwn(R) P				***

Too few RRs for analysis by factor

Table 2E18 - 4

IESLC - Meta-analysis of Ever Smoking (or Current if Ever not available), Mixed smokers
Squamous
Least adjusted

REF	NRR	X	SEX	AGEL	AGEH	RACE	YF	LC TYPE	LOC	START	ST	NLC	R	VB	P	H	AD	SM	DENOM	De
ALDERS	80	x	m	0	0	all	-	q+s	Eu:UK	1977	CC	1448	n	V	n	n	0	ev	nev any	st
BRESLO	9		c	0	0	all	-	not a	NAmer	1949	CC	518	n	bl	n	y	0	ev	nev+1	st
HAMMON	70	x	m	0	0	wh	0	not a	NAmer	1952	pr	448	n	bl	n	n	0	ev	nev any	st
LUBIN	17		m	0	0	all	-	KI	As:Chi	1984	CC	427	m	ot	y	n	0	ev	nev any	st
STASZE	15		m	0	0	all	-	q	Eu:est	1954	CC	281	n	bl	n	y	0	ev	nev any	ot
WYNDE7	60		m	0	0	all	-	KI	NAmer	1977	CC	2085	n	bl	n	y	0	ev	nev any	st

Table 2E18 - 5

IESLC - Meta-analysis of Ever Smoking (or Current if Ever not available), Mixed smokers
Squamous
Least adjusted

REF	NRR	SEX	AD	Number Exposed Case	Cont	Non-exposed Case	Cont	RR	95.00%CI
ALDERS	80	m	0	70	179	4	133	13.00	(4.63- 36.50)
BRESLO	9	c	0	146	154	15	56	3.54	(1.92- 6.53)
*HAMMON	70	m	0	103	156773	4	115884	19.03	(7.01- 51.68)
LUBIN	17	m	0	249	597	4	72	7.51	(2.71- 20.77)
STASZE	15	m	0	18	101	0	158	57.78~	(3.44- 969.38)
WYNDE7	60	m	0	223	614	22	918	15.16	(9.67- 23.76)
Totals				809	158418	49	117221		

*prospective study ~ With 0.5 adjustment for zero

REF	NRR	SEX	AD	Ys	Ws	Qs	Ps
ALDERS	80	m	0	2.57	3.61	0.23	0.0000
BRESLO	9	c	0	1.26	10.22	11.28	0.0001
*HAMMON	70	m	0	2.95	3.85	1.54	0.0000
LUBIN	17	m	0	2.02	3.71	0.33	0.0001
STASZE	15	m	0	4.06	0.48	1.47	0.0048
WYNDE7	60	m	0	2.72	18.99	3.10	0.0000

	N	6
	NS	6
	Wt	40.86
Het	Chi	17.93
Het	df	5
Het	P	**
Fixed	RR	10.12
	RRl	7.45
	RRu	13.75
	P	+++
Random	RR	10.62
	RRl	5.38
	RRu	20.94
	P	+++
Asymm	P	N.S.

Table 2E18 - 6

IESLC - Meta-analysis of Ever Smoking (or Current if Ever not available), Mixed smokers
Squamous
Least adjusted

	Sex combined	male	female	Total
N	1	5		6
NS	1	5		6
Wt	10.22	30.64		40.86
Het Chi	0.00	2.89		17.93
Het df	0	4		5
Het P	N.S.	N.S.		**
Fixed RR	3.54	14.37		10.12
RRl	1.92	10.08		7.45
RRu	6.53	20.47		13.75
P	+++	+++		+++
Random RR	3.54	14.37		10.62
RRl	1.92	10.08		5.38
RRu	6.53	20.47		20.94
P	+++	+++		+++
Between Chi				15.04
Between df				1
Between P				***
Btwn(F) P				*
Btwn(R) P				***

Table 2E18 - 7

IESLC - Meta-analysis of Ever Smoking (or Current if Ever not available), Mixed smokers
Squamous
Excluded studies (and stage at which they were excluded)

1 BOUCHA BUELL LAURIL MZILEN

2 ABRAHA AGUDO AKIBA AMANDU AMES ANDERS ARCHER AUSTIN AUVINE AXELSO AXELSS BAND BARBON BECHER BENSHL BERRIN
BLOHMK BLOT1 BLOT2 BLOT3 BLOT4 BRETT BROCKM BROSS BROWN1 BROWN2 BUFFLE BYERS1 BYERS2 CARPEN CASCO2 CASCOR
CHAN CHANG CHATZI CHEN CHEN2 CHEN3 CHIAZZ CHOI CHYOU COMSTO COOKSO CORREA CPSII DARBY DAVEYS DEAN3
DEKLER DESTE2 DESTEF DOCKER DOLL DORANT DORGAN DOSEME DROSTE DU DUNN EBELIN ENGELA ENSTRO ESAKI FAN
GAO GAO2 GARCIA GARDIN GARSHI GENG GER GILLIS GODLEY GOODMA GREGOR GSELL HAENSZ HAMMO2 HANSEN HEGMAN
HEIN HENNEK HINDS HIRAOK HIRAYA HITOSU HOLE HOROWI HORWIT HU HU2 HUANG HUMBLE ISHIMA JAHN JAIN
JARUP JARVHO JEDRYC JIANG JONES JUSSAW KAISE2 KAISER KANELL KATSOU KAUFMA KELLER KHUDER KIHARA KINLEN KJUUS
KNEKT KO KOHLME KOO KOULUM KREUZE KREYBE KUBIK LAMTH LAMWK LAMWK2 LANGE LAUSSM LEI LEMARC LETOUR
LEVIN LIAW LICKIN LIDDEL LIU LIU2 LIU3 LIU4 LIU5 LOMBA2 LUO MACLEN MAGNUS MARSH MARSH2 MARTIS
MASTRA MATOS MATSUD MCDUFF MCLAUG MILLER MILLS MOLLO MRFIT MRFITR MURATA NAM NOTAN2 NOTANI NOU ODRISC
ORMOS OSANN OSANN2 PARKIN PASTOR PAWLEG PERNU PERSH2 PERSHA PETO PEZZO2 PEZZOT PIKE PISANI POFFIJ POLEDN
PRESCO QIAO2 RACHTA RADZIK RANDIG REN RESTRE RIMING RONCO ROOTS ROTHSC SAARIK SANKAR SCHWAR SEGI SEGI2
SEOW SHAW SHIMIZ SIEMIA SIMARA SIMONA SITAS SOBUE SOBUE2 SPEIZE SPITZ STAYNE STOCKS STOCKW STUCKE SUN
SUZUK2 SUZUKI SVENSS TANG TANG2 TAO TENKAN TIZZAN TOKARS TOUSEY TSUGAN TULINI ULMER VANDER VEIERO VUTUC
WAKAI WALD WANG WANG2 WANG3 WANG4 WARSIN WATSON WICKLU WIGLE WILKIN WU WU2 WUNSCH WUWILL WYNDE2
WYNDE3 WYNDE4 WYNDE5 WYNDE6 WYNDE8 XU XU2 XU3 XU4 YAMAGU YONG YUAN ZHANG ZHENG ZHOU

5 HIRAY2 SCHWA2 TVERDA WYNDER

7 ABELIN ARMADA BENHAM BEST BOFFET BOUCOT CEDERL CHOW CPSI DAMBER DEAN DEAN2 DOLL2 DORN GOLLED GRAHAM
JOLY LOMBAR LUBIN2 MCCONN MIGRAN QIAO SADOWS XIANGZ

Table 2E18 - 8
Potentially overlapping studies

REF	REFGP	PRINC	.	OVERLAP/LINK
WYNDE7	WYNDE6	2		WYNDE5/6/7/8
LUBIN	XIANGZ	2		LUBIN/XIANGZ/QIAO

Table 2E19 -

IESLC - Meta-analysis of Current Smoking (or Ever if Current not available), Mixed smokers
Squamous

This analysis is restricted to results for:
1) Non-dose-response data
2) Mixed smokers (cigarettes and pipe/cigar)
3) Results complete enough for use in metaanalysis

Within each study, results are then selected (in the following order of preference, within each sex) for:
4) SMKSTA: current smokers, ever smokers
5) DENOM: never smoked anything, (never +1 = +long term ex)
6) Followup period (prospective studies): whole study (coded as 0) or longest available
7) LCtype: all or nearest available, at least Squamous and Adeno. (q = squamous, s = small,
l = large, a = adeno, mix = mixed, alv = alveolar)
8) Race: all or nearest available, otherwise by race (wh or w = white, bl or b = black, hi = hispanic
ch = chinese, jap = japanese, haw = hawaiian, w+o = white + oriental, sca = scandinavian, as = asian)
9) For overlapping studies: principal rather than subsidiary studies
Finally by Age: whole study (coded as 0) if available, otherwise by widest available age group
and then for single sex results (m, f) in preference to combined sex results (c).

Results adjusted (AD) for the most potential confounders are then chosen in Sections -1 to -3
(and those which actually differ from the adjusted results in Table 2E18- 1 are marked 'x' in Section -1)
and results adjusted for the least confounders in Sections -4 to -6. (Those least adjusted results which
actually differ from the most adjusted as marked 'x' in column X in Section -4)
(Results adjusted for an unknown number of confounder(s) are coded as 20.)

Section -7 shows excluded studies, together with the stage (as above) at which no qualifying
results were found.

Section -8 lists the potentially overlapping studies which have been included (1=principal, 2=subsidiary).

Section -9 lists any results which would have been included in preference except that they had data not complete
enough for use in meta-analysis, with their significance (yes/no), if known, and any further comment as entered
on the database.

In addition to those mentioned above, the following fields, levels and abbreviations are used:
* or nk = not known, n = no, y = yes, ot = other
ev = ever, cu = current, nev = never
REF: 6-character study reference
NRR: number of the RR on the database within the study
ST : study type (CC = case control, pr or prosp = prospective)
NLC: number of lung cancer cases in whole study
R : risky occupational population (n = no, m = mining, o = other risky)
VB : national cigarette type (V = at least 75% Virginia, bl = at least 75% blended, ot = other)
P : any proxy use
H : full histological confirmation
De : derivation of RR/CI (or = original, st = standard method, ot = other method of estimation)

Table 2E19 - 1

IESLC - Meta-analysis of Current Smoking (or Ever if Current not available), Mixed smokers
Squamous
Most adjusted

REF	NRR	2E18	SEX	AGEL	AGEH	RACE	YF	LC TYPE	LOC	START	ST	NLC	R	VB	P	H	AD	SM	DENOM	De
ALDERS	29		m	0	0	all	-	q+s	Eu:UK	1977	CC	1448	n	V	n	n	1	ev	nev any	ot
BRESLO	9		c	0	0	all	-	not a	NAmer	1949	CC	518	n	bl	n	y	0	ev	nev+1	st
HAMMON	57		m	0	0	wh	0	not a	NAmer	1952	pr	448	n	bl	n	n	1	ev	nev any	ot
LUBIN	17		m	0	0	all	-	KI	As:Chi	1984	CC	427	m	ot	y	n	0	ev	nev any	st
STASZE	15		m	0	0	all	-	q	Eu:est	1954	CC	281	n	bl	n	y	0	ev	nev any	ot
WYNDE7	23	x	m	0	0	all	-	KI	NAmer	1977	CC	2085	n	bl	n	y	0	cu	nev any	st

Table 2E19 - 2

IESLC - Meta-analysis of Current Smoking (or Ever if Current not available), Mixed smokers
Squamous
Most adjusted

REF	NRR	SEX	AD	Number Exposed Case	Exposed Cont	Non-exposed Case	Non-exposed Cont	RR	95.00%CI
ALDERS	29	m	1	-	-	-	-	7.58	(2.70- 21.28)
BRESLO	9	c	0	146	154	15	56	3.54	(1.92- 6.53)
*HAMMON	57	m	1	-	-	-	-	19.71	(7.26- 53.50)
LUBIN	17	m	0	249	597	4	72	7.51	(2.71- 20.77)
STASZE	15	m	0	18	101	0	158	57.78~	(3.44- 969.38)
WYNDE7	23	m	0	152	287	22	918	22.10	(13.86- 35.23)
Partial Totals				565	1139	41	1204		

*prospective study ~ With 0.5 adjustment for zero

REF	NRR	SEX	AD	Ys	Ws	Qs	Ps
ALDERS	29	m	1	2.03	3.61	0.57	0.0001
BRESLO	9	c	0	1.26	10.22	13.75	0.0001
*HAMMON	57	m	1	2.98	3.85	1.20	0.0000
LUBIN	17	m	0	2.02	3.71	0.62	0.0001
STASZE	15	m	0	4.06	0.48	1.29	0.0048
WYNDE7	23	m	0	3.10	17.67	7.97	0.0000

	N	6
	NS	6
	Wt	39.53
Het	Chi	25.39
Het	df	5
Het	P	***
Fixed	RR	11.29
	RRl	8.27
	RRu	15.42
	P	+++
Random	RR	10.90
	RRl	4.84
	RRu	24.53
	P	+++
Asymm	P	N.S.

Table 2E19 - 3

IESLC - Meta-analysis of Current Smoking (or Ever if Current not available), Mixed smokers
Squamous
Most adjusted

		combined	Sex male	female	Total
	N	1	5		6
	NS	1	5		6
	Wt	10.22	29.32		39.53
Het	Chi	0.00	6.85		25.39
Het	df	0	4		5
Het	P	N.S.	N.S.		***
Fixed	RR	3.54	16.91		11.29
	RRl	1.92	11.78		8.27
	RRu	6.53	24.29		15.42
	P	+++	+++		+++
Random	RR	3.54	14.82		10.90
	RRl	1.92	8.39		4.84
	RRu	6.53	26.19		24.53
	P	+++	+++		+++
Between	Chi				18.54
Between	df				1
Between	P				***
Btwn(F)	P				*
Btwn(R)	P				***

Too few RRs for analysis by factor

Table 2E19 - 4

IESLC - Meta-analysis of Current Smoking (or Ever if Current not available), Mixed smokers
Squamous
Least adjusted

REF	NRR	X	SEX	AGEL	AGEH	RACE	YF	LC TYPE	LOC	START	ST	NLC	R	VB	P	H	AD	SM	DENOM	De
ALDERS	80	x	m	0	0	all	-	q+s	Eu:UK	1977	CC	1448	n	V	n	n	0	ev	nev any	st
BRESLO	9		c	0	0	all	-	not a	NAmer	1949	CC	518	n	bl	n	y	0	ev	nev+1	st
HAMMON	70	x	m	0	0	wh	0	not a	NAmer	1952	pr	448	n	bl	n	n	0	ev	nev any	st
LUBIN	17		m	0	0	all	-	KI	As:Chi	1984	CC	427	m	ot	y	n	0	ev	nev any	st
STASZE	15		m	0	0	all	-	q	Eu:est	1954	CC	281	n	bl	n	y	0	ev	nev any	ot
WYNDE7	23		m	0	0	all	-	KI	NAmer	1977	CC	2085	n	bl	n	y	0	cu	nev any	st

Table 2E19 - 5

IESLC - Meta-analysis of Current Smoking (or Ever if Current not available), Mixed smokers
Squamous
Least adjusted

REF	NRR	SEX	AD	Number Exposed Case	Number Exposed Cont	Non-exposed Case	Non-exposed Cont	RR	95.00%CI
ALDERS	80	m	0	70	179	4	133	13.00	(4.63- 36.50)
BRESLO	9	c	0	146	154	15	56	3.54	(1.92- 6.53)
*HAMMON	70	m	0	103	156773	4	115884	19.03	(7.01- 51.68)
LUBIN	17	m	0	249	597	4	72	7.51	(2.71- 20.77)
STASZE	15	m	0	18	101	0	158	57.78~	(3.44- 969.38)
WYNDE7	23	m	0	152	287	22	918	22.10	(13.86- 35.23)
Totals				738	158091	49	117221		

*prospective study ~ With 0.5 adjustment for zero

REF	NRR	SEX	AD	Ys	Ws	Qs	Ps
ALDERS	80	m	0	2.57	3.61	0.03	0.0000
BRESLO	9	c	0	1.26	10.22	14.85	0.0001
*HAMMON	70	m	0	2.95	3.85	0.87	0.0000
LUBIN	17	m	0	2.02	3.71	0.76	0.0001
STASZE	15	m	0	4.06	0.48	1.22	0.0048
WYNDE7	23	m	0	3.10	17.67	6.92	0.0000

	N	6
	NS	6
	Wt	39.53
Het	Chi	24.66
Het	df	5
Het	P	***
Fixed	RR	11.82
	RRl	8.65
	RRu	16.14
	P	+++
Random	RR	11.85
	RRl	5.33
	RRu	26.37
	P	+++
Asymm	P	N.S.

Table 2E19 - 6

IESLC - Meta-analysis of Current Smoking (or Ever if Current not available), Mixed smokers
Squamous
Least adjusted

	combined	Sex male	female	Total
N	1	5		6
NS	1	5		6
Wt	10.22	29.31		39.53
Het Chi	0.00	4.63		24.66
Het df	0	4		5
Het P	N.S.	N.S.		***
Fixed RR	3.54	17.99		11.82
RRl	1.92	12.53		8.65
RRu	6.53	25.84		16.14
P	+++	+++		+++
Random RR	3.54	17.21		11.85
RRl	1.92	11.22		5.33
RRu	6.53	26.38		26.37
P	+++	+++		+++
Between Chi				20.03
Between df				1
Between P				***
Btwn(F) P				*
Btwn(R) P				***

Table 2E19 - 7

IESLC - Meta-analysis of Current Smoking (or Ever if Current not available), Mixed smokers
Squamous
Excluded studies (and stage at which they were excluded)

1 BOUCHA BUELL LAURIL MZILEN

2 ABRAHA AGUDO AKIBA AMANDU AMES ANDERS ARCHER AUSTIN AUVINE AXELSO AXELSS BAND BARBON BECHER BENSHL BERRIN
BLOHMK BLOT1 BLOT2 BLOT3 BLOT4 BRETT BROCKM BROSS BROWN1 BROWN2 BUFFLE BYERS1 BYERS2 CARPEN CASCO2 CASCOR
CHAN CHANG CHATZI CHEN CHEN2 CHEN3 CHIAZZ CHOI CHYOU COMSTO COOKSO CORREA CPSII DARBY DAVEYS DEAN3
DEKLER DESTE2 DESTEF DOCKER DOLL DORANT DORGAN DOSEME DROSTE DU DUNN EBELIN ENGELA ENSTRO ESAKI FAN
GAO GAO2 GARCIA GARDIN GARSHI GENG GER GILLIS GODLEY GOODMA GREGOR GSELL HAENSZ HAMMO2 HANSEN HEGMAN
HEIN HENNEK HINDS HIRAOK HIRAYA HITOSU HOLE HOROWI HORWIT HU HU2 HUANG HUMBLE ISHIMA JAHN JAIN
JARUP JARVHO JEDRYC JIANG JONES JUSSAW KAISE2 KAISER KANELL KATSOU KAUFMA KELLER KHUDER KIHARA KINLEN KJUUS
KNEKT KO KOHLME KOO KOULUM KREUZE KREYBE KUBIK LAMTH LAMWK LAMWK2 LANGE LAUSSM LEI LEMARC LETOUR
LEVIN LIAW LICKIN LIDDEL LIU LIU2 LIU3 LIU4 LIU5 LOMBA2 LUO MACLEN MAGNUS MARSH MARSH2 MARTIS
MASTRA MATOS MATSUD MCDUFF MCLAUG MILLER MILLS MOLLO MRFIT MRFITR MURATA NAM NOTAN2 NOTANI NOU ODRISC
ORMOS OSANN OSANN2 PARKIN PASTOR PAWLEG PERNU PERSH2 PERSHA PETO PEZZO2 PEZZOT PIKE PISANI POFFIJ POLEDN
PRESCO QIAO2 RACHTA RADZIK RANDIG REN RESTRE RIMING RONCO ROOTS ROTHSC SAARIK SANKAR SCHWAR SEGI SEGI2
SEOW SHAW SHIMIZ SIEMIA SIMARA SIMONA SITAS SOBUE SOBUE2 SPEIZE SPITZ STAYNE STOCKS STOCKW STUCKE SUN
SUZUK2 SUZUKI SVENSS TANG TANG2 TAO TENKAN TIZZAN TOKARS TOUSEY TSUGAN TULINI ULMER VANDER VEIERO VUTUC
WAKAI WALD WANG WANG2 WANG3 WANG4 WARSIN WATSON WICKLU WIGLE WILKIN WU WU2 WUNSCH WUWILL WYNDE2
WYNDE3 WYNDE4 WYNDE5 WYNDE6 WYNDE8 XU XU2 XU3 XU4 YAMAGU YONG YUAN ZHANG ZHENG ZHOU

5 HIRAY2 SCHWA2 TVERDA WYNDER

7 ABELIN ARMADA BENHAM BEST BOFFET BOUCOT CEDERL CHOW CPSI DAMBER DEAN DEAN2 DOLL2 DORN GOLLED GRAHAM
JOLY LOMBAR LUBIN2 MCCONN MIGRAN QIAO SADOWS XIANGZ

Table 2E19 - 8

Potentially overlapping studies

REF	REFGP	PRINC	.	OVERLAP/LINK
WYNDE7	WYNDE6	2		WYNDE5/6/7/8
LUBIN	XIANGZ	2		LUBIN/XIANGZ/QIAO

Table 2E20 -

IESLC - Meta-analysis of Ex Smoking, Mixed smokers
Squamous

This analysis is restricted to results for:
1) Non-dose-response data
2) Mixed smokers (cigarettes and pipe/cigar)
3) Ex smokers
4) Results complete enough for use in metaanalysis

Within each study, results are then selected (in the following order of preference, within each sex) for:
5) DENOM: never smoked anything, (never +1 = +long term ex)
6) Followup period (prospective studies): whole study (coded as 0) or longest available
7) LCtype: all or nearest available, at least Squamous and Adeno. (q = squamous, s = small,
l = large, a = adeno, mix = mixed, alv = alveolar)
8) Race: all or nearest available, otherwise by race (wh or w = white, bl or b = black, hi = hispanic
ch = chinese, jap = japanese, haw = hawaiian, w+o = white + oriental, sca = scandinavian, as = asian)
9) For overlapping studies: principal rather than subsidiary studies
Finally by Age: whole study (coded as 0) if available, otherwise by widest available age group
and then for single sex results (m, f) in preference to combined sex results (c).

Results adjusted (AD) for the most potential confounders are then chosen in Sections -1 to -3
and results adjusted for the least confounders in Sections -4 to -6. (Those least adjusted results which
actually differ from the most adjusted as marked 'x' in column X in Section -4)
(Results adjusted for an unknown number of confounder(s) are coded as 20.)

Section -7 shows excluded studies, together with the stage (as above) at which no qualifying
results were found.

Section -8 lists the potentially overlapping studies which have been included (1=principal, 2=subsidiary).

Section -9 lists any results which would have been included in preference except that they had data not complete
enough for use in meta-analysis, with their significance (yes/no), if known, and any further comment as entered
on the database.

In addition to those mentioned above, the following fields, levels and abbreviations are used:
* or nk = not known, n = no, y = yes, ot = other
nev = never
REF: 6-character study reference
NRR: number of the RR on the database within the study
ST : study type (CC = case control, pr or prosp = prospective)
NLC: number of lung cancer cases in whole study
R : risky occupational population (n = no, m = mining, o = other risky)
VB : national cigarette type (V = at least 75% Virginia, bl = at least 75% blended, ot = other)
P : any proxy use
H : full histological confirmation
De : derivation of RR/CI (or = original, st = standard method, ot = other method of estimation)

Table 2E20 - 1

IESLC - Meta-analysis of Ex Smoking, Mixed smokers
Squamous
Most adjusted

REF	NRR	SEX	AGEL	AGEH	RACE	YF	LC TYPE	LOC	START	ST	NLC	R	VB	P	H	AD	DENOM	De
WYNDE7	28	m	0	0	all	-	KI	NAmer	1977	CC	2085	n	bl	n	y	0	nev any	st

Table 2E20 - 2

IESLC - Meta-analysis of Ex Smoking, Mixed smokers
Squamous
Most adjusted

REF	NRR	SEX	AD	Number Case	Exposed Cont	Non-exposed Case	Cont	RR	95.00%CI
WYNDE7	28	m	0	71	327	22	918	9.06	(5.52- 14.86)
Totals				71	327	22	918		

*prospective study

REF	NRR	SEX	AD	Ys	Ws	Qs	Ps
WYNDE7	28	m	0	2.20	15.70	0.00	0.0000

	N	1
	NS	1
	Wt	15.70
Het	Chi	0.00
Het	df	0
Het	P	N.S.
Fixed	RR	9.06
	RRl	5.52
	RRu	14.86
	P	+++
Random	RR	9.06
	RRl	5.52
	RRu	14.86
	P	+++
Asymm	P	

Table 2E20 - 3

IESLC - Meta-analysis of Ex Smoking, Mixed smokers
Squamous
Most adjusted

	Sex combined	male	female	Total
N		1		1
NS		1		1
Wt		15.70		15.70
Het Chi		0.00		0.00
Het df		0		0
Het P		N.S.		N.S.
Fixed RR		9.06		9.06
RRl		5.52		5.52
RRu		14.86		14.86
P		+++		+++
Random RR		9.06		9.06
RRl		5.52		5.52
RRu		14.86		14.86
P		+++		+++
Between Chi				
Between df				
Between P				N.S.
Btwn(F) P				N.S.
Btwn(R) P				N.S.

Too few RRs for analysis by factor

Table 2E20 - 4

IESLC - Meta-analysis of Ex Smoking, Mixed smokers
Squamous
Least adjusted

REF	NRR	X	SEX	AGEL	AGEH	RACE	YF	LC TYPE	LOC	START	ST	NLC	R	VB	P	H	AD	DENOM	De
WYNDE7	28		m	0	0	all	-	KI	NAmer	1977	CC	2085	n	bl	n	y	0	nev any	st

Table 2E20 - 5

IESLC - Meta-analysis of Ex Smoking, Mixed smokers
Squamous
Least adjusted

REF	NRR	SEX	AD	Number Exposed Case	Cont	Non-exposed Case	Cont	RR	95.00%CI
WYNDE7	28	m	0	71	327	22	918	9.06	(5.52- 14.86)
Totals				71	327	22	918		

*prospective study

REF	NRR	SEX	AD	Ys	Ws	Qs	Ps
WYNDE7	28	m	0	2.20	15.70	0.00	0.0000

	N	1
	NS	1
	Wt	15.70
Het	Chi	0.00
Het	df	0
Het	P	N.S.
Fixed	RR	9.06
	RRl	5.52
	RRu	14.86
	P	+++
Random	RR	9.06
	RRl	5.52
	RRu	14.86
	P	+++
Asymm	P	

Table 2E20 - 6

IESLC - Meta-analysis of Ex Smoking, Mixed smokers
Squamous
Least adjusted

	Sex combined	male	female	Total
N		1		1
NS		1		1
Wt		15.70		15.70
Het Chi		0.00		0.00
Het df		0		0
Het P		N.S.		N.S.
Fixed RR		9.06		9.06
RRl		5.52		5.52
RRu		14.86		14.86
P		+++		+++
Random RR		9.06		9.06
RRl		5.52		5.52
RRu		14.86		14.86
P		+++		+++
Between Chi				
Between df				
Between P				N.S.
Btwn(F) P				N.S.
Btwn(R) P				N.S.

Table 2E20 - 7

IESLC - Meta-analysis of Ex Smoking, Mixed smokers
Squamous
Excluded studies (and stage at which they were excluded)

1	BOUCHA BUELL LAURIL MZILEN
2	ABRAHA AGUDO AKIBA AMANDU AMES ANDERS ARCHER AUSTIN AUVINE AXELSO AXELSS BAND BARBON BECHER BENSHL BERRIN BLOHMK BLOT1 BLOT2 BLOT3 BLOT4 BRETT BROCKM BROSS BROWN1 BROWN2 BUFFLE BYERS1 BYERS2 CARPEN CASCO2 CASCOR CHAN CHANG CHATZI CHEN CHEN2 CHEN3 CHIAZZ CHOI CHYOU COMSTO COOKSO CORREA CPSII DARBY DAVEYS DEAN3 DEKLER DESTE2 DESTEF DOCKER DOLL DORANT DORGAN DOSEME DROSTE DU DUNN EBELIN ENGELA ENSTRO ESAKI FAN GAO GAO2 GARCIA GARDIN GARSHI GENG GER GILLIS GODLEY GOODMA GREGOR GSELL HAENSZ HAMMO2 HANSEN HEGMAN HEIN HENNEK HINDS HIRAOK HIRAYA HITOSU HOLE HOROWI HORWIT HU HU2 HUANG HUMBLE ISHIMA JAHN JAIN JARUP JARVHO JEDRYC JIANG JONES JUSSAW KAISE2 KAISER KANELL KATSOU KAUFMA KELLER KHUDER KIHARA KINLEN KJUUS KNEKT KO KOHLME KOO KOULUM KREUZE KREYBE KUBIK LAMTH LAMWK LAMWK2 LANGE LAUSSM LEI LEMARC LETOUR LEVIN LIAW LICKIN LIDDEL LIU LIU2 LIU3 LIU4 LIU5 LOMBA2 LUO MACLEN MAGNUS MARSH MARSH2 MARTIS MASTRA MATOS MATSUD MCDUFF MCLAUG MILLER MILLS MOLLO MRFIT MRFITR MURATA NAM NOTAN2 NOTANI NOU ODRISC ORMOS OSANN OSANN2 PARKIN PASTOR PAWLEG PERNU PERSH2 PERSHA PETO PEZZO2 PEZZOT PIKE PISANI POFFIJ POLEDN PRESCO QIAO2 RACHTA RADZIK RANDIG REN RESTRE RIMING RONCO ROOTS ROTHSC SAARIK SANKAR SCHWAR SEGI SEGI2 SEOW SHAW SHIMIZ SIEMIA SIMARA SIMONA SITAS SOBUE SOBUE2 SPEIZE SPITZ STAYNE STOCKS STOCKW STUCKE SUN SUZUK2 SUZUKI SVENSS TANG TANG2 TAO TENKAN TIZZAN TOKARS TOUSEY TSUGAN TULINI ULMER VANDER VEIERO VUTUC WAKAI WALD WANG WANG2 WANG3 WANG4 WARSIN WATSON WICKLU WIGLE WILKIN WU WU2 WUNSCH WUWILL WYNDE2 WYNDE3 WYNDE4 WYNDE5 WYNDE6 WYNDE8 XU XU2 XU3 XU4 YAMAGU YONG YUAN ZHANG ZHENG ZHOU
3	ABELIN ALDERS ARMADA BENHAM BEST BOFFET BOUCOT BRESLO CEDERL CPSI DAMBER DEAN DEAN2 GOLLED HAMMON HIRAY2 JOLY LUBIN MCCONN MIGRAN QIAO SADOWS SCHWA2 STASZE TVERDA WYNDER XIANGZ
7	CHOW DOLL2 DORN GRAHAM LOMBAR LUBIN2

Table 2E20 - 8
Potentially overlapping studies

REF	REFGP	PRINC	.	OVERLAP/LINK
WYNDE7	WYNDE6	2		WYNDE5/6/7/8
