# Supplementary material for: Systematic review with meta-analysis of the epidemiological evidence in the 1900s relating smoking to lung cancer
Source: BMC Cancer. 2012 Sep 3;12:385. doi: 10.1186/1471-2407-12-385 (PMC3505152; doi:10.1186/1471-2407-12-385)
Supplement: Additional file 5 — Detailed Analysis Tables (Individual file names as described in Additional file 1: Methods, Table1). [file 1471-2407-12-385-S5.zip › PDF/1H.pdf]

Table 1H1 -

IESLC - Meta-analysis of Ever Smoking by Age started, Overview
All LC types, Any Product (or Cigarettes if Any not available)

This analysis is restricted to results for:
1) Ever smokers
2) Results by Age started
3) Categorical results by Age started
Results by Age started are grouped under 2 schemes (S1, S2). Each scheme has a set of "key values". An interval is allocated to the category whose key value it includes, and intervals which include none or more than one of the key values are excluded. (Open-ended intervals are coded as 999)

S1	key value	maximum range
1	26	19+
2	18	15-25
3	14	1-17

S2	key value	maximum range
1	30	27+
2	26	23-29
3	22	19-25
4	18	15-21
5	14	11-17
6	10	1-13

4) All LC types (or near equivalent)
5) Results complete enough for use in metaanalysis

Within each study, results are then selected (in the following order of preference, within each sex) for:
6) (not applicable)
7) PRODUCT: all/unspec, cigarettes regardless of other products, cigarettes only
8) CIGTYPE: all/unspecified, MC regardless of HR, MC only
9) (not applicable)
10) DENOM: never smoked anything, never smoked cigarettes, never any + low, never cigs + low
11) Followup period (YF, prospective studies): whole study (coded as 0) or longest available
12) LCtype: all or nearest available, at least Squamous and Adeno. (q = squamous, s = small,
l = large, a = adeno, mix = mixed, alv = alveolar)
13) Race: all or nearest available, otherwise by race (wh or w = white, bl or b = black, hi = hispanic
ch = chinese, jap = japanese, haw = hawaiian, w+o = white + oriental, sca = scandinavian, as = asian)
14) For overlapping studies: principal rather than subsidiary studies
Finally by Age: whole study (coded as 0) if available, otherwise by widest available age group and then for single sex results (m, f) in preference to results for both sexes combined (c).

Results adjusted (AD) for the most potential confounders are then chosen in Sections -1 to -3 and results adjusted for the least confounders in Sections -4 to -6. (Those least adjusted results which actually differ from the most adjusted are marked 'x' in column X in Section -4)

Section -7 shows excluded studies, together with the stage (as above) at which no qualifying results were found.

Section -8 lists the potentially overlapping studies which have been included (1=principal, 2=subsidiary).

Section -9 lists any results which would have been included in preference except that they had data not complete enough for use in meta-analysis, with their significance (yes/no), if known, and any further comment as entered on the database. It also lists as "gap" any categories for which no data were presented by the original authors.

In addition to those mentioned above, the following fields, levels and abbreviations are used:
* or nk = not known, n = no, y = yes, ot = other
nev = never
all/unspec = all or unspecified, cig+/-ot = cigarettes irrespective of other products (cigar, pipe etc)
MC = manufactured cigarettes, HR = hand-rolled cigarettes
exL, exH = range of exposure (low and high) in the smoking group, in terms of Age started
REF: 6-character study reference
NRR: number of the RR on the database within the study
ST : study type (CC = case control, pr or prosp = prospective)
NLC: number of lung cancer cases in whole study
R : risky occupational population (n = no, m = mining, o = other risky)
VB : national cigarette type (V = at least 75% Virginia, bl = at least 75% blended, ot = other)
P : any proxy use
H : full histological confirmation
De : derivation of RR/CI (or = original, st = standard method, ot = other method of estimation)

Table 1H1 - 1

IESLC - Meta-analysis of Ever Smoking by Age started, Overview
All LC types, Any Product (or Cigarettes if Any not available)
Most adjusted

REF	NRR	SEX	AGEL	AGEH	RACE	YF	LC TYPE	LOC	START	ST	NLC	R	VB	P	H	AD	PRODUCT	exL	exH	S1	S2	DENOM	De
AGUDO	504	f	0	0	all	-	all	Eu:wst	1989	CC	103	n	bl	n	n	3	cig only	24	999	1	0	nev cigs	or
AGUDO	505	f	0	0	all	-	all	Eu:wst	1989	CC	103	n	bl	n	n	3	cig only	1	23	0	0	nev cigs	or
ARMADA	511	m	0	0	all	-	all	Eu:wst	1986	CC	325	n	bl	n	y	0	cig+/-ot	17	45	0	0	nev cigs	st
ARMADA	512	m	0	0	all	-	all	Eu:wst	1986	CC	325	n	bl	n	y	0	cig+/-ot	7	16	3	0	nev cigs	st
AUVINE	520	c	0	0	all	-	all	Eu:Sca	1986	CC	517	n	bl	y	n	2	cig+/-ot	16	999	0	0	nev cigs	or
AUVINE	521	c	0	0	all	-	all	Eu:Sca	1986	CC	517	n	bl	y	n	2	cig+/-ot	1	15	3	0	nev cigs	or
BARBON	520	m	0	0	all	-	all	Eu:wst	1979	CC	755	n	bl	y	y	1	all/unsp	20	999	1	0	nev any	or
BARBON	521	m	0	0	all	-	all	Eu:wst	1979	CC	755	n	bl	y	y	1	all/unsp	15	19	2	4	nev any	or
BARBON	522	m	0	0	all	-	all	Eu:wst	1979	CC	755	n	bl	y	y	1	all/unsp	1	14	3	0	nev any	or
BRESLO	501	c	0	0	all	-	all	NAmer	1949	CC	518	n	bl	n	y	0	cig+/-ot	25	999	1	0	nev any	st
BRESLO	502	c	0	0	all	-	all	NAmer	1949	CC	518	n	bl	n	y	0	cig+/-ot	15	24	2	0	nev any	st
BRESLO	503	c	0	0	all	-	all	NAmer	1949	CC	518	n	bl	n	y	0	cig+/-ot	0	14	3	0	nev any	st
BUFFLE	517	f	0	0	w-hi	-	all	NAmer	1976	CC	943	n	bl	y	n	0	cig+/-ot	30	999	0	1	nev cigs	or
BUFFLE	518	f	0	0	w-hi	-	all	NAmer	1976	CC	943	n	bl	y	n	0	cig+/-ot	21	29	1	0	nev cigs	or
BUFFLE	519	f	0	0	w-hi	-	all	NAmer	1976	CC	943	n	bl	y	n	0	cig+/-ot	19	20	0	0	nev cigs	or
BUFFLE	520	f	0	0	w-hi	-	all	NAmer	1976	CC	943	n	bl	y	n	0	cig+/-ot	17	18	2	4	nev cigs	ot
BUFFLE	521	f	0	0	w-hi	-	all	NAmer	1976	CC	943	n	bl	y	n	0	cig+/-ot	6	16	3	0	nev cigs	or
CHEN2	517	m	0	0	all	-	all	As:Chi	1983	CC	193	n	ot	y	n	0	all/unsp	31	999	0	0	nev any	st
CHEN2	518	m	0	0	all	-	all	As:Chi	1983	CC	193	n	ot	y	n	0	all/unsp	20	30	1	0	nev any	st
CHEN2	519	m	0	0	all	-	all	As:Chi	1983	CC	193	n	ot	y	n	0	all/unsp	1	19	0	0	nev any	st
CHEN2	522	f	0	0	all	-	all	As:Chi	1983	CC	193	n	ot	y	n	0	all/unsp	31	999	0	0	nev any	st
CHEN2	523	f	0	0	all	-	all	As:Chi	1983	CC	193	n	ot	y	n	0	all/unsp	20	30	1	0	nev any	st
CHEN2	524	f	0	0	all	-	all	As:Chi	1983	CC	193	n	ot	y	n	0	all/unsp	1	19	0	0	nev any	st
CHIAZZ	501	m	0	0	all	-	all	NAmer	1940	CC	144	o	bl	y	n	2	cig+/-ot	20	999	1	0	nev cigs	or
CHIAZZ	502	m	0	0	all	-	all	NAmer	1940	CC	144	o	bl	y	n	2	cig+/-ot	1	19	0	0	nev cigs	or
CHOI	523	m	0	0	all	-	all	As:oth	1985	CC	375	n	bl	n	n	0	cig+/-ot	25	999	1	0	nev cigs	st
CHOI	524	m	0	0	all	-	all	As:oth	1985	CC	375	n	bl	n	n	0	cig+/-ot	20	24	0	3	nev cigs	st
CHOI	525	m	0	0	all	-	all	As:oth	1985	CC	375	n	bl	n	n	0	cig+/-ot	15	19	2	4	nev cigs	st
CHOI	526	m	0	0	all	-	all	As:oth	1985	CC	375	n	bl	n	n	0	cig+/-ot	1	14	3	0	nev cigs	st
CHOI	530	f	0	0	all	-	all	As:oth	1985	CC	375	n	bl	n	n	0	cig+/-ot	25	999	1	0	nev cigs	st
CHOI	531	f	0	0	all	-	all	As:oth	1985	CC	375	n	bl	n	n	0	cig+/-ot	1	24	0	0	nev cigs	st
DAMBER	501	m	0	0	all	-	all	Eu:Sca	1972	CC	579	n	bl	y	n	0	all/unsp	21	999	1	0	nev any	st
DAMBER	502	m	0	0	all	-	all	Eu:Sca	1972	CC	579	n	bl	y	n	0	all/unsp	16	20	2	4	nev any	st
DAMBER	503	m	0	0	all	-	all	Eu:Sca	1972	CC	579	n	bl	y	n	0	all/unsp	1	15	3	0	nev any	st
DOLL	501	m	0	0	all	-	all	Eu:UK	1948	CC	1465	n	V	n	n	0	all/unsp	40	999	0	0	nev any	st
DOLL	502	m	0	0	all	-	all	Eu:UK	1948	CC	1465	n	V	n	n	0	all/unsp	30	39	0	1	nev any	st
DOLL	503	m	0	0	all	-	all	Eu:UK	1948	CC	1465	n	V	n	n	0	all/unsp	20	29	1	0	nev any	st
DOLL	504	m	0	0	all	-	all	Eu:UK	1948	CC	1465	n	V	n	n	0	all/unsp	1	19	0	0	nev any	st
DOLL	508	f	0	0	all	-	all	Eu:UK	1948	CC	1465	n	V	n	n	0	all/unsp	40	999	0	0	nev any	st
DOLL	509	f	0	0	all	-	all	Eu:UK	1948	CC	1465	n	V	n	n	0	all/unsp	30	39	0	1	nev any	st
DOLL	510	f	0	0	all	-	all	Eu:UK	1948	CC	1465	n	V	n	n	0	all/unsp	20	29	1	0	nev any	st
DOLL	511	f	0	0	all	-	all	Eu:UK	1948	CC	1465	n	V	n	n	0	all/unsp	1	19	0	0	nev any	st
DORN	610	m	55	64	wh	8	all	NAmer	1954	pr	5097	n	bl	n	n	0	cig+/-ot	25	999	1	0	nev any	st
DORN	611	m	55	64	wh	8	all	NAmer	1954	pr	5097	n	bl	n	n	0	cig+/-ot	20	24	0	3	nev any	st
DORN	612	m	55	64	wh	8	all	NAmer	1954	pr	5097	n	bl	n	n	0	cig+/-ot	15	19	2	4	nev any	st
DORN	613	m	55	64	wh	8	all	NAmer	1954	pr	5097	n	bl	n	n	0	cig+/-ot	1	14	3	0	nev any	st
DORN	647	m	65	74	wh	8	all	NAmer	1954	pr	5097	n	bl	n	n	0	cig+/-ot	25	999	1	0	nev any	st
DORN	648	m	65	74	wh	8	all	NAmer	1954	pr	5097	n	bl	n	n	0	cig+/-ot	20	24	0	3	nev any	st
DORN	649	m	65	74	wh	8	all	NAmer	1954	pr	5097	n	bl	n	n	0	cig+/-ot	15	19	2	4	nev any	st
DORN	650	m	65	74	wh	8	all	NAmer	1954	pr	5097	n	bl	n	n	0	cig+/-ot	1	14	3	0	nev any	st
GAO	506	m	0	0	all	-	all	As:Chi	1984	CC	1405	n	ot	n	n	2	cig+/-ot	30	999	0	1	nev cigs	or
GAO	507	m	0	0	all	-	all	As:Chi	1984	CC	1405	n	ot	n	n	2	cig+/-ot	20	29	1	0	nev cigs	or
GAO	508	m	0	0	all	-	all	As:Chi	1984	CC	1405	n	ot	n	n	2	cig+/-ot	10	19	0	0	nev cigs	or
GAO	516	f	0	0	all	-	all	As:Chi	1984	CC	1405	n	ot	n	n	2	cig+/-ot	30	999	0	1	nev cigs	or
GAO	517	f	0	0	all	-	all	As:Chi	1984	CC	1405	n	ot	n	n	2	cig+/-ot	20	29	1	0	nev cigs	or
GAO	518	f	0	0	all	-	all	As:Chi	1984	CC	1405	n	ot	n	n	2	cig+/-ot	10	19	0	0	nev cigs	or
GENG	533	f	0	0	all	-	all	As:Chi	1985	CC	292	n	ot	*	n	1	cig+/-ot	21	999	1	0	nev any	st
GENG	534	f	0	0	all	-	all	As:Chi	1985	CC	292	n	ot	*	n	1	cig+/-ot	16	20	2	4	nev any	st
GENG	535	f	0	0	all	-	all	As:Chi	1985	CC	292	n	ot	*	n	1	cig+/-ot	1	15	3	0	nev any	st
HAENSZ	537	f	0	0	all	-	not alv	NAmer	1955	CC	158	n	bl	n	y	0	cig+/-ot	25	999	1	0	nev any	st
HAENSZ	538	f	0	0	all	-	not alv	NAmer	1955	CC	158	n	bl	n	y	0	cig+/-ot	1	24	0	0	nev any	st
HEGMAN	513	m	0	0	all	-	all	NAmer	1989	CC	282	n	bl	y	y	1	all/unsp	20	999	1	0	nev any	or
HEGMAN	514	m	0	0	all	-	all	NAmer	1989	CC	282	n	bl	y	y	1	all/unsp	1	19	0	0	nev any	or
HEGMAN	516	f	0	0	all	-	all	NAmer	1989	CC	282	n	bl	y	y	1	all/unsp	26	999	1	0	nev any	or
HEGMAN	517	f	0	0	all	-	all	NAmer	1989	CC	282	n	bl	y	y	1	all/unsp	1	25	0	0	nev any	or
HU	511	m	0	0	all	-	all	As:Chi	1985	CC	227	n	ot	n	y	0	cig+/-ot	30	999	0	1	nev cigs	st
HU	512	m	0	0	all	-	all	As:Chi	1985	CC	227	n	ot	n	y	0	cig+/-ot	16	29	0	0	nev cigs	st
HU	513	m	0	0	all	-	all	As:Chi	1985	CC	227	n	ot	n	y	0	cig+/-ot	1	15	3	0	nev cigs	st
HU	516	f	0	0	all	-	all	As:Chi	1985	CC	227	n	ot	n	y	0	cig+/-ot	30	999	0	1	nev cigs	st
HU	517	f	0	0	all	-	all	As:Chi	1985	CC	227	n	ot	n	y	0	cig+/-ot	16	29	0	0	nev cigs	st
HU	518	f	0	0	all	-	all	As:Chi	1985	CC	227	n	ot	n	y	0	cig+/-ot	1	15	3	0	nev cigs	st
HU2	501	c	0	0	all	-	all	As:Chi	1977	CC	523	n	ot	y	n	0	cig+/-ot	40	999	0	0	nev cigs	ot

Table 1H1 - 1

IESLC - Meta-analysis of Ever Smoking by Age started, Overview
All LC types, Any Product (or Cigarettes if Any not available)
Most adjusted

REF	NRR	SEX	AGEL	AGEH	RACE	YF	LC TYPE	LOC	START	ST	NLC	R	VB	P	H	AD	PRODUCT	exL	exH	S1	S2	DENOM	De
HU2	502	c	0	0	all	-	all	As:Chi	1977	CC	523	n	ot	y	n	0	cig+/-ot	30	39	0	1	nev cigs	st
HU2	503	c	0	0	all	-	all	As:Chi	1977	CC	523	n	ot	y	n	0	cig+/-ot	20	29	1	0	nev cigs	st
HU2	504	c	0	0	all	-	all	As:Chi	1977	CC	523	n	ot	y	n	0	cig+/-ot	1	19	0	0	nev cigs	or
JEDRYC	607	m	0	0	all	-	all	Eu:est	1980	CC	1630	n	bl	y	n	0	cig+/-ot	17	18	2	4	nev any	st
JEDRYC	608	m	0	0	all	-	all	Eu:est	1980	CC	1630	n	bl	y	n	0	cig+/-ot	1	16	3	0	nev any	st
JEDRYC	619	f	0	0	all	-	all	Eu:est	1980	CC	1630	n	bl	y	n	0	cig+/-ot	1	22	0	0	nev any	st
JOLY	543	m	0	0	all	-	all	SCAmer	1978	CC	826	n	bl	n	n	0	cig+/-ot	25	999	1	0	nev any	st
JOLY	544	m	0	0	all	-	all	SCAmer	1978	CC	826	n	bl	n	n	0	cig+/-ot	15	24	2	0	nev any	st
JOLY	545	m	0	0	all	-	all	SCAmer	1978	CC	826	n	bl	n	n	0	cig+/-ot	1	14	3	0	nev any	st
JOLY	533	f	0	0	all	-	all	SCAmer	1978	CC	826	n	bl	n	n	0	cig+/-ot	25	999	1	0	nev any	st
JOLY	534	f	0	0	all	-	all	SCAmer	1978	CC	826	n	bl	n	n	0	cig+/-ot	15	24	2	0	nev any	st
JOLY	535	f	0	0	all	-	all	SCAmer	1978	CC	826	n	bl	n	n	0	cig+/-ot	1	14	3	0	nev any	st
KHUDER	506	m	0	0	all	-	all	NAmer	1985	CC	482	n	bl	n	y	0	cig+/-ot	20	999	1	0	nev cigs	st
KHUDER	507	m	0	0	all	-	all	NAmer	1985	CC	482	n	bl	n	y	0	cig+/-ot	16	19	2	4	nev cigs	st
KHUDER	508	m	0	0	all	-	all	NAmer	1985	CC	482	n	bl	n	y	0	cig+/-ot	1	15	3	0	nev cigs	st
KOULUM	501	m	0	0	all	-	all	Eu:Sca	1936	CC	812	n	bl	n	n	0	all/unsp	31	999	0	0	nev any	st
KOULUM	502	m	0	0	all	-	all	Eu:Sca	1936	CC	812	n	bl	n	n	0	all/unsp	21	30	1	0	nev any	st
KOULUM	503	m	0	0	all	-	all	Eu:Sca	1936	CC	812	n	bl	n	n	0	all/unsp	16	20	2	4	nev any	st
KOULUM	504	m	0	0	all	-	all	Eu:Sca	1936	CC	812	n	bl	n	n	0	all/unsp	11	15	3	5	nev any	st
KOULUM	505	m	0	0	all	-	all	Eu:Sca	1936	CC	812	n	bl	n	n	0	all/unsp	0	10	0	6	nev any	st
LETOUR	501	c	0	0	all	-	all	NAmer	1983	CC	738	n	V	y	y	0	cig+/-ot	21	999	1	0	nev cigs	st
LETOUR	502	c	0	0	all	-	all	NAmer	1983	CC	738	n	V	y	y	0	cig+/-ot	15	20	2	4	nev cigs	st
LETOUR	503	c	0	0	all	-	all	NAmer	1983	CC	738	n	V	y	y	0	cig+/-ot	1	14	3	0	nev cigs	st
LIU3	504	m	0	0	all	-	all	As:Chi	1985	CC	110	n	ot	n	n	2	all/unsp	21	999	1	0	nev any	or
LIU3	505	m	0	0	all	-	all	As:Chi	1985	CC	110	n	ot	n	n	2	all/unsp	1	20	0	0	nev any	or
LIU4	501	m	35	69	all	-	all	As:Chi	1986	CC	1000-00	n	ot	y	n	2	all/unsp	25	999	1	0	nev any	ot
LIU4	502	m	35	69	all	-	all	As:Chi	1986	CC	1000-00	n	ot	y	n	2	all/unsp	20	24	0	3	nev any	ot
LIU4	503	m	35	69	all	-	all	As:Chi	1986	CC	1000-00	n	ot	y	n	2	all/unsp	0	19	0	0	nev any	ot
LIU5	501	c	0	0	all	-	all	As:Chi	1978	CC	111	n	ot	y	n	0	all/unsp	30	999	0	1	nev any	st
LIU5	502	c	0	0	all	-	all	As:Chi	1978	CC	111	n	ot	y	n	0	all/unsp	1	29	0	0	nev any	st
LUBIN	565	m	0	0	all	-	all	As:Chi	1984	CC	427	m	ot	y	n	0	cig+/-ot	27	999	0	1	nev any	st
LUBIN	566	m	0	0	all	-	all	As:Chi	1984	CC	427	m	ot	y	n	0	cig+/-ot	23	26	1	2	nev any	st
LUBIN	567	m	0	0	all	-	all	As:Chi	1984	CC	427	m	ot	y	n	0	cig+/-ot	20	22	0	3	nev any	st
LUBIN	568	m	0	0	all	-	all	As:Chi	1984	CC	427	m	ot	y	n	0	cig+/-ot	1	19	0	0	nev any	st
LUBIN2	1156	m	0	0	all	-	all	Eu:mul	1976	CC	7804	n	bl	n	y	1	cig+/-ot	31	999	0	0	nev cigs	st
LUBIN2	1157	m	0	0	all	-	all	Eu:mul	1976	CC	7804	n	bl	n	y	1	cig+/-ot	21	30	1	0	nev cigs	st
LUBIN2	1158	m	0	0	all	-	all	Eu:mul	1976	CC	7804	n	bl	n	y	1	cig+/-ot	17	20	2	4	nev cigs	st
LUBIN2	1159	m	0	0	all	-	all	Eu:mul	1976	CC	7804	n	bl	n	y	1	cig+/-ot	13	16	3	5	nev cigs	st
LUBIN2	1160	m	0	0	all	-	all	Eu:mul	1976	CC	7804	n	bl	n	y	1	cig+/-ot	1	12	0	6	nev cigs	st
MATOS	576	m	0	0	all	-	all	SCAmer	1994	CC	200	n	bl	n	n	2	cig+/-ot	20	999	1	0	nev any	or
MATOS	577	m	0	0	all	-	all	SCAmer	1994	CC	200	n	bl	n	n	2	cig+/-ot	15	19	2	4	nev any	or
MATOS	578	m	0	0	all	-	all	SCAmer	1994	CC	200	n	bl	n	n	2	cig+/-ot	1	14	3	0	nev any	or
PERNU	504	m	0	0	all	-	all	Eu:Sca	1944	CC	1606	n	bl	n	n	0	all/unsp	15	999	0	0	nev any	st
PERNU	505	m	0	0	all	-	all	Eu:Sca	1944	CC	1606	n	bl	n	n	0	all/unsp	1	14	3	0	nev any	st
PERNU	501	f	0	0	all	-	all	Eu:Sca	1944	CC	1606	n	bl	n	n	0	all/unsp	15	999	0	0	nev any	st
PERNU	502	f	0	0	all	-	all	Eu:Sca	1944	CC	1606	n	bl	n	n	0	all/unsp	1	14	3	0	nev any	ot
PEZZOT	570	m	0	0	all	-	all	SCAmer	1987	CC	215	n	bl	n	y	0	cig only	19	999	1	0	nev cigs	st
PEZZOT	571	m	0	0	all	-	all	SCAmer	1987	CC	215	n	bl	n	y	0	cig only	14	18	0	0	nev cigs	st
PEZZOT	572	m	0	0	all	-	all	SCAmer	1987	CC	215	n	bl	n	y	0	cig only	1	13	0	6	nev cigs	st
QIAO2	506	m	0	0	all	0	all	As:Chi	1992	pr	241	m	ot	n	n	1	all/unsp	21	999	1	0	nev any	or
QIAO2	507	m	0	0	all	0	all	As:Chi	1992	pr	241	m	ot	n	n	1	all/unsp	17	20	2	4	nev any	or
QIAO2	508	m	0	0	all	0	all	As:Chi	1992	pr	241	m	ot	n	n	1	all/unsp	1	16	3	0	nev any	or
RACHTA	506	f	0	0	all	-	all	Eu:est	1991	CC	118	n	bl	n	y	1	cig+/-ot	31	999	0	0	nev cigs	or
RACHTA	507	f	0	0	all	-	all	Eu:est	1991	CC	118	n	bl	n	y	1	cig+/-ot	20	30	1	0	nev cigs	or
RACHTA	508	f	0	0	all	-	all	Eu:est	1991	CC	118	n	bl	n	y	1	cig+/-ot	1	19	0	0	nev cigs	or
SOBUE	654	m	0	0	all	-	all	As:Jap	1986	CC	1376	n	bl	n	y	0	cig+/-ot	23	999	1	0	nev cigs	st
SOBUE	655	m	0	0	all	-	all	As:Jap	1986	CC	1376	n	bl	n	y	0	cig+/-ot	18	22	2	0	nev cigs	st
SOBUE	656	m	0	0	all	-	all	As:Jap	1986	CC	1376	n	bl	n	y	0	cig+/-ot	10	17	3	0	nev cigs	st
SUZUK2	501	c	0	0	all	-	all	SCAmer	1991	CC	123	n	bl	n	y	0	all/unsp	19	999	1	0	nev any	st
SUZUK2	502	c	0	0	all	-	all	SCAmer	1991	CC	123	n	bl	n	y	0	all/unsp	12	18	0	0	nev any	st
SUZUK2	503	c	0	0	all	-	all	SCAmer	1991	CC	123	n	bl	n	y	0	all/unsp	0	11	0	6	nev any	st
TIZZAN	506	m	0	0	all	-	all	Eu:wst	1959	CC	1358	n	bl	n	n	0	all/unsp	31	999	0	0	nev any	st
TIZZAN	507	m	0	0	all	-	all	Eu:wst	1959	CC	1358	n	bl	n	n	0	all/unsp	20	30	1	0	nev any	st
TIZZAN	508	m	0	0	all	-	all	Eu:wst	1959	CC	1358	n	bl	n	n	0	all/unsp	1	19	0	0	nev any	st
TIZZAN	519	f	0	0	all	-	all	Eu:wst	1959	CC	1358	n	bl	n	n	0	all/unsp	31	999	0	0	nev any	st
TIZZAN	520	f	0	0	all	-	all	Eu:wst	1959	CC	1358	n	bl	n	n	0	all/unsp	20	30	1	0	nev any	st
TIZZAN	521	f	0	0	all	-	all	Eu:wst	1959	CC	1358	n	bl	n	n	0	all/unsp	1	19	0	0	nev any	st
WYNDE6	759	m	0	0	wh	-	q+s+a	NAmer	1969	CC	4423	n	bl	n	y	0	cig+/-ot	21	999	1	0	nev cigs	st
WYNDE6	760	m	0	0	wh	-	q+s+a	NAmer	1969	CC	4423	n	bl	n	y	0	cig+/-ot	18	20	2	4	nev cigs	st

Table 1H1 - 1

IESLC - Meta-analysis of Ever Smoking by Age started, Overview
All LC types, Any Product (or Cigarettes if Any not available)
Most adjusted

REF	NRR	SEX	AGEL	AGEH	RACE	YF	LC TYPE	LOC	START	ST	NLC	R	VB	P	H	AD	PRODUCT	exL	exH	S1	S2	DENOM	De
WYNDE6	761	m	0	0	wh	-	q+s+a	NAmer	1969	CC	4423	n	bl	n	y	0	cig+/-ot	1	17	3	0	nev cigs	st
WYNDE6	767	f	0	0	wh	-	q+s+a	NAmer	1969	CC	4423	n	bl	n	y	0	cig+/-ot	21	999	1	0	nev cigs	st
WYNDE6	768	f	0	0	wh	-	q+s+a	NAmer	1969	CC	4423	n	bl	n	y	0	cig+/-ot	18	20	2	4	nev cigs	st
WYNDE6	769	f	0	0	wh	-	q+s+a	NAmer	1969	CC	4423	n	bl	n	y	0	cig+/-ot	1	17	3	0	nev cigs	st
ZHENG	563	m	0	0	all	-	all	As:Chi	1982	CC	540	n	ot	*	y	0	cig+/-ot	30	999	0	1	nev cigs	st
ZHENG	564	m	0	0	all	-	all	As:Chi	1982	CC	540	n	ot	*	y	0	cig+/-ot	20	29	1	0	nev cigs	st
ZHENG	565	m	0	0	all	-	all	As:Chi	1982	CC	540	n	ot	*	y	0	cig+/-ot	1	19	0	0	nev cigs	st
ZHENG	572	f	0	0	all	-	all	As:Chi	1982	CC	540	n	ot	*	y	0	cig+/-ot	30	999	0	1	nev cigs	st
ZHENG	573	f	0	0	all	-	all	As:Chi	1982	CC	540	n	ot	*	y	0	cig+/-ot	1	29	0	0	nev cigs	st

Cigarette type is all/unspec for all RRs

In this overview table, subtotals and Qs values may be invalid and should be ignored

Table 1H1 - 2

IESLC - Meta-analysis of Ever Smoking by Age started, Overview
All LC types, Any Product (or Cigarettes if Any not available)
Most adjusted

REF	NRR	SEX	AD	Number Exposed Case	Cont	Non-exposed Case	Cont	RR	95.00%CI
AGUDO	504	f	3	7	-	80	-	1.58	(0.59- 4.23)
AGUDO	505	f	3	16	-	80	-	10.76	(2.38- 48.73)
Subtotal AGUDO								2.80	(1.23- 6.39)
ARMADA	511	m	0	113	144	8	71	6.96	(3.22- 15.06)
ARMADA	512	m	0	204	110	8	71	16.46	(7.64- 35.44)
Subtotal ARMADA								10.73	(6.23- 18.49)
AUVINE	520	c	2	211	-	44	-	13.40	(7.62- 23.50)
AUVINE	521	c	2	55	-	44	-	47.60	(21.20- 107.00)
Subtotal AUVINE								20.26	(12.76- 32.17)
BARBON	520	m	1	200	-	22	-	8.20	(5.00- 13.30)
BARBON	521	m	1	395	-	22	-	9.90	(6.20- 15.80)
BARBON	522	m	1	138	-	22	-	50.80	(27.20- 95.00)
Subtotal BARBON								13.37	(9.93- 18.00)
BRESLO	501	c	0	32	35	19	56	2.69	(1.33- 5.47)
BRESLO	502	c	0	286	243	19	56	3.47	(2.01- 6.00)
BRESLO	503	c	0	166	116	19	56	4.22	(2.38- 7.47)
Subtotal BRESLO								3.51	(2.48- 4.95)
BUFFLE	517	f	0	23	23	12	112	9.33	(4.07- 21.40)
BUFFLE	518	f	0	47	34	12	112	12.90	(6.15- 27.07)
BUFFLE	519	f	0	40	29	12	112	12.87	(6.00- 27.62)
BUFFLE	520	f	0	53	33	12	112	14.99	(7.17- 31.33)
BUFFLE	521	f	0	78	41	12	112	17.76	(8.77- 35.94)
Subtotal BUFFLE								13.57	(9.69- 18.99)
CHEN2	517	m	0	8	5	9	33	5.87	(1.54- 22.37)
CHEN2	518	m	0	29	25	9	33	4.25	(1.71- 10.57)
CHEN2	519	m	0	84	67	9	33	4.60	(2.06- 10.27)
CHEN2	522	f	0	5	8	25	33	0.83	(0.24- 2.83)
CHEN2	523	f	0	9	8	25	33	1.49	(0.50- 4.39)
CHEN2	524	f	0	23	13	25	33	2.34	(0.99- 5.50)
Subtotal CHEN2								2.84	(1.90- 4.25)
CHIAZZ	501	m	2	-	-	4	-	3.00	(0.31- 28.84)
CHIAZZ	502	m	2	-	-	4	-	19.89	(2.66- 148.96)
Subtotal CHIAZZ								8.64	(1.92- 38.91)
CHOI	523	m	0	36	77	13	95	3.42	(1.69- 6.89)
CHOI	524	m	0	130	232	13	95	4.09	(2.21- 7.60)
CHOI	525	m	0	79	138	13	95	4.18	(2.20- 7.95)
CHOI	526	m	0	22	18	13	95	8.93	(3.81- 20.91)
CHOI	530	f	0	15	25	76	164	1.29	(0.65- 2.60)
CHOI	531	f	0	4	1	76	164	8.63	(0.95- 78.54)
Subtotal CHOI								3.57	(2.63- 4.85)
DAMBER	501	m	0	70	76	42	208	4.56	(2.87- 7.26)
DAMBER	502	m	0	261	190	42	208	6.80	(4.65- 9.95)
DAMBER	503	m	0	206	98	42	208	10.41	(6.91- 15.68)
Subtotal DAMBER								7.07	(5.57- 8.98)
DOLL	501	m	0	4	7	7	61	4.98	(1.16- 21.36)
DOLL	502	m	0	18	33	7	61	4.75	(1.80- 12.54)
DOLL	503	m	0	251	264	7	61	8.29	(3.72- 18.46)
DOLL	504	m	0	1077	992	7	61	9.46	(4.31- 20.78)
DOLL	508	f	0	15	15	40	59	1.48	(0.65- 3.35)
DOLL	509	f	0	10	7	40	59	2.11	(0.74- 6.00)
DOLL	510	f	0	23	15	40	59	2.26	(1.05- 4.86)
DOLL	511	f	0	20	12	40	59	2.46	(1.08- 5.58)
Subtotal DOLL								3.65	(2.67- 4.98)
*DORN	610	m	0	37	73050	25	213858	4.33	(2.61- 7.20)
*DORN	611	m	0	157	147948	25	213858	9.08	(5.95- 13.84)
*DORN	612	m	0	342	213156	25	213858	13.73	(9.14- 20.60)
*DORN	613	m	0	84	36304	25	213858	19.79	(12.67- 30.93)
*DORN	647	m	0	90	74464	49	171211	4.22	(2.98- 5.98)
*DORN	648	m	0	171	90036	49	171211	6.64	(4.83- 9.12)
*DORN	649	m	0	306	118234	49	171211	9.04	(6.69- 12.23)
*DORN	650	m	0	81	24616	49	171211	11.50	(8.07- 16.39)
Subtotal DORN								8.46	(7.42- 9.65)
GAO	506	m	2	45	-	62	-	1.20	(0.80- 1.90)
GAO	507	m	2	363	-	62	-	4.70	(3.30- 6.50)
GAO	508	m	2	262	-	62	-	5.10	(3.60- 7.20)
GAO	516	f	2	73	-	435	-	2.00	(1.40- 3.00)
GAO	517	f	2	87	-	435	-	3.80	(2.60- 5.80)
GAO	518	f	2	77	-	435	-	5.60	(3.40- 9.00)
Subtotal GAO								3.38	(2.88- 3.96)
GENG	533	f	1	28	-	54	-	1.55	(0.83- 2.89)
GENG	534	f	1	39	-	54	-	2.95	(1.57- 5.52)

Table 1H1 - 2

IESLC - Meta-analysis of Ever Smoking by Age started, Overview
All LC types, Any Product (or Cigarettes if Any not available)
Most adjusted

REF	NRR	SEX	AD	Number Exposed Case	Cont	Non-exposed Case	Cont	RR	95.00%CI
GENG	535	f	1	36	-	54	-	6.24	(2.85- 13.67)
Subtotal GENG								2.77	(1.88- 4.07)
HAENSZ	537	f	0	44	66	81	236	1.94	(1.23- 3.07)
HAENSZ	538	f	0	30	37	81	236	2.36	(1.37- 4.07)
Subtotal HAENSZ								2.11	(1.48- 2.99)
HEGMAN	513	m	1	26	-	-	-	9.40	(4.60- 19.30)
HEGMAN	514	m	1	146	-	-	-	22.30	(12.00- 41.40)
HEGMAN	516	f	1	2	-	-	-	4.80	(1.00- 22.10)
HEGMAN	517	f	1	81	-	-	-	26.80	(15.40- 46.80)
Subtotal HEGMAN								18.07	(12.75- 25.62)
HU	511	m	0	14	20	41	67	1.14	(0.52- 2.51)
HU	512	m	0	93	67	41	67	2.27	(1.38- 3.74)
HU	513	m	0	13	7	41	67	3.03	(1.12- 8.23)
HU	516	f	0	3	5	40	48	0.72	(0.16- 3.20)
HU	517	f	0	18	9	40	48	2.40	(0.97- 5.92)
HU	518	f	0	5	4	40	48	1.50	(0.38- 5.96)
Subtotal HU								1.92	(1.37- 2.69)
HU2	501	c	0	15	29	121	213	0.91	(0.47- 1.77)
HU2	502	c	0	29	54	121	213	0.95	(0.57- 1.56)
HU2	503	c	0	229	159	121	213	2.54	(1.88- 3.43)
HU2	504	c	0	129	68	121	213	3.34	(2.31- 4.83)
Subtotal HU2								2.14	(1.75- 2.61)
JEDRYC	607	m	0	239	146	49	219	7.32	(5.04- 10.61)
JEDRYC	608	m	0	135	66	49	219	9.14	(5.96- 14.02)
JEDRYC	619	f	0	63	11	78	166	12.19	(6.09- 24.42)
Subtotal JEDRYC								8.54	(6.58- 11.07)
JOLY	543	m	0	18	70	12	218	4.67	(2.14- 10.18)
JOLY	544	m	0	217	357	12	218	11.04	(6.03- 20.22)
JOLY	545	m	0	317	282	12	218	20.42	(11.18- 37.32)
JOLY	533	f	0	23	41	52	283	3.05	(1.69- 5.51)
JOLY	534	f	0	67	47	52	283	7.76	(4.82- 12.49)
JOLY	535	f	0	76	35	52	283	11.82	(7.18- 19.44)
Subtotal JOLY								8.56	(6.78- 10.80)
KHUDER	506	m	0	72	152	23	309	6.36	(3.83- 10.58)
KHUDER	507	m	0	161	338	23	309	6.40	(4.03- 10.17)
KHUDER	508	m	0	226	295	23	309	10.29	(6.51- 16.27)
Subtotal KHUDER								7.58	(5.76- 9.97)
KOULUM	501	m	0	8	8	5	54	10.80	(2.82- 41.31)
KOULUM	502	m	0	60	67	5	54	9.67	(3.63- 25.77)
KOULUM	503	m	0	267	103	5	54	28.00	(10.89- 71.96)
KOULUM	504	m	0	199	52	5	54	41.33	(15.74- 108.56)
KOULUM	505	m	0	143	16	5	54	96.53	(33.72- 276.34)
Subtotal KOULUM								27.41	(17.28- 43.49)
LETOUR	501	c	0	188	160	24	224	10.97	(6.85- 17.56)
LETOUR	502	c	0	309	241	24	224	11.97	(7.60- 18.83)
LETOUR	503	c	0	151	76	24	224	18.54	(11.21- 30.67)
Subtotal LETOUR								13.23	(10.06- 17.40)
LIU3	504	m	2	20	-	4	-	1.10	(0.25- 4.93)
LIU3	505	m	2	32	-	4	-	1.39	(0.32- 6.06)
Subtotal LIU3								1.24	(0.43- 3.53)
LIU4	501	m	2	-	-	-	-	2.41	(2.32- 2.49)
LIU4	502	m	2	-	-	-	-	2.86	(2.78- 2.95)
LIU4	503	m	2	-	-	-	-	3.81	(3.70- 3.93)
Subtotal LIU4								3.03	(2.98- 3.09)
LIU5	501	c	0	13	22	26	41	0.93	(0.40- 2.17)
LIU5	502	c	0	72	48	26	41	2.37	(1.28- 4.36)
Subtotal LIU5								1.72	(1.05- 2.82)
LUBIN	565	m	0	30	179	9	72	1.34	(0.61- 2.96)
LUBIN	566	m	0	65	146	9	72	3.56	(1.68- 7.56)
LUBIN	567	m	0	89	212	9	72	3.36	(1.61- 7.01)
LUBIN	568	m	0	178	251	9	72	5.67	(2.76- 11.64)
Subtotal LUBIN								3.20	(2.20- 4.66)
LUBIN2	1156	m	1	68	-	185	-	3.35	(2.42- 4.64)
LUBIN2	1157	m	1	564	-	185	-	4.97	(4.12- 6.00)
LUBIN2	1158	m	1	1796	-	185	-	5.43	(4.59- 6.41)
LUBIN2	1159	m	1	1312	-	185	-	6.74	(5.65- 8.04)
LUBIN2	1160	m	1	250	-	185	-	9.01	(6.92- 11.72)
Subtotal LUBIN2								5.76	(5.26- 6.31)
MATOS	576	m	2	28	-	11	-	3.90	(1.80- 8.30)
MATOS	577	m	2	91	-	11	-	7.80	(4.00- 15.50)
MATOS	578	m	2	69	-	11	-	7.80	(3.90- 15.70)

Table 1H1 - 2

IESLC - Meta-analysis of Ever Smoking by Age started, Overview
All LC types, Any Product (or Cigarettes if Any not available)
Most adjusted

REF	NRR	SEX	AD	Number Exposed Case	Cont	Non-exposed Case	Cont	RR	95.00%CI
Subtotal MATOS								6.39	(4.24- 9.63)
PERNU	504	m	0	1043	346	97	275	8.55	(6.58- 11.10)
PERNU	505	m	0	337	92	97	275	10.38	(7.49- 14.40)
PERNU	501	f	0	18	89	110	971	1.79	(1.04- 3.07)
PERNU	502	f	0	1	0	110	971	26.38~	(1.07- 651.38)
Subtotal PERNU								7.56	(6.25- 9.15)
PEZZOT	570	m	0	41	105	4	116	11.32	(3.92- 32.69)
PEZZOT	571	m	0	118	145	4	116	23.60	(8.46- 65.84)
PEZZOT	572	m	0	52	67	4	116	22.51	(7.79- 65.00)
Subtotal PEZZOT								18.29	(9.98- 33.50)
*QIAO2	506	m	1	52	-	10	-	1.32	(0.67- 2.60)
*QIAO2	507	m	1	75	-	10	-	1.47	(0.76- 2.84)
*QIAO2	508	m	1	104	-	10	-	1.81	(0.94- 3.48)
Subtotal QIAO2								1.53	(1.04- 2.24)
RACHTA	506	f	1	8	-	33	-	5.31	(1.48- 19.05)
RACHTA	507	f	1	25	-	33	-	5.33	(2.79- 10.20)
RACHTA	508	f	1	52	-	33	-	11.60	(5.04- 26.68)
Subtotal RACHTA								6.86	(4.27- 11.03)
SOBUE	654	m	0	110	121	29	126	3.95	(2.45- 6.38)
SOBUE	655	m	0	776	772	29	126	4.37	(2.88- 6.62)
SOBUE	656	m	0	137	62	29	126	9.60	(5.81- 15.88)
Subtotal SOBUE								5.28	(4.05- 6.89)
SUZUK2	501	c	0	16	22	11	53	3.50	(1.40- 8.75)
SUZUK2	502	c	0	64	38	11	53	8.11	(3.78- 17.41)
SUZUK2	503	c	0	31	10	11	53	14.94	(5.69- 39.18)
Subtotal SUZUK2								7.44	(4.51- 12.27)
TIZZAN	506	m	0	12	44	180	305	0.46	(0.24- 0.90)
TIZZAN	507	m	0	313	330	180	305	1.61	(1.26- 2.04)
TIZZAN	508	m	0	699	529	180	305	2.24	(1.80- 2.78)
TIZZAN	519	f	0	2	5	117	114	0.39	(0.07- 2.05)
TIZZAN	520	f	0	12	21	117	114	0.56	(0.26- 1.18)
TIZZAN	521	f	0	11	2	117	114	5.36	(1.16- 24.71)
Subtotal TIZZAN								1.70	(1.46- 1.98)
WYNDE6	759	m	0	111	92	51	589	13.93	(9.36- 20.74)
WYNDE6	760	m	0	223	139	51	589	18.53	(12.98- 26.45)
WYNDE6	761	m	0	611	301	51	589	23.44	(17.06- 32.21)
WYNDE6	767	f	0	127	90	73	673	13.01	(9.06- 18.69)
WYNDE6	768	f	0	200	94	73	673	19.62	(13.90- 27.67)
WYNDE6	769	f	0	291	91	73	673	29.48	(21.04- 41.31)
Subtotal WYNDE6								19.46	(16.87- 22.45)
ZHENG	563	m	0	28	66	33	94	1.21	(0.67- 2.19)
ZHENG	564	m	0	145	109	33	94	3.79	(2.37- 6.05)
ZHENG	565	m	0	106	43	33	94	7.02	(4.13- 11.95)
ZHENG	572	f	0	16	16	152	184	1.21	(0.59- 2.50)
ZHENG	573	f	0	60	28	152	184	2.59	(1.58- 4.27)
Subtotal ZHENG								2.86	(2.24- 3.65)
Partial Totals				21261	788757	7842	1558241		

*prospective study

~ With 0.5 adjustment for zero

Table 1H1 - 2

IESLC - Meta-analysis of Ever Smoking by Age started, Overview
All LC types, Any Product (or Cigarettes if Any not available)
Most adjusted

REF	NRR	SEX	AD	Ys	Ws	Qs	Ps
AGUDO	504	f	3	0.46	3.96	2.27	0.3627
AGUDO	505	f	3	2.38	1.69	2.27	0.0020
Subtotal AGUDO				1.03	5.65	4.54	
ARMADA	511	m	0	1.94	6.46	3.40	0.0000
ARMADA	512	m	0	2.80	6.53	16.42	0.0000
Subtotal ARMADA				2.37	12.99	19.82	
AUVINE	520	c	2	2.60	12.11	23.07	0.0000
AUVINE	521	c	2	3.86	5.86	41.10	0.0000
Subtotal AUVINE				3.01	17.98	64.16	
BARBON	520	m	1	2.10	16.05	12.68	0.0000
BARBON	521	m	1	2.29	17.56	20.37	0.0000
BARBON	522	m	1	3.93	9.82	72.28	0.0000
Subtotal BARBON				2.59	43.44	105.34	
BRESLO	501	c	0	0.99	7.67	0.39	0.0060
BRESLO	502	c	0	1.24	12.80	0.01	0.0000
BRESLO	503	c	0	1.44	11.75	0.59	0.0000
Subtotal BRESLO				1.25	32.22	0.98	
BUFFLE	517	f	0	2.23	5.58	5.79	0.0000
BUFFLE	518	f	0	2.56	7.00	12.60	0.0000
BUFFLE	519	f	0	2.56	6.59	11.83	0.0000
BUFFLE	520	f	0	2.71	7.07	15.74	0.0000
BUFFLE	521	f	0	2.88	7.72	21.32	0.0000
Subtotal BUFFLE				2.61	33.96	67.27	
CHEN2	517	m	0	1.77	2.14	0.66	0.0096
CHEN2	518	m	0	1.45	4.63	0.25	0.0018
CHEN2	519	m	0	1.53	5.94	0.57	0.0002
CHEN2	522	f	0	-0.19	2.53	5.01	0.7596
CHEN2	523	f	0	0.40	3.26	2.19	0.4750
CHEN2	524	f	0	0.85	5.24	0.71	0.0521
Subtotal CHEN2				1.04	23.76	9.39	
CHIAZZ	501	m	2	1.10	0.75	0.01	0.3421
CHIAZZ	502	m	2	2.99	0.95	2.99	0.0036
Subtotal CHIAZZ				2.16	1.70	3.00	
CHOI	523	m	0	1.23	7.80	0.00	0.0006
CHOI	524	m	0	1.41	10.06	0.38	0.0000
CHOI	525	m	0	1.43	9.31	0.43	0.0000
CHOI	526	m	0	2.19	5.31	5.04	0.0000
CHOI	530	f	0	0.26	7.94	7.27	0.4667
CHOI	531	f	0	2.16	0.79	0.70	0.0557
Subtotal CHOI				1.27	41.20	13.82	
DAMBER	501	m	0	1.52	17.84	1.63	0.0000
DAMBER	502	m	0	1.92	26.52	13.07	0.0000
DAMBER	503	m	0	2.34	22.90	29.10	0.0000
Subtotal DAMBER				1.96	67.25	43.80	
DOLL	501	m	0	1.61	1.81	0.28	0.0307
DOLL	502	m	0	1.56	4.08	0.48	0.0016
DOLL	503	m	0	2.11	5.99	4.84	0.0000
DOLL	504	m	0	2.25	6.20	6.60	0.0000
DOLL	508	f	0	0.39	5.71	3.90	0.3532
DOLL	509	f	0	0.75	3.51	0.78	0.1625
DOLL	510	f	0	0.82	6.57	1.05	0.0364
DOLL	511	f	0	0.90	5.71	0.57	0.0317
Subtotal DOLL				1.29	39.58	18.49	
*DORN	610	m	0	1.47	14.92	0.94	0.0000
*DORN	611	m	0	2.21	21.57	21.16	0.0000
*DORN	612	m	0	2.62	23.30	45.92	0.0000
*DORN	613	m	0	2.99	19.28	60.39	0.0000
*DORN	647	m	0	1.44	31.75	1.61	0.0000
*DORN	648	m	0	1.89	38.11	17.48	0.0000
*DORN	649	m	0	2.20	42.26	41.14	0.0000
*DORN	650	m	0	2.44	30.57	46.01	0.0000
Subtotal DORN				2.14	221.77	234.66	
GAO	506	m	2	0.18	20.54	21.92	0.4087
GAO	507	m	2	1.55	33.44	3.69	0.0000
GAO	508	m	2	1.63	31.98	5.48	0.0000
GAO	516	f	2	0.69	26.45	7.21	0.0004
GAO	517	f	2	1.34	23.87	0.34	0.0000
GAO	518	f	2	1.72	16.22	4.17	0.0000
Subtotal GAO				1.22	152.50	42.82	
GENG	533	f	1	0.44	9.87	5.96	0.1685
GENG	534	f	1	1.08	9.72	0.17	0.0007

Table 1H1 - 2

IESLC - Meta-analysis of Ever Smoking by Age started, Overview
All LC types, Any Product (or Cigarettes if Any not available)
Most adjusted

REF	NRR	SEX	AD	Ys	Ws	Qs	Ps
GENG	535	f	1	1.83	6.25	2.37	0.0000
Subtotal GENG				1.02	25.84	8.50	
HAENSZ	537	f	0	0.66	18.36	5.58	0.0044
HAENSZ	538	f	0	0.86	13.00	1.64	0.0019
Subtotal HAENSZ				0.75	31.36	7.23	
HEGMAN	513	m	1	2.24	7.47	7.86	0.0000
HEGMAN	514	m	1	3.10	10.02	35.76	0.0000
HEGMAN	516	f	1	1.57	1.60	0.20	0.0470
HEGMAN	517	f	1	3.29	12.44	53.45	0.0000
Subtotal HEGMAN				2.89	31.53	97.27	
HU	511	m	0	0.13	6.22	7.27	0.7374
HU	512	m	0	0.82	15.39	2.42	0.0013
HU	513	m	0	1.11	3.86	0.04	0.0292
HU	516	f	0	-0.33	1.73	4.12	0.6660
HU	517	f	0	0.88	4.71	0.54	0.0575
HU	518	f	0	0.41	2.02	1.32	0.5647
Subtotal HU				0.65	33.92	15.71	
HU2	501	c	0	-0.09	8.76	15.02	0.7814
HU2	502	c	0	-0.06	15.16	24.51	0.8268
HU2	503	c	0	0.93	42.35	3.44	0.0000
HU2	504	c	0	1.21	28.23	0.00	0.0000
Subtotal HU2				0.76	94.50	42.97	
JEDRYC	607	m	0	1.99	27.77	16.67	0.0000
JEDRYC	608	m	0	2.21	21.04	20.93	0.0000
JEDRYC	619	f	0	2.50	7.96	13.15	0.0000
Subtotal JEDRYC				2.14	56.77	50.75	
JOLY	543	m	0	1.54	6.34	0.67	0.0001
JOLY	544	m	0	2.40	10.49	14.76	0.0000
JOLY	545	m	0	3.02	10.57	34.29	0.0000
JOLY	533	f	0	1.12	11.03	0.11	0.0002
JOLY	534	f	0	2.05	16.96	11.78	0.0000
JOLY	535	f	0	2.47	15.51	24.39	0.0000
Subtotal JOLY				2.15	70.89	86.01	
KHUDER	506	m	0	1.85	14.88	6.01	0.0000
KHUDER	507	m	0	1.86	17.89	7.35	0.0000
KHUDER	508	m	0	2.33	18.34	22.84	0.0000
Subtotal KHUDER				2.03	51.12	36.20	
KOULUM	501	m	0	2.38	2.13	2.89	0.0005
KOULUM	502	m	0	2.27	4.00	4.44	0.0000
KOULUM	503	m	0	3.33	4.31	19.31	0.0000
KOULUM	504	m	0	3.72	4.12	25.87	0.0000
KOULUM	505	m	0	4.57	3.47	39.07	0.0000
Subtotal KOULUM				3.31	18.03	91.59	
LETOUR	501	c	0	2.39	17.33	24.11	0.0000
LETOUR	502	c	0	2.48	18.69	29.99	0.0000
LETOUR	503	c	0	2.92	15.17	44.09	0.0000
Subtotal LETOUR				2.58	51.19	98.19	
LIU3	504	m	2	0.10	1.73	2.17	0.9003
LIU3	505	m	2	0.33	1.78	1.39	0.6607
Subtotal LIU3				0.21	3.50	3.56	
LIU4	501	m	2	0.88	3072.74	346.35	0.0000
LIU4	502	m	2	1.05	4361.66	118.09	0.0000
LIU4	503	m	2	1.34	4224.99	63.16	0.0000
Subtotal LIU4				1.11	11659.40	527.60	
LIU5	501	c	0	-0.07	5.40	8.93	0.8697
LIU5	502	c	0	0.86	10.25	1.29	0.0058
Subtotal LIU5				0.54	15.65	10.22	
LUBIN	565	m	0	0.29	6.10	5.19	0.4689
LUBIN	566	m	0	1.27	6.79	0.02	0.0009
LUBIN	567	m	0	1.21	7.09	0.00	0.0013
LUBIN	568	m	0	1.74	7.43	2.01	0.0000
Subtotal LUBIN				1.16	27.42	7.22	
LUBIN2	1156	m	1	1.21	36.26	0.00	0.0000
LUBIN2	1157	m	1	1.60	108.74	16.38	0.0000
LUBIN2	1158	m	1	1.69	137.76	31.29	0.0000
LUBIN2	1159	m	1	1.91	123.47	59.24	0.0000
LUBIN2	1160	m	1	2.20	55.35	53.48	0.0000
Subtotal LUBIN2				1.75	461.59	160.39	
MATOS	576	m	2	1.36	6.58	0.14	0.0005
MATOS	577	m	2	2.05	8.37	5.89	0.0000
MATOS	578	m	2	2.05	7.92	5.57	0.0000

Table 1H1 - 2

IESLC - Meta-analysis of Ever Smoking by Age started, Overview
All LC types, Any Product (or Cigarettes if Any not available)
Most adjusted

REF	NRR	SEX	AD	Ys	Ws	Qs	Ps
Subtotal MATOS				1.85	22.87	11.60	
PERNU	504	m	0	2.15	56.20	48.62	0.0000
PERNU	505	m	0	2.34	35.99	45.55	0.0000
PERNU	501	f	0	0.58	13.00	5.26	0.0366
PERNU	502	f	0	3.27	0.37	1.58	0.0455
Subtotal PERNU				2.02	105.57	101.01	
PEZZOT	570	m	0	2.43	3.42	5.02	0.0000
PEZZOT	571	m	0	3.16	3.65	13.82	0.0000
PEZZOT	572	m	0	3.11	3.42	12.31	0.0000
Subtotal PEZZOT				2.91	10.48	31.15	
*QIAO2	506	m	1	0.28	8.36	7.35	0.4222
*QIAO2	507	m	1	0.39	8.84	6.09	0.2520
*QIAO2	508	m	1	0.59	8.97	3.47	0.0756
Subtotal QIAO2				0.42	26.17	16.91	
RACHTA	506	f	1	1.67	2.35	0.49	0.0104
RACHTA	507	f	1	1.67	9.14	1.92	0.0000
RACHTA	508	f	1	2.45	5.53	8.45	0.0000
Subtotal RACHTA				1.93	17.03	10.85	
SOBUE	654	m	0	1.37	16.73	0.42	0.0000
SOBUE	655	m	0	1.47	22.22	1.49	0.0000
SOBUE	656	m	0	2.26	15.19	16.63	0.0000
Subtotal SOBUE				1.66	54.14	18.54	
SUZUK2	501	c	0	1.25	4.59	0.01	0.0072
SUZUK2	502	c	0	2.09	6.59	5.08	0.0000
SUZUK2	503	c	0	2.70	4.13	9.15	0.0000
Subtotal SUZUK2				2.01	15.32	14.25	
TIZZAN	506	m	0	-0.77	8.70	34.37	0.0228
TIZZAN	507	m	0	0.47	66.40	36.45	0.0001
TIZZAN	508	m	0	0.81	82.27	13.79	0.0000
TIZZAN	519	f	0	-0.94	1.39	6.49	0.2659
TIZZAN	520	f	0	-0.59	6.74	21.87	0.1283
TIZZAN	521	f	0	1.68	1.64	0.35	0.0314
Subtotal TIZZAN				0.53	167.16	113.33	
WYNDE6	759	m	0	2.63	24.28	48.89	0.0000
WYNDE6	760	m	0	2.92	30.32	88.02	0.0000
WYNDE6	761	m	0	3.15	38.07	143.18	0.0000
WYNDE6	767	f	0	2.57	29.27	53.36	0.0000
WYNDE6	768	f	0	2.98	32.44	100.61	0.0000
WYNDE6	769	f	0	3.38	33.77	158.79	0.0000
Subtotal WYNDE6				2.97	188.15	592.86	
ZHENG	563	m	0	0.19	10.89	11.47	0.5320
ZHENG	564	m	0	1.33	17.54	0.24	0.0000
ZHENG	565	m	0	1.95	13.58	7.31	0.0000
ZHENG	572	f	0	0.19	7.30	7.66	0.6057
ZHENG	573	f	0	0.95	15.53	1.07	0.0002
Subtotal ZHENG				1.05	64.84	27.74	

N	150
NS	38

Table 1H1 - 3

IESLC - Meta-analysis of Ever Smoking by Age started, Overview
All LC types, Any Product (or Cigarettes if Any not available)
Most adjusted

	Sex			
	combined	male	female	Total
N	17	87	46	150
NS	6	27	17	50

In this overview table, other than the "N" rows, entries in the "absent" and "Total" columns may be invalid and should be ignored

	Age started (broad categories)				
	absent	19+k26	15-25k18	1-17k14	Total
N	63	41	20	26	150
NS	27	32	17	21	97
Wt	9323.69	3709.74	484.62	480.38	13998.42
Het Chi	786.07	424.64	146.86	203.73	2809.74
Het df	62	40	19	25	149
Het P	***	***	***	***	***
Fixed RR	3.35	2.63	7.63	11.26	3.37
RRl	3.28	2.55	6.98	10.30	3.32
RRu	3.42	2.72	8.34	12.32	3.43
P	+++	+++	+++	+++	+++
Random RR	3.98	3.87	7.80	11.80	5.36
RRl	3.51	3.15	5.98	8.92	4.87
RRu	4.53	4.75	10.16	15.61	5.91
P	+++	+++	+++	+++	+++

		Age started (narrow categories)						
	absent	27+k30	23-29k26	19-25k22	15-21k18	11-17k14	1-13k10	Total
N	110	12	1	5	16	2	4	150
NS	38	8	1	4	14	2	4	71
Wt	8824.07	112.96	6.79	4438.50	422.14	127.59	66.37	13998.42
Het Chi	1900.61	34.42	0.00	56.40	129.75	13.11	20.72	2809.74
Het df	109	11	0	4	15	1	3	149
Het P	***	***	N.S.	***	***	***	***	***
Fixed RR	3.46	1.53	3.56	2.90	7.97	7.15	11.03	3.37
RRl	3.39	1.27	1.68	2.82	7.24	6.01	8.67	3.32
RRu	3.53	1.84	7.56	2.99	8.77	8.50	14.03	3.43
P	+++	+++	+++	+++	+++	+++	+++	+++
Random RR	5.34	1.60	3.56	4.76	8.34	15.64	21.72	5.36
RRl	4.71	1.12	1.68	2.73	6.15	2.66	7.77	4.87
RRu	6.05	2.27	7.56	8.30	11.31	92.10	60.76	5.91
P	+++	++	+++	+++	+++	++	+++	+++

Table 1H1 - 3

IESLC - Meta-analysis of Ever Smoking by Age started, Overview
All LC types, Any Product (or Cigarettes if Any not available)
Most adjusted

MALES

		Age started (broad categories)				
		absent	19+k26	15-25k18	1-17k14	Total
	N	32	24	14	17	87
	NS	18	23	13	16	70
	Wt	9066.46	3499.17	386.94	381.95	13334.51
Het	Chi	553.44	259.69	92.60	129.89	1975.03
Het	df	31	23	13	16	86
Het	P	***	***	***	***	***
Fixed	RR	3.35	2.58	7.16	10.31	3.30
	RRl	3.28	2.50	6.48	9.32	3.25
	RRu	3.42	2.67	7.91	11.39	3.36
	P	+++	+++	+++	+++	+++
Random	RR	4.45	4.47	7.61	11.48	6.01
	RRl	3.80	3.46	5.68	8.39	5.37
	RRu	5.20	5.77	10.19	15.70	6.73
	P	+++	+++	+++	+++	+++

			Age started (narrow categories)					
		absent	27+k30	23-29k26	19-25k22	15-21k18	11-17k14	1-13k10	Total
	N	59	5	1	5	12	2	3	87
	NS	27	5	1	4	11	2	3	53
	Wt	8297.34	47.83	6.79	4438.50	354.22	127.59	62.24	13334.51
Het	Chi	1291.57	7.05	0.00	56.40	85.09	13.11	20.32	1975.03
Het	df	58	4	0	4	11	1	2	86
Het	P	***	N.S.	N.S.	***	***	***	***	***
Fixed	RR	3.37	1.36	3.56	2.90	7.29	7.15	10.81	3.30
	RRl	3.30	1.03	1.68	2.82	6.57	6.01	8.44	3.25
	RRu	3.44	1.81	7.56	2.99	8.09	8.50	13.86	3.36
	P	+++	+	+++	+++	+++	+++	+++	+++
Random	RR	6.04	1.44	3.56	4.76	7.77	15.64	25.30	6.01
	RRl	5.19	0.97	1.68	2.73	5.63	2.66	5.94	5.37
	RRu	7.03	2.16	7.56	8.30	10.72	92.10	107.72	6.73
	P	+++	(+)	+++	+++	+++	++	+++	+++

FEMALES

		Age started (broad categories)				
		absent	19+k26	15-25k18	1-17k14	Total
	N	23	13	4	6	46
	NS	14	13	4	6	37
	Wt	166.59	138.63	66.19	65.64	437.05
Het	Chi	144.87	114.08	30.36	30.46	497.96
Het	df	22	12	3	5	45
Het	P	***	***	***	***	***
Fixed	RR	3.60	3.61	11.38	17.60	5.45
	RRl	3.09	3.06	8.94	13.82	4.96
	RRu	4.19	4.27	14.48	22.42	5.98
	P	+++	+++	+++	+++	+++
Random	RR	3.50	2.81	9.17	11.06	4.10
	RRl	2.31	1.64	4.02	5.30	2.95
	RRu	5.31	4.82	20.89	23.12	5.70
	P	+++	+++	+++	+++	+++

Table 1H1 - 3

IESLC - Meta-analysis of Ever Smoking by Age started, Overview
All LC types, Any Product (or Cigarettes if Any not available)
Most adjusted

FEMALES

		Age started (narrow categories)							
	absent	27+k30	23-29k26	19-25k22	15-21k18	11-17k14	1-13k10	Total
N	38	5			3			46
NS	17	5			3			25
Wt	343.25	44.57			49.23			437.05
Het Chi	378.87	16.64			27.02			497.96
Het df	37	4			2			45
Het P	***	**			***			***
Fixed RR	5.42	2.16			12.98			5.45
RRl	4.88	1.61			9.82			4.96
RRu	6.03	2.89			17.17			5.98
P	+++	+++			+++			+++
Random RR	4.15	2.18			9.65			4.10
RRl	2.90	1.06			2.95			2.95
RRu	5.94	4.48			31.50			5.70
P	+++	+			+++			+++

Table 1H1 - 4

IESLC - Meta-analysis of Ever Smoking by Age started, Overview
All LC types, Any Product (or Cigarettes if Any not available)
Least adjusted

REF	NRR	X	SEX	AGEL	AGEH	RACE	YF	LC TYPE	LOC	START	ST	NLC	R	VB	P	H	AD	PRODUCT	exL	exH	S1	S2	DENOM	De
AGUDO	501	x	f	0	0	all	-	all	Eu:wst	1989	CC	103	n	bl	n	n	0	cig only	24	999	1	0	nev cigs	st
AGUDO	502	x	f	0	0	all	-	all	Eu:wst	1989	CC	103	n	bl	n	n	0	cig only	1	23	0	0	nev cigs	st
ARMADA	511		m	0	0	all	-	all	Eu:wst	1986	CC	325	n	bl	n	y	0	cig+/-ot	17	45	0	0	nev cigs	st
ARMADA	512		m	0	0	all	-	all	Eu:wst	1986	CC	325	n	bl	n	y	0	cig+/-ot	7	16	3	0	nev cigs	st
AUVINE	509	x	c	0	0	all	-	all	Eu:Sca	1986	CC	517	n	bl	y	n	0	cig+/-ot	21	999	1	0	nev cigs	st
AUVINE	510	x	c	0	0	all	-	all	Eu:Sca	1986	CC	517	n	bl	y	n	0	cig+/-ot	16	20	2	4	nev cigs	st
AUVINE	511	x	c	0	0	all	-	all	Eu:Sca	1986	CC	517	n	bl	y	n	0	cig+/-ot	1	15	3	0	nev cigs	st
BARBON	515	x	m	0	0	all	-	all	Eu:wst	1979	CC	755	n	bl	y	y	0	all/unsp	20	999	1	0	nev any	st
BARBON	516	x	m	0	0	all	-	all	Eu:wst	1979	CC	755	n	bl	y	y	0	all/unsp	15	19	2	4	nev any	st
BARBON	517	x	m	0	0	all	-	all	Eu:wst	1979	CC	755	n	bl	y	y	0	all/unsp	1	14	3	0	nev any	st
BRESLO	501		c	0	0	all	-	all	NAmer	1949	CC	518	n	bl	n	y	0	cig+/-ot	25	999	1	0	nev any	st
BRESLO	502		c	0	0	all	-	all	NAmer	1949	CC	518	n	bl	n	y	0	cig+/-ot	15	24	2	0	nev any	st
BRESLO	503		c	0	0	all	-	all	NAmer	1949	CC	518	n	bl	n	y	0	cig+/-ot	0	14	3	0	nev any	st
BUFFLE	517		f	0	0	w-hi	-	all	NAmer	1976	CC	943	n	bl	y	n	0	cig+/-ot	30	999	0	1	nev cigs	or
BUFFLE	518		f	0	0	w-hi	-	all	NAmer	1976	CC	943	n	bl	y	n	0	cig+/-ot	21	29	1	0	nev cigs	or
BUFFLE	519		f	0	0	w-hi	-	all	NAmer	1976	CC	943	n	bl	y	n	0	cig+/-ot	19	20	0	0	nev cigs	or
BUFFLE	520		f	0	0	w-hi	-	all	NAmer	1976	CC	943	n	bl	y	n	0	cig+/-ot	17	18	2	4	nev cigs	ot
BUFFLE	521		f	0	0	w-hi	-	all	NAmer	1976	CC	943	n	bl	y	n	0	cig+/-ot	6	16	3	0	nev cigs	or
CHEN2	517		m	0	0	all	-	all	As:Chi	1983	CC	193	n	ot	y	n	0	all/unsp	31	999	0	0	nev any	st
CHEN2	518		m	0	0	all	-	all	As:Chi	1983	CC	193	n	ot	y	n	0	all/unsp	20	30	1	0	nev any	st
CHEN2	519		m	0	0	all	-	all	As:Chi	1983	CC	193	n	ot	y	n	0	all/unsp	1	19	0	0	nev any	st
CHEN2	522		f	0	0	all	-	all	As:Chi	1983	CC	193	n	ot	y	n	0	all/unsp	31	999	0	0	nev any	st
CHEN2	523		f	0	0	all	-	all	As:Chi	1983	CC	193	n	ot	y	n	0	all/unsp	20	30	1	0	nev any	st
CHEN2	524		f	0	0	all	-	all	As:Chi	1983	CC	193	n	ot	y	n	0	all/unsp	1	19	0	0	nev any	st
CHIAZZ	501		m	0	0	all	-	all	NAmer	1940	CC	144	o	bl	y	n	2	cig+/-ot	20	999	1	0	nev cigs	or
CHIAZZ	502		m	0	0	all	-	all	NAmer	1940	CC	144	o	bl	y	n	2	cig+/-ot	1	19	0	0	nev cigs	or
CHOI	523		m	0	0	all	-	all	As:oth	1985	CC	375	n	bl	n	n	0	cig+/-ot	25	999	1	0	nev cigs	st
CHOI	524		m	0	0	all	-	all	As:oth	1985	CC	375	n	bl	n	n	0	cig+/-ot	20	24	0	3	nev cigs	st
CHOI	525		m	0	0	all	-	all	As:oth	1985	CC	375	n	bl	n	n	0	cig+/-ot	15	19	2	4	nev cigs	st
CHOI	526		m	0	0	all	-	all	As:oth	1985	CC	375	n	bl	n	n	0	cig+/-ot	1	14	3	0	nev cigs	st
CHOI	530		f	0	0	all	-	all	As:oth	1985	CC	375	n	bl	n	n	0	cig+/-ot	25	999	1	0	nev cigs	st
CHOI	531		f	0	0	all	-	all	As:oth	1985	CC	375	n	bl	n	n	0	cig+/-ot	1	24	0	0	nev cigs	st
DAMBER	501		m	0	0	all	-	all	Eu:Sca	1972	CC	579	n	bl	y	n	0	all/unsp	21	999	1	0	nev any	st
DAMBER	502		m	0	0	all	-	all	Eu:Sca	1972	CC	579	n	bl	y	n	0	all/unsp	16	20	2	4	nev any	st
DAMBER	503		m	0	0	all	-	all	Eu:Sca	1972	CC	579	n	bl	y	n	0	all/unsp	1	15	3	0	nev any	st
DOLL	501		m	0	0	all	-	all	Eu:UK	1948	CC	1465	n	V	n	n	0	all/unsp	40	999	0	0	nev any	st
DOLL	502		m	0	0	all	-	all	Eu:UK	1948	CC	1465	n	V	n	n	0	all/unsp	30	39	0	1	nev any	st
DOLL	503		m	0	0	all	-	all	Eu:UK	1948	CC	1465	n	V	n	n	0	all/unsp	20	29	1	0	nev any	st
DOLL	504		m	0	0	all	-	all	Eu:UK	1948	CC	1465	n	V	n	n	0	all/unsp	1	19	0	0	nev any	st
DOLL	508		f	0	0	all	-	all	Eu:UK	1948	CC	1465	n	V	n	n	0	all/unsp	40	999	0	0	nev any	st
DOLL	509		f	0	0	all	-	all	Eu:UK	1948	CC	1465	n	V	n	n	0	all/unsp	30	39	0	1	nev any	st
DOLL	510		f	0	0	all	-	all	Eu:UK	1948	CC	1465	n	V	n	n	0	all/unsp	20	29	1	0	nev any	st
DOLL	511		f	0	0	all	-	all	Eu:UK	1948	CC	1465	n	V	n	n	0	all/unsp	1	19	0	0	nev any	st
DORN	610		m	55	64	wh	8	all	NAmer	1954	pr	5097	n	bl	n	n	0	cig+/-ot	25	999	1	0	nev any	st
DORN	611		m	55	64	wh	8	all	NAmer	1954	pr	5097	n	bl	n	n	0	cig+/-ot	20	24	0	3	nev any	st
DORN	612		m	55	64	wh	8	all	NAmer	1954	pr	5097	n	bl	n	n	0	cig+/-ot	15	19	2	4	nev any	st
DORN	613		m	55	64	wh	8	all	NAmer	1954	pr	5097	n	bl	n	n	0	cig+/-ot	1	14	3	0	nev any	st
DORN	647		m	65	74	wh	8	all	NAmer	1954	pr	5097	n	bl	n	n	0	cig+/-ot	25	999	1	0	nev any	st
DORN	648		m	65	74	wh	8	all	NAmer	1954	pr	5097	n	bl	n	n	0	cig+/-ot	20	24	0	3	nev any	st
DORN	649		m	65	74	wh	8	all	NAmer	1954	pr	5097	n	bl	n	n	0	cig+/-ot	15	19	2	4	nev any	st
DORN	650		m	65	74	wh	8	all	NAmer	1954	pr	5097	n	bl	n	n	0	cig+/-ot	1	14	3	0	nev any	st
GAO	501	x	m	0	0	all	-	all	As:Chi	1984	CC	1405	n	ot	n	n	0	cig+/-ot	30	999	0	1	nev cigs	st
GAO	502	x	m	0	0	all	-	all	As:Chi	1984	CC	1405	n	ot	n	n	0	cig+/-ot	20	29	1	0	nev cigs	st
GAO	503	x	m	0	0	all	-	all	As:Chi	1984	CC	1405	n	ot	n	n	0	cig+/-ot	10	19	0	0	nev cigs	st
GAO	511	x	f	0	0	all	-	all	As:Chi	1984	CC	1405	n	ot	n	n	0	cig+/-ot	30	999	0	1	nev cigs	st
GAO	512	x	f	0	0	all	-	all	As:Chi	1984	CC	1405	n	ot	n	n	0	cig+/-ot	20	29	1	0	nev cigs	st
GAO	513	x	f	0	0	all	-	all	As:Chi	1984	CC	1405	n	ot	n	n	0	cig+/-ot	10	19	0	0	nev cigs	st
GENG	528	x	f	0	0	all	-	all	As:Chi	1985	CC	292	n	ot	*	n	0	cig+/-ot	21	999	1	0	nev any	st
GENG	529	x	f	0	0	all	-	all	As:Chi	1985	CC	292	n	ot	*	n	0	cig+/-ot	16	20	2	4	nev any	st
GENG	530	x	f	0	0	all	-	all	As:Chi	1985	CC	292	n	ot	*	n	0	cig+/-ot	1	15	3	0	nev any	st
HAENSZ	537		f	0	0	all	-	not alv	NAmer	1955	CC	158	n	bl	n	y	0	cig+/-ot	25	999	1	0	nev any	st
HAENSZ	538		f	0	0	all	-	not alv	NAmer	1955	CC	158	n	bl	n	y	0	cig+/-ot	1	24	0	0	nev any	st
HEGMAN	513		m	0	0	all	-	all	NAmer	1989	CC	282	n	bl	y	y	1	all/unsp	20	999	1	0	nev any	or
HEGMAN	514		m	0	0	all	-	all	NAmer	1989	CC	282	n	bl	y	y	1	all/unsp	1	19	0	0	nev any	or
HEGMAN	516		f	0	0	all	-	all	NAmer	1989	CC	282	n	bl	y	y	1	all/unsp	26	999	1	0	nev any	or
HEGMAN	517		f	0	0	all	-	all	NAmer	1989	CC	282	n	bl	y	y	1	all/unsp	1	25	0	0	nev any	or
HU	511		m	0	0	all	-	all	As:Chi	1985	CC	227	n	ot	n	y	0	cig+/-ot	30	999	0	1	nev cigs	st
HU	512		m	0	0	all	-	all	As:Chi	1985	CC	227	n	ot	n	y	0	cig+/-ot	16	29	0	0	nev cigs	st
HU	513		m	0	0	all	-	all	As:Chi	1985	CC	227	n	ot	n	y	0	cig+/-ot	1	15	3	0	nev cigs	st
HU	516		f	0	0	all	-	all	As:Chi	1985	CC	227	n	ot	n	y	0	cig+/-ot	30	999	0	1	nev cigs	st
HU	517		f	0	0	all	-	all	As:Chi	1985	CC	227	n	ot	n	y	0	cig+/-ot	16	29	0	0	nev cigs	st
HU	518		f	0	0	all	-	all	As:Chi	1985	CC	227	n	ot	n	y	0	cig+/-ot	1	15	3	0	nev cigs	st

Table 1H1 - 4

IESLC - Meta-analysis of Ever Smoking by Age started, Overview
All LC types, Any Product (or Cigarettes if Any not available)
Least adjusted

REF	NRR	X	SEX	AGEL	AGEH	RACE	YF	LC TYPE	LOC	START	ST	NLC	R	VB	P	H	AD	PRODUCT	exL	exH	S1	S2	DENOM	De
HU2	501		c	0	0	all	-	all	As:Chi	1977	CC	523	n	ot	y	n	0	cig+/-ot	40	999	0	0	nev cigs	ot
HU2	502		c	0	0	all	-	all	As:Chi	1977	CC	523	n	ot	y	n	0	cig+/-ot	30	39	0	1	nev cigs	st
HU2	503		c	0	0	all	-	all	As:Chi	1977	CC	523	n	ot	y	n	0	cig+/-ot	20	29	1	0	nev cigs	st
HU2	504		c	0	0	all	-	all	As:Chi	1977	CC	523	n	ot	y	n	0	cig+/-ot	1	19	0	0	nev cigs	or
JEDRYC	607		m	0	0	all	-	all	Eu:est	1980	CC	1630	n	bl	y	n	0	cig+/-ot	17	18	2	4	nev any	st
JEDRYC	608		m	0	0	all	-	all	Eu:est	1980	CC	1630	n	bl	y	n	0	cig+/-ot	1	16	3	0	nev any	st
JEDRYC	619		f	0	0	all	-	all	Eu:est	1980	CC	1630	n	bl	y	n	0	cig+/-ot	1	22	0	0	nev any	st
JOLY	543		m	0	0	all	-	all	SCAmer	1978	CC	826	n	bl	n	n	0	cig+/-ot	25	999	1	0	nev any	st
JOLY	544		m	0	0	all	-	all	SCAmer	1978	CC	826	n	bl	n	n	0	cig+/-ot	15	24	2	0	nev any	st
JOLY	545		m	0	0	all	-	all	SCAmer	1978	CC	826	n	bl	n	n	0	cig+/-ot	1	14	3	0	nev any	st
JOLY	533		f	0	0	all	-	all	SCAmer	1978	CC	826	n	bl	n	n	0	cig+/-ot	25	999	1	0	nev any	st
JOLY	534		f	0	0	all	-	all	SCAmer	1978	CC	826	n	bl	n	n	0	cig+/-ot	15	24	2	0	nev any	st
JOLY	535		f	0	0	all	-	all	SCAmer	1978	CC	826	n	bl	n	n	0	cig+/-ot	1	14	3	0	nev any	st
KHUDER	506		m	0	0	all	-	all	NAmer	1985	CC	482	n	bl	n	y	0	cig+/-ot	20	999	1	0	nev cigs	st
KHUDER	507		m	0	0	all	-	all	NAmer	1985	CC	482	n	bl	n	y	0	cig+/-ot	16	19	2	4	nev cigs	st
KHUDER	508		m	0	0	all	-	all	NAmer	1985	CC	482	n	bl	n	y	0	cig+/-ot	1	15	3	0	nev cigs	st
KOULUM	501		m	0	0	all	-	all	Eu:Sca	1936	CC	812	n	bl	n	n	0	all/unsp	31	999	0	0	nev any	st
KOULUM	502		m	0	0	all	-	all	Eu:Sca	1936	CC	812	n	bl	n	n	0	all/unsp	21	30	1	0	nev any	st
KOULUM	503		m	0	0	all	-	all	Eu:Sca	1936	CC	812	n	bl	n	n	0	all/unsp	16	20	2	4	nev any	st
KOULUM	504		m	0	0	all	-	all	Eu:Sca	1936	CC	812	n	bl	n	n	0	all/unsp	11	15	3	5	nev any	st
KOULUM	505		m	0	0	all	-	all	Eu:Sca	1936	CC	812	n	bl	n	n	0	all/unsp	0	10	0	6	nev any	st
LETOUR	501		c	0	0	all	-	all	NAmer	1983	CC	738	n	V	y	y	0	cig+/-ot	21	999	1	0	nev cigs	st
LETOUR	502		c	0	0	all	-	all	NAmer	1983	CC	738	n	V	y	y	0	cig+/-ot	15	20	2	4	nev cigs	st
LETOUR	503		c	0	0	all	-	all	NAmer	1983	CC	738	n	V	y	y	0	cig+/-ot	1	14	3	0	nev cigs	st
LIU3	501	x	m	0	0	all	-	all	As:Chi	1985	CC	110	n	ot	n	n	0	all/unsp	21	999	1	0	nev any	or
LIU3	502	x	m	0	0	all	-	all	As:Chi	1985	CC	110	n	ot	n	n	0	all/unsp	1	20	0	0	nev any	st
LIU4	501		m	35	69	all	-	all	As:Chi	1986	CC	1000-00	n	ot	y	n	2	all/unsp	25	999	1	0	nev any	ot
LIU4	502		m	35	69	all	-	all	As:Chi	1986	CC	1000-00	n	ot	y	n	2	all/unsp	20	24	0	3	nev any	ot
LIU4	503		m	35	69	all	-	all	As:Chi	1986	CC	1000-00	n	ot	y	n	2	all/unsp	0	19	0	0	nev any	ot
LIU5	501		c	0	0	all	-	all	As:Chi	1978	CC	111	n	ot	y	n	0	all/unsp	30	999	0	1	nev any	st
LIU5	502		c	0	0	all	-	all	As:Chi	1978	CC	111	n	ot	y	n	0	all/unsp	1	29	0	0	nev any	st
LUBIN	565		m	0	0	all	-	all	As:Chi	1984	CC	427	m	ot	y	n	0	cig+/-ot	27	999	0	1	nev any	st
LUBIN	566		m	0	0	all	-	all	As:Chi	1984	CC	427	m	ot	y	n	0	cig+/-ot	23	26	1	2	nev any	st
LUBIN	567		m	0	0	all	-	all	As:Chi	1984	CC	427	m	ot	y	n	0	cig+/-ot	20	22	0	3	nev any	st
LUBIN	568		m	0	0	all	-	all	As:Chi	1984	CC	427	m	ot	y	n	0	cig+/-ot	1	19	0	0	nev any	st
LUBIN2	1147	x	m	0	0	all	-	all	Eu:mul	1976	CC	7804	n	bl	n	y	0	cig+/-ot	31	999	0	0	nev cigs	st
LUBIN2	1148	x	m	0	0	all	-	all	Eu:mul	1976	CC	7804	n	bl	n	y	0	cig+/-ot	21	30	1	0	nev cigs	st
LUBIN2	1149	x	m	0	0	all	-	all	Eu:mul	1976	CC	7804	n	bl	n	y	0	cig+/-ot	17	20	2	4	nev cigs	st
LUBIN2	1150	x	m	0	0	all	-	all	Eu:mul	1976	CC	7804	n	bl	n	y	0	cig+/-ot	13	16	3	5	nev cigs	st
LUBIN2	1151	x	m	0	0	all	-	all	Eu:mul	1976	CC	7804	n	bl	n	y	0	cig+/-ot	1	12	0	6	nev cigs	st
MATOS	556	x	m	0	0	all	-	all	SCAmer	1994	CC	200	n	bl	n	n	0	cig+/-ot	20	999	1	0	nev any	st
MATOS	557	x	m	0	0	all	-	all	SCAmer	1994	CC	200	n	bl	n	n	0	cig+/-ot	15	19	2	4	nev any	st
MATOS	558	x	m	0	0	all	-	all	SCAmer	1994	CC	200	n	bl	n	n	0	cig+/-ot	1	14	3	0	nev any	st
PERNU	504		m	0	0	all	-	all	Eu:Sca	1944	CC	1606	n	bl	n	n	0	all/unsp	15	999	0	0	nev any	st
PERNU	505		m	0	0	all	-	all	Eu:Sca	1944	CC	1606	n	bl	n	n	0	all/unsp	1	14	3	0	nev any	st
PERNU	501		f	0	0	all	-	all	Eu:Sca	1944	CC	1606	n	bl	n	n	0	all/unsp	15	999	0	0	nev any	st
PERNU	502		f	0	0	all	-	all	Eu:Sca	1944	CC	1606	n	bl	n	n	0	all/unsp	1	14	3	0	nev any	ot
PEZZOT	570		m	0	0	all	-	all	SCAmer	1987	CC	215	n	bl	n	y	0	cig only	19	999	1	0	nev cigs	st
PEZZOT	571		m	0	0	all	-	all	SCAmer	1987	CC	215	n	bl	n	y	0	cig only	14	18	0	0	nev cigs	st
PEZZOT	572		m	0	0	all	-	all	SCAmer	1987	CC	215	n	bl	n	y	0	cig only	1	13	0	6	nev cigs	st
QIAO2	501	x	m	0	0	all	0	all	As:Chi	1992	pr	241	m	ot	n	n	0	all/unsp	21	999	1	0	nev any	st
QIAO2	502	x	m	0	0	all	0	all	As:Chi	1992	pr	241	m	ot	n	n	0	all/unsp	17	20	2	4	nev any	st
QIAO2	503	x	m	0	0	all	0	all	As:Chi	1992	pr	241	m	ot	n	n	0	all/unsp	1	16	3	0	nev any	st
RACHTA	501	x	f	0	0	all	-	all	Eu:est	1991	CC	118	n	bl	n	y	0	cig+/-ot	31	999	0	0	nev cigs	st
RACHTA	502	x	f	0	0	all	-	all	Eu:est	1991	CC	118	n	bl	n	y	0	cig+/-ot	20	30	1	0	nev cigs	st
RACHTA	503	x	f	0	0	all	-	all	Eu:est	1991	CC	118	n	bl	n	y	0	cig+/-ot	1	19	0	0	nev cigs	st
SOBUE	654		m	0	0	all	-	all	As:Jap	1986	CC	1376	n	bl	n	y	0	cig+/-ot	23	999	1	0	nev cigs	st
SOBUE	655		m	0	0	all	-	all	As:Jap	1986	CC	1376	n	bl	n	y	0	cig+/-ot	18	22	2	0	nev cigs	st
SOBUE	656		m	0	0	all	-	all	As:Jap	1986	CC	1376	n	bl	n	y	0	cig+/-ot	10	17	3	0	nev cigs	st
SUZUK2	501		c	0	0	all	-	all	SCAmer	1991	CC	123	n	bl	n	y	0	all/unsp	19	999	1	0	nev any	st
SUZUK2	502		c	0	0	all	-	all	SCAmer	1991	CC	123	n	bl	n	y	0	all/unsp	12	18	0	0	nev any	st
SUZUK2	503		c	0	0	all	-	all	SCAmer	1991	CC	123	n	bl	n	y	0	all/unsp	0	11	0	6	nev any	st
TIZZAN	506		m	0	0	all	-	all	Eu:wst	1959	CC	1358	n	bl	n	n	0	all/unsp	31	999	0	0	nev any	st
TIZZAN	507		m	0	0	all	-	all	Eu:wst	1959	CC	1358	n	bl	n	n	0	all/unsp	20	30	1	0	nev any	st
TIZZAN	508		m	0	0	all	-	all	Eu:wst	1959	CC	1358	n	bl	n	n	0	all/unsp	1	19	0	0	nev any	st
TIZZAN	519		f	0	0	all	-	all	Eu:wst	1959	CC	1358	n	bl	n	n	0	all/unsp	31	999	0	0	nev any	st
TIZZAN	520		f	0	0	all	-	all	Eu:wst	1959	CC	1358	n	bl	n	n	0	all/unsp	20	30	1	0	nev any	st
TIZZAN	521		f	0	0	all	-	all	Eu:wst	1959	CC	1358	n	bl	n	n	0	all/unsp	1	19	0	0	nev any	st
WYNDE6	759		m	0	0	wh	-	q+s+a	NAmer	1969	CC	4423	n	bl	n	y	0	cig+/-ot	21	999	1	0	nev cigs	st

Table 1H1 - 4

IESLC - Meta-analysis of Ever Smoking by Age started, Overview
All LC types, Any Product (or Cigarettes if Any not available)
Least adjusted

REF	NRR	X	SEX	AGEL	AGEH	RACE	YF	LC TYPE	LOC	START	ST	NLC	R	VB	P	H	AD	PRODUCT	exL	exH	S1	S2	DENOM	De
WYNDE6	760		m	0	0	wh	-	q+s+a	NAmer	1969	CC	4423	n	bl	n	y	0	cig+/-ot	18	20	2	4	nev cigs	st
WYNDE6	761		m	0	0	wh	-	q+s+a	NAmer	1969	CC	4423	n	bl	n	y	0	cig+/-ot	1	17	3	0	nev cigs	st
WYNDE6	767		f	0	0	wh	-	q+s+a	NAmer	1969	CC	4423	n	bl	n	y	0	cig+/-ot	21	999	1	0	nev cigs	st
WYNDE6	768		f	0	0	wh	-	q+s+a	NAmer	1969	CC	4423	n	bl	n	y	0	cig+/-ot	18	20	2	4	nev cigs	st
WYNDE6	769		f	0	0	wh	-	q+s+a	NAmer	1969	CC	4423	n	bl	n	y	0	cig+/-ot	1	17	3	0	nev cigs	st
ZHENG	563		m	0	0	all	-	all	As:Chi	1982	CC	540	n	ot	*	y	0	cig+/-ot	30	999	0	1	nev cigs	st
ZHENG	564		m	0	0	all	-	all	As:Chi	1982	CC	540	n	ot	*	y	0	cig+/-ot	20	29	1	0	nev cigs	st
ZHENG	565		m	0	0	all	-	all	As:Chi	1982	CC	540	n	ot	*	y	0	cig+/-ot	1	19	0	0	nev cigs	st
ZHENG	572		f	0	0	all	-	all	As:Chi	1982	CC	540	n	ot	*	y	0	cig+/-ot	30	999	0	1	nev cigs	st
ZHENG	573		f	0	0	all	-	all	As:Chi	1982	CC	540	n	ot	*	y	0	cig+/-ot	1	29	0	0	nev cigs	st

Cigarette type is all/unspec for all RRs

In this overview table, subtotals and Qs values may be invalid and should be ignored

Table 1H1 - 5

IESLC - Meta-analysis of Ever Smoking by Age started, Overview
All LC types, Any Product (or Cigarettes if Any not available)
Least adjusted

REF	NRR	SEX	AD	Number Exposed Case	Cont	Non-exposed Case	Cont	RR	95.00%CI
AGUDO	501	f	0	7	12	80	183	1.33	(0.51- 3.51)
AGUDO	502	f	0	16	11	80	183	3.33	(1.48- 7.49)
Subtotal AGUDO								2.28	(1.23- 4.25)
ARMADA	511	m	0	113	144	8	71	6.96	(3.22- 15.06)
ARMADA	512	m	0	204	110	8	71	16.46	(7.64- 35.44)
Subtotal ARMADA								10.73	(6.23- 18.49)
AUVINE	509	c	0	76	27	44	229	14.65	(8.50- 25.26)
AUVINE	510	c	0	135	47	44	229	14.95	(9.41- 23.75)
AUVINE	511	c	0	55	6	44	229	47.71	(19.35- 117.62)
Subtotal AUVINE								17.31	(12.46- 24.04)
BARBON	515	m	0	200	207	22	188	8.26	(5.10- 13.38)
BARBON	516	m	0	395	337	22	188	10.02	(6.29- 15.94)
BARBON	517	m	0	138	23	22	188	51.27	(27.46- 95.73)
Subtotal BARBON								13.42	(9.99- 18.02)
BRESLO	501	c	0	32	35	19	56	2.69	(1.33- 5.47)
BRESLO	502	c	0	286	243	19	56	3.47	(2.01- 6.00)
BRESLO	503	c	0	166	116	19	56	4.22	(2.38- 7.47)
Subtotal BRESLO								3.51	(2.48- 4.95)
BUFFLE	517	f	0	23	23	12	112	9.33	(4.07- 21.40)
BUFFLE	518	f	0	47	34	12	112	12.90	(6.15- 27.07)
BUFFLE	519	f	0	40	29	12	112	12.87	(6.00- 27.62)
BUFFLE	520	f	0	53	33	12	112	14.99	(7.17- 31.33)
BUFFLE	521	f	0	78	41	12	112	17.76	(8.77- 35.94)
Subtotal BUFFLE								13.57	(9.69- 18.99)
CHEN2	517	m	0	8	5	9	33	5.87	(1.54- 22.37)
CHEN2	518	m	0	29	25	9	33	4.25	(1.71- 10.57)
CHEN2	519	m	0	84	67	9	33	4.60	(2.06- 10.27)
CHEN2	522	f	0	5	8	25	33	0.83	(0.24- 2.83)
CHEN2	523	f	0	9	8	25	33	1.49	(0.50- 4.39)
CHEN2	524	f	0	23	13	25	33	2.34	(0.99- 5.50)
Subtotal CHEN2								2.84	(1.90- 4.25)
CHIAZZ	501	m	2	-	-	4	-	3.00	(0.31- 28.84)
CHIAZZ	502	m	2	-	-	4	-	19.89	(2.66- 148.96)
Subtotal CHIAZZ								8.64	(1.92- 38.91)
CHOI	523	m	0	36	77	13	95	3.42	(1.69- 6.89)
CHOI	524	m	0	130	232	13	95	4.09	(2.21- 7.60)
CHOI	525	m	0	79	138	13	95	4.18	(2.20- 7.95)
CHOI	526	m	0	22	18	13	95	8.93	(3.81- 20.91)
CHOI	530	f	0	15	25	76	164	1.29	(0.65- 2.60)
CHOI	531	f	0	4	1	76	164	8.63	(0.95- 78.54)
Subtotal CHOI								3.57	(2.63- 4.85)
DAMBER	501	m	0	70	76	42	208	4.56	(2.87- 7.26)
DAMBER	502	m	0	261	190	42	208	6.80	(4.65- 9.95)
DAMBER	503	m	0	206	98	42	208	10.41	(6.91- 15.68)
Subtotal DAMBER								7.07	(5.57- 8.98)
DOLL	501	m	0	4	7	7	61	4.98	(1.16- 21.36)
DOLL	502	m	0	18	33	7	61	4.75	(1.80- 12.54)
DOLL	503	m	0	251	264	7	61	8.29	(3.72- 18.46)
DOLL	504	m	0	1077	992	7	61	9.46	(4.31- 20.78)
DOLL	508	f	0	15	15	40	59	1.48	(0.65- 3.35)
DOLL	509	f	0	10	7	40	59	2.11	(0.74- 6.00)
DOLL	510	f	0	23	15	40	59	2.26	(1.05- 4.86)
DOLL	511	f	0	20	12	40	59	2.46	(1.08- 5.58)
Subtotal DOLL								3.65	(2.67- 4.98)
*DORN	610	m	0	37	73050	25	213858	4.33	(2.61- 7.20)
*DORN	611	m	0	157	147948	25	213858	9.08	(5.95- 13.84)
*DORN	612	m	0	342	213156	25	213858	13.73	(9.14- 20.60)
*DORN	613	m	0	84	36304	25	213858	19.79	(12.67- 30.93)
*DORN	647	m	0	90	74464	49	171211	4.22	(2.98- 5.98)
*DORN	648	m	0	171	90036	49	171211	6.64	(4.83- 9.12)
*DORN	649	m	0	306	118234	49	171211	9.04	(6.69- 12.23)
*DORN	650	m	0	81	24616	49	171211	11.50	(8.07- 16.39)
Subtotal DORN								8.46	(7.42- 9.65)
GAO	501	m	0	45	129	62	202	1.14	(0.73- 1.77)
GAO	502	m	0	363	262	62	202	4.51	(3.26- 6.25)
GAO	503	m	0	262	167	62	202	5.11	(3.62- 7.21)
GAO	511	f	0	73	64	435	605	1.59	(1.11- 2.27)
GAO	512	f	0	87	41	435	605	2.95	(2.00- 4.36)
GAO	513	f	0	77	25	435	605	4.28	(2.68- 6.84)
Subtotal GAO								2.99	(2.57- 3.50)
GENG	528	f	0	28	31	54	93	1.56	(0.84- 2.87)

Table 1H1 - 5

IESLC - Meta-analysis of Ever Smoking by Age started, Overview
All LC types, Any Product (or Cigarettes if Any not available)
Least adjusted

				Number Exposed		Non-exposed			
REF	NRR	SEX	AD	Case	Cont	Case	Cont	RR	95.00%CI
GENG	529	f	0	39	23	54	93	2.92	(1.58- 5.40)
GENG	530	f	0	36	10	54	93	6.20	(2.85- 13.48)
Subtotal GENG								2.74	(1.88- 4.00)
HAENSZ	537	f	0	44	66	81	236	1.94	(1.23- 3.07)
HAENSZ	538	f	0	30	37	81	236	2.36	(1.37- 4.07)
Subtotal HAENSZ								2.11	(1.48- 2.99)
HEGMAN	513	m	1	26	-	-	-	9.40	(4.60- 19.30)
HEGMAN	514	m	1	146	-	-	-	22.30	(12.00- 41.40)
HEGMAN	516	f	1	2	-	-	-	4.80	(1.00- 22.10)
HEGMAN	517	f	1	81	-	-	-	26.80	(15.40- 46.80)
Subtotal HEGMAN								18.07	(12.75- 25.62)
HU	511	m	0	14	20	41	67	1.14	(0.52- 2.51)
HU	512	m	0	93	67	41	67	2.27	(1.38- 3.74)
HU	513	m	0	13	7	41	67	3.03	(1.12- 8.23)
HU	516	f	0	3	5	40	48	0.72	(0.16- 3.20)
HU	517	f	0	18	9	40	48	2.40	(0.97- 5.92)
HU	518	f	0	5	4	40	48	1.50	(0.38- 5.96)
Subtotal HU								1.92	(1.37- 2.69)
HU2	501	c	0	15	29	121	213	0.91	(0.47- 1.77)
HU2	502	c	0	29	54	121	213	0.95	(0.57- 1.56)
HU2	503	c	0	229	159	121	213	2.54	(1.88- 3.43)
HU2	504	c	0	129	68	121	213	3.34	(2.31- 4.83)
Subtotal HU2								2.14	(1.75- 2.61)
JEDRYC	607	m	0	239	146	49	219	7.32	(5.04- 10.61)
JEDRYC	608	m	0	135	66	49	219	9.14	(5.96- 14.02)
JEDRYC	619	f	0	63	11	78	166	12.19	(6.09- 24.42)
Subtotal JEDRYC								8.54	(6.58- 11.07)
JOLY	543	m	0	18	70	12	218	4.67	(2.14- 10.18)
JOLY	544	m	0	217	357	12	218	11.04	(6.03- 20.22)
JOLY	545	m	0	317	282	12	218	20.42	(11.18- 37.32)
JOLY	533	f	0	23	41	52	283	3.05	(1.69- 5.51)
JOLY	534	f	0	67	47	52	283	7.76	(4.82- 12.49)
JOLY	535	f	0	76	35	52	283	11.82	(7.18- 19.44)
Subtotal JOLY								8.56	(6.78- 10.80)
KHUDER	506	m	0	72	152	23	309	6.36	(3.83- 10.58)
KHUDER	507	m	0	161	338	23	309	6.40	(4.03- 10.17)
KHUDER	508	m	0	226	295	23	309	10.29	(6.51- 16.27)
Subtotal KHUDER								7.58	(5.76- 9.97)
KOULUM	501	m	0	8	8	5	54	10.80	(2.82- 41.31)
KOULUM	502	m	0	60	67	5	54	9.67	(3.63- 25.77)
KOULUM	503	m	0	267	103	5	54	28.00	(10.89- 71.96)
KOULUM	504	m	0	199	52	5	54	41.33	(15.74- 108.56)
KOULUM	505	m	0	143	16	5	54	96.53	(33.72- 276.34)
Subtotal KOULUM								27.41	(17.28- 43.49)
LETOUR	501	c	0	188	160	24	224	10.97	(6.85- 17.56)
LETOUR	502	c	0	309	241	24	224	11.97	(7.60- 18.83)
LETOUR	503	c	0	151	76	24	224	18.54	(11.21- 30.67)
Subtotal LETOUR								13.23	(10.06- 17.40)
LIU3	501	m	0	20	80	4	19	1.19	(0.36- 3.88)
LIU3	502	m	0	32	125	4	19	1.22	(0.39- 3.83)
Subtotal LIU3								1.20	(0.53- 2.74)
LIU4	501	m	2	-	-	-	-	2.41	(2.32- 2.49)
LIU4	502	m	2	-	-	-	-	2.86	(2.78- 2.95)
LIU4	503	m	2	-	-	-	-	3.81	(3.70- 3.93)
Subtotal LIU4								3.03	(2.98- 3.09)
LIU5	501	c	0	13	22	26	41	0.93	(0.40- 2.17)
LIU5	502	c	0	72	48	26	41	2.37	(1.28- 4.36)
Subtotal LIU5								1.72	(1.05- 2.82)
LUBIN	565	m	0	30	179	9	72	1.34	(0.61- 2.96)
LUBIN	566	m	0	65	146	9	72	3.56	(1.68- 7.56)
LUBIN	567	m	0	89	212	9	72	3.36	(1.61- 7.01)
LUBIN	568	m	0	178	251	9	72	5.67	(2.76- 11.64)
Subtotal LUBIN								3.20	(2.20- 4.66)
LUBIN2	1147	m	0	68	221	185	1878	3.12	(2.29- 4.26)
LUBIN2	1148	m	0	564	1069	185	1878	5.36	(4.46- 6.43)
LUBIN2	1149	m	0	1796	3028	185	1878	6.02	(5.12- 7.08)
LUBIN2	1150	m	0	1312	1833	185	1878	7.27	(6.15- 8.59)
LUBIN2	1151	m	0	250	316	185	1878	8.03	(6.42- 10.05)
Subtotal LUBIN2								6.12	(5.61- 6.67)
MATOS	556	m	0	28	73	11	110	3.84	(1.80- 8.18)
MATOS	557	m	0	91	120	11	110	7.58	(3.85- 14.92)

Table 1H1 - 5

IESLC - Meta-analysis of Ever Smoking by Age started, Overview
All LC types, Any Product (or Cigarettes if Any not available)
Least adjusted

REF	NRR	SEX	AD	Number Exposed Case	Number Exposed Cont	Non-exposed Case	Non-exposed Cont	RR	95.00%CI
MATOS	558	m	0	69	90	11	110	7.67	(3.83- 15.36)
Subtotal MATOS								6.24	(4.15- 9.39)
PERNU	504	m	0	1043	346	97	275	8.55	(6.58- 11.10)
PERNU	505	m	0	337	92	97	275	10.38	(7.49- 14.40)
PERNU	501	f	0	18	89	110	971	1.79	(1.04- 3.07)
PERNU	502	f	0	1	0	110	971	26.38~	(1.07- 651.38)
Subtotal PERNU								7.56	(6.25- 9.15)
PEZZOT	570	m	0	41	105	4	116	11.32	(3.92- 32.69)
PEZZOT	571	m	0	118	145	4	116	23.60	(8.46- 65.84)
PEZZOT	572	m	0	52	67	4	116	22.51	(7.79- 65.00)
Subtotal PEZZOT								18.29	(9.98- 33.50)
*QIAO2	501	m	0	52	1947	10	709	1.89	(0.97- 3.71)
*QIAO2	502	m	0	75	2840	10	709	1.87	(0.97- 3.60)
*QIAO2	503	m	0	104	2130	10	709	3.46	(1.82- 6.59)
Subtotal QIAO2								2.33	(1.59- 3.40)
RACHTA	501	f	0	8	4	33	98	5.94	(1.68- 21.01)
RACHTA	502	f	0	25	18	33	98	4.12	(2.00- 8.50)
RACHTA	503	f	0	52	21	33	98	7.35	(3.87- 13.98)
Subtotal RACHTA								5.73	(3.66- 8.97)
SOBUE	654	m	0	110	121	29	126	3.95	(2.45- 6.38)
SOBUE	655	m	0	776	772	29	126	4.37	(2.88- 6.62)
SOBUE	656	m	0	137	62	29	126	9.60	(5.81- 15.88)
Subtotal SOBUE								5.28	(4.05- 6.89)
SUZUK2	501	c	0	16	22	11	53	3.50	(1.40- 8.75)
SUZUK2	502	c	0	64	38	11	53	8.11	(3.78- 17.41)
SUZUK2	503	c	0	31	10	11	53	14.94	(5.69- 39.18)
Subtotal SUZUK2								7.44	(4.51- 12.27)
TIZZAN	506	m	0	12	44	180	305	0.46	(0.24- 0.90)
TIZZAN	507	m	0	313	330	180	305	1.61	(1.26- 2.04)
TIZZAN	508	m	0	699	529	180	305	2.24	(1.80- 2.78)
TIZZAN	519	f	0	2	5	117	114	0.39	(0.07- 2.05)
TIZZAN	520	f	0	12	21	117	114	0.56	(0.26- 1.18)
TIZZAN	521	f	0	11	2	117	114	5.36	(1.16- 24.71)
Subtotal TIZZAN								1.70	(1.46- 1.98)
WYNDE6	759	m	0	111	92	51	589	13.93	(9.36- 20.74)
WYNDE6	760	m	0	223	139	51	589	18.53	(12.98- 26.45)
WYNDE6	761	m	0	611	301	51	589	23.44	(17.06- 32.21)
WYNDE6	767	f	0	127	90	73	673	13.01	(9.06- 18.69)
WYNDE6	768	f	0	200	94	73	673	19.62	(13.90- 27.67)
WYNDE6	769	f	0	291	91	73	673	29.48	(21.04- 41.31)
Subtotal WYNDE6								19.46	(16.87- 22.45)
ZHENG	563	m	0	28	66	33	94	1.21	(0.67- 2.19)
ZHENG	564	m	0	145	109	33	94	3.79	(2.37- 6.05)
ZHENG	565	m	0	106	43	33	94	7.02	(4.13- 11.95)
ZHENG	572	f	0	16	16	152	184	1.21	(0.59- 2.50)
ZHENG	573	f	0	60	28	152	184	2.59	(1.58- 4.27)
Subtotal ZHENG								2.86	(2.24- 3.65)
Partial Totals				21261	804094	7886	1574737		

*prospective study ~ With 0.5 adjustment for zero

Table 1H1 - 5

IESLC - Meta-analysis of Ever Smoking by Age started, Overview
All LC types, Any Product (or Cigarettes if Any not available)
Least adjusted

REF	NRR	SEX	AD	Ys	Ws	Qs	Ps
AGUDO	501	f	0	0.29	4.10	3.56	0.5594
AGUDO	502	f	0	1.20	5.84	0.00	0.0037
Subtotal AGUDO				0.83	9.93	3.56	
ARMADA	511	m	0	1.94	6.46	3.35	0.0000
ARMADA	512	m	0	2.80	6.53	16.32	0.0000
Subtotal ARMADA				2.37	12.99	19.67	
AUVINE	509	c	0	2.68	12.94	27.73	0.0000
AUVINE	510	c	0	2.70	17.93	39.50	0.0000
AUVINE	511	c	0	3.87	4.72	33.00	0.0000
Subtotal AUVINE				2.85	35.58	100.23	
BARBON	515	m	0	2.11	16.50	13.09	0.0000
BARBON	516	m	0	2.30	17.77	20.87	0.0000
BARBON	517	m	0	3.94	9.85	72.72	0.0000
Subtotal BARBON				2.60	44.12	106.68	
BRESLO	501	c	0	0.99	7.67	0.40	0.0060
BRESLO	502	c	0	1.24	12.80	0.01	0.0000
BRESLO	503	c	0	1.44	11.75	0.56	0.0000
Subtotal BRESLO				1.25	32.22	0.97	
BUFFLE	517	f	0	2.23	5.58	5.73	0.0000
BUFFLE	518	f	0	2.56	7.00	12.51	0.0000
BUFFLE	519	f	0	2.56	6.59	11.74	0.0000
BUFFLE	520	f	0	2.71	7.07	15.63	0.0000
BUFFLE	521	f	0	2.88	7.72	21.19	0.0000
Subtotal BUFFLE				2.61	33.96	66.80	
CHEN2	517	m	0	1.77	2.14	0.65	0.0096
CHEN2	518	m	0	1.45	4.63	0.24	0.0018
CHEN2	519	m	0	1.53	5.94	0.55	0.0002
CHEN2	522	f	0	-0.19	2.53	5.05	0.7596
CHEN2	523	f	0	0.40	3.26	2.22	0.4750
CHEN2	524	f	0	0.85	5.24	0.73	0.0521
Subtotal CHEN2				1.04	23.76	9.43	
CHIAZZ	501	m	2	1.10	0.75	0.01	0.3421
CHIAZZ	502	m	2	2.99	0.95	2.97	0.0036
Subtotal CHIAZZ				2.16	1.70	2.98	
CHOI	523	m	0	1.23	7.80	0.00	0.0006
CHOI	524	m	0	1.41	10.06	0.36	0.0000
CHOI	525	m	0	1.43	9.31	0.41	0.0000
CHOI	526	m	0	2.19	5.31	4.98	0.0000
CHOI	530	f	0	0.26	7.94	7.35	0.4667
CHOI	531	f	0	2.16	0.79	0.69	0.0557
Subtotal CHOI				1.27	41.20	13.80	
DAMBER	501	m	0	1.52	17.84	1.58	0.0000
DAMBER	502	m	0	1.92	26.52	12.88	0.0000
DAMBER	503	m	0	2.34	22.90	28.84	0.0000
Subtotal DAMBER				1.96	67.25	43.30	
DOLL	501	m	0	1.61	1.81	0.27	0.0307
DOLL	502	m	0	1.56	4.08	0.47	0.0016
DOLL	503	m	0	2.11	5.99	4.79	0.0000
DOLL	504	m	0	2.25	6.20	6.54	0.0000
DOLL	508	f	0	0.39	5.71	3.95	0.3532
DOLL	509	f	0	0.75	3.51	0.79	0.1625
DOLL	510	f	0	0.82	6.57	1.07	0.0364
DOLL	511	f	0	0.90	5.71	0.59	0.0317
Subtotal DOLL				1.29	39.58	18.46	
*DORN	610	m	0	1.47	14.92	0.90	0.0000
*DORN	611	m	0	2.21	21.57	20.95	0.0000
*DORN	612	m	0	2.62	23.30	45.60	0.0000
*DORN	613	m	0	2.99	19.28	60.05	0.0000
*DORN	647	m	0	1.44	31.75	1.54	0.0000
*DORN	648	m	0	1.89	38.11	17.22	0.0000
*DORN	649	m	0	2.20	42.26	40.72	0.0000
*DORN	650	m	0	2.44	30.57	45.64	0.0000
Subtotal DORN				2.14	221.77	232.62	
GAO	501	m	0	0.13	19.59	23.37	0.5711
GAO	502	m	0	1.51	36.16	2.98	0.0000
GAO	503	m	0	1.63	32.38	5.47	0.0000
GAO	511	f	0	0.46	30.05	17.31	0.0114
GAO	512	f	0	1.08	25.10	0.48	0.0000
GAO	513	f	0	1.45	17.56	0.97	0.0000
Subtotal GAO				1.10	160.85	50.57	
GENG	528	f	0	0.44	10.28	6.23	0.1565

Table 1H1 - 5

IESLC - Meta-analysis of Ever Smoking by Age started, Overview
All LC types, Any Product (or Cigarettes if Any not available)
Least adjusted

REF	NRR	SEX	AD	Ys	Ws	Qs	Ps
GENG	529	f	0	1.07	10.16	0.22	0.0006
GENG	530	f	0	1.82	6.37	2.32	0.0000
Subtotal GENG				1.01	26.81	8.78	
HAENSZ	537	f	0	0.66	18.36	5.69	0.0044
HAENSZ	538	f	0	0.86	13.00	1.69	0.0019
Subtotal HAENSZ				0.75	31.36	7.38	
HEGMAN	513	m	1	2.24	7.47	7.78	0.0000
HEGMAN	514	m	1	3.10	10.02	35.57	0.0000
HEGMAN	516	f	1	1.57	1.60	0.19	0.0470
HEGMAN	517	f	1	3.29	12.44	53.19	0.0000
Subtotal HEGMAN				2.89	31.53	96.74	
HU	511	m	0	0.13	6.22	7.34	0.7374
HU	512	m	0	0.82	15.39	2.48	0.0013
HU	513	m	0	1.11	3.86	0.05	0.0292
HU	516	f	0	-0.33	1.73	4.14	0.6660
HU	517	f	0	0.88	4.71	0.56	0.0575
HU	518	f	0	0.41	2.02	1.34	0.5647
Subtotal HU				0.65	33.92	15.90	
HU2	501	c	0	-0.09	8.76	15.13	0.7814
HU2	502	c	0	-0.06	15.16	24.71	0.8268
HU2	503	c	0	0.93	42.35	3.56	0.0000
HU2	504	c	0	1.21	28.23	0.01	0.0000
Subtotal HU2				0.76	94.50	43.41	
JEDRYC	607	m	0	1.99	27.77	16.45	0.0000
JEDRYC	608	m	0	2.21	21.04	20.72	0.0000
JEDRYC	619	f	0	2.50	7.96	13.04	0.0000
Subtotal JEDRYC				2.14	56.77	50.22	
JOLY	543	m	0	1.54	6.34	0.65	0.0001
JOLY	544	m	0	2.40	10.49	14.64	0.0000
JOLY	545	m	0	3.02	10.57	34.10	0.0000
JOLY	533	f	0	1.12	11.03	0.12	0.0002
JOLY	534	f	0	2.05	16.96	11.64	0.0000
JOLY	535	f	0	2.47	15.51	24.20	0.0000
Subtotal JOLY				2.15	70.89	85.35	
KHUDER	506	m	0	1.85	14.88	5.91	0.0000
KHUDER	507	m	0	1.86	17.89	7.23	0.0000
KHUDER	508	m	0	2.33	18.34	22.64	0.0000
Subtotal KHUDER				2.03	51.12	35.78	
KOULUM	501	m	0	2.38	2.13	2.87	0.0005
KOULUM	502	m	0	2.27	4.00	4.40	0.0000
KOULUM	503	m	0	3.33	4.31	19.22	0.0000
KOULUM	504	m	0	3.72	4.12	25.77	0.0000
KOULUM	505	m	0	4.57	3.47	38.95	0.0000
Subtotal KOULUM				3.31	18.03	91.21	
LETOUR	501	c	0	2.39	17.33	23.91	0.0000
LETOUR	502	c	0	2.48	18.69	29.75	0.0000
LETOUR	503	c	0	2.92	15.17	43.83	0.0000
Subtotal LETOUR				2.58	51.19	97.49	
LIU3	501	m	0	0.17	2.74	3.01	0.7761
LIU3	502	m	0	0.20	2.92	3.07	0.7380
Subtotal LIU3				0.18	5.66	6.08	
LIU4	501	m	2	0.88	3072.74	356.76	0.0000
LIU4	502	m	2	1.05	4361.66	125.38	0.0000
LIU4	503	m	2	1.34	4224.99	58.09	0.0000
Subtotal LIU4				1.11	11659.40	540.24	
LIU5	501	c	0	-0.07	5.40	9.00	0.8697
LIU5	502	c	0	0.86	10.25	1.32	0.0058
Subtotal LIU5				0.54	15.65	10.32	
LUBIN	565	m	0	0.29	6.10	5.24	0.4689
LUBIN	566	m	0	1.27	6.79	0.02	0.0009
LUBIN	567	m	0	1.21	7.09	0.00	0.0013
LUBIN	568	m	0	1.74	7.43	1.97	0.0000
Subtotal LUBIN				1.16	27.42	7.23	
LUBIN2	1147	m	0	1.14	39.73	0.26	0.0000
LUBIN2	1148	m	0	1.68	115.66	24.24	0.0000
LUBIN2	1149	m	0	1.80	146.52	48.43	0.0000
LUBIN2	1150	m	0	1.98	138.01	80.31	0.0000
LUBIN2	1151	m	0	2.08	76.32	56.84	0.0000
Subtotal LUBIN2				1.81	516.24	210.08	
MATOS	556	m	0	1.34	6.69	0.10	0.0005
MATOS	557	m	0	2.03	8.38	5.44	0.0000

Table 1H1 - 5

IESLC - Meta-analysis of Ever Smoking by Age started, Overview
All LC types, Any Product (or Cigarettes if Any not available)
Least adjusted

REF	NRR	SEX	AD	Ys	Ws	Qs	Ps
MATOS	558	m	0	2.04	7.96	5.31	0.0000
Subtotal MATOS				1.83	23.04	10.85	
PERNU	504	m	0	2.15	56.20	48.09	0.0000
PERNU	505	m	0	2.34	35.99	45.15	0.0000
PERNU	501	f	0	0.58	13.00	5.34	0.0366
PERNU	502	f	0	3.27	0.37	1.57	0.0455
Subtotal PERNU				2.02	105.57	100.16	
PEZZOT	570	m	0	2.43	3.42	4.98	0.0000
PEZZOT	571	m	0	3.16	3.65	13.75	0.0000
PEZZOT	572	m	0	3.11	3.42	12.25	0.0000
Subtotal PEZZOT				2.91	10.48	30.97	
*QIAO2	501	m	0	0.64	8.52	2.89	0.0623
*QIAO2	502	m	0	0.63	8.96	3.15	0.0604
*QIAO2	503	m	0	1.24	9.28	0.00	0.0002
Subtotal QIAO2				0.84	26.77	6.04	
RACHTA	501	f	0	1.78	2.41	0.76	0.0057
RACHTA	502	f	0	1.42	7.35	0.28	0.0001
RACHTA	503	f	0	2.00	9.31	5.59	0.0000
Subtotal RACHTA				1.75	19.07	6.63	
SOBUE	654	m	0	1.37	16.73	0.39	0.0000
SOBUE	655	m	0	1.47	22.22	1.43	0.0000
SOBUE	656	m	0	2.26	15.19	16.47	0.0000
Subtotal SOBUE				1.66	54.14	18.30	
SUZUK2	501	c	0	1.25	4.59	0.01	0.0072
SUZUK2	502	c	0	2.09	6.59	5.03	0.0000
SUZUK2	503	c	0	2.70	4.13	9.09	0.0000
Subtotal SUZUK2				2.01	15.32	14.12	
TIZZAN	506	m	0	-0.77	8.70	34.55	0.0228
TIZZAN	507	m	0	0.47	66.40	36.95	0.0001
TIZZAN	508	m	0	0.81	82.27	14.12	0.0000
TIZZAN	519	f	0	-0.94	1.39	6.52	0.2659
TIZZAN	520	f	0	-0.59	6.74	22.00	0.1283
TIZZAN	521	f	0	1.68	1.64	0.35	0.0314
Subtotal TIZZAN				0.53	167.16	114.48	
WYNDE6	759	m	0	2.63	24.28	48.55	0.0000
WYNDE6	760	m	0	2.92	30.32	87.51	0.0000
WYNDE6	761	m	0	3.15	38.07	142.44	0.0000
WYNDE6	767	f	0	2.57	29.27	52.97	0.0000
WYNDE6	768	f	0	2.98	32.44	100.03	0.0000
WYNDE6	769	f	0	3.38	33.77	158.06	0.0000
Subtotal WYNDE6				2.97	188.15	589.56	
ZHENG	563	m	0	0.19	10.89	11.58	0.5320
ZHENG	564	m	0	1.33	17.54	0.22	0.0000
ZHENG	565	m	0	1.95	13.58	7.21	0.0000
ZHENG	572	f	0	0.19	7.30	7.73	0.6057
ZHENG	573	f	0	0.95	15.53	1.11	0.0002
Subtotal ZHENG				1.05	64.84	27.85	

N	151
NS	38

Table 1H1 - 6

IESLC - Meta-analysis of Ever Smoking by Age started, Overview
All LC types, Any Product (or Cigarettes if Any not available)
Least adjusted

	Sex			
	combined	male	female	Total
N	18	87	46	151
NS	6	27	17	50

In this overview table, other than the "N" rows, entries in the "absent" and "Total" columns may be invalid and should be ignored

	Age started (broad categories)				
	absent	19+k26	15-25k18	1-17k14	Total
N	62	42	21	26	151
NS	26	33	18	21	98
Wt	9349.54	3734.05	512.09	494.27	14089.95
Het Chi	771.85	469.33	140.38	178.74	2894.27
Het df	61	41	20	25	150
Het P	***	***	***	***	***
Fixed RR	3.34	2.65	8.02	11.41	3.39
RRl	3.27	2.57	7.36	10.45	3.33
RRu	3.41	2.74	8.75	12.46	3.44
P	+++	+++	+++	+++	+++
Random RR	3.77	3.96	8.17	12.12	5.34
RRl	3.32	3.22	6.39	9.33	4.85
RRu	4.27	4.88	10.45	15.75	5.89
P	+++	+++	+++	+++	+++

		Age started (narrow categories)						
	absent	27+k30	23-29k26	19-25k22	15-21k18	11-17k14	1-13k10	Total
N	110	12	1	5	17	2	4	151
NS	38	8	1	4	15	2	4	72
Wt	8849.96	115.61	6.79	4438.50	449.62	142.13	87.34	14089.95
Het Chi	1893.21	32.65	0.00	56.40	121.05	12.09	24.20	2894.27
Het df	109	11	0	4	16	1	3	150
Het P	***	***	N.S.	***	***	***	***	***
Fixed RR	3.46	1.44	3.56	2.90	8.41	7.64	9.51	3.39
RRl	3.38	1.20	1.68	2.82	7.67	6.48	7.71	3.33
RRu	3.53	1.73	7.56	2.99	9.23	9.01	11.72	3.44
P	+++	+++	+++	+++	+++	+++	+++	+++
Random RR	5.26	1.54	3.56	4.76	8.80	16.19	21.16	5.34
RRl	4.65	1.10	1.68	2.73	6.68	2.96	7.08	4.85
RRu	5.95	2.17	7.56	8.30	11.61	88.51	63.23	5.89
P	+++	+	+++	+++	+++	++	+++	+++

Table 1H1 - 6

IESLC - Meta-analysis of Ever Smoking by Age started, Overview
All LC types, Any Product (or Cigarettes if Any not available)
Least adjusted

MALES

		Age started (broad categories)				
		absent	19+k26	15-25k18	1-17k14	Total
	N	32	24	14	17	87
	NS	18	23	13	16	70
	Wt	9091.49	3510.55	396.04	396.88	13394.95
Het	Chi	560.80	272.06	80.01	108.60	2023.85
Het	df	31	23	13	16	86
Het	P	***	***	***	***	***
Fixed	RR	3.35	2.59	7.43	10.56	3.32
	RRl	3.28	2.51	6.73	9.57	3.27
	RRu	3.42	2.68	8.20	11.65	3.38
	P	+++	+++	+++	+++	+++
Random	RR	4.37	4.51	7.78	11.95	6.06
	RRl	3.74	3.49	5.93	8.98	5.41
	RRu	5.12	5.84	10.21	15.90	6.78
	P	+++	+++	+++	+++	+++

			Age started (narrow categories)						
		absent	27+k30	23-29k26	19-25k22	15-21k18	11-17k14	1-13k10	Total
	N	59	5	1	5	12	2	3	87
	NS	27	5	1	4	11	2	3	53
	Wt	8314.12	46.88	6.79	4438.50	363.33	142.13	83.21	13394.95
Het	Chi	1295.21	7.34	0.00	56.40	71.93	12.09	23.32	2023.85
Het	df	58	4	0	4	11	1	2	86
Het	P	***	N.S.	N.S.	***	***	***	***	***
Fixed	RR	3.38	1.34	3.56	2.90	7.59	7.64	9.29	3.32
	RRl	3.31	1.00	1.68	2.82	6.84	6.48	7.50	3.27
	RRu	3.45	1.78	7.56	2.99	8.41	9.01	11.52	3.38
	P	+++	+	+++	+++	+++	+++	+++	+++
Random	RR	6.08	1.43	3.56	4.76	7.98	16.19	24.40	6.06
	RRl	5.22	0.95	1.68	2.73	5.94	2.96	5.26	5.41
	RRu	7.07	2.16	7.56	8.30	10.73	88.51	113.15	6.78
	P	+++	(+)	+++	+++	+++	++	+++	+++

FEMALES

		Age started (broad categories)				
		absent	19+k26	15-25k18	1-17k14	Total
	N	23	13	4	6	46
	NS	14	13	4	6	37
	Wt	179.52	138.62	66.64	65.76	450.53
Het	Chi	147.46	114.66	31.44	30.67	524.13
Het	df	22	12	3	5	45
Het	P	***	***	***	***	***
Fixed	RR	3.31	3.36	11.26	17.56	5.09
	RRl	2.86	2.85	8.86	13.79	4.64
	RRu	3.83	3.97	14.32	22.36	5.58
	P	+++	+++	+++	+++	+++
Random	RR	3.25	2.66	9.13	11.04	3.87
	RRl	2.17	1.54	3.97	5.28	2.78
	RRu	4.86	4.57	21.00	23.10	5.39
	P	+++	+++	+++	+++	+++

Table 1H1 - 6

IESLC - Meta-analysis of Ever Smoking by Age started, Overview
All LC types, Any Product (or Cigarettes if Any not available)
Least adjusted

FEMALES

		absent	Age started (narrow categories) 27+k30	23-29k26	19-25k22	15-21k18	11-17k14	1-13k10	Total
	N	38	5			3			46
	NS	17	5			3			25
	Wt	352.68	48.17			49.68			450.53
Het	Chi	386.40	18.23			28.28			524.13
Het	df	37	4			2			45
Het	P	***	**			***			***
Fixed	RR	5.13	1.86			12.79			5.09
	RRl	4.62	1.40			9.68			4.64
	RRu	5.69	2.46			16.89			5.58
	P	+++	+++			+++			+++
Random	RR	3.90	2.05			9.60			3.87
	RRl	2.74	0.97			2.89			2.78
	RRu	5.57	4.31			31.84			5.39
	P	+++	(+)			+++			+++

Table 1H1 - 7

IESLC - Meta-analysis of Ever Smoking by Age started, Overview
All LC types, Any Product (or Cigarettes if Any not available)
Excluded studies (and stage at which they were excluded)

1	AKIBA AMANDU AMES BECHER BENSHL BEST BLOT1 BROSS BROWN3 CARPEN CEDERL CHYOU CPSI CPSII DARBY DEAN2 DEAN3 DOLL2 ENGELA GAO2 GARCIA GILLIS GRAHAM GURSEL HAMMO2 HIRAYA HOLE HUMBLE JAHN JAIN KAISE2 KATSOU KAUFMA LAUSSM LIAW MCDUFF MIGRAN MRFITR PEZZO2 PISANI PRESCO QIAO SEGI2 SPEIZE SVENSS TVERDA WAKAI WATSON WIGLE WU WYNDE3 WYNDE8
2	AXELSS BOUCHA BOUCOT CHEN DESTEF DORGAN DOSEME FAN GARSHI GER HAMMON JUSSAW KOO KREUZE LEVIN MCCONN NOTAN2 OSANN2 RESTRE SADOWS VUTUC WANG2 WU2 WUWILL WYNDE2 XU ZHOU
3	GUO SPITZ STASZE ZHANG
4	LUO
5	CORREA YUAN
7	BOFFET WYNDE7
10	ALDERS
14	BENHAM

Table 1H1 - 8
Potentially overlapping studies

REF	REFGP	PRINC	.	OVERLAP/LINK
LUBIN2	LUBIN2	1		Lubin-combined
WYNDE6	WYNDE6	1		WYNDE5/6/7/8
LUBIN	XIANGZ	2		LUBIN/XIANGZ/QIAO

Table 1H1 - 9
Most adjusted - insufficient data for meta-analysis

REF	NRR	SEX	AGEL	AGEH	RACE	YF	LC TYPE	LOC	START	ST	NLC	R	VB	P	H	AD	PRODUCT	exL	exH	S1	S2	DENOM	De
CORREA	535	c	0	0	all	-	all	NAmer	1979	CC	1359	n	bl	y	n	2	cig+/-ot	21	999	1	0	nev cigs	or
CORREA	536	c	0	0	all	-	all	NAmer	1979	CC	1359	n	bl	y	n	2	cig+/-ot	16	20	2	4	nev cigs	or
CORREA	537	c	0	0	all	-	all	NAmer	1979	CC	1359	n	bl	y	n	2	cig+/-ot	1	15	3	0	nev cigs	or
JEDRYC	606	m	0	0	all	-	all	Eu:est	1980	CC	1630	n	bl	y	n	0	cig+/-ot	18	999	0	0	nev any	st
JEDRYC	618	f	0	0	all	-	all	Eu:est	1980	CC	1630	n	bl	y	n	0	cig+/-ot	23	999	1	0	nev any	st

REF	NRR	RR	SIG	RRDATA comment
CORREA	535	8.30		0
CORREA	536	17.40		0
CORREA	537	24.20		0
JEDRYC	606	*		0
JEDRYC	618	*		0

Table 1H2 -

IESLC - Meta-analysis of Ever Smoking, Age started, "Low"
All LC types, Any Product (or Cigarettes if Any not available)

This analysis is restricted to results for:
1) Ever smokers
2) Results by Age started
3) Categorical results by Age started
4) All LC types (or near equivalent)
5) Results complete enough for use in metaanalysis

Within each study, results are then selected (in the following order of preference, within each sex) for:
6) (not applicable)
7) PRODUCT: all/unspec, cigarettes regardless of other products, cigarettes only
8) CIGTYPE: all/unspecified, MC regardless of HR, MC only
9) (not applicable)
10) DENOM: never smoked anything, never smoked cigarettes, never any + low, never cigs + low
11) Followup period (YF, prospective studies): whole study (coded as 0) or longest available
12) LCtype: all or nearest available, at least Squamous and Adeno. (q = squamous, s = small,
l = large, a = adeno, mix = mixed, alv = alveolar)
13) Race: all or nearest available, otherwise by race (wh or w = white, bl or b = black, hi = hispanic
ch = chinese, jap = japanese, haw = hawaiian, w+o = white + oriental, sca = scandinavian, as = asian)
14) Age started "low" in key scheme 1 (key value 26, maximum range 19+)
15) For overlapping studies: principal rather than subsidiary studies
Finally by Age: whole study (coded as 0) if available, otherwise by widest available age group
and then for single sex results (m, f) in preference to results for both sexes combined (c).

Results adjusted (AD) for the most potential confounders are then chosen in Sections -1 to -3
and results adjusted for the least confounders in Sections -4 to -6. (Those least adjusted results which
actually differ from the most adjusted are marked 'x' in column X in Section -4)

Section -7 shows excluded studies, together with the stage (as above) at which no qualifying
results were found.

Section -8 lists the potentially overlapping studies which have been included (1=principal, 2=subsidiary).

Section -9 lists any results which would have been included in preference except that they had data not complete
enough for use in meta-analysis, with their significance (yes/no), if known, and any further comment as entered
on the database. It also lists as "gap" any categories for which no data were presented by the original authors.

In addition to those mentioned above, the following fields, levels and abbreviations are used:
* or nk = not known, n = no, y = yes, ot = other
nev = never
all/unspec = all or unspecified, cig+/-ot = cigarettes irrespective of other products (cigar, pipe etc)
MC = manufactured cigarettes, HR = hand-rolled cigarettes
exL, exH = range of exposure (low and high) in the smoking group, in terms of Age started
REF: 6-character study reference
NRR: number of the RR on the database within the study
ST : study type (CC = case control, pr or prosp = prospective)
NLC: number of lung cancer cases in whole study
R : risky occupational population (n = no, m = mining, o = other risky)
VB : national cigarette type (V = at least 75% Virginia, bl = at least 75% blended, ot = other)
P : any proxy use
H : full histological confirmation
De : derivation of RR/CI (or = original, st = standard method, ot = other method of estimation)

Table 1H2 - 1

IESLC - Meta-analysis of Ever Smoking, Age started, "Low"
All LC types, Any Product (or Cigarettes if Any not available)
Most adjusted

REF	NRR	SEX	AGEL	AGEH	RACE	YF	LC TYPE	LOC	START	ST	NLC	R	VB	P	H	AD	PRODUCT	exL	exH	DENOM	De
AGUDO	504	f	0	0	all	-	all	Eu:wst	1989	CC	103	n	bl	n	n	3	cig only	24	999	nev cigs	or
AUVINE	509	c	0	0	all	-	all	Eu:Sca	1986	CC	517	n	bl	y	n	0	cig+/-ot	21	999	nev cigs	st
BARBON	520	m	0	0	all	-	all	Eu:wst	1979	CC	755	n	bl	y	y	1	all/unsp	20	999	nev any	or
BRESLO	501	c	0	0	all	-	all	NAmer	1949	CC	518	n	bl	n	y	0	cig+/-ot	25	999	nev any	st
BUFFLE	518	f	0	0	w-hi	-	all	NAmer	1976	CC	943	n	bl	y	n	0	cig+/-ot	21	29	nev cigs	or
CHEN2	518	m	0	0	all	-	all	As:Chi	1983	CC	193	n	ot	y	n	0	all/unsp	20	30	nev any	st
CHEN2	523	f	0	0	all	-	all	As:Chi	1983	CC	193	n	ot	y	n	0	all/unsp	20	30	nev any	st
CHIAZZ	501	m	0	0	all	-	all	NAmer	1940	CC	144	o	bl	y	n	2	cig+/-ot	20	999	nev cigs	or
CHOI	523	m	0	0	all	-	all	As:oth	1985	CC	375	n	bl	n	n	0	cig+/-ot	25	999	nev cigs	st
CHOI	530	f	0	0	all	-	all	As:oth	1985	CC	375	n	bl	n	n	0	cig+/-ot	25	999	nev cigs	st
DAMBER	501	m	0	0	all	-	all	Eu:Sca	1972	CC	579	n	bl	y	n	0	all/unsp	21	999	nev any	st
DOLL	503	m	0	0	all	-	all	Eu:UK	1948	CC	1465	n	V	n	n	0	all/unsp	20	29	nev any	st
DOLL	510	f	0	0	all	-	all	Eu:UK	1948	CC	1465	n	V	n	n	0	all/unsp	20	29	nev any	st
DORN	610	m	55	64	wh	8	all	NAmer	1954	pr	5097	n	bl	n	n	0	cig+/-ot	25	999	nev any	st
DORN	647	m	65	74	wh	8	all	NAmer	1954	pr	5097	n	bl	n	n	0	cig+/-ot	25	999	nev any	st
GAO	507	m	0	0	all	-	all	As:Chi	1984	CC	1405	n	ot	n	n	2	cig+/-ot	20	29	nev cigs	or
GAO	517	f	0	0	all	-	all	As:Chi	1984	CC	1405	n	ot	n	n	2	cig+/-ot	20	29	nev cigs	or
GENG	533	f	0	0	all	-	all	As:Chi	1985	CC	292	n	ot	*	n	1	cig+/-ot	21	999	nev any	st
HAENSZ	537	f	0	0	all	-	not alv	NAmer	1955	CC	158	n	bl	n	y	0	cig+/-ot	25	999	nev any	st
HEGMAN	513	m	0	0	all	-	all	NAmer	1989	CC	282	n	bl	y	y	1	all/unsp	20	999	nev any	or
HEGMAN	516	f	0	0	all	-	all	NAmer	1989	CC	282	n	bl	y	y	1	all/unsp	26	999	nev any	or
HU2	503	c	0	0	all	-	all	As:Chi	1977	CC	523	n	ot	y	n	0	cig+/-ot	20	29	nev cigs	st
JOLY	543	m	0	0	all	-	all	SCAmer	1978	CC	826	n	bl	n	n	0	cig+/-ot	25	999	nev any	st
JOLY	533	f	0	0	all	-	all	SCAmer	1978	CC	826	n	bl	n	n	0	cig+/-ot	25	999	nev any	st
KHUDER	506	m	0	0	all	-	all	NAmer	1985	CC	482	n	bl	n	y	0	cig+/-ot	20	999	nev cigs	st
KOULUM	502	m	0	0	all	-	all	Eu:Sca	1936	CC	812	n	bl	n	n	0	all/unsp	21	30	nev any	st
LETOUR	501	c	0	0	all	-	all	NAmer	1983	CC	738	n	V	y	y	0	cig+/-ot	21	999	nev cigs	st
LIU3	504	m	0	0	all	-	all	As:Chi	1985	CC	110	n	ot	n	n	2	all/unsp	21	999	nev any	or
LIU4	501	m	35	69	all	-	all	As:Chi	1986	CC	1000-00	n	ot	y	n	2	all/unsp	25	999	nev any	ot
LUBIN	566	m	0	0	all	-	all	As:Chi	1984	CC	427	m	ot	y	n	0	cig+/-ot	23	26	nev any	st
LUBIN2	1157	m	0	0	all	-	all	Eu:mul	1976	CC	7804	n	bl	n	y	1	cig+/-ot	21	30	nev cigs	st
MATOS	576	m	0	0	all	-	all	SCAmer	1994	CC	200	n	bl	n	n	2	cig+/-ot	20	999	nev any	or
PEZZOT	570	m	0	0	all	-	all	SCAmer	1987	CC	215	n	bl	n	y	0	cig only	19	999	nev cigs	st
QIAO2	506	m	0	0	all	0	all	As:Chi	1992	pr	241	m	ot	n	n	1	all/unsp	21	999	nev any	or
RACHTA	507	f	0	0	all	-	all	Eu:est	1991	CC	118	n	bl	n	y	1	cig+/-ot	20	30	nev cigs	or
SOBUE	654	m	0	0	all	-	all	As:Jap	1986	CC	1376	n	bl	n	y	0	cig+/-ot	23	999	nev cigs	st
SUZUK2	501	c	0	0	all	-	all	SCAmer	1991	CC	123	n	bl	n	y	0	all/unsp	19	999	nev any	st
TIZZAN	507	m	0	0	all	-	all	Eu:wst	1959	CC	1358	n	bl	n	n	0	all/unsp	20	30	nev any	st
TIZZAN	520	f	0	0	all	-	all	Eu:wst	1959	CC	1358	n	bl	n	n	0	all/unsp	20	30	nev any	st
WYNDE6	759	m	0	0	wh	-	q+s+a	NAmer	1969	CC	4423	n	bl	n	y	0	cig+/-ot	21	999	nev cigs	st
WYNDE6	767	f	0	0	wh	-	q+s+a	NAmer	1969	CC	4423	n	bl	n	y	0	cig+/-ot	21	999	nev cigs	st
ZHENG	564	m	0	0	all	-	all	As:Chi	1982	CC	540	n	ot	*	y	0	cig+/-ot	20	29	nev cigs	st

Cigarette type is all/unspec for all RRs

Table 1H2 - 2

IESLC - Meta-analysis of Ever Smoking, Age started, "Low"
All LC types, Any Product (or Cigarettes if Any not available)
Most adjusted

REF	NRR	SEX	AD	Number Exposed Case	Cont	Non-exposed Case	Cont	RR	95.00%CI
AGUDO	504	f	3	7	-	80	-	1.58	(0.59- 4.23)
AUVINE	509	c	0	76	27	44	229	14.65	(8.50- 25.26)
BARBON	520	m	1	200	-	22	-	8.20	(5.00- 13.30)
BRESLO	501	c	0	32	35	19	56	2.69	(1.33- 5.47)
BUFFLE	518	f	0	47	34	12	112	12.90	(6.15- 27.07)
CHEN2	518	m	0	29	25	9	33	4.25	(1.71- 10.57)
CHEN2	523	f	0	9	8	25	33	1.49	(0.50- 4.39)
Subtotal CHEN2								2.75	(1.37- 5.53)
CHIAZZ	501	m	2	-	-	4	-	3.00	(0.31- 28.84)
CHOI	523	m	0	36	77	13	95	3.42	(1.69- 6.89)
CHOI	530	f	0	15	25	76	164	1.29	(0.65- 2.60)
Subtotal CHOI								2.09	(1.28- 3.43)
DAMBER	501	m	0	70	76	42	208	4.56	(2.87- 7.26)
DOLL	503	m	0	251	264	7	61	8.29	(3.72- 18.46)
DOLL	510	f	0	23	15	40	59	2.26	(1.05- 4.86)
Subtotal DOLL								4.20	(2.42- 7.30)
*DORN	610	m	0	37	73050	25	213858	4.33	(2.61- 7.20)
*DORN	647	m	0	90	74464	49	171211	4.22	(2.98- 5.98)
Subtotal DORN								4.26	(3.20- 5.67)
GAO	507	m	2	363	-	62	-	4.70	(3.30- 6.50)
GAO	517	f	2	87	-	435	-	3.80	(2.60- 5.80)
Subtotal GAO								4.30	(3.32- 5.57)
GENG	533	f	1	28	-	54	-	1.55	(0.83- 2.89)
HAENSZ	537	f	0	44	66	81	236	1.94	(1.23- 3.07)
HEGMAN	513	m	1	26	-	-	-	9.40	(4.60- 19.30)
HEGMAN	516	f	1	2	-	-	-	4.80	(1.00- 22.10)
Subtotal HEGMAN								8.35	(4.36- 16.00)
HU2	503	c	0	229	159	121	213	2.54	(1.88- 3.43)
JOLY	543	m	0	18	70	12	218	4.67	(2.14- 10.18)
JOLY	533	f	0	23	41	52	283	3.05	(1.69- 5.51)
Subtotal JOLY								3.57	(2.23- 5.71)
KHUDER	506	m	0	72	152	23	309	6.36	(3.83- 10.58)
KOULUM	502	m	0	60	67	5	54	9.67	(3.63- 25.77)
LETOUR	501	c	0	188	160	24	224	10.97	(6.85- 17.56)
LIU3	504	m	2	20	-	4	-	1.10	(0.25- 4.93)
LIU4	501	m	2	-	-	-	-	2.41	(2.32- 2.49)
LUBIN	566	m	0	65	146	9	72	3.56	(1.68- 7.56)
LUBIN2	1157	m	1	564	-	185	-	4.97	(4.12- 6.00)
MATOS	576	m	2	28	-	11	-	3.90	(1.80- 8.30)
PEZZOT	570	m	0	41	105	4	116	11.32	(3.92- 32.69)
*QIAO2	506	m	1	52	-	10	-	1.32	(0.67- 2.60)
RACHTA	507	f	1	25	-	33	-	5.33	(2.79- 10.20)
SOBUE	654	m	0	110	121	29	126	3.95	(2.45- 6.38)
SUZUK2	501	c	0	16	22	11	53	3.50	(1.40- 8.75)
TIZZAN	507	m	0	313	330	180	305	1.61	(1.26- 2.04)
TIZZAN	520	f	0	12	21	117	114	0.56	(0.26- 1.18)
Subtotal TIZZAN								1.46	(1.16- 1.83)
WYNDE6	759	m	0	111	92	51	589	13.93	(9.36- 20.74)
WYNDE6	767	f	0	127	90	73	673	13.01	(9.06- 18.69)
Subtotal WYNDE6								13.42	(10.27- 17.54)
ZHENG	564	m	0	145	109	33	94	3.79	(2.37- 6.05)
Partial Totals				3691	149851	2086	389798		

*prospective study

Table 1H2 - 2

IESLC - Meta-analysis of Ever Smoking, Age started, "Low"
All LC types, Any Product (or Cigarettes if Any not available)
Most adjusted

REF	NRR	SEX	AD	Ys	Ws	Qs	Ps
AGUDO	504	f	3	0.46	3.96	1.06	0.3627
AUVINE	509	c	0	2.68	12.94	37.83	0.0000
BARBON	520	m	1	2.10	16.05	20.49	0.0000
BRESLO	501	c	0	0.99	7.67	0.00	0.0060
BUFFLE	518	f	0	2.56	7.00	17.53	0.0000
CHEN2	518	m	0	1.45	4.63	1.04	0.0018
CHEN2	523	f	0	0.40	3.26	1.09	0.4750
Subtotal CHEN2				1.01	7.90	2.13	
CHIAZZ	501	m	2	1.10	0.75	0.01	0.3421
CHOI	523	m	0	1.23	7.80	0.50	0.0006
CHOI	530	f	0	0.26	7.94	4.07	0.4667
Subtotal CHOI				0.74	15.74	4.58	
DAMBER	501	m	0	1.52	17.84	5.26	0.0000
DOLL	503	m	0	2.11	5.99	7.78	0.0000
DOLL	510	f	0	0.82	6.57	0.16	0.0364
Subtotal DOLL				1.43	12.56	7.95	
*DORN	610	m	0	1.47	14.92	3.61	0.0000
*DORN	647	m	0	1.44	31.75	6.90	0.0000
Subtotal DORN				1.45	46.67	10.51	
GAO	507	m	2	1.55	33.44	10.98	0.0000
GAO	517	f	2	1.34	23.87	3.10	0.0000
Subtotal GAO				1.46	57.31	14.09	
GENG	533	f	1	0.44	9.87	2.84	0.1685
HAENSZ	537	f	0	0.66	18.36	1.77	0.0044
HEGMAN	513	m	1	2.24	7.47	11.98	0.0000
HEGMAN	516	f	1	1.57	1.60	0.57	0.0470
Subtotal HEGMAN				2.12	9.08	12.55	
HU2	503	c	0	0.93	42.35	0.08	0.0000
JOLY	543	m	0	1.54	6.34	2.04	0.0001
JOLY	533	f	0	1.12	11.03	0.22	0.0002
Subtotal JOLY				1.27	17.37	2.26	
KHUDER	506	m	0	1.85	14.88	11.43	0.0000
KOULUM	502	m	0	2.27	4.00	6.70	0.0000
LETOUR	501	c	0	2.39	17.33	34.97	0.0000
LIU3	504	m	2	0.10	1.73	1.34	0.9003
LIU4	501	m	2	0.88	3072.74	27.63	0.0000
LUBIN	566	m	0	1.27	6.79	0.59	0.0009
LUBIN2	1157	m	1	1.60	108.74	43.02	0.0000
MATOS	576	m	2	1.36	6.58	0.98	0.0005
PEZZOT	570	m	0	2.43	3.42	7.21	0.0000
*QIAO2	506	m	1	0.28	8.36	4.06	0.4222
RACHTA	507	f	1	1.67	9.14	4.47	0.0000
SOBUE	654	m	0	1.37	16.73	2.67	0.0000
SUZUK2	501	c	0	1.25	4.59	0.36	0.0072
TIZZAN	507	m	0	0.47	66.40	16.60	0.0001
TIZZAN	520	f	0	-0.59	6.74	16.41	0.1283
Subtotal TIZZAN				0.38	73.15	33.01	
WYNDE6	759	m	0	2.63	24.28	66.90	0.0000
WYNDE6	767	f	0	2.57	29.27	74.10	0.0000
Subtotal WYNDE6				2.60	53.55	141.00	
ZHENG	564	m	0	1.33	17.54	2.24	0.0000

	N	42
	NS	33
	Wt	3722.68
Het	Chi	462.60
Het	df	41
Het	P	***
Fixed	RR	2.65
	RRl	2.57
	RRu	2.74
	P	+++
Random	RR	4.00
	RRl	3.25
	RRu	4.93
	P	+++
Asymm	P	**

Table 1H2 - 3

IESLC - Meta-analysis of Ever Smoking, Age started, "Low"
All LC types, Any Product (or Cigarettes if Any not available)
Most adjusted

	Sex: combined	male	female	Total
N	5	24	13	42
NS	5	23	13	41
Wt	84.88	3499.17	138.63	3722.68
Het Chi	48.00	259.69	114.08	462.60
Het df	4	23	12	41
Het P	***	***	***	***
Fixed RR	4.57	2.58	3.61	2.65
RRl	3.70	2.50	3.06	2.57
RRu	5.65	2.67	4.27	2.74
P	+++	+++	+++	+++
Random RR	5.28	4.47	2.81	4.00
RRl	2.34	3.46	1.64	3.25
RRu	11.93	5.77	4.82	4.93
P	+++	+++	+++	+++
Between Chi				40.84
Between df				2
Between P				***
Btwn(F) P				N.S.
Btwn(R) P				N.S.

	Lung cancer type: all	other	Total
N	39	3	42
NS	31	2	33
Wt	3650.77	71.91	3722.68
Het Chi	318.03	51.15	462.60
Het df	38	2	41
Het P	***	***	***
Fixed RR	2.59	8.19	2.65
RRl	2.51	6.50	2.57
RRu	2.68	10.32	2.74
P	+++	+++	+++
Random RR	3.80	7.11	4.00
RRl	3.13	2.19	3.25
RRu	4.62	23.12	4.93
P	+++	++	+++
Between Chi			93.43
Between df			1
Between P			***
Btwn(F) P			**
Btwn(R) P			N.S.

	Location: NAmer	UK	Scand	othEur	China	Japan	othAs	other	Total
N	12	2	3	6	11	1	2	5	42
NS	9	1	3	5	9	1	1	4	33
Wt	175.29	12.56	34.77	211.05	3224.58	16.73	15.74	31.96	3722.68
Het Chi	78.79	5.28	10.45	91.51	32.47	0.00	3.70	4.73	462.60
Het df	11	1	2	5	10	0	1	4	41
Het P	***	*	**	***	***	N.S.	(*)	N.S.	***
Fixed RR	6.70	4.20	7.68	3.31	2.44	3.95	2.09	4.10	2.65
RRl	5.78	2.42	5.51	2.89	2.35	2.45	1.28	2.90	2.57
RRu	7.77	7.30	10.70	3.79	2.52	6.38	3.43	5.80	2.74
P	+++	+++	+++	+++	+++	+++	++	+++	+++
Random RR	6.33	4.30	8.47	2.71	2.76	3.95	2.10	4.18	4.00
RRl	4.13	1.21	3.71	1.36	2.19	2.45	0.81	2.85	3.25
RRu	9.71	15.36	19.31	5.40	3.48	6.38	5.44	6.12	4.93
P	+++	+	+++	++	+++	+++	N.S.	+++	+++
Between Chi									235.67
Between df									7
Between P									***
Btwn(F) P									***
Btwn(R) P									**

Table 1H2 - 3

IESLC - Meta-analysis of Ever Smoking, Age started, "Low"
All LC types, Any Product (or Cigarettes if Any not available)
Most adjusted

Detailed Country in "other Europe"

	multi	Germany	othWest	East	Balkans	Total
N	1		4	1		6
NS	1		3	1		5
Wt	108.74		93.16	9.14		211.05
Het Chi	0.00		46.36	0.00		91.51
Het df	0		3	0		5
Het P	N.S.		***	N.S.		***
Fixed RR	4.97		1.97	5.33		3.31
RRl	4.12		1.61	2.79		2.89
RRu	6.00		2.41	10.19		3.79
P	+++		+++	+++		+++
Random RR	4.97		1.90	5.33		2.71
RRl	4.12		0.67	2.79		1.36
RRu	6.00		5.39	10.19		5.40
P	+++		N.S.	+++		++
Between Chi						45.15
Between df						2
Between P						***
Btwn(F) P						N.S.
Btwn(R) P						N.S.

Detailed Country in "other Asia"

	India	HongKong	other	Total
N			2	2
NS			1	1
Wt			15.74	15.74
Het Chi			3.70	3.70
Het df			1	1
Het P			(*)	(*)
Fixed RR			2.09	2.09
RRl			1.28	1.28
RRu			3.43	3.43
P			++	++
Random RR			2.10	2.10
RRl			0.81	0.81
RRu			5.44	5.44
P			N.S.	N.S.
Between Chi				
Between df				
Between P				N.S.
Btwn(F) P				N.S.
Btwn(R) P				N.S.

Detailed other continent

	SCAmer	Total
N	5	5
NS	4	4
Wt	31.96	31.96
Het Chi	4.73	4.73
Het df	4	4
Het P	N.S.	N.S.
Fixed RR	4.10	4.10
RRl	2.90	2.90
RRu	5.80	5.80
P	+++	+++
Random RR	4.18	4.18
RRl	2.85	2.85
RRu	6.12	6.12
P	+++	+++
Between Chi		
Between df		
Between P		N.S.
Btwn(F) P		N.S.
Btwn(R) P		N.S.

Table 1H2 - 3

IESLC - Meta-analysis of Ever Smoking, Age started, "Low"
All LC types, Any Product (or Cigarettes if Any not available)
Most adjusted

	Start year of study					
	<1960	1960-69	1970-79	1980-89	1990+	Total
N	10	2	7	19	4	42
NS	7	1	6	15	4	33
Wt	163.16	53.55	209.35	3267.96	28.67	3722.68
Het Chi	58.36	0.06	30.23	152.59	9.19	462.60
Het df	9	1	6	18	3	41
Het P	***	N.S.	***	***	*	***
Fixed RR	2.40	13.42	4.49	2.50	3.09	2.65
RRl	2.06	10.27	3.92	2.42	2.14	2.57
RRu	2.80	17.54	5.15	2.59	4.45	2.74
P	+++	+++	+++	+++	+++	+++
Random RR	2.83	13.42	4.85	3.95	3.11	4.00
RRl	1.80	10.27	3.38	2.88	1.62	3.25
RRu	4.45	17.54	6.97	5.41	5.96	4.93
P	+++	+++	+++	+++	+++	+++
Between Chi						212.17
Between df						4
Between P						***
Btwn(F) P						***
Btwn(R) P						***

	Study type (1)		
	CC	other	Total
N	39	3	42
NS	31	2	33
Wt	3667.66	55.03	3722.68
Het Chi	447.97	9.73	462.60
Het df	38	2	41
Het P	***	**	***
Fixed RR	2.64	3.56	2.65
RRl	2.55	2.74	2.57
RRu	2.72	4.64	2.74
P	+++	+++	+++
Random RR	4.10	3.05	4.00
RRl	3.28	1.62	3.25
RRu	5.12	5.76	4.93
P	+++	+++	+++
Between Chi			4.91
Between df			1
Between P			*
Btwn(F) P			N.S.
Btwn(R) P			N.S.

	Study type (2)			
	CC	prosp	other	Total
N	39	3		42
NS	31	2		33
Wt	3667.66	55.03		3722.68
Het Chi	447.97	9.73		462.60
Het df	38	2		41
Het P	***	**		***
Fixed RR	2.64	3.56		2.65
RRl	2.55	2.74		2.57
RRu	2.72	4.64		2.74
P	+++	+++		+++
Random RR	4.10	3.05		4.00
RRl	3.28	1.62		3.25
RRu	5.12	5.76		4.93
P	+++	+++		+++
Between Chi				4.91
Between df				1
Between P				*
Btwn(F) P				N.S.
Btwn(R) P				N.S.

Table 1H2 - 3

IESLC - Meta-analysis of Ever Smoking, Age started, "Low"
All LC types, Any Product (or Cigarettes if Any not available)
Most adjusted

	Study size (number of LC cases) 100-249	250-499	500-999	1000+	Total
N	11	7	11	13	42
NS	10	5	10	8	33
Wt	64.78	56.37	160.09	3441.45	3722.68
Het Chi	23.38	27.17	64.27	275.86	462.60
Het df	10	6	10	12	41
Het P	**	***	***	***	***
Fixed RR	2.67	3.55	4.96	2.56	2.65
RRl	2.09	2.73	4.25	2.48	2.57
RRu	3.41	4.61	5.80	2.65	2.74
P	+++	+++	+++	+++	+++
Random RR	2.80	3.48	5.74	3.97	4.00
RRl	1.87	1.95	3.81	2.77	3.25
RRu	4.19	6.21	8.67	5.69	4.93
P	+++	+++	+++	+++	+++
Between Chi					71.93
Between df					3
Between P					***
Btwn(F) P					(*)
Btwn(R) P					N.S.

	Risky occupational population no	mining	othRisky	Total
N	39	2	1	42
NS	30	2	1	33
Wt	3706.78	15.15	0.75	3722.68
Het Chi	457.94	3.69	0.00	462.60
Het df	38	1	0	41
Het P	***	(*)	N.S.	***
Fixed RR	2.65	2.06	3.00	2.65
RRl	2.57	1.24	0.31	2.57
RRu	2.74	3.41	28.94	2.74
P	+++	++	N.S.	+++
Random RR	4.14	2.14	3.00	4.00
RRl	3.33	0.81	0.31	3.25
RRu	5.13	5.65	28.94	4.93
P	+++	N.S.	N.S.	+++
Between Chi				0.98
Between df				2
Between P				N.S.
Btwn(F) P				N.S.
Btwn(R) P				N.S.

	National cigarette tobacco type Virginia	blended	other	Total
N	3	28	11	42
NS	2	22	9	33
Wt	29.89	468.21	3224.58	3722.68
Het Chi	11.99	242.57	32.47	462.60
Het df	2	27	10	41
Het P	**	***	***	***
Fixed RR	7.33	4.42	2.44	2.65
RRl	5.12	4.03	2.35	2.57
RRu	10.48	4.83	2.52	2.74
P	+++	+++	+++	+++
Random RR	6.06	4.52	2.76	4.00
RRl	2.35	3.36	2.19	3.25
RRu	15.66	6.07	3.48	4.93
P	+++	+++	+++	+++
Between Chi				175.57
Between df				2
Between P				***
Btwn(F) P				***
Btwn(R) P				*

Table 1H2 - 3

IESLC - Meta-analysis of Ever Smoking, Age started, "Low"
All LC types, Any Product (or Cigarettes if Any not available)
Most adjusted

	Any proxy use No/nk	Yes	Total
N	29	13	42
NS	22	11	33
Wt	511.93	3210.75	3722.68
Het Chi	229.42	147.26	462.60
Het df	28	12	41
Het P	***	***	***
Fixed RR	3.88	2.49	2.65
RRl	3.55	2.41	2.57
RRu	4.23	2.58	2.74
P	+++	+++	+++
Random RR	3.55	5.29	4.00
RRl	2.72	3.40	3.25
RRu	4.64	8.23	4.93
P	+++	+++	+++
Between Chi			85.93
Between df			1
Between P			***
Btwn(F) P			**
Btwn(R) P			N.S.

	Full histological confirmation No	Yes	Total
N	27	15	42
NS	20	13	33
Wt	3425.59	297.09	3722.68
Het Chi	163.41	85.86	462.60
Het df	26	14	41
Het P	***	***	***
Fixed RR	2.47	5.97	2.65
RRl	2.39	5.33	2.57
RRu	2.55	6.69	2.74
P	+++	+++	+++
Random RR	3.17	5.99	4.00
RRl	2.56	4.37	3.25
RRu	3.93	8.22	4.93
P	+++	+++	+++
Between Chi			213.33
Between df			1
Between P			***
Btwn(F) P			***
Btwn(R) P			**

	Number of adjustment variables (1) 0	1	2+/+nk	Total
N	28	7	7	42
NS	21	6	6	33
Wt	418.37	161.24	3143.06	3722.68
Het Chi	259.11	34.67	22.84	462.60
Het df	27	6	6	41
Het P	***	***	***	***
Fixed RR	4.00	4.69	2.44	2.65
RRl	3.63	4.02	2.35	2.57
RRu	4.40	5.48	2.52	2.74
P	+++	+++	+++	+++
Random RR	4.26	4.13	3.04	4.00
RRl	3.12	2.50	2.12	3.25
RRu	5.81	6.83	4.37	4.93
P	+++	+++	+++	+++
Between Chi				145.98
Between df				2
Between P				***
Btwn(F) P				***
Btwn(R) P				N.S.

Table 1H2 - 3

IESLC - Meta-analysis of Ever Smoking, Age started, "Low"
All LC types, Any Product (or Cigarettes if Any not available)
Most adjusted

	Number of adjustment variables (2) 0	1	2	3-5	6+/+nk	Total
N	28	7	6	1		42
NS	21	6	5	1		33
Wt	418.37	161.24	3139.10	3.96		3722.68
Het Chi	259.11	34.67	22.10	0.00		462.60
Het df	27	6	5	0		41
Het P	***	***	***	N.S.		***
Fixed RR	4.00	4.69	2.44	1.58		2.65
RRl	3.63	4.02	2.35	0.59		2.57
RRu	4.40	5.48	2.52	4.23		2.74
P	+++	+++	+++	N.S.		+++
Random RR	4.26	4.13	3.25	1.58		4.00
RRl	3.12	2.50	2.20	0.59		3.25
RRu	5.81	6.83	4.80	4.23		4.93
P	+++	+++	+++	N.S.		+++
Between Chi						146.72
Between df						3
Between P						***
Btwn(F) P						**
Btwn(R) P						N.S.

	Product all/unsp	cig+/-ot	cig only	Total
N	15	25	2	42
NS	11	20	2	33
Wt	3227.99	487.31	7.38	3722.68
Het Chi	95.12	161.78	7.12	462.60
Het df	14	24	1	41
Het P	***	***	**	***
Fixed RR	2.42	4.79	3.93	2.65
RRl	2.34	4.38	1.91	2.57
RRu	2.51	5.24	8.10	2.74
P	+++	+++	+++	+++
Random RR	3.09	4.58	4.19	4.00
RRl	2.23	3.57	0.61	3.25
RRu	4.30	5.86	28.85	4.93
P	+++	+++	N.S.	+++
Between Chi				198.58
Between df				2
Between P				***
Btwn(F) P				***
Btwn(R) P				N.S.

	Denominator nev any	nev cigs	Total
N	24	18	42
NS	18	15	33
Wt	3341.31	381.37	3722.68
Het Chi	118.50	130.07	462.60
Het df	23	17	41
Het P	***	***	***
Fixed RR	2.44	5.39	2.65
RRl	2.36	4.87	2.57
RRu	2.53	5.96	2.74
P	+++	+++	+++
Random RR	3.09	5.48	4.00
RRl	2.48	4.04	3.25
RRu	3.87	7.44	4.93
P	+++	+++	+++
Between Chi			214.04
Between df			1
Between P			***
Btwn(F) P			***
Btwn(R) P			**

Table 1H2 - 3

IESLC - Meta-analysis of Ever Smoking, Age started, "Low"
All LC types, Any Product (or Cigarettes if Any not available)
Most adjusted

	Derivation of RR/CI			
	Orig	StdCalc	Other	Total
N	12	29	1	42
NS	10	22	1	33
Wt	119.95	529.99	3072.74	3722.68
Het Chi	39.10	263.45	0.00	462.60
Het df	11	28	0	41
Het P	***	***	N.S.	***
Fixed RR	4.63	4.05	2.41	2.65
RRl	3.87	3.72	2.33	2.57
RRu	5.54	4.41	2.50	2.74
P	+++	+++	+++	+++
Random RR	4.31	3.99	2.41	4.00
RRl	2.94	3.02	2.33	3.25
RRu	6.32	5.28	2.50	4.93
P	+++	+++	+++	+++
Between Chi				160.05
Between df				2
Between P				***
Btwn(F) P				***
Btwn(R) P				***

	Study LIU4		
	LIU4	others	Total
N	1	41	42
NS	1	32	33
Wt	3072.74	649.94	3722.68
Het Chi	0.00	304.35	462.60
Het df	0	40	41
Het P	N.S.	***	***
Fixed RR	2.41	4.15	2.65
RRl	2.33	3.84	2.57
RRu	2.50	4.48	2.74
P	+++	+++	+++
Random RR	2.41	4.06	4.00
RRl	2.33	3.23	3.25
RRu	2.50	5.10	4.93
P	+++	+++	+++
Between Chi			158.26
Between df			1
Between P			***
Btwn(F) P			***
Btwn(R) P			***

Table 1H2 - 4

IESLC - Meta-analysis of Ever Smoking, Age started, "Low"
All LC types, Any Product (or Cigarettes if Any not available)
Least adjusted

REF	NRR	X	SEX	AGEL	AGEH	RACE	YF	LC TYPE	LOC	START	ST	NLC	R	VB	P	H	AD	PRODUCT	exL	exH	DENOM	De
AGUDO	501	x	f	0	0	all	-	all	Eu:wst	1989	CC	103	n	bl	n	n	0	cig only	24	999	nev cigs	st
AUVINE	509		c	0	0	all	-	all	Eu:Sca	1986	CC	517	n	bl	y	n	0	cig+/-ot	21	999	nev cigs	st
BARBON	515	x	m	0	0	all	-	all	Eu:wst	1979	CC	755	n	bl	y	y	0	all/unsp	20	999	nev any	st
BRESLO	501		c	0	0	all	-	all	NAmer	1949	CC	518	n	bl	n	y	0	cig+/-ot	25	999	nev any	st
BUFFLE	518		f	0	0	w-hi	-	all	NAmer	1976	CC	943	n	bl	y	n	0	cig+/-ot	21	29	nev cigs	or
CHEN2	518		m	0	0	all	-	all	As:Chi	1983	CC	193	n	ot	y	n	0	all/unsp	20	30	nev any	st
CHEN2	523		f	0	0	all	-	all	As:Chi	1983	CC	193	n	ot	y	n	0	all/unsp	20	30	nev any	st
CHIAZZ	501		m	0	0	all	-	all	NAmer	1940	CC	144	o	bl	y	n	2	cig+/-ot	20	999	nev cigs	or
CHOI	523		m	0	0	all	-	all	As:oth	1985	CC	375	n	bl	n	n	0	cig+/-ot	25	999	nev cigs	st
CHOI	530		f	0	0	all	-	all	As:oth	1985	CC	375	n	bl	n	n	0	cig+/-ot	25	999	nev cigs	st
DAMBER	501		m	0	0	all	-	all	Eu:Sca	1972	CC	579	n	bl	y	n	0	all/unsp	21	999	nev any	st
DOLL	503		m	0	0	all	-	all	Eu:UK	1948	CC	1465	n	V	n	n	0	all/unsp	20	29	nev any	st
DOLL	510		f	0	0	all	-	all	Eu:UK	1948	CC	1465	n	V	n	n	0	all/unsp	20	29	nev any	st
DORN	610		m	55	64	wh	8	all	NAmer	1954	pr	5097	n	bl	n	n	0	cig+/-ot	25	999	nev any	st
DORN	647		m	65	74	wh	8	all	NAmer	1954	pr	5097	n	bl	n	n	0	cig+/-ot	25	999	nev any	st
GAO	502	x	m	0	0	all	-	all	As:Chi	1984	CC	1405	n	ot	n	n	0	cig+/-ot	20	29	nev cigs	st
GAO	512	x	f	0	0	all	-	all	As:Chi	1984	CC	1405	n	ot	n	n	0	cig+/-ot	20	29	nev cigs	st
GENG	528	x	f	0	0	all	-	all	As:Chi	1985	CC	292	n	ot	*	n	0	cig+/-ot	21	999	nev any	st
HAENSZ	537		f	0	0	all	-	not alv	NAmer	1955	CC	158	n	bl	n	y	0	cig+/-ot	25	999	nev any	st
HEGMAN	513		m	0	0	all	-	all	NAmer	1989	CC	282	n	bl	y	y	1	all/unsp	20	999	nev any	or
HEGMAN	516		f	0	0	all	-	all	NAmer	1989	CC	282	n	bl	y	y	1	all/unsp	26	999	nev any	or
HU2	503		c	0	0	all	-	all	As:Chi	1977	CC	523	n	ot	y	n	0	cig+/-ot	20	29	nev cigs	st
JOLY	543		m	0	0	all	-	all	SCAmer	1978	CC	826	n	bl	n	n	0	cig+/-ot	25	999	nev any	st
JOLY	533		f	0	0	all	-	all	SCAmer	1978	CC	826	n	bl	n	n	0	cig+/-ot	25	999	nev any	st
KHUDER	506		m	0	0	all	-	all	NAmer	1985	CC	482	n	bl	n	y	0	cig+/-ot	20	999	nev cigs	st
KOULUM	502		m	0	0	all	-	all	Eu:Sca	1936	CC	812	n	bl	n	n	0	all/unsp	21	30	nev any	st
LETOUR	501		c	0	0	all	-	all	NAmer	1983	CC	738	n	V	y	y	0	cig+/-ot	21	999	nev cigs	st
LIU3	501	x	m	0	0	all	-	all	As:Chi	1985	CC	110	n	ot	n	n	0	all/unsp	21	999	nev any	or
LIU4	501		m	35	69	all	-	all	As:Chi	1986	CC	1000-00	n	ot	y	n	2	all/unsp	25	999	nev any	ot
LUBIN	566		m	0	0	all	-	all	As:Chi	1984	CC	427	m	ot	y	n	0	cig+/-ot	23	26	nev any	st
LUBIN2	1148	x	m	0	0	all	-	all	Eu:mul	1976	CC	7804	n	bl	n	y	0	cig+/-ot	21	30	nev cigs	st
MATOS	556	x	m	0	0	all	-	all	SCAmer	1994	CC	200	n	bl	n	n	0	cig+/-ot	20	999	nev any	st
PEZZOT	570		m	0	0	all	-	all	SCAmer	1987	CC	215	n	bl	n	y	0	cig only	19	999	nev cigs	st
QIAO2	501	x	m	0	0	all	0	all	As:Chi	1992	pr	241	m	ot	n	n	0	all/unsp	21	999	nev any	st
RACHTA	502	x	f	0	0	all	-	all	Eu:est	1991	CC	118	n	bl	n	y	0	cig+/-ot	20	30	nev cigs	st
SOBUE	654		m	0	0	all	-	all	As:Jap	1986	CC	1376	n	bl	n	y	0	cig+/-ot	23	999	nev cigs	st
SUZUK2	501		c	0	0	all	-	all	SCAmer	1991	CC	123	n	bl	n	y	0	all/unsp	19	999	nev any	st
TIZZAN	507		m	0	0	all	-	all	Eu:wst	1959	CC	1358	n	bl	n	n	0	all/unsp	20	30	nev any	st
TIZZAN	520		f	0	0	all	-	all	Eu:wst	1959	CC	1358	n	bl	n	n	0	all/unsp	20	30	nev any	st
WYNDE6	759		m	0	0	wh	-	q+s+a	NAmer	1969	CC	4423	n	bl	n	y	0	cig+/-ot	21	999	nev cigs	st
WYNDE6	767		f	0	0	wh	-	q+s+a	NAmer	1969	CC	4423	n	bl	n	y	0	cig+/-ot	21	999	nev cigs	st
ZHENG	564		m	0	0	all	-	all	As:Chi	1982	CC	540	n	ot	*	y	0	cig+/-ot	20	29	nev cigs	st

Cigarette type is all/unspec for all RRs

Table 1H2 - 5

IESLC - Meta-analysis of Ever Smoking, Age started, "Low"
All LC types, Any Product (or Cigarettes if Any not available)
Least adjusted

REF	NRR	SEX	AD	Number Exposed Case	Cont	Non-exposed Case	Cont	RR	95.00%CI
AGUDO	501	f	0	7	12	80	183	1.33	(0.51- 3.51)
AUVINE	509	c	0	76	27	44	229	14.65	(8.50- 25.26)
BARBON	515	m	0	200	207	22	188	8.26	(5.10- 13.38)
BRESLO	501	c	0	32	35	19	56	2.69	(1.33- 5.47)
BUFFLE	518	f	0	47	34	12	112	12.90	(6.15- 27.07)
CHEN2	518	m	0	29	25	9	33	4.25	(1.71- 10.57)
CHEN2	523	f	0	9	8	25	33	1.49	(0.50- 4.39)
Subtotal CHEN2								2.75	(1.37- 5.53)
CHIAZZ	501	m	2	-	-	4	-	3.00	(0.31- 28.84)
CHOI	523	m	0	36	77	13	95	3.42	(1.69- 6.89)
CHOI	530	f	0	15	25	76	164	1.29	(0.65- 2.60)
Subtotal CHOI								2.09	(1.28- 3.43)
DAMBER	501	m	0	70	76	42	208	4.56	(2.87- 7.26)
DOLL	503	m	0	251	264	7	61	8.29	(3.72- 18.46)
DOLL	510	f	0	23	15	40	59	2.26	(1.05- 4.86)
Subtotal DOLL								4.20	(2.42- 7.30)
*DORN	610	m	0	37	73050	25	213858	4.33	(2.61- 7.20)
*DORN	647	m	0	90	74464	49	171211	4.22	(2.98- 5.98)
Subtotal DORN								4.26	(3.20- 5.67)
GAO	502	m	0	363	262	62	202	4.51	(3.26- 6.25)
GAO	512	f	0	87	41	435	605	2.95	(2.00- 4.36)
Subtotal GAO								3.79	(2.95- 4.87)
GENG	528	f	0	28	31	54	93	1.56	(0.84- 2.87)
HAENSZ	537	f	0	44	66	81	236	1.94	(1.23- 3.07)
HEGMAN	513	m	1	26	-	-	-	9.40	(4.60- 19.30)
HEGMAN	516	f	1	2	-	-	-	4.80	(1.00- 22.10)
Subtotal HEGMAN								8.35	(4.36- 16.00)
HU2	503	c	0	229	159	121	213	2.54	(1.88- 3.43)
JOLY	543	m	0	18	70	12	218	4.67	(2.14- 10.18)
JOLY	533	f	0	23	41	52	283	3.05	(1.69- 5.51)
Subtotal JOLY								3.57	(2.23- 5.71)
KHUDER	506	m	0	72	152	23	309	6.36	(3.83- 10.58)
KOULUM	502	m	0	60	67	5	54	9.67	(3.63- 25.77)
LETOUR	501	c	0	188	160	24	224	10.97	(6.85- 17.56)
LIU3	501	m	0	20	80	4	19	1.19	(0.36- 3.88)
LIU4	501	m	2	-	-	-	-	2.41	(2.32- 2.49)
LUBIN	566	m	0	65	146	9	72	3.56	(1.68- 7.56)
LUBIN2	1148	m	0	564	1069	185	1878	5.36	(4.46- 6.43)
MATOS	556	m	0	28	73	11	110	3.84	(1.80- 8.18)
PEZZOT	570	m	0	41	105	4	116	11.32	(3.92- 32.69)
*QIAO2	501	m	0	52	1947	10	709	1.89	(0.97- 3.71)
RACHTA	502	f	0	25	18	33	98	4.12	(2.00- 8.50)
SOBUE	654	m	0	110	121	29	126	3.95	(2.45- 6.38)
SUZUK2	501	c	0	16	22	11	53	3.50	(1.40- 8.75)
TIZZAN	507	m	0	313	330	180	305	1.61	(1.26- 2.04)
TIZZAN	520	f	0	12	21	117	114	0.56	(0.26- 1.18)
Subtotal TIZZAN								1.46	(1.16- 1.83)
WYNDE6	759	m	0	111	92	51	589	13.93	(9.36- 20.74)
WYNDE6	767	f	0	127	90	73	673	13.01	(9.06- 18.69)
Subtotal WYNDE6								13.42	(10.27- 17.54)
ZHENG	564	m	0	145	109	33	94	3.79	(2.37- 6.05)
Partial Totals				3691	153591	2086	393883		

*prospective study

Table 1H2 - 5

IESLC - Meta-analysis of Ever Smoking, Age started, "Low"
All LC types, Any Product (or Cigarettes if Any not available)
Least adjusted

REF	NRR	SEX	AD	Ys	Ws	Qs	Ps
AGUDO	501	f	0	0.29	4.10	1.94	0.5594
AUVINE	509	c	0	2.68	12.94	37.76	0.0000
BARBON	515	m	0	2.11	16.50	21.25	0.0000
BRESLO	501	c	0	0.99	7.67	0.00	0.0060
BUFFLE	518	f	0	2.56	7.00	17.49	0.0000
CHEN2	518	m	0	1.45	4.63	1.03	0.0018
CHEN2	523	f	0	0.40	3.26	1.10	0.4750
Subtotal CHEN2				1.01	7.90	2.13	
CHIAZZ	501	m	2	1.10	0.75	0.01	0.3421
CHOI	523	m	0	1.23	7.80	0.50	0.0006
CHOI	530	f	0	0.26	7.94	4.09	0.4667
Subtotal CHOI				0.74	15.74	4.59	
DAMBER	501	m	0	1.52	17.84	5.23	0.0000
DOLL	503	m	0	2.11	5.99	7.76	0.0000
DOLL	510	f	0	0.82	6.57	0.17	0.0364
Subtotal DOLL				1.43	12.56	7.93	
*DORN	610	m	0	1.47	14.92	3.59	0.0000
*DORN	647	m	0	1.44	31.75	6.85	0.0000
Subtotal DORN				1.45	46.67	10.44	
GAO	502	m	0	1.51	36.16	10.20	0.0000
GAO	512	f	0	1.08	25.10	0.28	0.0000
Subtotal GAO				1.33	61.27	10.49	
GENG	528	f	0	0.44	10.28	2.93	0.1565
HAENSZ	537	f	0	0.66	18.36	1.79	0.0044
HEGMAN	513	m	1	2.24	7.47	11.95	0.0000
HEGMAN	516	f	1	1.57	1.60	0.56	0.0470
Subtotal HEGMAN				2.12	9.08	12.51	
HU2	503	c	0	0.93	42.35	0.09	0.0000
JOLY	543	m	0	1.54	6.34	2.03	0.0001
JOLY	533	f	0	1.12	11.03	0.22	0.0002
Subtotal JOLY				1.27	17.37	2.24	
KHUDER	506	m	0	1.85	14.88	11.39	0.0000
KOULUM	502	m	0	2.27	4.00	6.69	0.0000
LETOUR	501	c	0	2.39	17.33	34.89	0.0000
LIU3	501	m	0	0.17	2.74	1.77	0.7761
LIU4	501	m	2	0.88	3072.74	28.57	0.0000
LUBIN	566	m	0	1.27	6.79	0.59	0.0009
LUBIN2	1148	m	0	1.68	115.66	57.02	0.0000
MATOS	556	m	0	1.34	6.69	0.91	0.0005
PEZZOT	570	m	0	2.43	3.42	7.20	0.0000
*QIAO2	501	m	0	0.64	8.52	0.97	0.0623
RACHTA	502	f	0	1.42	7.35	1.43	0.0001
SOBUE	654	m	0	1.37	16.73	2.65	0.0000
SUZUK2	501	c	0	1.25	4.59	0.35	0.0072
TIZZAN	507	m	0	0.47	66.40	16.71	0.0001
TIZZAN	520	f	0	-0.59	6.74	16.45	0.1283
Subtotal TIZZAN				0.38	73.15	33.15	
WYNDE6	759	m	0	2.63	24.28	66.77	0.0000
WYNDE6	767	f	0	2.57	29.27	73.95	0.0000
Subtotal WYNDE6				2.60	53.55	140.72	
ZHENG	564	m	0	1.33	17.54	2.22	0.0000

	N	42
	NS	33
	Wt	3734.05
Het	Chi	469.33
Het	df	41
Het	P	***
Fixed	RR	2.65
	RRl	2.57
	RRu	2.74
	P	+++
Random	RR	3.96
	RRl	3.22
	RRu	4.88
	P	+++
Asymm	P	**

Table 1H2 - 6

IESLC - Meta-analysis of Ever Smoking, Age started, "Low"
All LC types, Any Product (or Cigarettes if Any not available)
Least adjusted

	Sex			
	combined	male	female	Total
N	5	24	13	42
NS	5	23	13	41
Wt	84.88	3510.55	138.62	3734.05
Het Chi	48.00	272.06	114.66	469.33
Het df	4	23	12	41
Het P	***	***	***	***
Fixed RR	4.57	2.59	3.36	2.65
RRl	3.70	2.51	2.85	2.57
RRu	5.65	2.68	3.97	2.74
P	+++	+++	+++	+++
Random RR	5.28	4.51	2.66	3.96
RRl	2.34	3.49	1.54	3.22
RRu	11.93	5.84	4.57	4.88
P	+++	+++	+++	+++
Between Chi				34.61
Between df				2
Between P				***
Btwn(F) P				N.S.
Btwn(R) P				N.S.

Table 1H2 - 7

IESLC - Meta-analysis of Ever Smoking, Age started, "Low"
All LC types, Any Product (or Cigarettes if Any not available)
Excluded studies (and stage at which they were excluded)

1	AKIBA AMANDU AMES BECHER BENSHL BEST BLOT1 BROSS BROWN3 CARPEN CEDERL CHYOU CPSI CPSII DARBY DEAN2 DEAN3 DOLL2 ENGELA GAO2 GARCIA GILLIS GRAHAM GURSEL HAMMO2 HIRAYA HOLE HUMBLE JAHN JAIN KAISE2 KATSOU KAUFMA LAUSSM LIAW MCDUFF MIGRAN MRFITR PEZZO2 PISANI PRESCO QIAO SEGI2 SPEIZE SVENSS TVERDA WAKAI WATSON WIGLE WU WYNDE3 WYNDE8
2	AXELSS BOUCHA BOUCOT CHEN DESTEF DORGAN DOSEME FAN GARSHI GER HAMMON JUSSAW KOO KREUZE LEVIN MCCONN NOTAN2 OSANN2 RESTRE SADOWS VUTUC WANG2 WU2 WUWILL WYNDE2 XU ZHOU
3	GUO SPITZ STASZE ZHANG
4	LUO
5	CORREA YUAN
7	BOFFET WYNDE7
10	ALDERS
14	ARMADA HU JEDRYC LIU5 PERNU
15	BENHAM

Table 1H2 - 8
Potentially overlapping studies

REF	REFGP	PRINC	.	OVERLAP/LINK
LUBIN2	LUBIN2	1		Lubin-combined
WYNDE6	WYNDE6	1		WYNDE5/6/7/8
LUBIN	XIANGZ	2		LUBIN/XIANGZ/QIAO

Table 1H2 - 9
Most adjusted - insufficient data for meta-analysis

REF	NRR	SEX	AGEL	AGEH	RACE	YF	LC TYPE	LOC	START	ST	NLC	R	VB	P	H	AD	PRODUCT	exL	exH	DENOM	De
CORREA	535	c	0	0	all	-	all	NAmer	1979	CC	1359	n	bl	y	n	2	cig+/-ot	21	999	nev cigs	or
JEDRYC	618	f	0	0	all	-	all	Eu:est	1980	CC	1630	n	bl	y	n	0	cig+/-ot	23	999	nev any	st

REF	NRR	RR	SIG	RRDATA comment
CORREA	535	8.30		0
JEDRYC	618	*		0

Table 1H3 -

IESLC - Meta-analysis of Ever Smoking, Age started, "Mid"
All LC types, Any Product (or Cigarettes if Any not available)

This analysis is restricted to results for:
1) Ever smokers
2) Results by Age started
3) Categorical results by Age started
4) All LC types (or near equivalent)
5) Results complete enough for use in metaanalysis

Within each study, results are then selected (in the following order of preference, within each sex) for:
6) (not applicable)
7) PRODUCT: all/unspec, cigarettes regardless of other products, cigarettes only
8) CIGTYPE: all/unspecified, MC regardless of HR, MC only
9) (not applicable)
10) DENOM: never smoked anything, never smoked cigarettes, never any + low, never cigs + low
11) Followup period (YF, prospective studies): whole study (coded as 0) or longest available
12) LCtype: all or nearest available, at least Squamous and Adeno. (q = squamous, s = small,
l = large, a = adeno, mix = mixed, alv = alveolar)
13) Race: all or nearest available, otherwise by race (wh or w = white, bl or b = black, hi = hispanic
ch = chinese, jap = japanese, haw = hawaiian, w+o = white + oriental, sca = scandinavian, as = asian)
14) Age started "mid" in key scheme 1 (key value 18, maximum range 15-25)
15) For overlapping studies: principal rather than subsidiary studies
Finally by Age: whole study (coded as 0) if available, otherwise by widest available age group
and then for single sex results (m, f) in preference to results for both sexes combined (c).

Results adjusted (AD) for the most potential confounders are then chosen in Sections -1 to -3
and results adjusted for the least confounders in Sections -4 to -6. (Those least adjusted results which
actually differ from the most adjusted are marked 'x' in column X in Section -4)

Section -7 shows excluded studies, together with the stage (as above) at which no qualifying
results were found.

Section -8 lists the potentially overlapping studies which have been included (1=principal, 2=subsidiary).

Section -9 lists any results which would have been included in preference except that they had data not complete
enough for use in meta-analysis, with their significance (yes/no), if known, and any further comment as entered
on the database. It also lists as "gap" any categories for which no data were presented by the original authors.

In addition to those mentioned above, the following fields, levels and abbreviations are used:
* or nk = not known, n = no, y = yes, ot = other
nev = never
all/unspec = all or unspecified, cig+/-ot = cigarettes irrespective of other products (cigar, pipe etc)
MC = manufactured cigarettes, HR = hand-rolled cigarettes
exL, exH = range of exposure (low and high) in the smoking group, in terms of Age started
REF: 6-character study reference
NRR: number of the RR on the database within the study
ST : study type (CC = case control, pr or prosp = prospective)
NLC: number of lung cancer cases in whole study
R : risky occupational population (n = no, m = mining, o = other risky)
VB : national cigarette type (V = at least 75% Virginia, bl = at least 75% blended, ot = other)
P : any proxy use
H : full histological confirmation
De : derivation of RR/CI (or = original, st = standard method, ot = other method of estimation)

Table 1H3 - 1

IESLC - Meta-analysis of Ever Smoking, Age started, "Mid"
All LC types, Any Product (or Cigarettes if Any not available)
Most adjusted

REF	NRR	SEX	AGEL	AGEH	RACE	YF	LC TYPE	LOC	START	ST	NLC	R	VB	P	H	AD	PRODUCT	exL	exH	DENOM	De
AUVINE	510	c	0	0	all	-	all	Eu:Sca	1986	CC	517	n	bl	y	n	0	cig+/-ot	16	20	nev cigs	st
BARBON	521	m	0	0	all	-	all	Eu:wst	1979	CC	755	n	bl	y	y	1	all/unsp	15	19	nev any	or
BRESLO	502	c	0	0	all	-	all	NAmer	1949	CC	518	n	bl	n	y	0	cig+/-ot	15	24	nev any	st
BUFFLE	520	f	0	0	w-hi	-	all	NAmer	1976	CC	943	n	bl	y	n	0	cig+/-ot	17	18	nev cigs	ot
CHOI	525	m	0	0	all	-	all	As:oth	1985	CC	375	n	bl	n	n	0	cig+/-ot	15	19	nev cigs	st
DAMBER	502	m	0	0	all	-	all	Eu:Sca	1972	CC	579	n	bl	y	n	0	all/unsp	16	20	nev any	st
DORN	612	m	55	64	wh	8	all	NAmer	1954	pr	5097	n	bl	n	n	0	cig+/-ot	15	19	nev any	st
DORN	649	m	65	74	wh	8	all	NAmer	1954	pr	5097	n	bl	n	n	0	cig+/-ot	15	19	nev any	st
GENG	534	f	0	0	all	-	all	As:Chi	1985	CC	292	n	ot	*	n	1	cig+/-ot	16	20	nev any	st
JEDRYC	607	m	0	0	all	-	all	Eu:est	1980	CC	1630	n	bl	y	n	0	cig+/-ot	17	18	nev any	st
JOLY	544	m	0	0	all	-	all	SCAmer	1978	CC	826	n	bl	n	n	0	cig+/-ot	15	24	nev any	st
JOLY	534	f	0	0	all	-	all	SCAmer	1978	CC	826	n	bl	n	n	0	cig+/-ot	15	24	nev any	st
KHUDER	507	m	0	0	all	-	all	NAmer	1985	CC	482	n	bl	n	y	0	cig+/-ot	16	19	nev cigs	st
KOULUM	503	m	0	0	all	-	all	Eu:Sca	1936	CC	812	n	bl	n	n	0	all/unsp	16	20	nev any	st
LETOUR	502	c	0	0	all	-	all	NAmer	1983	CC	738	n	V	y	y	0	cig+/-ot	15	20	nev cigs	st
LUBIN2	1158	m	0	0	all	-	all	Eu:mul	1976	CC	7804	n	bl	n	y	1	cig+/-ot	17	20	nev cigs	st
MATOS	577	m	0	0	all	-	all	SCAmer	1994	CC	200	n	bl	n	n	2	cig+/-ot	15	19	nev any	or
QIAO2	507	m	0	0	all	0	all	As:Chi	1992	pr	241	m	ot	n	n	1	all/unsp	17	20	nev any	or
SOBUE	655	m	0	0	all	-	all	As:Jap	1986	CC	1376	n	bl	n	y	0	cig+/-ot	18	22	nev cigs	st
WYNDE6	760	m	0	0	wh	-	q+s+a	NAmer	1969	CC	4423	n	bl	n	y	0	cig+/-ot	18	20	nev cigs	st
WYNDE6	768	f	0	0	wh	-	q+s+a	NAmer	1969	CC	4423	n	bl	n	y	0	cig+/-ot	18	20	nev cigs	st

Cigarette type is all/unspec for all RRs

Table 1H3 - 2

IESLC - Meta-analysis of Ever Smoking, Age started, "Mid"
All LC types, Any Product (or Cigarettes if Any not available)
Most adjusted

REF	NRR	SEX	AD	Number Exposed Case	Number Exposed Cont	Non-exposed Case	Non-exposed Cont	RR	95.00%CI
AUVINE	510	c	0	135	47	44	229	14.95	(9.41- 23.75)
BARBON	521	m	1	395	-	22	-	9.90	(6.20- 15.80)
BRESLO	502	c	0	286	243	19	56	3.47	(2.01- 6.00)
BUFFLE	520	f	0	53	33	12	112	14.99	(7.17- 31.33)
CHOI	525	m	0	79	138	13	95	4.18	(2.20- 7.95)
DAMBER	502	m	0	261	190	42	208	6.80	(4.65- 9.95)
*DORN	612	m	0	342	213156	25	213858	13.73	(9.14- 20.60)
*DORN	649	m	0	306	118234	49	171211	9.04	(6.69- 12.23)
Subtotal DORN								10.49	(8.23- 13.36)
GENG	534	f	1	39	-	54	-	2.95	(1.57- 5.52)
JEDRYC	607	m	0	239	146	49	219	7.32	(5.04- 10.61)
JOLY	544	m	0	217	357	12	218	11.04	(6.03- 20.22)
JOLY	534	f	0	67	47	52	283	7.76	(4.82- 12.49)
Subtotal JOLY								8.88	(6.11- 12.91)
KHUDER	507	m	0	161	338	23	309	6.40	(4.03- 10.17)
KOULUM	503	m	0	267	103	5	54	28.00	(10.89- 71.96)
LETOUR	502	c	0	309	241	24	224	11.97	(7.60- 18.83)
LUBIN2	1158	m	1	1796	-	185	-	5.43	(4.59- 6.41)
MATOS	577	m	2	91	-	11	-	7.80	(4.00- 15.50)
*QIAO2	507	m	1	75	-	10	-	1.47	(0.76- 2.84)
SOBUE	655	m	0	776	772	29	126	4.37	(2.88- 6.62)
WYNDE6	760	m	0	223	139	51	589	18.53	(12.98- 26.45)
WYNDE6	768	f	0	200	94	73	673	19.62	(13.90- 27.67)
Subtotal WYNDE6								19.08	(14.90- 24.44)
Partial Totals				6317	334278	804	388464		

*prospective study

REF	NRR	SEX	AD	Ys	Ws	Qs	Ps
AUVINE	510	c	0	2.70	17.93	7.54	0.0000
BARBON	521	m	1	2.29	17.56	0.98	0.0000
BRESLO	502	c	0	1.24	12.80	8.45	0.0000
BUFFLE	520	f	0	2.71	7.07	3.00	0.0000
CHOI	525	m	0	1.43	9.31	3.64	0.0000
DAMBER	502	m	0	1.92	26.52	0.51	0.0000
*DORN	612	m	0	2.62	23.30	7.39	0.0000
*DORN	649	m	0	2.20	42.26	0.90	0.0000
Subtotal DORN				2.35	65.56	8.29	
GENG	534	f	1	1.08	9.72	9.23	0.0007
JEDRYC	607	m	0	1.99	27.77	0.12	0.0000
JOLY	544	m	0	2.40	10.49	1.25	0.0000
JOLY	534	f	0	2.05	16.96	0.00	0.0000
Subtotal JOLY				2.18	27.45	1.25	
KHUDER	507	m	0	1.86	17.89	0.71	0.0000
KOULUM	503	m	0	3.33	4.31	7.02	0.0000
LETOUR	502	c	0	2.48	18.69	3.39	0.0000
LUBIN2	1158	m	1	1.69	137.76	18.27	0.0000
MATOS	577	m	2	2.05	8.37	0.00	0.0000
*QIAO2	507	m	1	0.39	8.84	24.68	0.2520
SOBUE	655	m	0	1.47	22.22	7.52	0.0000
WYNDE6	760	m	0	2.92	30.32	22.59	0.0000
WYNDE6	768	f	0	2.98	32.44	27.47	0.0000
Subtotal WYNDE6				2.95	62.76	50.06	

Table 1H3 - 2

IESLC - Meta-analysis of Ever Smoking, Age started, "Mid"
All LC types, Any Product (or Cigarettes if Any not available)
Most adjusted

	N	21
	NS	18
	Wt	502.55
Het	Chi	154.68
Het	df	20
Het	P	***
Fixed	RR	7.82
	RRl	7.16
	RRu	8.53
	P	+++
Random	RR	8.05
	RRl	6.21
	RRu	10.43
	P	+++
Asymm	P	N.S.

Table 1H3 - 3

IESLC - Meta-analysis of Ever Smoking, Age started, "Mid"
All LC types, Any Product (or Cigarettes if Any not available)
Most adjusted

	Sex			
	combined	male	female	Total
N	3	14	4	21
NS	3	13	4	20
Wt	49.42	386.94	66.19	502.55
Het Chi	17.67	92.60	30.36	154.68
Het df	2	13	3	20
Het P	***	***	***	***
Fixed RR	9.41	7.16	11.38	7.82
RRl	7.12	6.48	8.94	7.16
RRu	12.44	7.91	14.48	8.53
P	+++	+++	+++	+++
Random RR	8.64	7.61	9.17	8.05
RRl	3.75	5.68	4.02	6.21
RRu	19.90	10.19	20.89	10.43
P	+++	+++	+++	+++
Between Chi				14.05
Between df				2
Between P				***
Btwn(F) P				N.S.
Btwn(R) P				N.S.

	Lung cancer type		
	all	other	Total
N	19	2	21
NS	17	1	18
Wt	439.79	62.76	502.55
Het Chi	97.47	0.05	154.68
Het df	18	1	20
Het P	***	N.S.	***
Fixed RR	6.88	19.08	7.82
RRl	6.27	14.90	7.16
RRu	7.55	24.44	8.53
P	+++	+++	+++
Random RR	7.26	19.08	8.05
RRl	5.73	14.90	6.21
RRu	9.21	24.44	10.43
P	+++	+++	+++
Between Chi			57.15
Between df			1
Between P			***
Btwn(F) P			**
Btwn(R) P			***

	Location								
	NAmer	UK	Scand	othEur	China	Japan	othAs	other	Total
N	8		3	3	2	1	1	3	21
NS	6		3	3	2	1	1	2	18
Wt	184.78		48.76	183.09	18.56	22.22	9.31	35.82	502.55
Het Chi	44.35		11.36	6.87	2.25	0.00	0.00	0.92	154.68
Het df	7		2	2	1	0	0	2	20
Het P	***		**	*	N.S.	N.S.	N.S.	N.S.	***
Fixed RR	11.66		10.30	6.02	2.12	4.37	4.18	8.61	7.82
RRl	10.09		7.78	5.21	1.34	2.88	2.20	6.21	7.16
RRu	13.47		13.64	6.96	3.34	6.62	7.95	11.95	8.53
P	+++		+++	+++	++	+++	+++	+++	+++
Random RR	10.89		12.97	6.95	2.10	4.37	4.18	8.61	8.05
RRl	7.49		6.14	4.90	1.06	2.88	2.20	6.21	6.21
RRu	15.84		27.36	9.86	4.15	6.62	7.95	11.95	10.43
P	+++		+++	+++	+	+++	+++	+++	+++
Between Chi									88.94
Between df									6
Between P									***
Btwn(F) P									*
Btwn(R) P									***

Table 1H3 - 3

IESLC - Meta-analysis of Ever Smoking, Age started, "Mid"
All LC types, Any Product (or Cigarettes if Any not available)
Most adjusted

Detailed Country in "other Europe"

	multi	Germany	othWest	East	Balkans	Total
N	1		1	1		3
NS	1		1	1		3
Wt	137.76		17.56	27.77		183.09
Het Chi	0.00		0.00	0.00		6.87
Het df	0		0	0		2
Het P	N.S.		N.S.	N.S.		*
Fixed RR	5.43		9.90	7.32		6.02
RRl	4.59		6.20	5.04		5.21
RRu	6.42		15.80	10.61		6.96
P	+++		+++	+++		+++
Random RR	5.43		9.90	7.32		6.95
RRl	4.59		6.20	5.04		4.90
RRu	6.42		15.80	10.61		9.86
P	+++		+++	+++		+++
Between Chi						6.87
Between df						2
Between P						*
Btwn(F) P						N.S.
Btwn(R) P						*

Detailed Country in "other Asia"

	India	HongKong	other	Total
N			1	1
NS			1	1
Wt			9.31	9.31
Het Chi			0.00	0.00
Het df			0	0
Het P			N.S.	N.S.
Fixed RR			4.18	4.18
RRl			2.20	2.20
RRu			7.95	7.95
P			+++	+++
Random RR			4.18	4.18
RRl			2.20	2.20
RRu			7.95	7.95
P			+++	+++
Between Chi				
Between df				
Between P				N.S.
Btwn(F) P				N.S.
Btwn(R) P				N.S.

Detailed other continent

	SCAmer	Total
N	3	3
NS	2	2
Wt	35.82	35.82
Het Chi	0.92	0.92
Het df	2	2
Het P	N.S.	N.S.
Fixed RR	8.61	8.61
RRl	6.21	6.21
RRu	11.95	11.95
P	+++	+++
Random RR	8.61	8.61
RRl	6.21	6.21
RRu	11.95	11.95
P	+++	+++
Between Chi		
Between df		
Between P		N.S.
Btwn(F) P		N.S.
Btwn(R) P		N.S.

Table 1H3 - 3

IESLC - Meta-analysis of Ever Smoking, Age started, "Mid"
All LC types, Any Product (or Cigarettes if Any not available)
Most adjusted

	Start year of study					
	<1960	1960-69	1970-79	1980-89	1990+	Total
N	4	2	6	7	2	21
NS	3	1	5	7	2	18
Wt	82.68	62.76	216.35	123.54	17.22	502.55
Het Chi	21.25	0.05	16.02	30.56	11.98	154.68
Het df	3	1	5	6	1	20
Het P	***	N.S.	**	***	***	***
Fixed RR	9.30	19.08	6.45	6.98	3.31	7.82
RRl	7.50	14.90	5.64	5.85	2.06	7.16
RRu	11.54	24.44	7.37	8.32	5.31	8.53
P	+++	+++	+++	+++	+++	+++
Random RR	9.82	19.08	8.09	6.59	3.38	8.05
RRl	5.16	14.90	5.96	4.39	0.66	6.21
RRu	18.67	24.44	10.98	9.88	17.34	10.43
P	+++	+++	+++	+++	N.S.	+++
Between Chi						74.81
Between df						4
Between P						***
Btwn(F) P						*
Btwn(R) P						***

	Study type (1)		
	CC	other	Total
N	18	3	21
NS	16	2	18
Wt	428.14	74.41	502.55
Het Chi	121.65	32.70	154.68
Het df	17	2	20
Het P	***	***	***
Fixed RR	7.73	8.30	7.82
RRl	7.03	6.62	7.16
RRu	8.50	10.42	8.53
P	+++	+++	+++
Random RR	8.42	5.92	8.05
RRl	6.40	2.17	6.21
RRu	11.07	16.16	10.43
P	+++	+++	+++
Between Chi			0.32
Between df			1
Between P			N.S.
Btwn(F) P			N.S.
Btwn(R) P			N.S.

	Study type (2)			
	CC	prosp	other	Total
N	18	3		21
NS	16	2		18
Wt	428.14	74.41		502.55
Het Chi	121.65	32.70		154.68
Het df	17	2		20
Het P	***	***		***
Fixed RR	7.73	8.30		7.82
RRl	7.03	6.62		7.16
RRu	8.50	10.42		8.53
P	+++	+++		+++
Random RR	8.42	5.92		8.05
RRl	6.40	2.17		6.21
RRu	11.07	16.16		10.43
P	+++	+++		+++
Between Chi				0.32
Between df				1
Between P				N.S.
Btwn(F) P				N.S.
Btwn(R) P				N.S.

Table 1H3 - 3

IESLC - Meta-analysis of Ever Smoking, Age started, "Mid"
All LC types, Any Product (or Cigarettes if Any not available)
Most adjusted

	Study size (number of LC cases) 100-249	250-499	500-999	1000+	Total
N	2	3	9	7	21
NS	2	3	8	5	18
Wt	17.22	36.93	132.32	316.08	502.55
Het Chi	11.98	3.94	28.05	83.93	154.68
Het df	1	2	8	6	20
Het P	***	N.S.	***	***	***
Fixed RR	3.31	4.69	9.32	8.08	7.82
RRl	2.06	3.40	7.86	7.23	7.16
RRu	5.31	6.47	11.05	9.02	8.53
P	+++	+++	+++	+++	+++
Random RR	3.38	4.47	9.88	9.62	8.05
RRl	0.66	2.81	7.11	6.18	6.21
RRu	17.34	7.11	13.74	14.99	10.43
P	N.S.	+++	+++	+++	+++
Between Chi					26.78
Between df					3
Between P					***
Btwn(F) P					N.S.
Btwn(R) P					*

	Risky occupational population no	mining	othRisky	Total
N	20	1		21
NS	17	1		18
Wt	493.70	8.84		502.55
Het Chi	129.55	0.00		154.68
Het df	19	0		20
Het P	***	N.S.		***
Fixed RR	8.05	1.47		7.82
RRl	7.37	0.76		7.16
RRu	8.80	2.84		8.53
P	+++	N.S.		+++
Random RR	8.67	1.47		8.05
RRl	6.78	0.76		6.21
RRu	11.10	2.84		10.43
P	+++	N.S.		+++
Between Chi				25.13
Between df				1
Between P				***
Btwn(F) P				(*)
Btwn(R) P				***

	National cigarette tobacco type Virginia	blended	other	Total
N	1	18	2	21
NS	1	15	2	18
Wt	18.69	465.30	18.56	502.55
Het Chi	0.00	116.80	2.25	154.68
Het df	0	17	1	20
Het P	N.S.	***	N.S.	***
Fixed RR	11.97	8.09	2.12	7.82
RRl	7.60	7.39	1.34	7.16
RRu	18.83	8.86	3.34	8.53
P	+++	+++	++	+++
Random RR	11.97	8.98	2.10	8.05
RRl	7.60	6.95	1.06	6.21
RRu	18.83	11.60	4.15	10.43
P	+++	+++	+	+++
Between Chi				35.63
Between df				2
Between P				***
Btwn(F) P				(*)
Btwn(R) P				***

Table 1H3 - 3

IESLC - Meta-analysis of Ever Smoking, Age started, "Mid"
All LC types, Any Product (or Cigarettes if Any not available)
Most adjusted

	Any proxy use No/nk	Yes	Total
N	15	6	21
NS	12	6	18
Wt	387.01	115.53	502.55
Het Chi	137.78	11.03	154.68
Het df	14	5	20
Het P	***	(*)	***
Fixed RR	7.37	9.52	7.82
RRl	6.67	7.94	7.16
RRu	8.14	11.43	8.53
P	+++	+++	+++
Random RR	7.27	9.97	8.05
RRl	5.17	7.55	6.21
RRu	10.22	13.17	10.43
P	+++	+++	+++
Between Chi			5.86
Between df			1
Between P			*
Btwn(F) P			N.S.
Btwn(R) P			N.S.

	Full histological confirmation No	Yes	Total
N	13	8	21
NS	11	7	18
Wt	212.86	289.68	502.55
Het Chi	64.91	89.12	154.68
Het df	12	7	20
Het P	***	***	***
Fixed RR	8.15	7.58	7.82
RRl	7.13	6.75	7.16
RRu	9.32	8.50	8.53
P	+++	+++	+++
Random RR	7.82	8.38	8.05
RRl	5.64	5.31	6.21
RRu	10.85	13.21	10.43
P	+++	+++	+++
Between Chi			0.65
Between df			1
Between P			N.S.
Btwn(F) P			N.S.
Btwn(R) P			N.S.

	Number of adjustment variables (1) 0	1	2+/+nk	Total
N	16	4	1	21
NS	13	4	1	18
Wt	320.29	173.88	8.37	502.55
Het Chi	86.10	24.77	0.00	154.68
Het df	15	3	0	20
Het P	***	***	N.S.	***
Fixed RR	9.73	5.22	7.80	7.82
RRl	8.72	4.50	3.96	7.16
RRu	10.86	6.05	15.35	8.53
P	+++	+++	+++	+++
Random RR	9.51	4.10	7.80	8.05
RRl	7.26	2.18	3.96	6.21
RRu	12.47	7.73	15.35	10.43
P	+++	+++	+++	+++
Between Chi				43.80
Between df				2
Between P				***
Btwn(F) P				*
Btwn(R) P				(*)

Table 1H3 - 3

IESLC - Meta-analysis of Ever Smoking, Age started, "Mid"
All LC types, Any Product (or Cigarettes if Any not available)
Most adjusted

	Number of adjustment variables (2) 0	1	2	3-5	6+/+nk	Total
N	16	4	1			21
NS	13	4	1			18
Wt	320.29	173.88	8.37			502.55
Het Chi	86.10	24.77	0.00			154.68
Het df	15	3	0			20
Het P	***	***	N.S.			***
Fixed RR	9.73	5.22	7.80			7.82
RRl	8.72	4.50	3.96			7.16
RRu	10.86	6.05	15.35			8.53
P	+++	+++	+++			+++
Random RR	9.51	4.10	7.80			8.05
RRl	7.26	2.18	3.96			6.21
RRu	12.47	7.73	15.35			10.43
P	+++	+++	+++			+++
Between Chi						43.80
Between df						2
Between P						***
Btwn(F) P						*
Btwn(R) P						(*)

	Product all/unsp	cig+/-ot	cig only	Total
N	4	17		21
NS	4	14		18
Wt	57.23	445.32		502.55
Het Chi	31.84	121.31		154.68
Het df	3	16		20
Het P	***	***		***
Fixed RR	6.70	7.97		7.82
RRl	5.17	7.26		7.16
RRu	8.68	8.75		8.53
P	+++	+++		+++
Random RR	7.00	8.35		8.05
RRl	2.81	6.35		6.21
RRu	17.41	10.99		10.43
P	+++	+++		+++
Between Chi				1.53
Between df				1
Between P				N.S.
Btwn(F) P				N.S.
Btwn(R) P				N.S.

	Denominator nev any	nev cigs	Total
N	12	9	21
NS	10	8	18
Wt	208.91	293.63	502.55
Het Chi	60.25	93.94	154.68
Het df	11	8	20
Het P	***	***	***
Fixed RR	7.53	8.02	7.82
RRl	6.58	7.16	7.16
RRu	8.63	9.00	8.53
P	+++	+++	+++
Random RR	7.07	9.48	8.05
RRl	5.08	6.11	6.21
RRu	9.84	14.72	10.43
P	+++	+++	+++
Between Chi			0.49
Between df			1
Between P			N.S.
Btwn(F) P			N.S.
Btwn(R) P			N.S.

Table 1H3 - 3

IESLC - Meta-analysis of Ever Smoking, Age started, "Mid"
All LC types, Any Product (or Cigarettes if Any not available)
Most adjusted

		Derivation of RR/CI			
		Orig	StdCalc	Other	Total
	N	3	17	1	21
	NS	3	14	1	18
	Wt	34.78	460.70	7.07	502.55
Het	Chi	22.41	125.93	0.00	154.68
Het	df	2	16	0	20
Het	P	***	***	N.S.	***
Fixed	RR	5.76	7.92	14.99	7.82
	RRl	4.13	7.23	7.17	7.16
	RRu	8.02	8.68	31.33	8.53
	P	+++	+++	+++	+++
Random	RR	4.90	8.43	14.99	8.05
	RRl	1.55	6.41	7.17	6.21
	RRu	15.53	11.09	31.33	10.43
	P	++	+++	+++	+++
Between	Chi				6.33
Between	df				2
Between	P				*
Btwn(F)	P				N.S.
Btwn(R)	P				N.S.

Table 1H3 - 4

IESLC - Meta-analysis of Ever Smoking, Age started, "Mid"
All LC types, Any Product (or Cigarettes if Any not available)
Least adjusted

REF	NRR	X	SEX	AGEL	AGEH	RACE	YF	LC TYPE	LOC	START	ST	NLC	R	VB	P	H	AD	PRODUCT	exL	exH	DENOM	De
AUVINE	510		c	0	0	all	-	all	Eu:Sca	1986	CC	517	n	bl	y	n	0	cig+/-ot	16	20	nev cigs	st
BARBON	516	x	m	0	0	all	-	all	Eu:wst	1979	CC	755	n	bl	y	y	0	all/unsp	15	19	nev any	st
BRESLO	502		c	0	0	all	-	all	NAmer	1949	CC	518	n	bl	n	y	0	cig+/-ot	15	24	nev any	st
BUFFLE	520		f	0	0	w-hi	-	all	NAmer	1976	CC	943	n	bl	y	n	0	cig+/-ot	17	18	nev cigs	ot
CHOI	525		m	0	0	all	-	all	As:oth	1985	CC	375	n	bl	n	n	0	cig+/-ot	15	19	nev cigs	st
DAMBER	502		m	0	0	all	-	all	Eu:Sca	1972	CC	579	n	bl	y	n	0	all/unsp	16	20	nev any	st
DORN	612		m	55	64	wh	8	all	NAmer	1954	pr	5097	n	bl	n	n	0	cig+/-ot	15	19	nev any	st
DORN	649		m	65	74	wh	8	all	NAmer	1954	pr	5097	n	bl	n	n	0	cig+/-ot	15	19	nev any	st
GENG	529	x	f	0	0	all	-	all	As:Chi	1985	CC	292	n	ot	*	n	0	cig+/-ot	16	20	nev any	st
JEDRYC	607		m	0	0	all	-	all	Eu:est	1980	CC	1630	n	bl	y	n	0	cig+/-ot	17	18	nev any	st
JOLY	544		m	0	0	all	-	all	SCAmer	1978	CC	826	n	bl	n	n	0	cig+/-ot	15	24	nev any	st
JOLY	534		f	0	0	all	-	all	SCAmer	1978	CC	826	n	bl	n	n	0	cig+/-ot	15	24	nev any	st
KHUDER	507		m	0	0	all	-	all	NAmer	1985	CC	482	n	bl	n	y	0	cig+/-ot	16	19	nev cigs	st
KOULUM	503		m	0	0	all	-	all	Eu:Sca	1936	CC	812	n	bl	n	n	0	all/unsp	16	20	nev any	st
LETOUR	502		c	0	0	all	-	all	NAmer	1983	CC	738	n	V	y	y	0	cig+/-ot	15	20	nev cigs	st
LUBIN2	1149	x	m	0	0	all	-	all	Eu:mul	1976	CC	7804	n	bl	n	y	0	cig+/-ot	17	20	nev cigs	st
MATOS	557	x	m	0	0	all	-	all	SCAmer	1994	CC	200	n	bl	n	n	0	cig+/-ot	15	19	nev any	st
QIAO2	502	x	m	0	0	all	0	all	As:Chi	1992	pr	241	m	ot	n	n	0	all/unsp	17	20	nev any	st
SOBUE	655		m	0	0	all	-	all	As:Jap	1986	CC	1376	n	bl	n	y	0	cig+/-ot	18	22	nev cigs	st
WYNDE6	760		m	0	0	wh	-	q+s+a	NAmer	1969	CC	4423	n	bl	n	y	0	cig+/-ot	18	20	nev cigs	st
WYNDE6	768		f	0	0	wh	-	q+s+a	NAmer	1969	CC	4423	n	bl	n	y	0	cig+/-ot	18	20	nev cigs	st

Cigarette type is all/unspec for all RRs

Table 1H3 - 5

IESLC - Meta-analysis of Ever Smoking, Age started, "Mid"
All LC types, Any Product (or Cigarettes if Any not available)
Least adjusted

REF	NRR	SEX	AD	Number Exposed Case	Number Exposed Cont	Non-exposed Case	Non-exposed Cont	RR	95.00%CI
AUVINE	510	c	0	135	47	44	229	14.95	(9.41- 23.75)
BARBON	516	m	0	395	337	22	188	10.02	(6.29- 15.94)
BRESLO	502	c	0	286	243	19	56	3.47	(2.01- 6.00)
BUFFLE	520	f	0	53	33	12	112	14.99	(7.17- 31.33)
CHOI	525	m	0	79	138	13	95	4.18	(2.20- 7.95)
DAMBER	502	m	0	261	190	42	208	6.80	(4.65- 9.95)
*DORN	612	m	0	342	213156	25	213858	13.73	(9.14- 20.60)
*DORN	649	m	0	306	118234	49	171211	9.04	(6.69- 12.23)
Subtotal DORN								10.49	(8.23- 13.36)
GENG	529	f	0	39	23	54	93	2.92	(1.58- 5.40)
JEDRYC	607	m	0	239	146	49	219	7.32	(5.04- 10.61)
JOLY	544	m	0	217	357	12	218	11.04	(6.03- 20.22)
JOLY	534	f	0	67	47	52	283	7.76	(4.82- 12.49)
Subtotal JOLY								8.88	(6.11- 12.91)
KHUDER	507	m	0	161	338	23	309	6.40	(4.03- 10.17)
KOULUM	503	m	0	267	103	5	54	28.00	(10.89- 71.96)
LETOUR	502	c	0	309	241	24	224	11.97	(7.60- 18.83)
LUBIN2	1149	m	0	1796	3028	185	1878	6.02	(5.12- 7.08)
MATOS	557	m	0	91	120	11	110	7.58	(3.85- 14.92)
*QIAO2	502	m	0	75	2840	10	709	1.87	(0.97- 3.60)
SOBUE	655	m	0	776	772	29	126	4.37	(2.88- 6.62)
WYNDE6	760	m	0	223	139	51	589	18.53	(12.98- 26.45)
WYNDE6	768	f	0	200	94	73	673	19.62	(13.90- 27.67)
Subtotal WYNDE6								19.08	(14.90- 24.44)
Totals				6317	340626	804	391442		

*prospective study

REF	NRR	SEX	AD	Ys	Ws	Qs	Ps
AUVINE	510	c	0	2.70	17.93	6.95	0.0000
BARBON	516	m	0	2.30	17.77	0.88	0.0000
BRESLO	502	c	0	1.24	12.80	9.00	0.0000
BUFFLE	520	f	0	2.71	7.07	2.76	0.0000
CHOI	525	m	0	1.43	9.31	3.95	0.0000
DAMBER	502	m	0	1.92	26.52	0.72	0.0000
*DORN	612	m	0	2.62	23.30	6.72	0.0000
*DORN	649	m	0	2.20	42.26	0.61	0.0000
Subtotal DORN				2.35	65.56	7.32	
GENG	529	f	0	1.07	10.16	10.38	0.0006
JEDRYC	607	m	0	1.99	27.77	0.24	0.0000
JOLY	544	m	0	2.40	10.49	1.07	0.0000
JOLY	534	f	0	2.05	16.96	0.02	0.0000
Subtotal JOLY				2.18	27.45	1.09	
KHUDER	507	m	0	1.86	17.89	0.91	0.0000
KOULUM	503	m	0	3.33	4.31	6.73	0.0000
LETOUR	502	c	0	2.48	18.69	2.99	0.0000
LUBIN2	1149	m	0	1.80	146.52	12.07	0.0000
MATOS	557	m	0	2.03	8.38	0.03	0.0000
*QIAO2	502	m	0	0.63	8.96	18.98	0.0604
SOBUE	655	m	0	1.47	22.22	8.22	0.0000
WYNDE6	760	m	0	2.92	30.32	21.24	0.0000
WYNDE6	768	f	0	2.98	32.44	25.93	0.0000
Subtotal WYNDE6				2.95	62.76	47.18	

Table 1H3 - 5

IESLC - Meta-analysis of Ever Smoking, Age started, "Mid"
All LC types, Any Product (or Cigarettes if Any not available)
Least adjusted

	N	21
	NS	18
	Wt	512.09
Het	Chi	140.38
Het	df	20
Het	P	***
Fixed	RR	8.02
	RRl	7.36
	RRu	8.75
	P	+++
Random	RR	8.17
	RRl	6.39
	RRu	10.45
	P	+++
Asymm	P	N.S.

Table 1H3 - 6

IESLC - Meta-analysis of Ever Smoking, Age started, "Mid"
All LC types, Any Product (or Cigarettes if Any not available)
Least adjusted

	Sex			
	combined	male	female	Total
N	3	14	4	21
NS	3	13	4	20
Wt	49.42	396.04	66.64	512.09
Het Chi	17.67	80.01	31.44	140.38
Het df	2	13	3	20
Het P	***	***	***	***
Fixed RR	9.41	7.43	11.26	8.02
RRl	7.12	6.73	8.86	7.36
RRu	12.44	8.20	14.32	8.75
P	+++	+++	+++	+++
Random RR	8.64	7.78	9.13	8.17
RRl	3.75	5.93	3.97	6.39
RRu	19.90	10.21	21.00	10.45
P	+++	+++	+++	+++
Between Chi				11.26
Between df				2
Between P				**
Btwn(F) P				N.S.
Btwn(R) P				N.S.

Table 1H3 - 7

IESLC - Meta-analysis of Ever Smoking, Age started, "Mid"
All LC types, Any Product (or Cigarettes if Any not available)
Excluded studies (and stage at which they were excluded)

1	AKIBA AMANDU AMES BECHER BENSHL BEST BLOT1 BROSS BROWN3 CARPEN CEDERL CHYOU CPSI CPSII DARBY DEAN2 DEAN3 DOLL2 ENGELA GAO2 GARCIA GILLIS GRAHAM GURSEL HAMMO2 HIRAYA HOLE HUMBLE JAHN JAIN KAISE2 KATSOU KAUFMA LAUSSM LIAW MCDUFF MIGRAN MRFITR PEZZO2 PISANI PRESCO QIAO SEGI2 SPEIZE SVENSS TVERDA WAKAI WATSON WIGLE WU WYNDE3 WYNDE8
2	AXELSS BOUCHA BOUCOT CHEN DESTEF DORGAN DOSEME FAN GARSHI GER HAMMON JUSSAW KOO KREUZE LEVIN MCCONN NOTAN2 OSANN2 RESTRE SADOWS VUTUC WANG2 WU2 WUWILL WYNDE2 XU ZHOU
3	GUO SPITZ STASZE ZHANG
4	LUO
5	CORREA YUAN
7	BOFFET WYNDE7
10	ALDERS
14	AGUDO ARMADA BENHAM CHEN2 CHIAZZ DOLL GAO HAENSZ HEGMAN HU HU2 LIU3 LIU4 LIU5 LUBIN PERNU PEZZOT RACHTA SUZUK2 TIZZAN ZHENG

Table 1H3 - 8
Potentially overlapping studies

REF	REFGP	PRINC	.	OVERLAP/LINK
LUBIN2	LUBIN2	1		Lubin-combined
WYNDE6	WYNDE6	1		WYNDE5/6/7/8

Table 1H3 - 9
Most adjusted - insufficient data for meta-analysis

REF	NRR	SEX	AGEL	AGEH	RACE	YF	LC TYPE	LOC	START	ST	NLC	R	VB	P	H	AD	PRODUCT	exL	exH	DENOM	De
CORREA	536	c	0	0	all	-	all	NAmer	1979	CC	1359	n	bl	y	n	2	cig+/-ot	16	20	nev cigs	or

REF	NRR	RR	SIG	RRDATA comment
CORREA	536	17.40		0

Table 1H4 -

IESLC - Meta-analysis of Ever Smoking, Age started, "High"
All LC types, Any Product (or Cigarettes if Any not available)

This analysis is restricted to results for:
1) Ever smokers
2) Results by Age started
3) Categorical results by Age started
4) All LC types (or near equivalent)
5) Results complete enough for use in metaanalysis

Within each study, results are then selected (in the following order of preference, within each sex) for:
6) PRODUCT: all/unspec, cigarettes regardless of other products, cigarettes only
7) CIGTYPE: all/unspecified, MC regardless of HR, MC only
8) (not applicable)
9) DENOM: never smoked anything, never smoked cigarettes, never any + low, never cigs + low
10) Followup period (YF, prospective studies): whole study (coded as 0) or longest available
11) LCtype: all or nearest available, at least Squamous and Adeno. (q = squamous, s = small,
l = large, a = adeno, mix = mixed, alv = alveolar)
12) Race: all or nearest available, otherwise by race (wh or w = white, bl or b = black, hi = hispanic
ch = chinese, jap = japanese, haw = hawaiian, w+o = white + oriental, sca = scandinavian, as = asian)
13) Age started "high" in key scheme 1 (key value 14, maximum range 1-17)
14) For overlapping studies: principal rather than subsidiary studies
Finally by Age: whole study (coded as 0) if available, otherwise by widest available age group
and then for single sex results (m, f) in preference to results for both sexes combined (c).

Results adjusted (AD) for the most potential confounders are then chosen in Sections -1 to -3
and results adjusted for the least confounders in Sections -4 to -6. (Those least adjusted results which
actually differ from the most adjusted are marked 'x' in column X in Section -4)

Section -7 shows excluded studies, together with the stage (as above) at which no qualifying
results were found.

Section -8 lists the potentially overlapping studies which have been included (1=principal, 2=subsidiary).

Section -9 lists any results which would have been included in preference except that they had data not complete
enough for use in meta-analysis, with their significance (yes/no), if known, and any further comment as entered
on the database. It also lists as "gap" any categories for which no data were presented by the original authors.

In addition to those mentioned above, the following fields, levels and abbreviations are used:
* or nk = not known, n = no, y = yes, ot = other
nev = never
all/unspec = all or unspecified, cig+/-ot = cigarettes irrespective of other products (cigar, pipe etc)
MC = manufactured cigarettes, HR = hand-rolled cigarettes
exL, exH = range of exposure (low and high) in the smoking group, in terms of Age started
REF: 6-character study reference
NRR: number of the RR on the database within the study
ST : study type (CC = case control, pr or prosp = prospective)
NLC: number of lung cancer cases in whole study
R : risky occupational population (n = no, m = mining, o = other risky)
VB : national cigarette type (V = at least 75% Virginia, bl = at least 75% blended, ot = other)
P : any proxy use
H : full histological confirmation
De : derivation of RR/CI (or = original, st = standard method, ot = other method of estimation)

Table 1H4 - 1

IESLC - Meta-analysis of Ever Smoking, Age started, "High"
All LC types, Any Product (or Cigarettes if Any not available)
Most adjusted

REF	NRR	SEX	AGEL	AGEH	RACE	YF	LC TYPE	LOC	START	ST	NLC	R	VB	P	H	AD	PRODUCT	exL	exH	DENOM	De
ARMADA	512	m	0	0	all	-	all	Eu:wst	1986	CC	325	n	bl	n	y	0	cig+/-ot	7	16	nev cigs	st
AUVINE	521	c	0	0	all	-	all	Eu:Sca	1986	CC	517	n	bl	y	n	2	cig+/-ot	1	15	nev cigs	or
BARBON	522	m	0	0	all	-	all	Eu:wst	1979	CC	755	n	bl	y	y	1	all/unsp	1	14	nev any	or
BRESLO	503	c	0	0	all	-	all	NAmer	1949	CC	518	n	bl	n	y	0	cig+/-ot	0	14	nev any	st
BUFFLE	521	f	0	0	w-hi	-	all	NAmer	1976	CC	943	n	bl	y	n	0	cig+/-ot	6	16	nev cigs	or
CHOI	526	m	0	0	all	-	all	As:oth	1985	CC	375	n	bl	n	n	0	cig+/-ot	1	14	nev cigs	st
DAMBER	503	m	0	0	all	-	all	Eu:Sca	1972	CC	579	n	bl	y	n	0	all/unsp	1	15	nev any	st
DORN	613	m	55	64	wh	8	all	NAmer	1954	pr	5097	n	bl	n	n	0	cig+/-ot	1	14	nev any	st
DORN	650	m	65	74	wh	8	all	NAmer	1954	pr	5097	n	bl	n	n	0	cig+/-ot	1	14	nev any	st
GENG	535	f	0	0	all	-	all	As:Chi	1985	CC	292	n	ot	*	n	1	cig+/-ot	1	15	nev any	st
HU	513	m	0	0	all	-	all	As:Chi	1985	CC	227	n	ot	n	y	0	cig+/-ot	1	15	nev cigs	st
HU	518	f	0	0	all	-	all	As:Chi	1985	CC	227	n	ot	n	y	0	cig+/-ot	1	15	nev cigs	st
JEDRYC	608	m	0	0	all	-	all	Eu:est	1980	CC	1630	n	bl	y	n	0	cig+/-ot	1	16	nev any	st
JOLY	545	m	0	0	all	-	all	SCAmer	1978	CC	826	n	bl	n	n	0	cig+/-ot	1	14	nev any	st
JOLY	535	f	0	0	all	-	all	SCAmer	1978	CC	826	n	bl	n	n	0	cig+/-ot	1	14	nev any	st
KHUDER	508	m	0	0	all	-	all	NAmer	1985	CC	482	n	bl	n	y	0	cig+/-ot	1	15	nev cigs	st
KOULUM	504	m	0	0	all	-	all	Eu:Sca	1936	CC	812	n	bl	n	n	0	all/unsp	11	15	nev any	st
LETOUR	503	c	0	0	all	-	all	NAmer	1983	CC	738	n	V	y	y	0	cig+/-ot	1	14	nev cigs	st
LUBIN2	1159	m	0	0	all	-	all	Eu:mul	1976	CC	7804	n	bl	n	y	1	cig+/-ot	13	16	nev cigs	st
MATOS	578	m	0	0	all	-	all	SCAmer	1994	CC	200	n	bl	n	n	2	cig+/-ot	1	14	nev any	or
PERNU	505	m	0	0	all	-	all	Eu:Sca	1944	CC	1606	n	bl	n	n	0	all/unsp	1	14	nev any	st
PERNU	502	f	0	0	all	-	all	Eu:Sca	1944	CC	1606	n	bl	n	n	0	all/unsp	1	14	nev any	ot
QIAO2	508	m	0	0	all	0	all	As:Chi	1992	pr	241	m	ot	n	n	1	all/unsp	1	16	nev any	or
SOBUE	656	m	0	0	all	-	all	As:Jap	1986	CC	1376	n	bl	n	y	0	cig+/-ot	10	17	nev cigs	st
WYNDE6	761	m	0	0	wh	-	q+s+a	NAmer	1969	CC	4423	n	bl	n	y	0	cig+/-ot	1	17	nev cigs	st
WYNDE6	769	f	0	0	wh	-	q+s+a	NAmer	1969	CC	4423	n	bl	n	y	0	cig+/-ot	1	17	nev cigs	st

Cigarette type is all/unspec for all RRs

Table 1H4 - 2

IESLC - Meta-analysis of Ever Smoking, Age started, "High"
All LC types, Any Product (or Cigarettes if Any not available)
Most adjusted

REF	NRR	SEX	AD	Number Exposed Case	Exposed Cont	Non-exposed Case	Non-exposed Cont	RR	95.00%CI
ARMADA	512	m	0	204	110	8	71	16.46	(7.64- 35.44)
AUVINE	521	c	2	55	-	44	-	47.60	(21.20- 107.00)
BARBON	522	m	1	138	-	22	-	50.80	(27.20- 95.00)
BRESLO	503	c	0	166	116	19	56	4.22	(2.38- 7.47)
BUFFLE	521	f	0	78	41	12	112	17.76	(8.77- 35.94)
CHOI	526	m	0	22	18	13	95	8.93	(3.81- 20.91)
DAMBER	503	m	0	206	98	42	208	10.41	(6.91- 15.68)
*DORN	613	m	0	84	36304	25	213858	19.79	(12.67- 30.93)
*DORN	650	m	0	81	24616	49	171211	11.50	(8.07- 16.39)
Subtotal DORN								14.18	(10.75- 18.72)
GENG	535	f	1	36	-	54	-	6.24	(2.85- 13.67)
HU	513	m	0	13	7	41	67	3.03	(1.12- 8.23)
HU	518	f	0	5	4	40	48	1.50	(0.38- 5.96)
Subtotal HU								2.38	(1.06- 5.35)
JEDRYC	608	m	0	135	66	49	219	9.14	(5.96- 14.02)
JOLY	545	m	0	317	282	12	218	20.42	(11.18- 37.32)
JOLY	535	f	0	76	35	52	283	11.82	(7.18- 19.44)
Subtotal JOLY								14.75	(10.05- 21.65)
KHUDER	508	m	0	226	295	23	309	10.29	(6.51- 16.27)
KOULUM	504	m	0	199	52	5	54	41.33	(15.74- 108.56)
LETOUR	503	c	0	151	76	24	224	18.54	(11.21- 30.67)
LUBIN2	1159	m	1	1312	-	185	-	6.74	(5.65- 8.04)
MATOS	578	m	2	69	-	11	-	7.80	(3.90- 15.70)
PERNU	505	m	0	337	92	97	275	10.38	(7.49- 14.40)
PERNU	502	f	0	1	0	110	971	26.38~	(1.07- 651.38)
Subtotal PERNU								10.48	(7.58- 14.51)
*QIAO2	508	m	1	104	-	10	-	1.81	(0.94- 3.48)
SOBUE	656	m	0	137	62	29	126	9.60	(5.81- 15.88)
WYNDE6	761	m	0	611	301	51	589	23.44	(17.06- 32.21)
WYNDE6	769	f	0	291	91	73	673	29.48	(21.04- 41.31)
Subtotal WYNDE6								26.11	(20.72- 32.90)
Partial Totals				5054	62666	1100	389667		

*prospective study ~ With 0.5 adjustment for zero

REF	NRR	SEX	AD	Ys	Ws	Qs	Ps
ARMADA	512	m	0	2.80	6.53	0.94	0.0000
AUVINE	521	c	2	3.86	5.86	12.18	0.0000
BARBON	522	m	1	3.93	9.82	22.30	0.0000
BRESLO	503	c	0	1.44	11.75	11.33	0.0000
BUFFLE	521	f	0	2.88	7.72	1.60	0.0000
CHOI	526	m	0	2.19	5.31	0.29	0.0000
DAMBER	503	m	0	2.34	22.90	0.14	0.0000
*DORN	613	m	0	2.99	19.28	6.13	0.0000
*DORN	650	m	0	2.44	30.57	0.01	0.0000
Subtotal DORN				2.65	49.85	6.14	
GENG	535	f	1	1.83	6.25	2.18	0.0000
HU	513	m	0	1.11	3.86	6.64	0.0292
HU	518	f	0	0.41	2.02	8.20	0.5647
Subtotal HU				0.87	5.88	14.83	
JEDRYC	608	m	0	2.21	21.04	0.91	0.0000
JOLY	545	m	0	3.02	10.57	3.74	0.0000
JOLY	535	f	0	2.47	15.51	0.04	0.0000
Subtotal JOLY				2.69	26.07	3.78	
KHUDER	508	m	0	2.33	18.34	0.15	0.0000
KOULUM	504	m	0	3.72	4.12	6.96	0.0000
LETOUR	503	c	0	2.92	15.17	3.77	0.0000
LUBIN2	1159	m	1	1.91	123.47	32.54	0.0000
MATOS	578	m	2	2.05	7.92	1.07	0.0000
PERNU	505	m	0	2.34	35.99	0.24	0.0000
PERNU	502	f	0	3.27	0.37	0.27	0.0455
Subtotal PERNU				2.35	36.37	0.51	
*QIAO2	508	m	1	0.59	8.97	29.97	0.0756
SOBUE	656	m	0	2.26	15.19	0.39	0.0000
WYNDE6	761	m	0	3.15	38.07	20.47	0.0000
WYNDE6	769	f	0	3.38	33.77	31.28	0.0000
Subtotal WYNDE6				3.26	71.85	51.75	

Table 1H4 - 2

IESLC - Meta-analysis of Ever Smoking, Age started, "High"
All LC types, Any Product (or Cigarettes if Any not available)
Most adjusted

	N	26
	NS	21
	Wt	480.38
Het	Chi	203.73
Het	df	25
Het	P	***
Fixed	RR	11.26
	RRl	10.30
	RRu	12.32
	P	+++
Random	RR	11.80
	RRl	8.92
	RRu	15.61
	P	+++
Asymm	P	N.S.

Table 1H4 - 3

IESLC - Meta-analysis of Ever Smoking, Age started, "High"
All LC types, Any Product (or Cigarettes if Any not available)
Most adjusted

	Sex: combined	male	female	Total
N	3	17	6	26
NS	3	16	6	25
Wt	32.78	381.95	65.64	480.38
Het Chi	26.67	129.89	30.46	203.73
Het df	2	16	5	25
Het P	***	***	***	***
Fixed RR	12.91	10.31	17.60	11.26
RRl	9.17	9.32	13.82	10.30
RRu	18.18	11.39	22.42	12.32
P	+++	+++	+++	+++
Random RR	15.15	11.48	11.06	11.80
RRl	4.15	8.39	5.30	8.92
RRu	55.30	15.70	23.12	15.61
P	+++	+++	+++	+++
Between Chi				16.71
Between df				2
Between P				***
Btwn(F) P				N.S.
Btwn(R) P				N.S.

	Lung cancer type: all	other	Total
N	24	2	26
NS	20	1	21
Wt	408.53	71.85	480.38
Het Chi	143.05	0.94	203.73
Het df	23	1	25
Het P	***	N.S.	***
Fixed RR	9.71	26.11	11.26
RRl	8.82	20.72	10.30
RRu	10.70	32.90	12.32
P	+++	+++	+++
Random RR	10.90	26.11	11.80
RRl	8.32	20.72	8.92
RRu	14.27	32.90	15.61
P	+++	+++	+++
Between Chi			59.74
Between df			1
Between P			***
Btwn(F) P			**
Btwn(R) P			***

	Location: NAmer	UK	Scand	othEur	China	Japan	othAs	other	Total
N	8		5	4	4	1	1	3	26
NS	6		4	4	3	1	1	2	21
Wt	174.68		69.25	160.86	21.10	15.19	5.31	34.00	480.38
Het Chi	46.83		18.52	40.84	6.53	0.00	0.00	4.35	203.73
Het df	7		4	3	3	0	0	2	25
Het P	***		***	***	(*)	N.S.	N.S.	N.S.	***
Fixed RR	16.80		12.90	8.23	2.82	9.60	8.93	12.72	11.26
RRl	14.48		10.19	7.05	1.84	5.81	3.81	9.09	10.30
RRu	19.48		16.33	9.60	4.32	15.88	20.91	17.80	12.32
P	+++		+++	+++	+++	+++	+++	+++	+++
Random RR	15.04		19.08	14.46	2.82	9.60	8.93	12.57	11.80
RRl	10.13		10.02	6.45	1.45	5.81	3.81	7.58	8.92
RRu	22.34		36.35	32.43	5.50	15.88	20.91	20.84	15.61
P	+++		+++	+++	++	+++	+++	+++	+++
Between Chi									86.66
Between df									6
Between P									***
Btwn(F) P									(*)
Btwn(R) P									**

Table 1H4 - 3

IESLC - Meta-analysis of Ever Smoking, Age started, "High"
All LC types, Any Product (or Cigarettes if Any not available)
Most adjusted

Detailed Country in "other Europe"

	multi	Germany	othWest	East	Balkans	Total
N	1		2	1		4
NS	1		2	1		4
Wt	123.47		16.36	21.04		160.86
Het Chi	0.00		4.98	0.00		40.84
Het df	0		1	0		3
Het P	N.S.		*	N.S.		***
Fixed RR	6.74		32.39	9.14		8.23
RRl	5.65		19.95	5.96		7.05
RRu	8.04		52.58	14.02		9.60
P	+++		+++	+++		+++
Random RR	6.74		29.58	9.14		14.46
RRl	5.65		9.81	5.96		6.45
RRu	8.04		89.18	14.02		32.43
P	+++		+++	+++		+++
Between Chi						35.86
Between df						2
Between P						***
Btwn(F) P						N.S.
Btwn(R) P						*

Detailed Country in "other Asia"

	India	HongKong	other	Total
N			1	1
NS			1	1
Wt			5.31	5.31
Het Chi			0.00	0.00
Het df			0	0
Het P			N.S.	N.S.
Fixed RR			8.93	8.93
RRl			3.81	3.81
RRu			20.91	20.91
P			+++	+++
Random RR			8.93	8.93
RRl			3.81	3.81
RRu			20.91	20.91
P			+++	+++
Between Chi				
Between df				
Between P				N.S.
Btwn(F) P				N.S.
Btwn(R) P				N.S.

Detailed other continent

	SCAmer	Total
N	3	3
NS	2	2
Wt	34.00	34.00
Het Chi	4.35	4.35
Het df	2	2
Het P	N.S.	N.S.
Fixed RR	12.72	12.72
RRl	9.09	9.09
RRu	17.80	17.80
P	+++	+++
Random RR	12.57	12.57
RRl	7.58	7.58
RRu	20.84	20.84
P	+++	+++
Between Chi		
Between df		
Between P		N.S.
Btwn(F) P		N.S.
Btwn(R) P		N.S.

Table 1H4 - 3

IESLC - Meta-analysis of Ever Smoking, Age started, "High"
All LC types, Any Product (or Cigarettes if Any not available)
Most adjusted

	Start year of study					
	<1960	1960-69	1970-79	1980-89	1990+	Total
N	6	2	6	10	2	26
NS	4	1	5	9	2	21
Wt	102.08	71.85	189.99	99.56	16.89	480.38
Het Chi	24.87	0.94	52.00	35.48	8.98	203.73
Het df	5	1	5	9	1	25
Het P	***	N.S.	***	***	**	***
Fixed RR	11.57	26.11	9.13	10.82	3.59	11.26
RRl	9.53	20.72	7.92	8.89	2.23	10.30
RRu	14.04	32.90	10.53	13.16	5.79	12.32
P	+++	+++	+++	+++	+++	+++
Random RR	12.53	26.11	15.16	10.00	3.74	11.80
RRl	7.57	20.72	8.51	6.60	0.89	8.92
RRu	20.75	32.90	27.01	15.16	15.65	15.61
P	+++	+++	+++	+++	(+)	+++
Between Chi						81.45
Between df						4
Between P						***
Btwn(F) P						*
Btwn(R) P						***

	Study type (1)		
	CC	other	Total
N	23	3	26
NS	19	2	21
Wt	421.55	58.82	480.38
Het Chi	167.55	35.71	203.73
Het df	22	2	25
Het P	***	***	***
Fixed RR	11.39	10.36	11.26
RRl	10.36	8.03	10.30
RRu	12.53	13.38	12.32
P	+++	+++	+++
Random RR	12.52	7.66	11.80
RRl	9.34	2.44	8.92
RRu	16.80	24.04	15.61
P	+++	+++	+++
Between Chi			0.46
Between df			1
Between P			N.S.
Btwn(F) P			N.S.
Btwn(R) P			N.S.

	Study type (2)			
	CC	prosp	other	Total
N	23	3		26
NS	19	2		21
Wt	421.55	58.82		480.38
Het Chi	167.55	35.71		203.73
Het df	22	2		25
Het P	***	***		***
Fixed RR	11.39	10.36		11.26
RRl	10.36	8.03		10.30
RRu	12.53	13.38		12.32
P	+++	+++		+++
Random RR	12.52	7.66		11.80
RRl	9.34	2.44		8.92
RRu	16.80	24.04		15.61
P	+++	+++		+++
Between Chi				0.46
Between df				1
Between P				N.S.
Btwn(F) P				N.S.
Btwn(R) P				N.S.

Table 1H4 - 3

IESLC - Meta-analysis of Ever Smoking, Age started, "High"
All LC types, Any Product (or Cigarettes if Any not available)
Most adjusted

	Study size (number of LC cases) 100-249	250-499	500-999	1000+	Total
N	4	4	9	9	26
NS	3	4	8	6	21
Wt	22.77	36.43	103.42	317.76	480.38
Het Chi	10.37	3.09	51.29	92.23	203.73
Het df	3	3	8	8	25
Het P	*	N.S.	***	***	***
Fixed RR	3.23	10.07	15.55	11.23	11.26
RRl	2.14	7.27	12.83	10.06	10.30
RRu	4.87	13.93	18.86	12.54	12.32
P	+++	+++	+++	+++	+++
Random RR	3.02	10.06	18.35	13.39	11.80
RRl	1.34	7.21	11.11	8.81	8.92
RRu	6.81	14.02	30.31	20.36	15.61
P	++	+++	+++	+++	+++
Between Chi					46.75
Between df					3
Between P					***
Btwn(F) P					N.S.
Btwn(R) P					**

	Risky occupational population no	mining	othRisky	Total
N	25	1		26
NS	20	1		21
Wt	471.41	8.97		480.38
Het Chi	173.18	0.00		203.73
Het df	24	0		25
Het P	***	N.S.		***
Fixed RR	11.66	1.81		11.26
RRl	10.65	0.94		10.30
RRu	12.76	3.48		12.32
P	+++	(+)		+++
Random RR	12.76	1.81		11.80
RRl	9.76	0.94		8.92
RRu	16.67	3.48		15.61
P	+++	(+)		+++
Between Chi				30.54
Between df				1
Between P				***
Btwn(F) P				(*)
Btwn(R) P				***

	National cigarette tobacco type Virginia	blended	other	Total
N	1	21	4	26
NS	1	17	3	21
Wt	15.17	444.11	21.10	480.38
Het Chi	0.00	151.92	6.53	203.73
Het df	0	20	3	25
Het P	N.S.	***	(*)	***
Fixed RR	18.54	11.82	2.82	11.26
RRl	11.21	10.77	1.84	10.30
RRu	30.67	12.98	4.32	12.32
P	+++	+++	+++	+++
Random RR	18.54	14.30	2.82	11.80
RRl	11.21	10.79	1.45	8.92
RRu	30.67	18.94	5.50	15.61
P	+++	+++	++	+++
Between Chi				45.28
Between df				2
Between P				***
Btwn(F) P				(*)
Btwn(R) P				***

Table 1H4 - 3

IESLC - Meta-analysis of Ever Smoking, Age started, "High"
All LC types, Any Product (or Cigarettes if Any not available)
Most adjusted

	Any proxy use No/nk	Yes	Total
N	20	6	26
NS	15	6	21
Wt	397.86	82.52	480.38
Het Chi	160.83	31.31	203.73
Het df	19	5	25
Het P	***	***	***
Fixed RR	10.49	15.84	11.26
RRl	9.51	12.77	10.30
RRu	11.58	19.65	12.32
P	+++	+++	+++
Random RR	10.02	19.63	11.80
RRl	7.27	11.21	8.92
RRu	13.82	34.36	15.61
P	+++	+++	+++
Between Chi			11.59
Between df			1
Between P			***
Btwn(F) P			N.S.
Btwn(R) P			*

	Full histological confirmation No	Yes	Total
N	15	11	26
NS	12	9	21
Wt	202.38	277.99	480.38
Het Chi	65.70	137.96	203.73
Het df	14	10	25
Het P	***	***	***
Fixed RR	11.42	11.15	11.26
RRl	9.95	9.91	10.30
RRu	13.11	12.54	12.32
P	+++	+++	+++
Random RR	11.89	11.31	11.80
RRl	8.62	6.87	8.92
RRu	16.41	18.62	15.61
P	+++	+++	+++
Between Chi			0.07
Between df			1
Between P			N.S.
Btwn(F) P			N.S.
Btwn(R) P			N.S.

	Number of adjustment variables (1) 0	1	2+/+nk	Total
N	20	4	2	26
NS	15	4	2	21
Wt	318.08	148.51	13.79	480.38
Het Chi	90.96	55.23	11.02	203.73
Het df	19	3	1	25
Het P	***	***	***	***
Fixed RR	13.73	7.09	16.83	11.26
RRl	12.30	6.04	9.93	10.30
RRu	15.33	8.33	28.54	12.32
P	+++	+++	+++	+++
Random RR	12.49	7.90	19.03	11.80
RRl	9.63	2.62	3.23	8.92
RRu	16.19	23.84	112.01	15.61
P	+++	+++	++	+++
Between Chi				46.51
Between df				2
Between P				***
Btwn(F) P				(*)
Btwn(R) P				N.S.

Table 1H4 - 3

IESLC - Meta-analysis of Ever Smoking, Age started, "High"
All LC types, Any Product (or Cigarettes if Any not available)
Most adjusted

	Number of adjustment variables (2) 0	1	2	3-5	6+/+nk	Total
N	20	4	2			26
NS	15	4	2			21
Wt	318.08	148.51	13.79			480.38
Het Chi	90.96	55.23	11.02			203.73
Het df	19	3	1			25
Het P	***	***	***			***
Fixed RR	13.73	7.09	16.83			11.26
RRl	12.30	6.04	9.93			10.30
RRu	15.33	8.33	28.54			12.32
P	+++	+++	+++			+++
Random RR	12.49	7.90	19.03			11.80
RRl	9.63	2.62	3.23			8.92
RRu	16.19	23.84	112.01			15.61
P	+++	+++	++			+++
Between Chi						46.51
Between df						2
Between P						***
Btwn(F) P						(*)
Btwn(R) P						N.S.

	Product all/unsp	cig+/-ot	cig only	Total
N	6	20		26
NS	5	16		21
Wt	82.18	398.20		480.38
Het Chi	59.87	143.85		203.73
Het df	5	19		25
Het P	***	***		***
Fixed RR	11.17	11.28		11.26
RRl	9.00	10.22		10.30
RRu	13.87	12.44		12.32
P	+++	+++		+++
Random RR	13.39	11.59		11.80
RRl	5.59	8.61		8.92
RRu	32.10	15.61		15.61
P	+++	+++		+++
Between Chi				0.01
Between df				1
Between P				N.S.
Btwn(F) P				N.S.
Btwn(R) P				N.S.

	Denominator nev any	nev cigs	Total
N	14	12	26
NS	11	10	21
Wt	205.06	275.32	480.38
Het Chi	85.19	118.35	203.73
Het df	13	11	25
Het P	***	***	***
Fixed RR	11.01	11.45	11.26
RRl	9.60	10.18	10.30
RRu	12.63	12.89	12.32
P	+++	+++	+++
Random RR	11.14	12.46	11.80
RRl	7.67	7.94	8.92
RRu	16.19	19.56	15.61
P	+++	+++	+++
Between Chi			0.18
Between df			1
Between P			N.S.
Btwn(F) P			N.S.
Btwn(R) P			N.S.

Table 1H4 - 3

IESLC - Meta-analysis of Ever Smoking, Age started, "High"
All LC types, Any Product (or Cigarettes if Any not available)
Most adjusted

		Derivation of RR/CI			
		Orig	StdCalc	Other	Total
	N	5	20	1	26
	NS	5	16	1	22
	Wt	40.30	439.70	0.37	480.38
Het	Chi	65.74	136.20	0.00	203.73
Het	df	4	19	0	25
Het	P	***	***	N.S.	***
Fixed	RR	13.55	11.06	26.38	11.26
	RRl	9.95	10.08	1.07	10.30
	RRu	18.45	12.15	651.38	12.32
	P	+++	+++	+	+++
Random	RR	14.27	11.36	26.38	11.80
	RRl	4.06	8.64	1.07	8.92
	RRu	50.13	14.94	651.38	15.61
	P	+++	+++	+	+++
Between	Chi				1.79
Between	df				2
Between	P				N.S.
Btwn(F)	P				N.S.
Btwn(R)	P				N.S.

Table 1H4 - 4

IESLC - Meta-analysis of Ever Smoking, Age started, "High"
All LC types, Any Product (or Cigarettes if Any not available)
Least adjusted

REF	NRR	X	SEX	AGEL	AGEH	RACE	YF	LC TYPE	LOC	START	ST	NLC	R	VB	P	H	AD	PRODUCT	exL	exH	DENOM	De
ARMADA	512		m	0	0	all	-	all	Eu:wst	1986	CC	325	n	bl	n	y	0	cig+/-ot	7	16	nev cigs	st
AUVINE	511	x	c	0	0	all	-	all	Eu:Sca	1986	CC	517	n	bl	y	n	0	cig+/-ot	1	15	nev cigs	st
BARBON	517	x	m	0	0	all	-	all	Eu:wst	1979	CC	755	n	bl	y	y	0	all/unsp	1	14	nev any	st
BRESLO	503		c	0	0	all	-	all	NAmer	1949	CC	518	n	bl	n	y	0	cig+/-ot	0	14	nev any	st
BUFFLE	521		f	0	0	w-hi	-	all	NAmer	1976	CC	943	n	bl	y	n	0	cig+/-ot	6	16	nev cigs	or
CHOI	526		m	0	0	all	-	all	As:oth	1985	CC	375	n	bl	n	n	0	cig+/-ot	1	14	nev cigs	st
DAMBER	503		m	0	0	all	-	all	Eu:Sca	1972	CC	579	n	bl	y	n	0	all/unsp	1	15	nev any	st
DORN	613		m	55	64	wh	8	all	NAmer	1954	pr	5097	n	bl	n	n	0	cig+/-ot	1	14	nev any	st
DORN	650		m	65	74	wh	8	all	NAmer	1954	pr	5097	n	bl	n	n	0	cig+/-ot	1	14	nev any	st
GENG	530	x	f	0	0	all	-	all	As:Chi	1985	CC	292	n	ot	*	n	0	cig+/-ot	1	15	nev any	st
HU	513		m	0	0	all	-	all	As:Chi	1985	CC	227	n	ot	n	y	0	cig+/-ot	1	15	nev cigs	st
HU	518		f	0	0	all	-	all	As:Chi	1985	CC	227	n	ot	n	y	0	cig+/-ot	1	15	nev cigs	st
JEDRYC	608		m	0	0	all	-	all	Eu:est	1980	CC	1630	n	bl	y	n	0	cig+/-ot	1	16	nev any	st
JOLY	545		m	0	0	all	-	all	SCAmer	1978	CC	826	n	bl	n	n	0	cig+/-ot	1	14	nev any	st
JOLY	535		f	0	0	all	-	all	SCAmer	1978	CC	826	n	bl	n	n	0	cig+/-ot	1	14	nev any	st
KHUDER	508		m	0	0	all	-	all	NAmer	1985	CC	482	n	bl	n	y	0	cig+/-ot	1	15	nev cigs	st
KOULUM	504		m	0	0	all	-	all	Eu:Sca	1936	CC	812	n	bl	n	n	0	all/unsp	11	15	nev any	st
LETOUR	503		c	0	0	all	-	all	NAmer	1983	CC	738	n	V	y	y	0	cig+/-ot	1	14	nev cigs	st
LUBIN2	1150	x	m	0	0	all	-	all	Eu:mul	1976	CC	7804	n	bl	n	y	0	cig+/-ot	13	16	nev cigs	st
MATOS	558	x	m	0	0	all	-	all	SCAmer	1994	CC	200	n	bl	n	n	0	cig+/-ot	1	14	nev any	st
PERNU	505		m	0	0	all	-	all	Eu:Sca	1944	CC	1606	n	bl	n	n	0	all/unsp	1	14	nev any	st
PERNU	502		f	0	0	all	-	all	Eu:Sca	1944	CC	1606	n	bl	n	n	0	all/unsp	1	14	nev any	ot
QIAO2	503	x	m	0	0	all	0	all	As:Chi	1992	pr	241	m	ot	n	n	0	all/unsp	1	16	nev any	st
SOBUE	656		m	0	0	all	-	all	As:Jap	1986	CC	1376	n	bl	n	y	0	cig+/-ot	10	17	nev cigs	st
WYNDE6	761		m	0	0	wh	-	q+s+a	NAmer	1969	CC	4423	n	bl	n	y	0	cig+/-ot	1	17	nev cigs	st
WYNDE6	769		f	0	0	wh	-	q+s+a	NAmer	1969	CC	4423	n	bl	n	y	0	cig+/-ot	1	17	nev cigs	st

Cigarette type is all/unspec for all RRs

Table 1H4 - 5

IESLC - Meta-analysis of Ever Smoking, Age started, "High"
All LC types, Any Product (or Cigarettes if Any not available)
Least adjusted

REF	NRR	SEX	AD	Number Exposed Case	Cont	Non-exposed Case	Cont	RR	95.00%CI
ARMADA	512	m	0	204	110	8	71	16.46	(7.64- 35.44)
AUVINE	511	c	0	55	6	44	229	47.71	(19.35- 117.62)
BARBON	517	m	0	138	23	22	188	51.27	(27.46- 95.73)
BRESLO	503	c	0	166	116	19	56	4.22	(2.38- 7.47)
BUFFLE	521	f	0	78	41	12	112	17.76	(8.77- 35.94)
CHOI	526	m	0	22	18	13	95	8.93	(3.81- 20.91)
DAMBER	503	m	0	206	98	42	208	10.41	(6.91- 15.68)
*DORN	613	m	0	84	36304	25	213858	19.79	(12.67- 30.93)
*DORN	650	m	0	81	24616	49	171211	11.50	(8.07- 16.39)
Subtotal DORN								14.18	(10.75- 18.72)
GENG	530	f	0	36	10	54	93	6.20	(2.85- 13.48)
HU	513	m	0	13	7	41	67	3.03	(1.12- 8.23)
HU	518	f	0	5	4	40	48	1.50	(0.38- 5.96)
Subtotal HU								2.38	(1.06- 5.35)
JEDRYC	608	m	0	135	66	49	219	9.14	(5.96- 14.02)
JOLY	545	m	0	317	282	12	218	20.42	(11.18- 37.32)
JOLY	535	f	0	76	35	52	283	11.82	(7.18- 19.44)
Subtotal JOLY								14.75	(10.05- 21.65)
KHUDER	508	m	0	226	295	23	309	10.29	(6.51- 16.27)
KOULUM	504	m	0	199	52	5	54	41.33	(15.74- 108.56)
LETOUR	503	c	0	151	76	24	224	18.54	(11.21- 30.67)
LUBIN2	1150	m	0	1312	1833	185	1878	7.27	(6.15- 8.59)
MATOS	558	m	0	69	90	11	110	7.67	(3.83- 15.36)
PERNU	505	m	0	337	92	97	275	10.38	(7.49- 14.40)
PERNU	502	f	0	1	0	110	971	26.38~	(1.07- 651.38)
Subtotal PERNU								10.48	(7.58- 14.51)
*QIAO2	503	m	0	104	2130	10	709	3.46	(1.82- 6.59)
SOBUE	656	m	0	137	62	29	126	9.60	(5.81- 15.88)
WYNDE6	761	m	0	611	301	51	589	23.44	(17.06- 32.21)
WYNDE6	769	f	0	291	91	73	673	29.48	(21.04- 41.31)
Subtotal WYNDE6								26.11	(20.72- 32.90)
Totals				5054	66758	1100	392874		

*prospective study ~ With 0.5 adjustment for zero

REF	NRR	SEX	AD	Ys	Ws	Qs	Ps
ARMADA	512	m	0	2.80	6.53	0.88	0.0000
AUVINE	511	c	0	3.87	4.72	9.65	0.0000
BARBON	517	m	0	3.94	9.85	22.24	0.0000
BRESLO	503	c	0	1.44	11.75	11.64	0.0000
BUFFLE	521	f	0	2.88	7.72	1.51	0.0000
CHOI	526	m	0	2.19	5.31	0.32	0.0000
DAMBER	503	m	0	2.34	22.90	0.19	0.0000
*DORN	613	m	0	2.99	19.28	5.84	0.0000
*DORN	650	m	0	2.44	30.57	0.00	0.0000
Subtotal DORN				2.65	49.85	5.85	
GENG	530	f	0	1.82	6.37	2.37	0.0000
HU	513	m	0	1.11	3.86	6.77	0.0292
HU	518	f	0	0.41	2.02	8.30	0.5647
Subtotal HU				0.87	5.88	15.08	
JEDRYC	608	m	0	2.21	21.04	1.04	0.0000
JOLY	545	m	0	3.02	10.57	3.58	0.0000
JOLY	535	f	0	2.47	15.51	0.02	0.0000
Subtotal JOLY				2.69	26.07	3.60	
KHUDER	508	m	0	2.33	18.34	0.20	0.0000
KOULUM	504	m	0	3.72	4.12	6.82	0.0000
LETOUR	503	c	0	2.92	15.17	3.58	0.0000
LUBIN2	1150	m	0	1.98	138.01	28.14	0.0000
MATOS	558	m	0	2.04	7.96	1.26	0.0000
PERNU	505	m	0	2.34	35.99	0.32	0.0000
PERNU	502	f	0	3.27	0.37	0.26	0.0455
Subtotal PERNU				2.35	36.37	0.58	
*QIAO2	503	m	0	1.24	9.28	13.21	0.0002
SOBUE	656	m	0	2.26	15.19	0.45	0.0000
WYNDE6	761	m	0	3.15	38.07	19.73	0.0000
WYNDE6	769	f	0	3.38	33.77	30.42	0.0000
Subtotal WYNDE6				3.26	71.85	50.15	

Table 1H4 - 5

IESLC - Meta-analysis of Ever Smoking, Age started, "High"
All LC types, Any Product (or Cigarettes if Any not available)
Least adjusted

	N	26
	NS	21
	Wt	494.27
Het	Chi	178.74
Het	df	25
Het	P	***
Fixed	RR	11.41
	RRl	10.45
	RRu	12.46
	P	+++
Random	RR	12.12
	RRl	9.33
	RRu	15.75
	P	+++
Asymm	P	N.S.

Table 1H4 - 6

IESLC - Meta-analysis of Ever Smoking, Age started, "High"
All LC types, Any Product (or Cigarettes if Any not available)
Least adjusted

	Sex			
	combined	male	female	Total
N	3	17	6	26
NS	3	16	6	25
Wt	31.64	396.88	65.76	494.27
Het Chi	24.68	108.60	30.67	178.74
Het df	2	16	5	25
Het P	***	***	***	***
Fixed RR	12.32	10.56	17.56	11.41
RRl	8.70	9.57	13.79	10.45
RRu	17.46	11.65	22.36	12.46
P	+++	+++	+++	+++
Random RR	14.99	11.95	11.04	12.12
RRl	4.14	8.98	5.28	9.33
RRu	54.29	15.90	23.10	15.75
P	+++	+++	+++	+++
Between Chi				14.78
Between df				2
Between P				***
Btwn(F) P				N.S.
Btwn(R) P				N.S.

Table 1H4 - 7

IESLC - Meta-analysis of Ever Smoking, Age started, "High"
All LC types, Any Product (or Cigarettes if Any not available)
Excluded studies (and stage at which they were excluded)

1	AKIBA AMANDU AMES BECHER BENSHL BEST BLOT1 BROSS BROWN3 CARPEN CEDERL CHYOU CPSI CPSII DARBY DEAN2 DEAN3 DOLL2 ENGELA GAO2 GARCIA GILLIS GRAHAM GURSEL HAMMO2 HIRAYA HOLE HUMBLE JAHN JAIN KAISE2 KATSOU KAUFMA LAUSSM LIAW MCDUFF MIGRAN MRFITR PEZZO2 PISANI PRESCO QIAO SEGI2 SPEIZE SVENSS TVERDA WAKAI WATSON WIGLE WU WYNDE3 WYNDE8
2	AXELSS BOUCHA BOUCOT CHEN DESTEF DORGAN DOSEME FAN GARSHI GER HAMMON JUSSAW KOO KREUZE LEVIN MCCONN NOTAN2 OSANN2 RESTRE SADOWS VUTUC WANG2 WU2 WUWILL WYNDE2 XU ZHOU
3	GUO SPITZ STASZE ZHANG
4	LUO
5	CORREA YUAN
7	BOFFET WYNDE7
10	ALDERS
14	AGUDO BENHAM CHEN2 CHIAZZ DOLL GAO HAENSZ HEGMAN HU2 LIU3 LIU4 LIU5 LUBIN PEZZOT RACHTA SUZUK2 TIZZAN ZHENG

Table 1H4 - 8
Potentially overlapping studies

REF	REFGP	PRINC	.	OVERLAP/LINK
LUBIN2	LUBIN2	1		Lubin-combined
WYNDE6	WYNDE6	1		WYNDE5/6/7/8

Table 1H4 - 9
Most adjusted - insufficient data for meta-analysis

REF	NRR	SEX	AGEL	AGEH	RACE	YF	LC TYPE	LOC	START	ST	NLC	R	VB	P	H	AD	PRODUCT	exL	exH	DENOM	De
CORREA	537	c	0	0	all	-	all	NAmer	1979	CC	1359	n	bl	y	n	2	cig+/-ot	1	15	nev cigs	or

REF	NRR	RR	SIG	RRDATA comment
CORREA	537	24.20		0

Table 1H5 -

IESLC - Meta-analysis of Ever Smoking, Age started, "Highest vs lowest"
All LC types, Any Product (or Cigarettes if Any not available)

This analysis is restricted to results for:
1) Ever smokers
2) Results by Age started
3) Categorical results by Age started
4) Denominator (unexposed) = "low"
5) All LC types (or near equivalent)
6) Results complete enough for use in metaanalysis

Within each study, results are then selected (in the following order of preference, within each sex) for:
7) (not applicable)
8) PRODUCT: all/unspec, cigarettes regardless of other products, cigarettes only
9) CIGTYPE: all/unspecified, MC regardless of HR, MC only
10) Results with least adjustment for other aspects of smoking (ADOS)
11) The highest vs lowest category
12) Followup period (YF, prospective studies): whole study (coded as 0) or longest available
13) LCtype: all or nearest available, at least Squamous and Adeno. (q = squamous, s = small,
l = large, a = adeno, mix = mixed, alv = alveolar)
14) Race: all or nearest available, otherwise by race (wh or w = white, bl or b = black, hi = hispanic
ch = chinese, jap = japanese, haw = hawaiian, w+o = white + oriental, sca = scandinavian, as = asian)
15) For overlapping studies: principal rather than subsidiary studies
Finally by Age: whole study (coded as 0) if available, otherwise by widest available age group
and then for single sex results (m, f) in preference to results for both sexes combined (c).

Results adjusted (AD) for the most potential confounders are then chosen in Sections -1 to -3
and results adjusted for the least confounders in Sections -4 to -6. (Those least adjusted results which
actually differ from the most adjusted are marked 'x' in column X in Section -4)

Section -7 shows excluded studies, together with the stage (as above) at which no qualifying
results were found.

Section -8 lists the potentially overlapping studies which have been included (1=principal, 2=subsidiary).

Section -9 lists any results which would have been included in preference except that they had data not complete
enough for use in meta-analysis, with their significance (yes/no), if known, and any further comment as entered
on the database. It also lists as "gap" any categories for which no data were presented by the original authors.

In addition to those mentioned above, the following fields, levels and abbreviations are used:
* or nk = not known, n = no, y = yes, ot = other
all/unspec = all or unspecified, cig+/-ot = cigarettes irrespective of other products (cigar, pipe etc)
MC = manufactured cigarettes, HR = hand-rolled cigarettes
exL, exH = range of exposure (low and high) in the "highest" group, in terms of Age started
unexL, unexH = range of exposure (low and high) in the "lowest" group, in terms of Age started
REF: 6-character study reference
NRR: number of the RR on the database within the study
ST : study type (CC = case control, pr or prosp = prospective)
NLC: number of lung cancer cases in whole study
R : risky occupational population (n = no, m = mining, o = other risky)
VB : national cigarette type (V = at least 75% Virginia, bl = at least 75% blended, ot = other)
P : any proxy use
H : full histological confirmation
De : derivation of RR/CI (or = original, st = standard method, ot = other method of estimation)

Table 1H5 - 1

IESLC - Meta-analysis of Ever Smoking, Age started, "Highest vs lowest"
All LC types, Any Product (or Cigarettes if Any not available)
Most adjusted

REF	NRR	SEX	AGEL	AGEH	RACE	YF	LC TYPE	LOC	START	ST	NLC	R	VB	P	H	AD	ADOS	PRODUCT	exL	exH	unexL	unexH	De
AGUDO	506	f	0	0	all	-	all	Eu:wst	1989	CC	103	n	bl	n	n	3	0	cig only	1	23	24	999	ot
ALDERS	503	m	0	0	all	-	all	Eu:UK	1977	CC	1448	n	V	n	n	2	1#	cig only	1	14	25	999	ot
ALDERS	506	f	0	0	all	-	all	Eu:UK	1977	CC	1448	n	V	n	n	2	1#	cig only	1	14	25	999	ot
ARMADA	513	m	0	0	all	-	all	Eu:wst	1986	CC	325	n	bl	n	y	0	0	cig+/-ot	7	16	17	45	st
AUVINE	513	c	0	0	all	-	all	Eu:Sca	1986	CC	517	n	bl	y	n	0	0	cig+/-ot	1	15	21	999	st
BARBON	524	m	0	0	all	-	all	Eu:wst	1979	CC	755	n	bl	y	y	1	0	all/unsp	1	14	20	999	ot
BRESLO	505	c	0	0	all	-	all	NAmer	1949	CC	518	n	bl	n	y	0	0	cig+/-ot	0	14	25	999	st
BUFFLE	525	f	0	0	w-hi	-	all	NAmer	1976	CC	943	n	bl	y	n	0	0	cig+/-ot	6	16	30	999	st
CHEN2	521	m	0	0	all	-	all	As:Chi	1983	CC	193	n	ot	y	n	0	0	all/unsp	1	19	31	999	st
CHEN2	526	f	0	0	all	-	all	As:Chi	1983	CC	193	n	ot	y	n	0	0	all/unsp	1	19	31	999	st
CHIAZZ	503	m	0	0	all	-	all	NAmer	1940	CC	144	o	bl	y	n	2	0	cig+/-ot	1	19	20	999	ot
CHOI	529	m	0	0	all	-	all	As:oth	1985	CC	375	n	bl	n	n	0	0	cig+/-ot	1	14	25	999	st
CHOI	532	f	0	0	all	-	all	As:oth	1985	CC	375	n	bl	n	n	0	0	cig+/-ot	1	24	25	999	st
DAMBER	505	m	0	0	all	-	all	Eu:Sca	1972	CC	579	n	bl	y	n	0	0	all/unsp	1	15	21	999	st
DOLL	507	m	0	0	all	-	all	Eu:UK	1948	CC	1465	n	V	n	n	0	0	all/unsp	1	19	40	999	st
DOLL	514	f	0	0	all	-	all	Eu:UK	1948	CC	1465	n	V	n	n	0	0	all/unsp	1	19	40	999	st
DORN	616	m	55	64	wh	8	all	NAmer	1954	pr	5097	n	bl	n	n	0	0	cig+/-ot	1	14	25	999	st
DORN	653	m	65	74	wh	8	all	NAmer	1954	pr	5097	n	bl	n	n	0	0	cig+/-ot	1	14	25	999	st
GAO	510	m	0	0	all	-	all	As:Chi	1984	CC	1405	n	ot	n	n	2	0	cig+/-ot	10	19	30	999	ot
GAO	520	f	0	0	all	-	all	As:Chi	1984	CC	1405	n	ot	n	n	2	0	cig+/-ot	10	19	30	999	ot
GENG	537	f	0	0	all	-	all	As:Chi	1985	CC	292	n	ot	*	n	1	0	cig+/-ot	1	15	21	999	st
HAENSZ	550	f	0	0	all	-	not alv	NAmer	1955	CC	158	n	bl	n	y	2	0	cig+/-ot	1	24	25	999	ot
HEGMAN	515	m	0	0	all	-	all	NAmer	1989	CC	282	n	bl	y	y	1	0	all/unsp	1	19	20	999	or
HEGMAN	518	f	0	0	all	-	all	NAmer	1989	CC	282	n	bl	y	y	1	0	all/unsp	1	25	26	999	or
HU	515	m	0	0	all	-	all	As:Chi	1985	CC	227	n	ot	n	y	0	0	cig+/-ot	1	15	30	999	st
HU	520	f	0	0	all	-	all	As:Chi	1985	CC	227	n	ot	n	y	0	0	cig+/-ot	1	15	30	999	st
HU2	507	c	0	0	all	-	all	As:Chi	1977	CC	523	n	ot	y	n	0	0	cig+/-ot	1	19	40	999	st
JEDRYC	610	m	0	0	all	-	all	Eu:est	1980	CC	1630	n	bl	y	n	5	2#	cig+/-ot	1	16	19	999	or
JEDRYC	620	f	0	0	all	-	all	Eu:est	1980	CC	1630	n	bl	y	n	5	2#	cig+/-ot	1	22	23	999	or
JOLY	547	m	0	0	all	-	all	SCAmer	1978	CC	826	n	bl	n	n	0	0	cig+/-ot	1	14	25	999	st
JOLY	537	f	0	0	all	-	all	SCAmer	1978	CC	826	n	bl	n	n	0	0	cig+/-ot	1	14	25	999	st
KHUDER	510	m	0	0	all	-	all	NAmer	1985	CC	482	n	bl	n	y	0	0	cig+/-ot	1	15	20	999	st
KOULUM	509	m	0	0	all	-	all	Eu:Sca	1936	CC	812	n	bl	n	n	0	0	all/unsp	0	10	31	999	st
LETOUR	505	c	0	0	all	-	all	NAmer	1983	CC	738	n	V	y	y	0	0	cig+/-ot	1	14	21	999	st
LIU3	506	m	0	0	all	-	all	As:Chi	1985	CC	110	n	ot	n	n	2	0	all/unsp	1	20	21	999	ot
LIU4	505	m	35	69	all	-	all	As:Chi	1986	CC	1000-00	n	ot	y	n	2	0	all/unsp	0	19	25	999	ot
LIU5	503	c	0	0	all	-	all	As:Chi	1978	CC	111	n	ot	y	n	0	0	all/unsp	1	29	30	999	st
LUBIN	571	m	0	0	all	-	all	As:Chi	1984	CC	427	m	ot	y	n	0	0	cig+/-ot	1	19	27	999	st
LUBIN2	1164	m	0	0	all	-	all	Eu:mul	1976	CC	7804	n	bl	n	y	1	0	cig+/-ot	1	12	31	999	st
MATOS	580	m	0	0	all	-	all	SCAmer	1994	CC	200	n	bl	n	n	2	0	cig+/-ot	1	14	20	999	ot
PERNU	506	m	0	0	all	-	all	Eu:Sca	1944	CC	1606	n	bl	n	n	0	0	all/unsp	1	14	15	999	st
PERNU	503	f	0	0	all	-	all	Eu:Sca	1944	CC	1606	n	bl	n	n	0	0	all/unsp	1	14	15	999	ot
PEZZOT	576	m	0	0	all	-	all	SCAmer	1987	CC	215	n	bl	n	y	2	0	cig only	1	13	19	999	ot
QIAO2	510	m	0	0	all	0	all	As:Chi	1992	pr	241	m	ot	n	n	1	0	all/unsp	1	16	21	999	ot
RACHTA	510	f	0	0	all	-	all	Eu:est	1991	CC	118	n	bl	n	y	1	0	cig+/-ot	1	19	31	999	ot
SOBUE	800	m	0	0	all	-	all	As:Jap	1986	CC	1376	n	bl	n	y	0	0	cig+/-ot	10	17	23	999	st
SUZUK2	523	c	0	0	all	-	all	SCAmer	1991	CC	123	n	bl	n	y	3	0	all/unsp	0	11	19	999	or
TIZZAN	510	m	0	0	all	-	all	Eu:wst	1959	CC	1358	n	bl	n	n	0	0	all/unsp	1	19	31	999	st
TIZZAN	523	f	0	0	all	-	all	Eu:wst	1959	CC	1358	n	bl	n	n	0	0	all/unsp	1	19	31	999	st
WYNDE6	763	m	0	0	wh	-	q+s+a	NAmer	1969	CC	4423	n	bl	n	y	0	0	cig+/-ot	1	17	21	999	st
WYNDE6	771	f	0	0	wh	-	q+s+a	NAmer	1969	CC	4423	n	bl	n	y	0	0	cig+/-ot	1	17	21	999	st
ZHENG	569	m	0	0	all	-	all	As:Chi	1982	CC	540	n	ot	*	y	1	0	cig+/-ot	1	19	30	999	ot
ZHENG	575	f	0	0	all	-	all	As:Chi	1982	CC	540	n	ot	*	y	1	0	cig+/-ot	1	29	30	999	ot

Comments on values in listings

ALDERS ADOS Number of cigs/day
ALDERS ADOS Number of cigs/day
JDERYC ADOS Number of cigs per day & time since quit
JEDRYC ADOS Number of cigs per day & time since quit

Cigarette type is all/unspec for all RRs
except for the following:

REF	NRR	CIGTYPE
ALDERS	503	MC only
ALDERS	506	MC only

Table 1H5 - 2

IESLC - Meta-analysis of Ever Smoking, Age started, "Highest vs lowest"
All LC types, Any Product (or Cigarettes if Any not available)
Most adjusted

REF	NRR	SEX	AD	Number Exposed Case	Number Exposed Cont	Non-exposed Case	Non-exposed Cont	RR	95.00%CI
AGUDO	506	f	3	16	-	7	-	6.81	(1.20- 38.56)
ALDERS	503	m	2	139	-	11	-	2.94	(1.38- 6.25)
ALDERS	506	f	2	78	-	97	-	2.08	(1.27- 3.41)
Subtotal ALDERS								2.31	(1.53- 3.49)
ARMADA	513	m	0	204	110	113	144	2.36	(1.69- 3.31)
AUVINE	513	c	0	55	6	76	27	3.26	(1.26- 8.42)
BARBON	524	m	1	138	-	200	-	6.20	(3.82- 10.05)
BRESLO	505	c	0	166	116	32	35	1.57	(0.92- 2.67)
BUFFLE	525	f	0	78	41	23	23	1.90	(0.95- 3.80)
CHEN2	521	m	0	84	67	8	5	0.78	(0.25- 2.51)
CHEN2	526	f	0	23	13	5	8	2.83	(0.77- 10.47)
Subtotal CHEN2								1.38	(0.58- 3.29)
CHIAZZ	503	m	2	-	-	-	-	6.63	(2.13- 20.63)
CHOI	529	m	0	22	18	36	77	2.61	(1.25- 5.47)
CHOI	532	f	0	4	1	15	25	6.67	(0.68- 65.37)
Subtotal CHOI								2.86	(1.42- 5.76)
DAMBER	505	m	0	206	98	70	76	2.28	(1.52- 3.42)
DOLL	507	m	0	1077	992	4	7	1.90	(0.55- 6.51)
DOLL	514	f	0	20	12	15	15	1.67	(0.61- 4.59)
Subtotal DOLL								1.76	(0.80- 3.84)
*DORN	616	m	0	84	36304	37	73050	4.57	(3.10- 6.72)
*DORN	653	m	0	81	24616	90	74464	2.72	(2.02- 3.67)
Subtotal DORN								3.31	(2.61- 4.19)
GAO	510	m	2	262	-	45	-	4.25	(2.93- 6.16)
GAO	520	f	2	77	-	73	-	2.80	(1.56- 5.04)
Subtotal GAO								3.77	(2.76- 5.16)
GENG	537	f	1	36	-	28	-	3.87	(1.62- 9.26)
HAENSZ	550	f	2	30	-	44	-	1.24	(0.67- 2.28)
HEGMAN	515	m	1	146	-	26	-	2.40	(1.50- 3.70)
HEGMAN	518	f	1	81	-	2	-	5.50	(1.20- 24.10)
Subtotal HEGMAN								2.57	(1.67- 3.96)
HU	515	m	0	13	7	14	20	2.65	(0.84- 8.34)
HU	520	f	0	5	4	3	5	2.08	(0.30- 14.55)
Subtotal HU								2.49	(0.93- 6.69)
HU2	507	c	0	129	68	15	29	3.67	(1.84- 7.31)
JEDRYC	610	m	5	135	-	49	-	1.66	(1.19- 2.32)
JEDRYC	620	f	5	63	-	-	-	1.77	(0.68- 4.60)
Subtotal JEDRYC								1.67	(1.22- 2.29)
JOLY	547	m	0	317	282	18	70	4.37	(2.54- 7.52)
JOLY	537	f	0	76	35	23	41	3.87	(2.02- 7.41)
Subtotal JOLY								4.16	(2.74- 6.30)
KHUDER	510	m	0	226	295	72	152	1.62	(1.16- 2.25)
KOULUM	509	m	0	143	16	8	8	8.94	(2.95- 27.06)
LETOUR	505	c	0	151	76	188	160	1.69	(1.20- 2.39)
LIU3	506	m	2	32	-	20	-	1.26	(0.58- 2.73)
LIU4	505	m	2	-	-	-	-	1.58	(1.53- 1.63)
LIU5	503	c	0	72	48	13	22	2.54	(1.17- 5.52)
LUBIN	571	m	0	178	251	30	179	4.23	(2.75- 6.52)
LUBIN2	1164	m	1	250	-	68	-	2.63	(1.79- 3.87)
MATOS	580	m	2	69	-	28	-	2.00	(1.18- 3.42)
PERNU	506	m	0	337	92	1043	346	1.22	(0.94- 1.58)
PERNU	503	f	0	1	0	18	89	14.51~	(0.57- 370.40)
Subtotal PERNU								1.23	(0.95- 1.60)
PEZZOT	576	m	2	52	-	41	-	2.00	(1.20- 3.33)
*QIAO2	510	m	1	104	-	52	-	1.37	(0.92- 2.03)
RACHTA	510	f	1	52	-	8	-	2.18	(0.54- 8.87)
SOBUE	800	m	0	137	92	110	121	1.64	(1.13- 2.37)
SUZUK2	523	c	3	31	-	16	-	4.80	(1.80- 13.00)
TIZZAN	510	m	0	699	529	12	44	4.84	(2.53- 9.26)
TIZZAN	523	f	0	11	2	2	5	13.75	(1.48- 127.47)
Subtotal TIZZAN								5.26	(2.82- 9.79)
WYNDE6	763	m	0	611	301	111	92	1.68	(1.24- 2.29)
WYNDE6	771	f	0	291	91	127	90	2.27	(1.58- 3.24)
Subtotal WYNDE6								1.91	(1.51- 2.41)
ZHENG	569	m	1	106	-	28	-	5.73	(3.25- 10.10)
ZHENG	575	f	1	60	-	16	-	1.79	(0.79- 4.06)
Subtotal ZHENG								3.93	(2.47- 6.26)
Partial Totals				7458	64583	3190	149429		

*prospective study

~ With 0.5 adjustment for zero

Table 1H5 - 2

IESLC - Meta-analysis of Ever Smoking, Age started, "Highest vs lowest"
All LC types, Any Product (or Cigarettes if Any not available)
Most adjusted

REF	NRR	SEX	AD	Ys	Ws	Qs	Ps
AGUDO	506	f	3	1.92	1.28	2.50	0.0302
ALDERS	503	m	2	1.08	6.73	2.11	0.0051
ALDERS	506	f	2	0.73	15.75	0.72	0.0037
Subtotal ALDERS				0.84	22.49	2.84	
ARMADA	513	m	0	0.86	33.57	3.93	0.0000
AUVINE	513	c	0	1.18	4.25	1.87	0.0149
BARBON	524	m	1	1.82	16.42	28.03	0.0000
BRESLO	505	c	0	0.45	13.43	0.07	0.1006
BUFFLE	525	f	0	0.64	8.05	0.13	0.0680
CHEN2	521	m	0	-0.24	2.84	1.65	0.6810
CHEN2	526	f	0	1.04	2.25	0.61	0.1190
Subtotal CHEN2				0.32	5.09	2.26	
CHIAZZ	503	m	2	1.89	2.98	5.62	0.0011
CHOI	529	m	0	0.96	7.05	1.38	0.0107
CHOI	532	f	0	1.90	0.74	1.40	0.1034
Subtotal CHOI				1.05	7.79	2.78	
DAMBER	505	m	0	0.83	23.53	2.22	0.0001
DOLL	507	m	0	0.64	2.53	0.04	0.3070
DOLL	514	f	0	0.51	3.75	0.00	0.3226
Subtotal DOLL				0.56	6.28	0.04	
*DORN	616	m	0	1.52	25.71	25.76	0.0000
*DORN	653	m	0	1.00	42.73	9.99	0.0000
Subtotal DORN				1.20	68.44	35.75	
GAO	510	m	2	1.45	27.83	24.01	0.0000
GAO	520	f	2	1.03	11.17	2.92	0.0006
Subtotal GAO				1.33	39.00	26.93	
GENG	537	f	1	1.35	5.06	3.53	0.0023
HAENSZ	550	f	2	0.22	10.25	0.94	0.4911
HEGMAN	515	m	1	0.88	18.85	2.41	0.0001
HEGMAN	518	f	1	1.70	1.71	2.40	0.0259
Subtotal HEGMAN				0.94	20.56	4.81	
HU	515	m	0	0.98	2.93	0.61	0.0948
HU	520	f	0	0.73	1.02	0.05	0.4592
Subtotal HU				0.91	3.95	0.66	
HU2	507	c	0	1.30	8.09	4.94	0.0002
JEDRYC	610	m	5	0.51	34.48	0.00	0.0029
JEDRYC	620	f	5	0.57	4.20	0.01	0.2417
Subtotal JEDRYC				0.51	38.68	0.02	
JOLY	547	m	0	1.48	13.06	11.97	0.0000
JOLY	537	f	0	1.35	9.12	6.37	0.0000
Subtotal JOLY				1.43	22.19	18.33	
KHUDER	510	m	0	0.48	35.36	0.05	0.0043
KOULUM	509	m	0	2.19	3.13	8.75	0.0001
LETOUR	505	c	0	0.53	31.90	0.00	0.0030
LIU3	506	m	2	0.23	6.40	0.53	0.5587
LIU4	505	m	2	0.46	3833.36	14.12	0.0000
LIU5	503	c	0	0.93	6.37	1.09	0.0188
LUBIN	571	m	0	1.44	20.61	17.61	0.0000
LUBIN2	1164	m	1	0.97	25.85	5.21	0.0000
MATOS	580	m	2	0.69	13.57	0.42	0.0107
PERNU	506	m	0	0.19	56.54	5.91	0.1428
PERNU	503	f	0	2.68	0.37	1.70	0.1056
Subtotal PERNU				0.21	56.91	7.61	
PEZZOT	576	m	2	0.69	14.75	0.45	0.0078
*QIAO2	510	m	1	0.31	24.53	1.01	0.1189
RACHTA	510	f	1	0.78	1.96	0.13	0.2751
SOBUE	800	m	0	0.49	28.15	0.02	0.0088
SUZUK2	523	c	3	1.57	3.93	4.34	0.0019
TIZZAN	510	m	0	1.58	9.14	10.27	0.0000
TIZZAN	523	f	0	2.62	0.77	3.43	0.0211
Subtotal TIZZAN				1.66	9.92	13.69	
WYNDE6	763	m	0	0.52	40.26	0.00	0.0010
WYNDE6	771	f	0	0.82	29.93	2.69	0.0000
Subtotal WYNDE6				0.65	70.19	2.69	
ZHENG	569	m	1	1.75	11.95	18.01	0.0000
ZHENG	575	f	1	0.58	5.73	0.02	0.1632
Subtotal ZHENG				1.37	17.69	18.03	

Table 1H5 - 2

IESLC - Meta-analysis of Ever Smoking, Age started, "Highest vs lowest"
All LC types, Any Product (or Cigarettes if Any not available)
Most adjusted

	N	53
	NS	39
	Wt	4565.94
Het	Chi	243.95
Het	df	52
Het	P	***
Fixed	RR	1.68
	RRl	1.63
	RRu	1.73
	P	+++
Random	RR	2.49
	RRl	2.17
	RRu	2.85
	P	+++
Asymm	P	***

Table 1H5 - 3

IESLC - Meta-analysis of Ever Smoking, Age started, "Highest vs lowest"
All LC types, Any Product (or Cigarettes if Any not available)
Most adjusted

	Sex: combined	male	female	Total
N	6	29	18	53
NS	6	28	18	52
Wt	67.97	4384.87	113.11	4565.94
Het Chi	8.91	201.45	17.11	243.95
Het df	5	28	17	52
Het P	N.S.	***	N.S.	***
Fixed RR	2.10	1.66	2.34	1.68
RRl	1.66	1.61	1.95	1.63
RRu	2.66	1.71	2.81	1.73
P	+++	+++	+++	+++
Random RR	2.36	2.51	2.34	2.49
RRl	1.65	2.09	1.94	2.17
RRu	3.36	3.00	2.82	2.85
P	+++	+++	+++	+++
Between Chi				16.48
Between df				2
Between P				***
Btwn(F) P				N.S.
Btwn(R) P				N.S.

	Lung cancer type: all	other	Total
N	50	3	53
NS	37	2	39
Wt	4485.50	80.44	4565.94
Het Chi	240.31	3.19	243.95
Het df	49	2	52
Het P	***	N.S.	***
Fixed RR	1.68	1.81	1.68
RRl	1.63	1.45	1.63
RRu	1.73	2.25	1.73
P	+++	+++	+++
Random RR	2.57	1.78	2.49
RRl	2.22	1.33	2.17
RRu	2.99	2.38	2.85
P	+++	+++	+++
Between Chi			0.45
Between df			1
Between P			N.S.
Btwn(F) P			N.S.
Btwn(R) P			*

	Location: NAmer	UK	Scand	othEur	China	Japan	othAs	other	Total
N	12	4	5	9	15	1	2	5	53
NS	9	2	4	7	11	1	1	4	39
Wt	261.16	28.77	87.82	127.67	3970.14	28.15	7.79	54.44	4565.94
Het Chi	34.29	0.96	20.39	27.17	84.26	0.00	0.58	7.86	243.95
Het df	11	3	4	8	14	0	1	4	52
Het P	***	N.S.	***	***	***	N.S.	N.S.	(*)	***
Fixed RR	2.15	2.17	1.64	2.64	1.61	1.64	2.86	2.87	1.68
RRl	1.90	1.51	1.33	2.22	1.56	1.13	1.42	2.20	1.63
RRu	2.42	3.13	2.02	3.14	1.66	2.37	5.76	3.74	1.73
P	+++	+++	+++	+++	+++	++	++	+++	+++
Random RR	2.19	2.17	2.73	3.04	2.52	1.64	2.86	2.98	2.49
RRl	1.73	1.51	1.40	2.07	1.82	1.13	1.42	2.03	2.17
RRu	2.76	3.13	5.36	4.45	3.48	2.37	5.76	4.38	2.85
P	+++	+++	++	+++	+++	++	++	+++	+++
Between Chi									68.43
Between df									7
Between P									***
Btwn(F) P									*
Btwn(R) P									N.S.

Table 1H5 - 3

IESLC - Meta-analysis of Ever Smoking, Age started, "Highest vs lowest"
All LC types, Any Product (or Cigarettes if Any not available)
Most adjusted

Detailed Country in "other Europe"

		multi	Germany	othWest	East	Balkans	Total
	N	1		5	3		9
	NS	1		4	2		7
	Wt	25.85		61.19	40.64		127.67
Het	Chi	0.00		13.51	0.15		27.17
Het	df	0		4	2		8
Het	P	N.S.		**	N.S.		***
Fixed	RR	2.63		3.56	1.69		2.64
	RRl	1.79		2.77	1.25		2.22
	RRu	3.87		4.58	2.30		3.14
	P	+++		+++	+++		+++
Random	RR	2.63		4.53	1.69		3.04
	RRl	1.79		2.51	1.25		2.07
	RRu	3.87		8.20	2.30		4.45
	P	+++		+++	+++		+++
Between	Chi						13.52
Between	df						2
Between	P						**
Btwn(F)	P						N.S.
Btwn(R)	P						**

Detailed Country in "other Asia"

		India	HongKong	other	Total
	N			2	2
	NS			1	1
	Wt			7.79	7.79
Het	Chi			0.58	0.58
Het	df			1	1
Het	P			N.S.	N.S.
Fixed	RR			2.86	2.86
	RRl			1.42	1.42
	RRu			5.76	5.76
	P			++	++
Random	RR			2.86	2.86
	RRl			1.42	1.42
	RRu			5.76	5.76
	P			++	++
Between	Chi				
Between	df				
Between	P				N.S.
Btwn(F)	P				N.S.
Btwn(R)	P				N.S.

Detailed other continent

		SCAmer	Total
	N	5	5
	NS	4	4
	Wt	54.44	54.44
Het	Chi	7.86	7.86
Het	df	4	4
Het	P	(*)	(*)
Fixed	RR	2.87	2.87
	RRl	2.20	2.20
	RRu	3.74	3.74
	P	+++	+++
Random	RR	2.98	2.98
	RRl	2.03	2.03
	RRu	4.38	4.38
	P	+++	+++
Between	Chi		
Between	df		
Between	P		N.S.
Btwn(F)	P		N.S.
Btwn(R)	P		N.S.

Table 1H5 - 3

IESLC - Meta-analysis of Ever Smoking, Age started, "Highest vs lowest"
All LC types, Any Product (or Cigarettes if Any not available)
Most adjusted

	Start year of study					
	<1960	1960-69	1970-79	1980-89	1990+	Total
N	12	2	10	25	4	53
NS	8	1	8	18	4	39
Wt	171.34	70.19	132.98	4147.44	43.99	4565.94
Het Chi	60.24	1.52	17.60	95.74	5.81	243.95
Het df	11	1	9	24	3	52
Het P	***	N.S.	*	***	N.S.	***
Fixed RR	2.19	1.91	3.01	1.63	1.76	1.68
RRl	1.89	1.51	2.54	1.58	1.31	1.63
RRu	2.55	2.41	3.56	1.68	2.36	1.73
P	+++	+++	+++	+++	+++	+++
Random RR	2.81	1.92	3.05	2.33	2.00	2.49
RRl	1.84	1.44	2.38	1.93	1.24	2.17
RRu	4.32	2.57	3.90	2.83	3.25	2.85
P	+++	+++	+++	+++	++	+++
Between Chi						63.04
Between df						4
Between P						***
Btwn(F) P						**
Btwn(R) P						N.S.

	Study type (1)		
	CC	other	Total
N	50	3	53
NS	37	2	39
Wt	4472.97	92.98	4565.94
Het Chi	206.80	18.32	243.95
Het df	49	2	52
Het P	***	***	***
Fixed RR	1.66	2.62	1.68
RRl	1.62	2.14	1.63
RRu	1.71	3.21	1.73
P	+++	+++	+++
Random RR	2.47	2.58	2.49
RRl	2.15	1.38	2.17
RRu	2.84	4.83	2.85
P	+++	++	+++
Between Chi			18.83
Between df			1
Between P			***
Btwn(F) P			*
Btwn(R) P			N.S.

	Study type (2)			
	CC	prosp	other	Total
N	50	3		53
NS	37	2		39
Wt	4472.97	92.98		4565.94
Het Chi	206.80	18.32		243.95
Het df	49	2		52
Het P	***	***		***
Fixed RR	1.66	2.62		1.68
RRl	1.62	2.14		1.63
RRu	1.71	3.21		1.73
P	+++	+++		+++
Random RR	2.47	2.58		2.49
RRl	2.15	1.38		2.17
RRu	2.84	4.83		2.85
P	+++	++		+++
Between Chi				18.83
Between df				1
Between P				***
Btwn(F) P				*
Btwn(R) P				N.S.

Table 1H5 - 3

IESLC - Meta-analysis of Ever Smoking, Age started, "Highest vs lowest"
All LC types, Any Product (or Cigarettes if Any not available)
Most adjusted

	Study size (number of LC cases) 100-249	250-499	500-999	1000+	Total
N	14	8	12	19	53
NS	12	6	10	11	39
Wt	95.05	122.94	148.68	4199.27	4565.94
Het Chi	19.15	15.25	40.91	104.67	243.95
Het df	13	7	11	18	52
Het P	N.S.	*	***	***	***
Fixed RR	1.84	2.45	2.85	1.63	1.68
RRl	1.51	2.05	2.42	1.58	1.63
RRu	2.25	2.92	3.34	1.68	1.73
P	+++	+++	+++	+++	+++
Random RR	1.97	2.68	3.12	2.31	2.49
RRl	1.51	1.98	2.25	1.89	2.17
RRu	2.59	3.62	4.32	2.84	2.85
P	+++	+++	+++	+++	+++
Between Chi					63.98
Between df					3
Between P					***
Btwn(F) P					**
Btwn(R) P					N.S.

	Risky occupational population no	mining	othRisky	Total
N	50	2	1	53
NS	36	2	1	39
Wt	4517.82	45.14	2.98	4565.94
Het Chi	219.63	14.24	0.00	243.95
Het df	49	1	0	52
Het P	***	***	N.S.	***
Fixed RR	1.67	2.29	6.63	1.68
RRl	1.62	1.71	2.13	1.63
RRu	1.72	3.07	20.63	1.73
P	+++	+++	++	+++
Random RR	2.46	2.40	6.63	2.49
RRl	2.14	0.79	2.13	2.17
RRu	2.83	7.25	20.63	2.85
P	+++	N.S.	++	+++
Between Chi				10.08
Between df				2
Between P				**
Btwn(F) P				N.S.
Btwn(R) P				N.S.

	National cigarette tobacco type Virginia	blended	other	Total
N	5	33	15	53
NS	3	25	11	39
Wt	60.67	535.13	3970.14	4565.94
Het Chi	1.91	106.96	84.26	243.95
Het df	4	32	14	52
Het P	N.S.	***	***	***
Fixed RR	1.90	2.23	1.61	1.68
RRl	1.48	2.05	1.56	1.63
RRu	2.45	2.43	1.66	1.73
P	+++	+++	+++	+++
Random RR	1.90	2.55	2.52	2.49
RRl	1.48	2.14	1.82	2.17
RRu	2.45	3.04	3.48	2.85
P	+++	+++	+++	+++
Between Chi				50.82
Between df				2
Between P				***
Btwn(F) P				**
Btwn(R) P				N.S.

Table 1H5 - 3

IESLC - Meta-analysis of Ever Smoking, Age started, "Highest vs lowest"
All LC types, Any Product (or Cigarettes if Any not available)
Most adjusted

	Any proxy use No/nk	Yes	Total
N	37	16	53
NS	26	13	39
Wt	546.06	4019.89	4565.94
Het Chi	114.24	76.33	243.95
Het df	36	15	52
Het P	***	***	***
Fixed RR	2.25	1.61	1.68
RRl	2.07	1.56	1.63
RRu	2.45	1.66	1.73
P	+++	+++	+++
Random RR	2.49	2.51	2.49
RRl	2.10	1.93	2.17
RRu	2.94	3.26	2.85
P	+++	+++	+++
Between Chi			53.38
Between df			1
Between P			***
Btwn(F) P			***
Btwn(R) P			N.S.

	Full histological confirmation No	Yes	Total
N	34	19	53
NS	24	15	39
Wt	4238.00	327.95	4565.94
Het Chi	173.07	49.67	243.95
Het df	33	18	52
Het P	***	***	***
Fixed RR	1.65	2.14	1.68
RRl	1.60	1.92	1.63
RRu	1.70	2.39	1.73
P	+++	+++	+++
Random RR	2.64	2.28	2.49
RRl	2.18	1.87	2.17
RRu	3.20	2.77	2.85
P	+++	+++	+++
Between Chi			21.21
Between df			1
Between P			***
Btwn(F) P			*
Btwn(R) P			N.S.

	Number of adjustment variables (1) 0	1	2+/+nk	Total
N	30	9	14	53
NS	21	7	11	39
Wt	467.20	112.06	3986.68	4565.94
Het Chi	89.72	32.16	50.20	243.95
Het df	29	8	13	52
Het P	***	***	***	***
Fixed RR	2.21	2.78	1.60	1.68
RRl	2.01	2.31	1.55	1.63
RRu	2.41	3.35	1.65	1.73
P	+++	+++	+++	+++
Random RR	2.47	3.01	2.24	2.49
RRl	2.06	2.00	1.74	2.17
RRu	2.97	4.53	2.87	2.85
P	+++	+++	+++	+++
Between Chi				71.86
Between df				2
Between P				***
Btwn(F) P				***
Btwn(R) P				N.S.

Table 1H5 - 3

IESLC - Meta-analysis of Ever Smoking, Age started, "Highest vs lowest"
All LC types, Any Product (or Cigarettes if Any not available)
Most adjusted

	Number of adjustment variables (2)					
	0	1	2	3-5	6+/+nk	Total
N	30	9	10	4		53
NS	21	7	8	3		39
Wt	467.20	112.06	3942.80	43.89		4565.94
Het Chi	89.72	32.16	42.71	6.10		243.95
Het df	29	8	9	3		52
Het P	***	***	***	N.S.		***
Fixed RR	2.21	2.78	1.60	1.91		1.68
RRl	2.01	2.31	1.55	1.42		1.63
RRu	2.41	3.35	1.65	2.57		1.73
P	+++	+++	+++	+++		+++
Random RR	2.47	3.01	2.22	2.46		2.49
RRl	2.06	2.00	1.64	1.33		2.17
RRu	2.97	4.53	3.01	4.57		2.85
P	+++	+++	+++	++		+++
Between Chi						73.25
Between df						3
Between P						***
Btwn(F) P						***
Btwn(R) P						N.S.

	Product			
	all/unsp	cig+/-ot	cig only	Total
N	18	31	4	53
NS	13	23	3	39
Wt	4016.43	511.00	38.51	4565.94
Het Chi	78.85	82.41	2.35	243.95
Het df	17	30	3	52
Het P	***	***	N.S.	***
Fixed RR	1.60	2.41	2.26	1.68
RRl	1.55	2.21	1.65	1.63
RRu	1.65	2.63	3.10	1.73
P	+++	+++	+++	+++
Random RR	2.40	2.54	2.26	2.49
RRl	1.83	2.16	1.65	2.17
RRu	3.14	2.97	3.10	2.85
P	+++	+++	+++	+++
Between Chi				80.35
Between df				2
Between P				***
Btwn(F) P				***
Btwn(R) P				N.S.

	Derivation of RR/CI			
	Orig	StdCalc	Other	Total
N	5	31	17	53
NS	3	23	14	40
Wt	63.17	497.73	4005.04	4565.94
Het Chi	6.60	90.73	97.24	243.95
Het df	4	30	16	52
Het P	N.S.	***	***	***
Fixed RR	2.05	2.24	1.62	1.68
RRl	1.60	2.05	1.57	1.63
RRu	2.63	2.44	1.67	1.73
P	+++	+++	+++	+++
Random RR	2.28	2.49	2.54	2.49
RRl	1.56	2.09	1.89	2.17
RRu	3.34	2.96	3.40	2.85
P	+++	+++	+++	+++
Between Chi				49.37
Between df				2
Between P				***
Btwn(F) P				**
Btwn(R) P				N.S.

Table 1H5 - 3

IESLC - Meta-analysis of Ever Smoking, Age started, "Highest vs lowest"
All LC types, Any Product (or Cigarettes if Any not available)
Most adjusted

	Study LIU4		
	LIU4	others	Total
N	1	52	53
NS	1	38	39
Wt	3833.36	732.58	4565.94
Het Chi	0.00	155.96	243.95
Het df	0	51	52
Het P	N.S.	***	***
Fixed RR	1.58	2.31	1.68
RRl	1.53	2.15	1.63
RRu	1.63	2.48	1.73
P	+++	+++	+++
Random RR	1.58	2.53	2.49
RRl	1.53	2.20	2.17
RRu	1.63	2.92	2.85
P	+++	+++	+++
Between Chi			87.99
Between df			1
Between P			***
Btwn(F) P			***
Btwn(R) P			***

Table 1H5 - 4

IESLC - Meta-analysis of Ever Smoking, Age started, "Highest vs lowest"
All LC types, Any Product (or Cigarettes if Any not available)
Least adjusted

REF	NRR	X	SEX	AGEL	AGEH	RACE	YF	LC TYPE	LOC	START	ST	NLC	R	VB	P	H	AD	ADOS	PRODUCT	exL	exH	unexL	unexH	De
AGUDO	503	x	f	0	0	all	-	all	Eu:wst	1989	CC	103	n	bl	n	n	0	0	cig only	1	23	24	999	st
ALDERS	503		m	0	0	all	-	all	Eu:UK	1977	CC	1448	n	V	n	n	2	1#	cig only	1	14	25	999	ot
ALDERS	506		f	0	0	all	-	all	Eu:UK	1977	CC	1448	n	V	n	n	2	1#	cig only	1	14	25	999	ot
ARMADA	513		m	0	0	all	-	all	Eu:wst	1986	CC	325	n	bl	n	y	0	0	cig+/-ot	7	16	17	45	st
AUVINE	513		c	0	0	all	-	all	Eu:Sca	1986	CC	517	n	bl	y	n	0	0	cig+/-ot	1	15	21	999	st
BARBON	519	x	m	0	0	all	-	all	Eu:wst	1979	CC	755	n	bl	y	y	0	0	all/unsp	1	14	20	999	st
BRESLO	505		c	0	0	all	-	all	NAmer	1949	CC	518	n	bl	n	y	0	0	cig+/-ot	0	14	25	999	st
BUFFLE	525		f	0	0	w-hi	-	all	NAmer	1976	CC	943	n	bl	y	n	0	0	cig+/-ot	6	16	30	999	st
CHEN2	521		m	0	0	all	-	all	As:Chi	1983	CC	193	n	ot	y	n	0	0	all/unsp	1	19	31	999	st
CHEN2	526		f	0	0	all	-	all	As:Chi	1983	CC	193	n	ot	y	n	0	0	all/unsp	1	19	31	999	st
CHIAZZ	503		m	0	0	all	-	all	NAmer	1940	CC	144	o	bl	y	n	2	0	cig+/-ot	1	19	20	999	ot
CHOI	529		m	0	0	all	-	all	As:oth	1985	CC	375	n	bl	n	n	0	0	cig+/-ot	1	14	25	999	st
CHOI	532		f	0	0	all	-	all	As:oth	1985	CC	375	n	bl	n	n	0	0	cig+/-ot	1	24	25	999	st
DAMBER	505		m	0	0	all	-	all	Eu:Sca	1972	CC	579	n	bl	y	n	0	0	all/unsp	1	15	21	999	st
DOLL	507		m	0	0	all	-	all	Eu:UK	1948	CC	1465	n	V	n	n	0	0	all/unsp	1	19	40	999	st
DOLL	514		f	0	0	all	-	all	Eu:UK	1948	CC	1465	n	V	n	n	0	0	all/unsp	1	19	40	999	st
DORN	616		m	55	64	wh	8	all	NAmer	1954	pr	5097	n	bl	n	n	0	0	cig+/-ot	1	14	25	999	st
DORN	653		m	65	74	wh	8	all	NAmer	1954	pr	5097	n	bl	n	n	0	0	cig+/-ot	1	14	25	999	st
GAO	505	x	m	0	0	all	-	all	As:Chi	1984	CC	1405	n	ot	n	n	0	0	cig+/-ot	10	19	30	999	st
GAO	515	x	f	0	0	all	-	all	As:Chi	1984	CC	1405	n	ot	n	n	0	0	cig+/-ot	10	19	30	999	st
GENG	532	x	f	0	0	all	-	all	As:Chi	1985	CC	292	n	ot	*	n	0	0	cig+/-ot	1	15	21	999	st
HAENSZ	541	x	f	0	0	all	-	not alv	NAmer	1955	CC	158	n	bl	n	y	0	0	cig+/-ot	1	24	25	999	st
HEGMAN	501	x	m	0	0	all	-	all	NAmer	1989	CC	282	n	bl	y	y	0	0	all/unsp	1	19	20	999	st
HEGMAN	502	x	f	0	0	all	-	all	NAmer	1989	CC	282	n	bl	y	y	0	0	all/unsp	1	25	26	999	st
HU	515		m	0	0	all	-	all	As:Chi	1985	CC	227	n	ot	n	y	0	0	cig+/-ot	1	15	30	999	st
HU	520		f	0	0	all	-	all	As:Chi	1985	CC	227	n	ot	n	y	0	0	cig+/-ot	1	15	30	999	st
HU2	507		c	0	0	all	-	all	As:Chi	1977	CC	523	n	ot	y	n	0	0	cig+/-ot	1	19	40	999	st
JEDRYC	610		m	0	0	all	-	all	Eu:est	1980	CC	1630	n	bl	y	n	5	2#	cig+/-ot	1	16	19	999	or
JEDRYC	620		f	0	0	all	-	all	Eu:est	1980	CC	1630	n	bl	y	n	5	2#	cig+/-ot	1	22	23	999	or
JOLY	547		m	0	0	all	-	all	SCAmer	1978	CC	826	n	bl	n	n	0	0	cig+/-ot	1	14	25	999	st
JOLY	537		f	0	0	all	-	all	SCAmer	1978	CC	826	n	bl	n	n	0	0	cig+/-ot	1	14	25	999	st
KHUDER	510		m	0	0	all	-	all	NAmer	1985	CC	482	n	bl	n	y	0	0	cig+/-ot	1	15	20	999	st
KOULUM	509		m	0	0	all	-	all	Eu:Sca	1936	CC	812	n	bl	n	n	0	0	all/unsp	0	10	31	999	st
LETOUR	505		c	0	0	all	-	all	NAmer	1983	CC	738	n	V	y	y	0	0	cig+/-ot	1	14	21	999	st
LIU3	503	x	m	0	0	all	-	all	As:Chi	1985	CC	110	n	ot	n	n	0	0	all/unsp	1	20	21	999	st
LIU4	505		m	35	69	all	-	all	As:Chi	1986	CC	1000-00	n	ot	y	n	2	0	all/unsp	0	19	25	999	ot
LIU5	503		c	0	0	all	-	all	As:Chi	1978	CC	111	n	ot	y	n	0	0	all/unsp	1	29	30	999	st
LUBIN	571		m	0	0	all	-	all	As:Chi	1984	CC	427	m	ot	y	n	0	0	cig+/-ot	1	19	27	999	st
LUBIN2	1155	x	m	0	0	all	-	all	Eu:mul	1976	CC	7804	n	bl	n	y	0	0	cig+/-ot	1	12	31	999	st
MATOS	560	x	m	0	0	all	-	all	SCAmer	1994	CC	200	n	bl	n	n	0	0	cig+/-ot	1	14	20	999	st
PERNU	506		m	0	0	all	-	all	Eu:Sca	1944	CC	1606	n	bl	n	n	0	0	all/unsp	1	14	15	999	st
PERNU	503		f	0	0	all	-	all	Eu:Sca	1944	CC	1606	n	bl	n	n	0	0	all/unsp	1	14	15	999	ot
PEZZOT	574	x	m	0	0	all	-	all	SCAmer	1987	CC	215	n	bl	n	y	0	0	cig only	1	13	19	999	st
QIAO2	505	x	m	0	0	all	0	all	As:Chi	1992	pr	241	m	ot	n	n	0	0	all/unsp	1	16	21	999	st
RACHTA	505	x	f	0	0	all	-	all	Eu:est	1991	CC	118	n	bl	n	y	0	0	cig+/-ot	1	19	31	999	st
SOBUE	800		m	0	0	all	-	all	As:Jap	1986	CC	1376	n	bl	n	y	0	0	cig+/-ot	10	17	23	999	st
SUZUK2	505	x	c	0	0	all	-	all	SCAmer	1991	CC	123	n	bl	n	y	0	0	all/unsp	0	11	19	999	st
TIZZAN	510		m	0	0	all	-	all	Eu:wst	1959	CC	1358	n	bl	n	n	0	0	all/unsp	1	19	31	999	st
TIZZAN	523		f	0	0	all	-	all	Eu:wst	1959	CC	1358	n	bl	n	n	0	0	all/unsp	1	19	31	999	st
WYNDE6	763		m	0	0	wh	-	q+s+a	NAmer	1969	CC	4423	n	bl	n	y	0	0	cig+/-ot	1	17	21	999	st
WYNDE6	771		f	0	0	wh	-	q+s+a	NAmer	1969	CC	4423	n	bl	n	y	0	0	cig+/-ot	1	17	21	999	st
ZHENG	567	x	m	0	0	all	-	all	As:Chi	1982	CC	540	n	ot	*	y	0	0	cig+/-ot	1	19	30	999	st
ZHENG	574	x	f	0	0	all	-	all	As:Chi	1982	CC	540	n	ot	*	y	0	0	cig+/-ot	1	29	30	999	st

Comments on values in listings

ALDERS ADOS Number of cigs/day
ALDERS ADOS Number of cigs/day
JEDRYC ADOS Number of cigs per day & time since quit
JDERYC ADOS Number of cigs per day & time since quit

Cigarette type is all/unspec for all RRs
except for the following:

REF	NRR	CIGTYPE
ALDERS	503	MC only
ALDERS	506	MC only

Table 1H5 - 5

IESLC - Meta-analysis of Ever Smoking, Age started, "Highest vs lowest"
All LC types, Any Product (or Cigarettes if Any not available)
Least adjusted

REF	NRR	SEX	AD	Number Exposed Case	Number Exposed Cont	Non-exposed Case	Non-exposed Cont	RR	95.00%CI
AGUDO	503	f	0	16	11	7	12	2.49	(0.75- 8.34)
ALDERS	503	m	2	139	-	11	-	2.94	(1.38- 6.25)
ALDERS	506	f	2	78	-	97	-	2.08	(1.27- 3.41)
Subtotal ALDERS								2.31	(1.53- 3.49)
ARMADA	513	m	0	204	110	113	144	2.36	(1.69- 3.31)
AUVINE	513	c	0	55	6	76	27	3.26	(1.26- 8.42)
BARBON	519	m	0	138	23	200	207	6.21	(3.83- 10.06)
BRESLO	505	c	0	166	116	32	35	1.57	(0.92- 2.67)
BUFFLE	525	f	0	78	41	23	23	1.90	(0.95- 3.80)
CHEN2	521	m	0	84	67	8	5	0.78	(0.25- 2.51)
CHEN2	526	f	0	23	13	5	8	2.83	(0.77- 10.47)
Subtotal CHEN2								1.38	(0.58- 3.29)
CHIAZZ	503	m	2	-	-	-	-	6.63	(2.13- 20.63)
CHOI	529	m	0	22	18	36	77	2.61	(1.25- 5.47)
CHOI	532	f	0	4	1	15	25	6.67	(0.68- 65.37)
Subtotal CHOI								2.86	(1.42- 5.76)
DAMBER	505	m	0	206	98	70	76	2.28	(1.52- 3.42)
DOLL	507	m	0	1077	992	4	7	1.90	(0.55- 6.51)
DOLL	514	f	0	20	12	15	15	1.67	(0.61- 4.59)
Subtotal DOLL								1.76	(0.80- 3.84)
*DORN	616	m	0	84	36304	37	73050	4.57	(3.10- 6.72)
*DORN	653	m	0	81	24616	90	74464	2.72	(2.02- 3.67)
Subtotal DORN								3.31	(2.61- 4.19)
GAO	505	m	0	262	167	45	129	4.50	(3.04- 6.65)
GAO	515	f	0	77	25	73	64	2.70	(1.54- 4.74)
Subtotal GAO								3.81	(2.76- 5.25)
GENG	532	f	0	36	10	28	31	3.99	(1.67- 9.49)
HAENSZ	541	f	0	30	37	44	66	1.22	(0.66- 2.25)
HEGMAN	501	m	0	146	716	26	289	2.27	(1.46- 3.52)
HEGMAN	502	f	0	81	169	2	28	6.71	(1.56- 28.86)
Subtotal HEGMAN								2.48	(1.63- 3.78)
HU	515	m	0	13	7	14	20	2.65	(0.84- 8.34)
HU	520	f	0	5	4	3	5	2.08	(0.30- 14.55)
Subtotal HU								2.49	(0.93- 6.69)
HU2	507	c	0	129	68	15	29	3.67	(1.84- 7.31)
JEDRYC	610	m	5	135	-	49	-	1.66	(1.19- 2.32)
JEDRYC	620	f	5	63	-	-	-	1.77	(0.68- 4.60)
Subtotal JEDRYC								1.67	(1.22- 2.29)
JOLY	547	m	0	317	282	18	70	4.37	(2.54- 7.52)
JOLY	537	f	0	76	35	23	41	3.87	(2.02- 7.41)
Subtotal JOLY								4.16	(2.74- 6.30)
KHUDER	510	m	0	226	295	72	152	1.62	(1.16- 2.25)
KOULUM	509	m	0	143	16	8	8	8.94	(2.95- 27.06)
LETOUR	505	c	0	151	76	188	160	1.69	(1.20- 2.39)
LIU3	503	m	0	32	125	20	80	1.02	(0.55- 1.91)
LIU4	505	m	2	-	-	-	-	1.58	(1.53- 1.63)
LIU5	503	c	0	72	48	13	22	2.54	(1.17- 5.52)
LUBIN	571	m	0	178	251	30	179	4.23	(2.75- 6.52)
LUBIN2	1155	m	0	250	316	68	221	2.57	(1.87- 3.54)
MATOS	560	m	0	69	90	28	73	2.00	(1.17- 3.42)
PERNU	506	m	0	337	92	1043	346	1.22	(0.94- 1.58)
PERNU	503	f	0	1	0	18	89	14.51~	(0.57- 370.40)
Subtotal PERNU								1.23	(0.95- 1.60)
PEZZOT	574	m	0	52	67	41	105	1.99	(1.19- 3.31)
*QIAO2	505	m	0	104	2130	52	1947	1.83	(1.32- 2.54)
RACHTA	505	f	0	52	21	8	4	1.24	(0.34- 4.56)
SOBUE	800	m	0	137	92	110	121	1.64	(1.13- 2.37)
SUZUK2	505	c	0	31	10	16	22	4.26	(1.63- 11.14)
TIZZAN	510	m	0	699	529	12	44	4.84	(2.53- 9.26)
TIZZAN	523	f	0	11	2	2	5	13.75	(1.48- 127.47)
Subtotal TIZZAN								5.26	(2.82- 9.79)
WYNDE6	763	m	0	611	301	111	92	1.68	(1.24- 2.29)
WYNDE6	771	f	0	291	91	127	90	2.27	(1.58- 3.24)
Subtotal WYNDE6								1.91	(1.51- 2.41)
ZHENG	567	m	0	106	43	28	66	5.81	(3.30- 10.24)
ZHENG	574	f	0	60	28	16	16	2.14	(0.94- 4.89)
Subtotal ZHENG								4.22	(2.65- 6.74)
Partial Totals				7458	68571	3190	152789		

*prospective study ~ With 0.5 adjustment for zero

Table 1H5 - 5

IESLC - Meta-analysis of Ever Smoking, Age started, "Highest vs lowest"
All LC types, Any Product (or Cigarettes if Any not available)
Least adjusted

REF	NRR	SEX	AD	Ys	Ws	Qs	Ps
AGUDO	503	f	0	0.91	2.63	0.41	0.1381
ALDERS	503	m	2	1.08	6.73	2.10	0.0051
ALDERS	506	f	2	0.73	15.75	0.71	0.0037
Subtotal ALDERS				0.84	22.49	2.81	
ARMADA	513	m	0	0.86	33.57	3.89	0.0000
AUVINE	513	c	0	1.18	4.25	1.86	0.0149
BARBON	519	m	0	1.83	16.51	28.19	0.0000
BRESLO	505	c	0	0.45	13.43	0.07	0.1006
BUFFLE	525	f	0	0.64	8.05	0.12	0.0680
CHEN2	521	m	0	-0.24	2.84	1.66	0.6810
CHEN2	526	f	0	1.04	2.25	0.61	0.1190
Subtotal CHEN2				0.32	5.09	2.27	
CHIAZZ	503	m	2	1.89	2.98	5.61	0.0011
CHOI	529	m	0	0.96	7.05	1.37	0.0107
CHOI	532	f	0	1.90	0.74	1.40	0.1034
Subtotal CHOI				1.05	7.79	2.77	
DAMBER	505	m	0	0.83	23.53	2.20	0.0001
DOLL	507	m	0	0.64	2.53	0.04	0.3070
DOLL	514	f	0	0.51	3.75	0.00	0.3226
Subtotal DOLL				0.56	6.28	0.04	
*DORN	616	m	0	1.52	25.71	25.68	0.0000
*DORN	653	m	0	1.00	42.73	9.92	0.0000
Subtotal DORN				1.20	68.44	35.60	
GAO	505	m	0	1.50	25.14	24.33	0.0000
GAO	515	f	0	0.99	12.15	2.73	0.0005
Subtotal GAO				1.34	37.29	27.06	
GENG	532	f	0	1.38	5.11	3.80	0.0018
HAENSZ	541	f	0	0.20	10.18	1.07	0.5323
HEGMAN	501	m	0	0.82	19.93	1.78	0.0003
HEGMAN	502	f	0	1.90	1.81	3.46	0.0105
Subtotal HEGMAN				0.91	21.74	5.23	
HU	515	m	0	0.98	2.93	0.61	0.0948
HU	520	f	0	0.73	1.02	0.05	0.4592
Subtotal HU				0.91	3.95	0.66	
HU2	507	c	0	1.30	8.09	4.92	0.0002
JEDRYC	610	m	5	0.51	34.48	0.01	0.0029
JEDRYC	620	f	5	0.57	4.20	0.01	0.2417
Subtotal JEDRYC				0.51	38.68	0.02	
JOLY	547	m	0	1.48	13.06	11.93	0.0000
JOLY	537	f	0	1.35	9.12	6.34	0.0000
Subtotal JOLY				1.43	22.19	18.27	
KHUDER	510	m	0	0.48	35.36	0.05	0.0043
KOULUM	509	m	0	2.19	3.13	8.73	0.0001
LETOUR	505	c	0	0.53	31.90	0.00	0.0030
LIU3	503	m	0	0.02	9.83	2.42	0.9407
LIU4	505	m	2	0.46	3833.36	14.86	0.0000
LIU5	503	c	0	0.93	6.37	1.08	0.0188
LUBIN	571	m	0	1.44	20.61	17.55	0.0000
LUBIN2	1155	m	0	0.94	37.89	6.83	0.0000
MATOS	560	m	0	0.69	13.33	0.40	0.0115
PERNU	506	m	0	0.19	56.54	5.97	0.1428
PERNU	503	f	0	2.68	0.37	1.70	0.1056
Subtotal PERNU				0.21	56.91	7.67	
PEZZOT	574	m	0	0.69	14.69	0.41	0.0085
*QIAO2	505	m	0	0.60	35.89	0.25	0.0003
RACHTA	505	f	0	0.21	2.26	0.21	0.7480
SOBUE	800	m	0	0.49	28.15	0.02	0.0088
SUZUK2	505	c	0	1.45	4.16	3.60	0.0031
TIZZAN	510	m	0	1.58	9.14	10.24	0.0000
TIZZAN	523	f	0	2.62	0.77	3.42	0.0211
Subtotal TIZZAN				1.66	9.92	13.66	
WYNDE6	763	m	0	0.52	40.26	0.00	0.0010
WYNDE6	771	f	0	0.82	29.93	2.66	0.0000
Subtotal WYNDE6				0.65	70.19	2.66	
ZHENG	567	m	0	1.76	11.97	18.40	0.0000
ZHENG	574	f	0	0.76	5.64	0.33	0.0704
Subtotal ZHENG				1.44	17.61	18.73	

Table 1H5 - 5

IESLC - Meta-analysis of Ever Smoking, Age started, "Highest vs lowest"
All LC types, Any Product (or Cigarettes if Any not available)
Least adjusted

	N	53
	NS	39
	Wt	4593.82
Het	Chi	246.01
Het	df	52
Het	P	***
Fixed	RR	1.68
	RRl	1.63
	RRu	1.73
	P	+++
Random	RR	2.47
	RRl	2.15
	RRu	2.82
	P	+++
Asymm	P	***

Table 1H5 - 6

IESLC - Meta-analysis of Ever Smoking, Age started, "Highest vs lowest"
All LC types, Any Product (or Cigarettes if Any not available)
Least adjusted

		combined	Sex male	female	Total
	N	6	29	18	53
	NS	6	28	18	52
	Wt	68.20	4409.89	115.73	4593.82
Het	Chi	8.31	204.84	17.23	246.01
Het	df	5	28	17	52
Het	P	N.S.	***	N.S.	***
Fixed	RR	2.09	1.66	2.31	1.68
	RRl	1.65	1.61	1.93	1.63
	RRu	2.65	1.71	2.78	1.73
	P	+++	+++	+++	+++
Random	RR	2.32	2.51	2.32	2.47
	RRl	1.65	2.10	1.93	2.15
	RRu	3.25	2.99	2.79	2.82
	P	+++	+++	+++	+++
Between	Chi				15.64
Between	df				2
Between	P				***
Btwn(F)	P				N.S.
Btwn(R)	P				N.S.

Table 1H5 - 7

IESLC - Meta-analysis of Ever Smoking, Age started, "Highest vs lowest"
All LC types, Any Product (or Cigarettes if Any not available)
Excluded studies (and stage at which they were excluded)

1	AKIBA AMANDU AMES BECHER BENSHL BEST BLOT1 BROSS BROWN3 CARPEN CEDERL CHYOU CPSI CPSII DARBY DEAN2 DEAN3 DOLL2 ENGELA GAO2 GARCIA GILLIS GRAHAM GURSEL HAMMO2 HIRAYA HOLE HUMBLE JAHN JAIN KAISE2 KATSOU KAUFMA LAUSSM LIAW MCDUFF MIGRAN MRFITR PEZZO2 PISANI PRESCO QIAO SEGI2 SPEIZE SVENSS TVERDA WAKAI WATSON WIGLE WU WYNDE3 WYNDE8
2	AXELSS BOUCHA BOUCOT CHEN DESTEF DORGAN DOSEME FAN GARSHI GER HAMMON JUSSAW KOO KREUZE LEVIN MCCONN NOTAN2 OSANN2 RESTRE SADOWS VUTUC WANG2 WU2 WUWILL WYNDE2 XU ZHOU
3	GUO SPITZ STASZE ZHANG
5	LUO
6	CORREA YUAN
8	BOFFET WYNDE7
15	BENHAM

Table 1H5 - 8
Potentially overlapping studies

REF	REFGP	PRINC	. OVERLAP/LINK
LUBIN2	LUBIN2	1	Lubin-combined
WYNDE6	WYNDE6	1	WYNDE5/6/7/8
LUBIN	XIANGZ	2	LUBIN/XIANGZ/QIAO

Table 1H5 - 9
Most adjusted - insufficient data for meta-analysis

REF	NRR	SEX	AGEL	AGEH	RACE	YF	LC TYPE	LOC	START	ST	NLC	R	VB	P	H	AD	ADOS	PRODUCT	exL	exH	unexL	unexH	De
CORREA	544	c	0	0	all	-	all	NAmer	1979	CC	1359	n	bl	y	n	2	0	cig+/-ot	1	15	21	999	st
YUAN	501	m	0	0	all	0	all	As:Chi	1986	pr	142	n	ot	n	n	3	1#	cig+/-ot	1	24	25	999	ot

Comments on values in listings

YUAN ADOS Amount smoked per day

REF	NRR	RR	SIG	RRDATA comment
CORREA	544	2.92		0
YUAN	501	2.40	y	Significant P<.001

Table 1H6 -

IESLC - Meta-analysis of Current Smoking by Age started, Overview
All LC types, Any Product (or Cigarettes if Any not available)

This analysis is restricted to results for:
1) Current smokers
2) Results by Age started
3) Categorical results by Age started
Results by Age started are grouped under 2 schemes (S1, S2). Each scheme has a set of "key values". An interval is allocated to the category whose key value it includes, and intervals which include none or more than one of the key values are excluded. (Open-ended intervals are coded as 999)

S1	key value	maximum range
1	26	19+
2	18	15-25
3	14	1-17

S2	key value	maximum range
1	30	27+
2	26	23-29
3	22	19-25
4	18	15-21
5	14	11-17
6	10	1-13

4) All LC types (or near equivalent)
5) Results complete enough for use in metaanalysis

Within each study, results are then selected (in the following order of preference, within each sex) for:
6) (not applicable)
7) PRODUCT: all/unspec, cigarettes regardless of other products, cigarettes only
8) CIGTYPE: all/unspecified, MC regardless of HR, MC only
9) (not applicable)
10) DENOM: never smoked anything, never smoked cigarettes, never any + low, never cigs + low
11) Followup period (YF, prospective studies): whole study (coded as 0) or longest available
12) LCtype: all or nearest available, at least Squamous and Adeno. (q = squamous, s = small, l = large, a = adeno, mix = mixed, alv = alveolar)
13) Race: all or nearest available, otherwise by race (wh or w = white, bl or b = black, hi = hispanic ch = chinese, jap = japanese, haw = hawaiian, w+o = white + oriental, sca = scandinavian, as = asian)
14) For overlapping studies: principal rather than subsidiary studies
Finally by Age: whole study (coded as 0) if available, otherwise by widest available age group and then for single sex results (m, f) in preference to results for both sexes combined (c).

Results adjusted (AD) for the most potential confounders are then chosen in Sections -1 to -3 and results adjusted for the least confounders in Sections -4 to -6. (Those least adjusted results which actually differ from the most adjusted are marked 'x' in column X in Section -4)

Section -7 shows excluded studies, together with the stage (as above) at which no qualifying results were found.

Section -8 lists the potentially overlapping studies which have been included (1=principal, 2=subsidiary).

Section -9 lists any results which would have been included in preference except that they had data not complete enough for use in meta-analysis, with their significance (yes/no), if known, and any further comment as entered on the database. It also lists as "gap" any categories for which no data were presented by the original authors.

In addition to those mentioned above, the following fields, levels and abbreviations are used:
* or nk = not known, n = no, y = yes, ot = other
nev = never
all/unspec = all or unspecified, cig+/-ot = cigarettes irrespective of other products (cigar, pipe etc)
MC = manufactured cigarettes, HR = hand-rolled cigarettes
exL, exH = range of exposure (low and high) in the smoking group, in terms of Age started
REF: 6-character study reference
NRR: number of the RR on the database within the study
ST : study type (CC = case control, pr or prosp = prospective)
NLC: number of lung cancer cases in whole study
R : risky occupational population (n = no, m = mining, o = other risky)
VB : national cigarette type (V = at least 75% Virginia, bl = at least 75% blended, ot = other)
P : any proxy use
H : full histological confirmation
De : derivation of RR/CI (or = original, st = standard method, ot = other method of estimation)

Table 1H6 - 1

IESLC - Meta-analysis of Current Smoking by Age started, Overview
All LC types, Any Product (or Cigarettes if Any not available)
Most adjusted

REF	NRR	SEX	AGEL	AGEH	RACE	YF	LC TYPE	LOC	START	ST	NLC	R	VB	P	H	AD	PRODUCT	exL	exH	S1	S2	DENOM	De
CEDERL	510	m	0	0	all	10	all	Eu:Sca	1963	pr	491	n	bl	n	n	1	cig only	19	999	1	0	nev any	ot
CEDERL	511	m	0	0	all	10	all	Eu:Sca	1963	pr	491	n	bl	n	n	1	cig only	17	18	2	4	nev any	ot
CEDERL	512	m	0	0	all	10	all	Eu:Sca	1963	pr	491	n	bl	n	n	1	cig only	1	16	3	0	nev any	ot
CEDERL	515	f	0	0	all	10	all	Eu:Sca	1963	pr	491	n	bl	n	n	0	cig only	19	999	1	0	nev any	st
CEDERL	516	f	0	0	all	10	all	Eu:Sca	1963	pr	491	n	bl	n	n	0	cig only	17	18	2	4	nev any	st
CEDERL	517	f	0	0	all	10	all	Eu:Sca	1963	pr	491	n	bl	n	n	0	cig only	1	16	3	0	nev any	st
CPSI	801	m	35	84	all	6	all	NAmer	1959	pr	5138	n	bl	n	n	1	cig+/-ot	25	999	1	0	nev any	ot
CPSI	802	m	35	84	all	6	all	NAmer	1959	pr	5138	n	bl	n	n	1	cig+/-ot	20	24	0	3	nev any	ot
CPSI	803	m	35	84	all	6	all	NAmer	1959	pr	5138	n	bl	n	n	1	cig+/-ot	15	19	2	4	nev any	ot
CPSI	804	m	35	84	all	6	all	NAmer	1959	pr	5138	n	bl	n	n	1	cig+/-ot	1	14	3	0	nev any	ot
CPSI	845	f	40	74	all	6	all	NAmer	1959	pr	5138	n	bl	n	n	1	cig only	25	999	1	0	nev any	ot
CPSI	846	f	40	74	all	6	all	NAmer	1959	pr	5138	n	bl	n	n	1	cig only	20	24	0	3	nev any	ot
CPSI	847	f	40	74	all	6	all	NAmer	1959	pr	5138	n	bl	n	n	1	cig only	15	19	2	4	nev any	ot
CPSI	848	f	40	74	all	6	all	NAmer	1959	pr	5138	n	bl	n	n	1	cig only	1	14	3	0	nev any	ot
DEAN3	564	m	0	0	all	-	all	Eu:UK	1969	CC	766	n	V	y	n	0	cig only	25	999	1	0	nev any	st
DEAN3	565	m	0	0	all	-	all	Eu:UK	1969	CC	766	n	V	y	n	0	cig only	20	24	0	3	nev any	st
DEAN3	566	m	0	0	all	-	all	Eu:UK	1969	CC	766	n	V	y	n	0	cig only	15	19	2	4	nev any	st
DEAN3	567	m	0	0	all	-	all	Eu:UK	1969	CC	766	n	V	y	n	0	cig only	1	14	3	0	nev any	st
DEAN3	583	f	0	0	all	-	all	Eu:UK	1969	CC	766	n	V	y	n	0	cig only	25	999	1	0	nev any	st
DEAN3	584	f	0	0	all	-	all	Eu:UK	1969	CC	766	n	V	y	n	0	cig only	20	24	0	3	nev any	st
DEAN3	585	f	0	0	all	-	all	Eu:UK	1969	CC	766	n	V	y	n	0	cig only	15	19	2	4	nev any	st
DEAN3	586	f	0	0	all	-	all	Eu:UK	1969	CC	766	n	V	y	n	0	cig only	1	14	3	0	nev any	st
DORN	583	m	55	64	wh	8	all	NAmer	1954	pr	5097	n	bl	n	n	0	cig+/-ot	25	999	1	0	nev any	st
DORN	584	m	55	64	wh	8	all	NAmer	1954	pr	5097	n	bl	n	n	0	cig+/-ot	20	24	0	3	nev any	st
DORN	585	m	55	64	wh	8	all	NAmer	1954	pr	5097	n	bl	n	n	0	cig+/-ot	15	19	2	4	nev any	st
DORN	586	m	55	64	wh	8	all	NAmer	1954	pr	5097	n	bl	n	n	0	cig+/-ot	1	14	3	0	nev any	st
DORN	620	m	65	74	wh	8	all	NAmer	1954	pr	5097	n	bl	n	n	0	cig+/-ot	25	999	1	0	nev any	st
DORN	621	m	65	74	wh	8	all	NAmer	1954	pr	5097	n	bl	n	n	0	cig+/-ot	20	24	0	3	nev any	st
DORN	622	m	65	74	wh	8	all	NAmer	1954	pr	5097	n	bl	n	n	0	cig+/-ot	15	19	2	4	nev any	st
DORN	623	m	65	74	wh	8	all	NAmer	1954	pr	5097	n	bl	n	n	0	cig+/-ot	1	14	3	0	nev any	st
ENGELA	501	m	0	0	all	0	all	Eu:Sca	1964	pr	435	n	bl	n	n	0	cig+/-ot	30	999	0	1	nev cigs	st
ENGELA	502	m	0	0	all	0	all	Eu:Sca	1964	pr	435	n	bl	n	n	0	cig+/-ot	20	29	1	0	nev cigs	st
ENGELA	503	m	0	0	all	0	all	Eu:Sca	1964	pr	435	n	bl	n	n	0	cig+/-ot	1	19	0	0	nev cigs	st
ENGELA	509	f	0	0	all	0	all	Eu:Sca	1964	pr	435	n	bl	n	n	0	cig+/-ot	30	999	0	1	nev cigs	st
ENGELA	510	f	0	0	all	0	all	Eu:Sca	1964	pr	435	n	bl	n	n	0	cig+/-ot	20	29	1	0	nev cigs	st
ENGELA	511	f	0	0	all	0	all	Eu:Sca	1964	pr	435	n	bl	n	n	0	cig+/-ot	1	19	0	0	nev cigs	st
GAO2	501	m	0	0	all	-	all	As:Jap	1988	CC	282	n	bl	n	n	0	cig+/-ot	30	999	0	1	nev cigs	or
GAO2	502	m	0	0	all	-	all	As:Jap	1988	CC	282	n	bl	n	n	0	cig+/-ot	20	29	1	0	nev cigs	st
GAO2	503	m	0	0	all	-	all	As:Jap	1988	CC	282	n	bl	n	n	0	cig+/-ot	1	19	0	0	nev cigs	or
HIRAYA	501	m	0	0	all	0	all	As:Jap	1965	pr	1917	n	bl	n	n	1	cig+/-ot	20	999	1	0	nev any	st
HIRAYA	502	m	0	0	all	0	all	As:Jap	1965	pr	1917	n	bl	n	n	1	cig+/-ot	1	19	0	0	nev any	st
HIRAYA	504	f	0	0	all	0	all	As:Jap	1965	pr	1917	n	bl	n	n	1	cig+/-ot	20	999	1	0	nev any	st
HIRAYA	505	f	0	0	all	0	all	As:Jap	1965	pr	1917	n	bl	n	n	1	cig+/-ot	1	19	0	0	nev any	st
LIAW	504	c	0	0	all	0	all	As:oth	1982	pr	127	n	ot	n	n	2	all/unsp	25	999	1	0	nev any	or
LIAW	505	c	0	0	all	0	all	As:oth	1982	pr	127	n	ot	n	n	2	all/unsp	21	24	0	3	nev any	or
LIAW	506	c	0	0	all	0	all	As:oth	1982	pr	127	n	ot	n	n	2	all/unsp	1	20	0	0	nev any	or
MATOS	561	m	0	0	all	-	all	SCAmer	1994	CC	200	n	bl	n	n	2	cig+/-ot	20	999	1	0	nev any	or
MATOS	562	m	0	0	all	-	all	SCAmer	1994	CC	200	n	bl	n	n	2	cig+/-ot	15	19	2	4	nev any	or
MATOS	563	m	0	0	all	-	all	SCAmer	1994	CC	200	n	bl	n	n	2	cig+/-ot	1	14	3	0	nev any	or
MIGRAN	501	m	0	0	all	0	all	Eu:UK	1964	pr	259	n	V	n	n	0	cig only	20	999	1	0	nev any	st
MIGRAN	503	m	0	0	all	0	all	Eu:UK	1964	pr	259	n	V	n	n	0	cig only	16	19	2	4	nev any	st
MIGRAN	505	m	0	0	all	0	all	Eu:UK	1964	pr	259	n	V	n	n	0	cig only	0	15	3	0	nev any	st
MIGRAN	511	f	0	0	all	0	all	Eu:UK	1964	pr	259	n	V	n	n	0	cig only	20	999	1	0	nev any	st
MIGRAN	513	f	0	0	all	0	all	Eu:UK	1964	pr	259	n	V	n	n	0	cig only	16	19	2	4	nev any	st
MIGRAN	515	f	0	0	all	0	all	Eu:UK	1964	pr	259	n	V	n	n	0	cig only	0	15	3	0	nev any	st
MRFITR	508	m	0	0	all	0	all	NAmer	1973	pr	119	n	bl	n	n	0	cig+/-ot	24	999	1	0	nev cigs	ot
MRFITR	509	m	0	0	all	0	all	NAmer	1973	pr	119	n	bl	n	n	0	cig+/-ot	22	23	0	3	nev cigs	ot
MRFITR	510	m	0	0	all	0	all	NAmer	1973	pr	119	n	bl	n	n	0	cig+/-ot	20	21	0	0	nev cigs	ot
MRFITR	511	m	0	0	all	0	all	NAmer	1973	pr	119	n	bl	n	n	0	cig+/-ot	18	19	2	4	nev cigs	ot
MRFITR	512	m	0	0	all	0	all	NAmer	1973	pr	119	n	bl	n	n	0	cig+/-ot	16	17	0	0	nev cigs	ot
MRFITR	513	m	0	0	all	0	all	NAmer	1973	pr	119	n	bl	n	n	0	cig+/-ot	1	15	3	0	nev cigs	ot
SEGI2	521	m	0	0	all	-	all	As:Jap	1962	CC	378	n	bl	n	n	1	cig+/-ot	23	999	1	0	nev any	ot
SEGI2	522	m	0	0	all	-	all	As:Jap	1962	CC	378	n	bl	n	n	1	cig+/-ot	20	22	0	3	nev any	ot
SEGI2	523	m	0	0	all	-	all	As:Jap	1962	CC	378	n	bl	n	n	1	cig+/-ot	1	19	0	0	nev any	ot
SOBUE	646	m	0	0	all	-	all	As:Jap	1986	CC	1376	n	bl	n	y	0	cig+/-ot	23	999	1	0	nev cigs	st
SOBUE	647	m	0	0	all	-	all	As:Jap	1986	CC	1376	n	bl	n	y	0	cig+/-ot	18	22	2	0	nev cigs	st
SOBUE	648	m	0	0	all	-	all	As:Jap	1986	CC	1376	n	bl	n	y	0	cig+/-ot	10	17	3	0	nev cigs	st
SVENSS	501	f	0	0	all	-	all	Eu:Sca	1983	CC	210	n	bl	n	n	0	all/unsp	26	999	1	0	nev any	st
SVENSS	502	f	0	0	all	-	all	Eu:Sca	1983	CC	210	n	bl	n	n	0	all/unsp	19	25	0	3	nev any	st
SVENSS	503	f	0	0	all	-	all	Eu:Sca	1983	CC	210	n	bl	n	n	0	all/unsp	0	18	0	0	nev any	st
WAKAI	501	m	0	0	all	-	all	As:Jap	1988	CC	333	n	bl	n	y	0	cig+/-ot	30	999	0	1	nev any	st
WAKAI	502	m	0	0	all	-	all	As:Jap	1988	CC	333	n	bl	n	y	0	cig+/-ot	20	29	1	0	nev any	st

Table 1H6 - 1

IESLC - Meta-analysis of Current Smoking by Age started, Overview
All LC types, Any Product (or Cigarettes if Any not available)
Most adjusted

REF	NRR	SEX	AGEL	AGEH	RACE	YF	LC TYPE	LOC	START	ST	NLC	R	VB	P	H	AD	PRODUCT	exL	exH	S1	S2	DENOM	De
WAKAI	503	m	0	0	all	-	all	As:Jap	1988	CC	333	n	bl	n	y	0	cig+/-ot	1	19	0	0	nev any	st
WU	541	f	0	0	wh	-	q+a	NAmer	1981	CC	220	n	bl	n	y	2	all/unsp	25	999	1	0	nev any	st
WU	542	f	0	0	wh	-	q+a	NAmer	1981	CC	220	n	bl	n	y	2	all/unsp	19	24	0	3	nev any	st
WU	543	f	0	0	wh	-	q+a	NAmer	1981	CC	220	n	bl	n	y	2	all/unsp	0	18	0	0	nev any	st

Cigarette type is all/unspec for all RRs
except for the following:

REF	NRR	CIGTYPE
DEAN3	564	MC only
DEAN3	565	MC only
DEAN3	566	MC only
DEAN3	567	MC only
DEAN3	583	MC only
DEAN3	584	MC only
DEAN3	585	MC only
DEAN3	586	MC only

In this overview table, subtotals and Qs values may be invalid and should be ignored

Table 1H6 - 2

IESLC - Meta-analysis of Current Smoking by Age started, Overview
All LC types, Any Product (or Cigarettes if Any not available)
Most adjusted

REF	NRR	SEX	AD	Number Case	Exposed Cont	Non-exposed Case	Cont	RR	95.00%CI
*CEDERL	510	m	1	11	-	7	-	6.50 (	2.52- 16.74)
*CEDERL	511	m	1	10	-	7	-	9.80 (	3.74- 25.69)
*CEDERL	512	m	1	7	-	7	-	6.40 (	2.25- 18.21)
*CEDERL	515	f	0	6	2806	19	17679	1.99 (	0.80- 4.98)
*CEDERL	516	f	0	2	1009	19	17679	1.84 (	0.43- 7.91)
*CEDERL	517	f	0	0	746	19	17679	0.61~(	0.04- 10.05)
Subtotal CEDERL								4.43 (	2.82- 6.96)
*CPSI	801	m	1	42	-	83	-	4.08 (	2.81- 5.91)
*CPSI	802	m	1	196	-	83	-	10.08 (	7.80- 13.03)
*CPSI	803	m	1	588	-	83	-	14.69 (	11.68- 18.49)
*CPSI	804	m	1	185	-	83	-	16.77 (	12.94- 21.73)
*CPSI	845	f	1	51	-	166	-	2.25 (	1.64- 3.08)
*CPSI	846	f	1	34	-	166	-	3.38 (	2.33- 4.88)
*CPSI	847	f	1	52	-	166	-	5.00 (	3.66- 6.83)
*CPSI	848	f	1	6	-	166	-	2.50 (	1.11- 5.65)
Subtotal CPSI								7.59 (	6.81- 8.46)
DEAN3	564	m	0	24	75	24	510	6.80 (	3.67- 12.58)
DEAN3	565	m	0	52	161	24	510	6.86 (	4.10- 11.49)
DEAN3	566	m	0	160	485	24	510	7.01 (	4.48- 10.96)
DEAN3	567	m	0	44	165	24	510	5.67 (	3.34- 9.60)
DEAN3	583	f	0	27	274	41	1538	3.70 (	2.24- 6.11)
DEAN3	584	f	0	18	229	41	1538	2.95 (	1.67- 5.22)
DEAN3	585	f	0	39	504	41	1538	2.90 (	1.85- 4.55)
DEAN3	586	f	0	7	109	41	1538	2.41 (	1.06- 5.50)
Subtotal DEAN3								4.59 (	3.81- 5.54)
*DORN	583	m	0	30	49537	25	213858	5.18 (	3.05- 8.81)
*DORN	584	m	0	133	102910	25	213858	11.06 (	7.21- 16.95)
*DORN	585	m	0	293	154204	25	213858	16.25 (	10.81- 24.45)
*DORN	586	m	0	70	25569	25	213858	23.42 (	14.84- 36.97)
*DORN	620	m	0	70	49386	49	171211	4.95 (	3.44- 7.13)
*DORN	621	m	0	138	60703	49	171211	7.94 (	5.73- 11.00)
*DORN	622	m	0	259	80043	49	171211	11.31 (	8.33- 15.34)
*DORN	623	m	0	65	16016	49	171211	14.18 (	9.79- 20.54)
Subtotal DORN								10.30 (	9.00- 11.79)
*ENGELA	501	m	0	17	9762	27	58716	3.79 (	2.07- 6.95)
*ENGELA	502	m	0	50	30195	27	58716	3.60 (	2.26- 5.75)
*ENGELA	503	m	0	173	50732	27	58716	7.42 (	4.94- 11.12)
*ENGELA	509	f	0	10	24560	31	207789	2.73 (	1.34- 5.57)
*ENGELA	510	f	0	36	29605	31	207789	8.15 (	5.04- 13.17)
*ENGELA	511	f	0	18	10687	31	207789	11.29 (	6.32- 20.17)
Subtotal ENGELA								5.83 (	4.73- 7.20)
GAO2	501	m	0	2	4	13	56	2.15 (	0.36- 13.05)
GAO2	502	m	0	127	85	13	56	6.44 (	3.32- 12.49)
GAO2	503	m	0	52	26	13	56	8.62 (	4.01- 18.52)
Subtotal GAO2								6.68 (	4.12- 10.83)
*HIRAYA	501	m	1	-	-	-	-	4.35 (	3.51- 5.39)
*HIRAYA	502	m	1	-	-	-	-	5.71 (	4.50- 7.25)
*HIRAYA	504	f	1	-	-	-	-	2.46 (	1.93- 3.13)
*HIRAYA	505	f	1	-	-	-	-	0.78 (	0.10- 6.10)
Subtotal HIRAYA								3.96 (	3.46- 4.52)
*LIAW	504	c	2	-	-	-	-	1.50 (	0.70- 3.30)
*LIAW	505	c	2	-	-	-	-	5.90 (	3.00- 11.30)
*LIAW	506	c	2	-	-	-	-	4.60 (	2.60- 8.10)
Subtotal LIAW								3.82 (	2.62- 5.58)
MATOS	561	m	2	18	-	11	-	5.30 (	2.30- 12.50)
MATOS	562	m	2	49	-	11	-	8.60 (	4.10- 18.20)
MATOS	563	m	2	45	-	11	-	11.30 (	5.30- 24.30)
Subtotal MATOS								8.25 (	5.26- 12.95)
*MIGRAN	501	m	0	24	668	4	867	7.79 (	2.72- 22.34)
*MIGRAN	503	m	0	59	1845	4	867	6.93 (	2.53- 19.02)
*MIGRAN	505	m	0	50	1081	4	867	10.03 (	3.64- 27.65)
*MIGRAN	511	f	0	11	1315	4	3814	7.98 (	2.54- 25.01)
*MIGRAN	513	f	0	9	1035	4	3814	8.29 (	2.56- 26.87)
*MIGRAN	515	f	0	2	266	4	3814	7.17 (	1.32- 38.96)
Subtotal MIGRAN								8.07 (	5.08- 12.80)
*MRFITR	508	m	0	3	544	0	1859	23.91~(	1.24- 462.09)
*MRFITR	509	m	0	6	402	0	1859	60.06~(	3.39-1063.94)
*MRFITR	510	m	0	7	1029	0	1859	27.09~(	1.55- 473.89)
*MRFITR	511	m	0	25	1876	0	1859	50.54~(	3.08- 829.51)
*MRFITR	512	m	0	40	2242	0	1859	67.17~(	4.13-1091.55)
*MRFITR	513	m	0	25	2065	0	1859	45.91~(	2.80- 753.64)

Table 1H6 - 2

IESLC - Meta-analysis of Current Smoking by Age started, Overview
All LC types, Any Product (or Cigarettes if Any not available)
Most adjusted

REF	NRR	SEX	AD	Number Exposed Case	Cont	Non-exposed Case	Cont	RR	95.00%CI
Subtotal MRFITR								43.20	(13.52- 138.02)
SEGI2	521	m	1	49	-	8	-	2.04	(0.89- 4.66)
SEGI2	522	m	1	125	-	8	-	3.33	(1.51- 7.37)
SEGI2	523	m	1	91	-	8	-	5.64	(2.49- 12.78)
Subtotal SEGI2								3.38	(2.12- 5.41)
SOBUE	646	m	0	75	75	29	126	4.34	(2.60- 7.27)
SOBUE	647	m	0	553	490	29	126	4.90	(3.22- 7.47)
SOBUE	648	m	0	109	68	29	126	6.96	(4.20- 11.54)
Subtotal SOBUE								5.25	(4.00- 6.91)
SVENSS	501	f	0	32	18	38	120	5.61	(2.84- 11.12)
SVENSS	502	f	0	58	14	38	120	13.08	(6.57- 26.04)
SVENSS	503	f	0	52	21	38	120	7.82	(4.19- 14.60)
Subtotal SVENSS								8.26	(5.63- 12.12)
WAKAI	501	m	0	8	25	10	65	2.08	(0.74- 5.87)
WAKAI	502	m	0	130	183	10	65	4.62	(2.29- 9.32)
WAKAI	503	m	0	42	74	10	65	3.69	(1.72- 7.94)
Subtotal WAKAI								3.63	(2.28- 5.77)
WU	541	f	2	14	-	31	-	1.55	(0.60- 3.98)
WU	542	f	2	40	-	31	-	3.57	(1.57- 8.14)
WU	543	f	2	106	-	31	-	10.32	(4.81- 22.13)
Subtotal WU								4.39	(2.71- 7.10)
Partial Totals				4961	715923	2248	2427318		

*prospective study ~ With 0.5 adjustment for zero

REF	NRR	SEX	AD	Ys	Ws	Qs	Ps
*CEDERL	510	m	1	1.87	4.29	0.01	0.0001
*CEDERL	511	m	1	2.28	4.14	0.84	0.0000
*CEDERL	512	m	1	1.86	3.51	0.00	0.0005
*CEDERL	515	f	0	0.69	4.57	5.97	0.1414
*CEDERL	516	f	0	0.61	1.81	2.69	0.4098
*CEDERL	517	f	0	-0.50	0.49	2.65	0.7276
Subtotal CEDERL				1.49	18.81	12.17	
*CPSI	801	m	1	1.41	27.80	5.03	0.0000
*CPSI	802	m	1	2.31	58.36	13.40	0.0000
*CPSI	803	m	1	2.69	72.82	53.34	0.0000
*CPSI	804	m	1	2.82	57.18	55.85	0.0000
*CPSI	845	f	1	0.81	38.69	40.28	0.0000
*CPSI	846	f	1	1.22	28.12	10.58	0.0000
*CPSI	847	f	1	1.61	39.48	1.94	0.0000
*CPSI	848	f	1	0.92	5.80	4.86	0.0273
Subtotal CPSI				2.03	328.25	185.28	
DEAN3	564	m	0	1.92	10.14	0.07	0.0000
DEAN3	565	m	0	1.93	14.48	0.13	0.0000
DEAN3	566	m	0	1.95	19.25	0.26	0.0000
DEAN3	567	m	0	1.73	13.81	0.13	0.0000
DEAN3	583	f	0	1.31	15.21	4.18	0.0000
DEAN3	584	f	0	1.08	11.77	6.62	0.0002
DEAN3	585	f	0	1.07	18.99	11.13	0.0000
DEAN3	586	f	0	0.88	5.65	5.12	0.0367
Subtotal DEAN3				1.52	109.30	27.64	
*DORN	583	m	0	1.64	13.64	0.47	0.0000
*DORN	584	m	0	2.40	21.05	6.88	0.0000
*DORN	585	m	0	2.79	23.04	21.10	0.0000
*DORN	586	m	0	3.15	18.44	32.23	0.0000
*DORN	620	m	0	1.60	28.85	1.54	0.0000
*DORN	621	m	0	2.07	36.19	2.10	0.0000
*DORN	622	m	0	2.43	41.24	14.55	0.0000
*DORN	623	m	0	2.65	27.99	18.85	0.0000
Subtotal DORN				2.33	210.43	97.73	
*ENGELA	501	m	0	1.33	10.44	2.61	0.0000
*ENGELA	502	m	0	1.28	17.55	5.31	0.0000
*ENGELA	503	m	0	2.00	23.38	0.69	0.0000
*ENGELA	509	f	0	1.00	7.56	5.18	0.0058
*ENGELA	510	f	0	2.10	16.67	1.19	0.0000
*ENGELA	511	f	0	2.42	11.40	4.00	0.0000
Subtotal ENGELA				1.76	87.00	18.98	
GAO2	501	m	0	0.77	1.18	1.34	0.4038
GAO2	502	m	0	1.86	8.74	0.01	0.0000
GAO2	503	m	0	2.15	6.56	0.68	0.0000
Subtotal GAO2				1.90	16.48	2.03	

Table 1H6 - 2

IESLC - Meta-analysis of Current Smoking by Age started, Overview
All LC types, Any Product (or Cigarettes if Any not available)
Most adjusted

REF	NRR	SEX	AD	Ys	Ws	Qs	Ps
*HIRAYA	501	m	1	1.47	83.52	10.89	0.0000
*HIRAYA	502	m	1	1.74	67.55	0.54	0.0000
*HIRAYA	504	f	1	0.90	65.73	56.99	0.0000
*HIRAYA	505	f	1	-0.25	0.91	3.93	0.8127
Subtotal HIRAYA				1.38	217.71	72.35	
*LIAW	504	c	2	0.41	6.39	12.99	0.3054
*LIAW	505	c	2	1.77	8.74	0.03	0.0000
*LIAW	506	c	2	1.53	11.90	1.11	0.0000
Subtotal LIAW				1.34	27.03	14.13	
MATOS	561	m	2	1.67	5.36	0.14	0.0001
MATOS	562	m	2	2.15	6.92	0.71	0.0000
MATOS	563	m	2	2.42	6.63	2.33	0.0000
Subtotal MATOS				2.11	18.91	3.19	
*MIGRAN	501	m	0	2.05	3.46	0.17	0.0001
*MIGRAN	503	m	0	1.94	3.77	0.04	0.0002
*MIGRAN	505	m	0	2.31	3.73	0.84	0.0000
*MIGRAN	511	f	0	2.08	2.94	0.18	0.0004
*MIGRAN	513	f	0	2.12	2.78	0.22	0.0004
*MIGRAN	515	f	0	1.97	1.34	0.03	0.0226
Subtotal MIGRAN				2.09	18.02	1.47	
*MRFITR	508	m	0	3.17	0.44	0.79	0.0357
*MRFITR	509	m	0	4.10	0.46	2.38	0.0052
*MRFITR	510	m	0	3.30	0.47	1.01	0.0238
*MRFITR	511	m	0	3.92	0.49	2.15	0.0060
*MRFITR	512	m	0	4.21	0.49	2.79	0.0031
*MRFITR	513	m	0	3.83	0.49	1.95	0.0074
Subtotal MRFITR				3.77	2.85	11.07	
SEGI2	521	m	1	0.71	5.61	7.01	0.0914
SEGI2	522	m	1	1.20	6.11	2.41	0.0029
SEGI2	523	m	1	1.73	5.74	0.06	0.0000
Subtotal SEGI2				1.22	17.46	9.49	
SOBUE	646	m	0	1.47	14.47	1.90	0.0000
SOBUE	647	m	0	1.59	21.61	1.26	0.0000
SOBUE	648	m	0	1.94	15.08	0.18	0.0000
Subtotal SOBUE				1.66	51.17	3.34	
SVENSS	501	f	0	1.73	8.23	0.09	0.0000
SVENSS	502	f	0	2.57	8.11	4.44	0.0000
SVENSS	503	f	0	2.06	9.85	0.50	0.0000
Subtotal SVENSS				2.11	26.19	5.03	
WAKAI	501	m	0	0.73	3.57	4.31	0.1666
WAKAI	502	m	0	1.53	7.78	0.71	0.0000
WAKAI	503	m	0	1.31	6.55	1.81	0.0008
Subtotal WAKAI				1.29	17.89	6.83	
WU	541	f	2	0.44	4.29	8.33	0.3639
WU	542	f	2	1.27	5.67	1.77	0.0024
WU	543	f	2	2.33	6.60	1.67	0.0000
Subtotal WU				1.48	16.56	11.77	

N	76
NS	16

Table 1H6 - 3

IESLC - Meta-analysis of Current Smoking by Age started, Overview
All LC types, Any Product (or Cigarettes if Any not available)
Most adjusted

		Sex		
	combined	male	female	Total
N	3	48	25	76
NS	1	13	8	22

In this overview table, other than the "N" rows, entries in the "absent" and "Total" columns may be invalid and should be ignored

	Age started (broad categories)				
	absent	19+k26	15-25k18	1-17k14	Total
N	27	23	13	13	76
NS	12	16	8	8	44
Wt	373.22	394.36	256.34	160.15	1184.07
Het Chi	82.61	65.25	80.94	63.44	482.50
Het df	26	22	12	12	75
Het P	***	***	***	***	***
Fixed RR	6.44	3.78	8.75	11.60	6.24
RRl	5.82	3.43	7.74	9.94	5.90
RRu	7.13	4.17	9.89	13.55	6.61
P	+++	+++	+++	+++	+++
Random RR	5.93	4.05	7.57	8.20	5.77
RRl	4.82	3.34	5.24	5.36	4.91
RRu	7.31	4.93	10.93	12.56	6.78
P	+++	+++	+++	+++	+++

			Age started (narrow categories)					
	absent	27+k30	23-29k26	19-25k22	15-21k18	11-17k14	1-13k10	Total
N	49	4		11	12			76
NS	16	3		8	7			34
Wt	727.52	22.76		199.06	234.73			1184.07
Het Chi	295.91	1.24		47.96	73.01			482.50
Het df	48	3		10	11			75
Het P	***	N.S.		***	***			***
Fixed RR	5.45	3.00		7.03	9.23			6.24
RRl	5.07	1.99		6.12	8.12			5.90
RRu	5.86	4.53		8.07	10.49			6.61
P	+++	+++		+++	+++			+++
Random RR	5.49	3.00		6.33	7.93			5.77
RRl	4.48	1.99		4.51	5.37			4.91
RRu	6.72	4.53		8.90	11.69			6.78
P	+++	+++		+++	+++			+++

Table 1H6 - 3

IESLC - Meta-analysis of Current Smoking by Age started, Overview
All LC types, Any Product (or Cigarettes if Any not available)
Most adjusted

MALES

	Age started (broad categories) absent	19+k26	15-25k18	1-17k14	Total
N	16	14	9	9	48
NS	9	13	8	8	38
Wt	262.59	231.64	193.28	146.87	834.38
Het Chi	38.52	11.10	30.05	28.56	243.13
Het df	15	13	8	8	47
Het P	***	N.S.	***	***	***
Fixed RR	7.13	4.56	11.11	13.29	7.78
RRl	6.32	4.01	9.65	11.30	7.27
RRu	8.04	5.18	12.79	15.62	8.33
P	+++	+++	+++	+++	+++
Random RR	6.55	4.56	9.86	11.53	7.15
RRl	5.16	4.01	7.12	8.05	6.00
RRu	8.31	5.18	13.64	16.52	8.52
P	+++	+++	+++	+++	+++

		Age started (narrow categories)						
	absent	27+k30	23-29k26	19-25k22	15-21k18	11-17k14	1-13k10	Total
N	31	3		6	8			48
NS	13	3		5	7			28
Wt	510.86	15.20		136.66	171.67			834.38
Het Chi	153.42	1.14		10.93	13.78			243.13
Het df	30	2		5	7			47
Het P	***	N.S.		(*)	(*)			***
Fixed RR	6.63	3.15		8.82	12.31			7.78
RRl	6.08	1.90		7.46	10.60			7.27
RRu	7.23	5.21		10.43	14.30			8.33
P	+++	+++		+++	+++			+++
Random RR	6.65	3.15		8.30	11.38			7.15
RRl	5.32	1.90		6.21	8.82			6.00
RRu	8.32	5.21		11.09	14.69			8.52
P	+++	+++		+++	+++			+++

FEMALES

	Age started (broad categories) absent	19+k26	15-25k18	1-17k14	Total
N	9	8	4	4	25
NS	6	8	4	4	22
Wt	89.99	156.33	63.06	13.28	322.66
Het Chi	34.64	31.79	6.30	2.45	93.90
Het df	8	7	3	3	24
Het P	***	***	(*)	N.S.	***
Fixed RR	5.05	2.98	4.22	2.60	3.67
RRl	4.11	2.55	3.30	1.52	3.29
RRu	6.21	3.49	5.40	4.45	4.10
P	+++	+++	+++	+++	+++
Random RR	5.26	3.45	4.02	2.60	4.05
RRl	3.31	2.33	2.53	1.52	3.17
RRu	8.35	5.11	6.37	4.45	5.18
P	+++	+++	+++	+++	+++

Table 1H6 - 3

IESLC - Meta-analysis of Current Smoking by Age started, Overview
All LC types, Any Product (or Cigarettes if Any not available)
Most adjusted

FEMALES

	absent	Age started (narrow categories) 27+k30	23-29k26	19-25k22	15-21k18	11-17k14	1-13k10	Total
N	16	1		4	4			25
NS	8	1		4	4			17
Wt	198.37	7.56		53.67	63.06			322.66
Het Chi	71.19	0.00		13.34	6.30			93.90
Het df	15	0		3	3			24
Het P	***	N.S.		**	(*)			***
Fixed RR	3.46	2.73		4.05	4.22			3.67
RRl	3.01	1.34		3.10	3.30			3.29
RRu	3.98	5.57		5.29	5.40			4.10
P	+++	++		+++	+++			+++
Random RR	4.01	2.73		4.54	4.02			4.05
RRl	2.80	1.34		2.45	2.53			3.17
RRu	5.73	5.57		8.42	6.37			5.18
P	+++	++		+++	+++			+++

Table 1H6 - 4

IESLC - Meta-analysis of Current Smoking by Age started, Overview
All LC types, Any Product (or Cigarettes if Any not available)
Least adjusted

REF	NRR	X	SEX	AGEL	AGEH	RACE	YF	LC TYPE	LOC	START	ST	NLC	R	VB	P	H	AD	PRODUCT	exL	exH	S1	S2	DENOM	De
CEDERL	510		m	0	0	all	10	all	Eu:Sca	1963	pr	491	n	bl	n	n	1	cig only	19	999	1	0	nev any	ot
CEDERL	511		m	0	0	all	10	all	Eu:Sca	1963	pr	491	n	bl	n	n	1	cig only	17	18	2	4	nev any	ot
CEDERL	512		m	0	0	all	10	all	Eu:Sca	1963	pr	491	n	bl	n	n	1	cig only	1	16	3	0	nev any	ot
CEDERL	515		f	0	0	all	10	all	Eu:Sca	1963	pr	491	n	bl	n	n	0	cig only	19	999	1	0	nev any	st
CEDERL	516		f	0	0	all	10	all	Eu:Sca	1963	pr	491	n	bl	n	n	0	cig only	17	18	2	4	nev any	st
CEDERL	517		f	0	0	all	10	all	Eu:Sca	1963	pr	491	n	bl	n	n	0	cig only	1	16	3	0	nev any	st
CPSI	801		m	35	84	all	6	all	NAmer	1959	pr	5138	n	bl	n	n	1	cig+/-ot	25	999	1	0	nev any	ot
CPSI	802		m	35	84	all	6	all	NAmer	1959	pr	5138	n	bl	n	n	1	cig+/-ot	20	24	0	3	nev any	ot
CPSI	803		m	35	84	all	6	all	NAmer	1959	pr	5138	n	bl	n	n	1	cig+/-ot	15	19	2	4	nev any	ot
CPSI	804		m	35	84	all	6	all	NAmer	1959	pr	5138	n	bl	n	n	1	cig+/-ot	1	14	3	0	nev any	ot
CPSI	845		f	40	74	all	6	all	NAmer	1959	pr	5138	n	bl	n	n	1	cig only	25	999	1	0	nev any	ot
CPSI	846		f	40	74	all	6	all	NAmer	1959	pr	5138	n	bl	n	n	1	cig only	20	24	0	3	nev any	ot
CPSI	847		f	40	74	all	6	all	NAmer	1959	pr	5138	n	bl	n	n	1	cig only	15	19	2	4	nev any	ot
CPSI	848		f	40	74	all	6	all	NAmer	1959	pr	5138	n	bl	n	n	1	cig only	1	14	3	0	nev any	ot
DEAN3	564		m	0	0	all	-	all	Eu:UK	1969	CC	766	n	V	y	n	0	cig only	25	999	1	0	nev any	st
DEAN3	565		m	0	0	all	-	all	Eu:UK	1969	CC	766	n	V	y	n	0	cig only	20	24	0	3	nev any	st
DEAN3	566		m	0	0	all	-	all	Eu:UK	1969	CC	766	n	V	y	n	0	cig only	15	19	2	4	nev any	st
DEAN3	567		m	0	0	all	-	all	Eu:UK	1969	CC	766	n	V	y	n	0	cig only	1	14	3	0	nev any	st
DEAN3	583		f	0	0	all	-	all	Eu:UK	1969	CC	766	n	V	y	n	0	cig only	25	999	1	0	nev any	st
DEAN3	584		f	0	0	all	-	all	Eu:UK	1969	CC	766	n	V	y	n	0	cig only	20	24	0	3	nev any	st
DEAN3	585		f	0	0	all	-	all	Eu:UK	1969	CC	766	n	V	y	n	0	cig only	15	19	2	4	nev any	st
DEAN3	586		f	0	0	all	-	all	Eu:UK	1969	CC	766	n	V	y	n	0	cig only	1	14	3	0	nev any	st
DORN	583		m	55	64	wh	8	all	NAmer	1954	pr	5097	n	bl	n	n	0	cig+/-ot	25	999	1	0	nev any	st
DORN	584		m	55	64	wh	8	all	NAmer	1954	pr	5097	n	bl	n	n	0	cig+/-ot	20	24	0	3	nev any	st
DORN	585		m	55	64	wh	8	all	NAmer	1954	pr	5097	n	bl	n	n	0	cig+/-ot	15	19	2	4	nev any	st
DORN	586		m	55	64	wh	8	all	NAmer	1954	pr	5097	n	bl	n	n	0	cig+/-ot	1	14	3	0	nev any	st
DORN	620		m	65	74	wh	8	all	NAmer	1954	pr	5097	n	bl	n	n	0	cig+/-ot	25	999	1	0	nev any	st
DORN	621		m	65	74	wh	8	all	NAmer	1954	pr	5097	n	bl	n	n	0	cig+/-ot	20	24	0	3	nev any	st
DORN	622		m	65	74	wh	8	all	NAmer	1954	pr	5097	n	bl	n	n	0	cig+/-ot	15	19	2	4	nev any	st
DORN	623		m	65	74	wh	8	all	NAmer	1954	pr	5097	n	bl	n	n	0	cig+/-ot	1	14	3	0	nev any	st
ENGELA	501		m	0	0	all	0	all	Eu:Sca	1964	pr	435	n	bl	n	n	0	cig+/-ot	30	999	0	1	nev cigs	st
ENGELA	502		m	0	0	all	0	all	Eu:Sca	1964	pr	435	n	bl	n	n	0	cig+/-ot	20	29	1	0	nev cigs	st
ENGELA	503		m	0	0	all	0	all	Eu:Sca	1964	pr	435	n	bl	n	n	0	cig+/-ot	1	19	0	0	nev cigs	st
ENGELA	509		f	0	0	all	0	all	Eu:Sca	1964	pr	435	n	bl	n	n	0	cig+/-ot	30	999	0	1	nev cigs	st
ENGELA	510		f	0	0	all	0	all	Eu:Sca	1964	pr	435	n	bl	n	n	0	cig+/-ot	20	29	1	0	nev cigs	st
ENGELA	511		f	0	0	all	0	all	Eu:Sca	1964	pr	435	n	bl	n	n	0	cig+/-ot	1	19	0	0	nev cigs	st
GAO2	501		m	0	0	all	-	all	As:Jap	1988	CC	282	n	bl	n	n	0	cig+/-ot	30	999	0	1	nev cigs	or
GAO2	502		m	0	0	all	-	all	As:Jap	1988	CC	282	n	bl	n	n	0	cig+/-ot	20	29	1	0	nev cigs	st
GAO2	503		m	0	0	all	-	all	As:Jap	1988	CC	282	n	bl	n	n	0	cig+/-ot	1	19	0	0	nev cigs	or
HIRAYA	501		m	0	0	all	0	all	As:Jap	1965	pr	1917	n	bl	n	n	1	cig+/-ot	20	999	1	0	nev any	st
HIRAYA	502		m	0	0	all	0	all	As:Jap	1965	pr	1917	n	bl	n	n	1	cig+/-ot	1	19	0	0	nev any	st
HIRAYA	504		f	0	0	all	0	all	As:Jap	1965	pr	1917	n	bl	n	n	1	cig+/-ot	20	999	1	0	nev any	st
HIRAYA	505		f	0	0	all	0	all	As:Jap	1965	pr	1917	n	bl	n	n	1	cig+/-ot	1	19	0	0	nev any	st
LIAW	504		c	0	0	all	0	all	As:oth	1982	pr	127	n	ot	n	n	2	all/unsp	25	999	1	0	nev any	or
LIAW	505		c	0	0	all	0	all	As:oth	1982	pr	127	n	ot	n	n	2	all/unsp	21	24	0	3	nev any	or
LIAW	506		c	0	0	all	0	all	As:oth	1982	pr	127	n	ot	n	n	2	all/unsp	1	20	0	0	nev any	or
MATOS	541	x	m	0	0	all	-	all	SCAmer	1994	CC	200	n	bl	n	n	0	cig+/-ot	20	999	1	0	nev any	st
MATOS	542	x	m	0	0	all	-	all	SCAmer	1994	CC	200	n	bl	n	n	0	cig+/-ot	15	19	2	4	nev any	st
MATOS	543	x	m	0	0	all	-	all	SCAmer	1994	CC	200	n	bl	n	n	0	cig+/-ot	1	14	3	0	nev any	st
MIGRAN	501		m	0	0	all	0	all	Eu:UK	1964	pr	259	n	V	n	n	0	cig only	20	999	1	0	nev any	st
MIGRAN	503		m	0	0	all	0	all	Eu:UK	1964	pr	259	n	V	n	n	0	cig only	16	19	2	4	nev any	st
MIGRAN	505		m	0	0	all	0	all	Eu:UK	1964	pr	259	n	V	n	n	0	cig only	0	15	3	0	nev any	st
MIGRAN	511		f	0	0	all	0	all	Eu:UK	1964	pr	259	n	V	n	n	0	cig only	20	999	1	0	nev any	st
MIGRAN	513		f	0	0	all	0	all	Eu:UK	1964	pr	259	n	V	n	n	0	cig only	16	19	2	4	nev any	st
MIGRAN	515		f	0	0	all	0	all	Eu:UK	1964	pr	259	n	V	n	n	0	cig only	0	15	3	0	nev any	st
MRFITR	508		m	0	0	all	0	all	NAmer	1973	pr	119	n	bl	n	n	0	cig+/-ot	24	999	1	0	nev cigs	ot
MRFITR	509		m	0	0	all	0	all	NAmer	1973	pr	119	n	bl	n	n	0	cig+/-ot	22	23	0	3	nev cigs	ot
MRFITR	510		m	0	0	all	0	all	NAmer	1973	pr	119	n	bl	n	n	0	cig+/-ot	20	21	0	0	nev cigs	ot
MRFITR	511		m	0	0	all	0	all	NAmer	1973	pr	119	n	bl	n	n	0	cig+/-ot	18	19	2	4	nev cigs	ot
MRFITR	512		m	0	0	all	0	all	NAmer	1973	pr	119	n	bl	n	n	0	cig+/-ot	16	17	0	0	nev cigs	ot
MRFITR	513		m	0	0	all	0	all	NAmer	1973	pr	119	n	bl	n	n	0	cig+/-ot	1	15	3	0	nev cigs	ot
SEGI2	516	x	m	0	0	all	-	all	As:Jap	1962	CC	378	n	bl	n	n	0	cig+/-ot	23	999	1	0	nev any	st
SEGI2	517	x	m	0	0	all	-	all	As:Jap	1962	CC	378	n	bl	n	n	0	cig+/-ot	20	22	0	3	nev any	st
SEGI2	518	x	m	0	0	all	-	all	As:Jap	1962	CC	378	n	bl	n	n	0	cig+/-ot	1	19	0	0	nev any	st
SOBUE	646		m	0	0	all	-	all	As:Jap	1986	CC	1376	n	bl	n	y	0	cig+/-ot	23	999	1	0	nev cigs	st
SOBUE	647		m	0	0	all	-	all	As:Jap	1986	CC	1376	n	bl	n	y	0	cig+/-ot	18	22	2	0	nev cigs	st
SOBUE	648		m	0	0	all	-	all	As:Jap	1986	CC	1376	n	bl	n	y	0	cig+/-ot	10	17	3	0	nev cigs	st
SVENSS	501		f	0	0	all	-	all	Eu:Sca	1983	CC	210	n	bl	n	n	0	all/unsp	26	999	1	0	nev any	st
SVENSS	502		f	0	0	all	-	all	Eu:Sca	1983	CC	210	n	bl	n	n	0	all/unsp	19	25	0	3	nev any	st
SVENSS	503		f	0	0	all	-	all	Eu:Sca	1983	CC	210	n	bl	n	n	0	all/unsp	0	18	0	0	nev any	st
WAKAI	501		m	0	0	all	-	all	As:Jap	1988	CC	333	n	bl	n	y	0	cig+/-ot	30	999	0	1	nev any	st
WAKAI	502		m	0	0	all	-	all	As:Jap	1988	CC	333	n	bl	n	y	0	cig+/-ot	20	29	1	0	nev any	st

Table 1H6 - 4

IESLC - Meta-analysis of Current Smoking by Age started, Overview
All LC types, Any Product (or Cigarettes if Any not available)
Least adjusted

REF	NRR	X	SEX	AGEL	AGEH	RACE	YF	LC TYPE	LOC	START	ST	NLC	R	VB	P	H	AD	PRODUCT	exL	exH	S1	S2	DENOM	De
WAKAI	503		m	0	0	all	-	all	As:Jap	1988	CC	333	n	bl	n	y	0	cig+/-ot	1	19	0	0	nev any	st
WU	517	x	f	0	0	wh	-	q+a	NAmer	1981	CC	220	n	bl	n	y	0	all/unsp	25	999	1	0	nev any	st
WU	518	x	f	0	0	wh	-	q+a	NAmer	1981	CC	220	n	bl	n	y	0	all/unsp	19	24	0	3	nev any	st
WU	519	x	f	0	0	wh	-	q+a	NAmer	1981	CC	220	n	bl	n	y	0	all/unsp	0	18	0	0	nev any	st

Cigarette type is all/unspec for all RRs
except for the following:

REF	NRR	CIGTYPE
DEAN3	564	MC only
DEAN3	565	MC only
DEAN3	566	MC only
DEAN3	567	MC only
DEAN3	583	MC only
DEAN3	584	MC only
DEAN3	585	MC only
DEAN3	586	MC only

In this overview table, subtotals and Qs values may be invalid and should be ignored

Table 1H6 - 5

IESLC - Meta-analysis of Current Smoking by Age started, Overview
All LC types, Any Product (or Cigarettes if Any not available)
Least adjusted

REF	NRR	SEX	AD	Number Case	Exposed Cont	Non-exposed Case	Cont	RR	95.00%CI
*CEDERL	510	m	1	11	-	7	-	6.50	(2.52- 16.74)
*CEDERL	511	m	1	10	-	7	-	9.80	(3.74- 25.69)
*CEDERL	512	m	1	7	-	7	-	6.40	(2.25- 18.21)
*CEDERL	515	f	0	6	2806	19	17679	1.99	(0.80- 4.98)
*CEDERL	516	f	0	2	1009	19	17679	1.84	(0.43- 7.91)
*CEDERL	517	f	0	0	746	19	17679	0.61~	(0.04- 10.05)
Subtotal CEDERL								4.43	(2.82- 6.96)
*CPSI	801	m	1	42	-	83	-	4.08	(2.81- 5.91)
*CPSI	802	m	1	196	-	83	-	10.08	(7.80- 13.03)
*CPSI	803	m	1	588	-	83	-	14.69	(11.68- 18.49)
*CPSI	804	m	1	185	-	83	-	16.77	(12.94- 21.73)
*CPSI	845	f	1	51	-	166	-	2.25	(1.64- 3.08)
*CPSI	846	f	1	34	-	166	-	3.38	(2.33- 4.88)
*CPSI	847	f	1	52	-	166	-	5.00	(3.66- 6.83)
*CPSI	848	f	1	6	-	166	-	2.50	(1.11- 5.65)
Subtotal CPSI								7.59	(6.81- 8.46)
DEAN3	564	m	0	24	75	24	510	6.80	(3.67- 12.58)
DEAN3	565	m	0	52	161	24	510	6.86	(4.10- 11.49)
DEAN3	566	m	0	160	485	24	510	7.01	(4.48- 10.96)
DEAN3	567	m	0	44	165	24	510	5.67	(3.34- 9.60)
DEAN3	583	f	0	27	274	41	1538	3.70	(2.24- 6.11)
DEAN3	584	f	0	18	229	41	1538	2.95	(1.67- 5.22)
DEAN3	585	f	0	39	504	41	1538	2.90	(1.85- 4.55)
DEAN3	586	f	0	7	109	41	1538	2.41	(1.06- 5.50)
Subtotal DEAN3								4.59	(3.81- 5.54)
*DORN	583	m	0	30	49537	25	213858	5.18	(3.05- 8.81)
*DORN	584	m	0	133	102910	25	213858	11.06	(7.21- 16.95)
*DORN	585	m	0	293	154204	25	213858	16.25	(10.81- 24.45)
*DORN	586	m	0	70	25569	25	213858	23.42	(14.84- 36.97)
*DORN	620	m	0	70	49386	49	171211	4.95	(3.44- 7.13)
*DORN	621	m	0	138	60703	49	171211	7.94	(5.73- 11.00)
*DORN	622	m	0	259	80043	49	171211	11.31	(8.33- 15.34)
*DORN	623	m	0	65	16016	49	171211	14.18	(9.79- 20.54)
Subtotal DORN								10.30	(9.00- 11.79)
*ENGELA	501	m	0	17	9762	27	58716	3.79	(2.07- 6.95)
*ENGELA	502	m	0	50	30195	27	58716	3.60	(2.26- 5.75)
*ENGELA	503	m	0	173	50732	27	58716	7.42	(4.94- 11.12)
*ENGELA	509	f	0	10	24560	31	207789	2.73	(1.34- 5.57)
*ENGELA	510	f	0	36	29605	31	207789	8.15	(5.04- 13.17)
*ENGELA	511	f	0	18	10687	31	207789	11.29	(6.32- 20.17)
Subtotal ENGELA								5.83	(4.73- 7.20)
GAO2	501	m	0	2	4	13	56	2.15	(0.36- 13.05)
GAO2	502	m	0	127	85	13	56	6.44	(3.32- 12.49)
GAO2	503	m	0	52	26	13	56	8.62	(4.01- 18.52)
Subtotal GAO2								6.68	(4.12- 10.83)
*HIRAYA	501	m	1	-	-	-	-	4.35	(3.51- 5.39)
*HIRAYA	502	m	1	-	-	-	-	5.71	(4.50- 7.25)
*HIRAYA	504	f	1	-	-	-	-	2.46	(1.93- 3.13)
*HIRAYA	505	f	1	-	-	-	-	0.78	(0.10- 6.10)
Subtotal HIRAYA								3.96	(3.46- 4.52)
*LIAW	504	c	2	-	-	-	-	1.50	(0.70- 3.30)
*LIAW	505	c	2	-	-	-	-	5.90	(3.00- 11.30)
*LIAW	506	c	2	-	-	-	-	4.60	(2.60- 8.10)
Subtotal LIAW								3.82	(2.62- 5.58)
MATOS	541	m	0	18	33	11	110	5.45	(2.34- 12.70)
MATOS	542	m	0	49	58	11	110	8.45	(4.08- 17.48)
MATOS	543	m	0	45	41	11	110	10.98	(5.18- 23.25)
Subtotal MATOS								8.20	(5.26- 12.79)
*MIGRAN	501	m	0	24	668	4	867	7.79	(2.72- 22.34)
*MIGRAN	503	m	0	59	1845	4	867	6.93	(2.53- 19.02)
*MIGRAN	505	m	0	50	1081	4	867	10.03	(3.64- 27.65)
*MIGRAN	511	f	0	11	1315	4	3814	7.98	(2.54- 25.01)
*MIGRAN	513	f	0	9	1035	4	3814	8.29	(2.56- 26.87)
*MIGRAN	515	f	0	2	266	4	3814	7.17	(1.32- 38.96)
Subtotal MIGRAN								8.07	(5.08- 12.80)
*MRFITR	508	m	0	3	544	0	1859	23.91~	(1.24- 462.09)
*MRFITR	509	m	0	6	402	0	1859	60.06~	(3.39-1063.94)
*MRFITR	510	m	0	7	1029	0	1859	27.09~	(1.55- 473.89)
*MRFITR	511	m	0	25	1876	0	1859	50.54~	(3.08- 829.51)
*MRFITR	512	m	0	40	2242	0	1859	67.17~	(4.13-1091.55)
*MRFITR	513	m	0	25	2065	0	1859	45.91~	(2.80- 753.64)

Table 1H6 - 5

IESLC - Meta-analysis of Current Smoking by Age started, Overview
All LC types, Any Product (or Cigarettes if Any not available)
Least adjusted

REF	NRR	SEX	AD	Number Exposed Case	Cont	Non-exposed Case	Cont	RR	95.00%CI
Subtotal MRFITR								43.20	(13.52- 138.02)
SEGI2	516	m	0	49	155	8	53	2.09	(0.93- 4.71)
SEGI2	517	m	0	125	224	8	53	3.70	(1.70- 8.02)
SEGI2	518	m	0	91	103	8	53	5.85	(2.64- 12.96)
Subtotal SEGI2								3.59	(2.27- 5.67)
SOBUE	646	m	0	75	75	29	126	4.34	(2.60- 7.27)
SOBUE	647	m	0	553	490	29	126	4.90	(3.22- 7.47)
SOBUE	648	m	0	109	68	29	126	6.96	(4.20- 11.54)
Subtotal SOBUE								5.25	(4.00- 6.91)
SVENSS	501	f	0	32	18	38	120	5.61	(2.84- 11.12)
SVENSS	502	f	0	58	14	38	120	13.08	(6.57- 26.04)
SVENSS	503	f	0	52	21	38	120	7.82	(4.19- 14.60)
Subtotal SVENSS								8.26	(5.63- 12.12)
WAKAI	501	m	0	8	25	10	65	2.08	(0.74- 5.87)
WAKAI	502	m	0	130	183	10	65	4.62	(2.29- 9.32)
WAKAI	503	m	0	42	74	10	65	3.69	(1.72- 7.94)
Subtotal WAKAI								3.63	(2.28- 5.77)
WU	517	f	0	14	19	31	92	2.19	(0.98- 4.87)
WU	518	f	0	40	22	31	92	5.40	(2.79- 10.45)
WU	519	f	0	106	32	31	92	9.83	(5.57- 17.34)
Subtotal WU								5.76	(3.94- 8.42)
Partial Totals				4961	716610	2248	2428083		

*prospective study ~ With 0.5 adjustment for zero

REF	NRR	SEX	AD	Ys	Ws	Qs	Ps
*CEDERL	510	m	1	1.87	4.29	0.01	0.0001
*CEDERL	511	m	1	2.28	4.14	0.83	0.0000
*CEDERL	512	m	1	1.86	3.51	0.00	0.0005
*CEDERL	515	f	0	0.69	4.57	6.01	0.1414
*CEDERL	516	f	0	0.61	1.81	2.71	0.4098
*CEDERL	517	f	0	-0.50	0.49	2.66	0.7276
Subtotal CEDERL				1.49	18.81	12.21	
*CPSI	801	m	1	1.41	27.80	5.11	0.0000
*CPSI	802	m	1	2.31	58.36	13.20	0.0000
*CPSI	803	m	1	2.69	72.82	52.89	0.0000
*CPSI	804	m	1	2.82	57.18	55.44	0.0000
*CPSI	845	f	1	0.81	38.69	40.57	0.0000
*CPSI	846	f	1	1.22	28.12	10.71	0.0000
*CPSI	847	f	1	1.61	39.48	2.01	0.0000
*CPSI	848	f	1	0.92	5.80	4.90	0.0273
Subtotal CPSI				2.03	328.25	184.82	
DEAN3	564	m	0	1.92	10.14	0.07	0.0000
DEAN3	565	m	0	1.93	14.48	0.12	0.0000
DEAN3	566	m	0	1.95	19.25	0.24	0.0000
DEAN3	567	m	0	1.73	13.81	0.14	0.0000
DEAN3	583	f	0	1.31	15.21	4.23	0.0000
DEAN3	584	f	0	1.08	11.77	6.68	0.0002
DEAN3	585	f	0	1.07	18.99	11.24	0.0000
DEAN3	586	f	0	0.88	5.65	5.16	0.0367
Subtotal DEAN3				1.52	109.30	27.89	
*DORN	583	m	0	1.64	13.64	0.49	0.0000
*DORN	584	m	0	2.40	21.05	6.79	0.0000
*DORN	585	m	0	2.79	23.04	20.94	0.0000
*DORN	586	m	0	3.15	18.44	32.06	0.0000
*DORN	620	m	0	1.60	28.85	1.59	0.0000
*DORN	621	m	0	2.07	36.19	2.04	0.0000
*DORN	622	m	0	2.43	41.24	14.37	0.0000
*DORN	623	m	0	2.65	27.99	18.68	0.0000
Subtotal DORN				2.33	210.43	96.97	
*ENGELA	501	m	0	1.33	10.44	2.65	0.0000
*ENGELA	502	m	0	1.28	17.55	5.38	0.0000
*ENGELA	503	m	0	2.00	23.38	0.67	0.0000
*ENGELA	509	f	0	1.00	7.56	5.22	0.0058
*ENGELA	510	f	0	2.10	16.67	1.15	0.0000
*ENGELA	511	f	0	2.42	11.40	3.95	0.0000
Subtotal ENGELA				1.76	87.00	19.02	
GAO2	501	m	0	0.77	1.18	1.35	0.4038
GAO2	502	m	0	1.86	8.74	0.01	0.0000
GAO2	503	m	0	2.15	6.56	0.67	0.0000
Subtotal GAO2				1.90	16.48	2.02	

Table 1H6 - 5

IESLC - Meta-analysis of Current Smoking by Age started, Overview
All LC types, Any Product (or Cigarettes if Any not available)
Least adjusted

REF	NRR	SEX	AD	Ys	Ws	Qs	Ps
*HIRAYA	501	m	1	1.47	83.52	11.11	0.0000
*HIRAYA	502	m	1	1.74	67.55	0.58	0.0000
*HIRAYA	504	f	1	0.90	65.73	57.43	0.0000
*HIRAYA	505	f	1	-0.25	0.91	3.95	0.8127
Subtotal HIRAYA				1.38	217.71	73.07	
*LIAW	504	c	2	0.41	6.39	13.06	0.3054
*LIAW	505	c	2	1.77	8.74	0.03	0.0000
*LIAW	506	c	2	1.53	11.90	1.14	0.0000
Subtotal LIAW				1.34	27.03	14.23	
MATOS	541	m	0	1.70	5.38	0.10	0.0001
MATOS	542	m	0	2.13	7.26	0.65	0.0000
MATOS	543	m	0	2.40	6.82	2.14	0.0000
Subtotal MATOS				2.10	19.47	2.90	
*MIGRAN	501	m	0	2.05	3.46	0.16	0.0001
*MIGRAN	503	m	0	1.94	3.77	0.04	0.0002
*MIGRAN	505	m	0	2.31	3.73	0.83	0.0000
*MIGRAN	511	f	0	2.08	2.94	0.17	0.0004
*MIGRAN	513	f	0	2.12	2.78	0.22	0.0004
*MIGRAN	515	f	0	1.97	1.34	0.02	0.0226
Subtotal MIGRAN				2.09	18.02	1.44	
*MRFITR	508	m	0	3.17	0.44	0.79	0.0357
*MRFITR	509	m	0	4.10	0.46	2.38	0.0052
*MRFITR	510	m	0	3.30	0.47	1.01	0.0238
*MRFITR	511	m	0	3.92	0.49	2.14	0.0060
*MRFITR	512	m	0	4.21	0.49	2.78	0.0031
*MRFITR	513	m	0	3.83	0.49	1.95	0.0074
Subtotal MRFITR				3.77	2.85	11.03	
SEGI2	516	m	0	0.74	5.86	7.03	0.0736
SEGI2	517	m	0	1.31	6.40	1.78	0.0009
SEGI2	518	m	0	1.77	6.08	0.03	0.0000
Subtotal SEGI2				1.28	18.33	8.84	
SOBUE	646	m	0	1.47	14.47	1.94	0.0000
SOBUE	647	m	0	1.59	21.61	1.30	0.0000
SOBUE	648	m	0	1.94	15.08	0.17	0.0000
Subtotal SOBUE				1.66	51.17	3.40	
SVENSS	501	f	0	1.73	8.23	0.10	0.0000
SVENSS	502	f	0	2.57	8.11	4.40	0.0000
SVENSS	503	f	0	2.06	9.85	0.48	0.0000
Subtotal SVENSS				2.11	26.19	4.98	
WAKAI	501	m	0	0.73	3.57	4.34	0.1666
WAKAI	502	m	0	1.53	7.78	0.72	0.0000
WAKAI	503	m	0	1.31	6.55	1.84	0.0008
Subtotal WAKAI				1.29	17.89	6.90	
WU	517	f	0	0.78	5.98	6.63	0.0557
WU	518	f	0	1.69	8.80	0.20	0.0000
WU	519	f	0	2.29	11.93	2.42	0.0000
Subtotal WU				1.75	26.72	9.24	

N	76
NS	16

Table 1H6 - 6

IESLC - Meta-analysis of Current Smoking by Age started, Overview
All LC types, Any Product (or Cigarettes if Any not available)
Least adjusted

	combined	Sex: male	female	Total
N	3	48	25	76
NS	1	13	8	22

In this overview table, other than the "N" rows, entries in the "absent" and "Total" columns may be invalid and should be ignored

	Age started (broad categories): absent	19+k26	15-25k18	1-17k14	Total
N	27	23	13	13	76
NS	12	16	8	8	44
Wt	382.30	396.32	256.69	160.34	1195.65
Het Chi	80.80	63.65	80.95	63.46	478.97
Het df	26	22	12	12	75
Het P	***	***	***	***	***
Fixed RR	6.51	3.79	8.75	11.59	6.26
RRl	5.89	3.43	7.74	9.93	5.92
RRu	7.20	4.18	9.89	13.53	6.63
P	+++	+++	+++	+++	+++
Random RR	6.06	4.08	7.56	8.18	5.83
RRl	4.95	3.37	5.23	5.35	4.97
RRu	7.42	4.94	10.92	12.52	6.83
P	+++	+++	+++	+++	+++

	absent	Age started (narrow categories): 27+k30	23-29k26	19-25k22	15-21k18	11-17k14	1-13k10	Total
N	49	4		11	12			76
NS	16	3		8	7			34
Wt	735.34	22.76		202.47	235.07			1195.65
Het Chi	295.35	1.24		45.18	73.04			478.97
Het df	48	3		10	11			75
Het P	***	N.S.		***	***			***
Fixed RR	5.47	3.00		7.10	9.23			6.26
RRl	5.09	1.99		6.18	8.12			5.92
RRu	5.88	4.53		8.14	10.49			6.63
P	+++	+++		+++	+++			+++
Random RR	5.52	3.00		6.56	7.91			5.83
RRl	4.52	1.99		4.74	5.37			4.97
RRu	6.75	4.53		9.08	11.66			6.83
P	+++	+++		+++	+++			+++

Table 1H6 - 6

IESLC - Meta-analysis of Current Smoking by Age started, Overview
All LC types, Any Product (or Cigarettes if Any not available)
Least adjusted

MALES

	Age started (broad categories) absent	19+k26	15-25k18	1-17k14	Total
N	16	14	9	9	48
NS	9	13	8	8	38
Wt	263.21	231.91	193.63	147.06	835.81
Het Chi	37.65	11.07	30.15	28.64	241.96
Het df	15	13	8	8	47
Het P	**	N.S.	***	***	***
Fixed RR	7.14	4.56	11.10	13.27	7.79
RRl	6.33	4.01	9.64	11.29	7.28
RRu	8.06	5.18	12.77	15.59	8.33
P	+++	+++	+++	+++	+++
Random RR	6.60	4.56	9.84	11.49	7.17
RRl	5.22	4.01	7.11	8.02	6.02
RRu	8.35	5.18	13.61	16.47	8.54
P	+++	+++	+++	+++	+++

	absent	Age started (narrow categories) 27+k30	23-29k26	19-25k22	15-21k18	11-17k14	1-13k10	Total
N	31	3		6	8			48
NS	13	3		5	7			28
Wt	511.66	15.20		136.94	172.02			835.81
Het Chi	153.14	1.14		9.97	13.92			241.96
Het df	30	2		5	7			47
Het P	***	N.S.		(*)	(*)			***
Fixed RR	6.63	3.15		8.85	12.29			7.79
RRl	6.08	1.90		7.48	10.59			7.28
RRu	7.23	5.21		10.46	14.28			8.33
P	+++	+++		+++	+++			+++
Random RR	6.66	3.15		8.40	11.35			7.17
RRl	5.33	1.90		6.39	8.79			6.02
RRu	8.32	5.21		11.05	14.65			8.54
P	+++	+++		+++	+++			+++

FEMALES

	Age started (broad categories) absent	19+k26	15-25k18	1-17k14	Total
N	9	8	4	4	25
NS	6	8	4	4	22
Wt	98.46	158.02	63.06	13.28	332.81
Het Chi	35.58	30.52	6.30	2.45	97.88
Het df	8	7	3	3	24
Het P	***	***	(*)	N.S.	***
Fixed RR	5.35	3.00	4.22	2.60	3.78
RRl	4.39	2.57	3.30	1.52	3.39
RRu	6.52	3.50	5.40	4.45	4.20
P	+++	+++	+++	+++	+++
Random RR	5.53	3.53	4.02	2.60	4.18
RRl	3.55	2.42	2.53	1.52	3.27
RRu	8.61	5.16	6.37	4.45	5.34
P	+++	+++	+++	+++	+++

Table 1H6 - 6

IESLC - Meta-analysis of Current Smoking by Age started, Overview
All LC types, Any Product (or Cigarettes if Any not available)
Least adjusted

FEMALES

	absent	Age started (narrow categories) 27+k30	23-29k26	19-25k22	15-21k18	11-17k14	1-13k10	Total
N	16	1		4	4			25
NS	8	1		4	4			17
Wt	205.39	7.56		56.80	63.06			332.81
Het Chi	74.63	0.00		13.80	6.30			97.88
Het df	15	0		3	3			24
Het P	***	N.S.		**	(*)			***
Fixed RR	3.57	2.73		4.29	4.22			3.78
RRl	3.11	1.34		3.30	3.30			3.39
RRu	4.09	5.57		5.56	5.40			4.20
P	+++	++		+++	+++			+++
Random RR	4.09	2.73		4.95	4.02			4.18
RRl	2.87	1.34		2.73	2.53			3.27
RRu	5.84	5.57		8.99	6.37			5.34
P	+++	++		+++	+++			+++

Table 1H6 - 7

IESLC - Meta-analysis of Current Smoking by Age started, Overview
All LC types, Any Product (or Cigarettes if Any not available)
Excluded studies (and stage at which they were excluded)

1	AGUDO ALDERS ARMADA AUVINE AXELSS BARBON BECHER BENHAM BLOT1 BOFFET BOUCHA BRESLO BROWN3 CARPEN CHEN CHEN2 CHIAZZ CHOI CHYOU CORREA DAMBER DARBY DESTEF DOLL DOLL2 DORGAN DOSEME FAN GAO GARCIA GARSHI GENG GER GRAHAM GUO GURSEL HAENSZ HAMMO2 HAMMON HEGMAN HU HU2 JAHN JAIN JEDRYC JOLY JUSSAW KHUDER KOO KOULUM KREUZE LAUSSM LETOUR LEVIN LIU3 LIU4 LIU5 LUBIN LUBIN2 LUO MCCONN NOTAN2 OSANN2 PERNU PEZZOT PRESCO QIAO QIAO2 RACHTA RESTRE SADOWS STASZE SUZUK2 TIZZAN TVERDA VUTUC WANG2 WIGLE WU2 WUWILL WYNDE2 WYNDE3 XU YUAN ZHANG ZHENG ZHOU
2	AKIBA AMANDU AMES BENSHL BEST BOUCOT BROSS BUFFLE CPSII DEAN2 GILLIS HUMBLE KAISE2 KATSOU KAUFMA PEZZO2 PISANI SPITZ WATSON WYNDE8
3	MCDUFF WYNDE6
5	HOLE
7	WYNDE7
10	SPEIZE

Table 1H6 - 8
Potentially overlapping studies

REF	REFGP	PRINC	.	OVERLAP/LINK
MRFITR	MRFIT	2		Subset of MRFIT
CPSI	CPSI	1		CPSI overall

Table 1H7 -

IESLC - Meta-analysis of Current Smoking, Age started, "Low"
All LC types, Any Product (or Cigarettes if Any not available)

This analysis is restricted to results for:
1) Current smokers
2) Results by Age started
3) Categorical results by Age started
4) All LC types (or near equivalent)
5) Results complete enough for use in metaanalysis

Within each study, results are then selected (in the following order of preference, within each sex) for:
6) (not applicable)
7) PRODUCT: all/unspec, cigarettes regardless of other products, cigarettes only
8) CIGTYPE: all/unspecified, MC regardless of HR, MC only
9) (not applicable)
10) DENOM: never smoked anything, never smoked cigarettes, never any + low, never cigs + low
11) Followup period (YF, prospective studies): whole study (coded as 0) or longest available
12) LCtype: all or nearest available, at least Squamous and Adeno. (q = squamous, s = small,
l = large, a = adeno, mix = mixed, alv = alveolar)
13) Race: all or nearest available, otherwise by race (wh or w = white, bl or b = black, hi = hispanic
ch = chinese, jap = japanese, haw = hawaiian, w+o = white + oriental, sca = scandinavian, as = asian)
14) Age started "low" in key scheme 1 (key value 26, maximum range 19+)
15) For overlapping studies: principal rather than subsidiary studies
Finally by Age: whole study (coded as 0) if available, otherwise by widest available age group
and then for single sex results (m, f) in preference to results for both sexes combined (c).

Results adjusted (AD) for the most potential confounders are then chosen in Sections -1 to -3
and results adjusted for the least confounders in Sections -4 to -6. (Those least adjusted results which
actually differ from the most adjusted are marked 'x' in column X in Section -4)

Section -7 shows excluded studies, together with the stage (as above) at which no qualifying
results were found.

Section -8 lists the potentially overlapping studies which have been included (1=principal, 2=subsidiary).

Section -9 lists any results which would have been included in preference except that they had data not complete
enough for use in meta-analysis, with their significance (yes/no), if known, and any further comment as entered
on the database. It also lists as "gap" any categories for which no data were presented by the original authors.

In addition to those mentioned above, the following fields, levels and abbreviations are used:
* or nk = not known, n = no, y = yes, ot = other
nev = never
all/unspec = all or unspecified, cig+/-ot = cigarettes irrespective of other products (cigar, pipe etc)
MC = manufactured cigarettes, HR = hand-rolled cigarettes
exL, exH = range of exposure (low and high) in the smoking group, in terms of Age started
REF: 6-character study reference
NRR: number of the RR on the database within the study
ST : study type (CC = case control, pr or prosp = prospective)
NLC: number of lung cancer cases in whole study
R : risky occupational population (n = no, m = mining, o = other risky)
VB : national cigarette type (V = at least 75% Virginia, bl = at least 75% blended, ot = other)
P : any proxy use
H : full histological confirmation
De : derivation of RR/CI (or = original, st = standard method, ot = other method of estimation)

Table 1H7 - 1

IESLC - Meta-analysis of Current Smoking, Age started, "Low"
All LC types, Any Product (or Cigarettes if Any not available)
Most adjusted

REF	NRR	SEX	AGEL	AGEH	RACE	YF	LC TYPE	LOC	START	ST	NLC	R	VB	P	H	AD	PRODUCT	exL	exH	DENOM	De
CEDERL	510	m	0	0	all	10	all	Eu:Sca	1963	pr	491	n	bl	n	n	1	cig only	19	999	nev any	ot
CEDERL	515	f	0	0	all	10	all	Eu:Sca	1963	pr	491	n	bl	n	n	0	cig only	19	999	nev any	st
CPSI	801	m	35	84	all	6	all	NAmer	1959	pr	5138	n	bl	n	n	1	cig+/-ot	25	999	nev any	ot
CPSI	845	f	40	74	all	6	all	NAmer	1959	pr	5138	n	bl	n	n	1	cig only	25	999	nev any	ot
DEAN3	564	m	0	0	all	-	all	Eu:UK	1969	CC	766	n	V	y	n	0	cig only	25	999	nev any	st
DEAN3	583	f	0	0	all	-	all	Eu:UK	1969	CC	766	n	V	y	n	0	cig only	25	999	nev any	st
DORN	583	m	55	64	wh	8	all	NAmer	1954	pr	5097	n	bl	n	n	0	cig+/-ot	25	999	nev any	st
DORN	620	m	65	74	wh	8	all	NAmer	1954	pr	5097	n	bl	n	n	0	cig+/-ot	25	999	nev any	st
ENGELA	502	m	0	0	all	0	all	Eu:Sca	1964	pr	435	n	bl	n	n	0	cig+/-ot	20	29	nev cigs	st
ENGELA	510	f	0	0	all	0	all	Eu:Sca	1964	pr	435	n	bl	n	n	0	cig+/-ot	20	29	nev cigs	st
GAO2	502	m	0	0	all	-	all	As:Jap	1988	CC	282	n	bl	n	n	0	cig+/-ot	20	29	nev cigs	st
HIRAYA	501	m	0	0	all	0	all	As:Jap	1965	pr	1917	n	bl	n	n	1	cig+/-ot	20	999	nev any	st
HIRAYA	504	f	0	0	all	0	all	As:Jap	1965	pr	1917	n	bl	n	n	1	cig+/-ot	20	999	nev any	st
LIAW	504	c	0	0	all	0	all	As:oth	1982	pr	127	n	ot	n	n	2	all/unsp	25	999	nev any	or
MATOS	561	m	0	0	all	-	all	SCAmer	1994	CC	200	n	bl	n	n	2	cig+/-ot	20	999	nev any	or
MIGRAN	501	m	0	0	all	0	all	Eu:UK	1964	pr	259	n	V	n	n	0	cig only	20	999	nev any	st
MIGRAN	511	f	0	0	all	0	all	Eu:UK	1964	pr	259	n	V	n	n	0	cig only	20	999	nev any	st
MRFITR	508	m	0	0	all	0	all	NAmer	1973	pr	119	n	bl	n	n	0	cig+/-ot	24	999	nev cigs	ot
SEGI2	521	m	0	0	all	-	all	As:Jap	1962	CC	378	n	bl	n	n	1	cig+/-ot	23	999	nev any	ot
SOBUE	646	m	0	0	all	-	all	As:Jap	1986	CC	1376	n	bl	n	y	0	cig+/-ot	23	999	nev cigs	st
SVENSS	501	f	0	0	all	-	all	Eu:Sca	1983	CC	210	n	bl	n	n	0	all/unsp	26	999	nev any	st
WAKAI	502	m	0	0	all	-	all	As:Jap	1988	CC	333	n	bl	n	y	0	cig+/-ot	20	29	nev any	st
WU	541	f	0	0	wh	-	q+a	NAmer	1981	CC	220	n	bl	n	y	2	all/unsp	25	999	nev any	st

Cigarette type is all/unspec for all RRs
except for the following:

REF	NRR	CIGTYPE
DEAN3	564	MC only
DEAN3	583	MC only

Table 1H7 - 2

IESLC - Meta-analysis of Current Smoking, Age started, "Low"
All LC types, Any Product (or Cigarettes if Any not available)
Most adjusted

REF	NRR	SEX	AD	Number Exposed Case	Cont	Non-exposed Case	Cont	RR	95.00%CI
*CEDERL	510	m	1	11	-	7	-	6.50	(2.52- 16.74)
*CEDERL	515	f	0	6	2806	19	17679	1.99	(0.80- 4.98)
Subtotal CEDERL								3.53	(1.83- 6.82)
*CPSI	801	m	1	42	-	83	-	4.08	(2.81- 5.91)
*CPSI	845	f	1	51	-	166	-	2.25	(1.64- 3.08)
Subtotal CPSI								2.89	(2.27- 3.67)
DEAN3	564	m	0	24	75	24	510	6.80	(3.67- 12.58)
DEAN3	583	f	0	27	274	41	1538	3.70	(2.24- 6.11)
Subtotal DEAN3								4.72	(3.20- 6.96)
*DORN	583	m	0	30	49537	25	213858	5.18	(3.05- 8.81)
*DORN	620	m	0	70	49386	49	171211	4.95	(3.44- 7.13)
Subtotal DORN								5.02	(3.72- 6.79)
*ENGELA	502	m	0	50	30195	27	58716	3.60	(2.26- 5.75)
*ENGELA	510	f	0	36	29605	31	207789	8.15	(5.04- 13.17)
Subtotal ENGELA								5.36	(3.83- 7.49)
GAO2	502	m	0	127	85	13	56	6.44	(3.32- 12.49)
*HIRAYA	501	m	1	-	-	-	-	4.35	(3.51- 5.39)
*HIRAYA	504	f	1	-	-	-	-	2.46	(1.93- 3.13)
Subtotal HIRAYA								3.38	(2.88- 3.97)
*LIAW	504	c	2	-	-	-	-	1.50	(0.70- 3.30)
MATOS	561	m	2	18	-	11	-	5.30	(2.30- 12.50)
*MIGRAN	501	m	0	24	668	4	867	7.79	(2.72- 22.34)
*MIGRAN	511	f	0	11	1315	4	3814	7.98	(2.54- 25.01)
Subtotal MIGRAN								7.87	(3.63- 17.08)
*MRFITR	508	m	0	3	544	0	1859	23.91~	(1.24- 462.09)
SEGI2	521	m	1	49	-	8	-	2.04	(0.89- 4.66)
SOBUE	646	m	0	75	75	29	126	4.34	(2.60- 7.27)
SVENSS	501	f	0	32	18	38	120	5.61	(2.84- 11.12)
WAKAI	502	m	0	130	183	10	65	4.62	(2.29- 9.32)
WU	541	f	2	14	-	31	-	1.55	(0.60- 3.98)
Partial Totals				830	164766	620	678208		

*prospective study ~ With 0.5 adjustment for zero

REF	NRR	SEX	AD	Ys	Ws	Qs	Ps
*CEDERL	510	m	1	1.87	4.29	1.26	0.0001
*CEDERL	515	f	0	0.69	4.57	1.88	0.1414
Subtotal CEDERL				1.26	8.85	3.14	
*CPSI	801	m	1	1.41	27.80	0.16	0.0000
*CPSI	845	f	1	0.81	38.69	10.43	0.0000
Subtotal CPSI				1.06	66.49	10.59	
DEAN3	564	m	0	1.92	10.14	3.49	0.0000
DEAN3	583	f	0	1.31	15.21	0.01	0.0000
Subtotal DEAN3				1.55	25.35	3.50	
*DORN	583	m	0	1.64	13.64	1.35	0.0000
*DORN	620	m	0	1.60	28.85	2.10	0.0000
Subtotal DORN				1.61	42.49	3.45	
*ENGELA	502	m	0	1.28	17.55	0.04	0.0000
*ENGELA	510	f	0	2.10	16.67	9.83	0.0000
Subtotal ENGELA				1.68	34.22	9.87	
GAO2	502	m	0	1.86	8.74	2.47	0.0000
*HIRAYA	501	m	1	1.47	83.52	1.63	0.0000
*HIRAYA	504	f	1	0.90	65.73	12.16	0.0000
Subtotal HIRAYA				1.22	149.25	13.79	
*LIAW	504	c	2	0.41	6.39	5.47	0.3054
MATOS	561	m	2	1.67	5.36	0.61	0.0001
*MIGRAN	501	m	0	2.05	3.46	1.80	0.0001
*MIGRAN	511	f	0	2.08	2.94	1.64	0.0004
Subtotal MIGRAN				2.06	6.40	3.44	
*MRFITR	508	m	0	3.17	0.44	1.49	0.0357
SEGI2	521	m	1	0.71	5.61	2.14	0.0914
SOBUE	646	m	0	1.47	14.47	0.28	0.0000
SVENSS	501	f	0	1.73	8.23	1.28	0.0000
WAKAI	502	m	0	1.53	7.78	0.31	0.0000
WU	541	f	2	0.44	4.29	3.42	0.3639

Table 1H7 - 2

IESLC - Meta-analysis of Current Smoking, Age started, "Low"
All LC types, Any Product (or Cigarettes if Any not available)
Most adjusted

	N	23
	NS	16
	Wt	394.36
Het	Chi	65.25
Het	df	22
Het	P	***
Fixed	RR	3.78
	RRl	3.43
	RRu	4.17
	P	+++
Random	RR	4.05
	RRl	3.34
	RRu	4.93
	P	+++
Asymm	P	N.S.

Table 1H7 - 3

IESLC - Meta-analysis of Current Smoking, Age started, "Low"
All LC types, Any Product (or Cigarettes if Any not available)
Most adjusted

	Sex: combined	male	female	Total
N	1	14	8	23
NS	1	13	8	22
Wt	6.39	231.64	156.33	394.36
Het Chi	0.00	11.10	31.79	65.25
Het df	0	13	7	22
Het P	N.S.	N.S.	***	***
Fixed RR	1.50	4.56	2.98	3.78
RRl	0.69	4.01	2.55	3.43
RRu	3.26	5.18	3.49	4.17
P	N.S.	+++	+++	+++
Random RR	1.50	4.56	3.45	4.05
RRl	0.69	4.01	2.33	3.34
RRu	3.26	5.18	5.11	4.93
P	N.S.	+++	+++	+++
Between Chi				22.36
Between df				2
Between P				***
Btwn(F) P				*
Btwn(R) P				*

	Lung cancer type: all	other	Total
N	22	1	23
NS	15	1	16
Wt	390.07	4.29	394.36
Het Chi	61.79	0.00	65.25
Het df	21	0	22
Het P	***	N.S.	***
Fixed RR	3.82	1.55	3.78
RRl	3.46	0.60	3.43
RRu	4.22	3.99	4.17
P	+++	N.S.	+++
Random RR	4.16	1.55	4.05
RRl	3.43	0.60	3.34
RRu	5.06	3.99	4.93
P	+++	N.S.	+++
Between Chi			3.45
Between df			1
Between P			(*)
Btwn(F) P			N.S.
Btwn(R) P			*

	Location: NAmer	UK	Scand	othEur	China	Japan	othAs	other	Total
N	6	4	5			6	1	1	23
NS	4	2	3			5	1	1	16
Wt	113.70	31.76	51.30			185.85	6.39	5.36	394.36
Het Chi	18.24	3.60	10.15			18.23	0.00	0.00	65.25
Het df	5	3	4			5	0	0	22
Het P	**	N.S.	*			**	N.S.	N.S.	***
Fixed RR	3.50	5.23	5.02			3.55	1.50	5.30	3.78
RRl	2.91	3.69	3.82			3.07	0.69	2.27	3.43
RRu	4.20	7.41	6.61			4.10	3.26	12.36	4.17
P	+++	+++	+++			+++	N.S.	+++	+++
Random RR	3.59	5.40	4.82			3.73	1.50	5.30	4.05
RRl	2.39	3.63	3.03			2.67	0.69	2.27	3.34
RRu	5.39	8.05	7.68			5.21	3.26	12.36	4.93
P	+++	+++	+++			+++	N.S.	+++	+++
Between Chi									15.01
Between df									5
Between P									*
Btwn(F) P									N.S.
Btwn(R) P									(*)

Table 1H7 - 3

IESLC - Meta-analysis of Current Smoking, Age started, "Low"
All LC types, Any Product (or Cigarettes if Any not available)
Most adjusted

Detailed Country in "other Europe"

	multi	Germany	othWest	East	Balkans	Total
N						
NS						
Wt						
Het Chi						
Het df						
Het P						**
Fixed RR						
RRl						
RRu						
P						+++
Random RR						
RRl						
RRu						
P						+++
Between Chi						
Between df						
Between P						N.S.
Btwn(F) P						N.S.
Btwn(R) P						N.S.

Detailed Country in "other Asia"

	India	HongKong	other	Total
N			1	1
NS			1	1
Wt			6.39	6.39
Het Chi			0.00	0.00
Het df			0	0
Het P			N.S.	N.S.
Fixed RR			1.50	1.50
RRl			0.69	0.69
RRu			3.26	3.26
P			N.S.	N.S.
Random RR			1.50	1.50
RRl			0.69	0.69
RRu			3.26	3.26
P			N.S.	N.S.
Between Chi				
Between df				
Between P				N.S.
Btwn(F) P				N.S.
Btwn(R) P				N.S.

Detailed other continent

	SCAmer	Total
N	1	1
NS	1	1
Wt	5.36	5.36
Het Chi	0.00	0.00
Het df	0	0
Het P	N.S.	N.S.
Fixed RR	5.30	5.30
RRl	2.27	2.27
RRu	12.36	12.36
P	+++	+++
Random RR	5.30	5.30
RRl	2.27	2.27
RRu	12.36	12.36
P	+++	+++
Between Chi		
Between df		
Between P		N.S.
Btwn(F) P		N.S.
Btwn(R) P		N.S.

Table 1H7 - 3

IESLC - Meta-analysis of Current Smoking, Age started, "Low"
All LC types, Any Product (or Cigarettes if Any not available)
Most adjusted

	Start year of study					
	<1960	1960-69	1970-79	1980-89	1990+	Total
N	4	11	1	6	1	23
NS	2	6	1	6	1	16
Wt	108.97	229.68	0.44	49.91	5.36	394.36
Het Chi	13.72	35.87	0.00	13.17	0.00	65.25
Het df	3	10	0	5	0	22
Het P	**	***	N.S.	*	N.S.	***
Fixed RR	3.58	3.81	23.91	3.91	5.30	3.78
RRl	2.97	3.35	1.24	2.97	2.27	3.43
RRu	4.32	4.33	462.09	5.17	12.36	4.17
P	+++	+++	+	+++	+++	+++
Random RR	3.84	4.23	23.91	3.66	5.30	4.05
RRl	2.54	3.15	1.24	2.30	2.27	3.34
RRu	5.79	5.69	462.09	5.80	12.36	4.93
P	+++	+++	+	+++	+++	+++
Between Chi						2.49
Between df						4
Between P						N.S.
Btwn(F) P						N.S.
Btwn(R) P						N.S.

	Study type (1)		
	CC	other	Total
N	9	14	23
NS	8	8	16
Wt	79.84	314.52	394.36
Het Chi	12.32	50.82	65.25
Het df	8	13	22
Het P	N.S.	***	***
Fixed RR	4.37	3.65	3.78
RRl	3.51	3.26	3.43
RRu	5.44	4.07	4.17
P	+++	+++	+++
Random RR	4.31	3.95	4.05
RRl	3.26	3.06	3.34
RRu	5.70	5.11	4.93
P	+++	+++	+++
Between Chi			2.11
Between df			1
Between P			N.S.
Btwn(F) P			N.S.
Btwn(R) P			N.S.

	Study type (2)			
	CC	prosp	other	Total
N	9	14		23
NS	8	8		16
Wt	79.84	314.52		394.36
Het Chi	12.32	50.82		65.25
Het df	8	13		22
Het P	N.S.	***		***
Fixed RR	4.37	3.65		3.78
RRl	3.51	3.26		3.43
RRu	5.44	4.07		4.17
P	+++	+++		+++
Random RR	4.31	3.95		4.05
RRl	3.26	3.06		3.34
RRu	5.70	5.11		4.93
P	+++	+++		+++
Between Chi				2.11
Between df				1
Between P				N.S.
Btwn(F) P				N.S.
Btwn(R) P				N.S.

Table 1H7 - 3

IESLC - Meta-analysis of Current Smoking, Age started, "Low"
All LC types, Any Product (or Cigarettes if Any not available)
Most adjusted

	Study size (number of LC cases) 100-249	250-499	500-999	1000+	Total
N	5	9	2	7	23
NS	5	6	1	4	16
Wt	24.72	71.60	25.35	272.69	394.36
Het Chi	11.66	16.46	2.26	26.57	65.25
Het df	4	8	1	6	22
Het P	*	*	N.S.	***	***
Fixed RR	3.23	4.91	4.72	3.51	3.78
RRl	2.18	3.90	3.20	3.12	3.43
RRu	4.80	6.20	6.96	3.95	4.17
P	+++	+++	+++	+++	+++
Random RR	3.28	4.81	4.88	3.68	4.05
RRl	1.58	3.38	2.69	2.82	3.34
RRu	6.83	6.85	8.85	4.80	4.93
P	++	+++	+++	+++	+++
Between Chi					8.30
Between df					3
Between P					*
Btwn(F) P					N.S.
Btwn(R) P					N.S.

	Risky occupational population no	mining	othRisky	Total
N	23			23
NS	16			16
Wt	394.36			394.36
Het Chi	65.25			65.25
Het df	22			22
Het P	***			***
Fixed RR	3.78			3.78
RRl	3.43			3.43
RRu	4.17			4.17
P	+++			+++
Random RR	4.05			4.05
RRl	3.34			3.34
RRu	4.93			4.93
P	+++			+++
Between Chi				
Between df				
Between P				N.S.
Btwn(F) P				N.S.
Btwn(R) P				N.S.

	National cigarette tobacco type Virginia	blended	other	Total
N	4	18	1	23
NS	2	13	1	16
Wt	31.76	356.21	6.39	394.36
Het Chi	3.60	52.79	0.00	65.25
Het df	3	17	0	22
Het P	N.S.	***	N.S.	***
Fixed RR	5.23	3.74	1.50	3.78
RRl	3.69	3.37	0.69	3.43
RRu	7.41	4.14	3.26	4.17
P	+++	+++	N.S.	+++
Random RR	5.40	3.99	1.50	4.05
RRl	3.63	3.23	0.69	3.34
RRu	8.05	4.92	3.26	4.93
P	+++	+++	N.S.	+++
Between Chi				8.86
Between df				2
Between P				*
Btwn(F) P				N.S.
Btwn(R) P				*

Table 1H7 - 3

IESLC - Meta-analysis of Current Smoking, Age started, "Low"
All LC types, Any Product (or Cigarettes if Any not available)
Most adjusted

	Any proxy use No/nk	Yes	Total
N	21	2	23
NS	15	1	16
Wt	369.01	25.35	394.36
Het Chi	61.66	2.26	65.25
Het df	20	1	22
Het P	***	N.S.	***
Fixed RR	3.73	4.72	3.78
RRl	3.36	3.20	3.43
RRu	4.13	6.96	4.17
P	+++	+++	+++
Random RR	3.98	4.88	4.05
RRl	3.23	2.69	3.34
RRu	4.89	8.85	4.93
P	+++	+++	+++
Between Chi			1.32
Between df			1
Between P			N.S.
Btwn(F) P			N.S.
Btwn(R) P			N.S.

	Full histological confirmation No	Yes	Total
N	20	3	23
NS	13	3	16
Wt	367.81	26.55	394.36
Het Chi	61.24	4.00	65.25
Het df	19	2	22
Het P	***	N.S.	***
Fixed RR	3.78	3.74	3.78
RRl	3.42	2.56	3.43
RRu	4.19	5.48	4.17
P	+++	+++	+++
Random RR	4.14	3.48	4.05
RRl	3.35	1.96	3.34
RRu	5.12	6.16	4.93
P	+++	+++	+++
Between Chi			0.00
Between df			1
Between P			N.S.
Btwn(F) P			N.S.
Btwn(R) P			N.S.

	Number of adjustment variables (1) 0	1	2+/+nk	Total
N	14	6	3	23
NS	10	4	3	17
Wt	152.69	225.62	16.04	394.36
Het Chi	15.51	22.13	5.57	65.25
Het df	13	5	2	22
Het P	N.S.	***	(*)	***
Fixed RR	5.03	3.23	2.31	3.78
RRl	4.29	2.83	1.41	3.43
RRu	5.90	3.68	3.76	4.17
P	+++	+++	+++	+++
Random RR	5.05	3.20	2.31	4.05
RRl	4.22	2.33	1.02	3.34
RRu	6.04	4.39	5.27	4.93
P	+++	+++	+	+++
Between Chi				22.04
Between df				2
Between P				***
Btwn(F) P				*
Btwn(R) P				*

Table 1H7 - 3

IESLC - Meta-analysis of Current Smoking, Age started, "Low"
All LC types, Any Product (or Cigarettes if Any not available)
Most adjusted

	Number of adjustment variables (2) 0	1	2	3-5	6+/+nk	Total
N	14	6	3			23
NS	10	4	3			17
Wt	152.69	225.62	16.04			394.36
Het Chi	15.51	22.13	5.57			65.25
Het df	13	5	2			22
Het P	N.S.	***	(*)			***
Fixed RR	5.03	3.23	2.31			3.78
RRl	4.29	2.83	1.41			3.43
RRu	5.90	3.68	3.76			4.17
P	+++	+++	+++			+++
Random RR	5.05	3.20	2.31			4.05
RRl	4.22	2.33	1.02			3.34
RRu	6.04	4.39	5.27			4.93
P	+++	+++	+			+++
Between Chi						22.04
Between df						2
Between P						***
Btwn(F) P						*
Btwn(R) P						*

	Product all/unsp	cig+/-ot	cig only	Total
N	3	13	7	23
NS	3	10	4	17
Wt	18.92	296.15	79.30	394.36
Het Chi	7.94	33.60	19.15	65.25
Het df	2	12	6	22
Het P	*	***	**	***
Fixed RR	2.68	4.00	3.32	3.78
RRl	1.71	3.57	2.66	3.43
RRu	4.21	4.49	4.13	4.17
P	+++	+++	+++	+++
Random RR	2.43	4.34	4.25	4.05
RRl	0.97	3.49	2.68	3.34
RRu	6.07	5.40	6.74	4.93
P	(+)	+++	+++	+++
Between Chi				4.55
Between df				2
Between P				N.S.
Btwn(F) P				N.S.
Btwn(R) P				N.S.

	Denominator nev any	nev cigs	Total
N	18	5	23
NS	12	4	16
Wt	336.49	57.87	394.36
Het Chi	50.02	7.60	65.25
Het df	17	4	22
Het P	***	N.S.	***
Fixed RR	3.57	5.29	3.78
RRl	3.21	4.09	3.43
RRu	3.97	6.84	4.17
P	+++	+++	+++
Random RR	3.75	5.42	4.05
RRl	3.03	3.69	3.34
RRu	4.64	7.96	4.93
P	+++	+++	+++
Between Chi			7.62
Between df			1
Between P			**
Btwn(F) P			N.S.
Btwn(R) P			N.S.

Table 1H7 - 3

IESLC - Meta-analysis of Current Smoking, Age started, "Low"
All LC types, Any Product (or Cigarettes if Any not available)
Most adjusted

	Derivation of RR/CI Orig	StdCalc	Other	Total
N	2	16	5	23
NS	2	11	4	17
Wt	11.75	305.79	76.82	394.36
Het Chi	4.65	42.05	11.11	65.25
Het df	1	15	4	22
Het P	*	***	*	***
Fixed RR	2.67	4.07	2.98	3.78
RRl	1.51	3.64	2.38	3.43
RRu	4.73	4.55	3.73	4.17
P	+++	+++	+++	+++
Random RR	2.79	4.43	3.32	4.05
RRl	0.81	3.58	2.06	3.34
RRu	9.60	5.50	5.34	4.93
P	N.S.	+++	+++	+++
Between Chi				7.44
Between df				2
Between P				*
Btwn(F) P				N.S.
Btwn(R) P				N.S.

Table 1H7 - 4

IESLC - Meta-analysis of Current Smoking, Age started, "Low"
All LC types, Any Product (or Cigarettes if Any not available)
Least adjusted

REF	NRR	X	SEX	AGEL	AGEH	RACE	YF	LC TYPE	LOC	START	ST	NLC	R	VB	P	H	AD	PRODUCT	exL	exH	DENOM	De
CEDERL	510		m	0	0	all	10	all	Eu:Sca	1963	pr	491	n	bl	n	n	1	cig only	19	999	nev any	ot
CEDERL	515		f	0	0	all	10	all	Eu:Sca	1963	pr	491	n	bl	n	n	0	cig only	19	999	nev any	st
CPSI	801		m	35	84	all	6	all	NAmer	1959	pr	5138	n	bl	n	n	1	cig+/-ot	25	999	nev any	ot
CPSI	845		f	40	74	all	6	all	NAmer	1959	pr	5138	n	bl	n	n	1	cig only	25	999	nev any	ot
DEAN3	564		m	0	0	all	-	all	Eu:UK	1969	CC	766	n	V	y	n	0	cig only	25	999	nev any	st
DEAN3	583		f	0	0	all	-	all	Eu:UK	1969	CC	766	n	V	y	n	0	cig only	25	999	nev any	st
DORN	583		m	55	64	wh	8	all	NAmer	1954	pr	5097	n	bl	n	n	0	cig+/-ot	25	999	nev any	st
DORN	620		m	65	74	wh	8	all	NAmer	1954	pr	5097	n	bl	n	n	0	cig+/-ot	25	999	nev any	st
ENGELA	502		m	0	0	all	0	all	Eu:Sca	1964	pr	435	n	bl	n	n	0	cig+/-ot	20	29	nev cigs	st
ENGELA	510		f	0	0	all	0	all	Eu:Sca	1964	pr	435	n	bl	n	n	0	cig+/-ot	20	29	nev cigs	st
GAO2	502		m	0	0	all	-	all	As:Jap	1988	CC	282	n	bl	n	n	0	cig+/-ot	20	29	nev cigs	st
HIRAYA	501		m	0	0	all	0	all	As:Jap	1965	pr	1917	n	bl	n	n	1	cig+/-ot	20	999	nev any	st
HIRAYA	504		f	0	0	all	0	all	As:Jap	1965	pr	1917	n	bl	n	n	1	cig+/-ot	20	999	nev any	st
LIAW	504		c	0	0	all	0	all	As:oth	1982	pr	127	n	ot	n	n	2	all/unsp	25	999	nev any	or
MATOS	541	x	m	0	0	all	-	all	SCAmer	1994	CC	200	n	bl	n	n	0	cig+/-ot	20	999	nev any	st
MIGRAN	501		m	0	0	all	0	all	Eu:UK	1964	pr	259	n	V	n	n	0	cig only	20	999	nev any	st
MIGRAN	511		f	0	0	all	0	all	Eu:UK	1964	pr	259	n	V	n	n	0	cig only	20	999	nev any	st
MRFITR	508		m	0	0	all	0	all	NAmer	1973	pr	119	n	bl	n	n	0	cig+/-ot	24	999	nev cigs	ot
SEGI2	516	x	m	0	0	all	-	all	As:Jap	1962	CC	378	n	bl	n	n	0	cig+/-ot	23	999	nev any	st
SOBUE	646		m	0	0	all	-	all	As:Jap	1986	CC	1376	n	bl	n	y	0	cig+/-ot	23	999	nev cigs	st
SVENSS	501		f	0	0	all	-	all	Eu:Sca	1983	CC	210	n	bl	n	n	0	all/unsp	26	999	nev any	st
WAKAI	502		m	0	0	all	-	all	As:Jap	1988	CC	333	n	bl	n	y	0	cig+/-ot	20	29	nev any	st
WU	517	x	f	0	0	wh	-	q+a	NAmer	1981	CC	220	n	bl	n	y	0	all/unsp	25	999	nev any	st

Cigarette type is all/unspec for all RRs
except for the following:

REF	NRR	CIGTYPE
DEAN3	564	MC only
DEAN3	583	MC only

Table 1H7 - 5

IESLC - Meta-analysis of Current Smoking, Age started, "Low"
All LC types, Any Product (or Cigarettes if Any not available)
Least adjusted

REF	NRR	SEX	AD	Number Exposed Case	Exposed Cont	Non-exposed Case	Non-exposed Cont	RR	95.00%CI
*CEDERL	510	m	1	11	-	7	-	6.50	(2.52- 16.74)
*CEDERL	515	f	0	6	2806	19	17679	1.99	(0.80- 4.98)
Subtotal CEDERL								3.53	(1.83- 6.82)
*CPSI	801	m	1	42	-	83	-	4.08	(2.81- 5.91)
*CPSI	845	f	1	51	-	166	-	2.25	(1.64- 3.08)
Subtotal CPSI								2.89	(2.27- 3.67)
DEAN3	564	m	0	24	75	24	510	6.80	(3.67- 12.58)
DEAN3	583	f	0	27	274	41	1538	3.70	(2.24- 6.11)
Subtotal DEAN3								4.72	(3.20- 6.96)
*DORN	583	m	0	30	49537	25	213858	5.18	(3.05- 8.81)
*DORN	620	m	0	70	49386	49	171211	4.95	(3.44- 7.13)
Subtotal DORN								5.02	(3.72- 6.79)
*ENGELA	502	m	0	50	30195	27	58716	3.60	(2.26- 5.75)
*ENGELA	510	f	0	36	29605	31	207789	8.15	(5.04- 13.17)
Subtotal ENGELA								5.36	(3.83- 7.49)
GAO2	502	m	0	127	85	13	56	6.44	(3.32- 12.49)
*HIRAYA	501	m	1	-	-	-	-	4.35	(3.51- 5.39)
*HIRAYA	504	f	1	-	-	-	-	2.46	(1.93- 3.13)
Subtotal HIRAYA								3.38	(2.88- 3.97)
*LIAW	504	c	2	-	-	-	-	1.50	(0.70- 3.30)
MATOS	541	m	0	18	33	11	110	5.45	(2.34- 12.70)
*MIGRAN	501	m	0	24	668	4	867	7.79	(2.72- 22.34)
*MIGRAN	511	f	0	11	1315	4	3814	7.98	(2.54- 25.01)
Subtotal MIGRAN								7.87	(3.63- 17.08)
*MRFITR	508	m	0	3	544	0	1859	23.91~	(1.24- 462.09)
SEGI2	516	m	0	49	155	8	53	2.09	(0.93- 4.71)
SOBUE	646	m	0	75	75	29	126	4.34	(2.60- 7.27)
SVENSS	501	f	0	32	18	38	120	5.61	(2.84- 11.12)
WAKAI	502	m	0	130	183	10	65	4.62	(2.29- 9.32)
WU	517	f	0	14	19	31	92	2.19	(0.98- 4.87)
Partial Totals				830	164973	620	678463		

*prospective study ~ With 0.5 adjustment for zero

REF	NRR	SEX	AD	Ys	Ws	Qs	Ps
*CEDERL	510	m	1	1.87	4.29	1.25	0.0001
*CEDERL	515	f	0	0.69	4.57	1.90	0.1414
Subtotal CEDERL				1.26	8.85	3.14	
*CPSI	801	m	1	1.41	27.80	0.15	0.0000
*CPSI	845	f	1	0.81	38.69	10.51	0.0000
Subtotal CPSI				1.06	66.49	10.66	
DEAN3	564	m	0	1.92	10.14	3.47	0.0000
DEAN3	583	f	0	1.31	15.21	0.01	0.0000
Subtotal DEAN3				1.55	25.35	3.48	
*DORN	583	m	0	1.64	13.64	1.34	0.0000
*DORN	620	m	0	1.60	28.85	2.07	0.0000
Subtotal DORN				1.61	42.49	3.40	
*ENGELA	502	m	0	1.28	17.55	0.05	0.0000
*ENGELA	510	f	0	2.10	16.67	9.78	0.0000
Subtotal ENGELA				1.68	34.22	9.83	
GAO2	502	m	0	1.86	8.74	2.45	0.0000
*HIRAYA	501	m	1	1.47	83.52	1.59	0.0000
*HIRAYA	504	f	1	0.90	65.73	12.26	0.0000
Subtotal HIRAYA				1.22	149.25	13.85	
*LIAW	504	c	2	0.41	6.39	5.49	0.3054
MATOS	541	m	0	1.70	5.38	0.71	0.0001
*MIGRAN	501	m	0	2.05	3.46	1.80	0.0001
*MIGRAN	511	f	0	2.08	2.94	1.63	0.0004
Subtotal MIGRAN				2.06	6.40	3.43	
*MRFITR	508	m	0	3.17	0.44	1.49	0.0357
SEGI2	516	m	0	0.74	5.86	2.06	0.0736
SOBUE	646	m	0	1.47	14.47	0.27	0.0000
SVENSS	501	f	0	1.73	8.23	1.27	0.0000
WAKAI	502	m	0	1.53	7.78	0.30	0.0000
WU	517	f	0	0.78	5.98	1.81	0.0557

Table 1H7 - 5

IESLC - Meta-analysis of Current Smoking, Age started, "Low"
All LC types, Any Product (or Cigarettes if Any not available)
Least adjusted

	N	23
	NS	16
	Wt	396.32
Het	Chi	63.65
Het	df	22
Het	P	***
Fixed	RR	3.79
	RRl	3.43
	RRu	4.18
	P	+++
Random	RR	4.08
	RRl	3.37
	RRu	4.94
	P	+++
Asymm	P	N.S.

Table 1H7 - 6

IESLC - Meta-analysis of Current Smoking, Age started, "Low"
All LC types, Any Product (or Cigarettes if Any not available)
Least adjusted

	combined	Sex<br>male	female	Total
N	1	14	8	23
NS	1	13	8	22
Wt	6.39	231.91	158.02	396.32
Het Chi	0.00	11.07	30.52	63.65
Het df	0	13	7	22
Het P	N.S.	N.S.	***	***
Fixed RR	1.50	4.56	3.00	3.79
RRl	0.69	4.01	2.57	3.43
RRu	3.26	5.18	3.50	4.18
P	N.S.	+++	+++	+++
Random RR	1.50	4.56	3.53	4.08
RRl	0.69	4.01	2.42	3.37
RRu	3.26	5.18	5.16	4.94
P	N.S.	+++	+++	+++
Between Chi				22.06
Between df				2
Between P				***
Btwn(F) P				*
Btwn(R) P				*

Table 1H7 - 7

IESLC - Meta-analysis of Current Smoking, Age started, "Low"
All LC types, Any Product (or Cigarettes if Any not available)
Excluded studies (and stage at which they were excluded)

1	AGUDO ALDERS ARMADA AUVINE AXELSS BARBON BECHER BENHAM BLOT1 BOFFET BOUCHA BRESLO BROWN3 CARPEN CHEN CHEN2 CHIAZZ CHOI CHYOU CORREA DAMBER DARBY DESTEF DOLL DOLL2 DORGAN DOSEME FAN GAO GARCIA GARSHI GENG GER GRAHAM GUO GURSEL HAENSZ HAMMO2 HAMMON HEGMAN HU HU2 JAHN JAIN JEDRYC JOLY JUSSAW KHUDER KOO KOULUM KREUZE LAUSSM LETOUR LEVIN LIU3 LIU4 LIU5 LUBIN LUBIN2 LUO MCCONN NOTAN2 OSANN2 PERNU PEZZOT PRESCO QIAO QIAO2 RACHTA RESTRE SADOWS STASZE SUZUK2 TIZZAN TVERDA VUTUC WANG2 WIGLE WU2 WUWILL WYNDE2 WYNDE3 XU YUAN ZHANG ZHENG ZHOU
2	AKIBA AMANDU AMES BENSHL BEST BOUCOT BROSS BUFFLE CPSII DEAN2 GILLIS HUMBLE KAISE2 KATSOU KAUFMA PEZZO2 PISANI SPITZ WATSON WYNDE8
3	MCDUFF WYNDE6
5	HOLE
7	WYNDE7
10	SPEIZE

Table 1H7 - 8
Potentially overlapping studies

REF	REFGP	PRINC	OVERLAP/LINK
MRFITR	MRFIT	2	Subset of MRFIT
CPSI	CPSI	1	CPSI overall

Table 1H8 -

IESLC - Meta-analysis of Current Smoking, Age started, "Mid"
All LC types, Any Product (or Cigarettes if Any not available)

This analysis is restricted to results for:
1) Current smokers
2) Results by Age started
3) Categorical results by Age started
4) All LC types (or near equivalent)
5) Results complete enough for use in metaanalysis

Within each study, results are then selected (in the following order of preference, within each sex) for:
6) (not applicable)
7) PRODUCT: all/unspec, cigarettes regardless of other products, cigarettes only
8) CIGTYPE: all/unspecified, MC regardless of HR, MC only
9) (not applicable)
10) DENOM: never smoked anything, never smoked cigarettes, never any + low, never cigs + low
11) Followup period (YF, prospective studies): whole study (coded as 0) or longest available
12) LCtype: all or nearest available, at least Squamous and Adeno. (q = squamous, s = small,
l = large, a = adeno, mix = mixed, alv = alveolar)
13) Race: all or nearest available, otherwise by race (wh or w = white, bl or b = black, hi = hispanic
ch = chinese, jap = japanese, haw = hawaiian, w+o = white + oriental, sca = scandinavian, as = asian)
14) Age started "mid" in key scheme 1 (key value 18, maximum range 15-25)
15) For overlapping studies: principal rather than subsidiary studies
Finally by Age: whole study (coded as 0) if available, otherwise by widest available age group
and then for single sex results (m, f) in preference to results for both sexes combined (c).

Results adjusted (AD) for the most potential confounders are then chosen in Sections -1 to -3
and results adjusted for the least confounders in Sections -4 to -6. (Those least adjusted results which
actually differ from the most adjusted are marked 'x' in column X in Section -4)

Section -7 shows excluded studies, together with the stage (as above) at which no qualifying
results were found.

Section -8 lists the potentially overlapping studies which have been included (1=principal, 2=subsidiary).

Section -9 lists any results which would have been included in preference except that they had data not complete
enough for use in meta-analysis, with their significance (yes/no), if known, and any further comment as entered
on the database. It also lists as "gap" any categories for which no data were presented by the original authors.

In addition to those mentioned above, the following fields, levels and abbreviations are used:
* or nk = not known, n = no, y = yes, ot = other
nev = never
all/unspec = all or unspecified, cig+/-ot = cigarettes irrespective of other products (cigar, pipe etc)
MC = manufactured cigarettes, HR = hand-rolled cigarettes
exL, exH = range of exposure (low and high) in the smoking group, in terms of Age started
REF: 6-character study reference
NRR: number of the RR on the database within the study
ST : study type (CC = case control, pr or prosp = prospective)
NLC: number of lung cancer cases in whole study
R : risky occupational population (n = no, m = mining, o = other risky)
VB : national cigarette type (V = at least 75% Virginia, bl = at least 75% blended, ot = other)
P : any proxy use
H : full histological confirmation
De : derivation of RR/CI (or = original, st = standard method, ot = other method of estimation)

Table 1H8 - 1

IESLC - Meta-analysis of Current Smoking, Age started, "Mid"
All LC types, Any Product (or Cigarettes if Any not available)
Most adjusted

REF	NRR	SEX	AGEL	AGEH	RACE	YF	LC TYPE	LOC	START	ST	NLC	R	VB	P	H	AD	PRODUCT	exL	exH	DENOM	De
CEDERL	511	m	0	0	all	10	all	Eu:Sca	1963	pr	491	n	bl	n	n	1	cig only	17	18	nev any	ot
CEDERL	516	f	0	0	all	10	all	Eu:Sca	1963	pr	491	n	bl	n	n	0	cig only	17	18	nev any	st
CPSI	803	m	35	84	all	6	all	NAmer	1959	pr	5138	n	bl	n	n	1	cig+/-ot	15	19	nev any	ot
CPSI	847	f	40	74	all	6	all	NAmer	1959	pr	5138	n	bl	n	n	1	cig only	15	19	nev any	ot
DEAN3	566	m	0	0	all	-	all	Eu:UK	1969	CC	766	n	V	y	n	0	cig only	15	19	nev any	st
DEAN3	585	f	0	0	all	-	all	Eu:UK	1969	CC	766	n	V	y	n	0	cig only	15	19	nev any	st
DORN	585	m	55	64	wh	8	all	NAmer	1954	pr	5097	n	bl	n	n	0	cig+/-ot	15	19	nev any	st
DORN	622	m	65	74	wh	8	all	NAmer	1954	pr	5097	n	bl	n	n	0	cig+/-ot	15	19	nev any	st
MATOS	562	m	0	0	all	-	all	SCAmer	1994	CC	200	n	bl	n	n	2	cig+/-ot	15	19	nev any	or
MIGRAN	503	m	0	0	all	0	all	Eu:UK	1964	pr	259	n	V	n	n	0	cig only	16	19	nev any	st
MIGRAN	513	f	0	0	all	0	all	Eu:UK	1964	pr	259	n	V	n	n	0	cig only	16	19	nev any	st
MRFITR	511	m	0	0	all	0	all	NAmer	1973	pr	119	n	bl	n	n	0	cig+/-ot	18	19	nev cigs	ot
SOBUE	647	m	0	0	all	-	all	As:Jap	1986	CC	1376	n	bl	n	y	0	cig+/-ot	18	22	nev cigs	st

Cigarette type is all/unspec for all RRs
except for the following:

REF	NRR	CIGTYPE
DEAN3	566	MC only
DEAN3	585	MC only

Table 1H8 - 2

IESLC - Meta-analysis of Current Smoking, Age started, "Mid"
All LC types, Any Product (or Cigarettes if Any not available)
Most adjusted

REF	NRR	SEX	AD	Number Exposed Case	Exposed Cont	Non-exposed Case	Non-exposed Cont	RR	95.00%CI
*CEDERL	511	m	1	10	-	7	-	9.80	(3.74- 25.69)
*CEDERL	516	f	0	2	1009	19	17679	1.84	(0.43- 7.91)
Subtotal CEDERL								5.89	(2.64- 13.16)
*CPSI	803	m	1	588	-	83	-	14.69	(11.68- 18.49)
*CPSI	847	f	1	52	-	166	-	5.00	(3.66- 6.83)
Subtotal CPSI								10.06	(8.36- 12.10)
DEAN3	566	m	0	160	485	24	510	7.01	(4.48- 10.96)
DEAN3	585	f	0	39	504	41	1538	2.90	(1.85- 4.55)
Subtotal DEAN3								4.52	(3.30- 6.21)
*DORN	585	m	0	293	154204	25	213858	16.25	(10.81- 24.45)
*DORN	622	m	0	259	80043	49	171211	11.31	(8.33- 15.34)
Subtotal DORN								12.88	(10.08- 16.44)
MATOS	562	m	2	49	-	11	-	8.60	(4.10- 18.20)
*MIGRAN	503	m	0	59	1845	4	867	6.93	(2.53- 19.02)
*MIGRAN	513	f	0	9	1035	4	3814	8.29	(2.56- 26.87)
Subtotal MIGRAN								7.48	(3.48- 16.09)
*MRFITR	511	m	0	25	1876	0	1859	50.54~	(3.08- 829.51)
SOBUE	647	m	0	553	490	29	126	4.90	(3.22- 7.47)
Partial Totals				2098	241491	462	411462		

*prospective study ~ With 0.5 adjustment for zero

REF	NRR	SEX	AD	Ys	Ws	Qs	Ps
*CEDERL	511	m	1	2.28	4.14	0.05	0.0000
*CEDERL	516	f	0	0.61	1.81	4.40	0.4098
Subtotal CEDERL				1.77	5.95	4.45	
*CPSI	803	m	1	2.69	72.82	19.52	0.0000
*CPSI	847	f	1	1.61	39.48	12.38	0.0000
Subtotal CPSI				2.31	112.30	31.90	
DEAN3	566	m	0	1.95	19.25	0.95	0.0000
DEAN3	585	f	0	1.07	18.99	23.13	0.0000
Subtotal DEAN3				1.51	38.24	24.08	
*DORN	585	m	0	2.79	23.04	8.83	0.0000
*DORN	622	m	0	2.43	41.24	2.70	0.0000
Subtotal DORN				2.56	64.28	11.53	
MATOS	562	m	2	2.15	6.92	0.00	0.0000
*MIGRAN	503	m	0	1.94	3.77	0.21	0.0002
*MIGRAN	513	f	0	2.12	2.78	0.01	0.0004
Subtotal MIGRAN				2.01	6.55	0.21	
*MRFITR	511	m	0	3.92	0.49	1.51	0.0060
SOBUE	647	m	0	1.59	21.61	7.26	0.0000

	N	13
	NS	8
	Wt	256.34
Het	Chi	80.94
Het	df	12
Het	P	***
Fixed	RR	8.75
	RRl	7.74
	RRu	9.89
	P	+++
Random	RR	7.57
	RRl	5.24
	RRu	10.93
	P	+++
Asymm	P	N.S.

Table 1H8 - 3

IESLC - Meta-analysis of Current Smoking, Age started, "Mid"
All LC types, Any Product (or Cigarettes if Any not available)
Most adjusted

	Sex			
	combined	male	female	Total
N		9	4	13
NS		8	4	12
Wt		193.28	63.06	256.34
Het Chi		30.05	6.30	80.94
Het df		8	3	12
Het P		***	(*)	***
Fixed RR		11.11	4.22	8.75
RRl		9.65	3.30	7.74
RRu		12.79	5.40	9.89
P		+++	+++	+++
Random RR		9.86	4.02	7.57
RRl		7.12	2.53	5.24
RRu		13.64	6.37	10.93
P		+++	+++	+++
Between Chi				44.58
Between df				1
Between P				***
Btwn(F) P				**
Btwn(R) P				**

	Lung cancer type		
	all	other	Total
N	13		13
NS	8		8
Wt	256.34		256.34
Het Chi	80.94		80.94
Het df	12		12
Het P	***		***
Fixed RR	8.75		8.75
RRl	7.74		7.74
RRu	9.89		9.89
P	+++		+++
Random RR	7.57		7.57
RRl	5.24		5.24
RRu	10.93		10.93
P	+++		+++
Between Chi			
Between df			
Between P			N.S.
Btwn(F) P			N.S.
Btwn(R) P			N.S.

	Location								
	NAmer	UK	Scand	othEur	China	Japan	othAs	other	Total
N	5	4	2			1		1	13
NS	3	2	1			1		1	8
Wt	177.07	44.79	5.95			21.61		6.92	256.34
Het Chi	35.32	8.90	3.52			0.00		0.00	80.94
Het df	4	3	1			0		0	12
Het P	***	*	(*)			N.S.		N.S.	***
Fixed RR	11.05	4.87	5.89			4.90		8.60	8.75
RRl	9.54	3.63	2.64			3.22		4.08	7.74
RRu	12.80	6.53	13.16			7.47		18.12	9.89
P	+++	+++	+++			+++		+++	+++
Random RR	11.24	5.36	4.66			4.90		8.60	7.57
RRl	6.77	3.00	0.92			3.22		4.08	5.24
RRu	18.66	9.56	23.73			7.47		18.12	10.93
P	+++	+++	(+)			+++		+++	+++
Between Chi									33.21
Between df									4
Between P									***
Btwn(F) P									N.S.
Btwn(R) P									N.S.

Table 1H8 - 3

IESLC - Meta-analysis of Current Smoking, Age started, "Mid"
All LC types, Any Product (or Cigarettes if Any not available)
Most adjusted

Detailed Country in "other Europe"

		multi	Germany	othWest	East	Balkans	Total
	N						
	NS						
	Wt						
Het	Chi						
Het	df						
Het	P						N.S.
Fixed	RR						
	RRl						
	RRu						
	P						+++
Random	RR						
	RRl						
	RRu						
	P						+++
Between	Chi						
Between	df						
Between	P						N.S.
Btwn(F)	P						N.S.
Btwn(R)	P						N.S.

Detailed Country in "other Asia"

		India	HongKong	other	Total
	N				
	NS				
	Wt				
Het	Chi				
Het	df				
Het	P				
Fixed	RR				
	RRl				
	RRu				
	P				
Random	RR				
	RRl				
	RRu				
	P				
Between	Chi				
Between	df				
Between	P				N.S.
Btwn(F)	P				N.S.
Btwn(R)	P				N.S.

Detailed other continent

		SCAmer	Total
	N	1	1
	NS	1	1
	Wt	6.92	6.92
Het	Chi	0.00	0.00
Het	df	0	0
Het	P	N.S.	N.S.
Fixed	RR	8.60	8.60
	RRl	4.08	4.08
	RRu	18.12	18.12
	P	+++	+++
Random	RR	8.60	8.60
	RRl	4.08	4.08
	RRu	18.12	18.12
	P	+++	+++
Between	Chi		
Between	df		
Between	P		N.S.
Btwn(F)	P		N.S.
Btwn(R)	P		N.S.

Table 1H8 - 3

IESLC - Meta-analysis of Current Smoking, Age started, "Mid"
All LC types, Any Product (or Cigarettes if Any not available)
Most adjusted

	Start year of study					
	<1960	1960-69	1970-79	1980-89	1990+	Total
N	4	6	1	1	1	13
NS	2	3	1	1	1	8
Wt	176.58	50.74	0.49	21.61	6.92	256.34
Het Chi	34.18	12.60	0.00	0.00	0.00	80.94
Het df	3	5	0	0	0	12
Het P	***	*	N.S.	N.S.	N.S.	***
Fixed RR	11.00	4.98	50.54	4.90	8.60	8.75
RRl	9.50	3.78	3.08	3.22	4.08	7.74
RRu	12.75	6.56	829.51	7.47	18.12	9.89
P	+++	+++	++	+++	+++	+++
Random RR	10.75	5.34	50.54	4.90	8.60	7.57
RRl	6.43	3.21	3.08	3.22	4.08	5.24
RRu	17.98	8.88	829.51	7.47	18.12	10.93
P	+++	+++	++	+++	+++	+++
Between Chi						34.16
Between df						4
Between P						***
Btwn(F) P						N.S.
Btwn(R) P						(*)

	Study type (1)		
	CC	other	Total
N	4	9	13
NS	3	5	8
Wt	66.77	189.57	256.34
Het Chi	9.85	42.03	80.94
Het df	3	8	12
Het P	*	***	***
Fixed RR	4.96	10.69	8.75
RRl	3.91	9.27	7.74
RRu	6.31	12.32	9.89
P	+++	+++	+++
Random RR	5.20	9.38	7.57
RRl	3.32	6.20	5.24
RRu	8.14	14.20	10.93
P	+++	+++	+++
Between Chi			29.06
Between df			1
Between P			***
Btwn(F) P			*
Btwn(R) P			(*)

	Study type (2)			
	CC	prosp	other	Total
N	4	9		13
NS	3	5		8
Wt	66.77	189.57		256.34
Het Chi	9.85	42.03		80.94
Het df	3	8		12
Het P	*	***		***
Fixed RR	4.96	10.69		8.75
RRl	3.91	9.27		7.74
RRu	6.31	12.32		9.89
P	+++	+++		+++
Random RR	5.20	9.38		7.57
RRl	3.32	6.20		5.24
RRu	8.14	14.20		10.93
P	+++	+++		+++
Between Chi				29.06
Between df				1
Between P				***
Btwn(F) P				*
Btwn(R) P				(*)

Table 1H8 - 3

IESLC - Meta-analysis of Current Smoking, Age started, "Mid"
All LC types, Any Product (or Cigarettes if Any not available)
Most adjusted

	Study size (number of LC cases) 100-249	250-499	500-999	1000+	Total
N	2	4	2	5	13
NS	2	2	1	3	8
Wt	7.41	12.50	38.24	198.19	256.34
Het Chi	1.44	3.75	7.43	46.76	80.94
Het df	1	3	1	4	12
Het P	N.S.	N.S.	**	***	***
Fixed RR	9.67	6.68	4.52	10.08	8.75
RRl	4.71	3.83	3.30	8.77	7.74
RRu	19.87	11.62	6.21	11.58	9.89
P	+++	+++	+++	+++	+++
Random RR	12.21	6.51	4.51	9.26	7.57
RRl	3.06	3.48	1.90	5.66	5.24
RRu	48.72	12.17	10.71	15.15	10.93
P	+++	+++	+++	+++	+++
Between Chi					21.56
Between df					3
Between P					***
Btwn(F) P					N.S.
Btwn(R) P					N.S.

	Risky occupational population no	mining	othRisky	Total
N	13			13
NS	8			8
Wt	256.34			256.34
Het Chi	80.94			80.94
Het df	12			12
Het P	***			***
Fixed RR	8.75			8.75
RRl	7.74			7.74
RRu	9.89			9.89
P	+++			+++
Random RR	7.57			7.57
RRl	5.24			5.24
RRu	10.93			10.93
P	+++			+++
Between Chi				
Between df				
Between P				N.S.
Btwn(F) P				N.S.
Btwn(R) P				N.S.

	National cigarette tobacco type Virginia	blended	other	Total
N	4	9		13
NS	2	6		8
Wt	44.79	211.55		256.34
Het Chi	8.90	53.38		80.94
Het df	3	8		12
Het P	*	***		***
Fixed RR	4.87	9.91		8.75
RRl	3.63	8.66		7.74
RRu	6.53	11.34		9.89
P	+++	+++		+++
Random RR	5.36	8.74		7.57
RRl	3.00	5.78		5.24
RRu	9.56	13.22		10.93
P	+++	+++		+++
Between Chi				18.66
Between df				1
Between P				***
Btwn(F) P				(*)
Btwn(R) P				N.S.

Table 1H8 - 3

IESLC - Meta-analysis of Current Smoking, Age started, "Mid"
All LC types, Any Product (or Cigarettes if Any not available)
Most adjusted

	Any proxy use No/nk	Yes	Total
N	11	2	13
NS	7	1	8
Wt	218.10	38.24	256.34
Het Chi	53.94	7.43	80.94
Het df	10	1	12
Het P	***	**	***
Fixed RR	9.83	4.52	8.75
RRl	8.61	3.30	7.74
RRu	11.22	6.21	9.89
P	+++	+++	+++
Random RR	8.59	4.51	7.57
RRl	5.91	1.90	5.24
RRu	12.48	10.71	10.93
P	+++	+++	+++
Between Chi			19.57
Between df			1
Between P			***
Btwn(F) P			(*)
Btwn(R) P			N.S.

	Full histological confirmation No	Yes	Total
N	12	1	13
NS	7	1	8
Wt	234.73	21.61	256.34
Het Chi	73.01	0.00	80.94
Het df	11	0	12
Het P	***	N.S.	***
Fixed RR	9.23	4.90	8.75
RRl	8.12	3.22	7.74
RRu	10.49	7.47	9.89
P	+++	+++	+++
Random RR	7.93	4.90	7.57
RRl	5.37	3.22	5.24
RRu	11.69	7.47	10.93
P	+++	+++	+++
Between Chi			7.93
Between df			1
Between P			**
Btwn(F) P			N.S.
Btwn(R) P			N.S.

	Number of adjustment variables (1) 0	1	2+/+nk	Total
N	9	3	1	13
NS	6	2	1	9
Wt	132.98	116.44	6.92	256.34
Het Chi	47.07	29.74	0.00	80.94
Het df	8	2	0	12
Het P	***	***	N.S.	***
Fixed RR	7.76	10.05	8.60	8.75
RRl	6.55	8.38	4.08	7.74
RRu	9.20	12.05	18.12	9.89
P	+++	+++	+++	+++
Random RR	6.96	8.91	8.60	7.57
RRl	4.31	3.82	4.08	5.24
RRu	11.25	20.79	18.12	10.93
P	+++	+++	+++	+++
Between Chi				4.13
Between df				2
Between P				N.S.
Btwn(F) P				N.S.
Btwn(R) P				N.S.

Table 1H8 - 3

IESLC - Meta-analysis of Current Smoking, Age started, "Mid"
All LC types, Any Product (or Cigarettes if Any not available)
Most adjusted

	Number of adjustment variables (2) 0	1	2	3-5	6+/+nk	Total
N	9	3	1			13
NS	6	2	1			9
Wt	132.98	116.44	6.92			256.34
Het Chi	47.07	29.74	0.00			80.94
Het df	8	2	0			12
Het P	***	***	N.S.			***
Fixed RR	7.76	10.05	8.60			8.75
RRl	6.55	8.38	4.08			7.74
RRu	9.20	12.05	18.12			9.89
P	+++	+++	+++			+++
Random RR	6.96	8.91	8.60			7.57
RRl	4.31	3.82	4.08			5.24
RRu	11.25	20.79	18.12			10.93
P	+++	+++	+++			+++
Between Chi						4.13
Between df						2
Between P						N.S.
Btwn(F) P						N.S.
Btwn(R) P						N.S.

	Product all/unsp	cig+/-ot	cig only	Total
N		6	7	13
NS		5	4	9
Wt		166.12	90.22	256.34
Het Chi		24.32	12.60	80.94
Het df		5	6	12
Het P		***	*	***
Fixed RR		11.88	4.99	8.75
RRl		10.20	4.06	7.74
RRu		13.83	6.13	9.89
P		+++	+++	+++
Random RR		10.87	5.19	7.57
RRl		7.30	3.63	5.24
RRu		16.19	7.41	10.93
P		+++	+++	+++
Between Chi				44.01
Between df				1
Between P				***
Btwn(F) P				**
Btwn(R) P				**

	Denominator nev any	nev cigs	Total
N	11	2	13
NS	6	2	8
Wt	234.24	22.10	256.34
Het Chi	71.59	2.61	80.94
Het df	10	1	12
Het P	***	N.S.	***
Fixed RR	9.20	5.16	8.75
RRl	8.09	3.40	7.74
RRu	10.46	7.84	9.89
P	+++	+++	+++
Random RR	7.68	10.27	7.57
RRl	5.20	1.22	5.24
RRu	11.36	86.21	10.93
P	+++	+	+++
Between Chi			6.74
Between df			1
Between P			**
Btwn(F) P			N.S.
Btwn(R) P			N.S.

Table 1H8 - 3

IESLC - Meta-analysis of Current Smoking, Age started, "Mid"
All LC types, Any Product (or Cigarettes if Any not available)
Most adjusted

	Derivation of RR/CI			
	Orig	StdCalc	Other	Total
N	1	8	4	13
NS	1	5	3	9
Wt	6.92	132.49	116.93	256.34
Het Chi	0.00	45.35	31.01	80.94
Het df	0	7	3	12
Het P	N.S.	***	***	***
Fixed RR	8.60	7.71	10.12	8.75
RRl	4.08	6.50	8.44	7.74
RRu	18.12	9.14	12.13	9.89
P	+++	+++	+++	+++
Random RR	8.60	6.62	10.04	7.57
RRl	4.08	4.09	4.44	5.24
RRu	18.12	10.73	22.72	10.93
P	+++	+++	+++	+++
Between Chi				4.58
Between df				2
Between P				N.S.
Btwn(F) P				N.S.
Btwn(R) P				N.S.

Table 1H8 - 4

IESLC - Meta-analysis of Current Smoking, Age started, "Mid"
All LC types, Any Product (or Cigarettes if Any not available)
Least adjusted

REF	NRR	X	SEX	AGEL	AGEH	RACE	YF	LC TYPE	LOC	START	ST	NLC	R	VB	P	H	AD	PRODUCT	exL	exH	DENOM	De
CEDERL	511		m	0	0	all	10	all	Eu:Sca	1963	pr	491	n	bl	n	n	1	cig only	17	18	nev any	ot
CEDERL	516		f	0	0	all	10	all	Eu:Sca	1963	pr	491	n	bl	n	n	0	cig only	17	18	nev any	st
CPSI	803		m	35	84	all	6	all	NAmer	1959	pr	5138	n	bl	n	n	1	cig+/-ot	15	19	nev any	ot
CPSI	847		f	40	74	all	6	all	NAmer	1959	pr	5138	n	bl	n	n	1	cig only	15	19	nev any	ot
DEAN3	566		m	0	0	all	-	all	Eu:UK	1969	CC	766	n	V	y	n	0	cig only	15	19	nev any	st
DEAN3	585		f	0	0	all	-	all	Eu:UK	1969	CC	766	n	V	y	n	0	cig only	15	19	nev any	st
DORN	585		m	55	64	wh	8	all	NAmer	1954	pr	5097	n	bl	n	n	0	cig+/-ot	15	19	nev any	st
DORN	622		m	65	74	wh	8	all	NAmer	1954	pr	5097	n	bl	n	n	0	cig+/-ot	15	19	nev any	st
MATOS	542	x	m	0	0	all	-	all	SCAmer	1994	CC	200	n	bl	n	n	0	cig+/-ot	15	19	nev any	st
MIGRAN	503		m	0	0	all	0	all	Eu:UK	1964	pr	259	n	V	n	n	0	cig only	16	19	nev any	st
MIGRAN	513		f	0	0	all	0	all	Eu:UK	1964	pr	259	n	V	n	n	0	cig only	16	19	nev any	st
MRFITR	511		m	0	0	all	0	all	NAmer	1973	pr	119	n	bl	n	n	0	cig+/-ot	18	19	nev cigs	ot
SOBUE	647		m	0	0	all	-	all	As:Jap	1986	CC	1376	n	bl	n	y	0	cig+/-ot	18	22	nev cigs	st

Cigarette type is all/unspec for all RRs
except for the following:

REF	NRR	CIGTYPE
DEAN3	566	MC only
DEAN3	585	MC only

Table 1H8 - 5

IESLC - Meta-analysis of Current Smoking, Age started, "Mid"
All LC types, Any Product (or Cigarettes if Any not available)
Least adjusted

REF	NRR	SEX	AD	Number Exposed Case	Exposed Cont	Non-exposed Case	Non-exposed Cont	RR	95.00%CI
*CEDERL	511	m	1	10	-	7	-	9.80	(3.74- 25.69)
*CEDERL	516	f	0	2	1009	19	17679	1.84	(0.43- 7.91)
Subtotal CEDERL								5.89	(2.64- 13.16)
*CPSI	803	m	1	588	-	83	-	14.69	(11.68- 18.49)
*CPSI	847	f	1	52	-	166	-	5.00	(3.66- 6.83)
Subtotal CPSI								10.06	(8.36- 12.10)
DEAN3	566	m	0	160	485	24	510	7.01	(4.48- 10.96)
DEAN3	585	f	0	39	504	41	1538	2.90	(1.85- 4.55)
Subtotal DEAN3								4.52	(3.30- 6.21)
*DORN	585	m	0	293	154204	25	213858	16.25	(10.81- 24.45)
*DORN	622	m	0	259	80043	49	171211	11.31	(8.33- 15.34)
Subtotal DORN								12.88	(10.08- 16.44)
MATOS	542	m	0	49	58	11	110	8.45	(4.08- 17.48)
*MIGRAN	503	m	0	59	1845	4	867	6.93	(2.53- 19.02)
*MIGRAN	513	f	0	9	1035	4	3814	8.29	(2.56- 26.87)
Subtotal MIGRAN								7.48	(3.48- 16.09)
*MRFITR	511	m	0	25	1876	0	1859	50.54~	(3.08- 829.51)
SOBUE	647	m	0	553	490	29	126	4.90	(3.22- 7.47)
Partial Totals				2098	241549	462	411572		

*prospective study ~ With 0.5 adjustment for zero

REF	NRR	SEX	AD	Ys	Ws	Qs	Ps
*CEDERL	511	m	1	2.28	4.14	0.05	0.0000
*CEDERL	516	f	0	0.61	1.81	4.39	0.4098
Subtotal CEDERL				1.77	5.95	4.45	
*CPSI	803	m	1	2.69	72.82	19.56	0.0000
*CPSI	847	f	1	1.61	39.48	12.36	0.0000
Subtotal CPSI				2.31	112.30	31.92	
DEAN3	566	m	0	1.95	19.25	0.94	0.0000
DEAN3	585	f	0	1.07	18.99	23.11	0.0000
Subtotal DEAN3				1.51	38.24	24.06	
*DORN	585	m	0	2.79	23.04	8.84	0.0000
*DORN	622	m	0	2.43	41.24	2.71	0.0000
Subtotal DORN				2.56	64.28	11.55	
MATOS	542	m	0	2.13	7.26	0.01	0.0000
*MIGRAN	503	m	0	1.94	3.77	0.20	0.0002
*MIGRAN	513	f	0	2.12	2.78	0.01	0.0004
Subtotal MIGRAN				2.01	6.55	0.21	
*MRFITR	511	m	0	3.92	0.49	1.51	0.0060
SOBUE	647	m	0	1.59	21.61	7.24	0.0000

	N	13
	NS	8
	Wt	256.69
Het	Chi	80.95
Het	df	12
Het	P	***
Fixed	RR	8.75
	RRl	7.74
	RRu	9.89
	P	+++
Random	RR	7.56
	RRl	5.23
	RRu	10.92
	P	+++
Asymm	P	N.S.

Table 1H8 - 6

IESLC - Meta-analysis of Current Smoking, Age started, "Mid"
All LC types, Any Product (or Cigarettes if Any not available)
Least adjusted

	Sex combined	male	female	Total
N		9	4	13
NS		8	4	12
Wt		193.63	63.06	256.69
Het Chi		30.15	6.30	80.95
Het df		8	3	12
Het P		***	(*)	***
Fixed RR		11.10	4.22	8.75
RRl		9.64	3.30	7.74
RRu		12.77	5.40	9.89
P		+++	+++	+++
Random RR		9.84	4.02	7.56
RRl		7.11	2.53	5.23
RRu		13.61	6.37	10.92
P		+++	+++	+++
Between Chi				44.50
Between df				1
Between P				***
Btwn(F) P				**
Btwn(R) P				**

Table 1H8 - 7

IESLC - Meta-analysis of Current Smoking, Age started, "Mid"
All LC types, Any Product (or Cigarettes if Any not available)
Excluded studies (and stage at which they were excluded)

1 AGUDO ALDERS ARMADA AUVINE AXELSS BARBON BECHER BENHAM BLOT1 BOFFET BOUCHA BRESLO BROWN3 CARPEN CHEN CHEN2
CHIAZZ CHOI CHYOU CORREA DAMBER DARBY DESTEF DOLL DOLL2 DORGAN DOSEME FAN GAO GARCIA GARSHI GENG
GER GRAHAM GUO GURSEL HAENSZ HAMMO2 HAMMON HEGMAN HU HU2 JAHN JAIN JEDRYC JOLY JUSSAW KHUDER
KOO KOULUM KREUZE LAUSSM LETOUR LEVIN LIU3 LIU4 LIU5 LUBIN LUBIN2 LUO MCCONN NOTAN2 OSANN2 PERNU
PEZZOT PRESCO QIAO QIAO2 RACHTA RESTRE SADOWS STASZE SUZUK2 TIZZAN TVERDA VUTUC WANG2 WIGLE WU2 WUWILL
WYNDE2 WYNDE3 XU YUAN ZHANG ZHENG ZHOU

2 AKIBA AMANDU AMES BENSHL BEST BOUCOT BROSS BUFFLE CPSII DEAN2 GILLIS HUMBLE KAISE2 KATSOU KAUFMA PEZZO2
PISANI SPITZ WATSON WYNDE8

3 MCDUFF WYNDE6

5 HOLE

7 WYNDE7

10 SPEIZE

14 ENGELA GAO2 HIRAYA LIAW SEGI2 SVENSS WAKAI WU

Table 1H8 - 8

Potentially overlapping studies

REF	REFGP	PRINC	OVERLAP/LINK
MRFITR	MRFIT	2	Subset of MRFIT
CPSI	CPSI	1	CPSI overall

Table 1H9 -

IESLC - Meta-analysis of Current Smoking, Age started, "High"
All LC types, Any Product (or Cigarettes if Any not available)

This analysis is restricted to results for:
1) Current smokers
2) Results by Age started
3) Categorical results by Age started
4) All LC types (or near equivalent)
5) Results complete enough for use in metaanalysis

Within each study, results are then selected (in the following order of preference, within each sex) for:
6) PRODUCT: all/unspec, cigarettes regardless of other products, cigarettes only
7) CIGTYPE: all/unspecified, MC regardless of HR, MC only
8) (not applicable)
9) DENOM: never smoked anything, never smoked cigarettes, never any + low, never cigs + low
10) Followup period (YF, prospective studies): whole study (coded as 0) or longest available
11) LCtype: all or nearest available, at least Squamous and Adeno. (q = squamous, s = small,
l = large, a = adeno, mix = mixed, alv = alveolar)
12) Race: all or nearest available, otherwise by race (wh or w = white, bl or b = black, hi = hispanic
ch = chinese, jap = japanese, haw = hawaiian, w+o = white + oriental, sca = scandinavian, as = asian)
13) Age started "high" in key scheme 1 (key value 14, maximum range 1-17)
14) For overlapping studies: principal rather than subsidiary studies
Finally by Age: whole study (coded as 0) if available, otherwise by widest available age group
and then for single sex results (m, f) in preference to results for both sexes combined (c).

Results adjusted (AD) for the most potential confounders are then chosen in Sections -1 to -3
and results adjusted for the least confounders in Sections -4 to -6. (Those least adjusted results which
actually differ from the most adjusted are marked 'x' in column X in Section -4)

Section -7 shows excluded studies, together with the stage (as above) at which no qualifying
results were found.

Section -8 lists the potentially overlapping studies which have been included (1=principal, 2=subsidiary).

Section -9 lists any results which would have been included in preference except that they had data not complete
enough for use in meta-analysis, with their significance (yes/no), if known, and any further comment as entered
on the database. It also lists as "gap" any categories for which no data were presented by the original authors.

In addition to those mentioned above, the following fields, levels and abbreviations are used:
* or nk = not known, n = no, y = yes, ot = other
nev = never
all/unspec = all or unspecified, cig+/-ot = cigarettes irrespective of other products (cigar, pipe etc)
MC = manufactured cigarettes, HR = hand-rolled cigarettes
exL, exH = range of exposure (low and high) in the smoking group, in terms of Age started
REF: 6-character study reference
NRR: number of the RR on the database within the study
ST : study type (CC = case control, pr or prosp = prospective)
NLC: number of lung cancer cases in whole study
R : risky occupational population (n = no, m = mining, o = other risky)
VB : national cigarette type (V = at least 75% Virginia, bl = at least 75% blended, ot = other)
P : any proxy use
H : full histological confirmation
De : derivation of RR/CI (or = original, st = standard method, ot = other method of estimation)

Table 1H9 - 1

IESLC - Meta-analysis of Current Smoking, Age started, "High"
All LC types, Any Product (or Cigarettes if Any not available)
Most adjusted

REF	NRR	SEX	AGEL	AGEH	RACE	YF	LC TYPE	LOC	START	ST	NLC	R	VB	P	H	AD	PRODUCT	exL	exH	DENOM	De
CEDERL	512	m	0	0	all	10	all	Eu:Sca	1963	pr	491	n	bl	n	n	1	cig only	1	16	nev any	ot
CEDERL	517	f	0	0	all	10	all	Eu:Sca	1963	pr	491	n	bl	n	n	0	cig only	1	16	nev any	ot
CPSI	804	m	35	84	all	6	all	NAmer	1959	pr	5138	n	bl	n	n	1	cig+/-ot	1	14	nev any	ot
CPSI	848	f	40	74	all	6	all	NAmer	1959	pr	5138	n	bl	n	n	1	cig only	1	14	nev any	ot
DEAN3	567	m	0	0	all	-	all	Eu:UK	1969	CC	766	n	V	y	n	0	cig only	1	14	nev any	st
DEAN3	586	f	0	0	all	-	all	Eu:UK	1969	CC	766	n	V	y	n	0	cig only	1	14	nev any	st
DORN	586	m	55	64	wh	8	all	NAmer	1954	pr	5097	n	bl	n	n	0	cig+/-ot	1	14	nev any	st
DORN	623	m	65	74	wh	8	all	NAmer	1954	pr	5097	n	bl	n	n	0	cig+/-ot	1	14	nev any	st
MATOS	563	m	0	0	all	-	all	SCAmer	1994	CC	200	n	bl	n	n	2	cig+/-ot	1	14	nev any	or
MIGRAN	505	m	0	0	all	0	all	Eu:UK	1964	pr	259	n	V	n	n	0	cig only	0	15	nev any	st
MIGRAN	515	f	0	0	all	0	all	Eu:UK	1964	pr	259	n	V	n	n	0	cig only	0	15	nev any	st
MRFITR	513	m	0	0	all	0	all	NAmer	1973	pr	119	n	bl	n	n	0	cig+/-ot	1	15	nev cigs	ot
SOBUE	648	m	0	0	all	-	all	As:Jap	1986	CC	1376	n	bl	n	y	0	cig+/-ot	10	17	nev cigs	st

Cigarette type is all/unspec for all RRs
except for the following:

REF	NRR	CIGTYPE
DEAN3	567	MC only
DEAN3	586	MC only

Table 1H9 - 2

IESLC - Meta-analysis of Current Smoking, Age started, "High"
All LC types, Any Product (or Cigarettes if Any not available)
Most adjusted

REF	NRR	SEX	AD	Number Exposed Case	Exposed Cont	Non-exposed Case	Non-exposed Cont	RR	95.00%CI
*CEDERL	512	m	1	7	-	7	-	6.40	(2.25- 18.21)
*CEDERL	517	f	0	0	746	19	17679	0.61~	(0.04- 10.05)
Subtotal CEDERL								4.80	(1.80- 12.79)
*CPSI	804	m	1	185	-	83	-	16.77	(12.94- 21.73)
*CPSI	848	f	1	6	-	166	-	2.50	(1.11- 5.65)
Subtotal CPSI								14.07	(10.99- 18.02)
DEAN3	567	m	0	44	165	24	510	5.67	(3.34- 9.60)
DEAN3	586	f	0	7	109	41	1538	2.41	(1.06- 5.50)
Subtotal DEAN3								4.42	(2.83- 6.89)
*DORN	586	m	0	70	25569	25	213858	23.42	(14.84- 36.97)
*DORN	623	m	0	65	16016	49	171211	14.18	(9.79- 20.54)
Subtotal DORN								17.31	(12.98- 23.07)
MATOS	563	m	2	45	-	11	-	11.30	(5.30- 24.30)
*MIGRAN	505	m	0	50	1081	4	867	10.03	(3.64- 27.65)
*MIGRAN	515	f	0	2	266	4	3814	7.17	(1.32- 38.96)
Subtotal MIGRAN								9.18	(3.84- 21.90)
*MRFITR	513	m	0	25	2065	0	1859	45.91~	(2.80- 753.64)
SOBUE	648	m	0	109	68	29	126	6.96	(4.20- 11.54)
Partial Totals				615	46085	462	411462		

*prospective study ~ With 0.5 adjustment for zero

REF	NRR	SEX	AD	Ys	Ws	Qs	Ps
*CEDERL	512	m	1	1.86	3.51	1.24	0.0005
*CEDERL	517	f	0	-0.50	0.49	4.25	0.7276
Subtotal CEDERL				1.57	4.00	5.49	
*CPSI	804	m	1	2.82	57.18	7.75	0.0000
*CPSI	848	f	1	0.92	5.80	13.67	0.0273
Subtotal CPSI				2.64	62.99	21.43	
DEAN3	567	m	0	1.73	13.81	7.10	0.0000
DEAN3	586	f	0	0.88	5.65	13.96	0.0367
Subtotal DEAN3				1.49	19.46	21.05	
*DORN	586	m	0	3.15	18.44	9.09	0.0000
*DORN	623	m	0	2.65	27.99	1.12	0.0000
Subtotal DORN				2.85	46.43	10.21	
MATOS	563	m	2	2.42	6.63	0.00	0.0000
*MIGRAN	505	m	0	2.31	3.73	0.08	0.0000
*MIGRAN	515	f	0	1.97	1.34	0.31	0.0226
Subtotal MIGRAN				2.22	5.07	0.39	
*MRFITR	513	m	0	3.83	0.49	0.93	0.0074
SOBUE	648	m	0	1.94	15.08	3.93	0.0000

	N	13
	NS	8
	Wt	160.15
Het	Chi	63.44
Het	df	12
Het	P	***
Fixed	RR	11.60
	RRl	9.94
	RRu	13.55
	P	+++
Random	RR	8.20
	RRl	5.36
	RRu	12.56
	P	+++
Asymm	P	(*)

Table 1H9 - 3

IESLC - Meta-analysis of Current Smoking, Age started, "High"
All LC types, Any Product (or Cigarettes if Any not available)
Most adjusted

	Sex			
	combined	male	female	Total
N		9	4	13
NS		8	4	12
Wt		146.87	13.28	160.15
Het Chi		28.56	2.45	63.44
Het df		8	3	12
Het P		***	N.S.	***
Fixed RR		13.29	2.60	11.60
RRl		11.30	1.52	9.94
RRu		15.62	4.45	13.55
P		+++	+++	+++
Random RR		11.53	2.60	8.20
RRl		8.05	1.52	5.36
RRu		16.52	4.45	12.56
P		+++	+++	+++
Between Chi				32.43
Between df				1
Between P				***
Btwn(F) P				**
Btwn(R) P				***

	Lung cancer type		
	all	other	Total
N	13		13
NS	8		8
Wt	160.15		160.15
Het Chi	63.44		63.44
Het df	12		12
Het P	***		***
Fixed RR	11.60		11.60
RRl	9.94		9.94
RRu	13.55		13.55
P	+++		+++
Random RR	8.20		8.20
RRl	5.36		5.36
RRu	12.56		12.56
P	+++		+++
Between Chi			
Between df			
Between P			N.S.
Btwn(F) P			N.S.
Btwn(R) P			N.S.

	Location								
	NAmer	UK	Scand	othEur	China	Japan	othAs	other	Total
N	5	4	2			1		1	13
NS	3	2	1			1		1	8
Wt	109.91	24.53	4.00			15.08		6.63	160.15
Het Chi	23.61	5.19	2.38			0.00		0.00	63.44
Het df	4	3	1			0		0	12
Het P	***	N.S.	N.S.			N.S.		N.S.	***
Fixed RR	15.44	5.14	4.80			6.96		11.30	11.60
RRl	12.81	3.46	1.80			4.20		5.28	9.94
RRu	18.61	7.64	12.79			11.54		24.20	13.55
P	+++	+++	++			+++		+++	+++
Random RR	12.78	5.18	2.87			6.96		11.30	8.20
RRl	7.27	2.88	0.32			4.20		5.28	5.36
RRu	22.44	9.32	25.57			11.54		24.20	12.56
P	+++	+++	N.S.			+++		+++	+++
Between Chi									32.27
Between df									4
Between P									***
Btwn(F) P									N.S.
Btwn(R) P									N.S.

Table 1H9 - 3

IESLC - Meta-analysis of Current Smoking, Age started, "High"
All LC types, Any Product (or Cigarettes if Any not available)
Most adjusted

Detailed Country in "other Europe"

	multi	Germany	othWest	East	Balkans	Total
N						
NS						
Wt						
Het Chi						
Het df						
Het P						N.S.
Fixed RR						
RRl						
RRu						
P						+++
Random RR						
RRl						
RRu						
P						+++
Between Chi						
Between df						
Between P						N.S.
Btwn(F) P						N.S.
Btwn(R) P						N.S.

Detailed Country in "other Asia"

	India	HongKong	other	Total
N				
NS				
Wt				
Het Chi				
Het df				
Het P				
Fixed RR				
RRl				
RRu				
P				
Random RR				
RRl				
RRu				
P				
Between Chi				
Between df				
Between P				N.S.
Btwn(F) P				N.S.
Btwn(R) P				N.S.

Detailed other continent

	SCAmer	Total
N	1	1
NS	1	1
Wt	6.63	6.63
Het Chi	0.00	0.00
Het df	0	0
Het P	N.S.	N.S.
Fixed RR	11.30	11.30
RRl	5.28	5.28
RRu	24.20	24.20
P	+++	+++
Random RR	11.30	11.30
RRl	5.28	5.28
RRu	24.20	24.20
P	+++	+++
Between Chi		
Between df		
Between P		N.S.
Btwn(F) P		N.S.
Btwn(R) P		N.S.

Table 1H9 - 3

IESLC - Meta-analysis of Current Smoking, Age started, "High"
All LC types, Any Product (or Cigarettes if Any not available)
Most adjusted

	Start year of study <1960	1960-69	1970-79	1980-89	1990+	Total
N	4	6	1	1	1	13
NS	2	3	1	1	1	8
Wt	109.41	28.53	0.49	15.08	6.63	160.15
Het Chi	23.02	7.58	0.00	0.00	0.00	63.44
Het df	3	5	0	0	0	12
Het P	***	N.S.	N.S.	N.S.	N.S.	***
Fixed RR	15.36	5.09	45.91	6.96	11.30	11.60
RRl	12.74	3.53	2.80	4.20	5.28	9.94
RRu	18.53	7.35	753.64	11.54	24.20	13.55
P	+++	+++	++	+++	+++	+++
Random RR	12.16	5.01	45.91	6.96	11.30	8.20
RRl	6.80	3.00	2.80	4.20	5.28	5.36
RRu	21.74	8.35	753.64	11.54	24.20	12.56
P	+++	+++	++	+++	+++	+++
Between Chi						32.84
Between df						4
Between P						***
Btwn(F) P						N.S.
Btwn(R) P						N.S.

	Study type (1) CC	other	Total
N	4	9	13
NS	3	5	8
Wt	41.17	118.98	160.15
Het Chi	7.73	32.48	63.44
Het df	3	8	12
Het P	(*)	***	***
Fixed RR	6.07	14.52	11.60
RRl	4.47	12.13	9.94
RRu	8.24	17.38	13.55
P	+++	+++	+++
Random RR	5.90	10.32	8.20
RRl	3.54	6.36	5.36
RRu	9.82	16.76	12.56
P	+++	+++	+++
Between Chi			23.23
Between df			1
Between P			***
Btwn(F) P			*
Btwn(R) P			N.S.

	Study type (2) CC	prosp	other	Total
N	4	9		13
NS	3	5		8
Wt	41.17	118.98		160.15
Het Chi	7.73	32.48		63.44
Het df	3	8		12
Het P	(*)	***		***
Fixed RR	6.07	14.52		11.60
RRl	4.47	12.13		9.94
RRu	8.24	17.38		13.55
P	+++	+++		+++
Random RR	5.90	10.32		8.20
RRl	3.54	6.36		5.36
RRu	9.82	16.76		12.56
P	+++	+++		+++
Between Chi				23.23
Between df				1
Between P				***
Btwn(F) P				*
Btwn(R) P				N.S.

Table 1H9 - 3

IESLC - Meta-analysis of Current Smoking, Age started, "High"
All LC types, Any Product (or Cigarettes if Any not available)
Most adjusted

	Study size (number of LC cases) 100-249	250-499	500-999	1000+	Total
N	2	4	2	5	13
NS	2	2	1	3	8
Wt	7.12	9.08	19.46	124.50	160.15
Het Chi	0.90	3.42	2.93	31.32	63.44
Het df	1	3	1	4	12
Het P	N.S.	N.S.	(*)	***	***
Fixed RR	12.45	6.90	4.42	13.96	11.60
RRl	5.97	3.60	2.83	11.71	9.94
RRu	25.95	13.22	6.89	16.64	13.55
P	+++	+++	+++	+++	+++
Random RR	12.45	6.71	3.93	10.84	8.20
RRl	5.97	3.27	1.71	6.33	5.36
RRu	25.95	13.75	9.00	18.55	12.56
P	+++	+++	++	+++	+++
Between Chi					24.86
Between df					3
Between P					***
Btwn(F) P					N.S.
Btwn(R) P					N.S.

	Risky occupational population no	mining	othRisky	Total
N	13			13
NS	8			8
Wt	160.15			160.15
Het Chi	63.44			63.44
Het df	12			12
Het P	***			***
Fixed RR	11.60			11.60
RRl	9.94			9.94
RRu	13.55			13.55
P	+++			+++
Random RR	8.20			8.20
RRl	5.36			5.36
RRu	12.56			12.56
P	+++			+++
Between Chi				
Between df				
Between P				N.S.
Btwn(F) P				N.S.
Btwn(R) P				N.S.

	National cigarette tobacco type Virginia	blended	other	Total
N	4	9		13
NS	2	6		8
Wt	24.53	135.62		160.15
Het Chi	5.19	39.05		63.44
Het df	3	8		12
Het P	N.S.	***		***
Fixed RR	5.14	13.45		11.60
RRl	3.46	11.36		9.94
RRu	7.64	15.91		13.55
P	+++	+++		+++
Random RR	5.18	10.04		8.20
RRl	2.88	6.34		5.36
RRu	9.32	15.89		12.56
P	+++	+++		+++
Between Chi				19.20
Between df				1
Between P				***
Btwn(F) P				(*)
Btwn(R) P				(*)

Table 1H9 - 3

IESLC - Meta-analysis of Current Smoking, Age started, "High"
All LC types, Any Product (or Cigarettes if Any not available)
Most adjusted

	Any proxy use No/nk	Yes	Total
N	11	2	13
NS	7	1	8
Wt	140.69	19.46	160.15
Het Chi	39.88	2.93	63.44
Het df	10	1	12
Het P	***	(*)	***
Fixed RR	13.26	4.42	11.60
RRl	11.24	2.83	9.94
RRu	15.64	6.89	13.55
P	+++	+++	+++
Random RR	9.96	3.93	8.20
RRl	6.59	1.71	5.36
RRu	15.07	9.00	12.56
P	+++	++	+++
Between Chi			20.63
Between df			1
Between P			***
Btwn(F) P			*
Btwn(R) P			*

	Full histological confirmation No	Yes	Total
N	12	1	13
NS	7	1	8
Wt	145.06	15.08	160.15
Het Chi	59.10	0.00	63.44
Het df	11	0	12
Het P	***	N.S.	***
Fixed RR	12.24	6.96	11.60
RRl	10.40	4.20	9.94
RRu	14.40	11.54	13.55
P	+++	+++	+++
Random RR	8.32	6.96	8.20
RRl	5.24	4.20	5.36
RRu	13.19	11.54	12.56
P	+++	+++	+++
Between Chi			4.34
Between df			1
Between P			*
Btwn(F) P			N.S.
Btwn(R) P			N.S.

	Number of adjustment variables (1) 0	1	2+/+nk	Total
N	9	3	1	13
NS	6	2	1	9
Wt	87.02	66.50	6.63	160.15
Het Chi	39.64	21.15	0.00	63.44
Het df	8	2	0	12
Het P	***	***	N.S.	***
Fixed RR	10.36	13.50	11.30	11.60
RRl	8.40	10.61	5.28	9.94
RRu	12.78	17.17	24.20	13.55
P	+++	+++	+++	+++
Random RR	8.22	6.72	11.30	8.20
RRl	4.70	1.85	5.28	5.36
RRu	14.38	24.43	24.20	12.56
P	+++	++	+++	+++
Between Chi				2.65
Between df				2
Between P				N.S.
Btwn(F) P				N.S.
Btwn(R) P				N.S.

Table 1H9 - 3

IESLC - Meta-analysis of Current Smoking, Age started, "High"
All LC types, Any Product (or Cigarettes if Any not available)
Most adjusted

Number of adjustment variables (2)

	0	1	2	3-5	6+/+nk	Total
N	9	3	1			13
NS	6	2	1			9
Wt	87.02	66.50	6.63			160.15
Het Chi	39.64	21.15	0.00			63.44
Het df	8	2	0			12
Het P	***	***	N.S.			***
Fixed RR	10.36	13.50	11.30			11.60
RRl	8.40	10.61	5.28			9.94
RRu	12.78	17.17	24.20			13.55
P	+++	+++	+++			+++
Random RR	8.22	6.72	11.30			8.20
RRl	4.70	1.85	5.28			5.36
RRu	14.38	24.43	24.20			12.56
P	+++	++	+++			+++
Between Chi						2.65
Between df						2
Between P						N.S.
Btwn(F) P						N.S.
Btwn(R) P						N.S.

Product

	all/unsp	cig+/-ot	cig only	Total
N		6	7	13
NS		5	4	9
Wt		125.81	34.33	160.15
Het Chi		14.48	10.02	63.44
Het df		5	6	12
Het P		*	N.S.	***
Fixed RR		15.01	4.52	11.60
RRl		12.61	3.23	9.94
RRu		17.88	6.31	13.55
P		+++	+++	+++
Random RR		14.19	4.40	8.20
RRl		10.00	2.73	5.36
RRu		20.14	7.11	12.56
P		+++	+++	+++
Between Chi				38.94
Between df				1
Between P				***
Btwn(F) P				**
Btwn(R) P				***

Denominator

	nev any	nev cigs	Total
N	11	2	13
NS	6	2	8
Wt	144.57	15.57	160.15
Het Chi	58.24	1.69	63.44
Het df	10	1	12
Het P	***	N.S.	***
Fixed RR	12.18	7.39	11.60
RRl	10.35	4.50	9.94
RRu	14.34	12.14	13.55
P	+++	+++	+++
Random RR	7.99	10.60	8.20
RRl	5.00	2.28	5.36
RRu	12.76	49.36	12.56
P	+++	++	+++
Between Chi			3.51
Between df			1
Between P			(*)
Btwn(F) P			N.S.
Btwn(R) P			N.S.

Table 1H9 - 3

IESLC - Meta-analysis of Current Smoking, Age started, "High"
All LC types, Any Product (or Cigarettes if Any not available)
Most adjusted

	Derivation of RR/CI Orig	StdCalc	Other	Total
N	1	7	5	13
NS	1	4	3	8
Wt	6.63	86.04	67.48	160.15
Het Chi	0.00	34.63	26.57	63.44
Het df	0	6	4	12
Het P	N.S.	***	***	***
Fixed RR	11.30	10.44	13.32	11.60
RRl	5.28	8.45	10.49	9.94
RRu	24.20	12.89	16.91	13.55
P	+++	+++	+++	+++
Random RR	11.30	8.50	6.37	8.20
RRl	5.28	4.86	1.97	5.36
RRu	24.20	14.87	20.62	12.56
P	+++	+++	++	+++
Between Chi				2.25
Between df				2
Between P				N.S.
Btwn(F) P				N.S.
Btwn(R) P				N.S.

Table 1H9 - 4

IESLC - Meta-analysis of Current Smoking, Age started, "High"
All LC types, Any Product (or Cigarettes if Any not available)
Least adjusted

REF	NRR	X	SEX	AGEL	AGEH	RACE	YF	LC TYPE	LOC	START	ST	NLC	R	VB	P	H	AD	PRODUCT	exL	exH	DENOM	De
CEDERL	512		m	0	0	all	10	all	Eu:Sca	1963	pr	491	n	bl	n	n	1	cig only	1	16	nev any	ot
CEDERL	517		f	0	0	all	10	all	Eu:Sca	1963	pr	491	n	bl	n	n	0	cig only	1	16	nev any	ot
CPSI	804		m	35	84	all	6	all	NAmer	1959	pr	5138	n	bl	n	n	1	cig+/-ot	1	14	nev any	ot
CPSI	848		f	40	74	all	6	all	NAmer	1959	pr	5138	n	bl	n	n	1	cig only	1	14	nev any	ot
DEAN3	567		m	0	0	all	-	all	Eu:UK	1969	CC	766	n	V	y	n	0	cig only	1	14	nev any	st
DEAN3	586		f	0	0	all	-	all	Eu:UK	1969	CC	766	n	V	y	n	0	cig only	1	14	nev any	st
DORN	586		m	55	64	wh	8	all	NAmer	1954	pr	5097	n	bl	n	n	0	cig+/-ot	1	14	nev any	st
DORN	623		m	65	74	wh	8	all	NAmer	1954	pr	5097	n	bl	n	n	0	cig+/-ot	1	14	nev any	st
MATOS	543	x	m	0	0	all	-	all	SCAmer	1994	CC	200	n	bl	n	n	0	cig+/-ot	1	14	nev any	st
MIGRAN	505		m	0	0	all	0	all	Eu:UK	1964	pr	259	n	V	n	n	0	cig only	0	15	nev any	st
MIGRAN	515		f	0	0	all	0	all	Eu:UK	1964	pr	259	n	V	n	n	0	cig only	0	15	nev any	st
MRFITR	513		m	0	0	all	0	all	NAmer	1973	pr	119	n	bl	n	n	0	cig+/-ot	1	15	nev cigs	ot
SOBUE	648		m	0	0	all	-	all	As:Jap	1986	CC	1376	n	bl	n	y	0	cig+/-ot	10	17	nev cigs	st

Cigarette type is all/unspec for all RRs
except for the following:

REF	NRR	CIGTYPE
DEAN3	567	MC only
DEAN3	586	MC only

Table 1H9 - 5

IESLC - Meta-analysis of Current Smoking, Age started, "High"
All LC types, Any Product (or Cigarettes if Any not available)
Least adjusted

REF	NRR	SEX	AD	Number Exposed Case	Number Exposed Cont	Non-exposed Case	Non-exposed Cont	RR	95.00%CI
*CEDERL	512	m	1	7	-	7	-	6.40	(2.25- 18.21)
*CEDERL	517	f	0	0	746	19	17679	0.61~	(0.04- 10.05)
Subtotal CEDERL								4.80	(1.80- 12.79)
*CPSI	804	m	1	185	-	83	-	16.77	(12.94- 21.73)
*CPSI	848	f	1	6	-	166	-	2.50	(1.11- 5.65)
Subtotal CPSI								14.07	(10.99- 18.02)
DEAN3	567	m	0	44	165	24	510	5.67	(3.34- 9.60)
DEAN3	586	f	0	7	109	41	1538	2.41	(1.06- 5.50)
Subtotal DEAN3								4.42	(2.83- 6.89)
*DORN	586	m	0	70	25569	25	213858	23.42	(14.84- 36.97)
*DORN	623	m	0	65	16016	49	171211	14.18	(9.79- 20.54)
Subtotal DORN								17.31	(12.98- 23.07)
MATOS	543	m	0	45	41	11	110	10.98	(5.18- 23.25)
*MIGRAN	505	m	0	50	1081	4	867	10.03	(3.64- 27.65)
*MIGRAN	515	f	0	2	266	4	3814	7.17	(1.32- 38.96)
Subtotal MIGRAN								9.18	(3.84- 21.90)
*MRFITR	513	m	0	25	2065	0	1859	45.91~	(2.80- 753.64)
SOBUE	648	m	0	109	68	29	126	6.96	(4.20- 11.54)
Partial Totals				615	46126	462	411572		

*prospective study ~ With 0.5 adjustment for zero

REF	NRR	SEX	AD	Ys	Ws	Qs	Ps
*CEDERL	512	m	1	1.86	3.51	1.24	0.0005
*CEDERL	517	f	0	-0.50	0.49	4.24	0.7276
Subtotal CEDERL				1.57	4.00	5.48	
*CPSI	804	m	1	2.82	57.18	7.81	0.0000
*CPSI	848	f	1	0.92	5.80	13.65	0.0273
Subtotal CPSI				2.64	62.99	21.46	
DEAN3	567	m	0	1.73	13.81	7.07	0.0000
DEAN3	586	f	0	0.88	5.65	13.94	0.0367
Subtotal DEAN3				1.49	19.46	21.01	
*DORN	586	m	0	3.15	18.44	9.12	0.0000
*DORN	623	m	0	2.65	27.99	1.14	0.0000
Subtotal DORN				2.85	46.43	10.26	
MATOS	543	m	0	2.40	6.82	0.02	0.0000
*MIGRAN	505	m	0	2.31	3.73	0.08	0.0000
*MIGRAN	515	f	0	1.97	1.34	0.31	0.0226
Subtotal MIGRAN				2.22	5.07	0.39	
*MRFITR	513	m	0	3.83	0.49	0.93	0.0074
SOBUE	648	m	0	1.94	15.08	3.91	0.0000

	N	13
	NS	8
	Wt	160.34
Het	Chi	63.46
Het	df	12
Het	P	***
Fixed	RR	11.59
	RRl	9.93
	RRu	13.53
	P	+++
Random	RR	8.18
	RRl	5.35
	RRu	12.52
	P	+++
Asymm	P	(*)

Table 1H9 - 6

IESLC - Meta-analysis of Current Smoking, Age started, "High"
All LC types, Any Product (or Cigarettes if Any not available)
Least adjusted

	Sex combined	male	female	Total
N		9	4	13
NS		8	4	12
Wt		147.06	13.28	160.34
Het Chi		28.64	2.45	63.46
Het df		8	3	12
Het P		***	N.S.	***
Fixed RR		13.27	2.60	11.59
RRl		11.29	1.52	9.93
RRu		15.59	4.45	13.53
P		+++	+++	+++
Random RR		11.49	2.60	8.18
RRl		8.02	1.52	5.35
RRu		16.47	4.45	12.52
P		+++	+++	+++
Between Chi				32.37
Between df				1
Between P				***
Btwn(F) P				**
Btwn(R) P				***

Table 1H9 - 7

IESLC - Meta-analysis of Current Smoking, Age started, "High"
All LC types, Any Product (or Cigarettes if Any not available)
Excluded studies (and stage at which they were excluded)

1	AGUDO ALDERS ARMADA AUVINE AXELSS BARBON BECHER BENHAM BLOT1 BOFFET BOUCHA BRESLO BROWN3 CARPEN CHEN CHEN2 CHIAZZ CHOI CHYOU CORREA DAMBER DARBY DESTEF DOLL DOLL2 DORGAN DOSEME FAN GAO GARCIA GARSHI GENG GER GRAHAM GUO GURSEL HAENSZ HAMMO2 HAMMON HEGMAN HU HU2 JAHN JAIN JEDRYC JOLY JUSSAW KHUDER KOO KOULUM KREUZE LAUSSM LETOUR LEVIN LIU3 LIU4 LIU5 LUBIN LUBIN2 LUO MCCONN NOTAN2 OSANN2 PERNU PEZZOT PRESCO QIAO QIAO2 RACHTA RESTRE SADOWS STASZE SUZUK2 TIZZAN TVERDA VUTUC WANG2 WIGLE WU2 WUWILL WYNDE2 WYNDE3 XU YUAN ZHANG ZHENG ZHOU
2	AKIBA AMANDU AMES BENSHL BEST BOUCOT BROSS BUFFLE CPSII DEAN2 GILLIS HUMBLE KAISE2 KATSOU KAUFMA PEZZO2 PISANI SPITZ WATSON WYNDE8
3	MCDUFF WYNDE6
5	HOLE
7	WYNDE7
10	SPEIZE
14	ENGELA GAO2 HIRAYA LIAW SEGI2 SVENSS WAKAI WU

Table 1H9 - 8

Potentially overlapping studies

REF	REFGP	PRINC	.	OVERLAP/LINK
MRFITR	MRFIT	2		Subset of MRFIT
CPSI	CPSI	1		CPSI overall

Table 1H10 -

IESLC - Meta-analysis of Current Smoking, Age started, "Highest vs lowest"
All LC types, Any Product (or Cigarettes if Any not available)

This analysis is restricted to results for:
1) Current smokers
2) Results by Age started
3) Categorical results by Age started
4) Denominator (unexposed) = "low"
5) All LC types (or near equivalent)
6) Results complete enough for use in metaanalysis

Within each study, results are then selected (in the following order of preference, within each sex) for:
7) (not applicable)
8) PRODUCT: all/unspec, cigarettes regardless of other products, cigarettes only
9) CIGTYPE: all/unspecified, MC regardless of HR, MC only
10) Results with least adjustment for other aspects of smoking (ADOS)
11) The highest vs lowest category
12) Followup period (YF, prospective studies): whole study (coded as 0) or longest available
13) LCtype: all or nearest available, at least Squamous and Adeno. (q = squamous, s = small,
l = large, a = adeno, mix = mixed, alv = alveolar)
14) Race: all or nearest available, otherwise by race (wh or w = white, bl or b = black, hi = hispanic
ch = chinese, jap = japanese, haw = hawaiian, w+o = white + oriental, sca = scandinavian, as = asian)
15) For overlapping studies: principal rather than subsidiary studies
Finally by Age: whole study (coded as 0) if available, otherwise by widest available age group
and then for single sex results (m, f) in preference to results for both sexes combined (c).

Results adjusted (AD) for the most potential confounders are then chosen in Sections -1 to -3
and results adjusted for the least confounders in Sections -4 to -6. (Those least adjusted results which
actually differ from the most adjusted are marked 'x' in column X in Section -4)

Section -7 shows excluded studies, together with the stage (as above) at which no qualifying
results were found.

Section -8 lists the potentially overlapping studies which have been included (1=principal, 2=subsidiary).

Section -9 lists any results which would have been included in preference except that they had data not complete
enough for use in meta-analysis, with their significance (yes/no), if known, and any further comment as entered
on the database. It also lists as "gap" any categories for which no data were presented by the original authors.

In addition to those mentioned above, the following fields, levels and abbreviations are used:
* or nk = not known, n = no, y = yes, ot = other
all/unspec = all or unspecified, cig+/-ot = cigarettes irrespective of other products (cigar, pipe etc)
MC = manufactured cigarettes, HR = hand-rolled cigarettes
exL, exH = range of exposure (low and high) in the "highest" group, in terms of Age started
unexL, unexH = range of exposure (low and high) in the "lowest" group, in terms of Age started
REF: 6-character study reference
NRR: number of the RR on the database within the study
ST : study type (CC = case control, pr or prosp = prospective)
NLC: number of lung cancer cases in whole study
R : risky occupational population (n = no, m = mining, o = other risky)
VB : national cigarette type (V = at least 75% Virginia, bl = at least 75% blended, ot = other)
P : any proxy use
H : full histological confirmation
De : derivation of RR/CI (or = original, st = standard method, ot = other method of estimation)

Table 1H10 - 1

IESLC - Meta-analysis of Current Smoking, Age started, "Highest vs lowest"
All LC types, Any Product (or Cigarettes if Any not available)
Most adjusted

REF	NRR	SEX	AGEL	AGEH	RACE	YF	LC TYPE	LOC	START	ST	NLC	R	VB	P	H	AD	ADOS	PRODUCT	exL	exH	unexL	unexH	De
CEDERL	556	m	0	0	all	16	all	Eu:Sca	1963	pr	491	n	bl	n	n	3	1#	all/unsp	1	19	25	999	ot
CEDERL	536	f	0	0	all	10	all	Eu:Sca	1963	pr	491	n	bl	n	n	0	0	cig only	1	16	19	999	ot
CPSI	822	m	35	84	all	6	all	NAmer	1959	pr	5138	n	bl	n	n	1	0	cig+/-ot	1	14	25	999	ot
CPSI	851	f	40	74	all	6	all	NAmer	1959	pr	5138	n	bl	n	n	1	0	cig only	1	14	25	999	ot
DEAN3	570	m	0	0	all	-	all	Eu:UK	1969	CC	766	n	V	y	n	0	0	cig only	1	14	25	999	st
DEAN3	589	f	0	0	all	-	all	Eu:UK	1969	CC	766	n	V	y	n	0	0	cig only	1	14	25	999	st
DORN	589	m	55	64	wh	8	all	NAmer	1954	pr	5097	n	bl	n	n	0	0	cig+/-ot	1	14	25	999	st
DORN	626	m	65	74	wh	8	all	NAmer	1954	pr	5097	n	bl	n	n	0	0	cig+/-ot	1	14	25	999	st
ENGELA	505	m	0	0	all	0	all	Eu:Sca	1964	pr	435	n	bl	n	n	0	0	cig+/-ot	1	19	30	999	st
ENGELA	513	f	0	0	all	0	all	Eu:Sca	1964	pr	435	n	bl	n	n	0	0	cig+/-ot	1	19	30	999	st
GAO2	505	m	0	0	all	-	all	As:Jap	1988	CC	282	n	bl	n	n	0	0	cig+/-ot	1	19	30	999	st
HIRAYA	564	m	0	0	all	5	all	As:Jap	1965	pr	1917	n	bl	n	n	1	0	cig+/-ot	1	19	25	999	ot
HIRAYA	506	f	0	0	all	0	all	As:Jap	1965	pr	1917	n	bl	n	n	1	0	cig+/-ot	1	19	20	999	ot
LIAW	510	c	0	0	all	0	all	As:oth	1982	pr	127	n	ot	n	n	2	0	all/unsp	1	20	25	999	ot
MATOS	565	m	0	0	all	-	all	SCAmer	1994	CC	200	n	bl	n	n	2	0	cig+/-ot	1	14	20	999	ot
MIGRAN	509	m	0	0	all	0	all	Eu:UK	1964	pr	259	n	V	n	n	0	0	cig only	0	15	20	999	st
MIGRAN	519	f	0	0	all	0	all	Eu:UK	1964	pr	259	n	V	n	n	0	0	cig only	0	15	20	999	st
MRFITR	518	m	0	0	all	0	all	NAmer	1973	pr	119	n	bl	n	n	0	0	cig+/-ot	1	15	24	999	st
SEGI2	525	m	0	0	all	-	all	As:Jap	1962	CC	378	n	bl	n	n	1	0	cig+/-ot	1	19	23	999	ot
SOBUE	650	m	0	0	all	-	all	As:Jap	1986	CC	1376	n	bl	n	y	0	0	cig+/-ot	10	17	23	999	st
SPEIZE	530	f	0	0	all	0	all	NAmer	1976	pr	593	n	bl	n	y	2	1#	cig+/-ot	18	19	22	999	st
SVENSS	505	f	0	0	all	-	all	Eu:Sca	1983	CC	210	n	bl	n	n	0	0	all/unsp	0	18	26	999	st
WAKAI	505	m	0	0	all	-	all	As:Jap	1988	CC	333	n	bl	n	y	0	0	cig+/-ot	1	19	30	999	st
WU	545	f	0	0	wh	-	q+a	NAmer	1981	CC	220	n	bl	n	y	2	0	all/unsp	0	18	25	999	ot

Comments on values in listings

CEDERL ADOS Amount smoked
SPEIZE ADOS Numbers of cigs/day

Cigarette type is all/unspec for all RRs
except for the following:

REF	NRR	CIGTYPE
DEAN3	570	MC only
DEAN3	589	MC only

Table 1H10 - 2

IESLC - Meta-analysis of Current Smoking, Age started, "Highest vs lowest"
All LC types, Any Product (or Cigarettes if Any not available)
Most adjusted

REF	NRR	SEX	AD	Number Exposed Case	Cont	Non-exposed Case	Cont	RR	95.00%CI
*CEDERL	556	m	3	183	-	6	-	4.70 (	2.08- 10.60)
*CEDERL	536	f	0	0	746	6	2806	0.29~(	0.02- 5.13)
Subtotal CEDERL								3.82 (	1.75- 8.36)
*CPSI	822	m	1	185	-	42	-	4.11 (	2.94- 5.75)
*CPSI	851	f	1	6	-	51	-	1.11 (	0.48- 2.59)
Subtotal CPSI								3.44 (	2.52- 4.69)
DEAN3	570	m	0	44	165	24	75	0.83 (	0.47- 1.47)
DEAN3	589	f	0	7	109	27	274	0.65 (	0.28- 1.54)
Subtotal DEAN3								0.77 (	0.48- 1.24)
*DORN	589	m	0	70	25569	30	49537	4.52 (	2.95- 6.93)
*DORN	626	m	0	65	16016	70	49386	2.86 (	2.04- 4.01)
Subtotal DORN								3.41 (	2.62- 4.45)
*ENGELA	505	m	0	173	50732	17	9762	1.96 (	1.19- 3.22)
*ENGELA	513	f	0	18	10687	10	24560	4.14 (	1.91- 8.96)
Subtotal ENGELA								2.44 (	1.60- 3.71)
GAO2	505	m	0	52	26	2	4	4.00 (	0.69- 23.28)
*HIRAYA	564	m	1	26	-	19	-	2.29 (	1.27- 4.14)
*HIRAYA	506	f	1	-	-	-	-	0.32 (	0.04- 2.42)
Subtotal HIRAYA								1.97 (	1.12- 3.47)
*LIAW	510	c	2	-	-	-	-	3.07 (	1.57- 6.01)
MATOS	565	m	2	45	-	18	-	2.13 (	1.06- 4.28)
*MIGRAN	509	m	0	50	1081	24	668	1.29 (	0.80- 2.07)
*MIGRAN	519	f	0	2	266	11	1315	0.90 (	0.20- 4.03)
Subtotal MIGRAN								1.25 (	0.79- 1.96)
*MRFITR	518	m	0	25	2065	3	544	2.20 (	0.67- 7.24)
SEGI2	525	m	1	91	-	49	-	2.80 (	1.82- 4.32)
SOBUE	650	m	0	109	68	75	75	1.60 (	1.03- 2.49)
*SPEIZE	530	f	2	-	-	-	-	1.25 (	0.91- 1.67)
SVENSS	505	f	0	52	21	32	18	1.39 (	0.65- 3.00)
WAKAI	505	m	0	42	74	8	25	1.77 (	0.73- 4.28)
WU	545	f	2	106	-	14	-	8.75 (	3.26- 23.45)
Partial Totals				1351	107625	538	139049		

*prospective study

~ With 0.5 adjustment for zero

REF	NRR	SEX	AD	Ys	Ws	Qs	Ps
*CEDERL	556	m	3	1.55	5.79	3.42	0.0002
*CEDERL	536	f	0	-1.24	0.46	1.90	0.3977
Subtotal CEDERL				1.34	6.26	5.31	
*CPSI	822	m	1	1.41	34.15	13.71	0.0000
*CPSI	851	f	1	0.10	5.41	2.47	0.8082
Subtotal CPSI				1.23	39.56	16.18	
DEAN3	570	m	0	-0.18	11.93	11.05	0.5288
DEAN3	589	f	0	-0.43	5.19	7.57	0.3294
Subtotal DEAN3				-0.26	17.12	18.62	
*DORN	589	m	0	1.51	21.03	11.17	0.0000
*DORN	626	m	0	1.05	33.80	2.51	0.0000
Subtotal DORN				1.23	54.82	13.68	
*ENGELA	505	m	0	0.67	15.51	0.18	0.0081
*ENGELA	513	f	0	1.42	6.43	2.64	0.0003
Subtotal ENGELA				0.89	21.94	2.82	
GAO2	505	m	0	1.39	1.24	0.46	0.1229
*HIRAYA	564	m	1	0.83	11.00	0.03	0.0060
*HIRAYA	506	f	1	-1.14	0.91	3.36	0.2763
Subtotal HIRAYA				0.68	11.92	3.39	
*LIAW	510	c	2	1.12	8.53	1.00	0.0011
MATOS	565	m	2	0.76	7.89	0.00	0.0337
*MIGRAN	509	m	0	0.25	16.88	4.69	0.2993
*MIGRAN	519	f	0	-0.11	1.71	1.34	0.8892
Subtotal MIGRAN				0.22	18.58	6.03	
*MRFITR	518	m	0	0.79	2.70	0.00	0.1967
SEGI2	525	m	1	1.03	20.56	1.28	0.0000
SOBUE	650	m	0	0.47	19.78	1.87	0.0358
*SPEIZE	530	f	2	0.22	41.69	12.91	0.1497
SVENSS	505	f	0	0.33	6.51	1.31	0.3979
WAKAI	505	m	0	0.57	4.94	0.21	0.2027
WU	545	f	2	2.17	3.95	7.62	0.0000

Table 1H10 - 2

IESLC - Meta-analysis of Current Smoking, Age started, "Highest vs lowest"
All LC types, Any Product (or Cigarettes if Any not available)
Most adjusted

	N	24
	NS	17
	Wt	287.99
Het	Chi	92.69
Het	df	23
Het	P	***
Fixed	RR	2.18
	RRl	1.94
	RRu	2.45
	P	+++
Random	RR	2.08
	RRl	1.60
	RRu	2.69
	P	+++
Asymm	P	N.S.

Table 1H10 - 3

IESLC - Meta-analysis of Current Smoking, Age started, "Highest vs lowest"
All LC types, Any Product (or Cigarettes if Any not available)
Most adjusted

		Sex			
		combined	male	female	Total
	N	1	14	9	24
	NS	1	13	9	23
	Wt	8.53	207.21	72.25	287.99
Het	Chi	0.00	46.87	28.12	92.69
Het	df	0	13	8	23
Het	P	N.S.	***	***	***
Fixed	RR	3.07	2.49	1.43	2.18
	RRl	1.57	2.18	1.13	1.94
	RRu	6.01	2.86	1.80	2.45
	P	++	+++	++	+++
Random	RR	3.07	2.33	1.47	2.08
	RRl	1.57	1.77	0.84	1.60
	RRu	6.01	3.08	2.58	2.69
	P	++	+++	N.S.	+++
Between	Chi				17.70
Between	df				2
Between	P				***
Btwn(F)	P				N.S.
Btwn(R)	P				N.S.

		Lung cancer type		
		all	other	Total
	N	23	1	24
	NS	16	1	17
	Wt	284.04	3.95	287.99
Het	Chi	84.96	0.00	92.69
Het	df	22	0	23
Het	P	***	N.S.	***
Fixed	RR	2.14	8.75	2.18
	RRl	1.90	3.26	1.94
	RRu	2.40	23.47	2.45
	P	+++	+++	+++
Random	RR	1.98	8.75	2.08
	RRl	1.53	3.26	1.60
	RRu	2.55	23.47	2.69
	P	+++	+++	+++
Between	Chi			7.72
Between	df			1
Between	P			**
Btwn(F)	P			N.S.
Btwn(R)	P			**

		Location								
		NAmer	UK	Scand	othEur	China	Japan	othAs	other	Total
	N	7	4	5			6	1	1	24
	NS	5	2	3			5	1	1	17
	Wt	142.71	35.71	34.71			58.45	8.53	7.89	287.99
Het	Chi	46.07	2.44	9.17			7.11	0.00	0.00	92.69
Het	df	6	3	4			5	0	0	23
Het	P	***	N.S.	(*)			N.S.	N.S.	N.S.	***
Fixed	RR	2.60	0.99	2.38			2.09	3.07	2.13	2.18
	RRl	2.20	0.71	1.71			1.62	1.57	1.06	1.94
	RRu	3.06	1.38	3.32			2.70	6.01	4.28	2.45
	P	+++	N.S.	+++			+++	++	+	+++
Random	RR	2.79	0.99	2.42			2.06	3.07	2.13	2.08
	RRl	1.68	0.71	1.38			1.46	1.57	1.06	1.60
	RRu	4.63	1.38	4.26			2.89	6.01	4.28	2.69
	P	+++	N.S.	++			+++	++	+	+++
Between	Chi									27.89
Between	df									5
Between	P									***
Btwn(F)	P									N.S.
Btwn(R)	P									**

Table 1H10 - 3

IESLC - Meta-analysis of Current Smoking, Age started, "Highest vs lowest"
All LC types, Any Product (or Cigarettes if Any not available)
Most adjusted

Detailed Country in "other Europe"

	multi	Germany	othWest	East	Balkans	Total
N						
NS						
Wt						
Het Chi						
Het df						
Het P						N.S.
Fixed RR						
RRl						
RRu						
P						+++
Random RR						
RRl						
RRu						
P						+++
Between Chi						
Between df						
Between P						N.S.
Btwn(F) P						N.S.
Btwn(R) P						N.S.

Detailed Country in "other Asia"

	India	HongKong	other	Total
N			1	1
NS			1	1
Wt			8.53	8.53
Het Chi			0.00	0.00
Het df			0	0
Het P			N.S.	N.S.
Fixed RR			3.07	3.07
RRl			1.57	1.57
RRu			6.01	6.01
P			++	++
Random RR			3.07	3.07
RRl			1.57	1.57
RRu			6.01	6.01
P			++	++
Between Chi				
Between df				
Between P				N.S.
Btwn(F) P				N.S.
Btwn(R) P				N.S.

Detailed other continent

	SCAmer	Total
N	1	1
NS	1	1
Wt	7.89	7.89
Het Chi	0.00	0.00
Het df	0	0
Het P	N.S.	N.S.
Fixed RR	2.13	2.13
RRl	1.06	1.06
RRu	4.28	4.28
P	+	+
Random RR	2.13	2.13
RRl	1.06	1.06
RRu	4.28	4.28
P	+	+
Between Chi		
Between df		
Between P		N.S.
Btwn(F) P		N.S.
Btwn(R) P		N.S.

Table 1H10 - 3

IESLC - Meta-analysis of Current Smoking, Age started, "Highest vs lowest"
All LC types, Any Product (or Cigarettes if Any not available)
Most adjusted

	Start year of study					
	<1960	1960-69	1970-79	1980-89	1990+	Total
N	4	11	2	6	1	24
NS	2	6	2	6	1	17
Wt	94.38	96.39	44.38	44.95	7.89	287.99
Het Chi	10.70	33.98	0.80	12.45	0.00	92.69
Het df	3	10	1	5	0	23
Het P	*	***	N.S.	*	N.S.	***
Fixed RR	3.42	1.80	1.29	2.14	2.13	2.18
RRl	2.80	1.48	0.96	1.60	1.06	1.94
RRu	4.19	2.20	1.74	2.86	4.28	2.45
P	+++	+++	(+)	+++	+	+++
Random RR	3.14	1.66	1.29	2.45	2.13	2.08
RRl	2.08	1.10	0.96	1.47	1.06	1.60
RRu	4.73	2.51	1.74	4.10	4.28	2.69
P	+++	+	(+)	+++	+	+++
Between Chi						34.75
Between df						4
Between P						***
Btwn(F) P						(*)
Btwn(R) P						**

	Study type (1)		
	CC	other	Total
N	9	15	24
NS	8	9	17
Wt	82.00	205.99	287.99
Het Chi	28.02	59.98	92.69
Het df	8	14	23
Het P	***	***	***
Fixed RR	1.78	2.36	2.18
RRl	1.43	2.06	1.94
RRu	2.21	2.71	2.45
P	+++	+++	+++
Random RR	1.82	2.25	2.08
RRl	1.17	1.63	1.60
RRu	2.83	3.11	2.69
P	++	+++	+++
Between Chi			4.69
Between df			1
Between P			*
Btwn(F) P			N.S.
Btwn(R) P			N.S.

	Study type (2)			
	CC	prosp	other	Total
N	9	15		24
NS	8	9		17
Wt	82.00	205.99		287.99
Het Chi	28.02	59.98		92.69
Het df	8	14		23
Het P	***	***		***
Fixed RR	1.78	2.36		2.18
RRl	1.43	2.06		1.94
RRu	2.21	2.71		2.45
P	+++	+++		+++
Random RR	1.82	2.25		2.08
RRl	1.17	1.63		1.60
RRu	2.83	3.11		2.69
P	++	+++		+++
Between Chi				4.69
Between df				1
Between P				*
Btwn(F) P				N.S.
Btwn(R) P				N.S.

Table 1H10 - 3

IESLC - Meta-analysis of Current Smoking, Age started, "Highest vs lowest"
All LC types, Any Product (or Cigarettes if Any not available)
Most adjusted

	Study size (number of LC cases) 100-249	250-499	500-999	1000+	Total
N	5	9	3	7	24
NS	5	6	2	4	17
Wt	29.57	73.53	58.81	126.08	287.99
Het Chi	8.97	16.11	3.01	25.29	92.69
Het df	4	8	2	6	23
Het P	(*)	*	N.S.	***	***
Fixed RR	2.61	2.19	1.09	2.88	2.18
RRl	1.82	1.75	0.84	2.42	1.94
RRu	3.74	2.76	1.40	3.43	2.45
P	+++	+++	N.S.	+++	+++
Random RR	2.71	2.24	1.01	2.44	2.08
RRl	1.55	1.54	0.70	1.64	1.60
RRu	4.73	3.24	1.46	3.64	2.69
P	+++	+++	N.S.	+++	+++
Between Chi					39.31
Between df					3
Between P					***
Btwn(F) P					*
Btwn(R) P					**

	Risky occupational population no	mining	othRisky	Total
N	24			24
NS	17			17
Wt	287.99			287.99
Het Chi	92.69			92.69
Het df	23			23
Het P	***			***
Fixed RR	2.18			2.18
RRl	1.94			1.94
RRu	2.45			2.45
P	+++			+++
Random RR	2.08			2.08
RRl	1.60			1.60
RRu	2.69			2.69
P	+++			+++
Between Chi				
Between df				
Between P				N.S.
Btwn(F) P				N.S.
Btwn(R) P				N.S.

	National cigarette tobacco type Virginia	blended	other	Total
N	4	19	1	24
NS	2	14	1	17
Wt	35.71	243.75	8.53	287.99
Het Chi	2.44	64.43	0.00	92.69
Het df	3	18	0	23
Het P	N.S.	***	N.S.	***
Fixed RR	0.99	2.42	3.07	2.18
RRl	0.71	2.13	1.57	1.94
RRu	1.38	2.74	6.01	2.45
P	N.S.	+++	++	+++
Random RR	0.99	2.41	3.07	2.08
RRl	0.71	1.84	1.57	1.60
RRu	1.38	3.15	6.01	2.69
P	N.S.	+++	++	+++
Between Chi				25.81
Between df				2
Between P				***
Btwn(F) P				*
Btwn(R) P				***

Table 1H10 - 3

IESLC - Meta-analysis of Current Smoking, Age started, "Highest vs lowest"
All LC types, Any Product (or Cigarettes if Any not available)
Most adjusted

	Any proxy use No/nk	Yes	Total
N	22	2	24
NS	16	1	17
Wt	270.86	17.12	287.99
Het Chi	72.91	0.22	92.69
Het df	21	1	23
Het P	***	N.S.	***
Fixed RR	2.33	0.77	2.18
RRl	2.07	0.48	1.94
RRu	2.62	1.24	2.45
P	+++	N.S.	+++
Random RR	2.30	0.77	2.08
RRl	1.79	0.48	1.60
RRu	2.96	1.24	2.69
P	+++	N.S.	+++
Between Chi			19.56
Between df			1
Between P			***
Btwn(F) P			*
Btwn(R) P			***

	Full histological confirmation No	Yes	Total
N	20	4	24
NS	13	4	17
Wt	217.63	70.36	287.99
Het Chi	67.24	13.85	92.69
Het df	19	3	23
Het P	***	**	***
Fixed RR	2.44	1.53	2.18
RRl	2.14	1.21	1.94
RRu	2.79	1.94	2.45
P	+++	+++	+++
Random RR	2.09	2.06	2.08
RRl	1.58	1.12	1.60
RRu	2.76	3.77	2.69
P	+++	+	+++
Between Chi			11.59
Between df			1
Between P			***
Btwn(F) P			(*)
Btwn(R) P			N.S.

	Number of adjustment variables (1) 0	1	2+/+nk	Total
N	14	5	5	24
NS	10	3	5	18
Wt	148.11	72.04	67.84	287.99
Het Chi	46.01	14.19	23.31	92.69
Het df	13	4	4	23
Het P	***	**	***	***
Fixed RR	2.02	2.96	1.87	2.18
RRl	1.72	2.35	1.47	1.94
RRu	2.37	3.72	2.37	2.45
P	+++	+++	+++	+++
Random RR	1.79	2.27	2.96	2.08
RRl	1.27	1.36	1.49	1.60
RRu	2.52	3.79	5.90	2.69
P	+++	++	++	+++
Between Chi				9.18
Between df				2
Between P				*
Btwn(F) P				N.S.
Btwn(R) P				N.S.

Table 1H10 - 3

IESLC - Meta-analysis of Current Smoking, Age started, "Highest vs lowest"
All LC types, Any Product (or Cigarettes if Any not available)
Most adjusted

	Number of adjustment variables (2) 0	1	2	3-5	6+/+nk	Total
N	14	5	4	1		24
NS	10	3	4	1		18
Wt	148.11	72.04	62.05	5.79		287.99
Het Chi	46.01	14.19	17.91	0.00		92.69
Het df	13	4	3	0		23
Het P	***	**	***	N.S.		***
Fixed RR	2.02	2.96	1.71	4.70		2.18
RRl	1.72	2.35	1.34	2.08		1.94
RRu	2.37	3.72	2.20	10.61		2.45
P	+++	+++	+++	+++		+++
Random RR	1.79	2.27	2.66	4.70		2.08
RRl	1.27	1.36	1.25	2.08		1.60
RRu	2.52	3.79	5.64	10.61		2.69
P	+++	++	+	+++		+++
Between Chi						14.58
Between df						3
Between P						**
Btwn(F) P						N.S.
Btwn(R) P						N.S.

	Product all/unsp	cig+/-ot	cig only	Total
N	4	14	6	24
NS	4	11	4	19
Wt	24.78	221.63	41.58	287.99
Het Chi	9.36	47.99	3.22	92.69
Het df	3	13	5	23
Het P	*	***	N.S.	***
Fixed RR	3.26	2.42	0.99	2.18
RRl	2.20	2.12	0.73	1.94
RRu	4.83	2.76	1.34	2.45
P	+++	+++	N.S.	+++
Random RR	3.49	2.41	0.99	2.08
RRl	1.72	1.81	0.73	1.60
RRu	7.07	3.19	1.34	2.69
P	+++	+++	N.S.	+++
Between Chi				32.12
Between df				2
Between P				***
Btwn(F) P				*
Btwn(R) P				***

	Derivation of RR/CI Orig	StdCalc	Other	Total
N		14	10	24
NS		10	7	17
Wt		189.33	98.66	287.99
Het Chi		51.92	23.32	92.69
Het df		13	9	23
Het P		***	**	***
Fixed RR		1.83	3.07	2.18
RRl		1.58	2.52	1.94
RRu		2.11	3.74	2.45
P		+++	+++	+++
Random RR		1.76	2.74	2.08
RRl		1.28	1.90	1.60
RRu		2.42	3.96	2.69
P		+++	+++	+++
Between Chi				17.45
Between df				1
Between P				***
Btwn(F) P				*
Btwn(R) P				(*)

Table 1H10 - 4

IESLC - Meta-analysis of Current Smoking, Age started, "Highest vs lowest"
All LC types, Any Product (or Cigarettes if Any not available)
Least adjusted

REF	NRR	X	SEX	AGEL	AGEH	RACE	YF	LC TYPE	LOC	START	ST	NLC	R	VB	P	H	AD	ADOS	PRODUCT	exL	exH	unexL	unexH	De
CEDERL	556		m	0	0	all	16	all	Eu:Sca	1963	pr	491	n	bl	n	n	3	1#	all/unsp	1	19	25	999	ot
CEDERL	536		f	0	0	all	10	all	Eu:Sca	1963	pr	491	n	bl	n	n	0	0	cig only	1	16	19	999	ot
CPSI	822		m	35	84	all	6	all	NAmer	1959	pr	5138	n	bl	n	n	1	0	cig+/-ot	1	14	25	999	ot
CPSI	851		f	40	74	all	6	all	NAmer	1959	pr	5138	n	bl	n	n	1	0	cig only	1	14	25	999	ot
DEAN3	570		m	0	0	all	-	all	Eu:UK	1969	CC	766	n	V	y	n	0	0	cig only	1	14	25	999	st
DEAN3	589		f	0	0	all	-	all	Eu:UK	1969	CC	766	n	V	y	n	0	0	cig only	1	14	25	999	st
DORN	589		m	55	64	wh	8	all	NAmer	1954	pr	5097	n	bl	n	n	0	0	cig+/-ot	1	14	25	999	st
DORN	626		m	65	74	wh	8	all	NAmer	1954	pr	5097	n	bl	n	n	0	0	cig+/-ot	1	14	25	999	st
ENGELA	505		m	0	0	all	0	all	Eu:Sca	1964	pr	435	n	bl	n	n	0	0	cig+/-ot	1	19	30	999	st
ENGELA	513		f	0	0	all	0	all	Eu:Sca	1964	pr	435	n	bl	n	n	0	0	cig+/-ot	1	19	30	999	st
GAO2	505		m	0	0	all	-	all	As:Jap	1988	CC	282	n	bl	n	n	0	0	cig+/-ot	1	19	30	999	st
HIRAYA	564		m	0	0	all	5	all	As:Jap	1965	pr	1917	n	bl	n	n	1	0	cig+/-ot	1	19	25	999	ot
HIRAYA	506		f	0	0	all	0	all	As:Jap	1965	pr	1917	n	bl	n	n	1	0	cig+/-ot	1	19	20	999	ot
LIAW	510		c	0	0	all	0	all	As:oth	1982	pr	127	n	ot	n	n	2	0	all/unsp	1	20	25	999	ot
MATOS	545	x	m	0	0	all	-	all	SCAmer	1994	CC	200	n	bl	n	n	0	0	cig+/-ot	1	14	20	999	st
MIGRAN	509		m	0	0	all	0	all	Eu:UK	1964	pr	259	n	V	n	n	0	0	cig only	0	15	20	999	st
MIGRAN	519		f	0	0	all	0	all	Eu:UK	1964	pr	259	n	V	n	n	0	0	cig only	0	15	20	999	st
MRFITR	518		m	0	0	all	0	all	NAmer	1973	pr	119	n	bl	n	n	0	0	cig+/-ot	1	15	24	999	st
SEGI2	520	x	m	0	0	all	-	all	As:Jap	1962	CC	378	n	bl	n	n	0	0	cig+/-ot	1	19	23	999	st
SOBUE	650		m	0	0	all	-	all	As:Jap	1986	CC	1376	n	bl	n	y	0	0	cig+/-ot	10	17	23	999	st
SPEIZE	530		f	0	0	all	0	all	NAmer	1976	pr	593	n	bl	n	y	2	1#	cig+/-ot	18	19	22	999	st
SVENSS	505		f	0	0	all	-	all	Eu:Sca	1983	CC	210	n	bl	n	n	0	0	all/unsp	0	18	26	999	st
WAKAI	505		m	0	0	all	-	all	As:Jap	1988	CC	333	n	bl	n	y	0	0	cig+/-ot	1	19	30	999	st
WU	521	x	f	0	0	wh	-	q+a	NAmer	1981	CC	220	n	bl	n	y	0	0	all/unsp	0	18	25	999	st

Comments on values in listings

CEDERL ADOS Amount smoked
SPEIZE 2 ADOS Numbers of cigs/day

Cigarette type is all/unspec for all RRs
except for the following:

REF	NRR	CIGTYPE
DEAN3	570	MC only
DEAN3	589	MC only

Table 1H10 - 5

IESLC - Meta-analysis of Current Smoking, Age started, "Highest vs lowest"
All LC types, Any Product (or Cigarettes if Any not available)
Least adjusted

REF	NRR	SEX	AD	Number Exposed Case	Number Exposed Cont	Non-exposed Case	Non-exposed Cont	RR	95.00%CI
*CEDERL	556	m	3	183	-	6	-	4.70	(2.08- 10.60)
*CEDERL	536	f	0	0	746	6	2806	0.29~	(0.02- 5.13)
Subtotal CEDERL								3.82	(1.75- 8.36)
*CPSI	822	m	1	185	-	42	-	4.11	(2.94- 5.75)
*CPSI	851	f	1	6	-	51	-	1.11	(0.48- 2.59)
Subtotal CPSI								3.44	(2.52- 4.69)
DEAN3	570	m	0	44	165	24	75	0.83	(0.47- 1.47)
DEAN3	589	f	0	7	109	27	274	0.65	(0.28- 1.54)
Subtotal DEAN3								0.77	(0.48- 1.24)
*DORN	589	m	0	70	25569	30	49537	4.52	(2.95- 6.93)
*DORN	626	m	0	65	16016	70	49386	2.86	(2.04- 4.01)
Subtotal DORN								3.41	(2.62- 4.45)
*ENGELA	505	m	0	173	50732	17	9762	1.96	(1.19- 3.22)
*ENGELA	513	f	0	18	10687	10	24560	4.14	(1.91- 8.96)
Subtotal ENGELA								2.44	(1.60- 3.71)
GAO2	505	m	0	52	26	2	4	4.00	(0.69- 23.28)
*HIRAYA	564	m	1	26	-	19	-	2.29	(1.27- 4.14)
*HIRAYA	506	f	1	-	-	-	-	0.32	(0.04- 2.42)
Subtotal HIRAYA								1.97	(1.12- 3.47)
*LIAW	510	c	2	-	-	-	-	3.07	(1.57- 6.01)
MATOS	545	m	0	45	41	18	33	2.01	(0.99- 4.11)
*MIGRAN	509	m	0	50	1081	24	668	1.29	(0.80- 2.07)
*MIGRAN	519	f	0	2	266	11	1315	0.90	(0.20- 4.03)
Subtotal MIGRAN								1.25	(0.79- 1.96)
*MRFITR	518	m	0	25	2065	3	544	2.20	(0.67- 7.24)
SEGI2	520	m	0	91	103	49	155	2.79	(1.82- 4.29)
SOBUE	650	m	0	109	68	75	75	1.60	(1.03- 2.49)
*SPEIZE	530	f	2	-	-	-	-	1.25	(0.91- 1.67)
SVENSS	505	f	0	52	21	32	18	1.39	(0.65- 3.00)
WAKAI	505	m	0	42	74	8	25	1.77	(0.73- 4.28)
WU	521	f	0	106	32	14	19	4.50	(2.03- 9.96)
Partial Totals				1351	107801	538	139256		

*prospective study ~ With 0.5 adjustment for zero

REF	NRR	SEX	AD	Ys	Ws	Qs	Ps
*CEDERL	556	m	3	1.55	5.79	3.46	0.0002
*CEDERL	536	f	0	-1.24	0.46	1.89	0.3977
Subtotal CEDERL				1.34	6.26	5.35	
*CPSI	822	m	1	1.41	34.15	13.93	0.0000
*CPSI	851	f	1	0.10	5.41	2.43	0.8082
Subtotal CPSI				1.23	39.56	16.36	
DEAN3	570	m	0	-0.18	11.93	10.93	0.5288
DEAN3	589	f	0	-0.43	5.19	7.51	0.3294
Subtotal DEAN3				-0.26	17.12	18.44	
*DORN	589	m	0	1.51	21.03	11.32	0.0000
*DORN	626	m	0	1.05	33.80	2.60	0.0000
Subtotal DORN				1.23	54.82	13.92	
*ENGELA	505	m	0	0.67	15.51	0.16	0.0081
*ENGELA	513	f	0	1.42	6.43	2.68	0.0003
Subtotal ENGELA				0.89	21.94	2.84	
GAO2	505	m	0	1.39	1.24	0.46	0.1229
*HIRAYA	564	m	1	0.83	11.00	0.03	0.0060
*HIRAYA	506	f	1	-1.14	0.91	3.34	0.2763
Subtotal HIRAYA				0.68	11.92	3.38	
*LIAW	510	c	2	1.12	8.53	1.03	0.0011
MATOS	545	m	0	0.70	7.55	0.04	0.0547
*MIGRAN	509	m	0	0.25	16.88	4.60	0.2993
*MIGRAN	519	f	0	-0.11	1.71	1.32	0.8892
Subtotal MIGRAN				0.22	18.58	5.93	
*MRFITR	518	m	0	0.79	2.70	0.00	0.1967
SEGI2	520	m	0	1.03	21.03	1.35	0.0000
SOBUE	650	m	0	0.47	19.78	1.82	0.0358
*SPEIZE	530	f	2	0.22	41.69	12.68	0.1497
SVENSS	505	f	0	0.33	6.51	1.28	0.3979
WAKAI	505	m	0	0.57	4.94	0.20	0.2027
WU	521	f	0	1.50	6.07	3.22	0.0002

Table 1H10 - 5

IESLC - Meta-analysis of Current Smoking, Age started, "Highest vs lowest"
All LC types, Any Product (or Cigarettes if Any not available)
Least adjusted

	N	24
	NS	17
	Wt	290.23
Het	Chi	88.29
Het	df	23
Het	P	***
Fixed	RR	2.17
	RRl	1.93
	RRu	2.43
	P	+++
Random	RR	2.04
	RRl	1.59
	RRu	2.62
	P	+++
Asymm	P	N.S.

Table 1H10 - 6

IESLC - Meta-analysis of Current Smoking, Age started, "Highest vs lowest"
All LC types, Any Product (or Cigarettes if Any not available)
Least adjusted

		Sex		
	combined	male	female	Total
N	1	14	9	24
NS	1	13	9	23
Wt	8.53	207.33	74.38	290.23
Het Chi	0.00	47.02	23.13	88.29
Het df	0	13	8	23
Het P	N.S.	***	**	***
Fixed RR	3.07	2.49	1.42	2.17
RRl	1.57	2.17	1.13	1.93
RRu	6.01	2.85	1.79	2.43
P	++	+++	++	+++
Random RR	3.07	2.32	1.42	2.04
RRl	1.57	1.76	0.87	1.59
RRu	6.01	3.07	2.34	2.62
P	++	+++	N.S.	+++
Between Chi				18.14
Between df				2
Between P				***
Btwn(F) P				(*)
Btwn(R) P				N.S.

Table 1H10 - 7

IESLC - Meta-analysis of Current Smoking, Age started, "Highest vs lowest"
All LC types, Any Product (or Cigarettes if Any not available)
Excluded studies (and stage at which they were excluded)

1	AGUDO ALDERS ARMADA AUVINE AXELSS BARBON BECHER BENHAM BLOT1 BOFFET BOUCHA BRESLO BROWN3 CARPEN CHEN CHEN2 CHIAZZ CHOI CHYOU CORREA DAMBER DARBY DESTEF DOLL DOLL2 DORGAN DOSEME FAN GAO GARCIA GARSHI GENG GER GRAHAM GUO GURSEL HAENSZ HAMMO2 HAMMON HEGMAN HU HU2 JAHN JAIN JEDRYC JOLY JUSSAW KHUDER KOO KOULUM KREUZE LAUSSM LETOUR LEVIN LIU3 LIU4 LIU5 LUBIN LUBIN2 LUO MCCONN NOTAN2 OSANN2 PERNU PEZZOT PRESCO QIAO QIAO2 RACHTA RESTRE SADOWS STASZE SUZUK2 TIZZAN TVERDA VUTUC WANG2 WIGLE WU2 WUWILL WYNDE2 WYNDE3 XU YUAN ZHANG ZHENG ZHOU
2	AKIBA AMANDU AMES BENSHL BEST BOUCOT BROSS BUFFLE CPSII DEAN2 GILLIS HUMBLE KAISE2 KATSOU KAUFMA PEZZO2 PISANI SPITZ WATSON WYNDE8
3	MCDUFF WYNDE6
6	HOLE
8	WYNDE7

Table 1H10 - 8
Potentially overlapping studies

REF	REFGP	PRINC	. OVERLAP/LINK
MRFITR	MRFIT	2	Subset of MRFIT
CPSI	CPSI	1	CPSI overall

Table 1H10 - 9
Most adjusted - insufficient data for meta-analysis

REF	NRR	SEX	AGEL	AGEH	RACE	YF	LC TYPE	LOC	START	ST	NLC	R	VB	P	H	AD	ADOS	PRODUCT	exL	exH	unexL	unexH	De
HIRAYA	548	m	0	0	all	8	all	As:Jap	1965	pr	1917	n	bl	n	n	1	0	cig+/-ot	1	19	25	999	st
HOLE	502	m	0	0	all	0	all	Eu:UK	1972	pr	225	n	V	n	n	3	1#	cig+/-ot	1	14	20	999	st
SPEIZE	533	f	0	0	all	0	all	NAmer	1976	pr	593	n	bl	n	y	2	1#	cig+/-ot	1	17	22	999	ot

Comments on values in listings

HOLEI	2	ADOS	Number smoked
SPEIZE	3	ADOS	Numbers of cigs/day

REF	NRR	RR	SIG	RRDATA comment
HIRAYA	548	1.55		0
HOLE	502	1.28		0
SPEIZE	533		*	gap Text page 478 SPEIZE1999 gives the risk for age start 1-17 vs 18-19 as 1.1(0.9-1.5)

Table 1H11 -

IESLC - Meta-analysis of Ever/current Smoking by Age started, Overview
All LC types, Any Product (or Cigarettes if Any not available)

This analysis is restricted to results for:
1) Ever/current smokers
2) Results by Age started
3) Categorical results by Age started
Results by Age started are grouped under 2 schemes (S1, S2). Each scheme has a set of "key values". An interval is allocated to the category whose key value it includes, and intervals which include none or more than one of the key values are excluded. (Open-ended intervals are coded as 999)

S1	key value	maximum range
1	26	19+
2	18	15-25
3	14	1-17

S2	key value	maximum range
1	30	27+
2	26	23-29
3	22	19-25
4	18	15-21
5	14	11-17
6	10	1-13

4) All LC types (or near equivalent)
5) Results complete enough for use in metaanalysis

Within each study, results are then selected (in the following order of preference, within each sex) for:
6) SMKSTA: ever, current
7) PRODUCT: all/unspec, cigarettes regardless of other products, cigarettes only
8) CIGTYPE: all/unspecified, MC regardless of HR, MC only
9) (not applicable)
10) DENOM: never smoked anything, never smoked cigarettes, never any + low, never cigs + low
11) Followup period (YF, prospective studies): whole study (coded as 0) or longest available
12) LCtype: all or nearest available, at least Squamous and Adeno. (q = squamous, s = small, l = large, a = adeno, mix = mixed, alv = alveolar)
13) Race: all or nearest available, otherwise by race (wh or w = white, bl or b = black, hi = hispanic ch = chinese, jap = japanese, haw = hawaiian, w+o = white + oriental, sca = scandinavian, as = asian)
14) For overlapping studies: principal rather than subsidiary studies
Finally by Age: whole study (coded as 0) if available, otherwise by widest available age group and then for single sex results (m, f) in preference to results for both sexes combined (c).

Results adjusted (AD) for the most potential confounders are then chosen in Sections -1 to -3 and results adjusted for the least confounders in Sections -4 to -6. (Those least adjusted results which actually differ from the most adjusted are marked 'x' in column X in Section -4)

Section -7 shows excluded studies, together with the stage (as above) at which no qualifying results were found.

Section -8 lists the potentially overlapping studies which have been included (1=principal, 2=subsidiary).

Section -9 lists any results which would have been included in preference except that they had data not complete enough for use in meta-analysis, with their significance (yes/no), if known, and any further comment as entered on the database. It also lists as "gap" any categories for which no data were presented by the original authors.

In addition to those mentioned above, the following fields, levels and abbreviations are used:
* or nk = not known, n = no, y = yes, ot = other
ev = ever, cu = current, nev = never
all/unspec = all or unspecified, cig+/-ot = cigarettes irrespective of other products (cigar, pipe etc)
MC = manufactured cigarettes, HR = hand-rolled cigarettes
exL, exH = range of exposure (low and high) in the smoking group, in terms of Age started
REF: 6-character study reference
NRR: number of the RR on the database within the study
ST : study type (CC = case control, pr or prosp = prospective)
NLC: number of lung cancer cases in whole study
R : risky occupational population (n = no, m = mining, o = other risky)
VB : national cigarette type (V = at least 75% Virginia, bl = at least 75% blended, ot = other)
P : any proxy use
H : full histological confirmation
De : derivation of RR/CI (or = original, st = standard method, ot = other method of estimation)

Table 1H11 - 1

IESLC - Meta-analysis of Ever/current Smoking by Age started, Overview
All LC types, Any Product (or Cigarettes if Any not available)
Most adjusted

REF	NRR	SEX	AGEL	AGEH	RACE	YF	LC TYPE	LOC	START	ST	NLC	R	VB	P	H	AD	SM	PRODUCT	exL	exH	S1	S2	DENOM	De
AGUDO	504	f	0	0	all	-	all	Eu:wst	1989	CC	103	n	bl	n	n	3	ev	cig only	24	999	1	0	nev cigs	or
AGUDO	505	f	0	0	all	-	all	Eu:wst	1989	CC	103	n	bl	n	n	3	ev	cig only	1	23	0	0	nev cigs	or
ARMADA	511	m	0	0	all	-	all	Eu:wst	1986	CC	325	n	bl	n	y	0	ev	cig+/-ot	17	45	0	0	nev cigs	st
ARMADA	512	m	0	0	all	-	all	Eu:wst	1986	CC	325	n	bl	n	y	0	ev	cig+/-ot	7	16	3	0	nev cigs	st
AUVINE	520	c	0	0	all	-	all	Eu:Sca	1986	CC	517	n	bl	y	n	2	ev	cig+/-ot	16	999	0	0	nev cigs	or
AUVINE	521	c	0	0	all	-	all	Eu:Sca	1986	CC	517	n	bl	y	n	2	ev	cig+/-ot	1	15	3	0	nev cigs	or
BARBON	520	m	0	0	all	-	all	Eu:wst	1979	CC	755	n	bl	y	y	1	ev	all/unsp	20	999	1	0	nev any	or
BARBON	521	m	0	0	all	-	all	Eu:wst	1979	CC	755	n	bl	y	y	1	ev	all/unsp	15	19	2	4	nev any	or
BARBON	522	m	0	0	all	-	all	Eu:wst	1979	CC	755	n	bl	y	y	1	ev	all/unsp	1	14	3	0	nev any	or
BRESLO	501	c	0	0	all	-	all	NAmer	1949	CC	518	n	bl	n	y	0	ev	cig+/-ot	25	999	1	0	nev any	st
BRESLO	502	c	0	0	all	-	all	NAmer	1949	CC	518	n	bl	n	y	0	ev	cig+/-ot	15	24	2	0	nev any	st
BRESLO	503	c	0	0	all	-	all	NAmer	1949	CC	518	n	bl	n	y	0	ev	cig+/-ot	0	14	3	0	nev any	st
BUFFLE	517	f	0	0	w-hi	-	all	NAmer	1976	CC	943	n	bl	y	n	0	ev	cig+/-ot	30	999	0	1	nev cigs	or
BUFFLE	518	f	0	0	w-hi	-	all	NAmer	1976	CC	943	n	bl	y	n	0	ev	cig+/-ot	21	29	1	0	nev cigs	or
BUFFLE	519	f	0	0	w-hi	-	all	NAmer	1976	CC	943	n	bl	y	n	0	ev	cig+/-ot	19	20	0	0	nev cigs	or
BUFFLE	520	f	0	0	w-hi	-	all	NAmer	1976	CC	943	n	bl	y	n	0	ev	cig+/-ot	17	18	2	4	nev cigs	ot
BUFFLE	521	f	0	0	w-hi	-	all	NAmer	1976	CC	943	n	bl	y	n	0	ev	cig+/-ot	6	16	3	0	nev cigs	or
CEDERL	510	m	0	0	all	10	all	Eu:Sca	1963	pr	491	n	bl	n	n	1	cu	cig only	19	999	1	0	nev any	ot
CEDERL	511	m	0	0	all	10	all	Eu:Sca	1963	pr	491	n	bl	n	n	1	cu	cig only	17	18	2	4	nev any	ot
CEDERL	512	m	0	0	all	10	all	Eu:Sca	1963	pr	491	n	bl	n	n	1	cu	cig only	1	16	3	0	nev any	ot
CEDERL	515	f	0	0	all	10	all	Eu:Sca	1963	pr	491	n	bl	n	n	0	cu	cig only	19	999	1	0	nev any	st
CEDERL	516	f	0	0	all	10	all	Eu:Sca	1963	pr	491	n	bl	n	n	0	cu	cig only	17	18	2	4	nev any	st
CEDERL	517	f	0	0	all	10	all	Eu:Sca	1963	pr	491	n	bl	n	n	0	cu	cig only	1	16	3	0	nev any	ot
CHEN2	517	m	0	0	all	-	all	As:Chi	1983	CC	193	n	ot	y	n	0	ev	all/unsp	31	999	0	0	nev any	st
CHEN2	518	m	0	0	all	-	all	As:Chi	1983	CC	193	n	ot	y	n	0	ev	all/unsp	20	30	1	0	nev any	st
CHEN2	519	m	0	0	all	-	all	As:Chi	1983	CC	193	n	ot	y	n	0	ev	all/unsp	1	19	0	0	nev any	st
CHEN2	522	f	0	0	all	-	all	As:Chi	1983	CC	193	n	ot	y	n	0	ev	all/unsp	31	999	0	0	nev any	st
CHEN2	523	f	0	0	all	-	all	As:Chi	1983	CC	193	n	ot	y	n	0	ev	all/unsp	20	30	1	0	nev any	st
CHEN2	524	f	0	0	all	-	all	As:Chi	1983	CC	193	n	ot	y	n	0	ev	all/unsp	1	19	0	0	nev any	st
CHIAZZ	501	m	0	0	all	-	all	NAmer	1940	CC	144	o	bl	y	n	2	ev	cig+/-ot	20	999	1	0	nev cigs	or
CHIAZZ	502	m	0	0	all	-	all	NAmer	1940	CC	144	o	bl	y	n	2	ev	cig+/-ot	1	19	0	0	nev cigs	or
CHOI	523	m	0	0	all	-	all	As:oth	1985	CC	375	n	bl	n	n	0	ev	cig+/-ot	25	999	1	0	nev cigs	st
CHOI	524	m	0	0	all	-	all	As:oth	1985	CC	375	n	bl	n	n	0	ev	cig+/-ot	20	24	0	3	nev cigs	st
CHOI	525	m	0	0	all	-	all	As:oth	1985	CC	375	n	bl	n	n	0	ev	cig+/-ot	15	19	2	4	nev cigs	st
CHOI	526	m	0	0	all	-	all	As:oth	1985	CC	375	n	bl	n	n	0	ev	cig+/-ot	1	14	3	0	nev cigs	st
CHOI	530	f	0	0	all	-	all	As:oth	1985	CC	375	n	bl	n	n	0	ev	cig+/-ot	25	999	1	0	nev cigs	st
CHOI	531	f	0	0	all	-	all	As:oth	1985	CC	375	n	bl	n	n	0	ev	cig+/-ot	1	24	0	0	nev cigs	st
CPSI	801	m	35	84	all	6	all	NAmer	1959	pr	5138	n	bl	n	n	1	cu	cig+/-ot	25	999	1	0	nev any	ot
CPSI	802	m	35	84	all	6	all	NAmer	1959	pr	5138	n	bl	n	n	1	cu	cig+/-ot	20	24	0	3	nev any	ot
CPSI	803	m	35	84	all	6	all	NAmer	1959	pr	5138	n	bl	n	n	1	cu	cig+/-ot	15	19	2	4	nev any	ot
CPSI	804	m	35	84	all	6	all	NAmer	1959	pr	5138	n	bl	n	n	1	cu	cig+/-ot	1	14	3	0	nev any	ot
CPSI	845	f	40	74	all	6	all	NAmer	1959	pr	5138	n	bl	n	n	1	cu	cig only	25	999	1	0	nev any	ot
CPSI	846	f	40	74	all	6	all	NAmer	1959	pr	5138	n	bl	n	n	1	cu	cig only	20	24	0	3	nev any	ot
CPSI	847	f	40	74	all	6	all	NAmer	1959	pr	5138	n	bl	n	n	1	cu	cig only	15	19	2	4	nev any	ot
CPSI	848	f	40	74	all	6	all	NAmer	1959	pr	5138	n	bl	n	n	1	cu	cig only	1	14	3	0	nev any	ot
DAMBER	501	m	0	0	all	-	all	Eu:Sca	1972	CC	579	n	bl	y	n	0	ev	all/unsp	21	999	1	0	nev any	st
DAMBER	502	m	0	0	all	-	all	Eu:Sca	1972	CC	579	n	bl	y	n	0	ev	all/unsp	16	20	2	4	nev any	st
DAMBER	503	m	0	0	all	-	all	Eu:Sca	1972	CC	579	n	bl	y	n	0	ev	all/unsp	1	15	3	0	nev any	st
DEAN3	564	m	0	0	all	-	all	Eu:UK	1969	CC	766	n	V	y	n	0	cu	cig only	25	999	1	0	nev any	st
DEAN3	565	m	0	0	all	-	all	Eu:UK	1969	CC	766	n	V	y	n	0	cu	cig only	20	24	0	3	nev any	st
DEAN3	566	m	0	0	all	-	all	Eu:UK	1969	CC	766	n	V	y	n	0	cu	cig only	15	19	2	4	nev any	st
DEAN3	567	m	0	0	all	-	all	Eu:UK	1969	CC	766	n	V	y	n	0	cu	cig only	1	14	3	0	nev any	st
DEAN3	583	f	0	0	all	-	all	Eu:UK	1969	CC	766	n	V	y	n	0	cu	cig only	25	999	1	0	nev any	st
DEAN3	584	f	0	0	all	-	all	Eu:UK	1969	CC	766	n	V	y	n	0	cu	cig only	20	24	0	3	nev any	st
DEAN3	585	f	0	0	all	-	all	Eu:UK	1969	CC	766	n	V	y	n	0	cu	cig only	15	19	2	4	nev any	st
DEAN3	586	f	0	0	all	-	all	Eu:UK	1969	CC	766	n	V	y	n	0	cu	cig only	1	14	3	0	nev any	st
DOLL	501	m	0	0	all	-	all	Eu:UK	1948	CC	1465	n	V	n	n	0	ev	all/unsp	40	999	0	0	nev any	st
DOLL	502	m	0	0	all	-	all	Eu:UK	1948	CC	1465	n	V	n	n	0	ev	all/unsp	30	39	0	1	nev any	st
DOLL	503	m	0	0	all	-	all	Eu:UK	1948	CC	1465	n	V	n	n	0	ev	all/unsp	20	29	1	0	nev any	st
DOLL	504	m	0	0	all	-	all	Eu:UK	1948	CC	1465	n	V	n	n	0	ev	all/unsp	1	19	0	0	nev any	st
DOLL	508	f	0	0	all	-	all	Eu:UK	1948	CC	1465	n	V	n	n	0	ev	all/unsp	40	999	0	0	nev any	st
DOLL	509	f	0	0	all	-	all	Eu:UK	1948	CC	1465	n	V	n	n	0	ev	all/unsp	30	39	0	1	nev any	st
DOLL	510	f	0	0	all	-	all	Eu:UK	1948	CC	1465	n	V	n	n	0	ev	all/unsp	20	29	1	0	nev any	st
DOLL	511	f	0	0	all	-	all	Eu:UK	1948	CC	1465	n	V	n	n	0	ev	all/unsp	1	19	0	0	nev any	st
DORN	610	m	55	64	wh	8	all	NAmer	1954	pr	5097	n	bl	n	n	0	ev	cig+/-ot	25	999	1	0	nev any	st
DORN	611	m	55	64	wh	8	all	NAmer	1954	pr	5097	n	bl	n	n	0	ev	cig+/-ot	20	24	0	3	nev any	st
DORN	612	m	55	64	wh	8	all	NAmer	1954	pr	5097	n	bl	n	n	0	ev	cig+/-ot	15	19	2	4	nev any	st
DORN	613	m	55	64	wh	8	all	NAmer	1954	pr	5097	n	bl	n	n	0	ev	cig+/-ot	1	14	3	0	nev any	st
DORN	647	m	65	74	wh	8	all	NAmer	1954	pr	5097	n	bl	n	n	0	ev	cig+/-ot	25	999	1	0	nev any	st
DORN	648	m	65	74	wh	8	all	NAmer	1954	pr	5097	n	bl	n	n	0	ev	cig+/-ot	20	24	0	3	nev any	st
DORN	649	m	65	74	wh	8	all	NAmer	1954	pr	5097	n	bl	n	n	0	ev	cig+/-ot	15	19	2	4	nev any	st
DORN	650	m	65	74	wh	8	all	NAmer	1954	pr	5097	n	bl	n	n	0	ev	cig+/-ot	1	14	3	0	nev any	st

Table 1H11 - 1

IESLC - Meta-analysis of Ever/current Smoking by Age started, Overview
All LC types, Any Product (or Cigarettes if Any not available)
Most adjusted

REF	NRR	SEX	AGEL	AGEH	RACE	YF	LC TYPE	LOC	START	ST	NLC	R	VB	P	H	AD	SM	PRODUCT	exL	exH	S1	S2	DENOM	De
ENGELA	501	m	0	0	all	0	all	Eu:Sca	1964	pr	435	n	bl	n	n	0	cu	cig+/-ot	30	999	0	1	nev cigs	st
ENGELA	502	m	0	0	all	0	all	Eu:Sca	1964	pr	435	n	bl	n	n	0	cu	cig+/-ot	20	29	1	0	nev cigs	st
ENGELA	503	m	0	0	all	0	all	Eu:Sca	1964	pr	435	n	bl	n	n	0	cu	cig+/-ot	1	19	0	0	nev cigs	st
ENGELA	509	f	0	0	all	0	all	Eu:Sca	1964	pr	435	n	bl	n	n	0	cu	cig+/-ot	30	999	0	1	nev cigs	st
ENGELA	510	f	0	0	all	0	all	Eu:Sca	1964	pr	435	n	bl	n	n	0	cu	cig+/-ot	20	29	1	0	nev cigs	st
ENGELA	511	f	0	0	all	0	all	Eu:Sca	1964	pr	435	n	bl	n	n	0	cu	cig+/-ot	1	19	0	0	nev cigs	st
GAO	506	m	0	0	all	-	all	As:Chi	1984	CC	1405	n	ot	n	n	2	ev	cig+/-ot	30	999	0	1	nev cigs	or
GAO	507	m	0	0	all	-	all	As:Chi	1984	CC	1405	n	ot	n	n	2	ev	cig+/-ot	20	29	1	0	nev cigs	or
GAO	508	m	0	0	all	-	all	As:Chi	1984	CC	1405	n	ot	n	n	2	ev	cig+/-ot	10	19	0	0	nev cigs	or
GAO	516	f	0	0	all	-	all	As:Chi	1984	CC	1405	n	ot	n	n	2	ev	cig+/-ot	30	999	0	1	nev cigs	or
GAO	517	f	0	0	all	-	all	As:Chi	1984	CC	1405	n	ot	n	n	2	ev	cig+/-ot	20	29	1	0	nev cigs	or
GAO	518	f	0	0	all	-	all	As:Chi	1984	CC	1405	n	ot	n	n	2	ev	cig+/-ot	10	19	0	0	nev cigs	or
GAO2	501	m	0	0	all	-	all	As:Jap	1988	CC	282	n	bl	n	n	0	cu	cig+/-ot	30	999	0	1	nev cigs	or
GAO2	502	m	0	0	all	-	all	As:Jap	1988	CC	282	n	bl	n	n	0	cu	cig+/-ot	20	29	1	0	nev cigs	st
GAO2	503	m	0	0	all	-	all	As:Jap	1988	CC	282	n	bl	n	n	0	cu	cig+/-ot	1	19	0	0	nev cigs	or
GENG	533	f	0	0	all	-	all	As:Chi	1985	CC	292	n	ot	*	n	1	ev	cig+/-ot	21	999	1	0	nev any	st
GENG	534	f	0	0	all	-	all	As:Chi	1985	CC	292	n	ot	*	n	1	ev	cig+/-ot	16	20	2	4	nev any	st
GENG	535	f	0	0	all	-	all	As:Chi	1985	CC	292	n	ot	*	n	1	ev	cig+/-ot	1	15	3	0	nev any	st
HAENSZ	537	f	0	0	all	-	not alv	NAmer	1955	CC	158	n	bl	n	y	0	ev	cig+/-ot	25	999	1	0	nev any	st
HAENSZ	538	f	0	0	all	-	not alv	NAmer	1955	CC	158	n	bl	n	y	0	ev	cig+/-ot	1	24	0	0	nev any	st
HEGMAN	513	m	0	0	all	-	all	NAmer	1989	CC	282	n	bl	y	y	1	ev	all/unsp	20	999	1	0	nev any	or
HEGMAN	514	m	0	0	all	-	all	NAmer	1989	CC	282	n	bl	y	y	1	ev	all/unsp	1	19	0	0	nev any	or
HEGMAN	516	f	0	0	all	-	all	NAmer	1989	CC	282	n	bl	y	y	1	ev	all/unsp	26	999	1	0	nev any	or
HEGMAN	517	f	0	0	all	-	all	NAmer	1989	CC	282	n	bl	y	y	1	ev	all/unsp	1	25	0	0	nev any	or
HIRAYA	501	m	0	0	all	0	all	As:Jap	1965	pr	1917	n	bl	n	n	1	cu	cig+/-ot	20	999	1	0	nev any	st
HIRAYA	502	m	0	0	all	0	all	As:Jap	1965	pr	1917	n	bl	n	n	1	cu	cig+/-ot	1	19	0	0	nev any	st
HIRAYA	504	f	0	0	all	0	all	As:Jap	1965	pr	1917	n	bl	n	n	1	cu	cig+/-ot	20	999	1	0	nev any	st
HIRAYA	505	f	0	0	all	0	all	As:Jap	1965	pr	1917	n	bl	n	n	1	cu	cig+/-ot	1	19	0	0	nev any	st
HU	511	m	0	0	all	-	all	As:Chi	1985	CC	227	n	ot	n	y	0	ev	cig+/-ot	30	999	0	1	nev cigs	st
HU	512	m	0	0	all	-	all	As:Chi	1985	CC	227	n	ot	n	y	0	ev	cig+/-ot	16	29	0	0	nev cigs	st
HU	513	m	0	0	all	-	all	As:Chi	1985	CC	227	n	ot	n	y	0	ev	cig+/-ot	1	15	3	0	nev cigs	st
HU	516	f	0	0	all	-	all	As:Chi	1985	CC	227	n	ot	n	y	0	ev	cig+/-ot	30	999	0	1	nev cigs	st
HU	517	f	0	0	all	-	all	As:Chi	1985	CC	227	n	ot	n	y	0	ev	cig+/-ot	16	29	0	0	nev cigs	st
HU	518	f	0	0	all	-	all	As:Chi	1985	CC	227	n	ot	n	y	0	ev	cig+/-ot	1	15	3	0	nev cigs	st
HU2	501	c	0	0	all	-	all	As:Chi	1977	CC	523	n	ot	y	n	0	ev	cig+/-ot	40	999	0	0	nev cigs	ot
HU2	502	c	0	0	all	-	all	As:Chi	1977	CC	523	n	ot	y	n	0	ev	cig+/-ot	30	39	0	1	nev cigs	st
HU2	503	c	0	0	all	-	all	As:Chi	1977	CC	523	n	ot	y	n	0	ev	cig+/-ot	20	29	1	0	nev cigs	st
HU2	504	c	0	0	all	-	all	As:Chi	1977	CC	523	n	ot	y	n	0	ev	cig+/-ot	1	19	0	0	nev cigs	or
JEDRYC	607	m	0	0	all	-	all	Eu:est	1980	CC	1630	n	bl	y	n	0	ev	cig+/-ot	17	18	2	4	nev any	st
JEDRYC	608	m	0	0	all	-	all	Eu:est	1980	CC	1630	n	bl	y	n	0	ev	cig+/-ot	1	16	3	0	nev any	st
JEDRYC	619	f	0	0	all	-	all	Eu:est	1980	CC	1630	n	bl	y	n	0	ev	cig+/-ot	1	22	0	0	nev any	st
JOLY	543	m	0	0	all	-	all	SCAmer	1978	CC	826	n	bl	n	n	0	ev	cig+/-ot	25	999	1	0	nev any	st
JOLY	544	m	0	0	all	-	all	SCAmer	1978	CC	826	n	bl	n	n	0	ev	cig+/-ot	15	24	2	0	nev any	st
JOLY	545	m	0	0	all	-	all	SCAmer	1978	CC	826	n	bl	n	n	0	ev	cig+/-ot	1	14	3	0	nev any	st
JOLY	533	f	0	0	all	-	all	SCAmer	1978	CC	826	n	bl	n	n	0	ev	cig+/-ot	25	999	1	0	nev any	st
JOLY	534	f	0	0	all	-	all	SCAmer	1978	CC	826	n	bl	n	n	0	ev	cig+/-ot	15	24	2	0	nev any	st
JOLY	535	f	0	0	all	-	all	SCAmer	1978	CC	826	n	bl	n	n	0	ev	cig+/-ot	1	14	3	0	nev any	st
KHUDER	506	m	0	0	all	-	all	NAmer	1985	CC	482	n	bl	n	y	0	ev	cig+/-ot	20	999	1	0	nev cigs	st
KHUDER	507	m	0	0	all	-	all	NAmer	1985	CC	482	n	bl	n	y	0	ev	cig+/-ot	16	19	2	4	nev cigs	st
KHUDER	508	m	0	0	all	-	all	NAmer	1985	CC	482	n	bl	n	y	0	ev	cig+/-ot	1	15	3	0	nev cigs	st
KOULUM	501	m	0	0	all	-	all	Eu:Sca	1936	CC	812	n	bl	n	n	0	ev	all/unsp	31	999	0	0	nev any	st
KOULUM	502	m	0	0	all	-	all	Eu:Sca	1936	CC	812	n	bl	n	n	0	ev	all/unsp	21	30	1	0	nev any	st
KOULUM	503	m	0	0	all	-	all	Eu:Sca	1936	CC	812	n	bl	n	n	0	ev	all/unsp	16	20	2	4	nev any	st
KOULUM	504	m	0	0	all	-	all	Eu:Sca	1936	CC	812	n	bl	n	n	0	ev	all/unsp	11	15	3	5	nev any	st
KOULUM	505	m	0	0	all	-	all	Eu:Sca	1936	CC	812	n	bl	n	n	0	ev	all/unsp	0	10	0	6	nev any	st
LETOUR	501	c	0	0	all	-	all	NAmer	1983	CC	738	n	V	y	y	0	ev	cig+/-ot	21	999	1	0	nev cigs	st
LETOUR	502	c	0	0	all	-	all	NAmer	1983	CC	738	n	V	y	y	0	ev	cig+/-ot	15	20	2	4	nev cigs	st
LETOUR	503	c	0	0	all	-	all	NAmer	1983	CC	738	n	V	y	y	0	ev	cig+/-ot	1	14	3	0	nev cigs	st
LIAW	504	c	0	0	all	0	all	As:oth	1982	pr	127	n	ot	n	n	2	cu	all/unsp	25	999	1	0	nev any	or
LIAW	505	c	0	0	all	0	all	As:oth	1982	pr	127	n	ot	n	n	2	cu	all/unsp	21	24	0	3	nev any	or
LIAW	506	c	0	0	all	0	all	As:oth	1982	pr	127	n	ot	n	n	2	cu	all/unsp	1	20	0	0	nev any	or
LIU3	504	m	0	0	all	-	all	As:Chi	1985	CC	110	n	ot	n	n	2	ev	all/unsp	21	999	1	0	nev any	or
LIU3	505	m	0	0	all	-	all	As:Chi	1985	CC	110	n	ot	n	n	2	ev	all/unsp	1	20	0	0	nev any	or
LIU4	501	m	35	69	all	-	all	As:Chi	1986	CC	1000-00	n	ot	y	n	2	ev	all/unsp	25	999	1	0	nev any	ot
LIU4	502	m	35	69	all	-	all	As:Chi	1986	CC	1000-00	n	ot	y	n	2	ev	all/unsp	20	24	0	3	nev any	ot
LIU4	503	m	35	69	all	-	all	As:Chi	1986	CC	1000-00	n	ot	y	n	2	ev	all/unsp	0	19	0	0	nev any	ot
LIU5	501	c	0	0	all	-	all	As:Chi	1978	CC	111	n	ot	y	n	0	ev	all/unsp	30	999	0	1	nev any	st
LIU5	502	c	0	0	all	-	all	As:Chi	1978	CC	111	n	ot	y	n	0	ev	all/unsp	1	29	0	0	nev any	st
LUBIN	565	m	0	0	all	-	all	As:Chi	1984	CC	427	m	ot	y	n	0	ev	cig+/-ot	27	999	0	1	nev any	st

Table 1H11 - 1

IESLC - Meta-analysis of Ever/current Smoking by Age started, Overview
All LC types, Any Product (or Cigarettes if Any not available)
Most adjusted

REF	NRR	SEX	AGEL	AGEH	RACE	YF	LC TYPE	LOC	START	ST	NLC	R	VB	P	H	AD	SM	PRODUCT	exL	exH	S1	S2	DENOM	De
LUBIN	566	m	0	0	all	-	all	As:Chi	1984	CC	427	m	ot	y	n	0	ev	cig+/-ot	23	26	1	2	nev any	st
LUBIN	567	m	0	0	all	-	all	As:Chi	1984	CC	427	m	ot	y	n	0	ev	cig+/-ot	20	22	0	3	nev any	st
LUBIN	568	m	0	0	all	-	all	As:Chi	1984	CC	427	m	ot	y	n	0	ev	cig+/-ot	1	19	0	0	nev any	st
LUBIN2	1156	m	0	0	all	-	all	Eu:mul	1976	CC	7804	n	bl	n	y	1	ev	cig+/-ot	31	999	0	0	nev cigs	st
LUBIN2	1157	m	0	0	all	-	all	Eu:mul	1976	CC	7804	n	bl	n	y	1	ev	cig+/-ot	21	30	1	0	nev cigs	st
LUBIN2	1158	m	0	0	all	-	all	Eu:mul	1976	CC	7804	n	bl	n	y	1	ev	cig+/-ot	17	20	2	4	nev cigs	st
LUBIN2	1159	m	0	0	all	-	all	Eu:mul	1976	CC	7804	n	bl	n	y	1	ev	cig+/-ot	13	16	3	5	nev cigs	st
LUBIN2	1160	m	0	0	all	-	all	Eu:mul	1976	CC	7804	n	bl	n	y	1	ev	cig+/-ot	1	12	0	6	nev cigs	st
MATOS	576	m	0	0	all	-	all	SCAmer	1994	CC	200	n	bl	n	n	2	ev	cig+/-ot	20	999	1	0	nev any	or
MATOS	577	m	0	0	all	-	all	SCAmer	1994	CC	200	n	bl	n	n	2	ev	cig+/-ot	15	19	2	4	nev any	or
MATOS	578	m	0	0	all	-	all	SCAmer	1994	CC	200	n	bl	n	n	2	ev	cig+/-ot	1	14	3	0	nev any	or
MIGRAN	501	m	0	0	all	0	all	Eu:UK	1964	pr	259	n	V	n	n	0	cu	cig only	20	999	1	0	nev any	st
MIGRAN	503	m	0	0	all	0	all	Eu:UK	1964	pr	259	n	V	n	n	0	cu	cig only	16	19	2	4	nev any	st
MIGRAN	505	m	0	0	all	0	all	Eu:UK	1964	pr	259	n	V	n	n	0	cu	cig only	0	15	3	0	nev any	st
MIGRAN	511	f	0	0	all	0	all	Eu:UK	1964	pr	259	n	V	n	n	0	cu	cig only	20	999	1	0	nev any	st
MIGRAN	513	f	0	0	all	0	all	Eu:UK	1964	pr	259	n	V	n	n	0	cu	cig only	16	19	2	4	nev any	st
MIGRAN	515	f	0	0	all	0	all	Eu:UK	1964	pr	259	n	V	n	n	0	cu	cig only	0	15	3	0	nev any	st
MRFITR	508	m	0	0	all	0	all	NAmer	1973	pr	119	n	bl	n	n	0	cu	cig+/-ot	24	999	1	0	nev cigs	ot
MRFITR	509	m	0	0	all	0	all	NAmer	1973	pr	119	n	bl	n	n	0	cu	cig+/-ot	22	23	0	3	nev cigs	ot
MRFITR	510	m	0	0	all	0	all	NAmer	1973	pr	119	n	bl	n	n	0	cu	cig+/-ot	20	21	0	0	nev cigs	ot
MRFITR	511	m	0	0	all	0	all	NAmer	1973	pr	119	n	bl	n	n	0	cu	cig+/-ot	18	19	2	4	nev cigs	ot
MRFITR	512	m	0	0	all	0	all	NAmer	1973	pr	119	n	bl	n	n	0	cu	cig+/-ot	16	17	0	0	nev cigs	ot
MRFITR	513	m	0	0	all	0	all	NAmer	1973	pr	119	n	bl	n	n	0	cu	cig+/-ot	1	15	3	0	nev cigs	ot
PERNU	504	m	0	0	all	-	all	Eu:Sca	1944	CC	1606	n	bl	n	n	0	ev	all/unsp	15	999	0	0	nev any	st
PERNU	505	m	0	0	all	-	all	Eu:Sca	1944	CC	1606	n	bl	n	n	0	ev	all/unsp	1	14	3	0	nev any	st
PERNU	501	f	0	0	all	-	all	Eu:Sca	1944	CC	1606	n	bl	n	n	0	ev	all/unsp	15	999	0	0	nev any	st
PERNU	502	f	0	0	all	-	all	Eu:Sca	1944	CC	1606	n	bl	n	n	0	ev	all/unsp	1	14	3	0	nev any	ot
PEZZOT	570	m	0	0	all	-	all	SCAmer	1987	CC	215	n	bl	n	y	0	ev	cig only	19	999	1	0	nev cigs	st
PEZZOT	571	m	0	0	all	-	all	SCAmer	1987	CC	215	n	bl	n	y	0	ev	cig only	14	18	0	0	nev cigs	st
PEZZOT	572	m	0	0	all	-	all	SCAmer	1987	CC	215	n	bl	n	y	0	ev	cig only	1	13	0	6	nev cigs	st
QIAO2	506	m	0	0	all	0	all	As:Chi	1992	pr	241	m	ot	n	n	1	ev	all/unsp	21	999	1	0	nev any	or
QIAO2	507	m	0	0	all	0	all	As:Chi	1992	pr	241	m	ot	n	n	1	ev	all/unsp	17	20	2	4	nev any	or
QIAO2	508	m	0	0	all	0	all	As:Chi	1992	pr	241	m	ot	n	n	1	ev	all/unsp	1	16	3	0	nev any	or
RACHTA	506	f	0	0	all	-	all	Eu:est	1991	CC	118	n	bl	n	y	1	ev	cig+/-ot	31	999	0	0	nev cigs	or
RACHTA	507	f	0	0	all	-	all	Eu:est	1991	CC	118	n	bl	n	y	1	ev	cig+/-ot	20	30	1	0	nev cigs	or
RACHTA	508	f	0	0	all	-	all	Eu:est	1991	CC	118	n	bl	n	y	1	ev	cig+/-ot	1	19	0	0	nev cigs	or
SEGI2	521	m	0	0	all	-	all	As:Jap	1962	CC	378	n	bl	n	n	1	cu	cig+/-ot	23	999	1	0	nev any	ot
SEGI2	522	m	0	0	all	-	all	As:Jap	1962	CC	378	n	bl	n	n	1	cu	cig+/-ot	20	22	0	3	nev any	ot
SEGI2	523	m	0	0	all	-	all	As:Jap	1962	CC	378	n	bl	n	n	1	cu	cig+/-ot	1	19	0	0	nev any	ot
SOBUE	654	m	0	0	all	-	all	As:Jap	1986	CC	1376	n	bl	n	y	0	ev	cig+/-ot	23	999	1	0	nev cigs	st
SOBUE	655	m	0	0	all	-	all	As:Jap	1986	CC	1376	n	bl	n	y	0	ev	cig+/-ot	18	22	2	0	nev cigs	st
SOBUE	656	m	0	0	all	-	all	As:Jap	1986	CC	1376	n	bl	n	y	0	ev	cig+/-ot	10	17	3	0	nev cigs	st
SUZUK2	501	c	0	0	all	-	all	SCAmer	1991	CC	123	n	bl	n	y	0	ev	all/unsp	19	999	1	0	nev any	st
SUZUK2	502	c	0	0	all	-	all	SCAmer	1991	CC	123	n	bl	n	y	0	ev	all/unsp	12	18	0	0	nev any	st
SUZUK2	503	c	0	0	all	-	all	SCAmer	1991	CC	123	n	bl	n	y	0	ev	all/unsp	0	11	0	6	nev any	st
SVENSS	501	f	0	0	all	-	all	Eu:Sca	1983	CC	210	n	bl	n	n	0	cu	all/unsp	26	999	1	0	nev any	st
SVENSS	502	f	0	0	all	-	all	Eu:Sca	1983	CC	210	n	bl	n	n	0	cu	all/unsp	19	25	0	3	nev any	st
SVENSS	503	f	0	0	all	-	all	Eu:Sca	1983	CC	210	n	bl	n	n	0	cu	all/unsp	0	18	0	0	nev any	st
TIZZAN	506	m	0	0	all	-	all	Eu:wst	1959	CC	1358	n	bl	n	n	0	ev	all/unsp	31	999	0	0	nev any	st
TIZZAN	507	m	0	0	all	-	all	Eu:wst	1959	CC	1358	n	bl	n	n	0	ev	all/unsp	20	30	1	0	nev any	st
TIZZAN	508	m	0	0	all	-	all	Eu:wst	1959	CC	1358	n	bl	n	n	0	ev	all/unsp	1	19	0	0	nev any	st
TIZZAN	519	f	0	0	all	-	all	Eu:wst	1959	CC	1358	n	bl	n	n	0	ev	all/unsp	31	999	0	0	nev any	st
TIZZAN	520	f	0	0	all	-	all	Eu:wst	1959	CC	1358	n	bl	n	n	0	ev	all/unsp	20	30	1	0	nev any	st
TIZZAN	521	f	0	0	all	-	all	Eu:wst	1959	CC	1358	n	bl	n	n	0	ev	all/unsp	1	19	0	0	nev any	st
WAKAI	501	m	0	0	all	-	all	As:Jap	1988	CC	333	n	bl	n	y	0	cu	cig+/-ot	30	999	0	1	nev any	st
WAKAI	502	m	0	0	all	-	all	As:Jap	1988	CC	333	n	bl	n	y	0	cu	cig+/-ot	20	29	1	0	nev any	st
WAKAI	503	m	0	0	all	-	all	As:Jap	1988	CC	333	n	bl	n	y	0	cu	cig+/-ot	1	19	0	0	nev any	st
WU	541	f	0	0	wh	-	q+a	NAmer	1981	CC	220	n	bl	n	y	2	cu	all/unsp	25	999	1	0	nev any	st
WU	542	f	0	0	wh	-	q+a	NAmer	1981	CC	220	n	bl	n	y	2	cu	all/unsp	19	24	0	3	nev any	st
WU	543	f	0	0	wh	-	q+a	NAmer	1981	CC	220	n	bl	n	y	2	cu	all/unsp	0	18	0	0	nev any	st
WYNDE6	759	m	0	0	wh	-	q+s+a	NAmer	1969	CC	4423	n	bl	n	y	0	ev	cig+/-ot	21	999	1	0	nev cigs	st
WYNDE6	760	m	0	0	wh	-	q+s+a	NAmer	1969	CC	4423	n	bl	n	y	0	ev	cig+/-ot	18	20	2	4	nev cigs	st
WYNDE6	761	m	0	0	wh	-	q+s+a	NAmer	1969	CC	4423	n	bl	n	y	0	ev	cig+/-ot	1	17	3	0	nev cigs	st
WYNDE6	767	f	0	0	wh	-	q+s+a	NAmer	1969	CC	4423	n	bl	n	y	0	ev	cig+/-ot	21	999	1	0	nev cigs	st
WYNDE6	768	f	0	0	wh	-	q+s+a	NAmer	1969	CC	4423	n	bl	n	y	0	ev	cig+/-ot	18	20	2	4	nev cigs	st
WYNDE6	769	f	0	0	wh	-	q+s+a	NAmer	1969	CC	4423	n	bl	n	y	0	ev	cig+/-ot	1	17	3	0	nev cigs	st
ZHENG	563	m	0	0	all	-	all	As:Chi	1982	CC	540	n	ot	*	y	0	ev	cig+/-ot	30	999	0	1	nev cigs	st
ZHENG	564	m	0	0	all	-	all	As:Chi	1982	CC	540	n	ot	*	y	0	ev	cig+/-ot	20	29	1	0	nev cigs	st
ZHENG	565	m	0	0	all	-	all	As:Chi	1982	CC	540	n	ot	*	y	0	ev	cig+/-ot	1	19	0	0	nev cigs	st
ZHENG	572	f	0	0	all	-	all	As:Chi	1982	CC	540	n	ot	*	y	0	ev	cig+/-ot	30	999	0	1	nev cigs	st
ZHENG	573	f	0	0	all	-	all	As:Chi	1982	CC	540	n	ot	*	y	0	ev	cig+/-ot	1	29	0	0	nev cigs	st

Table 1H11 - 1

IESLC - Meta-analysis of Ever/current Smoking by Age started, Overview
All LC types, Any Product (or Cigarettes if Any not available)
Most adjusted

Cigarette type is all/unspec for all RRs
except for the following:

REF	NRR	CIGTYPE
DEAN3	564	MC only
DEAN3	565	MC only
DEAN3	566	MC only
DEAN3	567	MC only
DEAN3	583	MC only
DEAN3	584	MC only
DEAN3	585	MC only
DEAN3	586	MC only

In this overview table, subtotals and Qs values may be invalid and should be ignored

Table 1H11 - 2

IESLC - Meta-analysis of Ever/current Smoking by Age started, Overview
All LC types, Any Product (or Cigarettes if Any not available)
Most adjusted

REF	NRR	SEX	AD	Number Case	Exposed Cont	Non-exposed Case	Cont	RR	95.00%CI
AGUDO	504	f	3	7	-	80	-	1.58	(0.59- 4.23)
AGUDO	505	f	3	16	-	80	-	10.76	(2.38- 48.73)
Subtotal AGUDO								2.80	(1.23- 6.39)
ARMADA	511	m	0	113	144	8	71	6.96	(3.22- 15.06)
ARMADA	512	m	0	204	110	8	71	16.46	(7.64- 35.44)
Subtotal ARMADA								10.73	(6.23- 18.49)
AUVINE	520	c	2	211	-	44	-	13.40	(7.62- 23.50)
AUVINE	521	c	2	55	-	44	-	47.60	(21.20- 107.00)
Subtotal AUVINE								20.26	(12.76- 32.17)
BARBON	520	m	1	200	-	22	-	8.20	(5.00- 13.30)
BARBON	521	m	1	395	-	22	-	9.90	(6.20- 15.80)
BARBON	522	m	1	138	-	22	-	50.80	(27.20- 95.00)
Subtotal BARBON								13.37	(9.93- 18.00)
BRESLO	501	c	0	32	35	19	56	2.69	(1.33- 5.47)
BRESLO	502	c	0	286	243	19	56	3.47	(2.01- 6.00)
BRESLO	503	c	0	166	116	19	56	4.22	(2.38- 7.47)
Subtotal BRESLO								3.51	(2.48- 4.95)
BUFFLE	517	f	0	23	23	12	112	9.33	(4.07- 21.40)
BUFFLE	518	f	0	47	34	12	112	12.90	(6.15- 27.07)
BUFFLE	519	f	0	40	29	12	112	12.87	(6.00- 27.62)
BUFFLE	520	f	0	53	33	12	112	14.99	(7.17- 31.33)
BUFFLE	521	f	0	78	41	12	112	17.76	(8.77- 35.94)
Subtotal BUFFLE								13.57	(9.69- 18.99)
*CEDERL	510	m	1	11	-	7	-	6.50	(2.52- 16.74)
*CEDERL	511	m	1	10	-	7	-	9.80	(3.74- 25.69)
*CEDERL	512	m	1	7	-	7	-	6.40	(2.25- 18.21)
*CEDERL	515	f	0	6	2806	19	17679	1.99	(0.80- 4.98)
*CEDERL	516	f	0	2	1009	19	17679	1.84	(0.43- 7.91)
*CEDERL	517	f	0	0	746	19	17679	0.61~	(0.04- 10.05)
Subtotal CEDERL								4.43	(2.82- 6.96)
CHEN2	517	m	0	8	5	9	33	5.87	(1.54- 22.37)
CHEN2	518	m	0	29	25	9	33	4.25	(1.71- 10.57)
CHEN2	519	m	0	84	67	9	33	4.60	(2.06- 10.27)
CHEN2	522	f	0	5	8	25	33	0.83	(0.24- 2.83)
CHEN2	523	f	0	9	8	25	33	1.49	(0.50- 4.39)
CHEN2	524	f	0	23	13	25	33	2.34	(0.99- 5.50)
Subtotal CHEN2								2.84	(1.90- 4.25)
CHIAZZ	501	m	2	-	-	4	-	3.00	(0.31- 28.84)
CHIAZZ	502	m	2	-	-	4	-	19.89	(2.66- 148.96)
Subtotal CHIAZZ								8.64	(1.92- 38.91)
CHOI	523	m	0	36	77	13	95	3.42	(1.69- 6.89)
CHOI	524	m	0	130	232	13	95	4.09	(2.21- 7.60)
CHOI	525	m	0	79	138	13	95	4.18	(2.20- 7.95)
CHOI	526	m	0	22	18	13	95	8.93	(3.81- 20.91)
CHOI	530	f	0	15	25	76	164	1.29	(0.65- 2.60)
CHOI	531	f	0	4	1	76	164	8.63	(0.95- 78.54)
Subtotal CHOI								3.57	(2.63- 4.85)
*CPSI	801	m	1	42	-	83	-	4.08	(2.81- 5.91)
*CPSI	802	m	1	196	-	83	-	10.08	(7.80- 13.03)
*CPSI	803	m	1	588	-	83	-	14.69	(11.68- 18.49)
*CPSI	804	m	1	185	-	83	-	16.77	(12.94- 21.73)
*CPSI	845	f	1	51	-	166	-	2.25	(1.64- 3.08)
*CPSI	846	f	1	34	-	166	-	3.38	(2.33- 4.88)
*CPSI	847	f	1	52	-	166	-	5.00	(3.66- 6.83)
*CPSI	848	f	1	6	-	166	-	2.50	(1.11- 5.65)
Subtotal CPSI								7.59	(6.81- 8.46)
DAMBER	501	m	0	70	76	42	208	4.56	(2.87- 7.26)
DAMBER	502	m	0	261	190	42	208	6.80	(4.65- 9.95)
DAMBER	503	m	0	206	98	42	208	10.41	(6.91- 15.68)
Subtotal DAMBER								7.07	(5.57- 8.98)
DEAN3	564	m	0	24	75	24	510	6.80	(3.67- 12.58)
DEAN3	565	m	0	52	161	24	510	6.86	(4.10- 11.49)
DEAN3	566	m	0	160	485	24	510	7.01	(4.48- 10.96)
DEAN3	567	m	0	44	165	24	510	5.67	(3.34- 9.60)
DEAN3	583	f	0	27	274	41	1538	3.70	(2.24- 6.11)
DEAN3	584	f	0	18	229	41	1538	2.95	(1.67- 5.22)
DEAN3	585	f	0	39	504	41	1538	2.90	(1.85- 4.55)
DEAN3	586	f	0	7	109	41	1538	2.41	(1.06- 5.50)
Subtotal DEAN3								4.59	(3.81- 5.54)
DOLL	501	m	0	4	7	7	61	4.98	(1.16- 21.36)
DOLL	502	m	0	18	33	7	61	4.75	(1.80- 12.54)

Table 1H11 - 2

IESLC - Meta-analysis of Ever/current Smoking by Age started, Overview
All LC types, Any Product (or Cigarettes if Any not available)
Most adjusted

REF	NRR	SEX	AD	Number Exposed Case	Cont	Non-exposed Case	Cont	RR	95.00%CI
DOLL	503	m	0	251	264	7	61	8.29	(3.72- 18.46)
DOLL	504	m	0	1077	992	7	61	9.46	(4.31- 20.78)
DOLL	508	f	0	15	15	40	59	1.48	(0.65- 3.35)
DOLL	509	f	0	10	7	40	59	2.11	(0.74- 6.00)
DOLL	510	f	0	23	15	40	59	2.26	(1.05- 4.86)
DOLL	511	f	0	20	12	40	59	2.46	(1.08- 5.58)
Subtotal DOLL								3.65	(2.67- 4.98)
*DORN	610	m	0	37	73050	25	213858	4.33	(2.61- 7.20)
*DORN	611	m	0	157	147948	25	213858	9.08	(5.95- 13.84)
*DORN	612	m	0	342	213156	25	213858	13.73	(9.14- 20.60)
*DORN	613	m	0	84	36304	25	213858	19.79	(12.67- 30.93)
*DORN	647	m	0	90	74464	49	171211	4.22	(2.98- 5.98)
*DORN	648	m	0	171	90036	49	171211	6.64	(4.83- 9.12)
*DORN	649	m	0	306	118234	49	171211	9.04	(6.69- 12.23)
*DORN	650	m	0	81	24616	49	171211	11.50	(8.07- 16.39)
Subtotal DORN								8.46	(7.42- 9.65)
*ENGELA	501	m	0	17	9762	27	58716	3.79	(2.07- 6.95)
*ENGELA	502	m	0	50	30195	27	58716	3.60	(2.26- 5.75)
*ENGELA	503	m	0	173	50732	27	58716	7.42	(4.94- 11.12)
*ENGELA	509	f	0	10	24560	31	207789	2.73	(1.34- 5.57)
*ENGELA	510	f	0	36	29605	31	207789	8.15	(5.04- 13.17)
*ENGELA	511	f	0	18	10687	31	207789	11.29	(6.32- 20.17)
Subtotal ENGELA								5.83	(4.73- 7.20)
GAO	506	m	2	45	-	62	-	1.20	(0.80- 1.90)
GAO	507	m	2	363	-	62	-	4.70	(3.30- 6.50)
GAO	508	m	2	262	-	62	-	5.10	(3.60- 7.20)
GAO	516	f	2	73	-	435	-	2.00	(1.40- 3.00)
GAO	517	f	2	87	-	435	-	3.80	(2.60- 5.80)
GAO	518	f	2	77	-	435	-	5.60	(3.40- 9.00)
Subtotal GAO								3.38	(2.88- 3.96)
GAO2	501	m	0	2	4	13	56	2.15	(0.36- 13.05)
GAO2	502	m	0	127	85	13	56	6.44	(3.32- 12.49)
GAO2	503	m	0	52	26	13	56	8.62	(4.01- 18.52)
Subtotal GAO2								6.68	(4.12- 10.83)
GENG	533	f	1	28	-	54	-	1.55	(0.83- 2.89)
GENG	534	f	1	39	-	54	-	2.95	(1.57- 5.52)
GENG	535	f	1	36	-	54	-	6.24	(2.85- 13.67)
Subtotal GENG								2.77	(1.88- 4.07)
HAENSZ	537	f	0	44	66	81	236	1.94	(1.23- 3.07)
HAENSZ	538	f	0	30	37	81	236	2.36	(1.37- 4.07)
Subtotal HAENSZ								2.11	(1.48- 2.99)
HEGMAN	513	m	1	26	-	-	-	9.40	(4.60- 19.30)
HEGMAN	514	m	1	146	-	-	-	22.30	(12.00- 41.40)
HEGMAN	516	f	1	2	-	-	-	4.80	(1.00- 22.10)
HEGMAN	517	f	1	81	-	-	-	26.80	(15.40- 46.80)
Subtotal HEGMAN								18.07	(12.75- 25.62)
*HIRAYA	501	m	1	-	-	-	-	4.35	(3.51- 5.39)
*HIRAYA	502	m	1	-	-	-	-	5.71	(4.50- 7.25)
*HIRAYA	504	f	1	-	-	-	-	2.46	(1.93- 3.13)
*HIRAYA	505	f	1	-	-	-	-	0.78	(0.10- 6.10)
Subtotal HIRAYA								3.96	(3.46- 4.52)
HU	511	m	0	14	20	41	67	1.14	(0.52- 2.51)
HU	512	m	0	93	67	41	67	2.27	(1.38- 3.74)
HU	513	m	0	13	7	41	67	3.03	(1.12- 8.23)
HU	516	f	0	3	5	40	48	0.72	(0.16- 3.20)
HU	517	f	0	18	9	40	48	2.40	(0.97- 5.92)
HU	518	f	0	5	4	40	48	1.50	(0.38- 5.96)
Subtotal HU								1.92	(1.37- 2.69)
HU2	501	c	0	15	29	121	213	0.91	(0.47- 1.77)
HU2	502	c	0	29	54	121	213	0.95	(0.57- 1.56)
HU2	503	c	0	229	159	121	213	2.54	(1.88- 3.43)
HU2	504	c	0	129	68	121	213	3.34	(2.31- 4.83)
Subtotal HU2								2.14	(1.75- 2.61)
JEDRYC	607	m	0	239	146	49	219	7.32	(5.04- 10.61)
JEDRYC	608	m	0	135	66	49	219	9.14	(5.96- 14.02)
JEDRYC	619	f	0	63	11	78	166	12.19	(6.09- 24.42)
Subtotal JEDRYC								8.54	(6.58- 11.07)
JOLY	543	m	0	18	70	12	218	4.67	(2.14- 10.18)
JOLY	544	m	0	217	357	12	218	11.04	(6.03- 20.22)
JOLY	545	m	0	317	282	12	218	20.42	(11.18- 37.32)
JOLY	533	f	0	23	41	52	283	3.05	(1.69- 5.51)

Table 1H11 - 2

IESLC - Meta-analysis of Ever/current Smoking by Age started, Overview
All LC types, Any Product (or Cigarettes if Any not available)
Most adjusted

REF	NRR	SEX	AD	Number Case	Exposed Cont	Non-exposed Case	Cont	RR	95.00%CI
JOLY	534	f	0	67	47	52	283	7.76	(4.82- 12.49)
JOLY	535	f	0	76	35	52	283	11.82	(7.18- 19.44)
Subtotal JOLY								8.56	(6.78- 10.80)
KHUDER	506	m	0	72	152	23	309	6.36	(3.83- 10.58)
KHUDER	507	m	0	161	338	23	309	6.40	(4.03- 10.17)
KHUDER	508	m	0	226	295	23	309	10.29	(6.51- 16.27)
Subtotal KHUDER								7.58	(5.76- 9.97)
KOULUM	501	m	0	8	8	5	54	10.80	(2.82- 41.31)
KOULUM	502	m	0	60	67	5	54	9.67	(3.63- 25.77)
KOULUM	503	m	0	267	103	5	54	28.00	(10.89- 71.96)
KOULUM	504	m	0	199	52	5	54	41.33	(15.74- 108.56)
KOULUM	505	m	0	143	16	5	54	96.53	(33.72- 276.34)
Subtotal KOULUM								27.41	(17.28- 43.49)
LETOUR	501	c	0	188	160	24	224	10.97	(6.85- 17.56)
LETOUR	502	c	0	309	241	24	224	11.97	(7.60- 18.83)
LETOUR	503	c	0	151	76	24	224	18.54	(11.21- 30.67)
Subtotal LETOUR								13.23	(10.06- 17.40)
*LIAW	504	c	2	-	-	-	-	1.50	(0.70- 3.30)
*LIAW	505	c	2	-	-	-	-	5.90	(3.00- 11.30)
*LIAW	506	c	2	-	-	-	-	4.60	(2.60- 8.10)
Subtotal LIAW								3.82	(2.62- 5.58)
LIU3	504	m	2	20	-	4	-	1.10	(0.25- 4.93)
LIU3	505	m	2	32	-	4	-	1.39	(0.32- 6.06)
Subtotal LIU3								1.24	(0.43- 3.53)
LIU4	501	m	2	-	-	-	-	2.41	(2.32- 2.49)
LIU4	502	m	2	-	-	-	-	2.86	(2.78- 2.95)
LIU4	503	m	2	-	-	-	-	3.81	(3.70- 3.93)
Subtotal LIU4								3.03	(2.98- 3.09)
LIU5	501	c	0	13	22	26	41	0.93	(0.40- 2.17)
LIU5	502	c	0	72	48	26	41	2.37	(1.28- 4.36)
Subtotal LIU5								1.72	(1.05- 2.82)
LUBIN	565	m	0	30	179	9	72	1.34	(0.61- 2.96)
LUBIN	566	m	0	65	146	9	72	3.56	(1.68- 7.56)
LUBIN	567	m	0	89	212	9	72	3.36	(1.61- 7.01)
LUBIN	568	m	0	178	251	9	72	5.67	(2.76- 11.64)
Subtotal LUBIN								3.20	(2.20- 4.66)
LUBIN2	1156	m	1	68	-	185	-	3.35	(2.42- 4.64)
LUBIN2	1157	m	1	564	-	185	-	4.97	(4.12- 6.00)
LUBIN2	1158	m	1	1796	-	185	-	5.43	(4.59- 6.41)
LUBIN2	1159	m	1	1312	-	185	-	6.74	(5.65- 8.04)
LUBIN2	1160	m	1	250	-	185	-	9.01	(6.92- 11.72)
Subtotal LUBIN2								5.76	(5.26- 6.31)
MATOS	576	m	2	28	-	11	-	3.90	(1.80- 8.30)
MATOS	577	m	2	91	-	11	-	7.80	(4.00- 15.50)
MATOS	578	m	2	69	-	11	-	7.80	(3.90- 15.70)
Subtotal MATOS								6.39	(4.24- 9.63)
*MIGRAN	501	m	0	24	668	4	867	7.79	(2.72- 22.34)
*MIGRAN	503	m	0	59	1845	4	867	6.93	(2.53- 19.02)
*MIGRAN	505	m	0	50	1081	4	867	10.03	(3.64- 27.65)
*MIGRAN	511	f	0	11	1315	4	3814	7.98	(2.54- 25.01)
*MIGRAN	513	f	0	9	1035	4	3814	8.29	(2.56- 26.87)
*MIGRAN	515	f	0	2	266	4	3814	7.17	(1.32- 38.96)
Subtotal MIGRAN								8.07	(5.08- 12.80)
*MRFITR	508	m	0	3	544	0	1859	23.91~	(1.24- 462.09)
*MRFITR	509	m	0	6	402	0	1859	60.06~	(3.39-1063.94)
*MRFITR	510	m	0	7	1029	0	1859	27.09~	(1.55- 473.89)
*MRFITR	511	m	0	25	1876	0	1859	50.54~	(3.08- 829.51)
*MRFITR	512	m	0	40	2242	0	1859	67.17~	(4.13-1091.55)
*MRFITR	513	m	0	25	2065	0	1859	45.91~	(2.80- 753.64)
Subtotal MRFITR								43.20	(13.52- 138.02)
PERNU	504	m	0	1043	346	97	275	8.55	(6.58- 11.10)
PERNU	505	m	0	337	92	97	275	10.38	(7.49- 14.40)
PERNU	501	f	0	18	89	110	971	1.79	(1.04- 3.07)
PERNU	502	f	0	1	0	110	971	26.38~	(1.07- 651.38)
Subtotal PERNU								7.56	(6.25- 9.15)
PEZZOT	570	m	0	41	105	4	116	11.32	(3.92- 32.69)
PEZZOT	571	m	0	118	145	4	116	23.60	(8.46- 65.84)
PEZZOT	572	m	0	52	67	4	116	22.51	(7.79- 65.00)
Subtotal PEZZOT								18.29	(9.98- 33.50)
*QIAO2	506	m	1	52	-	10	-	1.32	(0.67- 2.60)
*QIAO2	507	m	1	75	-	10	-	1.47	(0.76- 2.84)

Table 1H11 - 2

IESLC - Meta-analysis of Ever/current Smoking by Age started, Overview
All LC types, Any Product (or Cigarettes if Any not available)
Most adjusted

REF	NRR	SEX	AD	Number Exposed Case	Number Exposed Cont	Non-exposed Case	Non-exposed Cont	RR	95.00%CI
*QIAO2	508	m	1	104	-	10	-	1.81	(0.94- 3.48)
Subtotal QIAO2								1.53	(1.04- 2.24)
RACHTA	506	f	1	8	-	33	-	5.31	(1.48- 19.05)
RACHTA	507	f	1	25	-	33	-	5.33	(2.79- 10.20)
RACHTA	508	f	1	52	-	33	-	11.60	(5.04- 26.68)
Subtotal RACHTA								6.86	(4.27- 11.03)
SEGI2	521	m	1	49	-	8	-	2.04	(0.89- 4.66)
SEGI2	522	m	1	125	-	8	-	3.33	(1.51- 7.37)
SEGI2	523	m	1	91	-	8	-	5.64	(2.49- 12.78)
Subtotal SEGI2								3.38	(2.12- 5.41)
SOBUE	654	m	0	110	121	29	126	3.95	(2.45- 6.38)
SOBUE	655	m	0	776	772	29	126	4.37	(2.88- 6.62)
SOBUE	656	m	0	137	62	29	126	9.60	(5.81- 15.88)
Subtotal SOBUE								5.28	(4.05- 6.89)
SUZUK2	501	c	0	16	22	11	53	3.50	(1.40- 8.75)
SUZUK2	502	c	0	64	38	11	53	8.11	(3.78- 17.41)
SUZUK2	503	c	0	31	10	11	53	14.94	(5.69- 39.18)
Subtotal SUZUK2								7.44	(4.51- 12.27)
SVENSS	501	f	0	32	18	38	120	5.61	(2.84- 11.12)
SVENSS	502	f	0	58	14	38	120	13.08	(6.57- 26.04)
SVENSS	503	f	0	52	21	38	120	7.82	(4.19- 14.60)
Subtotal SVENSS								8.26	(5.63- 12.12)
TIZZAN	506	m	0	12	44	180	305	0.46	(0.24- 0.90)
TIZZAN	507	m	0	313	330	180	305	1.61	(1.26- 2.04)
TIZZAN	508	m	0	699	529	180	305	2.24	(1.80- 2.78)
TIZZAN	519	f	0	2	5	117	114	0.39	(0.07- 2.05)
TIZZAN	520	f	0	12	21	117	114	0.56	(0.26- 1.18)
TIZZAN	521	f	0	11	2	117	114	5.36	(1.16- 24.71)
Subtotal TIZZAN								1.70	(1.46- 1.98)
WAKAI	501	m	0	8	25	10	65	2.08	(0.74- 5.87)
WAKAI	502	m	0	130	183	10	65	4.62	(2.29- 9.32)
WAKAI	503	m	0	42	74	10	65	3.69	(1.72- 7.94)
Subtotal WAKAI								3.63	(2.28- 5.77)
WU	541	f	2	14	-	31	-	1.55	(0.60- 3.98)
WU	542	f	2	40	-	31	-	3.57	(1.57- 8.14)
WU	543	f	2	106	-	31	-	10.32	(4.81- 22.13)
Subtotal WU								4.39	(2.71- 7.10)
WYNDE6	759	m	0	111	92	51	589	13.93	(9.36- 20.74)
WYNDE6	760	m	0	223	139	51	589	18.53	(12.98- 26.45)
WYNDE6	761	m	0	611	301	51	589	23.44	(17.06- 32.21)
WYNDE6	767	f	0	127	90	73	673	13.01	(9.06- 18.69)
WYNDE6	768	f	0	200	94	73	673	19.62	(13.90- 27.67)
WYNDE6	769	f	0	291	91	73	673	29.48	(21.04- 41.31)
Subtotal WYNDE6								19.46	(16.87- 22.45)
ZHENG	563	m	0	28	66	33	94	1.21	(0.67- 2.19)
ZHENG	564	m	0	145	109	33	94	3.79	(2.37- 6.05)
ZHENG	565	m	0	106	43	33	94	7.02	(4.13- 11.95)
ZHENG	572	f	0	16	16	152	184	1.21	(0.59- 2.50)
ZHENG	573	f	0	60	28	152	184	2.59	(1.58- 4.27)
Subtotal ZHENG								2.86	(2.24- 3.65)
Partial Totals				24315	965679	9674	2444905		

*prospective study ~ With 0.5 adjustment for zero

Table 1H11 - 2

IESLC - Meta-analysis of Ever/current Smoking by Age started, Overview
All LC types, Any Product (or Cigarettes if Any not available)
Most adjusted

REF	NRR	SEX	AD	Ys	Ws	Qs	Ps
AGUDO	504	f	3	0.46	3.96	2.46	0.3627
AGUDO	505	f	3	2.38	1.69	2.15	0.0020
Subtotal AGUDO				1.03	5.65	4.61	
ARMADA	511	m	0	1.94	6.46	3.12	0.0000
ARMADA	512	m	0	2.80	6.53	15.80	0.0000
Subtotal ARMADA				2.37	12.99	18.91	
AUVINE	520	c	2	2.60	12.11	22.06	0.0000
AUVINE	521	c	2	3.86	5.86	40.16	0.0000
Subtotal AUVINE				3.01	17.98	62.21	
BARBON	520	m	1	2.10	16.05	11.83	0.0000
BARBON	521	m	1	2.29	17.56	19.24	0.0000
BARBON	522	m	1	3.93	9.82	70.66	0.0000
Subtotal BARBON				2.59	43.44	101.73	
BRESLO	501	c	0	0.99	7.67	0.50	0.0060
BRESLO	502	c	0	1.24	12.80	0.00	0.0000
BRESLO	503	c	0	1.44	11.75	0.44	0.0000
Subtotal BRESLO				1.25	32.22	0.94	
BUFFLE	517	f	0	2.23	5.58	5.44	0.0000
BUFFLE	518	f	0	2.56	7.00	12.03	0.0000
BUFFLE	519	f	0	2.56	6.59	11.30	0.0000
BUFFLE	520	f	0	2.71	7.07	15.10	0.0000
BUFFLE	521	f	0	2.88	7.72	20.54	0.0000
Subtotal BUFFLE				2.61	33.96	64.42	
*CEDERL	510	m	1	1.87	4.29	1.68	0.0001
*CEDERL	511	m	1	2.28	4.14	4.45	0.0000
*CEDERL	512	m	1	1.86	3.51	1.31	0.0005
*CEDERL	515	f	0	0.69	4.57	1.42	0.1414
*CEDERL	516	f	0	0.61	1.81	0.73	0.4098
*CEDERL	517	f	0	-0.50	0.49	1.48	0.7276
Subtotal CEDERL				1.49	18.81	11.07	
CHEN2	517	m	0	1.77	2.14	0.59	0.0096
CHEN2	518	m	0	1.45	4.63	0.19	0.0018
CHEN2	519	m	0	1.53	5.94	0.46	0.0002
CHEN2	522	f	0	-0.19	2.53	5.23	0.7596
CHEN2	523	f	0	0.40	3.26	2.36	0.4750
CHEN2	524	f	0	0.85	5.24	0.83	0.0521
Subtotal CHEN2				1.04	23.76	9.66	
CHIAZZ	501	m	2	1.10	0.75	0.02	0.3421
CHIAZZ	502	m	2	2.99	0.95	2.89	0.0036
Subtotal CHIAZZ				2.16	1.70	2.90	
CHOI	523	m	0	1.23	7.80	0.00	0.0006
CHOI	524	m	0	1.41	10.06	0.27	0.0000
CHOI	525	m	0	1.43	9.31	0.32	0.0000
CHOI	526	m	0	2.19	5.31	4.73	0.0000
CHOI	530	f	0	0.26	7.94	7.75	0.4667
CHOI	531	f	0	2.16	0.79	0.65	0.0557
Subtotal CHOI				1.27	41.20	13.71	
*CPSI	801	m	1	1.41	27.80	0.71	0.0000
*CPSI	802	m	1	2.31	58.36	66.15	0.0000
*CPSI	803	m	1	2.69	72.82	151.28	0.0000
*CPSI	804	m	1	2.82	57.18	141.62	0.0000
*CPSI	845	f	1	0.81	38.69	7.32	0.0000
*CPSI	846	f	1	1.22	28.12	0.02	0.0000
*CPSI	847	f	1	1.61	39.48	5.22	0.0000
*CPSI	848	f	1	0.92	5.80	0.63	0.0273
Subtotal CPSI				2.03	328.25	372.95	
DAMBER	501	m	0	1.52	17.84	1.32	0.0000
DAMBER	502	m	0	1.92	26.52	11.96	0.0000
DAMBER	503	m	0	2.34	22.90	27.55	0.0000
Subtotal DAMBER				1.96	67.25	40.82	
DEAN3	564	m	0	1.92	10.14	4.57	0.0000
DEAN3	565	m	0	1.93	14.48	6.70	0.0000
DEAN3	566	m	0	1.95	19.25	9.47	0.0000
DEAN3	567	m	0	1.73	13.81	3.30	0.0000
DEAN3	583	f	0	1.31	15.21	0.06	0.0000
DEAN3	584	f	0	1.08	11.77	0.32	0.0002
DEAN3	585	f	0	1.07	18.99	0.62	0.0000
DEAN3	586	f	0	0.88	5.65	0.76	0.0367
Subtotal DEAN3				1.52	109.30	25.79	
DOLL	501	m	0	1.61	1.81	0.23	0.0307
DOLL	502	m	0	1.56	4.08	0.40	0.0016

Table 1H11 - 2

IESLC - Meta-analysis of Ever/current Smoking by Age started, Overview
All LC types, Any Product (or Cigarettes if Any not available)
Most adjusted

REF	NRR	SEX	AD	Ys	Ws	Qs	Ps
DOLL	503	m	0	2.11	5.99	4.52	0.0000
DOLL	504	m	0	2.25	6.20	6.22	0.0000
DOLL	508	f	0	0.39	5.71	4.19	0.3532
DOLL	509	f	0	0.75	3.51	0.88	0.1625
DOLL	510	f	0	0.82	6.57	1.21	0.0364
DOLL	511	f	0	0.90	5.71	0.68	0.0317
Subtotal DOLL				1.29	39.58	18.34	
*DORN	610	m	0	1.47	14.92	0.72	0.0000
*DORN	611	m	0	2.21	21.57	19.88	0.0000
*DORN	612	m	0	2.62	23.30	43.95	0.0000
*DORN	613	m	0	2.99	19.28	58.33	0.0000
*DORN	647	m	0	1.44	31.75	1.20	0.0000
*DORN	648	m	0	1.89	38.11	15.94	0.0000
*DORN	649	m	0	2.20	42.26	38.63	0.0000
*DORN	650	m	0	2.44	30.57	43.75	0.0000
Subtotal DORN				2.14	221.77	222.41	
*ENGELA	501	m	0	1.33	10.44	0.08	0.0000
*ENGELA	502	m	0	1.28	17.55	0.02	0.0000
*ENGELA	503	m	0	2.00	23.38	13.42	0.0000
*ENGELA	509	f	0	1.00	7.56	0.44	0.0058
*ENGELA	510	f	0	2.10	16.67	12.11	0.0000
*ENGELA	511	f	0	2.42	11.40	15.82	0.0000
Subtotal ENGELA				1.76	87.00	41.89	
GAO	506	m	2	0.18	20.54	23.23	0.4087
GAO	507	m	2	1.55	33.44	3.04	0.0000
GAO	508	m	2	1.63	31.98	4.70	0.0000
GAO	516	f	2	0.69	26.45	8.08	0.0004
GAO	517	f	2	1.34	23.87	0.19	0.0000
GAO	518	f	2	1.72	16.22	3.69	0.0000
Subtotal GAO				1.22	152.50	42.93	
GAO2	501	m	0	0.77	1.18	0.27	0.4038
GAO2	502	m	0	1.86	8.74	3.32	0.0000
GAO2	503	m	0	2.15	6.56	5.40	0.0000
Subtotal GAO2				1.90	16.48	8.99	
GENG	533	f	1	0.44	9.87	6.44	0.1685
GENG	534	f	1	1.08	9.72	0.26	0.0007
GENG	535	f	1	1.83	6.25	2.14	0.0000
Subtotal GENG				1.02	25.84	8.84	
HAENSZ	537	f	0	0.66	18.36	6.22	0.0044
HAENSZ	538	f	0	0.86	13.00	1.94	0.0019
Subtotal HAENSZ				0.75	31.36	8.16	
HEGMAN	513	m	1	2.24	7.47	7.39	0.0000
HEGMAN	514	m	1	3.10	10.02	34.62	0.0000
HEGMAN	516	f	1	1.57	1.60	0.17	0.0470
HEGMAN	517	f	1	3.29	12.44	51.89	0.0000
Subtotal HEGMAN				2.89	31.53	94.07	
*HIRAYA	501	m	1	1.47	83.52	4.20	0.0000
*HIRAYA	502	m	1	1.74	67.55	16.64	0.0000
*HIRAYA	504	f	1	0.90	65.73	7.86	0.0000
*HIRAYA	505	f	1	-0.25	0.91	2.03	0.8127
Subtotal HIRAYA				1.38	217.71	30.73	
HU	511	m	0	0.13	6.22	7.68	0.7374
HU	512	m	0	0.82	15.39	2.80	0.0013
HU	513	m	0	1.11	3.86	0.07	0.0292
HU	516	f	0	-0.33	1.73	4.28	0.6660
HU	517	f	0	0.88	4.71	0.65	0.0575
HU	518	f	0	0.41	2.02	1.42	0.5647
Subtotal HU				0.65	33.92	16.91	
HU2	501	c	0	-0.09	8.76	15.73	0.7814
HU2	502	c	0	-0.06	15.16	25.70	0.8268
HU2	503	c	0	0.93	42.35	4.22	0.0000
HU2	504	c	0	1.21	28.23	0.05	0.0000
Subtotal HU2				0.76	94.50	45.69	
JEDRYC	607	m	0	1.99	27.77	15.38	0.0000
JEDRYC	608	m	0	2.21	21.04	19.67	0.0000
JEDRYC	619	f	0	2.50	7.96	12.53	0.0000
Subtotal JEDRYC				2.14	56.77	47.58	
JOLY	543	m	0	1.54	6.34	0.55	0.0001
JOLY	544	m	0	2.40	10.49	14.02	0.0000
JOLY	545	m	0	3.02	10.57	33.14	0.0000
JOLY	533	f	0	1.12	11.03	0.19	0.0002

Table 1H11 - 2

IESLC - Meta-analysis of Ever/current Smoking by Age started, Overview
All LC types, Any Product (or Cigarettes if Any not available)
Most adjusted

REF	NRR	SEX	AD	Ys	Ws	Qs	Ps
JOLY	534	f	0	2.05	16.96	10.93	0.0000
JOLY	535	f	0	2.47	15.51	23.22	0.0000
Subtotal JOLY				2.15	70.89	82.04	
KHUDER	506	m	0	1.85	14.88	5.44	0.0000
KHUDER	507	m	0	1.86	17.89	6.67	0.0000
KHUDER	508	m	0	2.33	18.34	21.61	0.0000
Subtotal KHUDER				2.03	51.12	33.72	
KOULUM	501	m	0	2.38	2.13	2.74	0.0005
KOULUM	502	m	0	2.27	4.00	4.19	0.0000
KOULUM	503	m	0	3.33	4.31	18.76	0.0000
KOULUM	504	m	0	3.72	4.12	25.25	0.0000
KOULUM	505	m	0	4.57	3.47	38.36	0.0000
Subtotal KOULUM				3.31	18.03	89.30	
LETOUR	501	c	0	2.39	17.33	22.88	0.0000
LETOUR	502	c	0	2.48	18.69	28.56	0.0000
LETOUR	503	c	0	2.92	15.17	42.53	0.0000
Subtotal LETOUR				2.58	51.19	93.97	
*LIAW	504	c	2	0.41	6.39	4.51	0.3054
*LIAW	505	c	2	1.77	8.74	2.45	0.0000
*LIAW	506	c	2	1.53	11.90	0.93	0.0000
Subtotal LIAW				1.34	27.03	7.89	
LIU3	504	m	2	0.10	1.73	2.29	0.9003
LIU3	505	m	2	0.33	1.78	1.49	0.6607
Subtotal LIU3				0.21	3.50	3.78	
LIU4	501	m	2	0.88	3072.74	412.17	0.0000
LIU4	502	m	2	1.05	4361.66	165.94	0.0000
LIU4	503	m	2	1.34	4224.99	35.57	0.0000
Subtotal LIU4				1.11	11659.40	613.68	
LIU5	501	c	0	-0.07	5.40	9.36	0.8697
LIU5	502	c	0	0.86	10.25	1.52	0.0058
Subtotal LIU5				0.54	15.65	10.88	
LUBIN	565	m	0	0.29	6.10	5.54	0.4689
LUBIN	566	m	0	1.27	6.79	0.00	0.0009
LUBIN	567	m	0	1.21	7.09	0.01	0.0013
LUBIN	568	m	0	1.74	7.43	1.78	0.0000
Subtotal LUBIN				1.16	27.42	7.33	
LUBIN2	1156	m	1	1.21	36.26	0.05	0.0000
LUBIN2	1157	m	1	1.60	108.74	13.90	0.0000
LUBIN2	1158	m	1	1.69	137.76	27.41	0.0000
LUBIN2	1159	m	1	1.91	123.47	54.14	0.0000
LUBIN2	1160	m	1	2.20	55.35	50.21	0.0000
Subtotal LUBIN2				1.75	461.59	145.72	
MATOS	576	m	2	1.36	6.58	0.09	0.0005
MATOS	577	m	2	2.05	8.37	5.47	0.0000
MATOS	578	m	2	2.05	7.92	5.18	0.0000
Subtotal MATOS				1.85	22.87	10.73	
*MIGRAN	501	m	0	2.05	3.46	2.25	0.0001
*MIGRAN	503	m	0	1.94	3.77	1.80	0.0002
*MIGRAN	505	m	0	2.31	3.73	4.19	0.0000
*MIGRAN	511	f	0	2.08	2.94	2.03	0.0004
*MIGRAN	513	f	0	2.12	2.78	2.10	0.0004
*MIGRAN	515	f	0	1.97	1.34	0.70	0.0226
Subtotal MIGRAN				2.09	18.02	13.07	
*MRFITR	508	m	0	3.17	0.44	1.63	0.0357
*MRFITR	509	m	0	4.10	0.46	3.77	0.0052
*MRFITR	510	m	0	3.30	0.47	1.98	0.0238
*MRFITR	511	m	0	3.92	0.49	3.52	0.0060
*MRFITR	512	m	0	4.21	0.49	4.33	0.0031
*MRFITR	513	m	0	3.83	0.49	3.27	0.0074
Subtotal MRFITR				3.77	2.85	18.50	
PERNU	504	m	0	2.15	56.20	45.48	0.0000
PERNU	505	m	0	2.34	35.99	43.12	0.0000
PERNU	501	f	0	0.58	13.00	5.77	0.0366
PERNU	502	f	0	3.27	0.37	1.53	0.0455
Subtotal PERNU				2.02	105.57	95.90	
PEZZOT	570	m	0	2.43	3.42	4.77	0.0000
PEZZOT	571	m	0	3.16	3.65	13.39	0.0000
PEZZOT	572	m	0	3.11	3.42	11.92	0.0000
Subtotal PEZZOT				2.91	10.48	30.08	
*QIAO2	506	m	1	0.28	8.36	7.83	0.4222
*QIAO2	507	m	1	0.39	8.84	6.55	0.2520

Table 1H11 - 2

IESLC - Meta-analysis of Ever/current Smoking by Age started, Overview
All LC types, Any Product (or Cigarettes if Any not available)
Most adjusted

REF	NRR	SEX	AD	Ys	Ws	Qs	Ps
*QIAO2	508	m	1	0.59	8.97	3.82	0.0756
Subtotal QIAO2				0.42	26.17	18.20	
RACHTA	506	f	1	1.67	2.35	0.42	0.0104
RACHTA	507	f	1	1.67	9.14	1.67	0.0000
RACHTA	508	f	1	2.45	5.53	8.04	0.0000
Subtotal RACHTA				1.93	17.03	10.13	
SEGI2	521	m	1	0.71	5.61	1.59	0.0914
SEGI2	522	m	1	1.20	6.11	0.01	0.0029
SEGI2	523	m	1	1.73	5.74	1.35	0.0000
Subtotal SEGI2				1.22	17.46	2.95	
SOBUE	654	m	0	1.37	16.73	0.27	0.0000
SOBUE	655	m	0	1.47	22.22	1.16	0.0000
SOBUE	656	m	0	2.26	15.19	15.68	0.0000
Subtotal SOBUE				1.66	54.14	17.11	
SUZUK2	501	c	0	1.25	4.59	0.00	0.0072
SUZUK2	502	c	0	2.09	6.59	4.74	0.0000
SUZUK2	503	c	0	2.70	4.13	8.78	0.0000
Subtotal SUZUK2				2.01	15.32	13.52	
SVENSS	501	f	0	1.73	8.23	1.89	0.0000
SVENSS	502	f	0	2.57	8.11	14.25	0.0000
SVENSS	503	f	0	2.06	9.85	6.48	0.0000
Subtotal SVENSS				2.11	26.19	22.61	
TIZZAN	506	m	0	-0.77	8.70	35.44	0.0228
TIZZAN	507	m	0	0.47	66.40	39.51	0.0001
TIZZAN	508	m	0	0.81	82.27	15.92	0.0000
TIZZAN	519	f	0	-0.94	1.39	6.67	0.2659
TIZZAN	520	f	0	-0.59	6.74	22.62	0.1283
TIZZAN	521	f	0	1.68	1.64	0.31	0.0314
Subtotal TIZZAN				0.53	167.16	120.47	
WAKAI	501	m	0	0.73	3.57	0.94	0.1666
WAKAI	502	m	0	1.53	7.78	0.63	0.0000
WAKAI	503	m	0	1.31	6.55	0.02	0.0008
Subtotal WAKAI				1.29	17.89	1.59	
WU	541	f	2	0.44	4.29	2.80	0.3639
WU	542	f	2	1.27	5.67	0.00	0.0024
WU	543	f	2	2.33	6.60	7.81	0.0000
Subtotal WU				1.48	16.56	10.62	
WYNDE6	759	m	0	2.63	24.28	46.81	0.0000
WYNDE6	760	m	0	2.92	30.32	84.90	0.0000
WYNDE6	761	m	0	3.15	38.07	138.71	0.0000
WYNDE6	767	f	0	2.57	29.27	50.98	0.0000
WYNDE6	768	f	0	2.98	32.44	97.15	0.0000
WYNDE6	769	f	0	3.38	33.77	154.36	0.0000
Subtotal WYNDE6				2.97	188.15	572.90	
ZHENG	563	m	0	0.19	10.89	12.16	0.5320
ZHENG	564	m	0	1.33	17.54	0.13	0.0000
ZHENG	565	m	0	1.95	13.58	6.71	0.0000
ZHENG	572	f	0	0.19	7.30	8.12	0.6057
ZHENG	573	f	0	0.95	15.53	1.33	0.0002
Subtotal ZHENG				1.05	64.84	28.46	

N	212
NS	51

Table 1H11 - 3

IESLC - Meta-analysis of Ever/current Smoking by Age started, Overview
All LC types, Any Product (or Cigarettes if Any not available)
Most adjusted

	Sex			
	combined	male	female	Total
N	20	121	71	212
NS	7	37	25	69

In this overview table, other than the "N" rows, entries in the "absent" and "Total" columns may be invalid and should be ignored

	Age started (broad categories)				
	absent	19+k26	15-25k18	1-17k14	Total
N	88	60	29	35	212
NS	38	45	22	26	131
Wt	9639.67	4041.78	648.15	572.38	14901.98
Het Chi	967.45	514.82	208.59	252.35	3391.42
Het df	87	59	28	34	211
Het P	***	***	***	***	***
Fixed RR	3.42	2.70	7.75	11.11	3.48
RRl	3.35	2.62	7.18	10.23	3.42
RRu	3.48	2.79	8.37	12.06	3.53
P	+++	+++	+++	+++	+++
Random RR	4.38	3.89	7.48	10.32	5.30
RRl	3.92	3.33	5.94	8.04	4.86
RRu	4.90	4.56	9.42	13.26	5.77
P	+++	+++	+++	+++	+++

	Age started (narrow categories)							
	absent	27+k30	23-29k26	19-25k22	15-21k18	11-17k14	1-13k10	Total
N	150	16	1	14	25	2	4	212
NS	51	11	1	11	19	2	4	99
Wt	9399.52	135.72	6.79	4580.31	585.68	127.59	66.37	14901.98
Het Chi	2192.36	44.33	0.00	183.11	191.04	13.11	20.72	3391.42
Het df	149	15	0	13	24	1	3	211
Het P	***	***	N.S.	***	***	***	***	***
Fixed RR	3.53	1.71	3.56	2.97	8.01	7.15	11.03	3.48
RRl	3.46	1.45	1.68	2.89	7.39	6.01	8.67	3.42
RRu	3.61	2.02	7.56	3.06	8.69	8.50	14.03	3.53
P	+++	+++	+++	+++	+++	+++	+++	+++
Random RR	5.25	1.80	3.56	5.31	7.78	15.64	21.72	5.30
RRl	4.71	1.32	1.68	3.66	6.04	2.66	7.77	4.86
RRu	5.86	2.47	7.56	7.71	10.03	92.10	60.76	5.77
P	+++	+++	+++	+++	+++	++	+++	+++

Table 1H11 - 3

IESLC - Meta-analysis of Ever/current Smoking by Age started, Overview
All LC types, Any Product (or Cigarettes if Any not available)
Most adjusted

MALES

	Age started (broad categories) absent	19+k26	15-25k18	1-17k14	Total
N	46	34	19	22	121
NS	26	33	18	21	98
Wt	9271.81	3668.49	487.41	460.68	13888.39
Het Chi	682.52	317.58	126.54	149.53	2470.09
Het df	45	33	18	21	120
Het P	***	***	***	***	***
Fixed RR	3.40	2.65	8.00	10.73	3.41
RRl	3.33	2.56	7.32	9.79	3.35
RRu	3.47	2.74	8.74	11.76	3.47
P	+++	+++	+++	+++	+++
Random RR	4.82	4.55	8.08	11.19	6.12
RRl	4.18	3.70	6.21	8.53	5.51
RRu	5.55	5.60	10.53	14.70	6.79
P	+++	+++	+++	+++	+++

	absent	Age started (narrow categories) 27+k30	23-29k26	19-25k22	15-21k18	11-17k14	1-13k10	Total
N	81	8	1	9	17	2	3	121
NS	37	8	1	8	16	2	3	75
Wt	8656.13	63.03	6.79	4517.91	454.70	127.59	62.24	13888.39
Het Chi	1516.56	16.28	0.00	160.41	117.10	13.11	20.32	2470.09
Het df	80	7	0	8	16	1	2	120
Het P	***	*	N.S.	***	***	***	***	***
Fixed RR	3.46	1.67	3.56	2.96	8.18	7.15	10.81	3.41
RRl	3.39	1.30	1.68	2.87	7.46	6.01	8.44	3.35
RRu	3.53	2.13	7.56	3.04	8.96	8.50	13.86	3.47
P	+++	+++	+++	+++	+++	+++	+++	+++
Random RR	6.08	1.80	3.56	5.66	8.28	15.64	25.30	6.12
RRl	5.31	1.20	1.68	3.39	6.24	2.66	5.94	5.51
RRu	6.97	2.72	7.56	9.45	10.98	92.10	107.72	6.79
P	+++	++	+++	+++	+++	++	+++	+++

FEMALES

	Age started (broad categories) absent	19+k26	15-25k18	1-17k14	Total
N	32	21	8	10	71
NS	20	21	8	10	59
Wt	256.58	294.96	129.25	78.92	759.71
Het Chi	186.19	148.58	68.47	73.34	620.60
Het df	31	20	7	9	70
Het P	***	***	***	***	***
Fixed RR	4.05	3.26	7.01	12.76	4.61
RRl	3.59	2.91	5.90	10.23	4.29
RRu	4.58	3.66	8.33	15.91	4.95
P	+++	+++	+++	+++	+++
Random RR	3.95	3.07	6.23	6.37	4.08
RRl	2.87	2.20	3.44	3.00	3.25
RRu	5.43	4.30	11.26	13.56	5.11
P	+++	+++	+++	+++	+++

Table 1H11 - 3

IESLC - Meta-analysis of Ever/current Smoking by Age started, Overview
All LC types, Any Product (or Cigarettes if Any not available)
Most adjusted

FEMALES

	absent	27+k30	23-29k26	19-25k22	15-21k18	11-17k14	1-13k10	Total
		Age started (narrow categories)						
N	54	6		4	7			71
NS	25	6		4	7			42
Wt	541.62	52.13		53.67	112.29			759.71
Het Chi	475.28	17.00		13.34	68.27			620.60
Het df	53	5		3	6			70
Het P	***	**		**	***			***
Fixed RR	4.60	2.23		4.05	6.91			4.61
RRl	4.23	1.70		3.10	5.74			4.29
RRu	5.01	2.93		5.29	8.31			4.95
P	+++	+++		+++	+++			+++
Random RR	4.09	2.29		4.54	5.97			4.08
RRl	3.12	1.29		2.45	2.97			3.25
RRu	5.36	4.07		8.42	11.99			5.11
P	+++	++		+++	+++			+++

Table 1H11 - 4

IESLC - Meta-analysis of Ever/current Smoking by Age started, Overview
All LC types, Any Product (or Cigarettes if Any not available)
Least adjusted

REF	NRR	X	SEX	AGEL	AGEH	RACE	YF	LC TYPE	LOC	START	ST	NLC	R	VB	P	H	AD	SM	PRODUCT	exL	exH	S1	S2	DENOM	De
AGUDO	501	x	f	0	0	all	-	all	Eu:wst	1989	CC	103	n	bl	n	n	0	ev	cig only	24	999	1	0	nev cigs	st
AGUDO	502	x	f	0	0	all	-	all	Eu:wst	1989	CC	103	n	bl	n	n	0	ev	cig only	1	23	0	0	nev cigs	st
ARMADA	511		m	0	0	all	-	all	Eu:wst	1986	CC	325	n	bl	n	y	0	ev	cig+/-ot	17	45	0	0	nev cigs	st
ARMADA	512		m	0	0	all	-	all	Eu:wst	1986	CC	325	n	bl	n	y	0	ev	cig+/-ot	7	16	3	0	nev cigs	st
AUVINE	509	x	c	0	0	all	-	all	Eu:Sca	1986	CC	517	n	bl	y	n	0	ev	cig+/-ot	21	999	1	0	nev cigs	st
AUVINE	510	x	c	0	0	all	-	all	Eu:Sca	1986	CC	517	n	bl	y	n	0	ev	cig+/-ot	16	20	2	4	nev cigs	st
AUVINE	511	x	c	0	0	all	-	all	Eu:Sca	1986	CC	517	n	bl	y	n	0	ev	cig+/-ot	1	15	3	0	nev cigs	st
BARBON	515	x	m	0	0	all	-	all	Eu:wst	1979	CC	755	n	bl	y	y	0	ev	all/unsp	20	999	1	0	nev any	st
BARBON	516	x	m	0	0	all	-	all	Eu:wst	1979	CC	755	n	bl	y	y	0	ev	all/unsp	15	19	2	4	nev any	st
BARBON	517	x	m	0	0	all	-	all	Eu:wst	1979	CC	755	n	bl	y	y	0	ev	all/unsp	1	14	3	0	nev any	st
BRESLO	501		c	0	0	all	-	all	NAmer	1949	CC	518	n	bl	n	y	0	ev	cig+/-ot	25	999	1	0	nev any	st
BRESLO	502		c	0	0	all	-	all	NAmer	1949	CC	518	n	bl	n	y	0	ev	cig+/-ot	15	24	2	0	nev any	st
BRESLO	503		c	0	0	all	-	all	NAmer	1949	CC	518	n	bl	n	y	0	ev	cig+/-ot	0	14	3	0	nev any	st
BUFFLE	517		f	0	0	w-hi	-	all	NAmer	1976	CC	943	n	bl	y	n	0	ev	cig+/-ot	30	999	0	1	nev cigs	or
BUFFLE	518		f	0	0	w-hi	-	all	NAmer	1976	CC	943	n	bl	y	n	0	ev	cig+/-ot	21	29	1	0	nev cigs	or
BUFFLE	519		f	0	0	w-hi	-	all	NAmer	1976	CC	943	n	bl	y	n	0	ev	cig+/-ot	19	20	0	0	nev cigs	or
BUFFLE	520		f	0	0	w-hi	-	all	NAmer	1976	CC	943	n	bl	y	n	0	ev	cig+/-ot	17	18	2	4	nev cigs	ot
BUFFLE	521		f	0	0	w-hi	-	all	NAmer	1976	CC	943	n	bl	y	n	0	ev	cig+/-ot	6	16	3	0	nev cigs	or
CEDERL	510		m	0	0	all	10	all	Eu:Sca	1963	pr	491	n	bl	n	n	1	cu	cig only	19	999	1	0	nev any	ot
CEDERL	511		m	0	0	all	10	all	Eu:Sca	1963	pr	491	n	bl	n	n	1	cu	cig only	17	18	2	4	nev any	ot
CEDERL	512		m	0	0	all	10	all	Eu:Sca	1963	pr	491	n	bl	n	n	1	cu	cig only	1	16	3	0	nev any	ot
CEDERL	515		f	0	0	all	10	all	Eu:Sca	1963	pr	491	n	bl	n	n	0	cu	cig only	19	999	1	0	nev any	st
CEDERL	516		f	0	0	all	10	all	Eu:Sca	1963	pr	491	n	bl	n	n	0	cu	cig only	17	18	2	4	nev any	st
CEDERL	517		f	0	0	all	10	all	Eu:Sca	1963	pr	491	n	bl	n	n	0	cu	cig only	1	16	3	0	nev any	ot
CHEN2	517		m	0	0	all	-	all	As:Chi	1983	CC	193	n	ot	y	n	0	ev	all/unsp	31	999	0	0	nev any	st
CHEN2	518		m	0	0	all	-	all	As:Chi	1983	CC	193	n	ot	y	n	0	ev	all/unsp	20	30	1	0	nev any	st
CHEN2	519		m	0	0	all	-	all	As:Chi	1983	CC	193	n	ot	y	n	0	ev	all/unsp	1	19	0	0	nev any	st
CHEN2	522		f	0	0	all	-	all	As:Chi	1983	CC	193	n	ot	y	n	0	ev	all/unsp	31	999	0	0	nev any	st
CHEN2	523		f	0	0	all	-	all	As:Chi	1983	CC	193	n	ot	y	n	0	ev	all/unsp	20	30	1	0	nev any	st
CHEN2	524		f	0	0	all	-	all	As:Chi	1983	CC	193	n	ot	y	n	0	ev	all/unsp	1	19	0	0	nev any	st
CHIAZZ	501		m	0	0	all	-	all	NAmer	1940	CC	144	o	bl	y	n	2	ev	cig+/-ot	20	999	1	0	nev cigs	or
CHIAZZ	502		m	0	0	all	-	all	NAmer	1940	CC	144	o	bl	y	n	2	ev	cig+/-ot	1	19	0	0	nev cigs	or
CHOI	523		m	0	0	all	-	all	As:oth	1985	CC	375	n	bl	n	n	0	ev	cig+/-ot	25	999	1	0	nev cigs	st
CHOI	524		m	0	0	all	-	all	As:oth	1985	CC	375	n	bl	n	n	0	ev	cig+/-ot	20	24	0	3	nev cigs	st
CHOI	525		m	0	0	all	-	all	As:oth	1985	CC	375	n	bl	n	n	0	ev	cig+/-ot	15	19	2	4	nev cigs	st
CHOI	526		m	0	0	all	-	all	As:oth	1985	CC	375	n	bl	n	n	0	ev	cig+/-ot	1	14	3	0	nev cigs	st
CHOI	530		f	0	0	all	-	all	As:oth	1985	CC	375	n	bl	n	n	0	ev	cig+/-ot	25	999	1	0	nev cigs	st
CHOI	531		f	0	0	all	-	all	As:oth	1985	CC	375	n	bl	n	n	0	ev	cig+/-ot	1	24	0	0	nev cigs	st
CPSI	801		m	35	84	all	6	all	NAmer	1959	pr	5138	n	bl	n	n	1	cu	cig+/-ot	25	999	1	0	nev any	ot
CPSI	802		m	35	84	all	6	all	NAmer	1959	pr	5138	n	bl	n	n	1	cu	cig+/-ot	20	24	0	3	nev any	ot
CPSI	803		m	35	84	all	6	all	NAmer	1959	pr	5138	n	bl	n	n	1	cu	cig+/-ot	15	19	2	4	nev any	ot
CPSI	804		m	35	84	all	6	all	NAmer	1959	pr	5138	n	bl	n	n	1	cu	cig+/-ot	1	14	3	0	nev any	ot
CPSI	845		f	40	74	all	6	all	NAmer	1959	pr	5138	n	bl	n	n	1	cu	cig only	25	999	1	0	nev any	ot
CPSI	846		f	40	74	all	6	all	NAmer	1959	pr	5138	n	bl	n	n	1	cu	cig only	20	24	0	3	nev any	ot
CPSI	847		f	40	74	all	6	all	NAmer	1959	pr	5138	n	bl	n	n	1	cu	cig only	15	19	2	4	nev any	ot
CPSI	848		f	40	74	all	6	all	NAmer	1959	pr	5138	n	bl	n	n	1	cu	cig only	1	14	3	0	nev any	ot
DAMBER	501		m	0	0	all	-	all	Eu:Sca	1972	CC	579	n	bl	y	n	0	ev	all/unsp	21	999	1	0	nev any	st
DAMBER	502		m	0	0	all	-	all	Eu:Sca	1972	CC	579	n	bl	y	n	0	ev	all/unsp	16	20	2	4	nev any	st
DAMBER	503		m	0	0	all	-	all	Eu:Sca	1972	CC	579	n	bl	y	n	0	ev	all/unsp	1	15	3	0	nev any	st
DEAN3	564		m	0	0	all	-	all	Eu:UK	1969	CC	766	n	V	y	n	0	cu	cig only	25	999	1	0	nev any	st
DEAN3	565		m	0	0	all	-	all	Eu:UK	1969	CC	766	n	V	y	n	0	cu	cig only	20	24	0	3	nev any	st
DEAN3	566		m	0	0	all	-	all	Eu:UK	1969	CC	766	n	V	y	n	0	cu	cig only	15	19	2	4	nev any	st
DEAN3	567		m	0	0	all	-	all	Eu:UK	1969	CC	766	n	V	y	n	0	cu	cig only	1	14	3	0	nev any	st
DEAN3	583		f	0	0	all	-	all	Eu:UK	1969	CC	766	n	V	y	n	0	cu	cig only	25	999	1	0	nev any	st
DEAN3	584		f	0	0	all	-	all	Eu:UK	1969	CC	766	n	V	y	n	0	cu	cig only	20	24	0	3	nev any	st
DEAN3	585		f	0	0	all	-	all	Eu:UK	1969	CC	766	n	V	y	n	0	cu	cig only	15	19	2	4	nev any	st
DEAN3	586		f	0	0	all	-	all	Eu:UK	1969	CC	766	n	V	y	n	0	cu	cig only	1	14	3	0	nev any	st
DOLL	501		m	0	0	all	-	all	Eu:UK	1948	CC	1465	n	V	n	n	0	ev	all/unsp	40	999	0	0	nev any	st
DOLL	502		m	0	0	all	-	all	Eu:UK	1948	CC	1465	n	V	n	n	0	ev	all/unsp	30	39	0	1	nev any	st
DOLL	503		m	0	0	all	-	all	Eu:UK	1948	CC	1465	n	V	n	n	0	ev	all/unsp	20	29	1	0	nev any	st
DOLL	504		m	0	0	all	-	all	Eu:UK	1948	CC	1465	n	V	n	n	0	ev	all/unsp	1	19	0	0	nev any	st
DOLL	508		f	0	0	all	-	all	Eu:UK	1948	CC	1465	n	V	n	n	0	ev	all/unsp	40	999	0	0	nev any	st
DOLL	509		f	0	0	all	-	all	Eu:UK	1948	CC	1465	n	V	n	n	0	ev	all/unsp	30	39	0	1	nev any	st
DOLL	510		f	0	0	all	-	all	Eu:UK	1948	CC	1465	n	V	n	n	0	ev	all/unsp	20	29	1	0	nev any	st
DOLL	511		f	0	0	all	-	all	Eu:UK	1948	CC	1465	n	V	n	n	0	ev	all/unsp	1	19	0	0	nev any	st
DORN	610		m	55	64	wh	8	all	NAmer	1954	pr	5097	n	bl	n	n	0	ev	cig+/-ot	25	999	1	0	nev any	st
DORN	611		m	55	64	wh	8	all	NAmer	1954	pr	5097	n	bl	n	n	0	ev	cig+/-ot	20	24	0	3	nev any	st
DORN	612		m	55	64	wh	8	all	NAmer	1954	pr	5097	n	bl	n	n	0	ev	cig+/-ot	15	19	2	4	nev any	st
DORN	613		m	55	64	wh	8	all	NAmer	1954	pr	5097	n	bl	n	n	0	ev	cig+/-ot	1	14	3	0	nev any	st
DORN	647		m	65	74	wh	8	all	NAmer	1954	pr	5097	n	bl	n	n	0	ev	cig+/-ot	25	999	1	0	nev any	st
DORN	648		m	65	74	wh	8	all	NAmer	1954	pr	5097	n	bl	n	n	0	ev	cig+/-ot	20	24	0	3	nev any	st
DORN	649		m	65	74	wh	8	all	NAmer	1954	pr	5097	n	bl	n	n	0	ev	cig+/-ot	15	19	2	4	nev any	st

Table 1H11 - 4

IESLC - Meta-analysis of Ever/current Smoking by Age started, Overview
All LC types, Any Product (or Cigarettes if Any not available)
Least adjusted

REF	NRR	X	SEX	AGEL	AGEH	RACE	YF	LC TYPE	LOC	START	ST	NLC	R	VB	P	H	AD	SM	PRODUCT	exL	exH	S1	S2	DENOM	De
DORN	650		m	65	74	wh	8	all	NAmer	1954	pr	5097	n	bl	n	n	0	ev	cig+/-ot	1	14	3	0	nev any	st
ENGELA	501		m	0	0	all	0	all	Eu:Sca	1964	pr	435	n	bl	n	n	0	cu	cig+/-ot	30	999	0	1	nev cigs	st
ENGELA	502		m	0	0	all	0	all	Eu:Sca	1964	pr	435	n	bl	n	n	0	cu	cig+/-ot	20	29	1	0	nev cigs	st
ENGELA	503		m	0	0	all	0	all	Eu:Sca	1964	pr	435	n	bl	n	n	0	cu	cig+/-ot	1	19	0	0	nev cigs	st
ENGELA	509		f	0	0	all	0	all	Eu:Sca	1964	pr	435	n	bl	n	n	0	cu	cig+/-ot	30	999	0	1	nev cigs	st
ENGELA	510		f	0	0	all	0	all	Eu:Sca	1964	pr	435	n	bl	n	n	0	cu	cig+/-ot	20	29	1	0	nev cigs	st
ENGELA	511		f	0	0	all	0	all	Eu:Sca	1964	pr	435	n	bl	n	n	0	cu	cig+/-ot	1	19	0	0	nev cigs	st
GAO	501	x	m	0	0	all	-	all	As:Chi	1984	CC	1405	n	ot	n	n	0	ev	cig+/-ot	30	999	0	1	nev cigs	st
GAO	502	x	m	0	0	all	-	all	As:Chi	1984	CC	1405	n	ot	n	n	0	ev	cig+/-ot	20	29	1	0	nev cigs	st
GAO	503	x	m	0	0	all	-	all	As:Chi	1984	CC	1405	n	ot	n	n	0	ev	cig+/-ot	10	19	0	0	nev cigs	st
GAO	511	x	f	0	0	all	-	all	As:Chi	1984	CC	1405	n	ot	n	n	0	ev	cig+/-ot	30	999	0	1	nev cigs	st
GAO	512	x	f	0	0	all	-	all	As:Chi	1984	CC	1405	n	ot	n	n	0	ev	cig+/-ot	20	29	1	0	nev cigs	st
GAO	513	x	f	0	0	all	-	all	As:Chi	1984	CC	1405	n	ot	n	n	0	ev	cig+/-ot	10	19	0	0	nev cigs	st
GAO2	501		m	0	0	all	-	all	As:Jap	1988	CC	282	n	bl	n	n	0	cu	cig+/-ot	30	999	0	1	nev cigs	or
GAO2	502		m	0	0	all	-	all	As:Jap	1988	CC	282	n	bl	n	n	0	cu	cig+/-ot	20	29	1	0	nev cigs	st
GAO2	503		m	0	0	all	-	all	As:Jap	1988	CC	282	n	bl	n	n	0	cu	cig+/-ot	1	19	0	0	nev cigs	or
GENG	528	x	f	0	0	all	-	all	As:Chi	1985	CC	292	n	ot	*	n	0	ev	cig+/-ot	21	999	1	0	nev any	st
GENG	529	x	f	0	0	all	-	all	As:Chi	1985	CC	292	n	ot	*	n	0	ev	cig+/-ot	16	20	2	4	nev any	st
GENG	530	x	f	0	0	all	-	all	As:Chi	1985	CC	292	n	ot	*	n	0	ev	cig+/-ot	1	15	3	0	nev any	st
HAENSZ	537		f	0	0	all	-	not alv	NAmer	1955	CC	158	n	bl	n	y	0	ev	cig+/-ot	25	999	1	0	nev any	st
HAENSZ	538		f	0	0	all	-	not alv	NAmer	1955	CC	158	n	bl	n	y	0	ev	cig+/-ot	1	24	0	0	nev any	st
HEGMAN	513		m	0	0	all	-	all	NAmer	1989	CC	282	n	bl	y	y	1	ev	all/unsp	20	999	1	0	nev any	or
HEGMAN	514		m	0	0	all	-	all	NAmer	1989	CC	282	n	bl	y	y	1	ev	all/unsp	1	19	0	0	nev any	or
HEGMAN	516		f	0	0	all	-	all	NAmer	1989	CC	282	n	bl	y	y	1	ev	all/unsp	26	999	1	0	nev any	or
HEGMAN	517		f	0	0	all	-	all	NAmer	1989	CC	282	n	bl	y	y	1	ev	all/unsp	1	25	0	0	nev any	or
HIRAYA	501		m	0	0	all	0	all	As:Jap	1965	pr	1917	n	bl	n	n	1	cu	cig+/-ot	20	999	1	0	nev any	st
HIRAYA	502		m	0	0	all	0	all	As:Jap	1965	pr	1917	n	bl	n	n	1	cu	cig+/-ot	1	19	0	0	nev any	st
HIRAYA	504		f	0	0	all	0	all	As:Jap	1965	pr	1917	n	bl	n	n	1	cu	cig+/-ot	20	999	1	0	nev any	st
HIRAYA	505		f	0	0	all	0	all	As:Jap	1965	pr	1917	n	bl	n	n	1	cu	cig+/-ot	1	19	0	0	nev any	st
HU	511		m	0	0	all	-	all	As:Chi	1985	CC	227	n	ot	n	y	0	ev	cig+/-ot	30	999	0	1	nev cigs	st
HU	512		m	0	0	all	-	all	As:Chi	1985	CC	227	n	ot	n	y	0	ev	cig+/-ot	16	29	0	0	nev cigs	st
HU	513		m	0	0	all	-	all	As:Chi	1985	CC	227	n	ot	n	y	0	ev	cig+/-ot	1	15	3	0	nev cigs	st
HU	516		f	0	0	all	-	all	As:Chi	1985	CC	227	n	ot	n	y	0	ev	cig+/-ot	30	999	0	1	nev cigs	st
HU	517		f	0	0	all	-	all	As:Chi	1985	CC	227	n	ot	n	y	0	ev	cig+/-ot	16	29	0	0	nev cigs	st
HU	518		f	0	0	all	-	all	As:Chi	1985	CC	227	n	ot	n	y	0	ev	cig+/-ot	1	15	3	0	nev cigs	st
HU2	501		c	0	0	all	-	all	As:Chi	1977	CC	523	n	ot	y	n	0	ev	cig+/-ot	40	999	0	0	nev cigs	ot
HU2	502		c	0	0	all	-	all	As:Chi	1977	CC	523	n	ot	y	n	0	ev	cig+/-ot	30	39	0	1	nev cigs	st
HU2	503		c	0	0	all	-	all	As:Chi	1977	CC	523	n	ot	y	n	0	ev	cig+/-ot	20	29	1	0	nev cigs	st
HU2	504		c	0	0	all	-	all	As:Chi	1977	CC	523	n	ot	y	n	0	ev	cig+/-ot	1	19	0	0	nev cigs	or
JEDRYC	607		m	0	0	all	-	all	Eu:est	1980	CC	1630	n	bl	y	n	0	ev	cig+/-ot	17	18	2	4	nev any	st
JEDRYC	608		m	0	0	all	-	all	Eu:est	1980	CC	1630	n	bl	y	n	0	ev	cig+/-ot	1	16	3	0	nev any	st
JEDRYC	619		f	0	0	all	-	all	Eu:est	1980	CC	1630	n	bl	y	n	0	ev	cig+/-ot	1	22	0	0	nev any	st
JOLY	543		m	0	0	all	-	all	SCAmer	1978	CC	826	n	bl	n	n	0	ev	cig+/-ot	25	999	1	0	nev any	st
JOLY	544		m	0	0	all	-	all	SCAmer	1978	CC	826	n	bl	n	n	0	ev	cig+/-ot	15	24	2	0	nev any	st
JOLY	545		m	0	0	all	-	all	SCAmer	1978	CC	826	n	bl	n	n	0	ev	cig+/-ot	1	14	3	0	nev any	st
JOLY	533		f	0	0	all	-	all	SCAmer	1978	CC	826	n	bl	n	n	0	ev	cig+/-ot	25	999	1	0	nev any	st
JOLY	534		f	0	0	all	-	all	SCAmer	1978	CC	826	n	bl	n	n	0	ev	cig+/-ot	15	24	2	0	nev any	st
JOLY	535		f	0	0	all	-	all	SCAmer	1978	CC	826	n	bl	n	n	0	ev	cig+/-ot	1	14	3	0	nev any	st
KHUDER	506		m	0	0	all	-	all	NAmer	1985	CC	482	n	bl	n	y	0	ev	cig+/-ot	20	999	1	0	nev cigs	st
KHUDER	507		m	0	0	all	-	all	NAmer	1985	CC	482	n	bl	n	y	0	ev	cig+/-ot	16	19	2	4	nev cigs	st
KHUDER	508		m	0	0	all	-	all	NAmer	1985	CC	482	n	bl	n	y	0	ev	cig+/-ot	1	15	3	0	nev cigs	st
KOULUM	501		m	0	0	all	-	all	Eu:Sca	1936	CC	812	n	bl	n	n	0	ev	all/unsp	31	999	0	0	nev any	st
KOULUM	502		m	0	0	all	-	all	Eu:Sca	1936	CC	812	n	bl	n	n	0	ev	all/unsp	21	30	1	0	nev any	st
KOULUM	503		m	0	0	all	-	all	Eu:Sca	1936	CC	812	n	bl	n	n	0	ev	all/unsp	16	20	2	4	nev any	st
KOULUM	504		m	0	0	all	-	all	Eu:Sca	1936	CC	812	n	bl	n	n	0	ev	all/unsp	11	15	3	5	nev any	st
KOULUM	505		m	0	0	all	-	all	Eu:Sca	1936	CC	812	n	bl	n	n	0	ev	all/unsp	0	10	0	6	nev any	st
LETOUR	501		c	0	0	all	-	all	NAmer	1983	CC	738	n	V	y	y	0	ev	cig+/-ot	21	999	1	0	nev cigs	st
LETOUR	502		c	0	0	all	-	all	NAmer	1983	CC	738	n	V	y	y	0	ev	cig+/-ot	15	20	2	4	nev cigs	st
LETOUR	503		c	0	0	all	-	all	NAmer	1983	CC	738	n	V	y	y	0	ev	cig+/-ot	1	14	3	0	nev cigs	st
LIAW	504		c	0	0	all	0	all	As:oth	1982	pr	127	n	ot	n	n	2	cu	all/unsp	25	999	1	0	nev any	or
LIAW	505		c	0	0	all	0	all	As:oth	1982	pr	127	n	ot	n	n	2	cu	all/unsp	21	24	0	3	nev any	or
LIAW	506		c	0	0	all	0	all	As:oth	1982	pr	127	n	ot	n	n	2	cu	all/unsp	1	20	0	0	nev any	or
LIU3	501	x	m	0	0	all	-	all	As:Chi	1985	CC	110	n	ot	n	n	0	ev	all/unsp	21	999	1	0	nev any	or
LIU3	502	x	m	0	0	all	-	all	As:Chi	1985	CC	110	n	ot	n	n	0	ev	all/unsp	1	20	0	0	nev any	st
LIU4	501		m	35	69	all	-	all	As:Chi	1986	CC	1000-00	n	ot	y	n	2	ev	all/unsp	25	999	1	0	nev any	ot
LIU4	502		m	35	69	all	-	all	As:Chi	1986	CC	1000-00	n	ot	y	n	2	ev	all/unsp	20	24	0	3	nev any	ot
LIU4	503		m	35	69	all	-	all	As:Chi	1986	CC	1000-00	n	ot	y	n	2	ev	all/unsp	0	19	0	0	nev any	ot
LIU5	501		c	0	0	all	-	all	As:Chi	1978	CC	111	n	ot	y	n	0	ev	all/unsp	30	999	0	1	nev any	st
LIU5	502		c	0	0	all	-	all	As:Chi	1978	CC	111	n	ot	y	n	0	ev	all/unsp	1	29	0	0	nev any	st

Table 1H11 - 4

IESLC - Meta-analysis of Ever/current Smoking by Age started, Overview
All LC types, Any Product (or Cigarettes if Any not available)
Least adjusted

REF	NRR	X	SEX	AGEL	AGEH	RACE	YF	LC TYPE	LOC	START	ST	NLC	R	VB	P	H	AD	SM	PRODUCT	exL	exH	S1	S2	DENOM	De
LUBIN	565		m	0	0	all	-	all	As:Chi	1984	CC	427	m	ot	y	n	0	ev	cig+/-ot	27	999	0	1	nev any	st
LUBIN	566		m	0	0	all	-	all	As:Chi	1984	CC	427	m	ot	y	n	0	ev	cig+/-ot	23	26	1	2	nev any	st
LUBIN	567		m	0	0	all	-	all	As:Chi	1984	CC	427	m	ot	y	n	0	ev	cig+/-ot	20	22	0	3	nev any	st
LUBIN	568		m	0	0	all	-	all	As:Chi	1984	CC	427	m	ot	y	n	0	ev	cig+/-ot	1	19	0	0	nev any	st
LUBIN2	1147	x	m	0	0	all	-	all	Eu:mul	1976	CC	7804	n	bl	n	y	0	ev	cig+/-ot	31	999	0	0	nev cigs	st
LUBIN2	1148	x	m	0	0	all	-	all	Eu:mul	1976	CC	7804	n	bl	n	y	0	ev	cig+/-ot	21	30	1	0	nev cigs	st
LUBIN2	1149	x	m	0	0	all	-	all	Eu:mul	1976	CC	7804	n	bl	n	y	0	ev	cig+/-ot	17	20	2	4	nev cigs	st
LUBIN2	1150	x	m	0	0	all	-	all	Eu:mul	1976	CC	7804	n	bl	n	y	0	ev	cig+/-ot	13	16	3	5	nev cigs	st
LUBIN2	1151	x	m	0	0	all	-	all	Eu:mul	1976	CC	7804	n	bl	n	y	0	ev	cig+/-ot	1	12	0	6	nev cigs	st
MATOS	556	x	m	0	0	all	-	all	SCAmer	1994	CC	200	n	bl	n	n	0	ev	cig+/-ot	20	999	1	0	nev any	st
MATOS	557	x	m	0	0	all	-	all	SCAmer	1994	CC	200	n	bl	n	n	0	ev	cig+/-ot	15	19	2	4	nev any	st
MATOS	558	x	m	0	0	all	-	all	SCAmer	1994	CC	200	n	bl	n	n	0	ev	cig+/-ot	1	14	3	0	nev any	st
MIGRAN	501		m	0	0	all	0	all	Eu:UK	1964	pr	259	n	V	n	n	0	cu	cig only	20	999	1	0	nev any	st
MIGRAN	503		m	0	0	all	0	all	Eu:UK	1964	pr	259	n	V	n	n	0	cu	cig only	16	19	2	4	nev any	st
MIGRAN	505		m	0	0	all	0	all	Eu:UK	1964	pr	259	n	V	n	n	0	cu	cig only	0	15	3	0	nev any	st
MIGRAN	511		f	0	0	all	0	all	Eu:UK	1964	pr	259	n	V	n	n	0	cu	cig only	20	999	1	0	nev any	st
MIGRAN	513		f	0	0	all	0	all	Eu:UK	1964	pr	259	n	V	n	n	0	cu	cig only	16	19	2	4	nev any	st
MIGRAN	515		f	0	0	all	0	all	Eu:UK	1964	pr	259	n	V	n	n	0	cu	cig only	0	15	3	0	nev any	st
MRFITR	508		m	0	0	all	0	all	NAmer	1973	pr	119	n	bl	n	n	0	cu	cig+/-ot	24	999	1	0	nev cigs	ot
MRFITR	509		m	0	0	all	0	all	NAmer	1973	pr	119	n	bl	n	n	0	cu	cig+/-ot	22	23	0	3	nev cigs	ot
MRFITR	510		m	0	0	all	0	all	NAmer	1973	pr	119	n	bl	n	n	0	cu	cig+/-ot	20	21	0	0	nev cigs	ot
MRFITR	511		m	0	0	all	0	all	NAmer	1973	pr	119	n	bl	n	n	0	cu	cig+/-ot	18	19	2	4	nev cigs	ot
MRFITR	512		m	0	0	all	0	all	NAmer	1973	pr	119	n	bl	n	n	0	cu	cig+/-ot	16	17	0	0	nev cigs	ot
MRFITR	513		m	0	0	all	0	all	NAmer	1973	pr	119	n	bl	n	n	0	cu	cig+/-ot	1	15	3	0	nev cigs	ot
PERNU	504		m	0	0	all	-	all	Eu:Sca	1944	CC	1606	n	bl	n	n	0	ev	all/unsp	15	999	0	0	nev any	st
PERNU	505		m	0	0	all	-	all	Eu:Sca	1944	CC	1606	n	bl	n	n	0	ev	all/unsp	1	14	3	0	nev any	st
PERNU	501		f	0	0	all	-	all	Eu:Sca	1944	CC	1606	n	bl	n	n	0	ev	all/unsp	15	999	0	0	nev any	st
PERNU	502		f	0	0	all	-	all	Eu:Sca	1944	CC	1606	n	bl	n	n	0	ev	all/unsp	1	14	3	0	nev any	ot
PEZZOT	570		m	0	0	all	-	all	SCAmer	1987	CC	215	n	bl	n	y	0	ev	cig only	19	999	1	0	nev cigs	st
PEZZOT	571		m	0	0	all	-	all	SCAmer	1987	CC	215	n	bl	n	y	0	ev	cig only	14	18	0	0	nev cigs	st
PEZZOT	572		m	0	0	all	-	all	SCAmer	1987	CC	215	n	bl	n	y	0	ev	cig only	1	13	0	6	nev cigs	st
QIAO2	501	x	m	0	0	all	0	all	As:Chi	1992	pr	241	m	ot	n	n	0	ev	all/unsp	21	999	1	0	nev any	st
QIAO2	502	x	m	0	0	all	0	all	As:Chi	1992	pr	241	m	ot	n	n	0	ev	all/unsp	17	20	2	4	nev any	st
QIAO2	503	x	m	0	0	all	0	all	As:Chi	1992	pr	241	m	ot	n	n	0	ev	all/unsp	1	16	3	0	nev any	st
RACHTA	501	x	f	0	0	all	-	all	Eu:est	1991	CC	118	n	bl	n	y	0	ev	cig+/-ot	31	999	0	0	nev cigs	st
RACHTA	502	x	f	0	0	all	-	all	Eu:est	1991	CC	118	n	bl	n	y	0	ev	cig+/-ot	20	30	1	0	nev cigs	st
RACHTA	503	x	f	0	0	all	-	all	Eu:est	1991	CC	118	n	bl	n	y	0	ev	cig+/-ot	1	19	0	0	nev cigs	st
SEGI2	516	x	m	0	0	all	-	all	As:Jap	1962	CC	378	n	bl	n	n	0	cu	cig+/-ot	23	999	1	0	nev any	st
SEGI2	517	x	m	0	0	all	-	all	As:Jap	1962	CC	378	n	bl	n	n	0	cu	cig+/-ot	20	22	0	3	nev any	st
SEGI2	518	x	m	0	0	all	-	all	As:Jap	1962	CC	378	n	bl	n	n	0	cu	cig+/-ot	1	19	0	0	nev any	st
SOBUE	654		m	0	0	all	-	all	As:Jap	1986	CC	1376	n	bl	n	y	0	ev	cig+/-ot	23	999	1	0	nev cigs	st
SOBUE	655		m	0	0	all	-	all	As:Jap	1986	CC	1376	n	bl	n	y	0	ev	cig+/-ot	18	22	2	0	nev cigs	st
SOBUE	656		m	0	0	all	-	all	As:Jap	1986	CC	1376	n	bl	n	y	0	ev	cig+/-ot	10	17	3	0	nev cigs	st
SUZUK2	501		c	0	0	all	-	all	SCAmer	1991	CC	123	n	bl	n	y	0	ev	all/unsp	19	999	1	0	nev any	st
SUZUK2	502		c	0	0	all	-	all	SCAmer	1991	CC	123	n	bl	n	y	0	ev	all/unsp	12	18	0	0	nev any	st
SUZUK2	503		c	0	0	all	-	all	SCAmer	1991	CC	123	n	bl	n	y	0	ev	all/unsp	0	11	0	6	nev any	st
SVENSS	501		f	0	0	all	-	all	Eu:Sca	1983	CC	210	n	bl	n	n	0	cu	all/unsp	26	999	1	0	nev any	st
SVENSS	502		f	0	0	all	-	all	Eu:Sca	1983	CC	210	n	bl	n	n	0	cu	all/unsp	19	25	0	3	nev any	st
SVENSS	503		f	0	0	all	-	all	Eu:Sca	1983	CC	210	n	bl	n	n	0	cu	all/unsp	0	18	0	0	nev any	st
TIZZAN	506		m	0	0	all	-	all	Eu:wst	1959	CC	1358	n	bl	n	n	0	ev	all/unsp	31	999	0	0	nev any	st
TIZZAN	507		m	0	0	all	-	all	Eu:wst	1959	CC	1358	n	bl	n	n	0	ev	all/unsp	20	30	1	0	nev any	st
TIZZAN	508		m	0	0	all	-	all	Eu:wst	1959	CC	1358	n	bl	n	n	0	ev	all/unsp	1	19	0	0	nev any	st
TIZZAN	519		f	0	0	all	-	all	Eu:wst	1959	CC	1358	n	bl	n	n	0	ev	all/unsp	31	999	0	0	nev any	st
TIZZAN	520		f	0	0	all	-	all	Eu:wst	1959	CC	1358	n	bl	n	n	0	ev	all/unsp	20	30	1	0	nev any	st
TIZZAN	521		f	0	0	all	-	all	Eu:wst	1959	CC	1358	n	bl	n	n	0	ev	all/unsp	1	19	0	0	nev any	st
WAKAI	501		m	0	0	all	-	all	As:Jap	1988	CC	333	n	bl	n	y	0	cu	cig+/-ot	30	999	0	1	nev any	st
WAKAI	502		m	0	0	all	-	all	As:Jap	1988	CC	333	n	bl	n	y	0	cu	cig+/-ot	20	29	1	0	nev any	st
WAKAI	503		m	0	0	all	-	all	As:Jap	1988	CC	333	n	bl	n	y	0	cu	cig+/-ot	1	19	0	0	nev any	st
WU	517	x	f	0	0	wh	-	q+a	NAmer	1981	CC	220	n	bl	n	y	0	cu	all/unsp	25	999	1	0	nev any	st
WU	518	x	f	0	0	wh	-	q+a	NAmer	1981	CC	220	n	bl	n	y	0	cu	all/unsp	19	24	0	3	nev any	st
WU	519	x	f	0	0	wh	-	q+a	NAmer	1981	CC	220	n	bl	n	y	0	cu	all/unsp	0	18	0	0	nev any	st
WYNDE6	759		m	0	0	wh	-	q+s+a	NAmer	1969	CC	4423	n	bl	n	y	0	ev	cig+/-ot	21	999	1	0	nev cigs	st
WYNDE6	760		m	0	0	wh	-	q+s+a	NAmer	1969	CC	4423	n	bl	n	y	0	ev	cig+/-ot	18	20	2	4	nev cigs	st
WYNDE6	761		m	0	0	wh	-	q+s+a	NAmer	1969	CC	4423	n	bl	n	y	0	ev	cig+/-ot	1	17	3	0	nev cigs	st
WYNDE6	767		f	0	0	wh	-	q+s+a	NAmer	1969	CC	4423	n	bl	n	y	0	ev	cig+/-ot	21	999	1	0	nev cigs	st
WYNDE6	768		f	0	0	wh	-	q+s+a	NAmer	1969	CC	4423	n	bl	n	y	0	ev	cig+/-ot	18	20	2	4	nev cigs	st
WYNDE6	769		f	0	0	wh	-	q+s+a	NAmer	1969	CC	4423	n	bl	n	y	0	ev	cig+/-ot	1	17	3	0	nev cigs	st
ZHENG	563		m	0	0	all	-	all	As:Chi	1982	CC	540	n	ot	*	y	0	ev	cig+/-ot	30	999	0	1	nev cigs	st
ZHENG	564		m	0	0	all	-	all	As:Chi	1982	CC	540	n	ot	*	y	0	ev	cig+/-ot	20	29	1	0	nev cigs	st
ZHENG	565		m	0	0	all	-	all	As:Chi	1982	CC	540	n	ot	*	y	0	ev	cig+/-ot	1	19	0	0	nev cigs	st
ZHENG	572		f	0	0	all	-	all	As:Chi	1982	CC	540	n	ot	*	y	0	ev	cig+/-ot	30	999	0	1	nev cigs	st
ZHENG	573		f	0	0	all	-	all	As:Chi	1982	CC	540	n	ot	*	y	0	ev	cig+/-ot	1	29	0	0	nev cigs	st

Table 1H11 - 4

IESLC - Meta-analysis of Ever/current Smoking by Age started, Overview
All LC types, Any Product (or Cigarettes if Any not available)
Least adjusted

Cigarette type is all/unspec for all RRs
except for the following:

REF	NRR	CIGTYPE
DEAN3	564	MC only
DEAN3	565	MC only
DEAN3	566	MC only
DEAN3	567	MC only
DEAN3	583	MC only
DEAN3	584	MC only
DEAN3	585	MC only
DEAN3	586	MC only

In this overview table, subtotals and Qs values may be invalid and should be ignored

Table 1H11 - 5

IESLC - Meta-analysis of Ever/current Smoking by Age started, Overview
All LC types, Any Product (or Cigarettes if Any not available)
Least adjusted

REF	NRR	SEX	AD	Number Exposed Case	Cont	Non-exposed Case	Cont	RR	95.00%CI
AGUDO	501	f	0	7	12	80	183	1.33	(0.51- 3.51)
AGUDO	502	f	0	16	11	80	183	3.33	(1.48- 7.49)
Subtotal AGUDO								2.28	(1.23- 4.25)
ARMADA	511	m	0	113	144	8	71	6.96	(3.22- 15.06)
ARMADA	512	m	0	204	110	8	71	16.46	(7.64- 35.44)
Subtotal ARMADA								10.73	(6.23- 18.49)
AUVINE	509	c	0	76	27	44	229	14.65	(8.50- 25.26)
AUVINE	510	c	0	135	47	44	229	14.95	(9.41- 23.75)
AUVINE	511	c	0	55	6	44	229	47.71	(19.35- 117.62)
Subtotal AUVINE								17.31	(12.46- 24.04)
BARBON	515	m	0	200	207	22	188	8.26	(5.10- 13.38)
BARBON	516	m	0	395	337	22	188	10.02	(6.29- 15.94)
BARBON	517	m	0	138	23	22	188	51.27	(27.46- 95.73)
Subtotal BARBON								13.42	(9.99- 18.02)
BRESLO	501	c	0	32	35	19	56	2.69	(1.33- 5.47)
BRESLO	502	c	0	286	243	19	56	3.47	(2.01- 6.00)
BRESLO	503	c	0	166	116	19	56	4.22	(2.38- 7.47)
Subtotal BRESLO								3.51	(2.48- 4.95)
BUFFLE	517	f	0	23	23	12	112	9.33	(4.07- 21.40)
BUFFLE	518	f	0	47	34	12	112	12.90	(6.15- 27.07)
BUFFLE	519	f	0	40	29	12	112	12.87	(6.00- 27.62)
BUFFLE	520	f	0	53	33	12	112	14.99	(7.17- 31.33)
BUFFLE	521	f	0	78	41	12	112	17.76	(8.77- 35.94)
Subtotal BUFFLE								13.57	(9.69- 18.99)
*CEDERL	510	m	1	11	-	7	-	6.50	(2.52- 16.74)
*CEDERL	511	m	1	10	-	7	-	9.80	(3.74- 25.69)
*CEDERL	512	m	1	7	-	7	-	6.40	(2.25- 18.21)
*CEDERL	515	f	0	6	2806	19	17679	1.99	(0.80- 4.98)
*CEDERL	516	f	0	2	1009	19	17679	1.84	(0.43- 7.91)
*CEDERL	517	f	0	0	746	19	17679	0.61~	(0.04- 10.05)
Subtotal CEDERL								4.43	(2.82- 6.96)
CHEN2	517	m	0	8	5	9	33	5.87	(1.54- 22.37)
CHEN2	518	m	0	29	25	9	33	4.25	(1.71- 10.57)
CHEN2	519	m	0	84	67	9	33	4.60	(2.06- 10.27)
CHEN2	522	f	0	5	8	25	33	0.83	(0.24- 2.83)
CHEN2	523	f	0	9	8	25	33	1.49	(0.50- 4.39)
CHEN2	524	f	0	23	13	25	33	2.34	(0.99- 5.50)
Subtotal CHEN2								2.84	(1.90- 4.25)
CHIAZZ	501	m	2	-	-	4	-	3.00	(0.31- 28.84)
CHIAZZ	502	m	2	-	-	4	-	19.89	(2.66- 148.96)
Subtotal CHIAZZ								8.64	(1.92- 38.91)
CHOI	523	m	0	36	77	13	95	3.42	(1.69- 6.89)
CHOI	524	m	0	130	232	13	95	4.09	(2.21- 7.60)
CHOI	525	m	0	79	138	13	95	4.18	(2.20- 7.95)
CHOI	526	m	0	22	18	13	95	8.93	(3.81- 20.91)
CHOI	530	f	0	15	25	76	164	1.29	(0.65- 2.60)
CHOI	531	f	0	4	1	76	164	8.63	(0.95- 78.54)
Subtotal CHOI								3.57	(2.63- 4.85)
*CPSI	801	m	1	42	-	83	-	4.08	(2.81- 5.91)
*CPSI	802	m	1	196	-	83	-	10.08	(7.80- 13.03)
*CPSI	803	m	1	588	-	83	-	14.69	(11.68- 18.49)
*CPSI	804	m	1	185	-	83	-	16.77	(12.94- 21.73)
*CPSI	845	f	1	51	-	166	-	2.25	(1.64- 3.08)
*CPSI	846	f	1	34	-	166	-	3.38	(2.33- 4.88)
*CPSI	847	f	1	52	-	166	-	5.00	(3.66- 6.83)
*CPSI	848	f	1	6	-	166	-	2.50	(1.11- 5.65)
Subtotal CPSI								7.59	(6.81- 8.46)
DAMBER	501	m	0	70	76	42	208	4.56	(2.87- 7.26)
DAMBER	502	m	0	261	190	42	208	6.80	(4.65- 9.95)
DAMBER	503	m	0	206	98	42	208	10.41	(6.91- 15.68)
Subtotal DAMBER								7.07	(5.57- 8.98)
DEAN3	564	m	0	24	75	24	510	6.80	(3.67- 12.58)
DEAN3	565	m	0	52	161	24	510	6.86	(4.10- 11.49)
DEAN3	566	m	0	160	485	24	510	7.01	(4.48- 10.96)
DEAN3	567	m	0	44	165	24	510	5.67	(3.34- 9.60)
DEAN3	583	f	0	27	274	41	1538	3.70	(2.24- 6.11)
DEAN3	584	f	0	18	229	41	1538	2.95	(1.67- 5.22)
DEAN3	585	f	0	39	504	41	1538	2.90	(1.85- 4.55)
DEAN3	586	f	0	7	109	41	1538	2.41	(1.06- 5.50)
Subtotal DEAN3								4.59	(3.81- 5.54)
DOLL	501	m	0	4	7	7	61	4.98	(1.16- 21.36)

Table 1H11 - 5

IESLC - Meta-analysis of Ever/current Smoking by Age started, Overview
All LC types, Any Product (or Cigarettes if Any not available)
Least adjusted

REF	NRR	SEX	AD	Number Exposed Case	Cont	Non-exposed Case	Cont	RR	95.00%CI
DOLL	502	m	0	18	33	7	61	4.75	(1.80- 12.54)
DOLL	503	m	0	251	264	7	61	8.29	(3.72- 18.46)
DOLL	504	m	0	1077	992	7	61	9.46	(4.31- 20.78)
DOLL	508	f	0	15	15	40	59	1.48	(0.65- 3.35)
DOLL	509	f	0	10	7	40	59	2.11	(0.74- 6.00)
DOLL	510	f	0	23	15	40	59	2.26	(1.05- 4.86)
DOLL	511	f	0	20	12	40	59	2.46	(1.08- 5.58)
Subtotal DOLL								3.65	(2.67- 4.98)
*DORN	610	m	0	37	73050	25	213858	4.33	(2.61- 7.20)
*DORN	611	m	0	157	147948	25	213858	9.08	(5.95- 13.84)
*DORN	612	m	0	342	213156	25	213858	13.73	(9.14- 20.60)
*DORN	613	m	0	84	36304	25	213858	19.79	(12.67- 30.93)
*DORN	647	m	0	90	74464	49	171211	4.22	(2.98- 5.98)
*DORN	648	m	0	171	90036	49	171211	6.64	(4.83- 9.12)
*DORN	649	m	0	306	118234	49	171211	9.04	(6.69- 12.23)
*DORN	650	m	0	81	24616	49	171211	11.50	(8.07- 16.39)
Subtotal DORN								8.46	(7.42- 9.65)
*ENGELA	501	m	0	17	9762	27	58716	3.79	(2.07- 6.95)
*ENGELA	502	m	0	50	30195	27	58716	3.60	(2.26- 5.75)
*ENGELA	503	m	0	173	50732	27	58716	7.42	(4.94- 11.12)
*ENGELA	509	f	0	10	24560	31	207789	2.73	(1.34- 5.57)
*ENGELA	510	f	0	36	29605	31	207789	8.15	(5.04- 13.17)
*ENGELA	511	f	0	18	10687	31	207789	11.29	(6.32- 20.17)
Subtotal ENGELA								5.83	(4.73- 7.20)
GAO	501	m	0	45	129	62	202	1.14	(0.73- 1.77)
GAO	502	m	0	363	262	62	202	4.51	(3.26- 6.25)
GAO	503	m	0	262	167	62	202	5.11	(3.62- 7.21)
GAO	511	f	0	73	64	435	605	1.59	(1.11- 2.27)
GAO	512	f	0	87	41	435	605	2.95	(2.00- 4.36)
GAO	513	f	0	77	25	435	605	4.28	(2.68- 6.84)
Subtotal GAO								2.99	(2.57- 3.50)
GAO2	501	m	0	2	4	13	56	2.15	(0.36- 13.05)
GAO2	502	m	0	127	85	13	56	6.44	(3.32- 12.49)
GAO2	503	m	0	52	26	13	56	8.62	(4.01- 18.52)
Subtotal GAO2								6.68	(4.12- 10.83)
GENG	528	f	0	28	31	54	93	1.56	(0.84- 2.87)
GENG	529	f	0	39	23	54	93	2.92	(1.58- 5.40)
GENG	530	f	0	36	10	54	93	6.20	(2.85- 13.48)
Subtotal GENG								2.74	(1.88- 4.00)
HAENSZ	537	f	0	44	66	81	236	1.94	(1.23- 3.07)
HAENSZ	538	f	0	30	37	81	236	2.36	(1.37- 4.07)
Subtotal HAENSZ								2.11	(1.48- 2.99)
HEGMAN	513	m	1	26	-	-	-	9.40	(4.60- 19.30)
HEGMAN	514	m	1	146	-	-	-	22.30	(12.00- 41.40)
HEGMAN	516	f	1	2	-	-	-	4.80	(1.00- 22.10)
HEGMAN	517	f	1	81	-	-	-	26.80	(15.40- 46.80)
Subtotal HEGMAN								18.07	(12.75- 25.62)
*HIRAYA	501	m	1	-	-	-	-	4.35	(3.51- 5.39)
*HIRAYA	502	m	1	-	-	-	-	5.71	(4.50- 7.25)
*HIRAYA	504	f	1	-	-	-	-	2.46	(1.93- 3.13)
*HIRAYA	505	f	1	-	-	-	-	0.78	(0.10- 6.10)
Subtotal HIRAYA								3.96	(3.46- 4.52)
HU	511	m	0	14	20	41	67	1.14	(0.52- 2.51)
HU	512	m	0	93	67	41	67	2.27	(1.38- 3.74)
HU	513	m	0	13	7	41	67	3.03	(1.12- 8.23)
HU	516	f	0	3	5	40	48	0.72	(0.16- 3.20)
HU	517	f	0	18	9	40	48	2.40	(0.97- 5.92)
HU	518	f	0	5	4	40	48	1.50	(0.38- 5.96)
Subtotal HU								1.92	(1.37- 2.69)
HU2	501	c	0	15	29	121	213	0.91	(0.47- 1.77)
HU2	502	c	0	29	54	121	213	0.95	(0.57- 1.56)
HU2	503	c	0	229	159	121	213	2.54	(1.88- 3.43)
HU2	504	c	0	129	68	121	213	3.34	(2.31- 4.83)
Subtotal HU2								2.14	(1.75- 2.61)
JEDRYC	607	m	0	239	146	49	219	7.32	(5.04- 10.61)
JEDRYC	608	m	0	135	66	49	219	9.14	(5.96- 14.02)
JEDRYC	619	f	0	63	11	78	166	12.19	(6.09- 24.42)
Subtotal JEDRYC								8.54	(6.58- 11.07)
JOLY	543	m	0	18	70	12	218	4.67	(2.14- 10.18)
JOLY	544	m	0	217	357	12	218	11.04	(6.03- 20.22)
JOLY	545	m	0	317	282	12	218	20.42	(11.18- 37.32)

Table 1H11 - 5

IESLC - Meta-analysis of Ever/current Smoking by Age started, Overview
All LC types, Any Product (or Cigarettes if Any not available)
Least adjusted

REF	NRR	SEX	AD	Number Exposed Case	Number Exposed Cont	Non-exposed Case	Non-exposed Cont	RR	95.00%CI
JOLY	533	f	0	23	41	52	283	3.05	(1.69- 5.51)
JOLY	534	f	0	67	47	52	283	7.76	(4.82- 12.49)
JOLY	535	f	0	76	35	52	283	11.82	(7.18- 19.44)
Subtotal JOLY								8.56	(6.78- 10.80)
KHUDER	506	m	0	72	152	23	309	6.36	(3.83- 10.58)
KHUDER	507	m	0	161	338	23	309	6.40	(4.03- 10.17)
KHUDER	508	m	0	226	295	23	309	10.29	(6.51- 16.27)
Subtotal KHUDER								7.58	(5.76- 9.97)
KOULUM	501	m	0	8	8	5	54	10.80	(2.82- 41.31)
KOULUM	502	m	0	60	67	5	54	9.67	(3.63- 25.77)
KOULUM	503	m	0	267	103	5	54	28.00	(10.89- 71.96)
KOULUM	504	m	0	199	52	5	54	41.33	(15.74- 108.56)
KOULUM	505	m	0	143	16	5	54	96.53	(33.72- 276.34)
Subtotal KOULUM								27.41	(17.28- 43.49)
LETOUR	501	c	0	188	160	24	224	10.97	(6.85- 17.56)
LETOUR	502	c	0	309	241	24	224	11.97	(7.60- 18.83)
LETOUR	503	c	0	151	76	24	224	18.54	(11.21- 30.67)
Subtotal LETOUR								13.23	(10.06- 17.40)
*LIAW	504	c	2	-	-	-	-	1.50	(0.70- 3.30)
*LIAW	505	c	2	-	-	-	-	5.90	(3.00- 11.30)
*LIAW	506	c	2	-	-	-	-	4.60	(2.60- 8.10)
Subtotal LIAW								3.82	(2.62- 5.58)
LIU3	501	m	0	20	80	4	19	1.19	(0.36- 3.88)
LIU3	502	m	0	32	125	4	19	1.22	(0.39- 3.83)
Subtotal LIU3								1.20	(0.53- 2.74)
LIU4	501	m	2	-	-	-	-	2.41	(2.32- 2.49)
LIU4	502	m	2	-	-	-	-	2.86	(2.78- 2.95)
LIU4	503	m	2	-	-	-	-	3.81	(3.70- 3.93)
Subtotal LIU4								3.03	(2.98- 3.09)
LIU5	501	c	0	13	22	26	41	0.93	(0.40- 2.17)
LIU5	502	c	0	72	48	26	41	2.37	(1.28- 4.36)
Subtotal LIU5								1.72	(1.05- 2.82)
LUBIN	565	m	0	30	179	9	72	1.34	(0.61- 2.96)
LUBIN	566	m	0	65	146	9	72	3.56	(1.68- 7.56)
LUBIN	567	m	0	89	212	9	72	3.36	(1.61- 7.01)
LUBIN	568	m	0	178	251	9	72	5.67	(2.76- 11.64)
Subtotal LUBIN								3.20	(2.20- 4.66)
LUBIN2	1147	m	0	68	221	185	1878	3.12	(2.29- 4.26)
LUBIN2	1148	m	0	564	1069	185	1878	5.36	(4.46- 6.43)
LUBIN2	1149	m	0	1796	3028	185	1878	6.02	(5.12- 7.08)
LUBIN2	1150	m	0	1312	1833	185	1878	7.27	(6.15- 8.59)
LUBIN2	1151	m	0	250	316	185	1878	8.03	(6.42- 10.05)
Subtotal LUBIN2								6.12	(5.61- 6.67)
MATOS	556	m	0	28	73	11	110	3.84	(1.80- 8.18)
MATOS	557	m	0	91	120	11	110	7.58	(3.85- 14.92)
MATOS	558	m	0	69	90	11	110	7.67	(3.83- 15.36)
Subtotal MATOS								6.24	(4.15- 9.39)
*MIGRAN	501	m	0	24	668	4	867	7.79	(2.72- 22.34)
*MIGRAN	503	m	0	59	1845	4	867	6.93	(2.53- 19.02)
*MIGRAN	505	m	0	50	1081	4	867	10.03	(3.64- 27.65)
*MIGRAN	511	f	0	11	1315	4	3814	7.98	(2.54- 25.01)
*MIGRAN	513	f	0	9	1035	4	3814	8.29	(2.56- 26.87)
*MIGRAN	515	f	0	2	266	4	3814	7.17	(1.32- 38.96)
Subtotal MIGRAN								8.07	(5.08- 12.80)
*MRFITR	508	m	0	3	544	0	1859	23.91~	(1.24- 462.09)
*MRFITR	509	m	0	6	402	0	1859	60.06~	(3.39-1063.94)
*MRFITR	510	m	0	7	1029	0	1859	27.09~	(1.55- 473.89)
*MRFITR	511	m	0	25	1876	0	1859	50.54~	(3.08- 829.51)
*MRFITR	512	m	0	40	2242	0	1859	67.17~	(4.13-1091.55)
*MRFITR	513	m	0	25	2065	0	1859	45.91~	(2.80- 753.64)
Subtotal MRFITR								43.20	(13.52- 138.02)
PERNU	504	m	0	1043	346	97	275	8.55	(6.58- 11.10)
PERNU	505	m	0	337	92	97	275	10.38	(7.49- 14.40)
PERNU	501	f	0	18	89	110	971	1.79	(1.04- 3.07)
PERNU	502	f	0	1	0	110	971	26.38~	(1.07- 651.38)
Subtotal PERNU								7.56	(6.25- 9.15)
PEZZOT	570	m	0	41	105	4	116	11.32	(3.92- 32.69)
PEZZOT	571	m	0	118	145	4	116	23.60	(8.46- 65.84)
PEZZOT	572	m	0	52	67	4	116	22.51	(7.79- 65.00)
Subtotal PEZZOT								18.29	(9.98- 33.50)
*QIAO2	501	m	0	52	1947	10	709	1.89	(0.97- 3.71)

Table 1H11 - 5

IESLC - Meta-analysis of Ever/current Smoking by Age started, Overview
All LC types, Any Product (or Cigarettes if Any not available)
Least adjusted

REF	NRR	SEX	AD	Number Exposed Case	Cont	Non-exposed Case	Cont	RR	95.00%CI
*QIAO2	502	m	0	75	2840	10	709	1.87	(0.97- 3.60)
*QIAO2	503	m	0	104	2130	10	709	3.46	(1.82- 6.59)
Subtotal QIAO2								2.33	(1.59- 3.40)
RACHTA	501	f	0	8	4	33	98	5.94	(1.68- 21.01)
RACHTA	502	f	0	25	18	33	98	4.12	(2.00- 8.50)
RACHTA	503	f	0	52	21	33	98	7.35	(3.87- 13.98)
Subtotal RACHTA								5.73	(3.66- 8.97)
SEGI2	516	m	0	49	155	8	53	2.09	(0.93- 4.71)
SEGI2	517	m	0	125	224	8	53	3.70	(1.70- 8.02)
SEGI2	518	m	0	91	103	8	53	5.85	(2.64- 12.96)
Subtotal SEGI2								3.59	(2.27- 5.67)
SOBUE	654	m	0	110	121	29	126	3.95	(2.45- 6.38)
SOBUE	655	m	0	776	772	29	126	4.37	(2.88- 6.62)
SOBUE	656	m	0	137	62	29	126	9.60	(5.81- 15.88)
Subtotal SOBUE								5.28	(4.05- 6.89)
SUZUK2	501	c	0	16	22	11	53	3.50	(1.40- 8.75)
SUZUK2	502	c	0	64	38	11	53	8.11	(3.78- 17.41)
SUZUK2	503	c	0	31	10	11	53	14.94	(5.69- 39.18)
Subtotal SUZUK2								7.44	(4.51- 12.27)
SVENSS	501	f	0	32	18	38	120	5.61	(2.84- 11.12)
SVENSS	502	f	0	58	14	38	120	13.08	(6.57- 26.04)
SVENSS	503	f	0	52	21	38	120	7.82	(4.19- 14.60)
Subtotal SVENSS								8.26	(5.63- 12.12)
TIZZAN	506	m	0	12	44	180	305	0.46	(0.24- 0.90)
TIZZAN	507	m	0	313	330	180	305	1.61	(1.26- 2.04)
TIZZAN	508	m	0	699	529	180	305	2.24	(1.80- 2.78)
TIZZAN	519	f	0	2	5	117	114	0.39	(0.07- 2.05)
TIZZAN	520	f	0	12	21	117	114	0.56	(0.26- 1.18)
TIZZAN	521	f	0	11	2	117	114	5.36	(1.16- 24.71)
Subtotal TIZZAN								1.70	(1.46- 1.98)
WAKAI	501	m	0	8	25	10	65	2.08	(0.74- 5.87)
WAKAI	502	m	0	130	183	10	65	4.62	(2.29- 9.32)
WAKAI	503	m	0	42	74	10	65	3.69	(1.72- 7.94)
Subtotal WAKAI								3.63	(2.28- 5.77)
WU	517	f	0	14	19	31	92	2.19	(0.98- 4.87)
WU	518	f	0	40	22	31	92	5.40	(2.79- 10.45)
WU	519	f	0	106	32	31	92	9.83	(5.57- 17.34)
Subtotal WU								5.76	(3.94- 8.42)
WYNDE6	759	m	0	111	92	51	589	13.93	(9.36- 20.74)
WYNDE6	760	m	0	223	139	51	589	18.53	(12.98- 26.45)
WYNDE6	761	m	0	611	301	51	589	23.44	(17.06- 32.21)
WYNDE6	767	f	0	127	90	73	673	13.01	(9.06- 18.69)
WYNDE6	768	f	0	200	94	73	673	19.62	(13.90- 27.67)
WYNDE6	769	f	0	291	91	73	673	29.48	(21.04- 41.31)
Subtotal WYNDE6								19.46	(16.87- 22.45)
ZHENG	563	m	0	28	66	33	94	1.21	(0.67- 2.19)
ZHENG	564	m	0	145	109	33	94	3.79	(2.37- 6.05)
ZHENG	565	m	0	106	43	33	94	7.02	(4.13- 11.95)
ZHENG	572	f	0	16	16	152	184	1.21	(0.59- 2.50)
ZHENG	573	f	0	60	28	152	184	2.59	(1.58- 4.27)
Subtotal ZHENG								2.86	(2.24- 3.65)
Partial Totals				24315	981571	9718	2461836		

*prospective study ~ With 0.5 adjustment for zero

Table 1H11 - 5

IESLC - Meta-analysis of Ever/current Smoking by Age started, Overview
All LC types, Any Product (or Cigarettes if Any not available)
Least adjusted

REF	NRR	SEX	AD	Ys	Ws	Qs	Ps
AGUDO	501	f	0	0.29	4.10	3.80	0.5594
AGUDO	502	f	0	1.20	5.84	0.01	0.0037
Subtotal AGUDO				0.83	9.93	3.81	
ARMADA	511	m	0	1.94	6.46	3.07	0.0000
ARMADA	512	m	0	2.80	6.53	15.69	0.0000
Subtotal ARMADA				2.37	12.99	18.76	
AUVINE	509	c	0	2.68	12.94	26.58	0.0000
AUVINE	510	c	0	2.70	17.93	37.88	0.0000
AUVINE	511	c	0	3.87	4.72	32.24	0.0000
Subtotal AUVINE				2.85	35.58	96.70	
BARBON	515	m	0	2.11	16.50	12.20	0.0000
BARBON	516	m	0	2.30	17.77	19.71	0.0000
BARBON	517	m	0	3.94	9.85	71.08	0.0000
Subtotal BARBON				2.60	44.12	102.99	
BRESLO	501	c	0	0.99	7.67	0.52	0.0060
BRESLO	502	c	0	1.24	12.80	0.00	0.0000
BRESLO	503	c	0	1.44	11.75	0.42	0.0000
Subtotal BRESLO				1.25	32.22	0.93	
BUFFLE	517	f	0	2.23	5.58	5.39	0.0000
BUFFLE	518	f	0	2.56	7.00	11.94	0.0000
BUFFLE	519	f	0	2.56	6.59	11.21	0.0000
BUFFLE	520	f	0	2.71	7.07	14.99	0.0000
BUFFLE	521	f	0	2.88	7.72	20.41	0.0000
Subtotal BUFFLE				2.61	33.96	63.94	
*CEDERL	510	m	1	1.87	4.29	1.65	0.0001
*CEDERL	511	m	1	2.28	4.14	4.40	0.0000
*CEDERL	512	m	1	1.86	3.51	1.29	0.0005
*CEDERL	515	f	0	0.69	4.57	1.45	0.1414
*CEDERL	516	f	0	0.61	1.81	0.74	0.4098
*CEDERL	517	f	0	-0.50	0.49	1.49	0.7276
Subtotal CEDERL				1.49	18.81	11.02	
CHEN2	517	m	0	1.77	2.14	0.58	0.0096
CHEN2	518	m	0	1.45	4.63	0.18	0.0018
CHEN2	519	m	0	1.53	5.94	0.45	0.0002
CHEN2	522	f	0	-0.19	2.53	5.27	0.7596
CHEN2	523	f	0	0.40	3.26	2.39	0.4750
CHEN2	524	f	0	0.85	5.24	0.85	0.0521
Subtotal CHEN2				1.04	23.76	9.71	
CHIAZZ	501	m	2	1.10	0.75	0.02	0.3421
CHIAZZ	502	m	2	2.99	0.95	2.87	0.0036
Subtotal CHIAZZ				2.16	1.70	2.89	
CHOI	523	m	0	1.23	7.80	0.00	0.0006
CHOI	524	m	0	1.41	10.06	0.25	0.0000
CHOI	525	m	0	1.43	9.31	0.30	0.0000
CHOI	526	m	0	2.19	5.31	4.67	0.0000
CHOI	530	f	0	0.26	7.94	7.83	0.4667
CHOI	531	f	0	2.16	0.79	0.64	0.0557
Subtotal CHOI				1.27	41.20	13.70	
*CPSI	801	m	1	1.41	27.80	0.67	0.0000
*CPSI	802	m	1	2.31	58.36	65.50	0.0000
*CPSI	803	m	1	2.69	72.82	150.18	0.0000
*CPSI	804	m	1	2.82	57.18	140.68	0.0000
*CPSI	845	f	1	0.81	38.69	7.50	0.0000
*CPSI	846	f	1	1.22	28.12	0.03	0.0000
*CPSI	847	f	1	1.61	39.48	5.07	0.0000
*CPSI	848	f	1	0.92	5.80	0.65	0.0273
Subtotal CPSI				2.03	328.25	370.28	
DAMBER	501	m	0	1.52	17.84	1.27	0.0000
DAMBER	502	m	0	1.92	26.52	11.77	0.0000
DAMBER	503	m	0	2.34	22.90	27.29	0.0000
Subtotal DAMBER				1.96	67.25	40.32	
DEAN3	564	m	0	1.92	10.14	4.49	0.0000
DEAN3	565	m	0	1.93	14.48	6.60	0.0000
DEAN3	566	m	0	1.95	19.25	9.33	0.0000
DEAN3	567	m	0	1.73	13.81	3.23	0.0000
DEAN3	583	f	0	1.31	15.21	0.05	0.0000
DEAN3	584	f	0	1.08	11.77	0.34	0.0002
DEAN3	585	f	0	1.07	18.99	0.65	0.0000
DEAN3	586	f	0	0.88	5.65	0.78	0.0367
Subtotal DEAN3				1.52	109.30	25.48	
DOLL	501	m	0	1.61	1.81	0.23	0.0307

Table 1H11 - 5

IESLC - Meta-analysis of Ever/current Smoking by Age started, Overview
All LC types, Any Product (or Cigarettes if Any not available)
Least adjusted

REF	NRR	SEX	AD	Ys	Ws	Qs	Ps
DOLL	502	m	0	1.56	4.08	0.39	0.0016
DOLL	503	m	0	2.11	5.99	4.46	0.0000
DOLL	504	m	0	2.25	6.20	6.16	0.0000
DOLL	508	f	0	0.39	5.71	4.24	0.3532
DOLL	509	f	0	0.75	3.51	0.90	0.1625
DOLL	510	f	0	0.82	6.57	1.24	0.0364
DOLL	511	f	0	0.90	5.71	0.71	0.0317
Subtotal DOLL				1.29	39.58	18.32	
*DORN	610	m	0	1.47	14.92	0.69	0.0000
*DORN	611	m	0	2.21	21.57	19.66	0.0000
*DORN	612	m	0	2.62	23.30	43.62	0.0000
*DORN	613	m	0	2.99	19.28	57.98	0.0000
*DORN	647	m	0	1.44	31.75	1.14	0.0000
*DORN	648	m	0	1.89	38.11	15.68	0.0000
*DORN	649	m	0	2.20	42.26	38.21	0.0000
*DORN	650	m	0	2.44	30.57	43.37	0.0000
Subtotal DORN				2.14	221.77	220.35	
*ENGELA	501	m	0	1.33	10.44	0.07	0.0000
*ENGELA	502	m	0	1.28	17.55	0.02	0.0000
*ENGELA	503	m	0	2.00	23.38	13.24	0.0000
*ENGELA	509	f	0	1.00	7.56	0.46	0.0058
*ENGELA	510	f	0	2.10	16.67	11.96	0.0000
*ENGELA	511	f	0	2.42	11.40	15.68	0.0000
Subtotal ENGELA				1.76	87.00	41.42	
GAO	501	m	0	0.13	19.59	24.71	0.5711
GAO	502	m	0	1.51	36.16	2.37	0.0000
GAO	503	m	0	1.63	32.38	4.68	0.0000
GAO	511	f	0	0.46	30.05	18.74	0.0114
GAO	512	f	0	1.08	25.10	0.72	0.0000
GAO	513	f	0	1.45	17.56	0.73	0.0000
Subtotal GAO				1.10	160.85	51.95	
GAO2	501	m	0	0.77	1.18	0.28	0.4038
GAO2	502	m	0	1.86	8.74	3.26	0.0000
GAO2	503	m	0	2.15	6.56	5.34	0.0000
Subtotal GAO2				1.90	16.48	8.88	
GENG	528	f	0	0.44	10.28	6.73	0.1565
GENG	529	f	0	1.07	10.16	0.33	0.0006
GENG	530	f	0	1.82	6.37	2.09	0.0000
Subtotal GENG				1.01	26.81	9.16	
HAENSZ	537	f	0	0.66	18.36	6.33	0.0044
HAENSZ	538	f	0	0.86	13.00	1.99	0.0019
Subtotal HAENSZ				0.75	31.36	8.32	
HEGMAN	513	m	1	2.24	7.47	7.32	0.0000
HEGMAN	514	m	1	3.10	10.02	34.42	0.0000
HEGMAN	516	f	1	1.57	1.60	0.16	0.0470
HEGMAN	517	f	1	3.29	12.44	51.62	0.0000
Subtotal HEGMAN				2.89	31.53	93.52	
*HIRAYA	501	m	1	1.47	83.52	4.01	0.0000
*HIRAYA	502	m	1	1.74	67.55	16.29	0.0000
*HIRAYA	504	f	1	0.90	65.73	8.09	0.0000
*HIRAYA	505	f	1	-0.25	0.91	2.04	0.8127
Subtotal HIRAYA				1.38	217.71	30.44	
HU	511	m	0	0.13	6.22	7.76	0.7374
HU	512	m	0	0.82	15.39	2.87	0.0013
HU	513	m	0	1.11	3.86	0.08	0.0292
HU	516	f	0	-0.33	1.73	4.31	0.6660
HU	517	f	0	0.88	4.71	0.66	0.0575
HU	518	f	0	0.41	2.02	1.44	0.5647
Subtotal HU				0.65	33.92	17.12	
HU2	501	c	0	-0.09	8.76	15.85	0.7814
HU2	502	c	0	-0.06	15.16	25.91	0.8268
HU2	503	c	0	0.93	42.35	4.36	0.0000
HU2	504	c	0	1.21	28.23	0.06	0.0000
Subtotal HU2				0.76	94.50	46.17	
JEDRYC	607	m	0	1.99	27.77	15.17	0.0000
JEDRYC	608	m	0	2.21	21.04	19.46	0.0000
JEDRYC	619	f	0	2.50	7.96	12.43	0.0000
Subtotal JEDRYC				2.14	56.77	47.05	
JOLY	543	m	0	1.54	6.34	0.53	0.0001
JOLY	544	m	0	2.40	10.49	13.89	0.0000
JOLY	545	m	0	3.02	10.57	32.94	0.0000

Table 1H11 - 5

IESLC - Meta-analysis of Ever/current Smoking by Age started, Overview
All LC types, Any Product (or Cigarettes if Any not available)
Least adjusted

REF	NRR	SEX	AD	Ys	Ws	Qs	Ps
JOLY	533	f	0	1.12	11.03	0.20	0.0002
JOLY	534	f	0	2.05	16.96	10.79	0.0000
JOLY	535	f	0	2.47	15.51	23.02	0.0000
Subtotal JOLY				2.15	70.89	81.38	
KHUDER	506	m	0	1.85	14.88	5.35	0.0000
KHUDER	507	m	0	1.86	17.89	6.55	0.0000
KHUDER	508	m	0	2.33	18.34	21.40	0.0000
Subtotal KHUDER				2.03	51.12	33.30	
KOULUM	501	m	0	2.38	2.13	2.72	0.0005
KOULUM	502	m	0	2.27	4.00	4.14	0.0000
KOULUM	503	m	0	3.33	4.31	18.67	0.0000
KOULUM	504	m	0	3.72	4.12	25.14	0.0000
KOULUM	505	m	0	4.57	3.47	38.24	0.0000
Subtotal KOULUM				3.31	18.03	88.91	
LETOUR	501	c	0	2.39	17.33	22.67	0.0000
LETOUR	502	c	0	2.48	18.69	28.32	0.0000
LETOUR	503	c	0	2.92	15.17	42.26	0.0000
Subtotal LETOUR				2.58	51.19	93.25	
*LIAW	504	c	2	0.41	6.39	4.57	0.3054
*LIAW	505	c	2	1.77	8.74	2.40	0.0000
*LIAW	506	c	2	1.53	11.90	0.90	0.0000
Subtotal LIAW				1.34	27.03	7.87	
LIU3	501	m	0	0.17	2.74	3.19	0.7761
LIU3	502	m	0	0.20	2.92	3.26	0.7380
Subtotal LIU3				0.18	5.66	6.45	
LIU4	501	m	2	0.88	3072.74	424.02	0.0000
LIU4	502	m	2	1.05	4361.66	174.96	0.0000
LIU4	503	m	2	1.34	4224.99	31.63	0.0000
Subtotal LIU4				1.11	11659.40	630.61	
LIU5	501	c	0	-0.07	5.40	9.43	0.8697
LIU5	502	c	0	0.86	10.25	1.56	0.0058
Subtotal LIU5				0.54	15.65	10.99	
LUBIN	565	m	0	0.29	6.10	5.60	0.4689
LUBIN	566	m	0	1.27	6.79	0.00	0.0009
LUBIN	567	m	0	1.21	7.09	0.01	0.0013
LUBIN	568	m	0	1.74	7.43	1.75	0.0000
Subtotal LUBIN				1.16	27.42	7.36	
LUBIN2	1147	m	0	1.14	39.73	0.50	0.0000
LUBIN2	1148	m	0	1.68	115.66	21.10	0.0000
LUBIN2	1149	m	0	1.80	146.52	43.39	0.0000
LUBIN2	1150	m	0	1.98	138.01	73.97	0.0000
LUBIN2	1151	m	0	2.08	76.32	52.86	0.0000
Subtotal LUBIN2				1.81	516.24	191.81	
MATOS	556	m	0	1.34	6.69	0.06	0.0005
MATOS	557	m	0	2.03	8.38	5.03	0.0000
MATOS	558	m	0	2.04	7.96	4.92	0.0000
Subtotal MATOS				1.83	23.04	10.01	
*MIGRAN	501	m	0	2.05	3.46	2.22	0.0001
*MIGRAN	503	m	0	1.94	3.77	1.77	0.0002
*MIGRAN	505	m	0	2.31	3.73	4.15	0.0000
*MIGRAN	511	f	0	2.08	2.94	2.00	0.0004
*MIGRAN	513	f	0	2.12	2.78	2.07	0.0004
*MIGRAN	515	f	0	1.97	1.34	0.69	0.0226
Subtotal MIGRAN				2.09	18.02	12.91	
*MRFITR	508	m	0	3.17	0.44	1.62	0.0357
*MRFITR	509	m	0	4.10	0.46	3.76	0.0052
*MRFITR	510	m	0	3.30	0.47	1.97	0.0238
*MRFITR	511	m	0	3.92	0.49	3.50	0.0060
*MRFITR	512	m	0	4.21	0.49	4.32	0.0031
*MRFITR	513	m	0	3.83	0.49	3.25	0.0074
Subtotal MRFITR				3.77	2.85	18.42	
PERNU	504	m	0	2.15	56.20	44.95	0.0000
PERNU	505	m	0	2.34	35.99	42.71	0.0000
PERNU	501	f	0	0.58	13.00	5.86	0.0366
PERNU	502	f	0	3.27	0.37	1.53	0.0455
Subtotal PERNU				2.02	105.57	95.05	
PEZZOT	570	m	0	2.43	3.42	4.73	0.0000
PEZZOT	571	m	0	3.16	3.65	13.32	0.0000
PEZZOT	572	m	0	3.11	3.42	11.85	0.0000
Subtotal PEZZOT				2.91	10.48	29.89	
*QIAO2	501	m	0	0.64	8.52	3.20	0.0623

Table 1H11 - 5

IESLC - Meta-analysis of Ever/current Smoking by Age started, Overview
All LC types, Any Product (or Cigarettes if Any not available)
Least adjusted

REF	NRR	SEX	AD	Ys	Ws	Qs	Ps
*QIAO2	502	m	0	0.63	8.96	3.49	0.0604
*QIAO2	503	m	0	1.24	9.28	0.00	0.0002
Subtotal QIAO2				0.84	26.77	6.69	
RACHTA	501	f	0	1.78	2.41	0.68	0.0057
RACHTA	502	f	0	1.42	7.35	0.20	0.0001
RACHTA	503	f	0	2.00	9.31	5.16	0.0000
Subtotal RACHTA				1.75	19.07	6.04	
SEGI2	516	m	0	0.74	5.86	1.53	0.0736
SEGI2	517	m	0	1.31	6.40	0.02	0.0009
SEGI2	518	m	0	1.77	6.08	1.62	0.0000
Subtotal SEGI2				1.28	18.33	3.17	
SOBUE	654	m	0	1.37	16.73	0.25	0.0000
SOBUE	655	m	0	1.47	22.22	1.11	0.0000
SOBUE	656	m	0	2.26	15.19	15.51	0.0000
Subtotal SOBUE				1.66	54.14	16.87	
SUZUK2	501	c	0	1.25	4.59	0.00	0.0072
SUZUK2	502	c	0	2.09	6.59	4.68	0.0000
SUZUK2	503	c	0	2.70	4.13	8.72	0.0000
Subtotal SUZUK2				2.01	15.32	13.40	
SVENSS	501	f	0	1.73	8.23	1.85	0.0000
SVENSS	502	f	0	2.57	8.11	14.13	0.0000
SVENSS	503	f	0	2.06	9.85	6.39	0.0000
Subtotal SVENSS				2.11	26.19	22.38	
TIZZAN	506	m	0	-0.77	8.70	35.62	0.0228
TIZZAN	507	m	0	0.47	66.40	40.05	0.0001
TIZZAN	508	m	0	0.81	82.27	16.30	0.0000
TIZZAN	519	f	0	-0.94	1.39	6.71	0.2659
TIZZAN	520	f	0	-0.59	6.74	22.75	0.1283
TIZZAN	521	f	0	1.68	1.64	0.30	0.0314
Subtotal TIZZAN				0.53	167.16	121.73	
WAKAI	501	m	0	0.73	3.57	0.96	0.1666
WAKAI	502	m	0	1.53	7.78	0.60	0.0000
WAKAI	503	m	0	1.31	6.55	0.02	0.0008
Subtotal WAKAI				1.29	17.89	1.58	
WU	517	f	0	0.78	5.98	1.31	0.0557
WU	518	f	0	1.69	8.80	1.66	0.0000
WU	519	f	0	2.29	11.93	12.77	0.0000
Subtotal WU				1.75	26.72	15.74	
WYNDE6	759	m	0	2.63	24.28	46.46	0.0000
WYNDE6	760	m	0	2.92	30.32	84.37	0.0000
WYNDE6	761	m	0	3.15	38.07	137.95	0.0000
WYNDE6	767	f	0	2.57	29.27	50.57	0.0000
WYNDE6	768	f	0	2.98	32.44	96.56	0.0000
WYNDE6	769	f	0	3.38	33.77	153.60	0.0000
Subtotal WYNDE6				2.97	188.15	569.52	
ZHENG	563	m	0	0.19	10.89	12.28	0.5320
ZHENG	564	m	0	1.33	17.54	0.12	0.0000
ZHENG	565	m	0	1.95	13.58	6.62	0.0000
ZHENG	572	f	0	0.19	7.30	8.20	0.6057
ZHENG	573	f	0	0.95	15.53	1.38	0.0002
Subtotal ZHENG				1.05	64.84	28.59	

N	213
NS	51

Table 1H11 - 6

IESLC - Meta-analysis of Ever/current Smoking by Age started, Overview
All LC types, Any Product (or Cigarettes if Any not available)
Least adjusted

	Sex			
	combined	male	female	Total
N	21	121	71	213
NS	7	37	25	69

In this overview table, other than the "N" rows, entries in the "absent" and "Total" columns may be invalid and should be ignored

	Age started (broad categories)				
	absent	19+k26	15-25k18	1-17k14	Total
N	87	61	30	35	213
NS	37	46	23	26	132
Wt	9674.60	4068.02	675.62	586.28	15004.53
Het Chi	961.62	556.97	201.65	227.55	3477.17
Het df	86	60	29	34	212
Het P	***	***	***	***	***
Fixed RR	3.41	2.72	8.05	11.24	3.49
RRl	3.34	2.64	7.46	10.36	3.44
RRu	3.48	2.81	8.68	12.18	3.55
P	+++	+++	+++	+++	+++
Random RR	4.24	3.97	7.76	10.57	5.30
RRl	3.79	3.39	6.24	8.34	4.87
RRu	4.74	4.66	9.65	13.41	5.78
P	+++	+++	+++	+++	+++

		Age started (narrow categories)						
	absent	27+k30	23-29k26	19-25k22	15-21k18	11-17k14	1-13k10	Total
N	150	16	1	14	26	2	4	213
NS	51	11	1	11	20	2	4	100
Wt	9433.02	138.37	6.79	4583.73	613.15	142.13	87.34	15004.53
Het Chi	2188.61	44.17	0.00	186.27	182.45	12.09	24.20	3477.17
Het df	149	15	0	13	25	1	3	212
Het P	***	***	N.S.	***	***	***	***	***
Fixed RR	3.54	1.62	3.56	2.97	8.34	7.64	9.51	3.49
RRl	3.47	1.38	1.68	2.89	7.70	6.48	7.71	3.44
RRu	3.61	1.92	7.56	3.06	9.02	9.01	11.72	3.55
P	+++	+++	+++	+++	+++	+++	+++	+++
Random RR	5.20	1.76	3.56	5.49	8.11	16.19	21.16	5.30
RRl	4.67	1.29	1.68	3.78	6.40	2.96	7.08	4.87
RRu	5.80	2.40	7.56	7.96	10.29	88.51	63.23	5.78
P	+++	+++	+++	+++	+++	++	+++	+++

Table 1H11 - 6

IESLC - Meta-analysis of Ever/current Smoking by Age started, Overview
All LC types, Any Product (or Cigarettes if Any not available)
Least adjusted

MALES

		Age started (broad categories)				
		absent	19+k26	15-25k18	1-17k14	Total
	N	46	34	19	22	121
	NS	26	33	18	21	98
	Wt	9297.46	3680.12	496.51	475.61	13949.69
Het	Chi	690.00	329.10	110.96	127.55	2514.87
Het	df	45	33	18	21	120
Het	P	***	***	***	***	***
Fixed	RR	3.41	2.66	8.22	10.94	3.43
	RRl	3.34	2.58	7.53	10.00	3.37
	RRu	3.48	2.75	8.98	11.97	3.48
	P	+++	+++	+++	+++	+++
Random	RR	4.77	4.59	8.22	11.58	6.16
	RRl	4.14	3.72	6.42	9.01	5.55
	RRu	5.49	5.65	10.53	14.88	6.83
	P	+++	+++	+++	+++	+++

			Age started (narrow categories)						
		absent	27+k30	23-29k26	19-25k22	15-21k18	11-17k14	1-13k10	Total
	N	81	8	1	9	17	2	3	121
	NS	37	8	1	8	16	2	3	75
	Wt	8673.49	62.08	6.79	4518.19	463.80	142.13	83.21	13949.69
Het	Chi	1519.67	16.93	0.00	160.64	100.90	12.09	23.32	2514.87
Het	df	80	7	0	8	16	1	2	120
Het	P	***	*	N.S.	***	***	***	***	***
Fixed	RR	3.46	1.65	3.56	2.96	8.42	7.64	9.29	3.43
	RRl	3.39	1.28	1.68	2.87	7.69	6.48	7.50	3.37
	RRu	3.54	2.11	7.56	3.04	9.22	9.01	11.52	3.48
	P	+++	+++	+++	+++	+++	+++	+++	+++
Random	RR	6.12	1.79	3.56	5.72	8.44	16.19	24.40	6.16
	RRl	5.34	1.18	1.68	3.42	6.50	2.96	5.26	5.55
	RRu	7.00	2.73	7.56	9.55	10.97	88.51	113.15	6.83
	P	+++	++	+++	+++	+++	++	+++	+++

FEMALES

		Age started (broad categories)				
		absent	19+k26	15-25k18	1-17k14	Total
	N	32	21	8	10	71
	NS	20	21	8	10	59
	Wt	277.97	296.64	129.70	79.04	783.34
Het	Chi	197.73	146.14	68.98	73.46	639.00
Het	df	31	20	7	9	70
Het	P	***	***	***	***	***
Fixed	RR	3.93	3.16	6.99	12.74	4.48
	RRl	3.49	2.82	5.88	10.22	4.18
	RRu	4.42	3.54	8.30	15.88	4.81
	P	+++	+++	+++	+++	+++
Random	RR	3.79	3.01	6.21	6.37	3.98
	RRl	2.77	2.16	3.43	2.99	3.17
	RRu	5.19	4.19	11.24	13.54	4.98
	P	+++	+++	+++	+++	+++

Table 1H11 - 6

IESLC - Meta-analysis of Ever/current Smoking by Age started, Overview
All LC types, Any Product (or Cigarettes if Any not available)
Least adjusted

FEMALES

	Age started (narrow categories)							
	absent	27+k30	23-29k26	19-25k22	15-21k18	11-17k14	1-13k10	Total
N	54	6		4	7			71
NS	25	6		4	7			42
Wt	558.08	55.73		56.80	112.74			783.34
Het Chi	478.12	19.21		13.80	68.76			639.00
Het df	53	5		3	6			70
Het P	***	**		**	***			***
Fixed RR	4.49	1.96		4.29	6.88			4.48
RRl	4.13	1.50		3.30	5.72			4.18
RRu	4.87	2.54		5.56	8.27			4.81
P	+++	+++		+++	+++			+++
Random RR	3.94	2.16		4.95	5.96			3.98
RRl	3.02	1.18		2.73	2.96			3.17
RRu	5.15	3.96		8.99	11.97			4.98
P	+++	+		+++	+++			+++

Table 1H11 - 7

IESLC - Meta-analysis of Ever/current Smoking by Age started, Overview
All LC types, Any Product (or Cigarettes if Any not available)
Excluded studies (and stage at which they were excluded)

1	BECHER BLOT1 BROWN3 CARPEN CHYOU DARBY DOLL2 GARCIA GRAHAM GURSEL HAMMO2 JAHN JAIN LAUSSM PRESCO QIAO TVERDA WIGLE WYNDE3
2	AKIBA AMANDU AMES AXELSS BENSHL BEST BOUCHA BOUCOT BROSS CHEN CPSII DEAN2 DESTEF DORGAN DOSEME FAN GARSHI GER GILLIS HAMMON HUMBLE JUSSAW KAISE2 KATSOU KAUFMA KOO KREUZE LEVIN MCCONN NOTAN2 OSANN2 PEZZO2 PISANI RESTRE SADOWS VUTUC WANG2 WATSON WU2 WUWILL WYNDE2 WYNDE8 XU ZHOU
3	GUO MCDUFF SPITZ STASZE ZHANG
4	LUO
5	CORREA HOLE YUAN
7	BOFFET WYNDE7
10	ALDERS SPEIZE
14	BENHAM

Table 1H11 - 8
Potentially overlapping studies

REF	REFGP	PRINC	. OVERLAP/LINK
LUBIN2	LUBIN2	1	Lubin-combined
MRFITR	MRFIT	2	Subset of MRFIT
WYNDE6	WYNDE6	1	WYNDE5/6/7/8
CPSI	CPSI	1	CPSI overall
LUBIN	XIANGZ	2	LUBIN/XIANGZ/QIAO

Table 1H11 - 9
Most adjusted - insufficient data for meta-analysis

REF	NRR	SEX	AGEL	AGEH	RACE	YF	LC TYPE	LOC	START	ST	NLC	R	VB	P	H	AD	SM	PRODUCT	exL	exH	S1	S2	DENOM	De
CORREA	535	c	0	0	all	-	all	NAmer	1979	CC	1359	n	bl	y	n	2	ev	cig+/-ot	21	999	1	0	nev cigs	or
CORREA	536	c	0	0	all	-	all	NAmer	1979	CC	1359	n	bl	y	n	2	ev	cig+/-ot	16	20	2	4	nev cigs	or
CORREA	537	c	0	0	all	-	all	NAmer	1979	CC	1359	n	bl	y	n	2	ev	cig+/-ot	1	15	3	0	nev cigs	or
JEDRYC	606	m	0	0	all	-	all	Eu:est	1980	CC	1630	n	bl	y	n	0	ev	cig+/-ot	18	999	0	0	nev any	st
JEDRYC	618	f	0	0	all	-	all	Eu:est	1980	CC	1630	n	bl	y	n	0	ev	cig+/-ot	23	999	1	0	nev any	st

REF	NRR	RR	SIG	RRDATA comment
CORREA	535	8.30		0
CORREA	536	17.40		0
CORREA	537	24.20		0
JEDRYC	606	*		0
JEDRYC	618	*		0

Table 1H12 -

IESLC - Meta-analysis of Ever/current Smoking, Age started, "Low"
All LC types, Any Product (or Cigarettes if Any not available)

This analysis is restricted to results for:
1) Ever/current smokers
2) Results by Age started
3) Categorical results by Age started
4) All LC types (or near equivalent)
5) Results complete enough for use in metaanalysis

Within each study, results are then selected (in the following order of preference, within each sex) for:
6) SMKSTA: ever, current
7) PRODUCT: all/unspec, cigarettes regardless of other products, cigarettes only
8) CIGTYPE: all/unspecified, MC regardless of HR, MC only
9) (not applicable)
10) DENOM: never smoked anything, never smoked cigarettes, never any + low, never cigs + low
11) Followup period (YF, prospective studies): whole study (coded as 0) or longest available
12) LCtype: all or nearest available, at least Squamous and Adeno. (q = squamous, s = small,
l = large, a = adeno, mix = mixed, alv = alveolar)
13) Race: all or nearest available, otherwise by race (wh or w = white, bl or b = black, hi = hispanic
ch = chinese, jap = japanese, haw = hawaiian, w+o = white + oriental, sca = scandinavian, as = asian)
14) Age started "low" in key scheme 1 (key value 26, maximum range 19+)
15) For overlapping studies: principal rather than subsidiary studies
Finally by Age: whole study (coded as 0) if available, otherwise by widest available age group
and then for single sex results (m, f) in preference to results for both sexes combined (c).

Results adjusted (AD) for the most potential confounders are then chosen in Sections -1 to -3
and results adjusted for the least confounders in Sections -4 to -6. (Those least adjusted results which
actually differ from the most adjusted are marked 'x' in column X in Section -4)

Section -7 shows excluded studies, together with the stage (as above) at which no qualifying
results were found.

Section -8 lists the potentially overlapping studies which have been included (1=principal, 2=subsidiary).

Section -9 lists any results which would have been included in preference except that they had data not complete
enough for use in meta-analysis, with their significance (yes/no), if known, and any further comment as entered
on the database. It also lists as "gap" any categories for which no data were presented by the original authors.

In addition to those mentioned above, the following fields, levels and abbreviations are used:
* or nk = not known, n = no, y = yes, ot = other
ev = ever, cu = current, nev = never
all/unspec = all or unspecified, cig+/-ot = cigarettes irrespective of other products (cigar, pipe etc)
MC = manufactured cigarettes, HR = hand-rolled cigarettes
exL, exH = range of exposure (low and high) in the smoking group, in terms of Age started
REF: 6-character study reference
NRR: number of the RR on the database within the study
ST : study type (CC = case control, pr or prosp = prospective)
NLC: number of lung cancer cases in whole study
R : risky occupational population (n = no, m = mining, o = other risky)
VB : national cigarette type (V = at least 75% Virginia, bl = at least 75% blended, ot = other)
P : any proxy use
H : full histological confirmation
De : derivation of RR/CI (or = original, st = standard method, ot = other method of estimation)

Table 1H12 - 1

IESLC - Meta-analysis of Ever/current Smoking, Age started, "Low"
All LC types, Any Product (or Cigarettes if Any not available)
Most adjusted

REF	NRR	SEX	AGEL	AGEH	RACE	YF	LC TYPE	LOC	START	ST	NLC	R	VB	P	H	AD	SM	PRODUCT	exL	exH	DENOM	De
AGUDO	504	f	0	0	all	-	all	Eu:wst	1989	CC	103	n	bl	n	n	3	ev	cig only	24	999	nev cigs	or
AUVINE	509	c	0	0	all	-	all	Eu:Sca	1986	CC	517	n	bl	y	n	0	ev	cig+/-ot	21	999	nev cigs	st
BARBON	520	m	0	0	all	-	all	Eu:wst	1979	CC	755	n	bl	y	y	1	ev	all/unsp	20	999	nev any	or
BRESLO	501	c	0	0	all	-	all	NAmer	1949	CC	518	n	bl	n	y	0	ev	cig+/-ot	25	999	nev any	st
BUFFLE	518	f	0	0	w-hi	-	all	NAmer	1976	CC	943	n	bl	y	n	0	ev	cig+/-ot	21	29	nev cigs	or
CEDERL	510	m	0	0	all	10	all	Eu:Sca	1963	pr	491	n	bl	n	n	1	cu	cig only	19	999	nev any	ot
CEDERL	515	f	0	0	all	10	all	Eu:Sca	1963	pr	491	n	bl	n	n	0	cu	cig only	19	999	nev any	st
CHEN2	518	m	0	0	all	-	all	As:Chi	1983	CC	193	n	ot	y	n	0	ev	all/unsp	20	30	nev any	st
CHEN2	523	f	0	0	all	-	all	As:Chi	1983	CC	193	n	ot	y	n	0	ev	all/unsp	20	30	nev any	st
CHIAZZ	501	m	0	0	all	-	all	NAmer	1940	CC	144	o	bl	y	n	2	ev	cig+/-ot	20	999	nev cigs	or
CHOI	523	m	0	0	all	-	all	As:oth	1985	CC	375	n	bl	n	n	0	ev	cig+/-ot	25	999	nev cigs	st
CHOI	530	f	0	0	all	-	all	As:oth	1985	CC	375	n	bl	n	n	0	ev	cig+/-ot	25	999	nev cigs	st
CPSI	801	m	35	84	all	6	all	NAmer	1959	pr	5138	n	bl	n	n	1	cu	cig+/-ot	25	999	nev any	ot
CPSI	845	f	40	74	all	6	all	NAmer	1959	pr	5138	n	bl	n	n	1	cu	cig only	25	999	nev any	ot
DAMBER	501	m	0	0	all	-	all	Eu:Sca	1972	CC	579	n	bl	y	n	0	ev	all/unsp	21	999	nev any	st
DEAN3	564	m	0	0	all	-	all	Eu:UK	1969	CC	766	n	V	y	n	0	cu	cig only	25	999	nev any	st
DEAN3	583	f	0	0	all	-	all	Eu:UK	1969	CC	766	n	V	y	n	0	cu	cig only	25	999	nev any	st
DOLL	503	m	0	0	all	-	all	Eu:UK	1948	CC	1465	n	V	n	n	0	ev	all/unsp	20	29	nev any	st
DOLL	510	f	0	0	all	-	all	Eu:UK	1948	CC	1465	n	V	n	n	0	ev	all/unsp	20	29	nev any	st
DORN	610	m	55	64	wh	8	all	NAmer	1954	pr	5097	n	bl	n	n	0	ev	cig+/-ot	25	999	nev any	st
DORN	647	m	65	74	wh	8	all	NAmer	1954	pr	5097	n	bl	n	n	0	ev	cig+/-ot	25	999	nev any	st
ENGELA	502	m	0	0	all	0	all	Eu:Sca	1964	pr	435	n	bl	n	n	0	cu	cig+/-ot	20	29	nev cigs	st
ENGELA	510	f	0	0	all	0	all	Eu:Sca	1964	pr	435	n	bl	n	n	0	cu	cig+/-ot	20	29	nev cigs	st
GAO	507	m	0	0	all	-	all	As:Chi	1984	CC	1405	n	ot	n	n	2	ev	cig+/-ot	20	29	nev cigs	or
GAO	517	f	0	0	all	-	all	As:Chi	1984	CC	1405	n	ot	n	n	2	ev	cig+/-ot	20	29	nev cigs	or
GAO2	502	m	0	0	all	-	all	As:Jap	1988	CC	282	n	bl	n	n	0	cu	cig+/-ot	20	29	nev cigs	st
GENG	533	f	0	0	all	-	all	As:Chi	1985	CC	292	n	ot	*	n	1	ev	cig+/-ot	21	999	nev any	st
HAENSZ	537	f	0	0	all	-	not alv	NAmer	1955	CC	158	n	bl	n	y	0	ev	cig+/-ot	25	999	nev any	st
HEGMAN	513	m	0	0	all	-	all	NAmer	1989	CC	282	n	bl	y	y	1	ev	all/unsp	20	999	nev any	or
HEGMAN	516	f	0	0	all	-	all	NAmer	1989	CC	282	n	bl	y	y	1	ev	all/unsp	26	999	nev any	or
HIRAYA	501	m	0	0	all	0	all	As:Jap	1965	pr	1917	n	bl	n	n	1	cu	cig+/-ot	20	999	nev any	st
HIRAYA	504	f	0	0	all	0	all	As:Jap	1965	pr	1917	n	bl	n	n	1	cu	cig+/-ot	20	999	nev any	st
HU2	503	c	0	0	all	-	all	As:Chi	1977	CC	523	n	ot	y	n	0	ev	cig+/-ot	20	29	nev cigs	st
JOLY	543	m	0	0	all	-	all	SCAmer	1978	CC	826	n	bl	n	n	0	ev	cig+/-ot	25	999	nev any	st
JOLY	533	f	0	0	all	-	all	SCAmer	1978	CC	826	n	bl	n	n	0	ev	cig+/-ot	25	999	nev any	st
KHUDER	506	m	0	0	all	-	all	NAmer	1985	CC	482	n	bl	n	y	0	ev	cig+/-ot	20	999	nev cigs	st
KOULUM	502	m	0	0	all	-	all	Eu:Sca	1936	CC	812	n	bl	n	n	0	ev	all/unsp	21	30	nev any	st
LETOUR	501	c	0	0	all	-	all	NAmer	1983	CC	738	n	V	y	y	0	ev	cig+/-ot	21	999	nev cigs	st
LIAW	504	c	0	0	all	0	all	As:oth	1982	pr	127	n	ot	n	n	2	cu	all/unsp	25	999	nev any	or
LIU3	504	m	0	0	all	-	all	As:Chi	1985	CC	110	n	ot	n	n	2	ev	all/unsp	21	999	nev any	or
LIU4	501	m	35	69	all	-	all	As:Chi	1986	CC	1000-00	n	ot	y	n	2	ev	all/unsp	25	999	nev any	ot
LUBIN	566	m	0	0	all	-	all	As:Chi	1984	CC	427	m	ot	y	n	0	ev	cig+/-ot	23	26	nev any	st
LUBIN2	1157	m	0	0	all	-	all	Eu:mul	1976	CC	7804	n	bl	n	y	1	ev	cig+/-ot	21	30	nev cigs	st
MATOS	576	m	0	0	all	-	all	SCAmer	1994	CC	200	n	bl	n	n	2	ev	cig+/-ot	20	999	nev any	or
MIGRAN	501	m	0	0	all	0	all	Eu:UK	1964	pr	259	n	V	n	n	0	cu	cig only	20	999	nev any	st
MIGRAN	511	f	0	0	all	0	all	Eu:UK	1964	pr	259	n	V	n	n	0	cu	cig only	20	999	nev any	st
MRFITR	508	m	0	0	all	0	all	NAmer	1973	pr	119	n	bl	n	n	0	cu	cig+/-ot	24	999	nev cigs	ot
PEZZOT	570	m	0	0	all	-	all	SCAmer	1987	CC	215	n	bl	n	y	0	ev	cig only	19	999	nev cigs	st
QIAO2	506	m	0	0	all	0	all	As:Chi	1992	pr	241	m	ot	n	n	1	ev	all/unsp	21	999	nev any	or
RACHTA	507	f	0	0	all	-	all	Eu:est	1991	CC	118	n	bl	n	y	1	ev	cig+/-ot	20	30	nev cigs	or
SEGI2	521	m	0	0	all	-	all	As:Jap	1962	CC	378	n	bl	n	n	1	cu	cig+/-ot	23	999	nev any	ot
SOBUE	654	m	0	0	all	-	all	As:Jap	1986	CC	1376	n	bl	n	y	0	ev	cig+/-ot	23	999	nev cigs	st
SUZUK2	501	c	0	0	all	-	all	SCAmer	1991	CC	123	n	bl	n	y	0	ev	all/unsp	19	999	nev any	st
SVENSS	501	f	0	0	all	-	all	Eu:Sca	1983	CC	210	n	bl	n	n	0	cu	all/unsp	26	999	nev any	st
TIZZAN	507	m	0	0	all	-	all	Eu:wst	1959	CC	1358	n	bl	n	n	0	ev	all/unsp	20	30	nev any	st
TIZZAN	520	f	0	0	all	-	all	Eu:wst	1959	CC	1358	n	bl	n	n	0	ev	all/unsp	20	30	nev any	st
WAKAI	502	m	0	0	all	-	all	As:Jap	1988	CC	333	n	bl	n	y	0	cu	cig+/-ot	20	29	nev any	st
WU	541	f	0	0	wh	-	q+a	NAmer	1981	CC	220	n	bl	n	y	2	cu	all/unsp	25	999	nev any	st
WYNDE6	759	m	0	0	wh	-	q+s+a	NAmer	1969	CC	4423	n	bl	n	y	0	ev	cig+/-ot	21	999	nev cigs	st
WYNDE6	767	f	0	0	wh	-	q+s+a	NAmer	1969	CC	4423	n	bl	n	y	0	ev	cig+/-ot	21	999	nev cigs	st
ZHENG	564	m	0	0	all	-	all	As:Chi	1982	CC	540	n	ot	*	y	0	ev	cig+/-ot	20	29	nev cigs	st

Cigarette type is all/unspec for all RRs
except for the following:

REF	NRR	CIGTYPE
DEAN3	564	MC only
DEAN3	583	MC only

Table 1H12 - 2

IESLC - Meta-analysis of Ever/current Smoking, Age started, "Low"
All LC types, Any Product (or Cigarettes if Any not available)
Most adjusted

REF	NRR	SEX	AD	Number Exposed Case	Cont	Non-exposed Case	Cont	RR	95.00%CI
AGUDO	504	f	3	7	-	80	-	1.58	(0.59- 4.23)
AUVINE	509	c	0	76	27	44	229	14.65	(8.50- 25.26)
BARBON	520	m	1	200	-	22	-	8.20	(5.00- 13.30)
BRESLO	501	c	0	32	35	19	56	2.69	(1.33- 5.47)
BUFFLE	518	f	0	47	34	12	112	12.90	(6.15- 27.07)
*CEDERL	510	m	1	11	-	7	-	6.50	(2.52- 16.74)
*CEDERL	515	f	0	6	2806	19	17679	1.99	(0.80- 4.98)
Subtotal CEDERL								3.53	(1.83- 6.82)
CHEN2	518	m	0	29	25	9	33	4.25	(1.71- 10.57)
CHEN2	523	f	0	9	8	25	33	1.49	(0.50- 4.39)
Subtotal CHEN2								2.75	(1.37- 5.53)
CHIAZZ	501	m	2	-	-	4	-	3.00	(0.31- 28.84)
CHOI	523	m	0	36	77	13	95	3.42	(1.69- 6.89)
CHOI	530	f	0	15	25	76	164	1.29	(0.65- 2.60)
Subtotal CHOI								2.09	(1.28- 3.43)
*CPSI	801	m	1	42	-	83	-	4.08	(2.81- 5.91)
*CPSI	845	f	1	51	-	166	-	2.25	(1.64- 3.08)
Subtotal CPSI								2.89	(2.27- 3.67)
DAMBER	501	m	0	70	76	42	208	4.56	(2.87- 7.26)
DEAN3	564	m	0	24	75	24	510	6.80	(3.67- 12.58)
DEAN3	583	f	0	27	274	41	1538	3.70	(2.24- 6.11)
Subtotal DEAN3								4.72	(3.20- 6.96)
DOLL	503	m	0	251	264	7	61	8.29	(3.72- 18.46)
DOLL	510	f	0	23	15	40	59	2.26	(1.05- 4.86)
Subtotal DOLL								4.20	(2.42- 7.30)
*DORN	610	m	0	37	73050	25	213858	4.33	(2.61- 7.20)
*DORN	647	m	0	90	74464	49	171211	4.22	(2.98- 5.98)
Subtotal DORN								4.26	(3.20- 5.67)
*ENGELA	502	m	0	50	30195	27	58716	3.60	(2.26- 5.75)
*ENGELA	510	f	0	36	29605	31	207789	8.15	(5.04- 13.17)
Subtotal ENGELA								5.36	(3.83- 7.49)
GAO	507	m	2	363	-	62	-	4.70	(3.30- 6.50)
GAO	517	f	2	87	-	435	-	3.80	(2.60- 5.80)
Subtotal GAO								4.30	(3.32- 5.57)
GAO2	502	m	0	127	85	13	56	6.44	(3.32- 12.49)
GENG	533	f	1	28	-	54	-	1.55	(0.83- 2.89)
HAENSZ	537	f	0	44	66	81	236	1.94	(1.23- 3.07)
HEGMAN	513	m	1	26	-	-	-	9.40	(4.60- 19.30)
HEGMAN	516	f	1	2	-	-	-	4.80	(1.00- 22.10)
Subtotal HEGMAN								8.35	(4.36- 16.00)
*HIRAYA	501	m	1	-	-	-	-	4.35	(3.51- 5.39)
*HIRAYA	504	f	1	-	-	-	-	2.46	(1.93- 3.13)
Subtotal HIRAYA								3.38	(2.88- 3.97)
HU2	503	c	0	229	159	121	213	2.54	(1.88- 3.43)
JOLY	543	m	0	18	70	12	218	4.67	(2.14- 10.18)
JOLY	533	f	0	23	41	52	283	3.05	(1.69- 5.51)
Subtotal JOLY								3.57	(2.23- 5.71)
KHUDER	506	m	0	72	152	23	309	6.36	(3.83- 10.58)
KOULUM	502	m	0	60	67	5	54	9.67	(3.63- 25.77)
LETOUR	501	c	0	188	160	24	224	10.97	(6.85- 17.56)
*LIAW	504	c	2	-	-	-	-	1.50	(0.70- 3.30)
LIU3	504	m	2	20	-	4	-	1.10	(0.25- 4.93)
LIU4	501	m	2	-	-	-	-	2.41	(2.32- 2.49)
LUBIN	566	m	0	65	146	9	72	3.56	(1.68- 7.56)
LUBIN2	1157	m	1	564	-	185	-	4.97	(4.12- 6.00)
MATOS	576	m	2	28	-	11	-	3.90	(1.80- 8.30)
*MIGRAN	501	m	0	24	668	4	867	7.79	(2.72- 22.34)
*MIGRAN	511	f	0	11	1315	4	3814	7.98	(2.54- 25.01)
Subtotal MIGRAN								7.87	(3.63- 17.08)
*MRFITR	508	m	0	3	544	0	1859	23.91~	(1.24- 462.09)
PEZZOT	570	m	0	41	105	4	116	11.32	(3.92- 32.69)
*QIAO2	506	m	1	52	-	10	-	1.32	(0.67- 2.60)
RACHTA	507	f	1	25	-	33	-	5.33	(2.79- 10.20)
SEGI2	521	m	1	49	-	8	-	2.04	(0.89- 4.66)
SOBUE	654	m	0	110	121	29	126	3.95	(2.45- 6.38)
SUZUK2	501	c	0	16	22	11	53	3.50	(1.40- 8.75)
SVENSS	501	f	0	32	18	38	120	5.61	(2.84- 11.12)
TIZZAN	507	m	0	313	330	180	305	1.61	(1.26- 2.04)
TIZZAN	520	f	0	12	21	117	114	0.56	(0.26- 1.18)
Subtotal TIZZAN								1.46	(1.16- 1.83)
WAKAI	502	m	0	130	183	10	65	4.62	(2.29- 9.32)

Table 1H12 - 2

IESLC - Meta-analysis of Ever/current Smoking, Age started, "Low"
All LC types, Any Product (or Cigarettes if Any not available)
Most adjusted

REF	NRR	SEX	AD	Number Exposed Case	Exposed Cont	Non-exposed Case	Non-exposed Cont	RR	95.00%CI
WU	541	f	2	14	-	31	-	1.55	(0.60- 3.98)
WYNDE6	759	m	0	111	92	51	589	13.93	(9.36- 20.74)
WYNDE6	767	f	0	127	90	73	673	13.01	(9.06- 18.69)
Subtotal WYNDE6								13.42	(10.27- 17.54)
ZHENG	564	m	0	145	109	33	94	3.79	(2.37- 6.05)
Partial Totals				4328	215619	2592	682811		

*prospective study ~ With 0.5 adjustment for zero

REF	NRR	SEX	AD	Ys	Ws	Qs	Ps
AGUDO	504	f	3	0.46	3.96	1.16	0.3627
AUVINE	509	c	0	2.68	12.94	36.72	0.0000
BARBON	520	m	1	2.10	16.05	19.58	0.0000
BRESLO	501	c	0	0.99	7.67	0.00	0.0060
BUFFLE	518	f	0	2.56	7.00	16.97	0.0000
*CEDERL	510	m	1	1.87	4.29	3.26	0.0001
*CEDERL	515	f	0	0.69	4.57	0.44	0.1414
Subtotal CEDERL				1.26	8.85	3.70	
CHEN2	518	m	0	1.45	4.63	0.93	0.0018
CHEN2	523	f	0	0.40	3.26	1.19	0.4750
Subtotal CHEN2				1.01	7.90	2.12	
CHIAZZ	501	m	2	1.10	0.75	0.01	0.3421
CHOI	523	m	0	1.23	7.80	0.41	0.0006
CHOI	530	f	0	0.26	7.94	4.36	0.4667
Subtotal CHOI				0.74	15.74	4.77	
*CPSI	801	m	1	1.41	27.80	4.59	0.0000
*CPSI	845	f	1	0.81	38.69	1.38	0.0000
Subtotal CPSI				1.06	66.49	5.97	
DAMBER	501	m	0	1.52	17.84	4.79	0.0000
DEAN3	564	m	0	1.92	10.14	8.53	0.0000
DEAN3	583	f	0	1.31	15.21	1.44	0.0000
Subtotal DEAN3				1.55	25.35	9.97	
DOLL	503	m	0	2.11	5.99	7.44	0.0000
DOLL	510	f	0	0.82	6.57	0.22	0.0364
Subtotal DOLL				1.43	12.56	7.66	
*DORN	610	m	0	1.47	14.92	3.25	0.0000
*DORN	647	m	0	1.44	31.75	6.17	0.0000
Subtotal DORN				1.45	46.67	9.42	
*ENGELA	502	m	0	1.28	17.55	1.39	0.0000
*ENGELA	510	f	0	2.10	16.67	20.11	0.0000
Subtotal ENGELA				1.68	34.22	21.50	
GAO	507	m	2	1.55	33.44	10.04	0.0000
GAO	517	f	2	1.34	23.87	2.68	0.0000
Subtotal GAO				1.46	57.31	12.72	
GAO2	502	m	0	1.86	8.74	6.50	0.0000
GENG	533	f	1	0.44	9.87	3.11	0.1685
HAENSZ	537	f	0	0.66	18.36	2.07	0.0044
HEGMAN	513	m	1	2.24	7.47	11.51	0.0000
HEGMAN	516	f	1	1.57	1.60	0.52	0.0470
Subtotal HEGMAN				2.12	9.08	12.03	
*HIRAYA	501	m	1	1.47	83.52	18.49	0.0000
*HIRAYA	504	f	1	0.90	65.73	0.65	0.0000
Subtotal HIRAYA				1.22	149.25	19.14	
HU2	503	c	0	0.93	42.35	0.20	0.0000
JOLY	543	m	0	1.54	6.34	1.86	0.0001
JOLY	533	f	0	1.12	11.03	0.15	0.0002
Subtotal JOLY				1.27	17.37	2.01	
KHUDER	506	m	0	1.85	14.88	10.78	0.0000
KOULUM	502	m	0	2.27	4.00	6.44	0.0000
LETOUR	501	c	0	2.39	17.33	33.74	0.0000
*LIAW	504	c	2	0.41	6.39	2.26	0.3054
LIU3	504	m	2	0.10	1.73	1.41	0.9003
LIU4	501	m	2	0.88	3072.74	44.28	0.0000
LUBIN	566	m	0	1.27	6.79	0.50	0.0009
LUBIN2	1157	m	1	1.60	108.74	39.64	0.0000
MATOS	576	m	2	1.36	6.58	0.86	0.0005
*MIGRAN	501	m	0	2.05	3.46	3.84	0.0001
*MIGRAN	511	f	0	2.08	2.94	3.41	0.0004
Subtotal MIGRAN				2.06	6.40	7.25	
*MRFITR	508	m	0	3.17	0.44	2.07	0.0357
PEZZOT	570	m	0	2.43	3.42	6.96	0.0000

Table 1H12 - 2

IESLC - Meta-analysis of Ever/current Smoking, Age started, "Low"
All LC types, Any Product (or Cigarettes if Any not available)
Most adjusted

REF	NRR	SEX	AD	Ys	Ws	Qs	Ps
*QIAO2	506	m	1	0.28	8.36	4.36	0.4222
RACHTA	507	f	1	1.67	9.14	4.15	0.0000
SEGI2	521	m	1	0.71	5.61	0.46	0.0914
SOBUE	654	m	0	1.37	16.73	2.34	0.0000
SUZUK2	501	c	0	1.25	4.59	0.30	0.0072
SVENSS	501	f	0	1.73	8.23	4.33	0.0000
TIZZAN	507	m	0	0.47	66.40	18.32	0.0001
TIZZAN	520	f	0	-0.59	6.74	16.95	0.1283
Subtotal TIZZAN				0.38	73.15	35.27	
WAKAI	502	m	0	1.53	7.78	2.19	0.0000
WU	541	f	2	0.44	4.29	1.35	0.3639
WYNDE6	759	m	0	2.63	24.28	64.88	0.0000
WYNDE6	767	f	0	2.57	29.27	71.77	0.0000
Subtotal WYNDE6				2.60	53.55	136.65	
ZHENG	564	m	0	1.33	17.54	1.94	0.0000

	N	61
	NS	46
	Wt	4054.72
Het	Chi	551.66
Het	df	60
Het	P	***
Fixed	RR	2.72
	RRl	2.64
	RRu	2.80
	P	+++
Random	RR	3.99
	RRl	3.40
	RRu	4.68
	P	+++
Asymm	P	***

Table 1H12 - 3

IESLC - Meta-analysis of Ever/current Smoking, Age started, "Low"
All LC types, Any Product (or Cigarettes if Any not available)
Most adjusted

	Sex			
	combined	male	female	Total
N	6	34	21	61
NS	6	33	21	60
Wt	91.27	3668.49	294.96	4054.72
Het Chi	55.38	317.58	148.58	551.66
Het df	5	33	20	60
Het P	***	***	***	***
Fixed RR	4.23	2.65	3.26	2.72
RRl	3.44	2.56	2.91	2.64
RRu	5.19	2.74	3.66	2.80
P	+++	+++	+++	+++
Random RR	4.33	4.55	3.07	3.99
RRl	2.05	3.70	2.20	3.40
RRu	9.17	5.60	4.30	4.68
P	+++	+++	+++	+++
Between Chi				30.12
Between df				2
Between P				***
Btwn(F) P				N.S.
Btwn(R) P				N.S.

	Lung cancer type		
	all	other	Total
N	57	4	61
NS	43	3	46
Wt	3978.52	76.20	4054.72
Het Chi	410.10	62.37	551.66
Het df	56	3	60
Het P	***	***	***
Fixed RR	2.67	7.46	2.72
RRl	2.58	5.96	2.64
RRu	2.75	9.34	2.80
P	+++	+++	+++
Random RR	3.88	5.06	3.99
RRl	3.34	1.72	3.40
RRu	4.52	14.90	4.68
P	+++	++	+++
Between Chi			79.19
Between df			1
Between P			***
Btwn(F) P			**
Btwn(R) P			N.S.

	Location								
	NAmer	UK	Scand	othEur	China	Japan	othAs	other	Total
N	16	6	8	6	11	6	3	5	61
NS	12	3	6	5	9	5	2	4	46
Wt	246.50	44.32	86.08	211.05	3224.58	188.10	22.13	31.96	4054.72
Het Chi	126.17	9.32	24.33	91.51	32.47	17.83	4.21	4.73	551.66
Het df	15	5	7	5	10	5	2	4	60
Het P	***	(*)	***	***	***	**	N.S.	N.S.	***
Fixed RR	5.22	4.91	5.96	3.31	2.44	3.53	1.90	4.10	2.72
RRl	4.60	3.66	4.83	2.89	2.35	3.06	1.25	2.90	2.64
RRu	5.91	6.60	7.37	3.79	2.52	4.07	2.88	5.80	2.80
P	+++	+++	+++	+++	+++	+++	++	+++	+++
Random RR	5.31	5.17	5.93	2.71	2.76	3.67	1.89	4.18	3.99
RRl	3.57	3.38	3.92	1.36	2.19	2.65	1.03	2.85	3.40
RRu	7.90	7.91	8.98	5.40	3.48	5.09	3.47	6.12	4.68
P	+++	+++	+++	++	+++	+++	+	+++	+++
Between Chi									241.10
Between df									7
Between P									***
Btwn(F) P									***
Btwn(R) P									**

Table 1H12 - 3

IESLC - Meta-analysis of Ever/current Smoking, Age started, "Low"
All LC types, Any Product (or Cigarettes if Any not available)
Most adjusted

Detailed Country in "other Europe"

	multi	Germany	othWest	East	Balkans	Total
N	1		4	1		6
NS	1		3	1		5
Wt	108.74		93.16	9.14		211.05
Het Chi	0.00		46.36	0.00		91.51
Het df	0		3	0		5
Het P	N.S.		***	N.S.		***
Fixed RR	4.97		1.97	5.33		3.31
RRl	4.12		1.61	2.79		2.89
RRu	6.00		2.41	10.19		3.79
P	+++		+++	+++		+++
Random RR	4.97		1.90	5.33		2.71
RRl	4.12		0.67	2.79		1.36
RRu	6.00		5.39	10.19		5.40
P	+++		N.S.	+++		++
Between Chi						45.15
Between df						2
Between P						***
Btwn(F) P						N.S.
Btwn(R) P						N.S.

Detailed Country in "other Asia"

	India	HongKong	other	Total
N			3	3
NS			2	2
Wt			22.13	22.13
Het Chi			4.21	4.21
Het df			2	2
Het P			N.S.	N.S.
Fixed RR			1.90	1.90
RRl			1.25	1.25
RRu			2.88	2.88
P			++	++
Random RR			1.89	1.89
RRl			1.03	1.03
RRu			3.47	3.47
P			+	+
Between Chi				
Between df				
Between P				N.S.
Btwn(F) P				N.S.
Btwn(R) P				N.S.

Detailed other continent

	SCAmer	Total
N	5	5
NS	4	4
Wt	31.96	31.96
Het Chi	4.73	4.73
Het df	4	4
Het P	N.S.	N.S.
Fixed RR	4.10	4.10
RRl	2.90	2.90
RRu	5.80	5.80
P	+++	+++
Random RR	4.18	4.18
RRl	2.85	2.85
RRu	6.12	6.12
P	+++	+++
Between Chi		
Between df		
Between P		N.S.
Btwn(F) P		N.S.
Btwn(R) P		N.S.

Table 1H12 - 3

IESLC - Meta-analysis of Ever/current Smoking, Age started, "Low"
All LC types, Any Product (or Cigarettes if Any not available)
Most adjusted

	Start year of study					
	<1960	1960-69	1970-79	1980-89	1990+	Total
N	12	13	8	24	4	61
NS	8	7	7	20	4	46
Wt	229.65	283.22	209.78	3303.39	28.67	4054.72
Het Chi	65.69	104.84	31.45	171.27	9.19	551.66
Het df	11	12	7	23	3	60
Het P	***	***	***	***	*	***
Fixed RR	2.53	4.83	4.51	2.51	3.09	2.72
RRl	2.22	4.30	3.94	2.43	2.14	2.64
RRu	2.88	5.43	5.16	2.60	4.45	2.80
P	+++	+++	+++	+++	+++	+++
Random RR	2.85	5.29	4.96	3.85	3.11	3.99
RRl	2.00	3.60	3.46	2.92	1.62	3.40
RRu	4.06	7.78	7.12	5.06	5.96	4.68
P	+++	+++	+++	+++	+++	+++
Between Chi						169.22
Between df						4
Between P						***
Btwn(F) P						***
Btwn(R) P						N.S.

	Study type (1)		
	CC	other	Total
N	46	15	61
NS	37	9	46
Wt	3727.66	327.06	4054.72
Het Chi	474.23	55.50	551.66
Het df	45	14	60
Het P	***	***	***
Fixed RR	2.66	3.48	2.72
RRl	2.57	3.13	2.64
RRu	2.75	3.88	2.80
P	+++	+++	+++
Random RR	4.09	3.60	3.99
RRl	3.35	2.80	3.40
RRu	5.01	4.63	4.68
P	+++	+++	+++
Between Chi			21.93
Between df			1
Between P			***
Btwn(F) P			N.S.
Btwn(R) P			N.S.

	Study type (2)			
	CC	prosp	other	Total
N	46	15		61
NS	37	9		46
Wt	3727.66	327.06		4054.72
Het Chi	474.23	55.50		551.66
Het df	45	14		60
Het P	***	***		***
Fixed RR	2.66	3.48		2.72
RRl	2.57	3.13		2.64
RRu	2.75	3.88		2.80
P	+++	+++		+++
Random RR	4.09	3.60		3.99
RRl	3.35	2.80		3.40
RRu	5.01	4.63		4.68
P	+++	+++		+++
Between Chi				21.93
Between df				1
Between P				***
Btwn(F) P				N.S.
Btwn(R) P				N.S.

Table 1H12 - 3

IESLC - Meta-analysis of Ever/current Smoking, Age started, "Low"
All LC types, Any Product (or Cigarettes if Any not available)
Most adjusted

	Study size (number of LC cases) 100-249	250-499	500-999	1000+	Total
N	15	16	13	17	61
NS	14	11	11	10	46
Wt	84.14	127.96	185.44	3657.18	4054.72
Het Chi	33.41	46.97	66.58	305.42	551.66
Het df	14	15	12	16	60
Het P	**	***	***	***	***
Fixed RR	2.70	4.26	4.93	2.60	2.72
RRl	2.18	3.58	4.27	2.51	2.64
RRu	3.35	5.06	5.69	2.68	2.80
P	+++	+++	+++	+++	+++
Random RR	2.79	4.15	5.60	3.76	3.99
RRl	1.95	3.01	3.93	2.88	3.40
RRu	3.99	5.72	7.97	4.91	4.68
P	+++	+++	+++	+++	+++
Between Chi					99.27
Between df					3
Between P					***
Btwn(F) P					**
Btwn(R) P					(*)

	Risky occupational population no	mining	othRisky	Total
N	58	2	1	61
NS	43	2	1	46
Wt	4038.82	15.15	0.75	4054.72
Het Chi	546.80	3.69	0.00	551.66
Het df	57	1	0	60
Het P	***	(*)	N.S.	***
Fixed RR	2.72	2.06	3.00	2.72
RRl	2.64	1.24	0.31	2.64
RRu	2.81	3.41	28.94	2.80
P	+++	++	N.S.	+++
Random RR	4.08	2.14	3.00	3.99
RRl	3.46	0.81	0.31	3.40
RRu	4.80	5.65	28.94	4.68
P	+++	N.S.	N.S.	+++
Between Chi				1.17
Between df				2
Between P				N.S.
Btwn(F) P				N.S.
Btwn(R) P				N.S.

	National cigarette tobacco type Virginia	blended	other	Total
N	7	42	12	61
NS	4	32	10	46
Wt	61.65	762.10	3230.97	4054.72
Het Chi	17.35	298.72	33.98	551.66
Het df	6	41	11	60
Het P	**	***	***	***
Fixed RR	6.16	4.05	2.43	2.72
RRl	4.80	3.77	2.35	2.64
RRu	7.90	4.35	2.52	2.80
P	+++	+++	+++	+++
Random RR	6.03	4.26	2.67	3.99
RRl	3.84	3.46	2.13	3.40
RRu	9.48	5.26	3.34	4.68
P	+++	+++	+++	+++
Between Chi				201.62
Between df				2
Between P				***
Btwn(F) P				***
Btwn(R) P				***

Table 1H12 - 3

IESLC - Meta-analysis of Ever/current Smoking, Age started, "Low"
All LC types, Any Product (or Cigarettes if Any not available)
Most adjusted

	Any proxy use No/nk	Yes	Total
N	46	15	61
NS	34	12	46
Wt	818.61	3236.11	4054.72
Het Chi	287.04	159.74	551.66
Het df	45	14	60
Het P	***	***	***
Fixed RR	3.74	2.51	2.72
RRl	3.49	2.42	2.64
RRu	4.01	2.59	2.80
P	+++	+++	+++
Random RR	3.65	5.25	3.99
RRl	3.02	3.54	3.40
RRu	4.42	7.78	4.68
P	+++	+++	+++
Between Chi			104.88
Between df			1
Between P			***
Btwn(F) P			***
Btwn(R) P			N.S.

	Full histological confirmation No	Yes	Total
N	44	17	61
NS	31	15	46
Wt	3745.55	309.17	4054.72
Het Chi	263.12	93.99	551.66
Het df	43	16	60
Het P	***	***	***
Fixed RR	2.55	5.82	2.72
RRl	2.47	5.21	2.64
RRu	2.63	6.51	2.80
P	+++	+++	+++
Random RR	3.48	5.56	3.99
RRl	2.96	4.10	3.40
RRu	4.08	7.52	4.68
P	+++	+++	+++
Between Chi			194.55
Between df			1
Between P			***
Btwn(F) P			***
Btwn(R) P			**

	Number of adjustment variables (1) 0	1	2+/+nk	Total
N	39	13	9	61
NS	29	10	8	47
Wt	514.10	386.87	3153.75	4054.72
Het Chi	279.21	69.98	25.21	551.66
Het df	38	12	8	60
Het P	***	***	**	***
Fixed RR	4.19	3.77	2.43	2.72
RRl	3.85	3.42	2.35	2.64
RRu	4.57	4.17	2.52	2.80
P	+++	+++	+++	+++
Random RR	4.52	3.65	2.71	3.99
RRl	3.53	2.76	1.97	3.40
RRu	5.79	4.83	3.71	4.68
P	+++	+++	+++	+++
Between Chi				177.26
Between df				2
Between P				***
Btwn(F) P				***
Btwn(R) P				*

Table 1H12 - 3

IESLC - Meta-analysis of Ever/current Smoking, Age started, "Low"
All LC types, Any Product (or Cigarettes if Any not available)
Most adjusted

	Number of adjustment variables (2) 0	1	2	3-5	6+/+nk	Total
N	39	13	8	1		61
NS	29	10	7	1		47
Wt	514.10	386.87	3149.79	3.96		4054.72
Het Chi	279.21	69.98	24.47	0.00		551.66
Het df	38	12	7	0		60
Het P	***	***	***	N.S.		***
Fixed RR	4.19	3.77	2.43	1.58		2.72
RRl	3.85	3.42	2.35	0.59		2.64
RRu	4.57	4.17	2.52	4.23		2.80
P	+++	+++	+++	N.S.		+++
Random RR	4.52	3.65	2.81	1.58		3.99
RRl	3.53	2.76	2.01	0.59		3.40
RRu	5.79	4.83	3.93	4.23		4.68
P	+++	+++	+++	N.S.		+++
Between Chi						178.00
Between df						3
Between P						***
Btwn(F) P						***
Btwn(R) P						*

	Smoking status ever	current	Total
N	42	19	61
NS	33	13	46
Wt	3722.68	332.04	4054.72
Het Chi	462.60	60.15	551.66
Het df	41	18	60
Het P	***	***	***
Fixed RR	2.65	3.61	2.72
RRl	2.57	3.24	2.64
RRu	2.74	4.01	2.80
P	+++	+++	+++
Random RR	4.00	3.88	3.99
RRl	3.25	3.09	3.40
RRu	4.93	4.89	4.68
P	+++	+++	+++
Between Chi			28.91
Between df			1
Between P			***
Btwn(F) P			(*)
Btwn(R) P			N.S.

	Product all/unsp	cig+/-ot	cig only	Total
N	18	34	9	61
NS	14	27	6	47
Wt	3246.91	721.14	86.67	4054.72
Het Chi	103.26	200.54	26.46	551.66
Het df	17	33	8	60
Het P	***	***	***	***
Fixed RR	2.42	4.44	3.37	2.72
RRl	2.34	4.13	2.73	2.64
RRu	2.51	4.78	4.15	2.80
P	+++	+++	+++	+++
Random RR	2.98	4.49	4.25	3.99
RRl	2.22	3.69	2.73	3.40
RRu	4.01	5.46	6.63	4.68
P	+++	+++	+++	+++
Between Chi				221.40
Between df				2
Between P				***
Btwn(F) P				***
Btwn(R) P				(*)

Table 1H12 - 3

IESLC - Meta-analysis of Ever/current Smoking, Age started, "Low"
All LC types, Any Product (or Cigarettes if Any not available)
Most adjusted

	Denominator nev any	nev cigs	Total
N	39	22	61
NS	28	18	46
Wt	3629.95	424.76	4054.72
Het Chi	189.21	137.01	551.66
Het df	38	21	60
Het P	***	***	***
Fixed RR	2.51	5.41	2.72
RRl	2.43	4.92	2.64
RRu	2.59	5.95	2.80
P	+++	+++	+++
Random RR	3.25	5.58	3.99
RRl	2.76	4.26	3.40
RRu	3.82	7.30	4.68
P	+++	+++	+++
Between Chi			225.44
Between df			1
Between P			***
Btwn(F) P			***
Btwn(R) P			***

	Derivation of RR/CI Orig	StdCalc	Other	Total
N	13	42	6	61
NS	11	31	5	47
Wt	126.34	778.82	3149.56	4054.72
Het Chi	46.82	303.33	14.48	551.66
Het df	12	41	5	60
Het P	***	***	*	***
Fixed RR	4.38	4.00	2.42	2.72
RRl	3.68	3.73	2.34	2.64
RRu	5.21	4.29	2.51	2.80
P	+++	+++	+++	+++
Random RR	3.94	4.12	2.89	3.99
RRl	2.68	3.35	2.16	3.40
RRu	5.79	5.06	3.87	4.68
P	+++	+++	+++	+++
Between Chi				187.03
Between df				2
Between P				***
Btwn(F) P				***
Btwn(R) P				N.S.

	Study LIU4 LIU4	others	Total
N	1	60	61
NS	1	45	46
Wt	3072.74	981.97	4054.72
Het Chi	0.00	368.83	551.66
Het df	0	59	60
Het P	N.S.	***	***
Fixed RR	2.41	3.96	2.72
RRl	2.33	3.72	2.64
RRu	2.50	4.21	2.80
P	+++	+++	+++
Random RR	2.41	4.03	3.99
RRl	2.33	3.40	3.40
RRu	2.50	4.78	4.68
P	+++	+++	+++
Between Chi			182.84
Between df			1
Between P			***
Btwn(F) P			***
Btwn(R) P			***

Table 1H12 - 4

IESLC - Meta-analysis of Ever/current Smoking, Age started, "Low"
All LC types, Any Product (or Cigarettes if Any not available)
Least adjusted

REF	NRR	X	SEX	AGEL	AGEH	RACE	YF	LC TYPE	LOC	START	ST	NLC	R	VB	P	H	AD	SM	PRODUCT	exL	exH	DENOM	De
AGUDO	501	x	f	0	0	all	-	all	Eu:wst	1989	CC	103	n	bl	n	n	0	ev	cig only	24	999	nev cigs	st
AUVINE	509		c	0	0	all	-	all	Eu:Sca	1986	CC	517	n	bl	y	n	0	ev	cig+/-ot	21	999	nev cigs	st
BARBON	515	x	m	0	0	all	-	all	Eu:wst	1979	CC	755	n	bl	y	y	0	ev	all/unsp	20	999	nev any	st
BRESLO	501		c	0	0	all	-	all	NAmer	1949	CC	518	n	bl	n	y	0	ev	cig+/-ot	25	999	nev any	st
BUFFLE	518		f	0	0	w-hi	-	all	NAmer	1976	CC	943	n	bl	y	n	0	ev	cig+/-ot	21	29	nev cigs	or
CEDERL	510		m	0	0	all	10	all	Eu:Sca	1963	pr	491	n	bl	n	n	1	cu	cig only	19	999	nev any	ot
CEDERL	515		f	0	0	all	10	all	Eu:Sca	1963	pr	491	n	bl	n	n	0	cu	cig only	19	999	nev any	st
CHEN2	518		m	0	0	all	-	all	As:Chi	1983	CC	193	n	ot	y	n	0	ev	all/unsp	20	30	nev any	st
CHEN2	523		f	0	0	all	-	all	As:Chi	1983	CC	193	n	ot	y	n	0	ev	all/unsp	20	30	nev any	st
CHIAZZ	501		m	0	0	all	-	all	NAmer	1940	CC	144	o	bl	y	n	2	ev	cig+/-ot	20	999	nev cigs	or
CHOI	523		m	0	0	all	-	all	As:oth	1985	CC	375	n	bl	n	n	0	ev	cig+/-ot	25	999	nev cigs	st
CHOI	530		f	0	0	all	-	all	As:oth	1985	CC	375	n	bl	n	n	0	ev	cig+/-ot	25	999	nev cigs	st
CPSI	801		m	35	84	all	6	all	NAmer	1959	pr	5138	n	bl	n	n	1	cu	cig+/-ot	25	999	nev any	ot
CPSI	845		f	40	74	all	6	all	NAmer	1959	pr	5138	n	bl	n	n	1	cu	cig only	25	999	nev any	ot
DAMBER	501		m	0	0	all	-	all	Eu:Sca	1972	CC	579	n	bl	y	n	0	ev	all/unsp	21	999	nev any	st
DEAN3	564		m	0	0	all	-	all	Eu:UK	1969	CC	766	n	V	y	n	0	cu	cig only	25	999	nev any	st
DEAN3	583		f	0	0	all	-	all	Eu:UK	1969	CC	766	n	V	y	n	0	cu	cig only	25	999	nev any	st
DOLL	503		m	0	0	all	-	all	Eu:UK	1948	CC	1465	n	V	n	n	0	ev	all/unsp	20	29	nev any	st
DOLL	510		f	0	0	all	-	all	Eu:UK	1948	CC	1465	n	V	n	n	0	ev	all/unsp	20	29	nev any	st
DORN	610		m	55	64	wh	8	all	NAmer	1954	pr	5097	n	bl	n	n	0	ev	cig+/-ot	25	999	nev any	st
DORN	647		m	65	74	wh	8	all	NAmer	1954	pr	5097	n	bl	n	n	0	ev	cig+/-ot	25	999	nev any	st
ENGELA	502		m	0	0	all	0	all	Eu:Sca	1964	pr	435	n	bl	n	n	0	cu	cig+/-ot	20	29	nev cigs	st
ENGELA	510		f	0	0	all	0	all	Eu:Sca	1964	pr	435	n	bl	n	n	0	cu	cig+/-ot	20	29	nev cigs	st
GAO	502	x	m	0	0	all	-	all	As:Chi	1984	CC	1405	n	ot	n	n	0	ev	cig+/-ot	20	29	nev cigs	st
GAO	512	x	f	0	0	all	-	all	As:Chi	1984	CC	1405	n	ot	n	n	0	ev	cig+/-ot	20	29	nev cigs	st
GAO2	502		m	0	0	all	-	all	As:Jap	1988	CC	282	n	bl	n	n	0	cu	cig+/-ot	20	29	nev cigs	st
GENG	528	x	f	0	0	all	-	all	As:Chi	1985	CC	292	n	ot	*	n	0	ev	cig+/-ot	21	999	nev any	st
HAENSZ	537		f	0	0	all	-	not alv	NAmer	1955	CC	158	n	bl	n	y	0	ev	cig+/-ot	25	999	nev any	st
HEGMAN	513		m	0	0	all	-	all	NAmer	1989	CC	282	n	bl	y	y	1	ev	all/unsp	20	999	nev any	or
HEGMAN	516		f	0	0	all	-	all	NAmer	1989	CC	282	n	bl	y	y	1	ev	all/unsp	26	999	nev any	or
HIRAYA	501		m	0	0	all	0	all	As:Jap	1965	pr	1917	n	bl	n	n	1	cu	cig+/-ot	20	999	nev any	st
HIRAYA	504		f	0	0	all	0	all	As:Jap	1965	pr	1917	n	bl	n	n	1	cu	cig+/-ot	20	999	nev any	st
HU2	503		c	0	0	all	-	all	As:Chi	1977	CC	523	n	ot	y	n	0	ev	cig+/-ot	20	29	nev cigs	st
JOLY	543		m	0	0	all	-	all	SCAmer	1978	CC	826	n	bl	n	n	0	ev	cig+/-ot	25	999	nev any	st
JOLY	533		f	0	0	all	-	all	SCAmer	1978	CC	826	n	bl	n	n	0	ev	cig+/-ot	25	999	nev any	st
KHUDER	506		m	0	0	all	-	all	NAmer	1985	CC	482	n	bl	n	y	0	ev	cig+/-ot	20	999	nev cigs	st
KOULUM	502		m	0	0	all	-	all	Eu:Sca	1936	CC	812	n	bl	n	n	0	ev	all/unsp	21	30	nev any	st
LETOUR	501		c	0	0	all	-	all	NAmer	1983	CC	738	n	V	y	y	0	ev	cig+/-ot	21	999	nev cigs	st
LIAW	504		c	0	0	all	0	all	As:oth	1982	pr	127	n	ot	n	n	2	cu	all/unsp	25	999	nev any	or
LIU3	501	x	m	0	0	all	-	all	As:Chi	1985	CC	110	n	ot	n	n	0	ev	all/unsp	21	999	nev any	or
LIU4	501		m	35	69	all	-	all	As:Chi	1986	CC	1000-00	n	ot	y	n	2	ev	all/unsp	25	999	nev any	ot
LUBIN	566		m	0	0	all	-	all	As:Chi	1984	CC	427	m	ot	y	n	0	ev	cig+/-ot	23	26	nev any	st
LUBIN2	1148	x	m	0	0	all	-	all	Eu:mul	1976	CC	7804	n	bl	n	y	0	ev	cig+/-ot	21	30	nev cigs	st
MATOS	556	x	m	0	0	all	-	all	SCAmer	1994	CC	200	n	bl	n	n	0	ev	cig+/-ot	20	999	nev any	st
MIGRAN	501		m	0	0	all	0	all	Eu:UK	1964	pr	259	n	V	n	n	0	cu	cig only	20	999	nev any	st
MIGRAN	511		f	0	0	all	0	all	Eu:UK	1964	pr	259	n	V	n	n	0	cu	cig only	20	999	nev any	st
MRFITR	508		m	0	0	all	0	all	NAmer	1973	pr	119	n	bl	n	n	0	cu	cig+/-ot	24	999	nev cigs	ot
PEZZOT	570		m	0	0	all	-	all	SCAmer	1987	CC	215	n	bl	n	y	0	ev	cig only	19	999	nev cigs	st
QIAO2	501	x	m	0	0	all	0	all	As:Chi	1992	pr	241	m	ot	n	n	0	ev	all/unsp	21	999	nev any	st
RACHTA	502	x	f	0	0	all	-	all	Eu:est	1991	CC	118	n	bl	n	y	0	ev	cig+/-ot	20	30	nev cigs	st
SEGI2	516	x	m	0	0	all	-	all	As:Jap	1962	CC	378	n	bl	n	n	0	cu	cig+/-ot	23	999	nev any	st
SOBUE	654		m	0	0	all	-	all	As:Jap	1986	CC	1376	n	bl	n	y	0	ev	cig+/-ot	23	999	nev cigs	st
SUZUK2	501		c	0	0	all	-	all	SCAmer	1991	CC	123	n	bl	n	y	0	ev	all/unsp	19	999	nev any	st
SVENSS	501		f	0	0	all	-	all	Eu:Sca	1983	CC	210	n	bl	n	n	0	cu	all/unsp	26	999	nev any	st
TIZZAN	507		m	0	0	all	-	all	Eu:wst	1959	CC	1358	n	bl	n	n	0	ev	all/unsp	20	30	nev any	st
TIZZAN	520		f	0	0	all	-	all	Eu:wst	1959	CC	1358	n	bl	n	n	0	ev	all/unsp	20	30	nev any	st
WAKAI	502		m	0	0	all	-	all	As:Jap	1988	CC	333	n	bl	n	y	0	cu	cig+/-ot	20	29	nev any	st
WU	517	x	f	0	0	wh	-	q+a	NAmer	1981	CC	220	n	bl	n	y	0	cu	all/unsp	25	999	nev any	st
WYNDE6	759		m	0	0	wh	-	q+s+a	NAmer	1969	CC	4423	n	bl	n	y	0	ev	cig+/-ot	21	999	nev cigs	st
WYNDE6	767		f	0	0	wh	-	q+s+a	NAmer	1969	CC	4423	n	bl	n	y	0	ev	cig+/-ot	21	999	nev cigs	st
ZHENG	564		m	0	0	all	-	all	As:Chi	1982	CC	540	n	ot	*	y	0	ev	cig+/-ot	20	29	nev cigs	st

Cigarette type is all/unspec for all RRs
except for the following:

REF	NRR	CIGTYPE
DEAN3	564	MC only
DEAN3	583	MC only

Table 1H12 - 5

IESLC - Meta-analysis of Ever/current Smoking, Age started, "Low"
All LC types, Any Product (or Cigarettes if Any not available)
Least adjusted

REF	NRR	SEX	AD	Number Exposed Case	Cont	Non-exposed Case	Cont	RR	95.00%CI
AGUDO	501	f	0	7	12	80	183	1.33	(0.51- 3.51)
AUVINE	509	c	0	76	27	44	229	14.65	(8.50- 25.26)
BARBON	515	m	0	200	207	22	188	8.26	(5.10- 13.38)
BRESLO	501	c	0	32	35	19	56	2.69	(1.33- 5.47)
BUFFLE	518	f	0	47	34	12	112	12.90	(6.15- 27.07)
*CEDERL	510	m	1	11	-	7	-	6.50	(2.52- 16.74)
*CEDERL	515	f	0	6	2806	19	17679	1.99	(0.80- 4.98)
Subtotal CEDERL								3.53	(1.83- 6.82)
CHEN2	518	m	0	29	25	9	33	4.25	(1.71- 10.57)
CHEN2	523	f	0	9	8	25	33	1.49	(0.50- 4.39)
Subtotal CHEN2								2.75	(1.37- 5.53)
CHIAZZ	501	m	2	-	-	4	-	3.00	(0.31- 28.84)
CHOI	523	m	0	36	77	13	95	3.42	(1.69- 6.89)
CHOI	530	f	0	15	25	76	164	1.29	(0.65- 2.60)
Subtotal CHOI								2.09	(1.28- 3.43)
*CPSI	801	m	1	42	-	83	-	4.08	(2.81- 5.91)
*CPSI	845	f	1	51	-	166	-	2.25	(1.64- 3.08)
Subtotal CPSI								2.89	(2.27- 3.67)
DAMBER	501	m	0	70	76	42	208	4.56	(2.87- 7.26)
DEAN3	564	m	0	24	75	24	510	6.80	(3.67- 12.58)
DEAN3	583	f	0	27	274	41	1538	3.70	(2.24- 6.11)
Subtotal DEAN3								4.72	(3.20- 6.96)
DOLL	503	m	0	251	264	7	61	8.29	(3.72- 18.46)
DOLL	510	f	0	23	15	40	59	2.26	(1.05- 4.86)
Subtotal DOLL								4.20	(2.42- 7.30)
*DORN	610	m	0	37	73050	25	213858	4.33	(2.61- 7.20)
*DORN	647	m	0	90	74464	49	171211	4.22	(2.98- 5.98)
Subtotal DORN								4.26	(3.20- 5.67)
*ENGELA	502	m	0	50	30195	27	58716	3.60	(2.26- 5.75)
*ENGELA	510	f	0	36	29605	31	207789	8.15	(5.04- 13.17)
Subtotal ENGELA								5.36	(3.83- 7.49)
GAO	502	m	0	363	262	62	202	4.51	(3.26- 6.25)
GAO	512	f	0	87	41	435	605	2.95	(2.00- 4.36)
Subtotal GAO								3.79	(2.95- 4.87)
GAO2	502	m	0	127	85	13	56	6.44	(3.32- 12.49)
GENG	528	f	0	28	31	54	93	1.56	(0.84- 2.87)
HAENSZ	537	f	0	44	66	81	236	1.94	(1.23- 3.07)
HEGMAN	513	m	1	26	-	-	-	9.40	(4.60- 19.30)
HEGMAN	516	f	1	2	-	-	-	4.80	(1.00- 22.10)
Subtotal HEGMAN								8.35	(4.36- 16.00)
*HIRAYA	501	m	1	-	-	-	-	4.35	(3.51- 5.39)
*HIRAYA	504	f	1	-	-	-	-	2.46	(1.93- 3.13)
Subtotal HIRAYA								3.38	(2.88- 3.97)
HU2	503	c	0	229	159	121	213	2.54	(1.88- 3.43)
JOLY	543	m	0	18	70	12	218	4.67	(2.14- 10.18)
JOLY	533	f	0	23	41	52	283	3.05	(1.69- 5.51)
Subtotal JOLY								3.57	(2.23- 5.71)
KHUDER	506	m	0	72	152	23	309	6.36	(3.83- 10.58)
KOULUM	502	m	0	60	67	5	54	9.67	(3.63- 25.77)
LETOUR	501	c	0	188	160	24	224	10.97	(6.85- 17.56)
*LIAW	504	c	2	-	-	-	-	1.50	(0.70- 3.30)
LIU3	501	m	0	20	80	4	19	1.19	(0.36- 3.88)
LIU4	501	m	2	-	-	-	-	2.41	(2.32- 2.49)
LUBIN	566	m	0	65	146	9	72	3.56	(1.68- 7.56)
LUBIN2	1148	m	0	564	1069	185	1878	5.36	(4.46- 6.43)
MATOS	556	m	0	28	73	11	110	3.84	(1.80- 8.18)
*MIGRAN	501	m	0	24	668	4	867	7.79	(2.72- 22.34)
*MIGRAN	511	f	0	11	1315	4	3814	7.98	(2.54- 25.01)
Subtotal MIGRAN								7.87	(3.63- 17.08)
*MRFITR	508	m	0	3	544	0	1859	23.91~	(1.24- 462.09)
PEZZOT	570	m	0	41	105	4	116	11.32	(3.92- 32.69)
*QIAO2	501	m	0	52	1947	10	709	1.89	(0.97- 3.71)
RACHTA	502	f	0	25	18	33	98	4.12	(2.00- 8.50)
SEGI2	516	m	0	49	155	8	53	2.09	(0.93- 4.71)
SOBUE	654	m	0	110	121	29	126	3.95	(2.45- 6.38)
SUZUK2	501	c	0	16	22	11	53	3.50	(1.40- 8.75)
SVENSS	501	f	0	32	18	38	120	5.61	(2.84- 11.12)
TIZZAN	507	m	0	313	330	180	305	1.61	(1.26- 2.04)
TIZZAN	520	f	0	12	21	117	114	0.56	(0.26- 1.18)
Subtotal TIZZAN								1.46	(1.16- 1.83)
WAKAI	502	m	0	130	183	10	65	4.62	(2.29- 9.32)

Table 1H12 - 5

IESLC - Meta-analysis of Ever/current Smoking, Age started, "Low"
All LC types, Any Product (or Cigarettes if Any not available)
Least adjusted

REF	NRR	SEX	AD	Number Exposed Case	Cont	Non-exposed Case	Cont	RR	95.00%CI
WU	517	f	0	14	19	31	92	2.19	(0.98- 4.87)
WYNDE6	759	m	0	111	92	51	589	13.93	(9.36- 20.74)
WYNDE6	767	f	0	127	90	73	673	13.01	(9.06- 18.69)
Subtotal WYNDE6								13.42	(10.27- 17.54)
ZHENG	564	m	0	145	109	33	94	3.79	(2.37- 6.05)
Partial Totals				4328	219533	2592	687041		

*prospective study ~ With 0.5 adjustment for zero

REF	NRR	SEX	AD	Ys	Ws	Qs	Ps
AGUDO	501	f	0	0.29	4.10	2.08	0.5594
AUVINE	509	c	0	2.68	12.94	36.65	0.0000
BARBON	515	m	0	2.11	16.50	20.32	0.0000
BRESLO	501	c	0	0.99	7.67	0.00	0.0060
BUFFLE	518	f	0	2.56	7.00	16.94	0.0000
*CEDERL	510	m	1	1.87	4.29	3.25	0.0001
*CEDERL	515	f	0	0.69	4.57	0.45	0.1414
Subtotal CEDERL				1.26	8.85	3.70	
CHEN2	518	m	0	1.45	4.63	0.92	0.0018
CHEN2	523	f	0	0.40	3.26	1.20	0.4750
Subtotal CHEN2				1.01	7.90	2.12	
CHIAZZ	501	m	2	1.10	0.75	0.01	0.3421
CHOI	523	m	0	1.23	7.80	0.40	0.0006
CHOI	530	f	0	0.26	7.94	4.38	0.4667
Subtotal CHOI				0.74	15.74	4.79	
*CPSI	801	m	1	1.41	27.80	4.55	0.0000
*CPSI	845	f	1	0.81	38.69	1.40	0.0000
Subtotal CPSI				1.06	66.49	5.96	
DAMBER	501	m	0	1.52	17.84	4.75	0.0000
DEAN3	564	m	0	1.92	10.14	8.50	0.0000
DEAN3	583	f	0	1.31	15.21	1.42	0.0000
Subtotal DEAN3				1.55	25.35	9.92	
DOLL	503	m	0	2.11	5.99	7.42	0.0000
DOLL	510	f	0	0.82	6.57	0.23	0.0364
Subtotal DOLL				1.43	12.56	7.64	
*DORN	610	m	0	1.47	14.92	3.22	0.0000
*DORN	647	m	0	1.44	31.75	6.12	0.0000
Subtotal DORN				1.45	46.67	9.35	
*ENGELA	502	m	0	1.28	17.55	1.37	0.0000
*ENGELA	510	f	0	2.10	16.67	20.05	0.0000
Subtotal ENGELA				1.68	34.22	21.42	
GAO	502	m	0	1.51	36.16	9.25	0.0000
GAO	512	f	0	1.08	25.10	0.16	0.0000
Subtotal GAO				1.33	61.27	9.42	
GAO2	502	m	0	1.86	8.74	6.47	0.0000
GENG	528	f	0	0.44	10.28	3.22	0.1565
HAENSZ	537	f	0	0.66	18.36	2.09	0.0044
HEGMAN	513	m	1	2.24	7.47	11.48	0.0000
HEGMAN	516	f	1	1.57	1.60	0.52	0.0470
Subtotal HEGMAN				2.12	9.08	11.99	
*HIRAYA	501	m	1	1.47	83.52	18.36	0.0000
*HIRAYA	504	f	1	0.90	65.73	0.67	0.0000
Subtotal HIRAYA				1.22	149.25	19.03	
HU2	503	c	0	0.93	42.35	0.21	0.0000
JOLY	543	m	0	1.54	6.34	1.85	0.0001
JOLY	533	f	0	1.12	11.03	0.15	0.0002
Subtotal JOLY				1.27	17.37	1.99	
KHUDER	506	m	0	1.85	14.88	10.74	0.0000
KOULUM	502	m	0	2.27	4.00	6.43	0.0000
LETOUR	501	c	0	2.39	17.33	33.65	0.0000
*LIAW	504	c	2	0.41	6.39	2.27	0.3054
LIU3	501	m	0	0.17	2.74	1.88	0.7761
LIU4	501	m	2	0.88	3072.74	45.53	0.0000
LUBIN	566	m	0	1.27	6.79	0.49	0.0009
LUBIN2	1148	m	0	1.68	115.66	52.98	0.0000
MATOS	556	m	0	1.34	6.69	0.79	0.0005
*MIGRAN	501	m	0	2.05	3.46	3.82	0.0001
*MIGRAN	511	f	0	2.08	2.94	3.40	0.0004
Subtotal MIGRAN				2.06	6.40	7.22	
*MRFITR	508	m	0	3.17	0.44	2.07	0.0357
PEZZOT	570	m	0	2.43	3.42	6.95	0.0000

Table 1H12 - 5

IESLC - Meta-analysis of Ever/current Smoking, Age started, "Low"
All LC types, Any Product (or Cigarettes if Any not available)
Least adjusted

REF	NRR	SEX	AD	Ys	Ws	Qs	Ps
*QIAO2	501	m	0	0.64	8.52	1.12	0.0623
RACHTA	502	f	0	1.42	7.35	1.27	0.0001
SEGI2	516	m	0	0.74	5.86	0.40	0.0736
SOBUE	654	m	0	1.37	16.73	2.32	0.0000
SUZUK2	501	c	0	1.25	4.59	0.29	0.0072
SVENSS	501	f	0	1.73	8.23	4.31	0.0000
TIZZAN	507	m	0	0.47	66.40	18.43	0.0001
TIZZAN	520	f	0	-0.59	6.74	16.99	0.1283
Subtotal TIZZAN				0.38	73.15	35.42	
WAKAI	502	m	0	1.53	7.78	2.17	0.0000
WU	517	f	0	0.78	5.98	0.29	0.0557
WYNDE6	759	m	0	2.63	24.28	64.75	0.0000
WYNDE6	767	f	0	2.57	29.27	71.62	0.0000
Subtotal WYNDE6				2.60	53.55	136.36	
ZHENG	564	m	0	1.33	17.54	1.92	0.0000

	N	61
	NS	46
	Wt	4068.02
Het	Chi	556.97
Het	df	60
Het	P	***
Fixed	RR	2.72
	RRl	2.64
	RRu	2.81
	P	+++
Random	RR	3.97
	RRl	3.39
	RRu	4.66
	P	+++
Asymm	P	***

Table 1H12 - 6

IESLC - Meta-analysis of Ever/current Smoking, Age started, "Low"
All LC types, Any Product (or Cigarettes if Any not available)
Least adjusted

	Sex combined	male	female	Total
N	6	34	21	61
NS	6	33	21	60
Wt	91.27	3680.12	296.64	4068.02
Het Chi	55.38	329.10	146.14	556.97
Het df	5	33	20	60
Het P	***	***	***	***
Fixed RR	4.23	2.66	3.16	2.72
RRl	3.44	2.58	2.82	2.64
RRu	5.19	2.75	3.54	2.81
P	+++	+++	+++	+++
Random RR	4.33	4.59	3.01	3.97
RRl	2.05	3.72	2.16	3.39
RRu	9.17	5.65	4.19	4.66
P	+++	+++	+++	+++
Between Chi				26.35
Between df				2
Between P				***
Btwn(F) P				N.S.
Btwn(R) P				N.S.

Table 1H12 - 7

IESLC - Meta-analysis of Ever/current Smoking, Age started, "Low"
All LC types, Any Product (or Cigarettes if Any not available)
Excluded studies (and stage at which they were excluded)

1	BECHER BLOT1 BROWN3 CARPEN CHYOU DARBY DOLL2 GARCIA GRAHAM GURSEL HAMMO2 JAHN JAIN LAUSSM PRESCO QIAO TVERDA WIGLE WYNDE3
2	AKIBA AMANDU AMES AXELSS BENSHL BEST BOUCHA BOUCOT BROSS CHEN CPSII DEAN2 DESTEF DORGAN DOSEME FAN GARSHI GER GILLIS HAMMON HUMBLE JUSSAW KAISE2 KATSOU KAUFMA KOO KREUZE LEVIN MCCONN NOTAN2 OSANN2 PEZZO2 PISANI RESTRE SADOWS VUTUC WANG2 WATSON WU2 WUWILL WYNDE2 WYNDE8 XU ZHOU
3	GUO MCDUFF SPITZ STASZE ZHANG
4	LUO
5	CORREA HOLE YUAN
7	BOFFET WYNDE7
10	ALDERS SPEIZE
14	ARMADA HU JEDRYC LIU5 PERNU
15	BENHAM

Table 1H12 - 8
Potentially overlapping studies

REF	REFGP	PRINC	.	OVERLAP/LINK
LUBIN2	LUBIN2	1		Lubin-combined
MRFITR	MRFIT	2		Subset of MRFIT
WYNDE6	WYNDE6	1		WYNDE5/6/7/8
CPSI	CPSI	1		CPSI overall
LUBIN	XIANGZ	2		LUBIN/XIANGZ/QIAO

Table 1H12 - 9
Most adjusted - insufficient data for meta-analysis

REF	NRR	SEX	AGEL	AGEH	RACE	YF	LC TYPE	LOC	START	ST	NLC	R	VB	P	H	AD	SM	PRODUCT	exL	exH	DENOM	De
CORREA	535	c	0	0	all	-	all	NAmer	1979	CC	1359	n	bl	y	n	2	ev	cig+/-ot	21	999	nev cigs	or
JEDRYC	618	f	0	0	all	-	all	Eu:est	1980	CC	1630	n	bl	y	n	0	ev	cig+/-ot	23	999	nev any	st

REF	NRR	RR	SIG	RRDATA comment
CORREA	535	8.30		0
JEDRYC	618	*		0

Table 1H13 -

IESLC - Meta-analysis of Ever/current Smoking, Age started, "Mid"
All LC types, Any Product (or Cigarettes if Any not available)

This analysis is restricted to results for:
1) Ever/current smokers
2) Results by Age started
3) Categorical results by Age started
4) All LC types (or near equivalent)
5) Results complete enough for use in metaanalysis

Within each study, results are then selected (in the following order of preference, within each sex) for:
6) SMKSTA: ever, current
7) PRODUCT: all/unspec, cigarettes regardless of other products, cigarettes only
8) CIGTYPE: all/unspecified, MC regardless of HR, MC only
9) (not applicable)
10) DENOM: never smoked anything, never smoked cigarettes, never any + low, never cigs + low
11) Followup period (YF, prospective studies): whole study (coded as 0) or longest available
12) LCtype: all or nearest available, at least Squamous and Adeno. (q = squamous, s = small,
l = large, a = adeno, mix = mixed, alv = alveolar)
13) Race: all or nearest available, otherwise by race (wh or w = white, bl or b = black, hi = hispanic
ch = chinese, jap = japanese, haw = hawaiian, w+o = white + oriental, sca = scandinavian, as = asian)
14) Age started "mid" in key scheme 1 (key value 18, maximum range 15-25)
15) For overlapping studies: principal rather than subsidiary studies
Finally by Age: whole study (coded as 0) if available, otherwise by widest available age group
and then for single sex results (m, f) in preference to results for both sexes combined (c).

Results adjusted (AD) for the most potential confounders are then chosen in Sections -1 to -3
and results adjusted for the least confounders in Sections -4 to -6. (Those least adjusted results which
actually differ from the most adjusted are marked 'x' in column X in Section -4)

Section -7 shows excluded studies, together with the stage (as above) at which no qualifying
results were found.

Section -8 lists the potentially overlapping studies which have been included (1=principal, 2=subsidiary).

Section -9 lists any results which would have been included in preference except that they had data not complete
enough for use in meta-analysis, with their significance (yes/no), if known, and any further comment as entered
on the database. It also lists as "gap" any categories for which no data were presented by the original authors.

In addition to those mentioned above, the following fields, levels and abbreviations are used:
* or nk = not known, n = no, y = yes, ot = other
ev = ever, cu = current, nev = never
all/unspec = all or unspecified, cig+/-ot = cigarettes irrespective of other products (cigar, pipe etc)
MC = manufactured cigarettes, HR = hand-rolled cigarettes
exL, exH = range of exposure (low and high) in the smoking group, in terms of Age started
REF: 6-character study reference
NRR: number of the RR on the database within the study
ST : study type (CC = case control, pr or prosp = prospective)
NLC: number of lung cancer cases in whole study
R : risky occupational population (n = no, m = mining, o = other risky)
VB : national cigarette type (V = at least 75% Virginia, bl = at least 75% blended, ot = other)
P : any proxy use
H : full histological confirmation
De : derivation of RR/CI (or = original, st = standard method, ot = other method of estimation)

Table 1H13 - 1

IESLC - Meta-analysis of Ever/current Smoking, Age started, "Mid"
All LC types, Any Product (or Cigarettes if Any not available)
Most adjusted

REF	NRR	SEX	AGEL	AGEH	RACE	YF	LC TYPE	LOC	START	ST	NLC	R	VB	P	H	AD	SM	PRODUCT	exL	exH	DENOM	De
AUVINE	510	c	0	0	all	-	all	Eu:Sca	1986	CC	517	n	bl	y	n	0	ev	cig+/-ot	16	20	nev cigs	st
BARBON	521	m	0	0	all	-	all	Eu:wst	1979	CC	755	n	bl	y	y	1	ev	all/unsp	15	19	nev any	or
BRESLO	502	c	0	0	all	-	all	NAmer	1949	CC	518	n	bl	n	y	0	ev	cig+/-ot	15	24	nev any	st
BUFFLE	520	f	0	0	w-hi	-	all	NAmer	1976	CC	943	n	bl	y	n	0	ev	cig+/-ot	17	18	nev cigs	ot
CEDERL	511	m	0	0	all	10	all	Eu:Sca	1963	pr	491	n	bl	n	n	1	cu	cig only	17	18	nev any	ot
CEDERL	516	f	0	0	all	10	all	Eu:Sca	1963	pr	491	n	bl	n	n	0	cu	cig only	17	18	nev any	st
CHOI	525	m	0	0	all	-	all	As:oth	1985	CC	375	n	bl	n	n	0	ev	cig+/-ot	15	19	nev cigs	st
CPSI	803	m	35	84	all	6	all	NAmer	1959	pr	5138	n	bl	n	n	1	cu	cig+/-ot	15	19	nev any	ot
CPSI	847	f	40	74	all	6	all	NAmer	1959	pr	5138	n	bl	n	n	1	cu	cig only	15	19	nev any	ot
DAMBER	502	m	0	0	all	-	all	Eu:Sca	1972	CC	579	n	bl	y	n	0	ev	all/unsp	16	20	nev any	st
DEAN3	566	m	0	0	all	-	all	Eu:UK	1969	CC	766	n	V	y	n	0	cu	cig only	15	19	nev any	st
DEAN3	585	f	0	0	all	-	all	Eu:UK	1969	CC	766	n	V	y	n	0	cu	cig only	15	19	nev any	st
DORN	612	m	55	64	wh	8	all	NAmer	1954	pr	5097	n	bl	n	n	0	ev	cig+/-ot	15	19	nev any	st
DORN	649	m	65	74	wh	8	all	NAmer	1954	pr	5097	n	bl	n	n	0	ev	cig+/-ot	15	19	nev any	st
GENG	534	f	0	0	all	-	all	As:Chi	1985	CC	292	n	ot	*	n	1	ev	cig+/-ot	16	20	nev any	st
JEDRYC	607	m	0	0	all	-	all	Eu:est	1980	CC	1630	n	bl	y	n	0	ev	cig+/-ot	17	18	nev any	st
JOLY	544	m	0	0	all	-	all	SCAmer	1978	CC	826	n	bl	n	n	0	ev	cig+/-ot	15	24	nev any	st
JOLY	534	f	0	0	all	-	all	SCAmer	1978	CC	826	n	bl	n	n	0	ev	cig+/-ot	15	24	nev any	st
KHUDER	507	m	0	0	all	-	all	NAmer	1985	CC	482	n	bl	n	y	0	ev	cig+/-ot	16	19	nev cigs	st
KOULUM	503	m	0	0	all	-	all	Eu:Sca	1936	CC	812	n	bl	n	n	0	ev	all/unsp	16	20	nev any	st
LETOUR	502	c	0	0	all	-	all	NAmer	1983	CC	738	n	V	y	y	0	ev	cig+/-ot	15	20	nev cigs	st
LUBIN2	1158	m	0	0	all	-	all	Eu:mul	1976	CC	7804	n	bl	n	y	1	ev	cig+/-ot	17	20	nev cigs	st
MATOS	577	m	0	0	all	-	all	SCAmer	1994	CC	200	n	bl	n	n	2	ev	cig+/-ot	15	19	nev any	or
MIGRAN	503	m	0	0	all	0	all	Eu:UK	1964	pr	259	n	V	n	n	0	cu	cig only	16	19	nev any	st
MIGRAN	513	f	0	0	all	0	all	Eu:UK	1964	pr	259	n	V	n	n	0	cu	cig only	16	19	nev any	st
MRFITR	511	m	0	0	all	0	all	NAmer	1973	pr	119	n	bl	n	n	0	cu	cig+/-ot	18	19	nev cigs	ot
QIAO2	507	m	0	0	all	0	all	As:Chi	1992	pr	241	m	ot	n	n	1	ev	all/unsp	17	20	nev any	or
SOBUE	655	m	0	0	all	-	all	As:Jap	1986	CC	1376	n	bl	n	y	0	ev	cig+/-ot	18	22	nev cigs	st
WYNDE6	760	m	0	0	wh	-	q+s+a	NAmer	1969	CC	4423	n	bl	n	y	0	ev	cig+/-ot	18	20	nev cigs	st
WYNDE6	768	f	0	0	wh	-	q+s+a	NAmer	1969	CC	4423	n	bl	n	y	0	ev	cig+/-ot	18	20	nev cigs	st

Cigarette type is all/unspec for all RRs
except for the following:

REF	NRR	CIGTYPE
DEAN3	566	MC only
DEAN3	585	MC only

Table 1H13 - 2

IESLC - Meta-analysis of Ever/current Smoking, Age started, "Mid"
All LC types, Any Product (or Cigarettes if Any not available)
Most adjusted

REF	NRR	SEX	AD	Number Exposed Case	Cont	Non-exposed Case	Cont	RR	95.00%CI
AUVINE	510	c	0	135	47	44	229	14.95	(9.41- 23.75)
BARBON	521	m	1	395	-	22	-	9.90	(6.20- 15.80)
BRESLO	502	c	0	286	243	19	56	3.47	(2.01- 6.00)
BUFFLE	520	f	0	53	33	12	112	14.99	(7.17- 31.33)
*CEDERL	511	m	1	10	-	7	-	9.80	(3.74- 25.69)
*CEDERL	516	f	0	2	1009	19	17679	1.84	(0.43- 7.91)
Subtotal CEDERL								5.89	(2.64- 13.16)
CHOI	525	m	0	79	138	13	95	4.18	(2.20- 7.95)
*CPSI	803	m	1	588	-	83	-	14.69	(11.68- 18.49)
*CPSI	847	f	1	52	-	166	-	5.00	(3.66- 6.83)
Subtotal CPSI								10.06	(8.36- 12.10)
DAMBER	502	m	0	261	190	42	208	6.80	(4.65- 9.95)
DEAN3	566	m	0	160	485	24	510	7.01	(4.48- 10.96)
DEAN3	585	f	0	39	504	41	1538	2.90	(1.85- 4.55)
Subtotal DEAN3								4.52	(3.30- 6.21)
*DORN	612	m	0	342	213156	25	213858	13.73	(9.14- 20.60)
*DORN	649	m	0	306	118234	49	171211	9.04	(6.69- 12.23)
Subtotal DORN								10.49	(8.23- 13.36)
GENG	534	f	1	39	-	54	-	2.95	(1.57- 5.52)
JEDRYC	607	m	0	239	146	49	219	7.32	(5.04- 10.61)
JOLY	544	m	0	217	357	12	218	11.04	(6.03- 20.22)
JOLY	534	f	0	67	47	52	283	7.76	(4.82- 12.49)
Subtotal JOLY								8.88	(6.11- 12.91)
KHUDER	507	m	0	161	338	23	309	6.40	(4.03- 10.17)
KOULUM	503	m	0	267	103	5	54	28.00	(10.89- 71.96)
LETOUR	502	c	0	309	241	24	224	11.97	(7.60- 18.83)
LUBIN2	1158	m	1	1796	-	185	-	5.43	(4.59- 6.41)
MATOS	577	m	2	91	-	11	-	7.80	(4.00- 15.50)
*MIGRAN	503	m	0	59	1845	4	867	6.93	(2.53- 19.02)
*MIGRAN	513	f	0	9	1035	4	3814	8.29	(2.56- 26.87)
Subtotal MIGRAN								7.48	(3.48- 16.09)
*MRFITR	511	m	0	25	1876	0	1859	50.54~	(3.08- 829.51)
*QIAO2	507	m	1	75	-	10	-	1.47	(0.76- 2.84)
SOBUE	655	m	0	776	772	29	126	4.37	(2.88- 6.62)
WYNDE6	760	m	0	223	139	51	589	18.53	(12.98- 26.45)
WYNDE6	768	f	0	200	94	73	673	19.62	(13.90- 27.67)
Subtotal WYNDE6								19.08	(14.90- 24.44)
Partial Totals				7261	341032	1152	414731		

*prospective study ~ With 0.5 adjustment for zero

REF	NRR	SEX	AD	Ys	Ws	Qs	Ps
AUVINE	510	c	0	2.70	17.93	7.32	0.0000
BARBON	521	m	1	2.29	17.56	0.90	0.0000
BRESLO	502	c	0	1.24	12.80	8.65	0.0000
BUFFLE	520	f	0	2.71	7.07	2.91	0.0000
*CEDERL	511	m	1	2.28	4.14	0.19	0.0000
*CEDERL	516	f	0	0.61	1.81	3.83	0.4098
Subtotal CEDERL				1.77	5.95	4.02	
CHOI	525	m	0	1.43	9.31	3.75	0.0000
*CPSI	803	m	1	2.69	72.82	28.13	0.0000
*CPSI	847	f	1	1.61	39.48	8.22	0.0000
Subtotal CPSI				2.31	112.30	36.35	
DAMBER	502	m	0	1.92	26.52	0.58	0.0000
DEAN3	566	m	0	1.95	19.25	0.27	0.0000
DEAN3	585	f	0	1.07	18.99	18.99	0.0000
Subtotal DEAN3				1.51	38.24	19.26	
*DORN	612	m	0	2.62	23.30	7.14	0.0000
*DORN	649	m	0	2.20	42.26	0.79	0.0000
Subtotal DORN				2.35	65.56	7.93	
GENG	534	f	1	1.08	9.72	9.41	0.0007
JEDRYC	607	m	0	1.99	27.77	0.16	0.0000
JOLY	544	m	0	2.40	10.49	1.18	0.0000
JOLY	534	f	0	2.05	16.96	0.00	0.0000
Subtotal JOLY				2.18	27.45	1.19	
KHUDER	507	m	0	1.86	17.89	0.78	0.0000
KOULUM	503	m	0	3.33	4.31	6.91	0.0000
LETOUR	502	c	0	2.48	18.69	3.24	0.0000
LUBIN2	1158	m	1	1.69	137.76	19.24	0.0000
MATOS	577	m	2	2.05	8.37	0.00	0.0000
*MIGRAN	503	m	0	1.94	3.77	0.06	0.0002

Table 1H13 - 2

IESLC - Meta-analysis of Ever/current Smoking, Age started, "Mid"
All LC types, Any Product (or Cigarettes if Any not available)
Most adjusted

REF	NRR	SEX	AD	Ys	Ws	Qs	Ps
*MIGRAN	513	f	0	2.12	2.78	0.01	0.0004
Subtotal MIGRAN				2.01	6.55	0.07	
*MRFITR	511	m	0	3.92	0.49	1.69	0.0060
*QIAO2	507	m	1	0.39	8.84	24.97	0.2520
SOBUE	655	m	0	1.47	22.22	7.77	0.0000
WYNDE6	760	m	0	2.92	30.32	22.09	0.0000
WYNDE6	768	f	0	2.98	32.44	26.91	0.0000
Subtotal WYNDE6				2.95	62.76	49.00	

	N	30
	NS	23
	Wt	666.08
Het	Chi	216.11
Het	df	29
Het	P	***
Fixed	RR	7.89
	RRl	7.31
	RRu	8.51
	P	+++
Random	RR	7.67
	RRl	6.12
	RRu	9.62
	P	+++
Asymm	P	N.S.

Table 1H13 - 3

IESLC - Meta-analysis of Ever/current Smoking, Age started, "Mid"
All LC types, Any Product (or Cigarettes if Any not available)
Most adjusted

	Sex			
	combined	male	female	Total
N	3	19	8	30
NS	3	18	8	29
Wt	49.42	487.41	129.25	666.08
Het Chi	17.67	126.54	68.47	216.11
Het df	2	18	7	29
Het P	***	***	***	***
Fixed RR	9.41	8.00	7.01	7.89
RRl	7.12	7.32	5.90	7.31
RRu	12.44	8.74	8.33	8.51
P	+++	+++	+++	+++
Random RR	8.64	8.08	6.23	7.67
RRl	3.75	6.21	3.44	6.12
RRu	19.90	10.53	11.26	9.62
P	+++	+++	+++	+++
Between Chi				3.43
Between df				2
Between P				N.S.
Btwn(F) P				N.S.
Btwn(R) P				N.S.

	Lung cancer type		
	all	other	Total
N	28	2	30
NS	22	1	23
Wt	603.32	62.76	666.08
Het Chi	162.02	0.05	216.11
Het df	27	1	29
Het P	***	N.S.	***
Fixed RR	7.20	19.08	7.89
RRl	6.65	14.90	7.31
RRu	7.80	24.44	8.51
P	+++	+++	+++
Random RR	7.09	19.08	7.67
RRl	5.71	14.90	6.12
RRu	8.81	24.44	9.62
P	+++	+++	+++
Between Chi			54.04
Between df			1
Between P			***
Btwn(F) P			**
Btwn(R) P			***

	Location								
	NAmer	UK	Scand	othEur	China	Japan	othAs	other	Total
N	11	4	5	3	2	1	1	3	30
NS	8	2	4	3	2	1	1	2	23
Wt	297.57	44.79	54.71	183.09	18.56	22.22	9.31	35.82	666.08
Het Chi	76.74	8.90	16.53	6.87	2.25	0.00	0.00	0.92	216.11
Het df	10	3	4	2	1	0	0	2	29
Het P	***	*	**	*	N.S.	N.S.	N.S.	N.S.	***
Fixed RR	11.05	4.87	9.69	6.02	2.12	4.37	4.18	8.61	7.89
RRl	9.87	3.63	7.44	5.21	1.34	2.88	2.20	6.21	7.31
RRu	12.38	6.53	12.63	6.96	3.34	6.62	7.95	11.95	8.51
P	+++	+++	+++	+++	++	+++	+++	+++	+++
Random RR	10.57	5.36	9.85	6.95	2.10	4.37	4.18	8.61	7.67
RRl	7.52	3.00	5.21	4.90	1.06	2.88	2.20	6.21	6.12
RRu	14.86	9.56	18.62	9.86	4.15	6.62	7.95	11.95	9.62
P	+++	+++	+++	+++	+	+++	+++	+++	+++
Between Chi									103.91
Between df									7
Between P									***
Btwn(F) P									*
Btwn(R) P									***

Table 1H13 - 3

IESLC - Meta-analysis of Ever/current Smoking, Age started, "Mid"
All LC types, Any Product (or Cigarettes if Any not available)
Most adjusted

Detailed Country in "other Europe"

	multi	Germany	othWest	East	Balkans	Total
N	1		1	1		3
NS	1		1	1		3
Wt	137.76		17.56	27.77		183.09
Het Chi	0.00		0.00	0.00		6.87
Het df	0		0	0		2
Het P	N.S.		N.S.	N.S.		*
Fixed RR	5.43		9.90	7.32		6.02
RRl	4.59		6.20	5.04		5.21
RRu	6.42		15.80	10.61		6.96
P	+++		+++	+++		+++
Random RR	5.43		9.90	7.32		6.95
RRl	4.59		6.20	5.04		4.90
RRu	6.42		15.80	10.61		9.86
P	+++		+++	+++		+++
Between Chi						6.87
Between df						2
Between P						*
Btwn(F) P						N.S.
Btwn(R) P						*

Detailed Country in "other Asia"

	India	HongKong	other	Total
N			1	1
NS			1	1
Wt			9.31	9.31
Het Chi			0.00	0.00
Het df			0	0
Het P			N.S.	N.S.
Fixed RR			4.18	4.18
RRl			2.20	2.20
RRu			7.95	7.95
P			+++	+++
Random RR			4.18	4.18
RRl			2.20	2.20
RRu			7.95	7.95
P			+++	+++
Between Chi				
Between df				
Between P				N.S.
Btwn(F) P				N.S.
Btwn(R) P				N.S.

Detailed other continent

	SCAmer	Total
N	3	3
NS	2	2
Wt	35.82	35.82
Het Chi	0.92	0.92
Het df	2	2
Het P	N.S.	N.S.
Fixed RR	8.61	8.61
RRl	6.21	6.21
RRu	11.95	11.95
P	+++	+++
Random RR	8.61	8.61
RRl	6.21	6.21
RRu	11.95	11.95
P	+++	+++
Between Chi		
Between df		
Between P		N.S.
Btwn(F) P		N.S.
Btwn(R) P		N.S.

Table 1H13 - 3

IESLC - Meta-analysis of Ever/current Smoking, Age started, "Mid"
All LC types, Any Product (or Cigarettes if Any not available)
Most adjusted

	Start year of study					
	<1960	1960-69	1970-79	1980-89	1990+	Total
N	6	8	7	7	2	30
NS	4	4	6	7	2	23
Wt	194.98	113.50	216.84	123.54	17.22	666.08
Het Chi	51.28	63.29	18.09	30.56	11.98	216.11
Het df	5	7	6	6	1	29
Het P	***	***	**	***	***	***
Fixed RR	9.73	10.47	6.48	6.98	3.31	7.89
RRl	8.46	8.71	5.67	5.85	2.06	7.31
RRu	11.20	12.58	7.40	8.32	5.31	8.51
P	+++	+++	+++	+++	+++	+++
Random RR	9.35	7.85	8.30	6.59	3.38	7.67
RRl	5.73	4.23	6.08	4.39	0.66	6.12
RRu	15.23	14.56	11.35	9.88	17.34	9.62
P	+++	+++	+++	+++	N.S.	+++
Between Chi						40.91
Between df						4
Between P						***
Btwn(F) P						N.S.
Btwn(R) P						N.S.

	Study type (1)		
	CC	other	Total
N	20	10	30
NS	17	6	23
Wt	466.38	199.70	666.08
Het Chi	139.17	70.56	216.11
Het df	19	9	29
Het P	***	***	***
Fixed RR	7.40	9.16	7.89
RRl	6.76	7.98	7.31
RRu	8.10	10.53	8.51
P	+++	+++	+++
Random RR	7.89	7.09	7.67
RRl	6.07	4.41	6.12
RRu	10.26	11.39	9.62
P	+++	+++	+++
Between Chi			6.38
Between df			1
Between P			*
Btwn(F) P			N.S.
Btwn(R) P			N.S.

	Study type (2)			
	CC	prosp	other	Total
N	20	10		30
NS	17	6		23
Wt	466.38	199.70		666.08
Het Chi	139.17	70.56		216.11
Het df	19	9		29
Het P	***	***		***
Fixed RR	7.40	9.16		7.89
RRl	6.76	7.98		7.31
RRu	8.10	10.53		8.51
P	+++	+++		+++
Random RR	7.89	7.09		7.67
RRl	6.07	4.41		6.12
RRu	10.26	11.39		9.62
P	+++	+++		+++
Between Chi				6.38
Between df				1
Between P				*
Btwn(F) P				N.S.
Btwn(R) P				N.S.

Table 1H13 - 3

IESLC - Meta-analysis of Ever/current Smoking, Age started, "Mid"
All LC types, Any Product (or Cigarettes if Any not available)
Most adjusted

	Study size (number of LC cases) 100-249	250-499	500-999	1000+	Total
N	3	7	11	9	30
NS	3	5	9	6	23
Wt	17.71	49.43	170.57	428.38	666.08
Het Chi	15.52	8.85	50.97	117.65	216.11
Het df	2	6	10	8	29
Het P	***	N.S.	***	***	***
Fixed RR	3.57	5.13	7.92	8.55	7.89
RRl	2.24	3.88	6.82	7.78	7.31
RRu	5.69	6.78	9.21	9.40	8.51
P	+++	+++	+++	+++	+++
Random RR	5.55	5.10	8.56	9.39	7.67
RRl	1.16	3.54	6.05	6.43	6.12
RRu	26.63	7.35	12.11	13.72	9.62
P	+	+++	+++	+++	+++
Between Chi					23.13
Between df					3
Between P					***
Btwn(F) P					N.S.
Btwn(R) P					(*)

	Risky occupational population no	mining	othRisky	Total
N	29	1		30
NS	22	1		23
Wt	657.24	8.84		666.08
Het Chi	190.81	0.00		216.11
Het df	28	0		29
Het P	***	N.S.		***
Fixed RR	8.07	1.47		7.89
RRl	7.48	0.76		7.31
RRu	8.71	2.84		8.51
P	+++	N.S.		+++
Random RR	8.10	1.47		7.67
RRl	6.51	0.76		6.12
RRu	10.09	2.84		9.62
P	+++	N.S.		+++
Between Chi				25.30
Between df				1
Between P				***
Btwn(F) P				(*)
Btwn(R) P				***

	National cigarette tobacco type Virginia	blended	other	Total
N	5	23	2	30
NS	3	18	2	23
Wt	63.48	584.04	18.56	666.08
Het Chi	19.55	156.66	2.25	216.11
Het df	4	22	1	29
Het P	***	***	N.S.	***
Fixed RR	6.35	8.42	2.12	7.89
RRl	4.96	7.77	1.34	7.31
RRu	8.12	9.14	3.34	8.51
P	+++	+++	++	+++
Random RR	6.59	8.81	2.10	7.67
RRl	3.63	6.94	1.06	6.12
RRu	11.96	11.19	4.15	9.62
P	+++	+++	+	+++
Between Chi				37.65
Between df				2
Between P				***
Btwn(F) P				(*)
Btwn(R) P				***

Table 1H13 - 3

IESLC - Meta-analysis of Ever/current Smoking, Age started, "Mid"
All LC types, Any Product (or Cigarettes if Any not available)
Most adjusted

Any proxy use

	No/nk	Yes	Total
N	22	8	30
NS	16	7	23
Wt	512.31	153.77	666.08
Het Chi	181.74	34.37	216.11
Het df	21	7	29
Het P	***	***	***
Fixed RR	7.88	7.91	7.89
RRl	7.23	6.76	7.31
RRu	8.60	9.27	8.51
P	+++	+++	+++
Random RR	7.40	8.29	7.67
RRl	5.55	5.81	6.12
RRu	9.86	11.84	9.62
P	+++	+++	+++
Between Chi			0.00
Between df			1
Between P			N.S.
Btwn(F) P			N.S.
Btwn(R) P			N.S.

Full histological confirmation

	No	Yes	Total
N	22	8	30
NS	16	7	23
Wt	376.40	289.68	666.08
Het Chi	126.16	89.12	216.11
Het df	21	7	29
Het P	***	***	***
Fixed RR	8.14	7.58	7.89
RRl	7.36	6.75	7.31
RRu	9.00	8.50	8.51
P	+++	+++	+++
Random RR	7.38	8.38	7.67
RRl	5.62	5.31	6.12
RRu	9.69	13.21	9.62
P	+++	+++	+++
Between Chi			0.84
Between df			1
Between P			N.S.
Btwn(F) P			N.S.
Btwn(R) P			N.S.

Number of adjustment variables (1)

	0	1	2+/+nk	Total
N	22	7	1	30
NS	17	6	1	24
Wt	367.38	290.32	8.37	666.08
Het Chi	119.81	84.46	0.00	216.11
Het df	21	6	0	29
Het P	***	***	N.S.	***
Fixed RR	8.89	6.79	7.80	7.89
RRl	8.03	6.05	3.96	7.31
RRu	9.85	7.61	15.35	8.51
P	+++	+++	+++	+++
Random RR	8.50	5.69	7.80	7.67
RRl	6.54	3.44	3.96	6.12
RRu	11.03	9.41	15.35	9.62
P	+++	+++	+++	+++
Between Chi				11.84
Between df				2
Between P				**
Btwn(F) P				N.S.
Btwn(R) P				N.S.

Table 1H13 - 3

IESLC - Meta-analysis of Ever/current Smoking, Age started, "Mid"
All LC types, Any Product (or Cigarettes if Any not available)
Most adjusted

	Number of adjustment variables (2) 0	1	2	3-5	6+/+nk	Total
N	22	7	1			30
NS	17	6	1			24
Wt	367.38	290.32	8.37			666.08
Het Chi	119.81	84.46	0.00			216.11
Het df	21	6	0			29
Het P	***	***	N.S.			***
Fixed RR	8.89	6.79	7.80			7.89
RRl	8.03	6.05	3.96			7.31
RRu	9.85	7.61	15.35			8.51
P	+++	+++	+++			+++
Random RR	8.50	5.69	7.80			7.67
RRl	6.54	3.44	3.96			6.12
RRu	11.03	9.41	15.35			9.62
P	+++	+++	+++			+++
Between Chi						11.84
Between df						2
Between P						**
Btwn(F) P						N.S.
Btwn(R) P						N.S.

	Smoking status ever	current	Total
N	21	9	30
NS	18	5	23
Wt	502.55	163.53	666.08
Het Chi	154.68	61.25	216.11
Het df	20	8	29
Het P	***	***	***
Fixed RR	7.82	8.13	7.89
RRl	7.16	6.97	7.31
RRu	8.53	9.47	8.51
P	+++	+++	+++
Random RR	8.05	6.65	7.67
RRl	6.21	3.91	6.12
RRu	10.43	11.31	9.62
P	+++	+++	+++
Between Chi			0.19
Between df			1
Between P			N.S.
Btwn(F) P			N.S.
Btwn(R) P			N.S.

	Product all/unsp	cig+/-ot	cig only	Total
N	4	19	7	30
NS	4	16	4	24
Wt	57.23	518.63	90.22	666.08
Het Chi	31.84	146.22	12.60	216.11
Het df	3	18	6	29
Het P	***	***	*	***
Fixed RR	6.70	8.70	4.99	7.89
RRl	5.17	7.98	4.06	7.31
RRu	8.68	9.48	6.13	8.51
P	+++	+++	+++	+++
Random RR	7.00	8.78	5.19	7.67
RRl	2.81	6.72	3.63	6.12
RRu	17.41	11.47	7.41	9.62
P	+++	+++	+++	+++
Between Chi				25.45
Between df				2
Between P				***
Btwn(F) P				N.S.
Btwn(R) P				(*)

Table 1H13 - 3

IESLC - Meta-analysis of Ever/current Smoking, Age started, "Mid"
All LC types, Any Product (or Cigarettes if Any not available)
Most adjusted

	Denominator nev any	nev cigs	Total
N	20	10	30
NS	14	9	23
Wt	371.96	294.12	666.08
Het Chi	120.31	95.60	216.11
Het df	19	9	29
Het P	***	***	***
Fixed RR	7.77	8.05	7.89
RRl	7.02	7.18	7.31
RRu	8.60	9.02	8.51
P	+++	+++	+++
Random RR	6.78	9.81	7.67
RRl	5.14	6.35	6.12
RRu	8.95	15.17	9.62
P	+++	+++	+++
Between Chi			0.21
Between df			1
Between P			N.S.
Btwn(F) P			N.S.
Btwn(R) P			N.S.

	Derivation of RR/CI Orig	StdCalc	Other	Total
N	3	22	5	30
NS	3	17	4	24
Wt	34.78	507.30	124.00	666.08
Het Chi	22.41	148.09	32.04	216.11
Het df	2	21	4	29
Het P	***	***	***	***
Fixed RR	5.76	7.55	10.35	7.89
RRl	4.13	6.92	8.68	7.31
RRu	8.02	8.23	12.34	8.51
P	+++	+++	+++	+++
Random RR	4.90	7.64	10.84	7.67
RRl	1.55	5.94	5.51	6.12
RRu	15.53	9.83	21.34	9.62
P	++	+++	+++	+++
Between Chi				13.57
Between df				2
Between P				**
Btwn(F) P				N.S.
Btwn(R) P				N.S.

Table 1H13 - 4

IESLC - Meta-analysis of Ever/current Smoking, Age started, "Mid"
All LC types, Any Product (or Cigarettes if Any not available)
Least adjusted

REF	NRR	X	SEX	AGEL	AGEH	RACE	YF	LC TYPE	LOC	START	ST	NLC	R	VB	P	H	AD	SM	PRODUCT	exL	exH	DENOM	De
AUVINE	510		c	0	0	all	-	all	Eu:Sca	1986	CC	517	n	bl	y	n	0	ev	cig+/-ot	16	20	nev cigs	st
BARBON	516	x	m	0	0	all	-	all	Eu:wst	1979	CC	755	n	bl	y	y	0	ev	all/unsp	15	19	nev any	st
BRESLO	502		c	0	0	all	-	all	NAmer	1949	CC	518	n	bl	n	y	0	ev	cig+/-ot	15	24	nev any	st
BUFFLE	520		f	0	0	w-hi	-	all	NAmer	1976	CC	943	n	bl	y	n	0	ev	cig+/-ot	17	18	nev cigs	ot
CEDERL	511		m	0	0	all	10	all	Eu:Sca	1963	pr	491	n	bl	n	n	1	cu	cig only	17	18	nev any	ot
CEDERL	516		f	0	0	all	10	all	Eu:Sca	1963	pr	491	n	bl	n	n	0	cu	cig only	17	18	nev any	st
CHOI	525		m	0	0	all	-	all	As:oth	1985	CC	375	n	bl	n	n	0	ev	cig+/-ot	15	19	nev cigs	st
CPSI	803		m	35	84	all	6	all	NAmer	1959	pr	5138	n	bl	n	n	1	cu	cig+/-ot	15	19	nev any	ot
CPSI	847		f	40	74	all	6	all	NAmer	1959	pr	5138	n	bl	n	n	1	cu	cig only	15	19	nev any	ot
DAMBER	502		m	0	0	all	-	all	Eu:Sca	1972	CC	579	n	bl	y	n	0	ev	all/unsp	16	20	nev any	st
DEAN3	566		m	0	0	all	-	all	Eu:UK	1969	CC	766	n	V	y	n	0	cu	cig only	15	19	nev any	st
DEAN3	585		f	0	0	all	-	all	Eu:UK	1969	CC	766	n	V	y	n	0	cu	cig only	15	19	nev any	st
DORN	612		m	55	64	wh	8	all	NAmer	1954	pr	5097	n	bl	n	n	0	ev	cig+/-ot	15	19	nev any	st
DORN	649		m	65	74	wh	8	all	NAmer	1954	pr	5097	n	bl	n	n	0	ev	cig+/-ot	15	19	nev any	st
GENG	529	x	f	0	0	all	-	all	As:Chi	1985	CC	292	n	ot	*	n	0	ev	cig+/-ot	16	20	nev any	st
JEDRYC	607		m	0	0	all	-	all	Eu:est	1980	CC	1630	n	bl	y	n	0	ev	cig+/-ot	17	18	nev any	st
JOLY	544		m	0	0	all	-	all	SCAmer	1978	CC	826	n	bl	n	n	0	ev	cig+/-ot	15	24	nev any	st
JOLY	534		f	0	0	all	-	all	SCAmer	1978	CC	826	n	bl	n	n	0	ev	cig+/-ot	15	24	nev any	st
KHUDER	507		m	0	0	all	-	all	NAmer	1985	CC	482	n	bl	n	y	0	ev	cig+/-ot	16	19	nev cigs	st
KOULUM	503		m	0	0	all	-	all	Eu:Sca	1936	CC	812	n	bl	n	n	0	ev	all/unsp	16	20	nev any	st
LETOUR	502		c	0	0	all	-	all	NAmer	1983	CC	738	n	V	y	y	0	ev	cig+/-ot	15	20	nev cigs	st
LUBIN2	1149	x	m	0	0	all	-	all	Eu:mul	1976	CC	7804	n	bl	n	y	0	ev	cig+/-ot	17	20	nev cigs	st
MATOS	557	x	m	0	0	all	-	all	SCAmer	1994	CC	200	n	bl	n	n	0	ev	cig+/-ot	15	19	nev any	st
MIGRAN	503		m	0	0	all	0	all	Eu:UK	1964	pr	259	n	V	n	n	0	cu	cig only	16	19	nev any	st
MIGRAN	513		f	0	0	all	0	all	Eu:UK	1964	pr	259	n	V	n	n	0	cu	cig only	16	19	nev any	st
MRFITR	511		m	0	0	all	0	all	NAmer	1973	pr	119	n	bl	n	n	0	cu	cig+/-ot	18	19	nev cigs	ot
QIAO2	502	x	m	0	0	all	0	all	As:Chi	1992	pr	241	m	ot	n	n	0	ev	all/unsp	17	20	nev any	st
SOBUE	655		m	0	0	all	-	all	As:Jap	1986	CC	1376	n	bl	n	y	0	ev	cig+/-ot	18	22	nev cigs	st
WYNDE6	760		m	0	0	wh	-	q+s+a	NAmer	1969	CC	4423	n	bl	n	y	0	ev	cig+/-ot	18	20	nev cigs	st
WYNDE6	768		f	0	0	wh	-	q+s+a	NAmer	1969	CC	4423	n	bl	n	y	0	ev	cig+/-ot	18	20	nev cigs	st

Cigarette type is all/unspec for all RRs
except for the following:

REF	NRR	CIGTYPE
DEAN3	566	MC only
DEAN3	585	MC only

Table 1H13 - 5

IESLC - Meta-analysis of Ever/current Smoking, Age started, "Mid"
All LC types, Any Product (or Cigarettes if Any not available)
Least adjusted

REF	NRR	SEX	AD	Number Exposed Case	Cont	Non-exposed Case	Cont	RR	95.00%CI
AUVINE	510	c	0	135	47	44	229	14.95	(9.41- 23.75)
BARBON	516	m	0	395	337	22	188	10.02	(6.29- 15.94)
BRESLO	502	c	0	286	243	19	56	3.47	(2.01- 6.00)
BUFFLE	520	f	0	53	33	12	112	14.99	(7.17- 31.33)
*CEDERL	511	m	1	10	-	7	-	9.80	(3.74- 25.69)
*CEDERL	516	f	0	2	1009	19	17679	1.84	(0.43- 7.91)
Subtotal CEDERL								5.89	(2.64- 13.16)
CHOI	525	m	0	79	138	13	95	4.18	(2.20- 7.95)
*CPSI	803	m	1	588	-	83	-	14.69	(11.68- 18.49)
*CPSI	847	f	1	52	-	166	-	5.00	(3.66- 6.83)
Subtotal CPSI								10.06	(8.36- 12.10)
DAMBER	502	m	0	261	190	42	208	6.80	(4.65- 9.95)
DEAN3	566	m	0	160	485	24	510	7.01	(4.48- 10.96)
DEAN3	585	f	0	39	504	41	1538	2.90	(1.85- 4.55)
Subtotal DEAN3								4.52	(3.30- 6.21)
*DORN	612	m	0	342	213156	25	213858	13.73	(9.14- 20.60)
*DORN	649	m	0	306	118234	49	171211	9.04	(6.69- 12.23)
Subtotal DORN								10.49	(8.23- 13.36)
GENG	529	f	0	39	23	54	93	2.92	(1.58- 5.40)
JEDRYC	607	m	0	239	146	49	219	7.32	(5.04- 10.61)
JOLY	544	m	0	217	357	12	218	11.04	(6.03- 20.22)
JOLY	534	f	0	67	47	52	283	7.76	(4.82- 12.49)
Subtotal JOLY								8.88	(6.11- 12.91)
KHUDER	507	m	0	161	338	23	309	6.40	(4.03- 10.17)
KOULUM	503	m	0	267	103	5	54	28.00	(10.89- 71.96)
LETOUR	502	c	0	309	241	24	224	11.97	(7.60- 18.83)
LUBIN2	1149	m	0	1796	3028	185	1878	6.02	(5.12- 7.08)
MATOS	557	m	0	91	120	11	110	7.58	(3.85- 14.92)
*MIGRAN	503	m	0	59	1845	4	867	6.93	(2.53- 19.02)
*MIGRAN	513	f	0	9	1035	4	3814	8.29	(2.56- 26.87)
Subtotal MIGRAN								7.48	(3.48- 16.09)
*MRFITR	511	m	0	25	1876	0	1859	50.54~	(3.08- 829.51)
*QIAO2	502	m	0	75	2840	10	709	1.87	(0.97- 3.60)
SOBUE	655	m	0	776	772	29	126	4.37	(2.88- 6.62)
WYNDE6	760	m	0	223	139	51	589	18.53	(12.98- 26.45)
WYNDE6	768	f	0	200	94	73	673	19.62	(13.90- 27.67)
Subtotal WYNDE6								19.08	(14.90- 24.44)
Partial Totals				7261	347380	1152	417709		

*prospective study ~ With 0.5 adjustment for zero

REF	NRR	SEX	AD	Ys	Ws	Qs	Ps
AUVINE	510	c	0	2.70	17.93	6.88	0.0000
BARBON	516	m	0	2.30	17.77	0.85	0.0000
BRESLO	502	c	0	1.24	12.80	9.07	0.0000
BUFFLE	520	f	0	2.71	7.07	2.74	0.0000
*CEDERL	511	m	1	2.28	4.14	0.16	0.0000
*CEDERL	516	f	0	0.61	1.81	3.93	0.4098
Subtotal CEDERL				1.77	5.95	4.10	
CHOI	525	m	0	1.43	9.31	3.99	0.0000
*CPSI	803	m	1	2.69	72.82	26.38	0.0000
*CPSI	847	f	1	1.61	39.48	8.94	0.0000
Subtotal CPSI				2.31	112.30	35.32	
DAMBER	502	m	0	1.92	26.52	0.75	0.0000
DEAN3	566	m	0	1.95	19.25	0.37	0.0000
DEAN3	585	f	0	1.07	18.99	19.74	0.0000
Subtotal DEAN3				1.51	38.24	20.11	
*DORN	612	m	0	2.62	23.30	6.64	0.0000
*DORN	649	m	0	2.20	42.26	0.58	0.0000
Subtotal DORN				2.35	65.56	7.22	
GENG	529	f	0	1.07	10.16	10.44	0.0006
JEDRYC	607	m	0	1.99	27.77	0.25	0.0000
JOLY	544	m	0	2.40	10.49	1.05	0.0000
JOLY	534	f	0	2.05	16.96	0.02	0.0000
Subtotal JOLY				2.18	27.45	1.07	
KHUDER	507	m	0	1.86	17.89	0.94	0.0000
KOULUM	503	m	0	3.33	4.31	6.70	0.0000
LETOUR	502	c	0	2.48	18.69	2.94	0.0000
LUBIN2	1149	m	0	1.80	146.52	12.33	0.0000
MATOS	557	m	0	2.03	8.38	0.03	0.0000
*MIGRAN	503	m	0	1.94	3.77	0.08	0.0002

Table 1H13 - 5

IESLC - Meta-analysis of Ever/current Smoking, Age started, "Mid"
All LC types, Any Product (or Cigarettes if Any not available)
Least adjusted

REF	NRR	SEX	AD	Ys	Ws	Qs	Ps
*MIGRAN	513	f	0	2.12	2.78	0.00	0.0004
Subtotal MIGRAN				2.01	6.55	0.09	
*MRFITR	511	m	0	3.92	0.49	1.66	0.0060
*QIAO2	502	m	0	0.63	8.96	19.06	0.0604
SOBUE	655	m	0	1.47	22.22	8.30	0.0000
WYNDE6	760	m	0	2.92	30.32	21.09	0.0000
WYNDE6	768	f	0	2.98	32.44	25.75	0.0000
Subtotal WYNDE6				2.95	62.76	46.84	

	N	30
	NS	23
	Wt	675.62
Het	Chi	201.65
Het	df	29
Het	P	***
Fixed	RR	8.05
	RRl	7.46
	RRu	8.68
	P	+++
Random	RR	7.76
	RRl	6.24
	RRu	9.65
	P	+++
Asymm	P	N.S.

Table 1H13 - 6

IESLC - Meta-analysis of Ever/current Smoking, Age started, "Mid"
All LC types, Any Product (or Cigarettes if Any not available)
Least adjusted

	Sex			
	combined	male	female	Total
N	3	19	8	30
NS	3	18	8	29
Wt	49.42	496.51	129.70	675.62
Het Chi	17.67	110.96	68.98	201.65
Het df	2	18	7	29
Het P	***	***	***	***
Fixed RR	9.41	8.22	6.99	8.05
RRl	7.12	7.53	5.88	7.46
RRu	12.44	8.98	8.30	8.68
P	+++	+++	+++	+++
Random RR	8.64	8.22	6.21	7.76
RRl	3.75	6.42	3.43	6.24
RRu	19.90	10.53	11.24	9.65
P	+++	+++	+++	+++
Between Chi				4.04
Between df				2
Between P				N.S.
Btwn(F) P				N.S.
Btwn(R) P				N.S.

Table 1H13 - 7

IESLC - Meta-analysis of Ever/current Smoking, Age started, "Mid"
All LC types, Any Product (or Cigarettes if Any not available)
Excluded studies (and stage at which they were excluded)

1	BECHER BLOT1 BROWN3 CARPEN CHYOU DARBY DOLL2 GARCIA GRAHAM GURSEL HAMMO2 JAHN JAIN LAUSSM PRESCO QIAO TVERDA WIGLE WYNDE3
2	AKIBA AMANDU AMES AXELSS BENSHL BEST BOUCHA BOUCOT BROSS CHEN CPSII DEAN2 DESTEF DORGAN DOSEME FAN GARSHI GER GILLIS HAMMON HUMBLE JUSSAW KAISE2 KATSOU KAUFMA KOO KREUZE LEVIN MCCONN NOTAN2 OSANN2 PEZZO2 PISANI RESTRE SADOWS VUTUC WANG2 WATSON WU2 WUWILL WYNDE2 WYNDE8 XU ZHOU
3	GUO MCDUFF SPITZ STASZE ZHANG
4	LUO
5	CORREA HOLE YUAN
7	BOFFET WYNDE7
10	ALDERS SPEIZE
14	AGUDO ARMADA BENHAM CHEN2 CHIAZZ DOLL ENGELA GAO GAO2 HAENSZ HEGMAN HIRAYA HU HU2 LIAW LIU3 LIU4 LIU5 LUBIN PERNU PEZZOT RACHTA SEGI2 SUZUK2 SVENSS TIZZAN WAKAI WU ZHENG

Table 1H13 - 8

Potentially overlapping studies

REF	REFGP	PRINC	.	OVERLAP/LINK
LUBIN2	LUBIN2	1		Lubin-combined
MRFITR	MRFIT	2		Subset of MRFIT
WYNDE6	WYNDE6	1		WYNDE5/6/7/8
CPSI	CPSI	1		CPSI overall

Table 1H13 - 9

Most adjusted - insufficient data for meta-analysis

REF	NRR	SEX	AGEL	AGEH	RACE	YF	LC TYPE	LOC	START	ST	NLC	R	VB	P	H	AD	SM	PRODUCT	exL	exH	DENOM	De
CORREA	536	c	0	0	all	-	all	NAmer	1979	CC	1359	n	bl	y	n	2	ev	cig+/-ot	16	20	nev cigs	or

REF	NRR	RR	SIG	RRDATA comment
CORREA	536	17.40		0

Table 1H14 -

IESLC - Meta-analysis of Ever/current Smoking, Age started, "High"
All LC types, Any Product (or Cigarettes if Any not available)

This analysis is restricted to results for:
1) Ever/current smokers
2) Results by Age started
3) Categorical results by Age started
4) All LC types (or near equivalent)
5) Results complete enough for use in metaanalysis

Within each study, results are then selected (in the following order of preference, within each sex) for:
6) PRODUCT: all/unspec, cigarettes regardless of other products, cigarettes only
7) CIGTYPE: all/unspecified, MC regardless of HR, MC only
8) (not applicable)
9) DENOM: never smoked anything, never smoked cigarettes, never any + low, never cigs + low
10) Followup period (YF, prospective studies): whole study (coded as 0) or longest available
11) LCtype: all or nearest available, at least Squamous and Adeno. (q = squamous, s = small,
l = large, a = adeno, mix = mixed, alv = alveolar)
12) Race: all or nearest available, otherwise by race (wh or w = white, bl or b = black, hi = hispanic
ch = chinese, jap = japanese, haw = hawaiian, w+o = white + oriental, sca = scandinavian, as = asian)
13) Age started "high" in key scheme 1 (key value 14, maximum range 1-17)
14) For overlapping studies: principal rather than subsidiary studies
Finally by Age: whole study (coded as 0) if available, otherwise by widest available age group
and then for single sex results (m, f) in preference to results for both sexes combined (c).

Results adjusted (AD) for the most potential confounders are then chosen in Sections -1 to -3
and results adjusted for the least confounders in Sections -4 to -6. (Those least adjusted results which
actually differ from the most adjusted are marked 'x' in column X in Section -4)

Section -7 shows excluded studies, together with the stage (as above) at which no qualifying
results were found.

Section -8 lists the potentially overlapping studies which have been included (1=principal, 2=subsidiary).

Section -9 lists any results which would have been included in preference except that they had data not complete
enough for use in meta-analysis, with their significance (yes/no), if known, and any further comment as entered
on the database. It also lists as "gap" any categories for which no data were presented by the original authors.

In addition to those mentioned above, the following fields, levels and abbreviations are used:
* or nk = not known, n = no, y = yes, ot = other
ev = ever, cu = current, nev = never
all/unspec = all or unspecified, cig+/-ot = cigarettes irrespective of other products (cigar, pipe etc)
MC = manufactured cigarettes, HR = hand-rolled cigarettes
exL, exH = range of exposure (low and high) in the smoking group, in terms of Age started
REF: 6-character study reference
NRR: number of the RR on the database within the study
ST : study type (CC = case control, pr or prosp = prospective)
NLC: number of lung cancer cases in whole study
R : risky occupational population (n = no, m = mining, o = other risky)
VB : national cigarette type (V = at least 75% Virginia, bl = at least 75% blended, ot = other)
P : any proxy use
H : full histological confirmation
De : derivation of RR/CI (or = original, st = standard method, ot = other method of estimation)

Table 1H14 - 1

IESLC - Meta-analysis of Ever/current Smoking, Age started, "High"
All LC types, Any Product (or Cigarettes if Any not available)
Most adjusted

REF	NRR	SEX	AGEL	AGEH	RACE	YF	LC TYPE	LOC	START	ST	NLC	R	VB	P	H	AD	SM	PRODUCT	exL	exH	DENOM	De
ARMADA	512	m	0	0	all	-	all	Eu:wst	1986	CC	325	n	bl	n	y	0	ev	cig+/-ot	7	16	nev cigs	st
AUVINE	521	c	0	0	all	-	all	Eu:Sca	1986	CC	517	n	bl	y	n	2	ev	cig+/-ot	1	15	nev cigs	or
BARBON	522	m	0	0	all	-	all	Eu:wst	1979	CC	755	n	bl	y	y	1	ev	all/unsp	1	14	nev any	or
BRESLO	503	c	0	0	all	-	all	NAmer	1949	CC	518	n	bl	n	y	0	ev	cig+/-ot	0	14	nev any	st
BUFFLE	521	f	0	0	w-hi	-	all	NAmer	1976	CC	943	n	bl	y	n	0	ev	cig+/-ot	6	16	nev cigs	or
CEDERL	512	m	0	0	all	10	all	Eu:Sca	1963	pr	491	n	bl	n	n	1	cu	cig only	1	16	nev any	ot
CEDERL	517	f	0	0	all	10	all	Eu:Sca	1963	pr	491	n	bl	n	n	0	cu	cig only	1	16	nev any	ot
CHOI	526	m	0	0	all	-	all	As:oth	1985	CC	375	n	bl	n	n	0	ev	cig+/-ot	1	14	nev cigs	st
CPSI	804	m	35	84	all	6	all	NAmer	1959	pr	5138	n	bl	n	n	1	cu	cig+/-ot	1	14	nev any	ot
CPSI	848	f	40	74	all	6	all	NAmer	1959	pr	5138	n	bl	n	n	1	cu	cig only	1	14	nev any	ot
DAMBER	503	m	0	0	all	-	all	Eu:Sca	1972	CC	579	n	bl	y	n	0	ev	all/unsp	1	15	nev any	st
DEAN3	567	m	0	0	all	-	all	Eu:UK	1969	CC	766	n	V	y	n	0	cu	cig only	1	14	nev any	st
DEAN3	586	f	0	0	all	-	all	Eu:UK	1969	CC	766	n	V	y	n	0	cu	cig only	1	14	nev any	st
DORN	613	m	55	64	wh	8	all	NAmer	1954	pr	5097	n	bl	n	n	0	ev	cig+/-ot	1	14	nev any	st
DORN	650	m	65	74	wh	8	all	NAmer	1954	pr	5097	n	bl	n	n	0	ev	cig+/-ot	1	14	nev any	st
GENG	535	f	0	0	all	-	all	As:Chi	1985	CC	292	n	ot	*	n	1	ev	cig+/-ot	1	15	nev any	st
HU	513	m	0	0	all	-	all	As:Chi	1985	CC	227	n	ot	n	y	0	ev	cig+/-ot	1	15	nev cigs	st
HU	518	f	0	0	all	-	all	As:Chi	1985	CC	227	n	ot	n	y	0	ev	cig+/-ot	1	15	nev cigs	st
JEDRYC	608	m	0	0	all	-	all	Eu:est	1980	CC	1630	n	bl	y	n	0	ev	cig+/-ot	1	16	nev any	st
JOLY	545	m	0	0	all	-	all	SCAmer	1978	CC	826	n	bl	n	n	0	ev	cig+/-ot	1	14	nev any	st
JOLY	535	f	0	0	all	-	all	SCAmer	1978	CC	826	n	bl	n	n	0	ev	cig+/-ot	1	14	nev any	st
KHUDER	508	m	0	0	all	-	all	NAmer	1985	CC	482	n	bl	n	y	0	ev	cig+/-ot	1	15	nev cigs	st
KOULUM	504	m	0	0	all	-	all	Eu:Sca	1936	CC	812	n	bl	n	n	0	ev	all/unsp	11	15	nev any	st
LETOUR	503	c	0	0	all	-	all	NAmer	1983	CC	738	n	V	y	y	0	ev	cig+/-ot	1	14	nev cigs	st
LUBIN2	1159	m	0	0	all	-	all	Eu:mul	1976	CC	7804	n	bl	n	y	1	ev	cig+/-ot	13	16	nev cigs	st
MATOS	578	m	0	0	all	-	all	SCAmer	1994	CC	200	n	bl	n	n	2	ev	cig+/-ot	1	14	nev any	or
MIGRAN	505	m	0	0	all	0	all	Eu:UK	1964	pr	259	n	V	n	n	0	cu	cig only	0	15	nev any	st
MIGRAN	515	f	0	0	all	0	all	Eu:UK	1964	pr	259	n	V	n	n	0	cu	cig only	0	15	nev any	st
MRFITR	513	m	0	0	all	0	all	NAmer	1973	pr	119	n	bl	n	n	0	cu	cig+/-ot	1	15	nev cigs	ot
PERNU	505	m	0	0	all	-	all	Eu:Sca	1944	CC	1606	n	bl	n	n	0	ev	all/unsp	1	14	nev any	st
PERNU	502	f	0	0	all	-	all	Eu:Sca	1944	CC	1606	n	bl	n	n	0	ev	all/unsp	1	14	nev any	ot
QIAO2	508	m	0	0	all	0	all	As:Chi	1992	pr	241	m	ot	n	n	1	ev	all/unsp	1	16	nev any	or
SOBUE	656	m	0	0	all	-	all	As:Jap	1986	CC	1376	n	bl	n	y	0	ev	cig+/-ot	10	17	nev cigs	st
WYNDE6	761	m	0	0	wh	-	q+s+a	NAmer	1969	CC	4423	n	bl	n	y	0	ev	cig+/-ot	1	17	nev cigs	st
WYNDE6	769	f	0	0	wh	-	q+s+a	NAmer	1969	CC	4423	n	bl	n	y	0	ev	cig+/-ot	1	17	nev cigs	st

Cigarette type is all/unspec for all RRs
except for the following:

REF	NRR	CIGTYPE
DEAN3	567	MC only
DEAN3	586	MC only

Table 1H14 - 2

IESLC - Meta-analysis of Ever/current Smoking, Age started, "High"
All LC types, Any Product (or Cigarettes if Any not available)
Most adjusted

REF	NRR	SEX	AD	Number Exposed Case	Number Exposed Cont	Non-exposed Case	Non-exposed Cont	RR	95.00%CI
ARMADA	512	m	0	204	110	8	71	16.46	(7.64- 35.44)
AUVINE	521	c	2	55	-	44	-	47.60	(21.20- 107.00)
BARBON	522	m	1	138	-	22	-	50.80	(27.20- 95.00)
BRESLO	503	c	0	166	116	19	56	4.22	(2.38- 7.47)
BUFFLE	521	f	0	78	41	12	112	17.76	(8.77- 35.94)
*CEDERL	512	m	1	7	-	7	-	6.40	(2.25- 18.21)
*CEDERL	517	f	0	0	746	19	17679	0.61~	(0.04- 10.05)
Subtotal CEDERL								4.80	(1.80- 12.79)
CHOI	526	m	0	22	18	13	95	8.93	(3.81- 20.91)
*CPSI	804	m	1	185	-	83	-	16.77	(12.94- 21.73)
*CPSI	848	f	1	6	-	166	-	2.50	(1.11- 5.65)
Subtotal CPSI								14.07	(10.99- 18.02)
DAMBER	503	m	0	206	98	42	208	10.41	(6.91- 15.68)
DEAN3	567	m	0	44	165	24	510	5.67	(3.34- 9.60)
DEAN3	586	f	0	7	109	41	1538	2.41	(1.06- 5.50)
Subtotal DEAN3								4.42	(2.83- 6.89)
*DORN	613	m	0	84	36304	25	213858	19.79	(12.67- 30.93)
*DORN	650	m	0	81	24616	49	171211	11.50	(8.07- 16.39)
Subtotal DORN								14.18	(10.75- 18.72)
GENG	535	f	1	36	-	54	-	6.24	(2.85- 13.67)
HU	513	m	0	13	7	41	67	3.03	(1.12- 8.23)
HU	518	f	0	5	4	40	48	1.50	(0.38- 5.96)
Subtotal HU								2.38	(1.06- 5.35)
JEDRYC	608	m	0	135	66	49	219	9.14	(5.96- 14.02)
JOLY	545	m	0	317	282	12	218	20.42	(11.18- 37.32)
JOLY	535	f	0	76	35	52	283	11.82	(7.18- 19.44)
Subtotal JOLY								14.75	(10.05- 21.65)
KHUDER	508	m	0	226	295	23	309	10.29	(6.51- 16.27)
KOULUM	504	m	0	199	52	5	54	41.33	(15.74- 108.56)
LETOUR	503	c	0	151	76	24	224	18.54	(11.21- 30.67)
LUBIN2	1159	m	1	1312	-	185	-	6.74	(5.65- 8.04)
MATOS	578	m	2	69	-	11	-	7.80	(3.90- 15.70)
*MIGRAN	505	m	0	50	1081	4	867	10.03	(3.64- 27.65)
*MIGRAN	515	f	0	2	266	4	3814	7.17	(1.32- 38.96)
Subtotal MIGRAN								9.18	(3.84- 21.90)
*MRFITR	513	m	0	25	2065	0	1859	45.91~	(2.80- 753.64)
PERNU	505	m	0	337	92	97	275	10.38	(7.49- 14.40)
PERNU	502	f	0	1	0	110	971	26.38~	(1.07- 651.38)
Subtotal PERNU								10.48	(7.58- 14.51)
*QIAO2	508	m	1	104	-	10	-	1.81	(0.94- 3.48)
SOBUE	656	m	0	137	62	29	126	9.60	(5.81- 15.88)
WYNDE6	761	m	0	611	301	51	589	23.44	(17.06- 32.21)
WYNDE6	769	f	0	291	91	73	673	29.48	(21.04- 41.31)
Subtotal WYNDE6								26.11	(20.72- 32.90)
Partial Totals				5380	67098	1448	415934		

*prospective study ~ With 0.5 adjustment for zero

REF	NRR	SEX	AD	Ys	Ws	Qs	Ps
ARMADA	512	m	0	2.80	6.53	1.01	0.0000
AUVINE	521	c	2	3.86	5.86	12.42	0.0000
BARBON	522	m	1	3.93	9.82	22.71	0.0000
BRESLO	503	c	0	1.44	11.75	11.01	0.0000
BUFFLE	521	f	0	2.88	7.72	1.70	0.0000
*CEDERL	512	m	1	1.86	3.51	1.07	0.0005
*CEDERL	517	f	0	-0.50	0.49	4.12	0.7276
Subtotal CEDERL				1.57	4.00	5.19	
CHOI	526	m	0	2.19	5.31	0.25	0.0000
*CPSI	804	m	1	2.82	57.18	9.71	0.0000
*CPSI	848	f	1	0.92	5.80	12.90	0.0273
Subtotal CPSI				2.64	62.99	22.61	
DAMBER	503	m	0	2.34	22.90	0.10	0.0000
DEAN3	567	m	0	1.73	13.81	6.25	0.0000
DEAN3	586	f	0	0.88	5.65	13.19	0.0367
Subtotal DEAN3				1.49	19.46	19.45	
*DORN	613	m	0	2.99	19.28	6.44	0.0000
*DORN	650	m	0	2.44	30.57	0.04	0.0000
Subtotal DORN				2.65	49.85	6.47	
GENG	535	f	1	1.83	6.25	2.08	0.0000
HU	513	m	0	1.11	3.86	6.50	0.0292
HU	518	f	0	0.41	2.02	8.08	0.5647

Table 1H14 - 2

IESLC - Meta-analysis of Ever/current Smoking, Age started, "High"
All LC types, Any Product (or Cigarettes if Any not available)
Most adjusted

REF	NRR	SEX	AD	Ys	Ws	Qs	Ps
Subtotal HU				0.87	5.88	14.58	
JEDRYC	608	m	0	2.21	21.04	0.80	0.0000
JOLY	545	m	0	3.02	10.57	3.92	0.0000
JOLY	535	f	0	2.47	15.51	0.06	0.0000
Subtotal JOLY				2.69	26.07	3.98	
KHUDER	508	m	0	2.33	18.34	0.11	0.0000
KOULUM	504	m	0	3.72	4.12	7.11	0.0000
LETOUR	503	c	0	2.92	15.17	3.99	0.0000
LUBIN2	1159	m	1	1.91	123.47	30.81	0.0000
MATOS	578	m	2	2.05	7.92	0.99	0.0000
*MIGRAN	505	m	0	2.31	3.73	0.04	0.0000
*MIGRAN	515	f	0	1.97	1.34	0.26	0.0226
Subtotal MIGRAN				2.22	5.07	0.30	
*MRFITR	513	m	0	3.83	0.49	0.99	0.0074
PERNU	505	m	0	2.34	35.99	0.16	0.0000
PERNU	502	f	0	3.27	0.37	0.28	0.0455
Subtotal PERNU				2.35	36.37	0.44	
*QIAO2	508	m	1	0.59	8.97	29.52	0.0756
SOBUE	656	m	0	2.26	15.19	0.32	0.0000
WYNDE6	761	m	0	3.15	38.07	21.25	0.0000
WYNDE6	769	f	0	3.38	33.77	32.18	0.0000
Subtotal WYNDE6				3.26	71.85	53.43	

	N	35
	NS	26
	Wt	572.38
Het	Chi	252.35
Het	df	34
Het	P	***
Fixed	RR	11.11
	RRl	10.23
	RRu	12.06
	P	+++
Random	RR	10.32
	RRl	8.04
	RRu	13.26
	P	+++
Asymm	P	N.S.

Table 1H14 - 3

IESLC - Meta-analysis of Ever/current Smoking, Age started, "High"
All LC types, Any Product (or Cigarettes if Any not available)
Most adjusted

	Sex			
	combined	male	female	Total
N	3	22	10	35
NS	3	21	10	34
Wt	32.78	460.68	78.92	572.38
Het Chi	26.67	149.53	73.34	252.35
Het df	2	21	9	34
Het P	***	***	***	***
Fixed RR	12.91	10.73	12.76	11.11
RRl	9.17	9.79	10.23	10.23
RRu	18.18	11.76	15.91	12.06
P	+++	+++	+++	+++
Random RR	15.15	11.19	6.37	10.32
RRl	4.15	8.53	3.00	8.04
RRu	55.30	14.70	13.56	13.26
P	+++	+++	+++	+++
Between Chi				2.81
Between df				2
Between P				N.S.
Btwn(F) P				N.S.
Btwn(R) P				N.S.

	Lung cancer type		
	all	other	Total
N	33	2	35
NS	25	1	26
Wt	500.54	71.85	572.38
Het Chi	191.39	0.94	252.35
Het df	32	1	34
Het P	***	N.S.	***
Fixed RR	9.82	26.11	11.11
RRl	9.00	20.72	10.23
RRu	10.72	32.90	12.06
P	+++	+++	+++
Random RR	9.62	26.11	10.32
RRl	7.54	20.72	8.04
RRu	12.29	32.90	13.26
P	+++	+++	+++
Between Chi			60.02
Between df			1
Between P			***
Btwn(F) P			**
Btwn(R) P			***

				Location					
	NAmer	UK	Scand	othEur	China	Japan	othAs	other	Total
N	11	4	7	4	4	1	1	3	35
NS	8	2	5	4	3	1	1	2	26
Wt	238.16	24.53	73.25	160.86	21.10	15.19	5.31	34.00	572.38
Het Chi	67.91	5.19	24.59	40.84	6.53	0.00	0.00	4.35	252.35
Het df	10	3	6	3	3	0	0	2	34
Het P	***	N.S.	***	***	(*)	N.S.	N.S.	N.S.	***
Fixed RR	16.06	5.14	12.22	8.23	2.82	9.60	8.93	12.72	11.11
RRl	14.15	3.46	9.72	7.05	1.84	5.81	3.81	9.09	10.23
RRu	18.24	7.64	15.37	9.60	4.32	15.88	20.91	17.80	12.06
P	+++	+++	+++	+++	+++	+++	+++	+++	+++
Random RR	13.53	5.18	14.35	14.46	2.82	9.60	8.93	12.57	10.32
RRl	9.44	2.88	7.78	6.45	1.45	5.81	3.81	7.58	8.04
RRu	19.41	9.32	26.46	32.43	5.50	15.88	20.91	20.84	13.26
P	+++	+++	+++	+++	++	+++	+++	+++	+++
Between Chi									102.95
Between df									7
Between P									***
Btwn(F) P									*
Btwn(R) P									***

Table 1H14 - 3

IESLC - Meta-analysis of Ever/current Smoking, Age started, "High"
All LC types, Any Product (or Cigarettes if Any not available)
Most adjusted

Detailed Country in "other Europe"

	multi	Germany	othWest	East	Balkans	Total
N	1		2	1		4
NS	1		2	1		4
Wt	123.47		16.36	21.04		160.86
Het Chi	0.00		4.98	0.00		40.84
Het df	0		1	0		3
Het P	N.S.		*	N.S.		***
Fixed RR	6.74		32.39	9.14		8.23
RRl	5.65		19.95	5.96		7.05
RRu	8.04		52.58	14.02		9.60
P	+++		+++	+++		+++
Random RR	6.74		29.58	9.14		14.46
RRl	5.65		9.81	5.96		6.45
RRu	8.04		89.18	14.02		32.43
P	+++		+++	+++		+++
Between Chi						35.86
Between df						2
Between P						***
Btwn(F) P						N.S.
Btwn(R) P						*

Detailed Country in "other Asia"

	India	HongKong	other	Total
N			1	1
NS			1	1
Wt			5.31	5.31
Het Chi			0.00	0.00
Het df			0	0
Het P			N.S.	N.S.
Fixed RR			8.93	8.93
RRl			3.81	3.81
RRu			20.91	20.91
P			+++	+++
Random RR			8.93	8.93
RRl			3.81	3.81
RRu			20.91	20.91
P			+++	+++
Between Chi				
Between df				
Between P				N.S.
Btwn(F) P				N.S.
Btwn(R) P				N.S.

Detailed other continent

	SCAmer	Total
N	3	3
NS	2	2
Wt	34.00	34.00
Het Chi	4.35	4.35
Het df	2	2
Het P	N.S.	N.S.
Fixed RR	12.72	12.72
RRl	9.09	9.09
RRu	17.80	17.80
P	+++	+++
Random RR	12.57	12.57
RRl	7.58	7.58
RRu	20.84	20.84
P	+++	+++
Between Chi		
Between df		
Between P		N.S.
Btwn(F) P		N.S.
Btwn(R) P		N.S.

Table 1H14 - 3

IESLC - Meta-analysis of Ever/current Smoking, Age started, "High"
All LC types, Any Product (or Cigarettes if Any not available)
Most adjusted

	Start year of study					
	<1960	1960-69	1970-79	1980-89	1990+	Total
N	8	8	7	10	2	35
NS	5	4	6	9	2	26
Wt	165.07	100.38	190.48	99.56	16.89	572.38
Het Chi	45.45	63.08	53.28	35.48	8.98	252.35
Het df	7	7	6	9	1	34
Het P	***	***	***	***	**	***
Fixed RR	12.47	16.41	9.17	10.82	3.59	11.11
RRl	10.70	13.49	7.96	8.89	2.23	10.23
RRu	14.52	19.95	10.57	13.16	5.79	12.06
P	+++	+++	+++	+++	+++	+++
Random RR	11.04	8.33	15.73	10.00	3.74	10.32
RRl	7.04	4.10	8.92	6.60	0.89	8.04
RRu	17.31	16.96	27.75	15.16	15.65	13.26
P	+++	+++	+++	+++	(+)	+++
Between Chi						46.08
Between df						4
Between P						***
Btwn(F) P						N.S.
Btwn(R) P						N.S.

	Study type (1)		
	CC	other	Total
N	25	10	35
NS	20	6	26
Wt	441.01	131.37	572.38
Het Chi	187.15	64.68	252.35
Het df	24	9	34
Het P	***	***	***
Fixed RR	10.93	11.73	11.11
RRl	9.95	9.89	10.23
RRu	12.00	13.92	12.06
P	+++	+++	+++
Random RR	11.38	7.45	10.32
RRl	8.53	4.18	8.04
RRu	15.18	13.29	13.26
P	+++	+++	+++
Between Chi			0.51
Between df			1
Between P			N.S.
Btwn(F) P			N.S.
Btwn(R) P			N.S.

	Study type (2)			
	CC	prosp	other	Total
N	25	10		35
NS	20	6		26
Wt	441.01	131.37		572.38
Het Chi	187.15	64.68		252.35
Het df	24	9		34
Het P	***	***		***
Fixed RR	10.93	11.73		11.11
RRl	9.95	9.89		10.23
RRu	12.00	13.92		12.06
P	+++	+++		+++
Random RR	11.38	7.45		10.32
RRl	8.53	4.18		8.04
RRu	15.18	13.29		13.26
P	+++	+++		+++
Between Chi				0.51
Between df				1
Between P				N.S.
Btwn(F) P				N.S.
Btwn(R) P				N.S.

Table 1H14 - 3

IESLC - Meta-analysis of Ever/current Smoking, Age started, "High"
All LC types, Any Product (or Cigarettes if Any not available)
Most adjusted

	Study size (number of LC cases) 100-249	250-499	500-999	1000+	Total
N	5	8	11	11	35
NS	4	6	9	7	26
Wt	23.26	45.50	122.87	380.75	572.38
Het Chi	13.75	7.56	80.13	113.99	252.35
Het df	4	7	10	10	34
Het P	**	N.S.	***	***	***
Fixed RR	3.42	9.33	12.74	11.66	11.11
RRl	2.28	6.98	10.68	10.54	10.23
RRu	5.13	12.48	15.21	12.89	12.06
P	+++	+++	+++	+++	+++
Random RR	3.64	9.23	13.95	12.13	10.32
RRl	1.55	6.75	8.36	8.33	8.04
RRu	8.58	12.62	23.28	17.66	13.26
P	++	+++	+++	+++	+++
Between Chi					36.92
Between df					3
Between P					***
Btwn(F) P					N.S.
Btwn(R) P					*

	Risky occupational population no	mining	othRisky	Total
N	34	1		35
NS	25	1		26
Wt	563.42	8.97		572.38
Het Chi	222.36	0.00		252.35
Het df	33	0		34
Het P	***	N.S.		***
Fixed RR	11.43	1.81		11.11
RRl	10.53	0.94		10.23
RRu	12.42	3.48		12.06
P	+++	(+)		+++
Random RR	10.96	1.81		10.32
RRl	8.60	0.94		8.04
RRu	13.96	3.48		13.26
P	+++	(+)		+++
Between Chi				29.99
Between df				1
Between P				***
Btwn(F) P				*
Btwn(R) P				***

	National cigarette tobacco type Virginia	blended	other	Total
N	5	26	4	35
NS	3	20	3	26
Wt	39.70	511.59	21.10	572.38
Het Chi	20.61	179.31	6.53	252.35
Het df	4	25	3	34
Het P	***	***	(*)	***
Fixed RR	8.39	12.01	2.82	11.11
RRl	6.15	11.01	1.84	10.23
RRu	11.46	13.10	4.32	12.06
P	+++	+++	+++	+++
Random RR	7.24	13.05	2.82	10.32
RRl	3.30	10.04	1.45	8.04
RRu	15.90	16.96	5.50	13.26
P	+++	+++	++	+++
Between Chi				45.89
Between df				2
Between P				***
Btwn(F) P				*
Btwn(R) P				***

Table 1H14 - 3

IESLC - Meta-analysis of Ever/current Smoking, Age started, "High"
All LC types, Any Product (or Cigarettes if Any not available)
Most adjusted

	Any proxy use No/nk	Yes	Total
N	27	8	35
NS	19	7	26
Wt	470.41	101.97	572.38
Het Chi	190.93	59.88	252.35
Het df	26	7	34
Het P	***	***	***
Fixed RR	10.84	12.42	11.11
RRl	9.90	10.23	10.23
RRu	11.87	15.08	12.06
P	+++	+++	+++
Random RR	9.48	13.34	10.32
RRl	7.13	7.44	8.04
RRu	12.60	23.93	13.26
P	+++	+++	+++
Between Chi			1.54
Between df			1
Between P			N.S.
Btwn(F) P			N.S.
Btwn(R) P			N.S.

	Full histological confirmation No	Yes	Total
N	24	11	35
NS	17	9	26
Wt	294.39	277.99	572.38
Het Chi	114.38	137.96	252.35
Het df	23	10	34
Het P	***	***	***
Fixed RR	11.07	11.15	11.11
RRl	9.87	9.91	10.23
RRu	12.41	12.54	12.06
P	+++	+++	+++
Random RR	9.74	11.31	10.32
RRl	7.30	6.87	8.04
RRu	12.99	18.62	13.26
P	+++	+++	+++
Between Chi			0.01
Between df			1
Between P			N.S.
Btwn(F) P			N.S.
Btwn(R) P			N.S.

	Number of adjustment variables (1) 0	1	2+/+nk	Total
N	26	7	2	35
NS	19	6	2	27
Wt	343.58	215.01	13.79	572.38
Het Chi	123.47	95.41	11.02	252.35
Het df	25	6	1	34
Het P	***	***	***	***
Fixed RR	12.77	8.65	16.83	11.11
RRl	11.49	7.57	9.93	10.23
RRu	14.19	9.89	28.54	12.06
P	+++	+++	+++	+++
Random RR	10.94	7.47	19.03	10.32
RRl	8.42	3.83	3.23	8.04
RRu	14.21	14.58	112.01	13.26
P	+++	+++	++	+++
Between Chi				22.45
Between df				2
Between P				***
Btwn(F) P				N.S.
Btwn(R) P				N.S.

Table 1H14 - 3

IESLC - Meta-analysis of Ever/current Smoking, Age started, "High"
All LC types, Any Product (or Cigarettes if Any not available)
Most adjusted

	Number of adjustment variables (2) 0	1	2	3-5	6+/+nk	Total
N	26	7	2			35
NS	19	6	2			27
Wt	343.58	215.01	13.79			572.38
Het Chi	123.47	95.41	11.02			252.35
Het df	25	6	1			34
Het P	***	***	***			***
Fixed RR	12.77	8.65	16.83			11.11
RRl	11.49	7.57	9.93			10.23
RRu	14.19	9.89	28.54			12.06
P	+++	+++	+++			+++
Random RR	10.94	7.47	19.03			10.32
RRl	8.42	3.83	3.23			8.04
RRu	14.21	14.58	112.01			13.26
P	+++	+++	++			+++
Between Chi						22.45
Between df						2
Between P						***
Btwn(F) P						N.S.
Btwn(R) P						N.S.

	Smoking status ever	current	Total
N	26	9	35
NS	21	5	26
Wt	480.38	92.01	572.38
Het Chi	203.73	48.05	252.35
Het df	25	8	34
Het P	***	***	***
Fixed RR	11.26	10.33	11.11
RRl	10.30	8.42	10.23
RRu	12.32	12.68	12.06
P	+++	+++	+++
Random RR	11.80	5.95	10.32
RRl	8.92	2.98	8.04
RRu	15.61	11.89	13.26
P	+++	+++	+++
Between Chi			0.57
Between df			1
Between P			N.S.
Btwn(F) P			N.S.
Btwn(R) P			(*)

	Product all/unsp	cig+/-ot	cig only	Total
N	6	22	7	35
NS	5	18	4	27
Wt	82.18	455.87	34.33	572.38
Het Chi	59.87	152.61	10.02	252.35
Het df	5	21	6	34
Het P	***	***	N.S.	***
Fixed RR	11.17	11.87	4.52	11.11
RRl	9.00	10.83	3.23	10.23
RRu	13.87	13.01	6.31	12.06
P	+++	+++	+++	+++
Random RR	13.39	12.02	4.40	10.32
RRl	5.59	9.12	2.73	8.04
RRu	32.10	15.84	7.11	13.26
P	+++	+++	+++	+++
Between Chi				29.84
Between df				2
Between P				***
Btwn(F) P				N.S.
Btwn(R) P				**

Table 1H14 - 3

IESLC - Meta-analysis of Ever/current Smoking, Age started, "High"
All LC types, Any Product (or Cigarettes if Any not available)
Most adjusted

	Denominator nev any	nev cigs	Total
N	22	13	35
NS	15	11	26
Wt	296.58	275.81	572.38
Het Chi	132.47	119.30	252.35
Het df	21	12	34
Het P	***	***	***
Fixed RR	10.77	11.48	11.11
RRl	9.61	10.20	10.23
RRu	12.07	12.92	12.06
P	+++	+++	+++
Random RR	9.03	12.79	10.32
RRl	6.58	8.19	8.04
RRu	12.39	19.98	13.26
P	+++	+++	+++
Between Chi			0.58
Between df			1
Between P			N.S.
Btwn(F) P			N.S.
Btwn(R) P			N.S.

	Derivation of RR/CI Orig	StdCalc	Other	Total
N	5	24	6	35
NS	5	18	4	27
Wt	40.30	464.23	67.85	572.38
Het Chi	65.74	155.03	26.74	252.35
Het df	4	23	5	34
Het P	***	***	***	***
Fixed RR	13.55	10.62	13.37	11.11
RRl	9.95	9.70	10.54	10.23
RRu	18.45	11.64	16.96	12.06
P	+++	+++	+++	+++
Random RR	14.27	10.28	7.18	10.32
RRl	4.06	7.91	2.40	8.04
RRu	50.13	13.34	21.45	13.26
P	+++	+++	+++	+++
Between Chi				4.84
Between df				2
Between P				(*)
Btwn(F) P				N.S.
Btwn(R) P				N.S.

Table 1H14 - 4

IESLC - Meta-analysis of Ever/current Smoking, Age started, "High"
All LC types, Any Product (or Cigarettes if Any not available)
Least adjusted

REF	NRR	X	SEX	AGEL	AGEH	RACE	YF	LC TYPE	LOC	START	ST	NLC	R	VB	P	H	AD	SM	PRODUCT	exL	exH	DENOM	De
ARMADA	512		m	0	0	all	-	all	Eu:wst	1986	CC	325	n	bl	n	y	0	ev	cig+/-ot	7	16	nev cigs	st
AUVINE	511	x	c	0	0	all	-	all	Eu:Sca	1986	CC	517	n	bl	y	n	0	ev	cig+/-ot	1	15	nev cigs	st
BARBON	517	x	m	0	0	all	-	all	Eu:wst	1979	CC	755	n	bl	y	y	0	ev	all/unsp	1	14	nev any	st
BRESLO	503		c	0	0	all	-	all	NAmer	1949	CC	518	n	bl	n	y	0	ev	cig+/-ot	0	14	nev any	st
BUFFLE	521		f	0	0	w-hi	-	all	NAmer	1976	CC	943	n	bl	y	n	0	ev	cig+/-ot	6	16	nev cigs	or
CEDERL	512		m	0	0	all	10	all	Eu:Sca	1963	pr	491	n	bl	n	n	1	cu	cig only	1	16	nev any	ot
CEDERL	517		f	0	0	all	10	all	Eu:Sca	1963	pr	491	n	bl	n	n	0	cu	cig only	1	16	nev any	ot
CHOI	526		m	0	0	all	-	all	As:oth	1985	CC	375	n	bl	n	n	0	ev	cig+/-ot	1	14	nev cigs	st
CPSI	804		m	35	84	all	6	all	NAmer	1959	pr	5138	n	bl	n	n	1	cu	cig+/-ot	1	14	nev any	ot
CPSI	848		f	40	74	all	6	all	NAmer	1959	pr	5138	n	bl	n	n	1	cu	cig only	1	14	nev any	ot
DAMBER	503		m	0	0	all	-	all	Eu:Sca	1972	CC	579	n	bl	y	n	0	ev	all/unsp	1	15	nev any	st
DEAN3	567		m	0	0	all	-	all	Eu:UK	1969	CC	766	n	V	y	n	0	cu	cig only	1	14	nev any	st
DEAN3	586		f	0	0	all	-	all	Eu:UK	1969	CC	766	n	V	y	n	0	cu	cig only	1	14	nev any	st
DORN	613		m	55	64	wh	8	all	NAmer	1954	pr	5097	n	bl	n	n	0	ev	cig+/-ot	1	14	nev any	st
DORN	650		m	65	74	wh	8	all	NAmer	1954	pr	5097	n	bl	n	n	0	ev	cig+/-ot	1	14	nev any	st
GENG	530	x	f	0	0	all	-	all	As:Chi	1985	CC	292	n	ot	*	n	0	ev	cig+/-ot	1	15	nev any	st
HU	513		m	0	0	all	-	all	As:Chi	1985	CC	227	n	ot	n	y	0	ev	cig+/-ot	1	15	nev cigs	st
HU	518		f	0	0	all	-	all	As:Chi	1985	CC	227	n	ot	n	y	0	ev	cig+/-ot	1	15	nev cigs	st
JEDRYC	608		m	0	0	all	-	all	Eu:est	1980	CC	1630	n	bl	y	n	0	ev	cig+/-ot	1	16	nev any	st
JOLY	545		m	0	0	all	-	all	SCAmer	1978	CC	826	n	bl	n	n	0	ev	cig+/-ot	1	14	nev any	st
JOLY	535		f	0	0	all	-	all	SCAmer	1978	CC	826	n	bl	n	n	0	ev	cig+/-ot	1	14	nev any	st
KHUDER	508		m	0	0	all	-	all	NAmer	1985	CC	482	n	bl	n	y	0	ev	cig+/-ot	1	15	nev cigs	st
KOULUM	504		m	0	0	all	-	all	Eu:Sca	1936	CC	812	n	bl	n	n	0	ev	all/unsp	11	15	nev any	st
LETOUR	503		c	0	0	all	-	all	NAmer	1983	CC	738	n	V	y	y	0	ev	cig+/-ot	1	14	nev cigs	st
LUBIN2	1150	x	m	0	0	all	-	all	Eu:mul	1976	CC	7804	n	bl	n	y	0	ev	cig+/-ot	13	16	nev cigs	st
MATOS	558	x	m	0	0	all	-	all	SCAmer	1994	CC	200	n	bl	n	n	0	ev	cig+/-ot	1	14	nev any	st
MIGRAN	505		m	0	0	all	0	all	Eu:UK	1964	pr	259	n	V	n	n	0	cu	cig only	0	15	nev any	st
MIGRAN	515		f	0	0	all	0	all	Eu:UK	1964	pr	259	n	V	n	n	0	cu	cig only	0	15	nev any	st
MRFITR	513		m	0	0	all	0	all	NAmer	1973	pr	119	n	bl	n	n	0	cu	cig+/-ot	1	15	nev cigs	ot
PERNU	505		m	0	0	all	-	all	Eu:Sca	1944	CC	1606	n	bl	n	n	0	ev	all/unsp	1	14	nev any	st
PERNU	502		f	0	0	all	-	all	Eu:Sca	1944	CC	1606	n	bl	n	n	0	ev	all/unsp	1	14	nev any	ot
QIAO2	503	x	m	0	0	all	0	all	As:Chi	1992	pr	241	m	ot	n	n	0	ev	all/unsp	1	16	nev any	st
SOBUE	656		m	0	0	all	-	all	As:Jap	1986	CC	1376	n	bl	n	y	0	ev	cig+/-ot	10	17	nev cigs	st
WYNDE6	761		m	0	0	wh	-	q+s+a	NAmer	1969	CC	4423	n	bl	n	y	0	ev	cig+/-ot	1	17	nev cigs	st
WYNDE6	769		f	0	0	wh	-	q+s+a	NAmer	1969	CC	4423	n	bl	n	y	0	ev	cig+/-ot	1	17	nev cigs	st

Cigarette type is all/unspec for all RRs
except for the following:

REF	NRR	CIGTYPE
DEAN3	567	MC only
DEAN3	586	MC only

Table 1H14 - 5

IESLC - Meta-analysis of Ever/current Smoking, Age started, "High"
All LC types, Any Product (or Cigarettes if Any not available)
Least adjusted

REF	NRR	SEX	AD	Number Exposed Case	Cont	Non-exposed Case	Cont	RR	95.00%CI
ARMADA	512	m	0	204	110	8	71	16.46	(7.64- 35.44)
AUVINE	511	c	0	55	6	44	229	47.71	(19.35- 117.62)
BARBON	517	m	0	138	23	22	188	51.27	(27.46- 95.73)
BRESLO	503	c	0	166	116	19	56	4.22	(2.38- 7.47)
BUFFLE	521	f	0	78	41	12	112	17.76	(8.77- 35.94)
*CEDERL	512	m	1	7	-	7	-	6.40	(2.25- 18.21)
*CEDERL	517	f	0	0	746	19	17679	0.61~	(0.04- 10.05)
Subtotal CEDERL								4.80	(1.80- 12.79)
CHOI	526	m	0	22	18	13	95	8.93	(3.81- 20.91)
*CPSI	804	m	1	185	-	83	-	16.77	(12.94- 21.73)
*CPSI	848	f	1	6	-	166	-	2.50	(1.11- 5.65)
Subtotal CPSI								14.07	(10.99- 18.02)
DAMBER	503	m	0	206	98	42	208	10.41	(6.91- 15.68)
DEAN3	567	m	0	44	165	24	510	5.67	(3.34- 9.60)
DEAN3	586	f	0	7	109	41	1538	2.41	(1.06- 5.50)
Subtotal DEAN3								4.42	(2.83- 6.89)
*DORN	613	m	0	84	36304	25	213858	19.79	(12.67- 30.93)
*DORN	650	m	0	81	24616	49	171211	11.50	(8.07- 16.39)
Subtotal DORN								14.18	(10.75- 18.72)
GENG	530	f	0	36	10	54	93	6.20	(2.85- 13.48)
HU	513	m	0	13	7	41	67	3.03	(1.12- 8.23)
HU	518	f	0	5	4	40	48	1.50	(0.38- 5.96)
Subtotal HU								2.38	(1.06- 5.35)
JEDRYC	608	m	0	135	66	49	219	9.14	(5.96- 14.02)
JOLY	545	m	0	317	282	12	218	20.42	(11.18- 37.32)
JOLY	535	f	0	76	35	52	283	11.82	(7.18- 19.44)
Subtotal JOLY								14.75	(10.05- 21.65)
KHUDER	508	m	0	226	295	23	309	10.29	(6.51- 16.27)
KOULUM	504	m	0	199	52	5	54	41.33	(15.74- 108.56)
LETOUR	503	c	0	151	76	24	224	18.54	(11.21- 30.67)
LUBIN2	1150	m	0	1312	1833	185	1878	7.27	(6.15- 8.59)
MATOS	558	m	0	69	90	11	110	7.67	(3.83- 15.36)
*MIGRAN	505	m	0	50	1081	4	867	10.03	(3.64- 27.65)
*MIGRAN	515	f	0	2	266	4	3814	7.17	(1.32- 38.96)
Subtotal MIGRAN								9.18	(3.84- 21.90)
*MRFITR	513	m	0	25	2065	0	1859	45.91~	(2.80- 753.64)
PERNU	505	m	0	337	92	97	275	10.38	(7.49- 14.40)
PERNU	502	f	0	1	0	110	971	26.38~	(1.07- 651.38)
Subtotal PERNU								10.48	(7.58- 14.51)
*QIAO2	503	m	0	104	2130	10	709	3.46	(1.82- 6.59)
SOBUE	656	m	0	137	62	29	126	9.60	(5.81- 15.88)
WYNDE6	761	m	0	611	301	51	589	23.44	(17.06- 32.21)
WYNDE6	769	f	0	291	91	73	673	29.48	(21.04- 41.31)
Subtotal WYNDE6								26.11	(20.72- 32.90)
Partial Totals				5380	71190	1448	419141		

*prospective study ~ With 0.5 adjustment for zero

REF	NRR	SEX	AD	Ys	Ws	Qs	Ps
ARMADA	512	m	0	2.80	6.53	0.95	0.0000
AUVINE	511	c	0	3.87	4.72	9.87	0.0000
BARBON	517	m	0	3.94	9.85	22.70	0.0000
BRESLO	503	c	0	1.44	11.75	11.28	0.0000
BUFFLE	521	f	0	2.88	7.72	1.62	0.0000
*CEDERL	512	m	1	1.86	3.51	1.11	0.0005
*CEDERL	517	f	0	-0.50	0.49	4.15	0.7276
Subtotal CEDERL				1.57	4.00	5.27	
CHOI	526	m	0	2.19	5.31	0.28	0.0000
*CPSI	804	m	1	2.82	57.18	9.17	0.0000
*CPSI	848	f	1	0.92	5.80	13.11	0.0273
Subtotal CPSI				2.64	62.99	22.28	
DAMBER	503	m	0	2.34	22.90	0.13	0.0000
DEAN3	567	m	0	1.73	13.81	6.47	0.0000
DEAN3	586	f	0	0.88	5.65	13.39	0.0367
Subtotal DEAN3				1.49	19.46	19.86	
*DORN	613	m	0	2.99	19.28	6.18	0.0000
*DORN	650	m	0	2.44	30.57	0.02	0.0000
Subtotal DORN				2.65	49.85	6.20	
GENG	530	f	0	1.82	6.37	2.25	0.0000
HU	513	m	0	1.11	3.86	6.61	0.0292
HU	518	f	0	0.41	2.02	8.18	0.5647

Table 1H14 - 5

IESLC - Meta-analysis of Ever/current Smoking, Age started, "High"
All LC types, Any Product (or Cigarettes if Any not available)
Least adjusted

REF	NRR	SEX	AD	Ys	Ws	Qs	Ps
Subtotal HU				0.87	5.88	14.79	
JEDRYC	608	m	0	2.21	21.04	0.89	0.0000
JOLY	545	m	0	3.02	10.57	3.77	0.0000
JOLY	535	f	0	2.47	15.51	0.04	0.0000
Subtotal JOLY				2.69	26.07	3.81	
KHUDER	508	m	0	2.33	18.34	0.14	0.0000
KOULUM	504	m	0	3.72	4.12	6.99	0.0000
LETOUR	503	c	0	2.92	15.17	3.81	0.0000
LUBIN2	1150	m	0	1.98	138.01	26.23	0.0000
MATOS	558	m	0	2.04	7.96	1.16	0.0000
*MIGRAN	505	m	0	2.31	3.73	0.05	0.0000
*MIGRAN	515	f	0	1.97	1.34	0.27	0.0226
Subtotal MIGRAN				2.22	5.07	0.32	
*MRFITR	513	m	0	3.83	0.49	0.97	0.0074
PERNU	505	m	0	2.34	35.99	0.22	0.0000
PERNU	502	f	0	3.27	0.37	0.27	0.0455
Subtotal PERNU				2.35	36.37	0.50	
*QIAO2	503	m	0	1.24	9.28	12.87	0.0002
SOBUE	656	m	0	2.26	15.19	0.38	0.0000
WYNDE6	761	m	0	3.15	38.07	20.59	0.0000
WYNDE6	769	f	0	3.38	33.77	31.43	0.0000
Subtotal WYNDE6				3.26	71.85	52.02	

	N	35
	NS	26
	Wt	586.28
Het	Chi	227.55
Het	df	34
Het	P	***
Fixed	RR	11.24
	RRl	10.36
	RRu	12.18
	P	+++
Random	RR	10.57
	RRl	8.34
	RRu	13.41
	P	+++
Asymm	P	N.S.

Table 1H14 - 6

IESLC - Meta-analysis of Ever/current Smoking, Age started, "High"
All LC types, Any Product (or Cigarettes if Any not available)
Least adjusted

		Sex			
		combined	male	female	Total
	N	3	22	10	35
	NS	3	21	10	34
	Wt	31.64	475.61	79.04	586.28
Het	Chi	24.68	127.55	73.46	227.55
Het	df	2	21	9	34
Het	P	***	***	***	***
Fixed	RR	12.32	10.94	12.74	11.24
	RRl	8.70	10.00	10.22	10.36
	RRu	17.46	11.97	15.88	12.18
	P	+++	+++	+++	+++
Random	RR	14.99	11.58	6.37	10.57
	RRl	4.14	9.01	2.99	8.34
	RRu	54.29	14.88	13.54	13.41
	P	+++	+++	+++	+++
Between	Chi				1.86
Between	df				2
Between	P				N.S.
Btwn(F)	P				N.S.
Btwn(R)	P				N.S.

Table 1H14 - 7

IESLC - Meta-analysis of Ever/current Smoking, Age started, "High"
All LC types, Any Product (or Cigarettes if Any not available)
Excluded studies (and stage at which they were excluded)

1	BECHER BLOT1 BROWN3 CARPEN CHYOU DARBY DOLL2 GARCIA GRAHAM GURSEL HAMMO2 JAHN JAIN LAUSSM PRESCO QIAO TVERDA WIGLE WYNDE3
2	AKIBA AMANDU AMES AXELSS BENSHL BEST BOUCHA BOUCOT BROSS CHEN CPSII DEAN2 DESTEF DORGAN DOSEME FAN GARSHI GER GILLIS HAMMON HUMBLE JUSSAW KAISE2 KATSOU KAUFMA KOO KREUZE LEVIN MCCONN NOTAN2 OSANN2 PEZZO2 PISANI RESTRE SADOWS VUTUC WANG2 WATSON WU2 WUWILL WYNDE2 WYNDE8 XU ZHOU
3	GUO MCDUFF SPITZ STASZE ZHANG
4	LUO
5	CORREA HOLE YUAN
7	BOFFET WYNDE7
10	ALDERS SPEIZE
14	AGUDO BENHAM CHEN2 CHIAZZ DOLL ENGELA GAO GAO2 HAENSZ HEGMAN HIRAYA HU2 LIAW LIU3 LIU4 LIU5 LUBIN PEZZOT RACHTA SEGI2 SUZUK2 SVENSS TIZZAN WAKAI WU ZHENG

Table 1H14 - 8

Potentially overlapping studies

REF	REFGP	PRINC	. OVERLAP/LINK
LUBIN2	LUBIN2	1	Lubin-combined
MRFITR	MRFIT	2	Subset of MRFIT
WYNDE6	WYNDE6	1	WYNDE5/6/7/8
CPSI	CPSI	1	CPSI overall

Table 1H14 - 9

Most adjusted - insufficient data for meta-analysis

REF	NRR	SEX	AGEL	AGEH	RACE	YF	LC TYPE	LOC	START	ST	NLC	R	VB	P	H	AD	SM	PRODUCT	exL	exH	DENOM	De
CORREA	537	c	0	0	all	-	all	NAmer	1979	CC	1359	n	bl	y	n	2	ev	cig+/-ot	1	15	nev cigs	or

REF	NRR	RR	SIG	RRDATA comment
CORREA	537	24.20		0

Table 1H15 -

IESLC - Meta-analysis of Ever/current Smoking, Age started, "Highest vs lowest"
All LC types, Any Product (or Cigarettes if Any not available)

This analysis is restricted to results for:
1) Ever/current smokers
2) Results by Age started
3) Categorical results by Age started
4) Denominator (unexposed) = "low"
5) All LC types (or near equivalent)
6) Results complete enough for use in metaanalysis

Within each study, results are then selected (in the following order of preference, within each sex) for:
7) SMKSTA: ever, current
8) PRODUCT: all/unspec, cigarettes regardless of other products, cigarettes only
9) CIGTYPE: all/unspecified, MC regardless of HR, MC only
10) Results with least adjustment for other aspects of smoking (ADOS)
11) The highest vs lowest category
12) Followup period (YF, prospective studies): whole study (coded as 0) or longest available
13) LCtype: all or nearest available, at least Squamous and Adeno. (q = squamous, s = small,
l = large, a = adeno, mix = mixed, alv = alveolar)
14) Race: all or nearest available, otherwise by race (wh or w = white, bl or b = black, hi = hispanic
ch = chinese, jap = japanese, haw = hawaiian, w+o = white + oriental, sca = scandinavian, as = asian)
15) For overlapping studies: principal rather than subsidiary studies
Finally by Age: whole study (coded as 0) if available, otherwise by widest available age group
and then for single sex results (m, f) in preference to results for both sexes combined (c).

Results adjusted (AD) for the most potential confounders are then chosen in Sections -1 to -3
and results adjusted for the least confounders in Sections -4 to -6. (Those least adjusted results which
actually differ from the most adjusted are marked 'x' in column X in Section -4)

Section -7 shows excluded studies, together with the stage (as above) at which no qualifying
results were found.

Section -8 lists the potentially overlapping studies which have been included (1=principal, 2=subsidiary).

Section -9 lists any results which would have been included in preference except that they had data not complete
enough for use in meta-analysis, with their significance (yes/no), if known, and any further comment as entered
on the database. It also lists as "gap" any categories for which no data were presented by the original authors.

In addition to those mentioned above, the following fields, levels and abbreviations are used:
* or nk = not known, n = no, y = yes, ot = other
all/unspec = all or unspecified, cig+/-ot = cigarettes irrespective of other products (cigar, pipe etc)
MC = manufactured cigarettes, HR = hand-rolled cigarettes
exL, exH = range of exposure (low and high) in the "highest" group, in terms of Age started
unexL, unexH = range of exposure (low and high) in the "lowest" group, in terms of Age started
REF: 6-character study reference
NRR: number of the RR on the database within the study
ST : study type (CC = case control, pr or prosp = prospective)
NLC: number of lung cancer cases in whole study
R : risky occupational population (n = no, m = mining, o = other risky)
VB : national cigarette type (V = at least 75% Virginia, bl = at least 75% blended, ot = other)
P : any proxy use
H : full histological confirmation
De : derivation of RR/CI (or = original, st = standard method, ot = other method of estimation)

Table 1H15 - 1

IESLC - Meta-analysis of Ever/current Smoking, Age started, "Highest vs lowest"
All LC types, Any Product (or Cigarettes if Any not available)
Most adjusted

REF	NRR	SEX	AGEL	AGEH	RACE	YF	LC TYPE	LOC	START	ST	NLC	R	VB	P	H	AD	ADOS	SM	PRODUCT	exL	exH	unexL	unexH	De
AGUDO	506	f	0	0	all	-	all	Eu:wst	1989	CC	103	n	bl	n	n	3	0	ev	cig only	1	23	24	999	ot
ALDERS	503	m	0	0	all	-	all	Eu:UK	1977	CC	1448	n	V	n	n	2	1#	ev	cig only	1	14	25	999	ot
ALDERS	506	f	0	0	all	-	all	Eu:UK	1977	CC	1448	n	V	n	n	2	1#	ev	cig only	1	14	25	999	ot
ARMADA	513	m	0	0	all	-	all	Eu:wst	1986	CC	325	n	bl	n	y	0	0	ev	cig+/-ot	7	16	17	45	st
AUVINE	513	c	0	0	all	-	all	Eu:Sca	1986	CC	517	n	bl	y	n	0	0	ev	cig+/-ot	1	15	21	999	st
BARBON	524	m	0	0	all	-	all	Eu:wst	1979	CC	755	n	bl	y	y	1	0	ev	all/unsp	1	14	20	999	ot
BRESLO	505	c	0	0	all	-	all	NAmer	1949	CC	518	n	bl	n	y	0	0	ev	cig+/-ot	0	14	25	999	st
BUFFLE	525	f	0	0	w-hi	-	all	NAmer	1976	CC	943	n	bl	y	n	0	0	ev	cig+/-ot	6	16	30	999	st
CEDERL	556	m	0	0	all	16	all	Eu:Sca	1963	pr	491	n	bl	n	n	3	1#	cu	all/unsp	1	19	25	999	ot
CEDERL	536	f	0	0	all	10	all	Eu:Sca	1963	pr	491	n	bl	n	n	0	0	cu	cig only	1	16	19	999	ot
CHEN2	521	m	0	0	all	-	all	As:Chi	1983	CC	193	n	ot	y	n	0	0	ev	all/unsp	1	19	31	999	st
CHEN2	526	f	0	0	all	-	all	As:Chi	1983	CC	193	n	ot	y	n	0	0	ev	all/unsp	1	19	31	999	st
CHIAZZ	503	m	0	0	all	-	all	NAmer	1940	CC	144	o	bl	y	n	2	0	ev	cig+/-ot	1	19	20	999	ot
CHOI	529	m	0	0	all	-	all	As:oth	1985	CC	375	n	bl	n	n	0	0	ev	cig+/-ot	1	14	25	999	st
CHOI	532	f	0	0	all	-	all	As:oth	1985	CC	375	n	bl	n	n	0	0	ev	cig+/-ot	1	24	25	999	st
CPSI	822	m	35	84	all	6	all	NAmer	1959	pr	5138	n	bl	n	n	1	0	cu	cig+/-ot	1	14	25	999	ot
CPSI	851	f	40	74	all	6	all	NAmer	1959	pr	5138	n	bl	n	n	1	0	cu	cig only	1	14	25	999	ot
DAMBER	505	m	0	0	all	-	all	Eu:Sca	1972	CC	579	n	bl	y	n	0	0	ev	all/unsp	1	15	21	999	st
DEAN3	570	m	0	0	all	-	all	Eu:UK	1969	CC	766	n	V	y	n	0	0	cu	cig only	1	14	25	999	st
DEAN3	589	f	0	0	all	-	all	Eu:UK	1969	CC	766	n	V	y	n	0	0	cu	cig only	1	14	25	999	st
DOLL	507	m	0	0	all	-	all	Eu:UK	1948	CC	1465	n	V	n	n	0	0	ev	all/unsp	1	19	40	999	st
DOLL	514	f	0	0	all	-	all	Eu:UK	1948	CC	1465	n	V	n	n	0	0	ev	all/unsp	1	19	40	999	st
DORN	616	m	55	64	wh	8	all	NAmer	1954	pr	5097	n	bl	n	n	0	0	ev	cig+/-ot	1	14	25	999	st
DORN	653	m	65	74	wh	8	all	NAmer	1954	pr	5097	n	bl	n	n	0	0	ev	cig+/-ot	1	14	25	999	st
ENGELA	505	m	0	0	all	0	all	Eu:Sca	1964	pr	435	n	bl	n	n	0	0	cu	cig+/-ot	1	19	30	999	st
ENGELA	513	f	0	0	all	0	all	Eu:Sca	1964	pr	435	n	bl	n	n	0	0	cu	cig+/-ot	1	19	30	999	st
GAO	510	m	0	0	all	-	all	As:Chi	1984	CC	1405	n	ot	n	n	2	0	ev	cig+/-ot	10	19	30	999	ot
GAO	520	f	0	0	all	-	all	As:Chi	1984	CC	1405	n	ot	n	n	2	0	ev	cig+/-ot	10	19	30	999	ot
GAO2	505	m	0	0	all	-	all	As:Jap	1988	CC	282	n	bl	n	n	0	0	cu	cig+/-ot	1	19	30	999	st
GENG	537	f	0	0	all	-	all	As:Chi	1985	CC	292	n	ot	*	n	1	0	ev	cig+/-ot	1	15	21	999	st
HAENSZ	550	f	0	0	all	-	not alv	NAmer	1955	CC	158	n	bl	n	y	2	0	ev	cig+/-ot	1	24	25	999	ot
HEGMAN	515	m	0	0	all	-	all	NAmer	1989	CC	282	n	bl	y	y	1	0	ev	all/unsp	1	19	20	999	or
HEGMAN	518	f	0	0	all	-	all	NAmer	1989	CC	282	n	bl	y	y	1	0	ev	all/unsp	1	25	26	999	or
HIRAYA	564	m	0	0	all	5	all	As:Jap	1965	pr	1917	n	bl	n	n	1	0	cu	cig+/-ot	1	19	25	999	ot
HIRAYA	506	f	0	0	all	0	all	As:Jap	1965	pr	1917	n	bl	n	n	1	0	cu	cig+/-ot	1	19	20	999	ot
HU	515	m	0	0	all	-	all	As:Chi	1985	CC	227	n	ot	n	y	0	0	ev	cig+/-ot	1	15	30	999	st
HU	520	f	0	0	all	-	all	As:Chi	1985	CC	227	n	ot	n	y	0	0	ev	cig+/-ot	1	15	30	999	st
HU2	507	c	0	0	all	-	all	As:Chi	1977	CC	523	n	ot	y	n	0	0	ev	cig+/-ot	1	19	40	999	st
JEDRYC	610	m	0	0	all	-	all	Eu:est	1980	CC	1630	n	bl	y	n	5	2#	ev	cig+/-ot	1	16	19	999	or
JEDRYC	620	f	0	0	all	-	all	Eu:est	1980	CC	1630	n	bl	y	n	5	2#	ev	cig+/-ot	1	22	23	999	or
JOLY	547	m	0	0	all	-	all	SCAmer	1978	CC	826	n	bl	n	n	0	0	ev	cig+/-ot	1	14	25	999	st
JOLY	537	f	0	0	all	-	all	SCAmer	1978	CC	826	n	bl	n	n	0	0	ev	cig+/-ot	1	14	25	999	st
KHUDER	510	m	0	0	all	-	all	NAmer	1985	CC	482	n	bl	n	y	0	0	ev	cig+/-ot	1	15	20	999	st
KOULUM	509	m	0	0	all	-	all	Eu:Sca	1936	CC	812	n	bl	n	n	0	0	ev	all/unsp	0	10	31	999	st
LETOUR	505	c	0	0	all	-	all	NAmer	1983	CC	738	n	V	y	y	0	0	ev	cig+/-ot	1	14	21	999	st
LIAW	510	c	0	0	all	0	all	As:oth	1982	pr	127	n	ot	n	n	2	0	cu	all/unsp	1	20	25	999	ot
LIU3	506	m	0	0	all	-	all	As:Chi	1985	CC	110	n	ot	n	n	2	0	ev	all/unsp	1	20	21	999	ot
LIU4	505	m	35	69	all	-	all	As:Chi	1986	CC	1000-00	n	ot	y	n	2	0	ev	all/unsp	0	19	25	999	ot
LIU5	503	c	0	0	all	-	all	As:Chi	1978	CC	111	n	ot	y	n	0	0	ev	all/unsp	1	29	30	999	st
LUBIN	571	m	0	0	all	-	all	As:Chi	1984	CC	427	m	ot	y	n	0	0	ev	cig+/-ot	1	19	27	999	st
LUBIN2	1164	m	0	0	all	-	all	Eu:mul	1976	CC	7804	n	bl	n	y	1	0	ev	cig+/-ot	1	12	31	999	st
MATOS	580	m	0	0	all	-	all	SCAmer	1994	CC	200	n	bl	n	n	2	0	ev	cig+/-ot	1	14	20	999	ot
MIGRAN	509	m	0	0	all	0	all	Eu:UK	1964	pr	259	n	V	n	n	0	0	cu	cig only	0	15	20	999	st
MIGRAN	519	f	0	0	all	0	all	Eu:UK	1964	pr	259	n	V	n	n	0	0	cu	cig only	0	15	20	999	st
MRFITR	518	m	0	0	all	0	all	NAmer	1973	pr	119	n	bl	n	n	0	0	cu	cig+/-ot	1	15	24	999	st
PERNU	506	m	0	0	all	-	all	Eu:Sca	1944	CC	1606	n	bl	n	n	0	0	ev	all/unsp	1	14	15	999	st
PERNU	503	f	0	0	all	-	all	Eu:Sca	1944	CC	1606	n	bl	n	n	0	0	ev	all/unsp	1	14	15	999	ot
PEZZOT	576	m	0	0	all	-	all	SCAmer	1987	CC	215	n	bl	n	y	2	0	ev	cig only	1	13	19	999	ot
QIAO2	510	m	0	0	all	0	all	As:Chi	1992	pr	241	m	ot	n	n	1	0	ev	all/unsp	1	16	21	999	ot
RACHTA	510	f	0	0	all	-	all	Eu:est	1991	CC	118	n	bl	n	y	1	0	ev	cig+/-ot	1	19	31	999	ot
SEGI2	525	m	0	0	all	-	all	As:Jap	1962	CC	378	n	bl	n	n	1	0	cu	cig+/-ot	1	19	23	999	ot
SOBUE	800	m	0	0	all	-	all	As:Jap	1986	CC	1376	n	bl	n	y	0	0	ev	cig+/-ot	10	17	23	999	st
SPEIZE	530	f	0	0	all	0	all	NAmer	1976	pr	593	n	bl	n	y	2	1#	cu	cig+/-ot	18	19	22	999	st
SUZUK2	523	c	0	0	all	-	all	SCAmer	1991	CC	123	n	bl	n	y	3	0	ev	all/unsp	0	11	19	999	or
SVENSS	505	f	0	0	all	-	all	Eu:Sca	1983	CC	210	n	bl	n	n	0	0	cu	all/unsp	0	18	26	999	st
TIZZAN	510	m	0	0	all	-	all	Eu:wst	1959	CC	1358	n	bl	n	n	0	0	ev	all/unsp	1	19	31	999	st
TIZZAN	523	f	0	0	all	-	all	Eu:wst	1959	CC	1358	n	bl	n	n	0	0	ev	all/unsp	1	19	31	999	st
WAKAI	505	m	0	0	all	-	all	As:Jap	1988	CC	333	n	bl	n	y	0	0	cu	cig+/-ot	1	19	30	999	st
WU	545	f	0	0	wh	-	q+a	NAmer	1981	CC	220	n	bl	n	y	2	0	cu	all/unsp	0	18	25	999	ot
WYNDE6	763	m	0	0	wh	-	q+s+a	NAmer	1969	CC	4423	n	bl	n	y	0	0	ev	cig+/-ot	1	17	21	999	st
WYNDE6	771	f	0	0	wh	-	q+s+a	NAmer	1969	CC	4423	n	bl	n	y	0	0	ev	cig+/-ot	1	17	21	999	st

Table 1H15 - 1

IESLC - Meta-analysis of Ever/current Smoking, Age started, "Highest vs lowest"
All LC types, Any Product (or Cigarettes if Any not available)
Most adjusted

REF	NRR	SEX	AGEL	AGEH	RACE	YF	LC TYPE	LOC	START	ST	NLC	R	VB	P	H	AD	ADOS	SM	PRODUCT	exL	exH	unexL	unexH	De
ZHENG	569	m	0	0	all	-	all	As:Chi	1982	CC	540	n	ot	*	y	1	0	ev	cig+/-ot	1	19	30	999	ot
ZHENG	575	f	0	0	all	-	all	As:Chi	1982	CC	540	n	ot	*	y	1	0	ev	cig+/-ot	1	29	30	999	ot

Comments on values in listings

ALDERS	ADOS	Number of cigs/day
ALDERS	ADOS	Number of cigs/day
CEDERL	ADOS	Amount smoked
JEDRYC	ADOS	Number of cigs per day & time since quit
JEDRYC	ADOS	Number of cigs per day & time since quit
SPEIZE	ADOS	Numbers of cigs/day

Cigarette type is all/unspec for all RRs
except for the following:

REF	NRR	CIGTYPE
ALDERS	503	MC only
ALDERS	506	MC only
DEAN3	570	MC only
DEAN3	589	MC only

Table 1H15 - 2

IESLC - Meta-analysis of Ever/current Smoking, Age started, "Highest vs lowest"
All LC types, Any Product (or Cigarettes if Any not available)
Most adjusted

REF	NRR	SEX	AD	Number Exposed Case	Number Exposed Cont	Non-exposed Case	Non-exposed Cont	RR	95.00%CI
AGUDO	506	f	3	16	-	7	-	6.81	(1.20- 38.56)
ALDERS	503	m	2	139	-	11	-	2.94	(1.38- 6.25)
ALDERS	506	f	2	78	-	97	-	2.08	(1.27- 3.41)
Subtotal ALDERS								2.31	(1.53- 3.49)
ARMADA	513	m	0	204	110	113	144	2.36	(1.69- 3.31)
AUVINE	513	c	0	55	6	76	27	3.26	(1.26- 8.42)
BARBON	524	m	1	138	-	200	-	6.20	(3.82- 10.05)
BRESLO	505	c	0	166	116	32	35	1.57	(0.92- 2.67)
BUFFLE	525	f	0	78	41	23	23	1.90	(0.95- 3.80)
*CEDERL	556	m	3	183	-	6	-	4.70	(2.08- 10.60)
*CEDERL	536	f	0	0	746	6	2806	0.29~	(0.02- 5.13)
Subtotal CEDERL								3.82	(1.75- 8.36)
CHEN2	521	m	0	84	67	8	5	0.78	(0.25- 2.51)
CHEN2	526	f	0	23	13	5	8	2.83	(0.77- 10.47)
Subtotal CHEN2								1.38	(0.58- 3.29)
CHIAZZ	503	m	2	-	-	-	-	6.63	(2.13- 20.63)
CHOI	529	m	0	22	18	36	77	2.61	(1.25- 5.47)
CHOI	532	f	0	4	1	15	25	6.67	(0.68- 65.37)
Subtotal CHOI								2.86	(1.42- 5.76)
*CPSI	822	m	1	185	-	42	-	4.11	(2.94- 5.75)
*CPSI	851	f	1	6	-	51	-	1.11	(0.48- 2.59)
Subtotal CPSI								3.44	(2.52- 4.69)
DAMBER	505	m	0	206	98	70	76	2.28	(1.52- 3.42)
DEAN3	570	m	0	44	165	24	75	0.83	(0.47- 1.47)
DEAN3	589	f	0	7	109	27	274	0.65	(0.28- 1.54)
Subtotal DEAN3								0.77	(0.48- 1.24)
DOLL	507	m	0	1077	992	4	7	1.90	(0.55- 6.51)
DOLL	514	f	0	20	12	15	15	1.67	(0.61- 4.59)
Subtotal DOLL								1.76	(0.80- 3.84)
*DORN	616	m	0	84	36304	37	73050	4.57	(3.10- 6.72)
*DORN	653	m	0	81	24616	90	74464	2.72	(2.02- 3.67)
Subtotal DORN								3.31	(2.61- 4.19)
*ENGELA	505	m	0	173	50732	17	9762	1.96	(1.19- 3.22)
*ENGELA	513	f	0	18	10687	10	24560	4.14	(1.91- 8.96)
Subtotal ENGELA								2.44	(1.60- 3.71)
GAO	510	m	2	262	-	45	-	4.25	(2.93- 6.16)
GAO	520	f	2	77	-	73	-	2.80	(1.56- 5.04)
Subtotal GAO								3.77	(2.76- 5.16)
GAO2	505	m	0	52	26	2	4	4.00	(0.69- 23.28)
GENG	537	f	1	36	-	28	-	3.87	(1.62- 9.26)
HAENSZ	550	f	2	30	-	44	-	1.24	(0.67- 2.28)
HEGMAN	515	m	1	146	-	26	-	2.40	(1.50- 3.70)
HEGMAN	518	f	1	81	-	2	-	5.50	(1.20- 24.10)
Subtotal HEGMAN								2.57	(1.67- 3.96)
*HIRAYA	564	m	1	26	-	19	-	2.29	(1.27- 4.14)
*HIRAYA	506	f	1	-	-	-	-	0.32	(0.04- 2.42)
Subtotal HIRAYA								1.97	(1.12- 3.47)
HU	515	m	0	13	7	14	20	2.65	(0.84- 8.34)
HU	520	f	0	5	4	3	5	2.08	(0.30- 14.55)
Subtotal HU								2.49	(0.93- 6.69)
HU2	507	c	0	129	68	15	29	3.67	(1.84- 7.31)
JEDRYC	610	m	5	135	-	49	-	1.66	(1.19- 2.32)
JEDRYC	620	f	5	63	-	-	-	1.77	(0.68- 4.60)
Subtotal JEDRYC								1.67	(1.22- 2.29)
JOLY	547	m	0	317	282	18	70	4.37	(2.54- 7.52)
JOLY	537	f	0	76	35	23	41	3.87	(2.02- 7.41)
Subtotal JOLY								4.16	(2.74- 6.30)
KHUDER	510	m	0	226	295	72	152	1.62	(1.16- 2.25)
KOULUM	509	m	0	143	16	8	8	8.94	(2.95- 27.06)
LETOUR	505	c	0	151	76	188	160	1.69	(1.20- 2.39)
*LIAW	510	c	2	-	-	-	-	3.07	(1.57- 6.01)
LIU3	506	m	2	32	-	20	-	1.26	(0.58- 2.73)
LIU4	505	m	2	-	-	-	-	1.58	(1.53- 1.63)
LIU5	503	c	0	72	48	13	22	2.54	(1.17- 5.52)
LUBIN	571	m	0	178	251	30	179	4.23	(2.75- 6.52)
LUBIN2	1164	m	1	250	-	68	-	2.63	(1.79- 3.87)
MATOS	580	m	2	69	-	28	-	2.00	(1.18- 3.42)
*MIGRAN	509	m	0	50	1081	24	668	1.29	(0.80- 2.07)
*MIGRAN	519	f	0	2	266	11	1315	0.90	(0.20- 4.03)
Subtotal MIGRAN								1.25	(0.79- 1.96)
*MRFITR	518	m	0	25	2065	3	544	2.20	(0.67- 7.24)

Table 1H15 - 2

IESLC - Meta-analysis of Ever/current Smoking, Age started, "Highest vs lowest"
All LC types, Any Product (or Cigarettes if Any not available)
Most adjusted

REF	NRR	SEX	AD	Number Case	Exposed Cont	Non-exposed Case	Cont	RR	95.00%CI
PERNU	506	m	0	337	92	1043	346	1.22	(0.94- 1.58)
PERNU	503	f	0	1	0	18	89	14.51~	(0.57- 370.40)
Subtotal PERNU								1.23	(0.95- 1.60)
PEZZOT	576	m	2	52	-	41	-	2.00	(1.20- 3.33)
*QIAO2	510	m	1	104	-	52	-	1.37	(0.92- 2.03)
RACHTA	510	f	1	52	-	8	-	2.18	(0.54- 8.87)
SEGI2	525	m	1	91	-	49	-	2.80	(1.82- 4.32)
SOBUE	800	m	0	137	92	110	121	1.64	(1.13- 2.37)
*SPEIZE	530	f	2	-	-	-	-	1.25	(0.91- 1.67)
SUZUK2	523	c	3	31	-	16	-	4.80	(1.80- 13.00)
SVENSS	505	f	0	52	21	32	18	1.39	(0.65- 3.00)
TIZZAN	510	m	0	699	529	12	44	4.84	(2.53- 9.26)
TIZZAN	523	f	0	11	2	2	5	13.75	(1.48- 127.47)
Subtotal TIZZAN								5.26	(2.82- 9.79)
WAKAI	505	m	0	42	74	8	25	1.77	(0.73- 4.28)
WU	545	f	2	106	-	14	-	8.75	(3.26- 23.45)
WYNDE6	763	m	0	611	301	111	92	1.68	(1.24- 2.29)
WYNDE6	771	f	0	291	91	127	90	2.27	(1.58- 3.24)
Subtotal WYNDE6								1.91	(1.51- 2.41)
ZHENG	569	m	1	106	-	28	-	5.73	(3.25- 10.10)
ZHENG	575	f	1	60	-	16	-	1.79	(0.79- 4.06)
Subtotal ZHENG								3.93	(2.47- 6.26)
Partial Totals				8520	130555	3535	189480		

*prospective study ~ With 0.5 adjustment for zero

REF	NRR	SEX	AD	Ys	Ws	Qs	Ps
AGUDO	506	f	3	1.92	1.28	2.48	0.0302
ALDERS	503	m	2	1.08	6.73	2.06	0.0051
ALDERS	506	f	2	0.73	15.75	0.67	0.0037
Subtotal ALDERS				0.84	22.49	2.73	
ARMADA	513	m	0	0.86	33.57	3.76	0.0000
AUVINE	513	c	0	1.18	4.25	1.83	0.0149
BARBON	524	m	1	1.82	16.42	27.71	0.0000
BRESLO	505	c	0	0.45	13.43	0.08	0.1006
BUFFLE	525	f	0	0.64	8.05	0.11	0.0680
*CEDERL	556	m	3	1.55	5.79	6.05	0.0002
*CEDERL	536	f	0	-1.24	0.46	1.45	0.3977
Subtotal CEDERL				1.34	6.26	7.50	
CHEN2	521	m	0	-0.24	2.84	1.68	0.6810
CHEN2	526	f	0	1.04	2.25	0.60	0.1190
Subtotal CHEN2				0.32	5.09	2.28	
CHIAZZ	503	m	2	1.89	2.98	5.56	0.0011
CHOI	529	m	0	0.96	7.05	1.34	0.0107
CHOI	532	f	0	1.90	0.74	1.39	0.1034
Subtotal CHOI				1.05	7.79	2.72	
*CPSI	822	m	1	1.41	34.15	26.92	0.0000
*CPSI	851	f	1	0.10	5.41	0.96	0.8082
Subtotal CPSI				1.23	39.56	27.88	
DAMBER	505	m	0	0.83	23.53	2.11	0.0001
DEAN3	570	m	0	-0.18	11.93	5.98	0.5288
DEAN3	589	f	0	-0.43	5.19	4.72	0.3294
Subtotal DEAN3				-0.26	17.12	10.70	
DOLL	507	m	0	0.64	2.53	0.03	0.3070
DOLL	514	f	0	0.51	3.75	0.00	0.3226
Subtotal DOLL				0.56	6.28	0.04	
*DORN	616	m	0	1.52	25.71	25.38	0.0000
*DORN	653	m	0	1.00	42.73	9.68	0.0000
Subtotal DORN				1.20	68.44	35.07	
*ENGELA	505	m	0	0.67	15.51	0.33	0.0081
*ENGELA	513	f	0	1.42	6.43	5.15	0.0003
Subtotal ENGELA				0.89	21.94	5.48	
GAO	510	m	2	1.45	27.83	23.62	0.0000
GAO	520	f	2	1.03	11.17	2.84	0.0006
Subtotal GAO				1.33	39.00	26.46	
GAO2	505	m	0	1.39	1.24	0.92	0.1229
GENG	537	f	1	1.35	5.06	3.46	0.0023
HAENSZ	550	f	2	0.22	10.25	0.99	0.4911
HEGMAN	515	m	1	0.88	18.85	2.31	0.0001
HEGMAN	518	f	1	1.70	1.71	2.37	0.0259
Subtotal HEGMAN				0.94	20.56	4.68	

Table 1H15 - 2

IESLC - Meta-analysis of Ever/current Smoking, Age started, "Highest vs lowest"
All LC types, Any Product (or Cigarettes if Any not available)
Most adjusted

REF	NRR	SEX	AD	Ys	Ws	Qs	Ps
*HIRAYA	564	m	1	0.83	11.00	1.01	0.0060
*HIRAYA	506	f	1	-1.14	0.91	2.53	0.2763
Subtotal HIRAYA				0.68	11.92	3.54	
HU	515	m	0	0.98	2.93	0.59	0.0948
HU	520	f	0	0.73	1.02	0.04	0.4592
Subtotal HU				0.91	3.95	0.64	
HU2	507	c	0	1.30	8.09	4.85	0.0002
JEDRYC	610	m	5	0.51	34.48	0.01	0.0029
JEDRYC	620	f	5	0.57	4.20	0.01	0.2417
Subtotal JEDRYC				0.51	38.68	0.02	
JOLY	547	m	0	1.48	13.06	11.78	0.0000
JOLY	537	f	0	1.35	9.12	6.25	0.0000
Subtotal JOLY				1.43	22.19	18.03	
KHUDER	510	m	0	0.48	35.36	0.07	0.0043
KOULUM	509	m	0	2.19	3.13	8.67	0.0001
LETOUR	505	c	0	0.53	31.90	0.00	0.0030
*LIAW	510	c	2	1.12	8.53	3.03	0.0011
LIU3	506	m	2	0.23	6.40	0.56	0.5587
LIU4	505	m	2	0.46	3833.36	17.79	0.0000
LIU5	503	c	0	0.93	6.37	1.05	0.0188
LUBIN	571	m	0	1.44	20.61	17.33	0.0000
LUBIN2	1164	m	1	0.97	25.85	5.04	0.0000
MATOS	580	m	2	0.69	13.57	0.38	0.0107
*MIGRAN	509	m	0	0.25	16.88	1.26	0.2993
*MIGRAN	519	f	0	-0.11	1.71	0.68	0.8892
Subtotal MIGRAN				0.22	18.58	1.94	
*MRFITR	518	m	0	0.79	2.70	0.18	0.1967
PERNU	506	m	0	0.19	56.54	6.18	0.1428
PERNU	503	f	0	2.68	0.37	1.69	0.1056
Subtotal PERNU				0.21	56.91	7.87	
PEZZOT	576	m	2	0.69	14.75	0.41	0.0078
*QIAO2	510	m	1	0.31	24.53	1.09	0.1189
RACHTA	510	f	1	0.78	1.96	0.13	0.2751
SEGI2	525	m	1	1.03	20.56	5.23	0.0000
SOBUE	800	m	0	0.49	28.15	0.03	0.0088
*SPEIZE	530	f	2	0.22	41.69	3.81	0.1497
SUZUK2	523	c	3	1.57	3.93	4.28	0.0019
SVENSS	505	f	0	0.33	6.51	0.25	0.3979
TIZZAN	510	m	0	1.58	9.14	10.13	0.0000
TIZZAN	523	f	0	2.62	0.77	3.40	0.0211
Subtotal TIZZAN				1.66	9.92	13.53	
WAKAI	505	m	0	0.57	4.94	0.01	0.2027
WU	545	f	2	2.17	3.95	10.66	0.0000
WYNDE6	763	m	0	0.52	40.26	0.00	0.0010
WYNDE6	771	f	0	0.82	29.93	2.56	0.0000
Subtotal WYNDE6				0.65	70.19	2.56	
ZHENG	569	m	1	1.75	11.95	17.79	0.0000
ZHENG	575	f	1	0.58	5.73	0.02	0.1632
Subtotal ZHENG				1.37	17.69	17.81	

	N	73
	NS	53
	Wt	4771.43
Het	Chi	325.33
Het	df	72
Het	P	***
Fixed	RR	1.69
	RRl	1.64
	RRu	1.74
	P	+++
Random	RR	2.35
	RRl	2.08
	RRu	2.65
	P	+++
Asymm	P	***

Table 1H15 - 3

IESLC - Meta-analysis of Ever/current Smoking, Age started, "Highest vs lowest"
All LC types, Any Product (or Cigarettes if Any not available)
Most adjusted

		Sex			
		combined	male	female	Total
	N	7	39	27	73
	NS	7	38	27	72
	Wt	76.50	4509.58	185.36	4771.43
Het	Chi	10.00	250.57	56.00	325.33
Het	df	6	38	26	72
Het	P	N.S.	***	***	***
Fixed	RR	2.19	1.67	1.93	1.69
	RRl	1.75	1.63	1.67	1.64
	RRu	2.74	1.72	2.23	1.74
	P	+++	+++	+++	+++
Random	RR	2.43	2.43	2.11	2.35
	RRl	1.77	2.08	1.65	2.08
	RRu	3.33	2.85	2.71	2.65
	P	+++	+++	+++	+++
Between	Chi				8.76
Between	df				2
Between	P				*
Btwn(F)	P				N.S.
Btwn(R)	P				N.S.

		Lung cancer type		
		all	other	Total
	N	69	4	73
	NS	50	3	53
	Wt	4687.05	84.38	4771.43
Het	Chi	311.09	12.55	325.33
Het	df	68	3	72
Het	P	***	**	***
Fixed	RR	1.69	1.95	1.69
	RRl	1.64	1.57	1.64
	RRu	1.74	2.41	1.74
	P	+++	+++	+++
Random	RR	2.36	2.19	2.35
	RRl	2.08	1.33	2.08
	RRu	2.69	3.61	2.65
	P	+++	++	+++
Between	Chi			1.69
Between	df			1
Between	P			N.S.
Btwn(F)	P			N.S.
Btwn(R)	P			N.S.

		Location								
		NAmer	UK	Scand	othEur	China	Japan	othAs	other	Total
	N	17	8	10	9	15	6	3	5	73
	NS	13	4	7	7	11	5	2	4	53
	Wt	349.04	64.48	122.53	127.67	3970.14	66.81	16.32	54.44	4771.43
Het	Chi	71.02	13.22	33.05	27.17	84.26	7.36	0.61	7.86	325.33
Het	df	16	7	9	8	14	5	2	4	72
Het	P	***	(*)	***	***	***	N.S.	N.S.	(*)	***
Fixed	RR	2.16	1.41	1.82	2.64	1.61	2.04	2.97	2.87	1.69
	RRl	1.94	1.10	1.52	2.22	1.56	1.61	1.83	2.20	1.64
	RRu	2.40	1.80	2.17	3.14	1.66	2.60	4.82	3.74	1.74
	P	+++	++	+++	+++	+++	+++	+++	+++	+++
Random	RR	2.26	1.39	2.51	3.04	2.52	2.04	2.97	2.98	2.35
	RRl	1.78	0.97	1.64	2.07	1.82	1.47	1.83	2.03	2.08
	RRu	2.88	2.01	3.85	4.45	3.48	2.85	4.82	4.38	2.65
	P	+++	(+)	+++	+++	+++	+++	+++	+++	+++
Between	Chi									80.77
Between	df									7
Between	P									***
Btwn(F)	P									**
Btwn(R)	P									(*)

Table 1H15 - 3

IESLC - Meta-analysis of Ever/current Smoking, Age started, "Highest vs lowest"
All LC types, Any Product (or Cigarettes if Any not available)
Most adjusted

Detailed Country in "other Europe"

	multi	Germany	othWest	East	Balkans	Total
N	1		5	3		9
NS	1		4	2		7
Wt	25.85		61.19	40.64		127.67
Het Chi	0.00		13.51	0.15		27.17
Het df	0		4	2		8
Het P	N.S.		**	N.S.		***
Fixed RR	2.63		3.56	1.69		2.64
RRl	1.79		2.77	1.25		2.22
RRu	3.87		4.58	2.30		3.14
P	+++		+++	+++		+++
Random RR	2.63		4.53	1.69		3.04
RRl	1.79		2.51	1.25		2.07
RRu	3.87		8.20	2.30		4.45
P	+++		+++	+++		+++
Between Chi						13.52
Between df						2
Between P						**
Btwn(F) P						N.S.
Btwn(R) P						**

Detailed Country in "other Asia"

	India	HongKong	other	Total
N			3	3
NS			2	2
Wt			16.32	16.32
Het Chi			0.61	0.61
Het df			2	2
Het P			N.S.	N.S.
Fixed RR			2.97	2.97
RRl			1.83	1.83
RRu			4.82	4.82
P			+++	+++
Random RR			2.97	2.97
RRl			1.83	1.83
RRu			4.82	4.82
P			+++	+++
Between Chi				
Between df				
Between P				N.S.
Btwn(F) P				N.S.
Btwn(R) P				N.S.

Detailed other continent

	SCAmer	Total
N	5	5
NS	4	4
Wt	54.44	54.44
Het Chi	7.86	7.86
Het df	4	4
Het P	(*)	(*)
Fixed RR	2.87	2.87
RRl	2.20	2.20
RRu	3.74	3.74
P	+++	+++
Random RR	2.98	2.98
RRl	2.03	2.03
RRu	4.38	4.38
P	+++	+++
Between Chi		
Between df		
Between P		N.S.
Btwn(F) P		N.S.
Btwn(R) P		N.S.

Table 1H15 - 3

IESLC - Meta-analysis of Ever/current Smoking, Age started, "Highest vs lowest"
All LC types, Any Product (or Cigarettes if Any not available)
Most adjusted

	Start year of study					
	<1960	1960-69	1970-79	1980-89	1990+	Total
N	14	13	12	30	4	73
NS	9	7	10	23	4	53
Wt	210.89	166.58	177.36	4172.60	43.99	4771.43
Het Chi	74.72	35.64	42.08	111.54	5.81	325.33
Het df	13	12	11	29	3	72
Het P	***	***	***	***	N.S.	***
Fixed RR	2.39	1.85	2.43	1.63	1.76	1.69
RRl	2.08	1.59	2.10	1.58	1.31	1.64
RRu	2.73	2.15	2.82	1.68	2.36	1.74
P	+++	+++	+++	+++	+++	+++
Random RR	2.73	1.76	2.73	2.39	2.00	2.35
RRl	1.86	1.31	2.01	1.99	1.24	2.08
RRu	4.01	2.38	3.71	2.87	3.25	2.65
P	+++	+++	+++	+++	++	+++
Between Chi						55.55
Between df						4
Between P						***
Btwn(F) P						*
Btwn(R) P						N.S.

	Study type (1)		
	CC	other	Total
N	57	16	73
NS	43	10	53
Wt	4527.29	244.14	4771.43
Het Chi	234.69	68.76	325.33
Het df	56	15	72
Het P	***	***	***
Fixed RR	1.66	2.26	1.69
RRl	1.62	2.00	1.64
RRu	1.71	2.57	1.74
P	+++	+++	+++
Random RR	2.39	2.16	2.35
RRl	2.09	1.59	2.08
RRu	2.74	2.93	2.65
P	+++	+++	+++
Between Chi			21.88
Between df			1
Between P			***
Btwn(F) P			*
Btwn(R) P			N.S.

	Study type (2)			
	CC	prosp	other	Total
N	57	16		73
NS	43	10		53
Wt	4527.29	244.14		4771.43
Het Chi	234.69	68.76		325.33
Het df	56	15		72
Het P	***	***		***
Fixed RR	1.66	2.26		1.69
RRl	1.62	2.00		1.64
RRu	1.71	2.57		1.74
P	+++	+++		+++
Random RR	2.39	2.16		2.35
RRl	2.09	1.59		2.08
RRu	2.74	2.93		2.65
P	+++	+++		+++
Between Chi				21.88
Between df				1
Between P				***
Btwn(F) P				*
Btwn(R) P				N.S.

Table 1H15 - 3

IESLC - Meta-analysis of Ever/current Smoking, Age started, "Highest vs lowest"
All LC types, Any Product (or Cigarettes if Any not available)
Most adjusted

	Study size (number of LC cases) 100-249	250-499	500-999	1000+	Total
N	18	17	15	23	73
NS	16	12	12	13	53
Wt	116.73	196.47	207.49	4250.74	4771.43
Het Chi	30.84	31.91	82.98	138.27	325.33
Het df	17	16	14	22	72
Het P	*	*	***	***	***
Fixed RR	1.99	2.35	2.17	1.64	1.69
RRl	1.66	2.04	1.89	1.59	1.64
RRu	2.39	2.70	2.48	1.69	1.74
P	+++	+++	+++	+++	+++
Random RR	2.20	2.47	2.44	2.31	2.35
RRl	1.68	1.97	1.72	1.89	2.08
RRu	2.88	3.10	3.45	2.82	2.65
P	+++	+++	+++	+++	+++
Between Chi					41.32
Between df					3
Between P					***
Btwn(F) P					*
Btwn(R) P					N.S.

	Risky occupational population no	mining	othRisky	Total
N	70	2	1	73
NS	50	2	1	53
Wt	4723.31	45.14	2.98	4771.43
Het Chi	301.28	14.24	0.00	325.33
Het df	69	1	0	72
Het P	***	***	N.S.	***
Fixed RR	1.69	2.29	6.63	1.69
RRl	1.64	1.71	2.13	1.64
RRu	1.73	3.07	20.63	1.74
P	+++	+++	++	+++
Random RR	2.33	2.40	6.63	2.35
RRl	2.06	0.79	2.13	2.08
RRu	2.63	7.25	20.63	2.65
P	+++	N.S.	++	+++
Between Chi				9.80
Between df				2
Between P				**
Btwn(F) P				N.S.
Btwn(R) P				N.S.

	National cigarette tobacco type Virginia	blended	other	Total
N	9	48	16	73
NS	5	36	12	53
Wt	96.38	696.39	3978.67	4771.43
Het Chi	13.94	158.20	87.79	325.33
Het df	8	47	15	72
Het P	(*)	***	***	***
Fixed RR	1.50	2.24	1.61	1.69
RRl	1.22	2.08	1.57	1.64
RRu	1.83	2.42	1.67	1.74
P	+++	+++	+++	+++
Random RR	1.45	2.50	2.55	2.35
RRl	1.08	2.14	1.87	2.08
RRu	1.95	2.92	3.48	2.65
P	+	+++	+++	+++
Between Chi				65.40
Between df				2
Between P				***
Btwn(F) P				***
Btwn(R) P				**

Table 1H15 - 3

IESLC - Meta-analysis of Ever/current Smoking, Age started, "Highest vs lowest"
All LC types, Any Product (or Cigarettes if Any not available)
Most adjusted

	Any proxy use No/nk	Yes	Total
N	55	18	73
NS	39	14	53
Wt	734.42	4037.01	4771.43
Het Chi	172.92	85.76	325.33
Het df	54	17	72
Het P	***	***	***
Fixed RR	2.23	1.61	1.69
RRl	2.08	1.56	1.64
RRu	2.40	1.66	1.74
P	+++	+++	+++
Random RR	2.41	2.21	2.35
RRl	2.08	1.72	2.08
RRu	2.79	2.83	2.65
P	+++	+++	+++
Between Chi			66.65
Between df			1
Between P			***
Btwn(F) P			***
Btwn(R) P			N.S.

	Full histological confirmation No	Yes	Total
N	51	22	73
NS	35	18	53
Wt	4392.91	378.52	4771.43
Het Chi	241.48	68.96	325.33
Het df	50	21	72
Het P	***	***	***
Fixed RR	1.66	2.05	1.69
RRl	1.62	1.85	1.64
RRu	1.71	2.26	1.74
P	+++	+++	+++
Random RR	2.39	2.26	2.35
RRl	2.04	1.85	2.08
RRu	2.81	2.77	2.65
P	+++	+++	+++
Between Chi			14.89
Between df			1
Between P			***
Btwn(F) P			(*)
Btwn(R) P			N.S.

	Number of adjustment variables (1) 0	1	2+/+nk	Total
N	41	14	18	73
NS	29	10	15	54
Wt	540.70	184.10	4046.64	4771.43
Het Chi	120.00	46.51	74.44	325.33
Het df	40	13	17	72
Het P	***	***	***	***
Fixed RR	2.08	2.85	1.61	1.69
RRl	1.92	2.47	1.56	1.64
RRu	2.27	3.29	1.66	1.74
P	+++	+++	+++	+++
Random RR	2.21	2.72	2.36	2.35
RRl	1.87	2.01	1.87	2.08
RRu	2.61	3.68	2.96	2.65
P	+++	+++	+++	+++
Between Chi				84.38
Between df				2
Between P				***
Btwn(F) P				***
Btwn(R) P				N.S.

Table 1H15 - 3

IESLC - Meta-analysis of Ever/current Smoking, Age started, "Highest vs lowest"
All LC types, Any Product (or Cigarettes if Any not available)
Most adjusted

	Number of adjustment variables (2) 0	1	2	3-5	6+/+nk	Total
N	41	14	13	5		73
NS	29	10	11	4		54
Wt	540.70	184.10	3996.96	49.68		4771.43
Het Chi	120.00	46.51	60.26	10.23		325.33
Het df	40	13	12	4		72
Het P	***	***	***	*		***
Fixed RR	2.08	2.85	1.60	2.13		1.69
RRl	1.92	2.47	1.55	1.61		1.64
RRu	2.27	3.29	1.65	2.81		1.74
P	+++	+++	+++	+++		+++
Random RR	2.21	2.72	2.27	2.89		2.35
RRl	1.87	2.01	1.74	1.59		2.08
RRu	2.61	3.68	2.96	5.25		2.65
P	+++	+++	+++	+++		+++
Between Chi						88.32
Between df						3
Between P						***
Btwn(F) P						***
Btwn(R) P						N.S.

	Smoking status ever	current	Total
N	53	20	73
NS	39	14	53
Wt	4565.94	205.49	4771.43
Het Chi	243.95	75.51	325.33
Het df	52	19	72
Het P	***	***	***
Fixed RR	1.68	2.00	1.69
RRl	1.63	1.74	1.64
RRu	1.73	2.29	1.74
P	+++	+++	+++
Random RR	2.49	1.94	2.35
RRl	2.17	1.43	2.08
RRu	2.85	2.64	2.65
P	+++	+++	+++
Between Chi			5.87
Between df			1
Between P			*
Btwn(F) P			N.S.
Btwn(R) P			N.S.

	Product all/unsp	cig+/-ot	cig only	Total
N	22	41	10	73
NS	17	31	7	55
Wt	4041.20	650.14	80.09	4771.43
Het Chi	100.67	117.55	19.17	325.33
Het df	21	40	9	72
Het P	***	***	*	***
Fixed RR	1.61	2.38	1.48	1.69
RRl	1.56	2.20	1.19	1.64
RRu	1.66	2.57	1.84	1.74
P	+++	+++	+++	+++
Random RR	2.58	2.49	1.44	2.35
RRl	2.00	2.15	1.01	2.08
RRu	3.33	2.88	2.05	2.65
P	+++	+++	+	+++
Between Chi				87.93
Between df				2
Between P				***
Btwn(F) P				***
Btwn(R) P				*

Table 1H15 - 3

IESLC - Meta-analysis of Ever/current Smoking, Age started, "Highest vs lowest"
All LC types, Any Product (or Cigarettes if Any not available)
Most adjusted

	Derivation of RR/CI			
	Orig	StdCalc	Other	Total
N	5	42	26	73
NS	3	31	20	54
Wt	63.17	612.45	4095.81	4771.43
Het Chi	6.60	130.80	159.62	325.33
Het df	4	41	25	72
Het P	N.S.	***	***	***
Fixed RR	2.05	2.04	1.64	1.69
RRl	1.60	1.89	1.59	1.64
RRu	2.63	2.21	1.69	1.74
P	+++	+++	+++	+++
Random RR	2.28	2.20	2.61	2.35
RRl	1.56	1.88	2.04	2.08
RRu	3.34	2.58	3.35	2.65
P	+++	+++	+++	+++
Between Chi				28.30
Between df				2
Between P				***
Btwn(F) P				*
Btwn(R) P				N.S.

	Study LIU4		
	LIU4	others	Total
N	1	72	73
NS	1	52	53
Wt	3833.36	938.07	4771.43
Het Chi	0.00	234.84	325.33
Het df	0	71	72
Het P	N.S.	***	***
Fixed RR	1.58	2.23	1.69
RRl	1.53	2.10	1.64
RRu	1.63	2.38	1.74
P	+++	+++	+++
Random RR	1.58	2.38	2.35
RRl	1.53	2.09	2.08
RRu	1.63	2.71	2.65
P	+++	+++	+++
Between Chi			90.49
Between df			1
Between P			***
Btwn(F) P			***
Btwn(R) P			***

Table 1H15 - 4

IESLC - Meta-analysis of Ever/current Smoking, Age started, "Highest vs lowest"
All LC types, Any Product (or Cigarettes if Any not available)
Least adjusted

REF	NRR	X	SEX	AGEL	AGEH	RACE	YF	LC TYPE	LOC	START	ST	NLC	R	VB	P	H	AD	ADOS	SM	PRODUCT	exL	exH	unexL	unexH	De
AGUDO	503	x	f	0	0	all	-	all	Eu:wst	1989	CC	103	n	bl	n	n	0	0	ev	cig only	1	23	24	999	st
ALDERS	503		m	0	0	all	-	all	Eu:UK	1977	CC	1448	n	V	n	n	2	1#	ev	cig only	1	14	25	999	ot
ALDERS	506		f	0	0	all	-	all	Eu:UK	1977	CC	1448	n	V	n	n	2	1#	ev	cig only	1	14	25	999	ot
ARMADA	513		m	0	0	all	-	all	Eu:wst	1986	CC	325	n	bl	n	y	0	0	ev	cig+/-ot	7	16	17	45	st
AUVINE	513		c	0	0	all	-	all	Eu:Sca	1986	CC	517	n	bl	y	n	0	0	ev	cig+/-ot	1	15	21	999	st
BARBON	519	x	m	0	0	all	-	all	Eu:wst	1979	CC	755	n	bl	y	y	0	0	ev	all/unsp	1	14	20	999	st
BRESLO	505		c	0	0	all	-	all	NAmer	1949	CC	518	n	bl	n	y	0	0	ev	cig+/-ot	0	14	25	999	st
BUFFLE	525		f	0	0	w-hi	-	all	NAmer	1976	CC	943	n	bl	y	n	0	0	ev	cig+/-ot	6	16	30	999	st
CEDERL	556		m	0	0	all	16	all	Eu:Sca	1963	pr	491	n	bl	n	n	3	1#	cu	all/unsp	1	19	25	999	ot
CEDERL	536		f	0	0	all	10	all	Eu:Sca	1963	pr	491	n	bl	n	n	0	0	cu	cig only	1	16	19	999	ot
CHEN2	521		m	0	0	all	-	all	As:Chi	1983	CC	193	n	ot	y	n	0	0	ev	all/unsp	1	19	31	999	st
CHEN2	526		f	0	0	all	-	all	As:Chi	1983	CC	193	n	ot	y	n	0	0	ev	all/unsp	1	19	31	999	st
CHIAZZ	503		m	0	0	all	-	all	NAmer	1940	CC	144	o	bl	y	n	2	0	ev	cig+/-ot	1	19	20	999	ot
CHOI	529		m	0	0	all	-	all	As:oth	1985	CC	375	n	bl	n	n	0	0	ev	cig+/-ot	1	14	25	999	st
CHOI	532		f	0	0	all	-	all	As:oth	1985	CC	375	n	bl	n	n	0	0	ev	cig+/-ot	1	24	25	999	st
CPSI	822		m	35	84	all	6	all	NAmer	1959	pr	5138	n	bl	n	n	1	0	cu	cig+/-ot	1	14	25	999	ot
CPSI	851		f	40	74	all	6	all	NAmer	1959	pr	5138	n	bl	n	n	1	0	cu	cig only	1	14	25	999	ot
DAMBER	505		m	0	0	all	-	all	Eu:Sca	1972	CC	579	n	bl	y	n	0	0	ev	all/unsp	1	15	21	999	st
DEAN3	570		m	0	0	all	-	all	Eu:UK	1969	CC	766	n	V	y	n	0	0	cu	cig only	1	14	25	999	st
DEAN3	589		f	0	0	all	-	all	Eu:UK	1969	CC	766	n	V	y	n	0	0	cu	cig only	1	14	25	999	st
DOLL	507		m	0	0	all	-	all	Eu:UK	1948	CC	1465	n	V	n	n	0	0	ev	all/unsp	1	19	40	999	st
DOLL	514		f	0	0	all	-	all	Eu:UK	1948	CC	1465	n	V	n	n	0	0	ev	all/unsp	1	19	40	999	st
DORN	616		m	55	64	wh	8	all	NAmer	1954	pr	5097	n	bl	n	n	0	0	ev	cig+/-ot	1	14	25	999	st
DORN	653		m	65	74	wh	8	all	NAmer	1954	pr	5097	n	bl	n	n	0	0	ev	cig+/-ot	1	14	25	999	st
ENGELA	505		m	0	0	all	0	all	Eu:Sca	1964	pr	435	n	bl	n	n	0	0	cu	cig+/-ot	1	19	30	999	st
ENGELA	513		f	0	0	all	0	all	Eu:Sca	1964	pr	435	n	bl	n	n	0	0	cu	cig+/-ot	1	19	30	999	st
GAO	505	x	m	0	0	all	-	all	As:Chi	1984	CC	1405	n	ot	n	n	0	0	ev	cig+/-ot	10	19	30	999	st
GAO	515	x	f	0	0	all	-	all	As:Chi	1984	CC	1405	n	ot	n	n	0	0	ev	cig+/-ot	10	19	30	999	st
GAO2	505		m	0	0	all	-	all	As:Jap	1988	CC	282	n	bl	n	n	0	0	cu	cig+/-ot	1	19	30	999	st
GENG	532	x	f	0	0	all	-	all	As:Chi	1985	CC	292	n	ot	*	n	0	0	ev	cig+/-ot	1	15	21	999	st
HAENSZ	541	x	f	0	0	all	-	not alv	NAmer	1955	CC	158	n	bl	n	y	0	0	ev	cig+/-ot	1	24	25	999	st
HEGMAN	501	x	m	0	0	all	-	all	NAmer	1989	CC	282	n	bl	y	y	0	0	ev	all/unsp	1	19	20	999	st
HEGMAN	502	x	f	0	0	all	-	all	NAmer	1989	CC	282	n	bl	y	y	0	0	ev	all/unsp	1	25	26	999	st
HIRAYA	564		m	0	0	all	5	all	As:Jap	1965	pr	1917	n	bl	n	n	1	0	cu	cig+/-ot	1	19	25	999	ot
HIRAYA	506		f	0	0	all	0	all	As:Jap	1965	pr	1917	n	bl	n	n	1	0	cu	cig+/-ot	1	19	20	999	ot
HU	515		m	0	0	all	-	all	As:Chi	1985	CC	227	n	ot	n	y	0	0	ev	cig+/-ot	1	15	30	999	st
HU	520		f	0	0	all	-	all	As:Chi	1985	CC	227	n	ot	n	y	0	0	ev	cig+/-ot	1	15	30	999	st
HU2	507		c	0	0	all	-	all	As:Chi	1977	CC	523	n	ot	y	n	0	0	ev	cig+/-ot	1	19	40	999	st
JEDRYC	610		m	0	0	all	-	all	Eu:est	1980	CC	1630	n	bl	y	n	5	2#	ev	cig+/-ot	1	16	19	999	or
JEDRYC	620		f	0	0	all	-	all	Eu:est	1980	CC	1630	n	bl	y	n	5	2#	ev	cig+/-ot	1	22	23	999	or
JOLY	547		m	0	0	all	-	all	SCAmer	1978	CC	826	n	bl	n	n	0	0	ev	cig+/-ot	1	14	25	999	st
JOLY	537		f	0	0	all	-	all	SCAmer	1978	CC	826	n	bl	n	n	0	0	ev	cig+/-ot	1	14	25	999	st
KHUDER	510		m	0	0	all	-	all	NAmer	1985	CC	482	n	bl	n	y	0	0	ev	cig+/-ot	1	15	20	999	st
KOULUM	509		m	0	0	all	-	all	Eu:Sca	1936	CC	812	n	bl	n	n	0	0	ev	all/unsp	0	10	31	999	st
LETOUR	505		c	0	0	all	-	all	NAmer	1983	CC	738	n	V	y	y	0	0	ev	cig+/-ot	1	14	21	999	st
LIAW	510		c	0	0	all	0	all	As:oth	1982	pr	127	n	ot	n	n	2	0	cu	all/unsp	1	20	25	999	ot
LIU3	503	x	m	0	0	all	-	all	As:Chi	1985	CC	110	n	ot	n	n	0	0	ev	all/unsp	1	20	21	999	st
LIU4	505		m	35	69	all	-	all	As:Chi	1986	CC	1000-00	n	ot	y	n	2	0	ev	all/unsp	0	19	25	999	ot
LIU5	503		c	0	0	all	-	all	As:Chi	1978	CC	111	n	ot	y	n	0	0	ev	all/unsp	1	29	30	999	st
LUBIN	571		m	0	0	all	-	all	As:Chi	1984	CC	427	m	ot	y	n	0	0	ev	cig+/-ot	1	19	27	999	st
LUBIN2	1155	x	m	0	0	all	-	all	Eu:mul	1976	CC	7804	n	bl	n	y	0	0	ev	cig+/-ot	1	12	31	999	st
MATOS	560	x	m	0	0	all	-	all	SCAmer	1994	CC	200	n	bl	n	n	0	0	ev	cig+/-ot	1	14	20	999	st
MIGRAN	509		m	0	0	all	0	all	Eu:UK	1964	pr	259	n	V	n	n	0	0	cu	cig only	0	15	20	999	st
MIGRAN	519		f	0	0	all	0	all	Eu:UK	1964	pr	259	n	V	n	n	0	0	cu	cig only	0	15	20	999	st
MRFITR	518		m	0	0	all	0	all	NAmer	1973	pr	119	n	bl	n	n	0	0	cu	cig+/-ot	1	15	24	999	st
PERNU	506		m	0	0	all	-	all	Eu:Sca	1944	CC	1606	n	bl	n	n	0	0	ev	all/unsp	1	14	15	999	st
PERNU	503		f	0	0	all	-	all	Eu:Sca	1944	CC	1606	n	bl	n	n	0	0	ev	all/unsp	1	14	15	999	ot
PEZZOT	574	x	m	0	0	all	-	all	SCAmer	1987	CC	215	n	bl	n	y	0	0	ev	cig only	1	13	19	999	st
QIAO2	505	x	m	0	0	all	0	all	As:Chi	1992	pr	241	m	ot	n	n	0	0	ev	all/unsp	1	16	21	999	st
RACHTA	505	x	f	0	0	all	-	all	Eu:est	1991	CC	118	n	bl	n	y	0	0	ev	cig+/-ot	1	19	31	999	st
SEGI2	520	x	m	0	0	all	-	all	As:Jap	1962	CC	378	n	bl	n	n	0	0	cu	cig+/-ot	1	19	23	999	st
SOBUE	800		m	0	0	all	-	all	As:Jap	1986	CC	1376	n	bl	n	y	0	0	ev	cig+/-ot	10	17	23	999	st
SPEIZE	530		f	0	0	all	0	all	NAmer	1976	pr	593	n	bl	n	y	2	1#	cu	cig+/-ot	18	19	22	999	st
SUZUK2	505	x	c	0	0	all	-	all	SCAmer	1991	CC	123	n	bl	n	y	0	0	ev	all/unsp	0	11	19	999	st
SVENSS	505		f	0	0	all	-	all	Eu:Sca	1983	CC	210	n	bl	n	n	0	0	cu	all/unsp	0	18	26	999	st
TIZZAN	510		m	0	0	all	-	all	Eu:wst	1959	CC	1358	n	bl	n	n	0	0	ev	all/unsp	1	19	31	999	st
TIZZAN	523		f	0	0	all	-	all	Eu:wst	1959	CC	1358	n	bl	n	n	0	0	ev	all/unsp	1	19	31	999	st
WAKAI	505		m	0	0	all	-	all	As:Jap	1988	CC	333	n	bl	n	y	0	0	cu	cig+/-ot	1	19	30	999	st
WU	521	x	f	0	0	wh	-	q+a	NAmer	1981	CC	220	n	bl	n	y	0	0	cu	all/unsp	0	18	25	999	st
WYNDE6	763		m	0	0	wh	-	q+s+a	NAmer	1969	CC	4423	n	bl	n	y	0	0	ev	cig+/-ot	1	17	21	999	st
WYNDE6	771		f	0	0	wh	-	q+s+a	NAmer	1969	CC	4423	n	bl	n	y	0	0	ev	cig+/-ot	1	17	21	999	st

Table 1H15 - 4

IESLC - Meta-analysis of Ever/current Smoking, Age started, "Highest vs lowest"
All LC types, Any Product (or Cigarettes if Any not available)
Least adjusted

REF	NRR	X	SEX	AGEL	AGEH	RACE	YF	LC TYPE	LOC	START	ST	NLC	R	VB	P	H	AD	ADOS	SM	PRODUCT	exL	exH	unexL	unexH	De
ZHENG	567	x	m	0	0	all	-	all	As:Chi	1982	CC	540	n	ot	*	y	0	0	ev	cig+/-ot	1	19	30	999	st
ZHENG	574	x	f	0	0	all	-	all	As:Chi	1982	CC	540	n	ot	*	y	0	0	ev	cig+/-ot	1	29	30	999	st

Comments on values in listings

ALDERS	ADOS	Number of cigs/day
ALDERS	ADOS	Number of cigs/day
CEDERL	ADOS	Amount smoked
JEDRYC	ADOS	Number of cigs per day & time since quit
JEDRYC	ADOS	Number of cigs per day & time since quit
SPEIZE	ADOS	Numbers of cigs/day

Cigarette type is all/unspec for all RRs
except for the following:

REF	NRR	CIGTYPE
ALDERS	503	MC only
ALDERS	506	MC only
DEAN3	570	MC only
DEAN3	589	MC only

Table 1H15 - 5

IESLC - Meta-analysis of Ever/current Smoking, Age started, "Highest vs lowest"
All LC types, Any Product (or Cigarettes if Any not available)
Least adjusted

REF	NRR	SEX	AD	Number Exposed Case	Cont	Non-exposed Case	Cont	RR	95.00%CI
AGUDO	503	f	0	16	11	7	12	2.49	(0.75- 8.34)
ALDERS	503	m	2	139	-	11	-	2.94	(1.38- 6.25)
ALDERS	506	f	2	78	-	97	-	2.08	(1.27- 3.41)
Subtotal ALDERS								2.31	(1.53- 3.49)
ARMADA	513	m	0	204	110	113	144	2.36	(1.69- 3.31)
AUVINE	513	c	0	55	6	76	27	3.26	(1.26- 8.42)
BARBON	519	m	0	138	23	200	207	6.21	(3.83- 10.06)
BRESLO	505	c	0	166	116	32	35	1.57	(0.92- 2.67)
BUFFLE	525	f	0	78	41	23	23	1.90	(0.95- 3.80)
*CEDERL	556	m	3	183	-	6	-	4.70	(2.08- 10.60)
*CEDERL	536	f	0	0	746	6	2806	0.29~	(0.02- 5.13)
Subtotal CEDERL								3.82	(1.75- 8.36)
CHEN2	521	m	0	84	67	8	5	0.78	(0.25- 2.51)
CHEN2	526	f	0	23	13	5	8	2.83	(0.77- 10.47)
Subtotal CHEN2								1.38	(0.58- 3.29)
CHIAZZ	503	m	2	-	-	-	-	6.63	(2.13- 20.63)
CHOI	529	m	0	22	18	36	77	2.61	(1.25- 5.47)
CHOI	532	f	0	4	1	15	25	6.67	(0.68- 65.37)
Subtotal CHOI								2.86	(1.42- 5.76)
*CPSI	822	m	1	185	-	42	-	4.11	(2.94- 5.75)
*CPSI	851	f	1	6	-	51	-	1.11	(0.48- 2.59)
Subtotal CPSI								3.44	(2.52- 4.69)
DAMBER	505	m	0	206	98	70	76	2.28	(1.52- 3.42)
DEAN3	570	m	0	44	165	24	75	0.83	(0.47- 1.47)
DEAN3	589	f	0	7	109	27	274	0.65	(0.28- 1.54)
Subtotal DEAN3								0.77	(0.48- 1.24)
DOLL	507	m	0	1077	992	4	7	1.90	(0.55- 6.51)
DOLL	514	f	0	20	12	15	15	1.67	(0.61- 4.59)
Subtotal DOLL								1.76	(0.80- 3.84)
*DORN	616	m	0	84	36304	37	73050	4.57	(3.10- 6.72)
*DORN	653	m	0	81	24616	90	74464	2.72	(2.02- 3.67)
Subtotal DORN								3.31	(2.61- 4.19)
*ENGELA	505	m	0	173	50732	17	9762	1.96	(1.19- 3.22)
*ENGELA	513	f	0	18	10687	10	24560	4.14	(1.91- 8.96)
Subtotal ENGELA								2.44	(1.60- 3.71)
GAO	505	m	0	262	167	45	129	4.50	(3.04- 6.65)
GAO	515	f	0	77	25	73	64	2.70	(1.54- 4.74)
Subtotal GAO								3.81	(2.76- 5.25)
GAO2	505	m	0	52	26	2	4	4.00	(0.69- 23.28)
GENG	532	f	0	36	10	28	31	3.99	(1.67- 9.49)
HAENSZ	541	f	0	30	37	44	66	1.22	(0.66- 2.25)
HEGMAN	501	m	0	146	716	26	289	2.27	(1.46- 3.52)
HEGMAN	502	f	0	81	169	2	28	6.71	(1.56- 28.86)
Subtotal HEGMAN								2.48	(1.63- 3.78)
*HIRAYA	564	m	1	26	-	19	-	2.29	(1.27- 4.14)
*HIRAYA	506	f	1	-	-	-	-	0.32	(0.04- 2.42)
Subtotal HIRAYA								1.97	(1.12- 3.47)
HU	515	m	0	13	7	14	20	2.65	(0.84- 8.34)
HU	520	f	0	5	4	3	5	2.08	(0.30- 14.55)
Subtotal HU								2.49	(0.93- 6.69)
HU2	507	c	0	129	68	15	29	3.67	(1.84- 7.31)
JEDRYC	610	m	5	135	-	49	-	1.66	(1.19- 2.32)
JEDRYC	620	f	5	63	-	-	-	1.77	(0.68- 4.60)
Subtotal JEDRYC								1.67	(1.22- 2.29)
JOLY	547	m	0	317	282	18	70	4.37	(2.54- 7.52)
JOLY	537	f	0	76	35	23	41	3.87	(2.02- 7.41)
Subtotal JOLY								4.16	(2.74- 6.30)
KHUDER	510	m	0	226	295	72	152	1.62	(1.16- 2.25)
KOULUM	509	m	0	143	16	8	8	8.94	(2.95- 27.06)
LETOUR	505	c	0	151	76	188	160	1.69	(1.20- 2.39)
*LIAW	510	c	2	-	-	-	-	3.07	(1.57- 6.01)
LIU3	503	m	0	32	125	20	80	1.02	(0.55- 1.91)
LIU4	505	m	2	-	-	-	-	1.58	(1.53- 1.63)
LIU5	503	c	0	72	48	13	22	2.54	(1.17- 5.52)
LUBIN	571	m	0	178	251	30	179	4.23	(2.75- 6.52)
LUBIN2	1155	m	0	250	316	68	221	2.57	(1.87- 3.54)
MATOS	560	m	0	69	90	28	73	2.00	(1.17- 3.42)
*MIGRAN	509	m	0	50	1081	24	668	1.29	(0.80- 2.07)
*MIGRAN	519	f	0	2	266	11	1315	0.90	(0.20- 4.03)
Subtotal MIGRAN								1.25	(0.79- 1.96)
*MRFITR	518	m	0	25	2065	3	544	2.20	(0.67- 7.24)

Table 1H15 - 5

IESLC - Meta-analysis of Ever/current Smoking, Age started, "Highest vs lowest"
All LC types, Any Product (or Cigarettes if Any not available)
Least adjusted

REF	NRR	SEX	AD	Number Exposed Case	Exposed Cont	Non-exposed Case	Non-exposed Cont	RR	95.00%CI
PERNU	506	m	0	337	92	1043	346	1.22 (	0.94- 1.58)
PERNU	503	f	0	1	0	18	89	14.51~(	0.57- 370.40)
Subtotal PERNU								1.23 (	0.95- 1.60)
PEZZOT	574	m	0	52	67	41	105	1.99 (	1.19- 3.31)
*QIAO2	505	m	0	104	2130	52	1947	1.83 (	1.32- 2.54)
RACHTA	505	f	0	52	21	8	4	1.24 (	0.34- 4.56)
SEGI2	520	m	0	91	103	49	155	2.79 (	1.82- 4.29)
SOBUE	800	m	0	137	92	110	121	1.64 (	1.13- 2.37)
*SPEIZE	530	f	2	-	-	-	-	1.25 (	0.91- 1.67)
SUZUK2	505	c	0	31	10	16	22	4.26 (	1.63- 11.14)
SVENSS	505	f	0	52	21	32	18	1.39 (	0.65- 3.00)
TIZZAN	510	m	0	699	529	12	44	4.84 (	2.53- 9.26)
TIZZAN	523	f	0	11	2	2	5	13.75 (	1.48- 127.47)
Subtotal TIZZAN								5.26 (	2.82- 9.79)
WAKAI	505	m	0	42	74	8	25	1.77 (	0.73- 4.28)
WU	521	f	0	106	32	14	19	4.50 (	2.03- 9.96)
WYNDE6	763	m	0	611	301	111	92	1.68 (	1.24- 2.29)
WYNDE6	771	f	0	291	91	127	90	2.27 (	1.58- 3.24)
Subtotal WYNDE6								1.91 (	1.51- 2.41)
ZHENG	567	m	0	106	43	28	66	5.81 (	3.30- 10.24)
ZHENG	574	f	0	60	28	16	16	2.14 (	0.94- 4.89)
Subtotal ZHENG								4.22 (	2.65- 6.74)
Partial Totals				8520	134678	3535	193014		

*prospective study ~ With 0.5 adjustment for zero

REF	NRR	SEX	AD	Ys	Ws	Qs	Ps
AGUDO	503	f	0	0.91	2.63	0.39	0.1381
ALDERS	503	m	2	1.08	6.73	2.05	0.0051
ALDERS	506	f	2	0.73	15.75	0.66	0.0037
Subtotal ALDERS				0.84	22.49	2.71	
ARMADA	513	m	0	0.86	33.57	3.73	0.0000
AUVINE	513	c	0	1.18	4.25	1.82	0.0149
BARBON	519	m	0	1.83	16.51	27.87	0.0000
BRESLO	505	c	0	0.45	13.43	0.08	0.1006
BUFFLE	525	f	0	0.64	8.05	0.11	0.0680
*CEDERL	556	m	3	1.55	5.79	6.04	0.0002
*CEDERL	536	f	0	-1.24	0.46	1.45	0.3977
Subtotal CEDERL				1.34	6.26	7.49	
CHEN2	521	m	0	-0.24	2.84	1.69	0.6810
CHEN2	526	f	0	1.04	2.25	0.59	0.1190
Subtotal CHEN2				0.32	5.09	2.28	
CHIAZZ	503	m	2	1.89	2.98	5.55	0.0011
CHOI	529	m	0	0.96	7.05	1.33	0.0107
CHOI	532	f	0	1.90	0.74	1.38	0.1034
Subtotal CHOI				1.05	7.79	2.71	
*CPSI	822	m	1	1.41	34.15	26.84	0.0000
*CPSI	851	f	1	0.10	5.41	0.97	0.8082
Subtotal CPSI				1.23	39.56	27.80	
DAMBER	505	m	0	0.83	23.53	2.09	0.0001
DEAN3	570	m	0	-0.18	11.93	6.00	0.5288
DEAN3	589	f	0	-0.43	5.19	4.73	0.3294
Subtotal DEAN3				-0.26	17.12	10.74	
DOLL	507	m	0	0.64	2.53	0.03	0.3070
DOLL	514	f	0	0.51	3.75	0.00	0.3226
Subtotal DOLL				0.56	6.28	0.03	
*DORN	616	m	0	1.52	25.71	25.31	0.0000
*DORN	653	m	0	1.00	42.73	9.63	0.0000
Subtotal DORN				1.20	68.44	34.94	
*ENGELA	505	m	0	0.67	15.51	0.33	0.0081
*ENGELA	513	f	0	1.42	6.43	5.13	0.0003
Subtotal ENGELA				0.89	21.94	5.46	
GAO	505	m	0	1.50	25.14	23.97	0.0000
GAO	515	f	0	0.99	12.15	2.64	0.0005
Subtotal GAO				1.34	37.29	26.62	
GAO2	505	m	0	1.39	1.24	0.91	0.1229
GENG	532	f	0	1.38	5.11	3.74	0.0018
HAENSZ	541	f	0	0.20	10.18	1.12	0.5323
HEGMAN	501	m	0	0.82	19.93	1.69	0.0003
HEGMAN	502	f	0	1.90	1.81	3.42	0.0105
Subtotal HEGMAN				0.91	21.74	5.11	

Table 1H15 - 5

IESLC - Meta-analysis of Ever/current Smoking, Age started, "Highest vs lowest"
All LC types, Any Product (or Cigarettes if Any not available)
Least adjusted

REF	NRR	SEX	AD	Ys	Ws	Qs	Ps
*HIRAYA	564	m	1	0.83	11.00	1.00	0.0060
*HIRAYA	506	f	1	-1.14	0.91	2.54	0.2763
Subtotal HIRAYA				0.68	11.92	3.54	
HU	515	m	0	0.98	2.93	0.59	0.0948
HU	520	f	0	0.73	1.02	0.04	0.4592
Subtotal HU				0.91	3.95	0.63	
HU2	507	c	0	1.30	8.09	4.83	0.0002
JEDRYC	610	m	5	0.51	34.48	0.01	0.0029
JEDRYC	620	f	5	0.57	4.20	0.01	0.2417
Subtotal JEDRYC				0.51	38.68	0.02	
JOLY	547	m	0	1.48	13.06	11.75	0.0000
JOLY	537	f	0	1.35	9.12	6.23	0.0000
Subtotal JOLY				1.43	22.19	17.98	
KHUDER	510	m	0	0.48	35.36	0.08	0.0043
KOULUM	509	m	0	2.19	3.13	8.66	0.0001
LETOUR	505	c	0	0.53	31.90	0.00	0.0030
*LIAW	510	c	2	1.12	8.53	3.02	0.0011
LIU3	503	m	0	0.02	9.83	2.49	0.9407
LIU4	505	m	2	0.46	3833.36	18.53	0.0000
LIU5	503	c	0	0.93	6.37	1.04	0.0188
LUBIN	571	m	0	1.44	20.61	17.28	0.0000
LUBIN2	1155	m	0	0.94	37.89	6.60	0.0000
MATOS	560	m	0	0.69	13.33	0.37	0.0115
*MIGRAN	509	m	0	0.25	16.88	1.27	0.2993
*MIGRAN	519	f	0	-0.11	1.71	0.68	0.8892
Subtotal MIGRAN				0.22	18.58	1.95	
*MRFITR	518	m	0	0.79	2.70	0.18	0.1967
PERNU	506	m	0	0.19	56.54	6.24	0.1428
PERNU	503	f	0	2.68	0.37	1.69	0.1056
Subtotal PERNU				0.21	56.91	7.92	
PEZZOT	574	m	0	0.69	14.69	0.38	0.0085
*QIAO2	505	m	0	0.60	35.89	0.21	0.0003
RACHTA	505	f	0	0.21	2.26	0.22	0.7480
SEGI2	520	m	0	1.03	21.03	5.27	0.0000
SOBUE	800	m	0	0.49	28.15	0.03	0.0088
*SPEIZE	530	f	2	0.22	41.69	3.85	0.1497
SUZUK2	505	c	0	1.45	4.16	3.55	0.0031
SVENSS	505	f	0	0.33	6.51	0.25	0.3979
TIZZAN	510	m	0	1.58	9.14	10.10	0.0000
TIZZAN	523	f	0	2.62	0.77	3.40	0.0211
Subtotal TIZZAN				1.66	9.92	13.50	
WAKAI	505	m	0	0.57	4.94	0.01	0.2027
WU	521	f	0	1.50	6.07	5.78	0.0002
WYNDE6	763	m	0	0.52	40.26	0.00	0.0010
WYNDE6	771	f	0	0.82	29.93	2.54	0.0000
Subtotal WYNDE6				0.65	70.19	2.54	
ZHENG	567	m	0	1.76	11.97	18.19	0.0000
ZHENG	574	f	0	0.76	5.64	0.31	0.0704
Subtotal ZHENG				1.44	17.61	18.50	

	N	73
	NS	53
	Wt	4801.90
Het	Chi	322.50
Het	df	72
Het	P	***
Fixed	RR	1.69
	RRl	1.65
	RRu	1.74
	P	+++
Random	RR	2.32
	RRl	2.06
	RRu	2.62
	P	+++
Asymm	P	***

Table 1H15 - 6

IESLC - Meta-analysis of Ever/current Smoking, Age started, "Highest vs lowest"
All LC types, Any Product (or Cigarettes if Any not available)
Least adjusted

		Sex			
		combined	male	female	Total
	N	7	39	27	73
	NS	7	38	27	72
	Wt	76.73	4535.06	190.11	4801.90
Het	Chi	9.43	253.88	51.06	322.50
Het	df	6	38	26	72
Het	P	N.S.	***	**	***
Fixed	RR	2.18	1.68	1.91	1.69
	RRl	1.74	1.63	1.66	1.65
	RRu	2.73	1.73	2.21	1.74
	P	+++	+++	+++	+++
Random	RR	2.39	2.43	2.04	2.32
	RRl	1.76	2.08	1.62	2.06
	RRu	3.25	2.85	2.57	2.62
	P	+++	+++	+++	+++
Between	Chi				8.14
Between	df				2
Between	P				*
Btwn(F)	P				N.S.
Btwn(R)	P				N.S.

Table 1H15 - 7

IESLC - Meta-analysis of Ever/current Smoking, Age started, "Highest vs lowest"
All LC types, Any Product (or Cigarettes if Any not available)
Excluded studies (and stage at which they were excluded)

1	BECHER BLOT1 BROWN3 CARPEN CHYOU DARBY DOLL2 GARCIA GRAHAM GURSEL HAMMO2 JAHN JAIN LAUSSM PRESCO QIAO TVERDA WIGLE WYNDE3
2	AKIBA AMANDU AMES AXELSS BENSHL BEST BOUCHA BOUCOT BROSS CHEN CPSII DEAN2 DESTEF DORGAN DOSEME FAN GARSHI GER GILLIS HAMMON HUMBLE JUSSAW KAISE2 KATSOU KAUFMA KOO KREUZE LEVIN MCCONN NOTAN2 OSANN2 PEZZO2 PISANI RESTRE SADOWS VUTUC WANG2 WATSON WU2 WUWILL WYNDE2 WYNDE8 XU ZHOU
3	GUO MCDUFF SPITZ STASZE ZHANG
5	LUO
6	CORREA HOLE YUAN
8	BOFFET WYNDE7
15	BENHAM

Table 1H15 - 8
Potentially overlapping studies

REF	REFGP	PRINC	.	OVERLAP/LINK
LUBIN2	LUBIN2	1		Lubin-combined
MRFITR	MRFIT	2		Subset of MRFIT
WYNDE6	WYNDE6	1		WYNDE5/6/7/8
CPSI	CPSI	1		CPSI overall
LUBIN	XIANGZ	2		LUBIN/XIANGZ/QIAO

Table 1H15 - 9
Most adjusted - insufficient data for meta-analysis

REF	NRR	SEX	AGEL	AGEH	RACE	YF	LC TYPE	LOC	START	ST	NLC	R	VB	P	H	AD	ADOS	SM	PRODUCT	exL	exH	unexL	unexH	De
CORREA	544	c	0	0	all	-	all	NAmer	1979	CC	1359	n	bl	y	n	2	0	ev	cig+/-ot	1	15	21	999	st
HIRAYA	548	m	0	0	all	8	all	As:Jap	1965	pr	1917	n	bl	n	n	1	0	cu	cig+/-ot	1	19	25	999	st
HOLE	502	m	0	0	all	0	all	Eu:UK	1972	pr	225	n	V	n	n	3	1#	cu	cig+/-ot	1	14	20	999	st
SPEIZE	533	f	0	0	all	0	all	NAmer	1976	pr	593	n	bl	n	y	2	1#	cu	cig+/-ot	1	17	22	999	ot
YUAN	501	m	0	0	all	0	all	As:Chi	1986	pr	142	n	ot	n	n	3	1#	ev	cig+/-ot	1	24	25	999	ot

Comments on values in listings

HOLE	ADOS	Number smoked
SPEIZE	ADOS	Numbers of cigs/day
YUAN	ADOS	Amount smoked per day

REF	NRR	RR	SIG	RRDATA comment
CORREA	544	2.92		0
HIRAYA	548	1.55		0
HOLE	502	1.28		0
SPEIZE	533	*	gap	Text page 478 SPEIZE1999 gives the risk for age start 1-17 vs 18-19 as 1.1(0.9-1.5)
YUAN	501	2.40	y	Significant P<.001

Table 1H16 -

IESLC - Meta-analysis of Ever/current Smoking by Age started, Overview
All LC types, Cigarettes (or Any Product if Cigarettes not available)

This analysis is restricted to results for:
1) Ever/current smokers
2) Results by Age started
3) Categorical results by Age started
Results by Age started are grouped under 2 schemes (S1, S2). Each scheme has a set of "key values". An interval is allocated to the category whose key value it includes, and intervals which include none or more than one of the key values are excluded. (Open-ended intervals are coded as 999)

S1	key value	maximum range
1	26	19+
2	18	15-25
3	14	1-17

S2	key value	maximum range
1	30	27+
2	26	23-29
3	22	19-25
4	18	15-21
5	14	11-17
6	10	1-13

4) All LC types (or near equivalent)
5) Results complete enough for use in metaanalysis

Within each study, results are then selected (in the following order of preference, within each sex) for:
6) SMKSTA: ever, current
7) PRODUCT: cigarettes regardless of other products, cigarettes only, all/unspec
8) CIGTYPE: all/unspecified, MC regardless of HR, MC only
9) (not applicable)
10) DENOM: never smoked anything, never smoked cigarettes, never any + low, never cigs + low
11) Followup period (YF, prospective studies): whole study (coded as 0) or longest available
12) LCtype: all or nearest available, at least Squamous and Adeno. (q = squamous, s = small, l = large, a = adeno, mix = mixed, alv = alveolar)
13) Race: all or nearest available, otherwise by race (wh or w = white, bl or b = black, hi = hispanic ch = chinese, jap = japanese, haw = hawaiian, w+o = white + oriental, sca = scandinavian, as = asian)
14) For overlapping studies: principal rather than subsidiary studies
Finally by Age: whole study (coded as 0) if available, otherwise by widest available age group and then for single sex results (m, f) in preference to results for both sexes combined (c).

Results adjusted (AD) for the most potential confounders are then chosen in Sections -1 to -3 (and those which actually differ from the adjusted results in Table 1H11 - 1 are marked 'x' in Section -1) and results adjusted for the least confounders in Sections -4 to -6. (Those least adjusted results which actually differ from the most adjusted are marked 'x' in column X in Section -4)

Section -7 shows excluded studies, together with the stage (as above) at which no qualifying results were found.

Section -8 lists the potentially overlapping studies which have been included (1=principal, 2=subsidiary).

Section -9 lists any results which would have been included in preference except that they had data not complete enough for use in meta-analysis, with their significance (yes/no), if known, and any further comment as entered on the database. It also lists as "gap" any categories for which no data were presented by the original authors.

In addition to those mentioned above, the following fields, levels and abbreviations are used:
* or nk = not known, n = no, y = yes, ot = other
ev = ever, cu = current, nev = never
all/unspec = all or unspecified, cig+/-ot = cigarettes irrespective of other products (cigar, pipe etc)
MC = manufactured cigarettes, HR = hand-rolled cigarettes
exL, exH = range of exposure (low and high) in the smoking group, in terms of Age started
REF: 6-character study reference
NRR: number of the RR on the database within the study
ST : study type (CC = case control, pr or prosp = prospective)
NLC: number of lung cancer cases in whole study
R : risky occupational population (n = no, m = mining, o = other risky)
VB : national cigarette type (V = at least 75% Virginia, bl = at least 75% blended, ot = other)
P : any proxy use
H : full histological confirmation
De : derivation of RR/CI (or = original, st = standard method, ot = other method of estimation)

Table 1H16 - 1

IESLC - Meta-analysis of Ever/current Smoking by Age started, Overview
All LC types, Cigarettes (or Any Product if Cigarettes not available)
Most adjusted

REF	NRR	1H11	SEX	AGEL	AGEH	RACE	YF	LC TYPE	LOC	START	ST	NLC	R	VB	P	H	AD	SM	PRODUCT	exL	exH	S1	S2	DENOM	De
AGUDO	504		f	0	0	all	-	all	Eu:wst	1989	CC	103	n	bl	n	n	3	ev	cig only	24	999	1	0	nev cigs	or
AGUDO	505		f	0	0	all	-	all	Eu:wst	1989	CC	103	n	bl	n	n	3	ev	cig only	1	23	0	0	nev cigs	or
ARMADA	511		m	0	0	all	-	all	Eu:wst	1986	CC	325	n	bl	n	y	0	ev	cig+/-ot	17	45	0	0	nev cigs	st
ARMADA	512		m	0	0	all	-	all	Eu:wst	1986	CC	325	n	bl	n	y	0	ev	cig+/-ot	7	16	3	0	nev cigs	st
AUVINE	520		c	0	0	all	-	all	Eu:Sca	1986	CC	517	n	bl	y	n	2	ev	cig+/-ot	16	999	0	0	nev cigs	or
AUVINE	521		c	0	0	all	-	all	Eu:Sca	1986	CC	517	n	bl	y	n	2	ev	cig+/-ot	1	15	3	0	nev cigs	or
BARBON	520		m	0	0	all	-	all	Eu:wst	1979	CC	755	n	bl	y	y	1	ev	all/unsp	20	999	1	0	nev any	or
BARBON	521		m	0	0	all	-	all	Eu:wst	1979	CC	755	n	bl	y	y	1	ev	all/unsp	15	19	2	4	nev any	or
BARBON	522		m	0	0	all	-	all	Eu:wst	1979	CC	755	n	bl	y	y	1	ev	all/unsp	1	14	3	0	nev any	or
BRESLO	501		c	0	0	all	-	all	NAmer	1949	CC	518	n	bl	n	y	0	ev	cig+/-ot	25	999	1	0	nev any	st
BRESLO	502		c	0	0	all	-	all	NAmer	1949	CC	518	n	bl	n	y	0	ev	cig+/-ot	15	24	2	0	nev any	st
BRESLO	503		c	0	0	all	-	all	NAmer	1949	CC	518	n	bl	n	y	0	ev	cig+/-ot	0	14	3	0	nev any	st
BUFFLE	517		f	0	0	w-hi	-	all	NAmer	1976	CC	943	n	bl	y	n	0	ev	cig+/-ot	30	999	0	1	nev cigs	or
BUFFLE	518		f	0	0	w-hi	-	all	NAmer	1976	CC	943	n	bl	y	n	0	ev	cig+/-ot	21	29	1	0	nev cigs	or
BUFFLE	519		f	0	0	w-hi	-	all	NAmer	1976	CC	943	n	bl	y	n	0	ev	cig+/-ot	19	20	0	0	nev cigs	or
BUFFLE	520		f	0	0	w-hi	-	all	NAmer	1976	CC	943	n	bl	y	n	0	ev	cig+/-ot	17	18	2	4	nev cigs	ot
BUFFLE	521		f	0	0	w-hi	-	all	NAmer	1976	CC	943	n	bl	y	n	0	ev	cig+/-ot	6	16	3	0	nev cigs	or
CEDERL	510		m	0	0	all	10	all	Eu:Sca	1963	pr	491	n	bl	n	n	1	cu	cig only	19	999	1	0	nev any	ot
CEDERL	511		m	0	0	all	10	all	Eu:Sca	1963	pr	491	n	bl	n	n	1	cu	cig only	17	18	2	4	nev any	ot
CEDERL	512		m	0	0	all	10	all	Eu:Sca	1963	pr	491	n	bl	n	n	1	cu	cig only	1	16	3	0	nev any	ot
CEDERL	515		f	0	0	all	10	all	Eu:Sca	1963	pr	491	n	bl	n	n	0	cu	cig only	19	999	1	0	nev any	st
CEDERL	516		f	0	0	all	10	all	Eu:Sca	1963	pr	491	n	bl	n	n	0	cu	cig only	17	18	2	4	nev any	st
CEDERL	517		f	0	0	all	10	all	Eu:Sca	1963	pr	491	n	bl	n	n	0	cu	cig only	1	16	3	0	nev any	ot
CHEN2	517		m	0	0	all	-	all	As:Chi	1983	CC	193	n	ot	y	n	0	ev	all/unsp	31	999	0	0	nev any	st
CHEN2	518		m	0	0	all	-	all	As:Chi	1983	CC	193	n	ot	y	n	0	ev	all/unsp	20	30	1	0	nev any	st
CHEN2	519		m	0	0	all	-	all	As:Chi	1983	CC	193	n	ot	y	n	0	ev	all/unsp	1	19	0	0	nev any	st
CHEN2	522		f	0	0	all	-	all	As:Chi	1983	CC	193	n	ot	y	n	0	ev	all/unsp	31	999	0	0	nev any	st
CHEN2	523		f	0	0	all	-	all	As:Chi	1983	CC	193	n	ot	y	n	0	ev	all/unsp	20	30	1	0	nev any	st
CHEN2	524		f	0	0	all	-	all	As:Chi	1983	CC	193	n	ot	y	n	0	ev	all/unsp	1	19	0	0	nev any	st
CHIAZZ	501		m	0	0	all	-	all	NAmer	1940	CC	144	o	bl	y	n	2	ev	cig+/-ot	20	999	1	0	nev cigs	or
CHIAZZ	502		m	0	0	all	-	all	NAmer	1940	CC	144	o	bl	y	n	2	ev	cig+/-ot	1	19	0	0	nev cigs	or
CHOI	523		m	0	0	all	-	all	As:oth	1985	CC	375	n	bl	n	n	0	ev	cig+/-ot	25	999	1	0	nev cigs	st
CHOI	524		m	0	0	all	-	all	As:oth	1985	CC	375	n	bl	n	n	0	ev	cig+/-ot	20	24	0	3	nev cigs	st
CHOI	525		m	0	0	all	-	all	As:oth	1985	CC	375	n	bl	n	n	0	ev	cig+/-ot	15	19	2	4	nev cigs	st
CHOI	526		m	0	0	all	-	all	As:oth	1985	CC	375	n	bl	n	n	0	ev	cig+/-ot	1	14	3	0	nev cigs	st
CHOI	530		f	0	0	all	-	all	As:oth	1985	CC	375	n	bl	n	n	0	ev	cig+/-ot	25	999	1	0	nev cigs	st
CHOI	531		f	0	0	all	-	all	As:oth	1985	CC	375	n	bl	n	n	0	ev	cig+/-ot	1	24	0	0	nev cigs	st
CPSI	801		m	35	84	all	6	all	NAmer	1959	pr	5138	n	bl	n	n	1	cu	cig+/-ot	25	999	1	0	nev any	ot
CPSI	802		m	35	84	all	6	all	NAmer	1959	pr	5138	n	bl	n	n	1	cu	cig+/-ot	20	24	0	3	nev any	ot
CPSI	803		m	35	84	all	6	all	NAmer	1959	pr	5138	n	bl	n	n	1	cu	cig+/-ot	15	19	2	4	nev any	ot
CPSI	804		m	35	84	all	6	all	NAmer	1959	pr	5138	n	bl	n	n	1	cu	cig+/-ot	1	14	3	0	nev any	ot
CPSI	845		f	40	74	all	6	all	NAmer	1959	pr	5138	n	bl	n	n	1	cu	cig only	25	999	1	0	nev any	ot
CPSI	846		f	40	74	all	6	all	NAmer	1959	pr	5138	n	bl	n	n	1	cu	cig only	20	24	0	3	nev any	ot
CPSI	847		f	40	74	all	6	all	NAmer	1959	pr	5138	n	bl	n	n	1	cu	cig only	15	19	2	4	nev any	ot
CPSI	848		f	40	74	all	6	all	NAmer	1959	pr	5138	n	bl	n	n	1	cu	cig only	1	14	3	0	nev any	ot
DAMBER	501		m	0	0	all	-	all	Eu:Sca	1972	CC	579	n	bl	y	n	0	ev	all/unsp	21	999	1	0	nev any	st
DAMBER	502		m	0	0	all	-	all	Eu:Sca	1972	CC	579	n	bl	y	n	0	ev	all/unsp	16	20	2	4	nev any	st
DAMBER	503		m	0	0	all	-	all	Eu:Sca	1972	CC	579	n	bl	y	n	0	ev	all/unsp	1	15	3	0	nev any	st
DEAN3	564		m	0	0	all	-	all	Eu:UK	1969	CC	766	n	V	y	n	0	cu	cig only	25	999	1	0	nev any	st
DEAN3	565		m	0	0	all	-	all	Eu:UK	1969	CC	766	n	V	y	n	0	cu	cig only	20	24	0	3	nev any	st
DEAN3	566		m	0	0	all	-	all	Eu:UK	1969	CC	766	n	V	y	n	0	cu	cig only	15	19	2	4	nev any	st
DEAN3	567		m	0	0	all	-	all	Eu:UK	1969	CC	766	n	V	y	n	0	cu	cig only	1	14	3	0	nev any	st
DEAN3	583		f	0	0	all	-	all	Eu:UK	1969	CC	766	n	V	y	n	0	cu	cig only	25	999	1	0	nev any	st
DEAN3	584		f	0	0	all	-	all	Eu:UK	1969	CC	766	n	V	y	n	0	cu	cig only	20	24	0	3	nev any	st
DEAN3	585		f	0	0	all	-	all	Eu:UK	1969	CC	766	n	V	y	n	0	cu	cig only	15	19	2	4	nev any	st
DEAN3	586		f	0	0	all	-	all	Eu:UK	1969	CC	766	n	V	y	n	0	cu	cig only	1	14	3	0	nev any	st
DOLL	501		m	0	0	all	-	all	Eu:UK	1948	CC	1465	n	V	n	n	0	ev	all/unsp	40	999	0	0	nev any	st
DOLL	502		m	0	0	all	-	all	Eu:UK	1948	CC	1465	n	V	n	n	0	ev	all/unsp	30	39	0	1	nev any	st
DOLL	503		m	0	0	all	-	all	Eu:UK	1948	CC	1465	n	V	n	n	0	ev	all/unsp	20	29	1	0	nev any	st
DOLL	504		m	0	0	all	-	all	Eu:UK	1948	CC	1465	n	V	n	n	0	ev	all/unsp	1	19	0	0	nev any	st
DOLL	508		f	0	0	all	-	all	Eu:UK	1948	CC	1465	n	V	n	n	0	ev	all/unsp	40	999	0	0	nev any	st
DOLL	509		f	0	0	all	-	all	Eu:UK	1948	CC	1465	n	V	n	n	0	ev	all/unsp	30	39	0	1	nev any	st
DOLL	510		f	0	0	all	-	all	Eu:UK	1948	CC	1465	n	V	n	n	0	ev	all/unsp	20	29	1	0	nev any	st
DOLL	511		f	0	0	all	-	all	Eu:UK	1948	CC	1465	n	V	n	n	0	ev	all/unsp	1	19	0	0	nev any	st
DORN	610		m	55	64	wh	8	all	NAmer	1954	pr	5097	n	bl	n	n	0	ev	cig+/-ot	25	999	1	0	nev any	st
DORN	611		m	55	64	wh	8	all	NAmer	1954	pr	5097	n	bl	n	n	0	ev	cig+/-ot	20	24	0	3	nev any	st
DORN	612		m	55	64	wh	8	all	NAmer	1954	pr	5097	n	bl	n	n	0	ev	cig+/-ot	15	19	2	4	nev any	st
DORN	613		m	55	64	wh	8	all	NAmer	1954	pr	5097	n	bl	n	n	0	ev	cig+/-ot	1	14	3	0	nev any	st
DORN	647		m	65	74	wh	8	all	NAmer	1954	pr	5097	n	bl	n	n	0	ev	cig+/-ot	25	999	1	0	nev any	st
DORN	648		m	65	74	wh	8	all	NAmer	1954	pr	5097	n	bl	n	n	0	ev	cig+/-ot	20	24	0	3	nev any	st
DORN	649		m	65	74	wh	8	all	NAmer	1954	pr	5097	n	bl	n	n	0	ev	cig+/-ot	15	19	2	4	nev any	st
DORN	650		m	65	74	wh	8	all	NAmer	1954	pr	5097	n	bl	n	n	0	ev	cig+/-ot	1	14	3	0	nev any	st

Table 1H16 - 1

IESLC - Meta-analysis of Ever/current Smoking by Age started, Overview
All LC types, Cigarettes (or Any Product if Cigarettes not available)
Most adjusted

REF	NRR	1H11	SEX	AGEL	AGEH	RACE	YF	LC TYPE	LOC	START	ST	NLC	R	VB	P	H	AD	SM	PRODUCT	exL	exH	S1	S2	DENOM	De
ENGELA	501		m	0	0	all	0	all	Eu:Sca	1964	pr	435	n	bl	n	n	0	cu	cig+/-ot	30	999	0	1	nev cigs	st
ENGELA	502		m	0	0	all	0	all	Eu:Sca	1964	pr	435	n	bl	n	n	0	cu	cig+/-ot	20	29	1	0	nev cigs	st
ENGELA	503		m	0	0	all	0	all	Eu:Sca	1964	pr	435	n	bl	n	n	0	cu	cig+/-ot	1	19	0	0	nev cigs	st
ENGELA	509		f	0	0	all	0	all	Eu:Sca	1964	pr	435	n	bl	n	n	0	cu	cig+/-ot	30	999	0	1	nev cigs	st
ENGELA	510		f	0	0	all	0	all	Eu:Sca	1964	pr	435	n	bl	n	n	0	cu	cig+/-ot	20	29	1	0	nev cigs	st
ENGELA	511		f	0	0	all	0	all	Eu:Sca	1964	pr	435	n	bl	n	n	0	cu	cig+/-ot	1	19	0	0	nev cigs	st
GAO	506		m	0	0	all	-	all	As:Chi	1984	CC	1405	n	ot	n	n	2	ev	cig+/-ot	30	999	0	1	nev cigs	or
GAO	507		m	0	0	all	-	all	As:Chi	1984	CC	1405	n	ot	n	n	2	ev	cig+/-ot	20	29	1	0	nev cigs	or
GAO	508		m	0	0	all	-	all	As:Chi	1984	CC	1405	n	ot	n	n	2	ev	cig+/-ot	10	19	0	0	nev cigs	or
GAO	516		f	0	0	all	-	all	As:Chi	1984	CC	1405	n	ot	n	n	2	ev	cig+/-ot	30	999	0	1	nev cigs	or
GAO	517		f	0	0	all	-	all	As:Chi	1984	CC	1405	n	ot	n	n	2	ev	cig+/-ot	20	29	1	0	nev cigs	or
GAO	518		f	0	0	all	-	all	As:Chi	1984	CC	1405	n	ot	n	n	2	ev	cig+/-ot	10	19	0	0	nev cigs	or
GAO2	501		m	0	0	all	-	all	As:Jap	1988	CC	282	n	bl	n	n	0	cu	cig+/-ot	30	999	0	1	nev cigs	or
GAO2	502		m	0	0	all	-	all	As:Jap	1988	CC	282	n	bl	n	n	0	cu	cig+/-ot	20	29	1	0	nev cigs	st
GAO2	503		m	0	0	all	-	all	As:Jap	1988	CC	282	n	bl	n	n	0	cu	cig+/-ot	1	19	0	0	nev cigs	or
GENG	533		f	0	0	all	-	all	As:Chi	1985	CC	292	n	ot	*	n	1	ev	cig+/-ot	21	999	1	0	nev any	st
GENG	534		f	0	0	all	-	all	As:Chi	1985	CC	292	n	ot	*	n	1	ev	cig+/-ot	16	20	2	4	nev any	st
GENG	535		f	0	0	all	-	all	As:Chi	1985	CC	292	n	ot	*	n	1	ev	cig+/-ot	1	15	3	0	nev any	st
HAENSZ	537		f	0	0	all	-	not alv	NAmer	1955	CC	158	n	bl	n	y	0	ev	cig+/-ot	25	999	1	0	nev any	st
HAENSZ	538		f	0	0	all	-	not alv	NAmer	1955	CC	158	n	bl	n	y	0	ev	cig+/-ot	1	24	0	0	nev any	st
HEGMAN	513		m	0	0	all	-	all	NAmer	1989	CC	282	n	bl	y	y	1	ev	all/unsp	20	999	1	0	nev any	or
HEGMAN	514		m	0	0	all	-	all	NAmer	1989	CC	282	n	bl	y	y	1	ev	all/unsp	1	19	0	0	nev any	or
HEGMAN	516		f	0	0	all	-	all	NAmer	1989	CC	282	n	bl	y	y	1	ev	all/unsp	26	999	1	0	nev any	or
HEGMAN	517		f	0	0	all	-	all	NAmer	1989	CC	282	n	bl	y	y	1	ev	all/unsp	1	25	0	0	nev any	or
HIRAYA	501		m	0	0	all	0	all	As:Jap	1965	pr	1917	n	bl	n	n	1	cu	cig+/-ot	20	999	1	0	nev any	st
HIRAYA	502		m	0	0	all	0	all	As:Jap	1965	pr	1917	n	bl	n	n	1	cu	cig+/-ot	1	19	0	0	nev any	st
HIRAYA	504		f	0	0	all	0	all	As:Jap	1965	pr	1917	n	bl	n	n	1	cu	cig+/-ot	20	999	1	0	nev any	st
HIRAYA	505		f	0	0	all	0	all	As:Jap	1965	pr	1917	n	bl	n	n	1	cu	cig+/-ot	1	19	0	0	nev any	st
HU	511		m	0	0	all	-	all	As:Chi	1985	CC	227	n	ot	n	y	0	ev	cig+/-ot	30	999	0	1	nev cigs	st
HU	512		m	0	0	all	-	all	As:Chi	1985	CC	227	n	ot	n	y	0	ev	cig+/-ot	16	29	0	0	nev cigs	st
HU	513		m	0	0	all	-	all	As:Chi	1985	CC	227	n	ot	n	y	0	ev	cig+/-ot	1	15	3	0	nev cigs	st
HU	516		f	0	0	all	-	all	As:Chi	1985	CC	227	n	ot	n	y	0	ev	cig+/-ot	30	999	0	1	nev cigs	st
HU	517		f	0	0	all	-	all	As:Chi	1985	CC	227	n	ot	n	y	0	ev	cig+/-ot	16	29	0	0	nev cigs	st
HU	518		f	0	0	all	-	all	As:Chi	1985	CC	227	n	ot	n	y	0	ev	cig+/-ot	1	15	3	0	nev cigs	st
HU2	501		c	0	0	all	-	all	As:Chi	1977	CC	523	n	ot	y	n	0	ev	cig+/-ot	40	999	0	0	nev cigs	ot
HU2	502		c	0	0	all	-	all	As:Chi	1977	CC	523	n	ot	y	n	0	ev	cig+/-ot	30	39	0	1	nev cigs	st
HU2	503		c	0	0	all	-	all	As:Chi	1977	CC	523	n	ot	y	n	0	ev	cig+/-ot	20	29	1	0	nev cigs	st
HU2	504		c	0	0	all	-	all	As:Chi	1977	CC	523	n	ot	y	n	0	ev	cig+/-ot	1	19	0	0	nev cigs	or
JEDRYC	607		m	0	0	all	-	all	Eu:est	1980	CC	1630	n	bl	y	n	0	ev	cig+/-ot	17	18	2	4	nev any	st
JEDRYC	608		m	0	0	all	-	all	Eu:est	1980	CC	1630	n	bl	y	n	0	ev	cig+/-ot	1	16	3	0	nev any	st
JEDRYC	619		f	0	0	all	-	all	Eu:est	1980	CC	1630	n	bl	y	n	0	ev	cig+/-ot	1	22	0	0	nev any	st
JOLY	543		m	0	0	all	-	all	SCAmer	1978	CC	826	n	bl	n	n	0	ev	cig+/-ot	25	999	1	0	nev any	st
JOLY	544		m	0	0	all	-	all	SCAmer	1978	CC	826	n	bl	n	n	0	ev	cig+/-ot	15	24	2	0	nev any	st
JOLY	545		m	0	0	all	-	all	SCAmer	1978	CC	826	n	bl	n	n	0	ev	cig+/-ot	1	14	3	0	nev any	st
JOLY	533		f	0	0	all	-	all	SCAmer	1978	CC	826	n	bl	n	n	0	ev	cig+/-ot	25	999	1	0	nev any	st
JOLY	534		f	0	0	all	-	all	SCAmer	1978	CC	826	n	bl	n	n	0	ev	cig+/-ot	15	24	2	0	nev any	st
JOLY	535		f	0	0	all	-	all	SCAmer	1978	CC	826	n	bl	n	n	0	ev	cig+/-ot	1	14	3	0	nev any	st
KHUDER	506		m	0	0	all	-	all	NAmer	1985	CC	482	n	bl	n	y	0	ev	cig+/-ot	20	999	1	0	nev cigs	st
KHUDER	507		m	0	0	all	-	all	NAmer	1985	CC	482	n	bl	n	y	0	ev	cig+/-ot	16	19	2	4	nev cigs	st
KHUDER	508		m	0	0	all	-	all	NAmer	1985	CC	482	n	bl	n	y	0	ev	cig+/-ot	1	15	3	0	nev cigs	st
KOULUM	501		m	0	0	all	-	all	Eu:Sca	1936	CC	812	n	bl	n	n	0	ev	all/unsp	31	999	0	0	nev any	st
KOULUM	502		m	0	0	all	-	all	Eu:Sca	1936	CC	812	n	bl	n	n	0	ev	all/unsp	21	30	1	0	nev any	st
KOULUM	503		m	0	0	all	-	all	Eu:Sca	1936	CC	812	n	bl	n	n	0	ev	all/unsp	16	20	2	4	nev any	st
KOULUM	504		m	0	0	all	-	all	Eu:Sca	1936	CC	812	n	bl	n	n	0	ev	all/unsp	11	15	3	5	nev any	st
KOULUM	505		m	0	0	all	-	all	Eu:Sca	1936	CC	812	n	bl	n	n	0	ev	all/unsp	0	10	0	6	nev any	st
LETOUR	501		c	0	0	all	-	all	NAmer	1983	CC	738	n	V	y	y	0	ev	cig+/-ot	21	999	1	0	nev cigs	st
LETOUR	502		c	0	0	all	-	all	NAmer	1983	CC	738	n	V	y	y	0	ev	cig+/-ot	15	20	2	4	nev cigs	st
LETOUR	503		c	0	0	all	-	all	NAmer	1983	CC	738	n	V	y	y	0	ev	cig+/-ot	1	14	3	0	nev cigs	st
LIAW	504		c	0	0	all	0	all	As:oth	1982	pr	127	n	ot	n	n	2	cu	all/unsp	25	999	1	0	nev any	or
LIAW	505		c	0	0	all	0	all	As:oth	1982	pr	127	n	ot	n	n	2	cu	all/unsp	21	24	0	3	nev any	or
LIAW	506		c	0	0	all	0	all	As:oth	1982	pr	127	n	ot	n	n	2	cu	all/unsp	1	20	0	0	nev any	or
LIU3	504		m	0	0	all	-	all	As:Chi	1985	CC	110	n	ot	n	n	2	ev	all/unsp	21	999	1	0	nev any	or
LIU3	505		m	0	0	all	-	all	As:Chi	1985	CC	110	n	ot	n	n	2	ev	all/unsp	1	20	0	0	nev any	or
LIU4	501		m	35	69	all	-	all	As:Chi	1986	CC	1000-00	n	ot	y	n	2	ev	all/unsp	25	999	1	0	nev any	ot
LIU4	502		m	35	69	all	-	all	As:Chi	1986	CC	1000-00	n	ot	y	n	2	ev	all/unsp	20	24	0	3	nev any	ot
LIU4	503		m	35	69	all	-	all	As:Chi	1986	CC	1000-00	n	ot	y	n	2	ev	all/unsp	0	19	0	0	nev any	ot
LIU5	501		c	0	0	all	-	all	As:Chi	1978	CC	111	n	ot	y	n	0	ev	all/unsp	30	999	0	1	nev any	st
LIU5	502		c	0	0	all	-	all	As:Chi	1978	CC	111	n	ot	y	n	0	ev	all/unsp	1	29	0	0	nev any	st
LUBIN	565		m	0	0	all	-	all	As:Chi	1984	CC	427	m	ot	y	n	0	ev	cig+/-ot	27	999	0	1	nev any	st

Table 1H16 - 1

IESLC - Meta-analysis of Ever/current Smoking by Age started, Overview
All LC types, Cigarettes (or Any Product if Cigarettes not available)
Most adjusted

REF	NRR	1H11	SEX	AGEL	AGEH	RACE	YF	LC TYPE	LOC	START	ST	NLC	R	VB	P	H	AD	SM	PRODUCT	exL	exH	S1	S2	DENOM	De
LUBIN	566		m	0	0	all	-	all	As:Chi	1984	CC	427	m	ot	y	n	0	ev	cig+/-ot	23	26	1	2	nev any	st
LUBIN	567		m	0	0	all	-	all	As:Chi	1984	CC	427	m	ot	y	n	0	ev	cig+/-ot	20	22	0	3	nev any	st
LUBIN	568		m	0	0	all	-	all	As:Chi	1984	CC	427	m	ot	y	n	0	ev	cig+/-ot	1	19	0	0	nev any	st
LUBIN2	1156		m	0	0	all	-	all	Eu:mul	1976	CC	7804	n	bl	n	y	1	ev	cig+/-ot	31	999	0	0	nev cigs	st
LUBIN2	1157		m	0	0	all	-	all	Eu:mul	1976	CC	7804	n	bl	n	y	1	ev	cig+/-ot	21	30	1	0	nev cigs	st
LUBIN2	1158		m	0	0	all	-	all	Eu:mul	1976	CC	7804	n	bl	n	y	1	ev	cig+/-ot	17	20	2	4	nev cigs	st
LUBIN2	1159		m	0	0	all	-	all	Eu:mul	1976	CC	7804	n	bl	n	y	1	ev	cig+/-ot	13	16	3	5	nev cigs	st
LUBIN2	1160		m	0	0	all	-	all	Eu:mul	1976	CC	7804	n	bl	n	y	1	ev	cig+/-ot	1	12	0	6	nev cigs	st
MATOS	576		m	0	0	all	-	all	SCAmer	1994	CC	200	n	bl	n	n	2	ev	cig+/-ot	20	999	1	0	nev any	or
MATOS	577		m	0	0	all	-	all	SCAmer	1994	CC	200	n	bl	n	n	2	ev	cig+/-ot	15	19	2	4	nev any	or
MATOS	578		m	0	0	all	-	all	SCAmer	1994	CC	200	n	bl	n	n	2	ev	cig+/-ot	1	14	3	0	nev any	or
MIGRAN	501		m	0	0	all	0	all	Eu:UK	1964	pr	259	n	V	n	n	0	cu	cig only	20	999	1	0	nev any	st
MIGRAN	503		m	0	0	all	0	all	Eu:UK	1964	pr	259	n	V	n	n	0	cu	cig only	16	19	2	4	nev any	st
MIGRAN	505		m	0	0	all	0	all	Eu:UK	1964	pr	259	n	V	n	n	0	cu	cig only	0	15	3	0	nev any	st
MIGRAN	511		f	0	0	all	0	all	Eu:UK	1964	pr	259	n	V	n	n	0	cu	cig only	20	999	1	0	nev any	st
MIGRAN	513		f	0	0	all	0	all	Eu:UK	1964	pr	259	n	V	n	n	0	cu	cig only	16	19	2	4	nev any	st
MIGRAN	515		f	0	0	all	0	all	Eu:UK	1964	pr	259	n	V	n	n	0	cu	cig only	0	15	3	0	nev any	st
MRFITR	508		m	0	0	all	0	all	NAmer	1973	pr	119	n	bl	n	n	0	cu	cig+/-ot	24	999	1	0	nev cigs	ot
MRFITR	509		m	0	0	all	0	all	NAmer	1973	pr	119	n	bl	n	n	0	cu	cig+/-ot	22	23	0	3	nev cigs	ot
MRFITR	510		m	0	0	all	0	all	NAmer	1973	pr	119	n	bl	n	n	0	cu	cig+/-ot	20	21	0	0	nev cigs	ot
MRFITR	511		m	0	0	all	0	all	NAmer	1973	pr	119	n	bl	n	n	0	cu	cig+/-ot	18	19	2	4	nev cigs	ot
MRFITR	512		m	0	0	all	0	all	NAmer	1973	pr	119	n	bl	n	n	0	cu	cig+/-ot	16	17	0	0	nev cigs	ot
MRFITR	513		m	0	0	all	0	all	NAmer	1973	pr	119	n	bl	n	n	0	cu	cig+/-ot	1	15	3	0	nev cigs	ot
PERNU	504		m	0	0	all	-	all	Eu:Sca	1944	CC	1606	n	bl	n	n	0	ev	all/unsp	15	999	0	0	nev any	st
PERNU	505		m	0	0	all	-	all	Eu:Sca	1944	CC	1606	n	bl	n	n	0	ev	all/unsp	1	14	3	0	nev any	st
PERNU	501		f	0	0	all	-	all	Eu:Sca	1944	CC	1606	n	bl	n	n	0	ev	all/unsp	15	999	0	0	nev any	st
PERNU	502		f	0	0	all	-	all	Eu:Sca	1944	CC	1606	n	bl	n	n	0	ev	all/unsp	1	14	3	0	nev any	ot
PEZZOT	570		m	0	0	all	-	all	SCAmer	1987	CC	215	n	bl	n	y	0	ev	cig only	19	999	1	0	nev cigs	st
PEZZOT	571		m	0	0	all	-	all	SCAmer	1987	CC	215	n	bl	n	y	0	ev	cig only	14	18	0	0	nev cigs	st
PEZZOT	572		m	0	0	all	-	all	SCAmer	1987	CC	215	n	bl	n	y	0	ev	cig only	1	13	0	6	nev cigs	st
QIAO2	506		m	0	0	all	0	all	As:Chi	1992	pr	241	m	ot	n	n	1	ev	all/unsp	21	999	1	0	nev any	or
QIAO2	507		m	0	0	all	0	all	As:Chi	1992	pr	241	m	ot	n	n	1	ev	all/unsp	17	20	2	4	nev any	or
QIAO2	508		m	0	0	all	0	all	As:Chi	1992	pr	241	m	ot	n	n	1	ev	all/unsp	1	16	3	0	nev any	or
RACHTA	506		f	0	0	all	-	all	Eu:est	1991	CC	118	n	bl	n	y	1	ev	cig+/-ot	31	999	0	0	nev cigs	or
RACHTA	507		f	0	0	all	-	all	Eu:est	1991	CC	118	n	bl	n	y	1	ev	cig+/-ot	20	30	1	0	nev cigs	or
RACHTA	508		f	0	0	all	-	all	Eu:est	1991	CC	118	n	bl	n	y	1	ev	cig+/-ot	1	19	0	0	nev cigs	or
SEGI2	521		m	0	0	all	-	all	As:Jap	1962	CC	378	n	bl	n	n	1	cu	cig+/-ot	23	999	1	0	nev any	ot
SEGI2	522		m	0	0	all	-	all	As:Jap	1962	CC	378	n	bl	n	n	1	cu	cig+/-ot	20	22	0	3	nev any	ot
SEGI2	523		m	0	0	all	-	all	As:Jap	1962	CC	378	n	bl	n	n	1	cu	cig+/-ot	1	19	0	0	nev any	ot
SOBUE	654		m	0	0	all	-	all	As:Jap	1986	CC	1376	n	bl	n	y	0	ev	cig+/-ot	23	999	1	0	nev cigs	st
SOBUE	655		m	0	0	all	-	all	As:Jap	1986	CC	1376	n	bl	n	y	0	ev	cig+/-ot	18	22	2	0	nev cigs	st
SOBUE	656		m	0	0	all	-	all	As:Jap	1986	CC	1376	n	bl	n	y	0	ev	cig+/-ot	10	17	3	0	nev cigs	st
SUZUK2	501		c	0	0	all	-	all	SCAmer	1991	CC	123	n	bl	n	y	0	ev	all/unsp	19	999	1	0	nev any	st
SUZUK2	502		c	0	0	all	-	all	SCAmer	1991	CC	123	n	bl	n	y	0	ev	all/unsp	12	18	0	0	nev any	st
SUZUK2	503		c	0	0	all	-	all	SCAmer	1991	CC	123	n	bl	n	y	0	ev	all/unsp	0	11	0	6	nev any	st
SVENSS	501		f	0	0	all	-	all	Eu:Sca	1983	CC	210	n	bl	n	n	0	cu	all/unsp	26	999	1	0	nev any	st
SVENSS	502		f	0	0	all	-	all	Eu:Sca	1983	CC	210	n	bl	n	n	0	cu	all/unsp	19	25	0	3	nev any	st
SVENSS	503		f	0	0	all	-	all	Eu:Sca	1983	CC	210	n	bl	n	n	0	cu	all/unsp	0	18	0	0	nev any	st
TIZZAN	506		m	0	0	all	-	all	Eu:wst	1959	CC	1358	n	bl	n	n	0	ev	all/unsp	31	999	0	0	nev any	st
TIZZAN	507		m	0	0	all	-	all	Eu:wst	1959	CC	1358	n	bl	n	n	0	ev	all/unsp	20	30	1	0	nev any	st
TIZZAN	508		m	0	0	all	-	all	Eu:wst	1959	CC	1358	n	bl	n	n	0	ev	all/unsp	1	19	0	0	nev any	st
TIZZAN	536	x	f	0	0	all	-	all	Eu:wst	1959	CC	1358	n	bl	n	n	0	ev	cig only	31	999	0	0	nev any	st
TIZZAN	537	x	f	0	0	all	-	all	Eu:wst	1959	CC	1358	n	bl	n	n	0	ev	cig only	20	30	1	0	nev any	st
TIZZAN	538	x	f	0	0	all	-	all	Eu:wst	1959	CC	1358	n	bl	n	n	0	ev	cig only	1	19	0	0	nev any	st
WAKAI	501		m	0	0	all	-	all	As:Jap	1988	CC	333	n	bl	n	y	0	cu	cig+/-ot	30	999	0	1	nev any	st
WAKAI	502		m	0	0	all	-	all	As:Jap	1988	CC	333	n	bl	n	y	0	cu	cig+/-ot	20	29	1	0	nev any	st
WAKAI	503		m	0	0	all	-	all	As:Jap	1988	CC	333	n	bl	n	y	0	cu	cig+/-ot	1	19	0	0	nev any	st
WU	541		f	0	0	wh	-	q+a	NAmer	1981	CC	220	n	bl	n	y	2	cu	all/unsp	25	999	1	0	nev any	st
WU	542		f	0	0	wh	-	q+a	NAmer	1981	CC	220	n	bl	n	y	2	cu	all/unsp	19	24	0	3	nev any	st
WU	543		f	0	0	wh	-	q+a	NAmer	1981	CC	220	n	bl	n	y	2	cu	all/unsp	0	18	0	0	nev any	st
WYNDE6	759		m	0	0	wh	-	q+s+a	NAmer	1969	CC	4423	n	bl	n	y	0	ev	cig+/-ot	21	999	1	0	nev cigs	st
WYNDE6	760		m	0	0	wh	-	q+s+a	NAmer	1969	CC	4423	n	bl	n	y	0	ev	cig+/-ot	18	20	2	4	nev cigs	st
WYNDE6	761		m	0	0	wh	-	q+s+a	NAmer	1969	CC	4423	n	bl	n	y	0	ev	cig+/-ot	1	17	3	0	nev cigs	st
WYNDE6	767		f	0	0	wh	-	q+s+a	NAmer	1969	CC	4423	n	bl	n	y	0	ev	cig+/-ot	21	999	1	0	nev cigs	st
WYNDE6	768		f	0	0	wh	-	q+s+a	NAmer	1969	CC	4423	n	bl	n	y	0	ev	cig+/-ot	18	20	2	4	nev cigs	st
WYNDE6	769		f	0	0	wh	-	q+s+a	NAmer	1969	CC	4423	n	bl	n	y	0	ev	cig+/-ot	1	17	3	0	nev cigs	st
ZHENG	563		m	0	0	all	-	all	As:Chi	1982	CC	540	n	ot	*	y	0	ev	cig+/-ot	30	999	0	1	nev cigs	st
ZHENG	564		m	0	0	all	-	all	As:Chi	1982	CC	540	n	ot	*	y	0	ev	cig+/-ot	20	29	1	0	nev cigs	st
ZHENG	565		m	0	0	all	-	all	As:Chi	1982	CC	540	n	ot	*	y	0	ev	cig+/-ot	1	19	0	0	nev cigs	st
ZHENG	572		f	0	0	all	-	all	As:Chi	1982	CC	540	n	ot	*	y	0	ev	cig+/-ot	30	999	0	1	nev cigs	st
ZHENG	573		f	0	0	all	-	all	As:Chi	1982	CC	540	n	ot	*	y	0	ev	cig+/-ot	1	29	0	0	nev cigs	st

Table 1H16 - 1

IESLC - Meta-analysis of Ever/current Smoking by Age started, Overview
All LC types, Cigarettes (or Any Product if Cigarettes not available)
Most adjusted

Cigarette type is all/unspec for all RRs
except for the following:

REF	NRR	CIGTYPE
DEAN3	564	MC only
DEAN3	565	MC only
DEAN3	566	MC only
DEAN3	567	MC only
DEAN3	583	MC only
DEAN3	584	MC only
DEAN3	585	MC only
DEAN3	586	MC only

In this overview table, subtotals and Qs values may be invalid and should be ignored

Table 1H16 - 2

IESLC - Meta-analysis of Ever/current Smoking by Age started, Overview
All LC types, Cigarettes (or Any Product if Cigarettes not available)
Most adjusted

REF	NRR	SEX	AD	Number Exposed Case	Number Exposed Cont	Non-exposed Case	Non-exposed Cont	RR	95.00%CI
AGUDO	504	f	3	7	-	80	-	1.58	(0.59- 4.23)
AGUDO	505	f	3	16	-	80	-	10.76	(2.38- 48.73)
Subtotal AGUDO								2.80	(1.23- 6.39)
ARMADA	511	m	0	113	144	8	71	6.96	(3.22- 15.06)
ARMADA	512	m	0	204	110	8	71	16.46	(7.64- 35.44)
Subtotal ARMADA								10.73	(6.23- 18.49)
AUVINE	520	c	2	211	-	44	-	13.40	(7.62- 23.50)
AUVINE	521	c	2	55	-	44	-	47.60	(21.20- 107.00)
Subtotal AUVINE								20.26	(12.76- 32.17)
BARBON	520	m	1	200	-	22	-	8.20	(5.00- 13.30)
BARBON	521	m	1	395	-	22	-	9.90	(6.20- 15.80)
BARBON	522	m	1	138	-	22	-	50.80	(27.20- 95.00)
Subtotal BARBON								13.37	(9.93- 18.00)
BRESLO	501	c	0	32	35	19	56	2.69	(1.33- 5.47)
BRESLO	502	c	0	286	243	19	56	3.47	(2.01- 6.00)
BRESLO	503	c	0	166	116	19	56	4.22	(2.38- 7.47)
Subtotal BRESLO								3.51	(2.48- 4.95)
BUFFLE	517	f	0	23	23	12	112	9.33	(4.07- 21.40)
BUFFLE	518	f	0	47	34	12	112	12.90	(6.15- 27.07)
BUFFLE	519	f	0	40	29	12	112	12.87	(6.00- 27.62)
BUFFLE	520	f	0	53	33	12	112	14.99	(7.17- 31.33)
BUFFLE	521	f	0	78	41	12	112	17.76	(8.77- 35.94)
Subtotal BUFFLE								13.57	(9.69- 18.99)
*CEDERL	510	m	1	11	-	7	-	6.50	(2.52- 16.74)
*CEDERL	511	m	1	10	-	7	-	9.80	(3.74- 25.69)
*CEDERL	512	m	1	7	-	7	-	6.40	(2.25- 18.21)
*CEDERL	515	f	0	6	2806	19	17679	1.99	(0.80- 4.98)
*CEDERL	516	f	0	2	1009	19	17679	1.84	(0.43- 7.91)
*CEDERL	517	f	0	0	746	19	17679	0.61~	(0.04- 10.05)
Subtotal CEDERL								4.43	(2.82- 6.96)
CHEN2	517	m	0	8	5	9	33	5.87	(1.54- 22.37)
CHEN2	518	m	0	29	25	9	33	4.25	(1.71- 10.57)
CHEN2	519	m	0	84	67	9	33	4.60	(2.06- 10.27)
CHEN2	522	f	0	5	8	25	33	0.83	(0.24- 2.83)
CHEN2	523	f	0	9	8	25	33	1.49	(0.50- 4.39)
CHEN2	524	f	0	23	13	25	33	2.34	(0.99- 5.50)
Subtotal CHEN2								2.84	(1.90- 4.25)
CHIAZZ	501	m	2	-	-	4	-	3.00	(0.31- 28.84)
CHIAZZ	502	m	2	-	-	4	-	19.89	(2.66- 148.96)
Subtotal CHIAZZ								8.64	(1.92- 38.91)
CHOI	523	m	0	36	77	13	95	3.42	(1.69- 6.89)
CHOI	524	m	0	130	232	13	95	4.09	(2.21- 7.60)
CHOI	525	m	0	79	138	13	95	4.18	(2.20- 7.95)
CHOI	526	m	0	22	18	13	95	8.93	(3.81- 20.91)
CHOI	530	f	0	15	25	76	164	1.29	(0.65- 2.60)
CHOI	531	f	0	4	1	76	164	8.63	(0.95- 78.54)
Subtotal CHOI								3.57	(2.63- 4.85)
*CPSI	801	m	1	42	-	83	-	4.08	(2.81- 5.91)
*CPSI	802	m	1	196	-	83	-	10.08	(7.80- 13.03)
*CPSI	803	m	1	588	-	83	-	14.69	(11.68- 18.49)
*CPSI	804	m	1	185	-	83	-	16.77	(12.94- 21.73)
*CPSI	845	f	1	51	-	166	-	2.25	(1.64- 3.08)
*CPSI	846	f	1	34	-	166	-	3.38	(2.33- 4.88)
*CPSI	847	f	1	52	-	166	-	5.00	(3.66- 6.83)
*CPSI	848	f	1	6	-	166	-	2.50	(1.11- 5.65)
Subtotal CPSI								7.59	(6.81- 8.46)
DAMBER	501	m	0	70	76	42	208	4.56	(2.87- 7.26)
DAMBER	502	m	0	261	190	42	208	6.80	(4.65- 9.95)
DAMBER	503	m	0	206	98	42	208	10.41	(6.91- 15.68)
Subtotal DAMBER								7.07	(5.57- 8.98)
DEAN3	564	m	0	24	75	24	510	6.80	(3.67- 12.58)
DEAN3	565	m	0	52	161	24	510	6.86	(4.10- 11.49)
DEAN3	566	m	0	160	485	24	510	7.01	(4.48- 10.96)
DEAN3	567	m	0	44	165	24	510	5.67	(3.34- 9.60)
DEAN3	583	f	0	27	274	41	1538	3.70	(2.24- 6.11)
DEAN3	584	f	0	18	229	41	1538	2.95	(1.67- 5.22)
DEAN3	585	f	0	39	504	41	1538	2.90	(1.85- 4.55)
DEAN3	586	f	0	7	109	41	1538	2.41	(1.06- 5.50)
Subtotal DEAN3								4.59	(3.81- 5.54)
DOLL	501	m	0	4	7	7	61	4.98	(1.16- 21.36)
DOLL	502	m	0	18	33	7	61	4.75	(1.80- 12.54)

Table 1H16 - 2

IESLC - Meta-analysis of Ever/current Smoking by Age started, Overview
All LC types, Cigarettes (or Any Product if Cigarettes not available)
Most adjusted

REF	NRR	SEX	AD	Number Exposed Case	Number Exposed Cont	Non-exposed Case	Non-exposed Cont	RR	95.00%CI
DOLL	503	m	0	251	264	7	61	8.29	(3.72- 18.46)
DOLL	504	m	0	1077	992	7	61	9.46	(4.31- 20.78)
DOLL	508	f	0	15	15	40	59	1.48	(0.65- 3.35)
DOLL	509	f	0	10	7	40	59	2.11	(0.74- 6.00)
DOLL	510	f	0	23	15	40	59	2.26	(1.05- 4.86)
DOLL	511	f	0	20	12	40	59	2.46	(1.08- 5.58)
Subtotal DOLL								3.65	(2.67- 4.98)
*DORN	610	m	0	37	73050	25	213858	4.33	(2.61- 7.20)
*DORN	611	m	0	157	147948	25	213858	9.08	(5.95- 13.84)
*DORN	612	m	0	342	213156	25	213858	13.73	(9.14- 20.60)
*DORN	613	m	0	84	36304	25	213858	19.79	(12.67- 30.93)
*DORN	647	m	0	90	74464	49	171211	4.22	(2.98- 5.98)
*DORN	648	m	0	171	90036	49	171211	6.64	(4.83- 9.12)
*DORN	649	m	0	306	118234	49	171211	9.04	(6.69- 12.23)
*DORN	650	m	0	81	24616	49	171211	11.50	(8.07- 16.39)
Subtotal DORN								8.46	(7.42- 9.65)
*ENGELA	501	m	0	17	9762	27	58716	3.79	(2.07- 6.95)
*ENGELA	502	m	0	50	30195	27	58716	3.60	(2.26- 5.75)
*ENGELA	503	m	0	173	50732	27	58716	7.42	(4.94- 11.12)
*ENGELA	509	f	0	10	24560	31	207789	2.73	(1.34- 5.57)
*ENGELA	510	f	0	36	29605	31	207789	8.15	(5.04- 13.17)
*ENGELA	511	f	0	18	10687	31	207789	11.29	(6.32- 20.17)
Subtotal ENGELA								5.83	(4.73- 7.20)
GAO	506	m	2	45	-	62	-	1.20	(0.80- 1.90)
GAO	507	m	2	363	-	62	-	4.70	(3.30- 6.50)
GAO	508	m	2	262	-	62	-	5.10	(3.60- 7.20)
GAO	516	f	2	73	-	435	-	2.00	(1.40- 3.00)
GAO	517	f	2	87	-	435	-	3.80	(2.60- 5.80)
GAO	518	f	2	77	-	435	-	5.60	(3.40- 9.00)
Subtotal GAO								3.38	(2.88- 3.96)
GAO2	501	m	0	2	4	13	56	2.15	(0.36- 13.05)
GAO2	502	m	0	127	85	13	56	6.44	(3.32- 12.49)
GAO2	503	m	0	52	26	13	56	8.62	(4.01- 18.52)
Subtotal GAO2								6.68	(4.12- 10.83)
GENG	533	f	1	28	-	54	-	1.55	(0.83- 2.89)
GENG	534	f	1	39	-	54	-	2.95	(1.57- 5.52)
GENG	535	f	1	36	-	54	-	6.24	(2.85- 13.67)
Subtotal GENG								2.77	(1.88- 4.07)
HAENSZ	537	f	0	44	66	81	236	1.94	(1.23- 3.07)
HAENSZ	538	f	0	30	37	81	236	2.36	(1.37- 4.07)
Subtotal HAENSZ								2.11	(1.48- 2.99)
HEGMAN	513	m	1	26	-	-	-	9.40	(4.60- 19.30)
HEGMAN	514	m	1	146	-	-	-	22.30	(12.00- 41.40)
HEGMAN	516	f	1	2	-	-	-	4.80	(1.00- 22.10)
HEGMAN	517	f	1	81	-	-	-	26.80	(15.40- 46.80)
Subtotal HEGMAN								18.07	(12.75- 25.62)
*HIRAYA	501	m	1	-	-	-	-	4.35	(3.51- 5.39)
*HIRAYA	502	m	1	-	-	-	-	5.71	(4.50- 7.25)
*HIRAYA	504	f	1	-	-	-	-	2.46	(1.93- 3.13)
*HIRAYA	505	f	1	-	-	-	-	0.78	(0.10- 6.10)
Subtotal HIRAYA								3.96	(3.46- 4.52)
HU	511	m	0	14	20	41	67	1.14	(0.52- 2.51)
HU	512	m	0	93	67	41	67	2.27	(1.38- 3.74)
HU	513	m	0	13	7	41	67	3.03	(1.12- 8.23)
HU	516	f	0	3	5	40	48	0.72	(0.16- 3.20)
HU	517	f	0	18	9	40	48	2.40	(0.97- 5.92)
HU	518	f	0	5	4	40	48	1.50	(0.38- 5.96)
Subtotal HU								1.92	(1.37- 2.69)
HU2	501	c	0	15	29	121	213	0.91	(0.47- 1.77)
HU2	502	c	0	29	54	121	213	0.95	(0.57- 1.56)
HU2	503	c	0	229	159	121	213	2.54	(1.88- 3.43)
HU2	504	c	0	129	68	121	213	3.34	(2.31- 4.83)
Subtotal HU2								2.14	(1.75- 2.61)
JEDRYC	607	m	0	239	146	49	219	7.32	(5.04- 10.61)
JEDRYC	608	m	0	135	66	49	219	9.14	(5.96- 14.02)
JEDRYC	619	f	0	63	11	78	166	12.19	(6.09- 24.42)
Subtotal JEDRYC								8.54	(6.58- 11.07)
JOLY	543	m	0	18	70	12	218	4.67	(2.14- 10.18)
JOLY	544	m	0	217	357	12	218	11.04	(6.03- 20.22)
JOLY	545	m	0	317	282	12	218	20.42	(11.18- 37.32)
JOLY	533	f	0	23	41	52	283	3.05	(1.69- 5.51)

Table 1H16 - 2

IESLC - Meta-analysis of Ever/current Smoking by Age started, Overview
All LC types, Cigarettes (or Any Product if Cigarettes not available)
Most adjusted

REF	NRR	SEX	AD	Number Exposed Case	Cont	Non-exposed Case	Cont	RR	95.00%CI
JOLY	534	f	0	67	47	52	283	7.76	(4.82- 12.49)
JOLY	535	f	0	76	35	52	283	11.82	(7.18- 19.44)
Subtotal JOLY								8.56	(6.78- 10.80)
KHUDER	506	m	0	72	152	23	309	6.36	(3.83- 10.58)
KHUDER	507	m	0	161	338	23	309	6.40	(4.03- 10.17)
KHUDER	508	m	0	226	295	23	309	10.29	(6.51- 16.27)
Subtotal KHUDER								7.58	(5.76- 9.97)
KOULUM	501	m	0	8	8	5	54	10.80	(2.82- 41.31)
KOULUM	502	m	0	60	67	5	54	9.67	(3.63- 25.77)
KOULUM	503	m	0	267	103	5	54	28.00	(10.89- 71.96)
KOULUM	504	m	0	199	52	5	54	41.33	(15.74- 108.56)
KOULUM	505	m	0	143	16	5	54	96.53	(33.72- 276.34)
Subtotal KOULUM								27.41	(17.28- 43.49)
LETOUR	501	c	0	188	160	24	224	10.97	(6.85- 17.56)
LETOUR	502	c	0	309	241	24	224	11.97	(7.60- 18.83)
LETOUR	503	c	0	151	76	24	224	18.54	(11.21- 30.67)
Subtotal LETOUR								13.23	(10.06- 17.40)
*LIAW	504	c	2	-	-	-	-	1.50	(0.70- 3.30)
*LIAW	505	c	2	-	-	-	-	5.90	(3.00- 11.30)
*LIAW	506	c	2	-	-	-	-	4.60	(2.60- 8.10)
Subtotal LIAW								3.82	(2.62- 5.58)
LIU3	504	m	2	20	-	4	-	1.10	(0.25- 4.93)
LIU3	505	m	2	32	-	4	-	1.39	(0.32- 6.06)
Subtotal LIU3								1.24	(0.43- 3.53)
LIU4	501	m	2	-	-	-	-	2.41	(2.32- 2.49)
LIU4	502	m	2	-	-	-	-	2.86	(2.78- 2.95)
LIU4	503	m	2	-	-	-	-	3.81	(3.70- 3.93)
Subtotal LIU4								3.03	(2.98- 3.09)
LIU5	501	c	0	13	22	26	41	0.93	(0.40- 2.17)
LIU5	502	c	0	72	48	26	41	2.37	(1.28- 4.36)
Subtotal LIU5								1.72	(1.05- 2.82)
LUBIN	565	m	0	30	179	9	72	1.34	(0.61- 2.96)
LUBIN	566	m	0	65	146	9	72	3.56	(1.68- 7.56)
LUBIN	567	m	0	89	212	9	72	3.36	(1.61- 7.01)
LUBIN	568	m	0	178	251	9	72	5.67	(2.76- 11.64)
Subtotal LUBIN								3.20	(2.20- 4.66)
LUBIN2	1156	m	1	68	-	185	-	3.35	(2.42- 4.64)
LUBIN2	1157	m	1	564	-	185	-	4.97	(4.12- 6.00)
LUBIN2	1158	m	1	1796	-	185	-	5.43	(4.59- 6.41)
LUBIN2	1159	m	1	1312	-	185	-	6.74	(5.65- 8.04)
LUBIN2	1160	m	1	250	-	185	-	9.01	(6.92- 11.72)
Subtotal LUBIN2								5.76	(5.26- 6.31)
MATOS	576	m	2	28	-	11	-	3.90	(1.80- 8.30)
MATOS	577	m	2	91	-	11	-	7.80	(4.00- 15.50)
MATOS	578	m	2	69	-	11	-	7.80	(3.90- 15.70)
Subtotal MATOS								6.39	(4.24- 9.63)
*MIGRAN	501	m	0	24	668	4	867	7.79	(2.72- 22.34)
*MIGRAN	503	m	0	59	1845	4	867	6.93	(2.53- 19.02)
*MIGRAN	505	m	0	50	1081	4	867	10.03	(3.64- 27.65)
*MIGRAN	511	f	0	11	1315	4	3814	7.98	(2.54- 25.01)
*MIGRAN	513	f	0	9	1035	4	3814	8.29	(2.56- 26.87)
*MIGRAN	515	f	0	2	266	4	3814	7.17	(1.32- 38.96)
Subtotal MIGRAN								8.07	(5.08- 12.80)
*MRFITR	508	m	0	3	544	0	1859	23.91~	(1.24- 462.09)
*MRFITR	509	m	0	6	402	0	1859	60.06~	(3.39-1063.94)
*MRFITR	510	m	0	7	1029	0	1859	27.09~	(1.55- 473.89)
*MRFITR	511	m	0	25	1876	0	1859	50.54~	(3.08- 829.51)
*MRFITR	512	m	0	40	2242	0	1859	67.17~	(4.13-1091.55)
*MRFITR	513	m	0	25	2065	0	1859	45.91~	(2.80- 753.64)
Subtotal MRFITR								43.20	(13.52- 138.02)
PERNU	504	m	0	1043	346	97	275	8.55	(6.58- 11.10)
PERNU	505	m	0	337	92	97	275	10.38	(7.49- 14.40)
PERNU	501	f	0	18	89	110	971	1.79	(1.04- 3.07)
PERNU	502	f	0	1	0	110	971	26.38~	(1.07- 651.38)
Subtotal PERNU								7.56	(6.25- 9.15)
PEZZOT	570	m	0	41	105	4	116	11.32	(3.92- 32.69)
PEZZOT	571	m	0	118	145	4	116	23.60	(8.46- 65.84)
PEZZOT	572	m	0	52	67	4	116	22.51	(7.79- 65.00)
Subtotal PEZZOT								18.29	(9.98- 33.50)
*QIAO2	506	m	1	52	-	10	-	1.32	(0.67- 2.60)
*QIAO2	507	m	1	75	-	10	-	1.47	(0.76- 2.84)

Table 1H16 - 2

IESLC - Meta-analysis of Ever/current Smoking by Age started, Overview
All LC types, Cigarettes (or Any Product if Cigarettes not available)
Most adjusted

REF	NRR	SEX	AD	Number Exposed Case	Number Exposed Cont	Non-exposed Case	Non-exposed Cont	RR	95.00%CI
*QIAO2	508	m	1	104	-	10	-	1.81	(0.94- 3.48)
Subtotal QIAO2								1.53	(1.04- 2.24)
RACHTA	506	f	1	8	-	33	-	5.31	(1.48- 19.05)
RACHTA	507	f	1	25	-	33	-	5.33	(2.79- 10.20)
RACHTA	508	f	1	52	-	33	-	11.60	(5.04- 26.68)
Subtotal RACHTA								6.86	(4.27- 11.03)
SEGI2	521	m	1	49	-	8	-	2.04	(0.89- 4.66)
SEGI2	522	m	1	125	-	8	-	3.33	(1.51- 7.37)
SEGI2	523	m	1	91	-	8	-	5.64	(2.49- 12.78)
Subtotal SEGI2								3.38	(2.12- 5.41)
SOBUE	654	m	0	110	121	29	126	3.95	(2.45- 6.38)
SOBUE	655	m	0	776	772	29	126	4.37	(2.88- 6.62)
SOBUE	656	m	0	137	62	29	126	9.60	(5.81- 15.88)
Subtotal SOBUE								5.28	(4.05- 6.89)
SUZUK2	501	c	0	16	22	11	53	3.50	(1.40- 8.75)
SUZUK2	502	c	0	64	38	11	53	8.11	(3.78- 17.41)
SUZUK2	503	c	0	31	10	11	53	14.94	(5.69- 39.18)
Subtotal SUZUK2								7.44	(4.51- 12.27)
SVENSS	501	f	0	32	18	38	120	5.61	(2.84- 11.12)
SVENSS	502	f	0	58	14	38	120	13.08	(6.57- 26.04)
SVENSS	503	f	0	52	21	38	120	7.82	(4.19- 14.60)
Subtotal SVENSS								8.26	(5.63- 12.12)
TIZZAN	506	m	0	12	44	180	305	0.46	(0.24- 0.90)
TIZZAN	507	m	0	313	330	180	305	1.61	(1.26- 2.04)
TIZZAN	508	m	0	699	529	180	305	2.24	(1.80- 2.78)
TIZZAN	536	f	0	2	5	117	114	0.39	(0.07- 2.05)
TIZZAN	537	f	0	12	21	117	114	0.56	(0.26- 1.18)
TIZZAN	538	f	0	11	2	117	114	5.36	(1.16- 24.71)
Subtotal TIZZAN								1.70	(1.46- 1.98)
WAKAI	501	m	0	8	25	10	65	2.08	(0.74- 5.87)
WAKAI	502	m	0	130	183	10	65	4.62	(2.29- 9.32)
WAKAI	503	m	0	42	74	10	65	3.69	(1.72- 7.94)
Subtotal WAKAI								3.63	(2.28- 5.77)
WU	541	f	2	14	-	31	-	1.55	(0.60- 3.98)
WU	542	f	2	40	-	31	-	3.57	(1.57- 8.14)
WU	543	f	2	106	-	31	-	10.32	(4.81- 22.13)
Subtotal WU								4.39	(2.71- 7.10)
WYNDE6	759	m	0	111	92	51	589	13.93	(9.36- 20.74)
WYNDE6	760	m	0	223	139	51	589	18.53	(12.98- 26.45)
WYNDE6	761	m	0	611	301	51	589	23.44	(17.06- 32.21)
WYNDE6	767	f	0	127	90	73	673	13.01	(9.06- 18.69)
WYNDE6	768	f	0	200	94	73	673	19.62	(13.90- 27.67)
WYNDE6	769	f	0	291	91	73	673	29.48	(21.04- 41.31)
Subtotal WYNDE6								19.46	(16.87- 22.45)
ZHENG	563	m	0	28	66	33	94	1.21	(0.67- 2.19)
ZHENG	564	m	0	145	109	33	94	3.79	(2.37- 6.05)
ZHENG	565	m	0	106	43	33	94	7.02	(4.13- 11.95)
ZHENG	572	f	0	16	16	152	184	1.21	(0.59- 2.50)
ZHENG	573	f	0	60	28	152	184	2.59	(1.58- 4.27)
Subtotal ZHENG								2.86	(2.24- 3.65)
Partial Totals				24315	965679	9674	2444905		

*prospective study ~ With 0.5 adjustment for zero

Table 1H16 - 2

IESLC - Meta-analysis of Ever/current Smoking by Age started, Overview
All LC types, Cigarettes (or Any Product if Cigarettes not available)
Most adjusted

REF	NRR	SEX	AD	Ys	Ws	Qs	Ps
AGUDO	504	f	3	0.46	3.96	2.46	0.3627
AGUDO	505	f	3	2.38	1.69	2.15	0.0020
Subtotal AGUDO				1.03	5.65	4.61	
ARMADA	511	m	0	1.94	6.46	3.12	0.0000
ARMADA	512	m	0	2.80	6.53	15.80	0.0000
Subtotal ARMADA				2.37	12.99	18.91	
AUVINE	520	c	2	2.60	12.11	22.06	0.0000
AUVINE	521	c	2	3.86	5.86	40.16	0.0000
Subtotal AUVINE				3.01	17.98	62.21	
BARBON	520	m	1	2.10	16.05	11.83	0.0000
BARBON	521	m	1	2.29	17.56	19.24	0.0000
BARBON	522	m	1	3.93	9.82	70.66	0.0000
Subtotal BARBON				2.59	43.44	101.73	
BRESLO	501	c	0	0.99	7.67	0.50	0.0060
BRESLO	502	c	0	1.24	12.80	0.00	0.0000
BRESLO	503	c	0	1.44	11.75	0.44	0.0000
Subtotal BRESLO				1.25	32.22	0.94	
BUFFLE	517	f	0	2.23	5.58	5.44	0.0000
BUFFLE	518	f	0	2.56	7.00	12.03	0.0000
BUFFLE	519	f	0	2.56	6.59	11.30	0.0000
BUFFLE	520	f	0	2.71	7.07	15.10	0.0000
BUFFLE	521	f	0	2.88	7.72	20.54	0.0000
Subtotal BUFFLE				2.61	33.96	64.42	
*CEDERL	510	m	1	1.87	4.29	1.68	0.0001
*CEDERL	511	m	1	2.28	4.14	4.45	0.0000
*CEDERL	512	m	1	1.86	3.51	1.31	0.0005
*CEDERL	515	f	0	0.69	4.57	1.42	0.1414
*CEDERL	516	f	0	0.61	1.81	0.73	0.4098
*CEDERL	517	f	0	-0.50	0.49	1.48	0.7276
Subtotal CEDERL				1.49	18.81	11.07	
CHEN2	517	m	0	1.77	2.14	0.59	0.0096
CHEN2	518	m	0	1.45	4.63	0.19	0.0018
CHEN2	519	m	0	1.53	5.94	0.46	0.0002
CHEN2	522	f	0	-0.19	2.53	5.23	0.7596
CHEN2	523	f	0	0.40	3.26	2.36	0.4750
CHEN2	524	f	0	0.85	5.24	0.83	0.0521
Subtotal CHEN2				1.04	23.76	9.66	
CHIAZZ	501	m	2	1.10	0.75	0.02	0.3421
CHIAZZ	502	m	2	2.99	0.95	2.89	0.0036
Subtotal CHIAZZ				2.16	1.70	2.90	
CHOI	523	m	0	1.23	7.80	0.00	0.0006
CHOI	524	m	0	1.41	10.06	0.27	0.0000
CHOI	525	m	0	1.43	9.31	0.32	0.0000
CHOI	526	m	0	2.19	5.31	4.73	0.0000
CHOI	530	f	0	0.26	7.94	7.75	0.4667
CHOI	531	f	0	2.16	0.79	0.65	0.0557
Subtotal CHOI				1.27	41.20	13.71	
*CPSI	801	m	1	1.41	27.80	0.71	0.0000
*CPSI	802	m	1	2.31	58.36	66.15	0.0000
*CPSI	803	m	1	2.69	72.82	151.28	0.0000
*CPSI	804	m	1	2.82	57.18	141.62	0.0000
*CPSI	845	f	1	0.81	38.69	7.32	0.0000
*CPSI	846	f	1	1.22	28.12	0.02	0.0000
*CPSI	847	f	1	1.61	39.48	5.22	0.0000
*CPSI	848	f	1	0.92	5.80	0.63	0.0273
Subtotal CPSI				2.03	328.25	372.95	
DAMBER	501	m	0	1.52	17.84	1.32	0.0000
DAMBER	502	m	0	1.92	26.52	11.96	0.0000
DAMBER	503	m	0	2.34	22.90	27.55	0.0000
Subtotal DAMBER				1.96	67.25	40.82	
DEAN3	564	m	0	1.92	10.14	4.57	0.0000
DEAN3	565	m	0	1.93	14.48	6.70	0.0000
DEAN3	566	m	0	1.95	19.25	9.47	0.0000
DEAN3	567	m	0	1.73	13.81	3.30	0.0000
DEAN3	583	f	0	1.31	15.21	0.06	0.0000
DEAN3	584	f	0	1.08	11.77	0.32	0.0002
DEAN3	585	f	0	1.07	18.99	0.62	0.0000
DEAN3	586	f	0	0.88	5.65	0.76	0.0367
Subtotal DEAN3				1.52	109.30	25.79	
DOLL	501	m	0	1.61	1.81	0.23	0.0307
DOLL	502	m	0	1.56	4.08	0.40	0.0016

Table 1H16 - 2

IESLC - Meta-analysis of Ever/current Smoking by Age started, Overview
All LC types, Cigarettes (or Any Product if Cigarettes not available)
Most adjusted

REF	NRR	SEX	AD	Ys	Ws	Qs	Ps
DOLL	503	m	0	2.11	5.99	4.52	0.0000
DOLL	504	m	0	2.25	6.20	6.22	0.0000
DOLL	508	f	0	0.39	5.71	4.19	0.3532
DOLL	509	f	0	0.75	3.51	0.88	0.1625
DOLL	510	f	0	0.82	6.57	1.21	0.0364
DOLL	511	f	0	0.90	5.71	0.68	0.0317
Subtotal DOLL				1.29	39.58	18.34	
*DORN	610	m	0	1.47	14.92	0.72	0.0000
*DORN	611	m	0	2.21	21.57	19.88	0.0000
*DORN	612	m	0	2.62	23.30	43.95	0.0000
*DORN	613	m	0	2.99	19.28	58.33	0.0000
*DORN	647	m	0	1.44	31.75	1.20	0.0000
*DORN	648	m	0	1.89	38.11	15.94	0.0000
*DORN	649	m	0	2.20	42.26	38.63	0.0000
*DORN	650	m	0	2.44	30.57	43.75	0.0000
Subtotal DORN				2.14	221.77	222.41	
*ENGELA	501	m	0	1.33	10.44	0.08	0.0000
*ENGELA	502	m	0	1.28	17.55	0.02	0.0000
*ENGELA	503	m	0	2.00	23.38	13.42	0.0000
*ENGELA	509	f	0	1.00	7.56	0.44	0.0058
*ENGELA	510	f	0	2.10	16.67	12.11	0.0000
*ENGELA	511	f	0	2.42	11.40	15.82	0.0000
Subtotal ENGELA				1.76	87.00	41.89	
GAO	506	m	2	0.18	20.54	23.23	0.4087
GAO	507	m	2	1.55	33.44	3.04	0.0000
GAO	508	m	2	1.63	31.98	4.70	0.0000
GAO	516	f	2	0.69	26.45	8.08	0.0004
GAO	517	f	2	1.34	23.87	0.19	0.0000
GAO	518	f	2	1.72	16.22	3.69	0.0000
Subtotal GAO				1.22	152.50	42.93	
GAO2	501	m	0	0.77	1.18	0.27	0.4038
GAO2	502	m	0	1.86	8.74	3.32	0.0000
GAO2	503	m	0	2.15	6.56	5.40	0.0000
Subtotal GAO2				1.90	16.48	8.99	
GENG	533	f	1	0.44	9.87	6.44	0.1685
GENG	534	f	1	1.08	9.72	0.26	0.0007
GENG	535	f	1	1.83	6.25	2.14	0.0000
Subtotal GENG				1.02	25.84	8.84	
HAENSZ	537	f	0	0.66	18.36	6.22	0.0044
HAENSZ	538	f	0	0.86	13.00	1.94	0.0019
Subtotal HAENSZ				0.75	31.36	8.16	
HEGMAN	513	m	1	2.24	7.47	7.39	0.0000
HEGMAN	514	m	1	3.10	10.02	34.62	0.0000
HEGMAN	516	f	1	1.57	1.60	0.17	0.0470
HEGMAN	517	f	1	3.29	12.44	51.89	0.0000
Subtotal HEGMAN				2.89	31.53	94.07	
*HIRAYA	501	m	1	1.47	83.52	4.20	0.0000
*HIRAYA	502	m	1	1.74	67.55	16.64	0.0000
*HIRAYA	504	f	1	0.90	65.73	7.86	0.0000
*HIRAYA	505	f	1	-0.25	0.91	2.03	0.8127
Subtotal HIRAYA				1.38	217.71	30.73	
HU	511	m	0	0.13	6.22	7.68	0.7374
HU	512	m	0	0.82	15.39	2.80	0.0013
HU	513	m	0	1.11	3.86	0.07	0.0292
HU	516	f	0	-0.33	1.73	4.28	0.6660
HU	517	f	0	0.88	4.71	0.65	0.0575
HU	518	f	0	0.41	2.02	1.42	0.5647
Subtotal HU				0.65	33.92	16.91	
HU2	501	c	0	-0.09	8.76	15.73	0.7814
HU2	502	c	0	-0.06	15.16	25.70	0.8268
HU2	503	c	0	0.93	42.35	4.22	0.0000
HU2	504	c	0	1.21	28.23	0.05	0.0000
Subtotal HU2				0.76	94.50	45.69	
JEDRYC	607	m	0	1.99	27.77	15.38	0.0000
JEDRYC	608	m	0	2.21	21.04	19.67	0.0000
JEDRYC	619	f	0	2.50	7.96	12.53	0.0000
Subtotal JEDRYC				2.14	56.77	47.58	
JOLY	543	m	0	1.54	6.34	0.55	0.0001
JOLY	544	m	0	2.40	10.49	14.02	0.0000
JOLY	545	m	0	3.02	10.57	33.14	0.0000
JOLY	533	f	0	1.12	11.03	0.19	0.0002

Table 1H16 - 2

IESLC - Meta-analysis of Ever/current Smoking by Age started, Overview
All LC types, Cigarettes (or Any Product if Cigarettes not available)
Most adjusted

REF	NRR	SEX	AD	Ys	Ws	Qs	Ps
JOLY	534	f	0	2.05	16.96	10.93	0.0000
JOLY	535	f	0	2.47	15.51	23.22	0.0000
Subtotal JOLY				2.15	70.89	82.04	
KHUDER	506	m	0	1.85	14.88	5.44	0.0000
KHUDER	507	m	0	1.86	17.89	6.67	0.0000
KHUDER	508	m	0	2.33	18.34	21.61	0.0000
Subtotal KHUDER				2.03	51.12	33.72	
KOULUM	501	m	0	2.38	2.13	2.74	0.0005
KOULUM	502	m	0	2.27	4.00	4.19	0.0000
KOULUM	503	m	0	3.33	4.31	18.76	0.0000
KOULUM	504	m	0	3.72	4.12	25.25	0.0000
KOULUM	505	m	0	4.57	3.47	38.36	0.0000
Subtotal KOULUM				3.31	18.03	89.30	
LETOUR	501	c	0	2.39	17.33	22.88	0.0000
LETOUR	502	c	0	2.48	18.69	28.56	0.0000
LETOUR	503	c	0	2.92	15.17	42.53	0.0000
Subtotal LETOUR				2.58	51.19	93.97	
*LIAW	504	c	2	0.41	6.39	4.51	0.3054
*LIAW	505	c	2	1.77	8.74	2.45	0.0000
*LIAW	506	c	2	1.53	11.90	0.93	0.0000
Subtotal LIAW				1.34	27.03	7.89	
LIU3	504	m	2	0.10	1.73	2.29	0.9003
LIU3	505	m	2	0.33	1.78	1.49	0.6607
Subtotal LIU3				0.21	3.50	3.78	
LIU4	501	m	2	0.88	3072.74	412.17	0.0000
LIU4	502	m	2	1.05	4361.66	165.94	0.0000
LIU4	503	m	2	1.34	4224.99	35.57	0.0000
Subtotal LIU4				1.11	11659.40	613.68	
LIU5	501	c	0	-0.07	5.40	9.36	0.8697
LIU5	502	c	0	0.86	10.25	1.52	0.0058
Subtotal LIU5				0.54	15.65	10.88	
LUBIN	565	m	0	0.29	6.10	5.54	0.4689
LUBIN	566	m	0	1.27	6.79	0.00	0.0009
LUBIN	567	m	0	1.21	7.09	0.01	0.0013
LUBIN	568	m	0	1.74	7.43	1.78	0.0000
Subtotal LUBIN				1.16	27.42	7.33	
LUBIN2	1156	m	1	1.21	36.26	0.05	0.0000
LUBIN2	1157	m	1	1.60	108.74	13.90	0.0000
LUBIN2	1158	m	1	1.69	137.76	27.41	0.0000
LUBIN2	1159	m	1	1.91	123.47	54.14	0.0000
LUBIN2	1160	m	1	2.20	55.35	50.21	0.0000
Subtotal LUBIN2				1.75	461.59	145.72	
MATOS	576	m	2	1.36	6.58	0.09	0.0005
MATOS	577	m	2	2.05	8.37	5.47	0.0000
MATOS	578	m	2	2.05	7.92	5.18	0.0000
Subtotal MATOS				1.85	22.87	10.73	
*MIGRAN	501	m	0	2.05	3.46	2.25	0.0001
*MIGRAN	503	m	0	1.94	3.77	1.80	0.0002
*MIGRAN	505	m	0	2.31	3.73	4.19	0.0000
*MIGRAN	511	f	0	2.08	2.94	2.03	0.0004
*MIGRAN	513	f	0	2.12	2.78	2.10	0.0004
*MIGRAN	515	f	0	1.97	1.34	0.70	0.0226
Subtotal MIGRAN				2.09	18.02	13.07	
*MRFITR	508	m	0	3.17	0.44	1.63	0.0357
*MRFITR	509	m	0	4.10	0.46	3.77	0.0052
*MRFITR	510	m	0	3.30	0.47	1.98	0.0238
*MRFITR	511	m	0	3.92	0.49	3.52	0.0060
*MRFITR	512	m	0	4.21	0.49	4.33	0.0031
*MRFITR	513	m	0	3.83	0.49	3.27	0.0074
Subtotal MRFITR				3.77	2.85	18.50	
PERNU	504	m	0	2.15	56.20	45.48	0.0000
PERNU	505	m	0	2.34	35.99	43.12	0.0000
PERNU	501	f	0	0.58	13.00	5.77	0.0366
PERNU	502	f	0	3.27	0.37	1.53	0.0455
Subtotal PERNU				2.02	105.57	95.90	
PEZZOT	570	m	0	2.43	3.42	4.77	0.0000
PEZZOT	571	m	0	3.16	3.65	13.39	0.0000
PEZZOT	572	m	0	3.11	3.42	11.92	0.0000
Subtotal PEZZOT				2.91	10.48	30.08	
*QIAO2	506	m	1	0.28	8.36	7.83	0.4222
*QIAO2	507	m	1	0.39	8.84	6.55	0.2520

Table 1H16 - 2

IESLC - Meta-analysis of Ever/current Smoking by Age started, Overview
All LC types, Cigarettes (or Any Product if Cigarettes not available)
Most adjusted

REF	NRR	SEX	AD	Ys	Ws	Qs	Ps
*QIAO2	508	m	1	0.59	8.97	3.82	0.0756
Subtotal QIAO2				0.42	26.17	18.20	
RACHTA	506	f	1	1.67	2.35	0.42	0.0104
RACHTA	507	f	1	1.67	9.14	1.67	0.0000
RACHTA	508	f	1	2.45	5.53	8.04	0.0000
Subtotal RACHTA				1.93	17.03	10.13	
SEGI2	521	m	1	0.71	5.61	1.59	0.0914
SEGI2	522	m	1	1.20	6.11	0.01	0.0029
SEGI2	523	m	1	1.73	5.74	1.35	0.0000
Subtotal SEGI2				1.22	17.46	2.95	
SOBUE	654	m	0	1.37	16.73	0.27	0.0000
SOBUE	655	m	0	1.47	22.22	1.16	0.0000
SOBUE	656	m	0	2.26	15.19	15.68	0.0000
Subtotal SOBUE				1.66	54.14	17.11	
SUZUK2	501	c	0	1.25	4.59	0.00	0.0072
SUZUK2	502	c	0	2.09	6.59	4.74	0.0000
SUZUK2	503	c	0	2.70	4.13	8.78	0.0000
Subtotal SUZUK2				2.01	15.32	13.52	
SVENSS	501	f	0	1.73	8.23	1.89	0.0000
SVENSS	502	f	0	2.57	8.11	14.25	0.0000
SVENSS	503	f	0	2.06	9.85	6.48	0.0000
Subtotal SVENSS				2.11	26.19	22.61	
TIZZAN	506	m	0	-0.77	8.70	35.44	0.0228
TIZZAN	507	m	0	0.47	66.40	39.51	0.0001
TIZZAN	508	m	0	0.81	82.27	15.92	0.0000
TIZZAN	536	f	0	-0.94	1.39	6.67	0.2659
TIZZAN	537	f	0	-0.59	6.74	22.62	0.1283
TIZZAN	538	f	0	1.68	1.64	0.31	0.0314
Subtotal TIZZAN				0.53	167.16	120.47	
WAKAI	501	m	0	0.73	3.57	0.94	0.1666
WAKAI	502	m	0	1.53	7.78	0.63	0.0000
WAKAI	503	m	0	1.31	6.55	0.02	0.0008
Subtotal WAKAI				1.29	17.89	1.59	
WU	541	f	2	0.44	4.29	2.80	0.3639
WU	542	f	2	1.27	5.67	0.00	0.0024
WU	543	f	2	2.33	6.60	7.81	0.0000
Subtotal WU				1.48	16.56	10.62	
WYNDE6	759	m	0	2.63	24.28	46.81	0.0000
WYNDE6	760	m	0	2.92	30.32	84.90	0.0000
WYNDE6	761	m	0	3.15	38.07	138.71	0.0000
WYNDE6	767	f	0	2.57	29.27	50.98	0.0000
WYNDE6	768	f	0	2.98	32.44	97.15	0.0000
WYNDE6	769	f	0	3.38	33.77	154.36	0.0000
Subtotal WYNDE6				2.97	188.15	572.90	
ZHENG	563	m	0	0.19	10.89	12.16	0.5320
ZHENG	564	m	0	1.33	17.54	0.13	0.0000
ZHENG	565	m	0	1.95	13.58	6.71	0.0000
ZHENG	572	f	0	0.19	7.30	8.12	0.6057
ZHENG	573	f	0	0.95	15.53	1.33	0.0002
Subtotal ZHENG				1.05	64.84	28.46	

N	212
NS	51

Table 1H16 - 3

IESLC - Meta-analysis of Ever/current Smoking by Age started, Overview
All LC types, Cigarettes (or Any Product if Cigarettes not available)
Most adjusted

	Sex			
	combined	male	female	Total
N	20	121	71	212
NS	7	37	25	69

In this overview table, other than the "N" rows, entries in the "absent" and "Total" columns may be invalid and should be ignored

	Age started (broad categories)				
	absent	19+k26	15-25k18	1-17k14	Total
N	88	60	29	35	212
NS	38	45	22	26	131
Wt	9639.67	4041.78	648.15	572.38	14901.98
Het Chi	967.45	514.82	208.59	252.35	3391.42
Het df	87	59	28	34	211
Het P	***	***	***	***	***
Fixed RR	3.42	2.70	7.75	11.11	3.48
RRl	3.35	2.62	7.18	10.23	3.42
RRu	3.48	2.79	8.37	12.06	3.53
P	+++	+++	+++	+++	+++
Random RR	4.38	3.89	7.48	10.32	5.30
RRl	3.92	3.33	5.94	8.04	4.86
RRu	4.90	4.56	9.42	13.26	5.77
P	+++	+++	+++	+++	+++

	Age started (narrow categories)							
	absent	27+k30	23-29k26	19-25k22	15-21k18	11-17k14	1-13k10	Total
N	150	16	1	14	25	2	4	212
NS	51	11	1	11	19	2	4	99
Wt	9399.52	135.72	6.79	4580.31	585.68	127.59	66.37	14901.98
Het Chi	2192.36	44.33	0.00	183.11	191.04	13.11	20.72	3391.42
Het df	149	15	0	13	24	1	3	211
Het P	***	***	N.S.	***	***	***	***	***
Fixed RR	3.53	1.71	3.56	2.97	8.01	7.15	11.03	3.48
RRl	3.46	1.45	1.68	2.89	7.39	6.01	8.67	3.42
RRu	3.61	2.02	7.56	3.06	8.69	8.50	14.03	3.53
P	+++	+++	+++	+++	+++	+++	+++	+++
Random RR	5.25	1.80	3.56	5.31	7.78	15.64	21.72	5.30
RRl	4.71	1.32	1.68	3.66	6.04	2.66	7.77	4.86
RRu	5.86	2.47	7.56	7.71	10.03	92.10	60.76	5.77
P	+++	+++	+++	+++	+++	++	+++	+++

Table 1H16 - 3

IESLC - Meta-analysis of Ever/current Smoking by Age started, Overview
All LC types, Cigarettes (or Any Product if Cigarettes not available)
Most adjusted

MALES

		Age started (broad categories)				
		absent	19+k26	15-25k18	1-17k14	Total
	N	46	34	19	22	121
	NS	26	33	18	21	98
	Wt	9271.81	3668.49	487.41	460.68	13888.39
Het	Chi	682.52	317.58	126.54	149.53	2470.09
Het	df	45	33	18	21	120
Het	P	***	***	***	***	***
Fixed	RR	3.40	2.65	8.00	10.73	3.41
	RRl	3.33	2.56	7.32	9.79	3.35
	RRu	3.47	2.74	8.74	11.76	3.47
	P	+++	+++	+++	+++	+++
Random	RR	4.82	4.55	8.08	11.19	6.12
	RRl	4.18	3.70	6.21	8.53	5.51
	RRu	5.55	5.60	10.53	14.70	6.79
	P	+++	+++	+++	+++	+++

			Age started (narrow categories)						
		absent	27+k30	23-29k26	19-25k22	15-21k18	11-17k14	1-13k10	Total
	N	81	8	1	9	17	2	3	121
	NS	37	8	1	8	16	2	3	75
	Wt	8656.13	63.03	6.79	4517.91	454.70	127.59	62.24	13888.39
Het	Chi	1516.56	16.28	0.00	160.41	117.10	13.11	20.32	2470.09
Het	df	80	7	0	8	16	1	2	120
Het	P	***	*	N.S.	***	***	***	***	***
Fixed	RR	3.46	1.67	3.56	2.96	8.18	7.15	10.81	3.41
	RRl	3.39	1.30	1.68	2.87	7.46	6.01	8.44	3.35
	RRu	3.53	2.13	7.56	3.04	8.96	8.50	13.86	3.47
	P	+++	+++	+++	+++	+++	+++	+++	+++
Random	RR	6.08	1.80	3.56	5.66	8.28	15.64	25.30	6.12
	RRl	5.31	1.20	1.68	3.39	6.24	2.66	5.94	5.51
	RRu	6.97	2.72	7.56	9.45	10.98	92.10	107.72	6.79
	P	+++	++	+++	+++	+++	++	+++	+++

FEMALES

		Age started (broad categories)				
		absent	19+k26	15-25k18	1-17k14	Total
	N	32	21	8	10	71
	NS	20	21	8	10	59
	Wt	256.58	294.96	129.25	78.92	759.71
Het	Chi	186.19	148.58	68.47	73.34	620.60
Het	df	31	20	7	9	70
Het	P	***	***	***	***	***
Fixed	RR	4.05	3.26	7.01	12.76	4.61
	RRl	3.59	2.91	5.90	10.23	4.29
	RRu	4.58	3.66	8.33	15.91	4.95
	P	+++	+++	+++	+++	+++
Random	RR	3.95	3.07	6.23	6.37	4.08
	RRl	2.87	2.20	3.44	3.00	3.25
	RRu	5.43	4.30	11.26	13.56	5.11
	P	+++	+++	+++	+++	+++

Table 1H16 - 3

IESLC - Meta-analysis of Ever/current Smoking by Age started, Overview
All LC types, Cigarettes (or Any Product if Cigarettes not available)
Most adjusted

FEMALES

	absent	Age started (narrow categories) 27+k30	23-29k26	19-25k22	15-21k18	11-17k14	1-13k10	Total
N	54	6		4	7			71
NS	25	6		4	7			42
Wt	541.62	52.13		53.67	112.29			759.71
Het Chi	475.28	17.00		13.34	68.27			620.60
Het df	53	5		3	6			70
Het P	***	**		**	***			***
Fixed RR	4.60	2.23		4.05	6.91			4.61
RRl	4.23	1.70		3.10	5.74			4.29
RRu	5.01	2.93		5.29	8.31			4.95
P	+++	+++		+++	+++			+++
Random RR	4.09	2.29		4.54	5.97			4.08
RRl	3.12	1.29		2.45	2.97			3.25
RRu	5.36	4.07		8.42	11.99			5.11
P	+++	++		+++	+++			+++

Table 1H16 - 4

IESLC - Meta-analysis of Ever/current Smoking by Age started, Overview
All LC types, Cigarettes (or Any Product if Cigarettes not available)
Least adjusted

REF	NRR	X	SEX	AGEL	AGEH	RACE	YF	LC TYPE	LOC	START	ST	NLC	R	VB	P	H	AD	SM	PRODUCT	exL	exH	S1	S2	DENOM	De
AGUDO	501	x	f	0	0	all	-	all	Eu:wst	1989	CC	103	n	bl	n	n	0	ev	cig only	24	999	1	0	nev cigs	st
AGUDO	502	x	f	0	0	all	-	all	Eu:wst	1989	CC	103	n	bl	n	n	0	ev	cig only	1	23	0	0	nev cigs	st
ARMADA	511		m	0	0	all	-	all	Eu:wst	1986	CC	325	n	bl	n	y	0	ev	cig+/-ot	17	45	0	0	nev cigs	st
ARMADA	512		m	0	0	all	-	all	Eu:wst	1986	CC	325	n	bl	n	y	0	ev	cig+/-ot	7	16	3	0	nev cigs	st
AUVINE	509	x	c	0	0	all	-	all	Eu:Sca	1986	CC	517	n	bl	y	n	0	ev	cig+/-ot	21	999	1	0	nev cigs	st
AUVINE	510	x	c	0	0	all	-	all	Eu:Sca	1986	CC	517	n	bl	y	n	0	ev	cig+/-ot	16	20	2	4	nev cigs	st
AUVINE	511	x	c	0	0	all	-	all	Eu:Sca	1986	CC	517	n	bl	y	n	0	ev	cig+/-ot	1	15	3	0	nev cigs	st
BARBON	515	x	m	0	0	all	-	all	Eu:wst	1979	CC	755	n	bl	y	y	0	ev	all/unsp	20	999	1	0	nev any	st
BARBON	516	x	m	0	0	all	-	all	Eu:wst	1979	CC	755	n	bl	y	y	0	ev	all/unsp	15	19	2	4	nev any	st
BARBON	517	x	m	0	0	all	-	all	Eu:wst	1979	CC	755	n	bl	y	y	0	ev	all/unsp	1	14	3	0	nev any	st
BRESLO	501		c	0	0	all	-	all	NAmer	1949	CC	518	n	bl	n	y	0	ev	cig+/-ot	25	999	1	0	nev any	st
BRESLO	502		c	0	0	all	-	all	NAmer	1949	CC	518	n	bl	n	y	0	ev	cig+/-ot	15	24	2	0	nev any	st
BRESLO	503		c	0	0	all	-	all	NAmer	1949	CC	518	n	bl	n	y	0	ev	cig+/-ot	0	14	3	0	nev any	st
BUFFLE	517		f	0	0	w-hi	-	all	NAmer	1976	CC	943	n	bl	y	n	0	ev	cig+/-ot	30	999	0	1	nev cigs	or
BUFFLE	518		f	0	0	w-hi	-	all	NAmer	1976	CC	943	n	bl	y	n	0	ev	cig+/-ot	21	29	1	0	nev cigs	or
BUFFLE	519		f	0	0	w-hi	-	all	NAmer	1976	CC	943	n	bl	y	n	0	ev	cig+/-ot	19	20	0	0	nev cigs	or
BUFFLE	520		f	0	0	w-hi	-	all	NAmer	1976	CC	943	n	bl	y	n	0	ev	cig+/-ot	17	18	2	4	nev cigs	ot
BUFFLE	521		f	0	0	w-hi	-	all	NAmer	1976	CC	943	n	bl	y	n	0	ev	cig+/-ot	6	16	3	0	nev cigs	or
CEDERL	510		m	0	0	all	10	all	Eu:Sca	1963	pr	491	n	bl	n	n	1	cu	cig only	19	999	1	0	nev any	ot
CEDERL	511		m	0	0	all	10	all	Eu:Sca	1963	pr	491	n	bl	n	n	1	cu	cig only	17	18	2	4	nev any	ot
CEDERL	512		m	0	0	all	10	all	Eu:Sca	1963	pr	491	n	bl	n	n	1	cu	cig only	1	16	3	0	nev any	ot
CEDERL	515		f	0	0	all	10	all	Eu:Sca	1963	pr	491	n	bl	n	n	0	cu	cig only	19	999	1	0	nev any	st
CEDERL	516		f	0	0	all	10	all	Eu:Sca	1963	pr	491	n	bl	n	n	0	cu	cig only	17	18	2	4	nev any	st
CEDERL	517		f	0	0	all	10	all	Eu:Sca	1963	pr	491	n	bl	n	n	0	cu	cig only	1	16	3	0	nev any	ot
CHEN2	517		m	0	0	all	-	all	As:Chi	1983	CC	193	n	ot	y	n	0	ev	all/unsp	31	999	0	0	nev any	st
CHEN2	518		m	0	0	all	-	all	As:Chi	1983	CC	193	n	ot	y	n	0	ev	all/unsp	20	30	1	0	nev any	st
CHEN2	519		m	0	0	all	-	all	As:Chi	1983	CC	193	n	ot	y	n	0	ev	all/unsp	1	19	0	0	nev any	st
CHEN2	522		f	0	0	all	-	all	As:Chi	1983	CC	193	n	ot	y	n	0	ev	all/unsp	31	999	0	0	nev any	st
CHEN2	523		f	0	0	all	-	all	As:Chi	1983	CC	193	n	ot	y	n	0	ev	all/unsp	20	30	1	0	nev any	st
CHEN2	524		f	0	0	all	-	all	As:Chi	1983	CC	193	n	ot	y	n	0	ev	all/unsp	1	19	0	0	nev any	st
CHIAZZ	501		m	0	0	all	-	all	NAmer	1940	CC	144	o	bl	y	n	2	ev	cig+/-ot	20	999	1	0	nev cigs	or
CHIAZZ	502		m	0	0	all	-	all	NAmer	1940	CC	144	o	bl	y	n	2	ev	cig+/-ot	1	19	0	0	nev cigs	or
CHOI	523		m	0	0	all	-	all	As:oth	1985	CC	375	n	bl	n	n	0	ev	cig+/-ot	25	999	1	0	nev cigs	st
CHOI	524		m	0	0	all	-	all	As:oth	1985	CC	375	n	bl	n	n	0	ev	cig+/-ot	20	24	0	3	nev cigs	st
CHOI	525		m	0	0	all	-	all	As:oth	1985	CC	375	n	bl	n	n	0	ev	cig+/-ot	15	19	2	4	nev cigs	st
CHOI	526		m	0	0	all	-	all	As:oth	1985	CC	375	n	bl	n	n	0	ev	cig+/-ot	1	14	3	0	nev cigs	st
CHOI	530		f	0	0	all	-	all	As:oth	1985	CC	375	n	bl	n	n	0	ev	cig+/-ot	25	999	1	0	nev cigs	st
CHOI	531		f	0	0	all	-	all	As:oth	1985	CC	375	n	bl	n	n	0	ev	cig+/-ot	1	24	0	0	nev cigs	st
CPSI	801		m	35	84	all	6	all	NAmer	1959	pr	5138	n	bl	n	n	1	cu	cig+/-ot	25	999	1	0	nev any	ot
CPSI	802		m	35	84	all	6	all	NAmer	1959	pr	5138	n	bl	n	n	1	cu	cig+/-ot	20	24	0	3	nev any	ot
CPSI	803		m	35	84	all	6	all	NAmer	1959	pr	5138	n	bl	n	n	1	cu	cig+/-ot	15	19	2	4	nev any	ot
CPSI	804		m	35	84	all	6	all	NAmer	1959	pr	5138	n	bl	n	n	1	cu	cig+/-ot	1	14	3	0	nev any	ot
CPSI	845		f	40	74	all	6	all	NAmer	1959	pr	5138	n	bl	n	n	1	cu	cig only	25	999	1	0	nev any	ot
CPSI	846		f	40	74	all	6	all	NAmer	1959	pr	5138	n	bl	n	n	1	cu	cig only	20	24	0	3	nev any	ot
CPSI	847		f	40	74	all	6	all	NAmer	1959	pr	5138	n	bl	n	n	1	cu	cig only	15	19	2	4	nev any	ot
CPSI	848		f	40	74	all	6	all	NAmer	1959	pr	5138	n	bl	n	n	1	cu	cig only	1	14	3	0	nev any	ot
DAMBER	501		m	0	0	all	-	all	Eu:Sca	1972	CC	579	n	bl	y	n	0	ev	all/unsp	21	999	1	0	nev any	st
DAMBER	502		m	0	0	all	-	all	Eu:Sca	1972	CC	579	n	bl	y	n	0	ev	all/unsp	16	20	2	4	nev any	st
DAMBER	503		m	0	0	all	-	all	Eu:Sca	1972	CC	579	n	bl	y	n	0	ev	all/unsp	1	15	3	0	nev any	st
DEAN3	564		m	0	0	all	-	all	Eu:UK	1969	CC	766	n	V	y	n	0	cu	cig only	25	999	1	0	nev any	st
DEAN3	565		m	0	0	all	-	all	Eu:UK	1969	CC	766	n	V	y	n	0	cu	cig only	20	24	0	3	nev any	st
DEAN3	566		m	0	0	all	-	all	Eu:UK	1969	CC	766	n	V	y	n	0	cu	cig only	15	19	2	4	nev any	st
DEAN3	567		m	0	0	all	-	all	Eu:UK	1969	CC	766	n	V	y	n	0	cu	cig only	1	14	3	0	nev any	st
DEAN3	583		f	0	0	all	-	all	Eu:UK	1969	CC	766	n	V	y	n	0	cu	cig only	25	999	1	0	nev any	st
DEAN3	584		f	0	0	all	-	all	Eu:UK	1969	CC	766	n	V	y	n	0	cu	cig only	20	24	0	3	nev any	st
DEAN3	585		f	0	0	all	-	all	Eu:UK	1969	CC	766	n	V	y	n	0	cu	cig only	15	19	2	4	nev any	st
DEAN3	586		f	0	0	all	-	all	Eu:UK	1969	CC	766	n	V	y	n	0	cu	cig only	1	14	3	0	nev any	st
DOLL	501		m	0	0	all	-	all	Eu:UK	1948	CC	1465	n	V	n	n	0	ev	all/unsp	40	999	0	0	nev any	st
DOLL	502		m	0	0	all	-	all	Eu:UK	1948	CC	1465	n	V	n	n	0	ev	all/unsp	30	39	0	1	nev any	st
DOLL	503		m	0	0	all	-	all	Eu:UK	1948	CC	1465	n	V	n	n	0	ev	all/unsp	20	29	1	0	nev any	st
DOLL	504		m	0	0	all	-	all	Eu:UK	1948	CC	1465	n	V	n	n	0	ev	all/unsp	1	19	0	0	nev any	st
DOLL	508		f	0	0	all	-	all	Eu:UK	1948	CC	1465	n	V	n	n	0	ev	all/unsp	40	999	0	0	nev any	st
DOLL	509		f	0	0	all	-	all	Eu:UK	1948	CC	1465	n	V	n	n	0	ev	all/unsp	30	39	0	1	nev any	st
DOLL	510		f	0	0	all	-	all	Eu:UK	1948	CC	1465	n	V	n	n	0	ev	all/unsp	20	29	1	0	nev any	st
DOLL	511		f	0	0	all	-	all	Eu:UK	1948	CC	1465	n	V	n	n	0	ev	all/unsp	1	19	0	0	nev any	st
DORN	610		m	55	64	wh	8	all	NAmer	1954	pr	5097	n	bl	n	n	0	ev	cig+/-ot	25	999	1	0	nev any	st
DORN	611		m	55	64	wh	8	all	NAmer	1954	pr	5097	n	bl	n	n	0	ev	cig+/-ot	20	24	0	3	nev any	st
DORN	612		m	55	64	wh	8	all	NAmer	1954	pr	5097	n	bl	n	n	0	ev	cig+/-ot	15	19	2	4	nev any	st
DORN	613		m	55	64	wh	8	all	NAmer	1954	pr	5097	n	bl	n	n	0	ev	cig+/-ot	1	14	3	0	nev any	st
DORN	647		m	65	74	wh	8	all	NAmer	1954	pr	5097	n	bl	n	n	0	ev	cig+/-ot	25	999	1	0	nev any	st
DORN	648		m	65	74	wh	8	all	NAmer	1954	pr	5097	n	bl	n	n	0	ev	cig+/-ot	20	24	0	3	nev any	st
DORN	649		m	65	74	wh	8	all	NAmer	1954	pr	5097	n	bl	n	n	0	ev	cig+/-ot	15	19	2	4	nev any	st

Table 1H16 - 4

IESLC - Meta-analysis of Ever/current Smoking by Age started, Overview
All LC types, Cigarettes (or Any Product if Cigarettes not available)
Least adjusted

REF	NRR	X	SEX	AGEL	AGEH	RACE	YF	LC TYPE	LOC	START	ST	NLC	R	VB	P	H	AD	SM	PRODUCT	exL	exH	S1	S2	DENOM	De
DORN	650		m	65	74	wh	8	all	NAmer	1954	pr	5097	n	bl	n	n	0	ev	cig+/-ot	1	14	3	0	nev any	st
ENGELA	501		m	0	0	all	0	all	Eu:Sca	1964	pr	435	n	bl	n	n	0	cu	cig+/-ot	30	999	0	1	nev cigs	st
ENGELA	502		m	0	0	all	0	all	Eu:Sca	1964	pr	435	n	bl	n	n	0	cu	cig+/-ot	20	29	1	0	nev cigs	st
ENGELA	503		m	0	0	all	0	all	Eu:Sca	1964	pr	435	n	bl	n	n	0	cu	cig+/-ot	1	19	0	0	nev cigs	st
ENGELA	509		f	0	0	all	0	all	Eu:Sca	1964	pr	435	n	bl	n	n	0	cu	cig+/-ot	30	999	0	1	nev cigs	st
ENGELA	510		f	0	0	all	0	all	Eu:Sca	1964	pr	435	n	bl	n	n	0	cu	cig+/-ot	20	29	1	0	nev cigs	st
ENGELA	511		f	0	0	all	0	all	Eu:Sca	1964	pr	435	n	bl	n	n	0	cu	cig+/-ot	1	19	0	0	nev cigs	st
GAO	501	x	m	0	0	all	-	all	As:Chi	1984	CC	1405	n	ot	n	n	0	ev	cig+/-ot	30	999	0	1	nev cigs	st
GAO	502	x	m	0	0	all	-	all	As:Chi	1984	CC	1405	n	ot	n	n	0	ev	cig+/-ot	20	29	1	0	nev cigs	st
GAO	503	x	m	0	0	all	-	all	As:Chi	1984	CC	1405	n	ot	n	n	0	ev	cig+/-ot	10	19	0	0	nev cigs	st
GAO	511	x	f	0	0	all	-	all	As:Chi	1984	CC	1405	n	ot	n	n	0	ev	cig+/-ot	30	999	0	1	nev cigs	st
GAO	512	x	f	0	0	all	-	all	As:Chi	1984	CC	1405	n	ot	n	n	0	ev	cig+/-ot	20	29	1	0	nev cigs	st
GAO	513	x	f	0	0	all	-	all	As:Chi	1984	CC	1405	n	ot	n	n	0	ev	cig+/-ot	10	19	0	0	nev cigs	st
GAO2	501		m	0	0	all	-	all	As:Jap	1988	CC	282	n	bl	n	n	0	cu	cig+/-ot	30	999	0	1	nev cigs	or
GAO2	502		m	0	0	all	-	all	As:Jap	1988	CC	282	n	bl	n	n	0	cu	cig+/-ot	20	29	1	0	nev cigs	st
GAO2	503		m	0	0	all	-	all	As:Jap	1988	CC	282	n	bl	n	n	0	cu	cig+/-ot	1	19	0	0	nev cigs	or
GENG	528	x	f	0	0	all	-	all	As:Chi	1985	CC	292	n	ot	*	n	0	ev	cig+/-ot	21	999	1	0	nev any	st
GENG	529	x	f	0	0	all	-	all	As:Chi	1985	CC	292	n	ot	*	n	0	ev	cig+/-ot	16	20	2	4	nev any	st
GENG	530	x	f	0	0	all	-	all	As:Chi	1985	CC	292	n	ot	*	n	0	ev	cig+/-ot	1	15	3	0	nev any	st
HAENSZ	537		f	0	0	all	-	not alv	NAmer	1955	CC	158	n	bl	n	y	0	ev	cig+/-ot	25	999	1	0	nev any	st
HAENSZ	538		f	0	0	all	-	not alv	NAmer	1955	CC	158	n	bl	n	y	0	ev	cig+/-ot	1	24	0	0	nev any	st
HEGMAN	513		m	0	0	all	-	all	NAmer	1989	CC	282	n	bl	y	y	1	ev	all/unsp	20	999	1	0	nev any	or
HEGMAN	514		m	0	0	all	-	all	NAmer	1989	CC	282	n	bl	y	y	1	ev	all/unsp	1	19	0	0	nev any	or
HEGMAN	516		f	0	0	all	-	all	NAmer	1989	CC	282	n	bl	y	y	1	ev	all/unsp	26	999	1	0	nev any	or
HEGMAN	517		f	0	0	all	-	all	NAmer	1989	CC	282	n	bl	y	y	1	ev	all/unsp	1	25	0	0	nev any	or
HIRAYA	501		m	0	0	all	0	all	As:Jap	1965	pr	1917	n	bl	n	n	1	cu	cig+/-ot	20	999	1	0	nev any	st
HIRAYA	502		m	0	0	all	0	all	As:Jap	1965	pr	1917	n	bl	n	n	1	cu	cig+/-ot	1	19	0	0	nev any	st
HIRAYA	504		f	0	0	all	0	all	As:Jap	1965	pr	1917	n	bl	n	n	1	cu	cig+/-ot	20	999	1	0	nev any	st
HIRAYA	505		f	0	0	all	0	all	As:Jap	1965	pr	1917	n	bl	n	n	1	cu	cig+/-ot	1	19	0	0	nev any	st
HU	511		m	0	0	all	-	all	As:Chi	1985	CC	227	n	ot	n	y	0	ev	cig+/-ot	30	999	0	1	nev cigs	st
HU	512		m	0	0	all	-	all	As:Chi	1985	CC	227	n	ot	n	y	0	ev	cig+/-ot	16	29	0	0	nev cigs	st
HU	513		m	0	0	all	-	all	As:Chi	1985	CC	227	n	ot	n	y	0	ev	cig+/-ot	1	15	3	0	nev cigs	st
HU	516		f	0	0	all	-	all	As:Chi	1985	CC	227	n	ot	n	y	0	ev	cig+/-ot	30	999	0	1	nev cigs	st
HU	517		f	0	0	all	-	all	As:Chi	1985	CC	227	n	ot	n	y	0	ev	cig+/-ot	16	29	0	0	nev cigs	st
HU	518		f	0	0	all	-	all	As:Chi	1985	CC	227	n	ot	n	y	0	ev	cig+/-ot	1	15	3	0	nev cigs	st
HU2	501		c	0	0	all	-	all	As:Chi	1977	CC	523	n	ot	y	n	0	ev	cig+/-ot	40	999	0	0	nev cigs	ot
HU2	502		c	0	0	all	-	all	As:Chi	1977	CC	523	n	ot	y	n	0	ev	cig+/-ot	30	39	0	1	nev cigs	st
HU2	503		c	0	0	all	-	all	As:Chi	1977	CC	523	n	ot	y	n	0	ev	cig+/-ot	20	29	1	0	nev cigs	st
HU2	504		c	0	0	all	-	all	As:Chi	1977	CC	523	n	ot	y	n	0	ev	cig+/-ot	1	19	0	0	nev cigs	or
JEDRYC	607		m	0	0	all	-	all	Eu:est	1980	CC	1630	n	bl	y	n	0	ev	cig+/-ot	17	18	2	4	nev any	st
JEDRYC	608		m	0	0	all	-	all	Eu:est	1980	CC	1630	n	bl	y	n	0	ev	cig+/-ot	1	16	3	0	nev any	st
JEDRYC	619		f	0	0	all	-	all	Eu:est	1980	CC	1630	n	bl	y	n	0	ev	cig+/-ot	1	22	0	0	nev any	st
JOLY	543		m	0	0	all	-	all	SCAmer	1978	CC	826	n	bl	n	n	0	ev	cig+/-ot	25	999	1	0	nev any	st
JOLY	544		m	0	0	all	-	all	SCAmer	1978	CC	826	n	bl	n	n	0	ev	cig+/-ot	15	24	2	0	nev any	st
JOLY	545		m	0	0	all	-	all	SCAmer	1978	CC	826	n	bl	n	n	0	ev	cig+/-ot	1	14	3	0	nev any	st
JOLY	533		f	0	0	all	-	all	SCAmer	1978	CC	826	n	bl	n	n	0	ev	cig+/-ot	25	999	1	0	nev any	st
JOLY	534		f	0	0	all	-	all	SCAmer	1978	CC	826	n	bl	n	n	0	ev	cig+/-ot	15	24	2	0	nev any	st
JOLY	535		f	0	0	all	-	all	SCAmer	1978	CC	826	n	bl	n	n	0	ev	cig+/-ot	1	14	3	0	nev any	st
KHUDER	506		m	0	0	all	-	all	NAmer	1985	CC	482	n	bl	n	y	0	ev	cig+/-ot	20	999	1	0	nev cigs	st
KHUDER	507		m	0	0	all	-	all	NAmer	1985	CC	482	n	bl	n	y	0	ev	cig+/-ot	16	19	2	4	nev cigs	st
KHUDER	508		m	0	0	all	-	all	NAmer	1985	CC	482	n	bl	n	y	0	ev	cig+/-ot	1	15	3	0	nev cigs	st
KOULUM	501		m	0	0	all	-	all	Eu:Sca	1936	CC	812	n	bl	n	n	0	ev	all/unsp	31	999	0	0	nev any	st
KOULUM	502		m	0	0	all	-	all	Eu:Sca	1936	CC	812	n	bl	n	n	0	ev	all/unsp	21	30	1	0	nev any	st
KOULUM	503		m	0	0	all	-	all	Eu:Sca	1936	CC	812	n	bl	n	n	0	ev	all/unsp	16	20	2	4	nev any	st
KOULUM	504		m	0	0	all	-	all	Eu:Sca	1936	CC	812	n	bl	n	n	0	ev	all/unsp	11	15	3	5	nev any	st
KOULUM	505		m	0	0	all	-	all	Eu:Sca	1936	CC	812	n	bl	n	n	0	ev	all/unsp	0	10	0	6	nev any	st
LETOUR	501		c	0	0	all	-	all	NAmer	1983	CC	738	n	V	y	y	0	ev	cig+/-ot	21	999	1	0	nev cigs	st
LETOUR	502		c	0	0	all	-	all	NAmer	1983	CC	738	n	V	y	y	0	ev	cig+/-ot	15	20	2	4	nev cigs	st
LETOUR	503		c	0	0	all	-	all	NAmer	1983	CC	738	n	V	y	y	0	ev	cig+/-ot	1	14	3	0	nev cigs	st
LIAW	504		c	0	0	all	0	all	As:oth	1982	pr	127	n	ot	n	n	2	cu	all/unsp	25	999	1	0	nev any	or
LIAW	505		c	0	0	all	0	all	As:oth	1982	pr	127	n	ot	n	n	2	cu	all/unsp	21	24	0	3	nev any	or
LIAW	506		c	0	0	all	0	all	As:oth	1982	pr	127	n	ot	n	n	2	cu	all/unsp	1	20	0	0	nev any	or
LIU3	501	x	m	0	0	all	-	all	As:Chi	1985	CC	110	n	ot	n	n	0	ev	all/unsp	21	999	1	0	nev any	or
LIU3	502	x	m	0	0	all	-	all	As:Chi	1985	CC	110	n	ot	n	n	0	ev	all/unsp	1	20	0	0	nev any	st
LIU4	501		m	35	69	all	-	all	As:Chi	1986	CC	1000-00	n	ot	y	n	2	ev	all/unsp	25	999	1	0	nev any	ot
LIU4	502		m	35	69	all	-	all	As:Chi	1986	CC	1000-00	n	ot	y	n	2	ev	all/unsp	20	24	0	3	nev any	ot
LIU4	503		m	35	69	all	-	all	As:Chi	1986	CC	1000-00	n	ot	y	n	2	ev	all/unsp	0	19	0	0	nev any	ot
LIU5	501		c	0	0	all	-	all	As:Chi	1978	CC	111	n	ot	y	n	0	ev	all/unsp	30	999	0	1	nev any	st
LIU5	502		c	0	0	all	-	all	As:Chi	1978	CC	111	n	ot	y	n	0	ev	all/unsp	1	29	0	0	nev any	st

Table 1H16 - 4

IESLC - Meta-analysis of Ever/current Smoking by Age started, Overview
All LC types, Cigarettes (or Any Product if Cigarettes not available)
Least adjusted

REF	NRR	X	SEX	AGEL	AGEH	RACE	YF	LC TYPE	LOC	START	ST	NLC	R	VB	P	H	AD	SM	PRODUCT	exL	exH	S1	S2	DENOM	De
LUBIN	565		m	0	0	all	-	all	As:Chi	1984	CC	427	m	ot	y	n	0	ev	cig+/-ot	27	999	0	1	nev any	st
LUBIN	566		m	0	0	all	-	all	As:Chi	1984	CC	427	m	ot	y	n	0	ev	cig+/-ot	23	26	1	2	nev any	st
LUBIN	567		m	0	0	all	-	all	As:Chi	1984	CC	427	m	ot	y	n	0	ev	cig+/-ot	20	22	0	3	nev any	st
LUBIN	568		m	0	0	all	-	all	As:Chi	1984	CC	427	m	ot	y	n	0	ev	cig+/-ot	1	19	0	0	nev any	st
LUBIN2	1147	x	m	0	0	all	-	all	Eu:mul	1976	CC	7804	n	bl	n	y	0	ev	cig+/-ot	31	999	0	0	nev cigs	st
LUBIN2	1148	x	m	0	0	all	-	all	Eu:mul	1976	CC	7804	n	bl	n	y	0	ev	cig+/-ot	21	30	1	0	nev cigs	st
LUBIN2	1149	x	m	0	0	all	-	all	Eu:mul	1976	CC	7804	n	bl	n	y	0	ev	cig+/-ot	17	20	2	4	nev cigs	st
LUBIN2	1150	x	m	0	0	all	-	all	Eu:mul	1976	CC	7804	n	bl	n	y	0	ev	cig+/-ot	13	16	3	5	nev cigs	st
LUBIN2	1151	x	m	0	0	all	-	all	Eu:mul	1976	CC	7804	n	bl	n	y	0	ev	cig+/-ot	1	12	0	6	nev cigs	st
MATOS	556	x	m	0	0	all	-	all	SCAmer	1994	CC	200	n	bl	n	n	0	ev	cig+/-ot	20	999	1	0	nev any	st
MATOS	557	x	m	0	0	all	-	all	SCAmer	1994	CC	200	n	bl	n	n	0	ev	cig+/-ot	15	19	2	4	nev any	st
MATOS	558	x	m	0	0	all	-	all	SCAmer	1994	CC	200	n	bl	n	n	0	ev	cig+/-ot	1	14	3	0	nev any	st
MIGRAN	501		m	0	0	all	0	all	Eu:UK	1964	pr	259	n	V	n	n	0	cu	cig only	20	999	1	0	nev any	st
MIGRAN	503		m	0	0	all	0	all	Eu:UK	1964	pr	259	n	V	n	n	0	cu	cig only	16	19	2	4	nev any	st
MIGRAN	505		m	0	0	all	0	all	Eu:UK	1964	pr	259	n	V	n	n	0	cu	cig only	0	15	3	0	nev any	st
MIGRAN	511		f	0	0	all	0	all	Eu:UK	1964	pr	259	n	V	n	n	0	cu	cig only	20	999	1	0	nev any	st
MIGRAN	513		f	0	0	all	0	all	Eu:UK	1964	pr	259	n	V	n	n	0	cu	cig only	16	19	2	4	nev any	st
MIGRAN	515		f	0	0	all	0	all	Eu:UK	1964	pr	259	n	V	n	n	0	cu	cig only	0	15	3	0	nev any	st
MRFITR	508		m	0	0	all	0	all	NAmer	1973	pr	119	n	bl	n	n	0	cu	cig+/-ot	24	999	1	0	nev cigs	ot
MRFITR	509		m	0	0	all	0	all	NAmer	1973	pr	119	n	bl	n	n	0	cu	cig+/-ot	22	23	0	3	nev cigs	ot
MRFITR	510		m	0	0	all	0	all	NAmer	1973	pr	119	n	bl	n	n	0	cu	cig+/-ot	20	21	0	0	nev cigs	ot
MRFITR	511		m	0	0	all	0	all	NAmer	1973	pr	119	n	bl	n	n	0	cu	cig+/-ot	18	19	2	4	nev cigs	ot
MRFITR	512		m	0	0	all	0	all	NAmer	1973	pr	119	n	bl	n	n	0	cu	cig+/-ot	16	17	0	0	nev cigs	ot
MRFITR	513		m	0	0	all	0	all	NAmer	1973	pr	119	n	bl	n	n	0	cu	cig+/-ot	1	15	3	0	nev cigs	ot
PERNU	504		m	0	0	all	-	all	Eu:Sca	1944	CC	1606	n	bl	n	n	0	ev	all/unsp	15	999	0	0	nev any	st
PERNU	505		m	0	0	all	-	all	Eu:Sca	1944	CC	1606	n	bl	n	n	0	ev	all/unsp	1	14	3	0	nev any	st
PERNU	501		f	0	0	all	-	all	Eu:Sca	1944	CC	1606	n	bl	n	n	0	ev	all/unsp	15	999	0	0	nev any	st
PERNU	502		f	0	0	all	-	all	Eu:Sca	1944	CC	1606	n	bl	n	n	0	ev	all/unsp	1	14	3	0	nev any	ot
PEZZOT	570		m	0	0	all	-	all	SCAmer	1987	CC	215	n	bl	n	y	0	ev	cig only	19	999	1	0	nev cigs	st
PEZZOT	571		m	0	0	all	-	all	SCAmer	1987	CC	215	n	bl	n	y	0	ev	cig only	14	18	0	0	nev cigs	st
PEZZOT	572		m	0	0	all	-	all	SCAmer	1987	CC	215	n	bl	n	y	0	ev	cig only	1	13	0	6	nev cigs	st
QIAO2	501	x	m	0	0	all	0	all	As:Chi	1992	pr	241	m	ot	n	n	0	ev	all/unsp	21	999	1	0	nev any	st
QIAO2	502	x	m	0	0	all	0	all	As:Chi	1992	pr	241	m	ot	n	n	0	ev	all/unsp	17	20	2	4	nev any	st
QIAO2	503	x	m	0	0	all	0	all	As:Chi	1992	pr	241	m	ot	n	n	0	ev	all/unsp	1	16	3	0	nev any	st
RACHTA	501	x	f	0	0	all	-	all	Eu:est	1991	CC	118	n	bl	n	y	0	ev	cig+/-ot	31	999	0	0	nev cigs	st
RACHTA	502	x	f	0	0	all	-	all	Eu:est	1991	CC	118	n	bl	n	y	0	ev	cig+/-ot	20	30	1	0	nev cigs	st
RACHTA	503	x	f	0	0	all	-	all	Eu:est	1991	CC	118	n	bl	n	y	0	ev	cig+/-ot	1	19	0	0	nev cigs	st
SEGI2	516	x	m	0	0	all	-	all	As:Jap	1962	CC	378	n	bl	n	n	0	cu	cig+/-ot	23	999	1	0	nev any	st
SEGI2	517	x	m	0	0	all	-	all	As:Jap	1962	CC	378	n	bl	n	n	0	cu	cig+/-ot	20	22	0	3	nev any	st
SEGI2	518	x	m	0	0	all	-	all	As:Jap	1962	CC	378	n	bl	n	n	0	cu	cig+/-ot	1	19	0	0	nev any	st
SOBUE	654		m	0	0	all	-	all	As:Jap	1986	CC	1376	n	bl	n	y	0	ev	cig+/-ot	23	999	1	0	nev cigs	st
SOBUE	655		m	0	0	all	-	all	As:Jap	1986	CC	1376	n	bl	n	y	0	ev	cig+/-ot	18	22	2	0	nev cigs	st
SOBUE	656		m	0	0	all	-	all	As:Jap	1986	CC	1376	n	bl	n	y	0	ev	cig+/-ot	10	17	3	0	nev cigs	st
SUZUK2	501		c	0	0	all	-	all	SCAmer	1991	CC	123	n	bl	n	y	0	ev	all/unsp	19	999	1	0	nev any	st
SUZUK2	502		c	0	0	all	-	all	SCAmer	1991	CC	123	n	bl	n	y	0	ev	all/unsp	12	18	0	0	nev any	st
SUZUK2	503		c	0	0	all	-	all	SCAmer	1991	CC	123	n	bl	n	y	0	ev	all/unsp	0	11	0	6	nev any	st
SVENSS	501		f	0	0	all	-	all	Eu:Sca	1983	CC	210	n	bl	n	n	0	cu	all/unsp	26	999	1	0	nev any	st
SVENSS	502		f	0	0	all	-	all	Eu:Sca	1983	CC	210	n	bl	n	n	0	cu	all/unsp	19	25	0	3	nev any	st
SVENSS	503		f	0	0	all	-	all	Eu:Sca	1983	CC	210	n	bl	n	n	0	cu	all/unsp	0	18	0	0	nev any	st
TIZZAN	506		m	0	0	all	-	all	Eu:wst	1959	CC	1358	n	bl	n	n	0	ev	all/unsp	31	999	0	0	nev any	st
TIZZAN	507		m	0	0	all	-	all	Eu:wst	1959	CC	1358	n	bl	n	n	0	ev	all/unsp	20	30	1	0	nev any	st
TIZZAN	508		m	0	0	all	-	all	Eu:wst	1959	CC	1358	n	bl	n	n	0	ev	all/unsp	1	19	0	0	nev any	st
TIZZAN	536		f	0	0	all	-	all	Eu:wst	1959	CC	1358	n	bl	n	n	0	ev	cig only	31	999	0	0	nev any	st
TIZZAN	537		f	0	0	all	-	all	Eu:wst	1959	CC	1358	n	bl	n	n	0	ev	cig only	20	30	1	0	nev any	st
TIZZAN	538		f	0	0	all	-	all	Eu:wst	1959	CC	1358	n	bl	n	n	0	ev	cig only	1	19	0	0	nev any	st
WAKAI	501		m	0	0	all	-	all	As:Jap	1988	CC	333	n	bl	n	y	0	cu	cig+/-ot	30	999	0	1	nev any	st
WAKAI	502		m	0	0	all	-	all	As:Jap	1988	CC	333	n	bl	n	y	0	cu	cig+/-ot	20	29	1	0	nev any	st
WAKAI	503		m	0	0	all	-	all	As:Jap	1988	CC	333	n	bl	n	y	0	cu	cig+/-ot	1	19	0	0	nev any	st
WU	517	x	f	0	0	wh	-	q+a	NAmer	1981	CC	220	n	bl	n	y	0	cu	all/unsp	25	999	1	0	nev any	st
WU	518	x	f	0	0	wh	-	q+a	NAmer	1981	CC	220	n	bl	n	y	0	cu	all/unsp	19	24	0	3	nev any	st
WU	519	x	f	0	0	wh	-	q+a	NAmer	1981	CC	220	n	bl	n	y	0	cu	all/unsp	0	18	0	0	nev any	st
WYNDE6	759		m	0	0	wh	-	q+s+a	NAmer	1969	CC	4423	n	bl	n	y	0	ev	cig+/-ot	21	999	1	0	nev cigs	st
WYNDE6	760		m	0	0	wh	-	q+s+a	NAmer	1969	CC	4423	n	bl	n	y	0	ev	cig+/-ot	18	20	2	4	nev cigs	st
WYNDE6	761		m	0	0	wh	-	q+s+a	NAmer	1969	CC	4423	n	bl	n	y	0	ev	cig+/-ot	1	17	3	0	nev cigs	st
WYNDE6	767		f	0	0	wh	-	q+s+a	NAmer	1969	CC	4423	n	bl	n	y	0	ev	cig+/-ot	21	999	1	0	nev cigs	st
WYNDE6	768		f	0	0	wh	-	q+s+a	NAmer	1969	CC	4423	n	bl	n	y	0	ev	cig+/-ot	18	20	2	4	nev cigs	st
WYNDE6	769		f	0	0	wh	-	q+s+a	NAmer	1969	CC	4423	n	bl	n	y	0	ev	cig+/-ot	1	17	3	0	nev cigs	st
ZHENG	563		m	0	0	all	-	all	As:Chi	1982	CC	540	n	ot	*	y	0	ev	cig+/-ot	30	999	0	1	nev cigs	st
ZHENG	564		m	0	0	all	-	all	As:Chi	1982	CC	540	n	ot	*	y	0	ev	cig+/-ot	20	29	1	0	nev cigs	st
ZHENG	565		m	0	0	all	-	all	As:Chi	1982	CC	540	n	ot	*	y	0	ev	cig+/-ot	1	19	0	0	nev cigs	st
ZHENG	572		f	0	0	all	-	all	As:Chi	1982	CC	540	n	ot	*	y	0	ev	cig+/-ot	30	999	0	1	nev cigs	st
ZHENG	573		f	0	0	all	-	all	As:Chi	1982	CC	540	n	ot	*	y	0	ev	cig+/-ot	1	29	0	0	nev cigs	st

Table 1H16 - 4

IESLC - Meta-analysis of Ever/current Smoking by Age started, Overview
All LC types, Cigarettes (or Any Product if Cigarettes not available)
Least adjusted

Cigarette type is all/unspec for all RRs
except for the following:

REF	NRR	CIGTYPE
DEAN3	564	MC only
DEAN3	565	MC only
DEAN3	566	MC only
DEAN3	567	MC only
DEAN3	583	MC only
DEAN3	584	MC only
DEAN3	585	MC only
DEAN3	586	MC only

In this overview table, subtotals and Qs values may be invalid and should be ignored

Table 1H16 - 5

IESLC - Meta-analysis of Ever/current Smoking by Age started, Overview
All LC types, Cigarettes (or Any Product if Cigarettes not available)
Least adjusted

REF	NRR	SEX	AD	Number Exposed Case	Number Exposed Cont	Non-exposed Case	Non-exposed Cont	RR	95.00%CI
AGUDO	501	f	0	7	12	80	183	1.33	(0.51- 3.51)
AGUDO	502	f	0	16	11	80	183	3.33	(1.48- 7.49)
Subtotal AGUDO								2.28	(1.23- 4.25)
ARMADA	511	m	0	113	144	8	71	6.96	(3.22- 15.06)
ARMADA	512	m	0	204	110	8	71	16.46	(7.64- 35.44)
Subtotal ARMADA								10.73	(6.23- 18.49)
AUVINE	509	c	0	76	27	44	229	14.65	(8.50- 25.26)
AUVINE	510	c	0	135	47	44	229	14.95	(9.41- 23.75)
AUVINE	511	c	0	55	6	44	229	47.71	(19.35- 117.62)
Subtotal AUVINE								17.31	(12.46- 24.04)
BARBON	515	m	0	200	207	22	188	8.26	(5.10- 13.38)
BARBON	516	m	0	395	337	22	188	10.02	(6.29- 15.94)
BARBON	517	m	0	138	23	22	188	51.27	(27.46- 95.73)
Subtotal BARBON								13.42	(9.99- 18.02)
BRESLO	501	c	0	32	35	19	56	2.69	(1.33- 5.47)
BRESLO	502	c	0	286	243	19	56	3.47	(2.01- 6.00)
BRESLO	503	c	0	166	116	19	56	4.22	(2.38- 7.47)
Subtotal BRESLO								3.51	(2.48- 4.95)
BUFFLE	517	f	0	23	23	12	112	9.33	(4.07- 21.40)
BUFFLE	518	f	0	47	34	12	112	12.90	(6.15- 27.07)
BUFFLE	519	f	0	40	29	12	112	12.87	(6.00- 27.62)
BUFFLE	520	f	0	53	33	12	112	14.99	(7.17- 31.33)
BUFFLE	521	f	0	78	41	12	112	17.76	(8.77- 35.94)
Subtotal BUFFLE								13.57	(9.69- 18.99)
*CEDERL	510	m	1	11	-	7	-	6.50	(2.52- 16.74)
*CEDERL	511	m	1	10	-	7	-	9.80	(3.74- 25.69)
*CEDERL	512	m	1	7	-	7	-	6.40	(2.25- 18.21)
*CEDERL	515	f	0	6	2806	19	17679	1.99	(0.80- 4.98)
*CEDERL	516	f	0	2	1009	19	17679	1.84	(0.43- 7.91)
*CEDERL	517	f	0	0	746	19	17679	0.61~	(0.04- 10.05)
Subtotal CEDERL								4.43	(2.82- 6.96)
CHEN2	517	m	0	8	5	9	33	5.87	(1.54- 22.37)
CHEN2	518	m	0	29	25	9	33	4.25	(1.71- 10.57)
CHEN2	519	m	0	84	67	9	33	4.60	(2.06- 10.27)
CHEN2	522	f	0	5	8	25	33	0.83	(0.24- 2.83)
CHEN2	523	f	0	9	8	25	33	1.49	(0.50- 4.39)
CHEN2	524	f	0	23	13	25	33	2.34	(0.99- 5.50)
Subtotal CHEN2								2.84	(1.90- 4.25)
CHIAZZ	501	m	2	-	-	4	-	3.00	(0.31- 28.84)
CHIAZZ	502	m	2	-	-	4	-	19.89	(2.66- 148.96)
Subtotal CHIAZZ								8.64	(1.92- 38.91)
CHOI	523	m	0	36	77	13	95	3.42	(1.69- 6.89)
CHOI	524	m	0	130	232	13	95	4.09	(2.21- 7.60)
CHOI	525	m	0	79	138	13	95	4.18	(2.20- 7.95)
CHOI	526	m	0	22	18	13	95	8.93	(3.81- 20.91)
CHOI	530	f	0	15	25	76	164	1.29	(0.65- 2.60)
CHOI	531	f	0	4	1	76	164	8.63	(0.95- 78.54)
Subtotal CHOI								3.57	(2.63- 4.85)
*CPSI	801	m	1	42	-	83	-	4.08	(2.81- 5.91)
*CPSI	802	m	1	196	-	83	-	10.08	(7.80- 13.03)
*CPSI	803	m	1	588	-	83	-	14.69	(11.68- 18.49)
*CPSI	804	m	1	185	-	83	-	16.77	(12.94- 21.73)
*CPSI	845	f	1	51	-	166	-	2.25	(1.64- 3.08)
*CPSI	846	f	1	34	-	166	-	3.38	(2.33- 4.88)
*CPSI	847	f	1	52	-	166	-	5.00	(3.66- 6.83)
*CPSI	848	f	1	6	-	166	-	2.50	(1.11- 5.65)
Subtotal CPSI								7.59	(6.81- 8.46)
DAMBER	501	m	0	70	76	42	208	4.56	(2.87- 7.26)
DAMBER	502	m	0	261	190	42	208	6.80	(4.65- 9.95)
DAMBER	503	m	0	206	98	42	208	10.41	(6.91- 15.68)
Subtotal DAMBER								7.07	(5.57- 8.98)
DEAN3	564	m	0	24	75	24	510	6.80	(3.67- 12.58)
DEAN3	565	m	0	52	161	24	510	6.86	(4.10- 11.49)
DEAN3	566	m	0	160	485	24	510	7.01	(4.48- 10.96)
DEAN3	567	m	0	44	165	24	510	5.67	(3.34- 9.60)
DEAN3	583	f	0	27	274	41	1538	3.70	(2.24- 6.11)
DEAN3	584	f	0	18	229	41	1538	2.95	(1.67- 5.22)
DEAN3	585	f	0	39	504	41	1538	2.90	(1.85- 4.55)
DEAN3	586	f	0	7	109	41	1538	2.41	(1.06- 5.50)
Subtotal DEAN3								4.59	(3.81- 5.54)
DOLL	501	m	0	4	7	7	61	4.98	(1.16- 21.36)

Table 1H16 - 5

IESLC - Meta-analysis of Ever/current Smoking by Age started, Overview
All LC types, Cigarettes (or Any Product if Cigarettes not available)
Least adjusted

REF	NRR	SEX	AD	Number Exposed Case	Number Exposed Cont	Non-exposed Case	Non-exposed Cont	RR	95.00%CI
DOLL	502	m	0	18	33	7	61	4.75	(1.80- 12.54)
DOLL	503	m	0	251	264	7	61	8.29	(3.72- 18.46)
DOLL	504	m	0	1077	992	7	61	9.46	(4.31- 20.78)
DOLL	508	f	0	15	15	40	59	1.48	(0.65- 3.35)
DOLL	509	f	0	10	7	40	59	2.11	(0.74- 6.00)
DOLL	510	f	0	23	15	40	59	2.26	(1.05- 4.86)
DOLL	511	f	0	20	12	40	59	2.46	(1.08- 5.58)
Subtotal DOLL								3.65	(2.67- 4.98)
*DORN	610	m	0	37	73050	25	213858	4.33	(2.61- 7.20)
*DORN	611	m	0	157	147948	25	213858	9.08	(5.95- 13.84)
*DORN	612	m	0	342	213156	25	213858	13.73	(9.14- 20.60)
*DORN	613	m	0	84	36304	25	213858	19.79	(12.67- 30.93)
*DORN	647	m	0	90	74464	49	171211	4.22	(2.98- 5.98)
*DORN	648	m	0	171	90036	49	171211	6.64	(4.83- 9.12)
*DORN	649	m	0	306	118234	49	171211	9.04	(6.69- 12.23)
*DORN	650	m	0	81	24616	49	171211	11.50	(8.07- 16.39)
Subtotal DORN								8.46	(7.42- 9.65)
*ENGELA	501	m	0	17	9762	27	58716	3.79	(2.07- 6.95)
*ENGELA	502	m	0	50	30195	27	58716	3.60	(2.26- 5.75)
*ENGELA	503	m	0	173	50732	27	58716	7.42	(4.94- 11.12)
*ENGELA	509	f	0	10	24560	31	207789	2.73	(1.34- 5.57)
*ENGELA	510	f	0	36	29605	31	207789	8.15	(5.04- 13.17)
*ENGELA	511	f	0	18	10687	31	207789	11.29	(6.32- 20.17)
Subtotal ENGELA								5.83	(4.73- 7.20)
GAO	501	m	0	45	129	62	202	1.14	(0.73- 1.77)
GAO	502	m	0	363	262	62	202	4.51	(3.26- 6.25)
GAO	503	m	0	262	167	62	202	5.11	(3.62- 7.21)
GAO	511	f	0	73	64	435	605	1.59	(1.11- 2.27)
GAO	512	f	0	87	41	435	605	2.95	(2.00- 4.36)
GAO	513	f	0	77	25	435	605	4.28	(2.68- 6.84)
Subtotal GAO								2.99	(2.57- 3.50)
GAO2	501	m	0	2	4	13	56	2.15	(0.36- 13.05)
GAO2	502	m	0	127	85	13	56	6.44	(3.32- 12.49)
GAO2	503	m	0	52	26	13	56	8.62	(4.01- 18.52)
Subtotal GAO2								6.68	(4.12- 10.83)
GENG	528	f	0	28	31	54	93	1.56	(0.84- 2.87)
GENG	529	f	0	39	23	54	93	2.92	(1.58- 5.40)
GENG	530	f	0	36	10	54	93	6.20	(2.85- 13.48)
Subtotal GENG								2.74	(1.88- 4.00)
HAENSZ	537	f	0	44	66	81	236	1.94	(1.23- 3.07)
HAENSZ	538	f	0	30	37	81	236	2.36	(1.37- 4.07)
Subtotal HAENSZ								2.11	(1.48- 2.99)
HEGMAN	513	m	1	26	-	-	-	9.40	(4.60- 19.30)
HEGMAN	514	m	1	146	-	-	-	22.30	(12.00- 41.40)
HEGMAN	516	f	1	2	-	-	-	4.80	(1.00- 22.10)
HEGMAN	517	f	1	81	-	-	-	26.80	(15.40- 46.80)
Subtotal HEGMAN								18.07	(12.75- 25.62)
*HIRAYA	501	m	1	-	-	-	-	4.35	(3.51- 5.39)
*HIRAYA	502	m	1	-	-	-	-	5.71	(4.50- 7.25)
*HIRAYA	504	f	1	-	-	-	-	2.46	(1.93- 3.13)
*HIRAYA	505	f	1	-	-	-	-	0.78	(0.10- 6.10)
Subtotal HIRAYA								3.96	(3.46- 4.52)
HU	511	m	0	14	20	41	67	1.14	(0.52- 2.51)
HU	512	m	0	93	67	41	67	2.27	(1.38- 3.74)
HU	513	m	0	13	7	41	67	3.03	(1.12- 8.23)
HU	516	f	0	3	5	40	48	0.72	(0.16- 3.20)
HU	517	f	0	18	9	40	48	2.40	(0.97- 5.92)
HU	518	f	0	5	4	40	48	1.50	(0.38- 5.96)
Subtotal HU								1.92	(1.37- 2.69)
HU2	501	c	0	15	29	121	213	0.91	(0.47- 1.77)
HU2	502	c	0	29	54	121	213	0.95	(0.57- 1.56)
HU2	503	c	0	229	159	121	213	2.54	(1.88- 3.43)
HU2	504	c	0	129	68	121	213	3.34	(2.31- 4.83)
Subtotal HU2								2.14	(1.75- 2.61)
JEDRYC	607	m	0	239	146	49	219	7.32	(5.04- 10.61)
JEDRYC	608	m	0	135	66	49	219	9.14	(5.96- 14.02)
JEDRYC	619	f	0	63	11	78	166	12.19	(6.09- 24.42)
Subtotal JEDRYC								8.54	(6.58- 11.07)
JOLY	543	m	0	18	70	12	218	4.67	(2.14- 10.18)
JOLY	544	m	0	217	357	12	218	11.04	(6.03- 20.22)
JOLY	545	m	0	317	282	12	218	20.42	(11.18- 37.32)

Table 1H16 - 5

IESLC - Meta-analysis of Ever/current Smoking by Age started, Overview
All LC types, Cigarettes (or Any Product if Cigarettes not available)
Least adjusted

REF	NRR	SEX	AD	Number Exposed Case	Cont	Non-exposed Case	Cont	RR	95.00%CI
JOLY	533	f	0	23	41	52	283	3.05	(1.69- 5.51)
JOLY	534	f	0	67	47	52	283	7.76	(4.82- 12.49)
JOLY	535	f	0	76	35	52	283	11.82	(7.18- 19.44)
Subtotal JOLY								8.56	(6.78- 10.80)
KHUDER	506	m	0	72	152	23	309	6.36	(3.83- 10.58)
KHUDER	507	m	0	161	338	23	309	6.40	(4.03- 10.17)
KHUDER	508	m	0	226	295	23	309	10.29	(6.51- 16.27)
Subtotal KHUDER								7.58	(5.76- 9.97)
KOULUM	501	m	0	8	8	5	54	10.80	(2.82- 41.31)
KOULUM	502	m	0	60	67	5	54	9.67	(3.63- 25.77)
KOULUM	503	m	0	267	103	5	54	28.00	(10.89- 71.96)
KOULUM	504	m	0	199	52	5	54	41.33	(15.74- 108.56)
KOULUM	505	m	0	143	16	5	54	96.53	(33.72- 276.34)
Subtotal KOULUM								27.41	(17.28- 43.49)
LETOUR	501	c	0	188	160	24	224	10.97	(6.85- 17.56)
LETOUR	502	c	0	309	241	24	224	11.97	(7.60- 18.83)
LETOUR	503	c	0	151	76	24	224	18.54	(11.21- 30.67)
Subtotal LETOUR								13.23	(10.06- 17.40)
*LIAW	504	c	2	-	-	-	-	1.50	(0.70- 3.30)
*LIAW	505	c	2	-	-	-	-	5.90	(3.00- 11.30)
*LIAW	506	c	2	-	-	-	-	4.60	(2.60- 8.10)
Subtotal LIAW								3.82	(2.62- 5.58)
LIU3	501	m	0	20	80	4	19	1.19	(0.36- 3.88)
LIU3	502	m	0	32	125	4	19	1.22	(0.39- 3.83)
Subtotal LIU3								1.20	(0.53- 2.74)
LIU4	501	m	2	-	-	-	-	2.41	(2.32- 2.49)
LIU4	502	m	2	-	-	-	-	2.86	(2.78- 2.95)
LIU4	503	m	2	-	-	-	-	3.81	(3.70- 3.93)
Subtotal LIU4								3.03	(2.98- 3.09)
LIU5	501	c	0	13	22	26	41	0.93	(0.40- 2.17)
LIU5	502	c	0	72	48	26	41	2.37	(1.28- 4.36)
Subtotal LIU5								1.72	(1.05- 2.82)
LUBIN	565	m	0	30	179	9	72	1.34	(0.61- 2.96)
LUBIN	566	m	0	65	146	9	72	3.56	(1.68- 7.56)
LUBIN	567	m	0	89	212	9	72	3.36	(1.61- 7.01)
LUBIN	568	m	0	178	251	9	72	5.67	(2.76- 11.64)
Subtotal LUBIN								3.20	(2.20- 4.66)
LUBIN2	1147	m	0	68	221	185	1878	3.12	(2.29- 4.26)
LUBIN2	1148	m	0	564	1069	185	1878	5.36	(4.46- 6.43)
LUBIN2	1149	m	0	1796	3028	185	1878	6.02	(5.12- 7.08)
LUBIN2	1150	m	0	1312	1833	185	1878	7.27	(6.15- 8.59)
LUBIN2	1151	m	0	250	316	185	1878	8.03	(6.42- 10.05)
Subtotal LUBIN2								6.12	(5.61- 6.67)
MATOS	556	m	0	28	73	11	110	3.84	(1.80- 8.18)
MATOS	557	m	0	91	120	11	110	7.58	(3.85- 14.92)
MATOS	558	m	0	69	90	11	110	7.67	(3.83- 15.36)
Subtotal MATOS								6.24	(4.15- 9.39)
*MIGRAN	501	m	0	24	668	4	867	7.79	(2.72- 22.34)
*MIGRAN	503	m	0	59	1845	4	867	6.93	(2.53- 19.02)
*MIGRAN	505	m	0	50	1081	4	867	10.03	(3.64- 27.65)
*MIGRAN	511	f	0	11	1315	4	3814	7.98	(2.54- 25.01)
*MIGRAN	513	f	0	9	1035	4	3814	8.29	(2.56- 26.87)
*MIGRAN	515	f	0	2	266	4	3814	7.17	(1.32- 38.96)
Subtotal MIGRAN								8.07	(5.08- 12.80)
*MRFITR	508	m	0	3	544	0	1859	23.91~	(1.24- 462.09)
*MRFITR	509	m	0	6	402	0	1859	60.06~	(3.39-1063.94)
*MRFITR	510	m	0	7	1029	0	1859	27.09~	(1.55- 473.89)
*MRFITR	511	m	0	25	1876	0	1859	50.54~	(3.08- 829.51)
*MRFITR	512	m	0	40	2242	0	1859	67.17~	(4.13-1091.55)
*MRFITR	513	m	0	25	2065	0	1859	45.91~	(2.80- 753.64)
Subtotal MRFITR								43.20	(13.52- 138.02)
PERNU	504	m	0	1043	346	97	275	8.55	(6.58- 11.10)
PERNU	505	m	0	337	92	97	275	10.38	(7.49- 14.40)
PERNU	501	f	0	18	89	110	971	1.79	(1.04- 3.07)
PERNU	502	f	0	1	0	110	971	26.38~	(1.07- 651.38)
Subtotal PERNU								7.56	(6.25- 9.15)
PEZZOT	570	m	0	41	105	4	116	11.32	(3.92- 32.69)
PEZZOT	571	m	0	118	145	4	116	23.60	(8.46- 65.84)
PEZZOT	572	m	0	52	67	4	116	22.51	(7.79- 65.00)
Subtotal PEZZOT								18.29	(9.98- 33.50)
*QIAO2	501	m	0	52	1947	10	709	1.89	(0.97- 3.71)

Table 1H16 - 5

IESLC - Meta-analysis of Ever/current Smoking by Age started, Overview
All LC types, Cigarettes (or Any Product if Cigarettes not available)
Least adjusted

REF	NRR	SEX	AD	Number Exposed Case	Number Exposed Cont	Non-exposed Case	Non-exposed Cont	RR	95.00%CI
*QIAO2	502	m	0	75	2840	10	709	1.87	(0.97- 3.60)
*QIAO2	503	m	0	104	2130	10	709	3.46	(1.82- 6.59)
Subtotal QIAO2								2.33	(1.59- 3.40)
RACHTA	501	f	0	8	4	33	98	5.94	(1.68- 21.01)
RACHTA	502	f	0	25	18	33	98	4.12	(2.00- 8.50)
RACHTA	503	f	0	52	21	33	98	7.35	(3.87- 13.98)
Subtotal RACHTA								5.73	(3.66- 8.97)
SEGI2	516	m	0	49	155	8	53	2.09	(0.93- 4.71)
SEGI2	517	m	0	125	224	8	53	3.70	(1.70- 8.02)
SEGI2	518	m	0	91	103	8	53	5.85	(2.64- 12.96)
Subtotal SEGI2								3.59	(2.27- 5.67)
SOBUE	654	m	0	110	121	29	126	3.95	(2.45- 6.38)
SOBUE	655	m	0	776	772	29	126	4.37	(2.88- 6.62)
SOBUE	656	m	0	137	62	29	126	9.60	(5.81- 15.88)
Subtotal SOBUE								5.28	(4.05- 6.89)
SUZUK2	501	c	0	16	22	11	53	3.50	(1.40- 8.75)
SUZUK2	502	c	0	64	38	11	53	8.11	(3.78- 17.41)
SUZUK2	503	c	0	31	10	11	53	14.94	(5.69- 39.18)
Subtotal SUZUK2								7.44	(4.51- 12.27)
SVENSS	501	f	0	32	18	38	120	5.61	(2.84- 11.12)
SVENSS	502	f	0	58	14	38	120	13.08	(6.57- 26.04)
SVENSS	503	f	0	52	21	38	120	7.82	(4.19- 14.60)
Subtotal SVENSS								8.26	(5.63- 12.12)
TIZZAN	506	m	0	12	44	180	305	0.46	(0.24- 0.90)
TIZZAN	507	m	0	313	330	180	305	1.61	(1.26- 2.04)
TIZZAN	508	m	0	699	529	180	305	2.24	(1.80- 2.78)
TIZZAN	536	f	0	2	5	117	114	0.39	(0.07- 2.05)
TIZZAN	537	f	0	12	21	117	114	0.56	(0.26- 1.18)
TIZZAN	538	f	0	11	2	117	114	5.36	(1.16- 24.71)
Subtotal TIZZAN								1.70	(1.46- 1.98)
WAKAI	501	m	0	8	25	10	65	2.08	(0.74- 5.87)
WAKAI	502	m	0	130	183	10	65	4.62	(2.29- 9.32)
WAKAI	503	m	0	42	74	10	65	3.69	(1.72- 7.94)
Subtotal WAKAI								3.63	(2.28- 5.77)
WU	517	f	0	14	19	31	92	2.19	(0.98- 4.87)
WU	518	f	0	40	22	31	92	5.40	(2.79- 10.45)
WU	519	f	0	106	32	31	92	9.83	(5.57- 17.34)
Subtotal WU								5.76	(3.94- 8.42)
WYNDE6	759	m	0	111	92	51	589	13.93	(9.36- 20.74)
WYNDE6	760	m	0	223	139	51	589	18.53	(12.98- 26.45)
WYNDE6	761	m	0	611	301	51	589	23.44	(17.06- 32.21)
WYNDE6	767	f	0	127	90	73	673	13.01	(9.06- 18.69)
WYNDE6	768	f	0	200	94	73	673	19.62	(13.90- 27.67)
WYNDE6	769	f	0	291	91	73	673	29.48	(21.04- 41.31)
Subtotal WYNDE6								19.46	(16.87- 22.45)
ZHENG	563	m	0	28	66	33	94	1.21	(0.67- 2.19)
ZHENG	564	m	0	145	109	33	94	3.79	(2.37- 6.05)
ZHENG	565	m	0	106	43	33	94	7.02	(4.13- 11.95)
ZHENG	572	f	0	16	16	152	184	1.21	(0.59- 2.50)
ZHENG	573	f	0	60	28	152	184	2.59	(1.58- 4.27)
Subtotal ZHENG								2.86	(2.24- 3.65)
Partial Totals				24315	981571	9718	2461836		

*prospective study ~ With 0.5 adjustment for zero

Table 1H16 - 5

IESLC - Meta-analysis of Ever/current Smoking by Age started, Overview
All LC types, Cigarettes (or Any Product if Cigarettes not available)
Least adjusted

REF	NRR	SEX	AD	Ys	Ws	Qs	Ps
AGUDO	501	f	0	0.29	4.10	3.80	0.5594
AGUDO	502	f	0	1.20	5.84	0.01	0.0037
Subtotal AGUDO				0.83	9.93	3.81	
ARMADA	511	m	0	1.94	6.46	3.07	0.0000
ARMADA	512	m	0	2.80	6.53	15.69	0.0000
Subtotal ARMADA				2.37	12.99	18.76	
AUVINE	509	c	0	2.68	12.94	26.58	0.0000
AUVINE	510	c	0	2.70	17.93	37.88	0.0000
AUVINE	511	c	0	3.87	4.72	32.24	0.0000
Subtotal AUVINE				2.85	35.58	96.70	
BARBON	515	m	0	2.11	16.50	12.20	0.0000
BARBON	516	m	0	2.30	17.77	19.71	0.0000
BARBON	517	m	0	3.94	9.85	71.08	0.0000
Subtotal BARBON				2.60	44.12	102.99	
BRESLO	501	c	0	0.99	7.67	0.52	0.0060
BRESLO	502	c	0	1.24	12.80	0.00	0.0000
BRESLO	503	c	0	1.44	11.75	0.42	0.0000
Subtotal BRESLO				1.25	32.22	0.93	
BUFFLE	517	f	0	2.23	5.58	5.39	0.0000
BUFFLE	518	f	0	2.56	7.00	11.94	0.0000
BUFFLE	519	f	0	2.56	6.59	11.21	0.0000
BUFFLE	520	f	0	2.71	7.07	14.99	0.0000
BUFFLE	521	f	0	2.88	7.72	20.41	0.0000
Subtotal BUFFLE				2.61	33.96	63.94	
*CEDERL	510	m	1	1.87	4.29	1.65	0.0001
*CEDERL	511	m	1	2.28	4.14	4.40	0.0000
*CEDERL	512	m	1	1.86	3.51	1.29	0.0005
*CEDERL	515	f	0	0.69	4.57	1.45	0.1414
*CEDERL	516	f	0	0.61	1.81	0.74	0.4098
*CEDERL	517	f	0	-0.50	0.49	1.49	0.7276
Subtotal CEDERL				1.49	18.81	11.02	
CHEN2	517	m	0	1.77	2.14	0.58	0.0096
CHEN2	518	m	0	1.45	4.63	0.18	0.0018
CHEN2	519	m	0	1.53	5.94	0.45	0.0002
CHEN2	522	f	0	-0.19	2.53	5.27	0.7596
CHEN2	523	f	0	0.40	3.26	2.39	0.4750
CHEN2	524	f	0	0.85	5.24	0.85	0.0521
Subtotal CHEN2				1.04	23.76	9.71	
CHIAZZ	501	m	2	1.10	0.75	0.02	0.3421
CHIAZZ	502	m	2	2.99	0.95	2.87	0.0036
Subtotal CHIAZZ				2.16	1.70	2.89	
CHOI	523	m	0	1.23	7.80	0.00	0.0006
CHOI	524	m	0	1.41	10.06	0.25	0.0000
CHOI	525	m	0	1.43	9.31	0.30	0.0000
CHOI	526	m	0	2.19	5.31	4.67	0.0000
CHOI	530	f	0	0.26	7.94	7.83	0.4667
CHOI	531	f	0	2.16	0.79	0.64	0.0557
Subtotal CHOI				1.27	41.20	13.70	
*CPSI	801	m	1	1.41	27.80	0.67	0.0000
*CPSI	802	m	1	2.31	58.36	65.50	0.0000
*CPSI	803	m	1	2.69	72.82	150.18	0.0000
*CPSI	804	m	1	2.82	57.18	140.68	0.0000
*CPSI	845	f	1	0.81	38.69	7.50	0.0000
*CPSI	846	f	1	1.22	28.12	0.03	0.0000
*CPSI	847	f	1	1.61	39.48	5.07	0.0000
*CPSI	848	f	1	0.92	5.80	0.65	0.0273
Subtotal CPSI				2.03	328.25	370.28	
DAMBER	501	m	0	1.52	17.84	1.27	0.0000
DAMBER	502	m	0	1.92	26.52	11.77	0.0000
DAMBER	503	m	0	2.34	22.90	27.29	0.0000
Subtotal DAMBER				1.96	67.25	40.32	
DEAN3	564	m	0	1.92	10.14	4.49	0.0000
DEAN3	565	m	0	1.93	14.48	6.60	0.0000
DEAN3	566	m	0	1.95	19.25	9.33	0.0000
DEAN3	567	m	0	1.73	13.81	3.23	0.0000
DEAN3	583	f	0	1.31	15.21	0.05	0.0000
DEAN3	584	f	0	1.08	11.77	0.34	0.0002
DEAN3	585	f	0	1.07	18.99	0.65	0.0000
DEAN3	586	f	0	0.88	5.65	0.78	0.0367
Subtotal DEAN3				1.52	109.30	25.48	
DOLL	501	m	0	1.61	1.81	0.23	0.0307

Table 1H16 - 5

IESLC - Meta-analysis of Ever/current Smoking by Age started, Overview
All LC types, Cigarettes (or Any Product if Cigarettes not available)
Least adjusted

REF	NRR	SEX	AD	Ys	Ws	Qs	Ps
DOLL	502	m	0	1.56	4.08	0.39	0.0016
DOLL	503	m	0	2.11	5.99	4.46	0.0000
DOLL	504	m	0	2.25	6.20	6.16	0.0000
DOLL	508	f	0	0.39	5.71	4.24	0.3532
DOLL	509	f	0	0.75	3.51	0.90	0.1625
DOLL	510	f	0	0.82	6.57	1.24	0.0364
DOLL	511	f	0	0.90	5.71	0.71	0.0317
Subtotal DOLL				1.29	39.58	18.32	
*DORN	610	m	0	1.47	14.92	0.69	0.0000
*DORN	611	m	0	2.21	21.57	19.66	0.0000
*DORN	612	m	0	2.62	23.30	43.62	0.0000
*DORN	613	m	0	2.99	19.28	57.98	0.0000
*DORN	647	m	0	1.44	31.75	1.14	0.0000
*DORN	648	m	0	1.89	38.11	15.68	0.0000
*DORN	649	m	0	2.20	42.26	38.21	0.0000
*DORN	650	m	0	2.44	30.57	43.37	0.0000
Subtotal DORN				2.14	221.77	220.35	
*ENGELA	501	m	0	1.33	10.44	0.07	0.0000
*ENGELA	502	m	0	1.28	17.55	0.02	0.0000
*ENGELA	503	m	0	2.00	23.38	13.24	0.0000
*ENGELA	509	f	0	1.00	7.56	0.46	0.0058
*ENGELA	510	f	0	2.10	16.67	11.96	0.0000
*ENGELA	511	f	0	2.42	11.40	15.68	0.0000
Subtotal ENGELA				1.76	87.00	41.42	
GAO	501	m	0	0.13	19.59	24.71	0.5711
GAO	502	m	0	1.51	36.16	2.37	0.0000
GAO	503	m	0	1.63	32.38	4.68	0.0000
GAO	511	f	0	0.46	30.05	18.74	0.0114
GAO	512	f	0	1.08	25.10	0.72	0.0000
GAO	513	f	0	1.45	17.56	0.73	0.0000
Subtotal GAO				1.10	160.85	51.95	
GAO2	501	m	0	0.77	1.18	0.28	0.4038
GAO2	502	m	0	1.86	8.74	3.26	0.0000
GAO2	503	m	0	2.15	6.56	5.34	0.0000
Subtotal GAO2				1.90	16.48	8.88	
GENG	528	f	0	0.44	10.28	6.73	0.1565
GENG	529	f	0	1.07	10.16	0.33	0.0006
GENG	530	f	0	1.82	6.37	2.09	0.0000
Subtotal GENG				1.01	26.81	9.16	
HAENSZ	537	f	0	0.66	18.36	6.33	0.0044
HAENSZ	538	f	0	0.86	13.00	1.99	0.0019
Subtotal HAENSZ				0.75	31.36	8.32	
HEGMAN	513	m	1	2.24	7.47	7.32	0.0000
HEGMAN	514	m	1	3.10	10.02	34.42	0.0000
HEGMAN	516	f	1	1.57	1.60	0.16	0.0470
HEGMAN	517	f	1	3.29	12.44	51.62	0.0000
Subtotal HEGMAN				2.89	31.53	93.52	
*HIRAYA	501	m	1	1.47	83.52	4.01	0.0000
*HIRAYA	502	m	1	1.74	67.55	16.29	0.0000
*HIRAYA	504	f	1	0.90	65.73	8.09	0.0000
*HIRAYA	505	f	1	-0.25	0.91	2.04	0.8127
Subtotal HIRAYA				1.38	217.71	30.44	
HU	511	m	0	0.13	6.22	7.76	0.7374
HU	512	m	0	0.82	15.39	2.87	0.0013
HU	513	m	0	1.11	3.86	0.08	0.0292
HU	516	f	0	-0.33	1.73	4.31	0.6660
HU	517	f	0	0.88	4.71	0.66	0.0575
HU	518	f	0	0.41	2.02	1.44	0.5647
Subtotal HU				0.65	33.92	17.12	
HU2	501	c	0	-0.09	8.76	15.85	0.7814
HU2	502	c	0	-0.06	15.16	25.91	0.8268
HU2	503	c	0	0.93	42.35	4.36	0.0000
HU2	504	c	0	1.21	28.23	0.06	0.0000
Subtotal HU2				0.76	94.50	46.17	
JEDRYC	607	m	0	1.99	27.77	15.17	0.0000
JEDRYC	608	m	0	2.21	21.04	19.46	0.0000
JEDRYC	619	f	0	2.50	7.96	12.43	0.0000
Subtotal JEDRYC				2.14	56.77	47.05	
JOLY	543	m	0	1.54	6.34	0.53	0.0001
JOLY	544	m	0	2.40	10.49	13.89	0.0000
JOLY	545	m	0	3.02	10.57	32.94	0.0000

Table 1H16 - 5

IESLC - Meta-analysis of Ever/current Smoking by Age started, Overview
All LC types, Cigarettes (or Any Product if Cigarettes not available)
Least adjusted

REF	NRR	SEX	AD	Ys	Ws	Qs	Ps
JOLY	533	f	0	1.12	11.03	0.20	0.0002
JOLY	534	f	0	2.05	16.96	10.79	0.0000
JOLY	535	f	0	2.47	15.51	23.02	0.0000
Subtotal JOLY				2.15	70.89	81.38	
KHUDER	506	m	0	1.85	14.88	5.35	0.0000
KHUDER	507	m	0	1.86	17.89	6.55	0.0000
KHUDER	508	m	0	2.33	18.34	21.40	0.0000
Subtotal KHUDER				2.03	51.12	33.30	
KOULUM	501	m	0	2.38	2.13	2.72	0.0005
KOULUM	502	m	0	2.27	4.00	4.14	0.0000
KOULUM	503	m	0	3.33	4.31	18.67	0.0000
KOULUM	504	m	0	3.72	4.12	25.14	0.0000
KOULUM	505	m	0	4.57	3.47	38.24	0.0000
Subtotal KOULUM				3.31	18.03	88.91	
LETOUR	501	c	0	2.39	17.33	22.67	0.0000
LETOUR	502	c	0	2.48	18.69	28.32	0.0000
LETOUR	503	c	0	2.92	15.17	42.26	0.0000
Subtotal LETOUR				2.58	51.19	93.25	
*LIAW	504	c	2	0.41	6.39	4.57	0.3054
*LIAW	505	c	2	1.77	8.74	2.40	0.0000
*LIAW	506	c	2	1.53	11.90	0.90	0.0000
Subtotal LIAW				1.34	27.03	7.87	
LIU3	501	m	0	0.17	2.74	3.19	0.7761
LIU3	502	m	0	0.20	2.92	3.26	0.7380
Subtotal LIU3				0.18	5.66	6.45	
LIU4	501	m	2	0.88	3072.74	424.02	0.0000
LIU4	502	m	2	1.05	4361.66	174.96	0.0000
LIU4	503	m	2	1.34	4224.99	31.63	0.0000
Subtotal LIU4				1.11	11659.40	630.61	
LIU5	501	c	0	-0.07	5.40	9.43	0.8697
LIU5	502	c	0	0.86	10.25	1.56	0.0058
Subtotal LIU5				0.54	15.65	10.99	
LUBIN	565	m	0	0.29	6.10	5.60	0.4689
LUBIN	566	m	0	1.27	6.79	0.00	0.0009
LUBIN	567	m	0	1.21	7.09	0.01	0.0013
LUBIN	568	m	0	1.74	7.43	1.75	0.0000
Subtotal LUBIN				1.16	27.42	7.36	
LUBIN2	1147	m	0	1.14	39.73	0.50	0.0000
LUBIN2	1148	m	0	1.68	115.66	21.10	0.0000
LUBIN2	1149	m	0	1.80	146.52	43.39	0.0000
LUBIN2	1150	m	0	1.98	138.01	73.97	0.0000
LUBIN2	1151	m	0	2.08	76.32	52.86	0.0000
Subtotal LUBIN2				1.81	516.24	191.81	
MATOS	556	m	0	1.34	6.69	0.06	0.0005
MATOS	557	m	0	2.03	8.38	5.03	0.0000
MATOS	558	m	0	2.04	7.96	4.92	0.0000
Subtotal MATOS				1.83	23.04	10.01	
*MIGRAN	501	m	0	2.05	3.46	2.22	0.0001
*MIGRAN	503	m	0	1.94	3.77	1.77	0.0002
*MIGRAN	505	m	0	2.31	3.73	4.15	0.0000
*MIGRAN	511	f	0	2.08	2.94	2.00	0.0004
*MIGRAN	513	f	0	2.12	2.78	2.07	0.0004
*MIGRAN	515	f	0	1.97	1.34	0.69	0.0226
Subtotal MIGRAN				2.09	18.02	12.91	
*MRFITR	508	m	0	3.17	0.44	1.62	0.0357
*MRFITR	509	m	0	4.10	0.46	3.76	0.0052
*MRFITR	510	m	0	3.30	0.47	1.97	0.0238
*MRFITR	511	m	0	3.92	0.49	3.50	0.0060
*MRFITR	512	m	0	4.21	0.49	4.32	0.0031
*MRFITR	513	m	0	3.83	0.49	3.25	0.0074
Subtotal MRFITR				3.77	2.85	18.42	
PERNU	504	m	0	2.15	56.20	44.95	0.0000
PERNU	505	m	0	2.34	35.99	42.71	0.0000
PERNU	501	f	0	0.58	13.00	5.86	0.0366
PERNU	502	f	0	3.27	0.37	1.53	0.0455
Subtotal PERNU				2.02	105.57	95.05	
PEZZOT	570	m	0	2.43	3.42	4.73	0.0000
PEZZOT	571	m	0	3.16	3.65	13.32	0.0000
PEZZOT	572	m	0	3.11	3.42	11.85	0.0000
Subtotal PEZZOT				2.91	10.48	29.89	
*QIAO2	501	m	0	0.64	8.52	3.20	0.0623

Table 1H16 - 5

IESLC - Meta-analysis of Ever/current Smoking by Age started, Overview
All LC types, Cigarettes (or Any Product if Cigarettes not available)
Least adjusted

REF	NRR	SEX	AD	Ys	Ws	Qs	Ps
*QIAO2	502	m	0	0.63	8.96	3.49	0.0604
*QIAO2	503	m	0	1.24	9.28	0.00	0.0002
Subtotal QIAO2				0.84	26.77	6.69	
RACHTA	501	f	0	1.78	2.41	0.68	0.0057
RACHTA	502	f	0	1.42	7.35	0.20	0.0001
RACHTA	503	f	0	2.00	9.31	5.16	0.0000
Subtotal RACHTA				1.75	19.07	6.04	
SEGI2	516	m	0	0.74	5.86	1.53	0.0736
SEGI2	517	m	0	1.31	6.40	0.02	0.0009
SEGI2	518	m	0	1.77	6.08	1.62	0.0000
Subtotal SEGI2				1.28	18.33	3.17	
SOBUE	654	m	0	1.37	16.73	0.25	0.0000
SOBUE	655	m	0	1.47	22.22	1.11	0.0000
SOBUE	656	m	0	2.26	15.19	15.51	0.0000
Subtotal SOBUE				1.66	54.14	16.87	
SUZUK2	501	c	0	1.25	4.59	0.00	0.0072
SUZUK2	502	c	0	2.09	6.59	4.68	0.0000
SUZUK2	503	c	0	2.70	4.13	8.72	0.0000
Subtotal SUZUK2				2.01	15.32	13.40	
SVENSS	501	f	0	1.73	8.23	1.85	0.0000
SVENSS	502	f	0	2.57	8.11	14.13	0.0000
SVENSS	503	f	0	2.06	9.85	6.39	0.0000
Subtotal SVENSS				2.11	26.19	22.38	
TIZZAN	506	m	0	-0.77	8.70	35.62	0.0228
TIZZAN	507	m	0	0.47	66.40	40.05	0.0001
TIZZAN	508	m	0	0.81	82.27	16.30	0.0000
TIZZAN	536	f	0	-0.94	1.39	6.71	0.2659
TIZZAN	537	f	0	-0.59	6.74	22.75	0.1283
TIZZAN	538	f	0	1.68	1.64	0.30	0.0314
Subtotal TIZZAN				0.53	167.16	121.73	
WAKAI	501	m	0	0.73	3.57	0.96	0.1666
WAKAI	502	m	0	1.53	7.78	0.60	0.0000
WAKAI	503	m	0	1.31	6.55	0.02	0.0008
Subtotal WAKAI				1.29	17.89	1.58	
WU	517	f	0	0.78	5.98	1.31	0.0557
WU	518	f	0	1.69	8.80	1.66	0.0000
WU	519	f	0	2.29	11.93	12.77	0.0000
Subtotal WU				1.75	26.72	15.74	
WYNDE6	759	m	0	2.63	24.28	46.46	0.0000
WYNDE6	760	m	0	2.92	30.32	84.37	0.0000
WYNDE6	761	m	0	3.15	38.07	137.95	0.0000
WYNDE6	767	f	0	2.57	29.27	50.57	0.0000
WYNDE6	768	f	0	2.98	32.44	96.56	0.0000
WYNDE6	769	f	0	3.38	33.77	153.60	0.0000
Subtotal WYNDE6				2.97	188.15	569.52	
ZHENG	563	m	0	0.19	10.89	12.28	0.5320
ZHENG	564	m	0	1.33	17.54	0.12	0.0000
ZHENG	565	m	0	1.95	13.58	6.62	0.0000
ZHENG	572	f	0	0.19	7.30	8.20	0.6057
ZHENG	573	f	0	0.95	15.53	1.38	0.0002
Subtotal ZHENG				1.05	64.84	28.59	

N	213
NS	51

Table 1H16 - 6

IESLC - Meta-analysis of Ever/current Smoking by Age started, Overview
All LC types, Cigarettes (or Any Product if Cigarettes not available)
Least adjusted

	Sex			
	combined	male	female	Total
N	21	121	71	213
NS	7	37	25	69

In this overview table, other than the "N" rows, entries in the "absent" and "Total" columns may be invalid and should be ignored

	Age started (broad categories)				
	absent	19+k26	15-25k18	1-17k14	Total
N	87	61	30	35	213
NS	37	46	23	26	132
Wt	9674.60	4068.02	675.62	586.28	15004.53
Het Chi	961.62	556.97	201.65	227.55	3477.17
Het df	86	60	29	34	212
Het P	***	***	***	***	***
Fixed RR	3.41	2.72	8.05	11.24	3.49
RRl	3.34	2.64	7.46	10.36	3.44
RRu	3.48	2.81	8.68	12.18	3.55
P	+++	+++	+++	+++	+++
Random RR	4.24	3.97	7.76	10.57	5.30
RRl	3.79	3.39	6.24	8.34	4.87
RRu	4.74	4.66	9.65	13.41	5.78
P	+++	+++	+++	+++	+++

	Age started (narrow categories)							
	absent	27+k30	23-29k26	19-25k22	15-21k18	11-17k14	1-13k10	Total
N	150	16	1	14	26	2	4	213
NS	51	11	1	11	20	2	4	100
Wt	9433.02	138.37	6.79	4583.73	613.15	142.13	87.34	15004.53
Het Chi	2188.61	44.17	0.00	186.27	182.45	12.09	24.20	3477.17
Het df	149	15	0	13	25	1	3	212
Het P	***	***	N.S.	***	***	***	***	***
Fixed RR	3.54	1.62	3.56	2.97	8.34	7.64	9.51	3.49
RRl	3.47	1.38	1.68	2.89	7.70	6.48	7.71	3.44
RRu	3.61	1.92	7.56	3.06	9.02	9.01	11.72	3.55
P	+++	+++	+++	+++	+++	+++	+++	+++
Random RR	5.20	1.76	3.56	5.49	8.11	16.19	21.16	5.30
RRl	4.67	1.29	1.68	3.78	6.40	2.96	7.08	4.87
RRu	5.80	2.40	7.56	7.96	10.29	88.51	63.23	5.78
P	+++	+++	+++	+++	+++	++	+++	+++

Table 1H16 - 6

IESLC - Meta-analysis of Ever/current Smoking by Age started, Overview
All LC types, Cigarettes (or Any Product if Cigarettes not available)
Least adjusted

MALES

	Age started (broad categories)				
	absent	19+k26	15-25k18	1-17k14	Total
N	46	34	19	22	121
NS	26	33	18	21	98
Wt	9297.46	3680.12	496.51	475.61	13949.69
Het Chi	690.00	329.10	110.96	127.55	2514.87
Het df	45	33	18	21	120
Het P	***	***	***	***	***
Fixed RR	3.41	2.66	8.22	10.94	3.43
RRl	3.34	2.58	7.53	10.00	3.37
RRu	3.48	2.75	8.98	11.97	3.48
P	+++	+++	+++	+++	+++
Random RR	4.77	4.59	8.22	11.58	6.16
RRl	4.14	3.72	6.42	9.01	5.55
RRu	5.49	5.65	10.53	14.88	6.83
P	+++	+++	+++	+++	+++

		Age started (narrow categories)						
	absent	27+k30	23-29k26	19-25k22	15-21k18	11-17k14	1-13k10	Total
N	81	8	1	9	17	2	3	121
NS	37	8	1	8	16	2	3	75
Wt	8673.49	62.08	6.79	4518.19	463.80	142.13	83.21	13949.69
Het Chi	1519.67	16.93	0.00	160.64	100.90	12.09	23.32	2514.87
Het df	80	7	0	8	16	1	2	120
Het P	***	*	N.S.	***	***	***	***	***
Fixed RR	3.46	1.65	3.56	2.96	8.42	7.64	9.29	3.43
RRl	3.39	1.28	1.68	2.87	7.69	6.48	7.50	3.37
RRu	3.54	2.11	7.56	3.04	9.22	9.01	11.52	3.48
P	+++	+++	+++	+++	+++	+++	+++	+++
Random RR	6.12	1.79	3.56	5.72	8.44	16.19	24.40	6.16
RRl	5.34	1.18	1.68	3.42	6.50	2.96	5.26	5.55
RRu	7.00	2.73	7.56	9.55	10.97	88.51	113.15	6.83
P	+++	++	+++	+++	+++	++	+++	+++

FEMALES

	Age started (broad categories)				
	absent	19+k26	15-25k18	1-17k14	Total
N	32	21	8	10	71
NS	20	21	8	10	59
Wt	277.97	296.64	129.70	79.04	783.34
Het Chi	197.73	146.14	68.98	73.46	639.00
Het df	31	20	7	9	70
Het P	***	***	***	***	***
Fixed RR	3.93	3.16	6.99	12.74	4.48
RRl	3.49	2.82	5.88	10.22	4.18
RRu	4.42	3.54	8.30	15.88	4.81
P	+++	+++	+++	+++	+++
Random RR	3.79	3.01	6.21	6.37	3.98
RRl	2.77	2.16	3.43	2.99	3.17
RRu	5.19	4.19	11.24	13.54	4.98
P	+++	+++	+++	+++	+++

Table 1H16 - 6

IESLC - Meta-analysis of Ever/current Smoking by Age started, Overview
All LC types, Cigarettes (or Any Product if Cigarettes not available)
Least adjusted

FEMALES

		Age started (narrow categories)						
	absent	27+k30	23-29k26	19-25k22	15-21k18	11-17k14	1-13k10	Total
N	54	6		4	7			71
NS	25	6		4	7			42
Wt	558.08	55.73		56.80	112.74			783.34
Het Chi	478.12	19.21		13.80	68.76			639.00
Het df	53	5		3	6			70
Het P	***	**		**	***			***
Fixed RR	4.49	1.96		4.29	6.88			4.48
RRl	4.13	1.50		3.30	5.72			4.18
RRu	4.87	2.54		5.56	8.27			4.81
P	+++	+++		+++	+++			+++
Random RR	3.94	2.16		4.95	5.96			3.98
RRl	3.02	1.18		2.73	2.96			3.17
RRu	5.15	3.96		8.99	11.97			4.98
P	+++	+		+++	+++			+++

Table 1H16 - 7

IESLC - Meta-analysis of Ever/current Smoking by Age started, Overview
All LC types, Cigarettes (or Any Product if Cigarettes not available)
Excluded studies (and stage at which they were excluded)

1	BECHER BLOT1 BROWN3 CARPEN CHYOU DARBY DOLL2 GARCIA GRAHAM GURSEL HAMMO2 JAHN JAIN LAUSSM PRESCO QIAO TVERDA WIGLE WYNDE3
2	AKIBA AMANDU AMES AXELSS BENSHL BEST BOUCHA BOUCOT BROSS CHEN CPSII DEAN2 DESTEF DORGAN DOSEME FAN GARSHI GER GILLIS HAMMON HUMBLE JUSSAW KAISE2 KATSOU KAUFMA KOO KREUZE LEVIN MCCONN NOTAN2 OSANN2 PEZZO2 PISANI RESTRE SADOWS VUTUC WANG2 WATSON WU2 WUWILL WYNDE2 WYNDE8 XU ZHOU
3	GUO MCDUFF SPITZ STASZE ZHANG
4	LUO
5	CORREA HOLE YUAN
7	BOFFET WYNDE7
10	ALDERS SPEIZE
14	BENHAM

Table 1H16 - 8
Potentially overlapping studies

REF	REFGP	PRINC	.	OVERLAP/LINK
LUBIN2	LUBIN2	1		Lubin-combined
MRFITR	MRFIT	2		Subset of MRFIT
WYNDE6	WYNDE6	1		WYNDE5/6/7/8
CPSI	CPSI	1		CPSI overall
LUBIN	XIANGZ	2		LUBIN/XIANGZ/QIAO

Table 1H16 - 9
Most adjusted - insufficient data for meta-analysis

REF	NRR	SEX	AGEL	AGEH	RACE	YF	LC TYPE	LOC	START	ST	NLC	R	VB	P	H	AD	SM	PRODUCT	exL	exH	S1	S2	DENOM	De
CORREA	535	c	0	0	all	-	all	NAmer	1979	CC	1359	n	bl	y	n	2	ev	cig+/-ot	21	999	1	0	nev cigs	or
CORREA	536	c	0	0	all	-	all	NAmer	1979	CC	1359	n	bl	y	n	2	ev	cig+/-ot	16	20	2	4	nev cigs	or
CORREA	537	c	0	0	all	-	all	NAmer	1979	CC	1359	n	bl	y	n	2	ev	cig+/-ot	1	15	3	0	nev cigs	or
JEDRYC	606	m	0	0	all	-	all	Eu:est	1980	CC	1630	n	bl	y	n	0	ev	cig+/-ot	18	999	0	0	nev any	st
JEDRYC	618	f	0	0	all	-	all	Eu:est	1980	CC	1630	n	bl	y	n	0	ev	cig+/-ot	23	999	1	0	nev any	st

REF	NRR	RR	SIG	RRDATA comment
CORREA	535	8.30		0
CORREA	536	17.40		0
CORREA	537	24.20		0
JEDRYC	606	*		0
JEDRYC	618	*		0

Table 1H17 -

IESLC - Meta-analysis of Ever/current Smoking, Age started, "Low"
All LC types, Cigarettes (or Any Product if Cigarettes not available)

This analysis is restricted to results for:
1) Ever/current smokers
2) Results by Age started
3) Categorical results by Age started
4) All LC types (or near equivalent)
5) Results complete enough for use in metaanalysis

Within each study, results are then selected (in the following order of preference, within each sex) for:
6) SMKSTA: ever, current
7) PRODUCT: cigarettes regardless of other products, cigarettes only, all/unspec
8) CIGTYPE: all/unspecified, MC regardless of HR, MC only
9) (not applicable)
10) DENOM: never smoked anything, never smoked cigarettes, never any + low, never cigs + low
11) Followup period (YF, prospective studies): whole study (coded as 0) or longest available
12) LCtype: all or nearest available, at least Squamous and Adeno. (q = squamous, s = small, l = large, a = adeno, mix = mixed, alv = alveolar)
13) Race: all or nearest available, otherwise by race (wh or w = white, bl or b = black, hi = hispanic ch = chinese, jap = japanese, haw = hawaiian, w+o = white + oriental, sca = scandinavian, as = asian)
14) Age started "low" in key scheme 1 (key value 26, maximum range 19+)
15) For overlapping studies: principal rather than subsidiary studies
Finally by Age: whole study (coded as 0) if available, otherwise by widest available age group and then for single sex results (m, f) in preference to results for both sexes combined (c).

Results adjusted (AD) for the most potential confounders are then chosen in Sections -1 to -3 (and those which actually differ from the adjusted results in Table 1H12 - 1 are marked 'x' in Section -1) and results adjusted for the least confounders in Sections -4 to -6. (Those least adjusted results which actually differ from the most adjusted are marked 'x' in column X in Section -4)

Section -7 shows excluded studies, together with the stage (as above) at which no qualifying results were found.

Section -8 lists the potentially overlapping studies which have been included (1=principal, 2=subsidiary).

Section -9 lists any results which would have been included in preference except that they had data not complete enough for use in meta-analysis, with their significance (yes/no), if known, and any further comment as entered on the database. It also lists as "gap" any categories for which no data were presented by the original authors.

In addition to those mentioned above, the following fields, levels and abbreviations are used:
* or nk = not known, n = no, y = yes, ot = other
ev = ever, cu = current, nev = never
all/unspec = all or unspecified, cig+/-ot = cigarettes irrespective of other products (cigar, pipe etc)
MC = manufactured cigarettes, HR = hand-rolled cigarettes
exL, exH = range of exposure (low and high) in the smoking group, in terms of Age started
REF: 6-character study reference
NRR: number of the RR on the database within the study
ST : study type (CC = case control, pr or prosp = prospective)
NLC: number of lung cancer cases in whole study
R : risky occupational population (n = no, m = mining, o = other risky)
VB : national cigarette type (V = at least 75% Virginia, bl = at least 75% blended, ot = other)
P : any proxy use
H : full histological confirmation
De : derivation of RR/CI (or = original, st = standard method, ot = other method of estimation)

Table 1H17 - 1

IESLC - Meta-analysis of Ever/current Smoking, Age started, "Low"
All LC types, Cigarettes (or Any Product if Cigarettes not available)
Most adjusted

REF	NRR	1H12	SEX	AGEL	AGEH	RACE	YF	LC TYPE	LOC	START	ST	NLC	R	VB	P	H	AD	SM	PRODUCT	exL	exH	DENOM	De
AGUDO	504		f	0	0	all	-	all	Eu:wst	1989	CC	103	n	bl	n	n	3	ev	cig only	24	999	nev cigs	or
AUVINE	509		c	0	0	all	-	all	Eu:Sca	1986	CC	517	n	bl	y	n	0	ev	cig+/-ot	21	999	nev cigs	st
BARBON	520		m	0	0	all	-	all	Eu:wst	1979	CC	755	n	bl	y	y	1	ev	all/unsp	20	999	nev any	or
BRESLO	501		c	0	0	all	-	all	NAmer	1949	CC	518	n	bl	n	y	0	ev	cig+/-ot	25	999	nev any	st
BUFFLE	518		f	0	0	w-hi	-	all	NAmer	1976	CC	943	n	bl	n	n	0	ev	cig+/-ot	21	29	nev cigs	or
CEDERL	510		m	0	0	all	10	all	Eu:Sca	1963	pr	491	n	bl	n	n	1	cu	cig only	19	999	nev any	ot
CEDERL	515		f	0	0	all	10	all	Eu:Sca	1963	pr	491	n	bl	n	n	0	cu	cig only	19	999	nev any	st
CHEN2	518		m	0	0	all	-	all	As:Chi	1983	CC	193	n	ot	y	n	0	ev	all/unsp	20	30	nev any	st
CHEN2	523		f	0	0	all	-	all	As:Chi	1983	CC	193	n	ot	y	n	0	ev	all/unsp	20	30	nev any	st
CHIAZZ	501		m	0	0	all	-	all	NAmer	1940	CC	144	o	bl	y	n	2	ev	cig+/-ot	20	999	nev cigs	or
CHOI	523		m	0	0	all	-	all	As:oth	1985	CC	375	n	bl	n	n	0	ev	cig+/-ot	25	999	nev cigs	st
CHOI	530		f	0	0	all	-	all	As:oth	1985	CC	375	n	bl	n	n	0	ev	cig+/-ot	25	999	nev cigs	st
CPSI	801		m	35	84	all	6	all	NAmer	1959	pr	5138	n	bl	n	n	1	cu	cig+/-ot	25	999	nev any	ot
CPSI	845		f	40	74	all	6	all	NAmer	1959	pr	5138	n	bl	n	n	1	cu	cig only	25	999	nev any	ot
DAMBER	501		m	0	0	all	-	all	Eu:Sca	1972	CC	579	n	bl	y	n	0	ev	all/unsp	21	999	nev any	st
DEAN3	564		m	0	0	all	-	all	Eu:UK	1969	CC	766	n	V	y	n	0	cu	cig only	25	999	nev any	st
DEAN3	583		f	0	0	all	-	all	Eu:UK	1969	CC	766	n	V	y	n	0	cu	cig only	25	999	nev any	st
DOLL	503		m	0	0	all	-	all	Eu:UK	1948	CC	1465	n	V	n	n	0	ev	all/unsp	20	29	nev any	st
DOLL	510		f	0	0	all	-	all	Eu:UK	1948	CC	1465	n	V	n	n	0	ev	all/unsp	20	29	nev any	st
DORN	610		m	55	64	wh	8	all	NAmer	1954	pr	5097	n	bl	n	n	0	ev	cig+/-ot	25	999	nev any	st
DORN	647		m	65	74	wh	8	all	NAmer	1954	pr	5097	n	bl	n	n	0	ev	cig+/-ot	25	999	nev any	st
ENGELA	502		m	0	0	all	0	all	Eu:Sca	1964	pr	435	n	bl	n	n	0	cu	cig+/-ot	20	29	nev cigs	st
ENGELA	510		f	0	0	all	0	all	Eu:Sca	1964	pr	435	n	bl	n	n	0	cu	cig+/-ot	20	29	nev cigs	st
GAO	507		m	0	0	all	-	all	As:Chi	1984	CC	1405	n	ot	n	n	2	ev	cig+/-ot	20	29	nev cigs	or
GAO	517		f	0	0	all	-	all	As:Chi	1984	CC	1405	n	ot	n	n	2	ev	cig+/-ot	20	29	nev cigs	or
GAO2	502		m	0	0	all	-	all	As:Jap	1988	CC	282	n	bl	n	n	0	cu	cig+/-ot	20	29	nev cigs	st
GENG	533		f	0	0	all	-	all	As:Chi	1985	CC	292	n	ot	*	n	1	ev	cig+/-ot	21	999	nev any	st
HAENSZ	537		f	0	0	all	-	not alv	NAmer	1955	CC	158	n	bl	n	y	0	ev	cig+/-ot	25	999	nev any	st
HEGMAN	513		m	0	0	all	-	all	NAmer	1989	CC	282	n	bl	y	y	1	ev	all/unsp	20	999	nev any	or
HEGMAN	516		f	0	0	all	-	all	NAmer	1989	CC	282	n	bl	y	y	1	ev	all/unsp	26	999	nev any	or
HIRAYA	501		m	0	0	all	0	all	As:Jap	1965	pr	1917	n	bl	n	n	1	cu	cig+/-ot	20	999	nev any	st
HIRAYA	504		f	0	0	all	0	all	As:Jap	1965	pr	1917	n	bl	n	n	1	cu	cig+/-ot	20	999	nev any	st
HU2	503		c	0	0	all	-	all	As:Chi	1977	CC	523	n	ot	y	n	0	ev	cig+/-ot	20	29	nev cigs	st
JOLY	543		m	0	0	all	-	all	SCAmer	1978	CC	826	n	bl	n	n	0	ev	cig+/-ot	25	999	nev any	st
JOLY	533		f	0	0	all	-	all	SCAmer	1978	CC	826	n	bl	n	n	0	ev	cig+/-ot	25	999	nev any	st
KHUDER	506		m	0	0	all	-	all	NAmer	1985	CC	482	n	bl	n	y	0	ev	cig+/-ot	20	999	nev cigs	st
KOULUM	502		m	0	0	all	-	all	Eu:Sca	1936	CC	812	n	bl	n	n	0	ev	all/unsp	21	30	nev any	st
LETOUR	501		c	0	0	all	-	all	NAmer	1983	CC	738	n	V	y	y	0	ev	cig+/-ot	21	999	nev cigs	st
LIAW	504		c	0	0	all	0	all	As:oth	1982	pr	127	n	ot	n	n	2	cu	all/unsp	25	999	nev any	or
LIU3	504		m	0	0	all	-	all	As:Chi	1985	CC	110	n	ot	n	n	2	ev	all/unsp	21	999	nev any	or
LIU4	501		m	35	69	all	-	all	As:Chi	1986	CC	1000-00	n	ot	y	n	2	ev	all/unsp	25	999	nev any	ot
LUBIN	566		m	0	0	all	-	all	As:Chi	1984	CC	427	m	ot	y	n	0	ev	cig+/-ot	23	26	nev any	st
LUBIN2	1157		m	0	0	all	-	all	Eu:mul	1976	CC	7804	n	bl	n	y	1	ev	cig+/-ot	21	30	nev cigs	st
MATOS	576		m	0	0	all	-	all	SCAmer	1994	CC	200	n	bl	n	n	2	ev	cig+/-ot	20	999	nev any	or
MIGRAN	501		m	0	0	all	0	all	Eu:UK	1964	pr	259	n	V	n	n	0	cu	cig only	20	999	nev any	st
MIGRAN	511		f	0	0	all	0	all	Eu:UK	1964	pr	259	n	V	n	n	0	cu	cig only	20	999	nev any	st
MRFITR	508		m	0	0	all	0	all	NAmer	1973	pr	119	n	bl	n	n	0	cu	cig+/-ot	24	999	nev cigs	ot
PEZZOT	570		m	0	0	all	-	all	SCAmer	1987	CC	215	n	bl	n	y	0	ev	cig only	19	999	nev cigs	st
QIAO2	506		m	0	0	all	0	all	As:Chi	1992	pr	241	m	ot	n	n	1	ev	all/unsp	21	999	nev any	or
RACHTA	507		f	0	0	all	-	all	Eu:est	1991	CC	118	n	bl	n	y	1	ev	cig+/-ot	20	30	nev cigs	or
SEGI2	521		m	0	0	all	-	all	As:Jap	1962	CC	378	n	bl	n	n	1	cu	cig+/-ot	23	999	nev any	or
SOBUE	654		m	0	0	all	-	all	As:Jap	1986	CC	1376	n	bl	n	y	0	ev	cig+/-ot	23	999	nev cigs	st
SUZUK2	501		c	0	0	all	-	all	SCAmer	1991	CC	123	n	bl	n	y	0	ev	all/unsp	19	999	nev any	st
SVENSS	501		f	0	0	all	-	all	Eu:Sca	1983	CC	210	n	bl	n	n	0	cu	all/unsp	26	999	nev any	st
TIZZAN	507		m	0	0	all	-	all	Eu:wst	1959	CC	1358	n	bl	n	n	0	ev	all/unsp	20	30	nev any	st
TIZZAN	537	x	f	0	0	all	-	all	Eu:wst	1959	CC	1358	n	bl	n	n	0	ev	cig only	20	30	nev any	st
WAKAI	502		m	0	0	all	-	all	As:Jap	1988	CC	333	n	bl	n	y	0	cu	cig+/-ot	20	29	nev any	st
WU	541		f	0	0	wh	-	q+a	NAmer	1981	CC	220	n	bl	n	y	2	cu	all/unsp	25	999	nev any	st
WYNDE6	759		m	0	0	wh	-	q+s+a	NAmer	1969	CC	4423	n	bl	n	y	0	ev	cig+/-ot	21	999	nev cigs	st
WYNDE6	767		f	0	0	wh	-	q+s+a	NAmer	1969	CC	4423	n	bl	n	y	0	ev	cig+/-ot	21	999	nev cigs	st
ZHENG	564		m	0	0	all	-	all	As:Chi	1982	CC	540	n	ot	*	y	0	ev	cig+/-ot	20	29	nev cigs	st

Cigarette type is all/unspec for all RRs
except for the following:

REF	NRR	CIGTYPE
DEAN3	564	MC only
DEAN3	583	MC only

Table 1H17 - 2

IESLC - Meta-analysis of Ever/current Smoking, Age started, "Low"
All LC types, Cigarettes (or Any Product if Cigarettes not available)
Most adjusted

REF	NRR	SEX	AD	Number Exposed Case	Exposed Cont	Non-exposed Case	Non-exposed Cont	RR	95.00%CI
AGUDO	504	f	3	7	-	80	-	1.58	(0.59- 4.23)
AUVINE	509	c	0	76	27	44	229	14.65	(8.50- 25.26)
BARBON	520	m	1	200	-	22	-	8.20	(5.00- 13.30)
BRESLO	501	c	0	32	35	19	56	2.69	(1.33- 5.47)
BUFFLE	518	f	0	47	34	12	112	12.90	(6.15- 27.07)
*CEDERL	510	m	1	11	-	7	-	6.50	(2.52- 16.74)
*CEDERL	515	f	0	6	2806	19	17679	1.99	(0.80- 4.98)
Subtotal CEDERL								3.53	(1.83- 6.82)
CHEN2	518	m	0	29	25	9	33	4.25	(1.71- 10.57)
CHEN2	523	f	0	9	8	25	33	1.49	(0.50- 4.39)
Subtotal CHEN2								2.75	(1.37- 5.53)
CHIAZZ	501	m	2	-	-	4	-	3.00	(0.31- 28.84)
CHOI	523	m	0	36	77	13	95	3.42	(1.69- 6.89)
CHOI	530	f	0	15	25	76	164	1.29	(0.65- 2.60)
Subtotal CHOI								2.09	(1.28- 3.43)
*CPSI	801	m	1	42	-	83	-	4.08	(2.81- 5.91)
*CPSI	845	f	1	51	-	166	-	2.25	(1.64- 3.08)
Subtotal CPSI								2.89	(2.27- 3.67)
DAMBER	501	m	0	70	76	42	208	4.56	(2.87- 7.26)
DEAN3	564	m	0	24	75	24	510	6.80	(3.67- 12.58)
DEAN3	583	f	0	27	274	41	1538	3.70	(2.24- 6.11)
Subtotal DEAN3								4.72	(3.20- 6.96)
DOLL	503	m	0	251	264	7	61	8.29	(3.72- 18.46)
DOLL	510	f	0	23	15	40	59	2.26	(1.05- 4.86)
Subtotal DOLL								4.20	(2.42- 7.30)
*DORN	610	m	0	37	73050	25	213858	4.33	(2.61- 7.20)
*DORN	647	m	0	90	74464	49	171211	4.22	(2.98- 5.98)
Subtotal DORN								4.26	(3.20- 5.67)
*ENGELA	502	m	0	50	30195	27	58716	3.60	(2.26- 5.75)
*ENGELA	510	f	0	36	29605	31	207789	8.15	(5.04- 13.17)
Subtotal ENGELA								5.36	(3.83- 7.49)
GAO	507	m	2	363	-	62	-	4.70	(3.30- 6.50)
GAO	517	f	2	87	-	435	-	3.80	(2.60- 5.80)
Subtotal GAO								4.30	(3.32- 5.57)
GAO2	502	m	0	127	85	13	56	6.44	(3.32- 12.49)
GENG	533	f	1	28	-	54	-	1.55	(0.83- 2.89)
HAENSZ	537	f	0	44	66	81	236	1.94	(1.23- 3.07)
HEGMAN	513	m	1	26	-	-	-	9.40	(4.60- 19.30)
HEGMAN	516	f	1	2	-	-	-	4.80	(1.00- 22.10)
Subtotal HEGMAN								8.35	(4.36- 16.00)
*HIRAYA	501	m	1	-	-	-	-	4.35	(3.51- 5.39)
*HIRAYA	504	f	1	-	-	-	-	2.46	(1.93- 3.13)
Subtotal HIRAYA								3.38	(2.88- 3.97)
HU2	503	c	0	229	159	121	213	2.54	(1.88- 3.43)
JOLY	543	m	0	18	70	12	218	4.67	(2.14- 10.18)
JOLY	533	f	0	23	41	52	283	3.05	(1.69- 5.51)
Subtotal JOLY								3.57	(2.23- 5.71)
KHUDER	506	m	0	72	152	23	309	6.36	(3.83- 10.58)
KOULUM	502	m	0	60	67	5	54	9.67	(3.63- 25.77)
LETOUR	501	c	0	188	160	24	224	10.97	(6.85- 17.56)
*LIAW	504	c	2	-	-	-	-	1.50	(0.70- 3.30)
LIU3	504	m	2	20	-	4	-	1.10	(0.25- 4.93)
LIU4	501	m	2	-	-	-	-	2.41	(2.32- 2.49)
LUBIN	566	m	0	65	146	9	72	3.56	(1.68- 7.56)
LUBIN2	1157	m	1	564	-	185	-	4.97	(4.12- 6.00)
MATOS	576	m	2	28	-	11	-	3.90	(1.80- 8.30)
*MIGRAN	501	m	0	24	668	4	867	7.79	(2.72- 22.34)
*MIGRAN	511	f	0	11	1315	4	3814	7.98	(2.54- 25.01)
Subtotal MIGRAN								7.87	(3.63- 17.08)
*MRFITR	508	m	0	3	544	0	1859	23.91~	(1.24- 462.09)
PEZZOT	570	m	0	41	105	4	116	11.32	(3.92- 32.69)
*QIAO2	506	m	1	52	-	10	-	1.32	(0.67- 2.60)
RACHTA	507	f	1	25	-	33	-	5.33	(2.79- 10.20)
SEGI2	521	m	1	49	-	8	-	2.04	(0.89- 4.66)
SOBUE	654	m	0	110	121	29	126	3.95	(2.45- 6.38)
SUZUK2	501	c	0	16	22	11	53	3.50	(1.40- 8.75)
SVENSS	501	f	0	32	18	38	120	5.61	(2.84- 11.12)
TIZZAN	507	m	0	313	330	180	305	1.61	(1.26- 2.04)
TIZZAN	537	f	0	12	21	117	114	0.56	(0.26- 1.18)
Subtotal TIZZAN								1.46	(1.16- 1.83)
WAKAI	502	m	0	130	183	10	65	4.62	(2.29- 9.32)

Table 1H17 - 2

IESLC - Meta-analysis of Ever/current Smoking, Age started, "Low"
All LC types, Cigarettes (or Any Product if Cigarettes not available)
Most adjusted

REF	NRR	SEX	AD	Number Exposed Case	Number Exposed Cont	Non-exposed Case	Non-exposed Cont	RR	95.00%CI
WU	541	f	2	14	-	31	-	1.55	(0.60- 3.98)
WYNDE6	759	m	0	111	92	51	589	13.93	(9.36- 20.74)
WYNDE6	767	f	0	127	90	73	673	13.01	(9.06- 18.69)
Subtotal WYNDE6								13.42	(10.27- 17.54)
ZHENG	564	m	0	145	109	33	94	3.79	(2.37- 6.05)
Partial Totals				4328	215619	2592	682811		

*prospective study ~ With 0.5 adjustment for zero

REF	NRR	SEX	AD	Ys	Ws	Qs	Ps
AGUDO	504	f	3	0.46	3.96	1.16	0.3627
AUVINE	509	c	0	2.68	12.94	36.72	0.0000
BARBON	520	m	1	2.10	16.05	19.58	0.0000
BRESLO	501	c	0	0.99	7.67	0.00	0.0060
BUFFLE	518	f	0	2.56	7.00	16.97	0.0000
*CEDERL	510	m	1	1.87	4.29	3.26	0.0001
*CEDERL	515	f	0	0.69	4.57	0.44	0.1414
Subtotal CEDERL				1.26	8.85	3.70	
CHEN2	518	m	0	1.45	4.63	0.93	0.0018
CHEN2	523	f	0	0.40	3.26	1.19	0.4750
Subtotal CHEN2				1.01	7.90	2.12	
CHIAZZ	501	m	2	1.10	0.75	0.01	0.3421
CHOI	523	m	0	1.23	7.80	0.41	0.0006
CHOI	530	f	0	0.26	7.94	4.36	0.4667
Subtotal CHOI				0.74	15.74	4.77	
*CPSI	801	m	1	1.41	27.80	4.59	0.0000
*CPSI	845	f	1	0.81	38.69	1.38	0.0000
Subtotal CPSI				1.06	66.49	5.97	
DAMBER	501	m	0	1.52	17.84	4.79	0.0000
DEAN3	564	m	0	1.92	10.14	8.53	0.0000
DEAN3	583	f	0	1.31	15.21	1.44	0.0000
Subtotal DEAN3				1.55	25.35	9.97	
DOLL	503	m	0	2.11	5.99	7.44	0.0000
DOLL	510	f	0	0.82	6.57	0.22	0.0364
Subtotal DOLL				1.43	12.56	7.66	
*DORN	610	m	0	1.47	14.92	3.25	0.0000
*DORN	647	m	0	1.44	31.75	6.17	0.0000
Subtotal DORN				1.45	46.67	9.42	
*ENGELA	502	m	0	1.28	17.55	1.39	0.0000
*ENGELA	510	f	0	2.10	16.67	20.11	0.0000
Subtotal ENGELA				1.68	34.22	21.50	
GAO	507	m	2	1.55	33.44	10.04	0.0000
GAO	517	f	2	1.34	23.87	2.68	0.0000
Subtotal GAO				1.46	57.31	12.72	
GAO2	502	m	0	1.86	8.74	6.50	0.0000
GENG	533	f	1	0.44	9.87	3.11	0.1685
HAENSZ	537	f	0	0.66	18.36	2.07	0.0044
HEGMAN	513	m	1	2.24	7.47	11.51	0.0000
HEGMAN	516	f	1	1.57	1.60	0.52	0.0470
Subtotal HEGMAN				2.12	9.08	12.03	
*HIRAYA	501	m	1	1.47	83.52	18.49	0.0000
*HIRAYA	504	f	1	0.90	65.73	0.65	0.0000
Subtotal HIRAYA				1.22	149.25	19.14	
HU2	503	c	0	0.93	42.35	0.20	0.0000
JOLY	543	m	0	1.54	6.34	1.86	0.0001
JOLY	533	f	0	1.12	11.03	0.15	0.0002
Subtotal JOLY				1.27	17.37	2.01	
KHUDER	506	m	0	1.85	14.88	10.78	0.0000
KOULUM	502	m	0	2.27	4.00	6.44	0.0000
LETOUR	501	c	0	2.39	17.33	33.74	0.0000
*LIAW	504	c	2	0.41	6.39	2.26	0.3054
LIU3	504	m	2	0.10	1.73	1.41	0.9003
LIU4	501	m	2	0.88	3072.74	44.28	0.0000
LUBIN	566	m	0	1.27	6.79	0.50	0.0009
LUBIN2	1157	m	1	1.60	108.74	39.64	0.0000
MATOS	576	m	2	1.36	6.58	0.86	0.0005
*MIGRAN	501	m	0	2.05	3.46	3.84	0.0001
*MIGRAN	511	f	0	2.08	2.94	3.41	0.0004
Subtotal MIGRAN				2.06	6.40	7.25	
*MRFITR	508	m	0	3.17	0.44	2.07	0.0357
PEZZOT	570	m	0	2.43	3.42	6.96	0.0000

Table 1H17 - 2

IESLC - Meta-analysis of Ever/current Smoking, Age started, "Low"
All LC types, Cigarettes (or Any Product if Cigarettes not available)
Most adjusted

REF	NRR	SEX	AD	Ys	Ws	Qs	Ps
*QIAO2	506	m	1	0.28	8.36	4.36	0.4222
RACHTA	507	f	1	1.67	9.14	4.15	0.0000
SEGI2	521	m	1	0.71	5.61	0.46	0.0914
SOBUE	654	m	0	1.37	16.73	2.34	0.0000
SUZUK2	501	c	0	1.25	4.59	0.30	0.0072
SVENSS	501	f	0	1.73	8.23	4.33	0.0000
TIZZAN	507	m	0	0.47	66.40	18.32	0.0001
TIZZAN	537	f	0	-0.59	6.74	16.95	0.1283
Subtotal TIZZAN				0.38	73.15	35.27	
WAKAI	502	m	0	1.53	7.78	2.19	0.0000
WU	541	f	2	0.44	4.29	1.35	0.3639
WYNDE6	759	m	0	2.63	24.28	64.88	0.0000
WYNDE6	767	f	0	2.57	29.27	71.77	0.0000
Subtotal WYNDE6				2.60	53.55	136.65	
ZHENG	564	m	0	1.33	17.54	1.94	0.0000

	N	61
	NS	46
	Wt	4054.72
Het	Chi	551.66
Het	df	60
Het	P	***
Fixed	RR	2.72
	RRl	2.64
	RRu	2.80
	P	+++
Random	RR	3.99
	RRl	3.40
	RRu	4.68
	P	+++
Asymm	P	***

Table 1H17 - 3

IESLC - Meta-analysis of Ever/current Smoking, Age started, "Low"
All LC types, Cigarettes (or Any Product if Cigarettes not available)
Most adjusted

	Sex combined	male	female	Total
N	6	34	21	61
NS	6	33	21	60
Wt	91.27	3668.49	294.96	4054.72
Het Chi	55.38	317.58	148.58	551.66
Het df	5	33	20	60
Het P	***	***	***	***
Fixed RR	4.23	2.65	3.26	2.72
RRl	3.44	2.56	2.91	2.64
RRu	5.19	2.74	3.66	2.80
P	+++	+++	+++	+++
Random RR	4.33	4.55	3.07	3.99
RRl	2.05	3.70	2.20	3.40
RRu	9.17	5.60	4.30	4.68
P	+++	+++	+++	+++
Between Chi				30.12
Between df				2
Between P				***
Btwn(F) P				N.S.
Btwn(R) P				N.S.

	Lung cancer type all	other	Total
N	57	4	61
NS	43	3	46
Wt	3978.52	76.20	4054.72
Het Chi	410.10	62.37	551.66
Het df	56	3	60
Het P	***	***	***
Fixed RR	2.67	7.46	2.72
RRl	2.58	5.96	2.64
RRu	2.75	9.34	2.80
P	+++	+++	+++
Random RR	3.88	5.06	3.99
RRl	3.34	1.72	3.40
RRu	4.52	14.90	4.68
P	+++	++	+++
Between Chi			79.19
Between df			1
Between P			***
Btwn(F) P			**
Btwn(R) P			N.S.

	Location								
	NAmer	UK	Scand	othEur	China	Japan	othAs	other	Total
N	16	6	8	6	11	6	3	5	61
NS	12	3	6	5	9	5	2	4	46
Wt	246.50	44.32	86.08	211.05	3224.58	188.10	22.13	31.96	4054.72
Het Chi	126.17	9.32	24.33	91.51	32.47	17.83	4.21	4.73	551.66
Het df	15	5	7	5	10	5	2	4	60
Het P	***	(*)	***	***	***	**	N.S.	N.S.	***
Fixed RR	5.22	4.91	5.96	3.31	2.44	3.53	1.90	4.10	2.72
RRl	4.60	3.66	4.83	2.89	2.35	3.06	1.25	2.90	2.64
RRu	5.91	6.60	7.37	3.79	2.52	4.07	2.88	5.80	2.80
P	+++	+++	+++	+++	+++	+++	++	+++	+++
Random RR	5.31	5.17	5.93	2.71	2.76	3.67	1.89	4.18	3.99
RRl	3.57	3.38	3.92	1.36	2.19	2.65	1.03	2.85	3.40
RRu	7.90	7.91	8.98	5.40	3.48	5.09	3.47	6.12	4.68
P	+++	+++	+++	++	+++	+++	+	+++	+++
Between Chi									241.10
Between df									7
Between P									***
Btwn(F) P									***
Btwn(R) P									**

Table 1H17 - 3

IESLC - Meta-analysis of Ever/current Smoking, Age started, "Low"
All LC types, Cigarettes (or Any Product if Cigarettes not available)
Most adjusted

Detailed Country in "other Europe"

	multi	Germany	othWest	East	Balkans	Total
N	1		4	1		6
NS	1		3	1		5
Wt	108.74		93.16	9.14		211.05
Het Chi	0.00		46.36	0.00		91.51
Het df	0		3	0		5
Het P	N.S.		***	N.S.		***
Fixed RR	4.97		1.97	5.33		3.31
RRl	4.12		1.61	2.79		2.89
RRu	6.00		2.41	10.19		3.79
P	+++		+++	+++		+++
Random RR	4.97		1.90	5.33		2.71
RRl	4.12		0.67	2.79		1.36
RRu	6.00		5.39	10.19		5.40
P	+++		N.S.	+++		++
Between Chi						45.15
Between df						2
Between P						***
Btwn(F) P						N.S.
Btwn(R) P						N.S.

Detailed Country in "other Asia"

	India	HongKong	other	Total
N			3	3
NS			2	2
Wt			22.13	22.13
Het Chi			4.21	4.21
Het df			2	2
Het P			N.S.	N.S.
Fixed RR			1.90	1.90
RRl			1.25	1.25
RRu			2.88	2.88
P			++	++
Random RR			1.89	1.89
RRl			1.03	1.03
RRu			3.47	3.47
P			+	+
Between Chi				
Between df				
Between P				N.S.
Btwn(F) P				N.S.
Btwn(R) P				N.S.

Detailed other continent

	SCAmer	Total
N	5	5
NS	4	4
Wt	31.96	31.96
Het Chi	4.73	4.73
Het df	4	4
Het P	N.S.	N.S.
Fixed RR	4.10	4.10
RRl	2.90	2.90
RRu	5.80	5.80
P	+++	+++
Random RR	4.18	4.18
RRl	2.85	2.85
RRu	6.12	6.12
P	+++	+++
Between Chi		
Between df		
Between P		N.S.
Btwn(F) P		N.S.
Btwn(R) P		N.S.

Table 1H17 - 3

IESLC - Meta-analysis of Ever/current Smoking, Age started, "Low"
All LC types, Cigarettes (or Any Product if Cigarettes not available)
Most adjusted

	Start year of study					
	<1960	1960-69	1970-79	1980-89	1990+	Total
N	12	13	8	24	4	61
NS	8	7	7	20	4	46
Wt	229.65	283.22	209.78	3303.39	28.67	4054.72
Het Chi	65.69	104.84	31.45	171.27	9.19	551.66
Het df	11	12	7	23	3	60
Het P	***	***	***	***	*	***
Fixed RR	2.53	4.83	4.51	2.51	3.09	2.72
RRl	2.22	4.30	3.94	2.43	2.14	2.64
RRu	2.88	5.43	5.16	2.60	4.45	2.80
P	+++	+++	+++	+++	+++	+++
Random RR	2.85	5.29	4.96	3.85	3.11	3.99
RRl	2.00	3.60	3.46	2.92	1.62	3.40
RRu	4.06	7.78	7.12	5.06	5.96	4.68
P	+++	+++	+++	+++	+++	+++
Between Chi						169.22
Between df						4
Between P						***
Btwn(F) P						***
Btwn(R) P						N.S.

	Study type (1)		
	CC	other	Total
N	46	15	61
NS	37	9	46
Wt	3727.66	327.06	4054.72
Het Chi	474.23	55.50	551.66
Het df	45	14	60
Het P	***	***	***
Fixed RR	2.66	3.48	2.72
RRl	2.57	3.13	2.64
RRu	2.75	3.88	2.80
P	+++	+++	+++
Random RR	4.09	3.60	3.99
RRl	3.35	2.80	3.40
RRu	5.01	4.63	4.68
P	+++	+++	+++
Between Chi			21.93
Between df			1
Between P			***
Btwn(F) P			N.S.
Btwn(R) P			N.S.

	Study type (2)			
	CC	prosp	other	Total
N	46	15		61
NS	37	9		46
Wt	3727.66	327.06		4054.72
Het Chi	474.23	55.50		551.66
Het df	45	14		60
Het P	***	***		***
Fixed RR	2.66	3.48		2.72
RRl	2.57	3.13		2.64
RRu	2.75	3.88		2.80
P	+++	+++		+++
Random RR	4.09	3.60		3.99
RRl	3.35	2.80		3.40
RRu	5.01	4.63		4.68
P	+++	+++		+++
Between Chi				21.93
Between df				1
Between P				***
Btwn(F) P				N.S.
Btwn(R) P				N.S.

Table 1H17 - 3

IESLC - Meta-analysis of Ever/current Smoking, Age started, "Low"
All LC types, Cigarettes (or Any Product if Cigarettes not available)
Most adjusted

	Study size (number of LC cases) 100-249	250-499	500-999	1000+	Total
N	15	16	13	17	61
NS	14	11	11	10	46
Wt	84.14	127.96	185.44	3657.18	4054.72
Het Chi	33.41	46.97	66.58	305.42	551.66
Het df	14	15	12	16	60
Het P	**	***	***	***	***
Fixed RR	2.70	4.26	4.93	2.60	2.72
RRl	2.18	3.58	4.27	2.51	2.64
RRu	3.35	5.06	5.69	2.68	2.80
P	+++	+++	+++	+++	+++
Random RR	2.79	4.15	5.60	3.76	3.99
RRl	1.95	3.01	3.93	2.88	3.40
RRu	3.99	5.72	7.97	4.91	4.68
P	+++	+++	+++	+++	+++
Between Chi					99.27
Between df					3
Between P					***
Btwn(F) P					**
Btwn(R) P					(*)

	Risky occupational population no	mining	othRisky	Total
N	58	2	1	61
NS	43	2	1	46
Wt	4038.82	15.15	0.75	4054.72
Het Chi	546.80	3.69	0.00	551.66
Het df	57	1	0	60
Het P	***	(*)	N.S.	***
Fixed RR	2.72	2.06	3.00	2.72
RRl	2.64	1.24	0.31	2.64
RRu	2.81	3.41	28.94	2.80
P	+++	++	N.S.	+++
Random RR	4.08	2.14	3.00	3.99
RRl	3.46	0.81	0.31	3.40
RRu	4.80	5.65	28.94	4.68
P	+++	N.S.	N.S.	+++
Between Chi				1.17
Between df				2
Between P				N.S.
Btwn(F) P				N.S.
Btwn(R) P				N.S.

	National cigarette tobacco type Virginia	blended	other	Total
N	7	42	12	61
NS	4	32	10	46
Wt	61.65	762.10	3230.97	4054.72
Het Chi	17.35	298.72	33.98	551.66
Het df	6	41	11	60
Het P	**	***	***	***
Fixed RR	6.16	4.05	2.43	2.72
RRl	4.80	3.77	2.35	2.64
RRu	7.90	4.35	2.52	2.80
P	+++	+++	+++	+++
Random RR	6.03	4.26	2.67	3.99
RRl	3.84	3.46	2.13	3.40
RRu	9.48	5.26	3.34	4.68
P	+++	+++	+++	+++
Between Chi				201.62
Between df				2
Between P				***
Btwn(F) P				***
Btwn(R) P				***

Table 1H17 - 3

IESLC - Meta-analysis of Ever/current Smoking, Age started, "Low"
All LC types, Cigarettes (or Any Product if Cigarettes not available)
Most adjusted

	Any proxy use No/nk	Yes	Total
N	46	15	61
NS	34	12	46
Wt	818.61	3236.11	4054.72
Het Chi	287.04	159.74	551.66
Het df	45	14	60
Het P	***	***	***
Fixed RR	3.74	2.51	2.72
RRl	3.49	2.42	2.64
RRu	4.01	2.59	2.80
P	+++	+++	+++
Random RR	3.65	5.25	3.99
RRl	3.02	3.54	3.40
RRu	4.42	7.78	4.68
P	+++	+++	+++
Between Chi			104.88
Between df			1
Between P			***
Btwn(F) P			***
Btwn(R) P			N.S.

	Full histological confirmation No	Yes	Total
N	44	17	61
NS	31	15	46
Wt	3745.55	309.17	4054.72
Het Chi	263.12	93.99	551.66
Het df	43	16	60
Het P	***	***	***
Fixed RR	2.55	5.82	2.72
RRl	2.47	5.21	2.64
RRu	2.63	6.51	2.80
P	+++	+++	+++
Random RR	3.48	5.56	3.99
RRl	2.96	4.10	3.40
RRu	4.08	7.52	4.68
P	+++	+++	+++
Between Chi			194.55
Between df			1
Between P			***
Btwn(F) P			***
Btwn(R) P			**

	Number of adjustment variables (1) 0	1	2+/+nk	Total
N	39	13	9	61
NS	29	10	8	47
Wt	514.10	386.87	3153.75	4054.72
Het Chi	279.21	69.98	25.21	551.66
Het df	38	12	8	60
Het P	***	***	**	***
Fixed RR	4.19	3.77	2.43	2.72
RRl	3.85	3.42	2.35	2.64
RRu	4.57	4.17	2.52	2.80
P	+++	+++	+++	+++
Random RR	4.52	3.65	2.71	3.99
RRl	3.53	2.76	1.97	3.40
RRu	5.79	4.83	3.71	4.68
P	+++	+++	+++	+++
Between Chi				177.26
Between df				2
Between P				***
Btwn(F) P				***
Btwn(R) P				*

Table 1H17 - 3

IESLC - Meta-analysis of Ever/current Smoking, Age started, "Low"
All LC types, Cigarettes (or Any Product if Cigarettes not available)
Most adjusted

	Number of adjustment variables (2) 0	1	2	3-5	6+/+nk	Total
N	39	13	8	1		61
NS	29	10	7	1		47
Wt	514.10	386.87	3149.79	3.96		4054.72
Het Chi	279.21	69.98	24.47	0.00		551.66
Het df	38	12	7	0		60
Het P	***	***	***	N.S.		***
Fixed RR	4.19	3.77	2.43	1.58		2.72
RRl	3.85	3.42	2.35	0.59		2.64
RRu	4.57	4.17	2.52	4.23		2.80
P	+++	+++	+++	N.S.		+++
Random RR	4.52	3.65	2.81	1.58		3.99
RRl	3.53	2.76	2.01	0.59		3.40
RRu	5.79	4.83	3.93	4.23		4.68
P	+++	+++	+++	N.S.		+++
Between Chi						178.00
Between df						3
Between P						***
Btwn(F) P						***
Btwn(R) P						*

	Smoking status ever	current	Total
N	42	19	61
NS	33	13	46
Wt	3722.68	332.04	4054.72
Het Chi	462.60	60.15	551.66
Het df	41	18	60
Het P	***	***	***
Fixed RR	2.65	3.61	2.72
RRl	2.57	3.24	2.64
RRu	2.74	4.01	2.80
P	+++	+++	+++
Random RR	4.00	3.88	3.99
RRl	3.25	3.09	3.40
RRu	4.93	4.89	4.68
P	+++	+++	+++
Between Chi			28.91
Between df			1
Between P			***
Btwn(F) P			(*)
Btwn(R) P			N.S.

	Product all/unsp	cig+/-ot	cig only	Total
N	17	34	10	61
NS	14	27	7	48
Wt	3240.16	721.14	93.42	4054.72
Het Chi	88.65	200.54	46.72	551.66
Het df	16	33	9	60
Het P	***	***	***	***
Fixed RR	2.43	4.44	2.96	2.72
RRl	2.35	4.13	2.41	2.64
RRu	2.51	4.78	3.62	2.80
P	+++	+++	+++	+++
Random RR	3.29	4.49	3.51	3.99
RRl	2.46	3.69	2.09	3.40
RRu	4.41	5.46	5.89	4.68
P	+++	+++	+++	+++
Between Chi				215.75
Between df				2
Between P				***
Btwn(F) P				***
Btwn(R) P				N.S.

Table 1H17 - 3

IESLC - Meta-analysis of Ever/current Smoking, Age started, "Low"
All LC types, Cigarettes (or Any Product if Cigarettes not available)
Most adjusted

	Denominator		
	nev any	nev cigs	Total
N	39	22	61
NS	28	18	46
Wt	3629.95	424.76	4054.72
Het Chi	189.21	137.01	551.66
Het df	38	21	60
Het P	***	***	***
Fixed RR	2.51	5.41	2.72
RRl	2.43	4.92	2.64
RRu	2.59	5.95	2.80
P	+++	+++	+++
Random RR	3.25	5.58	3.99
RRl	2.76	4.26	3.40
RRu	3.82	7.30	4.68
P	+++	+++	+++
Between Chi			225.44
Between df			1
Between P			***
Btwn(F) P			***
Btwn(R) P			***

	Derivation of RR/CI			
	Orig	StdCalc	Other	Total
N	13	42	6	61
NS	11	31	5	47
Wt	126.34	778.82	3149.56	4054.72
Het Chi	46.82	303.33	14.48	551.66
Het df	12	41	5	60
Het P	***	***	*	***
Fixed RR	4.38	4.00	2.42	2.72
RRl	3.68	3.73	2.34	2.64
RRu	5.21	4.29	2.51	2.80
P	+++	+++	+++	+++
Random RR	3.94	4.12	2.89	3.99
RRl	2.68	3.35	2.16	3.40
RRu	5.79	5.06	3.87	4.68
P	+++	+++	+++	+++
Between Chi				187.03
Between df				2
Between P				***
Btwn(F) P				***
Btwn(R) P				N.S.

	Study LIU4		
	LIU4	others	Total
N	1	60	61
NS	1	45	46
Wt	3072.74	981.97	4054.72
Het Chi	0.00	368.83	551.66
Het df	0	59	60
Het P	N.S.	***	***
Fixed RR	2.41	3.96	2.72
RRl	2.33	3.72	2.64
RRu	2.50	4.21	2.80
P	+++	+++	+++
Random RR	2.41	4.03	3.99
RRl	2.33	3.40	3.40
RRu	2.50	4.78	4.68
P	+++	+++	+++
Between Chi			182.84
Between df			1
Between P			***
Btwn(F) P			***
Btwn(R) P			***

Table 1H17 - 4

IESLC - Meta-analysis of Ever/current Smoking, Age started, "Low"
All LC types, Cigarettes (or Any Product if Cigarettes not available)
Least adjusted

REF	NRR	X	SEX	AGEL	AGEH	RACE	YF	LC TYPE	LOC	START	ST	NLC	R	VB	P	H	AD	SM	PRODUCT	exL	exH	DENOM	De
AGUDO	501	x	f	0	0	all	-	all	Eu:wst	1989	CC	103	n	bl	n	n	0	ev	cig only	24	999	nev cigs	st
AUVINE	509		c	0	0	all	-	all	Eu:Sca	1986	CC	517	n	bl	y	n	0	ev	cig+/-ot	21	999	nev cigs	st
BARBON	515	x	m	0	0	all	-	all	Eu:wst	1979	CC	755	n	bl	y	y	0	ev	all/unsp	20	999	nev any	st
BRESLO	501		c	0	0	all	-	all	NAmer	1949	CC	518	n	bl	n	y	0	ev	cig+/-ot	25	999	nev any	st
BUFFLE	518		f	0	0	w-hi	-	all	NAmer	1976	CC	943	n	bl	y	n	0	ev	cig+/-ot	21	29	nev cigs	or
CEDERL	510		m	0	0	all	10	all	Eu:Sca	1963	pr	491	n	bl	n	n	1	cu	cig only	19	999	nev any	ot
CEDERL	515		f	0	0	all	10	all	Eu:Sca	1963	pr	491	n	bl	n	n	0	cu	cig only	19	999	nev any	st
CHEN2	518		m	0	0	all	-	all	As:Chi	1983	CC	193	n	ot	y	n	0	ev	all/unsp	20	30	nev any	st
CHEN2	523		f	0	0	all	-	all	As:Chi	1983	CC	193	n	ot	y	n	0	ev	all/unsp	20	30	nev any	st
CHIAZZ	501		m	0	0	all	-	all	NAmer	1940	CC	144	o	bl	y	n	2	ev	cig+/-ot	20	999	nev cigs	or
CHOI	523		m	0	0	all	-	all	As:oth	1985	CC	375	n	bl	n	n	0	ev	cig+/-ot	25	999	nev cigs	st
CHOI	530		f	0	0	all	-	all	As:oth	1985	CC	375	n	bl	n	n	0	ev	cig+/-ot	25	999	nev cigs	st
CPSI	801		m	35	84	all	6	all	NAmer	1959	pr	5138	n	bl	n	n	1	cu	cig+/-ot	25	999	nev any	ot
CPSI	845		f	40	74	all	6	all	NAmer	1959	pr	5138	n	bl	n	n	1	cu	cig only	25	999	nev any	ot
DAMBER	501		m	0	0	all	-	all	Eu:Sca	1972	CC	579	n	bl	y	n	0	ev	all/unsp	21	999	nev any	st
DEAN3	564		m	0	0	all	-	all	Eu:UK	1969	CC	766	n	V	y	n	0	cu	cig only	25	999	nev any	st
DEAN3	583		f	0	0	all	-	all	Eu:UK	1969	CC	766	n	V	y	n	0	cu	cig only	25	999	nev any	st
DOLL	503		m	0	0	all	-	all	Eu:UK	1948	CC	1465	n	V	n	n	0	ev	all/unsp	20	29	nev any	st
DOLL	510		f	0	0	all	-	all	Eu:UK	1948	CC	1465	n	V	n	n	0	ev	all/unsp	20	29	nev any	st
DORN	610		m	55	64	wh	8	all	NAmer	1954	pr	5097	n	bl	n	n	0	ev	cig+/-ot	25	999	nev any	st
DORN	647		m	65	74	wh	8	all	NAmer	1954	pr	5097	n	bl	n	n	0	ev	cig+/-ot	25	999	nev any	st
ENGELA	502		m	0	0	all	0	all	Eu:Sca	1964	pr	435	n	bl	n	n	0	cu	cig+/-ot	20	29	nev cigs	st
ENGELA	510		f	0	0	all	0	all	Eu:Sca	1964	pr	435	n	bl	n	n	0	cu	cig+/-ot	20	29	nev cigs	st
GAO	502	x	m	0	0	all	-	all	As:Chi	1984	CC	1405	n	ot	n	n	0	ev	cig+/-ot	20	29	nev cigs	st
GAO	512	x	f	0	0	all	-	all	As:Chi	1984	CC	1405	n	ot	n	n	0	ev	cig+/-ot	20	29	nev cigs	st
GAO2	502		m	0	0	all	-	all	As:Jap	1988	CC	282	n	bl	n	n	0	cu	cig+/-ot	20	29	nev cigs	st
GENG	528	x	f	0	0	all	-	all	As:Chi	1985	CC	292	n	ot	*	n	0	ev	cig+/-ot	21	999	nev any	st
HAENSZ	537		f	0	0	all	-	not alv	NAmer	1955	CC	158	n	bl	n	y	0	ev	cig+/-ot	25	999	nev any	st
HEGMAN	513		m	0	0	all	-	all	NAmer	1989	CC	282	n	bl	y	y	1	ev	all/unsp	20	999	nev any	or
HEGMAN	516		f	0	0	all	-	all	NAmer	1989	CC	282	n	bl	y	y	1	ev	all/unsp	26	999	nev any	or
HIRAYA	501		m	0	0	all	0	all	As:Jap	1965	pr	1917	n	bl	n	n	1	cu	cig+/-ot	20	999	nev any	st
HIRAYA	504		f	0	0	all	0	all	As:Jap	1965	pr	1917	n	bl	n	n	1	cu	cig+/-ot	20	999	nev any	st
HU2	503		c	0	0	all	-	all	As:Chi	1977	CC	523	n	ot	y	n	0	ev	cig+/-ot	20	29	nev cigs	st
JOLY	543		m	0	0	all	-	all	SCAmer	1978	CC	826	n	bl	n	n	0	ev	cig+/-ot	25	999	nev any	st
JOLY	533		f	0	0	all	-	all	SCAmer	1978	CC	826	n	bl	n	n	0	ev	cig+/-ot	25	999	nev any	st
KHUDER	506		m	0	0	all	-	all	NAmer	1985	CC	482	n	bl	n	y	0	ev	cig+/-ot	20	999	nev cigs	st
KOULUM	502		m	0	0	all	-	all	Eu:Sca	1936	CC	812	n	bl	n	n	0	ev	all/unsp	21	30	nev any	st
LETOUR	501		c	0	0	all	-	all	NAmer	1983	CC	738	n	V	y	y	0	ev	cig+/-ot	21	999	nev cigs	st
LIAW	504		c	0	0	all	0	all	As:oth	1982	pr	127	n	ot	n	n	2	cu	all/unsp	25	999	nev any	or
LIU3	501	x	m	0	0	all	-	all	As:Chi	1985	CC	110	n	ot	n	n	0	ev	all/unsp	21	999	nev any	or
LIU4	501		m	35	69	all	-	all	As:Chi	1986	CC	1000-00	n	ot	y	n	2	ev	all/unsp	25	999	nev any	ot
LUBIN	566		m	0	0	all	-	all	As:Chi	1984	CC	427	m	ot	y	n	0	ev	cig+/-ot	23	26	nev any	st
LUBIN2	1148	x	m	0	0	all	-	all	Eu:mul	1976	CC	7804	n	bl	n	y	0	ev	cig+/-ot	21	30	nev cigs	st
MATOS	556	x	m	0	0	all	-	all	SCAmer	1994	CC	200	n	bl	n	n	0	ev	cig+/-ot	20	999	nev any	st
MIGRAN	501		m	0	0	all	0	all	Eu:UK	1964	pr	259	n	V	n	n	0	cu	cig only	20	999	nev any	st
MIGRAN	511		f	0	0	all	0	all	Eu:UK	1964	pr	259	n	V	n	n	0	cu	cig only	20	999	nev any	st
MRFITR	508		m	0	0	all	0	all	NAmer	1973	pr	119	n	bl	n	n	0	cu	cig+/-ot	24	999	nev cigs	ot
PEZZOT	570		m	0	0	all	-	all	SCAmer	1987	CC	215	n	bl	n	y	0	ev	cig only	19	999	nev cigs	st
QIAO2	501	x	m	0	0	all	0	all	As:Chi	1992	pr	241	m	ot	n	n	0	ev	all/unsp	21	999	nev any	st
RACHTA	502	x	f	0	0	all	-	all	Eu:est	1991	CC	118	n	bl	n	y	0	ev	cig+/-ot	20	30	nev cigs	st
SEGI2	516	x	m	0	0	all	-	all	As:Jap	1962	CC	378	n	bl	n	n	0	cu	cig+/-ot	23	999	nev any	st
SOBUE	654		m	0	0	all	-	all	As:Jap	1986	CC	1376	n	bl	n	y	0	ev	cig+/-ot	23	999	nev cigs	st
SUZUK2	501		c	0	0	all	-	all	SCAmer	1991	CC	123	n	bl	n	y	0	ev	all/unsp	19	999	nev any	st
SVENSS	501		f	0	0	all	-	all	Eu:Sca	1983	CC	210	n	bl	n	n	0	cu	all/unsp	26	999	nev any	st
TIZZAN	507		m	0	0	all	-	all	Eu:wst	1959	CC	1358	n	bl	n	n	0	ev	all/unsp	20	30	nev any	st
TIZZAN	537		f	0	0	all	-	all	Eu:wst	1959	CC	1358	n	bl	n	n	0	ev	cig only	20	30	nev any	st
WAKAI	502		m	0	0	all	-	all	As:Jap	1988	CC	333	n	bl	n	y	0	cu	cig+/-ot	20	29	nev any	st
WU	517	x	f	0	0	wh	-	q+a	NAmer	1981	CC	220	n	bl	n	y	0	cu	all/unsp	25	999	nev any	st
WYNDE6	759		m	0	0	wh	-	q+s+a	NAmer	1969	CC	4423	n	bl	n	y	0	ev	cig+/-ot	21	999	nev cigs	st
WYNDE6	767		f	0	0	wh	-	q+s+a	NAmer	1969	CC	4423	n	bl	n	y	0	ev	cig+/-ot	21	999	nev cigs	st
ZHENG	564		m	0	0	all	-	all	As:Chi	1982	CC	540	n	ot	*	y	0	ev	cig+/-ot	20	29	nev cigs	st

Cigarette type is all/unspec for all RRs
except for the following:

REF	NRR	CIGTYPE
DEAN3	564	MC only
DEAN3	583	MC only

Table 1H17 - 5

IESLC - Meta-analysis of Ever/current Smoking, Age started, "Low"
All LC types, Cigarettes (or Any Product if Cigarettes not available)
Least adjusted

REF	NRR	SEX	AD	Number Exposed Case	Number Exposed Cont	Non-exposed Case	Non-exposed Cont	RR	95.00%CI
AGUDO	501	f	0	7	12	80	183	1.33	(0.51- 3.51)
AUVINE	509	c	0	76	27	44	229	14.65	(8.50- 25.26)
BARBON	515	m	0	200	207	22	188	8.26	(5.10- 13.38)
BRESLO	501	c	0	32	35	19	56	2.69	(1.33- 5.47)
BUFFLE	518	f	0	47	34	12	112	12.90	(6.15- 27.07)
*CEDERL	510	m	1	11	-	7	-	6.50	(2.52- 16.74)
*CEDERL	515	f	0	6	2806	19	17679	1.99	(0.80- 4.98)
Subtotal CEDERL								3.53	(1.83- 6.82)
CHEN2	518	m	0	29	25	9	33	4.25	(1.71- 10.57)
CHEN2	523	f	0	9	8	25	33	1.49	(0.50- 4.39)
Subtotal CHEN2								2.75	(1.37- 5.53)
CHIAZZ	501	m	2	-	-	4	-	3.00	(0.31- 28.84)
CHOI	523	m	0	36	77	13	95	3.42	(1.69- 6.89)
CHOI	530	f	0	15	25	76	164	1.29	(0.65- 2.60)
Subtotal CHOI								2.09	(1.28- 3.43)
*CPSI	801	m	1	42	-	83	-	4.08	(2.81- 5.91)
*CPSI	845	f	1	51	-	166	-	2.25	(1.64- 3.08)
Subtotal CPSI								2.89	(2.27- 3.67)
DAMBER	501	m	0	70	76	42	208	4.56	(2.87- 7.26)
DEAN3	564	m	0	24	75	24	510	6.80	(3.67- 12.58)
DEAN3	583	f	0	27	274	41	1538	3.70	(2.24- 6.11)
Subtotal DEAN3								4.72	(3.20- 6.96)
DOLL	503	m	0	251	264	7	61	8.29	(3.72- 18.46)
DOLL	510	f	0	23	15	40	59	2.26	(1.05- 4.86)
Subtotal DOLL								4.20	(2.42- 7.30)
*DORN	610	m	0	37	73050	25	213858	4.33	(2.61- 7.20)
*DORN	647	m	0	90	74464	49	171211	4.22	(2.98- 5.98)
Subtotal DORN								4.26	(3.20- 5.67)
*ENGELA	502	m	0	50	30195	27	58716	3.60	(2.26- 5.75)
*ENGELA	510	f	0	36	29605	31	207789	8.15	(5.04- 13.17)
Subtotal ENGELA								5.36	(3.83- 7.49)
GAO	502	m	0	363	262	62	202	4.51	(3.26- 6.25)
GAO	512	f	0	87	41	435	605	2.95	(2.00- 4.36)
Subtotal GAO								3.79	(2.95- 4.87)
GAO2	502	m	0	127	85	13	56	6.44	(3.32- 12.49)
GENG	528	f	0	28	31	54	93	1.56	(0.84- 2.87)
HAENSZ	537	f	0	44	66	81	236	1.94	(1.23- 3.07)
HEGMAN	513	m	1	26	-	-	-	9.40	(4.60- 19.30)
HEGMAN	516	f	1	2	-	-	-	4.80	(1.00- 22.10)
Subtotal HEGMAN								8.35	(4.36- 16.00)
*HIRAYA	501	m	1	-	-	-	-	4.35	(3.51- 5.39)
*HIRAYA	504	f	1	-	-	-	-	2.46	(1.93- 3.13)
Subtotal HIRAYA								3.38	(2.88- 3.97)
HU2	503	c	0	229	159	121	213	2.54	(1.88- 3.43)
JOLY	543	m	0	18	70	12	218	4.67	(2.14- 10.18)
JOLY	533	f	0	23	41	52	283	3.05	(1.69- 5.51)
Subtotal JOLY								3.57	(2.23- 5.71)
KHUDER	506	m	0	72	152	23	309	6.36	(3.83- 10.58)
KOULUM	502	m	0	60	67	5	54	9.67	(3.63- 25.77)
LETOUR	501	c	0	188	160	24	224	10.97	(6.85- 17.56)
*LIAW	504	c	2	-	-	-	-	1.50	(0.70- 3.30)
LIU3	501	m	0	20	80	4	19	1.19	(0.36- 3.88)
LIU4	501	m	2	-	-	-	-	2.41	(2.32- 2.49)
LUBIN	566	m	0	65	146	9	72	3.56	(1.68- 7.56)
LUBIN2	1148	m	0	564	1069	185	1878	5.36	(4.46- 6.43)
MATOS	556	m	0	28	73	11	110	3.84	(1.80- 8.18)
*MIGRAN	501	m	0	24	668	4	867	7.79	(2.72- 22.34)
*MIGRAN	511	f	0	11	1315	4	3814	7.98	(2.54- 25.01)
Subtotal MIGRAN								7.87	(3.63- 17.08)
*MRFITR	508	m	0	3	544	0	1859	23.91~	(1.24- 462.09)
PEZZOT	570	m	0	41	105	4	116	11.32	(3.92- 32.69)
*QIAO2	501	m	0	52	1947	10	709	1.89	(0.97- 3.71)
RACHTA	502	f	0	25	18	33	98	4.12	(2.00- 8.50)
SEGI2	516	m	0	49	155	8	53	2.09	(0.93- 4.71)
SOBUE	654	m	0	110	121	29	126	3.95	(2.45- 6.38)
SUZUK2	501	c	0	16	22	11	53	3.50	(1.40- 8.75)
SVENSS	501	f	0	32	18	38	120	5.61	(2.84- 11.12)
TIZZAN	507	m	0	313	330	180	305	1.61	(1.26- 2.04)
TIZZAN	537	f	0	12	21	117	114	0.56	(0.26- 1.18)
Subtotal TIZZAN								1.46	(1.16- 1.83)
WAKAI	502	m	0	130	183	10	65	4.62	(2.29- 9.32)

Table 1H17 - 5

IESLC - Meta-analysis of Ever/current Smoking, Age started, "Low"
All LC types, Cigarettes (or Any Product if Cigarettes not available)
Least adjusted

REF	NRR	SEX	AD	Number Exposed Case	Cont	Non-exposed Case	Cont	RR	95.00%CI
WU	517	f	0	14	19	31	92	2.19	(0.98- 4.87)
WYNDE6	759	m	0	111	92	51	589	13.93	(9.36- 20.74)
WYNDE6	767	f	0	127	90	73	673	13.01	(9.06- 18.69)
Subtotal WYNDE6								13.42	(10.27- 17.54)
ZHENG	564	m	0	145	109	33	94	3.79	(2.37- 6.05)
Partial Totals				4328	219533	2592	687041		

*prospective study ~ With 0.5 adjustment for zero

REF	NRR	SEX	AD	Ys	Ws	Qs	Ps
AGUDO	501	f	0	0.29	4.10	2.08	0.5594
AUVINE	509	c	0	2.68	12.94	36.65	0.0000
BARBON	515	m	0	2.11	16.50	20.32	0.0000
BRESLO	501	c	0	0.99	7.67	0.00	0.0060
BUFFLE	518	f	0	2.56	7.00	16.94	0.0000
*CEDERL	510	m	1	1.87	4.29	3.25	0.0001
*CEDERL	515	f	0	0.69	4.57	0.45	0.1414
Subtotal CEDERL				1.26	8.85	3.70	
CHEN2	518	m	0	1.45	4.63	0.92	0.0018
CHEN2	523	f	0	0.40	3.26	1.20	0.4750
Subtotal CHEN2				1.01	7.90	2.12	
CHIAZZ	501	m	2	1.10	0.75	0.01	0.3421
CHOI	523	m	0	1.23	7.80	0.40	0.0006
CHOI	530	f	0	0.26	7.94	4.38	0.4667
Subtotal CHOI				0.74	15.74	4.79	
*CPSI	801	m	1	1.41	27.80	4.55	0.0000
*CPSI	845	f	1	0.81	38.69	1.40	0.0000
Subtotal CPSI				1.06	66.49	5.96	
DAMBER	501	m	0	1.52	17.84	4.75	0.0000
DEAN3	564	m	0	1.92	10.14	8.50	0.0000
DEAN3	583	f	0	1.31	15.21	1.42	0.0000
Subtotal DEAN3				1.55	25.35	9.92	
DOLL	503	m	0	2.11	5.99	7.42	0.0000
DOLL	510	f	0	0.82	6.57	0.23	0.0364
Subtotal DOLL				1.43	12.56	7.64	
*DORN	610	m	0	1.47	14.92	3.22	0.0000
*DORN	647	m	0	1.44	31.75	6.12	0.0000
Subtotal DORN				1.45	46.67	9.35	
*ENGELA	502	m	0	1.28	17.55	1.37	0.0000
*ENGELA	510	f	0	2.10	16.67	20.05	0.0000
Subtotal ENGELA				1.68	34.22	21.42	
GAO	502	m	0	1.51	36.16	9.25	0.0000
GAO	512	f	0	1.08	25.10	0.16	0.0000
Subtotal GAO				1.33	61.27	9.42	
GAO2	502	m	0	1.86	8.74	6.47	0.0000
GENG	528	f	0	0.44	10.28	3.22	0.1565
HAENSZ	537	f	0	0.66	18.36	2.09	0.0044
HEGMAN	513	m	1	2.24	7.47	11.48	0.0000
HEGMAN	516	f	1	1.57	1.60	0.52	0.0470
Subtotal HEGMAN				2.12	9.08	11.99	
*HIRAYA	501	m	1	1.47	83.52	18.36	0.0000
*HIRAYA	504	f	1	0.90	65.73	0.67	0.0000
Subtotal HIRAYA				1.22	149.25	19.03	
HU2	503	c	0	0.93	42.35	0.21	0.0000
JOLY	543	m	0	1.54	6.34	1.85	0.0001
JOLY	533	f	0	1.12	11.03	0.15	0.0002
Subtotal JOLY				1.27	17.37	1.99	
KHUDER	506	m	0	1.85	14.88	10.74	0.0000
KOULUM	502	m	0	2.27	4.00	6.43	0.0000
LETOUR	501	c	0	2.39	17.33	33.65	0.0000
*LIAW	504	c	2	0.41	6.39	2.27	0.3054
LIU3	501	m	0	0.17	2.74	1.88	0.7761
LIU4	501	m	2	0.88	3072.74	45.53	0.0000
LUBIN	566	m	0	1.27	6.79	0.49	0.0009
LUBIN2	1148	m	0	1.68	115.66	52.98	0.0000
MATOS	556	m	0	1.34	6.69	0.79	0.0005
*MIGRAN	501	m	0	2.05	3.46	3.82	0.0001
*MIGRAN	511	f	0	2.08	2.94	3.40	0.0004
Subtotal MIGRAN				2.06	6.40	7.22	
*MRFITR	508	m	0	3.17	0.44	2.07	0.0357
PEZZOT	570	m	0	2.43	3.42	6.95	0.0000

Table 1H17 - 5

IESLC - Meta-analysis of Ever/current Smoking, Age started, "Low"
All LC types, Cigarettes (or Any Product if Cigarettes not available)
Least adjusted

REF	NRR	SEX	AD	Ys	Ws	Qs	Ps
*QIAO2	501	m	0	0.64	8.52	1.12	0.0623
RACHTA	502	f	0	1.42	7.35	1.27	0.0001
SEGI2	516	m	0	0.74	5.86	0.40	0.0736
SOBUE	654	m	0	1.37	16.73	2.32	0.0000
SUZUK2	501	c	0	1.25	4.59	0.29	0.0072
SVENSS	501	f	0	1.73	8.23	4.31	0.0000
TIZZAN	507	m	0	0.47	66.40	18.43	0.0001
TIZZAN	537	f	0	-0.59	6.74	16.99	0.1283
Subtotal TIZZAN				0.38	73.15	35.42	
WAKAI	502	m	0	1.53	7.78	2.17	0.0000
WU	517	f	0	0.78	5.98	0.29	0.0557
WYNDE6	759	m	0	2.63	24.28	64.75	0.0000
WYNDE6	767	f	0	2.57	29.27	71.62	0.0000
Subtotal WYNDE6				2.60	53.55	136.36	
ZHENG	564	m	0	1.33	17.54	1.92	0.0000

	N	61
	NS	46
	Wt	4068.02
Het	Chi	556.97
Het	df	60
Het	P	***
Fixed	RR	2.72
	RRl	2.64
	RRu	2.81
	P	+++
Random	RR	3.97
	RRl	3.39
	RRu	4.66
	P	+++
Asymm	P	***

Table 1H17 - 6

IESLC - Meta-analysis of Ever/current Smoking, Age started, "Low"
All LC types, Cigarettes (or Any Product if Cigarettes not available)
Least adjusted

		combined	Sex male	female	Total
	N	6	34	21	61
	NS	6	33	21	60
	Wt	91.27	3680.12	296.64	4068.02
Het	Chi	55.38	329.10	146.14	556.97
Het	df	5	33	20	60
Het	P	***	***	***	***
Fixed	RR	4.23	2.66	3.16	2.72
	RRl	3.44	2.58	2.82	2.64
	RRu	5.19	2.75	3.54	2.81
	P	+++	+++	+++	+++
Random	RR	4.33	4.59	3.01	3.97
	RRl	2.05	3.72	2.16	3.39
	RRu	9.17	5.65	4.19	4.66
	P	+++	+++	+++	+++
Between	Chi				26.35
Between	df				2
Between	P				***
Btwn(F)	P				N.S.
Btwn(R)	P				N.S.

Table 1H17 - 7

IESLC - Meta-analysis of Ever/current Smoking, Age started, "Low"
All LC types, Cigarettes (or Any Product if Cigarettes not available)
Excluded studies (and stage at which they were excluded)

1	BECHER BLOT1 BROWN3 CARPEN CHYOU DARBY DOLL2 GARCIA GRAHAM GURSEL HAMMO2 JAHN JAIN LAUSSM PRESCO QIAO TVERDA WIGLE WYNDE3
2	AKIBA AMANDU AMES AXELSS BENSHL BEST BOUCHA BOUCOT BROSS CHEN CPSII DEAN2 DESTEF DORGAN DOSEME FAN GARSHI GER GILLIS HAMMON HUMBLE JUSSAW KAISE2 KATSOU KAUFMA KOO KREUZE LEVIN MCCONN NOTAN2 OSANN2 PEZZO2 PISANI RESTRE SADOWS VUTUC WANG2 WATSON WU2 WUWILL WYNDE2 WYNDE8 XU ZHOU
3	GUO MCDUFF SPITZ STASZE ZHANG
4	LUO
5	CORREA HOLE YUAN
7	BOFFET WYNDE7
10	ALDERS SPEIZE
14	ARMADA HU JEDRYC LIU5 PERNU
15	BENHAM

Table 1H17 - 8
Potentially overlapping studies

REF	REFGP	PRINC	.	OVERLAP/LINK
LUBIN2	LUBIN2	1		Lubin-combined
MRFITR	MRFIT	2		Subset of MRFIT
WYNDE6	WYNDE6	1		WYNDE5/6/7/8
CPSI	CPSI	1		CPSI overall
LUBIN	XIANGZ	2		LUBIN/XIANGZ/QIAO

Table 1H17 - 9
Most adjusted - insufficient data for meta-analysis

REF	NRR	SEX	AGEL	AGEH	RACE	YF	LC TYPE	LOC	START	ST	NLC	R	VB	P	H	AD	SM	PRODUCT	exL	exH	DENOM	De
CORREA	535	c	0	0	all	-	all	NAmer	1979	CC	1359	n	bl	y	n	2	ev	cig+/-ot	21	999	nev cigs	or
JEDRYC	618	f	0	0	all	-	all	Eu:est	1980	CC	1630	n	bl	y	n	0	ev	cig+/-ot	23	999	nev any	st

REF	NRR	RR	SIG	RRDATA comment
CORREA	535	8.30		0
JEDRYC	618	*		0

Table 1H18 -

IESLC - Meta-analysis of Ever/current Smoking, Age started, "Mid"
All LC types, Cigarettes (or Any Product if Cigarettes not available)

This analysis is restricted to results for:
1) Ever/current smokers
2) Results by Age started
3) Categorical results by Age started
4) All LC types (or near equivalent)
5) Results complete enough for use in metaanalysis

Within each study, results are then selected (in the following order of preference, within each sex) for:
6) SMKSTA: ever, current
7) PRODUCT: cigarettes regardless of other products, cigarettes only, all/unspec
8) CIGTYPE: all/unspecified, MC regardless of HR, MC only
9) (not applicable)
10) DENOM: never smoked anything, never smoked cigarettes, never any + low, never cigs + low
11) Followup period (YF, prospective studies): whole study (coded as 0) or longest available
12) LCtype: all or nearest available, at least Squamous and Adeno. (q = squamous, s = small,
l = large, a = adeno, mix = mixed, alv = alveolar)
13) Race: all or nearest available, otherwise by race (wh or w = white, bl or b = black, hi = hispanic
ch = chinese, jap = japanese, haw = hawaiian, w+o = white + oriental, sca = scandinavian, as = asian)
14) Age started "mid" in key scheme 1 (key value 18, maximum range 15-25)
15) For overlapping studies: principal rather than subsidiary studies
Finally by Age: whole study (coded as 0) if available, otherwise by widest available age group
and then for single sex results (m, f) in preference to results for both sexes combined (c).

Results adjusted (AD) for the most potential confounders are then chosen in Sections -1 to -3
(and those which actually differ from the adjusted results in Table 1H13 - 1 are marked 'x' in Section -1)
and results adjusted for the least confounders in Sections -4 to -6. (Those least adjusted results which
actually differ from the most adjusted are marked 'x' in column X in Section -4)

Section -7 shows excluded studies, together with the stage (as above) at which no qualifying
results were found.

Section -8 lists the potentially overlapping studies which have been included (1=principal, 2=subsidiary).

Section -9 lists any results which would have been included in preference except that they had data not complete
enough for use in meta-analysis, with their significance (yes/no), if known, and any further comment as entered
on the database. It also lists as "gap" any categories for which no data were presented by the original authors.

In addition to those mentioned above, the following fields, levels and abbreviations are used:
* or nk = not known, n = no, y = yes, ot = other
ev = ever, cu = current, nev = never
all/unspec = all or unspecified, cig+/-ot = cigarettes irrespective of other products (cigar, pipe etc)
MC = manufactured cigarettes, HR = hand-rolled cigarettes
exL, exH = range of exposure (low and high) in the smoking group, in terms of Age started
REF: 6-character study reference
NRR: number of the RR on the database within the study
ST : study type (CC = case control, pr or prosp = prospective)
NLC: number of lung cancer cases in whole study
R : risky occupational population (n = no, m = mining, o = other risky)
VB : national cigarette type (V = at least 75% Virginia, bl = at least 75% blended, ot = other)
P : any proxy use
H : full histological confirmation
De : derivation of RR/CI (or = original, st = standard method, ot = other method of estimation)

Table 1H18 - 1

IESLC - Meta-analysis of Ever/current Smoking, Age started, "Mid"
All LC types, Cigarettes (or Any Product if Cigarettes not available)
Most adjusted

REF	NRR	1H13	SEX	AGEL	AGEH	RACE	YF	LC TYPE	LOC	START	ST	NLC	R	VB	P	H	AD	SM	PRODUCT	exL	exH	DENOM	De
AUVINE	510		c	0	0	all	-	all	Eu:Sca	1986	CC	517	n	bl	y	n	0	ev	cig+/-ot	16	20	nev cigs	st
BARBON	521		m	0	0	all	-	all	Eu:wst	1979	CC	755	n	bl	y	y	1	ev	all/unsp	15	19	nev any	or
BRESLO	502		c	0	0	all	-	all	NAmer	1949	CC	518	n	bl	n	y	0	ev	cig+/-ot	15	24	nev any	st
BUFFLE	520		f	0	0	w-hi	-	all	NAmer	1976	CC	943	n	bl	y	n	0	ev	cig+/-ot	17	18	nev cigs	ot
CEDERL	511		m	0	0	all	10	all	Eu:Sca	1963	pr	491	n	bl	n	n	1	cu	cig only	17	18	nev any	ot
CEDERL	516		f	0	0	all	10	all	Eu:Sca	1963	pr	491	n	bl	n	n	0	cu	cig only	17	18	nev any	st
CHOI	525		m	0	0	all	-	all	As:oth	1985	CC	375	n	bl	n	n	0	ev	cig+/-ot	15	19	nev cigs	st
CPSI	803		m	35	84	all	6	all	NAmer	1959	pr	5138	n	bl	n	n	1	cu	cig+/-ot	15	19	nev any	ot
CPSI	847		f	40	74	all	6	all	NAmer	1959	pr	5138	n	bl	n	n	1	cu	cig only	15	19	nev any	ot
DAMBER	502		m	0	0	all	-	all	Eu:Sca	1972	CC	579	n	bl	y	n	0	ev	all/unsp	16	20	nev any	st
DEAN3	566		m	0	0	all	-	all	Eu:UK	1969	CC	766	n	V	y	n	0	cu	cig only	15	19	nev any	st
DEAN3	585		f	0	0	all	-	all	Eu:UK	1969	CC	766	n	V	y	n	0	cu	cig only	15	19	nev any	st
DORN	612		m	55	64	wh	8	all	NAmer	1954	pr	5097	n	bl	n	n	0	ev	cig+/-ot	15	19	nev any	st
DORN	649		m	65	74	wh	8	all	NAmer	1954	pr	5097	n	bl	n	n	0	ev	cig+/-ot	15	19	nev any	st
GENG	534		f	0	0	all	-	all	As:Chi	1985	CC	292	n	ot	*	n	1	ev	cig+/-ot	16	20	nev any	st
JEDRYC	607		m	0	0	all	-	all	Eu:est	1980	CC	1630	n	bl	y	n	0	ev	cig+/-ot	17	18	nev any	st
JOLY	544		m	0	0	all	-	all	SCAmer	1978	CC	826	n	bl	n	n	0	ev	cig+/-ot	15	24	nev any	st
JOLY	534		f	0	0	all	-	all	SCAmer	1978	CC	826	n	bl	n	n	0	ev	cig+/-ot	15	24	nev any	st
KHUDER	507		m	0	0	all	-	all	NAmer	1985	CC	482	n	bl	n	y	0	ev	cig+/-ot	16	19	nev cigs	st
KOULUM	503		m	0	0	all	-	all	Eu:Sca	1936	CC	812	n	bl	n	n	0	ev	all/unsp	16	20	nev any	st
LETOUR	502		c	0	0	all	-	all	NAmer	1983	CC	738	n	V	y	y	0	ev	cig+/-ot	15	20	nev cigs	st
LUBIN2	1158		m	0	0	all	-	all	Eu:mul	1976	CC	7804	n	bl	n	y	1	ev	cig+/-ot	17	20	nev cigs	st
MATOS	577		m	0	0	all	-	all	SCAmer	1994	CC	200	n	bl	n	n	2	ev	cig+/-ot	15	19	nev any	or
MIGRAN	503		m	0	0	all	0	all	Eu:UK	1964	pr	259	n	V	n	n	0	cu	cig only	16	19	nev any	st
MIGRAN	513		f	0	0	all	0	all	Eu:UK	1964	pr	259	n	V	n	n	0	cu	cig only	16	19	nev any	st
MRFITR	511		m	0	0	all	0	all	NAmer	1973	pr	119	n	bl	n	n	0	cu	cig+/-ot	18	19	nev cigs	ot
QIAO2	507		m	0	0	all	0	all	As:Chi	1992	pr	241	m	ot	n	n	1	ev	all/unsp	17	20	nev any	or
SOBUE	655		m	0	0	all	-	all	As:Jap	1986	CC	1376	n	bl	n	y	0	ev	cig+/-ot	18	22	nev cigs	st
WYNDE6	760		m	0	0	wh	-	q+s+a	NAmer	1969	CC	4423	n	bl	n	y	0	ev	cig+/-ot	18	20	nev cigs	st
WYNDE6	768		f	0	0	wh	-	q+s+a	NAmer	1969	CC	4423	n	bl	n	y	0	ev	cig+/-ot	18	20	nev cigs	st

Cigarette type is all/unspec for all RRs
except for the following:

REF	NRR	CIGTYPE
DEAN3	566	MC only
DEAN3	585	MC only

Table 1H18 - 2

IESLC - Meta-analysis of Ever/current Smoking, Age started, "Mid"
All LC types, Cigarettes (or Any Product if Cigarettes not available)
Most adjusted

REF	NRR	SEX	AD	Number Exposed Case	Number Exposed Cont	Non-exposed Case	Non-exposed Cont	RR	95.00%CI
AUVINE	510	c	0	135	47	44	229	14.95	(9.41- 23.75)
BARBON	521	m	1	395	-	22	-	9.90	(6.20- 15.80)
BRESLO	502	c	0	286	243	19	56	3.47	(2.01- 6.00)
BUFFLE	520	f	0	53	33	12	112	14.99	(7.17- 31.33)
*CEDERL	511	m	1	10	-	7	-	9.80	(3.74- 25.69)
*CEDERL	516	f	0	2	1009	19	17679	1.84	(0.43- 7.91)
Subtotal CEDERL								5.89	(2.64- 13.16)
CHOI	525	m	0	79	138	13	95	4.18	(2.20- 7.95)
*CPSI	803	m	1	588	-	83	-	14.69	(11.68- 18.49)
*CPSI	847	f	1	52	-	166	-	5.00	(3.66- 6.83)
Subtotal CPSI								10.06	(8.36- 12.10)
DAMBER	502	m	0	261	190	42	208	6.80	(4.65- 9.95)
DEAN3	566	m	0	160	485	24	510	7.01	(4.48- 10.96)
DEAN3	585	f	0	39	504	41	1538	2.90	(1.85- 4.55)
Subtotal DEAN3								4.52	(3.30- 6.21)
*DORN	612	m	0	342	213156	25	213858	13.73	(9.14- 20.60)
*DORN	649	m	0	306	118234	49	171211	9.04	(6.69- 12.23)
Subtotal DORN								10.49	(8.23- 13.36)
GENG	534	f	1	39	-	54	-	2.95	(1.57- 5.52)
JEDRYC	607	m	0	239	146	49	219	7.32	(5.04- 10.61)
JOLY	544	m	0	217	357	12	218	11.04	(6.03- 20.22)
JOLY	534	f	0	67	47	52	283	7.76	(4.82- 12.49)
Subtotal JOLY								8.88	(6.11- 12.91)
KHUDER	507	m	0	161	338	23	309	6.40	(4.03- 10.17)
KOULUM	503	m	0	267	103	5	54	28.00	(10.89- 71.96)
LETOUR	502	c	0	309	241	24	224	11.97	(7.60- 18.83)
LUBIN2	1158	m	1	1796	-	185	-	5.43	(4.59- 6.41)
MATOS	577	m	2	91	-	11	-	7.80	(4.00- 15.50)
*MIGRAN	503	m	0	59	1845	4	867	6.93	(2.53- 19.02)
*MIGRAN	513	f	0	9	1035	4	3814	8.29	(2.56- 26.87)
Subtotal MIGRAN								7.48	(3.48- 16.09)
*MRFITR	511	m	0	25	1876	0	1859	50.54~	(3.08- 829.51)
*QIAO2	507	m	1	75	-	10	-	1.47	(0.76- 2.84)
SOBUE	655	m	0	776	772	29	126	4.37	(2.88- 6.62)
WYNDE6	760	m	0	223	139	51	589	18.53	(12.98- 26.45)
WYNDE6	768	f	0	200	94	73	673	19.62	(13.90- 27.67)
Subtotal WYNDE6								19.08	(14.90- 24.44)
Partial Totals				7261	341032	1152	414731		

*prospective study ~ With 0.5 adjustment for zero

REF	NRR	SEX	AD	Ys	Ws	Qs	Ps
AUVINE	510	c	0	2.70	17.93	7.32	0.0000
BARBON	521	m	1	2.29	17.56	0.90	0.0000
BRESLO	502	c	0	1.24	12.80	8.65	0.0000
BUFFLE	520	f	0	2.71	7.07	2.91	0.0000
*CEDERL	511	m	1	2.28	4.14	0.19	0.0000
*CEDERL	516	f	0	0.61	1.81	3.83	0.4098
Subtotal CEDERL				1.77	5.95	4.02	
CHOI	525	m	0	1.43	9.31	3.75	0.0000
*CPSI	803	m	1	2.69	72.82	28.13	0.0000
*CPSI	847	f	1	1.61	39.48	8.22	0.0000
Subtotal CPSI				2.31	112.30	36.35	
DAMBER	502	m	0	1.92	26.52	0.58	0.0000
DEAN3	566	m	0	1.95	19.25	0.27	0.0000
DEAN3	585	f	0	1.07	18.99	18.99	0.0000
Subtotal DEAN3				1.51	38.24	19.26	
*DORN	612	m	0	2.62	23.30	7.14	0.0000
*DORN	649	m	0	2.20	42.26	0.79	0.0000
Subtotal DORN				2.35	65.56	7.93	
GENG	534	f	1	1.08	9.72	9.41	0.0007
JEDRYC	607	m	0	1.99	27.77	0.16	0.0000
JOLY	544	m	0	2.40	10.49	1.18	0.0000
JOLY	534	f	0	2.05	16.96	0.00	0.0000
Subtotal JOLY				2.18	27.45	1.19	
KHUDER	507	m	0	1.86	17.89	0.78	0.0000
KOULUM	503	m	0	3.33	4.31	6.91	0.0000
LETOUR	502	c	0	2.48	18.69	3.24	0.0000
LUBIN2	1158	m	1	1.69	137.76	19.24	0.0000
MATOS	577	m	2	2.05	8.37	0.00	0.0000
*MIGRAN	503	m	0	1.94	3.77	0.06	0.0002

Table 1H18 - 2

IESLC - Meta-analysis of Ever/current Smoking, Age started, "Mid"
All LC types, Cigarettes (or Any Product if Cigarettes not available)
Most adjusted

REF	NRR	SEX	AD	Ys	Ws	Qs	Ps
*MIGRAN	513	f	0	2.12	2.78	0.01	0.0004
Subtotal MIGRAN				2.01	6.55	0.07	
*MRFITR	511	m	0	3.92	0.49	1.69	0.0060
*QIAO2	507	m	1	0.39	8.84	24.97	0.2520
SOBUE	655	m	0	1.47	22.22	7.77	0.0000
WYNDE6	760	m	0	2.92	30.32	22.09	0.0000
WYNDE6	768	f	0	2.98	32.44	26.91	0.0000
Subtotal WYNDE6				2.95	62.76	49.00	

	N	30
	NS	23
	Wt	666.08
Het	Chi	216.11
Het	df	29
Het	P	***
Fixed	RR	7.89
	RRl	7.31
	RRu	8.51
	P	+++
Random	RR	7.67
	RRl	6.12
	RRu	9.62
	P	+++
Asymm	P	N.S.

Table 1H18 - 3

IESLC - Meta-analysis of Ever/current Smoking, Age started, "Mid"
All LC types, Cigarettes (or Any Product if Cigarettes not available)
Most adjusted

	Sex			
	combined	male	female	Total
N	3	19	8	30
NS	3	18	8	29
Wt	49.42	487.41	129.25	666.08
Het Chi	17.67	126.54	68.47	216.11
Het df	2	18	7	29
Het P	***	***	***	***
Fixed RR	9.41	8.00	7.01	7.89
RRl	7.12	7.32	5.90	7.31
RRu	12.44	8.74	8.33	8.51
P	+++	+++	+++	+++
Random RR	8.64	8.08	6.23	7.67
RRl	3.75	6.21	3.44	6.12
RRu	19.90	10.53	11.26	9.62
P	+++	+++	+++	+++
Between Chi				3.43
Between df				2
Between P				N.S.
Btwn(F) P				N.S.
Btwn(R) P				N.S.

	Lung cancer type		
	all	other	Total
N	28	2	30
NS	22	1	23
Wt	603.32	62.76	666.08
Het Chi	162.02	0.05	216.11
Het df	27	1	29
Het P	***	N.S.	***
Fixed RR	7.20	19.08	7.89
RRl	6.65	14.90	7.31
RRu	7.80	24.44	8.51
P	+++	+++	+++
Random RR	7.09	19.08	7.67
RRl	5.71	14.90	6.12
RRu	8.81	24.44	9.62
P	+++	+++	+++
Between Chi			54.04
Between df			1
Between P			***
Btwn(F) P			**
Btwn(R) P			***

	Location								
	NAmer	UK	Scand	othEur	China	Japan	othAs	other	Total
N	11	4	5	3	2	1	1	3	30
NS	8	2	4	3	2	1	1	2	23
Wt	297.57	44.79	54.71	183.09	18.56	22.22	9.31	35.82	666.08
Het Chi	76.74	8.90	16.53	6.87	2.25	0.00	0.00	0.92	216.11
Het df	10	3	4	2	1	0	0	2	29
Het P	***	*	**	*	N.S.	N.S.	N.S.	N.S.	***
Fixed RR	11.05	4.87	9.69	6.02	2.12	4.37	4.18	8.61	7.89
RRl	9.87	3.63	7.44	5.21	1.34	2.88	2.20	6.21	7.31
RRu	12.38	6.53	12.63	6.96	3.34	6.62	7.95	11.95	8.51
P	+++	+++	+++	+++	++	+++	+++	+++	+++
Random RR	10.57	5.36	9.85	6.95	2.10	4.37	4.18	8.61	7.67
RRl	7.52	3.00	5.21	4.90	1.06	2.88	2.20	6.21	6.12
RRu	14.86	9.56	18.62	9.86	4.15	6.62	7.95	11.95	9.62
P	+++	+++	+++	+++	+	+++	+++	+++	+++
Between Chi									103.91
Between df									7
Between P									***
Btwn(F) P									*
Btwn(R) P									***

Table 1H18 - 3

IESLC - Meta-analysis of Ever/current Smoking, Age started, "Mid"
All LC types, Cigarettes (or Any Product if Cigarettes not available)
Most adjusted

Detailed Country in "other Europe"

	multi	Germany	othWest	East	Balkans	Total
N	1		1	1		3
NS	1		1	1		3
Wt	137.76		17.56	27.77		183.09
Het Chi	0.00		0.00	0.00		6.87
Het df	0		0	0		2
Het P	N.S.		N.S.	N.S.		*
Fixed RR	5.43		9.90	7.32		6.02
RRl	4.59		6.20	5.04		5.21
RRu	6.42		15.80	10.61		6.96
P	+++		+++	+++		+++
Random RR	5.43		9.90	7.32		6.95
RRl	4.59		6.20	5.04		4.90
RRu	6.42		15.80	10.61		9.86
P	+++		+++	+++		+++
Between Chi						6.87
Between df						2
Between P						*
Btwn(F) P						N.S.
Btwn(R) P						*

Detailed Country in "other Asia"

	India	HongKong	other	Total
N			1	1
NS			1	1
Wt			9.31	9.31
Het Chi			0.00	0.00
Het df			0	0
Het P			N.S.	N.S.
Fixed RR			4.18	4.18
RRl			2.20	2.20
RRu			7.95	7.95
P			+++	+++
Random RR			4.18	4.18
RRl			2.20	2.20
RRu			7.95	7.95
P			+++	+++
Between Chi				
Between df				
Between P				N.S.
Btwn(F) P				N.S.
Btwn(R) P				N.S.

Detailed other continent

	SCAmer	Total
N	3	3
NS	2	2
Wt	35.82	35.82
Het Chi	0.92	0.92
Het df	2	2
Het P	N.S.	N.S.
Fixed RR	8.61	8.61
RRl	6.21	6.21
RRu	11.95	11.95
P	+++	+++
Random RR	8.61	8.61
RRl	6.21	6.21
RRu	11.95	11.95
P	+++	+++
Between Chi		
Between df		
Between P		N.S.
Btwn(F) P		N.S.
Btwn(R) P		N.S.

Table 1H18 - 3

IESLC - Meta-analysis of Ever/current Smoking, Age started, "Mid"
All LC types, Cigarettes (or Any Product if Cigarettes not available)
Most adjusted

	Start year of study					
	<1960	1960-69	1970-79	1980-89	1990+	Total
N	6	8	7	7	2	30
NS	4	4	6	7	2	23
Wt	194.98	113.50	216.84	123.54	17.22	666.08
Het Chi	51.28	63.29	18.09	30.56	11.98	216.11
Het df	5	7	6	6	1	29
Het P	***	***	**	***	***	***
Fixed RR	9.73	10.47	6.48	6.98	3.31	7.89
RRl	8.46	8.71	5.67	5.85	2.06	7.31
RRu	11.20	12.58	7.40	8.32	5.31	8.51
P	+++	+++	+++	+++	+++	+++
Random RR	9.35	7.85	8.30	6.59	3.38	7.67
RRl	5.73	4.23	6.08	4.39	0.66	6.12
RRu	15.23	14.56	11.35	9.88	17.34	9.62
P	+++	+++	+++	+++	N.S.	+++
Between Chi						40.91
Between df						4
Between P						***
Btwn(F) P						N.S.
Btwn(R) P						N.S.

	Study type (1)		
	CC	other	Total
N	20	10	30
NS	17	6	23
Wt	466.38	199.70	666.08
Het Chi	139.17	70.56	216.11
Het df	19	9	29
Het P	***	***	***
Fixed RR	7.40	9.16	7.89
RRl	6.76	7.98	7.31
RRu	8.10	10.53	8.51
P	+++	+++	+++
Random RR	7.89	7.09	7.67
RRl	6.07	4.41	6.12
RRu	10.26	11.39	9.62
P	+++	+++	+++
Between Chi			6.38
Between df			1
Between P			*
Btwn(F) P			N.S.
Btwn(R) P			N.S.

	Study type (2)			
	CC	prosp	other	Total
N	20	10		30
NS	17	6		23
Wt	466.38	199.70		666.08
Het Chi	139.17	70.56		216.11
Het df	19	9		29
Het P	***	***		***
Fixed RR	7.40	9.16		7.89
RRl	6.76	7.98		7.31
RRu	8.10	10.53		8.51
P	+++	+++		+++
Random RR	7.89	7.09		7.67
RRl	6.07	4.41		6.12
RRu	10.26	11.39		9.62
P	+++	+++		+++
Between Chi				6.38
Between df				1
Between P				*
Btwn(F) P				N.S.
Btwn(R) P				N.S.

Table 1H18 - 3

IESLC - Meta-analysis of Ever/current Smoking, Age started, "Mid"
All LC types, Cigarettes (or Any Product if Cigarettes not available)
Most adjusted

	Study size (number of LC cases) 100-249	250-499	500-999	1000+	Total
N	3	7	11	9	30
NS	3	5	9	6	23
Wt	17.71	49.43	170.57	428.38	666.08
Het Chi	15.52	8.85	50.97	117.65	216.11
Het df	2	6	10	8	29
Het P	***	N.S.	***	***	***
Fixed RR	3.57	5.13	7.92	8.55	7.89
RRl	2.24	3.88	6.82	7.78	7.31
RRu	5.69	6.78	9.21	9.40	8.51
P	+++	+++	+++	+++	+++
Random RR	5.55	5.10	8.56	9.39	7.67
RRl	1.16	3.54	6.05	6.43	6.12
RRu	26.63	7.35	12.11	13.72	9.62
P	+	+++	+++	+++	+++
Between Chi					23.13
Between df					3
Between P					***
Btwn(F) P					N.S.
Btwn(R) P					(*)

	Risky occupational population no	mining	othRisky	Total
N	29	1		30
NS	22	1		23
Wt	657.24	8.84		666.08
Het Chi	190.81	0.00		216.11
Het df	28	0		29
Het P	***	N.S.		***
Fixed RR	8.07	1.47		7.89
RRl	7.48	0.76		7.31
RRu	8.71	2.84		8.51
P	+++	N.S.		+++
Random RR	8.10	1.47		7.67
RRl	6.51	0.76		6.12
RRu	10.09	2.84		9.62
P	+++	N.S.		+++
Between Chi				25.30
Between df				1
Between P				***
Btwn(F) P				(*)
Btwn(R) P				***

	National cigarette tobacco type Virginia	blended	other	Total
N	5	23	2	30
NS	3	18	2	23
Wt	63.48	584.04	18.56	666.08
Het Chi	19.55	156.66	2.25	216.11
Het df	4	22	1	29
Het P	***	***	N.S.	***
Fixed RR	6.35	8.42	2.12	7.89
RRl	4.96	7.77	1.34	7.31
RRu	8.12	9.14	3.34	8.51
P	+++	+++	++	+++
Random RR	6.59	8.81	2.10	7.67
RRl	3.63	6.94	1.06	6.12
RRu	11.96	11.19	4.15	9.62
P	+++	+++	+	+++
Between Chi				37.65
Between df				2
Between P				***
Btwn(F) P				(*)
Btwn(R) P				***

Table 1H18 - 3

IESLC - Meta-analysis of Ever/current Smoking, Age started, "Mid"
All LC types, Cigarettes (or Any Product if Cigarettes not available)
Most adjusted

	Any proxy use No/nk	Yes	Total
N	22	8	30
NS	16	7	23
Wt	512.31	153.77	666.08
Het Chi	181.74	34.37	216.11
Het df	21	7	29
Het P	***	***	***
Fixed RR	7.88	7.91	7.89
RRl	7.23	6.76	7.31
RRu	8.60	9.27	8.51
P	+++	+++	+++
Random RR	7.40	8.29	7.67
RRl	5.55	5.81	6.12
RRu	9.86	11.84	9.62
P	+++	+++	+++
Between Chi			0.00
Between df			1
Between P			N.S.
Btwn(F) P			N.S.
Btwn(R) P			N.S.

	Full histological confirmation No	Yes	Total
N	22	8	30
NS	16	7	23
Wt	376.40	289.68	666.08
Het Chi	126.16	89.12	216.11
Het df	21	7	29
Het P	***	***	***
Fixed RR	8.14	7.58	7.89
RRl	7.36	6.75	7.31
RRu	9.00	8.50	8.51
P	+++	+++	+++
Random RR	7.38	8.38	7.67
RRl	5.62	5.31	6.12
RRu	9.69	13.21	9.62
P	+++	+++	+++
Between Chi			0.84
Between df			1
Between P			N.S.
Btwn(F) P			N.S.
Btwn(R) P			N.S.

	Number of adjustment variables (1) 0	1	2+/+nk	Total
N	22	7	1	30
NS	17	6	1	24
Wt	367.38	290.32	8.37	666.08
Het Chi	119.81	84.46	0.00	216.11
Het df	21	6	0	29
Het P	***	***	N.S.	***
Fixed RR	8.89	6.79	7.80	7.89
RRl	8.03	6.05	3.96	7.31
RRu	9.85	7.61	15.35	8.51
P	+++	+++	+++	+++
Random RR	8.50	5.69	7.80	7.67
RRl	6.54	3.44	3.96	6.12
RRu	11.03	9.41	15.35	9.62
P	+++	+++	+++	+++
Between Chi				11.84
Between df				2
Between P				**
Btwn(F) P				N.S.
Btwn(R) P				N.S.

Table 1H18 - 3

IESLC - Meta-analysis of Ever/current Smoking, Age started, "Mid"
All LC types, Cigarettes (or Any Product if Cigarettes not available)
Most adjusted

	Number of adjustment variables (2) 0	1	2	3-5	6+/+nk	Total
N	22	7	1			30
NS	17	6	1			24
Wt	367.38	290.32	8.37			666.08
Het Chi	119.81	84.46	0.00			216.11
Het df	21	6	0			29
Het P	***	***	N.S.			***
Fixed RR	8.89	6.79	7.80			7.89
RRl	8.03	6.05	3.96			7.31
RRu	9.85	7.61	15.35			8.51
P	+++	+++	+++			+++
Random RR	8.50	5.69	7.80			7.67
RRl	6.54	3.44	3.96			6.12
RRu	11.03	9.41	15.35			9.62
P	+++	+++	+++			+++
Between Chi						11.84
Between df						2
Between P						**
Btwn(F) P						N.S.
Btwn(R) P						N.S.

	Smoking status ever	current	Total
N	21	9	30
NS	18	5	23
Wt	502.55	163.53	666.08
Het Chi	154.68	61.25	216.11
Het df	20	8	29
Het P	***	***	***
Fixed RR	7.82	8.13	7.89
RRl	7.16	6.97	7.31
RRu	8.53	9.47	8.51
P	+++	+++	+++
Random RR	8.05	6.65	7.67
RRl	6.21	3.91	6.12
RRu	10.43	11.31	9.62
P	+++	+++	+++
Between Chi			0.19
Between df			1
Between P			N.S.
Btwn(F) P			N.S.
Btwn(R) P			N.S.

	Product all/unsp	cig+/-ot	cig only	Total
N	4	19	7	30
NS	4	16	4	24
Wt	57.23	518.63	90.22	666.08
Het Chi	31.84	146.22	12.60	216.11
Het df	3	18	6	29
Het P	***	***	*	***
Fixed RR	6.70	8.70	4.99	7.89
RRl	5.17	7.98	4.06	7.31
RRu	8.68	9.48	6.13	8.51
P	+++	+++	+++	+++
Random RR	7.00	8.78	5.19	7.67
RRl	2.81	6.72	3.63	6.12
RRu	17.41	11.47	7.41	9.62
P	+++	+++	+++	+++
Between Chi				25.45
Between df				2
Between P				***
Btwn(F) P				N.S.
Btwn(R) P				(*)

Table 1H18 - 3

IESLC - Meta-analysis of Ever/current Smoking, Age started, "Mid"
All LC types, Cigarettes (or Any Product if Cigarettes not available)
Most adjusted

	Denominator nev any	nev cigs	Total
N	20	10	30
NS	14	9	23
Wt	371.96	294.12	666.08
Het Chi	120.31	95.60	216.11
Het df	19	9	29
Het P	***	***	***
Fixed RR	7.77	8.05	7.89
RRl	7.02	7.18	7.31
RRu	8.60	9.02	8.51
P	+++	+++	+++
Random RR	6.78	9.81	7.67
RRl	5.14	6.35	6.12
RRu	8.95	15.17	9.62
P	+++	+++	+++
Between Chi			0.21
Between df			1
Between P			N.S.
Btwn(F) P			N.S.
Btwn(R) P			N.S.

	Derivation of RR/CI Orig	StdCalc	Other	Total
N	3	22	5	30
NS	3	17	4	24
Wt	34.78	507.30	124.00	666.08
Het Chi	22.41	148.09	32.04	216.11
Het df	2	21	4	29
Het P	***	***	***	***
Fixed RR	5.76	7.55	10.35	7.89
RRl	4.13	6.92	8.68	7.31
RRu	8.02	8.23	12.34	8.51
P	+++	+++	+++	+++
Random RR	4.90	7.64	10.84	7.67
RRl	1.55	5.94	5.51	6.12
RRu	15.53	9.83	21.34	9.62
P	++	+++	+++	+++
Between Chi				13.57
Between df				2
Between P				**
Btwn(F) P				N.S.
Btwn(R) P				N.S.

Table 1H18 - 4

IESLC - Meta-analysis of Ever/current Smoking, Age started, "Mid"
All LC types, Cigarettes (or Any Product if Cigarettes not available)
Least adjusted

REF	NRR	X	SEX	AGEL	AGEH	RACE	YF	LC TYPE	LOC	START	ST	NLC	R	VB	P	H	AD	SM	PRODUCT	exL	exH	DENOM	De
AUVINE	510		c	0	0	all	-	all	Eu:Sca	1986	CC	517	n	bl	y	n	0	ev	cig+/-ot	16	20	nev cigs	st
BARBON	516	x	m	0	0	all	-	all	Eu:wst	1979	CC	755	n	bl	y	y	0	ev	all/unsp	15	19	nev any	st
BRESLO	502		c	0	0	all	-	all	NAmer	1949	CC	518	n	bl	n	y	0	ev	cig+/-ot	15	24	nev any	st
BUFFLE	520		f	0	0	w-hi	-	all	NAmer	1976	CC	943	n	bl	y	n	0	ev	cig+/-ot	17	18	nev cigs	ot
CEDERL	511		m	0	0	all	10	all	Eu:Sca	1963	pr	491	n	bl	n	n	1	cu	cig only	17	18	nev any	ot
CEDERL	516		f	0	0	all	10	all	Eu:Sca	1963	pr	491	n	bl	n	n	0	cu	cig only	17	18	nev any	st
CHOI	525		m	0	0	all	-	all	As:oth	1985	CC	375	n	bl	n	n	0	ev	cig+/-ot	15	19	nev cigs	st
CPSI	803		m	35	84	all	6	all	NAmer	1959	pr	5138	n	bl	n	n	1	cu	cig+/-ot	15	19	nev any	ot
CPSI	847		f	40	74	all	6	all	NAmer	1959	pr	5138	n	bl	n	n	1	cu	cig only	15	19	nev any	ot
DAMBER	502		m	0	0	all	-	all	Eu:Sca	1972	CC	579	n	bl	y	n	0	ev	all/unsp	16	20	nev any	st
DEAN3	566		m	0	0	all	-	all	Eu:UK	1969	CC	766	n	V	y	n	0	cu	cig only	15	19	nev any	st
DEAN3	585		f	0	0	all	-	all	Eu:UK	1969	CC	766	n	V	y	n	0	cu	cig only	15	19	nev any	st
DORN	612		m	55	64	wh	8	all	NAmer	1954	pr	5097	n	bl	n	n	0	ev	cig+/-ot	15	19	nev any	st
DORN	649		m	65	74	wh	8	all	NAmer	1954	pr	5097	n	bl	n	n	0	ev	cig+/-ot	15	19	nev any	st
GENG	529	x	f	0	0	all	-	all	As:Chi	1985	CC	292	n	ot	*	n	0	ev	cig+/-ot	16	20	nev any	st
JEDRYC	607		m	0	0	all	-	all	Eu:est	1980	CC	1630	n	bl	y	n	0	ev	cig+/-ot	17	18	nev any	st
JOLY	544		m	0	0	all	-	all	SCAmer	1978	CC	826	n	bl	n	n	0	ev	cig+/-ot	15	24	nev any	st
JOLY	534		f	0	0	all	-	all	SCAmer	1978	CC	826	n	bl	n	n	0	ev	cig+/-ot	15	24	nev any	st
KHUDER	507		m	0	0	all	-	all	NAmer	1985	CC	482	n	bl	n	y	0	ev	cig+/-ot	16	19	nev cigs	st
KOULUM	503		m	0	0	all	-	all	Eu:Sca	1936	CC	812	n	bl	n	n	0	ev	all/unsp	16	20	nev any	st
LETOUR	502		c	0	0	all	-	all	NAmer	1983	CC	738	n	V	y	y	0	ev	cig+/-ot	15	20	nev cigs	st
LUBIN2	1149	x	m	0	0	all	-	all	Eu:mul	1976	CC	7804	n	bl	n	y	0	ev	cig+/-ot	17	20	nev cigs	st
MATOS	557	x	m	0	0	all	-	all	SCAmer	1994	CC	200	n	bl	n	n	0	ev	cig+/-ot	15	19	nev any	st
MIGRAN	503		m	0	0	all	0	all	Eu:UK	1964	pr	259	n	V	n	n	0	cu	cig only	16	19	nev any	st
MIGRAN	513		f	0	0	all	0	all	Eu:UK	1964	pr	259	n	V	n	n	0	cu	cig only	16	19	nev any	st
MRFITR	511		m	0	0	all	0	all	NAmer	1973	pr	119	n	bl	n	n	0	cu	cig+/-ot	18	19	nev cigs	ot
QIAO2	502	x	m	0	0	all	0	all	As:Chi	1992	pr	241	m	ot	n	n	0	ev	all/unsp	17	20	nev any	st
SOBUE	655		m	0	0	all	-	all	As:Jap	1986	CC	1376	n	bl	n	y	0	ev	cig+/-ot	18	22	nev cigs	st
WYNDE6	760		m	0	0	wh	-	q+s+a	NAmer	1969	CC	4423	n	bl	n	y	0	ev	cig+/-ot	18	20	nev cigs	st
WYNDE6	768		f	0	0	wh	-	q+s+a	NAmer	1969	CC	4423	n	bl	n	y	0	ev	cig+/-ot	18	20	nev cigs	st

Cigarette type is all/unspec for all RRs
except for the following:

REF	NRR	CIGTYPE
DEAN3	566	MC only
DEAN3	585	MC only

Table 1H18 - 5

IESLC - Meta-analysis of Ever/current Smoking, Age started, "Mid"
All LC types, Cigarettes (or Any Product if Cigarettes not available)
Least adjusted

REF	NRR	SEX	AD	Number Exposed Case	Cont	Non-exposed Case	Cont	RR	95.00%CI
AUVINE	510	c	0	135	47	44	229	14.95	(9.41- 23.75)
BARBON	516	m	0	395	337	22	188	10.02	(6.29- 15.94)
BRESLO	502	c	0	286	243	19	56	3.47	(2.01- 6.00)
BUFFLE	520	f	0	53	33	12	112	14.99	(7.17- 31.33)
*CEDERL	511	m	1	10	-	7	-	9.80	(3.74- 25.69)
*CEDERL	516	f	0	2	1009	19	17679	1.84	(0.43- 7.91)
Subtotal CEDERL								5.89	(2.64- 13.16)
CHOI	525	m	0	79	138	13	95	4.18	(2.20- 7.95)
*CPSI	803	m	1	588	-	83	-	14.69	(11.68- 18.49)
*CPSI	847	f	1	52	-	166	-	5.00	(3.66- 6.83)
Subtotal CPSI								10.06	(8.36- 12.10)
DAMBER	502	m	0	261	190	42	208	6.80	(4.65- 9.95)
DEAN3	566	m	0	160	485	24	510	7.01	(4.48- 10.96)
DEAN3	585	f	0	39	504	41	1538	2.90	(1.85- 4.55)
Subtotal DEAN3								4.52	(3.30- 6.21)
*DORN	612	m	0	342	213156	25	213858	13.73	(9.14- 20.60)
*DORN	649	m	0	306	118234	49	171211	9.04	(6.69- 12.23)
Subtotal DORN								10.49	(8.23- 13.36)
GENG	529	f	0	39	23	54	93	2.92	(1.58- 5.40)
JEDRYC	607	m	0	239	146	49	219	7.32	(5.04- 10.61)
JOLY	544	m	0	217	357	12	218	11.04	(6.03- 20.22)
JOLY	534	f	0	67	47	52	283	7.76	(4.82- 12.49)
Subtotal JOLY								8.88	(6.11- 12.91)
KHUDER	507	m	0	161	338	23	309	6.40	(4.03- 10.17)
KOULUM	503	m	0	267	103	5	54	28.00	(10.89- 71.96)
LETOUR	502	c	0	309	241	24	224	11.97	(7.60- 18.83)
LUBIN2	1149	m	0	1796	3028	185	1878	6.02	(5.12- 7.08)
MATOS	557	m	0	91	120	11	110	7.58	(3.85- 14.92)
*MIGRAN	503	m	0	59	1845	4	867	6.93	(2.53- 19.02)
*MIGRAN	513	f	0	9	1035	4	3814	8.29	(2.56- 26.87)
Subtotal MIGRAN								7.48	(3.48- 16.09)
*MRFITR	511	m	0	25	1876	0	1859	50.54~	(3.08- 829.51)
*QIAO2	502	m	0	75	2840	10	709	1.87	(0.97- 3.60)
SOBUE	655	m	0	776	772	29	126	4.37	(2.88- 6.62)
WYNDE6	760	m	0	223	139	51	589	18.53	(12.98- 26.45)
WYNDE6	768	f	0	200	94	73	673	19.62	(13.90- 27.67)
Subtotal WYNDE6								19.08	(14.90- 24.44)
Partial Totals				7261	347380	1152	417709		

*prospective study ~ With 0.5 adjustment for zero

REF	NRR	SEX	AD	Ys	Ws	Qs	Ps
AUVINE	510	c	0	2.70	17.93	6.88	0.0000
BARBON	516	m	0	2.30	17.77	0.85	0.0000
BRESLO	502	c	0	1.24	12.80	9.07	0.0000
BUFFLE	520	f	0	2.71	7.07	2.74	0.0000
*CEDERL	511	m	1	2.28	4.14	0.16	0.0000
*CEDERL	516	f	0	0.61	1.81	3.93	0.4098
Subtotal CEDERL				1.77	5.95	4.10	
CHOI	525	m	0	1.43	9.31	3.99	0.0000
*CPSI	803	m	1	2.69	72.82	26.38	0.0000
*CPSI	847	f	1	1.61	39.48	8.94	0.0000
Subtotal CPSI				2.31	112.30	35.32	
DAMBER	502	m	0	1.92	26.52	0.75	0.0000
DEAN3	566	m	0	1.95	19.25	0.37	0.0000
DEAN3	585	f	0	1.07	18.99	19.74	0.0000
Subtotal DEAN3				1.51	38.24	20.11	
*DORN	612	m	0	2.62	23.30	6.64	0.0000
*DORN	649	m	0	2.20	42.26	0.58	0.0000
Subtotal DORN				2.35	65.56	7.22	
GENG	529	f	0	1.07	10.16	10.44	0.0006
JEDRYC	607	m	0	1.99	27.77	0.25	0.0000
JOLY	544	m	0	2.40	10.49	1.05	0.0000
JOLY	534	f	0	2.05	16.96	0.02	0.0000
Subtotal JOLY				2.18	27.45	1.07	
KHUDER	507	m	0	1.86	17.89	0.94	0.0000
KOULUM	503	m	0	3.33	4.31	6.70	0.0000
LETOUR	502	c	0	2.48	18.69	2.94	0.0000
LUBIN2	1149	m	0	1.80	146.52	12.33	0.0000
MATOS	557	m	0	2.03	8.38	0.03	0.0000
*MIGRAN	503	m	0	1.94	3.77	0.08	0.0002

Table 1H18 - 5

IESLC - Meta-analysis of Ever/current Smoking, Age started, "Mid"
All LC types, Cigarettes (or Any Product if Cigarettes not available)
Least adjusted

REF	NRR	SEX	AD	Ys	Ws	Qs	Ps
*MIGRAN	513	f	0	2.12	2.78	0.00	0.0004
Subtotal MIGRAN				2.01	6.55	0.09	
*MRFITR	511	m	0	3.92	0.49	1.66	0.0060
*QIAO2	502	m	0	0.63	8.96	19.06	0.0604
SOBUE	655	m	0	1.47	22.22	8.30	0.0000
WYNDE6	760	m	0	2.92	30.32	21.09	0.0000
WYNDE6	768	f	0	2.98	32.44	25.75	0.0000
Subtotal WYNDE6				2.95	62.76	46.84	

	N	30
	NS	23
	Wt	675.62
Het	Chi	201.65
Het	df	29
Het	P	***
Fixed	RR	8.05
	RRl	7.46
	RRu	8.68
	P	+++
Random	RR	7.76
	RRl	6.24
	RRu	9.65
	P	+++
Asymm	P	N.S.

Table 1H18 - 6

IESLC - Meta-analysis of Ever/current Smoking, Age started, "Mid"
All LC types, Cigarettes (or Any Product if Cigarettes not available)
Least adjusted

	Sex			
	combined	male	female	Total
N	3	19	8	30
NS	3	18	8	29
Wt	49.42	496.51	129.70	675.62
Het Chi	17.67	110.96	68.98	201.65
Het df	2	18	7	29
Het P	***	***	***	***
Fixed RR	9.41	8.22	6.99	8.05
RRl	7.12	7.53	5.88	7.46
RRu	12.44	8.98	8.30	8.68
P	+++	+++	+++	+++
Random RR	8.64	8.22	6.21	7.76
RRl	3.75	6.42	3.43	6.24
RRu	19.90	10.53	11.24	9.65
P	+++	+++	+++	+++
Between Chi				4.04
Between df				2
Between P				N.S.
Btwn(F) P				N.S.
Btwn(R) P				N.S.

Table 1H18 - 7

IESLC - Meta-analysis of Ever/current Smoking, Age started, "Mid"
All LC types, Cigarettes (or Any Product if Cigarettes not available)
Excluded studies (and stage at which they were excluded)

1	BECHER BLOT1 BROWN3 CARPEN CHYOU DARBY DOLL2 GARCIA GRAHAM GURSEL HAMMO2 JAHN JAIN LAUSSM PRESCO QIAO TVERDA WIGLE WYNDE3
2	AKIBA AMANDU AMES AXELSS BENSHL BEST BOUCHA BOUCOT BROSS CHEN CPSII DEAN2 DESTEF DORGAN DOSEME FAN GARSHI GER GILLIS HAMMON HUMBLE JUSSAW KAISE2 KATSOU KAUFMA KOO KREUZE LEVIN MCCONN NOTAN2 OSANN2 PEZZO2 PISANI RESTRE SADOWS VUTUC WANG2 WATSON WU2 WUWILL WYNDE2 WYNDE8 XU ZHOU
3	GUO MCDUFF SPITZ STASZE ZHANG
4	LUO
5	CORREA HOLE YUAN
7	BOFFET WYNDE7
10	ALDERS SPEIZE
14	AGUDO ARMADA BENHAM CHEN2 CHIAZZ DOLL ENGELA GAO GAO2 HAENSZ HEGMAN HIRAYA HU HU2 LIAW LIU3 LIU4 LIU5 LUBIN PERNU PEZZOT RACHTA SEGI2 SUZUK2 SVENSS TIZZAN WAKAI WU ZHENG

Table 1H18 - 8

Potentially overlapping studies

REF	REFGP	PRINC	.	OVERLAP/LINK
LUBIN2	LUBIN2	1		Lubin-combined
MRFITR	MRFIT	2		Subset of MRFIT
WYNDE6	WYNDE6	1		WYNDE5/6/7/8
CPSI	CPSI	1		CPSI overall

Table 1H18 - 9

Most adjusted - insufficient data for meta-analysis

REF	NRR	SEX	AGEL	AGEH	RACE	YF	LC TYPE	LOC	START	ST	NLC	R	VB	P	H	AD	SM	PRODUCT	exL	exH	DENOM	De
CORREA	536	c	0	0	all	-	all	NAmer	1979	CC	1359	n	bl	y	n	2	ev	cig+/-ot	16	20	nev cigs	or

REF	NRR	RR	SIG	RRDATA comment
CORREA	536	17.40		0

Table 1H19 -

IESLC - Meta-analysis of Ever/current Smoking, Age started, "High"
All LC types, Cigarettes (or Any Product if Cigarettes not available)

This analysis is restricted to results for:
1) Ever/current smokers
2) Results by Age started
3) Categorical results by Age started
4) All LC types (or near equivalent)
5) Results complete enough for use in metaanalysis

Within each study, results are then selected (in the following order of preference, within each sex) for:
6) PRODUCT: cigarettes regardless of other products, cigarettes only, all/unspec
7) CIGTYPE: all/unspecified, MC regardless of HR, MC only
8) (not applicable)
9) DENOM: never smoked anything, never smoked cigarettes, never any + low, never cigs + low
10) Followup period (YF, prospective studies): whole study (coded as 0) or longest available
11) LCtype: all or nearest available, at least Squamous and Adeno. (q = squamous, s = small,
l = large, a = adeno, mix = mixed, alv = alveolar)
12) Race: all or nearest available, otherwise by race (wh or w = white, bl or b = black, hi = hispanic
ch = chinese, jap = japanese, haw = hawaiian, w+o = white + oriental, sca = scandinavian, as = asian)
13) Age started "high" in key scheme 1 (key value 14, maximum range 1-17)
14) For overlapping studies: principal rather than subsidiary studies
Finally by Age: whole study (coded as 0) if available, otherwise by widest available age group
and then for single sex results (m, f) in preference to results for both sexes combined (c).

Results adjusted (AD) for the most potential confounders are then chosen in Sections -1 to -3
(and those which actually differ from the adjusted results in Table 1H14 - 1 are marked 'x' in Section -1)
and results adjusted for the least confounders in Sections -4 to -6. (Those least adjusted results which
actually differ from the most adjusted are marked 'x' in column X in Section -4)

Section -7 shows excluded studies, together with the stage (as above) at which no qualifying
results were found.

Section -8 lists the potentially overlapping studies which have been included (1=principal, 2=subsidiary).

Section -9 lists any results which would have been included in preference except that they had data not complete
enough for use in meta-analysis, with their significance (yes/no), if known, and any further comment as entered
on the database. It also lists as "gap" any categories for which no data were presented by the original authors.

In addition to those mentioned above, the following fields, levels and abbreviations are used:
* or nk = not known, n = no, y = yes, ot = other
ev = ever, cu = current, nev = never
all/unspec = all or unspecified, cig+/-ot = cigarettes irrespective of other products (cigar, pipe etc)
MC = manufactured cigarettes, HR = hand-rolled cigarettes
exL, exH = range of exposure (low and high) in the smoking group, in terms of Age started
REF: 6-character study reference
NRR: number of the RR on the database within the study
ST : study type (CC = case control, pr or prosp = prospective)
NLC: number of lung cancer cases in whole study
R : risky occupational population (n = no, m = mining, o = other risky)
VB : national cigarette type (V = at least 75% Virginia, bl = at least 75% blended, ot = other)
P : any proxy use
H : full histological confirmation
De : derivation of RR/CI (or = original, st = standard method, ot = other method of estimation)

Table 1H19 - 1

IESLC - Meta-analysis of Ever/current Smoking, Age started, "High"
All LC types, Cigarettes (or Any Product if Cigarettes not available)
Most adjusted

REF	NRR	1H14	SEX	AGEL	AGEH	RACE	YF	LC TYPE	LOC	START	ST	NLC	R	VB	P	H	AD	SM	PRODUCT	exL	exH	DENOM	De
ARMADA	512		m	0	0	all	-	all	Eu:wst	1986	CC	325	n	bl	n	y	0	ev	cig+/-ot	7	16	nev cigs	st
AUVINE	521		c	0	0	all	-	all	Eu:Sca	1986	CC	517	n	bl	y	n	2	ev	cig+/-ot	1	15	nev cigs	or
BARBON	522		m	0	0	all	-	all	Eu:wst	1979	CC	755	n	bl	y	y	1	ev	all/unsp	1	14	nev any	or
BRESLO	503		c	0	0	all	-	all	NAmer	1949	CC	518	n	bl	n	y	0	ev	cig+/-ot	0	14	nev any	st
BUFFLE	521		f	0	0	w-hi	-	all	NAmer	1976	CC	943	n	bl	y	n	0	ev	cig+/-ot	6	16	nev cigs	or
CEDERL	512		m	0	0	all	10	all	Eu:Sca	1963	pr	491	n	bl	n	n	1	cu	cig only	1	16	nev any	ot
CEDERL	517		f	0	0	all	10	all	Eu:Sca	1963	pr	491	n	bl	n	n	0	cu	cig only	1	16	nev any	ot
CHOI	526		m	0	0	all	-	all	As:oth	1985	CC	375	n	bl	n	n	0	ev	cig+/-ot	1	14	nev cigs	st
CPSI	804		m	35	84	all	6	all	NAmer	1959	pr	5138	n	bl	n	n	1	cu	cig+/-ot	1	14	nev any	ot
CPSI	848		f	40	74	all	6	all	NAmer	1959	pr	5138	n	bl	n	n	1	cu	cig only	1	14	nev any	ot
DAMBER	503		m	0	0	all	-	all	Eu:Sca	1972	CC	579	n	bl	y	n	0	ev	all/unsp	1	15	nev any	st
DEAN3	567		m	0	0	all	-	all	Eu:UK	1969	CC	766	n	V	y	n	0	cu	cig only	1	14	nev any	st
DEAN3	586		f	0	0	all	-	all	Eu:UK	1969	CC	766	n	V	y	n	0	cu	cig only	1	14	nev any	st
DORN	613		m	55	64	wh	8	all	NAmer	1954	pr	5097	n	bl	n	n	0	ev	cig+/-ot	1	14	nev any	st
DORN	650		m	65	74	wh	8	all	NAmer	1954	pr	5097	n	bl	n	n	0	ev	cig+/-ot	1	14	nev any	st
GENG	535		f	0	0	all	-	all	As:Chi	1985	CC	292	n	ot	*	n	1	ev	cig+/-ot	1	15	nev any	st
HU	513		m	0	0	all	-	all	As:Chi	1985	CC	227	n	ot	n	y	0	ev	cig+/-ot	1	15	nev cigs	st
HU	518		f	0	0	all	-	all	As:Chi	1985	CC	227	n	ot	n	y	0	ev	cig+/-ot	1	15	nev cigs	st
JEDRYC	608		m	0	0	all	-	all	Eu:est	1980	CC	1630	n	bl	y	n	0	ev	cig+/-ot	1	16	nev any	st
JOLY	545		m	0	0	all	-	all	SCAmer	1978	CC	826	n	bl	n	n	0	ev	cig+/-ot	1	14	nev any	st
JOLY	535		f	0	0	all	-	all	SCAmer	1978	CC	826	n	bl	n	n	0	ev	cig+/-ot	1	14	nev any	st
KHUDER	508		m	0	0	all	-	all	NAmer	1985	CC	482	n	bl	n	y	0	ev	cig+/-ot	1	15	nev cigs	st
KOULUM	504		m	0	0	all	-	all	Eu:Sca	1936	CC	812	n	bl	n	n	0	ev	all/unsp	11	15	nev any	st
LETOUR	503		c	0	0	all	-	all	NAmer	1983	CC	738	n	V	y	y	0	ev	cig+/-ot	1	14	nev cigs	st
LUBIN2	1159		m	0	0	all	-	all	Eu:mul	1976	CC	7804	n	bl	n	y	1	ev	cig+/-ot	13	16	nev cigs	st
MATOS	578		m	0	0	all	-	all	SCAmer	1994	CC	200	n	bl	n	n	2	ev	cig+/-ot	1	14	nev any	or
MIGRAN	505		m	0	0	all	0	all	Eu:UK	1964	pr	259	n	V	n	n	0	cu	cig only	0	15	nev any	st
MIGRAN	515		f	0	0	all	0	all	Eu:UK	1964	pr	259	n	V	n	n	0	cu	cig only	0	15	nev any	st
MRFITR	513		m	0	0	all	0	all	NAmer	1973	pr	119	n	bl	n	n	0	cu	cig+/-ot	1	15	nev cigs	ot
PERNU	505		m	0	0	all	-	all	Eu:Sca	1944	CC	1606	n	bl	n	n	0	ev	all/unsp	1	14	nev any	st
PERNU	502		f	0	0	all	-	all	Eu:Sca	1944	CC	1606	n	bl	n	n	0	ev	all/unsp	1	14	nev any	ot
QIAO2	508		m	0	0	all	0	all	As:Chi	1992	pr	241	m	ot	n	n	1	ev	all/unsp	1	16	nev any	or
SOBUE	656		m	0	0	all	-	all	As:Jap	1986	CC	1376	n	bl	n	y	0	ev	cig+/-ot	10	17	nev cigs	st
WYNDE6	761		m	0	0	wh	-	q+s+a	NAmer	1969	CC	4423	n	bl	n	y	0	ev	cig+/-ot	1	17	nev cigs	st
WYNDE6	769		f	0	0	wh	-	q+s+a	NAmer	1969	CC	4423	n	bl	n	y	0	ev	cig+/-ot	1	17	nev cigs	st

Cigarette type is all/unspec for all RRs
except for the following:

REF	NRR	CIGTYPE
DEAN3	567	MC only
DEAN3	586	MC only

Table 1H19 - 2

IESLC - Meta-analysis of Ever/current Smoking, Age started, "High"
All LC types, Cigarettes (or Any Product if Cigarettes not available)
Most adjusted

REF	NRR	SEX	AD	Number Exposed Case	Number Exposed Cont	Non-exposed Case	Non-exposed Cont	RR	95.00%CI
ARMADA	512	m	0	204	110	8	71	16.46	(7.64- 35.44)
AUVINE	521	c	2	55	-	44	-	47.60	(21.20- 107.00)
BARBON	522	m	1	138	-	22	-	50.80	(27.20- 95.00)
BRESLO	503	c	0	166	116	19	56	4.22	(2.38- 7.47)
BUFFLE	521	f	0	78	41	12	112	17.76	(8.77- 35.94)
*CEDERL	512	m	1	7	-	7	-	6.40	(2.25- 18.21)
*CEDERL	517	f	0	0	746	19	17679	0.61~	(0.04- 10.05)
Subtotal CEDERL								4.80	(1.80- 12.79)
CHOI	526	m	0	22	18	13	95	8.93	(3.81- 20.91)
*CPSI	804	m	1	185	-	83	-	16.77	(12.94- 21.73)
*CPSI	848	f	1	6	-	166	-	2.50	(1.11- 5.65)
Subtotal CPSI								14.07	(10.99- 18.02)
DAMBER	503	m	0	206	98	42	208	10.41	(6.91- 15.68)
DEAN3	567	m	0	44	165	24	510	5.67	(3.34- 9.60)
DEAN3	586	f	0	7	109	41	1538	2.41	(1.06- 5.50)
Subtotal DEAN3								4.42	(2.83- 6.89)
*DORN	613	m	0	84	36304	25	213858	19.79	(12.67- 30.93)
*DORN	650	m	0	81	24616	49	171211	11.50	(8.07- 16.39)
Subtotal DORN								14.18	(10.75- 18.72)
GENG	535	f	1	36	-	54	-	6.24	(2.85- 13.67)
HU	513	m	0	13	7	41	67	3.03	(1.12- 8.23)
HU	518	f	0	5	4	40	48	1.50	(0.38- 5.96)
Subtotal HU								2.38	(1.06- 5.35)
JEDRYC	608	m	0	135	66	49	219	9.14	(5.96- 14.02)
JOLY	545	m	0	317	282	12	218	20.42	(11.18- 37.32)
JOLY	535	f	0	76	35	52	283	11.82	(7.18- 19.44)
Subtotal JOLY								14.75	(10.05- 21.65)
KHUDER	508	m	0	226	295	23	309	10.29	(6.51- 16.27)
KOULUM	504	m	0	199	52	5	54	41.33	(15.74- 108.56)
LETOUR	503	c	0	151	76	24	224	18.54	(11.21- 30.67)
LUBIN2	1159	m	1	1312	-	185	-	6.74	(5.65- 8.04)
MATOS	578	m	2	69	-	11	-	7.80	(3.90- 15.70)
*MIGRAN	505	m	0	50	1081	4	867	10.03	(3.64- 27.65)
*MIGRAN	515	f	0	2	266	4	3814	7.17	(1.32- 38.96)
Subtotal MIGRAN								9.18	(3.84- 21.90)
*MRFITR	513	m	0	25	2065	0	1859	45.91~	(2.80- 753.64)
PERNU	505	m	0	337	92	97	275	10.38	(7.49- 14.40)
PERNU	502	f	0	1	0	110	971	26.38~	(1.07- 651.38)
Subtotal PERNU								10.48	(7.58- 14.51)
*QIAO2	508	m	1	104	-	10	-	1.81	(0.94- 3.48)
SOBUE	656	m	0	137	62	29	126	9.60	(5.81- 15.88)
WYNDE6	761	m	0	611	301	51	589	23.44	(17.06- 32.21)
WYNDE6	769	f	0	291	91	73	673	29.48	(21.04- 41.31)
Subtotal WYNDE6								26.11	(20.72- 32.90)
Partial Totals				5380	67098	1448	415934		

*prospective study ~ With 0.5 adjustment for zero

REF	NRR	SEX	AD	Ys	Ws	Qs	Ps
ARMADA	512	m	0	2.80	6.53	1.01	0.0000
AUVINE	521	c	2	3.86	5.86	12.42	0.0000
BARBON	522	m	1	3.93	9.82	22.71	0.0000
BRESLO	503	c	0	1.44	11.75	11.01	0.0000
BUFFLE	521	f	0	2.88	7.72	1.70	0.0000
*CEDERL	512	m	1	1.86	3.51	1.07	0.0005
*CEDERL	517	f	0	-0.50	0.49	4.12	0.7276
Subtotal CEDERL				1.57	4.00	5.19	
CHOI	526	m	0	2.19	5.31	0.25	0.0000
*CPSI	804	m	1	2.82	57.18	9.71	0.0000
*CPSI	848	f	1	0.92	5.80	12.90	0.0273
Subtotal CPSI				2.64	62.99	22.61	
DAMBER	503	m	0	2.34	22.90	0.10	0.0000
DEAN3	567	m	0	1.73	13.81	6.25	0.0000
DEAN3	586	f	0	0.88	5.65	13.19	0.0367
Subtotal DEAN3				1.49	19.46	19.45	
*DORN	613	m	0	2.99	19.28	6.44	0.0000
*DORN	650	m	0	2.44	30.57	0.04	0.0000
Subtotal DORN				2.65	49.85	6.47	
GENG	535	f	1	1.83	6.25	2.08	0.0000
HU	513	m	0	1.11	3.86	6.50	0.0292
HU	518	f	0	0.41	2.02	8.08	0.5647

Table 1H19 - 2

IESLC - Meta-analysis of Ever/current Smoking, Age started, "High"
All LC types, Cigarettes (or Any Product if Cigarettes not available)
Most adjusted

REF	NRR	SEX	AD	Ys	Ws	Qs	Ps
Subtotal HU				0.87	5.88	14.58	
JEDRYC	608	m	0	2.21	21.04	0.80	0.0000
JOLY	545	m	0	3.02	10.57	3.92	0.0000
JOLY	535	f	0	2.47	15.51	0.06	0.0000
Subtotal JOLY				2.69	26.07	3.98	
KHUDER	508	m	0	2.33	18.34	0.11	0.0000
KOULUM	504	m	0	3.72	4.12	7.11	0.0000
LETOUR	503	c	0	2.92	15.17	3.99	0.0000
LUBIN2	1159	m	1	1.91	123.47	30.81	0.0000
MATOS	578	m	2	2.05	7.92	0.99	0.0000
*MIGRAN	505	m	0	2.31	3.73	0.04	0.0000
*MIGRAN	515	f	0	1.97	1.34	0.26	0.0226
Subtotal MIGRAN				2.22	5.07	0.30	
*MRFITR	513	m	0	3.83	0.49	0.99	0.0074
PERNU	505	m	0	2.34	35.99	0.16	0.0000
PERNU	502	f	0	3.27	0.37	0.28	0.0455
Subtotal PERNU				2.35	36.37	0.44	
*QIAO2	508	m	1	0.59	8.97	29.52	0.0756
SOBUE	656	m	0	2.26	15.19	0.32	0.0000
WYNDE6	761	m	0	3.15	38.07	21.25	0.0000
WYNDE6	769	f	0	3.38	33.77	32.18	0.0000
Subtotal WYNDE6				3.26	71.85	53.43	

	N	35
	NS	26
	Wt	572.38
Het	Chi	252.35
Het	df	34
Het	P	***
Fixed	RR	11.11
	RRl	10.23
	RRu	12.06
	P	+++
Random	RR	10.32
	RRl	8.04
	RRu	13.26
	P	+++
Asymm	P	N.S.

Table 1H19 - 3

IESLC - Meta-analysis of Ever/current Smoking, Age started, "High"
All LC types, Cigarettes (or Any Product if Cigarettes not available)
Most adjusted

	Sex			
	combined	male	female	Total
N	3	22	10	35
NS	3	21	10	34
Wt	32.78	460.68	78.92	572.38
Het Chi	26.67	149.53	73.34	252.35
Het df	2	21	9	34
Het P	***	***	***	***
Fixed RR	12.91	10.73	12.76	11.11
RRl	9.17	9.79	10.23	10.23
RRu	18.18	11.76	15.91	12.06
P	+++	+++	+++	+++
Random RR	15.15	11.19	6.37	10.32
RRl	4.15	8.53	3.00	8.04
RRu	55.30	14.70	13.56	13.26
P	+++	+++	+++	+++
Between Chi				2.81
Between df				2
Between P				N.S.
Btwn(F) P				N.S.
Btwn(R) P				N.S.

	Lung cancer type		
	all	other	Total
N	33	2	35
NS	25	1	26
Wt	500.54	71.85	572.38
Het Chi	191.39	0.94	252.35
Het df	32	1	34
Het P	***	N.S.	***
Fixed RR	9.82	26.11	11.11
RRl	9.00	20.72	10.23
RRu	10.72	32.90	12.06
P	+++	+++	+++
Random RR	9.62	26.11	10.32
RRl	7.54	20.72	8.04
RRu	12.29	32.90	13.26
P	+++	+++	+++
Between Chi			60.02
Between df			1
Between P			***
Btwn(F) P			**
Btwn(R) P			***

	Location								
	NAmer	UK	Scand	othEur	China	Japan	othAs	other	Total
N	11	4	7	4	4	1	1	3	35
NS	8	2	5	4	3	1	1	2	26
Wt	238.16	24.53	73.25	160.86	21.10	15.19	5.31	34.00	572.38
Het Chi	67.91	5.19	24.59	40.84	6.53	0.00	0.00	4.35	252.35
Het df	10	3	6	3	3	0	0	2	34
Het P	***	N.S.	***	***	(*)	N.S.	N.S.	N.S.	***
Fixed RR	16.06	5.14	12.22	8.23	2.82	9.60	8.93	12.72	11.11
RRl	14.15	3.46	9.72	7.05	1.84	5.81	3.81	9.09	10.23
RRu	18.24	7.64	15.37	9.60	4.32	15.88	20.91	17.80	12.06
P	+++	+++	+++	+++	+++	+++	+++	+++	+++
Random RR	13.53	5.18	14.35	14.46	2.82	9.60	8.93	12.57	10.32
RRl	9.44	2.88	7.78	6.45	1.45	5.81	3.81	7.58	8.04
RRu	19.41	9.32	26.46	32.43	5.50	15.88	20.91	20.84	13.26
P	+++	+++	+++	+++	++	+++	+++	+++	+++
Between Chi									102.95
Between df									7
Between P									***
Btwn(F) P									*
Btwn(R) P									***

Table 1H19 - 3

IESLC - Meta-analysis of Ever/current Smoking, Age started, "High"
All LC types, Cigarettes (or Any Product if Cigarettes not available)
Most adjusted

Detailed Country in "other Europe"

	multi	Germany	othWest	East	Balkans	Total
N	1		2	1		4
NS	1		2	1		4
Wt	123.47		16.36	21.04		160.86
Het Chi	0.00		4.98	0.00		40.84
Het df	0		1	0		3
Het P	N.S.		*	N.S.		***
Fixed RR	6.74		32.39	9.14		8.23
RRl	5.65		19.95	5.96		7.05
RRu	8.04		52.58	14.02		9.60
P	+++		+++	+++		+++
Random RR	6.74		29.58	9.14		14.46
RRl	5.65		9.81	5.96		6.45
RRu	8.04		89.18	14.02		32.43
P	+++		+++	+++		+++
Between Chi						35.86
Between df						2
Between P						***
Btwn(F) P						N.S.
Btwn(R) P						*

Detailed Country in "other Asia"

	India	HongKong	other	Total
N			1	1
NS			1	1
Wt			5.31	5.31
Het Chi			0.00	0.00
Het df			0	0
Het P			N.S.	N.S.
Fixed RR			8.93	8.93
RRl			3.81	3.81
RRu			20.91	20.91
P			+++	+++
Random RR			8.93	8.93
RRl			3.81	3.81
RRu			20.91	20.91
P			+++	+++
Between Chi				
Between df				
Between P				N.S.
Btwn(F) P				N.S.
Btwn(R) P				N.S.

Detailed other continent

	SCAmer	Total
N	3	3
NS	2	2
Wt	34.00	34.00
Het Chi	4.35	4.35
Het df	2	2
Het P	N.S.	N.S.
Fixed RR	12.72	12.72
RRl	9.09	9.09
RRu	17.80	17.80
P	+++	+++
Random RR	12.57	12.57
RRl	7.58	7.58
RRu	20.84	20.84
P	+++	+++
Between Chi		
Between df		
Between P		N.S.
Btwn(F) P		N.S.
Btwn(R) P		N.S.

Table 1H19 - 3

IESLC - Meta-analysis of Ever/current Smoking, Age started, "High"
All LC types, Cigarettes (or Any Product if Cigarettes not available)
Most adjusted

	Start year of study					
	<1960	1960-69	1970-79	1980-89	1990+	Total
N	8	8	7	10	2	35
NS	5	4	6	9	2	26
Wt	165.07	100.38	190.48	99.56	16.89	572.38
Het Chi	45.45	63.08	53.28	35.48	8.98	252.35
Het df	7	7	6	9	1	34
Het P	***	***	***	***	**	***
Fixed RR	12.47	16.41	9.17	10.82	3.59	11.11
RRl	10.70	13.49	7.96	8.89	2.23	10.23
RRu	14.52	19.95	10.57	13.16	5.79	12.06
P	+++	+++	+++	+++	+++	+++
Random RR	11.04	8.33	15.73	10.00	3.74	10.32
RRl	7.04	4.10	8.92	6.60	0.89	8.04
RRu	17.31	16.96	27.75	15.16	15.65	13.26
P	+++	+++	+++	+++	(+)	+++
Between Chi						46.08
Between df						4
Between P						***
Btwn(F) P						N.S.
Btwn(R) P						N.S.

	Study type (1)		
	CC	other	Total
N	25	10	35
NS	20	6	26
Wt	441.01	131.37	572.38
Het Chi	187.15	64.68	252.35
Het df	24	9	34
Het P	***	***	***
Fixed RR	10.93	11.73	11.11
RRl	9.95	9.89	10.23
RRu	12.00	13.92	12.06
P	+++	+++	+++
Random RR	11.38	7.45	10.32
RRl	8.53	4.18	8.04
RRu	15.18	13.29	13.26
P	+++	+++	+++
Between Chi			0.51
Between df			1
Between P			N.S.
Btwn(F) P			N.S.
Btwn(R) P			N.S.

	Study type (2)			
	CC	prosp	other	Total
N	25	10		35
NS	20	6		26
Wt	441.01	131.37		572.38
Het Chi	187.15	64.68		252.35
Het df	24	9		34
Het P	***	***		***
Fixed RR	10.93	11.73		11.11
RRl	9.95	9.89		10.23
RRu	12.00	13.92		12.06
P	+++	+++		+++
Random RR	11.38	7.45		10.32
RRl	8.53	4.18		8.04
RRu	15.18	13.29		13.26
P	+++	+++		+++
Between Chi				0.51
Between df				1
Between P				N.S.
Btwn(F) P				N.S.
Btwn(R) P				N.S.

Table 1H19 - 3

IESLC - Meta-analysis of Ever/current Smoking, Age started, "High"
All LC types, Cigarettes (or Any Product if Cigarettes not available)
Most adjusted

	Study size (number of LC cases) 100-249	250-499	500-999	1000+	Total
N	5	8	11	11	35
NS	4	6	9	7	26
Wt	23.26	45.50	122.87	380.75	572.38
Het Chi	13.75	7.56	80.13	113.99	252.35
Het df	4	7	10	10	34
Het P	**	N.S.	***	***	***
Fixed RR	3.42	9.33	12.74	11.66	11.11
RRl	2.28	6.98	10.68	10.54	10.23
RRu	5.13	12.48	15.21	12.89	12.06
P	+++	+++	+++	+++	+++
Random RR	3.64	9.23	13.95	12.13	10.32
RRl	1.55	6.75	8.36	8.33	8.04
RRu	8.58	12.62	23.28	17.66	13.26
P	++	+++	+++	+++	+++
Between Chi					36.92
Between df					3
Between P					***
Btwn(F) P					N.S.
Btwn(R) P					*

	Risky occupational population no	mining	othRisky	Total
N	34	1		35
NS	25	1		26
Wt	563.42	8.97		572.38
Het Chi	222.36	0.00		252.35
Het df	33	0		34
Het P	***	N.S.		***
Fixed RR	11.43	1.81		11.11
RRl	10.53	0.94		10.23
RRu	12.42	3.48		12.06
P	+++	(+)		+++
Random RR	10.96	1.81		10.32
RRl	8.60	0.94		8.04
RRu	13.96	3.48		13.26
P	+++	(+)		+++
Between Chi				29.99
Between df				1
Between P				***
Btwn(F) P				*
Btwn(R) P				***

	National cigarette tobacco type Virginia	blended	other	Total
N	5	26	4	35
NS	3	20	3	26
Wt	39.70	511.59	21.10	572.38
Het Chi	20.61	179.31	6.53	252.35
Het df	4	25	3	34
Het P	***	***	(*)	***
Fixed RR	8.39	12.01	2.82	11.11
RRl	6.15	11.01	1.84	10.23
RRu	11.46	13.10	4.32	12.06
P	+++	+++	+++	+++
Random RR	7.24	13.05	2.82	10.32
RRl	3.30	10.04	1.45	8.04
RRu	15.90	16.96	5.50	13.26
P	+++	+++	++	+++
Between Chi				45.89
Between df				2
Between P				***
Btwn(F) P				*
Btwn(R) P				***

Table 1H19 - 3

IESLC - Meta-analysis of Ever/current Smoking, Age started, "High"
All LC types, Cigarettes (or Any Product if Cigarettes not available)
Most adjusted

	Any proxy use No/nk	Yes	Total
N	27	8	35
NS	19	7	26
Wt	470.41	101.97	572.38
Het Chi	190.93	59.88	252.35
Het df	26	7	34
Het P	***	***	***
Fixed RR	10.84	12.42	11.11
RRl	9.90	10.23	10.23
RRu	11.87	15.08	12.06
P	+++	+++	+++
Random RR	9.48	13.34	10.32
RRl	7.13	7.44	8.04
RRu	12.60	23.93	13.26
P	+++	+++	+++
Between Chi			1.54
Between df			1
Between P			N.S.
Btwn(F) P			N.S.
Btwn(R) P			N.S.

	Full histological confirmation No	Yes	Total
N	24	11	35
NS	17	9	26
Wt	294.39	277.99	572.38
Het Chi	114.38	137.96	252.35
Het df	23	10	34
Het P	***	***	***
Fixed RR	11.07	11.15	11.11
RRl	9.87	9.91	10.23
RRu	12.41	12.54	12.06
P	+++	+++	+++
Random RR	9.74	11.31	10.32
RRl	7.30	6.87	8.04
RRu	12.99	18.62	13.26
P	+++	+++	+++
Between Chi			0.01
Between df			1
Between P			N.S.
Btwn(F) P			N.S.
Btwn(R) P			N.S.

	Number of adjustment variables (1) 0	1	2+/+nk	Total
N	26	7	2	35
NS	19	6	2	27
Wt	343.58	215.01	13.79	572.38
Het Chi	123.47	95.41	11.02	252.35
Het df	25	6	1	34
Het P	***	***	***	***
Fixed RR	12.77	8.65	16.83	11.11
RRl	11.49	7.57	9.93	10.23
RRu	14.19	9.89	28.54	12.06
P	+++	+++	+++	+++
Random RR	10.94	7.47	19.03	10.32
RRl	8.42	3.83	3.23	8.04
RRu	14.21	14.58	112.01	13.26
P	+++	+++	++	+++
Between Chi				22.45
Between df				2
Between P				***
Btwn(F) P				N.S.
Btwn(R) P				N.S.

Table 1H19 - 3

IESLC - Meta-analysis of Ever/current Smoking, Age started, "High"
All LC types, Cigarettes (or Any Product if Cigarettes not available)
Most adjusted

	Number of adjustment variables (2) 0	1	2	3-5	6+/+nk	Total
N	26	7	2			35
NS	19	6	2			27
Wt	343.58	215.01	13.79			572.38
Het Chi	123.47	95.41	11.02			252.35
Het df	25	6	1			34
Het P	***	***	***			***
Fixed RR	12.77	8.65	16.83			11.11
RRl	11.49	7.57	9.93			10.23
RRu	14.19	9.89	28.54			12.06
P	+++	+++	+++			+++
Random RR	10.94	7.47	19.03			10.32
RRl	8.42	3.83	3.23			8.04
RRu	14.21	14.58	112.01			13.26
P	+++	+++	++			+++
Between Chi						22.45
Between df						2
Between P						***
Btwn(F) P						N.S.
Btwn(R) P						N.S.

	Smoking status ever	current	Total
N	26	9	35
NS	21	5	26
Wt	480.38	92.01	572.38
Het Chi	203.73	48.05	252.35
Het df	25	8	34
Het P	***	***	***
Fixed RR	11.26	10.33	11.11
RRl	10.30	8.42	10.23
RRu	12.32	12.68	12.06
P	+++	+++	+++
Random RR	11.80	5.95	10.32
RRl	8.92	2.98	8.04
RRu	15.61	11.89	13.26
P	+++	+++	+++
Between Chi			0.57
Between df			1
Between P			N.S.
Btwn(F) P			N.S.
Btwn(R) P			(*)

	Product all/unsp	cig+/-ot	cig only	Total
N	6	22	7	35
NS	5	18	4	27
Wt	82.18	455.87	34.33	572.38
Het Chi	59.87	152.61	10.02	252.35
Het df	5	21	6	34
Het P	***	***	N.S.	***
Fixed RR	11.17	11.87	4.52	11.11
RRl	9.00	10.83	3.23	10.23
RRu	13.87	13.01	6.31	12.06
P	+++	+++	+++	+++
Random RR	13.39	12.02	4.40	10.32
RRl	5.59	9.12	2.73	8.04
RRu	32.10	15.84	7.11	13.26
P	+++	+++	+++	+++
Between Chi				29.84
Between df				2
Between P				***
Btwn(F) P				N.S.
Btwn(R) P				**

Table 1H19 - 3

IESLC - Meta-analysis of Ever/current Smoking, Age started, "High"
All LC types, Cigarettes (or Any Product if Cigarettes not available)
Most adjusted

		Denominator nev any	nev cigs	Total
	N	22	13	35
	NS	15	11	26
	Wt	296.58	275.81	572.38
Het	Chi	132.47	119.30	252.35
Het	df	21	12	34
Het	P	***	***	***
Fixed	RR	10.77	11.48	11.11
	RRl	9.61	10.20	10.23
	RRu	12.07	12.92	12.06
	P	+++	+++	+++
Random	RR	9.03	12.79	10.32
	RRl	6.58	8.19	8.04
	RRu	12.39	19.98	13.26
	P	+++	+++	+++
Between	Chi			0.58
Between	df			1
Between	P			N.S.
Btwn(F)	P			N.S.
Btwn(R)	P			N.S.

		Derivation of RR/CI Orig	StdCalc	Other	Total
	N	5	24	6	35
	NS	5	18	4	27
	Wt	40.30	464.23	67.85	572.38
Het	Chi	65.74	155.03	26.74	252.35
Het	df	4	23	5	34
Het	P	***	***	***	***
Fixed	RR	13.55	10.62	13.37	11.11
	RRl	9.95	9.70	10.54	10.23
	RRu	18.45	11.64	16.96	12.06
	P	+++	+++	+++	+++
Random	RR	14.27	10.28	7.18	10.32
	RRl	4.06	7.91	2.40	8.04
	RRu	50.13	13.34	21.45	13.26
	P	+++	+++	+++	+++
Between	Chi				4.84
Between	df				2
Between	P				(*)
Btwn(F)	P				N.S.
Btwn(R)	P				N.S.

Table 1H19 - 4

IESLC - Meta-analysis of Ever/current Smoking, Age started, "High"
All LC types, Cigarettes (or Any Product if Cigarettes not available)
Least adjusted

REF	NRR	X	SEX	AGEL	AGEH	RACE	YF	LC TYPE	LOC	START	ST	NLC	R	VB	P	H	AD	SM	PRODUCT	exL	exH	DENOM	De
ARMADA	512		m	0	0	all	-	all	Eu:wst	1986	CC	325	n	bl	n	y	0	ev	cig+/-ot	7	16	nev cigs	st
AUVINE	511	x	c	0	0	all	-	all	Eu:Sca	1986	CC	517	n	bl	y	n	0	ev	cig+/-ot	1	15	nev cigs	st
BARBON	517	x	m	0	0	all	-	all	Eu:wst	1979	CC	755	n	bl	y	y	0	ev	all/unsp	1	14	nev any	st
BRESLO	503		c	0	0	all	-	all	NAmer	1949	CC	518	n	bl	n	y	0	ev	cig+/-ot	0	14	nev any	st
BUFFLE	521		f	0	0	w-hi	-	all	NAmer	1976	CC	943	n	bl	y	n	0	ev	cig+/-ot	6	16	nev cigs	or
CEDERL	512		m	0	0	all	10	all	Eu:Sca	1963	pr	491	n	bl	n	n	1	cu	cig only	1	16	nev any	ot
CEDERL	517		f	0	0	all	10	all	Eu:Sca	1963	pr	491	n	bl	n	n	0	cu	cig only	1	16	nev any	ot
CHOI	526		m	0	0	all	-	all	As:oth	1985	CC	375	n	bl	n	n	0	ev	cig+/-ot	1	14	nev cigs	st
CPSI	804		m	35	84	all	6	all	NAmer	1959	pr	5138	n	bl	n	n	1	cu	cig+/-ot	1	14	nev any	ot
CPSI	848		f	40	74	all	6	all	NAmer	1959	pr	5138	n	bl	n	n	1	cu	cig only	1	14	nev any	ot
DAMBER	503		m	0	0	all	-	all	Eu:Sca	1972	CC	579	n	bl	y	n	0	ev	all/unsp	1	15	nev any	st
DEAN3	567		m	0	0	all	-	all	Eu:UK	1969	CC	766	n	V	y	n	0	cu	cig only	1	14	nev any	st
DEAN3	586		f	0	0	all	-	all	Eu:UK	1969	CC	766	n	V	y	n	0	cu	cig only	1	14	nev any	st
DORN	613		m	55	64	wh	8	all	NAmer	1954	pr	5097	n	bl	n	n	0	ev	cig+/-ot	1	14	nev any	st
DORN	650		m	65	74	wh	8	all	NAmer	1954	pr	5097	n	bl	n	n	0	ev	cig+/-ot	1	14	nev any	st
GENG	530	x	f	0	0	all	-	all	As:Chi	1985	CC	292	n	ot	*	n	0	ev	cig+/-ot	1	15	nev any	st
HU	513		m	0	0	all	-	all	As:Chi	1985	CC	227	n	ot	n	y	0	ev	cig+/-ot	1	15	nev cigs	st
HU	518		f	0	0	all	-	all	As:Chi	1985	CC	227	n	ot	n	y	0	ev	cig+/-ot	1	15	nev cigs	st
JEDRYC	608		m	0	0	all	-	all	Eu:est	1980	CC	1630	n	bl	y	n	0	ev	cig+/-ot	1	16	nev any	st
JOLY	545		m	0	0	all	-	all	SCAmer	1978	CC	826	n	bl	n	n	0	ev	cig+/-ot	1	14	nev any	st
JOLY	535		f	0	0	all	-	all	SCAmer	1978	CC	826	n	bl	n	n	0	ev	cig+/-ot	1	14	nev any	st
KHUDER	508		m	0	0	all	-	all	NAmer	1985	CC	482	n	bl	n	y	0	ev	cig+/-ot	1	15	nev cigs	st
KOULUM	504		m	0	0	all	-	all	Eu:Sca	1936	CC	812	n	bl	n	n	0	ev	all/unsp	11	15	nev any	st
LETOUR	503		c	0	0	all	-	all	NAmer	1983	CC	738	n	V	y	y	0	ev	cig+/-ot	1	14	nev cigs	st
LUBIN2	1150	x	m	0	0	all	-	all	Eu:mul	1976	CC	7804	n	bl	n	y	0	ev	cig+/-ot	13	16	nev cigs	st
MATOS	558	x	m	0	0	all	-	all	SCAmer	1994	CC	200	n	bl	n	n	0	ev	cig+/-ot	1	14	nev any	st
MIGRAN	505		m	0	0	all	0	all	Eu:UK	1964	pr	259	n	V	n	n	0	cu	cig only	0	15	nev any	st
MIGRAN	515		f	0	0	all	0	all	Eu:UK	1964	pr	259	n	V	n	n	0	cu	cig only	0	15	nev any	st
MRFITR	513		m	0	0	all	0	all	NAmer	1973	pr	119	n	bl	n	n	0	cu	cig+/-ot	1	15	nev cigs	ot
PERNU	505		m	0	0	all	-	all	Eu:Sca	1944	CC	1606	n	bl	n	n	0	ev	all/unsp	1	14	nev any	st
PERNU	502		f	0	0	all	-	all	Eu:Sca	1944	CC	1606	n	bl	n	n	0	ev	all/unsp	1	14	nev any	ot
QIAO2	503	x	m	0	0	all	0	all	As:Chi	1992	pr	241	m	ot	n	n	0	ev	all/unsp	1	16	nev any	st
SOBUE	656		m	0	0	all	-	all	As:Jap	1986	CC	1376	n	bl	n	y	0	ev	cig+/-ot	10	17	nev cigs	st
WYNDE6	761		m	0	0	wh	-	q+s+a	NAmer	1969	CC	4423	n	bl	n	y	0	ev	cig+/-ot	1	17	nev cigs	st
WYNDE6	769		f	0	0	wh	-	q+s+a	NAmer	1969	CC	4423	n	bl	n	y	0	ev	cig+/-ot	1	17	nev cigs	st

Cigarette type is all/unspec for all RRs
except for the following:

REF	NRR	CIGTYPE
DEAN3	567	MC only
DEAN3	586	MC only

Table 1H19 - 5

IESLC - Meta-analysis of Ever/current Smoking, Age started, "High"
All LC types, Cigarettes (or Any Product if Cigarettes not available)
Least adjusted

REF	NRR	SEX	AD	Number Exposed Case	Number Exposed Cont	Non-exposed Case	Non-exposed Cont	RR	95.00%CI
ARMADA	512	m	0	204	110	8	71	16.46	(7.64- 35.44)
AUVINE	511	c	0	55	6	44	229	47.71	(19.35- 117.62)
BARBON	517	m	0	138	23	22	188	51.27	(27.46- 95.73)
BRESLO	503	c	0	166	116	19	56	4.22	(2.38- 7.47)
BUFFLE	521	f	0	78	41	12	112	17.76	(8.77- 35.94)
*CEDERL	512	m	1	7	-	7	-	6.40	(2.25- 18.21)
*CEDERL	517	f	0	0	746	19	17679	0.61~	(0.04- 10.05)
Subtotal CEDERL								4.80	(1.80- 12.79)
CHOI	526	m	0	22	18	13	95	8.93	(3.81- 20.91)
*CPSI	804	m	1	185	-	83	-	16.77	(12.94- 21.73)
*CPSI	848	f	1	6	-	166	-	2.50	(1.11- 5.65)
Subtotal CPSI								14.07	(10.99- 18.02)
DAMBER	503	m	0	206	98	42	208	10.41	(6.91- 15.68)
DEAN3	567	m	0	44	165	24	510	5.67	(3.34- 9.60)
DEAN3	586	f	0	7	109	41	1538	2.41	(1.06- 5.50)
Subtotal DEAN3								4.42	(2.83- 6.89)
*DORN	613	m	0	84	36304	25	213858	19.79	(12.67- 30.93)
*DORN	650	m	0	81	24616	49	171211	11.50	(8.07- 16.39)
Subtotal DORN								14.18	(10.75- 18.72)
GENG	530	f	0	36	10	54	93	6.20	(2.85- 13.48)
HU	513	m	0	13	7	41	67	3.03	(1.12- 8.23)
HU	518	f	0	5	4	40	48	1.50	(0.38- 5.96)
Subtotal HU								2.38	(1.06- 5.35)
JEDRYC	608	m	0	135	66	49	219	9.14	(5.96- 14.02)
JOLY	545	m	0	317	282	12	218	20.42	(11.18- 37.32)
JOLY	535	f	0	76	35	52	283	11.82	(7.18- 19.44)
Subtotal JOLY								14.75	(10.05- 21.65)
KHUDER	508	m	0	226	295	23	309	10.29	(6.51- 16.27)
KOULUM	504	m	0	199	52	5	54	41.33	(15.74- 108.56)
LETOUR	503	c	0	151	76	24	224	18.54	(11.21- 30.67)
LUBIN2	1150	m	0	1312	1833	185	1878	7.27	(6.15- 8.59)
MATOS	558	m	0	69	90	11	110	7.67	(3.83- 15.36)
*MIGRAN	505	m	0	50	1081	4	867	10.03	(3.64- 27.65)
*MIGRAN	515	f	0	2	266	4	3814	7.17	(1.32- 38.96)
Subtotal MIGRAN								9.18	(3.84- 21.90)
*MRFITR	513	m	0	25	2065	0	1859	45.91~	(2.80- 753.64)
PERNU	505	m	0	337	92	97	275	10.38	(7.49- 14.40)
PERNU	502	f	0	1	0	110	971	26.38~	(1.07- 651.38)
Subtotal PERNU								10.48	(7.58- 14.51)
*QIAO2	503	m	0	104	2130	10	709	3.46	(1.82- 6.59)
SOBUE	656	m	0	137	62	29	126	9.60	(5.81- 15.88)
WYNDE6	761	m	0	611	301	51	589	23.44	(17.06- 32.21)
WYNDE6	769	f	0	291	91	73	673	29.48	(21.04- 41.31)
Subtotal WYNDE6								26.11	(20.72- 32.90)
Partial Totals				5380	71190	1448	419141		

*prospective study ~ With 0.5 adjustment for zero

REF	NRR	SEX	AD	Ys	Ws	Qs	Ps
ARMADA	512	m	0	2.80	6.53	0.95	0.0000
AUVINE	511	c	0	3.87	4.72	9.87	0.0000
BARBON	517	m	0	3.94	9.85	22.70	0.0000
BRESLO	503	c	0	1.44	11.75	11.28	0.0000
BUFFLE	521	f	0	2.88	7.72	1.62	0.0000
*CEDERL	512	m	1	1.86	3.51	1.11	0.0005
*CEDERL	517	f	0	-0.50	0.49	4.15	0.7276
Subtotal CEDERL				1.57	4.00	5.27	
CHOI	526	m	0	2.19	5.31	0.28	0.0000
*CPSI	804	m	1	2.82	57.18	9.17	0.0000
*CPSI	848	f	1	0.92	5.80	13.11	0.0273
Subtotal CPSI				2.64	62.99	22.28	
DAMBER	503	m	0	2.34	22.90	0.13	0.0000
DEAN3	567	m	0	1.73	13.81	6.47	0.0000
DEAN3	586	f	0	0.88	5.65	13.39	0.0367
Subtotal DEAN3				1.49	19.46	19.86	
*DORN	613	m	0	2.99	19.28	6.18	0.0000
*DORN	650	m	0	2.44	30.57	0.02	0.0000
Subtotal DORN				2.65	49.85	6.20	
GENG	530	f	0	1.82	6.37	2.25	0.0000
HU	513	m	0	1.11	3.86	6.61	0.0292
HU	518	f	0	0.41	2.02	8.18	0.5647

Table 1H19 - 5

IESLC - Meta-analysis of Ever/current Smoking, Age started, "High"
All LC types, Cigarettes (or Any Product if Cigarettes not available)
Least adjusted

REF	NRR	SEX	AD	Ys	Ws	Qs	Ps
Subtotal HU				0.87	5.88	14.79	
JEDRYC	608	m	0	2.21	21.04	0.89	0.0000
JOLY	545	m	0	3.02	10.57	3.77	0.0000
JOLY	535	f	0	2.47	15.51	0.04	0.0000
Subtotal JOLY				2.69	26.07	3.81	
KHUDER	508	m	0	2.33	18.34	0.14	0.0000
KOULUM	504	m	0	3.72	4.12	6.99	0.0000
LETOUR	503	c	0	2.92	15.17	3.81	0.0000
LUBIN2	1150	m	0	1.98	138.01	26.23	0.0000
MATOS	558	m	0	2.04	7.96	1.16	0.0000
*MIGRAN	505	m	0	2.31	3.73	0.05	0.0000
*MIGRAN	515	f	0	1.97	1.34	0.27	0.0226
Subtotal MIGRAN				2.22	5.07	0.32	
*MRFITR	513	m	0	3.83	0.49	0.97	0.0074
PERNU	505	m	0	2.34	35.99	0.22	0.0000
PERNU	502	f	0	3.27	0.37	0.27	0.0455
Subtotal PERNU				2.35	36.37	0.50	
*QIAO2	503	m	0	1.24	9.28	12.87	0.0002
SOBUE	656	m	0	2.26	15.19	0.38	0.0000
WYNDE6	761	m	0	3.15	38.07	20.59	0.0000
WYNDE6	769	f	0	3.38	33.77	31.43	0.0000
Subtotal WYNDE6				3.26	71.85	52.02	

	N	35
	NS	26
	Wt	586.28
Het	Chi	227.55
Het	df	34
Het	P	***
Fixed	RR	11.24
	RRl	10.36
	RRu	12.18
	P	+++
Random	RR	10.57
	RRl	8.34
	RRu	13.41
	P	+++
Asymm	P	N.S.

Table 1H19 - 6

IESLC - Meta-analysis of Ever/current Smoking, Age started, "High"
All LC types, Cigarettes (or Any Product if Cigarettes not available)
Least adjusted

		combined	Sex<br>male	female	Total
	N	3	22	10	35
	NS	3	21	10	34
	Wt	31.64	475.61	79.04	586.28
Het	Chi	24.68	127.55	73.46	227.55
Het	df	2	21	9	34
Het	P	***	***	***	***
Fixed	RR	12.32	10.94	12.74	11.24
	RRl	8.70	10.00	10.22	10.36
	RRu	17.46	11.97	15.88	12.18
	P	+++	+++	+++	+++
Random	RR	14.99	11.58	6.37	10.57
	RRl	4.14	9.01	2.99	8.34
	RRu	54.29	14.88	13.54	13.41
	P	+++	+++	+++	+++
Between	Chi				1.86
Between	df				2
Between	P				N.S.
Btwn(F)	P				N.S.
Btwn(R)	P				N.S.

Table 1H19 - 7

IESLC - Meta-analysis of Ever/current Smoking, Age started, "High"
All LC types, Cigarettes (or Any Product if Cigarettes not available)
Excluded studies (and stage at which they were excluded)

1	BECHER BLOT1 BROWN3 CARPEN CHYOU DARBY DOLL2 GARCIA GRAHAM GURSEL HAMMO2 JAHN JAIN LAUSSM PRESCO QIAO TVERDA WIGLE WYNDE3
2	AKIBA AMANDU AMES AXELSS BENSHL BEST BOUCHA BOUCOT BROSS CHEN CPSII DEAN2 DESTEF DORGAN DOSEME FAN GARSHI GER GILLIS HAMMON HUMBLE JUSSAW KAISE2 KATSOU KAUFMA KOO KREUZE LEVIN MCCONN NOTAN2 OSANN2 PEZZO2 PISANI RESTRE SADOWS VUTUC WANG2 WATSON WU2 WUWILL WYNDE2 WYNDE8 XU ZHOU
3	GUO MCDUFF SPITZ STASZE ZHANG
4	LUO
5	CORREA HOLE YUAN
7	BOFFET WYNDE7
10	ALDERS SPEIZE
14	AGUDO BENHAM CHEN2 CHIAZZ DOLL ENGELA GAO GAO2 HAENSZ HEGMAN HIRAYA HU2 LIAW LIU3 LIU4 LIU5 LUBIN PEZZOT RACHTA SEGI2 SUZUK2 SVENSS TIZZAN WAKAI WU ZHENG

Table 1H19 - 8
Potentially overlapping studies

REF	REFGP	PRINC	.	OVERLAP/LINK
LUBIN2	LUBIN2	1		Lubin-combined
MRFITR	MRFIT	2		Subset of MRFIT
WYNDE6	WYNDE6	1		WYNDE5/6/7/8
CPSI	CPSI	1		CPSI overall

Table 1H19 - 9
Most adjusted - insufficient data for meta-analysis

REF	NRR	SEX	AGEL	AGEH	RACE	YF	LC TYPE	LOC	START	ST	NLC	R	VB	P	H	AD	SM	PRODUCT	exL	exH	DENOM	De
CORREA	537	c	0	0	all	-	all	NAmer	1979	CC	1359	n	bl	y	n	2	ev	cig+/-ot	1	15	nev cigs	or

REF	NRR	RR	SIG	RRDATA comment
CORREA	537	24.20		0

Table 1H20 -

IESLC - Meta-analysis of Ever/current Smoking, Age started, "Highest vs lowest"
All LC types, Cigarettes (or Any Product if Cigarettes not available)

This analysis is restricted to results for:
1) Ever/current smokers
2) Results by Age started
3) Categorical results by Age started
4) Denominator (unexposed) = "low"
5) All LC types (or near equivalent)
6) Results complete enough for use in metaanalysis

Within each study, results are then selected (in the following order of preference, within each sex) for:
7) SMKSTA: ever, current
8) PRODUCT: cigarettes regardless of other products, cigarettes only, all/unspec
9) CIGTYPE: all/unspecified, MC regardless of HR, MC only
10) Results with least adjustment for other aspects of smoking (ADOS)
11) The highest vs lowest category
12) Followup period (YF, prospective studies): whole study (coded as 0) or longest available
13) LCtype: all or nearest available, at least Squamous and Adeno. (q = squamous, s = small,
l = large, a = adeno, mix = mixed, alv = alveolar)
14) Race: all or nearest available, otherwise by race (wh or w = white, bl or b = black, hi = hispanic
ch = chinese, jap = japanese, haw = hawaiian, w+o = white + oriental, sca = scandinavian, as = asian)
15) For overlapping studies: principal rather than subsidiary studies
Finally by Age: whole study (coded as 0) if available, otherwise by widest available age group
and then for single sex results (m, f) in preference to results for both sexes combined (c).

Results adjusted (AD) for the most potential confounders are then chosen in Sections -1 to -3
(and those which actually differ from the adjusted results in Table 1H15 - 1 are marked 'x' in Section -1)
and results adjusted for the least confounders in Sections -4 to -6. (Those least adjusted results which
actually differ from the most adjusted are marked 'x' in column X in Section -4)

Section -7 shows excluded studies, together with the stage (as above) at which no qualifying
results were found.

Section -8 lists the potentially overlapping studies which have been included (1=principal, 2=subsidiary).

Section -9 lists any results which would have been included in preference except that they had data not complete
enough for use in meta-analysis, with their significance (yes/no), if known, and any further comment as entered
on the database. It also lists as "gap" any categories for which no data were presented by the original authors.

In addition to those mentioned above, the following fields, levels and abbreviations are used:
* or nk = not known, n = no, y = yes, ot = other
all/unspec = all or unspecified, cig+/-ot = cigarettes irrespective of other products (cigar, pipe etc)
MC = manufactured cigarettes, HR = hand-rolled cigarettes
exL, exH = range of exposure (low and high) in the "highest" group, in terms of Age started
unexL, unexH = range of exposure (low and high) in the "lowest" group, in terms of Age started
REF: 6-character study reference
NRR: number of the RR on the database within the study
ST : study type (CC = case control, pr or prosp = prospective)
NLC: number of lung cancer cases in whole study
R : risky occupational population (n = no, m = mining, o = other risky)
VB : national cigarette type (V = at least 75% Virginia, bl = at least 75% blended, ot = other)
P : any proxy use
H : full histological confirmation
De : derivation of RR/CI (or = original, st = standard method, ot = other method of estimation)

Table 1H20 - 1

IESLC - Meta-analysis of Ever/current Smoking, Age started, "Highest vs lowest"
All LC types, Cigarettes (or Any Product if Cigarettes not available)
Most adjusted

REF	NRR	1H15	SEX	AGEL	AGEH	RACE	YF	LC TYPE	LOC	START	ST	NLC	R	VB	P	H	AD	ADOS	SM	PRODUCT	exL	exH	unexL	unexH	De
AGUDO	506		f	0	0	all	-	all	Eu:wst	1989	CC	103	n	bl	n	n	3	0	ev	cig only	1	23	24	999	ot
ALDERS	503		m	0	0	all	-	all	Eu:UK	1977	CC	1448	n	V	n	n	2	1#	ev	cig only	1	14	25	999	ot
ALDERS	506		f	0	0	all	-	all	Eu:UK	1977	CC	1448	n	V	n	n	2	1#	ev	cig only	1	14	25	999	ot
ARMADA	513		m	0	0	all	-	all	Eu:wst	1986	CC	325	n	bl	n	y	0	0	ev	cig+/-ot	7	16	17	45	st
AUVINE	513		c	0	0	all	-	all	Eu:Sca	1986	CC	517	n	bl	y	n	0	0	ev	cig+/-ot	1	15	21	999	st
BARBON	524		m	0	0	all	-	all	Eu:wst	1979	CC	755	n	bl	y	y	1	0	ev	all/unsp	1	14	20	999	ot
BRESLO	505		c	0	0	all	-	all	NAmer	1949	CC	518	n	bl	n	y	0	0	ev	cig+/-ot	0	14	25	999	st
BUFFLE	525		f	0	0	w-hi	-	all	NAmer	1976	CC	943	n	bl	y	n	0	0	ev	cig+/-ot	6	16	30	999	st
CEDERL	514	x	m	0	0	all	10	all	Eu:Sca	1963	pr	491	n	bl	n	n	1	0	cu	cig only	1	16	19	999	ot
CEDERL	536		f	0	0	all	10	all	Eu:Sca	1963	pr	491	n	bl	n	n	0	0	cu	cig only	1	16	19	999	ot
CHEN2	521		m	0	0	all	-	all	As:Chi	1983	CC	193	n	ot	y	n	0	0	ev	all/unsp	1	19	31	999	st
CHEN2	526		f	0	0	all	-	all	As:Chi	1983	CC	193	n	ot	y	n	0	0	ev	all/unsp	1	19	31	999	st
CHIAZZ	503		m	0	0	all	-	all	NAmer	1940	CC	144	o	bl	y	n	2	0	ev	cig+/-ot	1	19	20	999	ot
CHOI	529		m	0	0	all	-	all	As:oth	1985	CC	375	n	bl	n	n	0	0	ev	cig+/-ot	1	14	25	999	st
CHOI	532		f	0	0	all	-	all	As:oth	1985	CC	375	n	bl	n	n	0	0	ev	cig+/-ot	1	24	25	999	st
CPSI	822		m	35	84	all	6	all	NAmer	1959	pr	5138	n	bl	n	n	1	0	cu	cig+/-ot	1	14	25	999	ot
CPSI	851		f	40	74	all	6	all	NAmer	1959	pr	5138	n	bl	n	n	1	0	cu	cig only	1	14	25	999	ot
DAMBER	505		m	0	0	all	-	all	Eu:Sca	1972	CC	579	n	bl	y	n	0	0	ev	all/unsp	1	15	21	999	st
DEAN3	570		m	0	0	all	-	all	Eu:UK	1969	CC	766	n	V	y	n	0	0	cu	cig only	1	14	25	999	st
DEAN3	589		f	0	0	all	-	all	Eu:UK	1969	CC	766	n	V	y	n	0	0	cu	cig only	1	14	25	999	st
DOLL	507		m	0	0	all	-	all	Eu:UK	1948	CC	1465	n	V	n	n	0	0	ev	all/unsp	1	19	40	999	st
DOLL	514		f	0	0	all	-	all	Eu:UK	1948	CC	1465	n	V	n	n	0	0	ev	all/unsp	1	19	40	999	st
DORN	616		m	55	64	wh	8	all	NAmer	1954	pr	5097	n	bl	n	n	0	0	ev	cig+/-ot	1	14	25	999	st
DORN	653		m	65	74	wh	8	all	NAmer	1954	pr	5097	n	bl	n	n	0	0	ev	cig+/-ot	1	14	25	999	st
ENGELA	505		m	0	0	all	0	all	Eu:Sca	1964	pr	435	n	bl	n	n	0	0	cu	cig+/-ot	1	19	30	999	st
ENGELA	513		f	0	0	all	0	all	Eu:Sca	1964	pr	435	n	bl	n	n	0	0	cu	cig+/-ot	1	19	30	999	st
GAO	510		m	0	0	all	-	all	As:Chi	1984	CC	1405	n	ot	n	n	2	0	ev	cig+/-ot	10	19	30	999	ot
GAO	520		f	0	0	all	-	all	As:Chi	1984	CC	1405	n	ot	n	n	2	0	ev	cig+/-ot	10	19	30	999	ot
GAO2	505		m	0	0	all	-	all	As:Jap	1988	CC	282	n	bl	n	n	0	0	cu	cig+/-ot	1	19	30	999	st
GENG	537		f	0	0	all	-	all	As:Chi	1985	CC	292	n	ot	*	n	1	0	ev	cig+/-ot	1	15	21	999	st
HAENSZ	550		f	0	0	all	-	not alv	NAmer	1955	CC	158	n	bl	n	y	2	0	ev	cig+/-ot	1	24	25	999	ot
HEGMAN	515		m	0	0	all	-	all	NAmer	1989	CC	282	n	bl	y	y	1	0	ev	all/unsp	1	19	20	999	or
HEGMAN	518		f	0	0	all	-	all	NAmer	1989	CC	282	n	bl	y	y	1	0	ev	all/unsp	1	25	26	999	or
HIRAYA	564		m	0	0	all	5	all	As:Jap	1965	pr	1917	n	bl	n	n	1	0	cu	cig+/-ot	1	19	25	999	ot
HIRAYA	506		f	0	0	all	0	all	As:Jap	1965	pr	1917	n	bl	n	n	1	0	cu	cig+/-ot	1	19	20	999	ot
HU	515		m	0	0	all	-	all	As:Chi	1985	CC	227	n	ot	n	y	0	0	ev	cig+/-ot	1	15	30	999	st
HU	520		f	0	0	all	-	all	As:Chi	1985	CC	227	n	ot	n	y	0	0	ev	cig+/-ot	1	15	30	999	st
HU2	507		c	0	0	all	-	all	As:Chi	1977	CC	523	n	ot	y	n	0	0	ev	cig+/-ot	1	19	40	999	st
JEDRYC	610		m	0	0	all	-	all	Eu:est	1980	CC	1630	n	bl	y	n	5	2#	ev	cig+/-ot	1	16	19	999	or
JEDRYC	620		f	0	0	all	-	all	Eu:est	1980	CC	1630	n	bl	y	n	5	2#	ev	cig+/-ot	1	22	23	999	or
JOLY	547		m	0	0	all	-	all	SCAmer	1978	CC	826	n	bl	n	n	0	0	ev	cig+/-ot	1	14	25	999	st
JOLY	537		f	0	0	all	-	all	SCAmer	1978	CC	826	n	bl	n	n	0	0	ev	cig+/-ot	1	14	25	999	st
KHUDER	510		m	0	0	all	-	all	NAmer	1985	CC	482	n	bl	n	y	0	0	ev	cig+/-ot	1	15	20	999	st
KOULUM	509		m	0	0	all	-	all	Eu:Sca	1936	CC	812	n	bl	n	n	0	0	ev	all/unsp	0	10	31	999	st
LETOUR	505		c	0	0	all	-	all	NAmer	1983	CC	738	n	V	y	y	0	0	ev	cig+/-ot	1	14	21	999	st
LIAW	510		c	0	0	all	0	all	As:oth	1982	pr	127	n	ot	n	n	2	0	cu	all/unsp	1	20	25	999	ot
LIU3	506		m	0	0	all	-	all	As:Chi	1985	CC	110	n	ot	n	n	2	0	ev	all/unsp	1	20	21	999	ot
LIU4	505		m	35	69	all	-	all	As:Chi	1986	CC	1000-00	n	ot	y	n	2	0	ev	all/unsp	0	19	25	999	ot
LIU5	503		c	0	0	all	-	all	As:Chi	1978	CC	111	n	ot	y	n	0	0	ev	all/unsp	1	29	30	999	st
LUBIN	571		m	0	0	all	-	all	As:Chi	1984	CC	427	m	ot	y	n	0	0	ev	cig+/-ot	1	19	27	999	st
LUBIN2	1164		m	0	0	all	-	all	Eu:mul	1976	CC	7804	n	bl	n	y	1	0	ev	cig+/-ot	1	12	31	999	st
MATOS	580		m	0	0	all	-	all	SCAmer	1994	CC	200	n	bl	n	n	2	0	ev	cig+/-ot	1	14	20	999	ot
MIGRAN	509		m	0	0	all	0	all	Eu:UK	1964	pr	259	n	V	n	n	0	0	cu	cig only	0	15	20	999	st
MIGRAN	519		f	0	0	all	0	all	Eu:UK	1964	pr	259	n	V	n	n	0	0	cu	cig only	0	15	20	999	st
MRFITR	518		m	0	0	all	0	all	NAmer	1973	pr	119	n	bl	n	n	0	0	cu	cig+/-ot	1	15	24	999	st
PERNU	506		m	0	0	all	-	all	Eu:Sca	1944	CC	1606	n	bl	n	n	0	0	ev	all/unsp	1	14	15	999	st
PERNU	503		f	0	0	all	-	all	Eu:Sca	1944	CC	1606	n	bl	n	n	0	0	ev	all/unsp	1	14	15	999	ot
PEZZOT	576		m	0	0	all	-	all	SCAmer	1987	CC	215	n	bl	n	y	2	0	ev	cig only	1	13	19	999	ot
QIAO2	510		m	0	0	all	0	all	As:Chi	1992	pr	241	m	ot	n	n	1	0	ev	all/unsp	1	16	21	999	ot
RACHTA	510		f	0	0	all	-	all	Eu:est	1991	CC	118	n	bl	n	y	1	0	ev	cig+/-ot	1	19	31	999	ot
SEGI2	525		m	0	0	all	-	all	As:Jap	1962	CC	378	n	bl	n	n	1	0	cu	cig+/-ot	1	19	23	999	ot
SOBUE	800		m	0	0	all	-	all	As:Jap	1986	CC	1376	n	bl	n	y	0	0	ev	cig+/-ot	10	17	23	999	st
SPEIZE	530		f	0	0	all	0	all	NAmer	1976	pr	593	n	bl	n	y	2	1#	cu	cig+/-ot	18	19	22	999	st
SUZUK2	523		c	0	0	all	-	all	SCAmer	1991	CC	123	n	bl	n	y	3	0	ev	all/unsp	0	11	19	999	or
SVENSS	505		f	0	0	all	-	all	Eu:Sca	1983	CC	210	n	bl	n	n	0	0	cu	all/unsp	0	18	26	999	st
TIZZAN	510		m	0	0	all	-	all	Eu:wst	1959	CC	1358	n	bl	n	n	0	0	ev	all/unsp	1	19	31	999	st
TIZZAN	540	x	f	0	0	all	-	all	Eu:wst	1959	CC	1358	n	bl	n	n	0	0	ev	cig only	1	19	31	999	st
WAKAI	505		m	0	0	all	-	all	As:Jap	1988	CC	333	n	bl	n	y	0	0	cu	cig+/-ot	1	19	30	999	st
WU	545		f	0	0	wh	-	q+a	NAmer	1981	CC	220	n	bl	n	y	2	0	cu	all/unsp	0	18	25	999	ot
WYNDE6	763		m	0	0	wh	-	q+s+a	NAmer	1969	CC	4423	n	bl	n	y	0	0	ev	cig+/-ot	1	17	21	999	st
WYNDE6	771		f	0	0	wh	-	q+s+a	NAmer	1969	CC	4423	n	bl	n	y	0	0	ev	cig+/-ot	1	17	21	999	st

Table 1H20 - 1

IESLC - Meta-analysis of Ever/current Smoking, Age started, "Highest vs lowest"
All LC types, Cigarettes (or Any Product if Cigarettes not available)
Most adjusted

REF	NRR	1H15	SEX	AGEL	AGEH	RACE	YF	LC TYPE	LOC	START	ST	NLC	R	VB	P	H	AD	ADOS	SM	PRODUCT	exL	exH	unexL	unexH	De
ZHENG	569		m	0	0	all	-	all	As:Chi	1982	CC	540	n	ot	*	y	1	0	ev	cig+/-ot	1	19	30	999	ot
ZHENG	575		f	0	0	all	-	all	As:Chi	1982	CC	540	n	ot	*	y	1	0	ev	cig+/-ot	1	29	30	999	ot

Comments on values in listings

ALDERS	ADOS	Number of cigs/day
ALDERS	ADOS	Number of cigs/day
JEDRYC	ADOS	Number of cigs per day & time since quit
JEDRYC	ADOS	Number of cigs per day & time since quit
SPEIZE	ADOS	Numbers of cigs/day

Cigarette type is all/unspec for all RRs
except for the following:

REF	NRR	CIGTYPE
ALDERS	503	MC only
ALDERS	506	MC only
DEAN3	570	MC only
DEAN3	589	MC only

Table 1H20 - 2

IESLC - Meta-analysis of Ever/current Smoking, Age started, "Highest vs lowest"
All LC types, Cigarettes (or Any Product if Cigarettes not available)
Most adjusted

REF	NRR	SEX	AD	Number Case	Exposed Cont	Non-exposed Case	Cont	RR	95.00%CI
AGUDO	506	f	3	16	-	7	-	6.81 (	1.20- 38.56)
ALDERS	503	m	2	139	-	11	-	2.94 (	1.38- 6.25)
ALDERS	506	f	2	78	-	97	-	2.08 (	1.27- 3.41)
Subtotal ALDERS								2.31 (	1.53- 3.49)
ARMADA	513	m	0	204	110	113	144	2.36 (	1.69- 3.31)
AUVINE	513	c	0	55	6	76	27	3.26 (	1.26- 8.42)
BARBON	524	m	1	138	-	200	-	6.20 (	3.82- 10.05)
BRESLO	505	c	0	166	116	32	35	1.57 (	0.92- 2.67)
BUFFLE	525	f	0	78	41	23	23	1.90 (	0.95- 3.80)
*CEDERL	514	m	1	7	-	11	-	0.98 (	0.40- 2.45)
*CEDERL	536	f	0	0	746	6	2806	0.29~(	0.02- 5.13)
Subtotal CEDERL								0.88 (	0.37- 2.08)
CHEN2	521	m	0	84	67	8	5	0.78 (	0.25- 2.51)
CHEN2	526	f	0	23	13	5	8	2.83 (	0.77- 10.47)
Subtotal CHEN2								1.38 (	0.58- 3.29)
CHIAZZ	503	m	2	-	-	-	-	6.63 (	2.13- 20.63)
CHOI	529	m	0	22	18	36	77	2.61 (	1.25- 5.47)
CHOI	532	f	0	4	1	15	25	6.67 (	0.68- 65.37)
Subtotal CHOI								2.86 (	1.42- 5.76)
*CPSI	822	m	1	185	-	42	-	4.11 (	2.94- 5.75)
*CPSI	851	f	1	6	-	51	-	1.11 (	0.48- 2.59)
Subtotal CPSI								3.44 (	2.52- 4.69)
DAMBER	505	m	0	206	98	70	76	2.28 (	1.52- 3.42)
DEAN3	570	m	0	44	165	24	75	0.83 (	0.47- 1.47)
DEAN3	589	f	0	7	109	27	274	0.65 (	0.28- 1.54)
Subtotal DEAN3								0.77 (	0.48- 1.24)
DOLL	507	m	0	1077	992	4	7	1.90 (	0.55- 6.51)
DOLL	514	f	0	20	12	15	15	1.67 (	0.61- 4.59)
Subtotal DOLL								1.76 (	0.80- 3.84)
*DORN	616	m	0	84	36304	37	73050	4.57 (	3.10- 6.72)
*DORN	653	m	0	81	24616	90	74464	2.72 (	2.02- 3.67)
Subtotal DORN								3.31 (	2.61- 4.19)
*ENGELA	505	m	0	173	50732	17	9762	1.96 (	1.19- 3.22)
*ENGELA	513	f	0	18	10687	10	24560	4.14 (	1.91- 8.96)
Subtotal ENGELA								2.44 (	1.60- 3.71)
GAO	510	m	2	262	-	45	-	4.25 (	2.93- 6.16)
GAO	520	f	2	77	-	73	-	2.80 (	1.56- 5.04)
Subtotal GAO								3.77 (	2.76- 5.16)
GAO2	505	m	0	52	26	2	4	4.00 (	0.69- 23.28)
GENG	537	f	1	36	-	28	-	3.87 (	1.62- 9.26)
HAENSZ	550	f	2	30	-	44	-	1.24 (	0.67- 2.28)
HEGMAN	515	m	1	146	-	26	-	2.40 (	1.50- 3.70)
HEGMAN	518	f	1	81	-	2	-	5.50 (	1.20- 24.10)
Subtotal HEGMAN								2.57 (	1.67- 3.96)
*HIRAYA	564	m	1	26	-	19	-	2.29 (	1.27- 4.14)
*HIRAYA	506	f	1	-	-	-	-	0.32 (	0.04- 2.42)
Subtotal HIRAYA								1.97 (	1.12- 3.47)
HU	515	m	0	13	7	14	20	2.65 (	0.84- 8.34)
HU	520	f	0	5	4	3	5	2.08 (	0.30- 14.55)
Subtotal HU								2.49 (	0.93- 6.69)
HU2	507	c	0	129	68	15	29	3.67 (	1.84- 7.31)
JEDRYC	610	m	5	135	-	49	-	1.66 (	1.19- 2.32)
JEDRYC	620	f	5	63	-	-	-	1.77 (	0.68- 4.60)
Subtotal JEDRYC								1.67 (	1.22- 2.29)
JOLY	547	m	0	317	282	18	70	4.37 (	2.54- 7.52)
JOLY	537	f	0	76	35	23	41	3.87 (	2.02- 7.41)
Subtotal JOLY								4.16 (	2.74- 6.30)
KHUDER	510	m	0	226	295	72	152	1.62 (	1.16- 2.25)
KOULUM	509	m	0	143	16	8	8	8.94 (	2.95- 27.06)
LETOUR	505	c	0	151	76	188	160	1.69 (	1.20- 2.39)
*LIAW	510	c	2	-	-	-	-	3.07 (	1.57- 6.01)
LIU3	506	m	2	32	-	20	-	1.26 (	0.58- 2.73)
LIU4	505	m	2	-	-	-	-	1.58 (	1.53- 1.63)
LIU5	503	c	0	72	48	13	22	2.54 (	1.17- 5.52)
LUBIN	571	m	0	178	251	30	179	4.23 (	2.75- 6.52)
LUBIN2	1164	m	1	250	-	68	-	2.63 (	1.79- 3.87)
MATOS	580	m	2	69	-	28	-	2.00 (	1.18- 3.42)
*MIGRAN	509	m	0	50	1081	24	668	1.29 (	0.80- 2.07)
*MIGRAN	519	f	0	2	266	11	1315	0.90 (	0.20- 4.03)
Subtotal MIGRAN								1.25 (	0.79- 1.96)
*MRFITR	518	m	0	25	2065	3	544	2.20 (	0.67- 7.24)

Table 1H20 - 2

IESLC - Meta-analysis of Ever/current Smoking, Age started, "Highest vs lowest"
All LC types, Cigarettes (or Any Product if Cigarettes not available)
Most adjusted

REF	NRR	SEX	AD	Number Exposed Case	Cont	Non-exposed Case	Cont	RR	95.00%CI
PERNU	506	m	0	337	92	1043	346	1.22 (	0.94- 1.58)
PERNU	503	f	0	1	0	18	89	14.51~(	0.57- 370.40)
Subtotal PERNU								1.23 (	0.95- 1.60)
PEZZOT	576	m	2	52	-	41	-	2.00 (	1.20- 3.33)
*QIAO2	510	m	1	104	-	52	-	1.37 (	0.92- 2.03)
RACHTA	510	f	1	52	-	8	-	2.18 (	0.54- 8.87)
SEGI2	525	m	1	91	-	49	-	2.80 (	1.82- 4.32)
SOBUE	800	m	0	137	92	110	121	1.64 (	1.13- 2.37)
*SPEIZE	530	f	2	-	-	-	-	1.25 (	0.91- 1.67)
SUZUK2	523	c	3	31	-	16	-	4.80 (	1.80- 13.00)
SVENSS	505	f	0	52	21	32	18	1.39 (	0.65- 3.00)
TIZZAN	510	m	0	699	529	12	44	4.84 (	2.53- 9.26)
TIZZAN	540	f	0	11	2	2	5	13.75 (	1.48- 127.47)
Subtotal TIZZAN								5.26 (	2.82- 9.79)
WAKAI	505	m	0	42	74	8	25	1.77 (	0.73- 4.28)
WU	545	f	2	106	-	14	-	8.75 (	3.26- 23.45)
WYNDE6	763	m	0	611	301	111	92	1.68 (	1.24- 2.29)
WYNDE6	771	f	0	291	91	127	90	2.27 (	1.58- 3.24)
Subtotal WYNDE6								1.91 (	1.51- 2.41)
ZHENG	569	m	1	106	-	28	-	5.73 (	3.25- 10.10)
ZHENG	575	f	1	60	-	16	-	1.79 (	0.79- 4.06)
Subtotal ZHENG								3.93 (	2.47- 6.26)
Partial Totals				8344	130555	3540	189480		

*prospective study ~ With 0.5 adjustment for zero

REF	NRR	SEX	AD	Ys	Ws	Qs	Ps
AGUDO	506	f	3	1.92	1.28	2.48	0.0302
ALDERS	503	m	2	1.08	6.73	2.07	0.0051
ALDERS	506	f	2	0.73	15.75	0.69	0.0037
Subtotal ALDERS				0.84	22.49	2.76	
ARMADA	513	m	0	0.86	33.57	3.80	0.0000
AUVINE	513	c	0	1.18	4.25	1.84	0.0149
BARBON	524	m	1	1.82	16.42	27.79	0.0000
BRESLO	505	c	0	0.45	13.43	0.08	0.1006
BUFFLE	525	f	0	0.64	8.05	0.11	0.0680
*CEDERL	514	m	1	-0.02	4.68	1.38	0.9651
*CEDERL	536	f	0	-1.24	0.46	1.45	0.3977
Subtotal CEDERL				-0.13	5.14	2.83	
CHEN2	521	m	0	-0.24	2.84	1.67	0.6810
CHEN2	526	f	0	1.04	2.25	0.60	0.1190
Subtotal CHEN2				0.32	5.09	2.27	
CHIAZZ	503	m	2	1.89	2.98	5.58	0.0011
CHOI	529	m	0	0.96	7.05	1.35	0.0107
CHOI	532	f	0	1.90	0.74	1.39	0.1034
Subtotal CHOI				1.05	7.79	2.74	
*CPSI	822	m	1	1.41	34.15	27.03	0.0000
*CPSI	851	f	1	0.10	5.41	0.95	0.8082
Subtotal CPSI				1.23	39.56	27.98	
DAMBER	505	m	0	0.83	23.53	2.14	0.0001
DEAN3	570	m	0	-0.18	11.93	5.95	0.5288
DEAN3	589	f	0	-0.43	5.19	4.70	0.3294
Subtotal DEAN3				-0.26	17.12	10.65	
DOLL	507	m	0	0.64	2.53	0.04	0.3070
DOLL	514	f	0	0.51	3.75	0.00	0.3226
Subtotal DOLL				0.56	6.28	0.04	
*DORN	616	m	0	1.52	25.71	25.47	0.0000
*DORN	653	m	0	1.00	42.73	9.75	0.0000
Subtotal DORN				1.20	68.44	35.23	
*ENGELA	505	m	0	0.67	15.51	0.34	0.0081
*ENGELA	513	f	0	1.42	6.43	5.17	0.0003
Subtotal ENGELA				0.89	21.94	5.51	
GAO	510	m	2	1.45	27.83	23.72	0.0000
GAO	520	f	2	1.03	11.17	2.86	0.0006
Subtotal GAO				1.33	39.00	26.57	
GAO2	505	m	0	1.39	1.24	0.92	0.1229
GENG	537	f	1	1.35	5.06	3.48	0.0023
HAENSZ	550	f	2	0.22	10.25	0.98	0.4911
HEGMAN	515	m	1	0.88	18.85	2.33	0.0001
HEGMAN	518	f	1	1.70	1.71	2.38	0.0259
Subtotal HEGMAN				0.94	20.56	4.71	

Table 1H20 - 2

IESLC - Meta-analysis of Ever/current Smoking, Age started, "Highest vs lowest"
All LC types, Cigarettes (or Any Product if Cigarettes not available)
Most adjusted

REF	NRR	SEX	AD	Ys	Ws	Qs	Ps
*HIRAYA	564	m	1	0.83	11.00	1.02	0.0060
*HIRAYA	506	f	1	-1.14	0.91	2.53	0.2763
Subtotal HIRAYA				0.68	11.92	3.55	
HU	515	m	0	0.98	2.93	0.60	0.0948
HU	520	f	0	0.73	1.02	0.04	0.4592
Subtotal HU				0.91	3.95	0.64	
HU2	507	c	0	1.30	8.09	4.87	0.0002
JEDRYC	610	m	5	0.51	34.48	0.01	0.0029
JEDRYC	620	f	5	0.57	4.20	0.01	0.2417
Subtotal JEDRYC				0.51	38.68	0.02	
JOLY	547	m	0	1.48	13.06	11.82	0.0000
JOLY	537	f	0	1.35	9.12	6.28	0.0000
Subtotal JOLY				1.43	22.19	18.11	
KHUDER	510	m	0	0.48	35.36	0.07	0.0043
KOULUM	509	m	0	2.19	3.13	8.69	0.0001
LETOUR	505	c	0	0.53	31.90	0.00	0.0030
*LIAW	510	c	2	1.12	8.53	3.05	0.0011
LIU3	506	m	2	0.23	6.40	0.55	0.5587
LIU4	505	m	2	0.46	3833.36	16.87	0.0000
LIU5	503	c	0	0.93	6.37	1.06	0.0188
LUBIN	571	m	0	1.44	20.61	17.40	0.0000
LUBIN2	1164	m	1	0.97	25.85	5.08	0.0000
MATOS	580	m	2	0.69	13.57	0.39	0.0107
*MIGRAN	509	m	0	0.25	16.88	1.24	0.2993
*MIGRAN	519	f	0	-0.11	1.71	0.68	0.8892
Subtotal MIGRAN				0.22	18.58	1.92	
*MRFITR	518	m	0	0.79	2.70	0.19	0.1967
PERNU	506	m	0	0.19	56.54	6.12	0.1428
PERNU	503	f	0	2.68	0.37	1.69	0.1056
Subtotal PERNU				0.21	56.91	7.81	
PEZZOT	576	m	2	0.69	14.75	0.42	0.0078
*QIAO2	510	m	1	0.31	24.53	1.07	0.1189
RACHTA	510	f	1	0.78	1.96	0.13	0.2751
SEGI2	525	m	1	1.03	20.56	5.26	0.0000
SOBUE	800	m	0	0.49	28.15	0.03	0.0088
*SPEIZE	530	f	2	0.22	41.69	3.77	0.1497
SUZUK2	523	c	3	1.57	3.93	4.29	0.0019
SVENSS	505	f	0	0.33	6.51	0.24	0.3979
TIZZAN	510	m	0	1.58	9.14	10.16	0.0000
TIZZAN	540	f	0	2.62	0.77	3.41	0.0211
Subtotal TIZZAN				1.66	9.92	13.57	
WAKAI	505	m	0	0.57	4.94	0.01	0.2027
WU	545	f	2	2.17	3.95	10.68	0.0000
WYNDE6	763	m	0	0.52	40.26	0.00	0.0010
WYNDE6	771	f	0	0.82	29.93	2.59	0.0000
Subtotal WYNDE6				0.65	70.19	2.59	
ZHENG	569	m	1	1.75	11.95	17.85	0.0000
ZHENG	575	f	1	0.58	5.73	0.02	0.1632
Subtotal ZHENG				1.37	17.69	17.86	

	N	73
	NS	53
	Wt	4770.32
Het	Chi	320.65
Het	df	72
Het	P	***
Fixed	RR	1.69
	RRl	1.64
	RRu	1.74
	P	+++
Random	RR	2.31
	RRl	2.04
	RRu	2.60
	P	+++
Asymm	P	***

Table 1H20 - 3

IESLC - Meta-analysis of Ever/current Smoking, Age started, "Highest vs lowest"
All LC types, Cigarettes (or Any Product if Cigarettes not available)
Most adjusted

	Sex			
	combined	male	female	Total
N	7	39	27	73
NS	7	38	27	72
Wt	76.50	4508.46	185.36	4770.32
Het Chi	10.00	245.73	56.00	320.65
Het df	6	38	26	72
Het P	N.S.	***	***	***
Fixed RR	2.19	1.67	1.93	1.69
RRl	1.75	1.62	1.67	1.64
RRu	2.74	1.72	2.23	1.74
P	+++	+++	+++	+++
Random RR	2.43	2.37	2.11	2.31
RRl	1.77	2.02	1.65	2.04
RRu	3.33	2.77	2.71	2.60
P	+++	+++	+++	+++
Between Chi				8.92
Between df				2
Between P				*
Btwn(F) P				N.S.
Btwn(R) P				N.S.

	Lung cancer type		
	all	other	Total
N	69	4	73
NS	50	3	53
Wt	4685.93	84.38	4770.32
Het Chi	306.37	12.55	320.65
Het df	68	3	72
Het P	***	**	***
Fixed RR	1.68	1.95	1.69
RRl	1.64	1.57	1.64
RRu	1.73	2.41	1.74
P	+++	+++	+++
Random RR	2.32	2.19	2.31
RRl	2.04	1.33	2.04
RRu	2.63	3.61	2.60
P	+++	++	+++
Between Chi			1.74
Between df			1
Between P			N.S.
Btwn(F) P			N.S.
Btwn(R) P			N.S.

	Location								
	NAmer	UK	Scand	othEur	China	Japan	othAs	other	Total
N	17	8	10	9	15	6	3	5	73
NS	13	4	7	7	11	5	2	4	53
Wt	349.04	64.48	121.41	127.67	3970.14	66.81	16.32	54.44	4770.32
Het Chi	71.02	13.22	29.05	27.17	84.26	7.36	0.61	7.86	320.65
Het df	16	7	9	8	14	5	2	4	72
Het P	***	(*)	***	***	***	N.S.	N.S.	(*)	***
Fixed RR	2.16	1.41	1.70	2.64	1.61	2.04	2.97	2.87	1.69
RRl	1.94	1.10	1.42	2.22	1.56	1.61	1.83	2.20	1.64
RRu	2.40	1.80	2.03	3.14	1.66	2.60	4.82	3.74	1.74
P	+++	++	+++	+++	+++	+++	+++	+++	+++
Random RR	2.26	1.39	2.12	3.04	2.52	2.04	2.97	2.98	2.31
RRl	1.78	0.97	1.41	2.07	1.82	1.47	1.83	2.03	2.04
RRu	2.88	2.01	3.18	4.45	3.48	2.85	4.82	4.38	2.60
P	+++	(+)	+++	+++	+++	+++	+++	+++	+++
Between Chi									80.10
Between df									7
Between P									***
Btwn(F) P									**
Btwn(R) P									(*)

Table 1H20 - 3

IESLC - Meta-analysis of Ever/current Smoking, Age started, "Highest vs lowest"
All LC types, Cigarettes (or Any Product if Cigarettes not available)
Most adjusted

Detailed Country in "other Europe"

	multi	Germany	othWest	East	Balkans	Total
N	1		5	3		9
NS	1		4	2		7
Wt	25.85		61.19	40.64		127.67
Het Chi	0.00		13.51	0.15		27.17
Het df	0		4	2		8
Het P	N.S.		**	N.S.		***
Fixed RR	2.63		3.56	1.69		2.64
RRl	1.79		2.77	1.25		2.22
RRu	3.87		4.58	2.30		3.14
P	+++		+++	+++		+++
Random RR	2.63		4.53	1.69		3.04
RRl	1.79		2.51	1.25		2.07
RRu	3.87		8.20	2.30		4.45
P	+++		+++	+++		+++
Between Chi						13.52
Between df						2
Between P						**
Btwn(F) P						N.S.
Btwn(R) P						**

Detailed Country in "other Asia"

	India	HongKong	other	Total
N			3	3
NS			2	2
Wt			16.32	16.32
Het Chi			0.61	0.61
Het df			2	2
Het P			N.S.	N.S.
Fixed RR			2.97	2.97
RRl			1.83	1.83
RRu			4.82	4.82
P			+++	+++
Random RR			2.97	2.97
RRl			1.83	1.83
RRu			4.82	4.82
P			+++	+++
Between Chi				
Between df				
Between P				N.S.
Btwn(F) P				N.S.
Btwn(R) P				N.S.

Detailed other continent

	SCAmer	Total
N	5	5
NS	4	4
Wt	54.44	54.44
Het Chi	7.86	7.86
Het df	4	4
Het P	(*)	(*)
Fixed RR	2.87	2.87
RRl	2.20	2.20
RRu	3.74	3.74
P	+++	+++
Random RR	2.98	2.98
RRl	2.03	2.03
RRu	4.38	4.38
P	+++	+++
Between Chi		
Between df		
Between P		N.S.
Btwn(F) P		N.S.
Btwn(R) P		N.S.

Table 1H20 - 3

IESLC - Meta-analysis of Ever/current Smoking, Age started, "Highest vs lowest"
All LC types, Cigarettes (or Any Product if Cigarettes not available)
Most adjusted

	Start year of study					
	<1960	1960-69	1970-79	1980-89	1990+	Total
N	14	13	12	30	4	73
NS	9	7	10	23	4	53
Wt	210.89	165.47	177.36	4172.60	43.99	4770.32
Het Chi	74.72	32.05	42.08	111.54	5.81	320.65
Het df	13	12	11	29	3	72
Het P	***	**	***	***	N.S.	***
Fixed RR	2.39	1.76	2.43	1.63	1.76	1.69
RRl	2.08	1.51	2.10	1.58	1.31	1.64
RRu	2.73	2.05	2.82	1.68	2.36	1.74
P	+++	+++	+++	+++	+++	+++
Random RR	2.73	1.60	2.73	2.39	2.00	2.31
RRl	1.86	1.21	2.01	1.99	1.24	2.04
RRu	4.01	2.13	3.71	2.87	3.25	2.60
P	+++	++	+++	+++	++	+++
Between Chi						54.46
Between df						4
Between P						***
Btwn(F) P						*
Btwn(R) P						(*)

	Study type (1)		
	CC	other	Total
N	57	16	73
NS	43	10	53
Wt	4527.29	243.03	4770.32
Het Chi	234.69	68.67	320.65
Het df	56	15	72
Het P	***	***	***
Fixed RR	1.66	2.19	1.69
RRl	1.62	1.93	1.64
RRu	1.71	2.48	1.74
P	+++	+++	+++
Random RR	2.39	1.98	2.31
RRl	2.09	1.46	2.04
RRu	2.74	2.69	2.60
P	+++	+++	+++
Between Chi			17.29
Between df			1
Between P			***
Btwn(F) P			*
Btwn(R) P			N.S.

	Study type (2)			
	CC	prosp	other	Total
N	57	16		73
NS	43	10		53
Wt	4527.29	243.03		4770.32
Het Chi	234.69	68.67		320.65
Het df	56	15		72
Het P	***	***		***
Fixed RR	1.66	2.19		1.69
RRl	1.62	1.93		1.64
RRu	1.71	2.48		1.74
P	+++	+++		+++
Random RR	2.39	1.98		2.31
RRl	2.09	1.46		2.04
RRu	2.74	2.69		2.60
P	+++	+++		+++
Between Chi				17.29
Between df				1
Between P				***
Btwn(F) P				*
Btwn(R) P				N.S.

Table 1H20 - 3

IESLC - Meta-analysis of Ever/current Smoking, Age started, "Highest vs lowest"
All LC types, Cigarettes (or Any Product if Cigarettes not available)
Most adjusted

	Study size (number of LC cases) 100-249	250-499	500-999	1000+	Total
N	18	17	15	23	73
NS	16	12	12	13	53
Wt	116.73	195.36	207.49	4250.74	4770.32
Het Chi	30.84	32.36	82.98	138.27	320.65
Het df	17	16	14	22	72
Het P	*	**	***	***	***
Fixed RR	1.99	2.25	2.17	1.64	1.69
RRl	1.66	1.96	1.89	1.59	1.64
RRu	2.39	2.59	2.48	1.69	1.74
P	+++	+++	+++	+++	+++
Random RR	2.20	2.30	2.44	2.31	2.31
RRl	1.68	1.83	1.72	1.89	2.04
RRu	2.88	2.89	3.45	2.82	2.60
P	+++	+++	+++	+++	+++
Between Chi					36.20
Between df					3
Between P					***
Btwn(F) P					*
Btwn(R) P					N.S.

	Risky occupational population no	mining	othRisky	Total
N	70	2	1	73
NS	50	2	1	53
Wt	4722.20	45.14	2.98	4770.32
Het Chi	296.54	14.24	0.00	320.65
Het df	69	1	0	72
Het P	***	***	N.S.	***
Fixed RR	1.68	2.29	6.63	1.69
RRl	1.63	1.71	2.13	1.64
RRu	1.73	3.07	20.63	1.74
P	+++	+++	++	+++
Random RR	2.28	2.40	6.63	2.31
RRl	2.02	0.79	2.13	2.04
RRu	2.58	7.25	20.63	2.60
P	+++	N.S.	++	+++
Between Chi				9.87
Between df				2
Between P				**
Btwn(F) P				N.S.
Btwn(R) P				N.S.

	National cigarette tobacco type Virginia	blended	other	Total
N	9	48	16	73
NS	5	36	12	53
Wt	96.38	695.27	3978.67	4770.32
Het Chi	13.94	158.13	87.79	320.65
Het df	8	47	15	72
Het P	(*)	***	***	***
Fixed RR	1.50	2.22	1.61	1.69
RRl	1.22	2.06	1.57	1.64
RRu	1.83	2.39	1.67	1.74
P	+++	+++	+++	+++
Random RR	1.45	2.44	2.55	2.31
RRl	1.08	2.09	1.87	2.04
RRu	1.95	2.84	3.48	2.60
P	+	+++	+++	+++
Between Chi				60.79
Between df				2
Between P				***
Btwn(F) P				***
Btwn(R) P				**

Table 1H20 - 3

IESLC - Meta-analysis of Ever/current Smoking, Age started, "Highest vs lowest"
All LC types, Cigarettes (or Any Product if Cigarettes not available)
Most adjusted

	Any proxy use No/nk	Yes	Total
N	55	18	73
NS	39	14	53
Wt	733.31	4037.01	4770.32
Het Chi	172.78	85.76	320.65
Het df	54	17	72
Het P	***	***	***
Fixed RR	2.21	1.61	1.69
RRl	2.05	1.56	1.64
RRu	2.37	1.66	1.74
P	+++	+++	+++
Random RR	2.35	2.21	2.31
RRl	2.03	1.72	2.04
RRu	2.72	2.83	2.60
P	+++	+++	+++
Between Chi			62.11
Between df			1
Between P			***
Btwn(F) P			***
Btwn(R) P			N.S.

	Full histological confirmation No	Yes	Total
N	51	22	73
NS	35	18	53
Wt	4391.80	378.52	4770.32
Het Chi	236.53	68.96	320.65
Het df	50	21	72
Het P	***	***	***
Fixed RR	1.66	2.05	1.69
RRl	1.61	1.85	1.64
RRu	1.71	2.26	1.74
P	+++	+++	+++
Random RR	2.33	2.26	2.31
RRl	1.99	1.85	2.04
RRu	2.73	2.77	2.60
P	+++	+++	+++
Between Chi			15.17
Between df			1
Between P			***
Btwn(F) P			(*)
Btwn(R) P			N.S.

	Number of adjustment variables (1) 0	1	2+/+nk	Total
N	41	15	17	73
NS	29	11	14	54
Wt	540.70	188.78	4040.84	4770.32
Het Chi	120.00	51.71	67.75	320.65
Het df	40	14	16	72
Het P	***	***	***	***
Fixed RR	2.08	2.77	1.60	1.69
RRl	1.92	2.41	1.56	1.64
RRu	2.27	3.20	1.65	1.74
P	+++	+++	+++	+++
Random RR	2.21	2.56	2.27	2.31
RRl	1.87	1.89	1.81	2.04
RRu	2.61	3.47	2.86	2.60
P	+++	+++	+++	+++
Between Chi				81.20
Between df				2
Between P				***
Btwn(F) P				***
Btwn(R) P				N.S.

Table 1H20 - 3

IESLC - Meta-analysis of Ever/current Smoking, Age started, "Highest vs lowest"
All LC types, Cigarettes (or Any Product if Cigarettes not available)
Most adjusted

	Number of adjustment variables (2) 0	1	2	3-5	6+/+nk	Total
N	41	15	13	4		73
NS	29	11	11	3		54
Wt	540.70	188.78	3996.96	43.89		4770.32
Het Chi	120.00	51.71	60.26	6.10		320.65
Het df	40	14	12	3		72
Het P	***	***	***	N.S.		***
Fixed RR	2.08	2.77	1.60	1.91		1.69
RRl	1.92	2.41	1.55	1.42		1.64
RRu	2.27	3.20	1.65	2.57		1.74
P	+++	+++	+++	+++		+++
Random RR	2.21	2.56	2.27	2.46		2.31
RRl	1.87	1.89	1.74	1.33		2.04
RRu	2.61	3.47	2.96	4.57		2.60
P	+++	+++	+++	++		+++
Between Chi						82.58
Between df						3
Between P						***
Btwn(F) P						***
Btwn(R) P						N.S.

	Smoking status ever	current	Total
N	53	20	73
NS	39	14	53
Wt	4565.94	204.38	4770.32
Het Chi	243.95	73.29	320.65
Het df	52	19	72
Het P	***	***	***
Fixed RR	1.68	1.92	1.69
RRl	1.63	1.67	1.64
RRu	1.73	2.20	1.74
P	+++	+++	+++
Random RR	2.49	1.80	2.31
RRl	2.17	1.32	2.04
RRu	2.85	2.44	2.60
P	+++	+++	+++
Between Chi			3.42
Between df			1
Between P			(*)
Btwn(F) P			N.S.
Btwn(R) P			(*)

	Product all/unsp	cig+/-ot	cig only	Total
N	20	41	12	73
NS	16	31	8	55
Wt	4034.63	650.14	85.55	4770.32
Het Chi	90.40	117.55	23.82	320.65
Het df	19	40	11	72
Het P	***	***	*	***
Fixed RR	1.60	2.38	1.47	1.69
RRl	1.55	2.20	1.19	1.64
RRu	1.65	2.57	1.82	1.74
P	+++	+++	+++	+++
Random RR	2.45	2.49	1.46	2.31
RRl	1.90	2.15	1.03	2.04
RRu	3.16	2.88	2.08	2.60
P	+++	+++	+	+++
Between Chi				88.89
Between df				2
Between P				***
Btwn(F) P				***
Btwn(R) P				*

Table 1H20 - 3

IESLC - Meta-analysis of Ever/current Smoking, Age started, "Highest vs lowest"
All LC types, Cigarettes (or Any Product if Cigarettes not available)
Most adjusted

	Derivation of RR/CI			
	Orig	StdCalc	Other	Total
N	5	42	26	73
NS	3	31	20	54
Wt	63.17	612.45	4094.70	4770.32
Het Chi	6.60	130.80	154.41	320.65
Het df	4	41	25	72
Het P	N.S.	***	***	***
Fixed RR	2.05	2.04	1.64	1.69
RRl	1.60	1.89	1.59	1.64
RRu	2.63	2.21	1.69	1.74
P	+++	+++	+++	+++
Random RR	2.28	2.20	2.47	2.31
RRl	1.56	1.88	1.93	2.04
RRu	3.34	2.58	3.17	2.60
P	+++	+++	+++	+++
Between Chi				28.84
Between df				2
Between P				***
Btwn(F) P				*
Btwn(R) P				N.S.

	Study LIU4		
	LIU4	others	Total
N	1	72	73
NS	1	52	53
Wt	3833.36	936.96	4770.32
Het Chi	0.00	234.74	320.65
Het df	0	71	72
Het P	N.S.	***	***
Fixed RR	1.58	2.21	1.69
RRl	1.53	2.08	1.64
RRu	1.63	2.36	1.74
P	+++	+++	+++
Random RR	1.58	2.34	2.31
RRl	1.53	2.05	2.04
RRu	1.63	2.66	2.60
P	+++	+++	+++
Between Chi			85.92
Between df			1
Between P			***
Btwn(F) P			***
Btwn(R) P			***

Table 1H20 - 4

IESLC - Meta-analysis of Ever/current Smoking, Age started, "Highest vs lowest"
All LC types, Cigarettes (or Any Product if Cigarettes not available)
Least adjusted

REF	NRR	X	SEX	AGEL	AGEH	RACE	YF	LC TYPE	LOC	START	ST	NLC	R	VB	P	H	AD	ADOS	SM	PRODUCT	exL	exH	unexL	unexH	De
AGUDO	503	x	f	0	0	all	-	all	Eu:wst	1989	CC	103	n	bl	n	n	0	0	ev	cig only	1	23	24	999	st
ALDERS	503		m	0	0	all	-	all	Eu:UK	1977	CC	1448	n	V	n	n	2	1#	ev	cig only	1	14	25	999	ot
ALDERS	506		f	0	0	all	-	all	Eu:UK	1977	CC	1448	n	V	n	n	2	1#	ev	cig only	1	14	25	999	ot
ARMADA	513		m	0	0	all	-	all	Eu:wst	1986	CC	325	n	bl	n	y	0	0	ev	cig+/-ot	7	16	17	45	st
AUVINE	513		c	0	0	all	-	all	Eu:Sca	1986	CC	517	n	bl	y	n	0	0	ev	cig+/-ot	1	15	21	999	st
BARBON	519	x	m	0	0	all	-	all	Eu:wst	1979	CC	755	n	bl	y	y	0	0	ev	all/unsp	1	14	20	999	st
BRESLO	505		c	0	0	all	-	all	NAmer	1949	CC	518	n	bl	n	y	0	0	ev	cig+/-ot	0	14	25	999	st
BUFFLE	525		f	0	0	w-hi	-	all	NAmer	1976	CC	943	n	bl	y	n	0	0	ev	cig+/-ot	6	16	30	999	st
CEDERL	514		m	0	0	all	10	all	Eu:Sca	1963	pr	491	n	bl	n	n	1	0	cu	cig only	1	16	19	999	ot
CEDERL	536		f	0	0	all	10	all	Eu:Sca	1963	pr	491	n	bl	n	n	0	0	cu	cig only	1	16	19	999	ot
CHEN2	521		m	0	0	all	-	all	As:Chi	1983	CC	193	n	ot	y	n	0	0	ev	all/unsp	1	19	31	999	st
CHEN2	526		f	0	0	all	-	all	As:Chi	1983	CC	193	n	ot	y	n	0	0	ev	all/unsp	1	19	31	999	st
CHIAZZ	503		m	0	0	all	-	all	NAmer	1940	CC	144	o	bl	y	n	2	0	ev	cig+/-ot	1	19	20	999	ot
CHOI	529		m	0	0	all	-	all	As:oth	1985	CC	375	n	bl	n	n	0	0	ev	cig+/-ot	1	14	25	999	st
CHOI	532		f	0	0	all	-	all	As:oth	1985	CC	375	n	bl	n	n	0	0	ev	cig+/-ot	1	24	25	999	st
CPSI	822		m	35	84	all	6	all	NAmer	1959	pr	5138	n	bl	n	n	1	0	cu	cig+/-ot	1	14	25	999	ot
CPSI	851		f	40	74	all	6	all	NAmer	1959	pr	5138	n	bl	n	n	1	0	cu	cig only	1	14	25	999	ot
DAMBER	505		m	0	0	all	-	all	Eu:Sca	1972	CC	579	n	bl	y	n	0	0	ev	all/unsp	1	15	21	999	st
DEAN3	570		m	0	0	all	-	all	Eu:UK	1969	CC	766	n	V	y	n	0	0	cu	cig only	1	14	25	999	st
DEAN3	589		f	0	0	all	-	all	Eu:UK	1969	CC	766	n	V	y	n	0	0	cu	cig only	1	14	25	999	st
DOLL	507		m	0	0	all	-	all	Eu:UK	1948	CC	1465	n	V	n	n	0	0	ev	all/unsp	1	19	40	999	st
DOLL	514		f	0	0	all	-	all	Eu:UK	1948	CC	1465	n	V	n	n	0	0	ev	all/unsp	1	19	40	999	st
DORN	616		m	55	64	wh	8	all	NAmer	1954	pr	5097	n	bl	n	n	0	0	ev	cig+/-ot	1	14	25	999	st
DORN	653		m	65	74	wh	8	all	NAmer	1954	pr	5097	n	bl	n	n	0	0	ev	cig+/-ot	1	14	25	999	st
ENGELA	505		m	0	0	all	0	all	Eu:Sca	1964	pr	435	n	bl	n	n	0	0	cu	cig+/-ot	1	19	30	999	st
ENGELA	513		f	0	0	all	0	all	Eu:Sca	1964	pr	435	n	bl	n	n	0	0	cu	cig+/-ot	1	19	30	999	st
GAO	505	x	m	0	0	all	-	all	As:Chi	1984	CC	1405	n	ot	n	n	0	0	ev	cig+/-ot	10	19	30	999	st
GAO	515	x	f	0	0	all	-	all	As:Chi	1984	CC	1405	n	ot	n	n	0	0	ev	cig+/-ot	10	19	30	999	st
GAO2	505		m	0	0	all	-	all	As:Jap	1988	CC	282	n	bl	n	n	0	0	cu	cig+/-ot	1	19	30	999	st
GENG	532	x	f	0	0	all	-	all	As:Chi	1985	CC	292	n	ot	*	n	0	0	ev	cig+/-ot	1	15	21	999	st
HAENSZ	541	x	f	0	0	all	-	not alv	NAmer	1955	CC	158	n	bl	n	y	0	0	ev	cig+/-ot	1	24	25	999	st
HEGMAN	501	x	m	0	0	all	-	all	NAmer	1989	CC	282	n	bl	y	y	0	0	ev	all/unsp	1	19	20	999	st
HEGMAN	502	x	f	0	0	all	-	all	NAmer	1989	CC	282	n	bl	y	y	0	0	ev	all/unsp	1	25	26	999	st
HIRAYA	564		m	0	0	all	5	all	As:Jap	1965	pr	1917	n	bl	n	n	1	0	cu	cig+/-ot	1	19	25	999	ot
HIRAYA	506		f	0	0	all	0	all	As:Jap	1965	pr	1917	n	bl	n	n	1	0	cu	cig+/-ot	1	19	20	999	ot
HU	515		m	0	0	all	-	all	As:Chi	1985	CC	227	n	ot	n	y	0	0	ev	cig+/-ot	1	15	30	999	st
HU	520		f	0	0	all	-	all	As:Chi	1985	CC	227	n	ot	n	y	0	0	ev	cig+/-ot	1	15	30	999	st
HU2	507		c	0	0	all	-	all	As:Chi	1977	CC	523	n	ot	y	n	0	0	ev	cig+/-ot	1	19	40	999	st
JEDRYC	610		m	0	0	all	-	all	Eu:est	1980	CC	1630	n	bl	y	n	5	2#	ev	cig+/-ot	1	16	19	999	or
JEDRYC	620		f	0	0	all	-	all	Eu:est	1980	CC	1630	n	bl	y	n	5	2#	ev	cig+/-ot	1	22	23	999	or
JOLY	547		m	0	0	all	-	all	SCAmer	1978	CC	826	n	bl	n	n	0	0	ev	cig+/-ot	1	14	25	999	st
JOLY	537		f	0	0	all	-	all	SCAmer	1978	CC	826	n	bl	n	n	0	0	ev	cig+/-ot	1	14	25	999	st
KHUDER	510		m	0	0	all	-	all	NAmer	1985	CC	482	n	bl	n	y	0	0	ev	cig+/-ot	1	15	20	999	st
KOULUM	509		m	0	0	all	-	all	Eu:Sca	1936	CC	812	n	bl	n	n	0	0	ev	all/unsp	0	10	31	999	st
LETOUR	505		c	0	0	all	-	all	NAmer	1983	CC	738	n	V	y	y	0	0	ev	cig+/-ot	1	14	21	999	st
LIAW	510		c	0	0	all	0	all	As:oth	1982	pr	127	n	ot	n	n	2	0	cu	all/unsp	1	20	25	999	ot
LIU3	503	x	m	0	0	all	-	all	As:Chi	1985	CC	110	n	ot	n	n	0	0	ev	all/unsp	1	20	21	999	st
LIU4	505		m	35	69	all	-	all	As:Chi	1986	CC	1000-00	n	ot	y	n	2	0	ev	all/unsp	0	19	25	999	ot
LIU5	503		c	0	0	all	-	all	As:Chi	1978	CC	111	n	ot	y	n	0	0	ev	all/unsp	1	29	30	999	st
LUBIN	571		m	0	0	all	-	all	As:Chi	1984	CC	427	m	ot	y	n	0	0	ev	cig+/-ot	1	19	27	999	st
LUBIN2	1155	x	m	0	0	all	-	all	Eu:mul	1976	CC	7804	n	bl	n	y	0	0	ev	cig+/-ot	1	12	31	999	st
MATOS	560	x	m	0	0	all	-	all	SCAmer	1994	CC	200	n	bl	n	n	0	0	ev	cig+/-ot	1	14	20	999	st
MIGRAN	509		m	0	0	all	0	all	Eu:UK	1964	pr	259	n	V	n	n	0	0	cu	cig only	0	15	20	999	st
MIGRAN	519		f	0	0	all	0	all	Eu:UK	1964	pr	259	n	V	n	n	0	0	cu	cig only	0	15	20	999	st
MRFITR	518		m	0	0	all	0	all	NAmer	1973	pr	119	n	bl	n	n	0	0	cu	cig+/-ot	1	15	24	999	st
PERNU	506		m	0	0	all	-	all	Eu:Sca	1944	CC	1606	n	bl	n	n	0	0	ev	all/unsp	1	14	15	999	st
PERNU	503		f	0	0	all	-	all	Eu:Sca	1944	CC	1606	n	bl	n	n	0	0	ev	all/unsp	1	14	15	999	ot
PEZZOT	574	x	m	0	0	all	-	all	SCAmer	1987	CC	215	n	bl	n	y	0	0	ev	cig only	1	13	19	999	st
QIAO2	505	x	m	0	0	all	0	all	As:Chi	1992	pr	241	m	ot	n	n	0	0	ev	all/unsp	1	16	21	999	st
RACHTA	505	x	f	0	0	all	-	all	Eu:est	1991	CC	118	n	bl	n	y	0	0	ev	cig+/-ot	1	19	31	999	st
SEGI2	520	x	m	0	0	all	-	all	As:Jap	1962	CC	378	n	bl	n	n	0	0	cu	cig+/-ot	1	19	23	999	st
SOBUE	800		m	0	0	all	-	all	As:Jap	1986	CC	1376	n	bl	n	y	0	0	ev	cig+/-ot	10	17	23	999	st
SPEIZE	530		f	0	0	all	0	all	NAmer	1976	pr	593	n	bl	n	y	2	1#	cu	cig+/-ot	18	19	22	999	st
SUZUK2	505	x	c	0	0	all	-	all	SCAmer	1991	CC	123	n	bl	n	y	0	0	ev	all/unsp	0	11	19	999	st
SVENSS	505		f	0	0	all	-	all	Eu:Sca	1983	CC	210	n	bl	n	n	0	0	cu	all/unsp	0	18	26	999	st
TIZZAN	510		m	0	0	all	-	all	Eu:wst	1959	CC	1358	n	bl	n	n	0	0	ev	all/unsp	1	19	31	999	st
TIZZAN	540		f	0	0	all	-	all	Eu:wst	1959	CC	1358	n	bl	n	n	0	0	ev	cig only	1	19	31	999	st
WAKAI	505		m	0	0	all	-	all	As:Jap	1988	CC	333	n	bl	n	y	0	0	cu	cig+/-ot	1	19	30	999	st
WU	521	x	f	0	0	wh	-	q+a	NAmer	1981	CC	220	n	bl	n	y	0	0	cu	all/unsp	0	18	25	999	st
WYNDE6	763		m	0	0	wh	-	q+s+a	NAmer	1969	CC	4423	n	bl	n	y	0	0	ev	cig+/-ot	1	17	21	999	st
WYNDE6	771		f	0	0	wh	-	q+s+a	NAmer	1969	CC	4423	n	bl	n	y	0	0	ev	cig+/-ot	1	17	21	999	st

Table 1H20 - 4

IESLC - Meta-analysis of Ever/current Smoking, Age started, "Highest vs lowest"
All LC types, Cigarettes (or Any Product if Cigarettes not available)
Least adjusted

REF	NRR	X	SEX	AGEL	AGEH	RACE	YF	LC TYPE	LOC	START	ST	NLC	R	VB	P	H	AD	ADOS	SM	PRODUCT	exL	exH	unexL	unexH	De
ZHENG	567	x	m	0	0	all	-	all	As:Chi	1982	CC	540	n	ot	*	y	0	0	ev	cig+/-ot	1	19	30	999	st
ZHENG	574	x	f	0	0	all	-	all	As:Chi	1982	CC	540	n	ot	*	y	0	0	ev	cig+/-ot	1	29	30	999	st

Comments on values in listings

ALDERS	ADOS	Number of cigs/day
ALDERS	ADOS	Number of cigs/day
JEDRYC	ADOS	Number of cigs per day & time since quit
JEDRYC	ADOS	Number of cigs per day & time since quit
SPEIZE	ADOS	Numbers of cigs/day

Cigarette type is all/unspec for all RRs
except for the following:

REF	NRR	CIGTYPE
ALDERS	503	MC only
ALDERS	506	MC only
DEAN3	570	MC only
DEAN3	589	MC only

Table 1H20 - 5

IESLC - Meta-analysis of Ever/current Smoking, Age started, "Highest vs lowest"
All LC types, Cigarettes (or Any Product if Cigarettes not available)
Least adjusted

REF	NRR	SEX	AD	Number Exposed Case	Cont	Non-exposed Case	Cont	RR	95.00%CI
AGUDO	503	f	0	16	11	7	12	2.49 (	0.75- 8.34)
ALDERS	503	m	2	139	-	11	-	2.94 (	1.38- 6.25)
ALDERS	506	f	2	78	-	97	-	2.08 (	1.27- 3.41)
Subtotal ALDERS								2.31 (	1.53- 3.49)
ARMADA	513	m	0	204	110	113	144	2.36 (	1.69- 3.31)
AUVINE	513	c	0	55	6	76	27	3.26 (	1.26- 8.42)
BARBON	519	m	0	138	23	200	207	6.21 (	3.83- 10.06)
BRESLO	505	c	0	166	116	32	35	1.57 (	0.92- 2.67)
BUFFLE	525	f	0	78	41	23	23	1.90 (	0.95- 3.80)
*CEDERL	514	m	1	7	-	11	-	0.98 (	0.40- 2.45)
*CEDERL	536	f	0	0	746	6	2806	0.29~(	0.02- 5.13)
Subtotal CEDERL								0.88 (	0.37- 2.08)
CHEN2	521	m	0	84	67	8	5	0.78 (	0.25- 2.51)
CHEN2	526	f	0	23	13	5	8	2.83 (	0.77- 10.47)
Subtotal CHEN2								1.38 (	0.58- 3.29)
CHIAZZ	503	m	2	-	-	-	-	6.63 (	2.13- 20.63)
CHOI	529	m	0	22	18	36	77	2.61 (	1.25- 5.47)
CHOI	532	f	0	4	1	15	25	6.67 (	0.68- 65.37)
Subtotal CHOI								2.86 (	1.42- 5.76)
*CPSI	822	m	1	185	-	42	-	4.11 (	2.94- 5.75)
*CPSI	851	f	1	6	-	51	-	1.11 (	0.48- 2.59)
Subtotal CPSI								3.44 (	2.52- 4.69)
DAMBER	505	m	0	206	98	70	76	2.28 (	1.52- 3.42)
DEAN3	570	m	0	44	165	24	75	0.83 (	0.47- 1.47)
DEAN3	589	f	0	7	109	27	274	0.65 (	0.28- 1.54)
Subtotal DEAN3								0.77 (	0.48- 1.24)
DOLL	507	m	0	1077	992	4	7	1.90 (	0.55- 6.51)
DOLL	514	f	0	20	12	15	15	1.67 (	0.61- 4.59)
Subtotal DOLL								1.76 (	0.80- 3.84)
*DORN	616	m	0	84	36304	37	73050	4.57 (	3.10- 6.72)
*DORN	653	m	0	81	24616	90	74464	2.72 (	2.02- 3.67)
Subtotal DORN								3.31 (	2.61- 4.19)
*ENGELA	505	m	0	173	50732	17	9762	1.96 (	1.19- 3.22)
*ENGELA	513	f	0	18	10687	10	24560	4.14 (	1.91- 8.96)
Subtotal ENGELA								2.44 (	1.60- 3.71)
GAO	505	m	0	262	167	45	129	4.50 (	3.04- 6.65)
GAO	515	f	0	77	25	73	64	2.70 (	1.54- 4.74)
Subtotal GAO								3.81 (	2.76- 5.25)
GAO2	505	m	0	52	26	2	4	4.00 (	0.69- 23.28)
GENG	532	f	0	36	10	28	31	3.99 (	1.67- 9.49)
HAENSZ	541	f	0	30	37	44	66	1.22 (	0.66- 2.25)
HEGMAN	501	m	0	146	716	26	289	2.27 (	1.46- 3.52)
HEGMAN	502	f	0	81	169	2	28	6.71 (	1.56- 28.86)
Subtotal HEGMAN								2.48 (	1.63- 3.78)
*HIRAYA	564	m	1	26	-	19	-	2.29 (	1.27- 4.14)
*HIRAYA	506	f	1	-	-	-	-	0.32 (	0.04- 2.42)
Subtotal HIRAYA								1.97 (	1.12- 3.47)
HU	515	m	0	13	7	14	20	2.65 (	0.84- 8.34)
HU	520	f	0	5	4	3	5	2.08 (	0.30- 14.55)
Subtotal HU								2.49 (	0.93- 6.69)
HU2	507	c	0	129	68	15	29	3.67 (	1.84- 7.31)
JEDRYC	610	m	5	135	-	49	-	1.66 (	1.19- 2.32)
JEDRYC	620	f	5	63	-	-	-	1.77 (	0.68- 4.60)
Subtotal JEDRYC								1.67 (	1.22- 2.29)
JOLY	547	m	0	317	282	18	70	4.37 (	2.54- 7.52)
JOLY	537	f	0	76	35	23	41	3.87 (	2.02- 7.41)
Subtotal JOLY								4.16 (	2.74- 6.30)
KHUDER	510	m	0	226	295	72	152	1.62 (	1.16- 2.25)
KOULUM	509	m	0	143	16	8	8	8.94 (	2.95- 27.06)
LETOUR	505	c	0	151	76	188	160	1.69 (	1.20- 2.39)
*LIAW	510	c	2	-	-	-	-	3.07 (	1.57- 6.01)
LIU3	503	m	0	32	125	20	80	1.02 (	0.55- 1.91)
LIU4	505	m	2	-	-	-	-	1.58 (	1.53- 1.63)
LIU5	503	c	0	72	48	13	22	2.54 (	1.17- 5.52)
LUBIN	571	m	0	178	251	30	179	4.23 (	2.75- 6.52)
LUBIN2	1155	m	0	250	316	68	221	2.57 (	1.87- 3.54)
MATOS	560	m	0	69	90	28	73	2.00 (	1.17- 3.42)
*MIGRAN	509	m	0	50	1081	24	668	1.29 (	0.80- 2.07)
*MIGRAN	519	f	0	2	266	11	1315	0.90 (	0.20- 4.03)
Subtotal MIGRAN								1.25 (	0.79- 1.96)
*MRFITR	518	m	0	25	2065	3	544	2.20 (	0.67- 7.24)

Table 1H20 - 5

IESLC - Meta-analysis of Ever/current Smoking, Age started, "Highest vs lowest"
All LC types, Cigarettes (or Any Product if Cigarettes not available)
Least adjusted

REF	NRR	SEX	AD	Number Exposed Case	Cont	Non-exposed Case	Cont	RR	95.00%CI
PERNU	506	m	0	337	92	1043	346	1.22 (	0.94- 1.58)
PERNU	503	f	0	1	0	18	89	14.51~(	0.57- 370.40)
Subtotal PERNU								1.23 (	0.95- 1.60)
PEZZOT	574	m	0	52	67	41	105	1.99 (	1.19- 3.31)
*QIAO2	505	m	0	104	2130	52	1947	1.83 (	1.32- 2.54)
RACHTA	505	f	0	52	21	8	4	1.24 (	0.34- 4.56)
SEGI2	520	m	0	91	103	49	155	2.79 (	1.82- 4.29)
SOBUE	800	m	0	137	92	110	121	1.64 (	1.13- 2.37)
*SPEIZE	530	f	2	-	-	-	-	1.25 (	0.91- 1.67)
SUZUK2	505	c	0	31	10	16	22	4.26 (	1.63- 11.14)
SVENSS	505	f	0	52	21	32	18	1.39 (	0.65- 3.00)
TIZZAN	510	m	0	699	529	12	44	4.84 (	2.53- 9.26)
TIZZAN	540	f	0	11	2	2	5	13.75 (	1.48- 127.47)
Subtotal TIZZAN								5.26 (	2.82- 9.79)
WAKAI	505	m	0	42	74	8	25	1.77 (	0.73- 4.28)
WU	521	f	0	106	32	14	19	4.50 (	2.03- 9.96)
WYNDE6	763	m	0	611	301	111	92	1.68 (	1.24- 2.29)
WYNDE6	771	f	0	291	91	127	90	2.27 (	1.58- 3.24)
Subtotal WYNDE6								1.91 (	1.51- 2.41)
ZHENG	567	m	0	106	43	28	66	5.81 (	3.30- 10.24)
ZHENG	574	f	0	60	28	16	16	2.14 (	0.94- 4.89)
Subtotal ZHENG								4.22 (	2.65- 6.74)
Partial Totals				8344	134678	3540	193014		

*prospective study ~ With 0.5 adjustment for zero

REF	NRR	SEX	AD	Ys	Ws	Qs	Ps
AGUDO	503	f	0	0.91	2.63	0.40	0.1381
ALDERS	503	m	2	1.08	6.73	2.06	0.0051
ALDERS	506	f	2	0.73	15.75	0.68	0.0037
Subtotal ALDERS				0.84	22.49	2.74	
ARMADA	513	m	0	0.86	33.57	3.76	0.0000
AUVINE	513	c	0	1.18	4.25	1.83	0.0149
BARBON	519	m	0	1.83	16.51	27.95	0.0000
BRESLO	505	c	0	0.45	13.43	0.08	0.1006
BUFFLE	525	f	0	0.64	8.05	0.11	0.0680
*CEDERL	514	m	1	-0.02	4.68	1.39	0.9651
*CEDERL	536	f	0	-1.24	0.46	1.45	0.3977
Subtotal CEDERL				-0.13	5.14	2.84	
CHEN2	521	m	0	-0.24	2.84	1.68	0.6810
CHEN2	526	f	0	1.04	2.25	0.60	0.1190
Subtotal CHEN2				0.32	5.09	2.28	
CHIAZZ	503	m	2	1.89	2.98	5.56	0.0011
CHOI	529	m	0	0.96	7.05	1.34	0.0107
CHOI	532	f	0	1.90	0.74	1.39	0.1034
Subtotal CHOI				1.05	7.79	2.73	
*CPSI	822	m	1	1.41	34.15	26.94	0.0000
*CPSI	851	f	1	0.10	5.41	0.96	0.8082
Subtotal CPSI				1.23	39.56	27.90	
DAMBER	505	m	0	0.83	23.53	2.12	0.0001
DEAN3	570	m	0	-0.18	11.93	5.97	0.5288
DEAN3	589	f	0	-0.43	5.19	4.72	0.3294
Subtotal DEAN3				-0.26	17.12	10.69	
DOLL	507	m	0	0.64	2.53	0.03	0.3070
DOLL	514	f	0	0.51	3.75	0.00	0.3226
Subtotal DOLL				0.56	6.28	0.04	
*DORN	616	m	0	1.52	25.71	25.40	0.0000
*DORN	653	m	0	1.00	42.73	9.70	0.0000
Subtotal DORN				1.20	68.44	35.10	
*ENGELA	505	m	0	0.67	15.51	0.33	0.0081
*ENGELA	513	f	0	1.42	6.43	5.15	0.0003
Subtotal ENGELA				0.89	21.94	5.48	
GAO	505	m	0	1.50	25.14	24.06	0.0000
GAO	515	f	0	0.99	12.15	2.66	0.0005
Subtotal GAO				1.34	37.29	26.72	
GAO2	505	m	0	1.39	1.24	0.92	0.1229
GENG	532	f	0	1.38	5.11	3.76	0.0018
HAENSZ	541	f	0	0.20	10.18	1.10	0.5323
HEGMAN	501	m	0	0.82	19.93	1.71	0.0003
HEGMAN	502	f	0	1.90	1.81	3.43	0.0105
Subtotal HEGMAN				0.91	21.74	5.14	

Table 1H20 - 5

IESLC - Meta-analysis of Ever/current Smoking, Age started, "Highest vs lowest"
All LC types, Cigarettes (or Any Product if Cigarettes not available)
Least adjusted

REF	NRR	SEX	AD	Ys	Ws	Qs	Ps
*HIRAYA	564	m	1	0.83	11.00	1.01	0.0060
*HIRAYA	506	f	1	-1.14	0.91	2.53	0.2763
Subtotal HIRAYA				0.68	11.92	3.54	
HU	515	m	0	0.98	2.93	0.59	0.0948
HU	520	f	0	0.73	1.02	0.04	0.4592
Subtotal HU				0.91	3.95	0.64	
HU2	507	c	0	1.30	8.09	4.85	0.0002
JEDRYC	610	m	5	0.51	34.48	0.01	0.0029
JEDRYC	620	f	5	0.57	4.20	0.01	0.2417
Subtotal JEDRYC				0.51	38.68	0.02	
JOLY	547	m	0	1.48	13.06	11.79	0.0000
JOLY	537	f	0	1.35	9.12	6.26	0.0000
Subtotal JOLY				1.43	22.19	18.05	
KHUDER	510	m	0	0.48	35.36	0.07	0.0043
KOULUM	509	m	0	2.19	3.13	8.68	0.0001
LETOUR	505	c	0	0.53	31.90	0.00	0.0030
*LIAW	510	c	2	1.12	8.53	3.03	0.0011
LIU3	503	m	0	0.02	9.83	2.47	0.9407
LIU4	505	m	2	0.46	3833.36	17.60	0.0000
LIU5	503	c	0	0.93	6.37	1.05	0.0188
LUBIN	571	m	0	1.44	20.61	17.34	0.0000
LUBIN2	1155	m	0	0.94	37.89	6.66	0.0000
MATOS	560	m	0	0.69	13.33	0.37	0.0115
*MIGRAN	509	m	0	0.25	16.88	1.25	0.2993
*MIGRAN	519	f	0	-0.11	1.71	0.68	0.8892
Subtotal MIGRAN				0.22	18.58	1.93	
*MRFITR	518	m	0	0.79	2.70	0.18	0.1967
PERNU	506	m	0	0.19	56.54	6.17	0.1428
PERNU	503	f	0	2.68	0.37	1.69	0.1056
Subtotal PERNU				0.21	56.91	7.86	
PEZZOT	574	m	0	0.69	14.69	0.38	0.0085
*QIAO2	505	m	0	0.60	35.89	0.22	0.0003
RACHTA	505	f	0	0.21	2.26	0.22	0.7480
SEGI2	520	m	0	1.03	21.03	5.31	0.0000
SOBUE	800	m	0	0.49	28.15	0.03	0.0088
*SPEIZE	530	f	2	0.22	41.69	3.80	0.1497
SUZUK2	505	c	0	1.45	4.16	3.56	0.0031
SVENSS	505	f	0	0.33	6.51	0.24	0.3979
TIZZAN	510	m	0	1.58	9.14	10.13	0.0000
TIZZAN	540	f	0	2.62	0.77	3.40	0.0211
Subtotal TIZZAN				1.66	9.92	13.54	
WAKAI	505	m	0	0.57	4.94	0.01	0.2027
WU	521	f	0	1.50	6.07	5.80	0.0002
WYNDE6	763	m	0	0.52	40.26	0.00	0.0010
WYNDE6	771	f	0	0.82	29.93	2.57	0.0000
Subtotal WYNDE6				0.65	70.19	2.57	
ZHENG	567	m	0	1.76	11.97	18.24	0.0000
ZHENG	574	f	0	0.76	5.64	0.32	0.0704
Subtotal ZHENG				1.44	17.61	18.56	

	N	73
	NS	53
	Wt	4800.78
Het	Chi	317.85
Het	df	72
Het	P	***
Fixed	RR	1.69
	RRl	1.64
	RRu	1.74
	P	+++
Random	RR	2.28
	RRl	2.03
	RRu	2.57
	P	+++
Asymm	P	***

Table 1H20 - 6

IESLC - Meta-analysis of Ever/current Smoking, Age started, "Highest vs lowest"
All LC types, Cigarettes (or Any Product if Cigarettes not available)
Least adjusted

		Sex			
		combined	male	female	Total
	N	7	39	27	73
	NS	7	38	27	72
	Wt	76.73	4533.95	190.11	4800.78
Het	Chi	9.43	249.07	51.06	317.85
Het	df	6	38	26	72
Het	P	N.S.	***	**	***
Fixed	RR	2.18	1.67	1.91	1.69
	RRl	1.74	1.63	1.66	1.64
	RRu	2.73	1.72	2.21	1.74
	P	+++	+++	+++	+++
Random	RR	2.39	2.37	2.04	2.28
	RRl	1.76	2.02	1.62	2.03
	RRu	3.25	2.77	2.57	2.57
	P	+++	+++	+++	+++
Between	Chi				8.30
Between	df				2
Between	P				*
Btwn(F)	P				N.S.
Btwn(R)	P				N.S.

Table 1H20 - 7

IESLC - Meta-analysis of Ever/current Smoking, Age started, "Highest vs lowest"
All LC types, Cigarettes (or Any Product if Cigarettes not available)
Excluded studies (and stage at which they were excluded)

1	BECHER BLOT1 BROWN3 CARPEN CHYOU DARBY DOLL2 GARCIA GRAHAM GURSEL HAMMO2 JAHN JAIN LAUSSM PRESCO QIAO TVERDA WIGLE WYNDE3
2	AKIBA AMANDU AMES AXELSS BENSHL BEST BOUCHA BOUCOT BROSS CHEN CPSII DEAN2 DESTEF DORGAN DOSEME FAN GARSHI GER GILLIS HAMMON HUMBLE JUSSAW KAISE2 KATSOU KAUFMA KOO KREUZE LEVIN MCCONN NOTAN2 OSANN2 PEZZO2 PISANI RESTRE SADOWS VUTUC WANG2 WATSON WU2 WUWILL WYNDE2 WYNDE8 XU ZHOU
3	GUO MCDUFF SPITZ STASZE ZHANG
5	LUO
6	CORREA HOLE YUAN
8	BOFFET WYNDE7
15	BENHAM

Table 1H20 - 8
Potentially overlapping studies

REF	REFGP	PRINC	.	OVERLAP/LINK
LUBIN2	LUBIN2	1		Lubin-combined
MRFITR	MRFIT	2		Subset of MRFIT
WYNDE6	WYNDE6	1		WYNDE5/6/7/8
CPSI	CPSI	1		CPSI overall
LUBIN	XIANGZ	2		LUBIN/XIANGZ/QIAO

Table 1H20 - 9
Most adjusted - insufficient data for meta-analysis

REF	NRR	SEX	AGEL	AGEH	RACE	YF	LC TYPE	LOC	START	ST	NLC	R	VB	P	H	AD	ADOS	SM	PRODUCT	exL	exH	unexL	unexH	De
CORREA	544	c	0	0	all	-	all	NAmer	1979	CC	1359	n	bl	y	n	2	0	ev	cig+/-ot	1	15	21	999	st
HIRAYA	548	m	0	0	all	8	all	As:Jap	1965	pr	1917	n	bl	n	n	1	0	cu	cig+/-ot	1	19	25	999	st
HOLE	502	m	0	0	all	0	all	Eu:UK	1972	pr	225	n	V	n	n	3	1#	cu	cig+/-ot	1	14	20	999	st
SPEIZE	533	f	0	0	all	0	all	NAmer	1976	pr	593	n	bl	n	y	2	1#	cu	cig+/-ot	1	17	22	999	ot
YUAN	501	m	0	0	all	0	all	As:Chi	1986	pr	142	n	ot	n	n	3	1#	ev	cig+/-ot	1	24	25	999	ot

Comments on values in listings

HOLE	ADOS	Number smoked
SPIEZE	ADOS	Numbers of cigs/day
YUAN	ADOS	Amount smoked per day

REF	NRR	RR	SIG	RRDATA comment
CORREA	544	2.92		0
HIRAYA	548	1.55		0
HOLE	502	1.28		0
SPEIZE	533	*	gap	Text page 478 SPEIZE1999 gives the risk for age start 1-17 vs 18-19 as 1.1(0.9-1.5)
YUAN	501	2.40	y	Significant P<.001

Table 1H21 -

IESLC - Meta-analysis of Ever/current Smoking by Age started, Overview
All LC types, Cigarettes only

This analysis is restricted to results for:
1) Ever/current smokers
2) Results by Age started
3) Categorical results by Age started
Results by Age started are grouped under 2 schemes (S1, S2). Each scheme has a set of "key values". An interval is allocated to the category whose key value it includes, and intervals which include none or more than one of the key values are excluded. (Open-ended intervals are coded as 999)

S1	key value	maximum range
1	26	19+
2	18	15-25
3	14	1-17

S2	key value	maximum range
1	30	27+
2	26	23-29
3	22	19-25
4	18	15-21
5	14	11-17
6	10	1-13

4) All LC types (or near equivalent)
5) Results complete enough for use in metaanalysis

Within each study, results are then selected (in the following order of preference, within each sex) for:
6) SMKSTA: ever, current
7) PRODUCT: cigarettes only
8) CIGTYPE: all/unspecified, MC regardless of HR, MC only
9) (not applicable)
10) DENOM: never smoked anything, never smoked cigarettes, never any + low, never cigs + low
11) Followup period (YF, prospective studies): whole study (coded as 0) or longest available
12) LCtype: all or nearest available, at least Squamous and Adeno. (q = squamous, s = small, l = large, a = adeno, mix = mixed, alv = alveolar)
13) Race: all or nearest available, otherwise by race (wh or w = white, bl or b = black, hi = hispanic ch = chinese, jap = japanese, haw = hawaiian, w+o = white + oriental, sca = scandinavian, as = asian)
14) For overlapping studies: principal rather than subsidiary studies
Finally by Age: whole study (coded as 0) if available, otherwise by widest available age group and then for single sex results (m, f) in preference to results for both sexes combined (c).

Results adjusted (AD) for the most potential confounders are then chosen in Sections -1 to -3 (and those which actually differ from the adjusted results in Table 1H11 - 1 are marked 'x' in Section -1) and results adjusted for the least confounders in Sections -4 to -6. (Those least adjusted results which actually differ from the most adjusted are marked 'x' in column X in Section -4)

Section -7 shows excluded studies, together with the stage (as above) at which no qualifying results were found.

Section -8 lists the potentially overlapping studies which have been included (1=principal, 2=subsidiary).

Section -9 lists any results which would have been included in preference except that they had data not complete enough for use in meta-analysis, with their significance (yes/no), if known, and any further comment as entered on the database. It also lists as "gap" any categories for which no data were presented by the original authors.

In addition to those mentioned above, the following fields, levels and abbreviations are used:
* or nk = not known, n = no, y = yes, ot = other
ev = ever, cu = current, nev = never
all/unspec = all or unspecified, MC = manufactured cigarettes, HR = hand-rolled cigarettes
exL, exH = range of exposure (low and high) in the smoking group, in terms of Age started
REF: 6-character study reference
NRR: number of the RR on the database within the study
ST : study type (CC = case control, pr or prosp = prospective)
NLC: number of lung cancer cases in whole study
R : risky occupational population (n = no, m = mining, o = other risky)
VB : national cigarette type (V = at least 75% Virginia, bl = at least 75% blended, ot = other)
P : any proxy use
H : full histological confirmation
De : derivation of RR/CI (or = original, st = standard method, ot = other method of estimation)

Table 1H21 - 1

IESLC - Meta-analysis of Ever/current Smoking by Age started, Overview
All LC types, Cigarettes only
Most adjusted

REF	NRR	1H11	SEX	AGEL	AGEH	RACE	YF	LC TYPE	LOC	START	ST	NLC	R	VB	P	H	AD	SM	PRODUCT	exL	exH	S1	S2	DENOM	De
AGUDO	504		f	0	0	all	-	all	Eu:wst	1989	CC	103	n	bl	n	n	3	ev	cig only	24	999	1	0	nev cigs	or
AGUDO	505		f	0	0	all	-	all	Eu:wst	1989	CC	103	n	bl	n	n	3	ev	cig only	1	23	0	0	nev cigs	or
BENHAM	617	x	m	0	0	all	-	all	Eu:wst	1976	CC	1625	n	bl	n	y	3	ev	cig only	20	999	1	0	nev any	or
BENHAM	618	x	m	0	0	all	-	all	Eu:wst	1976	CC	1625	n	bl	n	y	3	ev	cig only	1	19	0	0	nev any	or
CEDERL	510		m	0	0	all	10	all	Eu:Sca	1963	pr	491	n	bl	n	n	1	cu	cig only	19	999	1	0	nev any	ot
CEDERL	511		m	0	0	all	10	all	Eu:Sca	1963	pr	491	n	bl	n	n	1	cu	cig only	17	18	2	4	nev any	ot
CEDERL	512		m	0	0	all	10	all	Eu:Sca	1963	pr	491	n	bl	n	n	1	cu	cig only	1	16	3	0	nev any	ot
CEDERL	515		f	0	0	all	10	all	Eu:Sca	1963	pr	491	n	bl	n	n	0	cu	cig only	19	999	1	0	nev any	st
CEDERL	516		f	0	0	all	10	all	Eu:Sca	1963	pr	491	n	bl	n	n	0	cu	cig only	17	18	2	4	nev any	st
CEDERL	517		f	0	0	all	10	all	Eu:Sca	1963	pr	491	n	bl	n	n	0	cu	cig only	1	16	3	0	nev any	ot
CPSI	892	x	m	35	84	all	4	all	NAmer	1959	pr	5138	n	bl	n	n	1	cu	cig only	25	999	1	0	nev any	ot
CPSI	893	x	m	35	84	all	4	all	NAmer	1959	pr	5138	n	bl	n	n	1	cu	cig only	20	24	0	3	nev any	ot
CPSI	894	x	m	35	84	all	4	all	NAmer	1959	pr	5138	n	bl	n	n	1	cu	cig only	15	19	2	4	nev any	ot
CPSI	895	x	m	35	84	all	4	all	NAmer	1959	pr	5138	n	bl	n	n	1	cu	cig only	1	14	3	0	nev any	ot
CPSI	845		f	40	74	all	6	all	NAmer	1959	pr	5138	n	bl	n	n	1	cu	cig only	25	999	1	0	nev any	ot
CPSI	846		f	40	74	all	6	all	NAmer	1959	pr	5138	n	bl	n	n	1	cu	cig only	20	24	0	3	nev any	ot
CPSI	847		f	40	74	all	6	all	NAmer	1959	pr	5138	n	bl	n	n	1	cu	cig only	15	19	2	4	nev any	ot
CPSI	848		f	40	74	all	6	all	NAmer	1959	pr	5138	n	bl	n	n	1	cu	cig only	1	14	3	0	nev any	ot
DEAN3	564		m	0	0	all	-	all	Eu:UK	1969	CC	766	n	V	y	n	0	cu	cig only	25	999	1	0	nev any	st
DEAN3	565		m	0	0	all	-	all	Eu:UK	1969	CC	766	n	V	y	n	0	cu	cig only	20	24	0	3	nev any	st
DEAN3	566		m	0	0	all	-	all	Eu:UK	1969	CC	766	n	V	y	n	0	cu	cig only	15	19	2	4	nev any	st
DEAN3	567		m	0	0	all	-	all	Eu:UK	1969	CC	766	n	V	y	n	0	cu	cig only	1	14	3	0	nev any	st
DEAN3	583		f	0	0	all	-	all	Eu:UK	1969	CC	766	n	V	y	n	0	cu	cig only	25	999	1	0	nev any	st
DEAN3	584		f	0	0	all	-	all	Eu:UK	1969	CC	766	n	V	y	n	0	cu	cig only	20	24	0	3	nev any	st
DEAN3	585		f	0	0	all	-	all	Eu:UK	1969	CC	766	n	V	y	n	0	cu	cig only	15	19	2	4	nev any	st
DEAN3	586		f	0	0	all	-	all	Eu:UK	1969	CC	766	n	V	y	n	0	cu	cig only	1	14	3	0	nev any	st
DORN	730	x	m	55	64	wh	8	all	NAmer	1954	pr	5097	n	bl	n	n	0	ev	cig only	25	999	1	0	nev any	st
DORN	731	x	m	55	64	wh	8	all	NAmer	1954	pr	5097	n	bl	n	n	0	ev	cig only	20	24	0	3	nev any	st
DORN	732	x	m	55	64	wh	8	all	NAmer	1954	pr	5097	n	bl	n	n	0	ev	cig only	15	19	2	4	nev any	st
DORN	733	x	m	55	64	wh	8	all	NAmer	1954	pr	5097	n	bl	n	n	0	ev	cig only	1	14	3	0	nev any	st
DORN	767	x	m	65	74	wh	8	all	NAmer	1954	pr	5097	n	bl	n	n	0	ev	cig only	25	999	1	0	nev any	st
DORN	768	x	m	65	74	wh	8	all	NAmer	1954	pr	5097	n	bl	n	n	0	ev	cig only	20	24	0	3	nev any	st
DORN	769	x	m	65	74	wh	8	all	NAmer	1954	pr	5097	n	bl	n	n	0	ev	cig only	15	19	2	4	nev any	st
DORN	770	x	m	65	74	wh	8	all	NAmer	1954	pr	5097	n	bl	n	n	0	ev	cig only	1	14	3	0	nev any	st
MIGRAN	501		m	0	0	all	0	all	Eu:UK	1964	pr	259	n	V	n	n	0	cu	cig only	20	999	1	0	nev any	st
MIGRAN	503		m	0	0	all	0	all	Eu:UK	1964	pr	259	n	V	n	n	0	cu	cig only	16	19	2	4	nev any	st
MIGRAN	505		m	0	0	all	0	all	Eu:UK	1964	pr	259	n	V	n	n	0	cu	cig only	0	15	3	0	nev any	st
MIGRAN	511		f	0	0	all	0	all	Eu:UK	1964	pr	259	n	V	n	n	0	cu	cig only	20	999	1	0	nev any	st
MIGRAN	513		f	0	0	all	0	all	Eu:UK	1964	pr	259	n	V	n	n	0	cu	cig only	16	19	2	4	nev any	st
MIGRAN	515		f	0	0	all	0	all	Eu:UK	1964	pr	259	n	V	n	n	0	cu	cig only	0	15	3	0	nev any	st
PEZZOT	570		m	0	0	all	-	all	SCAmer	1987	CC	215	n	bl	n	y	0	ev	cig only	19	999	1	0	nev cigs	st
PEZZOT	571		m	0	0	all	-	all	SCAmer	1987	CC	215	n	bl	n	y	0	ev	cig only	14	18	0	0	nev cigs	st
PEZZOT	572		m	0	0	all	-	all	SCAmer	1987	CC	215	n	bl	n	y	0	ev	cig only	1	13	0	6	nev cigs	st
TIZZAN	536	x	f	0	0	all	-	all	Eu:wst	1959	CC	1358	n	bl	n	n	0	ev	cig only	31	999	0	0	nev any	st
TIZZAN	537	x	f	0	0	all	-	all	Eu:wst	1959	CC	1358	n	bl	n	n	0	ev	cig only	20	30	1	0	nev any	st
TIZZAN	538	x	f	0	0	all	-	all	Eu:wst	1959	CC	1358	n	bl	n	n	0	ev	cig only	1	19	0	0	nev any	st

Cigarette type is all/unspec for all RRs
except for the following:

REF	NRR	CIGTYPE
DEAN3	564	MC only
DEAN3	565	MC only
DEAN3	566	MC only
DEAN3	567	MC only
DEAN3	583	MC only
DEAN3	584	MC only
DEAN3	585	MC only
DEAN3	586	MC only

In this overview table, subtotals and Qs values may be invalid and should be ignored

Table 1H21 - 2

IESLC - Meta-analysis of Ever/current Smoking by Age started, Overview
All LC types, Cigarettes only
Most adjusted

REF	NRR	SEX	AD	Number Exposed Case	Cont	Non-exposed Case	Cont	RR	95.00%CI
AGUDO	504	f	3	7	-	80	-	1.58	(0.59- 4.23)
AGUDO	505	f	3	16	-	80	-	10.76	(2.38- 48.73)
Subtotal AGUDO								2.80	(1.23- 6.39)
BENHAM	617	m	3	-	-	-	-	12.90	(8.70- 19.20)
BENHAM	618	m	3	-	-	-	-	15.50	(10.50- 23.00)
Subtotal BENHAM								14.15	(10.71- 18.70)
*CEDERL	510	m	1	11	-	7	-	6.50	(2.52- 16.74)
*CEDERL	511	m	1	10	-	7	-	9.80	(3.74- 25.69)
*CEDERL	512	m	1	7	-	7	-	6.40	(2.25- 18.21)
*CEDERL	515	f	0	6	2806	19	17679	1.99	(0.80- 4.98)
*CEDERL	516	f	0	2	1009	19	17679	1.84	(0.43- 7.91)
*CEDERL	517	f	0	0	746	19	17679	0.61~	(0.04- 10.05)
Subtotal CEDERL								4.43	(2.82- 6.96)
*CPSI	892	m	1	20	-	49	-	3.21	(1.91- 5.40)
*CPSI	893	m	1	110	-	49	-	9.72	(6.94- 13.61)
*CPSI	894	m	1	315	-	49	-	12.81	(9.48- 17.31)
*CPSI	895	m	1	101	-	49	-	15.10	(10.73- 21.24)
*CPSI	845	f	1	51	-	166	-	2.25	(1.64- 3.08)
*CPSI	846	f	1	34	-	166	-	3.38	(2.33- 4.88)
*CPSI	847	f	1	52	-	166	-	5.00	(3.66- 6.83)
*CPSI	848	f	1	6	-	166	-	2.50	(1.11- 5.65)
Subtotal CPSI								6.09	(5.36- 6.92)
DEAN3	564	m	0	24	75	24	510	6.80	(3.67- 12.58)
DEAN3	565	m	0	52	161	24	510	6.86	(4.10- 11.49)
DEAN3	566	m	0	160	485	24	510	7.01	(4.48- 10.96)
DEAN3	567	m	0	44	165	24	510	5.67	(3.34- 9.60)
DEAN3	583	f	0	27	274	41	1538	3.70	(2.24- 6.11)
DEAN3	584	f	0	18	229	41	1538	2.95	(1.67- 5.22)
DEAN3	585	f	0	39	504	41	1538	2.90	(1.85- 4.55)
DEAN3	586	f	0	7	109	41	1538	2.41	(1.06- 5.50)
Subtotal DEAN3								4.59	(3.81- 5.54)
*DORN	730	m	0	25	40397	25	213858	5.29	(3.04- 9.21)
*DORN	731	m	0	109	88553	25	213858	10.53	(6.82- 16.26)
*DORN	732	m	0	239	128130	25	213858	15.96	(10.57- 24.09)
*DORN	733	m	0	59	21349	25	213858	23.64	(14.81- 37.73)
*DORN	767	m	0	38	38849	49	171211	3.42	(2.24- 5.22)
*DORN	768	m	0	117	52388	49	171211	7.80	(5.59- 10.89)
*DORN	769	m	0	193	67425	49	171211	10.00	(7.31- 13.68)
*DORN	770	m	0	44	13616	49	171211	11.29	(7.52- 16.96)
Subtotal DORN								9.49	(8.24- 10.94)
*MIGRAN	501	m	0	24	668	4	867	7.79	(2.72- 22.34)
*MIGRAN	503	m	0	59	1845	4	867	6.93	(2.53- 19.02)
*MIGRAN	505	m	0	50	1081	4	867	10.03	(3.64- 27.65)
*MIGRAN	511	f	0	11	1315	4	3814	7.98	(2.54- 25.01)
*MIGRAN	513	f	0	9	1035	4	3814	8.29	(2.56- 26.87)
*MIGRAN	515	f	0	2	266	4	3814	7.17	(1.32- 38.96)
Subtotal MIGRAN								8.07	(5.08- 12.80)
PEZZOT	570	m	0	41	105	4	116	11.32	(3.92- 32.69)
PEZZOT	571	m	0	118	145	4	116	23.60	(8.46- 65.84)
PEZZOT	572	m	0	52	67	4	116	22.51	(7.79- 65.00)
Subtotal PEZZOT								18.29	(9.98- 33.50)
TIZZAN	536	f	0	2	5	117	114	0.39	(0.07- 2.05)
TIZZAN	537	f	0	12	21	117	114	0.56	(0.26- 1.18)
TIZZAN	538	f	0	11	2	117	114	5.36	(1.16- 24.71)
Subtotal TIZZAN								0.77	(0.41- 1.45)
Partial Totals				2334	463825	2041	1616238		

*prospective study ~ With 0.5 adjustment for zero

Table 1H21 - 2

IESLC - Meta-analysis of Ever/current Smoking by Age started, Overview
All LC types, Cigarettes only
Most adjusted

REF	NRR	SEX	AD	Ys	Ws	Qs	Ps
AGUDO	504	f	3	0.46	3.96	8.62	0.3627
AGUDO	505	f	3	2.38	1.69	0.33	0.0020
Subtotal AGUDO				1.03	5.65	8.95	
BENHAM	617	m	3	2.56	24.52	9.55	0.0000
BENHAM	618	m	3	2.74	24.99	16.30	0.0000
Subtotal BENHAM				2.65	49.51	25.85	
*CEDERL	510	m	1	1.87	4.29	0.02	0.0001
*CEDERL	511	m	1	2.28	4.14	0.50	0.0000
*CEDERL	512	m	1	1.86	3.51	0.02	0.0005
*CEDERL	515	f	0	0.69	4.57	7.08	0.1414
*CEDERL	516	f	0	0.61	1.81	3.16	0.4098
*CEDERL	517	f	0	-0.50	0.49	2.89	0.7276
Subtotal CEDERL				1.49	18.81	13.67	
*CPSI	892	m	1	1.17	14.23	8.37	0.0000
*CPSI	893	m	1	2.27	33.87	3.94	0.0000
*CPSI	894	m	1	2.55	42.39	16.14	0.0000
*CPSI	895	m	1	2.71	32.95	20.13	0.0000
*CPSI	845	f	1	0.81	38.69	48.72	0.0000
*CPSI	846	f	1	1.22	28.12	14.39	0.0000
*CPSI	847	f	1	1.61	39.48	4.14	0.0000
*CPSI	848	f	1	0.92	5.80	6.00	0.0273
Subtotal CPSI				1.81	235.52	121.82	
DEAN3	564	m	0	1.92	10.14	0.00	0.0000
DEAN3	565	m	0	1.93	14.48	0.00	0.0000
DEAN3	566	m	0	1.95	19.25	0.00	0.0000
DEAN3	567	m	0	1.73	13.81	0.54	0.0000
DEAN3	583	f	0	1.31	15.21	5.96	0.0000
DEAN3	584	f	0	1.08	11.77	8.54	0.0002
DEAN3	585	f	0	1.07	18.99	14.29	0.0000
DEAN3	586	f	0	0.88	5.65	6.27	0.0367
Subtotal DEAN3				1.52	109.30	35.61	
*DORN	730	m	0	1.67	12.50	0.89	0.0000
*DORN	731	m	0	2.35	20.34	3.61	0.0000
*DORN	732	m	0	2.77	22.64	15.85	0.0000
*DORN	733	m	0	3.16	17.58	26.58	0.0000
*DORN	767	m	0	1.23	21.42	10.62	0.0000
*DORN	768	m	0	2.05	34.57	0.51	0.0000
*DORN	769	m	0	2.30	39.11	5.34	0.0000
*DORN	770	m	0	2.42	23.23	5.60	0.0000
Subtotal DORN				2.25	191.38	68.99	
*MIGRAN	501	m	0	2.05	3.46	0.05	0.0001
*MIGRAN	503	m	0	1.94	3.77	0.00	0.0002
*MIGRAN	505	m	0	2.31	3.73	0.52	0.0000
*MIGRAN	511	f	0	2.08	2.94	0.06	0.0004
*MIGRAN	513	f	0	2.12	2.78	0.09	0.0004
*MIGRAN	515	f	0	1.97	1.34	0.00	0.0226
Subtotal MIGRAN				2.09	18.02	0.72	
PEZZOT	570	m	0	2.43	3.42	0.83	0.0000
PEZZOT	571	m	0	3.16	3.65	5.50	0.0000
PEZZOT	572	m	0	3.11	3.42	4.76	0.0000
Subtotal PEZZOT				2.91	10.48	11.10	
TIZZAN	536	f	0	-0.94	1.39	11.53	0.2659
TIZZAN	537	f	0	-0.59	6.74	42.79	0.1283
TIZZAN	538	f	0	1.68	1.64	0.11	0.0314
Subtotal TIZZAN				-0.26	9.78	54.42	

N	46
NS	9

Table 1H21 - 3

IESLC - Meta-analysis of Ever/current Smoking by Age started, Overview
All LC types, Cigarettes only
Most adjusted

	Sex			
	combined	male	female	Total
N		27	19	46
NS		7	6	13

In this overview table, other than the "N" rows, entries in the "absent" and "Total" columns may be invalid and should be ignored

	Age started (broad categories)				
	absent	19+k26	15-25k18	1-17k14	Total
N	12	14	10	10	46
NS	7	9	5	5	26
Wt	179.93	166.09	194.36	108.09	648.46
Het Chi	67.49	91.06	54.85	50.51	341.14
Het df	11	13	9	9	45
Het P	***	***	***	***	***
Fixed RR	7.69	3.94	8.07	10.40	6.91
RRl	6.64	3.38	7.01	8.61	6.40
RRu	8.89	4.59	9.29	12.56	7.46
P	+++	+++	+++	+++	+++
Random RR	7.46	4.01	7.35	7.26	6.05
RRl	4.96	2.60	4.98	4.35	4.81
RRu	11.22	6.17	10.85	12.13	7.62
P	+++	+++	+++	+++	+++

				Age started (narrow categories)				
	absent	27+k30	23-29k26	19-25k22	15-21k18	11-17k14	1-13k10	Total
N	29			6	10		1	46
NS	9			3	5		1	18
Wt	307.54			143.15	194.36		3.42	648.46
Het Chi	243.29			30.74	54.85		0.00	341.14
Het df	28			5	9		0	45
Het P	***			***	***		N.S.	***
Fixed RR	6.30			6.63	8.07		22.51	6.91
RRl	5.64			5.63	7.01		7.79	6.40
RRu	7.05			7.81	9.29		65.00	7.46
P	+++			+++	+++		+++	+++
Random RR	5.31			6.26	7.35		22.51	6.05
RRl	3.71			4.13	4.98		7.79	4.81
RRu	7.58			9.49	10.85		65.00	7.62
P	+++			+++	+++		+++	+++

Table 1H21 - 3

IESLC - Meta-analysis of Ever/current Smoking by Age started, Overview
All LC types, Cigarettes only
Most adjusted

MALES

	Age started (broad categories)				
	absent	19+k26	15-25k18	1-17k14	Total
N	7	8	6	6	27
NS	5	7	5	5	22
Wt	135.32	93.97	131.30	94.81	455.40
Het Chi	13.93	28.70	9.24	18.94	100.67
Het df	6	7	5	5	26
Het P	*	***	N.S.	**	***
Fixed RR	10.22	6.06	11.02	12.63	9.80
RRl	8.63	4.95	9.29	10.33	8.94
RRu	12.09	7.42	13.08	15.44	10.74
P	+++	+++	+++	+++	+++
Random RR	10.77	6.18	10.73	11.41	9.37
RRl	8.14	3.98	8.30	7.42	7.71
RRu	14.24	9.59	13.86	17.55	11.38
P	+++	+++	+++	+++	+++

		Age started (narrow categories)						
	absent	27+k30	23-29k26	19-25k22	15-21k18	11-17k14	1-13k10	Total
N	16			4	6		1	27
NS	7			3	5		1	16
Wt	217.42			103.26	131.30		3.42	455.40
Het Chi	83.33			2.38	9.24		0.00	100.67
Het df	15			3	5		0	26
Het P	***			N.S.	N.S.		N.S.	***
Fixed RR	9.51			8.74	11.02		22.51	9.80
RRl	8.33			7.20	9.29		7.79	8.94
RRu	10.87			10.60	13.08		65.00	10.74
P	+++			+++	+++		+++	+++
Random RR	8.88			8.74	10.73		22.51	9.37
RRl	6.36			7.20	8.30		7.79	7.71
RRu	12.39			10.60	13.86		65.00	11.38
P	+++			+++	+++		+++	+++

FEMALES

	Age started (broad categories)				
	absent	19+k26	15-25k18	1-17k14	Total
N	5	6	4	4	19
NS	4	6	4	4	18
Wt	44.61	72.12	63.06	13.28	193.06
Het Chi	9.25	22.18	6.30	2.45	54.05
Het df	4	5	3	3	18
Het P	(*)	***	(*)	N.S.	***
Fixed RR	3.24	2.25	4.22	2.60	3.03
RRl	2.41	1.78	3.30	1.52	2.63
RRu	4.34	2.83	5.40	4.45	3.49
P	+++	+++	+++	+++	+++
Random RR	3.14	2.14	4.02	2.60	2.82
RRl	1.72	1.20	2.53	1.52	2.11
RRu	5.74	3.82	6.37	4.45	3.77
P	+++	++	+++	+++	+++

Table 1H21 - 3

IESLC - Meta-analysis of Ever/current Smoking by Age started, Overview
All LC types, Cigarettes only
Most adjusted

FEMALES

		absent	Age started (narrow categories) 27+k30	23-29k26	19-25k22	15-21k18	11-17k14	1-13k10	Total
	N	13			2	4			19
	NS	6			2	4			12
	Wt	90.12			39.89	63.06			193.06
Het	Chi	34.43			0.15	6.30			54.05
Het	df	12			1	3			18
Het	P	***			N.S.	(*)			***
Fixed	RR	2.34			3.25	4.22			3.03
	RRl	1.90			2.38	3.30			2.63
	RRu	2.87			4.43	5.40			3.49
	P	+++			+++	+++			+++
Random	RR	2.38			3.25	4.02			2.82
	RRl	1.56			2.38	2.53			2.11
	RRu	3.65			4.43	6.37			3.77
	P	+++			+++	+++			+++

Table 1H21 - 4

IESLC - Meta-analysis of Ever/current Smoking by Age started, Overview
All LC types, Cigarettes only
Least adjusted

REF	NRR	X	SEX	AGEL	AGEH	RACE	YF	LC TYPE	LOC	START	ST	NLC	R	VB	P	H	AD	SM	PRODUCT	exL	exH	S1	S2	DENOM	De
AGUDO	501	x	f	0	0	all	-	all	Eu:wst	1989	CC	103	n	bl	n	n	0	ev	cig only	24	999	1	0	nev cigs	st
AGUDO	502	x	f	0	0	all	-	all	Eu:wst	1989	CC	103	n	bl	n	n	0	ev	cig only	1	23	0	0	nev cigs	st
BENHAM	617		m	0	0	all	-	all	Eu:wst	1976	CC	1625	n	bl	n	y	3	ev	cig only	20	999	1	0	nev any	or
BENHAM	618		m	0	0	all	-	all	Eu:wst	1976	CC	1625	n	bl	n	y	3	ev	cig only	1	19	0	0	nev any	or
CEDERL	510		m	0	0	all	10	all	Eu:Sca	1963	pr	491	n	bl	n	n	1	cu	cig only	19	999	1	0	nev any	ot
CEDERL	511		m	0	0	all	10	all	Eu:Sca	1963	pr	491	n	bl	n	n	1	cu	cig only	17	18	2	4	nev any	ot
CEDERL	512		m	0	0	all	10	all	Eu:Sca	1963	pr	491	n	bl	n	n	1	cu	cig only	1	16	3	0	nev any	ot
CEDERL	515		f	0	0	all	10	all	Eu:Sca	1963	pr	491	n	bl	n	n	0	cu	cig only	19	999	1	0	nev any	st
CEDERL	516		f	0	0	all	10	all	Eu:Sca	1963	pr	491	n	bl	n	n	0	cu	cig only	17	18	2	4	nev any	st
CEDERL	517		f	0	0	all	10	all	Eu:Sca	1963	pr	491	n	bl	n	n	0	cu	cig only	1	16	3	0	nev any	ot
CPSI	892		m	35	84	all	4	all	NAmer	1959	pr	5138	n	bl	n	n	1	cu	cig only	25	999	1	0	nev any	ot
CPSI	893		m	35	84	all	4	all	NAmer	1959	pr	5138	n	bl	n	n	1	cu	cig only	20	24	0	3	nev any	ot
CPSI	894		m	35	84	all	4	all	NAmer	1959	pr	5138	n	bl	n	n	1	cu	cig only	15	19	2	4	nev any	ot
CPSI	895		m	35	84	all	4	all	NAmer	1959	pr	5138	n	bl	n	n	1	cu	cig only	1	14	3	0	nev any	ot
CPSI	845		f	40	74	all	6	all	NAmer	1959	pr	5138	n	bl	n	n	1	cu	cig only	25	999	1	0	nev any	ot
CPSI	846		f	40	74	all	6	all	NAmer	1959	pr	5138	n	bl	n	n	1	cu	cig only	20	24	0	3	nev any	ot
CPSI	847		f	40	74	all	6	all	NAmer	1959	pr	5138	n	bl	n	n	1	cu	cig only	15	19	2	4	nev any	ot
CPSI	848		f	40	74	all	6	all	NAmer	1959	pr	5138	n	bl	n	n	1	cu	cig only	1	14	3	0	nev any	ot
DEAN3	564		m	0	0	all	-	all	Eu:UK	1969	CC	766	n	V	y	n	0	cu	cig only	25	999	1	0	nev any	st
DEAN3	565		m	0	0	all	-	all	Eu:UK	1969	CC	766	n	V	y	n	0	cu	cig only	20	24	0	3	nev any	st
DEAN3	566		m	0	0	all	-	all	Eu:UK	1969	CC	766	n	V	y	n	0	cu	cig only	15	19	2	4	nev any	st
DEAN3	567		m	0	0	all	-	all	Eu:UK	1969	CC	766	n	V	y	n	0	cu	cig only	1	14	3	0	nev any	st
DEAN3	583		f	0	0	all	-	all	Eu:UK	1969	CC	766	n	V	y	n	0	cu	cig only	25	999	1	0	nev any	st
DEAN3	584		f	0	0	all	-	all	Eu:UK	1969	CC	766	n	V	y	n	0	cu	cig only	20	24	0	3	nev any	st
DEAN3	585		f	0	0	all	-	all	Eu:UK	1969	CC	766	n	V	y	n	0	cu	cig only	15	19	2	4	nev any	st
DEAN3	586		f	0	0	all	-	all	Eu:UK	1969	CC	766	n	V	y	n	0	cu	cig only	1	14	3	0	nev any	st
DORN	730		m	55	64	wh	8	all	NAmer	1954	pr	5097	n	bl	n	n	0	ev	cig only	25	999	1	0	nev any	st
DORN	731		m	55	64	wh	8	all	NAmer	1954	pr	5097	n	bl	n	n	0	ev	cig only	20	24	0	3	nev any	st
DORN	732		m	55	64	wh	8	all	NAmer	1954	pr	5097	n	bl	n	n	0	ev	cig only	15	19	2	4	nev any	st
DORN	733		m	55	64	wh	8	all	NAmer	1954	pr	5097	n	bl	n	n	0	ev	cig only	1	14	3	0	nev any	st
DORN	767		m	65	74	wh	8	all	NAmer	1954	pr	5097	n	bl	n	n	0	ev	cig only	25	999	1	0	nev any	st
DORN	768		m	65	74	wh	8	all	NAmer	1954	pr	5097	n	bl	n	n	0	ev	cig only	20	24	0	3	nev any	st
DORN	769		m	65	74	wh	8	all	NAmer	1954	pr	5097	n	bl	n	n	0	ev	cig only	15	19	2	4	nev any	st
DORN	770		m	65	74	wh	8	all	NAmer	1954	pr	5097	n	bl	n	n	0	ev	cig only	1	14	3	0	nev any	st
MIGRAN	501		m	0	0	all	0	all	Eu:UK	1964	pr	259	n	V	n	n	0	cu	cig only	20	999	1	0	nev any	st
MIGRAN	503		m	0	0	all	0	all	Eu:UK	1964	pr	259	n	V	n	n	0	cu	cig only	16	19	2	4	nev any	st
MIGRAN	505		m	0	0	all	0	all	Eu:UK	1964	pr	259	n	V	n	n	0	cu	cig only	0	15	3	0	nev any	st
MIGRAN	511		f	0	0	all	0	all	Eu:UK	1964	pr	259	n	V	n	n	0	cu	cig only	20	999	1	0	nev any	st
MIGRAN	513		f	0	0	all	0	all	Eu:UK	1964	pr	259	n	V	n	n	0	cu	cig only	16	19	2	4	nev any	st
MIGRAN	515		f	0	0	all	0	all	Eu:UK	1964	pr	259	n	V	n	n	0	cu	cig only	0	15	3	0	nev any	st
PEZZOT	570		m	0	0	all	-	all	SCAmer	1987	CC	215	n	bl	n	y	0	ev	cig only	19	999	1	0	nev cigs	st
PEZZOT	571		m	0	0	all	-	all	SCAmer	1987	CC	215	n	bl	n	y	0	ev	cig only	14	18	0	0	nev cigs	st
PEZZOT	572		m	0	0	all	-	all	SCAmer	1987	CC	215	n	bl	n	y	0	ev	cig only	1	13	0	6	nev cigs	st
TIZZAN	536		f	0	0	all	-	all	Eu:wst	1959	CC	1358	n	bl	n	n	0	ev	cig only	31	999	0	0	nev any	st
TIZZAN	537		f	0	0	all	-	all	Eu:wst	1959	CC	1358	n	bl	n	n	0	ev	cig only	20	30	1	0	nev any	st
TIZZAN	538		f	0	0	all	-	all	Eu:wst	1959	CC	1358	n	bl	n	n	0	ev	cig only	1	19	0	0	nev any	st

Cigarette type is all/unspec for all RRs
except for the following:

REF	NRR	CIGTYPE
DEAN3	564	MC only
DEAN3	565	MC only
DEAN3	566	MC only
DEAN3	567	MC only
DEAN3	583	MC only
DEAN3	584	MC only
DEAN3	585	MC only
DEAN3	586	MC only

In this overview table, subtotals and Qs values may be invalid and should be ignored

Table 1H21 - 5

IESLC - Meta-analysis of Ever/current Smoking by Age started, Overview
All LC types, Cigarettes only
Least adjusted

REF	NRR	SEX	AD	Number Exposed Case	Cont	Non-exposed Case	Cont	RR	95.00%CI
AGUDO	501	f	0	7	12	80	183	1.33	(0.51- 3.51)
AGUDO	502	f	0	16	11	80	183	3.33	(1.48- 7.49)
Subtotal AGUDO								2.28	(1.23- 4.25)
BENHAM	617	m	3	-	-	-	-	12.90	(8.70- 19.20)
BENHAM	618	m	3	-	-	-	-	15.50	(10.50- 23.00)
Subtotal BENHAM								14.15	(10.71- 18.70)
*CEDERL	510	m	1	11	-	7	-	6.50	(2.52- 16.74)
*CEDERL	511	m	1	10	-	7	-	9.80	(3.74- 25.69)
*CEDERL	512	m	1	7	-	7	-	6.40	(2.25- 18.21)
*CEDERL	515	f	0	6	2806	19	17679	1.99	(0.80- 4.98)
*CEDERL	516	f	0	2	1009	19	17679	1.84	(0.43- 7.91)
*CEDERL	517	f	0	0	746	19	17679	0.61~	(0.04- 10.05)
Subtotal CEDERL								4.43	(2.82- 6.96)
*CPSI	892	m	1	20	-	49	-	3.21	(1.91- 5.40)
*CPSI	893	m	1	110	-	49	-	9.72	(6.94- 13.61)
*CPSI	894	m	1	315	-	49	-	12.81	(9.48- 17.31)
*CPSI	895	m	1	101	-	49	-	15.10	(10.73- 21.24)
*CPSI	845	f	1	51	-	166	-	2.25	(1.64- 3.08)
*CPSI	846	f	1	34	-	166	-	3.38	(2.33- 4.88)
*CPSI	847	f	1	52	-	166	-	5.00	(3.66- 6.83)
*CPSI	848	f	1	6	-	166	-	2.50	(1.11- 5.65)
Subtotal CPSI								6.09	(5.36- 6.92)
DEAN3	564	m	0	24	75	24	510	6.80	(3.67- 12.58)
DEAN3	565	m	0	52	161	24	510	6.86	(4.10- 11.49)
DEAN3	566	m	0	160	485	24	510	7.01	(4.48- 10.96)
DEAN3	567	m	0	44	165	24	510	5.67	(3.34- 9.60)
DEAN3	583	f	0	27	274	41	1538	3.70	(2.24- 6.11)
DEAN3	584	f	0	18	229	41	1538	2.95	(1.67- 5.22)
DEAN3	585	f	0	39	504	41	1538	2.90	(1.85- 4.55)
DEAN3	586	f	0	7	109	41	1538	2.41	(1.06- 5.50)
Subtotal DEAN3								4.59	(3.81- 5.54)
*DORN	730	m	0	25	40397	25	213858	5.29	(3.04- 9.21)
*DORN	731	m	0	109	88553	25	213858	10.53	(6.82- 16.26)
*DORN	732	m	0	239	128130	25	213858	15.96	(10.57- 24.09)
*DORN	733	m	0	59	21349	25	213858	23.64	(14.81- 37.73)
*DORN	767	m	0	38	38849	49	171211	3.42	(2.24- 5.22)
*DORN	768	m	0	117	52388	49	171211	7.80	(5.59- 10.89)
*DORN	769	m	0	193	67425	49	171211	10.00	(7.31- 13.68)
*DORN	770	m	0	44	13616	49	171211	11.29	(7.52- 16.96)
Subtotal DORN								9.49	(8.24- 10.94)
*MIGRAN	501	m	0	24	668	4	867	7.79	(2.72- 22.34)
*MIGRAN	503	m	0	59	1845	4	867	6.93	(2.53- 19.02)
*MIGRAN	505	m	0	50	1081	4	867	10.03	(3.64- 27.65)
*MIGRAN	511	f	0	11	1315	4	3814	7.98	(2.54- 25.01)
*MIGRAN	513	f	0	9	1035	4	3814	8.29	(2.56- 26.87)
*MIGRAN	515	f	0	2	266	4	3814	7.17	(1.32- 38.96)
Subtotal MIGRAN								8.07	(5.08- 12.80)
PEZZOT	570	m	0	41	105	4	116	11.32	(3.92- 32.69)
PEZZOT	571	m	0	118	145	4	116	23.60	(8.46- 65.84)
PEZZOT	572	m	0	52	67	4	116	22.51	(7.79- 65.00)
Subtotal PEZZOT								18.29	(9.98- 33.50)
TIZZAN	536	f	0	2	5	117	114	0.39	(0.07- 2.05)
TIZZAN	537	f	0	12	21	117	114	0.56	(0.26- 1.18)
TIZZAN	538	f	0	11	2	117	114	5.36	(1.16- 24.71)
Subtotal TIZZAN								0.77	(0.41- 1.45)
Partial Totals				2334	463848	2041	1616604		

*prospective study

~ With 0.5 adjustment for zero

Table 1H21 - 5

IESLC - Meta-analysis of Ever/current Smoking by Age started, Overview
All LC types, Cigarettes only
Least adjusted

REF	NRR	SEX	AD	Ys	Ws	Qs	Ps
AGUDO	501	f	0	0.29	4.10	10.96	0.5594
AGUDO	502	f	0	1.20	5.84	3.04	0.0037
Subtotal AGUDO				0.83	9.93	14.00	
BENHAM	617	m	3	2.56	24.52	9.83	0.0000
BENHAM	618	m	3	2.74	24.99	16.67	0.0000
Subtotal BENHAM				2.65	49.51	26.50	
*CEDERL	510	m	1	1.87	4.29	0.01	0.0001
*CEDERL	511	m	1	2.28	4.14	0.53	0.0000
*CEDERL	512	m	1	1.86	3.51	0.02	0.0005
*CEDERL	515	f	0	0.69	4.57	6.98	0.1414
*CEDERL	516	f	0	0.61	1.81	3.12	0.4098
*CEDERL	517	f	0	-0.50	0.49	2.86	0.7276
Subtotal CEDERL				1.49	18.81	13.53	
*CPSI	892	m	1	1.17	14.23	8.17	0.0000
*CPSI	893	m	1	2.27	33.87	4.15	0.0000
*CPSI	894	m	1	2.55	42.39	16.62	0.0000
*CPSI	895	m	1	2.71	32.95	20.60	0.0000
*CPSI	845	f	1	0.81	38.69	47.94	0.0000
*CPSI	846	f	1	1.22	28.12	14.02	0.0000
*CPSI	847	f	1	1.61	39.48	3.91	0.0000
*CPSI	848	f	1	0.92	5.80	5.89	0.0273
Subtotal CPSI				1.81	235.52	121.30	
DEAN3	564	m	0	1.92	10.14	0.00	0.0000
DEAN3	565	m	0	1.93	14.48	0.00	0.0000
DEAN3	566	m	0	1.95	19.25	0.01	0.0000
DEAN3	567	m	0	1.73	13.81	0.50	0.0000
DEAN3	583	f	0	1.31	15.21	5.79	0.0000
DEAN3	584	f	0	1.08	11.77	8.36	0.0002
DEAN3	585	f	0	1.07	18.99	13.99	0.0000
DEAN3	586	f	0	0.88	5.65	6.17	0.0367
Subtotal DEAN3				1.52	109.30	34.81	
*DORN	730	m	0	1.67	12.50	0.83	0.0000
*DORN	731	m	0	2.35	20.34	3.76	0.0000
*DORN	732	m	0	2.77	22.64	16.19	0.0000
*DORN	733	m	0	3.16	17.58	26.97	0.0000
*DORN	767	m	0	1.23	21.42	10.35	0.0000
*DORN	768	m	0	2.05	34.57	0.59	0.0000
*DORN	769	m	0	2.30	39.11	5.61	0.0000
*DORN	770	m	0	2.42	23.23	5.80	0.0000
Subtotal DORN				2.25	191.38	70.11	
*MIGRAN	501	m	0	2.05	3.46	0.06	0.0001
*MIGRAN	503	m	0	1.94	3.77	0.00	0.0002
*MIGRAN	505	m	0	2.31	3.73	0.54	0.0000
*MIGRAN	511	f	0	2.08	2.94	0.07	0.0004
*MIGRAN	513	f	0	2.12	2.78	0.10	0.0004
*MIGRAN	515	f	0	1.97	1.34	0.00	0.0226
Subtotal MIGRAN				2.09	18.02	0.77	
PEZZOT	570	m	0	2.43	3.42	0.86	0.0000
PEZZOT	571	m	0	3.16	3.65	5.59	0.0000
PEZZOT	572	m	0	3.11	3.42	4.83	0.0000
Subtotal PEZZOT				2.91	10.48	11.28	
TIZZAN	536	f	0	-0.94	1.39	11.45	0.2659
TIZZAN	537	f	0	-0.59	6.74	42.48	0.1283
TIZZAN	538	f	0	1.68	1.64	0.10	0.0314
Subtotal TIZZAN				-0.26	9.78	54.03	

N	46
NS	9

Table 1H21 - 6

IESLC - Meta-analysis of Ever/current Smoking by Age started, Overview
All LC types, Cigarettes only
Least adjusted

	Sex combined	male	female	Total
N		27	19	46
NS		7	6	13

In this overview table, other than the "N" rows, entries in the "absent" and "Total" columns may be invalid and should be ignored

	Age started (broad categories) absent	19+k26	15-25k18	1-17k14	Total
N	12	14	10	10	46
NS	7	9	5	5	26
Wt	184.08	166.22	194.36	108.09	652.75
Het Chi	71.22	92.55	54.85	50.51	346.33
Het df	11	13	9	9	45
Het P	***	***	***	***	***
Fixed RR	7.46	3.92	8.07	10.40	6.85
RRl	6.46	3.37	7.01	8.61	6.34
RRu	8.62	4.56	9.29	12.56	7.40
P	+++	+++	+++	+++	+++
Random RR	6.88	3.96	7.35	7.26	5.91
RRl	4.58	2.57	4.98	4.35	4.70
RRu	10.33	6.12	10.85	12.13	7.44
P	+++	+++	+++	+++	+++

			Age started (narrow categories)					
	absent	27+k30	23-29k26	19-25k22	15-21k18	11-17k14	1-13k10	Total
N	29			6	10		1	46
NS	9			3	5		1	18
Wt	311.83			143.15	194.36		3.42	652.75
Het Chi	247.38			30.74	54.85		0.00	346.33
Het df	28			5	9		0	45
Het P	***			***	***		N.S.	***
Fixed RR	6.19			6.63	8.07		22.51	6.85
RRl	5.54			5.63	7.01		7.79	6.34
RRu	6.92			7.81	9.29		65.00	7.40
P	+++			+++	+++		+++	+++
Random RR	5.10			6.26	7.35		22.51	5.91
RRl	3.57			4.13	4.98		7.79	4.70
RRu	7.27			9.49	10.85		65.00	7.44
P	+++			+++	+++		+++	+++

Table 1H21 - 6

IESLC - Meta-analysis of Ever/current Smoking by Age started, Overview
All LC types, Cigarettes only
Least adjusted

MALES

	Age started (broad categories) absent	19+k26	15-25k18	1-17k14	Total
N	7	8	6	6	27
NS	5	7	5	5	22
Wt	135.32	93.97	131.30	94.81	455.40
Het Chi	13.93	28.70	9.24	18.94	100.67
Het df	6	7	5	5	26
Het P	*	***	N.S.	**	***
Fixed RR	10.22	6.06	11.02	12.63	9.80
RRl	8.63	4.95	9.29	10.33	8.94
RRu	12.09	7.42	13.08	15.44	10.74
P	+++	+++	+++	+++	+++
Random RR	10.77	6.18	10.73	11.41	9.37
RRl	8.14	3.98	8.30	7.42	7.71
RRu	14.24	9.59	13.86	17.55	11.38
P	+++	+++	+++	+++	+++

	absent	27+k30	23-29k26	Age started (narrow categories) 19-25k22	15-21k18	11-17k14	1-13k10	Total
N	16			4	6		1	27
NS	7			3	5		1	16
Wt	217.42			103.26	131.30		3.42	455.40
Het Chi	83.33			2.38	9.24		0.00	100.67
Het df	15			3	5		0	26
Het P	***			N.S.	N.S.		N.S.	***
Fixed RR	9.51			8.74	11.02		22.51	9.80
RRl	8.33			7.20	9.29		7.79	8.94
RRu	10.87			10.60	13.08		65.00	10.74
P	+++			+++	+++		+++	+++
Random RR	8.88			8.74	10.73		22.51	9.37
RRl	6.36			7.20	8.30		7.79	7.71
RRu	12.39			10.60	13.86		65.00	11.38
P	+++			+++	+++		+++	+++

FEMALES

	Age started (broad categories) absent	19+k26	15-25k18	1-17k14	Total
N	5	6	4	4	19
NS	4	6	4	4	18
Wt	48.76	72.25	63.06	13.28	197.35
Het Chi	6.75	22.79	6.30	2.45	52.44
Het df	4	5	3	3	18
Het P	N.S.	***	(*)	N.S.	***
Fixed RR	3.12	2.22	4.22	2.60	3.00
RRl	2.35	1.77	3.30	1.52	2.61
RRu	4.13	2.80	5.40	4.45	3.45
P	+++	+++	+++	+++	+++
Random RR	2.93	2.09	4.02	2.60	2.73
RRl	1.88	1.17	2.53	1.52	2.06
RRu	4.56	3.75	6.37	4.45	3.61
P	+++	+	+++	+++	+++

Table 1H21 - 6

IESLC - Meta-analysis of Ever/current Smoking by Age started, Overview
All LC types, Cigarettes only
Least adjusted

FEMALES

	absent	Age started (narrow categories) 27+k30	23-29k26	19-25k22	15-21k18	11-17k14	1-13k10	Total
N	13			2	4			19
NS	6			2	4			12
Wt	94.40			39.89	63.06			197.35
Het Chi	31.89			0.15	6.30			52.44
Het df	12			1	3			18
Het P	**			N.S.	(*)			***
Fixed RR	2.31			3.25	4.22			3.00
RRl	1.88			2.38	3.30			2.61
RRu	2.82			4.43	5.40			3.45
P	+++			+++	+++			+++
Random RR	2.25			3.25	4.02			2.73
RRl	1.52			2.38	2.53			2.06
RRu	3.34			4.43	6.37			3.61
P	+++			+++	+++			+++

Table 1H21 - 7

IESLC - Meta-analysis of Ever/current Smoking by Age started, Overview
All LC types, Cigarettes only
Excluded studies (and stage at which they were excluded)

1	BECHER BLOT1 BROWN3 CARPEN CHYOU DARBY DOLL2 GARCIA GRAHAM GURSEL HAMMO2 JAHN JAIN LAUSSM PRESCO QIAO TVERDA WIGLE WYNDE3
2	AKIBA AMANDU AMES AXELSS BENSHL BEST BOUCHA BOUCOT BROSS CHEN CPSII DEAN2 DESTEF DORGAN DOSEME FAN GARSHI GER GILLIS HAMMON HUMBLE JUSSAW KAISE2 KATSOU KAUFMA KOO KREUZE LEVIN MCCONN NOTAN2 OSANN2 PEZZO2 PISANI RESTRE SADOWS VUTUC WANG2 WATSON WU2 WUWILL WYNDE2 WYNDE8 XU ZHOU
3	GUO MCDUFF SPITZ STASZE ZHANG
4	LUO
5	CORREA HOLE YUAN
7	ARMADA AUVINE BARBON BOFFET BRESLO BUFFLE CHEN2 CHIAZZ CHOI DAMBER DOLL ENGELA GAO GAO2 GENG HAENSZ HEGMAN HIRAYA HU HU2 JEDRYC JOLY KHUDER KOULUM LETOUR LIAW LIU3 LIU4 LIU5 LUBIN LUBIN2 MATOS MRFITR PERNU QIAO2 RACHTA SEGI2 SOBUE SPEIZE SUZUK2 SVENSS WAKAI WU WYNDE6 WYNDE7 ZHENG
10	ALDERS

Table 1H21 - 8

Potentially overlapping studies

REF	REFGP	PRINC.	OVERLAP/LINK
BENHAM	LUBIN2	2	Subset of Lubin2
CPSI	CPSI	1	CPSI overall

Table 1H22 -

IESLC - Meta-analysis of Ever/current Smoking, Age started, "Low"
All LC types, Cigarettes only

This analysis is restricted to results for:
1) Ever/current smokers
2) Results by Age started
3) Categorical results by Age started
4) All LC types (or near equivalent)
5) Results complete enough for use in metaanalysis

Within each study, results are then selected (in the following order of preference, within each sex) for:
6) SMKSTA: ever, current
7) PRODUCT: cigarettes only
8) CIGTYPE: all/unspecified, MC regardless of HR, MC only
9) (not applicable)
10) DENOM: never smoked anything, never smoked cigarettes, never any + low, never cigs + low
11) Followup period (YF, prospective studies): whole study (coded as 0) or longest available
12) LCtype: all or nearest available, at least Squamous and Adeno. (q = squamous, s = small,
l = large, a = adeno, mix = mixed, alv = alveolar)
13) Race: all or nearest available, otherwise by race (wh or w = white, bl or b = black, hi = hispanic
ch = chinese, jap = japanese, haw = hawaiian, w+o = white + oriental, sca = scandinavian, as = asian)
14) Age started "low" in key scheme 1 (key value 26, maximum range 19+)
15) For overlapping studies: principal rather than subsidiary studies
Finally by Age: whole study (coded as 0) if available, otherwise by widest available age group
and then for single sex results (m, f) in preference to results for both sexes combined (c).

Results adjusted (AD) for the most potential confounders are then chosen in Sections -1 to -3
(and those which actually differ from the adjusted results in Table 1H12 - 1 are marked 'x' in Section -1)
and results adjusted for the least confounders in Sections -4 to -6. (Those least adjusted results which
actually differ from the most adjusted are marked 'x' in column X in Section -4)

Section -7 shows excluded studies, together with the stage (as above) at which no qualifying
results were found.

Section -8 lists the potentially overlapping studies which have been included (1=principal, 2=subsidiary).

Section -9 lists any results which would have been included in preference except that they had data not complete
enough for use in meta-analysis, with their significance (yes/no), if known, and any further comment as entered
on the database. It also lists as "gap" any categories for which no data were presented by the original authors.

In addition to those mentioned above, the following fields, levels and abbreviations are used:
* or nk = not known, n = no, y = yes, ot = other
ev = ever, cu = current, nev = never
all/unspec = all or unspecified, MC = manufactured cigarettes, HR = hand-rolled cigarettes
exL, exH = range of exposure (low and high) in the smoking group, in terms of Age started
REF: 6-character study reference
NRR: number of the RR on the database within the study
ST : study type (CC = case control, pr or prosp = prospective)
NLC: number of lung cancer cases in whole study
R : risky occupational population (n = no, m = mining, o = other risky)
VB : national cigarette type (V = at least 75% Virginia, bl = at least 75% blended, ot = other)
P : any proxy use
H : full histological confirmation
De : derivation of RR/CI (or = original, st = standard method, ot = other method of estimation)

Table 1H22 - 1

IESLC - Meta-analysis of Ever/current Smoking, Age started, "Low"
All LC types, Cigarettes only
Most adjusted

REF	NRR	1H12	SEX	AGEL	AGEH	RACE	YF	LC TYPE	LOC	START	ST	NLC	R	VB	P	H	AD	SM	PRODUCT	exL	exH	DENOM	De
AGUDO	504		f	0	0	all	-	all	Eu:wst	1989	CC	103	n	bl	n	n	3	ev	cig only	24	999	nev cigs	or
BENHAM	617	x	m	0	0	all	-	all	Eu:wst	1976	CC	1625	n	bl	n	y	3	ev	cig only	20	999	nev any	or
CEDERL	510		m	0	0	all	10	all	Eu:Sca	1963	pr	491	n	bl	n	n	1	cu	cig only	19	999	nev any	ot
CEDERL	515		f	0	0	all	10	all	Eu:Sca	1963	pr	491	n	bl	n	n	0	cu	cig only	19	999	nev any	st
CPSI	892	x	m	35	84	all	4	all	NAmer	1959	pr	5138	n	bl	n	n	1	cu	cig only	25	999	nev any	ot
CPSI	845		f	40	74	all	6	all	NAmer	1959	pr	5138	n	bl	n	n	1	cu	cig only	25	999	nev any	ot
DEAN3	564		m	0	0	all	-	all	Eu:UK	1969	CC	766	n	V	y	n	0	cu	cig only	25	999	nev any	st
DEAN3	583		f	0	0	all	-	all	Eu:UK	1969	CC	766	n	V	y	n	0	cu	cig only	25	999	nev any	st
DORN	730	x	m	55	64	wh	8	all	NAmer	1954	pr	5097	n	bl	n	n	0	ev	cig only	25	999	nev any	st
DORN	767	x	m	65	74	wh	8	all	NAmer	1954	pr	5097	n	bl	n	n	0	ev	cig only	25	999	nev any	st
MIGRAN	501		m	0	0	all	0	all	Eu:UK	1964	pr	259	n	V	n	n	0	cu	cig only	20	999	nev any	st
MIGRAN	511		f	0	0	all	0	all	Eu:UK	1964	pr	259	n	V	n	n	0	cu	cig only	20	999	nev any	st
PEZZOT	570		m	0	0	all	-	all	SCAmer	1987	CC	215	n	bl	n	y	0	ev	cig only	19	999	nev cigs	st
TIZZAN	537	x	f	0	0	all	-	all	Eu:wst	1959	CC	1358	n	bl	n	n	0	ev	cig only	20	30	nev any	st

Cigarette type is all/unspec for all RRs
except for the following:

REF	NRR	CIGTYPE
DEAN3	564	MC only
DEAN3	583	MC only

Table 1H22 - 2

IESLC - Meta-analysis of Ever/current Smoking, Age started, "Low"
All LC types, Cigarettes only
Most adjusted

REF	NRR	SEX	AD	Number Exposed Case	Cont	Non-exposed Case	Cont	RR	95.00%CI
AGUDO	504	f	3	7	-	80	-	1.58	(0.59- 4.23)
BENHAM	617	m	3	-	-	-	-	12.90	(8.70- 19.20)
*CEDERL	510	m	1	11	-	7	-	6.50	(2.52- 16.74)
*CEDERL	515	f	0	6	2806	19	17679	1.99	(0.80- 4.98)
Subtotal CEDERL								3.53	(1.83- 6.82)
*CPSI	892	m	1	20	-	49	-	3.21	(1.91- 5.40)
*CPSI	845	f	1	51	-	166	-	2.25	(1.64- 3.08)
Subtotal CPSI								2.48	(1.89- 3.24)
DEAN3	564	m	0	24	75	24	510	6.80	(3.67- 12.58)
DEAN3	583	f	0	27	274	41	1538	3.70	(2.24- 6.11)
Subtotal DEAN3								4.72	(3.20- 6.96)
*DORN	730	m	0	25	40397	25	213858	5.29	(3.04- 9.21)
*DORN	767	m	0	38	38849	49	171211	3.42	(2.24- 5.22)
Subtotal DORN								4.02	(2.87- 5.62)
*MIGRAN	501	m	0	24	668	4	867	7.79	(2.72- 22.34)
*MIGRAN	511	f	0	11	1315	4	3814	7.98	(2.54- 25.01)
Subtotal MIGRAN								7.87	(3.63- 17.08)
PEZZOT	570	m	0	41	105	4	116	11.32	(3.92- 32.69)
TIZZAN	537	f	0	12	21	117	114	0.56	(0.26- 1.18)
Partial Totals				297	84510	589	409707		

*prospective study

REF	NRR	SEX	AD	Ys	Ws	Qs	Ps
AGUDO	504	f	3	0.46	3.96	3.30	0.3627
BENHAM	617	m	3	2.56	24.52	34.51	0.0000
*CEDERL	510	m	1	1.87	4.29	1.08	0.0001
*CEDERL	515	f	0	0.69	4.57	2.13	0.1414
Subtotal CEDERL				1.26	8.85	3.21	
*CPSI	892	m	1	1.17	14.23	0.60	0.0000
*CPSI	845	f	1	0.81	38.69	12.13	0.0000
Subtotal CPSI				0.91	52.91	12.73	
DEAN3	564	m	0	1.92	10.14	3.02	0.0000
DEAN3	583	f	0	1.31	15.21	0.06	0.0000
Subtotal DEAN3				1.55	25.35	3.08	
*DORN	730	m	0	1.67	12.50	1.09	0.0000
*DORN	767	m	0	1.23	21.42	0.43	0.0000
Subtotal DORN				1.39	33.92	1.52	
*MIGRAN	501	m	0	2.05	3.46	1.61	0.0001
*MIGRAN	511	f	0	2.08	2.94	1.46	0.0004
Subtotal MIGRAN				2.06	6.40	3.07	
PEZZOT	570	m	0	2.43	3.42	3.81	0.0000
TIZZAN	537	f	0	-0.59	6.74	25.82	0.1283

	N	14
	NS	9
	Wt	166.09
Het	Chi	91.06
Het	df	13
Het	P	***
Fixed	RR	3.94
	RRl	3.38
	RRu	4.59
	P	+++
Random	RR	4.01
	RRl	2.60
	RRu	6.17
	P	+++
Asymm	P	N.S.

Table 1H22 - 3

IESLC - Meta-analysis of Ever/current Smoking, Age started, "Low"
All LC types, Cigarettes only
Most adjusted

	Sex combined	male	female	Total
N		8	6	14
NS		7	6	13
Wt		93.97	72.12	166.09
Het Chi		28.70	22.18	91.06
Het df		7	5	13
Het P		***	***	***
Fixed RR		6.06	2.25	3.94
RRl		4.95	1.78	3.38
RRu		7.42	2.83	4.59
P		+++	+++	+++
Random RR		6.18	2.14	4.01
RRl		3.98	1.20	2.60
RRu		9.59	3.82	6.17
P		+++	++	+++
Between Chi				40.17
Between df				1
Between P				***
Btwn(F) P				**
Btwn(R) P				**

	Lung cancer type all	other	Total
N	14		14
NS	9		9
Wt	166.09		166.09
Het Chi	91.06		91.06
Het df	13		13
Het P	***		***
Fixed RR	3.94		3.94
RRl	3.38		3.38
RRu	4.59		4.59
P	+++		+++
Random RR	4.01		4.01
RRl	2.60		2.60
RRu	6.17		6.17
P	+++		+++
Between Chi			
Between df			
Between P			N.S.
Btwn(F) P			N.S.
Btwn(R) P			N.S.

	NAmer	UK	Scand	Location othEur	China	Japan	othAs	other	Total
N	4	4	2	3				1	14
NS	2	2	1	3				1	9
Wt	86.83	31.76	8.85	35.23				3.42	166.09
Het Chi	7.66	3.60	3.10	59.35				0.00	91.06
Het df	3	3	1	2				0	13
Het P	(*)	N.S.	(*)	***				N.S.	***
Fixed RR	2.99	5.23	3.53	5.58				11.32	3.94
RRl	2.42	3.69	1.83	4.01				3.92	3.38
RRu	3.69	7.41	6.82	7.77				32.69	4.59
P	+++	+++	+++	+++				+++	+++
Random RR	3.24	5.40	3.57	2.29				11.32	4.01
RRl	2.27	3.63	1.12	0.27				3.92	2.60
RRu	4.60	8.05	11.40	19.87				32.69	6.17
P	+++	+++	+	N.S.				+++	+++
Between Chi									17.34
Between df									4
Between P									**
Btwn(F) P									N.S.
Btwn(R) P									N.S.

Table 1H22 - 3

IESLC - Meta-analysis of Ever/current Smoking, Age started, "Low"
All LC types, Cigarettes only
Most adjusted

Detailed Country in "other Europe"

	multi	Germany	othWest	East	Balkans	Total
N			3			3
NS			3			3
Wt			35.23			35.23
Het Chi			59.35			59.35
Het df			2			2
Het P			***			***
Fixed RR			5.58			5.58
RRl			4.01			4.01
RRu			7.77			7.77
P			+++			+++
Random RR			2.29			2.29
RRl			0.27			0.27
RRu			19.87			19.87
P			N.S.			N.S.
Between Chi						
Between df						
Between P						N.S.
Btwn(F) P						N.S.
Btwn(R) P						N.S.

Detailed Country in "other Asia"

	India	HongKong	other	Total
N				
NS				
Wt				
Het Chi				
Het df				
Het P				
Fixed RR				
RRl				
RRu				
P				
Random RR				
RRl				
RRu				
P				
Between Chi				
Between df				
Between P				N.S.
Btwn(F) P				N.S.
Btwn(R) P				N.S.

Detailed other continent

	SCAmer	Total
N	1	1
NS	1	1
Wt	3.42	3.42
Het Chi	0.00	0.00
Het df	0	0
Het P	N.S.	N.S.
Fixed RR	11.32	11.32
RRl	3.92	3.92
RRu	32.69	32.69
P	+++	+++
Random RR	11.32	11.32
RRl	3.92	3.92
RRu	32.69	32.69
P	+++	+++
Between Chi		
Between df		
Between P		N.S.
Btwn(F) P		N.S.
Btwn(R) P		N.S.

Table 1H22 - 3

IESLC - Meta-analysis of Ever/current Smoking, Age started, "Low"
All LC types, Cigarettes only
Most adjusted

	Start year of study					
	<1960	1960-69	1970-79	1980-89	1990+	Total
N	5	6	1	2		14
NS	3	3	1	2		9
Wt	93.58	40.61	24.52	7.38		166.09
Het Chi	25.35	7.77	0.00	7.12		91.06
Het df	4	5	0	1		13
Het P	***	N.S.	N.S.	**		***
Fixed RR	2.65	4.80	12.90	3.93		3.94
RRl	2.16	3.53	8.68	1.91		3.38
RRu	3.24	6.53	19.16	8.10		4.59
P	+++	+++	+++	+++		+++
Random RR	2.47	4.95	12.90	4.19		4.01
RRl	1.44	3.29	8.68	0.61		2.60
RRu	4.25	7.45	19.16	28.85		6.17
P	++	+++	+++	N.S.		+++
Between Chi						50.82
Between df						3
Between P						***
Btwn(F) P						*
Btwn(R) P						***

	Study type (1)		
	CC	other	Total
N	6	8	14
NS	5	4	9
Wt	64.00	102.09	166.09
Het Chi	63.99	16.43	91.06
Het df	5	7	13
Het P	***	*	***
Fixed RR	5.42	3.22	3.94
RRl	4.24	2.66	3.38
RRu	6.93	3.91	4.59
P	+++	+++	+++
Random RR	3.90	3.75	4.01
RRl	1.53	2.67	2.60
RRu	9.93	5.25	6.17
P	++	+++	+++
Between Chi			10.64
Between df			1
Between P			**
Btwn(F) P			N.S.
Btwn(R) P			N.S.

	Study type (2)			
	CC	prosp	other	Total
N	6	8		14
NS	5	4		9
Wt	64.00	102.09		166.09
Het Chi	63.99	16.43		91.06
Het df	5	7		13
Het P	***	*		***
Fixed RR	5.42	3.22		3.94
RRl	4.24	2.66		3.38
RRu	6.93	3.91		4.59
P	+++	+++		+++
Random RR	3.90	3.75		4.01
RRl	1.53	2.67		2.60
RRu	9.93	5.25		6.17
P	++	+++		+++
Between Chi				10.64
Between df				1
Between P				**
Btwn(F) P				N.S.
Btwn(R) P				N.S.

Table 1H22 - 3

IESLC - Meta-analysis of Ever/current Smoking, Age started, "Low"
All LC types, Cigarettes only
Most adjusted

	Study size (number of LC cases) 100-249	250-499	500-999	1000+	Total
N	2	4	2	6	14
NS	2	2	1	4	9
Wt	7.38	15.26	25.35	118.10	166.09
Het Chi	7.12	5.49	2.26	74.03	91.06
Het df	1	3	1	5	13
Het P	**	N.S.	N.S.	***	***
Fixed RR	3.93	4.94	4.72	3.68	3.94
RRl	1.91	2.99	3.20	3.07	3.38
RRu	8.10	8.16	6.96	4.41	4.59
P	+++	+++	+++	+++	+++
Random RR	4.19	5.11	4.88	3.22	4.01
RRl	0.61	2.58	2.69	1.57	2.60
RRu	28.85	10.12	8.85	6.62	6.17
P	N.S.	+++	+++	++	+++
Between Chi					2.15
Between df					3
Between P					N.S.
Btwn(F) P					N.S.
Btwn(R) P					N.S.

	Risky occupational population no	mining	othRisky	Total
N	14			14
NS	9			9
Wt	166.09			166.09
Het Chi	91.06			91.06
Het df	13			13
Het P	***			***
Fixed RR	3.94			3.94
RRl	3.38			3.38
RRu	4.59			4.59
P	+++			+++
Random RR	4.01			4.01
RRl	2.60			2.60
RRu	6.17			6.17
P	+++			+++
Between Chi				
Between df				
Between P				N.S.
Btwn(F) P				N.S.
Btwn(R) P				N.S.

	National cigarette tobacco type Virginia	blended	other	Total
N	4	10		14
NS	2	7		9
Wt	31.76	134.33		166.09
Het Chi	3.60	84.30		91.06
Het df	3	9		13
Het P	N.S.	***		***
Fixed RR	5.23	3.68		3.94
RRl	3.69	3.11		3.38
RRu	7.41	4.36		4.59
P	+++	+++		+++
Random RR	5.40	3.43		4.01
RRl	3.63	1.97		2.60
RRu	8.05	5.99		6.17
P	+++	+++		+++
Between Chi				3.16
Between df				1
Between P				(*)
Btwn(F) P				N.S.
Btwn(R) P				N.S.

Table 1H22 - 3

IESLC - Meta-analysis of Ever/current Smoking, Age started, "Low"
All LC types, Cigarettes only
Most adjusted

	Any proxy use No/nk	Yes	Total
N	12	2	14
NS	8	1	9
Wt	140.73	25.35	166.09
Het Chi	87.83	2.26	91.06
Het df	11	1	13
Het P	***	N.S.	***
Fixed RR	3.81	4.72	3.94
RRl	3.23	3.20	3.38
RRu	4.50	6.96	4.59
P	+++	+++	+++
Random RR	3.86	4.88	4.01
RRl	2.32	2.69	2.60
RRu	6.40	8.85	6.17
P	+++	+++	+++
Between Chi			0.97
Between df			1
Between P			N.S.
Btwn(F) P			N.S.
Btwn(R) P			N.S.

	Full histological confirmation No	Yes	Total
N	12	2	14
NS	7	2	9
Wt	138.15	27.94	166.09
Het Chi	44.99	0.05	91.06
Het df	11	1	13
Het P	***	N.S.	***
Fixed RR	3.11	12.70	3.94
RRl	2.63	8.76	3.38
RRu	3.67	18.39	4.59
P	+++	+++	+++
Random RR	3.30	12.70	4.01
RRl	2.29	8.76	2.60
RRu	4.77	18.39	6.17
P	+++	+++	+++
Between Chi			46.01
Between df			1
Between P			***
Btwn(F) P			**
Btwn(R) P			***

	Number of adjustment variables (1) 0	1	2+/+nk	Total
N	9	3	2	14
NS	6	2	2	10
Wt	80.41	57.20	28.48	166.09
Het Chi	39.33	5.01	15.03	91.06
Het df	8	2	1	13
Het P	***	(*)	***	***
Fixed RR	3.79	2.66	9.63	3.94
RRl	3.05	2.05	6.67	3.38
RRu	4.72	3.45	13.91	4.59
P	+++	+++	+++	+++
Random RR	3.97	3.10	4.75	4.01
RRl	2.37	1.89	0.61	2.60
RRu	6.64	5.08	37.08	6.17
P	+++	+++	N.S.	+++
Between Chi				31.69
Between df				2
Between P				***
Btwn(F) P				(*)
Btwn(R) P				N.S.

Table 1H22 - 3

IESLC - Meta-analysis of Ever/current Smoking, Age started, "Low"
All LC types, Cigarettes only
Most adjusted

	Number of adjustment variables (2) 0	1	2	3-5	6+/+nk	Total
N	9	3		2		14
NS	6	2		2		10
Wt	80.41	57.20		28.48		166.09
Het Chi	39.33	5.01		15.03		91.06
Het df	8	2		1		13
Het P	***	(*)		***		***
Fixed RR	3.79	2.66		9.63		3.94
RRl	3.05	2.05		6.67		3.38
RRu	4.72	3.45		13.91		4.59
P	+++	+++		+++		+++
Random RR	3.97	3.10		4.75		4.01
RRl	2.37	1.89		0.61		2.60
RRu	6.64	5.08		37.08		6.17
P	+++	+++		N.S.		+++
Between Chi						31.69
Between df						2
Between P						***
Btwn(F) P						(*)
Btwn(R) P						N.S.

	Smoking status ever	current	Total
N	6	8	14
NS	5	4	9
Wt	72.57	93.52	166.09
Het Chi	65.20	19.16	91.06
Het df	5	7	13
Het P	***	**	***
Fixed RR	4.95	3.30	3.94
RRl	3.93	2.69	3.38
RRu	6.23	4.04	4.59
P	+++	+++	+++
Random RR	3.69	4.00	4.01
RRl	1.52	2.73	2.60
RRu	8.98	5.86	6.17
P	++	+++	+++
Between Chi			6.70
Between df			1
Between P			**
Btwn(F) P			N.S.
Btwn(R) P			N.S.

	Denominator nev any	nev cigs	Total
N	12	2	14
NS	7	2	9
Wt	158.71	7.38	166.09
Het Chi	83.94	7.12	91.06
Het df	11	1	13
Het P	***	**	***
Fixed RR	3.94	3.93	3.94
RRl	3.37	1.91	3.38
RRu	4.60	8.10	4.59
P	+++	+++	+++
Random RR	3.99	4.19	4.01
RRl	2.52	0.61	2.60
RRu	6.30	28.85	6.17
P	+++	N.S.	+++
Between Chi			0.00
Between df			1
Between P			N.S.
Btwn(F) P			N.S.
Btwn(R) P			N.S.

Table 1H22 - 3

IESLC - Meta-analysis of Ever/current Smoking, Age started, "Low"
All LC types, Cigarettes only
Most adjusted

	Derivation of RR/CI Orig	StdCalc	Other	Total
N	2	9	3	14
NS	2	6	2	10
Wt	28.48	80.41	57.20	166.09
Het Chi	15.03	39.33	5.01	91.06
Het df	1	8	2	13
Het P	***	***	(*)	***
Fixed RR	9.63	3.79	2.66	3.94
RRl	6.67	3.05	2.05	3.38
RRu	13.91	4.72	3.45	4.59
P	+++	+++	+++	+++
Random RR	4.75	3.97	3.10	4.01
RRl	0.61	2.37	1.89	2.60
RRu	37.08	6.64	5.08	6.17
P	N.S.	+++	+++	+++
Between Chi				31.69
Between df				2
Between P				***
Btwn(F) P				(*)
Btwn(R) P				N.S.

Table 1H22 - 4

IESLC - Meta-analysis of Ever/current Smoking, Age started, "Low"
All LC types, Cigarettes only
Least adjusted

REF	NRR	X	SEX	AGEL	AGEH	RACE	YF	LC TYPE	LOC	START	ST	NLC	R	VB	P	H	AD	SM	PRODUCT	exL	exH	DENOM	De
AGUDO	501	x	f	0	0	all	-	all	Eu:wst	1989	CC	103	n	bl	n	n	0	ev	cig only	24	999	nev cigs	st
BENHAM	617		m	0	0	all	-	all	Eu:wst	1976	CC	1625	n	bl	n	y	3	ev	cig only	20	999	nev any	or
CEDERL	510		m	0	0	all	10	all	Eu:Sca	1963	pr	491	n	bl	n	n	1	cu	cig only	19	999	nev any	ot
CEDERL	515		f	0	0	all	10	all	Eu:Sca	1963	pr	491	n	bl	n	n	0	cu	cig only	19	999	nev any	st
CPSI	892		m	35	84	all	4	all	NAmer	1959	pr	5138	n	bl	n	n	1	cu	cig only	25	999	nev any	ot
CPSI	845		f	40	74	all	6	all	NAmer	1959	pr	5138	n	bl	n	n	1	cu	cig only	25	999	nev any	ot
DEAN3	564		m	0	0	all	-	all	Eu:UK	1969	CC	766	n	V	y	n	0	cu	cig only	25	999	nev any	st
DEAN3	583		f	0	0	all	-	all	Eu:UK	1969	CC	766	n	V	y	n	0	cu	cig only	25	999	nev any	st
DORN	730		m	55	64	wh	8	all	NAmer	1954	pr	5097	n	bl	n	n	0	ev	cig only	25	999	nev any	st
DORN	767		m	65	74	wh	8	all	NAmer	1954	pr	5097	n	bl	n	n	0	ev	cig only	25	999	nev any	st
MIGRAN	501		m	0	0	all	0	all	Eu:UK	1964	pr	259	n	V	n	n	0	cu	cig only	20	999	nev any	st
MIGRAN	511		f	0	0	all	0	all	Eu:UK	1964	pr	259	n	V	n	n	0	cu	cig only	20	999	nev any	st
PEZZOT	570		m	0	0	all	-	all	SCAmer	1987	CC	215	n	bl	n	y	0	ev	cig only	19	999	nev cigs	st
TIZZAN	537		f	0	0	all	-	all	Eu:wst	1959	CC	1358	n	bl	n	n	0	ev	cig only	20	30	nev any	st

Cigarette type is all/unspec for all RRs
except for the following:

REF	NRR	CIGTYPE
DEAN3	564	MC only
DEAN3	583	MC only

Table 1H22 - 5

IESLC - Meta-analysis of Ever/current Smoking, Age started, "Low"
All LC types, Cigarettes only
Least adjusted

REF	NRR	SEX	AD	Number Exposed Case	Exposed Cont	Non-exposed Case	Non-exposed Cont	RR	95.00%CI
AGUDO	501	f	0	7	12	80	183	1.33	(0.51- 3.51)
BENHAM	617	m	3	-	-	-	-	12.90	(8.70- 19.20)
*CEDERL	510	m	1	11	-	7	-	6.50	(2.52- 16.74)
*CEDERL	515	f	0	6	2806	19	17679	1.99	(0.80- 4.98)
Subtotal CEDERL								3.53	(1.83- 6.82)
*CPSI	892	m	1	20	-	49	-	3.21	(1.91- 5.40)
*CPSI	845	f	1	51	-	166	-	2.25	(1.64- 3.08)
Subtotal CPSI								2.48	(1.89- 3.24)
DEAN3	564	m	0	24	75	24	510	6.80	(3.67- 12.58)
DEAN3	583	f	0	27	274	41	1538	3.70	(2.24- 6.11)
Subtotal DEAN3								4.72	(3.20- 6.96)
*DORN	730	m	0	25	40397	25	213858	5.29	(3.04- 9.21)
*DORN	767	m	0	38	38849	49	171211	3.42	(2.24- 5.22)
Subtotal DORN								4.02	(2.87- 5.62)
*MIGRAN	501	m	0	24	668	4	867	7.79	(2.72- 22.34)
*MIGRAN	511	f	0	11	1315	4	3814	7.98	(2.54- 25.01)
Subtotal MIGRAN								7.87	(3.63- 17.08)
PEZZOT	570	m	0	41	105	4	116	11.32	(3.92- 32.69)
TIZZAN	537	f	0	12	21	117	114	0.56	(0.26- 1.18)
Partial Totals				297	84522	589	409890		

*prospective study

REF	NRR	SEX	AD	Ys	Ws	Qs	Ps
AGUDO	501	f	0	0.29	4.10	4.76	0.5594
BENHAM	617	m	3	2.56	24.52	34.80	0.0000
*CEDERL	510	m	1	1.87	4.29	1.10	0.0001
*CEDERL	515	f	0	0.69	4.57	2.10	0.1414
Subtotal CEDERL				1.26	8.85	3.20	
*CPSI	892	m	1	1.17	14.23	0.57	0.0000
*CPSI	845	f	1	0.81	38.69	11.92	0.0000
Subtotal CPSI				0.91	52.91	12.49	
DEAN3	564	m	0	1.92	10.14	3.08	0.0000
DEAN3	583	f	0	1.31	15.21	0.05	0.0000
Subtotal DEAN3				1.55	25.35	3.13	
*DORN	730	m	0	1.67	12.50	1.13	0.0000
*DORN	767	m	0	1.23	21.42	0.40	0.0000
Subtotal DORN				1.39	33.92	1.53	
*MIGRAN	501	m	0	2.05	3.46	1.63	0.0001
*MIGRAN	511	f	0	2.08	2.94	1.48	0.0004
Subtotal MIGRAN				2.06	6.40	3.12	
PEZZOT	570	m	0	2.43	3.42	3.85	0.0000
TIZZAN	537	f	0	-0.59	6.74	25.69	0.1283

	N	14
	NS	9
	Wt	166.22
Het	Chi	92.55
Het	df	13
Het	P	***
Fixed	RR	3.92
	RRl	3.37
	RRu	4.56
	P	+++
Random	RR	3.96
	RRl	2.57
	RRu	6.12
	P	+++
Asymm	P	N.S.

Table 1H22 - 6

IESLC - Meta-analysis of Ever/current Smoking, Age started, "Low"
All LC types, Cigarettes only
Least adjusted

	Sex combined	male	female	Total
N		8	6	14
NS		7	6	13
Wt		93.97	72.25	166.22
Het Chi		28.70	22.79	92.55
Het df		7	5	13
Het P		***	***	***
Fixed RR		6.06	2.22	3.92
RRl		4.95	1.77	3.37
RRu		7.42	2.80	4.56
P		+++	+++	+++
Random RR		6.18	2.09	3.96
RRl		3.98	1.17	2.57
RRu		9.59	3.75	6.12
P		+++	+	+++
Between Chi				41.05
Between df				1
Between P				***
Btwn(F) P				**
Btwn(R) P				**

Table 1H22 - 7

IESLC - Meta-analysis of Ever/current Smoking, Age started, "Low"
All LC types, Cigarettes only
Excluded studies (and stage at which they were excluded)

1	BECHER BLOT1 BROWN3 CARPEN CHYOU DARBY DOLL2 GARCIA GRAHAM GURSEL HAMMO2 JAHN JAIN LAUSSM PRESCO QIAO TVERDA WIGLE WYNDE3
2	AKIBA AMANDU AMES AXELSS BENSHL BEST BOUCHA BOUCOT BROSS CHEN CPSII DEAN2 DESTEF DORGAN DOSEME FAN GARSHI GER GILLIS HAMMON HUMBLE JUSSAW KAISE2 KATSOU KAUFMA KOO KREUZE LEVIN MCCONN NOTAN2 OSANN2 PEZZO2 PISANI RESTRE SADOWS VUTUC WANG2 WATSON WU2 WUWILL WYNDE2 WYNDE8 XU ZHOU
3	GUO MCDUFF SPITZ STASZE ZHANG
4	LUO
5	CORREA HOLE YUAN
7	ARMADA AUVINE BARBON BOFFET BRESLO BUFFLE CHEN2 CHIAZZ CHOI DAMBER DOLL ENGELA GAO GAO2 GENG HAENSZ HEGMAN HIRAYA HU HU2 JEDRYC JOLY KHUDER KOULUM LETOUR LIAW LIU3 LIU4 LIU5 LUBIN LUBIN2 MATOS MRFITR PERNU QIAO2 RACHTA SEGI2 SOBUE SPEIZE SUZUK2 SVENSS WAKAI WU WYNDE6 WYNDE7 ZHENG
10	ALDERS

Table 1H22 - 8
Potentially overlapping studies

REF	REFGP	PRINC	. OVERLAP/LINK
BENHAM	LUBIN2	2	Subset of Lubin2
CPSI	CPSI	1	CPSI overall

Table 1H23 -

IESLC - Meta-analysis of Ever/current Smoking, Age started, "Mid"
All LC types, Cigarettes only

This analysis is restricted to results for:
1) Ever/current smokers
2) Results by Age started
3) Categorical results by Age started
4) All LC types (or near equivalent)
5) Results complete enough for use in metaanalysis

Within each study, results are then selected (in the following order of preference, within each sex) for:
6) SMKSTA: ever, current
7) PRODUCT: cigarettes only
8) CIGTYPE: all/unspecified, MC regardless of HR, MC only
9) (not applicable)
10) DENOM: never smoked anything, never smoked cigarettes, never any + low, never cigs + low
11) Followup period (YF, prospective studies): whole study (coded as 0) or longest available
12) LCtype: all or nearest available, at least Squamous and Adeno. (q = squamous, s = small,
l = large, a = adeno, mix = mixed, alv = alveolar)
13) Race: all or nearest available, otherwise by race (wh or w = white, bl or b = black, hi = hispanic
ch = chinese, jap = japanese, haw = hawaiian, w+o = white + oriental, sca = scandinavian, as = asian)
14) Age started "mid" in key scheme 1 (key value 18, maximum range 15-25)
15) For overlapping studies: principal rather than subsidiary studies
Finally by Age: whole study (coded as 0) if available, otherwise by widest available age group
and then for single sex results (m, f) in preference to results for both sexes combined (c).

Results adjusted (AD) for the most potential confounders are then chosen in Sections -1 to -3
(and those which actually differ from the adjusted results in Table 1H13 - 1 are marked 'x' in Section -1)
and results adjusted for the least confounders in Sections -4 to -6. (Those least adjusted results which
actually differ from the most adjusted are marked 'x' in column X in Section -4)

Section -7 shows excluded studies, together with the stage (as above) at which no qualifying
results were found.

Section -8 lists the potentially overlapping studies which have been included (1=principal, 2=subsidiary).

Section -9 lists any results which would have been included in preference except that they had data not complete
enough for use in meta-analysis, with their significance (yes/no), if known, and any further comment as entered
on the database. It also lists as "gap" any categories for which no data were presented by the original authors.

In addition to those mentioned above, the following fields, levels and abbreviations are used:
* or nk = not known, n = no, y = yes, ot = other
ev = ever, cu = current, nev = never
all/unspec = all or unspecified, MC = manufactured cigarettes, HR = hand-rolled cigarettes
exL, exH = range of exposure (low and high) in the smoking group, in terms of Age started
REF: 6-character study reference
NRR: number of the RR on the database within the study
ST : study type (CC = case control, pr or prosp = prospective)
NLC: number of lung cancer cases in whole study
R : risky occupational population (n = no, m = mining, o = other risky)
VB : national cigarette type (V = at least 75% Virginia, bl = at least 75% blended, ot = other)
P : any proxy use
H : full histological confirmation
De : derivation of RR/CI (or = original, st = standard method, ot = other method of estimation)

Table 1H23 - 1

IESLC - Meta-analysis of Ever/current Smoking, Age started, "Mid"
All LC types, Cigarettes only
Most adjusted

REF	NRR	1H13	SEX	AGEL	AGEH	RACE	YF	LC TYPE	LOC	START	ST	NLC	R	VB	P	H	AD	SM	PRODUCT	exL	exH	DENOM	De
CEDERL	511		m	0	0	all	10	all	Eu:Sca	1963	pr	491	n	bl	n	n	1	cu	cig only	17	18	nev any	ot
CEDERL	516		f	0	0	all	10	all	Eu:Sca	1963	pr	491	n	bl	n	n	0	cu	cig only	17	18	nev any	st
CPSI	894	x	m	35	84	all	4	all	NAmer	1959	pr	5138	n	bl	n	n	1	cu	cig only	15	19	nev any	ot
CPSI	847		f	40	74	all	6	all	NAmer	1959	pr	5138	n	bl	n	n	1	cu	cig only	15	19	nev any	ot
DEAN3	566		m	0	0	all	-	all	Eu:UK	1969	CC	766	n	V	y	n	0	cu	cig only	15	19	nev any	st
DEAN3	585		f	0	0	all	-	all	Eu:UK	1969	CC	766	n	V	y	n	0	cu	cig only	15	19	nev any	st
DORN	732	x	m	55	64	wh	8	all	NAmer	1954	pr	5097	n	bl	n	n	0	ev	cig only	15	19	nev any	st
DORN	769	x	m	65	74	wh	8	all	NAmer	1954	pr	5097	n	bl	n	n	0	ev	cig only	15	19	nev any	st
MIGRAN	503		m	0	0	all	0	all	Eu:UK	1964	pr	259	n	V	n	n	0	cu	cig only	16	19	nev any	st
MIGRAN	513		f	0	0	all	0	all	Eu:UK	1964	pr	259	n	V	n	n	0	cu	cig only	16	19	nev any	st

Cigarette type is all/unspec for all RRs
except for the following:

REF	NRR	CIGTYPE
DEAN3	566	MC only
DEAN3	585	MC only

Table 1H23 - 2

IESLC - Meta-analysis of Ever/current Smoking, Age started, "Mid"
All LC types, Cigarettes only
Most adjusted

REF	NRR	SEX	AD	Number Exposed Case	Cont	Non-exposed Case	Cont	RR	95.00%CI
*CEDERL	511	m	1	10	-	7	-	9.80	(3.74- 25.69)
*CEDERL	516	f	0	2	1009	19	17679	1.84	(0.43- 7.91)
Subtotal CEDERL								5.89	(2.64- 13.16)
*CPSI	894	m	1	315	-	49	-	12.81	(9.48- 17.31)
*CPSI	847	f	1	52	-	166	-	5.00	(3.66- 6.83)
Subtotal CPSI								8.14	(6.55- 10.11)
DEAN3	566	m	0	160	485	24	510	7.01	(4.48- 10.96)
DEAN3	585	f	0	39	504	41	1538	2.90	(1.85- 4.55)
Subtotal DEAN3								4.52	(3.30- 6.21)
*DORN	732	m	0	239	128130	25	213858	15.96	(10.57- 24.09)
*DORN	769	m	0	193	67425	49	171211	10.00	(7.31- 13.68)
Subtotal DORN								11.87	(9.25- 15.23)
*MIGRAN	503	m	0	59	1845	4	867	6.93	(2.53- 19.02)
*MIGRAN	513	f	0	9	1035	4	3814	8.29	(2.56- 26.87)
Subtotal MIGRAN								7.48	(3.48- 16.09)
Partial Totals				1078	200433	388	409477		

*prospective study

REF	NRR	SEX	AD	Ys	Ws	Qs	Ps
*CEDERL	511	m	1	2.28	4.14	0.16	0.0000
*CEDERL	516	f	0	0.61	1.81	3.95	0.4098
Subtotal CEDERL				1.77	5.95	4.11	
*CPSI	894	m	1	2.55	42.39	9.05	0.0000
*CPSI	847	f	1	1.61	39.48	9.05	0.0000
Subtotal CPSI				2.10	81.87	18.10	
DEAN3	566	m	0	1.95	19.25	0.38	0.0000
DEAN3	585	f	0	1.07	18.99	19.85	0.0000
Subtotal DEAN3				1.51	38.24	20.24	
*DORN	732	m	0	2.77	22.64	10.52	0.0000
*DORN	769	m	0	2.30	39.11	1.80	0.0000
Subtotal DORN				2.47	61.75	12.32	
*MIGRAN	503	m	0	1.94	3.77	0.09	0.0002
*MIGRAN	513	f	0	2.12	2.78	0.00	0.0004
Subtotal MIGRAN				2.01	6.55	0.09	

	N	10
	NS	5
	Wt	194.36
Het	Chi	54.85
Het	df	9
Het	P	***
Fixed	RR	8.07
	RRl	7.01
	RRu	9.29
	P	+++
Random	RR	7.35
	RRl	4.98
	RRu	10.85
	P	+++
Asymm	P	N.S.

Table 1H23 - 3

IESLC - Meta-analysis of Ever/current Smoking, Age started, "Mid"
All LC types, Cigarettes only
Most adjusted

	Sex			
	combined	male	female	Total
N		6	4	10
NS		5	4	9
Wt		131.30	63.06	194.36
Het Chi		9.24	6.30	54.85
Het df		5	3	9
Het P		N.S.	(*)	***
Fixed RR		11.02	4.22	8.07
RRl		9.29	3.30	7.01
RRu		13.08	5.40	9.29
P		+++	+++	+++
Random RR		10.73	4.02	7.35
RRl		8.30	2.53	4.98
RRu		13.86	6.37	10.85
P		+++	+++	+++
Between Chi				39.31
Between df				1
Between P				***
Btwn(F) P				**
Btwn(R) P				***

	Lung cancer type		
	all	other	Total
N	10		10
NS	5		5
Wt	194.36		194.36
Het Chi	54.85		54.85
Het df	9		9
Het P	***		***
Fixed RR	8.07		8.07
RRl	7.01		7.01
RRu	9.29		9.29
P	+++		+++
Random RR	7.35		7.35
RRl	4.98		4.98
RRu	10.85		10.85
P	+++		+++
Between Chi			
Between df			
Between P			N.S.
Btwn(F) P			N.S.
Btwn(R) P			N.S.

	Location								
	NAmer	UK	Scand	othEur	China	Japan	othAs	other	Total
N	4	4	2						10
NS	2	2	1						5
Wt	143.61	44.79	5.95						194.36
Het Chi	26.24	8.90	3.52						54.85
Het df	3	3	1						9
Het P	***	*	(*)						***
Fixed RR	9.57	4.87	5.89						8.07
RRl	8.13	3.63	2.64						7.01
RRu	11.27	6.53	13.16						9.29
P	+++	+++	+++						+++
Random RR	9.98	5.36	4.66						7.35
RRl	6.12	3.00	0.92						4.98
RRu	16.26	9.56	23.73						10.85
P	+++	+++	(+)						+++
Between Chi									16.20
Between df									2
Between P									***
Btwn(F) P									N.S.
Btwn(R) P									N.S.

Table 1H23 - 3

IESLC - Meta-analysis of Ever/current Smoking, Age started, "Mid"
All LC types, Cigarettes only
Most adjusted

Detailed Country in "other Europe"

	multi	Germany	othWest	East	Balkans	Total
N						
NS						
Wt						
Het Chi						
Het df						
Het P						
Fixed RR						
RRl						
RRu						
P						
Random RR						
RRl						
RRu						
P						
Between Chi						
Between df						
Between P						N.S.
Btwn(F) P						N.S.
Btwn(R) P						N.S.

Detailed Country in "other Asia"

	India	HongKong	other	Total
N				
NS				
Wt				
Het Chi				
Het df				
Het P				
Fixed RR				
RRl				
RRu				
P				
Random RR				
RRl				
RRu				
P				
Between Chi				
Between df				
Between P				N.S.
Btwn(F) P				N.S.
Btwn(R) P				N.S.

Detailed other continent

	SCAmer	Total
N		
NS		
Wt		
Het Chi		
Het df		
Het P		
Fixed RR		
RRl		
RRu		
P		
Random RR		
RRl		
RRu		
P		
Between Chi		
Between df		
Between P		N.S.
Btwn(F) P		N.S.
Btwn(R) P		N.S.

Table 1H23 - 3

IESLC - Meta-analysis of Ever/current Smoking, Age started, "Mid"
All LC types, Cigarettes only
Most adjusted

	Start year of study <1960	1960-69	1970-79	1980-89	1990+	Total
N	4	6				10
NS	2	3				5
Wt	143.61	50.74				194.36
Het Chi	26.24	12.60				54.85
Het df	3	5				9
Het P	***	*				***
Fixed RR	9.57	4.98				8.07
RRl	8.13	3.78				7.01
RRu	11.27	6.56				9.29
P	+++	+++				+++
Random RR	9.98	5.34				7.35
RRl	6.12	3.21				4.98
RRu	16.26	8.88				10.85
P	+++	+++				+++
Between Chi						16.01
Between df						1
Between P						***
Btwn(F) P						N.S.
Btwn(R) P						(*)

	Study type (1) CC	other	Total
N	2	8	10
NS	1	4	5
Wt	38.24	156.11	194.36
Het Chi	7.43	31.47	54.85
Het df	1	7	9
Het P	**	***	***
Fixed RR	4.52	9.30	8.07
RRl	3.30	7.95	7.01
RRu	6.21	10.88	9.29
P	+++	+++	+++
Random RR	4.51	8.70	7.35
RRl	1.90	5.87	4.98
RRu	10.71	12.89	10.85
P	+++	+++	+++
Between Chi			15.94
Between df			1
Between P			***
Btwn(F) P			N.S.
Btwn(R) P			N.S.

	Study type (2) CC	prosp	other	Total
N	2	8		10
NS	1	4		5
Wt	38.24	156.11		194.36
Het Chi	7.43	31.47		54.85
Het df	1	7		9
Het P	**	***		***
Fixed RR	4.52	9.30		8.07
RRl	3.30	7.95		7.01
RRu	6.21	10.88		9.29
P	+++	+++		+++
Random RR	4.51	8.70		7.35
RRl	1.90	5.87		4.98
RRu	10.71	12.89		10.85
P	+++	+++		+++
Between Chi				15.94
Between df				1
Between P				***
Btwn(F) P				N.S.
Btwn(R) P				N.S.

Table 1H23 - 3

IESLC - Meta-analysis of Ever/current Smoking, Age started, "Mid"
All LC types, Cigarettes only
Most adjusted

	Study size (number of LC cases)				
	100-249	250-499	500-999	1000+	Total
N		4	2	4	10
NS		2	1	2	5
Wt		12.50	38.24	143.61	194.36
Het Chi		3.75	7.43	26.24	54.85
Het df		3	1	3	9
Het P		N.S.	**	***	***
Fixed RR		6.68	4.52	9.57	8.07
RRl		3.83	3.30	8.13	7.01
RRu		11.62	6.21	11.27	9.29
P		+++	+++	+++	+++
Random RR		6.51	4.51	9.98	7.35
RRl		3.48	1.90	6.12	4.98
RRu		12.17	10.71	16.26	10.85
P		+++	+++	+++	+++
Between Chi					17.43
Between df					2
Between P					***
Btwn(F) P					N.S.
Btwn(R) P					N.S.

	Risky occupational population			
	no	mining	othRisky	Total
N	10			10
NS	5			5
Wt	194.36			194.36
Het Chi	54.85			54.85
Het df	9			9
Het P	***			***
Fixed RR	8.07			8.07
RRl	7.01			7.01
RRu	9.29			9.29
P	+++			+++
Random RR	7.35			7.35
RRl	4.98			4.98
RRu	10.85			10.85
P	+++			+++
Between Chi				
Between df				
Between P				N.S.
Btwn(F) P				N.S.
Btwn(R) P				N.S.

	National cigarette tobacco type			
	Virginia	blended	other	Total
N	4	6		10
NS	2	3		5
Wt	44.79	149.57		194.36
Het Chi	8.90	31.10		54.85
Het df	3	5		9
Het P	*	***		***
Fixed RR	4.87	9.39		8.07
RRl	3.63	8.00		7.01
RRu	6.53	11.02		9.29
P	+++	+++		+++
Random RR	5.36	8.88		7.35
RRl	3.00	5.65		4.98
RRu	9.56	13.95		10.85
P	+++	+++		+++
Between Chi				14.85
Between df				1
Between P				***
Btwn(F) P				N.S.
Btwn(R) P				N.S.

Table 1H23 - 3

IESLC - Meta-analysis of Ever/current Smoking, Age started, "Mid"
All LC types, Cigarettes only
Most adjusted

	Any proxy use No/nk	Yes	Total
N	8	2	10
NS	4	1	5
Wt	156.11	38.24	194.36
Het Chi	31.47	7.43	54.85
Het df	7	1	9
Het P	***	**	***
Fixed RR	9.30	4.52	8.07
RRl	7.95	3.30	7.01
RRu	10.88	6.21	9.29
P	+++	+++	+++
Random RR	8.70	4.51	7.35
RRl	5.87	1.90	4.98
RRu	12.89	10.71	10.85
P	+++	+++	+++
Between Chi			15.94
Between df			1
Between P			***
Btwn(F) P			N.S.
Btwn(R) P			N.S.

	Full histological confirmation No	Yes	Total
N	10		10
NS	5		5
Wt	194.36		194.36
Het Chi	54.85		54.85
Het df	9		9
Het P	***		***
Fixed RR	8.07		8.07
RRl	7.01		7.01
RRu	9.29		9.29
P	+++		+++
Random RR	7.35		7.35
RRl	4.98		4.98
RRu	10.85		10.85
P	+++		+++
Between Chi			
Between df			
Between P			N.S.
Btwn(F) P			N.S.
Btwn(R) P			N.S.

	Number of adjustment variables (1) 0	1	2+/+nk	Total
N	7	3		10
NS	4	2		6
Wt	108.35	86.00		194.36
Het Chi	36.57	18.23		54.85
Het df	6	2		9
Het P	***	***		***
Fixed RR	7.96	8.21		8.07
RRl	6.59	6.65		7.01
RRu	9.61	10.14		9.29
P	+++	+++		+++
Random RR	6.79	8.41		7.35
RRl	4.00	4.03		4.98
RRu	11.53	17.56		10.85
P	+++	+++		+++
Between Chi				0.05
Between df				1
Between P				N.S.
Btwn(F) P				N.S.
Btwn(R) P				N.S.

Table 1H23 - 3

IESLC - Meta-analysis of Ever/current Smoking, Age started, "Mid"
All LC types, Cigarettes only
Most adjusted

	Number of adjustment variables (2) 0	1	2	3-5	6+/+nk	Total
N	7	3				10
NS	4	2				6
Wt	108.35	86.00				194.36
Het Chi	36.57	18.23				54.85
Het df	6	2				9
Het P	***	***				***
Fixed RR	7.96	8.21				8.07
RRl	6.59	6.65				7.01
RRu	9.61	10.14				9.29
P	+++	+++				+++
Random RR	6.79	8.41				7.35
RRl	4.00	4.03				4.98
RRu	11.53	17.56				10.85
P	+++	+++				+++
Between Chi						0.05
Between df						1
Between P						N.S.
Btwn(F) P						N.S.
Btwn(R) P						N.S.

	Smoking status ever	current	Total
N	2	8	10
NS	1	4	5
Wt	61.75	132.61	194.36
Het Chi	3.13	38.25	54.85
Het df	1	7	9
Het P	(*)	***	***
Fixed RR	11.87	6.74	8.07
RRl	9.25	5.69	7.01
RRu	15.23	7.99	9.29
P	+++	+++	+++
Random RR	12.38	6.14	7.35
RRl	7.85	3.86	4.98
RRu	19.54	9.77	10.85
P	+++	+++	+++
Between Chi			13.47
Between df			1
Between P			***
Btwn(F) P			N.S.
Btwn(R) P			*

	Denominator nev any	nev cigs	Total
N	10		10
NS	5		5
Wt	194.36		194.36
Het Chi	54.85		54.85
Het df	9		9
Het P	***		***
Fixed RR	8.07		8.07
RRl	7.01		7.01
RRu	9.29		9.29
P	+++		+++
Random RR	7.35		7.35
RRl	4.98		4.98
RRu	10.85		10.85
P	+++		+++
Between Chi			
Between df			
Between P			N.S.
Btwn(F) P			N.S.
Btwn(R) P			N.S.

Table 1H23 - 3

IESLC - Meta-analysis of Ever/current Smoking, Age started, "Mid"
All LC types, Cigarettes only
Most adjusted

	Orig	StdCalc	Other	Total
	Derivation of RR/CI			
N		7	3	10
NS		4	2	6
Wt		108.35	86.00	194.36
Het Chi		36.57	18.23	54.85
Het df		6	2	9
Het P		***	***	***
Fixed RR		7.96	8.21	8.07
RRl		6.59	6.65	7.01
RRu		9.61	10.14	9.29
P		+++	+++	+++
Random RR		6.79	8.41	7.35
RRl		4.00	4.03	4.98
RRu		11.53	17.56	10.85
P		+++	+++	+++
Between Chi				0.05
Between df				1
Between P				N.S.
Btwn(F) P				N.S.
Btwn(R) P				N.S.

Table 1H23 - 4

IESLC - Meta-analysis of Ever/current Smoking, Age started, "Mid"
All LC types, Cigarettes only
Least adjusted

REF	NRR	X	SEX	AGEL	AGEH	RACE	YF	LC TYPE	LOC	START	ST	NLC	R	VB	P	H	AD	SM	PRODUCT	exL	exH	DENOM	De
CEDERL	511		m	0	0	all	10	all	Eu:Sca	1963	pr	491	n	bl	n	n	1	cu	cig only	17	18	nev any	ot
CEDERL	516		f	0	0	all	10	all	Eu:Sca	1963	pr	491	n	bl	n	n	0	cu	cig only	17	18	nev any	st
CPSI	894		m	35	84	all	4	all	NAmer	1959	pr	5138	n	bl	n	n	1	cu	cig only	15	19	nev any	ot
CPSI	847		f	40	74	all	6	all	NAmer	1959	pr	5138	n	bl	n	n	1	cu	cig only	15	19	nev any	ot
DEAN3	566		m	0	0	all	-	all	Eu:UK	1969	CC	766	n	V	y	n	0	cu	cig only	15	19	nev any	st
DEAN3	585		f	0	0	all	-	all	Eu:UK	1969	CC	766	n	V	y	n	0	cu	cig only	15	19	nev any	st
DORN	732		m	55	64	wh	8	all	NAmer	1954	pr	5097	n	bl	n	n	0	ev	cig only	15	19	nev any	st
DORN	769		m	65	74	wh	8	all	NAmer	1954	pr	5097	n	bl	n	n	0	ev	cig only	15	19	nev any	st
MIGRAN	503		m	0	0	all	0	all	Eu:UK	1964	pr	259	n	V	n	n	0	cu	cig only	16	19	nev any	st
MIGRAN	513		f	0	0	all	0	all	Eu:UK	1964	pr	259	n	V	n	n	0	cu	cig only	16	19	nev any	st

Cigarette type is all/unspec for all RRs
except for the following:

REF	NRR	CIGTYPE
DEAN3	566	MC only
DEAN3	585	MC only

Table 1H23 - 5

IESLC - Meta-analysis of Ever/current Smoking, Age started, "Mid"
All LC types, Cigarettes only
Least adjusted

REF	NRR	SEX	AD	Number Case	Exposed Cont	Non-exposed Case	Cont	RR	95.00%CI
*CEDERL	511	m	1	10	-	7	-	9.80	(3.74- 25.69)
*CEDERL	516	f	0	2	1009	19	17679	1.84	(0.43- 7.91)
Subtotal CEDERL								5.89	(2.64- 13.16)
*CPSI	894	m	1	315	-	49	-	12.81	(9.48- 17.31)
*CPSI	847	f	1	52	-	166	-	5.00	(3.66- 6.83)
Subtotal CPSI								8.14	(6.55- 10.11)
DEAN3	566	m	0	160	485	24	510	7.01	(4.48- 10.96)
DEAN3	585	f	0	39	504	41	1538	2.90	(1.85- 4.55)
Subtotal DEAN3								4.52	(3.30- 6.21)
*DORN	732	m	0	239	128130	25	213858	15.96	(10.57- 24.09)
*DORN	769	m	0	193	67425	49	171211	10.00	(7.31- 13.68)
Subtotal DORN								11.87	(9.25- 15.23)
*MIGRAN	503	m	0	59	1845	4	867	6.93	(2.53- 19.02)
*MIGRAN	513	f	0	9	1035	4	3814	8.29	(2.56- 26.87)
Subtotal MIGRAN								7.48	(3.48- 16.09)
Partial Totals				1078	200433	388	409477		

*prospective study

REF	NRR	SEX	AD	Ys	Ws	Qs	Ps
*CEDERL	511	m	1	2.28	4.14	0.16	0.0000
*CEDERL	516	f	0	0.61	1.81	3.95	0.4098
Subtotal CEDERL				1.77	5.95	4.11	
*CPSI	894	m	1	2.55	42.39	9.05	0.0000
*CPSI	847	f	1	1.61	39.48	9.05	0.0000
Subtotal CPSI				2.10	81.87	18.10	
DEAN3	566	m	0	1.95	19.25	0.38	0.0000
DEAN3	585	f	0	1.07	18.99	19.85	0.0000
Subtotal DEAN3				1.51	38.24	20.24	
*DORN	732	m	0	2.77	22.64	10.52	0.0000
*DORN	769	m	0	2.30	39.11	1.80	0.0000
Subtotal DORN				2.47	61.75	12.32	
*MIGRAN	503	m	0	1.94	3.77	0.09	0.0002
*MIGRAN	513	f	0	2.12	2.78	0.00	0.0004
Subtotal MIGRAN				2.01	6.55	0.09	

	N	10
	NS	5
	Wt	194.36
Het	Chi	54.85
Het	df	9
Het	P	***
Fixed	RR	8.07
	RRl	7.01
	RRu	9.29
	P	+++
Random	RR	7.35
	RRl	4.98
	RRu	10.85
	P	+++
Asymm	P	N.S.

Table 1H23 - 6

IESLC - Meta-analysis of Ever/current Smoking, Age started, "Mid"
All LC types, Cigarettes only
Least adjusted

	Sex combined	male	female	Total
N		6	4	10
NS		5	4	9
Wt		131.30	63.06	194.36
Het Chi		9.24	6.30	54.85
Het df		5	3	9
Het P		N.S.	(*)	***
Fixed RR		11.02	4.22	8.07
RRl		9.29	3.30	7.01
RRu		13.08	5.40	9.29
P		+++	+++	+++
Random RR		10.73	4.02	7.35
RRl		8.30	2.53	4.98
RRu		13.86	6.37	10.85
P		+++	+++	+++
Between Chi				39.31
Between df				1
Between P				***
Btwn(F) P				**
Btwn(R) P				***

Table 1H23 - 7

IESLC - Meta-analysis of Ever/current Smoking, Age started, "Mid"
All LC types, Cigarettes only
Excluded studies (and stage at which they were excluded)

1	BECHER BLOT1 BROWN3 CARPEN CHYOU DARBY DOLL2 GARCIA GRAHAM GURSEL HAMMO2 JAHN JAIN LAUSSM PRESCO QIAO TVERDA WIGLE WYNDE3
2	AKIBA AMANDU AMES AXELSS BENSHL BEST BOUCHA BOUCOT BROSS CHEN CPSII DEAN2 DESTEF DORGAN DOSEME FAN GARSHI GER GILLIS HAMMON HUMBLE JUSSAW KAISE2 KATSOU KAUFMA KOO KREUZE LEVIN MCCONN NOTAN2 OSANN2 PEZZO2 PISANI RESTRE SADOWS VUTUC WANG2 WATSON WU2 WUWILL WYNDE2 WYNDE8 XU ZHOU
3	GUO MCDUFF SPITZ STASZE ZHANG
4	LUO
5	CORREA HOLE YUAN
7	ARMADA AUVINE BARBON BOFFET BRESLO BUFFLE CHEN2 CHIAZZ CHOI DAMBER DOLL ENGELA GAO GAO2 GENG HAENSZ HEGMAN HIRAYA HU HU2 JEDRYC JOLY KHUDER KOULUM LETOUR LIAW LIU3 LIU4 LIU5 LUBIN LUBIN2 MATOS MRFITR PERNU QIAO2 RACHTA SEGI2 SOBUE SPEIZE SUZUK2 SVENSS WAKAI WU WYNDE6 WYNDE7 ZHENG
10	ALDERS
14	AGUDO BENHAM PEZZOT TIZZAN

Table 1H23 - 8
Potentially overlapping studies

REF	REFGP	PRINC	.	OVERLAP/LINK
CPSI	CPSI	1		CPSI overall

Table 1H24 -

IESLC - Meta-analysis of Ever/current Smoking, Age started, "High"
All LC types, Cigarettes only

This analysis is restricted to results for:
1) Ever/current smokers
2) Results by Age started
3) Categorical results by Age started
4) All LC types (or near equivalent)
5) Results complete enough for use in metaanalysis

Within each study, results are then selected (in the following order of preference, within each sex) for:
6) PRODUCT: cigarettes only
7) CIGTYPE: all/unspecified, MC regardless of HR, MC only
8) (not applicable)
9) DENOM: never smoked anything, never smoked cigarettes, never any + low, never cigs + low
10) Followup period (YF, prospective studies): whole study (coded as 0) or longest available
11) LCtype: all or nearest available, at least Squamous and Adeno. (q = squamous, s = small,
l = large, a = adeno, mix = mixed, alv = alveolar)
12) Race: all or nearest available, otherwise by race (wh or w = white, bl or b = black, hi = hispanic
ch = chinese, jap = japanese, haw = hawaiian, w+o = white + oriental, sca = scandinavian, as = asian)
13) Age started "high" in key scheme 1 (key value 14, maximum range 1-17)
14) For overlapping studies: principal rather than subsidiary studies
Finally by Age: whole study (coded as 0) if available, otherwise by widest available age group
and then for single sex results (m, f) in preference to results for both sexes combined (c).

Results adjusted (AD) for the most potential confounders are then chosen in Sections -1 to -3
(and those which actually differ from the adjusted results in Table 1H14 - 1 are marked 'x' in Section -1)
and results adjusted for the least confounders in Sections -4 to -6. (Those least adjusted results which
actually differ from the most adjusted are marked 'x' in column X in Section -4)

Section -7 shows excluded studies, together with the stage (as above) at which no qualifying
results were found.

Section -8 lists the potentially overlapping studies which have been included (1=principal, 2=subsidiary).

Section -9 lists any results which would have been included in preference except that they had data not complete
enough for use in meta-analysis, with their significance (yes/no), if known, and any further comment as entered
on the database. It also lists as "gap" any categories for which no data were presented by the original authors.

In addition to those mentioned above, the following fields, levels and abbreviations are used:
* or nk = not known, n = no, y = yes, ot = other
ev = ever, cu = current, nev = never
all/unspec = all or unspecified, MC = manufactured cigarettes, HR = hand-rolled cigarettes
exL, exH = range of exposure (low and high) in the smoking group, in terms of Age started
REF: 6-character study reference
NRR: number of the RR on the database within the study
ST : study type (CC = case control, pr or prosp = prospective)
NLC: number of lung cancer cases in whole study
R : risky occupational population (n = no, m = mining, o = other risky)
VB : national cigarette type (V = at least 75% Virginia, bl = at least 75% blended, ot = other)
P : any proxy use
H : full histological confirmation
De : derivation of RR/CI (or = original, st = standard method, ot = other method of estimation)

Table 1H24 - 1

IESLC - Meta-analysis of Ever/current Smoking, Age started, "High"
All LC types, Cigarettes only
Most adjusted

REF	NRR	1H14	SEX	AGEL	AGEH	RACE	YF	LC TYPE	LOC	START	ST	NLC	R	VB	P	H	AD	SM	PRODUCT	exL	exH	DENOM	De
CEDERL	512		m	0	0	all	10	all	Eu:Sca	1963	pr	491	n	bl	n	n	1	cu	cig only	1	16	nev any	ot
CEDERL	517		f	0	0	all	10	all	Eu:Sca	1963	pr	491	n	bl	n	n	0	cu	cig only	1	16	nev any	ot
CPSI	895	x	m	35	84	all	4	all	NAmer	1959	pr	5138	n	bl	n	n	1	cu	cig only	1	14	nev any	ot
CPSI	848		f	40	74	all	6	all	NAmer	1959	pr	5138	n	bl	n	n	1	cu	cig only	1	14	nev any	ot
DEAN3	567		m	0	0	all	-	all	Eu:UK	1969	CC	766	n	V	y	n	0	cu	cig only	1	14	nev any	st
DEAN3	586		f	0	0	all	-	all	Eu:UK	1969	CC	766	n	V	y	n	0	cu	cig only	1	14	nev any	st
DORN	733	x	m	55	64	wh	8	all	NAmer	1954	pr	5097	n	bl	n	n	0	ev	cig only	1	14	nev any	st
DORN	770	x	m	65	74	wh	8	all	NAmer	1954	pr	5097	n	bl	n	n	0	ev	cig only	1	14	nev any	st
MIGRAN	505		m	0	0	all	0	all	Eu:UK	1964	pr	259	n	V	n	n	0	cu	cig only	0	15	nev any	st
MIGRAN	515		f	0	0	all	0	all	Eu:UK	1964	pr	259	n	V	n	n	0	cu	cig only	0	15	nev any	st

Cigarette type is all/unspec for all RRs
except for the following:

REF	NRR	CIGTYPE
DEAN3	567	MC only
DEAN3	586	MC only

Table 1H24 - 2

IESLC - Meta-analysis of Ever/current Smoking, Age started, "High"
All LC types, Cigarettes only
Most adjusted

REF	NRR	SEX	AD	Number Exposed Case	Cont	Non-exposed Case	Cont	RR	95.00%CI
*CEDERL	512	m	1	7	-	7	-	6.40	(2.25- 18.21)
*CEDERL	517	f	0	0	746	19	17679	0.61~	(0.04- 10.05)
Subtotal CEDERL								4.80	(1.80- 12.79)
*CPSI	895	m	1	101	-	49	-	15.10	(10.73- 21.24)
*CPSI	848	f	1	6	-	166	-	2.50	(1.11- 5.65)
Subtotal CPSI								11.54	(8.42- 15.80)
DEAN3	567	m	0	44	165	24	510	5.67	(3.34- 9.60)
DEAN3	586	f	0	7	109	41	1538	2.41	(1.06- 5.50)
Subtotal DEAN3								4.42	(2.83- 6.89)
*DORN	733	m	0	59	21349	25	213858	23.64	(14.81- 37.73)
*DORN	770	m	0	44	13616	49	171211	11.29	(7.52- 16.96)
Subtotal DORN								15.52	(11.42- 21.10)
*MIGRAN	505	m	0	50	1081	4	867	10.03	(3.64- 27.65)
*MIGRAN	515	f	0	2	266	4	3814	7.17	(1.32- 38.96)
Subtotal MIGRAN								9.18	(3.84- 21.90)
Partial Totals				320	37332	388	409477		

*prospective study ~ With 0.5 adjustment for zero

REF	NRR	SEX	AD	Ys	Ws	Qs	Ps
*CEDERL	512	m	1	1.86	3.51	0.83	0.0005
*CEDERL	517	f	0	-0.50	0.49	3.94	0.7276
Subtotal CEDERL				1.57	4.00	4.76	
*CPSI	895	m	1	2.71	32.95	4.59	0.0000
*CPSI	848	f	1	0.92	5.80	11.79	0.0273
Subtotal CPSI				2.45	38.76	16.37	
DEAN3	567	m	0	1.73	13.81	5.09	0.0000
DEAN3	586	f	0	0.88	5.65	12.08	0.0367
Subtotal DEAN3				1.49	19.46	17.17	
*DORN	733	m	0	3.16	17.58	11.85	0.0000
*DORN	770	m	0	2.42	23.23	0.16	0.0000
Subtotal DORN				2.74	40.80	12.01	
*MIGRAN	505	m	0	2.31	3.73	0.00	0.0000
*MIGRAN	515	f	0	1.97	1.34	0.19	0.0226
Subtotal MIGRAN				2.22	5.07	0.19	

	N	10
	NS	5
	Wt	108.09
Het	Chi	50.51
Het	df	9
Het	P	***
Fixed	RR	10.40
	RRl	8.61
	RRu	12.56
	P	+++
Random	RR	7.26
	RRl	4.35
	RRu	12.13
	P	+++
Asymm	P	(*)

Table 1H24 - 3

IESLC - Meta-analysis of Ever/current Smoking, Age started, "High"
All LC types, Cigarettes only
Most adjusted

	Sex			
	combined	male	female	Total
N		6	4	10
NS		5	4	9
Wt		94.81	13.28	108.09
Het Chi		18.94	2.45	50.51
Het df		5	3	9
Het P		**	N.S.	***
Fixed RR		12.63	2.60	10.40
RRl		10.33	1.52	8.61
RRu		15.44	4.45	12.56
P		+++	+++	+++
Random RR		11.41	2.60	7.26
RRl		7.42	1.52	4.35
RRu		17.55	4.45	12.13
P		+++	+++	+++
Between Chi				29.11
Between df				1
Between P				***
Btwn(F) P				*
Btwn(R) P				***

	Lung cancer type		
	all	other	Total
N	10		10
NS	5		5
Wt	108.09		108.09
Het Chi	50.51		50.51
Het df	9		9
Het P	***		***
Fixed RR	10.40		10.40
RRl	8.61		8.61
RRu	12.56		12.56
P	+++		+++
Random RR	7.26		7.26
RRl	4.35		4.35
RRu	12.13		12.13
P	+++		+++
Between Chi			
Between df			
Between P			N.S.
Btwn(F) P			N.S.
Btwn(R) P			N.S.

	Location								
	NAmer	UK	Scand	othEur	China	Japan	othAs	other	Total
N	4	4	2						10
NS	2	2	1						5
Wt	79.56	24.53	4.00						108.09
Het Chi	23.17	5.19	2.38						50.51
Het df	3	3	1						9
Het P	***	N.S.	N.S.						***
Fixed RR	13.43	5.14	4.80						10.40
RRl	10.78	3.46	1.80						8.61
RRu	16.73	7.64	12.79						12.56
P	+++	+++	++						+++
Random RR	10.90	5.18	2.87						7.26
RRl	5.70	2.88	0.32						4.35
RRu	20.84	9.32	25.57						12.13
P	+++	+++	N.S.						+++
Between Chi									19.77
Between df									2
Between P									***
Btwn(F) P									N.S.
Btwn(R) P									N.S.

Table 1H24 - 3

IESLC - Meta-analysis of Ever/current Smoking, Age started, "High"
All LC types, Cigarettes only
Most adjusted

		Detailed Country in "other Europe"					
		multi	Germany	othWest	East	Balkans	Total
	N						
	NS						
	Wt						
Het	Chi						
Het	df						
Het	P						
Fixed	RR						
	RRl						
	RRu						
	P						
Random	RR						
	RRl						
	RRu						
	P						
Between	Chi						
Between	df						
Between	P						N.S.
Btwn(F)	P						N.S.
Btwn(R)	P						N.S.

		Detailed Country in "other Asia"			
		India	HongKong	other	Total
	N				
	NS				
	Wt				
Het	Chi				
Het	df				
Het	P				
Fixed	RR				
	RRl				
	RRu				
	P				
Random	RR				
	RRl				
	RRu				
	P				
Between	Chi				
Between	df				
Between	P				N.S.
Btwn(F)	P				N.S.
Btwn(R)	P				N.S.

		Detailed other continent	
		SCAmer	Total
	N		
	NS		
	Wt		
Het	Chi		
Het	df		
Het	P		
Fixed	RR		
	RRl		
	RRu		
	P		
Random	RR		
	RRl		
	RRu		
	P		
Between	Chi		
Between	df		
Between	P		N.S.
Btwn(F)	P		N.S.
Btwn(R)	P		N.S.

Table 1H24 - 3

IESLC - Meta-analysis of Ever/current Smoking, Age started, "High"
All LC types, Cigarettes only
Most adjusted

	Start year of study <1960	1960-69	1970-79	1980-89	1990+	Total
N	4	6				10
NS	2	3				5
Wt	79.56	28.53				108.09
Het Chi	23.17	7.58				50.51
Het df	3	5				9
Het P	***	N.S.				***
Fixed RR	13.43	5.09				10.40
RRl	10.78	3.53				8.61
RRu	16.73	7.35				12.56
P	+++	+++				+++
Random RR	10.90	5.01				7.26
RRl	5.70	3.00				4.35
RRu	20.84	8.35				12.13
P	+++	+++				+++
Between Chi						19.76
Between df						1
Between P						***
Btwn(F) P						(*)
Btwn(R) P						(*)

	Study type (1) CC	other	Total
N	2	8	10
NS	1	4	5
Wt	19.46	88.63	108.09
Het Chi	2.93	30.22	50.51
Het df	1	7	9
Het P	(*)	***	***
Fixed RR	4.42	12.55	10.40
RRl	2.83	10.19	8.61
RRu	6.89	15.45	12.56
P	+++	+++	+++
Random RR	3.93	9.10	7.26
RRl	1.71	5.39	4.35
RRu	9.00	15.36	12.13
P	++	+++	+++
Between Chi			17.36
Between df			1
Between P			***
Btwn(F) P			(*)
Btwn(R) P			(*)

	Study type (2) CC	prosp	other	Total
N	2	8		10
NS	1	4		5
Wt	19.46	88.63		108.09
Het Chi	2.93	30.22		50.51
Het df	1	7		9
Het P	(*)	***		***
Fixed RR	4.42	12.55		10.40
RRl	2.83	10.19		8.61
RRu	6.89	15.45		12.56
P	+++	+++		+++
Random RR	3.93	9.10		7.26
RRl	1.71	5.39		4.35
RRu	9.00	15.36		12.13
P	++	+++		+++
Between Chi				17.36
Between df				1
Between P				***
Btwn(F) P				(*)
Btwn(R) P				(*)

Table 1H24 - 3

IESLC - Meta-analysis of Ever/current Smoking, Age started, "High"
All LC types, Cigarettes only
Most adjusted

	Study size (number of LC cases) 100-249	250-499	500-999	1000+	Total
N		4	2	4	10
NS		2	1	2	5
Wt		9.08	19.46	79.56	108.09
Het Chi		3.42	2.93	23.17	50.51
Het df		3	1	3	9
Het P		N.S.	(*)	***	***
Fixed RR		6.90	4.42	13.43	10.40
RRl		3.60	2.83	10.78	8.61
RRu		13.22	6.89	16.73	12.56
P		+++	+++	+++	+++
Random RR		6.71	3.93	10.90	7.26
RRl		3.27	1.71	5.70	4.35
RRu		13.75	9.00	20.84	12.13
P		+++	++	+++	+++
Between Chi					20.98
Between df					2
Between P					***
Btwn(F) P					N.S.
Btwn(R) P					N.S.

	Risky occupational population no	mining	othRisky	Total
N	10			10
NS	5			5
Wt	108.09			108.09
Het Chi	50.51			50.51
Het df	9			9
Het P	***			***
Fixed RR	10.40			10.40
RRl	8.61			8.61
RRu	12.56			12.56
P	+++			+++
Random RR	7.26			7.26
RRl	4.35			4.35
RRu	12.13			12.13
P	+++			+++
Between Chi				
Between df				
Between P				N.S.
Btwn(F) P				N.S.
Btwn(R) P				N.S.

	National cigarette tobacco type Virginia	blended	other	Total
N	4	6		10
NS	2	3		5
Wt	24.53	83.56		108.09
Het Chi	5.19	29.58		50.51
Het df	3	5		9
Het P	N.S.	***		***
Fixed RR	5.14	12.79		10.40
RRl	3.46	10.32		8.61
RRu	7.64	15.84		12.56
P	+++	+++		+++
Random RR	5.18	8.96		7.26
RRl	2.88	4.85		4.35
RRu	9.32	16.57		12.13
P	+++	+++		+++
Between Chi				15.74
Between df				1
Between P				***
Btwn(F) P				(*)
Btwn(R) P				N.S.

Table 1H24 - 3

IESLC - Meta-analysis of Ever/current Smoking, Age started, "High"
All LC types, Cigarettes only
Most adjusted

	Any proxy use No/nk	Yes	Total
N	8	2	10
NS	4	1	5
Wt	88.63	19.46	108.09
Het Chi	30.22	2.93	50.51
Het df	7	1	9
Het P	***	(*)	***
Fixed RR	12.55	4.42	10.40
RRl	10.19	2.83	8.61
RRu	15.45	6.89	12.56
P	+++	+++	+++
Random RR	9.10	3.93	7.26
RRl	5.39	1.71	4.35
RRu	15.36	9.00	12.13
P	+++	++	+++
Between Chi			17.36
Between df			1
Between P			***
Btwn(F) P			(*)
Btwn(R) P			(*)

	Full histological confirmation No	Yes	Total
N	10		10
NS	5		5
Wt	108.09		108.09
Het Chi	50.51		50.51
Het df	9		9
Het P	***		***
Fixed RR	10.40		10.40
RRl	8.61		8.61
RRu	12.56		12.56
P	+++		+++
Random RR	7.26		7.26
RRl	4.35		4.35
RRu	12.13		12.13
P	+++		+++
Between Chi			
Between df			
Between P			N.S.
Btwn(F) P			N.S.
Btwn(R) P			N.S.

	Number of adjustment variables (1) 0	1	2+/+nk	Total
N	7	3		10
NS	4	2		6
Wt	65.82	42.27		108.09
Het Chi	33.23	17.08		50.51
Het df	6	2		9
Het P	***	***		***
Fixed RR	10.04	10.98		10.40
RRl	7.88	8.13		8.61
RRu	12.78	14.85		12.56
P	+++	+++		+++
Random RR	7.43	6.48		7.26
RRl	3.82	1.95		4.35
RRu	14.46	21.54		12.13
P	+++	++		+++
Between Chi				0.21
Between df				1
Between P				N.S.
Btwn(F) P				N.S.
Btwn(R) P				N.S.

Table 1H24 - 3

IESLC - Meta-analysis of Ever/current Smoking, Age started, "High"
All LC types, Cigarettes only
Most adjusted

	Number of adjustment variables (2) 0	1	2	3-5	6+/+nk	Total
N	7	3				10
NS	4	2				6
Wt	65.82	42.27				108.09
Het Chi	33.23	17.08				50.51
Het df	6	2				9
Het P	***	***				***
Fixed RR	10.04	10.98				10.40
RRl	7.88	8.13				8.61
RRu	12.78	14.85				12.56
P	+++	+++				+++
Random RR	7.43	6.48				7.26
RRl	3.82	1.95				4.35
RRu	14.46	21.54				12.13
P	+++	++				+++
Between Chi						0.21
Between df						1
Between P						N.S.
Btwn(F) P						N.S.
Btwn(R) P						N.S.

	Smoking status ever	current	Total
N	2	8	10
NS	1	4	5
Wt	40.80	67.29	108.09
Het Chi	5.46	34.53	50.51
Het df	1	7	9
Het P	*	***	***
Fixed RR	15.52	8.16	10.40
RRl	11.42	6.42	8.61
RRu	21.10	10.36	12.56
P	+++	+++	+++
Random RR	16.19	5.38	7.26
RRl	7.85	2.82	4.35
RRu	33.38	10.27	12.13
P	+++	+++	+++
Between Chi			10.52
Between df			1
Between P			**
Btwn(F) P			N.S.
Btwn(R) P			*

	Denominator nev any	nev cigs	Total
N	10		10
NS	5		5
Wt	108.09		108.09
Het Chi	50.51		50.51
Het df	9		9
Het P	***		***
Fixed RR	10.40		10.40
RRl	8.61		8.61
RRu	12.56		12.56
P	+++		+++
Random RR	7.26		7.26
RRl	4.35		4.35
RRu	12.13		12.13
P	+++		+++
Between Chi			
Between df			
Between P			N.S.
Btwn(F) P			N.S.
Btwn(R) P			N.S.

Table 1H24 - 3

IESLC - Meta-analysis of Ever/current Smoking, Age started, "High"
All LC types, Cigarettes only
Most adjusted

	Derivation of RR/CI Orig	StdCalc	Other	Total
N		6	4	10
NS		3	2	5
Wt		65.33	42.76	108.09
Het Chi		29.36	21.12	50.51
Het df		5	3	9
Het P		***	***	***
Fixed RR		10.25	10.63	10.40
RRl		8.04	7.88	8.61
RRu		13.06	14.34	12.56
P		+++	+++	+++
Random RR		8.38	4.88	7.26
RRl		4.35	1.48	4.35
RRu		16.12	16.16	12.13
P		+++	++	+++
Between Chi				0.03
Between df				1
Between P				N.S.
Btwn(F) P				N.S.
Btwn(R) P				N.S.

Table 1H24 - 4

IESLC - Meta-analysis of Ever/current Smoking, Age started, "High"
All LC types, Cigarettes only
Least adjusted

REF	NRR	X	SEX	AGEL	AGEH	RACE	YF	LC TYPE	LOC	START	ST	NLC	R	VB	P	H	AD	SM	PRODUCT	exL	exH	DENOM	De
CEDERL	512		m	0	0	all	10	all	Eu:Sca	1963	pr	491	n	bl	n	n	1	cu	cig only	1	16	nev any	ot
CEDERL	517		f	0	0	all	10	all	Eu:Sca	1963	pr	491	n	bl	n	n	0	cu	cig only	1	16	nev any	ot
CPSI	895		m	35	84	all	4	all	NAmer	1959	pr	5138	n	bl	n	n	1	cu	cig only	1	14	nev any	ot
CPSI	848		f	40	74	all	6	all	NAmer	1959	pr	5138	n	bl	n	n	1	cu	cig only	1	14	nev any	ot
DEAN3	567		m	0	0	all	-	all	Eu:UK	1969	CC	766	n	V	y	n	0	cu	cig only	1	14	nev any	st
DEAN3	586		f	0	0	all	-	all	Eu:UK	1969	CC	766	n	V	y	n	0	cu	cig only	1	14	nev any	st
DORN	733		m	55	64	wh	8	all	NAmer	1954	pr	5097	n	bl	n	n	0	ev	cig only	1	14	nev any	st
DORN	770		m	65	74	wh	8	all	NAmer	1954	pr	5097	n	bl	n	n	0	ev	cig only	1	14	nev any	st
MIGRAN	505		m	0	0	all	0	all	Eu:UK	1964	pr	259	n	V	n	n	0	cu	cig only	0	15	nev any	st
MIGRAN	515		f	0	0	all	0	all	Eu:UK	1964	pr	259	n	V	n	n	0	cu	cig only	0	15	nev any	st

Cigarette type is all/unspec for all RRs
except for the following:

REF	NRR	CIGTYPE
DEAN3	567	MC only
DEAN3	586	MC only

Table 1H24 - 5

IESLC - Meta-analysis of Ever/current Smoking, Age started, "High"
All LC types, Cigarettes only
Least adjusted

REF	NRR	SEX	AD	Number Case	Exposed Cont	Non-exposed Case	Cont	RR	95.00%CI
*CEDERL	512	m	1	7	-	7	-	6.40	(2.25- 18.21)
*CEDERL	517	f	0	0	746	19	17679	0.61~	(0.04- 10.05)
Subtotal CEDERL								4.80	(1.80- 12.79)
*CPSI	895	m	1	101	-	49	-	15.10	(10.73- 21.24)
*CPSI	848	f	1	6	-	166	-	2.50	(1.11- 5.65)
Subtotal CPSI								11.54	(8.42- 15.80)
DEAN3	567	m	0	44	165	24	510	5.67	(3.34- 9.60)
DEAN3	586	f	0	7	109	41	1538	2.41	(1.06- 5.50)
Subtotal DEAN3								4.42	(2.83- 6.89)
*DORN	733	m	0	59	21349	25	213858	23.64	(14.81- 37.73)
*DORN	770	m	0	44	13616	49	171211	11.29	(7.52- 16.96)
Subtotal DORN								15.52	(11.42- 21.10)
*MIGRAN	505	m	0	50	1081	4	867	10.03	(3.64- 27.65)
*MIGRAN	515	f	0	2	266	4	3814	7.17	(1.32- 38.96)
Subtotal MIGRAN								9.18	(3.84- 21.90)
Partial Totals				320	37332	388	409477		

*prospective study ~ With 0.5 adjustment for zero

REF	NRR	SEX	AD	Ys	Ws	Qs	Ps
*CEDERL	512	m	1	1.86	3.51	0.83	0.0005
*CEDERL	517	f	0	-0.50	0.49	3.94	0.7276
Subtotal CEDERL				1.57	4.00	4.76	
*CPSI	895	m	1	2.71	32.95	4.59	0.0000
*CPSI	848	f	1	0.92	5.80	11.79	0.0273
Subtotal CPSI				2.45	38.76	16.37	
DEAN3	567	m	0	1.73	13.81	5.09	0.0000
DEAN3	586	f	0	0.88	5.65	12.08	0.0367
Subtotal DEAN3				1.49	19.46	17.17	
*DORN	733	m	0	3.16	17.58	11.85	0.0000
*DORN	770	m	0	2.42	23.23	0.16	0.0000
Subtotal DORN				2.74	40.80	12.01	
*MIGRAN	505	m	0	2.31	3.73	0.00	0.0000
*MIGRAN	515	f	0	1.97	1.34	0.19	0.0226
Subtotal MIGRAN				2.22	5.07	0.19	

	N	10
	NS	5
	Wt	108.09
Het	Chi	50.51
Het	df	9
Het	P	***
Fixed	RR	10.40
	RRl	8.61
	RRu	12.56
	P	+++
Random	RR	7.26
	RRl	4.35
	RRu	12.13
	P	+++
Asymm	P	(*)

Table 1H24 - 6

IESLC - Meta-analysis of Ever/current Smoking, Age started, "High"
All LC types, Cigarettes only
Least adjusted

	Sex combined	male	female	Total
N		6	4	10
NS		5	4	9
Wt		94.81	13.28	108.09
Het Chi		18.94	2.45	50.51
Het df		5	3	9
Het P		**	N.S.	***
Fixed RR		12.63	2.60	10.40
RRl		10.33	1.52	8.61
RRu		15.44	4.45	12.56
P		+++	+++	+++
Random RR		11.41	2.60	7.26
RRl		7.42	1.52	4.35
RRu		17.55	4.45	12.13
P		+++	+++	+++
Between Chi				29.11
Between df				1
Between P				***
Btwn(F) P				*
Btwn(R) P				***

Table 1H24 - 7

IESLC - Meta-analysis of Ever/current Smoking, Age started, "High"
All LC types, Cigarettes only
Excluded studies (and stage at which they were excluded)

1	BECHER BLOT1 BROWN3 CARPEN CHYOU DARBY DOLL2 GARCIA GRAHAM GURSEL HAMMO2 JAHN JAIN LAUSSM PRESCO QIAO TVERDA WIGLE WYNDE3
2	AKIBA AMANDU AMES AXELSS BENSHL BEST BOUCHA BOUCOT BROSS CHEN CPSII DEAN2 DESTEF DORGAN DOSEME FAN GARSHI GER GILLIS HAMMON HUMBLE JUSSAW KAISE2 KATSOU KAUFMA KOO KREUZE LEVIN MCCONN NOTAN2 OSANN2 PEZZO2 PISANI RESTRE SADOWS VUTUC WANG2 WATSON WU2 WUWILL WYNDE2 WYNDE8 XU ZHOU
3	GUO MCDUFF SPITZ STASZE ZHANG
4	LUO
5	CORREA HOLE YUAN
7	ARMADA AUVINE BARBON BOFFET BRESLO BUFFLE CHEN2 CHIAZZ CHOI DAMBER DOLL ENGELA GAO GAO2 GENG HAENSZ HEGMAN HIRAYA HU HU2 JEDRYC JOLY KHUDER KOULUM LETOUR LIAW LIU3 LIU4 LIU5 LUBIN LUBIN2 MATOS MRFITR PERNU QIAO2 RACHTA SEGI2 SOBUE SPEIZE SUZUK2 SVENSS WAKAI WU WYNDE6 WYNDE7 ZHENG
10	ALDERS
14	AGUDO BENHAM PEZZOT TIZZAN

Table 1H24 - 8
Potentially overlapping studies

REF	REFGP	PRINC	.	OVERLAP/LINK
CPSI	CPSI	1		CPSI overall

Table 1H25 -

IESLC - Meta-analysis of Ever/current Smoking, Age started, "Highest vs lowest"
All LC types, Cigarettes only

This analysis is restricted to results for:
1) Ever/current smokers
2) Results by Age started
3) Categorical results by Age started
4) Denominator (unexposed) = "low"
5) All LC types (or near equivalent)
6) Results complete enough for use in metaanalysis

Within each study, results are then selected (in the following order of preference, within each sex) for:
7) SMKSTA: ever, current
8) PRODUCT: cigarettes only
9) CIGTYPE: all/unspecified, MC regardless of HR, MC only
10) Results with least adjustment for other aspects of smoking (ADOS)
11) The highest vs lowest category
12) Followup period (YF, prospective studies): whole study (coded as 0) or longest available
13) LCtype: all or nearest available, at least Squamous and Adeno. (q = squamous, s = small, l = large, a = adeno, mix = mixed, alv = alveolar)
14) Race: all or nearest available, otherwise by race (wh or w = white, bl or b = black, hi = hispanic ch = chinese, jap = japanese, haw = hawaiian, w+o = white + oriental, sca = scandinavian, as = asian)
15) For overlapping studies: principal rather than subsidiary studies
Finally by Age: whole study (coded as 0) if available, otherwise by widest available age group and then for single sex results (m, f) in preference to results for both sexes combined (c).

Results adjusted (AD) for the most potential confounders are then chosen in Sections -1 to -3 (and those which actually differ from the adjusted results in Table 1H15 - 1 are marked 'x' in Section -1) and results adjusted for the least confounders in Sections -4 to -6. (Those least adjusted results which actually differ from the most adjusted are marked 'x' in column X in Section -4)

Section -7 shows excluded studies, together with the stage (as above) at which no qualifying results were found.

Section -8 lists the potentially overlapping studies which have been included (1=principal, 2=subsidiary).

Section -9 lists any results which would have been included in preference except that they had data not complete enough for use in meta-analysis, with their significance (yes/no), if known, and any further comment as entered on the database. It also lists as "gap" any categories for which no data were presented by the original authors.

In addition to those mentioned above, the following fields, levels and abbreviations are used:
* or nk = not known, n = no, y = yes, ot = other
all/unspec = all or unspecified, MC = manufactured cigarettes, HR = hand-rolled cigarettes
exL, exH = range of exposure (low and high) in the "highest" group, in terms of Age started
unexL, unexH = range of exposure (low and high) in the "lowest" group, in terms of Age started
REF: 6-character study reference
NRR: number of the RR on the database within the study
ST : study type (CC = case control, pr or prosp = prospective)
NLC: number of lung cancer cases in whole study
R : risky occupational population (n = no, m = mining, o = other risky)
VB : national cigarette type (V = at least 75% Virginia, bl = at least 75% blended, ot = other)
P : any proxy use
H : full histological confirmation
De : derivation of RR/CI (or = original, st = standard method, ot = other method of estimation)

Table 1H25 - 1

IESLC - Meta-analysis of Ever/current Smoking, Age started, "Highest vs lowest"
All LC types, Cigarettes only
Most adjusted

REF	NRR	1H15	SEX	AGEL	AGEH	RACE	YF	LC TYPE	LOC	START	ST	NLC	R	VB	P	H	AD	ADOS	SM	PRODUCT	exL	exH	unexL	unexH	De
AGUDO	506		f	0	0	all	-	all	Eu:wst	1989	CC	103	n	bl	n	n	3	0	ev	cig only	1	23	24	999	ot
ALDERS	503		m	0	0	all	-	all	Eu:UK	1977	CC	1448	n	V	n	n	2	1#	ev	cig only	1	14	25	999	ot
ALDERS	506		f	0	0	all	-	all	Eu:UK	1977	CC	1448	n	V	n	n	2	1#	ev	cig only	1	14	25	999	ot
BENHAM	642	x	m	0	0	all	-	all	Eu:wst	1976	CC	1625	n	bl	n	y	3	0	ev	cig only	1	19	20	999	ot
CEDERL	514	x	m	0	0	all	10	all	Eu:Sca	1963	pr	491	n	bl	n	n	1	0	cu	cig only	1	16	19	999	ot
CEDERL	536		f	0	0	all	10	all	Eu:Sca	1963	pr	491	n	bl	n	n	0	0	cu	cig only	1	16	19	999	ot
CPSI	898	x	m	35	84	all	4	all	NAmer	1959	pr	5138	n	bl	n	n	1	0	cu	cig only	1	14	25	999	ot
CPSI	851		f	40	74	all	6	all	NAmer	1959	pr	5138	n	bl	n	n	1	0	cu	cig only	1	14	25	999	ot
DEAN3	570		m	0	0	all	-	all	Eu:UK	1969	CC	766	n	V	y	n	0	0	cu	cig only	1	14	25	999	st
DEAN3	589		f	0	0	all	-	all	Eu:UK	1969	CC	766	n	V	y	n	0	0	cu	cig only	1	14	25	999	st
DORN	736	x	m	55	64	wh	8	all	NAmer	1954	pr	5097	n	bl	n	n	0	0	ev	cig only	1	14	25	999	st
DORN	773	x	m	65	74	wh	8	all	NAmer	1954	pr	5097	n	bl	n	n	0	0	ev	cig only	1	14	25	999	st
MIGRAN	509		m	0	0	all	0	all	Eu:UK	1964	pr	259	n	V	n	n	0	0	cu	cig only	0	15	20	999	st
MIGRAN	519		f	0	0	all	0	all	Eu:UK	1964	pr	259	n	V	n	n	0	0	cu	cig only	0	15	20	999	st
PEZZOT	576		m	0	0	all	-	all	SCAmer	1987	CC	215	n	bl	n	y	2	0	ev	cig only	1	13	19	999	ot
TIZZAN	540	x	f	0	0	all	-	all	Eu:wst	1959	CC	1358	n	bl	n	n	0	0	ev	cig only	1	19	31	999	st

Comments on values in listings

ALDERS ADOS Number of cigs/day
ALDERS ADOS Number of cigs/day

Cigarette type is all/unspec for all RRs
except for the following:

REF	NRR	CIGTYPE
ALDERS	503	MC only
ALDERS	506	MC only
DEAN3	570	MC only
DEAN3	589	MC only

Table 1H25 - 2

IESLC - Meta-analysis of Ever/current Smoking, Age started, "Highest vs lowest"
All LC types, Cigarettes only
Most adjusted

REF	NRR	SEX	AD	Number Exposed Case	Cont	Non-exposed Case	Cont	RR	95.00%CI
AGUDO	506	f	3	16	-	7	-	6.81	(1.20- 38.56)
ALDERS	503	m	2	139	-	11	-	2.94	(1.38- 6.25)
ALDERS	506	f	2	78	-	97	-	2.08	(1.27- 3.41)
Subtotal ALDERS								2.31	(1.53- 3.49)
BENHAM	642	m	3	-	-	-	-	1.20	(1.03- 1.41)
*CEDERL	514	m	1	7	-	11	-	0.98	(0.40- 2.45)
*CEDERL	536	f	0	0	746	6	2806	0.29~	(0.02- 5.13)
Subtotal CEDERL								0.88	(0.37- 2.08)
*CPSI	898	m	1	101	-	20	-	4.70	(2.91- 7.60)
*CPSI	851	f	1	6	-	51	-	1.11	(0.48- 2.59)
Subtotal CPSI								3.30	(2.17- 5.01)
DEAN3	570	m	0	44	165	24	75	0.83	(0.47- 1.47)
DEAN3	589	f	0	7	109	27	274	0.65	(0.28- 1.54)
Subtotal DEAN3								0.77	(0.48- 1.24)
*DORN	736	m	0	59	21349	25	40397	4.47	(2.80- 7.13)
*DORN	773	m	0	44	13616	38	38849	3.30	(2.14- 5.10)
Subtotal DORN								3.80	(2.76- 5.22)
*MIGRAN	509	m	0	50	1081	24	668	1.29	(0.80- 2.07)
*MIGRAN	519	f	0	2	266	11	1315	0.90	(0.20- 4.03)
Subtotal MIGRAN								1.25	(0.79- 1.96)
PEZZOT	576	m	2	52	-	41	-	2.00	(1.20- 3.33)
TIZZAN	540	f	0	11	2	2	5	13.75	(1.48- 127.47)
Partial Totals				616	37334	395	84389		

*prospective study ~ With 0.5 adjustment for zero

REF	NRR	SEX	AD	Ys	Ws	Qs	Ps
AGUDO	506	f	3	1.92	1.28	2.69	0.0302
ALDERS	503	m	2	1.08	6.73	2.52	0.0051
ALDERS	506	f	2	0.73	15.75	1.12	0.0037
Subtotal ALDERS				0.84	22.49	3.64	
BENHAM	642	m	3	0.18	155.82	12.56	0.0229
*CEDERL	514	m	1	-0.02	4.68	1.11	0.9651
*CEDERL	536	f	0	-1.24	0.46	1.35	0.3977
Subtotal CEDERL				-0.13	5.14	2.46	
*CPSI	898	m	1	1.55	16.67	19.50	0.0000
*CPSI	851	f	1	0.10	5.41	0.71	0.8082
Subtotal CPSI				1.19	22.08	20.20	
DEAN3	570	m	0	-0.18	11.93	5.02	0.5288
DEAN3	589	f	0	-0.43	5.19	4.15	0.3294
Subtotal DEAN3				-0.26	17.12	9.17	
*DORN	736	m	0	1.50	17.58	18.66	0.0000
*DORN	773	m	0	1.20	20.43	10.85	0.0000
Subtotal DORN				1.33	38.01	29.51	
*MIGRAN	509	m	0	0.25	16.88	0.77	0.2993
*MIGRAN	519	f	0	-0.11	1.71	0.56	0.8892
Subtotal MIGRAN				0.22	18.58	1.33	
PEZZOT	576	m	2	0.69	14.75	0.76	0.0078
TIZZAN	540	f	0	2.62	0.77	3.60	0.0211

	N	16
	NS	10
	Wt	296.05
Het	Chi	85.92
Het	df	15
Het	P	***
Fixed	RR	1.59
	RRl	1.42
	RRu	1.79
	P	+++
Random	RR	1.86
	RRl	1.31
	RRu	2.65
	P	+++
Asymm	P	N.S.

Table 1H25 - 3

IESLC - Meta-analysis of Ever/current Smoking, Age started, "Highest vs lowest"
All LC types, Cigarettes only
Most adjusted

	Sex			
	combined	male	female	Total
N		9	7	16
NS		8	7	15
Wt		265.48	30.57	296.05
Het Chi		71.75	14.16	85.92
Het df		8	6	15
Het P		***	*	***
Fixed RR		1.60	1.56	1.59
RRl		1.42	1.09	1.42
RRu		1.80	2.22	1.79
P		+++	+	+++
Random RR		2.03	1.54	1.86
RRl		1.30	0.78	1.31
RRu		3.15	3.02	2.65
P		++	N.S.	+++
Between Chi				0.02
Between df				1
Between P				N.S.
Btwn(F) P				N.S.
Btwn(R) P				N.S.

	Lung cancer type		
	all	other	Total
N	16		16
NS	10		10
Wt	296.05		296.05
Het Chi	85.92		85.92
Het df	15		15
Het P	***		***
Fixed RR	1.59		1.59
RRl	1.42		1.42
RRu	1.79		1.79
P	+++		+++
Random RR	1.86		1.86
RRl	1.31		1.31
RRu	2.65		2.65
P	+++		+++
Between Chi			
Between df			
Between P			N.S.
Btwn(F) P			N.S.
Btwn(R) P			N.S.

	Location								
	NAmer	UK	Scand	othEur	China	Japan	othAs	other	Total
N	4	6	2	3				1	16
NS	2	3	1	3				1	10
Wt	60.09	58.19	5.14	157.87				14.75	296.05
Het Chi	9.64	12.85	0.63	8.35				0.00	85.92
Het df	3	5	1	2				0	15
Het P	*	*	N.S.	*				N.S.	***
Fixed RR	3.61	1.37	0.88	1.23				2.00	1.59
RRl	2.80	1.06	0.37	1.05				1.20	1.42
RRu	4.64	1.78	2.08	1.44				3.33	1.79
P	+++	+	N.S.	++				++	+++
Random RR	3.28	1.33	0.88	3.71				2.00	1.86
RRl	2.04	0.85	0.37	0.71				1.20	1.31
RRu	5.25	2.06	2.08	19.27				3.33	2.65
P	+++	N.S.	N.S.	N.S.				++	+++
Between Chi									54.45
Between df									4
Between P									***
Btwn(F) P									*
Btwn(R) P									*

Table 1H25 - 3

IESLC - Meta-analysis of Ever/current Smoking, Age started, "Highest vs lowest"
All LC types, Cigarettes only
Most adjusted

	Detailed Country in "other Europe" multi	Germany	othWest	East	Balkans	Total
N			3			3
NS			3			3
Wt			157.87			157.87
Het Chi			8.35			8.35
Het df			2			2
Het P			*			*
Fixed RR			1.23			1.23
RRl			1.05			1.05
RRu			1.44			1.44
P			++			++
Random RR			3.71			3.71
RRl			0.71			0.71
RRu			19.27			19.27
P			N.S.			N.S.
Between Chi						
Between df						
Between P						N.S.
Btwn(F) P						N.S.
Btwn(R) P						N.S.

	Detailed Country in "other Asia" India	HongKong	other	Total
N				
NS				
Wt				
Het Chi				
Het df				
Het P				
Fixed RR				
RRl				
RRu				
P				
Random RR				
RRl				
RRu				
P				
Between Chi				
Between df				
Between P				N.S.
Btwn(F) P				N.S.
Btwn(R) P				N.S.

	Detailed other continent SCAmer	Total
N	1	1
NS	1	1
Wt	14.75	14.75
Het Chi	0.00	0.00
Het df	0	0
Het P	N.S.	N.S.
Fixed RR	2.00	2.00
RRl	1.20	1.20
RRu	3.33	3.33
P	++	++
Random RR	2.00	2.00
RRl	1.20	1.20
RRu	3.33	3.33
P	++	++
Between Chi		
Between df		
Between P		N.S.
Btwn(F) P		N.S.
Btwn(R) P		N.S.

Table 1H25 - 3

IESLC - Meta-analysis of Ever/current Smoking, Age started, "Highest vs lowest"
All LC types, Cigarettes only
Most adjusted

	Start year of study					
	<1960	1960-69	1970-79	1980-89	1990+	Total
N	5	6	3	2		16
NS	3	3	2	2		10
Wt	60.87	40.85	178.30	16.03		296.05
Het Chi	11.01	3.14	8.96	1.76		85.92
Het df	4	5	2	1		15
Het P	*	N.S.	*	N.S.		***
Fixed RR	3.67	0.98	1.30	2.20		1.59
RRl	2.85	0.72	1.13	1.35		1.42
RRu	4.72	1.33	1.51	3.60		1.79
P	+++	N.S.	+++	++		+++
Random RR	3.46	0.98	1.78	2.76		1.86
RRl	2.17	0.72	1.03	0.96		1.31
RRu	5.52	1.33	3.07	7.92		2.65
P	+++	N.S.	+	(+)		+++
Between Chi						61.05
Between df						3
Between P						***
Btwn(F) P						**
Btwn(R) P						***

	Study type (1)		
	CC	other	Total
N	8	8	16
NS	6	4	10
Wt	212.23	83.82	296.05
Het Chi	24.33	33.03	85.92
Het df	7	7	15
Het P	***	***	***
Fixed RR	1.31	2.61	1.59
RRl	1.15	2.11	1.42
RRu	1.50	3.24	1.79
P	+++	+++	+++
Random RR	1.63	2.01	1.86
RRl	1.09	1.19	1.31
RRu	2.42	3.40	2.65
P	+	++	+++
Between Chi			28.56
Between df			1
Between P			***
Btwn(F) P			*
Btwn(R) P			N.S.

	Study type (2)			
	CC	prosp	other	Total
N	8	8		16
NS	6	4		10
Wt	212.23	83.82		296.05
Het Chi	24.33	33.03		85.92
Het df	7	7		15
Het P	***	***		***
Fixed RR	1.31	2.61		1.59
RRl	1.15	2.11		1.42
RRu	1.50	3.24		1.79
P	+++	+++		+++
Random RR	1.63	2.01		1.86
RRl	1.09	1.19		1.31
RRu	2.42	3.40		2.65
P	+	++		+++
Between Chi				28.56
Between df				1
Between P				***
Btwn(F) P				*
Btwn(R) P				N.S.

Table 1H25 - 3

IESLC - Meta-analysis of Ever/current Smoking, Age started, "Highest vs lowest"
All LC types, Cigarettes only
Most adjusted

	Study size (number of LC cases) 100-249	250-499	500-999	1000+	Total
N	2	4	2	8	16
NS	2	2	1	5	10
Wt	16.03	23.73	17.12	239.17	296.05
Het Chi	1.76	1.32	0.22	68.59	85.92
Het df	1	3	1	7	15
Het P	N.S.	N.S.	N.S.	***	***
Fixed RR	2.20	1.15	0.77	1.70	1.59
RRl	1.35	0.77	0.48	1.49	1.42
RRu	3.60	1.73	1.24	1.93	1.79
P	++	N.S.	N.S.	+++	+++
Random RR	2.76	1.15	0.77	2.68	1.86
RRl	0.96	0.77	0.48	1.57	1.31
RRu	7.92	1.73	1.24	4.57	2.65
P	(+)	N.S.	N.S.	+++	+++
Between Chi					14.03
Between df					3
Between P					**
Btwn(F) P					N.S.
Btwn(R) P					**

	Risky occupational population no	mining	othRisky	Total
N	16			16
NS	10			10
Wt	296.05			296.05
Het Chi	85.92			85.92
Het df	15			15
Het P	***			***
Fixed RR	1.59			1.59
RRl	1.42			1.42
RRu	1.79			1.79
P	+++			+++
Random RR	1.86			1.86
RRl	1.31			1.31
RRu	2.65			2.65
P	+++			+++
Between Chi				
Between df				
Between P				N.S.
Btwn(F) P				N.S.
Btwn(R) P				N.S.

	National cigarette tobacco type Virginia	blended	other	Total
N	6	10		16
NS	3	7		10
Wt	58.19	237.85		296.05
Het Chi	12.85	71.47		85.92
Het df	5	9		15
Het P	*	***		***
Fixed RR	1.37	1.65		1.59
RRl	1.06	1.46		1.42
RRu	1.78	1.88		1.79
P	+	+++		+++
Random RR	1.33	2.37		1.86
RRl	0.85	1.41		1.31
RRu	2.06	3.98		2.65
P	N.S.	++		+++
Between Chi				1.60
Between df				1
Between P				N.S.
Btwn(F) P				N.S.
Btwn(R) P				(*)

Table 1H25 - 3

IESLC - Meta-analysis of Ever/current Smoking, Age started, "Highest vs lowest"
All LC types, Cigarettes only
Most adjusted

	Any proxy use No/nk	Yes	Total
N	14	2	16
NS	9	1	10
Wt	278.92	17.12	296.05
Het Chi	76.20	0.22	85.92
Het df	13	1	15
Het P	***	N.S.	***
Fixed RR	1.67	0.77	1.59
RRl	1.48	0.48	1.42
RRu	1.87	1.24	1.79
P	+++	N.S.	+++
Random RR	2.15	0.77	1.86
RRl	1.47	0.48	1.31
RRu	3.15	1.24	2.65
P	+++	N.S.	+++
Between Chi			9.50
Between df			1
Between P			**
Btwn(F) P			N.S.
Btwn(R) P			***

	Full histological confirmation No	Yes	Total
N	14	2	16
NS	8	2	10
Wt	125.48	170.57	296.05
Het Chi	59.29	3.52	85.92
Het df	13	1	15
Het P	***	(*)	***
Fixed RR	2.21	1.25	1.59
RRl	1.85	1.08	1.42
RRu	2.63	1.46	1.79
P	+++	++	+++
Random RR	1.94	1.46	1.86
RRl	1.28	0.90	1.31
RRu	2.94	2.37	2.65
P	++	N.S.	+++
Between Chi			23.12
Between df			1
Between P			***
Btwn(F) P			*
Btwn(R) P			N.S.

	Number of adjustment variables (1) 0	1	2+/+nk	Total
N	8	3	5	16
NS	5	2	4	11
Wt	74.96	26.76	194.33	296.05
Het Chi	41.15	14.20	14.79	85.92
Het df	7	2	4	15
Het P	***	***	**	***
Fixed RR	2.00	2.67	1.36	1.59
RRl	1.59	1.83	1.18	1.42
RRu	2.50	3.90	1.57	1.79
P	+++	+++	+++	+++
Random RR	1.65	1.81	1.97	1.86
RRl	0.87	0.59	1.25	1.31
RRu	3.14	5.59	3.08	2.65
P	N.S.	N.S.	++	+++
Between Chi				15.79
Between df				2
Between P				***
Btwn(F) P				N.S.
Btwn(R) P				N.S.

Table 1H25 - 3

IESLC - Meta-analysis of Ever/current Smoking, Age started, "Highest vs lowest"
All LC types, Cigarettes only
Most adjusted

	Number of adjustment variables (2) 0	1	2	3-5	6+/+nk	Total
N	8	3	3	2		16
NS	5	2	2	2		11
Wt	74.96	26.76	37.24	157.09		296.05
Het Chi	41.15	14.20	0.75	3.82		85.92
Het df	7	2	2	1		15
Het P	***	***	N.S.	(*)		***
Fixed RR	2.00	2.67	2.18	1.22		1.59
RRl	1.59	1.83	1.58	1.04		1.42
RRu	2.50	3.90	3.01	1.42		1.79
P	+++	+++	+++	+		+++
Random RR	1.65	1.81	2.18	2.29		1.86
RRl	0.87	0.59	1.58	0.44		1.31
RRu	3.14	5.59	3.01	11.83		2.65
P	N.S.	N.S.	+++	N.S.		+++
Between Chi						26.02
Between df						3
Between P						***
Btwn(F) P						N.S.
Btwn(R) P						N.S.

	Smoking status ever	current	Total
N	8	8	16
NS	6	4	10
Wt	233.12	62.93	296.05
Het Chi	52.69	32.92	85.92
Het df	7	7	15
Het P	***	***	***
Fixed RR	1.62	1.50	1.59
RRl	1.43	1.17	1.42
RRu	1.84	1.92	1.79
P	+++	++	+++
Random RR	2.73	1.18	1.86
RRl	1.66	0.65	1.31
RRu	4.50	2.15	2.65
P	+++	N.S.	+++
Between Chi			0.32
Between df			1
Between P			N.S.
Btwn(F) P			N.S.
Btwn(R) P			*

	Derivation of RR/CI Orig	StdCalc	Other	Total
N		7	9	16
NS		4	6	10
Wt		74.50	221.55	296.05
Het Chi		39.40	40.90	85.92
Het df		6	8	15
Het P		***	***	***
Fixed RR		2.02	1.47	1.59
RRl		1.61	1.29	1.42
RRu		2.54	1.68	1.79
P		+++	+++	+++
Random RR		1.78	1.94	1.86
RRl		0.93	1.24	1.31
RRu		3.41	3.04	2.65
P		(+)	++	+++
Between Chi				5.63
Between df				1
Between P				*
Btwn(F) P				N.S.
Btwn(R) P				N.S.

Table 1H25 - 4

IESLC - Meta-analysis of Ever/current Smoking, Age started, "Highest vs lowest"
All LC types, Cigarettes only
Least adjusted

REF	NRR	X	SEX	AGEL	AGEH	RACE	YF	LC TYPE	LOC	START	ST	NLC	R	VB	P	H	AD	ADOS	SM	PRODUCT	exL	exH	unexL	unexH	De
AGUDO	503	x	f	0	0	all	-	all	Eu:wst	1989	CC	103	n	bl	n	n	0	0	ev	cig only	1	23	24	999	st
ALDERS	503		m	0	0	all	-	all	Eu:UK	1977	CC	1448	n	V	n	n	2	1#	ev	cig only	1	14	25	999	ot
ALDERS	506		f	0	0	all	-	all	Eu:UK	1977	CC	1448	n	V	n	n	2	1#	ev	cig only	1	14	25	999	ot
BENHAM	642		m	0	0	all	-	all	Eu:wst	1976	CC	1625	n	bl	n	y	3	0	ev	cig only	1	19	20	999	ot
CEDERL	514		m	0	0	all	10	all	Eu:Sca	1963	pr	491	n	bl	n	n	1	0	cu	cig only	1	16	19	999	ot
CEDERL	536		f	0	0	all	10	all	Eu:Sca	1963	pr	491	n	bl	n	n	0	0	cu	cig only	1	16	19	999	ot
CPSI	898		m	35	84	all	4	all	NAmer	1959	pr	5138	n	bl	n	n	1	0	cu	cig only	1	14	25	999	ot
CPSI	851		f	40	74	all	6	all	NAmer	1959	pr	5138	n	bl	n	n	1	0	cu	cig only	1	14	25	999	ot
DEAN3	570		m	0	0	all	-	all	Eu:UK	1969	CC	766	n	V	y	n	0	0	cu	cig only	1	14	25	999	st
DEAN3	589		f	0	0	all	-	all	Eu:UK	1969	CC	766	n	V	y	n	0	0	cu	cig only	1	14	25	999	st
DORN	736		m	55	64	wh	8	all	NAmer	1954	pr	5097	n	bl	n	n	0	0	ev	cig only	1	14	25	999	st
DORN	773		m	65	74	wh	8	all	NAmer	1954	pr	5097	n	bl	n	n	0	0	ev	cig only	1	14	25	999	st
MIGRAN	509		m	0	0	all	0	all	Eu:UK	1964	pr	259	n	V	n	n	0	0	cu	cig only	0	15	20	999	st
MIGRAN	519		f	0	0	all	0	all	Eu:UK	1964	pr	259	n	V	n	n	0	0	cu	cig only	0	15	20	999	st
PEZZOT	574	x	m	0	0	all	-	all	SCAmer	1987	CC	215	n	bl	n	y	0	0	ev	cig only	1	13	19	999	st
TIZZAN	540		f	0	0	all	-	all	Eu:wst	1959	CC	1358	n	bl	n	n	0	0	ev	cig only	1	19	31	999	st

Comments on values in listings

ALDERS ADOS Number of cigs/day
ALDERS ADOS Number of cigs/day

Cigarette type is all/unspec for all RRs
except for the following:

REF	NRR	CIGTYPE
ALDERS	503	MC only
ALDERS	506	MC only
DEAN3	570	MC only
DEAN3	589	MC only

Table 1H25 - 5

IESLC - Meta-analysis of Ever/current Smoking, Age started, "Highest vs lowest"
All LC types, Cigarettes only
Least adjusted

REF	NRR	SEX	AD	Number Exposed Case	Exposed Cont	Non-exposed Case	Non-exposed Cont	RR	95.00%CI
AGUDO	503	f	0	16	11	7	12	2.49	(0.75- 8.34)
ALDERS	503	m	2	139	-	11	-	2.94	(1.38- 6.25)
ALDERS	506	f	2	78	-	97	-	2.08	(1.27- 3.41)
Subtotal ALDERS								2.31	(1.53- 3.49)
BENHAM	642	m	3	-	-	-	-	1.20	(1.03- 1.41)
*CEDERL	514	m	1	7	-	11	-	0.98	(0.40- 2.45)
*CEDERL	536	f	0	0	746	6	2806	0.29~	(0.02- 5.13)
Subtotal CEDERL								0.88	(0.37- 2.08)
*CPSI	898	m	1	101	-	20	-	4.70	(2.91- 7.60)
*CPSI	851	f	1	6	-	51	-	1.11	(0.48- 2.59)
Subtotal CPSI								3.30	(2.17- 5.01)
DEAN3	570	m	0	44	165	24	75	0.83	(0.47- 1.47)
DEAN3	589	f	0	7	109	27	274	0.65	(0.28- 1.54)
Subtotal DEAN3								0.77	(0.48- 1.24)
*DORN	736	m	0	59	21349	25	40397	4.47	(2.80- 7.13)
*DORN	773	m	0	44	13616	38	38849	3.30	(2.14- 5.10)
Subtotal DORN								3.80	(2.76- 5.22)
*MIGRAN	509	m	0	50	1081	24	668	1.29	(0.80- 2.07)
*MIGRAN	519	f	0	2	266	11	1315	0.90	(0.20- 4.03)
Subtotal MIGRAN								1.25	(0.79- 1.96)
PEZZOT	574	m	0	52	67	41	105	1.99	(1.19- 3.31)
TIZZAN	540	f	0	11	2	2	5	13.75	(1.48- 127.47)
Partial Totals				616	37412	395	84506		

*prospective study ~ With 0.5 adjustment for zero

REF	NRR	SEX	AD	Ys	Ws	Qs	Ps
AGUDO	503	f	0	0.91	2.63	0.53	0.1381
ALDERS	503	m	2	1.08	6.73	2.55	0.0051
ALDERS	506	f	2	0.73	15.75	1.14	0.0037
Subtotal ALDERS				0.84	22.49	3.68	
BENHAM	642	m	3	0.18	155.82	12.33	0.0229
*CEDERL	514	m	1	-0.02	4.68	1.09	0.9651
*CEDERL	536	f	0	-1.24	0.46	1.35	0.3977
Subtotal CEDERL				-0.13	5.14	2.44	
*CPSI	898	m	1	1.55	16.67	19.59	0.0000
*CPSI	851	f	1	0.10	5.41	0.70	0.8082
Subtotal CPSI				1.19	22.08	20.29	
DEAN3	570	m	0	-0.18	11.93	4.98	0.5288
DEAN3	589	f	0	-0.43	5.19	4.13	0.3294
Subtotal DEAN3				-0.26	17.12	9.11	
*DORN	736	m	0	1.50	17.58	18.76	0.0000
*DORN	773	m	0	1.20	20.43	10.93	0.0000
Subtotal DORN				1.33	38.01	29.69	
*MIGRAN	509	m	0	0.25	16.88	0.75	0.2993
*MIGRAN	519	f	0	-0.11	1.71	0.55	0.8892
Subtotal MIGRAN				0.22	18.58	1.31	
PEZZOT	574	m	0	0.69	14.69	0.73	0.0085
TIZZAN	540	f	0	2.62	0.77	3.61	0.0211

	N	16
	NS	10
	Wt	297.35
Het	Chi	83.71
Het	df	15
Het	P	***
Fixed	RR	1.59
	RRl	1.42
	RRu	1.78
	P	+++
Random	RR	1.82
	RRl	1.29
	RRu	2.57
	P	+++
Asymm	P	N.S.

Table 1H25 - 6

IESLC - Meta-analysis of Ever/current Smoking, Age started, "Highest vs lowest"
All LC types, Cigarettes only
Least adjusted

		Sex		
	combined	male	female	Total
N		9	7	16
NS		8	7	15
Wt		265.42	31.93	297.35
Het Chi		71.70	11.96	83.71
Het df		8	6	15
Het P		***	(*)	***
Fixed RR		1.60	1.53	1.59
RRl		1.42	1.08	1.42
RRu		1.80	2.16	1.78
P		+++	+	+++
Random RR		2.03	1.43	1.82
RRl		1.30	0.79	1.29
RRu		3.15	2.57	2.57
P		++	N.S.	+++
Between Chi				0.05
Between df				1
Between P				N.S.
Btwn(F) P				N.S.
Btwn(R) P				N.S.

Table 1H25 - 7

IESLC - Meta-analysis of Ever/current Smoking, Age started, "Highest vs lowest"
All LC types, Cigarettes only
Excluded studies (and stage at which they were excluded)

Stage	Studies
1	BECHER BLOT1 BROWN3 CARPEN CHYOU DARBY DOLL2 GARCIA GRAHAM GURSEL HAMMO2 JAHN JAIN LAUSSM PRESCO QIAO TVERDA WIGLE WYNDE3
2	AKIBA AMANDU AMES AXELSS BENSHL BEST BOUCHA BOUCOT BROSS CHEN CPSII DEAN2 DESTEF DORGAN DOSEME FAN GARSHI GER GILLIS HAMMON HUMBLE JUSSAW KAISE2 KATSOU KAUFMA KOO KREUZE LEVIN MCCONN NOTAN2 OSANN2 PEZZO2 PISANI RESTRE SADOWS VUTUC WANG2 WATSON WU2 WUWILL WYNDE2 WYNDE8 XU ZHOU
3	GUO MCDUFF SPITZ STASZE ZHANG
5	LUO
6	CORREA HOLE YUAN
8	ARMADA AUVINE BARBON BOFFET BRESLO BUFFLE CHEN2 CHIAZZ CHOI DAMBER DOLL ENGELA GAO GAO2 GENG HAENSZ HEGMAN HIRAYA HU HU2 JEDRYC JOLY KHUDER KOULUM LETOUR LIAW LIU3 LIU4 LIU5 LUBIN LUBIN2 MATOS MRFITR PERNU QIAO2 RACHTA SEGI2 SOBUE SPEIZE SUZUK2 SVENSS WAKAI WU WYNDE6 WYNDE7 ZHENG

Table 1H25 - 8

Potentially overlapping studies

REF	REFGP	PRINC	.	OVERLAP/LINK
BENHAM	LUBIN2	2		Subset of Lubin2
CPSI	CPSI	1		CPSI overall

Table 1H26 -

IESLC - Meta-analysis of Ever Smoking by Age started, Overview
All LC types, Pipes and/or cigars (not cigs)

This analysis is restricted to results for:
1) Ever smokers
2) Results by Age started
3) Categorical results by Age started
Results by Age started are grouped under 2 schemes (S1, S2). Each scheme has a set of "key values". An interval is allocated to the category whose key value it includes, and intervals which include none or more than one of the key values are excluded. (Open-ended intervals are coded as 999)

S1	key value	maximum range
1	26	19+
2	18	15-25
3	14	1-17

S2	key value	maximum range
1	30	27+
2	26	23-29
3	22	19-25
4	18	15-21
5	14	11-17
6	10	1-13

4) All LC types (or near equivalent)
5) Results complete enough for use in metaanalysis
6) pipes and/or cigars (not cigs)

Within each study, results are then selected (in the following order of preference, within each sex) for:
7) (not applicable)
8) DENOM: never smoked anything, never any + low
9) Followup period (YF, prospective studies): whole study (coded as 0) or longest available
10) LCtype: all or nearest available, at least Squamous and Adeno. (q = squamous, s = small, l = large, a = adeno, mix = mixed, alv = alveolar)
11) Race: all or nearest available, otherwise by race (wh or w = white, bl or b = black, hi = hispanic ch = chinese, jap = japanese, haw = hawaiian, w+o = white + oriental, sca = scandinavian, as = asian)
12) For overlapping studies: principal rather than subsidiary studies
Finally by Age: whole study (coded as 0) if available, otherwise by widest available age group and then for single sex results (m, f) in preference to results for both sexes combined (c).

Results adjusted (AD) for the most potential confounders are then chosen in Sections -1 to -3 and results adjusted for the least confounders in Sections -4 to -6. (Those least adjusted results which actually differ from the most adjusted are marked 'x' in column X in Section -4)

Section -7 shows excluded studies, together with the stage (as above) at which no qualifying results were found.

Section -8 lists the potentially overlapping studies which have been included (1=principal, 2=subsidiary).

Section -9 lists any results which would have been included in preference except that they had data not complete enough for use in meta-analysis, with their significance (yes/no), if known, and any further comment as entered on the database. It also lists as "gap" any categories for which no data were presented by the original authors.

In addition to those mentioned above, the following fields, levels and abbreviations are used:
* or nk = not known, n = no, y = yes, ot = other
nev = never
all/unspec = all or unspecified, cig+/-ot = cigarettes irrespective of other products (cigar, pipe etc)
MC = manufactured cigarettes, HR = hand-rolled cigarettes
exL, exH = range of exposure (low and high) in the smoking group, in terms of Age started
REF: 6-character study reference
NRR: number of the RR on the database within the study
ST : study type (CC = case control, pr or prosp = prospective)
NLC: number of lung cancer cases in whole study
R : risky occupational population (n = no, m = mining, o = other risky)
VB : national cigarette type (V = at least 75% Virginia, bl = at least 75% blended, ot = other)
P : any proxy use
H : full histological confirmation
De : derivation of RR/CI (or = original, st = standard method, ot = other method of estimation)

Table 1H26 - 1

IESLC - Meta-analysis of Ever Smoking by Age started, Overview
All LC types, Pipes and/or cigars (not cigs)
Most adjusted

REF	NRR	SEX	AGEL	AGEH	RACE	YF	LC TYPE	LOC	START	ST	NLC	R	VB	P	H	AD	exL	exH	S1	S2	DENOM	De
WYNDE7	551	m	0	0	all	-	all	NAmer	1977	CC	2085	n	bl	n	y	0	30	999	0	1	nev any	st
WYNDE7	552	m	0	0	all	-	all	NAmer	1977	CC	2085	n	bl	n	y	0	25	29	1	2	nev any	st
WYNDE7	553	m	0	0	all	-	all	NAmer	1977	CC	2085	n	bl	n	y	0	20	24	0	3	nev any	st
WYNDE7	554	m	0	0	all	-	all	NAmer	1977	CC	2085	n	bl	n	y	0	15	19	2	4	nev any	st
WYNDE7	555	m	0	0	all	-	all	NAmer	1977	CC	2085	n	bl	n	y	0	1	14	3	0	nev any	st

In this overview table, subtotals and Qs values may be invalid and should be ignored

Table 1H26 - 2

IESLC - Meta-analysis of Ever Smoking by Age started, Overview
All LC types, Pipes and/or cigars (not cigs)
Most adjusted

REF	NRR	SEX	AD	Number Exposed Case	Cont	Non-exposed Case	Cont	RR	95.00%CI
WYNDE7	551	m	0	11	73	64	918	2.16	(1.09- 4.28)
WYNDE7	552	m	0	10	58	64	918	2.47	(1.21- 5.07)
WYNDE7	553	m	0	16	80	64	918	2.87	(1.58- 5.19)
WYNDE7	554	m	0	10	88	64	918	1.63	(0.81- 3.29)
WYNDE7	555	m	0	2	7	64	918	4.10	(0.83- 20.13)
Subtotal WYNDE7								2.34	(1.69- 3.25)
Totals				49	306	320	4590		

*prospective study

REF	NRR	SEX	AD	Ys	Ws	Qs	Ps
WYNDE7	551	m	0	0.77	8.24	0.05	0.0269
WYNDE7	552	m	0	0.91	7.47	0.02	0.0134
WYNDE7	553	m	0	1.05	10.90	0.45	0.0005
WYNDE7	554	m	0	0.49	7.81	1.02	0.1722
WYNDE7	555	m	0	1.41	1.52	0.48	0.0824
Subtotal WYNDE7				0.85	35.93	2.02	

N	5
NS	1

Table 1H26 - 3

IESLC - Meta-analysis of Ever Smoking by Age started, Overview
All LC types, Pipes and/or cigars (not cigs)
Most adjusted

	Sex			
	combined	male	female	Total
N		5		5
NS		1		1

In this overview table, other than the "N" rows, entries in the "absent" and "Total" columns may be invalid and should be ignored

	Age started (broad categories)				
	absent	19+k26	15-25k18	1-17k14	Total
N	2	1	1	1	5
NS	1	1	1	1	1
Wt	19.15	7.47	7.81	1.52	35.93
Het Chi	0.38	0.00	0.00	0.00	2.02
Het df	1	0	0	0	4
Het P	N.S.	N.S.	N.S.	N.S.	N.S.
Fixed RR	2.54	2.47	1.63	4.10	2.34
RRl	1.62	1.21	0.81	0.83	1.69
RRu	3.97	5.07	3.29	20.13	3.25
P	+++	+	N.S.	(+)	+++
Random RR	2.54	2.47	1.63	4.10	2.34
RRl	1.62	1.21	0.81	0.83	1.69
RRu	3.97	5.07	3.29	20.13	3.25
P	+++	+	N.S.	(+)	+++

	Age started (narrow categories)							
	absent	27+k30	23-29k26	19-25k22	15-21k18	11-17k14	1-13k10	Total
N	1	1	1	1	1			5
NS	1	1	1	1	1			1
Wt	1.52	8.24	7.47	10.90	7.81			35.93
Het Chi	0.00	0.00	0.00	0.00	0.00			2.02
Het df	0	0	0	0	0			4
Het P	N.S.	N.S.	N.S.	N.S.	N.S.			N.S.
Fixed RR	4.10	2.16	2.47	2.87	1.63			2.34
RRl	0.83	1.09	1.21	1.58	0.81			1.69
RRu	20.13	4.28	5.07	5.19	3.29			3.25
P	(+)	+	+	+++	N.S.			+++
Random RR	4.10	2.16	2.47	2.87	1.63			2.34
RRl	0.83	1.09	1.21	1.58	0.81			1.69
RRu	20.13	4.28	5.07	5.19	3.29			3.25
P	(+)	+	+	+++	N.S.			+++

Table 1H26 - 4

IESLC - Meta-analysis of Ever Smoking by Age started, Overview
All LC types, Pipes and/or cigars (not cigs)
Least adjusted

REF	NRR	X	SEX	AGEL	AGEH	RACE	YF	LC TYPE	LOC	START	ST	NLC	R	VB	P	H	AD	exL	exH	S1	S2	DENOM	De
WYNDE7	551		m	0	0	all	-	all	NAmer	1977	CC	2085	n	bl	n	y	0	30	999	0	1	nev any	st
WYNDE7	552		m	0	0	all	-	all	NAmer	1977	CC	2085	n	bl	n	y	0	25	29	1	2	nev any	st
WYNDE7	553		m	0	0	all	-	all	NAmer	1977	CC	2085	n	bl	n	y	0	20	24	0	3	nev any	st
WYNDE7	554		m	0	0	all	-	all	NAmer	1977	CC	2085	n	bl	n	y	0	15	19	2	4	nev any	st
WYNDE7	555		m	0	0	all	-	all	NAmer	1977	CC	2085	n	bl	n	y	0	1	14	3	0	nev any	st

In this overview table, subtotals and Qs values may be invalid and should be ignored

Table 1H26 - 5

IESLC - Meta-analysis of Ever Smoking by Age started, Overview
All LC types, Pipes and/or cigars (not cigs)
Least adjusted

REF	NRR	SEX	AD	Number Exposed Case	Cont	Non-exposed Case	Cont	RR	95.00%CI
WYNDE7	551	m	0	11	73	64	918	2.16	(1.09- 4.28)
WYNDE7	552	m	0	10	58	64	918	2.47	(1.21- 5.07)
WYNDE7	553	m	0	16	80	64	918	2.87	(1.58- 5.19)
WYNDE7	554	m	0	10	88	64	918	1.63	(0.81- 3.29)
WYNDE7	555	m	0	2	7	64	918	4.10	(0.83- 20.13)
Subtotal WYNDE7								2.34	(1.69- 3.25)
Totals				49	306	320	4590		

*prospective study

REF	NRR	SEX	AD	Ys	Ws	Qs	Ps
WYNDE7	551	m	0	0.77	8.24	0.05	0.0269
WYNDE7	552	m	0	0.91	7.47	0.02	0.0134
WYNDE7	553	m	0	1.05	10.90	0.45	0.0005
WYNDE7	554	m	0	0.49	7.81	1.02	0.1722
WYNDE7	555	m	0	1.41	1.52	0.48	0.0824
Subtotal WYNDE7				0.85	35.93	2.02	

N	5
NS	1

Table 1H26 - 6

IESLC - Meta-analysis of Ever Smoking by Age started, Overview
All LC types, Pipes and/or cigars (not cigs)
Least adjusted

	Sex combined	male	female	Total
N		5		5
NS		1		1

In this overview table, other than the "N" rows, entries in the "absent" and "Total" columns may be invalid and should be ignored

	Age started (broad categories) absent	19+k26	15-25k18	1-17k14	Total
N	2	1	1	1	5
NS	1	1	1	1	1
Wt	19.15	7.47	7.81	1.52	35.93
Het Chi	0.38	0.00	0.00	0.00	2.02
Het df	1	0	0	0	4
Het P	N.S.	N.S.	N.S.	N.S.	N.S.
Fixed RR	2.54	2.47	1.63	4.10	2.34
RRl	1.62	1.21	0.81	0.83	1.69
RRu	3.97	5.07	3.29	20.13	3.25
P	+++	+	N.S.	(+)	+++
Random RR	2.54	2.47	1.63	4.10	2.34
RRl	1.62	1.21	0.81	0.83	1.69
RRu	3.97	5.07	3.29	20.13	3.25
P	+++	+	N.S.	(+)	+++

	absent	27+k30	23-29k26	Age started (narrow categories) 19-25k22	15-21k18	11-17k14	1-13k10	Total
N	1	1	1	1	1			5
NS	1	1	1	1	1			1
Wt	1.52	8.24	7.47	10.90	7.81			35.93
Het Chi	0.00	0.00	0.00	0.00	0.00			2.02
Het df	0	0	0	0	0			4
Het P	N.S.	N.S.	N.S.	N.S.	N.S.			N.S.
Fixed RR	4.10	2.16	2.47	2.87	1.63			2.34
RRl	0.83	1.09	1.21	1.58	0.81			1.69
RRu	20.13	4.28	5.07	5.19	3.29			3.25
P	(+)	+	+	+++	N.S.			+++
Random RR	4.10	2.16	2.47	2.87	1.63			2.34
RRl	0.83	1.09	1.21	1.58	0.81			1.69
RRu	20.13	4.28	5.07	5.19	3.29			3.25
P	(+)	+	+	+++	N.S.			+++

Table 1H26 - 7

IESLC - Meta-analysis of Ever Smoking by Age started, Overview
All LC types, Pipes and/or cigars (not cigs)
Excluded studies (and stage at which they were excluded)

1	AKIBA AMANDU AMES BECHER BENSHL BEST BLOT1 BROSS BROWN3 CARPEN CEDERL CHYOU CPSI CPSII DARBY DEAN2 DEAN3 DOLL2 ENGELA GAO2 GARCIA GILLIS GRAHAM GURSEL HAMMO2 HIRAYA HOLE HUMBLE JAHN JAIN KAISE2 KATSOU KAUFMA LAUSSM LIAW MCDUFF MIGRAN MRFITR PEZZO2 PISANI PRESCO QIAO SEGI2 SPEIZE SVENSS TVERDA WAKAI WATSON WIGLE WU WYNDE3 WYNDE8
2	AXELSS BOUCHA BOUCOT CHEN DESTEF DORGAN DOSEME FAN GARSHI GER HAMMON JUSSAW KOO KREUZE LEVIN MCCONN NOTAN2 OSANN2 RESTRE SADOWS VUTUC WANG2 WU2 WUWILL WYNDE2 XU ZHOU
3	GUO SPITZ STASZE ZHANG
4	LUO
5	CORREA YUAN
7	AGUDO ALDERS ARMADA AUVINE BARBON BENHAM BOFFET BRESLO BUFFLE CHEN2 CHIAZZ CHOI DAMBER DOLL DORN GAO GENG HAENSZ HEGMAN HU HU2 JEDRYC JOLY KHUDER KOULUM LETOUR LIU3 LIU4 LIU5 LUBIN LUBIN2 MATOS PERNU PEZZOT QIAO2 RACHTA SOBUE SUZUK2 TIZZAN WYNDE6 ZHENG

Table 1H26 - 8

Potentially overlapping studies

REF	REFGP	PRINC	.	OVERLAP/LINK
WYNDE7	WYNDE6	2		WYNDE5/6/7/8

Table 1H27 -

IESLC - Meta-analysis of Ever Smoking, Age started, "Highest vs lowest"
All LC types, Pipes and/or cigars (not cigs)

This analysis is restricted to results for:
1) Ever smokers
2) Results by Age started
3) Categorical results by Age started
4) Denominator (unexposed) = "low"
5) All LC types (or near equivalent)
6) Results complete enough for use in metaanalysis
7) (not applicable)
8) PRODUCT: pipes and/or cigars (not cigs)

Within each study, results are then selected (in the following order of preference, within each sex) for:
9) Results with least adjustment for other aspects of smoking (ADOS)
10) The highest vs lowest category
11) Followup period (YF, prospective studies): whole study (coded as 0) or longest available
12) LCtype: all or nearest available, at least Squamous and Adeno. (q = squamous, s = small,
l = large, a = adeno, mix = mixed, alv = alveolar)
13) Race: all or nearest available, otherwise by race (wh or w = white, bl or b = black, hi = hispanic
ch = chinese, jap = japanese, haw = hawaiian, w+o = white + oriental, sca = scandinavian, as = asian)
14) For overlapping studies: principal rather than subsidiary studies
Finally by Age: whole study (coded as 0) if available, otherwise by widest available age group
and then for single sex results (m, f) in preference to results for both sexes combined (c).

Results adjusted (AD) for the most potential confounders are then chosen in Sections -1 to -3
and results adjusted for the least confounders in Sections -4 to -6. (Those least adjusted results which
actually differ from the most adjusted are marked 'x' in column X in Section -4)

Section -7 shows excluded studies, together with the stage (as above) at which no qualifying
results were found.

Section -8 lists the potentially overlapping studies which have been included (1=principal, 2=subsidiary).

Section -9 lists any results which would have been included in preference except that they had data not complete
enough for use in meta-analysis, with their significance (yes/no), if known, and any further comment as entered
on the database. It also lists as "gap" any categories for which no data were presented by the original authors.

In addition to those mentioned above, the following fields, levels and abbreviations are used:
* or nk = not known, n = no, y = yes, ot = other
all/unspec = all or unspecified, cig+/-ot = cigarettes irrespective of other products (cigar, pipe etc)
MC = manufactured cigarettes, HR = hand-rolled cigarettes
exL, exH = range of exposure (low and high) in the "highest" group, in terms of Age started
unexL, unexH = range of exposure (low and high) in the "lowest" group, in terms of Age started
REF: 6-character study reference
NRR: number of the RR on the database within the study
ST : study type (CC = case control, pr or prosp = prospective)
NLC: number of lung cancer cases in whole study
R : risky occupational population (n = no, m = mining, o = other risky)
VB : national cigarette type (V = at least 75% Virginia, bl = at least 75% blended, ot = other)
P : any proxy use
H : full histological confirmation
De : derivation of RR/CI (or = original, st = standard method, ot = other method of estimation)

Table 1H27 - 1

IESLC - Meta-analysis of Ever Smoking, Age started, "Highest vs lowest"
All LC types, Pipes and/or cigars (not cigs)
Most adjusted

REF	NRR	SEX	AGEL	AGEH	RACE	YF	LC TYPE	LOC	START	ST	NLC	R	VB	P	H	AD	ADOS	exL	exH	unexL	unexH	De
WYNDE7	559	m	0	0	all	-	all	NAmer	1977	CC	2085	n	bl	n	y	0	0	1	14	30	999	st

Table 1H27 - 2

IESLC - Meta-analysis of Ever Smoking, Age started, "Highest vs lowest"
All LC types, Pipes and/or cigars (not cigs)
Most adjusted

REF	NRR	SEX	AD	Number Exposed Case	Cont	Non-exposed Case	Cont	RR	95.00%CI
WYNDE7	559	m	0	2	7	11	73	1.90	(0.35- 10.32)
Totals				2	7	11	73		

*prospective study

REF	NRR	SEX	AD	Ys	Ws	Qs	Ps
WYNDE7	559	m	0	0.64	1.34	0.00	0.4593

	N	1
	NS	1
	Wt	1.34
Het	Chi	0.00
Het	df	0
Het	P	N.S.
Fixed	RR	1.90
	RRl	0.35
	RRu	10.32
	P	N.S.
Random	RR	1.90
	RRl	0.35
	RRu	10.32
	P	N.S.
Asymm	P	

Table 1H27 - 3

IESLC - Meta-analysis of Ever Smoking, Age started, "Highest vs lowest"
All LC types, Pipes and/or cigars (not cigs)
Most adjusted

	Sex combined	male	female	Total
N		1		1
NS		1		1
Wt		1.34		1.34
Het Chi		0.00		0.00
Het df		0		0
Het P		N.S.		N.S.
Fixed RR		1.90		1.90
RRl		0.35		0.35
RRu		10.32		10.32
P		N.S.		N.S.
Random RR		1.90		1.90
RRl		0.35		0.35
RRu		10.32		10.32
P		N.S.		N.S.
Between Chi				
Between df				
Between P				N.S.
Btwn(F) P				N.S.
Btwn(R) P				N.S.

MALES

Too few RRs for analysis by factor

Table 1H27 - 4

IESLC - Meta-analysis of Ever Smoking, Age started, "Highest vs lowest"
All LC types, Pipes and/or cigars (not cigs)
Least adjusted

REF	NRR	X	SEX	AGEL	AGEH	RACE	YF	LC TYPE	LOC	START	ST	NLC	R	VB	P	H	AD	ADOS	exL	exH	unexL	unexH	De
WYNDE7	559		m	0	0	all	-	all	NAmer	1977	CC	2085	n	bl	n	y	0	0	1	14	30	999	st

Table 1H27 - 5

IESLC - Meta-analysis of Ever Smoking, Age started, "Highest vs lowest"
All LC types, Pipes and/or cigars (not cigs)
Least adjusted

REF	NRR	SEX	AD	Number Exposed Case	Exposed Cont	Non-exposed Case	Non-exposed Cont	RR	95.00%CI
WYNDE7	559	m	0	2	7	11	73	1.90	(0.35- 10.32)
Totals				2	7	11	73		

*prospective study

REF	NRR	SEX	AD	Ys	Ws	Qs	Ps
WYNDE7	559	m	0	0.64	1.34	0.00	0.4593

N	1
NS	1
Wt	1.34
Het Chi	0.00
Het df	0
Het P	N.S.
Fixed RR	1.90
RRl	0.35
RRu	10.32
P	N.S.
Random RR	1.90
RRl	0.35
RRu	10.32
P	N.S.
Asymm P	

Table 1H27 - 6

IESLC - Meta-analysis of Ever Smoking, Age started, "Highest vs lowest"
All LC types, Pipes and/or cigars (not cigs)
Least adjusted

	Sex combined	male	female	Total
N		1		1
NS		1		1
Wt		1.34		1.34
Het Chi		0.00		0.00
Het df		0		0
Het P		N.S.		N.S.
Fixed RR		1.90		1.90
RRl		0.35		0.35
RRu		10.32		10.32
P		N.S.		N.S.
Random RR		1.90		1.90
RRl		0.35		0.35
RRu		10.32		10.32
P		N.S.		N.S.
Between Chi				
Between df				
Between P				N.S.
Btwn(F) P				N.S.
Btwn(R) P				N.S.

Table 1H27 - 7

IESLC - Meta-analysis of Ever Smoking, Age started, "Highest vs lowest"
All LC types, Pipes and/or cigars (not cigs)
Excluded studies (and stage at which they were excluded)

1	AKIBA AMANDU AMES BECHER BENSHL BEST BLOT1 BROSS BROWN3 CARPEN CEDERL CHYOU CPSI CPSII DARBY DEAN2 DEAN3 DOLL2 ENGELA GAO2 GARCIA GILLIS GRAHAM GURSEL HAMMO2 HIRAYA HOLE HUMBLE JAHN JAIN KAISE2 KATSOU KAUFMA LAUSSM LIAW MCDUFF MIGRAN MRFITR PEZZO2 PISANI PRESCO QIAO SEGI2 SPEIZE SVENSS TVERDA WAKAI WATSON WIGLE WU WYNDE3 WYNDE8
2	AXELSS BOUCHA BOUCOT CHEN DESTEF DORGAN DOSEME FAN GARSHI GER HAMMON JUSSAW KOO KREUZE LEVIN MCCONN NOTAN2 OSANN2 RESTRE SADOWS VUTUC WANG2 WU2 WUWILL WYNDE2 XU ZHOU
3	GUO SPITZ STASZE ZHANG
5	LUO
6	CORREA YUAN
8	AGUDO ALDERS ARMADA AUVINE BARBON BENHAM BOFFET BRESLO BUFFLE CHEN2 CHIAZZ CHOI DAMBER DOLL DORN GAO GENG HAENSZ HEGMAN HU HU2 JEDRYC JOLY KHUDER KOULUM LETOUR LIU3 LIU4 LIU5 LUBIN LUBIN2 MATOS PERNU PEZZOT QIAO2 RACHTA SOBUE SUZUK2 TIZZAN WYNDE6 ZHENG

Table 1H27 - 8

Potentially overlapping studies

REF	REFGP	PRINC	.	OVERLAP/LINK
WYNDE7	WYNDE6	2		WYNDE5/6/7/8

Table 1H28 -

IESLC - Meta-analysis of Current Smoking by Age started, Overview
All LC types, Pipes and/or cigars (not cigs)

This analysis is restricted to results for:
1) Current smokers
2) Results by Age started
3) Categorical results by Age started
Results by Age started are grouped under 2 schemes (S1, S2). Each scheme has a set of "key values". An interval is allocated to the category whose key value it includes, and intervals which include none or more than one of the key values are excluded. (Open-ended intervals are coded as 999)

S1	key value	maximum range
1	26	19+
2	18	15-25
3	14	1-17

S2	key value	maximum range
1	30	27+
2	26	23-29
3	22	19-25
4	18	15-21
5	14	11-17
6	10	1-13

4) All LC types (or near equivalent)
5) Results complete enough for use in metaanalysis
6) pipes and/or cigars (not cigs)

Within each study, results are then selected (in the following order of preference, within each sex) for:
7) (not applicable)
8) DENOM: never smoked anything, never any + low
9) Followup period (YF, prospective studies): whole study (coded as 0) or longest available
10) LCtype: all or nearest available, at least Squamous and Adeno. (q = squamous, s = small, l = large, a = adeno, mix = mixed, alv = alveolar)
11) Race: all or nearest available, otherwise by race (wh or w = white, bl or b = black, hi = hispanic ch = chinese, jap = japanese, haw = hawaiian, w+o = white + oriental, sca = scandinavian, as = asian)
12) For overlapping studies: principal rather than subsidiary studies
Finally by Age: whole study (coded as 0) if available, otherwise by widest available age group and then for single sex results (m, f) in preference to results for both sexes combined (c).

Results adjusted (AD) for the most potential confounders are then chosen in Sections -1 to -3 and results adjusted for the least confounders in Sections -4 to -6. (Those least adjusted results which actually differ from the most adjusted are marked 'x' in column X in Section -4)

Section -7 shows excluded studies, together with the stage (as above) at which no qualifying results were found.

Section -8 lists the potentially overlapping studies which have been included (1=principal, 2=subsidiary).

Section -9 lists any results which would have been included in preference except that they had data not complete enough for use in meta-analysis, with their significance (yes/no), if known, and any further comment as entered on the database. It also lists as "gap" any categories for which no data were presented by the original authors.

In addition to those mentioned above, the following fields, levels and abbreviations are used:
* or nk = not known, n = no, y = yes, ot = other
nev = never
all/unspec = all or unspecified, cig+/-ot = cigarettes irrespective of other products (cigar, pipe etc)
MC = manufactured cigarettes, HR = hand-rolled cigarettes
exL, exH = range of exposure (low and high) in the smoking group, in terms of Age started
REF: 6-character study reference
NRR: number of the RR on the database within the study
ST : study type (CC = case control, pr or prosp = prospective)
NLC: number of lung cancer cases in whole study
R : risky occupational population (n = no, m = mining, o = other risky)
VB : national cigarette type (V = at least 75% Virginia, bl = at least 75% blended, ot = other)
P : any proxy use
H : full histological confirmation
De : derivation of RR/CI (or = original, st = standard method, ot = other method of estimation)

Table 1H28 - 1

IESLC - Meta-analysis of Current Smoking by Age started, Overview
All LC types, Pipes and/or cigars (not cigs)
Most adjusted

REF	NRR	SEX	AGEL	AGEH	RACE	YF	LC TYPE	LOC	START	ST	NLC	R	VB	P	H	AD	exL	exH	S1	S2	DENOM	De
WYNDE7	530	m	0	0	all	-	all	NAmer	1977	CC	2085	n	bl	n	y	0	30	999	0	1	nev any	st
WYNDE7	531	m	0	0	all	-	all	NAmer	1977	CC	2085	n	bl	n	y	0	25	29	1	2	nev any	st
WYNDE7	532	m	0	0	all	-	all	NAmer	1977	CC	2085	n	bl	n	y	0	20	24	0	3	nev any	st
WYNDE7	533	m	0	0	all	-	all	NAmer	1977	CC	2085	n	bl	n	y	0	15	19	2	4	nev any	st
WYNDE7	534	m	0	0	all	-	all	NAmer	1977	CC	2085	n	bl	n	y	0	1	14	3	0	nev any	st

In this overview table, subtotals and Qs values may be invalid and should be ignored

Table 1H28 - 2

IESLC - Meta-analysis of Current Smoking by Age started, Overview
All LC types, Pipes and/or cigars (not cigs)
Most adjusted

REF	NRR	SEX	AD	Number Case	Exposed Cont	Non-exposed Case	Cont	RR	95.00%CI
WYNDE7	530	m	0	5	47	64	918	1.53	(0.59- 3.97)
WYNDE7	531	m	0	6	36	64	918	2.39	(0.97- 5.88)
WYNDE7	532	m	0	10	42	64	918	3.42	(1.64- 7.12)
WYNDE7	533	m	0	8	42	64	918	2.73	(1.23- 6.06)
WYNDE7	534	m	0	2	4	64	918	7.17	(1.29- 39.90)
Subtotal WYNDE7								2.71	(1.80- 4.06)
Totals				31	171	320	4590		

*prospective study

REF	NRR	SEX	AD	Ys	Ws	Qs	Ps
WYNDE7	530	m	0	0.42	4.20	1.38	0.3863
WYNDE7	531	m	0	0.87	4.74	0.07	0.0579
WYNDE7	532	m	0	1.23	7.12	0.39	0.0011
WYNDE7	533	m	0	1.01	6.04	0.00	0.0135
WYNDE7	534	m	0	1.97	1.30	1.24	0.0244
Subtotal WYNDE7				1.00	23.40	3.08	

N	5
NS	1

Table 1H28 - 3

IESLC - Meta-analysis of Current Smoking by Age started, Overview
All LC types, Pipes and/or cigars (not cigs)
Most adjusted

	Sex			
	combined	male	female	Total
N		5		5
NS		1		1

In this overview table, other than the "N" rows, entries in the "absent" and "Total" columns may be invalid and should be ignored

MALES

	Age started (broad categories)				
	absent	19+k26	15-25k18	1-17k14	Total
N	2	1	1	1	5
NS	1	1	1	1	1
Wt	11.32	4.74	6.04	1.30	23.40
Het Chi	1.71	0.00	0.00	0.00	3.08
Het df	1	0	0	0	4
Het P	N.S.	N.S.	N.S.	N.S.	N.S.
Fixed RR	2.53	2.39	2.73	7.17	2.71
RRl	1.41	0.97	1.23	1.29	1.80
RRu	4.53	5.88	6.06	39.90	4.06
P	++	(+)	+	+	+++
Random RR	2.43	2.39	2.73	7.17	2.71
RRl	1.11	0.97	1.23	1.29	1.80
RRu	5.29	5.88	6.06	39.90	4.06
P	+	(+)	+	+	+++

		Age started (narrow categories)						
	absent	27+k30	23-29k26	19-25k22	15-21k18	11-17k14	1-13k10	Total
N	1	1	1	1	1			5
NS	1	1	1	1	1			1
Wt	1.30	4.20	4.74	7.12	6.04			23.40
Het Chi	0.00	0.00	0.00	0.00	0.00			3.08
Het df	0	0	0	0	0			4
Het P	N.S.	N.S.	N.S.	N.S.	N.S.			N.S.
Fixed RR	7.17	1.53	2.39	3.42	2.73			2.71
RRl	1.29	0.59	0.97	1.64	1.23			1.80
RRu	39.90	3.97	5.88	7.12	6.06			4.06
P	+	N.S.	(+)	++	+			+++
Random RR	7.17	1.53	2.39	3.42	2.73			2.71
RRl	1.29	0.59	0.97	1.64	1.23			1.80
RRu	39.90	3.97	5.88	7.12	6.06			4.06
P	+	N.S.	(+)	++	+			+++

Table 1H28 - 4

IESLC - Meta-analysis of Current Smoking by Age started, Overview
All LC types, Pipes and/or cigars (not cigs)
Least adjusted

REF	NRR	X	SEX	AGEL	AGEH	RACE	YF	LC TYPE	LOC	START	ST	NLC	R	VB	P	H	AD	exL	exH	S1	S2	DENOM	De
WYNDE7	530		m	0	0	all	-	all	NAmer	1977	CC	2085	n	bl	n	y	0	30	999	0	1	nev any	st
WYNDE7	531		m	0	0	all	-	all	NAmer	1977	CC	2085	n	bl	n	y	0	25	29	1	2	nev any	st
WYNDE7	532		m	0	0	all	-	all	NAmer	1977	CC	2085	n	bl	n	y	0	20	24	0	3	nev any	st
WYNDE7	533		m	0	0	all	-	all	NAmer	1977	CC	2085	n	bl	n	y	0	15	19	2	4	nev any	st
WYNDE7	534		m	0	0	all	-	all	NAmer	1977	CC	2085	n	bl	n	y	0	1	14	3	0	nev any	st

In this overview table, subtotals and Qs values may be invalid and should be ignored

Table 1H28 - 5

IESLC - Meta-analysis of Current Smoking by Age started, Overview
All LC types, Pipes and/or cigars (not cigs)
Least adjusted

REF	NRR	SEX	AD	Number Case	Exposed Cont	Non-exposed Case	Cont	RR	95.00%CI
WYNDE7	530	m	0	5	47	64	918	1.53	(0.59- 3.97)
WYNDE7	531	m	0	6	36	64	918	2.39	(0.97- 5.88)
WYNDE7	532	m	0	10	42	64	918	3.42	(1.64- 7.12)
WYNDE7	533	m	0	8	42	64	918	2.73	(1.23- 6.06)
WYNDE7	534	m	0	2	4	64	918	7.17	(1.29- 39.90)
Subtotal WYNDE7								2.71	(1.80- 4.06)
Totals				31	171	320	4590		

*prospective study

REF	NRR	SEX	AD	Ys	Ws	Qs	Ps
WYNDE7	530	m	0	0.42	4.20	1.38	0.3863
WYNDE7	531	m	0	0.87	4.74	0.07	0.0579
WYNDE7	532	m	0	1.23	7.12	0.39	0.0011
WYNDE7	533	m	0	1.01	6.04	0.00	0.0135
WYNDE7	534	m	0	1.97	1.30	1.24	0.0244
Subtotal WYNDE7				1.00	23.40	3.08	

N	5
NS	1

Table 1H28 - 6

IESLC - Meta-analysis of Current Smoking by Age started, Overview
All LC types, Pipes and/or cigars (not cigs)
Least adjusted

	Sex combined	male	female	Total
N		5		5
NS		1		1

In this overview table, other than the "N" rows, entries in the "absent" and "Total" columns may be invalid and should be ignored

MALES

	Age started (broad categories) absent	19+k26	15-25k18	1-17k14	Total
N	2	1	1	1	5
NS	1	1	1	1	1
Wt	11.32	4.74	6.04	1.30	23.40
Het Chi	1.71	0.00	0.00	0.00	3.08
Het df	1	0	0	0	4
Het P	N.S.	N.S.	N.S.	N.S.	N.S.
Fixed RR	2.53	2.39	2.73	7.17	2.71
RRl	1.41	0.97	1.23	1.29	1.80
RRu	4.53	5.88	6.06	39.90	4.06
P	++	(+)	+	+	+++
Random RR	2.43	2.39	2.73	7.17	2.71
RRl	1.11	0.97	1.23	1.29	1.80
RRu	5.29	5.88	6.06	39.90	4.06
P	+	(+)	+	+	+++

	absent	Age started (narrow categories) 27+k30	23-29k26	19-25k22	15-21k18	11-17k14	1-13k10	Total
N	1	1	1	1	1			5
NS	1	1	1	1	1			1
Wt	1.30	4.20	4.74	7.12	6.04			23.40
Het Chi	0.00	0.00	0.00	0.00	0.00			3.08
Het df	0	0	0	0	0			4
Het P	N.S.	N.S.	N.S.	N.S.	N.S.			N.S.
Fixed RR	7.17	1.53	2.39	3.42	2.73			2.71
RRl	1.29	0.59	0.97	1.64	1.23			1.80
RRu	39.90	3.97	5.88	7.12	6.06			4.06
P	+	N.S.	(+)	++	+			+++
Random RR	7.17	1.53	2.39	3.42	2.73			2.71
RRl	1.29	0.59	0.97	1.64	1.23			1.80
RRu	39.90	3.97	5.88	7.12	6.06			4.06
P	+	N.S.	(+)	++	+			+++

Table 1H28 - 7

IESLC - Meta-analysis of Current Smoking by Age started, Overview
All LC types, Pipes and/or cigars (not cigs)
Excluded studies (and stage at which they were excluded)

1	AGUDO ALDERS ARMADA AUVINE AXELSS BARBON BECHER BENHAM BLOT1 BOFFET BOUCHA BRESLO BROWN3 CARPEN CHEN CHEN2 CHIAZZ CHOI CHYOU CORREA DAMBER DARBY DESTEF DOLL DOLL2 DORGAN DOSEME FAN GAO GARCIA GARSHI GENG GER GRAHAM GUO GURSEL HAENSZ HAMMO2 HAMMON HEGMAN HU HU2 JAHN JAIN JEDRYC JOLY JUSSAW KHUDER KOO KOULUM KREUZE LAUSSM LETOUR LEVIN LIU3 LIU4 LIU5 LUBIN LUBIN2 LUO MCCONN NOTAN2 OSANN2 PERNU PEZZOT PRESCO QIAO QIAO2 RACHTA RESTRE SADOWS STASZE SUZUK2 TIZZAN TVERDA VUTUC WANG2 WIGLE WU2 WUWILL WYNDE2 WYNDE3 XU YUAN ZHANG ZHENG ZHOU
2	AKIBA AMANDU AMES BENSHL BEST BOUCOT BROSS BUFFLE CPSII DEAN2 GILLIS HUMBLE KAISE2 KATSOU KAUFMA PEZZO2 PISANI SPITZ WATSON WYNDE8
3	MCDUFF WYNDE6
5	HOLE
7	CEDERL CPSI DEAN3 DORN ENGELA GAO2 HIRAYA LIAW MATOS MIGRAN MRFITR SEGI2 SOBUE SPEIZE SVENSS WAKAI WU

Table 1H28 - 8
Potentially overlapping studies

REF	REFGP	PRINC	.	OVERLAP/LINK
WYNDE7	WYNDE6	2		WYNDE5/6/7/8

Table 1H29 -

IESLC - Meta-analysis of Current Smoking, Age started, "Highest vs lowest"
All LC types, Pipes and/or cigars (not cigs)

This analysis is restricted to results for:
1) Current smokers
2) Results by Age started
3) Categorical results by Age started
4) Denominator (unexposed) = "low"
5) All LC types (or near equivalent)
6) Results complete enough for use in metaanalysis
7) (not applicable)
8) PRODUCT: pipes and/or cigars (not cigs)

Within each study, results are then selected (in the following order of preference, within each sex) for:
9) Results with least adjustment for other aspects of smoking (ADOS)
10) The highest vs lowest category
11) Followup period (YF, prospective studies): whole study (coded as 0) or longest available
12) LCtype: all or nearest available, at least Squamous and Adeno. (q = squamous, s = small,
l = large, a = adeno, mix = mixed, alv = alveolar)
13) Race: all or nearest available, otherwise by race (wh or w = white, bl or b = black, hi = hispanic
ch = chinese, jap = japanese, haw = hawaiian, w+o = white + oriental, sca = scandinavian, as = asian)
14) For overlapping studies: principal rather than subsidiary studies
Finally by Age: whole study (coded as 0) if available, otherwise by widest available age group
and then for single sex results (m, f) in preference to results for both sexes combined (c).

Results adjusted (AD) for the most potential confounders are then chosen in Sections -1 to -3
and results adjusted for the least confounders in Sections -4 to -6. (Those least adjusted results which
actually differ from the most adjusted are marked 'x' in column X in Section -4)

Section -7 shows excluded studies, together with the stage (as above) at which no qualifying
results were found.

Section -8 lists the potentially overlapping studies which have been included (1=principal, 2=subsidiary).

Section -9 lists any results which would have been included in preference except that they had data not complete
enough for use in meta-analysis, with their significance (yes/no), if known, and any further comment as entered
on the database. It also lists as "gap" any categories for which no data were presented by the original authors.

In addition to those mentioned above, the following fields, levels and abbreviations are used:
* or nk = not known, n = no, y = yes, ot = other
all/unspec = all or unspecified, cig+/-ot = cigarettes irrespective of other products (cigar, pipe etc)
MC = manufactured cigarettes, HR = hand-rolled cigarettes
exL, exH = range of exposure (low and high) in the "highest" group, in terms of Age started
unexL, unexH = range of exposure (low and high) in the "lowest" group, in terms of Age started
REF: 6-character study reference
NRR: number of the RR on the database within the study
ST : study type (CC = case control, pr or prosp = prospective)
NLC: number of lung cancer cases in whole study
R : risky occupational population (n = no, m = mining, o = other risky)
VB : national cigarette type (V = at least 75% Virginia, bl = at least 75% blended, ot = other)
P : any proxy use
H : full histological confirmation
De : derivation of RR/CI (or = original, st = standard method, ot = other method of estimation)

Table 1H29 - 1

IESLC - Meta-analysis of Current Smoking, Age started, "Highest vs lowest"
All LC types, Pipes and/or cigars (not cigs)
Most adjusted

REF	NRR	SEX	AGEL	AGEH	RACE	YF	LC TYPE	LOC	START	ST	NLC	R	VB	P	H	AD	ADOS	exL	exH	unexL	unexH	De
WYNDE7	543	m	0	0	all	-	all	NAmer	1977	CC	2085	n	bl	n	y	0	0	1	14	30	999	st

Table 1H29 - 2

IESLC - Meta-analysis of Current Smoking, Age started, "Highest vs lowest"
All LC types, Pipes and/or cigars (not cigs)
Most adjusted

REF	NRR	SEX	AD	Number Case	Exposed Cont	Non-exposed Case	Cont	RR	95.00%CI
WYNDE7	543	m	0	2	4	5	47	4.70	(0.68- 32.43)
Totals				2	4	5	47		

*prospective study

REF	NRR	SEX	AD	Ys	Ws	Qs	Ps
WYNDE7	543	m	0	1.55	1.03	0.00	0.1164

	N	1
	NS	1
	Wt	1.03
Het	Chi	0.00
Het	df	0
Het	P	N.S.
Fixed	RR	4.70
	RRl	0.68
	RRu	32.43
	P	N.S.
Random	RR	4.70
	RRl	0.68
	RRu	32.43
	P	N.S.
Asymm	P	

Table 1H29 - 3

IESLC - Meta-analysis of Current Smoking, Age started, "Highest vs lowest"
All LC types, Pipes and/or cigars (not cigs)
Most adjusted

	Sex combined	male	female	Total
N		1		1
NS		1		1
Wt		1.03		1.03
Het Chi		0.00		0.00
Het df		0		0
Het P		N.S.		N.S.
Fixed RR		4.70		4.70
RRl		0.68		0.68
RRu		32.43		32.43
P		N.S.		N.S.
Random RR		4.70		4.70
RRl		0.68		0.68
RRu		32.43		32.43
P		N.S.		N.S.
Between Chi				
Between df				
Between P				N.S.
Btwn(F) P				N.S.
Btwn(R) P				N.S.

MALES

Too few RRs for analysis by factor

Table 1H29 - 4

IESLC - Meta-analysis of Current Smoking, Age started, "Highest vs lowest"
All LC types, Pipes and/or cigars (not cigs)
Least adjusted

REF	NRR	X	SEX	AGEL	AGEH	RACE	YF	LC TYPE	LOC	START	ST	NLC	R	VB	P	H	AD	ADOS	exL	exH	unexL	unexH	De
WYNDE7	543		m	0	0	all	-	all	NAmer	1977	CC	2085	n	bl	n	y	0	0	1	14	30	999	st

Table 1H29 - 5

IESLC - Meta-analysis of Current Smoking, Age started, "Highest vs lowest"
All LC types, Pipes and/or cigars (not cigs)
Least adjusted

REF	NRR	SEX	AD	Number Case	Exposed Cont	Non-exposed Case	Cont	RR	95.00%CI
WYNDE7	543	m	0	2	4	5	47	4.70	(0.68- 32.43)
Totals				2	4	5	47		

*prospective study

REF	NRR	SEX	AD	Ys	Ws	Qs	Ps
WYNDE7	543	m	0	1.55	1.03	0.00	0.1164

	N	1
	NS	1
	Wt	1.03
Het	Chi	0.00
Het	df	0
Het	P	N.S.
Fixed	RR	4.70
	RRl	0.68
	RRu	32.43
	P	N.S.
Random	RR	4.70
	RRl	0.68
	RRu	32.43
	P	N.S.
Asymm	P	

Table 1H29 - 6

IESLC - Meta-analysis of Current Smoking, Age started, "Highest vs lowest"
All LC types, Pipes and/or cigars (not cigs)
Least adjusted

	Sex combined	male	female	Total
N		1		1
NS		1		1
Wt		1.03		1.03
Het Chi		0.00		0.00
Het df		0		0
Het P		N.S.		N.S.
Fixed RR		4.70		4.70
RRl		0.68		0.68
RRu		32.43		32.43
P		N.S.		N.S.
Random RR		4.70		4.70
RRl		0.68		0.68
RRu		32.43		32.43
P		N.S.		N.S.
Between Chi				
Between df				
Between P				N.S.
Btwn(F) P				N.S.
Btwn(R) P				N.S.

Table 1H29 - 7

IESLC - Meta-analysis of Current Smoking, Age started, "Highest vs lowest"
All LC types, Pipes and/or cigars (not cigs)
Excluded studies (and stage at which they were excluded)

1 AGUDO ALDERS ARMADA AUVINE AXELSS BARBON BECHER BENHAM BLOT1 BOFFET BOUCHA BRESLO BROWN3 CARPEN CHEN CHEN2 CHIAZZ CHOI CHYOU CORREA DAMBER DARBY DESTEF DOLL DOLL2 DORGAN DOSEME FAN GAO GARCIA GARSHI GENG GER GRAHAM GUO GURSEL HAENSZ HAMMO2 HAMMON HEGMAN HU HU2 JAHN JAIN JEDRYC JOLY JUSSAW KHUDER KOO KOULUM KREUZE LAUSSM LETOUR LEVIN LIU3 LIU4 LIU5 LUBIN LUBIN2 LUO MCCONN NOTAN2 OSANN2 PERNU PEZZOT PRESCO QIAO QIAO2 RACHTA RESTRE SADOWS STASZE SUZUK2 TIZZAN TVERDA VUTUC WANG2 WIGLE WU2 WUWILL WYNDE2 WYNDE3 XU YUAN ZHANG ZHENG ZHOU

2 AKIBA AMANDU AMES BENSHL BEST BOUCOT BROSS BUFFLE CPSII DEAN2 GILLIS HUMBLE KAISE2 KATSOU KAUFMA PEZZO2 PISANI SPITZ WATSON WYNDE8

3 MCDUFF WYNDE6

6 HOLE

8 CEDERL CPSI DEAN3 DORN ENGELA GAO2 HIRAYA LIAW MATOS MIGRAN MRFITR SEGI2 SOBUE SPEIZE SVENSS WAKAI WU

Table 1H29 - 8

Potentially overlapping studies

REF	REFGP	PRINC	.	OVERLAP/LINK
WYNDE7	WYNDE6	2		WYNDE5/6/7/8

Table 1H30 -

IESLC - Meta-analysis of Ever/current Smoking by Age started, Overview
All LC types, Pipes and/or cigars (not cigs)

This analysis is restricted to results for:
1) Ever/current smokers
2) Results by Age started
3) Categorical results by Age started
Results by Age started are grouped under 2 schemes (S1, S2). Each scheme has a set of "key values". An interval is allocated to the category whose key value it includes, and intervals which include none or more than one of the key values are excluded. (Open-ended intervals are coded as 999)

S1	key value	maximum range
1	26	19+
2	18	15-25
3	14	1-17

S2	key value	maximum range
1	30	27+
2	26	23-29
3	22	19-25
4	18	15-21
5	14	11-17
6	10	1-13

4) All LC types (or near equivalent)
5) Results complete enough for use in metaanalysis
6) pipes and/or cigars (not cigs)

Within each study, results are then selected (in the following order of preference, within each sex) for:
7) (not applicable)
8) DENOM: never smoked anything, never any + low
9) Followup period (YF, prospective studies): whole study (coded as 0) or longest available
10) LCtype: all or nearest available, at least Squamous and Adeno. (q = squamous, s = small,
l = large, a = adeno, mix = mixed, alv = alveolar)
11) Race: all or nearest available, otherwise by race (wh or w = white, bl or b = black, hi = hispanic
ch = chinese, jap = japanese, haw = hawaiian, w+o = white + oriental, sca = scandinavian, as = asian)
12) For overlapping studies: principal rather than subsidiary studies
Finally by Age: whole study (coded as 0) if available, otherwise by widest available age group and then for single sex results (m, f) in preference to results for both sexes combined (c).

Results adjusted (AD) for the most potential confounders are then chosen in Sections -1 to -3 and results adjusted for the least confounders in Sections -4 to -6. (Those least adjusted results which actually differ from the most adjusted are marked 'x' in column X in Section -4)

Section -7 shows excluded studies, together with the stage (as above) at which no qualifying results were found.

Section -8 lists the potentially overlapping studies which have been included (1=principal, 2=subsidiary).

Section -9 lists any results which would have been included in preference except that they had data not complete enough for use in meta-analysis, with their significance (yes/no), if known, and any further comment as entered on the database. It also lists as "gap" any categories for which no data were presented by the original authors.

In addition to those mentioned above, the following fields, levels and abbreviations are used:
* or nk = not known, n = no, y = yes, ot = other
ev = ever, cu = current, nev = never
all/unspec = all or unspecified, cig+/-ot = cigarettes irrespective of other products (cigar, pipe etc)
MC = manufactured cigarettes, HR = hand-rolled cigarettes
exL, exH = range of exposure (low and high) in the smoking group, in terms of Age started
REF: 6-character study reference
NRR: number of the RR on the database within the study
ST : study type (CC = case control, pr or prosp = prospective)
NLC: number of lung cancer cases in whole study
R : risky occupational population (n = no, m = mining, o = other risky)
VB : national cigarette type (V = at least 75% Virginia, bl = at least 75% blended, ot = other)
P : any proxy use
H : full histological confirmation
De : derivation of RR/CI (or = original, st = standard method, ot = other method of estimation)

Table 1H30 - 1

IESLC - Meta-analysis of Ever/current Smoking by Age started, Overview
All LC types, Pipes and/or cigars (not cigs)
Most adjusted

REF	NRR	SEX	AGEL	AGEH	RACE	YF	LC TYPE	LOC	START	ST	NLC	R	VB	P	H	AD	SM	exL	exH	S1	S2	DENOM	De
WYNDE7	551	m	0	0	all	-	all	NAmer	1977	CC	2085	n	bl	n	y	0	ev	30	999	0	1	nev any	st
WYNDE7	552	m	0	0	all	-	all	NAmer	1977	CC	2085	n	bl	n	y	0	ev	25	29	1	2	nev any	st
WYNDE7	553	m	0	0	all	-	all	NAmer	1977	CC	2085	n	bl	n	y	0	ev	20	24	0	3	nev any	st
WYNDE7	554	m	0	0	all	-	all	NAmer	1977	CC	2085	n	bl	n	y	0	ev	15	19	2	4	nev any	st
WYNDE7	555	m	0	0	all	-	all	NAmer	1977	CC	2085	n	bl	n	y	0	ev	1	14	3	0	nev any	st

In this overview table, subtotals and Qs values may be invalid and should be ignored

Table 1H30 - 2

IESLC - Meta-analysis of Ever/current Smoking by Age started, Overview
All LC types, Pipes and/or cigars (not cigs)
Most adjusted

REF	NRR	SEX	AD	Number Exposed Case	Cont	Non-exposed Case	Cont	RR	95.00%CI
WYNDE7	551	m	0	11	73	64	918	2.16	(1.09- 4.28)
WYNDE7	552	m	0	10	58	64	918	2.47	(1.21- 5.07)
WYNDE7	553	m	0	16	80	64	918	2.87	(1.58- 5.19)
WYNDE7	554	m	0	10	88	64	918	1.63	(0.81- 3.29)
WYNDE7	555	m	0	2	7	64	918	4.10	(0.83- 20.13)
Subtotal WYNDE7								2.34	(1.69- 3.25)
Totals				49	306	320	4590		

*prospective study

REF	NRR	SEX	AD	Ys	Ws	Qs	Ps
WYNDE7	551	m	0	0.77	8.24	0.05	0.0269
WYNDE7	552	m	0	0.91	7.47	0.02	0.0134
WYNDE7	553	m	0	1.05	10.90	0.45	0.0005
WYNDE7	554	m	0	0.49	7.81	1.02	0.1722
WYNDE7	555	m	0	1.41	1.52	0.48	0.0824
Subtotal WYNDE7				0.85	35.93	2.02	

N	5
NS	1

Table 1H30 - 3

IESLC - Meta-analysis of Ever/current Smoking by Age started, Overview
All LC types, Pipes and/or cigars (not cigs)
Most adjusted

	Sex			
	combined	male	female	Total
N		5		5
NS		1		1

In this overview table, other than the "N" rows, entries in the "absent" and "Total" columns may be invalid and should be ignored

MALES

	Age started (broad categories)				
	absent	19+k26	15-25k18	1-17k14	Total
N	2	1	1	1	5
NS	1	1	1	1	1
Wt	19.15	7.47	7.81	1.52	35.93
Het Chi	0.38	0.00	0.00	0.00	2.02
Het df	1	0	0	0	4
Het P	N.S.	N.S.	N.S.	N.S.	N.S.
Fixed RR	2.54	2.47	1.63	4.10	2.34
RRl	1.62	1.21	0.81	0.83	1.69
RRu	3.97	5.07	3.29	20.13	3.25
P	+++	+	N.S.	(+)	+++
Random RR	2.54	2.47	1.63	4.10	2.34
RRl	1.62	1.21	0.81	0.83	1.69
RRu	3.97	5.07	3.29	20.13	3.25
P	+++	+	N.S.	(+)	+++

	Age started (narrow categories)							
	absent	27+k30	23-29k26	19-25k22	15-21k18	11-17k14	1-13k10	Total
N	1	1	1	1	1			5
NS	1	1	1	1	1			1
Wt	1.52	8.24	7.47	10.90	7.81			35.93
Het Chi	0.00	0.00	0.00	0.00	0.00			2.02
Het df	0	0	0	0	0			4
Het P	N.S.	N.S.	N.S.	N.S.	N.S.			N.S.
Fixed RR	4.10	2.16	2.47	2.87	1.63			2.34
RRl	0.83	1.09	1.21	1.58	0.81			1.69
RRu	20.13	4.28	5.07	5.19	3.29			3.25
P	(+)	+	+	+++	N.S.			+++
Random RR	4.10	2.16	2.47	2.87	1.63			2.34
RRl	0.83	1.09	1.21	1.58	0.81			1.69
RRu	20.13	4.28	5.07	5.19	3.29			3.25
P	(+)	+	+	+++	N.S.			+++

Table 1H30 - 4

IESLC - Meta-analysis of Ever/current Smoking by Age started, Overview
All LC types, Pipes and/or cigars (not cigs)
Least adjusted

REF	NRR	X	SEX	AGEL	AGEH	RACE	YF	LC TYPE	LOC	START	ST	NLC	R	VB	P	H	AD	SM	exL	exH	S1	S2	DENOM	De
WYNDE7	551		m	0	0	all	-	all	NAmer	1977	CC	2085	n	bl	n	y	0	ev	30	999	0	1	nev any	st
WYNDE7	552		m	0	0	all	-	all	NAmer	1977	CC	2085	n	bl	n	y	0	ev	25	29	1	2	nev any	st
WYNDE7	553		m	0	0	all	-	all	NAmer	1977	CC	2085	n	bl	n	y	0	ev	20	24	0	3	nev any	st
WYNDE7	554		m	0	0	all	-	all	NAmer	1977	CC	2085	n	bl	n	y	0	ev	15	19	2	4	nev any	st
WYNDE7	555		m	0	0	all	-	all	NAmer	1977	CC	2085	n	bl	n	y	0	ev	1	14	3	0	nev any	st

In this overview table, subtotals and Qs values may be invalid and should be ignored

Table 1H30 - 5

IESLC - Meta-analysis of Ever/current Smoking by Age started, Overview
All LC types, Pipes and/or cigars (not cigs)
Least adjusted

REF	NRR	SEX	AD	Number Exposed Case	Cont	Non-exposed Case	Cont	RR	95.00%CI
WYNDE7	551	m	0	11	73	64	918	2.16	(1.09- 4.28)
WYNDE7	552	m	0	10	58	64	918	2.47	(1.21- 5.07)
WYNDE7	553	m	0	16	80	64	918	2.87	(1.58- 5.19)
WYNDE7	554	m	0	10	88	64	918	1.63	(0.81- 3.29)
WYNDE7	555	m	0	2	7	64	918	4.10	(0.83- 20.13)
Subtotal WYNDE7								2.34	(1.69- 3.25)
Totals				49	306	320	4590		

*prospective study

REF	NRR	SEX	AD	Ys	Ws	Qs	Ps
WYNDE7	551	m	0	0.77	8.24	0.05	0.0269
WYNDE7	552	m	0	0.91	7.47	0.02	0.0134
WYNDE7	553	m	0	1.05	10.90	0.45	0.0005
WYNDE7	554	m	0	0.49	7.81	1.02	0.1722
WYNDE7	555	m	0	1.41	1.52	0.48	0.0824
Subtotal WYNDE7				0.85	35.93	2.02	

N	5
NS	1

Table 1H30 - 6

IESLC - Meta-analysis of Ever/current Smoking by Age started, Overview
All LC types, Pipes and/or cigars (not cigs)
Least adjusted

	combined	Sex male	female	Total
N		5		5
NS		1		1

In this overview table, other than the "N" rows, entries in the "absent" and "Total" columns may be invalid and should be ignored

MALES

	Age started (broad categories) absent	19+k26	15-25k18	1-17k14	Total
N	2	1	1	1	5
NS	1	1	1	1	1
Wt	19.15	7.47	7.81	1.52	35.93
Het Chi	0.38	0.00	0.00	0.00	2.02
Het df	1	0	0	0	4
Het P	N.S.	N.S.	N.S.	N.S.	N.S.
Fixed RR	2.54	2.47	1.63	4.10	2.34
RRl	1.62	1.21	0.81	0.83	1.69
RRu	3.97	5.07	3.29	20.13	3.25
P	+++	+	N.S.	(+)	+++
Random RR	2.54	2.47	1.63	4.10	2.34
RRl	1.62	1.21	0.81	0.83	1.69
RRu	3.97	5.07	3.29	20.13	3.25
P	+++	+	N.S.	(+)	+++

	absent	Age started (narrow categories) 27+k30	23-29k26	19-25k22	15-21k18	11-17k14	1-13k10	Total
N	1	1	1	1	1			5
NS	1	1	1	1	1			1
Wt	1.52	8.24	7.47	10.90	7.81			35.93
Het Chi	0.00	0.00	0.00	0.00	0.00			2.02
Het df	0	0	0	0	0			4
Het P	N.S.	N.S.	N.S.	N.S.	N.S.			N.S.
Fixed RR	4.10	2.16	2.47	2.87	1.63			2.34
RRl	0.83	1.09	1.21	1.58	0.81			1.69
RRu	20.13	4.28	5.07	5.19	3.29			3.25
P	(+)	+	+	+++	N.S.			+++
Random RR	4.10	2.16	2.47	2.87	1.63			2.34
RRl	0.83	1.09	1.21	1.58	0.81			1.69
RRu	20.13	4.28	5.07	5.19	3.29			3.25
P	(+)	+	+	+++	N.S.			+++

Table 1H30 - 7

IESLC - Meta-analysis of Ever/current Smoking by Age started, Overview
All LC types, Pipes and/or cigars (not cigs)
Excluded studies (and stage at which they were excluded)

1	BECHER BLOT1 BROWN3 CARPEN CHYOU DARBY DOLL2 GARCIA GRAHAM GURSEL HAMMO2 JAHN JAIN LAUSSM PRESCO QIAO TVERDA WIGLE WYNDE3
2	AKIBA AMANDU AMES AXELSS BENSHL BEST BOUCHA BOUCOT BROSS CHEN CPSII DEAN2 DESTEF DORGAN DOSEME FAN GARSHI GER GILLIS HAMMON HUMBLE JUSSAW KAISE2 KATSOU KAUFMA KOO KREUZE LEVIN MCCONN NOTAN2 OSANN2 PEZZO2 PISANI RESTRE SADOWS VUTUC WANG2 WATSON WU2 WUWILL WYNDE2 WYNDE8 XU ZHOU
3	GUO MCDUFF SPITZ STASZE ZHANG
4	LUO
5	CORREA HOLE YUAN
7	AGUDO ALDERS ARMADA AUVINE BARBON BENHAM BOFFET BRESLO BUFFLE CEDERL CHEN2 CHIAZZ CHOI CPSI DAMBER DEAN3 DOLL DORN ENGELA GAO GAO2 GENG HAENSZ HEGMAN HIRAYA HU HU2 JEDRYC JOLY KHUDER KOULUM LETOUR LIAW LIU3 LIU4 LIU5 LUBIN LUBIN2 MATOS MIGRAN MRFITR PERNU PEZZOT QIAO2 RACHTA SEGI2 SOBUE SPEIZE SUZUK2 SVENSS TIZZAN WAKAI WU WYNDE6 ZHENG

Table 1H30 - 8

Potentially overlapping studies

REF	REFGP	PRINC	.	OVERLAP/LINK
WYNDE7	WYNDE6	2		WYNDE5/6/7/8

Table 1H31 -

IESLC - Meta-analysis of Ever/current Smoking, Age started, "Highest vs lowest"
All LC types, Pipes and/or cigars (not cigs)

This analysis is restricted to results for:
1) Ever/current smokers
2) Results by Age started
3) Categorical results by Age started
4) Denominator (unexposed) = "low"
5) All LC types (or near equivalent)
6) Results complete enough for use in metaanalysis
7) SMKSTA: ever, current
8) PRODUCT: pipes and/or cigars (not cigs)

Within each study, results are then selected (in the following order of preference, within each sex) for:
9) Results with least adjustment for other aspects of smoking (ADOS)
10) The highest vs lowest category
11) Followup period (YF, prospective studies): whole study (coded as 0) or longest available
12) LCtype: all or nearest available, at least Squamous and Adeno. (q = squamous, s = small,
l = large, a = adeno, mix = mixed, alv = alveolar)
13) Race: all or nearest available, otherwise by race (wh or w = white, bl or b = black, hi = hispanic
ch = chinese, jap = japanese, haw = hawaiian, w+o = white + oriental, sca = scandinavian, as = asian)
14) For overlapping studies: principal rather than subsidiary studies
Finally by Age: whole study (coded as 0) if available, otherwise by widest available age group
and then for single sex results (m, f) in preference to results for both sexes combined (c).

Results adjusted (AD) for the most potential confounders are then chosen in Sections -1 to -3
and results adjusted for the least confounders in Sections -4 to -6. (Those least adjusted results which
actually differ from the most adjusted are marked 'x' in column X in Section -4)

Section -7 shows excluded studies, together with the stage (as above) at which no qualifying
results were found.

Section -8 lists the potentially overlapping studies which have been included (1=principal, 2=subsidiary).

Section -9 lists any results which would have been included in preference except that they had data not complete
enough for use in meta-analysis, with their significance (yes/no), if known, and any further comment as entered
on the database. It also lists as "gap" any categories for which no data were presented by the original authors.

In addition to those mentioned above, the following fields, levels and abbreviations are used:
* or nk = not known, n = no, y = yes, ot = other
all/unspec = all or unspecified, cig+/-ot = cigarettes irrespective of other products (cigar, pipe etc)
MC = manufactured cigarettes, HR = hand-rolled cigarettes
exL, exH = range of exposure (low and high) in the "highest" group, in terms of Age started
unexL, unexH = range of exposure (low and high) in the "lowest" group, in terms of Age started
REF: 6-character study reference
NRR: number of the RR on the database within the study
ST : study type (CC = case control, pr or prosp = prospective)
NLC: number of lung cancer cases in whole study
R : risky occupational population (n = no, m = mining, o = other risky)
VB : national cigarette type (V = at least 75% Virginia, bl = at least 75% blended, ot = other)
P : any proxy use
H : full histological confirmation
De : derivation of RR/CI (or = original, st = standard method, ot = other method of estimation)

Table 1H31 - 1

IESLC - Meta-analysis of Ever/current Smoking, Age started, "Highest vs lowest"
All LC types, Pipes and/or cigars (not cigs)
Most adjusted

REF	NRR	SEX	AGEL	AGEH	RACE	YF	LC TYPE	LOC	START	ST	NLC	R	VB	P	H	AD	ADOS	SM	exL	exH	unexL	unexH	De
WYNDE7	559	m	0	0	all	-	all	NAmer	1977	CC	2085	n	bl	n	y	0	0	ev	1	14	30	999	st

Table 1H31 - 2

IESLC - Meta-analysis of Ever/current Smoking, Age started, "Highest vs lowest"
All LC types, Pipes and/or cigars (not cigs)
Most adjusted

REF	NRR	SEX	AD	Number Exposed Case	Cont	Non-exposed Case	Cont	RR	95.00%CI
WYNDE7	559	m	0	2	7	11	73	1.90	(0.35- 10.32)
Totals				2	7	11	73		

*prospective study

REF	NRR	SEX	AD	Ys	Ws	Qs	Ps
WYNDE7	559	m	0	0.64	1.34	0.00	0.4593

	N	1
	NS	1
	Wt	1.34
Het	Chi	0.00
Het	df	0
Het	P	N.S.
Fixed	RR	1.90
	RRl	0.35
	RRu	10.32
	P	N.S.
Random	RR	1.90
	RRl	0.35
	RRu	10.32
	P	N.S.
Asymm	P	

Table 1H31 - 3

IESLC - Meta-analysis of Ever/current Smoking, Age started, "Highest vs lowest"
All LC types, Pipes and/or cigars (not cigs)
Most adjusted

	Sex: combined	male	female	Total
N		1		1
NS		1		1
Wt		1.34		1.34
Het Chi		0.00		0.00
Het df		0		0
Het P		N.S.		N.S.
Fixed RR		1.90		1.90
RRl		0.35		0.35
RRu		10.32		10.32
P		N.S.		N.S.
Random RR		1.90		1.90
RRl		0.35		0.35
RRu		10.32		10.32
P		N.S.		N.S.
Between Chi				
Between df				
Between P				N.S.
Btwn(F) P				N.S.
Btwn(R) P				N.S.

MALES

Too few RRs for analysis by factor

Table 1H31 - 4

IESLC - Meta-analysis of Ever/current Smoking, Age started, "Highest vs lowest"
All LC types, Pipes and/or cigars (not cigs)
Least adjusted

REF	NRR	X	SEX	AGEL	AGEH	RACE	YF	LC TYPE	LOC	START	ST	NLC	R	VB	P	H	AD	ADOS	SM	exL	exH	unexL	unexH	De
WYNDE7	559		m	0	0	all	-	all	NAmer	1977	CC	2085	n	bl	n	y	0	0	ev	1	14	30	999	st

Table 1H31 - 5

IESLC - Meta-analysis of Ever/current Smoking, Age started, "Highest vs lowest"
All LC types, Pipes and/or cigars (not cigs)
Least adjusted

REF	NRR	SEX	AD	Number Exposed Case	Number Exposed Cont	Non-exposed Case	Non-exposed Cont	RR	95.00%CI
WYNDE7	559	m	0	2	7	11	73	1.90	(0.35- 10.32)
Totals				2	7	11	73		

*prospective study

REF	NRR	SEX	AD	Ys	Ws	Qs	Ps
WYNDE7	559	m	0	0.64	1.34	0.00	0.4593

	N	1
	NS	1
	Wt	1.34
Het	Chi	0.00
Het	df	0
Het	P	N.S.
Fixed	RR	1.90
	RRl	0.35
	RRu	10.32
	P	N.S.
Random	RR	1.90
	RRl	0.35
	RRu	10.32
	P	N.S.
Asymm	P	

Table 1H31 - 6

IESLC - Meta-analysis of Ever/current Smoking, Age started, "Highest vs lowest"
All LC types, Pipes and/or cigars (not cigs)
Least adjusted

	Sex			
	combined	male	female	Total
N		1		1
NS		1		1
Wt		1.34		1.34
Het Chi		0.00		0.00
Het df		0		0
Het P		N.S.		N.S.
Fixed RR		1.90		1.90
RRl		0.35		0.35
RRu		10.32		10.32
P		N.S.		N.S.
Random RR		1.90		1.90
RRl		0.35		0.35
RRu		10.32		10.32
P		N.S.		N.S.
Between Chi				
Between df				
Between P				N.S.
Btwn(F) P				N.S.
Btwn(R) P				N.S.

Table 1H31 - 7

IESLC - Meta-analysis of Ever/current Smoking, Age started, "Highest vs lowest"
All LC types, Pipes and/or cigars (not cigs)
Excluded studies (and stage at which they were excluded)

1	BECHER BLOT1 BROWN3 CARPEN CHYOU DARBY DOLL2 GARCIA GRAHAM GURSEL HAMMO2 JAHN JAIN LAUSSM PRESCO QIAO TVERDA WIGLE WYNDE3
2	AKIBA AMANDU AMES AXELSS BENSHL BEST BOUCHA BOUCOT BROSS CHEN CPSII DEAN2 DESTEF DORGAN DOSEME FAN GARSHI GER GILLIS HAMMON HUMBLE JUSSAW KAISE2 KATSOU KAUFMA KOO KREUZE LEVIN MCCONN NOTAN2 OSANN2 PEZZO2 PISANI RESTRE SADOWS VUTUC WANG2 WATSON WU2 WUWILL WYNDE2 WYNDE8 XU ZHOU
3	GUO MCDUFF SPITZ STASZE ZHANG
5	LUO
6	CORREA HOLE YUAN
8	AGUDO ALDERS ARMADA AUVINE BARBON BENHAM BOFFET BRESLO BUFFLE CEDERL CHEN2 CHIAZZ CHOI CPSI DAMBER DEAN3 DOLL DORN ENGELA GAO GAO2 GENG HAENSZ HEGMAN HIRAYA HU HU2 JEDRYC JOLY KHUDER KOULUM LETOUR LIAW LIU3 LIU4 LIU5 LUBIN LUBIN2 MATOS MIGRAN MRFITR PERNU PEZZOT QIAO2 RACHTA SEGI2 SOBUE SPEIZE SUZUK2 SVENSS TIZZAN WAKAI WU WYNDE6 ZHENG

Table 1H31 - 8

Potentially overlapping studies

REF	REFGP	PRINC	.	OVERLAP/LINK
WYNDE7	WYNDE6	2		WYNDE5/6/7/8

Table 1H32 -

IESLC - Meta-analysis of Ever/current Smoking by Age started, Overview
All LC types, Pipes only

This analysis is restricted to results for:
1) Ever/current smokers
2) Results by Age started
3) Categorical results by Age started
Results by Age started are grouped under 2 schemes (S1, S2). Each scheme has a set of "key values". An interval is allocated to the category whose key value it includes, and intervals which include none or more than one of the key values are excluded. (Open-ended intervals are coded as 999)

S1	key value	maximum range
1	26	19+
2	18	15-25
3	14	1-17

S2	key value	maximum range
1	30	27+
2	26	23-29
3	22	19-25
4	18	15-21
5	14	11-17
6	10	1-13

4) All LC types (or near equivalent)
5) Results complete enough for use in metaanalysis
6) pipes only

Within each study, results are then selected (in the following order of preference, within each sex) for:
7) (not applicable)
8) DENOM: never smoked anything, never any + low
9) Followup period (YF, prospective studies): whole study (coded as 0) or longest available
10) LCtype: all or nearest available, at least Squamous and Adeno. (q = squamous, s = small,
l = large, a = adeno, mix = mixed, alv = alveolar)
11) Race: all or nearest available, otherwise by race (wh or w = white, bl or b = black, hi = hispanic
ch = chinese, jap = japanese, haw = hawaiian, w+o = white + oriental, sca = scandinavian, as = asian)
12) For overlapping studies: principal rather than subsidiary studies
Finally by Age: whole study (coded as 0) if available, otherwise by widest available age group
and then for single sex results (m, f) in preference to results for both sexes combined (c).

Results adjusted (AD) for the most potential confounders are then chosen in Sections -1 to -3
and results adjusted for the least confounders in Sections -4 to -6. (Those least adjusted results which
actually differ from the most adjusted are marked 'x' in column X in Section -4)

Section -7 shows excluded studies, together with the stage (as above) at which no qualifying
results were found.

Section -8 lists the potentially overlapping studies which have been included (1=principal, 2=subsidiary).

Section -9 lists any results which would have been included in preference except that they had data not complete
enough for use in meta-analysis, with their significance (yes/no), if known, and any further comment as entered
on the database. It also lists as "gap" any categories for which no data were presented by the original authors.

In addition to those mentioned above, the following fields, levels and abbreviations are used:
* or nk = not known, n = no, y = yes, ot = other
ev = ever, cu = current, nev = never
all/unspec = all or unspecified, cig+/-ot = cigarettes irrespective of other products (cigar, pipe etc)
MC = manufactured cigarettes, HR = hand-rolled cigarettes
exL, exH = range of exposure (low and high) in the smoking group, in terms of Age started
REF: 6-character study reference
NRR: number of the RR on the database within the study
ST : study type (CC = case control, pr or prosp = prospective)
NLC: number of lung cancer cases in whole study
R : risky occupational population (n = no, m = mining, o = other risky)
VB : national cigarette type (V = at least 75% Virginia, bl = at least 75% blended, ot = other)
P : any proxy use
H : full histological confirmation
De : derivation of RR/CI (or = original, st = standard method, ot = other method of estimation)

Table 1H32 - 1

IESLC - Meta-analysis of Ever/current Smoking by Age started, Overview
All LC types, Pipes only
Most adjusted

REF	NRR	SEX	AGEL	AGEH	RACE	YF	LC TYPE	LOC	START	ST	NLC	R	VB	P	H	AD	SM	exL	exH	S1	S2	DENOM	De
BOFFET	544	m	0	0	all	-	all	Eu:mul	1988	CC	5621	n	bl	y	n	2	ev	27	999	0	1	nev any	or
BOFFET	545	m	0	0	all	-	all	Eu:mul	1988	CC	5621	n	bl	y	n	2	ev	20	26	1	0	nev any	or
BOFFET	546	m	0	0	all	-	all	Eu:mul	1988	CC	5621	n	bl	y	n	2	ev	1	19	0	0	nev any	or
CEDERL	523	m	0	0	all	10	all	Eu:Sca	1963	pr	491	n	bl	n	n	1	cu	19	999	1	0	nev any	ot
CEDERL	524	m	0	0	all	10	all	Eu:Sca	1963	pr	491	n	bl	n	n	1	cu	17	18	2	4	nev any	ot
CEDERL	525	m	0	0	all	10	all	Eu:Sca	1963	pr	491	n	bl	n	n	1	cu	1	16	3	0	nev any	ot
DEAN3	578	m	0	0	all	-	all	Eu:UK	1969	CC	766	n	V	y	n	0	cu	25	999	1	0	nev any	st
DEAN3	579	m	0	0	all	-	all	Eu:UK	1969	CC	766	n	V	y	n	0	cu	20	24	0	3	nev any	st
DEAN3	580	m	0	0	all	-	all	Eu:UK	1969	CC	766	n	V	y	n	0	cu	1	19	0	0	nev any	st

In this overview table, subtotals and Qs values may be invalid and should be ignored

Table 1H32 - 2

IESLC - Meta-analysis of Ever/current Smoking by Age started, Overview
All LC types, Pipes only
Most adjusted

REF	NRR	SEX	AD	Number Exposed Case	Exposed Cont	Non-exposed Case	Non-exposed Cont	RR	95.00%CI
BOFFET	544	m	2	14	-	117	-	8.20	(4.10- 16.30)
BOFFET	545	m	2	20	-	117	-	6.30	(3.50- 11.20)
BOFFET	546	m	2	27	-	117	-	9.60	(5.60- 16.70)
Subtotal BOFFET								7.96	(5.64- 11.24)
*CEDERL	523	m	1	8	-	7	-	4.40	(1.60- 12.11)
*CEDERL	524	m	1	11	-	7	-	9.20	(3.57- 23.68)
*CEDERL	525	m	1	11	-	7	-	9.90	(3.85- 25.47)
Subtotal CEDERL								7.54	(4.32- 13.18)
DEAN3	578	m	0	4	16	24	510	5.31	(1.65- 17.11)
DEAN3	579	m	0	3	21	24	510	3.04	(0.85- 10.89)
DEAN3	580	m	0	6	67	24	510	1.90	(0.75- 4.82)
Subtotal DEAN3								2.88	(1.53- 5.42)
Partial Totals				104	104	444	1530		

*prospective study

REF	NRR	SEX	AD	Ys	Ws	Qs	Ps
BOFFET	544	m	2	2.10	8.07	0.40	0.0000
BOFFET	545	m	2	1.84	11.36	0.02	0.0000
BOFFET	546	m	2	2.26	12.87	1.85	0.0000
Subtotal BOFFET				2.07	32.29	2.27	
*CEDERL	523	m	1	1.48	3.75	0.60	0.0041
*CEDERL	524	m	1	2.22	4.29	0.49	0.0000
*CEDERL	525	m	1	2.29	4.30	0.72	0.0000
Subtotal CEDERL				2.02	12.35	1.81	
DEAN3	578	m	0	1.67	2.81	0.13	0.0051
DEAN3	579	m	0	1.11	2.36	1.40	0.0883
DEAN3	580	m	0	0.64	4.44	6.81	0.1752
Subtotal DEAN3				1.06	9.60	8.34	

N	9
NS	3

Table 1H32 - 3

IESLC - Meta-analysis of Ever/current Smoking by Age started, Overview
All LC types, Pipes only
Most adjusted

	Sex combined	male	female	Total
N		9		9
NS		3		3

In this overview table, other than the "N" rows, entries in the "absent" and "Total" columns may be invalid and should be ignored

MALES

	Age started (broad categories) absent	19+k26	15-25k18	1-17k14	Total
N	4	3	1	1	9
NS	2	3	1	1	6
Wt	27.73	17.92	4.29	4.30	54.25
Het Chi	10.45	0.38	0.00	0.00	12.43
Het df	3	2	0	0	8
Het P	*	N.S.	N.S.	N.S.	N.S.
Fixed RR	6.42	5.69	9.20	9.90	6.57
RRl	4.42	3.58	3.57	3.85	5.03
RRu	9.31	9.04	23.69	25.46	8.57
P	+++	+++	+++	+++	+++
Random RR	5.12	5.69	9.20	9.90	6.23
RRl	2.41	3.58	3.57	3.85	4.40
RRu	10.86	9.04	23.69	25.46	8.82
P	+++	+++	+++	+++	+++

	absent	27+k30	23-29k26	Age started (narrow categories) 19-25k22	15-21k18	11-17k14	1-13k10	Total
N	6	1		1	1			9
NS	3	1		1	1			6
Wt	39.53	8.07		2.36	4.29			54.25
Het Chi	10.09	0.00		0.00	0.00			12.43
Het df	5	0		0	0			8
Het P	(*)	N.S.		N.S.	N.S.			N.S.
Fixed RR	6.34	8.20		3.04	9.20			6.57
RRl	4.64	4.11		0.85	3.57			5.03
RRu	8.65	16.35		10.89	23.69			8.57
P	+++	+++		(+)	+++			+++
Random RR	5.83	8.20		3.04	9.20			6.23
RRl	3.64	4.11		0.85	3.57			4.40
RRu	9.33	16.35		10.89	23.69			8.82
P	+++	+++		(+)	+++			+++

Table 1H32 - 4

IESLC - Meta-analysis of Ever/current Smoking by Age started, Overview
All LC types, Pipes only
Least adjusted

REF	NRR	X	SEX	AGEL	AGEH	RACE	YF	LC TYPE	LOC	START	ST	NLC	R	VB	P	H	AD	SM	exL	exH	S1	S2	DENOM	De
BOFFET	539	x	m	0	0	all	-	all	Eu:mul	1988	CC	5621	n	bl	y	n	0	ev	27	999	0	1	nev any	st
BOFFET	540	x	m	0	0	all	-	all	Eu:mul	1988	CC	5621	n	bl	y	n	0	ev	20	26	1	0	nev any	st
BOFFET	541	x	m	0	0	all	-	all	Eu:mul	1988	CC	5621	n	bl	y	n	0	ev	1	19	0	0	nev any	st
CEDERL	523		m	0	0	all	10	all	Eu:Sca	1963	pr	491	n	bl	n	n	1	cu	19	999	1	0	nev any	ot
CEDERL	524		m	0	0	all	10	all	Eu:Sca	1963	pr	491	n	bl	n	n	1	cu	17	18	2	4	nev any	ot
CEDERL	525		m	0	0	all	10	all	Eu:Sca	1963	pr	491	n	bl	n	n	1	cu	1	16	3	0	nev any	ot
DEAN3	578		m	0	0	all	-	all	Eu:UK	1969	CC	766	n	V	y	n	0	cu	25	999	1	0	nev any	st
DEAN3	579		m	0	0	all	-	all	Eu:UK	1969	CC	766	n	V	y	n	0	cu	20	24	0	3	nev any	st
DEAN3	580		m	0	0	all	-	all	Eu:UK	1969	CC	766	n	V	y	n	0	cu	1	19	0	0	nev any	st

In this overview table, subtotals and Qs values may be invalid and should be ignored

Table 1H32 - 5

IESLC - Meta-analysis of Ever/current Smoking by Age started, Overview
All LC types, Pipes only
Least adjusted

REF	NRR	SEX	AD	Number Exposed Case	Cont	Non-exposed Case	Cont	RR	95.00%CI
BOFFET	539	m	0	14	29	117	1750	7.22	(3.71- 14.04)
BOFFET	540	m	0	20	52	117	1750	5.75	(3.32- 9.96)
BOFFET	541	m	0	27	48	117	1750	8.41	(5.07- 13.97)
Subtotal BOFFET								7.10	(5.13- 9.82)
*CEDERL	523	m	1	8	-	7	-	4.40	(1.60- 12.11)
*CEDERL	524	m	1	11	-	7	-	9.20	(3.57- 23.68)
*CEDERL	525	m	1	11	-	7	-	9.90	(3.85- 25.47)
Subtotal CEDERL								7.54	(4.32- 13.18)
DEAN3	578	m	0	4	16	24	510	5.31	(1.65- 17.11)
DEAN3	579	m	0	3	21	24	510	3.04	(0.85- 10.89)
DEAN3	580	m	0	6	67	24	510	1.90	(0.75- 4.82)
Subtotal DEAN3								2.88	(1.53- 5.42)
Partial Totals				104	233	444	6780		

*prospective study

REF	NRR	SEX	AD	Ys	Ws	Qs	Ps
BOFFET	539	m	0	1.98	8.69	0.20	0.0000
BOFFET	540	m	0	1.75	12.76	0.07	0.0000
BOFFET	541	m	0	2.13	14.93	1.39	0.0000
Subtotal BOFFET				1.96	36.38	1.67	
*CEDERL	523	m	1	1.48	3.75	0.44	0.0041
*CEDERL	524	m	1	2.22	4.29	0.67	0.0000
*CEDERL	525	m	1	2.29	4.30	0.94	0.0000
Subtotal CEDERL				2.02	12.35	2.05	
DEAN3	578	m	0	1.67	2.81	0.07	0.0051
DEAN3	579	m	0	1.11	2.36	1.20	0.0883
DEAN3	580	m	0	0.64	4.44	6.19	0.1752
Subtotal DEAN3				1.06	9.60	7.46	

N	9
NS	3

Table 1H32 - 6

IESLC - Meta-analysis of Ever/current Smoking by Age started, Overview
All LC types, Pipes only
Least adjusted

	combined	Sex male	female	Total
N		9		9
NS		3		3

In this overview table, other than the "N" rows, entries in the "absent" and "Total" columns may be invalid and should be ignored

MALES

	Age started (broad categories) absent	19+k26	15-25k18	1-17k14	Total
N	4	3	1	1	9
NS	2	3	1	1	6
Wt	30.42	19.32	4.29	4.30	58.34
Het Chi	8.95	0.21	0.00	0.00	11.18
Het df	3	2	0	0	8
Het P	*	N.S.	N.S.	N.S.	N.S.
Fixed RR	5.99	5.40	9.20	9.90	6.20
RRl	4.20	3.46	3.57	3.85	4.80
RRu	8.55	8.43	23.69	25.46	8.01
P	+++	+++	+++	+++	+++
Random RR	4.87	5.40	9.20	9.90	5.98
RRl	2.48	3.46	3.57	3.85	4.34
RRu	9.57	8.43	23.69	25.46	8.22
P	+++	+++	+++	+++	+++

	absent	27+k30	Age started (narrow categories) 23-29k26	19-25k22	15-21k18	11-17k14	1-13k10	Total
N	6	1		1	1			9
NS	3	1		1	1			6
Wt	42.99	8.69		2.36	4.29			58.34
Het Chi	9.07	0.00		0.00	0.00			11.18
Het df	5	0		0	0			8
Het P	N.S.	N.S.		N.S.	N.S.			N.S.
Fixed RR	6.01	7.22		3.04	9.20			6.20
RRl	4.46	3.71		0.85	3.57			4.80
RRu	8.10	14.04		10.89	23.69			8.01
P	+++	+++		(+)	+++			+++
Random RR	5.61	7.22		3.04	9.20			5.98
RRl	3.64	3.71		0.85	3.57			4.34
RRu	8.66	14.04		10.89	23.69			8.22
P	+++	+++		(+)	+++			+++

Table 1H32 - 7

IESLC - Meta-analysis of Ever/current Smoking by Age started, Overview
All LC types, Pipes only
Excluded studies (and stage at which they were excluded)

1 BECHER BLOT1 BROWN3 CARPEN CHYOU DARBY DOLL2 GARCIA GRAHAM GURSEL HAMMO2 JAHN JAIN LAUSSM PRESCO QIAO
TVERDA WIGLE WYNDE3

2 AKIBA AMANDU AMES AXELSS BENSHL BEST BOUCHA BOUCOT BROSS CHEN CPSII DEAN2 DESTEF DORGAN DOSEME FAN
GARSHI GER GILLIS HAMMON HUMBLE JUSSAW KAISE2 KATSOU KAUFMA KOO KREUZE LEVIN MCCONN NOTAN2 OSANN2 PEZZO2
PISANI RESTRE SADOWS VUTUC WANG2 WATSON WU2 WUWILL WYNDE2 WYNDE8 XU ZHOU

3 GUO MCDUFF SPITZ STASZE ZHANG

4 LUO

5 CORREA HOLE YUAN

7 AGUDO ALDERS ARMADA AUVINE BARBON BENHAM BRESLO BUFFLE CHEN2 CHIAZZ CHOI CPSI DAMBER DOLL DORN ENGELA
GAO GAO2 GENG HAENSZ HEGMAN HIRAYA HU HU2 JEDRYC JOLY KHUDER KOULUM LETOUR LIAW LIU3 LIU4
LIU5 LUBIN LUBIN2 MATOS MIGRAN MRFITR PERNU PEZZOT QIAO2 RACHTA SEGI2 SOBUE SPEIZE SUZUK2 SVENSS TIZZAN
WAKAI WU WYNDE6 WYNDE7 ZHENG

Table 1H32 - 8

Potentially overlapping studies

REF	REFGP	PRINC	.	OVERLAP/LINK
BOFFET	BOFFET	1		BOFFET-combined

Table 1H33 -

IESLC - Meta-analysis of Ever/current Smoking, Age started, "Highest vs lowest"
All LC types, Pipes only

This analysis is restricted to results for:
1) Ever/current smokers
2) Results by Age started
3) Categorical results by Age started
4) Denominator (unexposed) = "low"
5) All LC types (or near equivalent)
6) Results complete enough for use in metaanalysis
7) SMKSTA: ever, current
8) PRODUCT: pipes only

Within each study, results are then selected (in the following order of preference, within each sex) for:
9) Results with least adjustment for other aspects of smoking (ADOS)
10) The highest vs lowest category
11) Followup period (YF, prospective studies): whole study (coded as 0) or longest available
12) LCtype: all or nearest available, at least Squamous and Adeno. (q = squamous, s = small,
l = large, a = adeno, mix = mixed, alv = alveolar)
13) Race: all or nearest available, otherwise by race (wh or w = white, bl or b = black, hi = hispanic
ch = chinese, jap = japanese, haw = hawaiian, w+o = white + oriental, sca = scandinavian, as = asian)
14) For overlapping studies: principal rather than subsidiary studies
Finally by Age: whole study (coded as 0) if available, otherwise by widest available age group
and then for single sex results (m, f) in preference to results for both sexes combined (c).

Results adjusted (AD) for the most potential confounders are then chosen in Sections -1 to -3
and results adjusted for the least confounders in Sections -4 to -6. (Those least adjusted results which
actually differ from the most adjusted are marked 'x' in column X in Section -4)

Section -7 shows excluded studies, together with the stage (as above) at which no qualifying
results were found.

Section -8 lists the potentially overlapping studies which have been included (1=principal, 2=subsidiary).

Section -9 lists any results which would have been included in preference except that they had data not complete
enough for use in meta-analysis, with their significance (yes/no), if known, and any further comment as entered
on the database. It also lists as "gap" any categories for which no data were presented by the original authors.

In addition to those mentioned above, the following fields, levels and abbreviations are used:
* or nk = not known, n = no, y = yes, ot = other
all/unspec = all or unspecified, cig+/-ot = cigarettes irrespective of other products (cigar, pipe etc)
MC = manufactured cigarettes, HR = hand-rolled cigarettes
exL, exH = range of exposure (low and high) in the "highest" group, in terms of Age started
unexL, unexH = range of exposure (low and high) in the "lowest" group, in terms of Age started
REF: 6-character study reference
NRR: number of the RR on the database within the study
ST : study type (CC = case control, pr or prosp = prospective)
NLC: number of lung cancer cases in whole study
R : risky occupational population (n = no, m = mining, o = other risky)
VB : national cigarette type (V = at least 75% Virginia, bl = at least 75% blended, ot = other)
P : any proxy use
H : full histological confirmation
De : derivation of RR/CI (or = original, st = standard method, ot = other method of estimation)

Table 1H33 - 1

IESLC - Meta-analysis of Ever/current Smoking, Age started, "Highest vs lowest"
All LC types, Pipes only
Most adjusted

REF	NRR	SEX	AGEL	AGEH	RACE	YF	LC TYPE	LOC	START	ST	NLC	R	VB	P	H	AD	ADOS	SM	exL	exH	unexL	unexH	De
BOFFET	548	m	0	0	all	-	all	Eu:mul	1988	CC	5621	n	bl	y	n	2	0	ev	1	19	27	999	ot
CEDERL	527	m	0	0	all	10	all	Eu:Sca	1963	pr	491	n	bl	n	n	1	0	cu	1	16	19	999	ot
DEAN3	582	m	0	0	all	-	all	Eu:UK	1969	CC	766	n	V	y	n	0	0	cu	1	19	25	999	st

Table 1H33 - 2

IESLC - Meta-analysis of Ever/current Smoking, Age started, "Highest vs lowest"
All LC types, Pipes only
Most adjusted

REF	NRR	SEX	AD	Number Exposed Case	Exposed Cont	Non-exposed Case	Non-exposed Cont	RR	95.00%CI
BOFFET	548	m	2	27	-	14	-	1.17	(0.51- 2.69)
*CEDERL	527	m	1	11	-	8	-	2.25	(0.87- 5.80)
DEAN3	582	m	0	6	67	4	16	0.36	(0.09- 1.42)
Partial Totals				44	67	26	16		

*prospective study

REF	NRR	SEX	AD	Ys	Ws	Qs	Ps
BOFFET	548	m	2	0.16	5.56	0.01	0.7113
*CEDERL	527	m	1	0.81	4.27	1.64	0.0938
DEAN3	582	m	0	-1.03	2.02	3.00	0.1441

	N	3
	NS	3
	Wt	11.85
Het	Chi	4.65
Het	df	2
Het	P	(*)
Fixed	RR	1.21
	RRl	0.68
	RRu	2.14
	P	N.S.
Random	RR	1.10
	RRl	0.45
	RRu	2.72
	P	N.S.
Asymm	P	N.S.

Table 1H33 - 3

IESLC - Meta-analysis of Ever/current Smoking, Age started, "Highest vs lowest"
All LC types, Pipes only
Most adjusted

	Sex combined	male	female	Total
N		3		3
NS		3		3
Wt		11.85		11.85
Het Chi		4.65		4.65
Het df		2		2
Het P		(*)		(*)
Fixed RR		1.21		1.21
RRl		0.68		0.68
RRu		2.14		2.14
P		N.S.		N.S.
Random RR		1.10		1.10
RRl		0.45		0.45
RRu		2.72		2.72
P		N.S.		N.S.
Between Chi				
Between df				
Between P				N.S.
Btwn(F) P				N.S.
Btwn(R) P				N.S.

MALES

Too few RRs for analysis by factor

Table 1H33 - 4

IESLC - Meta-analysis of Ever/current Smoking, Age started, "Highest vs lowest"
All LC types, Pipes only
Least adjusted

REF	NRR	X	SEX	AGEL	AGEH	RACE	YF	LC TYPE	LOC	START	ST	NLC	R	VB	P	H	AD	ADOS	SM	exL	exH	unexL	unexH	De
BOFFET	543	x	m	0	0	all	-	all	Eu:mul	1988	CC	5621	n	bl	y	n	0	0	ev	1	19	27	999	st
CEDERL	527		m	0	0	all	10	all	Eu:Sca	1963	pr	491	n	bl	n	n	1	0	cu	1	16	19	999	ot
DEAN3	582		m	0	0	all	-	all	Eu:UK	1969	CC	766	n	V	y	n	0	0	cu	1	19	25	999	st

Table 1H33 - 5

IESLC - Meta-analysis of Ever/current Smoking, Age started, "Highest vs lowest"
All LC types, Pipes only
Least adjusted

				Number Exposed		Non-exposed			
REF	NRR	SEX	AD	Case	Cont	Case	Cont	RR	95.00%CI
BOFFET	543	m	0	27	48	14	29	1.17	(0.53- 2.58)
*CEDERL	527	m	1	11	-	8	-	2.25	(0.87- 5.80)
DEAN3	582	m	0	6	67	4	16	0.36	(0.09- 1.42)
Partial Totals				44	115	26	45		

*prospective study

REF	NRR	SEX	AD	Ys	Ws	Qs	Ps
BOFFET	543	m	0	0.15	6.11	0.01	0.7056
*CEDERL	527	m	1	0.81	4.27	1.66	0.0938
DEAN3	582	m	0	-1.03	2.02	2.98	0.1441

	N	3
	NS	3
	Wt	12.40
Het	Chi	4.65
Het	df	2
Het	P	(*)
Fixed	RR	1.21
	RRl	0.69
	RRu	2.10
	P	N.S.
Random	RR	1.10
	RRl	0.45
	RRu	2.68
	P	N.S.
Asymm	P	N.S.

Table 1H33 - 6

IESLC - Meta-analysis of Ever/current Smoking, Age started, "Highest vs lowest"
All LC types, Pipes only
Least adjusted

	Sex combined	male	female	Total
N		3		3
NS		3		3
Wt		12.40		12.40
Het Chi		4.65		4.65
Het df		2		2
Het P		(*)		(*)
Fixed RR		1.21		1.21
RRl		0.69		0.69
RRu		2.10		2.10
P		N.S.		N.S.
Random RR		1.10		1.10
RRl		0.45		0.45
RRu		2.68		2.68
P		N.S.		N.S.
Between Chi				
Between df				
Between P				N.S.
Btwn(F) P				N.S.
Btwn(R) P				N.S.

Table 1H33 - 7

IESLC - Meta-analysis of Ever/current Smoking, Age started, "Highest vs lowest"
All LC types, Pipes only
Excluded studies (and stage at which they were excluded)

1 BECHER BLOT1 BROWN3 CARPEN CHYOU DARBY DOLL2 GARCIA GRAHAM GURSEL HAMMO2 JAHN JAIN LAUSSM PRESCO QIAO
TVERDA WIGLE WYNDE3

2 AKIBA AMANDU AMES AXELSS BENSHL BEST BOUCHA BOUCOT BROSS CHEN CPSII DEAN2 DESTEF DORGAN DOSEME FAN
GARSHI GER GILLIS HAMMON HUMBLE JUSSAW KAISE2 KATSOU KAUFMA KOO KREUZE LEVIN MCCONN NOTAN2 OSANN2 PEZZO2
PISANI RESTRE SADOWS VUTUC WANG2 WATSON WU2 WUWILL WYNDE2 WYNDE8 XU ZHOU

3 GUO MCDUFF SPITZ STASZE ZHANG

5 LUO

6 CORREA HOLE YUAN

8 AGUDO ALDERS ARMADA AUVINE BARBON BENHAM BRESLO BUFFLE CHEN2 CHIAZZ CHOI CPSI DAMBER DOLL DORN ENGELA
GAO GAO2 GENG HAENSZ HEGMAN HIRAYA HU HU2 JEDRYC JOLY KHUDER KOULUM LETOUR LIAW LIU3 LIU4
LIU5 LUBIN LUBIN2 MATOS MIGRAN MRFITR PERNU PEZZOT QIAO2 RACHTA SEGI2 SOBUE SPEIZE SUZUK2 SVENSS TIZZAN
WAKAI WU WYNDE6 WYNDE7 ZHENG

Table 1H33 - 8

Potentially overlapping studies

REF	REFGP	PRINC	.	OVERLAP/LINK
BOFFET	BOFFET	1		BOFFET-combined

Table 1H34 -

IESLC - Meta-analysis of Ever/current Smoking by Age started, Overview
All LC types, Cigars only

This analysis is restricted to results for:
1) Ever/current smokers
2) Results by Age started
3) Categorical results by Age started
Results by Age started are grouped under 2 schemes (S1, S2). Each scheme has a set of "key values". An interval is allocated to the category whose key value it includes, and intervals which include none or more than one of the key values are excluded. (Open-ended intervals are coded as 999)

S1	key value	maximum range
1	26	19+
2	18	15-25
3	14	1-17

S2	key value	maximum range
1	30	27+
2	26	23-29
3	22	19-25
4	18	15-21
5	14	11-17
6	10	1-13

4) All LC types (or near equivalent)
5) Results complete enough for use in metaanalysis
6) cigars only

Within each study, results are then selected (in the following order of preference, within each sex) for:
7) (not applicable)
8) DENOM: never smoked anything, never any + low
9) Followup period (YF, prospective studies): whole study (coded as 0) or longest available
10) LCtype: all or nearest available, at least Squamous and Adeno. (q = squamous, s = small, l = large, a = adeno, mix = mixed, alv = alveolar)
11) Race: all or nearest available, otherwise by race (wh or w = white, bl or b = black, hi = hispanic ch = chinese, jap = japanese, haw = hawaiian, w+o = white + oriental, sca = scandinavian, as = asian)
12) For overlapping studies: principal rather than subsidiary studies
Finally by Age: whole study (coded as 0) if available, otherwise by widest available age group and then for single sex results (m, f) in preference to results for both sexes combined (c).

Results adjusted (AD) for the most potential confounders are then chosen in Sections -1 to -3 and results adjusted for the least confounders in Sections -4 to -6. (Those least adjusted results which actually differ from the most adjusted are marked 'x' in column X in Section -4)

Section -7 shows excluded studies, together with the stage (as above) at which no qualifying results were found.

Section -8 lists the potentially overlapping studies which have been included (1=principal, 2=subsidiary).

Section -9 lists any results which would have been included in preference except that they had data not complete enough for use in meta-analysis, with their significance (yes/no), if known, and any further comment as entered on the database. It also lists as "gap" any categories for which no data were presented by the original authors.

In addition to those mentioned above, the following fields, levels and abbreviations are used:
* or nk = not known, n = no, y = yes, ot = other
ev = ever, cu = current, nev = never
all/unspec = all or unspecified, MC = manufactured cigarettes, HR = hand-rolled cigarettes
exL, exH = range of exposure (low and high) in the smoking group, in terms of Age started
REF: 6-character study reference
NRR: number of the RR on the database within the study
ST : study type (CC = case control, pr or prosp = prospective)
NLC: number of lung cancer cases in whole study
R : risky occupational population (n = no, m = mining, o = other risky)
VB : national cigarette type (V = at least 75% Virginia, bl = at least 75% blended, ot = other)
P : any proxy use
H : full histological confirmation
De : derivation of RR/CI (or = original, st = standard method, ot = other method of estimation)

Table 1H34 - 1

IESLC - Meta-analysis of Ever/current Smoking by Age started, Overview
All LC types, Cigars only
Most adjusted

REF	NRR	SEX	AGEL	AGEH	RACE	YF	LC TYPE	LOC	START	ST	NLC	R	VB	P	H	AD	SM	exL	exH	S1	S2	DENOM	De
BOFFET	520	m	0	0	all	-	all	Eu:mul	1988	CC	5621	n	bl	y	n	2	ev	27	999	0	1	nev any	or
BOFFET	521	m	0	0	all	-	all	Eu:mul	1988	CC	5621	n	bl	y	n	2	ev	20	26	1	0	nev any	or
BOFFET	522	m	0	0	all	-	all	Eu:mul	1988	CC	5621	n	bl	y	n	2	ev	1	19	0	0	nev any	or
JOLY	677	m	0	0	all	-	all	SCAmer	1978	CC	826	n	bl	n	n	0	ev	25	999	1	0	nev any	st
JOLY	678	m	0	0	all	-	all	SCAmer	1978	CC	826	n	bl	n	n	0	ev	15	24	2	0	nev any	st
JOLY	679	m	0	0	all	-	all	SCAmer	1978	CC	826	n	bl	n	n	0	ev	1	14	3	0	nev any	st

In this overview table, subtotals and Qs values may be invalid and should be ignored

Table 1H34 - 2

IESLC - Meta-analysis of Ever/current Smoking by Age started, Overview
All LC types, Cigars only
Most adjusted

REF	NRR	SEX	AD	Number Case	Exposed Cont	Non-exposed Case	Cont	RR	95.00%CI
BOFFET	520	m	2	7	-	117	-	3.40	(1.50- 8.00)
BOFFET	521	m	2	16	-	117	-	10.50	(5.30- 21.10)
BOFFET	522	m	2	20	-	117	-	17.00	(8.60- 33.40)
Subtotal BOFFET								9.51	(6.26- 14.46)
JOLY	677	m	0	7	34	12	218	3.74	(1.38- 10.16)
JOLY	678	m	0	17	91	12	218	3.39	(1.56- 7.39)
JOLY	679	m	0	19	54	12	218	6.39	(2.93- 13.97)
Subtotal JOLY								4.42	(2.73- 7.17)
Partial Totals				86	179	387	654		

*prospective study

REF	NRR	SEX	AD	Ys	Ws	Qs	Ps
BOFFET	520	m	2	1.22	5.48	2.68	0.0042
BOFFET	521	m	2	2.35	8.05	1.47	0.0000
BOFFET	522	m	2	2.83	8.35	6.91	0.0000
Subtotal BOFFET				2.25	21.88	11.07	
JOLY	677	m	0	1.32	3.84	1.40	0.0097
JOLY	678	m	0	1.22	6.34	3.12	0.0021
JOLY	679	m	0	1.86	6.29	0.03	0.0000
Subtotal JOLY				1.49	16.47	4.55	

N	6
NS	2

Table 1H34 - 3

IESLC - Meta-analysis of Ever/current Smoking by Age started, Overview
All LC types, Cigars only
Most adjusted

	Sex			
	combined	male	female	Total
N		6		6
NS		2		2

In this overview table, other than the "N" rows, entries in the "absent" and "Total" columns may be invalid and should be ignored

MALES

	Age started (broad categories)				
	absent	19+k26	15-25k18	1-17k14	Total
N	2	2	1	1	6
NS	1	2	1	1	2
Wt	13.83	11.89	6.34	6.29	38.35
Het Chi	8.57	2.77	0.00	0.00	15.62
Het df	1	1	0	0	5
Het P	**	(*)	N.S.	N.S.	**
Fixed RR	8.98	7.52	3.39	6.39	6.84
RRl	5.30	4.26	1.56	2.93	4.99
RRu	15.21	13.28	7.39	13.97	9.39
P	+++	+++	++	+++	+++
Random RR	7.75	6.69	3.39	6.39	6.30
RRl	1.60	2.45	1.56	2.93	3.58
RRu	37.51	18.25	7.39	13.97	11.09
P	+	+++	++	+++	+++

			Age started (narrow categories)					
	absent	27+k30	23-29k26	19-25k22	15-21k18	11-17k14	1-13k10	Total
N	5	1						6
NS	2	1						3
Wt	32.87	5.48						38.35
Het Chi	12.49	0.00						15.62
Het df	4	0						5
Het P	*	N.S.						**
Fixed RR	7.69	3.40						6.84
RRl	5.47	1.47						4.99
RRu	10.83	7.85						9.39
P	+++	++						+++
Random RR	7.09	3.40						6.30
RRl	3.85	1.47						3.58
RRu	13.08	7.85						11.09
P	+++	++						+++

Table 1H34 - 4

IESLC - Meta-analysis of Ever/current Smoking by Age started, Overview
All LC types, Cigars only
Least adjusted

REF	NRR	X	SEX	AGEL	AGEH	RACE	YF	LC TYPE	LOC	START	ST	NLC	R	VB	P	H	AD	SM	exL	exH	S1	S2	DENOM	De
BOFFET	515	x	m	0	0	all	-	all	Eu:mul	1988	CC	5621	n	bl	y	n	0	ev	27	999	0	1	nev any	st
BOFFET	516	x	m	0	0	all	-	all	Eu:mul	1988	CC	5621	n	bl	y	n	0	ev	20	26	1	0	nev any	st
BOFFET	517	x	m	0	0	all	-	all	Eu:mul	1988	CC	5621	n	bl	y	n	0	ev	1	19	0	0	nev any	st
JOLY	677		m	0	0	all	-	all	SCAmer	1978	CC	826	n	bl	n	n	0	ev	25	999	1	0	nev any	st
JOLY	678		m	0	0	all	-	all	SCAmer	1978	CC	826	n	bl	n	n	0	ev	15	24	2	0	nev any	st
JOLY	679		m	0	0	all	-	all	SCAmer	1978	CC	826	n	bl	n	n	0	ev	1	14	3	0	nev any	st

In this overview table, subtotals and Qs values may be invalid and should be ignored

Table 1H34 - 5

IESLC - Meta-analysis of Ever/current Smoking by Age started, Overview
All LC types, Cigars only
Least adjusted

REF	NRR	SEX	AD	Number Case	Exposed Cont	Non-exposed Case	Cont	RR	95.00%CI
BOFFET	515	m	0	7	34	117	1750	3.08	(1.34- 7.10)
BOFFET	516	m	0	16	23	117	1750	10.41	(5.35- 20.23)
BOFFET	517	m	0	20	20	117	1750	14.96	(7.83- 28.58)
Subtotal BOFFET								9.00	(6.00- 13.50)
JOLY	677	m	0	7	34	12	218	3.74	(1.38- 10.16)
JOLY	678	m	0	17	91	12	218	3.39	(1.56- 7.39)
JOLY	679	m	0	19	54	12	218	6.39	(2.93- 13.97)
Subtotal JOLY								4.42	(2.73- 7.17)
Totals				86	256	387	5904		

*prospective study

REF	NRR	SEX	AD	Ys	Ws	Qs	Ps
BOFFET	515	m	0	1.12	5.51	3.34	0.0083
BOFFET	516	m	0	2.34	8.69	1.67	0.0000
BOFFET	517	m	0	2.71	9.16	5.89	0.0000
Subtotal BOFFET				2.20	23.37	10.91	
JOLY	677	m	0	1.32	3.84	1.31	0.0097
JOLY	678	m	0	1.22	6.34	2.94	0.0021
JOLY	679	m	0	1.86	6.29	0.01	0.0000
Subtotal JOLY				1.49	16.47	4.27	

N	6
NS	2

Table 1H34 - 6

IESLC - Meta-analysis of Ever/current Smoking by Age started, Overview
All LC types, Cigars only
Least adjusted

	Sex combined	male	female	Total
N		6		6
NS		2		2

In this overview table, other than the "N" rows, entries in the "absent" and "Total" columns may be invalid and should be ignored

MALES

	Age started (broad categories) absent	19+k26	15-25k18	1-17k14	Total
N	2	2	1	1	6
NS	1	2	1	1	2
Wt	14.68	12.53	6.34	6.29	39.84
Het Chi	8.60	2.79	0.00	0.00	15.18
Het df	1	1	0	0	5
Het P	**	(*)	N.S.	N.S.	**
Fixed RR	8.26	7.60	3.39	6.39	6.71
RRl	4.95	4.37	1.56	2.93	4.92
RRu	13.78	13.23	7.39	13.97	9.15
P	+++	+++	++	+++	+++
Random RR	6.94	6.70	3.39	6.39	6.10
RRl	1.48	2.48	1.56	2.93	3.52
RRu	32.66	18.08	7.39	13.97	10.55
P	+	+++	++	+++	+++

	absent	27+k30	23-29k26	19-25k22	15-21k18	11-17k14	1-13k10	Total
			Age started (narrow categories)					
N	5	1						6
NS	2	1						3
Wt	34.32	5.51						39.84
Het Chi	11.30	0.00						15.18
Het df	4	0						5
Het P	*	N.S.						**
Fixed RR	7.60	3.08						6.71
RRl	5.44	1.34						4.92
RRu	10.62	7.10						9.15
P	+++	++						+++
Random RR	6.97	3.08						6.10
RRl	3.93	1.34						3.52
RRu	12.34	7.10						10.55
P	+++	++						+++

Table 1H34 - 7

IESLC - Meta-analysis of Ever/current Smoking by Age started, Overview
All LC types, Cigars only
Excluded studies (and stage at which they were excluded)

1	BECHER BLOT1 BROWN3 CARPEN CHYOU DARBY DOLL2 GARCIA GRAHAM GURSEL HAMMO2 JAHN JAIN LAUSSM PRESCO QIAO TVERDA WIGLE WYNDE3
2	AKIBA AMANDU AMES AXELSS BENSHL BEST BOUCHA BOUCOT BROSS CHEN CPSII DEAN2 DESTEF DORGAN DOSEME FAN GARSHI GER GILLIS HAMMON HUMBLE JUSSAW KAISE2 KATSOU KAUFMA KOO KREUZE LEVIN MCCONN NOTAN2 OSANN2 PEZZO2 PISANI RESTRE SADOWS VUTUC WANG2 WATSON WU2 WUWILL WYNDE2 WYNDE8 XU ZHOU
3	GUO MCDUFF SPITZ STASZE ZHANG
4	LUO
5	CORREA HOLE YUAN
7	AGUDO ALDERS ARMADA AUVINE BARBON BENHAM BRESLO BUFFLE CEDERL CHEN2 CHIAZZ CHOI CPSI DAMBER DEAN3 DOLL DORN ENGELA GAO GAO2 GENG HAENSZ HEGMAN HIRAYA HU HU2 JEDRYC KHUDER KOULUM LETOUR LIAW LIU3 LIU4 LIU5 LUBIN LUBIN2 MATOS MIGRAN MRFITR PERNU PEZZOT QIAO2 RACHTA SEGI2 SOBUE SPEIZE SUZUK2 SVENSS TIZZAN WAKAI WU WYNDE6 WYNDE7 ZHENG

Table 1H34 - 8

Potentially overlapping studies

REF	REFGP	PRINC	.	OVERLAP/LINK
BOFFET	BOFFET	1		BOFFET-combined

Table 1H35 -

IESLC - Meta-analysis of Ever/current Smoking, Age started, "Highest vs lowest"
All LC types, Cigars only

This analysis is restricted to results for:
1) Ever/current smokers
2) Results by Age started
3) Categorical results by Age started
4) Denominator (unexposed) = "low"
5) All LC types (or near equivalent)
6) Results complete enough for use in metaanalysis
7) SMKSTA: ever, current
8) PRODUCT: cigars only

Within each study, results are then selected (in the following order of preference, within each sex) for:
9) Results with least adjustment for other aspects of smoking (ADOS)
10) The highest vs lowest category
11) Followup period (YF, prospective studies): whole study (coded as 0) or longest available
12) LCtype: all or nearest available, at least Squamous and Adeno. (q = squamous, s = small,
l = large, a = adeno, mix = mixed, alv = alveolar)
13) Race: all or nearest available, otherwise by race (wh or w = white, bl or b = black, hi = hispanic
ch = chinese, jap = japanese, haw = hawaiian, w+o = white + oriental, sca = scandinavian, as = asian)
14) For overlapping studies: principal rather than subsidiary studies
Finally by Age: whole study (coded as 0) if available, otherwise by widest available age group
and then for single sex results (m, f) in preference to results for both sexes combined (c).

Results adjusted (AD) for the most potential confounders are then chosen in Sections -1 to -3
and results adjusted for the least confounders in Sections -4 to -6. (Those least adjusted results which
actually differ from the most adjusted are marked 'x' in column X in Section -4)

Section -7 shows excluded studies, together with the stage (as above) at which no qualifying
results were found.

Section -8 lists the potentially overlapping studies which have been included (1=principal, 2=subsidiary).

Section -9 lists any results which would have been included in preference except that they had data not complete
enough for use in meta-analysis, with their significance (yes/no), if known, and any further comment as entered
on the database. It also lists as "gap" any categories for which no data were presented by the original authors.

In addition to those mentioned above, the following fields, levels and abbreviations are used:
* or nk = not known, n = no, y = yes, ot = other
all/unspec = all or unspecified, MC = manufactured cigarettes, HR = hand-rolled cigarettes
exL, exH = range of exposure (low and high) in the "highest" group, in terms of Age started
unexL, unexH = range of exposure (low and high) in the "lowest" group, in terms of Age started
REF: 6-character study reference
NRR: number of the RR on the database within the study
ST : study type (CC = case control, pr or prosp = prospective)
NLC: number of lung cancer cases in whole study
R : risky occupational population (n = no, m = mining, o = other risky)
VB : national cigarette type (V = at least 75% Virginia, bl = at least 75% blended, ot = other)
P : any proxy use
H : full histological confirmation
De : derivation of RR/CI (or = original, st = standard method, ot = other method of estimation)

Table 1H35 - 1

IESLC - Meta-analysis of Ever/current Smoking, Age started, "Highest vs lowest"
All LC types, Cigars only
Most adjusted

REF	NRR	SEX	AGEL	AGEH	RACE	YF	LC TYPE	LOC	START	ST	NLC	R	VB	P	H	AD	ADOS	SM	exL	exH	unexL	unexH	De
BOFFET	524	m	0	0	all	-	all	Eu:mul	1988	CC	5621	n	bl	y	n	2	0	ev	1	19	27	999	ot
JOLY	681	m	0	0	all	-	all	SCAmer	1978	CC	826	n	bl	n	n	0	0	ev	1	14	25	999	st

Table 1H35 - 2

IESLC - Meta-analysis of Ever/current Smoking, Age started, "Highest vs lowest"
All LC types, Cigars only
Most adjusted

REF	NRR	SEX	AD	Number Case	Exposed Cont	Non-exposed Case	Cont	RR	95.00%CI
BOFFET	524	m	2	20	-	7	-	5.00	(1.76- 14.21)
JOLY	681	m	0	19	54	7	34	1.71	(0.65- 4.49)
Partial Totals				39	54	14	34		

*prospective study

REF	NRR	SEX	AD	Ys	Ws	Qs	Ps
BOFFET	524	m	2	1.61	3.52	1.18	0.0025
JOLY	681	m	0	0.54	4.11	1.01	0.2774

	N	2
	NS	2
	Wt	7.63
Het	Chi	2.19
Het	df	1
Het	P	N.S.
Fixed	RR	2.81
	RRl	1.38
	RRu	5.70
	P	++
Random	RR	2.87
	RRl	1.00
	RRu	8.21
	P	+
Asymm	P	

Table 1H35 - 3

IESLC - Meta-analysis of Ever/current Smoking, Age started, "Highest vs lowest"
All LC types, Cigars only
Most adjusted

	Sex combined	male	female	Total
N		2		2
NS		2		2
Wt		7.63		7.63
Het Chi		2.19		2.19
Het df		1		1
Het P		N.S.		N.S.
Fixed RR		2.81		2.81
RRl		1.38		1.38
RRu		5.70		5.70
P		++		++
Random RR		2.87		2.87
RRl		1.00		1.00
RRu		8.21		8.21
P		+		+
Between Chi				
Between df				
Between P				N.S.
Btwn(F) P				N.S.
Btwn(R) P				N.S.

MALES

Too few RRs for analysis by factor

Table 1H35 - 4

IESLC - Meta-analysis of Ever/current Smoking, Age started, "Highest vs lowest"
All LC types, Cigars only
Least adjusted

REF	NRR	X	SEX	AGEL	AGEH	RACE	YF	LC TYPE	LOC	START	ST	NLC	R	VB	P	H	AD	ADOS	SM	exL	exH	unexL	unexH	De
BOFFET	519	x	m	0	0	all	-	all	Eu:mul	1988	CC	5621	n	bl	y	n	0	0	ev	1	19	27	999	st
JOLY	681		m	0	0	all	-	all	SCAmer	1978	CC	826	n	bl	n	n	0	0	ev	1	14	25	999	st

Table 1H35 - 5

IESLC - Meta-analysis of Ever/current Smoking, Age started, "Highest vs lowest"
All LC types, Cigars only
Least adjusted

REF	NRR	SEX	AD	Number Case	Exposed Cont	Non-exposed Case	Cont	RR	95.00%CI
BOFFET	519	m	0	20	20	7	34	4.86	(1.75- 13.51)
JOLY	681	m	0	19	54	7	34	1.71	(0.65- 4.49)
Totals				39	74	14	68		

*prospective study

REF	NRR	SEX	AD	Ys	Ws	Qs	Ps
BOFFET	519	m	0	1.58	3.67	1.12	0.0025
JOLY	681	m	0	0.54	4.11	1.00	0.2774

	N	2
	NS	2
	Wt	7.78
Het	Chi	2.12
Het	df	1
Het	P	N.S.
Fixed	RR	2.80
	RRl	1.39
	RRu	5.65
	P	++
Random	RR	2.84
	RRl	1.02
	RRu	7.91
	P	+
Asymm	P	

Table 1H35 - 6

IESLC - Meta-analysis of Ever/current Smoking, Age started, "Highest vs lowest"
All LC types, Cigars only
Least adjusted

	Sex combined	male	female	Total
N		2		2
NS		2		2
Wt		7.78		7.78
Het Chi		2.12		2.12
Het df		1		1
Het P		N.S.		N.S.
Fixed RR		2.80		2.80
RRl		1.39		1.39
RRu		5.65		5.65
P		++		++
Random RR		2.84		2.84
RRl		1.02		1.02
RRu		7.91		7.91
P		+		+
Between Chi				
Between df				
Between P				N.S.
Btwn(F) P				N.S.
Btwn(R) P				N.S.

Table 1H35 - 7

IESLC - Meta-analysis of Ever/current Smoking, Age started, "Highest vs lowest"
All LC types, Cigars only
Excluded studies (and stage at which they were excluded)

1	BECHER BLOT1 BROWN3 CARPEN CHYOU DARBY DOLL2 GARCIA GRAHAM GURSEL HAMMO2 JAHN JAIN LAUSSM PRESCO QIAO TVERDA WIGLE WYNDE3
2	AKIBA AMANDU AMES AXELSS BENSHL BEST BOUCHA BOUCOT BROSS CHEN CPSII DEAN2 DESTEF DORGAN DOSEME FAN GARSHI GER GILLIS HAMMON HUMBLE JUSSAW KAISE2 KATSOU KAUFMA KOO KREUZE LEVIN MCCONN NOTAN2 OSANN2 PEZZO2 PISANI RESTRE SADOWS VUTUC WANG2 WATSON WU2 WUWILL WYNDE2 WYNDE8 XU ZHOU
3	GUO MCDUFF SPITZ STASZE ZHANG
5	LUO
6	CORREA HOLE YUAN
8	AGUDO ALDERS ARMADA AUVINE BARBON BENHAM BRESLO BUFFLE CEDERL CHEN2 CHIAZZ CHOI CPSI DAMBER DEAN3 DOLL DORN ENGELA GAO GAO2 GENG HAENSZ HEGMAN HIRAYA HU HU2 JEDRYC KHUDER KOULUM LETOUR LIAW LIU3 LIU4 LIU5 LUBIN LUBIN2 MATOS MIGRAN MRFITR PERNU PEZZOT QIAO2 RACHTA SEGI2 SOBUE SPEIZE SUZUK2 SVENSS TIZZAN WAKAI WU WYNDE6 WYNDE7 ZHENG

Table 1H35 - 8
Potentially overlapping studies

REF	REFGP	PRINC	.	OVERLAP/LINK
BOFFET	BOFFET	1		BOFFET-combined

Table 1H36 -

IESLC - Meta-analysis of Ever/current Smoking by Age started, Overview
All LC types, Mixed smokers

This analysis is restricted to results for:
1) Ever/current smokers
2) Results by Age started
3) Categorical results by Age started
Results by Age started are grouped under 2 schemes (S1, S2). Each scheme has a set of "key values". An interval is allocated to the category whose key value it includes, and intervals which include none or more than one of the key values are excluded. (Open-ended intervals are coded as 999)

S1	key value	maximum range
1	26	19+
2	18	15-25
3	14	1-17

S2	key value	maximum range
1	30	27+
2	26	23-29
3	22	19-25
4	18	15-21
5	14	11-17
6	10	1-13

4) All LC types (or near equivalent)
5) Results complete enough for use in metaanalysis
6) mixed smokers (cigarettes and pipe/cigar)

Within each study, results are then selected (in the following order of preference, within each sex) for:
7) (not applicable)
8) DENOM: never smoked anything, never any + low
9) Followup period (YF, prospective studies): whole study (coded as 0) or longest available
10) LCtype: all or nearest available, at least Squamous and Adeno. (q = squamous, s = small, l = large, a = adeno, mix = mixed, alv = alveolar)
11) Race: all or nearest available, otherwise by race (wh or w = white, bl or b = black, hi = hispanic ch = chinese, jap = japanese, haw = hawaiian, w+o = white + oriental, sca = scandinavian, as = asian)
12) For overlapping studies: principal rather than subsidiary studies
Finally by Age: whole study (coded as 0) if available, otherwise by widest available age group and then for single sex results (m, f) in preference to results for both sexes combined (c).

Results adjusted (AD) for the most potential confounders are then chosen in Sections -1 to -3 and results adjusted for the least confounders in Sections -4 to -6. (Those least adjusted results which actually differ from the most adjusted are marked 'x' in column X in Section -4)

Section -7 shows excluded studies, together with the stage (as above) at which no qualifying results were found.

Section -8 lists the potentially overlapping studies which have been included (1=principal, 2=subsidiary).

Section -9 lists any results which would have been included in preference except that they had data not complete enough for use in meta-analysis, with their significance (yes/no), if known, and any further comment as entered on the database. It also lists as "gap" any categories for which no data were presented by the original authors.

In addition to those mentioned above, the following fields, levels and abbreviations are used:
* or nk = not known, n = no, y = yes, ot = other
ev = ever, cu = current, nev = never
exL, exH = range of exposure (low and high) in the smoking group, in terms of Age started
REF: 6-character study reference
NRR: number of the RR on the database within the study
ST : study type (CC = case control, pr or prosp = prospective)
NLC: number of lung cancer cases in whole study
R : risky occupational population (n = no, m = mining, o = other risky)
VB : national cigarette type (V = at least 75% Virginia, bl = at least 75% blended, ot = other)
P : any proxy use
H : full histological confirmation
De : derivation of RR/CI (or = original, st = standard method, ot = other method of estimation)

Table 1H36 - 0

No RRs selected for this analysis

Table 1H36 - 7

IESLC - Meta-analysis of Ever/current Smoking by Age started, Overview
All LC types, Mixed smokers
Excluded studies (and stage at which they were excluded)

1 BECHER BLOT1 BROWN3 CARPEN CHYOU DARBY DOLL2 GARCIA GRAHAM GURSEL HAMMO2 JAHN JAIN LAUSSM PRESCO QIAO TVERDA WIGLE WYNDE3

2 AKIBA AMANDU AMES AXELSS BENSHL BEST BOUCHA BOUCOT BROSS CHEN CPSII DEAN2 DESTEF DORGAN DOSEME FAN GARSHI GER GILLIS HAMMON HUMBLE JUSSAW KAISE2 KATSOU KAUFMA KOO KREUZE LEVIN MCCONN NOTAN2 OSANN2 PEZZO2 PISANI RESTRE SADOWS VUTUC WANG2 WATSON WU2 WUWILL WYNDE2 WYNDE8 XU ZHOU

3 GUO MCDUFF SPITZ STASZE ZHANG

4 LUO

5 CORREA HOLE YUAN

7 AGUDO ALDERS ARMADA AUVINE BARBON BENHAM BOFFET BRESLO BUFFLE CEDERL CHEN2 CHIAZZ CHOI CPSI DAMBER DEAN3 DOLL DORN ENGELA GAO GAO2 GENG HAENSZ HEGMAN HIRAYA HU HU2 JEDRYC JOLY KHUDER KOULUM LETOUR LIAW LIU3 LIU4 LIU5 LUBIN LUBIN2 MATOS MIGRAN MRFITR PERNU PEZZOT QIAO2 RACHTA SEGI2 SOBUE SPEIZE SUZUK2 SVENSS TIZZAN WAKAI WU WYNDE6 WYNDE7 ZHENG

Table 1H37 -

IESLC - Meta-analysis of Ever/current Smoking, Age started, "Highest vs lowest"
All LC types, Mixed smokers

This analysis is restricted to results for:
1) Ever/current smokers
2) Results by Age started
3) Categorical results by Age started
4) Denominator (unexposed) = "low"
5) All LC types (or near equivalent)
6) Results complete enough for use in metaanalysis
7) SMKSTA: ever, current
8) PRODUCT: mixed smokers (cigarettes and pipe/cigar)

Within each study, results are then selected (in the following order of preference, within each sex) for:
9) Results with least adjustment for other aspects of smoking (ADOS)
10) The highest vs lowest category
11) Followup period (YF, prospective studies): whole study (coded as 0) or longest available
12) LCtype: all or nearest available, at least Squamous and Adeno. (q = squamous, s = small,
l = large, a = adeno, mix = mixed, alv = alveolar)
13) Race: all or nearest available, otherwise by race (wh or w = white, bl or b = black, hi = hispanic
ch = chinese, jap = japanese, haw = hawaiian, w+o = white + oriental, sca = scandinavian, as = asian)
14) For overlapping studies: principal rather than subsidiary studies
Finally by Age: whole study (coded as 0) if available, otherwise by widest available age group
and then for single sex results (m, f) in preference to results for both sexes combined (c).

Results adjusted (AD) for the most potential confounders are then chosen in Sections -1 to -3
and results adjusted for the least confounders in Sections -4 to -6. (Those least adjusted results which
actually differ from the most adjusted are marked 'x' in column X in Section -4)

Section -7 shows excluded studies, together with the stage (as above) at which no qualifying
results were found.

Section -8 lists the potentially overlapping studies which have been included (1=principal, 2=subsidiary).

Section -9 lists any results which would have been included in preference except that they had data not complete
enough for use in meta-analysis, with their significance (yes/no), if known, and any further comment as entered
on the database. It also lists as "gap" any categories for which no data were presented by the original authors.

In addition to those mentioned above, the following fields, levels and abbreviations are used:
* or nk = not known, n = no, y = yes, ot = other
exL, exH = range of exposure (low and high) in the "highest" group, in terms of Age started
unexL, unexH = range of exposure (low and high) in the "lowest" group, in terms of Age started
REF: 6-character study reference
NRR: number of the RR on the database within the study
ST : study type (CC = case control, pr or prosp = prospective)
NLC: number of lung cancer cases in whole study
R : risky occupational population (n = no, m = mining, o = other risky)
VB : national cigarette type (V = at least 75% Virginia, bl = at least 75% blended, ot = other)
P : any proxy use
H : full histological confirmation
De : derivation of RR/CI (or = original, st = standard method, ot = other method of estimation)

Table 1H37 - 0

No RRs selected for this analysis

Table 1H37 - 7

IESLC - Meta-analysis of Ever/current Smoking, Age started, "Highest vs lowest"
All LC types, Mixed smokers
Excluded studies (and stage at which they were excluded)

1 BECHER BLOT1 BROWN3 CARPEN CHYOU DARBY DOLL2 GARCIA GRAHAM GURSEL HAMMO2 JAHN JAIN LAUSSM PRESCO QIAO TVERDA WIGLE WYNDE3

2 AKIBA AMANDU AMES AXELSS BENSHL BEST BOUCHA BOUCOT BROSS CHEN CPSII DEAN2 DESTEF DORGAN DOSEME FAN GARSHI GER GILLIS HAMMON HUMBLE JUSSAW KAISE2 KATSOU KAUFMA KOO KREUZE LEVIN MCCONN NOTAN2 OSANN2 PEZZO2 PISANI RESTRE SADOWS VUTUC WANG2 WATSON WU2 WUWILL WYNDE2 WYNDE8 XU ZHOU

3 GUO MCDUFF SPITZ STASZE ZHANG

5 LUO

6 CORREA HOLE YUAN

8 AGUDO ALDERS ARMADA AUVINE BARBON BENHAM BOFFET BRESLO BUFFLE CEDERL CHEN2 CHIAZZ CHOI CPSI DAMBER DEAN3 DOLL DORN ENGELA GAO GAO2 GENG HAENSZ HEGMAN HIRAYA HU HU2 JEDRYC JOLY KHUDER KOULUM LETOUR LIAW LIU3 LIU4 LIU5 LUBIN LUBIN2 MATOS MIGRAN MRFITR PERNU PEZZOT QIAO2 RACHTA SEGI2 SOBUE SPEIZE SUZUK2 SVENSS TIZZAN WAKAI WU WYNDE6 WYNDE7 ZHENG
