# Supplementary material for: Systematic review with meta-analysis of the epidemiological evidence in the 1900s relating smoking to lung cancer
Source: BMC Cancer. 2012 Sep 3;12:385. doi: 10.1186/1471-2407-12-385 (PMC3505152; doi:10.1186/1471-2407-12-385)
Supplement: Additional file 5 — Detailed Analysis Tables (Individual file names as described in Additional file 1: Methods, Table1). [file 1471-2407-12-385-S5.zip › PDF/3K.pdf]

Table 3K1 -

IESLC - Meta-analysis of Ex Smoking by Years quit (vs current), Overview
Adenocarcinoma, Any Product (or Cigarettes if Any not available)

This analysis is restricted to results for:
1) Ex smokers
2) Results by Years quit (vs current)
3) Categorical results by Years quit (vs current)
Results by Years quit (vs current) are grouped under 2 schemes (S1, S2). Each scheme has a set of "key values". An interval is allocated to the category whose key value it includes, and intervals which include none or more than one of the key values are excluded. (Open-ended intervals are coded as 999)

S1	key value	maximum range
1	3	1-6
2	7	4-11
3	12	8+

S2	key value	maximum range
1	3	1-11
2	12	4-19
3	20	13+

4) Adenocarcinoma (or near equivalent)
5) Results complete enough for use in metaanalysis

Within each study, results are then selected (in the following order of preference, within each sex) for:
6) (not applicable)
7) PRODUCT: all/unspec, cigarettes regardless of other products, cigarettes only
8) CIGTYPE: all/unspecified, MC regardless of HR, MC only
9) Results with least adjustment for other aspects of smoking (ADOS)
10) DENOM: current smokers, current + recent smokers (up to number of m=months or y=years, max 2 years)
11) Followup period (YF, prospective studies): whole study (coded as 0) or longest available
12) LCtype: adeno or nearest available, but not squamous. (q = squamous, s = small,
a = adeno, l = large, KII = Kreyberg II, al = alveolar, br = bronchiolar, u = undifferentiated)
13) Race: all or nearest available, otherwise by race (wh or w = white, bl or b = black, hi = hispanic
ch = chinese, jap = japanese, haw = hawaiian, w+o = white + oriental, sca = scandinavian, as = asian)
14) For overlapping studies: principal rather than subsidiary studies
Finally by Age: whole study (coded as 0) if available, otherwise by widest available age group
and then for single sex results (m, f) in preference to results for both sexes combined (c).

Results adjusted (AD) for the most potential confounders are then chosen in Sections -1 to -3
and results adjusted for the least confounders in Sections -4 to -6. (Those least adjusted results which
actually differ from the most adjusted are marked 'x' in column X in Section -4)

Section -7 shows excluded studies, together with the stage (as above) at which no qualifying
results were found.

Section -8 lists the potentially overlapping studies which have been included (1=principal, 2=subsidiary).

Section -9 lists any results which would have been included in preference except that they had data not complete
enough for use in meta-analysis, with their significance (yes/no), if known, and any further comment as entered
on the database. It also lists as "gap" any categories for which no data were presented by the original authors.

In addition to those mentioned above, the following fields, levels and abbreviations are used:
* or nk = not known, n = no, y = yes, ot = other
nev = never
all/unspec = all or unspecified, cig+/-ot = cigarettes irrespective of other products (cigar, pipe etc)
MC = manufactured cigarettes, HR = hand-rolled cigarettes
exL, exH = range of exposure (low and high) in the smoking group, in terms of Years quit (vs current)
REF: 6-character study reference
NRR: number of the RR on the database within the study
ST : study type (CC = case control, pr or prosp = prospective)
NLC: number of lung cancer cases in whole study
R : risky occupational population (n = no, m = mining, o = other risky)
VB : national cigarette type (V = at least 75% Virginia, bl = at least 75% blended, ot = other)
P : any proxy use
H : full histological confirmation
De : derivation of RR/CI (or = original, st = standard method, ot = other method of estimation)

Table 3K1 - 1

IESLC - Meta-analysis of Ex Smoking by Years quit (vs current), Overview
Adenocarcinoma, Any Product (or Cigarettes if Any not available)
Most adjusted

REF	NRR	SEX	AGEL	AGEH	RACE	YF	LC TYPE	LOC	START	ST	NLC	R	VB	P	H	AD	ADOS	PRODUCT	exL	exH	S1	S2	DENOM	De
BARBON	764	m	0	0	all	-	a	Eu:wst	1979	CC	755	n	bl	y	y	1	0	all/unsp	0.1	4	1	1	current	ot
BARBON	765	m	0	0	all	-	a	Eu:wst	1979	CC	755	n	bl	y	y	1	0	all/unsp	5	14	0	2	current	ot
BARBON	766	m	0	0	all	-	a	Eu:wst	1979	CC	755	n	bl	y	y	1	0	all/unsp	15	24	0	3	current	ot
BARBON	767	m	0	0	all	-	a	Eu:wst	1979	CC	755	n	bl	y	y	1	0	all/unsp	25	999	0	0	current	ot
JAHN	651	m	0	0	all	-	a	Eu:Ger	1988	CC	1004	n	bl	n	n	0	0	cig+/-ot	0.1	0.9	0	0	current	st
JAHN	652	m	0	0	all	-	a	Eu:Ger	1988	CC	1004	n	bl	n	n	0	0	cig+/-ot	1.0	1.9	0	0	current	st
JAHN	653	m	0	0	all	-	a	Eu:Ger	1988	CC	1004	n	bl	n	n	0	0	cig+/-ot	2	5	1	1	current	st
JAHN	654	m	0	0	all	-	a	Eu:Ger	1988	CC	1004	n	bl	n	n	0	0	cig+/-ot	6	10	2	0	current	st
JAHN	655	m	0	0	all	-	a	Eu:Ger	1988	CC	1004	n	bl	n	n	0	0	cig+/-ot	11	20	3	0	current	st
JAHN	656	m	0	0	all	-	a	Eu:Ger	1988	CC	1004	n	bl	n	n	0	0	cig+/-ot	21	999	0	0	current	st
JAIN	540	m	0	0	all	-	a	NAmer	1981	CC	845	n	V	y	n	0	0	cig+/-ot	2	9	0	1	cur+2y	st
JAIN	541	m	0	0	all	-	a	NAmer	1981	CC	845	n	V	y	n	0	0	cig+/-ot	10	999	3	0	cur+2y	st
JAIN	504	f	0	0	all	-	a	NAmer	1981	CC	845	n	V	y	n	0	0	cig+/-ot	2	9	0	1	cur+2y	st
JAIN	505	f	0	0	all	-	a	NAmer	1981	CC	845	n	V	y	n	0	0	cig+/-ot	10	999	3	0	cur+2y	st
LUBIN2	871	m	0	0	all	-	a	Eu:mul	1976	CC	7804	n	bl	n	y	0	0	cig+/-ot	0.1	4	1	1	current	st
LUBIN2	872	m	0	0	all	-	a	Eu:mul	1976	CC	7804	n	bl	n	y	0	0	cig+/-ot	5	9	2	0	current	st
LUBIN2	873	m	0	0	all	-	a	Eu:mul	1976	CC	7804	n	bl	n	y	0	0	cig+/-ot	10	14	3	2	current	st
LUBIN2	874	m	0	0	all	-	a	Eu:mul	1976	CC	7804	n	bl	n	y	0	0	cig+/-ot	15	19	0	0	current	st
LUBIN2	875	m	0	0	all	-	a	Eu:mul	1976	CC	7804	n	bl	n	y	0	0	cig+/-ot	20	999	0	3	current	st
LUBIN2	971	f	0	0	all	-	a	Eu:mul	1976	CC	7804	n	bl	n	y	0	0	cig+/-ot	0.1	9	0	1	current	st
LUBIN2	972	f	0	0	all	-	a	Eu:mul	1976	CC	7804	n	bl	n	y	0	0	cig+/-ot	10	19	3	2	current	st
LUBIN2	973	f	0	0	all	-	a	Eu:mul	1976	CC	7804	n	bl	n	y	0	0	cig+/-ot	20	999	0	3	current	st
MATOS	676	m	0	0	all	-	a	SCAmer	1994	CC	200	n	bl	n	n	2	0	cig+/-ot	1.0	5	1	1	cur+1y	or
MATOS	677	m	0	0	all	-	a	SCAmer	1994	CC	200	n	bl	n	n	2	0	cig+/-ot	6	10	2	0	cur+1y	or
MATOS	678	m	0	0	all	-	a	SCAmer	1994	CC	200	n	bl	n	n	2	0	cig+/-ot	11	999	3	0	cur+1y	or
PEZZOT	588	m	0	0	all	-	a	SCAmer	1987	CC	215	n	bl	n	y	0	0	cig only	1.0	10	0	1	cur+1y	st
PEZZOT	589	m	0	0	all	-	a	SCAmer	1987	CC	215	n	bl	n	y	0	0	cig only	11	999	3	0	cur+1y	st
SOBUE	744	m	0	0	all	-	a	As:Jap	1986	CC	1376	n	bl	n	y	0	0	cig+/-ot	1.0	4	1	1	cur+1y	st
SOBUE	745	m	0	0	all	-	a	As:Jap	1986	CC	1376	n	bl	n	y	0	0	cig+/-ot	5	9	2	0	cur+1y	st
SOBUE	746	m	0	0	all	-	a	As:Jap	1986	CC	1376	n	bl	n	y	0	0	cig+/-ot	10	999	3	0	cur+1y	st
SVENSS	572	f	0	0	all	-	a	Eu:Sca	1983	CC	210	n	bl	n	n	0	0	all/unsp	3	10	0	1	cur+2y	st
SVENSS	573	f	0	0	all	-	a	Eu:Sca	1983	CC	210	n	bl	n	n	0	0	all/unsp	11	999	3	0	cur+2y	st
WYNDE3	528	m	0	0	all	-	KII	NAmer	1966	CC	350	n	bl	n	y	0	0	all/unsp	1.0	3	1	1	cur+1y	st
WYNDE3	529	m	0	0	all	-	KII	NAmer	1966	CC	350	n	bl	n	y	0	0	all/unsp	4	6	0	0	cur+1y	st
WYNDE3	530	m	0	0	all	-	KII	NAmer	1966	CC	350	n	bl	n	y	0	0	all/unsp	7	12	0	2	cur+1y	st
WYNDE3	531	m	0	0	all	-	KII	NAmer	1966	CC	350	n	bl	n	y	0	0	all/unsp	13	999	0	3	cur+1y	st

Cigarette type is all/unspec for all RRs

In this overview table, subtotals and Qs values may be invalid and should be ignored

Table 3K1 - 2

IESLC - Meta-analysis of Ex Smoking by Years quit (vs current), Overview
Adenocarcinoma, Any Product (or Cigarettes if Any not available)
Most adjusted

REF	NRR	SEX	AD	Number Exposed Case	Number Exposed Cont	Non-exposed Case	Non-exposed Cont	RR	95.00%CI
BARBON	764	m	1	7	-	109	-	1.15	(0.47- 2.78)
BARBON	765	m	1	23	-	109	-	0.89	(0.54- 1.47)
BARBON	766	m	1	7	-	109	-	0.56	(0.24- 1.29)
BARBON	767	m	1	4	-	109	-	0.22	(0.08- 0.62)
Subtotal BARBON								0.72	(0.50- 1.03)
JAHN	651	m	0	40	8	75	269	17.93	(8.05- 39.95)
JAHN	652	m	0	18	9	75	269	7.17	(3.10- 16.62)
JAHN	653	m	0	19	46	75	269	1.48	(0.82- 2.68)
JAHN	654	m	0	13	63	75	269	0.74	(0.39- 1.42)
JAHN	655	m	0	22	130	75	269	0.61	(0.36- 1.02)
JAHN	656	m	0	15	146	75	269	0.37	(0.20- 0.66)
Subtotal JAHN								1.22	(0.94- 1.59)
JAIN	540	m	0	16	46	60	118	0.68	(0.36- 1.31)
JAIN	541	m	0	14	113	60	118	0.24	(0.13- 0.46)
JAIN	504	f	0	14	36	69	99	0.56	(0.28- 1.11)
JAIN	505	f	0	3	61	69	99	0.07	(0.02- 0.23)
Subtotal JAIN								0.38	(0.26- 0.54)
LUBIN2	871	m	0	77	1047	454	6209	1.01	(0.78- 1.29)
LUBIN2	872	m	0	50	882	454	6209	0.78	(0.57- 1.05)
LUBIN2	873	m	0	30	693	454	6209	0.59	(0.41- 0.86)
LUBIN2	874	m	0	21	478	454	6209	0.60	(0.38- 0.94)
LUBIN2	875	m	0	35	1128	454	6209	0.42	(0.30- 0.60)
LUBIN2	971	f	0	13	95	69	410	0.81	(0.43- 1.53)
LUBIN2	972	f	0	3	33	69	410	0.54	(0.16- 1.81)
LUBIN2	973	f	0	1	29	69	410	0.20	(0.03- 1.53)
Subtotal LUBIN2								0.71	(0.62- 0.82)
MATOS	676	m	2	12	-	46	-	1.30	(0.60- 3.00)
MATOS	677	m	2	9	-	46	-	1.00	(0.40- 2.30)
MATOS	678	m	2	12	-	46	-	0.30	(0.20- 0.70)
Subtotal MATOS								0.61	(0.40- 0.94)
PEZZOT	588	m	0	11	21	42	38	0.47	(0.20- 1.11)
PEZZOT	589	m	0	7	31	42	38	0.20	(0.08- 0.52)
Subtotal PEZZOT								0.32	(0.17- 0.61)
SOBUE	744	m	0	44	116	270	633	0.89	(0.61- 1.29)
SOBUE	745	m	0	22	92	270	633	0.56	(0.34- 0.91)
SOBUE	746	m	0	49	144	270	633	0.80	(0.56- 1.14)
Subtotal SOBUE								0.77	(0.61- 0.97)
SVENSS	572	f	0	5	13	38	53	0.54	(0.18- 1.63)
SVENSS	573	f	0	7	24	38	53	0.41	(0.16- 1.04)
Subtotal SVENSS								0.46	(0.22- 0.94)
WYNDE3	528	m	0	3	22	56	207	0.50	(0.15- 1.75)
WYNDE3	529	m	0	3	17	56	207	0.65	(0.18- 2.31)
WYNDE3	530	m	0	3	31	56	207	0.36	(0.11- 1.21)
WYNDE3	531	m	0	3	55	56	207	0.20	(0.06- 0.67)
Subtotal WYNDE3								0.39	(0.21- 0.71)
Partial Totals				635	5609	4953	37232		

*prospective study

Table 3K1 - 2

IESLC - Meta-analysis of Ex Smoking by Years quit (vs current), Overview
Adenocarcinoma, Any Product (or Cigarettes if Any not available)
Most adjusted

REF	NRR	SEX	AD	Ys	Ws	Qs	Ps
BARBON	764	m	1	0.14	4.86	1.15	0.7579
BARBON	765	m	1	-0.12	15.32	0.81	0.6483
BARBON	766	m	1	-0.58	5.43	0.30	0.1765
BARBON	767	m	1	-1.51	3.66	5.00	0.0037
Subtotal BARBON				-0.33	29.28	7.25	
JAHN	651	m	0	2.89	5.99	62.57	0.0000
JAHN	652	m	0	1.97	5.44	29.21	0.0000
JAHN	653	m	0	0.39	10.94	5.98	0.1937
JAHN	654	m	0	-0.30	9.10	0.02	0.3638
JAHN	655	m	0	-0.50	14.25	0.33	0.0595
JAHN	656	m	0	-1.00	11.04	4.69	0.0009
Subtotal JAHN				0.20	56.76	102.81	
JAIN	540	m	0	-0.38	9.14	0.01	0.2509
JAIN	541	m	0	-1.41	9.49	10.77	0.0000
JAIN	504	f	0	-0.58	8.08	0.45	0.0973
JAIN	505	f	0	-2.65	2.67	14.19	0.0000
Subtotal JAIN				-0.98	29.38	25.43	
LUBIN2	871	m	0	0.01	61.33	7.61	0.9639
LUBIN2	872	m	0	-0.25	42.56	0.36	0.0968
LUBIN2	873	m	0	-0.52	26.93	0.85	0.0065
LUBIN2	874	m	0	-0.51	19.20	0.51	0.0256
LUBIN2	875	m	0	-0.86	31.43	8.20	0.0000
LUBIN2	971	f	0	-0.21	9.58	0.19	0.5220
LUBIN2	972	f	0	-0.62	2.63	0.19	0.3181
LUBIN2	973	f	0	-1.59	0.95	1.46	0.1221
Subtotal LUBIN2				-0.34	194.60	19.36	
MATOS	676	m	2	0.26	5.93	2.20	0.5228
MATOS	677	m	2	0.00	5.02	0.60	1.0000
MATOS	678	m	2	-1.20	9.79	7.20	0.0002
Subtotal MATOS				-0.49	20.74	10.00	
PEZZOT	588	m	0	-0.75	5.30	0.85	0.0856
PEZZOT	589	m	0	-1.59	4.44	6.85	0.0008
Subtotal PEZZOT				-1.13	9.74	7.70	
SOBUE	744	m	0	-0.12	27.30	1.43	0.5398
SOBUE	745	m	0	-0.58	16.23	0.88	0.0197
SOBUE	746	m	0	-0.23	30.64	0.44	0.2110
Subtotal SOBUE				-0.26	74.17	2.75	
SVENSS	572	f	0	-0.62	3.10	0.24	0.2725
SVENSS	573	f	0	-0.90	4.35	1.33	0.0606
Subtotal SVENSS				-0.78	7.46	1.57	
WYNDE3	528	m	0	-0.69	2.49	0.29	0.2796
WYNDE3	529	m	0	-0.43	2.41	0.02	0.5071
WYNDE3	530	m	0	-1.03	2.58	1.20	0.0990
WYNDE3	531	m	0	-1.60	2.67	4.21	0.0089
Subtotal WYNDE3				-0.95	10.15	5.71	

N	36
NS	9

Table 3K1 - 3

IESLC - Meta-analysis of Ex Smoking by Years quit (vs current), Overview
Adenocarcinoma, Any Product (or Cigarettes if Any not available)
Most adjusted

	Sex: combined	male	female	Total
N		29	7	36
NS		8	3	11

In this overview table, other than the "N" rows, entries in the "absent" and "Total" columns may be invalid and should be ignored

Years quit vs current (lower focus)

	absent	1-6k3	4-11k7	8+k12	Total
N	17	6	4	9	36
NS	7	6	4	7	24
Wt	141.33	112.85	72.92	105.18	432.28
Het Chi	119.58	3.71	1.78	29.45	182.58
Het df	16	5	3	8	35
Het P	***	N.S.	N.S.	***	***
Fixed RR	0.67	1.02	0.73	0.50	0.71
RRl	0.57	0.85	0.58	0.41	0.64
RRu	0.79	1.22	0.92	0.60	0.78
P	---	N.S.	--	---	---
Random RR	0.70	1.02	0.73	0.39	0.65
RRl	0.43	0.85	0.58	0.26	0.52
RRu	1.14	1.22	0.92	0.58	0.83
P	N.S.	N.S.	--	---	---

Years quit vs current (higher focus)

	absent	1-11k3	4-19k12	13+k20	Total
N	17	11	4	4	36
NS	9	9	3	3	24
Wt	196.29	148.06	47.45	40.48	432.28
Het Chi	144.29	11.04	2.75	2.37	182.58
Het df	16	10	3	3	35
Het P	***	N.S.	N.S.	N.S.	***
Fixed RR	0.67	0.91	0.65	0.41	0.71
RRl	0.58	0.77	0.49	0.30	0.64
RRu	0.77	1.07	0.87	0.56	0.78
P	---	N.S.	--	---	---
Random RR	0.63	0.89	0.65	0.41	0.65
RRl	0.41	0.75	0.49	0.30	0.52
RRu	0.99	1.07	0.87	0.56	0.83
P	-	N.S.	--	---	---

Table 3K1 - 3

IESLC - Meta-analysis of Ex Smoking by Years quit (vs current), Overview
Adenocarcinoma, Any Product (or Cigarettes if Any not available)
Most adjusted

MALES

		Years quit vs current (lower focus) absent	1-6k3	4-11k7	8+k12	Total
	N	13	6	4	6	29
	NS	6	6	4	6	22
	Wt	119.62	112.85	72.92	95.53	400.91
Het	Chi	117.43	3.71	1.78	18.65	164.18
Het	df	12	5	3	5	28
Het	P	***	N.S.	N.S.	**	***
Fixed	RR	0.68	1.02	0.73	0.53	0.73
	RRl	0.57	0.85	0.58	0.43	0.66
	RRu	0.82	1.22	0.92	0.65	0.80
	P	---	N.S.	--	---	---
Random	RR	0.76	1.02	0.73	0.44	0.71
	RRl	0.42	0.85	0.58	0.29	0.55
	RRu	1.37	1.22	0.92	0.67	0.92
	P	N.S.	N.S.	--	---	--

		Years quit vs current (higher focus) absent	1-11k3	4-19k12	13+k20	Total
	N	15	8	3	3	29
	NS	8	8	3	3	22
	Wt	189.27	127.29	44.82	39.53	400.91
Het	Chi	129.41	7.78	2.65	1.89	164.18
Het	df	14	7	2	2	28
Het	P	***	N.S.	N.S.	N.S.	***
Fixed	RR	0.70	0.96	0.66	0.42	0.73
	RRl	0.60	0.81	0.49	0.31	0.66
	RRu	0.80	1.14	0.89	0.57	0.80
	P	---	N.S.	--	---	---
Random	RR	0.73	0.95	0.66	0.42	0.71
	RRl	0.46	0.78	0.46	0.31	0.55
	RRu	1.15	1.16	0.96	0.57	0.92
	P	N.S.	N.S.	-	---	--

FEMALES

		Years quit vs current (lower focus) absent	1-6k3	4-11k7	8+k12	Total
	N	4			3	7
	NS	3			3	6
	Wt	21.71			9.65	31.37
Het	Chi	2.02			6.81	13.55
Het	df	3			2	6
Het	P	N.S.			*	*
Fixed	RR	0.63			0.27	0.48
	RRl	0.41			0.14	0.34
	RRu	0.95			0.51	0.69
	P	-			---	---
Random	RR	0.63			0.25	0.41
	RRl	0.41			0.08	0.23
	RRu	0.95			0.83	0.73
	P	-			-	--

Table 3K1 - 3

IESLC - Meta-analysis of Ex Smoking by Years quit (vs current), Overview
Adenocarcinoma, Any Product (or Cigarettes if Any not available)
Most adjusted

FEMALES

	Years quit vs current (higher focus)				
	absent	1-11k3	4-19k12	13+k20	Total
N	2	3	1	1	7
NS	2	3	1	1	6
Wt	7.02	20.76	2.63	0.95	31.37
Het Chi	5.08	0.78	0.00	0.00	13.55
Het df	1	2	0	0	6
Het P	*	N.S.	N.S.	N.S.	*
Fixed RR	0.21	0.66	0.54	0.20	0.48
RRl	0.10	0.43	0.16	0.03	0.34
RRu	0.44	1.01	1.81	1.53	0.69
P	---	(-)	N.S.	N.S.	---
Random RR	0.18	0.66	0.54	0.20	0.41
RRl	0.03	0.43	0.16	0.03	0.23
RRu	0.98	1.01	1.81	1.53	0.73
P	-	(-)	N.S.	N.S.	--

Table 3K1 - 4

IESLC - Meta-analysis of Ex Smoking by Years quit (vs current), Overview
Adenocarcinoma, Any Product (or Cigarettes if Any not available)
Least adjusted

REF	NRR	X	SEX	AGEL	AGEH	RACE	YF	LC TYPE	LOC	START	ST	NLC	R	VB	P	H	AD	ADOS	PRODUCT	exL	exH	S1	S2	DENOM	De
BARBON	749	x	m	0	0	all	-	a	Eu:wst	1979	CC	755	n	bl	y	y	0	0	all/unsp	0.1	4	1	1	current	st
BARBON	750	x	m	0	0	all	-	a	Eu:wst	1979	CC	755	n	bl	y	y	0	0	all/unsp	5	14	0	2	current	st
BARBON	751	x	m	0	0	all	-	a	Eu:wst	1979	CC	755	n	bl	y	y	0	0	all/unsp	15	24	0	3	current	st
BARBON	752	x	m	0	0	all	-	a	Eu:wst	1979	CC	755	n	bl	y	y	0	0	all/unsp	25	999	0	0	current	st
JAHN	651		m	0	0	all	-	a	Eu:Ger	1988	CC	1004	n	bl	n	n	0	0	cig+/-ot	0.1	0.9	0	0	current	st
JAHN	652		m	0	0	all	-	a	Eu:Ger	1988	CC	1004	n	bl	n	n	0	0	cig+/-ot	1.0	1.9	0	0	current	st
JAHN	653		m	0	0	all	-	a	Eu:Ger	1988	CC	1004	n	bl	n	n	0	0	cig+/-ot	2	5	1	1	current	st
JAHN	654		m	0	0	all	-	a	Eu:Ger	1988	CC	1004	n	bl	n	n	0	0	cig+/-ot	6	10	2	0	current	st
JAHN	655		m	0	0	all	-	a	Eu:Ger	1988	CC	1004	n	bl	n	n	0	0	cig+/-ot	11	20	3	0	current	st
JAHN	656		m	0	0	all	-	a	Eu:Ger	1988	CC	1004	n	bl	n	n	0	0	cig+/-ot	21	999	0	0	current	st
JAIN	540		m	0	0	all	-	a	NAmer	1981	CC	845	n	V	y	n	0	0	cig+/-ot	2	9	0	1	cur+2y	st
JAIN	541		m	0	0	all	-	a	NAmer	1981	CC	845	n	V	y	n	0	0	cig+/-ot	10	999	3	0	cur+2y	st
JAIN	504		f	0	0	all	-	a	NAmer	1981	CC	845	n	V	y	n	0	0	cig+/-ot	2	9	0	1	cur+2y	st
JAIN	505		f	0	0	all	-	a	NAmer	1981	CC	845	n	V	y	n	0	0	cig+/-ot	10	999	3	0	cur+2y	st
LUBIN2	871		m	0	0	all	-	a	Eu:mul	1976	CC	7804	n	bl	n	y	0	0	cig+/-ot	0.1	4	1	1	current	st
LUBIN2	872		m	0	0	all	-	a	Eu:mul	1976	CC	7804	n	bl	n	y	0	0	cig+/-ot	5	9	2	0	current	st
LUBIN2	873		m	0	0	all	-	a	Eu:mul	1976	CC	7804	n	bl	n	y	0	0	cig+/-ot	10	14	3	2	current	st
LUBIN2	874		m	0	0	all	-	a	Eu:mul	1976	CC	7804	n	bl	n	y	0	0	cig+/-ot	15	19	0	0	current	st
LUBIN2	875		m	0	0	all	-	a	Eu:mul	1976	CC	7804	n	bl	n	y	0	0	cig+/-ot	20	999	0	3	current	st
LUBIN2	971		f	0	0	all	-	a	Eu:mul	1976	CC	7804	n	bl	n	y	0	0	cig+/-ot	0.1	9	0	1	current	st
LUBIN2	972		f	0	0	all	-	a	Eu:mul	1976	CC	7804	n	bl	n	y	0	0	cig+/-ot	10	19	3	2	current	st
LUBIN2	973		f	0	0	all	-	a	Eu:mul	1976	CC	7804	n	bl	n	y	0	0	cig+/-ot	20	999	0	3	current	st
MATOS	666	x	m	0	0	all	-	a	SCAmer	1994	CC	200	n	bl	n	n	0	0	cig+/-ot	1.0	5	1	1	cur+1y	st
MATOS	667	x	m	0	0	all	-	a	SCAmer	1994	CC	200	n	bl	n	n	0	0	cig+/-ot	6	10	2	0	cur+1y	st
MATOS	668	x	m	0	0	all	-	a	SCAmer	1994	CC	200	n	bl	n	n	0	0	cig+/-ot	11	999	3	0	cur+1y	st
PEZZOT	588		m	0	0	all	-	a	SCAmer	1987	CC	215	n	bl	n	y	0	0	cig only	1.0	10	0	1	cur+1y	st
PEZZOT	589		m	0	0	all	-	a	SCAmer	1987	CC	215	n	bl	n	y	0	0	cig only	11	999	3	0	cur+1y	st
SOBUE	744		m	0	0	all	-	a	As:Jap	1986	CC	1376	n	bl	n	y	0	0	cig+/-ot	1.0	4	1	1	cur+1y	st
SOBUE	745		m	0	0	all	-	a	As:Jap	1986	CC	1376	n	bl	n	y	0	0	cig+/-ot	5	9	2	0	cur+1y	st
SOBUE	746		m	0	0	all	-	a	As:Jap	1986	CC	1376	n	bl	n	y	0	0	cig+/-ot	10	999	3	0	cur+1y	st
SVENSS	572		f	0	0	all	-	a	Eu:Sca	1983	CC	210	n	bl	n	n	0	0	all/unsp	3	10	0	1	cur+2y	st
SVENSS	573		f	0	0	all	-	a	Eu:Sca	1983	CC	210	n	bl	n	n	0	0	all/unsp	11	999	3	0	cur+2y	st
WYNDE3	528		m	0	0	all	-	KII	NAmer	1966	CC	350	n	bl	n	y	0	0	all/unsp	1.0	3	1	1	cur+1y	st
WYNDE3	529		m	0	0	all	-	KII	NAmer	1966	CC	350	n	bl	n	y	0	0	all/unsp	4	6	0	0	cur+1y	st
WYNDE3	530		m	0	0	all	-	KII	NAmer	1966	CC	350	n	bl	n	y	0	0	all/unsp	7	12	0	2	cur+1y	st
WYNDE3	531		m	0	0	all	-	KII	NAmer	1966	CC	350	n	bl	n	y	0	0	all/unsp	13	999	0	3	cur+1y	st

Cigarette type is all/unspec for all RRs

In this overview table, subtotals and Qs values may be invalid and should be ignored

Table 3K1 - 5

IESLC - Meta-analysis of Ex Smoking by Years quit (vs current), Overview
Adenocarcinoma, Any Product (or Cigarettes if Any not available)
Least adjusted

REF	NRR	SEX	AD	Number Exposed Case	Cont	Non-exposed Case	Cont	RR	95.00%CI
BARBON	749	m	0	7	20	109	362	1.16	(0.48- 2.82)
BARBON	750	m	0	23	85	109	362	0.90	(0.54- 1.49)
BARBON	751	m	0	7	41	109	362	0.57	(0.25- 1.30)
BARBON	752	m	0	4	59	109	362	0.23	(0.08- 0.63)
Subtotal BARBON								0.72	(0.50- 1.04)
JAHN	651	m	0	40	8	75	269	17.93	(8.05- 39.95)
JAHN	652	m	0	18	9	75	269	7.17	(3.10- 16.62)
JAHN	653	m	0	19	46	75	269	1.48	(0.82- 2.68)
JAHN	654	m	0	13	63	75	269	0.74	(0.39- 1.42)
JAHN	655	m	0	22	130	75	269	0.61	(0.36- 1.02)
JAHN	656	m	0	15	146	75	269	0.37	(0.20- 0.66)
Subtotal JAHN								1.22	(0.94- 1.59)
JAIN	540	m	0	16	46	60	118	0.68	(0.36- 1.31)
JAIN	541	m	0	14	113	60	118	0.24	(0.13- 0.46)
JAIN	504	f	0	14	36	69	99	0.56	(0.28- 1.11)
JAIN	505	f	0	3	61	69	99	0.07	(0.02- 0.23)
Subtotal JAIN								0.38	(0.26- 0.54)
LUBIN2	871	m	0	77	1047	454	6209	1.01	(0.78- 1.29)
LUBIN2	872	m	0	50	882	454	6209	0.78	(0.57- 1.05)
LUBIN2	873	m	0	30	693	454	6209	0.59	(0.41- 0.86)
LUBIN2	874	m	0	21	478	454	6209	0.60	(0.38- 0.94)
LUBIN2	875	m	0	35	1128	454	6209	0.42	(0.30- 0.60)
LUBIN2	971	f	0	13	95	69	410	0.81	(0.43- 1.53)
LUBIN2	972	f	0	3	33	69	410	0.54	(0.16- 1.81)
LUBIN2	973	f	0	1	29	69	410	0.20	(0.03- 1.53)
Subtotal LUBIN2								0.71	(0.62- 0.82)
MATOS	666	m	0	12	23	46	132	1.50	(0.69- 3.25)
MATOS	667	m	0	9	27	46	132	0.96	(0.42- 2.18)
MATOS	668	m	0	12	101	46	132	0.34	(0.17- 0.68)
Subtotal MATOS								0.73	(0.47- 1.12)
PEZZOT	588	m	0	11	21	42	38	0.47	(0.20- 1.11)
PEZZOT	589	m	0	7	31	42	38	0.20	(0.08- 0.52)
Subtotal PEZZOT								0.32	(0.17- 0.61)
SOBUE	744	m	0	44	116	270	633	0.89	(0.61- 1.29)
SOBUE	745	m	0	22	92	270	633	0.56	(0.34- 0.91)
SOBUE	746	m	0	49	144	270	633	0.80	(0.56- 1.14)
Subtotal SOBUE								0.77	(0.61- 0.97)
SVENSS	572	f	0	5	13	38	53	0.54	(0.18- 1.63)
SVENSS	573	f	0	7	24	38	53	0.41	(0.16- 1.04)
Subtotal SVENSS								0.46	(0.22- 0.94)
WYNDE3	528	m	0	3	22	56	207	0.50	(0.15- 1.75)
WYNDE3	529	m	0	3	17	56	207	0.65	(0.18- 2.31)
WYNDE3	530	m	0	3	31	56	207	0.36	(0.11- 1.21)
WYNDE3	531	m	0	3	55	56	207	0.20	(0.06- 0.67)
Subtotal WYNDE3								0.39	(0.21- 0.71)
Totals				635	5965	4953	39076		

*prospective study

Table 3K1 - 5

IESLC - Meta-analysis of Ex Smoking by Years quit (vs current), Overview
Adenocarcinoma, Any Product (or Cigarettes if Any not available)
Least adjusted

REF	NRR	SEX	AD	Ys	Ws	Qs	Ps
BARBON	749	m	0	0.15	4.88	1.16	0.7395
BARBON	750	m	0	-0.11	14.89	0.79	0.6801
BARBON	751	m	0	-0.57	5.58	0.30	0.1801
BARBON	752	m	0	-1.49	3.59	4.77	0.0048
Subtotal BARBON				-0.32	28.93	7.02	
JAHN	651	m	0	2.89	5.99	62.22	0.0000
JAHN	652	m	0	1.97	5.44	28.99	0.0000
JAHN	653	m	0	0.39	10.94	5.83	0.1937
JAHN	654	m	0	-0.30	9.10	0.01	0.3638
JAHN	655	m	0	-0.50	14.25	0.37	0.0595
JAHN	656	m	0	-1.00	11.04	4.82	0.0009
Subtotal JAHN				0.20	56.76	102.25	
JAIN	540	m	0	-0.38	9.14	0.02	0.2509
JAIN	541	m	0	-1.41	9.49	10.96	0.0000
JAIN	504	f	0	-0.58	8.08	0.49	0.0973
JAIN	505	f	0	-2.65	2.67	14.30	0.0000
Subtotal JAIN				-0.98	29.38	25.77	
LUBIN2	871	m	0	0.01	61.33	7.22	0.9639
LUBIN2	872	m	0	-0.25	42.56	0.29	0.0968
LUBIN2	873	m	0	-0.52	26.93	0.94	0.0065
LUBIN2	874	m	0	-0.51	19.20	0.57	0.0256
LUBIN2	875	m	0	-0.86	31.43	8.49	0.0000
LUBIN2	971	f	0	-0.21	9.58	0.16	0.5220
LUBIN2	972	f	0	-0.62	2.63	0.20	0.3181
LUBIN2	973	f	0	-1.59	0.95	1.48	0.1221
Subtotal LUBIN2				-0.34	194.60	19.36	
MATOS	666	m	0	0.40	6.41	3.52	0.3071
MATOS	667	m	0	-0.04	5.63	0.48	0.9160
MATOS	668	m	0	-1.08	8.16	4.45	0.0021
Subtotal MATOS				-0.32	20.20	8.45	
PEZZOT	588	m	0	-0.75	5.30	0.89	0.0856
PEZZOT	589	m	0	-1.59	4.44	6.95	0.0008
Subtotal PEZZOT				-1.13	9.74	7.84	
SOBUE	744	m	0	-0.12	27.30	1.32	0.5398
SOBUE	745	m	0	-0.58	16.23	0.95	0.0197
SOBUE	746	m	0	-0.23	30.64	0.38	0.2110
Subtotal SOBUE				-0.26	74.17	2.65	
SVENSS	572	f	0	-0.62	3.10	0.25	0.2725
SVENSS	573	f	0	-0.90	4.35	1.38	0.0606
Subtotal SVENSS				-0.78	7.46	1.63	
WYNDE3	528	m	0	-0.69	2.49	0.30	0.2796
WYNDE3	529	m	0	-0.43	2.41	0.02	0.5071
WYNDE3	530	m	0	-1.03	2.58	1.23	0.0990
WYNDE3	531	m	0	-1.60	2.67	4.27	0.0089
Subtotal WYNDE3				-0.95	10.15	5.82	

N	36
NS	9

Table 3K1 - 6

IESLC - Meta-analysis of Ex Smoking by Years quit (vs current), Overview
Adenocarcinoma, Any Product (or Cigarettes if Any not available)
Least adjusted

	Sex combined	male	female	Total
N		29	7	36
NS		8	3	11

In this overview table, other than the "N" rows, entries in the "absent" and "Total" columns may be invalid and should be ignored

Years quit vs current (lower focus)	absent	1-6k3	4-11k7	8+k12	Total
N	17	6	4	9	36
NS	7	6	4	7	24
Wt	140.97	113.34	73.53	103.55	431.39
Het Chi	119.33	4.31	1.70	28.06	180.78
Het df	16	5	3	8	35
Het P	***	N.S.	N.S.	***	***
Fixed RR	0.68	1.03	0.73	0.51	0.71
RRl	0.57	0.85	0.58	0.42	0.65
RRu	0.80	1.24	0.92	0.62	0.78
P	---	N.S.	--	---	---
Random RR	0.71	1.03	0.73	0.39	0.66
RRl	0.44	0.85	0.58	0.26	0.52
RRu	1.14	1.24	0.92	0.59	0.83
P	N.S.	N.S.	--	---	---

Years quit vs current (higher focus)	absent	1-11k3	4-19k12	13+k20	Total
N	17	11	4	4	36
NS	9	9	3	3	24
Wt	195.20	148.55	47.01	40.63	431.39
Het Chi	141.32	11.89	2.80	2.42	180.78
Het df	16	10	3	3	35
Het P	***	N.S.	N.S.	N.S.	***
Fixed RR	0.68	0.92	0.65	0.41	0.71
RRl	0.59	0.78	0.49	0.30	0.65
RRu	0.78	1.08	0.87	0.56	0.78
P	---	N.S.	--	---	---
Random RR	0.64	0.89	0.65	0.41	0.66
RRl	0.41	0.74	0.49	0.30	0.52
RRu	0.99	1.09	0.87	0.56	0.83
P	-	N.S.	--	---	---

Table 3K1 - 6

IESLC - Meta-analysis of Ex Smoking by Years quit (vs current), Overview
Adenocarcinoma, Any Product (or Cigarettes if Any not available)
Least adjusted

MALES

Years quit vs current (lower focus)

	absent	1-6k3	4-11k7	8+k12	Total
N	13	6	4	6	29
NS	6	6	4	6	22
Wt	119.25	113.34	73.53	93.90	400.02
Het Chi	117.17	4.31	1.70	17.01	162.14
Het df	12	5	3	5	28
Het P	***	N.S.	N.S.	**	***
Fixed RR	0.68	1.03	0.73	0.54	0.74
RRl	0.57	0.85	0.58	0.44	0.67
RRu	0.82	1.24	0.92	0.66	0.81
P	---	N.S.	--	---	---
Random RR	0.76	1.03	0.73	0.45	0.72
RRl	0.42	0.85	0.58	0.30	0.56
RRu	1.38	1.24	0.92	0.68	0.93
P	N.S.	N.S.	--	---	-

Years quit vs current (higher focus)

	absent	1-11k3	4-19k12	13+k20	Total
N	15	8	3	3	29
NS	8	8	3	3	22
Wt	188.17	127.79	44.39	39.68	400.02
Het Chi	126.21	8.51	2.70	1.95	162.14
Het df	14	7	2	2	28
Het P	***	N.S.	N.S.	N.S.	***
Fixed RR	0.71	0.97	0.66	0.42	0.74
RRl	0.61	0.81	0.49	0.31	0.67
RRu	0.81	1.15	0.89	0.57	0.81
P	---	N.S.	--	---	---
Random RR	0.74	0.96	0.66	0.42	0.72
RRl	0.47	0.77	0.46	0.31	0.56
RRu	1.16	1.19	0.96	0.57	0.93
P	N.S.	N.S.	-	---	-

FEMALES

Years quit vs current (lower focus)

	absent	1-6k3	4-11k7	8+k12	Total
N	4			3	7
NS	3			3	6
Wt	21.71			9.65	31.37
Het Chi	2.02			6.81	13.55
Het df	3			2	6
Het P	N.S.			*	*
Fixed RR	0.63			0.27	0.48
RRl	0.41			0.14	0.34
RRu	0.95			0.51	0.69
P	-			---	---
Random RR	0.63			0.25	0.41
RRl	0.41			0.08	0.23
RRu	0.95			0.83	0.73
P	-			-	--

Table 3K1 - 6

IESLC - Meta-analysis of Ex Smoking by Years quit (vs current), Overview
Adenocarcinoma, Any Product (or Cigarettes if Any not available)
Least adjusted

FEMALES

	Years quit vs current (higher focus)				
	absent	1-11k3	4-19k12	13+k20	Total
N	2	3	1	1	7
NS	2	3	1	1	6
Wt	7.02	20.76	2.63	0.95	31.37
Het Chi	5.08	0.78	0.00	0.00	13.55
Het df	1	2	0	0	6
Het P	*	N.S.	N.S.	N.S.	*
Fixed RR	0.21	0.66	0.54	0.20	0.48
RRl	0.10	0.43	0.16	0.03	0.34
RRu	0.44	1.01	1.81	1.53	0.69
P	---	(-)	N.S.	N.S.	---
Random RR	0.18	0.66	0.54	0.20	0.41
RRl	0.03	0.43	0.16	0.03	0.23
RRu	0.98	1.01	1.81	1.53	0.73
P	-	(-)	N.S.	N.S.	--

Table 3K1 - 7

IESLC - Meta-analysis of Ex Smoking by Years quit (vs current), Overview
Adenocarcinoma, Any Product (or Cigarettes if Any not available)
Excluded studies (and stage at which they were excluded)

1	AGUDO AKIBA AMANDU AMES AXELSS BEST BOUCHA BOUCOT BRESLO CHEN CHEN2 CHIAZZ DEAN2 DOSEME ENGELA FAN GENG GER GUO HAENSZ HEGMAN HOLE HU HU2 JUSSAW KATSOU KAUFMA KOO KOULUM KREUZE LETOUR LEVIN LIAW LIU3 LIU4 LIU5 MCCONN MIGRAN MRFITR NOTAN2 OSANN2 PERNU QIAO2 RACHTA RESTRE SADOWS SEGI2 STASZE TIZZAN VUTUC WATSON WU WUWILL WYNDE2 WYNDE8 XU YUAN ZHANG ZHENG ZHOU
2	AUVINE BENSHL BLOT1 BROWN3 BUFFLE GURSEL LAUSSM MCDUFF PISANI PRESCO SPITZ WU2 WYNDE7
4	ARMADA BECHER BOFFET BROSS CARPEN CEDERL CHOI CHYOU CORREA CPSI CPSII DAMBER DARBY DEAN3 DESTEF DOLL DOLL2 DORGAN DORN GAO GAO2 GARCIA GARSHI GILLIS GRAHAM HAMMO2 HAMMON HIRAYA HUMBLE JOLY KAISE2 KHUDER LUBIN LUO PEZZO2 QIAO SPEIZE SUZUK2 TVERDA WANG2 WIGLE
5	ALDERS
10	JEDRYC WAKAI WYNDE6
14	BENHAM

Table 3K1 - 8

Potentially overlapping studies

REF	REFGP	PRINC	. OVERLAP/LINK
LUBIN2	LUBIN2	1	Lubin-combined
JAHN	BOFFET	2	Subset of BOFFET

Table 3K1 - 9

Most adjusted - insufficient data for meta-analysis

REF	NRR	SEX	AGEL	AGEH	RACE	YF	LC TYPE	LOC	START	ST	NLC	R	VB	P	H	AD	ADOS	PRODUCT	exL	exH	S1	S2	DENOM	De
ALDERS	573	m	0	0	all	-	not q+s	Eu:UK	1977	CC	1448	n	V	n	n	1	0	cig only	0.1	2	0	0	current	ot
ALDERS	574	m	0	0	all	-	not q+s	Eu:UK	1977	CC	1448	n	V	n	n	1	0	cig only	3	9	0	1	current	ot
ALDERS	575	m	0	0	all	-	not q+s	Eu:UK	1977	CC	1448	n	V	n	n	1	0	cig only	10	999	3	0	current	ot
ALDERS	584	f	0	0	all	-	not q+s	Eu:UK	1977	CC	1448	n	V	n	n	1	0	cig only	0.1	2	0	0	current	ot
ALDERS	585	f	0	0	all	-	not q+s	Eu:UK	1977	CC	1448	n	V	n	n	1	0	cig only	3	9	0	1	current	ot
ALDERS	586	f	0	0	all	-	not q+s	Eu:UK	1977	CC	1448	n	V	n	n	1	0	cig only	10	999	3	0	current	ot

REF	NRR	RR	SIG	RRDATA comment
ALDERS	573	2.07	n	0
ALDERS	574	1.56	n	0
ALDERS	575	0.91	n	0
ALDERS	584	1.38	n	0
ALDERS	585	0.16	y	0.001<p<0.01
ALDERS	586	0.36	n	0

Table 3K2 -

IESLC - Meta-analysis of Ex Smoking, Years quit (vs current), "Low"
Adenocarcinoma, Any Product (or Cigarettes if Any not available)

This analysis is restricted to results for:
1) Ex smokers
2) Results by Years quit (vs current)
3) Categorical results by Years quit (vs current)
4) Adenocarcinoma (or near equivalent)
5) Results complete enough for use in metaanalysis

Within each study, results are then selected (in the following order of preference, within each sex) for:
6) (not applicable)
7) PRODUCT: all/unspec, cigarettes regardless of other products, cigarettes only
8) CIGTYPE: all/unspecified, MC regardless of HR, MC only
9) Results with least adjustment for other aspects of smoking (ADOS)
10) DENOM: current smokers, current + recent smokers (up to number of m=months or y=years, max 2 years)
11) Followup period (YF, prospective studies): whole study (coded as 0) or longest available
12) LCtype: adeno or nearest available, but not squamous. (q = squamous, s = small,
a = adeno, l = large, KII = Kreyberg II, al = alveolar, br = bronchiolar, u = undifferentiated)
13) Race: all or nearest available, otherwise by race (wh or w = white, bl or b = black, hi = hispanic
ch = chinese, jap = japanese, haw = hawaiian, w+o = white + oriental, sca = scandinavian, as = asian)
14) Years quit (vs current) "low" in key scheme 1 (key value 3, maximum range 1-6)
15) For overlapping studies: principal rather than subsidiary studies
Finally by Age: whole study (coded as 0) if available, otherwise by widest available age group
and then for single sex results (m, f) in preference to results for both sexes combined (c).

Results adjusted (AD) for the most potential confounders are then chosen in Sections -1 to -3
and results adjusted for the least confounders in Sections -4 to -6. (Those least adjusted results which
actually differ from the most adjusted are marked 'x' in column X in Section -4)

Section -7 shows excluded studies, together with the stage (as above) at which no qualifying
results were found.

Section -8 lists the potentially overlapping studies which have been included (1=principal, 2=subsidiary).

Section -9 lists any results which would have been included in preference except that they had data not complete
enough for use in meta-analysis, with their significance (yes/no), if known, and any further comment as entered
on the database. It also lists as "gap" any categories for which no data were presented by the original authors.

In addition to those mentioned above, the following fields, levels and abbreviations are used:
* or nk = not known, n = no, y = yes, ot = other
nev = never
all/unspec = all or unspecified, cig+/-ot = cigarettes irrespective of other products (cigar, pipe etc)
MC = manufactured cigarettes, HR = hand-rolled cigarettes
exL, exH = range of exposure (low and high) in the smoking group, in terms of Years quit (vs current)
REF: 6-character study reference
NRR: number of the RR on the database within the study
ST : study type (CC = case control, pr or prosp = prospective)
NLC: number of lung cancer cases in whole study
R : risky occupational population (n = no, m = mining, o = other risky)
VB : national cigarette type (V = at least 75% Virginia, bl = at least 75% blended, ot = other)
P : any proxy use
H : full histological confirmation
De : derivation of RR/CI (or = original, st = standard method, ot = other method of estimation)

Table 3K2 - 1

IESLC - Meta-analysis of Ex Smoking, Years quit (vs current), "Low"
Adenocarcinoma, Any Product (or Cigarettes if Any not available)
Most adjusted

REF	NRR	SEX	AGEL	AGEH	RACE	YF	LC TYPE	LOC	START	ST	NLC	R	VB	P	H	AD	ADOS	PRODUCT	exL	exH	DENOM	De
BARBON	764	m	0	0	all	-	a	Eu:wst	1979	CC	755	n	bl	y	y	1	0	all/unsp	0.1	4	current	ot
JAHN	653	m	0	0	all	-	a	Eu:Ger	1988	CC	1004	n	bl	n	n	0	0	cig+/-ot	2	5	current	st
LUBIN2	871	m	0	0	all	-	a	Eu:mul	1976	CC	7804	n	bl	n	y	0	0	cig+/-ot	0.1	4	current	st
LUBIN2	980	f	0	0	all	-	a	Eu:mul	1976	CC	7804	n	bl	n	y	2	1#	cig+/-ot	0.1	4	current	ot
MATOS	676	m	0	0	all	-	a	SCAmer	1994	CC	200	n	bl	n	n	2	0	cig+/-ot	1.0	5	cur+1y	or
SOBUE	744	m	0	0	all	-	a	As:Jap	1986	CC	1376	n	bl	n	y	0	0	cig+/-ot	1.0	4	cur+1y	st
WYNDE3	528	m	0	0	all	-	KII	NAmer	1966	CC	350	n	bl	n	y	0	0	all/unsp	1.0	3	cur+1y	st

Comments on values in listings

LUBIN2 ADOS Duration of smoking

Cigarette type is all/unspec for all RRs

Table 3K2 - 2

IESLC - Meta-analysis of Ex Smoking, Years quit (vs current), "Low"
Adenocarcinoma, Any Product (or Cigarettes if Any not available)
Most adjusted

REF	NRR	SEX	AD	Number Exposed Case	Cont	Non-exposed Case	Cont	RR	95.00%CI
BARBON	764	m	1	7	-	109	-	1.15	(0.47- 2.78)
JAHN	653	m	0	19	46	75	269	1.48	(0.82- 2.68)
LUBIN2	871	m	0	77	1047	454	6209	1.01	(0.78- 1.29)
LUBIN2	980	f	2	6	-	69	-	0.70	(0.29- 1.69)
Subtotal LUBIN2								0.98	(0.77- 1.25)
MATOS	676	m	2	12	-	46	-	1.30	(0.60- 3.00)
SOBUE	744	m	0	44	116	270	633	0.89	(0.61- 1.29)
WYNDE3	528	m	0	3	22	56	207	0.50	(0.15- 1.75)
Partial Totals				168	1231	1079	7318		

*prospective study

REF	NRR	SEX	AD	Ys	Ws	Qs	Ps
BARBON	764	m	1	0.14	4.86	0.09	0.7579
JAHN	653	m	0	0.39	10.94	1.67	0.1937
LUBIN2	871	m	0	0.01	61.33	0.00	0.9639
LUBIN2	980	f	2	-0.36	4.95	0.64	0.4276
Subtotal LUBIN2				-0.02	66.27	0.64	
MATOS	676	m	2	0.26	5.93	0.40	0.5228
SOBUE	744	m	0	-0.12	27.30	0.39	0.5398
WYNDE3	528	m	0	-0.69	2.49	1.18	0.2796

	N	7
	NS	6
	Wt	117.80
Het	Chi	4.37
Het	df	6
Het	P	N.S.
Fixed	RR	1.00
	RRl	0.84
	RRu	1.20
	P	N.S.
Random	RR	1.00
	RRl	0.84
	RRu	1.20
	P	N.S.
Asymm	P	N.S.

Table 3K2 - 3

IESLC - Meta-analysis of Ex Smoking, Years quit (vs current), "Low"
Adenocarcinoma, Any Product (or Cigarettes if Any not available)
Most adjusted

	Sex			
	combined	male	female	Total
N		6	1	7
NS		6	1	7
Wt		112.85	4.95	117.80
Het Chi		3.71	0.00	4.37
Het df		5	0	6
Het P		N.S.	N.S.	N.S.
Fixed RR		1.02	0.70	1.00
RRl		0.85	0.29	0.84
RRu		1.22	1.69	1.20
P		N.S.	N.S.	N.S.
Random RR		1.02	0.70	1.00
RRl		0.85	0.29	0.84
RRu		1.22	1.69	1.20
P		N.S.	N.S.	N.S.
Between Chi				0.66
Between df				1
Between P				N.S.
Btwn(F) P				N.S.
Btwn(R) P				N.S.

Too few RRs for analysis by factor

Table 3K2 - 4

IESLC - Meta-analysis of Ex Smoking, Years quit (vs current), "Low"
Adenocarcinoma, Any Product (or Cigarettes if Any not available)
Least adjusted

REF	NRR	X	SEX	AGEL	AGEH	RACE	YF	LC TYPE	LOC	START	ST	NLC	R	VB	P	H	AD	ADOS	PRODUCT	exL	exH	DENOM	De
BARBON	749	x	m	0	0	all	-	a	Eu:wst	1979	CC	755	n	bl	y	y	0	0	all/unsp	0.1	4	current	st
JAHN	653		m	0	0	all	-	a	Eu:Ger	1988	CC	1004	n	bl	n	n	0	0	cig+/-ot	2	5	current	st
LUBIN2	871		m	0	0	all	-	a	Eu:mul	1976	CC	7804	n	bl	n	y	0	0	cig+/-ot	0.1	4	current	st
LUBIN2	980		f	0	0	all	-	a	Eu:mul	1976	CC	7804	n	bl	n	y	2	1#	cig+/-ot	0.1	4	current	ot
MATOS	666	x	m	0	0	all	-	a	SCAmer	1994	CC	200	n	bl	n	n	0	0	cig+/-ot	1.0	5	cur+1y	st
SOBUE	744		m	0	0	all	-	a	As:Jap	1986	CC	1376	n	bl	n	y	0	0	cig+/-ot	1.0	4	cur+1y	st
WYNDE3	528		m	0	0	all	-	KII	NAmer	1966	CC	350	n	bl	n	y	0	0	all/unsp	1.0	3	cur+1y	st

Comments on values in listings

LUBIN2 ADOS Duration of smoking

Cigarette type is all/unspec for all RRs

Table 3K2 - 5

IESLC - Meta-analysis of Ex Smoking, Years quit (vs current), "Low"
Adenocarcinoma, Any Product (or Cigarettes if Any not available)
Least adjusted

REF	NRR	SEX	AD	Number Exposed Case	Cont	Non-exposed Case	Cont	RR	95.00%CI
BARBON	749	m	0	7	20	109	362	1.16	(0.48- 2.82)
JAHN	653	m	0	19	46	75	269	1.48	(0.82- 2.68)
LUBIN2	871	m	0	77	1047	454	6209	1.01	(0.78- 1.29)
LUBIN2	980	f	2	6	-	69	-	0.70	(0.29- 1.69)
Subtotal LUBIN2								0.98	(0.77- 1.25)
MATOS	666	m	0	12	23	46	132	1.50	(0.69- 3.25)
SOBUE	744	m	0	44	116	270	633	0.89	(0.61- 1.29)
WYNDE3	528	m	0	3	22	56	207	0.50	(0.15- 1.75)
Partial Totals				168	1274	1079	7812		

*prospective study

REF	NRR	SEX	AD	Ys	Ws	Qs	Ps
BARBON	749	m	0	0.15	4.88	0.10	0.7395
JAHN	653	m	0	0.39	10.94	1.60	0.1937
LUBIN2	871	m	0	0.01	61.33	0.00	0.9639
LUBIN2	980	f	2	-0.36	4.95	0.67	0.4276
Subtotal LUBIN2				-0.02	66.27	0.67	
MATOS	666	m	0	0.40	6.41	0.99	0.3071
SOBUE	744	m	0	-0.12	27.30	0.45	0.5398
WYNDE3	528	m	0	-0.69	2.49	1.21	0.2796

	N	7
	NS	6
	Wt	118.29
Het	Chi	5.01
Het	df	6
Het	P	N.S.
Fixed	RR	1.01
	RRl	0.84
	RRu	1.21
	P	N.S.
Random	RR	1.01
	RRl	0.84
	RRu	1.21
	P	N.S.
Asymm	P	N.S.

Table 3K2 - 6

IESLC - Meta-analysis of Ex Smoking, Years quit (vs current), "Low"
Adenocarcinoma, Any Product (or Cigarettes if Any not available)
Least adjusted

	Sex		
combined	male	female	Total
N	6	1	7
NS	6	1	7
Wt	113.34	4.95	118.29
Het Chi	4.31	0.00	5.01
Het df	5	0	6
Het P	N.S.	N.S.	N.S.
Fixed RR	1.03	0.70	1.01
RRl	0.85	0.29	0.84
RRu	1.24	1.69	1.21
P	N.S.	N.S.	N.S.
Random RR	1.03	0.70	1.01
RRl	0.85	0.29	0.84
RRu	1.24	1.69	1.21
P	N.S.	N.S.	N.S.
Between Chi			0.70
Between df			1
Between P			N.S.
Btwn(F) P			N.S.
Btwn(R) P			N.S.

Table 3K2 - 7

IESLC - Meta-analysis of Ex Smoking, Years quit (vs current), "Low"
Adenocarcinoma, Any Product (or Cigarettes if Any not available)
Excluded studies (and stage at which they were excluded)

1	AGUDO AKIBA AMANDU AMES AXELSS BEST BOUCHA BOUCOT BRESLO CHEN CHEN2 CHIAZZ DEAN2 DOSEME ENGELA FAN GENG GER GUO HAENSZ HEGMAN HOLE HU HU2 JUSSAW KATSOU KAUFMA KOO KOULUM KREUZE LETOUR LEVIN LIAW LIU3 LIU4 LIU5 MCCONN MIGRAN MRFITR NOTAN2 OSANN2 PERNU QIAO2 RACHTA RESTRE SADOWS SEGI2 STASZE TIZZAN VUTUC WATSON WU WUWILL WYNDE2 WYNDE8 XU YUAN ZHANG ZHENG ZHOU
2	AUVINE BENSHL BLOT1 BROWN3 BUFFLE GURSEL LAUSSM MCDUFF PISANI PRESCO SPITZ WU2 WYNDE7
4	ARMADA BECHER BOFFET BROSS CARPEN CEDERL CHOI CHYOU CORREA CPSI CPSII DAMBER DARBY DEAN3 DESTEF DOLL DOLL2 DORGAN DORN GAO GAO2 GARCIA GARSHI GILLIS GRAHAM HAMMO2 HAMMON HIRAYA HUMBLE JOLY KAISE2 KHUDER LUBIN LUO PEZZO2 QIAO SPEIZE SUZUK2 TVERDA WANG2 WIGLE
5	ALDERS
10	JEDRYC WAKAI WYNDE6
14	JAIN PEZZOT SVENSS
15	BENHAM

Table 3K2 - 8
Potentially overlapping studies

REF	REFGP	PRINC	OVERLAP/LINK
LUBIN2	LUBIN2	1	Lubin-combined
JAHN	BOFFET	2	Subset of BOFFET

Table 3K3 -

IESLC - Meta-analysis of Ex Smoking, Years quit (vs current), "Mid"
Adenocarcinoma, Any Product (or Cigarettes if Any not available)

This analysis is restricted to results for:
1) Ex smokers
2) Results by Years quit (vs current)
3) Categorical results by Years quit (vs current)
4) Adenocarcinoma (or near equivalent)
5) Results complete enough for use in metaanalysis

Within each study, results are then selected (in the following order of preference, within each sex) for:
6) (not applicable)
7) PRODUCT: all/unspec, cigarettes regardless of other products, cigarettes only
8) CIGTYPE: all/unspecified, MC regardless of HR, MC only
9) Results with least adjustment for other aspects of smoking (ADOS)
10) DENOM: current smokers, current + recent smokers (up to number of m=months or y=years, max 2 years)
11) Followup period (YF, prospective studies): whole study (coded as 0) or longest available
12) LCtype: adeno or nearest available, but not squamous. (q = squamous, s = small,
a = adeno, l = large, KII = Kreyberg II, al = alveolar, br = bronchiolar, u = undifferentiated)
13) Race: all or nearest available, otherwise by race (wh or w = white, bl or b = black, hi = hispanic
ch = chinese, jap = japanese, haw = hawaiian, w+o = white + oriental, sca = scandinavian, as = asian)
14) Years quit (vs current) "mid" in key scheme 1 (key value 7, maximum range 4-11)
15) For overlapping studies: principal rather than subsidiary studies
Finally by Age: whole study (coded as 0) if available, otherwise by widest available age group
and then for single sex results (m, f) in preference to results for both sexes combined (c).

Results adjusted (AD) for the most potential confounders are then chosen in Sections -1 to -3
and results adjusted for the least confounders in Sections -4 to -6. (Those least adjusted results which
actually differ from the most adjusted are marked 'x' in column X in Section -4)

Section -7 shows excluded studies, together with the stage (as above) at which no qualifying
results were found.

Section -8 lists the potentially overlapping studies which have been included (1=principal, 2=subsidiary).

Section -9 lists any results which would have been included in preference except that they had data not complete
enough for use in meta-analysis, with their significance (yes/no), if known, and any further comment as entered
on the database. It also lists as "gap" any categories for which no data were presented by the original authors.

In addition to those mentioned above, the following fields, levels and abbreviations are used:
* or nk = not known, n = no, y = yes, ot = other
nev = never
all/unspec = all or unspecified, cig+/-ot = cigarettes irrespective of other products (cigar, pipe etc)
MC = manufactured cigarettes, HR = hand-rolled cigarettes
exL, exH = range of exposure (low and high) in the smoking group, in terms of Years quit (vs current)
REF: 6-character study reference
NRR: number of the RR on the database within the study
ST : study type (CC = case control, pr or prosp = prospective)
NLC: number of lung cancer cases in whole study
R : risky occupational population (n = no, m = mining, o = other risky)
VB : national cigarette type (V = at least 75% Virginia, bl = at least 75% blended, ot = other)
P : any proxy use
H : full histological confirmation
De : derivation of RR/CI (or = original, st = standard method, ot = other method of estimation)

Table 3K3 - 1

IESLC - Meta-analysis of Ex Smoking, Years quit (vs current), "Mid"
Adenocarcinoma, Any Product (or Cigarettes if Any not available)
Most adjusted

REF	NRR	SEX	AGEL	AGEH	RACE	YF	LC TYPE	LOC	START	ST	NLC	R	VB	P	H	AD	ADOS	PRODUCT	exL	exH	DENOM	De
JAHN	654	m	0	0	all	-	a	Eu:Ger	1988	CC	1004	n	bl	n	n	0	0	cig+/-ot	6	10	current	st
LUBIN2	872	m	0	0	all	-	a	Eu:mul	1976	CC	7804	n	bl	n	y	0	0	cig+/-ot	5	9	current	st
LUBIN2	981	f	0	0	all	-	a	Eu:mul	1976	CC	7804	n	bl	n	y	2	1#	cig+/-ot	5	9	current	ot
MATOS	677	m	0	0	all	-	a	SCAmer	1994	CC	200	n	bl	n	n	2	0	cig+/-ot	6	10	cur+1y	or
SOBUE	745	m	0	0	all	-	a	As:Jap	1986	CC	1376	n	bl	n	y	0	0	cig+/-ot	5	9	cur+1y	st

Comments on values in listings

LUBIN2 ADOS Duration of smoking

Cigarette type is all/unspec for all RRs

Table 3K3 - 2

IESLC - Meta-analysis of Ex Smoking, Years quit (vs current), "Mid"
Adenocarcinoma, Any Product (or Cigarettes if Any not available)
Most adjusted

REF	NRR	SEX	AD	Number Exposed Case	Cont	Non-exposed Case	Cont	RR	95.00%CI
JAHN	654	m	0	13	63	75	269	0.74	(0.39- 1.42)
LUBIN2	872	m	0	50	882	454	6209	0.78	(0.57- 1.05)
LUBIN2	981	f	2	7	-	69	-	1.00	(0.43- 2.32)
Subtotal LUBIN2								0.80	(0.60- 1.06)
MATOS	677	m	2	9	-	46	-	1.00	(0.40- 2.30)
SOBUE	745	m	0	22	92	270	633	0.56	(0.34- 0.91)
Partial Totals				101	1037	914	7111		

*prospective study

REF	NRR	SEX	AD	Ys	Ws	Qs	Ps
JAHN	654	m	0	-0.30	9.10	0.00	0.3638
LUBIN2	872	m	0	-0.25	42.56	0.06	0.0968
LUBIN2	981	f	2	0.00	5.41	0.46	1.0000
Subtotal LUBIN2				-0.23	47.97	0.53	
MATOS	677	m	2	0.00	5.02	0.43	1.0000
SOBUE	745	m	0	-0.58	16.23	1.32	0.0197

	N	5
	NS	4
	Wt	78.32
Het	Chi	2.28
Het	df	4
Het	P	N.S.
Fixed	RR	0.75
	RRl	0.60
	RRu	0.93
	P	--
Random	RR	0.75
	RRl	0.60
	RRu	0.93
	P	--
Asymm	P	N.S.

Table 3K3 - 3

IESLC - Meta-analysis of Ex Smoking, Years quit (vs current), "Mid"
Adenocarcinoma, Any Product (or Cigarettes if Any not available)
Most adjusted

	Sex combined	male	female	Total
N		4	1	5
NS		4	1	5
Wt		72.92	5.41	78.32
Het Chi		1.78	0.00	2.28
Het df		3	0	4
Het P		N.S.	N.S.	N.S.
Fixed RR		0.73	1.00	0.75
RRl		0.58	0.43	0.60
RRu		0.92	2.32	0.93
P		--	N.S.	--
Random RR		0.73	1.00	0.75
RRl		0.58	0.43	0.60
RRu		0.92	2.32	0.93
P		--	N.S.	--
Between Chi				0.50
Between df				1
Between P				N.S.
Btwn(F) P				N.S.
Btwn(R) P				N.S.

Too few RRs for analysis by factor

Table 3K3 - 4

IESLC - Meta-analysis of Ex Smoking, Years quit (vs current), "Mid"
Adenocarcinoma, Any Product (or Cigarettes if Any not available)
Least adjusted

REF	NRR	X	SEX	AGEL	AGEH	RACE	YF	LC TYPE	LOC	START	ST	NLC	R	VB	P	H	AD	ADOS	PRODUCT	exL	exH	DENOM	De
JAHN	654		m	0	0	all	-	a	Eu:Ger	1988	CC	1004	n	bl	n	n	0	0	cig+/-ot	6	10	current	st
LUBIN2	872		m	0	0	all	-	a	Eu:mul	1976	CC	7804	n	bl	n	y	0	0	cig+/-ot	5	9	current	st
LUBIN2	981		f	0	0	all	-	a	Eu:mul	1976	CC	7804	n	bl	n	y	2	1#	cig+/-ot	5	9	current	ot
MATOS	667	x	m	0	0	all	-	a	SCAmer	1994	CC	200	n	bl	n	n	0	0	cig+/-ot	6	10	cur+1y	st
SOBUE	745		m	0	0	all	-	a	As:Jap	1986	CC	1376	n	bl	n	y	0	0	cig+/-ot	5	9	cur+1y	st

Comments on values in listings

LUBIN2 ADOS Duration of smoking

Cigarette type is all/unspec for all RRs

Table 3K3 - 5

IESLC - Meta-analysis of Ex Smoking, Years quit (vs current), "Mid"
Adenocarcinoma, Any Product (or Cigarettes if Any not available)
Least adjusted

				Number Exposed		Non-exposed			
REF	NRR	SEX	AD	Case	Cont	Case	Cont	RR	95.00%CI
JAHN	654	m	0	13	63	75	269	0.74	(0.39- 1.42)
LUBIN2	872	m	0	50	882	454	6209	0.78	(0.57- 1.05)
LUBIN2	981	f	2	7	-	69	-	1.00	(0.43- 2.32)
Subtotal LUBIN2								0.80	(0.60- 1.06)
MATOS	667	m	0	9	27	46	132	0.96	(0.42- 2.18)
SOBUE	745	m	0	22	92	270	633	0.56	(0.34- 0.91)
Partial Totals				101	1064	914	7243		

*prospective study

REF	NRR	SEX	AD	Ys	Ws	Qs	Ps
JAHN	654	m	0	-0.30	9.10	0.00	0.3638
LUBIN2	872	m	0	-0.25	42.56	0.07	0.0968
LUBIN2	981	f	2	0.00	5.41	0.47	1.0000
Subtotal LUBIN2				-0.23	47.97	0.53	
MATOS	667	m	0	-0.04	5.63	0.35	0.9160
SOBUE	745	m	0	-0.58	16.23	1.31	0.0197

	N	5
	NS	4
	Wt	78.94
Het	Chi	2.20
Het	df	4
Het	P	N.S.
Fixed	RR	0.75
	RRl	0.60
	RRu	0.93
	P	--
Random	RR	0.75
	RRl	0.60
	RRu	0.93
	P	--
Asymm	P	N.S.

Table 3K3 - 6

IESLC - Meta-analysis of Ex Smoking, Years quit (vs current), "Mid"
Adenocarcinoma, Any Product (or Cigarettes if Any not available)
Least adjusted

	Sex			
	combined	male	female	Total
N		4	1	5
NS		4	1	5
Wt		73.53	5.41	78.94
Het Chi		1.70	0.00	2.20
Het df		3	0	4
Het P		N.S.	N.S.	N.S.
Fixed RR		0.73	1.00	0.75
RRl		0.58	0.43	0.60
RRu		0.92	2.32	0.93
P		--	N.S.	--
Random RR		0.73	1.00	0.75
RRl		0.58	0.43	0.60
RRu		0.92	2.32	0.93
P		--	N.S.	--
Between Chi				0.50
Between df				1
Between P				N.S.
Btwn(F) P				N.S.
Btwn(R) P				N.S.

Table 3K3 - 7

IESLC - Meta-analysis of Ex Smoking, Years quit (vs current), "Mid"
Adenocarcinoma, Any Product (or Cigarettes if Any not available)
Excluded studies (and stage at which they were excluded)

1	AGUDO AKIBA AMANDU AMES AXELSS BEST BOUCHA BOUCOT BRESLO CHEN CHEN2 CHIAZZ DEAN2 DOSEME ENGELA FAN GENG GER GUO HAENSZ HEGMAN HOLE HU HU2 JUSSAW KATSOU KAUFMA KOO KOULUM KREUZE LETOUR LEVIN LIAW LIU3 LIU4 LIU5 MCCONN MIGRAN MRFITR NOTAN2 OSANN2 PERNU QIAO2 RACHTA RESTRE SADOWS SEGI2 STASZE TIZZAN VUTUC WATSON WU WUWILL WYNDE2 WYNDE8 XU YUAN ZHANG ZHENG ZHOU
2	AUVINE BENSHL BLOT1 BROWN3 BUFFLE GURSEL LAUSSM MCDUFF PISANI PRESCO SPITZ WU2 WYNDE7
4	ARMADA BECHER BOFFET BROSS CARPEN CEDERL CHOI CHYOU CORREA CPSI CPSII DAMBER DARBY DEAN3 DESTEF DOLL DOLL2 DORGAN DORN GAO GAO2 GARCIA GARSHI GILLIS GRAHAM HAMMO2 HAMMON HIRAYA HUMBLE JOLY KAISE2 KHUDER LUBIN LUO PEZZO2 QIAO SPEIZE SUZUK2 TVERDA WANG2 WIGLE
5	ALDERS
10	JEDRYC WAKAI WYNDE6
14	BARBON JAIN PEZZOT SVENSS WYNDE3
15	BENHAM

Table 3K3 - 8
Potentially overlapping studies

REF	REFGP	PRINC	OVERLAP/LINK
LUBIN2	LUBIN2	1	Lubin-combined
JAHN	BOFFET	2	Subset of BOFFET

Table 3K4 -

IESLC - Meta-analysis of Ex Smoking, Years quit (vs current), "High"
Adenocarcinoma, Any Product (or Cigarettes if Any not available)

This analysis is restricted to results for:
1) Ex smokers
2) Results by Years quit (vs current)
3) Categorical results by Years quit (vs current)
4) Adenocarcinoma (or near equivalent)
5) Results complete enough for use in metaanalysis

Within each study, results are then selected (in the following order of preference, within each sex) for:
6) PRODUCT: all/unspec, cigarettes regardless of other products, cigarettes only
7) CIGTYPE: all/unspecified, MC regardless of HR, MC only
8) Results with least adjustment for other aspects of smoking (ADOS)
9) DENOM: current smokers, current + recent smokers (up to number of m=months or y=years, max 2 years)
10) Followup period (YF, prospective studies): whole study (coded as 0) or longest available
11) LCtype: adeno or nearest available, but not squamous. (q = squamous, s = small,
a = adeno, l = large, KII = Kreyberg II, al = alveolar, br = bronchiolar, u = undifferentiated)
12) Race: all or nearest available, otherwise by race (wh or w = white, bl or b = black, hi = hispanic
ch = chinese, jap = japanese, haw = hawaiian, w+o = white + oriental, sca = scandinavian, as = asian)
13) Years quit (vs current) "high" in key scheme 1 (key value 12, maximum range 8+)
14) For overlapping studies: principal rather than subsidiary studies
Finally by Age: whole study (coded as 0) if available, otherwise by widest available age group
and then for single sex results (m, f) in preference to results for both sexes combined (c).

Results adjusted (AD) for the most potential confounders are then chosen in Sections -1 to -3
and results adjusted for the least confounders in Sections -4 to -6. (Those least adjusted results which
actually differ from the most adjusted are marked 'x' in column X in Section -4)

Section -7 shows excluded studies, together with the stage (as above) at which no qualifying
results were found.

Section -8 lists the potentially overlapping studies which have been included (1=principal, 2=subsidiary).

Section -9 lists any results which would have been included in preference except that they had data not complete
enough for use in meta-analysis, with their significance (yes/no), if known, and any further comment as entered
on the database. It also lists as "gap" any categories for which no data were presented by the original authors.

In addition to those mentioned above, the following fields, levels and abbreviations are used:
* or nk = not known, n = no, y = yes, ot = other
nev = never
all/unspec = all or unspecified, cig+/-ot = cigarettes irrespective of other products (cigar, pipe etc)
MC = manufactured cigarettes, HR = hand-rolled cigarettes
exL, exH = range of exposure (low and high) in the smoking group, in terms of Years quit (vs current)
REF: 6-character study reference
NRR: number of the RR on the database within the study
ST : study type (CC = case control, pr or prosp = prospective)
NLC: number of lung cancer cases in whole study
R : risky occupational population (n = no, m = mining, o = other risky)
VB : national cigarette type (V = at least 75% Virginia, bl = at least 75% blended, ot = other)
P : any proxy use
H : full histological confirmation
De : derivation of RR/CI (or = original, st = standard method, ot = other method of estimation)

Table 3K4 - 1

IESLC - Meta-analysis of Ex Smoking, Years quit (vs current), "High"
Adenocarcinoma, Any Product (or Cigarettes if Any not available)
Most adjusted

REF	NRR	SEX	AGEL	AGEH	RACE	YF	LC TYPE	LOC	START	ST	NLC	R	VB	P	H	AD	ADOS	PRODUCT	exL	exH	DENOM	De
JAHN	655	m	0	0	all	-	a	Eu:Ger	1988	CC	1004	n	bl	n	n	0	0	cig+/-ot	11	20	current	st
JAIN	541	m	0	0	all	-	a	NAmer	1981	CC	845	n	V	y	n	0	0	cig+/-ot	10	999	cur+2y	st
JAIN	505	f	0	0	all	-	a	NAmer	1981	CC	845	n	V	y	n	0	0	cig+/-ot	10	999	cur+2y	st
LUBIN2	873	m	0	0	all	-	a	Eu:mul	1976	CC	7804	n	bl	n	y	0	0	cig+/-ot	10	14	current	st
LUBIN2	972	f	0	0	all	-	a	Eu:mul	1976	CC	7804	n	bl	n	y	0	0	cig+/-ot	10	19	current	st
MATOS	678	m	0	0	all	-	a	SCAmer	1994	CC	200	n	bl	n	n	2	0	cig+/-ot	11	999	cur+1y	or
PEZZOT	589	m	0	0	all	-	a	SCAmer	1987	CC	215	n	bl	n	y	0	0	cig only	11	999	cur+1y	st
SOBUE	746	m	0	0	all	-	a	As:Jap	1986	CC	1376	n	bl	n	y	0	0	cig+/-ot	10	999	cur+1y	st
SVENSS	573	f	0	0	all	-	a	Eu:Sca	1983	CC	210	n	bl	n	n	0	0	all/unsp	11	999	cur+2y	st

Cigarette type is all/unspec for all RRs

Table 3K4 - 2

IESLC - Meta-analysis of Ex Smoking, Years quit (vs current), "High"
Adenocarcinoma, Any Product (or Cigarettes if Any not available)
Most adjusted

REF	NRR	SEX	AD	Number Exposed Case	Cont	Non-exposed Case	Cont	RR	95.00%CI
JAHN	655	m	0	22	130	75	269	0.61	(0.36- 1.02)
JAIN	541	m	0	14	113	60	118	0.24	(0.13- 0.46)
JAIN	505	f	0	3	61	69	99	0.07	(0.02- 0.23)
Subtotal JAIN								0.19	(0.11- 0.33)
LUBIN2	873	m	0	30	693	454	6209	0.59	(0.41- 0.86)
LUBIN2	972	f	0	3	33	69	410	0.54	(0.16- 1.81)
Subtotal LUBIN2								0.59	(0.41- 0.84)
MATOS	678	m	2	12	-	46	-	0.30	(0.20- 0.70)
PEZZOT	589	m	0	7	31	42	38	0.20	(0.08- 0.52)
SOBUE	746	m	0	49	144	270	633	0.80	(0.56- 1.14)
SVENSS	573	f	0	7	24	38	53	0.41	(0.16- 1.04)
Partial Totals				147	1229	1123	7829		

*prospective study

REF	NRR	SEX	AD	Ys	Ws	Qs	Ps
JAHN	655	m	0	-0.50	14.25	0.54	0.0595
JAIN	541	m	0	-1.41	9.49	4.89	0.0000
JAIN	505	f	0	-2.65	2.67	10.23	0.0000
Subtotal JAIN				-1.68	12.16	15.12	
LUBIN2	873	m	0	-0.52	26.93	0.78	0.0065
LUBIN2	972	f	0	-0.62	2.63	0.02	0.3181
Subtotal LUBIN2				-0.53	29.55	0.79	
MATOS	678	m	2	-1.20	9.79	2.55	0.0002
PEZZOT	589	m	0	-1.59	4.44	3.55	0.0008
SOBUE	746	m	0	-0.23	30.64	6.71	0.2110
SVENSS	573	f	0	-0.90	4.35	0.18	0.0606

	N	9
	NS	7
	Wt	105.18
Het	Chi	29.45
Het	df	8
Het	P	***
Fixed	RR	0.50
	RRl	0.41
	RRu	0.60
	P	---
Random	RR	0.39
	RRl	0.26
	RRu	0.58
	P	---
Asymm	P	*

Table 3K4 - 3

IESLC - Meta-analysis of Ex Smoking, Years quit (vs current), "High"
Adenocarcinoma, Any Product (or Cigarettes if Any not available)
Most adjusted

	Sex combined	male	female	Total
N		6	3	9
NS		6	3	9
Wt		95.53	9.65	105.18
Het Chi		18.65	6.81	29.45
Het df		5	2	8
Het P		**	*	***
Fixed RR		0.53	0.27	0.50
RRl		0.43	0.14	0.41
RRu		0.65	0.51	0.60
P		---	---	---
Random RR		0.44	0.25	0.39
RRl		0.29	0.08	0.26
RRu		0.67	0.83	0.58
P		---	-	---
Between Chi				3.99
Between df				1
Between P				*
Btwn(F) P				N.S.
Btwn(R) P				N.S.

Too few RRs for analysis by factor

Table 3K4 - 4

IESLC - Meta-analysis of Ex Smoking, Years quit (vs current), "High"
Adenocarcinoma, Any Product (or Cigarettes if Any not available)
Least adjusted

REF	NRR	X	SEX	AGEL	AGEH	RACE	YF	LC TYPE	LOC	START	ST	NLC	R	VB	P	H	AD	ADOS	PRODUCT	exL	exH	DENOM	De
JAHN	655		m	0	0	all	-	a	Eu:Ger	1988	CC	1004	n	bl	n	n	0	0	cig+/-ot	11	20	current	st
JAIN	541		m	0	0	all	-	a	NAmer	1981	CC	845	n	V	y	n	0	0	cig+/-ot	10	999	cur+2y	st
JAIN	505		f	0	0	all	-	a	NAmer	1981	CC	845	n	V	y	n	0	0	cig+/-ot	10	999	cur+2y	st
LUBIN2	873		m	0	0	all	-	a	Eu:mul	1976	CC	7804	n	bl	n	y	0	0	cig+/-ot	10	14	current	st
LUBIN2	972		f	0	0	all	-	a	Eu:mul	1976	CC	7804	n	bl	n	y	0	0	cig+/-ot	10	19	current	st
MATOS	668	x	m	0	0	all	-	a	SCAmer	1994	CC	200	n	bl	n	n	0	0	cig+/-ot	11	999	cur+1y	st
PEZZOT	589		m	0	0	all	-	a	SCAmer	1987	CC	215	n	bl	n	y	0	0	cig only	11	999	cur+1y	st
SOBUE	746		m	0	0	all	-	a	As:Jap	1986	CC	1376	n	bl	n	y	0	0	cig+/-ot	10	999	cur+1y	st
SVENSS	573		f	0	0	all	-	a	Eu:Sca	1983	CC	210	n	bl	n	n	0	0	all/unsp	11	999	cur+2y	st

Cigarette type is all/unspec for all RRs

Table 3K4 - 5

IESLC - Meta-analysis of Ex Smoking, Years quit (vs current), "High"
Adenocarcinoma, Any Product (or Cigarettes if Any not available)
Least adjusted

REF	NRR	SEX	AD	Number Exposed Case	Exposed Cont	Non-exposed Case	Non-exposed Cont	RR	95.00%CI
JAHN	655	m	0	22	130	75	269	0.61	(0.36- 1.02)
JAIN	541	m	0	14	113	60	118	0.24	(0.13- 0.46)
JAIN	505	f	0	3	61	69	99	0.07	(0.02- 0.23)
Subtotal JAIN								0.19	(0.11- 0.33)
LUBIN2	873	m	0	30	693	454	6209	0.59	(0.41- 0.86)
LUBIN2	972	f	0	3	33	69	410	0.54	(0.16- 1.81)
Subtotal LUBIN2								0.59	(0.41- 0.84)
MATOS	668	m	0	12	101	46	132	0.34	(0.17- 0.68)
PEZZOT	589	m	0	7	31	42	38	0.20	(0.08- 0.52)
SOBUE	746	m	0	49	144	270	633	0.80	(0.56- 1.14)
SVENSS	573	f	0	7	24	38	53	0.41	(0.16- 1.04)
Totals				147	1330	1123	7961		

*prospective study

REF	NRR	SEX	AD	Ys	Ws	Qs	Ps
JAHN	655	m	0	-0.50	14.25	0.44	0.0595
JAIN	541	m	0	-1.41	9.49	5.14	0.0000
JAIN	505	f	0	-2.65	2.67	10.42	0.0000
Subtotal JAIN				-1.68	12.16	15.56	
LUBIN2	873	m	0	-0.52	26.93	0.62	0.0065
LUBIN2	972	f	0	-0.62	2.63	0.01	0.3181
Subtotal LUBIN2				-0.53	29.55	0.63	
MATOS	668	m	0	-1.08	8.16	1.31	0.0021
PEZZOT	589	m	0	-1.59	4.44	3.69	0.0008
SOBUE	746	m	0	-0.23	30.64	6.20	0.2110
SVENSS	573	f	0	-0.90	4.35	0.22	0.0606

	N	9
	NS	7
	Wt	103.55
Het	Chi	28.06
Het	df	8
Het	P	***
Fixed	RR	0.51
	RRl	0.42
	RRu	0.62
	P	---
Random	RR	0.39
	RRl	0.26
	RRu	0.59
	P	---
Asymm	P	*

Table 3K4 - 6

IESLC - Meta-analysis of Ex Smoking, Years quit (vs current), "High"
Adenocarcinoma, Any Product (or Cigarettes if Any not available)
Least adjusted

	Sex		
combined	male	female	Total
N	6	3	9
NS	6	3	9
Wt	93.90	9.65	103.55
Het Chi	17.01	6.81	28.06
Het df	5	2	8
Het P	**	*	***
Fixed RR	0.54	0.27	0.51
RRl	0.44	0.14	0.42
RRu	0.66	0.51	0.62
P	---	---	---
Random RR	0.45	0.25	0.39
RRl	0.30	0.08	0.26
RRu	0.68	0.83	0.59
P	---	-	---
Between Chi			4.24
Between df			1
Between P			*
Btwn(F) P			N.S.
Btwn(R) P			N.S.

Table 3K4 - 7

IESLC - Meta-analysis of Ex Smoking, Years quit (vs current), "High"
Adenocarcinoma, Any Product (or Cigarettes if Any not available)
Excluded studies (and stage at which they were excluded)

1	AGUDO AKIBA AMANDU AMES AXELSS BEST BOUCHA BOUCOT BRESLO CHEN CHEN2 CHIAZZ DEAN2 DOSEME ENGELA FAN GENG GER GUO HAENSZ HEGMAN HOLE HU HU2 JUSSAW KATSOU KAUFMA KOO KOULUM KREUZE LETOUR LEVIN LIAW LIU3 LIU4 LIU5 MCCONN MIGRAN MRFITR NOTAN2 OSANN2 PERNU QIAO2 RACHTA RESTRE SADOWS SEGI2 STASZE TIZZAN VUTUC WATSON WU WUWILL WYNDE2 WYNDE8 XU YUAN ZHANG ZHENG ZHOU
2	AUVINE BENSHL BLOT1 BROWN3 BUFFLE GURSEL LAUSSM MCDUFF PISANI PRESCO SPITZ WU2 WYNDE7
4	ARMADA BECHER BOFFET BROSS CARPEN CEDERL CHOI CHYOU CORREA CPSI CPSII DAMBER DARBY DEAN3 DESTEF DOLL DOLL2 DORGAN DORN GAO GAO2 GARCIA GARSHI GILLIS GRAHAM HAMMO2 HAMMON HIRAYA HUMBLE JOLY KAISE2 KHUDER LUBIN LUO PEZZO2 QIAO SPEIZE SUZUK2 TVERDA WANG2 WIGLE
5	ALDERS
10	JEDRYC WAKAI WYNDE6
14	BARBON WYNDE3
15	BENHAM

Table 3K4 - 8
Potentially overlapping studies

REF	REFGP	PRINC	.	OVERLAP/LINK
LUBIN2	LUBIN2	1		Lubin-combined
JAHN	BOFFET	2		Subset of BOFFET

Table 3K4 - 9
Most adjusted - insufficient data for meta-analysis

REF	NRR	SEX	AGEL	AGEH	RACE	YF	LC TYPE	LOC	START	ST	NLC	R	VB	P	H	AD	ADOS	PRODUCT	exL	exH	DENOM	De
ALDERS	575	m	0	0	all	-	not q+s	Eu:UK	1977	CC	1448	n	V	n	n	1	0	cig only	10	999	current	ot
ALDERS	586	f	0	0	all	-	not q+s	Eu:UK	1977	CC	1448	n	V	n	n	1	0	cig only	10	999	current	ot

REF	NRR	RR	SIG	RRDATA comment
ALDERS	575	0.91	n	0
ALDERS	586	0.36	n	0

Table 3K5 -

IESLC - Meta-analysis of Ex Smoking, Years quit (vs current), "Highest vs lowest"
Adenocarcinoma, Any Product (or Cigarettes if Any not available)

This analysis is restricted to results for:
1) Ex smokers
2) Results by Years quit (vs current)
3) Categorical results by Years quit (vs current)
4) Denominator (unexposed) = "low"
5) Adenocarcinoma (or near equivalent)
6) Results complete enough for use in metaanalysis

Within each study, results are then selected (in the following order of preference, within each sex) for:
7) (not applicable)
8) PRODUCT: all/unspec, cigarettes regardless of other products, cigarettes only
9) CIGTYPE: all/unspecified, MC regardless of HR, MC only
10) Results with least adjustment for other aspects of smoking (ADOS)
11) The highest vs lowest category
12) Followup period (YF, prospective studies): whole study (coded as 0) or longest available
13) LCtype: adeno or nearest available, but not squamous. (q = squamous, s = small,
a = adeno, l = large, KII = Kreyberg II, al = alveolar, br = bronchiolar, u = undifferentiated)
14) Race: all or nearest available, otherwise by race (wh or w = white, bl or b = black, hi = hispanic
ch = chinese, jap = japanese, haw = hawaiian, w+o = white + oriental, sca = scandinavian, as = asian)
15) For overlapping studies: principal rather than subsidiary studies
Finally by Age: whole study (coded as 0) if available, otherwise by widest available age group
and then for single sex results (m, f) in preference to results for both sexes combined (c).

Results adjusted (AD) for the most potential confounders are then chosen in Sections -1 to -3
and results adjusted for the least confounders in Sections -4 to -6. (Those least adjusted results which
actually differ from the most adjusted are marked 'x' in column X in Section -4)

Section -7 shows excluded studies, together with the stage (as above) at which no qualifying
results were found.

Section -8 lists the potentially overlapping studies which have been included (1=principal, 2=subsidiary).

Section -9 lists any results which would have been included in preference except that they had data not complete
enough for use in meta-analysis, with their significance (yes/no), if known, and any further comment as entered
on the database. It also lists as "gap" any categories for which no data were presented by the original authors.

In addition to those mentioned above, the following fields, levels and abbreviations are used:
* or nk = not known, n = no, y = yes, ot = other
all/unspec = all or unspecified, cig+/-ot = cigarettes irrespective of other products (cigar, pipe etc)
MC = manufactured cigarettes, HR = hand-rolled cigarettes
exL, exH = range of exposure (low and high) in the "highest" group, in terms of Years quit (vs current)
unexL, unexH = range of exposure (low and high) in the "lowest" group, in terms of Years quit (vs current)
REF: 6-character study reference
NRR: number of the RR on the database within the study
ST : study type (CC = case control, pr or prosp = prospective)
NLC: number of lung cancer cases in whole study
R : risky occupational population (n = no, m = mining, o = other risky)
VB : national cigarette type (V = at least 75% Virginia, bl = at least 75% blended, ot = other)
P : any proxy use
H : full histological confirmation
De : derivation of RR/CI (or = original, st = standard method, ot = other method of estimation)

Table 3K5 - 1

IESLC - Meta-analysis of Ex Smoking, Years quit (vs current), "Highest vs lowest"
Adenocarcinoma, Any Product (or Cigarettes if Any not available)
Most adjusted

REF	NRR	SEX	AGEL	AGEH	RACE	YF	LC TYPE	LOC	START	ST	NLC	R	VB	P	H	AD	ADOS	PRODUCT	exL	exH	unexL	unexH	De
BARBON	770	m	0	0	all	-	a	Eu:wst	1979	CC	755	n	bl	y	y	1	0	all/unsp	25	999	0.1	4	ot
JAHN	661	m	0	0	all	-	a	Eu:Ger	1988	CC	1004	n	bl	n	n	0	0	cig+/-ot	21	999	0.1	0.9	st
JAIN	542	m	0	0	all	-	a	NAmer	1981	CC	845	n	V	y	n	0	0	cig+/-ot	10	999	2	9	st
JAIN	506	f	0	0	all	-	a	NAmer	1981	CC	845	n	V	y	n	0	0	cig+/-ot	10	999	2	9	st
LUBIN2	879	m	0	0	all	-	a	Eu:mul	1976	CC	7804	n	bl	n	y	0	0	cig+/-ot	20	999	0.1	4	st
LUBIN2	975	f	0	0	all	-	a	Eu:mul	1976	CC	7804	n	bl	n	y	0	0	cig+/-ot	20	999	0.1	9	st
MATOS	680	m	0	0	all	-	a	SCAmer	1994	CC	200	n	bl	n	n	2	0	cig+/-ot	11	999	1.0	5	ot
PEZZOT	590	m	0	0	all	-	a	SCAmer	1987	CC	215	n	bl	n	y	0	0	cig only	11	999	1.0	10	st
SOBUE	748	m	0	0	all	-	a	As:Jap	1986	CC	1376	n	bl	n	y	0	0	cig+/-ot	10	999	1.0	4	st
SVENSS	574	f	0	0	all	-	a	Eu:Sca	1983	CC	210	n	bl	n	n	0	0	all/unsp	11	999	3	10	st
WYNDE3	534	m	0	0	all	-	KII	NAmer	1966	CC	350	n	bl	n	y	0	0	all/unsp	13	999	1.0	3	st
WYNDE6	831	m	0	0	all	-	KII	NAmer	1969	CC	4423	n	bl	n	y	2	0	cig+/-ot	16	999	1.0	3	ot

Cigarette type is all/unspec for all RRs

Table 3K5 - 2

IESLC - Meta-analysis of Ex Smoking, Years quit (vs current), "Highest vs lowest"
Adenocarcinoma, Any Product (or Cigarettes if Any not available)
Most adjusted

REF	NRR	SEX	AD	Number Exposed Case	Cont	Non-exposed Case	Cont	RR	95.00%CI
BARBON	770	m	1	4	-	7	-	0.19	(0.05- 0.73)
JAHN	661	m	0	15	146	40	8	0.02	(0.01- 0.05)
JAIN	542	m	0	14	113	16	46	0.36	(0.16- 0.79)
JAIN	506	f	0	3	61	14	36	0.13	(0.03- 0.47)
Subtotal JAIN								0.27	(0.14- 0.53)
LUBIN2	879	m	0	35	1128	77	1047	0.42	(0.28- 0.63)
LUBIN2	975	f	0	1	29	13	95	0.25	(0.03- 2.01)
Subtotal LUBIN2								0.41	(0.28- 0.62)
MATOS	680	m	2	12	-	12	-	0.23	(0.09- 0.58)
PEZZOT	590	m	0	7	31	11	21	0.43	(0.14- 1.29)
SOBUE	748	m	0	49	144	44	116	0.90	(0.56- 1.44)
SVENSS	574	f	0	7	24	5	13	0.76	(0.20- 2.87)
WYNDE3	534	m	0	3	55	3	22	0.40	(0.07- 2.14)
WYNDE6	831	m	2	6	-	29	-	0.02	(0.01- 0.05)
Partial Totals				156	1731	271	1404		

*prospective study

REF	NRR	SEX	AD	Ys	Ws	Qs	Ps
BARBON	770	m	1	-1.66	2.14	0.42	0.0152
JAHN	661	m	0	-3.88	4.47	31.77	0.0000
JAIN	542	m	0	-1.03	6.08	0.21	0.0109
JAIN	506	f	0	-2.07	2.23	1.60	0.0020
Subtotal JAIN				-1.31	8.31	1.82	
LUBIN2	879	m	0	-0.86	23.04	2.94	0.0000
LUBIN2	975	f	0	-1.38	0.89	0.02	0.1932
Subtotal LUBIN2				-0.88	23.93	2.96	
MATOS	680	m	2	-1.47	4.43	0.28	0.0020
PEZZOT	590	m	0	-0.84	3.19	0.46	0.1330
SOBUE	748	m	0	-0.11	17.04	21.04	0.6540
SVENSS	574	f	0	-0.28	2.17	1.93	0.6838
WYNDE3	534	m	0	-0.92	1.37	0.13	0.2836
WYNDE6	831	m	2	-3.91	5.93	42.99	0.0000

	N	12
	NS	10
	Wt	72.97
Het	Chi	103.79
Het	df	11
Het	P	***
Fixed	RR	0.30
	RRl	0.23
	RRu	0.37
	P	---
Random	RR	0.21
	RRl	0.10
	RRu	0.46
	P	---
Asymm	P	N.S.

Table 3K5 - 3

IESLC - Meta-analysis of Ex Smoking, Years quit (vs current), "Highest vs lowest"
Adenocarcinoma, Any Product (or Cigarettes if Any not available)
Most adjusted

	Sex			
	combined	male	female	Total
N		9	3	12
NS		9	3	12
Wt		67.68	5.29	72.97
Het Chi		100.23	3.55	103.79
Het df		8	2	11
Het P		***	N.S.	***
Fixed RR		0.30	0.30	0.30
RRl		0.23	0.13	0.23
RRu		0.37	0.69	0.37
P		---	--	---
Random RR		0.20	0.29	0.21
RRl		0.08	0.09	0.10
RRu		0.49	0.95	0.46
P		---	-	---
Between Chi				0.00
Between df				1
Between P				N.S.
Btwn(F) P				N.S.
Btwn(R) P				N.S.

	Lung cancer type						
	a	a+l	a+al+br	KII	not q+u	not q+s	Total
N	10			2			12
NS	8			2			10
Wt	65.67			7.30			72.97
Het Chi	56.99			9.98			103.79
Het df	9			1			11
Het P	***			**			***
Fixed RR	0.37			0.04			0.30
RRl	0.29			0.02			0.23
RRu	0.48			0.07			0.37
P	---			---			---
Random RR	0.26			0.08			0.21
RRl	0.13			0.00			0.10
RRu	0.53			1.53			0.46
P	---			(-)			---
Between Chi							36.82
Between df							1
Between P							***
Btwn(F) P							*
Btwn(R) P							N.S.

	Location								
	NAmer	UK	Scand	othEur	China	Japan	othAs	other	Total
N	4		1	4		1		2	12
NS	3		1	3		1		2	10
Wt	15.61		2.17	30.54		17.04		7.61	72.97
Het Chi	27.91		0.00	34.40		0.00		0.73	103.79
Het df	3		0	3		0		1	11
Het P	***		N.S.	***		N.S.		N.S.	***
Fixed RR	0.10		0.76	0.25		0.90		0.30	0.30
RRl	0.06		0.20	0.18		0.56		0.15	0.23
RRu	0.17		2.87	0.36		1.44		0.61	0.37
P	---		N.S.	---		N.S.		---	---
Random RR	0.13		0.76	0.14		0.90		0.30	0.21
RRl	0.03		0.20	0.03		0.56		0.15	0.10
RRu	0.66		2.87	0.75		1.44		0.61	0.46
P	-		N.S.	-		N.S.		---	---
Between Chi									40.75
Between df									4
Between P									***
Btwn(F) P									N.S.
Btwn(R) P									*

Table 3K5 - 3

IESLC - Meta-analysis of Ex Smoking, Years quit (vs current), "Highest vs lowest"
Adenocarcinoma, Any Product (or Cigarettes if Any not available)
Most adjusted

Detailed Country in "other Europe"

	multi	Germany	othWest	East	Balkans	Total
N	2	1	1			4
NS	1	1	1			3
Wt	23.93	4.47	2.14			30.54
Het Chi	0.23	0.00	0.00			34.40
Het df	1	0	0			3
Het P	N.S.	N.S.	N.S.			***
Fixed RR	0.41	0.02	0.19			0.25
RRl	0.28	0.01	0.05			0.18
RRu	0.62	0.05	0.73			0.36
P	---	---	-			---
Random RR	0.41	0.02	0.19			0.14
RRl	0.28	0.01	0.05			0.03
RRu	0.62	0.05	0.73			0.75
P	---	---	-			-
Between Chi						34.17
Between df						2
Between P						***
Btwn(F) P						(*)
Btwn(R) P						***

Detailed Country in "other Asia"

	India	HongKong	other	Total
N				
NS				
Wt				
Het Chi				
Het df				
Het P				
Fixed RR				
RRl				
RRu				
P				
Random RR				
RRl				
RRu				
P				
Between Chi				
Between df				
Between P				N.S.
Btwn(F) P				N.S.
Btwn(R) P				N.S.

Detailed other continent

	SCAmer	Total
N	2	2
NS	2	2
Wt	7.61	7.61
Het Chi	0.73	0.73
Het df	1	1
Het P	N.S.	N.S.
Fixed RR	0.30	0.30
RRl	0.15	0.15
RRu	0.61	0.61
P	---	---
Random RR	0.30	0.30
RRl	0.15	0.15
RRu	0.61	0.61
P	---	---
Between Chi		
Between df		
Between P		N.S.
Btwn(F) P		N.S.
Btwn(R) P		N.S.

Table 3K5 - 3

IESLC - Meta-analysis of Ex Smoking, Years quit (vs current), "Highest vs lowest"
Adenocarcinoma, Any Product (or Cigarettes if Any not available)
Most adjusted

	<1960	Start year of study 1960-69	1970-79	1980-89	1990+	Total
N		2	3	6	1	12
NS		2	2	5	1	10
Wt		7.30	26.07	35.17	4.43	72.97
Het Chi		9.98	1.42	54.44	0.00	103.79
Het df		1	2	5	0	11
Het P		**	N.S.	***	N.S.	***
Fixed RR		0.04	0.39	0.39	0.23	0.30
RRl		0.02	0.26	0.28	0.09	0.23
RRu		0.07	0.57	0.54	0.58	0.37
P		---	---	---	--	---
Random RR		0.08	0.39	0.26	0.23	0.21
RRl		0.00	0.26	0.08	0.09	0.10
RRu		1.53	0.57	0.86	0.58	0.46
P		(-)	---	-	--	---
Between Chi						37.94
Between df						3
Between P						***
Btwn(F) P						N.S.
Btwn(R) P						N.S.

	Study type (1) CC	other	Total
N	12		12
NS	10		10
Wt	72.97		72.97
Het Chi	103.79		103.79
Het df	11		11
Het P	***		***
Fixed RR	0.30		0.30
RRl	0.23		0.23
RRu	0.37		0.37
P	---		---
Random RR	0.21		0.21
RRl	0.10		0.10
RRu	0.46		0.46
P	---		---
Between Chi			
Between df			
Between P			N.S.
Btwn(F) P			N.S.
Btwn(R) P			N.S.

	Study type (2) CC	prosp	other	Total
N	12			12
NS	10			10
Wt	72.97			72.97
Het Chi	103.79			103.79
Het df	11			11
Het P	***			***
Fixed RR	0.30			0.30
RRl	0.23			0.23
RRu	0.37			0.37
P	---			---
Random RR	0.21			0.21
RRl	0.10			0.10
RRu	0.46			0.46
P	---			---
Between Chi				
Between df				
Between P				N.S.
Btwn(F) P				N.S.
Btwn(R) P				N.S.

Table 3K5 - 3

IESLC - Meta-analysis of Ex Smoking, Years quit (vs current), "Highest vs lowest"
Adenocarcinoma, Any Product (or Cigarettes if Any not available)
Most adjusted

	Study size (number of LC cases) 100-249	250-499	500-999	1000+	Total
N	3	1	3	5	12
NS	3	1	2	4	10
Wt	9.78	1.37	10.44	51.37	72.97
Het Chi	2.19	0.00	1.96	98.75	103.79
Het df	2	0	2	4	11
Het P	N.S.	N.S.	N.S.	***	***
Fixed RR	0.37	0.40	0.25	0.29	0.30
RRl	0.20	0.07	0.14	0.22	0.23
RRu	0.69	2.14	0.46	0.38	0.37
P	--	N.S.	---	---	---
Random RR	0.37	0.40	0.25	0.13	0.21
RRl	0.19	0.07	0.14	0.03	0.10
RRu	0.72	2.14	0.46	0.61	0.46
P	--	N.S.	---	--	---
Between Chi					0.89
Between df					3
Between P					N.S.
Btwn(F) P					N.S.
Btwn(R) P					N.S.

	Risky occupational population no	mining	othRisky	Total
N	12			12
NS	10			10
Wt	72.97			72.97
Het Chi	103.79			103.79
Het df	11			11
Het P	***			***
Fixed RR	0.30			0.30
RRl	0.23			0.23
RRu	0.37			0.37
P	---			---
Random RR	0.21			0.21
RRl	0.10			0.10
RRu	0.46			0.46
P	---			---
Between Chi				
Between df				
Between P				N.S.
Btwn(F) P				N.S.
Btwn(R) P				N.S.

	National cigarette tobacco type Virginia	blended	other	Total
N	2	10		12
NS	1	9		10
Wt	8.31	64.66		72.97
Het Chi	1.75	101.96		103.79
Het df	1	9		11
Het P	N.S.	***		***
Fixed RR	0.27	0.30		0.30
RRl	0.14	0.23		0.23
RRu	0.53	0.38		0.37
P	---	---		---
Random RR	0.24	0.21		0.21
RRl	0.09	0.08		0.10
RRu	0.65	0.53		0.46
P	--	---		---
Between Chi				0.08
Between df				1
Between P				N.S.
Btwn(F) P				N.S.
Btwn(R) P				N.S.

Table 3K5 - 3

IESLC - Meta-analysis of Ex Smoking, Years quit (vs current), "Highest vs lowest"
Adenocarcinoma, Any Product (or Cigarettes if Any not available)
Most adjusted

	Any proxy use No/nk	Yes	Total
N	9	3	12
NS	8	2	10
Wt	62.53	10.44	72.97
Het Chi	101.51	1.96	103.79
Het df	8	2	11
Het P	***	N.S.	***
Fixed RR	0.30	0.25	0.30
RRl	0.24	0.14	0.23
RRu	0.39	0.46	0.37
P	---	---	---
Random RR	0.21	0.25	0.21
RRl	0.08	0.14	0.10
RRu	0.58	0.46	0.46
P	--	---	---
Between Chi			0.32
Between df			1
Between P			N.S.
Btwn(F) P			N.S.
Btwn(R) P			N.S.

	Full histological confirmation No	Yes	Total
N	5	7	12
NS	4	6	10
Wt	19.37	53.60	72.97
Het Chi	28.69	65.42	103.79
Het df	4	6	11
Het P	***	***	***
Fixed RR	0.16	0.37	0.30
RRl	0.10	0.28	0.23
RRu	0.25	0.48	0.37
P	---	---	---
Random RR	0.17	0.25	0.21
RRl	0.05	0.09	0.10
RRu	0.58	0.71	0.46
P	--	--	---
Between Chi			9.67
Between df			1
Between P			**
Btwn(F) P			N.S.
Btwn(R) P			N.S.

	Number of adjustment variables (1) 0	1	2+/+nk	Total
N	9	1	2	12
NS	7	1	2	10
Wt	60.47	2.14	10.36	72.97
Het Chi	54.74	0.00	15.12	103.79
Het df	8	0	1	11
Het P	***	N.S.	***	***
Fixed RR	0.40	0.19	0.06	0.30
RRl	0.31	0.05	0.03	0.23
RRu	0.51	0.73	0.10	0.37
P	---	-	---	---
Random RR	0.29	0.19	0.07	0.21
RRl	0.13	0.05	0.01	0.10
RRu	0.62	0.73	0.73	0.46
P	--	-	-	---
Between Chi				33.93
Between df				2
Between P				***
Btwn(F) P				N.S.
Btwn(R) P				N.S.

Table 3K5 - 3

IESLC - Meta-analysis of Ex Smoking, Years quit (vs current), "Highest vs lowest"
Adenocarcinoma, Any Product (or Cigarettes if Any not available)
Most adjusted

	Number of adjustment variables (2)					
	0	1	2	3-5	6+/+nk	Total
N	9	1	2			12
NS	7	1	2			10
Wt	60.47	2.14	10.36			72.97
Het Chi	54.74	0.00	15.12			103.79
Het df	8	0	1			11
Het P	***	N.S.	***			***
Fixed RR	0.40	0.19	0.06			0.30
RRl	0.31	0.05	0.03			0.23
RRu	0.51	0.73	0.10			0.37
P	---	-	---			---
Random RR	0.29	0.19	0.07			0.21
RRl	0.13	0.05	0.01			0.10
RRu	0.62	0.73	0.73			0.46
P	--	-	-			---
Between Chi						33.93
Between df						2
Between P						***
Btwn(F) P						N.S.
Btwn(R) P						N.S.

	Product			
	all/unsp	cig+/-ot	cig only	Total
N	3	8	1	12
NS	3	6	1	10
Wt	5.67	64.11	3.19	72.97
Het Chi	2.06	100.74	0.00	103.79
Het df	2	7	0	11
Het P	N.S.	***	N.S.	***
Fixed RR	0.39	0.28	0.43	0.30
RRl	0.17	0.22	0.14	0.23
RRu	0.88	0.36	1.29	0.37
P	-	---	N.S.	---
Random RR	0.39	0.16	0.43	0.21
RRl	0.17	0.06	0.14	0.10
RRu	0.89	0.45	1.29	0.46
P	-	---	N.S.	---
Between Chi				0.98
Between df				2
Between P				N.S.
Btwn(F) P				N.S.
Btwn(R) P				N.S.

	Derivation of RR/CI			
	Orig	StdCalc	Other	Total
N		9	3	12
NS		7	3	10
Wt		60.47	12.50	72.97
Het Chi		54.74	17.70	103.79
Het df		8	2	11
Het P		***	***	***
Fixed RR		0.40	0.07	0.30
RRl		0.31	0.04	0.23
RRu		0.51	0.12	0.37
P		---	---	---
Random RR		0.29	0.09	0.21
RRl		0.13	0.02	0.10
RRu		0.62	0.51	0.46
P		--	--	---
Between Chi				31.34
Between df				1
Between P				***
Btwn(F) P				(*)
Btwn(R) P				N.S.

Table 3K5 - 4

IESLC - Meta-analysis of Ex Smoking, Years quit (vs current), "Highest vs lowest"
Adenocarcinoma, Any Product (or Cigarettes if Any not available)
Least adjusted

REF	NRR	X	SEX	AGEL	AGEH	RACE	YF	LC TYPE	LOC	START	ST	NLC	R	VB	P	H	AD	ADOS	PRODUCT	exL	exH	unexL	unexH	De
BARBON	755	x	m	0	0	all	-	a	Eu:wst	1979	CC	755	n	bl	y	y	0	0	all/unsp	25	999	0.1	4	st
JAHN	661		m	0	0	all	-	a	Eu:Ger	1988	CC	1004	n	bl	n	n	0	0	cig+/-ot	21	999	0.1	0.9	st
JAIN	542		m	0	0	all	-	a	NAmer	1981	CC	845	n	V	y	n	0	0	cig+/-ot	10	999	2	9	st
JAIN	506		f	0	0	all	-	a	NAmer	1981	CC	845	n	V	y	n	0	0	cig+/-ot	10	999	2	9	st
LUBIN2	879		m	0	0	all	-	a	Eu:mul	1976	CC	7804	n	bl	n	y	0	0	cig+/-ot	20	999	0.1	4	st
LUBIN2	975		f	0	0	all	-	a	Eu:mul	1976	CC	7804	n	bl	n	y	0	0	cig+/-ot	20	999	0.1	9	st
MATOS	670	x	m	0	0	all	-	a	SCAmer	1994	CC	200	n	bl	n	n	0	0	cig+/-ot	11	999	1.0	5	st
PEZZOT	590		m	0	0	all	-	a	SCAmer	1987	CC	215	n	bl	n	y	0	0	cig only	11	999	1.0	10	st
SOBUE	748		m	0	0	all	-	a	As:Jap	1986	CC	1376	n	bl	n	y	0	0	cig+/-ot	10	999	1.0	4	st
SVENSS	574		f	0	0	all	-	a	Eu:Sca	1983	CC	210	n	bl	n	n	0	0	all/unsp	11	999	3	10	st
WYNDE3	534		m	0	0	all	-	KII	NAmer	1966	CC	350	n	bl	n	y	0	0	all/unsp	13	999	1.0	3	st
WYNDE6	816	x	m	0	0	all	-	KII	NAmer	1969	CC	4423	n	bl	n	y	0	0	cig+/-ot	16	999	1.0	3	st

Cigarette type is all/unspec for all RRs

Table 3K5 - 5

IESLC - Meta-analysis of Ex Smoking, Years quit (vs current), "Highest vs lowest"
Adenocarcinoma, Any Product (or Cigarettes if Any not available)
Least adjusted

REF	NRR	SEX	AD	Number Exposed Case	Number Exposed Cont	Non-exposed Case	Non-exposed Cont	RR	95.00%CI
BARBON	755	m	0	4	59	7	20	0.19	(0.05- 0.73)
JAHN	661	m	0	15	146	40	8	0.02	(0.01- 0.05)
JAIN	542	m	0	14	113	16	46	0.36	(0.16- 0.79)
JAIN	506	f	0	3	61	14	36	0.13	(0.03- 0.47)
Subtotal JAIN								0.27	(0.14- 0.53)
LUBIN2	879	m	0	35	1128	77	1047	0.42	(0.28- 0.63)
LUBIN2	975	f	0	1	29	13	95	0.25	(0.03- 2.01)
Subtotal LUBIN2								0.41	(0.28- 0.62)
MATOS	670	m	0	12	101	12	23	0.23	(0.09- 0.57)
PEZZOT	590	m	0	7	31	11	21	0.43	(0.14- 1.29)
SOBUE	748	m	0	49	144	44	116	0.90	(0.56- 1.44)
SVENSS	574	f	0	7	24	5	13	0.76	(0.20- 2.87)
WYNDE3	534	m	0	3	55	3	22	0.40	(0.07- 2.14)
WYNDE6	816	m	0	6	530	29	307	0.12	(0.05- 0.29)
Totals				156	2421	271	1754		

*prospective study

REF	NRR	SEX	AD	Ys	Ws	Qs	Ps
BARBON	755	m	0	-1.64	2.17	0.74	0.0155
JAHN	661	m	0	-3.88	4.47	35.71	0.0000
JAIN	542	m	0	-1.03	6.08	0.00	0.0109
JAIN	506	f	0	-2.07	2.23	2.26	0.0020
Subtotal JAIN				-1.31	8.31	2.27	
LUBIN2	879	m	0	-0.86	23.04	0.89	0.0000
LUBIN2	975	f	0	-1.38	0.89	0.09	0.1932
Subtotal LUBIN2				-0.88	23.93	0.98	
MATOS	670	m	0	-1.48	4.54	0.80	0.0016
PEZZOT	590	m	0	-0.84	3.19	0.15	0.1330
SOBUE	748	m	0	-0.11	17.04	15.41	0.6540
SVENSS	574	f	0	-0.28	2.17	1.33	0.6838
WYNDE3	534	m	0	-0.92	1.37	0.03	0.2836
WYNDE6	816	m	0	-2.12	4.85	5.47	0.0000

	N	12
	NS	10
	Wt	72.04
Het	Chi	62.89
Het	df	11
Het	P	***
Fixed	RR	0.35
	RRl	0.28
	RRu	0.44
	P	---
Random	RR	0.25
	RRl	0.14
	RRu	0.47
	P	---
Asymm	P	N.S.

Table 3K5 - 6

IESLC - Meta-analysis of Ex Smoking, Years quit (vs current), "Highest vs lowest"
Adenocarcinoma, Any Product (or Cigarettes if Any not available)
Least adjusted

	Sex		
combined	male	female	Total
N	9	3	12
NS	9	3	12
Wt	66.75	5.29	72.04
Het Chi	59.19	3.55	62.89
Het df	8	2	11
Het P	***	N.S.	***
Fixed RR	0.35	0.30	0.35
RRl	0.28	0.13	0.28
RRu	0.45	0.69	0.44
P	---	--	---
Random RR	0.24	0.29	0.25
RRl	0.12	0.09	0.14
RRu	0.50	0.95	0.47
P	---	-	---
Between Chi			0.14
Between df			1
Between P			N.S.
Btwn(F) P			N.S.
Btwn(R) P			N.S.

Table 3K5 - 7

IESLC - Meta-analysis of Ex Smoking, Years quit (vs current), "Highest vs lowest"
Adenocarcinoma, Any Product (or Cigarettes if Any not available)
Excluded studies (and stage at which they were excluded)

1	AGUDO AKIBA AMANDU AMES AXELSS BEST BOUCHA BOUCOT BRESLO CHEN CHEN2 CHIAZZ DEAN2 DOSEME ENGELA FAN GENG GER GUO HAENSZ HEGMAN HOLE HU HU2 JUSSAW KATSOU KAUFMA KOO KOULUM KREUZE LETOUR LEVIN LIAW LIU3 LIU4 LIU5 MCCONN MIGRAN MRFITR NOTAN2 OSANN2 PERNU QIAO2 RACHTA RESTRE SADOWS SEGI2 STASZE TIZZAN VUTUC WATSON WU WUWILL WYNDE2 WYNDE8 XU YUAN ZHANG ZHENG ZHOU
2	AUVINE BENSHL BLOT1 BROWN3 BUFFLE GURSEL LAUSSM MCDUFF PISANI PRESCO SPITZ WU2 WYNDE7
4	GARSHI JEDRYC LUO WAKAI
5	ARMADA BECHER BOFFET BROSS CARPEN CEDERL CHOI CHYOU CORREA CPSI CPSII DAMBER DARBY DEAN3 DESTEF DOLL DOLL2 DORGAN DORN GAO GAO2 GARCIA GILLIS GRAHAM HAMMO2 HAMMON HIRAYA HUMBLE JOLY KAISE2 KHUDER LUBIN PEZZO2 QIAO SPEIZE SUZUK2 TVERDA WANG2 WIGLE
6	ALDERS
15	BENHAM

Table 3K5 - 8
Potentially overlapping studies

REF	REFGP	PRINC	. OVERLAP/LINK
LUBIN2	LUBIN2	1	Lubin-combined
WYNDE6	WYNDE6	1	WYNDE5/6/7/8
JAHN	BOFFET	2	Subset of BOFFET

Table 3K5 - 9
Most adjusted - insufficient data for meta-analysis

REF	NRR	SEX	AGEL	AGEH	RACE	YF	LC TYPE	LOC	START	ST	NLC	R	VB	P	H	AD	ADOS	PRODUCT	exL	exH	unexL	unexH	De
ALDERS	577	m	0	0	all	-	not q+s	Eu:UK	1977	CC	1448	n	V	n	n	1	0	cig only	10	999	0.1	2	st
ALDERS	588	f	0	0	all	-	not q+s	Eu:UK	1977	CC	1448	n	V	n	n	1	0	cig only	10	999	0.1	2	st

REF	NRR	RR	SIG	RRDATA comment
ALDERS	577	0.44		0
ALDERS	588	0.26		0

Table 3K6 -

IESLC - Meta-analysis of Ex Smoking by Years quit (vs current), Overview
Adenocarcinoma, Cigarettes (or Any Product if Cigarettes not available)

This analysis is restricted to results for:
1) Ex smokers
2) Results by Years quit (vs current)
3) Categorical results by Years quit (vs current)
Results by Years quit (vs current) are grouped under 2 schemes (S1, S2). Each scheme has a set of "key values". An interval is allocated to the category whose key value it includes, and intervals which include none or more than one of the key values are excluded. (Open-ended intervals are coded as 999)

S1	key value	maximum range
1	3	1-6
2	7	4-11
3	12	8+

S2	key value	maximum range
1	3	1-11
2	12	4-19
3	20	13+

4) Adenocarcinoma (or near equivalent)
5) Results complete enough for use in metaanalysis

Within each study, results are then selected (in the following order of preference, within each sex) for:
6) (not applicable)
7) PRODUCT: cigarettes regardless of other products, cigarettes only, all/unspec
8) CIGTYPE: all/unspecified, MC regardless of HR, MC only
9) Results with least adjustment for other aspects of smoking (ADOS)
10) DENOM: current smokers, current + recent smokers (up to number of m=months or y=years, max 2 years)
11) Followup period (YF, prospective studies): whole study (coded as 0) or longest available
12) LCtype: adeno or nearest available, but not squamous. (q = squamous, s = small,
a = adeno, l = large, KII = Kreyberg II, al = alveolar, br = bronchiolar, u = undifferentiated)
13) Race: all or nearest available, otherwise by race (wh or w = white, bl or b = black, hi = hispanic
ch = chinese, jap = japanese, haw = hawaiian, w+o = white + oriental, sca = scandinavian, as = asian)
14) For overlapping studies: principal rather than subsidiary studies
Finally by Age: whole study (coded as 0) if available, otherwise by widest available age group
and then for single sex results (m, f) in preference to results for both sexes combined (c).

Results adjusted (AD) for the most potential confounders are then chosen in Sections -1 to -3
(and those which actually differ from the adjusted results in Table 3K1 - 1 are marked 'x' in Section -1)
and results adjusted for the least confounders in Sections -4 to -6. (Those least adjusted results which
actually differ from the most adjusted are marked 'x' in column X in Section -4)

Section -7 shows excluded studies, together with the stage (as above) at which no qualifying
results were found.

Section -8 lists the potentially overlapping studies which have been included (1=principal, 2=subsidiary).

Section -9 lists any results which would have been included in preference except that they had data not complete
enough for use in meta-analysis, with their significance (yes/no), if known, and any further comment as entered
on the database. It also lists as "gap" any categories for which no data were presented by the original authors.

In addition to those mentioned above, the following fields, levels and abbreviations are used:
* or nk = not known, n = no, y = yes, ot = other
nev = never
all/unspec = all or unspecified, cig+/-ot = cigarettes irrespective of other products (cigar, pipe etc)
MC = manufactured cigarettes, HR = hand-rolled cigarettes
exL, exH = range of exposure (low and high) in the smoking group, in terms of Years quit (vs current)
REF: 6-character study reference
NRR: number of the RR on the database within the study
ST : study type (CC = case control, pr or prosp = prospective)
NLC: number of lung cancer cases in whole study
R : risky occupational population (n = no, m = mining, o = other risky)
VB : national cigarette type (V = at least 75% Virginia, bl = at least 75% blended, ot = other)
P : any proxy use
H : full histological confirmation
De : derivation of RR/CI (or = original, st = standard method, ot = other method of estimation)

Table 3K6 - 1

IESLC - Meta-analysis of Ex Smoking by Years quit (vs current), Overview
Adenocarcinoma, Cigarettes (or Any Product if Cigarettes not available)
Most adjusted

REF	NRR	3K1	SEX	AGEL	AGEH	RACE	YF	LC TYPE	LOC	START	ST	NLC	R	VB	P	H	AD	ADOS	PRODUCT	exL	exH	S1	S2	DENOM	De
BARBON	764		m	0	0	all	-	a	Eu:wst	1979	CC	755	n	bl	y	y	1	0	all/unsp	0.1	4	1	1	current	ot
BARBON	765		m	0	0	all	-	a	Eu:wst	1979	CC	755	n	bl	y	y	1	0	all/unsp	5	14	0	2	current	ot
BARBON	766		m	0	0	all	-	a	Eu:wst	1979	CC	755	n	bl	y	y	1	0	all/unsp	15	24	0	3	current	ot
BARBON	767		m	0	0	all	-	a	Eu:wst	1979	CC	755	n	bl	y	y	1	0	all/unsp	25	999	0	0	current	ot
JAHN	651		m	0	0	all	-	a	Eu:Ger	1988	CC	1004	n	bl	n	n	0	0	cig+/-ot	0.1	0.9	0	0	current	st
JAHN	652		m	0	0	all	-	a	Eu:Ger	1988	CC	1004	n	bl	n	n	0	0	cig+/-ot	1.0	1.9	0	0	current	st
JAHN	653		m	0	0	all	-	a	Eu:Ger	1988	CC	1004	n	bl	n	n	0	0	cig+/-ot	2	5	1	1	current	st
JAHN	654		m	0	0	all	-	a	Eu:Ger	1988	CC	1004	n	bl	n	n	0	0	cig+/-ot	6	10	2	0	current	st
JAHN	655		m	0	0	all	-	a	Eu:Ger	1988	CC	1004	n	bl	n	n	0	0	cig+/-ot	11	20	3	0	current	st
JAHN	656		m	0	0	all	-	a	Eu:Ger	1988	CC	1004	n	bl	n	n	0	0	cig+/-ot	21	999	0	0	current	st
JAIN	540		m	0	0	all	-	a	NAmer	1981	CC	845	n	V	y	n	0	0	cig+/-ot	2	9	0	1	cur+2y	st
JAIN	541		m	0	0	all	-	a	NAmer	1981	CC	845	n	V	y	n	0	0	cig+/-ot	10	999	3	0	cur+2y	st
JAIN	504		f	0	0	all	-	a	NAmer	1981	CC	845	n	V	y	n	0	0	cig+/-ot	2	9	0	1	cur+2y	st
JAIN	505		f	0	0	all	-	a	NAmer	1981	CC	845	n	V	y	n	0	0	cig+/-ot	10	999	3	0	cur+2y	st
LUBIN2	871		m	0	0	all	-	a	Eu:mul	1976	CC	7804	n	bl	n	y	0	0	cig+/-ot	0.1	4	1	1	current	st
LUBIN2	872		m	0	0	all	-	a	Eu:mul	1976	CC	7804	n	bl	n	y	0	0	cig+/-ot	5	9	2	0	current	st
LUBIN2	873		m	0	0	all	-	a	Eu:mul	1976	CC	7804	n	bl	n	y	0	0	cig+/-ot	10	14	3	2	current	st
LUBIN2	874		m	0	0	all	-	a	Eu:mul	1976	CC	7804	n	bl	n	y	0	0	cig+/-ot	15	19	0	0	current	st
LUBIN2	875		m	0	0	all	-	a	Eu:mul	1976	CC	7804	n	bl	n	y	0	0	cig+/-ot	20	999	0	3	current	st
LUBIN2	971		f	0	0	all	-	a	Eu:mul	1976	CC	7804	n	bl	n	y	0	0	cig+/-ot	0.1	9	0	1	current	st
LUBIN2	972		f	0	0	all	-	a	Eu:mul	1976	CC	7804	n	bl	n	y	0	0	cig+/-ot	10	19	3	2	current	st
LUBIN2	973		f	0	0	all	-	a	Eu:mul	1976	CC	7804	n	bl	n	y	0	0	cig+/-ot	20	999	0	3	current	st
MATOS	676		m	0	0	all	-	a	SCAmer	1994	CC	200	n	bl	n	n	2	0	cig+/-ot	1.0	5	1	1	cur+1y	or
MATOS	677		m	0	0	all	-	a	SCAmer	1994	CC	200	n	bl	n	n	2	0	cig+/-ot	6	10	2	0	cur+1y	or
MATOS	678		m	0	0	all	-	a	SCAmer	1994	CC	200	n	bl	n	n	2	0	cig+/-ot	11	999	3	0	cur+1y	or
PEZZOT	588		m	0	0	all	-	a	SCAmer	1987	CC	215	n	bl	n	y	0	0	cig only	1.0	10	0	1	cur+1y	st
PEZZOT	589		m	0	0	all	-	a	SCAmer	1987	CC	215	n	bl	n	y	0	0	cig only	11	999	3	0	cur+1y	st
SOBUE	744		m	0	0	all	-	a	As:Jap	1986	CC	1376	n	bl	n	y	0	0	cig+/-ot	1.0	4	1	1	cur+1y	st
SOBUE	745		m	0	0	all	-	a	As:Jap	1986	CC	1376	n	bl	n	y	0	0	cig+/-ot	5	9	2	0	cur+1y	st
SOBUE	746		m	0	0	all	-	a	As:Jap	1986	CC	1376	n	bl	n	y	0	0	cig+/-ot	10	999	3	0	cur+1y	st
SVENSS	572		f	0	0	all	-	a	Eu:Sca	1983	CC	210	n	bl	n	n	0	0	all/unsp	3	10	0	1	cur+2y	st
SVENSS	573		f	0	0	all	-	a	Eu:Sca	1983	CC	210	n	bl	n	n	0	0	all/unsp	11	999	3	0	cur+2y	st
WYNDE3	528		m	0	0	all	-	KII	NAmer	1966	CC	350	n	bl	n	y	0	0	all/unsp	1.0	3	1	1	cur+1y	st
WYNDE3	529		m	0	0	all	-	KII	NAmer	1966	CC	350	n	bl	n	y	0	0	all/unsp	4	6	0	0	cur+1y	st
WYNDE3	530		m	0	0	all	-	KII	NAmer	1966	CC	350	n	bl	n	y	0	0	all/unsp	7	12	0	2	cur+1y	st
WYNDE3	531		m	0	0	all	-	KII	NAmer	1966	CC	350	n	bl	n	y	0	0	all/unsp	13	999	0	3	cur+1y	st

Cigarette type is all/unspec for all RRs

In this overview table, subtotals and Qs values may be invalid and should be ignored

Table 3K6 - 2

IESLC - Meta-analysis of Ex Smoking by Years quit (vs current), Overview
Adenocarcinoma, Cigarettes (or Any Product if Cigarettes not available)
Most adjusted

REF	NRR	SEX	AD	Number Exposed Case	Exposed Cont	Non-exposed Case	Non-exposed Cont	RR	95.00%CI
BARBON	764	m	1	7	-	109	-	1.15	(0.47- 2.78)
BARBON	765	m	1	23	-	109	-	0.89	(0.54- 1.47)
BARBON	766	m	1	7	-	109	-	0.56	(0.24- 1.29)
BARBON	767	m	1	4	-	109	-	0.22	(0.08- 0.62)
Subtotal BARBON								0.72	(0.50- 1.03)
JAHN	651	m	0	40	8	75	269	17.93	(8.05- 39.95)
JAHN	652	m	0	18	9	75	269	7.17	(3.10- 16.62)
JAHN	653	m	0	19	46	75	269	1.48	(0.82- 2.68)
JAHN	654	m	0	13	63	75	269	0.74	(0.39- 1.42)
JAHN	655	m	0	22	130	75	269	0.61	(0.36- 1.02)
JAHN	656	m	0	15	146	75	269	0.37	(0.20- 0.66)
Subtotal JAHN								1.22	(0.94- 1.59)
JAIN	540	m	0	16	46	60	118	0.68	(0.36- 1.31)
JAIN	541	m	0	14	113	60	118	0.24	(0.13- 0.46)
JAIN	504	f	0	14	36	69	99	0.56	(0.28- 1.11)
JAIN	505	f	0	3	61	69	99	0.07	(0.02- 0.23)
Subtotal JAIN								0.38	(0.26- 0.54)
LUBIN2	871	m	0	77	1047	454	6209	1.01	(0.78- 1.29)
LUBIN2	872	m	0	50	882	454	6209	0.78	(0.57- 1.05)
LUBIN2	873	m	0	30	693	454	6209	0.59	(0.41- 0.86)
LUBIN2	874	m	0	21	478	454	6209	0.60	(0.38- 0.94)
LUBIN2	875	m	0	35	1128	454	6209	0.42	(0.30- 0.60)
LUBIN2	971	f	0	13	95	69	410	0.81	(0.43- 1.53)
LUBIN2	972	f	0	3	33	69	410	0.54	(0.16- 1.81)
LUBIN2	973	f	0	1	29	69	410	0.20	(0.03- 1.53)
Subtotal LUBIN2								0.71	(0.62- 0.82)
MATOS	676	m	2	12	-	46	-	1.30	(0.60- 3.00)
MATOS	677	m	2	9	-	46	-	1.00	(0.40- 2.30)
MATOS	678	m	2	12	-	46	-	0.30	(0.20- 0.70)
Subtotal MATOS								0.61	(0.40- 0.94)
PEZZOT	588	m	0	11	21	42	38	0.47	(0.20- 1.11)
PEZZOT	589	m	0	7	31	42	38	0.20	(0.08- 0.52)
Subtotal PEZZOT								0.32	(0.17- 0.61)
SOBUE	744	m	0	44	116	270	633	0.89	(0.61- 1.29)
SOBUE	745	m	0	22	92	270	633	0.56	(0.34- 0.91)
SOBUE	746	m	0	49	144	270	633	0.80	(0.56- 1.14)
Subtotal SOBUE								0.77	(0.61- 0.97)
SVENSS	572	f	0	5	13	38	53	0.54	(0.18- 1.63)
SVENSS	573	f	0	7	24	38	53	0.41	(0.16- 1.04)
Subtotal SVENSS								0.46	(0.22- 0.94)
WYNDE3	528	m	0	3	22	56	207	0.50	(0.15- 1.75)
WYNDE3	529	m	0	3	17	56	207	0.65	(0.18- 2.31)
WYNDE3	530	m	0	3	31	56	207	0.36	(0.11- 1.21)
WYNDE3	531	m	0	3	55	56	207	0.20	(0.06- 0.67)
Subtotal WYNDE3								0.39	(0.21- 0.71)
Partial Totals				635	5609	4953	37232		

*prospective study

Table 3K6 - 2

IESLC - Meta-analysis of Ex Smoking by Years quit (vs current), Overview
Adenocarcinoma, Cigarettes (or Any Product if Cigarettes not available)
Most adjusted

REF	NRR	SEX	AD	Ys	Ws	Qs	Ps
BARBON	764	m	1	0.14	4.86	1.15	0.7579
BARBON	765	m	1	-0.12	15.32	0.81	0.6483
BARBON	766	m	1	-0.58	5.43	0.30	0.1765
BARBON	767	m	1	-1.51	3.66	5.00	0.0037
Subtotal BARBON				-0.33	29.28	7.25	
JAHN	651	m	0	2.89	5.99	62.57	0.0000
JAHN	652	m	0	1.97	5.44	29.21	0.0000
JAHN	653	m	0	0.39	10.94	5.98	0.1937
JAHN	654	m	0	-0.30	9.10	0.02	0.3638
JAHN	655	m	0	-0.50	14.25	0.33	0.0595
JAHN	656	m	0	-1.00	11.04	4.69	0.0009
Subtotal JAHN				0.20	56.76	102.81	
JAIN	540	m	0	-0.38	9.14	0.01	0.2509
JAIN	541	m	0	-1.41	9.49	10.77	0.0000
JAIN	504	f	0	-0.58	8.08	0.45	0.0973
JAIN	505	f	0	-2.65	2.67	14.19	0.0000
Subtotal JAIN				-0.98	29.38	25.43	
LUBIN2	871	m	0	0.01	61.33	7.61	0.9639
LUBIN2	872	m	0	-0.25	42.56	0.36	0.0968
LUBIN2	873	m	0	-0.52	26.93	0.85	0.0065
LUBIN2	874	m	0	-0.51	19.20	0.51	0.0256
LUBIN2	875	m	0	-0.86	31.43	8.20	0.0000
LUBIN2	971	f	0	-0.21	9.58	0.19	0.5220
LUBIN2	972	f	0	-0.62	2.63	0.19	0.3181
LUBIN2	973	f	0	-1.59	0.95	1.46	0.1221
Subtotal LUBIN2				-0.34	194.60	19.36	
MATOS	676	m	2	0.26	5.93	2.20	0.5228
MATOS	677	m	2	0.00	5.02	0.60	1.0000
MATOS	678	m	2	-1.20	9.79	7.20	0.0002
Subtotal MATOS				-0.49	20.74	10.00	
PEZZOT	588	m	0	-0.75	5.30	0.85	0.0856
PEZZOT	589	m	0	-1.59	4.44	6.85	0.0008
Subtotal PEZZOT				-1.13	9.74	7.70	
SOBUE	744	m	0	-0.12	27.30	1.43	0.5398
SOBUE	745	m	0	-0.58	16.23	0.88	0.0197
SOBUE	746	m	0	-0.23	30.64	0.44	0.2110
Subtotal SOBUE				-0.26	74.17	2.75	
SVENSS	572	f	0	-0.62	3.10	0.24	0.2725
SVENSS	573	f	0	-0.90	4.35	1.33	0.0606
Subtotal SVENSS				-0.78	7.46	1.57	
WYNDE3	528	m	0	-0.69	2.49	0.29	0.2796
WYNDE3	529	m	0	-0.43	2.41	0.02	0.5071
WYNDE3	530	m	0	-1.03	2.58	1.20	0.0990
WYNDE3	531	m	0	-1.60	2.67	4.21	0.0089
Subtotal WYNDE3				-0.95	10.15	5.71	

N	36
NS	9

Table 3K6 - 3

IESLC - Meta-analysis of Ex Smoking by Years quit (vs current), Overview
Adenocarcinoma, Cigarettes (or Any Product if Cigarettes not available)
Most adjusted

	Sex: combined	male	female	Total
N		29	7	36
NS		8	3	11

In this overview table, other than the "N" rows, entries in the "absent" and "Total" columns may be invalid and should be ignored

Years quit vs current (lower focus)	absent	1-6k3	4-11k7	8+k12	Total
N	17	6	4	9	36
NS	7	6	4	7	24
Wt	141.33	112.85	72.92	105.18	432.28
Het Chi	119.58	3.71	1.78	29.45	182.58
Het df	16	5	3	8	35
Het P	***	N.S.	N.S.	***	***
Fixed RR	0.67	1.02	0.73	0.50	0.71
RRl	0.57	0.85	0.58	0.41	0.64
RRu	0.79	1.22	0.92	0.60	0.78
P	---	N.S.	--	---	---
Random RR	0.70	1.02	0.73	0.39	0.65
RRl	0.43	0.85	0.58	0.26	0.52
RRu	1.14	1.22	0.92	0.58	0.83
P	N.S.	N.S.	--	---	---

Years quit vs current (higher focus)	absent	1-11k3	4-19k12	13+k20	Total
N	17	11	4	4	36
NS	9	9	3	3	24
Wt	196.29	148.06	47.45	40.48	432.28
Het Chi	144.29	11.04	2.75	2.37	182.58
Het df	16	10	3	3	35
Het P	***	N.S.	N.S.	N.S.	***
Fixed RR	0.67	0.91	0.65	0.41	0.71
RRl	0.58	0.77	0.49	0.30	0.64
RRu	0.77	1.07	0.87	0.56	0.78
P	---	N.S.	--	---	---
Random RR	0.63	0.89	0.65	0.41	0.65
RRl	0.41	0.75	0.49	0.30	0.52
RRu	0.99	1.07	0.87	0.56	0.83
P	-	N.S.	--	---	---

Table 3K6 - 3

IESLC - Meta-analysis of Ex Smoking by Years quit (vs current), Overview
Adenocarcinoma, Cigarettes (or Any Product if Cigarettes not available)
Most adjusted

MALES

		Years quit vs current (lower focus)				
		absent	1-6k3	4-11k7	8+k12	Total
	N	13	6	4	6	29
	NS	6	6	4	6	22
	Wt	119.62	112.85	72.92	95.53	400.91
Het	Chi	117.43	3.71	1.78	18.65	164.18
Het	df	12	5	3	5	28
Het	P	***	N.S.	N.S.	**	***
Fixed	RR	0.68	1.02	0.73	0.53	0.73
	RRl	0.57	0.85	0.58	0.43	0.66
	RRu	0.82	1.22	0.92	0.65	0.80
	P	---	N.S.	--	---	---
Random	RR	0.76	1.02	0.73	0.44	0.71
	RRl	0.42	0.85	0.58	0.29	0.55
	RRu	1.37	1.22	0.92	0.67	0.92
	P	N.S.	N.S.	--	---	--

		Years quit vs current (higher focus)				
		absent	1-11k3	4-19k12	13+k20	Total
	N	15	8	3	3	29
	NS	8	8	3	3	22
	Wt	189.27	127.29	44.82	39.53	400.91
Het	Chi	129.41	7.78	2.65	1.89	164.18
Het	df	14	7	2	2	28
Het	P	***	N.S.	N.S.	N.S.	***
Fixed	RR	0.70	0.96	0.66	0.42	0.73
	RRl	0.60	0.81	0.49	0.31	0.66
	RRu	0.80	1.14	0.89	0.57	0.80
	P	---	N.S.	--	---	---
Random	RR	0.73	0.95	0.66	0.42	0.71
	RRl	0.46	0.78	0.46	0.31	0.55
	RRu	1.15	1.16	0.96	0.57	0.92
	P	N.S.	N.S.	-	---	--

FEMALES

		Years quit vs current (lower focus)				
		absent	1-6k3	4-11k7	8+k12	Total
	N	4			3	7
	NS	3			3	6
	Wt	21.71			9.65	31.37
Het	Chi	2.02			6.81	13.55
Het	df	3			2	6
Het	P	N.S.			*	*
Fixed	RR	0.63			0.27	0.48
	RRl	0.41			0.14	0.34
	RRu	0.95			0.51	0.69
	P	-			---	---
Random	RR	0.63			0.25	0.41
	RRl	0.41			0.08	0.23
	RRu	0.95			0.83	0.73
	P	-			-	--

Table 3K6 - 3

IESLC - Meta-analysis of Ex Smoking by Years quit (vs current), Overview
Adenocarcinoma, Cigarettes (or Any Product if Cigarettes not available)
Most adjusted

FEMALES

	Years quit vs current (higher focus) absent	1-11k3	4-19k12	13+k20	Total
N	2	3	1	1	7
NS	2	3	1	1	6
Wt	7.02	20.76	2.63	0.95	31.37
Het Chi	5.08	0.78	0.00	0.00	13.55
Het df	1	2	0	0	6
Het P	*	N.S.	N.S.	N.S.	*
Fixed RR	0.21	0.66	0.54	0.20	0.48
RRl	0.10	0.43	0.16	0.03	0.34
RRu	0.44	1.01	1.81	1.53	0.69
P	---	(-)	N.S.	N.S.	---
Random RR	0.18	0.66	0.54	0.20	0.41
RRl	0.03	0.43	0.16	0.03	0.23
RRu	0.98	1.01	1.81	1.53	0.73
P	-	(-)	N.S.	N.S.	--

Table 3K6 - 4

IESLC - Meta-analysis of Ex Smoking by Years quit (vs current), Overview
Adenocarcinoma, Cigarettes (or Any Product if Cigarettes not available)
Least adjusted

REF	NRR	X	SEX	AGEL	AGEH	RACE	YF	LC TYPE	LOC	START	ST	NLC	R	VB	P	H	AD	ADOS	PRODUCT	exL	exH	S1	S2	DENOM	De
BARBON	749	x	m	0	0	all	-	a	Eu:wst	1979	CC	755	n	bl	y	y	0	0	all/unsp	0.1	4	1	1	current	st
BARBON	750	x	m	0	0	all	-	a	Eu:wst	1979	CC	755	n	bl	y	y	0	0	all/unsp	5	14	0	2	current	st
BARBON	751	x	m	0	0	all	-	a	Eu:wst	1979	CC	755	n	bl	y	y	0	0	all/unsp	15	24	0	3	current	st
BARBON	752	x	m	0	0	all	-	a	Eu:wst	1979	CC	755	n	bl	y	y	0	0	all/unsp	25	999	0	0	current	st
JAHN	651		m	0	0	all	-	a	Eu:Ger	1988	CC	1004	n	bl	n	n	0	0	cig+/-ot	0.1	0.9	0	0	current	st
JAHN	652		m	0	0	all	-	a	Eu:Ger	1988	CC	1004	n	bl	n	n	0	0	cig+/-ot	1.0	1.9	0	0	current	st
JAHN	653		m	0	0	all	-	a	Eu:Ger	1988	CC	1004	n	bl	n	n	0	0	cig+/-ot	2	5	1	1	current	st
JAHN	654		m	0	0	all	-	a	Eu:Ger	1988	CC	1004	n	bl	n	n	0	0	cig+/-ot	6	10	2	0	current	st
JAHN	655		m	0	0	all	-	a	Eu:Ger	1988	CC	1004	n	bl	n	n	0	0	cig+/-ot	11	20	3	0	current	st
JAHN	656		m	0	0	all	-	a	Eu:Ger	1988	CC	1004	n	bl	n	n	0	0	cig+/-ot	21	999	0	0	current	st
JAIN	540		m	0	0	all	-	a	NAmer	1981	CC	845	n	V	y	n	0	0	cig+/-ot	2	9	0	1	cur+2y	st
JAIN	541		m	0	0	all	-	a	NAmer	1981	CC	845	n	V	y	n	0	0	cig+/-ot	10	999	3	0	cur+2y	st
JAIN	504		f	0	0	all	-	a	NAmer	1981	CC	845	n	V	y	n	0	0	cig+/-ot	2	9	0	1	cur+2y	st
JAIN	505		f	0	0	all	-	a	NAmer	1981	CC	845	n	V	y	n	0	0	cig+/-ot	10	999	3	0	cur+2y	st
LUBIN2	871		m	0	0	all	-	a	Eu:mul	1976	CC	7804	n	bl	n	y	0	0	cig+/-ot	0.1	4	1	1	current	st
LUBIN2	872		m	0	0	all	-	a	Eu:mul	1976	CC	7804	n	bl	n	y	0	0	cig+/-ot	5	9	2	0	current	st
LUBIN2	873		m	0	0	all	-	a	Eu:mul	1976	CC	7804	n	bl	n	y	0	0	cig+/-ot	10	14	3	2	current	st
LUBIN2	874		m	0	0	all	-	a	Eu:mul	1976	CC	7804	n	bl	n	y	0	0	cig+/-ot	15	19	0	0	current	st
LUBIN2	875		m	0	0	all	-	a	Eu:mul	1976	CC	7804	n	bl	n	y	0	0	cig+/-ot	20	999	0	3	current	st
LUBIN2	971		f	0	0	all	-	a	Eu:mul	1976	CC	7804	n	bl	n	y	0	0	cig+/-ot	0.1	9	0	1	current	st
LUBIN2	972		f	0	0	all	-	a	Eu:mul	1976	CC	7804	n	bl	n	y	0	0	cig+/-ot	10	19	3	2	current	st
LUBIN2	973		f	0	0	all	-	a	Eu:mul	1976	CC	7804	n	bl	n	y	0	0	cig+/-ot	20	999	0	3	current	st
MATOS	666	x	m	0	0	all	-	a	SCAmer	1994	CC	200	n	bl	n	n	0	0	cig+/-ot	1.0	5	1	1	cur+1y	st
MATOS	667	x	m	0	0	all	-	a	SCAmer	1994	CC	200	n	bl	n	n	0	0	cig+/-ot	6	10	2	0	cur+1y	st
MATOS	668	x	m	0	0	all	-	a	SCAmer	1994	CC	200	n	bl	n	n	0	0	cig+/-ot	11	999	3	0	cur+1y	st
PEZZOT	588		m	0	0	all	-	a	SCAmer	1987	CC	215	n	bl	n	y	0	0	cig only	1.0	10	0	1	cur+1y	st
PEZZOT	589		m	0	0	all	-	a	SCAmer	1987	CC	215	n	bl	n	y	0	0	cig only	11	999	3	0	cur+1y	st
SOBUE	744		m	0	0	all	-	a	As:Jap	1986	CC	1376	n	bl	n	y	0	0	cig+/-ot	1.0	4	1	1	cur+1y	st
SOBUE	745		m	0	0	all	-	a	As:Jap	1986	CC	1376	n	bl	n	y	0	0	cig+/-ot	5	9	2	0	cur+1y	st
SOBUE	746		m	0	0	all	-	a	As:Jap	1986	CC	1376	n	bl	n	y	0	0	cig+/-ot	10	999	3	0	cur+1y	st
SVENSS	572		f	0	0	all	-	a	Eu:Sca	1983	CC	210	n	bl	n	n	0	0	all/unsp	3	10	0	1	cur+2y	st
SVENSS	573		f	0	0	all	-	a	Eu:Sca	1983	CC	210	n	bl	n	n	0	0	all/unsp	11	999	3	0	cur+2y	st
WYNDE3	528		m	0	0	all	-	KII	NAmer	1966	CC	350	n	bl	n	y	0	0	all/unsp	1.0	3	1	1	cur+1y	st
WYNDE3	529		m	0	0	all	-	KII	NAmer	1966	CC	350	n	bl	n	y	0	0	all/unsp	4	6	0	0	cur+1y	st
WYNDE3	530		m	0	0	all	-	KII	NAmer	1966	CC	350	n	bl	n	y	0	0	all/unsp	7	12	0	2	cur+1y	st
WYNDE3	531		m	0	0	all	-	KII	NAmer	1966	CC	350	n	bl	n	y	0	0	all/unsp	13	999	0	3	cur+1y	st

Cigarette type is all/unspec for all RRs

In this overview table, subtotals and Qs values may be invalid and should be ignored

Table 3K6 - 5

IESLC - Meta-analysis of Ex Smoking by Years quit (vs current), Overview
Adenocarcinoma, Cigarettes (or Any Product if Cigarettes not available)
Least adjusted

REF	NRR	SEX	AD	Number Exposed Case	Cont	Non-exposed Case	Cont	RR	95.00%CI
BARBON	749	m	0	7	20	109	362	1.16	(0.48- 2.82)
BARBON	750	m	0	23	85	109	362	0.90	(0.54- 1.49)
BARBON	751	m	0	7	41	109	362	0.57	(0.25- 1.30)
BARBON	752	m	0	4	59	109	362	0.23	(0.08- 0.63)
Subtotal BARBON								0.72	(0.50- 1.04)
JAHN	651	m	0	40	8	75	269	17.93	(8.05- 39.95)
JAHN	652	m	0	18	9	75	269	7.17	(3.10- 16.62)
JAHN	653	m	0	19	46	75	269	1.48	(0.82- 2.68)
JAHN	654	m	0	13	63	75	269	0.74	(0.39- 1.42)
JAHN	655	m	0	22	130	75	269	0.61	(0.36- 1.02)
JAHN	656	m	0	15	146	75	269	0.37	(0.20- 0.66)
Subtotal JAHN								1.22	(0.94- 1.59)
JAIN	540	m	0	16	46	60	118	0.68	(0.36- 1.31)
JAIN	541	m	0	14	113	60	118	0.24	(0.13- 0.46)
JAIN	504	f	0	14	36	69	99	0.56	(0.28- 1.11)
JAIN	505	f	0	3	61	69	99	0.07	(0.02- 0.23)
Subtotal JAIN								0.38	(0.26- 0.54)
LUBIN2	871	m	0	77	1047	454	6209	1.01	(0.78- 1.29)
LUBIN2	872	m	0	50	882	454	6209	0.78	(0.57- 1.05)
LUBIN2	873	m	0	30	693	454	6209	0.59	(0.41- 0.86)
LUBIN2	874	m	0	21	478	454	6209	0.60	(0.38- 0.94)
LUBIN2	875	m	0	35	1128	454	6209	0.42	(0.30- 0.60)
LUBIN2	971	f	0	13	95	69	410	0.81	(0.43- 1.53)
LUBIN2	972	f	0	3	33	69	410	0.54	(0.16- 1.81)
LUBIN2	973	f	0	1	29	69	410	0.20	(0.03- 1.53)
Subtotal LUBIN2								0.71	(0.62- 0.82)
MATOS	666	m	0	12	23	46	132	1.50	(0.69- 3.25)
MATOS	667	m	0	9	27	46	132	0.96	(0.42- 2.18)
MATOS	668	m	0	12	101	46	132	0.34	(0.17- 0.68)
Subtotal MATOS								0.73	(0.47- 1.12)
PEZZOT	588	m	0	11	21	42	38	0.47	(0.20- 1.11)
PEZZOT	589	m	0	7	31	42	38	0.20	(0.08- 0.52)
Subtotal PEZZOT								0.32	(0.17- 0.61)
SOBUE	744	m	0	44	116	270	633	0.89	(0.61- 1.29)
SOBUE	745	m	0	22	92	270	633	0.56	(0.34- 0.91)
SOBUE	746	m	0	49	144	270	633	0.80	(0.56- 1.14)
Subtotal SOBUE								0.77	(0.61- 0.97)
SVENSS	572	f	0	5	13	38	53	0.54	(0.18- 1.63)
SVENSS	573	f	0	7	24	38	53	0.41	(0.16- 1.04)
Subtotal SVENSS								0.46	(0.22- 0.94)
WYNDE3	528	m	0	3	22	56	207	0.50	(0.15- 1.75)
WYNDE3	529	m	0	3	17	56	207	0.65	(0.18- 2.31)
WYNDE3	530	m	0	3	31	56	207	0.36	(0.11- 1.21)
WYNDE3	531	m	0	3	55	56	207	0.20	(0.06- 0.67)
Subtotal WYNDE3								0.39	(0.21- 0.71)
Totals				635	5965	4953	39076		

*prospective study

Table 3K6 - 5

IESLC - Meta-analysis of Ex Smoking by Years quit (vs current), Overview
Adenocarcinoma, Cigarettes (or Any Product if Cigarettes not available)
Least adjusted

REF	NRR	SEX	AD	Ys	Ws	Qs	Ps
BARBON	749	m	0	0.15	4.88	1.16	0.7395
BARBON	750	m	0	-0.11	14.89	0.79	0.6801
BARBON	751	m	0	-0.57	5.58	0.30	0.1801
BARBON	752	m	0	-1.49	3.59	4.77	0.0048
Subtotal BARBON				-0.32	28.93	7.02	
JAHN	651	m	0	2.89	5.99	62.22	0.0000
JAHN	652	m	0	1.97	5.44	28.99	0.0000
JAHN	653	m	0	0.39	10.94	5.83	0.1937
JAHN	654	m	0	-0.30	9.10	0.01	0.3638
JAHN	655	m	0	-0.50	14.25	0.37	0.0595
JAHN	656	m	0	-1.00	11.04	4.82	0.0009
Subtotal JAHN				0.20	56.76	102.25	
JAIN	540	m	0	-0.38	9.14	0.02	0.2509
JAIN	541	m	0	-1.41	9.49	10.96	0.0000
JAIN	504	f	0	-0.58	8.08	0.49	0.0973
JAIN	505	f	0	-2.65	2.67	14.30	0.0000
Subtotal JAIN				-0.98	29.38	25.77	
LUBIN2	871	m	0	0.01	61.33	7.22	0.9639
LUBIN2	872	m	0	-0.25	42.56	0.29	0.0968
LUBIN2	873	m	0	-0.52	26.93	0.94	0.0065
LUBIN2	874	m	0	-0.51	19.20	0.57	0.0256
LUBIN2	875	m	0	-0.86	31.43	8.49	0.0000
LUBIN2	971	f	0	-0.21	9.58	0.16	0.5220
LUBIN2	972	f	0	-0.62	2.63	0.20	0.3181
LUBIN2	973	f	0	-1.59	0.95	1.48	0.1221
Subtotal LUBIN2				-0.34	194.60	19.36	
MATOS	666	m	0	0.40	6.41	3.52	0.3071
MATOS	667	m	0	-0.04	5.63	0.48	0.9160
MATOS	668	m	0	-1.08	8.16	4.45	0.0021
Subtotal MATOS				-0.32	20.20	8.45	
PEZZOT	588	m	0	-0.75	5.30	0.89	0.0856
PEZZOT	589	m	0	-1.59	4.44	6.95	0.0008
Subtotal PEZZOT				-1.13	9.74	7.84	
SOBUE	744	m	0	-0.12	27.30	1.32	0.5398
SOBUE	745	m	0	-0.58	16.23	0.95	0.0197
SOBUE	746	m	0	-0.23	30.64	0.38	0.2110
Subtotal SOBUE				-0.26	74.17	2.65	
SVENSS	572	f	0	-0.62	3.10	0.25	0.2725
SVENSS	573	f	0	-0.90	4.35	1.38	0.0606
Subtotal SVENSS				-0.78	7.46	1.63	
WYNDE3	528	m	0	-0.69	2.49	0.30	0.2796
WYNDE3	529	m	0	-0.43	2.41	0.02	0.5071
WYNDE3	530	m	0	-1.03	2.58	1.23	0.0990
WYNDE3	531	m	0	-1.60	2.67	4.27	0.0089
Subtotal WYNDE3				-0.95	10.15	5.82	

N	36
NS	9

Table 3K6 - 6

IESLC - Meta-analysis of Ex Smoking by Years quit (vs current), Overview
Adenocarcinoma, Cigarettes (or Any Product if Cigarettes not available)
Least adjusted

	Sex			
	combined	male	female	Total
N		29	7	36
NS		8	3	11

In this overview table, other than the "N" rows, entries in the "absent" and "Total" columns may be invalid and should be ignored

	Years quit vs current (lower focus)				
	absent	1-6k3	4-11k7	8+k12	Total
N	17	6	4	9	36
NS	7	6	4	7	24
Wt	140.97	113.34	73.53	103.55	431.39
Het Chi	119.33	4.31	1.70	28.06	180.78
Het df	16	5	3	8	35
Het P	***	N.S.	N.S.	***	***
Fixed RR	0.68	1.03	0.73	0.51	0.71
RRl	0.57	0.85	0.58	0.42	0.65
RRu	0.80	1.24	0.92	0.62	0.78
P	---	N.S.	--	---	---
Random RR	0.71	1.03	0.73	0.39	0.66
RRl	0.44	0.85	0.58	0.26	0.52
RRu	1.14	1.24	0.92	0.59	0.83
P	N.S.	N.S.	--	---	---

	Years quit vs current (higher focus)				
	absent	1-11k3	4-19k12	13+k20	Total
N	17	11	4	4	36
NS	9	9	3	3	24
Wt	195.20	148.55	47.01	40.63	431.39
Het Chi	141.32	11.89	2.80	2.42	180.78
Het df	16	10	3	3	35
Het P	***	N.S.	N.S.	N.S.	***
Fixed RR	0.68	0.92	0.65	0.41	0.71
RRl	0.59	0.78	0.49	0.30	0.65
RRu	0.78	1.08	0.87	0.56	0.78
P	---	N.S.	--	---	---
Random RR	0.64	0.89	0.65	0.41	0.66
RRl	0.41	0.74	0.49	0.30	0.52
RRu	0.99	1.09	0.87	0.56	0.83
P	-	N.S.	--	---	---

Table 3K6 - 6

IESLC - Meta-analysis of Ex Smoking by Years quit (vs current), Overview
Adenocarcinoma, Cigarettes (or Any Product if Cigarettes not available)
Least adjusted

MALES

Years quit vs current (lower focus)

		absent	1-6k3	4-11k7	8+k12	Total
	N	13	6	4	6	29
	NS	6	6	4	6	22
	Wt	119.25	113.34	73.53	93.90	400.02
Het	Chi	117.17	4.31	1.70	17.01	162.14
Het	df	12	5	3	5	28
Het	P	***	N.S.	N.S.	**	***
Fixed	RR	0.68	1.03	0.73	0.54	0.74
	RRl	0.57	0.85	0.58	0.44	0.67
	RRu	0.82	1.24	0.92	0.66	0.81
	P	---	N.S.	--	---	---
Random	RR	0.76	1.03	0.73	0.45	0.72
	RRl	0.42	0.85	0.58	0.30	0.56
	RRu	1.38	1.24	0.92	0.68	0.93
	P	N.S.	N.S.	--	---	-

Years quit vs current (higher focus)

		absent	1-11k3	4-19k12	13+k20	Total
	N	15	8	3	3	29
	NS	8	8	3	3	22
	Wt	188.17	127.79	44.39	39.68	400.02
Het	Chi	126.21	8.51	2.70	1.95	162.14
Het	df	14	7	2	2	28
Het	P	***	N.S.	N.S.	N.S.	***
Fixed	RR	0.71	0.97	0.66	0.42	0.74
	RRl	0.61	0.81	0.49	0.31	0.67
	RRu	0.81	1.15	0.89	0.57	0.81
	P	---	N.S.	--	---	---
Random	RR	0.74	0.96	0.66	0.42	0.72
	RRl	0.47	0.77	0.46	0.31	0.56
	RRu	1.16	1.19	0.96	0.57	0.93
	P	N.S.	N.S.	-	---	-

FEMALES

Years quit vs current (lower focus)

		absent	1-6k3	4-11k7	8+k12	Total
	N	4			3	7
	NS	3			3	6
	Wt	21.71			9.65	31.37
Het	Chi	2.02			6.81	13.55
Het	df	3			2	6
Het	P	N.S.			*	*
Fixed	RR	0.63			0.27	0.48
	RRl	0.41			0.14	0.34
	RRu	0.95			0.51	0.69
	P	-			---	---
Random	RR	0.63			0.25	0.41
	RRl	0.41			0.08	0.23
	RRu	0.95			0.83	0.73
	P	-			-	--

Table 3K6 - 6

IESLC - Meta-analysis of Ex Smoking by Years quit (vs current), Overview
Adenocarcinoma, Cigarettes (or Any Product if Cigarettes not available)
Least adjusted

FEMALES

	Years quit vs current (higher focus) absent	1-11k3	4-19k12	13+k20	Total
N	2	3	1	1	7
NS	2	3	1	1	6
Wt	7.02	20.76	2.63	0.95	31.37
Het Chi	5.08	0.78	0.00	0.00	13.55
Het df	1	2	0	0	6
Het P	*	N.S.	N.S.	N.S.	*
Fixed RR	0.21	0.66	0.54	0.20	0.48
RRl	0.10	0.43	0.16	0.03	0.34
RRu	0.44	1.01	1.81	1.53	0.69
P	---	(-)	N.S.	N.S.	---
Random RR	0.18	0.66	0.54	0.20	0.41
RRl	0.03	0.43	0.16	0.03	0.23
RRu	0.98	1.01	1.81	1.53	0.73
P	-	(-)	N.S.	N.S.	--

Table 3K6 - 7

IESLC - Meta-analysis of Ex Smoking by Years quit (vs current), Overview
Adenocarcinoma, Cigarettes (or Any Product if Cigarettes not available)
Excluded studies (and stage at which they were excluded)

1	AGUDO AKIBA AMANDU AMES AXELSS BEST BOUCHA BOUCOT BRESLO CHEN CHEN2 CHIAZZ DEAN2 DOSEME ENGELA FAN GENG GER GUO HAENSZ HEGMAN HOLE HU HU2 JUSSAW KATSOU KAUFMA KOO KOULUM KREUZE LETOUR LEVIN LIAW LIU3 LIU4 LIU5 MCCONN MIGRAN MRFITR NOTAN2 OSANN2 PERNU QIAO2 RACHTA RESTRE SADOWS SEGI2 STASZE TIZZAN VUTUC WATSON WU WUWILL WYNDE2 WYNDE8 XU YUAN ZHANG ZHENG ZHOU
2	AUVINE BENSHL BLOT1 BROWN3 BUFFLE GURSEL LAUSSM MCDUFF PISANI PRESCO SPITZ WU2 WYNDE7
4	ARMADA BECHER BOFFET BROSS CARPEN CEDERL CHOI CHYOU CORREA CPSI CPSII DAMBER DARBY DEAN3 DESTEF DOLL DOLL2 DORGAN DORN GAO GAO2 GARCIA GARSHI GILLIS GRAHAM HAMMO2 HAMMON HIRAYA HUMBLE JOLY KAISE2 KHUDER LUBIN LUO PEZZO2 QIAO SPEIZE SUZUK2 TVERDA WANG2 WIGLE
5	ALDERS
10	JEDRYC WAKAI WYNDE6
14	BENHAM

Table 3K6 - 8
Potentially overlapping studies

REF	REFGP	PRINC	. OVERLAP/LINK
LUBIN2	LUBIN2	1	Lubin-combined
JAHN	BOFFET	2	Subset of BOFFET

Table 3K6 - 9
Most adjusted - insufficient data for meta-analysis

REF	NRR	SEX	AGEL	AGEH	RACE	YF	LC TYPE	LOC	START	ST	NLC	R	VB	P	H	AD	ADOS	PRODUCT	exL	exH	S1	S2	DENOM	De
ALDERS	573	m	0	0	all	-	not q+s	Eu:UK	1977	CC	1448	n	V	n	n	1	0	cig only	0.1	2	0	0	current	ot
ALDERS	574	m	0	0	all	-	not q+s	Eu:UK	1977	CC	1448	n	V	n	n	1	0	cig only	3	9	0	1	current	ot
ALDERS	575	m	0	0	all	-	not q+s	Eu:UK	1977	CC	1448	n	V	n	n	1	0	cig only	10	999	3	0	current	ot
ALDERS	584	f	0	0	all	-	not q+s	Eu:UK	1977	CC	1448	n	V	n	n	1	0	cig only	0.1	2	0	0	current	ot
ALDERS	585	f	0	0	all	-	not q+s	Eu:UK	1977	CC	1448	n	V	n	n	1	0	cig only	3	9	0	1	current	ot
ALDERS	586	f	0	0	all	-	not q+s	Eu:UK	1977	CC	1448	n	V	n	n	1	0	cig only	10	999	3	0	current	ot

REF	NRR	RR	SIG	RRDATA comment
ALDERS	573	2.07	n	0
ALDERS	574	1.56	n	0
ALDERS	575	0.91	n	0
ALDERS	584	1.38	n	0
ALDERS	585	0.16	y	0.001<p<0.01
ALDERS	586	0.36	n	0

Table 3K7 -

IESLC - Meta-analysis of Ex Smoking, Years quit (vs current), "Low"
Adenocarcinoma, Cigarettes (or Any Product if Cigarettes not available)

This analysis is restricted to results for:
1) Ex smokers
2) Results by Years quit (vs current)
3) Categorical results by Years quit (vs current)
4) Adenocarcinoma (or near equivalent)
5) Results complete enough for use in metaanalysis

Within each study, results are then selected (in the following order of preference, within each sex) for:
6) (not applicable)
7) PRODUCT: cigarettes regardless of other products, cigarettes only, all/unspec
8) CIGTYPE: all/unspecified, MC regardless of HR, MC only
9) Results with least adjustment for other aspects of smoking (ADOS)
10) DENOM: current smokers, current + recent smokers (up to number of m=months or y=years, max 2 years)
11) Followup period (YF, prospective studies): whole study (coded as 0) or longest available
12) LCtype: adeno or nearest available, but not squamous. (q = squamous, s = small,
a = adeno, l = large, KII = Kreyberg II, al = alveolar, br = bronchiolar, u = undifferentiated)
13) Race: all or nearest available, otherwise by race (wh or w = white, bl or b = black, hi = hispanic
ch = chinese, jap = japanese, haw = hawaiian, w+o = white + oriental, sca = scandinavian, as = asian)
14) Years quit (vs current) "low" in key scheme 1 (key value 3, maximum range 1-6)
15) For overlapping studies: principal rather than subsidiary studies
Finally by Age: whole study (coded as 0) if available, otherwise by widest available age group
and then for single sex results (m, f) in preference to results for both sexes combined (c).

Results adjusted (AD) for the most potential confounders are then chosen in Sections -1 to -3
(and those which actually differ from the adjusted results in Table 3K2 - 1 are marked 'x' in Section -1)
and results adjusted for the least confounders in Sections -4 to -6. (Those least adjusted results which
actually differ from the most adjusted are marked 'x' in column X in Section -4)

Section -7 shows excluded studies, together with the stage (as above) at which no qualifying
results were found.

Section -8 lists the potentially overlapping studies which have been included (1=principal, 2=subsidiary).

Section -9 lists any results which would have been included in preference except that they had data not complete
enough for use in meta-analysis, with their significance (yes/no), if known, and any further comment as entered
on the database. It also lists as "gap" any categories for which no data were presented by the original authors.

In addition to those mentioned above, the following fields, levels and abbreviations are used:
* or nk = not known, n = no, y = yes, ot = other
nev = never
all/unspec = all or unspecified, cig+/-ot = cigarettes irrespective of other products (cigar, pipe etc)
MC = manufactured cigarettes, HR = hand-rolled cigarettes
exL, exH = range of exposure (low and high) in the smoking group, in terms of Years quit (vs current)
REF: 6-character study reference
NRR: number of the RR on the database within the study
ST : study type (CC = case control, pr or prosp = prospective)
NLC: number of lung cancer cases in whole study
R : risky occupational population (n = no, m = mining, o = other risky)
VB : national cigarette type (V = at least 75% Virginia, bl = at least 75% blended, ot = other)
P : any proxy use
H : full histological confirmation
De : derivation of RR/CI (or = original, st = standard method, ot = other method of estimation)

Table 3K7 - 1

IESLC - Meta-analysis of Ex Smoking, Years quit (vs current), "Low"
Adenocarcinoma, Cigarettes (or Any Product if Cigarettes not available)
Most adjusted

REF	NRR	3K2	SEX	AGEL	AGEH	RACE	YF	LC TYPE	LOC	START	ST	NLC	R	VB	P	H	AD	ADOS	PRODUCT	exL	exH	DENOM	De
BARBON	764		m	0	0	all	-	a	Eu:wst	1979	CC	755	n	bl	y	y	1	0	all/unsp	0.1	4	current	ot
JAHN	653		m	0	0	all	-	a	Eu:Ger	1988	CC	1004	n	bl	n	n	0	0	cig+/-ot	2	5	current	st
LUBIN2	871		m	0	0	all	-	a	Eu:mul	1976	CC	7804	n	bl	n	y	0	0	cig+/-ot	0.1	4	current	st
LUBIN2	980		f	0	0	all	-	a	Eu:mul	1976	CC	7804	n	bl	n	y	2	1#	cig+/-ot	0.1	4	current	ot
MATOS	676		m	0	0	all	-	a	SCAmer	1994	CC	200	n	bl	n	n	2	0	cig+/-ot	1.0	5	cur+1y	or
SOBUE	744		m	0	0	all	-	a	As:Jap	1986	CC	1376	n	bl	n	y	0	0	cig+/-ot	1.0	4	cur+1y	st
WYNDE3	528		m	0	0	all	-	KII	NAmer	1966	CC	350	n	bl	n	y	0	0	all/unsp	1.0	3	cur+1y	st

Comments on values in listings

LUBIN2 ADOS Duration of smoking

Cigarette type is all/unspec for all RRs

Table 3K7 - 2

IESLC - Meta-analysis of Ex Smoking, Years quit (vs current), "Low"
Adenocarcinoma, Cigarettes (or Any Product if Cigarettes not available)
Most adjusted

REF	NRR	SEX	AD	Number Case	Exposed Cont	Non-exposed Case	Cont	RR	95.00%CI
BARBON	764	m	1	7	-	109	-	1.15	(0.47- 2.78)
JAHN	653	m	0	19	46	75	269	1.48	(0.82- 2.68)
LUBIN2	871	m	0	77	1047	454	6209	1.01	(0.78- 1.29)
LUBIN2	980	f	2	6	-	69	-	0.70	(0.29- 1.69)
Subtotal LUBIN2								0.98	(0.77- 1.25)
MATOS	676	m	2	12	-	46	-	1.30	(0.60- 3.00)
SOBUE	744	m	0	44	116	270	633	0.89	(0.61- 1.29)
WYNDE3	528	m	0	3	22	56	207	0.50	(0.15- 1.75)
Partial Totals				168	1231	1079	7318		

*prospective study

REF	NRR	SEX	AD	Ys	Ws	Qs	Ps
BARBON	764	m	1	0.14	4.86	0.09	0.7579
JAHN	653	m	0	0.39	10.94	1.67	0.1937
LUBIN2	871	m	0	0.01	61.33	0.00	0.9639
LUBIN2	980	f	2	-0.36	4.95	0.64	0.4276
Subtotal LUBIN2				-0.02	66.27	0.64	
MATOS	676	m	2	0.26	5.93	0.40	0.5228
SOBUE	744	m	0	-0.12	27.30	0.39	0.5398
WYNDE3	528	m	0	-0.69	2.49	1.18	0.2796

	N	7
	NS	6
	Wt	117.80
Het	Chi	4.37
Het	df	6
Het	P	N.S.
Fixed	RR	1.00
	RRl	0.84
	RRu	1.20
	P	N.S.
Random	RR	1.00
	RRl	0.84
	RRu	1.20
	P	N.S.
Asymm	P	N.S.

Table 3K7 - 3

IESLC - Meta-analysis of Ex Smoking, Years quit (vs current), "Low"
Adenocarcinoma, Cigarettes (or Any Product if Cigarettes not available)
Most adjusted

	Sex combined	male	female	Total
N		6	1	7
NS		6	1	7
Wt		112.85	4.95	117.80
Het Chi		3.71	0.00	4.37
Het df		5	0	6
Het P		N.S.	N.S.	N.S.
Fixed RR		1.02	0.70	1.00
RRl		0.85	0.29	0.84
RRu		1.22	1.69	1.20
P		N.S.	N.S.	N.S.
Random RR		1.02	0.70	1.00
RRl		0.85	0.29	0.84
RRu		1.22	1.69	1.20
P		N.S.	N.S.	N.S.
Between Chi				0.66
Between df				1
Between P				N.S.
Btwn(F) P				N.S.
Btwn(R) P				N.S.

Too few RRs for analysis by factor

Table 3K7 - 4

IESLC - Meta-analysis of Ex Smoking, Years quit (vs current), "Low"
Adenocarcinoma, Cigarettes (or Any Product if Cigarettes not available)
Least adjusted

REF	NRR	X	SEX	AGEL	AGEH	RACE	YF	LC TYPE	LOC	START	ST	NLC	R	VB	P	H	AD	ADOS	PRODUCT	exL	exH	DENOM	De
BARBON	749	x	m	0	0	all	-	a	Eu:wst	1979	CC	755	n	bl	y	y	0	0	all/unsp	0.1	4	current	st
JAHN	653		m	0	0	all	-	a	Eu:Ger	1988	CC	1004	n	bl	n	n	0	0	cig+/-ot	2	5	current	st
LUBIN2	871		m	0	0	all	-	a	Eu:mul	1976	CC	7804	n	bl	n	y	0	0	cig+/-ot	0.1	4	current	st
LUBIN2	980		f	0	0	all	-	a	Eu:mul	1976	CC	7804	n	bl	n	y	2	1#	cig+/-ot	0.1	4	current	ot
MATOS	666	x	m	0	0	all	-	a	SCAmer	1994	CC	200	n	bl	n	n	0	0	cig+/-ot	1.0	5	cur+1y	st
SOBUE	744		m	0	0	all	-	a	As:Jap	1986	CC	1376	n	bl	n	y	0	0	cig+/-ot	1.0	4	cur+1y	st
WYNDE3	528		m	0	0	all	-	KII	NAmer	1966	CC	350	n	bl	n	y	0	0	all/unsp	1.0	3	cur+1y	st

Comments on values in listings

LUBIN2 ADOS Duration of smoking

Cigarette type is all/unspec for all RRs

Table 3K7 - 5

IESLC - Meta-analysis of Ex Smoking, Years quit (vs current), "Low"
Adenocarcinoma, Cigarettes (or Any Product if Cigarettes not available)
Least adjusted

REF	NRR	SEX	AD	Number Exposed Case	Cont	Non-exposed Case	Cont	RR	95.00%CI
BARBON	749	m	0	7	20	109	362	1.16	(0.48- 2.82)
JAHN	653	m	0	19	46	75	269	1.48	(0.82- 2.68)
LUBIN2	871	m	0	77	1047	454	6209	1.01	(0.78- 1.29)
LUBIN2	980	f	2	6	-	69	-	0.70	(0.29- 1.69)
Subtotal LUBIN2								0.98	(0.77- 1.25)
MATOS	666	m	0	12	23	46	132	1.50	(0.69- 3.25)
SOBUE	744	m	0	44	116	270	633	0.89	(0.61- 1.29)
WYNDE3	528	m	0	3	22	56	207	0.50	(0.15- 1.75)
Partial Totals				168	1274	1079	7812		

*prospective study

REF	NRR	SEX	AD	Ys	Ws	Qs	Ps
BARBON	749	m	0	0.15	4.88	0.10	0.7395
JAHN	653	m	0	0.39	10.94	1.60	0.1937
LUBIN2	871	m	0	0.01	61.33	0.00	0.9639
LUBIN2	980	f	2	-0.36	4.95	0.67	0.4276
Subtotal LUBIN2				-0.02	66.27	0.67	
MATOS	666	m	0	0.40	6.41	0.99	0.3071
SOBUE	744	m	0	-0.12	27.30	0.45	0.5398
WYNDE3	528	m	0	-0.69	2.49	1.21	0.2796

	N	7
	NS	6
	Wt	118.29
Het	Chi	5.01
Het	df	6
Het	P	N.S.
Fixed	RR	1.01
	RRl	0.84
	RRu	1.21
	P	N.S.
Random	RR	1.01
	RRl	0.84
	RRu	1.21
	P	N.S.
Asymm	P	N.S.

Table 3K7 - 6

IESLC - Meta-analysis of Ex Smoking, Years quit (vs current), "Low"
Adenocarcinoma, Cigarettes (or Any Product if Cigarettes not available)
Least adjusted

Sex combined	male	female	Total
N	6	1	7
NS	6	1	7
Wt	113.34	4.95	118.29
Het Chi	4.31	0.00	5.01
Het df	5	0	6
Het P	N.S.	N.S.	N.S.
Fixed RR	1.03	0.70	1.01
RRl	0.85	0.29	0.84
RRu	1.24	1.69	1.21
P	N.S.	N.S.	N.S.
Random RR	1.03	0.70	1.01
RRl	0.85	0.29	0.84
RRu	1.24	1.69	1.21
P	N.S.	N.S.	N.S.
Between Chi			0.70
Between df			1
Between P			N.S.
Btwn(F) P			N.S.
Btwn(R) P			N.S.

Table 3K7 - 7

IESLC - Meta-analysis of Ex Smoking, Years quit (vs current), "Low"
Adenocarcinoma, Cigarettes (or Any Product if Cigarettes not available)
Excluded studies (and stage at which they were excluded)

1	AGUDO AKIBA AMANDU AMES AXELSS BEST BOUCHA BOUCOT BRESLO CHEN CHEN2 CHIAZZ DEAN2 DOSEME ENGELA FAN GENG GER GUO HAENSZ HEGMAN HOLE HU HU2 JUSSAW KATSOU KAUFMA KOO KOULUM KREUZE LETOUR LEVIN LIAW LIU3 LIU4 LIU5 MCCONN MIGRAN MRFITR NOTAN2 OSANN2 PERNU QIAO2 RACHTA RESTRE SADOWS SEGI2 STASZE TIZZAN VUTUC WATSON WU WUWILL WYNDE2 WYNDE8 XU YUAN ZHANG ZHENG ZHOU
2	AUVINE BENSHL BLOT1 BROWN3 BUFFLE GURSEL LAUSSM MCDUFF PISANI PRESCO SPITZ WU2 WYNDE7
4	ARMADA BECHER BOFFET BROSS CARPEN CEDERL CHOI CHYOU CORREA CPSI CPSII DAMBER DARBY DEAN3 DESTEF DOLL DOLL2 DORGAN DORN GAO GAO2 GARCIA GARSHI GILLIS GRAHAM HAMMO2 HAMMON HIRAYA HUMBLE JOLY KAISE2 KHUDER LUBIN LUO PEZZO2 QIAO SPEIZE SUZUK2 TVERDA WANG2 WIGLE
5	ALDERS
10	JEDRYC WAKAI WYNDE6
14	JAIN PEZZOT SVENSS
15	BENHAM

Table 3K7 - 8
Potentially overlapping studies

REF	REFGP	PRINC	OVERLAP/LINK
LUBIN2	LUBIN2	1	Lubin-combined
JAHN	BOFFET	2	Subset of BOFFET

Table 3K8 -

IESLC - Meta-analysis of Ex Smoking, Years quit (vs current), "Mid"
Adenocarcinoma, Cigarettes (or Any Product if Cigarettes not available)

This analysis is restricted to results for:
1) Ex smokers
2) Results by Years quit (vs current)
3) Categorical results by Years quit (vs current)
4) Adenocarcinoma (or near equivalent)
5) Results complete enough for use in metaanalysis

Within each study, results are then selected (in the following order of preference, within each sex) for:
6) (not applicable)
7) PRODUCT: cigarettes regardless of other products, cigarettes only, all/unspec
8) CIGTYPE: all/unspecified, MC regardless of HR, MC only
9) Results with least adjustment for other aspects of smoking (ADOS)
10) DENOM: current smokers, current + recent smokers (up to number of m=months or y=years, max 2 years)
11) Followup period (YF, prospective studies): whole study (coded as 0) or longest available
12) LCtype: adeno or nearest available, but not squamous. (q = squamous, s = small,
a = adeno, l = large, KII = Kreyberg II, al = alveolar, br = bronchiolar, u = undifferentiated)
13) Race: all or nearest available, otherwise by race (wh or w = white, bl or b = black, hi = hispanic
ch = chinese, jap = japanese, haw = hawaiian, w+o = white + oriental, sca = scandinavian, as = asian)
14) Years quit (vs current) "mid" in key scheme 1 (key value 7, maximum range 4-11)
15) For overlapping studies: principal rather than subsidiary studies
Finally by Age: whole study (coded as 0) if available, otherwise by widest available age group
and then for single sex results (m, f) in preference to results for both sexes combined (c).

Results adjusted (AD) for the most potential confounders are then chosen in Sections -1 to -3
(and those which actually differ from the adjusted results in Table 3K3 - 1 are marked 'x' in Section -1)
and results adjusted for the least confounders in Sections -4 to -6. (Those least adjusted results which
actually differ from the most adjusted are marked 'x' in column X in Section -4)

Section -7 shows excluded studies, together with the stage (as above) at which no qualifying
results were found.

Section -8 lists the potentially overlapping studies which have been included (1=principal, 2=subsidiary).

Section -9 lists any results which would have been included in preference except that they had data not complete
enough for use in meta-analysis, with their significance (yes/no), if known, and any further comment as entered
on the database. It also lists as "gap" any categories for which no data were presented by the original authors.

In addition to those mentioned above, the following fields, levels and abbreviations are used:
* or nk = not known, n = no, y = yes, ot = other
nev = never
all/unspec = all or unspecified, cig+/-ot = cigarettes irrespective of other products (cigar, pipe etc)
MC = manufactured cigarettes, HR = hand-rolled cigarettes
exL, exH = range of exposure (low and high) in the smoking group, in terms of Years quit (vs current)
REF: 6-character study reference
NRR: number of the RR on the database within the study
ST : study type (CC = case control, pr or prosp = prospective)
NLC: number of lung cancer cases in whole study
R : risky occupational population (n = no, m = mining, o = other risky)
VB : national cigarette type (V = at least 75% Virginia, bl = at least 75% blended, ot = other)
P : any proxy use
H : full histological confirmation
De : derivation of RR/CI (or = original, st = standard method, ot = other method of estimation)

Table 3K8 - 1

IESLC - Meta-analysis of Ex Smoking, Years quit (vs current), "Mid"
Adenocarcinoma, Cigarettes (or Any Product if Cigarettes not available)
Most adjusted

REF	NRR	3K3	SEX	AGEL	AGEH	RACE	YF	LC TYPE	LOC	START	ST	NLC	R	VB	P	H	AD	ADOS	PRODUCT	exL	exH	DENOM	De
JAHN	654		m	0	0	all	-	a	Eu:Ger	1988	CC	1004	n	bl	n	n	0	0	cig+/-ot	6	10	current	st
LUBIN2	872		m	0	0	all	-	a	Eu:mul	1976	CC	7804	n	bl	n	y	0	0	cig+/-ot	5	9	current	st
LUBIN2	981		f	0	0	all	-	a	Eu:mul	1976	CC	7804	n	bl	n	y	2	1#	cig+/-ot	5	9	current	ot
MATOS	677		m	0	0	all	-	a	SCAmer	1994	CC	200	n	bl	n	n	2	0	cig+/-ot	6	10	cur+1y	or
SOBUE	745		m	0	0	all	-	a	As:Jap	1986	CC	1376	n	bl	n	y	0	0	cig+/-ot	5	9	cur+1y	st

Comments on values in listings

LUBIN2 ADOS Duration of smoking

Cigarette type is all/unspec for all RRs

Table 3K8 - 2

IESLC - Meta-analysis of Ex Smoking, Years quit (vs current), "Mid"
Adenocarcinoma, Cigarettes (or Any Product if Cigarettes not available)
Most adjusted

REF	NRR	SEX	AD	Number Exposed Case	Cont	Non-exposed Case	Cont	RR	95.00%CI
JAHN	654	m	0	13	63	75	269	0.74	(0.39- 1.42)
LUBIN2	872	m	0	50	882	454	6209	0.78	(0.57- 1.05)
LUBIN2	981	f	2	7	-	69	-	1.00	(0.43- 2.32)
Subtotal LUBIN2								0.80	(0.60- 1.06)
MATOS	677	m	2	9	-	46	-	1.00	(0.40- 2.30)
SOBUE	745	m	0	22	92	270	633	0.56	(0.34- 0.91)
Partial Totals				101	1037	914	7111		

*prospective study

REF	NRR	SEX	AD	Ys	Ws	Qs	Ps
JAHN	654	m	0	-0.30	9.10	0.00	0.3638
LUBIN2	872	m	0	-0.25	42.56	0.06	0.0968
LUBIN2	981	f	2	0.00	5.41	0.46	1.0000
Subtotal LUBIN2				-0.23	47.97	0.53	
MATOS	677	m	2	0.00	5.02	0.43	1.0000
SOBUE	745	m	0	-0.58	16.23	1.32	0.0197

	N	5
	NS	4
	Wt	78.32
Het	Chi	2.28
Het	df	4
Het	P	N.S.
Fixed	RR	0.75
	RRl	0.60
	RRu	0.93
	P	--
Random	RR	0.75
	RRl	0.60
	RRu	0.93
	P	--
Asymm	P	N.S.

Table 3K8 - 3

IESLC - Meta-analysis of Ex Smoking, Years quit (vs current), "Mid"
Adenocarcinoma, Cigarettes (or Any Product if Cigarettes not available)
Most adjusted

	Sex combined	male	female	Total
N		4	1	5
NS		4	1	5
Wt		72.92	5.41	78.32
Het Chi		1.78	0.00	2.28
Het df		3	0	4
Het P		N.S.	N.S.	N.S.
Fixed RR		0.73	1.00	0.75
RRl		0.58	0.43	0.60
RRu		0.92	2.32	0.93
P		--	N.S.	--
Random RR		0.73	1.00	0.75
RRl		0.58	0.43	0.60
RRu		0.92	2.32	0.93
P		--	N.S.	--
Between Chi				0.50
Between df				1
Between P				N.S.
Btwn(F) P				N.S.
Btwn(R) P				N.S.

Too few RRs for analysis by factor

Table 3K8 - 4

IESLC - Meta-analysis of Ex Smoking, Years quit (vs current), "Mid"
Adenocarcinoma, Cigarettes (or Any Product if Cigarettes not available)
Least adjusted

REF	NRR	X	SEX	AGEL	AGEH	RACE	YF	LC TYPE	LOC	START	ST	NLC	R	VB	P	H	AD	ADOS	PRODUCT	exL	exH	DENOM	De
JAHN	654		m	0	0	all	-	a	Eu:Ger	1988	CC	1004	n	bl	n	n	0	0	cig+/-ot	6	10	current	st
LUBIN2	872		m	0	0	all	-	a	Eu:mul	1976	CC	7804	n	bl	n	y	0	0	cig+/-ot	5	9	current	st
LUBIN2	981		f	0	0	all	-	a	Eu:mul	1976	CC	7804	n	bl	n	y	2	1#	cig+/-ot	5	9	current	ot
MATOS	667	x	m	0	0	all	-	a	SCAmer	1994	CC	200	n	bl	n	n	0	0	cig+/-ot	6	10	cur+1y	st
SOBUE	745		m	0	0	all	-	a	As:Jap	1986	CC	1376	n	bl	n	y	0	0	cig+/-ot	5	9	cur+1y	st

Comments on values in listings

LUBIN2 ADOS Duration of smoking

Cigarette type is all/unspec for all RRs

Table 3K8 - 5

IESLC - Meta-analysis of Ex Smoking, Years quit (vs current), "Mid"
Adenocarcinoma, Cigarettes (or Any Product if Cigarettes not available)
Least adjusted

REF	NRR	SEX	AD	Number Exposed Case	Cont	Non-exposed Case	Cont	RR	95.00%CI
JAHN	654	m	0	13	63	75	269	0.74	(0.39- 1.42)
LUBIN2	872	m	0	50	882	454	6209	0.78	(0.57- 1.05)
LUBIN2	981	f	2	7	-	69	-	1.00	(0.43- 2.32)
Subtotal LUBIN2								0.80	(0.60- 1.06)
MATOS	667	m	0	9	27	46	132	0.96	(0.42- 2.18)
SOBUE	745	m	0	22	92	270	633	0.56	(0.34- 0.91)
Partial Totals				101	1064	914	7243		

*prospective study

REF	NRR	SEX	AD	Ys	Ws	Qs	Ps
JAHN	654	m	0	-0.30	9.10	0.00	0.3638
LUBIN2	872	m	0	-0.25	42.56	0.07	0.0968
LUBIN2	981	f	2	0.00	5.41	0.47	1.0000
Subtotal LUBIN2				-0.23	47.97	0.53	
MATOS	667	m	0	-0.04	5.63	0.35	0.9160
SOBUE	745	m	0	-0.58	16.23	1.31	0.0197

	N	5
	NS	4
	Wt	78.94
Het	Chi	2.20
Het	df	4
Het	P	N.S.
Fixed	RR	0.75
	RRl	0.60
	RRu	0.93
	P	--
Random	RR	0.75
	RRl	0.60
	RRu	0.93
	P	--
Asymm	P	N.S.

Table 3K8 - 6

IESLC - Meta-analysis of Ex Smoking, Years quit (vs current), "Mid"
Adenocarcinoma, Cigarettes (or Any Product if Cigarettes not available)
Least adjusted

	Sex combined	male	female	Total
N		4	1	5
NS		4	1	5
Wt		73.53	5.41	78.94
Het Chi		1.70	0.00	2.20
Het df		3	0	4
Het P		N.S.	N.S.	N.S.
Fixed RR		0.73	1.00	0.75
RRl		0.58	0.43	0.60
RRu		0.92	2.32	0.93
P		--	N.S.	--
Random RR		0.73	1.00	0.75
RRl		0.58	0.43	0.60
RRu		0.92	2.32	0.93
P		--	N.S.	--
Between Chi				0.50
Between df				1
Between P				N.S.
Btwn(F) P				N.S.
Btwn(R) P				N.S.

Table 3K8 - 7

IESLC - Meta-analysis of Ex Smoking, Years quit (vs current), "Mid"
Adenocarcinoma, Cigarettes (or Any Product if Cigarettes not available)
Excluded studies (and stage at which they were excluded)

1	AGUDO AKIBA AMANDU AMES AXELSS BEST BOUCHA BOUCOT BRESLO CHEN CHEN2 CHIAZZ DEAN2 DOSEME ENGELA FAN GENG GER GUO HAENSZ HEGMAN HOLE HU HU2 JUSSAW KATSOU KAUFMA KOO KOULUM KREUZE LETOUR LEVIN LIAW LIU3 LIU4 LIU5 MCCONN MIGRAN MRFITR NOTAN2 OSANN2 PERNU QIAO2 RACHTA RESTRE SADOWS SEGI2 STASZE TIZZAN VUTUC WATSON WU WUWILL WYNDE2 WYNDE8 XU YUAN ZHANG ZHENG ZHOU
2	AUVINE BENSHL BLOT1 BROWN3 BUFFLE GURSEL LAUSSM MCDUFF PISANI PRESCO SPITZ WU2 WYNDE7
4	ARMADA BECHER BOFFET BROSS CARPEN CEDERL CHOI CHYOU CORREA CPSI CPSII DAMBER DARBY DEAN3 DESTEF DOLL DOLL2 DORGAN DORN GAO GAO2 GARCIA GARSHI GILLIS GRAHAM HAMMO2 HAMMON HIRAYA HUMBLE JOLY KAISE2 KHUDER LUBIN LUO PEZZO2 QIAO SPEIZE SUZUK2 TVERDA WANG2 WIGLE
5	ALDERS
10	JEDRYC WAKAI WYNDE6
14	BARBON JAIN PEZZOT SVENSS WYNDE3
15	BENHAM

Table 3K8 - 8
Potentially overlapping studies

REF	REFGP	PRINC	OVERLAP/LINK
LUBIN2	LUBIN2	1	Lubin-combined
JAHN	BOFFET	2	Subset of BOFFET

Table 3K9 -

IESLC - Meta-analysis of Ex Smoking, Years quit (vs current), "High"
Adenocarcinoma, Cigarettes (or Any Product if Cigarettes not available)

This analysis is restricted to results for:
1) Ex smokers
2) Results by Years quit (vs current)
3) Categorical results by Years quit (vs current)
4) Adenocarcinoma (or near equivalent)
5) Results complete enough for use in metaanalysis

Within each study, results are then selected (in the following order of preference, within each sex) for:
6) PRODUCT: cigarettes regardless of other products, cigarettes only, all/unspec
7) CIGTYPE: all/unspecified, MC regardless of HR, MC only
8) Results with least adjustment for other aspects of smoking (ADOS)
9) DENOM: current smokers, current + recent smokers (up to number of m=months or y=years, max 2 years)
10) Followup period (YF, prospective studies): whole study (coded as 0) or longest available
11) LCtype: adeno or nearest available, but not squamous. (q = squamous, s = small,
a = adeno, l = large, KII = Kreyberg II, al = alveolar, br = bronchiolar, u = undifferentiated)
12) Race: all or nearest available, otherwise by race (wh or w = white, bl or b = black, hi = hispanic
ch = chinese, jap = japanese, haw = hawaiian, w+o = white + oriental, sca = scandinavian, as = asian)
13) Years quit (vs current) "high" in key scheme 1 (key value 12, maximum range 8+)
14) For overlapping studies: principal rather than subsidiary studies
Finally by Age: whole study (coded as 0) if available, otherwise by widest available age group
and then for single sex results (m, f) in preference to results for both sexes combined (c).

Results adjusted (AD) for the most potential confounders are then chosen in Sections -1 to -3
(and those which actually differ from the adjusted results in Table 3K4 - 1 are marked 'x' in Section -1)
and results adjusted for the least confounders in Sections -4 to -6. (Those least adjusted results which
actually differ from the most adjusted are marked 'x' in column X in Section -4)

Section -7 shows excluded studies, together with the stage (as above) at which no qualifying
results were found.

Section -8 lists the potentially overlapping studies which have been included (1=principal, 2=subsidiary).

Section -9 lists any results which would have been included in preference except that they had data not complete
enough for use in meta-analysis, with their significance (yes/no), if known, and any further comment as entered
on the database. It also lists as "gap" any categories for which no data were presented by the original authors.

In addition to those mentioned above, the following fields, levels and abbreviations are used:
* or nk = not known, n = no, y = yes, ot = other
nev = never
all/unspec = all or unspecified, cig+/-ot = cigarettes irrespective of other products (cigar, pipe etc)
MC = manufactured cigarettes, HR = hand-rolled cigarettes
exL, exH = range of exposure (low and high) in the smoking group, in terms of Years quit (vs current)
REF: 6-character study reference
NRR: number of the RR on the database within the study
ST : study type (CC = case control, pr or prosp = prospective)
NLC: number of lung cancer cases in whole study
R : risky occupational population (n = no, m = mining, o = other risky)
VB : national cigarette type (V = at least 75% Virginia, bl = at least 75% blended, ot = other)
P : any proxy use
H : full histological confirmation
De : derivation of RR/CI (or = original, st = standard method, ot = other method of estimation)

Table 3K9 - 1

IESLC - Meta-analysis of Ex Smoking, Years quit (vs current), "High"
Adenocarcinoma, Cigarettes (or Any Product if Cigarettes not available)
Most adjusted

REF	NRR	3K4	SEX	AGEL	AGEH	RACE	YF	LC TYPE	LOC	START	ST	NLC	R	VB	P	H	AD	ADOS	PRODUCT	exL	exH	DENOM	De
JAHN	655		m	0	0	all	-	a	Eu:Ger	1988	CC	1004	n	bl	n	n	0	0	cig+/-ot	11	20	current	st
JAIN	541		m	0	0	all	-	a	NAmer	1981	CC	845	n	V	y	n	0	0	cig+/-ot	10	999	cur+2y	st
JAIN	505		f	0	0	all	-	a	NAmer	1981	CC	845	n	V	y	n	0	0	cig+/-ot	10	999	cur+2y	st
LUBIN2	873		m	0	0	all	-	a	Eu:mul	1976	CC	7804	n	bl	n	y	0	0	cig+/-ot	10	14	current	st
LUBIN2	972		f	0	0	all	-	a	Eu:mul	1976	CC	7804	n	bl	n	y	0	0	cig+/-ot	10	19	current	st
MATOS	678		m	0	0	all	-	a	SCAmer	1994	CC	200	n	bl	n	n	2	0	cig+/-ot	11	999	cur+1y	or
PEZZOT	589		m	0	0	all	-	a	SCAmer	1987	CC	215	n	bl	n	y	0	0	cig only	11	999	cur+1y	st
SOBUE	746		m	0	0	all	-	a	As:Jap	1986	CC	1376	n	bl	n	y	0	0	cig+/-ot	10	999	cur+1y	st
SVENSS	573		f	0	0	all	-	a	Eu:Sca	1983	CC	210	n	bl	n	n	0	0	all/unsp	11	999	cur+2y	st

Cigarette type is all/unspec for all RRs

Table 3K9 - 2

IESLC - Meta-analysis of Ex Smoking, Years quit (vs current), "High"
Adenocarcinoma, Cigarettes (or Any Product if Cigarettes not available)
Most adjusted

REF	NRR	SEX	AD	Number Exposed Case	Cont	Non-exposed Case	Cont	RR	95.00%CI
JAHN	655	m	0	22	130	75	269	0.61	(0.36- 1.02)
JAIN	541	m	0	14	113	60	118	0.24	(0.13- 0.46)
JAIN	505	f	0	3	61	69	99	0.07	(0.02- 0.23)
Subtotal JAIN								0.19	(0.11- 0.33)
LUBIN2	873	m	0	30	693	454	6209	0.59	(0.41- 0.86)
LUBIN2	972	f	0	3	33	69	410	0.54	(0.16- 1.81)
Subtotal LUBIN2								0.59	(0.41- 0.84)
MATOS	678	m	2	12	-	46	-	0.30	(0.20- 0.70)
PEZZOT	589	m	0	7	31	42	38	0.20	(0.08- 0.52)
SOBUE	746	m	0	49	144	270	633	0.80	(0.56- 1.14)
SVENSS	573	f	0	7	24	38	53	0.41	(0.16- 1.04)
Partial Totals				147	1229	1123	7829		

*prospective study

REF	NRR	SEX	AD	Ys	Ws	Qs	Ps
JAHN	655	m	0	-0.50	14.25	0.54	0.0595
JAIN	541	m	0	-1.41	9.49	4.89	0.0000
JAIN	505	f	0	-2.65	2.67	10.23	0.0000
Subtotal JAIN				-1.68	12.16	15.12	
LUBIN2	873	m	0	-0.52	26.93	0.78	0.0065
LUBIN2	972	f	0	-0.62	2.63	0.02	0.3181
Subtotal LUBIN2				-0.53	29.55	0.79	
MATOS	678	m	2	-1.20	9.79	2.55	0.0002
PEZZOT	589	m	0	-1.59	4.44	3.55	0.0008
SOBUE	746	m	0	-0.23	30.64	6.71	0.2110
SVENSS	573	f	0	-0.90	4.35	0.18	0.0606

	N	9
	NS	7
	Wt	105.18
Het	Chi	29.45
Het	df	8
Het	P	***
Fixed	RR	0.50
	RRl	0.41
	RRu	0.60
	P	---
Random	RR	0.39
	RRl	0.26
	RRu	0.58
	P	---
Asymm	P	*

Table 3K9 - 3

IESLC - Meta-analysis of Ex Smoking, Years quit (vs current), "High"
Adenocarcinoma, Cigarettes (or Any Product if Cigarettes not available)
Most adjusted

	Sex: combined male	female	Total
N	6	3	9
NS	6	3	9
Wt	95.53	9.65	105.18
Het Chi	18.65	6.81	29.45
Het df	5	2	8
Het P	**	*	***
Fixed RR	0.53	0.27	0.50
RRl	0.43	0.14	0.41
RRu	0.65	0.51	0.60
P	---	---	---
Random RR	0.44	0.25	0.39
RRl	0.29	0.08	0.26
RRu	0.67	0.83	0.58
P	---	-	---
Between Chi			3.99
Between df			1
Between P			*
Btwn(F) P			N.S.
Btwn(R) P			N.S.

Too few RRs for analysis by factor

Table 3K9 - 4

IESLC - Meta-analysis of Ex Smoking, Years quit (vs current), "High"
Adenocarcinoma, Cigarettes (or Any Product if Cigarettes not available)
Least adjusted

REF	NRR	X	SEX	AGEL	AGEH	RACE	YF	LC TYPE	LOC	START	ST	NLC	R	VB	P	H	AD	ADOS	PRODUCT	exL	exH	DENOM	De
JAHN	655		m	0	0	all	-	a	Eu:Ger	1988	CC	1004	n	bl	n	n	0	0	cig+/-ot	11	20	current	st
JAIN	541		m	0	0	all	-	a	NAmer	1981	CC	845	n	V	y	n	0	0	cig+/-ot	10	999	cur+2y	st
JAIN	505		f	0	0	all	-	a	NAmer	1981	CC	845	n	V	y	n	0	0	cig+/-ot	10	999	cur+2y	st
LUBIN2	873		m	0	0	all	-	a	Eu:mul	1976	CC	7804	n	bl	n	y	0	0	cig+/-ot	10	14	current	st
LUBIN2	972		f	0	0	all	-	a	Eu:mul	1976	CC	7804	n	bl	n	y	0	0	cig+/-ot	10	19	current	st
MATOS	668	x	m	0	0	all	-	a	SCAmer	1994	CC	200	n	bl	n	n	0	0	cig+/-ot	11	999	cur+1y	st
PEZZOT	589		m	0	0	all	-	a	SCAmer	1987	CC	215	n	bl	n	y	0	0	cig only	11	999	cur+1y	st
SOBUE	746		m	0	0	all	-	a	As:Jap	1986	CC	1376	n	bl	n	y	0	0	cig+/-ot	10	999	cur+1y	st
SVENSS	573		f	0	0	all	-	a	Eu:Sca	1983	CC	210	n	bl	n	n	0	0	all/unsp	11	999	cur+2y	st

Cigarette type is all/unspec for all RRs

Table 3K9 - 5

IESLC - Meta-analysis of Ex Smoking, Years quit (vs current), "High"
Adenocarcinoma, Cigarettes (or Any Product if Cigarettes not available)
Least adjusted

REF	NRR	SEX	AD	Number Exposed Case	Number Exposed Cont	Non-exposed Case	Non-exposed Cont	RR	95.00%CI
JAHN	655	m	0	22	130	75	269	0.61	(0.36- 1.02)
JAIN	541	m	0	14	113	60	118	0.24	(0.13- 0.46)
JAIN	505	f	0	3	61	69	99	0.07	(0.02- 0.23)
Subtotal JAIN								0.19	(0.11- 0.33)
LUBIN2	873	m	0	30	693	454	6209	0.59	(0.41- 0.86)
LUBIN2	972	f	0	3	33	69	410	0.54	(0.16- 1.81)
Subtotal LUBIN2								0.59	(0.41- 0.84)
MATOS	668	m	0	12	101	46	132	0.34	(0.17- 0.68)
PEZZOT	589	m	0	7	31	42	38	0.20	(0.08- 0.52)
SOBUE	746	m	0	49	144	270	633	0.80	(0.56- 1.14)
SVENSS	573	f	0	7	24	38	53	0.41	(0.16- 1.04)
Totals				147	1330	1123	7961		

*prospective study

REF	NRR	SEX	AD	Ys	Ws	Qs	Ps
JAHN	655	m	0	-0.50	14.25	0.44	0.0595
JAIN	541	m	0	-1.41	9.49	5.14	0.0000
JAIN	505	f	0	-2.65	2.67	10.42	0.0000
Subtotal JAIN				-1.68	12.16	15.56	
LUBIN2	873	m	0	-0.52	26.93	0.62	0.0065
LUBIN2	972	f	0	-0.62	2.63	0.01	0.3181
Subtotal LUBIN2				-0.53	29.55	0.63	
MATOS	668	m	0	-1.08	8.16	1.31	0.0021
PEZZOT	589	m	0	-1.59	4.44	3.69	0.0008
SOBUE	746	m	0	-0.23	30.64	6.20	0.2110
SVENSS	573	f	0	-0.90	4.35	0.22	0.0606

	N	9
	NS	7
	Wt	103.55
Het	Chi	28.06
Het	df	8
Het	P	***
Fixed	RR	0.51
	RRl	0.42
	RRu	0.62
	P	---
Random	RR	0.39
	RRl	0.26
	RRu	0.59
	P	---
Asymm	P	*

Table 3K9 - 6

IESLC - Meta-analysis of Ex Smoking, Years quit (vs current), "High"
Adenocarcinoma, Cigarettes (or Any Product if Cigarettes not available)
Least adjusted

	Sex combined	male	female	Total
N		6	3	9
NS		6	3	9
Wt		93.90	9.65	103.55
Het Chi		17.01	6.81	28.06
Het df		5	2	8
Het P		**	*	***
Fixed RR		0.54	0.27	0.51
RRl		0.44	0.14	0.42
RRu		0.66	0.51	0.62
P		---	---	---
Random RR		0.45	0.25	0.39
RRl		0.30	0.08	0.26
RRu		0.68	0.83	0.59
P		---	-	---
Between Chi				4.24
Between df				1
Between P				*
Btwn(F) P				N.S.
Btwn(R) P				N.S.

Table 3K9 - 7

IESLC - Meta-analysis of Ex Smoking, Years quit (vs current), "High"
Adenocarcinoma, Cigarettes (or Any Product if Cigarettes not available)
Excluded studies (and stage at which they were excluded)

1	AGUDO AKIBA AMANDU AMES AXELSS BEST BOUCHA BOUCOT BRESLO CHEN CHEN2 CHIAZZ DEAN2 DOSEME ENGELA FAN GENG GER GUO HAENSZ HEGMAN HOLE HU HU2 JUSSAW KATSOU KAUFMA KOO KOULUM KREUZE LETOUR LEVIN LIAW LIU3 LIU4 LIU5 MCCONN MIGRAN MRFITR NOTAN2 OSANN2 PERNU QIAO2 RACHTA RESTRE SADOWS SEGI2 STASZE TIZZAN VUTUC WATSON WU WUWILL WYNDE2 WYNDE8 XU YUAN ZHANG ZHENG ZHOU
2	AUVINE BENSHL BLOT1 BROWN3 BUFFLE GURSEL LAUSSM MCDUFF PISANI PRESCO SPITZ WU2 WYNDE7
4	ARMADA BECHER BOFFET BROSS CARPEN CEDERL CHOI CHYOU CORREA CPSI CPSII DAMBER DARBY DEAN3 DESTEF DOLL DOLL2 DORGAN DORN GAO GAO2 GARCIA GARSHI GILLIS GRAHAM HAMMO2 HAMMON HIRAYA HUMBLE JOLY KAISE2 KHUDER LUBIN LUO PEZZO2 QIAO SPEIZE SUZUK2 TVERDA WANG2 WIGLE
5	ALDERS
10	JEDRYC WAKAI WYNDE6
14	BARBON WYNDE3
15	BENHAM

Table 3K9 - 8
Potentially overlapping studies

REF	REFGP	PRINC	.	OVERLAP/LINK
LUBIN2	LUBIN2	1		Lubin-combined
JAHN	BOFFET	2		Subset of BOFFET

Table 3K9 - 9
Most adjusted - insufficient data for meta-analysis

REF	NRR	SEX	AGEL	AGEH	RACE	YF	LC TYPE	LOC	START	ST	NLC	R	VB	P	H	AD	ADOS	PRODUCT	exL	exH	DENOM	De
ALDERS	575	m	0	0	all	-	not q+s	Eu:UK	1977	CC	1448	n	V	n	n	1	0	cig only	10	999	current	ot
ALDERS	586	f	0	0	all	-	not q+s	Eu:UK	1977	CC	1448	n	V	n	n	1	0	cig only	10	999	current	ot

REF	NRR	RR	SIG	RRDATA comment
ALDERS	575	0.91	n	0
ALDERS	586	0.36	n	0

Table 3K10 -

IESLC - Meta-analysis of Ex Smoking, Years quit (vs current), "Highest vs lowest"
Adenocarcinoma, Cigarettes (or Any Product if Cigarettes not available)

This analysis is restricted to results for:
1) Ex smokers
2) Results by Years quit (vs current)
3) Categorical results by Years quit (vs current)
4) Denominator (unexposed) = "low"
5) Adenocarcinoma (or near equivalent)
6) Results complete enough for use in metaanalysis

Within each study, results are then selected (in the following order of preference, within each sex) for:
7) (not applicable)
8) PRODUCT: cigarettes regardless of other products, cigarettes only, all/unspec
9) CIGTYPE: all/unspecified, MC regardless of HR, MC only
10) Results with least adjustment for other aspects of smoking (ADOS)
11) The highest vs lowest category
12) Followup period (YF, prospective studies): whole study (coded as 0) or longest available
13) LCtype: adeno or nearest available, but not squamous. (q = squamous, s = small,
a = adeno, l = large, KII = Kreyberg II, al = alveolar, br = bronchiolar, u = undifferentiated)
14) Race: all or nearest available, otherwise by race (wh or w = white, bl or b = black, hi = hispanic
ch = chinese, jap = japanese, haw = hawaiian, w+o = white + oriental, sca = scandinavian, as = asian)
15) For overlapping studies: principal rather than subsidiary studies
Finally by Age: whole study (coded as 0) if available, otherwise by widest available age group
and then for single sex results (m, f) in preference to results for both sexes combined (c).

Results adjusted (AD) for the most potential confounders are then chosen in Sections -1 to -3
(and those which actually differ from the adjusted results in Table 3K5 - 1 are marked 'x' in Section -1)
and results adjusted for the least confounders in Sections -4 to -6. (Those least adjusted results which
actually differ from the most adjusted are marked 'x' in column X in Section -4)

Section -7 shows excluded studies, together with the stage (as above) at which no qualifying
results were found.

Section -8 lists the potentially overlapping studies which have been included (1=principal, 2=subsidiary).

Section -9 lists any results which would have been included in preference except that they had data not complete
enough for use in meta-analysis, with their significance (yes/no), if known, and any further comment as entered
on the database. It also lists as "gap" any categories for which no data were presented by the original authors.

In addition to those mentioned above, the following fields, levels and abbreviations are used:
* or nk = not known, n = no, y = yes, ot = other
all/unspec = all or unspecified, cig+/-ot = cigarettes irrespective of other products (cigar, pipe etc)
MC = manufactured cigarettes, HR = hand-rolled cigarettes
exL, exH = range of exposure (low and high) in the "highest" group, in terms of Years quit (vs current)
unexL, unexH = range of exposure (low and high) in the "lowest" group, in terms of Years quit (vs current)
REF: 6-character study reference
NRR: number of the RR on the database within the study
ST : study type (CC = case control, pr or prosp = prospective)
NLC: number of lung cancer cases in whole study
R : risky occupational population (n = no, m = mining, o = other risky)
VB : national cigarette type (V = at least 75% Virginia, bl = at least 75% blended, ot = other)
P : any proxy use
H : full histological confirmation
De : derivation of RR/CI (or = original, st = standard method, ot = other method of estimation)

Table 3K10 - 1

IESLC - Meta-analysis of Ex Smoking, Years quit (vs current), "Highest vs lowest"
Adenocarcinoma, Cigarettes (or Any Product if Cigarettes not available)
Most adjusted

REF	NRR	3K5	SEX	AGEL	AGEH	RACE	YF	LC TYPE	LOC	START	ST	NLC	R	VB	P	H	AD	ADOS	PRODUCT	exL	exH	unexL	unexH	De
BARBON	770		m	0	0	all	-	a	Eu:wst	1979	CC	755	n	bl	y	y	1	0	all/unsp	25	999	0.1	4	ot
JAHN	661		m	0	0	all	-	a	Eu:Ger	1988	CC	1004	n	bl	n	n	0	0	cig+/-ot	21	999	0.1	0.9	st
JAIN	542		m	0	0	all	-	a	NAmer	1981	CC	845	n	V	y	n	0	0	cig+/-ot	10	999	2	9	st
JAIN	506		f	0	0	all	-	a	NAmer	1981	CC	845	n	V	y	n	0	0	cig+/-ot	10	999	2	9	st
LUBIN2	879		m	0	0	all	-	a	Eu:mul	1976	CC	7804	n	bl	n	y	0	0	cig+/-ot	20	999	0.1	4	st
LUBIN2	975		f	0	0	all	-	a	Eu:mul	1976	CC	7804	n	bl	n	y	0	0	cig+/-ot	20	999	0.1	9	st
MATOS	680		m	0	0	all	-	a	SCAmer	1994	CC	200	n	bl	n	n	2	0	cig+/-ot	11	999	1.0	5	ot
PEZZOT	590		m	0	0	all	-	a	SCAmer	1987	CC	215	n	bl	n	y	0	0	cig only	11	999	1.0	10	st
SOBUE	748		m	0	0	all	-	a	As:Jap	1986	CC	1376	n	bl	n	y	0	0	cig+/-ot	10	999	1.0	4	st
SVENSS	574		f	0	0	all	-	a	Eu:Sca	1983	CC	210	n	bl	n	n	0	0	all/unsp	11	999	3	10	st
WYNDE3	534		m	0	0	all	-	KII	NAmer	1966	CC	350	n	bl	n	y	0	0	all/unsp	13	999	1.0	3	st
WYNDE6	831		m	0	0	all	-	KII	NAmer	1969	CC	4423	n	bl	n	y	2	0	cig+/-ot	16	999	1.0	3	ot

Cigarette type is all/unspec for all RRs

Table 3K10 - 2

IESLC - Meta-analysis of Ex Smoking, Years quit (vs current), "Highest vs lowest"
Adenocarcinoma, Cigarettes (or Any Product if Cigarettes not available)
Most adjusted

REF	NRR	SEX	AD	Number Case	Exposed Cont	Non-exposed Case	Cont	RR	95.00%CI
BARBON	770	m	1	4	-	7	-	0.19	(0.05- 0.73)
JAHN	661	m	0	15	146	40	8	0.02	(0.01- 0.05)
JAIN	542	m	0	14	113	16	46	0.36	(0.16- 0.79)
JAIN	506	f	0	3	61	14	36	0.13	(0.03- 0.47)
Subtotal JAIN								0.27	(0.14- 0.53)
LUBIN2	879	m	0	35	1128	77	1047	0.42	(0.28- 0.63)
LUBIN2	975	f	0	1	29	13	95	0.25	(0.03- 2.01)
Subtotal LUBIN2								0.41	(0.28- 0.62)
MATOS	680	m	2	12	-	12	-	0.23	(0.09- 0.58)
PEZZOT	590	m	0	7	31	11	21	0.43	(0.14- 1.29)
SOBUE	748	m	0	49	144	44	116	0.90	(0.56- 1.44)
SVENSS	574	f	0	7	24	5	13	0.76	(0.20- 2.87)
WYNDE3	534	m	0	3	55	3	22	0.40	(0.07- 2.14)
WYNDE6	831	m	2	6	-	29	-	0.02	(0.01- 0.05)
Partial Totals				156	1731	271	1404		

*prospective study

REF	NRR	SEX	AD	Ys	Ws	Qs	Ps
BARBON	770	m	1	-1.66	2.14	0.42	0.0152
JAHN	661	m	0	-3.88	4.47	31.77	0.0000
JAIN	542	m	0	-1.03	6.08	0.21	0.0109
JAIN	506	f	0	-2.07	2.23	1.60	0.0020
Subtotal JAIN				-1.31	8.31	1.82	
LUBIN2	879	m	0	-0.86	23.04	2.94	0.0000
LUBIN2	975	f	0	-1.38	0.89	0.02	0.1932
Subtotal LUBIN2				-0.88	23.93	2.96	
MATOS	680	m	2	-1.47	4.43	0.28	0.0020
PEZZOT	590	m	0	-0.84	3.19	0.46	0.1330
SOBUE	748	m	0	-0.11	17.04	21.04	0.6540
SVENSS	574	f	0	-0.28	2.17	1.93	0.6838
WYNDE3	534	m	0	-0.92	1.37	0.13	0.2836
WYNDE6	831	m	2	-3.91	5.93	42.99	0.0000

	N	12
	NS	10
	Wt	72.97
Het	Chi	103.79
Het	df	11
Het	P	***
Fixed	RR	0.30
	RRl	0.23
	RRu	0.37
	P	---
Random	RR	0.21
	RRl	0.10
	RRu	0.46
	P	---
Asymm	P	N.S.

Table 3K10 - 3

IESLC - Meta-analysis of Ex Smoking, Years quit (vs current), "Highest vs lowest"
Adenocarcinoma, Cigarettes (or Any Product if Cigarettes not available)
Most adjusted

		Sex		
	combined	male	female	Total
N		9	3	12
NS		9	3	12
Wt		67.68	5.29	72.97
Het Chi		100.23	3.55	103.79
Het df		8	2	11
Het P		***	N.S.	***
Fixed RR		0.30	0.30	0.30
RRl		0.23	0.13	0.23
RRu		0.37	0.69	0.37
P		---	--	---
Random RR		0.20	0.29	0.21
RRl		0.08	0.09	0.10
RRu		0.49	0.95	0.46
P		---	-	---
Between Chi				0.00
Between df				1
Between P				N.S.
Btwn(F) P				N.S.
Btwn(R) P				N.S.

			Lung cancer type				
	a	a+l	a+al+br	KII	not q+u	not q+s	Total
N	10			2			12
NS	8			2			10
Wt	65.67			7.30			72.97
Het Chi	56.99			9.98			103.79
Het df	9			1			11
Het P	***			**			***
Fixed RR	0.37			0.04			0.30
RRl	0.29			0.02			0.23
RRu	0.48			0.07			0.37
P	---			---			---
Random RR	0.26			0.08			0.21
RRl	0.13			0.00			0.10
RRu	0.53			1.53			0.46
P	---			(-)			---
Between Chi							36.82
Between df							1
Between P							***
Btwn(F) P							*
Btwn(R) P							N.S.

				Location					
	NAmer	UK	Scand	othEur	China	Japan	othAs	other	Total
N	4		1	4		1		2	12
NS	3		1	3		1		2	10
Wt	15.61		2.17	30.54		17.04		7.61	72.97
Het Chi	27.91		0.00	34.40		0.00		0.73	103.79
Het df	3		0	3		0		1	11
Het P	***		N.S.	***		N.S.		N.S.	***
Fixed RR	0.10		0.76	0.25		0.90		0.30	0.30
RRl	0.06		0.20	0.18		0.56		0.15	0.23
RRu	0.17		2.87	0.36		1.44		0.61	0.37
P	---		N.S.	---		N.S.		---	---
Random RR	0.13		0.76	0.14		0.90		0.30	0.21
RRl	0.03		0.20	0.03		0.56		0.15	0.10
RRu	0.66		2.87	0.75		1.44		0.61	0.46
P	-		N.S.	-		N.S.		---	---
Between Chi									40.75
Between df									4
Between P									***
Btwn(F) P									N.S.
Btwn(R) P									*

Table 3K10 - 3

IESLC - Meta-analysis of Ex Smoking, Years quit (vs current), "Highest vs lowest"
Adenocarcinoma, Cigarettes (or Any Product if Cigarettes not available)
Most adjusted

Detailed Country in "other Europe"

		multi	Germany	othWest	East	Balkans	Total
	N	2	1	1			4
	NS	1	1	1			3
	Wt	23.93	4.47	2.14			30.54
Het	Chi	0.23	0.00	0.00			34.40
Het	df	1	0	0			3
Het	P	N.S.	N.S.	N.S.			***
Fixed	RR	0.41	0.02	0.19			0.25
	RRl	0.28	0.01	0.05			0.18
	RRu	0.62	0.05	0.73			0.36
	P	---	---	-			---
Random	RR	0.41	0.02	0.19			0.14
	RRl	0.28	0.01	0.05			0.03
	RRu	0.62	0.05	0.73			0.75
	P	---	---	-			-
Between	Chi						34.17
Between	df						2
Between	P						***
Btwn(F)	P						(*)
Btwn(R)	P						***

Detailed Country in "other Asia"

		India	HongKong	other	Total
	N				
	NS				
	Wt				
Het	Chi				
Het	df				
Het	P				
Fixed	RR				
	RRl				
	RRu				
	P				
Random	RR				
	RRl				
	RRu				
	P				
Between	Chi				
Between	df				
Between	P				N.S.
Btwn(F)	P				N.S.
Btwn(R)	P				N.S.

Detailed other continent

		SCAmer	Total
	N	2	2
	NS	2	2
	Wt	7.61	7.61
Het	Chi	0.73	0.73
Het	df	1	1
Het	P	N.S.	N.S.
Fixed	RR	0.30	0.30
	RRl	0.15	0.15
	RRu	0.61	0.61
	P	---	---
Random	RR	0.30	0.30
	RRl	0.15	0.15
	RRu	0.61	0.61
	P	---	---
Between	Chi		
Between	df		
Between	P		N.S.
Btwn(F)	P		N.S.
Btwn(R)	P		N.S.

Table 3K10 - 3

IESLC - Meta-analysis of Ex Smoking, Years quit (vs current), "Highest vs lowest"
Adenocarcinoma, Cigarettes (or Any Product if Cigarettes not available)
Most adjusted

	<1960	1960-69	1970-79	1980-89	1990+	Total
		Start year of study				
N		2	3	6	1	12
NS		2	2	5	1	10
Wt		7.30	26.07	35.17	4.43	72.97
Het Chi		9.98	1.42	54.44	0.00	103.79
Het df		1	2	5	0	11
Het P		**	N.S.	***	N.S.	***
Fixed RR		0.04	0.39	0.39	0.23	0.30
RRl		0.02	0.26	0.28	0.09	0.23
RRu		0.07	0.57	0.54	0.58	0.37
P		---	---	---	--	---
Random RR		0.08	0.39	0.26	0.23	0.21
RRl		0.00	0.26	0.08	0.09	0.10
RRu		1.53	0.57	0.86	0.58	0.46
P		(-)	---	-	--	---
Between Chi						37.94
Between df						3
Between P						***
Btwn(F) P						N.S.
Btwn(R) P						N.S.

	CC	other	Total
	Study type (1)		
N	12		12
NS	10		10
Wt	72.97		72.97
Het Chi	103.79		103.79
Het df	11		11
Het P	***		***
Fixed RR	0.30		0.30
RRl	0.23		0.23
RRu	0.37		0.37
P	---		---
Random RR	0.21		0.21
RRl	0.10		0.10
RRu	0.46		0.46
P	---		---
Between Chi			
Between df			
Between P			N.S.
Btwn(F) P			N.S.
Btwn(R) P			N.S.

	CC	prosp	other	Total
	Study type (2)			
N	12			12
NS	10			10
Wt	72.97			72.97
Het Chi	103.79			103.79
Het df	11			11
Het P	***			***
Fixed RR	0.30			0.30
RRl	0.23			0.23
RRu	0.37			0.37
P	---			---
Random RR	0.21			0.21
RRl	0.10			0.10
RRu	0.46			0.46
P	---			---
Between Chi				
Between df				
Between P				N.S.
Btwn(F) P				N.S.
Btwn(R) P				N.S.

Table 3K10 - 3

IESLC - Meta-analysis of Ex Smoking, Years quit (vs current), "Highest vs lowest"
Adenocarcinoma, Cigarettes (or Any Product if Cigarettes not available)
Most adjusted

	Study size (number of LC cases)				
	100-249	250-499	500-999	1000+	Total
N	3	1	3	5	12
NS	3	1	2	4	10
Wt	9.78	1.37	10.44	51.37	72.97
Het Chi	2.19	0.00	1.96	98.75	103.79
Het df	2	0	2	4	11
Het P	N.S.	N.S.	N.S.	***	***
Fixed RR	0.37	0.40	0.25	0.29	0.30
RRl	0.20	0.07	0.14	0.22	0.23
RRu	0.69	2.14	0.46	0.38	0.37
P	--	N.S.	---	---	---
Random RR	0.37	0.40	0.25	0.13	0.21
RRl	0.19	0.07	0.14	0.03	0.10
RRu	0.72	2.14	0.46	0.61	0.46
P	--	N.S.	---	--	---
Between Chi					0.89
Between df					3
Between P					N.S.
Btwn(F) P					N.S.
Btwn(R) P					N.S.

	Risky occupational population			
	no	mining	othRisky	Total
N	12			12
NS	10			10
Wt	72.97			72.97
Het Chi	103.79			103.79
Het df	11			11
Het P	***			***
Fixed RR	0.30			0.30
RRl	0.23			0.23
RRu	0.37			0.37
P	---			---
Random RR	0.21			0.21
RRl	0.10			0.10
RRu	0.46			0.46
P	---			---
Between Chi				
Between df				
Between P				N.S.
Btwn(F) P				N.S.
Btwn(R) P				N.S.

	National cigarette tobacco type			
	Virginia	blended	other	Total
N	2	10		12
NS	1	9		10
Wt	8.31	64.66		72.97
Het Chi	1.75	101.96		103.79
Het df	1	9		11
Het P	N.S.	***		***
Fixed RR	0.27	0.30		0.30
RRl	0.14	0.23		0.23
RRu	0.53	0.38		0.37
P	---	---		---
Random RR	0.24	0.21		0.21
RRl	0.09	0.08		0.10
RRu	0.65	0.53		0.46
P	--	---		---
Between Chi				0.08
Between df				1
Between P				N.S.
Btwn(F) P				N.S.
Btwn(R) P				N.S.

Table 3K10 - 3

IESLC - Meta-analysis of Ex Smoking, Years quit (vs current), "Highest vs lowest"
Adenocarcinoma, Cigarettes (or Any Product if Cigarettes not available)
Most adjusted

	Any proxy use		
	No/nk	Yes	Total
N	9	3	12
NS	8	2	10
Wt	62.53	10.44	72.97
Het Chi	101.51	1.96	103.79
Het df	8	2	11
Het P	***	N.S.	***
Fixed RR	0.30	0.25	0.30
RRl	0.24	0.14	0.23
RRu	0.39	0.46	0.37
P	---	---	---
Random RR	0.21	0.25	0.21
RRl	0.08	0.14	0.10
RRu	0.58	0.46	0.46
P	--	---	---
Between Chi			0.32
Between df			1
Between P			N.S.
Btwn(F) P			N.S.
Btwn(R) P			N.S.

	Full histological confirmation		
	No	Yes	Total
N	5	7	12
NS	4	6	10
Wt	19.37	53.60	72.97
Het Chi	28.69	65.42	103.79
Het df	4	6	11
Het P	***	***	***
Fixed RR	0.16	0.37	0.30
RRl	0.10	0.28	0.23
RRu	0.25	0.48	0.37
P	---	---	---
Random RR	0.17	0.25	0.21
RRl	0.05	0.09	0.10
RRu	0.58	0.71	0.46
P	--	--	---
Between Chi			9.67
Between df			1
Between P			**
Btwn(F) P			N.S.
Btwn(R) P			N.S.

	Number of adjustment variables (1)			
	0	1	2+/+nk	Total
N	9	1	2	12
NS	7	1	2	10
Wt	60.47	2.14	10.36	72.97
Het Chi	54.74	0.00	15.12	103.79
Het df	8	0	1	11
Het P	***	N.S.	***	***
Fixed RR	0.40	0.19	0.06	0.30
RRl	0.31	0.05	0.03	0.23
RRu	0.51	0.73	0.10	0.37
P	---	-	---	---
Random RR	0.29	0.19	0.07	0.21
RRl	0.13	0.05	0.01	0.10
RRu	0.62	0.73	0.73	0.46
P	--	-	-	---
Between Chi				33.93
Between df				2
Between P				***
Btwn(F) P				N.S.
Btwn(R) P				N.S.

Table 3K10 - 3

IESLC - Meta-analysis of Ex Smoking, Years quit (vs current), "Highest vs lowest"
Adenocarcinoma, Cigarettes (or Any Product if Cigarettes not available)
Most adjusted

	Number of adjustment variables (2) 0	1	2	3-5	6+/+nk	Total
N	9	1	2			12
NS	7	1	2			10
Wt	60.47	2.14	10.36			72.97
Het Chi	54.74	0.00	15.12			103.79
Het df	8	0	1			11
Het P	***	N.S.	***			***
Fixed RR	0.40	0.19	0.06			0.30
RRl	0.31	0.05	0.03			0.23
RRu	0.51	0.73	0.10			0.37
P	---	-	---			---
Random RR	0.29	0.19	0.07			0.21
RRl	0.13	0.05	0.01			0.10
RRu	0.62	0.73	0.73			0.46
P	--	-	-			---
Between Chi						33.93
Between df						2
Between P						***
Btwn(F) P						N.S.
Btwn(R) P						N.S.

	Product all/unsp	cig+/-ot	cig only	Total
N	3	8	1	12
NS	3	6	1	10
Wt	5.67	64.11	3.19	72.97
Het Chi	2.06	100.74	0.00	103.79
Het df	2	7	0	11
Het P	N.S.	***	N.S.	***
Fixed RR	0.39	0.28	0.43	0.30
RRl	0.17	0.22	0.14	0.23
RRu	0.88	0.36	1.29	0.37
P	-	---	N.S.	---
Random RR	0.39	0.16	0.43	0.21
RRl	0.17	0.06	0.14	0.10
RRu	0.89	0.45	1.29	0.46
P	-	---	N.S.	---
Between Chi				0.98
Between df				2
Between P				N.S.
Btwn(F) P				N.S.
Btwn(R) P				N.S.

	Derivation of RR/CI Orig	StdCalc	Other	Total
N		9	3	12
NS		7	3	10
Wt		60.47	12.50	72.97
Het Chi		54.74	17.70	103.79
Het df		8	2	11
Het P		***	***	***
Fixed RR		0.40	0.07	0.30
RRl		0.31	0.04	0.23
RRu		0.51	0.12	0.37
P		---	---	---
Random RR		0.29	0.09	0.21
RRl		0.13	0.02	0.10
RRu		0.62	0.51	0.46
P		--	--	---
Between Chi				31.34
Between df				1
Between P				***
Btwn(F) P				(*)
Btwn(R) P				N.S.

Table 3K10 - 4

IESLC - Meta-analysis of Ex Smoking, Years quit (vs current), "Highest vs lowest"
Adenocarcinoma, Cigarettes (or Any Product if Cigarettes not available)
Least adjusted

REF	NRR	X	SEX	AGEL	AGEH	RACE	YF	LC TYPE	LOC	START	ST	NLC	R	VB	P	H	AD	ADOS	PRODUCT	exL	exH	unexL	unexH	De
BARBON	755	x	m	0	0	all	-	a	Eu:wst	1979	CC	755	n	bl	y	y	0	0	all/unsp	25	999	0.1	4	st
JAHN	661		m	0	0	all	-	a	Eu:Ger	1988	CC	1004	n	bl	n	n	0	0	cig+/-ot	21	999	0.1	0.9	st
JAIN	542		m	0	0	all	-	a	NAmer	1981	CC	845	n	V	y	n	0	0	cig+/-ot	10	999	2	9	st
JAIN	506		f	0	0	all	-	a	NAmer	1981	CC	845	n	V	y	n	0	0	cig+/-ot	10	999	2	9	st
LUBIN2	879		m	0	0	all	-	a	Eu:mul	1976	CC	7804	n	bl	n	y	0	0	cig+/-ot	20	999	0.1	4	st
LUBIN2	975		f	0	0	all	-	a	Eu:mul	1976	CC	7804	n	bl	n	y	0	0	cig+/-ot	20	999	0.1	9	st
MATOS	670	x	m	0	0	all	-	a	SCAmer	1994	CC	200	n	bl	n	n	0	0	cig+/-ot	11	999	1.0	5	st
PEZZOT	590		m	0	0	all	-	a	SCAmer	1987	CC	215	n	bl	n	y	0	0	cig only	11	999	1.0	10	st
SOBUE	748		m	0	0	all	-	a	As:Jap	1986	CC	1376	n	bl	n	y	0	0	cig+/-ot	10	999	1.0	4	st
SVENSS	574		f	0	0	all	-	a	Eu:Sca	1983	CC	210	n	bl	n	n	0	0	all/unsp	11	999	3	10	st
WYNDE3	534		m	0	0	all	-	KII	NAmer	1966	CC	350	n	bl	n	y	0	0	all/unsp	13	999	1.0	3	st
WYNDE6	816	x	m	0	0	all	-	KII	NAmer	1969	CC	4423	n	bl	n	y	0	0	cig+/-ot	16	999	1.0	3	st

Cigarette type is all/unspec for all RRs

Table 3K10 - 5

IESLC - Meta-analysis of Ex Smoking, Years quit (vs current), "Highest vs lowest"
Adenocarcinoma, Cigarettes (or Any Product if Cigarettes not available)
Least adjusted

REF	NRR	SEX	AD	Number Exposed Case	Cont	Non-exposed Case	Cont	RR	95.00%CI
BARBON	755	m	0	4	59	7	20	0.19	(0.05- 0.73)
JAHN	661	m	0	15	146	40	8	0.02	(0.01- 0.05)
JAIN	542	m	0	14	113	16	46	0.36	(0.16- 0.79)
JAIN	506	f	0	3	61	14	36	0.13	(0.03- 0.47)
Subtotal JAIN								0.27	(0.14- 0.53)
LUBIN2	879	m	0	35	1128	77	1047	0.42	(0.28- 0.63)
LUBIN2	975	f	0	1	29	13	95	0.25	(0.03- 2.01)
Subtotal LUBIN2								0.41	(0.28- 0.62)
MATOS	670	m	0	12	101	12	23	0.23	(0.09- 0.57)
PEZZOT	590	m	0	7	31	11	21	0.43	(0.14- 1.29)
SOBUE	748	m	0	49	144	44	116	0.90	(0.56- 1.44)
SVENSS	574	f	0	7	24	5	13	0.76	(0.20- 2.87)
WYNDE3	534	m	0	3	55	3	22	0.40	(0.07- 2.14)
WYNDE6	816	m	0	6	530	29	307	0.12	(0.05- 0.29)
Totals				156	2421	271	1754		

*prospective study

REF	NRR	SEX	AD	Ys	Ws	Qs	Ps
BARBON	755	m	0	-1.64	2.17	0.74	0.0155
JAHN	661	m	0	-3.88	4.47	35.71	0.0000
JAIN	542	m	0	-1.03	6.08	0.00	0.0109
JAIN	506	f	0	-2.07	2.23	2.26	0.0020
Subtotal JAIN				-1.31	8.31	2.27	
LUBIN2	879	m	0	-0.86	23.04	0.89	0.0000
LUBIN2	975	f	0	-1.38	0.89	0.09	0.1932
Subtotal LUBIN2				-0.88	23.93	0.98	
MATOS	670	m	0	-1.48	4.54	0.80	0.0016
PEZZOT	590	m	0	-0.84	3.19	0.15	0.1330
SOBUE	748	m	0	-0.11	17.04	15.41	0.6540
SVENSS	574	f	0	-0.28	2.17	1.33	0.6838
WYNDE3	534	m	0	-0.92	1.37	0.03	0.2836
WYNDE6	816	m	0	-2.12	4.85	5.47	0.0000

	N	12
	NS	10
	Wt	72.04
Het	Chi	62.89
Het	df	11
Het	P	***
Fixed	RR	0.35
	RRl	0.28
	RRu	0.44
	P	---
Random	RR	0.25
	RRl	0.14
	RRu	0.47
	P	---
Asymm	P	N.S.

Table 3K10 - 6

IESLC - Meta-analysis of Ex Smoking, Years quit (vs current), "Highest vs lowest"
Adenocarcinoma, Cigarettes (or Any Product if Cigarettes not available)
Least adjusted

	Sex		
combined	male	female	Total
N	9	3	12
NS	9	3	12
Wt	66.75	5.29	72.04
Het Chi	59.19	3.55	62.89
Het df	8	2	11
Het P	***	N.S.	***
Fixed RR	0.35	0.30	0.35
RRl	0.28	0.13	0.28
RRu	0.45	0.69	0.44
P	---	--	---
Random RR	0.24	0.29	0.25
RRl	0.12	0.09	0.14
RRu	0.50	0.95	0.47
P	---	-	---
Between Chi			0.14
Between df			1
Between P			N.S.
Btwn(F) P			N.S.
Btwn(R) P			N.S.

Table 3K10 - 7

IESLC - Meta-analysis of Ex Smoking, Years quit (vs current), "Highest vs lowest"
Adenocarcinoma, Cigarettes (or Any Product if Cigarettes not available)
Excluded studies (and stage at which they were excluded)

1	AGUDO AKIBA AMANDU AMES AXELSS BEST BOUCHA BOUCOT BRESLO CHEN CHEN2 CHIAZZ DEAN2 DOSEME ENGELA FAN GENG GER GUO HAENSZ HEGMAN HOLE HU HU2 JUSSAW KATSOU KAUFMA KOO KOULUM KREUZE LETOUR LEVIN LIAW LIU3 LIU4 LIU5 MCCONN MIGRAN MRFITR NOTAN2 OSANN2 PERNU QIAO2 RACHTA RESTRE SADOWS SEGI2 STASZE TIZZAN VUTUC WATSON WU WUWILL WYNDE2 WYNDE8 XU YUAN ZHANG ZHENG ZHOU
2	AUVINE BENSHL BLOT1 BROWN3 BUFFLE GURSEL LAUSSM MCDUFF PISANI PRESCO SPITZ WU2 WYNDE7
4	GARSHI JEDRYC LUO WAKAI
5	ARMADA BECHER BOFFET BROSS CARPEN CEDERL CHOI CHYOU CORREA CPSI CPSII DAMBER DARBY DEAN3 DESTEF DOLL DOLL2 DORGAN DORN GAO GAO2 GARCIA GILLIS GRAHAM HAMMO2 HAMMON HIRAYA HUMBLE JOLY KAISE2 KHUDER LUBIN PEZZO2 QIAO SPEIZE SUZUK2 TVERDA WANG2 WIGLE
6	ALDERS
15	BENHAM

Table 3K10 - 8
Potentially overlapping studies

REF	REFGP	PRINC	.	OVERLAP/LINK
LUBIN2	LUBIN2	1		Lubin-combined
WYNDE6	WYNDE6	1		WYNDE5/6/7/8
JAHN	BOFFET	2		Subset of BOFFET

Table 3K10 - 9
Most adjusted - insufficient data for meta-analysis

REF	NRR	SEX	AGEL	AGEH	RACE	YF	LC TYPE	LOC	START	ST	NLC	R	VB	P	H	AD	ADOS	PRODUCT	exL	exH	unexL	unexH	De
ALDERS	577	m	0	0	all	-	not q+s	Eu:UK	1977	CC	1448	n	V	n	n	1	0	cig only	10	999	0.1	2	st
ALDERS	588	f	0	0	all	-	not q+s	Eu:UK	1977	CC	1448	n	V	n	n	1	0	cig only	10	999	0.1	2	st

REF	NRR	RR	SIG	RRDATA comment
ALDERS	577	0.44		0
ALDERS	588	0.26		0

Table 3K11 -

IESLC - Meta-analysis of Ex Smoking by Years quit (vs current), Overview
Adenocarcinoma, Cigarettes only

This analysis is restricted to results for:
1) Ex smokers
2) Results by Years quit (vs current)
3) Categorical results by Years quit (vs current)
Results by Years quit (vs current) are grouped under 2 schemes (S1, S2). Each scheme has a set of "key values". An interval is allocated to the category whose key value it includes, and intervals which include none or more than one of the key values are excluded. (Open-ended intervals are coded as 999)

S1	key value	maximum range
1	3	1-6
2	7	4-11
3	12	8+

S2	key value	maximum range
1	3	1-11
2	12	4-19
3	20	13+

4) Adenocarcinoma (or near equivalent)
5) Results complete enough for use in metaanalysis

Within each study, results are then selected (in the following order of preference, within each sex) for:
6) (not applicable)
7) PRODUCT: cigarettes only
8) CIGTYPE: all/unspecified, MC regardless of HR, MC only
9) Results with least adjustment for other aspects of smoking (ADOS)
10) DENOM: current smokers, current + recent smokers (up to number of m=months or y=years, max 2 years)
11) Followup period (YF, prospective studies): whole study (coded as 0) or longest available
12) LCtype: adeno or nearest available, but not squamous. (q = squamous, s = small, a = adeno, l = large, KII = Kreyberg II, al = alveolar, br = bronchiolar, u = undifferentiated)
13) Race: all or nearest available, otherwise by race (wh or w = white, bl or b = black, hi = hispanic ch = chinese, jap = japanese, haw = hawaiian, w+o = white + oriental, sca = scandinavian, as = asian)
14) For overlapping studies: principal rather than subsidiary studies
Finally by Age: whole study (coded as 0) if available, otherwise by widest available age group and then for single sex results (m, f) in preference to results for both sexes combined (c).

Results adjusted (AD) for the most potential confounders are then chosen in Sections -1 to -3 (and those which actually differ from the adjusted results in Table 3K1 - 1 are marked 'x' in Section -1) and results adjusted for the least confounders in Sections -4 to -6. (Those least adjusted results which actually differ from the most adjusted are marked 'x' in column X in Section -4)

Section -7 shows excluded studies, together with the stage (as above) at which no qualifying results were found.

Section -8 lists the potentially overlapping studies which have been included (1=principal, 2=subsidiary).

Section -9 lists any results which would have been included in preference except that they had data not complete enough for use in meta-analysis, with their significance (yes/no), if known, and any further comment as entered on the database. It also lists as "gap" any categories for which no data were presented by the original authors.

In addition to those mentioned above, the following fields, levels and abbreviations are used:
* or nk = not known, n = no, y = yes, ot = other
nev = never
all/unspec = all or unspecified, MC = manufactured cigarettes, HR = hand-rolled cigarettes
exL, exH = range of exposure (low and high) in the smoking group, in terms of Years quit (vs current)
REF: 6-character study reference
NRR: number of the RR on the database within the study
ST : study type (CC = case control, pr or prosp = prospective)
NLC: number of lung cancer cases in whole study
R : risky occupational population (n = no, m = mining, o = other risky)
VB : national cigarette type (V = at least 75% Virginia, bl = at least 75% blended, ot = other)
P : any proxy use
H : full histological confirmation
De : derivation of RR/CI (or = original, st = standard method, ot = other method of estimation)

Table 3K11 - 1

IESLC - Meta-analysis of Ex Smoking by Years quit (vs current), Overview
Adenocarcinoma, Cigarettes only
Most adjusted

REF	NRR	3K1	SEX	AGEL	AGEH	RACE	YF	LC TYPE	LOC	START	ST	NLC	R	VB	P	H	AD	ADOS	PRODUCT	exL	exH	S1	S2	DENOM	De
BENHAM	556	x	m	0	0	all	-	KII	Eu:wst	1976	CC	1625	n	bl	n	y	0	0	cig only	1.0	3	1	1	cur+1y	st
BENHAM	557	x	m	0	0	all	-	KII	Eu:wst	1976	CC	1625	n	bl	n	y	0	0	cig only	4	10	2	0	cur+1y	st
BENHAM	558	x	m	0	0	all	-	KII	Eu:wst	1976	CC	1625	n	bl	n	y	0	0	cig only	11	999	3	0	cur+1y	st
PEZZOT	588		m	0	0	all	-	a	SCAmer	1987	CC	215	n	bl	n	y	0	0	cig only	1.0	10	0	1	cur+1y	st
PEZZOT	589		m	0	0	all	-	a	SCAmer	1987	CC	215	n	bl	n	y	0	0	cig only	11	999	3	0	cur+1y	st

Cigarette type is all/unspec for all RRs

In this overview table, subtotals and Qs values may be invalid and should be ignored

Table 3K11 - 2

IESLC - Meta-analysis of Ex Smoking by Years quit (vs current), Overview
Adenocarcinoma, Cigarettes only
Most adjusted

REF	NRR	SEX	AD	Number Exposed Case	Cont	Non-exposed Case	Cont	RR	95.00%CI
BENHAM	556	m	0	13	9	85	95	1.61	(0.66- 3.97)
BENHAM	557	m	0	8	18	85	95	0.50	(0.21- 1.20)
BENHAM	558	m	0	4	21	85	95	0.21	(0.07- 0.65)
Subtotal BENHAM								0.63	(0.36- 1.08)
PEZZOT	588	m	0	11	21	42	38	0.47	(0.20- 1.11)
PEZZOT	589	m	0	7	31	42	38	0.20	(0.08- 0.52)
Subtotal PEZZOT								0.32	(0.17- 0.61)
Totals				43	100	339	361		

*prospective study

REF	NRR	SEX	AD	Ys	Ws	Qs	Ps
BENHAM	556	m	0	0.48	4.75	7.23	0.2963
BENHAM	557	m	0	-0.70	4.93	0.01	0.1203
BENHAM	558	m	0	-1.55	3.13	1.96	0.0062
Subtotal BENHAM				-0.47	12.81	9.21	
PEZZOT	588	m	0	-0.75	5.30	0.00	0.0856
PEZZOT	589	m	0	-1.59	4.44	3.08	0.0008
Subtotal PEZZOT				-1.13	9.74	3.09	

N	5
NS	2

Table 3K11 - 3

IESLC - Meta-analysis of Ex Smoking by Years quit (vs current), Overview
Adenocarcinoma, Cigarettes only
Most adjusted

	Sex			
	combined	male	female	Total
N		5		5
NS		2		2

In this overview table, other than the "N" rows, entries in the "absent" and "Total" columns may be invalid and should be ignored

	Years quit vs current (lower focus)				
	absent	1-6k3	4-11k7	8+k12	Total
N	1	1	1	2	5
NS	1	1	1	2	3
Wt	5.30	4.75	4.93	7.57	22.55
Het Chi	0.00	0.00	0.00	0.00	12.30
Het df	0	0	0	1	4
Het P	N.S.	N.S.	N.S.	N.S.	*
Fixed RR	0.47	1.61	0.50	0.21	0.47
RRl	0.20	0.66	0.21	0.10	0.31
RRu	1.11	3.97	1.20	0.42	0.71
P	(-)	N.S.	N.S.	---	---
Random RR	0.47	1.61	0.50	0.21	0.45
RRl	0.20	0.66	0.21	0.10	0.22
RRu	1.11	3.97	1.20	0.42	0.93
P	(-)	N.S.	N.S.	---	-

	Years quit vs current (higher focus)				
	absent	1-11k3	4-19k12	13+k20	Total
N	3	2			5
NS	2	2			4
Wt	12.50	10.06			22.55
Het Chi	2.27	3.77			12.30
Het df	2	1			4
Het P	N.S.	(*)			*
Fixed RR	0.29	0.85			0.47
RRl	0.17	0.46			0.31
RRu	0.51	1.57			0.71
P	---	N.S.			---
Random RR	0.29	0.87			0.45
RRl	0.16	0.26			0.22
RRu	0.53	2.88			0.93
P	---	N.S.			-

Table 3K11 - 3

IESLC - Meta-analysis of Ex Smoking by Years quit (vs current), Overview
Adenocarcinoma, Cigarettes only
Most adjusted

MALES

	Years quit vs current (lower focus) absent	1-6k3	4-11k7	8+k12	Total
N	1	1	1	2	5
NS	1	1	1	2	3
Wt	5.30	4.75	4.93	7.57	22.55
Het Chi	0.00	0.00	0.00	0.00	12.30
Het df	0	0	0	1	4
Het P	N.S.	N.S.	N.S.	N.S.	*
Fixed RR	0.47	1.61	0.50	0.21	0.47
RRl	0.20	0.66	0.21	0.10	0.31
RRu	1.11	3.97	1.20	0.42	0.71
P	(-)	N.S.	N.S.	---	---
Random RR	0.47	1.61	0.50	0.21	0.45
RRl	0.20	0.66	0.21	0.10	0.22
RRu	1.11	3.97	1.20	0.42	0.93
P	(-)	N.S.	N.S.	---	-

	Years quit vs current (higher focus) absent	1-11k3	4-19k12	13+k20	Total
N	3	2			5
NS	2	2			4
Wt	12.50	10.06			22.55
Het Chi	2.27	3.77			12.30
Het df	2	1			4
Het P	N.S.	(*)			*
Fixed RR	0.29	0.85			0.47
RRl	0.17	0.46			0.31
RRu	0.51	1.57			0.71
P	---	N.S.			---
Random RR	0.29	0.87			0.45
RRl	0.16	0.26			0.22
RRu	0.53	2.88			0.93
P	---	N.S.			-

Table 3K11 - 4

IESLC - Meta-analysis of Ex Smoking by Years quit (vs current), Overview
Adenocarcinoma, Cigarettes only
Least adjusted

REF	NRR	X	SEX	AGEL	AGEH	RACE	YF	LC TYPE	LOC	START	ST	NLC	R	VB	P	H	AD	ADOS	PRODUCT	exL	exH	S1	S2	DENOM	De
BENHAM	556		m	0	0	all	-	KII	Eu:wst	1976	CC	1625	n	bl	n	y	0	0	cig only	1.0	3	1	1	cur+1y	st
BENHAM	557		m	0	0	all	-	KII	Eu:wst	1976	CC	1625	n	bl	n	y	0	0	cig only	4	10	2	0	cur+1y	st
BENHAM	558		m	0	0	all	-	KII	Eu:wst	1976	CC	1625	n	bl	n	y	0	0	cig only	11	999	3	0	cur+1y	st
PEZZOT	588		m	0	0	all	-	a	SCAmer	1987	CC	215	n	bl	n	y	0	0	cig only	1.0	10	0	1	cur+1y	st
PEZZOT	589		m	0	0	all	-	a	SCAmer	1987	CC	215	n	bl	n	y	0	0	cig only	11	999	3	0	cur+1y	st

Cigarette type is all/unspec for all RRs

In this overview table, subtotals and Qs values may be invalid and should be ignored

Table 3K11 - 5

IESLC - Meta-analysis of Ex Smoking by Years quit (vs current), Overview
Adenocarcinoma, Cigarettes only
Least adjusted

REF	NRR	SEX	AD	Number Exposed Case	Number Exposed Cont	Non-exposed Case	Non-exposed Cont	RR	95.00%CI
BENHAM	556	m	0	13	9	85	95	1.61	(0.66- 3.97)
BENHAM	557	m	0	8	18	85	95	0.50	(0.21- 1.20)
BENHAM	558	m	0	4	21	85	95	0.21	(0.07- 0.65)
Subtotal BENHAM								0.63	(0.36- 1.08)
PEZZOT	588	m	0	11	21	42	38	0.47	(0.20- 1.11)
PEZZOT	589	m	0	7	31	42	38	0.20	(0.08- 0.52)
Subtotal PEZZOT								0.32	(0.17- 0.61)
Totals				43	100	339	361		

*prospective study

REF	NRR	SEX	AD	Ys	Ws	Qs	Ps
BENHAM	556	m	0	0.48	4.75	7.23	0.2963
BENHAM	557	m	0	-0.70	4.93	0.01	0.1203
BENHAM	558	m	0	-1.55	3.13	1.96	0.0062
Subtotal BENHAM				-0.47	12.81	9.21	
PEZZOT	588	m	0	-0.75	5.30	0.00	0.0856
PEZZOT	589	m	0	-1.59	4.44	3.08	0.0008
Subtotal PEZZOT				-1.13	9.74	3.09	

N	5
NS	2

Table 3K11 - 6

IESLC - Meta-analysis of Ex Smoking by Years quit (vs current), Overview
Adenocarcinoma, Cigarettes only
Least adjusted

	Sex: combined	male	female	Total
N		5		5
NS		2		2

In this overview table, other than the "N" rows, entries in the "absent" and "Total" columns may be invalid and should be ignored

Years quit vs current (lower focus)	absent	1-6k3	4-11k7	8+k12	Total
N	1	1	1	2	5
NS	1	1	1	2	3
Wt	5.30	4.75	4.93	7.57	22.55
Het Chi	0.00	0.00	0.00	0.00	12.30
Het df	0	0	0	1	4
Het P	N.S.	N.S.	N.S.	N.S.	*
Fixed RR	0.47	1.61	0.50	0.21	0.47
RRl	0.20	0.66	0.21	0.10	0.31
RRu	1.11	3.97	1.20	0.42	0.71
P	(-)	N.S.	N.S.	---	---
Random RR	0.47	1.61	0.50	0.21	0.45
RRl	0.20	0.66	0.21	0.10	0.22
RRu	1.11	3.97	1.20	0.42	0.93
P	(-)	N.S.	N.S.	---	-

Years quit vs current (higher focus)	absent	1-11k3	4-19k12	13+k20	Total
N	3	2			5
NS	2	2			4
Wt	12.50	10.06			22.55
Het Chi	2.27	3.77			12.30
Het df	2	1			4
Het P	N.S.	(*)			*
Fixed RR	0.29	0.85			0.47
RRl	0.17	0.46			0.31
RRu	0.51	1.57			0.71
P	---	N.S.			---
Random RR	0.29	0.87			0.45
RRl	0.16	0.26			0.22
RRu	0.53	2.88			0.93
P	---	N.S.			-

Table 3K11 - 6

IESLC - Meta-analysis of Ex Smoking by Years quit (vs current), Overview
Adenocarcinoma, Cigarettes only
Least adjusted

MALES

		Years quit vs current (lower focus)				
		absent	1-6k3	4-11k7	8+k12	Total
	N	1	1	1	2	5
	NS	1	1	1	2	3
	Wt	5.30	4.75	4.93	7.57	22.55
Het	Chi	0.00	0.00	0.00	0.00	12.30
Het	df	0	0	0	1	4
Het	P	N.S.	N.S.	N.S.	N.S.	*
Fixed	RR	0.47	1.61	0.50	0.21	0.47
	RRl	0.20	0.66	0.21	0.10	0.31
	RRu	1.11	3.97	1.20	0.42	0.71
	P	(-)	N.S.	N.S.	---	---
Random	RR	0.47	1.61	0.50	0.21	0.45
	RRl	0.20	0.66	0.21	0.10	0.22
	RRu	1.11	3.97	1.20	0.42	0.93
	P	(-)	N.S.	N.S.	---	-

		Years quit vs current (higher focus)				
		absent	1-11k3	4-19k12	13+k20	Total
	N	3	2			5
	NS	2	2			4
	Wt	12.50	10.06			22.55
Het	Chi	2.27	3.77			12.30
Het	df	2	1			4
Het	P	N.S.	(*)			*
Fixed	RR	0.29	0.85			0.47
	RRl	0.17	0.46			0.31
	RRu	0.51	1.57			0.71
	P	---	N.S.			---
Random	RR	0.29	0.87			0.45
	RRl	0.16	0.26			0.22
	RRu	0.53	2.88			0.93
	P	---	N.S.			-

Table 3K11 - 7

IESLC - Meta-analysis of Ex Smoking by Years quit (vs current), Overview
Adenocarcinoma, Cigarettes only
Excluded studies (and stage at which they were excluded)

1	AGUDO AKIBA AMANDU AMES AXELSS BEST BOUCHA BOUCOT BRESLO CHEN CHEN2 CHIAZZ DEAN2 DOSEME ENGELA FAN GENG GER GUO HAENSZ HEGMAN HOLE HU HU2 JUSSAW KATSOU KAUFMA KOO KOULUM KREUZE LETOUR LEVIN LIAW LIU3 LIU4 LIU5 MCCONN MIGRAN MRFITR NOTAN2 OSANN2 PERNU QIAO2 RACHTA RESTRE SADOWS SEGI2 STASZE TIZZAN VUTUC WATSON WU WUWILL WYNDE2 WYNDE8 XU YUAN ZHANG ZHENG ZHOU
2	AUVINE BENSHL BLOT1 BROWN3 BUFFLE GURSEL LAUSSM MCDUFF PISANI PRESCO SPITZ WU2 WYNDE7
4	ARMADA BECHER BOFFET BROSS CARPEN CEDERL CHOI CHYOU CORREA CPSI CPSII DAMBER DARBY DEAN3 DESTEF DOLL DOLL2 DORGAN DORN GAO GAO2 GARCIA GARSHI GILLIS GRAHAM HAMMO2 HAMMON HIRAYA HUMBLE JOLY KAISE2 KHUDER LUBIN LUO PEZZO2 QIAO SPEIZE SUZUK2 TVERDA WANG2 WIGLE
5	ALDERS
7	BARBON JAHN JAIN JEDRYC LUBIN2 MATOS SOBUE SVENSS WAKAI WYNDE3 WYNDE6

Table 3K11 - 8

Potentially overlapping studies

REF	REFGP	PRINC	.	OVERLAP/LINK
BENHAM	LUBIN2	2		Subset of Lubin2

Table 3K11 - 9

Most adjusted - insufficient data for meta-analysis

REF	NRR	SEX	AGEL	AGEH	RACE	YF	LC TYPE	LOC	START	ST	NLC	R	VB	P	H	AD	ADOS	PRODUCT	exL	exH	S1	S2	DENOM	De
ALDERS	573	m	0	0	all	-	not q+s	Eu:UK	1977	CC	1448	n	V	n	n	1	0	cig only	0.1	2	0	0	current	ot
ALDERS	574	m	0	0	all	-	not q+s	Eu:UK	1977	CC	1448	n	V	n	n	1	0	cig only	3	9	0	1	current	ot
ALDERS	575	m	0	0	all	-	not q+s	Eu:UK	1977	CC	1448	n	V	n	n	1	0	cig only	10	999	3	0	current	ot
ALDERS	584	f	0	0	all	-	not q+s	Eu:UK	1977	CC	1448	n	V	n	n	1	0	cig only	0.1	2	0	0	current	ot
ALDERS	585	f	0	0	all	-	not q+s	Eu:UK	1977	CC	1448	n	V	n	n	1	0	cig only	3	9	0	1	current	ot
ALDERS	586	f	0	0	all	-	not q+s	Eu:UK	1977	CC	1448	n	V	n	n	1	0	cig only	10	999	3	0	current	ot

REF	NRR	RR	SIG	RRDATA comment
ALDERS	573	2.07	n	0
ALDERS	574	1.56	n	0
ALDERS	575	0.91	n	0
ALDERS	584	1.38	n	0
ALDERS	585	0.16	y	0.001<p<0.01
ALDERS	586	0.36	n	0

Table 3K12 -

IESLC - Meta-analysis of Ex Smoking, Years quit (vs current), "Low"
Adenocarcinoma, Cigarettes only

This analysis is restricted to results for:
1) Ex smokers
2) Results by Years quit (vs current)
3) Categorical results by Years quit (vs current)
4) Adenocarcinoma (or near equivalent)
5) Results complete enough for use in metaanalysis

Within each study, results are then selected (in the following order of preference, within each sex) for:
6) (not applicable)
7) PRODUCT: cigarettes only
8) CIGTYPE: all/unspecified, MC regardless of HR, MC only
9) Results with least adjustment for other aspects of smoking (ADOS)
10) DENOM: current smokers, current + recent smokers (up to number of m=months or y=years, max 2 years)
11) Followup period (YF, prospective studies): whole study (coded as 0) or longest available
12) LCtype: adeno or nearest available, but not squamous. (q = squamous, s = small,
a = adeno, l = large, KII = Kreyberg II, al = alveolar, br = bronchiolar, u = undifferentiated)
13) Race: all or nearest available, otherwise by race (wh or w = white, bl or b = black, hi = hispanic
ch = chinese, jap = japanese, haw = hawaiian, w+o = white + oriental, sca = scandinavian, as = asian)
14) Years quit (vs current) "low" in key scheme 1 (key value 3, maximum range 1-6)
15) For overlapping studies: principal rather than subsidiary studies
Finally by Age: whole study (coded as 0) if available, otherwise by widest available age group
and then for single sex results (m, f) in preference to results for both sexes combined (c).

Results adjusted (AD) for the most potential confounders are then chosen in Sections -1 to -3
(and those which actually differ from the adjusted results in Table 3K2 - 1 are marked 'x' in Section -1)
and results adjusted for the least confounders in Sections -4 to -6. (Those least adjusted results which
actually differ from the most adjusted are marked 'x' in column X in Section -4)

Section -7 shows excluded studies, together with the stage (as above) at which no qualifying
results were found.

Section -8 lists the potentially overlapping studies which have been included (1=principal, 2=subsidiary).

Section -9 lists any results which would have been included in preference except that they had data not complete
enough for use in meta-analysis, with their significance (yes/no), if known, and any further comment as entered
on the database. It also lists as "gap" any categories for which no data were presented by the original authors.

In addition to those mentioned above, the following fields, levels and abbreviations are used:
* or nk = not known, n = no, y = yes, ot = other
nev = never
all/unspec = all or unspecified, MC = manufactured cigarettes, HR = hand-rolled cigarettes
exL, exH = range of exposure (low and high) in the smoking group, in terms of Years quit (vs current)
REF: 6-character study reference
NRR: number of the RR on the database within the study
ST : study type (CC = case control, pr or prosp = prospective)
NLC: number of lung cancer cases in whole study
R : risky occupational population (n = no, m = mining, o = other risky)
VB : national cigarette type (V = at least 75% Virginia, bl = at least 75% blended, ot = other)
P : any proxy use
H : full histological confirmation
De : derivation of RR/CI (or = original, st = standard method, ot = other method of estimation)

Table 3K12 - 1

IESLC - Meta-analysis of Ex Smoking, Years quit (vs current), "Low"
Adenocarcinoma, Cigarettes only
Most adjusted

REF	NRR	3K2	SEX	AGEL	AGEH	RACE	YF	LC TYPE	LOC	START	ST	NLC	R	VB	P	H	AD	ADOS	PRODUCT	exL	exH	DENOM	De
BENHAM	556	x	m	0	0	all	-	KII	Eu:wst	1976	CC	1625	n	bl	n	y	0	0	cig only	1.0	3	cur+1y	st

Cigarette type is all/unspec for all RRs

Table 3K12 - 2

IESLC - Meta-analysis of Ex Smoking, Years quit (vs current), "Low"
Adenocarcinoma, Cigarettes only
Most adjusted

REF	NRR	SEX	AD	Number Exposed Case	Cont	Non-exposed Case	Cont	RR	95.00%CI
BENHAM	556	m	0	13	9	85	95	1.61	(0.66- 3.97)
Totals				13	9	85	95		

*prospective study

REF	NRR	SEX	AD	Ys	Ws	Qs	Ps
BENHAM	556	m	0	0.48	4.75	0.00	0.2963

	N	1
	NS	1
	Wt	4.75
Het	Chi	0.00
Het	df	0
Het	P	N.S.
Fixed	RR	1.61
	RRl	0.66
	RRu	3.97
	P	N.S.
Random	RR	1.61
	RRl	0.66
	RRu	3.97
	P	N.S.
Asymm	P	

Table 3K12 - 3

IESLC - Meta-analysis of Ex Smoking, Years quit (vs current), "Low"
Adenocarcinoma, Cigarettes only
Most adjusted

	Sex: combined	male	female	Total
N		1		1
NS		1		1
Wt		4.75		4.75
Het Chi		0.00		0.00
Het df		0		0
Het P		N.S.		N.S.
Fixed RR		1.61		1.61
RRl		0.66		0.66
RRu		3.97		3.97
P		N.S.		N.S.
Random RR		1.61		1.61
RRl		0.66		0.66
RRu		3.97		3.97
P		N.S.		N.S.
Between Chi				
Between df				
Between P				N.S.
Btwn(F) P				N.S.
Btwn(R) P				N.S.

Too few RRs for analysis by factor

Table 3K12 - 4

IESLC - Meta-analysis of Ex Smoking, Years quit (vs current), "Low"
Adenocarcinoma, Cigarettes only
Least adjusted

REF	NRR	X	SEX	AGEL	AGEH	RACE	YF	LC TYPE	LOC	START	ST	NLC	R	VB	P	H	AD	ADOS	PRODUCT	exL	exH	DENOM	De
BENHAM	556		m	0	0	all	-	KII	Eu:wst	1976	CC	1625	n	bl	n	y	0	0	cig only	1.0	3	cur+1y	st

Cigarette type is all/unspec for all RRs

Table 3K12 - 5

IESLC - Meta-analysis of Ex Smoking, Years quit (vs current), "Low"
Adenocarcinoma, Cigarettes only
Least adjusted

REF	NRR	SEX	AD	Number Exposed Case	Exposed Cont	Non-exposed Case	Non-exposed Cont	RR	95.00%CI
BENHAM	556	m	0	13	9	85	95	1.61	(0.66- 3.97)
Totals				13	9	85	95		

*prospective study

REF	NRR	SEX	AD	Ys	Ws	Qs	Ps
BENHAM	556	m	0	0.48	4.75	0.00	0.2963

	N	1
	NS	1
	Wt	4.75
Het	Chi	0.00
Het	df	0
Het	P	N.S.
Fixed	RR	1.61
	RRl	0.66
	RRu	3.97
	P	N.S.
Random	RR	1.61
	RRl	0.66
	RRu	3.97
	P	N.S.
Asymm	P	

Table 3K12 - 6

IESLC - Meta-analysis of Ex Smoking, Years quit (vs current), "Low"
Adenocarcinoma, Cigarettes only
Least adjusted

	Sex combined	male	female	Total
N		1		1
NS		1		1
Wt		4.75		4.75
Het Chi		0.00		0.00
Het df		0		0
Het P		N.S.		N.S.
Fixed RR		1.61		1.61
RRl		0.66		0.66
RRu		3.97		3.97
P		N.S.		N.S.
Random RR		1.61		1.61
RRl		0.66		0.66
RRu		3.97		3.97
P		N.S.		N.S.
Between Chi				
Between df				
Between P				N.S.
Btwn(F) P				N.S.
Btwn(R) P				N.S.

Table 3K12 - 7

IESLC - Meta-analysis of Ex Smoking, Years quit (vs current), "Low"
Adenocarcinoma, Cigarettes only
Excluded studies (and stage at which they were excluded)

1	AGUDO AKIBA AMANDU AMES AXELSS BEST BOUCHA BOUCOT BRESLO CHEN CHEN2 CHIAZZ DEAN2 DOSEME ENGELA FAN GENG GER GUO HAENSZ HEGMAN HOLE HU HU2 JUSSAW KATSOU KAUFMA KOO KOULUM KREUZE LETOUR LEVIN LIAW LIU3 LIU4 LIU5 MCCONN MIGRAN MRFITR NOTAN2 OSANN2 PERNU QIAO2 RACHTA RESTRE SADOWS SEGI2 STASZE TIZZAN VUTUC WATSON WU WUWILL WYNDE2 WYNDE8 XU YUAN ZHANG ZHENG ZHOU
2	AUVINE BENSHL BLOT1 BROWN3 BUFFLE GURSEL LAUSSM MCDUFF PISANI PRESCO SPITZ WU2 WYNDE7
4	ARMADA BECHER BOFFET BROSS CARPEN CEDERL CHOI CHYOU CORREA CPSI CPSII DAMBER DARBY DEAN3 DESTEF DOLL DOLL2 DORGAN DORN GAO GAO2 GARCIA GARSHI GILLIS GRAHAM HAMMO2 HAMMON HIRAYA HUMBLE JOLY KAISE2 KHUDER LUBIN LUO PEZZO2 QIAO SPEIZE SUZUK2 TVERDA WANG2 WIGLE
5	ALDERS
7	BARBON JAHN JAIN JEDRYC LUBIN2 MATOS SOBUE SVENSS WAKAI WYNDE3 WYNDE6
14	PEZZOT

Table 3K12 - 8

Potentially overlapping studies

REF	REFGP	PRINC	.	OVERLAP/LINK
BENHAM	LUBIN2	2		Subset of Lubin2

Table 3K13 -

IESLC - Meta-analysis of Ex Smoking, Years quit (vs current), "Mid"
Adenocarcinoma, Cigarettes only

This analysis is restricted to results for:
1) Ex smokers
2) Results by Years quit (vs current)
3) Categorical results by Years quit (vs current)
4) Adenocarcinoma (or near equivalent)
5) Results complete enough for use in metaanalysis

Within each study, results are then selected (in the following order of preference, within each sex) for:
6) (not applicable)
7) PRODUCT: cigarettes only
8) CIGTYPE: all/unspecified, MC regardless of HR, MC only
9) Results with least adjustment for other aspects of smoking (ADOS)
10) DENOM: current smokers, current + recent smokers (up to number of m=months or y=years, max 2 years)
11) Followup period (YF, prospective studies): whole study (coded as 0) or longest available
12) LCtype: adeno or nearest available, but not squamous. (q = squamous, s = small,
a = adeno, l = large, KII = Kreyberg II, al = alveolar, br = bronchiolar, u = undifferentiated)
13) Race: all or nearest available, otherwise by race (wh or w = white, bl or b = black, hi = hispanic
ch = chinese, jap = japanese, haw = hawaiian, w+o = white + oriental, sca = scandinavian, as = asian)
14) Years quit (vs current) "mid" in key scheme 1 (key value 7, maximum range 4-11)
15) For overlapping studies: principal rather than subsidiary studies
Finally by Age: whole study (coded as 0) if available, otherwise by widest available age group
and then for single sex results (m, f) in preference to results for both sexes combined (c).

Results adjusted (AD) for the most potential confounders are then chosen in Sections -1 to -3
(and those which actually differ from the adjusted results in Table 3K3 - 1 are marked 'x' in Section -1)
and results adjusted for the least confounders in Sections -4 to -6. (Those least adjusted results which
actually differ from the most adjusted are marked 'x' in column X in Section -4)

Section -7 shows excluded studies, together with the stage (as above) at which no qualifying
results were found.

Section -8 lists the potentially overlapping studies which have been included (1=principal, 2=subsidiary).

Section -9 lists any results which would have been included in preference except that they had data not complete
enough for use in meta-analysis, with their significance (yes/no), if known, and any further comment as entered
on the database. It also lists as "gap" any categories for which no data were presented by the original authors.

In addition to those mentioned above, the following fields, levels and abbreviations are used:
* or nk = not known, n = no, y = yes, ot = other
nev = never
all/unspec = all or unspecified, MC = manufactured cigarettes, HR = hand-rolled cigarettes
exL, exH = range of exposure (low and high) in the smoking group, in terms of Years quit (vs current)
REF: 6-character study reference
NRR: number of the RR on the database within the study
ST : study type (CC = case control, pr or prosp = prospective)
NLC: number of lung cancer cases in whole study
R : risky occupational population (n = no, m = mining, o = other risky)
VB : national cigarette type (V = at least 75% Virginia, bl = at least 75% blended, ot = other)
P : any proxy use
H : full histological confirmation
De : derivation of RR/CI (or = original, st = standard method, ot = other method of estimation)

Table 3K13 - 1

IESLC - Meta-analysis of Ex Smoking, Years quit (vs current), "Mid"
Adenocarcinoma, Cigarettes only
Most adjusted

REF	NRR	3K3	SEX	AGEL	AGEH	RACE	YF	LC TYPE	LOC	START	ST	NLC	R	VB	P	H	AD	ADOS	PRODUCT	exL	exH	DENOM	De
BENHAM	557	x	m	0	0	all	-	KII	Eu:wst	1976	CC	1625	n	bl	n	y	0	0	cig only	4	10	cur+1y	st

Cigarette type is all/unspec for all RRs

Table 3K13 - 2

IESLC - Meta-analysis of Ex Smoking, Years quit (vs current), "Mid"
Adenocarcinoma, Cigarettes only
Most adjusted

REF	NRR	SEX	AD	Number Exposed Case	Number Exposed Cont	Non-exposed Case	Non-exposed Cont	RR	95.00%CI
BENHAM	557	m	0	8	18	85	95	0.50	(0.21- 1.20)
Totals				8	18	85	95		

*prospective study

REF	NRR	SEX	AD	Ys	Ws	Qs	Ps
BENHAM	557	m	0	-0.70	4.93	0.00	0.1203

	N	1
	NS	1
	Wt	4.93
Het	Chi	0.00
Het	df	0
Het	P	N.S.
Fixed	RR	0.50
	RRl	0.21
	RRu	1.20
	P	N.S.
Random	RR	0.50
	RRl	0.21
	RRu	1.20
	P	N.S.
Asymm	P	

Table 3K13 - 3

IESLC - Meta-analysis of Ex Smoking, Years quit (vs current), "Mid"
Adenocarcinoma, Cigarettes only
Most adjusted

	Sex: combined	male	female	Total
N		1		1
NS		1		1
Wt		4.93		4.93
Het Chi		0.00		0.00
Het df		0		0
Het P		N.S.		N.S.
Fixed RR		0.50		0.50
RRl		0.21		0.21
RRu		1.20		1.20
P		N.S.		N.S.
Random RR		0.50		0.50
RRl		0.21		0.21
RRu		1.20		1.20
P		N.S.		N.S.
Between Chi				
Between df				
Between P				N.S.
Btwn(F) P				N.S.
Btwn(R) P				N.S.

Too few RRs for analysis by factor

Table 3K13 - 4

IESLC - Meta-analysis of Ex Smoking, Years quit (vs current), "Mid"
Adenocarcinoma, Cigarettes only
Least adjusted

REF	NRR	X	SEX	AGEL	AGEH	RACE	YF	LC TYPE	LOC	START	ST	NLC	R	VB	P	H	AD	ADOS	PRODUCT	exL	exH	DENOM	De
BENHAM	557		m	0	0	all	-		KII	Eu:wst	1976	CC	1625	n	bl	n	y	0	0	cig only	4	10	cur+1y	st

Cigarette type is all/unspec for all RRs

Table 3K13 - 5

IESLC - Meta-analysis of Ex Smoking, Years quit (vs current), "Mid"
Adenocarcinoma, Cigarettes only
Least adjusted

REF	NRR	SEX	AD	Number Exposed Case	Exposed Cont	Non-exposed Case	Non-exposed Cont	RR	95.00%CI
BENHAM	557	m	0	8	18	85	95	0.50	(0.21- 1.20)
Totals				8	18	85	95		

*prospective study

REF	NRR	SEX	AD	Ys	Ws	Qs	Ps
BENHAM	557	m	0	-0.70	4.93	0.00	0.1203

	N	1
	NS	1
	Wt	4.93
Het	Chi	0.00
Het	df	0
Het	P	N.S.
Fixed	RR	0.50
	RRl	0.21
	RRu	1.20
	P	N.S.
Random	RR	0.50
	RRl	0.21
	RRu	1.20
	P	N.S.
Asymm	P	

Table 3K13 - 6

IESLC - Meta-analysis of Ex Smoking, Years quit (vs current), "Mid"
Adenocarcinoma, Cigarettes only
Least adjusted

	Sex combined	male	female	Total
N		1		1
NS		1		1
Wt		4.93		4.93
Het Chi		0.00		0.00
Het df		0		0
Het P		N.S.		N.S.
Fixed RR		0.50		0.50
RRl		0.21		0.21
RRu		1.20		1.20
P		N.S.		N.S.
Random RR		0.50		0.50
RRl		0.21		0.21
RRu		1.20		1.20
P		N.S.		N.S.
Between Chi				
Between df				
Between P				N.S.
Btwn(F) P				N.S.
Btwn(R) P				N.S.

Table 3K13 - 7

IESLC - Meta-analysis of Ex Smoking, Years quit (vs current), "Mid"
Adenocarcinoma, Cigarettes only
Excluded studies (and stage at which they were excluded)

1	AGUDO AKIBA AMANDU AMES AXELSS BEST BOUCHA BOUCOT BRESLO CHEN CHEN2 CHIAZZ DEAN2 DOSEME ENGELA FAN GENG GER GUO HAENSZ HEGMAN HOLE HU HU2 JUSSAW KATSOU KAUFMA KOO KOULUM KREUZE LETOUR LEVIN LIAW LIU3 LIU4 LIU5 MCCONN MIGRAN MRFITR NOTAN2 OSANN2 PERNU QIAO2 RACHTA RESTRE SADOWS SEGI2 STASZE TIZZAN VUTUC WATSON WU WUWILL WYNDE2 WYNDE8 XU YUAN ZHANG ZHENG ZHOU
2	AUVINE BENSHL BLOT1 BROWN3 BUFFLE GURSEL LAUSSM MCDUFF PISANI PRESCO SPITZ WU2 WYNDE7
4	ARMADA BECHER BOFFET BROSS CARPEN CEDERL CHOI CHYOU CORREA CPSI CPSII DAMBER DARBY DEAN3 DESTEF DOLL DOLL2 DORGAN DORN GAO GAO2 GARCIA GARSHI GILLIS GRAHAM HAMMO2 HAMMON HIRAYA HUMBLE JOLY KAISE2 KHUDER LUBIN LUO PEZZO2 QIAO SPEIZE SUZUK2 TVERDA WANG2 WIGLE
5	ALDERS
7	BARBON JAHN JAIN JEDRYC LUBIN2 MATOS SOBUE SVENSS WAKAI WYNDE3 WYNDE6
14	PEZZOT

Table 3K13 - 8
Potentially overlapping studies

REF	REFGP	PRINC.	OVERLAP/LINK
BENHAM	LUBIN2	2	Subset of Lubin2

Table 3K14 -

IESLC - Meta-analysis of Ex Smoking, Years quit (vs current), "High"
Adenocarcinoma, Cigarettes only

This analysis is restricted to results for:
1) Ex smokers
2) Results by Years quit (vs current)
3) Categorical results by Years quit (vs current)
4) Adenocarcinoma (or near equivalent)
5) Results complete enough for use in metaanalysis

Within each study, results are then selected (in the following order of preference, within each sex) for:
6) PRODUCT: cigarettes only
7) CIGTYPE: all/unspecified, MC regardless of HR, MC only
8) Results with least adjustment for other aspects of smoking (ADOS)
9) DENOM: current smokers, current + recent smokers (up to number of m=months or y=years, max 2 years)
10) Followup period (YF, prospective studies): whole study (coded as 0) or longest available
11) LCtype: adeno or nearest available, but not squamous. (q = squamous, s = small,
a = adeno, l = large, KII = Kreyberg II, al = alveolar, br = bronchiolar, u = undifferentiated)
12) Race: all or nearest available, otherwise by race (wh or w = white, bl or b = black, hi = hispanic
ch = chinese, jap = japanese, haw = hawaiian, w+o = white + oriental, sca = scandinavian, as = asian)
13) Years quit (vs current) "high" in key scheme 1 (key value 12, maximum range 8+)
14) For overlapping studies: principal rather than subsidiary studies
Finally by Age: whole study (coded as 0) if available, otherwise by widest available age group
and then for single sex results (m, f) in preference to results for both sexes combined (c).

Results adjusted (AD) for the most potential confounders are then chosen in Sections -1 to -3
(and those which actually differ from the adjusted results in Table 3K4 - 1 are marked 'x' in Section -1)
and results adjusted for the least confounders in Sections -4 to -6. (Those least adjusted results which
actually differ from the most adjusted are marked 'x' in column X in Section -4)

Section -7 shows excluded studies, together with the stage (as above) at which no qualifying
results were found.

Section -8 lists the potentially overlapping studies which have been included (1=principal, 2=subsidiary).

Section -9 lists any results which would have been included in preference except that they had data not complete
enough for use in meta-analysis, with their significance (yes/no), if known, and any further comment as entered
on the database. It also lists as "gap" any categories for which no data were presented by the original authors.

In addition to those mentioned above, the following fields, levels and abbreviations are used:
* or nk = not known, n = no, y = yes, ot = other
nev = never
all/unspec = all or unspecified, MC = manufactured cigarettes, HR = hand-rolled cigarettes
exL, exH = range of exposure (low and high) in the smoking group, in terms of Years quit (vs current)
REF: 6-character study reference
NRR: number of the RR on the database within the study
ST : study type (CC = case control, pr or prosp = prospective)
NLC: number of lung cancer cases in whole study
R : risky occupational population (n = no, m = mining, o = other risky)
VB : national cigarette type (V = at least 75% Virginia, bl = at least 75% blended, ot = other)
P : any proxy use
H : full histological confirmation
De : derivation of RR/CI (or = original, st = standard method, ot = other method of estimation)

Table 3K14 - 1

IESLC - Meta-analysis of Ex Smoking, Years quit (vs current), "High"
Adenocarcinoma, Cigarettes only
Most adjusted

REF	NRR	3K4	SEX	AGEL	AGEH	RACE	YF	LC TYPE	LOC	START	ST	NLC	R	VB	P	H	AD	ADOS	PRODUCT	exL	exH	DENOM	De
BENHAM	558	x	m	0	0	all	-	KII	Eu:wst	1976	CC	1625	n	bl	n	y	0	0	cig only	11	999	cur+1y	st
PEZZOT	589		m	0	0	all	-	a	SCAmer	1987	CC	215	n	bl	n	y	0	0	cig only	11	999	cur+1y	st

Cigarette type is all/unspec for all RRs

Table 3K14 - 2

IESLC - Meta-analysis of Ex Smoking, Years quit (vs current), "High"
Adenocarcinoma, Cigarettes only
Most adjusted

REF	NRR	SEX	AD	Number Exposed Case	Cont	Non-exposed Case	Cont	RR	95.00%CI
BENHAM	558	m	0	4	21	85	95	0.21	(0.07- 0.65)
PEZZOT	589	m	0	7	31	42	38	0.20	(0.08- 0.52)
Totals				11	52	127	133		

*prospective study

REF	NRR	SEX	AD	Ys	Ws	Qs	Ps
BENHAM	558	m	0	-1.55	3.13	0.00	0.0062
PEZZOT	589	m	0	-1.59	4.44	0.00	0.0008

	N	2
	NS	2
	Wt	7.57
Het	Chi	0.00
Het	df	1
Het	P	N.S.
Fixed	RR	0.21
	RRl	0.10
	RRu	0.42
	P	---
Random	RR	0.21
	RRl	0.10
	RRu	0.42
	P	---
Asymm	P	

Table 3K14 - 3

IESLC - Meta-analysis of Ex Smoking, Years quit (vs current), "High"
Adenocarcinoma, Cigarettes only
Most adjusted

	Sex: combined	male	female	Total
N		2		2
NS		2		2
Wt		7.57		7.57
Het Chi		0.00		0.00
Het df		1		1
Het P		N.S.		N.S.
Fixed RR		0.21		0.21
RRl		0.10		0.10
RRu		0.42		0.42
P		---		---
Random RR		0.21		0.21
RRl		0.10		0.10
RRu		0.42		0.42
P		---		---
Between Chi				
Between df				
Between P				N.S.
Btwn(F) P				N.S.
Btwn(R) P				N.S.

Too few RRs for analysis by factor

Table 3K14 - 4

IESLC - Meta-analysis of Ex Smoking, Years quit (vs current), "High"
Adenocarcinoma, Cigarettes only
Least adjusted

REF	NRR	X	SEX	AGEL	AGEH	RACE	YF	LC TYPE	LOC	START	ST	NLC	R	VB	P	H	AD	ADOS	PRODUCT	exL	exH	DENOM	De
BENHAM	558		m	0	0	all	-	KII	Eu:wst	1976	CC	1625	n	bl	n	y	0	0	cig only	11	999	cur+1y	st
PEZZOT	589		m	0	0	all	-	a	SCAmer	1987	CC	215	n	bl	n	y	0	0	cig only	11	999	cur+1y	st

Cigarette type is all/unspec for all RRs

Table 3K14 - 5

IESLC - Meta-analysis of Ex Smoking, Years quit (vs current), "High"
Adenocarcinoma, Cigarettes only
Least adjusted

REF	NRR	SEX	AD	Number Exposed Case	Number Exposed Cont	Non-exposed Case	Non-exposed Cont	RR	95.00%CI
BENHAM	558	m	0	4	21	85	95	0.21	(0.07- 0.65)
PEZZOT	589	m	0	7	31	42	38	0.20	(0.08- 0.52)
Totals				11	52	127	133		

*prospective study

REF	NRR	SEX	AD	Ys	Ws	Qs	Ps
BENHAM	558	m	0	-1.55	3.13	0.00	0.0062
PEZZOT	589	m	0	-1.59	4.44	0.00	0.0008

N	2
NS	2
Wt	7.57
Het Chi	0.00
Het df	1
Het P	N.S.
Fixed RR	0.21
RRl	0.10
RRu	0.42
P	---
Random RR	0.21
RRl	0.10
RRu	0.42
P	---
Asymm P	

Table 3K14 - 6

IESLC - Meta-analysis of Ex Smoking, Years quit (vs current), "High"
Adenocarcinoma, Cigarettes only
Least adjusted

	Sex combined	male	female	Total
N		2		2
NS		2		2
Wt		7.57		7.57
Het Chi		0.00		0.00
Het df		1		1
Het P		N.S.		N.S.
Fixed RR		0.21		0.21
RRl		0.10		0.10
RRu		0.42		0.42
P		---		---
Random RR		0.21		0.21
RRl		0.10		0.10
RRu		0.42		0.42
P		---		---
Between Chi				
Between df				
Between P				N.S.
Btwn(F) P				N.S.
Btwn(R) P				N.S.

Table 3K14 - 7

IESLC - Meta-analysis of Ex Smoking, Years quit (vs current), "High"
Adenocarcinoma, Cigarettes only
Excluded studies (and stage at which they were excluded)

1	AGUDO AKIBA AMANDU AMES AXELSS BEST BOUCHA BOUCOT BRESLO CHEN CHEN2 CHIAZZ DEAN2 DOSEME ENGELA FAN GENG GER GUO HAENSZ HEGMAN HOLE HU HU2 JUSSAW KATSOU KAUFMA KOO KOULUM KREUZE LETOUR LEVIN LIAW LIU3 LIU4 LIU5 MCCONN MIGRAN MRFITR NOTAN2 OSANN2 PERNU QIAO2 RACHTA RESTRE SADOWS SEGI2 STASZE TIZZAN VUTUC WATSON WU WUWILL WYNDE2 WYNDE8 XU YUAN ZHANG ZHENG ZHOU
2	AUVINE BENSHL BLOT1 BROWN3 BUFFLE GURSEL LAUSSM MCDUFF PISANI PRESCO SPITZ WU2 WYNDE7
4	ARMADA BECHER BOFFET BROSS CARPEN CEDERL CHOI CHYOU CORREA CPSI CPSII DAMBER DARBY DEAN3 DESTEF DOLL DOLL2 DORGAN DORN GAO GAO2 GARCIA GARSHI GILLIS GRAHAM HAMMO2 HAMMON HIRAYA HUMBLE JOLY KAISE2 KHUDER LUBIN LUO PEZZO2 QIAO SPEIZE SUZUK2 TVERDA WANG2 WIGLE
5	ALDERS
7	BARBON JAHN JAIN JEDRYC LUBIN2 MATOS SOBUE SVENSS WAKAI WYNDE3 WYNDE6

Table 3K14 - 8
Potentially overlapping studies

REF	REFGP	PRINC	OVERLAP/LINK
BENHAM	LUBIN2	2	Subset of Lubin2

Table 3K14 - 9
Most adjusted - insufficient data for meta-analysis

REF	NRR	SEX	AGEL	AGEH	RACE	YF	LC TYPE	LOC	START	ST	NLC	R	VB	P	H	AD	ADOS	PRODUCT	exL	exH	DENOM	De
ALDERS	575	m	0	0	all	-	not q+s	Eu:UK	1977	CC	1448	n	V	n	n	1	0	cig only	10	999	current	ot
ALDERS	586	f	0	0	all	-	not q+s	Eu:UK	1977	CC	1448	n	V	n	n	1	0	cig only	10	999	current	ot

REF	NRR	RR	SIG	RRDATA comment
ALDERS	575	0.91	n	0
ALDERS	586	0.36	n	0

Table 3K15 -

IESLC - Meta-analysis of Ex Smoking, Years quit (vs current), "Highest vs lowest"
Adenocarcinoma, Cigarettes only

This analysis is restricted to results for:
1) Ex smokers
2) Results by Years quit (vs current)
3) Categorical results by Years quit (vs current)
4) Denominator (unexposed) = "low"
5) Adenocarcinoma (or near equivalent)
6) Results complete enough for use in metaanalysis

Within each study, results are then selected (in the following order of preference, within each sex) for:
7) (not applicable)
8) PRODUCT: cigarettes only
9) CIGTYPE: all/unspecified, MC regardless of HR, MC only
10) Results with least adjustment for other aspects of smoking (ADOS)
11) The highest vs lowest category
12) Followup period (YF, prospective studies): whole study (coded as 0) or longest available
13) LCtype: adeno or nearest available, but not squamous. (q = squamous, s = small,
a = adeno, l = large, KII = Kreyberg II, al = alveolar, br = bronchiolar, u = undifferentiated)
14) Race: all or nearest available, otherwise by race (wh or w = white, bl or b = black, hi = hispanic
ch = chinese, jap = japanese, haw = hawaiian, w+o = white + oriental, sca = scandinavian, as = asian)
15) For overlapping studies: principal rather than subsidiary studies
Finally by Age: whole study (coded as 0) if available, otherwise by widest available age group
and then for single sex results (m, f) in preference to results for both sexes combined (c).

Results adjusted (AD) for the most potential confounders are then chosen in Sections -1 to -3
(and those which actually differ from the adjusted results in Table 3K5 - 1 are marked 'x' in Section -1)
and results adjusted for the least confounders in Sections -4 to -6. (Those least adjusted results which
actually differ from the most adjusted are marked 'x' in column X in Section -4)

Section -7 shows excluded studies, together with the stage (as above) at which no qualifying
results were found.

Section -8 lists the potentially overlapping studies which have been included (1=principal, 2=subsidiary).

Section -9 lists any results which would have been included in preference except that they had data not complete
enough for use in meta-analysis, with their significance (yes/no), if known, and any further comment as entered
on the database. It also lists as "gap" any categories for which no data were presented by the original authors.

In addition to those mentioned above, the following fields, levels and abbreviations are used:
* or nk = not known, n = no, y = yes, ot = other
all/unspec = all or unspecified, MC = manufactured cigarettes, HR = hand-rolled cigarettes
exL, exH = range of exposure (low and high) in the "highest" group, in terms of Years quit (vs current)
unexL, unexH = range of exposure (low and high) in the "lowest" group, in terms of Years quit (vs current)
REF: 6-character study reference
NRR: number of the RR on the database within the study
ST : study type (CC = case control, pr or prosp = prospective)
NLC: number of lung cancer cases in whole study
R : risky occupational population (n = no, m = mining, o = other risky)
VB : national cigarette type (V = at least 75% Virginia, bl = at least 75% blended, ot = other)
P : any proxy use
H : full histological confirmation
De : derivation of RR/CI (or = original, st = standard method, ot = other method of estimation)

Table 3K15 - 1

IESLC - Meta-analysis of Ex Smoking, Years quit (vs current), "Highest vs lowest"
Adenocarcinoma, Cigarettes only
Most adjusted

REF	NRR	3K5	SEX	AGEL	AGEH	RACE	YF	LC TYPE	LOC	START	ST	NLC	R	VB	P	H	AD	ADOS	PRODUCT	exL	exH	unexL	unexH	De
BENHAM	560	x	m	0	0	all	-	KII	Eu:wst	1976	CC	1625	n	bl	n	y	0	0	cig only	11	999	1.0	3	st
PEZZOT	590		m	0	0	all	-	a	SCAmer	1987	CC	215	n	bl	n	y	0	0	cig only	11	999	1.0	10	st

Cigarette type is all/unspec for all RRs

Table 3K15 - 2

IESLC - Meta-analysis of Ex Smoking, Years quit (vs current), "Highest vs lowest"
Adenocarcinoma, Cigarettes only
Most adjusted

REF	NRR	SEX	AD	Number Exposed Case	Exposed Cont	Non-exposed Case	Non-exposed Cont	RR	95.00%CI
BENHAM	560	m	0	4	21	13	9	0.13	(0.03- 0.52)
PEZZOT	590	m	0	7	31	11	21	0.43	(0.14- 1.29)
Totals				11	52	24	30		

*prospective study

REF	NRR	SEX	AD	Ys	Ws	Qs	Ps
BENHAM	560	m	0	-2.03	2.06	1.07	0.0036
PEZZOT	590	m	0	-0.84	3.19	0.69	0.1330

	N	2
	NS	2
	Wt	5.25
Het	Chi	1.76
Het	df	1
Het	P	N.S.
Fixed	RR	0.27
	RRl	0.12
	RRu	0.64
	P	--
Random	RR	0.26
	RRl	0.08
	RRu	0.81
	P	-
Asymm	P	

Table 3K15 - 3

IESLC - Meta-analysis of Ex Smoking, Years quit (vs current), "Highest vs lowest"
Adenocarcinoma, Cigarettes only
Most adjusted

	Sex: combined	male	female	Total
N		2		2
NS		2		2
Wt		5.25		5.25
Het Chi		1.76		1.76
Het df		1		1
Het P		N.S.		N.S.
Fixed RR		0.27		0.27
RRl		0.12		0.12
RRu		0.64		0.64
P		--		--
Random RR		0.26		0.26
RRl		0.08		0.08
RRu		0.81		0.81
P		-		-
Between Chi				
Between df				
Between P				N.S.
Btwn(F) P				N.S.
Btwn(R) P				N.S.

Too few RRs for analysis by factor

Table 3K15 - 4

IESLC - Meta-analysis of Ex Smoking, Years quit (vs current), "Highest vs lowest"
Adenocarcinoma, Cigarettes only
Least adjusted

REF	NRR	X	SEX	AGEL	AGEH	RACE	YF	LC TYPE	LOC	START	ST	NLC	R	VB	P	H	AD	ADOS	PRODUCT	exL	exH	unexL	unexH	De
BENHAM	560		m	0	0	all	-	KII	Eu:wst	1976	CC	1625	n	bl	n	y	0	0	cig only	11	999	1.0	3	st
PEZZOT	590		m	0	0	all	-	a	SCAmer	1987	CC	215	n	bl	n	y	0	0	cig only	11	999	1.0	10	st

Cigarette type is all/unspec for all RRs

Table 3K15 - 5

IESLC - Meta-analysis of Ex Smoking, Years quit (vs current), "Highest vs lowest"
Adenocarcinoma, Cigarettes only
Least adjusted

REF	NRR	SEX	AD	Number Exposed Case	Exposed Cont	Non-exposed Case	Non-exposed Cont	RR	95.00%CI
BENHAM	560	m	0	4	21	13	9	0.13	(0.03- 0.52)
PEZZOT	590	m	0	7	31	11	21	0.43	(0.14- 1.29)
Totals				11	52	24	30		

*prospective study

REF	NRR	SEX	AD	Ys	Ws	Qs	Ps
BENHAM	560	m	0	-2.03	2.06	1.07	0.0036
PEZZOT	590	m	0	-0.84	3.19	0.69	0.1330

	N	2
	NS	2
	Wt	5.25
Het	Chi	1.76
Het	df	1
Het	P	N.S.
Fixed	RR	0.27
	RRl	0.12
	RRu	0.64
	P	--
Random	RR	0.26
	RRl	0.08
	RRu	0.81
	P	-
Asymm	P	

Table 3K15 - 6

IESLC - Meta-analysis of Ex Smoking, Years quit (vs current), "Highest vs lowest"
Adenocarcinoma, Cigarettes only
Least adjusted

	combined	Sex male	female	Total
N		2		2
NS		2		2
Wt		5.25		5.25
Het Chi		1.76		1.76
Het df		1		1
Het P		N.S.		N.S.
Fixed RR		0.27		0.27
RRl		0.12		0.12
RRu		0.64		0.64
P		--		--
Random RR		0.26		0.26
RRl		0.08		0.08
RRu		0.81		0.81
P		-		-
Between Chi				
Between df				
Between P				N.S.
Btwn(F) P				N.S.
Btwn(R) P				N.S.

Table 3K15 - 7

IESLC - Meta-analysis of Ex Smoking, Years quit (vs current), "Highest vs lowest"
Adenocarcinoma, Cigarettes only
Excluded studies (and stage at which they were excluded)

1	AGUDO AKIBA AMANDU AMES AXELSS BEST BOUCHA BOUCOT BRESLO CHEN CHEN2 CHIAZZ DEAN2 DOSEME ENGELA FAN
	GENG GER GUO HAENSZ HEGMAN HOLE HU HU2 JUSSAW KATSOU KAUFMA KOO KOULUM KREUZE LETOUR LEVIN
	LIAW LIU3 LIU4 LIU5 MCCONN MIGRAN MRFITR NOTAN2 OSANN2 PERNU QIAO2 RACHTA RESTRE SADOWS SEGI2 STASZE
	TIZZAN VUTUC WATSON WU WUWILL WYNDE2 WYNDE8 XU YUAN ZHANG ZHENG ZHOU
2	AUVINE BENSHL BLOT1 BROWN3 BUFFLE GURSEL LAUSSM MCDUFF PISANI PRESCO SPITZ WU2 WYNDE7
4	GARSHI JEDRYC LUO WAKAI
5	ARMADA BECHER BOFFET BROSS CARPEN CEDERL CHOI CHYOU CORREA CPSI CPSII DAMBER DARBY DEAN3 DESTEF DOLL
	DOLL2 DORGAN DORN GAO GAO2 GARCIA GILLIS GRAHAM HAMMO2 HAMMON HIRAYA HUMBLE JOLY KAISE2 KHUDER LUBIN
	PEZZO2 QIAO SPEIZE SUZUK2 TVERDA WANG2 WIGLE
6	ALDERS
8	BARBON JAHN JAIN LUBIN2 MATOS SOBUE SVENSS WYNDE3 WYNDE6

Table 3K15 - 8
Potentially overlapping studies

REF	REFGP	PRINC	.	OVERLAP/LINK
BENHAM	LUBIN2	2		Subset of Lubin2

Table 3K15 - 9
Most adjusted - insufficient data for meta-analysis

REF	NRR	SEX	AGEL	AGEH	RACE	YF	LC TYPE	LOC	START	ST	NLC	R	VB	P	H	AD	ADOS	PRODUCT	exL	exH	unexL	unexH	De
ALDERS	577	m	0	0	all	-	not q+s	Eu:UK	1977	CC	1448	n	V	n	n	1	0	cig only	10	999	0.1	2	st
ALDERS	588	f	0	0	all	-	not q+s	Eu:UK	1977	CC	1448	n	V	n	n	1	0	cig only	10	999	0.1	2	st

REF	NRR	RR	SIG	RRDATA comment
ALDERS	577	0.44		0
ALDERS	588	0.26		0
