# Supplementary material for: Systematic review with meta-analysis of the epidemiological evidence in the 1900s relating smoking to lung cancer
Source: BMC Cancer. 2012 Sep 3;12:385. doi: 10.1186/1471-2407-12-385 (PMC3505152; doi:10.1186/1471-2407-12-385)
Supplement: Additional file 5 — Detailed Analysis Tables (Individual file names as described in Additional file 1: Methods, Table1). [file 1471-2407-12-385-S5.zip › PDF/3AR.pdf]

Table 3A1R - 1

IESLC - Meta-regression of ever smoking, any product (or cigs if any not available)
Multiple regression of data from Table 3A1
Adenocarcinoma

Stepwise allowing only characteristics from the fixed model

Log Relative risk
WEIGHTED on Weight

		Deviance	(DF)				
Model 1		930.356	(106)				
		Estimate	S.E.	P	RR	95%CIl	95%CIu
Constant		1.234	0.026	+++	3.436	3.267	3.613

		Deviance	(DF)	Drop Dev	P		
Model 2		431.955	(99)	498.402	***		
		Estimate	S.E.	P	RR	95%CIl	95%CIu
Constant		1.748	0.035	+++	5.746	5.363	6.156
Location							
NAmer	40	Aliased			5.746	4.975	6.636
UK	4	-0.741	0.303	-	2.739	0.799	9.386
Scand	7	-1.036	0.162	---	2.038	1.065	3.902
othEur	15	-0.917	0.082	---	2.297	1.700	3.106
China	12	-1.359	0.080	---	1.476	1.101	1.979
Japan	11	-1.194	0.093	---	1.741	1.222	2.482
othAs	12	-1.118	0.100	---	1.879	1.280	2.760
other	6	-0.313	0.190	N.S.	4.201	1.955	9.026

		Deviance	(DF)	Drop Dev	P		
Model 3		369.648	(96)	62.307	**		
		Estimate	S.E.	P	RR	95%CIl	95%CIu
Constant		1.204	0.087	+++	3.332	2.810	3.952
Location							
NAmer	40	Aliased			5.383	4.675	6.198
UK	4	-0.852	0.303	--	2.295	0.719	7.332
Scand	7	-0.694	0.170	---	2.690	1.433	5.050
othEur	15	-0.952	0.082	---	2.079	1.557	2.775
China	12	-1.279	0.087	---	1.498	1.115	2.012
Japan	11	-0.999	0.097	---	1.983	1.408	2.792
othAs	12	-0.731	0.113	---	2.592	1.742	3.856
other	6	-0.060	0.198	N.S.	5.068	2.415	10.634
Study size (number of LC cases)							
100-249	27	Aliased			2.127	1.570	2.881
250-499	31	0.223	0.098	+	2.657	2.050	3.445
500-999	18	0.447	0.114	+++	3.324	2.425	4.557
1000+	31	0.656	0.090	+++	4.099	3.566	4.712

		Deviance	(DF)	Drop Dev	P		
Model 4		328.896	(92)	40.752	*		
		Estimate	S.E.	P	RR	95%CIl	95%CIu
Constant		0.455	0.157	++	1.575	1.158	2.143
Location							
NAmer	40	Aliased			5.348	4.625	6.183
UK	4	-0.422	0.314	N.S.	3.506	1.109	11.082
Scand	7	-0.489	0.175	--	3.278	1.761	6.103
othEur	15	-0.758	0.101	---	2.505	1.798	3.491
China	12	-1.393	0.093	---	1.328	0.983	1.794
Japan	11	-1.068	0.103	---	1.839	1.309	2.583
othAs	12	-0.780	0.116	---	2.450	1.661	3.616
other	6	-0.115	0.207	N.S.	4.765	2.285	9.936
Study size (number of LC cases)							
100-249	27	Aliased			2.192	1.623	2.961
250-499	31	0.199	0.099	+	2.675	2.042	3.506
500-999	18	0.498	0.119	+++	3.609	2.635	4.942
1000+	31	0.601	0.094	+++	4.000	3.479	4.599
Start year of study							
<1960	14	Aliased			1.586	0.959	2.622
1960-69	14	0.731	0.157	+++	3.296	2.454	4.427
1970-79	31	0.635	0.144	+++	2.993	2.331	3.842
1980-89	40	0.861	0.144	+++	3.752	3.267	4.309
1990+	8	1.013	0.187	+++	4.366	2.759	6.912

Table 3A1R - 1

IESLC - Meta-regression of ever smoking, any product (or cigs if any not available)
Multiple regression of data from Table 3A1
Adenocarcinoma

Log Relative risk
WEIGHTED on Weight

		Deviance	(DF)	Drop Dev	P		
Model 5		307.336	(90)	21.560	*		
		Estimate	S.E.	P	RR	95%CIl	95%CIu
Constant		0.555	0.170	++	1.741	1.247	2.432
Location							
NAmer	40	Aliased			5.382	4.668	6.205
UK	4	-0.319	0.315	N.S.	3.911	1.264	12.099
Scand	7	-0.480	0.176	--	3.332	1.806	6.146
othEur	15	-0.795	0.102	---	2.430	1.755	3.365
China	12	-1.413	0.093	---	1.311	0.977	1.759
Japan	11	-1.099	0.103	---	1.793	1.285	2.500
othAs	12	-0.752	0.117	---	2.537	1.730	3.721
other	6	-0.097	0.211	N.S.	4.883	2.351	10.140
Study size (number of LC cases)							
100-249	27	Aliased			2.328	1.729	3.136
250-499	31	0.143	0.100	N.S.	2.686	2.057	3.506
500-999	18	0.461	0.119	+++	3.692	2.714	5.023
1000+	31	0.523	0.096	+++	3.930	3.425	4.510
Start year of study							
<1960	14	Aliased			1.454	0.874	2.420
1960-69	14	0.808	0.163	+++	3.263	2.439	4.365
1970-79	31	0.707	0.149	+++	2.949	2.309	3.768
1980-89	40	0.985	0.152	+++	3.892	3.381	4.481
1990+	8	0.876	0.196	+++	3.493	1.975	6.179
Sex(RR)							
Male	51	Aliased			3.876	3.305	4.545
Female	45	-0.242	0.057	---	3.042	2.663	3.474
Combined	11	0.128	0.135	N.S.	4.406	2.825	6.872

Table 3A1R - 1

IESLC - Meta-regression of ever smoking, any product (or cigs if any not available)
Multiple regression of data from Table 3A1
Adenocarcinoma

Fixed model

Log Relative risk
WEIGHTED on Weight

		Deviance	(DF)				
Model 7		284.147	(87)				
		Estimate	S.E.	P	RR	95%CIl	95%CIu
Constant		0.368	0.178	+	1.445	1.020	2.047
Sex(RR)							
Male	51	Aliased			3.954	3.376	4.632
Female	45	-0.275	0.058	---	3.004	2.636	3.422
Combined	11	0.102	0.138	N.S.	4.378	2.815	6.811
Location							
NAmer	40	Aliased			5.273	4.555	6.104
UK	4	-0.535	0.324	N.S.	3.090	0.995	9.591
Scand	7	-0.630	0.190	--	2.808	1.482	5.318
othEur	15	-0.731	0.107	---	2.539	1.814	3.553
China	12	-1.356	0.094	---	1.359	1.017	1.817
Japan	11	-1.158	0.119	---	1.656	1.146	2.394
othAs	12	-0.557	0.127	---	3.022	2.023	4.514
other	6	-0.024	0.213	N.S.	5.148	2.511	10.555
Start year of study							
<1960	14	Aliased			1.585	0.959	2.622
1960-69	14	0.750	0.166	+++	3.355	2.468	4.561
1970-79	31	0.672	0.150	+++	3.106	2.433	3.965
1980-89	40	0.857	0.155	+++	3.735	3.238	4.309
1990+	8	0.892	0.200	+++	3.866	2.177	6.867
Study type (1)							
CC	98	Aliased			3.382	3.079	3.714
other	9	0.352	0.140	+	4.810	2.965	7.804
Study size (number of LC cases)							
100-249	27	Aliased			2.163	1.606	2.914
250-499	31	0.265	0.107	+	2.819	2.103	3.778
500-999	18	0.550	0.121	+++	3.748	2.764	5.084
1000+	31	0.594	0.100	+++	3.919	3.393	4.528
Number of adjustment variables (1)							
0	54	Aliased			2.861	2.373	3.449
1	20	0.391	0.110	+++	4.229	3.072	5.821
2+/+nk	33	0.272	0.078	+++	3.755	3.214	4.386

Table 3A1R - 2

IESLC - Meta-regression of ever smoking, any product (or cigs if any not available)
Multiple regression of data from Table 3A1
Adenocarcinoma
Effect of removing characteristics

Log Relative risk
WEIGHTED on Weight

		Deviance	(DF)				
Model 7		284.147	(87)				
		Estimate	S.E.	P	RR	95%CIl	95%CIu
Constant		0.368	0.178	+	1.445	1.020	2.047
Sex(RR)							
Male	51	Aliased			3.954	3.376	4.632
Female	45	-0.275	0.058	---	3.004	2.636	3.422
Combined	11	0.102	0.138	N.S.	4.378	2.815	6.811
Location							
NAmer	40	Aliased			5.273	4.555	6.104
UK	4	-0.535	0.324	N.S.	3.090	0.995	9.591
Scand	7	-0.630	0.190	--	2.808	1.482	5.318
othEur	15	-0.731	0.107	---	2.539	1.814	3.553
China	12	-1.356	0.094	---	1.359	1.017	1.817
Japan	11	-1.158	0.119	---	1.656	1.146	2.394
othAs	12	-0.557	0.127	---	3.022	2.023	4.514
other	6	-0.024	0.213	N.S.	5.148	2.511	10.555
Start year of study							
<1960	14	Aliased			1.585	0.959	2.622
1960-69	14	0.750	0.166	+++	3.355	2.468	4.561
1970-79	31	0.672	0.150	+++	3.106	2.433	3.965
1980-89	40	0.857	0.155	+++	3.735	3.238	4.309
1990+	8	0.892	0.200	+++	3.866	2.177	6.867
Study type (1)							
CC	98	Aliased			3.382	3.079	3.714
other	9	0.352	0.140	+	4.810	2.965	7.804
Study size (number of LC cases)							
100-249	27	Aliased			2.163	1.606	2.914
250-499	31	0.265	0.107	+	2.819	2.103	3.778
500-999	18	0.550	0.121	+++	3.748	2.764	5.084
1000+	31	0.594	0.100	+++	3.919	3.393	4.528
Number of adjustment variables (1)							
0	54	Aliased			2.861	2.373	3.449
1	20	0.391	0.110	+++	4.229	3.072	5.821
2+/+nk	33	0.272	0.078	+++	3.755	3.214	4.386

Omit Sex		Deviance	(DF)	Drop Dev	P		
Model 8		310.251	(89)	-26.104	*		
		Estimate	S.E.	P	RR	95%CIl	95%CIu
Constant		0.260	0.165	N.S.	1.297	0.940	1.791
Number of adjustment variables (1)							
0	54	Aliased			2.917	2.426	3.507
1	20	0.333	0.108	++	4.069	2.989	5.539
2+/+nk	33	0.247	0.077	++	3.735	3.206	4.352
Location							
NAmer	40	Aliased			5.232	4.533	6.039
UK	4	-0.606	0.322	(-)	2.854	0.937	8.693
Scand	7	-0.606	0.186	--	2.854	1.533	5.313
othEur	15	-0.698	0.106	---	2.604	1.869	3.626
China	12	-1.337	0.094	---	1.374	1.031	1.830
Japan	11	-1.104	0.117	---	1.734	1.209	2.489
othAs	12	-0.600	0.126	---	2.872	1.939	4.254
other	6	-0.037	0.208	N.S.	5.041	2.512	10.119
Start year of study							
<1960	14	Aliased			1.718	1.061	2.781
1960-69	14	0.686	0.158	+++	3.409	2.532	4.591
1970-79	31	0.604	0.145	+++	3.144	2.471	4.000
1980-89	40	0.742	0.148	+++	3.607	3.145	4.137
1990+	8	1.019	0.189	+++	4.759	3.055	7.413
Study type (1)							
CC	98	Aliased			3.386	3.086	3.714
other	9	0.326	0.140	+	4.689	2.907	7.563

Table 3A1R - 2

IESLC - Meta-regression of ever smoking, any product (or cigs if any not available)
Multiple regression of data from Table 3A1
Adenocarcinoma
Effect of removing characteristics

Log Relative risk
WEIGHTED on Weight

		Estimate	S.E.	P	RR	95%CIl	95%CIu
Study size (number of LC cases)							
100-249	27	Aliased			2.037	1.522	2.726
250-499	31	0.310	0.107	++	2.778	2.085	3.700
500-999	18	0.582	0.121	+++	3.644	2.698	4.922
1000+	31	0.676	0.098	+++	4.006	3.479	4.613

Omit Location		Deviance	(DF)	Drop Dev	P		
Model 8		573.654	(94)	-289.507	***		
		Estimate	S.E.	P	RR	95%CIl	95%CIu
Constant		-0.096	0.160	N.S.	0.909	0.664	1.244
Number of adjustment variables (1)							
0	54	Aliased			2.656	2.250	3.135
1	20	0.173	0.090	(+)	3.159	2.445	4.081
2+/+nk	33	0.478	0.071	+++	4.282	3.716	4.936
Sex(RR)							
Male	51	Aliased			3.767	3.243	4.375
Female	45	-0.186	0.057	--	3.127	2.760	3.541
Combined	11	0.106	0.131	N.S.	4.187	2.786	6.291
Start year of study							
<1960	14	Aliased			1.888	1.197	2.978
1960-69	14	1.007	0.155	+++	5.166	3.945	6.765
1970-79	31	0.426	0.146	++	2.891	2.349	3.558
1980-89	40	0.658	0.143	+++	3.647	3.202	4.153
1990+	8	0.237	0.184	N.S.	2.392	1.429	4.004
Study type (1)							
CC	98	Aliased			3.345	3.057	3.659
other	9	0.597	0.132	+++	6.077	3.923	9.414
Study size (number of LC cases)							
100-249	27	Aliased			2.005	1.533	2.622
250-499	31	0.322	0.104	++	2.767	2.160	3.543
500-999	18	0.275	0.111	+	2.640	2.033	3.427
1000+	31	0.762	0.089	+++	4.293	3.794	4.859

Omit Start year		Deviance	(DF)	Drop Dev	P		
Model 8		318.088	(91)	-33.941	*		
		Estimate	S.E.	P	RR	95%CIl	95%CIu
Constant		1.043	0.115	+++	2.838	2.267	3.553
Number of adjustment variables (1)							
0	54	Aliased			2.746	2.306	3.269
1	20	0.403	0.107	+++	4.109	3.049	5.536
2+/+nk	33	0.352	0.071	+++	3.905	3.389	4.498
Sex(RR)							
Male	51	Aliased			3.949	3.394	4.595
Female	45	-0.262	0.057	---	3.038	2.684	3.439
Combined	11	0.039	0.106	N.S.	4.107	2.953	5.712
Location							
NAmer	40	Aliased			5.238	4.589	5.978
UK	4	-0.910	0.311	--	2.107	0.723	6.140
Scand	7	-0.799	0.183	---	2.356	1.281	4.332
othEur	15	-0.856	0.093	---	2.225	1.661	2.980
China	12	-1.252	0.089	---	1.497	1.141	1.966
Japan	11	-1.068	0.109	---	1.800	1.279	2.535
othAs	12	-0.468	0.121	---	3.281	2.238	4.808
other	6	0.035	0.201	N.S.	5.427	2.766	10.646
Study type (1)							
CC	98	Aliased			3.370	3.075	3.694
other	9	0.428	0.139	++	5.168	3.233	8.261
Study size (number of LC cases)							
100-249	27	Aliased			2.066	1.561	2.734
250-499	31	0.317	0.106	++	2.835	2.140	3.757
500-999	18	0.532	0.117	+++	3.516	2.628	4.704
1000+	31	0.661	0.095	+++	3.999	3.483	4.592

Table 3A1R - 2

IESLC - Meta-regression of ever smoking, any product (or cigs if any not available)
Multiple regression of data from Table 3A1
Adenocarcinoma
Effect of removing characteristics

Log Relative risk
WEIGHTED on Weight

Omit Study type		Deviance	(DF)	Drop Dev	P		
Model 8		290.439	(88)	-6.292	N.S.		
		Estimate	S.E.	P	RR	95%CIl	95%CIu
Constant		0.399	0.177	+	1.491	1.053	2.110
Number of adjustment variables (1)							
0	54	Aliased			2.876	2.389	3.463
1	20	0.397	0.110	+++	4.279	3.116	5.876
2+/+nk	33	0.260	0.078	++	3.729	3.195	4.351
Sex(RR)							
Male	51	Aliased			3.947	3.373	4.619
Female	45	-0.271	0.058	---	3.011	2.645	3.428
Combined	11	0.096	0.138	N.S.	4.345	2.801	6.741
Location							
NAmer	40	Aliased			5.338	4.619	6.168
UK	4	-0.524	0.324	N.S.	3.162	1.026	9.748
Scand	7	-0.669	0.189	---	2.735	1.451	5.156
othEur	15	-0.693	0.105	---	2.669	1.925	3.700
China	12	-1.393	0.093	---	1.326	0.996	1.765
Japan	11	-1.204	0.117	---	1.601	1.113	2.302
othAs	12	-0.639	0.123	---	2.819	1.914	4.150
other	6	-0.073	0.212	N.S.	4.963	2.435	10.114
Start year of study							
<1960	14	Aliased			1.531	0.930	2.518
1960-69	14	0.782	0.165	+++	3.347	2.467	4.542
1970-79	31	0.700	0.149	+++	3.083	2.419	3.931
1980-89	40	0.901	0.154	+++	3.768	3.271	4.341
1990+	8	0.897	0.200	+++	3.752	2.123	6.633
Study size (number of LC cases)							
100-249	27	Aliased			2.240	1.672	3.000
250-499	31	0.294	0.107	++	3.005	2.278	3.964
500-999	18	0.539	0.121	+++	3.840	2.841	5.189
1000+	31	0.529	0.096	+++	3.802	3.315	4.359

Omit Study size		Deviance	(DF)	Drop Dev	P		
Model 8		321.689	(90)	-37.542	*		
		Estimate	S.E.	P	RR	95%CIl	95%CIu
Constant		0.837	0.156	+++	2.310	1.700	3.139
Number of adjustment variables (1)							
0	54	Aliased			2.881	2.423	3.426
1	20	0.410	0.104	+++	4.342	3.195	5.902
2+/+nk	33	0.253	0.073	+++	3.709	3.194	4.307
Sex(RR)							
Male	51	Aliased			4.098	3.515	4.778
Female	45	-0.333	0.057	---	2.939	2.587	3.338
Combined	11	0.053	0.137	N.S.	4.319	2.806	6.649
Location							
NAmer	40	Aliased			5.489	4.773	6.313
UK	4	-0.429	0.324	N.S.	3.576	1.181	10.830
Scand	7	-0.872	0.179	---	2.295	1.264	4.168
othEur	15	-0.674	0.103	---	2.797	2.054	3.808
China	12	-1.374	0.085	---	1.389	1.071	1.802
Japan	11	-1.317	0.114	---	1.470	1.034	2.090
othAs	12	-0.866	0.115	---	2.309	1.612	3.307
other	6	-0.127	0.205	N.S.	4.833	2.435	9.590
Start year of study							
<1960	14	Aliased			1.454	0.891	2.372
1960-69	14	0.743	0.165	+++	3.056	2.271	4.113
1970-79	31	0.750	0.149	+++	3.076	2.427	3.899
1980-89	40	0.992	0.152	+++	3.921	3.419	4.498
1990+	8	0.786	0.196	+++	3.192	1.844	5.524
Study type (1)							
CC	98	Aliased			3.416	3.116	3.744
other	9	0.129	0.129	N.S.	3.886	2.505	6.030

Table 3A1R - 2

IESLC - Meta-regression of ever smoking, any product (or cigs if any not available)
Multiple regression of data from Table 3A1
Adenocarcinoma
Effect of removing characteristics

Log Relative risk
WEIGHTED on Weight

Omit N adjustment vars		Deviance	(DF)	Drop Dev	P		
Model 8		301.449	(89)	-17.302	(*)		
		Estimate	S.E.	P	RR	95%CIl	95%CIu
Constant		0.533	0.171	++	1.703	1.219	2.380
Study size (number of LC cases)							
100-249	27	Aliased			2.255	1.684	3.018
250-499	31	0.111	0.100	N.S.	2.520	1.916	3.313
500-999	18	0.470	0.119	+++	3.607	2.674	4.866
1000+	31	0.585	0.100	+++	4.049	3.520	4.656
Sex(RR)							
Male	51	Aliased			3.887	3.331	4.534
Female	45	-0.248	0.057	---	3.034	2.668	3.451
Combined	11	0.128	0.135	N.S.	4.419	2.875	6.793
Location							
NAmer	40	Aliased			5.332	4.643	6.123
UK	4	-0.340	0.315	N.S.	3.796	1.273	11.324
Scand	7	-0.451	0.177	-	3.397	1.878	6.145
othEur	15	-0.835	0.103	---	2.312	1.674	3.193
China	12	-1.379	0.094	---	1.343	1.008	1.788
Japan	11	-1.064	0.104	---	1.840	1.331	2.543
othAs	12	-0.680	0.120	---	2.702	1.845	3.957
other	6	-0.055	0.211	N.S.	5.046	2.485	10.244
Start year of study							
<1960	14	Aliased			1.501	0.916	2.462
1960-69	14	0.772	0.164	+++	3.248	2.451	4.305
1970-79	31	0.680	0.150	+++	2.964	2.339	3.757
1980-89	40	0.946	0.153	+++	3.866	3.373	4.432
1990+	8	0.879	0.196	+++	3.617	2.079	6.291
Study type (1)							
CC	98	Aliased			3.384	3.084	3.712
other	9	0.340	0.140	+	4.752	2.950	7.654

Table 3A1R - 3

IESLC - Meta-regression of ever smoking, any product (or cigs if any not available)
Multiple regression of data from Table 3A1
Adenocarcinoma
Study outliers

Study Ref	NRR	LOGRR	FITVAL	SEFITV	STDRES
LOMBA2	3	-0.641	0.843	0.399	-3.721
BAND	2	1.411	2.091	0.285	-2.388
GER	9	0.095	1.077	0.627	-1.565
DORGAN	104	1.361	1.817	0.302	-1.506
LUBIN2	168	0.248	0.629	0.265	-1.439
SOBUE	109	0.344	0.777	0.315	-1.375
LAMWK2	3	-0.073	0.749	0.604	-1.359
CHOI	65	-0.441	0.658	0.925	-1.188
SOBUE	99	0.604	1.052	0.379	-1.180
OSANN2	31	0.916	1.586	0.621	-1.078
WYNDE4	56	-0.511	0.915	1.382	-1.031
WYNDE2	14	0.513	1.383	0.864	-1.006
CORREA	36	1.723	2.128	0.407	-0.996
JAIN	42	1.238	1.772	0.622	-0.858
CHOI	63	0.295	0.933	0.755	-0.845
BRESLO	35	0.241	1.020	0.967	-0.805
GAO	3	0.470	0.736	0.358	-0.743
NOU	8	-0.127	0.400	0.711	-0.742
DOLL	87	-0.051	0.819	1.274	-0.683
WYNDE3	135	0.642	1.108	0.685	-0.680
DAMBER	32	0.875	1.351	0.711	-0.669
ENGELA	76	0.846	1.377	0.832	-0.638
DORGAN	125	1.569	2.091	0.848	-0.616
WU	31	0.956	1.222	0.489	-0.545
DOSEME	4	0.956	1.176	0.415	-0.532
WYNDE4	42	0.414	0.918	0.965	-0.522
ABRAHA	2	0.874	1.212	0.671	-0.503
LUBIN	36	-0.342	0.134	0.962	-0.495
DESTE2	17	1.459	1.772	0.879	-0.357
WYNDE3	29	1.100	1.383	0.795	-0.356
HAENSZ	5	0.174	0.365	0.558	-0.342
JOLY	50	1.119	1.292	0.507	-0.340
TSUGAN	10	-0.073	0.154	0.699	-0.324
TSUGAN	11	-0.400	-0.121	1.022	-0.274
WAKAI	82	0.329	0.447	0.676	-0.175
JEDRYC	56	1.235	1.361	0.748	-0.167
GAO	13	0.405	0.461	0.341	-0.163
STAYNE	4	1.283	1.383	0.743	-0.134
BUFFLE	50	1.504	1.590	0.814	-0.106
KATSOU	33	0.542	0.610	0.697	-0.097
JOLY	51	1.488	1.567	0.831	-0.095
WAKAI	76	0.658	0.722	0.701	-0.093
LAMTH	3	0.626	0.658	0.385	-0.084
HINDS	24	1.358	1.379	0.407	-0.052
WUWILL	11	0.405	0.416	0.251	-0.043
SIEMIA	8	1.841	1.862	0.855	-0.025
ZHOU	26	0.270	0.279	0.480	-0.020
ZHENG	21	0.148	0.144	0.465	0.008
JAIN	47	2.079	2.047	1.420	0.023
ABRAHA	5	0.963	0.937	0.605	0.042
BROWN1	3	1.502	1.431	1.036	0.068
ALDERS	57	1.275	1.216	0.775	0.076
XU3	26	0.086	-0.015	1.301	0.078
BROWN2	4	2.104	2.091	0.157	0.081
SUZUK2	16	1.792	1.609	1.917	0.095
DOLL	89	0.678	0.544	1.042	0.129
SCHWAR	7	2.089	1.820	1.965	0.137
WYNDE6	69	1.749	1.712	0.256	0.144
HAMMON	92	1.192	0.985	1.304	0.159
FAN	4	0.329	0.271	0.361	0.163
BUFFLE	45	1.391	1.315	0.464	0.163
KOO	7	0.476	0.394	0.490	0.169

Table 3A1R - 3

IESLC - Meta-regression of ever smoking, any product (or cigs if any not available)
Multiple regression of data from Table 3A1
Adenocarcinoma
Study outliers

Study Ref	NRR	LOGRR	FITVAL	SEFITV	STDRES
LAMWK2	7	0.565	0.474	0.537	0.171
KREYBE	27	0.247	0.119	0.687	0.187
LUO	9	0.405	0.278	0.664	0.192
KIHARA	29	0.532	0.468	0.305	0.210
STASZE	4	0.082	-0.373	1.878	0.242
COMSTO	79	1.567	1.383	0.757	0.243
TIZZAN	19	0.452	0.334	0.418	0.282
BROWN1	4	1.374	1.156	0.735	0.296
COMSTO	67	2.065	1.658	1.311	0.311
CHAN	16	0.668	0.474	0.531	0.365
MATOS	69	1.826	1.508	0.847	0.376
ZHOU	27	0.358	0.004	0.838	0.421
ZHENG	10	0.604	0.419	0.432	0.427
ALDERS	54	1.962	1.372	1.365	0.431
TOKARS	8	1.459	1.113	0.741	0.466
KREYBE	8	0.892	0.394	1.057	0.471
SCHWAR	16	1.907	1.545	0.656	0.551
BYERS1	3	1.410	0.963	0.738	0.607
LUBIN2	148	1.058	0.904	0.252	0.612
SCHWAR	15	2.278	1.545	1.199	0.612
SVENSS	74	1.068	0.711	0.564	0.634
SEOW	2	0.860	0.428	0.670	0.645
ANDERS	12	1.806	1.567	0.360	0.665
LAMWK	4	0.747	0.394	0.493	0.717
PEZZOT	7	1.990	1.201	1.059	0.745
JAHN	47	1.613	1.089	0.668	0.785
KHUDER	27	2.093	1.490	0.727	0.830
ORMOS	21	1.912	-0.363	2.682	0.848
NOU	3	1.492	0.675	0.957	0.853
STASZE	21	2.153	-0.098	2.563	0.878
BECHER	12	2.382	0.610	1.910	0.928
SCHWAR	8	3.539	1.820	1.829	0.940
ISHIMA	8	2.708	0.333	2.524	0.941
BROWN2	3	1.932	1.817	0.119	0.966
CHAN	12	3.416	0.749	2.565	1.040
XU3	22	1.577	0.260	1.150	1.145
MATSUD	12	2.879	-0.040	2.551	1.145
BARBON	130	1.949	1.131	0.706	1.157
HEGMAN	4	2.259	1.592	0.505	1.322
OSANN	48	2.251	1.817	0.326	1.334
SUZUKI	15	0.784	-0.121	0.580	1.559
OSANN	47	2.885	2.091	0.503	1.578
JUSSAW	26	2.274	1.111	0.602	1.933
SUZUKI	11	1.511	0.154	0.551	2.463
WYNDE6	414	2.638	1.828	0.291	2.780

Table 3A1R - 4

IESLC - Meta-regression of ever smoking, any product (or cigs if any not available)
Multiple regression of data from Table 3A1
Adenocarcinoma
Effect of additional characteristics

Log Relative risk
WEIGHTED on Weight

		Deviance	(DF)				
Model 7		284.147	(87)				
		Estimate	S.E.	P	RR	95%CIl	95%CIu
Constant		0.368	0.178	+	1.445	1.020	2.047
Sex(RR)							
Male	51	Aliased			3.954	3.376	4.632
Female	45	-0.275	0.058	---	3.004	2.636	3.422
Combined	11	0.102	0.138	N.S.	4.378	2.815	6.811
Location							
NAmer	40	Aliased			5.273	4.555	6.104
UK	4	-0.535	0.324	N.S.	3.090	0.995	9.591
Scand	7	-0.630	0.190	--	2.808	1.482	5.318
othEur	15	-0.731	0.107	---	2.539	1.814	3.553
China	12	-1.356	0.094	---	1.359	1.017	1.817
Japan	11	-1.158	0.119	---	1.656	1.146	2.394
othAs	12	-0.557	0.127	---	3.022	2.023	4.514
other	6	-0.024	0.213	N.S.	5.148	2.511	10.555
Start year of study							
<1960	14	Aliased			1.585	0.959	2.622
1960-69	14	0.750	0.166	+++	3.355	2.468	4.561
1970-79	31	0.672	0.150	+++	3.106	2.433	3.965
1980-89	40	0.857	0.155	+++	3.735	3.238	4.309
1990+	8	0.892	0.200	+++	3.866	2.177	6.867
Study type (1)							
CC	98	Aliased			3.382	3.079	3.714
other	9	0.352	0.140	+	4.810	2.965	7.804
Study size (number of LC cases)							
100-249	27	Aliased			2.163	1.606	2.914
250-499	31	0.265	0.107	+	2.819	2.103	3.778
500-999	18	0.550	0.121	+++	3.748	2.764	5.084
1000+	31	0.594	0.100	+++	3.919	3.393	4.528
Number of adjustment variables (1)							
0	54	Aliased			2.861	2.373	3.449
1	20	0.391	0.110	+++	4.229	3.072	5.821
2+/+nk	33	0.272	0.078	+++	3.755	3.214	4.386

		Deviance	(DF)	Drop Dev	P		
Model 8		274.188	(83)	9.958	N.S.		
		Estimate	S.E.	P	RR	95%CIl	95%CIu
Constant		0.282	0.183	N.S.	1.326	0.926	1.899
Sex(RR)							
Male	51	Aliased			3.974	3.384	4.667
Female	45	-0.264	0.059	---	3.053	2.671	3.490
Combined	11	-0.024	0.149	N.S.	3.881	2.394	6.291
Location							
NAmer	40	Aliased			4.863	4.256	5.557
UK	4	-0.571	0.325	(-)	2.746	0.872	8.647
Scand	7	-0.657	0.191	---	2.521	1.321	4.810
othEur	15	Aliased			4.863	4.256	5.557
China	12	-1.350	0.095	---	1.261	0.941	1.689
Japan	11	-1.195	0.120	---	1.472	1.010	2.144
othAs	12	-0.602	0.130	---	2.662	1.757	4.033
other	6	-0.087	0.214	N.S.	4.458	2.146	9.261
Start year of study							
<1960	14	Aliased			1.417	0.818	2.456
1960-69	14	0.837	0.174	+++	3.272	2.390	4.479
1970-79	31	0.847	0.166	+++	3.304	2.555	4.274
1980-89	40	0.949	0.166	+++	3.662	3.164	4.238
1990+	8	1.101	0.224	+++	4.262	2.348	7.738
Study type (1)							
CC	98	Aliased			3.390	3.083	3.728
other	9	0.298	0.158	(+)	4.569	2.645	7.891

Table 3A1R - 4

IESLC - Meta-regression of ever smoking, any product (or cigs if any not available)
Multiple regression of data from Table 3A1
Adenocarcinoma
Effect of additional characteristics

WEIGHTED on Weight

		Estimate	S.E.	P	RR	95%CIl	95%CIu
Study size (number of LC cases)							
100-249	27	Aliased			2.174	1.607	2.940
250-499	31	0.276	0.108	+	2.866	2.124	3.867
500-999	18	0.501	0.124	+++	3.587	2.606	4.938
1000+	31	0.592	0.101	+++	3.931	3.393	4.554
Number of adjustment variables (1)							
0	54	Aliased			2.866	2.349	3.497
1	20	0.409	0.114	+++	4.315	3.113	5.980
2+/+nk	33	0.263	0.084	++	3.730	3.169	4.391
Detailed Country in othEur							
not o E	92	Aliased			3.770	3.401	4.178
multi	2	-0.922	0.149	---	1.499	0.908	2.475
Germany	2	-0.080	0.362	N.S.	3.480	0.963	12.578
othWest	2	-0.281	0.251	N.S.	2.846	1.179	6.872
East	7	-0.630	0.225	--	2.008	0.914	4.416
Balkans	2	-0.979	0.214	---	1.416	0.669	2.998

		Deviance	(DF)	Drop Dev	P		
Model 8		262.584	(85)	21.562	*		
		Estimate	S.E.	P	RR	95%CIl	95%CIu
Constant		0.439	0.178	+	1.551	1.093	2.200
Sex(RR)							
Male	51	Aliased			3.942	3.377	4.601
Female	45	-0.261	0.059	---	3.035	2.672	3.448
Combined	11	0.054	0.139	N.S.	4.161	2.703	6.406
Location							
NAmer	40	Aliased			5.115	4.490	5.827
UK	4	-0.589	0.325	(-)	2.838	0.943	8.540
Scand	7	-0.683	0.191	---	2.584	1.379	4.842
othEur	15	-0.738	0.107	---	2.445	1.762	3.394
China	12	-1.363	0.095	---	1.309	0.982	1.745
Japan	11	-1.237	0.120	---	1.484	1.027	2.145
othAs	12	Aliased			5.115	4.490	5.827
other	6	-0.064	0.214	N.S.	4.797	2.370	9.709
Start year of study							
<1960	14	Aliased			1.595	0.978	2.601
1960-69	14	0.644	0.168	+++	3.036	2.225	4.143
1970-79	31	0.677	0.150	+++	3.137	2.467	3.990
1980-89	40	0.853	0.155	+++	3.742	3.254	4.303
1990+	8	1.025	0.208	+++	4.445	2.459	8.035
Study type (1)							
CC	98	Aliased			3.382	3.087	3.704
other	9	0.353	0.141	+	4.812	3.002	7.714
Study size (number of LC cases)							
100-249	27	Aliased			2.284	1.704	3.063
250-499	31	0.235	0.108	+	2.890	2.157	3.874
500-999	18	0.423	0.127	++	3.488	2.561	4.751
1000+	31	0.536	0.101	+++	3.905	3.393	4.495
Number of adjustment variables (1)							
0	54	Aliased			2.854	2.378	3.425
1	20	0.427	0.110	+++	4.373	3.202	5.973
2+/+nk	33	0.268	0.078	+++	3.730	3.205	4.341
Detailed Country in othAsia							
not o A	95	Aliased			3.598	3.273	3.955
India	1	0.768	0.363	+	7.754	2.227	26.998
HongKong	7	-0.589	0.151	---	1.997	1.212	3.289
othAsia	4	-1.200	0.231	---	1.083	0.495	2.373

		Deviance	(DF)	Drop Dev	P		
Model 8		274.166	(86)	9.981	(*)		
		Estimate	S.E.	P	RR	95%CIl	95%CIu
Constant		0.508	0.183	++	1.662	1.160	2.379

Table 3A1R - 4

IESLC - Meta-regression of ever smoking, any product (or cigs if any not available)
Multiple regression of data from Table 3A1
Adenocarcinoma
Effect of additional characteristics

WEIGHTED on Weight

		Estimate	S.E.	P	RR	95%CIl	95%CIu
Sex(RR)							
Male	51	Aliased			4.041	3.451	4.733
Female	45	-0.305	0.059	---	2.978	2.617	3.389
Combined	11	0.043	0.139	N.S.	4.219	2.722	6.541
Location							
NAmer	40	Aliased			5.241	4.534	6.058
UK	4	-0.419	0.326	N.S.	3.448	1.119	10.630
Scand	7	-0.539	0.192	--	3.056	1.615	5.783
othEur	15	-0.737	0.107	---	2.509	1.800	3.498
China	12	-1.348	0.095	---	1.362	1.022	1.814
Japan	11	-1.166	0.119	---	1.633	1.135	2.351
othAs	12	-0.505	0.128	---	3.163	2.121	4.717
other	6	-0.061	0.213	N.S.	4.932	2.423	10.041
Start year of study							
<1960	14	Aliased			1.622	0.986	2.669
1960-69	14	0.869	0.170	+++	3.870	2.749	5.448
1970-79	31	0.633	0.150	+++	3.054	2.398	3.891
1980-89	40	0.812	0.155	+++	3.653	3.166	4.216
1990+	8	0.845	0.201	+++	3.776	2.139	6.664
Study type (1)							
CC	98	Aliased			3.386	3.087	3.715
other	9	0.322	0.141	+	4.671	2.893	7.542
Study size (number of LC cases)							
100-249	27	Aliased			2.244	1.667	3.021
250-499	31	0.214	0.108	(+)	2.779	2.080	3.713
500-999	18	0.506	0.122	+++	3.722	2.754	5.030
1000+	31	0.556	0.101	+++	3.913	3.393	4.512
Number of adjustment variables (1)							
0	54	Aliased			2.931	2.432	3.532
1	20	0.355	0.110	++	4.179	3.047	5.732
2+/+nk	33	0.232	0.079	++	3.696	3.167	4.315
Adeno (or nearest)							
a	87	Aliased			3.570	3.232	3.944
oth	20	-0.331	0.105	--	2.564	1.831	3.589

		Deviance	(DF)	Drop Dev	P		
Model 8		213.006	(82)	71.140	***		
		Estimate	S.E.	P	RR	95%CIl	95%CIu
Constant		0.811	0.194	+++	2.251	1.539	3.292
Sex(RR)							
Male	51	Aliased			3.947	3.413	4.564
Female	45	-0.263	0.060	---	3.033	2.696	3.412
Combined	11	0.053	0.140	N.S.	4.160	2.796	6.190
Location							
NAmer	40	Aliased			5.469	4.790	6.244
UK	4	-0.273	0.330	N.S.	4.161	1.488	11.640
Scand	7	-0.513	0.204	-	3.274	1.766	6.068
othEur	15	-0.822	0.113	---	2.405	1.750	3.306
China	12	-1.399	0.095	---	1.351	1.041	1.752
Japan	11	-1.185	0.119	---	1.672	1.202	2.325
othAs	12	-0.820	0.138	---	2.408	1.630	3.556
other	6	-0.171	0.214	N.S.	4.608	2.421	8.769
Start year of study							
<1960	14	Aliased			1.505	0.955	2.372
1960-69	14	1.120	0.174	+++	4.614	3.288	6.474
1970-79	31	0.639	0.155	+++	2.852	2.272	3.581
1980-89	40	0.888	0.157	+++	3.658	3.212	4.166
1990+	8	0.855	0.204	+++	3.538	2.107	5.942
Study type (1)							
CC	98	Aliased			3.408	3.134	3.705
other	9	0.181	0.142	N.S.	4.082	2.639	6.315

Table 3A1R - 4

IESLC - Meta-regression of ever smoking, any product (or cigs if any not available)
Multiple regression of data from Table 3A1
Adenocarcinoma
Effect of additional characteristics

WEIGHTED on Weight

		Estimate	S.E.	P	RR	95%CIl	95%CIu
Study size (number of LC cases)							
100-249	27	Aliased			2.820	2.113	3.764
250-499	31	-0.062	0.116	N.S.	2.652	2.033	3.459
500-999	18	0.275	0.130	+	3.713	2.808	4.909
1000+	31	0.297	0.107	++	3.794	3.333	4.319
Number of adjustment variables (1)							
0	54	Aliased			3.260	2.712	3.919
1	20	0.149	0.118	N.S.	3.786	2.828	5.067
2+/+nk	33	0.068	0.087	N.S.	3.491	3.013	4.044
Adeno (or nearest)							
a	87	Aliased			3.636	3.320	3.982
a+l	3	0.144	0.230	N.S.	4.201	2.049	8.616
a+al+br	1	-0.486	0.460	N.S.	2.236	0.524	9.536
KII	13	-0.387	0.146	--	2.470	1.600	3.813
not q+u	1	-2.309	0.281	---	0.361	0.151	0.862
not q+s	2	0.035	0.253	N.S.	3.766	1.693	8.373

		Deviance	(DF)	Drop Dev	P		
Model 8		283.898	(86)	0.248	N.S.		
		Estimate	S.E.	P	RR	95%CIl	95%CIu
Constant		0.368	0.178	+	1.445	1.020	2.047
Sex(RR)							
Male	51	Aliased			3.951	3.370	4.632
Female	45	-0.275	0.058	---	3.001	2.631	3.422
Combined	11	0.112	0.139	N.S.	4.419	2.820	6.924
Location							
NAmer	40	Aliased			5.270	4.549	6.106
UK	4	-0.537	0.324	N.S.	3.080	0.986	9.621
Scand	7	-0.637	0.190	--	2.789	1.464	5.310
othEur	15	-0.731	0.107	---	2.536	1.809	3.556
China	12	-1.352	0.095	---	1.364	1.018	1.828
Japan	11	-1.159	0.119	---	1.653	1.141	2.395
othAs	12	-0.554	0.127	---	3.030	2.023	4.538
other	6	-0.019	0.213	N.S.	5.171	2.511	10.650
Start year of study							
<1960	14	Aliased			1.583	0.954	2.625
1960-69	14	0.759	0.167	+++	3.380	2.471	4.624
1970-79	31	0.677	0.150	+++	3.114	2.434	3.984
1980-89	40	0.857	0.155	+++	3.729	3.229	4.307
1990+	8	0.885	0.201	+++	3.836	2.147	6.853
		Estimate					
Study type (1)							
CC	98	Aliased					
other	9	Aliased					
		Estimate	S.E.	P	RR	95%CIl	95%CIu
Study size (number of LC cases)							
100-249	27	Aliased			2.177	1.608	2.947
250-499	31	0.257	0.108	+	2.815	2.097	3.779
500-999	18	0.539	0.123	+++	3.733	2.743	5.078
1000+	31	0.588	0.101	+++	3.919	3.390	4.531
Number of adjustment variables (1)							
0	54	Aliased			2.851	2.358	3.446
1	20	0.398	0.111	+++	4.243	3.075	5.856
2+/+nk	33	0.277	0.079	+++	3.762	3.216	4.401
Study type (2)							
CC	98	Aliased			3.381	3.077	3.716
prosp	5	0.397	0.167	+	5.030	2.810	9.004
other	4	0.258	0.236	N.S.	4.375	1.899	10.076

		Deviance	(DF)	Drop Dev	P		
Model 8		275.837	(85)	8.309	N.S.		
		Estimate	S.E.	P	RR	95%CIl	95%CIu
Constant		0.282	0.182	N.S.	1.325	0.928	1.892

Table 3A1R - 4

IESLC - Meta-regression of ever smoking, any product (or cigs if any not available)
Multiple regression of data from Table 3A1
Adenocarcinoma
Effect of additional characteristics

WEIGHTED on Weight

		Estimate	S.E.	P	RR	95%CIl	95%CIu
Sex(RR)							
Male	51	Aliased			3.921	3.342	4.600
Female	45	-0.272	0.059	---	2.988	2.621	3.408
Combined	11	0.177	0.140	N.S.	4.680	2.990	7.325
Location							
NAmer	40	Aliased			5.153	4.435	5.987
UK	4	-0.486	0.325	N.S.	3.169	1.024	9.813
Scand	7	-0.544	0.193	--	2.989	1.565	5.710
othEur	15	-0.677	0.111	---	2.620	1.855	3.700
China	12	-1.310	0.097	---	1.390	1.038	1.861
Japan	11	-1.137	0.120	---	1.653	1.142	2.392
othAs	12	-0.470	0.131	---	3.221	2.135	4.860
other	6	-0.047	0.213	N.S.	4.916	2.398	10.079
Start year of study							
<1960	14	Aliased			1.541	0.932	2.548
1960-69	14	0.851	0.171	+++	3.608	2.607	4.991
1970-79	31	0.707	0.150	+++	3.123	2.447	3.987
1980-89	40	0.866	0.155	+++	3.664	3.171	4.234
1990+	8	0.962	0.205	+++	4.031	2.232	7.279
Study type (1)							
CC	98	Aliased			3.375	3.074	3.706
other	9	0.396	0.143	++	5.017	3.072	8.195
Study size (number of LC cases)							
100-249	27	Aliased			2.200	1.633	2.963
250-499	31	0.250	0.110	+	2.824	2.091	3.814
500-999	18	0.599	0.124	+++	4.005	2.903	5.523
1000+	31	0.560	0.101	+++	3.853	3.320	4.471
		Estimate					
Number of adjustment variables (1)							
0	54	Aliased					
1	20	Aliased					
2+/+nk	33	Aliased					
		Estimate	S.E.	P	RR	95%CIl	95%CIu
Number of adjustment variables (2)							
0	54	Aliased			2.745	2.261	3.332
1	20	0.427	0.111	+++	4.206	3.058	5.784
2	19	0.404	0.093	+++	4.111	3.364	5.025
3-5	10	0.199	0.108	(+)	3.349	2.384	4.703
6+/+nk	4	-0.222	0.234	N.S.	2.199	0.966	5.008

		Deviance	(DF)	Drop Dev	P		
Model 8		279.377	(86)	4.770	N.S.		
		Estimate	S.E.	P	RR	95%CIl	95%CIu
Constant		0.283	0.182	N.S.	1.327	0.929	1.895
Sex(RR)							
Male	51	Aliased			3.957	3.379	4.632
Female	45	-0.281	0.058	---	2.989	2.623	3.405
Combined	11	0.130	0.138	N.S.	4.505	2.893	7.017
Location							
NAmer	40	Aliased			5.257	4.543	6.084
UK	4	-0.500	0.325	N.S.	3.189	1.029	9.881
Scand	7	-0.652	0.190	---	2.740	1.448	5.188
othEur	15	-0.664	0.111	---	2.705	1.905	3.841
China	12	-1.356	0.094	---	1.355	1.015	1.810
Japan	11	-1.201	0.120	---	1.582	1.087	2.301
othAs	12	-0.587	0.128	---	2.923	1.951	4.377
other	6	0.039	0.215	N.S.	5.469	2.655	11.265
Start year of study							
<1960	14	Aliased			1.517	0.914	2.518
1960-69	14	0.835	0.170	+++	3.494	2.555	4.779
1970-79	31	0.686	0.150	+++	3.011	2.348	3.862
1980-89	40	0.913	0.157	+++	3.778	3.273	4.362
1990+	8	0.885	0.200	+++	3.675	2.061	6.556

Table 3A1R - 4

IESLC - Meta-regression of ever smoking, any product (or cigs if any not available)
Multiple regression of data from Table 3A1
Adenocarcinoma
Effect of additional characteristics

WEIGHTED on Weight

		Estimate	S.E.	P	RR	95%CIl	95%CIu
Study type (1)							
CC	98	Aliased			3.401	3.096	3.736
other	9	0.225	0.152	N.S.	4.259	2.529	7.172
Study size (number of LC cases)							
100-249	27	Aliased			2.216	1.642	2.990
250-499	31	0.231	0.108	+	2.791	2.083	3.739
500-999	18	0.546	0.121	+++	3.825	2.817	5.192
1000+	31	0.564	0.101	+++	3.896	3.373	4.501
Number of adjustment variables (1)							
0	54	Aliased			2.748	2.255	3.348
1	20	0.470	0.116	+++	4.398	3.178	6.086
2+/+nk	33	0.334	0.083	+++	3.836	3.272	4.496
RR adjusted for or study matched on age							
Yes	82	Aliased			3.340	3.017	3.697
No	25	0.239	0.110	+	4.243	2.980	6.041

		Deviance	(DF)	Drop Dev	P		
Model 8		279.603	(86)	4.543	N.S.		
		Estimate	S.E.	P	RR	95%CIl	95%CIu
Constant		0.526	0.193	++	1.692	1.160	2.468
Sex(RR)							
Male	51	Aliased			3.935	3.360	4.608
Female	45	-0.270	0.058	---	3.005	2.638	3.423
Combined	11	0.124	0.138	N.S.	4.455	2.864	6.930
Location							
NAmer	40	Aliased			5.253	4.539	6.081
UK	4	-0.590	0.325	(-)	2.913	0.937	9.057
Scand	7	-0.566	0.192	--	2.984	1.565	5.689
othEur	15	-0.765	0.108	---	2.444	1.738	3.438
China	12	-1.355	0.094	---	1.355	1.015	1.810
Japan	11	-1.075	0.125	---	1.792	1.213	2.648
othAs	12	-0.567	0.127	---	2.981	1.996	4.452
other	6	-0.030	0.213	N.S.	5.100	2.491	10.442
Start year of study							
<1960	14	Aliased			1.695	1.014	2.833
1960-69	14	0.661	0.171	+++	3.282	2.411	4.468
1970-79	31	0.601	0.153	+++	3.090	2.422	3.944
1980-89	40	0.797	0.157	+++	3.761	3.260	4.339
1990+	8	0.776	0.208	+++	3.680	2.063	6.566
Study type (1)							
CC	98	Aliased			3.373	3.072	3.704
other	9	0.407	0.143	++	5.066	3.102	8.272
Study size (number of LC cases)							
100-249	27	Aliased			2.190	1.626	2.950
250-499	31	0.271	0.107	+	2.871	2.140	3.851
500-999	18	0.506	0.123	+++	3.633	2.669	4.945
1000+	31	0.580	0.100	+++	3.914	3.389	4.519
Number of adjustment variables (1)							
0	54	Aliased			3.002	2.451	3.678
1	20	0.347	0.112	++	4.247	3.087	5.842
2+/+nk	33	0.185	0.088	+	3.613	3.055	4.273
RR adjusted for or matched on factor other than sex, age							
Yes	67	Aliased			3.581	3.196	4.013
No	40	-0.187	0.088	-	2.971	2.297	3.843

		Deviance	(DF)	Drop Dev	P		
Model 8		266.664	(85)	17.483	(*)		
		Estimate	S.E.	P	RR	95%CIl	95%CIu
Constant		0.429	0.179	+	1.536	1.082	2.180
Sex(RR)							
Male	51	Aliased			4.086	3.489	4.785
Female	45	-0.328	0.060	---	2.944	2.582	3.356
Combined	11	0.058	0.140	N.S.	4.330	2.783	6.735

Table 3A1R - 4

IESLC - Meta-regression of ever smoking, any product (or cigs if any not available)
Multiple regression of data from Table 3A1
Adenocarcinoma
Effect of additional characteristics

WEIGHTED on Weight

		Estimate	S.E.	P	RR	95%CIl	95%CIu
Location							
NAmer	40	Aliased			5.367	4.634	6.215
UK	4	-0.604	0.336	(-)	2.933	0.936	9.195
Scand	7	-0.680	0.195	---	2.719	1.436	5.149
othEur	15	-0.768	0.108	---	2.489	1.788	3.464
China	12	-1.387	0.095	---	1.340	1.005	1.787
Japan	11	-1.211	0.121	---	1.599	1.109	2.305
othAs	12	-0.601	0.133	---	2.941	1.959	4.416
other	6	0.019	0.213	N.S.	5.469	2.701	11.077
Start year of study							
<1960	14	Aliased			1.519	0.917	2.515
1960-69	14	0.741	0.166	+++	3.187	2.317	4.383
1970-79	31	0.705	0.154	+++	3.073	2.414	3.911
1980-89	40	0.916	0.158	+++	3.796	3.298	4.370
1990+	8	0.947	0.210	+++	3.914	2.203	6.956
Study type (1)							
CC	98	Aliased			3.379	3.082	3.704
other	9	0.369	0.142	+	4.887	3.027	7.890
Study size (number of LC cases)							
100-249	27	Aliased			2.160	1.604	2.908
250-499	31	0.244	0.108	+	2.757	2.043	3.719
500-999	18	0.539	0.122	+++	3.704	2.746	4.994
1000+	31	0.605	0.104	+++	3.956	3.417	4.580
Number of adjustment variables (1)							
0	54	Aliased			2.836	2.359	3.409
1	20	0.407	0.112	+++	4.259	3.102	5.848
2+/+nk	33	0.286	0.078	+++	3.774	3.240	4.395
Product							
all/unsp	55	Aliased			3.664	2.919	4.601
cig+/-ot	50	-0.064	0.084	N.S.	3.438	3.037	3.892
cig only	2	-0.731	0.181	---	1.763	1.005	3.095

		Deviance	(DF)	Drop Dev	P		
Model 8		281.383	(86)	2.763	N.S.		
		Estimate	S.E.	P	RR	95%CIl	95%CIu
Constant		0.355	0.178	+	1.426	1.006	2.020
Sex(RR)							
Male	51	Aliased			3.972	3.390	4.654
Female	45	-0.285	0.058	---	2.986	2.619	3.405
Combined	11	0.115	0.138	N.S.	4.454	2.858	6.942
Location							
NAmer	40	Aliased			5.180	4.453	6.027
UK	4	-0.415	0.332	N.S.	3.421	1.078	10.853
Scand	7	-0.561	0.194	--	2.955	1.545	5.653
othEur	15	-0.664	0.114	---	2.668	1.875	3.795
China	12	-1.369	0.095	---	1.318	0.979	1.775
Japan	11	-1.109	0.122	---	1.709	1.175	2.487
othAs	12	-0.520	0.129	---	3.081	2.057	4.614
other	6	0.046	0.217	N.S.	5.423	2.621	11.221
Start year of study							
<1960	14	Aliased			1.627	0.981	2.701
1960-69	14	0.756	0.166	+++	3.464	2.529	4.746
1970-79	31	0.655	0.150	+++	3.131	2.451	4.002
1980-89	40	0.825	0.156	+++	3.711	3.215	4.285
1990+	8	0.818	0.205	+++	3.687	2.057	6.611
Study type (1)							
CC	98	Aliased			3.390	3.086	3.724
other	9	0.298	0.144	+	4.569	2.780	7.507
Study size (number of LC cases)							
100-249	27	Aliased			2.225	1.641	3.016
250-499	31	0.264	0.107	+	2.899	2.149	3.910
500-999	18	0.511	0.123	+++	3.708	2.731	5.035
1000+	31	0.555	0.103	+++	3.874	3.346	4.485

Table 3A1R - 4

IESLC - Meta-regression of ever smoking, any product (or cigs if any not available)
Multiple regression of data from Table 3A1
Adenocarcinoma
Effect of additional characteristics

WEIGHTED on Weight

		Estimate	S.E.	P	RR	95%CIl	95%CIu
Number of adjustment variables (1)							
0	54	Aliased			2.899	2.399	3.503
1	20	0.336	0.115	++	4.059	2.913	5.654
2+/+nk	33	0.259	0.078	++	3.755	3.214	4.388
Denominator							
nev any	67	Aliased			3.202	2.686	3.817
nev cigs	40	0.125	0.075	N.S.	3.626	3.131	4.200

		Deviance	(DF)	Drop Dev	P		
Model 8		262.676	(85)	21.470	*		
		Estimate	S.E.	P	RR	95%CIl	95%CIu
Constant		-0.121	0.226	N.S.	0.886	0.569	1.381
Sex(RR)							
Male	51	Aliased			4.072	3.481	4.763
Female	45	-0.335	0.060	---	2.913	2.560	3.315
Combined	11	0.139	0.139	N.S.	4.677	3.031	7.218
Location							
NAmer	40	Aliased			4.391	3.470	5.558
UK	4	-0.071	0.353	N.S.	4.089	1.213	13.783
Scand	7	-0.618	0.190	--	2.368	1.246	4.498
othEur	15	-0.746	0.107	---	2.082	1.430	3.032
China	12	-0.006	0.413	N.S.	4.363	1.273	14.957
Japan	11	-1.206	0.119	---	1.314	0.865	1.995
othAs	12	-0.378	0.134	--	3.010	2.027	4.471
other	6	-0.122	0.214	N.S.	3.889	1.863	8.115
Start year of study							
<1960	14	Aliased			1.540	0.944	2.514
1960-69	14	0.793	0.166	+++	3.403	2.524	4.588
1970-79	31	0.688	0.150	+++	3.064	2.415	3.888
1980-89	40	0.875	0.155	+++	3.696	3.214	4.250
1990+	8	1.060	0.204	+++	4.447	2.510	7.879
Study type (1)							
CC	98	Aliased			3.377	3.082	3.699
other	9	0.384	0.141	++	4.957	3.091	7.949
Study size (number of LC cases)							
100-249	27	Aliased			2.160	1.615	2.889
250-499	31	0.201	0.109	(+)	2.641	1.977	3.529
500-999	18	0.649	0.124	+++	4.132	3.036	5.622
1000+	31	0.595	0.100	+++	3.918	3.404	4.510
Number of adjustment variables (1)							
0	54	Aliased			2.726	2.265	3.282
1	20	0.444	0.110	+++	4.248	3.113	5.797
2+/+nk	33	0.356	0.080	+++	3.893	3.338	4.539
National cigarette tobacco type							
Virginia	9	Aliased			2.660	1.626	4.353
blended	85	0.471	0.141	++	4.260	3.446	5.266
other	13	-0.961	0.421	-	1.018	0.300	3.454

		Deviance	(DF)	Drop Dev	P		
Model 8		264.249	(86)	19.897	*		
		Estimate	S.E.	P	RR	95%CIl	95%CIu
Constant		0.419	0.178	+	1.521	1.073	2.156
Sex(RR)							
Male	51	Aliased			3.996	3.427	4.659
Female	45	-0.318	0.059	---	2.908	2.556	3.308
Combined	11	0.247	0.142	(+)	5.115	3.278	7.982

Table 3A1R - 4

IESLC - Meta-regression of ever smoking, any product (or cigs if any not available)
Multiple regression of data from Table 3A1
Adenocarcinoma
Effect of additional characteristics

WEIGHTED on Weight

		Estimate	S.E.	P	RR	95%CIl	95%CIu
Location							
NAmer	40	Aliased			5.651	4.856	6.577
UK	4	-0.681	0.326	-	2.859	0.952	8.593
Scand	7	-0.648	0.190	---	2.957	1.590	5.502
othEur	15	-0.862	0.111	---	2.386	1.716	3.317
China	12	-1.489	0.099	---	1.274	0.958	1.696
Japan	11	-1.384	0.129	---	1.416	0.971	2.064
othAs	12	-0.690	0.130	---	2.834	1.915	4.196
other	6	-0.268	0.220	N.S.	4.322	2.126	8.784
Start year of study							
<1960	14	Aliased			1.429	0.872	2.343
1960-69	14	0.737	0.166	+++	2.987	2.189	4.076
1970-79	31	0.844	0.155	+++	3.323	2.607	4.236
1980-89	40	0.962	0.156	+++	3.740	3.256	4.296
1990+	8	1.043	0.203	+++	4.055	2.320	7.087
Study type (1)							
CC	98	Aliased			3.399	3.104	3.723
other	9	0.236	0.143	N.S.	4.303	2.670	6.934
Study size (number of LC cases)							
100-249	27	Aliased			2.195	1.644	2.931
250-499	31	0.272	0.107	+	2.881	2.167	3.829
500-999	18	0.635	0.122	+++	4.142	3.052	5.621
1000+	31	0.551	0.100	+++	3.806	3.303	4.386
Number of adjustment variables (1)							
0	54	Aliased			2.842	2.371	3.408
1	20	0.472	0.111	+++	4.555	3.324	6.242
2+/+nk	33	0.265	0.078	++	3.703	3.184	4.308
Any proxy use							
No/nk	79	Aliased			3.655	3.306	4.041
Yes	28	-0.396	0.089	---	2.461	1.875	3.229

		Deviance	(DF)	Drop Dev	P		
Model 8		281.797	(86)	2.349	N.S.		
		Estimate	S.E.	P	RR	95%CIl	95%CIu
Constant		0.280	0.187	N.S.	1.323	0.917	1.908
Sex(RR)							
Male	51	Aliased			3.926	3.348	4.604
Female	45	-0.269	0.058	---	3.001	2.633	3.420
Combined	11	0.144	0.141	N.S.	4.536	2.892	7.113
Location							
NAmer	40	Aliased			5.189	4.461	6.037
UK	4	-0.448	0.329	N.S.	3.315	1.054	10.431
Scand	7	-0.589	0.192	--	2.879	1.514	5.472
othEur	15	-0.722	0.107	---	2.522	1.800	3.532
China	12	-1.302	0.101	---	1.411	1.042	1.912
Japan	11	-1.158	0.119	---	1.630	1.125	2.362
othAs	12	-0.489	0.134	---	3.181	2.092	4.838
other	6	0.037	0.217	N.S.	5.386	2.604	11.140
Start year of study							
<1960	14	Aliased			1.603	0.968	2.655
1960-69	14	0.745	0.166	+++	3.375	2.481	4.591
1970-79	31	0.672	0.150	+++	3.138	2.454	4.013
1980-89	40	0.842	0.155	+++	3.719	3.222	4.293
1990+	8	0.867	0.201	+++	3.816	2.145	6.789
Study type (1)							
CC	98	Aliased			3.376	3.073	3.708
other	9	0.391	0.143	++	4.989	3.050	8.159
Study size (number of LC cases)							
100-249	27	Aliased			2.146	1.592	2.894
250-499	31	0.294	0.109	++	2.879	2.138	3.877
500-999	18	0.571	0.122	+++	3.800	2.796	5.165
1000+	31	0.595	0.100	+++	3.890	3.363	4.500

Table 3A1R - 4

IESLC - Meta-regression of ever smoking, any product (or cigs if any not available)
Multiple regression of data from Table 3A1
Adenocarcinoma
Effect of additional characteristics

WEIGHTED on Weight

		Estimate	S.E.	P	RR	95%CIl	95%CIu
Number of adjustment variables (1)							
0	54	Aliased			2.850	2.363	3.438
1	20	0.387	0.110	+++	4.196	3.045	5.781
2+/+nk	33	0.280	0.078	+++	3.773	3.227	4.411
Full histological confirmation							
No	59	Aliased			3.226	2.716	3.831
Yes	48	0.106	0.069	N.S.	3.588	3.133	4.111

		Deviance	(DF)	Drop Dev	P		
Model 8		282.281	(85)	1.865	N.S.		
		Estimate	S.E.	P	RR	95%CIl	95%CIu
Constant		0.405	0.180	+	1.499	1.053	2.133
Sex(RR)							
Male	51	Aliased			3.983	3.392	4.676
Female	45	-0.278	0.058	---	3.015	2.640	3.444
Combined	11	0.042	0.148	N.S.	4.153	2.573	6.702
Location							
NAmer	40	Aliased			5.319	4.573	6.187
UK	4	-0.567	0.326	(-)	3.019	0.960	9.492
Scand	7	-0.659	0.191	---	2.752	1.442	5.252
othEur	15	-0.758	0.110	---	2.493	1.765	3.523
China	12	-1.355	0.096	---	1.372	1.021	1.842
Japan	11	-1.178	0.120	---	1.637	1.128	2.376
othAs	12	-0.586	0.129	---	2.960	1.968	4.453
other	6	-0.054	0.215	N.S.	5.041	2.434	10.442
Start year of study							
<1960	14	Aliased			1.609	0.966	2.682
1960-69	14	0.704	0.171	+++	3.254	2.352	4.501
1970-79	31	0.663	0.150	+++	3.122	2.437	4.000
1980-89	40	0.842	0.156	+++	3.734	3.231	4.315
1990+	8	0.911	0.202	+++	4.003	2.197	7.295
Study type (1)							
CC	98	Aliased			3.389	3.083	3.726
other	9	0.302	0.147	+	4.584	2.752	7.636
Study size (number of LC cases)							
100-249	27	Aliased			2.148	1.586	2.909
250-499	31	0.287	0.109	++	2.862	2.123	3.858
500-999	18	0.553	0.122	+++	3.732	2.743	5.079
1000+	31	0.599	0.101	+++	3.911	3.380	4.525
Number of adjustment variables (1)							
0	54	Aliased			2.893	2.389	3.502
1	20	0.393	0.110	+++	4.286	3.097	5.931
2+/+nk	33	0.249	0.080	++	3.710	3.159	4.358
Risky occupational population							
No	105	Aliased			3.433	3.131	3.765
Mining	1	-0.521	0.553	N.S.	2.040	0.284	14.666
Other risky	1	0.515	0.508	N.S.	5.746	0.941	35.092

		Deviance	(DF)	Drop Dev	P		
Model 8		271.526	(84)	12.621	N.S.		
		Estimate	S.E.	P	RR	95%CIl	95%CIu
Constant		0.327	0.179	(+)	1.386	0.976	1.969
Sex(RR)							
Male	51	Aliased			3.954	3.377	4.630
Female	45	-0.269	0.058	---	3.022	2.654	3.442
Combined	11	0.065	0.139	N.S.	4.221	2.712	6.569

Table 3A1R - 4

IESLC - Meta-regression of ever smoking, any product (or cigs if any not available)
Multiple regression of data from Table 3A1
Adenocarcinoma
Effect of additional characteristics

WEIGHTED on Weight

		Estimate	S.E.	P	RR	95%CIl	95%CIu
Location							
NAmer	40	Aliased			5.111	4.395	5.944
UK	4	-0.455	0.334	N.S.	3.241	1.023	10.275
Scand	7	-0.521	0.193	--	3.034	1.596	5.770
othEur	15	-0.732	0.107	---	2.459	1.753	3.449
China	12	-1.235	0.117	---	1.486	1.042	2.121
Japan	11	-1.123	0.120	---	1.662	1.150	2.402
othAs	12	-0.445	0.133	--	3.276	2.153	4.984
other	6	0.018	0.214	N.S.	5.202	2.531	10.692
Start year of study							
<1960	14	Aliased			1.691	1.021	2.803
1960-69	14	0.781	0.166	+++	3.694	2.680	5.089
1970-79	31	0.657	0.150	+++	3.261	2.538	4.190
1980-89	40	0.744	0.159	+++	3.560	3.056	4.145
1990+	8	0.887	0.203	+++	4.106	2.287	7.370
Study type (1)							
CC	98	Aliased			3.417	3.110	3.754
other	9	0.123	0.160	N.S.	3.862	2.240	6.658
Study size (number of LC cases)							
100-249	27	Aliased			2.132	1.584	2.869
250-499	31	0.225	0.109	+	2.670	1.976	3.609
500-999	18	0.568	0.130	+++	3.762	2.657	5.325
1000+	31	0.627	0.101	+++	3.991	3.434	4.637
Number of adjustment variables (1)							
0	54	Aliased			2.690	2.203	3.286
1	20	0.409	0.112	+++	4.051	2.934	5.593
2+/+nk	33	0.392	0.087	+++	3.981	3.366	4.707
Lowest age in RR							
<25/unlim	76	Aliased			3.404	3.035	3.817
25-39	17	-0.166	0.119	N.S.	2.882	1.989	4.178
40+	12	0.498	0.163	++	5.603	3.244	9.680
unknown	2	0.114	0.246	N.S.	3.813	1.668	8.716

		Deviance	(DF)	Drop Dev	P		
Model 8		282.182	(83)	1.965	N.S.		
		Estimate	S.E.	P	RR	95%CIl	95%CIu
Constant		0.528	0.276	(+)	1.696	0.987	2.913
Sex(RR)							
Male	51	Aliased			3.941	3.351	4.635
Female	45	-0.269	0.059	---	3.012	2.635	3.443
Combined	11	0.102	0.139	N.S.	4.363	2.772	6.869
Location							
NAmer	40	Aliased			5.240	4.425	6.206
UK	4	-0.575	0.378	N.S.	2.948	0.789	11.013
Scand	7	-0.608	0.194	--	2.853	1.478	5.505
othEur	15	-0.715	0.109	---	2.564	1.813	3.625
China	12	-1.374	0.143	---	1.327	0.861	2.046
Japan	11	-1.151	0.126	---	1.658	1.120	2.452
othAs	12	-0.498	0.138	---	3.186	2.048	4.954
other	6	0.039	0.221	N.S.	5.450	2.561	11.599
Start year of study							
<1960	14	Aliased			1.619	0.965	2.717
1960-69	14	0.754	0.166	+++	3.439	2.485	4.761
1970-79	31	0.661	0.151	+++	3.135	2.416	4.070
1980-89	40	0.825	0.158	+++	3.694	3.169	4.306
1990+	8	0.881	0.207	+++	3.906	2.115	7.212
Study type (1)							
CC	98	Aliased			3.381	3.069	3.724
other	9	0.358	0.164	+	4.838	2.722	8.599

Table 3A1R - 4

IESLC - Meta-regression of ever smoking, any product (or cigs if any not available)
Multiple regression of data from Table 3A1
Adenocarcinoma
Effect of additional characteristics

WEIGHTED on Weight

		Estimate	S.E.	P	RR	95%CIl	95%CIu
Study size (number of LC cases)							
100-249	27	Aliased			2.111	1.546	2.884
250-499	31	0.289	0.110	+	2.820	2.078	3.826
500-999	18	0.529	0.130	+++	3.583	2.495	5.146
1000+	31	0.632	0.104	+++	3.974	3.395	4.651
Number of adjustment variables (1)							
0	54	Aliased			2.785	2.266	3.422
1	20	0.429	0.114	+++	4.279	3.061	5.980
2+/+nk	33	0.317	0.087	+++	3.822	3.222	4.535
Highest age in RR							
<65	6	Aliased			4.197	1.835	9.598
65-74	12	-0.194	0.281	N.S.	3.455	2.069	5.771
75-84	13	-0.121	0.258	N.S.	3.720	2.477	5.589
85+/unlim	74	-0.218	0.230	N.S.	3.374	2.957	3.851
unknown	2	0.008	0.345	N.S.	4.232	1.728	10.363

		Deviance	(DF)	Drop Dev	P		
Model 8		283.489	(86)	0.657	N.S.		
		Estimate	S.E.	P	RR	95%CIl	95%CIu
Constant		-0.044	0.538	N.S.	0.957	0.334	2.748
Sex(RR)							
Male	51	Aliased			3.942	3.361	4.623
Female	45	-0.272	0.058	---	3.004	2.635	3.425
Combined	11	0.118	0.139	N.S.	4.434	2.835	6.935
Location							
NAmer	40	Aliased			5.229	4.493	6.085
UK	4	-0.467	0.335	N.S.	3.278	1.020	10.534
Scand	7	-0.617	0.190	--	2.821	1.484	5.360
othEur	15	-0.718	0.108	---	2.551	1.819	3.578
China	12	-1.322	0.103	---	1.394	1.020	1.905
Japan	11	-1.144	0.120	---	1.665	1.149	2.413
othAs	12	-0.552	0.127	---	3.010	2.011	4.507
other	6	-0.024	0.213	N.S.	5.107	2.480	10.514
Start year of study							
<1960	14	Aliased			1.585	0.956	2.627
1960-69	14	0.764	0.167	+++	3.401	2.484	4.655
1970-79	31	0.676	0.150	+++	3.116	2.437	3.985
1980-89	40	0.854	0.155	+++	3.724	3.224	4.302
1990+	8	0.882	0.201	+++	3.830	2.147	6.830
Study type (1)							
CC	98	Aliased			3.383	3.079	3.717
other	9	0.345	0.141	+	4.776	2.935	7.774
Study size (number of LC cases)							
100-249	27	Aliased			2.195	1.616	2.981
250-499	31	0.249	0.109	+	2.815	2.098	3.779
500-999	18	0.541	0.121	+++	3.769	2.772	5.123
1000+	31	0.576	0.102	+++	3.905	3.375	4.518
Number of adjustment variables (1)							
0	54	Aliased			2.848	2.358	3.440
1	20	0.385	0.110	+++	4.186	3.027	5.788
2+/+nk	33	0.282	0.079	+++	3.778	3.224	4.426
Midpoint age in RR		0.007	0.009	N.S.	2.296	0.391	13.497

		Deviance	(DF)	Drop Dev	P		
Model 8		284.140	(86)	0.007	N.S.		
		Estimate	S.E.	P	RR	95%CIl	95%CIu
Constant		0.376	0.201	(+)	1.457	0.981	2.162
Sex(RR)							
Male	51	Aliased			3.957	3.365	4.655
Female	45	-0.276	0.059	---	3.003	2.633	3.425
Combined	11	0.099	0.141	N.S.	4.371	2.784	6.861

Table 3A1R - 4

IESLC - Meta-regression of ever smoking, any product (or cigs if any not available)
Multiple regression of data from Table 3A1
Adenocarcinoma
Effect of additional characteristics

WEIGHTED on Weight

		Estimate	S.E.	P	RR	95%CIl	95%CIu
Location							
NAmer	40	Aliased			5.274	4.550	6.113
UK	4	-0.536	0.325	N.S.	3.087	0.987	9.651
Scand	7	-0.632	0.191	--	2.804	1.470	5.347
othEur	15	-0.731	0.107	---	2.538	1.810	3.559
China	12	-1.356	0.095	---	1.358	1.014	1.821
Japan	11	-1.156	0.121	---	1.660	1.130	2.437
othAs	12	-0.560	0.132	---	3.014	1.981	4.586
other	6	-0.023	0.213	N.S.	5.153	2.500	10.618
Start year of study							
<1960	14	Aliased			1.587	0.954	2.640
1960-69	14	0.749	0.166	+++	3.359	2.459	4.587
1970-79	31	0.671	0.151	+++	3.105	2.429	3.971
1980-89	40	0.855	0.156	+++	3.734	3.231	4.315
1990+	8	0.891	0.200	+++	3.871	2.167	6.915
Study type (1)							
CC	98	Aliased			3.382	3.077	3.716
other	9	0.353	0.141	+	4.812	2.957	7.829
Study size (number of LC cases)							
100-249	27	Aliased			2.167	1.593	2.947
250-499	31	0.264	0.107	+	2.822	2.092	3.806
500-999	18	0.548	0.123	+++	3.747	2.757	5.093
1000+	31	0.592	0.103	+++	3.917	3.380	4.539
Number of adjustment variables (1)							
0	54	Aliased			2.865	2.354	3.486
1	20	0.391	0.110	+++	4.233	3.060	5.856
2+/+nk	33	0.269	0.084	++	3.750	3.177	4.426
Derivation of RR/CI							
Orig/2x2	40	Aliased			3.445	2.971	3.995
Other	67	-0.006	0.073	N.S.	3.424	2.889	4.059

		Deviance	(DF)	Drop Dev	P		
Model 8		283.397	(85)	0.749	N.S.		
		Estimate	S.E.	P	RR	95%CIl	95%CIu
Constant		0.488	0.240	+	1.629	1.017	2.609
Sex(RR)							
Male	51	Aliased			3.956	3.364	4.652
Female	45	-0.275	0.059	---	3.005	2.634	3.428
Combined	11	0.098	0.142	N.S.	4.362	2.761	6.893
Location							
NAmer	40	Aliased			5.246	4.517	6.092
UK	4	-0.529	0.325	N.S.	3.091	0.981	9.735
Scand	7	-0.617	0.190	--	2.830	1.482	5.402
othEur	15	-0.719	0.108	---	2.556	1.815	3.599
China	12	-1.351	0.095	---	1.359	1.013	1.823
Japan	11	-1.130	0.126	---	1.695	1.142	2.518
othAs	12	-0.561	0.128	---	2.992	1.983	4.514
other	6	0.005	0.216	N.S.	5.273	2.529	10.995
Start year of study							
<1960	14	Aliased			1.609	0.956	2.706
1960-69	14	0.763	0.168	+++	3.450	2.478	4.802
1970-79	31	0.656	0.153	+++	3.099	2.420	3.967
1980-89	40	0.839	0.159	+++	3.724	3.218	4.309
1990+	8	0.850	0.206	+++	3.765	2.067	6.859
Study type (1)							
CC	98	Aliased			3.384	3.078	3.721
other	9	0.334	0.142	+	4.728	2.885	7.749
Study size (number of LC cases)							
100-249	27	Aliased			2.184	1.599	2.984
250-499	31	0.261	0.108	+	2.835	2.104	3.820
500-999	18	0.527	0.124	+++	3.699	2.702	5.066
1000+	31	0.584	0.104	+++	3.916	3.375	4.543

Table 3A1R - 4

IESLC - Meta-regression of ever smoking, any product (or cigs if any not available)
Multiple regression of data from Table 3A1
Adenocarcinoma
Effect of additional characteristics

WEIGHTED on Weight

		Estimate	S.E.	P	RR	95%CIl	95%CIu
Number of adjustment variables (1)							
0	54	Aliased			3.022	2.249	4.061
1	20	0.296	0.158	(+)	4.064	2.781	5.939
2+/+nk	33	0.185	0.128	N.S.	3.635	2.949	4.481
Derivation of RR/CI							
Orig	26	Aliased			3.592	2.849	4.528
StcCalc	45	-0.115	0.136	N.S.	3.202	2.359	4.346
Other	36	-0.027	0.095	N.S.	3.496	2.739	4.463
