# Supplementary material for: Systematic review with meta-analysis of the epidemiological evidence in the 1900s relating smoking to lung cancer
Source: BMC Cancer. 2012 Sep 3;12:385. doi: 10.1186/1471-2407-12-385 (PMC3505152; doi:10.1186/1471-2407-12-385)
Supplement: Additional file 5 — Detailed Analysis Tables (Individual file names as described in Additional file 1: Methods, Table1). [file 1471-2407-12-385-S5.zip › PDF/3G.pdf]

Table 3G1 -

IESLC - Meta-analysis of Ever Smoking by Amount, Overview, Any product (or Cigarettes if Any not available)
Adenocarcinoma

This analysis is restricted to results for:
1) Results by Amount smoked
   Results by Amount smoked (in numbers of cigarettes or cigarette equivalents) are grouped under 2 schemes (S1, S2).
   Each scheme has a set of "key values". An interval is allocated to the category whose key value
   it includes and intervals which include none or more than one of the key values are excluded.
   (Open-ended intervals are coded as 99.)

S1	key value	maximum range	S2	key value	maximum range
1	5	1-19	1	1	1-9
2	20	6-44	2	10	2-19
3	45	21+	3	20	11-29
			4	30	21-39
			5	40	31-98
			6	99	41+

   For all/unspec product, the definition of cigarette equivalents is shown at the end of Sections -1 and -4.
2) Ever smokers
3) Results complete enough for use in metaanalysis

Within each study, results are then selected (in the following order of preference, within each sex) for:
4) PRODUCT: all/unspec, cigarettes regardless of other products, cigarettes only
5) CIGTYPE: all/unspecified, MC regardless of HR, MC only
6) DENOM: never smoked anything, never smoked cigarettes, (never +1 = +long term ex, +2 = +amount unknown,
       +3 = never cigs+long term ex)
7) Followup period (YF, prospective studies): whole study (coded as 0) or longest available
8) LCtype: adeno or nearest available, but not squamous. (q = squamous, s = small,
       a = adeno, l = large, KII = Kreyberg II, al = alveolar, br = bronchiolar, u = undifferentiated)
9) Race: all or nearest available, otherwise by race (wh or w = white, bl or b = black, hi = hispanic
       ch = chinese, jap = japanese, haw = hawaiian, w+o = white + oriental, sca = scandinavian, as = asian)
10) For overlapping studies: principal rather than subsidiary studies
Finally by Age: whole study (coded as 0) if available, otherwise by widest available age group
and then for single sex results (m, f) in preference to combined sex results (c).

Results adjusted (AD) for the most potential confounders are then chosen in Sections -1 to -3
and results adjusted for the least confounders in Sections -4 to -6. (Those least adjusted results which
actually differ from the most adjusted as marked 'x' in column X in Section -4)
(Results adjusted for an unknown number of confounder(s) are coded as 20.)

Section -7 shows excluded studies, together with the stage (as above) at which no qualifying
results were found.

Section -8 lists the potentially overlapping studies which have been included (1=principal, 2=subsidiary).

Section -9 lists any results which would have been included in preference except that they had data not complete
enough for use in meta-analysis, with their significance (yes/no), if known, and any further comment as entered
on the database.

In addition to those mentioned above, the following fields, levels and abbreviations are used:
* or nk = not known, n = no, y = yes, ot = other
nev = never
all/unspec = all or unspecified, cig+/-ot = cigarettes irrespective of other products (cigar, pipe etc)
MC = manufactured cigarettes, HR = hand-rolled cigarettes
exL, exH = range of exposure (low and high) in the smoking group, in terms of Amount smoked,
      cigarettes or cigarette equivalents
REF: 6-character study reference
NRR: number of the RR on the database within the study
ST : study type (CC = case control, pr or prosp = prospective)
NLC: number of lung cancer cases in whole study
R : risky occupational population (n = no, m = mining, o = other risky)
VB : national cigarette type (V = at least 75% Virginia, bl = at least 75% blended, ot = other)
P : any proxy use
H : full histological confirmation
De : derivation of RR/CI (or = original, st = standard method, ot = other method of estimation)

Table 3G1 - 1

IESLC - Meta-analysis of Ever Smoking by Amount, Overview, Any product (or Cigarettes if Any not available)
Adenocarcinoma
Most adjusted

REF	NRR	SEX	AGEL	AGEH	RACE	YF	LC TYPE	LOC	START	ST	NLC	R	VB	P	H	AD	PRODUCT	exL	exH	S1	S2	DENOM	De
ALDERS	46	m	0	0	all	-	not q+s	Eu:UK	1977	CC	1448	n	V	n	n	1	cig only	1	17	1	0	nev+2	ot
ALDERS	47	m	0	0	all	-	not q+s	Eu:UK	1977	CC	1448	n	V	n	n	1	cig only	18	27	2	3	nev+2	ot
ALDERS	48	m	0	0	all	-	not q+s	Eu:UK	1977	CC	1448	n	V	n	n	1	cig only	28	99	3	0	nev+2	ot
ALDERS	49	f	0	0	all	-	not q+s	Eu:UK	1977	CC	1448	n	V	n	n	1	cig only	1	17	1	0	nev+2	ot
ALDERS	50	f	0	0	all	-	not q+s	Eu:UK	1977	CC	1448	n	V	n	n	1	cig only	18	27	2	3	nev+2	ot
ALDERS	51	f	0	0	all	-	not q+s	Eu:UK	1977	CC	1448	n	V	n	n	1	cig only	28	99	3	0	nev+2	ot
BARBON	79	m	0	0	all	-	a	Eu:wst	1979	CC	755	n	bl	y	y	3	all/unsp	1	19	1	0	nev any	or
BARBON	80	m	0	0	all	-	a	Eu:wst	1979	CC	755	n	bl	y	y	3	all/unsp	20	39	2	0	nev any	or
BARBON	81	m	0	0	all	-	a	Eu:wst	1979	CC	755	n	bl	y	y	3	all/unsp	40	99	3	0	nev any	or
BROWN2	34	m	0	0	wh	-	a	NAmer	1984	CC	14596	n	bl	n	y	2	cig+/-ot	1	19	1	0	nev cigs	or
BROWN2	44	m	0	0	wh	-	a	NAmer	1984	CC	14596	n	bl	n	y	2	cig+/-ot	20	99	0	0	nev cigs	or
BROWN2	33	f	0	0	wh	-	a	NAmer	1984	CC	14596	n	bl	n	y	2	cig+/-ot	1	19	1	0	nev cigs	or
BROWN2	43	f	0	0	wh	-	a	NAmer	1984	CC	14596	n	bl	n	y	2	cig+/-ot	20	99	0	0	nev cigs	or
CHOI	51	m	0	0	all	-	a	As:oth	1985	CC	375	n	bl	n	n	0	cig+/-ot	1	10	1	0	nev cigs	st
CHOI	52	m	0	0	all	-	a	As:oth	1985	CC	375	n	bl	n	n	0	cig+/-ot	11	20	2	3	nev cigs	st
CHOI	53	m	0	0	all	-	a	As:oth	1985	CC	375	n	bl	n	n	0	cig+/-ot	21	30	0	4	nev cigs	st
CHOI	54	m	0	0	all	-	a	As:oth	1985	CC	375	n	bl	n	n	0	cig+/-ot	31	40	0	5	nev cigs	st
CHOI	55	m	0	0	all	-	a	As:oth	1985	CC	375	n	bl	n	n	0	cig+/-ot	41	99	3	6	nev cigs	st
CHOI	59	f	0	0	all	-	a	As:oth	1985	CC	375	n	bl	n	n	0	cig+/-ot	1	10	1	0	nev cigs	st
CHOI	60	f	0	0	all	-	a	As:oth	1985	CC	375	n	bl	n	n	0	cig+/-ot	11	30	2	0	nev cigs	ot
CHOI	61	f	0	0	all	-	a	As:oth	1985	CC	375	n	bl	n	n	0	cig+/-ot	31	99	3	0	nev cigs	st
DOLL	72	m	0	0	all	-	KII	Eu:UK	1948	CC	1465	n	V	n	n	1	all/unsp	1	4	0	1	nev any	ot
DOLL	73	m	0	0	all	-	KII	Eu:UK	1948	CC	1465	n	V	n	n	1	all/unsp	5	14	1	2	nev any	ot
DOLL	74	m	0	0	all	-	KII	Eu:UK	1948	CC	1465	n	V	n	n	1	all/unsp	15	24	2	3	nev any	ot
DOLL	75	m	0	0	all	-	KII	Eu:UK	1948	CC	1465	n	V	n	n	1	all/unsp	25	99	3	0	nev any	ot
DOLL	79	f	0	0	all	-	KII	Eu:UK	1948	CC	1465	n	V	n	n	1	all/unsp	1	4	0	1	nev any	ot
DOLL	80	f	0	0	all	-	KII	Eu:UK	1948	CC	1465	n	V	n	n	1	all/unsp	5	14	1	2	nev any	ot
DOLL	81	f	0	0	all	-	KII	Eu:UK	1948	CC	1465	n	V	n	n	1	all/unsp	15	99	0	0	nev any	ot
DORGAN	126	m	0	0	wh	-	a	NAmer	1980	CC	2026	n	bl	y	y	2	cig+/-ot	1	19	1	0	nev any	ot
DORGAN	127	m	0	0	wh	-	a	NAmer	1980	CC	2026	n	bl	y	y	2	cig+/-ot	20	99	0	0	nev any	ot
DORGAN	105	f	0	0	all	-	a	NAmer	1980	CC	2026	n	bl	y	y	3	cig+/-ot	1	19	1	0	nev any	ot
DORGAN	106	f	0	0	all	-	a	NAmer	1980	CC	2026	n	bl	y	y	3	cig+/-ot	20	99	0	0	nev any	ot
DOSEME	8	m	0	0	all	-	not q+s	Eu:bal	1979	CC	1210	n	bl	n	n	2	cig+/-ot	1	10	1	0	nev cigs	or
DOSEME	12	m	0	0	all	-	not q+s	Eu:bal	1979	CC	1210	n	bl	n	n	2	cig+/-ot	11	20	2	3	nev cigs	or
DOSEME	16	m	0	0	all	-	not q+s	Eu:bal	1979	CC	1210	n	bl	n	n	2	cig+/-ot	21	99	3	0	nev cigs	or
GER	10	c	0	0	all	-	a	As:oth	1990	CC	141	n	ot	y	n	8	all/unsp	1	10	1	0	nev any	ot
GER	11	c	0	0	all	-	a	As:oth	1990	CC	141	n	ot	y	n	8	all/unsp	11	20	2	3	nev any	ot
GER	12	c	0	0	all	-	a	As:oth	1990	CC	141	n	ot	y	n	8	all/unsp	21	99	3	0	nev any	ot
JEDRYC	36	m	0	0	all	-	a	Eu:est	1980	CC	1630	n	bl	y	n	3	cig+/-ot	1	19	1	0	nev any	or
JEDRYC	37	m	0	0	all	-	a	Eu:est	1980	CC	1630	n	bl	y	n	3	cig+/-ot	20	29	2	3	nev any	or
JEDRYC	38	m	0	0	all	-	a	Eu:est	1980	CC	1630	n	bl	y	n	3	cig+/-ot	30	99	3	0	nev any	or
KREYBE	5	m	0	0	all	-	KII	Eu:Sca	1948	CC	300	n	bl	n	y	1	all/unsp	1	14	1	0	nev any	ot
KREYBE	6	m	0	0	all	-	KII	Eu:Sca	1948	CC	300	n	bl	n	y	1	all/unsp	15	24	2	3	nev any	ot
KREYBE	7	m	0	0	all	-	KII	Eu:Sca	1948	CC	300	n	bl	n	y	1	all/unsp	25	99	3	0	nev any	ot
KREYBE	26	f	0	0	all	-	KII	Eu:Sca	1948	CC	300	n	bl	n	y	1	all/unsp	1	14	1	0	nev any	ot
KREYBE	61	f	0	0	all	-	KII	Eu:Sca	1948	CC	300	n	bl	n	y	1	all/unsp	15	99	0	0	nev any	ot
LAMTH	16	f	0	0	ch	-	a	As:HK	1983	CC	445	n	bl	n	n	0	all/unsp	1	10	1	0	nev any	or
LAMTH	17	f	0	0	ch	-	a	As:HK	1983	CC	445	n	bl	n	n	0	all/unsp	11	20	2	3	nev any	or
LAMTH	18	f	0	0	ch	-	a	As:HK	1983	CC	445	n	bl	n	n	0	all/unsp	21	99	3	0	nev any	or
LUBIN2	152	m	0	0	all	-	a	Eu:mul	1976	CC	7804	n	bl	n	y	0	cig+/-ot	1	9	1	1	nev any	st
LUBIN2	156	m	0	0	all	-	a	Eu:mul	1976	CC	7804	n	bl	n	y	0	cig+/-ot	10	19	0	2	nev any	st
LUBIN2	160	m	0	0	all	-	a	Eu:mul	1976	CC	7804	n	bl	n	y	0	cig+/-ot	20	29	2	3	nev any	st
LUBIN2	164	m	0	0	all	-	a	Eu:mul	1976	CC	7804	n	bl	n	y	0	cig+/-ot	30	99	3	0	nev any	st
LUBIN2	172	f	0	0	all	-	a	Eu:mul	1976	CC	7804	n	bl	n	y	0	cig+/-ot	1	9	1	1	nev any	st
LUBIN2	176	f	0	0	all	-	a	Eu:mul	1976	CC	7804	n	bl	n	y	0	cig+/-ot	10	19	0	2	nev any	st
LUBIN2	180	f	0	0	all	-	a	Eu:mul	1976	CC	7804	n	bl	n	y	0	cig+/-ot	20	29	2	3	nev any	st
LUBIN2	184	f	0	0	all	-	a	Eu:mul	1976	CC	7804	n	bl	n	y	0	cig+/-ot	30	99	3	0	nev any	st
MATOS	57	m	0	0	all	-	a	SCAmer	1994	CC	200	n	bl	n	n	2	cig+/-ot	1	14	1	0	nev any	or
MATOS	59	m	0	0	all	-	a	SCAmer	1994	CC	200	n	bl	n	n	2	cig+/-ot	15	24	2	3	nev any	or
MATOS	61	m	0	0	all	-	a	SCAmer	1994	CC	200	n	bl	n	n	2	cig+/-ot	25	99	3	0	nev any	or
MATSUD	7	m	0	0	all	-	a	As:Jap	1965	CC	179	n	bl	n	n	0	cig+/-ot	1	10	1	0	nev cigs	ot
MATSUD	8	m	0	0	all	-	a	As:Jap	1965	CC	179	n	bl	n	n	0	cig+/-ot	11	20	2	3	nev cigs	ot
MATSUD	9	m	0	0	all	-	a	As:Jap	1965	CC	179	n	bl	n	n	0	cig+/-ot	21	99	3	0	nev cigs	ot
ORMOS	22	m	0	0	all	-	KII	Eu:est	1947	CC	119	n	bl	y	y	0	cig+/-ot	1	15	1	0	nev any	st
ORMOS	23	m	0	0	all	-	KII	Eu:est	1947	CC	119	n	bl	y	y	0	cig+/-ot	16	30	2	0	nev any	st
ORMOS	24	m	0	0	all	-	KII	Eu:est	1947	CC	119	n	bl	y	y	0	cig+/-ot	31	99	3	0	nev any	st
OSANN	55	m	0	0	all	-	a	NAmer	1984	CC	1986	n	bl	n	n	2	cig+/-ot	1	39	0	0	nev cigs	or
OSANN	63	m	0	0	all	-	a	NAmer	1984	CC	1986	n	bl	n	n	2	cig+/-ot	40	99	3	0	nev cigs	or
OSANN	56	f	0	0	all	-	a	NAmer	1984	CC	1986	n	bl	n	n	2	cig+/-ot	1	39	0	0	nev cigs	or
OSANN	64	f	0	0	all	-	a	NAmer	1984	CC	1986	n	bl	n	n	2	cig+/-ot	40	99	3	0	nev cigs	or
OSANN2	34	f	0	0	all	-	KII	NAmer	1964	ot	217	n	bl	n	y	1	cig+/-ot	1	19	1	0	nev cigs	or
OSANN2	35	f	0	0	all	-	KII	NAmer	1964	ot	217	n	bl	n	y	1	cig+/-ot	20	99	0	0	nev cigs	or

Table 3G1 - 1

IESLC - Meta-analysis of Ever Smoking by Amount, Overview, Any product (or Cigarettes if Any not available)
Adenocarcinoma
Most adjusted

REF	NRR	SEX	AGEL	AGEH	RACE	YF	LC TYPE	LOC	START	ST	NLC	R	VB	P	H	AD	PRODUCT	exL	exH	S1	S2	DENOM	De
WUWILL	16	f	0	0	all	-	a	As:Chi	1985	CC	965	n	ot	n	n	3	cig+/-ot	1	19	1	0	nev cigs	ot
WUWILL	17	f	0	0	all	-	a	As:Chi	1985	CC	965	n	ot	n	n	3	cig+/-ot	20	99	0	0	nev cigs	ot
WYNDE2	10	m	0	0	all	-	KII	NAmer	1962	CC	404	n	bl	n	y	0	cig+/-ot	1	10	1	0	nev any	st
WYNDE2	11	m	0	0	all	-	KII	NAmer	1962	CC	404	n	bl	n	y	0	cig+/-ot	11	20	2	3	nev any	st
WYNDE2	12	m	0	0	all	-	KII	NAmer	1962	CC	404	n	bl	n	y	0	cig+/-ot	21	34	0	4	nev any	st
WYNDE2	13	m	0	0	all	-	KII	NAmer	1962	CC	404	n	bl	n	y	0	cig+/-ot	35	99	3	0	nev any	st
WYNDE3	24	m	0	0	all	-	KII	NAmer	1966	CC	350	n	bl	n	y	0	cig+/-ot	1	9	1	1	nev any	st
WYNDE3	25	m	0	0	all	-	KII	NAmer	1966	CC	350	n	bl	n	y	0	cig+/-ot	10	20	2	0	nev any	st
WYNDE3	26	m	0	0	all	-	KII	NAmer	1966	CC	350	n	bl	n	y	0	cig+/-ot	21	40	0	0	nev any	st
WYNDE3	27	m	0	0	all	-	KII	NAmer	1966	CC	350	n	bl	n	y	0	cig+/-ot	41	99	3	6	nev any	st
WYNDE3	71	f	0	0	all	-	KII	NAmer	1966	CC	350	n	bl	n	y	0	cig+/-ot	1	9	1	1	nev any	st
WYNDE3	72	f	0	0	all	-	KII	NAmer	1966	CC	350	n	bl	n	y	0	cig+/-ot	10	20	2	0	nev any	st
WYNDE3	73	f	0	0	all	-	KII	NAmer	1966	CC	350	n	bl	n	y	0	cig+/-ot	21	40	0	0	nev any	st
WYNDE3	74	f	0	0	all	-	KII	NAmer	1966	CC	350	n	bl	n	y	0	cig+/-ot	41	99	3	6	nev any	st
WYNDE4	37	m	0	0	all	-	a	NAmer	1948	CC	684	n	bl	y	n	0	all/unsp	1	9	1	1	nev any	st
WYNDE4	38	m	0	0	all	-	a	NAmer	1948	CC	684	n	bl	y	n	0	all/unsp	10	15	0	2	nev any	st
WYNDE4	39	m	0	0	all	-	a	NAmer	1948	CC	684	n	bl	y	n	0	all/unsp	16	20	2	3	nev any	st
WYNDE4	40	m	0	0	all	-	a	NAmer	1948	CC	684	n	bl	y	n	0	all/unsp	21	34	0	4	nev any	st
WYNDE4	41	m	0	0	all	-	a	NAmer	1948	CC	684	n	bl	y	n	0	all/unsp	35	99	3	0	nev any	st
WYNDE4	55	f	0	0	all	-	a	NAmer	1948	CC	684	n	bl	y	n	2	all/unsp	1	9	1	1	nev any	ot
ZHENG	6	m	0	0	all	-	a	As:Chi	1982	CC	540	n	ot	*	y	0	cig+/-ot	1	9	1	1	nev cigs	st
ZHENG	7	m	0	0	all	-	a	As:Chi	1982	CC	540	n	ot	*	y	0	cig+/-ot	10	19	0	2	nev cigs	st
ZHENG	8	m	0	0	all	-	a	As:Chi	1982	CC	540	n	ot	*	y	0	cig+/-ot	20	29	2	3	nev cigs	st
ZHENG	9	m	0	0	all	-	a	As:Chi	1982	CC	540	n	ot	*	y	0	cig+/-ot	30	99	3	0	nev cigs	st
ZHENG	19	f	0	0	all	-	a	As:Chi	1982	CC	540	n	ot	*	y	0	cig+/-ot	1	9	1	1	nev cigs	st
ZHENG	20	f	0	0	all	-	a	As:Chi	1982	CC	540	n	ot	*	y	0	cig+/-ot	10	99	0	0	nev cigs	st

Cigarette type is all/unspec for all RRs

except for the following:

REF	NRR	CIGTYPE
ALDERS	46	MC only
ALDERS	47	MC only
ALDERS	48	MC only
ALDERS	49	MC only
ALDERS	50	MC only
ALDERS	51	MC only

REF	NRR	Cigarette equivalent
ALDERS	46	-
ALDERS	47	-
ALDERS	48	-
ALDERS	49	-
ALDERS	50	-
ALDERS	51	-
BARBON	79	*
BARBON	80	*
BARBON	81	*
BROWN2	34	*
BROWN2	44	*
BROWN2	33	*
BROWN2	43	*
CHOI	51	*
CHOI	52	*
CHOI	53	*
CHOI	54	*
CHOI	55	*
CHOI	59	*
CHOI	60	*
CHOI	61	*
DOLL	72	grams
DOLL	73	grams
DOLL	74	grams
DOLL	75	grams
DOLL	79	grams
DOLL	80	grams
DOLL	81	grams
DORGAN	126	*
DORGAN	127	*

Table 3G1 - 1

IESLC - Meta-analysis of Ever Smoking by Amount, Overview, Any product (or Cigarettes if Any not available)
Adenocarcinoma
Most adjusted

REF	NRR	Cigarette equivalent
DORGAN	105	*
DORGAN	106	*
DOSEME	8	*
DOSEME	12	*
DOSEME	16	*
GER	10	*
GER	11	*
GER	12	*
JEDRYC	36	*
JEDRYC	37	*
JEDRYC	38	*
KREYBE	5	grams inc 1 cig=1
KREYBE	6	grams inc 1 cig=1
KREYBE	7	grams inc 1 cig=1
KREYBE	26	grams inc 1 cig=1
KREYBE	61	grams inc 1 cig=1
LAMTH	16	*
LAMTH	17	*
LAMTH	18	*
LUBIN2	152	*
LUBIN2	156	*
LUBIN2	160	*
LUBIN2	164	*
LUBIN2	172	*
LUBIN2	176	*
LUBIN2	180	*
LUBIN2	184	*
MATOS	57	*
MATOS	59	*
MATOS	61	*
MATSUD	7	*
MATSUD	8	*
MATSUD	9	*
ORMOS	22	*
ORMOS	23	*
ORMOS	24	*
OSANN	55	*
OSANN	63	*
OSANN	56	*
OSANN	64	*
OSANN2	34	*
OSANN2	35	*
WUWILL	16	*
WUWILL	17	*
WYNDE2	10	*
WYNDE2	11	*
WYNDE2	12	*
WYNDE2	13	*
WYNDE3	24	*
WYNDE3	25	*
WYNDE3	26	*
WYNDE3	27	*
WYNDE3	71	*
WYNDE3	72	*
WYNDE3	73	*
WYNDE3	74	*
WYNDE4	37	inc 1 cigar = 5 cigs, 1 pipe = 2.5 cigs
WYNDE4	38	inc 1 cigar = 5 cigs, 1 pipe = 2.5 cigs
WYNDE4	39	inc 1 cigar = 5 cigs, 1 pipe = 2.5 cigs
WYNDE4	40	inc 1 cigar = 5 cigs, 1 pipe = 2.5 cigs
WYNDE4	41	inc 1 cigar = 5 cigs, 1 pipe = 2.5 cigs
WYNDE4	55	inc 1 cigar = 5 cigs, 1 pipe = 2.5 cigs
ZHENG	6	*
ZHENG	7	*
ZHENG	8	*
ZHENG	9	*
ZHENG	19	*
ZHENG	20	*

In this overview table, subtotals and Qs values may be invalid and should be ignored

Table 3G1 - 2

IESLC - Meta-analysis of Ever Smoking by Amount, Overview, Any product (or Cigarettes if Any not available)
Adenocarcinoma
Most adjusted

REF	NRR	SEX	AD	Number Exposed Case	Number Exposed Cont	Non-exposed Case	Non-exposed Cont	RR	95.00%CI
ALDERS	46	m	1	-	-	-	-	2.80	(1.01- 7.75)
ALDERS	47	m	1	-	-	-	-	2.67	(0.99- 7.18)
ALDERS	48	m	1	-	-	-	-	3.32	(1.36- 8.10)
ALDERS	49	f	1	-	-	-	-	2.77	(1.63- 4.70)
ALDERS	50	f	1	-	-	-	-	4.58	(2.67- 7.85)
ALDERS	51	f	1	-	-	-	-	3.31	(1.80- 6.10)
Subtotal ALDERS								3.34	(2.53- 4.41)
BARBON	79	m	3	-	-	-	-	5.00	(2.20- 11.00)
BARBON	80	m	3	-	-	-	-	8.30	(3.70- 19.00)
BARBON	81	m	3	-	-	-	-	12.10	(5.10- 28.00)
Subtotal BARBON								7.82	(4.86- 12.59)
BROWN2	34	m	2	-	-	-	-	6.20	(4.90- 7.90)
BROWN2	44	m	2	-	-	-	-	10.70	(8.90- 13.00)
BROWN2	33	f	2	-	-	-	-	5.80	(4.70- 7.10)
BROWN2	43	f	2	-	-	-	-	8.60	(7.30- 10.10)
Subtotal BROWN2								7.91	(7.18- 8.72)
CHOI	51	m	0	4	90	7	95	0.60	(0.17- 2.13)
CHOI	52	m	0	27	281	7	95	1.30	(0.55- 3.09)
CHOI	53	m	0	11	49	7	95	3.05	(1.11- 8.35)
CHOI	54	m	0	2	39	7	95	0.70	(0.14- 3.50)
CHOI	55	m	0	2	6	7	95	4.52	(0.77- 26.69)
CHOI	59	f	0	4	16	49	164	0.84	(0.27- 2.62)
CHOI	60	f	0	0	9	49	164	0.17~	(0.01- 3.06)
CHOI	61	f	0	1	1	49	164	3.35	(0.21- 54.50)
Subtotal CHOI								1.32	(0.83- 2.09)
DOLL	72	m	1	-	-	-	-	0.50	(0.07- 3.63)
DOLL	73	m	1	-	-	-	-	0.80	(0.18- 3.60)
DOLL	74	m	1	-	-	-	-	1.20	(0.27- 5.35)
DOLL	75	m	1	-	-	-	-	1.10	(0.22- 5.60)
DOLL	79	f	1	-	-	-	-	1.10	(0.20- 6.05)
DOLL	80	f	1	-	-	-	-	2.30	(0.56- 9.48)
DOLL	81	f	1	-	-	-	-	4.10	(0.65- 25.88)
Subtotal DOLL								1.30	(0.71- 2.40)
DORGAN	126	m	2	-	-	-	-	3.23	(1.20- 8.70)
DORGAN	127	m	2	-	-	-	-	5.63	(2.22- 14.25)
DORGAN	105	f	3	-	-	-	-	2.87	(2.00- 4.11)
DORGAN	106	f	3	-	-	-	-	5.39	(3.73- 7.78)
Subtotal DORGAN								3.96	(3.11- 5.04)
DOSEME	8	m	2	-	-	-	-	1.80	(0.80- 4.10)
DOSEME	12	m	2	-	-	-	-	2.70	(1.60- 4.70)
DOSEME	16	m	2	-	-	-	-	3.20	(1.40- 7.00)
Subtotal DOSEME								2.56	(1.73- 3.79)
GER	10	c	8	-	-	-	-	0.62	(0.23- 1.72)
GER	11	c	8	-	-	-	-	2.10	(0.88- 5.04)
GER	12	c	8	-	-	-	-	0.88	(0.25- 3.03)
Subtotal GER								1.15	(0.64- 2.07)
JEDRYC	36	m	3	-	-	-	-	2.19	(0.84- 5.72)
JEDRYC	37	m	3	-	-	-	-	4.38	(1.87- 10.28)
JEDRYC	38	m	3	-	-	-	-	5.11	(2.09- 12.53)
Subtotal JEDRYC								3.77	(2.24- 6.33)
KREYBE	5	m	1	-	-	-	-	2.65	(0.81- 8.70)
KREYBE	6	m	1	-	-	-	-	1.43	(0.36- 5.72)
KREYBE	7	m	1	-	-	-	-	3.99	(0.95- 16.81)
KREYBE	26	f	1	-	-	-	-	1.30	(0.60- 2.80)
KREYBE	61	f	1	-	-	-	-	0.88	(0.12- 6.63)
Subtotal KREYBE								1.71	(1.01- 2.89)
LAMTH	16	f	0	36	29	131	158	1.50	(0.87- 2.57)
LAMTH	17	f	0	27	14	131	158	2.33	(1.17- 4.62)
LAMTH	18	f	0	9	5	131	158	2.17	(0.71- 6.64)
Subtotal LAMTH								1.82	(1.22- 2.71)
LUBIN2	152	m	0	66	2194	57	2616	1.38	(0.96- 1.98)
LUBIN2	156	m	0	204	3385	57	2616	2.77	(2.05- 3.73)
LUBIN2	160	m	0	234	3108	57	2616	3.46	(2.57- 4.64)
LUBIN2	164	m	0	151	1746	57	2616	3.97	(2.91- 5.42)
LUBIN2	172	f	0	20	184	138	1180	0.93	(0.57- 1.52)
LUBIN2	176	f	0	43	234	138	1180	1.57	(1.09- 2.27)
LUBIN2	180	f	0	13	110	138	1180	1.01	(0.55- 1.84)
LUBIN2	184	f	0	9	39	138	1180	1.97	(0.94- 4.16)
Subtotal LUBIN2								2.28	(1.99- 2.60)
MATOS	57	m	2	-	-	-	-	2.80	(0.90- 8.50)
MATOS	59	m	2	-	-	-	-	7.00	(2.60- 19.10)

Table 3G1 - 2

IESLC - Meta-analysis of Ever Smoking by Amount, Overview, Any product (or Cigarettes if Any not available)
Adenocarcinoma
Most adjusted

REF	NRR	SEX	AD	Number Exposed Case	Cont	Non-exposed Case	Cont	RR	95.00%CI
MATOS	61	m	2	-	-	-	-	8.40	(3.01- 22.20)
Subtotal MATOS								5.76	(3.17- 10.48)
MATSUD	7	m	0	6	1237	0	1255	13.19~	(0.74- 234.37)
MATSUD	8	m	0	13	1607	0	1255	21.09~	(1.25- 355.07)
MATSUD	9	m	0	4	470	0	1255	24.02~	(1.29- 446.92)
Subtotal MATSUD								18.81	(3.58- 98.87)
ORMOS	22	m	0	4	329	2	777	4.72	(0.86- 25.91)
ORMOS	23	m	0	3	577	2	777	2.02	(0.34- 12.13)
ORMOS	24	m	0	1	128	2	777	3.04	(0.27- 33.72)
Subtotal ORMOS								3.13	(1.04- 9.39)
OSANN	55	m	2	-	-	-	-	16.50	(9.30- 29.30)
OSANN	63	m	2	-	-	-	-	37.50	(21.30- 66.00)
OSANN	56	f	2	-	-	-	-	8.80	(6.10- 12.80)
OSANN	64	f	2	-	-	-	-	24.20	(15.80- 37.20)
Subtotal OSANN								16.57	(13.16- 20.85)
OSANN2	34	f	1	-	-	-	-	0.90	(0.30- 2.70)
OSANN2	35	f	1	-	-	-	-	3.80	(1.60- 8.80)
Subtotal OSANN2								2.21	(1.13- 4.34)
WUWILL	16	f	3	-	-	-	-	1.43	(1.07- 1.90)
WUWILL	17	f	3	-	-	-	-	2.26	(1.35- 3.78)
Subtotal WUWILL								1.59	(1.24- 2.05)
WYNDE2	10	m	0	2	114	5	105	0.37	(0.07- 1.94)
WYNDE2	11	m	0	14	203	5	105	1.45	(0.51- 4.13)
WYNDE2	12	m	0	14	83	5	105	3.54	(1.23- 10.23)
WYNDE2	13	m	0	16	112	5	105	3.00	(1.06- 8.48)
Subtotal WYNDE2								1.98	(1.12- 3.51)
WYNDE3	24	m	0	1	42	6	88	0.35	(0.04- 2.99)
WYNDE3	25	m	0	20	114	6	88	2.57	(0.99- 6.68)
WYNDE3	26	m	0	34	82	6	88	6.08	(2.43- 15.24)
WYNDE3	27	m	0	9	26	6	88	5.08	(1.65- 15.59)
WYNDE3	71	f	0	2	19	15	76	0.53	(0.11- 2.53)
WYNDE3	72	f	0	11	24	15	76	2.32	(0.94- 5.73)
WYNDE3	73	f	0	7	10	15	76	3.55	(1.16- 10.80)
WYNDE3	74	f	0	1	3	15	76	1.69	(0.16- 17.36)
Subtotal WYNDE3								2.82	(1.87- 4.25)
WYNDE4	37	m	0	3	82	4	115	1.05	(0.23- 4.83)
WYNDE4	38	m	0	6	147	4	115	1.17	(0.32- 4.26)
WYNDE4	39	m	0	15	274	4	115	1.57	(0.51- 4.84)
WYNDE4	40	m	0	4	98	4	115	1.17	(0.29- 4.82)
WYNDE4	41	m	0	7	64	4	115	3.14	(0.89- 11.15)
WYNDE4	55	f	2	-	-	-	-	1.33	(0.29- 6.07)
Subtotal WYNDE4								1.51	(0.88- 2.60)
ZHENG	6	m	0	18	40	29	94	1.46	(0.73- 2.92)
ZHENG	7	m	0	35	66	29	94	1.72	(0.96- 3.08)
ZHENG	8	m	0	53	89	29	94	1.93	(1.13- 3.30)
ZHENG	9	m	0	17	23	29	94	2.40	(1.13- 5.08)
ZHENG	19	f	0	13	29	119	184	0.69	(0.35- 1.39)
ZHENG	20	f	0	20	15	119	184	2.06	(1.02- 4.19)
Subtotal ZHENG								1.62	(1.24- 2.11)
Partial Totals				1216	17646	1839	25116		

*prospective study ~ With 0.5 adjustment for zero

REF	NRR	SEX	AD	Ys	Ws	Qs	Ps
ALDERS	46	m	1	1.03	3.70	0.56	0.0476
ALDERS	47	m	1	0.98	3.91	0.75	0.0520
ALDERS	48	m	1	1.20	4.83	0.23	0.0084
ALDERS	49	f	1	1.02	13.70	2.20	0.0002
ALDERS	50	f	1	1.52	13.21	0.14	0.0000
ALDERS	51	f	1	1.20	10.32	0.51	0.0001
Subtotal ALDERS				1.21	49.67	4.40	
BARBON	79	m	3	1.61	5.93	0.21	0.0001
BARBON	80	m	3	2.12	5.74	2.78	0.0000
BARBON	81	m	3	2.49	5.30	6.10	0.0000
Subtotal BARBON				2.06	16.97	9.10	
BROWN2	34	m	2	1.82	67.36	11.02	0.0000
BROWN2	44	m	2	2.37	107.03	96.65	0.0000
BROWN2	33	f	2	1.76	90.29	10.31	0.0000
BROWN2	43	f	2	2.15	145.78	78.06	0.0000
Subtotal BROWN2				2.07	410.46	196.04	
CHOI	51	m	0	-0.51	2.41	8.95	0.4323

Table 3G1 - 2

IESLC - Meta-analysis of Ever Smoking by Amount, Overview, Any product (or Cigarettes if Any not available)
Adenocarcinoma
Most adjusted

REF	NRR	SEX	AD	Ys	Ws	Qs	Ps
CHOI	52	m	0	0.27	5.16	6.87	0.5467
CHOI	53	m	0	1.11	3.78	0.35	0.0304
CHOI	54	m	0	-0.36	1.47	4.68	0.6600
CHOI	55	m	0	1.51	1.22	0.01	0.0956
CHOI	59	f	0	-0.18	2.95	7.53	0.7595
CHOI	60	f	0	-1.74	0.47	4.70	0.2324
CHOI	61	f	0	1.21	0.49	0.02	0.3961
Subtotal CHOI				0.27	17.95	33.11	
DOLL	72	m	1	-0.69	0.99	4.40	0.4914
DOLL	73	m	1	-0.22	1.71	4.62	0.7703
DOLL	74	m	1	0.18	1.72	2.64	0.8109
DOLL	75	m	1	0.10	1.47	2.57	0.9081
DOLL	79	f	1	0.10	1.32	2.32	0.9127
DOLL	80	f	1	0.83	1.92	0.66	0.2485
DOLL	81	f	1	1.41	1.13	0.00	0.1333
Subtotal DOLL				0.26	10.26	17.22	
DORGAN	126	m	2	1.17	3.92	0.24	0.0203
DORGAN	127	m	2	1.73	4.45	0.42	0.0003
DORGAN	105	f	3	1.05	29.62	3.96	0.0000
DORGAN	106	f	3	1.68	28.43	1.99	0.0000
Subtotal DORGAN				1.38	66.41	6.61	
DOSEME	8	m	2	0.59	5.75	3.99	0.1585
DOSEME	12	m	2	0.99	13.23	2.41	0.0003
DOSEME	16	m	2	1.16	5.93	0.39	0.0046
Subtotal DOSEME				0.94	24.92	6.79	
GER	10	c	8	-0.48	3.80	13.67	0.3517
GER	11	c	8	0.74	5.04	2.32	0.0956
GER	12	c	8	-0.13	2.47	5.91	0.8408
Subtotal GER				0.14	11.31	21.91	
JEDRYC	36	m	3	0.78	4.18	1.69	0.1092
JEDRYC	37	m	3	1.48	5.29	0.02	0.0007
JEDRYC	38	m	3	1.63	4.79	0.21	0.0004
Subtotal JEDRYC				1.33	14.26	1.92	
KREYBE	5	m	1	0.97	2.73	0.54	0.1076
KREYBE	6	m	1	0.36	2.01	2.27	0.6122
KREYBE	7	m	1	1.38	1.86	0.00	0.0590
KREYBE	26	f	1	0.26	6.48	8.68	0.5044
KREYBE	61	f	1	-0.13	0.95	2.29	0.9006
Subtotal KREYBE				0.54	14.03	13.78	
LAMTH	16	f	0	0.40	13.12	13.55	0.1438
LAMTH	17	f	0	0.84	8.17	2.71	0.0158
LAMTH	18	f	0	0.78	3.08	1.28	0.1740
Subtotal LAMTH				0.60	24.36	17.54	
LUBIN2	152	m	0	0.32	29.82	35.92	0.0782
LUBIN2	156	m	0	1.02	43.25	7.01	0.0000
LUBIN2	160	m	0	1.24	44.40	1.44	0.0000
LUBIN2	164	m	0	1.38	39.81	0.07	0.0000
LUBIN2	172	f	0	-0.07	15.74	35.10	0.7715
LUBIN2	176	f	0	0.45	28.07	26.31	0.0167
LUBIN2	180	f	0	0.01	10.63	21.11	0.9727
LUBIN2	184	f	0	0.68	6.90	3.78	0.0741
Subtotal LUBIN2				0.82	218.62	130.74	
MATOS	57	m	2	1.03	3.05	0.46	0.0723
MATOS	59	m	2	1.95	3.86	1.07	0.0001
MATOS	61	m	2	2.13	3.85	1.93	0.0000
Subtotal MATOS				1.75	10.76	3.46	
MATSUD	7	m	0	2.58	0.46	0.62	0.0789
MATSUD	8	m	0	3.05	0.48	1.28	0.0343
MATSUD	9	m	0	3.18	0.45	1.39	0.0331
Subtotal MATSUD				2.93	1.40	3.29	
ORMOS	22	m	0	1.55	1.33	0.02	0.0738
ORMOS	23	m	0	0.70	1.20	0.61	0.4420
ORMOS	24	m	0	1.11	0.66	0.06	0.3661
Subtotal ORMOS				1.14	3.18	0.70	
OSANN	55	m	2	2.80	11.67	22.33	0.0000
OSANN	63	m	2	3.62	12.01	58.38	0.0000
OSANN	56	f	2	2.17	27.97	15.93	0.0000
OSANN	64	f	2	3.19	20.96	65.38	0.0000
Subtotal OSANN				2.81	72.61	162.02	
OSANN2	34	f	1	-0.11	3.18	7.41	0.8509
OSANN2	35	f	1	1.34	5.29	0.04	0.0021

Table 3G1 - 2

IESLC - Meta-analysis of Ever Smoking by Amount, Overview, Any product (or Cigarettes if Any not available)
Adenocarcinoma
Most adjusted

REF	NRR	SEX	AD	Ys	Ws	Qs	Ps
Subtotal OSANN2				0.79	8.47	7.44	
WUWILL	16	f	3	0.36	46.61	52.60	0.0146
WUWILL	17	f	3	0.82	14.49	5.30	0.0019
Subtotal WUWILL				0.47	61.10	57.89	
WYNDE2	10	m	0	-1.00	1.39	8.14	0.2387
WYNDE2	11	m	0	0.37	3.50	3.85	0.4885
WYNDE2	12	m	0	1.26	3.41	0.08	0.0195
WYNDE2	13	m	0	1.10	3.56	0.37	0.0382
Subtotal WYNDE2				0.69	11.86	12.45	
WYNDE3	24	m	0	-1.05	0.83	5.08	0.3372
WYNDE3	25	m	0	0.95	4.22	0.95	0.0521
WYNDE3	26	m	0	1.81	4.55	0.68	0.0001
WYNDE3	27	m	0	1.62	3.05	0.13	0.0045
WYNDE3	71	f	0	-0.63	1.58	6.64	0.4293
WYNDE3	72	f	0	0.84	4.71	1.57	0.0675
WYNDE3	73	f	0	1.27	3.10	0.07	0.0258
WYNDE3	74	f	0	0.52	0.71	0.57	0.6593
Subtotal WYNDE3				1.04	22.76	15.69	
WYNDE4	37	m	0	0.05	1.66	3.10	0.9482
WYNDE4	38	m	0	0.16	2.31	3.67	0.8077
WYNDE4	39	m	0	0.45	3.04	2.84	0.4291
WYNDE4	40	m	0	0.16	1.93	3.06	0.8243
WYNDE4	41	m	0	1.15	2.40	0.18	0.0761
WYNDE4	55	f	2	0.29	1.66	2.14	0.7132
Subtotal WYNDE4				0.41	12.99	15.00	
ZHENG	6	m	0	0.38	7.96	8.65	0.2870
ZHENG	7	m	0	0.54	11.26	8.68	0.0692
ZHENG	8	m	0	0.66	13.29	7.73	0.0165
ZHENG	9	m	0	0.87	6.78	2.02	0.0229
ZHENG	19	f	0	-0.37	7.98	25.48	0.3003
ZHENG	20	f	0	0.72	7.66	3.72	0.0452
Subtotal ZHENG				0.48	54.94	56.28	

N	98
NS	22

Table 3G1 - 3

IESLC - Meta-analysis of Ever Smoking by Amount, Overview, Any product (or Cigarettes if Any not available)
Adenocarcinoma
Most adjusted

	Sex combined	male	female	Total
N	3	60	35	98
NS	1	18	14	33

In this overview table, other than the "N" rows, entries in the "absent" and "Total" columns may be invalid and should be ignored

Amount smoked (broad categories)

	absent	<20k5	6-44k20	>20k45	Total
N	23	31	21	23	98
NS	13	21	17	18	69
Wt	460.30	382.81	153.29	142.89	1139.28
Het Chi	223.92	219.94	43.31	135.03	793.38
Het df	22	30	20	22	97
Het P	***	***	**	***	***
Fixed RR	6.01	2.75	2.72	5.81	4.14
RRl	5.48	2.49	2.33	4.93	3.90
RRu	6.58	3.04	3.19	6.85	4.38
P	+++	+++	+++	+++	+++
Random RR	3.39	1.71	2.53	4.40	2.68
RRl	2.38	1.24	1.93	2.77	2.22
RRu	4.84	2.36	3.30	6.98	3.25
P	+++	+++	+++	+++	+++

Amount smoked (narrow categories)

	absent	<10k1	2-19k10	11-29k20	21-39k30	31-98k40	>40k99	Total
N	59	10	6	16	3	1	3	98
NS	22	5	4	14	3	1	2	50
Wt	828.70	69.54	88.52	136.95	9.12	1.47	4.98	1139.28
Het Chi	486.33	6.92	8.47	32.30	1.64	0.00	0.70	793.38
Het df	58	9	5	15	2	0	2	97
Het P	***	N.S.	N.S.	**	N.S.	N.S.	N.S.	***
Fixed RR	5.40	1.10	2.07	2.65	2.63	0.70	4.22	4.14
RRl	5.05	0.87	1.68	2.24	1.38	0.14	1.75	3.90
RRu	5.78	1.39	2.55	3.13	5.04	3.50	10.16	4.38
P	+++	N.S.	+++	+++	++	N.S.	++	+++
Random RR	3.47	1.10	1.89	2.43	2.63	0.70	4.22	2.68
RRl	2.75	0.87	1.35	1.83	1.38	0.14	1.75	2.22
RRu	4.36	1.39	2.64	3.22	5.04	3.50	10.16	3.25
P	+++	N.S.	+++	+++	++	N.S.	++	+++

MALES

Amount smoked (broad categories)

	absent	<20k5	6-44k20	>20k45	Total
N	12	17	15	16	60
NS	10	17	15	16	58
Wt	196.09	144.18	111.06	97.97	549.30
Het Chi	117.78	83.09	24.46	67.43	355.78
Het df	11	16	14	15	59
Het P	***	***	*	***	***
Fixed RR	6.28	3.15	2.94	5.37	4.37
RRl	5.46	2.67	2.44	4.40	4.02
RRu	7.23	3.71	3.54	6.54	4.75
P	+++	+++	+++	+++	+++
Random RR	3.20	2.01	2.77	5.01	3.07
RRl	1.76	1.25	2.07	3.06	2.42
RRu	5.80	3.23	3.71	8.22	3.90
P	+++	++	+++	+++	+++

Table 3G1 - 3

IESLC - Meta-analysis of Ever Smoking by Amount, Overview, Any product (or Cigarettes if Any not available)
Adenocarcinoma
Most adjusted

MALES

	Amount smoked (narrow categories)							
	absent	<10k1	2-19k10	11-29k20	21-39k30	31-98k40	>40k99	Total
N	33	5	4	12	3	1	2	60
NS	18	5	4	12	3	1	2	44
Wt	334.75	41.25	58.53	99.90	9.12	1.47	4.27	549.30
Het Chi	162.42	2.62	5.35	17.78	1.64	0.00	0.01	355.78
Het df	32	4	3	11	2	0	1	59
Het P	***	N.S.	N.S.	(*)	N.S.	N.S.	N.S.	***
Fixed RR	6.59	1.31	2.35	2.79	2.63	0.70	4.91	4.37
RRl	5.92	0.97	1.82	2.30	1.38	0.14	1.90	4.02
RRu	7.33	1.78	3.04	3.40	5.04	3.50	12.68	4.75
P	+++	(+)	+++	+++	++	N.S.	++	+++
Random RR	4.38	1.31	1.95	2.56	2.63	0.70	4.91	3.07
RRl	3.26	0.97	1.21	1.90	1.38	0.14	1.90	2.42
RRu	5.90	1.78	3.13	3.46	5.04	3.50	12.68	3.90
P	+++	(+)	++	+++	++	N.S.	++	+++

FEMALES

	Amount smoked (broad categories)				
	absent	<20k5	6-44k20	>20k45	Total
N	11	13	5	6	35
NS	10	13	5	6	34
Wt	264.21	234.83	37.18	42.45	578.67
Het Chi	105.46	124.96	16.56	54.53	413.64
Het df	10	12	4	5	34
Het P	***	***	**	***	***
Fixed RR	5.81	2.59	2.26	7.79	4.03
RRl	5.15	2.28	1.64	5.77	3.71
RRu	6.56	2.95	3.11	10.53	4.37
P	+++	+++	+++	+++	+++
Random RR	3.40	1.53	1.96	3.87	2.34
RRl	2.03	0.94	0.94	1.17	1.68
RRu	5.67	2.48	4.06	12.80	3.24
P	+++	(+)	(+)	+	+++

	Amount smoked (narrow categories)							
	absent	<10k1	2-19k10	11-29k20	21-39k30	31-98k40	>40k99	Total
N	24	5	2	3			1	35
NS	13	5	2	3			1	24
Wt	487.68	28.29	29.99	32.01			0.71	578.67
Het Chi	278.92	1.22	0.26	13.45			0.00	413.64
Het df	23	4	1	2			0	34
Het P	***	N.S.	N.S.	**			N.S.	***
Fixed RR	4.84	0.85	1.61	2.33			1.69	4.03
RRl	4.43	0.59	1.13	1.65			0.16	3.71
RRu	5.29	1.23	2.30	3.30			17.36	4.37
P	+++	N.S.	++	+++			N.S.	+++
Random RR	2.93	0.85	1.61	2.22			1.69	2.34
RRl	2.04	0.59	1.13	0.90			0.16	1.68
RRu	4.21	1.23	2.30	5.51			17.36	3.24
P	+++	N.S.	++	(+)			N.S.	+++

Table 3G1 - 4

IESLC - Meta-analysis of Ever Smoking by Amount, Overview, Any product (or Cigarettes if Any not available)
Adenocarcinoma
Least adjusted

REF	NRR	X	SEX	AGEL	AGEH	RACE	YF	LC TYPE	LOC	START	ST	NLC	R	VB	P	H	AD	PRODUCT	exL	exH	S1	S2	DENOM	De
ALDERS	46		m	0	0	all	-	not q+s	Eu:UK	1977	CC	1448	n	V	n	n	1	cig only	1	17	1	0	nev+2	ot
ALDERS	47		m	0	0	all	-	not q+s	Eu:UK	1977	CC	1448	n	V	n	n	1	cig only	18	27	2	3	nev+2	ot
ALDERS	48		m	0	0	all	-	not q+s	Eu:UK	1977	CC	1448	n	V	n	n	1	cig only	28	99	3	0	nev+2	ot
ALDERS	49		f	0	0	all	-	not q+s	Eu:UK	1977	CC	1448	n	V	n	n	1	cig only	1	17	1	0	nev+2	ot
ALDERS	50		f	0	0	all	-	not q+s	Eu:UK	1977	CC	1448	n	V	n	n	1	cig only	18	27	2	3	nev+2	ot
ALDERS	51		f	0	0	all	-	not q+s	Eu:UK	1977	CC	1448	n	V	n	n	1	cig only	28	99	3	0	nev+2	ot
BARBON	45	x	m	0	0	all	-	a	Eu:wst	1979	CC	755	n	bl	y	y	0	all/unsp	1	9	1	1	nev any	st
BARBON	46	x	m	0	0	all	-	a	Eu:wst	1979	CC	755	n	bl	y	y	0	all/unsp	10	19	0	2	nev any	st
BARBON	47	x	m	0	0	all	-	a	Eu:wst	1979	CC	755	n	bl	y	y	0	all/unsp	20	29	2	3	nev any	st
BARBON	48	x	m	0	0	all	-	a	Eu:wst	1979	CC	755	n	bl	y	y	0	all/unsp	30	39	0	4	nev any	st
BARBON	49	x	m	0	0	all	-	a	Eu:wst	1979	CC	755	n	bl	y	y	0	all/unsp	40	99	3	0	nev any	st
BROWN2	34		m	0	0	wh	-	a	NAmer	1984	CC	14596	n	bl	n	y	2	cig+/-ot	1	19	1	0	nev cigs	or
BROWN2	44		m	0	0	wh	-	a	NAmer	1984	CC	14596	n	bl	n	y	2	cig+/-ot	20	99	0	0	nev cigs	or
BROWN2	33		f	0	0	wh	-	a	NAmer	1984	CC	14596	n	bl	n	y	2	cig+/-ot	1	19	1	0	nev cigs	or
BROWN2	43		f	0	0	wh	-	a	NAmer	1984	CC	14596	n	bl	n	y	2	cig+/-ot	20	99	0	0	nev cigs	or
CHOI	51		m	0	0	all	-	a	As:oth	1985	CC	375	n	bl	n	n	0	cig+/-ot	1	10	1	0	nev cigs	st
CHOI	52		m	0	0	all	-	a	As:oth	1985	CC	375	n	bl	n	n	0	cig+/-ot	11	20	2	3	nev cigs	st
CHOI	53		m	0	0	all	-	a	As:oth	1985	CC	375	n	bl	n	n	0	cig+/-ot	21	30	0	4	nev cigs	st
CHOI	54		m	0	0	all	-	a	As:oth	1985	CC	375	n	bl	n	n	0	cig+/-ot	31	40	0	5	nev cigs	st
CHOI	55		m	0	0	all	-	a	As:oth	1985	CC	375	n	bl	n	n	0	cig+/-ot	41	99	3	6	nev cigs	st
CHOI	59		f	0	0	all	-	a	As:oth	1985	CC	375	n	bl	n	n	0	cig+/-ot	1	10	1	0	nev cigs	st
CHOI	60		f	0	0	all	-	a	As:oth	1985	CC	375	n	bl	n	n	0	cig+/-ot	11	30	2	0	nev cigs	ot
CHOI	61		f	0	0	all	-	a	As:oth	1985	CC	375	n	bl	n	n	0	cig+/-ot	31	99	3	0	nev cigs	st
DOLL	58	x	m	0	0	all	-	KII	Eu:UK	1948	CC	1465	n	V	n	n	0	all/unsp	1	4	0	1	nev any	st
DOLL	59	x	m	0	0	all	-	KII	Eu:UK	1948	CC	1465	n	V	n	n	0	all/unsp	5	14	1	2	nev any	st
DOLL	60	x	m	0	0	all	-	KII	Eu:UK	1948	CC	1465	n	V	n	n	0	all/unsp	15	24	2	3	nev any	st
DOLL	61	x	m	0	0	all	-	KII	Eu:UK	1948	CC	1465	n	V	n	n	0	all/unsp	25	99	3	0	nev any	st
DOLL	65	x	f	0	0	all	-	KII	Eu:UK	1948	CC	1465	n	V	n	n	0	all/unsp	1	4	0	1	nev any	st
DOLL	66	x	f	0	0	all	-	KII	Eu:UK	1948	CC	1465	n	V	n	n	0	all/unsp	5	14	1	2	nev any	st
DOLL	67	x	f	0	0	all	-	KII	Eu:UK	1948	CC	1465	n	V	n	n	0	all/unsp	15	99	0	0	nev any	st
DORGAN	126		m	0	0	wh	-	a	NAmer	1980	CC	2026	n	bl	y	y	2	cig+/-ot	1	19	1	0	nev any	ot
DORGAN	127		m	0	0	wh	-	a	NAmer	1980	CC	2026	n	bl	y	y	2	cig+/-ot	20	99	0	0	nev any	ot
DORGAN	105		f	0	0	all	-	a	NAmer	1980	CC	2026	n	bl	y	y	3	cig+/-ot	1	19	1	0	nev any	ot
DORGAN	106		f	0	0	all	-	a	NAmer	1980	CC	2026	n	bl	y	y	3	cig+/-ot	20	99	0	0	nev any	ot
DOSEME	8		m	0	0	all	-	not q+s	Eu:bal	1979	CC	1210	n	bl	n	n	2	cig+/-ot	1	10	1	0	nev cigs	or
DOSEME	12		m	0	0	all	-	not q+s	Eu:bal	1979	CC	1210	n	bl	n	n	2	cig+/-ot	11	20	2	3	nev cigs	or
DOSEME	16		m	0	0	all	-	not q+s	Eu:bal	1979	CC	1210	n	bl	n	n	2	cig+/-ot	21	99	3	0	nev cigs	or
GER	2	x	c	0	0	all	-	a	As:oth	1990	CC	141	n	ot	y	n	0	all/unsp	1	10	1	0	nev any	st
GER	3	x	c	0	0	all	-	a	As:oth	1990	CC	141	n	ot	y	n	0	all/unsp	11	20	2	3	nev any	st
GER	4	x	c	0	0	all	-	a	As:oth	1990	CC	141	n	ot	y	n	0	all/unsp	21	99	3	0	nev any	st
JEDRYC	15	x	m	0	0	all	-	a	Eu:est	1980	CC	1630	n	bl	y	n	0	cig+/-ot	1	9	1	1	nev any	st
JEDRYC	16	x	m	0	0	all	-	a	Eu:est	1980	CC	1630	n	bl	y	n	0	cig+/-ot	10	19	0	2	nev any	st
JEDRYC	17	x	m	0	0	all	-	a	Eu:est	1980	CC	1630	n	bl	y	n	0	cig+/-ot	20	29	2	3	nev any	st
JEDRYC	18	x	m	0	0	all	-	a	Eu:est	1980	CC	1630	n	bl	y	n	0	cig+/-ot	30	39	0	4	nev any	st
JEDRYC	19	x	m	0	0	all	-	a	Eu:est	1980	CC	1630	n	bl	y	n	0	cig+/-ot	40	99	3	0	nev any	st
KREYBE	17	x	m	0	0	all	-	KII	Eu:Sca	1948	CC	300	n	bl	n	y	0	all/unsp	1	14	1	0	nev any	st
KREYBE	18	x	m	0	0	all	-	KII	Eu:Sca	1948	CC	300	n	bl	n	y	0	all/unsp	15	24	2	3	nev any	st
KREYBE	19	x	m	0	0	all	-	KII	Eu:Sca	1948	CC	300	n	bl	n	y	0	all/unsp	25	99	3	0	nev any	st
KREYBE	34	x	f	0	0	all	-	KII	Eu:Sca	1948	CC	300	n	bl	n	y	0	all/unsp	1	14	1	0	nev any	st
KREYBE	35	x	f	0	0	all	-	KII	Eu:Sca	1948	CC	300	n	bl	n	y	0	all/unsp	15	99	0	0	nev any	st
LAMTH	16		f	0	0	ch	-	a	As:HK	1983	CC	445	n	bl	n	n	0	all/unsp	1	10	1	0	nev any	or
LAMTH	17		f	0	0	ch	-	a	As:HK	1983	CC	445	n	bl	n	n	0	all/unsp	11	20	2	3	nev any	or
LAMTH	18		f	0	0	ch	-	a	As:HK	1983	CC	445	n	bl	n	n	0	all/unsp	21	99	3	0	nev any	or
LUBIN2	152		m	0	0	all	-	a	Eu:mul	1976	CC	7804	n	bl	n	y	0	cig+/-ot	1	9	1	1	nev any	st
LUBIN2	156		m	0	0	all	-	a	Eu:mul	1976	CC	7804	n	bl	n	y	0	cig+/-ot	10	19	0	2	nev any	st
LUBIN2	160		m	0	0	all	-	a	Eu:mul	1976	CC	7804	n	bl	n	y	0	cig+/-ot	20	29	2	3	nev any	st
LUBIN2	164		m	0	0	all	-	a	Eu:mul	1976	CC	7804	n	bl	n	y	0	cig+/-ot	30	99	3	0	nev any	st
LUBIN2	172		f	0	0	all	-	a	Eu:mul	1976	CC	7804	n	bl	n	y	0	cig+/-ot	1	9	1	1	nev any	st
LUBIN2	176		f	0	0	all	-	a	Eu:mul	1976	CC	7804	n	bl	n	y	0	cig+/-ot	10	19	0	2	nev any	st
LUBIN2	180		f	0	0	all	-	a	Eu:mul	1976	CC	7804	n	bl	n	y	0	cig+/-ot	20	29	2	3	nev any	st
LUBIN2	184		f	0	0	all	-	a	Eu:mul	1976	CC	7804	n	bl	n	y	0	cig+/-ot	30	99	3	0	nev any	st
MATOS	56	x	m	0	0	all	-	a	SCAmer	1994	CC	200	n	bl	n	n	0	cig+/-ot	1	14	1	0	nev any	st
MATOS	58	x	m	0	0	all	-	a	SCAmer	1994	CC	200	n	bl	n	n	0	cig+/-ot	15	24	2	3	nev any	st
MATOS	60	x	m	0	0	all	-	a	SCAmer	1994	CC	200	n	bl	n	n	0	cig+/-ot	25	99	3	0	nev any	st
MATSUD	7		m	0	0	all	-	a	As:Jap	1965	CC	179	n	bl	n	n	0	cig+/-ot	1	10	1	0	nev cigs	ot
MATSUD	8		m	0	0	all	-	a	As:Jap	1965	CC	179	n	bl	n	n	0	cig+/-ot	11	20	2	3	nev cigs	ot
MATSUD	9		m	0	0	all	-	a	As:Jap	1965	CC	179	n	bl	n	n	0	cig+/-ot	21	99	3	0	nev cigs	ot
ORMOS	22		m	0	0	all	-	KII	Eu:est	1947	CC	119	n	bl	y	y	0	cig+/-ot	1	15	1	0	nev any	st
ORMOS	23		m	0	0	all	-	KII	Eu:est	1947	CC	119	n	bl	y	y	0	cig+/-ot	16	30	2	0	nev any	st
ORMOS	24		m	0	0	all	-	KII	Eu:est	1947	CC	119	n	bl	y	y	0	cig+/-ot	31	99	3	0	nev any	st
OSANN	55		m	0	0	all	-	a	NAmer	1984	CC	1986	n	bl	n	n	2	cig+/-ot	1	39	0	0	nev cigs	or
OSANN	63		m	0	0	all	-	a	NAmer	1984	CC	1986	n	bl	n	n	2	cig+/-ot	40	99	3	0	nev cigs	or

Table 3G1 - 4

IESLC - Meta-analysis of Ever Smoking by Amount, Overview, Any product (or Cigarettes if Any not available)
Adenocarcinoma
Least adjusted

REF	NRR	X	SEX	AGEL	AGEH	RACE	YF	LC TYPE	LOC	START	ST	NLC	R	VB	P	H	AD	PRODUCT	exL	exH	S1	S2	DENOM	De
OSANN	56		f	0	0	all	-	a	NAmer	1984	CC	1986	n	bl	n	n	2	cig+/-ot	1	39	0	0	nev cigs	or
OSANN	64		f	0	0	all	-	a	NAmer	1984	CC	1986	n	bl	n	n	2	cig+/-ot	40	99	3	0	nev cigs	or
OSANN2	16	x	f	0	0	all	-	KII	NAmer	1964	ot	217	n	bl	n	y	0	cig+/-ot	1	19	1	0	nev cigs	st
OSANN2	17	x	f	0	0	all	-	KII	NAmer	1964	ot	217	n	bl	n	y	0	cig+/-ot	20	99	0	0	nev cigs	st
WUWILL	22	x	f	0	0	all	-	a	As:Chi	1985	CC	965	n	ot	n	n	0	cig+/-ot	1	19	1	0	nev cigs	st
WUWILL	23	x	f	0	0	all	-	a	As:Chi	1985	CC	965	n	ot	n	n	0	cig+/-ot	20	99	0	0	nev cigs	st
WYNDE2	10		m	0	0	all	-	KII	NAmer	1962	CC	404	n	bl	n	y	0	cig+/-ot	1	10	1	0	nev any	st
WYNDE2	11		m	0	0	all	-	KII	NAmer	1962	CC	404	n	bl	n	y	0	cig+/-ot	11	20	2	3	nev any	st
WYNDE2	12		m	0	0	all	-	KII	NAmer	1962	CC	404	n	bl	n	y	0	cig+/-ot	21	34	0	4	nev any	st
WYNDE2	13		m	0	0	all	-	KII	NAmer	1962	CC	404	n	bl	n	y	0	cig+/-ot	35	99	3	0	nev any	st
WYNDE3	24		m	0	0	all	-	KII	NAmer	1966	CC	350	n	bl	n	y	0	cig+/-ot	1	9	1	1	nev any	st
WYNDE3	25		m	0	0	all	-	KII	NAmer	1966	CC	350	n	bl	n	y	0	cig+/-ot	10	20	2	0	nev any	st
WYNDE3	26		m	0	0	all	-	KII	NAmer	1966	CC	350	n	bl	n	y	0	cig+/-ot	21	40	0	0	nev any	st
WYNDE3	27		m	0	0	all	-	KII	NAmer	1966	CC	350	n	bl	n	y	0	cig+/-ot	41	99	3	6	nev any	st
WYNDE3	71		f	0	0	all	-	KII	NAmer	1966	CC	350	n	bl	n	y	0	cig+/-ot	1	9	1	1	nev any	st
WYNDE3	72		f	0	0	all	-	KII	NAmer	1966	CC	350	n	bl	n	y	0	cig+/-ot	10	20	2	0	nev any	st
WYNDE3	73		f	0	0	all	-	KII	NAmer	1966	CC	350	n	bl	n	y	0	cig+/-ot	21	40	0	0	nev any	st
WYNDE3	74		f	0	0	all	-	KII	NAmer	1966	CC	350	n	bl	n	y	0	cig+/-ot	41	99	3	6	nev any	st
WYNDE4	37		m	0	0	all	-	a	NAmer	1948	CC	684	n	bl	y	n	0	all/unsp	1	9	1	1	nev any	st
WYNDE4	38		m	0	0	all	-	a	NAmer	1948	CC	684	n	bl	y	n	0	all/unsp	10	15	0	2	nev any	st
WYNDE4	39		m	0	0	all	-	a	NAmer	1948	CC	684	n	bl	y	n	0	all/unsp	16	20	2	3	nev any	st
WYNDE4	40		m	0	0	all	-	a	NAmer	1948	CC	684	n	bl	y	n	0	all/unsp	21	34	0	4	nev any	st
WYNDE4	41		m	0	0	all	-	a	NAmer	1948	CC	684	n	bl	y	n	0	all/unsp	35	99	3	0	nev any	st
WYNDE4	55		f	0	0	all	-	a	NAmer	1948	CC	684	n	bl	y	n	2	all/unsp	1	9	1	1	nev any	ot
ZHENG	6		m	0	0	all	-	a	As:Chi	1982	CC	540	n	ot	*	y	0	cig+/-ot	1	9	1	1	nev cigs	st
ZHENG	7		m	0	0	all	-	a	As:Chi	1982	CC	540	n	ot	*	y	0	cig+/-ot	10	19	0	2	nev cigs	st
ZHENG	8		m	0	0	all	-	a	As:Chi	1982	CC	540	n	ot	*	y	0	cig+/-ot	20	29	2	3	nev cigs	st
ZHENG	9		m	0	0	all	-	a	As:Chi	1982	CC	540	n	ot	*	y	0	cig+/-ot	30	99	3	0	nev cigs	st
ZHENG	19		f	0	0	all	-	a	As:Chi	1982	CC	540	n	ot	*	y	0	cig+/-ot	1	9	1	1	nev cigs	st
ZHENG	20		f	0	0	all	-	a	As:Chi	1982	CC	540	n	ot	*	y	0	cig+/-ot	10	99	0	0	nev cigs	st

Cigarette type is all/unspec for all RRs

except for the following:

REF	NRR	CIGTYPE
ALDERS	46	MC only
ALDERS	47	MC only
ALDERS	48	MC only
ALDERS	49	MC only
ALDERS	50	MC only
ALDERS	51	MC only

REF	NRR	Cigarette equivalent
ALDERS	46	-
ALDERS	47	-
ALDERS	48	-
ALDERS	49	-
ALDERS	50	-
ALDERS	51	-
BARBON	45	*
BARBON	46	*
BARBON	47	*
BARBON	48	*
BARBON	49	*
BROWN2	34	*
BROWN2	44	*
BROWN2	33	*
BROWN2	43	*
CHOI	51	*
CHOI	52	*
CHOI	53	*
CHOI	54	*
CHOI	55	*
CHOI	59	*
CHOI	60	*
CHOI	61	*
DOLL	58	grams
DOLL	59	grams
DOLL	60	grams

Table 3G1 - 4

IESLC - Meta-analysis of Ever Smoking by Amount, Overview, Any product (or Cigarettes if Any not available)
Adenocarcinoma
Least adjusted

REF	NRR	Cigarette equivalent
DOLL	61	grams
DOLL	65	grams
DOLL	66	grams
DOLL	67	grams
DORGAN	126	*
DORGAN	127	*
DORGAN	105	*
DORGAN	106	*
DOSEME	8	*
DOSEME	12	*
DOSEME	16	*
GER	2	*
GER	3	*
GER	4	*
JEDRYC	15	*
JEDRYC	16	*
JEDRYC	17	*
JEDRYC	18	*
JEDRYC	19	*
KREYBE	17	grams inc 1 cig=1
KREYBE	18	grams inc 1 cig=1
KREYBE	19	grams inc 1 cig=1
KREYBE	34	grams inc 1 cig=1
KREYBE	35	grams inc 1 cig=1
LAMTH	16	*
LAMTH	17	*
LAMTH	18	*
LUBIN2	152	*
LUBIN2	156	*
LUBIN2	160	*
LUBIN2	164	*
LUBIN2	172	*
LUBIN2	176	*
LUBIN2	180	*
LUBIN2	184	*
MATOS	56	*
MATOS	58	*
MATOS	60	*
MATSUD	7	*
MATSUD	8	*
MATSUD	9	*
ORMOS	22	*
ORMOS	23	*
ORMOS	24	*
OSANN	55	*
OSANN	63	*
OSANN	56	*
OSANN	64	*
OSANN2	16	*
OSANN2	17	*
WUWILL	22	*
WUWILL	23	*
WYNDE2	10	*
WYNDE2	11	*
WYNDE2	12	*
WYNDE2	13	*
WYNDE3	24	*
WYNDE3	25	*
WYNDE3	26	*
WYNDE3	27	*
WYNDE3	71	*
WYNDE3	72	*
WYNDE3	73	*
WYNDE3	74	*
WYNDE4	37	inc 1 cigar = 5 cigs, 1 pipe = 2.5 cigs
WYNDE4	38	inc 1 cigar = 5 cigs, 1 pipe = 2.5 cigs
WYNDE4	39	inc 1 cigar = 5 cigs, 1 pipe = 2.5 cigs
WYNDE4	40	inc 1 cigar = 5 cigs, 1 pipe = 2.5 cigs
WYNDE4	41	inc 1 cigar = 5 cigs, 1 pipe = 2.5 cigs
WYNDE4	55	inc 1 cigar = 5 cigs, 1 pipe = 2.5 cigs
ZHENG	6	*
ZHENG	7	*

Table 3G1 - 4

IESLC - Meta-analysis of Ever Smoking by Amount, Overview, Any product (or Cigarettes if Any not available)
Adenocarcinoma
Least adjusted

REF	NRR	Cigarette equivalent
ZHENG	8	*
ZHENG	9	*
ZHENG	19	*
ZHENG	20	*

In this overview table, subtotals and Qs values may be invalid and should be ignored

Table 3G1 - 5

IESLC - Meta-analysis of Ever Smoking by Amount, Overview, Any product (or Cigarettes if Any not available)
Adenocarcinoma
Least adjusted

REF	NRR	SEX	AD	Number Exposed Case	Number Exposed Cont	Non-exposed Case	Non-exposed Cont	RR	95.00%CI
ALDERS	46	m	1	-	-	-	-	2.80	(1.01- 7.75)
ALDERS	47	m	1	-	-	-	-	2.67	(0.99- 7.18)
ALDERS	48	m	1	-	-	-	-	3.32	(1.36- 8.10)
ALDERS	49	f	1	-	-	-	-	2.77	(1.63- 4.70)
ALDERS	50	f	1	-	-	-	-	4.58	(2.67- 7.85)
ALDERS	51	f	1	-	-	-	-	3.31	(1.80- 6.10)
Subtotal ALDERS								3.34	(2.53- 4.41)
BARBON	45	m	0	7	87	7	188	2.16	(0.74- 6.35)
BARBON	46	m	0	31	111	7	188	7.50	(3.20- 17.60)
BARBON	47	m	0	43	176	7	188	6.56	(2.88- 14.97)
BARBON	48	m	0	30	82	7	188	9.83	(4.15- 23.28)
BARBON	49	m	0	40	111	7	188	9.68	(4.19- 22.34)
Subtotal BARBON								6.90	(4.66- 10.22)
BROWN2	34	m	2	-	-	-	-	6.20	(4.90- 7.90)
BROWN2	44	m	2	-	-	-	-	10.70	(8.90- 13.00)
BROWN2	33	f	2	-	-	-	-	5.80	(4.70- 7.10)
BROWN2	43	f	2	-	-	-	-	8.60	(7.30- 10.10)
Subtotal BROWN2								7.91	(7.18- 8.72)
CHOI	51	m	0	4	90	7	95	0.60	(0.17- 2.13)
CHOI	52	m	0	27	281	7	95	1.30	(0.55- 3.09)
CHOI	53	m	0	11	49	7	95	3.05	(1.11- 8.35)
CHOI	54	m	0	2	39	7	95	0.70	(0.14- 3.50)
CHOI	55	m	0	2	6	7	95	4.52	(0.77- 26.69)
CHOI	59	f	0	4	16	49	164	0.84	(0.27- 2.62)
CHOI	60	f	0	0	9	49	164	0.17~	(0.01- 3.06)
CHOI	61	f	0	1	1	49	164	3.35	(0.21- 54.50)
Subtotal CHOI								1.32	(0.83- 2.09)
DOLL	58	m	0	2	129	2	61	0.47	(0.07- 3.44)
DOLL	59	m	0	14	570	2	61	0.75	(0.17- 3.37)
DOLL	60	m	0	16	431	2	61	1.13	(0.25- 5.05)
DOLL	61	m	0	6	166	2	61	1.10	(0.22- 5.61)
DOLL	65	f	0	2	25	5	59	0.94	(0.17- 5.19)
DOLL	66	f	0	4	18	5	59	2.62	(0.64- 10.81)
DOLL	67	f	0	2	6	5	59	3.93	(0.62- 24.83)
Subtotal DOLL								1.27	(0.69- 2.35)
DORGAN	126	m	2	-	-	-	-	3.23	(1.20- 8.70)
DORGAN	127	m	2	-	-	-	-	5.63	(2.22- 14.25)
DORGAN	105	f	3	-	-	-	-	2.87	(2.00- 4.11)
DORGAN	106	f	3	-	-	-	-	5.39	(3.73- 7.78)
Subtotal DORGAN								3.96	(3.11- 5.04)
DOSEME	8	m	2	-	-	-	-	1.80	(0.80- 4.10)
DOSEME	12	m	2	-	-	-	-	2.70	(1.60- 4.70)
DOSEME	16	m	2	-	-	-	-	3.20	(1.40- 7.00)
Subtotal DOSEME								2.56	(1.73- 3.79)
GER	2	c	0	9	47	37	149	0.77	(0.35- 1.71)
GER	3	c	0	21	66	37	149	1.28	(0.70- 2.36)
GER	4	c	0	5	26	37	149	0.77	(0.28- 2.15)
Subtotal GER								1.00	(0.65- 1.55)
JEDRYC	15	m	0	2	67	7	289	1.23	(0.25- 6.07)
JEDRYC	16	m	0	11	199	7	289	2.28	(0.87- 5.99)
JEDRYC	17	m	0	52	434	7	289	4.95	(2.22- 11.04)
JEDRYC	18	m	0	19	118	7	289	6.65	(2.72- 16.23)
JEDRYC	19	m	0	9	82	7	289	4.53	(1.64- 12.54)
Subtotal JEDRYC								4.02	(2.59- 6.22)
KREYBE	17	m	0	31	2341	3	644	2.84	(0.87- 9.33)
KREYBE	18	m	0	6	925	3	644	1.39	(0.35- 5.59)
KREYBE	19	m	0	5	248	3	644	4.33	(1.03- 18.25)
KREYBE	34	f	0	9	286	27	657	0.77	(0.36- 1.65)
KREYBE	35	f	0	1	42	27	657	0.58	(0.08- 4.37)
Subtotal KREYBE								1.33	(0.79- 2.24)
LAMTH	16	f	0	36	29	131	158	1.50	(0.87- 2.57)
LAMTH	17	f	0	27	14	131	158	2.33	(1.17- 4.62)
LAMTH	18	f	0	9	5	131	158	2.17	(0.71- 6.64)
Subtotal LAMTH								1.82	(1.22- 2.71)
LUBIN2	152	m	0	66	2194	57	2616	1.38	(0.96- 1.98)
LUBIN2	156	m	0	204	3385	57	2616	2.77	(2.05- 3.73)
LUBIN2	160	m	0	234	3108	57	2616	3.46	(2.57- 4.64)
LUBIN2	164	m	0	151	1746	57	2616	3.97	(2.91- 5.42)
LUBIN2	172	f	0	20	184	138	1180	0.93	(0.57- 1.52)
LUBIN2	176	f	0	43	234	138	1180	1.57	(1.09- 2.27)
LUBIN2	180	f	0	13	110	138	1180	1.01	(0.55- 1.84)

Table 3G1 - 5

IESLC - Meta-analysis of Ever Smoking by Amount, Overview, Any product (or Cigarettes if Any not available)
Adenocarcinoma
Least adjusted

REF	NRR	SEX	AD	Number Exposed Case	Number Exposed Cont	Non-exposed Case	Non-exposed Cont	RR	95.00%CI
LUBIN2	184	f	0	9	39	138	1180	1.97	(0.94- 4.16)
Subtotal LUBIN2								2.28	(1.99- 2.60)
MATOS	56	m	0	11	88	5	110	2.75	(0.92- 8.21)
MATOS	58	m	0	28	90	5	110	6.84	(2.54- 18.45)
MATOS	60	m	0	40	105	5	110	8.38	(3.19- 22.05)
Subtotal MATOS								5.68	(3.16- 10.19)
MATSUD	7	m	0	6	1237	0	1255	13.19~	(0.74- 234.37)
MATSUD	8	m	0	13	1607	0	1255	21.09~	(1.25- 355.07)
MATSUD	9	m	0	4	470	0	1255	24.02~	(1.29- 446.92)
Subtotal MATSUD								18.81	(3.58- 98.87)
ORMOS	22	m	0	4	329	2	777	4.72	(0.86- 25.91)
ORMOS	23	m	0	3	577	2	777	2.02	(0.34- 12.13)
ORMOS	24	m	0	1	128	2	777	3.04	(0.27- 33.72)
Subtotal ORMOS								3.13	(1.04- 9.39)
OSANN	55	m	2	-	-	-	-	16.50	(9.30- 29.30)
OSANN	63	m	2	-	-	-	-	37.50	(21.30- 66.00)
OSANN	56	f	2	-	-	-	-	8.80	(6.10- 12.80)
OSANN	64	f	2	-	-	-	-	24.20	(15.80- 37.20)
Subtotal OSANN								16.57	(13.16- 20.85)
OSANN2	16	f	0	14	24	22	43	1.14	(0.49- 2.63)
OSANN2	17	f	0	47	15	22	43	6.12	(2.82- 13.30)
Subtotal OSANN2								2.81	(1.59- 4.97)
WUWILL	22	f	0	112	311	172	601	1.26	(0.96- 1.66)
WUWILL	23	f	0	26	40	172	601	2.27	(1.35- 3.83)
Subtotal WUWILL								1.43	(1.12- 1.82)
WYNDE2	10	m	0	2	114	5	105	0.37	(0.07- 1.94)
WYNDE2	11	m	0	14	203	5	105	1.45	(0.51- 4.13)
WYNDE2	12	m	0	14	83	5	105	3.54	(1.23- 10.23)
WYNDE2	13	m	0	16	112	5	105	3.00	(1.06- 8.48)
Subtotal WYNDE2								1.98	(1.12- 3.51)
WYNDE3	24	m	0	1	42	6	88	0.35	(0.04- 2.99)
WYNDE3	25	m	0	20	114	6	88	2.57	(0.99- 6.68)
WYNDE3	26	m	0	34	82	6	88	6.08	(2.43- 15.24)
WYNDE3	27	m	0	9	26	6	88	5.08	(1.65- 15.59)
WYNDE3	71	f	0	2	19	15	76	0.53	(0.11- 2.53)
WYNDE3	72	f	0	11	24	15	76	2.32	(0.94- 5.73)
WYNDE3	73	f	0	7	10	15	76	3.55	(1.16- 10.80)
WYNDE3	74	f	0	1	3	15	76	1.69	(0.16- 17.36)
Subtotal WYNDE3								2.82	(1.87- 4.25)
WYNDE4	37	m	0	3	82	4	115	1.05	(0.23- 4.83)
WYNDE4	38	m	0	6	147	4	115	1.17	(0.32- 4.26)
WYNDE4	39	m	0	15	274	4	115	1.57	(0.51- 4.84)
WYNDE4	40	m	0	4	98	4	115	1.17	(0.29- 4.82)
WYNDE4	41	m	0	7	64	4	115	3.14	(0.89- 11.15)
WYNDE4	55	f	2	-	-	-	-	1.33	(0.29- 6.07)
Subtotal WYNDE4								1.51	(0.88- 2.60)
ZHENG	6	m	0	18	40	29	94	1.46	(0.73- 2.92)
ZHENG	7	m	0	35	66	29	94	1.72	(0.96- 3.08)
ZHENG	8	m	0	53	89	29	94	1.93	(1.13- 3.30)
ZHENG	9	m	0	17	23	29	94	2.40	(1.13- 5.08)
ZHENG	19	f	0	13	29	119	184	0.69	(0.35- 1.39)
ZHENG	20	f	0	20	15	119	184	2.06	(1.02- 4.19)
Subtotal ZHENG								1.62	(1.24- 2.11)
Partial Totals				1871	25112	2509	33233		

*prospective study

~ With 0.5 adjustment for zero

Table 3G1 - 5

IESLC - Meta-analysis of Ever Smoking by Amount, Overview, Any product (or Cigarettes if Any not available)
Adenocarcinoma
Least adjusted

REF	NRR	SEX	AD	Ys	Ws	Qs	Ps
ALDERS	46	m	1	1.03	3.70	0.50	0.0476
ALDERS	47	m	1	0.98	3.91	0.68	0.0520
ALDERS	48	m	1	1.20	4.83	0.19	0.0084
ALDERS	49	f	1	1.02	13.70	1.97	0.0002
ALDERS	50	f	1	1.52	13.21	0.20	0.0000
ALDERS	51	f	1	1.20	10.32	0.42	0.0001
Subtotal ALDERS				1.21	49.67	3.97	
BARBON	45	m	0	0.77	3.31	1.30	0.1612
BARBON	46	m	0	2.01	5.28	2.01	0.0000
BARBON	47	m	0	1.88	5.65	1.32	0.0000
BARBON	48	m	0	2.29	5.16	4.06	0.0000
BARBON	49	m	0	2.27	5.49	4.17	0.0000
Subtotal BARBON				1.93	24.88	12.85	
BROWN2	34	m	2	1.82	67.36	12.23	0.0000
BROWN2	44	m	2	2.37	107.03	101.08	0.0000
BROWN2	33	f	2	1.76	90.29	11.66	0.0000
BROWN2	43	f	2	2.15	145.78	82.72	0.0000
Subtotal BROWN2				2.07	410.46	207.69	
CHOI	51	m	0	-0.51	2.41	8.75	0.4323
CHOI	52	m	0	0.27	5.16	6.62	0.5467
CHOI	53	m	0	1.11	3.78	0.31	0.0304
CHOI	54	m	0	-0.36	1.47	4.57	0.6600
CHOI	55	m	0	1.51	1.22	0.01	0.0956
CHOI	59	f	0	-0.18	2.95	7.33	0.7595
CHOI	60	f	0	-1.74	0.47	4.63	0.2324
CHOI	61	f	0	1.21	0.49	0.02	0.3961
Subtotal CHOI				0.27	17.95	32.23	
DOLL	58	m	0	-0.75	0.98	4.50	0.4593
DOLL	59	m	0	-0.29	1.70	4.83	0.7068
DOLL	60	m	0	0.12	1.72	2.79	0.8706
DOLL	61	m	0	0.10	1.45	2.46	0.9065
DOLL	65	f	0	-0.06	1.32	2.80	0.9472
DOLL	66	f	0	0.96	1.91	0.36	0.1823
DOLL	67	f	0	1.37	1.13	0.00	0.1451
Subtotal DOLL				0.24	10.21	17.74	
DORGAN	126	m	2	1.17	3.92	0.20	0.0203
DORGAN	127	m	2	1.73	4.45	0.48	0.0003
DORGAN	105	f	3	1.05	29.62	3.51	0.0000
DORGAN	106	f	3	1.68	28.43	2.33	0.0000
Subtotal DORGAN				1.38	66.41	6.52	
DOSEME	8	m	2	0.59	5.75	3.78	0.1585
DOSEME	12	m	2	0.99	13.23	2.17	0.0003
DOSEME	16	m	2	1.16	5.93	0.33	0.0046
Subtotal DOSEME				0.94	24.92	6.28	
GER	2	c	0	-0.26	6.02	16.55	0.5237
GER	3	c	0	0.25	10.36	13.72	0.4249
GER	4	c	0	-0.26	3.67	10.05	0.6242
Subtotal GER				0.00	20.06	40.32	
JEDRYC	15	m	0	0.21	1.51	2.14	0.7972
JEDRYC	16	m	0	0.83	4.13	1.36	0.0937
JEDRYC	17	m	0	1.60	5.96	0.24	0.0001
JEDRYC	18	m	0	1.89	4.82	1.19	0.0000
JEDRYC	19	m	0	1.51	3.71	0.05	0.0036
Subtotal JEDRYC				1.39	20.13	4.97	
KREYBE	17	m	0	1.04	2.72	0.34	0.0849
KREYBE	18	m	0	0.33	1.99	2.27	0.6405
KREYBE	19	m	0	1.47	1.86	0.01	0.0460
KREYBE	34	f	0	-0.27	6.53	18.11	0.4952
KREYBE	35	f	0	-0.55	0.94	3.56	0.5964
Subtotal KREYBE				0.28	14.04	24.28	
LAMTH	16	f	0	0.40	13.12	12.98	0.1438
LAMTH	17	f	0	0.84	8.17	2.51	0.0158
LAMTH	18	f	0	0.78	3.08	1.20	0.1740
Subtotal LAMTH				0.60	24.36	16.69	
LUBIN2	152	m	0	0.32	29.82	34.52	0.0782
LUBIN2	156	m	0	1.02	43.25	6.28	0.0000
LUBIN2	160	m	0	1.24	44.40	1.12	0.0000
LUBIN2	164	m	0	1.38	39.81	0.02	0.0000
LUBIN2	172	f	0	-0.07	15.74	34.09	0.7715
LUBIN2	176	f	0	0.45	28.07	25.15	0.0167
LUBIN2	180	f	0	0.01	10.63	20.47	0.9727

Table 3G1 - 5

IESLC - Meta-analysis of Ever Smoking by Amount, Overview, Any product (or Cigarettes if Any not available)
Adenocarcinoma
Least adjusted

REF	NRR	SEX	AD	Ys	Ws	Qs	Ps
LUBIN2	184	f	0	0.68	6.90	3.57	0.0741
Subtotal LUBIN2				0.82	218.62	125.21	
MATOS	56	m	0	1.01	3.21	0.48	0.0698
MATOS	58	m	0	1.92	3.91	1.08	0.0001
MATOS	60	m	0	2.13	4.10	2.17	0.0000
Subtotal MATOS				1.74	11.22	3.73	
MATSUD	7	m	0	2.58	0.46	0.65	0.0789
MATSUD	8	m	0	3.05	0.48	1.31	0.0343
MATSUD	9	m	0	3.18	0.45	1.42	0.0331
Subtotal MATSUD				2.93	1.40	3.38	
ORMOS	22	m	0	1.55	1.33	0.03	0.0738
ORMOS	23	m	0	0.70	1.20	0.58	0.4420
ORMOS	24	m	0	1.11	0.66	0.06	0.3661
Subtotal ORMOS				1.14	3.18	0.66	
OSANN	55	m	2	2.80	11.67	23.03	0.0000
OSANN	63	m	2	3.62	12.01	59.52	0.0000
OSANN	56	f	2	2.17	27.97	16.86	0.0000
OSANN	64	f	2	3.19	20.96	66.99	0.0000
Subtotal OSANN				2.81	72.61	166.40	
OSANN2	16	f	0	0.13	5.50	8.83	0.7584
OSANN2	17	f	0	1.81	6.38	1.09	0.0000
Subtotal OSANN2				1.03	11.88	9.93	
WUWILL	22	f	0	0.23	50.96	69.60	0.1009
WUWILL	23	f	0	0.82	14.10	4.71	0.0021
Subtotal WUWILL				0.36	65.06	74.31	
WYNDE2	10	m	0	-1.00	1.39	8.00	0.2387
WYNDE2	11	m	0	0.37	3.50	3.70	0.4885
WYNDE2	12	m	0	1.26	3.41	0.06	0.0195
WYNDE2	13	m	0	1.10	3.56	0.32	0.0382
Subtotal WYNDE2				0.69	11.86	12.08	
WYNDE3	24	m	0	-1.05	0.83	5.00	0.3372
WYNDE3	25	m	0	0.95	4.22	0.87	0.0521
WYNDE3	26	m	0	1.81	4.55	0.75	0.0001
WYNDE3	27	m	0	1.62	3.05	0.16	0.0045
WYNDE3	71	f	0	-0.63	1.58	6.50	0.4293
WYNDE3	72	f	0	0.84	4.71	1.46	0.0675
WYNDE3	73	f	0	1.27	3.10	0.05	0.0258
WYNDE3	74	f	0	0.52	0.71	0.54	0.6593
Subtotal WYNDE3				1.04	22.76	15.32	
WYNDE4	37	m	0	0.05	1.66	3.01	0.9482
WYNDE4	38	m	0	0.16	2.31	3.55	0.8077
WYNDE4	39	m	0	0.45	3.04	2.71	0.4291
WYNDE4	40	m	0	0.16	1.93	2.96	0.8243
WYNDE4	41	m	0	1.15	2.40	0.15	0.0761
WYNDE4	55	f	2	0.29	1.66	2.06	0.7132
Subtotal WYNDE4				0.41	12.99	14.44	
ZHENG	6	m	0	0.38	7.96	8.29	0.2870
ZHENG	7	m	0	0.54	11.26	8.26	0.0692
ZHENG	8	m	0	0.66	13.29	7.30	0.0165
ZHENG	9	m	0	0.87	6.78	1.87	0.0229
ZHENG	19	f	0	-0.37	7.98	24.87	0.3003
ZHENG	20	f	0	0.72	7.66	3.49	0.0452
Subtotal ZHENG				0.48	54.94	54.08	

N	102
NS	22

Table 3G1 - 6

IESLC - Meta-analysis of Ever Smoking by Amount, Overview, Any product (or Cigarettes if Any not available)
Adenocarcinoma
Least adjusted

	Sex combined	male	female	Total
N	3	64	35	102
NS	1	18	14	33

In this overview table, other than the "N" rows, entries in the "absent" and "Total" columns may be invalid and should be ignored

	Amount smoked (broad categories) absent	<20k5	6-44k20	>20k45	Total
N	27	31	21	23	102
NS	15	21	17	18	71
Wt	480.36	386.60	159.20	143.44	1169.61
Het Chi	230.31	237.75	46.82	139.66	853.10
Het df	26	30	20	22	101
Het P	***	***	***	***	***
Fixed RR	6.04	2.61	2.61	5.66	4.05
RRl	5.52	2.36	2.23	4.80	3.82
RRu	6.61	2.88	3.04	6.66	4.29
P	+++	+++	+++	+++	+++
Random RR	3.73	1.61	2.41	4.27	2.69
RRl	2.70	1.15	1.84	2.67	2.22
RRu	5.14	2.24	3.17	6.82	3.25
P	+++	++	+++	+++	+++

	absent	<10k1	2-19k10	11-29k20	21-39k30	31-98k40	>40k99	Total
		Amount smoked (narrow categories)						
N	56	12	8	17	5	1	3	102
NS	22	7	6	15	5	1	2	57
Wt	823.19	74.35	97.90	148.61	19.10	1.47	4.98	1169.61
Het Chi	531.66	8.50	17.00	43.22	8.08	0.00	0.70	853.10
Het df	55	11	7	16	4	0	2	101
Het P	***	N.S.	*	***	(*)	N.S.	N.S.	***
Fixed RR	5.26	1.13	2.23	2.64	4.75	0.70	4.22	4.05
RRl	4.91	0.90	1.83	2.25	3.03	0.14	1.75	3.82
RRu	5.63	1.42	2.72	3.11	7.44	3.50	10.16	4.29
P	+++	N.S.	+++	+++	+++	N.S.	++	+++
Random RR	3.33	1.13	2.19	2.48	4.33	0.70	4.22	2.69
RRl	2.60	0.90	1.49	1.84	2.26	0.14	1.75	2.22
RRu	4.26	1.42	3.22	3.34	8.28	3.50	10.16	3.25
P	+++	N.S.	+++	+++	+++	N.S.	++	+++

MALES

	Amount smoked (broad categories) absent	<20k5	6-44k20	>20k45	Total
N	16	17	15	16	64
NS	12	17	15	16	60
Wt	215.47	139.03	111.66	97.31	563.47
Het Chi	123.44	83.21	22.82	65.84	362.55
Het df	15	16	14	15	63
Het P	***	***	(*)	***	***
Fixed RR	6.26	3.06	2.92	5.30	4.38
RRl	5.48	2.59	2.43	4.35	4.04
RRu	7.16	3.62	3.52	6.47	4.76
P	+++	+++	+++	+++	+++
Random RR	3.79	1.83	2.75	4.92	3.13
RRl	2.34	1.12	2.08	3.01	2.49
RRu	6.12	2.99	3.64	8.05	3.94
P	+++	+	+++	+++	+++

Table 3G1 - 6

IESLC - Meta-analysis of Ever Smoking by Amount, Overview, Any product (or Cigarettes if Any not available)
Adenocarcinoma
Least adjusted

MALES

	Amount smoked (narrow categories)							
	absent	<10k1	2-19k10	11-29k20	21-39k30	31-98k40	>40k99	Total
N	30	7	6	13	5	1	2	64
NS	18	7	6	13	5	1	2	51
Wt	318.41	46.06	67.92	106.24	19.10	1.47	4.27	563.47
Het Chi	155.31	3.51	12.17	22.58	8.08	0.00	0.01	362.55
Het df	29	6	5	12	4	0	1	63
Het P	***	N.S.	*	*	(*)	N.S.	N.S.	***
Fixed RR	6.67	1.35	2.57	2.95	4.75	0.70	4.91	4.38
RRl	5.97	1.01	2.02	2.44	3.03	0.14	1.90	4.04
RRu	7.44	1.81	3.26	3.56	7.44	3.50	12.68	4.76
P	+++	+	+++	+++	+++	N.S.	++	+++
Random RR	4.31	1.35	2.36	2.77	4.33	0.70	4.91	3.13
RRl	3.14	1.01	1.45	2.03	2.26	0.14	1.90	2.49
RRu	5.92	1.81	3.84	3.79	8.28	3.50	12.68	3.94
P	+++	+	+++	+++	+++	N.S.	++	+++

FEMALES

	Amount smoked (broad categories)				
	absent	<20k5	6-44k20	>20k45	Total
N	11	13	5	6	35
NS	10	13	5	6	34
Wt	264.89	241.55	37.18	42.45	586.08
Het Chi	106.35	141.08	16.56	54.53	446.16
Het df	10	12	4	5	34
Het P	***	***	**	***	***
Fixed RR	5.87	2.45	2.26	7.79	3.93
RRl	5.20	2.16	1.64	5.77	3.63
RRu	6.62	2.78	3.11	10.53	4.27
P	+++	+++	+++	+++	+++
Random RR	3.46	1.47	1.96	3.87	2.30
RRl	2.07	0.89	0.94	1.17	1.65
RRu	5.79	2.43	4.06	12.80	3.22
P	+++	N.S.	(+)	+	+++

	Amount smoked (narrow categories)							
	absent	<10k1	2-19k10	11-29k20	21-39k30	31-98k40	>40k99	Total
N	24	5	2	3			1	35
NS	13	5	2	3			1	24
Wt	495.09	28.29	29.99	32.01			0.71	586.08
Het Chi	316.34	1.15	0.47	13.45			0.00	446.16
Het df	23	4	1	2			0	34
Het P	***	N.S.	N.S.	**			N.S.	***
Fixed RR	4.69	0.85	1.62	2.33			1.69	3.93
RRl	4.30	0.59	1.14	1.65			0.16	3.63
RRu	5.12	1.23	2.32	3.30			17.36	4.27
P	+++	N.S.	++	+++			N.S.	+++
Random RR	2.87	0.85	1.62	2.22			1.69	2.30
RRl	1.96	0.59	1.14	0.90			0.16	1.65
RRu	4.19	1.23	2.32	5.51			17.36	3.22
P	+++	N.S.	++	(+)			N.S.	+++

Table 3G1 - 7

IESLC - Meta-analysis of Ever Smoking by Amount, Overview, Any product (or Cigarettes if Any not available)
Adenocarcinoma
Excluded studies (and stage at which they were excluded)

1 ABELIN ABRAHA AMANDU AMES ANDERS AUSTIN AXELSO BAND BECHER BERRIN BLOHMK BLOT4 BROCKM BROWN1 BYERS1 BYERS2
CARPEN CASCO2 CASCOR CHAN CHEN3 CHIAZZ CHYOU DESTE2 DOCKER DROSTE DU GARCIA GARDIN GENG GODLEY GOODMA
GRAHAM GREGOR HEGMAN HEIN HENNEK HINDS HIRAOK HOROWI HORWIT HUANG ISHIMA JAHN JAIN JARVHO JIANG KELLER
KIHARA KJUUS KO KOHLME KUBIK LAMWK LAMWK2 LANGE LEI LEMARC LEVIN LIU LOMBA2 LOMBAR MAGNUS MARSH
MARSH2 MCDUFF MCLAUG MILLER MILLS NOTANI NOU ODRISC PAWLEG PERSHA POFFIJ QIAO QIAO2 RADZIK REN RONCO
ROOTS ROTHSC SAARIK SANKAR SCHWAR SEGI SEOW SHIMIZ SIMARA SIMONA SITAS SOBUE2 STASZE STAYNE STUCKE SUN
SUZUK2 SUZUKI TANG TAO TOKARS TOUSEY ULMER VEIERO VUTUC WALD WANG WANG3 WANG4 WICKLU WIGLE WILKIN
WU2 WUNSCH WYNDE8 XIANGZ XU XU2 XU4 YONG ZHANG

2 AKIBA ARCHER BENSHL BRETT BROSS CEDERL CHANG CHOW COMSTO DARBY DEAN3 DEKLER DORANT DORN ENGELA ENSTRO
GAO2 GILLIS HAENSZ HAMMO2 HIRAY2 HIRAYA HITOSU HOLE HUMBLE KAISE2 KAISER KANELL KATSOU KAUFMA KINLEN KNEKT
KOO LAURIL LIAW LICKIN LIDDEL MIGRAN MRFIT MRFITR MURATA NAM PARKIN PERSH2 PETO PEZZO2 PEZZOT PISANI
PRESCO RIMING SEGI2 SOBUE SPEIZE STOCKW SVENSS TANG2 TENKAN TSUGAN TULINI TVERDA WAKAI WARSIN WATSON WU
WYNDE5 WYNDER YAMAGU

3 BUELL CHEN MASTRA MZILEN RESTRE SADOWS

4 BEST BOFFET WYNDE7

6 BLOT1 BLOT2 BLOT3 BOUCHA JONES MOLLO SCHWA2 VANDER

8 AGUDO ARMADA AUVINE AXELSS BOUCOT BRESLO BUFFLE CHATZI CHEN2 COOKSO CORREA CPSI CPSII DAMBER DAVEYS DEAN
DEAN2 DESTEF DOLL2 DUNN EBELIN ESAKI FAN GAO GARSHI GOLLED GSELL HAMMON HANSEN HU HU2 JARUP
JOLY JUSSAW KHUDER KOULUM KREUZE LAUSSM LETOUR LIU2 LIU3 LIU4 LIU5 LUBIN LUO MACLEN MARTIS MCCONN
NOTAN2 PASTOR PERNU PIKE POLEDN RACHTA RANDIG SHAW SIEMIA SPITZ STOCKS TIZZAN WANG2 WYNDE6 XU3 YUAN
ZHOU

10 BENHAM

Table 3G1 - 8

Potentially overlapping studies

REF	REFGP	PRINC	.	OVERLAP/LINK
LUBIN2	LUBIN2	1		Lubin-combined
LAMTH	LAMTH	1		KOO/LAMTH/LAMWK
OSANN2	KAISER	2		KAISER/OSANN2
MATSUD	MATSUD	1		SOBUE2/MATSUD

Table 3G1 - 9

Most adjusted - insufficient data for metaanalysis

REF	NRR	SEX	AGEL	AGEH	RACE	YF	LC TYPE	LOC	START	ST	NLC	R	VB	P	H	AD	PRODUCT	exL	exH	S1	S2	DENOM	De
CHEN	15	c	0	0	all	-	a	As:oth	1987	CC	323	n	ot	n	y	2	cig+/-ot	1	10	1	0	nev cigs	ot
CHEN	14	c	0	0	all	-	a	As:oth	1987	CC	323	n	ot	n	y	2	cig+/-ot	11	20	2	3	nev cigs	ot
CHEN	13	c	0	0	all	-	a	As:oth	1987	CC	323	n	ot	n	y	2	cig+/-ot	21	30	0	4	nev cigs	ot
CHEN	12	c	0	0	all	-	a	As:oth	1987	CC	323	n	ot	n	y	2	cig+/-ot	31	99	3	0	nev cigs	ot

REF	NRR	RR	SIG	Cigarette equivalent	RRDATA comment
CHEN	15	1.21	n	*	0
CHEN	14	1.74	n	*	0
CHEN	13	2.34	n	*	0
CHEN	12	3.61	y	*	P < 0.001

Table 3G2 -

IESLC - Meta-analysis of Ever Smoking, Amount smoked, "Low", Any product (or Cigarettes if Any not available)
Adenocarcinoma

This analysis is restricted to results for:
1) Results by Amount smoked
2) Ever smokers
3) Results complete enough for use in metaanalysis

Within each study, results are then selected (in the following order of preference, within each sex) for:
4) PRODUCT: all/unspec, cigarettes regardless of other products, cigarettes only
5) CIGTYPE: all/unspecified, MC regardless of HR, MC only
6) DENOM: never smoked anything, never smoked cigarettes, (never +1 = +long term ex, +2 = +amount unknown,
+3 = never cigs+long term ex)
7) Followup period (YF, prospective studies): whole study (coded as 0) or longest available
8) LCtype: adeno or nearest available, but not squamous. (q = squamous, s = small,
a = adeno, l = large, KII = Kreyberg II, al = alveolar, br = bronchiolar, u = undifferentiated)
9) Race: all or nearest available, otherwise by race (wh or w = white, bl or b = black, hi = hispanic
ch = chinese, jap = japanese, haw = hawaiian, w+o = white + oriental, sca = scandinavian, as = asian)
10) Amount smoked "low" in key scheme 1 (key value 5, maximum range <20, in numbers of
cigarettes or cigarette equivalents)
11) For overlapping studies: principal rather than subsidiary studies
Finally by Age: whole study (coded as 0) if available, otherwise by widest available age group
and then for single sex results (m, f) in preference to combined sex results (c).

Results adjusted (AD) for the most potential confounders are then chosen in Sections -1 to -3
and results adjusted for the least confounders in Sections -4 to -6. (Those least adjusted results which
actually differ from the most adjusted as marked 'x' in column X in Section -4)
(Results adjusted for an unknown number of confounder(s) are coded as 20.)

Section -7 shows excluded studies, together with the stage (as above) at which no qualifying
results were found.

Section -8 lists the potentially overlapping studies which have been included (1=principal, 2=subsidiary).

Section -9 lists any results which would have been included in preference except that they had data not complete
enough for use in meta-analysis, with their significance (yes/no), if known, and any further comment as entered
on the database.

In addition to those mentioned above, the following fields, levels and abbreviations are used:
* or nk = not known, n = no, y = yes, ot = other
nev = never
all/unspec = all or unspecified, cig+/-ot = cigarettes irrespective of other products (cigar, pipe etc)
MC = manufactured cigarettes, HR = hand-rolled cigarettes
exL, exH = range of exposure (low and high) in the smoking group, in terms of Amount smoked,
cigarettes or cigarette equivalents
REF: 6-character study reference
NRR: number of the RR on the database within the study
ST : study type (CC = case control, pr or prosp = prospective)
NLC: number of lung cancer cases in whole study
R : risky occupational population (n = no, m = mining, o = other risky)
VB : national cigarette type (V = at least 75% Virginia, bl = at least 75% blended, ot = other)
P : any proxy use
H : full histological confirmation
De : derivation of RR/CI (or = original, st = standard method, ot = other method of estimation)

Table 3G2 - 1

IESLC - Meta-analysis of Ever Smoking, Amount smoked, "Low", Any product (or Cigarettes if Any not available)
Adenocarcinoma
Most adjusted

REF	NRR	SEX	AGEL	AGEH	RACE	YF	LC TYPE	LOC	START	ST	NLC	R	VB	P	H	AD	PRODUCT	exL	exH	DENOM	De
ALDERS	46	m	0	0	all	-	not q+s	Eu:UK	1977	CC	1448	n	V	n	n	1	cig only	1	17	nev+2	ot
ALDERS	49	f	0	0	all	-	not q+s	Eu:UK	1977	CC	1448	n	V	n	n	1	cig only	1	17	nev+2	ot
BARBON	79	m	0	0	all	-	a	Eu:wst	1979	CC	755	n	bl	y	y	3	all/unsp	1	19	nev any	or
BROWN2	34	m	0	0	wh	-	a	NAmer	1984	CC	14596	n	bl	n	y	2	cig+/-ot	1	19	nev cigs	or
BROWN2	33	f	0	0	wh	-	a	NAmer	1984	CC	14596	n	bl	n	y	2	cig+/-ot	1	19	nev cigs	or
CHOI	51	m	0	0	all	-	a	As:oth	1985	CC	375	n	bl	n	n	0	cig+/-ot	1	10	nev cigs	st
CHOI	59	f	0	0	all	-	a	As:oth	1985	CC	375	n	bl	n	n	0	cig+/-ot	1	10	nev cigs	st
DOLL	73	m	0	0	all	-	KII	Eu:UK	1948	CC	1465	n	V	n	n	1	all/unsp	5	14	nev any	ot
DOLL	80	f	0	0	all	-	KII	Eu:UK	1948	CC	1465	n	V	n	n	1	all/unsp	5	14	nev any	ot
DORGAN	126	m	0	0	wh	-	a	NAmer	1980	CC	2026	n	bl	y	y	2	cig+/-ot	1	19	nev any	ot
DORGAN	105	f	0	0	all	-	a	NAmer	1980	CC	2026	n	bl	y	y	3	cig+/-ot	1	19	nev any	ot
DOSEME	8	m	0	0	all	-	not q+s	Eu:bal	1979	CC	1210	n	bl	n	n	2	cig+/-ot	1	10	nev cigs	or
GER	10	c	0	0	all	-	a	As:oth	1990	CC	141	n	ot	y	n	8	all/unsp	1	10	nev any	ot
JEDRYC	36	m	0	0	all	-	a	Eu:est	1980	CC	1630	n	bl	y	n	3	cig+/-ot	1	19	nev any	or
KREYBE	5	m	0	0	all	-	KII	Eu:Sca	1948	CC	300	n	bl	n	y	1	all/unsp	1	14	nev any	ot
KREYBE	26	f	0	0	all	-	KII	Eu:Sca	1948	CC	300	n	bl	n	y	1	all/unsp	1	14	nev any	ot
LAMTH	16	f	0	0	ch	-	a	As:HK	1983	CC	445	n	bl	n	n	0	all/unsp	1	10	nev any	or
LUBIN2	152	m	0	0	all	-	a	Eu:mul	1976	CC	7804	n	bl	n	y	0	cig+/-ot	1	9	nev any	st
LUBIN2	172	f	0	0	all	-	a	Eu:mul	1976	CC	7804	n	bl	n	y	0	cig+/-ot	1	9	nev any	st
MATOS	57	m	0	0	all	-	a	SCAmer	1994	CC	200	n	bl	n	n	2	cig+/-ot	1	14	nev any	or
MATSUD	7	m	0	0	all	-	a	As:Jap	1965	CC	179	n	bl	n	n	0	cig+/-ot	1	10	nev cigs	ot
ORMOS	22	m	0	0	all	-	KII	Eu:est	1947	CC	119	n	bl	y	y	0	cig+/-ot	1	15	nev any	st
OSANN2	34	f	0	0	all	-	KII	NAmer	1964	ot	217	n	bl	n	y	1	cig+/-ot	1	19	nev cigs	or
WUWILL	16	f	0	0	all	-	a	As:Chi	1985	CC	965	n	ot	n	n	3	cig+/-ot	1	19	nev cigs	ot
WYNDE2	10	m	0	0	all	-	KII	NAmer	1962	CC	404	n	bl	n	y	0	cig+/-ot	1	10	nev any	st
WYNDE3	24	m	0	0	all	-	KII	NAmer	1966	CC	350	n	bl	n	y	0	cig+/-ot	1	9	nev any	st
WYNDE3	71	f	0	0	all	-	KII	NAmer	1966	CC	350	n	bl	n	y	0	cig+/-ot	1	9	nev any	st
WYNDE4	37	m	0	0	all	-	a	NAmer	1948	CC	684	n	bl	y	n	0	all/unsp	1	9	nev any	st
WYNDE4	55	f	0	0	all	-	a	NAmer	1948	CC	684	n	bl	y	n	2	all/unsp	1	9	nev any	ot
ZHENG	6	m	0	0	all	-	a	As:Chi	1982	CC	540	n	ot	*	y	0	cig+/-ot	1	9	nev cigs	st
ZHENG	19	f	0	0	all	-	a	As:Chi	1982	CC	540	n	ot	*	y	0	cig+/-ot	1	9	nev cigs	st

Cigarette type is all/unspec for all RRs

except for the following:

REF	NRR	CIGTYPE
ALDERS	46	MC only
ALDERS	49	MC only

Table 3G2 - 2

IESLC - Meta-analysis of Ever Smoking, Amount smoked, "Low", Any product (or Cigarettes if Any not available)
Adenocarcinoma
Most adjusted

REF	NRR	SEX	AD	Number Exposed Case	Number Exposed Cont	Non-exposed Case	Non-exposed Cont	RR	95.00%CI
ALDERS	46	m	1	-	-	-	-	2.80	(1.01- 7.75)
ALDERS	49	f	1	-	-	-	-	2.77	(1.63- 4.70)
Subtotal ALDERS								2.78	(1.74- 4.44)
BARBON	79	m	3	-	-	-	-	5.00	(2.20- 11.00)
BROWN2	34	m	2	-	-	-	-	6.20	(4.90- 7.90)
BROWN2	33	f	2	-	-	-	-	5.80	(4.70- 7.10)
Subtotal BROWN2								5.97	(5.11- 6.98)
CHOI	51	m	0	4	90	7	95	0.60	(0.17- 2.13)
CHOI	59	f	0	4	16	49	164	0.84	(0.27- 2.62)
Subtotal CHOI								0.72	(0.31- 1.68)
DOLL	73	m	1	-	-	-	-	0.80	(0.18- 3.60)
DOLL	80	f	1	-	-	-	-	2.30	(0.56- 9.48)
Subtotal DOLL								1.40	(0.50- 3.91)
DORGAN	126	m	2	-	-	-	-	3.23	(1.20- 8.70)
DORGAN	105	f	3	-	-	-	-	2.87	(2.00- 4.11)
Subtotal DORGAN								2.91	(2.07- 4.08)
DOSEME	8	m	2	-	-	-	-	1.80	(0.80- 4.10)
GER	10	c	8	-	-	-	-	0.62	(0.23- 1.72)
JEDRYC	36	m	3	-	-	-	-	2.19	(0.84- 5.72)
KREYBE	5	m	1	-	-	-	-	2.65	(0.81- 8.70)
KREYBE	26	f	1	-	-	-	-	1.30	(0.60- 2.80)
Subtotal KREYBE								1.61	(0.84- 3.06)
LAMTH	16	f	0	36	29	131	158	1.50	(0.87- 2.57)
LUBIN2	152	m	0	66	2194	57	2616	1.38	(0.96- 1.98)
LUBIN2	172	f	0	20	184	138	1180	0.93	(0.57- 1.52)
Subtotal LUBIN2								1.20	(0.90- 1.61)
MATOS	57	m	2	-	-	-	-	2.80	(0.90- 8.50)
MATSUD	7	m	0	6	1237	0	1255	13.19~	(0.74- 234.37)
ORMOS	22	m	0	4	329	2	777	4.72	(0.86- 25.91)
OSANN2	34	f	1	-	-	-	-	0.90	(0.30- 2.70)
WUWILL	16	f	3	-	-	-	-	1.43	(1.07- 1.90)
WYNDE2	10	m	0	2	114	5	105	0.37	(0.07- 1.94)
WYNDE3	24	m	0	1	42	6	88	0.35	(0.04- 2.99)
WYNDE3	71	f	0	2	19	15	76	0.53	(0.11- 2.53)
Subtotal WYNDE3								0.46	(0.13- 1.63)
WYNDE4	37	m	0	3	82	4	115	1.05	(0.23- 4.83)
WYNDE4	55	f	2	-	-	-	-	1.33	(0.29- 6.07)
Subtotal WYNDE4								1.18	(0.40- 3.47)
ZHENG	6	m	0	18	40	29	94	1.46	(0.73- 2.92)
ZHENG	19	f	0	13	29	119	184	0.69	(0.35- 1.39)
Subtotal ZHENG								1.00	(0.62- 1.64)
Partial Totals				179	4405	562	6907		

*prospective study ~ With 0.5 adjustment for zero

REF	NRR	SEX	AD	Ys	Ws	Qs	Ps
ALDERS	46	m	1	1.03	3.70	0.00	0.0476
ALDERS	49	f	1	1.02	13.70	0.00	0.0002
Subtotal ALDERS				1.02	17.40	0.00	
BARBON	79	m	3	1.61	5.93	2.12	0.0001
BROWN2	34	m	2	1.82	67.36	44.51	0.0000
BROWN2	33	f	2	1.76	90.29	50.27	0.0000
Subtotal BROWN2				1.79	157.65	94.78	
CHOI	51	m	0	-0.51	2.41	5.55	0.4323
CHOI	59	f	0	-0.18	2.95	4.18	0.7595
Subtotal CHOI				-0.33	5.36	9.73	
DOLL	73	m	1	-0.22	1.71	2.61	0.7703
DOLL	80	f	1	0.83	1.92	0.06	0.2485
Subtotal DOLL				0.34	3.63	2.67	
DORGAN	126	m	2	1.17	3.92	0.10	0.0203
DORGAN	105	f	3	1.05	29.62	0.05	0.0000
Subtotal DORGAN				1.07	33.53	0.16	
DOSEME	8	m	2	0.59	5.75	1.03	0.1585
GER	10	c	8	-0.48	3.80	8.42	0.3517
JEDRYC	36	m	3	0.78	4.18	0.22	0.1092
KREYBE	5	m	1	0.97	2.73	0.00	0.1076
KREYBE	26	f	1	0.26	6.48	3.64	0.5044
Subtotal KREYBE				0.47	9.20	3.64	
LAMTH	16	f	0	0.40	13.12	4.85	0.1438
LUBIN2	152	m	0	0.32	29.82	14.16	0.0782
LUBIN2	172	f	0	-0.07	15.74	18.53	0.7715

Table 3G2 - 2

IESLC - Meta-analysis of Ever Smoking, Amount smoked, "Low", Any product (or Cigarettes if Any not available)
Adenocarcinoma
Most adjusted

REF	NRR	SEX	AD	Ys	Ws	Qs	Ps
Subtotal LUBIN2				0.19	45.56	32.69	
MATOS	57	m	2	1.03	3.05	0.00	0.0723
MATSUD	7	m	0	2.58	0.46	1.14	0.0789
ORMOS	22	m	0	1.55	1.33	0.39	0.0738
OSANN2	34	f	1	-0.11	3.18	3.97	0.8509
WUWILL	16	f	3	0.36	46.61	19.93	0.0146
WYNDE2	10	m	0	-1.00	1.39	5.63	0.2387
WYNDE3	24	m	0	-1.05	0.83	3.54	0.3372
WYNDE3	71	f	0	-0.63	1.58	4.25	0.4293
Subtotal WYNDE3				-0.77	2.41	7.80	
WYNDE4	37	m	0	0.05	1.66	1.53	0.9482
WYNDE4	55	f	2	0.29	1.66	0.88	0.7132
Subtotal WYNDE4				0.17	3.32	2.41	
ZHENG	6	m	0	0.38	7.96	3.20	0.2870
ZHENG	19	f	0	-0.37	7.98	15.17	0.3003
Subtotal ZHENG				0.00	15.94	18.37	

	N	31
	NS	21
	Wt	382.81
Het	Chi	219.94
Het	df	30
Het	P	***
Fixed	RR	2.75
	RRl	2.49
	RRu	3.04
	P	+++
Random	RR	1.71
	RRl	1.24
	RRu	2.36
	P	+++
Asymm	P	**

Table 3G2 - 3

IESLC - Meta-analysis of Ever Smoking, Amount smoked, "Low", Any product (or Cigarettes if Any not available)
Adenocarcinoma
Most adjusted

	combined	Sex: male	female	Total
N	1	17	13	31
NS	1	17	13	31
Wt	3.80	144.18	234.83	382.81
Het Chi	0.00	83.09	124.96	219.94
Het df	0	16	12	30
Het P	N.S.	***	***	***
Fixed RR	0.62	3.15	2.59	2.75
RRl	0.23	2.67	2.28	2.49
RRu	1.70	3.71	2.95	3.04
P	N.S.	+++	+++	+++
Random RR	0.62	2.01	1.53	1.71
RRl	0.23	1.25	0.94	1.24
RRu	1.70	3.23	2.48	2.36
P	N.S.	++	(+)	+++
Between Chi				11.89
Between df				2
Between P				**
Btwn(F) P				N.S.
Btwn(R) P				N.S.

Table 3G2 - 4

IESLC - Meta-analysis of Ever Smoking, Amount smoked, "Low", Any product (or Cigarettes if Any not available)
Adenocarcinoma
Least adjusted

REF	NRR	X	SEX	AGEL	AGEH	RACE	YF	LC TYPE	LOC	START	ST	NLC	R	VB	P	H	AD	PRODUCT	exL	exH	DENOM	De
ALDERS	46		m	0	0	all	-	not q+s	Eu:UK	1977	CC	1448	n	V	n	n	1	cig only	1	17	nev+2	ot
ALDERS	49		f	0	0	all	-	not q+s	Eu:UK	1977	CC	1448	n	V	n	n	1	cig only	1	17	nev+2	ot
BARBON	45	x	m	0	0	all	-	a	Eu:wst	1979	CC	755	n	bl	y	y	0	all/unsp	1	9	nev any	st
BROWN2	34		m	0	0	wh	-	a	NAmer	1984	CC	14596	n	bl	n	y	2	cig+/-ot	1	19	nev cigs	or
BROWN2	33		f	0	0	wh	-	a	NAmer	1984	CC	14596	n	bl	n	y	2	cig+/-ot	1	19	nev cigs	or
CHOI	51		m	0	0	all	-	a	As:oth	1985	CC	375	n	bl	n	n	0	cig+/-ot	1	10	nev cigs	st
CHOI	59		f	0	0	all	-	a	As:oth	1985	CC	375	n	bl	n	n	0	cig+/-ot	1	10	nev cigs	st
DOLL	59	x	m	0	0	all	-	KII	Eu:UK	1948	CC	1465	n	V	n	n	0	all/unsp	5	14	nev any	st
DOLL	66	x	f	0	0	all	-	KII	Eu:UK	1948	CC	1465	n	V	n	n	0	all/unsp	5	14	nev any	st
DORGAN	126		m	0	0	wh	-	a	NAmer	1980	CC	2026	n	bl	y	y	2	cig+/-ot	1	19	nev any	ot
DORGAN	105		f	0	0	all	-	a	NAmer	1980	CC	2026	n	bl	y	y	3	cig+/-ot	1	19	nev any	ot
DOSEME	8		m	0	0	all	-	not q+s	Eu:bal	1979	CC	1210	n	bl	n	n	2	cig+/-ot	1	10	nev cigs	or
GER	2	x	c	0	0	all	-	a	As:oth	1990	CC	141	n	ot	y	n	0	all/unsp	1	10	nev any	st
JEDRYC	15	x	m	0	0	all	-	a	Eu:est	1980	CC	1630	n	bl	y	n	0	cig+/-ot	1	9	nev any	st
KREYBE	17	x	m	0	0	all	-	KII	Eu:Sca	1948	CC	300	n	bl	n	y	0	all/unsp	1	14	nev any	st
KREYBE	34	x	f	0	0	all	-	KII	Eu:Sca	1948	CC	300	n	bl	n	y	0	all/unsp	1	14	nev any	st
LAMTH	16		f	0	0	ch	-	a	As:HK	1983	CC	445	n	bl	n	n	0	all/unsp	1	10	nev any	or
LUBIN2	152		m	0	0	all	-	a	Eu:mul	1976	CC	7804	n	bl	n	y	0	cig+/-ot	1	9	nev any	st
LUBIN2	172		f	0	0	all	-	a	Eu:mul	1976	CC	7804	n	bl	n	y	0	cig+/-ot	1	9	nev any	st
MATOS	56	x	m	0	0	all	-	a	SCAmer	1994	CC	200	n	bl	n	n	0	cig+/-ot	1	14	nev any	st
MATSUD	7		m	0	0	all	-	a	As:Jap	1965	CC	179	n	bl	n	n	0	cig+/-ot	1	10	nev cigs	ot
ORMOS	22		m	0	0	all	-	KII	Eu:est	1947	CC	119	n	bl	y	y	0	cig+/-ot	1	15	nev any	st
OSANN2	16	x	f	0	0	all	-	KII	NAmer	1964	ot	217	n	bl	n	y	0	cig+/-ot	1	19	nev cigs	st
WUWILL	22	x	f	0	0	all	-	a	As:Chi	1985	CC	965	n	ot	n	n	0	cig+/-ot	1	19	nev cigs	st
WYNDE2	10		m	0	0	all	-	KII	NAmer	1962	CC	404	n	bl	n	y	0	cig+/-ot	1	10	nev any	st
WYNDE3	24		m	0	0	all	-	KII	NAmer	1966	CC	350	n	bl	n	y	0	cig+/-ot	1	9	nev any	st
WYNDE3	71		f	0	0	all	-	KII	NAmer	1966	CC	350	n	bl	n	y	0	cig+/-ot	1	9	nev any	st
WYNDE4	37		m	0	0	all	-	a	NAmer	1948	CC	684	n	bl	y	n	0	all/unsp	1	9	nev any	st
WYNDE4	55		f	0	0	all	-	a	NAmer	1948	CC	684	n	bl	y	n	2	all/unsp	1	9	nev any	ot
ZHENG	6		m	0	0	all	-	a	As:Chi	1982	CC	540	n	ot	*	y	0	cig+/-ot	1	9	nev cigs	st
ZHENG	19		f	0	0	all	-	a	As:Chi	1982	CC	540	n	ot	*	y	0	cig+/-ot	1	9	nev cigs	st

Cigarette type is all/unspec for all RRs

except for the following:

REF	NRR	CIGTYPE
ALDERS	46	MC only
ALDERS	49	MC only

Table 3G2 - 5

IESLC - Meta-analysis of Ever Smoking, Amount smoked, "Low", Any product (or Cigarettes if Any not available)
Adenocarcinoma
Least adjusted

REF	NRR	SEX	AD	Number Exposed Case	Number Exposed Cont	Non-exposed Case	Non-exposed Cont	RR	95.00%CI
ALDERS	46	m	1	-	-	-	-	2.80	(1.01- 7.75)
ALDERS	49	f	1	-	-	-	-	2.77	(1.63- 4.70)
Subtotal ALDERS								2.78	(1.74- 4.44)
BARBON	45	m	0	7	87	7	188	2.16	(0.74- 6.35)
BROWN2	34	m	2	-	-	-	-	6.20	(4.90- 7.90)
BROWN2	33	f	2	-	-	-	-	5.80	(4.70- 7.10)
Subtotal BROWN2								5.97	(5.11- 6.98)
CHOI	51	m	0	4	90	7	95	0.60	(0.17- 2.13)
CHOI	59	f	0	4	16	49	164	0.84	(0.27- 2.62)
Subtotal CHOI								0.72	(0.31- 1.68)
DOLL	59	m	0	14	570	2	61	0.75	(0.17- 3.37)
DOLL	66	f	0	4	18	5	59	2.62	(0.64- 10.81)
Subtotal DOLL								1.46	(0.52- 4.08)
DORGAN	126	m	2	-	-	-	-	3.23	(1.20- 8.70)
DORGAN	105	f	3	-	-	-	-	2.87	(2.00- 4.11)
Subtotal DORGAN								2.91	(2.07- 4.08)
DOSEME	8	m	2	-	-	-	-	1.80	(0.80- 4.10)
GER	2	c	0	9	47	37	149	0.77	(0.35- 1.71)
JEDRYC	15	m	0	2	67	7	289	1.23	(0.25- 6.07)
KREYBE	17	m	0	31	2341	3	644	2.84	(0.87- 9.33)
KREYBE	34	f	0	9	286	27	657	0.77	(0.36- 1.65)
Subtotal KREYBE								1.13	(0.59- 2.15)
LAMTH	16	f	0	36	29	131	158	1.50	(0.87- 2.57)
LUBIN2	152	m	0	66	2194	57	2616	1.38	(0.96- 1.98)
LUBIN2	172	f	0	20	184	138	1180	0.93	(0.57- 1.52)
Subtotal LUBIN2								1.20	(0.90- 1.61)
MATOS	56	m	0	11	88	5	110	2.75	(0.92- 8.21)
MATSUD	7	m	0	6	1237	0	1255	13.19~	(0.74- 234.37)
ORMOS	22	m	0	4	329	2	777	4.72	(0.86- 25.91)
OSANN2	16	f	0	14	24	22	43	1.14	(0.49- 2.63)
WUWILL	22	f	0	112	311	172	601	1.26	(0.96- 1.66)
WYNDE2	10	m	0	2	114	5	105	0.37	(0.07- 1.94)
WYNDE3	24	m	0	1	42	6	88	0.35	(0.04- 2.99)
WYNDE3	71	f	0	2	19	15	76	0.53	(0.11- 2.53)
Subtotal WYNDE3								0.46	(0.13- 1.63)
WYNDE4	37	m	0	3	82	4	115	1.05	(0.23- 4.83)
WYNDE4	55	f	2	-	-	-	-	1.33	(0.29- 6.07)
Subtotal WYNDE4								1.18	(0.40- 3.47)
ZHENG	6	m	0	18	40	29	94	1.46	(0.73- 2.92)
ZHENG	19	f	0	13	29	119	184	0.69	(0.35- 1.39)
Subtotal ZHENG								1.00	(0.62- 1.64)
Partial Totals				392	8244	849	9708		

*prospective study ~ With 0.5 adjustment for zero

REF	NRR	SEX	AD	Ys	Ws	Qs	Ps
ALDERS	46	m	1	1.03	3.70	0.02	0.0476
ALDERS	49	f	1	1.02	13.70	0.05	0.0002
Subtotal ALDERS				1.02	17.40	0.07	
BARBON	45	m	0	0.77	3.31	0.12	0.1612
BROWN2	34	m	2	1.82	67.36	50.49	0.0000
BROWN2	33	f	2	1.76	90.29	57.65	0.0000
Subtotal BROWN2				1.79	157.65	108.14	
CHOI	51	m	0	-0.51	2.41	5.17	0.4323
CHOI	59	f	0	-0.18	2.95	3.81	0.7595
Subtotal CHOI				-0.33	5.36	8.99	
DOLL	59	m	0	-0.29	1.70	2.64	0.7068
DOLL	66	f	0	0.96	1.91	0.00	0.1823
Subtotal DOLL				0.38	3.61	2.64	
DORGAN	126	m	2	1.17	3.92	0.18	0.0203
DORGAN	105	f	3	1.05	29.62	0.27	0.0000
Subtotal DORGAN				1.07	33.53	0.45	
DOSEME	8	m	2	0.59	5.75	0.79	0.1585
GER	2	c	0	-0.26	6.02	8.94	0.5237
JEDRYC	15	m	0	0.21	1.51	0.85	0.7972
KREYBE	17	m	0	1.04	2.72	0.02	0.0849
KREYBE	34	f	0	-0.27	6.53	9.81	0.4952
Subtotal KREYBE				0.12	9.25	9.83	
LAMTH	16	f	0	0.40	13.12	4.04	0.1438
LUBIN2	152	m	0	0.32	29.82	12.07	0.0782
LUBIN2	172	f	0	-0.07	15.74	16.76	0.7715

Table 3G2 - 5

IESLC - Meta-analysis of Ever Smoking, Amount smoked, "Low", Any product (or Cigarettes if Any not available)
Adenocarcinoma
Least adjusted

REF	NRR	SEX	AD	Ys	Ws	Qs	Ps
Subtotal LUBIN2				0.19	45.56	28.83	
MATOS	56	m	0	1.01	3.21	0.01	0.0698
MATSUD	7	m	0	2.58	0.46	1.22	0.0789
ORMOS	22	m	0	1.55	1.33	0.47	0.0738
OSANN2	16	f	0	0.13	5.50	3.77	0.7584
WUWILL	22	f	0	0.23	50.96	27.08	0.1009
WYNDE2	10	m	0	-1.00	1.39	5.33	0.2387
WYNDE3	24	m	0	-1.05	0.83	3.36	0.3372
WYNDE3	71	f	0	-0.63	1.58	3.98	0.4293
Subtotal WYNDE3				-0.77	2.41	7.35	
WYNDE4	37	m	0	0.05	1.66	1.37	0.9482
WYNDE4	55	f	2	0.29	1.66	0.75	0.7132
Subtotal WYNDE4				0.17	3.32	2.12	
ZHENG	6	m	0	0.38	7.96	2.69	0.2870
ZHENG	19	f	0	-0.37	7.98	14.02	0.3003
Subtotal ZHENG				0.00	15.94	16.71	

	N	31
	NS	21
	Wt	386.60
Het	Chi	237.75
Het	df	30
Het	P	***
Fixed	RR	2.61
	RRl	2.36
	RRu	2.88
	P	+++
Random	RR	1.61
	RRl	1.15
	RRu	2.24
	P	++
Asymm	P	**

Table 3G2 - 6

IESLC - Meta-analysis of Ever Smoking, Amount smoked, "Low", Any product (or Cigarettes if Any not available)
Adenocarcinoma
Least adjusted

		Sex		
	combined	male	female	Total
N	1	17	13	31
NS	1	17	13	31
Wt	6.02	139.03	241.55	386.60
Het Chi	0.00	83.21	141.08	237.75
Het df	0	16	12	30
Het P	N.S.	***	***	***
Fixed RR	0.77	3.06	2.45	2.61
RRl	0.35	2.59	2.16	2.36
RRu	1.71	3.62	2.78	2.88
P	N.S.	+++	+++	+++
Random RR	0.77	1.83	1.47	1.61
RRl	0.35	1.12	0.89	1.15
RRu	1.71	2.99	2.43	2.24
P	N.S.	+	N.S.	++
Between Chi				13.45
Between df				2
Between P				**
Btwn(F) P				N.S.
Btwn(R) P				N.S.

Table 3G2 - 7

IESLC - Meta-analysis of Ever Smoking, Amount smoked, "Low", Any product (or Cigarettes if Any not available)
Adenocarcinoma
Excluded studies (and stage at which they were excluded)

1 ABELIN ABRAHA AMANDU AMES ANDERS AUSTIN AXELSO BAND BECHER BERRIN BLOHMK BLOT4 BROCKM BROWN1 BYERS1 BYERS2 CARPEN CASCO2 CASCOR CHAN CHEN3 CHIAZZ CHYOU DESTE2 DOCKER DROSTE DU GARCIA GARDIN GENG GODLEY GOODMA GRAHAM GREGOR HEGMAN HEIN HENNEK HINDS HIRAOK HOROWI HORWIT HUANG ISHIMA JAHN JAIN JARVHO JIANG KELLER KIHARA KJUUS KO KOHLME KUBIK LAMWK LAMWK2 LANGE LEI LEMARC LEVIN LIU LOMBA2 LOMBAR MAGNUS MARSH MARSH2 MCDUFF MCLAUG MILLER MILLS NOTANI NOU ODRISC PAWLEG PERSHA POFFIJ QIAO QIAO2 RADZIK REN RONCO ROOTS ROTHSC SAARIK SANKAR SCHWAR SEGI SEOW SHIMIZ SIMARA SIMONA SITAS SOBUE2 STASZE STAYNE STUCKE SUN SUZUK2 SUZUKI TANG TAO TOKARS TOUSEY ULMER VEIERO VUTUC WALD WANG WANG3 WANG4 WICKLU WIGLE WILKIN WU2 WUNSCH WYNDE8 XIANGZ XU XU2 XU4 YONG ZHANG

2 AKIBA ARCHER BENSHL BRETT BROSS CEDERL CHANG CHOW COMSTO DARBY DEAN3 DEKLER DORANT DORN ENGELA ENSTRO GAO2 GILLIS HAENSZ HAMMO2 HIRAY2 HIRAYA HITOSU HOLE HUMBLE KAISE2 KAISER KANELL KATSOU KAUFMA KINLEN KNEKT KOO LAURIL LIAW LICKIN LIDDEL MIGRAN MRFIT MRFITR MURATA NAM PARKIN PERSH2 PETO PEZZO2 PEZZOT PISANI PRESCO RIMING SEGI2 SOBUE SPEIZE STOCKW SVENSS TANG2 TENKAN TSUGAN TULINI TVERDA WAKAI WARSIN WATSON WU WYNDE5 WYNDER YAMAGU

3 BUELL CHEN MASTRA MZILEN RESTRE SADOWS

4 BEST BOFFET WYNDE7

6 BLOT1 BLOT2 BLOT3 BOUCHA JONES MOLLO SCHWA2 VANDER

8 AGUDO ARMADA AUVINE AXELSS BOUCOT BRESLO BUFFLE CHATZI CHEN2 COOKSO CORREA CPSI CPSII DAMBER DAVEYS DEAN DEAN2 DESTEF DOLL2 DUNN EBELIN ESAKI FAN GAO GARSHI GOLLED GSELL HAMMON HANSEN HU HU2 JARUP JOLY JUSSAW KHUDER KOULUM KREUZE LAUSSM LETOUR LIU2 LIU3 LIU4 LIU5 LUBIN LUO MACLEN MARTIS MCCONN NOTAN2 PASTOR PERNU PIKE POLEDN RACHTA RANDIG SHAW SIEMIA SPITZ STOCKS TIZZAN WANG2 WYNDE6 XU3 YUAN ZHOU

10 OSANN

11 BENHAM

Table 3G2 - 8

Potentially overlapping studies

REF	REFGP	PRINC	.	OVERLAP/LINK
LUBIN2	LUBIN2	1		Lubin-combined
LAMTH	LAMTH	1		KOO/LAMTH/LAMWK
OSANN2	KAISER	2		KAISER/OSANN2
MATSUD	MATSUD	1		SOBUE2/MATSUD

Table 3G2 - 9

Most adjusted - insufficient data for metaanalysis

REF	NRR	SEX	AGEL	AGEH	RACE	YF	LC TYPE	LOC	START	ST	NLC	R	VB	P	H	AD	PRODUCT	exL	exH	DENOM	De
CHEN	15	c	0	0	all	-	a	As:oth	1987	CC	323	n	ot	n	y	2	cig+/-ot	1	10	nev cigs	ot

REF	NRR	RR	SIG	RRDATA comment
CHEN	15	1.21	n	0

Table 3G3 -

IESLC - Meta-analysis of Ever Smoking, Amount smoked, "Mid", Any product (or Cigarettes if Any not available)
Adenocarcinoma

This analysis is restricted to results for:
1) Results by Amount smoked
2) Ever smokers
3) Results complete enough for use in metaanalysis

Within each study, results are then selected (in the following order of preference, within each sex) for:
4) PRODUCT: all/unspec, cigarettes regardless of other products, cigarettes only
5) CIGTYPE: all/unspecified, MC regardless of HR, MC only
6) DENOM: never smoked anything, never smoked cigarettes, (never +1 = +long term ex, +2 = +amount unknown, +3 = never cigs+long term ex)
7) Followup period (YF, prospective studies): whole study (coded as 0) or longest available
8) LCtype: adeno or nearest available, but not squamous. (q = squamous, s = small, a = adeno, l = large, KII = Kreyberg II, al = alveolar, br = bronchiolar, u = undifferentiated)
9) Race: all or nearest available, otherwise by race (wh or w = white, bl or b = black, hi = hispanic ch = chinese, jap = japanese, haw = hawaiian, w+o = white + oriental, sca = scandinavian, as = asian)
10) Amount smoked "mid" in key scheme 1 (key value 20, maximum range 6-44, in numbers of cigarettes or cigarette equivalents)
11) For overlapping studies: principal rather than subsidiary studies
Finally by Age: whole study (coded as 0) if available, otherwise by widest available age group
and then for single sex results (m, f) in preference to combined sex results (c).

Results adjusted (AD) for the most potential confounders are then chosen in Sections -1 to -3
and results adjusted for the least confounders in Sections -4 to -6. (Those least adjusted results which actually differ from the most adjusted as marked 'x' in column X in Section -4)
(Results adjusted for an unknown number of confounder(s) are coded as 20.)

Section -7 shows excluded studies, together with the stage (as above) at which no qualifying results were found.

Section -8 lists the potentially overlapping studies which have been included (1=principal, 2=subsidiary).

Section -9 lists any results which would have been included in preference except that they had data not complete enough for use in meta-analysis, with their significance (yes/no), if known, and any further comment as entered on the database.

In addition to those mentioned above, the following fields, levels and abbreviations are used:
* or nk = not known, n = no, y = yes, ot = other
nev = never
all/unspec = all or unspecified, cig+/-ot = cigarettes irrespective of other products (cigar, pipe etc)
MC = manufactured cigarettes, HR = hand-rolled cigarettes
exL, exH = range of exposure (low and high) in the smoking group, in terms of Amount smoked, cigarettes or cigarette equivalents
REF: 6-character study reference
NRR: number of the RR on the database within the study
ST : study type (CC = case control, pr or prosp = prospective)
NLC: number of lung cancer cases in whole study
R : risky occupational population (n = no, m = mining, o = other risky)
VB : national cigarette type (V = at least 75% Virginia, bl = at least 75% blended, ot = other)
P : any proxy use
H : full histological confirmation
De : derivation of RR/CI (or = original, st = standard method, ot = other method of estimation)

Table 3G3 - 1

IESLC - Meta-analysis of Ever Smoking, Amount smoked, "Mid", Any product (or Cigarettes if Any not available)
Adenocarcinoma
Most adjusted

REF	NRR	SEX	AGEL	AGEH	RACE	YF	LC TYPE	LOC	START	ST	NLC	R	VB	P	H	AD	PRODUCT	exL	exH	DENOM	De
ALDERS	47	m	0	0	all	-	not q+s	Eu:UK	1977	CC	1448	n	V	n	n	1	cig only	18	27	nev+2	ot
ALDERS	50	f	0	0	all	-	not q+s	Eu:UK	1977	CC	1448	n	V	n	n	1	cig only	18	27	nev+2	ot
BARBON	80	m	0	0	all	-	a	Eu:wst	1979	CC	755	n	bl	y	y	3	all/unsp	20	39	nev any	or
CHOI	52	m	0	0	all	-	a	As:oth	1985	CC	375	n	bl	n	n	0	cig+/-ot	11	20	nev cigs	st
CHOI	60	f	0	0	all	-	a	As:oth	1985	CC	375	n	bl	n	n	0	cig+/-ot	11	30	nev cigs	ot
DOLL	74	m	0	0	all	-	KII	Eu:UK	1948	CC	1465	n	V	n	n	1	all/unsp	15	24	nev any	ot
DOSEME	12	m	0	0	all	-	not q+s	Eu:bal	1979	CC	1210	n	bl	n	n	2	cig+/-ot	11	20	nev cigs	or
GER	11	c	0	0	all	-	a	As:oth	1990	CC	141	n	ot	y	n	8	all/unsp	11	20	nev any	ot
JEDRYC	37	m	0	0	all	-	a	Eu:est	1980	CC	1630	n	bl	y	n	3	cig+/-ot	20	29	nev any	or
KREYBE	6	m	0	0	all	-	KII	Eu:Sca	1948	CC	300	n	bl	n	y	1	all/unsp	15	24	nev any	ot
LAMTH	17	f	0	0	ch	-	a	As:HK	1983	CC	445	n	bl	n	n	0	all/unsp	11	20	nev any	or
LUBIN2	160	m	0	0	all	-	a	Eu:mul	1976	CC	7804	n	bl	n	y	0	cig+/-ot	20	29	nev any	st
LUBIN2	180	f	0	0	all	-	a	Eu:mul	1976	CC	7804	n	bl	n	y	0	cig+/-ot	20	29	nev any	st
MATOS	59	m	0	0	all	-	a	SCAmer	1994	CC	200	n	bl	n	n	2	cig+/-ot	15	24	nev any	or
MATSUD	8	m	0	0	all	-	a	As:Jap	1965	CC	179	n	bl	n	n	0	cig+/-ot	11	20	nev cigs	ot
ORMOS	23	m	0	0	all	-	KII	Eu:est	1947	CC	119	n	bl	y	y	0	cig+/-ot	16	30	nev any	st
WYNDE2	11	m	0	0	all	-	KII	NAmer	1962	CC	404	n	bl	n	y	0	cig+/-ot	11	20	nev any	st
WYNDE3	25	m	0	0	all	-	KII	NAmer	1966	CC	350	n	bl	n	y	0	cig+/-ot	10	20	nev any	st
WYNDE3	72	f	0	0	all	-	KII	NAmer	1966	CC	350	n	bl	n	y	0	cig+/-ot	10	20	nev any	st
WYNDE4	39	m	0	0	all	-	a	NAmer	1948	CC	684	n	bl	y	n	0	all/unsp	16	20	nev any	st
ZHENG	8	m	0	0	all	-	a	As:Chi	1982	CC	540	n	ot	*	y	0	cig+/-ot	20	29	nev cigs	st

Cigarette type is all/unspec for all RRs

except for the following:

REF	NRR	CIGTYPE
ALDERS	47	MC only
ALDERS	50	MC only

Table 3G3 - 2

IESLC - Meta-analysis of Ever Smoking, Amount smoked, "Mid", Any product (or Cigarettes if Any not available)
Adenocarcinoma
Most adjusted

REF	NRR	SEX	AD	Number Exposed Case	Number Exposed Cont	Non-exposed Case	Non-exposed Cont	RR	95.00%CI
ALDERS	47	m	1	-	-	-	-	2.67	(0.99- 7.18)
ALDERS	50	f	1	-	-	-	-	4.58	(2.67- 7.85)
Subtotal ALDERS								4.05	(2.52- 6.50)
BARBON	80	m	3	-	-	-	-	8.30	(3.70- 19.00)
CHOI	52	m	0	27	281	7	95	1.30	(0.55- 3.09)
CHOI	60	f	0	0	9	49	164	0.17~	(0.01- 3.06)
Subtotal CHOI								1.10	(0.48- 2.52)
DOLL	74	m	1	-	-	-	-	1.20	(0.27- 5.35)
DOSEME	12	m	2	-	-	-	-	2.70	(1.60- 4.70)
GER	11	c	8	-	-	-	-	2.10	(0.88- 5.04)
JEDRYC	37	m	3	-	-	-	-	4.38	(1.87- 10.28)
KREYBE	6	m	1	-	-	-	-	1.43	(0.36- 5.72)
LAMTH	17	f	0	27	14	131	158	2.33	(1.17- 4.62)
LUBIN2	160	m	0	234	3108	57	2616	3.46	(2.57- 4.64)
LUBIN2	180	f	0	13	110	138	1180	1.01	(0.55- 1.84)
Subtotal LUBIN2								2.73	(2.09- 3.55)
MATOS	59	m	2	-	-	-	-	7.00	(2.60- 19.10)
MATSUD	8	m	0	13	1607	0	1255	21.09~	(1.25- 355.07)
ORMOS	23	m	0	3	577	2	777	2.02	(0.34- 12.13)
WYNDE2	11	m	0	14	203	5	105	1.45	(0.51- 4.13)
WYNDE3	25	m	0	20	114	6	88	2.57	(0.99- 6.68)
WYNDE3	72	f	0	11	24	15	76	2.32	(0.94- 5.73)
Subtotal WYNDE3								2.44	(1.27- 4.70)
WYNDE4	39	m	0	15	274	4	115	1.57	(0.51- 4.84)
ZHENG	8	m	0	53	89	29	94	1.93	(1.13- 3.30)
Partial Totals				430	6410	443	6723		

*prospective study

~ With 0.5 adjustment for zero

REF	NRR	SEX	AD	Ys	Ws	Qs	Ps
ALDERS	47	m	1	0.98	3.91	0.00	0.0520
ALDERS	50	f	1	1.52	13.21	3.56	0.0000
Subtotal ALDERS				1.40	17.13	3.57	
BARBON	80	m	3	2.12	5.74	7.12	0.0000
CHOI	52	m	0	0.27	5.16	2.80	0.5467
CHOI	60	f	0	-1.74	0.47	3.54	0.2324
Subtotal CHOI				0.10	5.62	6.34	
DOLL	74	m	1	0.18	1.72	1.16	0.8109
DOSEME	12	m	2	0.99	13.23	0.00	0.0003
GER	11	c	8	0.74	5.04	0.34	0.0956
JEDRYC	37	m	3	1.48	5.29	1.19	0.0007
KREYBE	6	m	1	0.36	2.01	0.83	0.6122
LAMTH	17	f	0	0.84	8.17	0.20	0.0158
LUBIN2	160	m	0	1.24	44.40	2.51	0.0000
LUBIN2	180	f	0	0.01	10.63	10.45	0.9727
Subtotal LUBIN2				1.00	55.03	12.96	
MATOS	59	m	2	1.95	3.86	3.44	0.0001
MATSUD	8	m	0	3.05	0.48	2.02	0.0343
ORMOS	23	m	0	0.70	1.20	0.11	0.4420
WYNDE2	11	m	0	0.37	3.50	1.40	0.4885
WYNDE3	25	m	0	0.95	4.22	0.01	0.0521
WYNDE3	72	f	0	0.84	4.71	0.12	0.0675
Subtotal WYNDE3				0.89	8.93	0.13	
WYNDE4	39	m	0	0.45	3.04	0.92	0.4291
ZHENG	8	m	0	0.66	13.29	1.58	0.0165

Table 3G3 - 2

IESLC - Meta-analysis of Ever Smoking, Amount smoked, "Mid", Any product (or Cigarettes if Any not available)
Adenocarcinoma
Most adjusted

	N	21
	NS	17
	Wt	153.29
Het	Chi	43.31
Het	df	20
Het	P	**
Fixed	RR	2.72
	RRl	2.33
	RRu	3.19
	P	+++
Random	RR	2.53
	RRl	1.93
	RRu	3.30
	P	+++
Asymm	P	N.S.

Table 3G3 - 3

IESLC - Meta-analysis of Ever Smoking, Amount smoked, "Mid", Any product (or Cigarettes if Any not available)
Adenocarcinoma
Most adjusted

		Sex		
	combined	male	female	Total
N	1	15	5	21
NS	1	15	5	21
Wt	5.04	111.06	37.18	153.29
Het Chi	0.00	24.46	16.56	43.31
Het df	0	14	4	20
Het P	N.S.	*	**	**
Fixed RR	2.10	2.94	2.26	2.72
RRl	0.88	2.44	1.64	2.33
RRu	5.03	3.54	3.11	3.19
P	(+)	+++	+++	+++
Random RR	2.10	2.77	1.96	2.53
RRl	0.88	2.07	0.94	1.93
RRu	5.03	3.71	4.06	3.30
P	(+)	+++	(+)	+++
Between Chi				2.29
Between df				2
Between P				N.S.
Btwn(F) P				N.S.
Btwn(R) P				N.S.

Table 3G3 - 4

IESLC - Meta-analysis of Ever Smoking, Amount smoked, "Mid", Any product (or Cigarettes if Any not available)
Adenocarcinoma
Least adjusted

REF	NRR	X	SEX	AGEL	AGEH	RACE	YF	LC TYPE	LOC	START	ST	NLC	R	VB	P	H	AD	PRODUCT	exL	exH	DENOM	De
ALDERS	47		m	0	0	all	-	not q+s	Eu:UK	1977	CC	1448	n	V	n	n	1	cig only	18	27	nev+2	ot
ALDERS	50		f	0	0	all	-	not q+s	Eu:UK	1977	CC	1448	n	V	n	n	1	cig only	18	27	nev+2	ot
BARBON	47	x	m	0	0	all	-	a	Eu:wst	1979	CC	755	n	bl	y	y	0	all/unsp	20	29	nev any	st
CHOI	52		m	0	0	all	-	a	As:oth	1985	CC	375	n	bl	n	n	0	cig+/-ot	11	20	nev cigs	st
CHOI	60		f	0	0	all	-	a	As:oth	1985	CC	375	n	bl	n	n	0	cig+/-ot	11	30	nev cigs	ot
DOLL	60	x	m	0	0	all	-	KII	Eu:UK	1948	CC	1465	n	V	n	n	0	all/unsp	15	24	nev any	st
DOSEME	12		m	0	0	all	-	not q+s	Eu:bal	1979	CC	1210	n	bl	n	n	2	cig+/-ot	11	20	nev cigs	or
GER	3	x	c	0	0	all	-	a	As:oth	1990	CC	141	n	ot	y	n	0	all/unsp	11	20	nev any	st
JEDRYC	17	x	m	0	0	all	-	a	Eu:est	1980	CC	1630	n	bl	y	n	0	cig+/-ot	20	29	nev any	st
KREYBE	18	x	m	0	0	all	-	KII	Eu:Sca	1948	CC	300	n	bl	n	y	0	all/unsp	15	24	nev any	st
LAMTH	17		f	0	0	ch	-	a	As:HK	1983	CC	445	n	bl	n	n	0	all/unsp	11	20	nev any	or
LUBIN2	160		m	0	0	all	-	a	Eu:mul	1976	CC	7804	n	bl	n	y	0	cig+/-ot	20	29	nev any	st
LUBIN2	180		f	0	0	all	-	a	Eu:mul	1976	CC	7804	n	bl	n	y	0	cig+/-ot	20	29	nev any	st
MATOS	58	x	m	0	0	all	-	a	SCAmer	1994	CC	200	n	bl	n	n	0	cig+/-ot	15	24	nev any	st
MATSUD	8		m	0	0	all	-	a	As:Jap	1965	CC	179	n	bl	n	n	0	cig+/-ot	11	20	nev cigs	ot
ORMOS	23		m	0	0	all	-	KII	Eu:est	1947	CC	119	n	bl	y	y	0	cig+/-ot	16	30	nev any	st
WYNDE2	11		m	0	0	all	-	KII	NAmer	1962	CC	404	n	bl	n	y	0	cig+/-ot	11	20	nev any	st
WYNDE3	25		m	0	0	all	-	KII	NAmer	1966	CC	350	n	bl	n	y	0	cig+/-ot	10	20	nev any	st
WYNDE3	72		f	0	0	all	-	KII	NAmer	1966	CC	350	n	bl	n	y	0	cig+/-ot	10	20	nev any	st
WYNDE4	39		m	0	0	all	-	a	NAmer	1948	CC	684	n	bl	y	n	0	all/unsp	16	20	nev any	st
ZHENG	8		m	0	0	all	-	a	As:Chi	1982	CC	540	n	ot	*	y	0	cig+/-ot	20	29	nev cigs	st

Cigarette type is all/unspec for all RRs

except for the following:

REF	NRR	CIGTYPE
ALDERS	47	MC only
ALDERS	50	MC only

Table 3G3 - 5

IESLC - Meta-analysis of Ever Smoking, Amount smoked, "Mid", Any product (or Cigarettes if Any not available)
Adenocarcinoma
Least adjusted

REF	NRR	SEX	AD	Number Exposed Case	Number Exposed Cont	Non-exposed Case	Non-exposed Cont	RR	95.00%CI
ALDERS	47	m	1	-	-	-	-	2.67	(0.99- 7.18)
ALDERS	50	f	1	-	-	-	-	4.58	(2.67- 7.85)
Subtotal ALDERS								4.05	(2.52- 6.50)
BARBON	47	m	0	43	176	7	188	6.56	(2.88- 14.97)
CHOI	52	m	0	27	281	7	95	1.30	(0.55- 3.09)
CHOI	60	f	0	0	9	49	164	0.17~	(0.01- 3.06)
Subtotal CHOI								1.10	(0.48- 2.52)
DOLL	60	m	0	16	431	2	61	1.13	(0.25- 5.05)
DOSEME	12	m	2	-	-	-	-	2.70	(1.60- 4.70)
GER	3	c	0	21	66	37	149	1.28	(0.70- 2.36)
JEDRYC	17	m	0	52	434	7	289	4.95	(2.22- 11.04)
KREYBE	18	m	0	6	925	3	644	1.39	(0.35- 5.59)
LAMTH	17	f	0	27	14	131	158	2.33	(1.17- 4.62)
LUBIN2	160	m	0	234	3108	57	2616	3.46	(2.57- 4.64)
LUBIN2	180	f	0	13	110	138	1180	1.01	(0.55- 1.84)
Subtotal LUBIN2								2.73	(2.09- 3.55)
MATOS	58	m	0	28	90	5	110	6.84	(2.54- 18.45)
MATSUD	8	m	0	13	1607	0	1255	21.09~	(1.25- 355.07)
ORMOS	23	m	0	3	577	2	777	2.02	(0.34- 12.13)
WYNDE2	11	m	0	14	203	5	105	1.45	(0.51- 4.13)
WYNDE3	25	m	0	20	114	6	88	2.57	(0.99- 6.68)
WYNDE3	72	f	0	11	24	15	76	2.32	(0.94- 5.73)
Subtotal WYNDE3								2.44	(1.27- 4.70)
WYNDE4	39	m	0	15	274	4	115	1.57	(0.51- 4.84)
ZHENG	8	m	0	53	89	29	94	1.93	(1.13- 3.30)
Partial Totals				596	8532	504	8164		

*prospective study

~ With 0.5 adjustment for zero

REF	NRR	SEX	AD	Ys	Ws	Qs	Ps
ALDERS	47	m	1	0.98	3.91	0.00	0.0520
ALDERS	50	f	1	1.52	13.21	4.20	0.0000
Subtotal ALDERS				1.40	17.13	4.20	
BARBON	47	m	0	1.88	5.65	4.81	0.0000
CHOI	52	m	0	0.27	5.16	2.47	0.5467
CHOI	60	f	0	-1.74	0.47	3.42	0.2324
Subtotal CHOI				0.10	5.62	5.90	
DOLL	60	m	0	0.12	1.72	1.20	0.8706
DOSEME	12	m	2	0.99	13.23	0.02	0.0003
GER	3	c	0	0.25	10.36	5.22	0.4249
JEDRYC	17	m	0	1.60	5.96	2.45	0.0001
KREYBE	18	m	0	0.33	1.99	0.78	0.6405
LAMTH	17	f	0	0.84	8.17	0.11	0.0158
LUBIN2	160	m	0	1.24	44.40	3.53	0.0000
LUBIN2	180	f	0	0.01	10.63	9.54	0.9727
Subtotal LUBIN2				1.00	55.03	13.07	
MATOS	58	m	0	1.92	3.91	3.64	0.0001
MATSUD	8	m	0	3.05	0.48	2.11	0.0343
ORMOS	23	m	0	0.70	1.20	0.08	0.4420
WYNDE2	11	m	0	0.37	3.50	1.21	0.4885
WYNDE3	25	m	0	0.95	4.22	0.00	0.0521
WYNDE3	72	f	0	0.84	4.71	0.06	0.0675
Subtotal WYNDE3				0.89	8.93	0.06	
WYNDE4	39	m	0	0.45	3.04	0.77	0.4291
ZHENG	8	m	0	0.66	13.29	1.20	0.0165

Table 3G3 - 5

IESLC - Meta-analysis of Ever Smoking, Amount smoked, "Mid", Any product (or Cigarettes if Any not available)
Adenocarcinoma
Least adjusted

	N	21
	NS	17
	Wt	159.20
Het	Chi	46.82
Het	df	20
Het	P	***
Fixed	RR	2.61
	RRl	2.23
	RRu	3.04
	P	+++
Random	RR	2.41
	RRl	1.84
	RRu	3.17
	P	+++
Asymm	P	N.S.

Table 3G3 - 6

IESLC - Meta-analysis of Ever Smoking, Amount smoked, "Mid", Any product (or Cigarettes if Any not available)
Adenocarcinoma
Least adjusted

	combined	Sex: male	female	Total
N	1	15	5	21
NS	1	15	5	21
Wt	10.36	111.66	37.18	159.20
Het Chi	0.00	22.82	16.56	46.82
Het df	0	14	4	20
Het P	N.S.	(*)	**	***
Fixed RR	1.28	2.92	2.26	2.61
RRl	0.70	2.43	1.64	2.23
RRu	2.36	3.52	3.11	3.04
P	N.S.	+++	+++	+++
Random RR	1.28	2.75	1.96	2.41
RRl	0.70	2.08	0.94	1.84
RRu	2.36	3.64	4.06	3.17
P	N.S.	+++	(+)	+++
Between Chi				7.44
Between df				2
Between P				*
Btwn(F) P				N.S.
Btwn(R) P				(*)

Table 3G3 - 7

IESLC - Meta-analysis of Ever Smoking, Amount smoked, "Mid", Any product (or Cigarettes if Any not available)
Adenocarcinoma
Excluded studies (and stage at which they were excluded)

1 ABELIN ABRAHA AMANDU AMES ANDERS AUSTIN AXELSO BAND BECHER BERRIN BLOHMK BLOT4 BROCKM BROWN1 BYERS1 BYERS2 CARPEN CASCO2 CASCOR CHAN CHEN3 CHIAZZ CHYOU DESTE2 DOCKER DROSTE DU GARCIA GARDIN GENG GODLEY GOODMA GRAHAM GREGOR HEGMAN HEIN HENNEK HINDS HIRAOK HOROWI HORWIT HUANG ISHIMA JAHN JAIN JARVHO JIANG KELLER KIHARA KJUUS KO KOHLME KUBIK LAMWK LAMWK2 LANGE LEI LEMARC LEVIN LIU LOMBA2 LOMBAR MAGNUS MARSH MARSH2 MCDUFF MCLAUG MILLER MILLS NOTANI NOU ODRISC PAWLEG PERSHA POFFIJ QIAO QIAO2 RADZIK REN RONCO ROOTS ROTHSC SAARIK SANKAR SCHWAR SEGI SEOW SHIMIZ SIMARA SIMONA SITAS SOBUE2 STASZE STAYNE STUCKE SUN SUZUK2 SUZUKI TANG TAO TOKARS TOUSEY ULMER VEIERO VUTUC WALD WANG WANG3 WANG4 WICKLU WIGLE WILKIN WU2 WUNSCH WYNDE8 XIANGZ XU XU2 XU4 YONG ZHANG

2 AKIBA ARCHER BENSHL BRETT BROSS CEDERL CHANG CHOW COMSTO DARBY DEAN3 DEKLER DORANT DORN ENGELA ENSTRO GAO2 GILLIS HAENSZ HAMMO2 HIRAY2 HIRAYA HITOSU HOLE HUMBLE KAISE2 KAISER KANELL KATSOU KAUFMA KINLEN KNEKT KOO LAURIL LIAW LICKIN LIDDEL MIGRAN MRFIT MRFITR MURATA NAM PARKIN PERSH2 PETO PEZZO2 PEZZOT PISANI PRESCO RIMING SEGI2 SOBUE SPEIZE STOCKW SVENSS TANG2 TENKAN TSUGAN TULINI TVERDA WAKAI WARSIN WATSON WU WYNDE5 WYNDER YAMAGU

3 BUELL CHEN MASTRA MZILEN RESTRE SADOWS

4 BEST BOFFET WYNDE7

6 BLOT1 BLOT2 BLOT3 BOUCHA JONES MOLLO SCHWA2 VANDER

8 AGUDO ARMADA AUVINE AXELSS BOUCOT BRESLO BUFFLE CHATZI CHEN2 COOKSO CORREA CPSI CPSII DAMBER DAVEYS DEAN DEAN2 DESTEF DOLL2 DUNN EBELIN ESAKI FAN GAO GARSHI GOLLED GSELL HAMMON HANSEN HU HU2 JARUP JOLY JUSSAW KHUDER KOULUM KREUZE LAUSSM LETOUR LIU2 LIU3 LIU4 LIU5 LUBIN LUO MACLEN MARTIS MCCONN NOTAN2 PASTOR PERNU PIKE POLEDN RACHTA RANDIG SHAW SIEMIA SPITZ STOCKS TIZZAN WANG2 WYNDE6 XU3 YUAN ZHOU

10 BROWN2 DORGAN OSANN OSANN2 WUWILL

11 BENHAM

Table 3G3 - 8
Potentially overlapping studies

REF	REFGP	PRINC	.	OVERLAP/LINK
LUBIN2	LUBIN2	1		Lubin-combined
LAMTH	LAMTH	1		KOO/LAMTH/LAMWK
MATSUD	MATSUD	1		SOBUE2/MATSUD

Table 3G3 - 9
Most adjusted - insufficient data for metaanalysis

REF	NRR	SEX	AGEL	AGEH	RACE	YF	LC TYPE	LOC	START	ST	NLC	R	VB	P	H	AD	PRODUCT	exL	exH	DENOM	De
CHEN	14	c	0	0	all	-	a	As:oth	1987	CC	323	n	ot	n	y	2	cig+/-ot	11	20	nev cigs	ot

REF	NRR	RR	SIG	RRDATA comment
CHEN	14	1.74	n	0

Table 3G4 -

IESLC - Meta-analysis of Ever Smoking, Amount smoked, "High", Any product (or Cigarettes if Any not available)
Adenocarcinoma

This analysis is restricted to results for:
1) Results by Amount smoked
2) Ever smokers
3) Results complete enough for use in metaanalysis

Within each study, results are then selected (in the following order of preference, within each sex) for:
4) PRODUCT: all/unspec, cigarettes regardless of other products, cigarettes only
5) CIGTYPE: all/unspecified, MC regardless of HR, MC only
6) DENOM: never smoked anything, never smoked cigarettes, (never +1 = +long term ex, +2 = +amount unknown,
+3 = never cigs+long term ex)
7) Followup period (YF, prospective studies): whole study (coded as 0) or longest available
8) LCtype: adeno or nearest available, but not squamous. (q = squamous, s = small,
a = adeno, l = large, KII = Kreyberg II, al = alveolar, br = bronchiolar, u = undifferentiated)
9) Race: all or nearest available, otherwise by race (wh or w = white, bl or b = black, hi = hispanic
ch = chinese, jap = japanese, haw = hawaiian, w+o = white + oriental, sca = scandinavian, as = asian)
10) Amount smoked "high" in key scheme 1 (key value 45, maximum range >20, in numbers of
cigarettes or cigarette equivalents)
11) For overlapping studies: principal rather than subsidiary studies
Finally by Age: whole study (coded as 0) if available, otherwise by widest available age group
and then for single sex results (m, f) in preference to combined sex results (c).

Results adjusted (AD) for the most potential confounders are then chosen in Sections -1 to -3
and results adjusted for the least confounders in Sections -4 to -6. (Those least adjusted results which
actually differ from the most adjusted as marked 'x' in column X in Section -4)
(Results adjusted for an unknown number of confounder(s) are coded as 20.)

Section -7 shows excluded studies, together with the stage (as above) at which no qualifying
results were found.

Section -8 lists the potentially overlapping studies which have been included (1=principal, 2=subsidiary).

Section -9 lists any results which would have been included in preference except that they had data not complete
enough for use in meta-analysis, with their significance (yes/no), if known, and any further comment as entered
on the database.

In addition to those mentioned above, the following fields, levels and abbreviations are used:
* or nk = not known, n = no, y = yes, ot = other
nev = never
all/unspec = all or unspecified, cig+/-ot = cigarettes irrespective of other products (cigar, pipe etc)
MC = manufactured cigarettes, HR = hand-rolled cigarettes
exL, exH = range of exposure (low and high) in the smoking group, in terms of Amount smoked,
cigarettes or cigarette equivalents
REF: 6-character study reference
NRR: number of the RR on the database within the study
ST : study type (CC = case control, pr or prosp = prospective)
NLC: number of lung cancer cases in whole study
R : risky occupational population (n = no, m = mining, o = other risky)
VB : national cigarette type (V = at least 75% Virginia, bl = at least 75% blended, ot = other)
P : any proxy use
H : full histological confirmation
De : derivation of RR/CI (or = original, st = standard method, ot = other method of estimation)

Table 3G4 - 1

IESLC - Meta-analysis of Ever Smoking, Amount smoked, "High", Any product (or Cigarettes if Any not available)
Adenocarcinoma
Most adjusted

REF	NRR	SEX	AGEL	AGEH	RACE	YF	LC TYPE	LOC	START	ST	NLC	R	VB	P	H	AD	PRODUCT	exL	exH	DENOM	De
ALDERS	48	m	0	0	all	-	not q+s	Eu:UK	1977	CC	1448	n	V	n	n	1	cig only	28	99	nev+2	ot
ALDERS	51	f	0	0	all	-	not q+s	Eu:UK	1977	CC	1448	n	V	n	n	1	cig only	28	99	nev+2	ot
BARBON	81	m	0	0	all	-	a	Eu:wst	1979	CC	755	n	bl	y	y	3	all/unsp	40	99	nev any	or
CHOI	55	m	0	0	all	-	a	As:oth	1985	CC	375	n	bl	n	n	0	cig+/-ot	41	99	nev cigs	st
CHOI	61	f	0	0	all	-	a	As:oth	1985	CC	375	n	bl	n	n	0	cig+/-ot	31	99	nev cigs	st
DOLL	75	m	0	0	all	-	KII	Eu:UK	1948	CC	1465	n	V	n	n	1	all/unsp	25	99	nev any	ot
DOSEME	16	m	0	0	all	-	not q+s	Eu:bal	1979	CC	1210	n	bl	n	n	2	cig+/-ot	21	99	nev cigs	or
GER	12	c	0	0	all	-	a	As:oth	1990	CC	141	n	ot	y	n	8	all/unsp	21	99	nev any	ot
JEDRYC	38	m	0	0	all	-	a	Eu:est	1980	CC	1630	n	bl	y	n	3	cig+/-ot	30	99	nev any	or
KREYBE	7	m	0	0	all	-	KII	Eu:Sca	1948	CC	300	n	bl	n	y	1	all/unsp	25	99	nev any	ot
LAMTH	18	f	0	0	ch	-	a	As:HK	1983	CC	445	n	bl	n	n	0	all/unsp	21	99	nev any	or
LUBIN2	164	m	0	0	all	-	a	Eu:mul	1976	CC	7804	n	bl	n	y	0	cig+/-ot	30	99	nev any	st
LUBIN2	184	f	0	0	all	-	a	Eu:mul	1976	CC	7804	n	bl	n	y	0	cig+/-ot	30	99	nev any	st
MATOS	61	m	0	0	all	-	a	SCAmer	1994	CC	200	n	bl	n	n	2	cig+/-ot	25	99	nev any	or
MATSUD	9	m	0	0	all	-	a	As:Jap	1965	CC	179	n	bl	n	n	0	cig+/-ot	21	99	nev cigs	ot
ORMOS	24	m	0	0	all	-	KII	Eu:est	1947	CC	119	n	bl	y	y	0	cig+/-ot	31	99	nev any	st
OSANN	63	m	0	0	all	-	a	NAmer	1984	CC	1986	n	bl	n	n	2	cig+/-ot	40	99	nev cigs	or
OSANN	64	f	0	0	all	-	a	NAmer	1984	CC	1986	n	bl	n	n	2	cig+/-ot	40	99	nev cigs	or
WYNDE2	13	m	0	0	all	-	KII	NAmer	1962	CC	404	n	bl	n	y	0	cig+/-ot	35	99	nev any	st
WYNDE3	27	m	0	0	all	-	KII	NAmer	1966	CC	350	n	bl	n	y	0	cig+/-ot	41	99	nev any	st
WYNDE3	74	f	0	0	all	-	KII	NAmer	1966	CC	350	n	bl	n	y	0	cig+/-ot	41	99	nev any	st
WYNDE4	41	m	0	0	all	-	a	NAmer	1948	CC	684	n	bl	y	n	0	all/unsp	35	99	nev any	st
ZHENG	9	m	0	0	all	-	a	As:Chi	1982	CC	540	n	ot	*	y	0	cig+/-ot	30	99	nev cigs	st

Cigarette type is all/unspec for all RRs

except for the following:

REF	NRR	CIGTYPE
ALDERS	48	MC only
ALDERS	51	MC only

Table 3G4 - 2

IESLC - Meta-analysis of Ever Smoking, Amount smoked, "High", Any product (or Cigarettes if Any not available)
Adenocarcinoma
Most adjusted

REF	NRR	SEX	AD	Number Exposed Case	Number Exposed Cont	Non-exposed Case	Non-exposed Cont	RR	95.00%CI
ALDERS	48	m	1	-	-	-	-	3.32	(1.36- 8.10)
ALDERS	51	f	1	-	-	-	-	3.31	(1.80- 6.10)
Subtotal ALDERS								3.31	(2.00- 5.48)
BARBON	81	m	3	-	-	-	-	12.10	(5.10- 28.00)
CHOI	55	m	0	2	6	7	95	4.52	(0.77- 26.69)
CHOI	61	f	0	1	1	49	164	3.35	(0.21- 54.50)
Subtotal CHOI								4.15	(0.93- 18.54)
DOLL	75	m	1	-	-	-	-	1.10	(0.22- 5.60)
DOSEME	16	m	2	-	-	-	-	3.20	(1.40- 7.00)
GER	12	c	8	-	-	-	-	0.88	(0.25- 3.03)
JEDRYC	38	m	3	-	-	-	-	5.11	(2.09- 12.53)
KREYBE	7	m	1	-	-	-	-	3.99	(0.95- 16.81)
LAMTH	18	f	0	9	5	131	158	2.17	(0.71- 6.64)
LUBIN2	164	m	0	151	1746	57	2616	3.97	(2.91- 5.42)
LUBIN2	184	f	0	9	39	138	1180	1.97	(0.94- 4.16)
Subtotal LUBIN2								3.58	(2.69- 4.77)
MATOS	61	m	2	-	-	-	-	8.40	(3.01- 22.20)
MATSUD	9	m	0	4	470	0	1255	24.02~	(1.29- 446.92)
ORMOS	24	m	0	1	128	2	777	3.04	(0.27- 33.72)
OSANN	63	m	2	-	-	-	-	37.50	(21.30- 66.00)
OSANN	64	f	2	-	-	-	-	24.20	(15.80- 37.20)
Subtotal OSANN								28.39	(20.18- 39.94)
WYNDE2	13	m	0	16	112	5	105	3.00	(1.06- 8.48)
WYNDE3	27	m	0	9	26	6	88	5.08	(1.65- 15.59)
WYNDE3	74	f	0	1	3	15	76	1.69	(0.16- 17.36)
Subtotal WYNDE3								4.13	(1.50- 11.34)
WYNDE4	41	m	0	7	64	4	115	3.14	(0.89- 11.15)
ZHENG	9	m	0	17	23	29	94	2.40	(1.13- 5.08)
Partial Totals				227	2623	443	6723		

*prospective study ~ With 0.5 adjustment for zero

REF	NRR	SEX	AD	Ys	Ws	Qs	Ps
ALDERS	48	m	1	1.20	4.83	1.51	0.0084
ALDERS	51	f	1	1.20	10.32	3.27	0.0001
Subtotal ALDERS				1.20	15.14	4.78	
BARBON	81	m	3	2.49	5.30	2.85	0.0000
CHOI	55	m	0	1.51	1.22	0.08	0.0956
CHOI	61	f	0	1.21	0.49	0.15	0.3961
Subtotal CHOI				1.42	1.71	0.23	
DOLL	75	m	1	0.10	1.47	4.06	0.9081
DOSEME	16	m	2	1.16	5.93	2.11	0.0046
GER	12	c	8	-0.13	2.47	8.79	0.8408
JEDRYC	38	m	3	1.63	4.79	0.08	0.0004
KREYBE	7	m	1	1.38	1.86	0.26	0.0590
LAMTH	18	f	0	0.78	3.08	2.98	0.1740
LUBIN2	164	m	0	1.38	39.81	5.78	0.0000
LUBIN2	184	f	0	0.68	6.90	8.05	0.0741
Subtotal LUBIN2				1.28	46.71	13.83	
MATOS	61	m	2	2.13	3.85	0.52	0.0000
MATSUD	9	m	0	3.18	0.45	0.90	0.0331
ORMOS	24	m	0	1.11	0.66	0.28	0.3661
OSANN	63	m	2	3.62	12.01	41.77	0.0000
OSANN	64	f	2	3.19	20.96	42.66	0.0000
Subtotal OSANN				3.35	32.97	84.43	
WYNDE2	13	m	0	1.10	3.56	1.56	0.0382
WYNDE3	27	m	0	1.62	3.05	0.06	0.0045
WYNDE3	74	f	0	0.52	0.71	1.08	0.6593
Subtotal WYNDE3				1.42	3.76	1.14	
WYNDE4	41	m	0	1.15	2.40	0.90	0.0761
ZHENG	9	m	0	0.87	6.78	5.32	0.0229

Table 3G4 - 2

IESLC - Meta-analysis of Ever Smoking, Amount smoked, "High", Any product (or Cigarettes if Any not available)
Adenocarcinoma
Most adjusted

	N	23
	NS	18
	Wt	142.89
Het	Chi	135.03
Het	df	22
Het	P	***
Fixed	RR	5.81
	RRl	4.93
	RRu	6.85
	P	+++
Random	RR	4.40
	RRl	2.77
	RRu	6.98
	P	+++
Asymm	P	N.S.

Table 3G4 - 3

IESLC - Meta-analysis of Ever Smoking, Amount smoked, "High", Any product (or Cigarettes if Any not available)
Adenocarcinoma
Most adjusted

	combined	Sex male	female	Total
N	1	16	6	23
NS	1	16	6	23
Wt	2.47	97.97	42.45	142.89
Het Chi	0.00	67.43	54.53	135.03
Het df	0	15	5	22
Het P	N.S.	***	***	***
Fixed RR	0.88	5.37	7.79	5.81
RRl	0.25	4.40	5.77	4.93
RRu	3.06	6.54	10.53	6.85
P	N.S.	+++	+++	+++
Random RR	0.88	5.01	3.87	4.40
RRl	0.25	3.06	1.17	2.77
RRu	3.06	8.22	12.80	6.98
P	N.S.	+++	+	+++
Between Chi				13.07
Between df				2
Between P				**
Btwn(F) P				N.S.
Btwn(R) P				*

Table 3G4 - 4

IESLC - Meta-analysis of Ever Smoking, Amount smoked, "High", Any product (or Cigarettes if Any not available)
Adenocarcinoma
Least adjusted

REF	NRR	X	SEX	AGEL	AGEH	RACE	YF	LC TYPE	LOC	START	ST	NLC	R	VB	P	H	AD	PRODUCT	exL	exH	DENOM	De
ALDERS	48		m	0	0	all	-	not q+s	Eu:UK	1977	CC	1448	n	V	n	n	1	cig only	28	99	nev+2	ot
ALDERS	51		f	0	0	all	-	not q+s	Eu:UK	1977	CC	1448	n	V	n	n	1	cig only	28	99	nev+2	ot
BARBON	49	x	m	0	0	all	-	a	Eu:wst	1979	CC	755	n	bl	y	y	0	all/unsp	40	99	nev any	st
CHOI	55		m	0	0	all	-	a	As:oth	1985	CC	375	n	bl	n	n	0	cig+/-ot	41	99	nev cigs	st
CHOI	61		f	0	0	all	-	a	As:oth	1985	CC	375	n	bl	n	n	0	cig+/-ot	31	99	nev cigs	st
DOLL	61	x	m	0	0	all	-	KII	Eu:UK	1948	CC	1465	n	V	n	n	0	all/unsp	25	99	nev any	st
DOSEME	16		m	0	0	all	-	not q+s	Eu:bal	1979	CC	1210	n	bl	n	n	2	cig+/-ot	21	99	nev cigs	or
GER	4	x	c	0	0	all	-	a	As:oth	1990	CC	141	n	ot	y	n	0	all/unsp	21	99	nev any	st
JEDRYC	19	x	m	0	0	all	-	a	Eu:est	1980	CC	1630	n	bl	y	n	0	cig+/-ot	40	99	nev any	st
KREYBE	19	x	m	0	0	all	-	KII	Eu:Sca	1948	CC	300	n	bl	n	y	0	all/unsp	25	99	nev any	st
LAMTH	18		f	0	0	ch	-	a	As:HK	1983	CC	445	n	bl	n	n	0	all/unsp	21	99	nev any	or
LUBIN2	164		m	0	0	all	-	a	Eu:mul	1976	CC	7804	n	bl	n	y	0	cig+/-ot	30	99	nev any	st
LUBIN2	184		f	0	0	all	-	a	Eu:mul	1976	CC	7804	n	bl	n	y	0	cig+/-ot	30	99	nev any	st
MATOS	60	x	m	0	0	all	-	a	SCAmer	1994	CC	200	n	bl	n	n	0	cig+/-ot	25	99	nev any	st
MATSUD	9		m	0	0	all	-	a	As:Jap	1965	CC	179	n	bl	n	n	0	cig+/-ot	21	99	nev cigs	ot
ORMOS	24		m	0	0	all	-	KII	Eu:est	1947	CC	119	n	bl	y	y	0	cig+/-ot	31	99	nev any	st
OSANN	63		m	0	0	all	-	a	NAmer	1984	CC	1986	n	bl	n	n	2	cig+/-ot	40	99	nev cigs	or
OSANN	64		f	0	0	all	-	a	NAmer	1984	CC	1986	n	bl	n	n	2	cig+/-ot	40	99	nev cigs	or
WYNDE2	13		m	0	0	all	-	KII	NAmer	1962	CC	404	n	bl	n	y	0	cig+/-ot	35	99	nev any	st
WYNDE3	27		m	0	0	all	-	KII	NAmer	1966	CC	350	n	bl	n	y	0	cig+/-ot	41	99	nev any	st
WYNDE3	74		f	0	0	all	-	KII	NAmer	1966	CC	350	n	bl	n	y	0	cig+/-ot	41	99	nev any	st
WYNDE4	41		m	0	0	all	-	a	NAmer	1948	CC	684	n	bl	y	n	0	all/unsp	35	99	nev any	st
ZHENG	9		m	0	0	all	-	a	As:Chi	1982	CC	540	n	ot	*	y	0	cig+/-ot	30	99	nev cigs	st

Cigarette type is all/unspec for all RRs

except for the following:

REF	NRR	CIGTYPE
ALDERS	48	MC only
ALDERS	51	MC only

Table 3G4 - 5

IESLC - Meta-analysis of Ever Smoking, Amount smoked, "High", Any product (or Cigarettes if Any not available)
Adenocarcinoma
Least adjusted

REF	NRR	SEX	AD	Number Exposed Case	Cont	Non-exposed Case	Cont	RR	95.00%CI
ALDERS	48	m	1	-	-	-	-	3.32	(1.36- 8.10)
ALDERS	51	f	1	-	-	-	-	3.31	(1.80- 6.10)
Subtotal ALDERS								3.31	(2.00- 5.48)
BARBON	49	m	0	40	111	7	188	9.68	(4.19- 22.34)
CHOI	55	m	0	2	6	7	95	4.52	(0.77- 26.69)
CHOI	61	f	0	1	1	49	164	3.35	(0.21- 54.50)
Subtotal CHOI								4.15	(0.93- 18.54)
DOLL	61	m	0	6	166	2	61	1.10	(0.22- 5.61)
DOSEME	16	m	2	-	-	-	-	3.20	(1.40- 7.00)
GER	4	c	0	5	26	37	149	0.77	(0.28- 2.15)
JEDRYC	19	m	0	9	82	7	289	4.53	(1.64- 12.54)
KREYBE	19	m	0	5	248	3	644	4.33	(1.03- 18.25)
LAMTH	18	f	0	9	5	131	158	2.17	(0.71- 6.64)
LUBIN2	164	m	0	151	1746	57	2616	3.97	(2.91- 5.42)
LUBIN2	184	f	0	9	39	138	1180	1.97	(0.94- 4.16)
Subtotal LUBIN2								3.58	(2.69- 4.77)
MATOS	60	m	0	40	105	5	110	8.38	(3.19- 22.05)
MATSUD	9	m	0	4	470	0	1255	24.02~	(1.29- 446.92)
ORMOS	24	m	0	1	128	2	777	3.04	(0.27- 33.72)
OSANN	63	m	2	-	-	-	-	37.50	(21.30- 66.00)
OSANN	64	f	2	-	-	-	-	24.20	(15.80- 37.20)
Subtotal OSANN								28.39	(20.18- 39.94)
WYNDE2	13	m	0	16	112	5	105	3.00	(1.06- 8.48)
WYNDE3	27	m	0	9	26	6	88	5.08	(1.65- 15.59)
WYNDE3	74	f	0	1	3	15	76	1.69	(0.16- 17.36)
Subtotal WYNDE3								4.13	(1.50- 11.34)
WYNDE4	41	m	0	7	64	4	115	3.14	(0.89- 11.15)
ZHENG	9	m	0	17	23	29	94	2.40	(1.13- 5.08)
Partial Totals				332	3361	504	8164		

*prospective study ~ With 0.5 adjustment for zero

REF	NRR	SEX	AD	Ys	Ws	Qs	Ps
ALDERS	48	m	1	1.20	4.83	1.37	0.0084
ALDERS	51	f	1	1.20	10.32	2.96	0.0001
Subtotal ALDERS				1.20	15.14	4.33	
BARBON	49	m	0	2.27	5.49	1.58	0.0000
CHOI	55	m	0	1.51	1.22	0.06	0.0956
CHOI	61	f	0	1.21	0.49	0.14	0.3961
Subtotal CHOI				1.42	1.71	0.20	
DOLL	61	m	0	0.10	1.45	3.88	0.9065
DOSEME	16	m	2	1.16	5.93	1.92	0.0046
GER	4	c	0	-0.26	3.67	14.52	0.6242
JEDRYC	19	m	0	1.51	3.71	0.18	0.0036
KREYBE	19	m	0	1.47	1.86	0.13	0.0460
LAMTH	18	f	0	0.78	3.08	2.82	0.1740
LUBIN2	164	m	0	1.38	39.81	4.99	0.0000
LUBIN2	184	f	0	0.68	6.90	7.65	0.0741
Subtotal LUBIN2				1.28	46.71	12.65	
MATOS	60	m	0	2.13	4.10	0.63	0.0000
MATSUD	9	m	0	3.18	0.45	0.94	0.0331
ORMOS	24	m	0	1.11	0.66	0.26	0.3661
OSANN	63	m	2	3.62	12.01	42.99	0.0000
OSANN	64	f	2	3.19	20.96	44.28	0.0000
Subtotal OSANN				3.35	32.97	87.27	
WYNDE2	13	m	0	1.10	3.56	1.43	0.0382
WYNDE3	27	m	0	1.62	3.05	0.04	0.0045
WYNDE3	74	f	0	0.52	0.71	1.03	0.6593
Subtotal WYNDE3				1.42	3.76	1.07	
WYNDE4	41	m	0	1.15	2.40	0.83	0.0761
ZHENG	9	m	0	0.87	6.78	5.00	0.0229

Table 3G4 - 5

IESLC - Meta-analysis of Ever Smoking, Amount smoked, "High", Any product (or Cigarettes if Any not available)
Adenocarcinoma
Least adjusted

	N	23
	NS	18
	Wt	143.44
Het	Chi	139.66
Het	df	22
Het	P	***
Fixed	RR	5.66
	RRl	4.80
	RRu	6.66
	P	+++
Random	RR	4.27
	RRl	2.67
	RRu	6.82
	P	+++
Asymm	P	N.S.

Table 3G4 - 6

IESLC - Meta-analysis of Ever Smoking, Amount smoked, "High", Any product (or Cigarettes if Any not available)
Adenocarcinoma
Least adjusted

	combined	Sex male	female	Total
N	1	16	6	23
NS	1	16	6	23
Wt	3.67	97.31	42.45	143.44
Het Chi	0.00	65.84	54.53	139.66
Het df	0	15	5	22
Het P	N.S.	***	***	***
Fixed RR	0.77	5.30	7.79	5.66
RRl	0.28	4.35	5.77	4.80
RRu	2.15	6.47	10.53	6.66
P	N.S.	+++	+++	+++
Random RR	0.77	4.92	3.87	4.27
RRl	0.28	3.01	1.17	2.67
RRu	2.15	8.05	12.80	6.82
P	N.S.	+++	+	+++
Between Chi				19.29
Between df				2
Between P				***
Btwn(F) P				N.S.
Btwn(R) P				**

Table 3G4 - 7

IESLC - Meta-analysis of Ever Smoking, Amount smoked, "High", Any product (or Cigarettes if Any not available)
Adenocarcinoma
Excluded studies (and stage at which they were excluded)

1 ABELIN ABRAHA AMANDU AMES ANDERS AUSTIN AXELSO BAND BECHER BERRIN BLOHMK BLOT4 BROCKM BROWN1 BYERS1 BYERS2 CARPEN CASCO2 CASCOR CHAN CHEN3 CHIAZZ CHYOU DESTE2 DOCKER DROSTE DU GARCIA GARDIN GENG GODLEY GOODMA GRAHAM GREGOR HEGMAN HEIN HENNEK HINDS HIRAOK HOROWI HORWIT HUANG ISHIMA JAHN JAIN JARVHO JIANG KELLER KIHARA KJUUS KO KOHLME KUBIK LAMWK LAMWK2 LANGE LEI LEMARC LEVIN LIU LOMBA2 LOMBAR MAGNUS MARSH MARSH2 MCDUFF MCLAUG MILLER MILLS NOTANI NOU ODRISC PAWLEG PERSHA POFFIJ QIAO QIAO2 RADZIK REN RONCO ROOTS ROTHSC SAARIK SANKAR SCHWAR SEGI SEOW SHIMIZ SIMARA SIMONA SITAS SOBUE2 STASZE STAYNE STUCKE SUN SUZUK2 SUZUKI TANG TAO TOKARS TOUSEY ULMER VEIERO VUTUC WALD WANG WANG3 WANG4 WICKLU WIGLE WILKIN WU2 WUNSCH WYNDE8 XIANGZ XU XU2 XU4 YONG ZHANG

2 AKIBA ARCHER BENSHL BRETT BROSS CEDERL CHANG CHOW COMSTO DARBY DEAN3 DEKLER DORANT DORN ENGELA ENSTRO GAO2 GILLIS HAENSZ HAMMO2 HIRAY2 HIRAYA HITOSU HOLE HUMBLE KAISE2 KAISER KANELL KATSOU KAUFMA KINLEN KNEKT KOO LAURIL LIAW LICKIN LIDDEL MIGRAN MRFIT MRFITR MURATA NAM PARKIN PERSH2 PETO PEZZO2 PEZZOT PISANI PRESCO RIMING SEGI2 SOBUE SPEIZE STOCKW SVENSS TANG2 TENKAN TSUGAN TULINI TVERDA WAKAI WARSIN WATSON WU WYNDE5 WYNDER YAMAGU

3 BUELL CHEN MASTRA MZILEN RESTRE SADOWS

4 BEST BOFFET WYNDE7

6 BLOT1 BLOT2 BLOT3 BOUCHA JONES MOLLO SCHWA2 VANDER

8 AGUDO ARMADA AUVINE AXELSS BOUCOT BRESLO BUFFLE CHATZI CHEN2 COOKSO CORREA CPSI CPSII DAMBER DAVEYS DEAN DEAN2 DESTEF DOLL2 DUNN EBELIN ESAKI FAN GAO GARSHI GOLLED GSELL HAMMON HANSEN HU HU2 JARUP JOLY JUSSAW KHUDER KOULUM KREUZE LAUSSM LETOUR LIU2 LIU3 LIU4 LIU5 LUBIN LUO MACLEN MARTIS MCCONN NOTAN2 PASTOR PERNU PIKE POLEDN RACHTA RANDIG SHAW SIEMIA SPITZ STOCKS TIZZAN WANG2 WYNDE6 XU3 YUAN ZHOU

10 BROWN2 DORGAN OSANN2 WUWILL

11 BENHAM

Table 3G4 - 8
Potentially overlapping studies

REF	REFGP	PRINC	.	OVERLAP/LINK
LUBIN2	LUBIN2	1		Lubin-combined
LAMTH	LAMTH	1		KOO/LAMTH/LAMWK
MATSUD	MATSUD	1		SOBUE2/MATSUD

Table 3G4 - 9
Most adjusted - insufficient data for metaanalysis

REF	NRR	SEX	AGEL	AGEH	RACE	YF	LC TYPE	LOC	START	ST	NLC	R	VB	P	H	AD	PRODUCT	exL	exH	DENOM	De
CHEN	12	c	0	0	all	-	a	As:oth	1987	CC	323	n	ot	n	y	2	cig+/-ot	31	99	nev cigs	ot

REF	NRR	RR	SIG	RRDATA comment
CHEN	12	3.61	y	P < 0.001

Table 3G6 -

IESLC - Meta-analysis of Current Smoking by Amount, Overview, Any product (or Cigarettes if Any not available)
Adenocarcinoma

This analysis is restricted to results for:
1) Results by Amount smoked
   Results by Amount smoked (in numbers of cigarettes or cigarette equivalents) are grouped under 2 schemes (S1, S2).
   Each scheme has a set of "key values". An interval is allocated to the category whose key value
   it includes and intervals which include none or more than one of the key values are excluded.
   (Open-ended intervals are coded as 99.)

S1	key value	maximum range	S2	key value	maximum range
1	5	1-19	1	1	1-9
2	20	6-44	2	10	2-19
3	45	21+	3	20	11-29
			4	30	21-39
			5	40	31-98
			6	99	41+

   For all/unspec product, the definition of cigarette equivalents is shown at the end of Sections -1 and -4.
2) Current smokers
3) Results complete enough for use in metaanalysis

Within each study, results are then selected (in the following order of preference, within each sex) for:
4) PRODUCT: all/unspec, cigarettes regardless of other products, cigarettes only
5) CIGTYPE: all/unspecified, MC regardless of HR, MC only
6) DENOM: never smoked anything, never smoked cigarettes, (never +1 = +long term ex, +2 = +amount unknown,
          +3 = never cigs+long term ex)
7) Followup period (YF, prospective studies): whole study (coded as 0) or longest available
8) LCtype: adeno or nearest available, but not squamous. (q = squamous, s = small,
          a = adeno, l = large, KII = Kreyberg II, al = alveolar, br = bronchiolar, u = undifferentiated)
9) Race: all or nearest available, otherwise by race (wh or w = white, bl or b = black, hi = hispanic
          ch = chinese, jap = japanese, haw = hawaiian, w+o = white + oriental, sca = scandinavian, as = asian)
10) For overlapping studies: principal rather than subsidiary studies
Finally by Age: whole study (coded as 0) if available, otherwise by widest available age group
and then for single sex results (m, f) in preference to combined sex results (c).

Results adjusted (AD) for the most potential confounders are then chosen in Sections -1 to -3
and results adjusted for the least confounders in Sections -4 to -6. (Those least adjusted results which
actually differ from the most adjusted as marked 'x' in column X in Section -4)
(Results adjusted for an unknown number of confounder(s) are coded as 20.)

Section -7 shows excluded studies, together with the stage (as above) at which no qualifying
results were found.

Section -8 lists the potentially overlapping studies which have been included (1=principal, 2=subsidiary).

Section -9 lists any results which would have been included in preference except that they had data not complete
enough for use in meta-analysis, with their significance (yes/no), if known, and any further comment as entered
on the database.

In addition to those mentioned above, the following fields, levels and abbreviations are used:
* or nk = not known, n = no, y = yes, ot = other
nev = never
all/unspec = all or unspecified, cig+/-ot = cigarettes irrespective of other products (cigar, pipe etc)
MC = manufactured cigarettes, HR = hand-rolled cigarettes
exL, exH = range of exposure (low and high) in the smoking group, in terms of Amount smoked,
      cigarettes or cigarette equivalents
REF: 6-character study reference
NRR: number of the RR on the database within the study
ST : study type (CC = case control, pr or prosp = prospective)
NLC: number of lung cancer cases in whole study
R : risky occupational population (n = no, m = mining, o = other risky)
VB : national cigarette type (V = at least 75% Virginia, bl = at least 75% blended, ot = other)
P : any proxy use
H : full histological confirmation
De : derivation of RR/CI (or = original, st = standard method, ot = other method of estimation)

Table 3G6 - 1

IESLC - Meta-analysis of Current Smoking by Amount, Overview, Any product (or Cigarettes if Any not available)
Adenocarcinoma
Most adjusted

REF	NRR	SEX	AGEL	AGEH	RACE	YF	LC TYPE	LOC	START	ST	NLC	R	VB	P	H	AD	PRODUCT	exL	exH	S1	S2	DENOM	De
BOUCOT	148	m	0	0	all	0	a	NAmer	1951	pr	121	n	bl	n	n	2	cig only	1	20	0	0	nev any	ot
BOUCOT	149	m	0	0	all	0	a	NAmer	1951	pr	121	n	bl	n	n	2	cig only	21	99	3	0	nev any	ot
CORREA	48	c	0	0	all	-	a	NAmer	1979	CC	1359	n	bl	y	n	1	cig+/-ot	1	20	0	0	nev cigs	or
CORREA	52	c	0	0	all	-	a	NAmer	1979	CC	1359	n	bl	y	n	1	cig+/-ot	21	99	3	0	nev cigs	or
ENGELA	71	m	0	0	all	0	a	Eu:Sca	1964	pr	435	n	bl	n	n	7	cig+/-ot	1	4	0	1	nev cigs	or
ENGELA	72	m	0	0	all	0	a	Eu:Sca	1964	pr	435	n	bl	n	n	7	cig+/-ot	5	9	1	0	nev cigs	or
ENGELA	73	m	0	0	all	0	a	Eu:Sca	1964	pr	435	n	bl	n	n	7	cig+/-ot	10	14	0	2	nev cigs	or
ENGELA	74	m	0	0	all	0	a	Eu:Sca	1964	pr	435	n	bl	n	n	7	cig+/-ot	15	19	0	0	nev cigs	or
ENGELA	75	m	0	0	all	0	a	Eu:Sca	1964	pr	435	n	bl	n	n	7	cig+/-ot	20	99	0	0	nev cigs	or
HAENSZ	35	f	0	0	all	-	a	NAmer	1955	CC	158	n	bl	n	y	0	cig+/-ot	1	20	0	0	nev any	or
HAENSZ	34	f	0	0	all	-	a	NAmer	1955	CC	158	n	bl	n	y	0	cig+/-ot	21	99	3	0	nev any	or
HAMMON	108	m	0	0	wh	0	a	NAmer	1952	pr	448	n	bl	n	n	1	cig only	1	9	1	1	nev any	ot
HAMMON	109	m	0	0	wh	0	a	NAmer	1952	pr	448	n	bl	n	n	1	cig only	10	20	2	0	nev any	ot
HAMMON	110	m	0	0	wh	0	a	NAmer	1952	pr	448	n	bl	n	n	1	cig only	21	39	0	4	nev any	ot
KATSOU	13	f	0	0	all	-	a	Eu:bal	1987	CC	101	n	bl	n	n	1	all/unsp	1	20	0	0	nev any	or
KATSOU	14	f	0	0	all	-	a	Eu:bal	1987	CC	101	n	bl	n	n	1	all/unsp	21	99	3	0	nev any	or
SOBUE	56	m	0	0	all	-	a	As:Jap	1986	CC	1376	n	bl	n	y	0	cig+/-ot	1	19	1	0	nev cigs	st
SOBUE	57	m	0	0	all	-	a	As:Jap	1986	CC	1376	n	bl	n	y	0	cig+/-ot	20	29	2	3	nev cigs	st
SOBUE	58	m	0	0	all	-	a	As:Jap	1986	CC	1376	n	bl	n	y	0	cig+/-ot	30	99	3	0	nev cigs	st
SVENSS	9	f	0	0	all	-	a	Eu:Sca	1983	CC	210	n	bl	n	n	1	all/unsp	1	10	1	0	nev any	or
SVENSS	14	f	0	0	all	-	a	Eu:Sca	1983	CC	210	n	bl	n	n	1	all/unsp	11	20	2	3	nev any	or
SVENSS	19	f	0	0	all	-	a	Eu:Sca	1983	CC	210	n	bl	n	n	1	all/unsp	21	99	3	0	nev any	ot
TSUGAN	4	m	0	0	all	-	a	As:Jap	1976	CC	134	n	bl	n	y	0	all/unsp	1	15	1	0	nev any	st
TSUGAN	5	m	0	0	all	-	a	As:Jap	1976	CC	134	n	bl	n	y	0	all/unsp	16	35	2	0	nev any	st
TSUGAN	6	m	0	0	all	-	a	As:Jap	1976	CC	134	n	bl	n	y	0	all/unsp	36	99	3	0	nev any	st
WAKAI	52	m	0	0	all	-	a	As:Jap	1988	CC	333	n	bl	n	y	1	cig+/-ot	1	19	1	0	nev any	or
WAKAI	53	m	0	0	all	-	a	As:Jap	1988	CC	333	n	bl	n	y	1	cig+/-ot	20	29	2	3	nev any	or
WAKAI	54	m	0	0	all	-	a	As:Jap	1988	CC	333	n	bl	n	y	1	cig+/-ot	30	99	3	0	nev any	or
WU	8	f	0	0	wh	-	a	NAmer	1981	CC	220	n	bl	n	y	2	all/unsp	1	20	0	0	nev any	or
WU	9	f	0	0	wh	-	a	NAmer	1981	CC	220	n	bl	n	y	2	all/unsp	21	99	3	0	nev any	or
WYNDE6	24	m	0	0	all	-	KII	NAmer	1969	CC	4423	n	bl	n	y	0	cig+/-ot	1	10	1	0	nev any	st
WYNDE6	33	m	0	0	all	-	KII	NAmer	1969	CC	4423	n	bl	n	y	0	cig+/-ot	11	20	2	3	nev any	st
WYNDE6	42	m	0	0	all	-	KII	NAmer	1969	CC	4423	n	bl	n	y	0	cig+/-ot	21	30	0	4	nev any	st
WYNDE6	51	m	0	0	all	-	KII	NAmer	1969	CC	4423	n	bl	n	y	0	cig+/-ot	31	99	3	0	nev any	st
WYNDE6	213	f	0	0	all	-	KII	NAmer	1969	CC	4423	n	bl	n	y	0	cig+/-ot	1	10	1	0	nev cigs	st
WYNDE6	222	f	0	0	all	-	KII	NAmer	1969	CC	4423	n	bl	n	y	0	cig+/-ot	11	20	2	3	nev cigs	st
WYNDE6	231	f	0	0	all	-	KII	NAmer	1969	CC	4423	n	bl	n	y	0	cig+/-ot	21	30	0	4	nev cigs	st
WYNDE6	240	f	0	0	all	-	KII	NAmer	1969	CC	4423	n	bl	n	y	0	cig+/-ot	30	99	3	0	nev cigs	st

Cigarette type is all/unspec for all RRs

REF	NRR	Cigarette equivalent
BOUCOT	148	-
BOUCOT	149	-
CORREA	48	*
CORREA	52	*
ENGELA	71	*
ENGELA	72	*
ENGELA	73	*
ENGELA	74	*
ENGELA	75	*
HAENSZ	35	*
HAENSZ	34	*
HAMMON	108	-
HAMMON	109	-
HAMMON	110	-
KATSOU	13	*
KATSOU	14	*
SOBUE	56	*
SOBUE	57	*
SOBUE	58	*
SVENSS	9	*
SVENSS	14	*
SVENSS	19	*
TSUGAN	4	*
TSUGAN	5	*
TSUGAN	6	*
WAKAI	52	*
WAKAI	53	*
WAKAI	54	*

Table 3G6 - 1

IESLC - Meta-analysis of Current Smoking by Amount, Overview, Any product (or Cigarettes if Any not available)
Adenocarcinoma
Most adjusted

REF	NRR	Cigarette equivalent
WU	8	*
WU	9	*
WYNDE6	24	*
WYNDE6	33	*
WYNDE6	42	*
WYNDE6	51	*
WYNDE6	213	*
WYNDE6	222	*
WYNDE6	231	*
WYNDE6	240	*

In this overview table, subtotals and Qs values may be invalid and should be ignored

Table 3G6 - 2

IESLC - Meta-analysis of Current Smoking by Amount, Overview, Any product (or Cigarettes if Any not available)
Adenocarcinoma
Most adjusted

REF	NRR	SEX	AD	Number Exposed Case	Number Exposed Cont	Non-exposed Case	Non-exposed Cont	RR	95.00%CI
*BOUCOT	148	m	2	-	-	-	-	8.22	(0.56- 166.83)
*BOUCOT	149	m	2	-	-	-	-	18.00	(1.01- 319.52)
Subtotal BOUCOT								12.11	(1.60- 91.75)
CORREA	48	c	1	-	-	-	-	4.30	(2.60- 7.20)
CORREA	52	c	1	-	-	-	-	12.00	(7.30- 19.70)
Subtotal CORREA								7.28	(5.10- 10.39)
*ENGELA	71	m	7	-	-	-	-	2.60	(0.60- 11.00)
*ENGELA	72	m	7	-	-	-	-	8.50	(2.50- 29.00)
*ENGELA	73	m	7	-	-	-	-	7.10	(2.10- 24.00)
*ENGELA	74	m	7	-	-	-	-	9.30	(2.10- 41.00)
*ENGELA	75	m	7	-	-	-	-	17.00	(5.10- 56.00)
Subtotal ENGELA								8.06	(4.51- 14.41)
HAENSZ	35	f	0	10	66	37	236	0.97	(0.46- 2.05)
HAENSZ	34	f	0	5	13	37	236	2.45	(0.83- 7.28)
Subtotal HAENSZ								1.30	(0.70- 2.42)
*HAMMON	108	m	1	-	-	-	-	1.83	(0.17- 20.22)
*HAMMON	109	m	1	-	-	-	-	2.83	(0.55- 14.60)
*HAMMON	110	m	1	-	-	-	-	6.44	(1.34- 31.02)
Subtotal HAMMON								3.71	(1.33- 10.33)
KATSOU	13	f	1	-	-	-	-	1.35	(0.52- 3.49)
KATSOU	14	f	1	-	-	-	-	2.95	(0.76- 11.41)
Subtotal KATSOU								1.75	(0.80- 3.81)
SOBUE	56	m	0	63	157	27	128	1.90	(1.15- 3.16)
SOBUE	57	m	0	95	222	27	128	2.03	(1.26- 3.28)
SOBUE	58	m	0	76	187	27	128	1.93	(1.18- 3.16)
Subtotal SOBUE								1.95	(1.47- 2.60)
SVENSS	9	f	1	-	-	-	-	2.20	(1.00- 5.80)
SVENSS	14	f	1	-	-	-	-	5.40	(2.40- 13.20)
SVENSS	19	f	1	-	-	-	-	19.70	(1.70- 228.29)
Subtotal SVENSS								3.87	(2.14- 7.00)
TSUGAN	4	m	0	12	14	18	17	0.81	(0.29- 2.24)
TSUGAN	5	m	0	23	23	18	17	0.94	(0.39- 2.28)
TSUGAN	6	m	0	10	13	18	17	0.73	(0.25- 2.09)
Subtotal TSUGAN								0.84	(0.48- 1.47)
WAKAI	52	m	1	-	-	-	-	1.30	(0.52- 3.21)
WAKAI	53	m	1	-	-	-	-	1.93	(0.84- 4.44)
WAKAI	54	m	1	-	-	-	-	4.53	(1.89- 10.90)
Subtotal WAKAI								2.27	(1.37- 3.75)
WU	8	f	2	-	-	-	-	2.70	(1.40- 5.40)
WU	9	f	2	-	-	-	-	6.50	(3.10- 13.90)
Subtotal WU								4.00	(2.42- 6.61)
WYNDE6	24	m	0	42	122	58	617	3.66	(2.35- 5.70)
WYNDE6	33	m	0	191	293	58	617	6.93	(5.01- 9.60)
WYNDE6	42	m	0	136	129	58	617	11.22	(7.81- 16.09)
WYNDE6	51	m	0	282	197	58	617	15.23	(11.00- 21.07)
WYNDE6	213	f	0	39	109	119	856	2.57	(1.70- 3.89)
WYNDE6	222	f	0	176	165	119	856	7.67	(5.76- 10.21)
WYNDE6	231	f	0	100	50	119	856	14.39	(9.74- 21.25)
WYNDE6	240	f	0	157	52	119	856	21.72	(15.04- 31.37)
Subtotal WYNDE6								8.99	(7.93- 10.19)
Partial Totals				1417	1812	917	6799		

*prospective study

Table 3G6 - 2

IESLC - Meta-analysis of Current Smoking by Amount, Overview, Any product (or Cigarettes if Any not available)
Adenocarcinoma
Most adjusted

REF	NRR	SEX	AD	Ys	Ws	Qs	Ps
*BOUCOT	148	m	2	2.11	0.47	0.06	0.1472
*BOUCOT	149	m	2	2.89	0.46	0.61	0.0491
Subtotal BOUCOT				2.49	0.94	0.67	
CORREA	48	c	1	1.46	14.81	1.22	0.0000
CORREA	52	c	1	2.48	15.59	8.52	0.0000
Subtotal CORREA				1.98	30.40	9.74	
*ENGELA	71	m	7	0.96	1.82	1.13	0.1979
*ENGELA	72	m	7	2.14	2.56	0.40	0.0006
*ENGELA	73	m	7	1.96	2.59	0.12	0.0016
*ENGELA	74	m	7	2.23	1.74	0.41	0.0033
*ENGELA	75	m	7	2.83	2.68	3.17	0.0000
Subtotal ENGELA				2.09	11.38	5.22	
HAENSZ	35	f	0	-0.03	6.83	21.63	0.9289
HAENSZ	34	f	0	0.90	3.24	2.33	0.1060
Subtotal HAENSZ				0.27	10.07	23.97	
*HAMMON	108	m	1	0.60	0.67	0.88	0.6201
*HAMMON	109	m	1	1.04	1.43	0.71	0.2136
*HAMMON	110	m	1	1.86	1.56	0.02	0.0201
Subtotal HAMMON				1.31	3.66	1.61	
KATSOU	13	f	1	0.30	4.24	8.86	0.5366
KATSOU	14	f	1	1.08	2.09	0.92	0.1175
Subtotal KATSOU				0.56	6.33	9.78	
SOBUE	56	m	0	0.64	14.90	18.12	0.0130
SOBUE	57	m	0	0.71	16.70	18.00	0.0038
SOBUE	58	m	0	0.66	15.78	18.75	0.0092
Subtotal SOBUE				0.67	47.39	54.87	
SVENSS	9	f	1	0.79	4.97	4.56	0.0787
SVENSS	14	f	1	1.69	5.29	0.02	0.0001
SVENSS	19	f	1	2.98	0.64	0.98	0.0171
Subtotal SVENSS				1.35	10.90	5.55	
TSUGAN	4	m	0	-0.21	3.72	14.23	0.6838
TSUGAN	5	m	0	-0.06	4.97	16.14	0.8986
TSUGAN	6	m	0	-0.32	3.43	14.64	0.5538
Subtotal TSUGAN				-0.18	12.12	45.01	
WAKAI	52	m	1	0.26	4.64	10.20	0.5721
WAKAI	53	m	1	0.66	5.54	6.56	0.1216
WAKAI	54	m	1	1.51	5.00	0.28	0.0007
Subtotal WAKAI				0.82	15.19	17.04	
WU	8	f	2	0.99	8.43	4.77	0.0039
WU	9	f	2	1.87	6.82	0.11	0.0000
Subtotal WU				1.39	15.26	4.88	
WYNDE6	24	m	0	1.30	19.66	3.94	0.0000
WYNDE6	33	m	0	1.94	36.35	1.32	0.0000
WYNDE6	42	m	0	2.42	29.44	13.28	0.0000
WYNDE6	51	m	0	2.72	36.38	34.76	0.0000
WYNDE6	213	f	0	0.95	22.53	14.43	0.0000
WYNDE6	222	f	0	2.04	46.92	4.00	0.0000
WYNDE6	231	f	0	2.67	25.27	21.42	0.0000
WYNDE6	240	f	0	3.08	28.43	50.48	0.0000
Subtotal WYNDE6				2.20	244.98	143.64	

N	38
NS	12

Table 3G6 - 3

IESLC - Meta-analysis of Current Smoking by Amount, Overview, Any product (or Cigarettes if Any not available)
Adenocarcinoma
Most adjusted

	Sex combined	male	female	Total
N	2	23	13	38
NS	1	7	5	13

In this overview table, other than the "N" rows, entries in the "absent" and "Total" columns may be invalid and should be ignored

	Amount smoked (broad categories) absent	<20k5	6-44k20	>20k45	Total
N	12	8	7	11	38
NS	8	7	6	10	31
Wt	99.87	73.65	117.19	117.90	408.61
Het Chi	73.07	14.67	45.32	104.29	321.99
Het df	11	7	6	10	37
Het P	***	*	***	***	***
Fixed RR	6.82	2.47	5.13	9.34	5.73
RRl	5.60	1.97	4.28	7.79	5.20
RRu	8.30	3.11	6.15	11.18	6.31
P	+++	+++	+++	+++	+++
Random RR	5.01	2.32	3.37	5.71	4.03
RRl	2.79	1.59	1.88	2.91	2.94
RRu	9.00	3.38	6.02	11.19	5.54
P	+++	+++	+++	+++	+++

	absent	Amount smoked (narrow categories) <10k1	2-19k10	11-29k20	21-39k30	31-98k40	>40k99	Total
N	27	2	1	5	3			38
NS	12	2	1	4	2			21
Wt	236.47	2.49	2.59	110.80	56.27			408.61
Het Chi	248.95	0.06	0.00	29.83	1.52			321.99
Het df	26	1	0	4	2			37
Het P	***	N.S.	N.S.	***	N.S.			***
Fixed RR	4.87	2.36	7.10	5.57	12.35			5.73
RRl	4.29	0.68	2.10	4.63	9.51			5.20
RRu	5.53	8.19	24.00	6.72	16.04			6.31
P	+++	N.S.	++	+++	+++			+++
Random RR	3.58	2.36	7.10	4.27	12.35			4.03
RRl	2.33	0.68	2.10	2.41	9.51			2.94
RRu	5.51	8.19	24.00	7.55	16.04			5.54
P	+++	N.S.	++	+++	+++			+++

MALES

	Amount smoked (broad categories) absent	<20k5	6-44k20	>20k45	Total
N	7	6	5	5	23
NS	4	6	5	5	20
Wt	40.29	46.15	64.99	61.07	212.50
Het Chi	5.06	14.57	32.02	67.19	172.94
Het df	6	5	4	4	22
Het P	N.S.	*	***	***	***
Fixed RR	10.14	2.45	3.82	6.82	4.93
RRl	7.45	1.84	2.99	5.31	4.31
RRu	13.81	3.27	4.87	8.76	5.64
P	+++	+++	+++	+++	+++
Random RR	10.14	2.23	2.43	3.94	3.60
RRl	7.45	1.24	1.06	1.08	2.34
RRu	13.81	3.99	5.57	14.39	5.54
P	+++	++	+	+	+++

Table 3G6 - 3

IESLC - Meta-analysis of Current Smoking by Amount, Overview, Any product (or Cigarettes if Any not available)
Adenocarcinoma
Most adjusted

MALES

		Amount smoked (narrow categories)						
	absent	<10k1	2-19k10	11-29k20	21-39k30	31-98k40	>40k99	Total
N	15	2	1	3	2			23
NS	7	2	1	3	2			15
Wt	117.83	2.49	2.59	58.59	31.00			212.50
Het Chi	126.73	0.06	0.00	21.28	0.45			172.94
Het df	14	1	0	2	1			22
Het P	***	N.S.	N.S.	***	N.S.			***
Fixed RR	4.30	2.36	7.10	4.33	10.91			4.93
RRl	3.59	0.68	2.10	3.35	7.67			4.31
RRu	5.15	8.19	24.00	5.59	15.51			5.64
P	+++	N.S.	++	+++	+++			+++
Random RR	3.32	2.36	7.10	3.12	10.91			3.60
RRl	1.79	0.68	2.10	1.20	7.67			2.34
RRu	6.18	8.19	24.00	8.10	15.51			5.54
P	+++	N.S.	++	+	+++			+++

FEMALES

	Amount smoked (broad categories)				
	absent	<20k5	6-44k20	>20k45	Total
N	4	2	2	5	13
NS	4	2	2	5	13
Wt	44.77	27.50	52.20	41.24	165.71
Het Chi	56.64	0.10	0.59	24.44	130.85
Het df	3	1	1	4	12
Het P	***	N.S.	N.S.	***	***
Fixed RR	5.56	2.50	7.40	13.52	6.65
RRl	4.15	1.72	5.65	9.96	5.71
RRu	7.45	3.64	9.71	18.34	7.74
P	+++	+++	+++	+++	+++
Random RR	2.75	2.50	7.40	7.09	4.33
RRl	0.67	1.72	5.65	2.53	2.48
RRu	11.31	3.64	9.71	19.84	7.56
P	N.S.	+++	+++	+++	+++

		Amount smoked (narrow categories)						
	absent	<10k1	2-19k10	11-29k20	21-39k30	31-98k40	>40k99	Total
N	10			2	1			13
NS	5			2	1			7
Wt	88.24			52.20	25.27			165.71
Het Chi	107.44			0.59	0.00			130.85
Het df	9			1	0			12
Het P	***			N.S.	N.S.			***
Fixed RR	5.00			7.40	14.39			6.65
RRl	4.06			5.65	9.74			5.71
RRu	6.16			9.71	21.25			7.74
P	+++			+++	+++			+++
Random RR	3.42			7.40	14.39			4.33
RRl	1.55			5.65	9.74			2.48
RRu	7.59			9.71	21.25			7.56
P	++			+++	+++			+++

Table 3G6 - 4

IESLC - Meta-analysis of Current Smoking by Amount, Overview, Any product (or Cigarettes if Any not available)
Adenocarcinoma
Least adjusted

REF	NRR	X	SEX	AGEL	AGEH	RACE	YF	LC TYPE	LOC	START	ST	NLC	R	VB	P	H	AD	PRODUCT	exL	exH	S1	S2	DENOM	De
BOUCOT	27	x	m	0	0	all	0	a	NAmer	1951	pr	121	n	bl	n	n	0	cig only	1	20	0	0	nev any	ot
BOUCOT	28	x	m	0	0	all	0	a	NAmer	1951	pr	121	n	bl	n	n	0	cig only	21	99	3	0	nev any	ot
CORREA	48		c	0	0	all	-	a	NAmer	1979	CC	1359	n	bl	y	n	1	cig+/-ot	1	20	0	0	nev cigs	or
CORREA	52		c	0	0	all	-	a	NAmer	1979	CC	1359	n	bl	y	n	1	cig+/-ot	21	99	3	0	nev cigs	or
ENGELA	71		m	0	0	all	0	a	Eu:Sca	1964	pr	435	n	bl	n	n	7	cig+/-ot	1	4	0	1	nev cigs	or
ENGELA	72		m	0	0	all	0	a	Eu:Sca	1964	pr	435	n	bl	n	n	7	cig+/-ot	5	9	1	0	nev cigs	or
ENGELA	73		m	0	0	all	0	a	Eu:Sca	1964	pr	435	n	bl	n	n	7	cig+/-ot	10	14	0	2	nev cigs	or
ENGELA	74		m	0	0	all	0	a	Eu:Sca	1964	pr	435	n	bl	n	n	7	cig+/-ot	15	19	0	0	nev cigs	or
ENGELA	75		m	0	0	all	0	a	Eu:Sca	1964	pr	435	n	bl	n	n	7	cig+/-ot	20	99	0	0	nev cigs	or
HAENSZ	35		f	0	0	all	-	a	NAmer	1955	CC	158	n	bl	n	y	0	cig+/-ot	1	20	0	0	nev any	or
HAENSZ	34		f	0	0	all	-	a	NAmer	1955	CC	158	n	bl	n	y	0	cig+/-ot	21	99	3	0	nev any	or
HAMMON	108		m	0	0	wh	0	a	NAmer	1952	pr	448	n	bl	n	n	1	cig only	1	9	1	1	nev any	ot
HAMMON	109		m	0	0	wh	0	a	NAmer	1952	pr	448	n	bl	n	n	1	cig only	10	20	2	0	nev any	ot
HAMMON	110		m	0	0	wh	0	a	NAmer	1952	pr	448	n	bl	n	n	1	cig only	21	39	0	4	nev any	ot
KATSOU	17	x	f	0	0	all	-	a	Eu:bal	1987	CC	101	n	bl	n	n	0	all/unsp	1	20	0	0	nev any	st
KATSOU	18	x	f	0	0	all	-	a	Eu:bal	1987	CC	101	n	bl	n	n	0	all/unsp	21	99	3	0	nev any	st
SOBUE	56		m	0	0	all	-	a	As:Jap	1986	CC	1376	n	bl	n	y	0	cig+/-ot	1	19	1	0	nev cigs	st
SOBUE	57		m	0	0	all	-	a	As:Jap	1986	CC	1376	n	bl	n	y	0	cig+/-ot	20	29	2	3	nev cigs	st
SOBUE	58		m	0	0	all	-	a	As:Jap	1986	CC	1376	n	bl	n	y	0	cig+/-ot	30	99	3	0	nev cigs	st
SVENSS	29	x	f	0	0	all	-	a	Eu:Sca	1983	CC	210	n	bl	n	n	0	all/unsp	1	10	1	0	nev any	st
SVENSS	34	x	f	0	0	all	-	a	Eu:Sca	1983	CC	210	n	bl	n	n	0	all/unsp	11	20	2	3	nev any	st
SVENSS	39	x	f	0	0	all	-	a	Eu:Sca	1983	CC	210	n	bl	n	n	0	all/unsp	21	99	3	0	nev any	st
TSUGAN	4		m	0	0	all	-	a	As:Jap	1976	CC	134	n	bl	n	y	0	all/unsp	1	15	1	0	nev any	st
TSUGAN	5		m	0	0	all	-	a	As:Jap	1976	CC	134	n	bl	n	y	0	all/unsp	16	35	2	0	nev any	st
TSUGAN	6		m	0	0	all	-	a	As:Jap	1976	CC	134	n	bl	n	y	0	all/unsp	36	99	3	0	nev any	st
WAKAI	49	x	m	0	0	all	-	a	As:Jap	1988	CC	333	n	bl	n	y	0	cig+/-ot	1	19	1	0	nev any	st
WAKAI	50	x	m	0	0	all	-	a	As:Jap	1988	CC	333	n	bl	n	y	0	cig+/-ot	20	29	2	3	nev any	st
WAKAI	51	x	m	0	0	all	-	a	As:Jap	1988	CC	333	n	bl	n	y	0	cig+/-ot	30	99	3	0	nev any	st
WU	3	x	f	0	0	wh	-	a	NAmer	1981	CC	220	n	bl	n	y	0	all/unsp	1	20	0	0	nev any	st
WU	4	x	f	0	0	wh	-	a	NAmer	1981	CC	220	n	bl	n	y	0	all/unsp	21	99	3	0	nev any	st
WYNDE6	24		m	0	0	all	-	KII	NAmer	1969	CC	4423	n	bl	n	y	0	cig+/-ot	1	10	1	0	nev any	st
WYNDE6	33		m	0	0	all	-	KII	NAmer	1969	CC	4423	n	bl	n	y	0	cig+/-ot	11	20	2	3	nev any	st
WYNDE6	42		m	0	0	all	-	KII	NAmer	1969	CC	4423	n	bl	n	y	0	cig+/-ot	21	30	0	4	nev any	st
WYNDE6	51		m	0	0	all	-	KII	NAmer	1969	CC	4423	n	bl	n	y	0	cig+/-ot	31	99	3	0	nev any	st
WYNDE6	213		f	0	0	all	-	KII	NAmer	1969	CC	4423	n	bl	n	y	0	cig+/-ot	1	10	1	0	nev cigs	st
WYNDE6	222		f	0	0	all	-	KII	NAmer	1969	CC	4423	n	bl	n	y	0	cig+/-ot	11	20	2	3	nev cigs	st
WYNDE6	231		f	0	0	all	-	KII	NAmer	1969	CC	4423	n	bl	n	y	0	cig+/-ot	21	30	0	4	nev cigs	st
WYNDE6	240		f	0	0	all	-	KII	NAmer	1969	CC	4423	n	bl	n	y	0	cig+/-ot	30	99	3	0	nev cigs	st

Cigarette type is all/unspec for all RRs

REF	NRR	Cigarette equivalent
BOUCOT	27	-
BOUCOT	28	-
CORREA	48	*
CORREA	52	*
ENGELA	71	*
ENGELA	72	*
ENGELA	73	*
ENGELA	74	*
ENGELA	75	*
HAENSZ	35	*
HAENSZ	34	*
HAMMON	108	-
HAMMON	109	-
HAMMON	110	-
KATSOU	17	*
KATSOU	18	*
SOBUE	56	*
SOBUE	57	*
SOBUE	58	*
SVENSS	29	*
SVENSS	34	*
SVENSS	39	*
TSUGAN	4	*
TSUGAN	5	*
TSUGAN	6	*
WAKAI	49	*
WAKAI	50	*
WAKAI	51	*

Table 3G6 - 4

IESLC - Meta-analysis of Current Smoking by Amount, Overview, Any product (or Cigarettes if Any not available)
Adenocarcinoma
Least adjusted

REF	NRR	Cigarette equivalent
WU	3	*
WU	4	*
WYNDE6	24	*
WYNDE6	33	*
WYNDE6	42	*
WYNDE6	51	*
WYNDE6	213	*
WYNDE6	222	*
WYNDE6	231	*
WYNDE6	240	*

In this overview table, subtotals and Qs values may be invalid and should be ignored

Table 3G6 - 5

IESLC - Meta-analysis of Current Smoking by Amount, Overview, Any product (or Cigarettes if Any not available)
Adenocarcinoma
Least adjusted

REF	NRR	SEX	AD	Number Exposed Case	Number Exposed Cont	Non-exposed Case	Non-exposed Cont	RR	95.00%CI
*BOUCOT	27	m	0	8	15208	0	7551	8.44~	(0.49- 146.22)
*BOUCOT	28	m	0	6	6940	0	7551	14.14~	(0.80- 251.03)
Subtotal BOUCOT								10.90	(1.44- 82.62)
CORREA	48	c	1	-	-	-	-	4.30	(2.60- 7.20)
CORREA	52	c	1	-	-	-	-	12.00	(7.30- 19.70)
Subtotal CORREA								7.28	(5.10- 10.39)
*ENGELA	71	m	7	-	-	-	-	2.60	(0.60- 11.00)
*ENGELA	72	m	7	-	-	-	-	8.50	(2.50- 29.00)
*ENGELA	73	m	7	-	-	-	-	7.10	(2.10- 24.00)
*ENGELA	74	m	7	-	-	-	-	9.30	(2.10- 41.00)
*ENGELA	75	m	7	-	-	-	-	17.00	(5.10- 56.00)
Subtotal ENGELA								8.06	(4.51- 14.41)
HAENSZ	35	f	0	10	66	37	236	0.97	(0.46- 2.05)
HAENSZ	34	f	0	5	13	37	236	2.45	(0.83- 7.28)
Subtotal HAENSZ								1.30	(0.70- 2.42)
*HAMMON	108	m	1	-	-	-	-	1.83	(0.17- 20.22)
*HAMMON	109	m	1	-	-	-	-	2.83	(0.55- 14.60)
*HAMMON	110	m	1	-	-	-	-	6.44	(1.34- 31.02)
Subtotal HAMMON								3.71	(1.33- 10.33)
KATSOU	17	f	0	9	14	30	67	1.44	(0.56- 3.68)
KATSOU	18	f	0	6	4	30	67	3.35	(0.88- 12.75)
Subtotal KATSOU								1.90	(0.88- 4.11)
SOBUE	56	m	0	63	157	27	128	1.90	(1.15- 3.16)
SOBUE	57	m	0	95	222	27	128	2.03	(1.26- 3.28)
SOBUE	58	m	0	76	187	27	128	1.93	(1.18- 3.16)
Subtotal SOBUE								1.95	(1.47- 2.60)
SVENSS	29	f	0	12	30	22	120	2.18	(0.97- 4.90)
SVENSS	34	f	0	22	22	22	120	5.45	(2.59- 11.50)
SVENSS	39	f	0	4	1	22	120	21.82	(2.33- 204.53)
Subtotal SVENSS								3.97	(2.33- 6.76)
TSUGAN	4	m	0	12	14	18	17	0.81	(0.29- 2.24)
TSUGAN	5	m	0	23	23	18	17	0.94	(0.39- 2.28)
TSUGAN	6	m	0	10	13	18	17	0.73	(0.25- 2.09)
Subtotal TSUGAN								0.84	(0.48- 1.47)
WAKAI	49	m	0	16	105	8	65	1.24	(0.50- 3.06)
WAKAI	50	m	0	30	129	8	65	1.89	(0.82- 4.35)
WAKAI	51	m	0	27	48	8	65	4.57	(1.91- 10.94)
Subtotal WAKAI								2.22	(1.34- 3.67)
WU	3	f	0	38	28	29	62	2.90	(1.50- 5.60)
WU	4	f	0	61	22	29	62	5.93	(3.07- 11.44)
Subtotal WU								4.15	(2.61- 6.60)
WYNDE6	24	m	0	42	122	58	617	3.66	(2.35- 5.70)
WYNDE6	33	m	0	191	293	58	617	6.93	(5.01- 9.60)
WYNDE6	42	m	0	136	129	58	617	11.22	(7.81- 16.09)
WYNDE6	51	m	0	282	197	58	617	15.23	(11.00- 21.07)
WYNDE6	213	f	0	39	109	119	856	2.57	(1.70- 3.89)
WYNDE6	222	f	0	176	165	119	856	7.67	(5.76- 10.21)
WYNDE6	231	f	0	100	50	119	856	14.39	(9.74- 21.25)
WYNDE6	240	f	0	157	52	119	856	21.72	(15.04- 31.37)
Subtotal WYNDE6								8.99	(7.93- 10.19)
Partial Totals				1656	24363	1125	22714		

*prospective study ~ With 0.5 adjustment for zero

Table 3G6 - 5

IESLC - Meta-analysis of Current Smoking by Amount, Overview, Any product (or Cigarettes if Any not available)
Adenocarcinoma
Least adjusted

REF	NRR	SEX	AD	Ys	Ws	Qs	Ps
*BOUCOT	27	m	0	2.13	0.47	0.07	0.1427
*BOUCOT	28	m	0	2.65	0.46	0.38	0.0710
Subtotal BOUCOT				2.39	0.94	0.45	
CORREA	48	c	1	1.46	14.81	1.20	0.0000
CORREA	52	c	1	2.48	15.59	8.58	0.0000
Subtotal CORREA				1.98	30.40	9.78	
*ENGELA	71	m	7	0.96	1.82	1.13	0.1979
*ENGELA	72	m	7	2.14	2.56	0.40	0.0006
*ENGELA	73	m	7	1.96	2.59	0.12	0.0016
*ENGELA	74	m	7	2.23	1.74	0.41	0.0033
*ENGELA	75	m	7	2.83	2.68	3.18	0.0000
Subtotal ENGELA				2.09	11.38	5.24	
HAENSZ	35	f	0	-0.03	6.83	21.57	0.9289
HAENSZ	34	f	0	0.90	3.24	2.32	0.1060
Subtotal HAENSZ				0.27	10.07	23.89	
*HAMMON	108	m	1	0.60	0.67	0.87	0.6201
*HAMMON	109	m	1	1.04	1.43	0.71	0.2136
*HAMMON	110	m	1	1.86	1.56	0.02	0.0201
Subtotal HAMMON				1.31	3.66	1.60	
KATSOU	17	f	0	0.36	4.33	8.27	0.4516
KATSOU	18	f	0	1.21	2.15	0.61	0.0762
Subtotal KATSOU				0.64	6.48	8.88	
SOBUE	56	m	0	0.64	14.90	18.03	0.0130
SOBUE	57	m	0	0.71	16.70	17.91	0.0038
SOBUE	58	m	0	0.66	15.78	18.66	0.0092
Subtotal SOBUE				0.67	47.39	54.61	
SVENSS	29	f	0	0.78	5.87	5.44	0.0588
SVENSS	34	f	0	1.70	6.91	0.02	0.0000
SVENSS	39	f	0	3.08	0.77	1.38	0.0069
Subtotal SVENSS				1.38	13.54	6.83	
TSUGAN	4	m	0	-0.21	3.72	14.19	0.6838
TSUGAN	5	m	0	-0.06	4.97	16.10	0.8986
TSUGAN	6	m	0	-0.32	3.43	14.60	0.5538
Subtotal TSUGAN				-0.18	12.12	44.89	
WAKAI	49	m	0	0.21	4.71	11.01	0.6431
WAKAI	50	m	0	0.64	5.51	6.75	0.1352
WAKAI	51	m	0	1.52	5.04	0.25	0.0006
Subtotal WAKAI				0.80	15.26	18.02	
WU	3	f	0	1.07	8.88	4.08	0.0015
WU	4	f	0	1.78	8.89	0.01	0.0000
Subtotal WU				1.42	17.77	4.09	
WYNDE6	24	m	0	1.30	19.66	3.89	0.0000
WYNDE6	33	m	0	1.94	36.35	1.36	0.0000
WYNDE6	42	m	0	2.42	29.44	13.38	0.0000
WYNDE6	51	m	0	2.72	36.38	34.95	0.0000
WYNDE6	213	f	0	0.95	22.53	14.34	0.0000
WYNDE6	222	f	0	2.04	46.92	4.07	0.0000
WYNDE6	231	f	0	2.67	25.27	21.54	0.0000
WYNDE6	240	f	0	3.08	28.43	50.68	0.0000
Subtotal WYNDE6				2.20	244.98	144.21	

N	38
NS	12

Table 3G6 - 6

IESLC - Meta-analysis of Current Smoking by Amount, Overview, Any product (or Cigarettes if Any not available)
Adenocarcinoma
Least adjusted

	Sex combined	male	female	Total
N	2	23	13	38
NS	1	7	5	13

In this overview table, other than the "N" rows, entries in the "absent" and "Total" columns may be invalid and should be ignored

	Amount smoked (broad categories) absent	<20k5	6-44k20	>20k45	Total
N	12	8	7	11	38
NS	8	7	6	10	31
Wt	100.41	74.61	118.78	120.19	414.00
Het Chi	71.72	15.03	45.53	104.73	322.50
Het df	11	7	6	10	37
Het P	***	*	***	***	***
Fixed RR	6.84	2.46	5.13	9.24	5.71
RRl	5.63	1.96	4.29	7.72	5.19
RRu	8.32	3.08	6.14	11.04	6.29
P	+++	+++	+++	+++	+++
Random RR	5.08	2.30	3.38	5.73	4.05
RRl	2.85	1.57	1.90	2.95	2.96
RRu	9.05	3.36	6.00	11.14	5.54
P	+++	+++	+++	+++	+++

	absent	<10k1	2-19k10	Amount smoked (narrow categories) 11-29k20	21-39k30	31-98k40	>40k99	Total
N	27	2	1	5	3			38
NS	12	2	1	4	2			21
Wt	240.26	2.49	2.59	112.39	56.27			414.00
Het Chi	249.03	0.06	0.00	30.04	1.52			322.50
Het df	26	1	0	4	2			37
Het P	***	N.S.	N.S.	***	N.S.			***
Fixed RR	4.86	2.36	7.10	5.57	12.35			5.71
RRl	4.28	0.68	2.10	4.63	9.51			5.19
RRu	5.52	8.19	24.00	6.70	16.04			6.29
P	+++	N.S.	++	+++	+++			+++
Random RR	3.60	2.36	7.10	4.28	12.35			4.05
RRl	2.35	0.68	2.10	2.44	9.51			2.96
RRu	5.52	8.19	24.00	7.51	16.04			5.54
P	+++	N.S.	++	+++	+++			+++

MALES

	Amount smoked (broad categories) absent	<20k5	6-44k20	>20k45	Total
N	7	6	5	5	23
NS	4	6	5	5	20
Wt	40.29	46.22	64.96	61.11	212.57
Het Chi	5.06	14.89	32.17	66.97	173.63
Het df	6	5	4	4	22
Het P	N.S.	*	***	***	***
Fixed RR	10.14	2.44	3.81	6.81	4.92
RRl	7.45	1.83	2.99	5.30	4.30
RRu	13.81	3.25	4.86	8.75	5.63
P	+++	+++	+++	+++	+++
Random RR	10.14	2.21	2.42	3.85	3.58
RRl	7.45	1.22	1.05	1.06	2.33
RRu	13.81	3.97	5.56	14.00	5.51
P	+++	++	+	+	+++

Table 3G6 - 6

IESLC - Meta-analysis of Current Smoking by Amount, Overview, Any product (or Cigarettes if Any not available)
Adenocarcinoma
Least adjusted

MALES

Amount smoked (narrow categories)

	absent	<10k1	2-19k10	11-29k20	21-39k30	31-98k40	>40k99	Total
N	15	2	1	3	2			23
NS	7	2	1	3	2			15
Wt	117.94	2.49	2.59	58.56	31.00			212.57
Het Chi	127.12	0.06	0.00	21.45	0.45			173.63
Het df	14	1	0	2	1			22
Het P	***	N.S.	N.S.	***	N.S.			***
Fixed RR	4.29	2.36	7.10	4.32	10.91			4.92
RRl	3.58	0.68	2.10	3.35	7.67			4.30
RRu	5.14	8.19	24.00	5.58	15.51			5.63
P	+++	N.S.	++	+++	+++			+++
Random RR	3.29	2.36	7.10	3.10	10.91			3.58
RRl	1.77	0.68	2.10	1.19	7.67			2.33
RRu	6.12	8.19	24.00	8.08	15.51			5.51
P	+++	N.S.	++	+	+++			+++

FEMALES

Amount smoked (broad categories)

	absent	<20k5	6-44k20	>20k45	Total
N	4	2	2	5	13
NS	4	2	2	5	13
Wt	45.31	28.40	53.83	43.49	171.02
Het Chi	55.44	0.13	0.70	26.15	130.82
Het df	3	1	1	4	12
Het P	***	N.S.	N.S.	***	***
Fixed RR	5.61	2.49	7.34	12.90	6.59
RRl	4.20	1.72	5.62	9.59	5.68
RRu	7.51	3.59	9.59	17.37	7.66
P	+++	+++	+++	+++	+++
Random RR	2.84	2.49	7.34	7.26	4.42
RRl	0.71	1.72	5.62	2.66	2.57
RRu	11.37	3.59	9.59	19.78	7.62
P	N.S.	+++	+++	+++	+++

Amount smoked (narrow categories)

	absent	<10k1	2-19k10	11-29k20	21-39k30	31-98k40	>40k99	Total
N	10			2	1			13
NS	5			2	1			7
Wt	91.92			53.83	25.27			171.02
Het Chi	107.04			0.70	0.00			130.82
Het df	9			1	0			12
Het P	***			N.S.	N.S.			***
Fixed RR	5.00			7.34	14.39			6.59
RRl	4.07			5.62	9.74			5.68
RRu	6.13			9.59	21.25			7.66
P	+++			+++	+++			+++
Random RR	3.52			7.34	14.39			4.42
RRl	1.63			5.62	9.74			2.57
RRu	7.62			9.59	21.25			7.62
P	++			+++	+++			+++

Table 3G6 - 7

IESLC - Meta-analysis of Current Smoking by Amount, Overview, Any product (or Cigarettes if Any not available)
Adenocarcinoma
Excluded studies (and stage at which they were excluded)

1 ABELIN ABRAHA AMANDU AMES ANDERS AUSTIN AXELSO BAND BECHER BERRIN BLOHMK BLOT4 BROCKM BROWN1 BYERS1 BYERS2
CARPEN CASCO2 CASCOR CHAN CHEN3 CHIAZZ CHYOU DESTE2 DOCKER DROSTE DU GARCIA GARDIN GENG GODLEY GOODMA
GRAHAM GREGOR HEGMAN HEIN HENNEK HINDS HIRAOK HOROWI HORWIT HUANG ISHIMA JAHN JAIN JARVHO JIANG KELLER
KIHARA KJUUS KO KOHLME KUBIK LAMWK LAMWK2 LANGE LEI LEMARC LEVIN LIU LOMBA2 LOMBAR MAGNUS MARSH
MARSH2 MCDUFF MCLAUG MILLER MILLS NOTANI NOU ODRISC PAWLEG PERSHA POFFIJ QIAO QIAO2 RADZIK REN RONCO
ROOTS ROTHSC SAARIK SANKAR SCHWAR SEGI SEOW SHIMIZ SIMARA SIMONA SITAS SOBUE2 STASZE STAYNE STUCKE SUN
SUZUK2 SUZUKI TANG TAO TOKARS TOUSEY ULMER VEIERO VUTUC WALD WANG WANG3 WANG4 WICKLU WIGLE WILKIN
WU2 WUNSCH WYNDE8 XIANGZ XU XU2 XU4 YONG ZHANG

2 AGUDO ALDERS ARMADA AUVINE BARBON BENHAM BLOT1 BLOT2 BLOT3 BOFFET BOUCHA BRESLO BROWN2 BUELL CHATZI CHEN
CHEN2 CHOI COOKSO DAMBER DAVEYS DEAN DEAN2 DOLL DOSEME DUNN EBELIN ESAKI FAN GAO GARSHI GER
GOLLED GSELL HANSEN HU HU2 JARUP JEDRYC JOLY JONES JUSSAW KHUDER KOULUM KREUZE KREYBE LAMTH LAUSSM
LETOUR LIU2 LIU3 LIU4 LIU5 LUBIN LUBIN2 LUO MARTIS MASTRA MATSUD MCCONN MOLLO MZILEN NOTAN2 ORMOS
OSANN OSANN2 PASTOR PERNU PIKE POLEDN RACHTA RANDIG RESTRE SADOWS SCHWA2 SIEMIA SPITZ STOCKS TIZZAN VANDER
WANG2 WUWILL WYNDE2 WYNDE3 WYNDE4 XU3 YUAN ZHENG ZHOU

3 PISANI

4 WYNDE7

5 RIMING TANG2 WYNDE5

6 DESTEF HIRAY2 LAURIL LICKIN MRFIT MURATA WARSIN WATSON WYNDER

8 AKIBA ARCHER AXELSS BENSHL BEST BRETT BROSS BUFFLE CEDERL CHANG CHOW COMSTO CPSI CPSII DARBY DEAN3
DEKLER DOLL2 DORANT DORGAN DORN ENSTRO GAO2 GILLIS HAMMO2 HIRAYA HITOSU HOLE HUMBLE KAISE2 KAISER KANELL
KAUFMA KINLEN KNEKT KOO LIAW LIDDEL MACLEN MATOS MIGRAN MRFITR NAM PARKIN PERSH2 PETO PEZZO2 PEZZOT
PRESCO SEGI2 SHAW SPEIZE STOCKW TENKAN TULINI TVERDA YAMAGU

Table 3G6 - 8

Potentially overlapping studies

REF	REFGP	PRINC	.	OVERLAP/LINK
WYNDE6	WYNDE6	1		WYNDE5/6/7/8

Table 3G6 - 9

Most adjusted - insufficient data for metaanalysis

REF	NRR	SEX	AGEL	AGEH	RACE	YF	LC TYPE	LOC	START	ST	NLC	R	VB	P	H	AD	PRODUCT	exL	exH	S1	S2	DENOM	De
GILLIS	17	m	0	0	all	-	a	Eu:UK	1977	CC	656	n	V	n	n	3	cig+/-ot	1	14	1	0	nev any	ot
GILLIS	18	m	0	0	all	-	a	Eu:UK	1977	CC	656	n	V	n	n	3	cig+/-ot	15	24	2	3	nev any	ot
GILLIS	19	m	0	0	all	-	a	Eu:UK	1977	CC	656	n	V	n	n	3	cig+/-ot	25	34	0	4	nev any	ot
GILLIS	20	m	0	0	all	-	a	Eu:UK	1977	CC	656	n	V	n	n	3	cig+/-ot	35	99	3	0	nev any	ot

REF	NRR	RR	SIG	Cigarette equivalent	RRDATA comment
GILLIS	17	1.82		*	0
GILLIS	18	1.44		*	0
GILLIS	19	3.31		*	0
GILLIS	20	2.65		*	0

Table 3G7 -

IESLC - Meta-analysis of Current Smoking, Amount smoked, "Low", Any product (or Cigarettes if Any not available)
Adenocarcinoma

This analysis is restricted to results for:
1) Results by Amount smoked
2) Current smokers
3) Results complete enough for use in metaanalysis

Within each study, results are then selected (in the following order of preference, within each sex) for:
4) PRODUCT: all/unspec, cigarettes regardless of other products, cigarettes only
5) CIGTYPE: all/unspecified, MC regardless of HR, MC only
6) DENOM: never smoked anything, never smoked cigarettes, (never +1 = +long term ex, +2 = +amount unknown,
+3 = never cigs+long term ex)
7) Followup period (YF, prospective studies): whole study (coded as 0) or longest available
8) LCtype: adeno or nearest available, but not squamous. (q = squamous, s = small,
a = adeno, l = large, KII = Kreyberg II, al = alveolar, br = bronchiolar, u = undifferentiated)
9) Race: all or nearest available, otherwise by race (wh or w = white, bl or b = black, hi = hispanic
ch = chinese, jap = japanese, haw = hawaiian, w+o = white + oriental, sca = scandinavian, as = asian)
10) Amount smoked "low" in key scheme 1 (key value 5, maximum range <20, in numbers of
cigarettes or cigarette equivalents)
11) For overlapping studies: principal rather than subsidiary studies
Finally by Age: whole study (coded as 0) if available, otherwise by widest available age group
and then for single sex results (m, f) in preference to combined sex results (c).

Results adjusted (AD) for the most potential confounders are then chosen in Sections -1 to -3
and results adjusted for the least confounders in Sections -4 to -6. (Those least adjusted results which
actually differ from the most adjusted as marked 'x' in column X in Section -4)
(Results adjusted for an unknown number of confounder(s) are coded as 20.)

Section -7 shows excluded studies, together with the stage (as above) at which no qualifying
results were found.

Section -8 lists the potentially overlapping studies which have been included (1=principal, 2=subsidiary).

Section -9 lists any results which would have been included in preference except that they had data not complete
enough for use in meta-analysis, with their significance (yes/no), if known, and any further comment as entered
on the database.

In addition to those mentioned above, the following fields, levels and abbreviations are used:
* or nk = not known, n = no, y = yes, ot = other
nev = never
all/unspec = all or unspecified, cig+/-ot = cigarettes irrespective of other products (cigar, pipe etc)
MC = manufactured cigarettes, HR = hand-rolled cigarettes
exL, exH = range of exposure (low and high) in the smoking group, in terms of Amount smoked,
cigarettes or cigarette equivalents
REF: 6-character study reference
NRR: number of the RR on the database within the study
ST : study type (CC = case control, pr or prosp = prospective)
NLC: number of lung cancer cases in whole study
R : risky occupational population (n = no, m = mining, o = other risky)
VB : national cigarette type (V = at least 75% Virginia, bl = at least 75% blended, ot = other)
P : any proxy use
H : full histological confirmation
De : derivation of RR/CI (or = original, st = standard method, ot = other method of estimation)

Table 3G7 - 1

IESLC - Meta-analysis of Current Smoking, Amount smoked, "Low", Any product (or Cigarettes if Any not available)
Adenocarcinoma
Most adjusted

REF	NRR	SEX	AGEL	AGEH	RACE	YF	LC TYPE	LOC	START	ST	NLC	R	VB	P	H	AD	PRODUCT	exL	exH	DENOM	De
ENGELA	72	m	0	0	all	0	a	Eu:Sca	1964	pr	435	n	bl	n	n	7	cig+/-ot	5	9	nev cigs	or
HAMMON	108	m	0	0	wh	0	a	NAmer	1952	pr	448	n	bl	n	n	1	cig only	1	9	nev any	ot
SOBUE	56	m	0	0	all	-	a	As:Jap	1986	CC	1376	n	bl	n	y	0	cig+/-ot	1	19	nev cigs	st
SVENSS	9	f	0	0	all	-	a	Eu:Sca	1983	CC	210	n	bl	n	n	1	all/unsp	1	10	nev any	or
TSUGAN	4	m	0	0	all	-	a	As:Jap	1976	CC	134	n	bl	n	y	0	all/unsp	1	15	nev any	st
WAKAI	52	m	0	0	all	-	a	As:Jap	1988	CC	333	n	bl	n	y	1	cig+/-ot	1	19	nev any	or
WYNDE6	24	m	0	0	all	-	KII	NAmer	1969	CC	4423	n	bl	n	y	0	cig+/-ot	1	10	nev any	st
WYNDE6	213	f	0	0	all	-	KII	NAmer	1969	CC	4423	n	bl	n	y	0	cig+/-ot	1	10	nev cigs	st

Cigarette type is all/unspec for all RRs

Table 3G7 - 2

IESLC - Meta-analysis of Current Smoking, Amount smoked, "Low", Any product (or Cigarettes if Any not available)
Adenocarcinoma
Most adjusted

REF	NRR	SEX	AD	Number Exposed Case	Cont	Non-exposed Case	Cont	RR	95.00%CI
*ENGELA	72	m	7	-	-	-	-	8.50	(2.50- 29.00)
*HAMMON	108	m	1	-	-	-	-	1.83	(0.17- 20.22)
SOBUE	56	m	0	63	157	27	128	1.90	(1.15- 3.16)
SVENSS	9	f	1	-	-	-	-	2.20	(1.00- 5.80)
TSUGAN	4	m	0	12	14	18	17	0.81	(0.29- 2.24)
WAKAI	52	m	1	-	-	-	-	1.30	(0.52- 3.21)
WYNDE6	24	m	0	42	122	58	617	3.66	(2.35- 5.70)
WYNDE6	213	f	0	39	109	119	856	2.57	(1.70- 3.89)
Subtotal WYNDE6								3.03	(2.24- 4.10)
Partial Totals				156	402	222	1618		

*prospective study

REF	NRR	SEX	AD	Ys	Ws	Qs	Ps
*ENGELA	72	m	7	2.14	2.56	3.90	0.0006
*HAMMON	108	m	1	0.60	0.67	0.06	0.6201
SOBUE	56	m	0	0.64	14.90	1.02	0.0130
SVENSS	9	f	1	0.79	4.97	0.07	0.0787
TSUGAN	4	m	0	-0.21	3.72	4.63	0.6838
WAKAI	52	m	1	0.26	4.64	1.91	0.5721
WYNDE6	24	m	0	1.30	19.66	3.04	0.0000
WYNDE6	213	f	0	0.95	22.53	0.04	0.0000
Subtotal WYNDE6				1.11	42.19	3.08	

	N	8
	NS	7
	Wt	73.65
Het	Chi	14.67
Het	df	7
Het	P	*
Fixed	RR	2.47
	RRl	1.97
	RRu	3.11
	P	+++
Random	RR	2.32
	RRl	1.59
	RRu	3.38
	P	+++
Asymm	P	N.S.

Table 3G7 - 3

IESLC - Meta-analysis of Current Smoking, Amount smoked, "Low", Any product (or Cigarettes if Any not available)
Adenocarcinoma
Most adjusted

	Sex: combined	male	female	Total
N		6	2	8
NS		6	2	8
Wt		46.15	27.50	73.65
Het Chi		14.57	0.10	14.67
Het df		5	1	7
Het P		*	N.S.	*
Fixed RR		2.45	2.50	2.47
RRl		1.84	1.72	1.97
RRu		3.27	3.64	3.11
P		+++	+++	+++
Random RR		2.23	2.50	2.32
RRl		1.24	1.72	1.59
RRu		3.99	3.64	3.38
P		++	+++	+++
Between Chi				0.01
Between df				1
Between P				N.S.
Btwn(F) P				N.S.
Btwn(R) P				N.S.

Too few RRs for analysis by factor

Table 3G7 - 4

IESLC - Meta-analysis of Current Smoking, Amount smoked, "Low", Any product (or Cigarettes if Any not available)
Adenocarcinoma
Least adjusted

REF	NRR	X	SEX	AGEL	AGEH	RACE	YF	LC TYPE	LOC	START	ST	NLC	R	VB	P	H	AD	PRODUCT	exL	exH	DENOM	De
ENGELA	72		m	0	0	all	0	a	Eu:Sca	1964	pr	435	n	bl	n	n	7	cig+/-ot	5	9	nev cigs	or
HAMMON	108		m	0	0	wh	0	a	NAmer	1952	pr	448	n	bl	n	n	1	cig only	1	9	nev any	ot
SOBUE	56		m	0	0	all	-	a	As:Jap	1986	CC	1376	n	bl	n	y	0	cig+/-ot	1	19	nev cigs	st
SVENSS	29	x	f	0	0	all	-	a	Eu:Sca	1983	CC	210	n	bl	n	n	0	all/unsp	1	10	nev any	st
TSUGAN	4		m	0	0	all	-	a	As:Jap	1976	CC	134	n	bl	n	y	0	all/unsp	1	15	nev any	st
WAKAI	49	x	m	0	0	all	-	a	As:Jap	1988	CC	333	n	bl	n	y	0	cig+/-ot	1	19	nev any	st
WYNDE6	24		m	0	0	all	-	KII	NAmer	1969	CC	4423	n	bl	n	y	0	cig+/-ot	1	10	nev any	st
WYNDE6	213		f	0	0	all	-	KII	NAmer	1969	CC	4423	n	bl	n	y	0	cig+/-ot	1	10	nev cigs	st

Cigarette type is all/unspec for all RRs

Table 3G7 - 5

IESLC - Meta-analysis of Current Smoking, Amount smoked, "Low", Any product (or Cigarettes if Any not available)
Adenocarcinoma
Least adjusted

REF	NRR	SEX	AD	Number Exposed Case	Number Exposed Cont	Non-exposed Case	Non-exposed Cont	RR	95.00%CI
*ENGELA	72	m	7	-	-	-	-	8.50	(2.50- 29.00)
*HAMMON	108	m	1	-	-	-	-	1.83	(0.17- 20.22)
SOBUE	56	m	0	63	157	27	128	1.90	(1.15- 3.16)
SVENSS	29	f	0	12	30	22	120	2.18	(0.97- 4.90)
TSUGAN	4	m	0	12	14	18	17	0.81	(0.29- 2.24)
WAKAI	49	m	0	16	105	8	65	1.24	(0.50- 3.06)
WYNDE6	24	m	0	42	122	58	617	3.66	(2.35- 5.70)
WYNDE6	213	f	0	39	109	119	856	2.57	(1.70- 3.89)
Subtotal WYNDE6								3.03	(2.24- 4.10)
Partial Totals				184	537	252	1803		

*prospective study

REF	NRR	SEX	AD	Ys	Ws	Qs	Ps
*ENGELA	72	m	7	2.14	2.56	3.94	0.0006
*HAMMON	108	m	1	0.60	0.67	0.06	0.6201
SOBUE	56	m	0	0.64	14.90	0.98	0.0130
SVENSS	29	f	0	0.78	5.87	0.08	0.0588
TSUGAN	4	m	0	-0.21	3.72	4.58	0.6838
WAKAI	49	m	0	0.21	4.71	2.21	0.6431
WYNDE6	24	m	0	1.30	19.66	3.13	0.0000
WYNDE6	213	f	0	0.95	22.53	0.05	0.0000
Subtotal WYNDE6				1.11	42.19	3.18	

	N	8
	NS	7
	Wt	74.61
Het	Chi	15.03
Het	df	7
Het	P	*
Fixed	RR	2.46
	RRl	1.96
	RRu	3.08
	P	+++
Random	RR	2.30
	RRl	1.57
	RRu	3.36
	P	+++
Asymm	P	N.S.

Table 3G7 - 6

IESLC - Meta-analysis of Current Smoking, Amount smoked, "Low", Any product (or Cigarettes if Any not available)
Adenocarcinoma
Least adjusted

	Sex combined	male	female	Total
N		6	2	8
NS		6	2	8
Wt		46.22	28.40	74.61
Het Chi		14.89	0.13	15.03
Het df		5	1	7
Het P		*	N.S.	*
Fixed RR		2.44	2.49	2.46
RRl		1.83	1.72	1.96
RRu		3.25	3.59	3.08
P		+++	+++	+++
Random RR		2.21	2.49	2.30
RRl		1.22	1.72	1.57
RRu		3.97	3.59	3.36
P		++	+++	+++
Between Chi				0.01
Between df				1
Between P				N.S.
Btwn(F) P				N.S.
Btwn(R) P				N.S.

Table 3G7 - 7

IESLC - Meta-analysis of Current Smoking, Amount smoked, "Low", Any product (or Cigarettes if Any not available)
Adenocarcinoma
Excluded studies (and stage at which they were excluded)

```
1      ABELIN ABRAHA AMANDU   AMES ANDERS AUSTIN AXELSO   BAND BECHER BERRIN BLOHMK  BLOT4 BROCKM BROWN1 BYERS1 BYERS2
       CARPEN CASCO2 CASCOR   CHAN  CHEN3 CHIAZZ  CHYOU DESTE2 DOCKER DROSTE     DU GARCIA GARDIN   GENG GODLEY GOODMA
       GRAHAM GREGOR HEGMAN   HEIN HENNEK  HINDS HIRAOK HOROWI HORWIT  HUANG ISHIMA   JAHN   JAIN JARVHO  JIANG KELLER
       KIHARA  KJUUS     KO KOHLME  KUBIK  LAMWK LAMWK2  LANGE    LEI LEMARC  LEVIN    LIU LOMBA2 LOMBAR MAGNUS  MARSH
       MARSH2 MCDUFF MCLAUG MILLER  MILLS NOTANI    NOU ODRISC PAWLEG PERSHA POFFIJ   QIAO  QIAO2 RADZIK    REN  RONCO
        ROOTS ROTHSC SAARIK SANKAR SCHWAR   SEGI   SEOW SHIMIZ SIMARA SIMONA  SITAS SOBUE2 STASZE STAYNE STUCKE    SUN
       SUZUK2 SUZUKI   TANG    TAO TOKARS TOUSEY  ULMER VEIERO  VUTUC   WALD   WANG  WANG3  WANG4 WICKLU  WIGLE WILKIN
          WU2 WUNSCH WYNDE8 XIANGZ     XU    XU2    XU4   YONG  ZHANG

2       AGUDO ALDERS ARMADA AUVINE BARBON BENHAM  BLOT1  BLOT2  BLOT3 BOFFET BOUCHA BRESLO BROWN2  BUELL CHATZI   CHEN
        CHEN2   CHOI COOKSO DAMBER DAVEYS   DEAN  DEAN2   DOLL DOSEME   DUNN EBELIN  ESAKI    FAN    GAO GARSHI    GER
       GOLLED  GSELL HANSEN     HU    HU2  JARUP JEDRYC   JOLY  JONES JUSSAW KHUDER KOULUM KREUZE KREYBE  LAMTH LAUSSM
       LETOUR   LIU2   LIU3   LIU4   LIU5  LUBIN LUBIN2    LUO MARTIS MASTRA MATSUD MCCONN  MOLLO MZILEN NOTAN2  ORMOS
        OSANN OSANN2 PASTOR  PERNU   PIKE POLEDN RACHTA RANDIG RESTRE SADOWS SCHWA2 SIEMIA  SPITZ STOCKS TIZZAN VANDER
        WANG2 WUWILL WYNDE2 WYNDE3 WYNDE4    XU3   YUAN  ZHENG   ZHOU

3      PISANI

4      WYNDE7

5      RIMING  TANG2 WYNDE5

6      DESTEF HIRAY2 LAURIL LICKIN  MRFIT MURATA WARSIN WATSON WYNDER

8       AKIBA ARCHER AXELSS BENSHL   BEST  BRETT  BROSS BUFFLE CEDERL  CHANG   CHOW COMSTO   CPSI  CPSII  DARBY  DEAN3
       DEKLER  DOLL2 DORANT DORGAN   DORN ENSTRO   GAO2 GILLIS HAMMO2 HIRAYA HITOSU   HOLE HUMBLE KAISE2 KAISER KANELL
       KAUFMA KINLEN  KNEKT    KOO   LIAW LIDDEL MACLEN  MATOS MIGRAN MRFITR    NAM PARKIN PERSH2   PETO PEZZO2 PEZZOT
       PRESCO  SEGI2   SHAW SPEIZE STOCKW TENKAN TULINI TVERDA YAMAGU

10     BOUCOT CORREA HAENSZ KATSOU     WU
```

Table 3G7 - 8

Potentially overlapping studies

REF	REFGP	PRINC	.	OVERLAP/LINK
WYNDE6	WYNDE6	1		WYNDE5/6/7/8

Table 3G7 - 9

Most adjusted - insufficient data for metaanalysis

REF	NRR	SEX	AGEL	AGEH	RACE	YF	LC TYPE	LOC	START	ST	NLC	R	VB	P	H	AD	PRODUCT	exL	exH	DENOM	De
GILLIS	17	m	0	0	all	-	a	Eu:UK	1977	CC	656	n	V	n	n	3	cig+/-ot	1	14	nev	any ot

REF	NRR	RR	SIG	RRDATA	comment
GILLIS	17	1.82		0	

Table 3G8 -

IESLC - Meta-analysis of Current Smoking, Amount smoked, "Mid", Any product (or Cigarettes if Any not available)
Adenocarcinoma

This analysis is restricted to results for:
1) Results by Amount smoked
2) Current smokers
3) Results complete enough for use in metaanalysis

Within each study, results are then selected (in the following order of preference, within each sex) for:
4) PRODUCT: all/unspec, cigarettes regardless of other products, cigarettes only
5) CIGTYPE: all/unspecified, MC regardless of HR, MC only
6) DENOM: never smoked anything, never smoked cigarettes, (never +1 = +long term ex, +2 = +amount unknown, +3 = never cigs+long term ex)
7) Followup period (YF, prospective studies): whole study (coded as 0) or longest available
8) LCtype: adeno or nearest available, but not squamous. (q = squamous, s = small, a = adeno, l = large, KII = Kreyberg II, al = alveolar, br = bronchiolar, u = undifferentiated)
9) Race: all or nearest available, otherwise by race (wh or w = white, bl or b = black, hi = hispanic ch = chinese, jap = japanese, haw = hawaiian, w+o = white + oriental, sca = scandinavian, as = asian)
10) Amount smoked "mid" in key scheme 1 (key value 20, maximum range 6-44, in numbers of cigarettes or cigarette equivalents)
11) For overlapping studies: principal rather than subsidiary studies
Finally by Age: whole study (coded as 0) if available, otherwise by widest available age group
and then for single sex results (m, f) in preference to combined sex results (c).

Results adjusted (AD) for the most potential confounders are then chosen in Sections -1 to -3
and results adjusted for the least confounders in Sections -4 to -6. (Those least adjusted results which actually differ from the most adjusted as marked 'x' in column X in Section -4)
(Results adjusted for an unknown number of confounder(s) are coded as 20.)

Section -7 shows excluded studies, together with the stage (as above) at which no qualifying results were found.

Section -8 lists the potentially overlapping studies which have been included (1=principal, 2=subsidiary).

Section -9 lists any results which would have been included in preference except that they had data not complete enough for use in meta-analysis, with their significance (yes/no), if known, and any further comment as entered on the database.

In addition to those mentioned above, the following fields, levels and abbreviations are used:
* or nk = not known, n = no, y = yes, ot = other
nev = never
all/unspec = all or unspecified, cig+/-ot = cigarettes irrespective of other products (cigar, pipe etc)
MC = manufactured cigarettes, HR = hand-rolled cigarettes
exL, exH = range of exposure (low and high) in the smoking group, in terms of Amount smoked, cigarettes or cigarette equivalents
REF: 6-character study reference
NRR: number of the RR on the database within the study
ST : study type (CC = case control, pr or prosp = prospective)
NLC: number of lung cancer cases in whole study
R : risky occupational population (n = no, m = mining, o = other risky)
VB : national cigarette type (V = at least 75% Virginia, bl = at least 75% blended, ot = other)
P : any proxy use
H : full histological confirmation
De : derivation of RR/CI (or = original, st = standard method, ot = other method of estimation)

Table 3G8 - 1

IESLC - Meta-analysis of Current Smoking, Amount smoked, "Mid", Any product (or Cigarettes if Any not available)
Adenocarcinoma
Most adjusted

REF	NRR	SEX	AGEL	AGEH	RACE	YF	LC TYPE	LOC	START	ST	NLC	R	VB	P	H	AD	PRODUCT	exL	exH	DENOM	De
HAMMON	109	m	0	0	wh	0	a	NAmer	1952	pr	448	n	bl	n	n	1	cig only	10	20	nev any	ot
SOBUE	57	m	0	0	all	-	a	As:Jap	1986	CC	1376	n	bl	n	y	0	cig+/-ot	20	29	nev cigs	st
SVENSS	14	f	0	0	all	-	a	Eu:Sca	1983	CC	210	n	bl	n	n	1	all/unsp	11	20	nev any	or
TSUGAN	5	m	0	0	all	-	a	As:Jap	1976	CC	134	n	bl	n	y	0	all/unsp	16	35	nev any	st
WAKAI	53	m	0	0	all	-	a	As:Jap	1988	CC	333	n	bl	n	y	1	cig+/-ot	20	29	nev any	or
WYNDE6	33	m	0	0	all	-	KII	NAmer	1969	CC	4423	n	bl	n	y	0	cig+/-ot	11	20	nev any	st
WYNDE6	222	f	0	0	all	-	KII	NAmer	1969	CC	4423	n	bl	n	y	0	cig+/-ot	11	20	nev cigs	st

Cigarette type is all/unspec for all RRs

Table 3G8 - 2

IESLC - Meta-analysis of Current Smoking, Amount smoked, "Mid", Any product (or Cigarettes if Any not available)
Adenocarcinoma
Most adjusted

REF	NRR	SEX	AD	Number Exposed Case	Number Exposed Cont	Non-exposed Case	Non-exposed Cont	RR	95.00%CI
*HAMMON	109	m	1	-	-	-	-	2.83	(0.55- 14.60)
SOBUE	57	m	0	95	222	27	128	2.03	(1.26- 3.28)
SVENSS	14	f	1	-	-	-	-	5.40	(2.40- 13.20)
TSUGAN	5	m	0	23	23	18	17	0.94	(0.39- 2.28)
WAKAI	53	m	1	-	-	-	-	1.93	(0.84- 4.44)
WYNDE6	33	m	0	191	293	58	617	6.93	(5.01- 9.60)
WYNDE6	222	f	0	176	165	119	856	7.67	(5.76- 10.21)
Subtotal WYNDE6								7.34	(5.92- 9.10)
Partial Totals				485	703	222	1618		

*prospective study

REF	NRR	SEX	AD	Ys	Ws	Qs	Ps
*HAMMON	109	m	1	1.04	1.43	0.50	0.2136
SOBUE	57	m	0	0.71	16.70	14.36	0.0038
SVENSS	14	f	1	1.69	5.29	0.01	0.0001
TSUGAN	5	m	0	-0.06	4.97	14.22	0.8986
WAKAI	53	m	1	0.66	5.54	5.29	0.1216
WYNDE6	33	m	0	1.94	36.35	3.31	0.0000
WYNDE6	222	f	0	2.04	46.92	7.62	0.0000
Subtotal WYNDE6				1.99	83.27	10.93	

	N	7
	NS	6
	Wt	117.19
Het	Chi	45.32
Het	df	6
Het	P	***
Fixed	RR	5.13
	RRl	4.28
	RRu	6.15
	P	+++
Random	RR	3.37
	RRl	1.88
	RRu	6.02
	P	+++
Asymm	P	(*)

Table 3G8 - 3

IESLC - Meta-analysis of Current Smoking, Amount smoked, "Mid", Any product (or Cigarettes if Any not available)
Adenocarcinoma
Most adjusted

	Sex combined	male	female	Total
N		5	2	7
NS		5	2	7
Wt		64.99	52.20	117.19
Het Chi		32.02	0.59	45.32
Het df		4	1	6
Het P		***	N.S.	***
Fixed RR		3.82	7.40	5.13
RRl		2.99	5.65	4.28
RRu		4.87	9.71	6.15
P		+++	+++	+++
Random RR		2.43	7.40	3.37
RRl		1.06	5.65	1.88
RRu		5.57	9.71	6.02
P		+	+++	+++
Between Chi				12.71
Between df				1
Between P				***
Btwn(F) P				N.S.
Btwn(R) P				*

Too few RRs for analysis by factor

Table 3G8 - 4

IESLC - Meta-analysis of Current Smoking, Amount smoked, "Mid", Any product (or Cigarettes if Any not available)
Adenocarcinoma
Least adjusted

REF	NRR	X	SEX	AGEL	AGEH	RACE	YF	LC TYPE	LOC	START	ST	NLC	R	VB	P	H	AD	PRODUCT	exL	exH	DENOM	De
HAMMON	109		m	0	0	wh	0	a	NAmer	1952	pr	448	n	bl	n	n	1	cig only	10	20	nev any	ot
SOBUE	57		m	0	0	all	-	a	As:Jap	1986	CC	1376	n	bl	n	y	0	cig+/-ot	20	29	nev cigs	st
SVENSS	34	x	f	0	0	all	-	a	Eu:Sca	1983	CC	210	n	bl	n	n	0	all/unsp	11	20	nev any	st
TSUGAN	5		m	0	0	all	-	a	As:Jap	1976	CC	134	n	bl	n	y	0	all/unsp	16	35	nev any	st
WAKAI	50	x	m	0	0	all	-	a	As:Jap	1988	CC	333	n	bl	n	y	0	cig+/-ot	20	29	nev any	st
WYNDE6	33		m	0	0	all	-	KII	NAmer	1969	CC	4423	n	bl	n	y	0	cig+/-ot	11	20	nev any	st
WYNDE6	222		f	0	0	all	-	KII	NAmer	1969	CC	4423	n	bl	n	y	0	cig+/-ot	11	20	nev cigs	st

Cigarette type is all/unspec for all RRs

Table 3G8 - 5

IESLC - Meta-analysis of Current Smoking, Amount smoked, "Mid", Any product (or Cigarettes if Any not available)
Adenocarcinoma
Least adjusted

REF	NRR	SEX	AD	Number Exposed Case	Number Exposed Cont	Non-exposed Case	Non-exposed Cont	RR	95.00%CI
*HAMMON	109	m	1	-	-	-	-	2.83	(0.55- 14.60)
SOBUE	57	m	0	95	222	27	128	2.03	(1.26- 3.28)
SVENSS	34	f	0	22	22	22	120	5.45	(2.59- 11.50)
TSUGAN	5	m	0	23	23	18	17	0.94	(0.39- 2.28)
WAKAI	50	m	0	30	129	8	65	1.89	(0.82- 4.35)
WYNDE6	33	m	0	191	293	58	617	6.93	(5.01- 9.60)
WYNDE6	222	f	0	176	165	119	856	7.67	(5.76- 10.21)
Subtotal WYNDE6								7.34	(5.92- 9.10)
Partial Totals				537	854	252	1803		

*prospective study

REF	NRR	SEX	AD	Ys	Ws	Qs	Ps
*HAMMON	109	m	1	1.04	1.43	0.51	0.2136
SOBUE	57	m	0	0.71	16.70	14.38	0.0038
SVENSS	34	f	0	1.70	6.91	0.03	0.0000
TSUGAN	5	m	0	-0.06	4.97	14.23	0.8986
WAKAI	50	m	0	0.64	5.51	5.50	0.1352
WYNDE6	33	m	0	1.94	36.35	3.30	0.0000
WYNDE6	222	f	0	2.04	46.92	7.60	0.0000
Subtotal WYNDE6				1.99	83.27	10.90	

	N	7
	NS	6
	Wt	118.78
Het	Chi	45.53
Het	df	6
Het	P	***
Fixed	RR	5.13
	RRl	4.29
	RRu	6.14
	P	+++
Random	RR	3.38
	RRl	1.90
	RRu	6.00
	P	+++
Asymm	P	(*)

Table 3G8 - 6

IESLC - Meta-analysis of Current Smoking, Amount smoked, "Mid", Any product (or Cigarettes if Any not available)
Adenocarcinoma
Least adjusted

	Sex combined	male	female	Total
N		5	2	7
NS		5	2	7
Wt		64.96	53.83	118.78
Het Chi		32.17	0.70	45.53
Het df		4	1	6
Het P		***	N.S.	***
Fixed RR		3.81	7.34	5.13
RRl		2.99	5.62	4.29
RRu		4.86	9.59	6.14
P		+++	+++	+++
Random RR		2.42	7.34	3.38
RRl		1.05	5.62	1.90
RRu		5.56	9.59	6.00
P		+	+++	+++
Between Chi				12.66
Between df				1
Between P				***
Btwn(F) P				N.S.
Btwn(R) P				*

Table 3G8 - 7

IESLC - Meta-analysis of Current Smoking, Amount smoked, "Mid", Any product (or Cigarettes if Any not available)
Adenocarcinoma
Excluded studies (and stage at which they were excluded)

1 ABELIN ABRAHA AMANDU AMES ANDERS AUSTIN AXELSO BAND BECHER BERRIN BLOHMK BLOT4 BROCKM BROWN1 BYERS1 BYERS2 CARPEN CASCO2 CASCOR CHAN CHEN3 CHIAZZ CHYOU DESTE2 DOCKER DROSTE DU GARCIA GARDIN GENG GODLEY GOODMA GRAHAM GREGOR HEGMAN HEIN HENNEK HINDS HIRAOK HOROWI HORWIT HUANG ISHIMA JAHN JAIN JARVHO JIANG KELLER KIHARA KJUUS KO KOHLME KUBIK LAMWK LAMWK2 LANGE LEI LEMARC LEVIN LIU LOMBA2 LOMBAR MAGNUS MARSH MARSH2 MCDUFF MCLAUG MILLER MILLS NOTANI NOU ODRISC PAWLEG PERSHA POFFIJ QIAO QIAO2 RADZIK REN RONCO ROOTS ROTHSC SAARIK SANKAR SCHWAR SEGI SEOW SHIMIZ SIMARA SIMONA SITAS SOBUE2 STASZE STAYNE STUCKE SUN SUZUK2 SUZUKI TANG TAO TOKARS TOUSEY ULMER VEIERO VUTUC WALD WANG WANG3 WANG4 WICKLU WIGLE WILKIN WU2 WUNSCH WYNDE8 XIANGZ XU XU2 XU4 YONG ZHANG

2 AGUDO ALDERS ARMADA AUVINE BARBON BENHAM BLOT1 BLOT2 BLOT3 BOFFET BOUCHA BRESLO BROWN2 BUELL CHATZI CHEN CHEN2 CHOI COOKSO DAMBER DAVEYS DEAN DEAN2 DOLL DOSEME DUNN EBELIN ESAKI FAN GAO GARSHI GER GOLLED GSELL HANSEN HU HU2 JARUP JEDRYC JOLY JONES JUSSAW KHUDER KOULUM KREUZE KREYBE LAMTH LAUSSM LETOUR LIU2 LIU3 LIU4 LIU5 LUBIN LUBIN2 LUO MARTIS MASTRA MATSUD MCCONN MOLLO MZILEN NOTAN2 ORMOS OSANN OSANN2 PASTOR PERNU PIKE POLEDN RACHTA RANDIG RESTRE SADOWS SCHWA2 SIEMIA SPITZ STOCKS TIZZAN VANDER WANG2 WUWILL WYNDE2 WYNDE3 WYNDE4 XU3 YUAN ZHENG ZHOU

3 PISANI

4 WYNDE7

5 RIMING TANG2 WYNDE5

6 DESTEF HIRAY2 LAURIL LICKIN MRFIT MURATA WARSIN WATSON WYNDER

8 AKIBA ARCHER AXELSS BENSHL BEST BRETT BROSS BUFFLE CEDERL CHANG CHOW COMSTO CPSI CPSII DARBY DEAN3 DEKLER DOLL2 DORANT DORGAN DORN ENSTRO GAO2 GILLIS HAMMO2 HIRAYA HITOSU HOLE HUMBLE KAISE2 KAISER KANELL KAUFMA KINLEN KNEKT KOO LIAW LIDDEL MACLEN MATOS MIGRAN MRFITR NAM PARKIN PERSH2 PETO PEZZO2 PEZZOT PRESCO SEGI2 SHAW SPEIZE STOCKW TENKAN TULINI TVERDA YAMAGU

10 BOUCOT CORREA ENGELA HAENSZ KATSOU WU

Table 3G8 - 8
Potentially overlapping studies

REF	REFGP	PRINC	.	OVERLAP/LINK
WYNDE6	WYNDE6	1		WYNDE5/6/7/8

Table 3G8 - 9
Most adjusted - insufficient data for metaanalysis

REF	NRR	SEX	AGEL	AGEH	RACE	YF	LC TYPE	LOC	START	ST	NLC	R	VB	P	H	AD	PRODUCT	exL	exH	DENOM	De
GILLIS	18	m	0	0	all	-	a	Eu:UK	1977	CC	656	n	V	n	n	3	cig+/-ot	15	24	nev any ot	

REF	NRR	RR	SIG	RRDATA comment
GILLIS	18	1.44		0

Table 3G9 -

IESLC - Meta-analysis of Current Smoking, Amount smoked, "High", Any product (or Cigarettes if Any not available)
Adenocarcinoma

This analysis is restricted to results for:
1) Results by Amount smoked
2) Current smokers
3) Results complete enough for use in metaanalysis

Within each study, results are then selected (in the following order of preference, within each sex) for:
4) PRODUCT: all/unspec, cigarettes regardless of other products, cigarettes only
5) CIGTYPE: all/unspecified, MC regardless of HR, MC only
6) DENOM: never smoked anything, never smoked cigarettes, (never +1 = +long term ex, +2 = +amount unknown,
+3 = never cigs+long term ex)
7) Followup period (YF, prospective studies): whole study (coded as 0) or longest available
8) LCtype: adeno or nearest available, but not squamous. (q = squamous, s = small,
a = adeno, l = large, KII = Kreyberg II, al = alveolar, br = bronchiolar, u = undifferentiated)
9) Race: all or nearest available, otherwise by race (wh or w = white, bl or b = black, hi = hispanic
ch = chinese, jap = japanese, haw = hawaiian, w+o = white + oriental, sca = scandinavian, as = asian)
10) Amount smoked "high" in key scheme 1 (key value 45, maximum range >20, in numbers of
cigarettes or cigarette equivalents)
11) For overlapping studies: principal rather than subsidiary studies
Finally by Age: whole study (coded as 0) if available, otherwise by widest available age group
and then for single sex results (m, f) in preference to combined sex results (c).

Results adjusted (AD) for the most potential confounders are then chosen in Sections -1 to -3
and results adjusted for the least confounders in Sections -4 to -6. (Those least adjusted results which
actually differ from the most adjusted as marked 'x' in column X in Section -4)
(Results adjusted for an unknown number of confounder(s) are coded as 20.)

Section -7 shows excluded studies, together with the stage (as above) at which no qualifying
results were found.

Section -8 lists the potentially overlapping studies which have been included (1=principal, 2=subsidiary).

Section -9 lists any results which would have been included in preference except that they had data not complete
enough for use in meta-analysis, with their significance (yes/no), if known, and any further comment as entered
on the database.

In addition to those mentioned above, the following fields, levels and abbreviations are used:
* or nk = not known, n = no, y = yes, ot = other
nev = never
all/unspec = all or unspecified, cig+/-ot = cigarettes irrespective of other products (cigar, pipe etc)
MC = manufactured cigarettes, HR = hand-rolled cigarettes
exL, exH = range of exposure (low and high) in the smoking group, in terms of Amount smoked,
cigarettes or cigarette equivalents
REF: 6-character study reference
NRR: number of the RR on the database within the study
ST : study type (CC = case control, pr or prosp = prospective)
NLC: number of lung cancer cases in whole study
R : risky occupational population (n = no, m = mining, o = other risky)
VB : national cigarette type (V = at least 75% Virginia, bl = at least 75% blended, ot = other)
P : any proxy use
H : full histological confirmation
De : derivation of RR/CI (or = original, st = standard method, ot = other method of estimation)

Table 3G9 - 1

IESLC - Meta-analysis of Current Smoking, Amount smoked, "High", Any product (or Cigarettes if Any not available)
Adenocarcinoma
Most adjusted

REF	NRR	SEX	AGEL	AGEH	RACE	YF	LC TYPE	LOC	START	ST	NLC	R	VB	P	H	AD	PRODUCT	exL	exH	DENOM	De
BOUCOT	149	m	0	0	all	0	a	NAmer	1951	pr	121	n	bl	n	n	2	cig only	21	99	nev any	ot
CORREA	52	c	0	0	all	-	a	NAmer	1979	CC	1359	n	bl	y	n	1	cig+/-ot	21	99	nev cigs	or
HAENSZ	34	f	0	0	all	-	a	NAmer	1955	CC	158	n	bl	n	y	0	cig+/-ot	21	99	nev any	or
KATSOU	14	f	0	0	all	-	a	Eu:bal	1987	CC	101	n	bl	n	n	1	all/unsp	21	99	nev any	or
SOBUE	58	m	0	0	all	-	a	As:Jap	1986	CC	1376	n	bl	n	y	0	cig+/-ot	30	99	nev cigs	st
SVENSS	19	f	0	0	all	-	a	Eu:Sca	1983	CC	210	n	bl	n	n	1	all/unsp	21	99	nev any	ot
TSUGAN	6	m	0	0	all	-	a	As:Jap	1976	CC	134	n	bl	n	y	0	all/unsp	36	99	nev any	st
WAKAI	54	m	0	0	all	-	a	As:Jap	1988	CC	333	n	bl	n	y	1	cig+/-ot	30	99	nev any	or
WU	9	f	0	0	wh	-	a	NAmer	1981	CC	220	n	bl	n	y	2	all/unsp	21	99	nev any	or
WYNDE6	51	m	0	0	all	-	KII	NAmer	1969	CC	4423	n	bl	n	y	0	cig+/-ot	31	99	nev any	st
WYNDE6	240	f	0	0	all	-	KII	NAmer	1969	CC	4423	n	bl	n	y	0	cig+/-ot	30	99	nev cigs	st

Cigarette type is all/unspec for all RRs

Table 3G9 - 2

IESLC - Meta-analysis of Current Smoking, Amount smoked, "High", Any product (or Cigarettes if Any not available)
Adenocarcinoma
Most adjusted

REF	NRR	SEX	AD	Number Exposed Case	Number Exposed Cont	Non-exposed Case	Non-exposed Cont	RR	95.00%CI
*BOUCOT	149	m	2	-	-	-	-	18.00	(1.01- 319.52)
CORREA	52	c	1	-	-	-	-	12.00	(7.30- 19.70)
HAENSZ	34	f	0	5	13	37	236	2.45	(0.83- 7.28)
KATSOU	14	f	1	-	-	-	-	2.95	(0.76- 11.41)
SOBUE	58	m	0	76	187	27	128	1.93	(1.18- 3.16)
SVENSS	19	f	1	-	-	-	-	19.70	(1.70- 228.29)
TSUGAN	6	m	0	10	13	18	17	0.73	(0.25- 2.09)
WAKAI	54	m	1	-	-	-	-	4.53	(1.89- 10.90)
WU	9	f	2	-	-	-	-	6.50	(3.10- 13.90)
WYNDE6	51	m	0	282	197	58	617	15.23	(11.00- 21.07)
WYNDE6	240	f	0	157	52	119	856	21.72	(15.04- 31.37)
Subtotal WYNDE6								17.79	(13.95- 22.70)
Partial Totals				530	462	259	1854		

*prospective study

REF	NRR	SEX	AD	Ys	Ws	Qs	Ps
*BOUCOT	149	m	2	2.89	0.46	0.20	0.0491
CORREA	52	c	1	2.48	15.59	0.98	0.0000
HAENSZ	34	f	0	0.90	3.24	5.79	0.1060
KATSOU	14	f	1	1.08	2.09	2.78	0.1175
SOBUE	58	m	0	0.66	15.78	39.30	0.0092
SVENSS	19	f	1	2.98	0.64	0.36	0.0171
TSUGAN	6	m	0	-0.32	3.43	22.38	0.5538
WAKAI	54	m	1	1.51	5.00	2.62	0.0007
WU	9	f	2	1.87	6.82	0.89	0.0000
WYNDE6	51	m	0	2.72	36.38	8.71	0.0000
WYNDE6	240	f	0	3.08	28.43	20.27	0.0000
Subtotal WYNDE6				2.88	64.82	28.98	

	N	11
	NS	10
	Wt	117.90
Het	Chi	104.29
Het	df	10
Het	P	***
Fixed	RR	9.34
	RRl	7.79
	RRu	11.18
	P	+++
Random	RR	5.71
	RRl	2.91
	RRu	11.19
	P	+++
Asymm	P	N.S.

Table 3G9 - 3

IESLC - Meta-analysis of Current Smoking, Amount smoked, "High", Any product (or Cigarettes if Any not available)
Adenocarcinoma
Most adjusted

		Sex		
	combined	male	female	Total
N	1	5	5	11
NS	1	5	5	11
Wt	15.59	61.07	41.24	117.90
Het Chi	0.00	67.19	24.44	104.29
Het df	0	4	4	10
Het P	N.S.	***	***	***
Fixed RR	12.00	6.82	13.52	9.34
RRl	7.30	5.31	9.96	7.79
RRu	19.71	8.76	18.34	11.18
P	+++	+++	+++	+++
Random RR	12.00	3.94	7.09	5.71
RRl	7.30	1.08	2.53	2.91
RRu	19.71	14.39	19.84	11.19
P	+++	+	+++	+++
Between Chi				12.66
Between df				2
Between P				**
Btwn(F) P				N.S.
Btwn(R) P				N.S.

Table 3G9 - 4

IESLC - Meta-analysis of Current Smoking, Amount smoked, "High", Any product (or Cigarettes if Any not available)
Adenocarcinoma
Least adjusted

REF	NRR	X	SEX	AGEL	AGEH	RACE	YF	LC TYPE	LOC	START	ST	NLC	R	VB	P	H	AD	PRODUCT	exL	exH	DENOM	De
BOUCOT	28	x	m	0	0	all	0	a	NAmer	1951	pr	121	n	bl	n	n	0	cig only	21	99	nev any	ot
CORREA	52		c	0	0	all	-	a	NAmer	1979	CC	1359	n	bl	y	n	1	cig+/-ot	21	99	nev cigs	or
HAENSZ	34		f	0	0	all	-	a	NAmer	1955	CC	158	n	bl	n	y	0	cig+/-ot	21	99	nev any	or
KATSOU	18	x	f	0	0	all	-	a	Eu:bal	1987	CC	101	n	bl	n	n	0	all/unsp	21	99	nev any	st
SOBUE	58		m	0	0	all	-	a	As:Jap	1986	CC	1376	n	bl	n	y	0	cig+/-ot	30	99	nev cigs	st
SVENSS	39	x	f	0	0	all	-	a	Eu:Sca	1983	CC	210	n	bl	n	n	0	all/unsp	21	99	nev any	st
TSUGAN	6		m	0	0	all	-	a	As:Jap	1976	CC	134	n	bl	n	y	0	all/unsp	36	99	nev any	st
WAKAI	51	x	m	0	0	all	-	a	As:Jap	1988	CC	333	n	bl	n	y	0	cig+/-ot	30	99	nev any	st
WU	4	x	f	0	0	wh	-	a	NAmer	1981	CC	220	n	bl	n	y	0	all/unsp	21	99	nev any	st
WYNDE6	51		m	0	0	all	-	KII	NAmer	1969	CC	4423	n	bl	n	y	0	cig+/-ot	31	99	nev any	st
WYNDE6	240		f	0	0	all	-	KII	NAmer	1969	CC	4423	n	bl	n	y	0	cig+/-ot	30	99	nev cigs	st

Cigarette type is all/unspec for all RRs

Table 3G9 - 5

IESLC - Meta-analysis of Current Smoking, Amount smoked, "High", Any product (or Cigarettes if Any not available)
Adenocarcinoma
Least adjusted

REF	NRR	SEX	AD	Number Exposed Case	Number Exposed Cont	Non-exposed Case	Non-exposed Cont	RR	95.00%CI
*BOUCOT	28	m	0	6	6940	0	7551	14.14~	(0.80- 251.03)
CORREA	52	c	1	-	-	-	-	12.00	(7.30- 19.70)
HAENSZ	34	f	0	5	13	37	236	2.45	(0.83- 7.28)
KATSOU	18	f	0	6	4	30	67	3.35	(0.88- 12.75)
SOBUE	58	m	0	76	187	27	128	1.93	(1.18- 3.16)
SVENSS	39	f	0	4	1	22	120	21.82	(2.33- 204.53)
TSUGAN	6	m	0	10	13	18	17	0.73	(0.25- 2.09)
WAKAI	51	m	0	27	48	8	65	4.57	(1.91- 10.94)
WU	4	f	0	61	22	29	62	5.93	(3.07- 11.44)
WYNDE6	51	m	0	282	197	58	617	15.23	(11.00- 21.07)
WYNDE6	240	f	0	157	52	119	856	21.72	(15.04- 31.37)
Subtotal WYNDE6								17.79	(13.95- 22.70)
Partial Totals				634	7477	348	9719		

*prospective study ~ With 0.5 adjustment for zero

REF	NRR	SEX	AD	Ys	Ws	Qs	Ps
*BOUCOT	28	m	0	2.65	0.46	0.08	0.0710
CORREA	52	c	1	2.48	15.59	1.07	0.0000
HAENSZ	34	f	0	0.90	3.24	5.70	0.1060
KATSOU	18	f	0	1.21	2.15	2.21	0.0762
SOBUE	58	m	0	0.66	15.78	38.77	0.0092
SVENSS	39	f	0	3.08	0.77	0.57	0.0069
TSUGAN	6	m	0	-0.32	3.43	22.19	0.5538
WAKAI	51	m	0	1.52	5.04	2.50	0.0006
WU	4	f	0	1.78	8.89	1.75	0.0000
WYNDE6	51	m	0	2.72	36.38	9.10	0.0000
WYNDE6	240	f	0	3.08	28.43	20.79	0.0000
Subtotal WYNDE6				2.88	64.82	29.88	

	N	11
	NS	10
	Wt	120.19
Het	Chi	104.73
Het	df	10
Het	P	***
Fixed	RR	9.24
	RRl	7.72
	RRu	11.04
	P	+++
Random	RR	5.73
	RRl	2.95
	RRu	11.14
	P	+++
Asymm	P	N.S.

Table 3G9 - 6

IESLC - Meta-analysis of Current Smoking, Amount smoked, "High", Any product (or Cigarettes if Any not available)
Adenocarcinoma
Least adjusted

	Sex combined	male	female	Total
N	1	5	5	11
NS	1	5	5	11
Wt	15.59	61.11	43.49	120.19
Het Chi	0.00	66.97	26.15	104.73
Het df	0	4	4	10
Het P	N.S.	***	***	***
Fixed RR	12.00	6.81	12.90	9.24
RRl	7.30	5.30	9.59	7.72
RRu	19.71	8.75	17.37	11.04
P	+++	+++	+++	+++
Random RR	12.00	3.85	7.26	5.73
RRl	7.30	1.06	2.66	2.95
RRu	19.71	14.00	19.78	11.14
P	+++	+	+++	+++
Between Chi				11.61
Between df				2
Between P				**
Btwn(F) P				N.S.
Btwn(R) P				N.S.

Table 3G9 - 7

IESLC - Meta-analysis of Current Smoking, Amount smoked, "High", Any product (or Cigarettes if Any not available)
Adenocarcinoma
Excluded studies (and stage at which they were excluded)

1 ABELIN ABRAHA AMANDU AMES ANDERS AUSTIN AXELSO BAND BECHER BERRIN BLOHMK BLOT4 BROCKM BROWN1 BYERS1 BYERS2
CARPEN CASCO2 CASCOR CHAN CHEN3 CHIAZZ CHYOU DESTE2 DOCKER DROSTE DU GARCIA GARDIN GENG GODLEY GOODMA
GRAHAM GREGOR HEGMAN HEIN HENNEK HINDS HIRAOK HOROWI HORWIT HUANG ISHIMA JAHN JAIN JARVHO JIANG KELLER
KIHARA KJUUS KO KOHLME KUBIK LAMWK LAMWK2 LANGE LEI LEMARC LEVIN LIU LOMBA2 LOMBAR MAGNUS MARSH
MARSH2 MCDUFF MCLAUG MILLER MILLS NOTANI NOU ODRISC PAWLEG PERSHA POFFIJ QIAO QIAO2 RADZIK REN RONCO
ROOTS ROTHSC SAARIK SANKAR SCHWAR SEGI SEOW SHIMIZ SIMARA SIMONA SITAS SOBUE2 STASZE STAYNE STUCKE SUN
SUZUK2 SUZUKI TANG TAO TOKARS TOUSEY ULMER VEIERO VUTUC WALD WANG WANG3 WANG4 WICKLU WIGLE WILKIN
WU2 WUNSCH WYNDE8 XIANGZ XU XU2 XU4 YONG ZHANG

2 AGUDO ALDERS ARMADA AUVINE BARBON BENHAM BLOT1 BLOT2 BLOT3 BOFFET BOUCHA BRESLO BROWN2 BUELL CHATZI CHEN
CHEN2 CHOI COOKSO DAMBER DAVEYS DEAN DEAN2 DOLL DOSEME DUNN EBELIN ESAKI FAN GAO GARSHI GER
GOLLED GSELL HANSEN HU HU2 JARUP JEDRYC JOLY JONES JUSSAW KHUDER KOULUM KREUZE KREYBE LAMTH LAUSSM
LETOUR LIU2 LIU3 LIU4 LIU5 LUBIN LUBIN2 LUO MARTIS MASTRA MATSUD MCCONN MOLLO MZILEN NOTAN2 ORMOS
OSANN OSANN2 PASTOR PERNU PIKE POLEDN RACHTA RANDIG RESTRE SADOWS SCHWA2 SIEMIA SPITZ STOCKS TIZZAN VANDER
WANG2 WUWILL WYNDE2 WYNDE3 WYNDE4 XU3 YUAN ZHENG ZHOU

3 PISANI

4 WYNDE7

5 RIMING TANG2 WYNDE5

6 DESTEF HIRAY2 LAURIL LICKIN MRFIT MURATA WARSIN WATSON WYNDER

8 AKIBA ARCHER AXELSS BENSHL BEST BRETT BROSS BUFFLE CEDERL CHANG CHOW COMSTO CPSI CPSII DARBY DEAN3
DEKLER DOLL2 DORANT DORGAN DORN ENSTRO GAO2 GILLIS HAMMO2 HIRAYA HITOSU HOLE HUMBLE KAISE2 KAISER KANELL
KAUFMA KINLEN KNEKT KOO LIAW LIDDEL MACLEN MATOS MIGRAN MRFITR NAM PARKIN PERSH2 PETO PEZZO2 PEZZOT
PRESCO SEGI2 SHAW SPEIZE STOCKW TENKAN TULINI TVERDA YAMAGU

10 ENGELA HAMMON

Table 3G9 - 8

Potentially overlapping studies

REF	REFGP	PRINC	.	OVERLAP/LINK
WYNDE6	WYNDE6	1		WYNDE5/6/7/8

Table 3G9 - 9

Most adjusted - insufficient data for metaanalysis

REF	NRR	SEX	AGEL	AGEH	RACE	YF	LC TYPE	LOC	START	ST	NLC	R	VB	P	H	AD	PRODUCT	exL	exH	DENOM	De
GILLIS	20	m	0	0	all	-	a	Eu:UK	1977	CC	656	n	V	n	n	3	cig+/-ot	35	99	nev any ot	

REF	NRR	RR	SIG	RRDATA comment
GILLIS	20	2.65		0

Table 3G11 -

IESLC - Meta-anal of Ever Smoking (or Curr if Ever not avail) by Amount, Overview, Any prod (or Cigs if Any not avail)
Adenocarcinoma

This analysis is restricted to results for:
1) Results by Amount smoked
Results by Amount smoked (in numbers of cigarettes or cigarette equivalents) are grouped under 2 schemes (S1, S2).
Each scheme has a set of "key values". An interval is allocated to the category whose key value
it includes and intervals which include none or more than one of the key values are excluded.
(Open-ended intervals are coded as 99.)

S1	key value	maximum range	S2	key value	maximum range
1	5	1-19	1	1	1-9
2	20	6-44	2	10	2-19
3	45	21+	3	20	11-29
			4	30	21-39
			5	40	31-98
			6	99	41+

For all/unspec product, the definition of cigarette equivalents is shown at the end of Sections -1 and -4.
2) Results complete enough for use in metaanalysis

Within each study, results are then selected (in the following order of preference, within each sex) for:
3) SMKSTA: ever smokers, current smokers
4) PRODUCT: all/unspec, cigarettes regardless of other products, cigarettes only
5) CIGTYPE: all/unspecified, MC regardless of HR, MC only
6) DENOM: never smoked anything, never smoked cigarettes, (never +1 = +long term ex, +2 = +amount unknown,
+3 = never cigs+long term ex)
7) Followup period (YF, prospective studies): whole study (coded as 0) or longest available
8) LCtype: adeno or nearest available, but not squamous. (q = squamous, s = small,
a = adeno, l = large, KII = Kreyberg II, al = alveolar, br = bronchiolar, u = undifferentiated)
9) Race: all or nearest available, otherwise by race (wh or w = white, bl or b = black, hi = hispanic
ch = chinese, jap = japanese, haw = hawaiian, w+o = white + oriental, sca = scandinavian, as = asian)
10) For overlapping studies: principal rather than subsidiary studies
Finally by Age: whole study (coded as 0) if available, otherwise by widest available age group
and then for single sex results (m, f) in preference to combined sex results (c).

Results adjusted (AD) for the most potential confounders are then chosen in Sections -1 to -3
and results adjusted for the least confounders in Sections -4 to -6. (Those least adjusted results which
actually differ from the most adjusted as marked 'x' in column X in Section -4)
(Results adjusted for an unknown number of confounder(s) are coded as 20.)

Section -7 shows excluded studies, together with the stage (as above) at which no qualifying
results were found.

Section -8 lists the potentially overlapping studies which have been included (1=principal, 2=subsidiary).

Section -9 lists any results which would have been included in preference except that they had data not complete
enough for use in meta-analysis, with their significance (yes/no), if known, and any further comment as entered
on the database.

In addition to those mentioned above, the following fields, levels and abbreviations are used:
* or nk = not known, n = no, y = yes, ot = other
ev = ever, cu = current, nev = never
all/unspec = all or unspecified, cig+/-ot = cigarettes irrespective of other products (cigar, pipe etc)
MC = manufactured cigarettes, HR = hand-rolled cigarettes
exL, exH = range of exposure (low and high) in the smoking group, in terms of Amount smoked,
cigarettes or cigarette equivalents
REF: 6-character study reference
NRR: number of the RR on the database within the study
ST : study type (CC = case control, pr or prosp = prospective)
NLC: number of lung cancer cases in whole study
R : risky occupational population (n = no, m = mining, o = other risky)
VB : national cigarette type (V = at least 75% Virginia, bl = at least 75% blended, ot = other)
P : any proxy use
H : full histological confirmation
De : derivation of RR/CI (or = original, st = standard method, ot = other method of estimation)

Table 3G11 - 1

IESLC - Meta-anal of Ever Smoking (or Curr if Ever not avail) by Amount, Overview, Any prod (or Cigs if Any not avail)
Adenocarcinoma
Most adjusted

REF	NRR	SEX	AGEL	AGEH	RACE	YF	LC TYPE	LOC	START	ST	NLC	R	VB	P	H	AD	SM	PRODUCT	exL	exH	S1	S2	DENOM	De
ALDERS	46	m	0	0	all	-	not q+s	Eu:UK	1977	CC	1448	n	V	n	n	1	ev	cig only	1	17	1	0	nev+2	ot
ALDERS	47	m	0	0	all	-	not q+s	Eu:UK	1977	CC	1448	n	V	n	n	1	ev	cig only	18	27	2	3	nev+2	ot
ALDERS	48	m	0	0	all	-	not q+s	Eu:UK	1977	CC	1448	n	V	n	n	1	ev	cig only	28	99	3	0	nev+2	ot
ALDERS	49	f	0	0	all	-	not q+s	Eu:UK	1977	CC	1448	n	V	n	n	1	ev	cig only	1	17	1	0	nev+2	ot
ALDERS	50	f	0	0	all	-	not q+s	Eu:UK	1977	CC	1448	n	V	n	n	1	ev	cig only	18	27	2	3	nev+2	ot
ALDERS	51	f	0	0	all	-	not q+s	Eu:UK	1977	CC	1448	n	V	n	n	1	ev	cig only	28	99	3	0	nev+2	ot
BARBON	79	m	0	0	all	-	a	Eu:wst	1979	CC	755	n	bl	y	y	3	ev	all/unsp	1	19	1	0	nev any	or
BARBON	80	m	0	0	all	-	a	Eu:wst	1979	CC	755	n	bl	y	y	3	ev	all/unsp	20	39	2	0	nev any	or
BARBON	81	m	0	0	all	-	a	Eu:wst	1979	CC	755	n	bl	y	y	3	ev	all/unsp	40	99	3	0	nev any	or
BOUCOT	148	m	0	0	all	0	a	NAmer	1951	pr	121	n	bl	n	n	2	cu	cig only	1	20	0	0	nev any	ot
BOUCOT	149	m	0	0	all	0	a	NAmer	1951	pr	121	n	bl	n	n	2	cu	cig only	21	99	3	0	nev any	ot
BROWN2	34	m	0	0	wh	-	a	NAmer	1984	CC	14596	n	bl	n	y	2	ev	cig+/-ot	1	19	1	0	nev cigs	or
BROWN2	44	m	0	0	wh	-	a	NAmer	1984	CC	14596	n	bl	n	y	2	ev	cig+/-ot	20	99	0	0	nev cigs	or
BROWN2	33	f	0	0	wh	-	a	NAmer	1984	CC	14596	n	bl	n	y	2	ev	cig+/-ot	1	19	1	0	nev cigs	or
BROWN2	43	f	0	0	wh	-	a	NAmer	1984	CC	14596	n	bl	n	y	2	ev	cig+/-ot	20	99	0	0	nev cigs	or
CHOI	51	m	0	0	all	-	a	As:oth	1985	CC	375	n	bl	n	n	0	ev	cig+/-ot	1	10	1	0	nev cigs	st
CHOI	52	m	0	0	all	-	a	As:oth	1985	CC	375	n	bl	n	n	0	ev	cig+/-ot	11	20	2	3	nev cigs	st
CHOI	53	m	0	0	all	-	a	As:oth	1985	CC	375	n	bl	n	n	0	ev	cig+/-ot	21	30	0	4	nev cigs	st
CHOI	54	m	0	0	all	-	a	As:oth	1985	CC	375	n	bl	n	n	0	ev	cig+/-ot	31	40	0	5	nev cigs	st
CHOI	55	m	0	0	all	-	a	As:oth	1985	CC	375	n	bl	n	n	0	ev	cig+/-ot	41	99	3	6	nev cigs	st
CHOI	59	f	0	0	all	-	a	As:oth	1985	CC	375	n	bl	n	n	0	ev	cig+/-ot	1	10	1	0	nev cigs	st
CHOI	60	f	0	0	all	-	a	As:oth	1985	CC	375	n	bl	n	n	0	ev	cig+/-ot	11	30	2	0	nev cigs	ot
CHOI	61	f	0	0	all	-	a	As:oth	1985	CC	375	n	bl	n	n	0	ev	cig+/-ot	31	99	3	0	nev cigs	st
CORREA	48	c	0	0	all	-	a	NAmer	1979	CC	1359	n	bl	y	n	1	cu	cig+/-ot	1	20	0	0	nev cigs	or
CORREA	52	c	0	0	all	-	a	NAmer	1979	CC	1359	n	bl	y	n	1	cu	cig+/-ot	21	99	3	0	nev cigs	or
DOLL	72	m	0	0	all	-	KII	Eu:UK	1948	CC	1465	n	V	n	n	1	ev	all/unsp	1	4	0	1	nev any	ot
DOLL	73	m	0	0	all	-	KII	Eu:UK	1948	CC	1465	n	V	n	n	1	ev	all/unsp	5	14	1	2	nev any	ot
DOLL	74	m	0	0	all	-	KII	Eu:UK	1948	CC	1465	n	V	n	n	1	ev	all/unsp	15	24	2	3	nev any	ot
DOLL	75	m	0	0	all	-	KII	Eu:UK	1948	CC	1465	n	V	n	n	1	ev	all/unsp	25	99	3	0	nev any	ot
DOLL	79	f	0	0	all	-	KII	Eu:UK	1948	CC	1465	n	V	n	n	1	ev	all/unsp	1	4	0	1	nev any	ot
DOLL	80	f	0	0	all	-	KII	Eu:UK	1948	CC	1465	n	V	n	n	1	ev	all/unsp	5	14	1	2	nev any	ot
DOLL	81	f	0	0	all	-	KII	Eu:UK	1948	CC	1465	n	V	n	n	1	ev	all/unsp	15	99	0	0	nev any	ot
DORGAN	126	m	0	0	wh	-	a	NAmer	1980	CC	2026	n	bl	y	y	2	ev	cig+/-ot	1	19	1	0	nev any	ot
DORGAN	127	m	0	0	wh	-	a	NAmer	1980	CC	2026	n	bl	y	y	2	ev	cig+/-ot	20	99	0	0	nev any	ot
DORGAN	105	f	0	0	all	-	a	NAmer	1980	CC	2026	n	bl	y	y	3	ev	cig+/-ot	1	19	1	0	nev any	ot
DORGAN	106	f	0	0	all	-	a	NAmer	1980	CC	2026	n	bl	y	y	3	ev	cig+/-ot	20	99	0	0	nev any	ot
DOSEME	8	m	0	0	all	-	not q+s	Eu:bal	1979	CC	1210	n	bl	n	n	2	ev	cig+/-ot	1	10	1	0	nev cigs	or
DOSEME	12	m	0	0	all	-	not q+s	Eu:bal	1979	CC	1210	n	bl	n	n	2	ev	cig+/-ot	11	20	2	3	nev cigs	or
DOSEME	16	m	0	0	all	-	not q+s	Eu:bal	1979	CC	1210	n	bl	n	n	2	ev	cig+/-ot	21	99	3	0	nev cigs	or
ENGELA	71	m	0	0	all	0	a	Eu:Sca	1964	pr	435	n	bl	n	n	7	cu	cig+/-ot	1	4	0	1	nev cigs	or
ENGELA	72	m	0	0	all	0	a	Eu:Sca	1964	pr	435	n	bl	n	n	7	cu	cig+/-ot	5	9	1	0	nev cigs	or
ENGELA	73	m	0	0	all	0	a	Eu:Sca	1964	pr	435	n	bl	n	n	7	cu	cig+/-ot	10	14	0	2	nev cigs	or
ENGELA	74	m	0	0	all	0	a	Eu:Sca	1964	pr	435	n	bl	n	n	7	cu	cig+/-ot	15	19	0	0	nev cigs	or
ENGELA	75	m	0	0	all	0	a	Eu:Sca	1964	pr	435	n	bl	n	n	7	cu	cig+/-ot	20	99	0	0	nev cigs	or
GER	10	c	0	0	all	-	a	As:oth	1990	CC	141	n	ot	y	n	8	ev	all/unsp	1	10	1	0	nev any	ot
GER	11	c	0	0	all	-	a	As:oth	1990	CC	141	n	ot	y	n	8	ev	all/unsp	11	20	2	3	nev any	ot
GER	12	c	0	0	all	-	a	As:oth	1990	CC	141	n	ot	y	n	8	ev	all/unsp	21	99	3	0	nev any	ot
HAENSZ	35	f	0	0	all	-	a	NAmer	1955	CC	158	n	bl	n	y	0	cu	cig+/-ot	1	20	0	0	nev any	or
HAENSZ	34	f	0	0	all	-	a	NAmer	1955	CC	158	n	bl	n	y	0	cu	cig+/-ot	21	99	3	0	nev any	or
HAMMON	108	m	0	0	wh	0	a	NAmer	1952	pr	448	n	bl	n	n	1	cu	cig only	1	9	1	1	nev any	ot
HAMMON	109	m	0	0	wh	0	a	NAmer	1952	pr	448	n	bl	n	n	1	cu	cig only	10	20	2	0	nev any	ot
HAMMON	110	m	0	0	wh	0	a	NAmer	1952	pr	448	n	bl	n	n	1	cu	cig only	21	39	0	4	nev any	ot
JEDRYC	36	m	0	0	all	-	a	Eu:est	1980	CC	1630	n	bl	y	n	3	ev	cig+/-ot	1	19	1	0	nev any	or
JEDRYC	37	m	0	0	all	-	a	Eu:est	1980	CC	1630	n	bl	y	n	3	ev	cig+/-ot	20	29	2	3	nev any	or
JEDRYC	38	m	0	0	all	-	a	Eu:est	1980	CC	1630	n	bl	y	n	3	ev	cig+/-ot	30	99	3	0	nev any	or
KATSOU	13	f	0	0	all	-	a	Eu:bal	1987	CC	101	n	bl	n	n	1	cu	all/unsp	1	20	0	0	nev any	or
KATSOU	14	f	0	0	all	-	a	Eu:bal	1987	CC	101	n	bl	n	n	1	cu	all/unsp	21	99	3	0	nev any	or
KREYBE	5	m	0	0	all	-	KII	Eu:Sca	1948	CC	300	n	bl	n	y	1	ev	all/unsp	1	14	1	0	nev any	ot
KREYBE	6	m	0	0	all	-	KII	Eu:Sca	1948	CC	300	n	bl	n	y	1	ev	all/unsp	15	24	2	3	nev any	ot
KREYBE	7	m	0	0	all	-	KII	Eu:Sca	1948	CC	300	n	bl	n	y	1	ev	all/unsp	25	99	3	0	nev any	ot
KREYBE	26	f	0	0	all	-	KII	Eu:Sca	1948	CC	300	n	bl	n	y	1	ev	all/unsp	1	14	1	0	nev any	ot
KREYBE	61	f	0	0	all	-	KII	Eu:Sca	1948	CC	300	n	bl	n	y	1	ev	all/unsp	15	99	0	0	nev any	ot
LAMTH	16	f	0	0	ch	-	a	As:HK	1983	CC	445	n	bl	n	n	0	ev	all/unsp	1	10	1	0	nev any	or
LAMTH	17	f	0	0	ch	-	a	As:HK	1983	CC	445	n	bl	n	n	0	ev	all/unsp	11	20	2	3	nev any	or
LAMTH	18	f	0	0	ch	-	a	As:HK	1983	CC	445	n	bl	n	n	0	ev	all/unsp	21	99	3	0	nev any	or
LUBIN2	152	m	0	0	all	-	a	Eu:mul	1976	CC	7804	n	bl	n	y	0	ev	cig+/-ot	1	9	1	1	nev any	st
LUBIN2	156	m	0	0	all	-	a	Eu:mul	1976	CC	7804	n	bl	n	y	0	ev	cig+/-ot	10	19	0	2	nev any	st
LUBIN2	160	m	0	0	all	-	a	Eu:mul	1976	CC	7804	n	bl	n	y	0	ev	cig+/-ot	20	29	2	3	nev any	st
LUBIN2	164	m	0	0	all	-	a	Eu:mul	1976	CC	7804	n	bl	n	y	0	ev	cig+/-ot	30	99	3	0	nev any	st
LUBIN2	172	f	0	0	all	-	a	Eu:mul	1976	CC	7804	n	bl	n	y	0	ev	cig+/-ot	1	9	1	1	nev any	st
LUBIN2	176	f	0	0	all	-	a	Eu:mul	1976	CC	7804	n	bl	n	y	0	ev	cig+/-ot	10	19	0	2	nev any	st
LUBIN2	180	f	0	0	all	-	a	Eu:mul	1976	CC	7804	n	bl	n	y	0	ev	cig+/-ot	20	29	2	3	nev any	st

Table 3G11 - 1

IESLC - Meta-anal of Ever Smoking (or Curr if Ever not avail) by Amount, Overview, Any prod (or Cigs if Any not avail)
Adenocarcinoma
Most adjusted

REF	NRR	SEX	AGEL	AGEH	RACE	YF	LC TYPE	LOC	START	ST	NLC	R	VB	P	H	AD	SM	PRODUCT	exL	exH	S1	S2	DENOM	De
LUBIN2	184	f	0	0	all	-	a	Eu:mul	1976	CC	7804	n	bl	n	y	0	ev	cig+/-ot	30	99	3	0	nev any	st
MATOS	57	m	0	0	all	-	a	SCAmer	1994	CC	200	n	bl	n	n	2	ev	cig+/-ot	1	14	1	0	nev any	or
MATOS	59	m	0	0	all	-	a	SCAmer	1994	CC	200	n	bl	n	n	2	ev	cig+/-ot	15	24	2	3	nev any	or
MATOS	61	m	0	0	all	-	a	SCAmer	1994	CC	200	n	bl	n	n	2	ev	cig+/-ot	25	99	3	0	nev any	or
MATSUD	7	m	0	0	all	-	a	As:Jap	1965	CC	179	n	bl	n	n	0	ev	cig+/-ot	1	10	1	0	nev cigs	ot
MATSUD	8	m	0	0	all	-	a	As:Jap	1965	CC	179	n	bl	n	n	0	ev	cig+/-ot	11	20	2	3	nev cigs	ot
MATSUD	9	m	0	0	all	-	a	As:Jap	1965	CC	179	n	bl	n	n	0	ev	cig+/-ot	21	99	3	0	nev cigs	ot
ORMOS	22	m	0	0	all	-	KII	Eu:est	1947	CC	119	n	bl	y	y	0	ev	cig+/-ot	1	15	1	0	nev any	st
ORMOS	23	m	0	0	all	-	KII	Eu:est	1947	CC	119	n	bl	y	y	0	ev	cig+/-ot	16	30	2	0	nev any	st
ORMOS	24	m	0	0	all	-	KII	Eu:est	1947	CC	119	n	bl	y	y	0	ev	cig+/-ot	31	99	3	0	nev any	st
OSANN	55	m	0	0	all	-	a	NAmer	1984	CC	1986	n	bl	n	n	2	ev	cig+/-ot	1	39	0	0	nev cigs	or
OSANN	63	m	0	0	all	-	a	NAmer	1984	CC	1986	n	bl	n	n	2	ev	cig+/-ot	40	99	3	0	nev cigs	or
OSANN	56	f	0	0	all	-	a	NAmer	1984	CC	1986	n	bl	n	n	2	ev	cig+/-ot	1	39	0	0	nev cigs	or
OSANN	64	f	0	0	all	-	a	NAmer	1984	CC	1986	n	bl	n	n	2	ev	cig+/-ot	40	99	3	0	nev cigs	or
OSANN2	34	f	0	0	all	-	KII	NAmer	1964	ot	217	n	bl	n	y	1	ev	cig+/-ot	1	19	1	0	nev cigs	or
OSANN2	35	f	0	0	all	-	KII	NAmer	1964	ot	217	n	bl	n	y	1	ev	cig+/-ot	20	99	0	0	nev cigs	or
SOBUE	56	m	0	0	all	-	a	As:Jap	1986	CC	1376	n	bl	n	y	0	cu	cig+/-ot	1	19	1	0	nev cigs	st
SOBUE	57	m	0	0	all	-	a	As:Jap	1986	CC	1376	n	bl	n	y	0	cu	cig+/-ot	20	29	2	3	nev cigs	st
SOBUE	58	m	0	0	all	-	a	As:Jap	1986	CC	1376	n	bl	n	y	0	cu	cig+/-ot	30	99	3	0	nev cigs	st
SVENSS	9	f	0	0	all	-	a	Eu:Sca	1983	CC	210	n	bl	n	n	1	cu	all/unsp	1	10	1	0	nev any	or
SVENSS	14	f	0	0	all	-	a	Eu:Sca	1983	CC	210	n	bl	n	n	1	cu	all/unsp	11	20	2	3	nev any	or
SVENSS	19	f	0	0	all	-	a	Eu:Sca	1983	CC	210	n	bl	n	n	1	cu	all/unsp	21	99	3	0	nev any	ot
TSUGAN	4	m	0	0	all	-	a	As:Jap	1976	CC	134	n	bl	n	y	0	cu	all/unsp	1	15	1	0	nev any	st
TSUGAN	5	m	0	0	all	-	a	As:Jap	1976	CC	134	n	bl	n	y	0	cu	all/unsp	16	35	2	0	nev any	st
TSUGAN	6	m	0	0	all	-	a	As:Jap	1976	CC	134	n	bl	n	y	0	cu	all/unsp	36	99	3	0	nev any	st
WAKAI	52	m	0	0	all	-	a	As:Jap	1988	CC	333	n	bl	n	y	1	cu	cig+/-ot	1	19	1	0	nev any	or
WAKAI	53	m	0	0	all	-	a	As:Jap	1988	CC	333	n	bl	n	y	1	cu	cig+/-ot	20	29	2	3	nev any	or
WAKAI	54	m	0	0	all	-	a	As:Jap	1988	CC	333	n	bl	n	y	1	cu	cig+/-ot	30	99	3	0	nev any	or
WU	8	f	0	0	wh	-	a	NAmer	1981	CC	220	n	bl	n	y	2	cu	all/unsp	1	20	0	0	nev any	or
WU	9	f	0	0	wh	-	a	NAmer	1981	CC	220	n	bl	n	y	2	cu	all/unsp	21	99	3	0	nev any	or
WUWILL	16	f	0	0	all	-	a	As:Chi	1985	CC	965	n	ot	n	n	3	ev	cig+/-ot	1	19	1	0	nev cigs	ot
WUWILL	17	f	0	0	all	-	a	As:Chi	1985	CC	965	n	ot	n	n	3	ev	cig+/-ot	20	99	0	0	nev cigs	ot
WYNDE2	10	m	0	0	all	-	KII	NAmer	1962	CC	404	n	bl	n	y	0	ev	cig+/-ot	1	10	1	0	nev any	st
WYNDE2	11	m	0	0	all	-	KII	NAmer	1962	CC	404	n	bl	n	y	0	ev	cig+/-ot	11	20	2	3	nev any	st
WYNDE2	12	m	0	0	all	-	KII	NAmer	1962	CC	404	n	bl	n	y	0	ev	cig+/-ot	21	34	0	4	nev any	st
WYNDE2	13	m	0	0	all	-	KII	NAmer	1962	CC	404	n	bl	n	y	0	ev	cig+/-ot	35	99	3	0	nev any	st
WYNDE3	24	m	0	0	all	-	KII	NAmer	1966	CC	350	n	bl	n	y	0	ev	cig+/-ot	1	9	1	1	nev any	st
WYNDE3	25	m	0	0	all	-	KII	NAmer	1966	CC	350	n	bl	n	y	0	ev	cig+/-ot	10	20	2	0	nev any	st
WYNDE3	26	m	0	0	all	-	KII	NAmer	1966	CC	350	n	bl	n	y	0	ev	cig+/-ot	21	40	0	0	nev any	st
WYNDE3	27	m	0	0	all	-	KII	NAmer	1966	CC	350	n	bl	n	y	0	ev	cig+/-ot	41	99	3	6	nev any	st
WYNDE3	71	f	0	0	all	-	KII	NAmer	1966	CC	350	n	bl	n	y	0	ev	cig+/-ot	1	9	1	1	nev any	st
WYNDE3	72	f	0	0	all	-	KII	NAmer	1966	CC	350	n	bl	n	y	0	ev	cig+/-ot	10	20	2	0	nev any	st
WYNDE3	73	f	0	0	all	-	KII	NAmer	1966	CC	350	n	bl	n	y	0	ev	cig+/-ot	21	40	0	0	nev any	st
WYNDE3	74	f	0	0	all	-	KII	NAmer	1966	CC	350	n	bl	n	y	0	ev	cig+/-ot	41	99	3	6	nev any	st
WYNDE4	37	m	0	0	all	-	a	NAmer	1948	CC	684	n	bl	y	n	0	ev	all/unsp	1	9	1	1	nev any	st
WYNDE4	38	m	0	0	all	-	a	NAmer	1948	CC	684	n	bl	y	n	0	ev	all/unsp	10	15	0	2	nev any	st
WYNDE4	39	m	0	0	all	-	a	NAmer	1948	CC	684	n	bl	y	n	0	ev	all/unsp	16	20	2	3	nev any	st
WYNDE4	40	m	0	0	all	-	a	NAmer	1948	CC	684	n	bl	y	n	0	ev	all/unsp	21	34	0	4	nev any	st
WYNDE4	41	m	0	0	all	-	a	NAmer	1948	CC	684	n	bl	y	n	0	ev	all/unsp	35	99	3	0	nev any	st
WYNDE4	55	f	0	0	all	-	a	NAmer	1948	CC	684	n	bl	y	n	2	ev	all/unsp	1	9	1	1	nev any	ot
WYNDE6	24	m	0	0	all	-	KII	NAmer	1969	CC	4423	n	bl	n	y	0	cu	cig+/-ot	1	10	1	0	nev any	st
WYNDE6	33	m	0	0	all	-	KII	NAmer	1969	CC	4423	n	bl	n	y	0	cu	cig+/-ot	11	20	2	3	nev any	st
WYNDE6	42	m	0	0	all	-	KII	NAmer	1969	CC	4423	n	bl	n	y	0	cu	cig+/-ot	21	30	0	4	nev any	st
WYNDE6	51	m	0	0	all	-	KII	NAmer	1969	CC	4423	n	bl	n	y	0	cu	cig+/-ot	31	99	3	0	nev any	st
WYNDE6	213	f	0	0	all	-	KII	NAmer	1969	CC	4423	n	bl	n	y	0	cu	cig+/-ot	1	10	1	0	nev cigs	st
WYNDE6	222	f	0	0	all	-	KII	NAmer	1969	CC	4423	n	bl	n	y	0	cu	cig+/-ot	11	20	2	3	nev cigs	st
WYNDE6	231	f	0	0	all	-	KII	NAmer	1969	CC	4423	n	bl	n	y	0	cu	cig+/-ot	21	30	0	4	nev cigs	st
WYNDE6	240	f	0	0	all	-	KII	NAmer	1969	CC	4423	n	bl	n	y	0	cu	cig+/-ot	30	99	3	0	nev cigs	st
ZHENG	6	m	0	0	all	-	a	As:Chi	1982	CC	540	n	ot	*	y	0	ev	cig+/-ot	1	9	1	1	nev cigs	st
ZHENG	7	m	0	0	all	-	a	As:Chi	1982	CC	540	n	ot	*	y	0	ev	cig+/-ot	10	19	0	2	nev cigs	st
ZHENG	8	m	0	0	all	-	a	As:Chi	1982	CC	540	n	ot	*	y	0	ev	cig+/-ot	20	29	2	3	nev cigs	st
ZHENG	9	m	0	0	all	-	a	As:Chi	1982	CC	540	n	ot	*	y	0	ev	cig+/-ot	30	99	3	0	nev cigs	st
ZHENG	19	f	0	0	all	-	a	As:Chi	1982	CC	540	n	ot	*	y	0	ev	cig+/-ot	1	9	1	1	nev cigs	st
ZHENG	20	f	0	0	all	-	a	As:Chi	1982	CC	540	n	ot	*	y	0	ev	cig+/-ot	10	99	0	0	nev cigs	st

Cigarette type is all/unspec for all RRs

except for the following:

Table 3G11 - 1

IESLC - Meta-anal of Ever Smoking (or Curr if Ever not avail) by Amount, Overview, Any prod (or Cigs if Any not avail)
Adenocarcinoma
Most adjusted

REF	NRR	CIGTYPE
ALDERS	46	MC only
ALDERS	47	MC only
ALDERS	48	MC only
ALDERS	49	MC only
ALDERS	50	MC only
ALDERS	51	MC only

REF	NRR	Cigarette equivalent
ALDERS	46	-
ALDERS	47	-
ALDERS	48	-
ALDERS	49	-
ALDERS	50	-
ALDERS	51	-
BARBON	79	*
BARBON	80	*
BARBON	81	*
BOUCOT	148	-
BOUCOT	149	-
BROWN2	34	*
BROWN2	44	*
BROWN2	33	*
BROWN2	43	*
CHOI	51	*
CHOI	52	*
CHOI	53	*
CHOI	54	*
CHOI	55	*
CHOI	59	*
CHOI	60	*
CHOI	61	*
CORREA	48	*
CORREA	52	*
DOLL	72	grams
DOLL	73	grams
DOLL	74	grams
DOLL	75	grams
DOLL	79	grams
DOLL	80	grams
DOLL	81	grams
DORGAN	126	*
DORGAN	127	*
DORGAN	105	*
DORGAN	106	*
DOSEME	8	*
DOSEME	12	*
DOSEME	16	*
ENGELA	71	*
ENGELA	72	*
ENGELA	73	*
ENGELA	74	*
ENGELA	75	*
GER	10	*
GER	11	*
GER	12	*
HAENSZ	35	*
HAENSZ	34	*
HAMMON	108	-
HAMMON	109	-
HAMMON	110	-
JEDRYC	36	*
JEDRYC	37	*
JEDRYC	38	*
KATSOU	13	*
KATSOU	14	*
KREYBE	5	grams inc 1 cig=1
KREYBE	6	grams inc 1 cig=1
KREYBE	7	grams inc 1 cig=1
KREYBE	26	grams inc 1 cig=1
KREYBE	61	grams inc 1 cig=1
LAMTH	16	*
LAMTH	17	*

Table 3G11 - 1

IESLC - Meta-anal of Ever Smoking (or Curr if Ever not avail) by Amount, Overview, Any prod (or Cigs if Any not avail)
Adenocarcinoma
Most adjusted

REF	NRR	Cigarette equivalent
LAMTH	18	*
LUBIN2	152	*
LUBIN2	156	*
LUBIN2	160	*
LUBIN2	164	*
LUBIN2	172	*
LUBIN2	176	*
LUBIN2	180	*
LUBIN2	184	*
MATOS	57	*
MATOS	59	*
MATOS	61	*
MATSUD	7	*
MATSUD	8	*
MATSUD	9	*
ORMOS	22	*
ORMOS	23	*
ORMOS	24	*
OSANN	55	*
OSANN	63	*
OSANN	56	*
OSANN	64	*
OSANN2	34	*
OSANN2	35	*
SOBUE	56	*
SOBUE	57	*
SOBUE	58	*
SVENSS	9	*
SVENSS	14	*
SVENSS	19	*
TSUGAN	4	*
TSUGAN	5	*
TSUGAN	6	*
WAKAI	52	*
WAKAI	53	*
WAKAI	54	*
WU	8	*
WU	9	*
WUWILL	16	*
WUWILL	17	*
WYNDE2	10	*
WYNDE2	11	*
WYNDE2	12	*
WYNDE2	13	*
WYNDE3	24	*
WYNDE3	25	*
WYNDE3	26	*
WYNDE3	27	*
WYNDE3	71	*
WYNDE3	72	*
WYNDE3	73	*
WYNDE3	74	*
WYNDE4	37	inc 1 cigar = 5 cigs, 1 pipe = 2.5 cigs
WYNDE4	38	inc 1 cigar = 5 cigs, 1 pipe = 2.5 cigs
WYNDE4	39	inc 1 cigar = 5 cigs, 1 pipe = 2.5 cigs
WYNDE4	40	inc 1 cigar = 5 cigs, 1 pipe = 2.5 cigs
WYNDE4	41	inc 1 cigar = 5 cigs, 1 pipe = 2.5 cigs
WYNDE4	55	inc 1 cigar = 5 cigs, 1 pipe = 2.5 cigs
WYNDE6	24	*
WYNDE6	33	*
WYNDE6	42	*
WYNDE6	51	*
WYNDE6	213	*
WYNDE6	222	*
WYNDE6	231	*
WYNDE6	240	*
ZHENG	6	*
ZHENG	7	*
ZHENG	8	*
ZHENG	9	*
ZHENG	19	*
ZHENG	20	*

Table 3G11 - 1

IESLC - Meta-anal of Ever Smoking (or Curr if Ever not avail) by Amount, Overview, Any prod (or Cigs if Any not avail)
Adenocarcinoma
Most adjusted

In this overview table, subtotals and Qs values may be invalid and should be ignored

Table 3G11 - 2

IESLC - Meta-anal of Ever Smoking (or Curr if Ever not avail) by Amount, Overview, Any prod (or Cigs if Any not avail)
Adenocarcinoma
Most adjusted

REF	NRR	SEX	AD	Number Exposed Case	Number Exposed Cont	Non-exposed Case	Non-exposed Cont	RR	95.00%CI
ALDERS	46	m	1	-	-	-	-	2.80	(1.01- 7.75)
ALDERS	47	m	1	-	-	-	-	2.67	(0.99- 7.18)
ALDERS	48	m	1	-	-	-	-	3.32	(1.36- 8.10)
ALDERS	49	f	1	-	-	-	-	2.77	(1.63- 4.70)
ALDERS	50	f	1	-	-	-	-	4.58	(2.67- 7.85)
ALDERS	51	f	1	-	-	-	-	3.31	(1.80- 6.10)
Subtotal ALDERS								3.34	(2.53- 4.41)
BARBON	79	m	3	-	-	-	-	5.00	(2.20- 11.00)
BARBON	80	m	3	-	-	-	-	8.30	(3.70- 19.00)
BARBON	81	m	3	-	-	-	-	12.10	(5.10- 28.00)
Subtotal BARBON								7.82	(4.86- 12.59)
*BOUCOT	148	m	2	-	-	-	-	8.22	(0.56- 166.83)
*BOUCOT	149	m	2	-	-	-	-	18.00	(1.01- 319.52)
Subtotal BOUCOT								12.11	(1.60- 91.75)
BROWN2	34	m	2	-	-	-	-	6.20	(4.90- 7.90)
BROWN2	44	m	2	-	-	-	-	10.70	(8.90- 13.00)
BROWN2	33	f	2	-	-	-	-	5.80	(4.70- 7.10)
BROWN2	43	f	2	-	-	-	-	8.60	(7.30- 10.10)
Subtotal BROWN2								7.91	(7.18- 8.72)
CHOI	51	m	0	4	90	7	95	0.60	(0.17- 2.13)
CHOI	52	m	0	27	281	7	95	1.30	(0.55- 3.09)
CHOI	53	m	0	11	49	7	95	3.05	(1.11- 8.35)
CHOI	54	m	0	2	39	7	95	0.70	(0.14- 3.50)
CHOI	55	m	0	2	6	7	95	4.52	(0.77- 26.69)
CHOI	59	f	0	4	16	49	164	0.84	(0.27- 2.62)
CHOI	60	f	0	0	9	49	164	0.17~	(0.01- 3.06)
CHOI	61	f	0	1	1	49	164	3.35	(0.21- 54.50)
Subtotal CHOI								1.32	(0.83- 2.09)
CORREA	48	c	1	-	-	-	-	4.30	(2.60- 7.20)
CORREA	52	c	1	-	-	-	-	12.00	(7.30- 19.70)
Subtotal CORREA								7.28	(5.10- 10.39)
DOLL	72	m	1	-	-	-	-	0.50	(0.07- 3.63)
DOLL	73	m	1	-	-	-	-	0.80	(0.18- 3.60)
DOLL	74	m	1	-	-	-	-	1.20	(0.27- 5.35)
DOLL	75	m	1	-	-	-	-	1.10	(0.22- 5.60)
DOLL	79	f	1	-	-	-	-	1.10	(0.20- 6.05)
DOLL	80	f	1	-	-	-	-	2.30	(0.56- 9.48)
DOLL	81	f	1	-	-	-	-	4.10	(0.65- 25.88)
Subtotal DOLL								1.30	(0.71- 2.40)
DORGAN	126	m	2	-	-	-	-	3.23	(1.20- 8.70)
DORGAN	127	m	2	-	-	-	-	5.63	(2.22- 14.25)
DORGAN	105	f	3	-	-	-	-	2.87	(2.00- 4.11)
DORGAN	106	f	3	-	-	-	-	5.39	(3.73- 7.78)
Subtotal DORGAN								3.96	(3.11- 5.04)
DOSEME	8	m	2	-	-	-	-	1.80	(0.80- 4.10)
DOSEME	12	m	2	-	-	-	-	2.70	(1.60- 4.70)
DOSEME	16	m	2	-	-	-	-	3.20	(1.40- 7.00)
Subtotal DOSEME								2.56	(1.73- 3.79)
*ENGELA	71	m	7	-	-	-	-	2.60	(0.60- 11.00)
*ENGELA	72	m	7	-	-	-	-	8.50	(2.50- 29.00)
*ENGELA	73	m	7	-	-	-	-	7.10	(2.10- 24.00)
*ENGELA	74	m	7	-	-	-	-	9.30	(2.10- 41.00)
*ENGELA	75	m	7	-	-	-	-	17.00	(5.10- 56.00)
Subtotal ENGELA								8.06	(4.51- 14.41)
GER	10	c	8	-	-	-	-	0.62	(0.23- 1.72)
GER	11	c	8	-	-	-	-	2.10	(0.88- 5.04)
GER	12	c	8	-	-	-	-	0.88	(0.25- 3.03)
Subtotal GER								1.15	(0.64- 2.07)
HAENSZ	35	f	0	10	66	37	236	0.97	(0.46- 2.05)
HAENSZ	34	f	0	5	13	37	236	2.45	(0.83- 7.28)
Subtotal HAENSZ								1.30	(0.70- 2.42)
*HAMMON	108	m	1	-	-	-	-	1.83	(0.17- 20.22)
*HAMMON	109	m	1	-	-	-	-	2.83	(0.55- 14.60)
*HAMMON	110	m	1	-	-	-	-	6.44	(1.34- 31.02)
Subtotal HAMMON								3.71	(1.33- 10.33)
JEDRYC	36	m	3	-	-	-	-	2.19	(0.84- 5.72)
JEDRYC	37	m	3	-	-	-	-	4.38	(1.87- 10.28)
JEDRYC	38	m	3	-	-	-	-	5.11	(2.09- 12.53)
Subtotal JEDRYC								3.77	(2.24- 6.33)
KATSOU	13	f	1	-	-	-	-	1.35	(0.52- 3.49)
KATSOU	14	f	1	-	-	-	-	2.95	(0.76- 11.41)

Table 3G11 - 2

IESLC - Meta-anal of Ever Smoking (or Curr if Ever not avail) by Amount, Overview, Any prod (or Cigs if Any not avail)
Adenocarcinoma
Most adjusted

REF	NRR	SEX	AD	Number Exposed Case	Cont	Non-exposed Case	Cont	RR	95.00%CI
Subtotal KATSOU								1.75	(0.80- 3.81)
KREYBE	5	m	1	-	-	-	-	2.65	(0.81- 8.70)
KREYBE	6	m	1	-	-	-	-	1.43	(0.36- 5.72)
KREYBE	7	m	1	-	-	-	-	3.99	(0.95- 16.81)
KREYBE	26	f	1	-	-	-	-	1.30	(0.60- 2.80)
KREYBE	61	f	1	-	-	-	-	0.88	(0.12- 6.63)
Subtotal KREYBE								1.71	(1.01- 2.89)
LAMTH	16	f	0	36	29	131	158	1.50	(0.87- 2.57)
LAMTH	17	f	0	27	14	131	158	2.33	(1.17- 4.62)
LAMTH	18	f	0	9	5	131	158	2.17	(0.71- 6.64)
Subtotal LAMTH								1.82	(1.22- 2.71)
LUBIN2	152	m	0	66	2194	57	2616	1.38	(0.96- 1.98)
LUBIN2	156	m	0	204	3385	57	2616	2.77	(2.05- 3.73)
LUBIN2	160	m	0	234	3108	57	2616	3.46	(2.57- 4.64)
LUBIN2	164	m	0	151	1746	57	2616	3.97	(2.91- 5.42)
LUBIN2	172	f	0	20	184	138	1180	0.93	(0.57- 1.52)
LUBIN2	176	f	0	43	234	138	1180	1.57	(1.09- 2.27)
LUBIN2	180	f	0	13	110	138	1180	1.01	(0.55- 1.84)
LUBIN2	184	f	0	9	39	138	1180	1.97	(0.94- 4.16)
Subtotal LUBIN2								2.28	(1.99- 2.60)
MATOS	57	m	2	-	-	-	-	2.80	(0.90- 8.50)
MATOS	59	m	2	-	-	-	-	7.00	(2.60- 19.10)
MATOS	61	m	2	-	-	-	-	8.40	(3.01- 22.20)
Subtotal MATOS								5.76	(3.17- 10.48)
MATSUD	7	m	0	6	1237	0	1255	13.19~	(0.74- 234.37)
MATSUD	8	m	0	13	1607	0	1255	21.09~	(1.25- 355.07)
MATSUD	9	m	0	4	470	0	1255	24.02~	(1.29- 446.92)
Subtotal MATSUD								18.81	(3.58- 98.87)
ORMOS	22	m	0	4	329	2	777	4.72	(0.86- 25.91)
ORMOS	23	m	0	3	577	2	777	2.02	(0.34- 12.13)
ORMOS	24	m	0	1	128	2	777	3.04	(0.27- 33.72)
Subtotal ORMOS								3.13	(1.04- 9.39)
OSANN	55	m	2	-	-	-	-	16.50	(9.30- 29.30)
OSANN	63	m	2	-	-	-	-	37.50	(21.30- 66.00)
OSANN	56	f	2	-	-	-	-	8.80	(6.10- 12.80)
OSANN	64	f	2	-	-	-	-	24.20	(15.80- 37.20)
Subtotal OSANN								16.57	(13.16- 20.85)
OSANN2	34	f	1	-	-	-	-	0.90	(0.30- 2.70)
OSANN2	35	f	1	-	-	-	-	3.80	(1.60- 8.80)
Subtotal OSANN2								2.21	(1.13- 4.34)
SOBUE	56	m	0	63	157	27	128	1.90	(1.15- 3.16)
SOBUE	57	m	0	95	222	27	128	2.03	(1.26- 3.28)
SOBUE	58	m	0	76	187	27	128	1.93	(1.18- 3.16)
Subtotal SOBUE								1.95	(1.47- 2.60)
SVENSS	9	f	1	-	-	-	-	2.20	(1.00- 5.80)
SVENSS	14	f	1	-	-	-	-	5.40	(2.40- 13.20)
SVENSS	19	f	1	-	-	-	-	19.70	(1.70- 228.29)
Subtotal SVENSS								3.87	(2.14- 7.00)
TSUGAN	4	m	0	12	14	18	17	0.81	(0.29- 2.24)
TSUGAN	5	m	0	23	23	18	17	0.94	(0.39- 2.28)
TSUGAN	6	m	0	10	13	18	17	0.73	(0.25- 2.09)
Subtotal TSUGAN								0.84	(0.48- 1.47)
WAKAI	52	m	1	-	-	-	-	1.30	(0.52- 3.21)
WAKAI	53	m	1	-	-	-	-	1.93	(0.84- 4.44)
WAKAI	54	m	1	-	-	-	-	4.53	(1.89- 10.90)
Subtotal WAKAI								2.27	(1.37- 3.75)
WU	8	f	2	-	-	-	-	2.70	(1.40- 5.40)
WU	9	f	2	-	-	-	-	6.50	(3.10- 13.90)
Subtotal WU								4.00	(2.42- 6.61)
WUWILL	16	f	3	-	-	-	-	1.43	(1.07- 1.90)
WUWILL	17	f	3	-	-	-	-	2.26	(1.35- 3.78)
Subtotal WUWILL								1.59	(1.24- 2.05)
WYNDE2	10	m	0	2	114	5	105	0.37	(0.07- 1.94)
WYNDE2	11	m	0	14	203	5	105	1.45	(0.51- 4.13)
WYNDE2	12	m	0	14	83	5	105	3.54	(1.23- 10.23)
WYNDE2	13	m	0	16	112	5	105	3.00	(1.06- 8.48)
Subtotal WYNDE2								1.98	(1.12- 3.51)
WYNDE3	24	m	0	1	42	6	88	0.35	(0.04- 2.99)
WYNDE3	25	m	0	20	114	6	88	2.57	(0.99- 6.68)
WYNDE3	26	m	0	34	82	6	88	6.08	(2.43- 15.24)
WYNDE3	27	m	0	9	26	6	88	5.08	(1.65- 15.59)

Table 3G11 - 2

IESLC - Meta-anal of Ever Smoking (or Curr if Ever not avail) by Amount, Overview, Any prod (or Cigs if Any not avail)
Adenocarcinoma
Most adjusted

REF	NRR	SEX	AD	Number Exposed Case	Number Exposed Cont	Non-exposed Case	Non-exposed Cont	RR	95.00%CI
WYNDE3	71	f	0	2	19	15	76	0.53	(0.11- 2.53)
WYNDE3	72	f	0	11	24	15	76	2.32	(0.94- 5.73)
WYNDE3	73	f	0	7	10	15	76	3.55	(1.16- 10.80)
WYNDE3	74	f	0	1	3	15	76	1.69	(0.16- 17.36)
Subtotal WYNDE3								2.82	(1.87- 4.25)
WYNDE4	37	m	0	3	82	4	115	1.05	(0.23- 4.83)
WYNDE4	38	m	0	6	147	4	115	1.17	(0.32- 4.26)
WYNDE4	39	m	0	15	274	4	115	1.57	(0.51- 4.84)
WYNDE4	40	m	0	4	98	4	115	1.17	(0.29- 4.82)
WYNDE4	41	m	0	7	64	4	115	3.14	(0.89- 11.15)
WYNDE4	55	f	2	-	-	-	-	1.33	(0.29- 6.07)
Subtotal WYNDE4								1.51	(0.88- 2.60)
WYNDE6	24	m	0	42	122	58	617	3.66	(2.35- 5.70)
WYNDE6	33	m	0	191	293	58	617	6.93	(5.01- 9.60)
WYNDE6	42	m	0	136	129	58	617	11.22	(7.81- 16.09)
WYNDE6	51	m	0	282	197	58	617	15.23	(11.00- 21.07)
WYNDE6	213	f	0	39	109	119	856	2.57	(1.70- 3.89)
WYNDE6	222	f	0	176	165	119	856	7.67	(5.76- 10.21)
WYNDE6	231	f	0	100	50	119	856	14.39	(9.74- 21.25)
WYNDE6	240	f	0	157	52	119	856	21.72	(15.04- 31.37)
Subtotal WYNDE6								8.99	(7.93- 10.19)
ZHENG	6	m	0	18	40	29	94	1.46	(0.73- 2.92)
ZHENG	7	m	0	35	66	29	94	1.72	(0.96- 3.08)
ZHENG	8	m	0	53	89	29	94	1.93	(1.13- 3.30)
ZHENG	9	m	0	17	23	29	94	2.40	(1.13- 5.08)
ZHENG	19	f	0	13	29	119	184	0.69	(0.35- 1.39)
ZHENG	20	f	0	20	15	119	184	2.06	(1.02- 4.19)
Subtotal ZHENG								1.62	(1.24- 2.11)
Partial Totals				2633	19458	2756	31915		

*prospective study ~ With 0.5 adjustment for zero

REF	NRR	SEX	AD	Ys	Ws	Qs	Ps
ALDERS	46	m	1	1.03	3.70	0.84	0.0476
ALDERS	47	m	1	0.98	3.91	1.07	0.0520
ALDERS	48	m	1	1.20	4.83	0.45	0.0084
ALDERS	49	f	1	1.02	13.70	3.25	0.0002
ALDERS	50	f	1	1.52	13.21	0.00	0.0000
ALDERS	51	f	1	1.20	10.32	0.99	0.0001
Subtotal ALDERS				1.21	49.67	6.61	
BARBON	79	m	3	1.61	5.93	0.06	0.0001
BARBON	80	m	3	2.12	5.74	2.14	0.0000
BARBON	81	m	3	2.49	5.30	5.16	0.0000
Subtotal BARBON				2.06	16.97	7.37	
*BOUCOT	148	m	2	2.11	0.47	0.17	0.1472
*BOUCOT	149	m	2	2.89	0.46	0.89	0.0491
Subtotal BOUCOT				2.49	0.94	1.06	
BROWN2	34	m	2	1.82	67.36	6.84	0.0000
BROWN2	44	m	2	2.37	107.03	79.95	0.0000
BROWN2	33	f	2	1.76	90.29	5.73	0.0000
BROWN2	43	f	2	2.15	145.78	60.80	0.0000
Subtotal BROWN2				2.07	410.46	153.31	
CHOI	51	m	0	-0.51	2.41	9.76	0.4323
CHOI	52	m	0	0.27	5.16	7.93	0.5467
CHOI	53	m	0	1.11	3.78	0.58	0.0304
CHOI	54	m	0	-0.36	1.47	5.14	0.6600
CHOI	55	m	0	1.51	1.22	0.00	0.0956
CHOI	59	f	0	-0.18	2.95	8.37	0.7595
CHOI	60	f	0	-1.74	0.47	4.95	0.2324
CHOI	61	f	0	1.21	0.49	0.04	0.3961
Subtotal CHOI				0.27	17.95	36.78	
CORREA	48	c	1	1.46	14.81	0.03	0.0000
CORREA	52	c	1	2.48	15.59	14.94	0.0000
Subtotal CORREA				1.98	30.40	14.97	
DOLL	72	m	1	-0.69	0.99	4.77	0.4914
DOLL	73	m	1	-0.22	1.71	5.12	0.7703
DOLL	74	m	1	0.18	1.72	3.02	0.8109
DOLL	75	m	1	0.10	1.47	2.92	0.9081
DOLL	79	f	1	0.10	1.32	2.63	0.9127
DOLL	80	f	1	0.83	1.92	0.87	0.2485
DOLL	81	f	1	1.41	1.13	0.01	0.1333

Table 3G11 - 2

IESLC - Meta-anal of Ever Smoking (or Curr if Ever not avail) by Amount, Overview, Any prod (or Cigs if Any not avail)
Adenocarcinoma
Most adjusted

REF	NRR	SEX	AD	Ys	Ws	Qs	Ps
Subtotal DOLL				0.26	10.26	19.33	
DORGAN	126	m	2	1.17	3.92	0.44	0.0203
DORGAN	127	m	2	1.73	4.45	0.22	0.0003
DORGAN	105	f	3	1.05	29.62	6.04	0.0000
DORGAN	106	f	3	1.68	28.43	0.91	0.0000
Subtotal DORGAN				1.38	66.41	7.60	
DOSEME	8	m	2	0.59	5.75	4.85	0.1585
DOSEME	12	m	2	0.99	13.23	3.48	0.0003
DOSEME	16	m	2	1.16	5.93	0.70	0.0046
Subtotal DOSEME				0.94	24.92	9.03	
*ENGELA	71	m	7	0.96	1.82	0.55	0.1979
*ENGELA	72	m	7	2.14	2.56	1.03	0.0006
*ENGELA	73	m	7	1.96	2.59	0.53	0.0016
*ENGELA	74	m	7	2.23	1.74	0.91	0.0033
*ENGELA	75	m	7	2.83	2.68	4.71	0.0000
Subtotal ENGELA				2.09	11.38	7.74	
GER	10	c	8	-0.48	3.80	14.94	0.3517
GER	11	c	8	0.74	5.04	2.94	0.0956
GER	12	c	8	-0.13	2.47	6.59	0.8408
Subtotal GER				0.14	11.31	24.48	
HAENSZ	35	f	0	-0.03	6.83	16.20	0.9289
HAENSZ	34	f	0	0.90	3.24	1.20	0.1060
Subtotal HAENSZ				0.27	10.07	17.40	
*HAMMON	108	m	1	0.60	0.67	0.55	0.6201
*HAMMON	109	m	1	1.04	1.43	0.31	0.2136
*HAMMON	110	m	1	1.86	1.56	0.20	0.0201
Subtotal HAMMON				1.31	3.66	1.05	
JEDRYC	36	m	3	0.78	4.18	2.18	0.1092
JEDRYC	37	m	3	1.48	5.29	0.00	0.0007
JEDRYC	38	m	3	1.63	4.79	0.08	0.0004
Subtotal JEDRYC				1.33	14.26	2.26	
KATSOU	13	f	1	0.30	4.24	6.16	0.5366
KATSOU	14	f	1	1.08	2.09	0.38	0.1175
Subtotal KATSOU				0.56	6.33	6.54	
KREYBE	5	m	1	0.97	2.73	0.77	0.1076
KREYBE	6	m	1	0.36	2.01	2.65	0.6122
KREYBE	7	m	1	1.38	1.86	0.03	0.0590
KREYBE	26	f	1	0.26	6.48	10.01	0.5044
KREYBE	61	f	1	-0.13	0.95	2.55	0.9006
Subtotal KREYBE				0.54	14.03	16.01	
LAMTH	16	f	0	0.40	13.12	15.94	0.1438
LAMTH	17	f	0	0.84	8.17	3.58	0.0158
LAMTH	18	f	0	0.78	3.08	1.64	0.1740
Subtotal LAMTH				0.60	24.36	21.16	
LUBIN2	152	m	0	0.32	29.82	41.77	0.0782
LUBIN2	156	m	0	1.02	43.25	10.32	0.0000
LUBIN2	160	m	0	1.24	44.40	3.14	0.0000
LUBIN2	164	m	0	1.38	39.81	0.65	0.0000
LUBIN2	172	f	0	-0.07	15.74	39.25	0.7715
LUBIN2	176	f	0	0.45	28.07	31.19	0.0167
LUBIN2	180	f	0	0.01	10.63	23.76	0.9727
LUBIN2	184	f	0	0.68	6.90	4.71	0.0741
Subtotal LUBIN2				0.82	218.62	154.80	
MATOS	57	m	2	1.03	3.05	0.69	0.0723
MATOS	59	m	2	1.95	3.86	0.75	0.0001
MATOS	61	m	2	2.13	3.85	1.49	0.0000
Subtotal MATOS				1.75	10.76	2.93	
MATSUD	7	m	0	2.58	0.46	0.53	0.0789
MATSUD	8	m	0	3.05	0.48	1.15	0.0343
MATSUD	9	m	0	3.18	0.45	1.26	0.0331
Subtotal MATSUD				2.93	1.40	2.94	
ORMOS	22	m	0	1.55	1.33	0.00	0.0738
ORMOS	23	m	0	0.70	1.20	0.77	0.4420
ORMOS	24	m	0	1.11	0.66	0.10	0.3661
Subtotal ORMOS				1.14	3.18	0.88	
OSANN	55	m	2	2.80	11.67	19.64	0.0000
OSANN	63	m	2	3.62	12.01	53.91	0.0000
OSANN	56	f	2	2.17	27.97	12.51	0.0000
OSANN	64	f	2	3.19	20.96	59.17	0.0000
Subtotal OSANN				2.81	72.61	145.24	
OSANN2	34	f	1	-0.11	3.18	8.26	0.8509

Table 3G11 - 2

IESLC - Meta-anal of Ever Smoking (or Curr if Ever not avail) by Amount, Overview, Any prod (or Cigs if Any not avail)
Adenocarcinoma
Most adjusted

REF	NRR	SEX	AD	Ys	Ws	Qs	Ps
OSANN2	35	f	1	1.34	5.29	0.15	0.0021
Subtotal OSANN2				0.79	8.47	8.42	
SOBUE	56	m	0	0.64	14.90	11.10	0.0130
SOBUE	57	m	0	0.71	16.70	10.65	0.0038
SOBUE	58	m	0	0.66	15.78	11.41	0.0092
Subtotal SOBUE				0.67	47.39	33.16	
SVENSS	9	f	1	0.79	4.97	2.56	0.0787
SVENSS	14	f	1	1.69	5.29	0.17	0.0001
SVENSS	19	f	1	2.98	0.64	1.39	0.0171
Subtotal SVENSS				1.35	10.90	4.12	
TSUGAN	4	m	0	-0.21	3.72	10.96	0.6838
TSUGAN	5	m	0	-0.06	4.97	12.14	0.8986
TSUGAN	6	m	0	-0.32	3.43	11.44	0.5538
Subtotal TSUGAN				-0.18	12.12	34.53	
WAKAI	52	m	1	0.26	4.64	7.17	0.5721
WAKAI	53	m	1	0.66	5.54	3.99	0.1216
WAKAI	54	m	1	1.51	5.00	0.00	0.0007
Subtotal WAKAI				0.82	15.19	11.16	
WU	8	f	2	0.99	8.43	2.22	0.0039
WU	9	f	2	1.87	6.82	0.91	0.0000
Subtotal WU				1.39	15.26	3.13	
WUWILL	16	f	3	0.36	46.61	61.45	0.0146
WUWILL	17	f	3	0.82	14.49	6.91	0.0019
Subtotal WUWILL				0.47	61.10	68.37	
WYNDE2	10	m	0	-1.00	1.39	8.73	0.2387
WYNDE2	11	m	0	0.37	3.50	4.51	0.4885
WYNDE2	12	m	0	1.26	3.41	0.20	0.0195
WYNDE2	13	m	0	1.10	3.56	0.59	0.0382
Subtotal WYNDE2				0.69	11.86	14.03	
WYNDE3	24	m	0	-1.05	0.83	5.44	0.3372
WYNDE3	25	m	0	0.95	4.22	1.33	0.0521
WYNDE3	26	m	0	1.81	4.55	0.41	0.0001
WYNDE3	27	m	0	1.62	3.05	0.04	0.0045
WYNDE3	71	f	0	-0.63	1.58	7.20	0.4293
WYNDE3	72	f	0	0.84	4.71	2.07	0.0675
WYNDE3	73	f	0	1.27	3.10	0.18	0.0258
WYNDE3	74	f	0	0.52	0.71	0.68	0.6593
Subtotal WYNDE3				1.04	22.76	17.36	
WYNDE4	37	m	0	0.05	1.66	3.51	0.9482
WYNDE4	38	m	0	0.16	2.31	4.19	0.8077
WYNDE4	39	m	0	0.45	3.04	3.37	0.4291
WYNDE4	40	m	0	0.16	1.93	3.49	0.8243
WYNDE4	41	m	0	1.15	2.40	0.31	0.0761
WYNDE4	55	f	2	0.29	1.66	2.48	0.7132
Subtotal WYNDE4				0.41	12.99	17.34	
WYNDE6	24	m	0	1.30	19.66	0.85	0.0000
WYNDE6	33	m	0	1.94	36.35	6.74	0.0000
WYNDE6	42	m	0	2.42	29.44	24.45	0.0000
WYNDE6	51	m	0	2.72	36.38	53.90	0.0000
WYNDE6	213	f	0	0.95	22.53	7.08	0.0000
WYNDE6	222	f	0	2.04	46.92	13.26	0.0000
WYNDE6	231	f	0	2.67	25.27	34.02	0.0000
WYNDE6	240	f	0	3.08	28.43	70.28	0.0000
Subtotal WYNDE6				2.20	244.98	210.59	
ZHENG	6	m	0	0.38	7.96	10.13	0.2870
ZHENG	7	m	0	0.54	11.26	10.47	0.0692
ZHENG	8	m	0	0.66	13.29	9.57	0.0165
ZHENG	9	m	0	0.87	6.78	2.71	0.0229
ZHENG	19	f	0	-0.37	7.98	28.00	0.3003
ZHENG	20	f	0	0.72	7.66	4.69	0.0452
Subtotal ZHENG				0.48	54.94	65.56	

N	136
NS	34

Table 3G11 - 3

IESLC - Meta-anal of Ever Smoking (or Curr if Ever not avail) by Amount, Overview, Any prod (or Cigs if Any not avail)
Adenocarcinoma
Most adjusted

	Sex			
	combined	male	female	Total
N	5	83	48	136
NS	2	25	19	46

In this overview table, other than the "N" rows, entries in the "absent" and "Total" columns may be invalid and should be ignored

	Amount smoked (broad categories)				
	absent	<20k5	6-44k20	>20k45	Total
N	35	39	28	34	136
NS	21	28	23	28	100
Wt	560.17	456.45	270.48	260.78	1547.89
Het Chi	298.30	235.32	115.19	253.85	1147.26
Het df	34	38	27	33	135
Het P	***	***	***	***	***
Fixed RR	6.15	2.70	3.58	7.20	4.51
RRl	5.66	2.47	3.18	6.38	4.29
RRu	6.68	2.96	4.04	8.13	4.74
P	+++	+++	+++	+++	+++
Random RR	3.85	1.83	2.73	4.80	3.01
RRl	2.87	1.40	2.06	3.29	2.56
RRu	5.16	2.39	3.61	7.01	3.55
P	+++	+++	+++	+++	+++

	Amount smoked (narrow categories)							
	absent	<10k1	2-19k10	11-29k20	21-39k30	31-98k40	>40k99	Total
N	86	12	7	21	6	1	3	136
NS	34	7	5	18	5	1	2	71
Wt	1065.17	72.03	91.11	247.75	65.39	1.47	4.98	1547.89
Het Chi	737.27	8.39	12.29	95.97	21.89	0.00	0.70	1147.26
Het df	85	11	6	20	5	0	2	135
Het P	***	N.S.	(*)	***	***	N.S.	N.S.	***
Fixed RR	5.28	1.13	2.14	3.70	9.96	0.70	4.22	4.51
RRl	4.97	0.90	1.75	3.26	7.81	0.14	1.75	4.29
RRu	5.60	1.42	2.63	4.19	12.69	3.50	10.16	4.74
P	+++	N.S.	+++	+++	+++	N.S.	++	+++
Random RR	3.48	1.13	2.04	2.84	5.96	0.70	4.22	3.01
RRl	2.84	0.90	1.40	2.09	3.16	0.14	1.75	2.56
RRu	4.25	1.42	2.97	3.86	11.24	3.50	10.16	3.55
P	+++	N.S.	+++	+++	+++	N.S.	++	+++

MALES

	Amount smoked (broad categories)				
	absent	<20k5	6-44k20	>20k45	Total
N	19	23	20	21	83
NS	14	23	20	21	78
Wt	236.38	190.33	176.05	159.04	761.80
Het Chi	130.50	99.84	59.31	136.78	530.96
Het df	18	22	19	20	82
Het P	***	***	***	***	***
Fixed RR	6.82	2.96	3.24	5.88	4.52
RRl	6.00	2.57	2.79	5.04	4.21
RRu	7.74	3.42	3.75	6.87	4.85
P	+++	+++	+++	+++	+++
Random RR	4.40	2.09	2.69	4.68	3.20
RRl	2.83	1.44	1.98	2.93	2.60
RRu	6.85	3.02	3.65	7.47	3.94
P	+++	+++	+++	+++	+++

Table 3G11 - 3

IESLC - Meta-anal of Ever Smoking (or Curr if Ever not avail) by Amount, Overview, Any prod (or Cigs if Any not avail)
Adenocarcinoma
Most adjusted

MALES

	absent	<10k1	2-19k10	11-29k20	21-39k30	31-98k40	>40k99	Total
		Amount smoked (narrow categories)						
N	48	7	5	15	5	1	2	83
NS	25	7	5	15	5	1	2	59
Wt	452.58	43.74	61.12	158.50	40.11	1.47	4.27	761.80
Het Chi	304.97	3.50	8.38	46.13	16.31	0.00	0.01	530.96
Het df	47	6	4	14	4	0	1	82
Het P	***	N.S.	(*)	***	**	N.S.	N.S.	***
Fixed RR	5.89	1.36	2.47	3.29	7.90	0.70	4.91	4.52
RRl	5.38	1.01	1.92	2.81	5.80	0.14	1.90	4.21
RRu	6.46	1.82	3.17	3.84	10.76	3.50	12.68	4.85
P	+++	+	+++	+++	+++	N.S.	++	+++
Random RR	3.94	1.36	2.20	2.69	4.29	0.70	4.91	3.20
RRl	2.99	1.01	1.32	1.93	1.79	0.14	1.90	2.60
RRu	5.18	1.82	3.67	3.75	10.32	3.50	12.68	3.94
P	+++	+	++	+++	++	N.S.	++	+++

FEMALES

	absent	<20k5	6-44k20	>20k45	Total
	Amount smoked (broad categories)				
N	15	15	7	11	48
NS	14	15	7	11	47
Wt	308.98	262.33	89.39	83.69	744.39
Het Chi	162.18	125.09	47.81	85.32	576.85
Het df	14	14	6	10	47
Het P	***	***	***	***	***
Fixed RR	5.78	2.58	4.52	10.22	4.50
RRl	5.17	2.29	3.67	8.25	4.19
RRu	6.46	2.92	5.56	12.67	4.84
P	+++	+++	+++	+++	+++
Random RR	3.22	1.65	2.83	5.21	2.77
RRl	2.03	1.08	1.40	2.50	2.09
RRu	5.11	2.50	5.72	10.86	3.68
P	+++	+	++	+++	+++

	absent	<10k1	2-19k10	11-29k20	21-39k30	31-98k40	>40k99	Total
		Amount smoked (narrow categories)						
N	34	5	2	5	1		1	48
NS	18	5	2	5	1		1	31
Wt	575.92	28.29	29.99	84.21	25.27		0.71	744.39
Het Chi	386.44	1.22	0.26	40.51	0.00		0.00	576.85
Het df	33	4	1	4	0		0	47
Het P	***	N.S.	N.S.	***	N.S.		N.S.	***
Fixed RR	4.86	0.85	1.61	4.77	14.39		1.69	4.50
RRl	4.48	0.59	1.13	3.86	9.74		0.16	4.19
RRu	5.28	1.23	2.30	5.91	21.25		17.36	4.84
P	+++	N.S.	++	+++	+++		N.S.	+++
Random RR	3.04	0.85	1.61	3.42	14.39		1.69	2.77
RRl	2.21	0.59	1.13	1.58	9.74		0.16	2.09
RRu	4.19	1.23	2.30	7.42	21.25		17.36	3.68
P	+++	N.S.	++	++	+++		N.S.	+++

Table 3G11 - 4

IESLC - Meta-anal of Ever Smoking (or Curr if Ever not avail) by Amount, Overview, Any prod (or Cigs if Any not avail)
Adenocarcinoma
Least adjusted

REF	NRR	X	SEX	AGEL	AGEH	RACE	YF	LC TYPE	LOC	START	ST	NLC	R	VB	P	H	AD	SM	PRODUCT	exL	exH	S1	S2	DENOM	De
ALDERS	46		m	0	0	all	-	not q+s	Eu:UK	1977	CC	1448	n	V	n	n	1	ev	cig only	1	17	1	0	nev+2	ot
ALDERS	47		m	0	0	all	-	not q+s	Eu:UK	1977	CC	1448	n	V	n	n	1	ev	cig only	18	27	2	3	nev+2	ot
ALDERS	48		m	0	0	all	-	not q+s	Eu:UK	1977	CC	1448	n	V	n	n	1	ev	cig only	28	99	3	0	nev+2	ot
ALDERS	49		f	0	0	all	-	not q+s	Eu:UK	1977	CC	1448	n	V	n	n	1	ev	cig only	1	17	1	0	nev+2	ot
ALDERS	50		f	0	0	all	-	not q+s	Eu:UK	1977	CC	1448	n	V	n	n	1	ev	cig only	18	27	2	3	nev+2	ot
ALDERS	51		f	0	0	all	-	not q+s	Eu:UK	1977	CC	1448	n	V	n	n	1	ev	cig only	28	99	3	0	nev+2	ot
BARBON	45	x	m	0	0	all	-	a	Eu:wst	1979	CC	755	n	bl	y	y	0	ev	all/unsp	1	9	1	1	nev any	st
BARBON	46	x	m	0	0	all	-	a	Eu:wst	1979	CC	755	n	bl	y	y	0	ev	all/unsp	10	19	0	2	nev any	st
BARBON	47	x	m	0	0	all	-	a	Eu:wst	1979	CC	755	n	bl	y	y	0	ev	all/unsp	20	29	2	3	nev any	st
BARBON	48	x	m	0	0	all	-	a	Eu:wst	1979	CC	755	n	bl	y	y	0	ev	all/unsp	30	39	0	4	nev any	st
BARBON	49	x	m	0	0	all	-	a	Eu:wst	1979	CC	755	n	bl	y	y	0	ev	all/unsp	40	99	3	0	nev any	st
BOUCOT	27	x	m	0	0	all	0	a	NAmer	1951	pr	121	n	bl	n	n	0	cu	cig only	1	20	0	0	nev any	ot
BOUCOT	28	x	m	0	0	all	0	a	NAmer	1951	pr	121	n	bl	n	n	0	cu	cig only	21	99	3	0	nev any	ot
BROWN2	34		m	0	0	wh	-	a	NAmer	1984	CC	14596	n	bl	n	y	2	ev	cig+/-ot	1	19	1	0	nev cigs	or
BROWN2	44		m	0	0	wh	-	a	NAmer	1984	CC	14596	n	bl	n	y	2	ev	cig+/-ot	20	99	0	0	nev cigs	or
BROWN2	33		f	0	0	wh	-	a	NAmer	1984	CC	14596	n	bl	n	y	2	ev	cig+/-ot	1	19	1	0	nev cigs	or
BROWN2	43		f	0	0	wh	-	a	NAmer	1984	CC	14596	n	bl	n	y	2	ev	cig+/-ot	20	99	0	0	nev cigs	or
CHOI	51		m	0	0	all	-	a	As:oth	1985	CC	375	n	bl	n	n	0	ev	cig+/-ot	1	10	1	0	nev cigs	st
CHOI	52		m	0	0	all	-	a	As:oth	1985	CC	375	n	bl	n	n	0	ev	cig+/-ot	11	20	2	3	nev cigs	st
CHOI	53		m	0	0	all	-	a	As:oth	1985	CC	375	n	bl	n	n	0	ev	cig+/-ot	21	30	0	4	nev cigs	st
CHOI	54		m	0	0	all	-	a	As:oth	1985	CC	375	n	bl	n	n	0	ev	cig+/-ot	31	40	0	5	nev cigs	st
CHOI	55		m	0	0	all	-	a	As:oth	1985	CC	375	n	bl	n	n	0	ev	cig+/-ot	41	99	3	6	nev cigs	st
CHOI	59		f	0	0	all	-	a	As:oth	1985	CC	375	n	bl	n	n	0	ev	cig+/-ot	1	10	1	0	nev cigs	st
CHOI	60		f	0	0	all	-	a	As:oth	1985	CC	375	n	bl	n	n	0	ev	cig+/-ot	11	30	2	0	nev cigs	ot
CHOI	61		f	0	0	all	-	a	As:oth	1985	CC	375	n	bl	n	n	0	ev	cig+/-ot	31	99	3	0	nev cigs	st
CORREA	48		c	0	0	all	-	a	NAmer	1979	CC	1359	n	bl	y	n	1	cu	cig+/-ot	1	20	0	0	nev cigs	or
CORREA	52		c	0	0	all	-	a	NAmer	1979	CC	1359	n	bl	y	n	1	cu	cig+/-ot	21	99	3	0	nev cigs	or
DOLL	58	x	m	0	0	all	-	KII	Eu:UK	1948	CC	1465	n	V	n	n	0	ev	all/unsp	1	4	0	1	nev any	st
DOLL	59	x	m	0	0	all	-	KII	Eu:UK	1948	CC	1465	n	V	n	n	0	ev	all/unsp	5	14	1	2	nev any	st
DOLL	60	x	m	0	0	all	-	KII	Eu:UK	1948	CC	1465	n	V	n	n	0	ev	all/unsp	15	24	2	3	nev any	st
DOLL	61	x	m	0	0	all	-	KII	Eu:UK	1948	CC	1465	n	V	n	n	0	ev	all/unsp	25	99	3	0	nev any	st
DOLL	65	x	f	0	0	all	-	KII	Eu:UK	1948	CC	1465	n	V	n	n	0	ev	all/unsp	1	4	0	1	nev any	st
DOLL	66	x	f	0	0	all	-	KII	Eu:UK	1948	CC	1465	n	V	n	n	0	ev	all/unsp	5	14	1	2	nev any	st
DOLL	67	x	f	0	0	all	-	KII	Eu:UK	1948	CC	1465	n	V	n	n	0	ev	all/unsp	15	99	0	0	nev any	st
DORGAN	126		m	0	0	wh	-	a	NAmer	1980	CC	2026	n	bl	y	y	2	ev	cig+/-ot	1	19	1	0	nev any	ot
DORGAN	127		m	0	0	wh	-	a	NAmer	1980	CC	2026	n	bl	y	y	2	ev	cig+/-ot	20	99	0	0	nev any	ot
DORGAN	105		f	0	0	all	-	a	NAmer	1980	CC	2026	n	bl	y	y	3	ev	cig+/-ot	1	19	1	0	nev any	ot
DORGAN	106		f	0	0	all	-	a	NAmer	1980	CC	2026	n	bl	y	y	3	ev	cig+/-ot	20	99	0	0	nev any	ot
DOSEME	8		m	0	0	all	-	not q+s	Eu:bal	1979	CC	1210	n	bl	n	n	2	ev	cig+/-ot	1	10	1	0	nev cigs	or
DOSEME	12		m	0	0	all	-	not q+s	Eu:bal	1979	CC	1210	n	bl	n	n	2	ev	cig+/-ot	11	20	2	3	nev cigs	or
DOSEME	16		m	0	0	all	-	not q+s	Eu:bal	1979	CC	1210	n	bl	n	n	2	ev	cig+/-ot	21	99	3	0	nev cigs	or
ENGELA	71		m	0	0	all	0	a	Eu:Sca	1964	pr	435	n	bl	n	n	7	cu	cig+/-ot	1	4	0	1	nev cigs	or
ENGELA	72		m	0	0	all	0	a	Eu:Sca	1964	pr	435	n	bl	n	n	7	cu	cig+/-ot	5	9	1	0	nev cigs	or
ENGELA	73		m	0	0	all	0	a	Eu:Sca	1964	pr	435	n	bl	n	n	7	cu	cig+/-ot	10	14	0	2	nev cigs	or
ENGELA	74		m	0	0	all	0	a	Eu:Sca	1964	pr	435	n	bl	n	n	7	cu	cig+/-ot	15	19	0	0	nev cigs	or
ENGELA	75		m	0	0	all	0	a	Eu:Sca	1964	pr	435	n	bl	n	n	7	cu	cig+/-ot	20	99	0	0	nev cigs	or
GER	2	x	c	0	0	all	-	a	As:oth	1990	CC	141	n	ot	y	n	0	ev	all/unsp	1	10	1	0	nev any	st
GER	3	x	c	0	0	all	-	a	As:oth	1990	CC	141	n	ot	y	n	0	ev	all/unsp	11	20	2	3	nev any	st
GER	4	x	c	0	0	all	-	a	As:oth	1990	CC	141	n	ot	y	n	0	ev	all/unsp	21	99	3	0	nev any	st
HAENSZ	35		f	0	0	all	-	a	NAmer	1955	CC	158	n	bl	n	y	0	cu	cig+/-ot	1	20	0	0	nev any	or
HAENSZ	34		f	0	0	all	-	a	NAmer	1955	CC	158	n	bl	n	y	0	cu	cig+/-ot	21	99	3	0	nev any	or
HAMMON	108		m	0	0	wh	0	a	NAmer	1952	pr	448	n	bl	n	n	1	cu	cig only	1	9	1	1	nev any	ot
HAMMON	109		m	0	0	wh	0	a	NAmer	1952	pr	448	n	bl	n	n	1	cu	cig only	10	20	2	0	nev any	ot
HAMMON	110		m	0	0	wh	0	a	NAmer	1952	pr	448	n	bl	n	n	1	cu	cig only	21	39	0	4	nev any	ot
JEDRYC	15	x	m	0	0	all	-	a	Eu:est	1980	CC	1630	n	bl	y	n	0	ev	cig+/-ot	1	9	1	1	nev any	st
JEDRYC	16	x	m	0	0	all	-	a	Eu:est	1980	CC	1630	n	bl	y	n	0	ev	cig+/-ot	10	19	0	2	nev any	st
JEDRYC	17	x	m	0	0	all	-	a	Eu:est	1980	CC	1630	n	bl	y	n	0	ev	cig+/-ot	20	29	2	3	nev any	st
JEDRYC	18	x	m	0	0	all	-	a	Eu:est	1980	CC	1630	n	bl	y	n	0	ev	cig+/-ot	30	39	0	4	nev any	st
JEDRYC	19	x	m	0	0	all	-	a	Eu:est	1980	CC	1630	n	bl	y	n	0	ev	cig+/-ot	40	99	3	0	nev any	st
KATSOU	17	x	f	0	0	all	-	a	Eu:bal	1987	CC	101	n	bl	n	n	0	cu	all/unsp	1	20	0	0	nev any	st
KATSOU	18	x	f	0	0	all	-	a	Eu:bal	1987	CC	101	n	bl	n	n	0	cu	all/unsp	21	99	3	0	nev any	st
KREYBE	17	x	m	0	0	all	-	KII	Eu:Sca	1948	CC	300	n	bl	n	y	0	ev	all/unsp	1	14	1	0	nev any	st
KREYBE	18	x	m	0	0	all	-	KII	Eu:Sca	1948	CC	300	n	bl	n	y	0	ev	all/unsp	15	24	2	3	nev any	st
KREYBE	19	x	m	0	0	all	-	KII	Eu:Sca	1948	CC	300	n	bl	n	y	0	ev	all/unsp	25	99	3	0	nev any	st
KREYBE	34	x	f	0	0	all	-	KII	Eu:Sca	1948	CC	300	n	bl	n	y	0	ev	all/unsp	1	14	1	0	nev any	st
KREYBE	35	x	f	0	0	all	-	KII	Eu:Sca	1948	CC	300	n	bl	n	y	0	ev	all/unsp	15	99	0	0	nev any	st
LAMTH	16		f	0	0	ch	-	a	As:HK	1983	CC	445	n	bl	n	n	0	ev	all/unsp	1	10	1	0	nev any	or
LAMTH	17		f	0	0	ch	-	a	As:HK	1983	CC	445	n	bl	n	n	0	ev	all/unsp	11	20	2	3	nev any	or
LAMTH	18		f	0	0	ch	-	a	As:HK	1983	CC	445	n	bl	n	n	0	ev	all/unsp	21	99	3	0	nev any	or
LUBIN2	152		m	0	0	all	-	a	Eu:mul	1976	CC	7804	n	bl	n	y	0	ev	cig+/-ot	1	9	1	1	nev any	st
LUBIN2	156		m	0	0	all	-	a	Eu:mul	1976	CC	7804	n	bl	n	y	0	ev	cig+/-ot	10	19	0	2	nev any	st
LUBIN2	160		m	0	0	all	-	a	Eu:mul	1976	CC	7804	n	bl	n	y	0	ev	cig+/-ot	20	29	2	3	nev any	st

Table 3G11 - 4

IESLC - Meta-anal of Ever Smoking (or Curr if Ever not avail) by Amount, Overview, Any prod (or Cigs if Any not avail)
Adenocarcinoma
Least adjusted

REF	NRR	X	SEX	AGEL	AGEH	RACE	YF	LC TYPE	LOC	START	ST	NLC	R	VB	P	H	AD	SM	PRODUCT	exL	exH	S1	S2	DENOM	De
LUBIN2	164		m	0	0	all	-	a	Eu:mul	1976	CC	7804	n	bl	n	y	0	ev	cig+/-ot	30	99	3	0	nev any	st
LUBIN2	172		f	0	0	all	-	a	Eu:mul	1976	CC	7804	n	bl	n	y	0	ev	cig+/-ot	1	9	1	1	nev any	st
LUBIN2	176		f	0	0	all	-	a	Eu:mul	1976	CC	7804	n	bl	n	y	0	ev	cig+/-ot	10	19	0	2	nev any	st
LUBIN2	180		f	0	0	all	-	a	Eu:mul	1976	CC	7804	n	bl	n	y	0	ev	cig+/-ot	20	29	2	3	nev any	st
LUBIN2	184		f	0	0	all	-	a	Eu:mul	1976	CC	7804	n	bl	n	y	0	ev	cig+/-ot	30	99	3	0	nev any	st
MATOS	56	x	m	0	0	all	-	a	SCAmer	1994	CC	200	n	bl	n	n	0	ev	cig+/-ot	1	14	1	0	nev any	st
MATOS	58	x	m	0	0	all	-	a	SCAmer	1994	CC	200	n	bl	n	n	0	ev	cig+/-ot	15	24	2	3	nev any	st
MATOS	60	x	m	0	0	all	-	a	SCAmer	1994	CC	200	n	bl	n	n	0	ev	cig+/-ot	25	99	3	0	nev any	st
MATSUD	7		m	0	0	all	-	a	As:Jap	1965	CC	179	n	bl	n	n	0	ev	cig+/-ot	1	10	1	0	nev cigs	ot
MATSUD	8		m	0	0	all	-	a	As:Jap	1965	CC	179	n	bl	n	n	0	ev	cig+/-ot	11	20	2	3	nev cigs	ot
MATSUD	9		m	0	0	all	-	a	As:Jap	1965	CC	179	n	bl	n	n	0	ev	cig+/-ot	21	99	3	0	nev cigs	ot
ORMOS	22		m	0	0	all	-	KII	Eu:est	1947	CC	119	n	bl	y	y	0	ev	cig+/-ot	1	15	1	0	nev any	st
ORMOS	23		m	0	0	all	-	KII	Eu:est	1947	CC	119	n	bl	y	y	0	ev	cig+/-ot	16	30	2	0	nev any	st
ORMOS	24		m	0	0	all	-	KII	Eu:est	1947	CC	119	n	bl	y	y	0	ev	cig+/-ot	31	99	3	0	nev any	st
OSANN	55		m	0	0	all	-	a	NAmer	1984	CC	1986	n	bl	n	n	2	ev	cig+/-ot	1	39	0	0	nev cigs	or
OSANN	63		m	0	0	all	-	a	NAmer	1984	CC	1986	n	bl	n	n	2	ev	cig+/-ot	40	99	3	0	nev cigs	or
OSANN	56		f	0	0	all	-	a	NAmer	1984	CC	1986	n	bl	n	n	2	ev	cig+/-ot	1	39	0	0	nev cigs	or
OSANN	64		f	0	0	all	-	a	NAmer	1984	CC	1986	n	bl	n	n	2	ev	cig+/-ot	40	99	3	0	nev cigs	or
OSANN2	16	x	f	0	0	all	-	KII	NAmer	1964	ot	217	n	bl	n	y	0	ev	cig+/-ot	1	19	1	0	nev cigs	st
OSANN2	17	x	f	0	0	all	-	KII	NAmer	1964	ot	217	n	bl	n	y	0	ev	cig+/-ot	20	99	0	0	nev cigs	st
SOBUE	56		m	0	0	all	-	a	As:Jap	1986	CC	1376	n	bl	n	y	0	cu	cig+/-ot	1	19	1	0	nev cigs	st
SOBUE	57		m	0	0	all	-	a	As:Jap	1986	CC	1376	n	bl	n	y	0	cu	cig+/-ot	20	29	2	3	nev cigs	st
SOBUE	58		m	0	0	all	-	a	As:Jap	1986	CC	1376	n	bl	n	y	0	cu	cig+/-ot	30	99	3	0	nev cigs	st
SVENSS	29	x	f	0	0	all	-	a	Eu:Sca	1983	CC	210	n	bl	n	n	0	cu	all/unsp	1	10	1	0	nev any	st
SVENSS	34	x	f	0	0	all	-	a	Eu:Sca	1983	CC	210	n	bl	n	n	0	cu	all/unsp	11	20	2	3	nev any	st
SVENSS	39	x	f	0	0	all	-	a	Eu:Sca	1983	CC	210	n	bl	n	n	0	cu	all/unsp	21	99	3	0	nev any	st
TSUGAN	4		m	0	0	all	-	a	As:Jap	1976	CC	134	n	bl	n	y	0	cu	all/unsp	1	15	1	0	nev any	st
TSUGAN	5		m	0	0	all	-	a	As:Jap	1976	CC	134	n	bl	n	y	0	cu	all/unsp	16	35	2	0	nev any	st
TSUGAN	6		m	0	0	all	-	a	As:Jap	1976	CC	134	n	bl	n	y	0	cu	all/unsp	36	99	3	0	nev any	st
WAKAI	49	x	m	0	0	all	-	a	As:Jap	1988	CC	333	n	bl	n	y	0	cu	cig+/-ot	1	19	1	0	nev any	st
WAKAI	50	x	m	0	0	all	-	a	As:Jap	1988	CC	333	n	bl	n	y	0	cu	cig+/-ot	20	29	2	3	nev any	st
WAKAI	51	x	m	0	0	all	-	a	As:Jap	1988	CC	333	n	bl	n	y	0	cu	cig+/-ot	30	99	3	0	nev any	st
WU	3	x	f	0	0	wh	-	a	NAmer	1981	CC	220	n	bl	n	y	0	cu	all/unsp	1	20	0	0	nev any	st
WU	4	x	f	0	0	wh	-	a	NAmer	1981	CC	220	n	bl	n	y	0	cu	all/unsp	21	99	3	0	nev any	st
WUWILL	22	x	f	0	0	all	-	a	As:Chi	1985	CC	965	n	ot	n	n	0	ev	cig+/-ot	1	19	1	0	nev cigs	st
WUWILL	23	x	f	0	0	all	-	a	As:Chi	1985	CC	965	n	ot	n	n	0	ev	cig+/-ot	20	99	0	0	nev cigs	st
WYNDE2	10		m	0	0	all	-	KII	NAmer	1962	CC	404	n	bl	n	y	0	ev	cig+/-ot	1	10	1	0	nev any	st
WYNDE2	11		m	0	0	all	-	KII	NAmer	1962	CC	404	n	bl	n	y	0	ev	cig+/-ot	11	20	2	3	nev any	st
WYNDE2	12		m	0	0	all	-	KII	NAmer	1962	CC	404	n	bl	n	y	0	ev	cig+/-ot	21	34	0	4	nev any	st
WYNDE2	13		m	0	0	all	-	KII	NAmer	1962	CC	404	n	bl	n	y	0	ev	cig+/-ot	35	99	3	0	nev any	st
WYNDE3	24		m	0	0	all	-	KII	NAmer	1966	CC	350	n	bl	n	y	0	ev	cig+/-ot	1	9	1	1	nev any	st
WYNDE3	25		m	0	0	all	-	KII	NAmer	1966	CC	350	n	bl	n	y	0	ev	cig+/-ot	10	20	2	0	nev any	st
WYNDE3	26		m	0	0	all	-	KII	NAmer	1966	CC	350	n	bl	n	y	0	ev	cig+/-ot	21	40	0	0	nev any	st
WYNDE3	27		m	0	0	all	-	KII	NAmer	1966	CC	350	n	bl	n	y	0	ev	cig+/-ot	41	99	3	6	nev any	st
WYNDE3	71		f	0	0	all	-	KII	NAmer	1966	CC	350	n	bl	n	y	0	ev	cig+/-ot	1	9	1	1	nev any	st
WYNDE3	72		f	0	0	all	-	KII	NAmer	1966	CC	350	n	bl	n	y	0	ev	cig+/-ot	10	20	2	0	nev any	st
WYNDE3	73		f	0	0	all	-	KII	NAmer	1966	CC	350	n	bl	n	y	0	ev	cig+/-ot	21	40	0	0	nev any	st
WYNDE3	74		f	0	0	all	-	KII	NAmer	1966	CC	350	n	bl	n	y	0	ev	cig+/-ot	41	99	3	6	nev any	st
WYNDE4	37		m	0	0	all	-	a	NAmer	1948	CC	684	n	bl	y	n	0	ev	all/unsp	1	9	1	1	nev any	st
WYNDE4	38		m	0	0	all	-	a	NAmer	1948	CC	684	n	bl	y	n	0	ev	all/unsp	10	15	0	2	nev any	st
WYNDE4	39		m	0	0	all	-	a	NAmer	1948	CC	684	n	bl	y	n	0	ev	all/unsp	16	20	2	3	nev any	st
WYNDE4	40		m	0	0	all	-	a	NAmer	1948	CC	684	n	bl	y	n	0	ev	all/unsp	21	34	0	4	nev any	st
WYNDE4	41		m	0	0	all	-	a	NAmer	1948	CC	684	n	bl	y	n	0	ev	all/unsp	35	99	3	0	nev any	st
WYNDE4	55		f	0	0	all	-	a	NAmer	1948	CC	684	n	bl	y	n	2	ev	all/unsp	1	9	1	1	nev any	ot
WYNDE6	24		m	0	0	all	-	KII	NAmer	1969	CC	4423	n	bl	n	y	0	cu	cig+/-ot	1	10	1	0	nev any	st
WYNDE6	33		m	0	0	all	-	KII	NAmer	1969	CC	4423	n	bl	n	y	0	cu	cig+/-ot	11	20	2	3	nev any	st
WYNDE6	42		m	0	0	all	-	KII	NAmer	1969	CC	4423	n	bl	n	y	0	cu	cig+/-ot	21	30	0	4	nev any	st
WYNDE6	51		m	0	0	all	-	KII	NAmer	1969	CC	4423	n	bl	n	y	0	cu	cig+/-ot	31	99	3	0	nev any	st
WYNDE6	213		f	0	0	all	-	KII	NAmer	1969	CC	4423	n	bl	n	y	0	cu	cig+/-ot	1	10	1	0	nev cigs	st
WYNDE6	222		f	0	0	all	-	KII	NAmer	1969	CC	4423	n	bl	n	y	0	cu	cig+/-ot	11	20	2	3	nev cigs	st
WYNDE6	231		f	0	0	all	-	KII	NAmer	1969	CC	4423	n	bl	n	y	0	cu	cig+/-ot	21	30	0	4	nev cigs	st
WYNDE6	240		f	0	0	all	-	KII	NAmer	1969	CC	4423	n	bl	n	y	0	cu	cig+/-ot	30	99	3	0	nev cigs	st
ZHENG	6		m	0	0	all	-	a	As:Chi	1982	CC	540	n	ot	*	y	0	ev	cig+/-ot	1	9	1	1	nev cigs	st
ZHENG	7		m	0	0	all	-	a	As:Chi	1982	CC	540	n	ot	*	y	0	ev	cig+/-ot	10	19	0	2	nev cigs	st
ZHENG	8		m	0	0	all	-	a	As:Chi	1982	CC	540	n	ot	*	y	0	ev	cig+/-ot	20	29	2	3	nev cigs	st
ZHENG	9		m	0	0	all	-	a	As:Chi	1982	CC	540	n	ot	*	y	0	ev	cig+/-ot	30	99	3	0	nev cigs	st
ZHENG	19		f	0	0	all	-	a	As:Chi	1982	CC	540	n	ot	*	y	0	ev	cig+/-ot	1	9	1	1	nev cigs	st
ZHENG	20		f	0	0	all	-	a	As:Chi	1982	CC	540	n	ot	*	y	0	ev	cig+/-ot	10	99	0	0	nev cigs	st

Cigarette type is all/unspec for all RRs

Table 3G11 - 4

IESLC - Meta-anal of Ever Smoking (or Curr if Ever not avail) by Amount, Overview, Any prod (or Cigs if Any not avail)
Adenocarcinoma
Least adjusted

except for the following:

REF	NRR	CIGTYPE
ALDERS	46	MC only
ALDERS	47	MC only
ALDERS	48	MC only
ALDERS	49	MC only
ALDERS	50	MC only
ALDERS	51	MC only

REF	NRR	Cigarette equivalent
ALDERS	46	-
ALDERS	47	-
ALDERS	48	-
ALDERS	49	-
ALDERS	50	-
ALDERS	51	-
BARBON	45	*
BARBON	46	*
BARBON	47	*
BARBON	48	*
BARBON	49	*
BOUCOT	27	-
BOUCOT	28	-
BROWN2	34	*
BROWN2	44	*
BROWN2	33	*
BROWN2	43	*
CHOI	51	*
CHOI	52	*
CHOI	53	*
CHOI	54	*
CHOI	55	*
CHOI	59	*
CHOI	60	*
CHOI	61	*
CORREA	48	*
CORREA	52	*
DOLL	58	grams
DOLL	59	grams
DOLL	60	grams
DOLL	61	grams
DOLL	65	grams
DOLL	66	grams
DOLL	67	grams
DORGAN	126	*
DORGAN	127	*
DORGAN	105	*
DORGAN	106	*
DOSEME	8	*
DOSEME	12	*
DOSEME	16	*
ENGELA	71	*
ENGELA	72	*
ENGELA	73	*
ENGELA	74	*
ENGELA	75	*
GER	2	*
GER	3	*
GER	4	*
HAENSZ	35	*
HAENSZ	34	*
HAMMON	108	-
HAMMON	109	-
HAMMON	110	-
JEDRYC	15	*
JEDRYC	16	*
JEDRYC	17	*
JEDRYC	18	*
JEDRYC	19	*
KATSOU	17	*
KATSOU	18	*
KREYBE	17	grams inc 1 cig=1
KREYBE	18	grams inc 1 cig=1

Table 3G11 - 4

IESLC - Meta-anal of Ever Smoking (or Curr if Ever not avail) by Amount, Overview, Any prod (or Cigs if Any not avail)
Adenocarcinoma
Least adjusted

REF	NRR	Cigarette equivalent
KREYBE	19	grams inc 1 cig=1
KREYBE	34	grams inc 1 cig=1
KREYBE	35	grams inc 1 cig=1
LAMTH	16	*
LAMTH	17	*
LAMTH	18	*
LUBIN2	152	*
LUBIN2	156	*
LUBIN2	160	*
LUBIN2	164	*
LUBIN2	172	*
LUBIN2	176	*
LUBIN2	180	*
LUBIN2	184	*
MATOS	56	*
MATOS	58	*
MATOS	60	*
MATSUD	7	*
MATSUD	8	*
MATSUD	9	*
ORMOS	22	*
ORMOS	23	*
ORMOS	24	*
OSANN	55	*
OSANN	63	*
OSANN	56	*
OSANN	64	*
OSANN2	16	*
OSANN2	17	*
SOBUE	56	*
SOBUE	57	*
SOBUE	58	*
SVENSS	29	*
SVENSS	34	*
SVENSS	39	*
TSUGAN	4	*
TSUGAN	5	*
TSUGAN	6	*
WAKAI	49	*
WAKAI	50	*
WAKAI	51	*
WU	3	*
WU	4	*
WUWILL	22	*
WUWILL	23	*
WYNDE2	10	*
WYNDE2	11	*
WYNDE2	12	*
WYNDE2	13	*
WYNDE3	24	*
WYNDE3	25	*
WYNDE3	26	*
WYNDE3	27	*
WYNDE3	71	*
WYNDE3	72	*
WYNDE3	73	*
WYNDE3	74	*
WYNDE4	37	inc 1 cigar = 5 cigs, 1 pipe = 2.5 cigs
WYNDE4	38	inc 1 cigar = 5 cigs, 1 pipe = 2.5 cigs
WYNDE4	39	inc 1 cigar = 5 cigs, 1 pipe = 2.5 cigs
WYNDE4	40	inc 1 cigar = 5 cigs, 1 pipe = 2.5 cigs
WYNDE4	41	inc 1 cigar = 5 cigs, 1 pipe = 2.5 cigs
WYNDE4	55	inc 1 cigar = 5 cigs, 1 pipe = 2.5 cigs
WYNDE6	24	*
WYNDE6	33	*
WYNDE6	42	*
WYNDE6	51	*
WYNDE6	213	*
WYNDE6	222	*
WYNDE6	231	*
WYNDE6	240	*
ZHENG	6	*

Table 3G11 - 4

IESLC - Meta-anal of Ever Smoking (or Curr if Ever not avail) by Amount, Overview, Any prod (or Cigs if Any not avail)
Adenocarcinoma
Least adjusted

REF	NRR	Cigarette equivalent
ZHENG	7	*
ZHENG	8	*
ZHENG	9	*
ZHENG	19	*
ZHENG	20	*

In this overview table, subtotals and Qs values may be invalid and should be ignored

Table 3G11 - 5

IESLC - Meta-anal of Ever Smoking (or Curr if Ever not avail) by Amount, Overview, Any prod (or Cigs if Any not avail)
Adenocarcinoma
Least adjusted

REF	NRR	SEX	AD	Number Exposed Case	Number Exposed Cont	Non-exposed Case	Non-exposed Cont	RR	95.00%CI
ALDERS	46	m	1	-	-	-	-	2.80	(1.01- 7.75)
ALDERS	47	m	1	-	-	-	-	2.67	(0.99- 7.18)
ALDERS	48	m	1	-	-	-	-	3.32	(1.36- 8.10)
ALDERS	49	f	1	-	-	-	-	2.77	(1.63- 4.70)
ALDERS	50	f	1	-	-	-	-	4.58	(2.67- 7.85)
ALDERS	51	f	1	-	-	-	-	3.31	(1.80- 6.10)
Subtotal ALDERS								3.34	(2.53- 4.41)
BARBON	45	m	0	7	87	7	188	2.16	(0.74- 6.35)
BARBON	46	m	0	31	111	7	188	7.50	(3.20- 17.60)
BARBON	47	m	0	43	176	7	188	6.56	(2.88- 14.97)
BARBON	48	m	0	30	82	7	188	9.83	(4.15- 23.28)
BARBON	49	m	0	40	111	7	188	9.68	(4.19- 22.34)
Subtotal BARBON								6.90	(4.66- 10.22)
*BOUCOT	27	m	0	8	15208	0	7551	8.44~	(0.49- 146.22)
*BOUCOT	28	m	0	6	6940	0	7551	14.14~	(0.80- 251.03)
Subtotal BOUCOT								10.90	(1.44- 82.62)
BROWN2	34	m	2	-	-	-	-	6.20	(4.90- 7.90)
BROWN2	44	m	2	-	-	-	-	10.70	(8.90- 13.00)
BROWN2	33	f	2	-	-	-	-	5.80	(4.70- 7.10)
BROWN2	43	f	2	-	-	-	-	8.60	(7.30- 10.10)
Subtotal BROWN2								7.91	(7.18- 8.72)
CHOI	51	m	0	4	90	7	95	0.60	(0.17- 2.13)
CHOI	52	m	0	27	281	7	95	1.30	(0.55- 3.09)
CHOI	53	m	0	11	49	7	95	3.05	(1.11- 8.35)
CHOI	54	m	0	2	39	7	95	0.70	(0.14- 3.50)
CHOI	55	m	0	2	6	7	95	4.52	(0.77- 26.69)
CHOI	59	f	0	4	16	49	164	0.84	(0.27- 2.62)
CHOI	60	f	0	0	9	49	164	0.17~	(0.01- 3.06)
CHOI	61	f	0	1	1	49	164	3.35	(0.21- 54.50)
Subtotal CHOI								1.32	(0.83- 2.09)
CORREA	48	c	1	-	-	-	-	4.30	(2.60- 7.20)
CORREA	52	c	1	-	-	-	-	12.00	(7.30- 19.70)
Subtotal CORREA								7.28	(5.10- 10.39)
DOLL	58	m	0	2	129	2	61	0.47	(0.07- 3.44)
DOLL	59	m	0	14	570	2	61	0.75	(0.17- 3.37)
DOLL	60	m	0	16	431	2	61	1.13	(0.25- 5.05)
DOLL	61	m	0	6	166	2	61	1.10	(0.22- 5.61)
DOLL	65	f	0	2	25	5	59	0.94	(0.17- 5.19)
DOLL	66	f	0	4	18	5	59	2.62	(0.64- 10.81)
DOLL	67	f	0	2	6	5	59	3.93	(0.62- 24.83)
Subtotal DOLL								1.27	(0.69- 2.35)
DORGAN	126	m	2	-	-	-	-	3.23	(1.20- 8.70)
DORGAN	127	m	2	-	-	-	-	5.63	(2.22- 14.25)
DORGAN	105	f	3	-	-	-	-	2.87	(2.00- 4.11)
DORGAN	106	f	3	-	-	-	-	5.39	(3.73- 7.78)
Subtotal DORGAN								3.96	(3.11- 5.04)
DOSEME	8	m	2	-	-	-	-	1.80	(0.80- 4.10)
DOSEME	12	m	2	-	-	-	-	2.70	(1.60- 4.70)
DOSEME	16	m	2	-	-	-	-	3.20	(1.40- 7.00)
Subtotal DOSEME								2.56	(1.73- 3.79)
*ENGELA	71	m	7	-	-	-	-	2.60	(0.60- 11.00)
*ENGELA	72	m	7	-	-	-	-	8.50	(2.50- 29.00)
*ENGELA	73	m	7	-	-	-	-	7.10	(2.10- 24.00)
*ENGELA	74	m	7	-	-	-	-	9.30	(2.10- 41.00)
*ENGELA	75	m	7	-	-	-	-	17.00	(5.10- 56.00)
Subtotal ENGELA								8.06	(4.51- 14.41)
GER	2	c	0	9	47	37	149	0.77	(0.35- 1.71)
GER	3	c	0	21	66	37	149	1.28	(0.70- 2.36)
GER	4	c	0	5	26	37	149	0.77	(0.28- 2.15)
Subtotal GER								1.00	(0.65- 1.55)
HAENSZ	35	f	0	10	66	37	236	0.97	(0.46- 2.05)
HAENSZ	34	f	0	5	13	37	236	2.45	(0.83- 7.28)
Subtotal HAENSZ								1.30	(0.70- 2.42)
*HAMMON	108	m	1	-	-	-	-	1.83	(0.17- 20.22)
*HAMMON	109	m	1	-	-	-	-	2.83	(0.55- 14.60)
*HAMMON	110	m	1	-	-	-	-	6.44	(1.34- 31.02)
Subtotal HAMMON								3.71	(1.33- 10.33)
JEDRYC	15	m	0	2	67	7	289	1.23	(0.25- 6.07)
JEDRYC	16	m	0	11	199	7	289	2.28	(0.87- 5.99)
JEDRYC	17	m	0	52	434	7	289	4.95	(2.22- 11.04)
JEDRYC	18	m	0	19	118	7	289	6.65	(2.72- 16.23)

Table 3G11 - 5

IESLC - Meta-anal of Ever Smoking (or Curr if Ever not avail) by Amount, Overview, Any prod (or Cigs if Any not avail)
Adenocarcinoma
Least adjusted

REF	NRR	SEX	AD	Number Exposed Case	Number Exposed Cont	Non-exposed Case	Non-exposed Cont	RR	95.00%CI
JEDRYC	19	m	0	9	82	7	289	4.53	(1.64- 12.54)
Subtotal JEDRYC								4.02	(2.59- 6.22)
KATSOU	17	f	0	9	14	30	67	1.44	(0.56- 3.68)
KATSOU	18	f	0	6	4	30	67	3.35	(0.88- 12.75)
Subtotal KATSOU								1.90	(0.88- 4.11)
KREYBE	17	m	0	31	2341	3	644	2.84	(0.87- 9.33)
KREYBE	18	m	0	6	925	3	644	1.39	(0.35- 5.59)
KREYBE	19	m	0	5	248	3	644	4.33	(1.03- 18.25)
KREYBE	34	f	0	9	286	27	657	0.77	(0.36- 1.65)
KREYBE	35	f	0	1	42	27	657	0.58	(0.08- 4.37)
Subtotal KREYBE								1.33	(0.79- 2.24)
LAMTH	16	f	0	36	29	131	158	1.50	(0.87- 2.57)
LAMTH	17	f	0	27	14	131	158	2.33	(1.17- 4.62)
LAMTH	18	f	0	9	5	131	158	2.17	(0.71- 6.64)
Subtotal LAMTH								1.82	(1.22- 2.71)
LUBIN2	152	m	0	66	2194	57	2616	1.38	(0.96- 1.98)
LUBIN2	156	m	0	204	3385	57	2616	2.77	(2.05- 3.73)
LUBIN2	160	m	0	234	3108	57	2616	3.46	(2.57- 4.64)
LUBIN2	164	m	0	151	1746	57	2616	3.97	(2.91- 5.42)
LUBIN2	172	f	0	20	184	138	1180	0.93	(0.57- 1.52)
LUBIN2	176	f	0	43	234	138	1180	1.57	(1.09- 2.27)
LUBIN2	180	f	0	13	110	138	1180	1.01	(0.55- 1.84)
LUBIN2	184	f	0	9	39	138	1180	1.97	(0.94- 4.16)
Subtotal LUBIN2								2.28	(1.99- 2.60)
MATOS	56	m	0	11	88	5	110	2.75	(0.92- 8.21)
MATOS	58	m	0	28	90	5	110	6.84	(2.54- 18.45)
MATOS	60	m	0	40	105	5	110	8.38	(3.19- 22.05)
Subtotal MATOS								5.68	(3.16- 10.19)
MATSUD	7	m	0	6	1237	0	1255	13.19~	(0.74- 234.37)
MATSUD	8	m	0	13	1607	0	1255	21.09~	(1.25- 355.07)
MATSUD	9	m	0	4	470	0	1255	24.02~	(1.29- 446.92)
Subtotal MATSUD								18.81	(3.58- 98.87)
ORMOS	22	m	0	4	329	2	777	4.72	(0.86- 25.91)
ORMOS	23	m	0	3	577	2	777	2.02	(0.34- 12.13)
ORMOS	24	m	0	1	128	2	777	3.04	(0.27- 33.72)
Subtotal ORMOS								3.13	(1.04- 9.39)
OSANN	55	m	2	-	-	-	-	16.50	(9.30- 29.30)
OSANN	63	m	2	-	-	-	-	37.50	(21.30- 66.00)
OSANN	56	f	2	-	-	-	-	8.80	(6.10- 12.80)
OSANN	64	f	2	-	-	-	-	24.20	(15.80- 37.20)
Subtotal OSANN								16.57	(13.16- 20.85)
OSANN2	16	f	0	14	24	22	43	1.14	(0.49- 2.63)
OSANN2	17	f	0	47	15	22	43	6.12	(2.82- 13.30)
Subtotal OSANN2								2.81	(1.59- 4.97)
SOBUE	56	m	0	63	157	27	128	1.90	(1.15- 3.16)
SOBUE	57	m	0	95	222	27	128	2.03	(1.26- 3.28)
SOBUE	58	m	0	76	187	27	128	1.93	(1.18- 3.16)
Subtotal SOBUE								1.95	(1.47- 2.60)
SVENSS	29	f	0	12	30	22	120	2.18	(0.97- 4.90)
SVENSS	34	f	0	22	22	22	120	5.45	(2.59- 11.50)
SVENSS	39	f	0	4	1	22	120	21.82	(2.33- 204.53)
Subtotal SVENSS								3.97	(2.33- 6.76)
TSUGAN	4	m	0	12	14	18	17	0.81	(0.29- 2.24)
TSUGAN	5	m	0	23	23	18	17	0.94	(0.39- 2.28)
TSUGAN	6	m	0	10	13	18	17	0.73	(0.25- 2.09)
Subtotal TSUGAN								0.84	(0.48- 1.47)
WAKAI	49	m	0	16	105	8	65	1.24	(0.50- 3.06)
WAKAI	50	m	0	30	129	8	65	1.89	(0.82- 4.35)
WAKAI	51	m	0	27	48	8	65	4.57	(1.91- 10.94)
Subtotal WAKAI								2.22	(1.34- 3.67)
WU	3	f	0	38	28	29	62	2.90	(1.50- 5.60)
WU	4	f	0	61	22	29	62	5.93	(3.07- 11.44)
Subtotal WU								4.15	(2.61- 6.60)
WUWILL	22	f	0	112	311	172	601	1.26	(0.96- 1.66)
WUWILL	23	f	0	26	40	172	601	2.27	(1.35- 3.83)
Subtotal WUWILL								1.43	(1.12- 1.82)
WYNDE2	10	m	0	2	114	5	105	0.37	(0.07- 1.94)
WYNDE2	11	m	0	14	203	5	105	1.45	(0.51- 4.13)
WYNDE2	12	m	0	14	83	5	105	3.54	(1.23- 10.23)
WYNDE2	13	m	0	16	112	5	105	3.00	(1.06- 8.48)
Subtotal WYNDE2								1.98	(1.12- 3.51)

Table 3G11 - 5

IESLC - Meta-anal of Ever Smoking (or Curr if Ever not avail) by Amount, Overview, Any prod (or Cigs if Any not avail)
Adenocarcinoma
Least adjusted

REF	NRR	SEX	AD	Number Exposed Case	Number Exposed Cont	Non-exposed Case	Non-exposed Cont	RR	95.00%CI
WYNDE3	24	m	0	1	42	6	88	0.35	(0.04- 2.99)
WYNDE3	25	m	0	20	114	6	88	2.57	(0.99- 6.68)
WYNDE3	26	m	0	34	82	6	88	6.08	(2.43- 15.24)
WYNDE3	27	m	0	9	26	6	88	5.08	(1.65- 15.59)
WYNDE3	71	f	0	2	19	15	76	0.53	(0.11- 2.53)
WYNDE3	72	f	0	11	24	15	76	2.32	(0.94- 5.73)
WYNDE3	73	f	0	7	10	15	76	3.55	(1.16- 10.80)
WYNDE3	74	f	0	1	3	15	76	1.69	(0.16- 17.36)
Subtotal WYNDE3								2.82	(1.87- 4.25)
WYNDE4	37	m	0	3	82	4	115	1.05	(0.23- 4.83)
WYNDE4	38	m	0	6	147	4	115	1.17	(0.32- 4.26)
WYNDE4	39	m	0	15	274	4	115	1.57	(0.51- 4.84)
WYNDE4	40	m	0	4	98	4	115	1.17	(0.29- 4.82)
WYNDE4	41	m	0	7	64	4	115	3.14	(0.89- 11.15)
WYNDE4	55	f	2	-	-	-	-	1.33	(0.29- 6.07)
Subtotal WYNDE4								1.51	(0.88- 2.60)
WYNDE6	24	m	0	42	122	58	617	3.66	(2.35- 5.70)
WYNDE6	33	m	0	191	293	58	617	6.93	(5.01- 9.60)
WYNDE6	42	m	0	136	129	58	617	11.22	(7.81- 16.09)
WYNDE6	51	m	0	282	197	58	617	15.23	(11.00- 21.07)
WYNDE6	213	f	0	39	109	119	856	2.57	(1.70- 3.89)
WYNDE6	222	f	0	176	165	119	856	7.67	(5.76- 10.21)
WYNDE6	231	f	0	100	50	119	856	14.39	(9.74- 21.25)
WYNDE6	240	f	0	157	52	119	856	21.72	(15.04- 31.37)
Subtotal WYNDE6								8.99	(7.93- 10.19)
ZHENG	6	m	0	18	40	29	94	1.46	(0.73- 2.92)
ZHENG	7	m	0	35	66	29	94	1.72	(0.96- 3.08)
ZHENG	8	m	0	53	89	29	94	1.93	(1.13- 3.30)
ZHENG	9	m	0	17	23	29	94	2.40	(1.13- 5.08)
ZHENG	19	f	0	13	29	119	184	0.69	(0.35- 1.39)
ZHENG	20	f	0	20	15	119	184	2.06	(1.02- 4.19)
Subtotal ZHENG								1.62	(1.24- 2.11)
Partial Totals				3527	49475	3634	55947		

*prospective study ~ With 0.5 adjustment for zero

REF	NRR	SEX	AD	Ys	Ws	Qs	Ps
ALDERS	46	m	1	1.03	3.70	0.78	0.0476
ALDERS	47	m	1	0.98	3.91	1.00	0.0520
ALDERS	48	m	1	1.20	4.83	0.40	0.0084
ALDERS	49	f	1	1.02	13.70	3.02	0.0002
ALDERS	50	f	1	1.52	13.21	0.01	0.0000
ALDERS	51	f	1	1.20	10.32	0.88	0.0001
Subtotal ALDERS				1.21	49.67	6.10	
BARBON	45	m	0	0.77	3.31	1.70	0.1612
BARBON	46	m	0	2.01	5.28	1.46	0.0000
BARBON	47	m	0	1.88	5.65	0.87	0.0000
BARBON	48	m	0	2.29	5.16	3.27	0.0000
BARBON	49	m	0	2.27	5.49	3.35	0.0000
Subtotal BARBON				1.93	24.88	10.66	
*BOUCOT	27	m	0	2.13	0.47	0.20	0.1427
*BOUCOT	28	m	0	2.65	0.46	0.63	0.0710
Subtotal BOUCOT				2.39	0.94	0.82	
BROWN2	34	m	2	1.82	67.36	7.60	0.0000
BROWN2	44	m	2	2.37	107.03	83.20	0.0000
BROWN2	33	f	2	1.76	90.29	6.55	0.0000
BROWN2	43	f	2	2.15	145.78	64.12	0.0000
Subtotal BROWN2				2.07	410.46	161.48	
CHOI	51	m	0	-0.51	2.41	9.59	0.4323
CHOI	52	m	0	0.27	5.16	7.71	0.5467
CHOI	53	m	0	1.11	3.78	0.53	0.0304
CHOI	54	m	0	-0.36	1.47	5.05	0.6600
CHOI	55	m	0	1.51	1.22	0.00	0.0956
CHOI	59	f	0	-0.18	2.95	8.20	0.7595
CHOI	60	f	0	-1.74	0.47	4.90	0.2324
CHOI	61	f	0	1.21	0.49	0.04	0.3961
Subtotal CHOI				0.27	17.95	36.02	
CORREA	48	c	1	1.46	14.81	0.01	0.0000
CORREA	52	c	1	2.48	15.59	15.48	0.0000
Subtotal CORREA				1.98	30.40	15.49	
DOLL	58	m	0	-0.75	0.98	4.89	0.4593

Table 3G11 - 5

IESLC - Meta-anal of Ever Smoking (or Curr if Ever not avail) by Amount, Overview, Any prod (or Cigs if Any not avail)
Adenocarcinoma
Least adjusted

REF	NRR	SEX	AD	Ys	Ws	Qs	Ps
DOLL	59	m	0	-0.29	1.70	5.36	0.7068
DOLL	60	m	0	0.12	1.72	3.20	0.8706
DOLL	61	m	0	0.10	1.45	2.81	0.9065
DOLL	65	f	0	-0.06	1.32	3.16	0.9472
DOLL	66	f	0	0.96	1.91	0.53	0.1823
DOLL	67	f	0	1.37	1.13	0.02	0.1451
Subtotal DOLL				0.24	10.21	19.96	
DORGAN	126	m	2	1.17	3.92	0.39	0.0203
DORGAN	127	m	2	1.73	4.45	0.26	0.0003
DORGAN	105	f	3	1.05	29.62	5.58	0.0000
DORGAN	106	f	3	1.68	28.43	1.09	0.0000
Subtotal DORGAN				1.38	66.41	7.32	
DOSEME	8	m	2	0.59	5.75	4.67	0.1585
DOSEME	12	m	2	0.99	13.23	3.25	0.0003
DOSEME	16	m	2	1.16	5.93	0.63	0.0046
Subtotal DOSEME				0.94	24.92	8.54	
*ENGELA	71	m	7	0.96	1.82	0.52	0.1979
*ENGELA	72	m	7	2.14	2.56	1.09	0.0006
*ENGELA	73	m	7	1.96	2.59	0.58	0.0016
*ENGELA	74	m	7	2.23	1.74	0.96	0.0033
*ENGELA	75	m	7	2.83	2.68	4.84	0.0000
Subtotal ENGELA				2.09	11.38	7.97	
GER	2	c	0	-0.26	6.02	18.40	0.5237
GER	3	c	0	0.25	10.36	15.95	0.4249
GER	4	c	0	-0.26	3.67	11.18	0.6242
Subtotal GER				0.00	20.06	45.53	
HAENSZ	35	f	0	-0.03	6.83	15.84	0.9289
HAENSZ	34	f	0	0.90	3.24	1.13	0.1060
Subtotal HAENSZ				0.27	10.07	16.97	
*HAMMON	108	m	1	0.60	0.67	0.53	0.6201
*HAMMON	109	m	1	1.04	1.43	0.29	0.2136
*HAMMON	110	m	1	1.86	1.56	0.22	0.0201
Subtotal HAMMON				1.31	3.66	1.03	
JEDRYC	15	m	0	0.21	1.51	2.48	0.7972
JEDRYC	16	m	0	0.83	4.13	1.82	0.0937
JEDRYC	17	m	0	1.60	5.96	0.07	0.0001
JEDRYC	18	m	0	1.89	4.82	0.79	0.0000
JEDRYC	19	m	0	1.51	3.71	0.00	0.0036
Subtotal JEDRYC				1.39	20.13	5.16	
KATSOU	17	f	0	0.36	4.33	5.50	0.4516
KATSOU	18	f	0	1.21	2.15	0.17	0.0762
Subtotal KATSOU				0.64	6.48	5.67	
KREYBE	17	m	0	1.04	2.72	0.54	0.0849
KREYBE	18	m	0	0.33	1.99	2.67	0.6405
KREYBE	19	m	0	1.47	1.86	0.00	0.0460
KREYBE	34	f	0	-0.27	6.53	20.12	0.4952
KREYBE	35	f	0	-0.55	0.94	3.90	0.5964
Subtotal KREYBE				0.28	14.04	27.22	
LAMTH	16	f	0	0.40	13.12	15.44	0.1438
LAMTH	17	f	0	0.84	8.17	3.39	0.0158
LAMTH	18	f	0	0.78	3.08	1.57	0.1740
Subtotal LAMTH				0.60	24.36	20.40	
LUBIN2	152	m	0	0.32	29.82	40.55	0.0782
LUBIN2	156	m	0	1.02	43.25	9.60	0.0000
LUBIN2	160	m	0	1.24	44.40	2.74	0.0000
LUBIN2	164	m	0	1.38	39.81	0.48	0.0000
LUBIN2	172	f	0	-0.07	15.74	38.39	0.7715
LUBIN2	176	f	0	0.45	28.07	30.17	0.0167
LUBIN2	180	f	0	0.01	10.63	23.21	0.9727
LUBIN2	184	f	0	0.68	6.90	4.52	0.0741
Subtotal LUBIN2				0.82	218.62	149.66	
MATOS	56	m	0	1.01	3.21	0.73	0.0698
MATOS	58	m	0	1.92	3.91	0.74	0.0001
MATOS	60	m	0	2.13	4.10	1.67	0.0000
Subtotal MATOS				1.74	11.22	3.14	
MATSUD	7	m	0	2.58	0.46	0.55	0.0789
MATSUD	8	m	0	3.05	0.48	1.17	0.0343
MATSUD	9	m	0	3.18	0.45	1.28	0.0331
Subtotal MATSUD				2.93	1.40	3.01	
ORMOS	22	m	0	1.55	1.33	0.01	0.0738
ORMOS	23	m	0	0.70	1.20	0.74	0.4420

Table 3G11 - 5

IESLC - Meta-anal of Ever Smoking (or Curr if Ever not avail) by Amount, Overview, Any prod (or Cigs if Any not avail)
Adenocarcinoma
Least adjusted

REF	NRR	SEX	AD	Ys	Ws	Qs	Ps
ORMOS	24	m	0	1.11	0.66	0.09	0.3661
Subtotal ORMOS				1.14	3.18	0.84	
OSANN	55	m	2	2.80	11.67	20.17	0.0000
OSANN	63	m	2	3.62	12.01	54.80	0.0000
OSANN	56	f	2	2.17	27.97	13.17	0.0000
OSANN	64	f	2	3.19	20.96	60.41	0.0000
Subtotal OSANN				2.81	72.61	148.55	
OSANN2	16	f	0	0.13	5.50	10.13	0.7584
OSANN2	17	f	0	1.81	6.38	0.67	0.0000
Subtotal OSANN2				1.03	11.88	10.80	
SOBUE	56	m	0	0.64	14.90	10.65	0.0130
SOBUE	57	m	0	0.71	16.70	10.19	0.0038
SOBUE	58	m	0	0.66	15.78	10.95	0.0092
Subtotal SOBUE				0.67	47.39	31.79	
SVENSS	29	f	0	0.78	5.87	2.94	0.0588
SVENSS	34	f	0	1.70	6.91	0.30	0.0000
SVENSS	39	f	0	3.08	0.77	1.95	0.0069
Subtotal SVENSS				1.38	13.54	5.19	
TSUGAN	4	m	0	-0.21	3.72	10.74	0.6838
TSUGAN	5	m	0	-0.06	4.97	11.87	0.8986
TSUGAN	6	m	0	-0.32	3.43	11.22	0.5538
Subtotal TSUGAN				-0.18	12.12	33.83	
WAKAI	49	m	0	0.21	4.71	7.65	0.6431
WAKAI	50	m	0	0.64	5.51	4.00	0.1352
WAKAI	51	m	0	1.52	5.04	0.00	0.0006
Subtotal WAKAI				0.80	15.26	11.66	
WU	3	f	0	1.07	8.88	1.59	0.0015
WU	4	f	0	1.78	8.89	0.75	0.0000
Subtotal WU				1.42	17.77	2.34	
WUWILL	22	f	0	0.23	50.96	80.75	0.1009
WUWILL	23	f	0	0.82	14.10	6.29	0.0021
Subtotal WUWILL				0.36	65.06	87.04	
WYNDE2	10	m	0	-1.00	1.39	8.61	0.2387
WYNDE2	11	m	0	0.37	3.50	4.37	0.4885
WYNDE2	12	m	0	1.26	3.41	0.17	0.0195
WYNDE2	13	m	0	1.10	3.56	0.54	0.0382
Subtotal WYNDE2				0.69	11.86	13.70	
WYNDE3	24	m	0	-1.05	0.83	5.37	0.3372
WYNDE3	25	m	0	0.95	4.22	1.25	0.0521
WYNDE3	26	m	0	1.81	4.55	0.46	0.0001
WYNDE3	27	m	0	1.62	3.05	0.06	0.0045
WYNDE3	71	f	0	-0.63	1.58	7.09	0.4293
WYNDE3	72	f	0	0.84	4.71	1.96	0.0675
WYNDE3	73	f	0	1.27	3.10	0.15	0.0258
WYNDE3	74	f	0	0.52	0.71	0.66	0.6593
Subtotal WYNDE3				1.04	22.76	16.99	
WYNDE4	37	m	0	0.05	1.66	3.42	0.9482
WYNDE4	38	m	0	0.16	2.31	4.08	0.8077
WYNDE4	39	m	0	0.45	3.04	3.26	0.4291
WYNDE4	40	m	0	0.16	1.93	3.40	0.8243
WYNDE4	41	m	0	1.15	2.40	0.28	0.0761
WYNDE4	55	f	2	0.29	1.66	2.41	0.7132
Subtotal WYNDE4				0.41	12.99	16.85	
WYNDE6	24	m	0	1.30	19.66	0.71	0.0000
WYNDE6	33	m	0	1.94	36.35	7.29	0.0000
WYNDE6	42	m	0	2.42	29.44	25.39	0.0000
WYNDE6	51	m	0	2.72	36.38	55.46	0.0000
WYNDE6	213	f	0	0.95	22.53	6.65	0.0000
WYNDE6	222	f	0	2.04	46.92	14.15	0.0000
WYNDE6	231	f	0	2.67	25.27	35.05	0.0000
WYNDE6	240	f	0	3.08	28.43	71.84	0.0000
Subtotal WYNDE6				2.20	244.98	216.55	
ZHENG	6	m	0	0.38	7.96	9.82	0.2870
ZHENG	7	m	0	0.54	11.26	10.09	0.0692
ZHENG	8	m	0	0.66	13.29	9.18	0.0165
ZHENG	9	m	0	0.87	6.78	2.56	0.0229
ZHENG	19	f	0	-0.37	7.98	27.48	0.3003
ZHENG	20	f	0	0.72	7.66	4.48	0.0452
Subtotal ZHENG				0.48	54.94	63.62	

Table 3G11 - 5

IESLC - Meta-anal of Ever Smoking (or Curr if Ever not avail) by Amount, Overview, Any prod (or Cigs if Any not avail)
Adenocarcinoma
Least adjusted

N	140
NS	34

Table 3G11 - 6

IESLC - Meta-anal of Ever Smoking (or Curr if Ever not avail) by Amount, Overview, Any prod (or Cigs if Any not avail)
Adenocarcinoma
Least adjusted

	combined	Sex: male	female	Total
N	5	87	48	140
NS	2	25	19	46

In this overview table, other than the "N" rows, entries in the "absent" and "Total" columns may be invalid and should be ignored

	Amount smoked (broad categories): absent	<20k5	6-44k20	>20k45	Total
N	39	39	28	34	140
NS	23	28	23	28	102
Wt	580.77	461.22	277.99	263.62	1583.60
Het Chi	303.31	253.00	123.57	260.11	1211.91
Het df	38	38	27	33	139
Het P	***	***	***	***	***
Fixed RR	6.17	2.58	3.48	7.07	4.43
RRl	5.69	2.36	3.09	6.27	4.22
RRu	6.70	2.83	3.92	7.98	4.65
P	+++	+++	+++	+++	+++
Random RR	4.07	1.74	2.64	4.72	3.01
RRl	3.10	1.32	1.99	3.23	2.56
RRu	5.35	2.29	3.52	6.90	3.54
P	+++	+++	+++	+++	+++

	absent	Amount smoked (narrow categories): <10k1	2-19k10	11-29k20	21-39k30	31-98k40	>40k99	Total
N	83	14	9	22	8	1	3	140
NS	34	9	7	19	7	1	2	78
Wt	1063.46	76.84	100.49	261.00	75.37	1.47	4.98	1583.60
Het Chi	781.86	9.86	20.38	108.79	22.62	0.00	0.70	1211.91
Het df	82	13	8	21	7	0	2	139
Het P	***	N.S.	**	***	**	N.S.	N.S.	***
Fixed RR	5.17	1.16	2.30	3.64	9.69	0.70	4.22	4.43
RRl	4.87	0.93	1.89	3.23	7.74	0.14	1.75	4.22
RRu	5.49	1.45	2.79	4.12	12.15	3.50	10.16	4.65
P	+++	N.S.	+++	+++	+++	N.S.	++	+++
Random RR	3.40	1.16	2.38	2.87	6.70	0.70	4.22	3.01
RRl	2.75	0.93	1.61	2.11	4.11	0.14	1.75	2.56
RRu	4.19	1.45	3.52	3.90	10.91	3.50	10.16	3.54
P	+++	N.S.	+++	+++	+++	N.S.	++	+++

MALES

	Amount smoked (broad categories): absent	<20k5	6-44k20	>20k45	Total
N	23	23	20	21	87
NS	16	23	20	21	80
Wt	255.76	185.25	176.61	158.42	776.04
Het Chi	136.39	99.91	57.90	135.16	538.22
Het df	22	22	19	20	86
Het P	***	***	***	***	***
Fixed RR	6.76	2.89	3.22	5.84	4.53
RRl	5.98	2.51	2.78	5.00	4.22
RRu	7.64	3.34	3.73	6.82	4.86
P	+++	+++	+++	+++	+++
Random RR	4.68	1.95	2.66	4.59	3.24
RRl	3.19	1.34	1.97	2.88	2.65
RRu	6.86	2.84	3.60	7.32	3.96
P	+++	+++	+++	+++	+++

Table 3G11 - 6

IESLC - Meta-anal of Ever Smoking (or Curr if Ever not avail) by Amount, Overview, Any prod (or Cigs if Any not avail)
Adenocarcinoma
Least adjusted

MALES

Amount smoked (narrow categories)

	absent	<10k1	2-19k10	11-29k20	21-39k30	31-98k40	>40k99	Total
N	45	9	7	16	7	1	2	87
NS	25	9	7	16	7	1	2	66
Wt	436.35	48.55	70.51	164.80	50.10	1.47	4.27	776.04
Het Chi	299.18	4.30	14.75	49.56	16.70	0.00	0.01	538.22
Het df	44	8	6	15	6	0	1	86
Het P	***	N.S.	*	***	*	N.S.	N.S.	***
Fixed RR	5.92	1.39	2.66	3.38	7.94	0.70	4.91	4.53
RRl	5.39	1.05	2.11	2.90	6.02	0.14	1.90	4.22
RRu	6.50	1.85	3.36	3.93	10.48	3.50	12.68	4.86
P	+++	+	+++	+++	+++	N.S.	++	+++
Random RR	3.86	1.39	2.62	2.86	5.51	0.70	4.91	3.24
RRl	2.89	1.05	1.62	2.07	3.11	0.14	1.90	2.65
RRu	5.15	1.85	4.23	3.95	9.77	3.50	12.68	3.96
P	+++	+	+++	+++	+++	N.S.	++	+++

FEMALES

Amount smoked (broad categories)

	absent	<20k5	6-44k20	>20k45	Total
N	15	15	7	11	48
NS	14	15	7	11	47
Wt	310.20	269.95	91.01	85.94	757.10
Het Chi	161.87	141.22	47.88	86.14	612.32
Het df	14	14	6	10	47
Het P	***	***	***	***	***
Fixed RR	5.83	2.46	4.53	10.06	4.42
RRl	5.21	2.18	3.69	8.14	4.12
RRu	6.51	2.77	5.57	12.43	4.75
P	+++	+++	+++	+++	+++
Random RR	3.29	1.59	2.86	5.29	2.77
RRl	2.08	1.03	1.43	2.57	2.08
RRu	5.21	2.45	5.70	10.91	3.69
P	+++	+	++	+++	+++

Amount smoked (narrow categories)

	absent	<10k1	2-19k10	11-29k20	21-39k30	31-98k40	>40k99	Total
N	34	5	2	5	1		1	48
NS	18	5	2	5	1		1	31
Wt	587.02	28.29	29.99	85.83	25.27		0.71	757.10
Het Chi	423.68	1.15	0.47	40.55	0.00		0.00	612.32
Het df	33	4	1	4	0		0	47
Het P	***	N.S.	N.S.	***	N.S.		N.S.	***
Fixed RR	4.74	0.85	1.62	4.79	14.39		1.69	4.42
RRl	4.37	0.59	1.14	3.88	9.74		0.16	4.12
RRu	5.14	1.23	2.32	5.92	21.25		17.36	4.75
P	+++	N.S.	++	+++	+++		N.S.	+++
Random RR	3.03	0.85	1.62	3.45	14.39		1.69	2.77
RRl	2.18	0.59	1.14	1.62	9.74		0.16	2.08
RRu	4.20	1.23	2.32	7.36	21.25		17.36	3.69
P	+++	N.S.	++	++	+++		N.S.	+++

Table 3G11 - 7

IESLC - Meta-anal of Ever Smoking (or Curr if Ever not avail) by Amount, Overview, Any prod (or Cigs if Any not avail)
Adenocarcinoma
Excluded studies (and stage at which they were excluded)

Stage																
1	ABELIN	ABRAHA	AMANDU	AMES	ANDERS	AUSTIN	AXELSO	BAND	BECHER	BERRIN	BLOHMK	BLOT4	BROCKM	BROWN1	BYERS1	BYERS2
	CARPEN	CASCO2	CASCOR	CHAN	CHEN3	CHIAZZ	CHYOU	DESTE2	DOCKER	DROSTE	DU	GARCIA	GARDIN	GENG	GODLEY	GOODMA
	GRAHAM	GREGOR	HEGMAN	HEIN	HENNEK	HINDS	HIRAOK	HOROWI	HORWIT	HUANG	ISHIMA	JAHN	JAIN	JARVHO	JIANG	KELLER
	KIHARA	KJUUS	KO	KOHLME	KUBIK	LAMWK	LAMWK2	LANGE	LEI	LEMARC	LEVIN	LIU	LOMBA2	LOMBAR	MAGNUS	MARSH
	MARSH2	MCDUFF	MCLAUG	MILLER	MILLS	NOTANI	NOU	ODRISC	PAWLEG	PERSHA	POFFIJ	QIAO	QIAO2	RADZIK	REN	RONCO
	ROOTS	ROTHSC	SAARIK	SANKAR	SCHWAR	SEGI	SEOW	SHIMIZ	SIMARA	SIMONA	SITAS	SOBUE2	STASZE	STAYNE	STUCKE	SUN
	SUZUK2	SUZUKI	TANG	TAO	TOKARS	TOUSEY	ULMER	VEIERO	VUTUC	WALD	WANG	WANG3	WANG4	WICKLU	WIGLE	WILKIN
	WU2	WUNSCH	WYNDE8	XIANGZ	XU	XU2	XU4	YONG	ZHANG							
2	BUELL	CHEN	MASTRA	MZILEN	PISANI	RESTRE	SADOWS									
4	BOFFET	WYNDE7														
5	RIMING	TANG2	WYNDE5													
6	BLOT1	BLOT2	BLOT3	BOUCHA	HIRAY2	JONES	LAURIL	LICKIN	MOLLO	MRFIT	MURATA	SCHWA2	VANDER	WARSIN	WATSON	WYNDER
8	AGUDO	AKIBA	ARCHER	ARMADA	AUVINE	AXELSS	BENSHL	BEST	BRESLO	BRETT	BROSS	BUFFLE	CEDERL	CHANG	CHATZI	CHEN2
	CHOW	COMSTO	COOKSO	CPSI	CPSII	DAMBER	DARBY	DAVEYS	DEAN	DEAN2	DEAN3	DEKLER	DESTEF	DOLL2	DORANT	DORN
	DUNN	EBELIN	ENSTRO	ESAKI	FAN	GAO	GAO2	GARSHI	GILLIS	GOLLED	GSELL	HAMMO2	HANSEN	HIRAYA	HITOSU	HOLE
	HU	HU2	HUMBLE	JARUP	JOLY	JUSSAW	KAISE2	KAISER	KANELL	KAUFMA	KHUDER	KINLEN	KNEKT	KOO	KOULUM	KREUZE
	LAUSSM	LETOUR	LIAW	LIDDEL	LIU2	LIU3	LIU4	LIU5	LUBIN	LUO	MACLEN	MARTIS	MCCONN	MIGRAN	MRFITR	NAM
	NOTAN2	PARKIN	PASTOR	PERNU	PERSH2	PETO	PEZZO2	PEZZOT	PIKE	POLEDN	PRESCO	RACHTA	RANDIG	SEGI2	SHAW	SIEMIA
	SPEIZE	SPITZ	STOCKS	STOCKW	TENKAN	TIZZAN	TULINI	TVERDA	WANG2	XU3	YAMAGU	YUAN	ZHOU			
10	BENHAM															

Table 3G11 - 8

Potentially overlapping studies

REF	REFGP	PRINC	. OVERLAP/LINK
LUBIN2	LUBIN2	1	Lubin-combined
LAMTH	LAMTH	1	KOO/LAMTH/LAMWK
OSANN2	KAISER	2	KAISER/OSANN2
WYNDE6	WYNDE6	1	WYNDE5/6/7/8
MATSUD	MATSUD	1	SOBUE2/MATSUD

Table 3G11 - 9

Most adjusted - insufficient data for metaanalysis

REF	NRR	SEX	AGEL	AGEH	RACE	YF	LC TYPE	LOC	START	ST	NLC	R	VB	P	H	AD	SM	PRODUCT	exL	exH	S1	S2	DENOM	De
CHEN	15	c	0	0	all	-	a	As:oth	1987	CC	323	n	ot	n	y	2	ev	cig+/-ot	1	10	1	0	nev cigs	ot
CHEN	14	c	0	0	all	-	a	As:oth	1987	CC	323	n	ot	n	y	2	ev	cig+/-ot	11	20	2	3	nev cigs	ot
CHEN	13	c	0	0	all	-	a	As:oth	1987	CC	323	n	ot	n	y	2	ev	cig+/-ot	21	30	0	4	nev cigs	ot
CHEN	12	c	0	0	all	-	a	As:oth	1987	CC	323	n	ot	n	y	2	ev	cig+/-ot	31	99	3	0	nev cigs	ot

REF	NRR	RR	SIG	Cigarette equivalent	RRDATA comment
CHEN	15	1.21	n	*	0
CHEN	14	1.74	n	*	0
CHEN	13	2.34	n	*	0
CHEN	12	3.61	y	*	P < 0.001

Table 3G12 -

IESLC - Meta-anal of Ever Smoking (or Current if ever not avail), Amount smoked, "Low", Any prod (or Cigs if Any not avail)
Adenocarcinoma

This analysis is restricted to results for:
1) Results by Amount smoked
2) Results complete enough for use in metaanalysis

Within each study, results are then selected (in the following order of preference, within each sex) for:
3) SMKSTA: ever smokers, current smokers
4) PRODUCT: all/unspec, cigarettes regardless of other products, cigarettes only
5) CIGTYPE: all/unspecified, MC regardless of HR, MC only
6) DENOM: never smoked anything, never smoked cigarettes, (never +1 = +long term ex, +2 = +amount unknown,
+3 = never cigs+long term ex)
7) Followup period (YF, prospective studies): whole study (coded as 0) or longest available
8) LCtype: adeno or nearest available, but not squamous. (q = squamous, s = small,
a = adeno, l = large, KII = Kreyberg II, al = alveolar, br = bronchiolar, u = undifferentiated)
9) Race: all or nearest available, otherwise by race (wh or w = white, bl or b = black, hi = hispanic
ch = chinese, jap = japanese, haw = hawaiian, w+o = white + oriental, sca = scandinavian, as = asian)
10) Amount smoked "low" in key scheme 1 (key value 5, maximum range <20, in numbers of
cigarettes or cigarette equivalents)
11) For overlapping studies: principal rather than subsidiary studies
Finally by Age: whole study (coded as 0) if available, otherwise by widest available age group
and then for single sex results (m, f) in preference to combined sex results (c).

Results adjusted (AD) for the most potential confounders are then chosen in Sections -1 to -3
and results adjusted for the least confounders in Sections -4 to -6. (Those least adjusted results which
actually differ from the most adjusted as marked 'x' in column X in Section -4)
(Results adjusted for an unknown number of confounder(s) are coded as 20.)

Section -7 shows excluded studies, together with the stage (as above) at which no qualifying
results were found.

Section -8 lists the potentially overlapping studies which have been included (1=principal, 2=subsidiary).

Section -9 lists any results which would have been included in preference except that they had data not complete
enough for use in meta-analysis, with their significance (yes/no), if known, and any further comment as entered
on the database.

In addition to those mentioned above, the following fields, levels and abbreviations are used:
* or nk = not known, n = no, y = yes, ot = other
ev = ever, cu = current, nev = never
all/unspec = all or unspecified, cig+/-ot = cigarettes irrespective of other products (cigar, pipe etc)
MC = manufactured cigarettes, HR = hand-rolled cigarettes
exL, exH = range of exposure (low and high) in the smoking group, in terms of Amount smoked,
cigarettes or cigarette equivalents
REF: 6-character study reference
NRR: number of the RR on the database within the study
ST : study type (CC = case control, pr or prosp = prospective)
NLC: number of lung cancer cases in whole study
R : risky occupational population (n = no, m = mining, o = other risky)
VB : national cigarette type (V = at least 75% Virginia, bl = at least 75% blended, ot = other)
P : any proxy use
H : full histological confirmation
De : derivation of RR/CI (or = original, st = standard method, ot = other method of estimation)

Table 3G12 - 1

IESLC - Meta-anal of Ever Smoking (or Current if ever not avail), Amount smoked, "Low", Any prod (or Cigs if Any not avail)
Adenocarcinoma
Most adjusted

REF	NRR	SEX	AGEL	AGEH	RACE	YF	LC TYPE	LOC	START	ST	NLC	R	VB	P	H	AD	SM	PRODUCT	exL	exH	DENOM	De
ALDERS	46	m	0	0	all	-	not q+s	Eu:UK	1977	CC	1448	n	V	n	n	1	ev	cig only	1	17	nev+2	ot
ALDERS	49	f	0	0	all	-	not q+s	Eu:UK	1977	CC	1448	n	V	n	n	1	ev	cig only	1	17	nev+2	ot
BARBON	79	m	0	0	all	-	a	Eu:wst	1979	CC	755	n	bl	y	y	3	ev	all/unsp	1	19	nev any	or
BROWN2	34	m	0	0	wh	-	a	NAmer	1984	CC	14596	n	bl	n	y	2	ev	cig+/-ot	1	19	nev cigs	or
BROWN2	33	f	0	0	wh	-	a	NAmer	1984	CC	14596	n	bl	n	y	2	ev	cig+/-ot	1	19	nev cigs	or
CHOI	51	m	0	0	all	-	a	As:oth	1985	CC	375	n	bl	n	n	0	ev	cig+/-ot	1	10	nev cigs	st
CHOI	59	f	0	0	all	-	a	As:oth	1985	CC	375	n	bl	n	n	0	ev	cig+/-ot	1	10	nev cigs	st
DOLL	73	m	0	0	all	-	KII	Eu:UK	1948	CC	1465	n	V	n	n	1	ev	all/unsp	5	14	nev any	ot
DOLL	80	f	0	0	all	-	KII	Eu:UK	1948	CC	1465	n	V	n	n	1	ev	all/unsp	5	14	nev any	ot
DORGAN	126	m	0	0	wh	-	a	NAmer	1980	CC	2026	n	bl	y	y	2	ev	cig+/-ot	1	19	nev any	ot
DORGAN	105	f	0	0	all	-	a	NAmer	1980	CC	2026	n	bl	y	y	3	ev	cig+/-ot	1	19	nev any	ot
DOSEME	8	m	0	0	all	-	not q+s	Eu:bal	1979	CC	1210	n	bl	n	n	2	ev	cig+/-ot	1	10	nev cigs	or
ENGELA	72	m	0	0	all	0	a	Eu:Sca	1964	pr	435	n	bl	n	n	7	cu	cig+/-ot	5	9	nev cigs	or
GER	10	c	0	0	all	-	a	As:oth	1990	CC	141	n	ot	y	n	8	ev	all/unsp	1	10	nev any	ot
HAMMON	108	m	0	0	wh	0	a	NAmer	1952	pr	448	n	bl	n	n	1	cu	cig only	1	9	nev any	ot
JEDRYC	36	m	0	0	all	-	a	Eu:est	1980	CC	1630	n	bl	y	n	3	ev	cig+/-ot	1	19	nev any	or
KREYBE	5	m	0	0	all	-	KII	Eu:Sca	1948	CC	300	n	bl	n	y	1	ev	all/unsp	1	14	nev any	ot
KREYBE	26	f	0	0	all	-	KII	Eu:Sca	1948	CC	300	n	bl	n	y	1	ev	all/unsp	1	14	nev any	ot
LAMTH	16	f	0	0	ch	-	a	As:HK	1983	CC	445	n	bl	n	n	0	ev	all/unsp	1	10	nev any	or
LUBIN2	152	m	0	0	all	-	a	Eu:mul	1976	CC	7804	n	bl	n	y	0	ev	cig+/-ot	1	9	nev any	st
LUBIN2	172	f	0	0	all	-	a	Eu:mul	1976	CC	7804	n	bl	n	y	0	ev	cig+/-ot	1	9	nev any	st
MATOS	57	m	0	0	all	-	a	SCAmer	1994	CC	200	n	bl	n	n	2	ev	cig+/-ot	1	14	nev any	or
MATSUD	7	m	0	0	all	-	a	As:Jap	1965	CC	179	n	bl	n	n	0	ev	cig+/-ot	1	10	nev cigs	ot
ORMOS	22	m	0	0	all	-	KII	Eu:est	1947	CC	119	n	bl	y	y	0	ev	cig+/-ot	1	15	nev any	st
OSANN2	34	f	0	0	all	-	KII	NAmer	1964	ot	217	n	bl	n	y	1	ev	cig+/-ot	1	19	nev cigs	or
SOBUE	56	m	0	0	all	-	a	As:Jap	1986	CC	1376	n	bl	n	y	0	cu	cig+/-ot	1	19	nev cigs	st
SVENSS	9	f	0	0	all	-	a	Eu:Sca	1983	CC	210	n	bl	n	n	1	cu	all/unsp	1	10	nev any	or
TSUGAN	4	m	0	0	all	-	a	As:Jap	1976	CC	134	n	bl	n	y	0	cu	all/unsp	1	15	nev any	st
WAKAI	52	m	0	0	all	-	a	As:Jap	1988	CC	333	n	bl	n	y	1	cu	cig+/-ot	1	19	nev any	or
WUWILL	16	f	0	0	all	-	a	As:Chi	1985	CC	965	n	ot	n	n	3	ev	cig+/-ot	1	19	nev cigs	ot
WYNDE2	10	m	0	0	all	-	KII	NAmer	1962	CC	404	n	bl	n	y	0	ev	cig+/-ot	1	10	nev any	st
WYNDE3	24	m	0	0	all	-	KII	NAmer	1966	CC	350	n	bl	n	y	0	ev	cig+/-ot	1	9	nev any	st
WYNDE3	71	f	0	0	all	-	KII	NAmer	1966	CC	350	n	bl	n	y	0	ev	cig+/-ot	1	9	nev any	st
WYNDE4	37	m	0	0	all	-	a	NAmer	1948	CC	684	n	bl	y	n	0	ev	all/unsp	1	9	nev any	st
WYNDE4	55	f	0	0	all	-	a	NAmer	1948	CC	684	n	bl	y	n	2	ev	all/unsp	1	9	nev any	ot
WYNDE6	24	m	0	0	all	-	KII	NAmer	1969	CC	4423	n	bl	n	y	0	cu	cig+/-ot	1	10	nev any	st
WYNDE6	213	f	0	0	all	-	KII	NAmer	1969	CC	4423	n	bl	n	y	0	cu	cig+/-ot	1	10	nev cigs	st
ZHENG	6	m	0	0	all	-	a	As:Chi	1982	CC	540	n	ot	*	y	0	ev	cig+/-ot	1	9	nev cigs	st
ZHENG	19	f	0	0	all	-	a	As:Chi	1982	CC	540	n	ot	*	y	0	ev	cig+/-ot	1	9	nev cigs	st

Cigarette type is all/unspec for all RRs

except for the following:

REF	NRR	CIGTYPE
ALDERS	46	MC only
ALDERS	49	MC only

Table 3G12 - 2

IESLC - Meta-anal of Ever Smoking (or Current if ever not avail), Amount smoked, "Low", Any prod (or Cigs if Any not avail)
Adenocarcinoma
Most adjusted

REF	NRR	SEX	AD	Number Exposed Case	Number Exposed Cont	Non-exposed Case	Non-exposed Cont	RR	95.00%CI
ALDERS	46	m	1	-	-	-	-	2.80	(1.01- 7.75)
ALDERS	49	f	1	-	-	-	-	2.77	(1.63- 4.70)
Subtotal ALDERS								2.78	(1.74- 4.44)
BARBON	79	m	3	-	-	-	-	5.00	(2.20- 11.00)
BROWN2	34	m	2	-	-	-	-	6.20	(4.90- 7.90)
BROWN2	33	f	2	-	-	-	-	5.80	(4.70- 7.10)
Subtotal BROWN2								5.97	(5.11- 6.98)
CHOI	51	m	0	4	90	7	95	0.60	(0.17- 2.13)
CHOI	59	f	0	4	16	49	164	0.84	(0.27- 2.62)
Subtotal CHOI								0.72	(0.31- 1.68)
DOLL	73	m	1	-	-	-	-	0.80	(0.18- 3.60)
DOLL	80	f	1	-	-	-	-	2.30	(0.56- 9.48)
Subtotal DOLL								1.40	(0.50- 3.91)
DORGAN	126	m	2	-	-	-	-	3.23	(1.20- 8.70)
DORGAN	105	f	3	-	-	-	-	2.87	(2.00- 4.11)
Subtotal DORGAN								2.91	(2.07- 4.08)
DOSEME	8	m	2	-	-	-	-	1.80	(0.80- 4.10)
*ENGELA	72	m	7	-	-	-	-	8.50	(2.50- 29.00)
GER	10	c	8	-	-	-	-	0.62	(0.23- 1.72)
*HAMMON	108	m	1	-	-	-	-	1.83	(0.17- 20.22)
JEDRYC	36	m	3	-	-	-	-	2.19	(0.84- 5.72)
KREYBE	5	m	1	-	-	-	-	2.65	(0.81- 8.70)
KREYBE	26	f	1	-	-	-	-	1.30	(0.60- 2.80)
Subtotal KREYBE								1.61	(0.84- 3.06)
LAMTH	16	f	0	36	29	131	158	1.50	(0.87- 2.57)
LUBIN2	152	m	0	66	2194	57	2616	1.38	(0.96- 1.98)
LUBIN2	172	f	0	20	184	138	1180	0.93	(0.57- 1.52)
Subtotal LUBIN2								1.20	(0.90- 1.61)
MATOS	57	m	2	-	-	-	-	2.80	(0.90- 8.50)
MATSUD	7	m	0	6	1237	0	1255	13.19~	(0.74- 234.37)
ORMOS	22	m	0	4	329	2	777	4.72	(0.86- 25.91)
OSANN2	34	f	1	-	-	-	-	0.90	(0.30- 2.70)
SOBUE	56	m	0	63	157	27	128	1.90	(1.15- 3.16)
SVENSS	9	f	1	-	-	-	-	2.20	(1.00- 5.80)
TSUGAN	4	m	0	12	14	18	17	0.81	(0.29- 2.24)
WAKAI	52	m	1	-	-	-	-	1.30	(0.52- 3.21)
WUWILL	16	f	3	-	-	-	-	1.43	(1.07- 1.90)
WYNDE2	10	m	0	2	114	5	105	0.37	(0.07- 1.94)
WYNDE3	24	m	0	1	42	6	88	0.35	(0.04- 2.99)
WYNDE3	71	f	0	2	19	15	76	0.53	(0.11- 2.53)
Subtotal WYNDE3								0.46	(0.13- 1.63)
WYNDE4	37	m	0	3	82	4	115	1.05	(0.23- 4.83)
WYNDE4	55	f	2	-	-	-	-	1.33	(0.29- 6.07)
Subtotal WYNDE4								1.18	(0.40- 3.47)
WYNDE6	24	m	0	42	122	58	617	3.66	(2.35- 5.70)
WYNDE6	213	f	0	39	109	119	856	2.57	(1.70- 3.89)
Subtotal WYNDE6								3.03	(2.24- 4.10)
ZHENG	6	m	0	18	40	29	94	1.46	(0.73- 2.92)
ZHENG	19	f	0	13	29	119	184	0.69	(0.35- 1.39)
Subtotal ZHENG								1.00	(0.62- 1.64)
Partial Totals				335	4807	784	8525		

*prospective study

~ With 0.5 adjustment for zero

Table 3G12 - 2

IESLC - Meta-anal of Ever Smoking (or Current if ever not avail), Amount smoked, "Low", Any prod (or Cigs if Any not avail)
Adenocarcinoma
Most adjusted

REF	NRR	SEX	AD	Ys	Ws	Qs	Ps
ALDERS	46	m	1	1.03	3.70	0.00	0.0476
ALDERS	49	f	1	1.02	13.70	0.01	0.0002
Subtotal ALDERS				1.02	17.40	0.01	
BARBON	79	m	3	1.61	5.93	2.24	0.0001
BROWN2	34	m	2	1.82	67.36	46.41	0.0000
BROWN2	33	f	2	1.76	90.29	52.62	0.0000
Subtotal BROWN2				1.79	157.65	99.04	
CHOI	51	m	0	-0.51	2.41	5.43	0.4323
CHOI	59	f	0	-0.18	2.95	4.06	0.7595
Subtotal CHOI				-0.33	5.36	9.48	
DOLL	73	m	1	-0.22	1.71	2.54	0.7703
DOLL	80	f	1	0.83	1.92	0.05	0.2485
Subtotal DOLL				0.34	3.63	2.59	
DORGAN	126	m	2	1.17	3.92	0.12	0.0203
DORGAN	105	f	3	1.05	29.62	0.11	0.0000
Subtotal DORGAN				1.07	33.53	0.23	
DOSEME	8	m	2	0.59	5.75	0.95	0.1585
*ENGELA	72	m	7	2.14	2.56	3.36	0.0006
GER	10	c	8	-0.48	3.80	8.23	0.3517
*HAMMON	108	m	1	0.60	0.67	0.10	0.6201
JEDRYC	36	m	3	0.78	4.18	0.19	0.1092
KREYBE	5	m	1	0.97	2.73	0.00	0.1076
KREYBE	26	f	1	0.26	6.48	3.47	0.5044
Subtotal KREYBE				0.47	9.20	3.47	
LAMTH	16	f	0	0.40	13.12	4.58	0.1438
LUBIN2	152	m	0	0.32	29.82	13.46	0.0782
LUBIN2	172	f	0	-0.07	15.74	17.94	0.7715
Subtotal LUBIN2				0.19	45.56	31.40	
MATOS	57	m	2	1.03	3.05	0.00	0.0723
MATSUD	7	m	0	2.58	0.46	1.17	0.0789
ORMOS	22	m	0	1.55	1.33	0.41	0.0738
OSANN2	34	f	1	-0.11	3.18	3.85	0.8509
SOBUE	56	m	0	0.64	14.90	1.84	0.0130
SVENSS	9	f	1	0.79	4.97	0.21	0.0787
TSUGAN	4	m	0	-0.21	3.72	5.40	0.6838
WAKAI	52	m	1	0.26	4.64	2.49	0.5721
WUWILL	16	f	3	0.36	46.61	18.90	0.0146
WYNDE2	10	m	0	-1.00	1.39	5.53	0.2387
WYNDE3	24	m	0	-1.05	0.83	3.48	0.3372
WYNDE3	71	f	0	-0.63	1.58	4.17	0.4293
Subtotal WYNDE3				-0.77	2.41	7.65	
WYNDE4	37	m	0	0.05	1.66	1.47	0.9482
WYNDE4	55	f	2	0.29	1.66	0.84	0.7132
Subtotal WYNDE4				0.17	3.32	2.31	
WYNDE6	24	m	0	1.30	19.66	1.81	0.0000
WYNDE6	213	f	0	0.95	22.53	0.05	0.0000
Subtotal WYNDE6				1.11	42.19	1.87	
ZHENG	6	m	0	0.38	7.96	3.03	0.2870
ZHENG	19	f	0	-0.37	7.98	14.79	0.3003
Subtotal ZHENG				0.00	15.94	17.82	

	N	39
	NS	28
	Wt	456.45
Het	Chi	235.32
Het	df	38
Het	P	***
Fixed	RR	2.70
	RRl	2.47
	RRu	2.96
	P	+++
Random	RR	1.83
	RRl	1.40
	RRu	2.39
	P	+++
Asymm	P	**

Table 3G12 - 3

IESLC - Meta-anal of Ever Smoking (or Current if ever not avail), Amount smoked, "Low", Any prod (or Cigs if Any not avail)
Adenocarcinoma
Most adjusted

	Sex			
	combined	male	female	Total
N	1	23	15	39
NS	1	23	15	39
Wt	3.80	190.33	262.33	456.45
Het Chi	0.00	99.84	125.09	235.32
Het df	0	22	14	38
Het P	N.S.	***	***	***
Fixed RR	0.62	2.96	2.58	2.70
RRl	0.23	2.57	2.29	2.47
RRu	1.70	3.42	2.92	2.96
P	N.S.	+++	+++	+++
Random RR	0.62	2.09	1.65	1.83
RRl	0.23	1.44	1.08	1.40
RRu	1.70	3.02	2.50	2.39
P	N.S.	+++	+	+++
Between Chi				10.39
Between df				2
Between P				**
Btwn(F) P				N.S.
Btwn(R) P				(*)

	Lung cancer type						
	a	a+l	a+al+br	KII	not q+u	not q+s	Total
N	25			11		3	39
NS	19			7		2	28
Wt	369.96			63.34		23.16	456.45
Het Chi	208.46			23.05		0.81	235.32
Het df	24			10		2	38
Het P	***			*		N.S.	***
Fixed RR	2.81			2.23		2.49	2.70
RRl	2.54			1.74		1.66	2.47
RRu	3.11			2.86		3.75	2.96
P	+++			+++		+++	+++
Random RR	1.87			1.63		2.49	1.83
RRl	1.32			1.03		1.66	1.40
RRu	2.66			2.59		3.75	2.39
P	+++			+		+++	+++
Between Chi							3.01
Between df							2
Between P							N.S.
Btwn(F) P							N.S.
Btwn(R) P							N.S.

	Location								
	NAmer	UK	Scand	othEur	China	Japan	othAs	other	Total
N	13	4	4	6	3	4	4	1	39
NS	8	2	3	5	2	4	3	1	28
Wt	244.34	21.03	16.73	62.75	62.55	23.72	22.28	3.05	456.45
Het Chi	62.98	2.42	6.54	14.92	3.68	4.44	3.58	0.00	235.32
Het df	12	3	3	5	2	3	3	0	38
Het P	***	N.S.	(*)	*	N.S.	N.S.	N.S.	N.S.	***
Fixed RR	4.39	2.47	2.27	1.53	1.31	1.60	1.08	2.80	2.70
RRl	3.88	1.61	1.41	1.20	1.02	1.07	0.71	0.91	2.47
RRu	4.98	3.78	3.67	1.96	1.67	2.40	1.64	8.60	2.96
P	+++	+++	+++	+++	+	+	N.S.	(+)	+++
Random RR	2.47	2.47	2.56	1.88	1.21	1.53	1.01	2.80	1.83
RRl	1.68	1.61	1.23	1.14	0.80	0.86	0.63	0.91	1.40
RRu	3.64	3.78	5.31	3.11	1.84	2.71	1.65	8.60	2.39
P	+++	+++	+	+	N.S.	N.S.	N.S.	(+)	+++
Between Chi									136.76
Between df									7
Between P									***
Btwn(F) P									***
Btwn(R) P									*

Table 3G12 - 3

IESLC - Meta-anal of Ever Smoking (or Current if ever not avail), Amount smoked, "Low", Any prod (or Cigs if Any not avail)
Adenocarcinoma
Most adjusted

	Detailed Country in "other Europe"					
	multi	Germany	othWest	East	Balkans	Total
N	2		1	2	1	6
NS	1		1	2	1	5
Wt	45.56		5.93	5.50	5.75	62.75
Het Chi	1.61		0.00	0.59	0.00	14.92
Het df	1		0	1	0	5
Het P	N.S.		N.S.	N.S.	N.S.	*
Fixed RR	1.20		5.00	2.64	1.80	1.53
RRl	0.90		2.24	1.14	0.80	1.20
RRu	1.61		11.18	6.08	4.07	1.96
P	N.S.		+++	+	N.S.	+++
Random RR	1.18		5.00	2.64	1.80	1.88
RRl	0.80		2.24	1.14	0.80	1.14
RRu	1.72		11.18	6.08	4.07	3.11
P	N.S.		+++	+	N.S.	+
Between Chi						12.71
Between df						3
Between P						**
Btwn(F) P						N.S.
Btwn(R) P						**

	Detailed Country in "other Asia"			
	India	HongKong	other	Total
N		1	3	4
NS		1	2	3
Wt		13.12	9.16	22.28
Het Chi		0.00	0.19	3.58
Het df		0	2	3
Het P		N.S.	N.S.	N.S.
Fixed RR		1.50	0.68	1.08
RRl		0.87	0.35	0.71
RRu		2.57	1.30	1.64
P		N.S.	N.S.	N.S.
Random RR		1.50	0.68	1.01
RRl		0.87	0.35	0.63
RRu		2.57	1.30	1.65
P		N.S.	N.S.	N.S.
Between Chi				3.39
Between df				1
Between P				(*)
Btwn(F) P				*
Btwn(R) P				(*)

	Detailed other continent			
	SCAmer	Auslia	Africa	Total
N	1			1
NS	1			1
Wt	3.05			3.05
Het Chi	0.00			0.00
Het df	0			0
Het P	N.S.			N.S.
Fixed RR	2.80			2.80
RRl	0.91			0.91
RRu	8.60			8.60
P	(+)			(+)
Random RR	2.80			2.80
RRl	0.91			0.91
RRu	8.60			8.60
P	(+)			(+)
Between Chi				
Between df				
Between P				N.S.
Btwn(F) P				N.S.
Btwn(R) P				N.S.

Table 3G12 - 3

IESLC - Meta-anal of Ever Smoking (or Current if ever not avail), Amount smoked, "Low", Any prod (or Cigs if Any not avail)
Adenocarcinoma
Most adjusted

	Start year of study <1960	1960-69	1970-79	1980-89	1990+	Total
N	8	8	7	14	2	39
NS	5	6	5	10	2	28
Wt	18.15	52.20	78.37	300.90	6.84	456.45
Het Chi	3.96	23.34	20.05	144.16	3.84	235.32
Het df	7	7	6	13	1	38
Het P	N.S.	**	**	***	*	***
Fixed RR	1.61	2.60	1.63	3.26	1.21	2.70
RRl	1.01	1.98	1.31	2.91	0.57	2.47
RRu	2.54	3.41	2.04	3.65	2.57	2.96
P	+	+++	+++	+++	N.S.	+++
Random RR	1.61	1.86	1.80	1.95	1.29	1.83
RRl	1.01	0.97	1.15	1.27	0.29	1.40
RRu	2.54	3.58	2.81	2.98	5.65	2.39
P	+	(+)	++	++	N.S.	+++
Between Chi						39.96
Between df						4
Between P						***
Btwn(F) P						N.S.
Btwn(R) P						N.S.

	Study type (1) CC	other	Total
N	36	3	39
NS	25	3	28
Wt	450.04	6.41	456.45
Het Chi	228.01	7.20	235.32
Het df	35	2	38
Het P	***	*	***
Fixed RR	2.71	2.37	2.70
RRl	2.47	1.09	2.47
RRu	2.97	5.15	2.96
P	+++	+	+++
Random RR	1.80	2.47	1.83
RRl	1.37	0.50	1.40
RRu	2.37	12.24	2.39
P	+++	N.S.	+++
Between Chi			0.11
Between df			1
Between P			N.S.
Btwn(F) P			N.S.
Btwn(R) P			N.S.

	Study type (2) CC	prosp	other	Total
N	36	2	1	39
NS	25	2	1	28
Wt	450.04	3.23	3.18	456.45
Het Chi	228.01	1.26	0.00	235.32
Het df	35	1	0	38
Het P	***	N.S.	N.S.	***
Fixed RR	2.71	6.17	0.90	2.70
RRl	2.47	2.07	0.30	2.47
RRu	2.97	18.37	2.70	2.96
P	+++	++	N.S.	+++
Random RR	1.80	5.63	0.90	1.83
RRl	1.37	1.49	0.30	1.40
RRu	2.37	21.36	2.70	2.39
P	+++	+	N.S.	+++
Between Chi				6.05
Between df				2
Between P				*
Btwn(F) P				N.S.
Btwn(R) P				N.S.

Table 3G12 - 3

IESLC - Meta-anal of Ever Smoking (or Current if ever not avail), Amount smoked, "Low", Any prod (or Cigs if Any not avail)
Adenocarcinoma
Most adjusted

	Study size (number of LC cases) 100-249	250-499	500-999	1000+	Total
N	7	11	6	15	39
NS	7	8	4	9	28
Wt	20.50	39.36	71.80	324.80	456.45
Het Chi	11.02	17.59	13.63	115.90	235.32
Het df	6	10	5	14	38
Het P	(*)	(*)	*	***	***
Fixed RR	1.43	1.35	1.45	3.51	2.70
RRl	0.93	0.99	1.15	3.15	2.47
RRu	2.21	1.84	1.83	3.91	2.96
P	N.S.	(+)	++	+++	+++
Random RR	1.54	1.27	1.50	2.52	1.83
RRl	0.83	0.80	0.90	1.77	1.40
RRu	2.84	2.02	2.49	3.59	2.39
P	N.S.	N.S.	N.S.	+++	+++
Between Chi					77.18
Between df					3
Between P					***
Btwn(F) P					**
Btwn(R) P					(*)

	Risky occupational population no	mining	othRisky	Total
N	39			39
NS	28			28
Wt	456.45			456.45
Het Chi	235.32			235.32
Het df	38			38
Het P	***			***
Fixed RR	2.70			2.70
RRl	2.47			2.47
RRu	2.96			2.96
P	+++			+++
Random RR	1.83			1.83
RRl	1.40			1.40
RRu	2.39			2.39
P	+++			+++
Between Chi				
Between df				
Between P				N.S.
Btwn(F) P				N.S.
Btwn(R) P				N.S.

	National cigarette tobacco type Virginia	blended	other	Total
N	4	31	4	39
NS	2	23	3	28
Wt	21.03	369.08	66.34	456.45
Het Chi	2.42	180.17	5.68	235.32
Het df	3	30	3	38
Het P	N.S.	***	N.S.	***
Fixed RR	2.47	3.12	1.25	2.70
RRl	1.61	2.82	0.98	2.47
RRu	3.78	3.46	1.59	2.96
P	+++	+++	(+)	+++
Random RR	2.47	1.98	1.10	1.83
RRl	1.61	1.47	0.72	1.40
RRu	3.78	2.67	1.67	2.39
P	+++	+++	N.S.	+++
Between Chi				47.05
Between df				2
Between P				***
Btwn(F) P				*
Btwn(R) P				*

Table 3G12 - 3

IESLC - Meta-anal of Ever Smoking (or Current if ever not avail), Amount smoked, "Low", Any prod (or Cigs if Any not avail)

Adenocarcinoma

Most adjusted

	Any proxy use No/nk	Yes	Total
N	31	8	39
NS	22	6	28
Wt	404.38	52.08	456.45
Het Chi	221.69	13.51	235.32
Het df	30	7	38
Het P	***	(*)	***
Fixed RR	2.72	2.58	2.70
RRl	2.47	1.97	2.47
RRu	3.00	3.39	2.96
P	+++	+++	+++
Random RR	1.74	2.30	1.83
RRl	1.28	1.44	1.40
RRu	2.37	3.67	2.39
P	+++	+++	+++
Between Chi			0.12
Between df			1
Between P			N.S.
Btwn(F) P			N.S.
Btwn(R) P			N.S.

	Full histological confirmation No	Yes	Total
N	18	21	39
NS	14	14	28
Wt	114.88	341.58	456.45
Het Chi	25.27	174.22	235.32
Het df	17	20	38
Het P	(*)	***	***
Fixed RR	1.67	3.18	2.70
RRl	1.39	2.86	2.47
RRu	2.00	3.54	2.96
P	+++	+++	+++
Random RR	1.74	1.88	1.83
RRl	1.33	1.31	1.40
RRu	2.28	2.70	2.39
P	+++	+++	+++
Between Chi			35.83
Between df			1
Between P			***
Btwn(F) P			*
Btwn(R) P			N.S.

	Number of adjustment variables (1) 0	1	2+/+nk	Total
N	17	10	12	39
NS	12	7	10	29
Wt	148.04	43.70	264.71	456.45
Het Chi	46.01	7.64	100.35	235.32
Het df	16	9	11	38
Het P	***	N.S.	***	***
Fixed RR	1.58	1.92	3.86	2.70
RRl	1.35	1.43	3.42	2.47
RRu	1.86	2.59	4.35	2.96
P	+++	+++	+++	+++
Random RR	1.35	1.92	2.87	1.83
RRl	0.98	1.43	1.85	1.40
RRu	1.86	2.59	4.46	2.39
P	(+)	+++	+++	+++
Between Chi				81.32
Between df				2
Between P				***
Btwn(F) P				***
Btwn(R) P				*

Table 3G12 - 3

IESLC - Meta-anal of Ever Smoking (or Current if ever not avail), Amount smoked, "Low", Any prod (or Cigs if Any not avail)
Adenocarcinoma
Most adjusted

	Number of adjustment variables (2) 0	1	2	3-5	6+/+nk	Total
N	17	10	6	4	2	39
NS	12	7	5	4	2	30
Wt	148.04	43.70	172.02	86.33	6.35	456.45
Het Chi	46.01	7.64	14.25	14.11	10.47	235.32
Het df	16	9	5	3	1	38
Het P	***	N.S.	*	**	**	***
Fixed RR	1.58	1.92	5.50	2.02	1.78	2.70
RRl	1.35	1.43	4.73	1.64	0.82	2.47
RRu	1.86	2.59	6.38	2.49	3.87	2.96
P	+++	+++	+++	+++	N.S.	+++
Random RR	1.35	1.92	4.21	2.45	2.24	1.83
RRl	0.98	1.43	2.93	1.42	0.17	1.40
RRu	1.86	2.59	6.06	4.23	29.13	2.39
P	(+)	+++	+++	++	N.S.	+++
Between Chi						142.84
Between df						4
Between P						***
Btwn(F) P						***
Btwn(R) P						***

	Product all/unsp	cig+/-ot	cig only	Total
N	11	25	3	39
NS	8	18	2	28
Wt	47.69	390.69	18.08	456.45
Het Chi	15.80	204.58	0.11	235.32
Het df	10	24	2	38
Het P	N.S.	***	N.S.	***
Fixed RR	1.60	2.88	2.73	2.70
RRl	1.20	2.61	1.72	2.47
RRu	2.12	3.18	4.33	2.96
P	++	+++	+++	+++
Random RR	1.56	1.89	2.73	1.83
RRl	1.07	1.34	1.72	1.40
RRu	2.29	2.65	4.33	2.39
P	+	+++	+++	+++
Between Chi				14.83
Between df				2
Between P				***
Btwn(F) P				N.S.
Btwn(R) P				N.S.

	Denominator nev any	nev cigs	Total
N	26	13	39
NS	19	10	29
Wt	181.51	274.95	456.45
Het Chi	56.44	141.65	235.32
Het df	25	12	38
Het P	***	***	***
Fixed RR	1.90	3.41	2.70
RRl	1.64	3.03	2.47
RRu	2.20	3.84	2.96
P	+++	+++	+++
Random RR	1.77	2.06	1.83
RRl	1.37	1.27	1.40
RRu	2.27	3.33	2.39
P	+++	++	+++
Between Chi			37.23
Between df			1
Between P			***
Btwn(F) P			*
Btwn(R) P			N.S.

Table 3G12 - 3

IESLC - Meta-anal of Ever Smoking (or Current if ever not avail), Amount smoked, "Low", Any prod (or Cigs if Any not avail)

Adenocarcinoma
Most adjusted

	Derivation of RR/CI			
	Orig	StdCalc	Other	Total
N	11	15	13	39
NS	10	10	9	29
Wt	205.03	134.46	116.97	456.45
Het Chi	55.02	43.88	21.70	235.32
Het df	10	14	12	38
Het P	***	***	*	***
Fixed RR	4.68	1.58	1.92	2.70
RRl	4.08	1.33	1.60	2.47
RRu	5.37	1.87	2.30	2.96
P	+++	+++	+++	+++
Random RR	2.89	1.29	1.94	1.83
RRl	1.92	0.91	1.42	1.40
RRu	4.36	1.82	2.65	2.39
P	+++	N.S.	+++	+++
Between Chi				114.72
Between df				2
Between P				***
Btwn(F) P				***
Btwn(R) P				*

	Smoking status		
	ever	current	Total
N	31	8	39
NS	21	7	28
Wt	382.81	73.65	456.45
Het Chi	219.94	14.67	235.32
Het df	30	7	38
Het P	***	*	***
Fixed RR	2.75	2.47	2.70
RRl	2.49	1.97	2.47
RRu	3.04	3.11	2.96
P	+++	+++	+++
Random RR	1.71	2.32	1.83
RRl	1.24	1.59	1.40
RRu	2.36	3.38	2.39
P	+++	+++	+++
Between Chi			0.71
Between df			1
Between P			N.S.
Btwn(F) P			N.S.
Btwn(R) P			N.S.

Table 3G12 - 4

IESLC - Meta-anal of Ever Smoking (or Current if ever not avail), Amount smoked, "Low", Any prod (or Cigs if Any not avail)
Adenocarcinoma
Least adjusted

REF	NRR	X	SEX	AGEL	AGEH	RACE	YF	LC TYPE	LOC	START	ST	NLC	R	VB	P	H	AD	SM	PRODUCT	exL	exH	DENOM	De
ALDERS	46		m	0	0	all	-	not q+s	Eu:UK	1977	CC	1448	n	V	n	n	1	ev	cig only	1	17	nev+2	ot
ALDERS	49		f	0	0	all	-	not q+s	Eu:UK	1977	CC	1448	n	V	n	n	1	ev	cig only	1	17	nev+2	ot
BARBON	45	x	m	0	0	all	-	a	Eu:wst	1979	CC	755	n	bl	y	y	0	ev	all/unsp	1	9	nev any	st
BROWN2	34		m	0	0	wh	-	a	NAmer	1984	CC	14596	n	bl	n	y	2	ev	cig+/-ot	1	19	nev cigs	or
BROWN2	33		f	0	0	wh	-	a	NAmer	1984	CC	14596	n	bl	n	y	2	ev	cig+/-ot	1	19	nev cigs	or
CHOI	51		m	0	0	all	-	a	As:oth	1985	CC	375	n	bl	n	n	0	ev	cig+/-ot	1	10	nev cigs	st
CHOI	59		f	0	0	all	-	a	As:oth	1985	CC	375	n	bl	n	n	0	ev	cig+/-ot	1	10	nev cigs	st
DOLL	59	x	m	0	0	all	-	KII	Eu:UK	1948	CC	1465	n	V	n	n	0	ev	all/unsp	5	14	nev any	st
DOLL	66	x	f	0	0	all	-	KII	Eu:UK	1948	CC	1465	n	V	n	n	0	ev	all/unsp	5	14	nev any	st
DORGAN	126		m	0	0	wh	-	a	NAmer	1980	CC	2026	n	bl	y	y	2	ev	cig+/-ot	1	19	nev any	ot
DORGAN	105		f	0	0	all	-	a	NAmer	1980	CC	2026	n	bl	y	y	3	ev	cig+/-ot	1	19	nev any	ot
DOSEME	8		m	0	0	all	-	not q+s	Eu:bal	1979	CC	1210	n	bl	n	n	2	ev	cig+/-ot	1	10	nev cigs	or
ENGELA	72		m	0	0	all	0	a	Eu:Sca	1964	pr	435	n	bl	n	n	7	cu	cig+/-ot	5	9	nev cigs	or
GER	2	x	c	0	0	all	-	a	As:oth	1990	CC	141	n	ot	y	n	0	ev	all/unsp	1	10	nev any	st
HAMMON	108		m	0	0	wh	0	a	NAmer	1952	pr	448	n	bl	n	n	1	cu	cig only	1	9	nev any	ot
JEDRYC	15	x	m	0	0	all	-	a	Eu:est	1980	CC	1630	n	bl	y	n	0	ev	cig+/-ot	1	9	nev any	st
KREYBE	17	x	m	0	0	all	-	KII	Eu:Sca	1948	CC	300	n	bl	n	y	0	ev	all/unsp	1	14	nev any	st
KREYBE	34	x	f	0	0	all	-	KII	Eu:Sca	1948	CC	300	n	bl	n	y	0	ev	all/unsp	1	14	nev any	st
LAMTH	16		f	0	0	ch	-	a	As:HK	1983	CC	445	n	bl	n	n	0	ev	all/unsp	1	10	nev any	or
LUBIN2	152		m	0	0	all	-	a	Eu:mul	1976	CC	7804	n	bl	n	y	0	ev	cig+/-ot	1	9	nev any	st
LUBIN2	172		f	0	0	all	-	a	Eu:mul	1976	CC	7804	n	bl	n	y	0	ev	cig+/-ot	1	9	nev any	st
MATOS	56	x	m	0	0	all	-	a	SCAmer	1994	CC	200	n	bl	n	n	0	ev	cig+/-ot	1	14	nev any	st
MATSUD	7		m	0	0	all	-	a	As:Jap	1965	CC	179	n	bl	n	n	0	ev	cig+/-ot	1	10	nev cigs	ot
ORMOS	22		m	0	0	all	-	KII	Eu:est	1947	CC	119	n	bl	y	y	0	ev	cig+/-ot	1	15	nev any	st
OSANN2	16	x	f	0	0	all	-	KII	NAmer	1964	ot	217	n	bl	n	y	0	ev	cig+/-ot	1	19	nev cigs	st
SOBUE	56		m	0	0	all	-	a	As:Jap	1986	CC	1376	n	bl	n	y	0	cu	cig+/-ot	1	19	nev cigs	st
SVENSS	29	x	f	0	0	all	-	a	Eu:Sca	1983	CC	210	n	bl	n	n	0	cu	all/unsp	1	10	nev any	st
TSUGAN	4		m	0	0	all	-	a	As:Jap	1976	CC	134	n	bl	n	y	0	cu	all/unsp	1	15	nev any	st
WAKAI	49	x	m	0	0	all	-	a	As:Jap	1988	CC	333	n	bl	n	y	0	cu	cig+/-ot	1	19	nev any	st
WUWILL	22	x	f	0	0	all	-	a	As:Chi	1985	CC	965	n	ot	n	n	0	ev	cig+/-ot	1	19	nev cigs	st
WYNDE2	10		m	0	0	all	-	KII	NAmer	1962	CC	404	n	bl	n	y	0	ev	cig+/-ot	1	10	nev any	st
WYNDE3	24		m	0	0	all	-	KII	NAmer	1966	CC	350	n	bl	n	y	0	ev	cig+/-ot	1	9	nev any	st
WYNDE3	71		f	0	0	all	-	KII	NAmer	1966	CC	350	n	bl	n	y	0	ev	cig+/-ot	1	9	nev any	st
WYNDE4	37		m	0	0	all	-	a	NAmer	1948	CC	684	n	bl	y	n	0	ev	all/unsp	1	9	nev any	st
WYNDE4	55		f	0	0	all	-	a	NAmer	1948	CC	684	n	bl	y	n	2	ev	all/unsp	1	9	nev any	ot
WYNDE6	24		m	0	0	all	-	KII	NAmer	1969	CC	4423	n	bl	n	y	0	cu	cig+/-ot	1	10	nev any	st
WYNDE6	213		f	0	0	all	-	KII	NAmer	1969	CC	4423	n	bl	n	y	0	cu	cig+/-ot	1	10	nev cigs	st
ZHENG	6		m	0	0	all	-	a	As:Chi	1982	CC	540	n	ot	*	y	0	ev	cig+/-ot	1	9	nev cigs	st
ZHENG	19		f	0	0	all	-	a	As:Chi	1982	CC	540	n	ot	*	y	0	ev	cig+/-ot	1	9	nev cigs	st

Cigarette type is all/unspec for all RRs

except for the following:

REF	NRR	CIGTYPE
ALDERS	46	MC only
ALDERS	49	MC only

Table 3G12 - 5

IESLC - Meta-anal of Ever Smoking (or Current if ever not avail), Amount smoked, "Low", Any prod (or Cigs if Any not avail)
Adenocarcinoma
Least adjusted

REF	NRR	SEX	AD	Number Exposed Case	Number Exposed Cont	Non-exposed Case	Non-exposed Cont	RR	95.00%CI
ALDERS	46	m	1	-	-	-	-	2.80	(1.01- 7.75)
ALDERS	49	f	1	-	-	-	-	2.77	(1.63- 4.70)
Subtotal ALDERS								2.78	(1.74- 4.44)
BARBON	45	m	0	7	87	7	188	2.16	(0.74- 6.35)
BROWN2	34	m	2	-	-	-	-	6.20	(4.90- 7.90)
BROWN2	33	f	2	-	-	-	-	5.80	(4.70- 7.10)
Subtotal BROWN2								5.97	(5.11- 6.98)
CHOI	51	m	0	4	90	7	95	0.60	(0.17- 2.13)
CHOI	59	f	0	4	16	49	164	0.84	(0.27- 2.62)
Subtotal CHOI								0.72	(0.31- 1.68)
DOLL	59	m	0	14	570	2	61	0.75	(0.17- 3.37)
DOLL	66	f	0	4	18	5	59	2.62	(0.64- 10.81)
Subtotal DOLL								1.46	(0.52- 4.08)
DORGAN	126	m	2	-	-	-	-	3.23	(1.20- 8.70)
DORGAN	105	f	3	-	-	-	-	2.87	(2.00- 4.11)
Subtotal DORGAN								2.91	(2.07- 4.08)
DOSEME	8	m	2	-	-	-	-	1.80	(0.80- 4.10)
*ENGELA	72	m	7	-	-	-	-	8.50	(2.50- 29.00)
GER	2	c	0	9	47	37	149	0.77	(0.35- 1.71)
*HAMMON	108	m	1	-	-	-	-	1.83	(0.17- 20.22)
JEDRYC	15	m	0	2	67	7	289	1.23	(0.25- 6.07)
KREYBE	17	m	0	31	2341	3	644	2.84	(0.87- 9.33)
KREYBE	34	f	0	9	286	27	657	0.77	(0.36- 1.65)
Subtotal KREYBE								1.13	(0.59- 2.15)
LAMTH	16	f	0	36	29	131	158	1.50	(0.87- 2.57)
LUBIN2	152	m	0	66	2194	57	2616	1.38	(0.96- 1.98)
LUBIN2	172	f	0	20	184	138	1180	0.93	(0.57- 1.52)
Subtotal LUBIN2								1.20	(0.90- 1.61)
MATOS	56	m	0	11	88	5	110	2.75	(0.92- 8.21)
MATSUD	7	m	0	6	1237	0	1255	13.19~	(0.74- 234.37)
ORMOS	22	m	0	4	329	2	777	4.72	(0.86- 25.91)
OSANN2	16	f	0	14	24	22	43	1.14	(0.49- 2.63)
SOBUE	56	m	0	63	157	27	128	1.90	(1.15- 3.16)
SVENSS	29	f	0	12	30	22	120	2.18	(0.97- 4.90)
TSUGAN	4	m	0	12	14	18	17	0.81	(0.29- 2.24)
WAKAI	49	m	0	16	105	8	65	1.24	(0.50- 3.06)
WUWILL	22	f	0	112	311	172	601	1.26	(0.96- 1.66)
WYNDE2	10	m	0	2	114	5	105	0.37	(0.07- 1.94)
WYNDE3	24	m	0	1	42	6	88	0.35	(0.04- 2.99)
WYNDE3	71	f	0	2	19	15	76	0.53	(0.11- 2.53)
Subtotal WYNDE3								0.46	(0.13- 1.63)
WYNDE4	37	m	0	3	82	4	115	1.05	(0.23- 4.83)
WYNDE4	55	f	2	-	-	-	-	1.33	(0.29- 6.07)
Subtotal WYNDE4								1.18	(0.40- 3.47)
WYNDE6	24	m	0	42	122	58	617	3.66	(2.35- 5.70)
WYNDE6	213	f	0	39	109	119	856	2.57	(1.70- 3.89)
Subtotal WYNDE6								3.03	(2.24- 4.10)
ZHENG	6	m	0	18	40	29	94	1.46	(0.73- 2.92)
ZHENG	19	f	0	13	29	119	184	0.69	(0.35- 1.39)
Subtotal ZHENG								1.00	(0.62- 1.64)
Partial Totals				576	8781	1101	11511		

*prospective study

~ With 0.5 adjustment for zero

Table 3G12 - 5

IESLC - Meta-anal of Ever Smoking (or Current if ever not avail), Amount smoked, "Low", Any prod (or Cigs if Any not avail)
Adenocarcinoma
Least adjusted

REF	NRR	SEX	AD	Ys	Ws	Qs	Ps
ALDERS	46	m	1	1.03	3.70	0.02	0.0476
ALDERS	49	f	1	1.02	13.70	0.07	0.0002
Subtotal ALDERS				1.02	17.40	0.09	
BARBON	45	m	0	0.77	3.31	0.11	0.1612
BROWN2	34	m	2	1.82	67.36	51.62	0.0000
BROWN2	33	f	2	1.76	90.29	59.06	0.0000
Subtotal BROWN2				1.79	157.65	110.68	
CHOI	51	m	0	-0.51	2.41	5.11	0.4323
CHOI	59	f	0	-0.18	2.95	3.75	0.7595
Subtotal CHOI				-0.33	5.36	8.85	
DOLL	59	m	0	-0.29	1.70	2.60	0.7068
DOLL	66	f	0	0.96	1.91	0.00	0.1823
Subtotal DOLL				0.38	3.61	2.60	
DORGAN	126	m	2	1.17	3.92	0.20	0.0203
DORGAN	105	f	3	1.05	29.62	0.33	0.0000
Subtotal DORGAN				1.07	33.53	0.52	
DOSEME	8	m	2	0.59	5.75	0.75	0.1585
*ENGELA	72	m	7	2.14	2.56	3.63	0.0006
GER	2	c	0	-0.26	6.02	8.80	0.5237
*HAMMON	108	m	1	0.60	0.67	0.08	0.6201
JEDRYC	15	m	0	0.21	1.51	0.83	0.7972
KREYBE	17	m	0	1.04	2.72	0.02	0.0849
KREYBE	34	f	0	-0.27	6.53	9.65	0.4952
Subtotal KREYBE				0.12	9.25	9.68	
LAMTH	16	f	0	0.40	13.12	3.90	0.1438
LUBIN2	152	m	0	0.32	29.82	11.71	0.0782
LUBIN2	172	f	0	-0.07	15.74	16.45	0.7715
Subtotal LUBIN2				0.19	45.56	28.16	
MATOS	56	m	0	1.01	3.21	0.01	0.0698
MATSUD	7	m	0	2.58	0.46	1.23	0.0789
ORMOS	22	m	0	1.55	1.33	0.48	0.0738
OSANN2	16	f	0	0.13	5.50	3.68	0.7584
SOBUE	56	m	0	0.64	14.90	1.40	0.0130
SVENSS	29	f	0	0.78	5.87	0.17	0.0588
TSUGAN	4	m	0	-0.21	3.72	5.00	0.6838
WAKAI	49	m	0	0.21	4.71	2.55	0.6431
WUWILL	22	f	0	0.23	50.96	26.37	0.1009
WYNDE2	10	m	0	-1.00	1.39	5.28	0.2387
WYNDE3	24	m	0	-1.05	0.83	3.33	0.3372
WYNDE3	71	f	0	-0.63	1.58	3.94	0.4293
Subtotal WYNDE3				-0.77	2.41	7.27	
WYNDE4	37	m	0	0.05	1.66	1.34	0.9482
WYNDE4	55	f	2	0.29	1.66	0.73	0.7132
Subtotal WYNDE4				0.17	3.32	2.07	
WYNDE6	24	m	0	1.30	19.66	2.39	0.0000
WYNDE6	213	f	0	0.95	22.53	0.00	0.0000
Subtotal WYNDE6				1.11	42.19	2.39	
ZHENG	6	m	0	0.38	7.96	2.60	0.2870
ZHENG	19	f	0	-0.37	7.98	13.82	0.3003
Subtotal ZHENG				0.00	15.94	16.42	

	N	39
	NS	28
	Wt	461.22
Het	Chi	253.00
Het	df	38
Het	P	***
Fixed	RR	2.58
	RRl	2.36
	RRu	2.83
	P	+++
Random	RR	1.74
	RRl	1.32
	RRu	2.29
	P	+++
Asymm	P	**

Table 3G12 - 6

IESLC - Meta-anal of Ever Smoking (or Current if ever not avail), Amount smoked, "Low", Any prod (or Cigs if Any not avail)
Adenocarcinoma
Least adjusted

	Sex			
	combined	male	female	Total
N	1	23	15	39
NS	1	23	15	39
Wt	6.02	185.25	269.95	461.22
Het Chi	0.00	99.91	141.22	253.00
Het df	0	22	14	38
Het P	N.S.	***	***	***
Fixed RR	0.77	2.89	2.46	2.58
RRl	0.35	2.51	2.18	2.36
RRu	1.71	3.34	2.77	2.83
P	N.S.	+++	+++	+++
Random RR	0.77	1.95	1.59	1.74
RRl	0.35	1.34	1.03	1.32
RRu	1.71	2.84	2.45	2.29
P	N.S.	+++	+	+++
Between Chi				11.88
Between df				2
Between P				**
Btwn(F) P				N.S.
Btwn(R) P				N.S.

Table 3G12 - 7

IESLC - Meta-anal of Ever Smoking (or Current if ever not avail), Amount smoked, "Low", Any prod (or Cigs if Any not avail)
Adenocarcinoma
Excluded studies (and stage at which they were excluded)

1 ABELIN ABRAHA AMANDU AMES ANDERS AUSTIN AXELSO BAND BECHER BERRIN BLOHMK BLOT4 BROCKM BROWN1 BYERS1 BYERS2
CARPEN CASCO2 CASCOR CHAN CHEN3 CHIAZZ CHYOU DESTE2 DOCKER DROSTE DU GARCIA GARDIN GENG GODLEY GOODMA
GRAHAM GREGOR HEGMAN HEIN HENNEK HINDS HIRAOK HOROWI HORWIT HUANG ISHIMA JAHN JAIN JARVHO JIANG KELLER
KIHARA KJUUS KO KOHLME KUBIK LAMWK LAMWK2 LANGE LEI LEMARC LEVIN LIU LOMBA2 LOMBAR MAGNUS MARSH
MARSH2 MCDUFF MCLAUG MILLER MILLS NOTANI NOU ODRISC PAWLEG PERSHA POFFIJ QIAO QIAO2 RADZIK REN RONCO
ROOTS ROTHSC SAARIK SANKAR SCHWAR SEGI SEOW SHIMIZ SIMARA SIMONA SITAS SOBUE2 STASZE STAYNE STUCKE SUN
SUZUK2 SUZUKI TANG TAO TOKARS TOUSEY ULMER VEIERO VUTUC WALD WANG WANG3 WANG4 WICKLU WIGLE WILKIN
WU2 WUNSCH WYNDE8 XIANGZ XU XU2 XU4 YONG ZHANG

2 BUELL CHEN MASTRA MZILEN PISANI RESTRE SADOWS

4 BOFFET WYNDE7

5 RIMING TANG2 WYNDE5

6 BLOT1 BLOT2 BLOT3 BOUCHA HIRAY2 JONES LAURIL LICKIN MOLLO MRFIT MURATA SCHWA2 VANDER WARSIN WATSON WYNDER

8 AGUDO AKIBA ARCHER ARMADA AUVINE AXELSS BENSHL BEST BRESLO BRETT BROSS BUFFLE CEDERL CHANG CHATZI CHEN2
CHOW COMSTO COOKSO CPSI CPSII DAMBER DARBY DAVEYS DEAN DEAN2 DEAN3 DEKLER DESTEF DOLL2 DORANT DORN
DUNN EBELIN ENSTRO ESAKI FAN GAO GAO2 GARSHI GILLIS GOLLED GSELL HAMMO2 HANSEN HIRAYA HITOSU HOLE
HU HU2 HUMBLE JARUP JOLY JUSSAW KAISE2 KAISER KANELL KAUFMA KHUDER KINLEN KNEKT KOO KOULUM KREUZE
LAUSSM LETOUR LIAW LIDDEL LIU2 LIU3 LIU4 LIU5 LUBIN LUO MACLEN MARTIS MCCONN MIGRAN MRFITR NAM
NOTAN2 PARKIN PASTOR PERNU PERSH2 PETO PEZZO2 PEZZOT PIKE POLEDN PRESCO RACHTA RANDIG SEGI2 SHAW SIEMIA
SPEIZE SPITZ STOCKS STOCKW TENKAN TIZZAN TULINI TVERDA WANG2 XU3 YAMAGU YUAN ZHOU

10 BOUCOT CORREA HAENSZ KATSOU OSANN WU

11 BENHAM

Table 3G12 - 8
Potentially overlapping studies

REF	REFGP	PRINC	.	OVERLAP/LINK
LUBIN2	LUBIN2	1		Lubin-combined
LAMTH	LAMTH	1		KOO/LAMTH/LAMWK
OSANN2	KAISER	2		KAISER/OSANN2
WYNDE6	WYNDE6	1		WYNDE5/6/7/8
MATSUD	MATSUD	1		SOBUE2/MATSUD

Table 3G12 - 9
Most adjusted - insufficient data for metaanalysis

REF	NRR	SEX	AGEL	AGEH	RACE	YF	LC TYPE	LOC	START	ST	NLC	R	VB	P	H	AD	SM	PRODUCT	exL	exH	DENOM	De
CHEN	15	c	0	0	all	-	a	As:oth	1987	CC	323	n	ot	n	y	2	ev	cig+/-ot	1	10	nev	cigs ot

REF	NRR	RR	SIG	RRDATA	comment
CHEN	15	1.21	n	0	

Table 3G13 -

IESLC - Meta-anal of Ever Smoking (or Current if ever not avail), Amount smoked, "Mid", Any prod (or Cigs if Any not avail)
Adenocarcinoma

This analysis is restricted to results for:
1) Results by Amount smoked
2) Results complete enough for use in metaanalysis

Within each study, results are then selected (in the following order of preference, within each sex) for:
3) SMKSTA: ever smokers, current smokers
4) PRODUCT: all/unspec, cigarettes regardless of other products, cigarettes only
5) CIGTYPE: all/unspecified, MC regardless of HR, MC only
6) DENOM: never smoked anything, never smoked cigarettes, (never +1 = +long term ex, +2 = +amount unknown,
+3 = never cigs+long term ex)
7) Followup period (YF, prospective studies): whole study (coded as 0) or longest available
8) LCtype: adeno or nearest available, but not squamous. (q = squamous, s = small,
a = adeno, l = large, KII = Kreyberg II, al = alveolar, br = bronchiolar, u = undifferentiated)
9) Race: all or nearest available, otherwise by race (wh or w = white, bl or b = black, hi = hispanic
ch = chinese, jap = japanese, haw = hawaiian, w+o = white + oriental, sca = scandinavian, as = asian)
10) Amount smoked "mid" in key scheme 1 (key value 20, maximum range 6-44, in numbers of
cigarettes or cigarette equivalents)
11) For overlapping studies: principal rather than subsidiary studies
Finally by Age: whole study (coded as 0) if available, otherwise by widest available age group
and then for single sex results (m, f) in preference to combined sex results (c).

Results adjusted (AD) for the most potential confounders are then chosen in Sections -1 to -3
and results adjusted for the least confounders in Sections -4 to -6. (Those least adjusted results which
actually differ from the most adjusted as marked 'x' in column X in Section -4)
(Results adjusted for an unknown number of confounder(s) are coded as 20.)

Section -7 shows excluded studies, together with the stage (as above) at which no qualifying
results were found.

Section -8 lists the potentially overlapping studies which have been included (1=principal, 2=subsidiary).

Section -9 lists any results which would have been included in preference except that they had data not complete
enough for use in meta-analysis, with their significance (yes/no), if known, and any further comment as entered
on the database.

In addition to those mentioned above, the following fields, levels and abbreviations are used:
* or nk = not known, n = no, y = yes, ot = other
ev = ever, cu = current, nev = never
all/unspec = all or unspecified, cig+/-ot = cigarettes irrespective of other products (cigar, pipe etc)
MC = manufactured cigarettes, HR = hand-rolled cigarettes
exL, exH = range of exposure (low and high) in the smoking group, in terms of Amount smoked,
cigarettes or cigarette equivalents
REF: 6-character study reference
NRR: number of the RR on the database within the study
ST : study type (CC = case control, pr or prosp = prospective)
NLC: number of lung cancer cases in whole study
R : risky occupational population (n = no, m = mining, o = other risky)
VB : national cigarette type (V = at least 75% Virginia, bl = at least 75% blended, ot = other)
P : any proxy use
H : full histological confirmation
De : derivation of RR/CI (or = original, st = standard method, ot = other method of estimation)

Table 3G13 - 1

IESLC - Meta-anal of Ever Smoking (or Current if ever not avail), Amount smoked, "Mid", Any prod (or Cigs if Any not avail)
Adenocarcinoma
Most adjusted

REF	NRR	SEX	AGEL	AGEH	RACE	YF	LC TYPE	LOC	START	ST	NLC	R	VB	P	H	AD	SM	PRODUCT	exL	exH	DENOM	De
ALDERS	47	m	0	0	all	-	not q+s	Eu:UK	1977	CC	1448	n	V	n	n	1	ev	cig only	18	27	nev+2	ot
ALDERS	50	f	0	0	all	-	not q+s	Eu:UK	1977	CC	1448	n	V	n	n	1	ev	cig only	18	27	nev+2	ot
BARBON	80	m	0	0	all	-	a	Eu:wst	1979	CC	755	n	bl	y	y	3	ev	all/unsp	20	39	nev any	or
CHOI	52	m	0	0	all	-	a	As:oth	1985	CC	375	n	bl	n	n	0	ev	cig+/-ot	11	20	nev cigs	st
CHOI	60	f	0	0	all	-	a	As:oth	1985	CC	375	n	bl	n	n	0	ev	cig+/-ot	11	30	nev cigs	ot
DOLL	74	m	0	0	all	-	KII	Eu:UK	1948	CC	1465	n	V	n	n	1	ev	all/unsp	15	24	nev any	ot
DOSEME	12	m	0	0	all	-	not q+s	Eu:bal	1979	CC	1210	n	bl	n	n	2	ev	cig+/-ot	11	20	nev cigs	or
GER	11	c	0	0	all	-	a	As:oth	1990	CC	141	n	ot	y	n	8	ev	all/unsp	11	20	nev any	ot
HAMMON	109	m	0	0	wh	0	a	NAmer	1952	pr	448	n	bl	n	n	1	cu	cig only	10	20	nev any	ot
JEDRYC	37	m	0	0	all	-	a	Eu:est	1980	CC	1630	n	bl	y	n	3	ev	cig+/-ot	20	29	nev any	or
KREYBE	6	m	0	0	all	-	KII	Eu:Sca	1948	CC	300	n	bl	n	y	1	ev	all/unsp	15	24	nev any	ot
LAMTH	17	f	0	0	ch	-	a	As:HK	1983	CC	445	n	bl	n	n	0	ev	all/unsp	11	20	nev any	or
LUBIN2	160	m	0	0	all	-	a	Eu:mul	1976	CC	7804	n	bl	n	y	0	ev	cig+/-ot	20	29	nev any	st
LUBIN2	180	f	0	0	all	-	a	Eu:mul	1976	CC	7804	n	bl	n	y	0	ev	cig+/-ot	20	29	nev any	st
MATOS	59	m	0	0	all	-	a	SCAmer	1994	CC	200	n	bl	n	n	2	ev	cig+/-ot	15	24	nev any	or
MATSUD	8	m	0	0	all	-	a	As:Jap	1965	CC	179	n	bl	n	n	0	ev	cig+/-ot	11	20	nev cigs	ot
ORMOS	23	m	0	0	all	-	KII	Eu:est	1947	CC	119	n	bl	y	y	0	ev	cig+/-ot	16	30	nev any	st
SOBUE	57	m	0	0	all	-	a	As:Jap	1986	CC	1376	n	bl	n	y	0	cu	cig+/-ot	20	29	nev cigs	st
SVENSS	14	f	0	0	all	-	a	Eu:Sca	1983	CC	210	n	bl	n	n	1	cu	all/unsp	11	20	nev any	or
TSUGAN	5	m	0	0	all	-	a	As:Jap	1976	CC	134	n	bl	n	y	0	cu	all/unsp	16	35	nev any	st
WAKAI	53	m	0	0	all	-	a	As:Jap	1988	CC	333	n	bl	n	y	1	cu	cig+/-ot	20	29	nev any	or
WYNDE2	11	m	0	0	all	-	KII	NAmer	1962	CC	404	n	bl	n	y	0	ev	cig+/-ot	11	20	nev any	st
WYNDE3	25	m	0	0	all	-	KII	NAmer	1966	CC	350	n	bl	n	y	0	ev	cig+/-ot	10	20	nev any	st
WYNDE3	72	f	0	0	all	-	KII	NAmer	1966	CC	350	n	bl	n	y	0	ev	cig+/-ot	10	20	nev any	st
WYNDE4	39	m	0	0	all	-	a	NAmer	1948	CC	684	n	bl	y	n	0	ev	all/unsp	16	20	nev any	st
WYNDE6	33	m	0	0	all	-	KII	NAmer	1969	CC	4423	n	bl	n	y	0	cu	cig+/-ot	11	20	nev any	st
WYNDE6	222	f	0	0	all	-	KII	NAmer	1969	CC	4423	n	bl	n	y	0	cu	cig+/-ot	11	20	nev cigs	st
ZHENG	8	m	0	0	all	-	a	As:Chi	1982	CC	540	n	ot	*	y	0	ev	cig+/-ot	20	29	nev cigs	st

Cigarette type is all/unspec for all RRs

except for the following:

REF	NRR	CIGTYPE
ALDERS	47	MC only
ALDERS	50	MC only

Table 3G13 - 2

IESLC - Meta-anal of Ever Smoking (or Current if ever not avail), Amount smoked, "Mid", Any prod (or Cigs if Any not avail)
Adenocarcinoma
Most adjusted

REF	NRR	SEX	AD	Number Exposed Case	Number Exposed Cont	Non-exposed Case	Non-exposed Cont	RR	95.00%CI
ALDERS	47	m	1	-	-	-	-	2.67	(0.99- 7.18)
ALDERS	50	f	1	-	-	-	-	4.58	(2.67- 7.85)
Subtotal ALDERS								4.05	(2.52- 6.50)
BARBON	80	m	3	-	-	-	-	8.30	(3.70- 19.00)
CHOI	52	m	0	27	281	7	95	1.30	(0.55- 3.09)
CHOI	60	f	0	0	9	49	164	0.17~	(0.01- 3.06)
Subtotal CHOI								1.10	(0.48- 2.52)
DOLL	74	m	1	-	-	-	-	1.20	(0.27- 5.35)
DOSEME	12	m	2	-	-	-	-	2.70	(1.60- 4.70)
GER	11	c	8	-	-	-	-	2.10	(0.88- 5.04)
*HAMMON	109	m	1	-	-	-	-	2.83	(0.55- 14.60)
JEDRYC	37	m	3	-	-	-	-	4.38	(1.87- 10.28)
KREYBE	6	m	1	-	-	-	-	1.43	(0.36- 5.72)
LAMTH	17	f	0	27	14	131	158	2.33	(1.17- 4.62)
LUBIN2	160	m	0	234	3108	57	2616	3.46	(2.57- 4.64)
LUBIN2	180	f	0	13	110	138	1180	1.01	(0.55- 1.84)
Subtotal LUBIN2								2.73	(2.09- 3.55)
MATOS	59	m	2	-	-	-	-	7.00	(2.60- 19.10)
MATSUD	8	m	0	13	1607	0	1255	21.09~	(1.25- 355.07)
ORMOS	23	m	0	3	577	2	777	2.02	(0.34- 12.13)
SOBUE	57	m	0	95	222	27	128	2.03	(1.26- 3.28)
SVENSS	14	f	1	-	-	-	-	5.40	(2.40- 13.20)
TSUGAN	5	m	0	23	23	18	17	0.94	(0.39- 2.28)
WAKAI	53	m	1	-	-	-	-	1.93	(0.84- 4.44)
WYNDE2	11	m	0	14	203	5	105	1.45	(0.51- 4.13)
WYNDE3	25	m	0	20	114	6	88	2.57	(0.99- 6.68)
WYNDE3	72	f	0	11	24	15	76	2.32	(0.94- 5.73)
Subtotal WYNDE3								2.44	(1.27- 4.70)
WYNDE4	39	m	0	15	274	4	115	1.57	(0.51- 4.84)
WYNDE6	33	m	0	191	293	58	617	6.93	(5.01- 9.60)
WYNDE6	222	f	0	176	165	119	856	7.67	(5.76- 10.21)
Subtotal WYNDE6								7.34	(5.92- 9.10)
ZHENG	8	m	0	53	89	29	94	1.93	(1.13- 3.30)
Partial Totals				915	7113	665	8341		

*prospective study ~ With 0.5 adjustment for zero

REF	NRR	SEX	AD	Ys	Ws	Qs	Ps
ALDERS	47	m	1	0.98	3.91	0.34	0.0520
ALDERS	50	f	1	1.52	13.21	0.80	0.0000
Subtotal ALDERS				1.40	17.13	1.13	
BARBON	80	m	3	2.12	5.74	4.05	0.0000
CHOI	52	m	0	0.27	5.16	5.27	0.5467
CHOI	60	f	0	-1.74	0.47	4.28	0.2324
Subtotal CHOI				0.10	5.62	9.55	
DOLL	74	m	1	0.18	1.72	2.06	0.8109
DOSEME	12	m	2	0.99	13.23	1.06	0.0003
GER	11	c	8	0.74	5.04	1.44	0.0956
*HAMMON	109	m	1	1.04	1.43	0.08	0.2136
JEDRYC	37	m	3	1.48	5.29	0.21	0.0007
KREYBE	6	m	1	0.36	2.01	1.70	0.6122
LAMTH	17	f	0	0.84	8.17	1.53	0.0158
LUBIN2	160	m	0	1.24	44.40	0.06	0.0000
LUBIN2	180	f	0	0.01	10.63	17.03	0.9727
Subtotal LUBIN2				1.00	55.03	17.08	
MATOS	59	m	2	1.95	3.86	1.73	0.0001
MATSUD	8	m	0	3.05	0.48	1.51	0.0343
ORMOS	23	m	0	0.70	1.20	0.39	0.4420
SOBUE	57	m	0	0.71	16.70	5.41	0.0038
SVENSS	14	f	1	1.69	5.29	0.89	0.0001
TSUGAN	5	m	0	-0.06	4.97	8.83	0.8986
WAKAI	53	m	1	0.66	5.54	2.12	0.1216
WYNDE2	11	m	0	0.37	3.50	2.87	0.4885
WYNDE3	25	m	0	0.95	4.22	0.46	0.0521
WYNDE3	72	f	0	0.84	4.71	0.89	0.0675
Subtotal WYNDE3				0.89	8.93	1.35	
WYNDE4	39	m	0	0.45	3.04	2.06	0.4291
WYNDE6	33	m	0	1.94	36.35	15.84	0.0000
WYNDE6	222	f	0	2.04	46.92	27.20	0.0000
Subtotal WYNDE6				1.99	83.27	43.04	
ZHENG	8	m	0	0.66	13.29	5.09	0.0165

Table 3G13 - 2

IESLC - Meta-anal of Ever Smoking (or Current if ever not avail), Amount smoked, "Mid", Any prod (or Cigs if Any not avail)
Adenocarcinoma
Most adjusted

	N	28
	NS	23
	Wt	270.48
Het	Chi	115.19
Het	df	27
Het	P	***
Fixed	RR	3.58
	RRl	3.18
	RRu	4.04
	P	+++
Random	RR	2.73
	RRl	2.06
	RRu	3.61
	P	+++
Asymm	P	**

Table 3G13 - 3

IESLC - Meta-anal of Ever Smoking (or Current if ever not avail), Amount smoked, "Mid", Any prod (or Cigs if Any not avail)
Adenocarcinoma
Most adjusted

	combined	Sex: male	female	Total
N	1	20	7	28
NS	1	20	7	28
Wt	5.04	176.05	89.39	270.48
Het Chi	0.00	59.31	47.81	115.19
Het df	0	19	6	27
Het P	N.S.	***	***	***
Fixed RR	2.10	3.24	4.52	3.58
RRl	0.88	2.79	3.67	3.18
RRu	5.03	3.75	5.56	4.04
P	(+)	+++	+++	+++
Random RR	2.10	2.69	2.83	2.73
RRl	0.88	1.98	1.40	2.06
RRu	5.03	3.65	5.72	3.61
P	(+)	+++	++	+++
Between Chi				8.07
Between df				2
Between P				*
Btwn(F) P				N.S.
Btwn(R) P				N.S.

	a	a+l	a+al+br	Lung cancer type: KII	not q+u	not q+s	Total
N	17			8		3	28
NS	15			6		2	23
Wt	139.50			100.62		30.36	270.48
Het Chi	45.72			27.85		2.10	115.19
Het df	16			7		2	27
Het P	***			***		N.S.	***
Fixed RR	2.56			5.81		3.39	3.58
RRl	2.17			4.78		2.38	3.18
RRu	3.02			7.07		4.84	4.04
P	+++			+++		+++	+++
Random RR	2.45			3.20		3.39	2.73
RRl	1.78			1.91		2.35	2.06
RRu	3.38			5.38		4.89	3.61
P	+++			+++		+++	+++
Between Chi							39.52
Between df							2
Between P							***
Btwn(F) P							**
Btwn(R) P							N.S.

	NAmer	UK	Scand	Location: othEur	China	Japan	othAs	other	Total
N	7	3	2	6	1	4	4	1	28
NS	5	2	2	5	1	4	3	1	23
Wt	100.16	18.85	7.30	80.49	13.29	27.69	18.84	3.86	270.48
Het Chi	24.17	3.19	2.57	20.50	0.00	5.24	3.74	0.00	115.19
Het df	6	2	1	5	0	3	3	0	27
Het P	***	N.S.	N.S.	**	N.S.	N.S.	N.S.	N.S.	***
Fixed RR	5.92	3.62	3.75	3.03	1.93	1.82	1.81	7.00	3.58
RRl	4.87	2.31	1.81	2.43	1.13	1.26	1.15	2.58	3.18
RRu	7.20	5.69	7.74	3.76	3.30	2.65	2.84	18.97	4.04
P	+++	+++	+++	+++	+	++	++	+++	+++
Random RR	3.57	3.14	3.12	2.99	1.93	1.81	1.76	7.00	2.73
RRl	2.15	1.61	0.87	1.75	1.13	1.01	1.04	2.58	2.06
RRu	5.93	6.14	11.25	5.12	3.30	3.23	2.98	18.97	3.61
P	+++	+++	(+)	+++	+	+	+	+++	+++
Between Chi									55.77
Between df									7
Between P									***
Btwn(F) P									*
Btwn(R) P									N.S.

Table 3G13 - 3

IESLC - Meta-anal of Ever Smoking (or Current if ever not avail), Amount smoked, "Mid", Any prod (or Cigs if Any not avail)
Adenocarcinoma
Most adjusted

	Detailed Country in "other Europe"					
	multi	Germany	othWest	East	Balkans	Total
N	2		1	2	1	6
NS	1		1	2	1	5
Wt	55.03		5.74	6.49	13.23	80.49
Het Chi	12.96		0.00	0.58	0.00	20.50
Het df	1		0	1	0	5
Het P	***		N.S.	N.S.	N.S.	**
Fixed RR	2.73		8.30	3.80	2.70	3.03
RRl	2.09		3.66	1.76	1.58	2.43
RRu	3.55		18.81	8.20	4.63	3.76
P	+++		+++	+++	+++	+++
Random RR	1.92		8.30	3.80	2.70	2.99
RRl	0.58		3.66	1.76	1.58	1.75
RRu	6.41		18.81	8.20	4.63	5.12
P	N.S.		+++	+++	+++	+++
Between Chi						6.95
Between df						3
Between P						(*)
Btwn(F) P						N.S.
Btwn(R) P						N.S.

	Detailed Country in "other Asia"			
	India	HongKong	other	Total
N		1	3	4
NS		1	2	3
Wt		8.17	10.67	18.84
Het Chi		0.00	2.84	3.74
Het df		0	2	3
Het P		N.S.	N.S.	N.S.
Fixed RR		2.33	1.50	1.81
RRl		1.17	0.82	1.15
RRu		4.62	2.72	2.84
P		+	N.S.	++
Random RR		2.33	1.41	1.76
RRl		1.17	0.65	1.04
RRu		4.62	3.09	2.98
P		+	N.S.	+
Between Chi				0.90
Between df				1
Between P				N.S.
Btwn(F) P				N.S.
Btwn(R) P				N.S.

	Detailed other continent			
	SCAmer	Auslia	Africa	Total
N	1			1
NS	1			1
Wt	3.86			3.86
Het Chi	0.00			0.00
Het df	0			0
Het P	N.S.			N.S.
Fixed RR	7.00			7.00
RRl	2.58			2.58
RRu	18.97			18.97
P	+++			+++
Random RR	7.00			7.00
RRl	2.58			2.58
RRu	18.97			18.97
P	+++			+++
Between Chi				
Between df				
Between P				N.S.
Btwn(F) P				N.S.
Btwn(R) P				N.S.

Table 3G13 - 3

IESLC - Meta-anal of Ever Smoking (or Current if ever not avail), Amount smoked, "Mid", Any prod (or Cigs if Any not avail)
Adenocarcinoma
Most adjusted

	Start year of study					
	<1960	1960-69	1970-79	1980-89	1990+	Total
N	5	6	7	8	2	28
NS	5	4	5	7	2	23
Wt	9.40	96.18	96.10	59.91	8.91	270.48
Het Chi	0.69	18.50	28.59	11.63	3.17	115.19
Het df	4	5	6	7	1	27
Het P	N.S.	**	***	N.S.	(*)	***
Fixed RR	1.66	6.28	2.96	2.24	3.54	3.58
RRl	0.87	5.14	2.42	1.74	1.84	3.18
RRu	3.14	7.67	3.61	2.89	6.83	4.04
P	N.S.	+++	+++	+++	+++	+++
Random RR	1.66	4.38	2.69	2.28	3.74	2.73
RRl	0.87	2.62	1.64	1.61	1.15	2.06
RRu	3.14	7.30	4.39	3.23	12.15	3.61
P	N.S.	+++	+++	+++	+	+++
Between Chi						52.61
Between df						4
Between P						***
Btwn(F) P						**
Btwn(R) P						N.S.

	Study type (1)		
	CC	other	Total
N	27	1	28
NS	22	1	23
Wt	269.05	1.43	270.48
Het Chi	115.11	0.00	115.19
Het df	26	0	27
Het P	***	N.S.	***
Fixed RR	3.59	2.83	3.58
RRl	3.18	0.55	3.18
RRu	4.04	14.58	4.04
P	+++	N.S.	+++
Random RR	2.72	2.83	2.73
RRl	2.05	0.55	2.06
RRu	3.62	14.58	3.61
P	+++	N.S.	+++
Between Chi			0.08
Between df			1
Between P			N.S.
Btwn(F) P			N.S.
Btwn(R) P			N.S.

	Study type (2)			
	CC	prosp	other	Total
N	27	1		28
NS	22	1		23
Wt	269.05	1.43		270.48
Het Chi	115.11	0.00		115.19
Het df	26	0		27
Het P	***	N.S.		***
Fixed RR	3.59	2.83		3.58
RRl	3.18	0.55		3.18
RRu	4.04	14.58		4.04
P	+++	N.S.		+++
Random RR	2.72	2.83		2.73
RRl	2.05	0.55		2.06
RRu	3.62	14.58		3.61
P	+++	N.S.		+++
Between Chi				0.08
Between df				1
Between P				N.S.
Btwn(F) P				N.S.
Btwn(R) P				N.S.

Table 3G13 - 3

IESLC - Meta-anal of Ever Smoking (or Current if ever not avail), Amount smoked, "Mid", Any prod (or Cigs if Any not avail)
Adenocarcinoma
Most adjusted

	Study size (number of LC cases)				
	100-249	250-499	500-999	1000+	Total
N	6	9	3	10	28
NS	6	7	3	7	23
Wt	20.84	35.20	22.07	192.37	270.48
Het Chi	13.87	4.96	9.62	64.45	115.19
Het df	5	8	2	9	27
Het P	*	N.S.	**	***	***
Fixed RR	2.90	1.90	2.74	4.25	3.58
RRl	1.89	1.36	1.81	3.69	3.18
RRu	4.46	2.64	4.16	4.89	4.04
P	+++	+++	+++	+++	+++
Random RR	3.14	1.90	2.97	3.29	2.73
RRl	1.45	1.36	1.08	2.17	2.06
RRu	6.80	2.64	8.22	4.98	3.61
P	++	+++	+	+++	+++
Between Chi					22.28
Between df					3
Between P					***
Btwn(F) P					N.S.
Btwn(R) P					N.S.

	Risky occupational population			
	no	mining	othRisky	Total
N	28			28
NS	23			23
Wt	270.48			270.48
Het Chi	115.19			115.19
Het df	27			27
Het P	***			***
Fixed RR	3.58			3.58
RRl	3.18			3.18
RRu	4.04			4.04
P	+++			+++
Random RR	2.73			2.73
RRl	2.06			2.06
RRu	3.61			3.61
P	+++			+++
Between Chi				
Between df				
Between P				N.S.
Btwn(F) P				N.S.
Btwn(R) P				N.S.

	National cigarette tobacco type			
	Virginia	blended	other	Total
N	3	23	2	28
NS	2	19	2	23
Wt	18.85	233.30	18.34	270.48
Het Chi	3.19	104.97	0.03	115.19
Het df	2	22	1	27
Het P	N.S.	***	N.S.	***
Fixed RR	3.62	3.75	1.98	3.58
RRl	2.31	3.30	1.25	3.18
RRu	5.69	4.27	3.12	4.04
P	+++	+++	++	+++
Random RR	3.14	2.78	1.98	2.73
RRl	1.61	2.01	1.25	2.06
RRu	6.14	3.85	3.12	3.61
P	+++	+++	++	+++
Between Chi				7.00
Between df				2
Between P				*
Btwn(F) P				N.S.
Btwn(R) P				N.S.

Table 3G13 - 3

IESLC - Meta-anal of Ever Smoking (or Current if ever not avail), Amount smoked, "Mid", Any prod (or Cigs if Any not avail)

Adenocarcinoma
Most adjusted

	Any proxy use		
	No/nk	Yes	Total
N	23	5	28
NS	18	5	23
Wt	250.17	20.31	270.48
Het Chi	107.03	8.15	115.19
Het df	22	4	27
Het P	***	(*)	***
Fixed RR	3.58	3.58	3.58
RRl	3.17	2.32	3.18
RRu	4.06	5.54	4.04
P	+++	+++	+++
Random RR	2.63	3.32	2.73
RRl	1.92	1.74	2.06
RRu	3.59	6.34	3.61
P	+++	+++	+++
Between Chi			
Between df			1
Between P			N.S.
Btwn(F) P			N.S.
Btwn(R) P			N.S.

	Full histological confirmation		
	No	Yes	Total
N	14	14	28
NS	12	11	23
Wt	70.31	200.17	270.48
Het Chi	21.01	91.14	115.19
Het df	13	13	27
Het P	(*)	***	***
Fixed RR	3.00	3.82	3.58
RRl	2.37	3.32	3.18
RRu	3.79	4.38	4.04
P	+++	+++	+++
Random RR	2.89	2.62	2.73
RRl	2.10	1.73	2.06
RRu	3.99	3.96	3.61
P	+++	+++	+++
Between Chi			3.03
Between df			1
Between P			(*)
Btwn(F) P			N.S.
Btwn(R) P			N.S.

	Number of adjustment variables (1)			
	0	1	2+/+nk	Total
N	16	7	5	28
NS	12	6	5	23
Wt	204.19	33.12	33.17	270.48
Het Chi	98.70	7.67	8.37	115.19
Het df	15	6	4	27
Het P	***	N.S.	(*)	***
Fixed RR	3.60	3.25	3.81	3.58
RRl	3.14	2.31	2.71	3.18
RRu	4.13	4.57	5.35	4.04
P	+++	+++	+++	+++
Random RR	2.32	3.04	4.09	2.73
RRl	1.54	2.02	2.45	2.06
RRu	3.49	4.59	6.84	3.61
P	+++	+++	+++	+++
Between Chi				0.45
Between df				2
Between P				N.S.
Btwn(F) P				N.S.
Btwn(R) P				N.S.

Table 3G13 - 3

IESLC - Meta-anal of Ever Smoking (or Current if ever not avail), Amount smoked, "Mid", Any prod (or Cigs if Any not avail)
Adenocarcinoma
Most adjusted

	Number of adjustment variables (2) 0	1	2	3-5	6+/+nk	Total
N	16	7	2	2	1	28
NS	12	6	2	2	1	23
Wt	204.19	33.12	17.10	11.03	5.04	270.48
Het Chi	98.70	7.67	2.71	1.12	0.00	115.19
Het df	15	6	1	1	0	27
Het P	***	N.S.	(*)	N.S.	N.S.	***
Fixed RR	3.60	3.25	3.35	6.11	2.10	3.58
RRl	3.14	2.31	2.08	3.39	0.88	3.18
RRu	4.13	4.57	5.38	11.02	5.03	4.04
P	+++	+++	+++	+++	(+)	+++
Random RR	2.32	3.04	3.95	6.10	2.10	2.73
RRl	1.54	2.02	1.58	3.26	0.88	2.06
RRu	3.49	4.59	9.85	11.41	5.03	3.61
P	+++	+++	++	+++	(+)	+++
Between Chi						4.98
Between df						4
Between P						N.S.
Btwn(F) P						N.S.
Btwn(R) P						N.S.

	Product all/unsp	cig+/-ot	cig only	Total
N	8	17	3	28
NS	8	13	2	23
Wt	35.98	215.95	18.56	270.48
Het Chi	18.49	90.93	1.05	115.19
Het df	7	16	2	27
Het P	**	***	N.S.	***
Fixed RR	2.56	3.76	3.94	3.58
RRl	1.85	3.29	2.50	3.18
RRu	3.55	4.30	6.21	4.04
P	+++	+++	+++	+++
Random RR	2.37	2.77	3.94	2.73
RRl	1.37	1.91	2.50	2.06
RRu	4.12	4.00	6.21	3.61
P	++	+++	+++	+++
Between Chi				4.72
Between df				2
Between P				(*)
Btwn(F) P				N.S.
Btwn(R) P				N.S.

	Denominator nev any	nev cigs	Total
N	21	7	28
NS	18	6	24
Wt	174.23	96.25	270.48
Het Chi	64.96	49.06	115.19
Het df	20	6	27
Het P	***	***	***
Fixed RR	3.41	3.91	3.58
RRl	2.94	3.20	3.18
RRu	3.96	4.78	4.04
P	+++	+++	+++
Random RR	2.78	2.56	2.73
RRl	2.05	1.26	2.06
RRu	3.77	5.22	3.61
P	+++	++	+++
Between Chi			1.16
Between df			1
Between P			N.S.
Btwn(F) P			N.S.
Btwn(R) P			N.S.

Table 3G13 - 3

IESLC - Meta-anal of Ever Smoking (or Current if ever not avail), Amount smoked, "Mid", Any prod (or Cigs if Any not avail)

Adenocarcinoma
Most adjusted

	Derivation of RR/CI: Orig	StdCalc	Other	Total
N	7	13	8	28
NS	7	10	7	24
Wt	47.13	195.07	28.28	270.48
Het Chi	11.59	91.25	11.23	115.19
Het df	6	12	7	27
Het P	(*)	***	N.S.	***
Fixed RR	3.58	3.68	2.98	3.58
RRl	2.69	3.20	2.06	3.18
RRu	4.76	4.24	4.30	4.04
P	+++	+++	+++	+++
Random RR	3.79	2.33	2.51	2.73
RRl	2.52	1.51	1.45	2.06
RRu	5.70	3.60	4.34	3.61
P	+++	+++	+++	+++
Between Chi				1.11
Between df				2
Between P				N.S.
Btwn(F) P				N.S.
Btwn(R) P				N.S.

	Smoking status: ever	current	Total
N	21	7	28
NS	17	6	23
Wt	153.29	117.19	270.48
Het Chi	43.31	45.32	115.19
Het df	20	6	27
Het P	**	***	***
Fixed RR	2.72	5.13	3.58
RRl	2.33	4.28	3.18
RRu	3.19	6.15	4.04
P	+++	+++	+++
Random RR	2.53	3.37	2.73
RRl	1.93	1.88	2.06
RRu	3.30	6.02	3.61
P	+++	+++	+++
Between Chi			26.56
Between df			1
Between P			***
Btwn(F) P			**
Btwn(R) P			N.S.

Table 3G13 - 4

IESLC - Meta-anal of Ever Smoking (or Current if ever not avail), Amount smoked, "Mid", Any prod (or Cigs if Any not avail)
Adenocarcinoma
Least adjusted

REF	NRR	X	SEX	AGEL	AGEH	RACE	YF	LC TYPE	LOC	START	ST	NLC	R	VB	P	H	AD	SM	PRODUCT	exL	exH	DENOM	De
ALDERS	47		m	0	0	all	-	not q+s	Eu:UK	1977	CC	1448	n	V	n	n	1	ev	cig only	18	27	nev+2	ot
ALDERS	50		f	0	0	all	-	not q+s	Eu:UK	1977	CC	1448	n	V	n	n	1	ev	cig only	18	27	nev+2	ot
BARBON	47	x	m	0	0	all	-	a	Eu:wst	1979	CC	755	n	bl	y	y	0	ev	all/unsp	20	29	nev any	st
CHOI	52		m	0	0	all	-	a	As:oth	1985	CC	375	n	bl	n	n	0	ev	cig+/-ot	11	20	nev cigs	st
CHOI	60		f	0	0	all	-	a	As:oth	1985	CC	375	n	bl	n	n	0	ev	cig+/-ot	11	30	nev cigs	ot
DOLL	60	x	m	0	0	all	-	KII	Eu:UK	1948	CC	1465	n	V	n	n	0	ev	all/unsp	15	24	nev any	st
DOSEME	12		m	0	0	all	-	not q+s	Eu:bal	1979	CC	1210	n	bl	n	n	2	ev	cig+/-ot	11	20	nev cigs	or
GER	3	x	c	0	0	all	-	a	As:oth	1990	CC	141	n	ot	y	n	0	ev	all/unsp	11	20	nev any	st
HAMMON	109		m	0	0	wh	0	a	NAmer	1952	pr	448	n	bl	n	n	1	cu	cig only	10	20	nev any	ot
JEDRYC	17	x	m	0	0	all	-	a	Eu:est	1980	CC	1630	n	bl	y	n	0	ev	cig+/-ot	20	29	nev any	st
KREYBE	18	x	m	0	0	all	-	KII	Eu:Sca	1948	CC	300	n	bl	n	y	0	ev	all/unsp	15	24	nev any	st
LAMTH	17		f	0	0	ch	-	a	As:HK	1983	CC	445	n	bl	n	n	0	ev	all/unsp	11	20	nev any	or
LUBIN2	160		m	0	0	all	-	a	Eu:mul	1976	CC	7804	n	bl	n	y	0	ev	cig+/-ot	20	29	nev any	st
LUBIN2	180		f	0	0	all	-	a	Eu:mul	1976	CC	7804	n	bl	n	y	0	ev	cig+/-ot	20	29	nev any	st
MATOS	58	x	m	0	0	all	-	a	SCAmer	1994	CC	200	n	bl	n	n	0	ev	cig+/-ot	15	24	nev any	st
MATSUD	8		m	0	0	all	-	a	As:Jap	1965	CC	179	n	bl	n	n	0	ev	cig+/-ot	11	20	nev cigs	ot
ORMOS	23		m	0	0	all	-	KII	Eu:est	1947	CC	119	n	bl	y	y	0	ev	cig+/-ot	16	30	nev any	st
SOBUE	57		m	0	0	all	-	a	As:Jap	1986	CC	1376	n	bl	n	y	0	cu	cig+/-ot	20	29	nev cigs	st
SVENSS	34	x	f	0	0	all	-	a	Eu:Sca	1983	CC	210	n	bl	n	n	0	cu	all/unsp	11	20	nev any	st
TSUGAN	5		m	0	0	all	-	a	As:Jap	1976	CC	134	n	bl	n	y	0	cu	all/unsp	16	35	nev any	st
WAKAI	50	x	m	0	0	all	-	a	As:Jap	1988	CC	333	n	bl	n	y	0	cu	cig+/-ot	20	29	nev any	st
WYNDE2	11		m	0	0	all	-	KII	NAmer	1962	CC	404	n	bl	n	y	0	ev	cig+/-ot	11	20	nev any	st
WYNDE3	25		m	0	0	all	-	KII	NAmer	1966	CC	350	n	bl	n	y	0	ev	cig+/-ot	10	20	nev any	st
WYNDE3	72		f	0	0	all	-	KII	NAmer	1966	CC	350	n	bl	n	y	0	ev	cig+/-ot	10	20	nev any	st
WYNDE4	39		m	0	0	all	-	a	NAmer	1948	CC	684	n	bl	y	n	0	ev	all/unsp	16	20	nev any	st
WYNDE6	33		m	0	0	all	-	KII	NAmer	1969	CC	4423	n	bl	n	y	0	cu	cig+/-ot	11	20	nev any	st
WYNDE6	222		f	0	0	all	-	KII	NAmer	1969	CC	4423	n	bl	n	y	0	cu	cig+/-ot	11	20	nev cigs	st
ZHENG	8		m	0	0	all	-	a	As:Chi	1982	CC	540	n	ot	*	y	0	ev	cig+/-ot	20	29	nev cigs	st

Cigarette type is all/unspec for all RRs

except for the following:

REF	NRR	CIGTYPE
ALDERS	47	MC only
ALDERS	50	MC only

Table 3G13 - 5

IESLC - Meta-anal of Ever Smoking (or Current if ever not avail), Amount smoked, "Mid", Any prod (or Cigs if Any not avail)
Adenocarcinoma
Least adjusted

REF	NRR	SEX	AD	Number Exposed Case	Cont	Non-exposed Case	Cont	RR	95.00%CI
ALDERS	47	m	1	-	-	-	-	2.67	(0.99- 7.18)
ALDERS	50	f	1	-	-	-	-	4.58	(2.67- 7.85)
Subtotal ALDERS								4.05	(2.52- 6.50)
BARBON	47	m	0	43	176	7	188	6.56	(2.88- 14.97)
CHOI	52	m	0	27	281	7	95	1.30	(0.55- 3.09)
CHOI	60	f	0	0	9	49	164	0.17~	(0.01- 3.06)
Subtotal CHOI								1.10	(0.48- 2.52)
DOLL	60	m	0	16	431	2	61	1.13	(0.25- 5.05)
DOSEME	12	m	2	-	-	-	-	2.70	(1.60- 4.70)
GER	3	c	0	21	66	37	149	1.28	(0.70- 2.36)
*HAMMON	109	m	1	-	-	-	-	2.83	(0.55- 14.60)
JEDRYC	17	m	0	52	434	7	289	4.95	(2.22- 11.04)
KREYBE	18	m	0	6	925	3	644	1.39	(0.35- 5.59)
LAMTH	17	f	0	27	14	131	158	2.33	(1.17- 4.62)
LUBIN2	160	m	0	234	3108	57	2616	3.46	(2.57- 4.64)
LUBIN2	180	f	0	13	110	138	1180	1.01	(0.55- 1.84)
Subtotal LUBIN2								2.73	(2.09- 3.55)
MATOS	58	m	0	28	90	5	110	6.84	(2.54- 18.45)
MATSUD	8	m	0	13	1607	0	1255	21.09~	(1.25- 355.07)
ORMOS	23	m	0	3	577	2	777	2.02	(0.34- 12.13)
SOBUE	57	m	0	95	222	27	128	2.03	(1.26- 3.28)
SVENSS	34	f	0	22	22	22	120	5.45	(2.59- 11.50)
TSUGAN	5	m	0	23	23	18	17	0.94	(0.39- 2.28)
WAKAI	50	m	0	30	129	8	65	1.89	(0.82- 4.35)
WYNDE2	11	m	0	14	203	5	105	1.45	(0.51- 4.13)
WYNDE3	25	m	0	20	114	6	88	2.57	(0.99- 6.68)
WYNDE3	72	f	0	11	24	15	76	2.32	(0.94- 5.73)
Subtotal WYNDE3								2.44	(1.27- 4.70)
WYNDE4	39	m	0	15	274	4	115	1.57	(0.51- 4.84)
WYNDE6	33	m	0	191	293	58	617	6.93	(5.01- 9.60)
WYNDE6	222	f	0	176	165	119	856	7.67	(5.76- 10.21)
Subtotal WYNDE6								7.34	(5.92- 9.10)
ZHENG	8	m	0	53	89	29	94	1.93	(1.13- 3.30)
Partial Totals				1133	9386	756	9967		

*prospective study ~ With 0.5 adjustment for zero

REF	NRR	SEX	AD	Ys	Ws	Qs	Ps
ALDERS	47	m	1	0.98	3.91	0.28	0.0520
ALDERS	50	f	1	1.52	13.21	0.99	0.0000
Subtotal ALDERS				1.40	17.13	1.27	
BARBON	47	m	0	1.88	5.65	2.27	0.0000
CHOI	52	m	0	0.27	5.16	4.97	0.5467
CHOI	60	f	0	-1.74	0.47	4.20	0.2324
Subtotal CHOI				0.10	5.62	9.17	
DOLL	60	m	0	0.12	1.72	2.17	0.8706
DOSEME	12	m	2	0.99	13.23	0.85	0.0003
GER	3	c	0	0.25	10.36	10.35	0.4249
*HAMMON	109	m	1	1.04	1.43	0.06	0.2136
JEDRYC	17	m	0	1.60	5.96	0.74	0.0001
KREYBE	18	m	0	0.33	1.99	1.67	0.6405
LAMTH	17	f	0	0.84	8.17	1.33	0.0158
LUBIN2	160	m	0	1.24	44.40	0.00	0.0000
LUBIN2	180	f	0	0.01	10.63	16.26	0.9727
Subtotal LUBIN2				1.00	55.03	16.26	
MATOS	58	m	0	1.92	3.91	1.79	0.0001
MATSUD	8	m	0	3.05	0.48	1.56	0.0343
ORMOS	23	m	0	0.70	1.20	0.35	0.4420
SOBUE	57	m	0	0.71	16.70	4.87	0.0038
SVENSS	34	f	0	1.70	6.91	1.39	0.0000
TSUGAN	5	m	0	-0.06	4.97	8.45	0.8986
WAKAI	50	m	0	0.64	5.51	2.06	0.1352
WYNDE2	11	m	0	0.37	3.50	2.69	0.4885
WYNDE3	25	m	0	0.95	4.22	0.39	0.0521
WYNDE3	72	f	0	0.84	4.71	0.77	0.0675
Subtotal WYNDE3				0.89	8.93	1.16	
WYNDE4	39	m	0	0.45	3.04	1.91	0.4291
WYNDE6	33	m	0	1.94	36.35	17.27	0.0000
WYNDE6	222	f	0	2.04	46.92	29.31	0.0000
Subtotal WYNDE6				1.99	83.27	46.58	
ZHENG	8	m	0	0.66	13.29	4.62	0.0165

Table 3G13 - 5

IESLC - Meta-anal of Ever Smoking (or Current if ever not avail), Amount smoked, "Mid", Any prod (or Cigs if Any not avail)
Adenocarcinoma
Least adjusted

	N	28
	NS	23
	Wt	277.99
Het	Chi	123.57
Het	df	27
Het	P	***
Fixed	RR	3.48
	RRl	3.09
	RRu	3.92
	P	+++
Random	RR	2.64
	RRl	1.99
	RRu	3.52
	P	+++
Asymm	P	**

Table 3G13 - 6

IESLC - Meta-anal of Ever Smoking (or Current if ever not avail), Amount smoked, "Mid", Any prod (or Cigs if Any not avail)
Adenocarcinoma
Least adjusted

	Sex combined	male	female	Total
N	1	20	7	28
NS	1	20	7	28
Wt	10.36	176.61	91.01	277.99
Het Chi	0.00	57.90	47.88	123.57
Het df	0	19	6	27
Het P	N.S.	***	***	***
Fixed RR	1.28	3.22	4.53	3.48
RRl	0.70	2.78	3.69	3.09
RRu	2.36	3.73	5.57	3.92
P	N.S.	+++	+++	+++
Random RR	1.28	2.66	2.86	2.64
RRl	0.70	1.97	1.43	1.99
RRu	2.36	3.60	5.70	3.52
P	N.S.	+++	++	+++
Between Chi				17.78
Between df				2
Between P				***
Btwn(F) P				N.S.
Btwn(R) P				(*)

Table 3G13 - 7

IESLC - Meta-anal of Ever Smoking (or Current if ever not avail), Amount smoked, "Mid", Any prod (or Cigs if Any not avail)
Adenocarcinoma
Excluded studies (and stage at which they were excluded)

1	ABELIN ABRAHA AMANDU AMES ANDERS AUSTIN AXELSO BAND BECHER BERRIN BLOHMK BLOT4 BROCKM BROWN1 BYERS1 BYERS2 CARPEN CASCO2 CASCOR CHAN CHEN3 CHIAZZ CHYOU DESTE2 DOCKER DROSTE DU GARCIA GARDIN GENG GODLEY GOODMA GRAHAM GREGOR HEGMAN HEIN HENNEK HINDS HIRAOK HOROWI HORWIT HUANG ISHIMA JAHN JAIN JARVHO JIANG KELLER KIHARA KJUUS KO KOHLME KUBIK LAMWK LAMWK2 LANGE LEI LEMARC LEVIN LIU LOMBA2 LOMBAR MAGNUS MARSH MARSH2 MCDUFF MCLAUG MILLER MILLS NOTANI NOU ODRISC PAWLEG PERSHA POFFIJ QIAO QIAO2 RADZIK REN RONCO ROOTS ROTHSC SAARIK SANKAR SCHWAR SEGI SEOW SHIMIZ SIMARA SIMONA SITAS SOBUE2 STASZE STAYNE STUCKE SUN SUZUK2 SUZUKI TANG TAO TOKARS TOUSEY ULMER VEIERO VUTUC WALD WANG WANG3 WANG4 WICKLU WIGLE WILKIN WU2 WUNSCH WYNDE8 XIANGZ XU XU2 XU4 YONG ZHANG
2	BUELL CHEN MASTRA MZILEN PISANI RESTRE SADOWS
4	BOFFET WYNDE7
5	RIMING TANG2 WYNDE5
6	BLOT1 BLOT2 BLOT3 BOUCHA HIRAY2 JONES LAURIL LICKIN MOLLO MRFIT MURATA SCHWA2 VANDER WARSIN WATSON WYNDER
8	AGUDO AKIBA ARCHER ARMADA AUVINE AXELSS BENSHL BEST BRESLO BRETT BROSS BUFFLE CEDERL CHANG CHATZI CHEN2 CHOW COMSTO COOKSO CPSI CPSII DAMBER DARBY DAVEYS DEAN DEAN2 DEAN3 DEKLER DESTEF DOLL2 DORANT DORN DUNN EBELIN ENSTRO ESAKI FAN GAO GAO2 GARSHI GILLIS GOLLED GSELL HAMMO2 HANSEN HIRAYA HITOSU HOLE HU HU2 HUMBLE JARUP JOLY JUSSAW KAISE2 KAISER KANELL KAUFMA KHUDER KINLEN KNEKT KOO KOULUM KREUZE LAUSSM LETOUR LIAW LIDDEL LIU2 LIU3 LIU4 LIU5 LUBIN LUO MACLEN MARTIS MCCONN MIGRAN MRFITR NAM NOTAN2 PARKIN PASTOR PERNU PERSH2 PETO PEZZO2 PEZZOT PIKE POLEDN PRESCO RACHTA RANDIG SEGI2 SHAW SIEMIA SPEIZE SPITZ STOCKS STOCKW TENKAN TIZZAN TULINI TVERDA WANG2 XU3 YAMAGU YUAN ZHOU
10	BOUCOT BROWN2 CORREA DORGAN ENGELA HAENSZ KATSOU OSANN OSANN2 WU WUWILL
11	BENHAM

Table 3G13 - 8
Potentially overlapping studies

REF	REFGP	PRINC	.	OVERLAP/LINK
LUBIN2	LUBIN2	1		Lubin-combined
LAMTH	LAMTH	1		KOO/LAMTH/LAMWK
WYNDE6	WYNDE6	1		WYNDE5/6/7/8
MATSUD	MATSUD	1		SOBUE2/MATSUD

Table 3G13 - 9
Most adjusted - insufficient data for metaanalysis

REF	NRR	SEX	AGEL	AGEH	RACE	YF	LC TYPE	LOC	START	ST	NLC	R	VB	P	H	AD	SM	PRODUCT	exL	exH	DENOM	De
CHEN	14	c	0	0	all	-	a	As:oth	1987	CC	323	n	ot	n	y	2	ev	cig+/-ot	11	20	nev cigs	ot

REF	NRR	RR	SIG	RRDATA comment
CHEN	14	1.74	n	0

Table 3G14 -

IESLC - Meta-anal of Ever Smoking (or Current if ev not avail), Amount smoked, “High”, Any prod (or Cigs if Any not avail)
Adenocarcinoma

This analysis is restricted to results for:
1) Results by Amount smoked
2) Results complete enough for use in metaanalysis

Within each study, results are then selected (in the following order of preference, within each sex) for:
3) SMKSTA: ever smokers, current smokers
4) PRODUCT: all/unspec, cigarettes regardless of other products, cigarettes only
5) CIGTYPE: all/unspecified, MC regardless of HR, MC only
6) DENOM: never smoked anything, never smoked cigarettes, (never +1 = +long term ex, +2 = +amount unknown,
+3 = never cigs+long term ex)
7) Followup period (YF, prospective studies): whole study (coded as 0) or longest available
8) LCtype: adeno or nearest available, but not squamous. (q = squamous, s = small,
a = adeno, l = large, KII = Kreyberg II, al = alveolar, br = bronchiolar, u = undifferentiated)
9) Race: all or nearest available, otherwise by race (wh or w = white, bl or b = black, hi = hispanic
ch = chinese, jap = japanese, haw = hawaiian, w+o = white + oriental, sca = scandinavian, as = asian)
10) Amount smoked "high" in key scheme 1 (key value 45, maximum range >20, in numbers of
cigarettes or cigarette equivalents)
11) For overlapping studies: principal rather than subsidiary studies
Finally by Age: whole study (coded as 0) if available, otherwise by widest available age group
and then for single sex results (m, f) in preference to combined sex results (c).

Results adjusted (AD) for the most potential confounders are then chosen in Sections -1 to -3
and results adjusted for the least confounders in Sections -4 to -6. (Those least adjusted results which
actually differ from the most adjusted as marked 'x' in column X in Section -4)
(Results adjusted for an unknown number of confounder(s) are coded as 20.)

Section -7 shows excluded studies, together with the stage (as above) at which no qualifying
results were found.

Section -8 lists the potentially overlapping studies which have been included (1=principal, 2=subsidiary).

Section -9 lists any results which would have been included in preference except that they had data not complete
enough for use in meta-analysis, with their significance (yes/no), if known, and any further comment as entered
on the database.

In addition to those mentioned above, the following fields, levels and abbreviations are used:
* or nk = not known, n = no, y = yes, ot = other
ev = ever, cu = current, nev = never
all/unspec = all or unspecified, cig+/-ot = cigarettes irrespective of other products (cigar, pipe etc)
MC = manufactured cigarettes, HR = hand-rolled cigarettes
exL, exH = range of exposure (low and high) in the smoking group, in terms of Amount smoked,
cigarettes or cigarette equivalents
REF: 6-character study reference
NRR: number of the RR on the database within the study
ST : study type (CC = case control, pr or prosp = prospective)
NLC: number of lung cancer cases in whole study
R : risky occupational population (n = no, m = mining, o = other risky)
VB : national cigarette type (V = at least 75% Virginia, bl = at least 75% blended, ot = other)
P : any proxy use
H : full histological confirmation
De : derivation of RR/CI (or = original, st = standard method, ot = other method of estimation)

Table 3G14 - 1

IESLC - Meta-anal of Ever Smoking (or Current if ev not avail), Amount smoked, “High”, Any prod (or Cigs if Any not avail)
Adenocarcinoma
Most adjusted

REF	NRR	SEX	AGEL	AGEH	RACE	YF	LC TYPE	LOC	START	ST	NLC	R	VB	P	H	AD	SM	PRODUCT	exL	exH	DENOM	De
ALDERS	48	m	0	0	all	-	not q+s	Eu:UK	1977	CC	1448	n	V	n	n	1	ev	cig only	28	99	nev+2	ot
ALDERS	51	f	0	0	all	-	not q+s	Eu:UK	1977	CC	1448	n	V	n	n	1	ev	cig only	28	99	nev+2	ot
BARBON	81	m	0	0	all	-	a	Eu:wst	1979	CC	755	n	bl	y	y	3	ev	all/unsp	40	99	nev any	or
BOUCOT	149	m	0	0	all	0	a	NAmer	1951	pr	121	n	bl	n	n	2	cu	cig only	21	99	nev any	ot
CHOI	55	m	0	0	all	-	a	As:oth	1985	CC	375	n	bl	n	n	0	ev	cig+/-ot	41	99	nev cigs	st
CHOI	61	f	0	0	all	-	a	As:oth	1985	CC	375	n	bl	n	n	0	ev	cig+/-ot	31	99	nev cigs	st
CORREA	52	c	0	0	all	-	a	NAmer	1979	CC	1359	n	bl	y	n	1	cu	cig+/-ot	21	99	nev cigs	or
DOLL	75	m	0	0	all	-	KII	Eu:UK	1948	CC	1465	n	V	n	n	1	ev	all/unsp	25	99	nev any	ot
DOSEME	16	m	0	0	all	-	not q+s	Eu:bal	1979	CC	1210	n	bl	n	n	2	ev	cig+/-ot	21	99	nev cigs	or
GER	12	c	0	0	all	-	a	As:oth	1990	CC	141	n	ot	y	n	8	ev	all/unsp	21	99	nev any	ot
HAENSZ	34	f	0	0	all	-	a	NAmer	1955	CC	158	n	bl	n	y	0	cu	cig+/-ot	21	99	nev any	or
JEDRYC	38	m	0	0	all	-	a	Eu:est	1980	CC	1630	n	bl	y	n	3	ev	cig+/-ot	30	99	nev any	or
KATSOU	14	f	0	0	all	-	a	Eu:bal	1987	CC	101	n	bl	n	n	1	cu	all/unsp	21	99	nev any	or
KREYBE	7	m	0	0	all	-	KII	Eu:Sca	1948	CC	300	n	bl	n	y	1	ev	all/unsp	25	99	nev any	ot
LAMTH	18	f	0	0	ch	-	a	As:HK	1983	CC	445	n	bl	n	n	0	ev	all/unsp	21	99	nev any	or
LUBIN2	164	m	0	0	all	-	a	Eu:mul	1976	CC	7804	n	bl	n	y	0	ev	cig+/-ot	30	99	nev any	st
LUBIN2	184	f	0	0	all	-	a	Eu:mul	1976	CC	7804	n	bl	n	y	0	ev	cig+/-ot	30	99	nev any	st
MATOS	61	m	0	0	all	-	a	SCAmer	1994	CC	200	n	bl	n	n	2	ev	cig+/-ot	25	99	nev any	or
MATSUD	9	m	0	0	all	-	a	As:Jap	1965	CC	179	n	bl	n	n	0	ev	cig+/-ot	21	99	nev cigs	ot
ORMOS	24	m	0	0	all	-	KII	Eu:est	1947	CC	119	n	bl	y	y	0	ev	cig+/-ot	31	99	nev any	st
OSANN	63	m	0	0	all	-	a	NAmer	1984	CC	1986	n	bl	n	n	2	ev	cig+/-ot	40	99	nev cigs	or
OSANN	64	f	0	0	all	-	a	NAmer	1984	CC	1986	n	bl	n	n	2	ev	cig+/-ot	40	99	nev cigs	or
SOBUE	58	m	0	0	all	-	a	As:Jap	1986	CC	1376	n	bl	n	y	0	cu	cig+/-ot	30	99	nev cigs	st
SVENSS	19	f	0	0	all	-	a	Eu:Sca	1983	CC	210	n	bl	n	n	1	cu	all/unsp	21	99	nev any	ot
TSUGAN	6	m	0	0	all	-	a	As:Jap	1976	CC	134	n	bl	n	y	0	cu	all/unsp	36	99	nev any	st
WAKAI	54	m	0	0	all	-	a	As:Jap	1988	CC	333	n	bl	n	y	1	cu	cig+/-ot	30	99	nev any	or
WU	9	f	0	0	wh	-	a	NAmer	1981	CC	220	n	bl	n	y	2	cu	all/unsp	21	99	nev any	or
WYNDE2	13	m	0	0	all	-	KII	NAmer	1962	CC	404	n	bl	n	y	0	ev	cig+/-ot	35	99	nev any	st
WYNDE3	27	m	0	0	all	-	KII	NAmer	1966	CC	350	n	bl	n	y	0	ev	cig+/-ot	41	99	nev any	st
WYNDE3	74	f	0	0	all	-	KII	NAmer	1966	CC	350	n	bl	n	y	0	ev	cig+/-ot	41	99	nev any	st
WYNDE4	41	m	0	0	all	-	a	NAmer	1948	CC	684	n	bl	y	n	0	ev	all/unsp	35	99	nev any	st
WYNDE6	51	m	0	0	all	-	KII	NAmer	1969	CC	4423	n	bl	n	y	0	cu	cig+/-ot	31	99	nev any	st
WYNDE6	240	f	0	0	all	-	KII	NAmer	1969	CC	4423	n	bl	n	y	0	cu	cig+/-ot	30	99	nev cigs	st
ZHENG	9	m	0	0	all	-	a	As:Chi	1982	CC	540	n	ot	*	y	0	ev	cig+/-ot	30	99	nev cigs	st

Cigarette type is all/unspec for all RRs

except for the following:

REF	NRR	CIGTYPE
ALDERS	48	MC only
ALDERS	51	MC only

Table 3G14 - 2

IESLC - Meta-anal of Ever Smoking (or Current if ev not avail), Amount smoked, “High”, Any prod (or Cigs if Any not avail)
Adenocarcinoma
Most adjusted

REF	NRR	SEX	AD	Number Exposed Case	Cont	Non-exposed Case	Cont	RR	95.00%CI
ALDERS	48	m	1	-	-	-	-	3.32	(1.36- 8.10)
ALDERS	51	f	1	-	-	-	-	3.31	(1.80- 6.10)
Subtotal ALDERS								3.31	(2.00- 5.48)
BARBON	81	m	3	-	-	-	-	12.10	(5.10- 28.00)
*BOUCOT	149	m	2	-	-	-	-	18.00	(1.01- 319.52)
CHOI	55	m	0	2	6	7	95	4.52	(0.77- 26.69)
CHOI	61	f	0	1	1	49	164	3.35	(0.21- 54.50)
Subtotal CHOI								4.15	(0.93- 18.54)
CORREA	52	c	1	-	-	-	-	12.00	(7.30- 19.70)
DOLL	75	m	1	-	-	-	-	1.10	(0.22- 5.60)
DOSEME	16	m	2	-	-	-	-	3.20	(1.40- 7.00)
GER	12	c	8	-	-	-	-	0.88	(0.25- 3.03)
HAENSZ	34	f	0	5	13	37	236	2.45	(0.83- 7.28)
JEDRYC	38	m	3	-	-	-	-	5.11	(2.09- 12.53)
KATSOU	14	f	1	-	-	-	-	2.95	(0.76- 11.41)
KREYBE	7	m	1	-	-	-	-	3.99	(0.95- 16.81)
LAMTH	18	f	0	9	5	131	158	2.17	(0.71- 6.64)
LUBIN2	164	m	0	151	1746	57	2616	3.97	(2.91- 5.42)
LUBIN2	184	f	0	9	39	138	1180	1.97	(0.94- 4.16)
Subtotal LUBIN2								3.58	(2.69- 4.77)
MATOS	61	m	2	-	-	-	-	8.40	(3.01- 22.20)
MATSUD	9	m	0	4	470	0	1255	24.02~	(1.29- 446.92)
ORMOS	24	m	0	1	128	2	777	3.04	(0.27- 33.72)
OSANN	63	m	2	-	-	-	-	37.50	(21.30- 66.00)
OSANN	64	f	2	-	-	-	-	24.20	(15.80- 37.20)
Subtotal OSANN								28.39	(20.18- 39.94)
SOBUE	58	m	0	76	187	27	128	1.93	(1.18- 3.16)
SVENSS	19	f	1	-	-	-	-	19.70	(1.70- 228.29)
TSUGAN	6	m	0	10	13	18	17	0.73	(0.25- 2.09)
WAKAI	54	m	1	-	-	-	-	4.53	(1.89- 10.90)
WU	9	f	2	-	-	-	-	6.50	(3.10- 13.90)
WYNDE2	13	m	0	16	112	5	105	3.00	(1.06- 8.48)
WYNDE3	27	m	0	9	26	6	88	5.08	(1.65- 15.59)
WYNDE3	74	f	0	1	3	15	76	1.69	(0.16- 17.36)
Subtotal WYNDE3								4.13	(1.50- 11.34)
WYNDE4	41	m	0	7	64	4	115	3.14	(0.89- 11.15)
WYNDE6	51	m	0	282	197	58	617	15.23	(11.00- 21.07)
WYNDE6	240	f	0	157	52	119	856	21.72	(15.04- 31.37)
Subtotal WYNDE6								17.79	(13.95- 22.70)
ZHENG	9	m	0	17	23	29	94	2.40	(1.13- 5.08)
Partial Totals				757	3085	702	8577		

*prospective study ~ With 0.5 adjustment for zero

REF	NRR	SEX	AD	Ys	Ws	Qs	Ps
ALDERS	48	m	1	1.20	4.83	2.89	0.0084
ALDERS	51	f	1	1.20	10.32	6.23	0.0001
Subtotal ALDERS				1.20	15.14	9.12	
BARBON	81	m	3	2.49	5.30	1.43	0.0000
*BOUCOT	149	m	2	2.89	0.46	0.39	0.0491
CHOI	55	m	0	1.51	1.22	0.26	0.0956
CHOI	61	f	0	1.21	0.49	0.29	0.3961
Subtotal CHOI				1.42	1.71	0.55	
CORREA	52	c	1	2.48	15.59	4.07	0.0000
DOLL	75	m	1	0.10	1.47	5.18	0.9081
DOSEME	16	m	2	1.16	5.93	3.90	0.0046
GER	12	c	8	-0.13	2.47	10.91	0.8408
HAENSZ	34	f	0	0.90	3.24	3.76	0.1060
JEDRYC	38	m	3	1.63	4.79	0.56	0.0004
KATSOU	14	f	1	1.08	2.09	1.67	0.1175
KREYBE	7	m	1	1.38	1.86	0.65	0.0590
LAMTH	18	f	0	0.78	3.08	4.42	0.1740
LUBIN2	164	m	0	1.38	39.81	14.11	0.0000
LUBIN2	184	f	0	0.68	6.90	11.57	0.0741
Subtotal LUBIN2				1.28	46.71	25.68	
MATOS	61	m	2	2.13	3.85	0.09	0.0000
MATSUD	9	m	0	3.18	0.45	0.65	0.0331
ORMOS	24	m	0	1.11	0.66	0.49	0.3661
OSANN	63	m	2	3.62	12.01	32.72	0.0000
OSANN	64	f	2	3.19	20.96	30.80	0.0000
Subtotal OSANN				3.35	32.97	63.52	

Table 3G14 - 2

IESLC - Meta-anal of Ever Smoking (or Current if ev not avail), Amount smoked, “High”, Any prod (or Cigs if Any not avail)
Adenocarcinoma
Most adjusted

REF	NRR	SEX	AD	Ys	Ws	Qs	Ps
SOBUE	58	m	0	0.66	15.78	27.43	0.0092
SVENSS	19	f	1	2.98	0.64	0.65	0.0171
TSUGAN	6	m	0	-0.32	3.43	18.06	0.5538
WAKAI	54	m	1	1.51	5.00	1.07	0.0007
WU	9	f	2	1.87	6.82	0.07	0.0000
WYNDE2	13	m	0	1.10	3.56	2.73	0.0382
WYNDE3	27	m	0	1.62	3.05	0.37	0.0045
WYNDE3	74	f	0	0.52	0.71	1.49	0.6593
Subtotal WYNDE3				1.42	3.76	1.86	
WYNDE4	41	m	0	1.15	2.40	1.64	0.0761
WYNDE6	51	m	0	2.72	36.38	20.42	0.0000
WYNDE6	240	f	0	3.08	28.43	34.66	0.0000
Subtotal WYNDE6				2.88	64.82	55.08	
ZHENG	9	m	0	0.87	6.78	8.21	0.0229

	N	34
	NS	28
	Wt	260.78
Het	Chi	253.85
Het	df	33
Het	P	***
Fixed	RR	7.20
	RRl	6.38
	RRu	8.13
	P	+++
Random	RR	4.80
	RRl	3.29
	RRu	7.01
	P	+++
Asymm	P	*

Table 3G14 - 3

IESLC - Meta-anal of Ever Smoking (or Current if ev not avail), Amount smoked, “High”, Any prod (or Cigs if Any not avail)
Adenocarcinoma
Most adjusted

	Sex: combined	male	female	Total
N	2	21	11	34
NS	2	21	11	34
Wt	18.06	159.04	83.69	260.78
Het Chi	14.55	136.78	85.32	253.85
Het df	1	20	10	33
Het P	***	***	***	***
Fixed RR	8.40	5.88	10.22	7.20
RRl	5.29	5.04	8.25	6.38
RRu	13.32	6.87	12.67	8.13
P	+++	+++	+++	+++
Random RR	3.47	4.68	5.21	4.80
RRl	0.27	2.93	2.50	3.29
RRu	44.75	7.47	10.86	7.01
P	N.S.	+++	+++	+++
Between Chi				17.20
Between df				2
Between P				***
Btwn(F) P				N.S.
Btwn(R) P				N.S.

	Lung cancer type: a	a+l	a+al+br	KII	not q+u	not q+s	Total
N	23			8		3	34
NS	20			6		2	28
Wt	163.59			76.13		21.07	260.78
Het Chi	168.40			34.43		0.01	253.85
Het df	22			7		2	33
Het P	***			***		N.S.	***
Fixed RR	5.90			13.71		3.28	7.20
RRl	5.07			10.95		2.14	6.38
RRu	6.88			17.16		5.03	8.13
P	+++			+++		+++	+++
Random RR	4.88			5.99		3.28	4.80
RRl	3.03			3.03		2.14	3.29
RRu	7.86			11.84		5.03	7.01
P	+++			+++		+++	+++
Between Chi							51.01
Between df							2
Between P							***
Btwn(F) P							*
Btwn(R) P							N.S.

	Location: NAmer	UK	Scand	othEur	China	Japan	othAs	other	Total
N	12	3	2	7	1	4	4	1	34
NS	9	2	2	6	1	4	3	1	28
Wt	133.63	16.61	2.50	65.49	6.78	24.67	7.26	3.85	260.78
Het Chi	56.96	1.63	1.21	10.77	0.00	9.61	2.59	0.00	253.85
Het df	11	2	1	6	0	3	3	0	33
Het P	***	N.S.	N.S.	(*)	N.S.	*	N.S.	N.S.	***
Fixed RR	15.32	3.01	6.00	3.98	2.40	2.09	1.86	8.40	7.20
RRl	12.93	1.86	1.74	3.13	1.13	1.41	0.90	3.09	6.38
RRu	18.15	4.86	20.73	5.07	5.08	3.11	3.85	22.81	8.13
P	+++	+++	++	+++	+	+++	(+)	+++	+++
Random RR	10.07	3.01	6.43	4.03	2.40	2.33	1.86	8.40	4.80
RRl	6.41	1.86	1.53	2.67	1.13	0.95	0.90	3.09	3.29
RRu	15.82	4.86	26.95	6.09	5.08	5.70	3.85	22.81	7.01
P	+++	+++	+	+++	+	(+)	(+)	+++	+++
Between Chi									171.08
Between df									7
Between P									***
Btwn(F) P									***
Btwn(R) P									***

Table 3G14 - 3

IESLC - Meta-anal of Ever Smoking (or Current if ev not avail), Amount smoked, “High”, Any prod (or Cigs if Any not avail)
Adenocarcinoma
Most adjusted

	Detailed Country in "other Europe"					
	multi	Germany	othWest	East	Balkans	Total
N	2		1	2	2	7
NS	1		1	2	2	6
Wt	46.71		5.30	5.45	8.03	65.49
Het Chi	2.87		0.00	0.16	0.01	10.77
Het df	1		0	1	1	6
Het P	(*)		N.S.	N.S.	N.S.	(*)
Fixed RR	3.58		12.10	4.80	3.13	3.98
RRl	2.69		5.16	2.07	1.57	3.13
RRu	4.77		28.35	11.10	6.26	5.07
P	+++		+++	+++	++	+++
Random RR	3.05		12.10	4.80	3.13	4.03
RRl	1.57		5.16	2.07	1.57	2.67
RRu	5.92		28.35	11.10	6.26	6.09
P	+++		+++	+++	++	+++
Between Chi						7.73
Between df						3
Between P						(*)
Btwn(F) P						N.S.
Btwn(R) P						(*)

	Detailed Country in "other Asia"			
	India	HongKong	other	Total
N		1	3	4
NS		1	2	3
Wt		3.08	4.18	7.26
Het Chi		0.00	2.46	2.59
Het df		0	2	3
Het P		N.S.	N.S.	N.S.
Fixed RR		2.17	1.66	1.86
RRl		0.71	0.64	0.90
RRu		6.64	4.33	3.85
P		N.S.	N.S.	(+)
Random RR		2.17	1.80	1.86
RRl		0.71	0.59	0.90
RRu		6.64	5.50	3.85
P		N.S.	N.S.	(+)
Between Chi				0.13
Between df				1
Between P				N.S.
Btwn(F) P				N.S.
Btwn(R) P				N.S.

	Detailed other continent			
	SCAmer	Auslia	Africa	Total
N	1			1
NS	1			1
Wt	3.85			3.85
Het Chi	0.00			0.00
Het df	0			0
Het P	N.S.			N.S.
Fixed RR	8.40			8.40
RRl	3.09			3.09
RRu	22.81			22.81
P	+++			+++
Random RR	8.40			8.40
RRl	3.09			3.09
RRu	22.81			22.81
P	+++			+++
Between Chi				
Between df				
Between P				N.S.
Btwn(F) P				N.S.
Btwn(R) P				N.S.

Table 3G14 - 3

IESLC - Meta-anal of Ever Smoking (or Current if ev not avail), Amount smoked, “High”, Any prod (or Cigs if Any not avail)
Adenocarcinoma
Most adjusted

	Start year of study					
	<1960	1960-69	1970-79	1980-89	1990+	Total
N	6	6	8	12	2	34
NS	6	4	6	10	2	28
Wt	10.10	72.59	92.11	79.68	6.32	260.78
Het Chi	3.21	20.17	38.92	107.64	7.65	253.85
Het df	5	5	7	11	1	33
Het P	N.S.	**	***	***	**	***
Fixed RR	2.82	15.15	4.35	7.81	3.48	7.20
RRl	1.52	12.03	3.55	6.27	1.59	6.38
RRu	5.22	19.06	5.34	9.72	7.59	8.13
P	++	+++	+++	+++	++	+++
Random RR	2.82	9.51	3.82	5.77	2.81	4.80
RRl	1.52	4.98	2.23	2.70	0.31	3.29
RRu	5.22	18.15	6.52	12.36	25.59	7.01
P	++	+++	+++	+++	N.S.	+++
Between Chi						76.25
Between df						4
Between P						***
Btwn(F) P						*
Btwn(R) P						(*)

	Study type (1)		
	CC	other	Total
N	33	1	34
NS	27	1	28
Wt	260.32	0.46	260.78
Het Chi	253.46	0.00	253.85
Het df	32	0	33
Het P	***	N.S.	***
Fixed RR	7.19	18.00	7.20
RRl	6.37	1.01	6.38
RRu	8.12	320.16	8.13
P	+++	+	+++
Random RR	4.72	18.00	4.80
RRl	3.22	1.01	3.29
RRu	6.92	320.16	7.01
P	+++	+	+++
Between Chi			0.39
Between df			1
Between P			N.S.
Btwn(F) P			N.S.
Btwn(R) P			N.S.

	Study type (2)			
	CC	prosp	other	Total
N	33	1		34
NS	27	1		28
Wt	260.32	0.46		260.78
Het Chi	253.46	0.00		253.85
Het df	32	0		33
Het P	***	N.S.		***
Fixed RR	7.19	18.00		7.20
RRl	6.37	1.01		6.38
RRu	8.12	320.16		8.13
P	+++	+		+++
Random RR	4.72	18.00		4.80
RRl	3.22	1.01		3.29
RRu	6.92	320.16		7.01
P	+++	+		+++
Between Chi				0.39
Between df				1
Between P				N.S.
Btwn(F) P				N.S.
Btwn(R) P				N.S.

Table 3G14 - 3

IESLC - Meta-anal of Ever Smoking (or Current if ev not avail), Amount smoked, “High”, Any prod (or Cigs if Any not avail)

Adenocarcinoma
Most adjusted

	Study size (number of LC cases)				
	100-249	250-499	500-999	1000+	Total
N	10	8	3	13	34
NS	10	6	3	9	28
Wt	24.13	18.97	14.48	203.20	260.78
Het Chi	24.06	2.02	8.19	187.64	253.85
Het df	9	7	2	12	33
Het P	**	N.S.	*	***	***
Fixed RR	3.49	3.58	4.53	8.66	7.20
RRl	2.34	2.28	2.71	7.54	6.38
RRu	5.20	5.61	7.59	9.93	8.13
P	+++	+++	+++	+++	+++
Random RR	3.63	3.58	4.57	6.36	4.80
RRl	1.76	2.28	1.54	3.59	3.29
RRu	7.52	5.61	13.58	11.28	7.01
P	+++	+++	++	+++	+++
Between Chi					31.94
Between df					3
Between P					***
Btwn(F) P					N.S.
Btwn(R) P					N.S.

	Risky occupational population			
	no	mining	othRisky	Total
N	34			34
NS	28			28
Wt	260.78			260.78
Het Chi	253.85			253.85
Het df	33			33
Het P	***			***
Fixed RR	7.20			7.20
RRl	6.38			6.38
RRu	8.13			8.13
P	+++			+++
Random RR	4.80			4.80
RRl	3.29			3.29
RRu	7.01			7.01
P	+++			+++
Between Chi				
Between df				
Between P				N.S.
Btwn(F) P				N.S.
Btwn(R) P				N.S.

	National cigarette tobacco type			
	Virginia	blended	other	Total
N	3	29	2	34
NS	2	24	2	28
Wt	16.61	234.92	9.25	260.78
Het Chi	1.63	217.29	1.82	253.85
Het df	2	28	1	33
Het P	N.S.	***	N.S.	***
Fixed RR	3.01	8.08	1.83	7.20
RRl	1.86	7.11	0.96	6.38
RRu	4.86	9.18	3.49	8.13
P	+++	+++	(+)	+++
Random RR	3.01	5.62	1.65	4.80
RRl	1.86	3.74	0.64	3.29
RRu	4.86	8.45	4.26	7.01
P	+++	+++	N.S.	+++
Between Chi				33.11
Between df				2
Between P				***
Btwn(F) P				N.S.
Btwn(R) P				*

Table 3G14 - 3

IESLC - Meta-anal of Ever Smoking (or Current if ev not avail), Amount smoked, “High”, Any prod (or Cigs if Any not avail)
Adenocarcinoma
Most adjusted

	Any proxy use No/nk	Yes	Total
N	28	6	34
NS	22	6	28
Wt	229.58	31.21	260.78
Het Chi	234.73	19.05	253.85
Het df	27	5	33
Het P	***	**	***
Fixed RR	7.16	7.51	7.20
RRl	6.29	5.29	6.38
RRu	8.15	10.67	8.13
P	+++	+++	+++
Random RR	4.78	4.99	4.80
RRl	3.11	2.25	3.29
RRu	7.36	11.06	7.01
P	+++	+++	+++
Between Chi			0.06
Between df			1
Between P			N.S.
Btwn(F) P			N.S.
Btwn(R) P			N.S.

	Full histological confirmation No	Yes	Total
N	18	16	34
NS	15	13	28
Wt	93.04	167.74	260.78
Het Chi	102.69	143.95	253.85
Het df	17	15	33
Het P	***	***	***
Fixed RR	9.00	6.36	7.20
RRl	7.35	5.47	6.38
RRu	11.03	7.40	8.13
P	+++	+++	+++
Random RR	5.55	4.16	4.80
RRl	3.15	2.45	3.29
RRu	9.79	7.05	7.01
P	+++	+++	+++
Between Chi			7.21
Between df			1
Between P			**
Btwn(F) P			N.S.
Btwn(R) P			N.S.

	Number of adjustment variables (1) 0	1	2+/+nk	Total
N	17	8	9	34
NS	13	7	8	28
Wt	156.39	41.80	62.60	260.78
Het Chi	146.42	19.41	59.40	253.85
Het df	16	7	8	33
Het P	***	**	***	***
Fixed RR	6.12	5.51	12.93	7.20
RRl	5.23	4.07	10.09	6.38
RRu	7.15	7.46	16.57	8.13
P	+++	+++	+++	+++
Random RR	3.65	4.44	8.25	4.80
RRl	2.08	2.48	3.94	3.29
RRu	6.42	7.95	17.27	7.01
P	+++	+++	+++	+++
Between Chi				28.62
Between df				2
Between P				***
Btwn(F) P				N.S.
Btwn(R) P				N.S.

Table 3G14 - 3

IESLC - Meta-anal of Ever Smoking (or Current if ev not avail), Amount smoked, “High”, Any prod (or Cigs if Any not avail)
Adenocarcinoma
Most adjusted

	Number of adjustment variables (2) 0	1	2	3-5	6+/+nk	Total
N	17	8	6	2	1	34
NS	13	7	5	2	1	28
Wt	156.39	41.80	50.04	10.09	2.47	260.78
Het Chi	146.42	19.41	34.80	1.87	0.00	253.85
Het df	16	7	5	1	0	33
Het P	***	**	***	N.S.	N.S.	***
Fixed RR	6.12	5.51	16.25	8.04	0.88	7.20
RRl	5.23	4.07	12.32	4.34	0.25	6.38
RRu	7.15	7.46	21.44	14.89	3.06	8.13
P	+++	+++	+++	+++	N.S.	+++
Random RR	3.65	4.44	11.80	7.96	0.88	4.80
RRl	2.08	2.48	5.15	3.42	0.25	3.29
RRu	6.42	7.95	27.04	18.51	3.06	7.01
P	+++	+++	+++	+++	N.S.	+++
Between Chi						51.35
Between df						4
Between P						***
Btwn(F) P						N.S.
Btwn(R) P						**

	Product all/unsp	cig+/-ot	cig only	Total
N	10	21	3	34
NS	10	16	2	28
Wt	29.56	215.62	15.60	260.78
Het Chi	28.65	194.59	1.29	253.85
Het df	9	20	2	33
Het P	***	***	N.S.	***
Fixed RR	3.45	8.39	3.48	7.20
RRl	2.40	7.34	2.12	6.38
RRu	4.94	9.59	5.72	8.13
P	+++	+++	+++	+++
Random RR	2.99	5.98	3.48	4.80
RRl	1.52	3.74	2.12	3.29
RRu	5.89	9.58	5.72	7.01
P	++	+++	+++	+++
Between Chi				29.31
Between df				2
Between P				***
Btwn(F) P				N.S.
Btwn(R) P				N.S.

	Denominator nev any	nev cigs	Total
N	24	10	34
NS	21	8	29
Wt	153.13	107.65	260.78
Het Chi	93.79	118.73	253.85
Het df	23	9	33
Het P	***	***	***
Fixed RR	5.16	11.57	7.20
RRl	4.40	9.58	6.38
RRu	6.04	13.98	8.13
P	+++	+++	+++
Random RR	3.79	8.37	4.80
RRl	2.61	3.86	3.29
RRu	5.51	18.14	7.01
P	+++	+++	+++
Between Chi			41.32
Between df			1
Between P			***
Btwn(F) P			*
Btwn(R) P			(*)

Table 3G14 - 3

IESLC - Meta-anal of Ever Smoking (or Current if ev not avail), Amount smoked, “High”, Any prod (or Cigs if Any not avail)

Adenocarcinoma
Most adjusted

	Derivation of RR/CI			
	Orig	StdCalc	Other	Total
N	12	14	8	34
NS	11	10	7	28
Wt	88.67	149.62	22.49	260.78
Het Chi	68.26	139.36	11.18	253.85
Het df	11	13	7	33
Het P	***	***	N.S.	***
Fixed RR	11.05	6.35	3.07	7.20
RRl	8.98	5.41	2.03	6.38
RRu	13.61	7.45	4.64	8.13
P	+++	+++	+++	+++
Random RR	7.28	3.69	3.17	4.80
RRl	4.21	1.99	1.71	3.29
RRu	12.58	6.85	5.86	7.01
P	+++	+++	+++	+++
Between Chi				35.04
Between df				2
Between P				***
Btwn(F) P				N.S.
Btwn(R) P				(*)

	Smoking status		
	ever	current	Total
N	23	11	34
NS	18	10	28
Wt	142.89	117.90	260.78
Het Chi	135.03	104.29	253.85
Het df	22	10	33
Het P	***	***	***
Fixed RR	5.81	9.34	7.20
RRl	4.93	7.79	6.38
RRu	6.85	11.18	8.13
P	+++	+++	+++
Random RR	4.40	5.71	4.80
RRl	2.77	2.91	3.29
RRu	6.98	11.19	7.01
P	+++	+++	+++
Between Chi			14.52
Between df			1
Between P			***
Btwn(F) P			N.S.
Btwn(R) P			N.S.

Table 3G14 - 4

IESLC - Meta-anal of Ever Smoking (or Current if ev not avail), Amount smoked, “High”, Any prod (or Cigs if Any not avail)
Adenocarcinoma
Least adjusted

REF	NRR	X	SEX	AGEL	AGEH	RACE	YF	LC TYPE	LOC	START	ST	NLC	R	VB	P	H	AD	SM	PRODUCT	exL	exH	DENOM	De
ALDERS	48		m	0	0	all	-	not q+s	Eu:UK	1977	CC	1448	n	V	n	n	1	ev	cig only	28	99	nev+2	ot
ALDERS	51		f	0	0	all	-	not q+s	Eu:UK	1977	CC	1448	n	V	n	n	1	ev	cig only	28	99	nev+2	ot
BARBON	49	x	m	0	0	all	-	a	Eu:wst	1979	CC	755	n	bl	y	y	0	ev	all/unsp	40	99	nev any	st
BOUCOT	28	x	m	0	0	all	0	a	NAmer	1951	pr	121	n	bl	n	n	0	cu	cig only	21	99	nev any	ot
CHOI	55		m	0	0	all	-	a	As:oth	1985	CC	375	n	bl	n	n	0	ev	cig+/-ot	41	99	nev cigs	st
CHOI	61		f	0	0	all	-	a	As:oth	1985	CC	375	n	bl	n	n	0	ev	cig+/-ot	31	99	nev cigs	st
CORREA	52		c	0	0	all	-	a	NAmer	1979	CC	1359	n	bl	y	n	1	cu	cig+/-ot	21	99	nev cigs	or
DOLL	61	x	m	0	0	all	-	KII	Eu:UK	1948	CC	1465	n	V	n	n	0	ev	all/unsp	25	99	nev any	st
DOSEME	16		m	0	0	all	-	not q+s	Eu:bal	1979	CC	1210	n	bl	n	n	2	ev	cig+/-ot	21	99	nev cigs	or
GER	4	x	c	0	0	all	-	a	As:oth	1990	CC	141	n	ot	y	n	0	ev	all/unsp	21	99	nev any	st
HAENSZ	34		f	0	0	all	-	a	NAmer	1955	CC	158	n	bl	n	y	0	cu	cig+/-ot	21	99	nev any	or
JEDRYC	19	x	m	0	0	all	-	a	Eu:est	1980	CC	1630	n	bl	y	n	0	ev	cig+/-ot	40	99	nev any	st
KATSOU	18	x	f	0	0	all	-	a	Eu:bal	1987	CC	101	n	bl	n	n	0	cu	all/unsp	21	99	nev any	st
KREYBE	19	x	m	0	0	all	-	KII	Eu:Sca	1948	CC	300	n	bl	n	y	0	ev	all/unsp	25	99	nev any	st
LAMTH	18		f	0	0	ch	-	a	As:HK	1983	CC	445	n	bl	n	n	0	ev	all/unsp	21	99	nev any	or
LUBIN2	164		m	0	0	all	-	a	Eu:mul	1976	CC	7804	n	bl	n	y	0	ev	cig+/-ot	30	99	nev any	st
LUBIN2	184		f	0	0	all	-	a	Eu:mul	1976	CC	7804	n	bl	n	y	0	ev	cig+/-ot	30	99	nev any	st
MATOS	60	x	m	0	0	all	-	a	SCAmer	1994	CC	200	n	bl	n	n	0	ev	cig+/-ot	25	99	nev any	st
MATSUD	9		m	0	0	all	-	a	As:Jap	1965	CC	179	n	bl	n	n	0	ev	cig+/-ot	21	99	nev cigs	ot
ORMOS	24		m	0	0	all	-	KII	Eu:est	1947	CC	119	n	bl	y	y	0	ev	cig+/-ot	31	99	nev any	st
OSANN	63		m	0	0	all	-	a	NAmer	1984	CC	1986	n	bl	n	n	2	ev	cig+/-ot	40	99	nev cigs	or
OSANN	64		f	0	0	all	-	a	NAmer	1984	CC	1986	n	bl	n	n	2	ev	cig+/-ot	40	99	nev cigs	or
SOBUE	58		m	0	0	all	-	a	As:Jap	1986	CC	1376	n	bl	n	y	0	cu	cig+/-ot	30	99	nev cigs	st
SVENSS	39	x	f	0	0	all	-	a	Eu:Sca	1983	CC	210	n	bl	n	n	0	cu	all/unsp	21	99	nev any	st
TSUGAN	6		m	0	0	all	-	a	As:Jap	1976	CC	134	n	bl	n	y	0	cu	all/unsp	36	99	nev any	st
WAKAI	51	x	m	0	0	all	-	a	As:Jap	1988	CC	333	n	bl	n	y	0	cu	cig+/-ot	30	99	nev any	st
WU	4	x	f	0	0	wh	-	a	NAmer	1981	CC	220	n	bl	n	y	0	cu	all/unsp	21	99	nev any	st
WYNDE2	13		m	0	0	all	-	KII	NAmer	1962	CC	404	n	bl	n	y	0	ev	cig+/-ot	35	99	nev any	st
WYNDE3	27		m	0	0	all	-	KII	NAmer	1966	CC	350	n	bl	n	y	0	ev	cig+/-ot	41	99	nev any	st
WYNDE3	74		f	0	0	all	-	KII	NAmer	1966	CC	350	n	bl	n	y	0	ev	cig+/-ot	41	99	nev any	st
WYNDE4	41		m	0	0	all	-	a	NAmer	1948	CC	684	n	bl	y	n	0	ev	all/unsp	35	99	nev any	st
WYNDE6	51		m	0	0	all	-	KII	NAmer	1969	CC	4423	n	bl	n	y	0	cu	cig+/-ot	31	99	nev any	st
WYNDE6	240		f	0	0	all	-	KII	NAmer	1969	CC	4423	n	bl	n	y	0	cu	cig+/-ot	30	99	nev cigs	st
ZHENG	9		m	0	0	all	-	a	As:Chi	1982	CC	540	n	ot	*	y	0	ev	cig+/-ot	30	99	nev cigs	st

Cigarette type is all/unspec for all RRs

except for the following:

REF	NRR	CIGTYPE
ALDERS	48	MC only
ALDERS	51	MC only

Table 3G14 - 5

IESLC - Meta-anal of Ever Smoking (or Current if ev not avail), Amount smoked, “High”, Any prod (or Cigs if Any not avail)
Adenocarcinoma
Least adjusted

REF	NRR	SEX	AD	Number Exposed Case	Cont	Non-exposed Case	Cont	RR	95.00%CI
ALDERS	48	m	1	-	-	-	-	3.32	(1.36- 8.10)
ALDERS	51	f	1	-	-	-	-	3.31	(1.80- 6.10)
Subtotal ALDERS								3.31	(2.00- 5.48)
BARBON	49	m	0	40	111	7	188	9.68	(4.19- 22.34)
*BOUCOT	28	m	0	6	6940	0	7551	14.14~	(0.80- 251.03)
CHOI	55	m	0	2	6	7	95	4.52	(0.77- 26.69)
CHOI	61	f	0	1	1	49	164	3.35	(0.21- 54.50)
Subtotal CHOI								4.15	(0.93- 18.54)
CORREA	52	c	1	-	-	-	-	12.00	(7.30- 19.70)
DOLL	61	m	0	6	166	2	61	1.10	(0.22- 5.61)
DOSEME	16	m	2	-	-	-	-	3.20	(1.40- 7.00)
GER	4	c	0	5	26	37	149	0.77	(0.28- 2.15)
HAENSZ	34	f	0	5	13	37	236	2.45	(0.83- 7.28)
JEDRYC	19	m	0	9	82	7	289	4.53	(1.64- 12.54)
KATSOU	18	f	0	6	4	30	67	3.35	(0.88- 12.75)
KREYBE	19	m	0	5	248	3	644	4.33	(1.03- 18.25)
LAMTH	18	f	0	9	5	131	158	2.17	(0.71- 6.64)
LUBIN2	164	m	0	151	1746	57	2616	3.97	(2.91- 5.42)
LUBIN2	184	f	0	9	39	138	1180	1.97	(0.94- 4.16)
Subtotal LUBIN2								3.58	(2.69- 4.77)
MATOS	60	m	0	40	105	5	110	8.38	(3.19- 22.05)
MATSUD	9	m	0	4	470	0	1255	24.02~	(1.29- 446.92)
ORMOS	24	m	0	1	128	2	777	3.04	(0.27- 33.72)
OSANN	63	m	2	-	-	-	-	37.50	(21.30- 66.00)
OSANN	64	f	2	-	-	-	-	24.20	(15.80- 37.20)
Subtotal OSANN								28.39	(20.18- 39.94)
SOBUE	58	m	0	76	187	27	128	1.93	(1.18- 3.16)
SVENSS	39	f	0	4	1	22	120	21.82	(2.33- 204.53)
TSUGAN	6	m	0	10	13	18	17	0.73	(0.25- 2.09)
WAKAI	51	m	0	27	48	8	65	4.57	(1.91- 10.94)
WU	4	f	0	61	22	29	62	5.93	(3.07- 11.44)
WYNDE2	13	m	0	16	112	5	105	3.00	(1.06- 8.48)
WYNDE3	27	m	0	9	26	6	88	5.08	(1.65- 15.59)
WYNDE3	74	f	0	1	3	15	76	1.69	(0.16- 17.36)
Subtotal WYNDE3								4.13	(1.50- 11.34)
WYNDE4	41	m	0	7	64	4	115	3.14	(0.89- 11.15)
WYNDE6	51	m	0	282	197	58	617	15.23	(11.00- 21.07)
WYNDE6	240	f	0	157	52	119	856	21.72	(15.04- 31.37)
Subtotal WYNDE6								17.79	(13.95- 22.70)
ZHENG	9	m	0	17	23	29	94	2.40	(1.13- 5.08)
Partial Totals				966	10838	852	17883		

*prospective study ~ With 0.5 adjustment for zero

REF	NRR	SEX	AD	Ys	Ws	Qs	Ps
ALDERS	48	m	1	1.20	4.83	2.76	0.0084
ALDERS	51	f	1	1.20	10.32	5.95	0.0001
Subtotal ALDERS				1.20	15.14	8.71	
BARBON	49	m	0	2.27	5.49	0.54	0.0000
*BOUCOT	28	m	0	2.65	0.46	0.22	0.0710
CHOI	55	m	0	1.51	1.22	0.24	0.0956
CHOI	61	f	0	1.21	0.49	0.28	0.3961
Subtotal CHOI				1.42	1.71	0.52	
CORREA	52	c	1	2.48	15.59	4.36	0.0000
DOLL	61	m	0	0.10	1.45	5.01	0.9065
DOSEME	16	m	2	1.16	5.93	3.73	0.0046
GER	4	c	0	-0.26	3.67	17.97	0.6242
HAENSZ	34	f	0	0.90	3.24	3.64	0.1060
JEDRYC	19	m	0	1.51	3.71	0.74	0.0036
KATSOU	18	f	0	1.21	2.15	1.20	0.0762
KREYBE	19	m	0	1.47	1.86	0.45	0.0460
LAMTH	18	f	0	0.78	3.08	4.29	0.1740
LUBIN2	164	m	0	1.38	39.81	13.29	0.0000
LUBIN2	184	f	0	0.68	6.90	11.25	0.0741
Subtotal LUBIN2				1.28	46.71	24.54	
MATOS	60	m	0	2.13	4.10	0.12	0.0000
MATSUD	9	m	0	3.18	0.45	0.67	0.0331
ORMOS	24	m	0	1.11	0.66	0.47	0.3661
OSANN	63	m	2	3.62	12.01	33.43	0.0000
OSANN	64	f	2	3.19	20.96	31.71	0.0000
Subtotal OSANN				3.35	32.97	65.14	

Table 3G14 - 5

IESLC - Meta-anal of Ever Smoking (or Current if ev not avail), Amount smoked, “High”, Any prod (or Cigs if Any not avail)
Adenocarcinoma
Least adjusted

REF	NRR	SEX	AD	Ys	Ws	Qs	Ps
SOBUE	58	m	0	0.66	15.78	26.69	0.0092
SVENSS	39	f	0	3.08	0.77	0.97	0.0069
TSUGAN	6	m	0	-0.32	3.43	17.78	0.5538
WAKAI	51	m	0	1.52	5.04	0.96	0.0006
WU	4	f	0	1.78	8.89	0.28	0.0000
WYNDE2	13	m	0	1.10	3.56	2.62	0.0382
WYNDE3	27	m	0	1.62	3.05	0.34	0.0045
WYNDE3	74	f	0	0.52	0.71	1.45	0.6593
Subtotal WYNDE3				1.42	3.76	1.79	
WYNDE4	41	m	0	1.15	2.40	1.58	0.0761
WYNDE6	51	m	0	2.72	36.38	21.40	0.0000
WYNDE6	240	f	0	3.08	28.43	35.78	0.0000
Subtotal WYNDE6				2.88	64.82	57.18	
ZHENG	9	m	0	0.87	6.78	7.95	0.0229

	N	34
	NS	28
	Wt	263.62
Het	Chi	260.11
Het	df	33
Het	P	***
Fixed	RR	7.07
	RRl	6.27
	RRu	7.98
	P	+++
Random	RR	4.72
	RRl	3.23
	RRu	6.90
	P	+++
Asymm	P	*

Table 3G14 - 6

IESLC - Meta-anal of Ever Smoking (or Current if ev not avail), Amount smoked, “High”, Any prod (or Cigs if Any not avail)
Adenocarcinoma
Least adjusted

		Sex		
	combined	male	female	Total
N	2	21	11	34
NS	2	21	11	34
Wt	19.27	158.42	85.94	263.62
Het Chi	22.33	135.16	86.14	260.11
Het df	1	20	10	33
Het P	***	***	***	***
Fixed RR	7.12	5.84	10.06	7.07
RRl	4.55	5.00	8.14	6.27
RRu	11.12	6.82	12.43	7.98
P	+++	+++	+++	+++
Random RR	3.17	4.59	5.29	4.72
RRl	0.22	2.88	2.57	3.23
RRu	46.40	7.32	10.91	6.90
P	N.S.	+++	+++	+++
Between Chi				16.48
Between df				2
Between P				***
Btwn(F) P				N.S.
Btwn(R) P				N.S.

Table 3G14 - 7

IESLC - Meta-anal of Ever Smoking (or Current if ev not avail), Amount smoked, “High”, Any prod (or Cigs if Any not avail)
Adenocarcinoma
Excluded studies (and stage at which they were excluded)

1 ABELIN ABRAHA AMANDU AMES ANDERS AUSTIN AXELSO BAND BECHER BERRIN BLOHMK BLOT4 BROCKM BROWN1 BYERS1 BYERS2
CARPEN CASCO2 CASCOR CHAN CHEN3 CHIAZZ CHYOU DESTE2 DOCKER DROSTE DU GARCIA GARDIN GENG GODLEY GOODMA
GRAHAM GREGOR HEGMAN HEIN HENNEK HINDS HIRAOK HOROWI HORWIT HUANG ISHIMA JAHN JAIN JARVHO JIANG KELLER
KIHARA KJUUS KO KOHLME KUBIK LAMWK LAMWK2 LANGE LEI LEMARC LEVIN LIU LOMBA2 LOMBAR MAGNUS MARSH
MARSH2 MCDUFF MCLAUG MILLER MILLS NOTANI NOU ODRISC PAWLEG PERSHA POFFIJ QIAO QIAO2 RADZIK REN RONCO
ROOTS ROTHSC SAARIK SANKAR SCHWAR SEGI SEOW SHIMIZ SIMARA SIMONA SITAS SOBUE2 STASZE STAYNE STUCKE SUN
SUZUK2 SUZUKI TANG TAO TOKARS TOUSEY ULMER VEIERO VUTUC WALD WANG WANG3 WANG4 WICKLU WIGLE WILKIN
WU2 WUNSCH WYNDE8 XIANGZ XU XU2 XU4 YONG ZHANG

2 BUELL CHEN MASTRA MZILEN PISANI RESTRE SADOWS

4 BOFFET WYNDE7

5 RIMING TANG2 WYNDE5

6 BLOT1 BLOT2 BLOT3 BOUCHA HIRAY2 JONES LAURIL LICKIN MOLLO MRFIT MURATA SCHWA2 VANDER WARSIN WATSON WYNDER

8 AGUDO AKIBA ARCHER ARMADA AUVINE AXELSS BENSHL BEST BRESLO BRETT BROSS BUFFLE CEDERL CHANG CHATZI CHEN2
CHOW COMSTO COOKSO CPSI CPSII DAMBER DARBY DAVEYS DEAN DEAN2 DEAN3 DEKLER DESTEF DOLL2 DORANT DORN
DUNN EBELIN ENSTRO ESAKI FAN GAO GAO2 GARSHI GILLIS GOLLED GSELL HAMMO2 HANSEN HIRAYA HITOSU HOLE
HU HU2 HUMBLE JARUP JOLY JUSSAW KAISE2 KAISER KANELL KAUFMA KHUDER KINLEN KNEKT KOO KOULUM KREUZE
LAUSSM LETOUR LIAW LIDDEL LIU2 LIU3 LIU4 LIU5 LUBIN LUO MACLEN MARTIS MCCONN MIGRAN MRFITR NAM
NOTAN2 PARKIN PASTOR PERNU PERSH2 PETO PEZZO2 PEZZOT PIKE POLEDN PRESCO RACHTA RANDIG SEGI2 SHAW SIEMIA
SPEIZE SPITZ STOCKS STOCKW TENKAN TIZZAN TULINI TVERDA WANG2 XU3 YAMAGU YUAN ZHOU

10 BROWN2 DORGAN ENGELA HAMMON OSANN2 WUWILL

11 BENHAM

Table 3G14 - 8
Potentially overlapping studies

REF	REFGP	PRINC	.	OVERLAP/LINK
LUBIN2	LUBIN2	1		Lubin-combined
LAMTH	LAMTH	1		KOO/LAMTH/LAMWK
WYNDE6	WYNDE6	1		WYNDE5/6/7/8
MATSUD	MATSUD	1		SOBUE2/MATSUD

Table 3G14 - 9
Most adjusted - insufficient data for metaanalysis

REF	NRR	SEX	AGEL	AGEH	RACE	YF	LC TYPE	LOC	START	ST	NLC	R	VB	P	H	AD	SM	PRODUCT	exL	exH	DENOM	De
CHEN	12	c	0	0	all	-	a	As:oth	1987	CC	323	n	ot	n	y	2	ev	cig+/-ot	31	99	nev cigs	ot

REF	NRR	RR	SIG	RRDATA comment
CHEN	12	3.61	y	P < 0.001

Table 3G16 -

IESLC - Meta-anal of Ever Smoking (or Curr if Ever not avail) by Amount, Overview, Cigs (or Any Prod if Cigs not avail)
Adenocarcinoma

This analysis is restricted to results for:
1) Results by Amount smoked
Results by Amount smoked (in numbers of cigarettes or cigarette equivalents) are grouped under 2 schemes (S1, S2).
Each scheme has a set of "key values". An interval is allocated to the category whose key value
it includes and intervals which include none or more than one of the key values are excluded.
(Open-ended intervals are coded as 99.)

S1	key value	maximum range	S2	key value	maximum range
1	5	1-19	1	1	1-9
2	20	6-44	2	10	2-19
3	45	21+	3	20	11-29
			4	30	21-39
			5	40	31-98
			6	99	41+

For all/unspec product, the definition of cigarette equivalents is shown at the end of Sections -1 and -4.
2) Results complete enough for use in metaanalysis

Within each study, results are then selected (in the following order of preference, within each sex) for:
3) SMKSTA: ever smokers, current smokers
4) PRODUCT: cigarettes regardless of other products, cigarettes only, all/unspec
5) CIGTYPE: all/unspecified, MC regardless of HR, MC only
6) DENOM: never smoked anything, never smoked cigarettes, (never +1 = +long term ex, +2 = +amount unknown,
+3 = never cigs+long term ex)
7) Followup period (YF, prospective studies): whole study (coded as 0) or longest available
8) LCtype: adeno or nearest available, but not squamous. (q = squamous, s = small,
a = adeno, l = large, KII = Kreyberg II, al = alveolar, br = bronchiolar, u = undifferentiated)
9) Race: all or nearest available, otherwise by race (wh or w = white, bl or b = black, hi = hispanic
ch = chinese, jap = japanese, haw = hawaiian, w+o = white + oriental, sca = scandinavian, as = asian)
10) For overlapping studies: principal rather than subsidiary studies
Finally by Age: whole study (coded as 0) if available, otherwise by widest available age group
and then for single sex results (m, f) in preference to combined sex results (c).

Results adjusted (AD) for the most potential confounders are then chosen in Sections -1 to -3
(and those which actually differ from the adjusted results in Table 3G11 - 1 are marked 'x' in Section -1)
and results adjusted for the least confounders in Sections -4 to -6. (Those least adjusted results which
actually differ from the most adjusted as marked 'x' in column X in Section -4)
(Results adjusted for an unknown number of confounder(s) are coded as 20.)

Section -7 shows excluded studies, together with the stage (as above) at which no qualifying
results were found.

Section -8 lists the potentially overlapping studies which have been included (1=principal, 2=subsidiary).

Section -9 lists any results which would have been included in preference except that they had data not complete
enough for use in meta-analysis, with their significance (yes/no), if known, and any further comment as entered
on the database.

In addition to those mentioned above, the following fields, levels and abbreviations are used:
* or nk = not known, n = no, y = yes, ot = other
ev = ever, cu = current, nev = never
all/unspec = all or unspecified, cig+/-ot = cigarettes irrespective of other products (cigar, pipe etc)
MC = manufactured cigarettes, HR = hand-rolled cigarettes
exL, exH = range of exposure (low and high) in the smoking group, in terms of Amount smoked,
cigarettes or cigarette equivalents
REF: 6-character study reference
NRR: number of the RR on the database within the study
ST : study type (CC = case control, pr or prosp = prospective)
NLC: number of lung cancer cases in whole study
R : risky occupational population (n = no, m = mining, o = other risky)
VB : national cigarette type (V = at least 75% Virginia, bl = at least 75% blended, ot = other)
P : any proxy use
H : full histological confirmation
De : derivation of RR/CI (or = original, st = standard method, ot = other method of estimation)

Table 3G16 - 1

IESLC - Meta-anal of Ever Smoking (or Curr if Ever not avail) by Amount, Overview, Cigs (or Any Prod if Cigs not avail)
Adenocarcinoma
Most adjusted

REF	NRR	3G11	SEX	AGEL	AGEH	RACE	YF	LC TYPE	LOC	START	ST	NLC	R	VB	P	H	AD	SM	PRODUCT	exL	exH	S1	S2	DENOM	De
ALDERS	46		m	0	0	all	-	not q+s	Eu:UK	1977	CC	1448	n	V	n	n	1	ev	cig only	1	17	1	0	nev+2	ot
ALDERS	47		m	0	0	all	-	not q+s	Eu:UK	1977	CC	1448	n	V	n	n	1	ev	cig only	18	27	2	3	nev+2	ot
ALDERS	48		m	0	0	all	-	not q+s	Eu:UK	1977	CC	1448	n	V	n	n	1	ev	cig only	28	99	3	0	nev+2	ot
ALDERS	49		f	0	0	all	-	not q+s	Eu:UK	1977	CC	1448	n	V	n	n	1	ev	cig only	1	17	1	0	nev+2	ot
ALDERS	50		f	0	0	all	-	not q+s	Eu:UK	1977	CC	1448	n	V	n	n	1	ev	cig only	18	27	2	3	nev+2	ot
ALDERS	51		f	0	0	all	-	not q+s	Eu:UK	1977	CC	1448	n	V	n	n	1	ev	cig only	28	99	3	0	nev+2	ot
BARBON	79		m	0	0	all	-	a	Eu:wst	1979	CC	755	n	bl	y	y	3	ev	all/unsp	1	19	1	0	nev any	or
BARBON	80		m	0	0	all	-	a	Eu:wst	1979	CC	755	n	bl	y	y	3	ev	all/unsp	20	39	2	0	nev any	or
BARBON	81		m	0	0	all	-	a	Eu:wst	1979	CC	755	n	bl	y	y	3	ev	all/unsp	40	99	3	0	nev any	or
BOUCOT	148		m	0	0	all	0	a	NAmer	1951	pr	121	n	bl	n	n	2	cu	cig only	1	20	0	0	nev any	ot
BOUCOT	149		m	0	0	all	0	a	NAmer	1951	pr	121	n	bl	n	n	2	cu	cig only	21	99	3	0	nev any	ot
BROWN2	34		m	0	0	wh	-	a	NAmer	1984	CC	14596	n	bl	n	y	2	ev	cig+/-ot	1	19	1	0	nev cigs	or
BROWN2	44		m	0	0	wh	-	a	NAmer	1984	CC	14596	n	bl	n	y	2	ev	cig+/-ot	20	99	0	0	nev cigs	or
BROWN2	33		f	0	0	wh	-	a	NAmer	1984	CC	14596	n	bl	n	y	2	ev	cig+/-ot	1	19	1	0	nev cigs	or
BROWN2	43		f	0	0	wh	-	a	NAmer	1984	CC	14596	n	bl	n	y	2	ev	cig+/-ot	20	99	0	0	nev cigs	or
CHOI	51		m	0	0	all	-	a	As:oth	1985	CC	375	n	bl	n	n	0	ev	cig+/-ot	1	10	1	0	nev cigs	st
CHOI	52		m	0	0	all	-	a	As:oth	1985	CC	375	n	bl	n	n	0	ev	cig+/-ot	11	20	2	3	nev cigs	st
CHOI	53		m	0	0	all	-	a	As:oth	1985	CC	375	n	bl	n	n	0	ev	cig+/-ot	21	30	0	4	nev cigs	st
CHOI	54		m	0	0	all	-	a	As:oth	1985	CC	375	n	bl	n	n	0	ev	cig+/-ot	31	40	0	5	nev cigs	st
CHOI	55		m	0	0	all	-	a	As:oth	1985	CC	375	n	bl	n	n	0	ev	cig+/-ot	41	99	3	6	nev cigs	st
CHOI	59		f	0	0	all	-	a	As:oth	1985	CC	375	n	bl	n	n	0	ev	cig+/-ot	1	10	1	0	nev cigs	st
CHOI	60		f	0	0	all	-	a	As:oth	1985	CC	375	n	bl	n	n	0	ev	cig+/-ot	11	30	2	0	nev cigs	st
CHOI	61		f	0	0	all	-	a	As:oth	1985	CC	375	n	bl	n	n	0	ev	cig+/-ot	31	99	3	0	nev cigs	st
CORREA	48		c	0	0	all	-	a	NAmer	1979	CC	1359	n	bl	y	n	1	cu	cig+/-ot	1	20	0	0	nev cigs	or
CORREA	52		c	0	0	all	-	a	NAmer	1979	CC	1359	n	bl	y	n	1	cu	cig+/-ot	21	99	3	0	nev cigs	or
DOLL	72		m	0	0	all	-	KII	Eu:UK	1948	CC	1465	n	V	n	n	1	ev	all/unsp	1	4	0	1	nev any	ot
DOLL	73		m	0	0	all	-	KII	Eu:UK	1948	CC	1465	n	V	n	n	1	ev	all/unsp	5	14	1	2	nev any	ot
DOLL	74		m	0	0	all	-	KII	Eu:UK	1948	CC	1465	n	V	n	n	1	ev	all/unsp	15	24	2	3	nev any	ot
DOLL	75		m	0	0	all	-	KII	Eu:UK	1948	CC	1465	n	V	n	n	1	ev	all/unsp	25	99	3	0	nev any	ot
DOLL	79		f	0	0	all	-	KII	Eu:UK	1948	CC	1465	n	V	n	n	1	ev	all/unsp	1	4	0	1	nev any	ot
DOLL	80		f	0	0	all	-	KII	Eu:UK	1948	CC	1465	n	V	n	n	1	ev	all/unsp	5	14	1	2	nev any	ot
DOLL	81		f	0	0	all	-	KII	Eu:UK	1948	CC	1465	n	V	n	n	1	ev	all/unsp	15	99	0	0	nev any	ot
DORGAN	126		m	0	0	wh	-	a	NAmer	1980	CC	2026	n	bl	y	y	2	ev	cig+/-ot	1	19	1	0	nev any	ot
DORGAN	127		m	0	0	wh	-	a	NAmer	1980	CC	2026	n	bl	y	y	2	ev	cig+/-ot	20	99	0	0	nev any	ot
DORGAN	105		f	0	0	all	-	a	NAmer	1980	CC	2026	n	bl	y	y	3	ev	cig+/-ot	1	19	1	0	nev any	ot
DORGAN	106		f	0	0	all	-	a	NAmer	1980	CC	2026	n	bl	y	y	3	ev	cig+/-ot	20	99	0	0	nev any	ot
DOSEME	8		m	0	0	all	-	not q+s	Eu:bal	1979	CC	1210	n	bl	n	n	2	ev	cig+/-ot	1	10	1	0	nev cigs	or
DOSEME	12		m	0	0	all	-	not q+s	Eu:bal	1979	CC	1210	n	bl	n	n	2	ev	cig+/-ot	11	20	2	3	nev cigs	or
DOSEME	16		m	0	0	all	-	not q+s	Eu:bal	1979	CC	1210	n	bl	n	n	2	ev	cig+/-ot	21	99	3	0	nev cigs	or
ENGELA	71		m	0	0	all	0	a	Eu:Sca	1964	pr	435	n	bl	n	n	7	cu	cig+/-ot	1	4	0	1	nev cigs	or
ENGELA	72		m	0	0	all	0	a	Eu:Sca	1964	pr	435	n	bl	n	n	7	cu	cig+/-ot	5	9	1	0	nev cigs	or
ENGELA	73		m	0	0	all	0	a	Eu:Sca	1964	pr	435	n	bl	n	n	7	cu	cig+/-ot	10	14	0	2	nev cigs	or
ENGELA	74		m	0	0	all	0	a	Eu:Sca	1964	pr	435	n	bl	n	n	7	cu	cig+/-ot	15	19	0	0	nev cigs	or
ENGELA	75		m	0	0	all	0	a	Eu:Sca	1964	pr	435	n	bl	n	n	7	cu	cig+/-ot	20	99	0	0	nev cigs	or
GER	10		c	0	0	all	-	a	As:oth	1990	CC	141	n	ot	y	n	8	ev	all/unsp	1	10	1	0	nev any	ot
GER	11		c	0	0	all	-	a	As:oth	1990	CC	141	n	ot	y	n	8	ev	all/unsp	11	20	2	3	nev any	ot
GER	12		c	0	0	all	-	a	As:oth	1990	CC	141	n	ot	y	n	8	ev	all/unsp	21	99	3	0	nev any	ot
HAENSZ	35		f	0	0	all	-	a	NAmer	1955	CC	158	n	bl	n	y	0	cu	cig+/-ot	1	20	0	0	nev any	or
HAENSZ	34		f	0	0	all	-	a	NAmer	1955	CC	158	n	bl	n	y	0	cu	cig+/-ot	21	99	3	0	nev any	or
HAMMON	108		m	0	0	wh	0	a	NAmer	1952	pr	448	n	bl	n	n	1	cu	cig only	1	9	1	1	nev any	ot
HAMMON	109		m	0	0	wh	0	a	NAmer	1952	pr	448	n	bl	n	n	1	cu	cig only	10	20	2	0	nev any	ot
HAMMON	110		m	0	0	wh	0	a	NAmer	1952	pr	448	n	bl	n	n	1	cu	cig only	21	39	0	4	nev any	ot
JEDRYC	36		m	0	0	all	-	a	Eu:est	1980	CC	1630	n	bl	y	n	3	ev	cig+/-ot	1	19	1	0	nev any	or
JEDRYC	37		m	0	0	all	-	a	Eu:est	1980	CC	1630	n	bl	y	n	3	ev	cig+/-ot	20	29	2	3	nev any	or
JEDRYC	38		m	0	0	all	-	a	Eu:est	1980	CC	1630	n	bl	y	n	3	ev	cig+/-ot	30	99	3	0	nev any	or
KATSOU	13		f	0	0	all	-	a	Eu:bal	1987	CC	101	n	bl	n	n	1	cu	all/unsp	1	20	0	0	nev any	or
KATSOU	14		f	0	0	all	-	a	Eu:bal	1987	CC	101	n	bl	n	n	1	cu	all/unsp	21	99	3	0	nev any	or
KREYBE	5		m	0	0	all	-	KII	Eu:Sca	1948	CC	300	n	bl	n	y	1	ev	all/unsp	1	14	1	0	nev any	ot
KREYBE	6		m	0	0	all	-	KII	Eu:Sca	1948	CC	300	n	bl	n	y	1	ev	all/unsp	15	24	2	3	nev any	ot
KREYBE	7		m	0	0	all	-	KII	Eu:Sca	1948	CC	300	n	bl	n	y	1	ev	all/unsp	25	99	3	0	nev any	ot
KREYBE	26		f	0	0	all	-	KII	Eu:Sca	1948	CC	300	n	bl	n	y	1	ev	all/unsp	1	14	1	0	nev any	ot
KREYBE	61		f	0	0	all	-	KII	Eu:Sca	1948	CC	300	n	bl	n	y	1	ev	all/unsp	15	99	0	0	nev any	ot
LAMTH	16		f	0	0	ch	-	a	As:HK	1983	CC	445	n	bl	n	n	0	ev	all/unsp	1	10	1	0	nev any	or
LAMTH	17		f	0	0	ch	-	a	As:HK	1983	CC	445	n	bl	n	n	0	ev	all/unsp	11	20	2	3	nev any	or
LAMTH	18		f	0	0	ch	-	a	As:HK	1983	CC	445	n	bl	n	n	0	ev	all/unsp	21	99	3	0	nev any	or
LUBIN2	152		m	0	0	all	-	a	Eu:mul	1976	CC	7804	n	bl	n	y	0	ev	cig+/-ot	1	9	1	1	nev any	st
LUBIN2	156		m	0	0	all	-	a	Eu:mul	1976	CC	7804	n	bl	n	y	0	ev	cig+/-ot	10	19	0	2	nev any	st
LUBIN2	160		m	0	0	all	-	a	Eu:mul	1976	CC	7804	n	bl	n	y	0	ev	cig+/-ot	20	29	2	3	nev any	st
LUBIN2	164		m	0	0	all	-	a	Eu:mul	1976	CC	7804	n	bl	n	y	0	ev	cig+/-ot	30	99	3	0	nev any	st
LUBIN2	172		f	0	0	all	-	a	Eu:mul	1976	CC	7804	n	bl	n	y	0	ev	cig+/-ot	1	9	1	1	nev any	st
LUBIN2	176		f	0	0	all	-	a	Eu:mul	1976	CC	7804	n	bl	n	y	0	ev	cig+/-ot	10	19	0	2	nev any	st
LUBIN2	180		f	0	0	all	-	a	Eu:mul	1976	CC	7804	n	bl	n	y	0	ev	cig+/-ot	20	29	2	3	nev any	st

Table 3G16 - 1

IESLC - Meta-anal of Ever Smoking (or Curr if Ever not avail) by Amount, Overview, Cigs (or Any Prod if Cigs not avail)
Adenocarcinoma
Most adjusted

REF	NRR	3G11	SEX	AGEL	AGEH	RACE	YF	LC TYPE	LOC	START	ST	NLC	R	VB	P	H	AD	SM	PRODUCT	exL	exH	S1	S2	DENOM	De
LUBIN2	184		f	0	0	all	-	a	Eu:mul	1976	CC	7804	n	bl	n	y	0	ev	cig+/-ot	30	99	3	0	nev any	st
MATOS	57		m	0	0	all	-	a	SCAmer	1994	CC	200	n	bl	n	n	2	ev	cig+/-ot	1	14	1	0	nev any	or
MATOS	59		m	0	0	all	-	a	SCAmer	1994	CC	200	n	bl	n	n	2	ev	cig+/-ot	15	24	2	3	nev any	or
MATOS	61		m	0	0	all	-	a	SCAmer	1994	CC	200	n	bl	n	n	2	ev	cig+/-ot	25	99	3	0	nev any	or
MATSUD	7		m	0	0	all	-	a	As:Jap	1965	CC	179	n	bl	n	n	0	ev	cig+/-ot	1	10	1	0	nev cigs	ot
MATSUD	8		m	0	0	all	-	a	As:Jap	1965	CC	179	n	bl	n	n	0	ev	cig+/-ot	11	20	2	3	nev cigs	ot
MATSUD	9		m	0	0	all	-	a	As:Jap	1965	CC	179	n	bl	n	n	0	ev	cig+/-ot	21	99	3	0	nev cigs	ot
ORMOS	22		m	0	0	all	-	KII	Eu:est	1947	CC	119	n	bl	y	y	0	ev	cig+/-ot	1	15	1	0	nev any	st
ORMOS	23		m	0	0	all	-	KII	Eu:est	1947	CC	119	n	bl	y	y	0	ev	cig+/-ot	16	30	2	0	nev any	st
ORMOS	24		m	0	0	all	-	KII	Eu:est	1947	CC	119	n	bl	y	y	0	ev	cig+/-ot	31	99	3	0	nev any	st
OSANN	55		m	0	0	all	-	a	NAmer	1984	CC	1986	n	bl	n	n	2	ev	cig+/-ot	1	39	0	0	nev cigs	or
OSANN	63		m	0	0	all	-	a	NAmer	1984	CC	1986	n	bl	n	n	2	ev	cig+/-ot	40	99	3	0	nev cigs	or
OSANN	56		f	0	0	all	-	a	NAmer	1984	CC	1986	n	bl	n	n	2	ev	cig+/-ot	1	39	0	0	nev cigs	or
OSANN	64		f	0	0	all	-	a	NAmer	1984	CC	1986	n	bl	n	n	2	ev	cig+/-ot	40	99	3	0	nev cigs	or
OSANN2	34		f	0	0	all	-	KII	NAmer	1964	ot	217	n	bl	n	y	1	ev	cig+/-ot	1	19	1	0	nev cigs	or
OSANN2	35		f	0	0	all	-	KII	NAmer	1964	ot	217	n	bl	n	y	1	ev	cig+/-ot	20	99	0	0	nev cigs	or
SOBUE	56		m	0	0	all	-	a	As:Jap	1986	CC	1376	n	bl	n	y	0	cu	cig+/-ot	1	19	1	0	nev cigs	st
SOBUE	57		m	0	0	all	-	a	As:Jap	1986	CC	1376	n	bl	n	y	0	cu	cig+/-ot	20	29	2	3	nev cigs	st
SOBUE	58		m	0	0	all	-	a	As:Jap	1986	CC	1376	n	bl	n	y	0	cu	cig+/-ot	30	99	3	0	nev cigs	st
SVENSS	9		f	0	0	all	-	a	Eu:Sca	1983	CC	210	n	bl	n	n	1	cu	all/unsp	1	10	1	0	nev any	or
SVENSS	14		f	0	0	all	-	a	Eu:Sca	1983	CC	210	n	bl	n	n	1	cu	all/unsp	11	20	2	3	nev any	or
SVENSS	19		f	0	0	all	-	a	Eu:Sca	1983	CC	210	n	bl	n	n	1	cu	all/unsp	21	99	3	0	nev any	ot
TSUGAN	4		m	0	0	all	-	a	As:Jap	1976	CC	134	n	bl	n	y	0	cu	all/unsp	1	15	1	0	nev any	st
TSUGAN	5		m	0	0	all	-	a	As:Jap	1976	CC	134	n	bl	n	y	0	cu	all/unsp	16	35	2	0	nev any	st
TSUGAN	6		m	0	0	all	-	a	As:Jap	1976	CC	134	n	bl	n	y	0	cu	all/unsp	36	99	3	0	nev any	st
WAKAI	52		m	0	0	all	-	a	As:Jap	1988	CC	333	n	bl	n	y	1	cu	cig+/-ot	1	19	1	0	nev any	or
WAKAI	53		m	0	0	all	-	a	As:Jap	1988	CC	333	n	bl	n	y	1	cu	cig+/-ot	20	29	2	3	nev any	or
WAKAI	54		m	0	0	all	-	a	As:Jap	1988	CC	333	n	bl	n	y	1	cu	cig+/-ot	30	99	3	0	nev any	or
WU	8		f	0	0	wh	-	a	NAmer	1981	CC	220	n	bl	n	y	2	cu	all/unsp	1	20	0	0	nev any	or
WU	9		f	0	0	wh	-	a	NAmer	1981	CC	220	n	bl	n	y	2	cu	all/unsp	21	99	3	0	nev any	or
WUWILL	16		f	0	0	all	-	a	As:Chi	1985	CC	965	n	ot	n	n	3	ev	cig+/-ot	1	19	1	0	nev cigs	ot
WUWILL	17		f	0	0	all	-	a	As:Chi	1985	CC	965	n	ot	n	n	3	ev	cig+/-ot	20	99	0	0	nev cigs	ot
WYNDE2	10		m	0	0	all	-	KII	NAmer	1962	CC	404	n	bl	n	y	0	ev	cig+/-ot	1	10	1	0	nev any	st
WYNDE2	11		m	0	0	all	-	KII	NAmer	1962	CC	404	n	bl	n	y	0	ev	cig+/-ot	11	20	2	3	nev any	st
WYNDE2	12		m	0	0	all	-	KII	NAmer	1962	CC	404	n	bl	n	y	0	ev	cig+/-ot	21	34	0	4	nev any	st
WYNDE2	13		m	0	0	all	-	KII	NAmer	1962	CC	404	n	bl	n	y	0	ev	cig+/-ot	35	99	3	0	nev any	st
WYNDE3	24		m	0	0	all	-	KII	NAmer	1966	CC	350	n	bl	n	y	0	ev	cig+/-ot	1	9	1	1	nev any	st
WYNDE3	25		m	0	0	all	-	KII	NAmer	1966	CC	350	n	bl	n	y	0	ev	cig+/-ot	10	20	2	0	nev any	st
WYNDE3	26		m	0	0	all	-	KII	NAmer	1966	CC	350	n	bl	n	y	0	ev	cig+/-ot	21	40	0	0	nev any	st
WYNDE3	27		m	0	0	all	-	KII	NAmer	1966	CC	350	n	bl	n	y	0	ev	cig+/-ot	41	99	3	6	nev any	st
WYNDE3	71		f	0	0	all	-	KII	NAmer	1966	CC	350	n	bl	n	y	0	ev	cig+/-ot	1	9	1	1	nev any	st
WYNDE3	72		f	0	0	all	-	KII	NAmer	1966	CC	350	n	bl	n	y	0	ev	cig+/-ot	10	20	2	0	nev any	st
WYNDE3	73		f	0	0	all	-	KII	NAmer	1966	CC	350	n	bl	n	y	0	ev	cig+/-ot	21	40	0	0	nev any	st
WYNDE3	74		f	0	0	all	-	KII	NAmer	1966	CC	350	n	bl	n	y	0	ev	cig+/-ot	41	99	3	6	nev any	st
WYNDE4	37		m	0	0	all	-	a	NAmer	1948	CC	684	n	bl	y	n	0	ev	all/unsp	1	9	1	1	nev any	st
WYNDE4	38		m	0	0	all	-	a	NAmer	1948	CC	684	n	bl	y	n	0	ev	all/unsp	10	15	0	2	nev any	st
WYNDE4	39		m	0	0	all	-	a	NAmer	1948	CC	684	n	bl	y	n	0	ev	all/unsp	16	20	2	3	nev any	st
WYNDE4	40		m	0	0	all	-	a	NAmer	1948	CC	684	n	bl	y	n	0	ev	all/unsp	21	34	0	4	nev any	st
WYNDE4	41		m	0	0	all	-	a	NAmer	1948	CC	684	n	bl	y	n	0	ev	all/unsp	35	99	3	0	nev any	st
WYNDE4	55		f	0	0	all	-	a	NAmer	1948	CC	684	n	bl	y	n	2	ev	all/unsp	1	9	1	1	nev any	ot
WYNDE6	24		m	0	0	all	-	KII	NAmer	1969	CC	4423	n	bl	n	y	0	cu	cig+/-ot	1	10	1	0	nev any	st
WYNDE6	33		m	0	0	all	-	KII	NAmer	1969	CC	4423	n	bl	n	y	0	cu	cig+/-ot	11	20	2	3	nev any	st
WYNDE6	42		m	0	0	all	-	KII	NAmer	1969	CC	4423	n	bl	n	y	0	cu	cig+/-ot	21	30	0	4	nev any	st
WYNDE6	51		m	0	0	all	-	KII	NAmer	1969	CC	4423	n	bl	n	y	0	cu	cig+/-ot	31	99	3	0	nev any	st
WYNDE6	213		f	0	0	all	-	KII	NAmer	1969	CC	4423	n	bl	n	y	0	cu	cig+/-ot	1	10	1	0	nev cigs	st
WYNDE6	222		f	0	0	all	-	KII	NAmer	1969	CC	4423	n	bl	n	y	0	cu	cig+/-ot	11	20	2	3	nev cigs	st
WYNDE6	231		f	0	0	all	-	KII	NAmer	1969	CC	4423	n	bl	n	y	0	cu	cig+/-ot	21	30	0	4	nev cigs	st
WYNDE6	240		f	0	0	all	-	KII	NAmer	1969	CC	4423	n	bl	n	y	0	cu	cig+/-ot	30	99	3	0	nev cigs	st
ZHENG	6		m	0	0	all	-	a	As:Chi	1982	CC	540	n	ot	*	y	0	ev	cig+/-ot	1	9	1	1	nev cigs	st
ZHENG	7		m	0	0	all	-	a	As:Chi	1982	CC	540	n	ot	*	y	0	ev	cig+/-ot	10	19	0	2	nev cigs	st
ZHENG	8		m	0	0	all	-	a	As:Chi	1982	CC	540	n	ot	*	y	0	ev	cig+/-ot	20	29	2	3	nev cigs	st
ZHENG	9		m	0	0	all	-	a	As:Chi	1982	CC	540	n	ot	*	y	0	ev	cig+/-ot	30	99	3	0	nev cigs	st
ZHENG	19		f	0	0	all	-	a	As:Chi	1982	CC	540	n	ot	*	y	0	ev	cig+/-ot	1	9	1	1	nev cigs	st
ZHENG	20		f	0	0	all	-	a	As:Chi	1982	CC	540	n	ot	*	y	0	ev	cig+/-ot	10	99	0	0	nev cigs	st

Cigarette type is all/unspec for all RRs

except for the following:

Table 3G16 - 1

IESLC - Meta-anal of Ever Smoking (or Curr if Ever not avail) by Amount, Overview, Cigs (or Any Prod if Cigs not avail)
Adenocarcinoma
Most adjusted

REF	NRR	CIGTYPE
ALDERS	46	MC only
ALDERS	47	MC only
ALDERS	48	MC only
ALDERS	49	MC only
ALDERS	50	MC only
ALDERS	51	MC only

REF	NRR	Cigarette equivalent
ALDERS	46	-
ALDERS	47	-
ALDERS	48	-
ALDERS	49	-
ALDERS	50	-
ALDERS	51	-
BARBON	79	*
BARBON	80	*
BARBON	81	*
BOUCOT	148	-
BOUCOT	149	-
BROWN2	34	*
BROWN2	44	*
BROWN2	33	*
BROWN2	43	*
CHOI	51	*
CHOI	52	*
CHOI	53	*
CHOI	54	*
CHOI	55	*
CHOI	59	*
CHOI	60	*
CHOI	61	*
CORREA	48	*
CORREA	52	*
DOLL	72	grams
DOLL	73	grams
DOLL	74	grams
DOLL	75	grams
DOLL	79	grams
DOLL	80	grams
DOLL	81	grams
DORGAN	126	*
DORGAN	127	*
DORGAN	105	*
DORGAN	106	*
DOSEME	8	*
DOSEME	12	*
DOSEME	16	*
ENGELA	71	*
ENGELA	72	*
ENGELA	73	*
ENGELA	74	*
ENGELA	75	*
GER	10	*
GER	11	*
GER	12	*
HAENSZ	35	*
HAENSZ	34	*
HAMMON	108	-
HAMMON	109	-
HAMMON	110	-
JEDRYC	36	*
JEDRYC	37	*
JEDRYC	38	*
KATSOU	13	*
KATSOU	14	*
KREYBE	5	grams inc 1 cig=1
KREYBE	6	grams inc 1 cig=1
KREYBE	7	grams inc 1 cig=1
KREYBE	26	grams inc 1 cig=1
KREYBE	61	grams inc 1 cig=1
LAMTH	16	*
LAMTH	17	*

Table 3G16 - 1

IESLC - Meta-anal of Ever Smoking (or Curr if Ever not avail) by Amount, Overview, Cigs (or Any Prod if Cigs not avail)
Adenocarcinoma
Most adjusted

REF	NRR	Cigarette equivalent
LAMTH	18	*
LUBIN2	152	*
LUBIN2	156	*
LUBIN2	160	*
LUBIN2	164	*
LUBIN2	172	*
LUBIN2	176	*
LUBIN2	180	*
LUBIN2	184	*
MATOS	57	*
MATOS	59	*
MATOS	61	*
MATSUD	7	*
MATSUD	8	*
MATSUD	9	*
ORMOS	22	*
ORMOS	23	*
ORMOS	24	*
OSANN	55	*
OSANN	63	*
OSANN	56	*
OSANN	64	*
OSANN2	34	*
OSANN2	35	*
SOBUE	56	*
SOBUE	57	*
SOBUE	58	*
SVENSS	9	*
SVENSS	14	*
SVENSS	19	*
TSUGAN	4	*
TSUGAN	5	*
TSUGAN	6	*
WAKAI	52	*
WAKAI	53	*
WAKAI	54	*
WU	8	*
WU	9	*
WUWILL	16	*
WUWILL	17	*
WYNDE2	10	*
WYNDE2	11	*
WYNDE2	12	*
WYNDE2	13	*
WYNDE3	24	*
WYNDE3	25	*
WYNDE3	26	*
WYNDE3	27	*
WYNDE3	71	*
WYNDE3	72	*
WYNDE3	73	*
WYNDE3	74	*
WYNDE4	37	inc 1 cigar = 5 cigs, 1 pipe = 2.5 cigs
WYNDE4	38	inc 1 cigar = 5 cigs, 1 pipe = 2.5 cigs
WYNDE4	39	inc 1 cigar = 5 cigs, 1 pipe = 2.5 cigs
WYNDE4	40	inc 1 cigar = 5 cigs, 1 pipe = 2.5 cigs
WYNDE4	41	inc 1 cigar = 5 cigs, 1 pipe = 2.5 cigs
WYNDE4	55	inc 1 cigar = 5 cigs, 1 pipe = 2.5 cigs
WYNDE6	24	*
WYNDE6	33	*
WYNDE6	42	*
WYNDE6	51	*
WYNDE6	213	*
WYNDE6	222	*
WYNDE6	231	*
WYNDE6	240	*
ZHENG	6	*
ZHENG	7	*
ZHENG	8	*
ZHENG	9	*
ZHENG	19	*
ZHENG	20	*

Table 3G16 - 1

IESLC - Meta-anal of Ever Smoking (or Curr if Ever not avail) by Amount, Overview, Cigs (or Any Prod if Cigs not avail)
Adenocarcinoma
Most adjusted

In this overview table, subtotals and Qs values may be invalid and should be ignored

Table 3G16 - 2

IESLC - Meta-anal of Ever Smoking (or Curr if Ever not avail) by Amount, Overview, Cigs (or Any Prod if Cigs not avail)
Adenocarcinoma
Most adjusted

REF	NRR	SEX	AD	Number Exposed Case	Number Exposed Cont	Non-exposed Case	Non-exposed Cont	RR	95.00%CI
ALDERS	46	m	1	-	-	-	-	2.80	(1.01- 7.75)
ALDERS	47	m	1	-	-	-	-	2.67	(0.99- 7.18)
ALDERS	48	m	1	-	-	-	-	3.32	(1.36- 8.10)
ALDERS	49	f	1	-	-	-	-	2.77	(1.63- 4.70)
ALDERS	50	f	1	-	-	-	-	4.58	(2.67- 7.85)
ALDERS	51	f	1	-	-	-	-	3.31	(1.80- 6.10)
Subtotal ALDERS								3.34	(2.53- 4.41)
BARBON	79	m	3	-	-	-	-	5.00	(2.20- 11.00)
BARBON	80	m	3	-	-	-	-	8.30	(3.70- 19.00)
BARBON	81	m	3	-	-	-	-	12.10	(5.10- 28.00)
Subtotal BARBON								7.82	(4.86- 12.59)
*BOUCOT	148	m	2	-	-	-	-	8.22	(0.56- 166.83)
*BOUCOT	149	m	2	-	-	-	-	18.00	(1.01- 319.52)
Subtotal BOUCOT								12.11	(1.60- 91.75)
BROWN2	34	m	2	-	-	-	-	6.20	(4.90- 7.90)
BROWN2	44	m	2	-	-	-	-	10.70	(8.90- 13.00)
BROWN2	33	f	2	-	-	-	-	5.80	(4.70- 7.10)
BROWN2	43	f	2	-	-	-	-	8.60	(7.30- 10.10)
Subtotal BROWN2								7.91	(7.18- 8.72)
CHOI	51	m	0	4	90	7	95	0.60	(0.17- 2.13)
CHOI	52	m	0	27	281	7	95	1.30	(0.55- 3.09)
CHOI	53	m	0	11	49	7	95	3.05	(1.11- 8.35)
CHOI	54	m	0	2	39	7	95	0.70	(0.14- 3.50)
CHOI	55	m	0	2	6	7	95	4.52	(0.77- 26.69)
CHOI	59	f	0	4	16	49	164	0.84	(0.27- 2.62)
CHOI	60	f	0	0	9	49	164	0.17~	(0.01- 3.06)
CHOI	61	f	0	1	1	49	164	3.35	(0.21- 54.50)
Subtotal CHOI								1.32	(0.83- 2.09)
CORREA	48	c	1	-	-	-	-	4.30	(2.60- 7.20)
CORREA	52	c	1	-	-	-	-	12.00	(7.30- 19.70)
Subtotal CORREA								7.28	(5.10- 10.39)
DOLL	72	m	1	-	-	-	-	0.50	(0.07- 3.63)
DOLL	73	m	1	-	-	-	-	0.80	(0.18- 3.60)
DOLL	74	m	1	-	-	-	-	1.20	(0.27- 5.35)
DOLL	75	m	1	-	-	-	-	1.10	(0.22- 5.60)
DOLL	79	f	1	-	-	-	-	1.10	(0.20- 6.05)
DOLL	80	f	1	-	-	-	-	2.30	(0.56- 9.48)
DOLL	81	f	1	-	-	-	-	4.10	(0.65- 25.88)
Subtotal DOLL								1.30	(0.71- 2.40)
DORGAN	126	m	2	-	-	-	-	3.23	(1.20- 8.70)
DORGAN	127	m	2	-	-	-	-	5.63	(2.22- 14.25)
DORGAN	105	f	3	-	-	-	-	2.87	(2.00- 4.11)
DORGAN	106	f	3	-	-	-	-	5.39	(3.73- 7.78)
Subtotal DORGAN								3.96	(3.11- 5.04)
DOSEME	8	m	2	-	-	-	-	1.80	(0.80- 4.10)
DOSEME	12	m	2	-	-	-	-	2.70	(1.60- 4.70)
DOSEME	16	m	2	-	-	-	-	3.20	(1.40- 7.00)
Subtotal DOSEME								2.56	(1.73- 3.79)
*ENGELA	71	m	7	-	-	-	-	2.60	(0.60- 11.00)
*ENGELA	72	m	7	-	-	-	-	8.50	(2.50- 29.00)
*ENGELA	73	m	7	-	-	-	-	7.10	(2.10- 24.00)
*ENGELA	74	m	7	-	-	-	-	9.30	(2.10- 41.00)
*ENGELA	75	m	7	-	-	-	-	17.00	(5.10- 56.00)
Subtotal ENGELA								8.06	(4.51- 14.41)
GER	10	c	8	-	-	-	-	0.62	(0.23- 1.72)
GER	11	c	8	-	-	-	-	2.10	(0.88- 5.04)
GER	12	c	8	-	-	-	-	0.88	(0.25- 3.03)
Subtotal GER								1.15	(0.64- 2.07)
HAENSZ	35	f	0	10	66	37	236	0.97	(0.46- 2.05)
HAENSZ	34	f	0	5	13	37	236	2.45	(0.83- 7.28)
Subtotal HAENSZ								1.30	(0.70- 2.42)
*HAMMON	108	m	1	-	-	-	-	1.83	(0.17- 20.22)
*HAMMON	109	m	1	-	-	-	-	2.83	(0.55- 14.60)
*HAMMON	110	m	1	-	-	-	-	6.44	(1.34- 31.02)
Subtotal HAMMON								3.71	(1.33- 10.33)
JEDRYC	36	m	3	-	-	-	-	2.19	(0.84- 5.72)
JEDRYC	37	m	3	-	-	-	-	4.38	(1.87- 10.28)
JEDRYC	38	m	3	-	-	-	-	5.11	(2.09- 12.53)
Subtotal JEDRYC								3.77	(2.24- 6.33)
KATSOU	13	f	1	-	-	-	-	1.35	(0.52- 3.49)
KATSOU	14	f	1	-	-	-	-	2.95	(0.76- 11.41)

Table 3G16 - 2

IESLC - Meta-anal of Ever Smoking (or Curr if Ever not avail) by Amount, Overview, Cigs (or Any Prod if Cigs not avail)
Adenocarcinoma
Most adjusted

REF	NRR	SEX	AD	Number Exposed Case	Number Exposed Cont	Non-exposed Case	Non-exposed Cont	RR	95.00%CI
Subtotal KATSOU								1.75	(0.80- 3.81)
KREYBE	5	m	1	-	-	-	-	2.65	(0.81- 8.70)
KREYBE	6	m	1	-	-	-	-	1.43	(0.36- 5.72)
KREYBE	7	m	1	-	-	-	-	3.99	(0.95- 16.81)
KREYBE	26	f	1	-	-	-	-	1.30	(0.60- 2.80)
KREYBE	61	f	1	-	-	-	-	0.88	(0.12- 6.63)
Subtotal KREYBE								1.71	(1.01- 2.89)
LAMTH	16	f	0	36	29	131	158	1.50	(0.87- 2.57)
LAMTH	17	f	0	27	14	131	158	2.33	(1.17- 4.62)
LAMTH	18	f	0	9	5	131	158	2.17	(0.71- 6.64)
Subtotal LAMTH								1.82	(1.22- 2.71)
LUBIN2	152	m	0	66	2194	57	2616	1.38	(0.96- 1.98)
LUBIN2	156	m	0	204	3385	57	2616	2.77	(2.05- 3.73)
LUBIN2	160	m	0	234	3108	57	2616	3.46	(2.57- 4.64)
LUBIN2	164	m	0	151	1746	57	2616	3.97	(2.91- 5.42)
LUBIN2	172	f	0	20	184	138	1180	0.93	(0.57- 1.52)
LUBIN2	176	f	0	43	234	138	1180	1.57	(1.09- 2.27)
LUBIN2	180	f	0	13	110	138	1180	1.01	(0.55- 1.84)
LUBIN2	184	f	0	9	39	138	1180	1.97	(0.94- 4.16)
Subtotal LUBIN2								2.28	(1.99- 2.60)
MATOS	57	m	2	-	-	-	-	2.80	(0.90- 8.50)
MATOS	59	m	2	-	-	-	-	7.00	(2.60- 19.10)
MATOS	61	m	2	-	-	-	-	8.40	(3.01- 22.20)
Subtotal MATOS								5.76	(3.17- 10.48)
MATSUD	7	m	0	6	1237	0	1255	13.19~	(0.74- 234.37)
MATSUD	8	m	0	13	1607	0	1255	21.09~	(1.25- 355.07)
MATSUD	9	m	0	4	470	0	1255	24.02~	(1.29- 446.92)
Subtotal MATSUD								18.81	(3.58- 98.87)
ORMOS	22	m	0	4	329	2	777	4.72	(0.86- 25.91)
ORMOS	23	m	0	3	577	2	777	2.02	(0.34- 12.13)
ORMOS	24	m	0	1	128	2	777	3.04	(0.27- 33.72)
Subtotal ORMOS								3.13	(1.04- 9.39)
OSANN	55	m	2	-	-	-	-	16.50	(9.30- 29.30)
OSANN	63	m	2	-	-	-	-	37.50	(21.30- 66.00)
OSANN	56	f	2	-	-	-	-	8.80	(6.10- 12.80)
OSANN	64	f	2	-	-	-	-	24.20	(15.80- 37.20)
Subtotal OSANN								16.57	(13.16- 20.85)
OSANN2	34	f	1	-	-	-	-	0.90	(0.30- 2.70)
OSANN2	35	f	1	-	-	-	-	3.80	(1.60- 8.80)
Subtotal OSANN2								2.21	(1.13- 4.34)
SOBUE	56	m	0	63	157	27	128	1.90	(1.15- 3.16)
SOBUE	57	m	0	95	222	27	128	2.03	(1.26- 3.28)
SOBUE	58	m	0	76	187	27	128	1.93	(1.18- 3.16)
Subtotal SOBUE								1.95	(1.47- 2.60)
SVENSS	9	f	1	-	-	-	-	2.20	(1.00- 5.80)
SVENSS	14	f	1	-	-	-	-	5.40	(2.40- 13.20)
SVENSS	19	f	1	-	-	-	-	19.70	(1.70- 228.29)
Subtotal SVENSS								3.87	(2.14- 7.00)
TSUGAN	4	m	0	12	14	18	17	0.81	(0.29- 2.24)
TSUGAN	5	m	0	23	23	18	17	0.94	(0.39- 2.28)
TSUGAN	6	m	0	10	13	18	17	0.73	(0.25- 2.09)
Subtotal TSUGAN								0.84	(0.48- 1.47)
WAKAI	52	m	1	-	-	-	-	1.30	(0.52- 3.21)
WAKAI	53	m	1	-	-	-	-	1.93	(0.84- 4.44)
WAKAI	54	m	1	-	-	-	-	4.53	(1.89- 10.90)
Subtotal WAKAI								2.27	(1.37- 3.75)
WU	8	f	2	-	-	-	-	2.70	(1.40- 5.40)
WU	9	f	2	-	-	-	-	6.50	(3.10- 13.90)
Subtotal WU								4.00	(2.42- 6.61)
WUWILL	16	f	3	-	-	-	-	1.43	(1.07- 1.90)
WUWILL	17	f	3	-	-	-	-	2.26	(1.35- 3.78)
Subtotal WUWILL								1.59	(1.24- 2.05)
WYNDE2	10	m	0	2	114	5	105	0.37	(0.07- 1.94)
WYNDE2	11	m	0	14	203	5	105	1.45	(0.51- 4.13)
WYNDE2	12	m	0	14	83	5	105	3.54	(1.23- 10.23)
WYNDE2	13	m	0	16	112	5	105	3.00	(1.06- 8.48)
Subtotal WYNDE2								1.98	(1.12- 3.51)
WYNDE3	24	m	0	1	42	6	88	0.35	(0.04- 2.99)
WYNDE3	25	m	0	20	114	6	88	2.57	(0.99- 6.68)
WYNDE3	26	m	0	34	82	6	88	6.08	(2.43- 15.24)
WYNDE3	27	m	0	9	26	6	88	5.08	(1.65- 15.59)

Table 3G16 - 2

IESLC - Meta-anal of Ever Smoking (or Curr if Ever not avail) by Amount, Overview, Cigs (or Any Prod if Cigs not avail)
Adenocarcinoma
Most adjusted

REF	NRR	SEX	AD	Number Exposed Case	Number Exposed Cont	Non-exposed Case	Non-exposed Cont	RR	95.00%CI
WYNDE3	71	f	0	2	19	15	76	0.53	(0.11- 2.53)
WYNDE3	72	f	0	11	24	15	76	2.32	(0.94- 5.73)
WYNDE3	73	f	0	7	10	15	76	3.55	(1.16- 10.80)
WYNDE3	74	f	0	1	3	15	76	1.69	(0.16- 17.36)
Subtotal WYNDE3								2.82	(1.87- 4.25)
WYNDE4	37	m	0	3	82	4	115	1.05	(0.23- 4.83)
WYNDE4	38	m	0	6	147	4	115	1.17	(0.32- 4.26)
WYNDE4	39	m	0	15	274	4	115	1.57	(0.51- 4.84)
WYNDE4	40	m	0	4	98	4	115	1.17	(0.29- 4.82)
WYNDE4	41	m	0	7	64	4	115	3.14	(0.89- 11.15)
WYNDE4	55	f	2	-	-	-	-	1.33	(0.29- 6.07)
Subtotal WYNDE4								1.51	(0.88- 2.60)
WYNDE6	24	m	0	42	122	58	617	3.66	(2.35- 5.70)
WYNDE6	33	m	0	191	293	58	617	6.93	(5.01- 9.60)
WYNDE6	42	m	0	136	129	58	617	11.22	(7.81- 16.09)
WYNDE6	51	m	0	282	197	58	617	15.23	(11.00- 21.07)
WYNDE6	213	f	0	39	109	119	856	2.57	(1.70- 3.89)
WYNDE6	222	f	0	176	165	119	856	7.67	(5.76- 10.21)
WYNDE6	231	f	0	100	50	119	856	14.39	(9.74- 21.25)
WYNDE6	240	f	0	157	52	119	856	21.72	(15.04- 31.37)
Subtotal WYNDE6								8.99	(7.93- 10.19)
ZHENG	6	m	0	18	40	29	94	1.46	(0.73- 2.92)
ZHENG	7	m	0	35	66	29	94	1.72	(0.96- 3.08)
ZHENG	8	m	0	53	89	29	94	1.93	(1.13- 3.30)
ZHENG	9	m	0	17	23	29	94	2.40	(1.13- 5.08)
ZHENG	19	f	0	13	29	119	184	0.69	(0.35- 1.39)
ZHENG	20	f	0	20	15	119	184	2.06	(1.02- 4.19)
Subtotal ZHENG								1.62	(1.24- 2.11)
Partial Totals				2633	19458	2756	31915		

*prospective study ~ With 0.5 adjustment for zero

REF	NRR	SEX	AD	Ys	Ws	Qs	Ps
ALDERS	46	m	1	1.03	3.70	0.84	0.0476
ALDERS	47	m	1	0.98	3.91	1.07	0.0520
ALDERS	48	m	1	1.20	4.83	0.45	0.0084
ALDERS	49	f	1	1.02	13.70	3.25	0.0002
ALDERS	50	f	1	1.52	13.21	0.00	0.0000
ALDERS	51	f	1	1.20	10.32	0.99	0.0001
Subtotal ALDERS				1.21	49.67	6.61	
BARBON	79	m	3	1.61	5.93	0.06	0.0001
BARBON	80	m	3	2.12	5.74	2.14	0.0000
BARBON	81	m	3	2.49	5.30	5.16	0.0000
Subtotal BARBON				2.06	16.97	7.37	
*BOUCOT	148	m	2	2.11	0.47	0.17	0.1472
*BOUCOT	149	m	2	2.89	0.46	0.89	0.0491
Subtotal BOUCOT				2.49	0.94	1.06	
BROWN2	34	m	2	1.82	67.36	6.84	0.0000
BROWN2	44	m	2	2.37	107.03	79.95	0.0000
BROWN2	33	f	2	1.76	90.29	5.73	0.0000
BROWN2	43	f	2	2.15	145.78	60.80	0.0000
Subtotal BROWN2				2.07	410.46	153.31	
CHOI	51	m	0	-0.51	2.41	9.76	0.4323
CHOI	52	m	0	0.27	5.16	7.93	0.5467
CHOI	53	m	0	1.11	3.78	0.58	0.0304
CHOI	54	m	0	-0.36	1.47	5.14	0.6600
CHOI	55	m	0	1.51	1.22	0.00	0.0956
CHOI	59	f	0	-0.18	2.95	8.37	0.7595
CHOI	60	f	0	-1.74	0.47	4.95	0.2324
CHOI	61	f	0	1.21	0.49	0.04	0.3961
Subtotal CHOI				0.27	17.95	36.78	
CORREA	48	c	1	1.46	14.81	0.03	0.0000
CORREA	52	c	1	2.48	15.59	14.94	0.0000
Subtotal CORREA				1.98	30.40	14.97	
DOLL	72	m	1	-0.69	0.99	4.77	0.4914
DOLL	73	m	1	-0.22	1.71	5.12	0.7703
DOLL	74	m	1	0.18	1.72	3.02	0.8109
DOLL	75	m	1	0.10	1.47	2.92	0.9081
DOLL	79	f	1	0.10	1.32	2.63	0.9127
DOLL	80	f	1	0.83	1.92	0.87	0.2485
DOLL	81	f	1	1.41	1.13	0.01	0.1333

Table 3G16 - 2

IESLC - Meta-anal of Ever Smoking (or Curr if Ever not avail) by Amount, Overview, Cigs (or Any Prod if Cigs not avail)
Adenocarcinoma
Most adjusted

REF	NRR	SEX	AD	Ys	Ws	Qs	Ps
Subtotal DOLL				0.26	10.26	19.33	
DORGAN	126	m	2	1.17	3.92	0.44	0.0203
DORGAN	127	m	2	1.73	4.45	0.22	0.0003
DORGAN	105	f	3	1.05	29.62	6.04	0.0000
DORGAN	106	f	3	1.68	28.43	0.91	0.0000
Subtotal DORGAN				1.38	66.41	7.60	
DOSEME	8	m	2	0.59	5.75	4.85	0.1585
DOSEME	12	m	2	0.99	13.23	3.48	0.0003
DOSEME	16	m	2	1.16	5.93	0.70	0.0046
Subtotal DOSEME				0.94	24.92	9.03	
*ENGELA	71	m	7	0.96	1.82	0.55	0.1979
*ENGELA	72	m	7	2.14	2.56	1.03	0.0006
*ENGELA	73	m	7	1.96	2.59	0.53	0.0016
*ENGELA	74	m	7	2.23	1.74	0.91	0.0033
*ENGELA	75	m	7	2.83	2.68	4.71	0.0000
Subtotal ENGELA				2.09	11.38	7.74	
GER	10	c	8	-0.48	3.80	14.94	0.3517
GER	11	c	8	0.74	5.04	2.94	0.0956
GER	12	c	8	-0.13	2.47	6.59	0.8408
Subtotal GER				0.14	11.31	24.48	
HAENSZ	35	f	0	-0.03	6.83	16.20	0.9289
HAENSZ	34	f	0	0.90	3.24	1.20	0.1060
Subtotal HAENSZ				0.27	10.07	17.40	
*HAMMON	108	m	1	0.60	0.67	0.55	0.6201
*HAMMON	109	m	1	1.04	1.43	0.31	0.2136
*HAMMON	110	m	1	1.86	1.56	0.20	0.0201
Subtotal HAMMON				1.31	3.66	1.05	
JEDRYC	36	m	3	0.78	4.18	2.18	0.1092
JEDRYC	37	m	3	1.48	5.29	0.00	0.0007
JEDRYC	38	m	3	1.63	4.79	0.08	0.0004
Subtotal JEDRYC				1.33	14.26	2.26	
KATSOU	13	f	1	0.30	4.24	6.16	0.5366
KATSOU	14	f	1	1.08	2.09	0.38	0.1175
Subtotal KATSOU				0.56	6.33	6.54	
KREYBE	5	m	1	0.97	2.73	0.77	0.1076
KREYBE	6	m	1	0.36	2.01	2.65	0.6122
KREYBE	7	m	1	1.38	1.86	0.03	0.0590
KREYBE	26	f	1	0.26	6.48	10.01	0.5044
KREYBE	61	f	1	-0.13	0.95	2.55	0.9006
Subtotal KREYBE				0.54	14.03	16.01	
LAMTH	16	f	0	0.40	13.12	15.94	0.1438
LAMTH	17	f	0	0.84	8.17	3.58	0.0158
LAMTH	18	f	0	0.78	3.08	1.64	0.1740
Subtotal LAMTH				0.60	24.36	21.16	
LUBIN2	152	m	0	0.32	29.82	41.77	0.0782
LUBIN2	156	m	0	1.02	43.25	10.32	0.0000
LUBIN2	160	m	0	1.24	44.40	3.14	0.0000
LUBIN2	164	m	0	1.38	39.81	0.65	0.0000
LUBIN2	172	f	0	-0.07	15.74	39.25	0.7715
LUBIN2	176	f	0	0.45	28.07	31.19	0.0167
LUBIN2	180	f	0	0.01	10.63	23.76	0.9727
LUBIN2	184	f	0	0.68	6.90	4.71	0.0741
Subtotal LUBIN2				0.82	218.62	154.80	
MATOS	57	m	2	1.03	3.05	0.69	0.0723
MATOS	59	m	2	1.95	3.86	0.75	0.0001
MATOS	61	m	2	2.13	3.85	1.49	0.0000
Subtotal MATOS				1.75	10.76	2.93	
MATSUD	7	m	0	2.58	0.46	0.53	0.0789
MATSUD	8	m	0	3.05	0.48	1.15	0.0343
MATSUD	9	m	0	3.18	0.45	1.26	0.0331
Subtotal MATSUD				2.93	1.40	2.94	
ORMOS	22	m	0	1.55	1.33	0.00	0.0738
ORMOS	23	m	0	0.70	1.20	0.77	0.4420
ORMOS	24	m	0	1.11	0.66	0.10	0.3661
Subtotal ORMOS				1.14	3.18	0.88	
OSANN	55	m	2	2.80	11.67	19.64	0.0000
OSANN	63	m	2	3.62	12.01	53.91	0.0000
OSANN	56	f	2	2.17	27.97	12.51	0.0000
OSANN	64	f	2	3.19	20.96	59.17	0.0000
Subtotal OSANN				2.81	72.61	145.24	
OSANN2	34	f	1	-0.11	3.18	8.26	0.8509

Table 3G16 - 2

IESLC - Meta-anal of Ever Smoking (or Curr if Ever not avail) by Amount, Overview, Cigs (or Any Prod if Cigs not avail)
Adenocarcinoma
Most adjusted

REF	NRR	SEX	AD	Ys	Ws	Qs	Ps
OSANN2	35	f	1	1.34	5.29	0.15	0.0021
Subtotal OSANN2				0.79	8.47	8.42	
SOBUE	56	m	0	0.64	14.90	11.10	0.0130
SOBUE	57	m	0	0.71	16.70	10.65	0.0038
SOBUE	58	m	0	0.66	15.78	11.41	0.0092
Subtotal SOBUE				0.67	47.39	33.16	
SVENSS	9	f	1	0.79	4.97	2.56	0.0787
SVENSS	14	f	1	1.69	5.29	0.17	0.0001
SVENSS	19	f	1	2.98	0.64	1.39	0.0171
Subtotal SVENSS				1.35	10.90	4.12	
TSUGAN	4	m	0	-0.21	3.72	10.96	0.6838
TSUGAN	5	m	0	-0.06	4.97	12.14	0.8986
TSUGAN	6	m	0	-0.32	3.43	11.44	0.5538
Subtotal TSUGAN				-0.18	12.12	34.53	
WAKAI	52	m	1	0.26	4.64	7.17	0.5721
WAKAI	53	m	1	0.66	5.54	3.99	0.1216
WAKAI	54	m	1	1.51	5.00	0.00	0.0007
Subtotal WAKAI				0.82	15.19	11.16	
WU	8	f	2	0.99	8.43	2.22	0.0039
WU	9	f	2	1.87	6.82	0.91	0.0000
Subtotal WU				1.39	15.26	3.13	
WUWILL	16	f	3	0.36	46.61	61.45	0.0146
WUWILL	17	f	3	0.82	14.49	6.91	0.0019
Subtotal WUWILL				0.47	61.10	68.37	
WYNDE2	10	m	0	-1.00	1.39	8.73	0.2387
WYNDE2	11	m	0	0.37	3.50	4.51	0.4885
WYNDE2	12	m	0	1.26	3.41	0.20	0.0195
WYNDE2	13	m	0	1.10	3.56	0.59	0.0382
Subtotal WYNDE2				0.69	11.86	14.03	
WYNDE3	24	m	0	-1.05	0.83	5.44	0.3372
WYNDE3	25	m	0	0.95	4.22	1.33	0.0521
WYNDE3	26	m	0	1.81	4.55	0.41	0.0001
WYNDE3	27	m	0	1.62	3.05	0.04	0.0045
WYNDE3	71	f	0	-0.63	1.58	7.20	0.4293
WYNDE3	72	f	0	0.84	4.71	2.07	0.0675
WYNDE3	73	f	0	1.27	3.10	0.18	0.0258
WYNDE3	74	f	0	0.52	0.71	0.68	0.6593
Subtotal WYNDE3				1.04	22.76	17.36	
WYNDE4	37	m	0	0.05	1.66	3.51	0.9482
WYNDE4	38	m	0	0.16	2.31	4.19	0.8077
WYNDE4	39	m	0	0.45	3.04	3.37	0.4291
WYNDE4	40	m	0	0.16	1.93	3.49	0.8243
WYNDE4	41	m	0	1.15	2.40	0.31	0.0761
WYNDE4	55	f	2	0.29	1.66	2.48	0.7132
Subtotal WYNDE4				0.41	12.99	17.34	
WYNDE6	24	m	0	1.30	19.66	0.85	0.0000
WYNDE6	33	m	0	1.94	36.35	6.74	0.0000
WYNDE6	42	m	0	2.42	29.44	24.45	0.0000
WYNDE6	51	m	0	2.72	36.38	53.90	0.0000
WYNDE6	213	f	0	0.95	22.53	7.08	0.0000
WYNDE6	222	f	0	2.04	46.92	13.26	0.0000
WYNDE6	231	f	0	2.67	25.27	34.02	0.0000
WYNDE6	240	f	0	3.08	28.43	70.28	0.0000
Subtotal WYNDE6				2.20	244.98	210.59	
ZHENG	6	m	0	0.38	7.96	10.13	0.2870
ZHENG	7	m	0	0.54	11.26	10.47	0.0692
ZHENG	8	m	0	0.66	13.29	9.57	0.0165
ZHENG	9	m	0	0.87	6.78	2.71	0.0229
ZHENG	19	f	0	-0.37	7.98	28.00	0.3003
ZHENG	20	f	0	0.72	7.66	4.69	0.0452
Subtotal ZHENG				0.48	54.94	65.56	

N	136
NS	34

Table 3G16 - 3

IESLC - Meta-anal of Ever Smoking (or Curr if Ever not avail) by Amount, Overview, Cigs (or Any Prod if Cigs not avail)
Adenocarcinoma
Most adjusted

	Sex			
	combined	male	female	Total
N	5	83	48	136
NS	2	25	19	46

In this overview table, other than the "N" rows, entries in the "absent" and "Total" columns may be invalid and should be ignored

	Amount smoked (broad categories)				
	absent	<20k5	6-44k20	>20k45	Total
N	35	39	28	34	136
NS	21	28	23	28	100
Wt	560.17	456.45	270.48	260.78	1547.89
Het Chi	298.30	235.32	115.19	253.85	1147.26
Het df	34	38	27	33	135
Het P	***	***	***	***	***
Fixed RR	6.15	2.70	3.58	7.20	4.51
RRl	5.66	2.47	3.18	6.38	4.29
RRu	6.68	2.96	4.04	8.13	4.74
P	+++	+++	+++	+++	+++
Random RR	3.85	1.83	2.73	4.80	3.01
RRl	2.87	1.40	2.06	3.29	2.56
RRu	5.16	2.39	3.61	7.01	3.55
P	+++	+++	+++	+++	+++

	Amount smoked (narrow categories)							
	absent	<10k1	2-19k10	11-29k20	21-39k30	31-98k40	>40k99	Total
N	86	12	7	21	6	1	3	136
NS	34	7	5	18	5	1	2	71
Wt	1065.17	72.03	91.11	247.75	65.39	1.47	4.98	1547.89
Het Chi	737.27	8.39	12.29	95.97	21.89	0.00	0.70	1147.26
Het df	85	11	6	20	5	0	2	135
Het P	***	N.S.	(*)	***	***	N.S.	N.S.	***
Fixed RR	5.28	1.13	2.14	3.70	9.96	0.70	4.22	4.51
RRl	4.97	0.90	1.75	3.26	7.81	0.14	1.75	4.29
RRu	5.60	1.42	2.63	4.19	12.69	3.50	10.16	4.74
P	+++	N.S.	+++	+++	+++	N.S.	++	+++
Random RR	3.48	1.13	2.04	2.84	5.96	0.70	4.22	3.01
RRl	2.84	0.90	1.40	2.09	3.16	0.14	1.75	2.56
RRu	4.25	1.42	2.97	3.86	11.24	3.50	10.16	3.55
P	+++	N.S.	+++	+++	+++	N.S.	++	+++

MALES

	Amount smoked (broad categories)				
	absent	<20k5	6-44k20	>20k45	Total
N	19	23	20	21	83
NS	14	23	20	21	78
Wt	236.38	190.33	176.05	159.04	761.80
Het Chi	130.50	99.84	59.31	136.78	530.96
Het df	18	22	19	20	82
Het P	***	***	***	***	***
Fixed RR	6.82	2.96	3.24	5.88	4.52
RRl	6.00	2.57	2.79	5.04	4.21
RRu	7.74	3.42	3.75	6.87	4.85
P	+++	+++	+++	+++	+++
Random RR	4.40	2.09	2.69	4.68	3.20
RRl	2.83	1.44	1.98	2.93	2.60
RRu	6.85	3.02	3.65	7.47	3.94
P	+++	+++	+++	+++	+++

Table 3G16 - 3

IESLC - Meta-anal of Ever Smoking (or Curr if Ever not avail) by Amount, Overview, Cigs (or Any Prod if Cigs not avail)
Adenocarcinoma
Most adjusted

MALES

	absent	<10k1	2-19k10	11-29k20	21-39k30	31-98k40	>40k99	Total
	Amount smoked (narrow categories)							
N	48	7	5	15	5	1	2	83
NS	25	7	5	15	5	1	2	59
Wt	452.58	43.74	61.12	158.50	40.11	1.47	4.27	761.80
Het Chi	304.97	3.50	8.38	46.13	16.31	0.00	0.01	530.96
Het df	47	6	4	14	4	0	1	82
Het P	***	N.S.	(*)	***	**	N.S.	N.S.	***
Fixed RR	5.89	1.36	2.47	3.29	7.90	0.70	4.91	4.52
RRl	5.38	1.01	1.92	2.81	5.80	0.14	1.90	4.21
RRu	6.46	1.82	3.17	3.84	10.76	3.50	12.68	4.85
P	+++	+	+++	+++	+++	N.S.	++	+++
Random RR	3.94	1.36	2.20	2.69	4.29	0.70	4.91	3.20
RRl	2.99	1.01	1.32	1.93	1.79	0.14	1.90	2.60
RRu	5.18	1.82	3.67	3.75	10.32	3.50	12.68	3.94
P	+++	+	++	+++	++	N.S.	++	+++

FEMALES

	absent	<20k5	6-44k20	>20k45	Total
	Amount smoked (broad categories)				
N	15	15	7	11	48
NS	14	15	7	11	47
Wt	308.98	262.33	89.39	83.69	744.39
Het Chi	162.18	125.09	47.81	85.32	576.85
Het df	14	14	6	10	47
Het P	***	***	***	***	***
Fixed RR	5.78	2.58	4.52	10.22	4.50
RRl	5.17	2.29	3.67	8.25	4.19
RRu	6.46	2.92	5.56	12.67	4.84
P	+++	+++	+++	+++	+++
Random RR	3.22	1.65	2.83	5.21	2.77
RRl	2.03	1.08	1.40	2.50	2.09
RRu	5.11	2.50	5.72	10.86	3.68
P	+++	+	++	+++	+++

	absent	<10k1	2-19k10	11-29k20	21-39k30	31-98k40	>40k99	Total
	Amount smoked (narrow categories)							
N	34	5	2	5	1		1	48
NS	18	5	2	5	1		1	31
Wt	575.92	28.29	29.99	84.21	25.27		0.71	744.39
Het Chi	386.44	1.22	0.26	40.51	0.00		0.00	576.85
Het df	33	4	1	4	0		0	47
Het P	***	N.S.	N.S.	***	N.S.		N.S.	***
Fixed RR	4.86	0.85	1.61	4.77	14.39		1.69	4.50
RRl	4.48	0.59	1.13	3.86	9.74		0.16	4.19
RRu	5.28	1.23	2.30	5.91	21.25		17.36	4.84
P	+++	N.S.	++	+++	+++		N.S.	+++
Random RR	3.04	0.85	1.61	3.42	14.39		1.69	2.77
RRl	2.21	0.59	1.13	1.58	9.74		0.16	2.09
RRu	4.19	1.23	2.30	7.42	21.25		17.36	3.68
P	+++	N.S.	++	++	+++		N.S.	+++

Table 3G16 - 4

IESLC - Meta-anal of Ever Smoking (or Curr if Ever not avail) by Amount, Overview, Cigs (or Any Prod if Cigs not avail)
Adenocarcinoma
Least adjusted

REF	NRR	X	SEX	AGEL	AGEH	RACE	YF	LC TYPE	LOC	START	ST	NLC	R	VB	P	H	AD	SM	PRODUCT	exL	exH	S1	S2	DENOM	De
ALDERS	46		m	0	0	all	-	not q+s	Eu:UK	1977	CC	1448	n	V	n	n	1	ev	cig only	1	17	1	0	nev+2	ot
ALDERS	47		m	0	0	all	-	not q+s	Eu:UK	1977	CC	1448	n	V	n	n	1	ev	cig only	18	27	2	3	nev+2	ot
ALDERS	48		m	0	0	all	-	not q+s	Eu:UK	1977	CC	1448	n	V	n	n	1	ev	cig only	28	99	3	0	nev+2	ot
ALDERS	49		f	0	0	all	-	not q+s	Eu:UK	1977	CC	1448	n	V	n	n	1	ev	cig only	1	17	1	0	nev+2	ot
ALDERS	50		f	0	0	all	-	not q+s	Eu:UK	1977	CC	1448	n	V	n	n	1	ev	cig only	18	27	2	3	nev+2	ot
ALDERS	51		f	0	0	all	-	not q+s	Eu:UK	1977	CC	1448	n	V	n	n	1	ev	cig only	28	99	3	0	nev+2	ot
BARBON	45	x	m	0	0	all	-	a	Eu:wst	1979	CC	755	n	bl	y	y	0	ev	all/unsp	1	9	1	1	nev any	st
BARBON	46	x	m	0	0	all	-	a	Eu:wst	1979	CC	755	n	bl	y	y	0	ev	all/unsp	10	19	0	2	nev any	st
BARBON	47	x	m	0	0	all	-	a	Eu:wst	1979	CC	755	n	bl	y	y	0	ev	all/unsp	20	29	2	3	nev any	st
BARBON	48	x	m	0	0	all	-	a	Eu:wst	1979	CC	755	n	bl	y	y	0	ev	all/unsp	30	39	0	4	nev any	st
BARBON	49	x	m	0	0	all	-	a	Eu:wst	1979	CC	755	n	bl	y	y	0	ev	all/unsp	40	99	3	0	nev any	st
BOUCOT	27	x	m	0	0	all	0	a	NAmer	1951	pr	121	n	bl	n	n	0	cu	cig only	1	20	0	0	nev any	ot
BOUCOT	28	x	m	0	0	all	0	a	NAmer	1951	pr	121	n	bl	n	n	0	cu	cig only	21	99	3	0	nev any	ot
BROWN2	34		m	0	0	wh	-	a	NAmer	1984	CC	14596	n	bl	n	y	2	ev	cig+/-ot	1	19	1	0	nev cigs	or
BROWN2	44		m	0	0	wh	-	a	NAmer	1984	CC	14596	n	bl	n	y	2	ev	cig+/-ot	20	99	0	0	nev cigs	or
BROWN2	33		f	0	0	wh	-	a	NAmer	1984	CC	14596	n	bl	n	y	2	ev	cig+/-ot	1	19	1	0	nev cigs	or
BROWN2	43		f	0	0	wh	-	a	NAmer	1984	CC	14596	n	bl	n	y	2	ev	cig+/-ot	20	99	0	0	nev cigs	or
CHOI	51		m	0	0	all	-	a	As:oth	1985	CC	375	n	bl	n	n	0	ev	cig+/-ot	1	10	1	0	nev cigs	st
CHOI	52		m	0	0	all	-	a	As:oth	1985	CC	375	n	bl	n	n	0	ev	cig+/-ot	11	20	2	3	nev cigs	st
CHOI	53		m	0	0	all	-	a	As:oth	1985	CC	375	n	bl	n	n	0	ev	cig+/-ot	21	30	0	4	nev cigs	st
CHOI	54		m	0	0	all	-	a	As:oth	1985	CC	375	n	bl	n	n	0	ev	cig+/-ot	31	40	0	5	nev cigs	st
CHOI	55		m	0	0	all	-	a	As:oth	1985	CC	375	n	bl	n	n	0	ev	cig+/-ot	41	99	3	6	nev cigs	st
CHOI	59		f	0	0	all	-	a	As:oth	1985	CC	375	n	bl	n	n	0	ev	cig+/-ot	1	10	1	0	nev cigs	st
CHOI	60		f	0	0	all	-	a	As:oth	1985	CC	375	n	bl	n	n	0	ev	cig+/-ot	11	30	2	0	nev cigs	ot
CHOI	61		f	0	0	all	-	a	As:oth	1985	CC	375	n	bl	n	n	0	ev	cig+/-ot	31	99	3	0	nev cigs	st
CORREA	48		c	0	0	all	-	a	NAmer	1979	CC	1359	n	bl	y	n	1	cu	cig+/-ot	1	20	0	0	nev cigs	or
CORREA	52		c	0	0	all	-	a	NAmer	1979	CC	1359	n	bl	y	n	1	cu	cig+/-ot	21	99	3	0	nev cigs	or
DOLL	58	x	m	0	0	all	-	KII	Eu:UK	1948	CC	1465	n	V	n	n	0	ev	all/unsp	1	4	0	1	nev any	st
DOLL	59	x	m	0	0	all	-	KII	Eu:UK	1948	CC	1465	n	V	n	n	0	ev	all/unsp	5	14	1	2	nev any	st
DOLL	60	x	m	0	0	all	-	KII	Eu:UK	1948	CC	1465	n	V	n	n	0	ev	all/unsp	15	24	2	3	nev any	st
DOLL	61	x	m	0	0	all	-	KII	Eu:UK	1948	CC	1465	n	V	n	n	0	ev	all/unsp	25	99	3	0	nev any	st
DOLL	65	x	f	0	0	all	-	KII	Eu:UK	1948	CC	1465	n	V	n	n	0	ev	all/unsp	1	4	0	1	nev any	st
DOLL	66	x	f	0	0	all	-	KII	Eu:UK	1948	CC	1465	n	V	n	n	0	ev	all/unsp	5	14	1	2	nev any	st
DOLL	67	x	f	0	0	all	-	KII	Eu:UK	1948	CC	1465	n	V	n	n	0	ev	all/unsp	15	99	0	0	nev any	st
DORGAN	126		m	0	0	wh	-	a	NAmer	1980	CC	2026	n	bl	y	y	2	ev	cig+/-ot	1	19	1	0	nev any	ot
DORGAN	127		m	0	0	wh	-	a	NAmer	1980	CC	2026	n	bl	y	y	2	ev	cig+/-ot	20	99	0	0	nev any	ot
DORGAN	105		f	0	0	all	-	a	NAmer	1980	CC	2026	n	bl	y	y	3	ev	cig+/-ot	1	19	1	0	nev any	ot
DORGAN	106		f	0	0	all	-	a	NAmer	1980	CC	2026	n	bl	y	y	3	ev	cig+/-ot	20	99	0	0	nev any	ot
DOSEME	8		m	0	0	all	-	not q+s	Eu:bal	1979	CC	1210	n	bl	n	n	2	ev	cig+/-ot	1	10	1	0	nev cigs	or
DOSEME	12		m	0	0	all	-	not q+s	Eu:bal	1979	CC	1210	n	bl	n	n	2	ev	cig+/-ot	11	20	2	3	nev cigs	or
DOSEME	16		m	0	0	all	-	not q+s	Eu:bal	1979	CC	1210	n	bl	n	n	2	ev	cig+/-ot	21	99	3	0	nev cigs	or
ENGELA	71		m	0	0	all	0	a	Eu:Sca	1964	pr	435	n	bl	n	n	7	cu	cig+/-ot	1	4	0	1	nev cigs	or
ENGELA	72		m	0	0	all	0	a	Eu:Sca	1964	pr	435	n	bl	n	n	7	cu	cig+/-ot	5	9	1	0	nev cigs	or
ENGELA	73		m	0	0	all	0	a	Eu:Sca	1964	pr	435	n	bl	n	n	7	cu	cig+/-ot	10	14	0	2	nev cigs	or
ENGELA	74		m	0	0	all	0	a	Eu:Sca	1964	pr	435	n	bl	n	n	7	cu	cig+/-ot	15	19	0	0	nev cigs	or
ENGELA	75		m	0	0	all	0	a	Eu:Sca	1964	pr	435	n	bl	n	n	7	cu	cig+/-ot	20	99	0	0	nev cigs	or
GER	2	x	c	0	0	all	-	a	As:oth	1990	CC	141	n	ot	y	n	0	ev	all/unsp	1	10	1	0	nev any	st
GER	3	x	c	0	0	all	-	a	As:oth	1990	CC	141	n	ot	y	n	0	ev	all/unsp	11	20	2	3	nev any	st
GER	4	x	c	0	0	all	-	a	As:oth	1990	CC	141	n	ot	y	n	0	ev	all/unsp	21	99	3	0	nev any	st
HAENSZ	35		f	0	0	all	-	a	NAmer	1955	CC	158	n	bl	n	y	0	cu	cig+/-ot	1	20	0	0	nev any	or
HAENSZ	34		f	0	0	all	-	a	NAmer	1955	CC	158	n	bl	n	y	0	cu	cig+/-ot	21	99	3	0	nev any	or
HAMMON	108		m	0	0	wh	0	a	NAmer	1952	pr	448	n	bl	n	n	1	cu	cig only	1	9	1	1	nev any	ot
HAMMON	109		m	0	0	wh	0	a	NAmer	1952	pr	448	n	bl	n	n	1	cu	cig only	10	20	2	0	nev any	ot
HAMMON	110		m	0	0	wh	0	a	NAmer	1952	pr	448	n	bl	n	n	1	cu	cig only	21	39	0	4	nev any	ot
JEDRYC	15	x	m	0	0	all	-	a	Eu:est	1980	CC	1630	n	bl	y	n	0	ev	cig+/-ot	1	9	1	1	nev any	st
JEDRYC	16	x	m	0	0	all	-	a	Eu:est	1980	CC	1630	n	bl	y	n	0	ev	cig+/-ot	10	19	0	2	nev any	st
JEDRYC	17	x	m	0	0	all	-	a	Eu:est	1980	CC	1630	n	bl	y	n	0	ev	cig+/-ot	20	29	2	3	nev any	st
JEDRYC	18	x	m	0	0	all	-	a	Eu:est	1980	CC	1630	n	bl	y	n	0	ev	cig+/-ot	30	39	0	4	nev any	st
JEDRYC	19	x	m	0	0	all	-	a	Eu:est	1980	CC	1630	n	bl	y	n	0	ev	cig+/-ot	40	99	3	0	nev any	st
KATSOU	17	x	f	0	0	all	-	a	Eu:bal	1987	CC	101	n	bl	n	n	0	cu	all/unsp	1	20	0	0	nev any	st
KATSOU	18	x	f	0	0	all	-	a	Eu:bal	1987	CC	101	n	bl	n	n	0	cu	all/unsp	21	99	3	0	nev any	st
KREYBE	17	x	m	0	0	all	-	KII	Eu:Sca	1948	CC	300	n	bl	n	y	0	ev	all/unsp	1	14	1	0	nev any	st
KREYBE	18	x	m	0	0	all	-	KII	Eu:Sca	1948	CC	300	n	bl	n	y	0	ev	all/unsp	15	24	2	3	nev any	st
KREYBE	19	x	m	0	0	all	-	KII	Eu:Sca	1948	CC	300	n	bl	n	y	0	ev	all/unsp	25	99	3	0	nev any	st
KREYBE	34	x	f	0	0	all	-	KII	Eu:Sca	1948	CC	300	n	bl	n	y	0	ev	all/unsp	1	14	1	0	nev any	st
KREYBE	35	x	f	0	0	all	-	KII	Eu:Sca	1948	CC	300	n	bl	n	y	0	ev	all/unsp	15	99	0	0	nev any	st
LAMTH	16		f	0	0	ch	-	a	As:HK	1983	CC	445	n	bl	n	n	0	ev	all/unsp	1	10	1	0	nev any	or
LAMTH	17		f	0	0	ch	-	a	As:HK	1983	CC	445	n	bl	n	n	0	ev	all/unsp	11	20	2	3	nev any	or
LAMTH	18		f	0	0	ch	-	a	As:HK	1983	CC	445	n	bl	n	n	0	ev	all/unsp	21	99	3	0	nev any	or
LUBIN2	152		m	0	0	all	-	a	Eu:mul	1976	CC	7804	n	bl	n	y	0	ev	cig+/-ot	1	9	1	1	nev any	st
LUBIN2	156		m	0	0	all	-	a	Eu:mul	1976	CC	7804	n	bl	n	y	0	ev	cig+/-ot	10	19	0	2	nev any	st
LUBIN2	160		m	0	0	all	-	a	Eu:mul	1976	CC	7804	n	bl	n	y	0	ev	cig+/-ot	20	29	2	3	nev any	st

Table 3G16 - 4

IESLC - Meta-anal of Ever Smoking (or Curr if Ever not avail) by Amount, Overview, Cigs (or Any Prod if Cigs not avail)
Adenocarcinoma
Least adjusted

REF	NRR	X	SEX	AGEL	AGEH	RACE	YF	LC TYPE	LOC	START	ST	NLC	R	VB	P	H	AD	SM	PRODUCT	exL	exH	S1	S2	DENOM	De
LUBIN2	164		m	0	0	all	-	a	Eu:mul	1976	CC	7804	n	bl	n	y	0	ev	cig+/-ot	30	99	3	0	nev any	st
LUBIN2	172		f	0	0	all	-	a	Eu:mul	1976	CC	7804	n	bl	n	y	0	ev	cig+/-ot	1	9	1	1	nev any	st
LUBIN2	176		f	0	0	all	-	a	Eu:mul	1976	CC	7804	n	bl	n	y	0	ev	cig+/-ot	10	19	0	2	nev any	st
LUBIN2	180		f	0	0	all	-	a	Eu:mul	1976	CC	7804	n	bl	n	y	0	ev	cig+/-ot	20	29	2	3	nev any	st
LUBIN2	184		f	0	0	all	-	a	Eu:mul	1976	CC	7804	n	bl	n	y	0	ev	cig+/-ot	30	99	3	0	nev any	st
MATOS	56	x	m	0	0	all	-	a	SCAmer	1994	CC	200	n	bl	n	n	0	ev	cig+/-ot	1	14	1	0	nev any	st
MATOS	58	x	m	0	0	all	-	a	SCAmer	1994	CC	200	n	bl	n	n	0	ev	cig+/-ot	15	24	2	3	nev any	st
MATOS	60	x	m	0	0	all	-	a	SCAmer	1994	CC	200	n	bl	n	n	0	ev	cig+/-ot	25	99	3	0	nev any	st
MATSUD	7		m	0	0	all	-	a	As:Jap	1965	CC	179	n	bl	n	n	0	ev	cig+/-ot	1	10	1	0	nev cigs	ot
MATSUD	8		m	0	0	all	-	a	As:Jap	1965	CC	179	n	bl	n	n	0	ev	cig+/-ot	11	20	2	3	nev cigs	ot
MATSUD	9		m	0	0	all	-	a	As:Jap	1965	CC	179	n	bl	n	n	0	ev	cig+/-ot	21	99	3	0	nev cigs	ot
ORMOS	22		m	0	0	all	-	KII	Eu:est	1947	CC	119	n	bl	y	y	0	ev	cig+/-ot	1	15	1	0	nev any	st
ORMOS	23		m	0	0	all	-	KII	Eu:est	1947	CC	119	n	bl	y	y	0	ev	cig+/-ot	16	30	2	0	nev any	st
ORMOS	24		m	0	0	all	-	KII	Eu:est	1947	CC	119	n	bl	y	y	0	ev	cig+/-ot	31	99	3	0	nev any	st
OSANN	55		m	0	0	all	-	a	NAmer	1984	CC	1986	n	bl	n	n	2	ev	cig+/-ot	1	39	0	0	nev cigs	or
OSANN	63		m	0	0	all	-	a	NAmer	1984	CC	1986	n	bl	n	n	2	ev	cig+/-ot	40	99	3	0	nev cigs	or
OSANN	56		f	0	0	all	-	a	NAmer	1984	CC	1986	n	bl	n	n	2	ev	cig+/-ot	1	39	0	0	nev cigs	or
OSANN	64		f	0	0	all	-	a	NAmer	1984	CC	1986	n	bl	n	n	2	ev	cig+/-ot	40	99	3	0	nev cigs	or
OSANN2	16	x	f	0	0	all	-	KII	NAmer	1964	ot	217	n	bl	n	y	0	ev	cig+/-ot	1	19	1	0	nev cigs	st
OSANN2	17	x	f	0	0	all	-	KII	NAmer	1964	ot	217	n	bl	n	y	0	ev	cig+/-ot	20	99	0	0	nev cigs	st
SOBUE	56		m	0	0	all	-	a	As:Jap	1986	CC	1376	n	bl	n	y	0	cu	cig+/-ot	1	19	1	0	nev cigs	st
SOBUE	57		m	0	0	all	-	a	As:Jap	1986	CC	1376	n	bl	n	y	0	cu	cig+/-ot	20	29	2	3	nev cigs	st
SOBUE	58		m	0	0	all	-	a	As:Jap	1986	CC	1376	n	bl	n	y	0	cu	cig+/-ot	30	99	3	0	nev cigs	st
SVENSS	29	x	f	0	0	all	-	a	Eu:Sca	1983	CC	210	n	bl	n	n	0	cu	all/unsp	1	10	1	0	nev any	st
SVENSS	34	x	f	0	0	all	-	a	Eu:Sca	1983	CC	210	n	bl	n	n	0	cu	all/unsp	11	20	2	3	nev any	st
SVENSS	39	x	f	0	0	all	-	a	Eu:Sca	1983	CC	210	n	bl	n	n	0	cu	all/unsp	21	99	3	0	nev any	st
TSUGAN	4		m	0	0	all	-	a	As:Jap	1976	CC	134	n	bl	n	y	0	cu	all/unsp	1	15	1	0	nev any	st
TSUGAN	5		m	0	0	all	-	a	As:Jap	1976	CC	134	n	bl	n	y	0	cu	all/unsp	16	35	2	0	nev any	st
TSUGAN	6		m	0	0	all	-	a	As:Jap	1976	CC	134	n	bl	n	y	0	cu	all/unsp	36	99	3	0	nev any	st
WAKAI	49	x	m	0	0	all	-	a	As:Jap	1988	CC	333	n	bl	n	y	0	cu	cig+/-ot	1	19	1	0	nev any	st
WAKAI	50	x	m	0	0	all	-	a	As:Jap	1988	CC	333	n	bl	n	y	0	cu	cig+/-ot	20	29	2	3	nev any	st
WAKAI	51	x	m	0	0	all	-	a	As:Jap	1988	CC	333	n	bl	n	y	0	cu	cig+/-ot	30	99	3	0	nev any	st
WU	3	x	f	0	0	wh	-	a	NAmer	1981	CC	220	n	bl	n	y	0	cu	all/unsp	1	20	0	0	nev any	st
WU	4	x	f	0	0	wh	-	a	NAmer	1981	CC	220	n	bl	n	y	0	cu	all/unsp	21	99	3	0	nev any	st
WUWILL	22	x	f	0	0	all	-	a	As:Chi	1985	CC	965	n	ot	n	n	0	ev	cig+/-ot	1	19	1	0	nev cigs	st
WUWILL	23	x	f	0	0	all	-	a	As:Chi	1985	CC	965	n	ot	n	n	0	ev	cig+/-ot	20	99	0	0	nev cigs	st
WYNDE2	10		m	0	0	all	-	KII	NAmer	1962	CC	404	n	bl	n	y	0	ev	cig+/-ot	1	10	1	0	nev any	st
WYNDE2	11		m	0	0	all	-	KII	NAmer	1962	CC	404	n	bl	n	y	0	ev	cig+/-ot	11	20	2	3	nev any	st
WYNDE2	12		m	0	0	all	-	KII	NAmer	1962	CC	404	n	bl	n	y	0	ev	cig+/-ot	21	34	0	4	nev any	st
WYNDE2	13		m	0	0	all	-	KII	NAmer	1962	CC	404	n	bl	n	y	0	ev	cig+/-ot	35	99	3	0	nev any	st
WYNDE3	24		m	0	0	all	-	KII	NAmer	1966	CC	350	n	bl	n	y	0	ev	cig+/-ot	1	9	1	1	nev any	st
WYNDE3	25		m	0	0	all	-	KII	NAmer	1966	CC	350	n	bl	n	y	0	ev	cig+/-ot	10	20	2	0	nev any	st
WYNDE3	26		m	0	0	all	-	KII	NAmer	1966	CC	350	n	bl	n	y	0	ev	cig+/-ot	21	40	0	0	nev any	st
WYNDE3	27		m	0	0	all	-	KII	NAmer	1966	CC	350	n	bl	n	y	0	ev	cig+/-ot	41	99	3	6	nev any	st
WYNDE3	71		f	0	0	all	-	KII	NAmer	1966	CC	350	n	bl	n	y	0	ev	cig+/-ot	1	9	1	1	nev any	st
WYNDE3	72		f	0	0	all	-	KII	NAmer	1966	CC	350	n	bl	n	y	0	ev	cig+/-ot	10	20	2	0	nev any	st
WYNDE3	73		f	0	0	all	-	KII	NAmer	1966	CC	350	n	bl	n	y	0	ev	cig+/-ot	21	40	0	0	nev any	st
WYNDE3	74		f	0	0	all	-	KII	NAmer	1966	CC	350	n	bl	n	y	0	ev	cig+/-ot	41	99	3	6	nev any	st
WYNDE4	37		m	0	0	all	-	a	NAmer	1948	CC	684	n	bl	y	n	0	ev	all/unsp	1	9	1	1	nev any	st
WYNDE4	38		m	0	0	all	-	a	NAmer	1948	CC	684	n	bl	y	n	0	ev	all/unsp	10	15	0	2	nev any	st
WYNDE4	39		m	0	0	all	-	a	NAmer	1948	CC	684	n	bl	y	n	0	ev	all/unsp	16	20	2	3	nev any	st
WYNDE4	40		m	0	0	all	-	a	NAmer	1948	CC	684	n	bl	y	n	0	ev	all/unsp	21	34	0	4	nev any	st
WYNDE4	41		m	0	0	all	-	a	NAmer	1948	CC	684	n	bl	y	n	0	ev	all/unsp	35	99	3	0	nev any	st
WYNDE4	55		f	0	0	all	-	a	NAmer	1948	CC	684	n	bl	y	n	2	ev	all/unsp	1	9	1	1	nev any	ot
WYNDE6	24		m	0	0	all	-	KII	NAmer	1969	CC	4423	n	bl	n	y	0	cu	cig+/-ot	1	10	1	0	nev any	st
WYNDE6	33		m	0	0	all	-	KII	NAmer	1969	CC	4423	n	bl	n	y	0	cu	cig+/-ot	11	20	2	3	nev any	st
WYNDE6	42		m	0	0	all	-	KII	NAmer	1969	CC	4423	n	bl	n	y	0	cu	cig+/-ot	21	30	0	4	nev any	st
WYNDE6	51		m	0	0	all	-	KII	NAmer	1969	CC	4423	n	bl	n	y	0	cu	cig+/-ot	31	99	3	0	nev any	st
WYNDE6	213		f	0	0	all	-	KII	NAmer	1969	CC	4423	n	bl	n	y	0	cu	cig+/-ot	1	10	1	0	nev cigs	st
WYNDE6	222		f	0	0	all	-	KII	NAmer	1969	CC	4423	n	bl	n	y	0	cu	cig+/-ot	11	20	2	3	nev cigs	st
WYNDE6	231		f	0	0	all	-	KII	NAmer	1969	CC	4423	n	bl	n	y	0	cu	cig+/-ot	21	30	0	4	nev cigs	st
WYNDE6	240		f	0	0	all	-	KII	NAmer	1969	CC	4423	n	bl	n	y	0	cu	cig+/-ot	30	99	3	0	nev cigs	st
ZHENG	6		m	0	0	all	-	a	As:Chi	1982	CC	540	n	ot	*	y	0	ev	cig+/-ot	1	9	1	1	nev cigs	st
ZHENG	7		m	0	0	all	-	a	As:Chi	1982	CC	540	n	ot	*	y	0	ev	cig+/-ot	10	19	0	2	nev cigs	st
ZHENG	8		m	0	0	all	-	a	As:Chi	1982	CC	540	n	ot	*	y	0	ev	cig+/-ot	20	29	2	3	nev cigs	st
ZHENG	9		m	0	0	all	-	a	As:Chi	1982	CC	540	n	ot	*	y	0	ev	cig+/-ot	30	99	3	0	nev cigs	st
ZHENG	19		f	0	0	all	-	a	As:Chi	1982	CC	540	n	ot	*	y	0	ev	cig+/-ot	1	9	1	1	nev cigs	st
ZHENG	20		f	0	0	all	-	a	As:Chi	1982	CC	540	n	ot	*	y	0	ev	cig+/-ot	10	99	0	0	nev cigs	st

Cigarette type is all/unspec for all RRs

Table 3G16 - 4

IESLC - Meta-anal of Ever Smoking (or Curr if Ever not avail) by Amount, Overview, Cigs (or Any Prod if Cigs not avail)
Adenocarcinoma
Least adjusted

except for the following:

REF	NRR	CIGTYPE
ALDERS	46	MC only
ALDERS	47	MC only
ALDERS	48	MC only
ALDERS	49	MC only
ALDERS	50	MC only
ALDERS	51	MC only

REF	NRR	Cigarette equivalent
ALDERS	46	-
ALDERS	47	-
ALDERS	48	-
ALDERS	49	-
ALDERS	50	-
ALDERS	51	-
BARBON	45	*
BARBON	46	*
BARBON	47	*
BARBON	48	*
BARBON	49	*
BOUCOT	27	-
BOUCOT	28	-
BROWN2	34	*
BROWN2	44	*
BROWN2	33	*
BROWN2	43	*
CHOI	51	*
CHOI	52	*
CHOI	53	*
CHOI	54	*
CHOI	55	*
CHOI	59	*
CHOI	60	*
CHOI	61	*
CORREA	48	*
CORREA	52	*
DOLL	58	grams
DOLL	59	grams
DOLL	60	grams
DOLL	61	grams
DOLL	65	grams
DOLL	66	grams
DOLL	67	grams
DORGAN	126	*
DORGAN	127	*
DORGAN	105	*
DORGAN	106	*
DOSEME	8	*
DOSEME	12	*
DOSEME	16	*
ENGELA	71	*
ENGELA	72	*
ENGELA	73	*
ENGELA	74	*
ENGELA	75	*
GER	2	*
GER	3	*
GER	4	*
HAENSZ	35	*
HAENSZ	34	*
HAMMON	108	-
HAMMON	109	-
HAMMON	110	-
JEDRYC	15	*
JEDRYC	16	*
JEDRYC	17	*
JEDRYC	18	*
JEDRYC	19	*
KATSOU	17	*
KATSOU	18	*
KREYBE	17	grams inc 1 cig=1
KREYBE	18	grams inc 1 cig=1

Table 3G16 - 4

IESLC - Meta-anal of Ever Smoking (or Curr if Ever not avail) by Amount, Overview, Cigs (or Any Prod if Cigs not avail)
Adenocarcinoma
Least adjusted

REF	NRR	Cigarette equivalent
KREYBE	19	grams inc 1 cig=1
KREYBE	34	grams inc 1 cig=1
KREYBE	35	grams inc 1 cig=1
LAMTH	16	*
LAMTH	17	*
LAMTH	18	*
LUBIN2	152	*
LUBIN2	156	*
LUBIN2	160	*
LUBIN2	164	*
LUBIN2	172	*
LUBIN2	176	*
LUBIN2	180	*
LUBIN2	184	*
MATOS	56	*
MATOS	58	*
MATOS	60	*
MATSUD	7	*
MATSUD	8	*
MATSUD	9	*
ORMOS	22	*
ORMOS	23	*
ORMOS	24	*
OSANN	55	*
OSANN	63	*
OSANN	56	*
OSANN	64	*
OSANN2	16	*
OSANN2	17	*
SOBUE	56	*
SOBUE	57	*
SOBUE	58	*
SVENSS	29	*
SVENSS	34	*
SVENSS	39	*
TSUGAN	4	*
TSUGAN	5	*
TSUGAN	6	*
WAKAI	49	*
WAKAI	50	*
WAKAI	51	*
WU	3	*
WU	4	*
WUWILL	22	*
WUWILL	23	*
WYNDE2	10	*
WYNDE2	11	*
WYNDE2	12	*
WYNDE2	13	*
WYNDE3	24	*
WYNDE3	25	*
WYNDE3	26	*
WYNDE3	27	*
WYNDE3	71	*
WYNDE3	72	*
WYNDE3	73	*
WYNDE3	74	*
WYNDE4	37	inc 1 cigar = 5 cigs, 1 pipe = 2.5 cigs
WYNDE4	38	inc 1 cigar = 5 cigs, 1 pipe = 2.5 cigs
WYNDE4	39	inc 1 cigar = 5 cigs, 1 pipe = 2.5 cigs
WYNDE4	40	inc 1 cigar = 5 cigs, 1 pipe = 2.5 cigs
WYNDE4	41	inc 1 cigar = 5 cigs, 1 pipe = 2.5 cigs
WYNDE4	55	inc 1 cigar = 5 cigs, 1 pipe = 2.5 cigs
WYNDE6	24	*
WYNDE6	33	*
WYNDE6	42	*
WYNDE6	51	*
WYNDE6	213	*
WYNDE6	222	*
WYNDE6	231	*
WYNDE6	240	*
ZHENG	6	*

Table 3G16 - 4

IESLC - Meta-anal of Ever Smoking (or Curr if Ever not avail) by Amount, Overview, Cigs (or Any Prod if Cigs not avail)
Adenocarcinoma
Least adjusted

REF	NRR	Cigarette equivalent
ZHENG	7	*
ZHENG	8	*
ZHENG	9	*
ZHENG	19	*
ZHENG	20	*

In this overview table, subtotals and Qs values may be invalid and should be ignored

Table 3G16 - 5

IESLC - Meta-anal of Ever Smoking (or Curr if Ever not avail) by Amount, Overview, Cigs (or Any Prod if Cigs not avail)
Adenocarcinoma
Least adjusted

REF	NRR	SEX	AD	Number Exposed Case	Number Exposed Cont	Non-exposed Case	Non-exposed Cont	RR	95.00%CI
ALDERS	46	m	1	-	-	-	-	2.80	(1.01- 7.75)
ALDERS	47	m	1	-	-	-	-	2.67	(0.99- 7.18)
ALDERS	48	m	1	-	-	-	-	3.32	(1.36- 8.10)
ALDERS	49	f	1	-	-	-	-	2.77	(1.63- 4.70)
ALDERS	50	f	1	-	-	-	-	4.58	(2.67- 7.85)
ALDERS	51	f	1	-	-	-	-	3.31	(1.80- 6.10)
Subtotal ALDERS								3.34	(2.53- 4.41)
BARBON	45	m	0	7	87	7	188	2.16	(0.74- 6.35)
BARBON	46	m	0	31	111	7	188	7.50	(3.20- 17.60)
BARBON	47	m	0	43	176	7	188	6.56	(2.88- 14.97)
BARBON	48	m	0	30	82	7	188	9.83	(4.15- 23.28)
BARBON	49	m	0	40	111	7	188	9.68	(4.19- 22.34)
Subtotal BARBON								6.90	(4.66- 10.22)
*BOUCOT	27	m	0	8	15208	0	7551	8.44~	(0.49- 146.22)
*BOUCOT	28	m	0	6	6940	0	7551	14.14~	(0.80- 251.03)
Subtotal BOUCOT								10.90	(1.44- 82.62)
BROWN2	34	m	2	-	-	-	-	6.20	(4.90- 7.90)
BROWN2	44	m	2	-	-	-	-	10.70	(8.90- 13.00)
BROWN2	33	f	2	-	-	-	-	5.80	(4.70- 7.10)
BROWN2	43	f	2	-	-	-	-	8.60	(7.30- 10.10)
Subtotal BROWN2								7.91	(7.18- 8.72)
CHOI	51	m	0	4	90	7	95	0.60	(0.17- 2.13)
CHOI	52	m	0	27	281	7	95	1.30	(0.55- 3.09)
CHOI	53	m	0	11	49	7	95	3.05	(1.11- 8.35)
CHOI	54	m	0	2	39	7	95	0.70	(0.14- 3.50)
CHOI	55	m	0	2	6	7	95	4.52	(0.77- 26.69)
CHOI	59	f	0	4	16	49	164	0.84	(0.27- 2.62)
CHOI	60	f	0	0	9	49	164	0.17~	(0.01- 3.06)
CHOI	61	f	0	1	1	49	164	3.35	(0.21- 54.50)
Subtotal CHOI								1.32	(0.83- 2.09)
CORREA	48	c	1	-	-	-	-	4.30	(2.60- 7.20)
CORREA	52	c	1	-	-	-	-	12.00	(7.30- 19.70)
Subtotal CORREA								7.28	(5.10- 10.39)
DOLL	58	m	0	2	129	2	61	0.47	(0.07- 3.44)
DOLL	59	m	0	14	570	2	61	0.75	(0.17- 3.37)
DOLL	60	m	0	16	431	2	61	1.13	(0.25- 5.05)
DOLL	61	m	0	6	166	2	61	1.10	(0.22- 5.61)
DOLL	65	f	0	2	25	5	59	0.94	(0.17- 5.19)
DOLL	66	f	0	4	18	5	59	2.62	(0.64- 10.81)
DOLL	67	f	0	2	6	5	59	3.93	(0.62- 24.83)
Subtotal DOLL								1.27	(0.69- 2.35)
DORGAN	126	m	2	-	-	-	-	3.23	(1.20- 8.70)
DORGAN	127	m	2	-	-	-	-	5.63	(2.22- 14.25)
DORGAN	105	f	3	-	-	-	-	2.87	(2.00- 4.11)
DORGAN	106	f	3	-	-	-	-	5.39	(3.73- 7.78)
Subtotal DORGAN								3.96	(3.11- 5.04)
DOSEME	8	m	2	-	-	-	-	1.80	(0.80- 4.10)
DOSEME	12	m	2	-	-	-	-	2.70	(1.60- 4.70)
DOSEME	16	m	2	-	-	-	-	3.20	(1.40- 7.00)
Subtotal DOSEME								2.56	(1.73- 3.79)
*ENGELA	71	m	7	-	-	-	-	2.60	(0.60- 11.00)
*ENGELA	72	m	7	-	-	-	-	8.50	(2.50- 29.00)
*ENGELA	73	m	7	-	-	-	-	7.10	(2.10- 24.00)
*ENGELA	74	m	7	-	-	-	-	9.30	(2.10- 41.00)
*ENGELA	75	m	7	-	-	-	-	17.00	(5.10- 56.00)
Subtotal ENGELA								8.06	(4.51- 14.41)
GER	2	c	0	9	47	37	149	0.77	(0.35- 1.71)
GER	3	c	0	21	66	37	149	1.28	(0.70- 2.36)
GER	4	c	0	5	26	37	149	0.77	(0.28- 2.15)
Subtotal GER								1.00	(0.65- 1.55)
HAENSZ	35	f	0	10	66	37	236	0.97	(0.46- 2.05)
HAENSZ	34	f	0	5	13	37	236	2.45	(0.83- 7.28)
Subtotal HAENSZ								1.30	(0.70- 2.42)
*HAMMON	108	m	1	-	-	-	-	1.83	(0.17- 20.22)
*HAMMON	109	m	1	-	-	-	-	2.83	(0.55- 14.60)
*HAMMON	110	m	1	-	-	-	-	6.44	(1.34- 31.02)
Subtotal HAMMON								3.71	(1.33- 10.33)
JEDRYC	15	m	0	2	67	7	289	1.23	(0.25- 6.07)
JEDRYC	16	m	0	11	199	7	289	2.28	(0.87- 5.99)
JEDRYC	17	m	0	52	434	7	289	4.95	(2.22- 11.04)
JEDRYC	18	m	0	19	118	7	289	6.65	(2.72- 16.23)

Table 3G16 - 5

IESLC - Meta-anal of Ever Smoking (or Curr if Ever not avail) by Amount, Overview, Cigs (or Any Prod if Cigs not avail)
Adenocarcinoma
Least adjusted

REF	NRR	SEX	AD	Number Exposed Case	Number Exposed Cont	Non-exposed Case	Non-exposed Cont	RR	95.00%CI
JEDRYC	19	m	0	9	82	7	289	4.53	(1.64- 12.54)
Subtotal JEDRYC								4.02	(2.59- 6.22)
KATSOU	17	f	0	9	14	30	67	1.44	(0.56- 3.68)
KATSOU	18	f	0	6	4	30	67	3.35	(0.88- 12.75)
Subtotal KATSOU								1.90	(0.88- 4.11)
KREYBE	17	m	0	31	2341	3	644	2.84	(0.87- 9.33)
KREYBE	18	m	0	6	925	3	644	1.39	(0.35- 5.59)
KREYBE	19	m	0	5	248	3	644	4.33	(1.03- 18.25)
KREYBE	34	f	0	9	286	27	657	0.77	(0.36- 1.65)
KREYBE	35	f	0	1	42	27	657	0.58	(0.08- 4.37)
Subtotal KREYBE								1.33	(0.79- 2.24)
LAMTH	16	f	0	36	29	131	158	1.50	(0.87- 2.57)
LAMTH	17	f	0	27	14	131	158	2.33	(1.17- 4.62)
LAMTH	18	f	0	9	5	131	158	2.17	(0.71- 6.64)
Subtotal LAMTH								1.82	(1.22- 2.71)
LUBIN2	152	m	0	66	2194	57	2616	1.38	(0.96- 1.98)
LUBIN2	156	m	0	204	3385	57	2616	2.77	(2.05- 3.73)
LUBIN2	160	m	0	234	3108	57	2616	3.46	(2.57- 4.64)
LUBIN2	164	m	0	151	1746	57	2616	3.97	(2.91- 5.42)
LUBIN2	172	f	0	20	184	138	1180	0.93	(0.57- 1.52)
LUBIN2	176	f	0	43	234	138	1180	1.57	(1.09- 2.27)
LUBIN2	180	f	0	13	110	138	1180	1.01	(0.55- 1.84)
LUBIN2	184	f	0	9	39	138	1180	1.97	(0.94- 4.16)
Subtotal LUBIN2								2.28	(1.99- 2.60)
MATOS	56	m	0	11	88	5	110	2.75	(0.92- 8.21)
MATOS	58	m	0	28	90	5	110	6.84	(2.54- 18.45)
MATOS	60	m	0	40	105	5	110	8.38	(3.19- 22.05)
Subtotal MATOS								5.68	(3.16- 10.19)
MATSUD	7	m	0	6	1237	0	1255	13.19~	(0.74- 234.37)
MATSUD	8	m	0	13	1607	0	1255	21.09~	(1.25- 355.07)
MATSUD	9	m	0	4	470	0	1255	24.02~	(1.29- 446.92)
Subtotal MATSUD								18.81	(3.58- 98.87)
ORMOS	22	m	0	4	329	2	777	4.72	(0.86- 25.91)
ORMOS	23	m	0	3	577	2	777	2.02	(0.34- 12.13)
ORMOS	24	m	0	1	128	2	777	3.04	(0.27- 33.72)
Subtotal ORMOS								3.13	(1.04- 9.39)
OSANN	55	m	2	-	-	-	-	16.50	(9.30- 29.30)
OSANN	63	m	2	-	-	-	-	37.50	(21.30- 66.00)
OSANN	56	f	2	-	-	-	-	8.80	(6.10- 12.80)
OSANN	64	f	2	-	-	-	-	24.20	(15.80- 37.20)
Subtotal OSANN								16.57	(13.16- 20.85)
OSANN2	16	f	0	14	24	22	43	1.14	(0.49- 2.63)
OSANN2	17	f	0	47	15	22	43	6.12	(2.82- 13.30)
Subtotal OSANN2								2.81	(1.59- 4.97)
SOBUE	56	m	0	63	157	27	128	1.90	(1.15- 3.16)
SOBUE	57	m	0	95	222	27	128	2.03	(1.26- 3.28)
SOBUE	58	m	0	76	187	27	128	1.93	(1.18- 3.16)
Subtotal SOBUE								1.95	(1.47- 2.60)
SVENSS	29	f	0	12	30	22	120	2.18	(0.97- 4.90)
SVENSS	34	f	0	22	22	22	120	5.45	(2.59- 11.50)
SVENSS	39	f	0	4	1	22	120	21.82	(2.33- 204.53)
Subtotal SVENSS								3.97	(2.33- 6.76)
TSUGAN	4	m	0	12	14	18	17	0.81	(0.29- 2.24)
TSUGAN	5	m	0	23	23	18	17	0.94	(0.39- 2.28)
TSUGAN	6	m	0	10	13	18	17	0.73	(0.25- 2.09)
Subtotal TSUGAN								0.84	(0.48- 1.47)
WAKAI	49	m	0	16	105	8	65	1.24	(0.50- 3.06)
WAKAI	50	m	0	30	129	8	65	1.89	(0.82- 4.35)
WAKAI	51	m	0	27	48	8	65	4.57	(1.91- 10.94)
Subtotal WAKAI								2.22	(1.34- 3.67)
WU	3	f	0	38	28	29	62	2.90	(1.50- 5.60)
WU	4	f	0	61	22	29	62	5.93	(3.07- 11.44)
Subtotal WU								4.15	(2.61- 6.60)
WUWILL	22	f	0	112	311	172	601	1.26	(0.96- 1.66)
WUWILL	23	f	0	26	40	172	601	2.27	(1.35- 3.83)
Subtotal WUWILL								1.43	(1.12- 1.82)
WYNDE2	10	m	0	2	114	5	105	0.37	(0.07- 1.94)
WYNDE2	11	m	0	14	203	5	105	1.45	(0.51- 4.13)
WYNDE2	12	m	0	14	83	5	105	3.54	(1.23- 10.23)
WYNDE2	13	m	0	16	112	5	105	3.00	(1.06- 8.48)
Subtotal WYNDE2								1.98	(1.12- 3.51)

Table 3G16 - 5

IESLC - Meta-anal of Ever Smoking (or Curr if Ever not avail) by Amount, Overview, Cigs (or Any Prod if Cigs not avail)
Adenocarcinoma
Least adjusted

REF	NRR	SEX	AD	Number Exposed Case	Cont	Non-exposed Case	Cont	RR	95.00%CI
WYNDE3	24	m	0	1	42	6	88	0.35	(0.04- 2.99)
WYNDE3	25	m	0	20	114	6	88	2.57	(0.99- 6.68)
WYNDE3	26	m	0	34	82	6	88	6.08	(2.43- 15.24)
WYNDE3	27	m	0	9	26	6	88	5.08	(1.65- 15.59)
WYNDE3	71	f	0	2	19	15	76	0.53	(0.11- 2.53)
WYNDE3	72	f	0	11	24	15	76	2.32	(0.94- 5.73)
WYNDE3	73	f	0	7	10	15	76	3.55	(1.16- 10.80)
WYNDE3	74	f	0	1	3	15	76	1.69	(0.16- 17.36)
Subtotal WYNDE3								2.82	(1.87- 4.25)
WYNDE4	37	m	0	3	82	4	115	1.05	(0.23- 4.83)
WYNDE4	38	m	0	6	147	4	115	1.17	(0.32- 4.26)
WYNDE4	39	m	0	15	274	4	115	1.57	(0.51- 4.84)
WYNDE4	40	m	0	4	98	4	115	1.17	(0.29- 4.82)
WYNDE4	41	m	0	7	64	4	115	3.14	(0.89- 11.15)
WYNDE4	55	f	2	-	-	-	-	1.33	(0.29- 6.07)
Subtotal WYNDE4								1.51	(0.88- 2.60)
WYNDE6	24	m	0	42	122	58	617	3.66	(2.35- 5.70)
WYNDE6	33	m	0	191	293	58	617	6.93	(5.01- 9.60)
WYNDE6	42	m	0	136	129	58	617	11.22	(7.81- 16.09)
WYNDE6	51	m	0	282	197	58	617	15.23	(11.00- 21.07)
WYNDE6	213	f	0	39	109	119	856	2.57	(1.70- 3.89)
WYNDE6	222	f	0	176	165	119	856	7.67	(5.76- 10.21)
WYNDE6	231	f	0	100	50	119	856	14.39	(9.74- 21.25)
WYNDE6	240	f	0	157	52	119	856	21.72	(15.04- 31.37)
Subtotal WYNDE6								8.99	(7.93- 10.19)
ZHENG	6	m	0	18	40	29	94	1.46	(0.73- 2.92)
ZHENG	7	m	0	35	66	29	94	1.72	(0.96- 3.08)
ZHENG	8	m	0	53	89	29	94	1.93	(1.13- 3.30)
ZHENG	9	m	0	17	23	29	94	2.40	(1.13- 5.08)
ZHENG	19	f	0	13	29	119	184	0.69	(0.35- 1.39)
ZHENG	20	f	0	20	15	119	184	2.06	(1.02- 4.19)
Subtotal ZHENG								1.62	(1.24- 2.11)
Partial Totals				3527	49475	3634	55947		

*prospective study ~ With 0.5 adjustment for zero

REF	NRR	SEX	AD	Ys	Ws	Qs	Ps
ALDERS	46	m	1	1.03	3.70	0.78	0.0476
ALDERS	47	m	1	0.98	3.91	1.00	0.0520
ALDERS	48	m	1	1.20	4.83	0.40	0.0084
ALDERS	49	f	1	1.02	13.70	3.02	0.0002
ALDERS	50	f	1	1.52	13.21	0.01	0.0000
ALDERS	51	f	1	1.20	10.32	0.88	0.0001
Subtotal ALDERS				1.21	49.67	6.10	
BARBON	45	m	0	0.77	3.31	1.70	0.1612
BARBON	46	m	0	2.01	5.28	1.46	0.0000
BARBON	47	m	0	1.88	5.65	0.87	0.0000
BARBON	48	m	0	2.29	5.16	3.27	0.0000
BARBON	49	m	0	2.27	5.49	3.35	0.0000
Subtotal BARBON				1.93	24.88	10.66	
*BOUCOT	27	m	0	2.13	0.47	0.20	0.1427
*BOUCOT	28	m	0	2.65	0.46	0.63	0.0710
Subtotal BOUCOT				2.39	0.94	0.82	
BROWN2	34	m	2	1.82	67.36	7.60	0.0000
BROWN2	44	m	2	2.37	107.03	83.20	0.0000
BROWN2	33	f	2	1.76	90.29	6.55	0.0000
BROWN2	43	f	2	2.15	145.78	64.12	0.0000
Subtotal BROWN2				2.07	410.46	161.48	
CHOI	51	m	0	-0.51	2.41	9.59	0.4323
CHOI	52	m	0	0.27	5.16	7.71	0.5467
CHOI	53	m	0	1.11	3.78	0.53	0.0304
CHOI	54	m	0	-0.36	1.47	5.05	0.6600
CHOI	55	m	0	1.51	1.22	0.00	0.0956
CHOI	59	f	0	-0.18	2.95	8.20	0.7595
CHOI	60	f	0	-1.74	0.47	4.90	0.2324
CHOI	61	f	0	1.21	0.49	0.04	0.3961
Subtotal CHOI				0.27	17.95	36.02	
CORREA	48	c	1	1.46	14.81	0.01	0.0000
CORREA	52	c	1	2.48	15.59	15.48	0.0000
Subtotal CORREA				1.98	30.40	15.49	
DOLL	58	m	0	-0.75	0.98	4.89	0.4593

Table 3G16 - 5

IESLC - Meta-anal of Ever Smoking (or Curr if Ever not avail) by Amount, Overview, Cigs (or Any Prod if Cigs not avail)
Adenocarcinoma
Least adjusted

REF	NRR	SEX	AD	Ys	Ws	Qs	Ps
DOLL	59	m	0	-0.29	1.70	5.36	0.7068
DOLL	60	m	0	0.12	1.72	3.20	0.8706
DOLL	61	m	0	0.10	1.45	2.81	0.9065
DOLL	65	f	0	-0.06	1.32	3.16	0.9472
DOLL	66	f	0	0.96	1.91	0.53	0.1823
DOLL	67	f	0	1.37	1.13	0.02	0.1451
Subtotal DOLL				0.24	10.21	19.96	
DORGAN	126	m	2	1.17	3.92	0.39	0.0203
DORGAN	127	m	2	1.73	4.45	0.26	0.0003
DORGAN	105	f	3	1.05	29.62	5.58	0.0000
DORGAN	106	f	3	1.68	28.43	1.09	0.0000
Subtotal DORGAN				1.38	66.41	7.32	
DOSEME	8	m	2	0.59	5.75	4.67	0.1585
DOSEME	12	m	2	0.99	13.23	3.25	0.0003
DOSEME	16	m	2	1.16	5.93	0.63	0.0046
Subtotal DOSEME				0.94	24.92	8.54	
*ENGELA	71	m	7	0.96	1.82	0.52	0.1979
*ENGELA	72	m	7	2.14	2.56	1.09	0.0006
*ENGELA	73	m	7	1.96	2.59	0.58	0.0016
*ENGELA	74	m	7	2.23	1.74	0.96	0.0033
*ENGELA	75	m	7	2.83	2.68	4.84	0.0000
Subtotal ENGELA				2.09	11.38	7.97	
GER	2	c	0	-0.26	6.02	18.40	0.5237
GER	3	c	0	0.25	10.36	15.95	0.4249
GER	4	c	0	-0.26	3.67	11.18	0.6242
Subtotal GER				0.00	20.06	45.53	
HAENSZ	35	f	0	-0.03	6.83	15.84	0.9289
HAENSZ	34	f	0	0.90	3.24	1.13	0.1060
Subtotal HAENSZ				0.27	10.07	16.97	
*HAMMON	108	m	1	0.60	0.67	0.53	0.6201
*HAMMON	109	m	1	1.04	1.43	0.29	0.2136
*HAMMON	110	m	1	1.86	1.56	0.22	0.0201
Subtotal HAMMON				1.31	3.66	1.03	
JEDRYC	15	m	0	0.21	1.51	2.48	0.7972
JEDRYC	16	m	0	0.83	4.13	1.82	0.0937
JEDRYC	17	m	0	1.60	5.96	0.07	0.0001
JEDRYC	18	m	0	1.89	4.82	0.79	0.0000
JEDRYC	19	m	0	1.51	3.71	0.00	0.0036
Subtotal JEDRYC				1.39	20.13	5.16	
KATSOU	17	f	0	0.36	4.33	5.50	0.4516
KATSOU	18	f	0	1.21	2.15	0.17	0.0762
Subtotal KATSOU				0.64	6.48	5.67	
KREYBE	17	m	0	1.04	2.72	0.54	0.0849
KREYBE	18	m	0	0.33	1.99	2.67	0.6405
KREYBE	19	m	0	1.47	1.86	0.00	0.0460
KREYBE	34	f	0	-0.27	6.53	20.12	0.4952
KREYBE	35	f	0	-0.55	0.94	3.90	0.5964
Subtotal KREYBE				0.28	14.04	27.22	
LAMTH	16	f	0	0.40	13.12	15.44	0.1438
LAMTH	17	f	0	0.84	8.17	3.39	0.0158
LAMTH	18	f	0	0.78	3.08	1.57	0.1740
Subtotal LAMTH				0.60	24.36	20.40	
LUBIN2	152	m	0	0.32	29.82	40.55	0.0782
LUBIN2	156	m	0	1.02	43.25	9.60	0.0000
LUBIN2	160	m	0	1.24	44.40	2.74	0.0000
LUBIN2	164	m	0	1.38	39.81	0.48	0.0000
LUBIN2	172	f	0	-0.07	15.74	38.39	0.7715
LUBIN2	176	f	0	0.45	28.07	30.17	0.0167
LUBIN2	180	f	0	0.01	10.63	23.21	0.9727
LUBIN2	184	f	0	0.68	6.90	4.52	0.0741
Subtotal LUBIN2				0.82	218.62	149.66	
MATOS	56	m	0	1.01	3.21	0.73	0.0698
MATOS	58	m	0	1.92	3.91	0.74	0.0001
MATOS	60	m	0	2.13	4.10	1.67	0.0000
Subtotal MATOS				1.74	11.22	3.14	
MATSUD	7	m	0	2.58	0.46	0.55	0.0789
MATSUD	8	m	0	3.05	0.48	1.17	0.0343
MATSUD	9	m	0	3.18	0.45	1.28	0.0331
Subtotal MATSUD				2.93	1.40	3.01	
ORMOS	22	m	0	1.55	1.33	0.01	0.0738
ORMOS	23	m	0	0.70	1.20	0.74	0.4420

Table 3G16 - 5

IESLC - Meta-anal of Ever Smoking (or Curr if Ever not avail) by Amount, Overview, Cigs (or Any Prod if Cigs not avail)
Adenocarcinoma
Least adjusted

REF	NRR	SEX	AD	Ys	Ws	Qs	Ps
ORMOS	24	m	0	1.11	0.66	0.09	0.3661
Subtotal ORMOS				1.14	3.18	0.84	
OSANN	55	m	2	2.80	11.67	20.17	0.0000
OSANN	63	m	2	3.62	12.01	54.80	0.0000
OSANN	56	f	2	2.17	27.97	13.17	0.0000
OSANN	64	f	2	3.19	20.96	60.41	0.0000
Subtotal OSANN				2.81	72.61	148.55	
OSANN2	16	f	0	0.13	5.50	10.13	0.7584
OSANN2	17	f	0	1.81	6.38	0.67	0.0000
Subtotal OSANN2				1.03	11.88	10.80	
SOBUE	56	m	0	0.64	14.90	10.65	0.0130
SOBUE	57	m	0	0.71	16.70	10.19	0.0038
SOBUE	58	m	0	0.66	15.78	10.95	0.0092
Subtotal SOBUE				0.67	47.39	31.79	
SVENSS	29	f	0	0.78	5.87	2.94	0.0588
SVENSS	34	f	0	1.70	6.91	0.30	0.0000
SVENSS	39	f	0	3.08	0.77	1.95	0.0069
Subtotal SVENSS				1.38	13.54	5.19	
TSUGAN	4	m	0	-0.21	3.72	10.74	0.6838
TSUGAN	5	m	0	-0.06	4.97	11.87	0.8986
TSUGAN	6	m	0	-0.32	3.43	11.22	0.5538
Subtotal TSUGAN				-0.18	12.12	33.83	
WAKAI	49	m	0	0.21	4.71	7.65	0.6431
WAKAI	50	m	0	0.64	5.51	4.00	0.1352
WAKAI	51	m	0	1.52	5.04	0.00	0.0006
Subtotal WAKAI				0.80	15.26	11.66	
WU	3	f	0	1.07	8.88	1.59	0.0015
WU	4	f	0	1.78	8.89	0.75	0.0000
Subtotal WU				1.42	17.77	2.34	
WUWILL	22	f	0	0.23	50.96	80.75	0.1009
WUWILL	23	f	0	0.82	14.10	6.29	0.0021
Subtotal WUWILL				0.36	65.06	87.04	
WYNDE2	10	m	0	-1.00	1.39	8.61	0.2387
WYNDE2	11	m	0	0.37	3.50	4.37	0.4885
WYNDE2	12	m	0	1.26	3.41	0.17	0.0195
WYNDE2	13	m	0	1.10	3.56	0.54	0.0382
Subtotal WYNDE2				0.69	11.86	13.70	
WYNDE3	24	m	0	-1.05	0.83	5.37	0.3372
WYNDE3	25	m	0	0.95	4.22	1.25	0.0521
WYNDE3	26	m	0	1.81	4.55	0.46	0.0001
WYNDE3	27	m	0	1.62	3.05	0.06	0.0045
WYNDE3	71	f	0	-0.63	1.58	7.09	0.4293
WYNDE3	72	f	0	0.84	4.71	1.96	0.0675
WYNDE3	73	f	0	1.27	3.10	0.15	0.0258
WYNDE3	74	f	0	0.52	0.71	0.66	0.6593
Subtotal WYNDE3				1.04	22.76	16.99	
WYNDE4	37	m	0	0.05	1.66	3.42	0.9482
WYNDE4	38	m	0	0.16	2.31	4.08	0.8077
WYNDE4	39	m	0	0.45	3.04	3.26	0.4291
WYNDE4	40	m	0	0.16	1.93	3.40	0.8243
WYNDE4	41	m	0	1.15	2.40	0.28	0.0761
WYNDE4	55	f	2	0.29	1.66	2.41	0.7132
Subtotal WYNDE4				0.41	12.99	16.85	
WYNDE6	24	m	0	1.30	19.66	0.71	0.0000
WYNDE6	33	m	0	1.94	36.35	7.29	0.0000
WYNDE6	42	m	0	2.42	29.44	25.39	0.0000
WYNDE6	51	m	0	2.72	36.38	55.46	0.0000
WYNDE6	213	f	0	0.95	22.53	6.65	0.0000
WYNDE6	222	f	0	2.04	46.92	14.15	0.0000
WYNDE6	231	f	0	2.67	25.27	35.05	0.0000
WYNDE6	240	f	0	3.08	28.43	71.84	0.0000
Subtotal WYNDE6				2.20	244.98	216.55	
ZHENG	6	m	0	0.38	7.96	9.82	0.2870
ZHENG	7	m	0	0.54	11.26	10.09	0.0692
ZHENG	8	m	0	0.66	13.29	9.18	0.0165
ZHENG	9	m	0	0.87	6.78	2.56	0.0229
ZHENG	19	f	0	-0.37	7.98	27.48	0.3003
ZHENG	20	f	0	0.72	7.66	4.48	0.0452
Subtotal ZHENG				0.48	54.94	63.62	

Table 3G16 - 5

IESLC - Meta-anal of Ever Smoking (or Curr if Ever not avail) by Amount, Overview, Cigs (or Any Prod if Cigs not avail)
Adenocarcinoma
Least adjusted

N	140
NS	34

Table 3G16 - 6

IESLC - Meta-anal of Ever Smoking (or Curr if Ever not avail) by Amount, Overview, Cigs (or Any Prod if Cigs not avail)
Adenocarcinoma
Least adjusted

	Sex combined	male	female	Total
N	5	87	48	140
NS	2	25	19	46

In this overview table, other than the "N" rows, entries in the "absent" and "Total" columns may be invalid and should be ignored

		Amount smoked (broad categories) absent	<20k5	6-44k20	>20k45	Total
	N	39	39	28	34	140
	NS	23	28	23	28	102
	Wt	580.77	461.22	277.99	263.62	1583.60
Het	Chi	303.31	253.00	123.57	260.11	1211.91
Het	df	38	38	27	33	139
Het	P	***	***	***	***	***
Fixed	RR	6.17	2.58	3.48	7.07	4.43
	RRl	5.69	2.36	3.09	6.27	4.22
	RRu	6.70	2.83	3.92	7.98	4.65
	P	+++	+++	+++	+++	+++
Random	RR	4.07	1.74	2.64	4.72	3.01
	RRl	3.10	1.32	1.99	3.23	2.56
	RRu	5.35	2.29	3.52	6.90	3.54
	P	+++	+++	+++	+++	+++

		absent	<10k1	Amount smoked (narrow categories) 2-19k10	11-29k20	21-39k30	31-98k40	>40k99	Total
	N	83	14	9	22	8	1	3	140
	NS	34	9	7	19	7	1	2	78
	Wt	1063.46	76.84	100.49	261.00	75.37	1.47	4.98	1583.60
Het	Chi	781.86	9.86	20.38	108.79	22.62	0.00	0.70	1211.91
Het	df	82	13	8	21	7	0	2	139
Het	P	***	N.S.	**	***	**	N.S.	N.S.	***
Fixed	RR	5.17	1.16	2.30	3.64	9.69	0.70	4.22	4.43
	RRl	4.87	0.93	1.89	3.23	7.74	0.14	1.75	4.22
	RRu	5.49	1.45	2.79	4.12	12.15	3.50	10.16	4.65
	P	+++	N.S.	+++	+++	+++	N.S.	++	+++
Random	RR	3.40	1.16	2.38	2.87	6.70	0.70	4.22	3.01
	RRl	2.75	0.93	1.61	2.11	4.11	0.14	1.75	2.56
	RRu	4.19	1.45	3.52	3.90	10.91	3.50	10.16	3.54
	P	+++	N.S.	+++	+++	+++	N.S.	++	+++

MALES

		Amount smoked (broad categories) absent	<20k5	6-44k20	>20k45	Total
	N	23	23	20	21	87
	NS	16	23	20	21	80
	Wt	255.76	185.25	176.61	158.42	776.04
Het	Chi	136.39	99.91	57.90	135.16	538.22
Het	df	22	22	19	20	86
Het	P	***	***	***	***	***
Fixed	RR	6.76	2.89	3.22	5.84	4.53
	RRl	5.98	2.51	2.78	5.00	4.22
	RRu	7.64	3.34	3.73	6.82	4.86
	P	+++	+++	+++	+++	+++
Random	RR	4.68	1.95	2.66	4.59	3.24
	RRl	3.19	1.34	1.97	2.88	2.65
	RRu	6.86	2.84	3.60	7.32	3.96
	P	+++	+++	+++	+++	+++

Table 3G16 - 6

IESLC - Meta-anal of Ever Smoking (or Curr if Ever not avail) by Amount, Overview, Cigs (or Any Prod if Cigs not avail)
Adenocarcinoma
Least adjusted

MALES

Amount smoked (narrow categories)

	absent	<10k1	2-19k10	11-29k20	21-39k30	31-98k40	>40k99	Total
N	45	9	7	16	7	1	2	87
NS	25	9	7	16	7	1	2	66
Wt	436.35	48.55	70.51	164.80	50.10	1.47	4.27	776.04
Het Chi	299.18	4.30	14.75	49.56	16.70	0.00	0.01	538.22
Het df	44	8	6	15	6	0	1	86
Het P	***	N.S.	*	***	*	N.S.	N.S.	***
Fixed RR	5.92	1.39	2.66	3.38	7.94	0.70	4.91	4.53
RRl	5.39	1.05	2.11	2.90	6.02	0.14	1.90	4.22
RRu	6.50	1.85	3.36	3.93	10.48	3.50	12.68	4.86
P	+++	+	+++	+++	+++	N.S.	++	+++
Random RR	3.86	1.39	2.62	2.86	5.51	0.70	4.91	3.24
RRl	2.89	1.05	1.62	2.07	3.11	0.14	1.90	2.65
RRu	5.15	1.85	4.23	3.95	9.77	3.50	12.68	3.96
P	+++	+	+++	+++	+++	N.S.	++	+++

FEMALES

Amount smoked (broad categories)

	absent	<20k5	6-44k20	>20k45	Total
N	15	15	7	11	48
NS	14	15	7	11	47
Wt	310.20	269.95	91.01	85.94	757.10
Het Chi	161.87	141.22	47.88	86.14	612.32
Het df	14	14	6	10	47
Het P	***	***	***	***	***
Fixed RR	5.83	2.46	4.53	10.06	4.42
RRl	5.21	2.18	3.69	8.14	4.12
RRu	6.51	2.77	5.57	12.43	4.75
P	+++	+++	+++	+++	+++
Random RR	3.29	1.59	2.86	5.29	2.77
RRl	2.08	1.03	1.43	2.57	2.08
RRu	5.21	2.45	5.70	10.91	3.69
P	+++	+	++	+++	+++

Amount smoked (narrow categories)

	absent	<10k1	2-19k10	11-29k20	21-39k30	31-98k40	>40k99	Total
N	34	5	2	5	1		1	48
NS	18	5	2	5	1		1	31
Wt	587.02	28.29	29.99	85.83	25.27		0.71	757.10
Het Chi	423.68	1.15	0.47	40.55	0.00		0.00	612.32
Het df	33	4	1	4	0		0	47
Het P	***	N.S.	N.S.	***	N.S.		N.S.	***
Fixed RR	4.74	0.85	1.62	4.79	14.39		1.69	4.42
RRl	4.37	0.59	1.14	3.88	9.74		0.16	4.12
RRu	5.14	1.23	2.32	5.92	21.25		17.36	4.75
P	+++	N.S.	++	+++	+++		N.S.	+++
Random RR	3.03	0.85	1.62	3.45	14.39		1.69	2.77
RRl	2.18	0.59	1.14	1.62	9.74		0.16	2.08
RRu	4.20	1.23	2.32	7.36	21.25		17.36	3.69
P	+++	N.S.	++	++	+++		N.S.	+++

Table 3G16 - 7

IESLC - Meta-anal of Ever Smoking (or Curr if Ever not avail) by Amount, Overview, Cigs (or Any Prod if Cigs not avail)
Adenocarcinoma
Excluded studies (and stage at which they were excluded)

1	ABELIN ABRAHA AMANDU AMES ANDERS AUSTIN AXELSO BAND BECHER BERRIN BLOHMK BLOT4 BROCKM BROWN1 BYERS1 BYERS2 CARPEN CASCO2 CASCOR CHAN CHEN3 CHIAZZ CHYOU DESTE2 DOCKER DROSTE DU GARCIA GARDIN GENG GODLEY GOODMA GRAHAM GREGOR HEGMAN HEIN HENNEK HINDS HIRAOK HOROWI HORWIT HUANG ISHIMA JAHN JAIN JARVHO JIANG KELLER KIHARA KJUUS KO KOHLME KUBIK LAMWK LAMWK2 LANGE LEI LEMARC LEVIN LIU LOMBA2 LOMBAR MAGNUS MARSH MARSH2 MCDUFF MCLAUG MILLER MILLS NOTANI NOU ODRISC PAWLEG PERSHA POFFIJ QIAO QIAO2 RADZIK REN RONCO ROOTS ROTHSC SAARIK SANKAR SCHWAR SEGI SEOW SHIMIZ SIMARA SIMONA SITAS SOBUE2 STASZE STAYNE STUCKE SUN SUZUK2 SUZUKI TANG TAO TOKARS TOUSEY ULMER VEIERO VUTUC WALD WANG WANG3 WANG4 WICKLU WIGLE WILKIN WU2 WUNSCH WYNDE8 XIANGZ XU XU2 XU4 YONG ZHANG
2	BUELL CHEN MASTRA MZILEN PISANI RESTRE SADOWS
4	BOFFET WYNDE7
5	RIMING TANG2 WYNDE5
6	BLOT1 BLOT2 BLOT3 BOUCHA HIRAY2 JONES LAURIL LICKIN MOLLO MRFIT MURATA SCHWA2 VANDER WARSIN WATSON WYNDER
8	AGUDO AKIBA ARCHER ARMADA AUVINE AXELSS BENSHL BEST BRESLO BRETT BROSS BUFFLE CEDERL CHANG CHATZI CHEN2 CHOW COMSTO COOKSO CPSI CPSII DAMBER DARBY DAVEYS DEAN DEAN2 DEAN3 DEKLER DESTEF DOLL2 DORANT DORN DUNN EBELIN ENSTRO ESAKI FAN GAO GAO2 GARSHI GILLIS GOLLED GSELL HAMMO2 HANSEN HIRAYA HITOSU HOLE HU HU2 HUMBLE JARUP JOLY JUSSAW KAISE2 KAISER KANELL KAUFMA KHUDER KINLEN KNEKT KOO KOULUM KREUZE LAUSSM LETOUR LIAW LIDDEL LIU2 LIU3 LIU4 LIU5 LUBIN LUO MACLEN MARTIS MCCONN MIGRAN MRFITR NAM NOTAN2 PARKIN PASTOR PERNU PERSH2 PETO PEZZO2 PEZZOT PIKE POLEDN PRESCO RACHTA RANDIG SEGI2 SHAW SIEMIA SPEIZE SPITZ STOCKS STOCKW TENKAN TIZZAN TULINI TVERDA WANG2 XU3 YAMAGU YUAN ZHOU
10	BENHAM

Table 3G16 - 8

Potentially overlapping studies

REF	REFGP	PRINC	.	OVERLAP/LINK
LUBIN2	LUBIN2	1		Lubin-combined
LAMTH	LAMTH	1		KOO/LAMTH/LAMWK
OSANN2	KAISER	2		KAISER/OSANN2
WYNDE6	WYNDE6	1		WYNDE5/6/7/8
MATSUD	MATSUD	1		SOBUE2/MATSUD

Table 3G16 - 9

Most adjusted - insufficient data for metaanalysis

REF	NRR	SEX	AGEL	AGEH	RACE	YF	LC TYPE	LOC	START	ST	NLC	R	VB	P	H	AD	SM	PRODUCT	exL	exH	S1	S2	DENOM	De
CHEN	15	c	0	0	all	-	a	As:oth	1987	CC	323	n	ot	n	y	2	ev	cig+/-ot	1	10	1	0	nev cigs	ot
CHEN	14	c	0	0	all	-	a	As:oth	1987	CC	323	n	ot	n	y	2	ev	cig+/-ot	11	20	2	3	nev cigs	ot
CHEN	13	c	0	0	all	-	a	As:oth	1987	CC	323	n	ot	n	y	2	ev	cig+/-ot	21	30	0	4	nev cigs	ot
CHEN	12	c	0	0	all	-	a	As:oth	1987	CC	323	n	ot	n	y	2	ev	cig+/-ot	31	99	3	0	nev cigs	ot

REF	NRR	RR	SIG	Cigarette equivalent	RRDATA comment
CHEN	15	1.21	n	*	0
CHEN	14	1.74	n	*	0
CHEN	13	2.34	n	*	0
CHEN	12	3.61	y	*	P < 0.001

Table 3G17 -

IESLC - Meta-anal of Ever Smoking (or Current if ev not avail), Amount smoked, “Low”, Cigs (or Any Prod if Cigs not avail)
Adenocarcinoma

This analysis is restricted to results for:
1) Results by Amount smoked
2) Results complete enough for use in metaanalysis

Within each study, results are then selected (in the following order of preference, within each sex) for:
3) SMKSTA: ever smokers, current smokers
4) PRODUCT: cigarettes regardless of other products, cigarettes only, all/unspec
5) CIGTYPE: all/unspecified, MC regardless of HR, MC only
6) DENOM: never smoked anything, never smoked cigarettes, (never +1 = +long term ex, +2 = +amount unknown,
+3 = never cigs+long term ex)
7) Followup period (YF, prospective studies): whole study (coded as 0) or longest available
8) LCtype: adeno or nearest available, but not squamous. (q = squamous, s = small,
a = adeno, l = large, KII = Kreyberg II, al = alveolar, br = bronchiolar, u = undifferentiated)
9) Race: all or nearest available, otherwise by race (wh or w = white, bl or b = black, hi = hispanic
ch = chinese, jap = japanese, haw = hawaiian, w+o = white + oriental, sca = scandinavian, as = asian)
10) Amount smoked "low" in key scheme 1 (key value 5, maximum range <20, in numbers of
cigarettes or cigarette equivalents)
11) For overlapping studies: principal rather than subsidiary studies
Finally by Age: whole study (coded as 0) if available, otherwise by widest available age group
and then for single sex results (m, f) in preference to combined sex results (c).

Results adjusted (AD) for the most potential confounders are then chosen in Sections -1 to -3
(and those which actually differ from the adjusted results in Table 3G12 - 1 are marked 'x' in Section -1)
and results adjusted for the least confounders in Sections -4 to -6. (Those least adjusted results which
actually differ from the most adjusted as marked 'x' in column X in Section -4)
(Results adjusted for an unknown number of confounder(s) are coded as 20.)

Section -7 shows excluded studies, together with the stage (as above) at which no qualifying
results were found.

Section -8 lists the potentially overlapping studies which have been included (1=principal, 2=subsidiary).

Section -9 lists any results which would have been included in preference except that they had data not complete
enough for use in meta-analysis, with their significance (yes/no), if known, and any further comment as entered
on the database.

In addition to those mentioned above, the following fields, levels and abbreviations are used:
* or nk = not known, n = no, y = yes, ot = other
ev = ever, cu = current, nev = never
all/unspec = all or unspecified, cig+/-ot = cigarettes irrespective of other products (cigar, pipe etc)
MC = manufactured cigarettes, HR = hand-rolled cigarettes
exL, exH = range of exposure (low and high) in the smoking group, in terms of Amount smoked,
cigarettes or cigarette equivalents
REF: 6-character study reference
NRR: number of the RR on the database within the study
ST : study type (CC = case control, pr or prosp = prospective)
NLC: number of lung cancer cases in whole study
R : risky occupational population (n = no, m = mining, o = other risky)
VB : national cigarette type (V = at least 75% Virginia, bl = at least 75% blended, ot = other)
P : any proxy use
H : full histological confirmation
De : derivation of RR/CI (or = original, st = standard method, ot = other method of estimation)

Table 3G17 - 1

IESLC - Meta-anal of Ever Smoking (or Current if ev not avail), Amount smoked, “Low”, Cigs (or Any Prod if Cigs not avail)
Adenocarcinoma
Most adjusted

REF	NRR	3G12	SEX	AGEL	AGEH	RACE	YF	LC TYPE	LOC	START	ST	NLC	R	VB	P	H	AD	SM	PRODUCT	exL	exH	DENOM	De
ALDERS	46		m	0	0	all	-	not q+s	Eu:UK	1977	CC	1448	n	V	n	n	1	ev	cig only	1	17	nev+2	ot
ALDERS	49		f	0	0	all	-	not q+s	Eu:UK	1977	CC	1448	n	V	n	n	1	ev	cig only	1	17	nev+2	ot
BARBON	79		m	0	0	all	-	a	Eu:wst	1979	CC	755	n	bl	y	y	3	ev	all/unsp	1	19	nev any	or
BROWN2	34		m	0	0	wh	-	a	NAmer	1984	CC	14596	n	bl	n	y	2	ev	cig+/-ot	1	19	nev cigs	or
BROWN2	33		f	0	0	wh	-	a	NAmer	1984	CC	14596	n	bl	n	y	2	ev	cig+/-ot	1	19	nev cigs	or
CHOI	51		m	0	0	all	-	a	As:oth	1985	CC	375	n	bl	n	n	0	ev	cig+/-ot	1	10	nev cigs	st
CHOI	59		f	0	0	all	-	a	As:oth	1985	CC	375	n	bl	n	n	0	ev	cig+/-ot	1	10	nev cigs	st
DOLL	73		m	0	0	all	-	KII	Eu:UK	1948	CC	1465	n	V	n	n	1	ev	all/unsp	5	14	nev any	ot
DOLL	80		f	0	0	all	-	KII	Eu:UK	1948	CC	1465	n	V	n	n	1	ev	all/unsp	5	14	nev any	ot
DORGAN	126		m	0	0	wh	-	a	NAmer	1980	CC	2026	n	bl	y	y	2	ev	cig+/-ot	1	19	nev any	ot
DORGAN	105		f	0	0	all	-	a	NAmer	1980	CC	2026	n	bl	y	y	3	ev	cig+/-ot	1	19	nev any	ot
DOSEME	8		m	0	0	all	-	not q+s	Eu:bal	1979	CC	1210	n	bl	n	n	2	ev	cig+/-ot	1	10	nev cigs	or
ENGELA	72		m	0	0	all	0	a	Eu:Sca	1964	pr	435	n	bl	n	n	7	cu	cig+/-ot	5	9	nev cigs	or
GER	10		c	0	0	all	-	a	As:oth	1990	CC	141	n	ot	y	n	8	ev	all/unsp	1	10	nev any	ot
HAMMON	108		m	0	0	wh	0	a	NAmer	1952	pr	448	n	bl	n	n	1	cu	cig only	1	9	nev any	ot
JEDRYC	36		m	0	0	all	-	a	Eu:est	1980	CC	1630	n	bl	y	n	3	ev	cig+/-ot	1	19	nev any	or
KREYBE	5		m	0	0	all	-	KII	Eu:Sca	1948	CC	300	n	bl	n	y	1	ev	all/unsp	1	14	nev any	ot
KREYBE	26		f	0	0	all	-	KII	Eu:Sca	1948	CC	300	n	bl	n	y	1	ev	all/unsp	1	14	nev any	ot
LAMTH	16		f	0	0	ch	-	a	As:HK	1983	CC	445	n	bl	n	n	0	ev	all/unsp	1	10	nev any	or
LUBIN2	152		m	0	0	all	-	a	Eu:mul	1976	CC	7804	n	bl	n	y	0	ev	cig+/-ot	1	9	nev any	st
LUBIN2	172		f	0	0	all	-	a	Eu:mul	1976	CC	7804	n	bl	n	y	0	ev	cig+/-ot	1	9	nev any	st
MATOS	57		m	0	0	all	-	a	SCAmer	1994	CC	200	n	bl	n	n	2	ev	cig+/-ot	1	14	nev any	or
MATSUD	7		m	0	0	all	-	a	As:Jap	1965	CC	179	n	bl	n	n	0	ev	cig+/-ot	1	10	nev cigs	ot
ORMOS	22		m	0	0	all	-	KII	Eu:est	1947	CC	119	n	bl	y	y	0	ev	cig+/-ot	1	15	nev any	st
OSANN2	34		f	0	0	all	-	KII	NAmer	1964	ot	217	n	bl	n	y	1	ev	cig+/-ot	1	19	nev cigs	or
SOBUE	56		m	0	0	all	-	a	As:Jap	1986	CC	1376	n	bl	n	y	0	cu	cig+/-ot	1	19	nev cigs	st
SVENSS	9		f	0	0	all	-	a	Eu:Sca	1983	CC	210	n	bl	n	n	1	cu	all/unsp	1	10	nev any	or
TSUGAN	4		m	0	0	all	-	a	As:Jap	1976	CC	134	n	bl	n	y	0	cu	all/unsp	1	15	nev any	st
WAKAI	52		m	0	0	all	-	a	As:Jap	1988	CC	333	n	bl	n	y	1	cu	cig+/-ot	1	19	nev any	or
WUWILL	16		f	0	0	all	-	a	As:Chi	1985	CC	965	n	ot	n	n	3	ev	cig+/-ot	1	19	nev cigs	ot
WYNDE2	10		m	0	0	all	-	KII	NAmer	1962	CC	404	n	bl	n	y	0	ev	cig+/-ot	1	10	nev any	st
WYNDE3	24		m	0	0	all	-	KII	NAmer	1966	CC	350	n	bl	n	y	0	ev	cig+/-ot	1	9	nev any	st
WYNDE3	71		f	0	0	all	-	KII	NAmer	1966	CC	350	n	bl	n	y	0	ev	cig+/-ot	1	9	nev any	st
WYNDE4	37		m	0	0	all	-	a	NAmer	1948	CC	684	n	bl	y	n	0	ev	all/unsp	1	9	nev any	st
WYNDE4	55		f	0	0	all	-	a	NAmer	1948	CC	684	n	bl	y	n	2	ev	all/unsp	1	9	nev any	ot
WYNDE6	24		m	0	0	all	-	KII	NAmer	1969	CC	4423	n	bl	n	y	0	cu	cig+/-ot	1	10	nev any	st
WYNDE6	213		f	0	0	all	-	KII	NAmer	1969	CC	4423	n	bl	n	y	0	cu	cig+/-ot	1	10	nev cigs	st
ZHENG	6		m	0	0	all	-	a	As:Chi	1982	CC	540	n	ot	*	y	0	ev	cig+/-ot	1	9	nev cigs	st
ZHENG	19		f	0	0	all	-	a	As:Chi	1982	CC	540	n	ot	*	y	0	ev	cig+/-ot	1	9	nev cigs	st

Cigarette type is all/unspec for all RRs

except for the following:

REF	NRR	CIGTYPE
ALDERS	46	MC only
ALDERS	49	MC only

Table 3G17 - 2

IESLC - Meta-anal of Ever Smoking (or Current if ev not avail), Amount smoked, “Low”, Cigs (or Any Prod if Cigs not avail)
Adenocarcinoma
Most adjusted

REF	NRR	SEX	AD	Number Exposed Case	Number Exposed Cont	Non-exposed Case	Non-exposed Cont	RR	95.00%CI
ALDERS	46	m	1	-	-	-	-	2.80	(1.01- 7.75)
ALDERS	49	f	1	-	-	-	-	2.77	(1.63- 4.70)
Subtotal ALDERS								2.78	(1.74- 4.44)
BARBON	79	m	3	-	-	-	-	5.00	(2.20- 11.00)
BROWN2	34	m	2	-	-	-	-	6.20	(4.90- 7.90)
BROWN2	33	f	2	-	-	-	-	5.80	(4.70- 7.10)
Subtotal BROWN2								5.97	(5.11- 6.98)
CHOI	51	m	0	4	90	7	95	0.60	(0.17- 2.13)
CHOI	59	f	0	4	16	49	164	0.84	(0.27- 2.62)
Subtotal CHOI								0.72	(0.31- 1.68)
DOLL	73	m	1	-	-	-	-	0.80	(0.18- 3.60)
DOLL	80	f	1	-	-	-	-	2.30	(0.56- 9.48)
Subtotal DOLL								1.40	(0.50- 3.91)
DORGAN	126	m	2	-	-	-	-	3.23	(1.20- 8.70)
DORGAN	105	f	3	-	-	-	-	2.87	(2.00- 4.11)
Subtotal DORGAN								2.91	(2.07- 4.08)
DOSEME	8	m	2	-	-	-	-	1.80	(0.80- 4.10)
*ENGELA	72	m	7	-	-	-	-	8.50	(2.50- 29.00)
GER	10	c	8	-	-	-	-	0.62	(0.23- 1.72)
*HAMMON	108	m	1	-	-	-	-	1.83	(0.17- 20.22)
JEDRYC	36	m	3	-	-	-	-	2.19	(0.84- 5.72)
KREYBE	5	m	1	-	-	-	-	2.65	(0.81- 8.70)
KREYBE	26	f	1	-	-	-	-	1.30	(0.60- 2.80)
Subtotal KREYBE								1.61	(0.84- 3.06)
LAMTH	16	f	0	36	29	131	158	1.50	(0.87- 2.57)
LUBIN2	152	m	0	66	2194	57	2616	1.38	(0.96- 1.98)
LUBIN2	172	f	0	20	184	138	1180	0.93	(0.57- 1.52)
Subtotal LUBIN2								1.20	(0.90- 1.61)
MATOS	57	m	2	-	-	-	-	2.80	(0.90- 8.50)
MATSUD	7	m	0	6	1237	0	1255	13.19~	(0.74- 234.37)
ORMOS	22	m	0	4	329	2	777	4.72	(0.86- 25.91)
OSANN2	34	f	1	-	-	-	-	0.90	(0.30- 2.70)
SOBUE	56	m	0	63	157	27	128	1.90	(1.15- 3.16)
SVENSS	9	f	1	-	-	-	-	2.20	(1.00- 5.80)
TSUGAN	4	m	0	12	14	18	17	0.81	(0.29- 2.24)
WAKAI	52	m	1	-	-	-	-	1.30	(0.52- 3.21)
WUWILL	16	f	3	-	-	-	-	1.43	(1.07- 1.90)
WYNDE2	10	m	0	2	114	5	105	0.37	(0.07- 1.94)
WYNDE3	24	m	0	1	42	6	88	0.35	(0.04- 2.99)
WYNDE3	71	f	0	2	19	15	76	0.53	(0.11- 2.53)
Subtotal WYNDE3								0.46	(0.13- 1.63)
WYNDE4	37	m	0	3	82	4	115	1.05	(0.23- 4.83)
WYNDE4	55	f	2	-	-	-	-	1.33	(0.29- 6.07)
Subtotal WYNDE4								1.18	(0.40- 3.47)
WYNDE6	24	m	0	42	122	58	617	3.66	(2.35- 5.70)
WYNDE6	213	f	0	39	109	119	856	2.57	(1.70- 3.89)
Subtotal WYNDE6								3.03	(2.24- 4.10)
ZHENG	6	m	0	18	40	29	94	1.46	(0.73- 2.92)
ZHENG	19	f	0	13	29	119	184	0.69	(0.35- 1.39)
Subtotal ZHENG								1.00	(0.62- 1.64)
Partial Totals				335	4807	784	8525		

*prospective study

~ With 0.5 adjustment for zero

Table 3G17 - 2

IESLC - Meta-anal of Ever Smoking (or Current if ev not avail), Amount smoked, “Low”, Cigs (or Any Prod if Cigs not avail)
Adenocarcinoma
Most adjusted

REF	NRR	SEX	AD	Ys	Ws	Qs	Ps
ALDERS	46	m	1	1.03	3.70	0.00	0.0476
ALDERS	49	f	1	1.02	13.70	0.01	0.0002
Subtotal ALDERS				1.02	17.40	0.01	
BARBON	79	m	3	1.61	5.93	2.24	0.0001
BROWN2	34	m	2	1.82	67.36	46.41	0.0000
BROWN2	33	f	2	1.76	90.29	52.62	0.0000
Subtotal BROWN2				1.79	157.65	99.04	
CHOI	51	m	0	-0.51	2.41	5.43	0.4323
CHOI	59	f	0	-0.18	2.95	4.06	0.7595
Subtotal CHOI				-0.33	5.36	9.48	
DOLL	73	m	1	-0.22	1.71	2.54	0.7703
DOLL	80	f	1	0.83	1.92	0.05	0.2485
Subtotal DOLL				0.34	3.63	2.59	
DORGAN	126	m	2	1.17	3.92	0.12	0.0203
DORGAN	105	f	3	1.05	29.62	0.11	0.0000
Subtotal DORGAN				1.07	33.53	0.23	
DOSEME	8	m	2	0.59	5.75	0.95	0.1585
*ENGELA	72	m	7	2.14	2.56	3.36	0.0006
GER	10	c	8	-0.48	3.80	8.23	0.3517
*HAMMON	108	m	1	0.60	0.67	0.10	0.6201
JEDRYC	36	m	3	0.78	4.18	0.19	0.1092
KREYBE	5	m	1	0.97	2.73	0.00	0.1076
KREYBE	26	f	1	0.26	6.48	3.47	0.5044
Subtotal KREYBE				0.47	9.20	3.47	
LAMTH	16	f	0	0.40	13.12	4.58	0.1438
LUBIN2	152	m	0	0.32	29.82	13.46	0.0782
LUBIN2	172	f	0	-0.07	15.74	17.94	0.7715
Subtotal LUBIN2				0.19	45.56	31.40	
MATOS	57	m	2	1.03	3.05	0.00	0.0723
MATSUD	7	m	0	2.58	0.46	1.17	0.0789
ORMOS	22	m	0	1.55	1.33	0.41	0.0738
OSANN2	34	f	1	-0.11	3.18	3.85	0.8509
SOBUE	56	m	0	0.64	14.90	1.84	0.0130
SVENSS	9	f	1	0.79	4.97	0.21	0.0787
TSUGAN	4	m	0	-0.21	3.72	5.40	0.6838
WAKAI	52	m	1	0.26	4.64	2.49	0.5721
WUWILL	16	f	3	0.36	46.61	18.90	0.0146
WYNDE2	10	m	0	-1.00	1.39	5.53	0.2387
WYNDE3	24	m	0	-1.05	0.83	3.48	0.3372
WYNDE3	71	f	0	-0.63	1.58	4.17	0.4293
Subtotal WYNDE3				-0.77	2.41	7.65	
WYNDE4	37	m	0	0.05	1.66	1.47	0.9482
WYNDE4	55	f	2	0.29	1.66	0.84	0.7132
Subtotal WYNDE4				0.17	3.32	2.31	
WYNDE6	24	m	0	1.30	19.66	1.81	0.0000
WYNDE6	213	f	0	0.95	22.53	0.05	0.0000
Subtotal WYNDE6				1.11	42.19	1.87	
ZHENG	6	m	0	0.38	7.96	3.03	0.2870
ZHENG	19	f	0	-0.37	7.98	14.79	0.3003
Subtotal ZHENG				0.00	15.94	17.82	

	N	39
	NS	28
	Wt	456.45
Het	Chi	235.32
Het	df	38
Het	P	***
Fixed	RR	2.70
	RRl	2.47
	RRu	2.96
	P	+++
Random	RR	1.83
	RRl	1.40
	RRu	2.39
	P	+++
Asymm	P	**

Table 3G17 - 3

IESLC - Meta-anal of Ever Smoking (or Current if ev not avail), Amount smoked, “Low”, Cigs (or Any Prod if Cigs not avail)
Adenocarcinoma
Most adjusted

	combined	Sex male	female	Total
N	1	23	15	39
NS	1	23	15	39
Wt	3.80	190.33	262.33	456.45
Het Chi	0.00	99.84	125.09	235.32
Het df	0	22	14	38
Het P	N.S.	***	***	***
Fixed RR	0.62	2.96	2.58	2.70
RRl	0.23	2.57	2.29	2.47
RRu	1.70	3.42	2.92	2.96
P	N.S.	+++	+++	+++
Random RR	0.62	2.09	1.65	1.83
RRl	0.23	1.44	1.08	1.40
RRu	1.70	3.02	2.50	2.39
P	N.S.	+++	+	+++
Between Chi				10.39
Between df				2
Between P				**
Btwn(F) P				N.S.
Btwn(R) P				(*)

Table 3G17 - 4

IESLC - Meta-anal of Ever Smoking (or Current if ev not avail), Amount smoked, “Low”, Cigs (or Any Prod if Cigs not avail)
Adenocarcinoma
Least adjusted

REF	NRR	X	SEX	AGEL	AGEH	RACE	YF	LC TYPE	LOC	START	ST	NLC	R	VB	P	H	AD	SM	PRODUCT	exL	exH	DENOM	De
ALDERS	46		m	0	0	all	-	not q+s	Eu:UK	1977	CC	1448	n	V	n	n	1	ev	cig only	1	17	nev+2	ot
ALDERS	49		f	0	0	all	-	not q+s	Eu:UK	1977	CC	1448	n	V	n	n	1	ev	cig only	1	17	nev+2	ot
BARBON	45	x	m	0	0	all	-	a	Eu:wst	1979	CC	755	n	bl	y	y	0	ev	all/unsp	1	9	nev any	st
BROWN2	34		m	0	0	wh	-	a	NAmer	1984	CC	14596	n	bl	n	y	2	ev	cig+/-ot	1	19	nev cigs	or
BROWN2	33		f	0	0	wh	-	a	NAmer	1984	CC	14596	n	bl	n	y	2	ev	cig+/-ot	1	19	nev cigs	or
CHOI	51		m	0	0	all	-	a	As:oth	1985	CC	375	n	bl	n	n	0	ev	cig+/-ot	1	10	nev cigs	st
CHOI	59		f	0	0	all	-	a	As:oth	1985	CC	375	n	bl	n	n	0	ev	cig+/-ot	1	10	nev cigs	st
DOLL	59	x	m	0	0	all	-	KII	Eu:UK	1948	CC	1465	n	V	n	n	0	ev	all/unsp	5	14	nev any	st
DOLL	66	x	f	0	0	all	-	KII	Eu:UK	1948	CC	1465	n	V	n	n	0	ev	all/unsp	5	14	nev any	st
DORGAN	126		m	0	0	wh	-	a	NAmer	1980	CC	2026	n	bl	y	y	2	ev	cig+/-ot	1	19	nev any	ot
DORGAN	105		f	0	0	all	-	a	NAmer	1980	CC	2026	n	bl	y	y	3	ev	cig+/-ot	1	19	nev any	ot
DOSEME	8		m	0	0	all	-	not q+s	Eu:bal	1979	CC	1210	n	bl	n	n	2	ev	cig+/-ot	1	10	nev cigs	or
ENGELA	72		m	0	0	all	0	a	Eu:Sca	1964	pr	435	n	bl	n	n	7	cu	cig+/-ot	5	9	nev cigs	or
GER	2	x	c	0	0	all	-	a	As:oth	1990	CC	141	n	ot	y	n	0	ev	all/unsp	1	10	nev any	st
HAMMON	108		m	0	0	wh	0	a	NAmer	1952	pr	448	n	bl	n	n	1	cu	cig only	1	9	nev any	ot
JEDRYC	15	x	m	0	0	all	-	a	Eu:est	1980	CC	1630	n	bl	y	n	0	ev	cig+/-ot	1	9	nev any	st
KREYBE	17	x	m	0	0	all	-	KII	Eu:Sca	1948	CC	300	n	bl	n	y	0	ev	all/unsp	1	14	nev any	st
KREYBE	34	x	f	0	0	all	-	KII	Eu:Sca	1948	CC	300	n	bl	n	y	0	ev	all/unsp	1	14	nev any	st
LAMTH	16		f	0	0	ch	-	a	As:HK	1983	CC	445	n	bl	n	n	0	ev	all/unsp	1	10	nev any	or
LUBIN2	152		m	0	0	all	-	a	Eu:mul	1976	CC	7804	n	bl	n	y	0	ev	cig+/-ot	1	9	nev any	st
LUBIN2	172		f	0	0	all	-	a	Eu:mul	1976	CC	7804	n	bl	n	y	0	ev	cig+/-ot	1	9	nev any	st
MATOS	56	x	m	0	0	all	-	a	SCAmer	1994	CC	200	n	bl	n	n	0	ev	cig+/-ot	1	14	nev any	st
MATSUD	7		m	0	0	all	-	a	As:Jap	1965	CC	179	n	bl	n	n	0	ev	cig+/-ot	1	10	nev cigs	ot
ORMOS	22		m	0	0	all	-	KII	Eu:est	1947	CC	119	n	bl	y	y	0	ev	cig+/-ot	1	15	nev any	st
OSANN2	16	x	f	0	0	all	-	KII	NAmer	1964	ot	217	n	bl	n	y	0	ev	cig+/-ot	1	19	nev cigs	st
SOBUE	56		m	0	0	all	-	a	As:Jap	1986	CC	1376	n	bl	n	y	0	cu	cig+/-ot	1	19	nev cigs	st
SVENSS	29	x	f	0	0	all	-	a	Eu:Sca	1983	CC	210	n	bl	n	n	0	cu	all/unsp	1	10	nev any	st
TSUGAN	4		m	0	0	all	-	a	As:Jap	1976	CC	134	n	bl	n	y	0	cu	all/unsp	1	15	nev any	st
WAKAI	49	x	m	0	0	all	-	a	As:Jap	1988	CC	333	n	bl	n	y	0	cu	cig+/-ot	1	19	nev any	st
WUWILL	22	x	f	0	0	all	-	a	As:Chi	1985	CC	965	n	ot	n	n	0	ev	cig+/-ot	1	19	nev cigs	st
WYNDE2	10		m	0	0	all	-	KII	NAmer	1962	CC	404	n	bl	n	y	0	ev	cig+/-ot	1	10	nev any	st
WYNDE3	24		m	0	0	all	-	KII	NAmer	1966	CC	350	n	bl	n	y	0	ev	cig+/-ot	1	9	nev any	st
WYNDE3	71		f	0	0	all	-	KII	NAmer	1966	CC	350	n	bl	n	y	0	ev	cig+/-ot	1	9	nev any	st
WYNDE4	37		m	0	0	all	-	a	NAmer	1948	CC	684	n	bl	y	n	0	ev	all/unsp	1	9	nev any	st
WYNDE4	55		f	0	0	all	-	a	NAmer	1948	CC	684	n	bl	y	n	2	ev	all/unsp	1	9	nev any	ot
WYNDE6	24		m	0	0	all	-	KII	NAmer	1969	CC	4423	n	bl	n	y	0	cu	cig+/-ot	1	10	nev any	st
WYNDE6	213		f	0	0	all	-	KII	NAmer	1969	CC	4423	n	bl	n	y	0	cu	cig+/-ot	1	10	nev cigs	st
ZHENG	6		m	0	0	all	-	a	As:Chi	1982	CC	540	n	ot	*	y	0	ev	cig+/-ot	1	9	nev cigs	st
ZHENG	19		f	0	0	all	-	a	As:Chi	1982	CC	540	n	ot	*	y	0	ev	cig+/-ot	1	9	nev cigs	st

Cigarette type is all/unspec for all RRs

except for the following:

REF	NRR	CIGTYPE
ALDERS	46	MC only
ALDERS	49	MC only

Table 3G17 - 5

IESLC - Meta-anal of Ever Smoking (or Current if ev not avail), Amount smoked, “Low”, Cigs (or Any Prod if Cigs not avail)
Adenocarcinoma
Least adjusted

REF	NRR	SEX	AD	Number Exposed Case	Number Exposed Cont	Non-exposed Case	Non-exposed Cont	RR	95.00%CI
ALDERS	46	m	1	-	-	-	-	2.80	(1.01- 7.75)
ALDERS	49	f	1	-	-	-	-	2.77	(1.63- 4.70)
Subtotal ALDERS								2.78	(1.74- 4.44)
BARBON	45	m	0	7	87	7	188	2.16	(0.74- 6.35)
BROWN2	34	m	2	-	-	-	-	6.20	(4.90- 7.90)
BROWN2	33	f	2	-	-	-	-	5.80	(4.70- 7.10)
Subtotal BROWN2								5.97	(5.11- 6.98)
CHOI	51	m	0	4	90	7	95	0.60	(0.17- 2.13)
CHOI	59	f	0	4	16	49	164	0.84	(0.27- 2.62)
Subtotal CHOI								0.72	(0.31- 1.68)
DOLL	59	m	0	14	570	2	61	0.75	(0.17- 3.37)
DOLL	66	f	0	4	18	5	59	2.62	(0.64- 10.81)
Subtotal DOLL								1.46	(0.52- 4.08)
DORGAN	126	m	2	-	-	-	-	3.23	(1.20- 8.70)
DORGAN	105	f	3	-	-	-	-	2.87	(2.00- 4.11)
Subtotal DORGAN								2.91	(2.07- 4.08)
DOSEME	8	m	2	-	-	-	-	1.80	(0.80- 4.10)
*ENGELA	72	m	7	-	-	-	-	8.50	(2.50- 29.00)
GER	2	c	0	9	47	37	149	0.77	(0.35- 1.71)
*HAMMON	108	m	1	-	-	-	-	1.83	(0.17- 20.22)
JEDRYC	15	m	0	2	67	7	289	1.23	(0.25- 6.07)
KREYBE	17	m	0	31	2341	3	644	2.84	(0.87- 9.33)
KREYBE	34	f	0	9	286	27	657	0.77	(0.36- 1.65)
Subtotal KREYBE								1.13	(0.59- 2.15)
LAMTH	16	f	0	36	29	131	158	1.50	(0.87- 2.57)
LUBIN2	152	m	0	66	2194	57	2616	1.38	(0.96- 1.98)
LUBIN2	172	f	0	20	184	138	1180	0.93	(0.57- 1.52)
Subtotal LUBIN2								1.20	(0.90- 1.61)
MATOS	56	m	0	11	88	5	110	2.75	(0.92- 8.21)
MATSUD	7	m	0	6	1237	0	1255	13.19~	(0.74- 234.37)
ORMOS	22	m	0	4	329	2	777	4.72	(0.86- 25.91)
OSANN2	16	f	0	14	24	22	43	1.14	(0.49- 2.63)
SOBUE	56	m	0	63	157	27	128	1.90	(1.15- 3.16)
SVENSS	29	f	0	12	30	22	120	2.18	(0.97- 4.90)
TSUGAN	4	m	0	12	14	18	17	0.81	(0.29- 2.24)
WAKAI	49	m	0	16	105	8	65	1.24	(0.50- 3.06)
WUWILL	22	f	0	112	311	172	601	1.26	(0.96- 1.66)
WYNDE2	10	m	0	2	114	5	105	0.37	(0.07- 1.94)
WYNDE3	24	m	0	1	42	6	88	0.35	(0.04- 2.99)
WYNDE3	71	f	0	2	19	15	76	0.53	(0.11- 2.53)
Subtotal WYNDE3								0.46	(0.13- 1.63)
WYNDE4	37	m	0	3	82	4	115	1.05	(0.23- 4.83)
WYNDE4	55	f	2	-	-	-	-	1.33	(0.29- 6.07)
Subtotal WYNDE4								1.18	(0.40- 3.47)
WYNDE6	24	m	0	42	122	58	617	3.66	(2.35- 5.70)
WYNDE6	213	f	0	39	109	119	856	2.57	(1.70- 3.89)
Subtotal WYNDE6								3.03	(2.24- 4.10)
ZHENG	6	m	0	18	40	29	94	1.46	(0.73- 2.92)
ZHENG	19	f	0	13	29	119	184	0.69	(0.35- 1.39)
Subtotal ZHENG								1.00	(0.62- 1.64)
Partial Totals				576	8781	1101	11511		

*prospective study ~ With 0.5 adjustment for zero

Table 3G17 - 5

IESLC - Meta-anal of Ever Smoking (or Current if ev not avail), Amount smoked, "Low", Cigs (or Any Prod if Cigs not avail)
Adenocarcinoma
Least adjusted

REF	NRR	SEX	AD	Ys	Ws	Qs	Ps
ALDERS	46	m	1	1.03	3.70	0.02	0.0476
ALDERS	49	f	1	1.02	13.70	0.07	0.0002
Subtotal ALDERS				1.02	17.40	0.09	
BARBON	45	m	0	0.77	3.31	0.11	0.1612
BROWN2	34	m	2	1.82	67.36	51.62	0.0000
BROWN2	33	f	2	1.76	90.29	59.06	0.0000
Subtotal BROWN2				1.79	157.65	110.68	
CHOI	51	m	0	-0.51	2.41	5.11	0.4323
CHOI	59	f	0	-0.18	2.95	3.75	0.7595
Subtotal CHOI				-0.33	5.36	8.85	
DOLL	59	m	0	-0.29	1.70	2.60	0.7068
DOLL	66	f	0	0.96	1.91	0.00	0.1823
Subtotal DOLL				0.38	3.61	2.60	
DORGAN	126	m	2	1.17	3.92	0.20	0.0203
DORGAN	105	f	3	1.05	29.62	0.33	0.0000
Subtotal DORGAN				1.07	33.53	0.52	
DOSEME	8	m	2	0.59	5.75	0.75	0.1585
*ENGELA	72	m	7	2.14	2.56	3.63	0.0006
GER	2	c	0	-0.26	6.02	8.80	0.5237
*HAMMON	108	m	1	0.60	0.67	0.08	0.6201
JEDRYC	15	m	0	0.21	1.51	0.83	0.7972
KREYBE	17	m	0	1.04	2.72	0.02	0.0849
KREYBE	34	f	0	-0.27	6.53	9.65	0.4952
Subtotal KREYBE				0.12	9.25	9.68	
LAMTH	16	f	0	0.40	13.12	3.90	0.1438
LUBIN2	152	m	0	0.32	29.82	11.71	0.0782
LUBIN2	172	f	0	-0.07	15.74	16.45	0.7715
Subtotal LUBIN2				0.19	45.56	28.16	
MATOS	56	m	0	1.01	3.21	0.01	0.0698
MATSUD	7	m	0	2.58	0.46	1.23	0.0789
ORMOS	22	m	0	1.55	1.33	0.48	0.0738
OSANN2	16	f	0	0.13	5.50	3.68	0.7584
SOBUE	56	m	0	0.64	14.90	1.40	0.0130
SVENSS	29	f	0	0.78	5.87	0.17	0.0588
TSUGAN	4	m	0	-0.21	3.72	5.00	0.6838
WAKAI	49	m	0	0.21	4.71	2.55	0.6431
WUWILL	22	f	0	0.23	50.96	26.37	0.1009
WYNDE2	10	m	0	-1.00	1.39	5.28	0.2387
WYNDE3	24	m	0	-1.05	0.83	3.33	0.3372
WYNDE3	71	f	0	-0.63	1.58	3.94	0.4293
Subtotal WYNDE3				-0.77	2.41	7.27	
WYNDE4	37	m	0	0.05	1.66	1.34	0.9482
WYNDE4	55	f	2	0.29	1.66	0.73	0.7132
Subtotal WYNDE4				0.17	3.32	2.07	
WYNDE6	24	m	0	1.30	19.66	2.39	0.0000
WYNDE6	213	f	0	0.95	22.53	0.00	0.0000
Subtotal WYNDE6				1.11	42.19	2.39	
ZHENG	6	m	0	0.38	7.96	2.60	0.2870
ZHENG	19	f	0	-0.37	7.98	13.82	0.3003
Subtotal ZHENG				0.00	15.94	16.42	

	N	39
	NS	28
	Wt	461.22
Het	Chi	253.00
Het	df	38
Het	P	***
Fixed	RR	2.58
	RRl	2.36
	RRu	2.83
	P	+++
Random	RR	1.74
	RRl	1.32
	RRu	2.29
	P	+++
Asymm	P	**

Table 3G17 - 6

IESLC - Meta-anal of Ever Smoking (or Current if ev not avail), Amount smoked, “Low”, Cigs (or Any Prod if Cigs not avail)
Adenocarcinoma
Least adjusted

	combined	Sex male	female	Total
N	1	23	15	39
NS	1	23	15	39
Wt	6.02	185.25	269.95	461.22
Het Chi	0.00	99.91	141.22	253.00
Het df	0	22	14	38
Het P	N.S.	***	***	***
Fixed RR	0.77	2.89	2.46	2.58
RRl	0.35	2.51	2.18	2.36
RRu	1.71	3.34	2.77	2.83
P	N.S.	+++	+++	+++
Random RR	0.77	1.95	1.59	1.74
RRl	0.35	1.34	1.03	1.32
RRu	1.71	2.84	2.45	2.29
P	N.S.	+++	+	+++
Between Chi				11.88
Between df				2
Between P				**
Btwn(F) P				N.S.
Btwn(R) P				N.S.

Table 3G17 - 7

IESLC - Meta-anal of Ever Smoking (or Current if ev not avail), Amount smoked, “Low”, Cigs (or Any Prod if Cigs not avail)
Adenocarcinoma
Excluded studies (and stage at which they were excluded)

1 ABELIN ABRAHA AMANDU AMES ANDERS AUSTIN AXELSO BAND BECHER BERRIN BLOHMK BLOT4 BROCKM BROWN1 BYERS1 BYERS2
CARPEN CASCO2 CASCOR CHAN CHEN3 CHIAZZ CHYOU DESTE2 DOCKER DROSTE DU GARCIA GARDIN GENG GODLEY GOODMA
GRAHAM GREGOR HEGMAN HEIN HENNEK HINDS HIRAOK HOROWI HORWIT HUANG ISHIMA JAHN JAIN JARVHO JIANG KELLER
KIHARA KJUUS KO KOHLME KUBIK LAMWK LAMWK2 LANGE LEI LEMARC LEVIN LIU LOMBA2 LOMBAR MAGNUS MARSH
MARSH2 MCDUFF MCLAUG MILLER MILLS NOTANI NOU ODRISC PAWLEG PERSHA POFFIJ QIAO QIAO2 RADZIK REN RONCO
ROOTS ROTHSC SAARIK SANKAR SCHWAR SEGI SEOW SHIMIZ SIMARA SIMONA SITAS SOBUE2 STASZE STAYNE STUCKE SUN
SUZUK2 SUZUKI TANG TAO TOKARS TOUSEY ULMER VEIERO VUTUC WALD WANG WANG3 WANG4 WICKLU WIGLE WILKIN
WU2 WUNSCH WYNDE8 XIANGZ XU XU2 XU4 YONG ZHANG

2 BUELL CHEN MASTRA MZILEN PISANI RESTRE SADOWS

4 BOFFET WYNDE7

5 RIMING TANG2 WYNDE5

6 BLOT1 BLOT2 BLOT3 BOUCHA HIRAY2 JONES LAURIL LICKIN MOLLO MRFIT MURATA SCHWA2 VANDER WARSIN WATSON WYNDER

8 AGUDO AKIBA ARCHER ARMADA AUVINE AXELSS BENSHL BEST BRESLO BRETT BROSS BUFFLE CEDERL CHANG CHATZI CHEN2
CHOW COMSTO COOKSO CPSI CPSII DAMBER DARBY DAVEYS DEAN DEAN2 DEAN3 DEKLER DESTEF DOLL2 DORANT DORN
DUNN EBELIN ENSTRO ESAKI FAN GAO GAO2 GARSHI GILLIS GOLLED GSELL HAMMO2 HANSEN HIRAYA HITOSU HOLE
HU HU2 HUMBLE JARUP JOLY JUSSAW KAISE2 KAISER KANELL KAUFMA KHUDER KINLEN KNEKT KOO KOULUM KREUZE
LAUSSM LETOUR LIAW LIDDEL LIU2 LIU3 LIU4 LIU5 LUBIN LUO MACLEN MARTIS MCCONN MIGRAN MRFITR NAM
NOTAN2 PARKIN PASTOR PERNU PERSH2 PETO PEZZO2 PEZZOT PIKE POLEDN PRESCO RACHTA RANDIG SEGI2 SHAW SIEMIA
SPEIZE SPITZ STOCKS STOCKW TENKAN TIZZAN TULINI TVERDA WANG2 XU3 YAMAGU YUAN ZHOU

10 BOUCOT CORREA HAENSZ KATSOU OSANN WU

11 BENHAM

Table 3G17 - 8

Potentially overlapping studies

REF	REFGP	PRINC	.	OVERLAP/LINK
LUBIN2	LUBIN2	1		Lubin-combined
LAMTH	LAMTH	1		KOO/LAMTH/LAMWK
OSANN2	KAISER	2		KAISER/OSANN2
WYNDE6	WYNDE6	1		WYNDE5/6/7/8
MATSUD	MATSUD	1		SOBUE2/MATSUD

Table 3G17 - 9

Most adjusted - insufficient data for metaanalysis

REF	NRR	SEX	AGEL	AGEH	RACE	YF	LC TYPE	LOC	START	ST	NLC	R	VB	P	H	AD	SM	PRODUCT	exL	exH	DENOM	De
CHEN	15	c	0	0	all	-	a	As:oth	1987	CC	323	n	ot	n	y	2	ev	cig+/-ot	1	10	nev cigs	ot

REF	NRR	RR	SIG	RRDATA comment
CHEN	15	1.21	n	0

Table 3G18 -

IESLC - Meta-anal of Ever Smoking (or Current if ev not avail), Amount smoked, “Mid”, Cigs (or Any Prod if Cigs not avail)
Adenocarcinoma

This analysis is restricted to results for:
1) Results by Amount smoked
2) Results complete enough for use in metaanalysis

Within each study, results are then selected (in the following order of preference, within each sex) for:
3) SMKSTA: ever smokers, current smokers
4) PRODUCT: cigarettes regardless of other products, cigarettes only, all/unspec
5) CIGTYPE: all/unspecified, MC regardless of HR, MC only
6) DENOM: never smoked anything, never smoked cigarettes, (never +1 = +long term ex, +2 = +amount unknown,
+3 = never cigs+long term ex)
7) Followup period (YF, prospective studies): whole study (coded as 0) or longest available
8) LCtype: adeno or nearest available, but not squamous. (q = squamous, s = small,
a = adeno, l = large, KII = Kreyberg II, al = alveolar, br = bronchiolar, u = undifferentiated)
9) Race: all or nearest available, otherwise by race (wh or w = white, bl or b = black, hi = hispanic
ch = chinese, jap = japanese, haw = hawaiian, w+o = white + oriental, sca = scandinavian, as = asian)
10) Amount smoked "mid" in key scheme 1 (key value 20, maximum range 6-44, in numbers of
cigarettes or cigarette equivalents)
11) For overlapping studies: principal rather than subsidiary studies
Finally by Age: whole study (coded as 0) if available, otherwise by widest available age group
and then for single sex results (m, f) in preference to combined sex results (c).

Results adjusted (AD) for the most potential confounders are then chosen in Sections -1 to -3
(and those which actually differ from the adjusted results in Table 3G13 - 1 are marked 'x' in Section -1)
and results adjusted for the least confounders in Sections -4 to -6. (Those least adjusted results which
actually differ from the most adjusted as marked 'x' in column X in Section -4)
(Results adjusted for an unknown number of confounder(s) are coded as 20.)

Section -7 shows excluded studies, together with the stage (as above) at which no qualifying
results were found.

Section -8 lists the potentially overlapping studies which have been included (1=principal, 2=subsidiary).

Section -9 lists any results which would have been included in preference except that they had data not complete
enough for use in meta-analysis, with their significance (yes/no), if known, and any further comment as entered
on the database.

In addition to those mentioned above, the following fields, levels and abbreviations are used:
* or nk = not known, n = no, y = yes, ot = other
ev = ever, cu = current, nev = never
all/unspec = all or unspecified, cig+/-ot = cigarettes irrespective of other products (cigar, pipe etc)
MC = manufactured cigarettes, HR = hand-rolled cigarettes
exL, exH = range of exposure (low and high) in the smoking group, in terms of Amount smoked,
cigarettes or cigarette equivalents
REF: 6-character study reference
NRR: number of the RR on the database within the study
ST : study type (CC = case control, pr or prosp = prospective)
NLC: number of lung cancer cases in whole study
R : risky occupational population (n = no, m = mining, o = other risky)
VB : national cigarette type (V = at least 75% Virginia, bl = at least 75% blended, ot = other)
P : any proxy use
H : full histological confirmation
De : derivation of RR/CI (or = original, st = standard method, ot = other method of estimation)

Table 3G18 - 1

IESLC - Meta-anal of Ever Smoking (or Current if ev not avail), Amount smoked, “Mid”, Cigs (or Any Prod if Cigs not avail)
Adenocarcinoma
Most adjusted

REF	NRR	3G13	SEX	AGEL	AGEH	RACE	YF	LC TYPE	LOC	START	ST	NLC	R	VB	P	H	AD	SM	PRODUCT	exL	exH	DENOM	De
ALDERS	47		m	0	0	all	-	not q+s	Eu:UK	1977	CC	1448	n	V	n	n	1	ev	cig only	18	27	nev+2	ot
ALDERS	50		f	0	0	all	-	not q+s	Eu:UK	1977	CC	1448	n	V	n	n	1	ev	cig only	18	27	nev+2	ot
BARBON	80		m	0	0	all	-	a	Eu:wst	1979	CC	755	n	bl	y	y	3	ev	all/unsp	20	39	nev any	or
CHOI	52		m	0	0	all	-	a	As:oth	1985	CC	375	n	bl	n	n	0	ev	cig+/-ot	11	20	nev cigs	st
CHOI	60		f	0	0	all	-	a	As:oth	1985	CC	375	n	bl	n	n	0	ev	cig+/-ot	11	30	nev cigs	ot
DOLL	74		m	0	0	all	-	KII	Eu:UK	1948	CC	1465	n	V	n	n	1	ev	all/unsp	15	24	nev any	ot
DOSEME	12		m	0	0	all	-	not q+s	Eu:bal	1979	CC	1210	n	bl	n	n	2	ev	cig+/-ot	11	20	nev cigs	or
GER	11		c	0	0	all	-	a	As:oth	1990	CC	141	n	ot	y	n	8	ev	all/unsp	11	20	nev any	ot
HAMMON	109		m	0	0	wh	0	a	NAmer	1952	pr	448	n	bl	n	n	1	cu	cig only	10	20	nev any	ot
JEDRYC	37		m	0	0	all	-	a	Eu:est	1980	CC	1630	n	bl	y	n	3	ev	cig+/-ot	20	29	nev any	or
KREYBE	6		m	0	0	all	-	KII	Eu:Sca	1948	CC	300	n	bl	n	y	1	ev	all/unsp	15	24	nev any	ot
LAMTH	17		f	0	0	ch	-	a	As:HK	1983	CC	445	n	bl	n	n	0	ev	all/unsp	11	20	nev any	or
LUBIN2	160		m	0	0	all	-	a	Eu:mul	1976	CC	7804	n	bl	n	y	0	ev	cig+/-ot	20	29	nev any	st
LUBIN2	180		f	0	0	all	-	a	Eu:mul	1976	CC	7804	n	bl	n	y	0	ev	cig+/-ot	20	29	nev any	st
MATOS	59		m	0	0	all	-	a	SCAmer	1994	CC	200	n	bl	n	n	2	ev	cig+/-ot	15	24	nev any	or
MATSUD	8		m	0	0	all	-	a	As:Jap	1965	CC	179	n	bl	n	n	0	ev	cig+/-ot	11	20	nev cigs	ot
ORMOS	23		m	0	0	all	-	KII	Eu:est	1947	CC	119	n	bl	y	y	0	ev	cig+/-ot	16	30	nev any	st
SOBUE	57		m	0	0	all	-	a	As:Jap	1986	CC	1376	n	bl	n	y	0	cu	cig+/-ot	20	29	nev cigs	st
SVENSS	14		f	0	0	all	-	a	Eu:Sca	1983	CC	210	n	bl	n	n	1	cu	all/unsp	11	20	nev any	or
TSUGAN	5		m	0	0	all	-	a	As:Jap	1976	CC	134	n	bl	n	y	0	cu	all/unsp	16	35	nev any	st
WAKAI	53		m	0	0	all	-	a	As:Jap	1988	CC	333	n	bl	n	y	1	cu	cig+/-ot	20	29	nev any	or
WYNDE2	11		m	0	0	all	-	KII	NAmer	1962	CC	404	n	bl	n	y	0	ev	cig+/-ot	11	20	nev any	st
WYNDE3	25		m	0	0	all	-	KII	NAmer	1966	CC	350	n	bl	n	y	0	ev	cig+/-ot	10	20	nev any	st
WYNDE3	72		f	0	0	all	-	KII	NAmer	1966	CC	350	n	bl	n	y	0	ev	cig+/-ot	10	20	nev any	st
WYNDE4	39		m	0	0	all	-	a	NAmer	1948	CC	684	n	bl	y	n	0	ev	all/unsp	16	20	nev any	st
WYNDE6	33		m	0	0	all	-	KII	NAmer	1969	CC	4423	n	bl	n	y	0	cu	cig+/-ot	11	20	nev any	st
WYNDE6	222		f	0	0	all	-	KII	NAmer	1969	CC	4423	n	bl	n	y	0	cu	cig+/-ot	11	20	nev cigs	st
ZHENG	8		m	0	0	all	-	a	As:Chi	1982	CC	540	n	ot	*	y	0	ev	cig+/-ot	20	29	nev cigs	st

Cigarette type is all/unspec for all RRs

except for the following:

REF	NRR	CIGTYPE
ALDERS	47	MC only
ALDERS	50	MC only

Table 3G18 - 2

IESLC - Meta-anal of Ever Smoking (or Current if ev not avail), Amount smoked, “Mid”, Cigs (or Any Prod if Cigs not avail)
Adenocarcinoma
Most adjusted

REF	NRR	SEX	AD	Number Exposed Case	Cont	Non-exposed Case	Cont	RR	95.00%CI
ALDERS	47	m	1	-	-	-	-	2.67	(0.99- 7.18)
ALDERS	50	f	1	-	-	-	-	4.58	(2.67- 7.85)
Subtotal ALDERS								4.05	(2.52- 6.50)
BARBON	80	m	3	-	-	-	-	8.30	(3.70- 19.00)
CHOI	52	m	0	27	281	7	95	1.30	(0.55- 3.09)
CHOI	60	f	0	0	9	49	164	0.17~	(0.01- 3.06)
Subtotal CHOI								1.10	(0.48- 2.52)
DOLL	74	m	1	-	-	-	-	1.20	(0.27- 5.35)
DOSEME	12	m	2	-	-	-	-	2.70	(1.60- 4.70)
GER	11	c	8	-	-	-	-	2.10	(0.88- 5.04)
*HAMMON	109	m	1	-	-	-	-	2.83	(0.55- 14.60)
JEDRYC	37	m	3	-	-	-	-	4.38	(1.87- 10.28)
KREYBE	6	m	1	-	-	-	-	1.43	(0.36- 5.72)
LAMTH	17	f	0	27	14	131	158	2.33	(1.17- 4.62)
LUBIN2	160	m	0	234	3108	57	2616	3.46	(2.57- 4.64)
LUBIN2	180	f	0	13	110	138	1180	1.01	(0.55- 1.84)
Subtotal LUBIN2								2.73	(2.09- 3.55)
MATOS	59	m	2	-	-	-	-	7.00	(2.60- 19.10)
MATSUD	8	m	0	13	1607	0	1255	21.09~	(1.25- 355.07)
ORMOS	23	m	0	3	577	2	777	2.02	(0.34- 12.13)
SOBUE	57	m	0	95	222	27	128	2.03	(1.26- 3.28)
SVENSS	14	f	1	-	-	-	-	5.40	(2.40- 13.20)
TSUGAN	5	m	0	23	23	18	17	0.94	(0.39- 2.28)
WAKAI	53	m	1	-	-	-	-	1.93	(0.84- 4.44)
WYNDE2	11	m	0	14	203	5	105	1.45	(0.51- 4.13)
WYNDE3	25	m	0	20	114	6	88	2.57	(0.99- 6.68)
WYNDE3	72	f	0	11	24	15	76	2.32	(0.94- 5.73)
Subtotal WYNDE3								2.44	(1.27- 4.70)
WYNDE4	39	m	0	15	274	4	115	1.57	(0.51- 4.84)
WYNDE6	33	m	0	191	293	58	617	6.93	(5.01- 9.60)
WYNDE6	222	f	0	176	165	119	856	7.67	(5.76- 10.21)
Subtotal WYNDE6								7.34	(5.92- 9.10)
ZHENG	8	m	0	53	89	29	94	1.93	(1.13- 3.30)
Partial Totals				915	7113	665	8341		

*prospective study ~ With 0.5 adjustment for zero

REF	NRR	SEX	AD	Ys	Ws	Qs	Ps
ALDERS	47	m	1	0.98	3.91	0.34	0.0520
ALDERS	50	f	1	1.52	13.21	0.80	0.0000
Subtotal ALDERS				1.40	17.13	1.13	
BARBON	80	m	3	2.12	5.74	4.05	0.0000
CHOI	52	m	0	0.27	5.16	5.27	0.5467
CHOI	60	f	0	-1.74	0.47	4.28	0.2324
Subtotal CHOI				0.10	5.62	9.55	
DOLL	74	m	1	0.18	1.72	2.06	0.8109
DOSEME	12	m	2	0.99	13.23	1.06	0.0003
GER	11	c	8	0.74	5.04	1.44	0.0956
*HAMMON	109	m	1	1.04	1.43	0.08	0.2136
JEDRYC	37	m	3	1.48	5.29	0.21	0.0007
KREYBE	6	m	1	0.36	2.01	1.70	0.6122
LAMTH	17	f	0	0.84	8.17	1.53	0.0158
LUBIN2	160	m	0	1.24	44.40	0.06	0.0000
LUBIN2	180	f	0	0.01	10.63	17.03	0.9727
Subtotal LUBIN2				1.00	55.03	17.08	
MATOS	59	m	2	1.95	3.86	1.73	0.0001
MATSUD	8	m	0	3.05	0.48	1.51	0.0343
ORMOS	23	m	0	0.70	1.20	0.39	0.4420
SOBUE	57	m	0	0.71	16.70	5.41	0.0038
SVENSS	14	f	1	1.69	5.29	0.89	0.0001
TSUGAN	5	m	0	-0.06	4.97	8.83	0.8986
WAKAI	53	m	1	0.66	5.54	2.12	0.1216
WYNDE2	11	m	0	0.37	3.50	2.87	0.4885
WYNDE3	25	m	0	0.95	4.22	0.46	0.0521
WYNDE3	72	f	0	0.84	4.71	0.89	0.0675
Subtotal WYNDE3				0.89	8.93	1.35	
WYNDE4	39	m	0	0.45	3.04	2.06	0.4291
WYNDE6	33	m	0	1.94	36.35	15.84	0.0000
WYNDE6	222	f	0	2.04	46.92	27.20	0.0000
Subtotal WYNDE6				1.99	83.27	43.04	
ZHENG	8	m	0	0.66	13.29	5.09	0.0165

Table 3G18 - 2

IESLC - Meta-anal of Ever Smoking (or Current if ev not avail), Amount smoked, “Mid”, Cigs (or Any Prod if Cigs not avail)
Adenocarcinoma
Most adjusted

	N	28
	NS	23
	Wt	270.48
Het	Chi	115.19
Het	df	27
Het	P	***
Fixed	RR	3.58
	RRl	3.18
	RRu	4.04
	P	+++
Random	RR	2.73
	RRl	2.06
	RRu	3.61
	P	+++
Asymm	P	**

Table 3G18 - 3

IESLC - Meta-anal of Ever Smoking (or Current if ev not avail), Amount smoked, “Mid”, Cigs (or Any Prod if Cigs not avail)
Adenocarcinoma
Most adjusted

	Sex			
	combined	male	female	Total
N	1	20	7	28
NS	1	20	7	28
Wt	5.04	176.05	89.39	270.48
Het Chi	0.00	59.31	47.81	115.19
Het df	0	19	6	27
Het P	N.S.	***	***	***
Fixed RR	2.10	3.24	4.52	3.58
RRl	0.88	2.79	3.67	3.18
RRu	5.03	3.75	5.56	4.04
P	(+)	+++	+++	+++
Random RR	2.10	2.69	2.83	2.73
RRl	0.88	1.98	1.40	2.06
RRu	5.03	3.65	5.72	3.61
P	(+)	+++	++	+++
Between Chi				8.07
Between df				2
Between P				*
Btwn(F) P				N.S.
Btwn(R) P				N.S.

Table 3G18 - 4

IESLC - Meta-anal of Ever Smoking (or Current if ev not avail), Amount smoked, “Mid”, Cigs (or Any Prod if Cigs not avail)
Adenocarcinoma
Least adjusted

REF	NRR	X	SEX	AGEL	AGEH	RACE	YF	LC TYPE	LOC	START	ST	NLC	R	VB	P	H	AD	SM	PRODUCT	exL	exH	DENOM	De
ALDERS	47		m	0	0	all	-	not q+s	Eu:UK	1977	CC	1448	n	V	n	n	1	ev	cig only	18	27	nev+2	ot
ALDERS	50		f	0	0	all	-	not q+s	Eu:UK	1977	CC	1448	n	V	n	n	1	ev	cig only	18	27	nev+2	ot
BARBON	47	x	m	0	0	all	-	a	Eu:wst	1979	CC	755	n	bl	y	y	0	ev	all/unsp	20	29	nev any	st
CHOI	52		m	0	0	all	-	a	As:oth	1985	CC	375	n	bl	n	n	0	ev	cig+/-ot	11	20	nev cigs	st
CHOI	60		f	0	0	all	-	a	As:oth	1985	CC	375	n	bl	n	n	0	ev	cig+/-ot	11	30	nev cigs	ot
DOLL	60	x	m	0	0	all	-	KII	Eu:UK	1948	CC	1465	n	V	n	n	0	ev	all/unsp	15	24	nev any	st
DOSEME	12		m	0	0	all	-	not q+s	Eu:bal	1979	CC	1210	n	bl	n	n	2	ev	cig+/-ot	11	20	nev cigs	or
GER	3	x	c	0	0	all	-	a	As:oth	1990	CC	141	n	ot	y	n	0	ev	all/unsp	11	20	nev any	st
HAMMON	109		m	0	0	wh	0	a	NAmer	1952	pr	448	n	bl	n	n	1	cu	cig only	10	20	nev any	ot
JEDRYC	17	x	m	0	0	all	-	a	Eu:est	1980	CC	1630	n	bl	y	n	0	ev	cig+/-ot	20	29	nev any	st
KREYBE	18	x	m	0	0	all	-	KII	Eu:Sca	1948	CC	300	n	bl	n	y	0	ev	all/unsp	15	24	nev any	st
LAMTH	17		f	0	0	ch	-	a	As:HK	1983	CC	445	n	bl	n	n	0	ev	all/unsp	11	20	nev any	or
LUBIN2	160		m	0	0	all	-	a	Eu:mul	1976	CC	7804	n	bl	n	y	0	ev	cig+/-ot	20	29	nev any	st
LUBIN2	180		f	0	0	all	-	a	Eu:mul	1976	CC	7804	n	bl	n	y	0	ev	cig+/-ot	20	29	nev any	st
MATOS	58	x	m	0	0	all	-	a	SCAmer	1994	CC	200	n	bl	n	n	0	ev	cig+/-ot	15	24	nev any	st
MATSUD	8		m	0	0	all	-	a	As:Jap	1965	CC	179	n	bl	n	n	0	ev	cig+/-ot	11	20	nev cigs	ot
ORMOS	23		m	0	0	all	-	KII	Eu:est	1947	CC	119	n	bl	y	y	0	ev	cig+/-ot	16	30	nev any	st
SOBUE	57		m	0	0	all	-	a	As:Jap	1986	CC	1376	n	bl	n	y	0	cu	cig+/-ot	20	29	nev cigs	st
SVENSS	34	x	f	0	0	all	-	a	Eu:Sca	1983	CC	210	n	bl	n	n	0	cu	all/unsp	11	20	nev any	st
TSUGAN	5		m	0	0	all	-	a	As:Jap	1976	CC	134	n	bl	n	y	0	cu	all/unsp	16	35	nev any	st
WAKAI	50	x	m	0	0	all	-	a	As:Jap	1988	CC	333	n	bl	n	y	0	cu	cig+/-ot	20	29	nev any	st
WYNDE2	11		m	0	0	all	-	KII	NAmer	1962	CC	404	n	bl	n	y	0	ev	cig+/-ot	11	20	nev any	st
WYNDE3	25		m	0	0	all	-	KII	NAmer	1966	CC	350	n	bl	n	y	0	ev	cig+/-ot	10	20	nev any	st
WYNDE3	72		f	0	0	all	-	KII	NAmer	1966	CC	350	n	bl	n	y	0	ev	cig+/-ot	10	20	nev any	st
WYNDE4	39		m	0	0	all	-	a	NAmer	1948	CC	684	n	bl	y	n	0	ev	all/unsp	16	20	nev any	st
WYNDE6	33		m	0	0	all	-	KII	NAmer	1969	CC	4423	n	bl	n	y	0	cu	cig+/-ot	11	20	nev any	st
WYNDE6	222		f	0	0	all	-	KII	NAmer	1969	CC	4423	n	bl	n	y	0	cu	cig+/-ot	11	20	nev cigs	st
ZHENG	8		m	0	0	all	-	a	As:Chi	1982	CC	540	n	ot	*	y	0	ev	cig+/-ot	20	29	nev cigs	st

Cigarette type is all/unspec for all RRs

except for the following:

REF	NRR	CIGTYPE
ALDERS	47	MC only
ALDERS	50	MC only

Table 3G18 - 5

IESLC - Meta-anal of Ever Smoking (or Current if ev not avail), Amount smoked, “Mid”, Cigs (or Any Prod if Cigs not avail)
Adenocarcinoma
Least adjusted

REF	NRR	SEX	AD	Number Exposed Case	Cont	Non-exposed Case	Cont	RR	95.00%CI
ALDERS	47	m	1	-	-	-	-	2.67	(0.99- 7.18)
ALDERS	50	f	1	-	-	-	-	4.58	(2.67- 7.85)
Subtotal ALDERS								4.05	(2.52- 6.50)
BARBON	47	m	0	43	176	7	188	6.56	(2.88- 14.97)
CHOI	52	m	0	27	281	7	95	1.30	(0.55- 3.09)
CHOI	60	f	0	0	9	49	164	0.17~	(0.01- 3.06)
Subtotal CHOI								1.10	(0.48- 2.52)
DOLL	60	m	0	16	431	2	61	1.13	(0.25- 5.05)
DOSEME	12	m	2	-	-	-	-	2.70	(1.60- 4.70)
GER	3	c	0	21	66	37	149	1.28	(0.70- 2.36)
*HAMMON	109	m	1	-	-	-	-	2.83	(0.55- 14.60)
JEDRYC	17	m	0	52	434	7	289	4.95	(2.22- 11.04)
KREYBE	18	m	0	6	925	3	644	1.39	(0.35- 5.59)
LAMTH	17	f	0	27	14	131	158	2.33	(1.17- 4.62)
LUBIN2	160	m	0	234	3108	57	2616	3.46	(2.57- 4.64)
LUBIN2	180	f	0	13	110	138	1180	1.01	(0.55- 1.84)
Subtotal LUBIN2								2.73	(2.09- 3.55)
MATOS	58	m	0	28	90	5	110	6.84	(2.54- 18.45)
MATSUD	8	m	0	13	1607	0	1255	21.09~	(1.25- 355.07)
ORMOS	23	m	0	3	577	2	777	2.02	(0.34- 12.13)
SOBUE	57	m	0	95	222	27	128	2.03	(1.26- 3.28)
SVENSS	34	f	0	22	22	22	120	5.45	(2.59- 11.50)
TSUGAN	5	m	0	23	23	18	17	0.94	(0.39- 2.28)
WAKAI	50	m	0	30	129	8	65	1.89	(0.82- 4.35)
WYNDE2	11	m	0	14	203	5	105	1.45	(0.51- 4.13)
WYNDE3	25	m	0	20	114	6	88	2.57	(0.99- 6.68)
WYNDE3	72	f	0	11	24	15	76	2.32	(0.94- 5.73)
Subtotal WYNDE3								2.44	(1.27- 4.70)
WYNDE4	39	m	0	15	274	4	115	1.57	(0.51- 4.84)
WYNDE6	33	m	0	191	293	58	617	6.93	(5.01- 9.60)
WYNDE6	222	f	0	176	165	119	856	7.67	(5.76- 10.21)
Subtotal WYNDE6								7.34	(5.92- 9.10)
ZHENG	8	m	0	53	89	29	94	1.93	(1.13- 3.30)
Partial Totals				1133	9386	756	9967		

*prospective study ~ With 0.5 adjustment for zero

REF	NRR	SEX	AD	Ys	Ws	Qs	Ps
ALDERS	47	m	1	0.98	3.91	0.28	0.0520
ALDERS	50	f	1	1.52	13.21	0.99	0.0000
Subtotal ALDERS				1.40	17.13	1.27	
BARBON	47	m	0	1.88	5.65	2.27	0.0000
CHOI	52	m	0	0.27	5.16	4.97	0.5467
CHOI	60	f	0	-1.74	0.47	4.20	0.2324
Subtotal CHOI				0.10	5.62	9.17	
DOLL	60	m	0	0.12	1.72	2.17	0.8706
DOSEME	12	m	2	0.99	13.23	0.85	0.0003
GER	3	c	0	0.25	10.36	10.35	0.4249
*HAMMON	109	m	1	1.04	1.43	0.06	0.2136
JEDRYC	17	m	0	1.60	5.96	0.74	0.0001
KREYBE	18	m	0	0.33	1.99	1.67	0.6405
LAMTH	17	f	0	0.84	8.17	1.33	0.0158
LUBIN2	160	m	0	1.24	44.40	0.00	0.0000
LUBIN2	180	f	0	0.01	10.63	16.26	0.9727
Subtotal LUBIN2				1.00	55.03	16.26	
MATOS	58	m	0	1.92	3.91	1.79	0.0001
MATSUD	8	m	0	3.05	0.48	1.56	0.0343
ORMOS	23	m	0	0.70	1.20	0.35	0.4420
SOBUE	57	m	0	0.71	16.70	4.87	0.0038
SVENSS	34	f	0	1.70	6.91	1.39	0.0000
TSUGAN	5	m	0	-0.06	4.97	8.45	0.8986
WAKAI	50	m	0	0.64	5.51	2.06	0.1352
WYNDE2	11	m	0	0.37	3.50	2.69	0.4885
WYNDE3	25	m	0	0.95	4.22	0.39	0.0521
WYNDE3	72	f	0	0.84	4.71	0.77	0.0675
Subtotal WYNDE3				0.89	8.93	1.16	
WYNDE4	39	m	0	0.45	3.04	1.91	0.4291
WYNDE6	33	m	0	1.94	36.35	17.27	0.0000
WYNDE6	222	f	0	2.04	46.92	29.31	0.0000
Subtotal WYNDE6				1.99	83.27	46.58	
ZHENG	8	m	0	0.66	13.29	4.62	0.0165

Table 3G18 - 5

IESLC - Meta-anal of Ever Smoking (or Current if ev not avail), Amount smoked, “Mid”, Cigs (or Any Prod if Cigs not avail)
Adenocarcinoma
Least adjusted

	N	28
	NS	23
	Wt	277.99
Het	Chi	123.57
Het	df	27
Het	P	***
Fixed	RR	3.48
	RRl	3.09
	RRu	3.92
	P	+++
Random	RR	2.64
	RRl	1.99
	RRu	3.52
	P	+++
Asymm	P	**

Table 3G18 - 6

IESLC - Meta-anal of Ever Smoking (or Current if ev not avail), Amount smoked, “Mid”, Cigs (or Any Prod if Cigs not avail)
Adenocarcinoma
Least adjusted

	combined	Sex: male	female	Total
N	1	20	7	28
NS	1	20	7	28
Wt	10.36	176.61	91.01	277.99
Het Chi	0.00	57.90	47.88	123.57
Het df	0	19	6	27
Het P	N.S.	***	***	***
Fixed RR	1.28	3.22	4.53	3.48
RRl	0.70	2.78	3.69	3.09
RRu	2.36	3.73	5.57	3.92
P	N.S.	+++	+++	+++
Random RR	1.28	2.66	2.86	2.64
RRl	0.70	1.97	1.43	1.99
RRu	2.36	3.60	5.70	3.52
P	N.S.	+++	++	+++
Between Chi				17.78
Between df				2
Between P				***
Btwn(F) P				N.S.
Btwn(R) P				(*)

Table 3G18 - 7

IESLC - Meta-anal of Ever Smoking (or Current if ev not avail), Amount smoked, “Mid”, Cigs (or Any Prod if Cigs not avail)
Adenocarcinoma
Excluded studies (and stage at which they were excluded)

```
1      ABELIN ABRAHA AMANDU   AMES ANDERS AUSTIN AXELSO   BAND BECHER BERRIN BLOHMK  BLOT4 BROCKM BROWN1 BYERS1 BYERS2
       CARPEN CASCO2 CASCOR   CHAN  CHEN3 CHIAZZ  CHYOU DESTE2 DOCKER DROSTE     DU GARCIA GARDIN   GENG GODLEY GOODMA
       GRAHAM GREGOR HEGMAN   HEIN HENNEK  HINDS HIRAOK HOROWI HORWIT  HUANG ISHIMA   JAHN   JAIN JARVHO  JIANG KELLER
       KIHARA  KJUUS     KO KOHLME  KUBIK  LAMWK LAMWK2  LANGE    LEI LEMARC  LEVIN    LIU LOMBA2 LOMBAR MAGNUS  MARSH
       MARSH2 MCDUFF MCLAUG MILLER  MILLS NOTANI    NOU ODRISC PAWLEG PERSHA POFFIJ   QIAO  QIAO2 RADZIK    REN  RONCO
        ROOTS ROTHSC SAARIK SANKAR SCHWAR   SEGI   SEOW SHIMIZ SIMARA SIMONA  SITAS SOBUE2 STASZE STAYNE STUCKE    SUN
       SUZUK2 SUZUKI   TANG    TAO TOKARS TOUSEY  ULMER VEIERO  VUTUC   WALD   WANG  WANG3  WANG4 WICKLU  WIGLE WILKIN
          WU2 WUNSCH WYNDE8 XIANGZ     XU    XU2    XU4   YONG  ZHANG

2       BUELL   CHEN MASTRA MZILEN PISANI RESTRE SADOWS

4      BOFFET WYNDE7

5      RIMING  TANG2 WYNDE5

6       BLOT1  BLOT2  BLOT3 BOUCHA HIRAY2  JONES LAURIL LICKIN  MOLLO  MRFIT MURATA SCHWA2 VANDER WARSIN WATSON WYNDER

8       AGUDO  AKIBA ARCHER ARMADA AUVINE AXELSS BENSHL   BEST BRESLO  BRETT  BROSS BUFFLE CEDERL  CHANG CHATZI  CHEN2
         CHOW COMSTO COOKSO   CPSI  CPSII DAMBER  DARBY DAVEYS   DEAN  DEAN2  DEAN3 DEKLER DESTEF  DOLL2 DORANT   DORN
         DUNN EBELIN ENSTRO  ESAKI    FAN    GAO   GAO2 GARSHI GILLIS GOLLED  GSELL HAMMO2 HANSEN HIRAYA HITOSU   HOLE
           HU    HU2 HUMBLE  JARUP   JOLY JUSSAW KAISE2 KAISER KANELL KAUFMA KHUDER KINLEN  KNEKT    KOO KOULUM KREUZE
       LAUSSM LETOUR   LIAW LIDDEL   LIU2   LIU3   LIU4   LIU5  LUBIN    LUO MACLEN MARTIS MCCONN MIGRAN MRFITR    NAM
       NOTAN2 PARKIN PASTOR  PERNU PERSH2   PETO PEZZO2 PEZZOT   PIKE POLEDN PRESCO RACHTA RANDIG  SEGI2   SHAW SIEMIA
       SPEIZE  SPITZ STOCKS STOCKW TENKAN TIZZAN TULINI TVERDA  WANG2    XU3 YAMAGU   YUAN   ZHOU

10     BOUCOT BROWN2 CORREA DORGAN ENGELA HAENSZ KATSOU  OSANN OSANN2     WU WUWILL

11     BENHAM
```

Table 3G18 - 8

Potentially overlapping studies

REF	REFGP	PRINC	.	OVERLAP/LINK
LUBIN2	LUBIN2	1		Lubin-combined
LAMTH	LAMTH	1		KOO/LAMTH/LAMWK
WYNDE6	WYNDE6	1		WYNDE5/6/7/8
MATSUD	MATSUD	1		SOBUE2/MATSUD

Table 3G18 - 9

Most adjusted - insufficient data for metaanalysis

REF	NRR	SEX	AGEL	AGEH	RACE	YF	LC TYPE	LOC	START	ST	NLC	R	VB	P	H	AD	SM	PRODUCT	exL	exH	DENOM	De
CHEN	14	c	0	0	all	-	a	As:oth	1987	CC	323	n	ot	n	y	2	ev	cig+/-ot	11	20	nev cigs	ot

REF	NRR	RR	SIG	RRDATA	comment
CHEN	14	1.74	n	0	

Table 3G19 -

IESLC - Meta-anal of Ever Smoking (or Current if ev not avail), Amount smoked, “High”, Cigs (or Any Prod if Cigs not avail)
Adenocarcinoma

This analysis is restricted to results for:
1) Results by Amount smoked
2) Results complete enough for use in metaanalysis

Within each study, results are then selected (in the following order of preference, within each sex) for:
3) SMKSTA: ever smokers, current smokers
4) PRODUCT: cigarettes regardless of other products, cigarettes only, all/unspec
5) CIGTYPE: all/unspecified, MC regardless of HR, MC only
6) DENOM: never smoked anything, never smoked cigarettes, (never +1 = +long term ex, +2 = +amount unknown,
+3 = never cigs+long term ex)
7) Followup period (YF, prospective studies): whole study (coded as 0) or longest available
8) LCtype: adeno or nearest available, but not squamous. (q = squamous, s = small,
a = adeno, l = large, KII = Kreyberg II, al = alveolar, br = bronchiolar, u = undifferentiated)
9) Race: all or nearest available, otherwise by race (wh or w = white, bl or b = black, hi = hispanic
ch = chinese, jap = japanese, haw = hawaiian, w+o = white + oriental, sca = scandinavian, as = asian)
10) Amount smoked "high" in key scheme 1 (key value 45, maximum range >20, in numbers of
cigarettes or cigarette equivalents)
11) For overlapping studies: principal rather than subsidiary studies
Finally by Age: whole study (coded as 0) if available, otherwise by widest available age group
and then for single sex results (m, f) in preference to combined sex results (c).

Results adjusted (AD) for the most potential confounders are then chosen in Sections -1 to -3
(and those which actually differ from the adjusted results in Table 3G14 - 1 are marked 'x' in Section -1)
and results adjusted for the least confounders in Sections -4 to -6. (Those least adjusted results which
actually differ from the most adjusted as marked 'x' in column X in Section -4)
(Results adjusted for an unknown number of confounder(s) are coded as 20.)

Section -7 shows excluded studies, together with the stage (as above) at which no qualifying
results were found.

Section -8 lists the potentially overlapping studies which have been included (1=principal, 2=subsidiary).

Section -9 lists any results which would have been included in preference except that they had data not complete
enough for use in meta-analysis, with their significance (yes/no), if known, and any further comment as entered
on the database.

In addition to those mentioned above, the following fields, levels and abbreviations are used:
* or nk = not known, n = no, y = yes, ot = other
ev = ever, cu = current, nev = never
all/unspec = all or unspecified, cig+/-ot = cigarettes irrespective of other products (cigar, pipe etc)
MC = manufactured cigarettes, HR = hand-rolled cigarettes
exL, exH = range of exposure (low and high) in the smoking group, in terms of Amount smoked,
cigarettes or cigarette equivalents
REF: 6-character study reference
NRR: number of the RR on the database within the study
ST : study type (CC = case control, pr or prosp = prospective)
NLC: number of lung cancer cases in whole study
R : risky occupational population (n = no, m = mining, o = other risky)
VB : national cigarette type (V = at least 75% Virginia, bl = at least 75% blended, ot = other)
P : any proxy use
H : full histological confirmation
De : derivation of RR/CI (or = original, st = standard method, ot = other method of estimation)

Table 3G19 - 1

IESLC - Meta-anal of Ever Smoking (or Current if ev not avail), Amount smoked, “High”, Cigs (or Any Prod if Cigs not avail)
Adenocarcinoma
Most adjusted

REF	NRR	3G14	SEX	AGEL	AGEH	RACE	YF	LC TYPE	LOC	START	ST	NLC	R	VB	P	H	AD	SM	PRODUCT	exL	exH	DENOM	De
ALDERS	48		m	0	0	all	-	not q+s	Eu:UK	1977	CC	1448	n	V	n	n	1	ev	cig only	28	99	nev+2	ot
ALDERS	51		f	0	0	all	-	not q+s	Eu:UK	1977	CC	1448	n	V	n	n	1	ev	cig only	28	99	nev+2	ot
BARBON	81		m	0	0	all	-	a	Eu:wst	1979	CC	755	n	bl	y	y	3	ev	all/unsp	40	99	nev any	or
BOUCOT	149		m	0	0	all	0	a	NAmer	1951	pr	121	n	bl	n	n	2	cu	cig only	21	99	nev any	ot
CHOI	55		m	0	0	all	-	a	As:oth	1985	CC	375	n	bl	n	n	0	ev	cig+/-ot	41	99	nev cigs	st
CHOI	61		f	0	0	all	-	a	As:oth	1985	CC	375	n	bl	n	n	0	ev	cig+/-ot	31	99	nev cigs	st
CORREA	52		c	0	0	all	-	a	NAmer	1979	CC	1359	n	bl	y	n	1	cu	cig+/-ot	21	99	nev cigs	or
DOLL	75		m	0	0	all	-	KII	Eu:UK	1948	CC	1465	n	V	n	n	1	ev	all/unsp	25	99	nev any	ot
DOSEME	16		m	0	0	all	-	not q+s	Eu:bal	1979	CC	1210	n	bl	n	n	2	ev	cig+/-ot	21	99	nev cigs	or
GER	12		c	0	0	all	-	a	As:oth	1990	CC	141	n	ot	y	n	8	ev	all/unsp	21	99	nev any	ot
HAENSZ	34		f	0	0	all	-	a	NAmer	1955	CC	158	n	bl	n	y	0	cu	cig+/-ot	21	99	nev any	or
JEDRYC	38		m	0	0	all	-	a	Eu:est	1980	CC	1630	n	bl	y	n	3	ev	cig+/-ot	30	99	nev any	or
KATSOU	14		f	0	0	all	-	a	Eu:bal	1987	CC	101	n	bl	n	n	1	cu	all/unsp	21	99	nev any	or
KREYBE	7		m	0	0	all	-	KII	Eu:Sca	1948	CC	300	n	bl	n	y	1	ev	all/unsp	25	99	nev any	ot
LAMTH	18		f	0	0	ch	-	a	As:HK	1983	CC	445	n	bl	n	n	0	ev	all/unsp	21	99	nev any	or
LUBIN2	164		m	0	0	all	-	a	Eu:mul	1976	CC	7804	n	bl	n	y	0	ev	cig+/-ot	30	99	nev any	st
LUBIN2	184		f	0	0	all	-	a	Eu:mul	1976	CC	7804	n	bl	n	y	0	ev	cig+/-ot	30	99	nev any	st
MATOS	61		m	0	0	all	-	a	SCAmer	1994	CC	200	n	bl	n	n	2	ev	cig+/-ot	25	99	nev any	or
MATSUD	9		m	0	0	all	-	a	As:Jap	1965	CC	179	n	bl	n	n	0	ev	cig+/-ot	21	99	nev cigs	ot
ORMOS	24		m	0	0	all	-	KII	Eu:est	1947	CC	119	n	bl	y	y	0	ev	cig+/-ot	31	99	nev any	st
OSANN	63		m	0	0	all	-	a	NAmer	1984	CC	1986	n	bl	n	n	2	ev	cig+/-ot	40	99	nev cigs	or
OSANN	64		f	0	0	all	-	a	NAmer	1984	CC	1986	n	bl	n	n	2	ev	cig+/-ot	40	99	nev cigs	or
SOBUE	58		m	0	0	all	-	a	As:Jap	1986	CC	1376	n	bl	n	y	0	cu	cig+/-ot	30	99	nev cigs	st
SVENSS	19		f	0	0	all	-	a	Eu:Sca	1983	CC	210	n	bl	n	n	1	cu	all/unsp	21	99	nev any	ot
TSUGAN	6		m	0	0	all	-	a	As:Jap	1976	CC	134	n	bl	n	y	0	cu	all/unsp	36	99	nev any	st
WAKAI	54		m	0	0	all	-	a	As:Jap	1988	CC	333	n	bl	n	y	1	cu	cig+/-ot	30	99	nev any	or
WU	9		f	0	0	wh	-	a	NAmer	1981	CC	220	n	bl	n	y	2	cu	all/unsp	21	99	nev any	or
WYNDE2	13		m	0	0	all	-	KII	NAmer	1962	CC	404	n	bl	n	y	0	ev	cig+/-ot	35	99	nev any	st
WYNDE3	27		m	0	0	all	-	KII	NAmer	1966	CC	350	n	bl	n	y	0	ev	cig+/-ot	41	99	nev any	st
WYNDE3	74		f	0	0	all	-	KII	NAmer	1966	CC	350	n	bl	n	y	0	ev	cig+/-ot	41	99	nev any	st
WYNDE4	41		m	0	0	all	-	a	NAmer	1948	CC	684	n	bl	y	n	0	ev	all/unsp	35	99	nev any	st
WYNDE6	51		m	0	0	all	-	KII	NAmer	1969	CC	4423	n	bl	n	y	0	cu	cig+/-ot	31	99	nev any	st
WYNDE6	240		f	0	0	all	-	KII	NAmer	1969	CC	4423	n	bl	n	y	0	cu	cig+/-ot	30	99	nev cigs	st
ZHENG	9		m	0	0	all	-	a	As:Chi	1982	CC	540	n	ot	*	y	0	ev	cig+/-ot	30	99	nev cigs	st

Cigarette type is all/unspec for all RRs

except for the following:

REF	NRR	CIGTYPE
ALDERS	48	MC only
ALDERS	51	MC only

Table 3G19 - 2

IESLC - Meta-anal of Ever Smoking (or Current if ev not avail), Amount smoked, “High”, Cigs (or Any Prod if Cigs not avail)
Adenocarcinoma
Most adjusted

REF	NRR	SEX	AD	Number Exposed Case	Cont	Non-exposed Case	Cont	RR	95.00%CI
ALDERS	48	m	1	-	-	-	-	3.32	(1.36- 8.10)
ALDERS	51	f	1	-	-	-	-	3.31	(1.80- 6.10)
Subtotal ALDERS								3.31	(2.00- 5.48)
BARBON	81	m	3	-	-	-	-	12.10	(5.10- 28.00)
*BOUCOT	149	m	2	-	-	-	-	18.00	(1.01- 319.52)
CHOI	55	m	0	2	6	7	95	4.52	(0.77- 26.69)
CHOI	61	f	0	1	1	49	164	3.35	(0.21- 54.50)
Subtotal CHOI								4.15	(0.93- 18.54)
CORREA	52	c	1	-	-	-	-	12.00	(7.30- 19.70)
DOLL	75	m	1	-	-	-	-	1.10	(0.22- 5.60)
DOSEME	16	m	2	-	-	-	-	3.20	(1.40- 7.00)
GER	12	c	8	-	-	-	-	0.88	(0.25- 3.03)
HAENSZ	34	f	0	5	13	37	236	2.45	(0.83- 7.28)
JEDRYC	38	m	3	-	-	-	-	5.11	(2.09- 12.53)
KATSOU	14	f	1	-	-	-	-	2.95	(0.76- 11.41)
KREYBE	7	m	1	-	-	-	-	3.99	(0.95- 16.81)
LAMTH	18	f	0	9	5	131	158	2.17	(0.71- 6.64)
LUBIN2	164	m	0	151	1746	57	2616	3.97	(2.91- 5.42)
LUBIN2	184	f	0	9	39	138	1180	1.97	(0.94- 4.16)
Subtotal LUBIN2								3.58	(2.69- 4.77)
MATOS	61	m	2	-	-	-	-	8.40	(3.01- 22.20)
MATSUD	9	m	0	4	470	0	1255	24.02~	(1.29- 446.92)
ORMOS	24	m	0	1	128	2	777	3.04	(0.27- 33.72)
OSANN	63	m	2	-	-	-	-	37.50	(21.30- 66.00)
OSANN	64	f	2	-	-	-	-	24.20	(15.80- 37.20)
Subtotal OSANN								28.39	(20.18- 39.94)
SOBUE	58	m	0	76	187	27	128	1.93	(1.18- 3.16)
SVENSS	19	f	1	-	-	-	-	19.70	(1.70- 228.29)
TSUGAN	6	m	0	10	13	18	17	0.73	(0.25- 2.09)
WAKAI	54	m	1	-	-	-	-	4.53	(1.89- 10.90)
WU	9	f	2	-	-	-	-	6.50	(3.10- 13.90)
WYNDE2	13	m	0	16	112	5	105	3.00	(1.06- 8.48)
WYNDE3	27	m	0	9	26	6	88	5.08	(1.65- 15.59)
WYNDE3	74	f	0	1	3	15	76	1.69	(0.16- 17.36)
Subtotal WYNDE3								4.13	(1.50- 11.34)
WYNDE4	41	m	0	7	64	4	115	3.14	(0.89- 11.15)
WYNDE6	51	m	0	282	197	58	617	15.23	(11.00- 21.07)
WYNDE6	240	f	0	157	52	119	856	21.72	(15.04- 31.37)
Subtotal WYNDE6								17.79	(13.95- 22.70)
ZHENG	9	m	0	17	23	29	94	2.40	(1.13- 5.08)
Partial Totals				757	3085	702	8577		

*prospective study ~ With 0.5 adjustment for zero

REF	NRR	SEX	AD	Ys	Ws	Qs	Ps
ALDERS	48	m	1	1.20	4.83	2.89	0.0084
ALDERS	51	f	1	1.20	10.32	6.23	0.0001
Subtotal ALDERS				1.20	15.14	9.12	
BARBON	81	m	3	2.49	5.30	1.43	0.0000
*BOUCOT	149	m	2	2.89	0.46	0.39	0.0491
CHOI	55	m	0	1.51	1.22	0.26	0.0956
CHOI	61	f	0	1.21	0.49	0.29	0.3961
Subtotal CHOI				1.42	1.71	0.55	
CORREA	52	c	1	2.48	15.59	4.07	0.0000
DOLL	75	m	1	0.10	1.47	5.18	0.9081
DOSEME	16	m	2	1.16	5.93	3.90	0.0046
GER	12	c	8	-0.13	2.47	10.91	0.8408
HAENSZ	34	f	0	0.90	3.24	3.76	0.1060
JEDRYC	38	m	3	1.63	4.79	0.56	0.0004
KATSOU	14	f	1	1.08	2.09	1.67	0.1175
KREYBE	7	m	1	1.38	1.86	0.65	0.0590
LAMTH	18	f	0	0.78	3.08	4.42	0.1740
LUBIN2	164	m	0	1.38	39.81	14.11	0.0000
LUBIN2	184	f	0	0.68	6.90	11.57	0.0741
Subtotal LUBIN2				1.28	46.71	25.68	
MATOS	61	m	2	2.13	3.85	0.09	0.0000
MATSUD	9	m	0	3.18	0.45	0.65	0.0331
ORMOS	24	m	0	1.11	0.66	0.49	0.3661
OSANN	63	m	2	3.62	12.01	32.72	0.0000
OSANN	64	f	2	3.19	20.96	30.80	0.0000
Subtotal OSANN				3.35	32.97	63.52	

Table 3G19 - 2

IESLC - Meta-anal of Ever Smoking (or Current if ev not avail), Amount smoked, “High”, Cigs (or Any Prod if Cigs not avail)
Adenocarcinoma
Most adjusted

REF	NRR	SEX	AD	Ys	Ws	Qs	Ps
SOBUE	58	m	0	0.66	15.78	27.43	0.0092
SVENSS	19	f	1	2.98	0.64	0.65	0.0171
TSUGAN	6	m	0	-0.32	3.43	18.06	0.5538
WAKAI	54	m	1	1.51	5.00	1.07	0.0007
WU	9	f	2	1.87	6.82	0.07	0.0000
WYNDE2	13	m	0	1.10	3.56	2.73	0.0382
WYNDE3	27	m	0	1.62	3.05	0.37	0.0045
WYNDE3	74	f	0	0.52	0.71	1.49	0.6593
Subtotal WYNDE3				1.42	3.76	1.86	
WYNDE4	41	m	0	1.15	2.40	1.64	0.0761
WYNDE6	51	m	0	2.72	36.38	20.42	0.0000
WYNDE6	240	f	0	3.08	28.43	34.66	0.0000
Subtotal WYNDE6				2.88	64.82	55.08	
ZHENG	9	m	0	0.87	6.78	8.21	0.0229

	N	34
	NS	28
	Wt	260.78
Het	Chi	253.85
Het	df	33
Het	P	***
Fixed	RR	7.20
	RRl	6.38
	RRu	8.13
	P	+++
Random	RR	4.80
	RRl	3.29
	RRu	7.01
	P	+++
Asymm	P	*

Table 3G19 - 3

IESLC - Meta-anal of Ever Smoking (or Current if ev not avail), Amount smoked, “High”, Cigs (or Any Prod if Cigs not avail)
Adenocarcinoma
Most adjusted

	Sex combined	male	female	Total
N	2	21	11	34
NS	2	21	11	34
Wt	18.06	159.04	83.69	260.78
Het Chi	14.55	136.78	85.32	253.85
Het df	1	20	10	33
Het P	***	***	***	***
Fixed RR	8.40	5.88	10.22	7.20
RRl	5.29	5.04	8.25	6.38
RRu	13.32	6.87	12.67	8.13
P	+++	+++	+++	+++
Random RR	3.47	4.68	5.21	4.80
RRl	0.27	2.93	2.50	3.29
RRu	44.75	7.47	10.86	7.01
P	N.S.	+++	+++	+++
Between Chi				17.20
Between df				2
Between P				***
Btwn(F) P				N.S.
Btwn(R) P				N.S.

Table 3G19 - 4

IESLC - Meta-anal of Ever Smoking (or Current if ev not avail), Amount smoked, “High”, Cigs (or Any Prod if Cigs not avail)
Adenocarcinoma
Least adjusted

REF	NRR	X	SEX	AGEL	AGEH	RACE	YF	LC TYPE	LOC	START	ST	NLC	R	VB	P	H	AD	SM	PRODUCT	exL	exH	DENOM	De
ALDERS	48		m	0	0	all	-	not q+s	Eu:UK	1977	CC	1448	n	V	n	n	1	ev	cig only	28	99	nev+2	ot
ALDERS	51		f	0	0	all	-	not q+s	Eu:UK	1977	CC	1448	n	V	n	n	1	ev	cig only	28	99	nev+2	ot
BARBON	49	x	m	0	0	all	-	a	Eu:wst	1979	CC	755	n	bl	y	y	0	ev	all/unsp	40	99	nev any	st
BOUCOT	28	x	m	0	0	all	0	a	NAmer	1951	pr	121	n	bl	n	n	0	cu	cig only	21	99	nev any	ot
CHOI	55		m	0	0	all	-	a	As:oth	1985	CC	375	n	bl	n	n	0	ev	cig+/-ot	41	99	nev cigs	st
CHOI	61		f	0	0	all	-	a	As:oth	1985	CC	375	n	bl	n	n	0	ev	cig+/-ot	31	99	nev cigs	st
CORREA	52		c	0	0	all	-	a	NAmer	1979	CC	1359	n	bl	y	n	1	cu	cig+/-ot	21	99	nev cigs	or
DOLL	61	x	m	0	0	all	-	KII	Eu:UK	1948	CC	1465	n	V	n	n	0	ev	all/unsp	25	99	nev any	st
DOSEME	16		m	0	0	all	-	not q+s	Eu:bal	1979	CC	1210	n	bl	n	n	2	ev	cig+/-ot	21	99	nev cigs	or
GER	4	x	c	0	0	all	-	a	As:oth	1990	CC	141	n	ot	y	n	0	ev	all/unsp	21	99	nev any	st
HAENSZ	34		f	0	0	all	-	a	NAmer	1955	CC	158	n	bl	n	y	0	cu	cig+/-ot	21	99	nev any	or
JEDRYC	19	x	m	0	0	all	-	a	Eu:est	1980	CC	1630	n	bl	y	n	0	ev	cig+/-ot	40	99	nev any	st
KATSOU	18	x	f	0	0	all	-	a	Eu:bal	1987	CC	101	n	bl	n	n	0	cu	all/unsp	21	99	nev any	st
KREYBE	19	x	m	0	0	all	-	KII	Eu:Sca	1948	CC	300	n	bl	n	y	0	ev	all/unsp	25	99	nev any	st
LAMTH	18		f	0	0	ch	-	a	As:HK	1983	CC	445	n	bl	n	n	0	ev	all/unsp	21	99	nev any	or
LUBIN2	164		m	0	0	all	-	a	Eu:mul	1976	CC	7804	n	bl	n	y	0	ev	cig+/-ot	30	99	nev any	st
LUBIN2	184		f	0	0	all	-	a	Eu:mul	1976	CC	7804	n	bl	n	y	0	ev	cig+/-ot	30	99	nev any	st
MATOS	60	x	m	0	0	all	-	a	SCAmer	1994	CC	200	n	bl	n	n	0	ev	cig+/-ot	25	99	nev any	st
MATSUD	9		m	0	0	all	-	a	As:Jap	1965	CC	179	n	bl	n	n	0	ev	cig+/-ot	21	99	nev cigs	ot
ORMOS	24		m	0	0	all	-	KII	Eu:est	1947	CC	119	n	bl	y	y	0	ev	cig+/-ot	31	99	nev any	st
OSANN	63		m	0	0	all	-	a	NAmer	1984	CC	1986	n	bl	n	n	2	ev	cig+/-ot	40	99	nev cigs	or
OSANN	64		f	0	0	all	-	a	NAmer	1984	CC	1986	n	bl	n	n	2	ev	cig+/-ot	40	99	nev cigs	or
SOBUE	58		m	0	0	all	-	a	As:Jap	1986	CC	1376	n	bl	n	y	0	cu	cig+/-ot	30	99	nev cigs	st
SVENSS	39	x	f	0	0	all	-	a	Eu:Sca	1983	CC	210	n	bl	n	n	0	cu	all/unsp	21	99	nev any	st
TSUGAN	6		m	0	0	all	-	a	As:Jap	1976	CC	134	n	bl	n	y	0	cu	all/unsp	36	99	nev any	st
WAKAI	51	x	m	0	0	all	-	a	As:Jap	1988	CC	333	n	bl	n	y	0	cu	cig+/-ot	30	99	nev any	st
WU	4	x	f	0	0	wh	-	a	NAmer	1981	CC	220	n	bl	n	y	0	cu	all/unsp	21	99	nev any	st
WYNDE2	13		m	0	0	all	-	KII	NAmer	1962	CC	404	n	bl	n	y	0	ev	cig+/-ot	35	99	nev any	st
WYNDE3	27		m	0	0	all	-	KII	NAmer	1966	CC	350	n	bl	n	y	0	ev	cig+/-ot	41	99	nev any	st
WYNDE3	74		f	0	0	all	-	KII	NAmer	1966	CC	350	n	bl	n	y	0	ev	cig+/-ot	41	99	nev any	st
WYNDE4	41		m	0	0	all	-	a	NAmer	1948	CC	684	n	bl	y	n	0	ev	all/unsp	35	99	nev any	st
WYNDE6	51		m	0	0	all	-	KII	NAmer	1969	CC	4423	n	bl	n	y	0	cu	cig+/-ot	31	99	nev any	st
WYNDE6	240		f	0	0	all	-	KII	NAmer	1969	CC	4423	n	bl	n	y	0	cu	cig+/-ot	30	99	nev cigs	st
ZHENG	9		m	0	0	all	-	a	As:Chi	1982	CC	540	n	ot	*	y	0	ev	cig+/-ot	30	99	nev cigs	st

Cigarette type is all/unspec for all RRs

except for the following:

REF	NRR	CIGTYPE
ALDERS	48	MC only
ALDERS	51	MC only

Table 3G19 - 5

IESLC - Meta-anal of Ever Smoking (or Current if ev not avail), Amount smoked, “High”, Cigs (or Any Prod if Cigs not avail)
Adenocarcinoma
Least adjusted

REF	NRR	SEX	AD	Number Case	Exposed Cont	Non-exposed Case	Cont	RR	95.00%CI
ALDERS	48	m	1	-	-	-	-	3.32	(1.36- 8.10)
ALDERS	51	f	1	-	-	-	-	3.31	(1.80- 6.10)
Subtotal ALDERS								3.31	(2.00- 5.48)
BARBON	49	m	0	40	111	7	188	9.68	(4.19- 22.34)
*BOUCOT	28	m	0	6	6940	0	7551	14.14~	(0.80- 251.03)
CHOI	55	m	0	2	6	7	95	4.52	(0.77- 26.69)
CHOI	61	f	0	1	1	49	164	3.35	(0.21- 54.50)
Subtotal CHOI								4.15	(0.93- 18.54)
CORREA	52	c	1	-	-	-	-	12.00	(7.30- 19.70)
DOLL	61	m	0	6	166	2	61	1.10	(0.22- 5.61)
DOSEME	16	m	2	-	-	-	-	3.20	(1.40- 7.00)
GER	4	c	0	5	26	37	149	0.77	(0.28- 2.15)
HAENSZ	34	f	0	5	13	37	236	2.45	(0.83- 7.28)
JEDRYC	19	m	0	9	82	7	289	4.53	(1.64- 12.54)
KATSOU	18	f	0	6	4	30	67	3.35	(0.88- 12.75)
KREYBE	19	m	0	5	248	3	644	4.33	(1.03- 18.25)
LAMTH	18	f	0	9	5	131	158	2.17	(0.71- 6.64)
LUBIN2	164	m	0	151	1746	57	2616	3.97	(2.91- 5.42)
LUBIN2	184	f	0	9	39	138	1180	1.97	(0.94- 4.16)
Subtotal LUBIN2								3.58	(2.69- 4.77)
MATOS	60	m	0	40	105	5	110	8.38	(3.19- 22.05)
MATSUD	9	m	0	4	470	0	1255	24.02~	(1.29- 446.92)
ORMOS	24	m	0	1	128	2	777	3.04	(0.27- 33.72)
OSANN	63	m	2	-	-	-	-	37.50	(21.30- 66.00)
OSANN	64	f	2	-	-	-	-	24.20	(15.80- 37.20)
Subtotal OSANN								28.39	(20.18- 39.94)
SOBUE	58	m	0	76	187	27	128	1.93	(1.18- 3.16)
SVENSS	39	f	0	4	1	22	120	21.82	(2.33- 204.53)
TSUGAN	6	m	0	10	13	18	17	0.73	(0.25- 2.09)
WAKAI	51	m	0	27	48	8	65	4.57	(1.91- 10.94)
WU	4	f	0	61	22	29	62	5.93	(3.07- 11.44)
WYNDE2	13	m	0	16	112	5	105	3.00	(1.06- 8.48)
WYNDE3	27	m	0	9	26	6	88	5.08	(1.65- 15.59)
WYNDE3	74	f	0	1	3	15	76	1.69	(0.16- 17.36)
Subtotal WYNDE3								4.13	(1.50- 11.34)
WYNDE4	41	m	0	7	64	4	115	3.14	(0.89- 11.15)
WYNDE6	51	m	0	282	197	58	617	15.23	(11.00- 21.07)
WYNDE6	240	f	0	157	52	119	856	21.72	(15.04- 31.37)
Subtotal WYNDE6								17.79	(13.95- 22.70)
ZHENG	9	m	0	17	23	29	94	2.40	(1.13- 5.08)
Partial Totals				966	10838	852	17883		

*prospective study ~ With 0.5 adjustment for zero

REF	NRR	SEX	AD	Ys	Ws	Qs	Ps
ALDERS	48	m	1	1.20	4.83	2.76	0.0084
ALDERS	51	f	1	1.20	10.32	5.95	0.0001
Subtotal ALDERS				1.20	15.14	8.71	
BARBON	49	m	0	2.27	5.49	0.54	0.0000
*BOUCOT	28	m	0	2.65	0.46	0.22	0.0710
CHOI	55	m	0	1.51	1.22	0.24	0.0956
CHOI	61	f	0	1.21	0.49	0.28	0.3961
Subtotal CHOI				1.42	1.71	0.52	
CORREA	52	c	1	2.48	15.59	4.36	0.0000
DOLL	61	m	0	0.10	1.45	5.01	0.9065
DOSEME	16	m	2	1.16	5.93	3.73	0.0046
GER	4	c	0	-0.26	3.67	17.97	0.6242
HAENSZ	34	f	0	0.90	3.24	3.64	0.1060
JEDRYC	19	m	0	1.51	3.71	0.74	0.0036
KATSOU	18	f	0	1.21	2.15	1.20	0.0762
KREYBE	19	m	0	1.47	1.86	0.45	0.0460
LAMTH	18	f	0	0.78	3.08	4.29	0.1740
LUBIN2	164	m	0	1.38	39.81	13.29	0.0000
LUBIN2	184	f	0	0.68	6.90	11.25	0.0741
Subtotal LUBIN2				1.28	46.71	24.54	
MATOS	60	m	0	2.13	4.10	0.12	0.0000
MATSUD	9	m	0	3.18	0.45	0.67	0.0331
ORMOS	24	m	0	1.11	0.66	0.47	0.3661
OSANN	63	m	2	3.62	12.01	33.43	0.0000
OSANN	64	f	2	3.19	20.96	31.71	0.0000
Subtotal OSANN				3.35	32.97	65.14	

Table 3G19 - 5

IESLC - Meta-anal of Ever Smoking (or Current if ev not avail), Amount smoked, “High”, Cigs (or Any Prod if Cigs not avail)
Adenocarcinoma
Least adjusted

REF	NRR	SEX	AD	Ys	Ws	Qs	Ps
SOBUE	58	m	0	0.66	15.78	26.69	0.0092
SVENSS	39	f	0	3.08	0.77	0.97	0.0069
TSUGAN	6	m	0	-0.32	3.43	17.78	0.5538
WAKAI	51	m	0	1.52	5.04	0.96	0.0006
WU	4	f	0	1.78	8.89	0.28	0.0000
WYNDE2	13	m	0	1.10	3.56	2.62	0.0382
WYNDE3	27	m	0	1.62	3.05	0.34	0.0045
WYNDE3	74	f	0	0.52	0.71	1.45	0.6593
Subtotal WYNDE3				1.42	3.76	1.79	
WYNDE4	41	m	0	1.15	2.40	1.58	0.0761
WYNDE6	51	m	0	2.72	36.38	21.40	0.0000
WYNDE6	240	f	0	3.08	28.43	35.78	0.0000
Subtotal WYNDE6				2.88	64.82	57.18	
ZHENG	9	m	0	0.87	6.78	7.95	0.0229

	N	34
	NS	28
	Wt	263.62
Het	Chi	260.11
Het	df	33
Het	P	***
Fixed	RR	7.07
	RRl	6.27
	RRu	7.98
	P	+++
Random	RR	4.72
	RRl	3.23
	RRu	6.90
	P	+++
Asymm	P	*

Table 3G19 - 6

IESLC - Meta-anal of Ever Smoking (or Current if ev not avail), Amount smoked, “High”, Cigs (or Any Prod if Cigs not avail)
Adenocarcinoma
Least adjusted

	Sex: combined	male	female	Total
N	2	21	11	34
NS	2	21	11	34
Wt	19.27	158.42	85.94	263.62
Het Chi	22.33	135.16	86.14	260.11
Het df	1	20	10	33
Het P	***	***	***	***
Fixed RR	7.12	5.84	10.06	7.07
RRl	4.55	5.00	8.14	6.27
RRu	11.12	6.82	12.43	7.98
P	+++	+++	+++	+++
Random RR	3.17	4.59	5.29	4.72
RRl	0.22	2.88	2.57	3.23
RRu	46.40	7.32	10.91	6.90
P	N.S.	+++	+++	+++
Between Chi				16.48
Between df				2
Between P				***
Btwn(F) P				N.S.
Btwn(R) P				N.S.

Table 3G19 - 7

IESLC - Meta-anal of Ever Smoking (or Current if ev not avail), Amount smoked, “High”, Cigs (or Any Prod if Cigs not avail)
Adenocarcinoma
Excluded studies (and stage at which they were excluded)

```
1      ABELIN ABRAHA AMANDU   AMES ANDERS AUSTIN AXELSO   BAND BECHER BERRIN BLOHMK  BLOT4 BROCKM BROWN1 BYERS1 BYERS2
       CARPEN CASCO2 CASCOR   CHAN  CHEN3 CHIAZZ  CHYOU DESTE2 DOCKER DROSTE     DU GARCIA GARDIN   GENG GODLEY GOODMA
       GRAHAM GREGOR HEGMAN   HEIN HENNEK  HINDS HIRAOK HOROWI HORWIT  HUANG ISHIMA   JAHN   JAIN JARVHO  JIANG KELLER
       KIHARA  KJUUS     KO KOHLME  KUBIK  LAMWK LAMWK2  LANGE    LEI LEMARC  LEVIN    LIU LOMBA2 LOMBAR MAGNUS  MARSH
       MARSH2 MCDUFF MCLAUG MILLER  MILLS NOTANI    NOU ODRISC PAWLEG PERSHA POFFIJ   QIAO  QIAO2 RADZIK    REN  RONCO
        ROOTS ROTHSC SAARIK SANKAR SCHWAR   SEGI   SEOW SHIMIZ SIMARA SIMONA  SITAS SOBUE2 STASZE STAYNE STUCKE    SUN
       SUZUK2 SUZUKI   TANG    TAO TOKARS TOUSEY  ULMER VEIERO  VUTUC   WALD   WANG  WANG3  WANG4 WICKLU  WIGLE WILKIN
          WU2 WUNSCH WYNDE8 XIANGZ     XU    XU2    XU4   YONG  ZHANG

2       BUELL   CHEN MASTRA MZILEN PISANI RESTRE SADOWS

4      BOFFET WYNDE7

5      RIMING  TANG2 WYNDE5

6       BLOT1  BLOT2  BLOT3 BOUCHA HIRAY2  JONES LAURIL LICKIN  MOLLO  MRFIT MURATA SCHWA2 VANDER WARSIN WATSON WYNDER

8       AGUDO  AKIBA ARCHER ARMADA AUVINE AXELSS BENSHL   BEST BRESLO  BRETT  BROSS BUFFLE CEDERL  CHANG CHATZI  CHEN2
         CHOW COMSTO COOKSO   CPSI  CPSII DAMBER  DARBY DAVEYS   DEAN  DEAN2  DEAN3 DEKLER DESTEF  DOLL2 DORANT   DORN
         DUNN EBELIN ENSTRO  ESAKI    FAN    GAO   GAO2 GARSHI GILLIS GOLLED  GSELL HAMMO2 HANSEN HIRAYA HITOSU   HOLE
           HU    HU2 HUMBLE  JARUP   JOLY JUSSAW KAISE2 KAISER KANELL KAUFMA KHUDER KINLEN  KNEKT    KOO KOULUM KREUZE
       LAUSSM LETOUR   LIAW LIDDEL   LIU2   LIU3   LIU4   LIU5  LUBIN    LUO MACLEN MARTIS MCCONN MIGRAN MRFITR    NAM
       NOTAN2 PARKIN PASTOR  PERNU PERSH2   PETO PEZZO2 PEZZOT   PIKE POLEDN PRESCO RACHTA RANDIG  SEGI2   SHAW SIEMIA
       SPEIZE  SPITZ STOCKS STOCKW TENKAN TIZZAN TULINI TVERDA  WANG2    XU3 YAMAGU   YUAN   ZHOU

10     BROWN2 DORGAN ENGELA HAMMON OSANN2 WUWILL

11     BENHAM
```

Table 3G19 - 8
Potentially overlapping studies

REF	REFGP	PRINC	.	OVERLAP/LINK
LUBIN2	LUBIN2	1		Lubin-combined
LAMTH	LAMTH	1		KOO/LAMTH/LAMWK
WYNDE6	WYNDE6	1		WYNDE5/6/7/8
MATSUD	MATSUD	1		SOBUE2/MATSUD

Table 3G19 - 9
Most adjusted - insufficient data for metaanalysis

REF	NRR	SEX	AGEL	AGEH	RACE	YF	LC TYPE	LOC	START	ST	NLC	R	VB	P	H	AD	SM	PRODUCT	exL	exH	DENOM	De
CHEN	12	c	0	0	all	-	a	As:oth	1987	CC	323	n	ot	n	y	2	ev	cig+/-ot	31	99	nev cigs	ot

REF	NRR	RR	SIG	RRDATA comment
CHEN	12	3.61	y	P < 0.001

Table 3G21 -

IESLC - Meta-anal of Ever Smoking (or Curr if Ever not avail) by Amount, Overview, Cigarettes only
Adenocarcinoma

This analysis is restricted to results for:
1) Results by Amount smoked
Results by Amount smoked (in numbers of cigarettes) are grouped under 2 schemes (S1, S2).
Each scheme has a set of "key values". An interval is allocated to the category whose key value it includes and intervals which include none or more than one of the key values are excluded.
(Open-ended intervals are coded as 99.)

S1	key value	maximum range	S2	key value	maximum range
1	5	1-19	1	1	1-9
2	20	6-44	2	10	2-19
3	45	21+	3	20	11-29
			4	30	21-39
			5	40	31-98
			6	99	41+

2) Results complete enough for use in metaanalysis

Within each study, results are then selected (in the following order of preference, within each sex) for:
3) SMKSTA: ever smokers, current smokers
4) PRODUCT: cigarettes only
5) CIGTYPE: all/unspecified, MC regardless of HR, MC only
6) DENOM: never smoked anything, never smoked cigarettes, (never +1 = +long term ex, +2 = +amount unknown, +3 = never cigs+long term ex)
7) Followup period (YF, prospective studies): whole study (coded as 0) or longest available
8) LCtype: adeno or nearest available, but not squamous. (q = squamous, s = small, a = adeno, l = large, KII = Kreyberg II, al = alveolar, br = bronchiolar, u = undifferentiated)
9) Race: all or nearest available, otherwise by race (wh or w = white, bl or b = black, hi = hispanic ch = chinese, jap = japanese, haw = hawaiian, w+o = white + oriental, sca = scandinavian, as = asian)
10) For overlapping studies: principal rather than subsidiary studies
Finally by Age: whole study (coded as 0) if available, otherwise by widest available age group and then for single sex results (m, f) in preference to combined sex results (c).

Results adjusted (AD) for the most potential confounders are then chosen in Sections -1 to -3 (and those which actually differ from the adjusted results in Table 3G11 - 1 are marked 'x' in Section -1) and results adjusted for the least confounders in Sections -4 to -6. (Those least adjusted results which actually differ from the most adjusted as marked 'x' in column X in Section -4)
(Results adjusted for an unknown number of confounder(s) are coded as 20.)

Section -7 shows excluded studies, together with the stage (as above) at which no qualifying results were found.

Section -8 lists the potentially overlapping studies which have been included (1=principal, 2=subsidiary).

Section -9 lists any results which would have been included in preference except that they had data not complete enough for use in meta-analysis, with their significance (yes/no), if known, and any further comment as entered on the database.

In addition to those mentioned above, the following fields, levels and abbreviations are used:
* or nk = not known, n = no, y = yes, ot = other
ev = ever, cu = current, nev = never
all/unspec = all or unspecified, MC = manufactured cigarettes, HR = hand-rolled cigarettes
exL, exH = range of exposure (low and high) in the smoking group, in terms of Amount smoked, cigarettes
REF: 6-character study reference
NRR: number of the RR on the database within the study
ST : study type (CC = case control, pr or prosp = prospective)
NLC: number of lung cancer cases in whole study
R : risky occupational population (n = no, m = mining, o = other risky)
VB : national cigarette type (V = at least 75% Virginia, bl = at least 75% blended, ot = other)
P : any proxy use
H : full histological confirmation
De : derivation of RR/CI (or = original, st = standard method, ot = other method of estimation)

Table 3G21 - 1

IESLC - Meta-anal of Ever Smoking (or Curr if Ever not avail) by Amount, Overview, Cigarettes only
Adenocarcinoma
Most adjusted

REF	NRR	3G11	SEX	AGEL	AGEH	RACE	YF	LC TYPE	LOC	START	ST	NLC	R	VB	P	H	AD	SM	PRODUCT	exL	exH	S1	S2	DENOM	De
ALDERS	46		m	0	0	all	-	not q+s	Eu:UK	1977	CC	1448	n	V	n	n	1	ev	cig only	1	17	1	0	nev+2	ot
ALDERS	47		m	0	0	all	-	not q+s	Eu:UK	1977	CC	1448	n	V	n	n	1	ev	cig only	18	27	2	3	nev+2	ot
ALDERS	48		m	0	0	all	-	not q+s	Eu:UK	1977	CC	1448	n	V	n	n	1	ev	cig only	28	99	3	0	nev+2	ot
ALDERS	49		f	0	0	all	-	not q+s	Eu:UK	1977	CC	1448	n	V	n	n	1	ev	cig only	1	17	1	0	nev+2	ot
ALDERS	50		f	0	0	all	-	not q+s	Eu:UK	1977	CC	1448	n	V	n	n	1	ev	cig only	18	27	2	3	nev+2	ot
ALDERS	51		f	0	0	all	-	not q+s	Eu:UK	1977	CC	1448	n	V	n	n	1	ev	cig only	28	99	3	0	nev+2	ot
BENHAM	16	x	m	0	0	all	-	KII	Eu:wst	1976	CC	1625	n	bl	n	y	0	ev	cig only	1	14	1	0	nev any	st
BENHAM	17	x	m	0	0	all	-	KII	Eu:wst	1976	CC	1625	n	bl	n	y	0	ev	cig only	15	20	2	3	nev any	st
BENHAM	18	x	m	0	0	all	-	KII	Eu:wst	1976	CC	1625	n	bl	n	y	0	ev	cig only	21	99	3	0	nev any	st
BOUCOT	148		m	0	0	all	0	a	NAmer	1951	pr	121	n	bl	n	n	2	cu	cig only	1	20	0	0	nev any	ot
BOUCOT	149		m	0	0	all	0	a	NAmer	1951	pr	121	n	bl	n	n	2	cu	cig only	21	99	3	0	nev any	ot
HAMMON	108		m	0	0	wh	0	a	NAmer	1952	pr	448	n	bl	n	n	1	cu	cig only	1	9	1	1	nev any	ot
HAMMON	109		m	0	0	wh	0	a	NAmer	1952	pr	448	n	bl	n	n	1	cu	cig only	10	20	2	0	nev any	ot
HAMMON	110		m	0	0	wh	0	a	NAmer	1952	pr	448	n	bl	n	n	1	cu	cig only	21	39	0	4	nev any	ot

Cigarette type is all/unspec for all RRs

except for the following:

REF	NRR	CIGTYPE
ALDERS	46	MC only
ALDERS	47	MC only
ALDERS	48	MC only
ALDERS	49	MC only
ALDERS	50	MC only
ALDERS	51	MC only

In this overview table, subtotals and Qs values may be invalid and should be ignored

Table 3G21 - 2

IESLC - Meta-anal of Ever Smoking (or Curr if Ever not avail) by Amount, Overview, Cigarettes only
Adenocarcinoma
Most adjusted

REF	NRR	SEX	AD	Number Exposed Case	Number Exposed Cont	Non-exposed Case	Non-exposed Cont	RR	95.00%CI
ALDERS	46	m	1	-	-	-	-	2.80	(1.01- 7.75)
ALDERS	47	m	1	-	-	-	-	2.67	(0.99- 7.18)
ALDERS	48	m	1	-	-	-	-	3.32	(1.36- 8.10)
ALDERS	49	f	1	-	-	-	-	2.77	(1.63- 4.70)
ALDERS	50	f	1	-	-	-	-	4.58	(2.67- 7.85)
ALDERS	51	f	1	-	-	-	-	3.31	(1.80- 6.10)
Subtotal ALDERS								3.34	(2.53- 4.41)
BENHAM	16	m	0	28	45	9	42	2.90	(1.23- 6.87)
BENHAM	17	m	0	41	65	9	42	2.94	(1.30- 6.68)
BENHAM	18	m	0	41	33	9	42	5.80	(2.47- 13.61)
Subtotal BENHAM								3.66	(2.25- 5.95)
*BOUCOT	148	m	2	-	-	-	-	8.22	(0.56- 166.83)
*BOUCOT	149	m	2	-	-	-	-	18.00	(1.01- 319.52)
Subtotal BOUCOT								12.11	(1.60- 91.75)
*HAMMON	108	m	1	-	-	-	-	1.83	(0.17- 20.22)
*HAMMON	109	m	1	-	-	-	-	2.83	(0.55- 14.60)
*HAMMON	110	m	1	-	-	-	-	6.44	(1.34- 31.02)
Subtotal HAMMON								3.71	(1.33- 10.33)
Partial Totals				110	143	27	126		

*prospective study

REF	NRR	SEX	AD	Ys	Ws	Qs	Ps
ALDERS	46	m	1	1.03	3.70	0.18	0.0476
ALDERS	47	m	1	0.98	3.91	0.28	0.0520
ALDERS	48	m	1	1.20	4.83	0.01	0.0084
ALDERS	49	f	1	1.02	13.70	0.72	0.0002
ALDERS	50	f	1	1.52	13.21	0.99	0.0000
ALDERS	51	f	1	1.20	10.32	0.03	0.0001
Subtotal ALDERS				1.21	49.67	2.20	
BENHAM	16	m	0	1.07	5.19	0.17	0.0152
BENHAM	17	m	0	1.08	5.72	0.16	0.0098
BENHAM	18	m	0	1.76	5.27	1.37	0.0001
Subtotal BENHAM				1.30	16.18	1.70	
*BOUCOT	148	m	2	2.11	0.47	0.35	0.1472
*BOUCOT	149	m	2	2.89	0.46	1.25	0.0491
Subtotal BOUCOT				2.49	0.94	1.60	
*HAMMON	108	m	1	0.60	0.67	0.28	0.6201
*HAMMON	109	m	1	1.04	1.43	0.06	0.2136
*HAMMON	110	m	1	1.86	1.56	0.59	0.0201
Subtotal HAMMON				1.31	3.66	0.93	

N	14
NS	4

Table 3G21 - 3

IESLC - Meta-anal of Ever Smoking (or Curr if Ever not avail) by Amount, Overview, Cigarettes only
Adenocarcinoma
Most adjusted

	Sex combined	male	female	Total
N		11	3	14
NS		4	1	5

In this overview table, other than the "N" rows, entries in the "absent" and "Total" columns may be invalid and should be ignored

	Amount smoked (broad categories) absent	<20k5	6-44k20	>20k45	Total
N	2	4	4	4	14
NS	2	3	3	3	11
Wt	2.03	23.26	24.28	20.88	70.45
Het Chi	0.02	0.13	1.42	2.31	6.43
Het df	1	3	3	3	13
Het P	N.S.	N.S.	N.S.	N.S.	N.S.
Fixed RR	6.82	2.77	3.68	3.96	3.49
RRl	1.72	1.85	2.47	2.58	2.76
RRu	26.98	4.16	5.47	6.08	4.40
P	++	+++	+++	+++	+++
Random RR	6.82	2.77	3.68	3.96	3.49
RRl	1.72	1.85	2.47	2.58	2.76
RRu	26.98	4.16	5.47	6.08	4.40
P	++	+++	+++	+++	+++

	absent	<10k1	2-19k10	Amount smoked (narrow categories) 11-29k20	21-39k30	31-98k40	>40k99	Total
N	9	1		3	1			14
NS	4	1		2	1			7
Wt	45.37	0.67		22.85	1.56			70.45
Het Chi	4.04	0.00		1.32	0.00			6.43
Het df	8	0		2	0			13
Het P	N.S.	N.S.		N.S.	N.S.			N.S.
Fixed RR	3.33	1.83		3.74	6.44			3.49
RRl	2.49	0.17		2.48	1.34			2.76
RRu	4.45	19.96		5.63	30.99			4.40
P	+++	N.S.		+++	+			+++
Random RR	3.33	1.83		3.74	6.44			3.49
RRl	2.49	0.17		2.48	1.34			2.76
RRu	4.45	19.96		5.63	30.99			4.40
P	+++	N.S.		+++	+			+++

MALES

	Amount smoked (broad categories) absent	<20k5	6-44k20	>20k45	Total
N	2	3	3	3	11
NS	2	3	3	3	11
Wt	2.03	9.56	11.07	10.56	33.22
Het Chi	0.02	0.13	0.02	1.65	4.70
Het df	1	2	2	2	10
Het P	N.S.	N.S.	N.S.	N.S.	N.S.
Fixed RR	6.82	2.77	2.83	4.72	3.49
RRl	1.72	1.47	1.57	2.58	2.49
RRu	26.98	5.22	5.10	8.63	4.91
P	++	++	+++	+++	+++
Random RR	6.82	2.77	2.83	4.72	3.49
RRl	1.72	1.47	1.57	2.58	2.49
RRu	26.98	5.22	5.10	8.63	4.91
P	++	++	+++	+++	+++

Table 3G21 - 3

IESLC - Meta-anal of Ever Smoking (or Curr if Ever not avail) by Amount, Overview, Cigarettes only
Adenocarcinoma
Most adjusted

MALES

	absent	<10k1	2-19k10	11-29k20	21-39k30	31-98k40	>40k99	Total
	Amount smoked (narrow categories)							
N	7	1		2	1			11
NS	4	1		2	1			7
Wt	21.35	0.67		9.64	1.56			33.22
Het Chi	3.27	0.00		0.02	0.00			4.70
Het df	6	0		1	0			10
Het P	N.S.	N.S.		N.S.	N.S.			N.S.
Fixed RR	3.75	1.83		2.83	6.44			3.49
RRl	2.45	0.17		1.50	1.34			2.49
RRu	5.73	19.96		5.32	30.99			4.91
P	+++	N.S.		++	+			+++
Random RR	3.75	1.83		2.83	6.44			3.49
RRl	2.45	0.17		1.50	1.34			2.49
RRu	5.73	19.96		5.32	30.99			4.91
P	+++	N.S.		++	+			+++

FEMALES

	absent	<20k5	6-44k20	>20k45	Total
	Amount smoked (broad categories)				
N		1	1	1	3
NS		1	1	1	1
Wt		13.70	13.21	10.32	37.23
Het Chi		0.00	0.00	0.00	1.74
Het df		0	0	0	2
Het P		N.S.	N.S.	N.S.	N.S.
Fixed RR		2.77	4.58	3.31	3.48
RRl		1.63	2.67	1.80	2.52
RRu		4.70	7.85	6.09	4.80
P		+++	+++	+++	+++
Random RR		2.77	4.58	3.31	3.48
RRl		1.63	2.67	1.80	2.52
RRu		4.70	7.85	6.09	4.80
P		+++	+++	+++	+++

	absent	<10k1	2-19k10	11-29k20	21-39k30	31-98k40	>40k99	Total
	Amount smoked (narrow categories)							
N	2			1				3
NS	1			1				1
Wt	24.02			13.21				37.23
Het Chi	0.19			0.00				1.74
Het df	1			0				2
Het P	N.S.			N.S.				N.S.
Fixed RR	2.99			4.58				3.48
RRl	2.00			2.67				2.52
RRu	4.46			7.85				4.80
P	+++			+++				+++
Random RR	2.99			4.58				3.48
RRl	2.00			2.67				2.52
RRu	4.46			7.85				4.80
P	+++			+++				+++

Table 3G21 - 4

IESLC - Meta-anal of Ever Smoking (or Curr if Ever not avail) by Amount, Overview, Cigarettes only
Adenocarcinoma
Least adjusted

REF	NRR	X	SEX	AGEL	AGEH	RACE	YF	LC TYPE	LOC	START	ST	NLC	R	VB	P	H	AD	SM	PRODUCT	exL	exH	S1	S2	DENOM	De
ALDERS	46		m	0	0	all	-	not q+s	Eu:UK	1977	CC	1448	n	V	n	n	1	ev	cig only	1	17	1	0	nev+2	ot
ALDERS	47		m	0	0	all	-	not q+s	Eu:UK	1977	CC	1448	n	V	n	n	1	ev	cig only	18	27	2	3	nev+2	ot
ALDERS	48		m	0	0	all	-	not q+s	Eu:UK	1977	CC	1448	n	V	n	n	1	ev	cig only	28	99	3	0	nev+2	ot
ALDERS	49		f	0	0	all	-	not q+s	Eu:UK	1977	CC	1448	n	V	n	n	1	ev	cig only	1	17	1	0	nev+2	ot
ALDERS	50		f	0	0	all	-	not q+s	Eu:UK	1977	CC	1448	n	V	n	n	1	ev	cig only	18	27	2	3	nev+2	ot
ALDERS	51		f	0	0	all	-	not q+s	Eu:UK	1977	CC	1448	n	V	n	n	1	ev	cig only	28	99	3	0	nev+2	ot
BENHAM	16		m	0	0	all	-	KII	Eu:wst	1976	CC	1625	n	bl	n	y	0	ev	cig only	1	14	1	0	nev any	st
BENHAM	17		m	0	0	all	-	KII	Eu:wst	1976	CC	1625	n	bl	n	y	0	ev	cig only	15	20	2	3	nev any	st
BENHAM	18		m	0	0	all	-	KII	Eu:wst	1976	CC	1625	n	bl	n	y	0	ev	cig only	21	99	3	0	nev any	st
BOUCOT	27	x	m	0	0	all	0	a	NAmer	1951	pr	121	n	bl	n	n	0	cu	cig only	1	20	0	0	nev any	ot
BOUCOT	28	x	m	0	0	all	0	a	NAmer	1951	pr	121	n	bl	n	n	0	cu	cig only	21	99	3	0	nev any	ot
HAMMON	108		m	0	0	wh	0	a	NAmer	1952	pr	448	n	bl	n	n	1	cu	cig only	1	9	1	1	nev any	ot
HAMMON	109		m	0	0	wh	0	a	NAmer	1952	pr	448	n	bl	n	n	1	cu	cig only	10	20	2	0	nev any	ot
HAMMON	110		m	0	0	wh	0	a	NAmer	1952	pr	448	n	bl	n	n	1	cu	cig only	21	39	0	4	nev any	ot

Cigarette type is all/unspec for all RRs

except for the following:

REF	NRR	CIGTYPE
ALDERS	46	MC only
ALDERS	47	MC only
ALDERS	48	MC only
ALDERS	49	MC only
ALDERS	50	MC only
ALDERS	51	MC only

In this overview table, subtotals and Qs values may be invalid and should be ignored

Table 3G21 - 5

IESLC - Meta-anal of Ever Smoking (or Curr if Ever not avail) by Amount, Overview, Cigarettes only
Adenocarcinoma
Least adjusted

REF	NRR	SEX	AD	Number Exposed Case	Number Exposed Cont	Non-exposed Case	Non-exposed Cont	RR	95.00%CI
ALDERS	46	m	1	-	-	-	-	2.80	(1.01- 7.75)
ALDERS	47	m	1	-	-	-	-	2.67	(0.99- 7.18)
ALDERS	48	m	1	-	-	-	-	3.32	(1.36- 8.10)
ALDERS	49	f	1	-	-	-	-	2.77	(1.63- 4.70)
ALDERS	50	f	1	-	-	-	-	4.58	(2.67- 7.85)
ALDERS	51	f	1	-	-	-	-	3.31	(1.80- 6.10)
Subtotal ALDERS								3.34	(2.53- 4.41)
BENHAM	16	m	0	28	45	9	42	2.90	(1.23- 6.87)
BENHAM	17	m	0	41	65	9	42	2.94	(1.30- 6.68)
BENHAM	18	m	0	41	33	9	42	5.80	(2.47- 13.61)
Subtotal BENHAM								3.66	(2.25- 5.95)
*BOUCOT	27	m	0	8	15208	0	7551	8.44~	(0.49- 146.22)
*BOUCOT	28	m	0	6	6940	0	7551	14.14~	(0.80- 251.03)
Subtotal BOUCOT								10.90	(1.44- 82.62)
*HAMMON	108	m	1	-	-	-	-	1.83	(0.17- 20.22)
*HAMMON	109	m	1	-	-	-	-	2.83	(0.55- 14.60)
*HAMMON	110	m	1	-	-	-	-	6.44	(1.34- 31.02)
Subtotal HAMMON								3.71	(1.33- 10.33)
Partial Totals				124	22291	27	15228		

*prospective study

~ With 0.5 adjustment for zero

REF	NRR	SEX	AD	Ys	Ws	Qs	Ps
ALDERS	46	m	1	1.03	3.70	0.18	0.0476
ALDERS	47	m	1	0.98	3.91	0.28	0.0520
ALDERS	48	m	1	1.20	4.83	0.01	0.0084
ALDERS	49	f	1	1.02	13.70	0.71	0.0002
ALDERS	50	f	1	1.52	13.21	1.00	0.0000
ALDERS	51	f	1	1.20	10.32	0.03	0.0001
Subtotal ALDERS				1.21	49.67	2.20	
BENHAM	16	m	0	1.07	5.19	0.17	0.0152
BENHAM	17	m	0	1.08	5.72	0.16	0.0098
BENHAM	18	m	0	1.76	5.27	1.37	0.0001
Subtotal BENHAM				1.30	16.18	1.70	
*BOUCOT	27	m	0	2.13	0.47	0.37	0.1427
*BOUCOT	28	m	0	2.65	0.46	0.91	0.0710
Subtotal BOUCOT				2.39	0.94	1.28	
*HAMMON	108	m	1	0.60	0.67	0.28	0.6201
*HAMMON	109	m	1	1.04	1.43	0.06	0.2136
*HAMMON	110	m	1	1.86	1.56	0.59	0.0201
Subtotal HAMMON				1.31	3.66	0.93	

N	14
NS	4

Table 3G21 - 6

IESLC - Meta-anal of Ever Smoking (or Curr if Ever not avail) by Amount, Overview, Cigarettes only
Adenocarcinoma
Least adjusted

	combined	Sex male	female	Total
N		11	3	14
NS		4	1	5

In this overview table, other than the "N" rows, entries in the "absent" and "Total" columns may be invalid and should be ignored

	Amount smoked (broad categories) absent	<20k5	6-44k20	>20k45	Total
N	2	4	4	4	14
NS	2	3	3	3	11
Wt	2.03	23.26	24.28	20.88	70.45
Het Chi	0.03	0.13	1.42	2.00	6.11
Het df	1	3	3	3	13
Het P	N.S.	N.S.	N.S.	N.S.	N.S.
Fixed RR	6.86	2.77	3.68	3.94	3.48
RRl	1.73	1.85	2.47	2.57	2.76
RRu	27.15	4.16	5.47	6.05	4.40
P	++	+++	+++	+++	+++
Random RR	6.86	2.77	3.68	3.94	3.48
RRl	1.73	1.85	2.47	2.57	2.76
RRu	27.15	4.16	5.47	6.05	4.40
P	++	+++	+++	+++	+++

	absent	<10k1	2-19k10	Amount smoked (narrow categories) 11-29k20	21-39k30	31-98k40	>40k99	Total
N	9	1		3	1			14
NS	4	1		2	1			7
Wt	45.37	0.67		22.85	1.56			70.45
Het Chi	3.71	0.00		1.32	0.00			6.11
Het df	8	0		2	0			13
Het P	N.S.	N.S.		N.S.	N.S.			N.S.
Fixed RR	3.32	1.83		3.74	6.44			3.48
RRl	2.48	0.17		2.48	1.34			2.76
RRu	4.44	19.96		5.63	30.99			4.40
P	+++	N.S.		+++	+			+++
Random RR	3.32	1.83		3.74	6.44			3.48
RRl	2.48	0.17		2.48	1.34			2.76
RRu	4.44	19.96		5.63	30.99			4.40
P	+++	N.S.		+++	+			+++

MALES

	Amount smoked (broad categories) absent	<20k5	6-44k20	>20k45	Total
N	2	3	3	3	11
NS	2	3	3	3	11
Wt	2.03	9.56	11.07	10.56	33.22
Het Chi	0.03	0.13	0.02	1.38	4.38
Het df	1	2	2	2	10
Het P	N.S.	N.S.	N.S.	N.S.	N.S.
Fixed RR	6.86	2.77	2.83	4.67	3.48
RRl	1.73	1.47	1.57	2.56	2.48
RRu	27.15	5.22	5.10	8.54	4.89
P	++	++	+++	+++	+++
Random RR	6.86	2.77	2.83	4.67	3.48
RRl	1.73	1.47	1.57	2.56	2.48
RRu	27.15	5.22	5.10	8.54	4.89
P	++	++	+++	+++	+++

Table 3G21 - 6

IESLC - Meta-anal of Ever Smoking (or Curr if Ever not avail) by Amount, Overview, Cigarettes only
Adenocarcinoma
Least adjusted

MALES

Amount smoked (narrow categories)

	absent	<10k1	2-19k10	11-29k20	21-39k30	31-98k40	>40k99	Total
N	7	1		2	1			11
NS	4	1		2	1			7
Wt	21.35	0.67		9.64	1.56			33.22
Het Chi	2.97	0.00		0.02	0.00			4.38
Het df	6	0		1	0			10
Het P	N.S.	N.S.		N.S.	N.S.			N.S.
Fixed RR	3.73	1.83		2.83	6.44			3.48
RRl	2.44	0.17		1.50	1.34			2.48
RRu	5.70	19.96		5.32	30.99			4.89
P	+++	N.S.		++	+			+++
Random RR	3.73	1.83		2.83	6.44			3.48
RRl	2.44	0.17		1.50	1.34			2.48
RRu	5.70	19.96		5.32	30.99			4.89
P	+++	N.S.		++	+			+++

FEMALES

Amount smoked (broad categories)

	absent	<20k5	6-44k20	>20k45	Total
N		1	1	1	3
NS		1	1	1	1
Wt		13.70	13.21	10.32	37.23
Het Chi		0.00	0.00	0.00	1.74
Het df		0	0	0	2
Het P		N.S.	N.S.	N.S.	N.S.
Fixed RR		2.77	4.58	3.31	3.48
RRl		1.63	2.67	1.80	2.52
RRu		4.70	7.85	6.09	4.80
P		+++	+++	+++	+++
Random RR		2.77	4.58	3.31	3.48
RRl		1.63	2.67	1.80	2.52
RRu		4.70	7.85	6.09	4.80
P		+++	+++	+++	+++

Amount smoked (narrow categories)

	absent	<10k1	2-19k10	11-29k20	21-39k30	31-98k40	>40k99	Total
N	2			1				3
NS	1			1				1
Wt	24.02			13.21				37.23
Het Chi	0.19			0.00				1.74
Het df	1			0				2
Het P	N.S.			N.S.				N.S.
Fixed RR	2.99			4.58				3.48
RRl	2.00			2.67				2.52
RRu	4.46			7.85				4.80
P	+++			+++				+++
Random RR	2.99			4.58				3.48
RRl	2.00			2.67				2.52
RRu	4.46			7.85				4.80
P	+++			+++				+++

Table 3G21 - 7

IESLC - Meta-anal of Ever Smoking (or Curr if Ever not avail) by Amount, Overview, Cigarettes only
Adenocarcinoma
Excluded studies (and stage at which they were excluded)

1 ABELIN ABRAHA AMANDU AMES ANDERS AUSTIN AXELSO BAND BECHER BERRIN BLOHMK BLOT4 BROCKM BROWN1 BYERS1 BYERS2
CARPEN CASCO2 CASCOR CHAN CHEN3 CHIAZZ CHYOU DESTE2 DOCKER DROSTE DU GARCIA GARDIN GENG GODLEY GOODMA
GRAHAM GREGOR HEGMAN HEIN HENNEK HINDS HIRAOK HOROWI HORWIT HUANG ISHIMA JAHN JAIN JARVHO JIANG KELLER
KIHARA KJUUS KO KOHLME KUBIK LAMWK LAMWK2 LANGE LEI LEMARC LEVIN LIU LOMBA2 LOMBAR MAGNUS MARSH
MARSH2 MCDUFF MCLAUG MILLER MILLS NOTANI NOU ODRISC PAWLEG PERSHA POFFIJ QIAO QIAO2 RADZIK REN RONCO
ROOTS ROTHSC SAARIK SANKAR SCHWAR SEGI SEOW SHIMIZ SIMARA SIMONA SITAS SOBUE2 STASZE STAYNE STUCKE SUN
SUZUK2 SUZUKI TANG TAO TOKARS TOUSEY ULMER VEIERO VUTUC WALD WANG WANG3 WANG4 WICKLU WIGLE WILKIN
WU2 WUNSCH WYNDE8 XIANGZ XU XU2 XU4 YONG ZHANG

2 BUELL CHEN MASTRA MZILEN PISANI RESTRE SADOWS

4 AKIBA ARCHER ARMADA AUVINE AXELSS BARBON BENSHL BLOT1 BLOT2 BLOT3 BOFFET BOUCHA BRESLO BRETT BROSS BROWN2
BUFFLE CHANG CHATZI CHEN2 CHOI CHOW COMSTO COOKSO CORREA DARBY DAVEYS DEKLER DESTEF DOLL DORANT DORGAN
DOSEME DUNN EBELIN ESAKI FAN GAO GAO2 GARSHI GER GILLIS GSELL HAENSZ HAMMO2 HANSEN HIRAYA HITOSU
HOLE HU HU2 HUMBLE JARUP JEDRYC JOLY JONES KAISER KANELL KATSOU KAUFMA KHUDER KINLEN KNEKT KOO
KOULUM KREUZE KREYBE LAMTH LAURIL LAUSSM LETOUR LIAW LICKIN LIDDEL LIU2 LIU3 LIU5 LUBIN2 LUO MACLEN
MARTIS MATOS MATSUD MOLLO MRFIT MRFITR MURATA NAM ORMOS OSANN OSANN2 PARKIN PASTOR PERNU PERSH2 PETO
PEZZO2 PIKE POLEDN PRESCO RACHTA RANDIG SEGI2 SHAW SIEMIA SOBUE SPEIZE SPITZ STOCKS STOCKW SVENSS TENKAN
TSUGAN TULINI VANDER WAKAI WANG2 WARSIN WATSON WU WUWILL WYNDE2 WYNDE3 WYNDE4 WYNDE5 WYNDE6 WYNDE7 WYNDER
XU3 YAMAGU YUAN ZHENG ZHOU

5 RIMING TANG2

6 HIRAY2 SCHWA2

8 AGUDO BEST CEDERL CPSI CPSII DAMBER DEAN DEAN2 DEAN3 DOLL2 DORN ENGELA ENSTRO GOLLED JUSSAW KAISE2
LIU4 LUBIN MCCONN MIGRAN NOTAN2 PEZZOT TIZZAN TVERDA

Table 3G21 - 8
Potentially overlapping studies

REF	REFGP	PRINC	. OVERLAP/LINK
BENHAM	LUBIN2	2	Subset of Lubin2

Table 3G22 -

IESLC - Meta-anal of Ever Smoking (or Current if ever not avail), Amount smoked, "Low", Cigarettes only
Adenocarcinoma

This analysis is restricted to results for:
1) Results by Amount smoked
2) Results complete enough for use in metaanalysis

Within each study, results are then selected (in the following order of preference, within each sex) for:
3) SMKSTA: ever smokers, current smokers
4) PRODUCT: cigarettes only
5) CIGTYPE: all/unspecified, MC regardless of HR, MC only
6) DENOM: never smoked anything, never smoked cigarettes, (never +1 = +long term ex, +2 = +amount unknown, +3 = never cigs+long term ex)
7) Followup period (YF, prospective studies): whole study (coded as 0) or longest available
8) LCtype: adeno or nearest available, but not squamous. (q = squamous, s = small, a = adeno, l = large, KII = Kreyberg II, al = alveolar, br = bronchiolar, u = undifferentiated)
9) Race: all or nearest available, otherwise by race (wh or w = white, bl or b = black, hi = hispanic ch = chinese, jap = japanese, haw = hawaiian, w+o = white + oriental, sca = scandinavian, as = asian)
10) Amount smoked "low" in key scheme 1 (key value 5, maximum range <20, in numbers of cigarettes)
11) For overlapping studies: principal rather than subsidiary studies
Finally by Age: whole study (coded as 0) if available, otherwise by widest available age group
and then for single sex results (m, f) in preference to combined sex results (c).

Results adjusted (AD) for the most potential confounders are then chosen in Sections -1 to -3
(and those which actually differ from the adjusted results in Table 3G12 - 1 are marked 'x' in Section -1)
and results adjusted for the least confounders in Sections -4 to -6. (Those least adjusted results which
actually differ from the most adjusted as marked 'x' in column X in Section -4)
(Results adjusted for an unknown number of confounder(s) are coded as 20.)

Section -7 shows excluded studies, together with the stage (as above) at which no qualifying
results were found.

Section -8 lists the potentially overlapping studies which have been included (1=principal, 2=subsidiary).

Section -9 lists any results which would have been included in preference except that they had data not complete
enough for use in meta-analysis, with their significance (yes/no), if known, and any further comment as entered
on the database.

In addition to those mentioned above, the following fields, levels and abbreviations are used:
* or nk = not known, n = no, y = yes, ot = other
ev = ever, cu = current, nev = never
all/unspec = all or unspecified, MC = manufactured cigarettes, HR = hand-rolled cigarettes
exL, exH = range of exposure (low and high) in the smoking group, in terms of Amount smoked, cigarettes
REF: 6-character study reference
NRR: number of the RR on the database within the study
ST : study type (CC = case control, pr or prosp = prospective)
NLC: number of lung cancer cases in whole study
R : risky occupational population (n = no, m = mining, o = other risky)
VB : national cigarette type (V = at least 75% Virginia, bl = at least 75% blended, ot = other)
P : any proxy use
H : full histological confirmation
De : derivation of RR/CI (or = original, st = standard method, ot = other method of estimation)

Table 3G22 - 1

IESLC - Meta-anal of Ever Smoking (or Current if ever not avail), Amount smoked, "Low", Cigarettes only
Adenocarcinoma
Most adjusted

REF	NRR	3G12	SEX	AGEL	AGEH	RACE	YF	LC TYPE	LOC	START	ST	NLC	R	VB	P	H	AD	SM	PRODUCT	exL	exH	DENOM	De
ALDERS	46		m	0	0	all	-	not q+s	Eu:UK	1977	CC	1448	n	V	n	n	1	ev	cig only	1	17	nev+2	ot
ALDERS	49		f	0	0	all	-	not q+s	Eu:UK	1977	CC	1448	n	V	n	n	1	ev	cig only	1	17	nev+2	ot
BENHAM	16	x	m	0	0	all	-	KII	Eu:wst	1976	CC	1625	n	bl	n	y	0	ev	cig only	1	14	nev any	st
HAMMON	108		m	0	0	wh	0	a	NAmer	1952	pr	448	n	bl	n	n	1	cu	cig only	1	9	nev any	ot

Cigarette type is all/unspec for all RRs

except for the following:

REF	NRR	CIGTYPE
ALDERS	46	MC only
ALDERS	49	MC only

Table 3G22 - 2

IESLC - Meta-anal of Ever Smoking (or Current if ever not avail), Amount smoked, "Low", Cigarettes only
Adenocarcinoma
Most adjusted

REF	NRR	SEX	AD	Number Exposed Case	Cont	Non-exposed Case	Cont	RR	95.00%CI
ALDERS	46	m	1	-	-	-	-	2.80	(1.01- 7.75)
ALDERS	49	f	1	-	-	-	-	2.77	(1.63- 4.70)
Subtotal ALDERS								2.78	(1.74- 4.44)
BENHAM	16	m	0	28	45	9	42	2.90	(1.23- 6.87)
*HAMMON	108	m	1	-	-	-	-	1.83	(0.17- 20.22)
Partial Totals				28	45	9	42		

*prospective study

REF	NRR	SEX	AD	Ys	Ws	Qs	Ps
ALDERS	46	m	1	1.03	3.70	0.00	0.0476
ALDERS	49	f	1	1.02	13.70	0.00	0.0002
Subtotal ALDERS				1.02	17.40	0.00	
BENHAM	16	m	0	1.07	5.19	0.01	0.0152
*HAMMON	108	m	1	0.60	0.67	0.12	0.6201

	N	4
	NS	3
	Wt	23.26
Het	Chi	0.13
Het	df	3
Het	P	N.S.
Fixed	RR	2.77
	RRl	1.85
	RRu	4.16
	P	+++
Random	RR	2.77
	RRl	1.85
	RRu	4.16
	P	+++
Asymm	P	N.S.

Table 3G22 - 3

IESLC - Meta-anal of Ever Smoking (or Current if ever not avail), Amount smoked, "Low", Cigarettes only
Adenocarcinoma
Most adjusted

	Sex combined	male	female	Total
N		3	1	4
NS		3	1	4
Wt		9.56	13.70	23.26
Het Chi		0.13	0.00	0.13
Het df		2	0	3
Het P		N.S.	N.S.	N.S.
Fixed RR		2.77	2.77	2.77
RRl		1.47	1.63	1.85
RRu		5.22	4.70	4.16
P		++	+++	+++
Random RR		2.77	2.77	2.77
RRl		1.47	1.63	1.85
RRu		5.22	4.70	4.16
P		++	+++	+++
Between Chi				0.00
Between df				1
Between P				N.S.
Btwn(F) P				N.S.
Btwn(R) P				N.S.

Too few RRs for analysis by factor

Table 3G22 - 4

IESLC - Meta-anal of Ever Smoking (or Current if ever not avail), Amount smoked, "Low", Cigarettes only
Adenocarcinoma
Least adjusted

REF	NRR	X	SEX	AGEL	AGEH	RACE	YF	LC TYPE	LOC	START	ST	NLC	R	VB	P	H	AD	SM	PRODUCT	exL	exH	DENOM	De
ALDERS	46		m	0	0	all	-	not q+s	Eu:UK	1977	CC	1448	n	V	n	n	1	ev	cig only	1	17	nev+2	ot
ALDERS	49		f	0	0	all	-	not q+s	Eu:UK	1977	CC	1448	n	V	n	n	1	ev	cig only	1	17	nev+2	ot
BENHAM	16		m	0	0	all	-	KII	Eu:wst	1976	CC	1625	n	bl	n	y	0	ev	cig only	1	14	nev any	st
HAMMON	108		m	0	0	wh	0	a	NAmer	1952	pr	448	n	bl	n	n	1	cu	cig only	1	9	nev any	ot

Cigarette type is all/unspec for all RRs

except for the following:

REF	NRR	CIGTYPE
ALDERS	46	MC only
ALDERS	49	MC only

Table 3G22 - 5

IESLC - Meta-anal of Ever Smoking (or Current if ever not avail), Amount smoked, "Low", Cigarettes only
Adenocarcinoma
Least adjusted

REF	NRR	SEX	AD	Number Exposed Case	Cont	Non-exposed Case	Cont	RR	95.00%CI
ALDERS	46	m	1	-	-	-	-	2.80	(1.01- 7.75)
ALDERS	49	f	1	-	-	-	-	2.77	(1.63- 4.70)
Subtotal ALDERS								2.78	(1.74- 4.44)
BENHAM	16	m	0	28	45	9	42	2.90	(1.23- 6.87)
*HAMMON	108	m	1	-	-	-	-	1.83	(0.17- 20.22)
Partial Totals				28	45	9	42		

*prospective study

REF	NRR	SEX	AD	Ys	Ws	Qs	Ps
ALDERS	46	m	1	1.03	3.70	0.00	0.0476
ALDERS	49	f	1	1.02	13.70	0.00	0.0002
Subtotal ALDERS				1.02	17.40	0.00	
BENHAM	16	m	0	1.07	5.19	0.01	0.0152
*HAMMON	108	m	1	0.60	0.67	0.12	0.6201

	N	4
	NS	3
	Wt	23.26
Het	Chi	0.13
Het	df	3
Het	P	N.S.
Fixed	RR	2.77
	RRl	1.85
	RRu	4.16
	P	+++
Random	RR	2.77
	RRl	1.85
	RRu	4.16
	P	+++
Asymm	P	N.S.

Table 3G22 - 6

IESLC - Meta-anal of Ever Smoking (or Current if ever not avail), Amount smoked, "Low", Cigarettes only
Adenocarcinoma
Least adjusted

	Sex combined	male	female	Total
N		3	1	4
NS		3	1	4
Wt		9.56	13.70	23.26
Het Chi		0.13	0.00	0.13
Het df		2	0	3
Het P		N.S.	N.S.	N.S.
Fixed RR		2.77	2.77	2.77
RRl		1.47	1.63	1.85
RRu		5.22	4.70	4.16
P		++	+++	+++
Random RR		2.77	2.77	2.77
RRl		1.47	1.63	1.85
RRu		5.22	4.70	4.16
P		++	+++	+++
Between Chi				0.00
Between df				1
Between P				N.S.
Btwn(F) P				N.S.
Btwn(R) P				N.S.

Table 3G22 - 7

IESLC - Meta-anal of Ever Smoking (or Current if ever not avail), Amount smoked, "Low", Cigarettes only
Adenocarcinoma
Excluded studies (and stage at which they were excluded)

```
1      ABELIN ABRAHA AMANDU   AMES ANDERS AUSTIN AXELSO   BAND BECHER BERRIN BLOHMK  BLOT4 BROCKM BROWN1 BYERS1 BYERS2
       CARPEN CASCO2 CASCOR   CHAN  CHEN3 CHIAZZ  CHYOU DESTE2 DOCKER DROSTE     DU GARCIA GARDIN   GENG GODLEY GOODMA
       GRAHAM GREGOR HEGMAN   HEIN HENNEK  HINDS HIRAOK HOROWI HORWIT  HUANG ISHIMA   JAHN   JAIN JARVHO  JIANG KELLER
       KIHARA  KJUUS     KO KOHLME  KUBIK  LAMWK LAMWK2  LANGE    LEI LEMARC  LEVIN    LIU LOMBA2 LOMBAR MAGNUS  MARSH
       MARSH2 MCDUFF MCLAUG MILLER  MILLS NOTANI    NOU ODRISC PAWLEG PERSHA POFFIJ   QIAO  QIAO2 RADZIK    REN  RONCO
        ROOTS ROTHSC SAARIK SANKAR SCHWAR   SEGI   SEOW SHIMIZ SIMARA SIMONA  SITAS SOBUE2 STASZE STAYNE STUCKE    SUN
       SUZUK2 SUZUKI   TANG    TAO TOKARS TOUSEY  ULMER VEIERO  VUTUC   WALD   WANG  WANG3  WANG4 WICKLU  WIGLE WILKIN
          WU2 WUNSCH WYNDE8 XIANGZ     XU    XU2    XU4   YONG  ZHANG

2       BUELL   CHEN MASTRA MZILEN PISANI RESTRE SADOWS

4       AKIBA ARCHER ARMADA AUVINE AXELSS BARBON BENSHL  BLOT1  BLOT2  BLOT3 BOFFET BOUCHA BRESLO  BRETT  BROSS BROWN2
       BUFFLE  CHANG CHATZI  CHEN2   CHOI   CHOW COMSTO COOKSO CORREA  DARBY DAVEYS DEKLER DESTEF   DOLL DORANT DORGAN
       DOSEME   DUNN EBELIN  ESAKI    FAN    GAO   GAO2 GARSHI    GER GILLIS  GSELL HAENSZ HAMMO2 HANSEN HIRAYA HITOSU
         HOLE     HU    HU2 HUMBLE  JARUP JEDRYC   JOLY  JONES KAISER KANELL KATSOU KAUFMA KHUDER KINLEN  KNEKT    KOO
       KOULUM KREUZE KREYBE  LAMTH LAURIL LAUSSM LETOUR   LIAW LICKIN LIDDEL   LIU2   LIU3   LIU5 LUBIN2    LUO MACLEN
       MARTIS  MATOS MATSUD  MOLLO  MRFIT MRFITR MURATA    NAM  ORMOS  OSANN OSANN2 PARKIN PASTOR  PERNU PERSH2   PETO
       PEZZO2   PIKE POLEDN PRESCO RACHTA RANDIG  SEGI2   SHAW SIEMIA  SOBUE SPEIZE  SPITZ STOCKS STOCKW SVENSS TENKAN
       TSUGAN TULINI VANDER  WAKAI  WANG2 WARSIN WATSON     WU WUWILL WYNDE2 WYNDE3 WYNDE4 WYNDE5 WYNDE6 WYNDE7 WYNDER
          XU3 YAMAGU   YUAN  ZHENG   ZHOU

5      RIMING  TANG2

6      HIRAY2 SCHWA2

8       AGUDO   BEST CEDERL   CPSI  CPSII DAMBER   DEAN  DEAN2  DEAN3  DOLL2   DORN ENGELA ENSTRO GOLLED JUSSAW KAISE2
         LIU4  LUBIN MCCONN MIGRAN NOTAN2 PEZZOT TIZZAN TVERDA

10     BOUCOT
```

Table 3G22 - 8

Potentially overlapping studies

REF	REFGP	PRINC	. OVERLAP/LINK
BENHAM	LUBIN2	2	Subset of Lubin2

Table 3G23 -

IESLC - Meta-anal of Ever Smoking (or Current if ever not avail), Amount smoked, "Mid", Cigarettes only
Adenocarcinoma

This analysis is restricted to results for:
1) Results by Amount smoked
2) Results complete enough for use in metaanalysis

Within each study, results are then selected (in the following order of preference, within each sex) for:
3) SMKSTA: ever smokers, current smokers
4) PRODUCT: cigarettes only
5) CIGTYPE: all/unspecified, MC regardless of HR, MC only
6) DENOM: never smoked anything, never smoked cigarettes, (never +1 = +long term ex, +2 = +amount unknown, +3 = never cigs+long term ex)
7) Followup period (YF, prospective studies): whole study (coded as 0) or longest available
8) LCtype: adeno or nearest available, but not squamous. (q = squamous, s = small, a = adeno, l = large, KII = Kreyberg II, al = alveolar, br = bronchiolar, u = undifferentiated)
9) Race: all or nearest available, otherwise by race (wh or w = white, bl or b = black, hi = hispanic ch = chinese, jap = japanese, haw = hawaiian, w+o = white + oriental, sca = scandinavian, as = asian)
10) Amount smoked "mid" in key scheme 1 (key value 20, maximum range 6-44, in numbers of cigarettes)
11) For overlapping studies: principal rather than subsidiary studies
Finally by Age: whole study (coded as 0) if available, otherwise by widest available age group and then for single sex results (m, f) in preference to combined sex results (c).

Results adjusted (AD) for the most potential confounders are then chosen in Sections -1 to -3 (and those which actually differ from the adjusted results in Table 3G13 - 1 are marked 'x' in Section -1) and results adjusted for the least confounders in Sections -4 to -6. (Those least adjusted results which actually differ from the most adjusted as marked 'x' in column X in Section -4) (Results adjusted for an unknown number of confounder(s) are coded as 20.)

Section -7 shows excluded studies, together with the stage (as above) at which no qualifying results were found.

Section -8 lists the potentially overlapping studies which have been included (1=principal, 2=subsidiary).

Section -9 lists any results which would have been included in preference except that they had data not complete enough for use in meta-analysis, with their significance (yes/no), if known, and any further comment as entered on the database.

In addition to those mentioned above, the following fields, levels and abbreviations are used:
* or nk = not known, n = no, y = yes, ot = other
ev = ever, cu = current, nev = never
all/unspec = all or unspecified, MC = manufactured cigarettes, HR = hand-rolled cigarettes
exL, exH = range of exposure (low and high) in the smoking group, in terms of Amount smoked, cigarettes
REF: 6-character study reference
NRR: number of the RR on the database within the study
ST : study type (CC = case control, pr or prosp = prospective)
NLC: number of lung cancer cases in whole study
R : risky occupational population (n = no, m = mining, o = other risky)
VB : national cigarette type (V = at least 75% Virginia, bl = at least 75% blended, ot = other)
P : any proxy use
H : full histological confirmation
De : derivation of RR/CI (or = original, st = standard method, ot = other method of estimation)

Table 3G23 - 1

IESLC - Meta-anal of Ever Smoking (or Current if ever not avail), Amount smoked, "Mid", Cigarettes only
Adenocarcinoma
Most adjusted

REF	NRR	3G13	SEX	AGEL	AGEH	RACE	YF	LC TYPE	LOC	START	ST	NLC	R	VB	P	H	AD	SM	PRODUCT	exL	exH	DENOM	De
ALDERS	47		m	0	0	all	-	not q+s	Eu:UK	1977	CC	1448	n	V	n	n	1	ev	cig only	18	27	nev+2	ot
ALDERS	50		f	0	0	all	-	not q+s	Eu:UK	1977	CC	1448	n	V	n	n	1	ev	cig only	18	27	nev+2	ot
BENHAM	17	x	m	0	0	all	-	KII	Eu:wst	1976	CC	1625	n	bl	n	y	0	ev	cig only	15	20	nev any	st
HAMMON	109		m	0	0	wh	0	a	NAmer	1952	pr	448	n	bl	n	n	1	cu	cig only	10	20	nev any	ot

Cigarette type is all/unspec for all RRs

except for the following:

REF	NRR	CIGTYPE
ALDERS	47	MC only
ALDERS	50	MC only

Table 3G23 - 2

IESLC - Meta-anal of Ever Smoking (or Current if ever not avail), Amount smoked, "Mid", Cigarettes only
Adenocarcinoma
Most adjusted

REF	NRR	SEX	AD	Number Exposed Case	Number Exposed Cont	Non-exposed Case	Non-exposed Cont	RR	95.00%CI
ALDERS	47	m	1	-	-	-	-	2.67	(0.99- 7.18)
ALDERS	50	f	1	-	-	-	-	4.58	(2.67- 7.85)
Subtotal ALDERS								4.05	(2.52- 6.50)
BENHAM	17	m	0	41	65	9	42	2.94	(1.30- 6.68)
*HAMMON	109	m	1	-	-	-	-	2.83	(0.55- 14.60)
Partial Totals				41	65	9	42		

*prospective study

REF	NRR	SEX	AD	Ys	Ws	Qs	Ps
ALDERS	47	m	1	0.98	3.91	0.40	0.0520
ALDERS	50	f	1	1.52	13.21	0.64	0.0000
Subtotal ALDERS				1.40	17.13	1.04	
BENHAM	17	m	0	1.08	5.72	0.28	0.0098
*HAMMON	109	m	1	1.04	1.43	0.10	0.2136

	N	4
	NS	3
	Wt	24.28
Het	Chi	1.42
Het	df	3
Het	P	N.S.
Fixed	RR	3.68
	RRl	2.47
	RRu	5.47
	P	+++
Random	RR	3.68
	RRl	2.47
	RRu	5.47
	P	+++
Asymm	P	N.S.

Table 3G23 - 3

IESLC - Meta-anal of Ever Smoking (or Current if ever not avail), Amount smoked, "Mid", Cigarettes only
Adenocarcinoma
Most adjusted

	combined	Sex male	female	Total
N		3	1	4
NS		3	1	4
Wt		11.07	13.21	24.28
Het Chi		0.02	0.00	1.42
Het df		2	0	3
Het P		N.S.	N.S.	N.S.
Fixed RR		2.83	4.58	3.68
RRl		1.57	2.67	2.47
RRu		5.10	7.85	5.47
P		+++	+++	+++
Random RR		2.83	4.58	3.68
RRl		1.57	2.67	2.47
RRu		5.10	7.85	5.47
P		+++	+++	+++
Between Chi				1.40
Between df				1
Between P				N.S.
Btwn(F) P				**
Btwn(R) P				N.S.

Too few RRs for analysis by factor

Table 3G23 - 4

IESLC - Meta-anal of Ever Smoking (or Current if ever not avail), Amount smoked, "Mid", Cigarettes only
Adenocarcinoma
Least adjusted

REF	NRR	X	SEX	AGEL	AGEH	RACE	YF	LC TYPE	LOC	START	ST	NLC	R	VB	P	H	AD	SM	PRODUCT	exL	exH	DENOM	De
ALDERS	47		m	0	0	all	-	not q+s	Eu:UK	1977	CC	1448	n	V	n	n	1	ev	cig only	18	27	nev+2	ot
ALDERS	50		f	0	0	all	-	not q+s	Eu:UK	1977	CC	1448	n	V	n	n	1	ev	cig only	18	27	nev+2	ot
BENHAM	17		m	0	0	all	-	KII	Eu:wst	1976	CC	1625	n	bl	n	y	0	ev	cig only	15	20	nev any	st
HAMMON	109		m	0	0	wh	0	a	NAmer	1952	pr	448	n	bl	n	n	1	cu	cig only	10	20	nev any	ot

Cigarette type is all/unspec for all RRs

except for the following:

REF	NRR	CIGTYPE
ALDERS	47	MC only
ALDERS	50	MC only

Table 3G23 - 5

IESLC - Meta-anal of Ever Smoking (or Current if ever not avail), Amount smoked, "Mid", Cigarettes only
Adenocarcinoma
Least adjusted

REF	NRR	SEX	AD	Number Exposed Case	Number Exposed Cont	Non-exposed Case	Non-exposed Cont	RR	95.00%CI
ALDERS	47	m	1	-	-	-	-	2.67	(0.99- 7.18)
ALDERS	50	f	1	-	-	-	-	4.58	(2.67- 7.85)
Subtotal ALDERS								4.05	(2.52- 6.50)
BENHAM	17	m	0	41	65	9	42	2.94	(1.30- 6.68)
*HAMMON	109	m	1	-	-	-	-	2.83	(0.55- 14.60)
Partial Totals				41	65	9	42		

*prospective study

REF	NRR	SEX	AD	Ys	Ws	Qs	Ps
ALDERS	47	m	1	0.98	3.91	0.40	0.0520
ALDERS	50	f	1	1.52	13.21	0.64	0.0000
Subtotal ALDERS				1.40	17.13	1.04	
BENHAM	17	m	0	1.08	5.72	0.28	0.0098
*HAMMON	109	m	1	1.04	1.43	0.10	0.2136

	N	4
	NS	3
	Wt	24.28
Het	Chi	1.42
Het	df	3
Het	P	N.S.
Fixed	RR	3.68
	RRl	2.47
	RRu	5.47
	P	+++
Random	RR	3.68
	RRl	2.47
	RRu	5.47
	P	+++
Asymm	P	N.S.

Table 3G23 - 6

IESLC - Meta-anal of Ever Smoking (or Current if ever not avail), Amount smoked, "Mid", Cigarettes only
Adenocarcinoma
Least adjusted

	Sex combined	male	female	Total
N		3	1	4
NS		3	1	4
Wt		11.07	13.21	24.28
Het Chi		0.02	0.00	1.42
Het df		2	0	3
Het P		N.S.	N.S.	N.S.
Fixed RR		2.83	4.58	3.68
RRl		1.57	2.67	2.47
RRu		5.10	7.85	5.47
P		+++	+++	+++
Random RR		2.83	4.58	3.68
RRl		1.57	2.67	2.47
RRu		5.10	7.85	5.47
P		+++	+++	+++
Between Chi				1.40
Between df				1
Between P				N.S.
Btwn(F) P				**
Btwn(R) P				N.S.

Table 3G23 - 7

IESLC - Meta-anal of Ever Smoking (or Current if ever not avail), Amount smoked, "Mid", Cigarettes only
Adenocarcinoma
Excluded studies (and stage at which they were excluded)

1	ABELIN ABRAHA AMANDU AMES ANDERS AUSTIN AXELSO BAND BECHER BERRIN BLOHMK BLOT4 BROCKM BROWN1 BYERS1 BYERS2 CARPEN CASCO2 CASCOR CHAN CHEN3 CHIAZZ CHYOU DESTE2 DOCKER DROSTE DU GARCIA GARDIN GENG GODLEY GOODMA GRAHAM GREGOR HEGMAN HEIN HENNEK HINDS HIRAOK HOROWI HORWIT HUANG ISHIMA JAHN JAIN JARVHO JIANG KELLER KIHARA KJUUS KO KOHLME KUBIK LAMWK LAMWK2 LANGE LEI LEMARC LEVIN LIU LOMBA2 LOMBAR MAGNUS MARSH MARSH2 MCDUFF MCLAUG MILLER MILLS NOTANI NOU ODRISC PAWLEG PERSHA POFFIJ QIAO QIAO2 RADZIK REN RONCO ROOTS ROTHSC SAARIK SANKAR SCHWAR SEGI SEOW SHIMIZ SIMARA SIMONA SITAS SOBUE2 STASZE STAYNE STUCKE SUN SUZUK2 SUZUKI TANG TAO TOKARS TOUSEY ULMER VEIERO VUTUC WALD WANG WANG3 WANG4 WICKLU WIGLE WILKIN WU2 WUNSCH WYNDE8 XIANGZ XU XU2 XU4 YONG ZHANG
2	BUELL CHEN MASTRA MZILEN PISANI RESTRE SADOWS
4	AKIBA ARCHER ARMADA AUVINE AXELSS BARBON BENSHL BLOT1 BLOT2 BLOT3 BOFFET BOUCHA BRESLO BRETT BROSS BROWN2 BUFFLE CHANG CHATZI CHEN2 CHOI CHOW COMSTO COOKSO CORREA DARBY DAVEYS DEKLER DESTEF DOLL DORANT DORGAN DOSEME DUNN EBELIN ESAKI FAN GAO GAO2 GARSHI GER GILLIS GSELL HAENSZ HAMMO2 HANSEN HIRAYA HITOSU HOLE HU HU2 HUMBLE JARUP JEDRYC JOLY JONES KAISER KANELL KATSOU KAUFMA KHUDER KINLEN KNEKT KOO KOULUM KREUZE KREYBE LAMTH LAURIL LAUSSM LETOUR LIAW LICKIN LIDDEL LIU2 LIU3 LIU5 LUBIN2 LUO MACLEN MARTIS MATOS MATSUD MOLLO MRFIT MRFITR MURATA NAM ORMOS OSANN OSANN2 PARKIN PASTOR PERNU PERSH2 PETO PEZZO2 PIKE POLEDN PRESCO RACHTA RANDIG SEGI2 SHAW SIEMIA SOBUE SPEIZE SPITZ STOCKS STOCKW SVENSS TENKAN TSUGAN TULINI VANDER WAKAI WANG2 WARSIN WATSON WU WUWILL WYNDE2 WYNDE3 WYNDE4 WYNDE5 WYNDE6 WYNDE7 WYNDER XU3 YAMAGU YUAN ZHENG ZHOU
5	RIMING TANG2
6	HIRAY2 SCHWA2
8	AGUDO BEST CEDERL CPSI CPSII DAMBER DEAN DEAN2 DEAN3 DOLL2 DORN ENGELA ENSTRO GOLLED JUSSAW KAISE2 LIU4 LUBIN MCCONN MIGRAN NOTAN2 PEZZOT TIZZAN TVERDA
10	BOUCOT

Table 3G23 - 8
Potentially overlapping studies

REF	REFGP	PRINC	OVERLAP/LINK
BENHAM	LUBIN2	2	Subset of Lubin2

Table 3G24 -

IESLC - Meta-anal of Ever Smoking (or Current if ever not avail), Amount smoked, "High", Cigarettes only
Adenocarcinoma

This analysis is restricted to results for:
1) Results by Amount smoked
2) Results complete enough for use in metaanalysis

Within each study, results are then selected (in the following order of preference, within each sex) for:
3) SMKSTA: ever smokers, current smokers
4) PRODUCT: cigarettes only
5) CIGTYPE: all/unspecified, MC regardless of HR, MC only
6) DENOM: never smoked anything, never smoked cigarettes, (never +1 = +long term ex, +2 = +amount unknown, +3 = never cigs+long term ex)
7) Followup period (YF, prospective studies): whole study (coded as 0) or longest available
8) LCtype: adeno or nearest available, but not squamous. (q = squamous, s = small, a = adeno, l = large, KII = Kreyberg II, al = alveolar, br = bronchiolar, u = undifferentiated)
9) Race: all or nearest available, otherwise by race (wh or w = white, bl or b = black, hi = hispanic ch = chinese, jap = japanese, haw = hawaiian, w+o = white + oriental, sca = scandinavian, as = asian)
10) Amount smoked "high" in key scheme 1 (key value 45, maximum range >20, in numbers of cigarettes)
11) For overlapping studies: principal rather than subsidiary studies
Finally by Age: whole study (coded as 0) if available, otherwise by widest available age group
and then for single sex results (m, f) in preference to combined sex results (c).

Results adjusted (AD) for the most potential confounders are then chosen in Sections -1 to -3
(and those which actually differ from the adjusted results in Table 3G14 - 1 are marked 'x' in Section -1)
and results adjusted for the least confounders in Sections -4 to -6. (Those least adjusted results which
actually differ from the most adjusted as marked 'x' in column X in Section -4)
(Results adjusted for an unknown number of confounder(s) are coded as 20.)

Section -7 shows excluded studies, together with the stage (as above) at which no qualifying
results were found.

Section -8 lists the potentially overlapping studies which have been included (1=principal, 2=subsidiary).

Section -9 lists any results which would have been included in preference except that they had data not complete
enough for use in meta-analysis, with their significance (yes/no), if known, and any further comment as entered
on the database.

In addition to those mentioned above, the following fields, levels and abbreviations are used:
* or nk = not known, n = no, y = yes, ot = other
ev = ever, cu = current, nev = never
all/unspec = all or unspecified, MC = manufactured cigarettes, HR = hand-rolled cigarettes
exL, exH = range of exposure (low and high) in the smoking group, in terms of Amount smoked, cigarettes
REF: 6-character study reference
NRR: number of the RR on the database within the study
ST : study type (CC = case control, pr or prosp = prospective)
NLC: number of lung cancer cases in whole study
R : risky occupational population (n = no, m = mining, o = other risky)
VB : national cigarette type (V = at least 75% Virginia, bl = at least 75% blended, ot = other)
P : any proxy use
H : full histological confirmation
De : derivation of RR/CI (or = original, st = standard method, ot = other method of estimation)

Table 3G24 - 1

IESLC - Meta-anal of Ever Smoking (or Current if ever not avail), Amount smoked, "High", Cigarettes only
Adenocarcinoma
Most adjusted

REF	NRR	3G14	SEX	AGEL	AGEH	RACE	YF	LC TYPE	LOC	START	ST	NLC	R	VB	P	H	AD	SM	PRODUCT	exL	exH	DENOM	De
ALDERS	48		m	0	0	all	-	not q+s	Eu:UK	1977	CC	1448	n	V	n	n	1	ev	cig only	28	99	nev+2	ot
ALDERS	51		f	0	0	all	-	not q+s	Eu:UK	1977	CC	1448	n	V	n	n	1	ev	cig only	28	99	nev+2	ot
BENHAM	18	x	m	0	0	all	-	KII	Eu:wst	1976	CC	1625	n	bl	n	y	0	ev	cig only	21	99	nev any	st
BOUCOT	149		m	0	0	all	0	a	NAmer	1951	pr	121	n	bl	n	n	2	cu	cig only	21	99	nev any	ot

Cigarette type is all/unspec for all RRs

except for the following:

REF	NRR	CIGTYPE
ALDERS	48	MC only
ALDERS	51	MC only

Table 3G24 - 2

IESLC - Meta-anal of Ever Smoking (or Current if ever not avail), Amount smoked, "High", Cigarettes only
Adenocarcinoma
Most adjusted

REF	NRR	SEX	AD	Number Exposed Case	Cont	Non-exposed Case	Cont	RR	95.00%CI
ALDERS	48	m	1	-	-	-	-	3.32	(1.36- 8.10)
ALDERS	51	f	1	-	-	-	-	3.31	(1.80- 6.10)
Subtotal ALDERS								3.31	(2.00- 5.48)
BENHAM	18	m	0	41	33	9	42	5.80	(2.47- 13.61)
*BOUCOT	149	m	2	-	-	-	-	18.00	(1.01- 319.52)
Partial Totals				41	33	9	42		

*prospective study

REF	NRR	SEX	AD	Ys	Ws	Qs	Ps
ALDERS	48	m	1	1.20	4.83	0.15	0.0084
ALDERS	51	f	1	1.20	10.32	0.33	0.0001
Subtotal ALDERS				1.20	15.14	0.48	
BENHAM	18	m	0	1.76	5.27	0.76	0.0001
*BOUCOT	149	m	2	2.89	0.46	1.06	0.0491

	N	4
	NS	3
	Wt	20.88
Het	Chi	2.31
Het	df	3
Het	P	N.S.
Fixed	RR	3.96
	RRl	2.58
	RRu	6.08
	P	+++
Random	RR	3.96
	RRl	2.58
	RRu	6.08
	P	+++
Asymm	P	N.S.

Table 3G24 - 3

IESLC - Meta-anal of Ever Smoking (or Current if ever not avail), Amount smoked, "High", Cigarettes only
Adenocarcinoma
Most adjusted

	combined	Sex male	female	Total
N		3	1	4
NS		3	1	4
Wt		10.56	10.32	20.88
Het Chi		1.65	0.00	2.31
Het df		2	0	3
Het P		N.S.	N.S.	N.S.
Fixed RR		4.72	3.31	3.96
RRl		2.58	1.80	2.58
RRu		8.63	6.09	6.08
P		+++	+++	+++
Random RR		4.72	3.31	3.96
RRl		2.58	1.80	2.58
RRu		8.63	6.09	6.08
P		+++	+++	+++
Between Chi				0.66
Between df				1
Between P				N.S.
Btwn(F) P				N.S.
Btwn(R) P				N.S.

Too few RRs for analysis by factor

Table 3G24 - 4

IESLC - Meta-anal of Ever Smoking (or Current if ever not avail), Amount smoked, "High", Cigarettes only
Adenocarcinoma
Least adjusted

REF	NRR	X	SEX	AGEL	AGEH	RACE	YF	LC TYPE	LOC	START	ST	NLC	R	VB	P	H	AD	SM	PRODUCT	exL	exH	DENOM	De
ALDERS	48		m	0	0	all	-	not q+s	Eu:UK	1977	CC	1448	n	V	n	n	1	ev	cig only	28	99	nev+2	ot
ALDERS	51		f	0	0	all	-	not q+s	Eu:UK	1977	CC	1448	n	V	n	n	1	ev	cig only	28	99	nev+2	ot
BENHAM	18		m	0	0	all	-	KII	Eu:wst	1976	CC	1625	n	bl	n	y	0	ev	cig only	21	99	nev any	st
BOUCOT	28	x	m	0	0	all	0	a	NAmer	1951	pr	121	n	bl	n	n	0	cu	cig only	21	99	nev any	ot

Cigarette type is all/unspec for all RRs

except for the following:

REF	NRR	CIGTYPE
ALDERS	48	MC only
ALDERS	51	MC only

Table 3G24 - 5

IESLC - Meta-anal of Ever Smoking (or Current if ever not avail), Amount smoked, "High", Cigarettes only
Adenocarcinoma
Least adjusted

REF	NRR	SEX	AD	Number Exposed Case	Number Exposed Cont	Non-exposed Case	Non-exposed Cont	RR	95.00%CI
ALDERS	48	m	1	-	-	-	-	3.32	(1.36- 8.10)
ALDERS	51	f	1	-	-	-	-	3.31	(1.80- 6.10)
Subtotal ALDERS								3.31	(2.00- 5.48)
BENHAM	18	m	0	41	33	9	42	5.80	(2.47- 13.61)
*BOUCOT	28	m	0	6	6940	0	7551	14.14~	(0.80- 251.03)
Partial Totals				47	6973	9	7593		

*prospective study ~ With 0.5 adjustment for zero

REF	NRR	SEX	AD	Ys	Ws	Qs	Ps
ALDERS	48	m	1	1.20	4.83	0.14	0.0084
ALDERS	51	f	1	1.20	10.32	0.31	0.0001
Subtotal ALDERS				1.20	15.14	0.46	
BENHAM	18	m	0	1.76	5.27	0.79	0.0001
*BOUCOT	28	m	0	2.65	0.46	0.76	0.0710

	N	4
	NS	3
	Wt	20.88
Het	Chi	2.00
Het	df	3
Het	P	N.S.
Fixed	RR	3.94
	RRl	2.57
	RRu	6.05
	P	+++
Random	RR	3.94
	RRl	2.57
	RRu	6.05
	P	+++
Asymm	P	N.S.

Table 3G24 - 6

IESLC - Meta-anal of Ever Smoking (or Current if ever not avail), Amount smoked, "High", Cigarettes only
Adenocarcinoma
Least adjusted

	Sex combined	male	female	Total
N		3	1	4
NS		3	1	4
Wt		10.56	10.32	20.88
Het Chi		1.38	0.00	2.00
Het df		2	0	3
Het P		N.S.	N.S.	N.S.
Fixed RR		4.67	3.31	3.94
RRl		2.56	1.80	2.57
RRu		8.54	6.09	6.05
P		+++	+++	+++
Random RR		4.67	3.31	3.94
RRl		2.56	1.80	2.57
RRu		8.54	6.09	6.05
P		+++	+++	+++
Between Chi				0.62
Between df				1
Between P				N.S.
Btwn(F) P				N.S.
Btwn(R) P				N.S.

Table 3G24 - 7

IESLC - Meta-anal of Ever Smoking (or Current if ever not avail), Amount smoked, "High", Cigarettes only
Adenocarcinoma
Excluded studies (and stage at which they were excluded)

1 ABELIN ABRAHA AMANDU AMES ANDERS AUSTIN AXELSO BAND BECHER BERRIN BLOHMK BLOT4 BROCKM BROWN1 BYERS1 BYERS2 CARPEN CASCO2 CASCOR CHAN CHEN3 CHIAZZ CHYOU DESTE2 DOCKER DROSTE DU GARCIA GARDIN GENG GODLEY GOODMA GRAHAM GREGOR HEGMAN HEIN HENNEK HINDS HIRAOK HOROWI HORWIT HUANG ISHIMA JAHN JAIN JARVHO JIANG KELLER KIHARA KJUUS KO KOHLME KUBIK LAMWK LAMWK2 LANGE LEI LEMARC LEVIN LIU LOMBA2 LOMBAR MAGNUS MARSH MARSH2 MCDUFF MCLAUG MILLER MILLS NOTANI NOU ODRISC PAWLEG PERSHA POFFIJ QIAO QIAO2 RADZIK REN RONCO ROOTS ROTHSC SAARIK SANKAR SCHWAR SEGI SEOW SHIMIZ SIMARA SIMONA SITAS SOBUE2 STASZE STAYNE STUCKE SUN SUZUK2 SUZUKI TANG TAO TOKARS TOUSEY ULMER VEIERO VUTUC WALD WANG WANG3 WANG4 WICKLU WIGLE WILKIN WU2 WUNSCH WYNDE8 XIANGZ XU XU2 XU4 YONG ZHANG

2 BUELL CHEN MASTRA MZILEN PISANI RESTRE SADOWS

4 AKIBA ARCHER ARMADA AUVINE AXELSS BARBON BENSHL BLOT1 BLOT2 BLOT3 BOFFET BOUCHA BRESLO BRETT BROSS BROWN2 BUFFLE CHANG CHATZI CHEN2 CHOI CHOW COMSTO COOKSO CORREA DARBY DAVEYS DEKLER DESTEF DOLL DORANT DORGAN DOSEME DUNN EBELIN ESAKI FAN GAO GAO2 GARSHI GER GILLIS GSELL HAENSZ HAMMO2 HANSEN HIRAYA HITOSU HOLE HU HU2 HUMBLE JARUP JEDRYC JOLY JONES KAISER KANELL KATSOU KAUFMA KHUDER KINLEN KNEKT KOO KOULUM KREUZE KREYBE LAMTH LAURIL LAUSSM LETOUR LIAW LICKIN LIDDEL LIU2 LIU3 LIU5 LUBIN2 LUO MACLEN MARTIS MATOS MATSUD MOLLO MRFIT MRFITR MURATA NAM ORMOS OSANN OSANN2 PARKIN PASTOR PERNU PERSH2 PETO PEZZO2 PIKE POLEDN PRESCO RACHTA RANDIG SEGI2 SHAW SIEMIA SOBUE SPEIZE SPITZ STOCKS STOCKW SVENSS TENKAN TSUGAN TULINI VANDER WAKAI WANG2 WARSIN WATSON WU WUWILL WYNDE2 WYNDE3 WYNDE4 WYNDE5 WYNDE6 WYNDE7 WYNDER XU3 YAMAGU YUAN ZHENG ZHOU

5 RIMING TANG2

6 HIRAY2 SCHWA2

8 AGUDO BEST CEDERL CPSI CPSII DAMBER DEAN DEAN2 DEAN3 DOLL2 DORN ENGELA ENSTRO GOLLED JUSSAW KAISE2 LIU4 LUBIN MCCONN MIGRAN NOTAN2 PEZZOT TIZZAN TVERDA

10 HAMMON

Table 3G24 - 8

Potentially overlapping studies

REF	REFGP	PRINC	OVERLAP/LINK
BENHAM	LUBIN2	2	Subset of Lubin2

Table 3G26 -

IESLC - Meta-analysis of Ever Smoking by Amount, Overview, Pipe and/or Cigars (not cigs)
Adenocarcinoma

This analysis is restricted to results for:
1) Results by Amount smoked
Results by Amount smoked are grouped under 2 schemes (S1, S2). Each scheme has a set of "key values". An interval is allocated to the category whose key value it includes and intervals which include none or more than one of the key values are excluded. (Open-ended intervals are coded as 99). Amounts are usually coded as number of cigarette equivalents, defined as shown at the end of Sections -1 and -4.

S1	key value	maximum range	S2	key value	maximum range
1	1	1-98	1	1	1-9
2	99	2-99	2	10	2-98
			3	99	11+

Thus the two levels in scheme S1 correspond to the lowest and highest intervals, irrespective of their values. In scheme C2, the lowest and highest intervals are chosen provided they do not include the value 10, and the interval including the value 10 is also chosen.
2) Smokers of pipe and/or cigars (but not cigarettes)
3) Ever smokers
4) Results complete enough for use in metaanalysis

Within each study, results are then selected (in the following order of preference, within each sex) for:
5) DENOM: never smoked anything, (never +1 = +long term ex)
6) Followup period (prospective studies): whole study (coded as 0) or longest available
7) LCtype: all or nearest available, at least Squamous and Adeno. (q = squamous, s = small,
l = large, a = adeno, mix = mixed, alv = alveolar)
8) Race: all or nearest available, otherwise by race (wh or w = white, bl or b = black, hi = hispanic
ch = chinese, jap = japanese, haw = hawaiian, w+o = white + oriental, sca = scandinavian, as = asian)
9) For overlapping studies: principal rather than subsidiary studies
Finally by Age: whole study (coded as 0) if available, otherwise by widest available age group
and then for single sex results (m, f) in preference to combined sex results (c).

Results adjusted (AD) for the most potential confounders are then chosen in Sections -1 to -3
and results adjusted for the least confounders in Sections -4 to -6. (Those least adjusted results which
actually differ from the most adjusted as marked 'x' in column X in Section -4)
(Results adjusted for an unknown number of confounder(s) are coded as 20.)

Section -7 shows excluded studies, together with the stage (as above) at which no qualifying
results were found.

Section -8 lists the potentially overlapping studies which have been included (1=principal, 2=subsidiary).

Section -9 lists any results which would have been included in preference except that they had data not complete
enough for use in meta-analysis, with their significance (yes/no), if known, and any further comment as entered
on the database.

In addition to those mentioned above, the following fields, levels and abbreviations are used:
* or nk = not known, n = no, y = yes, ot = other
nev = never
exL, exH = range of exposure (low and high) in the smoking group, in terms of Amount smoked,
cigarette equivalents
REF: 6-character study reference
NRR: number of the RR on the database within the study
ST : study type (CC = case control, pr or prosp = prospective)
NLC: number of lung cancer cases in whole study
R : risky occupational population (n = no, m = mining, o = other risky)
VB : national cigarette type (V = at least 75% Virginia, bl = at least 75% blended, ot = other)
P : any proxy use
H : full histological confirmation
De : derivation of RR/CI (or = original, st = standard method, ot = other method of estimation)

Table 3G26 - 0

No RRs selected for this analysis

Table 3G26 - 7

IESLC - Meta-analysis of Ever Smoking by Amount, Overview, Pipe and/or Cigars (not cigs)
Adenocarcinoma
Excluded studies (and stage at which they were excluded)

1 ABELIN ABRAHA AMANDU AMES ANDERS AUSTIN AXELSO BAND BECHER BERRIN BLOHMK BLOT4 BROCKM BROWN1 BYERS1 BYERS2 CARPEN
CASCO2 CASCOR CHAN CHEN3 CHIAZZ CHYOU DESTE2 DOCKER DROSTE DU GARCIA GARDIN GENG GODLEY GOODMA GRAHAM GREGOR
HEGMAN HEIN HENNEK HINDS HIRAOK HOROWI HORWIT HUANG ISHIMA JAHN JAIN JARVHO JIANG KELLER KIHARA KJUUS KO
KOHLME KUBIK LAMWK LAMWK2 LANGE LEI LEMARC LEVIN LIU LOMBA2 LOMBAR MAGNUS MARSH MARSH2 MCDUFF MCLAUG MILLER
MILLS NOTANI NOU ODRISC PAWLEG PERSHA POFFIJ QIAO QIAO2 RADZIK REN RONCO ROOTS ROTHSC SAARIK SANKAR SCHWAR
SEGI SEOW SHIMIZ SIMARA SIMONA SITAS SOBUE2 STASZE STAYNE STUCKE SUN SUZUK2 SUZUKI TANG TAO TOKARS TOUSEY
ULMER VEIERO VUTUC WALD WANG WANG3 WANG4 WICKLU WIGLE WILKIN WU2 WUNSCH WYNDE8 XIANGZ XU XU2 XU4
YONG ZHANG

2 AGUDO AKIBA ALDERS ARCHER ARMADA AUVINE AXELSS BARBON BENHAM BENSHL BEST BLOT1 BLOT2 BLOT3 BOFFET BOUCHA BOUCOT
BRESLO BRETT BROSS BROWN2 BUELL BUFFLE CEDERL CHANG CHATZI CHEN CHEN2 CHOI CHOW COMSTO COOKSO CORREA CPSI
CPSII DAMBER DARBY DAVEYS DEAN DEAN2 DEAN3 DEKLER DESTEF DOLL DORANT DORGAN DORN DOSEME DUNN EBELIN ENGELA
ENSTRO ESAKI FAN GAO GAO2 GARSHI GER GILLIS GOLLED GSELL HAENSZ HAMMO2 HAMMON HANSEN HIRAY2 HIRAYA HITOSU
HOLE HU HU2 HUMBLE JARUP JEDRYC JOLY JONES JUSSAW KAISE2 KAISER KANELL KATSOU KAUFMA KHUDER KINLEN KNEKT
KOO KOULUM KREUZE KREYBE LAMTH LAURIL LAUSSM LETOUR LIAW LICKIN LIDDEL LIU2 LIU3 LIU4 LIU5 LUBIN LUBIN2
LUO MACLEN MARTIS MASTRA MATOS MATSUD MCCONN MIGRAN MOLLO MRFIT MRFITR MURATA MZILEN NAM NOTAN2 ORMOS OSANN
OSANN2 PARKIN PASTOR PERNU PERSH2 PETO PEZZO2 PEZZOT PIKE PISANI POLEDN PRESCO RACHTA RANDIG RESTRE RIMING SADOWS
SCHWA2 SEGI2 SHAW SIEMIA SOBUE SPEIZE SPITZ STOCKS STOCKW SVENSS TANG2 TENKAN TIZZAN TSUGAN TULINI TVERDA VANDER
WAKAI WANG2 WARSIN WATSON WU WUWILL WYNDE2 WYNDE3 WYNDE4 WYNDE5 WYNDE6 WYNDER XU3 YAMAGU YUAN ZHENG ZHOU

3 DOLL2

7 WYNDE7

Table 3G28 -

IESLC - Meta-analysis of Current Smoking by Amount, Overview, Pipe and/or Cigars (not cigs)
Adenocarcinoma

This analysis is restricted to results for:
1) Results by Amount smoked
Results by Amount smoked are grouped under 2 schemes (S1, S2). Each scheme has a set of "key values". An interval is allocated to the category whose key value it includes and intervals which include none or more than one of the key values are excluded. (Open-ended intervals are coded as 99). Amounts are usually coded as number of cigarette equivalents, defined as shown at the end of Sections -1 and -4.

S1	key value	maximum range	S2	key value	maximum range
1	1	1-98	1	1	1-9
2	99	2-99	2	10	2-98
			3	99	11+

Thus the two levels in scheme S1 correspond to the lowest and highest intervals, irrespective of their values. In scheme C2, the lowest and highest intervals are chosen provided they do not include the value 10, and the interval including the value 10 is also chosen.
2) Smokers of pipe and/or cigars (but not cigarettes)
3) Current smokers
4) Results complete enough for use in metaanalysis

Within each study, results are then selected (in the following order of preference, within each sex) for:
5) DENOM: never smoked anything, (never +1 = +long term ex)
6) Followup period (prospective studies): whole study (coded as 0) or longest available
7) LCtype: all or nearest available, at least Squamous and Adeno. (q = squamous, s = small, l = large, a = adeno, mix = mixed, alv = alveolar)
8) Race: all or nearest available, otherwise by race (wh or w = white, bl or b = black, hi = hispanic ch = chinese, jap = japanese, haw = hawaiian, w+o = white + oriental, sca = scandinavian, as = asian)
9) For overlapping studies: principal rather than subsidiary studies
Finally by Age: whole study (coded as 0) if available, otherwise by widest available age group and then for single sex results (m, f) in preference to combined sex results (c).

Results adjusted (AD) for the most potential confounders are then chosen in Sections -1 to -3 and results adjusted for the least confounders in Sections -4 to -6. (Those least adjusted results which actually differ from the most adjusted as marked 'x' in column X in Section -4)
(Results adjusted for an unknown number of confounder(s) are coded as 20.)

Section -7 shows excluded studies, together with the stage (as above) at which no qualifying results were found.

Section -8 lists the potentially overlapping studies which have been included (1=principal, 2=subsidiary).

Section -9 lists any results which would have been included in preference except that they had data not complete enough for use in meta-analysis, with their significance (yes/no), if known, and any further comment as entered on the database.

In addition to those mentioned above, the following fields, levels and abbreviations are used:
* or nk = not known, n = no, y = yes, ot = other
nev = never
exL, exH = range of exposure (low and high) in the smoking group, in terms of Amount smoked, cigarette equivalents
REF: 6-character study reference
NRR: number of the RR on the database within the study
ST : study type (CC = case control, pr or prosp = prospective)
NLC: number of lung cancer cases in whole study
R : risky occupational population (n = no, m = mining, o = other risky)
VB : national cigarette type (V = at least 75% Virginia, bl = at least 75% blended, ot = other)
P : any proxy use
H : full histological confirmation
De : derivation of RR/CI (or = original, st = standard method, ot = other method of estimation)

Table 3G28 - 0

No RRs selected for this analysis

Table 3G28 - 7

IESLC - Meta-analysis of Current Smoking by Amount, Overview, Pipe and/or Cigars (not cigs)
Adenocarcinoma
Excluded studies (and stage at which they were excluded)

1 ABELIN ABRAHA AMANDU AMES ANDERS AUSTIN AXELSO BAND BECHER BERRIN BLOHMK BLOT4 BROCKM BROWN1 BYERS1 BYERS2 CARPEN
CASCO2 CASCOR CHAN CHEN3 CHIAZZ CHYOU DESTE2 DOCKER DROSTE DU GARCIA GARDIN GENG GODLEY GOODMA GRAHAM GREGOR
HEGMAN HEIN HENNEK HINDS HIRAOK HOROWI HORWIT HUANG ISHIMA JAHN JAIN JARVHO JIANG KELLER KIHARA KJUUS KO
KOHLME KUBIK LAMWK LAMWK2 LANGE LEI LEMARC LEVIN LIU LOMBA2 LOMBAR MAGNUS MARSH MARSH2 MCDUFF MCLAUG MILLER
MILLS NOTANI NOU ODRISC PAWLEG PERSHA POFFIJ QIAO QIAO2 RADZIK REN RONCO ROOTS ROTHSC SAARIK SANKAR SCHWAR
SEGI SEOW SHIMIZ SIMARA SIMONA SITAS SOBUE2 STASZE STAYNE STUCKE SUN SUZUK2 SUZUKI TANG TAO TOKARS TOUSEY
ULMER VEIERO VUTUC WALD WANG WANG3 WANG4 WICKLU WIGLE WILKIN WU2 WUNSCH WYNDE8 XIANGZ XU XU2 XU4
YONG ZHANG

2 AGUDO AKIBA ALDERS ARCHER ARMADA AUVINE AXELSS BARBON BENHAM BENSHL BEST BLOT1 BLOT2 BLOT3 BOFFET BOUCHA BOUCOT
BRESLO BRETT BROSS BROWN2 BUELL BUFFLE CEDERL CHANG CHATZI CHEN CHEN2 CHOI CHOW COMSTO COOKSO CORREA CPSI
CPSII DAMBER DARBY DAVEYS DEAN DEAN2 DEAN3 DEKLER DESTEF DOLL DORANT DORGAN DORN DOSEME DUNN EBELIN ENGELA
ENSTRO ESAKI FAN GAO GAO2 GARSHI GER GILLIS GOLLED GSELL HAENSZ HAMMO2 HAMMON HANSEN HIRAY2 HIRAYA HITOSU
HOLE HU HU2 HUMBLE JARUP JEDRYC JOLY JONES JUSSAW KAISE2 KAISER KANELL KATSOU KAUFMA KHUDER KINLEN KNEKT
KOO KOULUM KREUZE KREYBE LAMTH LAURIL LAUSSM LETOUR LIAW LICKIN LIDDEL LIU2 LIU3 LIU4 LIU5 LUBIN LUBIN2
LUO MACLEN MARTIS MASTRA MATOS MATSUD MCCONN MIGRAN MOLLO MRFIT MRFITR MURATA MZILEN NAM NOTAN2 ORMOS OSANN
OSANN2 PARKIN PASTOR PERNU PERSH2 PETO PEZZO2 PEZZOT PIKE PISANI POLEDN PRESCO RACHTA RANDIG RESTRE RIMING SADOWS
SCHWA2 SEGI2 SHAW SIEMIA SOBUE SPEIZE SPITZ STOCKS STOCKW SVENSS TANG2 TENKAN TIZZAN TSUGAN TULINI TVERDA VANDER
WAKAI WANG2 WARSIN WATSON WU WUWILL WYNDE2 WYNDE3 WYNDE4 WYNDE5 WYNDE6 WYNDER XU3 YAMAGU YUAN ZHENG ZHOU

7 DOLL2 WYNDE7

Table 3G30 -

IESLC - Meta-analysis of Ever smoking (or Current if Ever not avail) by Amount, Overview, Pipe and/or Cigars (not cigs)
Adenocarcinoma

This analysis is restricted to results for:
1) Results by Amount smoked
Results by Amount smoked are grouped under 2 schemes (S1, S2). Each scheme has a set of "key values". An interval is allocated to the category whose key value it includes and intervals which include none or more than one of the key values are excluded. (Open-ended intervals are coded as 99). Amounts are usually coded as number of cigarette equivalents, defined as shown at the end of Sections -1 and -4.

S1	key value	maximum range	S2	key value	maximum range
1	1	1-98	1	1	1-9
2	99	2-99	2	10	2-98
			3	99	11+

Thus the two levels in scheme S1 correspond to the lowest and highest intervals, irrespective of their values. In scheme C2, the lowest and highest intervals are chosen provided they do not include the value 10, and the interval including the value 10 is also chosen.
2) Smokers of pipe and/or cigars (but not cigarettes)
3) Results complete enough for use in metaanalysis

Within each study, results are then selected (in the following order of preference, within each sex) for:
4) SMKSTA: ever smokers, current smokers
5) DENOM: never smoked anything, (never +1 = +long term ex)
6) Followup period (prospective studies): whole study (coded as 0) or longest available
7) LCtype: all or nearest available, at least Squamous and Adeno. (q = squamous, s = small, l = large, a = adeno, mix = mixed, alv = alveolar)
8) Race: all or nearest available, otherwise by race (wh or w = white, bl or b = black, hi = hispanic ch = chinese, jap = japanese, haw = hawaiian, w+o = white + oriental, sca = scandinavian, as = asian)
9) For overlapping studies: principal rather than subsidiary studies
Finally by Age: whole study (coded as 0) if available, otherwise by widest available age group and then for single sex results (m, f) in preference to combined sex results (c).

Results adjusted (AD) for the most potential confounders are then chosen in Sections -1 to -3 and results adjusted for the least confounders in Sections -4 to -6. (Those least adjusted results which actually differ from the most adjusted as marked 'x' in column X in Section -4)
(Results adjusted for an unknown number of confounder(s) are coded as 20.)

Section -7 shows excluded studies, together with the stage (as above) at which no qualifying results were found.

Section -8 lists the potentially overlapping studies which have been included (1=principal, 2=subsidiary).

Section -9 lists any results which would have been included in preference except that they had data not complete enough for use in meta-analysis, with their significance (yes/no), if known, and any further comment as entered on the database.

In addition to those mentioned above, the following fields, levels and abbreviations are used:
* or nk = not known, n = no, y = yes, ot = other
ev = ever, cu = current, nev = never
exL, exH = range of exposure (low and high) in the smoking group, in terms of Amount smoked, cigarette equivalents
REF: 6-character study reference
NRR: number of the RR on the database within the study
ST : study type (CC = case control, pr or prosp = prospective)
NLC: number of lung cancer cases in whole study
R : risky occupational population (n = no, m = mining, o = other risky)
VB : national cigarette type (V = at least 75% Virginia, bl = at least 75% blended, ot = other)
P : any proxy use
H : full histological confirmation
De : derivation of RR/CI (or = original, st = standard method, ot = other method of estimation)

Table 3G30 - 0

No RRs selected for this analysis

Table 3G30 - 7

IESLC - Meta-analysis of Ever smoking (or Current if Ever not avail) by Amount, Overview, Pipe and/or Cigars (not cigs)
Adenocarcinoma
Excluded studies (and stage at which they were excluded)

1 ABELIN ABRAHA AMANDU AMES ANDERS AUSTIN AXELSO BAND BECHER BERRIN BLOHMK BLOT4 BROCKM BROWN1 BYERS1 BYERS2 CARPEN
CASCO2 CASCOR CHAN CHEN3 CHIAZZ CHYOU DESTE2 DOCKER DROSTE DU GARCIA GARDIN GENG GODLEY GOODMA GRAHAM GREGOR
HEGMAN HEIN HENNEK HINDS HIRAOK HOROWI HORWIT HUANG ISHIMA JAHN JAIN JARVHO JIANG KELLER KIHARA KJUUS KO
KOHLME KUBIK LAMWK LAMWK2 LANGE LEI LEMARC LEVIN LIU LOMBA2 LOMBAR MAGNUS MARSH MARSH2 MCDUFF MCLAUG MILLER
MILLS NOTANI NOU ODRISC PAWLEG PERSHA POFFIJ QIAO QIAO2 RADZIK REN RONCO ROOTS ROTHSC SAARIK SANKAR SCHWAR
SEGI SEOW SHIMIZ SIMARA SIMONA SITAS SOBUE2 STASZE STAYNE STUCKE SUN SUZUK2 SUZUKI TANG TAO TOKARS TOUSEY
ULMER VEIERO VUTUC WALD WANG WANG3 WANG4 WICKLU WIGLE WILKIN WU2 WUNSCH WYNDE8 XIANGZ XU XU2 XU4
YONG ZHANG

2 AGUDO AKIBA ALDERS ARCHER ARMADA AUVINE AXELSS BARBON BENHAM BENSHL BEST BLOT1 BLOT2 BLOT3 BOFFET BOUCHA BOUCOT
BRESLO BRETT BROSS BROWN2 BUELL BUFFLE CEDERL CHANG CHATZI CHEN CHEN2 CHOI CHOW COMSTO COOKSO CORREA CPSI
CPSII DAMBER DARBY DAVEYS DEAN DEAN2 DEAN3 DEKLER DESTEF DOLL DORANT DORGAN DORN DOSEME DUNN EBELIN ENGELA
ENSTRO ESAKI FAN GAO GAO2 GARSHI GER GILLIS GOLLED GSELL HAENSZ HAMMO2 HAMMON HANSEN HIRAY2 HIRAYA HITOSU
HOLE HU HU2 HUMBLE JARUP JEDRYC JOLY JONES JUSSAW KAISE2 KAISER KANELL KATSOU KAUFMA KHUDER KINLEN KNEKT
KOO KOULUM KREUZE KREYBE LAMTH LAURIL LAUSSM LETOUR LIAW LICKIN LIDDEL LIU2 LIU3 LIU4 LIU5 LUBIN LUBIN2
LUO MACLEN MARTIS MASTRA MATOS MATSUD MCCONN MIGRAN MOLLO MRFIT MRFITR MURATA MZILEN NAM NOTAN2 ORMOS OSANN
OSANN2 PARKIN PASTOR PERNU PERSH2 PETO PEZZO2 PEZZOT PIKE PISANI POLEDN PRESCO RACHTA RANDIG RESTRE RIMING SADOWS
SCHWA2 SEGI2 SHAW SIEMIA SOBUE SPEIZE SPITZ STOCKS STOCKW SVENSS TANG2 TENKAN TIZZAN TSUGAN TULINI TVERDA VANDER
WAKAI WANG2 WARSIN WATSON WU WUWILL WYNDE2 WYNDE3 WYNDE4 WYNDE5 WYNDE6 WYNDER XU3 YAMAGU YUAN ZHENG ZHOU

7 DOLL2 WYNDE7

Table 3G32 -

IESLC - Meta-analysis of Ever Smoking (or Current if ever not available) by Amount, Overview, Pipe only
Adenocarcinoma

This analysis is restricted to results for:
1) Results by Amount smoked
   Results by Amount smoked are grouped under 2 schemes (S1, S2). Each scheme has a set of "key values". An interval is allocated to the category whose key value it includes and intervals which include none or more than one of the key values are excluded. (Open-ended intervals are coded as 99). Amounts are usually coded as number of cigarette equivalents, defined as shown at the end of Sections -1 and -4.

S1	key value	maximum range	S2	key value	maximum range
1	1	1-98	1	1	1-9
2	99	2-99	2	10	2-98
			3	99	11+

   Thus the two levels in scheme S1 correspond to the lowest and highest intervals, irrespective of their values. In scheme C2, the lowest and highest intervals are chosen provided they do not include the value 10, and the interval including the value 10 is also chosen.
2) Smokers of pipe only
3) Results complete enough for use in metaanalysis

Within each study, results are then selected (in the following order of preference, within each sex) for:
4) SMKSTA: ever smokers, current smokers
5) DENOM: never smoked anything, (never +1 = +long term ex)
6) Followup period (prospective studies): whole study (coded as 0) or longest available
7) LCtype: all or nearest available, at least Squamous and Adeno. (q = squamous, s = small,
   l = large, a = adeno, mix = mixed, alv = alveolar)
8) Race: all or nearest available, otherwise by race (wh or w = white, bl or b = black, hi = hispanic
   ch = chinese, jap = japanese, haw = hawaiian, w+o = white + oriental, sca = scandinavian, as = asian)
9) For overlapping studies: principal rather than subsidiary studies
Finally by Age: whole study (coded as 0) if available, otherwise by widest available age group
and then for single sex results (m, f) in preference to combined sex results (c).

Results adjusted (AD) for the most potential confounders are then chosen in Sections -1 to -3
and results adjusted for the least confounders in Sections -4 to -6. (Those least adjusted results which
actually differ from the most adjusted as marked 'x' in column X in Section -4)
(Results adjusted for an unknown number of confounder(s) are coded as 20.)

Section -7 shows excluded studies, together with the stage (as above) at which no qualifying
results were found.

Section -8 lists the potentially overlapping studies which have been included (1=principal, 2=subsidiary).

Section -9 lists any results which would have been included in preference except that they had data not complete
enough for use in meta-analysis, with their significance (yes/no), if known, and any further comment as entered
on the database.

In addition to those mentioned above, the following fields, levels and abbreviations are used:
* or nk = not known, n = no, y = yes, ot = other
ev = ever, cu = current, nev = never
exL, exH = range of exposure (low and high) in the smoking group, in terms of Amount smoked,
    cigarette equivalents
REF: 6-character study reference
NRR: number of the RR on the database within the study
ST : study type (CC = case control, pr or prosp = prospective)
NLC: number of lung cancer cases in whole study
R : risky occupational population (n = no, m = mining, o = other risky)
VB : national cigarette type (V = at least 75% Virginia, bl = at least 75% blended, ot = other)
P : any proxy use
H : full histological confirmation
De : derivation of RR/CI (or = original, st = standard method, ot = other method of estimation)

Table 3G32 - 0

No RRs selected for this analysis

Table 3G32 - 7

IESLC - Meta-analysis of Ever Smoking (or Current if ever not available) by Amount, Overview, Pipe only
Adenocarcinoma
Excluded studies (and stage at which they were excluded)

1 ABELIN ABRAHA AMANDU AMES ANDERS AUSTIN AXELSO BAND BECHER BERRIN BLOHMK BLOT4 BROCKM BROWN1 BYERS1 BYERS2 CARPEN
CASCO2 CASCOR CHAN CHEN3 CHIAZZ CHYOU DESTE2 DOCKER DROSTE DU GARCIA GARDIN GENG GODLEY GOODMA GRAHAM GREGOR
HEGMAN HEIN HENNEK HINDS HIRAOK HOROWI HORWIT HUANG ISHIMA JAHN JAIN JARVHO JIANG KELLER KIHARA KJUUS KO
KOHLME KUBIK LAMWK LAMWK2 LANGE LEI LEMARC LEVIN LIU LOMBA2 LOMBAR MAGNUS MARSH MARSH2 MCDUFF MCLAUG MILLER
MILLS NOTANI NOU ODRISC PAWLEG PERSHA POFFIJ QIAO QIAO2 RADZIK REN RONCO ROOTS ROTHSC SAARIK SANKAR SCHWAR
SEGI SEOW SHIMIZ SIMARA SIMONA SITAS SOBUE2 STASZE STAYNE STUCKE SUN SUZUK2 SUZUKI TANG TAO TOKARS TOUSEY
ULMER VEIERO VUTUC WALD WANG WANG3 WANG4 WICKLU WIGLE WILKIN WU2 WUNSCH WYNDE8 XIANGZ XU XU2 XU4
YONG ZHANG

2 AGUDO AKIBA ALDERS ARCHER ARMADA AUVINE AXELSS BARBON BENHAM BENSHL BLOT1 BLOT2 BLOT3 BOUCHA BOUCOT BRESLO BRETT
BROSS BROWN2 BUELL BUFFLE CHANG CHATZI CHEN CHEN2 CHOI CHOW COMSTO COOKSO CORREA CPSI CPSII DARBY DAVEYS
DEAN DEAN2 DEKLER DESTEF DOLL2 DORANT DORGAN DOSEME DUNN EBELIN ENGELA ENSTRO ESAKI FAN GAO GAO2 GARSHI
GER GILLIS GOLLED GSELL HAENSZ HAMMO2 HAMMON HANSEN HIRAYA HITOSU HOLE HU HU2 HUMBLE JARUP JEDRYC JOLY
JONES JUSSAW KAISE2 KAISER KANELL KATSOU KAUFMA KHUDER KINLEN KNEKT KOO KOULUM KREUZE KREYBE LAMTH LAURIL LAUSSM
LETOUR LIAW LICKIN LIDDEL LIU2 LIU3 LIU4 LIU5 LUBIN LUO MACLEN MARTIS MASTRA MATOS MATSUD MCCONN MIGRAN
MOLLO MRFIT MRFITR MURATA MZILEN NAM NOTAN2 ORMOS OSANN OSANN2 PARKIN PASTOR PERNU PERSH2 PETO PEZZO2 PEZZOT
PIKE PISANI POLEDN PRESCO RACHTA RANDIG RESTRE RIMING SCHWA2 SEGI2 SHAW SIEMIA SOBUE SPEIZE SPITZ STOCKS STOCKW
SVENSS TANG2 TENKAN TIZZAN TSUGAN TULINI TVERDA VANDER WAKAI WANG2 WARSIN WATSON WU WUWILL WYNDE2 WYNDE3 WYNDE4
WYNDE5 WYNDE6 WYNDER XU3 YAMAGU YUAN ZHENG ZHOU

3 SADOWS

5 HIRAY2

7 BEST BOFFET CEDERL DAMBER DEAN3 DOLL DORN LUBIN2 WYNDE7

Table 3G34 -

IESLC - Meta-analysis of Ever Smoking (or Current if Ever not available) by Amount, Overview, Cigars only
Adenocarcinoma

This analysis is restricted to results for:
1) Results by Amount smoked
Results by Amount smoked are grouped under 2 schemes (S1, S2). Each scheme has a set of "key values". An interval is allocated to the category whose key value it includes and intervals which include none or more than one of the key values are excluded. (Open-ended intervals are coded as 99). Amounts are usually coded as number of cigarette equivalents, defined as shown at the end of Sections -1 and -4; if originally given as numbers of cigars, we use our own assumption (OA) of 1 cigar = 5 cigarettes.

S1	key value	maximum range	S2	key value	maximum range
1	1	1-98	1	1	1-9
2	99	2-99	2	10	2-98
			3	99	11+

Thus the two levels in scheme S1 correspond to the lowest and highest intervals, irrespective of their values. In scheme C2, the lowest and highest intervals are chosen provided they do not include the value 10, and the interval including the value 10 is also chosen.
2) Smokers of cigars only
3) Results complete enough for use in metaanalysis

Within each study, results are then selected (in the following order of preference, within each sex) for:
4) SMKSTA: ever smokers, current smokers
5) DENOM: never smoked anything, (never +1 = +long term ex)
6) Followup period (prospective studies): whole study (coded as 0) or longest available
7) LCtype: all or nearest available, at least Squamous and Adeno. (q = squamous, s = small, l = large, a = adeno, mix = mixed, alv = alveolar)
8) Race: all or nearest available, otherwise by race (wh or w = white, bl or b = black, hi = hispanic ch = chinese, jap = japanese, haw = hawaiian, w+o = white + oriental, sca = scandinavian, as = asian)
9) For overlapping studies: principal rather than subsidiary studies
Finally by Age: whole study (coded as 0) if available, otherwise by widest available age group and then for single sex results (m, f) in preference to combined sex results (c).

Results adjusted (AD) for the most potential confounders are then chosen in Sections -1 to -3 and results adjusted for the least confounders in Sections -4 to -6. (Those least adjusted results which actually differ from the most adjusted as marked 'x' in column X in Section -4)
(Results adjusted for an unknown number of confounder(s) are coded as 20.)

Section -7 shows excluded studies, together with the stage (as above) at which no qualifying results were found.

Section -8 lists the potentially overlapping studies which have been included (1=principal, 2=subsidiary).

Section -9 lists any results which would have been included in preference except that they had data not complete enough for use in meta-analysis, with their significance (yes/no), if known, and any further comment as entered on the database.

In addition to those mentioned above, the following fields, levels and abbreviations are used:
* or nk = not known, n = no, y = yes, ot = other
ev = ever, cu = current, nev = never
exL, exH = range of exposure (low and high) in the smoking group, in terms of Amount smoked, cigarette equivalents
REF: 6-character study reference
NRR: number of the RR on the database within the study
ST : study type (CC = case control, pr or prosp = prospective)
NLC: number of lung cancer cases in whole study
R : risky occupational population (n = no, m = mining, o = other risky)
VB : national cigarette type (V = at least 75% Virginia, bl = at least 75% blended, ot = other)
P : any proxy use
H : full histological confirmation
De : derivation of RR/CI (or = original, st = standard method, ot = other method of estimation)

Table 3G34 - 0

No RRs selected for this analysis

Table 3G34 - 7

IESLC - Meta-analysis of Ever Smoking (or Current if Ever not available) by Amount, Overview, Cigars only
Adenocarcinoma
Excluded studies (and stage at which they were excluded)

1 ABELIN ABRAHA AMANDU AMES ANDERS AUSTIN AXELSO BAND BECHER BERRIN BLOHMK BLOT4 BROCKM BROWN1 BYERS1 BYERS2 CARPEN
CASCO2 CASCOR CHAN CHEN3 CHIAZZ CHYOU DESTE2 DOCKER DROSTE DU GARCIA GARDIN GENG GODLEY GOODMA GRAHAM GREGOR
HEGMAN HEIN HENNEK HINDS HIRAOK HOROWI HORWIT HUANG ISHIMA JAHN JAIN JARVHO JIANG KELLER KIHARA KJUUS KO
KOHLME KUBIK LAMWK LAMWK2 LANGE LEI LEMARC LEVIN LIU LOMBA2 LOMBAR MAGNUS MARSH MARSH2 MCDUFF MCLAUG MILLER
MILLS NOTANI NOU ODRISC PAWLEG PERSHA POFFIJ QIAO QIAO2 RADZIK REN RONCO ROOTS ROTHSC SAARIK SANKAR SCHWAR
SEGI SEOW SHIMIZ SIMARA SIMONA SITAS SOBUE2 STASZE STAYNE STUCKE SUN SUZUK2 SUZUKI TANG TAO TOKARS TOUSEY
ULMER VEIERO VUTUC WALD WANG WANG3 WANG4 WICKLU WIGLE WILKIN WU2 WUNSCH WYNDE8 XIANGZ XU XU2 XU4
YONG ZHANG

2 AGUDO AKIBA ALDERS ARCHER ARMADA AUVINE AXELSS BARBON BENHAM BENSHL BEST BLOT1 BLOT2 BLOT3 BOUCHA BOUCOT BRESLO
BRETT BROSS BROWN2 BUELL BUFFLE CEDERL CHANG CHATZI CHEN CHEN2 CHOI CHOW COMSTO COOKSO CORREA CPSII DAMBER
DARBY DAVEYS DEAN DEAN2 DEAN3 DEKLER DESTEF DOLL DOLL2 DORANT DORGAN DOSEME DUNN EBELIN ENGELA ENSTRO ESAKI
FAN GAO GAO2 GARSHI GER GILLIS GOLLED GSELL HAENSZ HAMMO2 HAMMON HANSEN HIRAY2 HIRAYA HITOSU HOLE HU
HU2 HUMBLE JARUP JEDRYC JONES JUSSAW KAISE2 KAISER KANELL KATSOU KAUFMA KHUDER KINLEN KNEKT KOO KOULUM KREUZE
KREYBE LAMTH LAURIL LAUSSM LETOUR LIAW LICKIN LIDDEL LIU2 LIU3 LIU4 LIU5 LUBIN LUO MACLEN MARTIS MASTRA
MATOS MATSUD MCCONN MIGRAN MOLLO MRFIT MRFITR MURATA MZILEN NAM NOTAN2 ORMOS OSANN OSANN2 PARKIN PASTOR PERNU
PERSH2 PETO PEZZO2 PEZZOT PIKE PISANI POLEDN PRESCO RACHTA RANDIG RESTRE RIMING SCHWA2 SEGI2 SHAW SIEMIA SOBUE
SPEIZE SPITZ STOCKS STOCKW SVENSS TANG2 TENKAN TIZZAN TSUGAN TULINI TVERDA VANDER WAKAI WANG2 WARSIN WATSON WU
WUWILL WYNDE2 WYNDE3 WYNDE4 WYNDE5 WYNDE6 WYNDER XU3 YAMAGU YUAN ZHENG ZHOU

3 SADOWS

7 BOFFET CPSI DORN JOLY LUBIN2 WYNDE7

Table 3G36 -

IESLC - Meta-analysis of Ever Smoking (or Current if Ever not available) by Amount, Overview, Mixed smokers
Adenocarcinoma

This analysis is restricted to results for:
1) Results by Amount smoked
   Results by Amount smoked are grouped under 2 schemes (S1, S2). Each scheme has a set of "key values". An interval is allocated to the category whose key value it includes and intervals which include none or more than one of the key values are excluded. (Open-ended intervals are coded as 99). Amounts are usually coded as number of cigarette equivalents, defined as shown at the end of Sections -1 and -4; if originally given as numbers of cigars, we use our own assumption (OA) of 1 cigar = 5 cigarettes.

S1	key value	maximum range	S2	key value	maximum range
1	1	1-98	1	1	1-9
2	99	2-99	2	10	2-98
			3	99	11+

   Thus the two levels in scheme S1 correspond to the lowest and highest intervals, irrespective of their values. In scheme C2, the lowest and highest intervals are chosen provided they do not include the value 10, and the interval including the value 10 is also chosen.
2) Mixed smokers (cigarettes and pipe/cigar)
3) Results complete enough for use in metaanalysis

Within each study, results are then selected (in the following order of preference, within each sex) for:
4) SMKSTA: ever smokers, current smokers
5) DENOM: never smoked anything, (never +1 = +long term ex)
6) Followup period (prospective studies): whole study (coded as 0) or longest available
7) LCtype: all or nearest available, at least Squamous and Adeno. (q = squamous, s = small,
   l = large, a = adeno, mix = mixed, alv = alveolar)
8) Race: all or nearest available, otherwise by race (wh or w = white, bl or b = black, hi = hispanic
   ch = chinese, jap = japanese, haw = hawaiian, w+o = white + oriental, sca = scandinavian, as = asian)
9) For overlapping studies: principal rather than subsidiary studies
Finally by Age: whole study (coded as 0) if available, otherwise by widest available age group and then for single sex results (m, f) in preference to combined sex results (c).

Results adjusted (AD) for the most potential confounders are then chosen in Sections -1 to -3 and results adjusted for the least confounders in Sections -4 to -6. (Those least adjusted results which actually differ from the most adjusted as marked 'x' in column X in Section -4)
(Results adjusted for an unknown number of confounder(s) are coded as 20.)

Section -7 shows excluded studies, together with the stage (as above) at which no qualifying results were found.

Section -8 lists the potentially overlapping studies which have been included (1=principal, 2=subsidiary).

Section -9 lists any results which would have been included in preference except that they had data not complete enough for use in meta-analysis, with their significance (yes/no), if known, and any further comment as entered on the database.

In addition to those mentioned above, the following fields, levels and abbreviations are used:
* or nk = not known, n = no, y = yes, ot = other
ev = ever, cu = current, nev = never
exL, exH = range of exposure (low and high) in the smoking group, in terms of Amount smoked,
   cigarette equivalents
REF: 6-character study reference
NRR: number of the RR on the database within the study
ST : study type (CC = case control, pr or prosp = prospective)
NLC: number of lung cancer cases in whole study
R : risky occupational population (n = no, m = mining, o = other risky)
VB : national cigarette type (V = at least 75% Virginia, bl = at least 75% blended, ot = other)
P : any proxy use
H : full histological confirmation
De : derivation of RR/CI (or = original, st = standard method, ot = other method of estimation)

Table 3G36 - 0

No RRs selected for this analysis

Table 3G36 - 7

IESLC - Meta-analysis of Ever Smoking (or Current if Ever not available) by Amount, Overview, Mixed smokers
Adenocarcinoma
Excluded studies (and stage at which they were excluded)

1	ABELIN	ABRAHA	AMANDU	AMES	ANDERS	AUSTIN	AXELSO	BAND	BECHER	BERRIN	BLOHMK	BLOT4	BROCKM	BROWN1	BYERS1	BYERS2	CARPEN
	CASCO2	CASCOR	CHAN	CHEN3	CHIAZZ	CHYOU	DESTE2	DOCKER	DROSTE	DU	GARCIA	GARDIN	GENG	GODLEY	GOODMA	GRAHAM	GREGOR
	HEGMAN	HEIN	HENNEK	HINDS	HIRAOK	HOROWI	HORWIT	HUANG	ISHIMA	JAHN	JAIN	JARVHO	JIANG	KELLER	KIHARA	KJUUS	KO
	KOHLME	KUBIK	LAMWK	LAMWK2	LANGE	LEI	LEMARC	LEVIN	LIU	LOMBA2	LOMBAR	MAGNUS	MARSH	MARSH2	MCDUFF	MCLAUG	MILLER
	MILLS	NOTANI	NOU	ODRISC	PAWLEG	PERSHA	POFFIJ	QIAO	QIAO2	RADZIK	REN	RONCO	ROOTS	ROTHSC	SAARIK	SANKAR	SCHWAR
	SEGI	SEOW	SHIMIZ	SIMARA	SIMONA	SITAS	SOBUE2	STASZE	STAYNE	STUCKE	SUN	SUZUK2	SUZUKI	TANG	TAO	TOKARS	TOUSEY
	ULMER	VEIERO	VUTUC	WALD	WANG	WANG3	WANG4	WICKLU	WIGLE	WILKIN	WU2	WUNSCH	WYNDE8	XIANGZ	XU	XU2	XU4
	YONG	ZHANG															
2	AGUDO	AKIBA	ALDERS	ARCHER	ARMADA	AUVINE	AXELSS	BARBON	BENHAM	BENSHL	BEST	BLOT1	BLOT2	BLOT3	BOFFET	BOUCHA	BRESLO
	BRETT	BROSS	BROWN2	BUELL	BUFFLE	CEDERL	CHANG	CHATZI	CHEN	CHEN2	CHOI	CHOW	COMSTO	COOKSO	CORREA	CPSI	CPSII
	DAMBER	DARBY	DAVEYS	DEAN	DEAN2	DEAN3	DEKLER	DESTEF	DOLL	DOLL2	DORANT	DORGAN	DOSEME	DUNN	EBELIN	ENGELA	ENSTRO
	ESAKI	FAN	GAO	GAO2	GARSHI	GER	GILLIS	GOLLED	GSELL	HAENSZ	HAMMO2	HAMMON	HANSEN	HIRAY2	HIRAYA	HITOSU	HOLE
	HU	HU2	HUMBLE	JARUP	JEDRYC	JOLY	JONES	JUSSAW	KAISE2	KAISER	KANELL	KATSOU	KAUFMA	KHUDER	KINLEN	KNEKT	KOO
	KOULUM	KREUZE	KREYBE	LAMTH	LAURIL	LAUSSM	LETOUR	LIAW	LICKIN	LIDDEL	LIU2	LIU3	LIU4	LIU5	LUBIN	LUBIN2	LUO
	MACLEN	MARTIS	MASTRA	MATOS	MATSUD	MCCONN	MIGRAN	MOLLO	MRFIT	MRFITR	MURATA	MZILEN	NAM	NOTAN2	ORMOS	OSANN	OSANN2
	PARKIN	PASTOR	PERNU	PERSH2	PETO	PEZZO2	PEZZOT	PIKE	PISANI	POLEDN	PRESCO	RACHTA	RANDIG	RESTRE	RIMING	SADOWS	SCHWA2
	SEGI2	SHAW	SIEMIA	SOBUE	SPEIZE	SPITZ	STOCKS	STOCKW	SVENSS	TANG2	TENKAN	TIZZAN	TSUGAN	TULINI	TVERDA	VANDER	WAKAI
	WANG2	WARSIN	WATSON	WU	WUWILL	WYNDE2	WYNDE3	WYNDE4	WYNDE5	WYNDE6	WYNDE7	WYNDER	XU3	YAMAGU	YUAN	ZHENG	ZHOU
7	BOUCOT	DORN															
