# Supplementary material for: Systematic review with meta-analysis of the epidemiological evidence in the 1900s relating smoking to lung cancer
Source: BMC Cancer. 2012 Sep 3;12:385. doi: 10.1186/1471-2407-12-385 (PMC3505152; doi:10.1186/1471-2407-12-385)
Supplement: Additional file 5 — Detailed Analysis Tables (Individual file names as described in Additional file 1: Methods, Table1). [file 1471-2407-12-385-S5.zip › PDF/3M.pdf]

Table 3M1 -
IESLC - Meta-analysis of Ever Smoking, Butt length or Fraction smoked, "Highest vs lowest"
Adenocarcinoma, Cigarettes (or Any Product if Cigarettes not available)

This analysis is restricted to results for:
1) Ever smokers
2) Results by Butt length or Fraction smoked
3) Categorical results by Butt length or Fraction smoked
4) Denominator (unexposed) = "low"
5) Adenocarcinoma (or near equivalent)
6) Results complete enough for use in metaanalysis

Within each study, results are then selected (in the following order of preference, within each sex) for:
7) (not applicable)
8) PRODUCT: cigarettes regardless of other products, cigarettes only, all/unspec
9) CIGTYPE: all/unspecified, MC regardless of HR, MC only
10) Results with least adjustment for other aspects of smoking (ADOS)
11) The highest vs lowest category
12) Followup period (YF, prospective studies): whole study (coded as 0) or longest available
13) LCtype: adeno or nearest available, but not squamous. (q = squamous, s = small,
a = adeno, l = large, KII = Kreyberg II, al = alveolar, br = bronchiolar, u = undifferentiated)
14) Race: all or nearest available, otherwise by race (wh or w = white, bl or b = black, hi = hispanic
ch = chinese, jap = japanese, haw = hawaiian, w+o = white + oriental, sca = scandinavian, as = asian)
15) For overlapping studies: principal rather than subsidiary studies
Finally by Age: whole study (coded as 0) if available, otherwise by widest available age group
and then for single sex results (m, f) in preference to results for both sexes combined (c).

Results adjusted (AD) for the most potential confounders are then chosen in Sections -1 to -3
and results adjusted for the least confounders in Sections -4 to -6. (Those least adjusted results which
actually differ from the most adjusted are marked 'x' in column X in Section -4)

Section -7 shows excluded studies, together with the stage (as above) at which no qualifying
results were found.

Section -8 lists the potentially overlapping studies which have been included (1=principal, 2=subsidiary).

Section -9 lists any results which would have been included in preference except that they had data not complete
enough for use in meta-analysis, with their significance (yes/no), if known, and any further comment as entered
on the database. It also lists as "gap" any categories for which no data were presented by the original authors.

In addition to those mentioned above, the following fields, levels and abbreviations are used:
* or nk = not known, n = no, y = yes, ot = other
all/unspec = all or unspecified, cig+/-ot = cigarettes irrespective of other products (cigar, pipe etc)
MC = manufactured cigarettes, HR = hand-rolled cigarettes
exL, exH = range of exposure (low and high) in the "highest" group, in terms of Butt length or Fraction smoked
unexL, unexH = range of exposure (low and high) in the "lowest" group, in terms of Butt length or Fraction smoked
REF: 6-character study reference
NRR: number of the RR on the database within the study
ST : study type (CC = case control, pr or prosp = prospective)
NLC: number of lung cancer cases in whole study
R : risky occupational population (n = no, m = mining, o = other risky)
VB : national cigarette type (V = at least 75% Virginia, bl = at least 75% blended, ot = other)
P : any proxy use
H : full histological confirmation
De : derivation of RR/CI (or = original, st = standard method, ot = other method of estimation)

Table 3M1 - 1

IESLC - Meta-analysis of Ever Smoking, Butt length or Fraction smoked, "Highest vs lowest"
Adenocarcinoma, Cigarettes (or Any Product if Cigarettes not available)
Most adjusted

REF	NRR	SEX	AGEL	AGEH	RACE	YF	LC TYPE	LOC	START	ST	NLC	R	VB	P	H	AD	ADOS	PRODUCT	exL	exH	unexL	unexH	De
LUBIN2	1014	m	0	0	all	-	a	Eu:mul	1976	CC	7804	n	bl	n	y	0	0	cig+/-ot	76	100	1	50	st
LUBIN2	1042	f	0	0	all	-	a	Eu:mul	1976	CC	7804	n	bl	n	y	0	0	cig+/-ot	76	100	1	50	st
WYNDE6	604	m	0	0	wh	-	a	NAmer	1969	CC	4423	n	bl	n	y	0	0	cig+/-ot	50	100	1	49	st
WYNDE6	628	f	0	0	wh	-	a	NAmer	1969	CC	4423	n	bl	n	y	0	0	cig+/-ot	50	100	1	49	st

Cigarette type is all/unspec for all RRs

exL, exH, unexL, unexH refer to fraction of cigarette smoked (%) for all RRs

Table 3M1 - 2

IESLC - Meta-analysis of Ever Smoking, Butt length or Fraction smoked, "Highest vs lowest"
Adenocarcinoma, Cigarettes (or Any Product if Cigarettes not available)
Most adjusted

REF	NRR	SEX	AD	Number Exposed Case	Cont	Non-exposed Case	Cont	RR	95.00%CI
LUBIN2	1014	m	0	330	5390	25	518	1.27	(0.84- 1.92)
LUBIN2	1042	f	0	28	168	4	44	1.83	(0.61- 5.50)
Subtotal LUBIN2								1.33	(0.90- 1.96)
WYNDE6	604	m	0	110	138	117	161	1.10	(0.78- 1.55)
WYNDE6	628	f	0	102	81	98	101	1.30	(0.87- 1.94)
Subtotal WYNDE6								1.18	(0.91- 1.53)
Totals				570	5777	244	824		

*prospective study

REF	NRR	SEX	AD	Ys	Ws	Qs	Ps
LUBIN2	1014	m	0	0.24	22.15	0.03	0.2629
LUBIN2	1042	f	0	0.61	3.18	0.52	0.2797
Subtotal LUBIN2				0.28	25.33	0.55	
WYNDE6	604	m	0	0.09	32.16	0.38	0.6001
WYNDE6	628	f	0	0.26	23.67	0.08	0.2048
Subtotal WYNDE6				0.16	55.83	0.46	

	N	4
	NS	2
	Wt	81.16
Het	Chi	1.02
Het	df	3
Het	P	N.S.
Fixed	RR	1.22
	RRl	0.98
	RRu	1.52
	P	(+)
Random	RR	1.22
	RRl	0.98
	RRu	1.52
	P	(+)
Asymm	P	N.S.

Table 3M1 - 3

IESLC - Meta-analysis of Ever Smoking, Butt length or Fraction smoked, "Highest vs lowest"
Adenocarcinoma, Cigarettes (or Any Product if Cigarettes not available)
Most adjusted

	combined	Sex male	female	Total
N		2	2	4
NS		2	2	4
Wt		54.31	26.85	81.16
Het Chi		0.28	0.33	1.02
Het df		1	1	3
Het P		N.S.	N.S.	N.S.
Fixed RR		1.16	1.35	1.22
RRl		0.89	0.93	0.98
RRu		1.52	1.97	1.52
P		N.S.	N.S.	(+)
Random RR		1.16	1.35	1.22
RRl		0.89	0.93	0.98
RRu		1.52	1.97	1.52
P		N.S.	N.S.	(+)
Between Chi				0.40
Between df				1
Between P				N.S.
Btwn(F) P				N.S.
Btwn(R) P				N.S.

Too few RRs for analysis by factor

Table 3M1 - 4

IESLC - Meta-analysis of Ever Smoking, Butt length or Fraction smoked, "Highest vs lowest"
Adenocarcinoma, Cigarettes (or Any Product if Cigarettes not available)
Least adjusted

REF	NRR	X	SEX	AGEL	AGEH	RACE	YF	LC TYPE	LOC	START	ST	NLC	R	VB	P	H	AD	ADOS	PRODUCT	exL	exH	unexL	unexH	De
LUBIN2	1014		m	0	0	all	-	a	Eu:mul	1976	CC	7804	n	bl	n	y	0	0	cig+/-ot	76	100	1	50	st
LUBIN2	1042		f	0	0	all	-	a	Eu:mul	1976	CC	7804	n	bl	n	y	0	0	cig+/-ot	76	100	1	50	st
WYNDE6	604		m	0	0	wh	-	a	NAmer	1969	CC	4423	n	bl	n	y	0	0	cig+/-ot	50	100	1	49	st
WYNDE6	628		f	0	0	wh	-	a	NAmer	1969	CC	4423	n	bl	n	y	0	0	cig+/-ot	50	100	1	49	st

Cigarette type is all/unspec for all RRs

exL, exH, unexL, unexH refer to fraction of cigarette smoked (%) for all RRs

Table 3M1 - 5

IESLC - Meta-analysis of Ever Smoking, Butt length or Fraction smoked, "Highest vs lowest"
Adenocarcinoma, Cigarettes (or Any Product if Cigarettes not available)
Least adjusted

REF	NRR	SEX	AD	Number Exposed Case	Cont	Non-exposed Case	Cont	RR	95.00%CI
LUBIN2	1014	m	0	330	5390	25	518	1.27	(0.84- 1.92)
LUBIN2	1042	f	0	28	168	4	44	1.83	(0.61- 5.50)
Subtotal LUBIN2								1.33	(0.90- 1.96)
WYNDE6	604	m	0	110	138	117	161	1.10	(0.78- 1.55)
WYNDE6	628	f	0	102	81	98	101	1.30	(0.87- 1.94)
Subtotal WYNDE6								1.18	(0.91- 1.53)
Totals				570	5777	244	824		

*prospective study

REF	NRR	SEX	AD	Ys	Ws	Qs	Ps
LUBIN2	1014	m	0	0.24	22.15	0.03	0.2629
LUBIN2	1042	f	0	0.61	3.18	0.52	0.2797
Subtotal LUBIN2				0.28	25.33	0.55	
WYNDE6	604	m	0	0.09	32.16	0.38	0.6001
WYNDE6	628	f	0	0.26	23.67	0.08	0.2048
Subtotal WYNDE6				0.16	55.83	0.46	

	N	4
	NS	2
	Wt	81.16
Het	Chi	1.02
Het	df	3
Het	P	N.S.
Fixed	RR	1.22
	RRl	0.98
	RRu	1.52
	P	(+)
Random	RR	1.22
	RRl	0.98
	RRu	1.52
	P	(+)
Asymm	P	N.S.

Table 3M1 - 6

IESLC - Meta-analysis of Ever Smoking, Butt length or Fraction smoked, "Highest vs lowest"
Adenocarcinoma, Cigarettes (or Any Product if Cigarettes not available)
Least adjusted

	Sex combined	male	female	Total
N		2	2	4
NS		2	2	4
Wt		54.31	26.85	81.16
Het Chi		0.28	0.33	1.02
Het df		1	1	3
Het P		N.S.	N.S.	N.S.
Fixed RR		1.16	1.35	1.22
RRl		0.89	0.93	0.98
RRu		1.52	1.97	1.52
P		N.S.	N.S.	(+)
Random RR		1.16	1.35	1.22
RRl		0.89	0.93	0.98
RRu		1.52	1.97	1.52
P		N.S.	N.S.	(+)
Between Chi				0.40
Between df				1
Between P				N.S.
Btwn(F) P				N.S.
Btwn(R) P				N.S.

Table 3M1 - 7

IESLC - Meta-analysis of Ever Smoking, Butt length or Fraction smoked, "Highest vs lowest"
Adenocarcinoma, Cigarettes (or Any Product if Cigarettes not available)
Excluded studies (and stage at which they were excluded)

1	AKIBA	AMANDU	AMES	BECHER	BENSHL	BEST	BLOT1	BROSS	BROWN3	CARPEN	CEDERL	CHYOU	CPSI	CPSII	DARBY	DEAN2
	DEAN3	DOLL2	ENGELA	GAO2	GARCIA	GILLIS	GRAHAM	GURSEL	HAMMO2	HIRAYA	HOLE	HUMBLE	JAHN	JAIN	KAISE2	KATSOU
	KAUFMA	LAUSSM	LIAW	MCDUFF	MIGRAN	MRFITR	PEZZO2	PISANI	PRESCO	QIAO	SEGI2	SPEIZE	SVENSS	TVERDA	WAKAI	WATSON
	WIGLE	WU	WYNDE3	WYNDE8												
2	AGUDO	ALDERS	ARMADA	AUVINE	AXELSS	BARBON	BENHAM	BOFFET	BOUCHA	BOUCOT	BRESLO	BUFFLE	CHEN	CHEN2	CHIAZZ	CORREA
	DAMBER	DESTEF	DOLL	DORGAN	DORN	DOSEME	FAN	GAO	GARSHI	GENG	GER	GUO	HAENSZ	HAMMON	HEGMAN	HU
	HU2	JEDRYC	JUSSAW	KHUDER	KOULUM	KREUZE	LETOUR	LEVIN	LIU3	LIU4	LIU5	LUBIN	LUO	MATOS	MCCONN	NOTAN2
	OSANN2	PERNU	PEZZOT	QIAO2	RACHTA	SADOWS	SOBUE	SPITZ	STASZE	SUZUK2	VUTUC	WANG2	WU2	WUWILL	WYNDE7	XU
	YUAN	ZHANG	ZHENG	ZHOU												
3	JOLY															
5	CHOI	KOO	RESTRE	TIZZAN	WYNDE2											

Table 3M1 - 8
Potentially overlapping studies

REF	REFGP	PRINC	.	OVERLAP/LINK
LUBIN2	LUBIN2	1		Lubin-combined
WYNDE6	WYNDE6	1		WYNDE5/6/7/8

Table 3M2 -

IESLC - Meta-analysis of Current Smoking, Butt length or Fraction smoked, "Highest vs lowest"
Adenocarcinoma, Cigarettes (or Any Product if Cigarettes not available)

This analysis is restricted to results for:
1) Current smokers
2) Results by Butt length or Fraction smoked
3) Categorical results by Butt length or Fraction smoked
4) Denominator (unexposed) = "low"
5) Adenocarcinoma (or near equivalent)
6) Results complete enough for use in metaanalysis

Within each study, results are then selected (in the following order of preference, within each sex) for:
7) (not applicable)
8) PRODUCT: cigarettes regardless of other products, cigarettes only, all/unspec
9) CIGTYPE: all/unspecified, MC regardless of HR, MC only
10) Results with least adjustment for other aspects of smoking (ADOS)
11) The highest vs lowest category
12) Followup period (YF, prospective studies): whole study (coded as 0) or longest available
13) LCtype: adeno or nearest available, but not squamous. (q = squamous, s = small,
a = adeno, l = large, KII = Kreyberg II, al = alveolar, br = bronchiolar, u = undifferentiated)
14) Race: all or nearest available, otherwise by race (wh or w = white, bl or b = black, hi = hispanic
ch = chinese, jap = japanese, haw = hawaiian, w+o = white + oriental, sca = scandinavian, as = asian)
15) For overlapping studies: principal rather than subsidiary studies
Finally by Age: whole study (coded as 0) if available, otherwise by widest available age group
and then for single sex results (m, f) in preference to results for both sexes combined (c).

Results adjusted (AD) for the most potential confounders are then chosen in Sections -1 to -3
and results adjusted for the least confounders in Sections -4 to -6. (Those least adjusted results which
actually differ from the most adjusted are marked 'x' in column X in Section -4)

Section -7 shows excluded studies, together with the stage (as above) at which no qualifying
results were found.

Section -8 lists the potentially overlapping studies which have been included (1=principal, 2=subsidiary).

Section -9 lists any results which would have been included in preference except that they had data not complete
enough for use in meta-analysis, with their significance (yes/no), if known, and any further comment as entered
on the database. It also lists as "gap" any categories for which no data were presented by the original authors.

In addition to those mentioned above, the following fields, levels and abbreviations are used:
* or nk = not known, n = no, y = yes, ot = other
all/unspec = all or unspecified, cig+/-ot = cigarettes irrespective of other products (cigar, pipe etc)
MC = manufactured cigarettes, HR = hand-rolled cigarettes
exL, exH = range of exposure (low and high) in the "highest" group, in terms of Butt length or Fraction smoked
unexL, unexH = range of exposure (low and high) in the "lowest" group, in terms of Butt length or Fraction smoked
REF: 6-character study reference
NRR: number of the RR on the database within the study
ST : study type (CC = case control, pr or prosp = prospective)
NLC: number of lung cancer cases in whole study
R : risky occupational population (n = no, m = mining, o = other risky)
VB : national cigarette type (V = at least 75% Virginia, bl = at least 75% blended, ot = other)
P : any proxy use
H : full histological confirmation
De : derivation of RR/CI (or = original, st = standard method, ot = other method of estimation)

Table 3M2 - 1

IESLC - Meta-analysis of Current Smoking, Butt length or Fraction smoked, "Highest vs lowest"
Adenocarcinoma, Cigarettes (or Any Product if Cigarettes not available)
Most adjusted

REF	NRR	SEX	AGEL	AGEH	RACE	YF	LC TYPE	LOC	START	ST	NLC	R	VB	P	H	AD	ADOS	PRODUCT	exL	exH	unexL	unexH	De
SOBUE	580	m	0	0	all	-	a	As:Jap	1986	CC	1376	n	bl	n	y	0	0	cig+/-ot	67	100	1	49	st
WAKAI	603	m	0	0	all	-	a	As:Jap	1988	CC	333	n	bl	n	y	0	0	cig+/-ot	76	100	1	49	st

Cigarette type is all/unspec for all RRs

exL, exH, unexL, unexH refer to fraction of cigarette smoked (%) for all RRs

Table 3M2 - 2

IESLC - Meta-analysis of Current Smoking, Butt length or Fraction smoked, "Highest vs lowest"
Adenocarcinoma, Cigarettes (or Any Product if Cigarettes not available)
Most adjusted

REF	NRR	SEX	AD	Number Exposed Case	Cont	Non-exposed Case	Cont	RR	95.00%CI
SOBUE	580	m	0	52	87	48	148	1.84	(1.15- 2.96)
WAKAI	603	m	0	20	91	6	26	0.95	(0.35- 2.62)
Totals				72	178	54	174		

*prospective study

REF	NRR	SEX	AD	Ys	Ws	Qs	Ps
SOBUE	580	m	0	0.61	17.15	0.24	0.0114
WAKAI	603	m	0	-0.05	3.76	1.10	0.9246

	N	2
	NS	2
	Wt	20.91
Het	Chi	1.34
Het	df	1
Het	P	N.S.
Fixed	RR	1.64
	RRl	1.07
	RRu	2.51
	P	+
Random	RR	1.55
	RRl	0.88
	RRu	2.74
	P	N.S.
Asymm	P	

Table 3M2 - 3

IESLC - Meta-analysis of Current Smoking, Butt length or Fraction smoked, "Highest vs lowest"
Adenocarcinoma, Cigarettes (or Any Product if Cigarettes not available)
Most adjusted

	Sex combined	male	female	Total
N		2		2
NS		2		2
Wt		20.91		20.91
Het Chi		1.34		1.34
Het df		1		1
Het P		N.S.		N.S.
Fixed RR		1.64		1.64
RRl		1.07		1.07
RRu		2.51		2.51
P		+		+
Random RR		1.55		1.55
RRl		0.88		0.88
RRu		2.74		2.74
P		N.S.		N.S.
Between Chi				
Between df				
Between P				N.S.
Btwn(F) P				N.S.
Btwn(R) P				N.S.

Too few RRs for analysis by factor

Table 3M2 - 4

IESLC - Meta-analysis of Current Smoking, Butt length or Fraction smoked, "Highest vs lowest"
Adenocarcinoma, Cigarettes (or Any Product if Cigarettes not available)
Least adjusted

REF	NRR	X	SEX	AGEL	AGEH	RACE	YF	LC TYPE	LOC	START	ST	NLC	R	VB	P	H	AD	ADOS	PRODUCT	exL	exH	unexL	unexH	De
SOBUE	580		m	0	0	all	-	a	As:Jap	1986	CC	1376	n	bl	n	y	0	0	cig+/-ot	67	100	1	49	st
WAKAI	603		m	0	0	all	-	a	As:Jap	1988	CC	333	n	bl	n	y	0	0	cig+/-ot	76	100	1	49	st

Cigarette type is all/unspec for all RRs

exL, exH, unexL, unexH refer to fraction of cigarette smoked (%) for all RRs

Table 3M2 - 5

IESLC - Meta-analysis of Current Smoking, Butt length or Fraction smoked, "Highest vs lowest"
Adenocarcinoma, Cigarettes (or Any Product if Cigarettes not available)
Least adjusted

REF	NRR	SEX	AD	Number Exposed Case	Number Exposed Cont	Non-exposed Case	Non-exposed Cont	RR	95.00%CI
SOBUE	580	m	0	52	87	48	148	1.84	(1.15- 2.96)
WAKAI	603	m	0	20	91	6	26	0.95	(0.35- 2.62)
Totals				72	178	54	174		

*prospective study

REF	NRR	SEX	AD	Ys	Ws	Qs	Ps
SOBUE	580	m	0	0.61	17.15	0.24	0.0114
WAKAI	603	m	0	-0.05	3.76	1.10	0.9246

	N	2
	NS	2
	Wt	20.91
Het	Chi	1.34
Het	df	1
Het	P	N.S.
Fixed	RR	1.64
	RRl	1.07
	RRu	2.51
	P	+
Random	RR	1.55
	RRl	0.88
	RRu	2.74
	P	N.S.
Asymm	P	

Table 3M2 - 6

IESLC - Meta-analysis of Current Smoking, Butt length or Fraction smoked, "Highest vs lowest"
Adenocarcinoma, Cigarettes (or Any Product if Cigarettes not available)
Least adjusted

	Sex combined	male	female	Total
N		2		2
NS		2		2
Wt		20.91		20.91
Het Chi		1.34		1.34
Het df		1		1
Het P		N.S.		N.S.
Fixed RR		1.64		1.64
RRl		1.07		1.07
RRu		2.51		2.51
P		+		+
Random RR		1.55		1.55
RRl		0.88		0.88
RRu		2.74		2.74
P		N.S.		N.S.
Between Chi				
Between df				
Between P				N.S.
Btwn(F) P				N.S.
Btwn(R) P				N.S.

Table 3M2 - 7

IESLC - Meta-analysis of Current Smoking, Butt length or Fraction smoked, "Highest vs lowest"
Adenocarcinoma, Cigarettes (or Any Product if Cigarettes not available)
Excluded studies (and stage at which they were excluded)

1	AGUDO ALDERS ARMADA AUVINE AXELSS BARBON BECHER BENHAM BLOT1 BOFFET BOUCHA BRESLO BROWN3 CARPEN CHEN CHEN2 CHIAZZ CHOI CHYOU CORREA DAMBER DARBY DESTEF DOLL DOLL2 DORGAN DOSEME FAN GAO GARCIA GARSHI GENG GER GRAHAM GUO GURSEL HAENSZ HAMMO2 HAMMON HEGMAN HU HU2 JAHN JAIN JEDRYC JOLY JUSSAW KHUDER KOO KOULUM KREUZE LAUSSM LETOUR LEVIN LIU3 LIU4 LIU5 LUBIN LUBIN2 LUO MCCONN NOTAN2 OSANN2 PERNU PEZZOT PRESCO QIAO QIAO2 RACHTA RESTRE SADOWS STASZE SUZUK2 TIZZAN TVERDA VUTUC WANG2 WIGLE WU2 WUWILL WYNDE2 WYNDE3 XU YUAN ZHANG ZHENG ZHOU
2	AKIBA AMANDU AMES BENSHL BEST BOUCOT BROSS BUFFLE CEDERL CPSI CPSII DEAN2 DEAN3 DORN ENGELA GAO2 GILLIS HIRAYA HOLE HUMBLE KATSOU KAUFMA LIAW MATOS MCDUFF MIGRAN MRFITR PEZZO2 PISANI SEGI2 SPEIZE SPITZ SVENSS WATSON WU WYNDE6 WYNDE7 WYNDE8
5	KAISE2

Table 3M3 -

IESLC - Meta-analysis of Ever/current Smoking, Butt length or Fraction smoked, "Highest vs lowest"
Adenocarcinoma, Cigarettes (or Any Product if Cigarettes not available)

This analysis is restricted to results for:
1) Ever/current smokers
2) Results by Butt length or Fraction smoked
3) Categorical results by Butt length or Fraction smoked
4) Denominator (unexposed) = "low"
5) Adenocarcinoma (or near equivalent)
6) Results complete enough for use in metaanalysis

Within each study, results are then selected (in the following order of preference, within each sex) for:
7) SMKSTA: ever, current
8) PRODUCT: cigarettes regardless of other products, cigarettes only, all/unspec
9) CIGTYPE: all/unspecified, MC regardless of HR, MC only
10) Results with least adjustment for other aspects of smoking (ADOS)
11) The highest vs lowest category
12) Followup period (YF, prospective studies): whole study (coded as 0) or longest available
13) LCtype: adeno or nearest available, but not squamous. (q = squamous, s = small,
a = adeno, l = large, KII = Kreyberg II, al = alveolar, br = bronchiolar, u = undifferentiated)
14) Race: all or nearest available, otherwise by race (wh or w = white, bl or b = black, hi = hispanic
ch = chinese, jap = japanese, haw = hawaiian, w+o = white + oriental, sca = scandinavian, as = asian)
15) For overlapping studies: principal rather than subsidiary studies
Finally by Age: whole study (coded as 0) if available, otherwise by widest available age group
and then for single sex results (m, f) in preference to results for both sexes combined (c).

Results adjusted (AD) for the most potential confounders are then chosen in Sections -1 to -3
and results adjusted for the least confounders in Sections -4 to -6. (Those least adjusted results which
actually differ from the most adjusted are marked 'x' in column X in Section -4)

Section -7 shows excluded studies, together with the stage (as above) at which no qualifying
results were found.

Section -8 lists the potentially overlapping studies which have been included (1=principal, 2=subsidiary).

Section -9 lists any results which would have been included in preference except that they had data not complete
enough for use in meta-analysis, with their significance (yes/no), if known, and any further comment as entered
on the database. It also lists as "gap" any categories for which no data were presented by the original authors.

In addition to those mentioned above, the following fields, levels and abbreviations are used:
* or nk = not known, n = no, y = yes, ot = other
all/unspec = all or unspecified, cig+/-ot = cigarettes irrespective of other products (cigar, pipe etc)
MC = manufactured cigarettes, HR = hand-rolled cigarettes
exL, exH = range of exposure (low and high) in the "highest" group, in terms of Butt length or Fraction smoked
unexL, unexH = range of exposure (low and high) in the "lowest" group, in terms of Butt length or Fraction smoked
REF: 6-character study reference
NRR: number of the RR on the database within the study
ST : study type (CC = case control, pr or prosp = prospective)
NLC: number of lung cancer cases in whole study
R : risky occupational population (n = no, m = mining, o = other risky)
VB : national cigarette type (V = at least 75% Virginia, bl = at least 75% blended, ot = other)
P : any proxy use
H : full histological confirmation
De : derivation of RR/CI (or = original, st = standard method, ot = other method of estimation)

Table 3M3 - 1

IESLC - Meta-analysis of Ever/current Smoking, Butt length or Fraction smoked, "Highest vs lowest"
Adenocarcinoma, Cigarettes (or Any Product if Cigarettes not available)
Most adjusted

REF	NRR	SEX	AGEL	AGEH	RACE	YF	LC TYPE	LOC	START	ST	NLC	R	VB	P	H	AD	ADOS	SM	PRODUCT	exL	exH	unexL	unexH	De
LUBIN2	1014	m	0	0	all	-	a	Eu:mul	1976	CC	7804	n	bl	n	y	0	0	ev	cig+/-ot	76	100	1	50	st
LUBIN2	1042	f	0	0	all	-	a	Eu:mul	1976	CC	7804	n	bl	n	y	0	0	ev	cig+/-ot	76	100	1	50	st
SOBUE	580	m	0	0	all	-	a	As:Jap	1986	CC	1376	n	bl	n	y	0	0	cu	cig+/-ot	67	100	1	49	st
WAKAI	603	m	0	0	all	-	a	As:Jap	1988	CC	333	n	bl	n	y	0	0	cu	cig+/-ot	76	100	1	49	st
WYNDE6	604	m	0	0	wh	-	a	NAmer	1969	CC	4423	n	bl	n	y	0	0	ev	cig+/-ot	50	100	1	49	st
WYNDE6	628	f	0	0	wh	-	a	NAmer	1969	CC	4423	n	bl	n	y	0	0	ev	cig+/-ot	50	100	1	49	st

Cigarette type is all/unspec for all RRs

exL, exH, unexL, unexH refer to fraction of cigarette smoked (%) for all RRs

Table 3M3 - 2

IESLC - Meta-analysis of Ever/current Smoking, Butt length or Fraction smoked, "Highest vs lowest"
Adenocarcinoma, Cigarettes (or Any Product if Cigarettes not available)
Most adjusted

REF	NRR	SEX	AD	Number Exposed Case	Number Exposed Cont	Non-exposed Case	Non-exposed Cont	RR	95.00%CI
LUBIN2	1014	m	0	330	5390	25	518	1.27	(0.84- 1.92)
LUBIN2	1042	f	0	28	168	4	44	1.83	(0.61- 5.50)
Subtotal LUBIN2								1.33	(0.90- 1.96)
SOBUE	580	m	0	52	87	48	148	1.84	(1.15- 2.96)
WAKAI	603	m	0	20	91	6	26	0.95	(0.35- 2.62)
WYNDE6	604	m	0	110	138	117	161	1.10	(0.78- 1.55)
WYNDE6	628	f	0	102	81	98	101	1.30	(0.87- 1.94)
Subtotal WYNDE6								1.18	(0.91- 1.53)
Totals				642	5955	298	998		

*prospective study

REF	NRR	SEX	AD	Ys	Ws	Qs	Ps
LUBIN2	1014	m	0	0.24	22.15	0.01	0.2629
LUBIN2	1042	f	0	0.61	3.18	0.38	0.2797
Subtotal LUBIN2				0.28	25.33	0.39	
SOBUE	580	m	0	0.61	17.15	2.10	0.0114
WAKAI	603	m	0	-0.05	3.76	0.36	0.9246
WYNDE6	604	m	0	0.09	32.16	0.91	0.6001
WYNDE6	628	f	0	0.26	23.67	0.00	0.2048
Subtotal WYNDE6				0.16	55.83	0.91	

	N	6
	NS	4
	Wt	102.06
Het	Chi	3.77
Het	df	5
Het	P	N.S.
Fixed	RR	1.30
	RRl	1.07
	RRu	1.58
	P	++
Random	RR	1.30
	RRl	1.07
	RRu	1.58
	P	++
Asymm	P	N.S.

Table 3M3 - 3

IESLC - Meta-analysis of Ever/current Smoking, Butt length or Fraction smoked, "Highest vs lowest"
Adenocarcinoma, Cigarettes (or Any Product if Cigarettes not available)
Most adjusted

	Sex combined	male	female	Total
N		4	2	6
NS		4	2	6
Wt		75.22	26.85	102.06
Het Chi		3.38	0.33	3.77
Het df		3	1	5
Het P		N.S.	N.S.	N.S.
Fixed RR		1.28	1.35	1.30
RRl		1.02	0.93	1.07
RRu		1.60	1.97	1.58
P		+	N.S.	++
Random RR		1.29	1.35	1.30
RRl		1.01	0.93	1.07
RRu		1.64	1.97	1.58
P		+	N.S.	++
Between Chi				0.06
Between df				1
Between P				N.S.
Btwn(F) P				N.S.
Btwn(R) P				N.S.

Too few RRs for analysis by factor

Table 3M3 - 4

IESLC - Meta-analysis of Ever/current Smoking, Butt length or Fraction smoked, "Highest vs lowest"
Adenocarcinoma, Cigarettes (or Any Product if Cigarettes not available)
Least adjusted

REF	NRR	X	SEX	AGEL	AGEH	RACE	YF	LC TYPE	LOC	START	ST	NLC	R	VB	P	H	AD	ADOS	SM	PRODUCT	exL	exH	unexL	unexH	De
LUBIN2	1014		m	0	0	all	-	a	Eu:mul	1976	CC	7804	n	bl	n	y	0	0	ev	cig+/-ot	76	100	1	50	st
LUBIN2	1042		f	0	0	all	-	a	Eu:mul	1976	CC	7804	n	bl	n	y	0	0	ev	cig+/-ot	76	100	1	50	st
SOBUE	580		m	0	0	all	-	a	As:Jap	1986	CC	1376	n	bl	n	y	0	0	cu	cig+/-ot	67	100	1	49	st
WAKAI	603		m	0	0	all	-	a	As:Jap	1988	CC	333	n	bl	n	y	0	0	cu	cig+/-ot	76	100	1	49	st
WYNDE6	604		m	0	0	wh	-	a	NAmer	1969	CC	4423	n	bl	n	y	0	0	ev	cig+/-ot	50	100	1	49	st
WYNDE6	628		f	0	0	wh	-	a	NAmer	1969	CC	4423	n	bl	n	y	0	0	ev	cig+/-ot	50	100	1	49	st

Cigarette type is all/unspec for all RRs

exL, exH, unexL, unexH refer to fraction of cigarette smoked (%) for all RRs

Table 3M3 - 5

IESLC - Meta-analysis of Ever/current Smoking, Butt length or Fraction smoked, "Highest vs lowest"
Adenocarcinoma, Cigarettes (or Any Product if Cigarettes not available)
Least adjusted

REF	NRR	SEX	AD	Number Exposed Case	Cont	Non-exposed Case	Cont	RR	95.00%CI
LUBIN2	1014	m	0	330	5390	25	518	1.27	(0.84- 1.92)
LUBIN2	1042	f	0	28	168	4	44	1.83	(0.61- 5.50)
Subtotal LUBIN2								1.33	(0.90- 1.96)
SOBUE	580	m	0	52	87	48	148	1.84	(1.15- 2.96)
WAKAI	603	m	0	20	91	6	26	0.95	(0.35- 2.62)
WYNDE6	604	m	0	110	138	117	161	1.10	(0.78- 1.55)
WYNDE6	628	f	0	102	81	98	101	1.30	(0.87- 1.94)
Subtotal WYNDE6								1.18	(0.91- 1.53)
Totals				642	5955	298	998		

*prospective study

REF	NRR	SEX	AD	Ys	Ws	Qs	Ps
LUBIN2	1014	m	0	0.24	22.15	0.01	0.2629
LUBIN2	1042	f	0	0.61	3.18	0.38	0.2797
Subtotal LUBIN2				0.28	25.33	0.39	
SOBUE	580	m	0	0.61	17.15	2.10	0.0114
WAKAI	603	m	0	-0.05	3.76	0.36	0.9246
WYNDE6	604	m	0	0.09	32.16	0.91	0.6001
WYNDE6	628	f	0	0.26	23.67	0.00	0.2048
Subtotal WYNDE6				0.16	55.83	0.91	

	N	6
	NS	4
	Wt	102.06
Het	Chi	3.77
Het	df	5
Het	P	N.S.
Fixed	RR	1.30
	RRl	1.07
	RRu	1.58
	P	++
Random	RR	1.30
	RRl	1.07
	RRu	1.58
	P	++
Asymm	P	N.S.

Table 3M3 - 6

IESLC - Meta-analysis of Ever/current Smoking, Butt length or Fraction smoked, "Highest vs lowest"
Adenocarcinoma, Cigarettes (or Any Product if Cigarettes not available)
Least adjusted

	Sex combined	Sex male	Sex female	Total
N		4	2	6
NS		4	2	6
Wt		75.22	26.85	102.06
Het Chi		3.38	0.33	3.77
Het df		3	1	5
Het P		N.S.	N.S.	N.S.
Fixed RR		1.28	1.35	1.30
RRl		1.02	0.93	1.07
RRu		1.60	1.97	1.58
P		+	N.S.	++
Random RR		1.29	1.35	1.30
RRl		1.01	0.93	1.07
RRu		1.64	1.97	1.58
P		+	N.S.	++
Between Chi				0.06
Between df				1
Between P				N.S.
Btwn(F) P				N.S.
Btwn(R) P				N.S.

Table 3M3 - 7

IESLC - Meta-analysis of Ever/current Smoking, Butt length or Fraction smoked, "Highest vs lowest"
Adenocarcinoma, Cigarettes (or Any Product if Cigarettes not available)
Excluded studies (and stage at which they were excluded)

1	BECHER BLOT1 BROWN3 CARPEN CHYOU DARBY DOLL2 GARCIA GRAHAM GURSEL HAMMO2 JAHN JAIN LAUSSM PRESCO QIAO TVERDA WIGLE WYNDE3
2	AGUDO AKIBA ALDERS AMANDU AMES ARMADA AUVINE AXELSS BARBON BENHAM BENSHL BEST BOFFET BOUCHA BOUCOT BRESLO BROSS BUFFLE CEDERL CHEN CHEN2 CHIAZZ CORREA CPSI CPSII DAMBER DEAN2 DEAN3 DESTEF DOLL DORGAN DORN DOSEME ENGELA FAN GAO GAO2 GARSHI GENG GER GILLIS GUO HAENSZ HAMMON HEGMAN HIRAYA HOLE HU HU2 HUMBLE JEDRYC JUSSAW KATSOU KAUFMA KHUDER KOULUM KREUZE LETOUR LEVIN LIAW LIU3 LIU4 LIU5 LUBIN LUO MATOS MCCONN MCDUFF MIGRAN MRFITR NOTAN2 OSANN2 PERNU PEZZO2 PEZZOT PISANI QIAO2 RACHTA SADOWS SEGI2 SPEIZE SPITZ STASZE SUZUK2 SVENSS VUTUC WANG2 WATSON WU WU2 WUWILL WYNDE7 WYNDE8 XU YUAN ZHANG ZHENG ZHOU
3	JOLY
5	CHOI KAISE2 KOO RESTRE TIZZAN WYNDE2

Table 3M3 - 8

Potentially overlapping studies

REF	REFGP	PRINC	. OVERLAP/LINK
LUBIN2	LUBIN2	1	Lubin-combined
WYNDE6	WYNDE6	1	WYNDE5/6/7/8

Table 3M4 -

IESLC - Meta-analysis of Ever Smoking, Butt length or Fraction smoked, "Highest vs lowest"
Adenocarcinoma, Cigarettes only

This analysis is restricted to results for:
1) Ever smokers
2) Results by Butt length or Fraction smoked
3) Categorical results by Butt length or Fraction smoked
4) Denominator (unexposed) = "low"
5) Adenocarcinoma (or near equivalent)
6) Results complete enough for use in metaanalysis

Within each study, results are then selected (in the following order of preference, within each sex) for:
7) (not applicable)
8) PRODUCT: cigarettes only
9) CIGTYPE: all/unspecified, MC regardless of HR, MC only
10) Results with least adjustment for other aspects of smoking (ADOS)
11) The highest vs lowest category
12) Followup period (YF, prospective studies): whole study (coded as 0) or longest available
13) LCtype: adeno or nearest available, but not squamous. (q = squamous, s = small,
a = adeno, l = large, KII = Kreyberg II, al = alveolar, br = bronchiolar, u = undifferentiated)
14) Race: all or nearest available, otherwise by race (wh or w = white, bl or b = black, hi = hispanic
ch = chinese, jap = japanese, haw = hawaiian, w+o = white + oriental, sca = scandinavian, as = asian)
15) For overlapping studies: principal rather than subsidiary studies
Finally by Age: whole study (coded as 0) if available, otherwise by widest available age group
and then for single sex results (m, f) in preference to results for both sexes combined (c).

Results adjusted (AD) for the most potential confounders are then chosen in Sections -1 to -3
and results adjusted for the least confounders in Sections -4 to -6. (Those least adjusted results which
actually differ from the most adjusted are marked 'x' in column X in Section -4)

Section -7 shows excluded studies, together with the stage (as above) at which no qualifying
results were found.

Section -8 lists the potentially overlapping studies which have been included (1=principal, 2=subsidiary).

Section -9 lists any results which would have been included in preference except that they had data not complete
enough for use in meta-analysis, with their significance (yes/no), if known, and any further comment as entered
on the database. It also lists as "gap" any categories for which no data were presented by the original authors.

In addition to those mentioned above, the following fields, levels and abbreviations are used:
* or nk = not known, n = no, y = yes, ot = other
all/unspec = all or unspecified, MC = manufactured cigarettes, HR = hand-rolled cigarettes
exL, exH = range of exposure (low and high) in the "highest" group, in terms of Butt length or Fraction smoked
unexL, unexH = range of exposure (low and high) in the "lowest" group, in terms of Butt length or Fraction smoked
REF: 6-character study reference
NRR: number of the RR on the database within the study
ST : study type (CC = case control, pr or prosp = prospective)
NLC: number of lung cancer cases in whole study
R : risky occupational population (n = no, m = mining, o = other risky)
VB : national cigarette type (V = at least 75% Virginia, bl = at least 75% blended, ot = other)
P : any proxy use
H : full histological confirmation
De : derivation of RR/CI (or = original, st = standard method, ot = other method of estimation)

Table 3M4 - 0

No RRs selected for this analysis

Table 3M4 - 7

IESLC - Meta-analysis of Ever Smoking, Butt length or Fraction smoked, "Highest vs lowest"
Adenocarcinoma, Cigarettes only
Excluded studies (and stage at which they were excluded)

1 AKIBA AMANDU AMES BECHER BENSHL BEST BLOT1 BROSS BROWN3 CARPEN CEDERL CHYOU CPSI CPSII DARBY DEAN2 DEAN3
DOLL2 ENGELA GAO2 GARCIA GILLIS GRAHAM GURSEL HAMMO2 HIRAYA HOLE HUMBLE JAHN JAIN KAISE2 KATSOU KAUFMA LAUSSM
LIAW MCDUFF MIGRAN MRFITR PEZZO2 PISANI PRESCO QIAO SEGI2 SPEIZE SVENSS TVERDA WAKAI WATSON WIGLE WU WYNDE3
WYNDE8

2 AGUDO ALDERS ARMADA AUVINE AXELSS BARBON BENHAM BOFFET BOUCHA BOUCOT BRESLO BUFFLE CHEN CHEN2 CHIAZZ CORREA DAMBER
DESTEF DOLL DORGAN DORN DOSEME FAN GAO GARSHI GENG GER GUO HAENSZ HAMMON HEGMAN HU HU2 JEDRYC
JUSSAW KHUDER KOULUM KREUZE LETOUR LEVIN LIU3 LIU4 LIU5 LUBIN LUO MATOS MCCONN NOTAN2 OSANN2 PERNU PEZZOT
QIAO2 RACHTA SADOWS SOBUE SPITZ STASZE SUZUK2 VUTUC WANG2 WU2 WUWILL WYNDE7 XU YUAN ZHANG ZHENG ZHOU

3 JOLY

5 CHOI KOO RESTRE TIZZAN WYNDE2

8 LUBIN2 WYNDE6

Table 3M5 -

IESLC - Meta-analysis of Current Smoking, Butt length or Fraction smoked, "Highest vs lowest"
Adenocarcinoma, Cigarettes only

This analysis is restricted to results for:
1) Current smokers
2) Results by Butt length or Fraction smoked
3) Categorical results by Butt length or Fraction smoked
4) Denominator (unexposed) = "low"
5) Adenocarcinoma (or near equivalent)
6) Results complete enough for use in metaanalysis

Within each study, results are then selected (in the following order of preference, within each sex) for:
7) (not applicable)
8) PRODUCT: cigarettes only
9) CIGTYPE: all/unspecified, MC regardless of HR, MC only
10) Results with least adjustment for other aspects of smoking (ADOS)
11) The highest vs lowest category
12) Followup period (YF, prospective studies): whole study (coded as 0) or longest available
13) LCtype: adeno or nearest available, but not squamous. (q = squamous, s = small,
a = adeno, l = large, KII = Kreyberg II, al = alveolar, br = bronchiolar, u = undifferentiated)
14) Race: all or nearest available, otherwise by race (wh or w = white, bl or b = black, hi = hispanic
ch = chinese, jap = japanese, haw = hawaiian, w+o = white + oriental, sca = scandinavian, as = asian)
15) For overlapping studies: principal rather than subsidiary studies
Finally by Age: whole study (coded as 0) if available, otherwise by widest available age group
and then for single sex results (m, f) in preference to results for both sexes combined (c).

Results adjusted (AD) for the most potential confounders are then chosen in Sections -1 to -3
and results adjusted for the least confounders in Sections -4 to -6. (Those least adjusted results which
actually differ from the most adjusted are marked 'x' in column X in Section -4)

Section -7 shows excluded studies, together with the stage (as above) at which no qualifying
results were found.

Section -8 lists the potentially overlapping studies which have been included (1=principal, 2=subsidiary).

Section -9 lists any results which would have been included in preference except that they had data not complete
enough for use in meta-analysis, with their significance (yes/no), if known, and any further comment as entered
on the database. It also lists as "gap" any categories for which no data were presented by the original authors.

In addition to those mentioned above, the following fields, levels and abbreviations are used:
* or nk = not known, n = no, y = yes, ot = other
all/unspec = all or unspecified, MC = manufactured cigarettes, HR = hand-rolled cigarettes
exL, exH = range of exposure (low and high) in the "highest" group, in terms of Butt length or Fraction smoked
unexL, unexH = range of exposure (low and high) in the "lowest" group, in terms of Butt length or Fraction smoked
REF: 6-character study reference
NRR: number of the RR on the database within the study
ST : study type (CC = case control, pr or prosp = prospective)
NLC: number of lung cancer cases in whole study
R : risky occupational population (n = no, m = mining, o = other risky)
VB : national cigarette type (V = at least 75% Virginia, bl = at least 75% blended, ot = other)
P : any proxy use
H : full histological confirmation
De : derivation of RR/CI (or = original, st = standard method, ot = other method of estimation)

Table 3M5 - 0

No RRs selected for this analysis

Table 3M5 - 7

IESLC - Meta-analysis of Current Smoking, Butt length or Fraction smoked, "Highest vs lowest"
Adenocarcinoma, Cigarettes only
Excluded studies (and stage at which they were excluded)

1 AGUDO ALDERS ARMADA AUVINE AXELSS BARBON BECHER BENHAM BLOT1 BOFFET BOUCHA BRESLO BROWN3 CARPEN CHEN CHEN2 CHIAZZ CHOI CHYOU CORREA DAMBER DARBY DESTEF DOLL DOLL2 DORGAN DOSEME FAN GAO GARCIA GARSHI GENG GER GRAHAM GUO GURSEL HAENSZ HAMMO2 HAMMON HEGMAN HU HU2 JAHN JAIN JEDRYC JOLY JUSSAW KHUDER KOO KOULUM KREUZE LAUSSM LETOUR LEVIN LIU3 LIU4 LIU5 LUBIN LUBIN2 LUO MCCONN NOTAN2 OSANN2 PERNU PEZZOT PRESCO QIAO QIAO2 RACHTA RESTRE SADOWS STASZE SUZUK2 TIZZAN TVERDA VUTUC WANG2 WIGLE WU2 WUWILL WYNDE2 WYNDE3 XU YUAN ZHANG ZHENG ZHOU

2 AKIBA AMANDU AMES BENSHL BEST BOUCOT BROSS BUFFLE CEDERL CPSI CPSII DEAN2 DEAN3 DORN ENGELA GAO2 GILLIS HIRAYA HOLE HUMBLE KATSOU KAUFMA LIAW MATOS MCDUFF MIGRAN MRFITR PEZZO2 PISANI SEGI2 SPEIZE SPITZ SVENSS WATSON WU WYNDE6 WYNDE7 WYNDE8

5 KAISE2

8 SOBUE WAKAI

Table 3M6 -

IESLC - Meta-analysis of Ever/current Smoking, Butt length or Fraction smoked, "Highest vs lowest"
Adenocarcinoma, Cigarettes only

This analysis is restricted to results for:
1) Ever/current smokers
2) Results by Butt length or Fraction smoked
3) Categorical results by Butt length or Fraction smoked
4) Denominator (unexposed) = "low"
5) Adenocarcinoma (or near equivalent)
6) Results complete enough for use in metaanalysis

Within each study, results are then selected (in the following order of preference, within each sex) for:
7) SMKSTA: ever, current
8) PRODUCT: cigarettes only
9) CIGTYPE: all/unspecified, MC regardless of HR, MC only
10) Results with least adjustment for other aspects of smoking (ADOS)
11) The highest vs lowest category
12) Followup period (YF, prospective studies): whole study (coded as 0) or longest available
13) LCtype: adeno or nearest available, but not squamous. (q = squamous, s = small,
a = adeno, l = large, KII = Kreyberg II, al = alveolar, br = bronchiolar, u = undifferentiated)
14) Race: all or nearest available, otherwise by race (wh or w = white, bl or b = black, hi = hispanic
ch = chinese, jap = japanese, haw = hawaiian, w+o = white + oriental, sca = scandinavian, as = asian)
15) For overlapping studies: principal rather than subsidiary studies
Finally by Age: whole study (coded as 0) if available, otherwise by widest available age group
and then for single sex results (m, f) in preference to results for both sexes combined (c).

Results adjusted (AD) for the most potential confounders are then chosen in Sections -1 to -3
(and those which actually differ from the adjusted results in Table 3M3 - 1 are marked 'x' in Section -1)
and results adjusted for the least confounders in Sections -4 to -6. (Those least adjusted results which
actually differ from the most adjusted are marked 'x' in column X in Section -4)

Section -7 shows excluded studies, together with the stage (as above) at which no qualifying
results were found.

Section -8 lists the potentially overlapping studies which have been included (1=principal, 2=subsidiary).

Section -9 lists any results which would have been included in preference except that they had data not complete
enough for use in meta-analysis, with their significance (yes/no), if known, and any further comment as entered
on the database. It also lists as "gap" any categories for which no data were presented by the original authors.

In addition to those mentioned above, the following fields, levels and abbreviations are used:
* or nk = not known, n = no, y = yes, ot = other
all/unspec = all or unspecified, MC = manufactured cigarettes, HR = hand-rolled cigarettes
exL, exH = range of exposure (low and high) in the "highest" group, in terms of Butt length or Fraction smoked
unexL, unexH = range of exposure (low and high) in the "lowest" group, in terms of Butt length or Fraction smoked
REF: 6-character study reference
NRR: number of the RR on the database within the study
ST : study type (CC = case control, pr or prosp = prospective)
NLC: number of lung cancer cases in whole study
R : risky occupational population (n = no, m = mining, o = other risky)
VB : national cigarette type (V = at least 75% Virginia, bl = at least 75% blended, ot = other)
P : any proxy use
H : full histological confirmation
De : derivation of RR/CI (or = original, st = standard method, ot = other method of estimation)

Table 3M6 - 0

No RRs selected for this analysis

Table 3M6 - 7

IESLC - Meta-analysis of Ever/current Smoking, Butt length or Fraction smoked, "Highest vs lowest"
Adenocarcinoma, Cigarettes only
Excluded studies (and stage at which they were excluded)

1 BECHER BLOT1 BROWN3 CARPEN CHYOU DARBY DOLL2 GARCIA GRAHAM GURSEL HAMMO2 JAHN JAIN LAUSSM PRESCO QIAO TVERDA
WIGLE WYNDE3

2 AGUDO AKIBA ALDERS AMANDU AMES ARMADA AUVINE AXELSS BARBON BENHAM BENSHL BEST BOFFET BOUCHA BOUCOT BRESLO BROSS
BUFFLE CEDERL CHEN CHEN2 CHIAZZ CORREA CPSI CPSII DAMBER DEAN2 DEAN3 DESTEF DOLL DORGAN DORN DOSEME ENGELA
FAN GAO GAO2 GARSHI GENG GER GILLIS GUO HAENSZ HAMMON HEGMAN HIRAYA HOLE HU HU2 HUMBLE JEDRYC
JUSSAW KATSOU KAUFMA KHUDER KOULUM KREUZE LETOUR LEVIN LIAW LIU3 LIU4 LIU5 LUBIN LUO MATOS MCCONN MCDUFF
MIGRAN MRFITR NOTAN2 OSANN2 PERNU PEZZO2 PEZZOT PISANI QIAO2 RACHTA SADOWS SEGI2 SPEIZE SPITZ STASZE SUZUK2 SVENSS
VUTUC WANG2 WATSON WU WU2 WUWILL WYNDE7 WYNDE8 XU YUAN ZHANG ZHENG ZHOU

3 JOLY

5 CHOI KAISE2 KOO RESTRE TIZZAN WYNDE2

8 LUBIN2 SOBUE WAKAI WYNDE6
