# Supplementary material for: Systematic review with meta-analysis of the epidemiological evidence in the 1900s relating smoking to lung cancer
Source: BMC Cancer. 2012 Sep 3;12:385. doi: 10.1186/1471-2407-12-385 (PMC3505152; doi:10.1186/1471-2407-12-385)
Supplement: Additional file 5 — Detailed Analysis Tables (Individual file names as described in Additional file 1: Methods, Table1). [file 1471-2407-12-385-S5.zip › PDF/3BR.pdf]

Table 3B1R - 1

IESLC - Meta-regression of current smoking, any product (or cigs if any not available)
Multiple regression of data from Table 3B1
Adenocarcinoma

Stepwise allowing only characteristics from the fixed model

Log Relative risk
WEIGHTED on Weight

| | | | | | | | | |
|---|---|---|---|---|---|---|---|---|
| | | Deviance | (DF) | | | | | |
| Model 1 | | 528.128 | (43) | | | | | |
| | | Estimate | S.E. | P | RR | 95%CIl | 95%CIu | |
| Constant | | 1.562 | 0.031 | +++ | 4.769 | 4.485 | 5.071 | |

| | | | | | | | |
|---|---|---|---|---|---|---|---|
| | | Deviance | (DF) | Drop Dev | P | | |
| Model 2 | | 170.181 | (39) | 357.947 | *** | | |
| | | Estimate | S.E. | P | RR | 95%CIl | 95%CIu |
| Constant | | 2.032 | 0.040 | +++ | 7.626 | 7.046 | 8.253 |
| Location | | | | | | | |
| NAmer | 24 | Aliased | | | 7.626 | 6.464 | 8.995 |
| UK | 0 | Aliased | | | 7.626 | 6.464 | 8.995 |
| Scand | 2 | -0.493 | 0.287 | (-) | 4.657 | 1.454 | 14.909 |
| othEur | 6 | -1.058 | 0.102 | --- | 2.647 | 1.804 | 3.885 |
| China | 0 | Aliased | | | 7.626 | 6.464 | 8.995 |
| Japan | 11 | -1.286 | 0.073 | --- | 2.107 | 1.644 | 2.699 |
| othAs | 0 | Aliased | | | 7.626 | 6.464 | 8.995 |
| other | 1 | 0.035 | 0.497 | N.S. | 7.900 | 1.040 | 59.996 |

| | | | | | | | |
|---|---|---|---|---|---|---|---|
| | | Deviance | (DF) | Drop Dev | P | | |
| Model 3 | | 128.397 | (37) | 41.785 | ** | | |
| | | Estimate | S.E. | P | RR | 95%CIl | 95%CIu |
| Constant | | 2.296 | 0.057 | +++ | 9.933 | 8.876 | 11.118 |
| Location | | | | | | | |
| NAmer | 24 | Aliased | | | 7.813 | 6.736 | 9.063 |
| UK | 0 | Aliased | | | 7.813 | 6.736 | 9.063 |
| Scand | 2 | -0.476 | 0.287 | N.S. | 4.853 | 1.718 | 13.708 |
| othEur | 6 | -1.153 | 0.103 | --- | 2.466 | 1.746 | 3.482 |
| China | 0 | Aliased | | | 7.813 | 6.736 | 9.063 |
| Japan | 11 | -1.334 | 0.074 | --- | 2.058 | 1.644 | 2.576 |
| othAs | 0 | Aliased | | | 7.813 | 6.736 | 9.063 |
| other | 1 | -0.229 | 0.499 | N.S. | 6.214 | 1.014 | 38.087 |
| Sex(RR) | | | | | | | |
| Male | 22 | Aliased | | | 6.063 | 5.076 | 7.243 |
| Female | 20 | -0.422 | 0.066 | --- | 3.974 | 3.391 | 4.658 |
| Combined | 2 | -0.338 | 0.153 | - | 4.324 | 2.548 | 7.338 |

| | | | | | | | |
|---|---|---|---|---|---|---|---|
| | | Deviance | (DF) | Drop Dev | P | | |
| Model 4 | | 89.243 | (33) | 39.153 | * | | |
| | | Estimate | S.E. | P | RR | 95%CIl | 95%CIu |
| Constant | | 1.367 | 0.165 | +++ | 3.923 | 2.839 | 5.421 |
| Location | | | | | | | |
| NAmer | 24 | Aliased | | | 8.087 | 6.970 | 9.383 |
| UK | 0 | Aliased | | | 8.087 | 6.970 | 9.383 |
| Scand | 2 | -0.555 | 0.287 | (-) | 4.645 | 1.853 | 11.645 |
| othEur | 6 | -1.151 | 0.154 | --- | 2.559 | 1.653 | 3.960 |
| China | 0 | Aliased | | | 8.087 | 6.970 | 9.383 |
| Japan | 11 | -1.457 | 0.087 | --- | 1.884 | 1.512 | 2.348 |
| othAs | 0 | Aliased | | | 8.087 | 6.970 | 9.383 |
| other | 1 | -0.447 | 0.608 | N.S. | 5.172 | 0.753 | 35.543 |
| Sex(RR) | | | | | | | |
| Male | 22 | Aliased | | | 6.220 | 5.287 | 7.319 |
| Female | 20 | -0.465 | 0.066 | --- | 3.907 | 3.383 | 4.512 |
| Combined | 2 | -0.399 | 0.274 | N.S. | 4.176 | 1.808 | 9.643 |
| Start year of study | | | | | | | |
| <1960 | 5 | Aliased | | | 1.774 | 1.027 | 3.063 |
| 1960-69 | 7 | 1.118 | 0.181 | +++ | 5.426 | 4.383 | 6.719 |
| 1970-79 | 11 | 0.934 | 0.218 | +++ | 4.513 | 3.092 | 6.587 |
| 1980-89 | 19 | 0.995 | 0.171 | +++ | 4.795 | 4.044 | 5.686 |
| 1990+ | 2 | 1.147 | 0.387 | ++ | 5.585 | 1.974 | 15.802 |

Table 3B1R - 1

IESLC - Meta-regression of current smoking, any product (or cigs if any not available)
Multiple regression of data from Table 3B1
Adenocarcinoma

Fixed model

Log Relative risk
WEIGHTED on Weight

| | | Deviance | (DF) | | | | |
|---|---|---|---|---|---|---|---|
| Model 7 | | 65.727 | (27) | | | | |
| | | Estimate | S.E. | P | RR | 95%CIl | 95%CIu |
| Constant | | 0.816 | 0.239 | ++ | 2.261 | 1.414 | 3.614 |
| Sex(RR) | | | | | | | |
| Male | 22 | Aliased | | | 6.201 | 5.284 | 7.279 |
| Female | 20 | -0.440 | 0.067 | --- | 3.995 | 3.464 | 4.607 |
| Combined | 2 | -0.612 | 0.378 | N.S. | 3.362 | 1.122 | 10.075 |
| Location | | | | | | | |
| NAmer | 24 | Aliased | | | 8.237 | 6.907 | 9.822 |
| UK | 0 | Aliased | | | 8.237 | 6.907 | 9.822 |
| Scand | 2 | -0.298 | 0.320 | N.S. | 6.113 | 2.346 | 15.930 |
| othEur | 6 | -1.157 | 0.178 | --- | 2.589 | 1.607 | 4.171 |
| China | 0 | Aliased | | | 8.237 | 6.907 | 9.822 |
| Japan | 11 | -1.525 | 0.136 | --- | 1.792 | 1.322 | 2.429 |
| othAs | 0 | Aliased | | | 8.237 | 6.907 | 9.822 |
| other | 1 | -1.014 | 0.754 | N.S. | 2.988 | 0.323 | 27.641 |
| Start year of study | | | | | | | |
| <1960 | 5 | Aliased | | | 1.673 | 0.875 | 3.199 |
| 1960-69 | 7 | 1.179 | 0.231 | +++ | 5.438 | 4.252 | 6.956 |
| 1970-79 | 11 | 1.142 | 0.258 | +++ | 5.241 | 3.412 | 8.051 |
| 1980-89 | 19 | 0.957 | 0.224 | +++ | 4.356 | 3.392 | 5.593 |
| 1990+ | 2 | 1.982 | 0.575 | ++ | 12.148 | 2.626 | 56.185 |
| Study type (1) | | | | | | | |
| CC | 34 | Aliased | | | 4.721 | 4.256 | 5.237 |
| other | 10 | 0.178 | 0.224 | N.S. | 5.638 | 2.932 | 10.841 |
| Study size (number of LC cases) | | | | | | | |
| 100-249 | 11 | Aliased | | | 3.487 | 2.277 | 5.342 |
| 250-499 | 9 | -0.002 | 0.195 | N.S. | 3.480 | 2.280 | 5.312 |
| 500-999 | 4 | 0.646 | 0.232 | ++ | 6.651 | 3.658 | 12.095 |
| 1000+ | 20 | 0.362 | 0.149 | + | 5.007 | 4.473 | 5.606 |
| Number of adjustment variables (1) | | | | | | | |
| 0 | 17 | Aliased | | | 3.965 | 2.932 | 5.361 |
| 1 | 14 | 0.195 | 0.213 | N.S. | 4.818 | 3.104 | 7.479 |
| 2+/+nk | 13 | 0.283 | 0.125 | + | 5.260 | 4.512 | 6.133 |

Table 3B1R - 2

IESLC - Meta-regression of current smoking, any product (or cigs if any not available)
Multiple regression of data from Table 3B1
Adenocarcinoma
Effect of removing characteristics

Log Relative risk
WEIGHTED on Weight

| | | Deviance | (DF) | | | | |
|---|---|---|---|---|---|---|---|
| Model 7 | | 65.727 | (27) | | | | |
| | | Estimate | S.E. | P | RR | 95%CIl | 95%CIu |
| Constant | | 0.816 | 0.239 | ++ | 2.261 | 1.414 | 3.614 |
| Sex(RR) | | | | | | | |
| Male | 22 | Aliased | | | 6.201 | 5.284 | 7.279 |
| Female | 20 | -0.440 | 0.067 | --- | 3.995 | 3.464 | 4.607 |
| Combined | 2 | -0.612 | 0.378 | N.S. | 3.362 | 1.122 | 10.075 |
| Location | | | | | | | |
| NAmer | 24 | Aliased | | | 8.237 | 6.907 | 9.822 |
| UK | 0 | Aliased | | | 8.237 | 6.907 | 9.822 |
| Scand | 2 | -0.298 | 0.320 | N.S. | 6.113 | 2.346 | 15.930 |
| othEur | 6 | -1.157 | 0.178 | --- | 2.589 | 1.607 | 4.171 |
| China | 0 | Aliased | | | 8.237 | 6.907 | 9.822 |
| Japan | 11 | -1.525 | 0.136 | --- | 1.792 | 1.322 | 2.429 |
| othAs | 0 | Aliased | | | 8.237 | 6.907 | 9.822 |
| other | 1 | -1.014 | 0.754 | N.S. | 2.988 | 0.323 | 27.641 |
| Start year of study | | | | | | | |
| <1960 | 5 | Aliased | | | 1.673 | 0.875 | 3.199 |
| 1960-69 | 7 | 1.179 | 0.231 | +++ | 5.438 | 4.252 | 6.956 |
| 1970-79 | 11 | 1.142 | 0.258 | +++ | 5.241 | 3.412 | 8.051 |
| 1980-89 | 19 | 0.957 | 0.224 | +++ | 4.356 | 3.392 | 5.593 |
| 1990+ | 2 | 1.982 | 0.575 | ++ | 12.148 | 2.626 | 56.185 |
| Study type (1) | | | | | | | |
| CC | 34 | Aliased | | | 4.721 | 4.256 | 5.237 |
| other | 10 | 0.178 | 0.224 | N.S. | 5.638 | 2.932 | 10.841 |
| Study size (number of LC cases) | | | | | | | |
| 100-249 | 11 | Aliased | | | 3.487 | 2.277 | 5.342 |
| 250-499 | 9 | -0.002 | 0.195 | N.S. | 3.480 | 2.280 | 5.312 |
| 500-999 | 4 | 0.646 | 0.232 | ++ | 6.651 | 3.658 | 12.095 |
| 1000+ | 20 | 0.362 | 0.149 | + | 5.007 | 4.473 | 5.606 |
| Number of adjustment variables (1) | | | | | | | |
| 0 | 17 | Aliased | | | 3.965 | 2.932 | 5.361 |
| 1 | 14 | 0.195 | 0.213 | N.S. | 4.818 | 3.104 | 7.479 |
| 2+/+nk | 13 | 0.283 | 0.125 | + | 5.260 | 4.512 | 6.133 |

| Omit Sex | | Deviance | (DF) | Drop Dev | P | | |
|---|---|---|---|---|---|---|---|
| Model 8 | | 109.266 | (29) | -43.540 | *** | | |
| | | Estimate | S.E. | P | RR | 95%CIl | 95%CIu |
| Constant | | 0.590 | 0.232 | + | 1.804 | 1.145 | 2.843 |
| Number of adjustment variables (1) | | | | | | | |
| 0 | 17 | Aliased | | | 4.190 | 3.228 | 5.438 |
| 1 | 14 | 0.029 | 0.164 | N.S. | 4.314 | 3.115 | 5.975 |
| 2+/+nk | 13 | 0.226 | 0.117 | (+) | 5.250 | 4.528 | 6.088 |
| Location | | | | | | | |
| NAmer | 24 | Aliased | | | 7.707 | 6.697 | 8.871 |
| UK | 0 | Aliased | | | 7.707 | 6.697 | 8.871 |
| Scand | 2 | -0.191 | 0.318 | N.S. | 6.370 | 2.529 | 16.046 |
| othEur | 6 | -0.996 | 0.154 | --- | 2.846 | 1.853 | 4.372 |
| China | 0 | Aliased | | | 7.707 | 6.697 | 8.871 |
| Japan | 11 | -1.364 | 0.114 | --- | 1.971 | 1.513 | 2.568 |
| othAs | 0 | Aliased | | | 7.707 | 6.697 | 8.871 |
| other | 1 | -0.098 | 0.594 | N.S. | 6.991 | 1.244 | 39.272 |
| Start year of study | | | | | | | |
| <1960 | 5 | Aliased | | | 1.953 | 1.053 | 3.623 |
| 1960-69 | 7 | 1.009 | 0.228 | +++ | 5.357 | 4.225 | 6.792 |
| 1970-79 | 11 | 0.930 | 0.234 | +++ | 4.948 | 3.545 | 6.906 |
| 1980-89 | 19 | 0.850 | 0.221 | +++ | 4.569 | 3.816 | 5.469 |
| 1990+ | 2 | 1.349 | 0.347 | +++ | 7.524 | 3.564 | 15.883 |
| Study type (1) | | | | | | | |
| CC | 34 | Aliased | | | 4.677 | 4.239 | 5.160 |
| other | 10 | 0.339 | 0.196 | (+) | 6.562 | 3.773 | 11.411 |

Table 3B1R - 2

IESLC - Meta-regression of current smoking, any product (or cigs if any not available)
Multiple regression of data from Table 3B1
Adenocarcinoma
Effect of removing characteristics

Log Relative risk
WEIGHTED on Weight

| | | Estimate | S.E. | P | RR | 95%CIl | 95%CIu |
|---|---|---|---|---|---|---|---|
| Study size (number of LC cases) | | | | | | | |
| 100-249 | 11 | Aliased | | | 3.165 | 2.126 | 4.710 |
| 250-499 | 9 | 0.083 | 0.190 | N.S. | 3.440 | 2.288 | 5.171 |
| 500-999 | 4 | 0.727 | 0.232 | ++ | 6.544 | 3.691 | 11.601 |
| 1000+ | 20 | 0.469 | 0.143 | ++ | 5.060 | 4.540 | 5.639 |

| Omit Location | | Deviance | (DF) | Drop Dev | P | | |
|---|---|---|---|---|---|---|---|
| Model 8 | | 243.881 | (31) | -178.154 | *** | | |
| | | Estimate | S.E. | P | RR | 95%CIl | 95%CIu |
| Constant | | 1.075 | 0.238 | +++ | 2.931 | 1.838 | 4.674 |
| Number of adjustment variables (1) | | | | | | | |
| 0 | 17 | Aliased | | | 6.981 | 5.714 | 8.528 |
| 1 | 14 | -1.364 | 0.136 | --- | 1.784 | 1.322 | 2.407 |
| 2+/+nk | 13 | -0.338 | 0.091 | --- | 4.978 | 4.358 | 5.686 |
| Sex(RR) | | | | | | | |
| Male | 22 | Aliased | | | 5.381 | 4.678 | 6.190 |
| Female | 20 | -0.364 | 0.067 | --- | 3.738 | 3.281 | 4.258 |
| Combined | 2 | 1.493 | 0.254 | +++ | 23.954 | 11.921 | 48.136 |
| Start year of study | | | | | | | |
| <1960 | 5 | Aliased | | | 2.073 | 1.150 | 3.735 |
| 1960-69 | 7 | 0.626 | 0.216 | ++ | 3.875 | 3.247 | 4.626 |
| 1970-79 | 11 | 0.071 | 0.221 | N.S. | 2.226 | 1.695 | 2.922 |
| 1980-89 | 19 | 1.377 | 0.219 | +++ | 8.213 | 7.046 | 9.574 |
| 1990+ | 2 | -1.037 | 0.373 | -- | 0.735 | 0.313 | 1.722 |
| Study type (1) | | | | | | | |
| CC | 34 | Aliased | | | 4.355 | 3.962 | 4.787 |
| other | 10 | 1.579 | 0.187 | +++ | 21.127 | 12.664 | 35.245 |
| Study size (number of LC cases) | | | | | | | |
| 100-249 | 11 | Aliased | | | 4.473 | 3.114 | 6.425 |
| 250-499 | 9 | -0.555 | 0.186 | -- | 2.567 | 1.763 | 3.737 |
| 500-999 | 4 | 0.598 | 0.228 | + | 8.135 | 4.701 | 14.078 |
| 1000+ | 20 | 0.115 | 0.133 | N.S. | 5.020 | 4.532 | 5.560 |

| Omit Start Year | | Deviance | (DF) | Drop Dev | P | | |
|---|---|---|---|---|---|---|---|
| Model 8 | | 92.853 | (31) | -27.126 | * | | |
| | | Estimate | S.E. | P | RR | 95%CIl | 95%CIu |
| Constant | | 1.736 | 0.153 | +++ | 5.676 | 4.202 | 7.666 |
| Number of adjustment variables (1) | | | | | | | |
| 0 | 17 | Aliased | | | 4.454 | 3.685 | 5.383 |
| 1 | 14 | -0.167 | 0.122 | N.S. | 3.769 | 2.833 | 5.014 |
| 2+/+nk | 13 | 0.166 | 0.088 | (+) | 5.256 | 4.592 | 6.016 |
| Sex(RR) | | | | | | | |
| Male | 22 | Aliased | | | 5.927 | 5.149 | 6.824 |
| Female | 20 | -0.405 | 0.067 | --- | 3.953 | 3.487 | 4.482 |
| Combined | 2 | -0.060 | 0.178 | N.S. | 5.583 | 3.450 | 9.036 |
| Location | | | | | | | |
| NAmer | 24 | Aliased | | | 7.560 | 6.709 | 8.519 |
| UK | 0 | Aliased | | | 7.560 | 6.709 | 8.519 |
| Scand | 2 | 0.075 | 0.307 | N.S. | 8.148 | 3.437 | 19.316 |
| othEur | 6 | -1.064 | 0.119 | --- | 2.608 | 1.907 | 3.568 |
| China | 0 | Aliased | | | 7.560 | 6.709 | 8.519 |
| Japan | 11 | -1.279 | 0.080 | --- | 2.104 | 1.746 | 2.536 |
| othAs | 0 | Aliased | | | 7.560 | 6.709 | 8.519 |
| other | 1 | 0.165 | 0.517 | N.S. | 8.916 | 2.054 | 38.702 |
| Study type (1) | | | | | | | |
| CC | 34 | Aliased | | | 4.781 | 4.354 | 5.250 |
| other | 10 | -0.043 | 0.165 | N.S. | 4.581 | 2.910 | 7.212 |
| Study size (number of LC cases) | | | | | | | |
| 100-249 | 11 | Aliased | | | 3.078 | 2.146 | 4.413 |
| 250-499 | 9 | 0.201 | 0.176 | N.S. | 3.764 | 2.634 | 5.377 |
| 500-999 | 4 | 0.789 | 0.228 | ++ | 6.778 | 3.926 | 11.700 |
| 1000+ | 20 | 0.489 | 0.133 | +++ | 5.019 | 4.533 | 5.557 |

Table 3B1R - 2

IESLC - Meta-regression of current smoking, any product (or cigs if any not available)
Multiple regression of data from Table 3B1
Adenocarcinoma
Effect of removing characteristics

Log Relative risk
WEIGHTED on Weight

| Omit Study type | | Deviance | (DF) | Drop Dev | P | | |
|---|---|---|---|---|---|---|---|
| Model 8 | | 66.352 | (28) | -0.625 | N.S. | | |
| | | Estimate | S.E. | P | RR | 95%CIl | 95%CIu |
| Constant | | 0.862 | 0.232 | +++ | 2.367 | 1.502 | 3.732 |
| Number of adjustment variables (1) | | | | | | | |
| 0 | 17 | Aliased | | | 3.834 | 2.934 | 5.009 |
| 1 | 14 | 0.297 | 0.169 | (+) | 5.162 | 3.661 | 7.278 |
| 2+/+nk | 13 | 0.317 | 0.117 | + | 5.265 | 4.528 | 6.121 |
| Sex(RR) | | | | | | | |
| Male | 22 | Aliased | | | 6.254 | 5.361 | 7.295 |
| Female | 20 | -0.442 | 0.067 | --- | 4.018 | 3.499 | 4.614 |
| Combined | 2 | -0.757 | 0.331 | - | 2.932 | 1.141 | 7.535 |
| Location | | | | | | | |
| NAmer | 24 | Aliased | | | 8.405 | 7.198 | 9.813 |
| UK | 0 | Aliased | | | 8.405 | 7.198 | 9.813 |
| Scand | 2 | -0.300 | 0.320 | N.S. | 6.226 | 2.437 | 15.905 |
| othEur | 6 | -1.197 | 0.170 | --- | 2.540 | 1.600 | 4.034 |
| China | 0 | Aliased | | | 8.405 | 7.198 | 9.813 |
| Japan | 11 | -1.581 | 0.117 | --- | 1.729 | 1.325 | 2.256 |
| othAs | 0 | Aliased | | | 8.405 | 7.198 | 9.813 |
| other | 1 | -1.210 | 0.712 | N.S. | 2.506 | 0.313 | 20.068 |
| Start year of study | | | | | | | |
| <1960 | 5 | Aliased | | | 1.772 | 0.974 | 3.223 |
| 1960-69 | 7 | 1.134 | 0.224 | +++ | 5.504 | 4.341 | 6.978 |
| 1970-79 | 11 | 1.123 | 0.257 | +++ | 5.446 | 3.666 | 8.089 |
| 1980-89 | 19 | 0.865 | 0.192 | +++ | 4.207 | 3.420 | 5.176 |
| 1990+ | 2 | 2.098 | 0.556 | +++ | 14.435 | 3.728 | 55.892 |
| Study size (number of LC cases) | | | | | | | |
| 100-249 | 11 | Aliased | | | 3.431 | 2.267 | 5.192 |
| 250-499 | 9 | 0.038 | 0.188 | N.S. | 3.563 | 2.375 | 5.345 |
| 500-999 | 4 | 0.651 | 0.232 | ++ | 6.576 | 3.661 | 11.811 |
| 1000+ | 20 | 0.377 | 0.147 | + | 5.004 | 4.479 | 5.591 |

| Omit Study size | | Deviance | (DF) | Drop Dev | P | | |
|---|---|---|---|---|---|---|---|
| Model 8 | | 80.053 | (30) | -14.326 | N.S. | | |
| | | Estimate | S.E. | P | RR | 95%CIl | 95%CIu |
| Constant | | 1.100 | 0.218 | +++ | 3.003 | 1.958 | 4.605 |
| Number of adjustment variables (1) | | | | | | | |
| 0 | 17 | Aliased | | | 3.875 | 2.936 | 5.115 |
| 1 | 14 | 0.244 | 0.209 | N.S. | 4.947 | 3.279 | 7.465 |
| 2+/+nk | 13 | 0.311 | 0.119 | + | 5.291 | 4.594 | 6.093 |
| Sex(RR) | | | | | | | |
| Male | 22 | Aliased | | | 6.246 | 5.366 | 7.271 |
| Female | 20 | -0.460 | 0.067 | --- | 3.941 | 3.450 | 4.502 |
| Combined | 2 | -0.535 | 0.368 | N.S. | 3.659 | 1.335 | 10.025 |
| Location | | | | | | | |
| NAmer | 24 | Aliased | | | 8.319 | 7.047 | 9.820 |
| UK | 0 | Aliased | | | 8.319 | 7.047 | 9.820 |
| Scand | 2 | -0.645 | 0.301 | - | 4.365 | 1.864 | 10.226 |
| othEur | 6 | -1.044 | 0.166 | --- | 2.929 | 1.931 | 4.445 |
| China | 0 | Aliased | | | 8.319 | 7.047 | 9.820 |
| Japan | 11 | -1.594 | 0.135 | --- | 1.690 | 1.273 | 2.243 |
| othAs | 0 | Aliased | | | 8.319 | 7.047 | 9.820 |
| other | 1 | -1.032 | 0.747 | N.S. | 2.965 | 0.368 | 23.907 |
| Start year of study | | | | | | | |
| <1960 | 5 | Aliased | | | 1.628 | 0.895 | 2.960 |
| 1960-69 | 7 | 1.275 | 0.224 | +++ | 5.826 | 4.688 | 7.240 |
| 1970-79 | 11 | 1.093 | 0.255 | +++ | 4.858 | 3.330 | 7.088 |
| 1980-89 | 19 | 0.992 | 0.215 | +++ | 4.390 | 3.477 | 5.544 |
| 1990+ | 2 | 1.688 | 0.560 | ++ | 8.802 | 2.137 | 36.255 |
| Study type (1) | | | | | | | |
| CC | 34 | Aliased | | | 4.739 | 4.297 | 5.226 |
| other | 10 | 0.112 | 0.216 | N.S. | 5.300 | 2.916 | 9.633 |

Table 3B1R - 2

IESLC - Meta-regression of current smoking, any product (or cigs if any not available)
Multiple regression of data from Table 3B1
Adenocarcinoma
Effect of removing characteristics

Log Relative risk
WEIGHTED on Weight

| Omit N adjustment vars | | Deviance | (DF) | Drop Dev | P | | |
|---|---|---|---|---|---|---|---|
| Model 8 | | 72.055 | (29) | -6.328 | N.S. | | |
| | | Estimate | S.E. | P | RR | 95%CIl | 95%CIu |
| Constant | | 0.896 | 0.232 | +++ | 2.450 | 1.556 | 3.857 |
| Study size (number of LC cases) | | | | | | | |
| 100-249 | 11 | Aliased | | | 3.403 | 2.262 | 5.120 |
| 250-499 | 9 | -0.038 | 0.192 | N.S. | 3.277 | 2.198 | 4.884 |
| 500-999 | 4 | 0.551 | 0.229 | + | 5.904 | 3.380 | 10.315 |
| 1000+ | 20 | 0.399 | 0.147 | + | 5.071 | 4.552 | 5.649 |
| Sex(RR) | | | | | | | |
| Male | 22 | Aliased | | | 6.173 | 5.316 | 7.168 |
| Female | 20 | -0.438 | 0.067 | --- | 3.983 | 3.484 | 4.554 |
| Combined | 2 | -0.536 | 0.285 | (-) | 3.612 | 1.626 | 8.025 |
| Location | | | | | | | |
| NAmer | 24 | Aliased | | | 8.046 | 7.008 | 9.237 |
| UK | 0 | Aliased | | | 8.046 | 7.008 | 9.237 |
| Scand | 2 | -0.239 | 0.311 | N.S. | 6.338 | 2.533 | 15.855 |
| othEur | 6 | -1.268 | 0.164 | --- | 2.264 | 1.474 | 3.477 |
| China | 0 | Aliased | | | 8.046 | 7.008 | 9.237 |
| Japan | 11 | -1.401 | 0.089 | --- | 1.982 | 1.613 | 2.435 |
| othAs | 0 | Aliased | | | 8.046 | 7.008 | 9.237 |
| other | 1 | -0.567 | 0.637 | N.S. | 4.566 | 0.720 | 28.959 |
| Start year of study | | | | | | | |
| <1960 | 5 | Aliased | | | 1.593 | 0.861 | 2.947 |
| 1960-69 | 7 | 1.156 | 0.223 | +++ | 5.061 | 4.110 | 6.231 |
| 1970-79 | 11 | 1.143 | 0.243 | +++ | 4.997 | 3.421 | 7.299 |
| 1980-89 | 19 | 1.086 | 0.217 | +++ | 4.718 | 4.010 | 5.550 |
| 1990+ | 2 | 1.737 | 0.438 | +++ | 9.054 | 3.118 | 26.294 |
| Study type (1) | | | | | | | |
| CC | 34 | Aliased | | | 4.707 | 4.271 | 5.187 |
| other | 10 | 0.229 | 0.175 | N.S. | 5.916 | 3.603 | 9.715 |

Table 3B1R - 3

IESLC - Meta-regression of current smoking, any product (or cigs if any not available)
Multiple regression of data from Table 3B1
Adenocarcinoma
Study outliers

| Study Ref | NRR | LOGRR | FITVAL | SEFITV | STDRES |
|---|---|---|---|---|---|
| BROWN2 | 14 | 2.208 | 2.417 | 0.140 | -1.496 |
| LUBIN2 | 264 | 0.364 | 0.722 | 0.245 | -1.460 |
| OSANN2 | 32 | 1.163 | 1.927 | 0.571 | -1.338 |
| TSUGAN | 3 | -0.163 | 0.432 | 0.607 | -0.980 |
| WYNDE3 | 30 | 1.378 | 1.992 | 0.689 | -0.892 |
| CPSI | 406 | 0.358 | 1.110 | 0.876 | -0.859 |
| TSUGAN | 9 | -0.598 | -0.008 | 0.874 | -0.675 |
| HAENSZ | 37 | 0.082 | 0.376 | 0.503 | -0.584 |
| WYNDE6 | 15 | 2.235 | 2.356 | 0.229 | -0.530 |
| SOBUE | 36 | 0.642 | 0.804 | 0.330 | -0.490 |
| SOBUE2 | 6 | 0.588 | 0.674 | 0.179 | -0.479 |
| WU | 7 | 1.411 | 1.615 | 0.466 | -0.439 |
| JAIN | 12 | 1.827 | 1.978 | 0.406 | -0.373 |
| WU2 | 1 | 1.504 | 1.613 | 0.328 | -0.333 |
| SOBUE | 46 | 0.262 | 0.364 | 0.315 | -0.322 |
| ENGELA | 70 | 1.954 | 2.154 | 0.719 | -0.278 |
| BUFFLE | 71 | 2.097 | 2.163 | 0.643 | -0.103 |
| JAIN | 17 | 2.380 | 2.418 | 0.817 | -0.047 |
| CPSI | 404 | 1.522 | 1.550 | 0.760 | -0.037 |
| BROWN2 | 13 | 1.974 | 1.977 | 0.116 | -0.027 |
| CORREA | 44 | 1.902 | 1.902 | 0.349 | -0.000 |
| MATOS | 53 | 2.067 | 2.067 | 0.671 | -0.000 |
| KIHARA | 5 | 0.658 | 0.658 | 0.278 | 0.000 |
| CPSII | 118 | 2.108 | 2.067 | 0.499 | 0.082 |
| SOBUE2 | 2 | 1.131 | 1.114 | 0.172 | 0.104 |
| SVENSS | 99 | 1.330 | 1.229 | 0.518 | 0.194 |
| WAKAI | 30 | 0.131 | 0.000 | 0.655 | 0.200 |
| LUBIN2 | 252 | 1.211 | 1.162 | 0.221 | 0.220 |
| COMSTO | 31 | 1.850 | 1.693 | 0.672 | 0.233 |
| KATSOU | 12 | 0.531 | 0.370 | 0.634 | 0.253 |
| COMSTO | 24 | 2.534 | 2.133 | 1.129 | 0.355 |
| BOUCOT | 147 | 2.393 | 1.276 | 2.202 | 0.508 |
| KHUDER | 17 | 2.104 | 1.770 | 0.641 | 0.521 |
| CPSII | 115 | 2.956 | 2.507 | 0.855 | 0.525 |
| WAKAI | 12 | 0.779 | 0.440 | 0.611 | 0.556 |
| DORN | 340 | 1.783 | 1.550 | 0.343 | 0.681 |
| BARBON | 100 | 2.067 | 1.641 | 0.599 | 0.711 |
| JAHN | 8 | 1.571 | 0.977 | 0.591 | 1.004 |
| SUZUKI | 14 | 0.875 | 0.275 | 0.553 | 1.087 |
| JEDRYC | 26 | 1.694 | 0.977 | 0.618 | 1.161 |
| OSANN | 39 | 3.077 | 2.417 | 0.459 | 1.439 |
| WYNDE6 | 204 | 2.201 | 1.916 | 0.186 | 1.527 |
| OSANN | 40 | 2.451 | 1.977 | 0.275 | 1.720 |
| SUZUKI | 10 | 1.609 | 0.715 | 0.483 | 1.853 |

Table 3B1R - 4

IESLC - Meta-regression of current smoking, any product (or cigs if any not available)
Multiple regression of data from Table 3B1
Adenocarcinoma
Effect of additional characteristics

Log Relative risk
WEIGHTED on Weight

| | | Deviance | (DF) | | | | |
|---|---|---|---|---|---|---|---|
| Model 7 | | 65.727 | (27) | | | | |
| | | Estimate | S.E. | P | RR | 95%CIl | 95%CIu |
| Constant | | 0.816 | 0.239 | ++ | 2.261 | 1.414 | 3.614 |
| Sex(RR) | | | | | | | |
| Male | 22 | Aliased | | | 6.201 | 5.284 | 7.279 |
| Female | 20 | -0.440 | 0.067 | --- | 3.995 | 3.464 | 4.607 |
| Combined | 2 | -0.612 | 0.378 | N.S. | 3.362 | 1.122 | 10.075 |
| Location | | | | | | | |
| NAmer | 24 | Aliased | | | 8.237 | 6.907 | 9.822 |
| UK | 0 | Aliased | | | 8.237 | 6.907 | 9.822 |
| Scand | 2 | -0.298 | 0.320 | N.S. | 6.113 | 2.346 | 15.930 |
| othEur | 6 | -1.157 | 0.178 | --- | 2.589 | 1.607 | 4.171 |
| China | 0 | Aliased | | | 8.237 | 6.907 | 9.822 |
| Japan | 11 | -1.525 | 0.136 | --- | 1.792 | 1.322 | 2.429 |
| othAs | 0 | Aliased | | | 8.237 | 6.907 | 9.822 |
| other | 1 | -1.014 | 0.754 | N.S. | 2.988 | 0.323 | 27.641 |
| Start year of study | | | | | | | |
| <1960 | 5 | Aliased | | | 1.673 | 0.875 | 3.199 |
| 1960-69 | 7 | 1.179 | 0.231 | +++ | 5.438 | 4.252 | 6.956 |
| 1970-79 | 11 | 1.142 | 0.258 | +++ | 5.241 | 3.412 | 8.051 |
| 1980-89 | 19 | 0.957 | 0.224 | +++ | 4.356 | 3.392 | 5.593 |
| 1990+ | 2 | 1.982 | 0.575 | ++ | 12.148 | 2.626 | 56.185 |
| Study type (1) | | | | | | | |
| CC | 34 | Aliased | | | 4.721 | 4.256 | 5.237 |
| other | 10 | 0.178 | 0.224 | N.S. | 5.638 | 2.932 | 10.841 |
| Study size (number of LC cases) | | | | | | | |
| 100-249 | 11 | Aliased | | | 3.487 | 2.277 | 5.342 |
| 250-499 | 9 | -0.002 | 0.195 | N.S. | 3.480 | 2.280 | 5.312 |
| 500-999 | 4 | 0.646 | 0.232 | ++ | 6.651 | 3.658 | 12.095 |
| 1000+ | 20 | 0.362 | 0.149 | + | 5.007 | 4.473 | 5.606 |
| Number of adjustment variables (1) | | | | | | | |
| 0 | 17 | Aliased | | | 3.965 | 2.932 | 5.361 |
| 1 | 14 | 0.195 | 0.213 | N.S. | 4.818 | 3.104 | 7.479 |
| 2+/+nk | 13 | 0.283 | 0.125 | + | 5.260 | 4.512 | 6.133 |

| | | Deviance | (DF) | Drop Dev | P | | |
|---|---|---|---|---|---|---|---|
| Model 8 | | 51.160 | (23) | 14.567 | N.S. | | |
| | | Estimate | S.E. | P | RR | 95%CIl | 95%CIu |
| Constant | | 0.633 | 0.255 | + | 1.884 | 1.143 | 3.105 |
| Sex(RR) | | | | | | | |
| Male | 22 | Aliased | | | 6.306 | 5.360 | 7.419 |
| Female | 20 | -0.403 | 0.068 | --- | 4.214 | 3.638 | 4.881 |
| Combined | 2 | -1.359 | 0.527 | - | 1.619 | 0.375 | 6.994 |
| Location | | | | | | | |
| NAmer | 24 | Aliased | | | 7.537 | 6.241 | 9.103 |
| UK | 0 | Aliased | | | 7.537 | 6.241 | 9.103 |
| Scand | 2 | -0.150 | 0.341 | N.S. | 6.490 | 2.512 | 16.770 |
| othEur | 6 | Aliased | | | 7.537 | 6.241 | 9.103 |
| China | 0 | Aliased | | | 7.537 | 6.241 | 9.103 |
| Japan | 11 | -1.674 | 0.195 | --- | 1.414 | 0.934 | 2.140 |
| othAs | 0 | Aliased | | | 7.537 | 6.241 | 9.103 |
| other | 1 | -1.755 | 0.946 | (-) | 1.304 | 0.093 | 18.328 |
| Start year of study | | | | | | | |
| <1960 | 5 | Aliased | | | 1.708 | 0.914 | 3.194 |
| 1960-69 | 7 | 1.107 | 0.245 | +++ | 5.170 | 3.896 | 6.861 |
| 1970-79 | 11 | 1.590 | 0.307 | +++ | 8.377 | 4.493 | 15.617 |
| 1980-89 | 19 | 0.761 | 0.239 | ++ | 3.656 | 2.525 | 5.295 |
| 1990+ | 2 | 2.819 | 0.774 | ++ | 28.624 | 3.612 | 226.865 |
| Study type (1) | | | | | | | |
| CC | 34 | Aliased | | | 4.788 | 4.317 | 5.310 |
| other | 10 | -0.069 | 0.286 | N.S. | 4.470 | 2.024 | 9.872 |

Table 3B1R - 4

IESLC - Meta-regression of current smoking, any product (or cigs if any not available)
Multiple regression of data from Table 3B1
Adenocarcinoma
Effect of additional characteristics

WEIGHTED on Weight

| | | Estimate | S.E. | P | RR | 95%CIl | 95%CIu |
|---|---|---|---|---|---|---|---|
| Study size (number of LC cases) | | | | | | | |
| 100-249 | 11 | Aliased | | | 2.721 | 1.672 | 4.426 |
| 250-499 | 9 | 0.240 | 0.215 | N.S. | 3.457 | 2.280 | 5.243 |
| 500-999 | 4 | 0.724 | 0.286 | + | 5.610 | 2.727 | 11.543 |
| 1000+ | 20 | 0.639 | 0.179 | ++ | 5.153 | 4.598 | 5.776 |
| Number of adjustment variables (1) | | | | | | | |
| 0 | 17 | Aliased | | | 3.670 | 2.361 | 5.705 |
| 1 | 14 | 0.400 | 0.345 | N.S. | 5.474 | 2.935 | 10.209 |
| 2+/+nk | 13 | 0.370 | 0.185 | (+) | 5.311 | 4.524 | 6.235 |
| Detailed Country in othEur | | | | | | | |
| not o E | 38 | Aliased | | | 5.709 | 5.076 | 6.422 |
| multi | 2 | -1.850 | 0.265 | --- | 0.898 | 0.441 | 1.827 |
| Germany | 1 | -0.462 | 0.430 | N.S. | 3.596 | 1.020 | 12.677 |
| othWest | 1 | -1.280 | 0.711 | (-) | 1.588 | 0.205 | 12.298 |
| East | 1 | -0.339 | 0.446 | N.S. | 4.069 | 1.103 | 15.009 |
| Balkans | 1 | -0.860 | 0.489 | (-) | 2.416 | 0.579 | 10.081 |

| | | Deviance | (DF) | Drop Dev | P | | |
|---|---|---|---|---|---|---|---|
| Model 8 | | 65.727 | (27) | 0.000 | N.S. | | |
| | | Estimate | S.E. | P | RR | 95%CIl | 95%CIu |
| Constant | | 0.816 | 0.239 | ++ | 2.261 | 1.414 | 3.614 |
| Sex(RR) | | | | | | | |
| Male | 22 | Aliased | | | 6.201 | 5.284 | 7.279 |
| Female | 20 | -0.440 | 0.067 | --- | 3.995 | 3.464 | 4.607 |
| Combined | 2 | -0.612 | 0.378 | N.S. | 3.362 | 1.122 | 10.075 |
| Location | | | | | | | |
| NAmer | 24 | Aliased | | | 8.237 | 6.907 | 9.822 |
| UK | 0 | Aliased | | | 8.237 | 6.907 | 9.822 |
| Scand | 2 | -0.298 | 0.320 | N.S. | 6.113 | 2.346 | 15.930 |
| othEur | 6 | -1.157 | 0.178 | --- | 2.589 | 1.607 | 4.171 |
| China | 0 | Aliased | | | 8.237 | 6.907 | 9.822 |
| Japan | 11 | -1.525 | 0.136 | --- | 1.792 | 1.322 | 2.429 |
| othAs | 0 | Aliased | | | 8.237 | 6.907 | 9.822 |
| other | 1 | -1.014 | 0.754 | N.S. | 2.988 | 0.323 | 27.641 |
| Start year of study | | | | | | | |
| <1960 | 5 | Aliased | | | 1.673 | 0.875 | 3.199 |
| 1960-69 | 7 | 1.179 | 0.231 | +++ | 5.438 | 4.252 | 6.956 |
| 1970-79 | 11 | 1.142 | 0.258 | +++ | 5.241 | 3.412 | 8.051 |
| 1980-89 | 19 | 0.957 | 0.224 | +++ | 4.356 | 3.392 | 5.593 |
| 1990+ | 2 | 1.982 | 0.575 | ++ | 12.148 | 2.626 | 56.185 |
| Study type (1) | | | | | | | |
| CC | 34 | Aliased | | | 4.721 | 4.256 | 5.237 |
| other | 10 | 0.178 | 0.224 | N.S. | 5.638 | 2.932 | 10.841 |
| Study size (number of LC cases) | | | | | | | |
| 100-249 | 11 | Aliased | | | 3.487 | 2.277 | 5.342 |
| 250-499 | 9 | -0.002 | 0.195 | N.S. | 3.480 | 2.280 | 5.312 |
| 500-999 | 4 | 0.646 | 0.232 | ++ | 6.651 | 3.658 | 12.095 |
| 1000+ | 20 | 0.362 | 0.149 | + | 5.007 | 4.473 | 5.606 |
| Number of adjustment variables (1) | | | | | | | |
| 0 | 17 | Aliased | | | 3.965 | 2.932 | 5.361 |
| 1 | 14 | 0.195 | 0.213 | N.S. | 4.818 | 3.104 | 7.479 |
| 2+/+nk | 13 | 0.283 | 0.125 | + | 5.260 | 4.512 | 6.133 |
| | | Estimate | | | | | |
| Detailed Country in othAsia | | | | | | | |
| not o A | 44 | Aliased | | | | | |

| | | Deviance | (DF) | Drop Dev | P | | |
|---|---|---|---|---|---|---|---|
| Model 8 | | 58.996 | (26) | 6.731 | (*) | | |
| | | Estimate | S.E. | P | RR | 95%CIl | 95%CIu |
| Constant | | 0.597 | 0.254 | + | 1.818 | 1.105 | 2.989 |
| Sex(RR) | | | | | | | |
| Male | 22 | Aliased | | | 5.839 | 4.930 | 6.915 |
| Female | 20 | -0.415 | 0.068 | --- | 3.855 | 3.340 | 4.450 |
| Combined | 2 | 0.362 | 0.533 | N.S. | 8.384 | 1.900 | 36.992 |

Table 3B1R - 4

IESLC - Meta-regression of current smoking, any product (or cigs if any not available)
Multiple regression of data from Table 3B1
Adenocarcinoma
Effect of additional characteristics

WEIGHTED on Weight

| | | Estimate | S.E. | P | RR | 95%CIl | 95%CIu |
|---|---|---|---|---|---|---|---|
| Location | | | | | | | |
| NAmer | 24 | Aliased | | | 6.264 | 4.392 | 8.933 |
| UK | 0 | Aliased | | | 6.264 | 4.392 | 8.933 |
| Scand | 2 | 0.322 | 0.399 | N.S. | 8.642 | 3.163 | 23.612 |
| othEur | 6 | -0.537 | 0.298 | (-) | 3.661 | 1.997 | 6.712 |
| China | 0 | Aliased | | | 6.264 | 4.392 | 8.933 |
| Japan | 11 | -0.814 | 0.306 | - | 2.776 | 1.557 | 4.952 |
| othAs | 0 | Aliased | | | 6.264 | 4.392 | 8.933 |
| other | 1 | 0.463 | 0.945 | N.S. | 9.947 | 0.779 | 126.994 |
| Start year of study | | | | | | | |
| <1960 | 5 | Aliased | | | 2.969 | 1.202 | 7.333 |
| 1960-69 | 7 | 0.142 | 0.461 | N.S. | 3.423 | 1.921 | 6.101 |
| 1970-79 | 11 | 0.573 | 0.339 | N.S. | 5.266 | 3.480 | 7.971 |
| 1980-89 | 19 | 0.677 | 0.249 | + | 5.843 | 3.869 | 8.824 |
| 1990+ | 2 | 0.366 | 0.848 | N.S. | 4.280 | 0.642 | 28.507 |
| Study type (1) | | | | | | | |
| CC | 34 | Aliased | | | 4.597 | 4.141 | 5.104 |
| other | 10 | 0.637 | 0.286 | + | 8.694 | 3.903 | 19.366 |
| Study size (number of LC cases) | | | | | | | |
| 100-249 | 11 | Aliased | | | 3.059 | 1.974 | 4.740 |
| 250-499 | 9 | 0.147 | 0.203 | N.S. | 3.544 | 2.355 | 5.334 |
| 500-999 | 4 | 1.178 | 0.310 | +++ | 9.938 | 4.759 | 20.752 |
| 1000+ | 20 | 0.488 | 0.156 | ++ | 4.985 | 4.470 | 5.559 |
| Number of adjustment variables (1) | | | | | | | |
| 0 | 17 | Aliased | | | 3.396 | 2.416 | 4.774 |
| 1 | 14 | -0.119 | 0.245 | N.S. | 3.016 | 1.526 | 5.962 |
| 2+/+nk | 13 | 0.641 | 0.186 | ++ | 6.448 | 4.898 | 8.489 |
| Adeno (or nearest) | | | | | | | |
| a | 40 | Aliased | | | 4.125 | 3.414 | 4.985 |
| oth | 4 | 1.157 | 0.446 | + | 13.115 | 4.133 | 41.623 |

| | | Deviance | (DF) | Drop Dev | P | | |
|---|---|---|---|---|---|---|---|
| Model 8 | | 58.996 | (26) | 6.731 | (*) | | |
| | | Estimate | S.E. | P | RR | 95%CIl | 95%CIu |
| Constant | | 0.597 | 0.254 | + | 1.818 | 1.105 | 2.989 |
| Sex(RR) | | | | | | | |
| Male | 22 | Aliased | | | 5.839 | 4.930 | 6.915 |
| Female | 20 | -0.415 | 0.068 | --- | 3.855 | 3.340 | 4.450 |
| Combined | 2 | 0.362 | 0.533 | N.S. | 8.384 | 1.900 | 36.992 |
| Location | | | | | | | |
| NAmer | 24 | Aliased | | | 6.264 | 4.392 | 8.933 |
| UK | 0 | Aliased | | | 6.264 | 4.392 | 8.933 |
| Scand | 2 | 0.322 | 0.399 | N.S. | 8.642 | 3.163 | 23.612 |
| othEur | 6 | -0.537 | 0.298 | (-) | 3.661 | 1.997 | 6.712 |
| China | 0 | Aliased | | | 6.264 | 4.392 | 8.933 |
| Japan | 11 | -0.814 | 0.306 | - | 2.776 | 1.557 | 4.952 |
| othAs | 0 | Aliased | | | 6.264 | 4.392 | 8.933 |
| other | 1 | 0.463 | 0.945 | N.S. | 9.947 | 0.779 | 126.994 |
| Start year of study | | | | | | | |
| <1960 | 5 | Aliased | | | 2.969 | 1.202 | 7.333 |
| 1960-69 | 7 | 0.142 | 0.461 | N.S. | 3.423 | 1.921 | 6.101 |
| 1970-79 | 11 | 0.573 | 0.339 | N.S. | 5.266 | 3.480 | 7.971 |
| 1980-89 | 19 | 0.677 | 0.249 | + | 5.843 | 3.869 | 8.824 |
| 1990+ | 2 | 0.366 | 0.848 | N.S. | 4.280 | 0.642 | 28.507 |
| Study type (1) | | | | | | | |
| CC | 34 | Aliased | | | 4.597 | 4.141 | 5.104 |
| other | 10 | 0.637 | 0.286 | + | 8.694 | 3.903 | 19.366 |
| Study size (number of LC cases) | | | | | | | |
| 100-249 | 11 | Aliased | | | 3.059 | 1.974 | 4.740 |
| 250-499 | 9 | 0.147 | 0.203 | N.S. | 3.544 | 2.355 | 5.334 |
| 500-999 | 4 | 1.178 | 0.310 | +++ | 9.938 | 4.759 | 20.752 |
| 1000+ | 20 | 0.488 | 0.156 | ++ | 4.985 | 4.470 | 5.559 |

Table 3B1R - 4

IESLC - Meta-regression of current smoking, any product (or cigs if any not available)
Multiple regression of data from Table 3B1
Adenocarcinoma
Effect of additional characteristics

WEIGHTED on Weight

| | | Estimate | S.E. | P | RR | 95%CIl | 95%CIu |
|---|---|---|---|---|---|---|---|
| Number of adjustment variables (1) | | | | | | | |
| 0 | 17 | Aliased | | | 3.396 | 2.416 | 4.774 |
| 1 | 14 | -0.119 | 0.245 | N.S. | 3.016 | 1.526 | 5.962 |
| 2+/+nk | 13 | 0.641 | 0.186 | ++ | 6.448 | 4.898 | 8.489 |
| Adeno (or nearest) | | | | | | | |
| a | 40 | Aliased | | | 4.125 | 3.414 | 4.985 |
| a+l | 0 | Aliased | | | 4.125 | 3.414 | 4.985 |
| a+al+br | 0 | Aliased | | | 4.125 | 3.414 | 4.985 |
| KII | 4 | 1.157 | 0.446 | + | 13.115 | 4.133 | 41.623 |

| | | Deviance | (DF) | Drop Dev | P | | |
|---|---|---|---|---|---|---|---|
| Model 8 | | 63.600 | (26) | 2.127 | N.S. | | |
| | | Estimate | S.E. | P | RR | 95%CIl | 95%CIu |
| Constant | | 0.723 | 0.248 | ++ | 2.061 | 1.269 | 3.349 |
| Sex(RR) | | | | | | | |
| Male | 22 | Aliased | | | 6.179 | 5.261 | 7.256 |
| Female | 20 | -0.433 | 0.067 | --- | 4.008 | 3.473 | 4.625 |
| Combined | 2 | -0.613 | 0.378 | N.S. | 3.348 | 1.114 | 10.059 |
| Location | | | | | | | |
| NAmer | 24 | Aliased | | | 8.299 | 6.951 | 9.907 |
| UK | 0 | Aliased | | | 8.299 | 6.951 | 9.907 |
| Scand | 2 | -0.430 | 0.332 | N.S. | 5.397 | 1.995 | 14.600 |
| othEur | 6 | -1.170 | 0.178 | --- | 2.577 | 1.598 | 4.156 |
| China | 0 | Aliased | | | 8.299 | 6.951 | 9.907 |
| Japan | 11 | -1.541 | 0.137 | --- | 1.776 | 1.309 | 2.411 |
| othAs | 0 | Aliased | | | 8.299 | 6.951 | 9.907 |
| other | 1 | -1.062 | 0.755 | N.S. | 2.869 | 0.308 | 26.728 |
| Start year of study | | | | | | | |
| <1960 | 5 | Aliased | | | 1.419 | 0.680 | 2.963 |
| 1960-69 | 7 | 1.361 | 0.263 | +++ | 5.537 | 4.314 | 7.107 |
| 1970-79 | 11 | 1.322 | 0.286 | +++ | 5.324 | 3.458 | 8.197 |
| 1980-89 | 19 | 1.120 | 0.250 | +++ | 4.349 | 3.385 | 5.588 |
| 1990+ | 2 | 2.127 | 0.584 | ++ | 11.906 | 2.563 | 55.303 |
| | | Estimate | | | | | |
| Study type (1) | | | | | | | |
| CC | 34 | Aliased | | | | | |
| other | 10 | Aliased | | | | | |
| | | Estimate | S.E. | P | RR | 95%CIl | 95%CIu |
| Study size (number of LC cases) | | | | | | | |
| 100-249 | 11 | Aliased | | | 3.738 | 2.379 | 5.871 |
| 250-499 | 9 | -0.038 | 0.196 | N.S. | 3.600 | 2.342 | 5.534 |
| 500-999 | 4 | 0.568 | 0.238 | + | 6.594 | 3.620 | 12.012 |
| 1000+ | 20 | 0.283 | 0.158 | (+) | 4.961 | 4.422 | 5.564 |
| Number of adjustment variables (1) | | | | | | | |
| 0 | 17 | Aliased | | | 3.979 | 2.940 | 5.385 |
| 1 | 14 | 0.186 | 0.213 | N.S. | 4.793 | 3.084 | 7.450 |
| 2+/+nk | 13 | 0.279 | 0.125 | + | 5.257 | 4.508 | 6.132 |
| Study type (2) | | | | | | | |
| CC | 34 | Aliased | | | 4.700 | 4.235 | 5.217 |
| prosp | 7 | 0.384 | 0.265 | N.S. | 6.903 | 3.160 | 15.082 |
| other | 3 | -0.171 | 0.328 | N.S. | 3.960 | 1.471 | 10.661 |

| | | Deviance | (DF) | Drop Dev | P | | |
|---|---|---|---|---|---|---|---|
| Model 8 | | 65.388 | (26) | 0.339 | N.S. | | |
| | | Estimate | S.E. | P | RR | 95%CIl | 95%CIu |
| Constant | | 0.794 | 0.242 | ++ | 2.213 | 1.376 | 3.557 |
| Sex(RR) | | | | | | | |
| Male | 22 | Aliased | | | 6.197 | 5.266 | 7.293 |
| Female | 20 | -0.443 | 0.067 | --- | 3.981 | 3.440 | 4.607 |
| Combined | 2 | -0.568 | 0.386 | N.S. | 3.510 | 1.124 | 10.964 |

Table 3B1R - 4

IESLC - Meta-regression of current smoking, any product (or cigs if any not available)
Multiple regression of data from Table 3B1
Adenocarcinoma
Effect of additional characteristics

WEIGHTED on Weight

| | | Estimate | S.E. | P | RR | 95%CIl | 95%CIu |
|---|---|---|---|---|---|---|---|
| Location | | | | | | | |
| NAmer | 24 | Aliased | | | 8.179 | 6.812 | 9.820 |
| UK | 0 | Aliased | | | 8.179 | 6.812 | 9.820 |
| Scand | 2 | -0.153 | 0.405 | N.S. | 7.017 | 2.071 | 23.780 |
| othEur | 6 | -1.142 | 0.179 | --- | 2.610 | 1.605 | 4.245 |
| China | 0 | Aliased | | | 8.179 | 6.812 | 9.820 |
| Japan | 11 | -1.513 | 0.138 | --- | 1.801 | 1.320 | 2.456 |
| othAs | 0 | Aliased | | | 8.179 | 6.812 | 9.820 |
| other | 1 | -0.931 | 0.767 | N.S. | 3.224 | 0.324 | 32.063 |
| Start year of study | | | | | | | |
| <1960 | 5 | Aliased | | | 1.665 | 0.861 | 3.219 |
| 1960-69 | 7 | 1.186 | 0.231 | +++ | 5.453 | 4.244 | 7.006 |
| 1970-79 | 11 | 1.140 | 0.258 | +++ | 5.206 | 3.360 | 8.066 |
| 1980-89 | 19 | 0.967 | 0.225 | +++ | 4.380 | 3.391 | 5.657 |
| 1990+ | 2 | 1.920 | 0.585 | ++ | 11.357 | 2.299 | 56.115 |
| Study type (1) | | | | | | | |
| CC | 34 | Aliased | | | 4.707 | 4.232 | 5.236 |
| other | 10 | 0.228 | 0.240 | N.S. | 5.911 | 2.903 | 12.034 |
| Study size (number of LC cases) | | | | | | | |
| 100-249 | 11 | Aliased | | | 3.448 | 2.225 | 5.341 |
| 250-499 | 9 | 0.026 | 0.200 | N.S. | 3.538 | 2.281 | 5.486 |
| 500-999 | 4 | 0.667 | 0.235 | ++ | 6.716 | 3.649 | 12.359 |
| 1000+ | 20 | 0.372 | 0.150 | + | 5.002 | 4.459 | 5.611 |
| | | Estimate | | | | | |
| Number of adjustment variables (1) | | | | | | | |
| 0 | 17 | Aliased | | | | | |
| 1 | 14 | Aliased | | | | | |
| 2+/+nk | 13 | Aliased | | | | | |
| | | Estimate | S.E. | P | RR | 95%CIl | 95%CIu |
| Number of adjustment variables (2) | | | | | | | |
| 0 | 17 | Aliased | | | 3.987 | 2.930 | 5.426 |
| 1 | 14 | 0.164 | 0.219 | N.S. | 4.697 | 2.944 | 7.493 |
| 2 | 12 | 0.284 | 0.125 | + | 5.294 | 4.513 | 6.211 |
| 3-5 | 0 | Aliased | | | 3.987 | 2.930 | 5.426 |
| 6+/+nk | 1 | -0.127 | 0.714 | N.S. | 3.513 | 0.405 | 30.482 |

| | | Deviance | (DF) | Drop Dev | P | | |
|---|---|---|---|---|---|---|---|
| Model 8 | | 65.727 | (27) | -0.000 | N.S. | | |
| | | Estimate | S.E. | P | RR | 95%CIl | 95%CIu |
| Constant | | 0.816 | 0.239 | ++ | 2.261 | 1.414 | 3.614 |
| Sex(RR) | | | | | | | |
| Male | 22 | Aliased | | | 6.201 | 5.284 | 7.279 |
| Female | 20 | -0.440 | 0.067 | --- | 3.995 | 3.464 | 4.607 |
| Combined | 2 | -0.612 | 0.378 | N.S. | 3.362 | 1.122 | 10.075 |
| Location | | | | | | | |
| NAmer | 24 | Aliased | | | 8.203 | 6.909 | 9.740 |
| UK | 0 | Aliased | | | 8.203 | 6.909 | 9.740 |
| Scand | 2 | -0.298 | 0.320 | N.S. | 6.089 | 2.335 | 15.874 |
| othEur | 6 | -1.157 | 0.178 | --- | 2.579 | 1.599 | 4.158 |
| China | 0 | Aliased | | | 8.203 | 6.909 | 9.740 |
| Japan | 11 | -1.525 | 0.136 | --- | 1.784 | 1.311 | 2.429 |
| othAs | 0 | Aliased | | | 8.203 | 6.909 | 9.740 |
| other | 1 | Aliased | | | 8.203 | 6.909 | 9.740 |
| Start year of study | | | | | | | |
| <1960 | 5 | Aliased | | | 1.732 | 0.912 | 3.287 |
| 1960-69 | 7 | 1.179 | 0.231 | +++ | 5.628 | 4.298 | 7.370 |
| 1970-79 | 11 | 1.142 | 0.258 | +++ | 5.424 | 3.421 | 8.601 |
| 1980-89 | 19 | 0.957 | 0.224 | +++ | 4.508 | 3.624 | 5.609 |
| 1990+ | 2 | 0.968 | 0.553 | (+) | 4.561 | 0.974 | 21.360 |
| Study type (1) | | | | | | | |
| CC | 34 | Aliased | | | 4.721 | 4.256 | 5.237 |
| other | 10 | 0.178 | 0.224 | N.S. | 5.638 | 2.932 | 10.841 |

Table 3B1R - 4

IESLC - Meta-regression of current smoking, any product (or cigs if any not available)
Multiple regression of data from Table 3B1
Adenocarcinoma
Effect of additional characteristics

WEIGHTED on Weight

| | | Estimate | S.E. | P | RR | 95%CIl | 95%CIu |
|---|---|---|---|---|---|---|---|
| Study size (number of LC cases) | | | | | | | |
| 100-249 | 11 | Aliased | | | 3.487 | 2.277 | 5.342 |
| 250-499 | 9 | -0.002 | 0.195 | N.S. | 3.480 | 2.280 | 5.312 |
| 500-999 | 4 | 0.646 | 0.232 | ++ | 6.651 | 3.658 | 12.095 |
| 1000+ | 20 | 0.362 | 0.149 | + | 5.007 | 4.473 | 5.606 |
| Number of adjustment variables (1) | | | | | | | |
| 0 | 17 | Aliased | | | 3.965 | 2.932 | 5.361 |
| 1 | 14 | 0.195 | 0.213 | N.S. | 4.818 | 3.104 | 7.479 |
| 2+/+nk | 13 | 0.283 | 0.125 | + | 5.260 | 4.512 | 6.133 |
| RR adjusted for or study matched on age | | | | | | | |
| Yes | 43 | Aliased | | | 4.627 | 4.112 | 5.207 |
| No | 1 | 1.014 | 0.754 | N.S. | 12.755 | 1.360 | 119.653 |

| | | Deviance | (DF) | Drop Dev | P | | |
|---|---|---|---|---|---|---|---|
| Model 8 | | 63.188 | (26) | 2.539 | N.S. | | |
| | | Estimate | S.E. | P | RR | 95%CIl | 95%CIu |
| Constant | | 1.056 | 0.283 | +++ | 2.874 | 1.651 | 5.004 |
| Sex(RR) | | | | | | | |
| Male | 22 | Aliased | | | 6.240 | 5.315 | 7.326 |
| Female | 20 | -0.435 | 0.067 | --- | 4.040 | 3.498 | 4.666 |
| Combined | 2 | -0.795 | 0.395 | (-) | 2.818 | 0.894 | 8.878 |
| Location | | | | | | | |
| NAmer | 24 | Aliased | | | 8.127 | 6.803 | 9.708 |
| UK | 0 | Aliased | | | 8.127 | 6.803 | 9.708 |
| Scand | 2 | -0.112 | 0.340 | N.S. | 7.268 | 2.640 | 20.010 |
| othEur | 6 | -1.243 | 0.185 | --- | 2.345 | 1.404 | 3.916 |
| China | 0 | Aliased | | | 8.127 | 6.803 | 9.708 |
| Japan | 11 | -1.443 | 0.146 | --- | 1.920 | 1.378 | 2.676 |
| othAs | 0 | Aliased | | | 8.127 | 6.803 | 9.708 |
| other | 1 | -1.379 | 0.788 | (-) | 2.047 | 0.197 | 21.210 |
| Start year of study | | | | | | | |
| <1960 | 5 | Aliased | | | 2.042 | 0.963 | 4.332 |
| 1960-69 | 7 | 0.896 | 0.291 | ++ | 5.003 | 3.731 | 6.709 |
| 1970-79 | 11 | 0.918 | 0.294 | ++ | 5.112 | 3.320 | 7.871 |
| 1980-89 | 19 | 0.769 | 0.253 | ++ | 4.405 | 3.428 | 5.661 |
| 1990+ | 2 | 2.214 | 0.593 | +++ | 18.698 | 3.284 | 106.463 |
| Study type (1) | | | | | | | |
| CC | 34 | Aliased | | | 4.721 | 4.257 | 5.237 |
| other | 10 | 0.176 | 0.224 | N.S. | 5.629 | 2.929 | 10.819 |
| Study size (number of LC cases) | | | | | | | |
| 100-249 | 11 | Aliased | | | 3.329 | 2.154 | 5.145 |
| 250-499 | 9 | -0.008 | 0.195 | N.S. | 3.303 | 2.140 | 5.100 |
| 500-999 | 4 | 0.600 | 0.234 | + | 6.066 | 3.254 | 11.311 |
| 1000+ | 20 | 0.421 | 0.153 | + | 5.072 | 4.519 | 5.692 |
| Number of adjustment variables (1) | | | | | | | |
| 0 | 17 | Aliased | | | 4.144 | 3.030 | 5.669 |
| 1 | 14 | 0.303 | 0.223 | N.S. | 5.610 | 3.310 | 9.506 |
| 2+/+nk | 13 | 0.176 | 0.142 | N.S. | 4.940 | 4.065 | 6.004 |
| RR adjusted for or matched on factor other than sex, age | | | | | | | |
| Yes | 33 | Aliased | | | 5.014 | 4.378 | 5.743 |
| No | 11 | -0.366 | 0.230 | N.S. | 3.477 | 1.883 | 6.422 |

| | | Deviance | (DF) | Drop Dev | P | | |
|---|---|---|---|---|---|---|---|
| Model 8 | | 58.011 | (25) | 7.716 | N.S. | | |
| | | Estimate | S.E. | P | RR | 95%CIl | 95%CIu |
| Constant | | 0.054 | 0.364 | N.S. | 1.056 | 0.517 | 2.154 |
| Sex(RR) | | | | | | | |
| Male | 22 | Aliased | | | 6.124 | 5.226 | 7.176 |
| Female | 20 | -0.413 | 0.068 | --- | 4.051 | 3.521 | 4.661 |
| Combined | 2 | -0.643 | 0.390 | N.S. | 3.219 | 1.069 | 9.696 |

Table 3B1R - 4

IESLC - Meta-regression of current smoking, any product (or cigs if any not available)
Multiple regression of data from Table 3B1
Adenocarcinoma
Effect of additional characteristics

WEIGHTED on Weight

| | | Estimate | S.E. | P | RR | 95%CIl | 95%CIu |
|---|---|---|---|---|---|---|---|
| Location | | | | | | | |
| NAmer | 24 | Aliased | | | 8.332 | 6.994 | 9.925 |
| UK | 0 | Aliased | | | 8.332 | 6.994 | 9.925 |
| Scand | 2 | -0.303 | 0.320 | N.S. | 6.156 | 2.416 | 15.690 |
| othEur | 6 | -1.042 | 0.183 | --- | 2.938 | 1.795 | 4.808 |
| China | 0 | Aliased | | | 8.332 | 6.994 | 9.925 |
| Japan | 11 | -1.607 | 0.145 | --- | 1.671 | 1.214 | 2.300 |
| othAs | 0 | Aliased | | | 8.332 | 6.994 | 9.925 |
| other | 1 | -1.627 | 0.831 | (-) | 1.637 | 0.149 | 17.971 |
| Start year of study | | | | | | | |
| <1960 | 5 | Aliased | | | 0.936 | 0.366 | 2.396 |
| 1960-69 | 7 | 1.834 | 0.343 | +++ | 5.858 | 4.541 | 7.558 |
| 1970-79 | 11 | 1.675 | 0.339 | +++ | 4.999 | 3.277 | 7.626 |
| 1980-89 | 19 | 1.537 | 0.331 | +++ | 4.353 | 3.373 | 5.616 |
| 1990+ | 2 | 2.791 | 0.650 | +++ | 15.267 | 3.044 | 76.564 |
| Study type (1) | | | | | | | |
| CC | 34 | Aliased | | | 4.861 | 4.371 | 5.406 |
| other | 10 | -0.331 | 0.292 | N.S. | 3.492 | 1.529 | 7.976 |
| Study size (number of LC cases) | | | | | | | |
| 100-249 | 11 | Aliased | | | 4.856 | 2.772 | 8.508 |
| 250-499 | 9 | 0.063 | 0.203 | N.S. | 5.171 | 2.684 | 9.963 |
| 500-999 | 4 | 0.370 | 0.253 | N.S. | 7.031 | 3.897 | 12.685 |
| 1000+ | 20 | -0.042 | 0.215 | N.S. | 4.657 | 4.043 | 5.364 |
| Number of adjustment variables (1) | | | | | | | |
| 0 | 17 | Aliased | | | 3.758 | 2.725 | 5.184 |
| 1 | 14 | 0.349 | 0.245 | N.S. | 5.328 | 3.271 | 8.679 |
| 2+/+nk | 13 | 0.340 | 0.133 | + | 5.279 | 4.543 | 6.135 |
| Product | | | | | | | |
| all/unsp | 11 | Aliased | | | 2.931 | 1.530 | 5.614 |
| cig+/-ot | 28 | 0.509 | 0.235 | + | 4.875 | 4.335 | 5.483 |
| cig only | 5 | 1.589 | 0.576 | + | 14.359 | 3.853 | 53.508 |

| | | Deviance | (DF) | Drop Dev | P | | |
|---|---|---|---|---|---|---|---|
| Model 8 | | 65.291 | (26) | 0.436 | N.S. | | |
| | | Estimate | S.E. | P | RR | 95%CIl | 95%CIu |
| Constant | | 0.862 | 0.249 | ++ | 2.368 | 1.452 | 3.861 |
| Sex(RR) | | | | | | | |
| Male | 22 | Aliased | | | 6.237 | 5.289 | 7.356 |
| Female | 20 | -0.450 | 0.069 | --- | 3.976 | 3.434 | 4.603 |
| Combined | 2 | -0.618 | 0.379 | N.S. | 3.363 | 1.103 | 10.252 |
| Location | | | | | | | |
| NAmer | 24 | Aliased | | | 8.091 | 6.640 | 9.858 |
| UK | 0 | Aliased | | | 8.091 | 6.640 | 9.858 |
| Scand | 2 | -0.292 | 0.320 | N.S. | 6.042 | 2.281 | 16.007 |
| othEur | 6 | -1.068 | 0.223 | --- | 2.781 | 1.543 | 5.012 |
| China | 0 | Aliased | | | 8.091 | 6.640 | 9.858 |
| Japan | 11 | -1.497 | 0.143 | --- | 1.812 | 1.324 | 2.478 |
| othAs | 0 | Aliased | | | 8.091 | 6.640 | 9.858 |
| other | 1 | -1.002 | 0.754 | N.S. | 2.970 | 0.310 | 28.457 |
| Start year of study | | | | | | | |
| <1960 | 5 | Aliased | | | 1.809 | 0.851 | 3.847 |
| 1960-69 | 7 | 1.130 | 0.243 | +++ | 5.599 | 4.210 | 7.446 |
| 1970-79 | 11 | 1.054 | 0.291 | ++ | 5.191 | 3.349 | 8.046 |
| 1980-89 | 19 | 0.859 | 0.269 | ++ | 4.271 | 3.260 | 5.596 |
| 1990+ | 2 | 1.904 | 0.587 | ++ | 12.146 | 2.564 | 57.543 |
| Study type (1) | | | | | | | |
| CC | 34 | Aliased | | | 4.726 | 4.253 | 5.252 |
| other | 10 | 0.158 | 0.226 | N.S. | 5.537 | 2.835 | 10.815 |
| Study size (number of LC cases) | | | | | | | |
| 100-249 | 11 | Aliased | | | 3.619 | 2.269 | 5.772 |
| 250-499 | 9 | 0.007 | 0.195 | N.S. | 3.643 | 2.253 | 5.892 |
| 500-999 | 4 | 0.612 | 0.238 | + | 6.672 | 3.634 | 12.249 |
| 1000+ | 20 | 0.317 | 0.164 | (+) | 4.967 | 4.401 | 5.605 |

Table 3B1R - 4

IESLC - Meta-regression of current smoking, any product (or cigs if any not available)
Multiple regression of data from Table 3B1
Adenocarcinoma
Effect of additional characteristics

WEIGHTED on Weight

| | | Estimate | S.E. | P | RR | 95%CIl | 95%CIu |
|---|---|---|---|---|---|---|---|
| Number of adjustment variables (1) | | | | | | | |
| 0 | 17 | Aliased | | | 3.918 | 2.869 | 5.350 |
| 1 | 14 | 0.201 | 0.213 | N.S. | 4.788 | 3.060 | 7.491 |
| 2+/+nk | 13 | 0.303 | 0.128 | + | 5.303 | 4.517 | 6.227 |
| Denominator | | | | | | | |
| nev any | 27 | Aliased | | | 4.555 | 3.592 | 5.775 |
| nev cigs | 17 | 0.087 | 0.131 | N.S. | 4.968 | 4.007 | 6.159 |

| | | Deviance | (DF) | Drop Dev | P | | |
|---|---|---|---|---|---|---|---|
| Model 8 | | 64.489 | (26) | 1.238 | N.S. | | |
| | | Estimate | S.E. | P | RR | 95%CIl | 95%CIu |
| Constant | | 0.325 | 0.502 | N.S. | 1.384 | 0.517 | 3.702 |
| Sex(RR) | | | | | | | |
| Male | 22 | Aliased | | | 6.115 | 5.178 | 7.222 |
| Female | 20 | -0.435 | 0.067 | --- | 3.957 | 3.419 | 4.581 |
| Combined | 2 | -0.373 | 0.435 | N.S. | 4.212 | 1.181 | 15.023 |
| Location | | | | | | | |
| NAmer | 24 | Aliased | | | 7.989 | 6.561 | 9.727 |
| UK | 0 | Aliased | | | 7.989 | 6.561 | 9.727 |
| Scand | 2 | -0.257 | 0.322 | N.S. | 6.180 | 2.349 | 16.257 |
| othEur | 6 | -1.119 | 0.181 | --- | 2.608 | 1.611 | 4.222 |
| China | 0 | Aliased | | | 7.989 | 6.561 | 9.727 |
| Japan | 11 | -1.435 | 0.159 | --- | 1.903 | 1.342 | 2.698 |
| othAs | 0 | Aliased | | | 7.989 | 6.561 | 9.727 |
| other | 1 | -0.639 | 0.826 | N.S. | 4.217 | 0.367 | 48.408 |
| Start year of study | | | | | | | |
| <1960 | 5 | Aliased | | | 1.694 | 0.880 | 3.261 |
| 1960-69 | 7 | 1.141 | 0.233 | +++ | 5.299 | 4.091 | 6.863 |
| 1970-79 | 11 | 1.042 | 0.273 | +++ | 4.802 | 2.923 | 7.891 |
| 1980-89 | 19 | 1.008 | 0.229 | +++ | 4.642 | 3.412 | 6.314 |
| 1990+ | 2 | 1.641 | 0.652 | + | 8.735 | 1.449 | 52.658 |
| Study type (1) | | | | | | | |
| CC | 34 | Aliased | | | 4.689 | 4.216 | 5.215 |
| other | 10 | 0.294 | 0.248 | N.S. | 6.294 | 3.041 | 13.028 |
| Study size (number of LC cases) | | | | | | | |
| 100-249 | 11 | Aliased | | | 3.526 | 2.290 | 5.428 |
| 250-499 | 9 | -0.045 | 0.198 | N.S. | 3.372 | 2.181 | 5.214 |
| 500-999 | 4 | 0.953 | 0.360 | + | 9.140 | 3.139 | 26.611 |
| 1000+ | 20 | 0.342 | 0.150 | + | 4.963 | 4.416 | 5.576 |
| Number of adjustment variables (1) | | | | | | | |
| 0 | 17 | Aliased | | | 4.267 | 2.958 | 6.155 |
| 1 | 14 | 0.021 | 0.264 | N.S. | 4.355 | 2.577 | 7.362 |
| 2+/+nk | 13 | 0.195 | 0.147 | N.S. | 5.186 | 4.419 | 6.085 |
| National cigarette tobacco type | | | | | | | |
| Virginia | 2 | Aliased | | | 2.790 | 0.628 | 12.390 |
| blended | 42 | 0.546 | 0.490 | N.S. | 4.814 | 4.355 | 5.322 |

| | | Deviance | (DF) | Drop Dev | P | | |
|---|---|---|---|---|---|---|---|
| Model 8 | | 61.546 | (26) | 4.180 | N.S. | | |
| | | Estimate | S.E. | P | RR | 95%CIl | 95%CIu |
| Constant | | 0.760 | 0.241 | ++ | 2.138 | 1.333 | 3.429 |
| Sex(RR) | | | | | | | |
| Male | 22 | Aliased | | | 6.544 | 5.484 | 7.809 |
| Female | 20 | -0.430 | 0.067 | --- | 4.258 | 3.595 | 5.043 |
| Combined | 2 | -1.832 | 0.707 | - | 1.047 | 0.137 | 7.994 |
| Location | | | | | | | |
| NAmer | 24 | Aliased | | | 8.623 | 7.157 | 10.389 |
| UK | 0 | Aliased | | | 8.623 | 7.157 | 10.389 |
| Scand | 2 | -0.315 | 0.320 | N.S. | 6.292 | 2.445 | 16.193 |
| othEur | 6 | -1.316 | 0.194 | --- | 2.312 | 1.404 | 3.808 |
| China | 0 | Aliased | | | 8.623 | 7.157 | 10.389 |
| Japan | 11 | -1.609 | 0.142 | --- | 1.725 | 1.271 | 2.340 |
| othAs | 0 | Aliased | | | 8.623 | 7.157 | 10.389 |
| other | 1 | -2.337 | 0.993 | - | 0.833 | 0.046 | 15.013 |

Table 3B1R - 4

IESLC - Meta-regression of current smoking, any product (or cigs if any not available)
Multiple regression of data from Table 3B1
Adenocarcinoma
Effect of additional characteristics

WEIGHTED on Weight

| | | Estimate | S.E. | P | RR | 95%CIl | 95%CIu |
|---|---|---|---|---|---|---|---|
| Start year of study | | | | | | | |
| <1960 | 5 | Aliased | | | 1.605 | 0.845 | 3.050 |
| 1960-69 | 7 | 1.198 | 0.231 | +++ | 5.318 | 4.162 | 6.794 |
| 1970-79 | 11 | 1.280 | 0.267 | +++ | 5.774 | 3.694 | 9.026 |
| 1980-89 | 19 | 0.898 | 0.226 | +++ | 3.939 | 2.954 | 5.252 |
| 1990+ | 2 | 3.292 | 0.861 | +++ | 43.182 | 3.901 | 477.965 |
| Study type (1) | | | | | | | |
| CC | 34 | Aliased | | | 4.752 | 4.288 | 5.266 |
| other | 10 | 0.063 | 0.231 | N.S. | 5.059 | 2.604 | 9.831 |
| Study size (number of LC cases) | | | | | | | |
| 100-249 | 11 | Aliased | | | 3.450 | 2.265 | 5.256 |
| 250-499 | 9 | 0.048 | 0.196 | N.S. | 3.620 | 2.376 | 5.515 |
| 500-999 | 4 | -0.273 | 0.506 | N.S. | 2.625 | 0.590 | 11.677 |
| 1000+ | 20 | 0.402 | 0.150 | + | 5.159 | 4.577 | 5.815 |
| Number of adjustment variables (1) | | | | | | | |
| 0 | 17 | Aliased | | | 3.738 | 2.742 | 5.097 |
| 1 | 14 | 0.342 | 0.225 | N.S. | 5.261 | 3.346 | 8.273 |
| 2+/+nk | 13 | 0.351 | 0.129 | + | 5.312 | 4.563 | 6.184 |
| Any proxy use | | | | | | | |
| No/nk | 38 | Aliased | | | 4.533 | 4.018 | 5.114 |
| Yes | 6 | 0.950 | 0.465 | (+) | 11.724 | 3.101 | 44.330 |

| | | Deviance | (DF) | Drop Dev | P | | |
|---|---|---|---|---|---|---|---|
| Model 8 | | 51.756 | (26) | 13.971 | * | | |
| | | Estimate | S.E. | P | RR | 95%CIl | 95%CIu |
| Constant | | 1.180 | 0.259 | +++ | 3.256 | 1.962 | 5.404 |
| Sex(RR) | | | | | | | |
| Male | 22 | Aliased | | | 6.639 | 5.695 | 7.740 |
| Female | 20 | -0.463 | 0.067 | --- | 4.178 | 3.657 | 4.774 |
| Combined | 2 | -1.767 | 0.489 | -- | 1.134 | 0.316 | 4.068 |
| Location | | | | | | | |
| NAmer | 24 | Aliased | | | 9.653 | 7.921 | 11.764 |
| UK | 0 | Aliased | | | 9.653 | 7.921 | 11.764 |
| Scand | 2 | -0.768 | 0.344 | - | 4.478 | 1.828 | 10.972 |
| othEur | 6 | -1.538 | 0.205 | --- | 2.073 | 1.307 | 3.288 |
| China | 0 | Aliased | | | 9.653 | 7.921 | 11.764 |
| Japan | 11 | -1.915 | 0.172 | --- | 1.422 | 1.029 | 1.966 |
| othAs | 0 | Aliased | | | 9.653 | 7.921 | 11.764 |
| other | 1 | -2.476 | 0.849 | -- | 0.812 | 0.087 | 7.556 |
| Start year of study | | | | | | | |
| <1960 | 5 | Aliased | | | 1.382 | 0.757 | 2.526 |
| 1960-69 | 7 | 1.296 | 0.233 | +++ | 5.053 | 4.018 | 6.354 |
| 1970-79 | 11 | 1.624 | 0.289 | +++ | 7.014 | 4.499 | 10.934 |
| 1980-89 | 19 | 1.051 | 0.225 | +++ | 3.953 | 3.118 | 5.012 |
| 1990+ | 2 | 3.108 | 0.649 | +++ | 30.923 | 6.578 | 145.383 |
| Study type (1) | | | | | | | |
| CC | 34 | Aliased | | | 4.894 | 4.439 | 5.395 |
| other | 10 | -0.447 | 0.280 | N.S. | 3.129 | 1.502 | 6.522 |
| Study size (number of LC cases) | | | | | | | |
| 100-249 | 11 | Aliased | | | 3.580 | 2.433 | 5.267 |
| 250-499 | 9 | 0.052 | 0.195 | N.S. | 3.772 | 2.561 | 5.555 |
| 500-999 | 4 | 0.154 | 0.267 | N.S. | 4.177 | 2.200 | 7.929 |
| 1000+ | 20 | 0.341 | 0.149 | + | 5.036 | 4.547 | 5.578 |
| Number of adjustment variables (1) | | | | | | | |
| 0 | 17 | Aliased | | | 3.845 | 2.924 | 5.056 |
| 1 | 14 | 0.523 | 0.230 | + | 6.489 | 4.118 | 10.224 |
| 2+/+nk | 13 | 0.254 | 0.125 | (+) | 4.959 | 4.287 | 5.735 |
| Full histological confirmation | | | | | | | |
| No | 23 | Aliased | | | 6.420 | 5.069 | 8.132 |
| Yes | 21 | -0.457 | 0.122 | --- | 4.065 | 3.511 | 4.707 |

| | | Deviance | (DF) | Drop Dev | P |
|---|---|---|---|---|---|
| Model 8 | | 65.727 | (27) | 0.000 | N.S. |

Table 3B1R - 4

IESLC - Meta-regression of current smoking, any product (or cigs if any not available)
Multiple regression of data from Table 3B1
Adenocarcinoma
Effect of additional characteristics

WEIGHTED on Weight

| | | Estimate | S.E. | P | RR | 95%CIl | 95%CIu |
|---|---|---|---|---|---|---|---|
| Constant | | 0.816 | 0.239 | ++ | 2.261 | 1.414 | 3.614 |
| Sex(RR) | | | | | | | |
| Male | 22 | Aliased | | | 6.201 | 5.284 | 7.279 |
| Female | 20 | -0.440 | 0.067 | --- | 3.995 | 3.464 | 4.607 |
| Combined | 2 | -0.612 | 0.378 | N.S. | 3.362 | 1.122 | 10.075 |
| Location | | | | | | | |
| NAmer | 24 | Aliased | | | 8.237 | 6.907 | 9.822 |
| UK | 0 | Aliased | | | 8.237 | 6.907 | 9.822 |
| Scand | 2 | -0.298 | 0.320 | N.S. | 6.113 | 2.346 | 15.930 |
| othEur | 6 | -1.157 | 0.178 | --- | 2.589 | 1.607 | 4.171 |
| China | 0 | Aliased | | | 8.237 | 6.907 | 9.822 |
| Japan | 11 | -1.525 | 0.136 | --- | 1.792 | 1.322 | 2.429 |
| othAs | 0 | Aliased | | | 8.237 | 6.907 | 9.822 |
| other | 1 | -1.014 | 0.754 | N.S. | 2.988 | 0.323 | 27.641 |
| Start year of study | | | | | | | |
| <1960 | 5 | Aliased | | | 1.673 | 0.875 | 3.199 |
| 1960-69 | 7 | 1.179 | 0.231 | +++ | 5.438 | 4.252 | 6.956 |
| 1970-79 | 11 | 1.142 | 0.258 | +++ | 5.241 | 3.412 | 8.051 |
| 1980-89 | 19 | 0.957 | 0.224 | +++ | 4.356 | 3.392 | 5.593 |
| 1990+ | 2 | 1.982 | 0.575 | ++ | 12.148 | 2.626 | 56.185 |
| Study type (1) | | | | | | | |
| CC | 34 | Aliased | | | 4.721 | 4.256 | 5.237 |
| other | 10 | 0.178 | 0.224 | N.S. | 5.638 | 2.932 | 10.841 |
| Study size (number of LC cases) | | | | | | | |
| 100-249 | 11 | Aliased | | | 3.487 | 2.277 | 5.342 |
| 250-499 | 9 | -0.002 | 0.195 | N.S. | 3.480 | 2.280 | 5.312 |
| 500-999 | 4 | 0.646 | 0.232 | ++ | 6.651 | 3.658 | 12.095 |
| 1000+ | 20 | 0.362 | 0.149 | + | 5.007 | 4.473 | 5.606 |
| Number of adjustment variables (1) | | | | | | | |
| 0 | 17 | Aliased | | | 3.965 | 2.932 | 5.361 |
| 1 | 14 | 0.195 | 0.213 | N.S. | 4.818 | 3.104 | 7.479 |
| 2+/+nk | 13 | 0.283 | 0.125 | + | 5.260 | 4.512 | 6.133 |
| | | Estimate | | | | | |
| Risky occupational population | | | | | | | |
| No | 44 | Aliased | | | | | |

| | | Deviance | (DF) | Drop Dev | P | | |
|---|---|---|---|---|---|---|---|
| Model 8 | | 63.353 | (25) | 2.373 | N.S. | | |
| | | Estimate | S.E. | P | RR | 95%CIl | 95%CIu |
| Constant | | 0.878 | 0.251 | ++ | 2.406 | 1.472 | 3.932 |
| Sex(RR) | | | | | | | |
| Male | 22 | Aliased | | | 6.161 | 5.229 | 7.260 |
| Female | 20 | -0.434 | 0.067 | --- | 3.990 | 3.450 | 4.615 |
| Combined | 2 | -0.537 | 0.382 | N.S. | 3.601 | 1.165 | 11.129 |
| Location | | | | | | | |
| NAmer | 24 | Aliased | | | 8.199 | 6.836 | 9.834 |
| UK | 0 | Aliased | | | 8.199 | 6.836 | 9.834 |
| Scand | 2 | -0.241 | 0.322 | N.S. | 6.445 | 2.409 | 17.243 |
| othEur | 6 | -1.171 | 0.178 | --- | 2.542 | 1.555 | 4.155 |
| China | 0 | Aliased | | | 8.199 | 6.836 | 9.834 |
| Japan | 11 | -1.507 | 0.141 | --- | 1.817 | 1.318 | 2.506 |
| othAs | 0 | Aliased | | | 8.199 | 6.836 | 9.834 |
| other | 1 | -0.924 | 0.775 | N.S. | 3.255 | 0.316 | 33.556 |
| Start year of study | | | | | | | |
| <1960 | 5 | Aliased | | | 1.760 | 0.878 | 3.527 |
| 1960-69 | 7 | 1.110 | 0.247 | +++ | 5.343 | 4.126 | 6.919 |
| 1970-79 | 11 | 1.089 | 0.268 | +++ | 5.228 | 3.373 | 8.102 |
| 1980-89 | 19 | 0.919 | 0.230 | +++ | 4.412 | 3.397 | 5.730 |
| 1990+ | 2 | 1.849 | 0.619 | ++ | 11.184 | 2.167 | 57.716 |
| Study type (1) | | | | | | | |
| CC | 34 | Aliased | | | 4.678 | 4.199 | 5.211 |
| other | 10 | 0.335 | 0.254 | N.S. | 6.541 | 3.081 | 13.888 |

Table 3B1R - 4

IESLC - Meta-regression of current smoking, any product (or cigs if any not available)
Multiple regression of data from Table 3B1
Adenocarcinoma
Effect of additional characteristics

WEIGHTED on Weight

| | | Estimate | S.E. | P | RR | 95%CIl | 95%CIu |
|---|---|---|---|---|---|---|---|
| Study size (number of LC cases) | | | | | | | |
| 100-249 | 11 | Aliased | | | 3.475 | 2.243 | 5.384 |
| 250-499 | 9 | -0.025 | 0.224 | N.S. | 3.390 | 2.006 | 5.727 |
| 500-999 | 4 | 0.779 | 0.267 | ++ | 7.571 | 3.704 | 15.474 |
| 1000+ | 20 | 0.364 | 0.149 | + | 5.000 | 4.432 | 5.640 |
| Number of adjustment variables (1) | | | | | | | |
| 0 | 17 | Aliased | | | 4.056 | 2.962 | 5.554 |
| 1 | 14 | 0.109 | 0.220 | N.S. | 4.522 | 2.835 | 7.213 |
| 2+/+nk | 13 | 0.264 | 0.127 | + | 5.280 | 4.504 | 6.190 |
| Lowest age in RR | | | | | | | |
| <25/unlim | 29 | Aliased | | | 4.829 | 4.310 | 5.412 |
| 25-39 | 12 | -0.170 | 0.175 | N.S. | 4.076 | 2.473 | 6.718 |
| 40+ | 3 | 0.330 | 0.365 | N.S. | 6.715 | 2.198 | 20.513 |

| | | Deviance | (DF) | Drop Dev | P | | |
|---|---|---|---|---|---|---|---|
| Model 8 | | 60.442 | (24) | 5.285 | N.S. | | |
| | | Estimate | S.E. | P | RR | 95%CIl | 95%CIu |
| Constant | | 0.015 | 0.434 | N.S. | 1.015 | 0.433 | 2.376 |
| Sex(RR) | | | | | | | |
| Male | 22 | Aliased | | | 6.239 | 5.294 | 7.352 |
| Female | 20 | -0.454 | 0.068 | --- | 3.961 | 3.420 | 4.588 |
| Combined | 2 | -0.578 | 0.398 | N.S. | 3.499 | 1.079 | 11.346 |
| Location | | | | | | | |
| NAmer | 24 | Aliased | | | 8.094 | 6.717 | 9.752 |
| UK | 0 | Aliased | | | 8.094 | 6.717 | 9.752 |
| Scand | 2 | -0.198 | 0.406 | N.S. | 6.639 | 1.952 | 22.579 |
| othEur | 6 | -1.260 | 0.192 | --- | 2.296 | 1.353 | 3.894 |
| China | 0 | Aliased | | | 8.094 | 6.717 | 9.752 |
| Japan | 11 | -1.424 | 0.145 | --- | 1.949 | 1.400 | 2.714 |
| othAs | 0 | Aliased | | | 8.094 | 6.717 | 9.752 |
| other | 1 | -0.890 | 0.784 | N.S. | 3.325 | 0.318 | 34.734 |
| Start year of study | | | | | | | |
| <1960 | 5 | Aliased | | | 1.589 | 0.783 | 3.222 |
| 1960-69 | 7 | 1.172 | 0.245 | +++ | 5.130 | 3.936 | 6.688 |
| 1970-79 | 11 | 1.252 | 0.290 | +++ | 5.558 | 3.486 | 8.862 |
| 1980-89 | 19 | 1.040 | 0.234 | +++ | 4.493 | 3.441 | 5.866 |
| 1990+ | 2 | 1.866 | 0.640 | ++ | 10.267 | 1.877 | 56.172 |
| Study type (1) | | | | | | | |
| CC | 34 | Aliased | | | 4.694 | 4.218 | 5.225 |
| other | 10 | 0.274 | 0.248 | N.S. | 6.177 | 2.967 | 12.859 |
| Study size (number of LC cases) | | | | | | | |
| 100-249 | 11 | Aliased | | | 3.763 | 2.377 | 5.959 |
| 250-499 | 9 | -0.116 | 0.213 | N.S. | 3.352 | 2.079 | 5.404 |
| 500-999 | 4 | 0.502 | 0.268 | (+) | 6.216 | 2.982 | 12.960 |
| 1000+ | 20 | 0.285 | 0.158 | (+) | 5.005 | 4.435 | 5.649 |
| Number of adjustment variables (1) | | | | | | | |
| 0 | 17 | Aliased | | | 4.294 | 3.095 | 5.958 |
| 1 | 14 | 0.033 | 0.233 | N.S. | 4.437 | 2.713 | 7.256 |
| 2+/+nk | 13 | 0.180 | 0.133 | N.S. | 5.143 | 4.370 | 6.054 |
| Highest age in RR | | | | | | | |
| <65 | 2 | Aliased | | | 1.963 | 0.563 | 6.839 |
| 65-74 | 1 | 0.626 | 0.849 | N.S. | 3.671 | 0.402 | 33.573 |
| 75-84 | 7 | 0.922 | 0.437 | + | 4.934 | 3.070 | 7.930 |
| 85+/unlim | 34 | 0.895 | 0.403 | + | 4.804 | 4.315 | 5.348 |

| | | Deviance | (DF) | Drop Dev | P | | |
|---|---|---|---|---|---|---|---|
| Model 8 | | 64.425 | (26) | 1.301 | N.S. | | |
| | | Estimate | S.E. | P | RR | 95%CIl | 95%CIu |
| Constant | | 0.020 | 0.738 | N.S. | 1.020 | 0.240 | 4.330 |
| Sex(RR) | | | | | | | |
| Male | 22 | Aliased | | | 6.171 | 5.248 | 7.258 |
| Female | 20 | -0.442 | 0.067 | --- | 3.968 | 3.432 | 4.587 |
| Combined | 2 | -0.491 | 0.393 | N.S. | 3.777 | 1.195 | 11.941 |

Table 3B1R - 4

IESLC - Meta-regression of current smoking, any product (or cigs if any not available)
Multiple regression of data from Table 3B1
Adenocarcinoma
Effect of additional characteristics

WEIGHTED on Weight

| | | Estimate | S.E. | P | RR | 95%CIl | 95%CIu |
|---|---|---|---|---|---|---|---|
| Location | | | | | | | |
| NAmer | 24 | Aliased | | | 8.097 | 6.739 | 9.728 |
| UK | 0 | Aliased | | | 8.097 | 6.739 | 9.728 |
| Scand | 2 | -0.283 | 0.320 | N.S. | 6.102 | 2.322 | 16.036 |
| othEur | 6 | -1.130 | 0.179 | --- | 2.616 | 1.616 | 4.236 |
| China | 0 | Aliased | | | 8.097 | 6.739 | 9.728 |
| Japan | 11 | -1.476 | 0.143 | --- | 1.850 | 1.345 | 2.545 |
| othAs | 0 | Aliased | | | 8.097 | 6.739 | 9.728 |
| other | 1 | -0.863 | 0.766 | N.S. | 3.418 | 0.352 | 33.208 |
| Start year of study | | | | | | | |
| <1960 | 5 | Aliased | | | 1.678 | 0.872 | 3.227 |
| 1960-69 | 7 | 1.173 | 0.231 | +++ | 5.424 | 4.230 | 6.953 |
| 1970-79 | 11 | 1.116 | 0.259 | +++ | 5.122 | 3.307 | 7.934 |
| 1980-89 | 19 | 0.976 | 0.225 | +++ | 4.453 | 3.436 | 5.770 |
| 1990+ | 2 | 1.782 | 0.601 | ++ | 9.967 | 1.943 | 51.136 |
| Study type (1) | | | | | | | |
| CC | 34 | Aliased | | | 4.713 | 4.244 | 5.233 |
| other | 10 | 0.207 | 0.226 | N.S. | 5.794 | 2.983 | 11.253 |
| Study size (number of LC cases) | | | | | | | |
| 100-249 | 11 | Aliased | | | 3.778 | 2.334 | 6.115 |
| 250-499 | 9 | -0.066 | 0.203 | N.S. | 3.536 | 2.303 | 5.430 |
| 500-999 | 4 | 0.604 | 0.235 | + | 6.909 | 3.747 | 12.742 |
| 1000+ | 20 | 0.272 | 0.168 | N.S. | 4.957 | 4.409 | 5.573 |
| Number of adjustment variables (1) | | | | | | | |
| 0 | 17 | Aliased | | | 4.123 | 2.987 | 5.691 |
| 1 | 14 | 0.095 | 0.230 | N.S. | 4.536 | 2.827 | 7.277 |
| 2+/+nk | 13 | 0.238 | 0.131 | (+) | 5.229 | 4.475 | 6.110 |
| Midpoint age in RR | | 0.016 | 0.014 | N.S. | 1.981 | 0.184 | 21.363 |

| | | Deviance | (DF) | Drop Dev | P | | |
|---|---|---|---|---|---|---|---|
| Model 8 | | 63.541 | (26) | 2.186 | N.S. | | |
| | | Estimate | S.E. | P | RR | 95%CIl | 95%CIu |
| Constant | | 0.437 | 0.351 | N.S. | 1.547 | 0.778 | 3.078 |
| Sex(RR) | | | | | | | |
| Male | 22 | Aliased | | | 6.149 | 5.232 | 7.226 |
| Female | 20 | -0.432 | 0.067 | --- | 3.990 | 3.459 | 4.603 |
| Combined | 2 | -0.516 | 0.384 | N.S. | 3.669 | 1.204 | 11.181 |
| Location | | | | | | | |
| NAmer | 24 | Aliased | | | 7.898 | 6.487 | 9.615 |
| UK | 0 | Aliased | | | 7.898 | 6.487 | 9.615 |
| Scand | 2 | -0.502 | 0.348 | N.S. | 4.779 | 1.612 | 14.170 |
| othEur | 6 | -0.971 | 0.218 | --- | 2.990 | 1.703 | 5.251 |
| China | 0 | Aliased | | | 7.898 | 6.487 | 9.615 |
| Japan | 11 | -1.443 | 0.147 | --- | 1.866 | 1.360 | 2.561 |
| othAs | 0 | Aliased | | | 7.898 | 6.487 | 9.615 |
| other | 1 | -0.652 | 0.793 | N.S. | 4.116 | 0.402 | 42.125 |
| Start year of study | | | | | | | |
| <1960 | 5 | Aliased | | | 1.502 | 0.756 | 2.985 |
| 1960-69 | 7 | 1.239 | 0.234 | +++ | 5.188 | 3.980 | 6.764 |
| 1970-79 | 11 | 1.311 | 0.283 | +++ | 5.574 | 3.559 | 8.729 |
| 1980-89 | 19 | 1.101 | 0.244 | +++ | 4.518 | 3.478 | 5.871 |
| 1990+ | 2 | 1.792 | 0.589 | ++ | 9.019 | 1.725 | 47.149 |
| Study type (1) | | | | | | | |
| CC | 34 | Aliased | | | 4.730 | 4.263 | 5.248 |
| other | 10 | 0.145 | 0.226 | N.S. | 5.467 | 2.830 | 10.559 |
| Study size (number of LC cases) | | | | | | | |
| 100-249 | 11 | Aliased | | | 3.416 | 2.223 | 5.248 |
| 250-499 | 9 | 0.074 | 0.201 | N.S. | 3.677 | 2.371 | 5.703 |
| 500-999 | 4 | 0.618 | 0.233 | + | 6.335 | 3.451 | 11.631 |
| 1000+ | 20 | 0.380 | 0.149 | + | 4.996 | 4.462 | 5.595 |

Table 3B1R - 4

IESLC - Meta-regression of current smoking, any product (or cigs if any not available)
Multiple regression of data from Table 3B1
Adenocarcinoma
Effect of additional characteristics

WEIGHTED on Weight

| | | Estimate | S.E. | P | RR | 95%CIl | 95%CIu |
|---|---|---|---|---|---|---|---|
| Number of adjustment variables (1) | | | | | | | |
| 0 | 17 | Aliased | | | 3.486 | 2.330 | 5.217 |
| 1 | 14 | 0.291 | 0.223 | N.S. | 4.662 | 2.985 | 7.281 |
| 2+/+nk | 13 | 0.490 | 0.187 | + | 5.689 | 4.549 | 7.116 |
| Derivation of RR/CI | | | | | | | |
| Orig/2x2 | 28 | Aliased | | | 4.431 | 3.701 | 5.306 |
| Other | 16 | 0.315 | 0.213 | N.S. | 6.071 | 3.648 | 10.103 |

| | | Deviance | (DF) | Drop Dev | P | | |
|---|---|---|---|---|---|---|---|
| Model 8 | | 62.740 | (25) | 2.987 | N.S. | | |
| | | Estimate | S.E. | P | RR | 95%CIl | 95%CIu |
| Constant | | 0.202 | 0.435 | N.S. | 1.224 | 0.522 | 2.871 |
| Sex(RR) | | | | | | | |
| Male | 22 | Aliased | | | 6.130 | 5.199 | 7.227 |
| Female | 20 | -0.432 | 0.068 | --- | 3.980 | 3.442 | 4.602 |
| Combined | 2 | -0.459 | 0.389 | N.S. | 3.873 | 1.234 | 12.156 |
| Location | | | | | | | |
| NAmer | 24 | Aliased | | | 8.209 | 6.864 | 9.817 |
| UK | 0 | Aliased | | | 8.209 | 6.864 | 9.817 |
| Scand | 2 | -1.133 | 0.600 | (-) | 2.645 | 0.413 | 16.941 |
| othEur | 6 | -1.149 | 0.178 | --- | 2.601 | 1.599 | 4.232 |
| China | 0 | Aliased | | | 8.209 | 6.864 | 9.817 |
| Japan | 11 | -1.482 | 0.139 | --- | 1.865 | 1.358 | 2.562 |
| othAs | 0 | Aliased | | | 8.209 | 6.864 | 9.817 |
| other | 1 | -0.789 | 0.765 | N.S. | 3.730 | 0.376 | 37.017 |
| Start year of study | | | | | | | |
| <1960 | 5 | Aliased | | | 1.095 | 0.364 | 3.297 |
| 1960-69 | 7 | 1.614 | 0.373 | +++ | 5.502 | 4.279 | 7.075 |
| 1970-79 | 11 | 1.550 | 0.374 | +++ | 5.158 | 3.327 | 7.996 |
| 1980-89 | 19 | 1.429 | 0.386 | ++ | 4.575 | 3.491 | 5.994 |
| 1990+ | 2 | 2.125 | 0.605 | ++ | 9.175 | 1.786 | 47.142 |
| Study type (1) | | | | | | | |
| CC | 34 | Aliased | | | 4.779 | 4.289 | 5.324 |
| other | 10 | -0.035 | 0.263 | N.S. | 4.616 | 2.125 | 10.030 |
| Study size (number of LC cases) | | | | | | | |
| 100-249 | 11 | Aliased | | | 3.868 | 2.411 | 6.205 |
| 250-499 | 9 | -0.040 | 0.201 | N.S. | 3.715 | 2.355 | 5.862 |
| 500-999 | 4 | 0.523 | 0.243 | + | 6.527 | 3.553 | 11.990 |
| 1000+ | 20 | 0.243 | 0.164 | N.S. | 4.931 | 4.382 | 5.549 |
| Number of adjustment variables (1) | | | | | | | |
| 0 | 17 | Aliased | | | 3.376 | 1.784 | 6.391 |
| 1 | 14 | 0.366 | 0.321 | N.S. | 4.871 | 2.988 | 7.942 |
| 2+/+nk | 13 | 0.528 | 0.292 | (+) | 5.725 | 4.228 | 7.753 |
| Derivation of RR/CI | | | | | | | |
| Orig | 21 | Aliased | | | 4.188 | 3.044 | 5.762 |
| StcCalc | 17 | 0.312 | 0.286 | N.S. | 5.721 | 3.137 | 10.434 |
| Other | 6 | 0.870 | 0.521 | N.S. | 9.994 | 2.299 | 43.444 |
