# Supplementary material for: Systematic review with meta-analysis of the epidemiological evidence in the 1900s relating smoking to lung cancer
Source: BMC Cancer. 2012 Sep 3;12:385. doi: 10.1186/1471-2407-12-385 (PMC3505152; doi:10.1186/1471-2407-12-385)
Supplement: Additional file 5 — Detailed Analysis Tables (Individual file names as described in Additional file 1: Methods, Table1). [file 1471-2407-12-385-S5.zip › PDF/2G.pdf]

Table 2G1 -

IESLC - Meta-analysis of Ever Smoking by Amount, Overview, Any product (or Cigarettes if Any not available)
Squamous

This analysis is restricted to results for:
1) Results by Amount smoked
Results by Amount smoked (in numbers of cigarettes or cigarette equivalents) are grouped under 2 schemes (S1, S2).
Each scheme has a set of "key values". An interval is allocated to the category whose key value
it includes and intervals which include none or more than one of the key values are excluded.
(Open-ended intervals are coded as 99.)

S1	key value	maximum range	S2	key value	maximum range
1	5	1-19	1	1	1-9
2	20	6-44	2	10	2-19
3	45	21+	3	20	11-29
			4	30	21-39
			5	40	31-98
			6	99	41+

For all/unspec product, the definition of cigarette equivalents is shown at the end of Sections -1 and -4.
2) Ever smokers
3) Results complete enough for use in metaanalysis

Within each study, results are then selected (in the following order of preference, within each sex) for:
4) PRODUCT: all/unspec, cigarettes regardless of other products, cigarettes only
5) CIGTYPE: all/unspecified, MC regardless of HR, MC only
6) DENOM: never smoked anything, never smoked cigarettes, (never +1 = +long term ex, +2 = +amount unknown,
+3 = never cigs+long term ex)
7) Followup period (YF, prospective studies): whole study (coded as 0) or longest available
8) LCtype: squamous or nearest available, but not adeno. (q = squamous, s = small,
a = adeno, KI = Kreyberg I, u = undifferentiated)
9) Race: all or nearest available, otherwise by race (wh or w = white, bl or b = black, hi = hispanic
ch = chinese, jap = japanese, haw = hawaiian, w+o = white + oriental, sca = scandinavian, as = asian)
10) For overlapping studies: principal rather than subsidiary studies
Finally by Age: whole study (coded as 0) if available, otherwise by widest available age group
and then for single sex results (m, f) in preference to combined sex results (c).

Results adjusted (AD) for the most potential confounders are then chosen in Sections -1 to -3
and results adjusted for the least confounders in Sections -4 to -6. (Those least adjusted results which
actually differ from the most adjusted as marked 'x' in column X in Section -4)
(Results adjusted for an unknown number of confounder(s) are coded as 20.)

Section -7 shows excluded studies, together with the stage (as above) at which no qualifying
results were found.

Section -8 lists the potentially overlapping studies which have been included (1=principal, 2=subsidiary).

Section -9 lists any results which would have been included in preference except that they had data not complete
enough for use in meta-analysis, with their significance (yes/no), if known, and any further comment as entered
on the database.

In addition to those mentioned above, the following fields, levels and abbreviations are used:
* or nk = not known, n = no, y = yes, ot = other
nev = never
all/unspec = all or unspecified, cig+/-ot = cigarettes irrespective of other products (cigar, pipe etc)
MC = manufactured cigarettes, HR = hand-rolled cigarettes
exL, exH = range of exposure (low and high) in the smoking group, in terms of Amount smoked,
cigarettes or cigarette equivalents
REF: 6-character study reference
NRR: number of the RR on the database within the study
ST : study type (CC = case control, pr or prosp = prospective)
NLC: number of lung cancer cases in whole study
R : risky occupational population (n = no, m = mining, o = other risky)
VB : national cigarette type (V = at least 75% Virginia, bl = at least 75% blended, ot = other)
P : any proxy use
H : full histological confirmation
De : derivation of RR/CI (or = original, st = standard method, ot = other method of estimation)

Table 2G1 - 1

IESLC - Meta-analysis of Ever Smoking by Amount, Overview, Any product (or Cigarettes if Any not available)
Squamous
Most adjusted

REF	NRR	SEX	AGEL	AGEH	RACE	YF	LC TYPE	LOC	START	ST	NLC	R	VB	P	H	AD	PRODUCT	exL	exH	S1	S2	DENOM	De
ALDERS	34	m	0	0	all	-	q+s	Eu:UK	1977	CC	1448	n	V	n	n	1	cig only	1	17	1	0	nev+2	ot
ALDERS	35	m	0	0	all	-	q+s	Eu:UK	1977	CC	1448	n	V	n	n	1	cig only	18	27	2	3	nev+2	ot
ALDERS	36	m	0	0	all	-	q+s	Eu:UK	1977	CC	1448	n	V	n	n	1	cig only	28	99	3	0	nev+2	ot
ALDERS	37	f	0	0	all	-	q+s	Eu:UK	1977	CC	1448	n	V	n	n	1	cig only	1	17	1	0	nev+2	ot
ALDERS	38	f	0	0	all	-	q+s	Eu:UK	1977	CC	1448	n	V	n	n	1	cig only	18	27	2	3	nev+2	ot
ALDERS	39	f	0	0	all	-	q+s	Eu:UK	1977	CC	1448	n	V	n	n	1	cig only	28	99	3	0	nev+2	ot
BARBON	70	m	0	0	all	-	q	Eu:wst	1979	CC	755	n	bl	y	y	3	all/unsp	1	19	1	0	nev any	or
BARBON	71	m	0	0	all	-	q	Eu:wst	1979	CC	755	n	bl	y	y	3	all/unsp	20	39	2	0	nev any	or
BARBON	72	m	0	0	all	-	q	Eu:wst	1979	CC	755	n	bl	y	y	3	all/unsp	40	99	3	0	nev any	or
BROWN2	36	m	0	0	wh	-	q	NAmer	1984	CC	14596	n	bl	n	y	2	cig+/-ot	1	19	1	0	nev cigs	or
BROWN2	46	m	0	0	wh	-	q	NAmer	1984	CC	14596	n	bl	n	y	2	cig+/-ot	20	99	0	0	nev cigs	or
BROWN2	35	f	0	0	wh	-	q	NAmer	1984	CC	14596	n	bl	n	y	2	cig+/-ot	1	19	1	0	nev cigs	or
BROWN2	45	f	0	0	wh	-	q	NAmer	1984	CC	14596	n	bl	n	y	2	cig+/-ot	20	99	0	0	nev cigs	or
CHOI	46	m	0	0	all	-	q	As:oth	1985	CC	375	n	bl	n	n	0	cig+/-ot	1	10	1	0	nev cigs	st
CHOI	47	m	0	0	all	-	q	As:oth	1985	CC	375	n	bl	n	n	0	cig+/-ot	11	20	2	3	nev cigs	st
CHOI	48	m	0	0	all	-	q	As:oth	1985	CC	375	n	bl	n	n	0	cig+/-ot	21	30	0	4	nev cigs	st
CHOI	49	m	0	0	all	-	q	As:oth	1985	CC	375	n	bl	n	n	0	cig+/-ot	31	40	0	5	nev cigs	st
CHOI	50	m	0	0	all	-	q	As:oth	1985	CC	375	n	bl	n	n	0	cig+/-ot	41	99	3	6	nev cigs	st
CHOI	56	f	0	0	all	-	q	As:oth	1985	CC	375	n	bl	n	n	0	cig+/-ot	1	10	1	0	nev cigs	st
CHOI	57	f	0	0	all	-	q	As:oth	1985	CC	375	n	bl	n	n	0	cig+/-ot	11	30	2	0	nev cigs	st
CHOI	58	f	0	0	all	-	q	As:oth	1985	CC	375	n	bl	n	n	0	cig+/-ot	31	99	3	0	nev cigs	st
DOLL	68	m	0	0	all	-	KI	Eu:UK	1948	CC	1465	n	V	n	n	1	all/unsp	1	4	0	1	nev any	ot
DOLL	69	m	0	0	all	-	KI	Eu:UK	1948	CC	1465	n	V	n	n	1	all/unsp	5	14	1	2	nev any	ot
DOLL	70	m	0	0	all	-	KI	Eu:UK	1948	CC	1465	n	V	n	n	1	all/unsp	15	24	2	3	nev any	ot
DOLL	71	m	0	0	all	-	KI	Eu:UK	1948	CC	1465	n	V	n	n	1	all/unsp	25	99	3	0	nev any	ot
DOLL	76	f	0	0	all	-	KI	Eu:UK	1948	CC	1465	n	V	n	n	1	all/unsp	1	4	0	1	nev any	ot
DOLL	77	f	0	0	all	-	KI	Eu:UK	1948	CC	1465	n	V	n	n	1	all/unsp	5	14	1	2	nev any	ot
DOLL	78	f	0	0	all	-	KI	Eu:UK	1948	CC	1465	n	V	n	n	1	all/unsp	15	99	0	0	nev any	ot
DORGAN	114	m	0	0	wh	-	q	NAmer	1980	CC	2026	n	bl	y	y	2	cig+/-ot	1	19	1	0	nev any	ot
DORGAN	115	m	0	0	wh	-	q	NAmer	1980	CC	2026	n	bl	y	y	2	cig+/-ot	20	99	0	0	nev any	ot
DORGAN	99	f	0	0	all	-	q	NAmer	1980	CC	2026	n	bl	y	y	3	cig+/-ot	1	19	1	0	nev any	ot
DORGAN	100	f	0	0	all	-	q	NAmer	1980	CC	2026	n	bl	y	y	3	cig+/-ot	20	99	0	0	nev any	ot
DOSEME	7	m	0	0	all	-	q	Eu:bal	1979	CC	1210	n	bl	n	n	2	cig+/-ot	1	10	1	0	nev cigs	or
DOSEME	11	m	0	0	all	-	q	Eu:bal	1979	CC	1210	n	bl	n	n	2	cig+/-ot	11	20	2	3	nev cigs	or
DOSEME	15	m	0	0	all	-	q	Eu:bal	1979	CC	1210	n	bl	n	n	2	cig+/-ot	21	99	3	0	nev cigs	or
GER	14	c	0	0	all	-	q+s	As:oth	1990	CC	141	n	ot	y	n	10	all/unsp	1	10	1	0	nev any	ot
GER	15	c	0	0	all	-	q+s	As:oth	1990	CC	141	n	ot	y	n	10	all/unsp	11	20	2	3	nev any	ot
GER	16	c	0	0	all	-	q+s	As:oth	1990	CC	141	n	ot	y	n	10	all/unsp	21	99	3	0	nev any	ot
JEDRYC	28	m	0	0	all	-	q	Eu:est	1980	CC	1630	n	bl	y	n	3	cig+/-ot	1	19	1	0	nev any	or
JEDRYC	29	m	0	0	all	-	q	Eu:est	1980	CC	1630	n	bl	y	n	3	cig+/-ot	20	29	2	3	nev any	or
JEDRYC	30	m	0	0	all	-	q	Eu:est	1980	CC	1630	n	bl	y	n	3	cig+/-ot	30	99	3	0	nev any	or
KREYBE	1	m	0	0	all	-	KI	Eu:Sca	1948	CC	300	n	bl	n	y	1	all/unsp	1	14	1	0	nev any	ot
KREYBE	2	m	0	0	all	-	KI	Eu:Sca	1948	CC	300	n	bl	n	y	1	all/unsp	15	24	2	3	nev any	ot
KREYBE	3	m	0	0	all	-	KI	Eu:Sca	1948	CC	300	n	bl	n	y	1	all/unsp	25	99	3	0	nev any	ot
KREYBE	31	f	0	0	all	-	KI	Eu:Sca	1948	CC	300	n	bl	n	y	0	all/unsp	1	14	1	0	nev any	st
KREYBE	32	f	0	0	all	-	KI	Eu:Sca	1948	CC	300	n	bl	n	y	0	all/unsp	15	99	0	0	nev any	st
LAMTH	10	f	0	0	ch	-	q	As:HK	1983	CC	445	n	bl	n	n	0	all/unsp	1	10	1	0	nev any	or
LAMTH	11	f	0	0	ch	-	q	As:HK	1983	CC	445	n	bl	n	n	0	all/unsp	11	20	2	3	nev any	or
LAMTH	12	f	0	0	ch	-	q	As:HK	1983	CC	445	n	bl	n	n	0	all/unsp	21	99	3	0	nev any	st
LUBIN2	149	m	0	0	all	-	q	Eu:mul	1976	CC	7804	n	bl	n	y	0	cig+/-ot	1	9	1	1	nev any	st
LUBIN2	153	m	0	0	all	-	q	Eu:mul	1976	CC	7804	n	bl	n	y	0	cig+/-ot	10	19	0	2	nev any	st
LUBIN2	157	m	0	0	all	-	q	Eu:mul	1976	CC	7804	n	bl	n	y	0	cig+/-ot	20	29	2	3	nev any	st
LUBIN2	161	m	0	0	all	-	q	Eu:mul	1976	CC	7804	n	bl	n	y	0	cig+/-ot	30	99	3	0	nev any	st
LUBIN2	169	f	0	0	all	-	q	Eu:mul	1976	CC	7804	n	bl	n	y	0	cig+/-ot	1	9	1	1	nev any	st
LUBIN2	173	f	0	0	all	-	q	Eu:mul	1976	CC	7804	n	bl	n	y	0	cig+/-ot	10	19	0	2	nev any	st
LUBIN2	177	f	0	0	all	-	q	Eu:mul	1976	CC	7804	n	bl	n	y	0	cig+/-ot	20	29	2	3	nev any	st
LUBIN2	181	f	0	0	all	-	q	Eu:mul	1976	CC	7804	n	bl	n	y	0	cig+/-ot	30	99	3	0	nev any	st
LUO	10	c	0	0	all	-	q	As:Chi	1990	CC	102	n	ot	n	y	20	cig+/-ot	1	19	1	0	nev cigs	or
LUO	11	c	0	0	all	-	q	As:Chi	1990	CC	102	n	ot	n	y	20	cig+/-ot	20	29	2	3	nev cigs	or
LUO	12	c	0	0	all	-	q	As:Chi	1990	CC	102	n	ot	n	y	20	cig+/-ot	30	99	3	0	nev cigs	or
MATOS	43	m	0	0	all	-	q	SCAmer	1994	CC	200	n	bl	n	n	2	cig+/-ot	1	14	1	0	nev any	or
MATOS	45	m	0	0	all	-	q	SCAmer	1994	CC	200	n	bl	n	n	2	cig+/-ot	15	24	2	3	nev any	or
MATOS	47	m	0	0	all	-	q	SCAmer	1994	CC	200	n	bl	n	n	2	cig+/-ot	25	99	3	0	nev any	or
MATSUD	4	m	0	0	all	-	q	As:Jap	1965	CC	179	n	bl	n	n	0	cig+/-ot	1	10	1	0	nev cigs	st
MATSUD	5	m	0	0	all	-	q	As:Jap	1965	CC	179	n	bl	n	n	0	cig+/-ot	11	20	2	3	nev cigs	st
MATSUD	6	m	0	0	all	-	q	As:Jap	1965	CC	179	n	bl	n	n	0	cig+/-ot	21	99	3	0	nev cigs	st
ORMOS	5	m	0	0	all	-	q	Eu:est	1947	CC	119	n	bl	y	y	0	cig+/-ot	1	15	1	0	nev any	st
ORMOS	6	m	0	0	all	-	q	Eu:est	1947	CC	119	n	bl	y	y	0	cig+/-ot	16	30	2	0	nev any	st
ORMOS	7	m	0	0	all	-	q	Eu:est	1947	CC	119	n	bl	y	y	0	cig+/-ot	31	99	3	0	nev any	st
OSANN	51	m	0	0	all	-	q	NAmer	1984	CC	1986	n	bl	n	n	2	cig+/-ot	1	39	0	0	nev cigs	or
OSANN	59	m	0	0	all	-	q	NAmer	1984	CC	1986	n	bl	n	n	2	cig+/-ot	40	99	3	0	nev cigs	or
OSANN	52	f	0	0	all	-	q	NAmer	1984	CC	1986	n	bl	n	n	2	cig+/-ot	1	39	0	0	nev cigs	or

Table 2G1 - 1

IESLC - Meta-analysis of Ever Smoking by Amount, Overview, Any product (or Cigarettes if Any not available)
Squamous
Most adjusted

REF	NRR	SEX	AGEL	AGEH	RACE	YF	LC TYPE	LOC	START	ST	NLC	R	VB	P	H	AD	PRODUCT	exL	exH	S1	S2	DENOM	De
OSANN	60	f	0	0	all	-	q	NAmer	1984	CC	1986	n	bl	n	n	2	cig+/-ot	40	99	3	0	nev cigs	or
OSANN2	28	f	0	0	all	-	KI	NAmer	1964	ot	217	n	bl	n	y	1	cig+/-ot	1	19	1	0	nev cigs	or
OSANN2	29	f	0	0	all	-	KI	NAmer	1964	ot	217	n	bl	n	y	1	cig+/-ot	20	99	0	0	nev cigs	or
WUWILL	14	f	0	0	all	-	q+s	As:Chi	1985	CC	965	n	ot	n	n	3	cig+/-ot	1	19	1	0	nev cigs	ot
WUWILL	15	f	0	0	all	-	q+s	As:Chi	1985	CC	965	n	ot	n	n	3	cig+/-ot	20	99	0	0	nev cigs	ot
WYNDE2	3	m	0	0	all	-	KI	NAmer	1962	CC	404	n	bl	n	y	0	cig+/-ot	1	10	1	0	nev any	st
WYNDE2	4	m	0	0	all	-	KI	NAmer	1962	CC	404	n	bl	n	y	0	cig+/-ot	11	20	2	3	nev any	st
WYNDE2	5	m	0	0	all	-	KI	NAmer	1962	CC	404	n	bl	n	y	0	cig+/-ot	21	34	0	4	nev any	st
WYNDE2	6	m	0	0	all	-	KI	NAmer	1962	CC	404	n	bl	n	y	0	cig+/-ot	35	99	3	0	nev any	st
WYNDE3	4	m	0	0	all	-	KI	NAmer	1966	CC	350	n	bl	n	y	0	cig+/-ot	1	9	1	1	nev any	st
WYNDE3	5	m	0	0	all	-	KI	NAmer	1966	CC	350	n	bl	n	y	0	cig+/-ot	10	20	2	0	nev any	st
WYNDE3	6	m	0	0	all	-	KI	NAmer	1966	CC	350	n	bl	n	y	0	cig+/-ot	21	40	0	0	nev any	st
WYNDE3	7	m	0	0	all	-	KI	NAmer	1966	CC	350	n	bl	n	y	0	cig+/-ot	41	99	3	6	nev any	st
WYNDE3	63	f	0	0	all	-	KI	NAmer	1966	CC	350	n	bl	n	y	0	cig+/-ot	1	9	1	1	nev any	st
WYNDE3	64	f	0	0	all	-	KI	NAmer	1966	CC	350	n	bl	n	y	0	cig+/-ot	10	20	2	0	nev any	st
WYNDE3	65	f	0	0	all	-	KI	NAmer	1966	CC	350	n	bl	n	y	0	cig+/-ot	21	40	0	0	nev any	st
WYNDE3	66	f	0	0	all	-	KI	NAmer	1966	CC	350	n	bl	n	y	0	cig+/-ot	41	99	3	6	nev any	st
WYNDE4	63	m	0	0	all	-	not a	NAmer	1948	CC	684	n	bl	y	n	2	all/unsp	1	9	1	1	nev any	ot
WYNDE4	64	m	0	0	all	-	not a	NAmer	1948	CC	684	n	bl	y	n	2	all/unsp	10	15	0	2	nev any	ot
WYNDE4	65	m	0	0	all	-	not a	NAmer	1948	CC	684	n	bl	y	n	2	all/unsp	16	20	2	3	nev any	ot
WYNDE4	66	m	0	0	all	-	not a	NAmer	1948	CC	684	n	bl	y	n	2	all/unsp	21	34	0	4	nev any	ot
WYNDE4	67	m	0	0	all	-	not a	NAmer	1948	CC	684	n	bl	y	n	2	all/unsp	35	99	3	0	nev any	ot
WYNDE4	49	f	0	0	all	-	not a	NAmer	1948	CC	684	n	bl	y	n	2	all/unsp	1	9	1	1	nev any	ot
WYNDE4	50	f	0	0	all	-	not a	NAmer	1948	CC	684	n	bl	y	n	2	all/unsp	10	15	0	2	nev any	ot
WYNDE4	51	f	0	0	all	-	not a	NAmer	1948	CC	684	n	bl	y	n	2	all/unsp	16	20	2	3	nev any	ot
WYNDE4	52	f	0	0	all	-	not a	NAmer	1948	CC	684	n	bl	y	n	2	all/unsp	21	34	0	4	nev any	ot
WYNDE4	53	f	0	0	all	-	not a	NAmer	1948	CC	684	n	bl	y	n	2	all/unsp	35	99	3	0	nev any	ot
ZHENG	1	m	0	0	all	-	q	As:Chi	1982	CC	540	n	ot	*	y	0	cig+/-ot	1	9	1	1	nev cigs	st
ZHENG	2	m	0	0	all	-	q	As:Chi	1982	CC	540	n	ot	*	y	0	cig+/-ot	10	19	0	2	nev cigs	st
ZHENG	3	m	0	0	all	-	q	As:Chi	1982	CC	540	n	ot	*	y	0	cig+/-ot	20	29	2	3	nev cigs	st
ZHENG	4	m	0	0	all	-	q	As:Chi	1982	CC	540	n	ot	*	y	0	cig+/-ot	30	99	3	0	nev cigs	st
ZHENG	16	f	0	0	all	-	q	As:Chi	1982	CC	540	n	ot	*	y	0	cig+/-ot	1	9	1	1	nev cigs	st
ZHENG	17	f	0	0	all	-	q	As:Chi	1982	CC	540	n	ot	*	y	0	cig+/-ot	10	99	0	0	nev cigs	st
ZHOU	10	c	0	0	all	-	q	As:Chi	1978	CC	1360	n	ot	n	n	0	all/unsp	1	9	1	1	nev any	st
ZHOU	11	c	0	0	all	-	q	As:Chi	1978	CC	1360	n	ot	n	n	0	all/unsp	10	19	0	2	nev any	st
ZHOU	12	c	0	0	all	-	q	As:Chi	1978	CC	1360	n	ot	n	n	0	all/unsp	20	99	0	0	nev any	st

Cigarette type is all/unspec for all RRs

except for the following:

REF	NRR	CIGTYPE
ALDERS	34	MC only
ALDERS	35	MC only
ALDERS	36	MC only
ALDERS	37	MC only
ALDERS	38	MC only
ALDERS	39	MC only

REF	NRR	Cigarette equivalent
ALDERS	34	-
ALDERS	35	-
ALDERS	36	-
ALDERS	37	-
ALDERS	38	-
ALDERS	39	-
BARBON	70	*
BARBON	71	*
BARBON	72	*
BROWN2	36	*
BROWN2	46	*
BROWN2	35	*
BROWN2	45	*
CHOI	46	*
CHOI	47	*
CHOI	48	*
CHOI	49	*
CHOI	50	*
CHOI	56	*
CHOI	57	*

Table 2G1 - 1

IESLC - Meta-analysis of Ever Smoking by Amount, Overview, Any product (or Cigarettes if Any not available)
Squamous
Most adjusted

REF	NRR	Cigarette equivalent
CHOI	58	*
DOLL	68	grams
DOLL	69	grams
DOLL	70	grams
DOLL	71	grams
DOLL	76	grams
DOLL	77	grams
DOLL	78	grams
DORGAN	114	*
DORGAN	115	*
DORGAN	99	*
DORGAN	100	*
DOSEME	7	*
DOSEME	11	*
DOSEME	15	*
GER	14	*
GER	15	*
GER	16	*
JEDRYC	28	*
JEDRYC	29	*
JEDRYC	30	*
KREYBE	1	grams inc 1 cig=1
KREYBE	2	grams inc 1 cig=1
KREYBE	3	grams inc 1 cig=1
KREYBE	31	grams inc 1 cig=1
KREYBE	32	grams inc 1 cig=1
LAMTH	10	*
LAMTH	11	*
LAMTH	12	*
LUBIN2	149	*
LUBIN2	153	*
LUBIN2	157	*
LUBIN2	161	*
LUBIN2	169	*
LUBIN2	173	*
LUBIN2	177	*
LUBIN2	181	*
LUO	10	*
LUO	11	*
LUO	12	*
MATOS	43	*
MATOS	45	*
MATOS	47	*
MATSUD	4	*
MATSUD	5	*
MATSUD	6	*
ORMOS	5	*
ORMOS	6	*
ORMOS	7	*
OSANN	51	*
OSANN	59	*
OSANN	52	*
OSANN	60	*
OSANN2	28	*
OSANN2	29	*
WUWILL	14	*
WUWILL	15	*
WYNDE2	3	*
WYNDE2	4	*
WYNDE2	5	*
WYNDE2	6	*
WYNDE3	4	*
WYNDE3	5	*
WYNDE3	6	*
WYNDE3	7	*
WYNDE3	63	*
WYNDE3	64	*
WYNDE3	65	*
WYNDE3	66	*
WYNDE4	63	*
WYNDE4	64	*
WYNDE4	65	*

Table 2G1 - 1

IESLC - Meta-analysis of Ever Smoking by Amount, Overview, Any product (or Cigarettes if Any not available)
Squamous
Most adjusted

REF	NRR	Cigarette equivalent
WYNDE4	66	*
WYNDE4	67	*
WYNDE4	49	inc 1 cigar = 5 cigs, 1 pipe = 2.5 cigs
WYNDE4	50	inc 1 cigar = 5 cigs, 1 pipe = 2.5 cigs
WYNDE4	51	inc 1 cigar = 5 cigs, 1 pipe = 2.5 cigs
WYNDE4	52	inc 1 cigar = 5 cigs, 1 pipe = 2.5 cigs
WYNDE4	53	inc 1 cigar = 5 cigs, 1 pipe = 2.5 cigs
ZHENG	1	*
ZHENG	2	*
ZHENG	3	*
ZHENG	4	*
ZHENG	16	*
ZHENG	17	*
ZHOU	10	*
ZHOU	11	*
ZHOU	12	*

In this overview table, subtotals and Qs values may be invalid and should be ignored

Table 2G1 - 2

IESLC - Meta-analysis of Ever Smoking by Amount, Overview, Any product (or Cigarettes if Any not available)
Squamous
Most adjusted

REF	NRR	SEX	AD	Number Exposed Case	Number Exposed Cont	Non-exposed Case	Non-exposed Cont	RR	95.00%CI
ALDERS	34	m	1	-	-	-	-	3.79	(1.30- 11.02)
ALDERS	35	m	1	-	-	-	-	7.19	(2.75- 18.79)
ALDERS	36	m	1	-	-	-	-	8.78	(3.46- 22.31)
ALDERS	37	f	1	-	-	-	-	2.55	(1.42- 4.57)
ALDERS	38	f	1	-	-	-	-	9.24	(5.31- 16.09)
ALDERS	39	f	1	-	-	-	-	14.52	(7.93- 26.58)
Subtotal ALDERS								6.82	(5.12- 9.11)
BARBON	70	m	3	-	-	-	-	8.50	(3.60- 20.00)
BARBON	71	m	3	-	-	-	-	16.30	(7.00- 38.00)
BARBON	72	m	3	-	-	-	-	28.60	(12.00- 69.00)
Subtotal BARBON								15.71	(9.57- 25.79)
BROWN2	36	m	2	-	-	-	-	7.60	(6.20- 9.40)
BROWN2	46	m	2	-	-	-	-	17.20	(14.60- 20.30)
BROWN2	35	f	2	-	-	-	-	11.70	(8.70- 15.80)
BROWN2	45	f	2	-	-	-	-	26.10	(20.70- 32.80)
Subtotal BROWN2								14.51	(13.06- 16.12)
CHOI	46	m	0	12	90	6	95	2.11	(0.76- 5.86)
CHOI	47	m	0	84	281	6	95	4.73	(2.00- 11.19)
CHOI	48	m	0	30	49	6	95	9.69	(3.78- 24.86)
CHOI	49	m	0	25	39	6	95	10.15	(3.86- 26.66)
CHOI	50	m	0	9	6	6	95	23.75	(6.33- 89.09)
CHOI	56	f	0	4	16	10	164	4.10	(1.15- 14.57)
CHOI	57	f	0	5	9	10	164	9.11	(2.57- 32.31)
CHOI	58	f	0	2	1	10	164	32.80	(2.74- 393.20)
Subtotal CHOI								6.86	(4.63- 10.16)
DOLL	68	m	1	-	-	-	-	4.70	(1.38- 16.03)
DOLL	69	m	1	-	-	-	-	10.60	(3.30- 34.07)
DOLL	70	m	1	-	-	-	-	14.30	(4.45- 46.00)
DOLL	71	m	1	-	-	-	-	25.40	(7.83- 82.40)
DOLL	76	f	1	-	-	-	-	1.00	(0.38- 2.64)
DOLL	77	f	1	-	-	-	-	1.70	(0.64- 4.50)
DOLL	78	f	1	-	-	-	-	8.30	(2.77- 24.84)
Subtotal DOLL								5.02	(3.32- 7.61)
DORGAN	114	m	2	-	-	-	-	11.50	(4.10- 32.24)
DORGAN	115	m	2	-	-	-	-	23.29	(8.55- 63.49)
DORGAN	99	f	3	-	-	-	-	7.78	(4.86- 12.44)
DORGAN	100	f	3	-	-	-	-	16.38	(10.22- 26.26)
Subtotal DORGAN								12.06	(8.92- 16.31)
DOSEME	7	m	2	-	-	-	-	2.60	(1.50- 4.60)
DOSEME	11	m	2	-	-	-	-	3.20	(2.20- 4.60)
DOSEME	15	m	2	-	-	-	-	7.00	(4.10- 12.00)
Subtotal DOSEME								3.71	(2.84- 4.84)
GER	14	c	10	-	-	-	-	1.43	(0.36- 5.61)
GER	15	c	10	-	-	-	-	2.20	(0.65- 7.48)
GER	16	c	10	-	-	-	-	16.04	(4.22- 60.93)
Subtotal GER								3.64	(1.71- 7.73)
JEDRYC	28	m	3	-	-	-	-	7.51	(3.09- 18.27)
JEDRYC	29	m	3	-	-	-	-	13.46	(5.76- 31.47)
JEDRYC	30	m	3	-	-	-	-	21.42	(9.05- 50.68)
Subtotal JEDRYC								13.09	(7.94- 21.57)
KREYBE	1	m	1	-	-	-	-	9.00	(2.85- 28.38)
KREYBE	2	m	1	-	-	-	-	11.02	(3.42- 35.51)
KREYBE	3	m	1	-	-	-	-	24.63	(7.54- 80.53)
KREYBE	31	f	0	1	286	3	657	0.77	(0.08- 7.39)
KREYBE	32	f	0	1	42	3	657	5.21	(0.53- 51.21)
Subtotal KREYBE								10.04	(5.39- 18.69)
LAMTH	10	f	0	23	11	28	72	5.38	(2.32- 12.46)
LAMTH	11	f	0	28	6	28	72	12.00	(4.49- 32.10)
LAMTH	12	f	0	10	1	28	72	25.71	(3.14- 210.29)
Subtotal LAMTH								8.37	(4.54- 15.43)
LUBIN2	149	m	0	418	2194	54	2616	9.23	(6.91- 12.32)
LUBIN2	153	m	0	1022	3385	54	2616	14.63	(11.07- 19.32)
LUBIN2	157	m	0	1298	3108	54	2616	20.23	(15.33- 26.69)
LUBIN2	161	m	0	849	1746	54	2616	23.56	(17.77- 31.22)
LUBIN2	169	f	0	30	184	72	1180	2.67	(1.70- 4.20)
LUBIN2	173	f	0	91	234	72	1180	6.37	(4.54- 8.95)
LUBIN2	177	f	0	61	110	72	1180	9.09	(6.13- 13.46)
LUBIN2	181	f	0	18	39	72	1180	7.56	(4.12- 13.88)
Subtotal LUBIN2								11.82	(10.51- 13.28)
LUO	10	c	20	-	-	-	-	1.20	(0.10- 10.00)
LUO	11	c	20	-	-	-	-	24.60	(4.20- 145.70)

Table 2G1 - 2

IESLC - Meta-analysis of Ever Smoking by Amount, Overview, Any product (or Cigarettes if Any not available)
Squamous
Most adjusted

REF	NRR	SEX	AD	Number Exposed Case	Number Exposed Cont	Non-exposed Case	Non-exposed Cont	RR	95.00%CI
LUO	12	c	20	-	-	-	-	38.70	(5.20- 290.20)
Subtotal LUO								13.41	(4.24- 42.41)
MATOS	43	m	2	-	-	-	-	1.40	(0.30- 6.90)
MATOS	45	m	2	-	-	-	-	7.80	(2.20- 27.40)
MATOS	47	m	2	-	-	-	-	9.70	(2.80- 33.20)
Subtotal MATOS								5.61	(2.60- 12.11)
MATSUD	4	m	0	21	1237	1	1255	21.31	(2.86- 158.63)
MATSUD	5	m	0	43	1607	1	1255	33.58	(4.62- 244.19)
MATSUD	6	m	0	39	470	1	1255	104.14	(14.27- 760.12)
Subtotal MATSUD								42.26	(13.37- 133.55)
ORMOS	5	m	0	13	329	2	777	15.35	(3.44- 68.41)
ORMOS	6	m	0	10	577	2	777	6.73	(1.47- 30.85)
ORMOS	7	m	0	4	128	2	777	12.14	(2.20- 66.97)
Subtotal ORMOS								10.74	(4.35- 26.54)
OSANN	51	m	2	-	-	-	-	35.30	(17.00- 73.30)
OSANN	59	m	2	-	-	-	-	76.00	(36.80- 157.00)
OSANN	52	f	2	-	-	-	-	24.00	(12.70- 45.50)
OSANN	60	f	2	-	-	-	-	72.30	(36.80- 142.00)
Subtotal OSANN								45.20	(32.03- 63.79)
OSANN2	28	f	1	-	-	-	-	12.10	(1.50- 96.30)
OSANN2	29	f	1	-	-	-	-	71.20	(8.30- 609.00)
Subtotal OSANN2								28.54	(6.40- 127.22)
WUWILL	14	f	3	-	-	-	-	3.21	(2.39- 4.30)
WUWILL	15	f	3	-	-	-	-	5.08	(3.07- 8.39)
Subtotal WUWILL								3.61	(2.80- 4.65)
WYNDE2	3	m	0	15	114	3	105	4.61	(1.30- 16.36)
WYNDE2	4	m	0	108	203	3	105	18.62	(5.77- 60.06)
WYNDE2	5	m	0	74	83	3	105	31.20	(9.50- 102.54)
WYNDE2	6	m	0	139	112	3	105	43.44	(13.42- 140.56)
Subtotal WYNDE2								19.37	(10.64- 35.27)
WYNDE3	4	m	0	7	42	3	88	4.89	(1.20- 19.86)
WYNDE3	5	m	0	57	114	3	88	14.67	(4.44- 48.40)
WYNDE3	6	m	0	74	82	3	88	26.47	(8.03- 87.26)
WYNDE3	7	m	0	59	26	3	88	66.56	(19.27- 229.96)
WYNDE3	63	f	0	1	19	5	76	0.80	(0.09- 7.26)
WYNDE3	64	f	0	13	24	5	76	8.23	(2.66- 25.46)
WYNDE3	65	f	0	8	10	5	76	12.16	(3.32- 44.50)
WYNDE3	66	f	0	3	3	5	76	15.20	(2.42- 95.56)
Subtotal WYNDE3								13.67	(8.51- 21.97)
WYNDE4	63	m	2	-	-	-	-	2.22	(0.89- 5.53)
WYNDE4	64	m	2	-	-	-	-	5.86	(2.70- 12.74)
WYNDE4	65	m	2	-	-	-	-	10.92	(5.22- 22.86)
WYNDE4	66	m	2	-	-	-	-	29.52	(13.81- 63.11)
WYNDE4	67	m	2	-	-	-	-	29.54	(13.53- 64.50)
WYNDE4	49	f	2	-	-	-	-	0.87	(0.11- 6.90)
WYNDE4	50	f	2	-	-	-	-	4.61	(1.38- 15.41)
WYNDE4	51	f	2	-	-	-	-	14.92	(4.88- 45.67)
WYNDE4	52	f	2	-	-	-	-	26.53	(4.12- 171.09)
WYNDE4	53	f	2	-	-	-	-	26.53	(4.12- 171.09)
Subtotal WYNDE4								10.91	(8.00- 14.88)
ZHENG	1	m	0	7	40	4	94	4.11	(1.14- 14.84)
ZHENG	2	m	0	25	66	4	94	8.90	(2.96- 26.78)
ZHENG	3	m	0	75	89	4	94	19.80	(6.95- 56.41)
ZHENG	4	m	0	49	23	4	94	50.07	(16.39- 152.91)
ZHENG	16	f	0	11	29	33	184	2.11	(0.96- 4.64)
ZHENG	17	f	0	32	15	33	184	11.89	(5.81- 24.35)
Subtotal ZHENG								8.76	(5.95- 12.88)
ZHOU	10	c	0	15	5	138	68	1.48	(0.52- 4.24)
ZHOU	11	c	0	78	14	138	68	2.75	(1.45- 5.20)
ZHOU	12	c	0	285	29	138	68	4.84	(3.00- 7.82)
Subtotal ZHOU								3.52	(2.45- 5.04)
Partial Totals				5286	17327	1203	25801		

*prospective study

Table 2G1 - 2

IESLC - Meta-analysis of Ever Smoking by Amount, Overview, Any product (or Cigarettes if Any not available)
Squamous
Most adjusted

REF	NRR	SEX	AD	Ys	Ws	Qs	Ps
ALDERS	34	m	1	1.33	3.36	3.55	0.0145
ALDERS	35	m	1	1.97	4.16	0.62	0.0001
ALDERS	36	m	1	2.17	4.42	0.15	0.0000
ALDERS	37	f	1	0.94	11.25	22.79	0.0017
ALDERS	38	f	1	2.22	12.50	0.23	0.0000
ALDERS	39	f	1	2.68	10.50	1.05	0.0000
Subtotal ALDERS				1.92	46.20	28.40	
BARBON	70	m	3	2.14	5.23	0.25	0.0000
BARBON	71	m	3	2.79	5.37	1.00	0.0000
BARBON	72	m	3	3.35	5.02	4.96	0.0000
Subtotal BARBON				2.75	15.62	6.21	
BROWN2	36	m	2	2.03	88.72	9.75	0.0000
BROWN2	46	m	2	2.84	141.44	33.32	0.0000
BROWN2	35	f	2	2.46	43.16	0.43	0.0000
BROWN2	45	f	2	3.26	72.52	59.05	0.0000
Subtotal BROWN2				2.67	345.85	102.55	
CHOI	46	m	0	0.75	3.68	9.57	0.1517
CHOI	47	m	0	1.55	5.19	3.36	0.0004
CHOI	48	m	0	2.27	4.33	0.03	0.0000
CHOI	49	m	0	2.32	4.12	0.01	0.0000
CHOI	50	m	0	3.17	2.20	1.44	0.0000
CHOI	56	f	0	1.41	2.39	2.15	0.0292
CHOI	57	f	0	2.21	2.40	0.05	0.0006
CHOI	58	f	0	3.49	0.62	0.80	0.0059
Subtotal CHOI				1.93	24.93	17.41	
DOLL	68	m	1	1.55	2.55	1.68	0.0134
DOLL	69	m	1	2.36	2.82	0.00	0.0001
DOLL	70	m	1	2.66	2.82	0.25	0.0000
DOLL	71	m	1	3.23	2.77	2.12	0.0000
DOLL	76	f	1	0.00	4.09	22.77	1.0000
DOLL	77	f	1	0.53	4.04	13.51	0.2862
DOLL	78	f	1	2.12	3.19	0.19	0.0002
Subtotal DOLL				1.61	22.29	40.53	
DORGAN	114	m	2	2.44	3.61	0.02	0.0000
DORGAN	115	m	2	3.15	3.82	2.38	0.0000
DORGAN	99	f	3	2.05	17.39	1.65	0.0000
DORGAN	100	f	3	2.80	17.25	3.29	0.0000
Subtotal DORGAN				2.49	42.08	7.34	
DOSEME	7	m	2	0.96	12.24	24.12	0.0008
DOSEME	11	m	2	1.16	28.24	40.43	0.0000
DOSEME	15	m	2	1.95	13.32	2.28	0.0000
Subtotal DOSEME				1.31	53.80	66.83	
GER	14	c	10	0.36	2.04	8.17	0.6097
GER	15	c	10	0.79	2.57	6.36	0.2058
GER	16	c	10	2.78	2.16	0.37	0.0000
Subtotal GER				1.29	6.77	14.89	
JEDRYC	28	m	3	2.02	4.87	0.57	0.0000
JEDRYC	29	m	3	2.60	5.33	0.31	0.0000
JEDRYC	30	m	3	3.06	5.18	2.57	0.0000
Subtotal JEDRYC				2.57	15.37	3.45	
KREYBE	1	m	1	2.20	2.91	0.08	0.0002
KREYBE	2	m	1	2.40	2.81	0.00	0.0001
KREYBE	3	m	1	3.20	2.74	1.95	0.0000
KREYBE	31	f	0	-0.27	0.75	5.15	0.8175
KREYBE	32	f	0	1.65	0.74	0.37	0.1566
Subtotal KREYBE				2.31	9.94	7.56	
LAMTH	10	f	0	1.68	5.44	2.49	0.0001
LAMTH	11	f	0	2.48	3.97	0.06	0.0000
LAMTH	12	f	0	3.25	0.87	0.69	0.0025
Subtotal LAMTH				2.12	10.27	3.24	
LUBIN2	149	m	0	2.22	45.98	0.86	0.0000
LUBIN2	153	m	0	2.68	49.57	5.18	0.0000
LUBIN2	157	m	0	3.01	50.02	20.98	0.0000
LUBIN2	161	m	0	3.16	48.42	30.98	0.0000
LUBIN2	169	f	0	0.98	18.69	35.42	0.0000
LUBIN2	173	f	0	1.85	33.33	8.58	0.0000
LUBIN2	177	f	0	2.21	24.86	0.58	0.0000
LUBIN2	181	f	0	2.02	10.42	1.18	0.0000
Subtotal LUBIN2				2.47	281.30	103.77	
LUO	10	c	20	0.18	0.72	3.43	0.8767
LUO	11	c	20	3.20	1.22	0.87	0.0004

Table 2G1 - 2

IESLC - Meta-analysis of Ever Smoking by Amount, Overview, Any product (or Cigarettes if Any not available)
Squamous
Most adjusted

REF	NRR	SEX	AD	Ys	Ws	Qs	Ps
LUO	12	c	20	3.66	0.95	1.60	0.0004
Subtotal LUO				2.60	2.90	5.90	
MATOS	43	m	2	0.34	1.56	6.40	0.6740
MATOS	45	m	2	2.05	2.42	0.23	0.0014
MATOS	47	m	2	2.27	2.51	0.02	0.0003
Subtotal MATOS				1.72	6.49	6.64	
MATSUD	4	m	0	3.06	0.95	0.47	0.0028
MATSUD	5	m	0	3.51	0.98	1.30	0.0005
MATSUD	6	m	0	4.65	0.97	5.08	0.0000
Subtotal MATSUD				3.74	2.90	6.85	
ORMOS	5	m	0	2.73	1.72	0.24	0.0003
ORMOS	6	m	0	1.91	1.66	0.34	0.0141
ORMOS	7	m	0	2.50	1.32	0.02	0.0042
Subtotal ORMOS				2.37	4.70	0.60	
OSANN	51	m	2	3.56	7.20	10.44	0.0000
OSANN	59	m	2	4.33	7.30	28.37	0.0000
OSANN	52	f	2	3.18	9.44	6.32	0.0000
OSANN	60	f	2	4.28	8.43	31.11	0.0000
Subtotal OSANN				3.81	32.36	76.23	
OSANN2	28	f	1	2.49	0.89	0.02	0.0189
OSANN2	29	f	1	4.27	0.83	3.03	0.0001
Subtotal OSANN2				3.35	1.72	3.04	
WUWILL	14	f	3	1.17	44.55	63.43	0.0000
WUWILL	15	f	3	1.63	15.20	8.20	0.0000
Subtotal WUWILL				1.28	59.75	71.63	
WYNDE2	3	m	0	1.53	2.39	1.66	0.0182
WYNDE2	4	m	0	2.92	2.80	0.89	0.0000
WYNDE2	5	m	0	3.44	2.71	3.17	0.0000
WYNDE2	6	m	0	3.77	2.79	5.55	0.0000
Subtotal WYNDE2				2.96	10.69	11.27	
WYNDE3	4	m	0	1.59	1.96	1.17	0.0265
WYNDE3	5	m	0	2.69	2.70	0.29	0.0000
WYNDE3	6	m	0	3.28	2.70	2.27	0.0000
WYNDE3	7	m	0	4.20	2.50	8.45	0.0000
WYNDE3	63	f	0	-0.22	0.79	5.27	0.8428
WYNDE3	64	f	0	2.11	3.01	0.19	0.0003
WYNDE3	65	f	0	2.50	2.28	0.04	0.0002
WYNDE3	66	f	0	2.72	1.14	0.15	0.0037
Subtotal WYNDE3				2.62	17.07	17.82	
WYNDE4	63	m	2	0.80	4.60	11.24	0.0870
WYNDE4	64	m	2	1.77	6.38	2.23	0.0000
WYNDE4	65	m	2	2.39	7.04	0.01	0.0000
WYNDE4	66	m	2	3.39	6.66	7.00	0.0000
WYNDE4	67	m	2	3.39	6.30	6.63	0.0000
WYNDE4	49	f	2	-0.14	0.90	5.60	0.8951
WYNDE4	50	f	2	1.53	2.64	1.82	0.0130
WYNDE4	51	f	2	2.70	3.07	0.36	0.0000
WYNDE4	52	f	2	3.28	1.11	0.93	0.0006
WYNDE4	53	f	2	3.28	1.11	0.93	0.0006
Subtotal WYNDE4				2.39	39.81	36.76	
ZHENG	1	m	0	1.41	2.33	2.09	0.0308
ZHENG	2	m	0	2.19	3.17	0.10	0.0001
ZHENG	3	m	0	2.99	3.51	1.38	0.0000
ZHENG	4	m	0	3.91	3.08	7.44	0.0000
ZHENG	16	f	0	0.75	6.21	16.10	0.0620
ZHENG	17	f	0	2.48	7.48	0.10	0.0000
Subtotal ZHENG				2.17	25.78	27.20	
ZHOU	10	c	0	0.39	3.46	13.43	0.4669
ZHOU	11	c	0	1.01	9.42	17.15	0.0019
ZHOU	12	c	0	1.58	16.68	10.20	0.0000
Subtotal ZHOU				1.26	29.56	40.79	

N	108
NS	24

Table 2G1 - 3

IESLC - Meta-analysis of Ever Smoking by Amount, Overview, Any product (or Cigarettes if Any not available)
Squamous
Most adjusted

	combined	Sex male	female	Total
N	9	60	39	108
NS	3	18	14	35

In this overview table, other than the "N" rows, entries in the "absent" and "Total" columns may be invalid and should be ignored

	Amount smoked (broad categories) absent	<20k5	6-44k20	>20k45	Total
N	27	33	23	25	108
NS	14	23	18	19	74
Wt	430.86	351.59	178.64	147.05	1108.14
Het Chi	176.91	136.03	79.88	71.00	706.91
Het df	26	32	22	24	107
Het P	***	***	***	***	***
Fixed RR	13.96	5.70	10.23	21.61	10.59
RRl	12.70	5.13	8.83	18.39	9.98
RRu	15.34	6.32	11.84	25.40	11.23
P	+++	+++	+++	+++	+++
Random RR	11.01	4.30	10.20	23.54	9.61
RRl	8.17	3.29	7.37	16.96	8.08
RRu	14.83	5.62	14.13	32.68	11.44
P	+++	+++	+++	+++	+++

	absent	<10k1	2-19k10	Amount smoked (narrow categories) 11-29k20	21-39k30	31-98k40	>40k99	Total
N	63	11	8	18	4	1	3	108
NS	24	6	5	15	3	1	2	55
Wt	716.94	91.57	111.37	163.51	14.81	4.12	5.83	1108.14
Het Chi	431.04	52.93	41.79	77.88	3.84	0.00	2.15	706.91
Het df	62	10	7	17	3	0	2	107
Het P	***	***	***	***	N.S.	N.S.	N.S.	***
Fixed RR	12.07	4.63	8.27	10.11	21.36	10.15	33.86	10.59
RRl	11.21	3.77	6.87	8.67	12.84	3.86	15.04	9.98
RRu	12.98	5.68	9.96	11.79	35.55	26.66	76.22	11.23
P	+++	+++	+++	+++	+++	+++	+++	+++
Random RR	11.55	2.62	5.85	10.10	21.22	10.15	33.54	9.61
RRl	9.19	1.45	3.39	6.89	11.64	3.86	14.41	8.08
RRu	14.51	4.73	10.09	14.80	38.68	26.66	78.06	11.44
P	+++	++	+++	+++	+++	+++	+++	+++

MALES

	Amount smoked (broad categories) absent	<20k5	6-44k20	>20k45	Total
N	12	17	15	16	60
NS	10	17	15	16	58
Wt	234.65	188.94	125.03	110.85	659.47
Het Chi	23.78	38.13	71.36	44.52	301.75
Het df	11	16	14	15	59
Het P	*	**	***	***	***
Fixed RR	16.32	6.93	10.75	22.57	12.46
RRl	14.36	6.01	9.02	18.73	11.54
RRu	18.55	7.99	12.81	27.18	13.45
P	+++	+++	+++	+++	+++
Random RR	15.54	5.77	10.96	24.76	12.13
RRl	11.75	4.26	6.85	16.86	9.88
RRu	20.55	7.80	17.52	36.36	14.90
P	+++	+++	+++	+++	+++

Table 2G1 - 3

IESLC - Meta-analysis of Ever Smoking by Amount, Overview, Any product (or Cigarettes if Any not available)
Squamous
Most adjusted

MALES

	Amount smoked (narrow categories)							
	absent	<10k1	2-19k10	11-29k20	21-39k30	31-98k40	>40k99	Total
N	33	5	4	12	3	1	2	60
NS	18	5	4	12	3	1	2	44
Wt	402.28	57.43	61.94	115.31	13.70	4.12	4.70	659.47
Het Chi	179.99	10.56	5.30	69.76	3.79	0.00	1.24	301.75
Het df	32	4	3	11	2	0	1	59
Het P	***	*	N.S.	***	N.S.	N.S.	N.S.	***
Fixed RR	13.57	7.57	12.79	10.54	20.99	10.15	41.10	12.46
RRl	12.31	5.84	9.97	8.78	12.36	3.86	16.64	11.54
RRu	14.97	9.80	16.40	12.64	35.65	26.66	101.52	13.45
P	+++	+++	+++	+++	+++	+++	+++	+++
Random RR	13.68	4.94	10.51	10.71	20.62	10.15	40.83	12.13
RRl	10.23	2.53	6.45	6.18	9.72	3.86	14.89	9.88
RRu	18.27	9.64	17.12	18.55	43.74	26.66	111.95	14.90
P	+++	+++	+++	+++	+++	+++	+++	+++

FEMALES

	Amount smoked (broad categories)				
	absent	<20k5	6-44k20	>20k45	Total
N	13	13	6	7	39
NS	11	13	6	7	37
Wt	170.11	156.43	49.82	33.09	409.45
Het Chi	103.69	73.50	0.96	25.09	314.71
Het df	12	12	5	6	38
Het P	***	***	N.S.	***	***
Fixed RR	13.65	4.75	9.56	18.75	8.96
RRl	11.75	4.06	7.25	13.33	8.13
RRu	15.87	5.55	12.63	26.36	9.87
P	+++	+++	+++	+++	+++
Random RR	9.96	3.48	9.56	21.10	7.79
RRl	5.80	2.17	7.25	9.14	5.66
RRu	17.11	5.58	12.63	48.71	10.73
P	+++	+++	+++	+++	+++

	Amount smoked (narrow categories)							
	absent	<10k1	2-19k10	11-29k20	21-39k30	31-98k40	>40k99	Total
N	25	5	3	4	1		1	39
NS	14	5	3	4	1		1	27
Wt	292.11	30.67	40.01	44.41	1.11		1.14	409.45
Het Chi	214.33	4.77	6.37	0.88	0.00		0.00	314.71
Het df	24	4	2	3	0		0	38
Het P	***	N.S.	*	N.S.	N.S.		N.S.	***
Fixed RR	10.97	2.10	5.46	9.69	26.53		15.20	8.96
RRl	9.78	1.47	4.00	7.22	4.12		2.42	8.13
RRu	12.30	2.99	7.44	13.00	170.96		95.56	9.87
P	+++	+++	+++	+++	+++		++	+++
Random RR	10.39	1.94	3.97	9.69	26.53		15.20	7.79
RRl	6.95	1.26	1.71	7.22	4.12		2.42	5.66
RRu	15.52	3.01	9.23	13.00	170.96		95.56	10.73
P	+++	++	++	+++	+++		++	+++

Table 2G1 - 4

IESLC - Meta-analysis of Ever Smoking by Amount, Overview, Any product (or Cigarettes if Any not available)
Squamous
Least adjusted

REF	NRR	X	SEX	AGEL	AGEH	RACE	YF	LC TYPE	LOC	START	ST	NLC	R	VB	P	H	AD	PRODUCT	exL	exH	S1	S2	DENOM	De
ALDERS	34		m	0	0	all	-	q+s	Eu:UK	1977	CC	1448	n	V	n	n	1	cig only	1	17	1	0	nev+2	ot
ALDERS	35		m	0	0	all	-	q+s	Eu:UK	1977	CC	1448	n	V	n	n	1	cig only	18	27	2	3	nev+2	ot
ALDERS	36		m	0	0	all	-	q+s	Eu:UK	1977	CC	1448	n	V	n	n	1	cig only	28	99	3	0	nev+2	ot
ALDERS	37		f	0	0	all	-	q+s	Eu:UK	1977	CC	1448	n	V	n	n	1	cig only	1	17	1	0	nev+2	ot
ALDERS	38		f	0	0	all	-	q+s	Eu:UK	1977	CC	1448	n	V	n	n	1	cig only	18	27	2	3	nev+2	ot
ALDERS	39		f	0	0	all	-	q+s	Eu:UK	1977	CC	1448	n	V	n	n	1	cig only	28	99	3	0	nev+2	ot
BARBON	19	x	m	0	0	all	-	q	Eu:wst	1979	CC	755	n	bl	y	y	0	all/unsp	1	9	1	1	nev any	st
BARBON	21	x	m	0	0	all	-	q	Eu:wst	1979	CC	755	n	bl	y	y	0	all/unsp	10	19	0	2	nev any	st
BARBON	23	x	m	0	0	all	-	q	Eu:wst	1979	CC	755	n	bl	y	y	0	all/unsp	20	29	2	3	nev any	st
BARBON	25	x	m	0	0	all	-	q	Eu:wst	1979	CC	755	n	bl	y	y	0	all/unsp	30	39	0	4	nev any	st
BARBON	27	x	m	0	0	all	-	q	Eu:wst	1979	CC	755	n	bl	y	y	0	all/unsp	40	99	3	0	nev any	st
BROWN2	36		m	0	0	wh	-	q	NAmer	1984	CC	14596	n	bl	n	y	2	cig+/-ot	1	19	1	0	nev cigs	or
BROWN2	46		m	0	0	wh	-	q	NAmer	1984	CC	14596	n	bl	n	y	2	cig+/-ot	20	99	0	0	nev cigs	or
BROWN2	35		f	0	0	wh	-	q	NAmer	1984	CC	14596	n	bl	n	y	2	cig+/-ot	1	19	1	0	nev cigs	or
BROWN2	45		f	0	0	wh	-	q	NAmer	1984	CC	14596	n	bl	n	y	2	cig+/-ot	20	99	0	0	nev cigs	or
CHOI	46		m	0	0	all	-	q	As:oth	1985	CC	375	n	bl	n	n	0	cig+/-ot	1	10	1	0	nev cigs	st
CHOI	47		m	0	0	all	-	q	As:oth	1985	CC	375	n	bl	n	n	0	cig+/-ot	11	20	2	3	nev cigs	st
CHOI	48		m	0	0	all	-	q	As:oth	1985	CC	375	n	bl	n	n	0	cig+/-ot	21	30	0	4	nev cigs	st
CHOI	49		m	0	0	all	-	q	As:oth	1985	CC	375	n	bl	n	n	0	cig+/-ot	31	40	0	5	nev cigs	st
CHOI	50		m	0	0	all	-	q	As:oth	1985	CC	375	n	bl	n	n	0	cig+/-ot	41	99	3	6	nev cigs	st
CHOI	56		f	0	0	all	-	q	As:oth	1985	CC	375	n	bl	n	n	0	cig+/-ot	1	10	1	0	nev cigs	st
CHOI	57		f	0	0	all	-	q	As:oth	1985	CC	375	n	bl	n	n	0	cig+/-ot	11	30	2	0	nev cigs	st
CHOI	58		f	0	0	all	-	q	As:oth	1985	CC	375	n	bl	n	n	0	cig+/-ot	31	99	3	0	nev cigs	st
DOLL	54	x	m	0	0	all	-	KI	Eu:UK	1948	CC	1465	n	V	n	n	0	all/unsp	1	4	0	1	nev any	st
DOLL	55	x	m	0	0	all	-	KI	Eu:UK	1948	CC	1465	n	V	n	n	0	all/unsp	5	14	1	2	nev any	st
DOLL	56	x	m	0	0	all	-	KI	Eu:UK	1948	CC	1465	n	V	n	n	0	all/unsp	15	24	2	3	nev any	st
DOLL	57	x	m	0	0	all	-	KI	Eu:UK	1948	CC	1465	n	V	n	n	0	all/unsp	25	99	3	0	nev any	st
DOLL	62	x	f	0	0	all	-	KI	Eu:UK	1948	CC	1465	n	V	n	n	0	all/unsp	1	4	0	1	nev any	st
DOLL	63	x	f	0	0	all	-	KI	Eu:UK	1948	CC	1465	n	V	n	n	0	all/unsp	5	14	1	2	nev any	st
DOLL	64	x	f	0	0	all	-	KI	Eu:UK	1948	CC	1465	n	V	n	n	0	all/unsp	15	99	0	0	nev any	st
DORGAN	114		m	0	0	wh	-	q	NAmer	1980	CC	2026	n	bl	y	y	2	cig+/-ot	1	19	1	0	nev any	ot
DORGAN	115		m	0	0	wh	-	q	NAmer	1980	CC	2026	n	bl	y	y	2	cig+/-ot	20	99	0	0	nev any	ot
DORGAN	99		f	0	0	all	-	q	NAmer	1980	CC	2026	n	bl	y	y	3	cig+/-ot	1	19	1	0	nev any	ot
DORGAN	100		f	0	0	all	-	q	NAmer	1980	CC	2026	n	bl	y	y	3	cig+/-ot	20	99	0	0	nev any	ot
DOSEME	7		m	0	0	all	-	q	Eu:bal	1979	CC	1210	n	bl	n	n	2	cig+/-ot	1	10	1	0	nev cigs	or
DOSEME	11		m	0	0	all	-	q	Eu:bal	1979	CC	1210	n	bl	n	n	2	cig+/-ot	11	20	2	3	nev cigs	or
DOSEME	15		m	0	0	all	-	q	Eu:bal	1979	CC	1210	n	bl	n	n	2	cig+/-ot	21	99	3	0	nev cigs	or
GER	6	x	c	0	0	all	-	q+s	As:oth	1990	CC	141	n	ot	y	n	0	all/unsp	1	10	1	0	nev any	st
GER	7	x	c	0	0	all	-	q+s	As:oth	1990	CC	141	n	ot	y	n	0	all/unsp	11	20	2	3	nev any	st
GER	8	x	c	0	0	all	-	q+s	As:oth	1990	CC	141	n	ot	y	n	0	all/unsp	21	99	3	0	nev any	st
JEDRYC	1	x	m	0	0	all	-	q	Eu:est	1980	CC	1630	n	bl	y	n	0	cig+/-ot	1	9	1	1	nev any	st
JEDRYC	2	x	m	0	0	all	-	q	Eu:est	1980	CC	1630	n	bl	y	n	0	cig+/-ot	10	19	0	2	nev any	st
JEDRYC	3	x	m	0	0	all	-	q	Eu:est	1980	CC	1630	n	bl	y	n	0	cig+/-ot	20	29	2	3	nev any	st
JEDRYC	4	x	m	0	0	all	-	q	Eu:est	1980	CC	1630	n	bl	y	n	0	cig+/-ot	30	39	0	4	nev any	st
JEDRYC	5	x	m	0	0	all	-	q	Eu:est	1980	CC	1630	n	bl	y	n	0	cig+/-ot	40	99	3	0	nev any	st
KREYBE	13	x	m	0	0	all	-	KI	Eu:Sca	1948	CC	300	n	bl	n	y	0	all/unsp	1	14	1	0	nev any	st
KREYBE	14	x	m	0	0	all	-	KI	Eu:Sca	1948	CC	300	n	bl	n	y	0	all/unsp	15	24	2	3	nev any	st
KREYBE	15	x	m	0	0	all	-	KI	Eu:Sca	1948	CC	300	n	bl	n	y	0	all/unsp	25	99	3	0	nev any	st
KREYBE	31		f	0	0	all	-	KI	Eu:Sca	1948	CC	300	n	bl	n	y	0	all/unsp	1	14	1	0	nev any	st
KREYBE	32		f	0	0	all	-	KI	Eu:Sca	1948	CC	300	n	bl	n	y	0	all/unsp	15	99	0	0	nev any	st
LAMTH	10		f	0	0	ch	-	q	As:HK	1983	CC	445	n	bl	n	n	0	all/unsp	1	10	1	0	nev any	or
LAMTH	11		f	0	0	ch	-	q	As:HK	1983	CC	445	n	bl	n	n	0	all/unsp	11	20	2	3	nev any	or
LAMTH	12		f	0	0	ch	-	q	As:HK	1983	CC	445	n	bl	n	n	0	all/unsp	21	99	3	0	nev any	st
LUBIN2	149		m	0	0	all	-	q	Eu:mul	1976	CC	7804	n	bl	n	y	0	cig+/-ot	1	9	1	1	nev any	st
LUBIN2	153		m	0	0	all	-	q	Eu:mul	1976	CC	7804	n	bl	n	y	0	cig+/-ot	10	19	0	2	nev any	st
LUBIN2	157		m	0	0	all	-	q	Eu:mul	1976	CC	7804	n	bl	n	y	0	cig+/-ot	20	29	2	3	nev any	st
LUBIN2	161		m	0	0	all	-	q	Eu:mul	1976	CC	7804	n	bl	n	y	0	cig+/-ot	30	99	3	0	nev any	st
LUBIN2	169		f	0	0	all	-	q	Eu:mul	1976	CC	7804	n	bl	n	y	0	cig+/-ot	1	9	1	1	nev any	st
LUBIN2	173		f	0	0	all	-	q	Eu:mul	1976	CC	7804	n	bl	n	y	0	cig+/-ot	10	19	0	2	nev any	st
LUBIN2	177		f	0	0	all	-	q	Eu:mul	1976	CC	7804	n	bl	n	y	0	cig+/-ot	20	29	2	3	nev any	st
LUBIN2	181		f	0	0	all	-	q	Eu:mul	1976	CC	7804	n	bl	n	y	0	cig+/-ot	30	99	3	0	nev any	st
LUO	4	x	c	0	0	all	-	q	As:Chi	1990	CC	102	n	ot	n	y	0	cig+/-ot	1	19	1	0	nev cigs	st
LUO	5	x	c	0	0	all	-	q	As:Chi	1990	CC	102	n	ot	n	y	0	cig+/-ot	20	29	2	3	nev cigs	st
LUO	6	x	c	0	0	all	-	q	As:Chi	1990	CC	102	n	ot	n	y	0	cig+/-ot	30	99	3	0	nev cigs	st
MATOS	42	x	m	0	0	all	-	q	SCAmer	1994	CC	200	n	bl	n	n	0	cig+/-ot	1	14	1	0	nev any	st
MATOS	44	x	m	0	0	all	-	q	SCAmer	1994	CC	200	n	bl	n	n	0	cig+/-ot	15	24	2	3	nev any	st
MATOS	46	x	m	0	0	all	-	q	SCAmer	1994	CC	200	n	bl	n	n	0	cig+/-ot	25	99	3	0	nev any	st
MATSUD	4		m	0	0	all	-	q	As:Jap	1965	CC	179	n	bl	n	n	0	cig+/-ot	1	10	1	0	nev cigs	st
MATSUD	5		m	0	0	all	-	q	As:Jap	1965	CC	179	n	bl	n	n	0	cig+/-ot	11	20	2	3	nev cigs	st
MATSUD	6		m	0	0	all	-	q	As:Jap	1965	CC	179	n	bl	n	n	0	cig+/-ot	21	99	3	0	nev cigs	st
ORMOS	5		m	0	0	all	-	q	Eu:est	1947	CC	119	n	bl	y	y	0	cig+/-ot	1	15	1	0	nev any	st
ORMOS	6		m	0	0	all	-	q	Eu:est	1947	CC	119	n	bl	y	y	0	cig+/-ot	16	30	2	0	nev any	st

Table 2G1 - 4

IESLC - Meta-analysis of Ever Smoking by Amount, Overview, Any product (or Cigarettes if Any not available)
Squamous
Least adjusted

REF	NRR	X	SEX	AGEL	AGEH	RACE	YF	LC TYPE	LOC	START	ST	NLC	R	VB	P	H	AD	PRODUCT	exL	exH	S1	S2	DENOM	De
ORMOS	7		m	0	0	all	-	q	Eu:est	1947	CC	119	n	bl	y	y	0	cig+/-ot	31	99	3	0	nev any	st
OSANN	51		m	0	0	all	-	q	NAmer	1984	CC	1986	n	bl	n	n	2	cig+/-ot	1	39	0	0	nev cigs	or
OSANN	59		m	0	0	all	-	q	NAmer	1984	CC	1986	n	bl	n	n	2	cig+/-ot	40	99	3	0	nev cigs	or
OSANN	52		f	0	0	all	-	q	NAmer	1984	CC	1986	n	bl	n	n	2	cig+/-ot	1	39	0	0	nev cigs	or
OSANN	60		f	0	0	all	-	q	NAmer	1984	CC	1986	n	bl	n	n	2	cig+/-ot	40	99	3	0	nev cigs	or
OSANN2	10	x	f	0	0	all	-	KI	NAmer	1964	ot	217	n	bl	n	y	0	cig+/-ot	1	19	1	0	nev cigs	st
OSANN2	11	x	f	0	0	all	-	KI	NAmer	1964	ot	217	n	bl	n	y	0	cig+/-ot	20	99	0	0	nev cigs	st
WUWILL	20	x	f	0	0	all	-	q+s	As:Chi	1985	CC	965	n	ot	n	n	0	cig+/-ot	1	19	1	0	nev cigs	st
WUWILL	21	x	f	0	0	all	-	q+s	As:Chi	1985	CC	965	n	ot	n	n	0	cig+/-ot	20	99	0	0	nev cigs	st
WYNDE2	3		m	0	0	all	-	KI	NAmer	1962	CC	404	n	bl	n	y	0	cig+/-ot	1	10	1	0	nev any	st
WYNDE2	4		m	0	0	all	-	KI	NAmer	1962	CC	404	n	bl	n	y	0	cig+/-ot	11	20	2	3	nev any	st
WYNDE2	5		m	0	0	all	-	KI	NAmer	1962	CC	404	n	bl	n	y	0	cig+/-ot	21	34	0	4	nev any	st
WYNDE2	6		m	0	0	all	-	KI	NAmer	1962	CC	404	n	bl	n	y	0	cig+/-ot	35	99	3	0	nev any	st
WYNDE3	4		m	0	0	all	-	KI	NAmer	1966	CC	350	n	bl	n	y	0	cig+/-ot	1	9	1	1	nev any	st
WYNDE3	5		m	0	0	all	-	KI	NAmer	1966	CC	350	n	bl	n	y	0	cig+/-ot	10	20	2	0	nev any	st
WYNDE3	6		m	0	0	all	-	KI	NAmer	1966	CC	350	n	bl	n	y	0	cig+/-ot	21	40	0	0	nev any	st
WYNDE3	7		m	0	0	all	-	KI	NAmer	1966	CC	350	n	bl	n	y	0	cig+/-ot	41	99	3	6	nev any	st
WYNDE3	63		f	0	0	all	-	KI	NAmer	1966	CC	350	n	bl	n	y	0	cig+/-ot	1	9	1	1	nev any	st
WYNDE3	64		f	0	0	all	-	KI	NAmer	1966	CC	350	n	bl	n	y	0	cig+/-ot	10	20	2	0	nev any	st
WYNDE3	65		f	0	0	all	-	KI	NAmer	1966	CC	350	n	bl	n	y	0	cig+/-ot	21	40	0	0	nev any	st
WYNDE3	66		f	0	0	all	-	KI	NAmer	1966	CC	350	n	bl	n	y	0	cig+/-ot	41	99	3	6	nev any	st
WYNDE4	5	x	m	0	0	all	-	not a	NAmer	1948	CC	684	n	bl	y	n	0	all/unsp	1	9	1	1	nev any	st
WYNDE4	11	x	m	0	0	all	-	not a	NAmer	1948	CC	684	n	bl	y	n	0	all/unsp	10	15	0	2	nev any	st
WYNDE4	17	x	m	0	0	all	-	not a	NAmer	1948	CC	684	n	bl	y	n	0	all/unsp	16	20	2	3	nev any	st
WYNDE4	23	x	m	0	0	all	-	not a	NAmer	1948	CC	684	n	bl	y	n	0	all/unsp	21	34	0	4	nev any	st
WYNDE4	29	x	m	0	0	all	-	not a	NAmer	1948	CC	684	n	bl	y	n	0	all/unsp	35	99	3	0	nev any	st
WYNDE4	49		f	0	0	all	-	not a	NAmer	1948	CC	684	n	bl	y	n	2	all/unsp	1	9	1	1	nev any	ot
WYNDE4	50		f	0	0	all	-	not a	NAmer	1948	CC	684	n	bl	y	n	2	all/unsp	10	15	0	2	nev any	ot
WYNDE4	51		f	0	0	all	-	not a	NAmer	1948	CC	684	n	bl	y	n	2	all/unsp	16	20	2	3	nev any	ot
WYNDE4	52		f	0	0	all	-	not a	NAmer	1948	CC	684	n	bl	y	n	2	all/unsp	21	34	0	4	nev any	ot
WYNDE4	53		f	0	0	all	-	not a	NAmer	1948	CC	684	n	bl	y	n	2	all/unsp	35	99	3	0	nev any	ot
ZHENG	1		m	0	0	all	-	q	As:Chi	1982	CC	540	n	ot	*	y	0	cig+/-ot	1	9	1	1	nev cigs	st
ZHENG	2		m	0	0	all	-	q	As:Chi	1982	CC	540	n	ot	*	y	0	cig+/-ot	10	19	0	2	nev cigs	st
ZHENG	3		m	0	0	all	-	q	As:Chi	1982	CC	540	n	ot	*	y	0	cig+/-ot	20	29	2	3	nev cigs	st
ZHENG	4		m	0	0	all	-	q	As:Chi	1982	CC	540	n	ot	*	y	0	cig+/-ot	30	99	3	0	nev cigs	st
ZHENG	16		f	0	0	all	-	q	As:Chi	1982	CC	540	n	ot	*	y	0	cig+/-ot	1	9	1	1	nev cigs	st
ZHENG	17		f	0	0	all	-	q	As:Chi	1982	CC	540	n	ot	*	y	0	cig+/-ot	10	99	0	0	nev cigs	st
ZHOU	10		c	0	0	all	-	q	As:Chi	1978	CC	1360	n	ot	n	n	0	all/unsp	1	9	1	1	nev any	st
ZHOU	11		c	0	0	all	-	q	As:Chi	1978	CC	1360	n	ot	n	n	0	all/unsp	10	19	0	2	nev any	st
ZHOU	12		c	0	0	all	-	q	As:Chi	1978	CC	1360	n	ot	n	n	0	all/unsp	20	99	0	0	nev any	st

Cigarette type is all/unspec for all RRs

except for the following:

REF	NRR	CIGTYPE
ALDERS	34	MC only
ALDERS	35	MC only
ALDERS	36	MC only
ALDERS	37	MC only
ALDERS	38	MC only
ALDERS	39	MC only

REF	NRR	Cigarette equivalent
ALDERS	34	-
ALDERS	35	-
ALDERS	36	-
ALDERS	37	-
ALDERS	38	-
ALDERS	39	-
BARBON	19	*
BARBON	21	*
BARBON	23	*
BARBON	25	*
BARBON	27	*
BROWN2	36	*
BROWN2	46	*
BROWN2	35	*
BROWN2	45	*
CHOI	46	*

Table 2G1 - 4

IESLC - Meta-analysis of Ever Smoking by Amount, Overview, Any product (or Cigarettes if Any not available)
Squamous
Least adjusted

REF	NRR	Cigarette equivalent
CHOI	47	*
CHOI	48	*
CHOI	49	*
CHOI	50	*
CHOI	56	*
CHOI	57	*
CHOI	58	*
DOLL	54	grams
DOLL	55	grams
DOLL	56	grams
DOLL	57	grams
DOLL	62	grams
DOLL	63	grams
DOLL	64	grams
DORGAN	114	*
DORGAN	115	*
DORGAN	99	*
DORGAN	100	*
DOSEME	7	*
DOSEME	11	*
DOSEME	15	*
GER	6	*
GER	7	*
GER	8	*
JEDRYC	1	*
JEDRYC	2	*
JEDRYC	3	*
JEDRYC	4	*
JEDRYC	5	*
KREYBE	13	grams inc 1 cig=1
KREYBE	14	grams inc 1 cig=1
KREYBE	15	grams inc 1 cig=1
KREYBE	31	grams inc 1 cig=1
KREYBE	32	grams inc 1 cig=1
LAMTH	10	*
LAMTH	11	*
LAMTH	12	*
LUBIN2	149	*
LUBIN2	153	*
LUBIN2	157	*
LUBIN2	161	*
LUBIN2	169	*
LUBIN2	173	*
LUBIN2	177	*
LUBIN2	181	*
LUO	4	*
LUO	5	*
LUO	6	*
MATOS	42	*
MATOS	44	*
MATOS	46	*
MATSUD	4	*
MATSUD	5	*
MATSUD	6	*
ORMOS	5	*
ORMOS	6	*
ORMOS	7	*
OSANN	51	*
OSANN	59	*
OSANN	52	*
OSANN	60	*
OSANN2	10	*
OSANN2	11	*
WUWILL	20	*
WUWILL	21	*
WYNDE2	3	*
WYNDE2	4	*
WYNDE2	5	*
WYNDE2	6	*
WYNDE3	4	*
WYNDE3	5	*
WYNDE3	6	*

Table 2G1 - 4

IESLC - Meta-analysis of Ever Smoking by Amount, Overview, Any product (or Cigarettes if Any not available)
Squamous
Least adjusted

REF	NRR	Cigarette equivalent
WYNDE3	7	*
WYNDE3	63	*
WYNDE3	64	*
WYNDE3	65	*
WYNDE3	66	*
WYNDE4	5	inc 1 cigar = 5 cigs, 1 pipe = 2.5 cigs
WYNDE4	11	inc 1 cigar = 5 cigs, 1 pipe = 2.5 cigs
WYNDE4	17	inc 1 cigar = 5 cigs, 1 pipe = 2.5 cigs
WYNDE4	23	inc 1 cigar = 5 cigs, 1 pipe = 2.5 cigs
WYNDE4	29	inc 1 cigar = 5 cigs, 1 pipe = 2.5 cigs
WYNDE4	49	inc 1 cigar = 5 cigs, 1 pipe = 2.5 cigs
WYNDE4	50	inc 1 cigar = 5 cigs, 1 pipe = 2.5 cigs
WYNDE4	51	inc 1 cigar = 5 cigs, 1 pipe = 2.5 cigs
WYNDE4	52	inc 1 cigar = 5 cigs, 1 pipe = 2.5 cigs
WYNDE4	53	inc 1 cigar = 5 cigs, 1 pipe = 2.5 cigs
ZHENG	1	*
ZHENG	2	*
ZHENG	3	*
ZHENG	4	*
ZHENG	16	*
ZHENG	17	*
ZHOU	10	*
ZHOU	11	*
ZHOU	12	*

In this overview table, subtotals and Qs values may be invalid and should be ignored

Table 2G1 - 5

IESLC - Meta-analysis of Ever Smoking by Amount, Overview, Any product (or Cigarettes if Any not available)
Squamous
Least adjusted

REF	NRR	SEX	AD	Number Exposed Case	Number Exposed Cont	Non-exposed Case	Non-exposed Cont	RR	95.00%CI
ALDERS	34	m	1	-	-	-	-	3.79	(1.30- 11.02)
ALDERS	35	m	1	-	-	-	-	7.19	(2.75- 18.79)
ALDERS	36	m	1	-	-	-	-	8.78	(3.46- 22.31)
ALDERS	37	f	1	-	-	-	-	2.55	(1.42- 4.57)
ALDERS	38	f	1	-	-	-	-	9.24	(5.31- 16.09)
ALDERS	39	f	1	-	-	-	-	14.52	(7.93- 26.58)
Subtotal ALDERS								6.82	(5.12- 9.11)
BARBON	19	m	0	11	87	6	188	3.96	(1.42- 11.06)
BARBON	21	m	0	45	111	6	188	12.70	(5.25- 30.73)
BARBON	23	m	0	81	176	6	188	14.42	(6.14- 33.89)
BARBON	25	m	0	46	82	6	188	17.58	(7.22- 42.78)
BARBON	27	m	0	78	111	6	188	22.02	(9.29- 52.18)
Subtotal BARBON								13.16	(8.81- 19.65)
BROWN2	36	m	2	-	-	-	-	7.60	(6.20- 9.40)
BROWN2	46	m	2	-	-	-	-	17.20	(14.60- 20.30)
BROWN2	35	f	2	-	-	-	-	11.70	(8.70- 15.80)
BROWN2	45	f	2	-	-	-	-	26.10	(20.70- 32.80)
Subtotal BROWN2								14.51	(13.06- 16.12)
CHOI	46	m	0	12	90	6	95	2.11	(0.76- 5.86)
CHOI	47	m	0	84	281	6	95	4.73	(2.00- 11.19)
CHOI	48	m	0	30	49	6	95	9.69	(3.78- 24.86)
CHOI	49	m	0	25	39	6	95	10.15	(3.86- 26.66)
CHOI	50	m	0	9	6	6	95	23.75	(6.33- 89.09)
CHOI	56	f	0	4	16	10	164	4.10	(1.15- 14.57)
CHOI	57	f	0	5	9	10	164	9.11	(2.57- 32.31)
CHOI	58	f	0	2	1	10	164	32.80	(2.74- 393.20)
Subtotal CHOI								6.86	(4.63- 10.16)
DOLL	54	m	0	29	129	3	61	4.57	(1.34- 15.59)
DOLL	55	m	0	291	570	3	61	10.38	(3.23- 33.37)
DOLL	56	m	0	301	431	3	61	14.20	(4.41- 45.68)
DOLL	57	m	0	208	166	3	61	25.48	(7.85- 82.66)
DOLL	62	f	0	8	25	16	59	1.18	(0.45- 3.11)
DOLL	63	f	0	9	18	16	59	1.84	(0.70- 4.88)
DOLL	64	f	0	15	6	16	59	9.22	(3.08- 27.59)
Subtotal DOLL								5.29	(3.50- 8.02)
DORGAN	114	m	2	-	-	-	-	11.50	(4.10- 32.24)
DORGAN	115	m	2	-	-	-	-	23.29	(8.55- 63.49)
DORGAN	99	f	3	-	-	-	-	7.78	(4.86- 12.44)
DORGAN	100	f	3	-	-	-	-	16.38	(10.22- 26.26)
Subtotal DORGAN								12.06	(8.92- 16.31)
DOSEME	7	m	2	-	-	-	-	2.60	(1.50- 4.60)
DOSEME	11	m	2	-	-	-	-	3.20	(2.20- 4.60)
DOSEME	15	m	2	-	-	-	-	7.00	(4.10- 12.00)
Subtotal DOSEME								3.71	(2.84- 4.84)
GER	6	c	0	9	56	11	80	1.17	(0.45- 3.01)
GER	7	c	0	20	87	11	80	1.67	(0.75- 3.71)
GER	8	c	0	19	13	11	80	10.63	(4.13- 27.37)
Subtotal GER								2.59	(1.55- 4.32)
JEDRYC	1	m	0	1	67	6	289	0.72	(0.09- 6.07)
JEDRYC	2	m	0	38	199	6	289	9.20	(3.82- 22.17)
JEDRYC	3	m	0	152	434	6	289	16.87	(7.36- 38.66)
JEDRYC	4	m	0	61	118	6	289	24.90	(10.48- 59.17)
JEDRYC	5	m	0	57	82	6	289	33.48	(13.94- 80.42)
Subtotal JEDRYC								16.68	(10.93- 25.44)
KREYBE	13	m	0	123	2341	3	644	11.28	(3.58- 35.57)
KREYBE	14	m	0	49	925	3	644	11.37	(3.53- 36.64)
KREYBE	15	m	0	38	248	3	644	32.89	(10.06- 107.53)
KREYBE	31	f	0	1	286	3	657	0.77	(0.08- 7.39)
KREYBE	32	f	0	1	42	3	657	5.21	(0.53- 51.21)
Subtotal KREYBE								11.71	(6.29- 21.81)
LAMTH	10	f	0	23	11	28	72	5.38	(2.32- 12.46)
LAMTH	11	f	0	28	6	28	72	12.00	(4.49- 32.10)
LAMTH	12	f	0	10	1	28	72	25.71	(3.14- 210.29)
Subtotal LAMTH								8.37	(4.54- 15.43)
LUBIN2	149	m	0	418	2194	54	2616	9.23	(6.91- 12.32)
LUBIN2	153	m	0	1022	3385	54	2616	14.63	(11.07- 19.32)
LUBIN2	157	m	0	1298	3108	54	2616	20.23	(15.33- 26.69)
LUBIN2	161	m	0	849	1746	54	2616	23.56	(17.77- 31.22)
LUBIN2	169	f	0	30	184	72	1180	2.67	(1.70- 4.20)
LUBIN2	173	f	0	91	234	72	1180	6.37	(4.54- 8.95)
LUBIN2	177	f	0	61	110	72	1180	9.09	(6.13- 13.46)

Table 2G1 - 5

IESLC - Meta-analysis of Ever Smoking by Amount, Overview, Any product (or Cigarettes if Any not available)
Squamous
Least adjusted

REF	NRR	SEX	AD	Number Exposed Case	Number Exposed Cont	Non-exposed Case	Non-exposed Cont	RR	95.00%CI
LUBIN2	181	f	0	18	39	72	1180	7.56	(4.12- 13.88)
Subtotal LUBIN2								11.82	(10.51- 13.28)
LUO	4	c	0	3	39	5	51	0.78	(0.18- 3.48)
LUO	5	c	0	22	23	5	51	9.76	(3.28- 28.98)
LUO	6	c	0	9	4	5	51	22.95	(5.15- 102.20)
Subtotal LUO								6.34	(2.97- 13.53)
MATOS	42	m	0	3	88	3	110	1.25	(0.25- 6.35)
MATOS	44	m	0	18	90	3	110	7.33	(2.09- 25.69)
MATOS	46	m	0	26	105	3	110	9.08	(2.67- 30.90)
Subtotal MATOS								5.36	(2.48- 11.58)
MATSUD	4	m	0	21	1237	1	1255	21.31	(2.86- 158.63)
MATSUD	5	m	0	43	1607	1	1255	33.58	(4.62- 244.19)
MATSUD	6	m	0	39	470	1	1255	104.14	(14.27- 760.12)
Subtotal MATSUD								42.26	(13.37- 133.55)
ORMOS	5	m	0	13	329	2	777	15.35	(3.44- 68.41)
ORMOS	6	m	0	10	577	2	777	6.73	(1.47- 30.85)
ORMOS	7	m	0	4	128	2	777	12.14	(2.20- 66.97)
Subtotal ORMOS								10.74	(4.35- 26.54)
OSANN	51	m	2	-	-	-	-	35.30	(17.00- 73.30)
OSANN	59	m	2	-	-	-	-	76.00	(36.80- 157.00)
OSANN	52	f	2	-	-	-	-	24.00	(12.70- 45.50)
OSANN	60	f	2	-	-	-	-	72.30	(36.80- 142.00)
Subtotal OSANN								45.20	(32.03- 63.79)
OSANN2	10	f	0	18	31	7	58	4.81	(1.81- 12.77)
OSANN2	11	f	0	93	29	7	58	26.57	(10.93- 64.58)
Subtotal OSANN2								12.25	(6.35- 23.63)
WUWILL	20	f	0	168	311	117	601	2.77	(2.11- 3.65)
WUWILL	21	f	0	33	40	117	601	4.24	(2.57- 7.00)
Subtotal WUWILL								3.06	(2.41- 3.88)
WYNDE2	3	m	0	15	114	3	105	4.61	(1.30- 16.36)
WYNDE2	4	m	0	108	203	3	105	18.62	(5.77- 60.06)
WYNDE2	5	m	0	74	83	3	105	31.20	(9.50- 102.54)
WYNDE2	6	m	0	139	112	3	105	43.44	(13.42- 140.56)
Subtotal WYNDE2								19.37	(10.64- 35.27)
WYNDE3	4	m	0	7	42	3	88	4.89	(1.20- 19.86)
WYNDE3	5	m	0	57	114	3	88	14.67	(4.44- 48.40)
WYNDE3	6	m	0	74	82	3	88	26.47	(8.03- 87.26)
WYNDE3	7	m	0	59	26	3	88	66.56	(19.27- 229.96)
WYNDE3	63	f	0	1	19	5	76	0.80	(0.09- 7.26)
WYNDE3	64	f	0	13	24	5	76	8.23	(2.66- 25.46)
WYNDE3	65	f	0	8	10	5	76	12.16	(3.32- 44.50)
WYNDE3	66	f	0	3	3	5	76	15.20	(2.42- 95.56)
Subtotal WYNDE3								13.67	(8.51- 21.97)
WYNDE4	5	m	0	14	82	8	115	2.45	(0.98- 6.12)
WYNDE4	11	m	0	61	147	8	115	5.97	(2.74- 12.97)
WYNDE4	17	m	0	213	274	8	115	11.17	(5.34- 23.39)
WYNDE4	23	m	0	186	98	8	115	27.28	(12.79- 58.18)
WYNDE4	29	m	0	123	64	8	115	27.63	(12.69- 60.13)
WYNDE4	49	f	2	-	-	-	-	0.87	(0.11- 6.90)
WYNDE4	50	f	2	-	-	-	-	4.61	(1.38- 15.41)
WYNDE4	51	f	2	-	-	-	-	14.92	(4.88- 45.67)
WYNDE4	52	f	2	-	-	-	-	26.53	(4.12- 171.09)
WYNDE4	53	f	2	-	-	-	-	26.53	(4.12- 171.09)
Subtotal WYNDE4								10.88	(7.98- 14.84)
ZHENG	1	m	0	7	40	4	94	4.11	(1.14- 14.84)
ZHENG	2	m	0	25	66	4	94	8.90	(2.96- 26.78)
ZHENG	3	m	0	75	89	4	94	19.80	(6.95- 56.41)
ZHENG	4	m	0	49	23	4	94	50.07	(16.39- 152.91)
ZHENG	16	f	0	11	29	33	184	2.11	(0.96- 4.64)
ZHENG	17	f	0	32	15	33	184	11.89	(5.81- 24.35)
Subtotal ZHENG								8.76	(5.95- 12.88)
ZHOU	10	c	0	15	5	138	68	1.48	(0.52- 4.24)
ZHOU	11	c	0	78	14	138	68	2.75	(1.45- 5.20)
ZHOU	12	c	0	285	29	138	68	4.84	(3.00- 7.82)
Subtotal ZHOU								3.52	(2.45- 5.04)
Partial Totals				7965	25234	1677	33155		

*prospective study

Table 2G1 - 5

IESLC - Meta-analysis of Ever Smoking by Amount, Overview, Any product (or Cigarettes if Any not available)
Squamous
Least adjusted

REF	NRR	SEX	AD	Ys	Ws	Qs	Ps
ALDERS	34	m	1	1.33	3.36	3.38	0.0145
ALDERS	35	m	1	1.97	4.16	0.54	0.0001
ALDERS	36	m	1	2.17	4.42	0.12	0.0000
ALDERS	37	f	1	0.94	11.25	21.99	0.0017
ALDERS	38	f	1	2.22	12.50	0.15	0.0000
ALDERS	39	f	1	2.68	10.50	1.22	0.0000
Subtotal ALDERS				1.92	46.20	27.41	
BARBON	19	m	0	1.38	3.64	3.34	0.0086
BARBON	21	m	0	2.54	4.92	0.21	0.0000
BARBON	23	m	0	2.67	5.26	0.59	0.0000
BARBON	25	m	0	2.87	4.86	1.38	0.0000
BARBON	27	m	0	3.09	5.16	2.96	0.0000
Subtotal BARBON				2.58	23.84	8.48	
BROWN2	36	m	2	2.03	88.72	8.32	0.0000
BROWN2	46	m	2	2.84	141.44	36.86	0.0000
BROWN2	35	f	2	2.46	43.16	0.68	0.0000
BROWN2	45	f	2	3.26	72.52	62.39	0.0000
Subtotal BROWN2				2.67	345.85	108.24	
CHOI	46	m	0	0.75	3.68	9.27	0.1517
CHOI	47	m	0	1.55	5.19	3.16	0.0004
CHOI	48	m	0	2.27	4.33	0.02	0.0000
CHOI	49	m	0	2.32	4.12	0.00	0.0000
CHOI	50	m	0	3.17	2.20	1.53	0.0000
CHOI	56	f	0	1.41	2.39	2.04	0.0292
CHOI	57	f	0	2.21	2.40	0.04	0.0006
CHOI	58	f	0	3.49	0.62	0.83	0.0059
Subtotal CHOI				1.93	24.93	16.88	
DOLL	54	m	0	1.52	2.55	1.69	0.0152
DOLL	55	m	0	2.34	2.82	0.00	0.0001
DOLL	56	m	0	2.65	2.81	0.29	0.0000
DOLL	57	m	0	3.24	2.77	2.26	0.0000
DOLL	62	f	0	0.17	4.09	19.24	0.7378
DOLL	63	f	0	0.61	4.06	12.06	0.2175
DOLL	64	f	0	2.22	3.20	0.04	0.0001
Subtotal DOLL				1.67	22.31	35.59	
DORGAN	114	m	2	2.44	3.61	0.04	0.0000
DORGAN	115	m	2	3.15	3.82	2.53	0.0000
DORGAN	99	f	3	2.05	17.39	1.39	0.0000
DORGAN	100	f	3	2.80	17.25	3.68	0.0000
Subtotal DORGAN				2.49	42.08	7.64	
DOSEME	7	m	2	0.96	12.24	23.27	0.0008
DOSEME	11	m	2	1.16	28.24	38.75	0.0000
DOSEME	15	m	2	1.95	13.32	2.01	0.0000
Subtotal DOSEME				1.31	53.80	64.03	
GER	6	c	0	0.16	4.30	20.42	0.7462
GER	7	c	0	0.51	6.06	20.10	0.2056
GER	8	c	0	2.36	4.29	0.00	0.0000
Subtotal GER				0.95	14.66	40.52	
JEDRYC	1	m	0	-0.33	0.84	5.99	0.7618
JEDRYC	2	m	0	2.22	4.96	0.07	0.0000
JEDRYC	3	m	0	2.83	5.59	1.35	0.0000
JEDRYC	4	m	0	3.21	5.13	3.98	0.0000
JEDRYC	5	m	0	3.51	5.00	6.93	0.0000
Subtotal JEDRYC				2.81	21.53	18.31	
KREYBE	13	m	0	2.42	2.91	0.02	0.0000
KREYBE	14	m	0	2.43	2.81	0.03	0.0000
KREYBE	15	m	0	3.49	2.74	3.68	0.0000
KREYBE	31	f	0	-0.27	0.75	5.06	0.8175
KREYBE	32	f	0	1.65	0.74	0.34	0.1566
Subtotal KREYBE				2.46	9.94	9.13	
LAMTH	10	f	0	1.68	5.44	2.31	0.0001
LAMTH	11	f	0	2.48	3.97	0.09	0.0000
LAMTH	12	f	0	3.25	0.87	0.72	0.0025
Subtotal LAMTH				2.12	10.27	3.13	
LUBIN2	149	m	0	2.22	45.98	0.58	0.0000
LUBIN2	153	m	0	2.68	49.57	6.02	0.0000
LUBIN2	157	m	0	3.01	50.02	22.64	0.0000
LUBIN2	161	m	0	3.16	48.42	32.95	0.0000
LUBIN2	169	f	0	0.98	18.69	34.14	0.0000
LUBIN2	173	f	0	1.85	33.33	7.75	0.0000
LUBIN2	177	f	0	2.21	24.86	0.40	0.0000

Table 2G1 - 5

IESLC - Meta-analysis of Ever Smoking by Amount, Overview, Any product (or Cigarettes if Any not available)
Squamous
Least adjusted

REF	NRR	SEX	AD	Ys	Ws	Qs	Ps
LUBIN2	181	f	0	2.02	10.42	1.01	0.0000
Subtotal LUBIN2				2.47	281.30	105.50	
LUO	4	c	0	-0.24	1.73	11.48	0.7498
LUO	5	c	0	2.28	3.24	0.01	0.0000
LUO	6	c	0	3.13	1.72	1.10	0.0000
Subtotal LUO				1.85	6.69	12.59	
MATOS	42	m	0	0.22	1.46	6.49	0.7878
MATOS	44	m	0	1.99	2.44	0.29	0.0018
MATOS	46	m	0	2.21	2.56	0.04	0.0004
Subtotal MATOS				1.68	6.46	6.82	
MATSUD	4	m	0	3.06	0.95	0.50	0.0028
MATSUD	5	m	0	3.51	0.98	1.36	0.0005
MATSUD	6	m	0	4.65	0.97	5.19	0.0000
Subtotal MATSUD				3.74	2.90	7.05	
ORMOS	5	m	0	2.73	1.72	0.27	0.0003
ORMOS	6	m	0	1.91	1.66	0.30	0.0141
ORMOS	7	m	0	2.50	1.32	0.03	0.0042
Subtotal ORMOS				2.37	4.70	0.61	
OSANN	51	m	2	3.56	7.20	10.88	0.0000
OSANN	59	m	2	4.33	7.30	29.09	0.0000
OSANN	52	f	2	3.18	9.44	6.72	0.0000
OSANN	60	f	2	4.28	8.43	31.92	0.0000
Subtotal OSANN				3.81	32.36	78.61	
OSANN2	10	f	0	1.57	4.03	2.35	0.0016
OSANN2	11	f	0	3.28	4.87	4.35	0.0000
Subtotal OSANN2				2.51	8.90	6.70	
WUWILL	20	f	0	1.02	51.60	89.08	0.0000
WUWILL	21	f	0	1.44	15.26	12.10	0.0000
Subtotal WUWILL				1.12	66.87	101.18	
WYNDE2	3	m	0	1.53	2.39	1.56	0.0182
WYNDE2	4	m	0	2.92	2.80	0.97	0.0000
WYNDE2	5	m	0	3.44	2.71	3.32	0.0000
WYNDE2	6	m	0	3.77	2.79	5.75	0.0000
Subtotal WYNDE2				2.96	10.69	11.60	
WYNDE3	4	m	0	1.59	1.96	1.09	0.0265
WYNDE3	5	m	0	2.69	2.70	0.33	0.0000
WYNDE3	6	m	0	3.28	2.70	2.39	0.0000
WYNDE3	7	m	0	4.20	2.50	8.68	0.0000
WYNDE3	63	f	0	-0.22	0.79	5.17	0.8428
WYNDE3	64	f	0	2.11	3.01	0.15	0.0003
WYNDE3	65	f	0	2.50	2.28	0.06	0.0002
WYNDE3	66	f	0	2.72	1.14	0.17	0.0037
Subtotal WYNDE3				2.62	17.07	18.05	
WYNDE4	5	m	0	0.90	4.60	9.50	0.0541
WYNDE4	11	m	0	1.79	6.37	1.92	0.0000
WYNDE4	17	m	0	2.41	7.04	0.04	0.0000
WYNDE4	23	m	0	3.31	6.70	6.33	0.0000
WYNDE4	29	m	0	3.32	6.35	6.15	0.0000
WYNDE4	49	f	2	-0.14	0.90	5.49	0.8951
WYNDE4	50	f	2	1.53	2.64	1.72	0.0130
WYNDE4	51	f	2	2.70	3.07	0.42	0.0000
WYNDE4	52	f	2	3.28	1.11	0.99	0.0006
WYNDE4	53	f	2	3.28	1.11	0.99	0.0006
Subtotal WYNDE4				2.39	39.89	33.53	
ZHENG	1	m	0	1.41	2.33	1.98	0.0308
ZHENG	2	m	0	2.19	3.17	0.07	0.0001
ZHENG	3	m	0	2.99	3.51	1.49	0.0000
ZHENG	4	m	0	3.91	3.08	7.68	0.0000
ZHENG	16	f	0	0.75	6.21	15.60	0.0620
ZHENG	17	f	0	2.48	7.48	0.15	0.0000
Subtotal ZHENG				2.17	25.78	26.97	
ZHOU	10	c	0	0.39	3.46	13.09	0.4669
ZHOU	11	c	0	1.01	9.42	16.52	0.0019
ZHOU	12	c	0	1.58	16.68	9.56	0.0000
Subtotal ZHOU				1.26	29.56	39.17	

Table 2G1 - 5

IESLC - Meta-analysis of Ever Smoking by Amount, Overview, Any product (or Cigarettes if Any not available)
Squamous
Least adjusted

N	112
NS	24

Table 2G1 - 6

IESLC - Meta-analysis of Ever Smoking by Amount, Overview, Any product (or Cigarettes if Any not available)
Squamous
Least adjusted

	Sex combined	male	female	Total
N	9	64	39	112
NS	3	18	14	35

In this overview table, other than the "N" rows, entries in the "absent" and "Total" columns may be invalid and should be ignored

	Amount smoked (broad categories) absent	<20k5	6-44k20	>20k45	Total
N	31	33	23	25	112
NS	16	23	18	19	76
Wt	454.87	359.37	184.33	150.02	1148.58
Het Chi	181.22	160.24	93.02	73.72	787.72
Het df	30	32	22	24	111
Het P	***	***	***	***	***
Fixed RR	13.99	5.32	9.82	21.36	10.32
RRl	12.76	4.80	8.50	18.20	9.74
RRu	15.34	5.90	11.34	25.07	10.94
P	+++	+++	+++	+++	+++
Random RR	11.49	3.81	9.72	23.09	9.34
RRl	8.79	2.87	6.93	16.64	7.85
RRu	15.03	5.05	13.64	32.04	11.12
P	+++	+++	+++	+++	+++

	absent	Amount smoked (narrow categories) <10k1	2-19k10	11-29k20	21-39k30	31-98k40	>40k99	Total
N	60	13	10	19	6	1	3	112
NS	24	8	7	16	5	1	2	62
Wt	721.92	96.05	121.26	174.56	24.83	4.12	5.83	1148.58
Het Chi	495.12	53.34	41.61	92.24	3.83	0.00	2.15	787.72
Het df	59	12	9	18	5	0	2	111
Het P	***	***	***	***	N.S.	N.S.	N.S.	***
Fixed RR	11.64	4.58	8.48	9.83	20.79	10.15	33.86	10.32
RRl	10.82	3.75	7.09	8.47	14.03	3.86	15.04	9.74
RRu	12.52	5.59	10.13	11.40	30.81	26.66	76.22	10.94
P	+++	+++	+++	+++	+++	+++	+++	+++
Random RR	11.08	2.65	6.66	9.80	20.79	10.15	33.54	9.34
RRl	8.67	1.56	4.23	6.70	14.03	3.86	14.41	7.85
RRu	14.14	4.49	10.48	14.33	30.81	26.66	78.06	11.12
P	+++	+++	+++	+++	+++	+++	+++	+++

MALES

	Amount smoked (broad categories) absent	<20k5	6-44k20	>20k45	Total
N	16	17	15	16	64
NS	12	17	15	16	60
Wt	254.55	183.22	125.20	110.91	673.89
Het Chi	26.02	42.96	71.86	45.50	315.02
Het df	15	16	14	15	63
Het P	*	***	***	***	***
Fixed RR	16.20	6.77	10.81	22.79	12.54
RRl	14.32	5.86	9.07	18.92	11.62
RRu	18.31	7.82	12.88	27.45	13.52
P	+++	+++	+++	+++	+++
Random RR	15.44	5.21	11.04	25.28	12.17
RRl	12.27	3.74	6.90	17.16	9.96
RRu	19.42	7.27	17.68	37.26	14.87
P	+++	+++	+++	+++	+++

Table 2G1 - 6

IESLC - Meta-analysis of Ever Smoking by Amount, Overview, Any product (or Cigarettes if Any not available)
Squamous
Least adjusted

MALES

	Amount smoked (narrow categories)							
	absent	<10k1	2-19k10	11-29k20	21-39k30	31-98k40	>40k99	Total
N	30	7	6	13	5	1	2	64
NS	18	7	6	13	5	1	2	51
Wt	386.78	61.91	71.81	120.85	23.73	4.12	4.70	673.89
Het Chi	178.82	15.49	5.64	71.24	3.76	0.00	1.24	315.02
Het df	29	6	5	12	4	0	1	63
Het P	***	*	N.S.	***	N.S.	N.S.	N.S.	***
Fixed RR	13.79	7.10	12.51	10.80	20.55	10.15	41.10	12.54
RRl	12.48	5.53	9.92	9.04	13.75	3.86	16.64	11.62
RRu	15.24	9.11	15.76	12.91	30.74	26.66	101.52	13.52
P	+++	+++	+++	+++	+++	+++	+++	+++
Random RR	14.39	4.27	11.74	11.16	20.55	10.15	40.83	12.17
RRl	10.52	2.33	8.82	6.68	13.75	3.86	14.89	9.96
RRu	19.68	7.84	15.63	18.63	30.74	26.66	111.95	14.87
P	+++	+++	+++	+++	+++	+++	+++	+++

FEMALES

	Amount smoked (broad categories)				
	absent	<20k5	6-44k20	>20k45	Total
N	13	13	6	7	39
NS	11	13	6	7	37
Wt	174.22	166.65	49.82	33.09	423.78
Het Chi	105.89	79.47	0.96	25.09	341.56
Het df	12	12	5	6	38
Het P	***	***	N.S.	***	***
Fixed RR	13.66	4.45	9.56	18.75	8.64
RRl	11.77	3.83	7.25	13.33	7.85
RRu	15.84	5.18	12.63	26.36	9.50
P	+++	+++	+++	+++	+++
Random RR	10.02	3.37	9.56	21.10	7.66
RRl	5.92	2.11	7.25	9.14	5.55
RRu	16.97	5.38	12.63	48.71	10.58
P	+++	+++	+++	+++	+++

	Amount smoked (narrow categories)							
	absent	<10k1	2-19k10	11-29k20	21-39k30	31-98k40	>40k99	Total
N	25	5	3	4	1		1	39
NS	14	5	3	4	1		1	27
Wt	306.42	30.67	40.04	44.41	1.11		1.14	423.78
Het Chi	251.61	3.86	5.66	0.88	0.00		0.00	341.56
Het df	24	4	2	3	0		0	38
Het P	***	N.S.	(*)	N.S.	N.S.		N.S.	***
Fixed RR	10.30	2.14	5.50	9.69	26.53		15.20	8.64
RRl	9.21	1.50	4.04	7.22	4.12		2.42	7.85
RRu	11.52	3.05	7.50	13.00	170.96		95.56	9.50
P	+++	+++	+++	+++	+++		++	+++
Random RR	9.90	2.14	4.12	9.69	26.53		15.20	7.66
RRl	6.54	1.50	1.87	7.22	4.12		2.42	5.55
RRu	14.97	3.05	9.08	13.00	170.96		95.56	10.58
P	+++	+++	+++	+++	+++		++	+++

Table 2G1 - 7

IESLC - Meta-analysis of Ever Smoking by Amount, Overview, Any product (or Cigarettes if Any not available)
Squamous
Excluded studies (and stage at which they were excluded)

1	ABELIN ABRAHA AMANDU AMES ANDERS AUSTIN AXELSO BAND BECHER BERRIN BLOHMK BLOT4 BROCKM BROWN1 BYERS1 BYERS2
	CARPEN CASCO2 CASCOR CHAN CHEN3 CHIAZZ CHYOU DESTE2 DOCKER DROSTE DU GARCIA GARDIN GENG GODLEY GOODMA
	GRAHAM GREGOR HEGMAN HEIN HENNEK HINDS HIRAOK HOROWI HORWIT HUANG ISHIMA JAHN JAIN JARVHO JIANG KELLER
	KIHARA KJUUS KO KOHLME KUBIK LAMWK LAMWK2 LANGE LEI LEMARC LEVIN LIU LOMBA2 LOMBAR MAGNUS MARSH
	MARSH2 MCDUFF MCLAUG MILLER MILLS NOTANI NOU ODRISC PAWLEG PERSHA POFFIJ QIAO QIAO2 RADZIK REN RONCO
	ROOTS ROTHSC SAARIK SANKAR SCHWAR SEGI SEOW SHIMIZ SIMARA SIMONA SITAS SOBUE2 STASZE STAYNE STUCKE SUN
	SUZUK2 SUZUKI TANG TAO TOKARS TOUSEY ULMER VEIERO VUTUC WALD WANG WANG3 WANG4 WICKLU WIGLE WILKIN
	WU2 WUNSCH WYNDE8 XIANGZ XU XU2 XU4 YONG ZHANG
2	AKIBA ARCHER BENSHL BRETT BROSS CEDERL CHANG CHOW COMSTO DARBY DEAN3 DEKLER DORANT DORN ENGELA ENSTRO
	GAO2 GILLIS HAENSZ HAMMO2 HIRAY2 HIRAYA HITOSU HOLE HUMBLE KAISE2 KAISER KANELL KATSOU KAUFMA KINLEN KNEKT
	KOO LAURIL LIAW LICKIN LIDDEL MIGRAN MRFIT MRFITR MURATA NAM PARKIN PERSH2 PETO PEZZO2 PEZZOT PISANI
	PRESCO RIMING SEGI2 SOBUE SPEIZE STOCKW SVENSS TANG2 TENKAN TSUGAN TULINI TVERDA WAKAI WARSIN WATSON WU
	WYNDE5 WYNDER YAMAGU
3	BUELL CHEN MASTRA MZILEN RESTRE SADOWS
4	BEST BOFFET WYNDE7
6	BLOT1 BLOT2 BLOT3 BOUCHA JONES MOLLO SCHWA2 VANDER
8	AGUDO ARMADA AUVINE AXELSS BOUCOT BRESLO BUFFLE CHATZI CHEN2 COOKSO CORREA CPSI CPSII DAMBER DAVEYS DEAN
	DEAN2 DESTEF DOLL2 DUNN EBELIN ESAKI FAN GAO GARSHI GOLLED GSELL HAMMON HANSEN HU HU2 JARUP
	JOLY JUSSAW KHUDER KOULUM KREUZE LAUSSM LETOUR LIU2 LIU3 LIU4 LIU5 LUBIN MACLEN MARTIS MCCONN NOTAN2
	PASTOR PERNU PIKE POLEDN RACHTA RANDIG SHAW SIEMIA SPITZ STOCKS TIZZAN WANG2 WYNDE6 XU3 YUAN
10	BENHAM

Table 2G1 - 8
Potentially overlapping studies

REF	REFGP	PRINC	.	OVERLAP/LINK
LUBIN2	LUBIN2	1		Lubin-combined
LAMTH	LAMTH	1		KOO/LAMTH/LAMWK
OSANN2	KAISER	2		KAISER/OSANN2
MATSUD	MATSUD	1		SOBUE2/MATSUD

Table 2G1 - 9
Most adjusted - insufficient data for metaanalysis

REF	NRR	SEX	AGEL	AGEH	RACE	YF	LC TYPE	LOC	START	ST	NLC	R	VB	P	H	AD	PRODUCT	exL	exH	S1	S2	DENOM	De
CHEN	5	c	0	0	all	-	q	As:oth	1987	CC	323	n	ot	n	y	2	cig+/-ot	1	10	1	0	nev cigs	ot
CHEN	4	c	0	0	all	-	q	As:oth	1987	CC	323	n	ot	n	y	2	cig+/-ot	11	20	2	3	nev cigs	ot
CHEN	3	c	0	0	all	-	q	As:oth	1987	CC	323	n	ot	n	y	2	cig+/-ot	21	30	0	4	nev cigs	ot
CHEN	2	c	0	0	all	-	q	As:oth	1987	CC	323	n	ot	n	y	2	cig+/-ot	31	99	3	0	nev cigs	ot

REF	NRR	RR	SIG	Cigarette equivalent	RRDATA comment
CHEN	5	2.59	n	*	0
CHEN	4	7.05	n	*	0
CHEN	3	7.61	n	*	0
CHEN	2	11.11	y	*	P < 0.001

Table 2G2 -

IESLC - Meta-analysis of Ever Smoking, Amount smoked, "Low", Any product (or Cigarettes if Any not available)
Squamous

This analysis is restricted to results for:
1) Results by Amount smoked
2) Ever smokers
3) Results complete enough for use in metaanalysis

Within each study, results are then selected (in the following order of preference, within each sex) for:
4) PRODUCT: all/unspec, cigarettes regardless of other products, cigarettes only
5) CIGTYPE: all/unspecified, MC regardless of HR, MC only
6) DENOM: never smoked anything, never smoked cigarettes, (never +1 = +long term ex, +2 = +amount unknown, +3 = never cigs+long term ex)
7) Followup period (YF, prospective studies): whole study (coded as 0) or longest available
8) LCtype: squamous or nearest available, but not adeno. (q = squamous, s = small, a = adeno, KI = Kreyberg I, u = undifferentiated)
9) Race: all or nearest available, otherwise by race (wh or w = white, bl or b = black, hi = hispanic ch = chinese, jap = japanese, haw = hawaiian, w+o = white + oriental, sca = scandinavian, as = asian)
10) Amount smoked "low" in key scheme 1 (key value 5, maximum range <20, in numbers of cigarettes or cigarette equivalents)
11) For overlapping studies: principal rather than subsidiary studies
Finally by Age: whole study (coded as 0) if available, otherwise by widest available age group and then for single sex results (m, f) in preference to combined sex results (c).

Results adjusted (AD) for the most potential confounders are then chosen in Sections -1 to -3 and results adjusted for the least confounders in Sections -4 to -6. (Those least adjusted results which actually differ from the most adjusted as marked 'x' in column X in Section -4)
(Results adjusted for an unknown number of confounder(s) are coded as 20.)

Section -7 shows excluded studies, together with the stage (as above) at which no qualifying results were found.

Section -8 lists the potentially overlapping studies which have been included (1=principal, 2=subsidiary).

Section -9 lists any results which would have been included in preference except that they had data not complete enough for use in meta-analysis, with their significance (yes/no), if known, and any further comment as entered on the database.

In addition to those mentioned above, the following fields, levels and abbreviations are used:
* or nk = not known, n = no, y = yes, ot = other
nev = never
all/unspec = all or unspecified, cig+/-ot = cigarettes irrespective of other products (cigar, pipe etc)
MC = manufactured cigarettes, HR = hand-rolled cigarettes
exL, exH = range of exposure (low and high) in the smoking group, in terms of Amount smoked, cigarettes or cigarette equivalents
REF: 6-character study reference
NRR: number of the RR on the database within the study
ST : study type (CC = case control, pr or prosp = prospective)
NLC: number of lung cancer cases in whole study
R : risky occupational population (n = no, m = mining, o = other risky)
VB : national cigarette type (V = at least 75% Virginia, bl = at least 75% blended, ot = other)
P : any proxy use
H : full histological confirmation
De : derivation of RR/CI (or = original, st = standard method, ot = other method of estimation)

Table 2G2 - 1

IESLC - Meta-analysis of Ever Smoking, Amount smoked, "Low", Any product (or Cigarettes if Any not available)
Squamous
Most adjusted

REF	NRR	SEX	AGEL	AGEH	RACE	YF	LC TYPE	LOC	START	ST	NLC	R	VB	P	H	AD	PRODUCT	exL	exH	DENOM	De
ALDERS	34	m	0	0	all	-	q+s	Eu:UK	1977	CC	1448	n	V	n	n	1	cig only	1	17	nev+2	ot
ALDERS	37	f	0	0	all	-	q+s	Eu:UK	1977	CC	1448	n	V	n	n	1	cig only	1	17	nev+2	ot
BARBON	70	m	0	0	all	-	q	Eu:wst	1979	CC	755	n	bl	y	y	3	all/unsp	1	19	nev any	or
BROWN2	36	m	0	0	wh	-	q	NAmer	1984	CC	14596	n	bl	n	y	2	cig+/-ot	1	19	nev cigs	or
BROWN2	35	f	0	0	wh	-	q	NAmer	1984	CC	14596	n	bl	n	y	2	cig+/-ot	1	19	nev cigs	or
CHOI	46	m	0	0	all	-	q	As:oth	1985	CC	375	n	bl	n	n	0	cig+/-ot	1	10	nev cigs	st
CHOI	56	f	0	0	all	-	q	As:oth	1985	CC	375	n	bl	n	n	0	cig+/-ot	1	10	nev cigs	st
DOLL	69	m	0	0	all	-	KI	Eu:UK	1948	CC	1465	n	V	n	n	1	all/unsp	5	14	nev any	ot
DOLL	77	f	0	0	all	-	KI	Eu:UK	1948	CC	1465	n	V	n	n	1	all/unsp	5	14	nev any	ot
DORGAN	114	m	0	0	wh	-	q	NAmer	1980	CC	2026	n	bl	y	y	2	cig+/-ot	1	19	nev any	ot
DORGAN	99	f	0	0	all	-	q	NAmer	1980	CC	2026	n	bl	y	y	3	cig+/-ot	1	19	nev any	ot
DOSEME	7	m	0	0	all	-	q	Eu:bal	1979	CC	1210	n	bl	n	n	2	cig+/-ot	1	10	nev cigs	or
GER	14	c	0	0	all	-	q+s	As:oth	1990	CC	141	n	ot	y	n	10	all/unsp	1	10	nev any	ot
JEDRYC	28	m	0	0	all	-	q	Eu:est	1980	CC	1630	n	bl	y	n	3	cig+/-ot	1	19	nev any	or
KREYBE	1	m	0	0	all	-	KI	Eu:Sca	1948	CC	300	n	bl	n	y	1	all/unsp	1	14	nev any	ot
KREYBE	31	f	0	0	all	-	KI	Eu:Sca	1948	CC	300	n	bl	n	y	0	all/unsp	1	14	nev any	st
LAMTH	10	f	0	0	ch	-	q	As:HK	1983	CC	445	n	bl	n	n	0	all/unsp	1	10	nev any	or
LUBIN2	149	m	0	0	all	-	q	Eu:mul	1976	CC	7804	n	bl	n	y	0	cig+/-ot	1	9	nev any	st
LUBIN2	169	f	0	0	all	-	q	Eu:mul	1976	CC	7804	n	bl	n	y	0	cig+/-ot	1	9	nev any	st
LUO	10	c	0	0	all	-	q	As:Chi	1990	CC	102	n	ot	n	y	20	cig+/-ot	1	19	nev cigs	or
MATOS	43	m	0	0	all	-	q	SCAmer	1994	CC	200	n	bl	n	n	2	cig+/-ot	1	14	nev any	or
MATSUD	4	m	0	0	all	-	q	As:Jap	1965	CC	179	n	bl	n	n	0	cig+/-ot	1	10	nev cigs	st
ORMOS	5	m	0	0	all	-	q	Eu:est	1947	CC	119	n	bl	y	y	0	cig+/-ot	1	15	nev any	st
OSANN2	28	f	0	0	all	-	KI	NAmer	1964	ot	217	n	bl	n	y	1	cig+/-ot	1	19	nev cigs	or
WUWILL	14	f	0	0	all	-	q+s	As:Chi	1985	CC	965	n	ot	n	n	3	cig+/-ot	1	19	nev cigs	ot
WYNDE2	3	m	0	0	all	-	KI	NAmer	1962	CC	404	n	bl	n	y	0	cig+/-ot	1	10	nev any	st
WYNDE3	4	m	0	0	all	-	KI	NAmer	1966	CC	350	n	bl	n	y	0	cig+/-ot	1	9	nev any	st
WYNDE3	63	f	0	0	all	-	KI	NAmer	1966	CC	350	n	bl	n	y	0	cig+/-ot	1	9	nev any	st
WYNDE4	63	m	0	0	all	-	not a	NAmer	1948	CC	684	n	bl	y	n	2	all/unsp	1	9	nev any	ot
WYNDE4	49	f	0	0	all	-	not a	NAmer	1948	CC	684	n	bl	y	n	2	all/unsp	1	9	nev any	ot
ZHENG	1	m	0	0	all	-	q	As:Chi	1982	CC	540	n	ot	*	y	0	cig+/-ot	1	9	nev cigs	st
ZHENG	16	f	0	0	all	-	q	As:Chi	1982	CC	540	n	ot	*	y	0	cig+/-ot	1	9	nev cigs	st
ZHOU	10	c	0	0	all	-	q	As:Chi	1978	CC	1360	n	ot	n	n	0	all/unsp	1	9	nev any	st

Cigarette type is all/unspec for all RRs

except for the following:

REF	NRR	CIGTYPE
ALDERS	34	MC only
ALDERS	37	MC only

Table 2G2 - 2

IESLC - Meta-analysis of Ever Smoking, Amount smoked, "Low", Any product (or Cigarettes if Any not available)
Squamous
Most adjusted

REF	NRR	SEX	AD	Number Exposed Case	Number Exposed Cont	Non-exposed Case	Non-exposed Cont	RR	95.00%CI
ALDERS	34	m	1	-	-	-	-	3.79	(1.30- 11.02)
ALDERS	37	f	1	-	-	-	-	2.55	(1.42- 4.57)
Subtotal ALDERS								2.79	(1.67- 4.66)
BARBON	70	m	3	-	-	-	-	8.50	(3.60- 20.00)
BROWN2	36	m	2	-	-	-	-	7.60	(6.20- 9.40)
BROWN2	35	f	2	-	-	-	-	11.70	(8.70- 15.80)
Subtotal BROWN2								8.75	(7.38- 10.38)
CHOI	46	m	0	12	90	6	95	2.11	(0.76- 5.86)
CHOI	56	f	0	4	16	10	164	4.10	(1.15- 14.57)
Subtotal CHOI								2.74	(1.24- 6.07)
DOLL	69	m	1	-	-	-	-	10.60	(3.30- 34.07)
DOLL	77	f	1	-	-	-	-	1.70	(0.64- 4.50)
Subtotal DOLL								3.61	(1.71- 7.62)
DORGAN	114	m	2	-	-	-	-	11.50	(4.10- 32.24)
DORGAN	99	f	3	-	-	-	-	7.78	(4.86- 12.44)
Subtotal DORGAN								8.32	(5.43- 12.76)
DOSEME	7	m	2	-	-	-	-	2.60	(1.50- 4.60)
GER	14	c	10	-	-	-	-	1.43	(0.36- 5.61)
JEDRYC	28	m	3	-	-	-	-	7.51	(3.09- 18.27)
KREYBE	1	m	1	-	-	-	-	9.00	(2.85- 28.38)
KREYBE	31	f	0	1	286	3	657	0.77	(0.08- 7.39)
Subtotal KREYBE								5.44	(1.95- 15.16)
LAMTH	10	f	0	23	11	28	72	5.38	(2.32- 12.46)
LUBIN2	149	m	0	418	2194	54	2616	9.23	(6.91- 12.32)
LUBIN2	169	f	0	30	184	72	1180	2.67	(1.70- 4.20)
Subtotal LUBIN2								6.45	(5.06- 8.23)
LUO	10	c	20	-	-	-	-	1.20	(0.10- 10.00)
MATOS	43	m	2	-	-	-	-	1.40	(0.30- 6.90)
MATSUD	4	m	0	21	1237	1	1255	21.31	(2.86- 158.63)
ORMOS	5	m	0	13	329	2	777	15.35	(3.44- 68.41)
OSANN2	28	f	1	-	-	-	-	12.10	(1.50- 96.30)
WUWILL	14	f	3	-	-	-	-	3.21	(2.39- 4.30)
WYNDE2	3	m	0	15	114	3	105	4.61	(1.30- 16.36)
WYNDE3	4	m	0	7	42	3	88	4.89	(1.20- 19.86)
WYNDE3	63	f	0	1	19	5	76	0.80	(0.09- 7.26)
Subtotal WYNDE3								2.90	(0.89- 9.48)
WYNDE4	63	m	2	-	-	-	-	2.22	(0.89- 5.53)
WYNDE4	49	f	2	-	-	-	-	0.87	(0.11- 6.90)
Subtotal WYNDE4								1.91	(0.83- 4.39)
ZHENG	1	m	0	7	40	4	94	4.11	(1.14- 14.84)
ZHENG	16	f	0	11	29	33	184	2.11	(0.96- 4.64)
Subtotal ZHENG								2.54	(1.30- 4.96)
ZHOU	10	c	0	15	5	138	68	1.48	(0.52- 4.24)
Partial Totals				578	4596	362	7431		

*prospective study

REF	NRR	SEX	AD	Ys	Ws	Qs	Ps
ALDERS	34	m	1	1.33	3.36	0.56	0.0145
ALDERS	37	f	1	0.94	11.25	7.27	0.0017
Subtotal ALDERS				1.03	14.61	7.82	
BARBON	70	m	3	2.14	5.23	0.84	0.0000
BROWN2	36	m	2	2.03	88.72	7.38	0.0000
BROWN2	35	f	2	2.46	43.16	22.36	0.0000
Subtotal BROWN2				2.17	131.88	29.73	
CHOI	46	m	0	0.75	3.68	3.63	0.1517
CHOI	56	f	0	1.41	2.39	0.26	0.0292
Subtotal CHOI				1.01	6.07	3.89	
DOLL	69	m	1	2.36	2.82	1.09	0.0001
DOLL	77	f	1	0.53	4.04	5.91	0.2862
Subtotal DOLL				1.28	6.86	6.99	
DORGAN	114	m	2	2.44	3.61	1.78	0.0000
DORGAN	99	f	3	2.05	17.39	1.69	0.0000
Subtotal DORGAN				2.12	21.01	3.47	
DOSEME	7	m	2	0.96	12.24	7.53	0.0008
GER	14	c	10	0.36	2.04	3.89	0.6097
JEDRYC	28	m	3	2.02	4.87	0.37	0.0000
KREYBE	1	m	1	2.20	2.91	0.61	0.0002
KREYBE	31	f	0	-0.27	0.75	3.01	0.8175
Subtotal KREYBE				1.69	3.66	3.62	
LAMTH	10	f	0	1.68	5.44	0.02	0.0001

Table 2G2 - 2

IESLC - Meta-analysis of Ever Smoking, Amount smoked, "Low", Any product (or Cigarettes if Any not available)
Squamous
Most adjusted

REF	NRR	SEX	AD	Ys	Ws	Qs	Ps
LUBIN2	149	m	0	2.22	45.98	10.71	0.0000
LUBIN2	169	f	0	0.98	18.69	10.71	0.0000
Subtotal LUBIN2				1.86	64.67	21.42	
LUO	10	c	20	0.18	0.72	1.76	0.8767
MATOS	43	m	2	0.34	1.56	3.08	0.6740
MATSUD	4	m	0	3.06	0.95	1.66	0.0028
ORMOS	5	m	0	2.73	1.72	1.69	0.0003
OSANN2	28	f	1	2.49	0.89	0.50	0.0189
WUWILL	14	f	3	1.17	44.55	14.65	0.0000
WYNDE2	3	m	0	1.53	2.39	0.11	0.0182
WYNDE3	4	m	0	1.59	1.96	0.05	0.0265
WYNDE3	63	f	0	-0.22	0.79	3.04	0.8428
Subtotal WYNDE3				1.07	2.75	3.09	
WYNDE4	63	m	2	0.80	4.60	4.09	0.0870
WYNDE4	49	f	2	-0.14	0.90	3.17	0.8951
Subtotal WYNDE4				0.64	5.50	7.26	
ZHENG	1	m	0	1.41	2.33	0.25	0.0308
ZHENG	16	f	0	0.75	6.21	6.09	0.0620
Subtotal ZHENG				0.93	8.54	6.34	
ZHOU	10	c	0	0.39	3.46	6.30	0.4669

	N	33
	NS	23
	Wt	351.59
Het	Chi	136.03
Het	df	32
Het	P	***
Fixed	RR	5.70
	RRl	5.13
	RRu	6.32
	P	+++
Random	RR	4.30
	RRl	3.29
	RRu	5.62
	P	+++
Asymm	P	*

Table 2G2 - 3

IESLC - Meta-analysis of Ever Smoking, Amount smoked, "Low", Any product (or Cigarettes if Any not available)

Squamous

Most adjusted

	combined	Sex male	female	Total
N	3	17	13	33
NS	3	17	13	33
Wt	6.23	188.94	156.43	351.59
Het Chi	0.03	38.13	73.50	136.03
Het df	2	16	12	32
Het P	N.S.	**	***	***
Fixed RR	1.43	6.93	4.75	5.70
RRl	0.65	6.01	4.06	5.13
RRu	3.13	7.99	5.55	6.32
P	N.S.	+++	+++	+++
Random RR	1.43	5.77	3.48	4.30
RRl	0.65	4.26	2.17	3.29
RRu	3.13	7.80	5.58	5.62
P	N.S.	+++	+++	+++
Between Chi				24.38
Between df				2
Between P				***
Btwn(F) P				(*)
Btwn(R) P				**

Table 2G2 - 4

IESLC - Meta-analysis of Ever Smoking, Amount smoked, "Low", Any product (or Cigarettes if Any not available)
Squamous
Least adjusted

REF	NRR	X	SEX	AGEL	AGEH	RACE	YF	LC TYPE	LOC	START	ST	NLC	R	VB	P	H	AD	PRODUCT	exL	exH	DENOM	De
ALDERS	34		m	0	0	all	-	q+s	Eu:UK	1977	CC	1448	n	V	n	n	1	cig only	1	17	nev+2	ot
ALDERS	37		f	0	0	all	-	q+s	Eu:UK	1977	CC	1448	n	V	n	n	1	cig only	1	17	nev+2	ot
BARBON	19	x	m	0	0	all	-	q	Eu:wst	1979	CC	755	n	bl	y	y	0	all/unsp	1	9	nev any	st
BROWN2	36		m	0	0	wh	-	q	NAmer	1984	CC	14596	n	bl	n	y	2	cig+/-ot	1	19	nev cigs	or
BROWN2	35		f	0	0	wh	-	q	NAmer	1984	CC	14596	n	bl	n	y	2	cig+/-ot	1	19	nev cigs	or
CHOI	46		m	0	0	all	-	q	As:oth	1985	CC	375	n	bl	n	n	0	cig+/-ot	1	10	nev cigs	st
CHOI	56		f	0	0	all	-	q	As:oth	1985	CC	375	n	bl	n	n	0	cig+/-ot	1	10	nev cigs	st
DOLL	55	x	m	0	0	all	-	KI	Eu:UK	1948	CC	1465	n	V	n	n	0	all/unsp	5	14	nev any	st
DOLL	63	x	f	0	0	all	-	KI	Eu:UK	1948	CC	1465	n	V	n	n	0	all/unsp	5	14	nev any	st
DORGAN	114		m	0	0	wh	-	q	NAmer	1980	CC	2026	n	bl	y	y	2	cig+/-ot	1	19	nev any	ot
DORGAN	99		f	0	0	all	-	q	NAmer	1980	CC	2026	n	bl	y	y	3	cig+/-ot	1	19	nev any	ot
DOSEME	7		m	0	0	all	-	q	Eu:bal	1979	CC	1210	n	bl	n	n	2	cig+/-ot	1	10	nev cigs	or
GER	6	x	c	0	0	all	-	q+s	As:oth	1990	CC	141	n	ot	y	n	0	all/unsp	1	10	nev any	st
JEDRYC	1	x	m	0	0	all	-	q	Eu:est	1980	CC	1630	n	bl	y	n	0	cig+/-ot	1	9	nev any	st
KREYBE	13	x	m	0	0	all	-	KI	Eu:Sca	1948	CC	300	n	bl	n	y	0	all/unsp	1	14	nev any	st
KREYBE	31		f	0	0	all	-	KI	Eu:Sca	1948	CC	300	n	bl	n	y	0	all/unsp	1	14	nev any	st
LAMTH	10		f	0	0	ch	-	q	As:HK	1983	CC	445	n	bl	n	n	0	all/unsp	1	10	nev any	or
LUBIN2	149		m	0	0	all	-	q	Eu:mul	1976	CC	7804	n	bl	n	y	0	cig+/-ot	1	9	nev any	st
LUBIN2	169		f	0	0	all	-	q	Eu:mul	1976	CC	7804	n	bl	n	y	0	cig+/-ot	1	9	nev any	st
LUO	4	x	c	0	0	all	-	q	As:Chi	1990	CC	102	n	ot	n	y	0	cig+/-ot	1	19	nev cigs	st
MATOS	42	x	m	0	0	all	-	q	SCAmer	1994	CC	200	n	bl	n	n	0	cig+/-ot	1	14	nev any	st
MATSUD	4		m	0	0	all	-	q	As:Jap	1965	CC	179	n	bl	n	n	0	cig+/-ot	1	10	nev cigs	st
ORMOS	5		m	0	0	all	-	q	Eu:est	1947	CC	119	n	bl	y	y	0	cig+/-ot	1	15	nev any	st
OSANN2	10	x	f	0	0	all	-	KI	NAmer	1964	ot	217	n	bl	n	y	0	cig+/-ot	1	19	nev cigs	st
WUWILL	20	x	f	0	0	all	-	q+s	As:Chi	1985	CC	965	n	ot	n	n	0	cig+/-ot	1	19	nev cigs	st
WYNDE2	3		m	0	0	all	-	KI	NAmer	1962	CC	404	n	bl	n	y	0	cig+/-ot	1	10	nev any	st
WYNDE3	4		m	0	0	all	-	KI	NAmer	1966	CC	350	n	bl	n	y	0	cig+/-ot	1	9	nev any	st
WYNDE3	63		f	0	0	all	-	KI	NAmer	1966	CC	350	n	bl	n	y	0	cig+/-ot	1	9	nev any	st
WYNDE4	5	x	m	0	0	all	-	not a	NAmer	1948	CC	684	n	bl	y	n	0	all/unsp	1	9	nev any	st
WYNDE4	49		f	0	0	all	-	not a	NAmer	1948	CC	684	n	bl	y	n	2	all/unsp	1	9	nev any	ot
ZHENG	1		m	0	0	all	-	q	As:Chi	1982	CC	540	n	ot	*	y	0	cig+/-ot	1	9	nev cigs	st
ZHENG	16		f	0	0	all	-	q	As:Chi	1982	CC	540	n	ot	*	y	0	cig+/-ot	1	9	nev cigs	st
ZHOU	10		c	0	0	all	-	q	As:Chi	1978	CC	1360	n	ot	n	n	0	all/unsp	1	9	nev any	st

Cigarette type is all/unspec for all RRs

except for the following:

REF	NRR	CIGTYPE
ALDERS	34	MC only
ALDERS	37	MC only

Table 2G2 - 5

IESLC - Meta-analysis of Ever Smoking, Amount smoked, "Low", Any product (or Cigarettes if Any not available)
Squamous
Least adjusted

REF	NRR	SEX	AD	Number Exposed Case	Cont	Non-exposed Case	Cont	RR	95.00%CI
ALDERS	34	m	1	-	-	-	-	3.79	(1.30- 11.02)
ALDERS	37	f	1	-	-	-	-	2.55	(1.42- 4.57)
Subtotal ALDERS								2.79	(1.67- 4.66)
BARBON	19	m	0	11	87	6	188	3.96	(1.42- 11.06)
BROWN2	36	m	2	-	-	-	-	7.60	(6.20- 9.40)
BROWN2	35	f	2	-	-	-	-	11.70	(8.70- 15.80)
Subtotal BROWN2								8.75	(7.38- 10.38)
CHOI	46	m	0	12	90	6	95	2.11	(0.76- 5.86)
CHOI	56	f	0	4	16	10	164	4.10	(1.15- 14.57)
Subtotal CHOI								2.74	(1.24- 6.07)
DOLL	55	m	0	291	570	3	61	10.38	(3.23- 33.37)
DOLL	63	f	0	9	18	16	59	1.84	(0.70- 4.88)
Subtotal DOLL								3.74	(1.77- 7.90)
DORGAN	114	m	2	-	-	-	-	11.50	(4.10- 32.24)
DORGAN	99	f	3	-	-	-	-	7.78	(4.86- 12.44)
Subtotal DORGAN								8.32	(5.43- 12.76)
DOSEME	7	m	2	-	-	-	-	2.60	(1.50- 4.60)
GER	6	c	0	9	56	11	80	1.17	(0.45- 3.01)
JEDRYC	1	m	0	1	67	6	289	0.72	(0.09- 6.07)
KREYBE	13	m	0	123	2341	3	644	11.28	(3.58- 35.57)
KREYBE	31	f	0	1	286	3	657	0.77	(0.08- 7.39)
Subtotal KREYBE								6.51	(2.34- 18.14)
LAMTH	10	f	0	23	11	28	72	5.38	(2.32- 12.46)
LUBIN2	149	m	0	418	2194	54	2616	9.23	(6.91- 12.32)
LUBIN2	169	f	0	30	184	72	1180	2.67	(1.70- 4.20)
Subtotal LUBIN2								6.45	(5.06- 8.23)
LUO	4	c	0	3	39	5	51	0.78	(0.18- 3.48)
MATOS	42	m	0	3	88	3	110	1.25	(0.25- 6.35)
MATSUD	4	m	0	21	1237	1	1255	21.31	(2.86- 158.63)
ORMOS	5	m	0	13	329	2	777	15.35	(3.44- 68.41)
OSANN2	10	f	0	18	31	7	58	4.81	(1.81- 12.77)
WUWILL	20	f	0	168	311	117	601	2.77	(2.11- 3.65)
WYNDE2	3	m	0	15	114	3	105	4.61	(1.30- 16.36)
WYNDE3	4	m	0	7	42	3	88	4.89	(1.20- 19.86)
WYNDE3	63	f	0	1	19	5	76	0.80	(0.09- 7.26)
Subtotal WYNDE3								2.90	(0.89- 9.48)
WYNDE4	5	m	0	14	82	8	115	2.45	(0.98- 6.12)
WYNDE4	49	f	2	-	-	-	-	0.87	(0.11- 6.90)
Subtotal WYNDE4								2.07	(0.90- 4.78)
ZHENG	1	m	0	7	40	4	94	4.11	(1.14- 14.84)
ZHENG	16	f	0	11	29	33	184	2.11	(0.96- 4.64)
Subtotal ZHENG								2.54	(1.30- 4.96)
ZHOU	10	c	0	15	5	138	68	1.48	(0.52- 4.24)
Partial Totals				1228	8286	547	9687		

*prospective study

REF	NRR	SEX	AD	Ys	Ws	Qs	Ps
ALDERS	34	m	1	1.33	3.36	0.39	0.0145
ALDERS	37	f	1	0.94	11.25	6.09	0.0017
Subtotal ALDERS				1.03	14.61	6.48	
BARBON	19	m	0	1.38	3.64	0.32	0.0086
BROWN2	36	m	2	2.03	88.72	11.25	0.0000
BROWN2	35	f	2	2.46	43.16	26.77	0.0000
Subtotal BROWN2				2.17	131.88	38.02	
CHOI	46	m	0	0.75	3.68	3.15	0.1517
CHOI	56	f	0	1.41	2.39	0.16	0.0292
Subtotal CHOI				1.01	6.07	3.31	
DOLL	55	m	0	2.34	2.82	1.26	0.0001
DOLL	63	f	0	0.61	4.06	4.57	0.2175
Subtotal DOLL				1.32	6.88	5.82	
DORGAN	114	m	2	2.44	3.61	2.14	0.0000
DORGAN	99	f	3	2.05	17.39	2.51	0.0000
Subtotal DORGAN				2.12	21.01	4.65	
DOSEME	7	m	2	0.96	12.24	6.28	0.0008
GER	6	c	0	0.16	4.30	9.89	0.7462
JEDRYC	1	m	0	-0.33	0.84	3.38	0.7618
KREYBE	13	m	0	2.42	2.91	1.64	0.0000
KREYBE	31	f	0	-0.27	0.75	2.81	0.8175
Subtotal KREYBE				1.87	3.66	4.45	
LAMTH	10	f	0	1.68	5.44	0.00	0.0001

Table 2G2 - 5

IESLC - Meta-analysis of Ever Smoking, Amount smoked, "Low", Any product (or Cigarettes if Any not available)
Squamous
Least adjusted

REF	NRR	SEX	AD	Ys	Ws	Qs	Ps
LUBIN2	149	m	0	2.22	45.98	13.93	0.0000
LUBIN2	169	f	0	0.98	18.69	8.88	0.0000
Subtotal LUBIN2				1.86	64.67	22.81	
LUO	4	c	0	-0.24	1.73	6.34	0.7498
MATOS	42	m	0	0.22	1.46	3.06	0.7878
MATSUD	4	m	0	3.06	0.95	1.83	0.0028
ORMOS	5	m	0	2.73	1.72	1.93	0.0003
OSANN2	10	f	0	1.57	4.03	0.04	0.0016
WUWILL	20	f	0	1.02	51.60	21.90	0.0000
WYNDE2	3	m	0	1.53	2.39	0.05	0.0182
WYNDE3	4	m	0	1.59	1.96	0.01	0.0265
WYNDE3	63	f	0	-0.22	0.79	2.84	0.8428
Subtotal WYNDE3				1.07	2.75	2.85	
WYNDE4	5	m	0	0.90	4.60	2.76	0.0541
WYNDE4	49	f	2	-0.14	0.90	2.94	0.8951
Subtotal WYNDE4				0.73	5.50	5.70	
ZHENG	1	m	0	1.41	2.33	0.16	0.0308
ZHENG	16	f	0	0.75	6.21	5.29	0.0620
Subtotal ZHENG				0.93	8.54	5.44	
ZHOU	10	c	0	0.39	3.46	5.69	0.4669

	N	33
	NS	23
	Wt	359.37
Het	Chi	160.24
Het	df	32
Het	P	***
Fixed	RR	5.32
	RRl	4.80
	RRu	5.90
	P	+++
Random	RR	3.81
	RRl	2.87
	RRu	5.05
	P	+++
Asymm	P	*

Table 2G2 - 6

IESLC - Meta-analysis of Ever Smoking, Amount smoked, "Low", Any product (or Cigarettes if Any not available)
Squamous
Least adjusted

	combined	Sex male	female	Total
N	3	17	13	33
NS	3	17	13	33
Wt	9.50	183.22	166.65	359.37
Het Chi	0.46	42.96	79.47	160.24
Het df	2	16	12	32
Het P	N.S.	***	***	***
Fixed RR	1.18	6.77	4.45	5.32
RRl	0.63	5.86	3.83	4.80
RRu	2.24	7.82	5.18	5.90
P	N.S.	+++	+++	+++
Random RR	1.18	5.21	3.37	3.81
RRl	0.63	3.74	2.11	2.87
RRu	2.24	7.27	5.38	5.05
P	N.S.	+++	+++	+++
Between Chi				37.35
Between df				2
Between P				***
Btwn(F) P				*
Btwn(R) P				***

Table 2G2 - 7

IESLC - Meta-analysis of Ever Smoking, Amount smoked, "Low", Any product (or Cigarettes if Any not available)
Squamous
Excluded studies (and stage at which they were excluded)

1 ABELIN ABRAHA AMANDU AMES ANDERS AUSTIN AXELSO BAND BECHER BERRIN BLOHMK BLOT4 BROCKM BROWN1 BYERS1 BYERS2
CARPEN CASCO2 CASCOR CHAN CHEN3 CHIAZZ CHYOU DESTE2 DOCKER DROSTE DU GARCIA GARDIN GENG GODLEY GOODMA
GRAHAM GREGOR HEGMAN HEIN HENNEK HINDS HIRAOK HOROWI HORWIT HUANG ISHIMA JAHN JAIN JARVHO JIANG KELLER
KIHARA KJUUS KO KOHLME KUBIK LAMWK LAMWK2 LANGE LEI LEMARC LEVIN LIU LOMBA2 LOMBAR MAGNUS MARSH
MARSH2 MCDUFF MCLAUG MILLER MILLS NOTANI NOU ODRISC PAWLEG PERSHA POFFIJ QIAO QIAO2 RADZIK REN RONCO
ROOTS ROTHSC SAARIK SANKAR SCHWAR SEGI SEOW SHIMIZ SIMARA SIMONA SITAS SOBUE2 STASZE STAYNE STUCKE SUN
SUZUK2 SUZUKI TANG TAO TOKARS TOUSEY ULMER VEIERO VUTUC WALD WANG WANG3 WANG4 WICKLU WIGLE WILKIN
WU2 WUNSCH WYNDE8 XIANGZ XU XU2 XU4 YONG ZHANG

2 AKIBA ARCHER BENSHL BRETT BROSS CEDERL CHANG CHOW COMSTO DARBY DEAN3 DEKLER DORANT DORN ENGELA ENSTRO
GAO2 GILLIS HAENSZ HAMMO2 HIRAY2 HIRAYA HITOSU HOLE HUMBLE KAISE2 KAISER KANELL KATSOU KAUFMA KINLEN KNEKT
KOO LAURIL LIAW LICKIN LIDDEL MIGRAN MRFIT MRFITR MURATA NAM PARKIN PERSH2 PETO PEZZO2 PEZZOT PISANI
PRESCO RIMING SEGI2 SOBUE SPEIZE STOCKW SVENSS TANG2 TENKAN TSUGAN TULINI TVERDA WAKAI WARSIN WATSON WU
WYNDE5 WYNDER YAMAGU

3 BUELL CHEN MASTRA MZILEN RESTRE SADOWS

4 BEST BOFFET WYNDE7

6 BLOT1 BLOT2 BLOT3 BOUCHA JONES MOLLO SCHWA2 VANDER

8 AGUDO ARMADA AUVINE AXELSS BOUCOT BRESLO BUFFLE CHATZI CHEN2 COOKSO CORREA CPSI CPSII DAMBER DAVEYS DEAN
DEAN2 DESTEF DOLL2 DUNN EBELIN ESAKI FAN GAO GARSHI GOLLED GSELL HAMMON HANSEN HU HU2 JARUP
JOLY JUSSAW KHUDER KOULUM KREUZE LAUSSM LETOUR LIU2 LIU3 LIU4 LIU5 LUBIN MACLEN MARTIS MCCONN NOTAN2
PASTOR PERNU PIKE POLEDN RACHTA RANDIG SHAW SIEMIA SPITZ STOCKS TIZZAN WANG2 WYNDE6 XU3 YUAN

10 OSANN

11 BENHAM

Table 2G2 - 8
Potentially overlapping studies

REF	REFGP	PRINC	. OVERLAP/LINK
LUBIN2	LUBIN2	1	Lubin-combined
LAMTH	LAMTH	1	KOO/LAMTH/LAMWK
OSANN2	KAISER	2	KAISER/OSANN2
MATSUD	MATSUD	1	SOBUE2/MATSUD

Table 2G2 - 9
Most adjusted - insufficient data for metaanalysis

REF	NRR	SEX	AGEL	AGEH	RACE	YF	LC TYPE	LOC	START	ST	NLC	R	VB	P	H	AD	PRODUCT	exL	exH	DENOM	De
CHEN	5	c	0	0	all	-	q	As:oth	1987	CC	323	n	ot	n	y	2	cig+/-ot	1	10	nev cigs	ot

REF	NRR	RR	SIG	RRDATA comment
CHEN	5	2.59	n	0

Table 2G3 -

IESLC - Meta-analysis of Ever Smoking, Amount smoked, "Mid", Any product (or Cigarettes if Any not available)
Squamous

This analysis is restricted to results for:
1) Results by Amount smoked
2) Ever smokers
3) Results complete enough for use in metaanalysis

Within each study, results are then selected (in the following order of preference, within each sex) for:
4) PRODUCT: all/unspec, cigarettes regardless of other products, cigarettes only
5) CIGTYPE: all/unspecified, MC regardless of HR, MC only
6) DENOM: never smoked anything, never smoked cigarettes, (never +1 = +long term ex, +2 = +amount unknown,
+3 = never cigs+long term ex)
7) Followup period (YF, prospective studies): whole study (coded as 0) or longest available
8) LCtype: squamous or nearest available, but not adeno. (q = squamous, s = small,
a = adeno, KI = Kreyberg I, u = undifferentiated)
9) Race: all or nearest available, otherwise by race (wh or w = white, bl or b = black, hi = hispanic
ch = chinese, jap = japanese, haw = hawaiian, w+o = white + oriental, sca = scandinavian, as = asian)
10) Amount smoked "mid" in key scheme 1 (key value 20, maximum range 6-44, in numbers of
cigarettes or cigarette equivalents)
11) For overlapping studies: principal rather than subsidiary studies
Finally by Age: whole study (coded as 0) if available, otherwise by widest available age group
and then for single sex results (m, f) in preference to combined sex results (c).

Results adjusted (AD) for the most potential confounders are then chosen in Sections -1 to -3
and results adjusted for the least confounders in Sections -4 to -6. (Those least adjusted results which
actually differ from the most adjusted as marked 'x' in column X in Section -4)
(Results adjusted for an unknown number of confounder(s) are coded as 20.)

Section -7 shows excluded studies, together with the stage (as above) at which no qualifying
results were found.

Section -8 lists the potentially overlapping studies which have been included (1=principal, 2=subsidiary).

Section -9 lists any results which would have been included in preference except that they had data not complete
enough for use in meta-analysis, with their significance (yes/no), if known, and any further comment as entered
on the database.

In addition to those mentioned above, the following fields, levels and abbreviations are used:
* or nk = not known, n = no, y = yes, ot = other
nev = never
all/unspec = all or unspecified, cig+/-ot = cigarettes irrespective of other products (cigar, pipe etc)
MC = manufactured cigarettes, HR = hand-rolled cigarettes
exL, exH = range of exposure (low and high) in the smoking group, in terms of Amount smoked,
cigarettes or cigarette equivalents
REF: 6-character study reference
NRR: number of the RR on the database within the study
ST : study type (CC = case control, pr or prosp = prospective)
NLC: number of lung cancer cases in whole study
R : risky occupational population (n = no, m = mining, o = other risky)
VB : national cigarette type (V = at least 75% Virginia, bl = at least 75% blended, ot = other)
P : any proxy use
H : full histological confirmation
De : derivation of RR/CI (or = original, st = standard method, ot = other method of estimation)

Table 2G3 - 1

IESLC - Meta-analysis of Ever Smoking, Amount smoked, "Mid", Any product (or Cigarettes if Any not available)
Squamous
Most adjusted

REF	NRR	SEX	AGEL	AGEH	RACE	YF	LC TYPE	LOC	START	ST	NLC	R	VB	P	H	AD	PRODUCT	exL	exH	DENOM	De
ALDERS	35	m	0	0	all	-	q+s	Eu:UK	1977	CC	1448	n	V	n	n	1	cig only	18	27	nev+2	ot
ALDERS	38	f	0	0	all	-	q+s	Eu:UK	1977	CC	1448	n	V	n	n	1	cig only	18	27	nev+2	ot
BARBON	71	m	0	0	all	-	q	Eu:wst	1979	CC	755	n	bl	y	y	3	all/unsp	20	39	nev any	or
CHOI	47	m	0	0	all	-	q	As:oth	1985	CC	375	n	bl	n	n	0	cig+/-ot	11	20	nev cigs	st
CHOI	57	f	0	0	all	-	q	As:oth	1985	CC	375	n	bl	n	n	0	cig+/-ot	11	30	nev cigs	st
DOLL	70	m	0	0	all	-	KI	Eu:UK	1948	CC	1465	n	V	n	n	1	all/unsp	15	24	nev any	ot
DOSEME	11	m	0	0	all	-	q	Eu:bal	1979	CC	1210	n	bl	n	n	2	cig+/-ot	11	20	nev cigs	or
GER	15	c	0	0	all	-	q+s	As:oth	1990	CC	141	n	ot	y	n	10	all/unsp	11	20	nev any	ot
JEDRYC	29	m	0	0	all	-	q	Eu:est	1980	CC	1630	n	bl	y	n	3	cig+/-ot	20	29	nev any	or
KREYBE	2	m	0	0	all	-	KI	Eu:Sca	1948	CC	300	n	bl	n	y	1	all/unsp	15	24	nev any	ot
LAMTH	11	f	0	0	ch	-	q	As:HK	1983	CC	445	n	bl	n	n	0	all/unsp	11	20	nev any	or
LUBIN2	157	m	0	0	all	-	q	Eu:mul	1976	CC	7804	n	bl	n	y	0	cig+/-ot	20	29	nev any	st
LUBIN2	177	f	0	0	all	-	q	Eu:mul	1976	CC	7804	n	bl	n	y	0	cig+/-ot	20	29	nev any	st
LUO	11	c	0	0	all	-	q	As:Chi	1990	CC	102	n	ot	n	y	20	cig+/-ot	20	29	nev cigs	or
MATOS	45	m	0	0	all	-	q	SCAmer	1994	CC	200	n	bl	n	n	2	cig+/-ot	15	24	nev any	or
MATSUD	5	m	0	0	all	-	q	As:Jap	1965	CC	179	n	bl	n	n	0	cig+/-ot	11	20	nev cigs	st
ORMOS	6	m	0	0	all	-	q	Eu:est	1947	CC	119	n	bl	y	y	0	cig+/-ot	16	30	nev any	st
WYNDE2	4	m	0	0	all	-	KI	NAmer	1962	CC	404	n	bl	n	y	0	cig+/-ot	11	20	nev any	st
WYNDE3	5	m	0	0	all	-	KI	NAmer	1966	CC	350	n	bl	n	y	0	cig+/-ot	10	20	nev any	st
WYNDE3	64	f	0	0	all	-	KI	NAmer	1966	CC	350	n	bl	n	y	0	cig+/-ot	10	20	nev any	st
WYNDE4	65	m	0	0	all	-	not a	NAmer	1948	CC	684	n	bl	y	n	2	all/unsp	16	20	nev any	ot
WYNDE4	51	f	0	0	all	-	not a	NAmer	1948	CC	684	n	bl	y	n	2	all/unsp	16	20	nev any	ot
ZHENG	3	m	0	0	all	-	q	As:Chi	1982	CC	540	n	ot	*	y	0	cig+/-ot	20	29	nev cigs	st

Cigarette type is all/unspec for all RRs

except for the following:

REF	NRR	CIGTYPE
ALDERS	35	MC only
ALDERS	38	MC only

Table 2G3 - 2

IESLC - Meta-analysis of Ever Smoking, Amount smoked, "Mid", Any product (or Cigarettes if Any not available)
Squamous
Most adjusted

REF	NRR	SEX	AD	Number Exposed Case	Number Exposed Cont	Non-exposed Case	Non-exposed Cont	RR	95.00%CI
ALDERS	35	m	1	-	-	-	-	7.19	(2.75- 18.79)
ALDERS	38	f	1	-	-	-	-	9.24	(5.31- 16.09)
Subtotal ALDERS								8.68	(5.37- 14.03)
BARBON	71	m	3	-	-	-	-	16.30	(7.00- 38.00)
CHOI	47	m	0	84	281	6	95	4.73	(2.00- 11.19)
CHOI	57	f	0	5	9	10	164	9.11	(2.57- 32.31)
Subtotal CHOI								5.82	(2.86- 11.86)
DOLL	70	m	1	-	-	-	-	14.30	(4.45- 46.00)
DOSEME	11	m	2	-	-	-	-	3.20	(2.20- 4.60)
GER	15	c	10	-	-	-	-	2.20	(0.65- 7.48)
JEDRYC	29	m	3	-	-	-	-	13.46	(5.76- 31.47)
KREYBE	2	m	1	-	-	-	-	11.02	(3.42- 35.51)
LAMTH	11	f	0	28	6	28	72	12.00	(4.49- 32.10)
LUBIN2	157	m	0	1298	3108	54	2616	20.23	(15.33- 26.69)
LUBIN2	177	f	0	61	110	72	1180	9.09	(6.13- 13.46)
Subtotal LUBIN2								15.51	(12.37- 19.45)
LUO	11	c	20	-	-	-	-	24.60	(4.20- 145.70)
MATOS	45	m	2	-	-	-	-	7.80	(2.20- 27.40)
MATSUD	5	m	0	43	1607	1	1255	33.58	(4.62- 244.19)
ORMOS	6	m	0	10	577	2	777	6.73	(1.47- 30.85)
WYNDE2	4	m	0	108	203	3	105	18.62	(5.77- 60.06)
WYNDE3	5	m	0	57	114	3	88	14.67	(4.44- 48.40)
WYNDE3	64	f	0	13	24	5	76	8.23	(2.66- 25.46)
Subtotal WYNDE3								10.81	(4.76- 24.56)
WYNDE4	65	m	2	-	-	-	-	10.92	(5.22- 22.86)
WYNDE4	51	f	2	-	-	-	-	14.92	(4.88- 45.67)
Subtotal WYNDE4								12.01	(6.48- 22.23)
ZHENG	3	m	0	75	89	4	94	19.80	(6.95- 56.41)
Partial Totals				1782	6128	188	6522		

*prospective study

REF	NRR	SEX	AD	Ys	Ws	Qs	Ps
ALDERS	35	m	1	1.97	4.16	0.52	0.0001
ALDERS	38	f	1	2.22	12.50	0.13	0.0000
Subtotal ALDERS				2.16	16.66	0.64	
BARBON	71	m	3	2.79	5.37	1.17	0.0000
CHOI	47	m	0	1.55	5.19	3.08	0.0004
CHOI	57	f	0	2.21	2.40	0.03	0.0006
Subtotal CHOI				1.76	7.59	3.11	
DOLL	70	m	1	2.66	2.82	0.32	0.0000
DOSEME	11	m	2	1.16	28.24	38.12	0.0000
GER	15	c	10	0.79	2.57	6.08	0.2058
JEDRYC	29	m	3	2.60	5.33	0.40	0.0000
KREYBE	2	m	1	2.40	2.81	0.02	0.0001
LAMTH	11	f	0	2.48	3.97	0.10	0.0000
LUBIN2	157	m	0	3.01	50.02	23.29	0.0000
LUBIN2	177	f	0	2.21	24.86	0.35	0.0000
Subtotal LUBIN2				2.74	74.88	23.63	
LUO	11	c	20	3.20	1.22	0.94	0.0004
MATOS	45	m	2	2.05	2.42	0.18	0.0014
MATSUD	5	m	0	3.51	0.98	1.38	0.0005
ORMOS	6	m	0	1.91	1.66	0.29	0.0141
WYNDE2	4	m	0	2.92	2.80	1.01	0.0000
WYNDE3	5	m	0	2.69	2.70	0.35	0.0000
WYNDE3	64	f	0	2.11	3.01	0.14	0.0003
Subtotal WYNDE3				2.38	5.71	0.49	
WYNDE4	65	m	2	2.39	7.04	0.03	0.0000
WYNDE4	51	f	2	2.70	3.07	0.44	0.0000
Subtotal WYNDE4				2.49	10.12	0.47	
ZHENG	3	m	0	2.99	3.51	1.53	0.0000

Table 2G3 - 2

IESLC - Meta-analysis of Ever Smoking, Amount smoked, "Mid", Any product (or Cigarettes if Any not available)
Squamous
Most adjusted

	N	23
	NS	18
	Wt	178.64
Het	Chi	79.88
Het	df	22
Het	P	***
Fixed	RR	10.23
	RRl	8.83
	RRu	11.84
	P	+++
Random	RR	10.20
	RRl	7.37
	RRu	14.13
	P	+++
Asymm	P	N.S.

Table 2G3 - 3

IESLC - Meta-analysis of Ever Smoking, Amount smoked, "Mid", Any product (or Cigarettes if Any not available)
Squamous
Most adjusted

	combined	Sex male	female	Total
N	2	15	6	23
NS	2	15	6	23
Wt	3.80	125.03	49.82	178.64
Het Chi	4.83	71.36	0.96	79.88
Het df	1	14	5	22
Het P	*	***	N.S.	***
Fixed RR	4.78	10.75	9.56	10.23
RRl	1.75	9.02	7.25	8.83
RRu	13.08	12.81	12.63	11.84
P	++	+++	+++	+++
Random RR	6.73	10.96	9.56	10.20
RRl	0.64	6.85	7.25	7.37
RRu	71.24	17.52	12.63	14.13
P	N.S.	+++	+++	+++
Between Chi				2.72
Between df				2
Between P				N.S.
Btwn(F) P				N.S.
Btwn(R) P				N.S.

Table 2G3 - 4

IESLC - Meta-analysis of Ever Smoking, Amount smoked, "Mid", Any product (or Cigarettes if Any not available)
Squamous
Least adjusted

REF	NRR	X	SEX	AGEL	AGEH	RACE	YF	LC TYPE	LOC	START	ST	NLC	R	VB	P	H	AD	PRODUCT	exL	exH	DENOM	De
ALDERS	35		m	0	0	all	-	q+s	Eu:UK	1977	CC	1448	n	V	n	n	1	cig only	18	27	nev+2	ot
ALDERS	38		f	0	0	all	-	q+s	Eu:UK	1977	CC	1448	n	V	n	n	1	cig only	18	27	nev+2	ot
BARBON	23	x	m	0	0	all	-	q	Eu:wst	1979	CC	755	n	bl	y	y	0	all/unsp	20	29	nev any	st
CHOI	47		m	0	0	all	-	q	As:oth	1985	CC	375	n	bl	n	n	0	cig+/-ot	11	20	nev cigs	st
CHOI	57		f	0	0	all	-	q	As:oth	1985	CC	375	n	bl	n	n	0	cig+/-ot	11	30	nev cigs	st
DOLL	56	x	m	0	0	all	-	KI	Eu:UK	1948	CC	1465	n	V	n	n	0	all/unsp	15	24	nev any	st
DOSEME	11		m	0	0	all	-	q	Eu:bal	1979	CC	1210	n	bl	n	n	2	cig+/-ot	11	20	nev cigs	or
GER	7	x	c	0	0	all	-	q+s	As:oth	1990	CC	141	n	ot	y	n	0	all/unsp	11	20	nev any	st
JEDRYC	3	x	m	0	0	all	-	q	Eu:est	1980	CC	1630	n	bl	y	n	0	cig+/-ot	20	29	nev any	st
KREYBE	14	x	m	0	0	all	-	KI	Eu:Sca	1948	CC	300	n	bl	n	y	0	all/unsp	15	24	nev any	st
LAMTH	11		f	0	0	ch	-	q	As:HK	1983	CC	445	n	bl	n	n	0	all/unsp	11	20	nev any	or
LUBIN2	157		m	0	0	all	-	q	Eu:mul	1976	CC	7804	n	bl	n	y	0	cig+/-ot	20	29	nev any	st
LUBIN2	177		f	0	0	all	-	q	Eu:mul	1976	CC	7804	n	bl	n	y	0	cig+/-ot	20	29	nev any	st
LUO	5	x	c	0	0	all	-	q	As:Chi	1990	CC	102	n	ot	n	y	0	cig+/-ot	20	29	nev cigs	st
MATOS	44	x	m	0	0	all	-	q	SCAmer	1994	CC	200	n	bl	n	n	0	cig+/-ot	15	24	nev any	st
MATSUD	5		m	0	0	all	-	q	As:Jap	1965	CC	179	n	bl	n	n	0	cig+/-ot	11	20	nev cigs	st
ORMOS	6		m	0	0	all	-	q	Eu:est	1947	CC	119	n	bl	y	y	0	cig+/-ot	16	30	nev any	st
WYNDE2	4		m	0	0	all	-	KI	NAmer	1962	CC	404	n	bl	n	y	0	cig+/-ot	11	20	nev any	st
WYNDE3	5		m	0	0	all	-	KI	NAmer	1966	CC	350	n	bl	n	y	0	cig+/-ot	10	20	nev any	st
WYNDE3	64		f	0	0	all	-	KI	NAmer	1966	CC	350	n	bl	n	y	0	cig+/-ot	10	20	nev any	st
WYNDE4	17	x	m	0	0	all	-	not a	NAmer	1948	CC	684	n	bl	y	n	0	all/unsp	16	20	nev any	st
WYNDE4	51		f	0	0	all	-	not a	NAmer	1948	CC	684	n	bl	y	n	2	all/unsp	16	20	nev any	ot
ZHENG	3		m	0	0	all	-	q	As:Chi	1982	CC	540	n	ot	*	y	0	cig+/-ot	20	29	nev cigs	st

Cigarette type is all/unspec for all RRs

except for the following:

REF	NRR	CIGTYPE
ALDERS	35	MC only
ALDERS	38	MC only

Table 2G3 - 5

IESLC - Meta-analysis of Ever Smoking, Amount smoked, "Mid", Any product (or Cigarettes if Any not available)
Squamous
Least adjusted

REF	NRR	SEX	AD	Number Exposed Case	Number Exposed Cont	Non-exposed Case	Non-exposed Cont	RR	95.00%CI
ALDERS	35	m	1	-	-	-	-	7.19	(2.75- 18.79)
ALDERS	38	f	1	-	-	-	-	9.24	(5.31- 16.09)
Subtotal ALDERS								8.68	(5.37- 14.03)
BARBON	23	m	0	81	176	6	188	14.42	(6.14- 33.89)
CHOI	47	m	0	84	281	6	95	4.73	(2.00- 11.19)
CHOI	57	f	0	5	9	10	164	9.11	(2.57- 32.31)
Subtotal CHOI								5.82	(2.86- 11.86)
DOLL	56	m	0	301	431	3	61	14.20	(4.41- 45.68)
DOSEME	11	m	2	-	-	-	-	3.20	(2.20- 4.60)
GER	7	c	0	20	87	11	80	1.67	(0.75- 3.71)
JEDRYC	3	m	0	152	434	6	289	16.87	(7.36- 38.66)
KREYBE	14	m	0	49	925	3	644	11.37	(3.53- 36.64)
LAMTH	11	f	0	28	6	28	72	12.00	(4.49- 32.10)
LUBIN2	157	m	0	1298	3108	54	2616	20.23	(15.33- 26.69)
LUBIN2	177	f	0	61	110	72	1180	9.09	(6.13- 13.46)
Subtotal LUBIN2								15.51	(12.37- 19.45)
LUO	5	c	0	22	23	5	51	9.76	(3.28- 28.98)
MATOS	44	m	0	18	90	3	110	7.33	(2.09- 25.69)
MATSUD	5	m	0	43	1607	1	1255	33.58	(4.62- 244.19)
ORMOS	6	m	0	10	577	2	777	6.73	(1.47- 30.85)
WYNDE2	4	m	0	108	203	3	105	18.62	(5.77- 60.06)
WYNDE3	5	m	0	57	114	3	88	14.67	(4.44- 48.40)
WYNDE3	64	f	0	13	24	5	76	8.23	(2.66- 25.46)
Subtotal WYNDE3								10.81	(4.76- 24.56)
WYNDE4	17	m	0	213	274	8	115	11.17	(5.34- 23.39)
WYNDE4	51	f	2	-	-	-	-	14.92	(4.88- 45.67)
Subtotal WYNDE4								12.20	(6.59- 22.60)
ZHENG	3	m	0	75	89	4	94	19.80	(6.95- 56.41)
Partial Totals				2638	8568	233	8060		

*prospective study

REF	NRR	SEX	AD	Ys	Ws	Qs	Ps
ALDERS	35	m	1	1.97	4.16	0.40	0.0001
ALDERS	38	f	1	2.22	12.50	0.05	0.0000
Subtotal ALDERS				2.16	16.66	0.45	
BARBON	23	m	0	2.67	5.26	0.78	0.0000
CHOI	47	m	0	1.55	5.19	2.76	0.0004
CHOI	57	f	0	2.21	2.40	0.01	0.0006
Subtotal CHOI				1.76	7.59	2.78	
DOLL	56	m	0	2.65	2.81	0.38	0.0000
DOSEME	11	m	2	1.16	28.24	35.48	0.0000
GER	7	c	0	0.51	6.06	19.00	0.2056
JEDRYC	3	m	0	2.83	5.59	1.64	0.0000
KREYBE	14	m	0	2.43	2.81	0.06	0.0000
LAMTH	11	f	0	2.48	3.97	0.16	0.0000
LUBIN2	157	m	0	3.01	50.02	26.16	0.0000
LUBIN2	177	f	0	2.21	24.86	0.15	0.0000
Subtotal LUBIN2				2.74	74.88	26.31	
LUO	5	c	0	2.28	3.24	0.00	0.0000
MATOS	44	m	0	1.99	2.44	0.21	0.0018
MATSUD	5	m	0	3.51	0.98	1.48	0.0005
ORMOS	6	m	0	1.91	1.66	0.24	0.0141
WYNDE2	4	m	0	2.92	2.80	1.15	0.0000
WYNDE3	5	m	0	2.69	2.70	0.43	0.0000
WYNDE3	64	f	0	2.11	3.01	0.09	0.0003
Subtotal WYNDE3				2.38	5.71	0.53	
WYNDE4	17	m	0	2.41	7.04	0.12	0.0000
WYNDE4	51	f	2	2.70	3.07	0.54	0.0000
Subtotal WYNDE4				2.50	10.11	0.66	
ZHENG	3	m	0	2.99	3.51	1.73	0.0000

Table 2G3 - 5

IESLC - Meta-analysis of Ever Smoking, Amount smoked, "Mid", Any product (or Cigarettes if Any not available)
Squamous
Least adjusted

	N	23
	NS	18
	Wt	184.33
Het	Chi	93.02
Het	df	22
Het	P	***
Fixed	RR	9.82
	RRl	8.50
	RRu	11.34
	P	+++
Random	RR	9.72
	RRl	6.93
	RRu	13.64
	P	+++
Asymm	P	N.S.

Table 2G3 - 6

IESLC - Meta-analysis of Ever Smoking, Amount smoked, "Mid", Any product (or Cigarettes if Any not available)
Squamous
Least adjusted

	Sex			
	combined	male	female	Total
N	2	15	6	23
NS	2	15	6	23
Wt	9.31	125.20	49.82	184.33
Het Chi	6.57	71.86	0.96	93.02
Het df	1	14	5	22
Het P	*	***	N.S.	***
Fixed RR	3.09	10.81	9.56	9.82
RRl	1.63	9.07	7.25	8.50
RRu	5.88	12.88	12.63	11.34
P	+++	+++	+++	+++
Random RR	3.88	11.04	9.56	9.72
RRl	0.69	6.90	7.25	6.93
RRu	21.80	17.68	12.63	13.64
P	N.S.	+++	+++	+++
Between Chi				13.62
Between df				2
Between P				**
Btwn(F) P				N.S.
Btwn(R) P				N.S.

Table 2G3 - 7

IESLC - Meta-analysis of Ever Smoking, Amount smoked, "Mid", Any product (or Cigarettes if Any not available)
Squamous
Excluded studies (and stage at which they were excluded)

1	ABELIN ABRAHA AMANDU AMES ANDERS AUSTIN AXELSO BAND BECHER BERRIN BLOHMK BLOT4 BROCKM BROWN1 BYERS1 BYERS2 CARPEN CASCO2 CASCOR CHAN CHEN3 CHIAZZ CHYOU DESTE2 DOCKER DROSTE DU GARCIA GARDIN GENG GODLEY GOODMA GRAHAM GREGOR HEGMAN HEIN HENNEK HINDS HIRAOK HOROWI HORWIT HUANG ISHIMA JAHN JAIN JARVHO JIANG KELLER KIHARA KJUUS KO KOHLME KUBIK LAMWK LAMWK2 LANGE LEI LEMARC LEVIN LIU LOMBA2 LOMBAR MAGNUS MARSH MARSH2 MCDUFF MCLAUG MILLER MILLS NOTANI NOU ODRISC PAWLEG PERSHA POFFIJ QIAO QIAO2 RADZIK REN RONCO ROOTS ROTHSC SAARIK SANKAR SCHWAR SEGI SEOW SHIMIZ SIMARA SIMONA SITAS SOBUE2 STASZE STAYNE STUCKE SUN SUZUK2 SUZUKI TANG TAO TOKARS TOUSEY ULMER VEIERO VUTUC WALD WANG WANG3 WANG4 WICKLU WIGLE WILKIN WU2 WUNSCH WYNDE8 XIANGZ XU XU2 XU4 YONG ZHANG
2	AKIBA ARCHER BENSHL BRETT BROSS CEDERL CHANG CHOW COMSTO DARBY DEAN3 DEKLER DORANT DORN ENGELA ENSTRO GAO2 GILLIS HAENSZ HAMMO2 HIRAY2 HIRAYA HITOSU HOLE HUMBLE KAISE2 KAISER KANELL KATSOU KAUFMA KINLEN KNEKT KOO LAURIL LIAW LICKIN LIDDEL MIGRAN MRFIT MRFITR MURATA NAM PARKIN PERSH2 PETO PEZZO2 PEZZOT PISANI PRESCO RIMING SEGI2 SOBUE SPEIZE STOCKW SVENSS TANG2 TENKAN TSUGAN TULINI TVERDA WAKAI WARSIN WATSON WU WYNDE5 WYNDER YAMAGU
3	BUELL CHEN MASTRA MZILEN RESTRE SADOWS
4	BEST BOFFET WYNDE7
6	BLOT1 BLOT2 BLOT3 BOUCHA JONES MOLLO SCHWA2 VANDER
8	AGUDO ARMADA AUVINE AXELSS BOUCOT BRESLO BUFFLE CHATZI CHEN2 COOKSO CORREA CPSI CPSII DAMBER DAVEYS DEAN DEAN2 DESTEF DOLL2 DUNN EBELIN ESAKI FAN GAO GARSHI GOLLED GSELL HAMMON HANSEN HU HU2 JARUP JOLY JUSSAW KHUDER KOULUM KREUZE LAUSSM LETOUR LIU2 LIU3 LIU4 LIU5 LUBIN MACLEN MARTIS MCCONN NOTAN2 PASTOR PERNU PIKE POLEDN RACHTA RANDIG SHAW SIEMIA SPITZ STOCKS TIZZAN WANG2 WYNDE6 XU3 YUAN
10	BROWN2 DORGAN OSANN OSANN2 WUWILL ZHOU
11	BENHAM

Table 2G3 - 8
Potentially overlapping studies

REF	REFGP	PRINC	OVERLAP/LINK
LUBIN2	LUBIN2	1	Lubin-combined
LAMTH	LAMTH	1	KOO/LAMTH/LAMWK
MATSUD	MATSUD	1	SOBUE2/MATSUD

Table 2G3 - 9
Most adjusted - insufficient data for metaanalysis

REF	NRR	SEX	AGEL	AGEH	RACE	YF	LC TYPE	LOC	START	ST	NLC	R	VB	P	H	AD	PRODUCT	exL	exH	DENOM	De
CHEN	4	c	0	0	all	-	q	As:oth	1987	CC	323	n	ot	n	y	2	cig+/-ot	11	20	nev cigs	ot

REF	NRR	RR	SIG	RRDATA comment
CHEN	4	7.05	n	0

Table 2G4 -

IESLC - Meta-analysis of Ever Smoking, Amount smoked, "High", Any product (or Cigarettes if Any not available)
Squamous

This analysis is restricted to results for:
1) Results by Amount smoked
2) Ever smokers
3) Results complete enough for use in metaanalysis

Within each study, results are then selected (in the following order of preference, within each sex) for:
4) PRODUCT: all/unspec, cigarettes regardless of other products, cigarettes only
5) CIGTYPE: all/unspecified, MC regardless of HR, MC only
6) DENOM: never smoked anything, never smoked cigarettes, (never +1 = +long term ex, +2 = +amount unknown, +3 = never cigs+long term ex)
7) Followup period (YF, prospective studies): whole study (coded as 0) or longest available
8) LCtype: squamous or nearest available, but not adeno. (q = squamous, s = small, a = adeno, KI = Kreyberg I, u = undifferentiated)
9) Race: all or nearest available, otherwise by race (wh or w = white, bl or b = black, hi = hispanic ch = chinese, jap = japanese, haw = hawaiian, w+o = white + oriental, sca = scandinavian, as = asian)
10) Amount smoked "high" in key scheme 1 (key value 45, maximum range >20, in numbers of cigarettes or cigarette equivalents)
11) For overlapping studies: principal rather than subsidiary studies
Finally by Age: whole study (coded as 0) if available, otherwise by widest available age group and then for single sex results (m, f) in preference to combined sex results (c).

Results adjusted (AD) for the most potential confounders are then chosen in Sections -1 to -3 and results adjusted for the least confounders in Sections -4 to -6. (Those least adjusted results which actually differ from the most adjusted as marked 'x' in column X in Section -4) (Results adjusted for an unknown number of confounder(s) are coded as 20.)

Section -7 shows excluded studies, together with the stage (as above) at which no qualifying results were found.

Section -8 lists the potentially overlapping studies which have been included (1=principal, 2=subsidiary).

Section -9 lists any results which would have been included in preference except that they had data not complete enough for use in meta-analysis, with their significance (yes/no), if known, and any further comment as entered on the database.

In addition to those mentioned above, the following fields, levels and abbreviations are used:
* or nk = not known, n = no, y = yes, ot = other
nev = never
all/unspec = all or unspecified, cig+/-ot = cigarettes irrespective of other products (cigar, pipe etc)
MC = manufactured cigarettes, HR = hand-rolled cigarettes
exL, exH = range of exposure (low and high) in the smoking group, in terms of Amount smoked, cigarettes or cigarette equivalents
REF: 6-character study reference
NRR: number of the RR on the database within the study
ST : study type (CC = case control, pr or prosp = prospective)
NLC: number of lung cancer cases in whole study
R : risky occupational population (n = no, m = mining, o = other risky)
VB : national cigarette type (V = at least 75% Virginia, bl = at least 75% blended, ot = other)
P : any proxy use
H : full histological confirmation
De : derivation of RR/CI (or = original, st = standard method, ot = other method of estimation)

Table 2G4 - 1

IESLC - Meta-analysis of Ever Smoking, Amount smoked, "High", Any product (or Cigarettes if Any not available)
Squamous
Most adjusted

REF	NRR	SEX	AGEL	AGEH	RACE	YF	LC TYPE	LOC	START	ST	NLC	R	VB	P	H	AD	PRODUCT	exL	exH	DENOM	De
ALDERS	36	m	0	0	all	-	q+s	Eu:UK	1977	CC	1448	n	V	n	n	1	cig only	28	99	nev+2	ot
ALDERS	39	f	0	0	all	-	q+s	Eu:UK	1977	CC	1448	n	V	n	n	1	cig only	28	99	nev+2	ot
BARBON	72	m	0	0	all	-	q	Eu:wst	1979	CC	755	n	bl	y	y	3	all/unsp	40	99	nev any	or
CHOI	50	m	0	0	all	-	q	As:oth	1985	CC	375	n	bl	n	n	0	cig+/-ot	41	99	nev cigs	st
CHOI	58	f	0	0	all	-	q	As:oth	1985	CC	375	n	bl	n	n	0	cig+/-ot	31	99	nev cigs	st
DOLL	71	m	0	0	all	-	KI	Eu:UK	1948	CC	1465	n	V	n	n	1	all/unsp	25	99	nev any	ot
DOSEME	15	m	0	0	all	-	q	Eu:bal	1979	CC	1210	n	bl	n	n	2	cig+/-ot	21	99	nev cigs	or
GER	16	c	0	0	all	-	q+s	As:oth	1990	CC	141	n	ot	y	n	10	all/unsp	21	99	nev any	ot
JEDRYC	30	m	0	0	all	-	q	Eu:est	1980	CC	1630	n	bl	y	n	3	cig+/-ot	30	99	nev any	or
KREYBE	3	m	0	0	all	-	KI	Eu:Sca	1948	CC	300	n	bl	n	y	1	all/unsp	25	99	nev any	ot
LAMTH	12	f	0	0	ch	-	q	As:HK	1983	CC	445	n	bl	n	n	0	all/unsp	21	99	nev any	st
LUBIN2	161	m	0	0	all	-	q	Eu:mul	1976	CC	7804	n	bl	n	y	0	cig+/-ot	30	99	nev any	st
LUBIN2	181	f	0	0	all	-	q	Eu:mul	1976	CC	7804	n	bl	n	y	0	cig+/-ot	30	99	nev any	st
LUO	12	c	0	0	all	-	q	As:Chi	1990	CC	102	n	ot	n	y	20	cig+/-ot	30	99	nev cigs	or
MATOS	47	m	0	0	all	-	q	SCAmer	1994	CC	200	n	bl	n	n	2	cig+/-ot	25	99	nev any	or
MATSUD	6	m	0	0	all	-	q	As:Jap	1965	CC	179	n	bl	n	n	0	cig+/-ot	21	99	nev cigs	st
ORMOS	7	m	0	0	all	-	q	Eu:est	1947	CC	119	n	bl	y	y	0	cig+/-ot	31	99	nev any	st
OSANN	59	m	0	0	all	-	q	NAmer	1984	CC	1986	n	bl	n	n	2	cig+/-ot	40	99	nev cigs	or
OSANN	60	f	0	0	all	-	q	NAmer	1984	CC	1986	n	bl	n	n	2	cig+/-ot	40	99	nev cigs	or
WYNDE2	6	m	0	0	all	-	KI	NAmer	1962	CC	404	n	bl	n	y	0	cig+/-ot	35	99	nev any	st
WYNDE3	7	m	0	0	all	-	KI	NAmer	1966	CC	350	n	bl	n	y	0	cig+/-ot	41	99	nev any	st
WYNDE3	66	f	0	0	all	-	KI	NAmer	1966	CC	350	n	bl	n	y	0	cig+/-ot	41	99	nev any	st
WYNDE4	67	m	0	0	all	-	not a	NAmer	1948	CC	684	n	bl	y	n	2	all/unsp	35	99	nev any	ot
WYNDE4	53	f	0	0	all	-	not a	NAmer	1948	CC	684	n	bl	y	n	2	all/unsp	35	99	nev any	ot
ZHENG	4	m	0	0	all	-	q	As:Chi	1982	CC	540	n	ot	*	y	0	cig+/-ot	30	99	nev cigs	st

Cigarette type is all/unspec for all RRs

except for the following:

REF	NRR	CIGTYPE
ALDERS	36	MC only
ALDERS	39	MC only

Table 2G4 - 2

IESLC - Meta-analysis of Ever Smoking, Amount smoked, "High", Any product (or Cigarettes if Any not available)
Squamous
Most adjusted

REF	NRR	SEX	AD	Number Exposed Case	Cont	Non-exposed Case	Cont	RR	95.00%CI
ALDERS	36	m	1	-	-	-	-	8.78	(3.46- 22.31)
ALDERS	39	f	1	-	-	-	-	14.52	(7.93- 26.58)
Subtotal ALDERS								12.51	(7.53- 20.77)
BARBON	72	m	3	-	-	-	-	28.60	(12.00- 69.00)
CHOI	50	m	0	9	6	6	95	23.75	(6.33- 89.09)
CHOI	58	f	0	2	1	10	164	32.80	(2.74- 393.20)
Subtotal CHOI								25.50	(7.94- 81.93)
DOLL	71	m	1	-	-	-	-	25.40	(7.83- 82.40)
DOSEME	15	m	2	-	-	-	-	7.00	(4.10- 12.00)
GER	16	c	10	-	-	-	-	16.04	(4.22- 60.93)
JEDRYC	30	m	3	-	-	-	-	21.42	(9.05- 50.68)
KREYBE	3	m	1	-	-	-	-	24.63	(7.54- 80.53)
LAMTH	12	f	0	10	1	28	72	25.71	(3.14- 210.29)
LUBIN2	161	m	0	849	1746	54	2616	23.56	(17.77- 31.22)
LUBIN2	181	f	0	18	39	72	1180	7.56	(4.12- 13.88)
Subtotal LUBIN2								19.26	(14.92- 24.87)
LUO	12	c	20	-	-	-	-	38.70	(5.20- 290.20)
MATOS	47	m	2	-	-	-	-	9.70	(2.80- 33.20)
MATSUD	6	m	0	39	470	1	1255	104.14	(14.27- 760.12)
ORMOS	7	m	0	4	128	2	777	12.14	(2.20- 66.97)
OSANN	59	m	2	-	-	-	-	76.00	(36.80- 157.00)
OSANN	60	f	2	-	-	-	-	72.30	(36.80- 142.00)
Subtotal OSANN								73.99	(45.14- 121.29)
WYNDE2	6	m	0	139	112	3	105	43.44	(13.42- 140.56)
WYNDE3	7	m	0	59	26	3	88	66.56	(19.27- 229.96)
WYNDE3	66	f	0	3	3	5	76	15.20	(2.42- 95.56)
Subtotal WYNDE3								41.95	(15.01- 117.26)
WYNDE4	67	m	2	-	-	-	-	29.54	(13.53- 64.50)
WYNDE4	53	f	2	-	-	-	-	26.53	(4.12- 171.09)
Subtotal WYNDE4								29.07	(14.15- 59.73)
ZHENG	4	m	0	49	23	4	94	50.07	(16.39- 152.91)
Partial Totals				1181	2555	188	6522		

*prospective study

REF	NRR	SEX	AD	Ys	Ws	Qs	Ps
ALDERS	36	m	1	2.17	4.42	3.59	0.0000
ALDERS	39	f	1	2.68	10.50	1.66	0.0000
Subtotal ALDERS				2.53	14.93	5.25	
BARBON	72	m	3	3.35	5.02	0.39	0.0000
CHOI	50	m	0	3.17	2.20	0.02	0.0000
CHOI	58	f	0	3.49	0.62	0.11	0.0059
Subtotal CHOI				3.24	2.82	0.13	
DOLL	71	m	1	3.23	2.77	0.07	0.0000
DOSEME	15	m	2	1.95	13.32	16.93	0.0000
GER	16	c	10	2.78	2.16	0.19	0.0000
JEDRYC	30	m	3	3.06	5.18	0.00	0.0000
KREYBE	3	m	1	3.20	2.74	0.05	0.0000
LAMTH	12	f	0	3.25	0.87	0.03	0.0025
LUBIN2	161	m	0	3.16	48.42	0.36	0.0000
LUBIN2	181	f	0	2.02	10.42	11.49	0.0000
Subtotal LUBIN2				2.96	58.85	11.85	
LUO	12	c	20	3.66	0.95	0.32	0.0004
MATOS	47	m	2	2.27	2.51	1.61	0.0003
MATSUD	6	m	0	4.65	0.97	2.40	0.0000
ORMOS	7	m	0	2.50	1.32	0.44	0.0042
OSANN	59	m	2	4.33	7.30	11.55	0.0000
OSANN	60	f	2	4.28	8.43	12.29	0.0000
Subtotal OSANN				4.30	15.73	23.83	
WYNDE2	6	m	0	3.77	2.79	1.36	0.0000
WYNDE3	7	m	0	4.20	2.50	3.16	0.0000
WYNDE3	66	f	0	2.72	1.14	0.14	0.0037
Subtotal WYNDE3				3.74	3.64	3.30	
WYNDE4	67	m	2	3.39	6.30	0.62	0.0000
WYNDE4	53	f	2	3.28	1.11	0.05	0.0006
Subtotal WYNDE4				3.37	7.41	0.66	
ZHENG	4	m	0	3.91	3.08	2.17	0.0000

Table 2G4 - 2

IESLC - Meta-analysis of Ever Smoking, Amount smoked, "High", Any product (or Cigarettes if Any not available)
Squamous
Most adjusted

	N	25
	NS	19
	Wt	147.05
Het	Chi	71.00
Het	df	24
Het	P	***
Fixed	RR	21.61
	RRl	18.39
	RRu	25.40
	P	+++
Random	RR	23.54
	RRl	16.96
	RRu	32.68
	P	+++
Asymm	P	N.S.

Table 2G4 - 3

IESLC - Meta-analysis of Ever Smoking, Amount smoked, "High", Any product (or Cigarettes if Any not available)
Squamous
Most adjusted

	combined	Sex male	female	Total
N	2	16	7	25
NS	2	16	7	25
Wt	3.11	110.85	33.09	147.05
Het Chi	0.51	44.52	25.09	71.00
Het df	1	15	6	24
Het P	N.S.	***	***	***
Fixed RR	21.00	22.57	18.75	21.61
RRl	6.91	18.73	13.33	18.39
RRu	63.86	27.18	26.36	25.40
P	+++	+++	+++	+++
Random RR	21.00	24.76	21.10	23.54
RRl	6.91	16.86	9.14	16.96
RRu	63.86	36.36	48.71	32.68
P	+++	+++	+++	+++
Between Chi				0.88
Between df				2
Between P				N.S.
Btwn(F) P				N.S.
Btwn(R) P				N.S.

Table 2G4 - 4

IESLC - Meta-analysis of Ever Smoking, Amount smoked, "High", Any product (or Cigarettes if Any not available)
Squamous
Least adjusted

REF	NRR	X	SEX	AGEL	AGEH	RACE	YF	LC TYPE	LOC	START	ST	NLC	R	VB	P	H	AD	PRODUCT	exL	exH	DENOM	De
ALDERS	36		m	0	0	all	-	q+s	Eu:UK	1977	CC	1448	n	V	n	n	1	cig only	28	99	nev+2	ot
ALDERS	39		f	0	0	all	-	q+s	Eu:UK	1977	CC	1448	n	V	n	n	1	cig only	28	99	nev+2	ot
BARBON	27	x	m	0	0	all	-	q	Eu:wst	1979	CC	755	n	bl	y	y	0	all/unsp	40	99	nev any	st
CHOI	50		m	0	0	all	-	q	As:oth	1985	CC	375	n	bl	n	n	0	cig+/-ot	41	99	nev cigs	st
CHOI	58		f	0	0	all	-	q	As:oth	1985	CC	375	n	bl	n	n	0	cig+/-ot	31	99	nev cigs	st
DOLL	57	x	m	0	0	all	-	KI	Eu:UK	1948	CC	1465	n	V	n	n	0	all/unsp	25	99	nev any	st
DOSEME	15		m	0	0	all	-	q	Eu:bal	1979	CC	1210	n	bl	n	n	2	cig+/-ot	21	99	nev cigs	or
GER	8	x	c	0	0	all	-	q+s	As:oth	1990	CC	141	n	ot	y	n	0	all/unsp	21	99	nev any	st
JEDRYC	5	x	m	0	0	all	-	q	Eu:est	1980	CC	1630	n	bl	y	n	0	cig+/-ot	40	99	nev any	st
KREYBE	15	x	m	0	0	all	-	KI	Eu:Sca	1948	CC	300	n	bl	n	y	0	all/unsp	25	99	nev any	st
LAMTH	12		f	0	0	ch	-	q	As:HK	1983	CC	445	n	bl	n	n	0	all/unsp	21	99	nev any	st
LUBIN2	161		m	0	0	all	-	q	Eu:mul	1976	CC	7804	n	bl	n	y	0	cig+/-ot	30	99	nev any	st
LUBIN2	181		f	0	0	all	-	q	Eu:mul	1976	CC	7804	n	bl	n	y	0	cig+/-ot	30	99	nev any	st
LUO	6	x	c	0	0	all	-	q	As:Chi	1990	CC	102	n	ot	n	y	0	cig+/-ot	30	99	nev cigs	st
MATOS	46	x	m	0	0	all	-	q	SCAmer	1994	CC	200	n	bl	n	n	0	cig+/-ot	25	99	nev any	st
MATSUD	6		m	0	0	all	-	q	As:Jap	1965	CC	179	n	bl	n	n	0	cig+/-ot	21	99	nev cigs	st
ORMOS	7		m	0	0	all	-	q	Eu:est	1947	CC	119	n	bl	y	y	0	cig+/-ot	31	99	nev any	st
OSANN	59		m	0	0	all	-	q	NAmer	1984	CC	1986	n	bl	n	n	2	cig+/-ot	40	99	nev cigs	or
OSANN	60		f	0	0	all	-	q	NAmer	1984	CC	1986	n	bl	n	n	2	cig+/-ot	40	99	nev cigs	or
WYNDE2	6		m	0	0	all	-	KI	NAmer	1962	CC	404	n	bl	n	y	0	cig+/-ot	35	99	nev any	st
WYNDE3	7		m	0	0	all	-	KI	NAmer	1966	CC	350	n	bl	n	y	0	cig+/-ot	41	99	nev any	st
WYNDE3	66		f	0	0	all	-	KI	NAmer	1966	CC	350	n	bl	n	y	0	cig+/-ot	41	99	nev any	st
WYNDE4	29	x	m	0	0	all	-	not a	NAmer	1948	CC	684	n	bl	y	n	0	all/unsp	35	99	nev any	st
WYNDE4	53		f	0	0	all	-	not a	NAmer	1948	CC	684	n	bl	y	n	2	all/unsp	35	99	nev any	ot
ZHENG	4		m	0	0	all	-	q	As:Chi	1982	CC	540	n	ot	*	y	0	cig+/-ot	30	99	nev cigs	st

Cigarette type is all/unspec for all RRs

except for the following:

REF	NRR	CIGTYPE
ALDERS	36	MC only
ALDERS	39	MC only

Table 2G4 - 5

IESLC - Meta-analysis of Ever Smoking, Amount smoked, "High", Any product (or Cigarettes if Any not available)
Squamous
Least adjusted

REF	NRR	SEX	AD	Number Exposed Case	Number Exposed Cont	Non-exposed Case	Non-exposed Cont	RR	95.00%CI
ALDERS	36	m	1	-	-	-	-	8.78	(3.46- 22.31)
ALDERS	39	f	1	-	-	-	-	14.52	(7.93- 26.58)
Subtotal ALDERS								12.51	(7.53- 20.77)
BARBON	27	m	0	78	111	6	188	22.02	(9.29- 52.18)
CHOI	50	m	0	9	6	6	95	23.75	(6.33- 89.09)
CHOI	58	f	0	2	1	10	164	32.80	(2.74- 393.20)
Subtotal CHOI								25.50	(7.94- 81.93)
DOLL	57	m	0	208	166	3	61	25.48	(7.85- 82.66)
DOSEME	15	m	2	-	-	-	-	7.00	(4.10- 12.00)
GER	8	c	0	19	13	11	80	10.63	(4.13- 27.37)
JEDRYC	5	m	0	57	82	6	289	33.48	(13.94- 80.42)
KREYBE	15	m	0	38	248	3	644	32.89	(10.06- 107.53)
LAMTH	12	f	0	10	1	28	72	25.71	(3.14- 210.29)
LUBIN2	161	m	0	849	1746	54	2616	23.56	(17.77- 31.22)
LUBIN2	181	f	0	18	39	72	1180	7.56	(4.12- 13.88)
Subtotal LUBIN2								19.26	(14.92- 24.87)
LUO	6	c	0	9	4	5	51	22.95	(5.15- 102.20)
MATOS	46	m	0	26	105	3	110	9.08	(2.67- 30.90)
MATSUD	6	m	0	39	470	1	1255	104.14	(14.27- 760.12)
ORMOS	7	m	0	4	128	2	777	12.14	(2.20- 66.97)
OSANN	59	m	2	-	-	-	-	76.00	(36.80- 157.00)
OSANN	60	f	2	-	-	-	-	72.30	(36.80- 142.00)
Subtotal OSANN								73.99	(45.14- 121.29)
WYNDE2	6	m	0	139	112	3	105	43.44	(13.42- 140.56)
WYNDE3	7	m	0	59	26	3	88	66.56	(19.27- 229.96)
WYNDE3	66	f	0	3	3	5	76	15.20	(2.42- 95.56)
Subtotal WYNDE3								41.95	(15.01- 117.26)
WYNDE4	29	m	0	123	64	8	115	27.63	(12.69- 60.13)
WYNDE4	53	f	2	-	-	-	-	26.53	(4.12- 171.09)
Subtotal WYNDE4								27.46	(13.40- 56.29)
ZHENG	4	m	0	49	23	4	94	50.07	(16.39- 152.91)
Partial Totals				1739	3348	233	8060		

*prospective study

REF	NRR	SEX	AD	Ys	Ws	Qs	Ps
ALDERS	36	m	1	2.17	4.42	3.50	0.0000
ALDERS	39	f	1	2.68	10.50	1.57	0.0000
Subtotal ALDERS				2.53	14.93	5.06	
BARBON	27	m	0	3.09	5.16	0.00	0.0000
CHOI	50	m	0	3.17	2.20	0.02	0.0000
CHOI	58	f	0	3.49	0.62	0.11	0.0059
Subtotal CHOI				3.24	2.82	0.14	
DOLL	57	m	0	3.24	2.77	0.09	0.0000
DOSEME	15	m	2	1.95	13.32	16.58	0.0000
GER	8	c	0	2.36	4.29	2.09	0.0000
JEDRYC	5	m	0	3.51	5.00	1.01	0.0000
KREYBE	15	m	0	3.49	2.74	0.51	0.0000
LAMTH	12	f	0	3.25	0.87	0.03	0.0025
LUBIN2	161	m	0	3.16	48.42	0.46	0.0000
LUBIN2	181	f	0	2.02	10.42	11.23	0.0000
Subtotal LUBIN2				2.96	58.85	11.70	
LUO	6	c	0	3.13	1.72	0.01	0.0000
MATOS	46	m	0	2.21	2.56	1.87	0.0004
MATSUD	6	m	0	4.65	0.97	2.44	0.0000
ORMOS	7	m	0	2.50	1.32	0.42	0.0042
OSANN	59	m	2	4.33	7.30	11.76	0.0000
OSANN	60	f	2	4.28	8.43	12.53	0.0000
Subtotal OSANN				4.30	15.73	24.29	
WYNDE2	6	m	0	3.77	2.79	1.40	0.0000
WYNDE3	7	m	0	4.20	2.50	3.23	0.0000
WYNDE3	66	f	0	2.72	1.14	0.13	0.0037
Subtotal WYNDE3				3.74	3.64	3.36	
WYNDE4	29	m	0	3.32	6.35	0.42	0.0000
WYNDE4	53	f	2	3.28	1.11	0.05	0.0006
Subtotal WYNDE4				3.31	7.46	0.47	
ZHENG	4	m	0	3.91	3.08	2.24	0.0000

Table 2G4 - 5

IESLC - Meta-analysis of Ever Smoking, Amount smoked, "High", Any product (or Cigarettes if Any not available)
Squamous
Least adjusted

	N	25
	NS	19
	Wt	150.02
Het	Chi	73.72
Het	df	24
Het	P	***
Fixed	RR	21.36
	RRl	18.20
	RRu	25.07
	P	+++
Random	RR	23.09
	RRl	16.64
	RRu	32.04
	P	+++
Asymm	P	N.S.

Table 2G4 - 6

IESLC - Meta-analysis of Ever Smoking, Amount smoked, "High", Any product (or Cigarettes if Any not available)
Squamous
Least adjusted

	Sex combined	male	female	Total
N	2	16	7	25
NS	2	16	7	25
Wt	6.01	110.91	33.09	150.02
Het Chi	0.73	45.50	25.09	73.72
Het df	1	15	6	24
Het P	N.S.	***	***	***
Fixed RR	13.25	22.79	18.75	21.36
RRl	5.96	18.92	13.33	18.20
RRu	29.46	27.45	26.36	25.07
P	+++	+++	+++	+++
Random RR	13.25	25.28	21.10	23.09
RRl	5.96	17.16	9.14	16.64
RRu	29.46	37.26	48.71	32.04
P	+++	+++	+++	+++
Between Chi				2.40
Between df				2
Between P				N.S.
Btwn(F) P				N.S.
Btwn(R) P				N.S.

Table 2G4 - 7

IESLC - Meta-analysis of Ever Smoking, Amount smoked, "High", Any product (or Cigarettes if Any not available)
Squamous
Excluded studies (and stage at which they were excluded)

1	ABELIN ABRAHA AMANDU AMES ANDERS AUSTIN AXELSO BAND BECHER BERRIN BLOHMK BLOT4 BROCKM BROWN1 BYERS1 BYERS2 CARPEN CASCO2 CASCOR CHAN CHEN3 CHIAZZ CHYOU DESTE2 DOCKER DROSTE DU GARCIA GARDIN GENG GODLEY GOODMA GRAHAM GREGOR HEGMAN HEIN HENNEK HINDS HIRAOK HOROWI HORWIT HUANG ISHIMA JAHN JAIN JARVHO JIANG KELLER KIHARA KJUUS KO KOHLME KUBIK LAMWK LAMWK2 LANGE LEI LEMARC LEVIN LIU LOMBA2 LOMBAR MAGNUS MARSH MARSH2 MCDUFF MCLAUG MILLER MILLS NOTANI NOU ODRISC PAWLEG PERSHA POFFIJ QIAO QIAO2 RADZIK REN RONCO ROOTS ROTHSC SAARIK SANKAR SCHWAR SEGI SEOW SHIMIZ SIMARA SIMONA SITAS SOBUE2 STASZE STAYNE STUCKE SUN SUZUK2 SUZUKI TANG TAO TOKARS TOUSEY ULMER VEIERO VUTUC WALD WANG WANG3 WANG4 WICKLU WIGLE WILKIN WU2 WUNSCH WYNDE8 XIANGZ XU XU2 XU4 YONG ZHANG
2	AKIBA ARCHER BENSHL BRETT BROSS CEDERL CHANG CHOW COMSTO DARBY DEAN3 DEKLER DORANT DORN ENGELA ENSTRO GAO2 GILLIS HAENSZ HAMMO2 HIRAY2 HIRAYA HITOSU HOLE HUMBLE KAISE2 KAISER KANELL KATSOU KAUFMA KINLEN KNEKT KOO LAURIL LIAW LICKIN LIDDEL MIGRAN MRFIT MRFITR MURATA NAM PARKIN PERSH2 PETO PEZZO2 PEZZOT PISANI PRESCO RIMING SEGI2 SOBUE SPEIZE STOCKW SVENSS TANG2 TENKAN TSUGAN TULINI TVERDA WAKAI WARSIN WATSON WU WYNDE5 WYNDER YAMAGU
3	BUELL CHEN MASTRA MZILEN RESTRE SADOWS
4	BEST BOFFET WYNDE7
6	BLOT1 BLOT2 BLOT3 BOUCHA JONES MOLLO SCHWA2 VANDER
8	AGUDO ARMADA AUVINE AXELSS BOUCOT BRESLO BUFFLE CHATZI CHEN2 COOKSO CORREA CPSI CPSII DAMBER DAVEYS DEAN DEAN2 DESTEF DOLL2 DUNN EBELIN ESAKI FAN GAO GARSHI GOLLED GSELL HAMMON HANSEN HU HU2 JARUP JOLY JUSSAW KHUDER KOULUM KREUZE LAUSSM LETOUR LIU2 LIU3 LIU4 LIU5 LUBIN MACLEN MARTIS MCCONN NOTAN2 PASTOR PERNU PIKE POLEDN RACHTA RANDIG SHAW SIEMIA SPITZ STOCKS TIZZAN WANG2 WYNDE6 XU3 YUAN
10	BROWN2 DORGAN OSANN2 WUWILL ZHOU
11	BENHAM

Table 2G4 - 8
Potentially overlapping studies

REF	REFGP	PRINC	. OVERLAP/LINK
LUBIN2	LUBIN2	1	Lubin-combined
LAMTH	LAMTH	1	KOO/LAMTH/LAMWK
MATSUD	MATSUD	1	SOBUE2/MATSUD

Table 2G4 - 9
Most adjusted - insufficient data for metaanalysis

REF	NRR	SEX	AGEL	AGEH	RACE	YF	LC TYPE	LOC	START	ST	NLC	R	VB	P	H	AD	PRODUCT	exL	exH	DENOM	De
CHEN	2	c	0	0	all	-	q	As:oth	1987	CC	323	n	ot	n	y	2	cig+/-ot	31	99	nev cigs	ot

REF	NRR	RR	SIG	RRDATA comment
CHEN	2	11.11	y	P < 0.001

Table 2G6 -

IESLC - Meta-analysis of Current Smoking by Amount, Overview, Any product (or Cigarettes if Any not available)
Squamous

This analysis is restricted to results for:
1) Results by Amount smoked
Results by Amount smoked (in numbers of cigarettes or cigarette equivalents) are grouped under 2 schemes (S1, S2).
Each scheme has a set of "key values". An interval is allocated to the category whose key value
it includes and intervals which include none or more than one of the key values are excluded.
(Open-ended intervals are coded as 99.)

S1	key value	maximum range	S2	key value	maximum range
1	5	1-19	1	1	1-9
2	20	6-44	2	10	2-19
3	45	21+	3	20	11-29
			4	30	21-39
			5	40	31-98
			6	99	41+

For all/unspec product, the definition of cigarette equivalents is shown at the end of Sections -1 and -4.
2) Current smokers
3) Results complete enough for use in metaanalysis

Within each study, results are then selected (in the following order of preference, within each sex) for:
4) PRODUCT: all/unspec, cigarettes regardless of other products, cigarettes only
5) CIGTYPE: all/unspecified, MC regardless of HR, MC only
6) DENOM: never smoked anything, never smoked cigarettes, (never +1 = +long term ex, +2 = +amount unknown,
+3 = never cigs+long term ex)
7) Followup period (YF, prospective studies): whole study (coded as 0) or longest available
8) LCtype: squamous or nearest available, but not adeno. (q = squamous, s = small,
a = adeno, KI = Kreyberg I, u = undifferentiated)
9) Race: all or nearest available, otherwise by race (wh or w = white, bl or b = black, hi = hispanic
ch = chinese, jap = japanese, haw = hawaiian, w+o = white + oriental, sca = scandinavian, as = asian)
10) For overlapping studies: principal rather than subsidiary studies
Finally by Age: whole study (coded as 0) if available, otherwise by widest available age group
and then for single sex results (m, f) in preference to combined sex results (c).

Results adjusted (AD) for the most potential confounders are then chosen in Sections -1 to -3
and results adjusted for the least confounders in Sections -4 to -6. (Those least adjusted results which
actually differ from the most adjusted as marked 'x' in column X in Section -4)
(Results adjusted for an unknown number of confounder(s) are coded as 20.)

Section -7 shows excluded studies, together with the stage (as above) at which no qualifying
results were found.

Section -8 lists the potentially overlapping studies which have been included (1=principal, 2=subsidiary).

Section -9 lists any results which would have been included in preference except that they had data not complete
enough for use in meta-analysis, with their significance (yes/no), if known, and any further comment as entered
on the database.

In addition to those mentioned above, the following fields, levels and abbreviations are used:
* or nk = not known, n = no, y = yes, ot = other
nev = never
all/unspec = all or unspecified, cig+/-ot = cigarettes irrespective of other products (cigar, pipe etc)
MC = manufactured cigarettes, HR = hand-rolled cigarettes
exL, exH = range of exposure (low and high) in the smoking group, in terms of Amount smoked,
cigarettes or cigarette equivalents
REF: 6-character study reference
NRR: number of the RR on the database within the study
ST : study type (CC = case control, pr or prosp = prospective)
NLC: number of lung cancer cases in whole study
R : risky occupational population (n = no, m = mining, o = other risky)
VB : national cigarette type (V = at least 75% Virginia, bl = at least 75% blended, ot = other)
P : any proxy use
H : full histological confirmation
De : derivation of RR/CI (or = original, st = standard method, ot = other method of estimation)

Table 2G6 - 1

IESLC - Meta-analysis of Current Smoking by Amount, Overview, Any product (or Cigarettes if Any not available)
Squamous
Most adjusted

REF	NRR	SEX	AGEL	AGEH	RACE	YF	LC TYPE	LOC	START	ST	NLC	R	VB	P	H	AD	PRODUCT	exL	exH	S1	S2	DENOM	De
BOUCOT	142	m	0	0	all	0	q	NAmer	1951	pr	121	n	bl	n	n	2	cig only	1	20	0	0	nev any	ot
BOUCOT	143	m	0	0	all	0	q	NAmer	1951	pr	121	n	bl	n	n	2	cig only	21	99	3	0	nev any	ot
CORREA	47	c	0	0	all	-	q+s	NAmer	1979	CC	1359	n	bl	y	n	1	cig+/-ot	1	20	0	0	nev cigs	or
CORREA	51	c	0	0	all	-	q+s	NAmer	1979	CC	1359	n	bl	y	n	1	cig+/-ot	21	99	3	0	nev cigs	or
ENGELA	57	m	0	0	all	0	q	Eu:Sca	1964	pr	435	n	bl	n	n	7	cig+/-ot	1	4	0	1	nev cigs	or
ENGELA	58	m	0	0	all	0	q	Eu:Sca	1964	pr	435	n	bl	n	n	7	cig+/-ot	5	9	1	0	nev cigs	or
ENGELA	59	m	0	0	all	0	q	Eu:Sca	1964	pr	435	n	bl	n	n	7	cig+/-ot	10	14	0	2	nev cigs	or
ENGELA	60	m	0	0	all	0	q	Eu:Sca	1964	pr	435	n	bl	n	n	7	cig+/-ot	15	19	0	0	nev cigs	or
ENGELA	61	m	0	0	all	0	q	Eu:Sca	1964	pr	435	n	bl	n	n	7	cig+/-ot	20	99	0	0	nev cigs	or
HAENSZ	18	f	0	0	all	-	q+u	NAmer	1955	CC	158	n	bl	n	y	0	cig+/-ot	1	20	0	0	nev any	or
HAENSZ	17	f	0	0	all	-	q+u	NAmer	1955	CC	158	n	bl	n	y	0	cig+/-ot	21	99	3	0	nev any	or
HAMMON	98	m	0	0	wh	0	not a	NAmer	1952	pr	448	n	bl	n	n	1	cig only	1	9	1	1	nev any	ot
HAMMON	99	m	0	0	wh	0	not a	NAmer	1952	pr	448	n	bl	n	n	1	cig only	10	20	2	0	nev any	ot
HAMMON	100	m	0	0	wh	0	not a	NAmer	1952	pr	448	n	bl	n	n	1	cig only	21	39	0	4	nev any	ot
HAMMON	101	m	0	0	wh	0	not a	NAmer	1952	pr	448	n	bl	n	n	1	cig only	40	99	3	0	nev any	ot
KATSOU	21	f	0	0	all	-	KI	Eu:bal	1987	CC	101	n	bl	n	n	1	all/unsp	1	20	0	0	nev any	or
KATSOU	22	f	0	0	all	-	KI	Eu:bal	1987	CC	101	n	bl	n	n	1	all/unsp	21	99	3	0	nev any	or
SOBUE	53	m	0	0	all	-	q	As:Jap	1986	CC	1376	n	bl	n	y	0	cig+/-ot	1	19	1	0	nev cigs	st
SOBUE	54	m	0	0	all	-	q	As:Jap	1986	CC	1376	n	bl	n	y	0	cig+/-ot	20	29	2	3	nev cigs	st
SOBUE	55	m	0	0	all	-	q	As:Jap	1986	CC	1376	n	bl	n	y	0	cig+/-ot	30	99	3	0	nev cigs	st
SVENSS	7	f	0	0	all	-	q	Eu:Sca	1983	CC	210	n	bl	n	n	1	all/unsp	1	10	1	0	nev any	or
SVENSS	12	f	0	0	all	-	q	Eu:Sca	1983	CC	210	n	bl	n	n	1	all/unsp	11	20	2	3	nev any	or
SVENSS	17	f	0	0	all	-	q	Eu:Sca	1983	CC	210	n	bl	n	n	1	all/unsp	21	99	3	0	nev any	ot
TSUGAN	15	m	0	0	all	-	q	As:Jap	1976	CC	134	n	bl	n	y	0	all/unsp	1	15	1	0	nev any	ot
TSUGAN	16	m	0	0	all	-	q	As:Jap	1976	CC	134	n	bl	n	y	0	all/unsp	16	35	2	0	nev any	ot
TSUGAN	17	m	0	0	all	-	q	As:Jap	1976	CC	134	n	bl	n	y	0	all/unsp	36	99	3	0	nev any	ot
WAKAI	46	m	0	0	all	-	q	As:Jap	1988	CC	333	n	bl	n	y	1	cig+/-ot	1	19	1	0	nev any	or
WAKAI	47	m	0	0	all	-	q	As:Jap	1988	CC	333	n	bl	n	y	1	cig+/-ot	20	29	2	3	nev any	or
WAKAI	48	m	0	0	all	-	q	As:Jap	1988	CC	333	n	bl	n	y	1	cig+/-ot	30	99	3	0	nev any	or
WU	17	f	0	0	wh	-	q	NAmer	1981	CC	220	n	bl	n	y	2	all/unsp	1	20	0	0	nev any	or
WU	18	f	0	0	wh	-	q	NAmer	1981	CC	220	n	bl	n	y	2	all/unsp	21	99	3	0	nev any	or
WYNDE6	21	m	0	0	all	-	KI	NAmer	1969	CC	4423	n	bl	n	y	0	cig+/-ot	1	10	1	0	nev any	st
WYNDE6	30	m	0	0	all	-	KI	NAmer	1969	CC	4423	n	bl	n	y	0	cig+/-ot	11	20	2	3	nev any	st
WYNDE6	39	m	0	0	all	-	KI	NAmer	1969	CC	4423	n	bl	n	y	0	cig+/-ot	21	30	0	4	nev any	st
WYNDE6	48	m	0	0	all	-	KI	NAmer	1969	CC	4423	n	bl	n	y	0	cig+/-ot	31	99	3	0	nev any	st
WYNDE6	210	f	0	0	all	-	KI	NAmer	1969	CC	4423	n	bl	n	y	0	cig+/-ot	1	10	1	0	nev cigs	st
WYNDE6	219	f	0	0	all	-	KI	NAmer	1969	CC	4423	n	bl	n	y	0	cig+/-ot	11	20	2	3	nev cigs	st
WYNDE6	228	f	0	0	all	-	KI	NAmer	1969	CC	4423	n	bl	n	y	0	cig+/-ot	21	30	0	4	nev cigs	st
WYNDE6	237	f	0	0	all	-	KI	NAmer	1969	CC	4423	n	bl	n	y	0	cig+/-ot	30	99	3	0	nev cigs	st

Cigarette type is all/unspec for all RRs

REF	NRR	Cigarette equivalent
BOUCOT	142	-
BOUCOT	143	-
CORREA	47	*
CORREA	51	*
ENGELA	57	*
ENGELA	58	*
ENGELA	59	*
ENGELA	60	*
ENGELA	61	*
HAENSZ	18	*
HAENSZ	17	*
HAMMON	98	-
HAMMON	99	-
HAMMON	100	-
HAMMON	101	-
KATSOU	21	*
KATSOU	22	*
SOBUE	53	*
SOBUE	54	*
SOBUE	55	*
SVENSS	7	*
SVENSS	12	*
SVENSS	17	*
TSUGAN	15	*
TSUGAN	16	*
TSUGAN	17	*
WAKAI	46	*

Table 2G6 - 1

IESLC - Meta-analysis of Current Smoking by Amount, Overview, Any product (or Cigarettes if Any not available)
Squamous
Most adjusted

REF	NRR	Cigarette equivalent
WAKAI	47	*
WAKAI	48	*
WU	17	*
WU	18	*
WYNDE6	21	*
WYNDE6	30	*
WYNDE6	39	*
WYNDE6	48	*
WYNDE6	210	*
WYNDE6	219	*
WYNDE6	228	*
WYNDE6	237	*

In this overview table, subtotals and Qs values may be invalid and should be ignored

Table 2G6 - 2

IESLC - Meta-analysis of Current Smoking by Amount, Overview, Any product (or Cigarettes if Any not available)
Squamous
Most adjusted

REF	NRR	SEX	AD	Number Exposed Case	Cont	Non-exposed Case	Cont	RR	95.00%CI
*BOUCOT	142	m	2	-	-	-	-	21.41	(1.40- 385.13)
*BOUCOT	143	m	2	-	-	-	-	46.64	(2.80- 775.69)
Subtotal BOUCOT								31.58	(4.33- 230.41)
CORREA	47	c	1	-	-	-	-	23.20	(14.60- 37.00)
CORREA	51	c	1	-	-	-	-	54.80	(35.60- 89.20)
Subtotal CORREA								35.84	(25.85- 49.70)
*ENGELA	57	m	7	-	-	-	-	4.30	(1.00- 19.00)
*ENGELA	58	m	7	-	-	-	-	7.70	(1.90- 31.00)
*ENGELA	59	m	7	-	-	-	-	15.00	(3.90- 60.00)
*ENGELA	60	m	7	-	-	-	-	30.00	(7.40- 120.00)
*ENGELA	61	m	7	-	-	-	-	24.00	(5.90- 94.00)
Subtotal ENGELA								13.25	(7.08- 24.79)
HAENSZ	18	f	0	30	66	44	236	2.44	(1.42- 4.18)
HAENSZ	17	f	0	18	13	44	236	7.43	(3.40- 16.24)
Subtotal HAENSZ								3.49	(2.24- 5.43)
*HAMMON	98	m	1	-	-	-	-	15.12	(4.93- 46.36)
*HAMMON	99	m	1	-	-	-	-	17.44	(6.30- 48.29)
*HAMMON	100	m	1	-	-	-	-	42.32	(15.38- 116.45)
*HAMMON	101	m	1	-	-	-	-	63.91	(22.02- 185.47)
Subtotal HAMMON								29.45	(17.41- 49.82)
KATSOU	21	f	1	-	-	-	-	3.17	(1.14- 8.85)
KATSOU	22	f	1	-	-	-	-	19.53	(5.36- 71.11)
Subtotal KATSOU								6.39	(2.86- 14.27)
SOBUE	53	m	0	57	157	3	128	15.49	(4.74- 50.62)
SOBUE	54	m	0	103	222	3	128	19.80	(6.15- 63.68)
SOBUE	55	m	0	87	187	3	128	19.85	(6.14- 64.13)
Subtotal SOBUE								18.28	(9.28- 36.03)
SVENSS	7	f	1	-	-	-	-	9.70	(2.90- 45.90)
SVENSS	12	f	1	-	-	-	-	36.20	(12.00- 168.90)
SVENSS	17	f	1	-	-	-	-	96.00	(6.90-1335.65)
Subtotal SVENSS								23.24	(9.47- 57.03)
TSUGAN	15	m	0	2	5	0	5	5.00~	(0.19- 130.02)
TSUGAN	16	m	0	7	7	0	5	11.00~	(0.51- 236.22)
TSUGAN	17	m	0	9	1	0	5	69.67~	(2.40-2022.74)
Subtotal TSUGAN								14.94	(2.32- 96.11)
WAKAI	46	m	1	-	-	-	-	3.95	(0.86- 18.10)
WAKAI	47	m	1	-	-	-	-	10.40	(2.43- 44.30)
WAKAI	48	m	1	-	-	-	-	24.00	(5.46- 105.00)
Subtotal WAKAI								10.14	(4.31- 23.88)
WU	17	f	2	-	-	-	-	17.70	(2.30- 138.20)
WU	18	f	2	-	-	-	-	94.40	(9.90- 904.60)
Subtotal WU								37.68	(8.27- 171.75)
WYNDE6	21	m	0	75	122	29	617	13.08	(8.17- 20.94)
WYNDE6	30	m	0	270	293	29	617	19.61	(13.04- 29.47)
WYNDE6	39	m	0	179	129	29	617	29.52	(19.09- 45.65)
WYNDE6	48	m	0	502	197	29	617	54.22	(36.08- 81.47)
WYNDE6	210	f	0	37	109	40	856	7.26	(4.45- 11.85)
WYNDE6	219	f	0	191	165	40	856	24.77	(16.95- 36.20)
WYNDE6	228	f	0	101	50	40	856	43.23	(27.18- 68.76)
WYNDE6	237	f	0	221	52	40	856	90.95	(58.70- 140.93)
Subtotal WYNDE6								28.23	(24.23- 32.90)
Partial Totals				1889	1775	373	6763		

*prospective study ~ With 0.5 adjustment for zero

Table 2G6 - 2

IESLC - Meta-analysis of Current Smoking by Amount, Overview, Any product (or Cigarettes if Any not available)
Squamous
Most adjusted

REF	NRR	SEX	AD	Ys	Ws	Qs	Ps
*BOUCOT	142	m	2	3.06	0.49	0.00	0.0325
*BOUCOT	143	m	2	3.84	0.49	0.25	0.0074
Subtotal BOUCOT				3.45	0.97	0.25	
CORREA	47	c	1	3.14	17.77	0.01	0.0000
CORREA	51	c	1	4.00	18.21	13.99	0.0000
Subtotal CORREA				3.58	35.98	13.99	
*ENGELA	57	m	7	1.46	1.77	4.93	0.0522
*ENGELA	58	m	7	2.04	1.97	2.32	0.0042
*ENGELA	59	m	7	2.71	2.06	0.36	0.0001
*ENGELA	60	m	7	3.40	1.98	0.15	0.0000
*ENGELA	61	m	7	3.18	2.01	0.01	0.0000
Subtotal ENGELA				2.58	9.78	7.77	
HAENSZ	18	f	0	0.89	13.25	66.27	0.0012
HAENSZ	17	f	0	2.01	6.27	7.90	0.0000
Subtotal HAENSZ				1.25	19.53	74.17	
*HAMMON	98	m	1	2.72	3.06	0.52	0.0000
*HAMMON	99	m	1	2.86	3.70	0.27	0.0000
*HAMMON	100	m	1	3.75	3.75	1.43	0.0000
*HAMMON	101	m	1	4.16	3.38	3.59	0.0000
Subtotal HAMMON				3.38	13.90	5.81	
KATSOU	21	f	1	1.15	3.66	14.25	0.0273
KATSOU	22	f	1	2.97	2.30	0.06	0.0000
Subtotal KATSOU				1.86	5.96	14.30	
SOBUE	53	m	0	2.74	2.74	0.41	0.0000
SOBUE	54	m	0	2.99	2.81	0.06	0.0000
SOBUE	55	m	0	2.99	2.79	0.05	0.0000
Subtotal SOBUE				2.91	8.35	0.52	
SVENSS	7	f	1	2.27	2.01	1.47	0.0013
SVENSS	12	f	1	3.59	2.20	0.47	0.0000
SVENSS	17	f	1	4.56	0.55	1.14	0.0007
Subtotal SVENSS				3.15	4.77	3.09	
TSUGAN	15	m	0	1.61	0.36	0.83	0.3330
TSUGAN	16	m	0	2.40	0.41	0.22	0.1254
TSUGAN	17	m	0	4.24	0.34	0.42	0.0135
Subtotal TSUGAN				2.70	1.11	1.47	
WAKAI	46	m	1	1.37	1.66	5.09	0.0772
WAKAI	47	m	1	2.34	1.82	1.12	0.0016
WAKAI	48	m	1	3.18	1.76	0.00	0.0000
Subtotal WAKAI				2.32	5.24	6.22	
WU	17	f	2	2.87	0.92	0.06	0.0060
WU	18	f	2	4.55	0.75	1.52	0.0001
Subtotal WU				3.63	1.67	1.58	
WYNDE6	21	m	0	2.57	17.35	5.37	0.0000
WYNDE6	30	m	0	2.98	23.14	0.53	0.0000
WYNDE6	39	m	0	3.39	20.23	1.35	0.0000
WYNDE6	48	m	0	3.99	23.16	17.36	0.0000
WYNDE6	210	f	0	1.98	16.03	20.99	0.0000
WYNDE6	219	f	0	3.21	26.69	0.18	0.0000
WYNDE6	228	f	0	3.77	17.84	7.29	0.0000
WYNDE6	237	f	0	4.51	20.03	38.32	0.0000
Subtotal WYNDE6				3.34	164.47	91.38	

N	39
NS	12

Table 2G6 - 3

IESLC - Meta-analysis of Current Smoking by Amount, Overview, Any product (or Cigarettes if Any not available)
Squamous
Most adjusted

	Sex			
	combined	male	female	Total
N	2	24	13	39
NS	1	7	5	13

In this overview table, other than the "N" rows, entries in the "absent" and "Total" columns may be invalid and should be ignored

	Amount smoked (broad categories)				
	absent	<20k5	6-44k20	>20k45	Total
N	12	8	7	12	39
NS	8	7	6	11	32
Wt	85.71	45.19	60.78	80.04	271.72
Het Chi	90.92	5.67	2.65	36.42	220.56
Het df	11	7	6	11	38
Het P	***	N.S.	N.S.	***	***
Fixed RR	17.84	9.92	21.57	49.56	22.81
RRl	14.44	7.41	16.77	39.81	20.25
RRu	22.05	13.28	27.73	61.69	25.69
P	+++	+++	+++	+++	+++
Random RR	15.23	9.92	21.57	39.16	19.03
RRl	7.62	7.41	16.77	23.67	13.68
RRu	30.44	13.28	27.73	64.79	26.47
P	+++	+++	+++	+++	+++

	Amount smoked (narrow categories)							
	absent	<10k1	2-19k10	11-29k20	21-39k30	31-98k40	>40k99	Total
N	28	2	1	5	3			39
NS	12	2	1	4	2			21
Wt	166.35	4.83	2.06	56.66	41.81			271.72
Het Chi	201.49	1.77	0.00	2.28	1.49			220.56
Het df	27	1	0	4	2			38
Het P	***	N.S.	N.S.	N.S.	N.S.			***
Fixed RR	21.26	9.53	15.00	21.97	35.88			22.81
RRl	18.26	3.91	3.82	16.94	26.50			20.25
RRu	24.75	23.25	58.83	28.51	48.58			25.69
P	+++	+++	+++	+++	+++			+++
Random RR	18.09	8.86	15.00	21.97	35.88			19.03
RRl	11.21	2.62	3.82	16.94	26.50			13.68
RRu	29.19	29.96	58.83	28.51	48.58			26.47
P	+++	+++	+++	+++	+++			+++

MALES

	Amount smoked (broad categories)				
	absent	<20k5	6-44k20	>20k45	Total
N	7	6	5	6	24
NS	4	6	5	6	21
Wt	32.28	27.14	31.89	31.92	123.22
Het Chi	7.65	3.18	0.82	3.69	46.51
Het df	6	5	4	5	23
Het P	N.S.	N.S.	N.S.	N.S.	**
Fixed RR	26.08	11.94	18.53	48.33	23.58
RRl	18.47	8.20	13.10	34.16	19.77
RRu	36.83	17.40	26.22	68.37	28.14
P	+++	+++	+++	+++	+++
Random RR	23.96	11.94	18.53	48.33	20.42
RRl	14.94	8.20	13.10	34.16	15.02
RRu	38.43	17.40	26.22	68.37	27.76
P	+++	+++	+++	+++	+++

Table 2G6 - 3

IESLC - Meta-analysis of Current Smoking by Amount, Overview, Any product (or Cigarettes if Any not available)
Squamous
Most adjusted

MALES

	Amount smoked (narrow categories)							
	absent	<10k1	2-19k10	11-29k20	21-39k30	31-98k40	>40k99	Total
N	16	2	1	3	2			24
NS	7	2	1	3	2			15
Wt	64.59	4.83	2.06	27.77	23.98			123.22
Het Chi	35.59	1.77	0.00	0.69	0.41			46.51
Het df	15	1	0	2	1			23
Het P	**	N.S.	N.S.	N.S.	N.S.			**
Fixed RR	25.42	9.53	15.00	18.82	31.23			23.58
RRl	19.92	3.91	3.82	12.98	20.93			19.77
RRu	32.44	23.25	58.83	27.31	46.61			28.14
P	+++	+++	+++	+++	+++			+++
Random RR	20.84	8.86	15.00	18.82	31.23			20.42
RRl	12.95	2.62	3.82	12.98	20.93			15.02
RRu	33.52	29.96	58.83	27.31	46.61			27.76
P	+++	+++	+++	+++	+++			+++

FEMALES

	Amount smoked (broad categories)				
	absent	<20k5	6-44k20	>20k45	Total
N	4	2	2	5	13
NS	4	2	2	5	13
Wt	35.66	18.05	28.89	29.91	112.51
Het Chi	69.36	0.15	0.29	32.49	156.23
Het df	3	1	1	4	12
Het P	***	N.S.	N.S.	***	***
Fixed RR	11.10	7.50	25.50	47.88	19.03
RRl	7.99	4.73	17.71	33.46	15.82
RRu	15.41	11.90	36.72	68.51	22.90
P	+++	+++	+++	+++	+++
Random RR	8.53	7.50	25.50	36.19	17.07
RRl	1.39	4.73	17.71	9.06	8.10
RRu	52.35	11.90	36.72	144.51	36.01
P	+	+++	+++	+++	+++

	Amount smoked (narrow categories)							
	absent	<10k1	2-19k10	11-29k20	21-39k30	31-98k40	>40k99	Total
N	10			2	1			13
NS	5			2	1			7
Wt	65.79			28.89	17.84			112.51
Het Chi	133.38			0.29	0.00			156.23
Het df	9			1	0			12
Het P	***			N.S.	N.S.			***
Fixed RR	13.40			25.50	43.23			19.03
RRl	10.53			17.71	27.18			15.82
RRu	17.07			36.72	68.76			22.90
P	+++			+++	+++			+++
Random RR	14.06			25.50	43.23			17.07
RRl	4.92			17.71	27.18			8.10
RRu	40.12			36.72	68.76			36.01
P	+++			+++	+++			+++

Table 2G6 - 4

IESLC - Meta-analysis of Current Smoking by Amount, Overview, Any product (or Cigarettes if Any not available)
Squamous
Least adjusted

REF	NRR	X	SEX	AGEL	AGEH	RACE	YF	LC TYPE	LOC	START	ST	NLC	R	VB	P	H	AD	PRODUCT	exL	exH	S1	S2	DENOM	De
BOUCOT	21	x	m	0	0	all	0	q	NAmer	1951	pr	121	n	bl	n	n	0	cig only	1	20	0	0	nev any	ot
BOUCOT	22	x	m	0	0	all	0	q	NAmer	1951	pr	121	n	bl	n	n	0	cig only	21	99	3	0	nev any	ot
CORREA	47		c	0	0	all	-	q+s	NAmer	1979	CC	1359	n	bl	y	n	1	cig+/-ot	1	20	0	0	nev cigs	or
CORREA	51		c	0	0	all	-	q+s	NAmer	1979	CC	1359	n	bl	y	n	1	cig+/-ot	21	99	3	0	nev cigs	or
ENGELA	57		m	0	0	all	0	q	Eu:Sca	1964	pr	435	n	bl	n	n	7	cig+/-ot	1	4	0	1	nev cigs	or
ENGELA	58		m	0	0	all	0	q	Eu:Sca	1964	pr	435	n	bl	n	n	7	cig+/-ot	5	9	1	0	nev cigs	or
ENGELA	59		m	0	0	all	0	q	Eu:Sca	1964	pr	435	n	bl	n	n	7	cig+/-ot	10	14	0	2	nev cigs	or
ENGELA	60		m	0	0	all	0	q	Eu:Sca	1964	pr	435	n	bl	n	n	7	cig+/-ot	15	19	0	0	nev cigs	or
ENGELA	61		m	0	0	all	0	q	Eu:Sca	1964	pr	435	n	bl	n	n	7	cig+/-ot	20	99	0	0	nev cigs	or
HAENSZ	18		f	0	0	all	-	q+u	NAmer	1955	CC	158	n	bl	n	y	0	cig+/-ot	1	20	0	0	nev any	or
HAENSZ	17		f	0	0	all	-	q+u	NAmer	1955	CC	158	n	bl	n	y	0	cig+/-ot	21	99	3	0	nev any	or
HAMMON	98		m	0	0	wh	0	not a	NAmer	1952	pr	448	n	bl	n	n	1	cig only	1	9	1	1	nev any	ot
HAMMON	99		m	0	0	wh	0	not a	NAmer	1952	pr	448	n	bl	n	n	1	cig only	10	20	2	0	nev any	ot
HAMMON	100		m	0	0	wh	0	not a	NAmer	1952	pr	448	n	bl	n	n	1	cig only	21	39	0	4	nev any	ot
HAMMON	101		m	0	0	wh	0	not a	NAmer	1952	pr	448	n	bl	n	n	1	cig only	40	99	3	0	nev any	ot
KATSOU	25	x	f	0	0	all	-	KI	Eu:bal	1987	CC	101	n	bl	n	n	0	all/unsp	1	20	0	0	nev any	st
KATSOU	26	x	f	0	0	all	-	KI	Eu:bal	1987	CC	101	n	bl	n	n	0	all/unsp	21	99	3	0	nev any	st
SOBUE	53		m	0	0	all	-	q	As:Jap	1986	CC	1376	n	bl	n	y	0	cig+/-ot	1	19	1	0	nev cigs	st
SOBUE	54		m	0	0	all	-	q	As:Jap	1986	CC	1376	n	bl	n	y	0	cig+/-ot	20	29	2	3	nev cigs	st
SOBUE	55		m	0	0	all	-	q	As:Jap	1986	CC	1376	n	bl	n	y	0	cig+/-ot	30	99	3	0	nev cigs	st
SVENSS	27	x	f	0	0	all	-	q	Eu:Sca	1983	CC	210	n	bl	n	n	0	all/unsp	1	10	1	0	nev any	st
SVENSS	32	x	f	0	0	all	-	q	Eu:Sca	1983	CC	210	n	bl	n	n	0	all/unsp	11	20	2	3	nev any	st
SVENSS	37	x	f	0	0	all	-	q	Eu:Sca	1983	CC	210	n	bl	n	n	0	all/unsp	21	99	3	0	nev any	st
TSUGAN	15		m	0	0	all	-	q	As:Jap	1976	CC	134	n	bl	n	y	0	all/unsp	1	15	1	0	nev any	ot
TSUGAN	16		m	0	0	all	-	q	As:Jap	1976	CC	134	n	bl	n	y	0	all/unsp	16	35	2	0	nev any	ot
TSUGAN	17		m	0	0	all	-	q	As:Jap	1976	CC	134	n	bl	n	y	0	all/unsp	36	99	3	0	nev any	ot
WAKAI	43	x	m	0	0	all	-	q	As:Jap	1988	CC	333	n	bl	n	y	0	cig+/-ot	1	19	1	0	nev any	st
WAKAI	44	x	m	0	0	all	-	q	As:Jap	1988	CC	333	n	bl	n	y	0	cig+/-ot	20	29	2	3	nev any	st
WAKAI	45	x	m	0	0	all	-	q	As:Jap	1988	CC	333	n	bl	n	y	0	cig+/-ot	30	99	3	0	nev any	st
WU	12	x	f	0	0	wh	-	q	NAmer	1981	CC	220	n	bl	n	y	0	all/unsp	1	20	0	0	nev any	st
WU	13	x	f	0	0	wh	-	q	NAmer	1981	CC	220	n	bl	n	y	0	all/unsp	21	99	3	0	nev any	st
WYNDE6	21		m	0	0	all	-	KI	NAmer	1969	CC	4423	n	bl	n	y	0	cig+/-ot	1	10	1	0	nev any	st
WYNDE6	30		m	0	0	all	-	KI	NAmer	1969	CC	4423	n	bl	n	y	0	cig+/-ot	11	20	2	3	nev any	st
WYNDE6	39		m	0	0	all	-	KI	NAmer	1969	CC	4423	n	bl	n	y	0	cig+/-ot	21	30	0	4	nev any	st
WYNDE6	48		m	0	0	all	-	KI	NAmer	1969	CC	4423	n	bl	n	y	0	cig+/-ot	31	99	3	0	nev any	st
WYNDE6	210		f	0	0	all	-	KI	NAmer	1969	CC	4423	n	bl	n	y	0	cig+/-ot	1	10	1	0	nev cigs	st
WYNDE6	219		f	0	0	all	-	KI	NAmer	1969	CC	4423	n	bl	n	y	0	cig+/-ot	11	20	2	3	nev cigs	st
WYNDE6	228		f	0	0	all	-	KI	NAmer	1969	CC	4423	n	bl	n	y	0	cig+/-ot	21	30	0	4	nev cigs	st
WYNDE6	237		f	0	0	all	-	KI	NAmer	1969	CC	4423	n	bl	n	y	0	cig+/-ot	30	99	3	0	nev cigs	st

Cigarette type is all/unspec for all RRs

REF	NRR	Cigarette equivalent
BOUCOT	21	-
BOUCOT	22	-
CORREA	47	*
CORREA	51	*
ENGELA	57	*
ENGELA	58	*
ENGELA	59	*
ENGELA	60	*
ENGELA	61	*
HAENSZ	18	*
HAENSZ	17	*
HAMMON	98	-
HAMMON	99	-
HAMMON	100	-
HAMMON	101	-
KATSOU	25	*
KATSOU	26	*
SOBUE	53	*
SOBUE	54	*
SOBUE	55	*
SVENSS	27	*
SVENSS	32	*
SVENSS	37	*
TSUGAN	15	*
TSUGAN	16	*
TSUGAN	17	*
WAKAI	43	*

Table 2G6 - 4

IESLC - Meta-analysis of Current Smoking by Amount, Overview, Any product (or Cigarettes if Any not available)
Squamous
Least adjusted

REF	NRR	Cigarette equivalent
WAKAI	44	*
WAKAI	45	*
WU	12	*
WU	13	*
WYNDE6	21	*
WYNDE6	30	*
WYNDE6	39	*
WYNDE6	48	*
WYNDE6	210	*
WYNDE6	219	*
WYNDE6	228	*
WYNDE6	237	*

In this overview table, subtotals and Qs values may be invalid and should be ignored

Table 2G6 - 5

IESLC - Meta-analysis of Current Smoking by Amount, Overview, Any product (or Cigarettes if Any not available)
Squamous
Least adjusted

REF	NRR	SEX	AD	Number Exposed Case	Number Exposed Cont	Non-exposed Case	Non-exposed Cont	RR	95.00%CI
*BOUCOT	21	m	0	21	15208	0	7551	21.35~	(1.29- 352.41)
*BOUCOT	22	m	0	17	6940	0	7551	38.08~	(2.29- 633.12)
Subtotal BOUCOT								28.49	(3.91- 207.42)
CORREA	47	c	1	-	-	-	-	23.20	(14.60- 37.00)
CORREA	51	c	1	-	-	-	-	54.80	(35.60- 89.20)
Subtotal CORREA								35.84	(25.85- 49.70)
*ENGELA	57	m	7	-	-	-	-	4.30	(1.00- 19.00)
*ENGELA	58	m	7	-	-	-	-	7.70	(1.90- 31.00)
*ENGELA	59	m	7	-	-	-	-	15.00	(3.90- 60.00)
*ENGELA	60	m	7	-	-	-	-	30.00	(7.40- 120.00)
*ENGELA	61	m	7	-	-	-	-	24.00	(5.90- 94.00)
Subtotal ENGELA								13.25	(7.08- 24.79)
HAENSZ	18	f	0	30	66	44	236	2.44	(1.42- 4.18)
HAENSZ	17	f	0	18	13	44	236	7.43	(3.40- 16.24)
Subtotal HAENSZ								3.49	(2.24- 5.43)
*HAMMON	98	m	1	-	-	-	-	15.12	(4.93- 46.36)
*HAMMON	99	m	1	-	-	-	-	17.44	(6.30- 48.29)
*HAMMON	100	m	1	-	-	-	-	42.32	(15.38- 116.45)
*HAMMON	101	m	1	-	-	-	-	63.91	(22.02- 185.47)
Subtotal HAMMON								29.45	(17.41- 49.82)
KATSOU	25	f	0	9	14	14	67	3.08	(1.11- 8.50)
KATSOU	26	f	0	15	4	14	67	17.95	(5.17- 62.28)
Subtotal KATSOU								6.23	(2.84- 13.69)
SOBUE	53	m	0	57	157	3	128	15.49	(4.74- 50.62)
SOBUE	54	m	0	103	222	3	128	19.80	(6.15- 63.68)
SOBUE	55	m	0	87	187	3	128	19.85	(6.14- 64.13)
Subtotal SOBUE								18.28	(9.28- 36.03)
SVENSS	27	f	0	10	30	5	120	8.00	(2.54- 25.16)
SVENSS	32	f	0	28	22	5	120	30.55	(10.64- 87.69)
SVENSS	37	f	0	4	1	5	120	96.00	(9.00-1023.75)
Subtotal SVENSS								19.60	(9.38- 40.97)
TSUGAN	15	m	0	2	5	0	5	5.00~	(0.19- 130.02)
TSUGAN	16	m	0	7	7	0	5	11.00~	(0.51- 236.22)
TSUGAN	17	m	0	9	1	0	5	69.67~	(2.40-2022.74)
Subtotal TSUGAN								14.94	(2.32- 96.11)
WAKAI	43	m	0	13	105	2	65	4.02	(0.88- 18.41)
WAKAI	44	m	0	40	129	2	65	10.08	(2.36- 43.01)
WAKAI	45	m	0	33	48	2	65	22.34	(5.11- 97.69)
Subtotal WAKAI								9.85	(4.19- 23.17)
WU	12	f	0	19	14	2	30	20.36	(4.15- 99.74)
WU	13	f	0	42	9	2	30	70.00	(14.10- 347.48)
Subtotal WU								37.56	(12.15- 116.07)
WYNDE6	21	m	0	75	122	29	617	13.08	(8.17- 20.94)
WYNDE6	30	m	0	270	293	29	617	19.61	(13.04- 29.47)
WYNDE6	39	m	0	179	129	29	617	29.52	(19.09- 45.65)
WYNDE6	48	m	0	502	197	29	617	54.22	(36.08- 81.47)
WYNDE6	210	f	0	37	109	40	856	7.26	(4.45- 11.85)
WYNDE6	219	f	0	191	165	40	856	24.77	(16.95- 36.20)
WYNDE6	228	f	0	101	50	40	856	43.23	(27.18- 68.76)
WYNDE6	237	f	0	221	52	40	856	90.95	(58.70- 140.93)
Subtotal WYNDE6								28.23	(24.23- 32.90)
Partial Totals				2140	24299	426	22614		

*prospective study

~ With 0.5 adjustment for zero

Table 2G6 - 5

IESLC - Meta-analysis of Current Smoking by Amount, Overview, Any product (or Cigarettes if Any not available)
Squamous
Least adjusted

REF	NRR	SEX	AD	Ys	Ws	Qs	Ps
*BOUCOT	21	m	0	3.06	0.49	0.00	0.0324
*BOUCOT	22	m	0	3.64	0.49	0.13	0.0112
Subtotal BOUCOT				3.35	0.97	0.13	
CORREA	47	c	1	3.14	17.77	0.01	0.0000
CORREA	51	c	1	4.00	18.21	14.13	0.0000
Subtotal CORREA				3.58	35.98	14.14	
*ENGELA	57	m	7	1.46	1.77	4.91	0.0522
*ENGELA	58	m	7	2.04	1.97	2.31	0.0042
*ENGELA	59	m	7	2.71	2.06	0.35	0.0001
*ENGELA	60	m	7	3.40	1.98	0.15	0.0000
*ENGELA	61	m	7	3.18	2.01	0.01	0.0000
Subtotal ENGELA				2.58	9.78	7.73	
HAENSZ	18	f	0	0.89	13.25	66.01	0.0012
HAENSZ	17	f	0	2.01	6.27	7.84	0.0000
Subtotal HAENSZ				1.25	19.53	73.84	
*HAMMON	98	m	1	2.72	3.06	0.51	0.0000
*HAMMON	99	m	1	2.86	3.70	0.26	0.0000
*HAMMON	100	m	1	3.75	3.75	1.45	0.0000
*HAMMON	101	m	1	4.16	3.38	3.62	0.0000
Subtotal HAMMON				3.38	13.90	5.84	
KATSOU	25	f	0	1.12	3.72	14.86	0.0302
KATSOU	26	f	0	2.89	2.48	0.14	0.0000
Subtotal KATSOU				1.83	6.20	15.00	
SOBUE	53	m	0	2.74	2.74	0.40	0.0000
SOBUE	54	m	0	2.99	2.81	0.05	0.0000
SOBUE	55	m	0	2.99	2.79	0.05	0.0000
Subtotal SOBUE				2.91	8.35	0.50	
SVENSS	27	f	0	2.08	2.93	3.19	0.0004
SVENSS	32	f	0	3.42	3.45	0.30	0.0000
SVENSS	37	f	0	4.56	0.69	1.42	0.0002
Subtotal SVENSS				2.98	7.07	4.91	
TSUGAN	15	m	0	1.61	0.36	0.83	0.3330
TSUGAN	16	m	0	2.40	0.41	0.21	0.1254
TSUGAN	17	m	0	4.24	0.34	0.43	0.0135
Subtotal TSUGAN				2.70	1.11	1.47	
WAKAI	43	m	0	1.39	1.66	4.98	0.0727
WAKAI	44	m	0	2.31	1.82	1.20	0.0018
WAKAI	45	m	0	3.11	1.77	0.00	0.0000
Subtotal WAKAI				2.29	5.25	6.18	
WU	12	f	0	3.01	1.52	0.02	0.0002
WU	13	f	0	4.25	1.50	1.90	0.0000
Subtotal WU				3.63	3.02	1.91	
WYNDE6	21	m	0	2.57	17.35	5.28	0.0000
WYNDE6	30	m	0	2.98	23.14	0.50	0.0000
WYNDE6	39	m	0	3.39	20.23	1.39	0.0000
WYNDE6	48	m	0	3.99	23.16	17.54	0.0000
WYNDE6	210	f	0	1.98	16.03	20.83	0.0000
WYNDE6	219	f	0	3.21	26.69	0.20	0.0000
WYNDE6	228	f	0	3.77	17.84	7.39	0.0000
WYNDE6	237	f	0	4.51	20.03	38.56	0.0000
Subtotal WYNDE6				3.34	164.47	91.70	

N	39
NS	12

Table 2G6 - 6

IESLC - Meta-analysis of Current Smoking by Amount, Overview, Any product (or Cigarettes if Any not available)
Squamous
Least adjusted

	Sex			
	combined	male	female	Total
N	2	24	13	39
NS	1	7	5	13

In this overview table, other than the "N" rows, entries in the "absent" and "Total" columns may be invalid and should be ignored

	Amount smoked (broad categories)				
	absent	<20k5	6-44k20	>20k45	Total
N	12	8	7	12	39
NS	8	7	6	11	32
Wt	86.38	46.10	62.03	81.11	275.62
Het Chi	91.52	5.75	2.57	37.14	223.36
Het df	11	7	6	11	38
Het P	***	N.S.	N.S.	***	***
Fixed RR	17.84	9.80	21.57	49.26	22.71
RRl	14.45	7.34	16.82	39.62	20.18
RRu	22.03	13.08	27.66	61.23	25.56
P	+++	+++	+++	+++	+++
Random RR	15.36	9.80	21.57	38.69	18.90
RRl	7.73	7.34	16.82	23.54	13.63
RRu	30.53	13.08	27.66	63.59	26.20
P	+++	+++	+++	+++	+++

		Amount smoked (narrow categories)						
	absent	<10k1	2-19k10	11-29k20	21-39k30	31-98k40	>40k99	Total
N	28	2	1	5	3			39
NS	12	2	1	4	2			21
Wt	169.00	4.83	2.06	57.92	41.81			275.62
Het Chi	204.22	1.77	0.00	2.20	1.49			223.36
Het df	27	1	0	4	2			38
Het P	***	N.S.	N.S.	N.S.	N.S.			***
Fixed RR	21.13	9.53	15.00	21.97	35.88			22.71
RRl	18.18	3.91	3.82	16.98	26.50			20.18
RRu	24.57	23.25	58.83	28.42	48.58			25.56
P	+++	+++	+++	+++	+++			+++
Random RR	17.97	8.86	15.00	21.97	35.88			18.90
RRl	11.20	2.62	3.82	16.98	26.50			13.63
RRu	28.82	29.96	58.83	28.42	48.58			26.20
P	+++	+++	+++	+++	+++			+++

MALES

	Amount smoked (broad categories)				
	absent	<20k5	6-44k20	>20k45	Total
N	7	6	5	6	24
NS	4	6	5	6	21
Wt	32.28	27.14	31.89	31.93	123.24
Het Chi	7.65	3.12	0.89	3.90	46.41
Het df	6	5	4	5	23
Het P	N.S.	N.S.	N.S.	N.S.	**
Fixed RR	26.08	11.95	18.50	47.98	23.53
RRl	18.47	8.21	13.07	33.92	19.72
RRu	36.83	17.41	26.17	67.87	28.08
P	+++	+++	+++	+++	+++
Random RR	23.96	11.95	18.50	47.98	20.32
RRl	14.94	8.21	13.07	33.92	14.95
RRu	38.43	17.41	26.17	67.87	27.62
P	+++	+++	+++	+++	+++

Table 2G6 - 6

IESLC - Meta-analysis of Current Smoking by Amount, Overview, Any product (or Cigarettes if Any not available)
Squamous
Least adjusted

MALES

	Amount smoked (narrow categories)							
	absent	<10k1	2-19k10	11-29k20	21-39k30	31-98k40	>40k99	Total
N	16	2	1	3	2			24
NS	7	2	1	3	2			15
Wt	64.60	4.83	2.06	27.78	23.98			123.24
Het Chi	35.42	1.77	0.00	0.76	0.41			46.41
Het df	15	1	0	2	1			23
Het P	**	N.S.	N.S.	N.S.	N.S.			**
Fixed RR	25.33	9.53	15.00	18.79	31.23			23.53
RRl	19.85	3.91	3.82	12.95	20.93			19.72
RRu	32.33	23.25	58.83	27.25	46.61			28.08
P	+++	+++	+++	+++	+++			+++
Random RR	20.67	8.86	15.00	18.79	31.23			20.32
RRl	12.86	2.62	3.82	12.95	20.93			14.95
RRu	33.22	29.96	58.83	27.25	46.61			27.62
P	+++	+++	+++	+++	+++			+++

FEMALES

	Amount smoked (broad categories)				
	absent	<20k5	6-44k20	>20k45	Total
N	4	2	2	5	13
NS	4	2	2	5	13
Wt	36.33	18.96	30.15	30.97	116.40
Het Chi	70.10	0.02	0.13	32.96	158.93
Het df	3	1	1	4	12
Het P	***	N.S.	N.S.	***	***
Fixed RR	11.19	7.37	25.37	47.53	18.99
RRl	8.09	4.70	17.76	33.42	15.84
RRu	15.50	11.56	36.26	67.60	22.78
P	+++	+++	+++	+++	+++
Random RR	8.88	7.37	25.37	35.16	16.88
RRl	1.51	4.70	17.76	9.54	8.20
RRu	52.07	11.56	36.26	129.57	34.73
P	+	+++	+++	+++	+++

	Amount smoked (narrow categories)							
	absent	<10k1	2-19k10	11-29k20	21-39k30	31-98k40	>40k99	Total
N	10			2	1			13
NS	5			2	1			7
Wt	68.42			30.15	17.84			116.40
Het Chi	136.20			0.13	0.00			158.93
Het df	9			1	0			12
Het P	***			N.S.	N.S.			***
Fixed RR	13.49			25.37	43.23			18.99
RRl	10.64			17.76	27.18			15.84
RRu	17.10			36.26	68.76			22.78
P	+++			+++	+++			+++
Random RR	13.99			25.37	43.23			16.88
RRl	5.08			17.76	27.18			8.20
RRu	38.50			36.26	68.76			34.73
P	+++			+++	+++			+++

Table 2G6 - 7

IESLC - Meta-analysis of Current Smoking by Amount, Overview, Any product (or Cigarettes if Any not available)
Squamous
Excluded studies (and stage at which they were excluded)

1 ABELIN ABRAHA AMANDU AMES ANDERS AUSTIN AXELSO BAND BECHER BERRIN BLOHMK BLOT4 BROCKM BROWN1 BYERS1 BYERS2
CARPEN CASCO2 CASCOR CHAN CHEN3 CHIAZZ CHYOU DESTE2 DOCKER DROSTE DU GARCIA GARDIN GENG GODLEY GOODMA
GRAHAM GREGOR HEGMAN HEIN HENNEK HINDS HIRAOK HOROWI HORWIT HUANG ISHIMA JAHN JAIN JARVHO JIANG KELLER
KIHARA KJUUS KO KOHLME KUBIK LAMWK LAMWK2 LANGE LEI LEMARC LEVIN LIU LOMBA2 LOMBAR MAGNUS MARSH
MARSH2 MCDUFF MCLAUG MILLER MILLS NOTANI NOU ODRISC PAWLEG PERSHA POFFIJ QIAO QIAO2 RADZIK REN RONCO
ROOTS ROTHSC SAARIK SANKAR SCHWAR SEGI SEOW SHIMIZ SIMARA SIMONA SITAS SOBUE2 STASZE STAYNE STUCKE SUN
SUZUK2 SUZUKI TANG TAO TOKARS TOUSEY ULMER VEIERO VUTUC WALD WANG WANG3 WANG4 WICKLU WIGLE WILKIN
WU2 WUNSCH WYNDE8 XIANGZ XU XU2 XU4 YONG ZHANG

2 AGUDO ALDERS ARMADA AUVINE BARBON BENHAM BLOT1 BLOT2 BLOT3 BOFFET BOUCHA BRESLO BROWN2 BUELL CHATZI CHEN
CHEN2 CHOI COOKSO DAMBER DAVEYS DEAN DEAN2 DOLL DOSEME DUNN EBELIN ESAKI FAN GAO GARSHI GER
GOLLED GSELL HANSEN HU HU2 JARUP JEDRYC JOLY JONES JUSSAW KHUDER KOULUM KREUZE KREYBE LAMTH LAUSSM
LETOUR LIU2 LIU3 LIU4 LIU5 LUBIN LUBIN2 LUO MARTIS MASTRA MATSUD MCCONN MOLLO MZILEN NOTAN2 ORMOS
OSANN OSANN2 PASTOR PERNU PIKE POLEDN RACHTA RANDIG RESTRE SADOWS SCHWA2 SIEMIA SPITZ STOCKS TIZZAN VANDER
WANG2 WUWILL WYNDE2 WYNDE3 WYNDE4 XU3 YUAN ZHENG ZHOU

3 PISANI

4 WYNDE7

5 RIMING TANG2 WYNDE5

6 DESTEF HIRAY2 LAURIL LICKIN MRFIT MURATA WARSIN WATSON WYNDER

8 AKIBA ARCHER AXELSS BENSHL BEST BRETT BROSS BUFFLE CEDERL CHANG CHOW COMSTO CPSI CPSII DARBY DEAN3
DEKLER DOLL2 DORANT DORGAN DORN ENSTRO GAO2 GILLIS HAMMO2 HIRAYA HITOSU HOLE HUMBLE KAISE2 KAISER KANELL
KAUFMA KINLEN KNEKT KOO LIAW LIDDEL MACLEN MATOS MIGRAN MRFITR NAM PARKIN PERSH2 PETO PEZZO2 PEZZOT
PRESCO SEGI2 SHAW SPEIZE STOCKW TENKAN TULINI TVERDA YAMAGU

Table 2G6 - 8

Potentially overlapping studies

REF	REFGP	PRINC	.	OVERLAP/LINK
WYNDE6	WYNDE6	1		WYNDE5/6/7/8

Table 2G6 - 9

Most adjusted - insufficient data for metaanalysis

REF	NRR	SEX	AGEL	AGEH	RACE	YF	LC TYPE	LOC	START	ST	NLC	R	VB	P	H	AD	PRODUCT	exL	exH	S1	S2	DENOM	De
GILLIS	9	m	0	0	all	-	q	Eu:UK	1977	CC	656	n	V	n	n	3	cig+/-ot	1	14	1	0	nev any	ot
GILLIS	10	m	0	0	all	-	q	Eu:UK	1977	CC	656	n	V	n	n	3	cig+/-ot	15	24	2	3	nev any	ot
GILLIS	11	m	0	0	all	-	q	Eu:UK	1977	CC	656	n	V	n	n	3	cig+/-ot	25	34	0	4	nev any	ot
GILLIS	12	m	0	0	all	-	q	Eu:UK	1977	CC	656	n	V	n	n	3	cig+/-ot	35	99	3	0	nev any	ot

REF	NRR	RR	SIG	Cigarette equivalent	RRDATA comment
GILLIS	9	4.52		*	0
GILLIS	10	8.36		*	0
GILLIS	11	9.79		*	0
GILLIS	12	10.51		*	0

Table 2G7 -

IESLC - Meta-analysis of Current Smoking, Amount smoked, "Low", Any product (or Cigarettes if Any not available)
Squamous

This analysis is restricted to results for:
1) Results by Amount smoked
2) Current smokers
3) Results complete enough for use in metaanalysis

Within each study, results are then selected (in the following order of preference, within each sex) for:
4) PRODUCT: all/unspec, cigarettes regardless of other products, cigarettes only
5) CIGTYPE: all/unspecified, MC regardless of HR, MC only
6) DENOM: never smoked anything, never smoked cigarettes, (never +1 = +long term ex, +2 = +amount unknown, +3 = never cigs+long term ex)
7) Followup period (YF, prospective studies): whole study (coded as 0) or longest available
8) LCtype: squamous or nearest available, but not adeno. (q = squamous, s = small, a = adeno, KI = Kreyberg I, u = undifferentiated)
9) Race: all or nearest available, otherwise by race (wh or w = white, bl or b = black, hi = hispanic ch = chinese, jap = japanese, haw = hawaiian, w+o = white + oriental, sca = scandinavian, as = asian)
10) Amount smoked "low" in key scheme 1 (key value 5, maximum range <20, in numbers of cigarettes or cigarette equivalents)
11) For overlapping studies: principal rather than subsidiary studies
Finally by Age: whole study (coded as 0) if available, otherwise by widest available age group
and then for single sex results (m, f) in preference to combined sex results (c).

Results adjusted (AD) for the most potential confounders are then chosen in Sections -1 to -3
and results adjusted for the least confounders in Sections -4 to -6. (Those least adjusted results which actually differ from the most adjusted as marked 'x' in column X in Section -4)
(Results adjusted for an unknown number of confounder(s) are coded as 20.)

Section -7 shows excluded studies, together with the stage (as above) at which no qualifying results were found.

Section -8 lists the potentially overlapping studies which have been included (1=principal, 2=subsidiary).

Section -9 lists any results which would have been included in preference except that they had data not complete enough for use in meta-analysis, with their significance (yes/no), if known, and any further comment as entered on the database.

In addition to those mentioned above, the following fields, levels and abbreviations are used:
* or nk = not known, n = no, y = yes, ot = other
nev = never
all/unspec = all or unspecified, cig+/-ot = cigarettes irrespective of other products (cigar, pipe etc)
MC = manufactured cigarettes, HR = hand-rolled cigarettes
exL, exH = range of exposure (low and high) in the smoking group, in terms of Amount smoked, cigarettes or cigarette equivalents
REF: 6-character study reference
NRR: number of the RR on the database within the study
ST : study type (CC = case control, pr or prosp = prospective)
NLC: number of lung cancer cases in whole study
R : risky occupational population (n = no, m = mining, o = other risky)
VB : national cigarette type (V = at least 75% Virginia, bl = at least 75% blended, ot = other)
P : any proxy use
H : full histological confirmation
De : derivation of RR/CI (or = original, st = standard method, ot = other method of estimation)

Table 2G7 - 1

IESLC - Meta-analysis of Current Smoking, Amount smoked, "Low", Any product (or Cigarettes if Any not available)
Squamous
Most adjusted

REF	NRR	SEX	AGEL	AGEH	RACE	YF	LC TYPE	LOC	START	ST	NLC	R	VB	P	H	AD	PRODUCT	exL	exH	DENOM	De
ENGELA	58	m	0	0	all	0	q	Eu:Sca	1964	pr	435	n	bl	n	n	7	cig+/-ot	5	9	nev cigs	or
HAMMON	98	m	0	0	wh	0	not a	NAmer	1952	pr	448	n	bl	n	n	1	cig only	1	9	nev any	ot
SOBUE	53	m	0	0	all	-	q	As:Jap	1986	CC	1376	n	bl	n	y	0	cig+/-ot	1	19	nev cigs	st
SVENSS	7	f	0	0	all	-	q	Eu:Sca	1983	CC	210	n	bl	n	n	1	all/unsp	1	10	nev any	or
TSUGAN	15	m	0	0	all	-	q	As:Jap	1976	CC	134	n	bl	n	y	0	all/unsp	1	15	nev any	ot
WAKAI	46	m	0	0	all	-	q	As:Jap	1988	CC	333	n	bl	n	y	1	cig+/-ot	1	19	nev any	or
WYNDE6	21	m	0	0	all	-	KI	NAmer	1969	CC	4423	n	bl	n	y	0	cig+/-ot	1	10	nev any	st
WYNDE6	210	f	0	0	all	-	KI	NAmer	1969	CC	4423	n	bl	n	y	0	cig+/-ot	1	10	nev cigs	st

Cigarette type is all/unspec for all RRs

Table 2G7 - 2

IESLC - Meta-analysis of Current Smoking, Amount smoked, "Low", Any product (or Cigarettes if Any not available)
Squamous
Most adjusted

REF	NRR	SEX	AD	Number Exposed Case	Number Exposed Cont	Non-exposed Case	Non-exposed Cont	RR	95.00%CI
*ENGELA	58	m	7	-	-	-	-	7.70	(1.90- 31.00)
*HAMMON	98	m	1	-	-	-	-	15.12	(4.93- 46.36)
SOBUE	53	m	0	57	157	3	128	15.49	(4.74- 50.62)
SVENSS	7	f	1	-	-	-	-	9.70	(2.90- 45.90)
TSUGAN	15	m	0	2	5	0	5	5.00~	(0.19- 130.02)
WAKAI	46	m	1	-	-	-	-	3.95	(0.86- 18.10)
WYNDE6	21	m	0	75	122	29	617	13.08	(8.17- 20.94)
WYNDE6	210	f	0	37	109	40	856	7.26	(4.45- 11.85)
Subtotal WYNDE6								9.86	(7.02- 13.84)
Partial Totals				171	393	72	1606		

*prospective study

~ With 0.5 adjustment for zero

REF	NRR	SEX	AD	Ys	Ws	Qs	Ps
*ENGELA	58	m	7	2.04	1.97	0.13	0.0042
*HAMMON	98	m	1	2.72	3.06	0.54	0.0000
SOBUE	53	m	0	2.74	2.74	0.54	0.0000
SVENSS	7	f	1	2.27	2.01	0.00	0.0013
TSUGAN	15	m	0	1.61	0.36	0.17	0.3330
WAKAI	46	m	1	1.37	1.66	1.40	0.0772
WYNDE6	21	m	0	2.57	17.35	1.33	0.0000
WYNDE6	210	f	0	1.98	16.03	1.56	0.0000
Subtotal WYNDE6				2.29	33.38	2.88	

	N	8
	NS	7
	Wt	45.19
Het	Chi	5.67
Het	df	7
Het	P	N.S.
Fixed	RR	9.92
	RRl	7.41
	RRu	13.28
	P	+++
Random	RR	9.92
	RRl	7.41
	RRu	13.28
	P	+++
Asymm	P	N.S.

Table 2G7 - 3

IESLC - Meta-analysis of Current Smoking, Amount smoked, "Low", Any product (or Cigarettes if Any not available)
Squamous
Most adjusted

	combined	Sex male	female	Total
N		6	2	8
NS		6	2	8
Wt		27.14	18.05	45.19
Het Chi		3.18	0.15	5.67
Het df		5	1	7
Het P		N.S.	N.S.	N.S.
Fixed RR		11.94	7.50	9.92
RRl		8.20	4.73	7.41
RRu		17.40	11.90	13.28
P		+++	+++	+++
Random RR		11.94	7.50	9.92
RRl		8.20	4.73	7.41
RRu		17.40	11.90	13.28
P		+++	+++	+++
Between Chi				2.34
Between df				1
Between P				N.S.
Btwn(F) P				(*)
Btwn(R) P				N.S.

Too few RRs for analysis by factor

Table 2G7 - 4

IESLC - Meta-analysis of Current Smoking, Amount smoked, "Low", Any product (or Cigarettes if Any not available)
Squamous
Least adjusted

REF	NRR	X	SEX	AGEL	AGEH	RACE	YF	LC TYPE	LOC	START	ST	NLC	R	VB	P	H	AD	PRODUCT	exL	exH	DENOM	De
ENGELA	58		m	0	0	all	0	q	Eu:Sca	1964	pr	435	n	bl	n	n	7	cig+/-ot	5	9	nev cigs	or
HAMMON	98		m	0	0	wh	0	not a	NAmer	1952	pr	448	n	bl	n	n	1	cig only	1	9	nev any	ot
SOBUE	53		m	0	0	all	-	q	As:Jap	1986	CC	1376	n	bl	n	y	0	cig+/-ot	1	19	nev cigs	st
SVENSS	27	x	f	0	0	all	-	q	Eu:Sca	1983	CC	210	n	bl	n	n	0	all/unsp	1	10	nev any	st
TSUGAN	15		m	0	0	all	-	q	As:Jap	1976	CC	134	n	bl	n	y	0	all/unsp	1	15	nev any	ot
WAKAI	43	x	m	0	0	all	-	q	As:Jap	1988	CC	333	n	bl	n	y	0	cig+/-ot	1	19	nev any	st
WYNDE6	21		m	0	0	all	-	KI	NAmer	1969	CC	4423	n	bl	n	y	0	cig+/-ot	1	10	nev any	st
WYNDE6	210		f	0	0	all	-	KI	NAmer	1969	CC	4423	n	bl	n	y	0	cig+/-ot	1	10	nev cigs	st

Cigarette type is all/unspec for all RRs

Table 2G7 - 5

IESLC - Meta-analysis of Current Smoking, Amount smoked, "Low", Any product (or Cigarettes if Any not available)
Squamous
Least adjusted

REF	NRR	SEX	AD	Number Exposed Case	Number Exposed Cont	Non-exposed Case	Non-exposed Cont	RR	95.00%CI
*ENGELA	58	m	7	-	-	-	-	7.70	(1.90- 31.00)
*HAMMON	98	m	1	-	-	-	-	15.12	(4.93- 46.36)
SOBUE	53	m	0	57	157	3	128	15.49	(4.74- 50.62)
SVENSS	27	f	0	10	30	5	120	8.00	(2.54- 25.16)
TSUGAN	15	m	0	2	5	0	5	5.00~	(0.19- 130.02)
WAKAI	43	m	0	13	105	2	65	4.02	(0.88- 18.41)
WYNDE6	21	m	0	75	122	29	617	13.08	(8.17- 20.94)
WYNDE6	210	f	0	37	109	40	856	7.26	(4.45- 11.85)
Subtotal WYNDE6								9.86	(7.02- 13.84)
Partial Totals				194	528	79	1791		

*prospective study ~ With 0.5 adjustment for zero

REF	NRR	SEX	AD	Ys	Ws	Qs	Ps
*ENGELA	58	m	7	2.04	1.97	0.11	0.0042
*HAMMON	98	m	1	2.72	3.06	0.58	0.0000
SOBUE	53	m	0	2.74	2.74	0.57	0.0000
SVENSS	27	f	0	2.08	2.93	0.12	0.0004
TSUGAN	15	m	0	1.61	0.36	0.16	0.3330
WAKAI	43	m	0	1.39	1.66	1.32	0.0727
WYNDE6	21	m	0	2.57	17.35	1.45	0.0000
WYNDE6	210	f	0	1.98	16.03	1.44	0.0000
Subtotal WYNDE6				2.29	33.38	2.88	

	N	8
	NS	7
	Wt	46.10
Het	Chi	5.75
Het	df	7
Het	P	N.S.
Fixed	RR	9.80
	RRl	7.34
	RRu	13.08
	P	+++
Random	RR	9.80
	RRl	7.34
	RRu	13.08
	P	+++
Asymm	P	N.S.

Table 2G7 - 6

IESLC - Meta-analysis of Current Smoking, Amount smoked, "Low", Any product (or Cigarettes if Any not available)

Squamous

Least adjusted

	combined	Sex male	female	Total
N		6	2	8
NS		6	2	8
Wt		27.14	18.96	46.10
Het Chi		3.12	0.02	5.75
Het df		5	1	7
Het P		N.S.	N.S.	N.S.
Fixed RR		11.95	7.37	9.80
RRl		8.21	4.70	7.34
RRu		17.41	11.56	13.08
P		+++	+++	+++
Random RR		11.95	7.37	9.80
RRl		8.21	4.70	7.34
RRu		17.41	11.56	13.08
P		+++	+++	+++
Between Chi				2.61
Between df				1
Between P				N.S.
Btwn(F) P				(*)
Btwn(R) P				N.S.

Table 2G7 - 7

IESLC - Meta-analysis of Current Smoking, Amount smoked, "Low", Any product (or Cigarettes if Any not available)
Squamous
Excluded studies (and stage at which they were excluded)

1	ABELIN ABRAHA AMANDU AMES ANDERS AUSTIN AXELSO BAND BECHER BERRIN BLOHMK BLOT4 BROCKM BROWN1 BYERS1 BYERS2 CARPEN CASCO2 CASCOR CHAN CHEN3 CHIAZZ CHYOU DESTE2 DOCKER DROSTE DU GARCIA GARDIN GENG GODLEY GOODMA GRAHAM GREGOR HEGMAN HEIN HENNEK HINDS HIRAOK HOROWI HORWIT HUANG ISHIMA JAHN JAIN JARVHO JIANG KELLER KIHARA KJUUS KO KOHLME KUBIK LAMWK LAMWK2 LANGE LEI LEMARC LEVIN LIU LOMBA2 LOMBAR MAGNUS MARSH MARSH2 MCDUFF MCLAUG MILLER MILLS NOTANI NOU ODRISC PAWLEG PERSHA POFFIJ QIAO QIAO2 RADZIK REN RONCO ROOTS ROTHSC SAARIK SANKAR SCHWAR SEGI SEOW SHIMIZ SIMARA SIMONA SITAS SOBUE2 STASZE STAYNE STUCKE SUN SUZUK2 SUZUKI TANG TAO TOKARS TOUSEY ULMER VEIERO VUTUC WALD WANG WANG3 WANG4 WICKLU WIGLE WILKIN WU2 WUNSCH WYNDE8 XIANGZ XU XU2 XU4 YONG ZHANG
2	AGUDO ALDERS ARMADA AUVINE BARBON BENHAM BLOT1 BLOT2 BLOT3 BOFFET BOUCHA BRESLO BROWN2 BUELL CHATZI CHEN CHEN2 CHOI COOKSO DAMBER DAVEYS DEAN DEAN2 DOLL DOSEME DUNN EBELIN ESAKI FAN GAO GARSHI GER GOLLED GSELL HANSEN HU HU2 JARUP JEDRYC JOLY JONES JUSSAW KHUDER KOULUM KREUZE KREYBE LAMTH LAUSSM LETOUR LIU2 LIU3 LIU4 LIU5 LUBIN LUBIN2 LUO MARTIS MASTRA MATSUD MCCONN MOLLO MZILEN NOTAN2 ORMOS OSANN OSANN2 PASTOR PERNU PIKE POLEDN RACHTA RANDIG RESTRE SADOWS SCHWA2 SIEMIA SPITZ STOCKS TIZZAN VANDER WANG2 WUWILL WYNDE2 WYNDE3 WYNDE4 XU3 YUAN ZHENG ZHOU
3	PISANI
4	WYNDE7
5	RIMING TANG2 WYNDE5
6	DESTEF HIRAY2 LAURIL LICKIN MRFIT MURATA WARSIN WATSON WYNDER
8	AKIBA ARCHER AXELSS BENSHL BEST BRETT BROSS BUFFLE CEDERL CHANG CHOW COMSTO CPSI CPSII DARBY DEAN3 DEKLER DOLL2 DORANT DORGAN DORN ENSTRO GAO2 GILLIS HAMMO2 HIRAYA HITOSU HOLE HUMBLE KAISE2 KAISER KANELL KAUFMA KINLEN KNEKT KOO LIAW LIDDEL MACLEN MATOS MIGRAN MRFITR NAM PARKIN PERSH2 PETO PEZZO2 PEZZOT PRESCO SEGI2 SHAW SPEIZE STOCKW TENKAN TULINI TVERDA YAMAGU
10	BOUCOT CORREA HAENSZ KATSOU WU

Table 2G7 - 8

Potentially overlapping studies

REF	REFGP	PRINC	.	OVERLAP/LINK
WYNDE6	WYNDE6	1		WYNDE5/6/7/8

Table 2G7 - 9

Most adjusted - insufficient data for metaanalysis

REF	NRR	SEX	AGEL	AGEH	RACE	YF	LC TYPE	LOC	START	ST	NLC	R	VB	P	H	AD	PRODUCT	exL	exH	DENOM	De
GILLIS	9	m	0	0	all	-	q	Eu:UK	1977	CC	656	n	V	n	n	3	cig+/-ot	1	14	nev	any ot

REF	NRR	RR	SIG	RRDATA comment
GILLIS	9	4.52		0

Table 2G8 -

IESLC - Meta-analysis of Current Smoking, Amount smoked, "Mid", Any product (or Cigarettes if Any not available)
Squamous

This analysis is restricted to results for:
1) Results by Amount smoked
2) Current smokers
3) Results complete enough for use in metaanalysis

Within each study, results are then selected (in the following order of preference, within each sex) for:
4) PRODUCT: all/unspec, cigarettes regardless of other products, cigarettes only
5) CIGTYPE: all/unspecified, MC regardless of HR, MC only
6) DENOM: never smoked anything, never smoked cigarettes, (never +1 = +long term ex, +2 = +amount unknown,
+3 = never cigs+long term ex)
7) Followup period (YF, prospective studies): whole study (coded as 0) or longest available
8) LCtype: squamous or nearest available, but not adeno. (q = squamous, s = small,
a = adeno, KI = Kreyberg I, u = undifferentiated)
9) Race: all or nearest available, otherwise by race (wh or w = white, bl or b = black, hi = hispanic
ch = chinese, jap = japanese, haw = hawaiian, w+o = white + oriental, sca = scandinavian, as = asian)
10) Amount smoked "mid" in key scheme 1 (key value 20, maximum range 6-44, in numbers of
cigarettes or cigarette equivalents)
11) For overlapping studies: principal rather than subsidiary studies
Finally by Age: whole study (coded as 0) if available, otherwise by widest available age group
and then for single sex results (m, f) in preference to combined sex results (c).

Results adjusted (AD) for the most potential confounders are then chosen in Sections -1 to -3
and results adjusted for the least confounders in Sections -4 to -6. (Those least adjusted results which
actually differ from the most adjusted as marked 'x' in column X in Section -4)
(Results adjusted for an unknown number of confounder(s) are coded as 20.)

Section -7 shows excluded studies, together with the stage (as above) at which no qualifying
results were found.

Section -8 lists the potentially overlapping studies which have been included (1=principal, 2=subsidiary).

Section -9 lists any results which would have been included in preference except that they had data not complete
enough for use in meta-analysis, with their significance (yes/no), if known, and any further comment as entered
on the database.

In addition to those mentioned above, the following fields, levels and abbreviations are used:
* or nk = not known, n = no, y = yes, ot = other
nev = never
all/unspec = all or unspecified, cig+/-ot = cigarettes irrespective of other products (cigar, pipe etc)
MC = manufactured cigarettes, HR = hand-rolled cigarettes
exL, exH = range of exposure (low and high) in the smoking group, in terms of Amount smoked,
cigarettes or cigarette equivalents
REF: 6-character study reference
NRR: number of the RR on the database within the study
ST : study type (CC = case control, pr or prosp = prospective)
NLC: number of lung cancer cases in whole study
R : risky occupational population (n = no, m = mining, o = other risky)
VB : national cigarette type (V = at least 75% Virginia, bl = at least 75% blended, ot = other)
P : any proxy use
H : full histological confirmation
De : derivation of RR/CI (or = original, st = standard method, ot = other method of estimation)

Table 2G8 - 1

IESLC - Meta-analysis of Current Smoking, Amount smoked, "Mid", Any product (or Cigarettes if Any not available)
Squamous
Most adjusted

REF	NRR	SEX	AGEL	AGEH	RACE	YF	LC TYPE	LOC	START	ST	NLC	R	VB	P	H	AD	PRODUCT	exL	exH	DENOM	De
HAMMON	99	m	0	0	wh	0	not a	NAmer	1952	pr	448	n	bl	n	n	1	cig only	10	20	nev any	ot
SOBUE	54	m	0	0	all	-	q	As:Jap	1986	CC	1376	n	bl	n	y	0	cig+/-ot	20	29	nev cigs	st
SVENSS	12	f	0	0	all	-	q	Eu:Sca	1983	CC	210	n	bl	n	n	1	all/unsp	11	20	nev any	or
TSUGAN	16	m	0	0	all	-	q	As:Jap	1976	CC	134	n	bl	n	y	0	all/unsp	16	35	nev any	ot
WAKAI	47	m	0	0	all	-	q	As:Jap	1988	CC	333	n	bl	n	y	1	cig+/-ot	20	29	nev any	or
WYNDE6	30	m	0	0	all	-	KI	NAmer	1969	CC	4423	n	bl	n	y	0	cig+/-ot	11	20	nev any	st
WYNDE6	219	f	0	0	all	-	KI	NAmer	1969	CC	4423	n	bl	n	y	0	cig+/-ot	11	20	nev cigs	st

Cigarette type is all/unspec for all RRs

Table 2G8 - 2

IESLC - Meta-analysis of Current Smoking, Amount smoked, "Mid", Any product (or Cigarettes if Any not available)
Squamous
Most adjusted

REF	NRR	SEX	AD	Number Exposed Case	Number Exposed Cont	Non-exposed Case	Non-exposed Cont	RR	95.00%CI
*HAMMON	99	m	1	-	-	-	-	17.44	(6.30- 48.29)
SOBUE	54	m	0	103	222	3	128	19.80	(6.15- 63.68)
SVENSS	12	f	1	-	-	-	-	36.20	(12.00- 168.90)
TSUGAN	16	m	0	7	7	0	5	11.00~	(0.51- 236.22)
WAKAI	47	m	1	-	-	-	-	10.40	(2.43- 44.30)
WYNDE6	30	m	0	270	293	29	617	19.61	(13.04- 29.47)
WYNDE6	219	f	0	191	165	40	856	24.77	(16.95- 36.20)
Subtotal WYNDE6								22.22	(16.83- 29.33)
Partial Totals				571	687	72	1606		

*prospective study ~ With 0.5 adjustment for zero

REF	NRR	SEX	AD	Ys	Ws	Qs	Ps
*HAMMON	99	m	1	2.86	3.70	0.17	0.0000
SOBUE	54	m	0	2.99	2.81	0.02	0.0000
SVENSS	12	f	1	3.59	2.20	0.59	0.0000
TSUGAN	16	m	0	2.40	0.41	0.19	0.1254
WAKAI	47	m	1	2.34	1.82	0.97	0.0016
WYNDE6	30	m	0	2.98	23.14	0.21	0.0000
WYNDE6	219	f	0	3.21	26.69	0.51	0.0000
Subtotal WYNDE6				3.10	49.83	0.72	

	N	7
	NS	6
	Wt	60.78
Het	Chi	2.65
Het	df	6
Het	P	N.S.
Fixed	RR	21.57
	RRl	16.77
	RRu	27.73
	P	+++
Random	RR	21.57
	RRl	16.77
	RRu	27.73
	P	+++
Asymm	P	N.S.

Table 2G8 - 3

IESLC - Meta-analysis of Current Smoking, Amount smoked, "Mid", Any product (or Cigarettes if Any not available)
Squamous
Most adjusted

	combined	Sex male	female	Total
N		5	2	7
NS		5	2	7
Wt		31.89	28.89	60.78
Het Chi		0.82	0.29	2.65
Het df		4	1	6
Het P		N.S.	N.S.	N.S.
Fixed RR		18.53	25.50	21.57
RRl		13.10	17.71	16.77
RRu		26.22	36.72	27.73
P		+++	+++	+++
Random RR		18.53	25.50	21.57
RRl		13.10	17.71	16.77
RRu		26.22	36.72	27.73
P		+++	+++	+++
Between Chi				1.54
Between df				1
Between P				N.S.
Btwn(F) P				*
Btwn(R) P				N.S.

Too few RRs for analysis by factor

Table 2G8 - 4

IESLC - Meta-analysis of Current Smoking, Amount smoked, "Mid", Any product (or Cigarettes if Any not available)
Squamous
Least adjusted

REF	NRR	X	SEX	AGEL	AGEH	RACE	YF	LC TYPE	LOC	START	ST	NLC	R	VB	P	H	AD	PRODUCT	exL	exH	DENOM	De
HAMMON	99		m	0	0	wh	0	not a	NAmer	1952	pr	448	n	bl	n	n	1	cig only	10	20	nev any	ot
SOBUE	54		m	0	0	all	-	q	As:Jap	1986	CC	1376	n	bl	n	y	0	cig+/-ot	20	29	nev cigs	st
SVENSS	32	x	f	0	0	all	-	q	Eu:Sca	1983	CC	210	n	bl	n	n	0	all/unsp	11	20	nev any	st
TSUGAN	16		m	0	0	all	-	q	As:Jap	1976	CC	134	n	bl	n	y	0	all/unsp	16	35	nev any	ot
WAKAI	44	x	m	0	0	all	-	q	As:Jap	1988	CC	333	n	bl	n	y	0	cig+/-ot	20	29	nev any	st
WYNDE6	30		m	0	0	all	-	KI	NAmer	1969	CC	4423	n	bl	n	y	0	cig+/-ot	11	20	nev any	st
WYNDE6	219		f	0	0	all	-	KI	NAmer	1969	CC	4423	n	bl	n	y	0	cig+/-ot	11	20	nev cigs	st

Cigarette type is all/unspec for all RRs

Table 2G8 - 5

IESLC - Meta-analysis of Current Smoking, Amount smoked, "Mid", Any product (or Cigarettes if Any not available)
Squamous
Least adjusted

REF	NRR	SEX	AD	Number Exposed Case	Number Exposed Cont	Non-exposed Case	Non-exposed Cont	RR	95.00%CI
*HAMMON	99	m	1	-	-	-	-	17.44	(6.30- 48.29)
SOBUE	54	m	0	103	222	3	128	19.80	(6.15- 63.68)
SVENSS	32	f	0	28	22	5	120	30.55	(10.64- 87.69)
TSUGAN	16	m	0	7	7	0	5	11.00~	(0.51- 236.22)
WAKAI	44	m	0	40	129	2	65	10.08	(2.36- 43.01)
WYNDE6	30	m	0	270	293	29	617	19.61	(13.04- 29.47)
WYNDE6	219	f	0	191	165	40	856	24.77	(16.95- 36.20)
Subtotal WYNDE6								22.22	(16.83- 29.33)
Partial Totals				639	838	79	1791		

*prospective study ~ With 0.5 adjustment for zero

REF	NRR	SEX	AD	Ys	Ws	Qs	Ps
*HAMMON	99	m	1	2.86	3.70	0.17	0.0000
SOBUE	54	m	0	2.99	2.81	0.02	0.0000
SVENSS	32	f	0	3.42	3.45	0.42	0.0000
TSUGAN	16	m	0	2.40	0.41	0.19	0.1254
WAKAI	44	m	0	2.31	1.82	1.06	0.0018
WYNDE6	30	m	0	2.98	23.14	0.21	0.0000
WYNDE6	219	f	0	3.21	26.69	0.51	0.0000
Subtotal WYNDE6				3.10	49.83	0.72	

	N	7
	NS	6
	Wt	62.03
Het	Chi	2.57
Het	df	6
Het	P	N.S.
Fixed	RR	21.57
	RRl	16.82
	RRu	27.66
	P	+++
Random	RR	21.57
	RRl	16.82
	RRu	27.66
	P	+++
Asymm	P	N.S.

Table 2G8 - 6

IESLC - Meta-analysis of Current Smoking, Amount smoked, "Mid", Any product (or Cigarettes if Any not available)

Squamous

Least adjusted

	Sex combined	male	female	Total
N		5	2	7
NS		5	2	7
Wt		31.89	30.15	62.03
Het Chi		0.89	0.13	2.57
Het df		4	1	6
Het P		N.S.	N.S.	N.S.
Fixed RR		18.50	25.37	21.57
RRl		13.07	17.76	16.82
RRu		26.17	36.26	27.66
P		+++	+++	+++
Random RR		18.50	25.37	21.57
RRl		13.07	17.76	16.82
RRu		26.17	36.26	27.66
P		+++	+++	+++
Between Chi				1.55
Between df				1
Between P				N.S.
Btwn(F) P				*
Btwn(R) P				N.S.

Table 2G8 - 7

IESLC - Meta-analysis of Current Smoking, Amount smoked, "Mid", Any product (or Cigarettes if Any not available)
Squamous
Excluded studies (and stage at which they were excluded)

1 ABELIN ABRAHA AMANDU AMES ANDERS AUSTIN AXELSO BAND BECHER BERRIN BLOHMK BLOT4 BROCKM BROWN1 BYERS1 BYERS2
CARPEN CASCO2 CASCOR CHAN CHEN3 CHIAZZ CHYOU DESTE2 DOCKER DROSTE DU GARCIA GARDIN GENG GODLEY GOODMA
GRAHAM GREGOR HEGMAN HEIN HENNEK HINDS HIRAOK HOROWI HORWIT HUANG ISHIMA JAHN JAIN JARVHO JIANG KELLER
KIHARA KJUUS KO KOHLME KUBIK LAMWK LAMWK2 LANGE LEI LEMARC LEVIN LIU LOMBA2 LOMBAR MAGNUS MARSH
MARSH2 MCDUFF MCLAUG MILLER MILLS NOTANI NOU ODRISC PAWLEG PERSHA POFFIJ QIAO QIAO2 RADZIK REN RONCO
ROOTS ROTHSC SAARIK SANKAR SCHWAR SEGI SEOW SHIMIZ SIMARA SIMONA SITAS SOBUE2 STASZE STAYNE STUCKE SUN
SUZUK2 SUZUKI TANG TAO TOKARS TOUSEY ULMER VEIERO VUTUC WALD WANG WANG3 WANG4 WICKLU WIGLE WILKIN
WU2 WUNSCH WYNDE8 XIANGZ XU XU2 XU4 YONG ZHANG

2 AGUDO ALDERS ARMADA AUVINE BARBON BENHAM BLOT1 BLOT2 BLOT3 BOFFET BOUCHA BRESLO BROWN2 BUELL CHATZI CHEN
CHEN2 CHOI COOKSO DAMBER DAVEYS DEAN DEAN2 DOLL DOSEME DUNN EBELIN ESAKI FAN GAO GARSHI GER
GOLLED GSELL HANSEN HU HU2 JARUP JEDRYC JOLY JONES JUSSAW KHUDER KOULUM KREUZE KREYBE LAMTH LAUSSM
LETOUR LIU2 LIU3 LIU4 LIU5 LUBIN LUBIN2 LUO MARTIS MASTRA MATSUD MCCONN MOLLO MZILEN NOTAN2 ORMOS
OSANN OSANN2 PASTOR PERNU PIKE POLEDN RACHTA RANDIG RESTRE SADOWS SCHWA2 SIEMIA SPITZ STOCKS TIZZAN VANDER
WANG2 WUWILL WYNDE2 WYNDE3 WYNDE4 XU3 YUAN ZHENG ZHOU

3 PISANI

4 WYNDE7

5 RIMING TANG2 WYNDE5

6 DESTEF HIRAY2 LAURIL LICKIN MRFIT MURATA WARSIN WATSON WYNDER

8 AKIBA ARCHER AXELSS BENSHL BEST BRETT BROSS BUFFLE CEDERL CHANG CHOW COMSTO CPSI CPSII DARBY DEAN3
DEKLER DOLL2 DORANT DORGAN DORN ENSTRO GAO2 GILLIS HAMMO2 HIRAYA HITOSU HOLE HUMBLE KAISE2 KAISER KANELL
KAUFMA KINLEN KNEKT KOO LIAW LIDDEL MACLEN MATOS MIGRAN MRFITR NAM PARKIN PERSH2 PETO PEZZO2 PEZZOT
PRESCO SEGI2 SHAW SPEIZE STOCKW TENKAN TULINI TVERDA YAMAGU

10 BOUCOT CORREA ENGELA HAENSZ KATSOU WU

Table 2G8 - 8

Potentially overlapping studies

REF	REFGP	PRINC	.	OVERLAP/LINK
WYNDE6	WYNDE6	1		WYNDE5/6/7/8

Table 2G8 - 9

Most adjusted - insufficient data for metaanalysis

REF	NRR	SEX	AGEL	AGEH	RACE	YF	LC TYPE	LOC	START	ST	NLC	R	VB	P	H	AD	PRODUCT	exL	exH	DENOM	De
GILLIS	10	m	0	0	all	-	q	Eu:UK	1977	CC	656	n	V	n	n	3	cig+/-ot	15	24	nev any	ot

REF	NRR	RR	SIG	RRDATA comment
GILLIS	10	8.36		0

Table 2G9 -

IESLC - Meta-analysis of Current Smoking, Amount smoked, "High", Any product (or Cigarettes if Any not available)
Squamous

This analysis is restricted to results for:
1) Results by Amount smoked
2) Current smokers
3) Results complete enough for use in metaanalysis

Within each study, results are then selected (in the following order of preference, within each sex) for:
4) PRODUCT: all/unspec, cigarettes regardless of other products, cigarettes only
5) CIGTYPE: all/unspecified, MC regardless of HR, MC only
6) DENOM: never smoked anything, never smoked cigarettes, (never +1 = +long term ex, +2 = +amount unknown,
+3 = never cigs+long term ex)
7) Followup period (YF, prospective studies): whole study (coded as 0) or longest available
8) LCtype: squamous or nearest available, but not adeno. (q = squamous, s = small,
a = adeno, KI = Kreyberg I, u = undifferentiated)
9) Race: all or nearest available, otherwise by race (wh or w = white, bl or b = black, hi = hispanic
ch = chinese, jap = japanese, haw = hawaiian, w+o = white + oriental, sca = scandinavian, as = asian)
10) Amount smoked "high" in key scheme 1 (key value 45, maximum range >20, in numbers of
cigarettes or cigarette equivalents)
11) For overlapping studies: principal rather than subsidiary studies
Finally by Age: whole study (coded as 0) if available, otherwise by widest available age group
and then for single sex results (m, f) in preference to combined sex results (c).

Results adjusted (AD) for the most potential confounders are then chosen in Sections -1 to -3
and results adjusted for the least confounders in Sections -4 to -6. (Those least adjusted results which
actually differ from the most adjusted as marked 'x' in column X in Section -4)
(Results adjusted for an unknown number of confounder(s) are coded as 20.)

Section -7 shows excluded studies, together with the stage (as above) at which no qualifying
results were found.

Section -8 lists the potentially overlapping studies which have been included (1=principal, 2=subsidiary).

Section -9 lists any results which would have been included in preference except that they had data not complete
enough for use in meta-analysis, with their significance (yes/no), if known, and any further comment as entered
on the database.

In addition to those mentioned above, the following fields, levels and abbreviations are used:
* or nk = not known, n = no, y = yes, ot = other
nev = never
all/unspec = all or unspecified, cig+/-ot = cigarettes irrespective of other products (cigar, pipe etc)
MC = manufactured cigarettes, HR = hand-rolled cigarettes
exL, exH = range of exposure (low and high) in the smoking group, in terms of Amount smoked,
cigarettes or cigarette equivalents
REF: 6-character study reference
NRR: number of the RR on the database within the study
ST : study type (CC = case control, pr or prosp = prospective)
NLC: number of lung cancer cases in whole study
R : risky occupational population (n = no, m = mining, o = other risky)
VB : national cigarette type (V = at least 75% Virginia, bl = at least 75% blended, ot = other)
P : any proxy use
H : full histological confirmation
De : derivation of RR/CI (or = original, st = standard method, ot = other method of estimation)

Table 2G9 - 1

IESLC - Meta-analysis of Current Smoking, Amount smoked, "High", Any product (or Cigarettes if Any not available)
Squamous
Most adjusted

REF	NRR	SEX	AGEL	AGEH	RACE	YF	LC TYPE	LOC	START	ST	NLC	R	VB	P	H	AD	PRODUCT	exL	exH	DENOM	De
BOUCOT	143	m	0	0	all	0	q	NAmer	1951	pr	121	n	bl	n	n	2	cig only	21	99	nev any	ot
CORREA	51	c	0	0	all	-	q+s	NAmer	1979	CC	1359	n	bl	y	n	1	cig+/-ot	21	99	nev cigs	or
HAENSZ	17	f	0	0	all	-	q+u	NAmer	1955	CC	158	n	bl	n	y	0	cig+/-ot	21	99	nev any	or
HAMMON	101	m	0	0	wh	0	not a	NAmer	1952	pr	448	n	bl	n	n	1	cig only	40	99	nev any	ot
KATSOU	22	f	0	0	all	-	KI	Eu:bal	1987	CC	101	n	bl	n	n	1	all/unsp	21	99	nev any	or
SOBUE	55	m	0	0	all	-	q	As:Jap	1986	CC	1376	n	bl	n	y	0	cig+/-ot	30	99	nev cigs	st
SVENSS	17	f	0	0	all	-	q	Eu:Sca	1983	CC	210	n	bl	n	n	1	all/unsp	21	99	nev any	ot
TSUGAN	17	m	0	0	all	-	q	As:Jap	1976	CC	134	n	bl	n	y	0	all/unsp	36	99	nev any	ot
WAKAI	48	m	0	0	all	-	q	As:Jap	1988	CC	333	n	bl	n	y	1	cig+/-ot	30	99	nev any	or
WU	18	f	0	0	wh	-	q	NAmer	1981	CC	220	n	bl	n	y	2	all/unsp	21	99	nev any	or
WYNDE6	48	m	0	0	all	-	KI	NAmer	1969	CC	4423	n	bl	n	y	0	cig+/-ot	31	99	nev any	st
WYNDE6	237	f	0	0	all	-	KI	NAmer	1969	CC	4423	n	bl	n	y	0	cig+/-ot	30	99	nev cigs	st

Cigarette type is all/unspec for all RRs

Table 2G9 - 2

IESLC - Meta-analysis of Current Smoking, Amount smoked, "High", Any product (or Cigarettes if Any not available)
Squamous
Most adjusted

REF	NRR	SEX	AD	Number Exposed Case	Number Exposed Cont	Non-exposed Case	Non-exposed Cont	RR	95.00%CI
*BOUCOT	143	m	2	-	-	-	-	46.64	(2.80- 775.69)
CORREA	51	c	1	-	-	-	-	54.80	(35.60- 89.20)
HAENSZ	17	f	0	18	13	44	236	7.43	(3.40- 16.24)
*HAMMON	101	m	1	-	-	-	-	63.91	(22.02- 185.47)
KATSOU	22	f	1	-	-	-	-	19.53	(5.36- 71.11)
SOBUE	55	m	0	87	187	3	128	19.85	(6.14- 64.13)
SVENSS	17	f	1	-	-	-	-	96.00	(6.90-1335.65)
TSUGAN	17	m	0	9	1	0	5	69.67~	(2.40-2022.74)
WAKAI	48	m	1	-	-	-	-	24.00	(5.46- 105.00)
WU	18	f	2	-	-	-	-	94.40	(9.90- 904.60)
WYNDE6	48	m	0	502	197	29	617	54.22	(36.08- 81.47)
WYNDE6	237	f	0	221	52	40	856	90.95	(58.70- 140.93)
Subtotal WYNDE6								68.92	(51.14- 92.86)
Partial Totals				837	450	116	1842		

*prospective study ~ With 0.5 adjustment for zero

REF	NRR	SEX	AD	Ys	Ws	Qs	Ps
*BOUCOT	143	m	2	3.84	0.49	0.00	0.0074
CORREA	51	c	1	4.00	18.21	0.18	0.0000
HAENSZ	17	f	0	2.01	6.27	22.59	0.0000
*HAMMON	101	m	1	4.16	3.38	0.22	0.0000
KATSOU	22	f	1	2.97	2.30	1.99	0.0000
SOBUE	55	m	0	2.99	2.79	2.34	0.0000
SVENSS	17	f	1	4.56	0.55	0.24	0.0007
TSUGAN	17	m	0	4.24	0.34	0.04	0.0135
WAKAI	48	m	1	3.18	1.76	0.92	0.0000
WU	18	f	2	4.55	0.75	0.31	0.0001
WYNDE6	48	m	0	3.99	23.16	0.19	0.0000
WYNDE6	237	f	0	4.51	20.03	7.39	0.0000
Subtotal WYNDE6				4.23	43.19	7.57	

	N	12
	NS	11
	Wt	80.04
Het	Chi	36.42
Het	df	11
Het	P	***
Fixed	RR	49.56
	RRl	39.81
	RRu	61.69
	P	+++
Random	RR	39.16
	RRl	23.67
	RRu	64.79
	P	+++
Asymm	P	N.S.

Table 2G9 - 3

IESLC - Meta-analysis of Current Smoking, Amount smoked, "High", Any product (or Cigarettes if Any not available)
Squamous
Most adjusted

	Sex combined	male	female	Total
N	1	6	5	12
NS	1	6	5	12
Wt	18.21	31.92	29.91	80.04
Het Chi	0.00	3.69	32.49	36.42
Het df	0	5	4	11
Het P	N.S.	N.S.	***	***
Fixed RR	54.80	48.33	47.88	49.56
RRl	34.62	34.16	33.46	39.81
RRu	86.74	68.37	68.51	61.69
P	+++	+++	+++	+++
Random RR	54.80	48.33	36.19	39.16
RRl	34.62	34.16	9.06	23.67
RRu	86.74	68.37	144.51	64.79
P	+++	+++	+++	+++
Between Chi				0.24
Between df				2
Between P				N.S.
Btwn(F) P				N.S.
Btwn(R) P				N.S.

Table 2G9 - 4

IESLC - Meta-analysis of Current Smoking, Amount smoked, "High", Any product (or Cigarettes if Any not available)
Squamous
Least adjusted

REF	NRR	X	SEX	AGEL	AGEH	RACE	YF	LC TYPE	LOC	START	ST	NLC	R	VB	P	H	AD	PRODUCT	exL	exH	DENOM	De
BOUCOT	22	x	m	0	0	all	0	q	NAmer	1951	pr	121	n	bl	n	n	0	cig only	21	99	nev any	ot
CORREA	51		c	0	0	all	-	q+s	NAmer	1979	CC	1359	n	bl	y	n	1	cig+/-ot	21	99	nev cigs	or
HAENSZ	17		f	0	0	all	-	q+u	NAmer	1955	CC	158	n	bl	n	y	0	cig+/-ot	21	99	nev any	or
HAMMON	101		m	0	0	wh	0	not a	NAmer	1952	pr	448	n	bl	n	n	1	cig only	40	99	nev any	ot
KATSOU	26	x	f	0	0	all	-	KI	Eu:bal	1987	CC	101	n	bl	n	n	0	all/unsp	21	99	nev any	st
SOBUE	55		m	0	0	all	-	q	As:Jap	1986	CC	1376	n	bl	n	y	0	cig+/-ot	30	99	nev cigs	st
SVENSS	37	x	f	0	0	all	-	q	Eu:Sca	1983	CC	210	n	bl	n	n	0	all/unsp	21	99	nev any	st
TSUGAN	17		m	0	0	all	-	q	As:Jap	1976	CC	134	n	bl	n	y	0	all/unsp	36	99	nev any	ot
WAKAI	45	x	m	0	0	all	-	q	As:Jap	1988	CC	333	n	bl	n	y	0	cig+/-ot	30	99	nev any	st
WU	13	x	f	0	0	wh	-	q	NAmer	1981	CC	220	n	bl	n	y	0	all/unsp	21	99	nev any	st
WYNDE6	48		m	0	0	all	-	KI	NAmer	1969	CC	4423	n	bl	n	y	0	cig+/-ot	31	99	nev any	st
WYNDE6	237		f	0	0	all	-	KI	NAmer	1969	CC	4423	n	bl	n	y	0	cig+/-ot	30	99	nev cigs	st

Cigarette type is all/unspec for all RRs

Table 2G9 - 5

IESLC - Meta-analysis of Current Smoking, Amount smoked, "High", Any product (or Cigarettes if Any not available)
Squamous
Least adjusted

REF	NRR	SEX	AD	Number Exposed Case	Number Exposed Cont	Non-exposed Case	Non-exposed Cont	RR	95.00%CI
*BOUCOT	22	m	0	17	6940	0	7551	38.08~	(2.29- 633.12)
CORREA	51	c	1	-	-	-	-	54.80	(35.60- 89.20)
HAENSZ	17	f	0	18	13	44	236	7.43	(3.40- 16.24)
*HAMMON	101	m	1	-	-	-	-	63.91	(22.02- 185.47)
KATSOU	26	f	0	15	4	14	67	17.95	(5.17- 62.28)
SOBUE	55	m	0	87	187	3	128	19.85	(6.14- 64.13)
SVENSS	37	f	0	4	1	5	120	96.00	(9.00-1023.75)
TSUGAN	17	m	0	9	1	0	5	69.67~	(2.40-2022.74)
WAKAI	45	m	0	33	48	2	65	22.34	(5.11- 97.69)
WU	13	f	0	42	9	2	30	70.00	(14.10- 347.48)
WYNDE6	48	m	0	502	197	29	617	54.22	(36.08- 81.47)
WYNDE6	237	f	0	221	52	40	856	90.95	(58.70- 140.93)
Subtotal WYNDE6								68.92	(51.14- 92.86)
Partial Totals				948	7452	139	9675		

*prospective study ~ With 0.5 adjustment for zero

REF	NRR	SEX	AD	Ys	Ws	Qs	Ps
*BOUCOT	22	m	0	3.64	0.49	0.03	0.0112
CORREA	51	c	1	4.00	18.21	0.21	0.0000
HAENSZ	17	f	0	2.01	6.27	22.45	0.0000
*HAMMON	101	m	1	4.16	3.38	0.23	0.0000
KATSOU	26	f	0	2.89	2.48	2.53	0.0000
SOBUE	55	m	0	2.99	2.79	2.31	0.0000
SVENSS	37	f	0	4.56	0.69	0.31	0.0002
TSUGAN	17	m	0	4.24	0.34	0.04	0.0135
WAKAI	45	m	0	3.11	1.77	1.10	0.0000
WU	13	f	0	4.25	1.50	0.18	0.0000
WYNDE6	48	m	0	3.99	23.16	0.21	0.0000
WYNDE6	237	f	0	4.51	20.03	7.53	0.0000
Subtotal WYNDE6				4.23	43.19	7.75	

	N	12
	NS	11
	Wt	81.11
Het	Chi	37.14
Het	df	11
Het	P	***
Fixed	RR	49.26
	RRl	39.62
	RRu	61.23
	P	+++
Random	RR	38.69
	RRl	23.54
	RRu	63.59
	P	+++
Asymm	P	N.S.

Table 2G9 - 6

IESLC - Meta-analysis of Current Smoking, Amount smoked, "High", Any product (or Cigarettes if Any not available)
Squamous
Least adjusted

	Sex combined	male	female	Total
N	1	6	5	12
NS	1	6	5	12
Wt	18.21	31.93	30.97	81.11
Het Chi	0.00	3.90	32.96	37.14
Het df	0	5	4	11
Het P	N.S.	N.S.	***	***
Fixed RR	54.80	47.98	47.53	49.26
RRl	34.62	33.92	33.42	39.62
RRu	86.74	67.87	67.60	61.23
P	+++	+++	+++	+++
Random RR	54.80	47.98	35.16	38.69
RRl	34.62	33.92	9.54	23.54
RRu	86.74	67.87	129.57	63.59
P	+++	+++	+++	+++
Between Chi				0.27
Between df				2
Between P				N.S.
Btwn(F) P				N.S.
Btwn(R) P				N.S.

Table 2G9 - 7

IESLC - Meta-analysis of Current Smoking, Amount smoked, "High", Any product (or Cigarettes if Any not available)
Squamous
Excluded studies (and stage at which they were excluded)

1 ABELIN ABRAHA AMANDU AMES ANDERS AUSTIN AXELSO BAND BECHER BERRIN BLOHMK BLOT4 BROCKM BROWN1 BYERS1 BYERS2
CARPEN CASCO2 CASCOR CHAN CHEN3 CHIAZZ CHYOU DESTE2 DOCKER DROSTE DU GARCIA GARDIN GENG GODLEY GOODMA
GRAHAM GREGOR HEGMAN HEIN HENNEK HINDS HIRAOK HOROWI HORWIT HUANG ISHIMA JAHN JAIN JARVHO JIANG KELLER
KIHARA KJUUS KO KOHLME KUBIK LAMWK LAMWK2 LANGE LEI LEMARC LEVIN LIU LOMBA2 LOMBAR MAGNUS MARSH
MARSH2 MCDUFF MCLAUG MILLER MILLS NOTANI NOU ODRISC PAWLEG PERSHA POFFIJ QIAO QIAO2 RADZIK REN RONCO
ROOTS ROTHSC SAARIK SANKAR SCHWAR SEGI SEOW SHIMIZ SIMARA SIMONA SITAS SOBUE2 STASZE STAYNE STUCKE SUN
SUZUK2 SUZUKI TANG TAO TOKARS TOUSEY ULMER VEIERO VUTUC WALD WANG WANG3 WANG4 WICKLU WIGLE WILKIN
WU2 WUNSCH WYNDE8 XIANGZ XU XU2 XU4 YONG ZHANG

2 AGUDO ALDERS ARMADA AUVINE BARBON BENHAM BLOT1 BLOT2 BLOT3 BOFFET BOUCHA BRESLO BROWN2 BUELL CHATZI CHEN
CHEN2 CHOI COOKSO DAMBER DAVEYS DEAN DEAN2 DOLL DOSEME DUNN EBELIN ESAKI FAN GAO GARSHI GER
GOLLED GSELL HANSEN HU HU2 JARUP JEDRYC JOLY JONES JUSSAW KHUDER KOULUM KREUZE KREYBE LAMTH LAUSSM
LETOUR LIU2 LIU3 LIU4 LIU5 LUBIN LUBIN2 LUO MARTIS MASTRA MATSUD MCCONN MOLLO MZILEN NOTAN2 ORMOS
OSANN OSANN2 PASTOR PERNU PIKE POLEDN RACHTA RANDIG RESTRE SADOWS SCHWA2 SIEMIA SPITZ STOCKS TIZZAN VANDER
WANG2 WUWILL WYNDE2 WYNDE3 WYNDE4 XU3 YUAN ZHENG ZHOU

3 PISANI

4 WYNDE7

5 RIMING TANG2 WYNDE5

6 DESTEF HIRAY2 LAURIL LICKIN MRFIT MURATA WARSIN WATSON WYNDER

8 AKIBA ARCHER AXELSS BENSHL BEST BRETT BROSS BUFFLE CEDERL CHANG CHOW COMSTO CPSI CPSII DARBY DEAN3
DEKLER DOLL2 DORANT DORGAN DORN ENSTRO GAO2 GILLIS HAMMO2 HIRAYA HITOSU HOLE HUMBLE KAISE2 KAISER KANELL
KAUFMA KINLEN KNEKT KOO LIAW LIDDEL MACLEN MATOS MIGRAN MRFITR NAM PARKIN PERSH2 PETO PEZZO2 PEZZOT
PRESCO SEGI2 SHAW SPEIZE STOCKW TENKAN TULINI TVERDA YAMAGU

10 ENGELA

Table 2G9 - 8
Potentially overlapping studies

REF	REFGP	PRINC	.	OVERLAP/LINK
WYNDE6	WYNDE6	1		WYNDE5/6/7/8

Table 2G9 - 9
Most adjusted - insufficient data for metaanalysis

REF	NRR	SEX	AGEL	AGEH	RACE	YF	LC TYPE	LOC	START	ST	NLC	R	VB	P	H	AD	PRODUCT	exL	exH	DENOM	De
GILLIS	12	m	0	0	all	-	q	Eu:UK	1977	CC	656	n	V	n	n	3	cig+/-ot	35	99	nev any	ot

REF	NRR	RR	SIG	RRDATA comment
GILLIS	12	10.51		0

Table 2G11 -

IESLC - Meta-anal of Ever Smoking (or Curr if Ever not avail) by Amount, Overview, Any prod (or Cigs if Any not avail)
Squamous

This analysis is restricted to results for:
1) Results by Amount smoked
   Results by Amount smoked (in numbers of cigarettes or cigarette equivalents) are grouped under 2 schemes (S1, S2).
   Each scheme has a set of "key values". An interval is allocated to the category whose key value
   it includes and intervals which include none or more than one of the key values are excluded.
   (Open-ended intervals are coded as 99.)

S1	key value	maximum range	S2	key value	maximum range
1	5	1-19	1	1	1-9
2	20	6-44	2	10	2-19
3	45	21+	3	20	11-29
			4	30	21-39
			5	40	31-98
			6	99	41+

   For all/unspec product, the definition of cigarette equivalents is shown at the end of Sections -1 and -4.
2) Results complete enough for use in metaanalysis

Within each study, results are then selected (in the following order of preference, within each sex) for:
3) SMKSTA: ever smokers, current smokers
4) PRODUCT: all/unspec, cigarettes regardless of other products, cigarettes only
5) CIGTYPE: all/unspecified, MC regardless of HR, MC only
6) DENOM: never smoked anything, never smoked cigarettes, (never +1 = +long term ex, +2 = +amount unknown,
   +3 = never cigs+long term ex)
7) Followup period (YF, prospective studies): whole study (coded as 0) or longest available
8) LCtype: squamous or nearest available, but not adeno. (q = squamous, s = small,
   a = adeno, KI = Kreyberg I, u = undifferentiated)
9) Race: all or nearest available, otherwise by race (wh or w = white, bl or b = black, hi = hispanic
   ch = chinese, jap = japanese, haw = hawaiian, w+o = white + oriental, sca = scandinavian, as = asian)
10) For overlapping studies: principal rather than subsidiary studies
Finally by Age: whole study (coded as 0) if available, otherwise by widest available age group
and then for single sex results (m, f) in preference to combined sex results (c).

Results adjusted (AD) for the most potential confounders are then chosen in Sections -1 to -3
and results adjusted for the least confounders in Sections -4 to -6. (Those least adjusted results which
actually differ from the most adjusted as marked 'x' in column X in Section -4)
(Results adjusted for an unknown number of confounder(s) are coded as 20.)

Section -7 shows excluded studies, together with the stage (as above) at which no qualifying
results were found.

Section -8 lists the potentially overlapping studies which have been included (1=principal, 2=subsidiary).

Section -9 lists any results which would have been included in preference except that they had data not complete
enough for use in meta-analysis, with their significance (yes/no), if known, and any further comment as entered
on the database.

In addition to those mentioned above, the following fields, levels and abbreviations are used:
* or nk = not known, n = no, y = yes, ot = other
ev = ever, cu = current, nev = never
all/unspec = all or unspecified, cig+/-ot = cigarettes irrespective of other products (cigar, pipe etc)
MC = manufactured cigarettes, HR = hand-rolled cigarettes
exL, exH = range of exposure (low and high) in the smoking group, in terms of Amount smoked,
   cigarettes or cigarette equivalents
REF: 6-character study reference
NRR: number of the RR on the database within the study
ST : study type (CC = case control, pr or prosp = prospective)
NLC: number of lung cancer cases in whole study
R : risky occupational population (n = no, m = mining, o = other risky)
VB : national cigarette type (V = at least 75% Virginia, bl = at least 75% blended, ot = other)
P : any proxy use
H : full histological confirmation
De : derivation of RR/CI (or = original, st = standard method, ot = other method of estimation)

Table 2G11 - 1

IESLC - Meta-anal of Ever Smoking (or Curr if Ever not avail) by Amount, Overview, Any prod (or Cigs if Any not avail)
Squamous
Most adjusted

REF	NRR	SEX	AGEL	AGEH	RACE	YF	LC TYPE	LOC	START	ST	NLC	R	VB	P	H	AD	SM	PRODUCT	exL	exH	S1	S2	DENOM	De
ALDERS	34	m	0	0	all	-	q+s	Eu:UK	1977	CC	1448	n	V	n	n	1	ev	cig only	1	17	1	0	nev+2	ot
ALDERS	35	m	0	0	all	-	q+s	Eu:UK	1977	CC	1448	n	V	n	n	1	ev	cig only	18	27	2	3	nev+2	ot
ALDERS	36	m	0	0	all	-	q+s	Eu:UK	1977	CC	1448	n	V	n	n	1	ev	cig only	28	99	3	0	nev+2	ot
ALDERS	37	f	0	0	all	-	q+s	Eu:UK	1977	CC	1448	n	V	n	n	1	ev	cig only	1	17	1	0	nev+2	ot
ALDERS	38	f	0	0	all	-	q+s	Eu:UK	1977	CC	1448	n	V	n	n	1	ev	cig only	18	27	2	3	nev+2	ot
ALDERS	39	f	0	0	all	-	q+s	Eu:UK	1977	CC	1448	n	V	n	n	1	ev	cig only	28	99	3	0	nev+2	ot
BARBON	70	m	0	0	all	-	q	Eu:wst	1979	CC	755	n	bl	y	y	3	ev	all/unsp	1	19	1	0	nev any	or
BARBON	71	m	0	0	all	-	q	Eu:wst	1979	CC	755	n	bl	y	y	3	ev	all/unsp	20	39	2	0	nev any	or
BARBON	72	m	0	0	all	-	q	Eu:wst	1979	CC	755	n	bl	y	y	3	ev	all/unsp	40	99	3	0	nev any	or
BOUCOT	142	m	0	0	all	0	q	NAmer	1951	pr	121	n	bl	n	n	2	cu	cig only	1	20	0	0	nev any	ot
BOUCOT	143	m	0	0	all	0	q	NAmer	1951	pr	121	n	bl	n	n	2	cu	cig only	21	99	3	0	nev any	ot
BROWN2	36	m	0	0	wh	-	q	NAmer	1984	CC	14596	n	bl	n	y	2	ev	cig+/-ot	1	19	1	0	nev cigs	or
BROWN2	46	m	0	0	wh	-	q	NAmer	1984	CC	14596	n	bl	n	y	2	ev	cig+/-ot	20	99	0	0	nev cigs	or
BROWN2	35	f	0	0	wh	-	q	NAmer	1984	CC	14596	n	bl	n	y	2	ev	cig+/-ot	1	19	1	0	nev cigs	or
BROWN2	45	f	0	0	wh	-	q	NAmer	1984	CC	14596	n	bl	n	y	2	ev	cig+/-ot	20	99	0	0	nev cigs	or
CHOI	46	m	0	0	all	-	q	As:oth	1985	CC	375	n	bl	n	n	0	ev	cig+/-ot	1	10	1	0	nev cigs	st
CHOI	47	m	0	0	all	-	q	As:oth	1985	CC	375	n	bl	n	n	0	ev	cig+/-ot	11	20	2	3	nev cigs	st
CHOI	48	m	0	0	all	-	q	As:oth	1985	CC	375	n	bl	n	n	0	ev	cig+/-ot	21	30	0	4	nev cigs	st
CHOI	49	m	0	0	all	-	q	As:oth	1985	CC	375	n	bl	n	n	0	ev	cig+/-ot	31	40	0	5	nev cigs	st
CHOI	50	m	0	0	all	-	q	As:oth	1985	CC	375	n	bl	n	n	0	ev	cig+/-ot	41	99	3	6	nev cigs	st
CHOI	56	f	0	0	all	-	q	As:oth	1985	CC	375	n	bl	n	n	0	ev	cig+/-ot	1	10	1	0	nev cigs	st
CHOI	57	f	0	0	all	-	q	As:oth	1985	CC	375	n	bl	n	n	0	ev	cig+/-ot	11	30	2	0	nev cigs	st
CHOI	58	f	0	0	all	-	q	As:oth	1985	CC	375	n	bl	n	n	0	ev	cig+/-ot	31	99	3	0	nev cigs	st
CORREA	47	c	0	0	all	-	q+s	NAmer	1979	CC	1359	n	bl	y	n	1	cu	cig+/-ot	1	20	0	0	nev cigs	or
CORREA	51	c	0	0	all	-	q+s	NAmer	1979	CC	1359	n	bl	y	n	1	cu	cig+/-ot	21	99	3	0	nev cigs	or
DOLL	68	m	0	0	all	-	KI	Eu:UK	1948	CC	1465	n	V	n	n	1	ev	all/unsp	1	4	0	1	nev any	ot
DOLL	69	m	0	0	all	-	KI	Eu:UK	1948	CC	1465	n	V	n	n	1	ev	all/unsp	5	14	1	2	nev any	ot
DOLL	70	m	0	0	all	-	KI	Eu:UK	1948	CC	1465	n	V	n	n	1	ev	all/unsp	15	24	2	3	nev any	ot
DOLL	71	m	0	0	all	-	KI	Eu:UK	1948	CC	1465	n	V	n	n	1	ev	all/unsp	25	99	3	0	nev any	ot
DOLL	76	f	0	0	all	-	KI	Eu:UK	1948	CC	1465	n	V	n	n	1	ev	all/unsp	1	4	0	1	nev any	ot
DOLL	77	f	0	0	all	-	KI	Eu:UK	1948	CC	1465	n	V	n	n	1	ev	all/unsp	5	14	1	2	nev any	ot
DOLL	78	f	0	0	all	-	KI	Eu:UK	1948	CC	1465	n	V	n	n	1	ev	all/unsp	15	99	0	0	nev any	ot
DORGAN	114	m	0	0	wh	-	q	NAmer	1980	CC	2026	n	bl	y	y	2	ev	cig+/-ot	1	19	1	0	nev any	ot
DORGAN	115	m	0	0	wh	-	q	NAmer	1980	CC	2026	n	bl	y	y	2	ev	cig+/-ot	20	99	0	0	nev any	ot
DORGAN	99	f	0	0	all	-	q	NAmer	1980	CC	2026	n	bl	y	y	3	ev	cig+/-ot	1	19	1	0	nev any	ot
DORGAN	100	f	0	0	all	-	q	NAmer	1980	CC	2026	n	bl	y	y	3	ev	cig+/-ot	20	99	0	0	nev any	ot
DOSEME	7	m	0	0	all	-	q	Eu:bal	1979	CC	1210	n	bl	n	n	2	ev	cig+/-ot	1	10	1	0	nev cigs	or
DOSEME	11	m	0	0	all	-	q	Eu:bal	1979	CC	1210	n	bl	n	n	2	ev	cig+/-ot	11	20	2	3	nev cigs	or
DOSEME	15	m	0	0	all	-	q	Eu:bal	1979	CC	1210	n	bl	n	n	2	ev	cig+/-ot	21	99	3	0	nev cigs	or
ENGELA	57	m	0	0	all	0	q	Eu:Sca	1964	pr	435	n	bl	n	n	7	cu	cig+/-ot	1	4	0	1	nev cigs	or
ENGELA	58	m	0	0	all	0	q	Eu:Sca	1964	pr	435	n	bl	n	n	7	cu	cig+/-ot	5	9	1	0	nev cigs	or
ENGELA	59	m	0	0	all	0	q	Eu:Sca	1964	pr	435	n	bl	n	n	7	cu	cig+/-ot	10	14	0	2	nev cigs	or
ENGELA	60	m	0	0	all	0	q	Eu:Sca	1964	pr	435	n	bl	n	n	7	cu	cig+/-ot	15	19	0	0	nev cigs	or
ENGELA	61	m	0	0	all	0	q	Eu:Sca	1964	pr	435	n	bl	n	n	7	cu	cig+/-ot	20	99	0	0	nev cigs	or
GER	14	c	0	0	all	-	q+s	As:oth	1990	CC	141	n	ot	y	n	10	ev	all/unsp	1	10	1	0	nev any	ot
GER	15	c	0	0	all	-	q+s	As:oth	1990	CC	141	n	ot	y	n	10	ev	all/unsp	11	20	2	3	nev any	ot
GER	16	c	0	0	all	-	q+s	As:oth	1990	CC	141	n	ot	y	n	10	ev	all/unsp	21	99	3	0	nev any	ot
HAENSZ	18	f	0	0	all	-	q+u	NAmer	1955	CC	158	n	bl	n	y	0	cu	cig+/-ot	1	20	0	0	nev any	or
HAENSZ	17	f	0	0	all	-	q+u	NAmer	1955	CC	158	n	bl	n	y	0	cu	cig+/-ot	21	99	3	0	nev any	or
HAMMON	98	m	0	0	wh	0	not a	NAmer	1952	pr	448	n	bl	n	n	1	cu	cig only	1	9	1	1	nev any	ot
HAMMON	99	m	0	0	wh	0	not a	NAmer	1952	pr	448	n	bl	n	n	1	cu	cig only	10	20	2	0	nev any	ot
HAMMON	100	m	0	0	wh	0	not a	NAmer	1952	pr	448	n	bl	n	n	1	cu	cig only	21	39	0	4	nev any	ot
HAMMON	101	m	0	0	wh	0	not a	NAmer	1952	pr	448	n	bl	n	n	1	cu	cig only	40	99	3	0	nev any	ot
JEDRYC	28	m	0	0	all	-	q	Eu:est	1980	CC	1630	n	bl	y	n	3	ev	cig+/-ot	1	19	1	0	nev any	or
JEDRYC	29	m	0	0	all	-	q	Eu:est	1980	CC	1630	n	bl	y	n	3	ev	cig+/-ot	20	29	2	3	nev any	or
JEDRYC	30	m	0	0	all	-	q	Eu:est	1980	CC	1630	n	bl	y	n	3	ev	cig+/-ot	30	99	3	0	nev any	or
KATSOU	21	f	0	0	all	-	KI	Eu:bal	1987	CC	101	n	bl	n	n	1	cu	all/unsp	1	20	0	0	nev any	or
KATSOU	22	f	0	0	all	-	KI	Eu:bal	1987	CC	101	n	bl	n	n	1	cu	all/unsp	21	99	3	0	nev any	or
KREYBE	1	m	0	0	all	-	KI	Eu:Sca	1948	CC	300	n	bl	n	y	1	ev	all/unsp	1	14	1	0	nev any	ot
KREYBE	2	m	0	0	all	-	KI	Eu:Sca	1948	CC	300	n	bl	n	y	1	ev	all/unsp	15	24	2	3	nev any	ot
KREYBE	3	m	0	0	all	-	KI	Eu:Sca	1948	CC	300	n	bl	n	y	1	ev	all/unsp	25	99	3	0	nev any	ot
KREYBE	31	f	0	0	all	-	KI	Eu:Sca	1948	CC	300	n	bl	n	y	0	ev	all/unsp	1	14	1	0	nev any	st
KREYBE	32	f	0	0	all	-	KI	Eu:Sca	1948	CC	300	n	bl	n	y	0	ev	all/unsp	15	99	0	0	nev any	st
LAMTH	10	f	0	0	ch	-	q	As:HK	1983	CC	445	n	bl	n	n	0	ev	all/unsp	1	10	1	0	nev any	or
LAMTH	11	f	0	0	ch	-	q	As:HK	1983	CC	445	n	bl	n	n	0	ev	all/unsp	11	20	2	3	nev any	or
LAMTH	12	f	0	0	ch	-	q	As:HK	1983	CC	445	n	bl	n	n	0	ev	all/unsp	21	99	3	0	nev any	st
LUBIN2	149	m	0	0	all	-	q	Eu:mul	1976	CC	7804	n	bl	n	y	0	ev	cig+/-ot	1	9	1	1	nev any	st
LUBIN2	153	m	0	0	all	-	q	Eu:mul	1976	CC	7804	n	bl	n	y	0	ev	cig+/-ot	10	19	0	2	nev any	st
LUBIN2	157	m	0	0	all	-	q	Eu:mul	1976	CC	7804	n	bl	n	y	0	ev	cig+/-ot	20	29	2	3	nev any	st
LUBIN2	161	m	0	0	all	-	q	Eu:mul	1976	CC	7804	n	bl	n	y	0	ev	cig+/-ot	30	99	3	0	nev any	st
LUBIN2	169	f	0	0	all	-	q	Eu:mul	1976	CC	7804	n	bl	n	y	0	ev	cig+/-ot	1	9	1	1	nev any	st
LUBIN2	173	f	0	0	all	-	q	Eu:mul	1976	CC	7804	n	bl	n	y	0	ev	cig+/-ot	10	19	0	2	nev any	st

Table 2G11 - 1

IESLC - Meta-anal of Ever Smoking (or Curr if Ever not avail) by Amount, Overview, Any prod (or Cigs if Any not avail)
Squamous
Most adjusted

REF	NRR	SEX	AGEL	AGEH	RACE	YF	LC TYPE	LOC	START	ST	NLC	R	VB	P	H	AD	SM	PRODUCT	exL	exH	S1	S2	DENOM	De
LUBIN2	177	f	0	0	all	-	q	Eu:mul	1976	CC	7804	n	bl	n	y	0	ev	cig+/-ot	20	29	2	3	nev any	st
LUBIN2	181	f	0	0	all	-	q	Eu:mul	1976	CC	7804	n	bl	n	y	0	ev	cig+/-ot	30	99	3	0	nev any	st
LUO	10	c	0	0	all	-	q	As:Chi	1990	CC	102	n	ot	n	y	20	ev	cig+/-ot	1	19	1	0	nev cigs	or
LUO	11	c	0	0	all	-	q	As:Chi	1990	CC	102	n	ot	n	y	20	ev	cig+/-ot	20	29	2	3	nev cigs	or
LUO	12	c	0	0	all	-	q	As:Chi	1990	CC	102	n	ot	n	y	20	ev	cig+/-ot	30	99	3	0	nev cigs	or
MATOS	43	m	0	0	all	-	q	SCAmer	1994	CC	200	n	bl	n	n	2	ev	cig+/-ot	1	14	1	0	nev any	or
MATOS	45	m	0	0	all	-	q	SCAmer	1994	CC	200	n	bl	n	n	2	ev	cig+/-ot	15	24	2	3	nev any	or
MATOS	47	m	0	0	all	-	q	SCAmer	1994	CC	200	n	bl	n	n	2	ev	cig+/-ot	25	99	3	0	nev any	or
MATSUD	4	m	0	0	all	-	q	As:Jap	1965	CC	179	n	bl	n	n	0	ev	cig+/-ot	1	10	1	0	nev cigs	st
MATSUD	5	m	0	0	all	-	q	As:Jap	1965	CC	179	n	bl	n	n	0	ev	cig+/-ot	11	20	2	3	nev cigs	st
MATSUD	6	m	0	0	all	-	q	As:Jap	1965	CC	179	n	bl	n	n	0	ev	cig+/-ot	21	99	3	0	nev cigs	st
ORMOS	5	m	0	0	all	-	q	Eu:est	1947	CC	119	n	bl	y	y	0	ev	cig+/-ot	1	15	1	0	nev any	st
ORMOS	6	m	0	0	all	-	q	Eu:est	1947	CC	119	n	bl	y	y	0	ev	cig+/-ot	16	30	2	0	nev any	st
ORMOS	7	m	0	0	all	-	q	Eu:est	1947	CC	119	n	bl	y	y	0	ev	cig+/-ot	31	99	3	0	nev any	st
OSANN	51	m	0	0	all	-	q	NAmer	1984	CC	1986	n	bl	n	n	2	ev	cig+/-ot	1	39	0	0	nev cigs	or
OSANN	59	m	0	0	all	-	q	NAmer	1984	CC	1986	n	bl	n	n	2	ev	cig+/-ot	40	99	3	0	nev cigs	or
OSANN	52	f	0	0	all	-	q	NAmer	1984	CC	1986	n	bl	n	n	2	ev	cig+/-ot	1	39	0	0	nev cigs	or
OSANN	60	f	0	0	all	-	q	NAmer	1984	CC	1986	n	bl	n	n	2	ev	cig+/-ot	40	99	3	0	nev cigs	or
OSANN2	28	f	0	0	all	-	KI	NAmer	1964	ot	217	n	bl	n	y	1	ev	cig+/-ot	1	19	1	0	nev cigs	or
OSANN2	29	f	0	0	all	-	KI	NAmer	1964	ot	217	n	bl	n	y	1	ev	cig+/-ot	20	99	0	0	nev cigs	or
SOBUE	53	m	0	0	all	-	q	As:Jap	1986	CC	1376	n	bl	n	y	0	cu	cig+/-ot	1	19	1	0	nev cigs	st
SOBUE	54	m	0	0	all	-	q	As:Jap	1986	CC	1376	n	bl	n	y	0	cu	cig+/-ot	20	29	2	3	nev cigs	st
SOBUE	55	m	0	0	all	-	q	As:Jap	1986	CC	1376	n	bl	n	y	0	cu	cig+/-ot	30	99	3	0	nev cigs	st
SVENSS	7	f	0	0	all	-	q	Eu:Sca	1983	CC	210	n	bl	n	n	1	cu	all/unsp	1	10	1	0	nev any	or
SVENSS	12	f	0	0	all	-	q	Eu:Sca	1983	CC	210	n	bl	n	n	1	cu	all/unsp	11	20	2	3	nev any	or
SVENSS	17	f	0	0	all	-	q	Eu:Sca	1983	CC	210	n	bl	n	n	1	cu	all/unsp	21	99	3	0	nev any	ot
TSUGAN	15	m	0	0	all	-	q	As:Jap	1976	CC	134	n	bl	n	y	0	cu	all/unsp	1	15	1	0	nev any	ot
TSUGAN	16	m	0	0	all	-	q	As:Jap	1976	CC	134	n	bl	n	y	0	cu	all/unsp	16	35	2	0	nev any	ot
TSUGAN	17	m	0	0	all	-	q	As:Jap	1976	CC	134	n	bl	n	y	0	cu	all/unsp	36	99	3	0	nev any	ot
WAKAI	46	m	0	0	all	-	q	As:Jap	1988	CC	333	n	bl	n	y	1	cu	cig+/-ot	1	19	1	0	nev any	or
WAKAI	47	m	0	0	all	-	q	As:Jap	1988	CC	333	n	bl	n	y	1	cu	cig+/-ot	20	29	2	3	nev any	or
WAKAI	48	m	0	0	all	-	q	As:Jap	1988	CC	333	n	bl	n	y	1	cu	cig+/-ot	30	99	3	0	nev any	or
WU	17	f	0	0	wh	-	q	NAmer	1981	CC	220	n	bl	n	y	2	cu	all/unsp	1	20	0	0	nev any	or
WU	18	f	0	0	wh	-	q	NAmer	1981	CC	220	n	bl	n	y	2	cu	all/unsp	21	99	3	0	nev any	or
WUWILL	14	f	0	0	all	-	q+s	As:Chi	1985	CC	965	n	ot	n	n	3	ev	cig+/-ot	1	19	1	0	nev cigs	ot
WUWILL	15	f	0	0	all	-	q+s	As:Chi	1985	CC	965	n	ot	n	n	3	ev	cig+/-ot	20	99	0	0	nev cigs	ot
WYNDE2	3	m	0	0	all	-	KI	NAmer	1962	CC	404	n	bl	n	y	0	ev	cig+/-ot	1	10	1	0	nev any	st
WYNDE2	4	m	0	0	all	-	KI	NAmer	1962	CC	404	n	bl	n	y	0	ev	cig+/-ot	11	20	2	3	nev any	st
WYNDE2	5	m	0	0	all	-	KI	NAmer	1962	CC	404	n	bl	n	y	0	ev	cig+/-ot	21	34	0	4	nev any	st
WYNDE2	6	m	0	0	all	-	KI	NAmer	1962	CC	404	n	bl	n	y	0	ev	cig+/-ot	35	99	3	0	nev any	st
WYNDE3	4	m	0	0	all	-	KI	NAmer	1966	CC	350	n	bl	n	y	0	ev	cig+/-ot	1	9	1	1	nev any	st
WYNDE3	5	m	0	0	all	-	KI	NAmer	1966	CC	350	n	bl	n	y	0	ev	cig+/-ot	10	20	2	0	nev any	st
WYNDE3	6	m	0	0	all	-	KI	NAmer	1966	CC	350	n	bl	n	y	0	ev	cig+/-ot	21	40	0	0	nev any	st
WYNDE3	7	m	0	0	all	-	KI	NAmer	1966	CC	350	n	bl	n	y	0	ev	cig+/-ot	41	99	3	6	nev any	st
WYNDE3	63	f	0	0	all	-	KI	NAmer	1966	CC	350	n	bl	n	y	0	ev	cig+/-ot	1	9	1	1	nev any	st
WYNDE3	64	f	0	0	all	-	KI	NAmer	1966	CC	350	n	bl	n	y	0	ev	cig+/-ot	10	20	2	0	nev any	st
WYNDE3	65	f	0	0	all	-	KI	NAmer	1966	CC	350	n	bl	n	y	0	ev	cig+/-ot	21	40	0	0	nev any	st
WYNDE3	66	f	0	0	all	-	KI	NAmer	1966	CC	350	n	bl	n	y	0	ev	cig+/-ot	41	99	3	6	nev any	st
WYNDE4	63	m	0	0	all	-	not a	NAmer	1948	CC	684	n	bl	y	n	2	ev	all/unsp	1	9	1	1	nev any	ot
WYNDE4	64	m	0	0	all	-	not a	NAmer	1948	CC	684	n	bl	y	n	2	ev	all/unsp	10	15	0	2	nev any	ot
WYNDE4	65	m	0	0	all	-	not a	NAmer	1948	CC	684	n	bl	y	n	2	ev	all/unsp	16	20	2	3	nev any	ot
WYNDE4	66	m	0	0	all	-	not a	NAmer	1948	CC	684	n	bl	y	n	2	ev	all/unsp	21	34	0	4	nev any	ot
WYNDE4	67	m	0	0	all	-	not a	NAmer	1948	CC	684	n	bl	y	n	2	ev	all/unsp	35	99	3	0	nev any	ot
WYNDE4	49	f	0	0	all	-	not a	NAmer	1948	CC	684	n	bl	y	n	2	ev	all/unsp	1	9	1	1	nev any	ot
WYNDE4	50	f	0	0	all	-	not a	NAmer	1948	CC	684	n	bl	y	n	2	ev	all/unsp	10	15	0	2	nev any	ot
WYNDE4	51	f	0	0	all	-	not a	NAmer	1948	CC	684	n	bl	y	n	2	ev	all/unsp	16	20	2	3	nev any	ot
WYNDE4	52	f	0	0	all	-	not a	NAmer	1948	CC	684	n	bl	y	n	2	ev	all/unsp	21	34	0	4	nev any	ot
WYNDE4	53	f	0	0	all	-	not a	NAmer	1948	CC	684	n	bl	y	n	2	ev	all/unsp	35	99	3	0	nev any	ot
WYNDE6	21	m	0	0	all	-	KI	NAmer	1969	CC	4423	n	bl	n	y	0	cu	cig+/-ot	1	10	1	0	nev any	st
WYNDE6	30	m	0	0	all	-	KI	NAmer	1969	CC	4423	n	bl	n	y	0	cu	cig+/-ot	11	20	2	3	nev any	st
WYNDE6	39	m	0	0	all	-	KI	NAmer	1969	CC	4423	n	bl	n	y	0	cu	cig+/-ot	21	30	0	4	nev any	st
WYNDE6	48	m	0	0	all	-	KI	NAmer	1969	CC	4423	n	bl	n	y	0	cu	cig+/-ot	31	99	3	0	nev any	st
WYNDE6	210	f	0	0	all	-	KI	NAmer	1969	CC	4423	n	bl	n	y	0	cu	cig+/-ot	1	10	1	0	nev cigs	st
WYNDE6	219	f	0	0	all	-	KI	NAmer	1969	CC	4423	n	bl	n	y	0	cu	cig+/-ot	11	20	2	3	nev cigs	st
WYNDE6	228	f	0	0	all	-	KI	NAmer	1969	CC	4423	n	bl	n	y	0	cu	cig+/-ot	21	30	0	4	nev cigs	st
WYNDE6	237	f	0	0	all	-	KI	NAmer	1969	CC	4423	n	bl	n	y	0	cu	cig+/-ot	30	99	3	0	nev cigs	st
ZHENG	1	m	0	0	all	-	q	As:Chi	1982	CC	540	n	ot	*	y	0	ev	cig+/-ot	1	9	1	1	nev cigs	st
ZHENG	2	m	0	0	all	-	q	As:Chi	1982	CC	540	n	ot	*	y	0	ev	cig+/-ot	10	19	0	2	nev cigs	st
ZHENG	3	m	0	0	all	-	q	As:Chi	1982	CC	540	n	ot	*	y	0	ev	cig+/-ot	20	29	2	3	nev cigs	st
ZHENG	4	m	0	0	all	-	q	As:Chi	1982	CC	540	n	ot	*	y	0	ev	cig+/-ot	30	99	3	0	nev cigs	st
ZHENG	16	f	0	0	all	-	q	As:Chi	1982	CC	540	n	ot	*	y	0	ev	cig+/-ot	1	9	1	1	nev cigs	st
ZHENG	17	f	0	0	all	-	q	As:Chi	1982	CC	540	n	ot	*	y	0	ev	cig+/-ot	10	99	0	0	nev cigs	st

Table 2G11 - 1

IESLC - Meta-anal of Ever Smoking (or Curr if Ever not avail) by Amount, Overview, Any prod (or Cigs if Any not avail)
Squamous
Most adjusted

REF	NRR	SEX	AGEL	AGEH	RACE	YF	LC TYPE	LOC	START	ST	NLC	R	VB	P	H	AD	SM	PRODUCT	exL	exH	S1	S2	DENOM	De
ZHOU	10	c	0	0	all	-	q	As:Chi	1978	CC	1360	n	ot	n	n	0	ev	all/unsp	1	9	1	1	nev any	st
ZHOU	11	c	0	0	all	-	q	As:Chi	1978	CC	1360	n	ot	n	n	0	ev	all/unsp	10	19	0	2	nev any	st
ZHOU	12	c	0	0	all	-	q	As:Chi	1978	CC	1360	n	ot	n	n	0	ev	all/unsp	20	99	0	0	nev any	st

Cigarette type is all/unspec for all RRs

except for the following:

REF	NRR	CIGTYPE
ALDERS	34	MC only
ALDERS	35	MC only
ALDERS	36	MC only
ALDERS	37	MC only
ALDERS	38	MC only
ALDERS	39	MC only

REF	NRR	Cigarette equivalent
ALDERS	34	-
ALDERS	35	-
ALDERS	36	-
ALDERS	37	-
ALDERS	38	-
ALDERS	39	-
BARBON	70	*
BARBON	71	*
BARBON	72	*
BOUCOT	142	-
BOUCOT	143	-
BROWN2	36	*
BROWN2	46	*
BROWN2	35	*
BROWN2	45	*
CHOI	46	*
CHOI	47	*
CHOI	48	*
CHOI	49	*
CHOI	50	*
CHOI	56	*
CHOI	57	*
CHOI	58	*
CORREA	47	*
CORREA	51	*
DOLL	68	grams
DOLL	69	grams
DOLL	70	grams
DOLL	71	grams
DOLL	76	grams
DOLL	77	grams
DOLL	78	grams
DORGAN	114	*
DORGAN	115	*
DORGAN	99	*
DORGAN	100	*
DOSEME	7	*
DOSEME	11	*
DOSEME	15	*
ENGELA	57	*
ENGELA	58	*
ENGELA	59	*
ENGELA	60	*
ENGELA	61	*
GER	14	*
GER	15	*
GER	16	*
HAENSZ	18	*
HAENSZ	17	*
HAMMON	98	-
HAMMON	99	-
HAMMON	100	-
HAMMON	101	-

Table 2G11 - 1

IESLC - Meta-anal of Ever Smoking (or Curr if Ever not avail) by Amount, Overview, Any prod (or Cigs if Any not avail)
Squamous
Most adjusted

REF	NRR	Cigarette equivalent
JEDRYC	28	*
JEDRYC	29	*
JEDRYC	30	*
KATSOU	21	*
KATSOU	22	*
KREYBE	1	grams inc 1 cig=1
KREYBE	2	grams inc 1 cig=1
KREYBE	3	grams inc 1 cig=1
KREYBE	31	grams inc 1 cig=1
KREYBE	32	grams inc 1 cig=1
LAMTH	10	*
LAMTH	11	*
LAMTH	12	*
LUBIN2	149	*
LUBIN2	153	*
LUBIN2	157	*
LUBIN2	161	*
LUBIN2	169	*
LUBIN2	173	*
LUBIN2	177	*
LUBIN2	181	*
LUO	10	*
LUO	11	*
LUO	12	*
MATOS	43	*
MATOS	45	*
MATOS	47	*
MATSUD	4	*
MATSUD	5	*
MATSUD	6	*
ORMOS	5	*
ORMOS	6	*
ORMOS	7	*
OSANN	51	*
OSANN	59	*
OSANN	52	*
OSANN	60	*
OSANN2	28	*
OSANN2	29	*
SOBUE	53	*
SOBUE	54	*
SOBUE	55	*
SVENSS	7	*
SVENSS	12	*
SVENSS	17	*
TSUGAN	15	*
TSUGAN	16	*
TSUGAN	17	*
WAKAI	46	*
WAKAI	47	*
WAKAI	48	*
WU	17	*
WU	18	*
WUWILL	14	*
WUWILL	15	*
WYNDE2	3	*
WYNDE2	4	*
WYNDE2	5	*
WYNDE2	6	*
WYNDE3	4	*
WYNDE3	5	*
WYNDE3	6	*
WYNDE3	7	*
WYNDE3	63	*
WYNDE3	64	*
WYNDE3	65	*
WYNDE3	66	*
WYNDE4	63	*
WYNDE4	64	*
WYNDE4	65	*
WYNDE4	66	*
WYNDE4	67	*

Table 2G11 - 1

IESLC - Meta-anal of Ever Smoking (or Curr if Ever not avail) by Amount, Overview, Any prod (or Cigs if Any not avail)
Squamous
Most adjusted

REF	NRR	Cigarette equivalent
WYNDE4	49	inc 1 cigar = 5 cigs, 1 pipe = 2.5 cigs
WYNDE4	50	inc 1 cigar = 5 cigs, 1 pipe = 2.5 cigs
WYNDE4	51	inc 1 cigar = 5 cigs, 1 pipe = 2.5 cigs
WYNDE4	52	inc 1 cigar = 5 cigs, 1 pipe = 2.5 cigs
WYNDE4	53	inc 1 cigar = 5 cigs, 1 pipe = 2.5 cigs
WYNDE6	21	*
WYNDE6	30	*
WYNDE6	39	*
WYNDE6	48	*
WYNDE6	210	*
WYNDE6	219	*
WYNDE6	228	*
WYNDE6	237	*
ZHENG	1	*
ZHENG	2	*
ZHENG	3	*
ZHENG	4	*
ZHENG	16	*
ZHENG	17	*
ZHOU	10	*
ZHOU	11	*
ZHOU	12	*

In this overview table, subtotals and Qs values may be invalid and should be ignored

Table 2G11 - 2

IESLC - Meta-anal of Ever Smoking (or Curr if Ever not avail) by Amount, Overview, Any prod (or Cigs if Any not avail)
Squamous
Most adjusted

REF	NRR	SEX	AD	Number Exposed Case	Number Exposed Cont	Non-exposed Case	Non-exposed Cont	RR	95.00%CI
ALDERS	34	m	1	-	-	-	-	3.79	(1.30- 11.02)
ALDERS	35	m	1	-	-	-	-	7.19	(2.75- 18.79)
ALDERS	36	m	1	-	-	-	-	8.78	(3.46- 22.31)
ALDERS	37	f	1	-	-	-	-	2.55	(1.42- 4.57)
ALDERS	38	f	1	-	-	-	-	9.24	(5.31- 16.09)
ALDERS	39	f	1	-	-	-	-	14.52	(7.93- 26.58)
Subtotal ALDERS								6.82	(5.12- 9.11)
BARBON	70	m	3	-	-	-	-	8.50	(3.60- 20.00)
BARBON	71	m	3	-	-	-	-	16.30	(7.00- 38.00)
BARBON	72	m	3	-	-	-	-	28.60	(12.00- 69.00)
Subtotal BARBON								15.71	(9.57- 25.79)
*BOUCOT	142	m	2	-	-	-	-	21.41	(1.40- 385.13)
*BOUCOT	143	m	2	-	-	-	-	46.64	(2.80- 775.69)
Subtotal BOUCOT								31.58	(4.33- 230.41)
BROWN2	36	m	2	-	-	-	-	7.60	(6.20- 9.40)
BROWN2	46	m	2	-	-	-	-	17.20	(14.60- 20.30)
BROWN2	35	f	2	-	-	-	-	11.70	(8.70- 15.80)
BROWN2	45	f	2	-	-	-	-	26.10	(20.70- 32.80)
Subtotal BROWN2								14.51	(13.06- 16.12)
CHOI	46	m	0	12	90	6	95	2.11	(0.76- 5.86)
CHOI	47	m	0	84	281	6	95	4.73	(2.00- 11.19)
CHOI	48	m	0	30	49	6	95	9.69	(3.78- 24.86)
CHOI	49	m	0	25	39	6	95	10.15	(3.86- 26.66)
CHOI	50	m	0	9	6	6	95	23.75	(6.33- 89.09)
CHOI	56	f	0	4	16	10	164	4.10	(1.15- 14.57)
CHOI	57	f	0	5	9	10	164	9.11	(2.57- 32.31)
CHOI	58	f	0	2	1	10	164	32.80	(2.74- 393.20)
Subtotal CHOI								6.86	(4.63- 10.16)
CORREA	47	c	1	-	-	-	-	23.20	(14.60- 37.00)
CORREA	51	c	1	-	-	-	-	54.80	(35.60- 89.20)
Subtotal CORREA								35.84	(25.85- 49.70)
DOLL	68	m	1	-	-	-	-	4.70	(1.38- 16.03)
DOLL	69	m	1	-	-	-	-	10.60	(3.30- 34.07)
DOLL	70	m	1	-	-	-	-	14.30	(4.45- 46.00)
DOLL	71	m	1	-	-	-	-	25.40	(7.83- 82.40)
DOLL	76	f	1	-	-	-	-	1.00	(0.38- 2.64)
DOLL	77	f	1	-	-	-	-	1.70	(0.64- 4.50)
DOLL	78	f	1	-	-	-	-	8.30	(2.77- 24.84)
Subtotal DOLL								5.02	(3.32- 7.61)
DORGAN	114	m	2	-	-	-	-	11.50	(4.10- 32.24)
DORGAN	115	m	2	-	-	-	-	23.29	(8.55- 63.49)
DORGAN	99	f	3	-	-	-	-	7.78	(4.86- 12.44)
DORGAN	100	f	3	-	-	-	-	16.38	(10.22- 26.26)
Subtotal DORGAN								12.06	(8.92- 16.31)
DOSEME	7	m	2	-	-	-	-	2.60	(1.50- 4.60)
DOSEME	11	m	2	-	-	-	-	3.20	(2.20- 4.60)
DOSEME	15	m	2	-	-	-	-	7.00	(4.10- 12.00)
Subtotal DOSEME								3.71	(2.84- 4.84)
*ENGELA	57	m	7	-	-	-	-	4.30	(1.00- 19.00)
*ENGELA	58	m	7	-	-	-	-	7.70	(1.90- 31.00)
*ENGELA	59	m	7	-	-	-	-	15.00	(3.90- 60.00)
*ENGELA	60	m	7	-	-	-	-	30.00	(7.40- 120.00)
*ENGELA	61	m	7	-	-	-	-	24.00	(5.90- 94.00)
Subtotal ENGELA								13.25	(7.08- 24.79)
GER	14	c	10	-	-	-	-	1.43	(0.36- 5.61)
GER	15	c	10	-	-	-	-	2.20	(0.65- 7.48)
GER	16	c	10	-	-	-	-	16.04	(4.22- 60.93)
Subtotal GER								3.64	(1.71- 7.73)
HAENSZ	18	f	0	30	66	44	236	2.44	(1.42- 4.18)
HAENSZ	17	f	0	18	13	44	236	7.43	(3.40- 16.24)
Subtotal HAENSZ								3.49	(2.24- 5.43)
*HAMMON	98	m	1	-	-	-	-	15.12	(4.93- 46.36)
*HAMMON	99	m	1	-	-	-	-	17.44	(6.30- 48.29)
*HAMMON	100	m	1	-	-	-	-	42.32	(15.38- 116.45)
*HAMMON	101	m	1	-	-	-	-	63.91	(22.02- 185.47)
Subtotal HAMMON								29.45	(17.41- 49.82)
JEDRYC	28	m	3	-	-	-	-	7.51	(3.09- 18.27)
JEDRYC	29	m	3	-	-	-	-	13.46	(5.76- 31.47)
JEDRYC	30	m	3	-	-	-	-	21.42	(9.05- 50.68)
Subtotal JEDRYC								13.09	(7.94- 21.57)
KATSOU	21	f	1	-	-	-	-	3.17	(1.14- 8.85)

Table 2G11 - 2

IESLC - Meta-anal of Ever Smoking (or Curr if Ever not avail) by Amount, Overview, Any prod (or Cigs if Any not avail)
Squamous
Most adjusted

REF	NRR	SEX	AD	Number Exposed Case	Number Exposed Cont	Non-exposed Case	Non-exposed Cont	RR	95.00%CI
KATSOU	22	f	1	-	-	-	-	19.53	(5.36- 71.11)
Subtotal KATSOU								6.39	(2.86- 14.27)
KREYBE	1	m	1	-	-	-	-	9.00	(2.85- 28.38)
KREYBE	2	m	1	-	-	-	-	11.02	(3.42- 35.51)
KREYBE	3	m	1	-	-	-	-	24.63	(7.54- 80.53)
KREYBE	31	f	0	1	286	3	657	0.77	(0.08- 7.39)
KREYBE	32	f	0	1	42	3	657	5.21	(0.53- 51.21)
Subtotal KREYBE								10.04	(5.39- 18.69)
LAMTH	10	f	0	23	11	28	72	5.38	(2.32- 12.46)
LAMTH	11	f	0	28	6	28	72	12.00	(4.49- 32.10)
LAMTH	12	f	0	10	1	28	72	25.71	(3.14- 210.29)
Subtotal LAMTH								8.37	(4.54- 15.43)
LUBIN2	149	m	0	418	2194	54	2616	9.23	(6.91- 12.32)
LUBIN2	153	m	0	1022	3385	54	2616	14.63	(11.07- 19.32)
LUBIN2	157	m	0	1298	3108	54	2616	20.23	(15.33- 26.69)
LUBIN2	161	m	0	849	1746	54	2616	23.56	(17.77- 31.22)
LUBIN2	169	f	0	30	184	72	1180	2.67	(1.70- 4.20)
LUBIN2	173	f	0	91	234	72	1180	6.37	(4.54- 8.95)
LUBIN2	177	f	0	61	110	72	1180	9.09	(6.13- 13.46)
LUBIN2	181	f	0	18	39	72	1180	7.56	(4.12- 13.88)
Subtotal LUBIN2								11.82	(10.51- 13.28)
LUO	10	c	20	-	-	-	-	1.20	(0.10- 10.00)
LUO	11	c	20	-	-	-	-	24.60	(4.20- 145.70)
LUO	12	c	20	-	-	-	-	38.70	(5.20- 290.20)
Subtotal LUO								13.41	(4.24- 42.41)
MATOS	43	m	2	-	-	-	-	1.40	(0.30- 6.90)
MATOS	45	m	2	-	-	-	-	7.80	(2.20- 27.40)
MATOS	47	m	2	-	-	-	-	9.70	(2.80- 33.20)
Subtotal MATOS								5.61	(2.60- 12.11)
MATSUD	4	m	0	21	1237	1	1255	21.31	(2.86- 158.63)
MATSUD	5	m	0	43	1607	1	1255	33.58	(4.62- 244.19)
MATSUD	6	m	0	39	470	1	1255	104.14	(14.27- 760.12)
Subtotal MATSUD								42.26	(13.37- 133.55)
ORMOS	5	m	0	13	329	2	777	15.35	(3.44- 68.41)
ORMOS	6	m	0	10	577	2	777	6.73	(1.47- 30.85)
ORMOS	7	m	0	4	128	2	777	12.14	(2.20- 66.97)
Subtotal ORMOS								10.74	(4.35- 26.54)
OSANN	51	m	2	-	-	-	-	35.30	(17.00- 73.30)
OSANN	59	m	2	-	-	-	-	76.00	(36.80- 157.00)
OSANN	52	f	2	-	-	-	-	24.00	(12.70- 45.50)
OSANN	60	f	2	-	-	-	-	72.30	(36.80- 142.00)
Subtotal OSANN								45.20	(32.03- 63.79)
OSANN2	28	f	1	-	-	-	-	12.10	(1.50- 96.30)
OSANN2	29	f	1	-	-	-	-	71.20	(8.30- 609.00)
Subtotal OSANN2								28.54	(6.40- 127.22)
SOBUE	53	m	0	57	157	3	128	15.49	(4.74- 50.62)
SOBUE	54	m	0	103	222	3	128	19.80	(6.15- 63.68)
SOBUE	55	m	0	87	187	3	128	19.85	(6.14- 64.13)
Subtotal SOBUE								18.28	(9.28- 36.03)
SVENSS	7	f	1	-	-	-	-	9.70	(2.90- 45.90)
SVENSS	12	f	1	-	-	-	-	36.20	(12.00- 168.90)
SVENSS	17	f	1	-	-	-	-	96.00	(6.90-1335.65)
Subtotal SVENSS								23.24	(9.47- 57.03)
TSUGAN	15	m	0	2	5	0	5	5.00~	(0.19- 130.02)
TSUGAN	16	m	0	7	7	0	5	11.00~	(0.51- 236.22)
TSUGAN	17	m	0	9	1	0	5	69.67~	(2.40-2022.74)
Subtotal TSUGAN								14.94	(2.32- 96.11)
WAKAI	46	m	1	-	-	-	-	3.95	(0.86- 18.10)
WAKAI	47	m	1	-	-	-	-	10.40	(2.43- 44.30)
WAKAI	48	m	1	-	-	-	-	24.00	(5.46- 105.00)
Subtotal WAKAI								10.14	(4.31- 23.88)
WU	17	f	2	-	-	-	-	17.70	(2.30- 138.20)
WU	18	f	2	-	-	-	-	94.40	(9.90- 904.60)
Subtotal WU								37.68	(8.27- 171.75)
WUWILL	14	f	3	-	-	-	-	3.21	(2.39- 4.30)
WUWILL	15	f	3	-	-	-	-	5.08	(3.07- 8.39)
Subtotal WUWILL								3.61	(2.80- 4.65)
WYNDE2	3	m	0	15	114	3	105	4.61	(1.30- 16.36)
WYNDE2	4	m	0	108	203	3	105	18.62	(5.77- 60.06)
WYNDE2	5	m	0	74	83	3	105	31.20	(9.50- 102.54)
WYNDE2	6	m	0	139	112	3	105	43.44	(13.42- 140.56)

Table 2G11 - 2

IESLC - Meta-anal of Ever Smoking (or Curr if Ever not avail) by Amount, Overview, Any prod (or Cigs if Any not avail)
Squamous
Most adjusted

REF	NRR	SEX	AD	Number Exposed Case	Number Exposed Cont	Non-exposed Case	Non-exposed Cont	RR	95.00%CI
Subtotal WYNDE2								19.37	(10.64- 35.27)
WYNDE3	4	m	0	7	42	3	88	4.89	(1.20- 19.86)
WYNDE3	5	m	0	57	114	3	88	14.67	(4.44- 48.40)
WYNDE3	6	m	0	74	82	3	88	26.47	(8.03- 87.26)
WYNDE3	7	m	0	59	26	3	88	66.56	(19.27- 229.96)
WYNDE3	63	f	0	1	19	5	76	0.80	(0.09- 7.26)
WYNDE3	64	f	0	13	24	5	76	8.23	(2.66- 25.46)
WYNDE3	65	f	0	8	10	5	76	12.16	(3.32- 44.50)
WYNDE3	66	f	0	3	3	5	76	15.20	(2.42- 95.56)
Subtotal WYNDE3								13.67	(8.51- 21.97)
WYNDE4	63	m	2	-	-	-	-	2.22	(0.89- 5.53)
WYNDE4	64	m	2	-	-	-	-	5.86	(2.70- 12.74)
WYNDE4	65	m	2	-	-	-	-	10.92	(5.22- 22.86)
WYNDE4	66	m	2	-	-	-	-	29.52	(13.81- 63.11)
WYNDE4	67	m	2	-	-	-	-	29.54	(13.53- 64.50)
WYNDE4	49	f	2	-	-	-	-	0.87	(0.11- 6.90)
WYNDE4	50	f	2	-	-	-	-	4.61	(1.38- 15.41)
WYNDE4	51	f	2	-	-	-	-	14.92	(4.88- 45.67)
WYNDE4	52	f	2	-	-	-	-	26.53	(4.12- 171.09)
WYNDE4	53	f	2	-	-	-	-	26.53	(4.12- 171.09)
Subtotal WYNDE4								10.91	(8.00- 14.88)
WYNDE6	21	m	0	75	122	29	617	13.08	(8.17- 20.94)
WYNDE6	30	m	0	270	293	29	617	19.61	(13.04- 29.47)
WYNDE6	39	m	0	179	129	29	617	29.52	(19.09- 45.65)
WYNDE6	48	m	0	502	197	29	617	54.22	(36.08- 81.47)
WYNDE6	210	f	0	37	109	40	856	7.26	(4.45- 11.85)
WYNDE6	219	f	0	191	165	40	856	24.77	(16.95- 36.20)
WYNDE6	228	f	0	101	50	40	856	43.23	(27.18- 68.76)
WYNDE6	237	f	0	221	52	40	856	90.95	(58.70- 140.93)
Subtotal WYNDE6								28.23	(24.23- 32.90)
ZHENG	1	m	0	7	40	4	94	4.11	(1.14- 14.84)
ZHENG	2	m	0	25	66	4	94	8.90	(2.96- 26.78)
ZHENG	3	m	0	75	89	4	94	19.80	(6.95- 56.41)
ZHENG	4	m	0	49	23	4	94	50.07	(16.39- 152.91)
ZHENG	16	f	0	11	29	33	184	2.11	(0.96- 4.64)
ZHENG	17	f	0	32	15	33	184	11.89	(5.81- 24.35)
Subtotal ZHENG								8.76	(5.95- 12.88)
ZHOU	10	c	0	15	5	138	68	1.48	(0.52- 4.24)
ZHOU	11	c	0	78	14	138	68	2.75	(1.45- 5.20)
ZHOU	12	c	0	285	29	138	68	4.84	(3.00- 7.82)
Subtotal ZHOU								3.52	(2.45- 5.04)
Partial Totals				7175	19102	1576	32564		

*prospective study ~ With 0.5 adjustment for zero

REF	NRR	SEX	AD	Ys	Ws	Qs	Ps
ALDERS	34	m	1	1.33	3.36	4.67	0.0145
ALDERS	35	m	1	1.97	4.16	1.20	0.0001
ALDERS	36	m	1	2.17	4.42	0.51	0.0000
ALDERS	37	f	1	0.94	11.25	27.89	0.0017
ALDERS	38	f	1	2.22	12.50	1.03	0.0000
ALDERS	39	f	1	2.68	10.50	0.29	0.0000
Subtotal ALDERS				1.92	46.20	35.58	
BARBON	70	m	3	2.14	5.23	0.72	0.0000
BARBON	71	m	3	2.79	5.37	0.42	0.0000
BARBON	72	m	3	3.35	5.02	3.57	0.0000
Subtotal BARBON				2.75	15.62	4.71	
*BOUCOT	142	m	2	3.06	0.49	0.15	0.0325
*BOUCOT	143	m	2	3.84	0.49	0.86	0.0074
Subtotal BOUCOT				3.45	0.97	1.01	
BROWN2	36	m	2	2.03	88.72	20.66	0.0000
BROWN2	46	m	2	2.84	141.44	15.80	0.0000
BROWN2	35	f	2	2.46	43.16	0.11	0.0000
BROWN2	45	f	2	3.26	72.52	40.93	0.0000
Subtotal BROWN2				2.67	345.85	77.50	
CHOI	46	m	0	0.75	3.68	11.45	0.1517
CHOI	47	m	0	1.55	5.19	4.75	0.0004
CHOI	48	m	0	2.27	4.33	0.25	0.0000
CHOI	49	m	0	2.32	4.12	0.15	0.0000
CHOI	50	m	0	3.17	2.20	0.95	0.0000
CHOI	56	f	0	1.41	2.39	2.89	0.0292

Table 2G11 - 2

IESLC - Meta-anal of Ever Smoking (or Curr if Ever not avail) by Amount, Overview, Any prod (or Cigs if Any not avail)
Squamous
Most adjusted

REF	NRR	SEX	AD	Ys	Ws	Qs	Ps
CHOI	57	f	0	2.21	2.40	0.22	0.0006
CHOI	58	f	0	3.49	0.62	0.60	0.0059
Subtotal CHOI				1.93	24.93	21.25	
CORREA	47	c	1	3.14	17.77	7.13	0.0000
CORREA	51	c	1	4.00	18.21	40.59	0.0000
Subtotal CORREA				3.58	35.98	47.72	
DOLL	68	m	1	1.55	2.55	2.37	0.0134
DOLL	69	m	1	2.36	2.82	0.06	0.0001
DOLL	70	m	1	2.66	2.82	0.06	0.0000
DOLL	71	m	1	3.23	2.77	1.45	0.0000
DOLL	76	f	1	0.00	4.09	25.78	1.0000
DOLL	77	f	1	0.53	4.04	15.84	0.2862
DOLL	78	f	1	2.12	3.19	0.50	0.0002
Subtotal DOLL				1.61	22.29	46.07	
DORGAN	114	m	2	2.44	3.61	0.02	0.0000
DORGAN	115	m	2	3.15	3.82	1.55	0.0000
DORGAN	99	f	3	2.05	17.39	3.67	0.0000
DORGAN	100	f	3	2.80	17.25	1.40	0.0000
Subtotal DORGAN				2.49	42.08	6.64	
DOSEME	7	m	2	0.96	12.24	29.60	0.0008
DOSEME	11	m	2	1.16	28.24	51.29	0.0000
DOSEME	15	m	2	1.95	13.32	4.25	0.0000
Subtotal DOSEME				1.31	53.80	85.14	
*ENGELA	57	m	7	1.46	1.77	1.96	0.0522
*ENGELA	58	m	7	2.04	1.97	0.43	0.0042
*ENGELA	59	m	7	2.71	2.06	0.08	0.0001
*ENGELA	60	m	7	3.40	1.98	1.57	0.0000
*ENGELA	61	m	7	3.18	2.01	0.89	0.0000
Subtotal ENGELA				2.58	9.78	4.94	
GER	14	c	10	0.36	2.04	9.45	0.6097
GER	15	c	10	0.79	2.57	7.64	0.2058
GER	16	c	10	2.78	2.16	0.15	0.0000
Subtotal GER				1.29	6.77	17.23	
HAENSZ	18	f	0	0.89	13.25	34.76	0.0012
HAENSZ	17	f	0	2.01	6.27	1.60	0.0000
Subtotal HAENSZ				1.25	19.53	36.37	
*HAMMON	98	m	1	2.72	3.06	0.13	0.0000
*HAMMON	99	m	1	2.86	3.70	0.45	0.0000
*HAMMON	100	m	1	3.75	3.75	5.71	0.0000
*HAMMON	101	m	1	4.16	3.38	9.18	0.0000
Subtotal HAMMON				3.38	13.90	15.47	
JEDRYC	28	m	3	2.02	4.87	1.19	0.0000
JEDRYC	29	m	3	2.60	5.33	0.04	0.0000
JEDRYC	30	m	3	3.06	5.18	1.59	0.0000
Subtotal JEDRYC				2.57	15.37	2.82	
KATSOU	21	f	1	1.15	3.66	6.74	0.0273
KATSOU	22	f	1	2.97	2.30	0.49	0.0000
Subtotal KATSOU				1.86	5.96	7.23	
KREYBE	1	m	1	2.20	2.91	0.29	0.0002
KREYBE	2	m	1	2.40	2.81	0.03	0.0001
KREYBE	3	m	1	3.20	2.74	1.32	0.0000
KREYBE	31	f	0	-0.27	0.75	5.76	0.8175
KREYBE	32	f	0	1.65	0.74	0.54	0.1566
Subtotal KREYBE				2.31	9.94	7.95	
LAMTH	10	f	0	1.68	5.44	3.73	0.0001
LAMTH	11	f	0	2.48	3.97	0.00	0.0000
LAMTH	12	f	0	3.25	0.87	0.47	0.0025
Subtotal LAMTH				2.12	10.27	4.21	
LUBIN2	149	m	0	2.22	45.98	3.82	0.0000
LUBIN2	153	m	0	2.68	49.57	1.47	0.0000
LUBIN2	157	m	0	3.01	50.02	12.33	0.0000
LUBIN2	161	m	0	3.16	48.42	20.37	0.0000
LUBIN2	169	f	0	0.98	18.69	43.63	0.0000
LUBIN2	173	f	0	1.85	33.33	14.46	0.0000
LUBIN2	177	f	0	2.21	24.86	2.29	0.0000
LUBIN2	181	f	0	2.02	10.42	2.48	0.0000
Subtotal LUBIN2				2.47	281.30	100.85	
LUO	10	c	20	0.18	0.72	3.93	0.8767
LUO	11	c	20	3.20	1.22	0.59	0.0004
LUO	12	c	20	3.66	0.95	1.25	0.0004
Subtotal LUO				2.60	2.90	5.76	

Table 2G11 - 2

IESLC - Meta-anal of Ever Smoking (or Curr if Ever not avail) by Amount, Overview, Any prod (or Cigs if Any not avail)
Squamous
Most adjusted

REF	NRR	SEX	AD	Ys	Ws	Qs	Ps
MATOS	43	m	2	0.34	1.56	7.39	0.6740
MATOS	45	m	2	2.05	2.42	0.50	0.0014
MATOS	47	m	2	2.27	2.51	0.14	0.0003
Subtotal MATOS				1.72	6.49	8.04	
MATSUD	4	m	0	3.06	0.95	0.29	0.0028
MATSUD	5	m	0	3.51	0.98	0.98	0.0005
MATSUD	6	m	0	4.65	0.97	4.43	0.0000
Subtotal MATSUD				3.74	2.90	5.70	
ORMOS	5	m	0	2.73	1.72	0.08	0.0003
ORMOS	6	m	0	1.91	1.66	0.60	0.0141
ORMOS	7	m	0	2.50	1.32	0.00	0.0042
Subtotal ORMOS				2.37	4.70	0.69	
OSANN	51	m	2	3.56	7.20	7.98	0.0000
OSANN	59	m	2	4.33	7.30	24.18	0.0000
OSANN	52	f	2	3.18	9.44	4.20	0.0000
OSANN	60	f	2	4.28	8.43	26.40	0.0000
Subtotal OSANN				3.81	32.36	62.77	
OSANN2	28	f	1	2.49	0.89	0.00	0.0189
OSANN2	29	f	1	4.27	0.83	2.56	0.0001
Subtotal OSANN2				3.35	1.72	2.56	
SOBUE	53	m	0	2.74	2.74	0.14	0.0000
SOBUE	54	m	0	2.99	2.81	0.63	0.0000
SOBUE	55	m	0	2.99	2.79	0.64	0.0000
Subtotal SOBUE				2.91	8.35	1.42	
SVENSS	7	f	1	2.27	2.01	0.11	0.0013
SVENSS	12	f	1	3.59	2.20	2.56	0.0000
SVENSS	17	f	1	4.56	0.55	2.34	0.0007
Subtotal SVENSS				3.15	4.77	5.01	
TSUGAN	15	m	0	1.61	0.36	0.29	0.3330
TSUGAN	16	m	0	2.40	0.41	0.01	0.1254
TSUGAN	17	m	0	4.24	0.34	1.02	0.0135
Subtotal TSUGAN				2.70	1.11	1.32	
WAKAI	46	m	1	1.37	1.66	2.14	0.0772
WAKAI	47	m	1	2.34	1.82	0.05	0.0016
WAKAI	48	m	1	3.18	1.76	0.78	0.0000
Subtotal WAKAI				2.32	5.24	2.97	
WU	17	f	2	2.87	0.92	0.12	0.0060
WU	18	f	2	4.55	0.75	3.13	0.0001
Subtotal WU				3.63	1.67	3.25	
WUWILL	14	f	3	1.17	44.55	80.52	0.0000
WUWILL	15	f	3	1.63	15.20	11.92	0.0000
Subtotal WUWILL				1.28	59.75	92.44	
WYNDE2	3	m	0	1.53	2.39	2.31	0.0182
WYNDE2	4	m	0	2.92	2.80	0.48	0.0000
WYNDE2	5	m	0	3.44	2.71	2.35	0.0000
WYNDE2	6	m	0	3.77	2.79	4.43	0.0000
Subtotal WYNDE2				2.96	10.69	9.56	
WYNDE3	4	m	0	1.59	1.96	1.67	0.0265
WYNDE3	5	m	0	2.69	2.70	0.08	0.0000
WYNDE3	6	m	0	3.28	2.70	1.58	0.0000
WYNDE3	7	m	0	4.20	2.50	7.12	0.0000
WYNDE3	63	f	0	-0.22	0.79	5.90	0.8428
WYNDE3	64	f	0	2.11	3.01	0.49	0.0003
WYNDE3	65	f	0	2.50	2.28	0.00	0.0002
WYNDE3	66	f	0	2.72	1.14	0.05	0.0037
Subtotal WYNDE3				2.62	17.07	16.89	
WYNDE4	63	m	2	0.80	4.60	13.52	0.0870
WYNDE4	64	m	2	1.77	6.38	3.52	0.0000
WYNDE4	65	m	2	2.39	7.04	0.10	0.0000
WYNDE4	66	m	2	3.39	6.66	5.09	0.0000
WYNDE4	67	m	2	3.39	6.30	4.82	0.0000
WYNDE4	49	f	2	-0.14	0.90	6.30	0.8951
WYNDE4	50	f	2	1.53	2.64	2.55	0.0130
WYNDE4	51	f	2	2.70	3.07	0.11	0.0000
WYNDE4	52	f	2	3.28	1.11	0.65	0.0006
WYNDE4	53	f	2	3.28	1.11	0.65	0.0006
Subtotal WYNDE4				2.39	39.81	37.31	
WYNDE6	21	m	0	2.57	17.35	0.06	0.0000
WYNDE6	30	m	0	2.98	23.14	5.00	0.0000
WYNDE6	39	m	0	3.39	20.23	15.46	0.0000
WYNDE6	48	m	0	3.99	23.16	50.89	0.0000

Table 2G11 - 2

IESLC - Meta-anal of Ever Smoking (or Curr if Ever not avail) by Amount, Overview, Any prod (or Cigs if Any not avail)
Squamous
Most adjusted

REF	NRR	SEX	AD	Ys	Ws	Qs	Ps
WYNDE6	210	f	0	1.98	16.03	4.47	0.0000
WYNDE6	219	f	0	3.21	26.69	13.04	0.0000
WYNDE6	228	f	0	3.77	17.84	28.12	0.0000
WYNDE6	237	f	0	4.51	20.03	80.09	0.0000
Subtotal WYNDE6				3.34	164.47	197.14	
ZHENG	1	m	0	1.41	2.33	2.81	0.0308
ZHENG	2	m	0	2.19	3.17	0.33	0.0001
ZHENG	3	m	0	2.99	3.51	0.79	0.0000
ZHENG	4	m	0	3.91	3.08	6.06	0.0000
ZHENG	16	f	0	0.75	6.21	19.26	0.0620
ZHENG	17	f	0	2.48	7.48	0.01	0.0000
Subtotal ZHENG				2.17	25.78	29.26	
ZHOU	10	c	0	0.39	3.46	15.57	0.4669
ZHOU	11	c	0	1.01	9.42	21.21	0.0019
ZHOU	12	c	0	1.58	16.68	14.53	0.0000
Subtotal ZHOU				1.26	29.56	51.31	

N	147
NS	36

Table 2G11 - 3

IESLC - Meta-anal of Ever Smoking (or Curr if Ever not avail) by Amount, Overview, Any prod (or Cigs if Any not avail)
Squamous
Most adjusted

	combined	Sex male	female	Total
N	11	84	52	147
NS	4	25	19	48

In this overview table, other than the "N" rows, entries in the "absent" and "Total" columns may be invalid and should be ignored

	Amount smoked (broad categories) absent	<20k5	6-44k20	>20k45	Total
N	39	41	30	37	147
NS	22	30	24	30	106
Wt	516.57	396.78	239.42	227.09	1379.86
Het Chi	272.14	154.02	107.78	143.12	1056.07
Het df	38	40	29	36	146
Het P	***	***	***	***	***
Fixed RR	14.54	6.07	12.36	28.95	12.31
RRl	13.34	5.50	10.89	25.42	11.68
RRu	15.85	6.70	14.03	32.98	12.98
P	+++	+++	+++	+++	+++
Random RR	12.08	4.98	11.86	27.65	11.39
RRl	9.20	3.93	8.92	20.42	9.70
RRu	15.88	6.31	15.76	37.44	13.39
P	+++	+++	+++	+++	+++

	absent	Amount smoked (narrow categories) <10k1	2-19k10	11-29k20	21-39k30	31-98k40	>40k99	Total
N	91	13	9	23	7	1	3	147
NS	36	8	6	19	5	1	2	76
Wt	883.30	96.40	113.42	220.17	56.62	4.12	5.83	1379.86
Het Chi	675.83	57.10	42.50	105.52	8.27	0.00	2.15	1056.07
Het df	90	12	8	22	6	0	2	146
Het P	***	***	***	***	N.S.	N.S.	N.S.	***
Fixed RR	13.42	4.80	8.36	12.35	31.33	10.15	33.86	12.31
RRl	12.57	3.93	6.95	10.82	24.14	3.86	15.04	11.68
RRu	14.34	5.86	10.05	14.09	40.65	26.66	76.22	12.98
P	+++	+++	+++	+++	+++	+++	+++	+++
Random RR	13.07	3.11	6.29	11.88	30.14	10.15	33.54	11.39
RRl	10.59	1.81	3.75	8.52	21.51	3.86	14.41	9.70
RRu	16.13	5.33	10.54	16.58	42.24	26.66	78.06	13.39
P	+++	+++	+++	+++	+++	+++	+++	+++

MALES

	Amount smoked (broad categories) absent	<20k5	6-44k20	>20k45	Total
N	19	23	20	22	84
NS	14	23	20	22	79
Wt	266.93	216.08	156.92	142.77	782.69
Het Chi	37.66	48.34	79.72	62.58	390.54
Het df	18	22	19	21	83
Het P	**	***	***	***	***
Fixed RR	17.28	7.42	12.01	26.76	13.78
RRl	15.32	6.49	10.27	22.71	12.84
RRu	19.48	8.48	14.04	31.52	14.78
P	+++	+++	+++	+++	+++
Random RR	17.31	6.62	11.96	27.70	13.64
RRl	13.46	5.08	8.18	19.71	11.38
RRu	22.25	8.63	17.49	38.93	16.35
P	+++	+++	+++	+++	+++

Table 2G11 - 3

IESLC - Meta-anal of Ever Smoking (or Curr if Ever not avail) by Amount, Overview, Any prod (or Cigs if Any not avail)
Squamous
Most adjusted

MALES

	Amount smoked (narrow categories)							
	absent	<10k1	2-19k10	11-29k20	21-39k30	31-98k40	>40k99	Total
N	49	7	5	15	5	1	2	84
NS	25	7	5	15	5	1	2	59
Wt	466.87	62.26	63.99	143.08	37.67	4.12	4.70	782.69
Het Chi	237.48	12.57	5.35	77.99	5.57	0.00	1.24	390.54
Het df	48	6	4	14	4	0	1	83
Het P	***	(*)	N.S.	***	N.S.	N.S.	N.S.	***
Fixed RR	14.80	7.70	12.85	11.79	27.03	10.15	41.10	13.78
RRl	13.52	6.01	10.06	10.01	19.64	3.86	16.64	12.84
RRu	16.21	9.87	16.42	13.89	37.20	26.66	101.52	14.78
P	+++	+++	+++	+++	+++	+++	+++	+++
Random RR	15.23	5.77	11.33	11.64	26.31	10.15	40.83	13.64
RRl	11.82	3.38	7.66	7.39	17.36	3.86	14.89	11.38
RRu	19.61	9.84	16.76	18.33	39.88	26.66	111.95	16.35
P	+++	+++	+++	+++	+++	+++	+++	+++

FEMALES

	Amount smoked (broad categories)				
	absent	<20k5	6-44k20	>20k45	Total
N	17	15	8	12	52
NS	15	15	8	12	50
Wt	205.78	174.47	78.71	63.00	521.96
Het Chi	174.32	77.04	18.84	71.40	521.04
Het df	16	14	7	11	51
Het P	***	***	**	***	***
Fixed RR	13.17	4.98	13.71	29.26	10.54
RRl	11.49	4.29	10.99	22.86	9.67
RRu	15.10	5.78	17.10	37.45	11.48
P	+++	+++	+++	+++	+++
Random RR	9.58	3.95	13.14	26.18	9.40
RRl	5.66	2.60	8.57	12.49	6.91
RRu	16.21	6.00	20.13	54.88	12.79
P	+++	+++	+++	+++	+++

	Amount smoked (narrow categories)							
	absent	<10k1	2-19k10	11-29k20	21-39k30	31-98k40	>40k99	Total
N	35	5	3	6	2		1	52
NS	19	5	3	6	2		1	36
Wt	357.90	30.67	40.01	73.30	18.94		1.14	521.96
Het Chi	349.86	4.77	6.37	17.57	0.25		0.00	521.04
Het df	34	4	2	5	1		0	51
Het P	***	N.S.	*	**	N.S.		N.S.	***
Fixed RR	11.38	2.10	5.46	14.19	42.01		15.20	10.54
RRl	10.26	1.47	4.00	11.28	26.78		2.42	9.67
RRu	12.62	2.99	7.44	17.84	65.91		95.56	11.48
P	+++	+++	+++	+++	+++		++	+++
Random RR	11.04	1.94	3.97	14.35	42.01		15.20	9.40
RRl	7.55	1.26	1.71	8.73	26.78		2.42	6.91
RRu	16.15	3.01	9.23	23.57	65.91		95.56	12.79
P	+++	++	++	+++	+++		++	+++

Table 2G11 - 4

IESLC - Meta-anal of Ever Smoking (or Curr if Ever not avail) by Amount, Overview, Any prod (or Cigs if Any not avail)
Squamous
Least adjusted

REF	NRR	X	SEX	AGEL	AGEH	RACE	YF	LC TYPE	LOC	START	ST	NLC	R	VB	P	H	AD	SM	PRODUCT	exL	exH	S1	S2	DENOM	De
ALDERS	34		m	0	0	all	-	q+s	Eu:UK	1977	CC	1448	n	V	n	n	1	ev	cig only	1	17	1	0	nev+2	ot
ALDERS	35		m	0	0	all	-	q+s	Eu:UK	1977	CC	1448	n	V	n	n	1	ev	cig only	18	27	2	3	nev+2	ot
ALDERS	36		m	0	0	all	-	q+s	Eu:UK	1977	CC	1448	n	V	n	n	1	ev	cig only	28	99	3	0	nev+2	ot
ALDERS	37		f	0	0	all	-	q+s	Eu:UK	1977	CC	1448	n	V	n	n	1	ev	cig only	1	17	1	0	nev+2	ot
ALDERS	38		f	0	0	all	-	q+s	Eu:UK	1977	CC	1448	n	V	n	n	1	ev	cig only	18	27	2	3	nev+2	ot
ALDERS	39		f	0	0	all	-	q+s	Eu:UK	1977	CC	1448	n	V	n	n	1	ev	cig only	28	99	3	0	nev+2	ot
BARBON	19	x	m	0	0	all	-	q	Eu:wst	1979	CC	755	n	bl	y	y	0	ev	all/unsp	1	9	1	1	nev any	st
BARBON	21	x	m	0	0	all	-	q	Eu:wst	1979	CC	755	n	bl	y	y	0	ev	all/unsp	10	19	0	2	nev any	st
BARBON	23	x	m	0	0	all	-	q	Eu:wst	1979	CC	755	n	bl	y	y	0	ev	all/unsp	20	29	2	3	nev any	st
BARBON	25	x	m	0	0	all	-	q	Eu:wst	1979	CC	755	n	bl	y	y	0	ev	all/unsp	30	39	0	4	nev any	st
BARBON	27	x	m	0	0	all	-	q	Eu:wst	1979	CC	755	n	bl	y	y	0	ev	all/unsp	40	99	3	0	nev any	st
BOUCOT	21	x	m	0	0	all	0	q	NAmer	1951	pr	121	n	bl	n	n	0	cu	cig only	1	20	0	0	nev any	ot
BOUCOT	22	x	m	0	0	all	0	q	NAmer	1951	pr	121	n	bl	n	n	0	cu	cig only	21	99	3	0	nev any	ot
BROWN2	36		m	0	0	wh	-	q	NAmer	1984	CC	14596	n	bl	n	y	2	ev	cig+/-ot	1	19	1	0	nev cigs	or
BROWN2	46		m	0	0	wh	-	q	NAmer	1984	CC	14596	n	bl	n	y	2	ev	cig+/-ot	20	99	0	0	nev cigs	or
BROWN2	35		f	0	0	wh	-	q	NAmer	1984	CC	14596	n	bl	n	y	2	ev	cig+/-ot	1	19	1	0	nev cigs	or
BROWN2	45		f	0	0	wh	-	q	NAmer	1984	CC	14596	n	bl	n	y	2	ev	cig+/-ot	20	99	0	0	nev cigs	or
CHOI	46		m	0	0	all	-	q	As:oth	1985	CC	375	n	bl	n	n	0	ev	cig+/-ot	1	10	1	0	nev cigs	st
CHOI	47		m	0	0	all	-	q	As:oth	1985	CC	375	n	bl	n	n	0	ev	cig+/-ot	11	20	2	3	nev cigs	st
CHOI	48		m	0	0	all	-	q	As:oth	1985	CC	375	n	bl	n	n	0	ev	cig+/-ot	21	30	0	4	nev cigs	st
CHOI	49		m	0	0	all	-	q	As:oth	1985	CC	375	n	bl	n	n	0	ev	cig+/-ot	31	40	0	5	nev cigs	st
CHOI	50		m	0	0	all	-	q	As:oth	1985	CC	375	n	bl	n	n	0	ev	cig+/-ot	41	99	3	6	nev cigs	st
CHOI	56		f	0	0	all	-	q	As:oth	1985	CC	375	n	bl	n	n	0	ev	cig+/-ot	1	10	1	0	nev cigs	st
CHOI	57		f	0	0	all	-	q	As:oth	1985	CC	375	n	bl	n	n	0	ev	cig+/-ot	11	30	2	0	nev cigs	st
CHOI	58		f	0	0	all	-	q	As:oth	1985	CC	375	n	bl	n	n	0	ev	cig+/-ot	31	99	3	0	nev cigs	st
CORREA	47		c	0	0	all	-	q+s	NAmer	1979	CC	1359	n	bl	y	n	1	cu	cig+/-ot	1	20	0	0	nev cigs	or
CORREA	51		c	0	0	all	-	q+s	NAmer	1979	CC	1359	n	bl	y	n	1	cu	cig+/-ot	21	99	3	0	nev cigs	or
DOLL	54	x	m	0	0	all	-	KI	Eu:UK	1948	CC	1465	n	V	n	n	0	ev	all/unsp	1	4	0	1	nev any	st
DOLL	55	x	m	0	0	all	-	KI	Eu:UK	1948	CC	1465	n	V	n	n	0	ev	all/unsp	5	14	1	2	nev any	st
DOLL	56	x	m	0	0	all	-	KI	Eu:UK	1948	CC	1465	n	V	n	n	0	ev	all/unsp	15	24	2	3	nev any	st
DOLL	57	x	m	0	0	all	-	KI	Eu:UK	1948	CC	1465	n	V	n	n	0	ev	all/unsp	25	99	3	0	nev any	st
DOLL	62	x	f	0	0	all	-	KI	Eu:UK	1948	CC	1465	n	V	n	n	0	ev	all/unsp	1	4	0	1	nev any	st
DOLL	63	x	f	0	0	all	-	KI	Eu:UK	1948	CC	1465	n	V	n	n	0	ev	all/unsp	5	14	1	2	nev any	st
DOLL	64	x	f	0	0	all	-	KI	Eu:UK	1948	CC	1465	n	V	n	n	0	ev	all/unsp	15	99	0	0	nev any	st
DORGAN	114		m	0	0	wh	-	q	NAmer	1980	CC	2026	n	bl	y	y	2	ev	cig+/-ot	1	19	1	0	nev any	ot
DORGAN	115		m	0	0	wh	-	q	NAmer	1980	CC	2026	n	bl	y	y	2	ev	cig+/-ot	20	99	0	0	nev any	ot
DORGAN	99		f	0	0	all	-	q	NAmer	1980	CC	2026	n	bl	y	y	3	ev	cig+/-ot	1	19	1	0	nev any	ot
DORGAN	100		f	0	0	all	-	q	NAmer	1980	CC	2026	n	bl	y	y	3	ev	cig+/-ot	20	99	0	0	nev any	ot
DOSEME	7		m	0	0	all	-	q	Eu:bal	1979	CC	1210	n	bl	n	n	2	ev	cig+/-ot	1	10	1	0	nev cigs	or
DOSEME	11		m	0	0	all	-	q	Eu:bal	1979	CC	1210	n	bl	n	n	2	ev	cig+/-ot	11	20	2	3	nev cigs	or
DOSEME	15		m	0	0	all	-	q	Eu:bal	1979	CC	1210	n	bl	n	n	2	ev	cig+/-ot	21	99	3	0	nev cigs	or
ENGELA	57		m	0	0	all	0	q	Eu:Sca	1964	pr	435	n	bl	n	n	7	cu	cig+/-ot	1	4	0	1	nev cigs	or
ENGELA	58		m	0	0	all	0	q	Eu:Sca	1964	pr	435	n	bl	n	n	7	cu	cig+/-ot	5	9	1	0	nev cigs	or
ENGELA	59		m	0	0	all	0	q	Eu:Sca	1964	pr	435	n	bl	n	n	7	cu	cig+/-ot	10	14	0	2	nev cigs	or
ENGELA	60		m	0	0	all	0	q	Eu:Sca	1964	pr	435	n	bl	n	n	7	cu	cig+/-ot	15	19	0	0	nev cigs	or
ENGELA	61		m	0	0	all	0	q	Eu:Sca	1964	pr	435	n	bl	n	n	7	cu	cig+/-ot	20	99	0	0	nev cigs	or
GER	6	x	c	0	0	all	-	q+s	As:oth	1990	CC	141	n	ot	y	n	0	ev	all/unsp	1	10	1	0	nev any	st
GER	7	x	c	0	0	all	-	q+s	As:oth	1990	CC	141	n	ot	y	n	0	ev	all/unsp	11	20	2	3	nev any	st
GER	8	x	c	0	0	all	-	q+s	As:oth	1990	CC	141	n	ot	y	n	0	ev	all/unsp	21	99	3	0	nev any	st
HAENSZ	18		f	0	0	all	-	q+u	NAmer	1955	CC	158	n	bl	n	y	0	cu	cig+/-ot	1	20	0	0	nev any	or
HAENSZ	17		f	0	0	all	-	q+u	NAmer	1955	CC	158	n	bl	n	y	0	cu	cig+/-ot	21	99	3	0	nev any	or
HAMMON	98		m	0	0	wh	0	not a	NAmer	1952	pr	448	n	bl	n	n	1	cu	cig only	1	9	1	1	nev any	ot
HAMMON	99		m	0	0	wh	0	not a	NAmer	1952	pr	448	n	bl	n	n	1	cu	cig only	10	20	2	0	nev any	ot
HAMMON	100		m	0	0	wh	0	not a	NAmer	1952	pr	448	n	bl	n	n	1	cu	cig only	21	39	0	4	nev any	ot
HAMMON	101		m	0	0	wh	0	not a	NAmer	1952	pr	448	n	bl	n	n	1	cu	cig only	40	99	3	0	nev any	ot
JEDRYC	1	x	m	0	0	all	-	q	Eu:est	1980	CC	1630	n	bl	y	n	0	ev	cig+/-ot	1	9	1	1	nev any	st
JEDRYC	2	x	m	0	0	all	-	q	Eu:est	1980	CC	1630	n	bl	y	n	0	ev	cig+/-ot	10	19	0	2	nev any	st
JEDRYC	3	x	m	0	0	all	-	q	Eu:est	1980	CC	1630	n	bl	y	n	0	ev	cig+/-ot	20	29	2	3	nev any	st
JEDRYC	4	x	m	0	0	all	-	q	Eu:est	1980	CC	1630	n	bl	y	n	0	ev	cig+/-ot	30	39	0	4	nev any	st
JEDRYC	5	x	m	0	0	all	-	q	Eu:est	1980	CC	1630	n	bl	y	n	0	ev	cig+/-ot	40	99	3	0	nev any	st
KATSOU	25	x	f	0	0	all	-	KI	Eu:bal	1987	CC	101	n	bl	n	n	0	cu	all/unsp	1	20	0	0	nev any	st
KATSOU	26	x	f	0	0	all	-	KI	Eu:bal	1987	CC	101	n	bl	n	n	0	cu	all/unsp	21	99	3	0	nev any	st
KREYBE	13	x	m	0	0	all	-	KI	Eu:Sca	1948	CC	300	n	bl	n	y	0	ev	all/unsp	1	14	1	0	nev any	st
KREYBE	14	x	m	0	0	all	-	KI	Eu:Sca	1948	CC	300	n	bl	n	y	0	ev	all/unsp	15	24	2	3	nev any	st
KREYBE	15	x	m	0	0	all	-	KI	Eu:Sca	1948	CC	300	n	bl	n	y	0	ev	all/unsp	25	99	3	0	nev any	st
KREYBE	31		f	0	0	all	-	KI	Eu:Sca	1948	CC	300	n	bl	n	y	0	ev	all/unsp	1	14	1	0	nev any	st
KREYBE	32		f	0	0	all	-	KI	Eu:Sca	1948	CC	300	n	bl	n	y	0	ev	all/unsp	15	99	0	0	nev any	st
LAMTH	10		f	0	0	ch	-	q	As:HK	1983	CC	445	n	bl	n	n	0	ev	all/unsp	1	10	1	0	nev any	or
LAMTH	11		f	0	0	ch	-	q	As:HK	1983	CC	445	n	bl	n	n	0	ev	all/unsp	11	20	2	3	nev any	or
LAMTH	12		f	0	0	ch	-	q	As:HK	1983	CC	445	n	bl	n	n	0	ev	all/unsp	21	99	3	0	nev any	st
LUBIN2	149		m	0	0	all	-	q	Eu:mul	1976	CC	7804	n	bl	n	y	0	ev	cig+/-ot	1	9	1	1	nev any	st
LUBIN2	153		m	0	0	all	-	q	Eu:mul	1976	CC	7804	n	bl	n	y	0	ev	cig+/-ot	10	19	0	2	nev any	st

Table 2G11 - 4

IESLC - Meta-anal of Ever Smoking (or Curr if Ever not avail) by Amount, Overview, Any prod (or Cigs if Any not avail)
Squamous
Least adjusted

REF	NRR	X	SEX	AGEL	AGEH	RACE	YF	LC TYPE	LOC	START	ST	NLC	R	VB	P	H	AD	SM	PRODUCT	exL	exH	S1	S2	DENOM	De
LUBIN2	157		m	0	0	all	-	q	Eu:mul	1976	CC	7804	n	bl	n	y	0	ev	cig+/-ot	20	29	2	3	nev any	st
LUBIN2	161		m	0	0	all	-	q	Eu:mul	1976	CC	7804	n	bl	n	y	0	ev	cig+/-ot	30	99	3	0	nev any	st
LUBIN2	169		f	0	0	all	-	q	Eu:mul	1976	CC	7804	n	bl	n	y	0	ev	cig+/-ot	1	9	1	1	nev any	st
LUBIN2	173		f	0	0	all	-	q	Eu:mul	1976	CC	7804	n	bl	n	y	0	ev	cig+/-ot	10	19	0	2	nev any	st
LUBIN2	177		f	0	0	all	-	q	Eu:mul	1976	CC	7804	n	bl	n	y	0	ev	cig+/-ot	20	29	2	3	nev any	st
LUBIN2	181		f	0	0	all	-	q	Eu:mul	1976	CC	7804	n	bl	n	y	0	ev	cig+/-ot	30	99	3	0	nev any	st
LUO	4	x	c	0	0	all	-	q	As:Chi	1990	CC	102	n	ot	n	y	0	ev	cig+/-ot	1	19	1	0	nev cigs	st
LUO	5	x	c	0	0	all	-	q	As:Chi	1990	CC	102	n	ot	n	y	0	ev	cig+/-ot	20	29	2	3	nev cigs	st
LUO	6	x	c	0	0	all	-	q	As:Chi	1990	CC	102	n	ot	n	y	0	ev	cig+/-ot	30	99	3	0	nev cigs	st
MATOS	42	x	m	0	0	all	-	q	SCAmer	1994	CC	200	n	bl	n	n	0	ev	cig+/-ot	1	14	1	0	nev any	st
MATOS	44	x	m	0	0	all	-	q	SCAmer	1994	CC	200	n	bl	n	n	0	ev	cig+/-ot	15	24	2	3	nev any	st
MATOS	46	x	m	0	0	all	-	q	SCAmer	1994	CC	200	n	bl	n	n	0	ev	cig+/-ot	25	99	3	0	nev any	st
MATSUD	4		m	0	0	all	-	q	As:Jap	1965	CC	179	n	bl	n	n	0	ev	cig+/-ot	1	10	1	0	nev cigs	st
MATSUD	5		m	0	0	all	-	q	As:Jap	1965	CC	179	n	bl	n	n	0	ev	cig+/-ot	11	20	2	3	nev cigs	st
MATSUD	6		m	0	0	all	-	q	As:Jap	1965	CC	179	n	bl	n	n	0	ev	cig+/-ot	21	99	3	0	nev cigs	st
ORMOS	5		m	0	0	all	-	q	Eu:est	1947	CC	119	n	bl	y	y	0	ev	cig+/-ot	1	15	1	0	nev any	st
ORMOS	6		m	0	0	all	-	q	Eu:est	1947	CC	119	n	bl	y	y	0	ev	cig+/-ot	16	30	2	0	nev any	st
ORMOS	7		m	0	0	all	-	q	Eu:est	1947	CC	119	n	bl	y	y	0	ev	cig+/-ot	31	99	3	0	nev any	st
OSANN	51		m	0	0	all	-	q	NAmer	1984	CC	1986	n	bl	n	n	2	ev	cig+/-ot	1	39	0	0	nev cigs	or
OSANN	59		m	0	0	all	-	q	NAmer	1984	CC	1986	n	bl	n	n	2	ev	cig+/-ot	40	99	3	0	nev cigs	or
OSANN	52		f	0	0	all	-	q	NAmer	1984	CC	1986	n	bl	n	n	2	ev	cig+/-ot	1	39	0	0	nev cigs	or
OSANN	60		f	0	0	all	-	q	NAmer	1984	CC	1986	n	bl	n	n	2	ev	cig+/-ot	40	99	3	0	nev cigs	or
OSANN2	10	x	f	0	0	all	-	KI	NAmer	1964	ot	217	n	bl	n	y	0	ev	cig+/-ot	1	19	1	0	nev cigs	st
OSANN2	11	x	f	0	0	all	-	KI	NAmer	1964	ot	217	n	bl	n	y	0	ev	cig+/-ot	20	99	0	0	nev cigs	st
SOBUE	53		m	0	0	all	-	q	As:Jap	1986	CC	1376	n	bl	n	y	0	cu	cig+/-ot	1	19	1	0	nev cigs	st
SOBUE	54		m	0	0	all	-	q	As:Jap	1986	CC	1376	n	bl	n	y	0	cu	cig+/-ot	20	29	2	3	nev cigs	st
SOBUE	55		m	0	0	all	-	q	As:Jap	1986	CC	1376	n	bl	n	y	0	cu	cig+/-ot	30	99	3	0	nev cigs	st
SVENSS	27	x	f	0	0	all	-	q	Eu:Sca	1983	CC	210	n	bl	n	n	0	cu	all/unsp	1	10	1	0	nev any	st
SVENSS	32	x	f	0	0	all	-	q	Eu:Sca	1983	CC	210	n	bl	n	n	0	cu	all/unsp	11	20	2	3	nev any	st
SVENSS	37	x	f	0	0	all	-	q	Eu:Sca	1983	CC	210	n	bl	n	n	0	cu	all/unsp	21	99	3	0	nev any	st
TSUGAN	15		m	0	0	all	-	q	As:Jap	1976	CC	134	n	bl	n	y	0	cu	all/unsp	1	15	1	0	nev any	ot
TSUGAN	16		m	0	0	all	-	q	As:Jap	1976	CC	134	n	bl	n	y	0	cu	all/unsp	16	35	2	0	nev any	ot
TSUGAN	17		m	0	0	all	-	q	As:Jap	1976	CC	134	n	bl	n	y	0	cu	all/unsp	36	99	3	0	nev any	ot
WAKAI	43	x	m	0	0	all	-	q	As:Jap	1988	CC	333	n	bl	n	y	0	cu	cig+/-ot	1	19	1	0	nev any	st
WAKAI	44	x	m	0	0	all	-	q	As:Jap	1988	CC	333	n	bl	n	y	0	cu	cig+/-ot	20	29	2	3	nev any	st
WAKAI	45	x	m	0	0	all	-	q	As:Jap	1988	CC	333	n	bl	n	y	0	cu	cig+/-ot	30	99	3	0	nev any	st
WU	12	x	f	0	0	wh	-	q	NAmer	1981	CC	220	n	bl	n	y	0	cu	all/unsp	1	20	0	0	nev any	st
WU	13	x	f	0	0	wh	-	q	NAmer	1981	CC	220	n	bl	n	y	0	cu	all/unsp	21	99	3	0	nev any	st
WUWILL	20	x	f	0	0	all	-	q+s	As:Chi	1985	CC	965	n	ot	n	n	0	ev	cig+/-ot	1	19	1	0	nev cigs	st
WUWILL	21	x	f	0	0	all	-	q+s	As:Chi	1985	CC	965	n	ot	n	n	0	ev	cig+/-ot	20	99	0	0	nev cigs	st
WYNDE2	3		m	0	0	all	-	KI	NAmer	1962	CC	404	n	bl	n	y	0	ev	cig+/-ot	1	10	1	0	nev any	st
WYNDE2	4		m	0	0	all	-	KI	NAmer	1962	CC	404	n	bl	n	y	0	ev	cig+/-ot	11	20	2	3	nev any	st
WYNDE2	5		m	0	0	all	-	KI	NAmer	1962	CC	404	n	bl	n	y	0	ev	cig+/-ot	21	34	0	4	nev any	st
WYNDE2	6		m	0	0	all	-	KI	NAmer	1962	CC	404	n	bl	n	y	0	ev	cig+/-ot	35	99	3	0	nev any	st
WYNDE3	4		m	0	0	all	-	KI	NAmer	1966	CC	350	n	bl	n	y	0	ev	cig+/-ot	1	9	1	1	nev any	st
WYNDE3	5		m	0	0	all	-	KI	NAmer	1966	CC	350	n	bl	n	y	0	ev	cig+/-ot	10	20	2	0	nev any	st
WYNDE3	6		m	0	0	all	-	KI	NAmer	1966	CC	350	n	bl	n	y	0	ev	cig+/-ot	21	40	0	0	nev any	st
WYNDE3	7		m	0	0	all	-	KI	NAmer	1966	CC	350	n	bl	n	y	0	ev	cig+/-ot	41	99	3	6	nev any	st
WYNDE3	63		f	0	0	all	-	KI	NAmer	1966	CC	350	n	bl	n	y	0	ev	cig+/-ot	1	9	1	1	nev any	st
WYNDE3	64		f	0	0	all	-	KI	NAmer	1966	CC	350	n	bl	n	y	0	ev	cig+/-ot	10	20	2	0	nev any	st
WYNDE3	65		f	0	0	all	-	KI	NAmer	1966	CC	350	n	bl	n	y	0	ev	cig+/-ot	21	40	0	0	nev any	st
WYNDE3	66		f	0	0	all	-	KI	NAmer	1966	CC	350	n	bl	n	y	0	ev	cig+/-ot	41	99	3	6	nev any	st
WYNDE4	5	x	m	0	0	all	-	not a	NAmer	1948	CC	684	n	bl	y	n	0	ev	all/unsp	1	9	1	1	nev any	st
WYNDE4	11	x	m	0	0	all	-	not a	NAmer	1948	CC	684	n	bl	y	n	0	ev	all/unsp	10	15	0	2	nev any	st
WYNDE4	17	x	m	0	0	all	-	not a	NAmer	1948	CC	684	n	bl	y	n	0	ev	all/unsp	16	20	2	3	nev any	st
WYNDE4	23	x	m	0	0	all	-	not a	NAmer	1948	CC	684	n	bl	y	n	0	ev	all/unsp	21	34	0	4	nev any	st
WYNDE4	29	x	m	0	0	all	-	not a	NAmer	1948	CC	684	n	bl	y	n	0	ev	all/unsp	35	99	3	0	nev any	st
WYNDE4	49		f	0	0	all	-	not a	NAmer	1948	CC	684	n	bl	y	n	2	ev	all/unsp	1	9	1	1	nev any	ot
WYNDE4	50		f	0	0	all	-	not a	NAmer	1948	CC	684	n	bl	y	n	2	ev	all/unsp	10	15	0	2	nev any	ot
WYNDE4	51		f	0	0	all	-	not a	NAmer	1948	CC	684	n	bl	y	n	2	ev	all/unsp	16	20	2	3	nev any	ot
WYNDE4	52		f	0	0	all	-	not a	NAmer	1948	CC	684	n	bl	y	n	2	ev	all/unsp	21	34	0	4	nev any	ot
WYNDE4	53		f	0	0	all	-	not a	NAmer	1948	CC	684	n	bl	y	n	2	ev	all/unsp	35	99	3	0	nev any	ot
WYNDE6	21		m	0	0	all	-	KI	NAmer	1969	CC	4423	n	bl	n	y	0	cu	cig+/-ot	1	10	1	0	nev any	st
WYNDE6	30		m	0	0	all	-	KI	NAmer	1969	CC	4423	n	bl	n	y	0	cu	cig+/-ot	11	20	2	3	nev any	st
WYNDE6	39		m	0	0	all	-	KI	NAmer	1969	CC	4423	n	bl	n	y	0	cu	cig+/-ot	21	30	0	4	nev any	st
WYNDE6	48		m	0	0	all	-	KI	NAmer	1969	CC	4423	n	bl	n	y	0	cu	cig+/-ot	31	99	3	0	nev any	st
WYNDE6	210		f	0	0	all	-	KI	NAmer	1969	CC	4423	n	bl	n	y	0	cu	cig+/-ot	1	10	1	0	nev cigs	st
WYNDE6	219		f	0	0	all	-	KI	NAmer	1969	CC	4423	n	bl	n	y	0	cu	cig+/-ot	11	20	2	3	nev cigs	st
WYNDE6	228		f	0	0	all	-	KI	NAmer	1969	CC	4423	n	bl	n	y	0	cu	cig+/-ot	21	30	0	4	nev cigs	st
WYNDE6	237		f	0	0	all	-	KI	NAmer	1969	CC	4423	n	bl	n	y	0	cu	cig+/-ot	30	99	3	0	nev cigs	st
ZHENG	1		m	0	0	all	-	q	As:Chi	1982	CC	540	n	ot	*	y	0	ev	cig+/-ot	1	9	1	1	nev cigs	st
ZHENG	2		m	0	0	all	-	q	As:Chi	1982	CC	540	n	ot	*	y	0	ev	cig+/-ot	10	19	0	2	nev cigs	st

Table 2G11 - 4

IESLC - Meta-anal of Ever Smoking (or Curr if Ever not avail) by Amount, Overview, Any prod (or Cigs if Any not avail)
Squamous
Least adjusted

REF	NRR	X	SEX	AGEL	AGEH	RACE	YF	LC TYPE	LOC	START	ST	NLC	R	VB	P	H	AD	SM	PRODUCT	exL	exH	S1	S2	DENOM	De
ZHENG	3		m	0	0	all	-	q	As:Chi	1982	CC	540	n	ot	*	y	0	ev	cig+/-ot	20	29	2	3	nev cigs	st
ZHENG	4		m	0	0	all	-	q	As:Chi	1982	CC	540	n	ot	*	y	0	ev	cig+/-ot	30	99	3	0	nev cigs	st
ZHENG	16		f	0	0	all	-	q	As:Chi	1982	CC	540	n	ot	*	y	0	ev	cig+/-ot	1	9	1	1	nev cigs	st
ZHENG	17		f	0	0	all	-	q	As:Chi	1982	CC	540	n	ot	*	y	0	ev	cig+/-ot	10	99	0	0	nev cigs	st
ZHOU	10		c	0	0	all	-	q	As:Chi	1978	CC	1360	n	ot	n	n	0	ev	all/unsp	1	9	1	1	nev any	st
ZHOU	11		c	0	0	all	-	q	As:Chi	1978	CC	1360	n	ot	n	n	0	ev	all/unsp	10	19	0	2	nev any	st
ZHOU	12		c	0	0	all	-	q	As:Chi	1978	CC	1360	n	ot	n	n	0	ev	all/unsp	20	99	0	0	nev any	st

Cigarette type is all/unspec for all RRs

except for the following:

REF	NRR	CIGTYPE
ALDERS	34	MC only
ALDERS	35	MC only
ALDERS	36	MC only
ALDERS	37	MC only
ALDERS	38	MC only
ALDERS	39	MC only

REF	NRR	Cigarette equivalent
ALDERS	34	-
ALDERS	35	-
ALDERS	36	-
ALDERS	37	-
ALDERS	38	-
ALDERS	39	-
BARBON	19	*
BARBON	21	*
BARBON	23	*
BARBON	25	*
BARBON	27	*
BOUCOT	21	-
BOUCOT	22	-
BROWN2	36	*
BROWN2	46	*
BROWN2	35	*
BROWN2	45	*
CHOI	46	*
CHOI	47	*
CHOI	48	*
CHOI	49	*
CHOI	50	*
CHOI	56	*
CHOI	57	*
CHOI	58	*
CORREA	47	*
CORREA	51	*
DOLL	54	grams
DOLL	55	grams
DOLL	56	grams
DOLL	57	grams
DOLL	62	grams
DOLL	63	grams
DOLL	64	grams
DORGAN	114	*
DORGAN	115	*
DORGAN	99	*
DORGAN	100	*
DOSEME	7	*
DOSEME	11	*
DOSEME	15	*
ENGELA	57	*
ENGELA	58	*
ENGELA	59	*
ENGELA	60	*
ENGELA	61	*
GER	6	*
GER	7	*
GER	8	*

Table 2G11 - 4

IESLC - Meta-anal of Ever Smoking (or Curr if Ever not avail) by Amount, Overview, Any prod (or Cigs if Any not avail)
Squamous
Least adjusted

REF	NRR	Cigarette equivalent
HAENSZ	18	*
HAENSZ	17	*
HAMMON	98	-
HAMMON	99	-
HAMMON	100	-
HAMMON	101	-
JEDRYC	1	*
JEDRYC	2	*
JEDRYC	3	*
JEDRYC	4	*
JEDRYC	5	*
KATSOU	25	*
KATSOU	26	*
KREYBE	13	grams inc 1 cig=1
KREYBE	14	grams inc 1 cig=1
KREYBE	15	grams inc 1 cig=1
KREYBE	31	grams inc 1 cig=1
KREYBE	32	grams inc 1 cig=1
LAMTH	10	*
LAMTH	11	*
LAMTH	12	*
LUBIN2	149	*
LUBIN2	153	*
LUBIN2	157	*
LUBIN2	161	*
LUBIN2	169	*
LUBIN2	173	*
LUBIN2	177	*
LUBIN2	181	*
LUO	4	*
LUO	5	*
LUO	6	*
MATOS	42	*
MATOS	44	*
MATOS	46	*
MATSUD	4	*
MATSUD	5	*
MATSUD	6	*
ORMOS	5	*
ORMOS	6	*
ORMOS	7	*
OSANN	51	*
OSANN	59	*
OSANN	52	*
OSANN	60	*
OSANN2	10	*
OSANN2	11	*
SOBUE	53	*
SOBUE	54	*
SOBUE	55	*
SVENSS	27	*
SVENSS	32	*
SVENSS	37	*
TSUGAN	15	*
TSUGAN	16	*
TSUGAN	17	*
WAKAI	43	*
WAKAI	44	*
WAKAI	45	*
WU	12	*
WU	13	*
WUWILL	20	*
WUWILL	21	*
WYNDE2	3	*
WYNDE2	4	*
WYNDE2	5	*
WYNDE2	6	*
WYNDE3	4	*
WYNDE3	5	*
WYNDE3	6	*
WYNDE3	7	*
WYNDE3	63	*

Table 2G11 - 4

IESLC - Meta-anal of Ever Smoking (or Curr if Ever not avail) by Amount, Overview, Any prod (or Cigs if Any not avail)
Squamous
Least adjusted

REF	NRR	Cigarette equivalent
WYNDE3	64	*
WYNDE3	65	*
WYNDE3	66	*
WYNDE4	5	inc 1 cigar = 5 cigs, 1 pipe = 2.5 cigs
WYNDE4	11	inc 1 cigar = 5 cigs, 1 pipe = 2.5 cigs
WYNDE4	17	inc 1 cigar = 5 cigs, 1 pipe = 2.5 cigs
WYNDE4	23	inc 1 cigar = 5 cigs, 1 pipe = 2.5 cigs
WYNDE4	29	inc 1 cigar = 5 cigs, 1 pipe = 2.5 cigs
WYNDE4	49	inc 1 cigar = 5 cigs, 1 pipe = 2.5 cigs
WYNDE4	50	inc 1 cigar = 5 cigs, 1 pipe = 2.5 cigs
WYNDE4	51	inc 1 cigar = 5 cigs, 1 pipe = 2.5 cigs
WYNDE4	52	inc 1 cigar = 5 cigs, 1 pipe = 2.5 cigs
WYNDE4	53	inc 1 cigar = 5 cigs, 1 pipe = 2.5 cigs
WYNDE6	21	*
WYNDE6	30	*
WYNDE6	39	*
WYNDE6	48	*
WYNDE6	210	*
WYNDE6	219	*
WYNDE6	228	*
WYNDE6	237	*
ZHENG	1	*
ZHENG	2	*
ZHENG	3	*
ZHENG	4	*
ZHENG	16	*
ZHENG	17	*
ZHOU	10	*
ZHOU	11	*
ZHOU	12	*

In this overview table, subtotals and Qs values may be invalid and should be ignored

Table 2G11 - 5

IESLC - Meta-anal of Ever Smoking (or Curr if Ever not avail) by Amount, Overview, Any prod (or Cigs if Any not avail)
Squamous
Least adjusted

REF	NRR	SEX	AD	Number Exposed Case	Number Exposed Cont	Non-exposed Case	Non-exposed Cont	RR	95.00%CI
ALDERS	34	m	1	-	-	-	-	3.79	(1.30- 11.02)
ALDERS	35	m	1	-	-	-	-	7.19	(2.75- 18.79)
ALDERS	36	m	1	-	-	-	-	8.78	(3.46- 22.31)
ALDERS	37	f	1	-	-	-	-	2.55	(1.42- 4.57)
ALDERS	38	f	1	-	-	-	-	9.24	(5.31- 16.09)
ALDERS	39	f	1	-	-	-	-	14.52	(7.93- 26.58)
Subtotal ALDERS								6.82	(5.12- 9.11)
BARBON	19	m	0	11	87	6	188	3.96	(1.42- 11.06)
BARBON	21	m	0	45	111	6	188	12.70	(5.25- 30.73)
BARBON	23	m	0	81	176	6	188	14.42	(6.14- 33.89)
BARBON	25	m	0	46	82	6	188	17.58	(7.22- 42.78)
BARBON	27	m	0	78	111	6	188	22.02	(9.29- 52.18)
Subtotal BARBON								13.16	(8.81- 19.65)
*BOUCOT	21	m	0	21	15208	0	7551	21.35~	(1.29- 352.41)
*BOUCOT	22	m	0	17	6940	0	7551	38.08~	(2.29- 633.12)
Subtotal BOUCOT								28.49	(3.91- 207.42)
BROWN2	36	m	2	-	-	-	-	7.60	(6.20- 9.40)
BROWN2	46	m	2	-	-	-	-	17.20	(14.60- 20.30)
BROWN2	35	f	2	-	-	-	-	11.70	(8.70- 15.80)
BROWN2	45	f	2	-	-	-	-	26.10	(20.70- 32.80)
Subtotal BROWN2								14.51	(13.06- 16.12)
CHOI	46	m	0	12	90	6	95	2.11	(0.76- 5.86)
CHOI	47	m	0	84	281	6	95	4.73	(2.00- 11.19)
CHOI	48	m	0	30	49	6	95	9.69	(3.78- 24.86)
CHOI	49	m	0	25	39	6	95	10.15	(3.86- 26.66)
CHOI	50	m	0	9	6	6	95	23.75	(6.33- 89.09)
CHOI	56	f	0	4	16	10	164	4.10	(1.15- 14.57)
CHOI	57	f	0	5	9	10	164	9.11	(2.57- 32.31)
CHOI	58	f	0	2	1	10	164	32.80	(2.74- 393.20)
Subtotal CHOI								6.86	(4.63- 10.16)
CORREA	47	c	1	-	-	-	-	23.20	(14.60- 37.00)
CORREA	51	c	1	-	-	-	-	54.80	(35.60- 89.20)
Subtotal CORREA								35.84	(25.85- 49.70)
DOLL	54	m	0	29	129	3	61	4.57	(1.34- 15.59)
DOLL	55	m	0	291	570	3	61	10.38	(3.23- 33.37)
DOLL	56	m	0	301	431	3	61	14.20	(4.41- 45.68)
DOLL	57	m	0	208	166	3	61	25.48	(7.85- 82.66)
DOLL	62	f	0	8	25	16	59	1.18	(0.45- 3.11)
DOLL	63	f	0	9	18	16	59	1.84	(0.70- 4.88)
DOLL	64	f	0	15	6	16	59	9.22	(3.08- 27.59)
Subtotal DOLL								5.29	(3.50- 8.02)
DORGAN	114	m	2	-	-	-	-	11.50	(4.10- 32.24)
DORGAN	115	m	2	-	-	-	-	23.29	(8.55- 63.49)
DORGAN	99	f	3	-	-	-	-	7.78	(4.86- 12.44)
DORGAN	100	f	3	-	-	-	-	16.38	(10.22- 26.26)
Subtotal DORGAN								12.06	(8.92- 16.31)
DOSEME	7	m	2	-	-	-	-	2.60	(1.50- 4.60)
DOSEME	11	m	2	-	-	-	-	3.20	(2.20- 4.60)
DOSEME	15	m	2	-	-	-	-	7.00	(4.10- 12.00)
Subtotal DOSEME								3.71	(2.84- 4.84)
*ENGELA	57	m	7	-	-	-	-	4.30	(1.00- 19.00)
*ENGELA	58	m	7	-	-	-	-	7.70	(1.90- 31.00)
*ENGELA	59	m	7	-	-	-	-	15.00	(3.90- 60.00)
*ENGELA	60	m	7	-	-	-	-	30.00	(7.40- 120.00)
*ENGELA	61	m	7	-	-	-	-	24.00	(5.90- 94.00)
Subtotal ENGELA								13.25	(7.08- 24.79)
GER	6	c	0	9	56	11	80	1.17	(0.45- 3.01)
GER	7	c	0	20	87	11	80	1.67	(0.75- 3.71)
GER	8	c	0	19	13	11	80	10.63	(4.13- 27.37)
Subtotal GER								2.59	(1.55- 4.32)
HAENSZ	18	f	0	30	66	44	236	2.44	(1.42- 4.18)
HAENSZ	17	f	0	18	13	44	236	7.43	(3.40- 16.24)
Subtotal HAENSZ								3.49	(2.24- 5.43)
*HAMMON	98	m	1	-	-	-	-	15.12	(4.93- 46.36)
*HAMMON	99	m	1	-	-	-	-	17.44	(6.30- 48.29)
*HAMMON	100	m	1	-	-	-	-	42.32	(15.38- 116.45)
*HAMMON	101	m	1	-	-	-	-	63.91	(22.02- 185.47)
Subtotal HAMMON								29.45	(17.41- 49.82)
JEDRYC	1	m	0	1	67	6	289	0.72	(0.09- 6.07)
JEDRYC	2	m	0	38	199	6	289	9.20	(3.82- 22.17)
JEDRYC	3	m	0	152	434	6	289	16.87	(7.36- 38.66)

Table 2G11 - 5

IESLC - Meta-anal of Ever Smoking (or Curr if Ever not avail) by Amount, Overview, Any prod (or Cigs if Any not avail)
Squamous
Least adjusted

REF	NRR	SEX	AD	Number Exposed Case	Number Exposed Cont	Non-exposed Case	Non-exposed Cont	RR	95.00%CI
JEDRYC	4	m	0	61	118	6	289	24.90	(10.48- 59.17)
JEDRYC	5	m	0	57	82	6	289	33.48	(13.94- 80.42)
Subtotal JEDRYC								16.68	(10.93- 25.44)
KATSOU	25	f	0	9	14	14	67	3.08	(1.11- 8.50)
KATSOU	26	f	0	15	4	14	67	17.95	(5.17- 62.28)
Subtotal KATSOU								6.23	(2.84- 13.69)
KREYBE	13	m	0	123	2341	3	644	11.28	(3.58- 35.57)
KREYBE	14	m	0	49	925	3	644	11.37	(3.53- 36.64)
KREYBE	15	m	0	38	248	3	644	32.89	(10.06- 107.53)
KREYBE	31	f	0	1	286	3	657	0.77	(0.08- 7.39)
KREYBE	32	f	0	1	42	3	657	5.21	(0.53- 51.21)
Subtotal KREYBE								11.71	(6.29- 21.81)
LAMTH	10	f	0	23	11	28	72	5.38	(2.32- 12.46)
LAMTH	11	f	0	28	6	28	72	12.00	(4.49- 32.10)
LAMTH	12	f	0	10	1	28	72	25.71	(3.14- 210.29)
Subtotal LAMTH								8.37	(4.54- 15.43)
LUBIN2	149	m	0	418	2194	54	2616	9.23	(6.91- 12.32)
LUBIN2	153	m	0	1022	3385	54	2616	14.63	(11.07- 19.32)
LUBIN2	157	m	0	1298	3108	54	2616	20.23	(15.33- 26.69)
LUBIN2	161	m	0	849	1746	54	2616	23.56	(17.77- 31.22)
LUBIN2	169	f	0	30	184	72	1180	2.67	(1.70- 4.20)
LUBIN2	173	f	0	91	234	72	1180	6.37	(4.54- 8.95)
LUBIN2	177	f	0	61	110	72	1180	9.09	(6.13- 13.46)
LUBIN2	181	f	0	18	39	72	1180	7.56	(4.12- 13.88)
Subtotal LUBIN2								11.82	(10.51- 13.28)
LUO	4	c	0	3	39	5	51	0.78	(0.18- 3.48)
LUO	5	c	0	22	23	5	51	9.76	(3.28- 28.98)
LUO	6	c	0	9	4	5	51	22.95	(5.15- 102.20)
Subtotal LUO								6.34	(2.97- 13.53)
MATOS	42	m	0	3	88	3	110	1.25	(0.25- 6.35)
MATOS	44	m	0	18	90	3	110	7.33	(2.09- 25.69)
MATOS	46	m	0	26	105	3	110	9.08	(2.67- 30.90)
Subtotal MATOS								5.36	(2.48- 11.58)
MATSUD	4	m	0	21	1237	1	1255	21.31	(2.86- 158.63)
MATSUD	5	m	0	43	1607	1	1255	33.58	(4.62- 244.19)
MATSUD	6	m	0	39	470	1	1255	104.14	(14.27- 760.12)
Subtotal MATSUD								42.26	(13.37- 133.55)
ORMOS	5	m	0	13	329	2	777	15.35	(3.44- 68.41)
ORMOS	6	m	0	10	577	2	777	6.73	(1.47- 30.85)
ORMOS	7	m	0	4	128	2	777	12.14	(2.20- 66.97)
Subtotal ORMOS								10.74	(4.35- 26.54)
OSANN	51	m	2	-	-	-	-	35.30	(17.00- 73.30)
OSANN	59	m	2	-	-	-	-	76.00	(36.80- 157.00)
OSANN	52	f	2	-	-	-	-	24.00	(12.70- 45.50)
OSANN	60	f	2	-	-	-	-	72.30	(36.80- 142.00)
Subtotal OSANN								45.20	(32.03- 63.79)
OSANN2	10	f	0	18	31	7	58	4.81	(1.81- 12.77)
OSANN2	11	f	0	93	29	7	58	26.57	(10.93- 64.58)
Subtotal OSANN2								12.25	(6.35- 23.63)
SOBUE	53	m	0	57	157	3	128	15.49	(4.74- 50.62)
SOBUE	54	m	0	103	222	3	128	19.80	(6.15- 63.68)
SOBUE	55	m	0	87	187	3	128	19.85	(6.14- 64.13)
Subtotal SOBUE								18.28	(9.28- 36.03)
SVENSS	27	f	0	10	30	5	120	8.00	(2.54- 25.16)
SVENSS	32	f	0	28	22	5	120	30.55	(10.64- 87.69)
SVENSS	37	f	0	4	1	5	120	96.00	(9.00-1023.75)
Subtotal SVENSS								19.60	(9.38- 40.97)
TSUGAN	15	m	0	2	5	0	5	5.00~	(0.19- 130.02)
TSUGAN	16	m	0	7	7	0	5	11.00~	(0.51- 236.22)
TSUGAN	17	m	0	9	1	0	5	69.67~	(2.40-2022.74)
Subtotal TSUGAN								14.94	(2.32- 96.11)
WAKAI	43	m	0	13	105	2	65	4.02	(0.88- 18.41)
WAKAI	44	m	0	40	129	2	65	10.08	(2.36- 43.01)
WAKAI	45	m	0	33	48	2	65	22.34	(5.11- 97.69)
Subtotal WAKAI								9.85	(4.19- 23.17)
WU	12	f	0	19	14	2	30	20.36	(4.15- 99.74)
WU	13	f	0	42	9	2	30	70.00	(14.10- 347.48)
Subtotal WU								37.56	(12.15- 116.07)
WUWILL	20	f	0	168	311	117	601	2.77	(2.11- 3.65)
WUWILL	21	f	0	33	40	117	601	4.24	(2.57- 7.00)
Subtotal WUWILL								3.06	(2.41- 3.88)

Table 2G11 - 5

IESLC - Meta-anal of Ever Smoking (or Curr if Ever not avail) by Amount, Overview, Any prod (or Cigs if Any not avail)
Squamous
Least adjusted

REF	NRR	SEX	AD	Number Exposed Case	Exposed Cont	Non-exposed Case	Non-exposed Cont	RR	95.00%CI
WYNDE2	3	m	0	15	114	3	105	4.61	(1.30- 16.36)
WYNDE2	4	m	0	108	203	3	105	18.62	(5.77- 60.06)
WYNDE2	5	m	0	74	83	3	105	31.20	(9.50- 102.54)
WYNDE2	6	m	0	139	112	3	105	43.44	(13.42- 140.56)
Subtotal WYNDE2								19.37	(10.64- 35.27)
WYNDE3	4	m	0	7	42	3	88	4.89	(1.20- 19.86)
WYNDE3	5	m	0	57	114	3	88	14.67	(4.44- 48.40)
WYNDE3	6	m	0	74	82	3	88	26.47	(8.03- 87.26)
WYNDE3	7	m	0	59	26	3	88	66.56	(19.27- 229.96)
WYNDE3	63	f	0	1	19	5	76	0.80	(0.09- 7.26)
WYNDE3	64	f	0	13	24	5	76	8.23	(2.66- 25.46)
WYNDE3	65	f	0	8	10	5	76	12.16	(3.32- 44.50)
WYNDE3	66	f	0	3	3	5	76	15.20	(2.42- 95.56)
Subtotal WYNDE3								13.67	(8.51- 21.97)
WYNDE4	5	m	0	14	82	8	115	2.45	(0.98- 6.12)
WYNDE4	11	m	0	61	147	8	115	5.97	(2.74- 12.97)
WYNDE4	17	m	0	213	274	8	115	11.17	(5.34- 23.39)
WYNDE4	23	m	0	186	98	8	115	27.28	(12.79- 58.18)
WYNDE4	29	m	0	123	64	8	115	27.63	(12.69- 60.13)
WYNDE4	49	f	2	-	-	-	-	0.87	(0.11- 6.90)
WYNDE4	50	f	2	-	-	-	-	4.61	(1.38- 15.41)
WYNDE4	51	f	2	-	-	-	-	14.92	(4.88- 45.67)
WYNDE4	52	f	2	-	-	-	-	26.53	(4.12- 171.09)
WYNDE4	53	f	2	-	-	-	-	26.53	(4.12- 171.09)
Subtotal WYNDE4								10.88	(7.98- 14.84)
WYNDE6	21	m	0	75	122	29	617	13.08	(8.17- 20.94)
WYNDE6	30	m	0	270	293	29	617	19.61	(13.04- 29.47)
WYNDE6	39	m	0	179	129	29	617	29.52	(19.09- 45.65)
WYNDE6	48	m	0	502	197	29	617	54.22	(36.08- 81.47)
WYNDE6	210	f	0	37	109	40	856	7.26	(4.45- 11.85)
WYNDE6	219	f	0	191	165	40	856	24.77	(16.95- 36.20)
WYNDE6	228	f	0	101	50	40	856	43.23	(27.18- 68.76)
WYNDE6	237	f	0	221	52	40	856	90.95	(58.70- 140.93)
Subtotal WYNDE6								28.23	(24.23- 32.90)
ZHENG	1	m	0	7	40	4	94	4.11	(1.14- 14.84)
ZHENG	2	m	0	25	66	4	94	8.90	(2.96- 26.78)
ZHENG	3	m	0	75	89	4	94	19.80	(6.95- 56.41)
ZHENG	4	m	0	49	23	4	94	50.07	(16.39- 152.91)
ZHENG	16	f	0	11	29	33	184	2.11	(0.96- 4.64)
ZHENG	17	f	0	32	15	33	184	11.89	(5.81- 24.35)
Subtotal ZHENG								8.76	(5.95- 12.88)
ZHOU	10	c	0	15	5	138	68	1.48	(0.52- 4.24)
ZHOU	11	c	0	78	14	138	68	2.75	(1.45- 5.20)
ZHOU	12	c	0	285	29	138	68	4.84	(3.00- 7.82)
Subtotal ZHOU								3.52	(2.45- 5.04)
Partial Totals				10105	49533	2103	55769		

*prospective study ~ With 0.5 adjustment for zero

REF	NRR	SEX	AD	Ys	Ws	Qs	Ps
ALDERS	34	m	1	1.33	3.36	4.48	0.0145
ALDERS	35	m	1	1.97	4.16	1.10	0.0001
ALDERS	36	m	1	2.17	4.42	0.44	0.0000
ALDERS	37	f	1	0.94	11.25	27.05	0.0017
ALDERS	38	f	1	2.22	12.50	0.87	0.0000
ALDERS	39	f	1	2.68	10.50	0.37	0.0000
Subtotal ALDERS				1.92	46.20	34.32	
BARBON	19	m	0	1.38	3.64	4.49	0.0086
BARBON	21	m	0	2.54	4.92	0.01	0.0000
BARBON	23	m	0	2.67	5.26	0.17	0.0000
BARBON	25	m	0	2.87	4.86	0.70	0.0000
BARBON	27	m	0	3.09	5.16	1.89	0.0000
Subtotal BARBON				2.58	23.84	7.27	
*BOUCOT	21	m	0	3.06	0.49	0.16	0.0324
*BOUCOT	22	m	0	3.64	0.49	0.65	0.0112
Subtotal BOUCOT				3.35	0.97	0.81	
BROWN2	36	m	2	2.03	88.72	18.68	0.0000
BROWN2	46	m	2	2.84	141.44	18.12	0.0000
BROWN2	35	f	2	2.46	43.16	0.03	0.0000
BROWN2	45	f	2	3.26	72.52	43.55	0.0000
Subtotal BROWN2				2.67	345.85	80.38	

Table 2G11 - 5

IESLC - Meta-anal of Ever Smoking (or Curr if Ever not avail) by Amount, Overview, Any prod (or Cigs if Any not avail)
Squamous
Least adjusted

REF	NRR	SEX	AD	Ys	Ws	Qs	Ps
CHOI	46	m	0	0.75	3.68	11.14	0.1517
CHOI	47	m	0	1.55	5.19	4.51	0.0004
CHOI	48	m	0	2.27	4.33	0.20	0.0000
CHOI	49	m	0	2.32	4.12	0.12	0.0000
CHOI	50	m	0	3.17	2.20	1.02	0.0000
CHOI	56	f	0	1.41	2.39	2.77	0.0292
CHOI	57	f	0	2.21	2.40	0.18	0.0006
CHOI	58	f	0	3.49	0.62	0.63	0.0059
Subtotal CHOI				1.93	24.93	20.57	
CORREA	47	c	1	3.14	17.77	7.67	0.0000
CORREA	51	c	1	4.00	18.21	41.89	0.0000
Subtotal CORREA				3.58	35.98	49.57	
DOLL	54	m	0	1.52	2.55	2.39	0.0152
DOLL	55	m	0	2.34	2.82	0.06	0.0001
DOLL	56	m	0	2.65	2.81	0.08	0.0000
DOLL	57	m	0	3.24	2.77	1.56	0.0000
DOLL	62	f	0	0.17	4.09	22.05	0.7378
DOLL	63	f	0	0.61	4.06	14.29	0.2175
DOLL	64	f	0	2.22	3.20	0.23	0.0001
Subtotal DOLL				1.67	22.31	40.65	
DORGAN	114	m	2	2.44	3.61	0.01	0.0000
DORGAN	115	m	2	3.15	3.82	1.67	0.0000
DORGAN	99	f	3	2.05	17.39	3.30	0.0000
DORGAN	100	f	3	2.80	17.25	1.65	0.0000
Subtotal DORGAN				2.49	42.08	6.62	
DOSEME	7	m	2	0.96	12.24	28.70	0.0008
DOSEME	11	m	2	1.16	28.24	49.50	0.0000
DOSEME	15	m	2	1.95	13.32	3.90	0.0000
Subtotal DOSEME				1.31	53.80	82.10	
*ENGELA	57	m	7	1.46	1.77	1.87	0.0522
*ENGELA	58	m	7	2.04	1.97	0.39	0.0042
*ENGELA	59	m	7	2.71	2.06	0.10	0.0001
*ENGELA	60	m	7	3.40	1.98	1.65	0.0000
*ENGELA	61	m	7	3.18	2.01	0.96	0.0000
Subtotal ENGELA				2.58	9.78	4.98	
GER	6	c	0	0.16	4.30	23.38	0.7462
GER	7	c	0	0.51	6.06	23.61	0.2056
GER	8	c	0	2.36	4.29	0.07	0.0000
Subtotal GER				0.95	14.66	47.06	
HAENSZ	18	f	0	0.89	13.25	33.75	0.0012
HAENSZ	17	f	0	2.01	6.27	1.46	0.0000
Subtotal HAENSZ				1.25	19.53	35.21	
*HAMMON	98	m	1	2.72	3.06	0.16	0.0000
*HAMMON	99	m	1	2.86	3.70	0.51	0.0000
*HAMMON	100	m	1	3.75	3.75	5.94	0.0000
*HAMMON	101	m	1	4.16	3.38	9.44	0.0000
Subtotal HAMMON				3.38	13.90	16.05	
JEDRYC	1	m	0	-0.33	0.84	6.70	0.7618
JEDRYC	2	m	0	2.22	4.96	0.36	0.0000
JEDRYC	3	m	0	2.83	5.59	0.64	0.0000
JEDRYC	4	m	0	3.21	5.13	2.72	0.0000
JEDRYC	5	m	0	3.51	5.00	5.25	0.0000
Subtotal JEDRYC				2.81	21.53	15.66	
KATSOU	25	f	0	1.12	3.72	6.91	0.0302
KATSOU	26	f	0	2.89	2.48	0.40	0.0000
Subtotal KATSOU				1.83	6.20	7.31	
KREYBE	13	m	0	2.42	2.91	0.01	0.0000
KREYBE	14	m	0	2.43	2.81	0.01	0.0000
KREYBE	15	m	0	3.49	2.74	2.77	0.0000
KREYBE	31	f	0	-0.27	0.75	5.67	0.8175
KREYBE	32	f	0	1.65	0.74	0.51	0.1566
Subtotal KREYBE				2.46	9.94	8.97	
LAMTH	10	f	0	1.68	5.44	3.52	0.0001
LAMTH	11	f	0	2.48	3.97	0.00	0.0000
LAMTH	12	f	0	3.25	0.87	0.50	0.0025
Subtotal LAMTH				2.12	10.27	4.02	
LUBIN2	149	m	0	2.22	45.98	3.22	0.0000
LUBIN2	153	m	0	2.68	49.57	1.90	0.0000
LUBIN2	157	m	0	3.01	50.02	13.54	0.0000
LUBIN2	161	m	0	3.16	48.42	21.89	0.0000
LUBIN2	169	f	0	0.98	18.69	42.29	0.0000

Table 2G11 - 5

IESLC - Meta-anal of Ever Smoking (or Curr if Ever not avail) by Amount, Overview, Any prod (or Cigs if Any not avail)
Squamous
Least adjusted

REF	NRR	SEX	AD	Ys	Ws	Qs	Ps
LUBIN2	173	f	0	1.85	33.33	13.44	0.0000
LUBIN2	177	f	0	2.21	24.86	1.95	0.0000
LUBIN2	181	f	0	2.02	10.42	2.24	0.0000
Subtotal LUBIN2				2.47	281.30	100.46	
LUO	4	c	0	-0.24	1.73	12.88	0.7498
LUO	5	c	0	2.28	3.24	0.14	0.0000
LUO	6	c	0	3.13	1.72	0.72	0.0000
Subtotal LUO				1.85	6.69	13.74	
MATOS	42	m	0	0.22	1.46	7.46	0.7878
MATOS	44	m	0	1.99	2.44	0.60	0.0018
MATOS	46	m	0	2.21	2.56	0.20	0.0004
Subtotal MATOS				1.68	6.46	8.26	
MATSUD	4	m	0	3.06	0.95	0.31	0.0028
MATSUD	5	m	0	3.51	0.98	1.03	0.0005
MATSUD	6	m	0	4.65	0.97	4.53	0.0000
Subtotal MATSUD				3.74	2.90	5.87	
ORMOS	5	m	0	2.73	1.72	0.10	0.0003
ORMOS	6	m	0	1.91	1.66	0.56	0.0141
ORMOS	7	m	0	2.50	1.32	0.00	0.0042
Subtotal ORMOS				2.37	4.70	0.66	
OSANN	51	m	2	3.56	7.20	8.34	0.0000
OSANN	59	m	2	4.33	7.30	24.82	0.0000
OSANN	52	f	2	3.18	9.44	4.51	0.0000
OSANN	60	f	2	4.28	8.43	27.12	0.0000
Subtotal OSANN				3.81	32.36	64.78	
OSANN2	10	f	0	1.57	4.03	3.39	0.0016
OSANN2	11	f	0	3.28	4.87	3.06	0.0000
Subtotal OSANN2				2.51	8.90	6.45	
SOBUE	53	m	0	2.74	2.74	0.18	0.0000
SOBUE	54	m	0	2.99	2.81	0.70	0.0000
SOBUE	55	m	0	2.99	2.79	0.70	0.0000
Subtotal SOBUE				2.91	8.35	1.58	
SVENSS	27	f	0	2.08	2.93	0.49	0.0004
SVENSS	32	f	0	3.42	3.45	3.00	0.0000
SVENSS	37	f	0	4.56	0.69	2.96	0.0002
Subtotal SVENSS				2.98	7.07	6.45	
TSUGAN	15	m	0	1.61	0.36	0.28	0.3330
TSUGAN	16	m	0	2.40	0.41	0.00	0.1254
TSUGAN	17	m	0	4.24	0.34	1.04	0.0135
Subtotal TSUGAN				2.70	1.11	1.33	
WAKAI	43	m	0	1.39	1.66	1.99	0.0727
WAKAI	44	m	0	2.31	1.82	0.06	0.0018
WAKAI	45	m	0	3.11	1.77	0.68	0.0000
Subtotal WAKAI				2.29	5.25	2.73	
WU	12	f	0	3.01	1.52	0.42	0.0002
WU	13	f	0	4.25	1.50	4.64	0.0000
Subtotal WU				3.63	3.02	5.06	
WUWILL	20	f	0	1.02	51.60	110.97	0.0000
WUWILL	21	f	0	1.44	15.26	16.60	0.0000
Subtotal WUWILL				1.12	66.87	127.57	
WYNDE2	3	m	0	1.53	2.39	2.20	0.0182
WYNDE2	4	m	0	2.92	2.80	0.54	0.0000
WYNDE2	5	m	0	3.44	2.71	2.47	0.0000
WYNDE2	6	m	0	3.77	2.79	4.59	0.0000
Subtotal WYNDE2				2.96	10.69	9.80	
WYNDE3	4	m	0	1.59	1.96	1.58	0.0265
WYNDE3	5	m	0	2.69	2.70	0.11	0.0000
WYNDE3	6	m	0	3.28	2.70	1.68	0.0000
WYNDE3	7	m	0	4.20	2.50	7.32	0.0000
WYNDE3	63	f	0	-0.22	0.79	5.80	0.8428
WYNDE3	64	f	0	2.11	3.01	0.43	0.0003
WYNDE3	65	f	0	2.50	2.28	0.00	0.0002
WYNDE3	66	f	0	2.72	1.14	0.06	0.0037
Subtotal WYNDE3				2.62	17.07	16.99	
WYNDE4	5	m	0	0.90	4.60	11.62	0.0541
WYNDE4	11	m	0	1.79	6.37	3.13	0.0000
WYNDE4	17	m	0	2.41	7.04	0.04	0.0000
WYNDE4	23	m	0	3.31	6.70	4.50	0.0000
WYNDE4	29	m	0	3.32	6.35	4.39	0.0000
WYNDE4	49	f	2	-0.14	0.90	6.19	0.8951
WYNDE4	50	f	2	1.53	2.64	2.43	0.0130

Table 2G11 - 5

IESLC - Meta-anal of Ever Smoking (or Curr if Ever not avail) by Amount, Overview, Any prod (or Cigs if Any not avail)
Squamous
Least adjusted

REF	NRR	SEX	AD	Ys	Ws	Qs	Ps
WYNDE4	51	f	2	2.70	3.07	0.14	0.0000
WYNDE4	52	f	2	3.28	1.11	0.69	0.0006
WYNDE4	53	f	2	3.28	1.11	0.69	0.0006
Subtotal WYNDE4				2.39	39.89	33.82	
WYNDE6	21	m	0	2.57	17.35	0.12	0.0000
WYNDE6	30	m	0	2.98	23.14	5.53	0.0000
WYNDE6	39	m	0	3.39	20.23	16.31	0.0000
WYNDE6	48	m	0	3.99	23.16	52.53	0.0000
WYNDE6	210	f	0	1.98	16.03	4.07	0.0000
WYNDE6	219	f	0	3.21	26.69	13.94	0.0000
WYNDE6	228	f	0	3.77	17.84	29.20	0.0000
WYNDE6	237	f	0	4.51	20.03	82.00	0.0000
Subtotal WYNDE6				3.34	164.47	203.71	
ZHENG	1	m	0	1.41	2.33	2.69	0.0308
ZHENG	2	m	0	2.19	3.17	0.29	0.0001
ZHENG	3	m	0	2.99	3.51	0.87	0.0000
ZHENG	4	m	0	3.91	3.08	6.27	0.0000
ZHENG	16	f	0	0.75	6.21	18.75	0.0620
ZHENG	17	f	0	2.48	7.48	0.00	0.0000
Subtotal ZHENG				2.17	25.78	28.86	
ZHOU	10	c	0	0.39	3.46	15.22	0.4669
ZHOU	11	c	0	1.01	9.42	20.54	0.0019
ZHOU	12	c	0	1.58	16.68	13.80	0.0000
Subtotal ZHOU				1.26	29.56	49.57	

N	151
NS	36

Table 2G11 - 6

IESLC - Meta-anal of Ever Smoking (or Curr if Ever not avail) by Amount, Overview, Any prod (or Cigs if Any not avail)
Squamous
Least adjusted

	Sex			
	combined	male	female	Total
N	11	88	52	151
NS	4	25	19	48

In this overview table, other than the "N" rows, entries in the "absent" and "Total" columns may be invalid and should be ignored

	Amount smoked (broad categories)				
	absent	<20k5	6-44k20	>20k45	Total
N	43	41	30	37	151
NS	24	30	24	30	108
Wt	541.24	405.48	246.36	231.12	1424.21
Het Chi	277.02	181.21	124.35	147.60	1149.23
Het df	42	40	29	36	150
Het P	***	***	***	***	***
Fixed RR	14.54	5.71	11.97	28.64	12.03
RRl	13.37	5.18	10.56	25.17	11.42
RRu	15.82	6.29	13.56	32.58	12.67
P	+++	+++	+++	+++	+++
Random RR	12.37	4.50	11.37	27.18	11.07
RRl	9.62	3.50	8.46	20.11	9.42
RRu	15.92	5.78	15.27	36.76	13.00
P	+++	+++	+++	+++	+++

	Amount smoked (narrow categories)							
	absent	<10k1	2-19k10	11-29k20	21-39k30	31-98k40	>40k99	Total
N	88	15	11	24	9	1	3	151
NS	36	10	8	20	7	1	2	83
Wt	890.93	100.88	123.32	232.48	66.64	4.12	5.83	1424.21
Het Chi	748.13	57.59	42.27	122.56	9.96	0.00	2.15	1149.23
Het df	87	14	10	23	8	0	2	150
Het P	***	***	***	***	N.S.	N.S.	N.S.	***
Fixed RR	13.03	4.74	8.56	12.01	29.28	10.15	33.86	12.03
RRl	12.20	3.90	7.17	10.56	23.03	3.86	15.04	11.42
RRu	13.91	5.76	10.21	13.66	37.22	26.66	76.22	12.67
P	+++	+++	+++	+++	+++	+++	+++	+++
Random RR	12.72	3.07	6.99	11.47	28.21	10.15	33.54	11.07
RRl	10.20	1.88	4.53	8.22	21.18	3.86	14.41	9.42
RRu	15.87	5.01	10.79	16.00	37.56	26.66	78.06	13.00
P	+++	+++	+++	+++	+++	+++	+++	+++

MALES

	Amount smoked (broad categories)				
	absent	<20k5	6-44k20	>20k45	Total
N	23	23	20	22	88
NS	16	23	20	22	81
Wt	286.83	210.37	157.09	142.84	797.13
Het Chi	40.17	53.72	80.09	63.14	402.75
Het df	22	22	19	21	87
Het P	*	***	***	***	***
Fixed RR	17.09	7.28	12.05	26.92	13.82
RRl	15.22	6.36	10.31	22.85	12.89
RRu	19.19	8.34	14.09	31.72	14.81
P	+++	+++	+++	+++	+++
Random RR	16.89	6.17	12.02	28.00	13.58
RRl	13.59	4.63	8.21	19.90	11.37
RRu	20.99	8.23	17.58	39.40	16.22
P	+++	+++	+++	+++	+++

Table 2G11 - 6

IESLC - Meta-anal of Ever Smoking (or Curr if Ever not avail) by Amount, Overview, Any prod (or Cigs if Any not avail)
Squamous
Least adjusted

MALES

	Amount smoked (narrow categories)							
	absent	<10k1	2-19k10	11-29k20	21-39k30	31-98k40	>40k99	Total
N	46	9	7	16	7	1	2	88
NS	25	9	7	16	7	1	2	66
Wt	451.38	66.74	73.87	148.62	47.70	4.12	4.70	797.13
Het Chi	234.71	17.65	5.71	78.91	6.26	0.00	1.24	402.75
Het df	45	8	6	15	6	0	1	87
Het P	***	*	N.S.	***	N.S.	N.S.	N.S.	***
Fixed RR	15.05	7.25	12.57	11.98	25.36	10.15	41.10	13.82
RRl	13.72	5.70	10.01	10.20	19.10	3.86	16.64	12.89
RRu	16.50	9.22	15.79	14.07	33.69	26.66	101.52	14.81
P	+++	+++	+++	+++	+++	+++	+++	+++
Random RR	15.89	5.02	12.57	11.96	25.20	10.15	40.83	13.58
RRl	12.16	3.01	10.01	7.78	18.77	3.86	14.89	11.37
RRu	20.76	8.36	15.79	18.38	33.84	26.66	111.95	16.22
P	+++	+++	+++	+++	+++	+++	+++	+++

FEMALES

	Amount smoked (broad categories)				
	absent	<20k5	6-44k20	>20k45	Total
N	17	15	8	12	52
NS	15	15	8	12	50
Wt	210.55	185.61	79.96	64.06	540.18
Het Chi	177.18	83.83	18.98	71.90	557.15
Het df	16	14	7	11	51
Het P	***	***	**	***	***
Fixed RR	13.20	4.69	13.82	29.39	10.24
RRl	11.53	4.06	11.10	23.01	9.41
RRu	15.10	5.41	17.20	37.55	11.14
P	+++	+++	+++	+++	+++
Random RR	9.67	3.82	13.24	26.24	9.30
RRl	5.79	2.52	8.70	12.70	6.83
RRu	16.16	5.79	20.16	54.22	12.64
P	+++	+++	+++	+++	+++

	Amount smoked (narrow categories)							
	absent	<10k1	2-19k10	11-29k20	21-39k30	31-98k40	>40k99	Total
N	35	5	3	6	2		1	52
NS	19	5	3	6	2		1	36
Wt	374.84	30.67	40.04	74.55	18.94		1.14	540.18
Het Chi	391.89	3.86	5.66	17.67	0.25		0.00	557.15
Het df	34	4	2	5	1		0	51
Het P	***	N.S.	(*)	**	N.S.		N.S.	***
Fixed RR	10.82	2.14	5.50	14.30	42.01		15.20	10.24
RRl	9.78	1.50	4.04	11.39	26.78		2.42	9.41
RRu	11.97	3.05	7.50	17.94	65.91		95.56	11.14
P	+++	+++	+++	+++	+++		++	+++
Random RR	10.72	2.14	4.12	14.43	42.01		15.20	9.30
RRl	7.30	1.50	1.87	8.87	26.78		2.42	6.83
RRu	15.73	3.05	9.08	23.46	65.91		95.56	12.64
P	+++	+++	+++	+++	+++		++	+++

Table 2G11 - 7

IESLC - Meta-anal of Ever Smoking (or Curr if Ever not avail) by Amount, Overview, Any prod (or Cigs if Any not avail)
Squamous
Excluded studies (and stage at which they were excluded)

Stage	Studies
1	ABELIN ABRAHA AMANDU AMES ANDERS AUSTIN AXELSO BAND BECHER BERRIN BLOHMK BLOT4 BROCKM BROWN1 BYERS1 BYERS2 CARPEN CASCO2 CASCOR CHAN CHEN3 CHIAZZ CHYOU DESTE2 DOCKER DROSTE DU GARCIA GARDIN GENG GODLEY GOODMA GRAHAM GREGOR HEGMAN HEIN HENNEK HINDS HIRAOK HOROWI HORWIT HUANG ISHIMA JAHN JAIN JARVHO JIANG KELLER KIHARA KJUUS KO KOHLME KUBIK LAMWK LAMWK2 LANGE LEI LEMARC LEVIN LIU LOMBA2 LOMBAR MAGNUS MARSH MARSH2 MCDUFF MCLAUG MILLER MILLS NOTANI NOU ODRISC PAWLEG PERSHA POFFIJ QIAO QIAO2 RADZIK REN RONCO ROOTS ROTHSC SAARIK SANKAR SCHWAR SEGI SEOW SHIMIZ SIMARA SIMONA SITAS SOBUE2 STASZE STAYNE STUCKE SUN SUZUK2 SUZUKI TANG TAO TOKARS TOUSEY ULMER VEIERO VUTUC WALD WANG WANG3 WANG4 WICKLU WIGLE WILKIN WU2 WUNSCH WYNDE8 XIANGZ XU XU2 XU4 YONG ZHANG
2	BUELL CHEN MASTRA MZILEN PISANI RESTRE SADOWS
4	BOFFET WYNDE7
5	RIMING TANG2 WYNDE5
6	BLOT1 BLOT2 BLOT3 BOUCHA HIRAY2 JONES LAURIL LICKIN MOLLO MRFIT MURATA SCHWA2 VANDER WARSIN WATSON WYNDER
8	AGUDO AKIBA ARCHER ARMADA AUVINE AXELSS BENSHL BEST BRESLO BRETT BROSS BUFFLE CEDERL CHANG CHATZI CHEN2 CHOW COMSTO COOKSO CPSI CPSII DAMBER DARBY DAVEYS DEAN DEAN2 DEAN3 DEKLER DESTEF DOLL2 DORANT DORN DUNN EBELIN ENSTRO ESAKI FAN GAO GAO2 GARSHI GILLIS GOLLED GSELL HAMMO2 HANSEN HIRAYA HITOSU HOLE HU HU2 HUMBLE JARUP JOLY JUSSAW KAISE2 KAISER KANELL KAUFMA KHUDER KINLEN KNEKT KOO KOULUM KREUZE LAUSSM LETOUR LIAW LIDDEL LIU2 LIU3 LIU4 LIU5 LUBIN MACLEN MARTIS MCCONN MIGRAN MRFITR NAM NOTAN2 PARKIN PASTOR PERNU PERSH2 PETO PEZZO2 PEZZOT PIKE POLEDN PRESCO RACHTA RANDIG SEGI2 SHAW SIEMIA SPEIZE SPITZ STOCKS STOCKW TENKAN TIZZAN TULINI TVERDA WANG2 XU3 YAMAGU YUAN
10	BENHAM

Table 2G11 - 8

Potentially overlapping studies

REF	REFGP	PRINC	OVERLAP/LINK
LUBIN2	LUBIN2	1	Lubin-combined
LAMTH	LAMTH	1	KOO/LAMTH/LAMWK
OSANN2	KAISER	2	KAISER/OSANN2
WYNDE6	WYNDE6	1	WYNDE5/6/7/8
MATSUD	MATSUD	1	SOBUE2/MATSUD

Table 2G11 - 9

Most adjusted - insufficient data for metaanalysis

REF	NRR	SEX	AGEL	AGEH	RACE	YF	LC TYPE	LOC	START	ST	NLC	R	VB	P	H	AD	SM	PRODUCT	exL	exH	S1	S2	DENOM	De
CHEN	5	c	0	0	all	-	q	As:oth	1987	CC	323	n	ot	n	y	2	ev	cig+/-ot	1	10	1	0	nev cigs	ot
CHEN	4	c	0	0	all	-	q	As:oth	1987	CC	323	n	ot	n	y	2	ev	cig+/-ot	11	20	2	3	nev cigs	ot
CHEN	3	c	0	0	all	-	q	As:oth	1987	CC	323	n	ot	n	y	2	ev	cig+/-ot	21	30	0	4	nev cigs	ot
CHEN	2	c	0	0	all	-	q	As:oth	1987	CC	323	n	ot	n	y	2	ev	cig+/-ot	31	99	3	0	nev cigs	ot

REF	NRR	RR	SIG	Cigarette equivalent	RRDATA comment
CHEN	5	2.59	n	*	0
CHEN	4	7.05	n	*	0
CHEN	3	7.61	n	*	0
CHEN	2	11.11	y	*	P < 0.001

Table 2G12 -

IESLC - Meta-anal of Ever Smoking (or Current if ever not avail), Amount smoked, "Low", Any prod (or Cigs if Any not avail)
Squamous

This analysis is restricted to results for:
1) Results by Amount smoked
2) Results complete enough for use in metaanalysis

Within each study, results are then selected (in the following order of preference, within each sex) for:
3) SMKSTA: ever smokers, current smokers
4) PRODUCT: all/unspec, cigarettes regardless of other products, cigarettes only
5) CIGTYPE: all/unspecified, MC regardless of HR, MC only
6) DENOM: never smoked anything, never smoked cigarettes, (never +1 = +long term ex, +2 = +amount unknown, +3 = never cigs+long term ex)
7) Followup period (YF, prospective studies): whole study (coded as 0) or longest available
8) LCtype: squamous or nearest available, but not adeno. (q = squamous, s = small, a = adeno, KI = Kreyberg I, u = undifferentiated)
9) Race: all or nearest available, otherwise by race (wh or w = white, bl or b = black, hi = hispanic ch = chinese, jap = japanese, haw = hawaiian, w+o = white + oriental, sca = scandinavian, as = asian)
10) Amount smoked "low" in key scheme 1 (key value 5, maximum range <20, in numbers of cigarettes or cigarette equivalents)
11) For overlapping studies: principal rather than subsidiary studies
Finally by Age: whole study (coded as 0) if available, otherwise by widest available age group and then for single sex results (m, f) in preference to combined sex results (c).

Results adjusted (AD) for the most potential confounders are then chosen in Sections -1 to -3 and results adjusted for the least confounders in Sections -4 to -6. (Those least adjusted results which actually differ from the most adjusted as marked 'x' in column X in Section -4)
(Results adjusted for an unknown number of confounder(s) are coded as 20.)

Section -7 shows excluded studies, together with the stage (as above) at which no qualifying results were found.

Section -8 lists the potentially overlapping studies which have been included (1=principal, 2=subsidiary).

Section -9 lists any results which would have been included in preference except that they had data not complete enough for use in meta-analysis, with their significance (yes/no), if known, and any further comment as entered on the database.

In addition to those mentioned above, the following fields, levels and abbreviations are used:
* or nk = not known, n = no, y = yes, ot = other
ev = ever, cu = current, nev = never
all/unspec = all or unspecified, cig+/-ot = cigarettes irrespective of other products (cigar, pipe etc)
MC = manufactured cigarettes, HR = hand-rolled cigarettes
exL, exH = range of exposure (low and high) in the smoking group, in terms of Amount smoked, cigarettes or cigarette equivalents
REF: 6-character study reference
NRR: number of the RR on the database within the study
ST : study type (CC = case control, pr or prosp = prospective)
NLC: number of lung cancer cases in whole study
R : risky occupational population (n = no, m = mining, o = other risky)
VB : national cigarette type (V = at least 75% Virginia, bl = at least 75% blended, ot = other)
P : any proxy use
H : full histological confirmation
De : derivation of RR/CI (or = original, st = standard method, ot = other method of estimation)

Table 2G12 - 1

IESLC - Meta-anal of Ever Smoking (or Current if ever not avail), Amount smoked, "Low", Any prod (or Cigs if Any not avail)
Squamous
Most adjusted

REF	NRR	SEX	AGEL	AGEH	RACE	YF	LC TYPE	LOC	START	ST	NLC	R	VB	P	H	AD	SM	PRODUCT	exL	exH	DENOM	De
ALDERS	34	m	0	0	all	-	q+s	Eu:UK	1977	CC	1448	n	V	n	n	1	ev	cig only	1	17	nev+2	ot
ALDERS	37	f	0	0	all	-	q+s	Eu:UK	1977	CC	1448	n	V	n	n	1	ev	cig only	1	17	nev+2	ot
BARBON	70	m	0	0	all	-	q	Eu:wst	1979	CC	755	n	bl	y	y	3	ev	all/unsp	1	19	nev any	or
BROWN2	36	m	0	0	wh	-	q	NAmer	1984	CC	14596	n	bl	n	y	2	ev	cig+/-ot	1	19	nev cigs	or
BROWN2	35	f	0	0	wh	-	q	NAmer	1984	CC	14596	n	bl	n	y	2	ev	cig+/-ot	1	19	nev cigs	or
CHOI	46	m	0	0	all	-	q	As:oth	1985	CC	375	n	bl	n	n	0	ev	cig+/-ot	1	10	nev cigs	st
CHOI	56	f	0	0	all	-	q	As:oth	1985	CC	375	n	bl	n	n	0	ev	cig+/-ot	1	10	nev cigs	st
DOLL	69	m	0	0	all	-	KI	Eu:UK	1948	CC	1465	n	V	n	n	1	ev	all/unsp	5	14	nev any	ot
DOLL	77	f	0	0	all	-	KI	Eu:UK	1948	CC	1465	n	V	n	n	1	ev	all/unsp	5	14	nev any	ot
DORGAN	114	m	0	0	wh	-	q	NAmer	1980	CC	2026	n	bl	y	y	2	ev	cig+/-ot	1	19	nev any	ot
DORGAN	99	f	0	0	all	-	q	NAmer	1980	CC	2026	n	bl	y	y	3	ev	cig+/-ot	1	19	nev any	ot
DOSEME	7	m	0	0	all	-	q	Eu:bal	1979	CC	1210	n	bl	n	n	2	ev	cig+/-ot	1	10	nev cigs	or
ENGELA	58	m	0	0	all	0	q	Eu:Sca	1964	pr	435	n	bl	n	n	7	cu	cig+/-ot	5	9	nev cigs	or
GER	14	c	0	0	all	-	q+s	As:oth	1990	CC	141	n	ot	y	n	10	ev	all/unsp	1	10	nev any	ot
HAMMON	98	m	0	0	wh	0	not a	NAmer	1952	pr	448	n	bl	n	n	1	cu	cig only	1	9	nev any	ot
JEDRYC	28	m	0	0	all	-	q	Eu:est	1980	CC	1630	n	bl	y	n	3	ev	cig+/-ot	1	19	nev any	or
KREYBE	1	m	0	0	all	-	KI	Eu:Sca	1948	CC	300	n	bl	n	y	1	ev	all/unsp	1	14	nev any	or
KREYBE	31	f	0	0	all	-	KI	Eu:Sca	1948	CC	300	n	bl	n	y	0	ev	all/unsp	1	14	nev any	st
LAMTH	10	f	0	0	ch	-	q	As:HK	1983	CC	445	n	bl	n	n	0	ev	all/unsp	1	10	nev any	or
LUBIN2	149	m	0	0	all	-	q	Eu:mul	1976	CC	7804	n	bl	n	y	0	ev	cig+/-ot	1	9	nev any	st
LUBIN2	169	f	0	0	all	-	q	Eu:mul	1976	CC	7804	n	bl	n	y	0	ev	cig+/-ot	1	9	nev any	st
LUO	10	c	0	0	all	-	q	As:Chi	1990	CC	102	n	ot	n	y	20	ev	cig+/-ot	1	19	nev cigs	or
MATOS	43	m	0	0	all	-	q	SCAmer	1994	CC	200	n	bl	n	n	2	ev	cig+/-ot	1	14	nev any	or
MATSUD	4	m	0	0	all	-	q	As:Jap	1965	CC	179	n	bl	n	n	0	ev	cig+/-ot	1	10	nev cigs	st
ORMOS	5	m	0	0	all	-	q	Eu:est	1947	CC	119	n	bl	y	y	0	ev	cig+/-ot	1	15	nev any	st
OSANN2	28	f	0	0	all	-	KI	NAmer	1964	ot	217	n	bl	n	y	1	ev	cig+/-ot	1	19	nev cigs	or
SOBUE	53	m	0	0	all	-	q	As:Jap	1986	CC	1376	n	bl	n	y	0	cu	cig+/-ot	1	19	nev cigs	st
SVENSS	7	f	0	0	all	-	q	Eu:Sca	1983	CC	210	n	bl	n	n	1	cu	all/unsp	1	10	nev any	or
TSUGAN	15	m	0	0	all	-	q	As:Jap	1976	CC	134	n	bl	n	y	0	cu	all/unsp	1	15	nev any	ot
WAKAI	46	m	0	0	all	-	q	As:Jap	1988	CC	333	n	bl	n	y	1	cu	cig+/-ot	1	19	nev any	or
WUWILL	14	f	0	0	all	-	q+s	As:Chi	1985	CC	965	n	ot	n	n	3	ev	cig+/-ot	1	19	nev cigs	ot
WYNDE2	3	m	0	0	all	-	KI	NAmer	1962	CC	404	n	bl	n	y	0	ev	cig+/-ot	1	10	nev any	st
WYNDE3	4	m	0	0	all	-	KI	NAmer	1966	CC	350	n	bl	n	y	0	ev	cig+/-ot	1	9	nev any	st
WYNDE3	63	f	0	0	all	-	KI	NAmer	1966	CC	350	n	bl	n	y	0	ev	cig+/-ot	1	9	nev any	st
WYNDE4	63	m	0	0	all	-	not a	NAmer	1948	CC	684	n	bl	y	n	2	ev	all/unsp	1	9	nev any	ot
WYNDE4	49	f	0	0	all	-	not a	NAmer	1948	CC	684	n	bl	y	n	2	ev	all/unsp	1	9	nev any	ot
WYNDE6	21	m	0	0	all	-	KI	NAmer	1969	CC	4423	n	bl	n	y	0	cu	cig+/-ot	1	10	nev any	st
WYNDE6	210	f	0	0	all	-	KI	NAmer	1969	CC	4423	n	bl	n	y	0	cu	cig+/-ot	1	10	nev cigs	st
ZHENG	1	m	0	0	all	-	q	As:Chi	1982	CC	540	n	ot	*	y	0	ev	cig+/-ot	1	9	nev cigs	st
ZHENG	16	f	0	0	all	-	q	As:Chi	1982	CC	540	n	ot	*	y	0	ev	cig+/-ot	1	9	nev cigs	st
ZHOU	10	c	0	0	all	-	q	As:Chi	1978	CC	1360	n	ot	n	n	0	ev	all/unsp	1	9	nev any	st

Cigarette type is all/unspec for all RRs

except for the following:

REF	NRR	CIGTYPE
ALDERS	34	MC only
ALDERS	37	MC only

Table 2G12 - 2

IESLC - Meta-anal of Ever Smoking (or Current if ever not avail), Amount smoked, "Low", Any prod (or Cigs if Any not avail)
Squamous
Most adjusted

REF	NRR	SEX	AD	Number Exposed Case	Cont	Non-exposed Case	Cont	RR	95.00%CI
ALDERS	34	m	1	-	-	-	-	3.79	(1.30- 11.02)
ALDERS	37	f	1	-	-	-	-	2.55	(1.42- 4.57)
Subtotal ALDERS								2.79	(1.67- 4.66)
BARBON	70	m	3	-	-	-	-	8.50	(3.60- 20.00)
BROWN2	36	m	2	-	-	-	-	7.60	(6.20- 9.40)
BROWN2	35	f	2	-	-	-	-	11.70	(8.70- 15.80)
Subtotal BROWN2								8.75	(7.38- 10.38)
CHOI	46	m	0	12	90	6	95	2.11	(0.76- 5.86)
CHOI	56	f	0	4	16	10	164	4.10	(1.15- 14.57)
Subtotal CHOI								2.74	(1.24- 6.07)
DOLL	69	m	1	-	-	-	-	10.60	(3.30- 34.07)
DOLL	77	f	1	-	-	-	-	1.70	(0.64- 4.50)
Subtotal DOLL								3.61	(1.71- 7.62)
DORGAN	114	m	2	-	-	-	-	11.50	(4.10- 32.24)
DORGAN	99	f	3	-	-	-	-	7.78	(4.86- 12.44)
Subtotal DORGAN								8.32	(5.43- 12.76)
DOSEME	7	m	2	-	-	-	-	2.60	(1.50- 4.60)
*ENGELA	58	m	7	-	-	-	-	7.70	(1.90- 31.00)
GER	14	c	10	-	-	-	-	1.43	(0.36- 5.61)
*HAMMON	98	m	1	-	-	-	-	15.12	(4.93- 46.36)
JEDRYC	28	m	3	-	-	-	-	7.51	(3.09- 18.27)
KREYBE	1	m	1	-	-	-	-	9.00	(2.85- 28.38)
KREYBE	31	f	0	1	286	3	657	0.77	(0.08- 7.39)
Subtotal KREYBE								5.44	(1.95- 15.16)
LAMTH	10	f	0	23	11	28	72	5.38	(2.32- 12.46)
LUBIN2	149	m	0	418	2194	54	2616	9.23	(6.91- 12.32)
LUBIN2	169	f	0	30	184	72	1180	2.67	(1.70- 4.20)
Subtotal LUBIN2								6.45	(5.06- 8.23)
LUO	10	c	20	-	-	-	-	1.20	(0.10- 10.00)
MATOS	43	m	2	-	-	-	-	1.40	(0.30- 6.90)
MATSUD	4	m	0	21	1237	1	1255	21.31	(2.86- 158.63)
ORMOS	5	m	0	13	329	2	777	15.35	(3.44- 68.41)
OSANN2	28	f	1	-	-	-	-	12.10	(1.50- 96.30)
SOBUE	53	m	0	57	157	3	128	15.49	(4.74- 50.62)
SVENSS	7	f	1	-	-	-	-	9.70	(2.90- 45.90)
TSUGAN	15	m	0	2	5	0	5	5.00~	(0.19- 130.02)
WAKAI	46	m	1	-	-	-	-	3.95	(0.86- 18.10)
WUWILL	14	f	3	-	-	-	-	3.21	(2.39- 4.30)
WYNDE2	3	m	0	15	114	3	105	4.61	(1.30- 16.36)
WYNDE3	4	m	0	7	42	3	88	4.89	(1.20- 19.86)
WYNDE3	63	f	0	1	19	5	76	0.80	(0.09- 7.26)
Subtotal WYNDE3								2.90	(0.89- 9.48)
WYNDE4	63	m	2	-	-	-	-	2.22	(0.89- 5.53)
WYNDE4	49	f	2	-	-	-	-	0.87	(0.11- 6.90)
Subtotal WYNDE4								1.91	(0.83- 4.39)
WYNDE6	21	m	0	75	122	29	617	13.08	(8.17- 20.94)
WYNDE6	210	f	0	37	109	40	856	7.26	(4.45- 11.85)
Subtotal WYNDE6								9.86	(7.02- 13.84)
ZHENG	1	m	0	7	40	4	94	4.11	(1.14- 14.84)
ZHENG	16	f	0	11	29	33	184	2.11	(0.96- 4.64)
Subtotal ZHENG								2.54	(1.30- 4.96)
ZHOU	10	c	0	15	5	138	68	1.48	(0.52- 4.24)
Partial Totals				749	4989	434	9037		

*prospective study

~ With 0.5 adjustment for zero

Table 2G12 - 2

IESLC - Meta-anal of Ever Smoking (or Current if ever not avail), Amount smoked, "Low", Any prod (or Cigs if Any not avail)
Squamous
Most adjusted

REF	NRR	SEX	AD	Ys	Ws	Qs	Ps
ALDERS	34	m	1	1.33	3.36	0.74	0.0145
ALDERS	37	f	1	0.94	11.25	8.45	0.0017
Subtotal ALDERS				1.03	14.61	9.20	
BARBON	70	m	3	2.14	5.23	0.59	0.0000
BROWN2	36	m	2	2.03	88.72	4.50	0.0000
BROWN2	35	f	2	2.46	43.16	18.61	0.0000
Subtotal BROWN2				2.17	131.88	23.11	
CHOI	46	m	0	0.75	3.68	4.10	0.1517
CHOI	56	f	0	1.41	2.39	0.37	0.0292
Subtotal CHOI				1.01	6.07	4.47	
DOLL	69	m	1	2.36	2.82	0.88	0.0001
DOLL	77	f	1	0.53	4.04	6.54	0.2862
Subtotal DOLL				1.28	6.86	7.42	
DORGAN	114	m	2	2.44	3.61	1.48	0.0000
DORGAN	99	f	3	2.05	17.39	1.07	0.0000
Subtotal DORGAN				2.12	21.01	2.55	
DOSEME	7	m	2	0.96	12.24	8.79	0.0008
*ENGELA	58	m	7	2.04	1.97	0.11	0.0042
GER	14	c	10	0.36	2.04	4.26	0.6097
*HAMMON	98	m	1	2.72	3.06	2.55	0.0000
JEDRYC	28	m	3	2.02	4.87	0.22	0.0000
KREYBE	1	m	1	2.20	2.91	0.45	0.0002
KREYBE	31	f	0	-0.27	0.75	3.20	0.8175
Subtotal KREYBE				1.69	3.66	3.65	
LAMTH	10	f	0	1.68	5.44	0.08	0.0001
LUBIN2	149	m	0	2.22	45.98	8.09	0.0000
LUBIN2	169	f	0	0.98	18.69	12.57	0.0000
Subtotal LUBIN2				1.86	64.67	20.66	
LUO	10	c	20	0.18	0.72	1.90	0.8767
MATOS	43	m	2	0.34	1.56	3.36	0.6740
MATSUD	4	m	0	3.06	0.95	1.50	0.0028
ORMOS	5	m	0	2.73	1.72	1.48	0.0003
OSANN2	28	f	1	2.49	0.89	0.42	0.0189
SOBUE	53	m	0	2.74	2.74	2.41	0.0000
SVENSS	7	f	1	2.27	2.01	0.44	0.0013
TSUGAN	15	m	0	1.61	0.36	0.01	0.3330
WAKAI	46	m	1	1.37	1.66	0.31	0.0772
WUWILL	14	f	3	1.17	44.55	18.06	0.0000
WYNDE2	3	m	0	1.53	2.39	0.18	0.0182
WYNDE3	4	m	0	1.59	1.96	0.09	0.0265
WYNDE3	63	f	0	-0.22	0.79	3.24	0.8428
Subtotal WYNDE3				1.07	2.75	3.33	
WYNDE4	63	m	2	0.80	4.60	4.66	0.0870
WYNDE4	49	f	2	-0.14	0.90	3.38	0.8951
Subtotal WYNDE4				0.64	5.50	8.04	
WYNDE6	21	m	0	2.57	17.35	10.24	0.0000
WYNDE6	210	f	0	1.98	16.03	0.52	0.0000
Subtotal WYNDE6				2.29	33.38	10.76	
ZHENG	1	m	0	1.41	2.33	0.35	0.0308
ZHENG	16	f	0	0.75	6.21	6.89	0.0620
Subtotal ZHENG				0.93	8.54	7.25	
ZHOU	10	c	0	0.39	3.46	6.91	0.4669

Table 2G12 - 2

IESLC - Meta-anal of Ever Smoking (or Current if ever not avail), Amount smoked, "Low", Any prod (or Cigs if Any not avail)
Squamous
Most adjusted

	N	41
	NS	30
	Wt	396.78
Het	Chi	154.02
Het	df	40
Het	P	***
Fixed	RR	6.07
	RRl	5.50
	RRu	6.70
	P	+++
Random	RR	4.98
	RRl	3.93
	RRu	6.31
	P	+++
Asymm	P	(*)

Table 2G12 - 3

IESLC - Meta-anal of Ever Smoking (or Current if ever not avail), Amount smoked, "Low", Any prod (or Cigs if Any not avail)
Squamous
Most adjusted

	Sex			
	combined	male	female	Total
N	3	23	15	41
NS	3	23	15	41
Wt	6.23	216.08	174.47	396.78
Het Chi	0.03	48.34	77.04	154.02
Het df	2	22	14	40
Het P	N.S.	***	***	***
Fixed RR	1.43	7.42	4.98	6.07
RRl	0.65	6.49	4.29	5.50
RRu	3.13	8.48	5.78	6.70
P	N.S.	+++	+++	+++
Random RR	1.43	6.62	3.95	4.98
RRl	0.65	5.08	2.60	3.93
RRu	3.13	8.63	6.00	6.31
P	N.S.	+++	+++	+++
Between Chi				28.63
Between df				2
Between P				***
Btwn(F) P				*
Btwn(R) P				***

	Lung cancer type					
	q	q+s	q+u	KI	not a	Total
N	24	4		10	3	41
NS	19	3		6	2	30
Wt	277.10	61.19		49.92	8.56	396.78
Het Chi	81.47	1.80		23.69	9.09	154.02
Het df	23	3		9	2	40
Het P	***	N.S.		**	*	***
Fixed RR	6.91	3.02		7.44	3.99	6.07
RRl	6.14	2.35		5.64	2.04	5.50
RRu	7.77	3.88		9.82	7.81	6.70
P	+++	+++		+++	+++	+++
Random RR	5.52	3.02		5.56	3.52	4.98
RRl	4.16	2.35		3.22	0.73	3.93
RRu	7.34	3.88		9.58	16.95	6.31
P	+++	+++		+++	N.S.	+++
Between Chi						37.97
Between df						3
Between P						***
Btwn(F) P						*
Btwn(R) P						*

				Location					
	NAmer	UK	Scand	othEur	China	Japan	othAs	other	Total
N	13	4	4	6	5	4	4	1	41
NS	8	2	3	5	4	4	3	1	30
Wt	200.86	21.47	7.64	88.72	57.27	5.71	13.54	1.56	396.78
Het Chi	29.57	6.27	4.07	31.70	3.50	2.67	3.56	0.00	154.02
Het df	12	3	3	5	4	3	3	0	40
Het P	**	(*)	N.S.	***	N.S.	N.S.	N.S.	N.S.	***
Fixed RR	8.41	3.03	6.93	5.93	2.92	10.23	3.26	1.40	6.07
RRl	7.32	1.99	3.41	4.82	2.25	4.51	1.91	0.29	5.50
RRu	9.65	4.63	14.08	7.30	3.78	23.24	5.55	6.71	6.70
P	+++	+++	+++	+++	+++	+++	+++	N.S.	+++
Random RR	7.65	3.33	6.53	5.73	2.92	10.23	3.18	1.40	4.98
RRl	5.71	1.71	2.80	3.08	2.25	4.51	1.76	0.29	3.93
RRu	10.24	6.46	15.23	10.66	3.78	23.24	5.73	6.71	6.31
P	+++	+++	+++	+++	+++	+++	+++	N.S.	+++
Between Chi									72.67
Between df									7
Between P									***
Btwn(F) P									**
Btwn(R) P									***

Table 2G12 - 3

IESLC - Meta-anal of Ever Smoking (or Current if ever not avail), Amount smoked, "Low", Any prod (or Cigs if Any not avail)

Squamous

Most adjusted

	Detailed Country in "other Europe"					
	multi	Germany	othWest	East	Balkans	Total
N	2		1	2	1	6
NS	1		1	2	1	5
Wt	64.67		5.23	6.59	12.24	88.72
Het Chi	20.42		0.00	0.65	0.00	31.70
Het df	1		0	1	0	5
Het P	***		N.S.	N.S.	N.S.	***
Fixed RR	6.45		8.50	9.05	2.60	5.93
RRl	5.06		3.61	4.22	1.48	4.82
RRu	8.23		20.03	19.43	4.55	7.30
P	+++		+++	+++	+++	+++
Random RR	5.03		8.50	9.05	2.60	5.73
RRl	1.49		3.61	4.22	1.48	3.08
RRu	16.94		20.03	19.43	4.55	10.66
P	++		+++	+++	+++	+++
Between Chi						10.63
Between df						3
Between P						*
Btwn(F) P						N.S.
Btwn(R) P						*

	Detailed Country in "other Asia"			
	India	HongKong	other	Total
N		1	3	4
NS		1	2	3
Wt		5.44	8.11	13.54
Het Chi		0.00	1.28	3.56
Het df		0	2	3
Het P		N.S.	N.S.	N.S.
Fixed RR		5.38	2.33	3.26
RRl		2.32	1.17	1.91
RRu		12.46	4.63	5.55
P		+++	+	+++
Random RR		5.38	2.33	3.18
RRl		2.32	1.17	1.76
RRu		12.46	4.63	5.73
P		+++	+	+++
Between Chi				2.28
Between df				1
Between P				N.S.
Btwn(F) P				N.S.
Btwn(R) P				N.S.

	Detailed other continent			
	SCAmer	Auslia	Africa	Total
N	1			1
NS	1			1
Wt	1.56			1.56
Het Chi	0.00			0.00
Het df	0			0
Het P	N.S.			N.S.
Fixed RR	1.40			1.40
RRl	0.29			0.29
RRu	6.71			6.71
P	N.S.			N.S.
Random RR	1.40			1.40
RRl	0.29			0.29
RRu	6.71			6.71
P	N.S.			N.S.
Between Chi				
Between df				
Between P				N.S.
Btwn(F) P				N.S.
Btwn(R) P				N.S.

Table 2G12 - 3

IESLC - Meta-anal of Ever Smoking (or Current if ever not avail), Amount smoked, "Low", Any prod (or Cigs if Any not avail)
Squamous
Most adjusted

	Start year of study <1960	1960-69	1970-79	1980-89	1990+	Total
N	8	8	8	14	3	41
NS	5	6	6	10	3	30
Wt	20.80	42.33	100.57	228.75	4.32	396.78
Het Chi	21.44	10.41	41.80	57.55	0.02	154.02
Het df	7	7	7	13	2	40
Het P	**	N.S.	***	***	N.S.	***
Fixed RR	4.56	8.81	4.93	6.55	1.38	6.07
RRl	2.97	6.52	4.05	5.75	0.54	5.50
RRu	7.01	11.91	5.99	7.46	3.54	6.70
P	+++	+++	+++	+++	N.S.	+++
Random RR	4.50	7.96	3.73	5.89	1.38	4.98
RRl	2.04	5.05	2.11	4.16	0.54	3.93
RRu	9.90	12.52	6.60	8.33	3.54	6.31
P	+++	+++	+++	+++	N.S.	+++
Between Chi						22.81
Between df						4
Between P						***
Btwn(F) P						N.S.
Btwn(R) P						*

	Study type (1) CC	other	Total
N	38	3	41
NS	27	3	30
Wt	390.86	5.92	396.78
Het Chi	150.90	0.55	154.02
Het df	37	2	40
Het P	***	N.S.	***
Fixed RR	6.01	11.68	6.07
RRl	5.44	5.22	5.50
RRu	6.63	26.14	6.70
P	+++	+++	+++
Random RR	4.76	11.68	4.98
RRl	3.72	5.22	3.93
RRu	6.08	26.14	6.31
P	+++	+++	+++
Between Chi			2.58
Between df			1
Between P			N.S.
Btwn(F) P			N.S.
Btwn(R) P			*

	Study type (2) CC	prosp	other	Total
N	38	2	1	41
NS	27	2	1	30
Wt	390.86	5.03	0.89	396.78
Het Chi	150.90	0.55	0.00	154.02
Het df	37	1	0	40
Het P	***	N.S.	N.S.	***
Fixed RR	6.01	11.61	12.10	6.07
RRl	5.44	4.84	1.51	5.50
RRu	6.63	27.81	96.95	6.70
P	+++	+++	+	+++
Random RR	4.76	11.61	12.10	4.98
RRl	3.72	4.84	1.51	3.93
RRu	6.08	27.81	96.95	6.31
P	+++	+++	+	+++
Between Chi				2.58
Between df				2
Between P				N.S.
Btwn(F) P				N.S.
Btwn(R) P				N.S.

Table 2G12 - 3

IESLC - Meta-anal of Ever Smoking (or Current if ever not avail), Amount smoked, "Low", Any prod (or Cigs if Any not avail)

Squamous

Most adjusted

	Study size (number of LC cases)				
	100-249	250-499	500-999	1000+	Total
N	8	11	6	16	41
NS	8	8	4	10	30
Wt	10.26	26.98	63.81	295.72	396.78
Het Chi	12.94	13.35	8.33	84.04	154.02
Het df	7	10	5	15	40
Het P	(*)	N.S.	N.S.	***	***
Fixed RR	4.93	4.86	3.22	7.15	6.07
RRl	2.67	3.33	2.52	6.38	5.50
RRu	9.09	7.08	4.12	8.01	6.70
P	+++	+++	+++	+++	+++
Random RR	5.05	4.73	3.20	5.91	4.98
RRl	2.13	3.02	2.08	4.34	3.93
RRu	11.98	7.41	4.93	8.05	6.31
P	+++	+++	+++	+++	+++
Between Chi					35.36
Between df					3
Between P					***
Btwn(F) P					*
Btwn(R) P					N.S.

	Risky occupational population			
	no	mining	othRisky	Total
N	41			41
NS	30			30
Wt	396.78			396.78
Het Chi	154.02			154.02
Het df	40			40
Het P	***			***
Fixed RR	6.07			6.07
RRl	5.50			5.50
RRu	6.70			6.70
P	+++			+++
Random RR	4.98			4.98
RRl	3.93			3.93
RRu	6.31			6.31
P	+++			+++
Between Chi				
Between df				
Between P				N.S.
Btwn(F) P				N.S.
Btwn(R) P				N.S.

	National cigarette tobacco type			
	Virginia	blended	other	Total
N	4	31	6	41
NS	2	23	5	30
Wt	21.47	315.99	59.31	396.78
Het Chi	6.27	87.75	4.50	154.02
Het df	3	30	5	40
Het P	(*)	***	N.S.	***
Fixed RR	3.03	7.33	2.85	6.07
RRl	1.99	6.56	2.21	5.50
RRu	4.63	8.18	3.68	6.70
P	+++	+++	+++	+++
Random RR	3.33	6.23	2.85	4.98
RRl	1.71	4.89	2.21	3.93
RRu	6.46	7.94	3.68	6.31
P	+++	+++	+++	+++
Between Chi				55.50
Between df				2
Between P				***
Btwn(F) P				***
Btwn(R) P				***

Table 2G12 - 3

IESLC - Meta-anal of Ever Smoking (or Current if ever not avail), Amount smoked, "Low", Any prod (or Cigs if Any not avail)
Squamous
Most adjusted

	Any proxy use No/nk	Yes	Total
N	33	8	41
NS	24	6	30
Wt	356.42	40.36	396.78
Het Chi	136.87	17.07	154.02
Het df	32	7	40
Het P	***	*	***
Fixed RR	6.04	6.33	6.07
RRl	5.44	4.65	5.50
RRu	6.70	8.62	6.70
P	+++	+++	+++
Random RR	4.84	5.53	4.98
RRl	3.70	3.20	3.93
RRu	6.34	9.56	6.31
P	+++	+++	+++
Between Chi			0.08
Between df			1
Between P			N.S.
Btwn(F) P			N.S.
Btwn(R) P			N.S.

	Full histological confirmation No	Yes	Total
N	19	22	41
NS	15	15	30
Wt	115.19	281.59	396.78
Het Chi	33.87	61.62	154.02
Het df	18	21	40
Het P	*	***	***
Fixed RR	3.33	7.76	6.07
RRl	2.77	6.90	5.50
RRu	4.00	8.72	6.70
P	+++	+++	+++
Random RR	3.55	6.75	4.98
RRl	2.63	5.18	3.93
RRu	4.78	8.78	6.31
P	+++	+++	+++
Between Chi			58.53
Between df			1
Between P			***
Btwn(F) P			***
Btwn(R) P			**

	Number of adjustment variables (1) 0	1	2+/+nk	Total
N	18	9	14	41
NS	13	7	11	31
Wt	133.22	31.99	231.56	396.78
Het Chi	62.16	17.59	70.80	154.02
Het df	17	8	13	40
Het P	***	*	***	***
Fixed RR	6.23	4.42	6.25	6.07
RRl	5.25	3.13	5.49	5.50
RRu	7.38	6.26	7.10	6.70
P	+++	+++	+++	+++
Random RR	4.87	5.39	4.86	4.98
RRl	3.26	3.09	3.31	3.93
RRu	7.25	9.40	7.14	6.31
P	+++	+++	+++	+++
Between Chi				3.48
Between df				2
Between P				N.S.
Btwn(F) P				N.S.
Btwn(R) P				N.S.

Table 2G12 - 3

IESLC - Meta-anal of Ever Smoking (or Current if ever not avail), Amount smoked, "Low", Any prod (or Cigs if Any not avail)
Squamous
Most adjusted

	Number of adjustment variables (2) 0	1	2	3-5	6+/+nk	Total
N	18	9	7	4	3	41
NS	13	7	5	4	3	32
Wt	133.22	31.99	154.80	72.03	4.73	396.78
Het Chi	62.16	17.59	38.32	13.69	3.46	154.02
Het df	17	8	6	3	2	40
Het P	***	*	***	**	N.S.	***
Fixed RR	6.23	4.42	7.44	4.52	2.81	6.07
RRl	5.25	3.13	6.36	3.59	1.14	5.50
RRu	7.38	6.26	8.71	5.69	6.91	6.70
P	+++	+++	+++	+++	+	+++
Random RR	4.87	5.39	4.74	5.93	2.67	4.98
RRl	3.26	3.09	2.73	3.26	0.78	3.93
RRu	7.25	9.40	8.22	10.79	9.12	6.31
P	+++	+++	+++	+++	N.S.	+++
Between Chi						18.81
Between df						4
Between P						***
Btwn(F) P						N.S.
Btwn(R) P						N.S.

	Product all/unsp	cig+/-ot	cig only	Total
N	12	26	3	41
NS	9	19	2	30
Wt	34.56	344.55	17.67	396.78
Het Chi	23.81	109.09	7.62	154.02
Het df	11	25	2	40
Het P	*	***	*	***
Fixed RR	3.80	6.52	3.74	6.07
RRl	2.72	5.87	2.35	5.50
RRu	5.30	7.25	5.97	6.70
P	+++	+++	+++	+++
Random RR	3.57	5.65	4.88	4.98
RRl	2.12	4.28	1.74	3.93
RRu	6.02	7.44	13.63	6.31
P	+++	+++	++	+++
Between Chi				13.49
Between df				2
Between P				**
Btwn(F) P				N.S.
Btwn(R) P				N.S.

	Denominator nev any	nev cigs	Total
N	27	14	41
NS	20	11	31
Wt	170.19	226.58	396.78
Het Chi	85.40	68.47	154.02
Het df	26	13	40
Het P	***	***	***
Fixed RR	5.93	6.17	6.07
RRl	5.10	5.42	5.50
RRu	6.89	7.03	6.70
P	+++	+++	+++
Random RR	4.79	5.21	4.98
RRl	3.48	3.52	3.93
RRu	6.60	7.71	6.31
P	+++	+++	+++
Between Chi			0.15
Between df			1
Between P			N.S.
Btwn(F) P			N.S.
Btwn(R) P			N.S.

Table 2G12 - 3

IESLC - Meta-anal of Ever Smoking (or Current if ever not avail), Amount smoked, "Low", Any prod (or Cigs if Any not avail)

Squamous

Most adjusted

	Derivation of RR/CI Orig	StdCalc	Other	Total
N	12	16	13	41
NS	11	11	9	31
Wt	168.46	127.43	100.89	396.78
Het Chi	30.79	62.02	34.99	154.02
Het df	11	15	12	40
Het P	**	***	***	***
Fixed RR	7.60	6.27	4.00	6.07
RRl	6.54	5.27	3.29	5.50
RRu	8.84	7.46	4.86	6.70
P	+++	+++	+++	+++
Random RR	6.17	4.80	4.30	4.98
RRl	4.28	3.12	2.84	3.93
RRu	8.89	7.39	6.50	6.31
P	+++	+++	+++	+++
Between Chi				26.22
Between df				2
Between P				***
Btwn(F) P				*
Btwn(R) P				N.S.

	Smoking status ever	current	Total
N	33	8	41
NS	23	7	30
Wt	351.59	45.19	396.78
Het Chi	136.03	5.67	154.02
Het df	32	7	40
Het P	***	N.S.	***
Fixed RR	5.70	9.92	6.07
RRl	5.13	7.41	5.50
RRu	6.32	13.28	6.70
P	+++	+++	+++
Random RR	4.30	9.92	4.98
RRl	3.29	7.41	3.93
RRu	5.62	13.28	6.31
P	+++	+++	+++
Between Chi			12.32
Between df			1
Between P			***
Btwn(F) P			(*)
Btwn(R) P			***

Table 2G12 - 4

IESLC - Meta-anal of Ever Smoking (or Current if ever not avail), Amount smoked, "Low", Any prod (or Cigs if Any not avail)
Squamous
Least adjusted

REF	NRR	X	SEX	AGEL	AGEH	RACE	YF	LC TYPE	LOC	START	ST	NLC	R	VB	P	H	AD	SM	PRODUCT	exL	exH	DENOM	De
ALDERS	34		m	0	0	all	-	q+s	Eu:UK	1977	CC	1448	n	V	n	n	1	ev	cig only	1	17	nev+2	ot
ALDERS	37		f	0	0	all	-	q+s	Eu:UK	1977	CC	1448	n	V	n	n	1	ev	cig only	1	17	nev+2	ot
BARBON	19	x	m	0	0	all	-	q	Eu:wst	1979	CC	755	n	bl	y	y	0	ev	all/unsp	1	9	nev any	st
BROWN2	36		m	0	0	wh	-	q	NAmer	1984	CC	14596	n	bl	n	y	2	ev	cig+/-ot	1	19	nev cigs	or
BROWN2	35		f	0	0	wh	-	q	NAmer	1984	CC	14596	n	bl	n	y	2	ev	cig+/-ot	1	19	nev cigs	or
CHOI	46		m	0	0	all	-	q	As:oth	1985	CC	375	n	bl	n	n	0	ev	cig+/-ot	1	10	nev cigs	st
CHOI	56		f	0	0	all	-	q	As:oth	1985	CC	375	n	bl	n	n	0	ev	cig+/-ot	1	10	nev cigs	st
DOLL	55	x	m	0	0	all	-	KI	Eu:UK	1948	CC	1465	n	V	n	n	0	ev	all/unsp	5	14	nev any	st
DOLL	63	x	f	0	0	all	-	KI	Eu:UK	1948	CC	1465	n	V	n	n	0	ev	all/unsp	5	14	nev any	st
DORGAN	114		m	0	0	wh	-	q	NAmer	1980	CC	2026	n	bl	y	y	2	ev	cig+/-ot	1	19	nev any	ot
DORGAN	99		f	0	0	all	-	q	NAmer	1980	CC	2026	n	bl	y	y	3	ev	cig+/-ot	1	19	nev any	ot
DOSEME	7		m	0	0	all	-	q	Eu:bal	1979	CC	1210	n	bl	n	n	2	ev	cig+/-ot	1	10	nev cigs	or
ENGELA	58		m	0	0	all	0	q	Eu:Sca	1964	pr	435	n	bl	n	n	7	cu	cig+/-ot	5	9	nev cigs	or
GER	6	x	c	0	0	all	-	q+s	As:oth	1990	CC	141	n	ot	y	n	0	ev	all/unsp	1	10	nev any	st
HAMMON	98		m	0	0	wh	0	not a	NAmer	1952	pr	448	n	bl	n	n	1	cu	cig only	1	9	nev any	ot
JEDRYC	1	x	m	0	0	all	-	q	Eu:est	1980	CC	1630	n	bl	y	n	0	ev	cig+/-ot	1	9	nev any	st
KREYBE	13	x	m	0	0	all	-	KI	Eu:Sca	1948	CC	300	n	bl	n	y	0	ev	all/unsp	1	14	nev any	st
KREYBE	31		f	0	0	all	-	KI	Eu:Sca	1948	CC	300	n	bl	n	y	0	ev	all/unsp	1	14	nev any	st
LAMTH	10		f	0	0	ch	-	q	As:HK	1983	CC	445	n	bl	n	n	0	ev	all/unsp	1	10	nev any	or
LUBIN2	149		m	0	0	all	-	q	Eu:mul	1976	CC	7804	n	bl	n	y	0	ev	cig+/-ot	1	9	nev any	st
LUBIN2	169		f	0	0	all	-	q	Eu:mul	1976	CC	7804	n	bl	n	y	0	ev	cig+/-ot	1	9	nev any	st
LUO	4	x	c	0	0	all	-	q	As:Chi	1990	CC	102	n	ot	n	y	0	ev	cig+/-ot	1	19	nev cigs	st
MATOS	42	x	m	0	0	all	-	q	SCAmer	1994	CC	200	n	bl	n	n	0	ev	cig+/-ot	1	14	nev any	st
MATSUD	4		m	0	0	all	-	q	As:Jap	1965	CC	179	n	bl	n	n	0	ev	cig+/-ot	1	10	nev cigs	st
ORMOS	5		m	0	0	all	-	q	Eu:est	1947	CC	119	n	bl	y	y	0	ev	cig+/-ot	1	15	nev any	st
OSANN2	10	x	f	0	0	all	-	KI	NAmer	1964	ot	217	n	bl	n	y	0	ev	cig+/-ot	1	19	nev cigs	st
SOBUE	53		m	0	0	all	-	q	As:Jap	1986	CC	1376	n	bl	n	y	0	cu	cig+/-ot	1	19	nev cigs	st
SVENSS	27	x	f	0	0	all	-	q	Eu:Sca	1983	CC	210	n	bl	n	n	0	cu	all/unsp	1	10	nev any	st
TSUGAN	15		m	0	0	all	-	q	As:Jap	1976	CC	134	n	bl	n	y	0	cu	all/unsp	1	15	nev any	ot
WAKAI	43	x	m	0	0	all	-	q	As:Jap	1988	CC	333	n	bl	n	y	0	cu	cig+/-ot	1	19	nev any	st
WUWILL	20	x	f	0	0	all	-	q+s	As:Chi	1985	CC	965	n	ot	n	n	0	ev	cig+/-ot	1	19	nev cigs	st
WYNDE2	3		m	0	0	all	-	KI	NAmer	1962	CC	404	n	bl	n	y	0	ev	cig+/-ot	1	10	nev any	st
WYNDE3	4		m	0	0	all	-	KI	NAmer	1966	CC	350	n	bl	n	y	0	ev	cig+/-ot	1	9	nev any	st
WYNDE3	63		f	0	0	all	-	KI	NAmer	1966	CC	350	n	bl	n	y	0	ev	cig+/-ot	1	9	nev any	st
WYNDE4	5	x	m	0	0	all	-	not a	NAmer	1948	CC	684	n	bl	y	n	0	ev	all/unsp	1	9	nev any	st
WYNDE4	49		f	0	0	all	-	not a	NAmer	1948	CC	684	n	bl	y	n	2	ev	all/unsp	1	9	nev any	ot
WYNDE6	21		m	0	0	all	-	KI	NAmer	1969	CC	4423	n	bl	n	y	0	cu	cig+/-ot	1	10	nev any	st
WYNDE6	210		f	0	0	all	-	KI	NAmer	1969	CC	4423	n	bl	n	y	0	cu	cig+/-ot	1	10	nev cigs	st
ZHENG	1		m	0	0	all	-	q	As:Chi	1982	CC	540	n	ot	*	y	0	ev	cig+/-ot	1	9	nev cigs	st
ZHENG	16		f	0	0	all	-	q	As:Chi	1982	CC	540	n	ot	*	y	0	ev	cig+/-ot	1	9	nev cigs	st
ZHOU	10		c	0	0	all	-	q	As:Chi	1978	CC	1360	n	ot	n	n	0	ev	all/unsp	1	9	nev any	st

Cigarette type is all/unspec for all RRs

except for the following:

REF	NRR	CIGTYPE
ALDERS	34	MC only
ALDERS	37	MC only

Table 2G12 - 5

IESLC - Meta-anal of Ever Smoking (or Current if ever not avail), Amount smoked, "Low", Any prod (or Cigs if Any not avail)
Squamous
Least adjusted

REF	NRR	SEX	AD	Number Exposed Case	Number Exposed Cont	Non-exposed Case	Non-exposed Cont	RR	95.00%CI
ALDERS	34	m	1	-	-	-	-	3.79	(1.30- 11.02)
ALDERS	37	f	1	-	-	-	-	2.55	(1.42- 4.57)
Subtotal ALDERS								2.79	(1.67- 4.66)
BARBON	19	m	0	11	87	6	188	3.96	(1.42- 11.06)
BROWN2	36	m	2	-	-	-	-	7.60	(6.20- 9.40)
BROWN2	35	f	2	-	-	-	-	11.70	(8.70- 15.80)
Subtotal BROWN2								8.75	(7.38- 10.38)
CHOI	46	m	0	12	90	6	95	2.11	(0.76- 5.86)
CHOI	56	f	0	4	16	10	164	4.10	(1.15- 14.57)
Subtotal CHOI								2.74	(1.24- 6.07)
DOLL	55	m	0	291	570	3	61	10.38	(3.23- 33.37)
DOLL	63	f	0	9	18	16	59	1.84	(0.70- 4.88)
Subtotal DOLL								3.74	(1.77- 7.90)
DORGAN	114	m	2	-	-	-	-	11.50	(4.10- 32.24)
DORGAN	99	f	3	-	-	-	-	7.78	(4.86- 12.44)
Subtotal DORGAN								8.32	(5.43- 12.76)
DOSEME	7	m	2	-	-	-	-	2.60	(1.50- 4.60)
*ENGELA	58	m	7	-	-	-	-	7.70	(1.90- 31.00)
GER	6	c	0	9	56	11	80	1.17	(0.45- 3.01)
*HAMMON	98	m	1	-	-	-	-	15.12	(4.93- 46.36)
JEDRYC	1	m	0	1	67	6	289	0.72	(0.09- 6.07)
KREYBE	13	m	0	123	2341	3	644	11.28	(3.58- 35.57)
KREYBE	31	f	0	1	286	3	657	0.77	(0.08- 7.39)
Subtotal KREYBE								6.51	(2.34- 18.14)
LAMTH	10	f	0	23	11	28	72	5.38	(2.32- 12.46)
LUBIN2	149	m	0	418	2194	54	2616	9.23	(6.91- 12.32)
LUBIN2	169	f	0	30	184	72	1180	2.67	(1.70- 4.20)
Subtotal LUBIN2								6.45	(5.06- 8.23)
LUO	4	c	0	3	39	5	51	0.78	(0.18- 3.48)
MATOS	42	m	0	3	88	3	110	1.25	(0.25- 6.35)
MATSUD	4	m	0	21	1237	1	1255	21.31	(2.86- 158.63)
ORMOS	5	m	0	13	329	2	777	15.35	(3.44- 68.41)
OSANN2	10	f	0	18	31	7	58	4.81	(1.81- 12.77)
SOBUE	53	m	0	57	157	3	128	15.49	(4.74- 50.62)
SVENSS	27	f	0	10	30	5	120	8.00	(2.54- 25.16)
TSUGAN	15	m	0	2	5	0	5	5.00~	(0.19- 130.02)
WAKAI	43	m	0	13	105	2	65	4.02	(0.88- 18.41)
WUWILL	20	f	0	168	311	117	601	2.77	(2.11- 3.65)
WYNDE2	3	m	0	15	114	3	105	4.61	(1.30- 16.36)
WYNDE3	4	m	0	7	42	3	88	4.89	(1.20- 19.86)
WYNDE3	63	f	0	1	19	5	76	0.80	(0.09- 7.26)
Subtotal WYNDE3								2.90	(0.89- 9.48)
WYNDE4	5	m	0	14	82	8	115	2.45	(0.98- 6.12)
WYNDE4	49	f	2	-	-	-	-	0.87	(0.11- 6.90)
Subtotal WYNDE4								2.07	(0.90- 4.78)
WYNDE6	21	m	0	75	122	29	617	13.08	(8.17- 20.94)
WYNDE6	210	f	0	37	109	40	856	7.26	(4.45- 11.85)
Subtotal WYNDE6								9.86	(7.02- 13.84)
ZHENG	1	m	0	7	40	4	94	4.11	(1.14- 14.84)
ZHENG	16	f	0	11	29	33	184	2.11	(0.96- 4.64)
Subtotal ZHENG								2.54	(1.30- 4.96)
ZHOU	10	c	0	15	5	138	68	1.48	(0.52- 4.24)
Partial Totals				1422	8814	626	11478		

*prospective study

~ With 0.5 adjustment for zero

Table 2G12 - 5

IESLC - Meta-anal of Ever Smoking (or Current if ever not avail), Amount smoked, "Low", Any prod (or Cigs if Any not avail)
Squamous
Least adjusted

REF	NRR	SEX	AD	Ys	Ws	Qs	Ps
ALDERS	34	m	1	1.33	3.36	0.56	0.0145
ALDERS	37	f	1	0.94	11.25	7.29	0.0017
Subtotal ALDERS				1.03	14.61	7.86	
BARBON	19	m	0	1.38	3.64	0.48	0.0086
BROWN2	36	m	2	2.03	88.72	7.29	0.0000
BROWN2	35	f	2	2.46	43.16	22.26	0.0000
Subtotal BROWN2				2.17	131.88	29.56	
CHOI	46	m	0	0.75	3.68	3.64	0.1517
CHOI	56	f	0	1.41	2.39	0.26	0.0292
Subtotal CHOI				1.01	6.07	3.90	
DOLL	55	m	0	2.34	2.82	1.01	0.0001
DOLL	63	f	0	0.61	4.06	5.18	0.2175
Subtotal DOLL				1.32	6.88	6.19	
DORGAN	114	m	2	2.44	3.61	1.78	0.0000
DORGAN	99	f	3	2.05	17.39	1.67	0.0000
Subtotal DORGAN				2.12	21.01	3.45	
DOSEME	7	m	2	0.96	12.24	7.56	0.0008
*ENGELA	58	m	7	2.04	1.97	0.18	0.0042
GER	6	c	0	0.16	4.30	10.82	0.7462
*HAMMON	98	m	1	2.72	3.06	2.91	0.0000
JEDRYC	1	m	0	-0.33	0.84	3.62	0.7618
KREYBE	13	m	0	2.42	2.91	1.35	0.0000
KREYBE	31	f	0	-0.27	0.75	3.01	0.8175
Subtotal KREYBE				1.87	3.66	4.37	
LAMTH	10	f	0	1.68	5.44	0.02	0.0001
LUBIN2	149	m	0	2.22	45.98	10.64	0.0000
LUBIN2	169	f	0	0.98	18.69	10.75	0.0000
Subtotal LUBIN2				1.86	64.67	21.39	
LUO	4	c	0	-0.24	1.73	6.80	0.7498
MATOS	42	m	0	0.22	1.46	3.35	0.7878
MATSUD	4	m	0	3.06	0.95	1.65	0.0028
ORMOS	5	m	0	2.73	1.72	1.69	0.0003
OSANN2	10	f	0	1.57	4.03	0.12	0.0016
SOBUE	53	m	0	2.74	2.74	2.73	0.0000
SVENSS	27	f	0	2.08	2.93	0.33	0.0004
TSUGAN	15	m	0	1.61	0.36	0.01	0.3330
WAKAI	43	m	0	1.39	1.66	0.20	0.0727
WUWILL	20	f	0	1.02	51.60	26.81	0.0000
WYNDE2	3	m	0	1.53	2.39	0.11	0.0182
WYNDE3	4	m	0	1.59	1.96	0.05	0.0265
WYNDE3	63	f	0	-0.22	0.79	3.05	0.8428
Subtotal WYNDE3				1.07	2.75	3.10	
WYNDE4	5	m	0	0.90	4.60	3.27	0.0541
WYNDE4	49	f	2	-0.14	0.90	3.17	0.8951
Subtotal WYNDE4				0.73	5.50	6.45	
WYNDE6	21	m	0	2.57	17.35	11.94	0.0000
WYNDE6	210	f	0	1.98	16.03	0.94	0.0000
Subtotal WYNDE6				2.29	33.38	12.88	
ZHENG	1	m	0	1.41	2.33	0.25	0.0308
ZHENG	16	f	0	0.75	6.21	6.11	0.0620
Subtotal ZHENG				0.93	8.54	6.36	
ZHOU	10	c	0	0.39	3.46	6.32	0.4669

Table 2G12 - 5

IESLC - Meta-anal of Ever Smoking (or Current if ever not avail), Amount smoked, "Low", Any prod (or Cigs if Any not avail)
Squamous
Least adjusted

	N	41
	NS	30
	Wt	405.48
Het	Chi	181.21
Het	df	40
Het	P	***
Fixed	RR	5.71
	RRl	5.18
	RRu	6.29
	P	+++
Random	RR	4.50
	RRl	3.50
	RRu	5.78
	P	+++
Asymm	P	*

Table 2G12 - 6

IESLC - Meta-anal of Ever Smoking (or Current if ever not avail), Amount smoked, "Low", Any prod (or Cigs if Any not avail)
Squamous
Least adjusted

	Sex			
	combined	male	female	Total
N	3	23	15	41
NS	3	23	15	41
Wt	9.50	210.37	185.61	405.48
Het Chi	0.46	53.72	83.83	181.21
Het df	2	22	14	40
Het P	N.S.	***	***	***
Fixed RR	1.18	7.28	4.69	5.71
RRl	0.63	6.36	4.06	5.18
RRu	2.24	8.34	5.41	6.29
P	N.S.	+++	+++	+++
Random RR	1.18	6.17	3.82	4.50
RRl	0.63	4.63	2.52	3.50
RRu	2.24	8.23	5.79	5.78
P	N.S.	+++	+++	+++
Between Chi				43.20
Between df				2
Between P				***
Btwn(F) P				**
Btwn(R) P				***

Table 2G12 - 7

IESLC - Meta-anal of Ever Smoking (or Current if ever not avail), Amount smoked, "Low", Any prod (or Cigs if Any not avail)
Squamous
Excluded studies (and stage at which they were excluded)

1	ABELIN ABRAHA AMANDU AMES ANDERS AUSTIN AXELSO BAND BECHER BERRIN BLOHMK BLOT4 BROCKM BROWN1 BYERS1 BYERS2 CARPEN CASCO2 CASCOR CHAN CHEN3 CHIAZZ CHYOU DESTE2 DOCKER DROSTE DU GARCIA GARDIN GENG GODLEY GOODMA GRAHAM GREGOR HEGMAN HEIN HENNEK HINDS HIRAOK HOROWI HORWIT HUANG ISHIMA JAHN JAIN JARVHO JIANG KELLER KIHARA KJUUS KO KOHLME KUBIK LAMWK LAMWK2 LANGE LEI LEMARC LEVIN LIU LOMBA2 LOMBAR MAGNUS MARSH MARSH2 MCDUFF MCLAUG MILLER MILLS NOTANI NOU ODRISC PAWLEG PERSHA POFFIJ QIAO QIAO2 RADZIK REN RONCO ROOTS ROTHSC SAARIK SANKAR SCHWAR SEGI SEOW SHIMIZ SIMARA SIMONA SITAS SOBUE2 STASZE STAYNE STUCKE SUN SUZUK2 SUZUKI TANG TAO TOKARS TOUSEY ULMER VEIERO VUTUC WALD WANG WANG3 WANG4 WICKLU WIGLE WILKIN WU2 WUNSCH WYNDE8 XIANGZ XU XU2 XU4 YONG ZHANG
2	BUELL CHEN MASTRA MZILEN PISANI RESTRE SADOWS
4	BOFFET WYNDE7
5	RIMING TANG2 WYNDE5
6	BLOT1 BLOT2 BLOT3 BOUCHA HIRAY2 JONES LAURIL LICKIN MOLLO MRFIT MURATA SCHWA2 VANDER WARSIN WATSON WYNDER
8	AGUDO AKIBA ARCHER ARMADA AUVINE AXELSS BENSHL BEST BRESLO BRETT BROSS BUFFLE CEDERL CHANG CHATZI CHEN2 CHOW COMSTO COOKSO CPSI CPSII DAMBER DARBY DAVEYS DEAN DEAN2 DEAN3 DEKLER DESTEF DOLL2 DORANT DORN DUNN EBELIN ENSTRO ESAKI FAN GAO GAO2 GARSHI GILLIS GOLLED GSELL HAMMO2 HANSEN HIRAYA HITOSU HOLE HU HU2 HUMBLE JARUP JOLY JUSSAW KAISE2 KAISER KANELL KAUFMA KHUDER KINLEN KNEKT KOO KOULUM KREUZE LAUSSM LETOUR LIAW LIDDEL LIU2 LIU3 LIU4 LIU5 LUBIN MACLEN MARTIS MCCONN MIGRAN MRFITR NAM NOTAN2 PARKIN PASTOR PERNU PERSH2 PETO PEZZO2 PEZZOT PIKE POLEDN PRESCO RACHTA RANDIG SEGI2 SHAW SIEMIA SPEIZE SPITZ STOCKS STOCKW TENKAN TIZZAN TULINI TVERDA WANG2 XU3 YAMAGU YUAN
10	BOUCOT CORREA HAENSZ KATSOU OSANN WU
11	BENHAM

Table 2G12 - 8
Potentially overlapping studies

REF	REFGP	PRINC	.	OVERLAP/LINK
LUBIN2	LUBIN2	1		Lubin-combined
LAMTH	LAMTH	1		KOO/LAMTH/LAMWK
OSANN2	KAISER	2		KAISER/OSANN2
WYNDE6	WYNDE6	1		WYNDE5/6/7/8
MATSUD	MATSUD	1		SOBUE2/MATSUD

Table 2G12 - 9
Most adjusted - insufficient data for metaanalysis

REF	NRR	SEX	AGEL	AGEH	RACE	YF	LC TYPE	LOC	START	ST	NLC	R	VB	P	H	AD	SM	PRODUCT	exL	exH	DENOM	De
CHEN	5	c	0	0	all	-	q	As:oth	1987	CC	323	n	ot	n	y	2	ev	cig+/-ot	1	10	nev cigs ot	

REF	NRR	RR	SIG	RRDATA comment
CHEN	5	2.59	n	0

Table 2G13 -

IESLC - Meta-anal of Ever Smoking (or Current if ever not avail), Amount smoked, "Mid", Any prod (or Cigs if Any not avail)
Squamous

This analysis is restricted to results for:
1) Results by Amount smoked
2) Results complete enough for use in metaanalysis

Within each study, results are then selected (in the following order of preference, within each sex) for:
3) SMKSTA: ever smokers, current smokers
4) PRODUCT: all/unspec, cigarettes regardless of other products, cigarettes only
5) CIGTYPE: all/unspecified, MC regardless of HR, MC only
6) DENOM: never smoked anything, never smoked cigarettes, (never +1 = +long term ex, +2 = +amount unknown, +3 = never cigs+long term ex)
7) Followup period (YF, prospective studies): whole study (coded as 0) or longest available
8) LCtype: squamous or nearest available, but not adeno. (q = squamous, s = small, a = adeno, KI = Kreyberg I, u = undifferentiated)
9) Race: all or nearest available, otherwise by race (wh or w = white, bl or b = black, hi = hispanic ch = chinese, jap = japanese, haw = hawaiian, w+o = white + oriental, sca = scandinavian, as = asian)
10) Amount smoked "mid" in key scheme 1 (key value 20, maximum range 6-44, in numbers of cigarettes or cigarette equivalents)
11) For overlapping studies: principal rather than subsidiary studies
Finally by Age: whole study (coded as 0) if available, otherwise by widest available age group
and then for single sex results (m, f) in preference to combined sex results (c).

Results adjusted (AD) for the most potential confounders are then chosen in Sections -1 to -3
and results adjusted for the least confounders in Sections -4 to -6. (Those least adjusted results which
actually differ from the most adjusted as marked 'x' in column X in Section -4)
(Results adjusted for an unknown number of confounder(s) are coded as 20.)

Section -7 shows excluded studies, together with the stage (as above) at which no qualifying
results were found.

Section -8 lists the potentially overlapping studies which have been included (1=principal, 2=subsidiary).

Section -9 lists any results which would have been included in preference except that they had data not complete
enough for use in meta-analysis, with their significance (yes/no), if known, and any further comment as entered
on the database.

In addition to those mentioned above, the following fields, levels and abbreviations are used:
* or nk = not known, n = no, y = yes, ot = other
ev = ever, cu = current, nev = never
all/unspec = all or unspecified, cig+/-ot = cigarettes irrespective of other products (cigar, pipe etc)
MC = manufactured cigarettes, HR = hand-rolled cigarettes
exL, exH = range of exposure (low and high) in the smoking group, in terms of Amount smoked, cigarettes or cigarette equivalents
REF: 6-character study reference
NRR: number of the RR on the database within the study
ST : study type (CC = case control, pr or prosp = prospective)
NLC: number of lung cancer cases in whole study
R : risky occupational population (n = no, m = mining, o = other risky)
VB : national cigarette type (V = at least 75% Virginia, bl = at least 75% blended, ot = other)
P : any proxy use
H : full histological confirmation
De : derivation of RR/CI (or = original, st = standard method, ot = other method of estimation)

Table 2G13 - 1

IESLC - Meta-anal of Ever Smoking (or Current if ever not avail), Amount smoked, "Mid", Any prod (or Cigs if Any not avail)
Squamous
Most adjusted

REF	NRR	SEX	AGEL	AGEH	RACE	YF	LC TYPE	LOC	START	ST	NLC	R	VB	P	H	AD	SM	PRODUCT	exL	exH	DENOM	De
ALDERS	35	m	0	0	all	-	q+s	Eu:UK	1977	CC	1448	n	V	n	n	1	ev	cig only	18	27	nev+2	ot
ALDERS	38	f	0	0	all	-	q+s	Eu:UK	1977	CC	1448	n	V	n	n	1	ev	cig only	18	27	nev+2	ot
BARBON	71	m	0	0	all	-	q	Eu:wst	1979	CC	755	n	bl	y	y	3	ev	all/unsp	20	39	nev any	or
CHOI	47	m	0	0	all	-	q	As:oth	1985	CC	375	n	bl	n	n	0	ev	cig+/-ot	11	20	nev cigs	st
CHOI	57	f	0	0	all	-	q	As:oth	1985	CC	375	n	bl	n	n	0	ev	cig+/-ot	11	30	nev cigs	st
DOLL	70	m	0	0	all	-	KI	Eu:UK	1948	CC	1465	n	V	n	n	1	ev	all/unsp	15	24	nev any	ot
DOSEME	11	m	0	0	all	-	q	Eu:bal	1979	CC	1210	n	bl	n	n	2	ev	cig+/-ot	11	20	nev cigs	or
GER	15	c	0	0	all	-	q+s	As:oth	1990	CC	141	n	ot	y	n	10	ev	all/unsp	11	20	nev any	ot
HAMMON	99	m	0	0	wh	0	not a	NAmer	1952	pr	448	n	bl	n	n	1	cu	cig only	10	20	nev any	ot
JEDRYC	29	m	0	0	all	-	q	Eu:est	1980	CC	1630	n	bl	y	n	3	ev	cig+/-ot	20	29	nev any	or
KREYBE	2	m	0	0	all	-	KI	Eu:Sca	1948	CC	300	n	bl	n	y	1	ev	all/unsp	15	24	nev any	ot
LAMTH	11	f	0	0	ch	-	q	As:HK	1983	CC	445	n	bl	n	n	0	ev	all/unsp	11	20	nev any	or
LUBIN2	157	m	0	0	all	-	q	Eu:mul	1976	CC	7804	n	bl	n	y	0	ev	cig+/-ot	20	29	nev any	st
LUBIN2	177	f	0	0	all	-	q	Eu:mul	1976	CC	7804	n	bl	n	y	0	ev	cig+/-ot	20	29	nev any	st
LUO	11	c	0	0	all	-	q	As:Chi	1990	CC	102	n	ot	n	y	20	ev	cig+/-ot	20	29	nev cigs	or
MATOS	45	m	0	0	all	-	q	SCAmer	1994	CC	200	n	bl	n	n	2	ev	cig+/-ot	15	24	nev any	or
MATSUD	5	m	0	0	all	-	q	As:Jap	1965	CC	179	n	bl	n	n	0	ev	cig+/-ot	11	20	nev cigs	st
ORMOS	6	m	0	0	all	-	q	Eu:est	1947	CC	119	n	bl	y	y	0	ev	cig+/-ot	16	30	nev any	st
SOBUE	54	m	0	0	all	-	q	As:Jap	1986	CC	1376	n	bl	n	y	0	cu	cig+/-ot	20	29	nev cigs	st
SVENSS	12	f	0	0	all	-	q	Eu:Sca	1983	CC	210	n	bl	n	n	1	cu	all/unsp	11	20	nev any	or
TSUGAN	16	m	0	0	all	-	q	As:Jap	1976	CC	134	n	bl	n	y	0	cu	all/unsp	16	35	nev any	ot
WAKAI	47	m	0	0	all	-	q	As:Jap	1988	CC	333	n	bl	n	y	1	cu	cig+/-ot	20	29	nev any	or
WYNDE2	4	m	0	0	all	-	KI	NAmer	1962	CC	404	n	bl	n	y	0	ev	cig+/-ot	11	20	nev any	st
WYNDE3	5	m	0	0	all	-	KI	NAmer	1966	CC	350	n	bl	n	y	0	ev	cig+/-ot	10	20	nev any	st
WYNDE3	64	f	0	0	all	-	KI	NAmer	1966	CC	350	n	bl	n	y	0	ev	cig+/-ot	10	20	nev any	st
WYNDE4	65	m	0	0	all	-	not a	NAmer	1948	CC	684	n	bl	y	n	2	ev	all/unsp	16	20	nev any	ot
WYNDE4	51	f	0	0	all	-	not a	NAmer	1948	CC	684	n	bl	y	n	2	ev	all/unsp	16	20	nev any	ot
WYNDE6	30	m	0	0	all	-	KI	NAmer	1969	CC	4423	n	bl	n	y	0	cu	cig+/-ot	11	20	nev any	st
WYNDE6	219	f	0	0	all	-	KI	NAmer	1969	CC	4423	n	bl	n	y	0	cu	cig+/-ot	11	20	nev cigs	st
ZHENG	3	m	0	0	all	-	q	As:Chi	1982	CC	540	n	ot	*	y	0	ev	cig+/-ot	20	29	nev cigs	st

Cigarette type is all/unspec for all RRs

except for the following:

REF	NRR	CIGTYPE
ALDERS	35	MC only
ALDERS	38	MC only

Table 2G13 - 2

IESLC - Meta-anal of Ever Smoking (or Current if ever not avail), Amount smoked, "Mid", Any prod (or Cigs if Any not avail)
Squamous
Most adjusted

REF	NRR	SEX	AD	Number Exposed Case	Number Exposed Cont	Non-exposed Case	Non-exposed Cont	RR	95.00%CI
ALDERS	35	m	1	-	-	-	-	7.19	(2.75- 18.79)
ALDERS	38	f	1	-	-	-	-	9.24	(5.31- 16.09)
Subtotal ALDERS								8.68	(5.37- 14.03)
BARBON	71	m	3	-	-	-	-	16.30	(7.00- 38.00)
CHOI	47	m	0	84	281	6	95	4.73	(2.00- 11.19)
CHOI	57	f	0	5	9	10	164	9.11	(2.57- 32.31)
Subtotal CHOI								5.82	(2.86- 11.86)
DOLL	70	m	1	-	-	-	-	14.30	(4.45- 46.00)
DOSEME	11	m	2	-	-	-	-	3.20	(2.20- 4.60)
GER	15	c	10	-	-	-	-	2.20	(0.65- 7.48)
*HAMMON	99	m	1	-	-	-	-	17.44	(6.30- 48.29)
JEDRYC	29	m	3	-	-	-	-	13.46	(5.76- 31.47)
KREYBE	2	m	1	-	-	-	-	11.02	(3.42- 35.51)
LAMTH	11	f	0	28	6	28	72	12.00	(4.49- 32.10)
LUBIN2	157	m	0	1298	3108	54	2616	20.23	(15.33- 26.69)
LUBIN2	177	f	0	61	110	72	1180	9.09	(6.13- 13.46)
Subtotal LUBIN2								15.51	(12.37- 19.45)
LUO	11	c	20	-	-	-	-	24.60	(4.20- 145.70)
MATOS	45	m	2	-	-	-	-	7.80	(2.20- 27.40)
MATSUD	5	m	0	43	1607	1	1255	33.58	(4.62- 244.19)
ORMOS	6	m	0	10	577	2	777	6.73	(1.47- 30.85)
SOBUE	54	m	0	103	222	3	128	19.80	(6.15- 63.68)
SVENSS	12	f	1	-	-	-	-	36.20	(12.00- 168.90)
TSUGAN	16	m	0	7	7	0	5	11.00~	(0.51- 236.22)
WAKAI	47	m	1	-	-	-	-	10.40	(2.43- 44.30)
WYNDE2	4	m	0	108	203	3	105	18.62	(5.77- 60.06)
WYNDE3	5	m	0	57	114	3	88	14.67	(4.44- 48.40)
WYNDE3	64	f	0	13	24	5	76	8.23	(2.66- 25.46)
Subtotal WYNDE3								10.81	(4.76- 24.56)
WYNDE4	65	m	2	-	-	-	-	10.92	(5.22- 22.86)
WYNDE4	51	f	2	-	-	-	-	14.92	(4.88- 45.67)
Subtotal WYNDE4								12.01	(6.48- 22.23)
WYNDE6	30	m	0	270	293	29	617	19.61	(13.04- 29.47)
WYNDE6	219	f	0	191	165	40	856	24.77	(16.95- 36.20)
Subtotal WYNDE6								22.22	(16.83- 29.33)
ZHENG	3	m	0	75	89	4	94	19.80	(6.95- 56.41)
Partial Totals				2353	6815	260	8128		

*prospective study ~ With 0.5 adjustment for zero

REF	NRR	SEX	AD	Ys	Ws	Qs	Ps
ALDERS	35	m	1	1.97	4.16	1.22	0.0001
ALDERS	38	f	1	2.22	12.50	1.06	0.0000
Subtotal ALDERS				2.16	16.66	2.28	
BARBON	71	m	3	2.79	5.37	0.41	0.0000
CHOI	47	m	0	1.55	5.19	4.78	0.0004
CHOI	57	f	0	2.21	2.40	0.22	0.0006
Subtotal CHOI				1.76	7.59	5.00	
DOLL	70	m	1	2.66	2.82	0.06	0.0000
DOSEME	11	m	2	1.16	28.24	51.57	0.0000
GER	15	c	10	0.79	2.57	7.67	0.2058
*HAMMON	99	m	1	2.86	3.70	0.44	0.0000
JEDRYC	29	m	3	2.60	5.33	0.04	0.00060
KREYBE	2	m	1	2.40	2.81	0.04	0.0001
LAMTH	11	f	0	2.48	3.97	0.00	0.0000
LUBIN2	157	m	0	3.01	50.02	12.15	0.0000
LUBIN2	177	f	0	2.21	24.86	2.35	0.0000
Subtotal LUBIN2				2.74	74.88	14.50	
LUO	11	c	20	3.20	1.22	0.58	0.0004
MATOS	45	m	2	2.05	2.42	0.51	0.0014
MATSUD	5	m	0	3.51	0.98	0.98	0.0005
ORMOS	6	m	0	1.91	1.66	0.61	0.0141
SOBUE	54	m	0	2.99	2.81	0.62	0.0000
SVENSS	12	f	1	3.59	2.20	2.54	0.0000
TSUGAN	16	m	0	2.40	0.41	0.01	0.1254
WAKAI	47	m	1	2.34	1.82	0.05	0.0016
WYNDE2	4	m	0	2.92	2.80	0.47	0.0000
WYNDE3	5	m	0	2.69	2.70	0.08	0.0000
WYNDE3	64	f	0	2.11	3.01	0.50	0.0003
Subtotal WYNDE3				2.38	5.71	0.58	
WYNDE4	65	m	2	2.39	7.04	0.11	0.0000

Table 2G13 - 2

IESLC - Meta-anal of Ever Smoking (or Current if ever not avail), Amount smoked, "Mid", Any prod (or Cigs if Any not avail)
Squamous
Most adjusted

REF	NRR	SEX	AD	Ys	Ws	Qs	Ps
WYNDE4	51	f	2	2.70	3.07	0.11	0.0000
Subtotal WYNDE4				2.49	10.12	0.22	
WYNDE6	30	m	0	2.98	23.14	4.93	0.0000
WYNDE6	219	f	0	3.21	26.69	12.91	0.0000
Subtotal WYNDE6				3.10	49.83	17.83	
ZHENG	3	m	0	2.99	3.51	0.78	0.0000

	N	30
	NS	24
	Wt	239.42
Het	Chi	107.78
Het	df	29
Het	P	***
Fixed	RR	12.36
	RRl	10.89
	RRu	14.03
	P	+++
Random	RR	11.86
	RRl	8.92
	RRu	15.76
	P	+++
Asymm	P	N.S.

Table 2G13 - 3

IESLC - Meta-anal of Ever Smoking (or Current if ever not avail), Amount smoked, "Mid", Any prod (or Cigs if Any not avail)
Squamous
Most adjusted

	Sex			
	combined	male	female	Total
N	2	20	8	30
NS	2	20	8	30
Wt	3.80	156.92	78.71	239.42
Het Chi	4.83	79.72	18.84	107.78
Het df	1	19	7	29
Het P	*	***	**	***
Fixed RR	4.78	12.01	13.71	12.36
RRl	1.75	10.27	10.99	10.89
RRu	13.08	14.04	17.10	14.03
P	++	+++	+++	+++
Random RR	6.73	11.96	13.14	11.86
RRl	0.64	8.18	8.57	8.92
RRu	71.24	17.49	20.13	15.76
P	N.S.	+++	+++	+++
Between Chi				4.39
Between df				2
Between P				N.S.
Btwn(F) P				N.S.
Btwn(R) P				N.S.

	Lung cancer type					
	q	q+s	q+u	KI	not a	Total
N	17	3		7	3	30
NS	15	2		5	2	24
Wt	142.40	19.24		63.96	13.82	239.42
Het Chi	75.29	4.40		5.18	0.59	107.78
Het df	16	2		6	2	29
Het P	***	N.S.		N.S.	N.S.	***
Fixed RR	10.71	7.22		19.67	13.27	12.36
RRl	9.09	4.62		15.39	7.83	10.89
RRu	12.62	11.29		25.13	22.48	14.03
P	+++	+++		+++	+++	+++
Random RR	11.69	6.11		19.67	13.27	11.86
RRl	7.55	2.87		15.39	7.83	8.92
RRu	18.11	12.99		25.13	22.48	15.76
P	+++	+++		+++	+++	+++
Between Chi						22.33
Between df						3
Between P						***
Btwn(F) P						N.S.
Btwn(R) P						**

	Location								
	NAmer	UK	Scand	othEur	China	Japan	othAs	other	Total
N	8	3	2	6	2	4	4	1	30
NS	5	2	2	5	2	4	3	1	24
Wt	72.16	19.48	5.00	115.48	4.73	6.02	14.13	2.42	239.42
Het Chi	6.56	0.80	1.74	63.59	0.04	1.04	5.21	0.00	107.78
Het df	7	2	1	5	1	3	3	0	29
Het P	N.S.	N.S.	N.S.	***	N.S.	N.S.	N.S.	N.S.	***
Fixed RR	18.89	9.33	18.58	10.37	20.94	17.05	5.97	7.80	12.36
RRl	15.00	5.98	7.74	8.64	8.50	7.67	3.55	2.21	10.89
RRu	23.79	14.54	44.63	12.45	51.59	37.91	10.06	27.53	14.03
P	+++	+++	+++	+++	+++	+++	+++	++	+++
Random RR	18.89	9.33	19.16	9.84	20.94	17.05	5.92	7.80	11.86
RRl	15.00	5.98	5.99	4.67	8.50	7.67	2.94	2.21	8.92
RRu	23.79	14.54	61.29	20.71	51.59	37.91	11.94	27.53	15.76
P	+++	+++	+++	+++	+++	+++	+++	++	+++
Between Chi									28.81
Between df									7
Between P									***
Btwn(F) P									N.S.
Btwn(R) P									*

Table 2G13 - 3

IESLC - Meta-anal of Ever Smoking (or Current if ever not avail), Amount smoked, "Mid", Any prod (or Cigs if Any not avail)
Squamous
Most adjusted

	Detailed Country in "other Europe"					
	multi	Germany	othWest	East	Balkans	Total
N	2		1	2	1	6
NS	1		1	2	1	5
Wt	74.88		5.37	6.99	28.24	115.48
Het Chi	10.64		0.00	0.61	0.00	63.59
Het df	1		0	1	0	5
Het P	**		N.S.	N.S.	N.S.	***
Fixed RR	15.51		16.30	11.42	3.20	10.37
RRl	12.37		7.00	5.44	2.21	8.64
RRu	19.45		37.98	23.97	4.63	12.45
P	+++		+++	+++	+++	+++
Random RR	13.73		16.30	11.42	3.20	9.84
RRl	6.27		7.00	5.44	2.21	4.67
RRu	30.07		37.98	23.97	4.63	20.71
P	+++		+++	+++	+++	+++
Between Chi						52.35
Between df						3
Between P						***
Btwn(F) P						N.S.
Btwn(R) P						***

	Detailed Country in "other Asia"			
	India	HongKong	other	Total
N		1	3	4
NS		1	2	3
Wt		3.97	10.16	14.13
Het Chi		0.00	2.52	5.21
Het df		0	2	3
Het P		N.S.	N.S.	N.S.
Fixed RR		12.00	4.55	5.97
RRl		4.49	2.46	3.55
RRu		32.10	8.41	10.06
P		+++	+++	+++
Random RR		12.00	4.54	5.92
RRl		4.49	2.25	2.94
RRu		32.10	9.19	11.94
P		+++	+++	+++
Between Chi				2.69
Between df				1
Between P				N.S.
Btwn(F) P				N.S.
Btwn(R) P				N.S.

	Detailed other continent			
	SCAmer	Auslia	Africa	Total
N	1			1
NS	1			1
Wt	2.42			2.42
Het Chi	0.00			0.00
Het df	0			0
Het P	N.S.			N.S.
Fixed RR	7.80			7.80
RRl	2.21			2.21
RRu	27.53			27.53
P	++			++
Random RR	7.80			7.80
RRl	2.21			2.21
RRu	27.53			27.53
P	++			++
Between Chi				
Between df				
Between P				N.S.
Btwn(F) P				N.S.
Btwn(R) P				N.S.

Table 2G13 - 3

IESLC - Meta-anal of Ever Smoking (or Current if ever not avail), Amount smoked, "Mid", Any prod (or Cigs if Any not avail)

Squamous

Most adjusted

	Start year of study <1960	1960-69	1970-79	1980-89	1990+	Total
N	6	6	7	8	3	30
NS	5	4	5	7	3	24
Wt	21.10	59.32	125.56	27.23	6.21	239.42
Het Chi	1.36	4.07	63.54	9.04	5.18	107.78
Het df	5	5	6	7	2	29
Het P	N.S.	N.S.	***	N.S.	(*)	***
Fixed RR	12.40	20.70	10.08	12.20	5.79	12.36
RRl	8.09	16.05	8.46	8.38	2.64	10.89
RRu	18.99	26.70	12.01	17.76	12.70	14.03
P	+++	+++	+++	+++	+++	+++
Random RR	12.40	20.70	9.29	12.50	6.68	11.86
RRl	8.09	16.05	4.79	8.11	1.82	8.92
RRu	18.99	26.70	18.02	19.27	24.46	15.76
P	+++	+++	+++	+++	++	+++
Between Chi						24.59
Between df						4
Between P						***
Btwn(F) P						N.S.
Btwn(R) P						*

	Study type (1) CC	other	Total
N	29	1	30
NS	23	1	24
Wt	235.72	3.70	239.42
Het Chi	107.34	0.00	107.78
Het df	28	0	29
Het P	***	N.S.	***
Fixed RR	12.29	17.44	12.36
RRl	10.82	6.30	10.89
RRu	13.97	48.28	14.03
P	+++	+++	+++
Random RR	11.70	17.44	11.86
RRl	8.74	6.30	8.92
RRu	15.67	48.28	15.76
P	+++	+++	+++
Between Chi			0.45
Between df			1
Between P			N.S.
Btwn(F) P			N.S.
Btwn(R) P			N.S.

	Study type (2) CC	prosp	other	Total
N	29	1		30
NS	23	1		24
Wt	235.72	3.70		239.42
Het Chi	107.34	0.00		107.78
Het df	28	0		29
Het P	***	N.S.		***
Fixed RR	12.29	17.44		12.36
RRl	10.82	6.30		10.89
RRu	13.97	48.28		14.03
P	+++	+++		+++
Random RR	11.70	17.44		11.86
RRl	8.74	6.30		8.92
RRu	15.67	48.28		15.76
P	+++	+++		+++
Between Chi				0.45
Between df				1
Between P				N.S.
Btwn(F) P				N.S.
Btwn(R) P				N.S.

Table 2G13 - 3

IESLC - Meta-anal of Ever Smoking (or Current if ever not avail), Amount smoked, "Mid", Any prod (or Cigs if Any not avail)

Squamous
Most adjusted

	Study size (number of LC cases)				
	100-249	250-499	500-999	1000+	Total
N	7	9	4	10	30
NS	7	7	3	7	24
Wt	11.45	28.40	18.99	180.58	239.42
Het Chi	12.37	5.77	0.98	86.79	107.78
Het df	6	8	3	9	29
Het P	(*)	N.S.	N.S.	***	***
Fixed RR	9.99	10.44	14.36	12.66	12.36
RRl	5.60	7.22	9.16	10.94	10.89
RRu	17.84	15.07	22.51	14.65	14.03
P	+++	+++	+++	+++	+++
Random RR	11.08	10.44	14.36	12.07	11.86
RRl	4.66	7.22	9.16	7.37	8.92
RRu	26.35	15.07	22.51	19.76	15.76
P	+++	+++	+++	+++	+++
Between Chi					1.86
Between df					3
Between P					N.S.
Btwn(F) P					N.S.
Btwn(R) P					N.S.

	Risky occupational population			
	no	mining	othRisky	Total
N	30			30
NS	24			24
Wt	239.42			239.42
Het Chi	107.78			107.78
Het df	29			29
Het P	***			***
Fixed RR	12.36			12.36
RRl	10.89			10.89
RRu	14.03			14.03
P	+++			+++
Random RR	11.86			11.86
RRl	8.92			8.92
RRu	15.76			15.76
P	+++			+++
Between Chi				
Between df				
Between P				N.S.
Btwn(F) P				N.S.
Btwn(R) P				N.S.

	National cigarette tobacco type			
	Virginia	blended	other	Total
N	3	24	3	30
NS	2	19	3	24
Wt	19.48	212.64	7.30	239.42
Het Chi	0.80	96.16	8.51	107.78
Het df	2	23	2	29
Het P	N.S.	***	*	***
Fixed RR	9.33	12.80	9.46	12.36
RRl	5.98	11.19	4.58	10.89
RRu	14.54	14.64	19.54	14.03
P	+++	+++	+++	+++
Random RR	9.33	12.48	9.79	11.86
RRl	5.98	9.04	2.04	8.92
RRu	14.54	17.25	46.87	15.76
P	+++	+++	++	+++
Between Chi				2.32
Between df				2
Between P				N.S.
Btwn(F) P				N.S.
Btwn(R) P				N.S.

Table 2G13 - 3

IESLC - Meta-anal of Ever Smoking (or Current if ever not avail), Amount smoked, "Mid", Any prod (or Cigs if Any not avail)

Squamous

Most adjusted

	Any proxy use		
	No/nk	Yes	Total
N	24	6	30
NS	19	5	24
Wt	214.37	25.05	239.42
Het Chi	98.77	8.37	107.78
Het df	23	5	29
Het P	***	N.S.	***
Fixed RR	12.58	10.62	12.36
RRl	11.00	7.18	10.89
RRu	14.38	15.71	14.03
P	+++	+++	+++
Random RR	12.47	10.05	11.86
RRl	8.99	5.95	8.92
RRu	17.31	16.98	15.76
P	+++	+++	+++
Between Chi			0.64
Between df			1
Between P			N.S.
Btwn(F) P			N.S.
Btwn(R) P			N.S.

	Full histological confirmation		
	No	Yes	Total
N	15	15	30
NS	12	12	24
Wt	86.59	152.83	239.42
Het Chi	42.14	19.96	107.78
Het df	14	14	29
Het P	***	N.S.	***
Fixed RR	6.92	17.17	12.36
RRl	5.60	14.65	10.89
RRu	8.54	20.12	14.03
P	+++	+++	+++
Random RR	9.24	16.31	11.86
RRl	6.14	12.97	8.92
RRu	13.89	20.52	15.76
P	+++	+++	+++
Between Chi			45.68
Between df			1
Between P			***
Btwn(F) P			***
Btwn(R) P			*

	Number of adjustment variables (1)			
	0	1	2+/+nk	Total
N	15	7	8	30
NS	11	6	7	24
Wt	154.14	30.01	55.27	239.42
Het Chi	28.99	5.20	30.24	107.78
Het df	14	6	7	29
Het P	*	N.S.	***	***
Fixed RR	16.41	11.38	5.86	12.36
RRl	14.01	7.96	4.50	10.89
RRu	19.22	16.27	7.63	14.03
P	+++	+++	+++	+++
Random RR	14.51	11.38	8.61	11.86
RRl	10.91	7.96	4.51	8.92
RRu	19.30	16.27	16.43	15.76
P	+++	+++	+++	+++
Between Chi				43.35
Between df				2
Between P				***
Btwn(F) P				***
Btwn(R) P				N.S.

Table 2G13 - 3

IESLC - Meta-anal of Ever Smoking (or Current if ever not avail), Amount smoked, "Mid", Any prod (or Cigs if Any not avail)

Squamous

Most adjusted

	Number of adjustment variables (2) 0	1	2	3-5	6+/+nk	Total
N	15	7	4	2	2	30
NS	11	6	3	2	2	24
Wt	154.14	30.01	40.78	10.70	3.80	239.42
Het Chi	28.99	5.20	13.90	0.10	4.83	107.78
Het df	14	6	3	1	1	29
Het P	*	N.S.	**	N.S.	*	***
Fixed RR	16.41	11.38	4.68	14.82	4.78	12.36
RRl	14.01	7.96	3.45	8.14	1.75	10.89
RRu	19.22	16.27	6.37	26.98	13.08	14.03
P	+++	+++	+++	+++	++	+++
Random RR	14.51	11.38	7.39	14.82	6.73	11.86
RRl	10.91	7.96	3.15	8.14	0.64	8.92
RRu	19.30	16.27	17.31	26.98	71.24	15.76
P	+++	+++	+++	+++	N.S.	+++
Between Chi						54.76
Between df						4
Between P						***
Btwn(F) P						**
Btwn(R) P						N.S.

	Product all/unsp	cig+/-ot	cig only	Total
N	9	18	3	30
NS	8	14	2	24
Wt	30.26	188.79	20.37	239.42
Het Chi	10.91	93.96	1.67	107.78
Het df	8	17	2	29
Het P	N.S.	***	N.S.	***
Fixed RR	11.97	12.73	9.85	12.36
RRl	8.38	11.04	6.38	10.89
RRu	17.10	14.68	15.21	14.03
P	+++	+++	+++	+++
Random RR	11.92	12.20	9.85	11.86
RRl	7.75	8.20	6.38	8.92
RRu	18.34	18.14	15.21	15.76
P	+++	+++	+++	+++
Between Chi				1.24
Between df				2
Between P				N.S.
Btwn(F) P				N.S.
Btwn(R) P				N.S.

	Denominator nev any	nev cigs	Total
N	22	8	30
NS	18	7	25
Wt	168.38	71.04	239.42
Het Chi	32.85	66.51	107.78
Het df	21	7	29
Het P	*	***	***
Fixed RR	13.96	9.26	12.36
RRl	12.00	7.34	10.89
RRu	16.24	11.68	14.03
P	+++	+++	+++
Random RR	12.57	12.37	11.86
RRl	10.04	5.22	8.92
RRu	15.73	29.30	15.76
P	+++	+++	+++
Between Chi			8.43
Between df			1
Between P			**
Btwn(F) P			N.S.
Btwn(R) P			N.S.

Table 2G13 - 3

IESLC - Meta-anal of Ever Smoking (or Current if ever not avail), Amount smoked, "Mid", Any prod (or Cigs if Any not avail)
Squamous
Most adjusted

	Derivation of RR/CI			
	Orig	StdCalc	Other	Total
N	8	13	9	30
NS	8	9	7	24
Wt	50.57	149.76	39.09	239.42
Het Chi	32.08	28.53	8.49	107.78
Het df	7	12	8	29
Het P	***	**	N.S.	***
Fixed RR	6.24	16.56	9.74	12.36
RRl	4.74	14.11	7.12	10.89
RRu	8.22	19.44	13.33	14.03
P	+++	+++	+++	+++
Random RR	11.48	14.63	9.74	11.86
RRl	5.59	10.76	7.01	8.92
RRu	23.55	19.89	13.54	15.76
P	+++	+++	+++	+++
Between Chi				38.69
Between df				2
Between P				***
Btwn(F) P				**
Btwn(R) P				N.S.

	Smoking status		
	ever	current	Total
N	23	7	30
NS	18	6	24
Wt	178.64	60.78	239.42
Het Chi	79.88	2.65	107.78
Het df	22	6	29
Het P	***	N.S.	***
Fixed RR	10.23	21.57	12.36
RRl	8.83	16.77	10.89
RRu	11.84	27.73	14.03
P	+++	+++	+++
Random RR	10.20	21.57	11.86
RRl	7.37	16.77	8.92
RRu	14.13	27.73	15.76
P	+++	+++	+++
Between Chi			25.25
Between df			1
Between P			***
Btwn(F) P			**
Btwn(R) P			***

Table 2G13 - 4

IESLC - Meta-anal of Ever Smoking (or Current if ever not avail), Amount smoked, "Mid", Any prod (or Cigs if Any not avail)
Squamous
Least adjusted

REF	NRR	X	SEX	AGEL	AGEH	RACE	YF	LC TYPE	LOC	START	ST	NLC	R	VB	P	H	AD	SM	PRODUCT	exL	exH	DENOM	De
ALDERS	35		m	0	0	all	-	q+s	Eu:UK	1977	CC	1448	n	V	n	n	1	ev	cig only	18	27	nev+2	ot
ALDERS	38		f	0	0	all	-	q+s	Eu:UK	1977	CC	1448	n	V	n	n	1	ev	cig only	18	27	nev+2	ot
BARBON	23	x	m	0	0	all	-	q	Eu:wst	1979	CC	755	n	bl	y	y	0	ev	all/unsp	20	29	nev any	st
CHOI	47		m	0	0	all	-	q	As:oth	1985	CC	375	n	bl	n	n	0	ev	cig+/-ot	11	20	nev cigs	st
CHOI	57		f	0	0	all	-	q	As:oth	1985	CC	375	n	bl	n	n	0	ev	cig+/-ot	11	30	nev cigs	st
DOLL	56	x	m	0	0	all	-	KI	Eu:UK	1948	CC	1465	n	V	n	n	0	ev	all/unsp	15	24	nev any	st
DOSEME	11		m	0	0	all	-	q	Eu:bal	1979	CC	1210	n	bl	n	n	2	ev	cig+/-ot	11	20	nev cigs	or
GER	7	x	c	0	0	all	-	q+s	As:oth	1990	CC	141	n	ot	y	n	0	ev	all/unsp	11	20	nev any	st
HAMMON	99		m	0	0	wh	0	not a	NAmer	1952	pr	448	n	bl	n	n	1	cu	cig only	10	20	nev any	ot
JEDRYC	3	x	m	0	0	all	-	q	Eu:est	1980	CC	1630	n	bl	y	n	0	ev	cig+/-ot	20	29	nev any	st
KREYBE	14	x	m	0	0	all	-	KI	Eu:Sca	1948	CC	300	n	bl	n	y	0	ev	all/unsp	15	24	nev any	st
LAMTH	11		f	0	0	ch	-	q	As:HK	1983	CC	445	n	bl	n	n	0	ev	all/unsp	11	20	nev any	or
LUBIN2	157		m	0	0	all	-	q	Eu:mul	1976	CC	7804	n	bl	n	y	0	ev	cig+/-ot	20	29	nev any	st
LUBIN2	177		f	0	0	all	-	q	Eu:mul	1976	CC	7804	n	bl	n	y	0	ev	cig+/-ot	20	29	nev any	st
LUO	5	x	c	0	0	all	-	q	As:Chi	1990	CC	102	n	ot	n	y	0	ev	cig+/-ot	20	29	nev cigs	st
MATOS	44	x	m	0	0	all	-	q	SCAmer	1994	CC	200	n	bl	n	n	0	ev	cig+/-ot	15	24	nev any	st
MATSUD	5		m	0	0	all	-	q	As:Jap	1965	CC	179	n	bl	n	n	0	ev	cig+/-ot	11	20	nev cigs	st
ORMOS	6		m	0	0	all	-	q	Eu:est	1947	CC	119	n	bl	y	y	0	ev	cig+/-ot	16	30	nev any	st
SOBUE	54		m	0	0	all	-	q	As:Jap	1986	CC	1376	n	bl	n	y	0	cu	cig+/-ot	20	29	nev cigs	st
SVENSS	32	x	f	0	0	all	-	q	Eu:Sca	1983	CC	210	n	bl	n	n	0	cu	all/unsp	11	20	nev any	st
TSUGAN	16		m	0	0	all	-	q	As:Jap	1976	CC	134	n	bl	n	y	0	cu	all/unsp	16	35	nev any	ot
WAKAI	44	x	m	0	0	all	-	q	As:Jap	1988	CC	333	n	bl	n	y	0	cu	cig+/-ot	20	29	nev any	st
WYNDE2	4		m	0	0	all	-	KI	NAmer	1962	CC	404	n	bl	n	y	0	ev	cig+/-ot	11	20	nev any	st
WYNDE3	5		m	0	0	all	-	KI	NAmer	1966	CC	350	n	bl	n	y	0	ev	cig+/-ot	10	20	nev any	st
WYNDE3	64		f	0	0	all	-	KI	NAmer	1966	CC	350	n	bl	n	y	0	ev	cig+/-ot	10	20	nev any	st
WYNDE4	17	x	m	0	0	all	-	not a	NAmer	1948	CC	684	n	bl	y	n	0	ev	all/unsp	16	20	nev any	st
WYNDE4	51		f	0	0	all	-	not a	NAmer	1948	CC	684	n	bl	y	n	2	ev	all/unsp	16	20	nev any	ot
WYNDE6	30		m	0	0	all	-	KI	NAmer	1969	CC	4423	n	bl	n	y	0	cu	cig+/-ot	11	20	nev any	st
WYNDE6	219		f	0	0	all	-	KI	NAmer	1969	CC	4423	n	bl	n	y	0	cu	cig+/-ot	11	20	nev cigs	st
ZHENG	3		m	0	0	all	-	q	As:Chi	1982	CC	540	n	ot	*	y	0	ev	cig+/-ot	20	29	nev cigs	st

Cigarette type is all/unspec for all RRs

except for the following:

REF	NRR	CIGTYPE
ALDERS	35	MC only
ALDERS	38	MC only

Table 2G13 - 5

IESLC - Meta-anal of Ever Smoking (or Current if ever not avail), Amount smoked, "Mid", Any prod (or Cigs if Any not avail)
Squamous
Least adjusted

REF	NRR	SEX	AD	Number Exposed Case	Cont	Non-exposed Case	Cont	RR	95.00%CI
ALDERS	35	m	1	-	-	-	-	7.19	(2.75- 18.79)
ALDERS	38	f	1	-	-	-	-	9.24	(5.31- 16.09)
Subtotal ALDERS								8.68	(5.37- 14.03)
BARBON	23	m	0	81	176	6	188	14.42	(6.14- 33.89)
CHOI	47	m	0	84	281	6	95	4.73	(2.00- 11.19)
CHOI	57	f	0	5	9	10	164	9.11	(2.57- 32.31)
Subtotal CHOI								5.82	(2.86- 11.86)
DOLL	56	m	0	301	431	3	61	14.20	(4.41- 45.68)
DOSEME	11	m	2	-	-	-	-	3.20	(2.20- 4.60)
GER	7	c	0	20	87	11	80	1.67	(0.75- 3.71)
*HAMMON	99	m	1	-	-	-	-	17.44	(6.30- 48.29)
JEDRYC	3	m	0	152	434	6	289	16.87	(7.36- 38.66)
KREYBE	14	m	0	49	925	3	644	11.37	(3.53- 36.64)
LAMTH	11	f	0	28	6	28	72	12.00	(4.49- 32.10)
LUBIN2	157	m	0	1298	3108	54	2616	20.23	(15.33- 26.69)
LUBIN2	177	f	0	61	110	72	1180	9.09	(6.13- 13.46)
Subtotal LUBIN2								15.51	(12.37- 19.45)
LUO	5	c	0	22	23	5	51	9.76	(3.28- 28.98)
MATOS	44	m	0	18	90	3	110	7.33	(2.09- 25.69)
MATSUD	5	m	0	43	1607	1	1255	33.58	(4.62- 244.19)
ORMOS	6	m	0	10	577	2	777	6.73	(1.47- 30.85)
SOBUE	54	m	0	103	222	3	128	19.80	(6.15- 63.68)
SVENSS	32	f	0	28	22	5	120	30.55	(10.64- 87.69)
TSUGAN	16	m	0	7	7	0	5	11.00~	(0.51- 236.22)
WAKAI	44	m	0	40	129	2	65	10.08	(2.36- 43.01)
WYNDE2	4	m	0	108	203	3	105	18.62	(5.77- 60.06)
WYNDE3	5	m	0	57	114	3	88	14.67	(4.44- 48.40)
WYNDE3	64	f	0	13	24	5	76	8.23	(2.66- 25.46)
Subtotal WYNDE3								10.81	(4.76- 24.56)
WYNDE4	17	m	0	213	274	8	115	11.17	(5.34- 23.39)
WYNDE4	51	f	2	-	-	-	-	14.92	(4.88- 45.67)
Subtotal WYNDE4								12.20	(6.59- 22.60)
WYNDE6	30	m	0	270	293	29	617	19.61	(13.04- 29.47)
WYNDE6	219	f	0	191	165	40	856	24.77	(16.95- 36.20)
Subtotal WYNDE6								22.22	(16.83- 29.33)
ZHENG	3	m	0	75	89	4	94	19.80	(6.95- 56.41)
Partial Totals				3277	9406	312	9851		

*prospective study ~ With 0.5 adjustment for zero

REF	NRR	SEX	AD	Ys	Ws	Qs	Ps
ALDERS	35	m	1	1.97	4.16	1.08	0.0001
ALDERS	38	f	1	2.22	12.50	0.84	0.0000
Subtotal ALDERS				2.16	16.66	1.92	
BARBON	23	m	0	2.67	5.26	0.18	0.0000
CHOI	47	m	0	1.55	5.19	4.47	0.0004
CHOI	57	f	0	2.21	2.40	0.18	0.0006
Subtotal CHOI				1.76	7.59	4.65	
DOLL	56	m	0	2.65	2.81	0.08	0.0000
DOSEME	11	m	2	1.16	28.24	49.14	0.0000
GER	7	c	0	0.51	6.06	23.49	0.2056
*HAMMON	99	m	1	2.86	3.70	0.53	0.0000
JEDRYC	3	m	0	2.83	5.59	0.66	0.0000
KREYBE	14	m	0	2.43	2.81	0.01	0.0000
LAMTH	11	f	0	2.48	3.97	0.00	0.0000
LUBIN2	157	m	0	3.01	50.02	13.79	0.0000
LUBIN2	177	f	0	2.21	24.86	1.88	0.0000
Subtotal LUBIN2				2.74	74.88	15.67	
LUO	5	c	0	2.28	3.24	0.14	0.0000
MATOS	44	m	0	1.99	2.44	0.59	0.0018
MATSUD	5	m	0	3.51	0.98	1.04	0.0005
ORMOS	6	m	0	1.91	1.66	0.55	0.0141
SOBUE	54	m	0	2.99	2.81	0.71	0.0000
SVENSS	32	f	0	3.42	3.45	3.03	0.0000
TSUGAN	16	m	0	2.40	0.41	0.00	0.1254
WAKAI	44	m	0	2.31	1.82	0.05	0.0018
WYNDE2	4	m	0	2.92	2.80	0.55	0.0000
WYNDE3	5	m	0	2.69	2.70	0.11	0.0000
WYNDE3	64	f	0	2.11	3.01	0.42	0.0003
Subtotal WYNDE3				2.38	5.71	0.53	
WYNDE4	17	m	0	2.41	7.04	0.03	0.0000

Table 2G13 - 5

IESLC - Meta-anal of Ever Smoking (or Current if ever not avail), Amount smoked, "Mid", Any prod (or Cigs if Any not avail)
Squamous
Least adjusted

REF	NRR	SEX	AD	Ys	Ws	Qs	Ps
WYNDE4	51	f	2	2.70	3.07	0.15	0.0000
Subtotal WYNDE4				2.50	10.11	0.18	
WYNDE6	30	m	0	2.98	23.14	5.64	0.0000
WYNDE6	219	f	0	3.21	26.69	14.13	0.0000
Subtotal WYNDE6				3.10	49.83	19.76	
ZHENG	3	m	0	2.99	3.51	0.89	0.0000

	N	30
	NS	24
	Wt	246.36
Het	Chi	124.35
Het	df	29
Het	P	***
Fixed	RR	11.97
	RRl	10.56
	RRu	13.56
	P	+++
Random	RR	11.37
	RRl	8.46
	RRu	15.27
	P	+++
Asymm	P	N.S.

Table 2G13 - 6

IESLC - Meta-anal of Ever Smoking (or Current if ever not avail), Amount smoked, "Mid", Any prod (or Cigs if Any not avail)
Squamous
Least adjusted

	combined	Sex male	female	Total
N	2	20	8	30
NS	2	20	8	30
Wt	9.31	157.09	79.96	246.36
Het Chi	6.57	80.09	18.98	124.35
Het df	1	19	7	29
Het P	*	***	**	***
Fixed RR	3.09	12.05	13.82	11.97
RRl	1.63	10.31	11.10	10.56
RRu	5.88	14.09	17.20	13.56
P	+++	+++	+++	+++
Random RR	3.88	12.02	13.24	11.37
RRl	0.69	8.21	8.70	8.46
RRu	21.80	17.58	20.16	15.27
P	N.S.	+++	+++	+++
Between Chi				18.71
Between df				2
Between P				***
Btwn(F) P				N.S.
Btwn(R) P				N.S.

Table 2G13 - 7

IESLC - Meta-anal of Ever Smoking (or Current if ever not avail), Amount smoked, "Mid", Any prod (or Cigs if Any not avail)
Squamous
Excluded studies (and stage at which they were excluded)

1 ABELIN ABRAHA AMANDU AMES ANDERS AUSTIN AXELSO BAND BECHER BERRIN BLOHMK BLOT4 BROCKM BROWN1 BYERS1 BYERS2
CARPEN CASCO2 CASCOR CHAN CHEN3 CHIAZZ CHYOU DESTE2 DOCKER DROSTE DU GARCIA GARDIN GENG GODLEY GOODMA
GRAHAM GREGOR HEGMAN HEIN HENNEK HINDS HIRAOK HOROWI HORWIT HUANG ISHIMA JAHN JAIN JARVHO JIANG KELLER
KIHARA KJUUS KO KOHLME KUBIK LAMWK LAMWK2 LANGE LEI LEMARC LEVIN LIU LOMBA2 LOMBAR MAGNUS MARSH
MARSH2 MCDUFF MCLAUG MILLER MILLS NOTANI NOU ODRISC PAWLEG PERSHA POFFIJ QIAO QIAO2 RADZIK REN RONCO
ROOTS ROTHSC SAARIK SANKAR SCHWAR SEGI SEOW SHIMIZ SIMARA SIMONA SITAS SOBUE2 STASZE STAYNE STUCKE SUN
SUZUK2 SUZUKI TANG TAO TOKARS TOUSEY ULMER VEIERO VUTUC WALD WANG WANG3 WANG4 WICKLU WIGLE WILKIN
WU2 WUNSCH WYNDE8 XIANGZ XU XU2 XU4 YONG ZHANG

2 BUELL CHEN MASTRA MZILEN PISANI RESTRE SADOWS

4 BOFFET WYNDE7

5 RIMING TANG2 WYNDE5

6 BLOT1 BLOT2 BLOT3 BOUCHA HIRAY2 JONES LAURIL LICKIN MOLLO MRFIT MURATA SCHWA2 VANDER WARSIN WATSON WYNDER

8 AGUDO AKIBA ARCHER ARMADA AUVINE AXELSS BENSHL BEST BRESLO BRETT BROSS BUFFLE CEDERL CHANG CHATZI CHEN2
CHOW COMSTO COOKSO CPSI CPSII DAMBER DARBY DAVEYS DEAN DEAN2 DEAN3 DEKLER DESTEF DOLL2 DORANT DORN
DUNN EBELIN ENSTRO ESAKI FAN GAO GAO2 GARSHI GILLIS GOLLED GSELL HAMMO2 HANSEN HIRAYA HITOSU HOLE
HU HU2 HUMBLE JARUP JOLY JUSSAW KAISE2 KAISER KANELL KAUFMA KHUDER KINLEN KNEKT KOO KOULUM KREUZE
LAUSSM LETOUR LIAW LIDDEL LIU2 LIU3 LIU4 LIU5 LUBIN MACLEN MARTIS MCCONN MIGRAN MRFITR NAM NOTAN2
PARKIN PASTOR PERNU PERSH2 PETO PEZZO2 PEZZOT PIKE POLEDN PRESCO RACHTA RANDIG SEGI2 SHAW SIEMIA SPEIZE
SPITZ STOCKS STOCKW TENKAN TIZZAN TULINI TVERDA WANG2 XU3 YAMAGU YUAN

10 BOUCOT BROWN2 CORREA DORGAN ENGELA HAENSZ KATSOU OSANN OSANN2 WU WUWILL ZHOU

11 BENHAM

Table 2G13 - 8

Potentially overlapping studies

REF	REFGP	PRINC	.	OVERLAP/LINK
LUBIN2	LUBIN2	1		Lubin-combined
LAMTH	LAMTH	1		KOO/LAMTH/LAMWK
WYNDE6	WYNDE6	1		WYNDE5/6/7/8
MATSUD	MATSUD	1		SOBUE2/MATSUD

Table 2G13 - 9

Most adjusted - insufficient data for metaanalysis

REF	NRR	SEX	AGEL	AGEH	RACE	YF	LC TYPE	LOC	START	ST	NLC	R	VB	P	H	AD	SM	PRODUCT	exL	exH	DENOM	De
CHEN	4	c	0	0	all	-	q	As:oth	1987	CC	323	n	ot	n	y	2	ev	cig+/-ot	11	20	nev cigs	ot

REF	NRR	RR	SIG	RRDATA comment
CHEN	4	7.05	n	0

Table 2G14 -

IESLC - Meta-anal of Ever Smoking (or Current if ev not avail), Amount smoked, "High", Any prod (or Cigs if Any not avail)
Squamous

This analysis is restricted to results for:
1) Results by Amount smoked
2) Results complete enough for use in metaanalysis

Within each study, results are then selected (in the following order of preference, within each sex) for:
3) SMKSTA: ever smokers, current smokers
4) PRODUCT: all/unspec, cigarettes regardless of other products, cigarettes only
5) CIGTYPE: all/unspecified, MC regardless of HR, MC only
6) DENOM: never smoked anything, never smoked cigarettes, (never +1 = +long term ex, +2 = +amount unknown, +3 = never cigs+long term ex)
7) Followup period (YF, prospective studies): whole study (coded as 0) or longest available
8) LCtype: squamous or nearest available, but not adeno. (q = squamous, s = small, a = adeno, KI = Kreyberg I, u = undifferentiated)
9) Race: all or nearest available, otherwise by race (wh or w = white, bl or b = black, hi = hispanic ch = chinese, jap = japanese, haw = hawaiian, w+o = white + oriental, sca = scandinavian, as = asian)
10) Amount smoked "high" in key scheme 1 (key value 45, maximum range >20, in numbers of cigarettes or cigarette equivalents)
11) For overlapping studies: principal rather than subsidiary studies
Finally by Age: whole study (coded as 0) if available, otherwise by widest available age group
and then for single sex results (m, f) in preference to combined sex results (c).

Results adjusted (AD) for the most potential confounders are then chosen in Sections -1 to -3
and results adjusted for the least confounders in Sections -4 to -6. (Those least adjusted results which
actually differ from the most adjusted as marked 'x' in column X in Section -4)
(Results adjusted for an unknown number of confounder(s) are coded as 20.)

Section -7 shows excluded studies, together with the stage (as above) at which no qualifying
results were found.

Section -8 lists the potentially overlapping studies which have been included (1=principal, 2=subsidiary).

Section -9 lists any results which would have been included in preference except that they had data not complete
enough for use in meta-analysis, with their significance (yes/no), if known, and any further comment as entered
on the database.

In addition to those mentioned above, the following fields, levels and abbreviations are used:
* or nk = not known, n = no, y = yes, ot = other
ev = ever, cu = current, nev = never
all/unspec = all or unspecified, cig+/-ot = cigarettes irrespective of other products (cigar, pipe etc)
MC = manufactured cigarettes, HR = hand-rolled cigarettes
exL, exH = range of exposure (low and high) in the smoking group, in terms of Amount smoked, cigarettes or cigarette equivalents
REF: 6-character study reference
NRR: number of the RR on the database within the study
ST : study type (CC = case control, pr or prosp = prospective)
NLC: number of lung cancer cases in whole study
R : risky occupational population (n = no, m = mining, o = other risky)
VB : national cigarette type (V = at least 75% Virginia, bl = at least 75% blended, ot = other)
P : any proxy use
H : full histological confirmation
De : derivation of RR/CI (or = original, st = standard method, ot = other method of estimation)

Table 2G14 - 1

IESLC - Meta-anal of Ever Smoking (or Current if ev not avail), Amount smoked, “High”, Any prod (or Cigs if Any not avail)
Squamous
Most adjusted

REF	NRR	SEX	AGEL	AGEH	RACE	YF	LC TYPE	LOC	START	ST	NLC	R	VB	P	H	AD	SM	PRODUCT	exL	exH	DENOM	De
ALDERS	36	m	0	0	all	-	q+s	Eu:UK	1977	CC	1448	n	V	n	n	1	ev	cig only	28	99	nev+2	ot
ALDERS	39	f	0	0	all	-	q+s	Eu:UK	1977	CC	1448	n	V	n	n	1	ev	cig only	28	99	nev+2	ot
BARBON	72	m	0	0	all	-	q	Eu:wst	1979	CC	755	n	bl	y	y	3	ev	all/unsp	40	99	nev any	or
BOUCOT	143	m	0	0	all	0	q	NAmer	1951	pr	121	n	bl	n	n	2	cu	cig only	21	99	nev any	ot
CHOI	50	m	0	0	all	-	q	As:oth	1985	CC	375	n	bl	n	n	0	ev	cig+/-ot	41	99	nev cigs	st
CHOI	58	f	0	0	all	-	q	As:oth	1985	CC	375	n	bl	n	n	0	ev	cig+/-ot	31	99	nev cigs	st
CORREA	51	c	0	0	all	-	q+s	NAmer	1979	CC	1359	n	bl	y	n	1	cu	cig+/-ot	21	99	nev cigs	or
DOLL	71	m	0	0	all	-	KI	Eu:UK	1948	CC	1465	n	V	n	n	1	ev	all/unsp	25	99	nev any	ot
DOSEME	15	m	0	0	all	-	q	Eu:bal	1979	CC	1210	n	bl	n	n	2	ev	cig+/-ot	21	99	nev cigs	or
GER	16	c	0	0	all	-	q+s	As:oth	1990	CC	141	n	ot	y	n	10	ev	all/unsp	21	99	nev any	ot
HAENSZ	17	f	0	0	all	-	q+u	NAmer	1955	CC	158	n	bl	n	y	0	cu	cig+/-ot	21	99	nev any	or
HAMMON	101	m	0	0	wh	0	not a	NAmer	1952	pr	448	n	bl	n	n	1	cu	cig only	40	99	nev any	ot
JEDRYC	30	m	0	0	all	-	q	Eu:est	1980	CC	1630	n	bl	y	n	3	ev	cig+/-ot	30	99	nev any	or
KATSOU	22	f	0	0	all	-	KI	Eu:bal	1987	CC	101	n	bl	n	n	1	cu	all/unsp	21	99	nev any	or
KREYBE	3	m	0	0	all	-	KI	Eu:Sca	1948	CC	300	n	bl	n	y	1	ev	all/unsp	25	99	nev any	ot
LAMTH	12	f	0	0	ch	-	q	As:HK	1983	CC	445	n	bl	n	n	0	ev	all/unsp	21	99	nev any	st
LUBIN2	161	m	0	0	all	-	q	Eu:mul	1976	CC	7804	n	bl	n	y	0	ev	cig+/-ot	30	99	nev any	st
LUBIN2	181	f	0	0	all	-	q	Eu:mul	1976	CC	7804	n	bl	n	y	0	ev	cig+/-ot	30	99	nev any	st
LUO	12	c	0	0	all	-	q	As:Chi	1990	CC	102	n	ot	n	y	20	ev	cig+/-ot	30	99	nev cigs	or
MATOS	47	m	0	0	all	-	q	SCAmer	1994	CC	200	n	bl	n	n	2	ev	cig+/-ot	25	99	nev any	or
MATSUD	6	m	0	0	all	-	q	As:Jap	1965	CC	179	n	bl	n	n	0	ev	cig+/-ot	21	99	nev cigs	st
ORMOS	7	m	0	0	all	-	q	Eu:est	1947	CC	119	n	bl	y	y	0	ev	cig+/-ot	31	99	nev any	st
OSANN	59	m	0	0	all	-	q	NAmer	1984	CC	1986	n	bl	n	n	2	ev	cig+/-ot	40	99	nev cigs	or
OSANN	60	f	0	0	all	-	q	NAmer	1984	CC	1986	n	bl	n	n	2	ev	cig+/-ot	40	99	nev cigs	or
SOBUE	55	m	0	0	all	-	q	As:Jap	1986	CC	1376	n	bl	n	y	0	cu	cig+/-ot	30	99	nev cigs	st
SVENSS	17	f	0	0	all	-	q	Eu:Sca	1983	CC	210	n	bl	n	n	1	cu	all/unsp	21	99	nev any	ot
TSUGAN	17	m	0	0	all	-	q	As:Jap	1976	CC	134	n	bl	n	y	0	cu	all/unsp	36	99	nev any	ot
WAKAI	48	m	0	0	all	-	q	As:Jap	1988	CC	333	n	bl	n	y	1	cu	cig+/-ot	30	99	nev any	or
WU	18	f	0	0	wh	-	q	NAmer	1981	CC	220	n	bl	n	y	2	cu	all/unsp	21	99	nev any	or
WYNDE2	6	m	0	0	all	-	KI	NAmer	1962	CC	404	n	bl	n	y	0	ev	cig+/-ot	35	99	nev any	st
WYNDE3	7	m	0	0	all	-	KI	NAmer	1966	CC	350	n	bl	n	y	0	ev	cig+/-ot	41	99	nev any	st
WYNDE3	66	f	0	0	all	-	KI	NAmer	1966	CC	350	n	bl	n	y	0	ev	cig+/-ot	41	99	nev any	st
WYNDE4	67	m	0	0	all	-	not a	NAmer	1948	CC	684	n	bl	y	n	2	ev	all/unsp	35	99	nev any	ot
WYNDE4	53	f	0	0	all	-	not a	NAmer	1948	CC	684	n	bl	y	n	2	ev	all/unsp	35	99	nev any	ot
WYNDE6	48	m	0	0	all	-	KI	NAmer	1969	CC	4423	n	bl	n	y	0	cu	cig+/-ot	31	99	nev any	st
WYNDE6	237	f	0	0	all	-	KI	NAmer	1969	CC	4423	n	bl	n	y	0	cu	cig+/-ot	30	99	nev cigs	st
ZHENG	4	m	0	0	all	-	q	As:Chi	1982	CC	540	n	ot	*	y	0	ev	cig+/-ot	30	99	nev cigs	st

Cigarette type is all/unspec for all RRs

except for the following:

REF	NRR	CIGTYPE
ALDERS	36	MC only
ALDERS	39	MC only

Table 2G14 - 2

IESLC - Meta-anal of Ever Smoking (or Current if ev not avail), Amount smoked, “High”, Any prod (or Cigs if Any not avail)
Squamous
Most adjusted

REF	NRR	SEX	AD	Number Case	Exposed Cont	Non-exposed Case	Cont	RR	95.00%CI
ALDERS	36	m	1	-	-	-	-	8.78	(3.46- 22.31)
ALDERS	39	f	1	-	-	-	-	14.52	(7.93- 26.58)
Subtotal ALDERS								12.51	(7.53- 20.77)
BARBON	72	m	3	-	-	-	-	28.60	(12.00- 69.00)
*BOUCOT	143	m	2	-	-	-	-	46.64	(2.80- 775.69)
CHOI	50	m	0	9	6	6	95	23.75	(6.33- 89.09)
CHOI	58	f	0	2	1	10	164	32.80	(2.74- 393.20)
Subtotal CHOI								25.50	(7.94- 81.93)
CORREA	51	c	1	-	-	-	-	54.80	(35.60- 89.20)
DOLL	71	m	1	-	-	-	-	25.40	(7.83- 82.40)
DOSEME	15	m	2	-	-	-	-	7.00	(4.10- 12.00)
GER	16	c	10	-	-	-	-	16.04	(4.22- 60.93)
HAENSZ	17	f	0	18	13	44	236	7.43	(3.40- 16.24)
*HAMMON	101	m	1	-	-	-	-	63.91	(22.02- 185.47)
JEDRYC	30	m	3	-	-	-	-	21.42	(9.05- 50.68)
KATSOU	22	f	1	-	-	-	-	19.53	(5.36- 71.11)
KREYBE	3	m	1	-	-	-	-	24.63	(7.54- 80.53)
LAMTH	12	f	0	10	1	28	72	25.71	(3.14- 210.29)
LUBIN2	161	m	0	849	1746	54	2616	23.56	(17.77- 31.22)
LUBIN2	181	f	0	18	39	72	1180	7.56	(4.12- 13.88)
Subtotal LUBIN2								19.26	(14.92- 24.87)
LUO	12	c	20	-	-	-	-	38.70	(5.20- 290.20)
MATOS	47	m	2	-	-	-	-	9.70	(2.80- 33.20)
MATSUD	6	m	0	39	470	1	1255	104.14	(14.27- 760.12)
ORMOS	7	m	0	4	128	2	777	12.14	(2.20- 66.97)
OSANN	59	m	2	-	-	-	-	76.00	(36.80- 157.00)
OSANN	60	f	2	-	-	-	-	72.30	(36.80- 142.00)
Subtotal OSANN								73.99	(45.14- 121.29)
SOBUE	55	m	0	87	187	3	128	19.85	(6.14- 64.13)
SVENSS	17	f	1	-	-	-	-	96.00	(6.90-1335.65)
TSUGAN	17	m	0	9	1	0	5	69.67~	(2.40-2022.74)
WAKAI	48	m	1	-	-	-	-	24.00	(5.46- 105.00)
WU	18	f	2	-	-	-	-	94.40	(9.90- 904.60)
WYNDE2	6	m	0	139	112	3	105	43.44	(13.42- 140.56)
WYNDE3	7	m	0	59	26	3	88	66.56	(19.27- 229.96)
WYNDE3	66	f	0	3	3	5	76	15.20	(2.42- 95.56)
Subtotal WYNDE3								41.95	(15.01- 117.26)
WYNDE4	67	m	2	-	-	-	-	29.54	(13.53- 64.50)
WYNDE4	53	f	2	-	-	-	-	26.53	(4.12- 171.09)
Subtotal WYNDE4								29.07	(14.15- 59.73)
WYNDE6	48	m	0	502	197	29	617	54.22	(36.08- 81.47)
WYNDE6	237	f	0	221	52	40	856	90.95	(58.70- 140.93)
Subtotal WYNDE6								68.92	(51.14- 92.86)
ZHENG	4	m	0	49	23	4	94	50.07	(16.39- 152.91)
Partial Totals				2018	3005	304	8364		

*prospective study ~ With 0.5 adjustment for zero

REF	NRR	SEX	AD	Ys	Ws	Qs	Ps
ALDERS	36	m	1	2.17	4.42	6.30	0.0000
ALDERS	39	f	1	2.68	10.50	5.00	0.0000
Subtotal ALDERS				2.53	14.93	11.30	
BARBON	72	m	3	3.35	5.02	0.00	0.0000
*BOUCOT	143	m	2	3.84	0.49	0.11	0.0074
CHOI	50	m	0	3.17	2.20	0.09	0.0000
CHOI	58	f	0	3.49	0.62	0.01	0.0059
Subtotal CHOI				3.24	2.82	0.10	
CORREA	51	c	1	4.00	18.21	7.41	0.0000
DOLL	71	m	1	3.23	2.77	0.05	0.0000
DOSEME	15	m	2	1.95	13.32	26.86	0.0000
GER	16	c	10	2.78	2.16	0.75	0.0000
HAENSZ	17	f	0	2.01	6.27	11.61	0.0000
*HAMMON	101	m	1	4.16	3.38	2.12	0.0000
JEDRYC	30	m	3	3.06	5.18	0.47	0.0000
KATSOU	22	f	1	2.97	2.30	0.36	0.0000
KREYBE	3	m	1	3.20	2.74	0.07	0.0000
LAMTH	12	f	0	3.25	0.87	0.01	0.0025
LUBIN2	161	m	0	3.16	48.42	2.06	0.0000
LUBIN2	181	f	0	2.02	10.42	18.78	0.0000
Subtotal LUBIN2				2.96	58.85	20.84	
LUO	12	c	20	3.66	0.95	0.08	0.0004

Table 2G14 - 2

IESLC - Meta-anal of Ever Smoking (or Current if ev not avail), Amount smoked, “High”, Any prod (or Cigs if Any not avail)
Squamous
Most adjusted

REF	NRR	SEX	AD	Ys	Ws	Qs	Ps
MATOS	47	m	2	2.27	2.51	3.01	0.0003
MATSUD	6	m	0	4.65	0.97	1.59	0.0000
ORMOS	7	m	0	2.50	1.32	1.00	0.0042
OSANN	59	m	2	4.33	7.30	6.80	0.0000
OSANN	60	f	2	4.28	8.43	7.06	0.0000
Subtotal OSANN				4.30	15.73	13.86	
SOBUE	55	m	0	2.99	2.79	0.40	0.0000
SVENSS	17	f	1	4.56	0.55	0.80	0.0007
TSUGAN	17	m	0	4.24	0.34	0.26	0.0135
WAKAI	48	m	1	3.18	1.76	0.06	0.0000
WU	18	f	2	4.55	0.75	1.05	0.0001
WYNDE2	6	m	0	3.77	2.79	0.46	0.0000
WYNDE3	7	m	0	4.20	2.50	1.73	0.0000
WYNDE3	66	f	0	2.72	1.14	0.47	0.0037
Subtotal WYNDE3				3.74	3.64	2.20	
WYNDE4	67	m	2	3.39	6.30	0.00	0.0000
WYNDE4	53	f	2	3.28	1.11	0.01	0.0006
Subtotal WYNDE4				3.37	7.41	0.01	
WYNDE6	48	m	0	3.99	23.16	9.11	0.0000
WYNDE6	237	f	0	4.51	20.03	26.24	0.0000
Subtotal WYNDE6				4.23	43.19	35.35	
ZHENG	4	m	0	3.91	3.08	0.92	0.0000

	N	37
	NS	30
	Wt	227.09
Het	Chi	143.12
Het	df	36
Het	P	***
Fixed	RR	28.95
	RRl	25.42
	RRu	32.98
	P	+++
Random	RR	27.65
	RRl	20.42
	RRu	37.44
	P	+++
Asymm	P	N.S.

Table 2G14 - 3

IESLC - Meta-anal of Ever Smoking (or Current if ev not avail), Amount smoked, “High”, Any prod (or Cigs if Any not avail)
Squamous
Most adjusted

	Sex			
	combined	male	female	Total
N	3	22	12	37
NS	3	22	12	37
Wt	21.32	142.77	63.00	227.09
Het Chi	2.95	62.58	71.40	143.12
Het df	2	21	11	36
Het P	N.S.	***	***	***
Fixed RR	47.65	26.76	29.26	28.95
RRl	31.17	22.71	22.86	25.42
RRu	72.85	31.52	37.45	32.98
P	+++	+++	+++	+++
Random RR	39.52	27.70	26.18	27.65
RRl	18.61	19.71	12.49	20.42
RRu	83.95	38.93	54.88	37.44
P	+++	+++	+++	+++
Between Chi				6.19
Between df				2
Between P				*
Btwn(F) P				N.S.
Btwn(R) P				N.S.

	Lung cancer type					
	q	q+s	q+u	KI	not a	Total
N	21	4	1	8	3	37
NS	18	3	1	6	2	30
Wt	117.30	35.30	6.27	57.43	10.79	227.09
Het Chi	63.63	19.33	0.00	13.05	1.45	143.12
Het df	20	3	0	7	2	36
Het P	***	***	N.S.	(*)	N.S.	***
Fixed RR	22.38	27.22	7.43	56.33	37.22	28.95
RRl	18.68	19.57	3.40	43.50	20.49	25.42
RRu	26.82	37.86	16.24	72.96	67.59	32.98
P	+++	+++	+++	+++	+++	+++
Random RR	26.27	19.31	7.43	45.80	37.22	27.65
RRl	17.39	7.53	3.40	29.73	20.49	20.42
RRu	39.67	49.57	16.24	70.55	67.59	37.44
P	+++	+++	+++	+++	+++	+++
Between Chi						45.65
Between df						4
Between P						***
Btwn(F) P						*
Btwn(R) P						**

	Location								
	NAmer	UK	Scand	othEur	China	Japan	othAs	other	Total
N	14	3	2	7	2	4	4	1	37
NS	10	2	2	6	2	4	3	1	30
Wt	101.86	17.70	3.29	85.99	4.03	5.86	5.85	2.51	227.09
Het Chi	36.93	1.96	0.85	24.30	0.05	2.31	0.35	0.00	143.12
Het df	13	2	1	6	1	3	3	0	36
Het P	***	N.S.	N.S.	***	N.S.	N.S.	N.S.	N.S.	***
Fixed RR	53.01	13.98	30.97	16.85	47.12	29.74	21.52	9.70	28.95
RRl	43.66	8.77	10.52	13.64	17.75	13.24	9.57	2.82	25.42
RRu	64.38	22.27	91.18	20.81	125.06	66.82	48.41	33.40	32.98
P	+++	+++	+++	+++	+++	+++	+++	+++	+++
Random RR	46.63	13.98	30.97	14.84	47.12	29.74	21.52	9.70	27.65
RRl	31.84	8.77	10.52	8.72	17.75	13.24	9.57	2.82	20.42
RRu	68.27	22.27	91.18	25.27	125.06	66.82	48.41	33.40	37.44
P	+++	+++	+++	+++	+++	+++	+++	+++	+++
Between Chi									76.37
Between df									7
Between P									***
Btwn(F) P									**
Btwn(R) P									**

Table 2G14 - 3

IESLC - Meta-anal of Ever Smoking (or Current if ev not avail), Amount smoked, “High”, Any prod (or Cigs if Any not avail)

Squamous

Most adjusted

	Detailed Country in "other Europe"					
	multi	Germany	othWest	East	Balkans	Total
N	2		1	2	2	7
NS	1		1	2	2	6
Wt	58.85		5.02	6.49	15.62	85.99
Het Chi	11.07		0.00	0.34	2.06	24.30
Het df	1		0	1	1	6
Het P	***		N.S.	N.S.	N.S.	***
Fixed RR	19.26		28.60	19.09	8.14	16.85
RRl	14.92		11.93	8.85	4.96	13.64
RRu	24.87		68.58	41.19	13.37	20.81
P	+++		+++	+++	+++	+++
Random RR	13.80		28.60	19.09	9.81	14.84
RRl	4.54		11.93	8.85	3.81	8.72
RRu	41.93		68.58	41.19	25.24	25.27
P	+++		+++	+++	+++	+++
Between Chi						10.83
Between df						3
Between P						*
Btwn(F) P						N.S.
Btwn(R) P						N.S.

	Detailed Country in "other Asia"			
	India	HongKong	other	Total
N		1	3	4
NS		1	2	3
Wt		0.87	4.98	5.85
Het Chi		0.00	0.31	0.35
Het df		0	2	3
Het P		N.S.	N.S.	N.S.
Fixed RR		25.71	20.86	21.52
RRl		3.14	8.67	9.57
RRu		210.29	50.23	48.41
P		++	+++	+++
Random RR		25.71	20.86	21.52
RRl		3.14	8.67	9.57
RRu		210.29	50.23	48.41
P		++	+++	+++
Between Chi				0.03
Between df				1
Between P				N.S.
Btwn(F) P				N.S.
Btwn(R) P				N.S.

	Detailed other continent			
	SCAmer	Auslia	Africa	Total
N	1			1
NS	1			1
Wt	2.51			2.51
Het Chi	0.00			0.00
Het df	0			0
Het P	N.S.			N.S.
Fixed RR	9.70			9.70
RRl	2.82			2.82
RRu	33.40			33.40
P	+++			+++
Random RR	9.70			9.70
RRl	2.82			2.82
RRu	33.40			33.40
P	+++			+++
Between Chi				
Between df				
Between P				N.S.
Btwn(F) P				N.S.
Btwn(R) P				N.S.

Table 2G14 - 3

IESLC - Meta-anal of Ever Smoking (or Current if ev not avail), Amount smoked, "High", Any prod (or Cigs if Any not avail)

Squamous

Most adjusted

	Start year of study <1960	1960-69	1970-79	1980-89	1990+	Total
N	8	6	8	12	3	37
NS	7	4	6	10	3	30
Wt	24.38	50.59	110.67	35.84	5.62	227.09
Het Chi	12.63	6.09	49.52	12.80	1.34	143.12
Het df	7	5	7	11	2	36
Het P	(*)	N.S.	***	N.S.	N.S.	***
Fixed RR	21.25	65.35	19.53	42.42	14.87	28.95
RRl	14.29	49.61	16.21	30.58	6.50	25.42
RRu	31.60	86.08	23.53	58.86	33.99	32.98
P	+++	+++	+++	+++	+++	+++
Random RR	22.70	64.04	16.74	40.53	14.87	27.65
RRl	12.76	45.20	9.42	27.99	6.50	20.42
RRu	40.38	90.74	29.74	58.70	33.99	37.44
P	+++	+++	+++	+++	+++	+++
Between Chi						60.74
Between df						4
Between P						***
Btwn(F) P						**
Btwn(R) P						***

	Study type (1) CC	other	Total
N	35	2	37
NS	28	2	30
Wt	223.22	3.87	227.09
Het Chi	140.85	0.04	143.12
Het df	34	1	36
Het P	***	N.S.	***
Fixed RR	28.58	61.43	28.95
RRl	25.07	22.68	25.42
RRu	32.59	166.38	32.98
P	+++	+++	+++
Random RR	26.82	61.43	27.65
RRl	19.65	22.68	20.42
RRu	36.60	166.38	37.44
P	+++	+++	+++
Between Chi			2.23
Between df			1
Between P			N.S.
Btwn(F) P			N.S.
Btwn(R) P			N.S.

	Study type (2) CC	prosp	other	Total
N	35	2		37
NS	28	2		30
Wt	223.22	3.87		227.09
Het Chi	140.85	0.04		143.12
Het df	34	1		36
Het P	***	N.S.		***
Fixed RR	28.58	61.43		28.95
RRl	25.07	22.68		25.42
RRu	32.59	166.38		32.98
P	+++	+++		+++
Random RR	26.82	61.43		27.65
RRl	19.65	22.68		20.42
RRu	36.60	166.38		37.44
P	+++	+++		+++
Between Chi				2.23
Between df				1
Between P				N.S.
Btwn(F) P				N.S.
Btwn(R) P				N.S.

Table 2G14 - 3

IESLC - Meta-anal of Ever Smoking (or Current if ev not avail), Amount smoked, “High”, Any prod (or Cigs if Any not avail)

Squamous
Most adjusted

	Study size (number of LC cases) 100-249	250-499	500-999	1000+	Total
N	11	9	4	13	37
NS	11	7	3	9	30
Wt	18.61	17.99	15.51	174.98	227.09
Het Chi	14.11	4.17	0.76	116.37	143.12
Het df	10	8	3	12	36
Het P	N.S.	N.S.	N.S.	***	***
Fixed RR	16.03	36.01	32.21	29.87	28.95
RRl	10.18	22.68	19.58	25.75	25.42
RRu	25.25	57.16	52.99	34.64	32.98
P	+++	+++	+++	+++	+++
Random RR	20.05	36.01	32.21	26.67	27.65
RRl	11.10	22.68	19.58	16.31	20.42
RRu	36.21	57.16	52.99	43.62	37.44
P	+++	+++	+++	+++	+++
Between Chi					7.70
Between df					3
Between P					(*)
Btwn(F) P					N.S.
Btwn(R) P					N.S.

	Risky occupational population no	mining	othRisky	Total
N	37			37
NS	30			30
Wt	227.09			227.09
Het Chi	143.12			143.12
Het df	36			36
Het P	***			***
Fixed RR	28.95			28.95
RRl	25.42			25.42
RRu	32.98			32.98
P	+++			+++
Random RR	27.65			27.65
RRl	20.42			20.42
RRu	37.44			37.44
P	+++			+++
Between Chi				
Between df				
Between P				N.S.
Btwn(F) P				N.S.
Btwn(R) P				N.S.

	National cigarette tobacco type Virginia	blended	other	Total
N	3	31	3	37
NS	2	25	3	30
Wt	17.70	203.20	6.19	227.09
Het Chi	1.96	129.28	1.68	143.12
Het df	2	30	2	36
Het P	N.S.	***	N.S.	***
Fixed RR	13.98	30.75	32.37	28.95
RRl	8.77	26.80	14.72	25.42
RRu	22.27	35.28	71.18	32.98
P	+++	+++	+++	+++
Random RR	13.98	29.58	32.37	27.65
RRl	8.77	21.09	14.72	20.42
RRu	22.27	41.49	71.18	37.44
P	+++	+++	+++	+++
Between Chi				10.20
Between df				2
Between P				**
Btwn(F) P				N.S.
Btwn(R) P				*

Table 2G14 - 3

IESLC - Meta-anal of Ever Smoking (or Current if ev not avail), Amount smoked, “High”, Any prod (or Cigs if Any not avail)
Squamous
Most adjusted

	Any proxy use No/nk	Yes	Total
N	30	7	37
NS	24	6	30
Wt	187.80	39.29	227.09
Het Chi	133.17	8.17	143.12
Het df	29	6	36
Het P	***	N.S.	***
Fixed RR	27.81	35.14	28.95
RRl	24.10	25.70	25.42
RRu	32.08	48.04	32.98
P	+++	+++	+++
Random RR	28.03	30.95	27.65
RRl	19.50	20.62	20.42
RRu	40.30	46.48	37.44
P	+++	+++	+++
Between Chi			1.78
Between df			1
Between P			N.S.
Btwn(F) P			N.S.
Btwn(R) P			N.S.

	Full histological confirmation No	Yes	Total
N	20	17	37
NS	16	14	30
Wt	93.60	133.49	227.09
Het Chi	68.19	73.89	143.12
Het df	19	16	36
Het P	***	***	***
Fixed RR	26.72	30.64	28.95
RRl	21.82	25.86	25.42
RRu	32.72	36.30	32.98
P	+++	+++	+++
Random RR	26.97	28.53	27.65
RRl	17.49	18.18	20.42
RRu	41.59	44.76	37.44
P	+++	+++	+++
Between Chi			1.03
Between df			1
Between P			N.S.
Btwn(F) P			N.S.
Btwn(R) P			N.S.

	Number of adjustment variables (1) 0	1	2+/+nk	Total
N	16	9	12	37
NS	12	8	10	30
Wt	126.93	46.65	53.51	227.09
Het Chi	74.25	22.17	44.89	143.12
Het df	15	8	11	36
Het P	***	**	***	***
Fixed RR	30.83	29.20	24.76	28.95
RRl	25.91	21.92	18.94	25.42
RRu	36.69	38.90	32.37	32.98
P	+++	+++	+++	+++
Random RR	28.76	26.18	27.63	27.65
RRl	17.62	15.10	15.10	20.42
RRu	46.95	45.39	50.57	37.44
P	+++	+++	+++	+++
Between Chi				1.82
Between df				2
Between P				N.S.
Btwn(F) P				N.S.
Btwn(R) P				N.S.

Table 2G14 - 3

IESLC - Meta-anal of Ever Smoking (or Current if ev not avail), Amount smoked, “High”, Any prod (or Cigs if Any not avail)

Squamous
Most adjusted

	Number of adjustment variables (2) 0	1	2	3-5	6+/+nk	Total
N	16	9	8	2	2	37
NS	12	8	6	2	2	30
Wt	126.93	46.65	40.21	10.20	3.11	227.09
Het Chi	74.25	22.17	44.07	0.21	0.51	143.12
Het df	15	8	7	1	1	36
Het P	***	**	***	N.S.	N.S.	***
Fixed RR	30.83	29.20	25.09	24.70	21.00	28.95
RRl	25.91	21.92	18.42	13.37	6.91	25.42
RRu	36.69	38.90	34.18	45.62	63.86	32.98
P	+++	+++	+++	+++	+++	+++
Random RR	28.76	26.18	30.19	24.70	21.00	27.65
RRl	17.62	15.10	12.46	13.37	6.91	20.42
RRu	46.95	45.39	73.14	45.62	63.86	37.44
P	+++	+++	+++	+++	+++	+++
Between Chi						1.91
Between df						4
Between P						N.S.
Btwn(F) P						N.S.
Btwn(R) P						N.S.

	Product all/unsp	cig+/-ot	cig only	Total
N	11	22	4	37
NS	10	17	3	30
Wt	24.91	183.38	18.80	227.09
Het Chi	3.30	125.58	8.61	143.12
Het df	10	21	3	36
Het P	N.S.	***	*	***
Fixed RR	27.52	30.73	17.36	28.95
RRl	18.58	26.59	11.05	25.42
RRu	40.75	35.51	27.28	32.98
P	+++	+++	+++	+++
Random RR	27.52	28.60	20.51	27.65
RRl	18.58	19.02	8.32	20.42
RRu	40.75	42.99	50.57	37.44
P	+++	+++	+++	+++
Between Chi				5.63
Between df				2
Between P				(*)
Btwn(F) P				N.S.
Btwn(R) P				N.S.

	Denominator nev any	nev cigs	Total
N	26	11	37
NS	22	9	31
Wt	149.18	77.91	227.09
Het Chi	58.72	64.17	143.12
Het df	25	10	36
Het P	***	***	***
Fixed RR	23.34	43.76	28.95
RRl	19.88	35.05	25.42
RRu	27.40	54.64	32.98
P	+++	+++	+++
Random RR	22.44	41.14	27.65
RRl	16.59	21.65	20.42
RRu	30.35	78.18	37.44
P	+++	+++	+++
Between Chi			20.23
Between df			1
Between P			***
Btwn(F) P			*
Btwn(R) P			(*)

Table 2G14 - 3

IESLC - Meta-anal of Ever Smoking (or Current if ev not avail), Amount smoked, “High”, Any prod (or Cigs if Any not avail)

Squamous
Most adjusted

	Derivation of RR/CI: Orig	StdCalc	Other	Total
N	12	14	11	37
NS	11	10	9	30
Wt	72.01	120.32	34.76	227.09
Het Chi	64.34	60.69	12.18	143.12
Het df	11	13	10	36
Het P	***	***	N.S.	***
Fixed RR	26.82	33.13	21.28	28.95
RRl	21.29	27.71	15.26	25.42
RRu	33.79	39.62	29.67	32.98
P	+++	+++	+++	+++
Random RR	25.87	32.08	22.32	27.65
RRl	14.03	19.59	15.07	20.42
RRu	47.68	52.52	33.05	37.44
P	+++	+++	+++	+++
Between Chi				5.91
Between df				2
Between P				(*)
Btwn(F) P				N.S.
Btwn(R) P				N.S.

	Smoking status: ever	current	Total
N	25	12	37
NS	19	11	30
Wt	147.05	80.04	227.09
Het Chi	71.00	36.42	143.12
Het df	24	11	36
Het P	***	***	***
Fixed RR	21.61	49.56	28.95
RRl	18.39	39.81	25.42
RRu	25.40	61.69	32.98
P	+++	+++	+++
Random RR	23.54	39.16	27.65
RRl	16.96	23.67	20.42
RRu	32.68	64.79	37.44
P	+++	+++	+++
Between Chi			35.70
Between df			1
Between P			***
Btwn(F) P			**
Btwn(R) P			(*)

Table 2G14 - 4

IESLC - Meta-anal of Ever Smoking (or Current if ev not avail), Amount smoked, “High”, Any prod (or Cigs if Any not avail)
Squamous
Least adjusted

REF	NRR	X	SEX	AGEL	AGEH	RACE	YF	LC TYPE	LOC	START	ST	NLC	R	VB	P	H	AD	SM	PRODUCT	exL	exH	DENOM	De
ALDERS	36		m	0	0	all	-	q+s	Eu:UK	1977	CC	1448	n	V	n	n	1	ev	cig only	28	99	nev+2	ot
ALDERS	39		f	0	0	all	-	q+s	Eu:UK	1977	CC	1448	n	V	n	n	1	ev	cig only	28	99	nev+2	ot
BARBON	27	x	m	0	0	all	-	q	Eu:wst	1979	CC	755	n	bl	y	y	0	ev	all/unsp	40	99	nev any	st
BOUCOT	22	x	m	0	0	all	0	q	NAmer	1951	pr	121	n	bl	n	n	0	cu	cig only	21	99	nev any	ot
CHOI	50		m	0	0	all	-	q	As:oth	1985	CC	375	n	bl	n	n	0	ev	cig+/-ot	41	99	nev cigs	st
CHOI	58		f	0	0	all	-	q	As:oth	1985	CC	375	n	bl	n	n	0	ev	cig+/-ot	31	99	nev cigs	st
CORREA	51		c	0	0	all	-	q+s	NAmer	1979	CC	1359	n	bl	y	n	1	cu	cig+/-ot	21	99	nev cigs	or
DOLL	57	x	m	0	0	all	-	KI	Eu:UK	1948	CC	1465	n	V	n	n	0	ev	all/unsp	25	99	nev any	st
DOSEME	15		m	0	0	all	-	q	Eu:bal	1979	CC	1210	n	bl	n	n	2	ev	cig+/-ot	21	99	nev cigs	or
GER	8	x	c	0	0	all	-	q+s	As:oth	1990	CC	141	n	ot	y	n	0	ev	all/unsp	21	99	nev any	st
HAENSZ	17		f	0	0	all	-	q+u	NAmer	1955	CC	158	n	bl	n	y	0	cu	cig+/-ot	21	99	nev any	or
HAMMON	101		m	0	0	wh	0	not a	NAmer	1952	pr	448	n	bl	n	n	1	cu	cig only	40	99	nev any	ot
JEDRYC	5	x	m	0	0	all	-	q	Eu:est	1980	CC	1630	n	bl	y	n	0	ev	cig+/-ot	40	99	nev any	st
KATSOU	26	x	f	0	0	all	-	KI	Eu:bal	1987	CC	101	n	bl	n	n	0	cu	all/unsp	21	99	nev any	st
KREYBE	15	x	m	0	0	all	-	KI	Eu:Sca	1948	CC	300	n	bl	n	y	0	ev	all/unsp	25	99	nev any	st
LAMTH	12		f	0	0	ch	-	q	As:HK	1983	CC	445	n	bl	n	n	0	ev	all/unsp	21	99	nev any	st
LUBIN2	161		m	0	0	all	-	q	Eu:mul	1976	CC	7804	n	bl	n	y	0	ev	cig+/-ot	30	99	nev any	st
LUBIN2	181		f	0	0	all	-	q	Eu:mul	1976	CC	7804	n	bl	n	y	0	ev	cig+/-ot	30	99	nev any	st
LUO	6	x	c	0	0	all	-	q	As:Chi	1990	CC	102	n	ot	n	y	0	ev	cig+/-ot	30	99	nev cigs	st
MATOS	46	x	m	0	0	all	-	q	SCAmer	1994	CC	200	n	bl	n	n	0	ev	cig+/-ot	25	99	nev any	st
MATSUD	6		m	0	0	all	-	q	As:Jap	1965	CC	179	n	bl	n	n	0	ev	cig+/-ot	21	99	nev cigs	st
ORMOS	7		m	0	0	all	-	q	Eu:est	1947	CC	119	n	bl	y	y	0	ev	cig+/-ot	31	99	nev any	st
OSANN	59		m	0	0	all	-	q	NAmer	1984	CC	1986	n	bl	n	n	2	ev	cig+/-ot	40	99	nev cigs	or
OSANN	60		f	0	0	all	-	q	NAmer	1984	CC	1986	n	bl	n	n	2	ev	cig+/-ot	40	99	nev cigs	or
SOBUE	55		m	0	0	all	-	q	As:Jap	1986	CC	1376	n	bl	n	y	0	cu	cig+/-ot	30	99	nev cigs	st
SVENSS	37	x	f	0	0	all	-	q	Eu:Sca	1983	CC	210	n	bl	n	n	0	cu	all/unsp	21	99	nev any	st
TSUGAN	17		m	0	0	all	-	q	As:Jap	1976	CC	134	n	bl	n	y	0	cu	all/unsp	36	99	nev any	ot
WAKAI	45	x	m	0	0	all	-	q	As:Jap	1988	CC	333	n	bl	n	y	0	cu	cig+/-ot	30	99	nev any	st
WU	13	x	f	0	0	wh	-	q	NAmer	1981	CC	220	n	bl	n	y	0	cu	all/unsp	21	99	nev any	st
WYNDE2	6		m	0	0	all	-	KI	NAmer	1962	CC	404	n	bl	n	y	0	ev	cig+/-ot	35	99	nev any	st
WYNDE3	7		m	0	0	all	-	KI	NAmer	1966	CC	350	n	bl	n	y	0	ev	cig+/-ot	41	99	nev any	st
WYNDE3	66		f	0	0	all	-	KI	NAmer	1966	CC	350	n	bl	n	y	0	ev	cig+/-ot	41	99	nev any	st
WYNDE4	29	x	m	0	0	all	-	not a	NAmer	1948	CC	684	n	bl	y	n	0	ev	all/unsp	35	99	nev any	st
WYNDE4	53		f	0	0	all	-	not a	NAmer	1948	CC	684	n	bl	y	n	2	ev	all/unsp	35	99	nev any	ot
WYNDE6	48		m	0	0	all	-	KI	NAmer	1969	CC	4423	n	bl	n	y	0	cu	cig+/-ot	31	99	nev any	st
WYNDE6	237		f	0	0	all	-	KI	NAmer	1969	CC	4423	n	bl	n	y	0	cu	cig+/-ot	30	99	nev cigs	st
ZHENG	4		m	0	0	all	-	q	As:Chi	1982	CC	540	n	ot	*	y	0	ev	cig+/-ot	30	99	nev cigs	st

Cigarette type is all/unspec for all RRs

except for the following:

REF	NRR	CIGTYPE
ALDERS	36	MC only
ALDERS	39	MC only

Table 2G14 - 5

IESLC - Meta-anal of Ever Smoking (or Current if ev not avail), Amount smoked, “High”, Any prod (or Cigs if Any not avail)
Squamous
Least adjusted

REF	NRR	SEX	AD	Number Exposed Case	Number Exposed Cont	Non-exposed Case	Non-exposed Cont	RR	95.00%CI
ALDERS	36	m	1	-	-	-	-	8.78	(3.46- 22.31)
ALDERS	39	f	1	-	-	-	-	14.52	(7.93- 26.58)
Subtotal ALDERS								12.51	(7.53- 20.77)
BARBON	27	m	0	78	111	6	188	22.02	(9.29- 52.18)
*BOUCOT	22	m	0	17	6940	0	7551	38.08~	(2.29- 633.12)
CHOI	50	m	0	9	6	6	95	23.75	(6.33- 89.09)
CHOI	58	f	0	2	1	10	164	32.80	(2.74- 393.20)
Subtotal CHOI								25.50	(7.94- 81.93)
CORREA	51	c	1	-	-	-	-	54.80	(35.60- 89.20)
DOLL	57	m	0	208	166	3	61	25.48	(7.85- 82.66)
DOSEME	15	m	2	-	-	-	-	7.00	(4.10- 12.00)
GER	8	c	0	19	13	11	80	10.63	(4.13- 27.37)
HAENSZ	17	f	0	18	13	44	236	7.43	(3.40- 16.24)
*HAMMON	101	m	1	-	-	-	-	63.91	(22.02- 185.47)
JEDRYC	5	m	0	57	82	6	289	33.48	(13.94- 80.42)
KATSOU	26	f	0	15	4	14	67	17.95	(5.17- 62.28)
KREYBE	15	m	0	38	248	3	644	32.89	(10.06- 107.53)
LAMTH	12	f	0	10	1	28	72	25.71	(3.14- 210.29)
LUBIN2	161	m	0	849	1746	54	2616	23.56	(17.77- 31.22)
LUBIN2	181	f	0	18	39	72	1180	7.56	(4.12- 13.88)
Subtotal LUBIN2								19.26	(14.92- 24.87)
LUO	6	c	0	9	4	5	51	22.95	(5.15- 102.20)
MATOS	46	m	0	26	105	3	110	9.08	(2.67- 30.90)
MATSUD	6	m	0	39	470	1	1255	104.14	(14.27- 760.12)
ORMOS	7	m	0	4	128	2	777	12.14	(2.20- 66.97)
OSANN	59	m	2	-	-	-	-	76.00	(36.80- 157.00)
OSANN	60	f	2	-	-	-	-	72.30	(36.80- 142.00)
Subtotal OSANN								73.99	(45.14- 121.29)
SOBUE	55	m	0	87	187	3	128	19.85	(6.14- 64.13)
SVENSS	37	f	0	4	1	5	120	96.00	(9.00-1023.75)
TSUGAN	17	m	0	9	1	0	5	69.67~	(2.40-2022.74)
WAKAI	45	m	0	33	48	2	65	22.34	(5.11- 97.69)
WU	13	f	0	42	9	2	30	70.00	(14.10- 347.48)
WYNDE2	6	m	0	139	112	3	105	43.44	(13.42- 140.56)
WYNDE3	7	m	0	59	26	3	88	66.56	(19.27- 229.96)
WYNDE3	66	f	0	3	3	5	76	15.20	(2.42- 95.56)
Subtotal WYNDE3								41.95	(15.01- 117.26)
WYNDE4	29	m	0	123	64	8	115	27.63	(12.69- 60.13)
WYNDE4	53	f	2	-	-	-	-	26.53	(4.12- 171.09)
Subtotal WYNDE4								27.46	(13.40- 56.29)
WYNDE6	48	m	0	502	197	29	617	54.22	(36.08- 81.47)
WYNDE6	237	f	0	221	52	40	856	90.95	(58.70- 140.93)
Subtotal WYNDE6								68.92	(51.14- 92.86)
ZHENG	4	m	0	49	23	4	94	50.07	(16.39- 152.91)
Partial Totals				2687	10800	372	17735		

*prospective study ~ With 0.5 adjustment for zero

REF	NRR	SEX	AD	Ys	Ws	Qs	Ps
ALDERS	36	m	1	2.17	4.42	6.18	0.0000
ALDERS	39	f	1	2.68	10.50	4.85	0.0000
Subtotal ALDERS				2.53	14.93	11.03	
BARBON	27	m	0	3.09	5.16	0.36	0.0000
*BOUCOT	22	m	0	3.64	0.49	0.04	0.0112
CHOI	50	m	0	3.17	2.20	0.08	0.0000
CHOI	58	f	0	3.49	0.62	0.01	0.0059
Subtotal CHOI				3.24	2.82	0.09	
CORREA	51	c	1	4.00	18.21	7.67	0.0000
DOLL	57	m	0	3.24	2.77	0.04	0.0000
DOSEME	15	m	2	1.95	13.32	26.45	0.0000
GER	8	c	0	2.36	4.29	4.22	0.0000
HAENSZ	17	f	0	2.01	6.27	11.43	0.0000
*HAMMON	101	m	1	4.16	3.38	2.18	0.0000
JEDRYC	5	m	0	3.51	5.00	0.12	0.0000
KATSOU	26	f	0	2.89	2.48	0.54	0.0000
KREYBE	15	m	0	3.49	2.74	0.05	0.0000
LAMTH	12	f	0	3.25	0.87	0.01	0.0025
LUBIN2	161	m	0	3.16	48.42	1.85	0.0000
LUBIN2	181	f	0	2.02	10.42	18.48	0.0000
Subtotal LUBIN2				2.96	58.85	20.32	
LUO	6	c	0	3.13	1.72	0.08	0.0000

Table 2G14 - 5

IESLC - Meta-anal of Ever Smoking (or Current if ev not avail), Amount smoked, “High”, Any prod (or Cigs if Any not avail)
Squamous
Least adjusted

REF	NRR	SEX	AD	Ys	Ws	Qs	Ps
MATOS	46	m	0	2.21	2.56	3.38	0.0004
MATSUD	6	m	0	4.65	0.97	1.62	0.0000
ORMOS	7	m	0	2.50	1.32	0.97	0.0042
OSANN	59	m	2	4.33	7.30	6.95	0.0000
OSANN	60	f	2	4.28	8.43	7.23	0.0000
Subtotal OSANN				4.30	15.73	14.18	
SOBUE	55	m	0	2.99	2.79	0.38	0.0000
SVENSS	37	f	0	4.56	0.69	1.00	0.0002
TSUGAN	17	m	0	4.24	0.34	0.27	0.0135
WAKAI	45	m	0	3.11	1.77	0.11	0.0000
WU	13	f	0	4.25	1.50	1.20	0.0000
WYNDE2	6	m	0	3.77	2.79	0.48	0.0000
WYNDE3	7	m	0	4.20	2.50	1.78	0.0000
WYNDE3	66	f	0	2.72	1.14	0.46	0.0037
Subtotal WYNDE3				3.74	3.64	2.23	
WYNDE4	29	m	0	3.32	6.35	0.01	0.0000
WYNDE4	53	f	2	3.28	1.11	0.01	0.0006
Subtotal WYNDE4				3.31	7.46	0.01	
WYNDE6	48	m	0	3.99	23.16	9.43	0.0000
WYNDE6	237	f	0	4.51	20.03	26.75	0.0000
Subtotal WYNDE6				4.23	43.19	36.18	
ZHENG	4	m	0	3.91	3.08	0.96	0.0000

	N	37
	NS	30
	Wt	231.12
Het	Chi	147.60
Het	df	36
Het	P	***
Fixed	RR	28.64
	RRl	25.17
	RRu	32.58
	P	+++
Random	RR	27.18
	RRl	20.11
	RRu	36.76
	P	+++
Asymm	P	N.S.

Table 2G14 - 6

IESLC - Meta-anal of Ever Smoking (or Current if ev not avail), Amount smoked, “High”, Any prod (or Cigs if Any not avail)

Squamous
Least adjusted

	Sex			
	combined	male	female	Total
N	3	22	12	37
NS	3	22	12	37
Wt	24.23	142.84	64.06	231.12
Het Chi	9.84	63.14	71.90	147.60
Het df	2	21	11	36
Het P	**	***	***	***
Fixed RR	38.52	26.92	29.39	28.64
RRl	25.87	22.85	23.01	25.17
RRu	57.37	31.72	37.55	32.58
P	+++	+++	+++	+++
Random RR	25.23	28.00	26.24	27.18
RRl	7.86	19.90	12.70	20.11
RRu	80.91	39.40	54.22	36.76
P	+++	+++	+++	+++
Between Chi				2.72
Between df				2
Between P				N.S.
Btwn(F) P				N.S.
Btwn(R) P				N.S.

Table 2G14 - 7

IESLC - Meta-anal of Ever Smoking (or Current if ev not avail), Amount smoked, “High”, Any prod (or Cigs if Any not avail)
Squamous
Excluded studies (and stage at which they were excluded)

1 ABELIN ABRAHA AMANDU AMES ANDERS AUSTIN AXELSO BAND BECHER BERRIN BLOHMK BLOT4 BROCKM BROWN1 BYERS1 BYERS2
CARPEN CASCO2 CASCOR CHAN CHEN3 CHIAZZ CHYOU DESTE2 DOCKER DROSTE DU GARCIA GARDIN GENG GODLEY GOODMA
GRAHAM GREGOR HEGMAN HEIN HENNEK HINDS HIRAOK HOROWI HORWIT HUANG ISHIMA JAHN JAIN JARVHO JIANG KELLER
KIHARA KJUUS KO KOHLME KUBIK LAMWK LAMWK2 LANGE LEI LEMARC LEVIN LIU LOMBA2 LOMBAR MAGNUS MARSH
MARSH2 MCDUFF MCLAUG MILLER MILLS NOTANI NOU ODRISC PAWLEG PERSHA POFFIJ QIAO QIAO2 RADZIK REN RONCO
ROOTS ROTHSC SAARIK SANKAR SCHWAR SEGI SEOW SHIMIZ SIMARA SIMONA SITAS SOBUE2 STASZE STAYNE STUCKE SUN
SUZUK2 SUZUKI TANG TAO TOKARS TOUSEY ULMER VEIERO VUTUC WALD WANG WANG3 WANG4 WICKLU WIGLE WILKIN
WU2 WUNSCH WYNDE8 XIANGZ XU XU2 XU4 YONG ZHANG

2 BUELL CHEN MASTRA MZILEN PISANI RESTRE SADOWS

4 BOFFET WYNDE7

5 RIMING TANG2 WYNDE5

6 BLOT1 BLOT2 BLOT3 BOUCHA HIRAY2 JONES LAURIL LICKIN MOLLO MRFIT MURATA SCHWA2 VANDER WARSIN WATSON WYNDER

8 AGUDO AKIBA ARCHER ARMADA AUVINE AXELSS BENSHL BEST BRESLO BRETT BROSS BUFFLE CEDERL CHANG CHATZI CHEN2
CHOW COMSTO COOKSO CPSI CPSII DAMBER DARBY DAVEYS DEAN DEAN2 DEAN3 DEKLER DESTEF DOLL2 DORANT DORN
DUNN EBELIN ENSTRO ESAKI FAN GAO GAO2 GARSHI GILLIS GOLLED GSELL HAMMO2 HANSEN HIRAYA HITOSU HOLE
HU HU2 HUMBLE JARUP JOLY JUSSAW KAISE2 KAISER KANELL KAUFMA KHUDER KINLEN KNEKT KOO KOULUM KREUZE
LAUSSM LETOUR LIAW LIDDEL LIU2 LIU3 LIU4 LIU5 LUBIN MACLEN MARTIS MCCONN MIGRAN MRFITR NAM NOTAN2
PARKIN PASTOR PERNU PERSH2 PETO PEZZO2 PEZZOT PIKE POLEDN PRESCO RACHTA RANDIG SEGI2 SHAW SIEMIA SPEIZE
SPITZ STOCKS STOCKW TENKAN TIZZAN TULINI TVERDA WANG2 XU3 YAMAGU YUAN

10 BROWN2 DORGAN ENGELA OSANN2 WUWILL ZHOU

11 BENHAM

Table 2G14 - 8
Potentially overlapping studies

REF	REFGP	PRINC	. OVERLAP/LINK
LUBIN2	LUBIN2	1	Lubin-combined
LAMTH	LAMTH	1	KOO/LAMTH/LAMWK
WYNDE6	WYNDE6	1	WYNDE5/6/7/8
MATSUD	MATSUD	1	SOBUE2/MATSUD

Table 2G14 - 9
Most adjusted - insufficient data for metaanalysis

REF	NRR	SEX	AGEL	AGEH	RACE	YF	LC TYPE	LOC	START	ST	NLC	R	VB	P	H	AD	SM	PRODUCT	exL	exH	DENOM	De
CHEN	2	c	0	0	all	-	q	As:oth	1987	CC	323	n	ot	n	y	2	ev	cig+/-ot	31	99	nev cigs	ot

REF	NRR	RR	SIG	RRDATA comment
CHEN	2	11.11	y	P < 0.001

Table 2G16 -

IESLC - Meta-anal of Ever Smoking (or Curr if Ever not avail) by Amount, Overview, Cigs (or Any Prod if Cigs not avail)
Squamous

This analysis is restricted to results for:
1) Results by Amount smoked
Results by Amount smoked (in numbers of cigarettes or cigarette equivalents) are grouped under 2 schemes (S1, S2).
Each scheme has a set of "key values". An interval is allocated to the category whose key value it includes and intervals which include none or more than one of the key values are excluded.
(Open-ended intervals are coded as 99.)

S1	key value	maximum range	S2	key value	maximum range
1	5	1-19	1	1	1-9
2	20	6-44	2	10	2-19
3	45	21+	3	20	11-29
			4	30	21-39
			5	40	31-98
			6	99	41+

For all/unspec product, the definition of cigarette equivalents is shown at the end of Sections -1 and -4.
2) Results complete enough for use in metaanalysis

Within each study, results are then selected (in the following order of preference, within each sex) for:
3) SMKSTA: ever smokers, current smokers
4) PRODUCT: cigarettes regardless of other products, cigarettes only, all/unspec
5) CIGTYPE: all/unspecified, MC regardless of HR, MC only
6) DENOM: never smoked anything, never smoked cigarettes, (never +1 = +long term ex, +2 = +amount unknown,
+3 = never cigs+long term ex)
7) Followup period (YF, prospective studies): whole study (coded as 0) or longest available
8) LCtype: squamous or nearest available, but not adeno. (q = squamous, s = small,
a = adeno, KI = Kreyberg I, u = undifferentiated)
9) Race: all or nearest available, otherwise by race (wh or w = white, bl or b = black, hi = hispanic
ch = chinese, jap = japanese, haw = hawaiian, w+o = white + oriental, sca = scandinavian, as = asian)
10) For overlapping studies: principal rather than subsidiary studies
Finally by Age: whole study (coded as 0) if available, otherwise by widest available age group
and then for single sex results (m, f) in preference to combined sex results (c).

Results adjusted (AD) for the most potential confounders are then chosen in Sections -1 to -3
(and those which actually differ from the adjusted results in Table 2G11 - 1 are marked 'x' in Section -1)
and results adjusted for the least confounders in Sections -4 to -6. (Those least adjusted results which
actually differ from the most adjusted as marked 'x' in column X in Section -4)
(Results adjusted for an unknown number of confounder(s) are coded as 20.)

Section -7 shows excluded studies, together with the stage (as above) at which no qualifying
results were found.

Section -8 lists the potentially overlapping studies which have been included (1=principal, 2=subsidiary).

Section -9 lists any results which would have been included in preference except that they had data not complete
enough for use in meta-analysis, with their significance (yes/no), if known, and any further comment as entered
on the database.

In addition to those mentioned above, the following fields, levels and abbreviations are used:
* or nk = not known, n = no, y = yes, ot = other
ev = ever, cu = current, nev = never
all/unspec = all or unspecified, cig+/-ot = cigarettes irrespective of other products (cigar, pipe etc)
MC = manufactured cigarettes, HR = hand-rolled cigarettes
exL, exH = range of exposure (low and high) in the smoking group, in terms of Amount smoked,
cigarettes or cigarette equivalents
REF: 6-character study reference
NRR: number of the RR on the database within the study
ST : study type (CC = case control, pr or prosp = prospective)
NLC: number of lung cancer cases in whole study
R : risky occupational population (n = no, m = mining, o = other risky)
VB : national cigarette type (V = at least 75% Virginia, bl = at least 75% blended, ot = other)
P : any proxy use
H : full histological confirmation
De : derivation of RR/CI (or = original, st = standard method, ot = other method of estimation)

Table 2G16 - 1

IESLC - Meta-anal of Ever Smoking (or Curr if Ever not avail) by Amount, Overview, Cigs (or Any Prod if Cigs not avail)
Squamous
Most adjusted

REF	NRR	2G11	SEX	AGEL	AGEH	RACE	YF	LC TYPE	LOC	START	ST	NLC	R	VB	P	H	AD	SM	PRODUCT	exL	exH	S1	S2	DENOM	De
ALDERS	34		m	0	0	all	-	q+s	Eu:UK	1977	CC	1448	n	V	n	n	1	ev	cig only	1	17	1	0	nev+2	ot
ALDERS	35		m	0	0	all	-	q+s	Eu:UK	1977	CC	1448	n	V	n	n	1	ev	cig only	18	27	2	3	nev+2	ot
ALDERS	36		m	0	0	all	-	q+s	Eu:UK	1977	CC	1448	n	V	n	n	1	ev	cig only	28	99	3	0	nev+2	ot
ALDERS	37		f	0	0	all	-	q+s	Eu:UK	1977	CC	1448	n	V	n	n	1	ev	cig only	1	17	1	0	nev+2	ot
ALDERS	38		f	0	0	all	-	q+s	Eu:UK	1977	CC	1448	n	V	n	n	1	ev	cig only	18	27	2	3	nev+2	ot
ALDERS	39		f	0	0	all	-	q+s	Eu:UK	1977	CC	1448	n	V	n	n	1	ev	cig only	28	99	3	0	nev+2	ot
BARBON	70		m	0	0	all	-	q	Eu:wst	1979	CC	755	n	bl	y	y	3	ev	all/unsp	1	19	1	0	nev any	or
BARBON	71		m	0	0	all	-	q	Eu:wst	1979	CC	755	n	bl	y	y	3	ev	all/unsp	20	39	2	0	nev any	or
BARBON	72		m	0	0	all	-	q	Eu:wst	1979	CC	755	n	bl	y	y	3	ev	all/unsp	40	99	3	0	nev any	or
BOUCOT	142		m	0	0	all	0	q	NAmer	1951	pr	121	n	bl	n	n	2	cu	cig only	1	20	0	0	nev any	ot
BOUCOT	143		m	0	0	all	0	q	NAmer	1951	pr	121	n	bl	n	n	2	cu	cig only	21	99	3	0	nev any	ot
BROWN2	36		m	0	0	wh	-	q	NAmer	1984	CC	14596	n	bl	n	y	2	ev	cig+/-ot	1	19	1	0	nev cigs	or
BROWN2	46		m	0	0	wh	-	q	NAmer	1984	CC	14596	n	bl	n	y	2	ev	cig+/-ot	20	99	0	0	nev cigs	or
BROWN2	35		f	0	0	wh	-	q	NAmer	1984	CC	14596	n	bl	n	y	2	ev	cig+/-ot	1	19	1	0	nev cigs	or
BROWN2	45		f	0	0	wh	-	q	NAmer	1984	CC	14596	n	bl	n	y	2	ev	cig+/-ot	20	99	0	0	nev cigs	or
CHOI	46		m	0	0	all	-	q	As:oth	1985	CC	375	n	bl	n	n	0	ev	cig+/-ot	1	10	1	0	nev cigs	st
CHOI	47		m	0	0	all	-	q	As:oth	1985	CC	375	n	bl	n	n	0	ev	cig+/-ot	11	20	2	3	nev cigs	st
CHOI	48		m	0	0	all	-	q	As:oth	1985	CC	375	n	bl	n	n	0	ev	cig+/-ot	21	30	0	4	nev cigs	st
CHOI	49		m	0	0	all	-	q	As:oth	1985	CC	375	n	bl	n	n	0	ev	cig+/-ot	31	40	0	5	nev cigs	st
CHOI	50		m	0	0	all	-	q	As:oth	1985	CC	375	n	bl	n	n	0	ev	cig+/-ot	41	99	3	6	nev cigs	st
CHOI	56		f	0	0	all	-	q	As:oth	1985	CC	375	n	bl	n	n	0	ev	cig+/-ot	1	10	1	0	nev cigs	st
CHOI	57		f	0	0	all	-	q	As:oth	1985	CC	375	n	bl	n	n	0	ev	cig+/-ot	11	30	2	0	nev cigs	st
CHOI	58		f	0	0	all	-	q	As:oth	1985	CC	375	n	bl	n	n	0	ev	cig+/-ot	31	99	3	0	nev cigs	st
CORREA	47		c	0	0	all	-	q+s	NAmer	1979	CC	1359	n	bl	y	n	1	cu	cig+/-ot	1	20	0	0	nev cigs	or
CORREA	51		c	0	0	all	-	q+s	NAmer	1979	CC	1359	n	bl	y	n	1	cu	cig+/-ot	21	99	3	0	nev cigs	or
DOLL	68		m	0	0	all	-	KI	Eu:UK	1948	CC	1465	n	V	n	n	1	ev	all/unsp	1	4	0	1	nev any	ot
DOLL	69		m	0	0	all	-	KI	Eu:UK	1948	CC	1465	n	V	n	n	1	ev	all/unsp	5	14	1	2	nev any	ot
DOLL	70		m	0	0	all	-	KI	Eu:UK	1948	CC	1465	n	V	n	n	1	ev	all/unsp	15	24	2	3	nev any	ot
DOLL	71		m	0	0	all	-	KI	Eu:UK	1948	CC	1465	n	V	n	n	1	ev	all/unsp	25	99	3	0	nev any	ot
DOLL	76		f	0	0	all	-	KI	Eu:UK	1948	CC	1465	n	V	n	n	1	ev	all/unsp	1	4	0	1	nev any	ot
DOLL	77		f	0	0	all	-	KI	Eu:UK	1948	CC	1465	n	V	n	n	1	ev	all/unsp	5	14	1	2	nev any	ot
DOLL	78		f	0	0	all	-	KI	Eu:UK	1948	CC	1465	n	V	n	n	1	ev	all/unsp	15	99	0	0	nev any	ot
DORGAN	114		m	0	0	wh	-	q	NAmer	1980	CC	2026	n	bl	y	y	2	ev	cig+/-ot	1	19	1	0	nev any	ot
DORGAN	115		m	0	0	wh	-	q	NAmer	1980	CC	2026	n	bl	y	y	2	ev	cig+/-ot	20	99	0	0	nev any	ot
DORGAN	99		f	0	0	all	-	q	NAmer	1980	CC	2026	n	bl	y	y	3	ev	cig+/-ot	1	19	1	0	nev any	ot
DORGAN	100		f	0	0	all	-	q	NAmer	1980	CC	2026	n	bl	y	y	3	ev	cig+/-ot	20	99	0	0	nev any	ot
DOSEME	7		m	0	0	all	-	q	Eu:bal	1979	CC	1210	n	bl	n	n	2	ev	cig+/-ot	1	10	1	0	nev cigs	or
DOSEME	11		m	0	0	all	-	q	Eu:bal	1979	CC	1210	n	bl	n	n	2	ev	cig+/-ot	11	20	2	3	nev cigs	or
DOSEME	15		m	0	0	all	-	q	Eu:bal	1979	CC	1210	n	bl	n	n	2	ev	cig+/-ot	21	99	3	0	nev cigs	or
ENGELA	57		m	0	0	all	0	q	Eu:Sca	1964	pr	435	n	bl	n	n	7	cu	cig+/-ot	1	4	0	1	nev cigs	or
ENGELA	58		m	0	0	all	0	q	Eu:Sca	1964	pr	435	n	bl	n	n	7	cu	cig+/-ot	5	9	1	0	nev cigs	or
ENGELA	59		m	0	0	all	0	q	Eu:Sca	1964	pr	435	n	bl	n	n	7	cu	cig+/-ot	10	14	0	2	nev cigs	or
ENGELA	60		m	0	0	all	0	q	Eu:Sca	1964	pr	435	n	bl	n	n	7	cu	cig+/-ot	15	19	0	0	nev cigs	or
ENGELA	61		m	0	0	all	0	q	Eu:Sca	1964	pr	435	n	bl	n	n	7	cu	cig+/-ot	20	99	0	0	nev cigs	or
GER	14		c	0	0	all	-	q+s	As:oth	1990	CC	141	n	ot	y	n	10	ev	all/unsp	1	10	1	0	nev any	ot
GER	15		c	0	0	all	-	q+s	As:oth	1990	CC	141	n	ot	y	n	10	ev	all/unsp	11	20	2	3	nev any	ot
GER	16		c	0	0	all	-	q+s	As:oth	1990	CC	141	n	ot	y	n	10	ev	all/unsp	21	99	3	0	nev any	ot
HAENSZ	18		f	0	0	all	-	q+u	NAmer	1955	CC	158	n	bl	n	y	0	cu	cig+/-ot	1	20	0	0	nev any	or
HAENSZ	17		f	0	0	all	-	q+u	NAmer	1955	CC	158	n	bl	n	y	0	cu	cig+/-ot	21	99	3	0	nev any	or
HAMMON	98		m	0	0	wh	0	not a	NAmer	1952	pr	448	n	bl	n	n	1	cu	cig only	1	9	1	1	nev any	ot
HAMMON	99		m	0	0	wh	0	not a	NAmer	1952	pr	448	n	bl	n	n	1	cu	cig only	10	20	2	0	nev any	ot
HAMMON	100		m	0	0	wh	0	not a	NAmer	1952	pr	448	n	bl	n	n	1	cu	cig only	21	39	0	4	nev any	ot
HAMMON	101		m	0	0	wh	0	not a	NAmer	1952	pr	448	n	bl	n	n	1	cu	cig only	40	99	3	0	nev any	ot
JEDRYC	28		m	0	0	all	-	q	Eu:est	1980	CC	1630	n	bl	y	n	3	ev	cig+/-ot	1	19	1	0	nev any	or
JEDRYC	29		m	0	0	all	-	q	Eu:est	1980	CC	1630	n	bl	y	n	3	ev	cig+/-ot	20	29	2	3	nev any	or
JEDRYC	30		m	0	0	all	-	q	Eu:est	1980	CC	1630	n	bl	y	n	3	ev	cig+/-ot	30	99	3	0	nev any	or
KATSOU	21		f	0	0	all	-	KI	Eu:bal	1987	CC	101	n	bl	n	n	1	cu	all/unsp	1	20	0	0	nev any	or
KATSOU	22		f	0	0	all	-	KI	Eu:bal	1987	CC	101	n	bl	n	n	1	cu	all/unsp	21	99	3	0	nev any	or
KREYBE	1		m	0	0	all	-	KI	Eu:Sca	1948	CC	300	n	bl	n	y	1	ev	all/unsp	1	14	1	0	nev any	ot
KREYBE	2		m	0	0	all	-	KI	Eu:Sca	1948	CC	300	n	bl	n	y	1	ev	all/unsp	15	24	2	3	nev any	ot
KREYBE	3		m	0	0	all	-	KI	Eu:Sca	1948	CC	300	n	bl	n	y	1	ev	all/unsp	25	99	3	0	nev any	ot
KREYBE	31		f	0	0	all	-	KI	Eu:Sca	1948	CC	300	n	bl	n	y	0	ev	all/unsp	1	14	1	0	nev any	st
KREYBE	32		f	0	0	all	-	KI	Eu:Sca	1948	CC	300	n	bl	n	y	0	ev	all/unsp	15	99	0	0	nev any	st
LAMTH	10		f	0	0	ch	-	q	As:HK	1983	CC	445	n	bl	n	n	0	ev	all/unsp	1	10	1	0	nev any	or
LAMTH	11		f	0	0	ch	-	q	As:HK	1983	CC	445	n	bl	n	n	0	ev	all/unsp	11	20	2	3	nev any	or
LAMTH	12		f	0	0	ch	-	q	As:HK	1983	CC	445	n	bl	n	n	0	ev	all/unsp	21	99	3	0	nev any	st
LUBIN2	149		m	0	0	all	-	q	Eu:mul	1976	CC	7804	n	bl	n	y	0	ev	cig+/-ot	1	9	1	1	nev any	st
LUBIN2	153		m	0	0	all	-	q	Eu:mul	1976	CC	7804	n	bl	n	y	0	ev	cig+/-ot	10	19	0	2	nev any	st
LUBIN2	157		m	0	0	all	-	q	Eu:mul	1976	CC	7804	n	bl	n	y	0	ev	cig+/-ot	20	29	2	3	nev any	st
LUBIN2	161		m	0	0	all	-	q	Eu:mul	1976	CC	7804	n	bl	n	y	0	ev	cig+/-ot	30	99	3	0	nev any	st
LUBIN2	169		f	0	0	all	-	q	Eu:mul	1976	CC	7804	n	bl	n	y	0	ev	cig+/-ot	1	9	1	1	nev any	st
LUBIN2	173		f	0	0	all	-	q	Eu:mul	1976	CC	7804	n	bl	n	y	0	ev	cig+/-ot	10	19	0	2	nev any	st

Table 2G16 - 1

IESLC - Meta-anal of Ever Smoking (or Curr if Ever not avail) by Amount, Overview, Cigs (or Any Prod if Cigs not avail)
Squamous
Most adjusted

REF	NRR	2G11	SEX	AGEL	AGEH	RACE	YF	LC TYPE	LOC	START	ST	NLC	R	VB	P	H	AD	SM	PRODUCT	exL	exH	S1	S2	DENOM	De
LUBIN2	177		f	0	0	all	-	q	Eu:mul	1976	CC	7804	n	bl	n	y	0	ev	cig+/-ot	20	29	2	3	nev any	st
LUBIN2	181		f	0	0	all	-	q	Eu:mul	1976	CC	7804	n	bl	n	y	0	ev	cig+/-ot	30	99	3	0	nev any	st
LUO	10		c	0	0	all	-	q	As:Chi	1990	CC	102	n	ot	n	y	20	ev	cig+/-ot	1	19	1	0	nev cigs	or
LUO	11		c	0	0	all	-	q	As:Chi	1990	CC	102	n	ot	n	y	20	ev	cig+/-ot	20	29	2	3	nev cigs	or
LUO	12		c	0	0	all	-	q	As:Chi	1990	CC	102	n	ot	n	y	20	ev	cig+/-ot	30	99	3	0	nev cigs	or
MATOS	43		m	0	0	all	-	q	SCAmer	1994	CC	200	n	bl	n	n	2	ev	cig+/-ot	1	14	1	0	nev any	or
MATOS	45		m	0	0	all	-	q	SCAmer	1994	CC	200	n	bl	n	n	2	ev	cig+/-ot	15	24	2	3	nev any	or
MATOS	47		m	0	0	all	-	q	SCAmer	1994	CC	200	n	bl	n	n	2	ev	cig+/-ot	25	99	3	0	nev any	or
MATSUD	4		m	0	0	all	-	q	As:Jap	1965	CC	179	n	bl	n	n	0	ev	cig+/-ot	1	10	1	0	nev cigs	st
MATSUD	5		m	0	0	all	-	q	As:Jap	1965	CC	179	n	bl	n	n	0	ev	cig+/-ot	11	20	2	3	nev cigs	st
MATSUD	6		m	0	0	all	-	q	As:Jap	1965	CC	179	n	bl	n	n	0	ev	cig+/-ot	21	99	3	0	nev cigs	st
ORMOS	5		m	0	0	all	-	q	Eu:est	1947	CC	119	n	bl	y	y	0	ev	cig+/-ot	1	15	1	0	nev any	st
ORMOS	6		m	0	0	all	-	q	Eu:est	1947	CC	119	n	bl	y	y	0	ev	cig+/-ot	16	30	2	0	nev any	st
ORMOS	7		m	0	0	all	-	q	Eu:est	1947	CC	119	n	bl	y	y	0	ev	cig+/-ot	31	99	3	0	nev any	st
OSANN	51		m	0	0	all	-	q	NAmer	1984	CC	1986	n	bl	n	n	2	ev	cig+/-ot	1	39	0	0	nev cigs	or
OSANN	59		m	0	0	all	-	q	NAmer	1984	CC	1986	n	bl	n	n	2	ev	cig+/-ot	40	99	3	0	nev cigs	or
OSANN	52		f	0	0	all	-	q	NAmer	1984	CC	1986	n	bl	n	n	2	ev	cig+/-ot	1	39	0	0	nev cigs	or
OSANN	60		f	0	0	all	-	q	NAmer	1984	CC	1986	n	bl	n	n	2	ev	cig+/-ot	40	99	3	0	nev cigs	or
OSANN2	28		f	0	0	all	-	KI	NAmer	1964	ot	217	n	bl	n	y	1	ev	cig+/-ot	1	19	1	0	nev cigs	or
OSANN2	29		f	0	0	all	-	KI	NAmer	1964	ot	217	n	bl	n	y	1	ev	cig+/-ot	20	99	0	0	nev cigs	or
SOBUE	53		m	0	0	all	-	q	As:Jap	1986	CC	1376	n	bl	n	y	0	cu	cig+/-ot	1	19	1	0	nev cigs	st
SOBUE	54		m	0	0	all	-	q	As:Jap	1986	CC	1376	n	bl	n	y	0	cu	cig+/-ot	20	29	2	3	nev cigs	st
SOBUE	55		m	0	0	all	-	q	As:Jap	1986	CC	1376	n	bl	n	y	0	cu	cig+/-ot	30	99	3	0	nev cigs	st
SVENSS	7		f	0	0	all	-	q	Eu:Sca	1983	CC	210	n	bl	n	n	1	cu	all/unsp	1	10	1	0	nev any	or
SVENSS	12		f	0	0	all	-	q	Eu:Sca	1983	CC	210	n	bl	n	n	1	cu	all/unsp	11	20	2	3	nev any	or
SVENSS	17		f	0	0	all	-	q	Eu:Sca	1983	CC	210	n	bl	n	n	1	cu	all/unsp	21	99	3	0	nev any	ot
TSUGAN	15		m	0	0	all	-	q	As:Jap	1976	CC	134	n	bl	n	y	0	cu	all/unsp	1	15	1	0	nev any	ot
TSUGAN	16		m	0	0	all	-	q	As:Jap	1976	CC	134	n	bl	n	y	0	cu	all/unsp	16	35	2	0	nev any	ot
TSUGAN	17		m	0	0	all	-	q	As:Jap	1976	CC	134	n	bl	n	y	0	cu	all/unsp	36	99	3	0	nev any	ot
WAKAI	46		m	0	0	all	-	q	As:Jap	1988	CC	333	n	bl	n	y	1	cu	cig+/-ot	1	19	1	0	nev any	or
WAKAI	47		m	0	0	all	-	q	As:Jap	1988	CC	333	n	bl	n	y	1	cu	cig+/-ot	20	29	2	3	nev any	or
WAKAI	48		m	0	0	all	-	q	As:Jap	1988	CC	333	n	bl	n	y	1	cu	cig+/-ot	30	99	3	0	nev any	or
WU	17		f	0	0	wh	-	q	NAmer	1981	CC	220	n	bl	n	y	2	cu	all/unsp	1	20	0	0	nev any	or
WU	18		f	0	0	wh	-	q	NAmer	1981	CC	220	n	bl	n	y	2	cu	all/unsp	21	99	3	0	nev any	or
WUWILL	14		f	0	0	all	-	q+s	As:Chi	1985	CC	965	n	ot	n	n	3	ev	cig+/-ot	1	19	1	0	nev cigs	ot
WUWILL	15		f	0	0	all	-	q+s	As:Chi	1985	CC	965	n	ot	n	n	3	ev	cig+/-ot	20	99	0	0	nev cigs	ot
WYNDE2	3		m	0	0	all	-	KI	NAmer	1962	CC	404	n	bl	n	y	0	ev	cig+/-ot	1	10	1	0	nev any	st
WYNDE2	4		m	0	0	all	-	KI	NAmer	1962	CC	404	n	bl	n	y	0	ev	cig+/-ot	11	20	2	3	nev any	st
WYNDE2	5		m	0	0	all	-	KI	NAmer	1962	CC	404	n	bl	n	y	0	ev	cig+/-ot	21	34	0	4	nev any	st
WYNDE2	6		m	0	0	all	-	KI	NAmer	1962	CC	404	n	bl	n	y	0	ev	cig+/-ot	35	99	3	0	nev any	st
WYNDE3	4		m	0	0	all	-	KI	NAmer	1966	CC	350	n	bl	n	y	0	ev	cig+/-ot	1	9	1	1	nev any	st
WYNDE3	5		m	0	0	all	-	KI	NAmer	1966	CC	350	n	bl	n	y	0	ev	cig+/-ot	10	20	2	0	nev any	st
WYNDE3	6		m	0	0	all	-	KI	NAmer	1966	CC	350	n	bl	n	y	0	ev	cig+/-ot	21	40	0	0	nev any	st
WYNDE3	7		m	0	0	all	-	KI	NAmer	1966	CC	350	n	bl	n	y	0	ev	cig+/-ot	41	99	3	6	nev any	st
WYNDE3	63		f	0	0	all	-	KI	NAmer	1966	CC	350	n	bl	n	y	0	ev	cig+/-ot	1	9	1	1	nev any	st
WYNDE3	64		f	0	0	all	-	KI	NAmer	1966	CC	350	n	bl	n	y	0	ev	cig+/-ot	10	20	2	0	nev any	st
WYNDE3	65		f	0	0	all	-	KI	NAmer	1966	CC	350	n	bl	n	y	0	ev	cig+/-ot	21	40	0	0	nev any	st
WYNDE3	66		f	0	0	all	-	KI	NAmer	1966	CC	350	n	bl	n	y	0	ev	cig+/-ot	41	99	3	6	nev any	st
WYNDE4	63		m	0	0	all	-	not a	NAmer	1948	CC	684	n	bl	y	n	2	ev	all/unsp	1	9	1	1	nev any	ot
WYNDE4	64		m	0	0	all	-	not a	NAmer	1948	CC	684	n	bl	y	n	2	ev	all/unsp	10	15	0	2	nev any	ot
WYNDE4	65		m	0	0	all	-	not a	NAmer	1948	CC	684	n	bl	y	n	2	ev	all/unsp	16	20	2	3	nev any	ot
WYNDE4	66		m	0	0	all	-	not a	NAmer	1948	CC	684	n	bl	y	n	2	ev	all/unsp	21	34	0	4	nev any	ot
WYNDE4	67		m	0	0	all	-	not a	NAmer	1948	CC	684	n	bl	y	n	2	ev	all/unsp	35	99	3	0	nev any	ot
WYNDE4	49		f	0	0	all	-	not a	NAmer	1948	CC	684	n	bl	y	n	2	ev	all/unsp	1	9	1	1	nev any	ot
WYNDE4	50		f	0	0	all	-	not a	NAmer	1948	CC	684	n	bl	y	n	2	ev	all/unsp	10	15	0	2	nev any	ot
WYNDE4	51		f	0	0	all	-	not a	NAmer	1948	CC	684	n	bl	y	n	2	ev	all/unsp	16	20	2	3	nev any	ot
WYNDE4	52		f	0	0	all	-	not a	NAmer	1948	CC	684	n	bl	y	n	2	ev	all/unsp	21	34	0	4	nev any	ot
WYNDE4	53		f	0	0	all	-	not a	NAmer	1948	CC	684	n	bl	y	n	2	ev	all/unsp	35	99	3	0	nev any	ot
WYNDE6	21		m	0	0	all	-	KI	NAmer	1969	CC	4423	n	bl	n	y	0	cu	cig+/-ot	1	10	1	0	nev any	st
WYNDE6	30		m	0	0	all	-	KI	NAmer	1969	CC	4423	n	bl	n	y	0	cu	cig+/-ot	11	20	2	3	nev any	st
WYNDE6	39		m	0	0	all	-	KI	NAmer	1969	CC	4423	n	bl	n	y	0	cu	cig+/-ot	21	30	0	4	nev any	st
WYNDE6	48		m	0	0	all	-	KI	NAmer	1969	CC	4423	n	bl	n	y	0	cu	cig+/-ot	31	99	3	0	nev any	st
WYNDE6	210		f	0	0	all	-	KI	NAmer	1969	CC	4423	n	bl	n	y	0	cu	cig+/-ot	1	10	1	0	nev cigs	st
WYNDE6	219		f	0	0	all	-	KI	NAmer	1969	CC	4423	n	bl	n	y	0	cu	cig+/-ot	11	20	2	3	nev cigs	st
WYNDE6	228		f	0	0	all	-	KI	NAmer	1969	CC	4423	n	bl	n	y	0	cu	cig+/-ot	21	30	0	4	nev cigs	st
WYNDE6	237		f	0	0	all	-	KI	NAmer	1969	CC	4423	n	bl	n	y	0	cu	cig+/-ot	30	99	3	0	nev cigs	st
ZHENG	1		m	0	0	all	-	q	As:Chi	1982	CC	540	n	ot	*	y	0	ev	cig+/-ot	1	9	1	1	nev cigs	st
ZHENG	2		m	0	0	all	-	q	As:Chi	1982	CC	540	n	ot	*	y	0	ev	cig+/-ot	10	19	0	2	nev cigs	st
ZHENG	3		m	0	0	all	-	q	As:Chi	1982	CC	540	n	ot	*	y	0	ev	cig+/-ot	20	29	2	3	nev cigs	st
ZHENG	4		m	0	0	all	-	q	As:Chi	1982	CC	540	n	ot	*	y	0	ev	cig+/-ot	30	99	3	0	nev cigs	st
ZHENG	16		f	0	0	all	-	q	As:Chi	1982	CC	540	n	ot	*	y	0	ev	cig+/-ot	1	9	1	1	nev cigs	st
ZHENG	17		f	0	0	all	-	q	As:Chi	1982	CC	540	n	ot	*	y	0	ev	cig+/-ot	10	99	0	0	nev cigs	st

Table 2G16 - 1

IESLC - Meta-anal of Ever Smoking (or Curr if Ever not avail) by Amount, Overview, Cigs (or Any Prod if Cigs not avail)
Squamous
Most adjusted

REF	NRR	2G11	SEX	AGEL	AGEH	RACE	YF	LC TYPE	LOC	START	ST	NLC	R	VB	P	H	AD	SM	PRODUCT	exL	exH	S1	S2	DENOM	De
ZHOU	10		c	0	0	all	-	q	As:Chi	1978	CC	1360	n	ot	n	n	0	ev	all/unsp	1	9	1	1	nev any	st
ZHOU	11		c	0	0	all	-	q	As:Chi	1978	CC	1360	n	ot	n	n	0	ev	all/unsp	10	19	0	2	nev any	st
ZHOU	12		c	0	0	all	-	q	As:Chi	1978	CC	1360	n	ot	n	n	0	ev	all/unsp	20	99	0	0	nev any	st

Cigarette type is all/unspec for all RRs

except for the following:

REF	NRR	CIGTYPE
ALDERS	34	MC only
ALDERS	35	MC only
ALDERS	36	MC only
ALDERS	37	MC only
ALDERS	38	MC only
ALDERS	39	MC only

REF	NRR	Cigarette equivalent
ALDERS	34	-
ALDERS	35	-
ALDERS	36	-
ALDERS	37	-
ALDERS	38	-
ALDERS	39	-
BARBON	70	*
BARBON	71	*
BARBON	72	*
BOUCOT	142	-
BOUCOT	143	-
BROWN2	36	*
BROWN2	46	*
BROWN2	35	*
BROWN2	45	*
CHOI	46	*
CHOI	47	*
CHOI	48	*
CHOI	49	*
CHOI	50	*
CHOI	56	*
CHOI	57	*
CHOI	58	*
CORREA	47	*
CORREA	51	*
DOLL	68	grams
DOLL	69	grams
DOLL	70	grams
DOLL	71	grams
DOLL	76	grams
DOLL	77	grams
DOLL	78	grams
DORGAN	114	*
DORGAN	115	*
DORGAN	99	*
DORGAN	100	*
DOSEME	7	*
DOSEME	11	*
DOSEME	15	*
ENGELA	57	*
ENGELA	58	*
ENGELA	59	*
ENGELA	60	*
ENGELA	61	*
GER	14	*
GER	15	*
GER	16	*
HAENSZ	18	*
HAENSZ	17	*
HAMMON	98	-
HAMMON	99	-
HAMMON	100	-
HAMMON	101	-

Table 2G16 - 1

IESLC - Meta-anal of Ever Smoking (or Curr if Ever not avail) by Amount, Overview, Cigs (or Any Prod if Cigs not avail)
Squamous
Most adjusted

REF	NRR	Cigarette equivalent
JEDRYC	28	*
JEDRYC	29	*
JEDRYC	30	*
KATSOU	21	*
KATSOU	22	*
KREYBE	1	grams inc 1 cig=1
KREYBE	2	grams inc 1 cig=1
KREYBE	3	grams inc 1 cig=1
KREYBE	31	grams inc 1 cig=1
KREYBE	32	grams inc 1 cig=1
LAMTH	10	*
LAMTH	11	*
LAMTH	12	*
LUBIN2	149	*
LUBIN2	153	*
LUBIN2	157	*
LUBIN2	161	*
LUBIN2	169	*
LUBIN2	173	*
LUBIN2	177	*
LUBIN2	181	*
LUO	10	*
LUO	11	*
LUO	12	*
MATOS	43	*
MATOS	45	*
MATOS	47	*
MATSUD	4	*
MATSUD	5	*
MATSUD	6	*
ORMOS	5	*
ORMOS	6	*
ORMOS	7	*
OSANN	51	*
OSANN	59	*
OSANN	52	*
OSANN	60	*
OSANN2	28	*
OSANN2	29	*
SOBUE	53	*
SOBUE	54	*
SOBUE	55	*
SVENSS	7	*
SVENSS	12	*
SVENSS	17	*
TSUGAN	15	*
TSUGAN	16	*
TSUGAN	17	*
WAKAI	46	*
WAKAI	47	*
WAKAI	48	*
WU	17	*
WU	18	*
WUWILL	14	*
WUWILL	15	*
WYNDE2	3	*
WYNDE2	4	*
WYNDE2	5	*
WYNDE2	6	*
WYNDE3	4	*
WYNDE3	5	*
WYNDE3	6	*
WYNDE3	7	*
WYNDE3	63	*
WYNDE3	64	*
WYNDE3	65	*
WYNDE3	66	*
WYNDE4	63	*
WYNDE4	64	*
WYNDE4	65	*
WYNDE4	66	*
WYNDE4	67	*

Table 2G16 - 1

IESLC - Meta-anal of Ever Smoking (or Curr if Ever not avail) by Amount, Overview, Cigs (or Any Prod if Cigs not avail)
Squamous
Most adjusted

REF	NRR	Cigarette equivalent
WYNDE4	49	inc 1 cigar = 5 cigs, 1 pipe = 2.5 cigs
WYNDE4	50	inc 1 cigar = 5 cigs, 1 pipe = 2.5 cigs
WYNDE4	51	inc 1 cigar = 5 cigs, 1 pipe = 2.5 cigs
WYNDE4	52	inc 1 cigar = 5 cigs, 1 pipe = 2.5 cigs
WYNDE4	53	inc 1 cigar = 5 cigs, 1 pipe = 2.5 cigs
WYNDE6	21	*
WYNDE6	30	*
WYNDE6	39	*
WYNDE6	48	*
WYNDE6	210	*
WYNDE6	219	*
WYNDE6	228	*
WYNDE6	237	*
ZHENG	1	*
ZHENG	2	*
ZHENG	3	*
ZHENG	4	*
ZHENG	16	*
ZHENG	17	*
ZHOU	10	*
ZHOU	11	*
ZHOU	12	*

In this overview table, subtotals and Qs values may be invalid and should be ignored

Table 2G16 - 2

IESLC - Meta-anal of Ever Smoking (or Curr if Ever not avail) by Amount, Overview, Cigs (or Any Prod if Cigs not avail)
Squamous
Most adjusted

REF	NRR	SEX	AD	Number Exposed Case	Number Exposed Cont	Non-exposed Case	Non-exposed Cont	RR	95.00%CI
ALDERS	34	m	1	-	-	-	-	3.79	(1.30- 11.02)
ALDERS	35	m	1	-	-	-	-	7.19	(2.75- 18.79)
ALDERS	36	m	1	-	-	-	-	8.78	(3.46- 22.31)
ALDERS	37	f	1	-	-	-	-	2.55	(1.42- 4.57)
ALDERS	38	f	1	-	-	-	-	9.24	(5.31- 16.09)
ALDERS	39	f	1	-	-	-	-	14.52	(7.93- 26.58)
Subtotal ALDERS								6.82	(5.12- 9.11)
BARBON	70	m	3	-	-	-	-	8.50	(3.60- 20.00)
BARBON	71	m	3	-	-	-	-	16.30	(7.00- 38.00)
BARBON	72	m	3	-	-	-	-	28.60	(12.00- 69.00)
Subtotal BARBON								15.71	(9.57- 25.79)
*BOUCOT	142	m	2	-	-	-	-	21.41	(1.40- 385.13)
*BOUCOT	143	m	2	-	-	-	-	46.64	(2.80- 775.69)
Subtotal BOUCOT								31.58	(4.33- 230.41)
BROWN2	36	m	2	-	-	-	-	7.60	(6.20- 9.40)
BROWN2	46	m	2	-	-	-	-	17.20	(14.60- 20.30)
BROWN2	35	f	2	-	-	-	-	11.70	(8.70- 15.80)
BROWN2	45	f	2	-	-	-	-	26.10	(20.70- 32.80)
Subtotal BROWN2								14.51	(13.06- 16.12)
CHOI	46	m	0	12	90	6	95	2.11	(0.76- 5.86)
CHOI	47	m	0	84	281	6	95	4.73	(2.00- 11.19)
CHOI	48	m	0	30	49	6	95	9.69	(3.78- 24.86)
CHOI	49	m	0	25	39	6	95	10.15	(3.86- 26.66)
CHOI	50	m	0	9	6	6	95	23.75	(6.33- 89.09)
CHOI	56	f	0	4	16	10	164	4.10	(1.15- 14.57)
CHOI	57	f	0	5	9	10	164	9.11	(2.57- 32.31)
CHOI	58	f	0	2	1	10	164	32.80	(2.74- 393.20)
Subtotal CHOI								6.86	(4.63- 10.16)
CORREA	47	c	1	-	-	-	-	23.20	(14.60- 37.00)
CORREA	51	c	1	-	-	-	-	54.80	(35.60- 89.20)
Subtotal CORREA								35.84	(25.85- 49.70)
DOLL	68	m	1	-	-	-	-	4.70	(1.38- 16.03)
DOLL	69	m	1	-	-	-	-	10.60	(3.30- 34.07)
DOLL	70	m	1	-	-	-	-	14.30	(4.45- 46.00)
DOLL	71	m	1	-	-	-	-	25.40	(7.83- 82.40)
DOLL	76	f	1	-	-	-	-	1.00	(0.38- 2.64)
DOLL	77	f	1	-	-	-	-	1.70	(0.64- 4.50)
DOLL	78	f	1	-	-	-	-	8.30	(2.77- 24.84)
Subtotal DOLL								5.02	(3.32- 7.61)
DORGAN	114	m	2	-	-	-	-	11.50	(4.10- 32.24)
DORGAN	115	m	2	-	-	-	-	23.29	(8.55- 63.49)
DORGAN	99	f	3	-	-	-	-	7.78	(4.86- 12.44)
DORGAN	100	f	3	-	-	-	-	16.38	(10.22- 26.26)
Subtotal DORGAN								12.06	(8.92- 16.31)
DOSEME	7	m	2	-	-	-	-	2.60	(1.50- 4.60)
DOSEME	11	m	2	-	-	-	-	3.20	(2.20- 4.60)
DOSEME	15	m	2	-	-	-	-	7.00	(4.10- 12.00)
Subtotal DOSEME								3.71	(2.84- 4.84)
*ENGELA	57	m	7	-	-	-	-	4.30	(1.00- 19.00)
*ENGELA	58	m	7	-	-	-	-	7.70	(1.90- 31.00)
*ENGELA	59	m	7	-	-	-	-	15.00	(3.90- 60.00)
*ENGELA	60	m	7	-	-	-	-	30.00	(7.40- 120.00)
*ENGELA	61	m	7	-	-	-	-	24.00	(5.90- 94.00)
Subtotal ENGELA								13.25	(7.08- 24.79)
GER	14	c	10	-	-	-	-	1.43	(0.36- 5.61)
GER	15	c	10	-	-	-	-	2.20	(0.65- 7.48)
GER	16	c	10	-	-	-	-	16.04	(4.22- 60.93)
Subtotal GER								3.64	(1.71- 7.73)
HAENSZ	18	f	0	30	66	44	236	2.44	(1.42- 4.18)
HAENSZ	17	f	0	18	13	44	236	7.43	(3.40- 16.24)
Subtotal HAENSZ								3.49	(2.24- 5.43)
*HAMMON	98	m	1	-	-	-	-	15.12	(4.93- 46.36)
*HAMMON	99	m	1	-	-	-	-	17.44	(6.30- 48.29)
*HAMMON	100	m	1	-	-	-	-	42.32	(15.38- 116.45)
*HAMMON	101	m	1	-	-	-	-	63.91	(22.02- 185.47)
Subtotal HAMMON								29.45	(17.41- 49.82)
JEDRYC	28	m	3	-	-	-	-	7.51	(3.09- 18.27)
JEDRYC	29	m	3	-	-	-	-	13.46	(5.76- 31.47)
JEDRYC	30	m	3	-	-	-	-	21.42	(9.05- 50.68)
Subtotal JEDRYC								13.09	(7.94- 21.57)
KATSOU	21	f	1	-	-	-	-	3.17	(1.14- 8.85)

Table 2G16 - 2

IESLC - Meta-anal of Ever Smoking (or Curr if Ever not avail) by Amount, Overview, Cigs (or Any Prod if Cigs not avail)
Squamous
Most adjusted

REF	NRR	SEX	AD	Number Exposed Case	Number Exposed Cont	Non-exposed Case	Non-exposed Cont	RR	95.00%CI
KATSOU	22	f	1	-	-	-	-	19.53	(5.36- 71.11)
Subtotal KATSOU								6.39	(2.86- 14.27)
KREYBE	1	m	1	-	-	-	-	9.00	(2.85- 28.38)
KREYBE	2	m	1	-	-	-	-	11.02	(3.42- 35.51)
KREYBE	3	m	1	-	-	-	-	24.63	(7.54- 80.53)
KREYBE	31	f	0	1	286	3	657	0.77	(0.08- 7.39)
KREYBE	32	f	0	1	42	3	657	5.21	(0.53- 51.21)
Subtotal KREYBE								10.04	(5.39- 18.69)
LAMTH	10	f	0	23	11	28	72	5.38	(2.32- 12.46)
LAMTH	11	f	0	28	6	28	72	12.00	(4.49- 32.10)
LAMTH	12	f	0	10	1	28	72	25.71	(3.14- 210.29)
Subtotal LAMTH								8.37	(4.54- 15.43)
LUBIN2	149	m	0	418	2194	54	2616	9.23	(6.91- 12.32)
LUBIN2	153	m	0	1022	3385	54	2616	14.63	(11.07- 19.32)
LUBIN2	157	m	0	1298	3108	54	2616	20.23	(15.33- 26.69)
LUBIN2	161	m	0	849	1746	54	2616	23.56	(17.77- 31.22)
LUBIN2	169	f	0	30	184	72	1180	2.67	(1.70- 4.20)
LUBIN2	173	f	0	91	234	72	1180	6.37	(4.54- 8.95)
LUBIN2	177	f	0	61	110	72	1180	9.09	(6.13- 13.46)
LUBIN2	181	f	0	18	39	72	1180	7.56	(4.12- 13.88)
Subtotal LUBIN2								11.82	(10.51- 13.28)
LUO	10	c	20	-	-	-	-	1.20	(0.10- 10.00)
LUO	11	c	20	-	-	-	-	24.60	(4.20- 145.70)
LUO	12	c	20	-	-	-	-	38.70	(5.20- 290.20)
Subtotal LUO								13.41	(4.24- 42.41)
MATOS	43	m	2	-	-	-	-	1.40	(0.30- 6.90)
MATOS	45	m	2	-	-	-	-	7.80	(2.20- 27.40)
MATOS	47	m	2	-	-	-	-	9.70	(2.80- 33.20)
Subtotal MATOS								5.61	(2.60- 12.11)
MATSUD	4	m	0	21	1237	1	1255	21.31	(2.86- 158.63)
MATSUD	5	m	0	43	1607	1	1255	33.58	(4.62- 244.19)
MATSUD	6	m	0	39	470	1	1255	104.14	(14.27- 760.12)
Subtotal MATSUD								42.26	(13.37- 133.55)
ORMOS	5	m	0	13	329	2	777	15.35	(3.44- 68.41)
ORMOS	6	m	0	10	577	2	777	6.73	(1.47- 30.85)
ORMOS	7	m	0	4	128	2	777	12.14	(2.20- 66.97)
Subtotal ORMOS								10.74	(4.35- 26.54)
OSANN	51	m	2	-	-	-	-	35.30	(17.00- 73.30)
OSANN	59	m	2	-	-	-	-	76.00	(36.80- 157.00)
OSANN	52	f	2	-	-	-	-	24.00	(12.70- 45.50)
OSANN	60	f	2	-	-	-	-	72.30	(36.80- 142.00)
Subtotal OSANN								45.20	(32.03- 63.79)
OSANN2	28	f	1	-	-	-	-	12.10	(1.50- 96.30)
OSANN2	29	f	1	-	-	-	-	71.20	(8.30- 609.00)
Subtotal OSANN2								28.54	(6.40- 127.22)
SOBUE	53	m	0	57	157	3	128	15.49	(4.74- 50.62)
SOBUE	54	m	0	103	222	3	128	19.80	(6.15- 63.68)
SOBUE	55	m	0	87	187	3	128	19.85	(6.14- 64.13)
Subtotal SOBUE								18.28	(9.28- 36.03)
SVENSS	7	f	1	-	-	-	-	9.70	(2.90- 45.90)
SVENSS	12	f	1	-	-	-	-	36.20	(12.00- 168.90)
SVENSS	17	f	1	-	-	-	-	96.00	(6.90-1335.65)
Subtotal SVENSS								23.24	(9.47- 57.03)
TSUGAN	15	m	0	2	5	0	5	5.00~	(0.19- 130.02)
TSUGAN	16	m	0	7	7	0	5	11.00~	(0.51- 236.22)
TSUGAN	17	m	0	9	1	0	5	69.67~	(2.40-2022.74)
Subtotal TSUGAN								14.94	(2.32- 96.11)
WAKAI	46	m	1	-	-	-	-	3.95	(0.86- 18.10)
WAKAI	47	m	1	-	-	-	-	10.40	(2.43- 44.30)
WAKAI	48	m	1	-	-	-	-	24.00	(5.46- 105.00)
Subtotal WAKAI								10.14	(4.31- 23.88)
WU	17	f	2	-	-	-	-	17.70	(2.30- 138.20)
WU	18	f	2	-	-	-	-	94.40	(9.90- 904.60)
Subtotal WU								37.68	(8.27- 171.75)
WUWILL	14	f	3	-	-	-	-	3.21	(2.39- 4.30)
WUWILL	15	f	3	-	-	-	-	5.08	(3.07- 8.39)
Subtotal WUWILL								3.61	(2.80- 4.65)
WYNDE2	3	m	0	15	114	3	105	4.61	(1.30- 16.36)
WYNDE2	4	m	0	108	203	3	105	18.62	(5.77- 60.06)
WYNDE2	5	m	0	74	83	3	105	31.20	(9.50- 102.54)
WYNDE2	6	m	0	139	112	3	105	43.44	(13.42- 140.56)

Table 2G16 - 2

IESLC - Meta-anal of Ever Smoking (or Curr if Ever not avail) by Amount, Overview, Cigs (or Any Prod if Cigs not avail)
Squamous
Most adjusted

REF	NRR	SEX	AD	Number Exposed Case	Number Exposed Cont	Non-exposed Case	Non-exposed Cont	RR	95.00%CI
Subtotal WYNDE2								19.37	(10.64- 35.27)
WYNDE3	4	m	0	7	42	3	88	4.89	(1.20- 19.86)
WYNDE3	5	m	0	57	114	3	88	14.67	(4.44- 48.40)
WYNDE3	6	m	0	74	82	3	88	26.47	(8.03- 87.26)
WYNDE3	7	m	0	59	26	3	88	66.56	(19.27- 229.96)
WYNDE3	63	f	0	1	19	5	76	0.80	(0.09- 7.26)
WYNDE3	64	f	0	13	24	5	76	8.23	(2.66- 25.46)
WYNDE3	65	f	0	8	10	5	76	12.16	(3.32- 44.50)
WYNDE3	66	f	0	3	3	5	76	15.20	(2.42- 95.56)
Subtotal WYNDE3								13.67	(8.51- 21.97)
WYNDE4	63	m	2	-	-	-	-	2.22	(0.89- 5.53)
WYNDE4	64	m	2	-	-	-	-	5.86	(2.70- 12.74)
WYNDE4	65	m	2	-	-	-	-	10.92	(5.22- 22.86)
WYNDE4	66	m	2	-	-	-	-	29.52	(13.81- 63.11)
WYNDE4	67	m	2	-	-	-	-	29.54	(13.53- 64.50)
WYNDE4	49	f	2	-	-	-	-	0.87	(0.11- 6.90)
WYNDE4	50	f	2	-	-	-	-	4.61	(1.38- 15.41)
WYNDE4	51	f	2	-	-	-	-	14.92	(4.88- 45.67)
WYNDE4	52	f	2	-	-	-	-	26.53	(4.12- 171.09)
WYNDE4	53	f	2	-	-	-	-	26.53	(4.12- 171.09)
Subtotal WYNDE4								10.91	(8.00- 14.88)
WYNDE6	21	m	0	75	122	29	617	13.08	(8.17- 20.94)
WYNDE6	30	m	0	270	293	29	617	19.61	(13.04- 29.47)
WYNDE6	39	m	0	179	129	29	617	29.52	(19.09- 45.65)
WYNDE6	48	m	0	502	197	29	617	54.22	(36.08- 81.47)
WYNDE6	210	f	0	37	109	40	856	7.26	(4.45- 11.85)
WYNDE6	219	f	0	191	165	40	856	24.77	(16.95- 36.20)
WYNDE6	228	f	0	101	50	40	856	43.23	(27.18- 68.76)
WYNDE6	237	f	0	221	52	40	856	90.95	(58.70- 140.93)
Subtotal WYNDE6								28.23	(24.23- 32.90)
ZHENG	1	m	0	7	40	4	94	4.11	(1.14- 14.84)
ZHENG	2	m	0	25	66	4	94	8.90	(2.96- 26.78)
ZHENG	3	m	0	75	89	4	94	19.80	(6.95- 56.41)
ZHENG	4	m	0	49	23	4	94	50.07	(16.39- 152.91)
ZHENG	16	f	0	11	29	33	184	2.11	(0.96- 4.64)
ZHENG	17	f	0	32	15	33	184	11.89	(5.81- 24.35)
Subtotal ZHENG								8.76	(5.95- 12.88)
ZHOU	10	c	0	15	5	138	68	1.48	(0.52- 4.24)
ZHOU	11	c	0	78	14	138	68	2.75	(1.45- 5.20)
ZHOU	12	c	0	285	29	138	68	4.84	(3.00- 7.82)
Subtotal ZHOU								3.52	(2.45- 5.04)
Partial Totals				7175	19102	1576	32564		

*prospective study ~ With 0.5 adjustment for zero

REF	NRR	SEX	AD	Ys	Ws	Qs	Ps
ALDERS	34	m	1	1.33	3.36	4.67	0.0145
ALDERS	35	m	1	1.97	4.16	1.20	0.0001
ALDERS	36	m	1	2.17	4.42	0.51	0.0000
ALDERS	37	f	1	0.94	11.25	27.89	0.0017
ALDERS	38	f	1	2.22	12.50	1.03	0.0000
ALDERS	39	f	1	2.68	10.50	0.29	0.0000
Subtotal ALDERS				1.92	46.20	35.58	
BARBON	70	m	3	2.14	5.23	0.72	0.0000
BARBON	71	m	3	2.79	5.37	0.42	0.0000
BARBON	72	m	3	3.35	5.02	3.57	0.0000
Subtotal BARBON				2.75	15.62	4.71	
*BOUCOT	142	m	2	3.06	0.49	0.15	0.0325
*BOUCOT	143	m	2	3.84	0.49	0.86	0.0074
Subtotal BOUCOT				3.45	0.97	1.01	
BROWN2	36	m	2	2.03	88.72	20.66	0.0000
BROWN2	46	m	2	2.84	141.44	15.80	0.0000
BROWN2	35	f	2	2.46	43.16	0.11	0.0000
BROWN2	45	f	2	3.26	72.52	40.93	0.0000
Subtotal BROWN2				2.67	345.85	77.50	
CHOI	46	m	0	0.75	3.68	11.45	0.1517
CHOI	47	m	0	1.55	5.19	4.75	0.0004
CHOI	48	m	0	2.27	4.33	0.25	0.0000
CHOI	49	m	0	2.32	4.12	0.15	0.0000
CHOI	50	m	0	3.17	2.20	0.95	0.0000
CHOI	56	f	0	1.41	2.39	2.89	0.0292

Table 2G16 - 2

IESLC - Meta-anal of Ever Smoking (or Curr if Ever not avail) by Amount, Overview, Cigs (or Any Prod if Cigs not avail)
Squamous
Most adjusted

REF	NRR	SEX	AD	Ys	Ws	Qs	Ps
CHOI	57	f	0	2.21	2.40	0.22	0.0006
CHOI	58	f	0	3.49	0.62	0.60	0.0059
Subtotal CHOI				1.93	24.93	21.25	
CORREA	47	c	1	3.14	17.77	7.13	0.0000
CORREA	51	c	1	4.00	18.21	40.59	0.0000
Subtotal CORREA				3.58	35.98	47.72	
DOLL	68	m	1	1.55	2.55	2.37	0.0134
DOLL	69	m	1	2.36	2.82	0.06	0.0001
DOLL	70	m	1	2.66	2.82	0.06	0.0000
DOLL	71	m	1	3.23	2.77	1.45	0.0000
DOLL	76	f	1	0.00	4.09	25.78	1.0000
DOLL	77	f	1	0.53	4.04	15.84	0.2862
DOLL	78	f	1	2.12	3.19	0.50	0.0002
Subtotal DOLL				1.61	22.29	46.07	
DORGAN	114	m	2	2.44	3.61	0.02	0.0000
DORGAN	115	m	2	3.15	3.82	1.55	0.0000
DORGAN	99	f	3	2.05	17.39	3.67	0.0000
DORGAN	100	f	3	2.80	17.25	1.40	0.0000
Subtotal DORGAN				2.49	42.08	6.64	
DOSEME	7	m	2	0.96	12.24	29.60	0.0008
DOSEME	11	m	2	1.16	28.24	51.29	0.0000
DOSEME	15	m	2	1.95	13.32	4.25	0.0000
Subtotal DOSEME				1.31	53.80	85.14	
*ENGELA	57	m	7	1.46	1.77	1.96	0.0522
*ENGELA	58	m	7	2.04	1.97	0.43	0.0042
*ENGELA	59	m	7	2.71	2.06	0.08	0.0001
*ENGELA	60	m	7	3.40	1.98	1.57	0.0000
*ENGELA	61	m	7	3.18	2.01	0.89	0.0000
Subtotal ENGELA				2.58	9.78	4.94	
GER	14	c	10	0.36	2.04	9.45	0.6097
GER	15	c	10	0.79	2.57	7.64	0.2058
GER	16	c	10	2.78	2.16	0.15	0.0000
Subtotal GER				1.29	6.77	17.23	
HAENSZ	18	f	0	0.89	13.25	34.76	0.0012
HAENSZ	17	f	0	2.01	6.27	1.60	0.0000
Subtotal HAENSZ				1.25	19.53	36.37	
*HAMMON	98	m	1	2.72	3.06	0.13	0.0000
*HAMMON	99	m	1	2.86	3.70	0.45	0.0000
*HAMMON	100	m	1	3.75	3.75	5.71	0.0000
*HAMMON	101	m	1	4.16	3.38	9.18	0.0000
Subtotal HAMMON				3.38	13.90	15.47	
JEDRYC	28	m	3	2.02	4.87	1.19	0.0000
JEDRYC	29	m	3	2.60	5.33	0.04	0.0000
JEDRYC	30	m	3	3.06	5.18	1.59	0.0000
Subtotal JEDRYC				2.57	15.37	2.82	
KATSOU	21	f	1	1.15	3.66	6.74	0.0273
KATSOU	22	f	1	2.97	2.30	0.49	0.0000
Subtotal KATSOU				1.86	5.96	7.23	
KREYBE	1	m	1	2.20	2.91	0.29	0.0002
KREYBE	2	m	1	2.40	2.81	0.03	0.0001
KREYBE	3	m	1	3.20	2.74	1.32	0.0000
KREYBE	31	f	0	-0.27	0.75	5.76	0.8175
KREYBE	32	f	0	1.65	0.74	0.54	0.1566
Subtotal KREYBE				2.31	9.94	7.95	
LAMTH	10	f	0	1.68	5.44	3.73	0.0001
LAMTH	11	f	0	2.48	3.97	0.00	0.0000
LAMTH	12	f	0	3.25	0.87	0.47	0.0025
Subtotal LAMTH				2.12	10.27	4.21	
LUBIN2	149	m	0	2.22	45.98	3.82	0.0000
LUBIN2	153	m	0	2.68	49.57	1.47	0.0000
LUBIN2	157	m	0	3.01	50.02	12.33	0.0000
LUBIN2	161	m	0	3.16	48.42	20.37	0.0000
LUBIN2	169	f	0	0.98	18.69	43.63	0.0000
LUBIN2	173	f	0	1.85	33.33	14.46	0.0000
LUBIN2	177	f	0	2.21	24.86	2.29	0.0000
LUBIN2	181	f	0	2.02	10.42	2.48	0.0000
Subtotal LUBIN2				2.47	281.30	100.85	
LUO	10	c	20	0.18	0.72	3.93	0.8767
LUO	11	c	20	3.20	1.22	0.59	0.0004
LUO	12	c	20	3.66	0.95	1.25	0.0004
Subtotal LUO				2.60	2.90	5.76	

Table 2G16 - 2

IESLC - Meta-anal of Ever Smoking (or Curr if Ever not avail) by Amount, Overview, Cigs (or Any Prod if Cigs not avail)
Squamous
Most adjusted

REF	NRR	SEX	AD	Ys	Ws	Qs	Ps
MATOS	43	m	2	0.34	1.56	7.39	0.6740
MATOS	45	m	2	2.05	2.42	0.50	0.0014
MATOS	47	m	2	2.27	2.51	0.14	0.0003
Subtotal MATOS				1.72	6.49	8.04	
MATSUD	4	m	0	3.06	0.95	0.29	0.0028
MATSUD	5	m	0	3.51	0.98	0.98	0.0005
MATSUD	6	m	0	4.65	0.97	4.43	0.0000
Subtotal MATSUD				3.74	2.90	5.70	
ORMOS	5	m	0	2.73	1.72	0.08	0.0003
ORMOS	6	m	0	1.91	1.66	0.60	0.0141
ORMOS	7	m	0	2.50	1.32	0.00	0.0042
Subtotal ORMOS				2.37	4.70	0.69	
OSANN	51	m	2	3.56	7.20	7.98	0.0000
OSANN	59	m	2	4.33	7.30	24.18	0.0000
OSANN	52	f	2	3.18	9.44	4.20	0.0000
OSANN	60	f	2	4.28	8.43	26.40	0.0000
Subtotal OSANN				3.81	32.36	62.77	
OSANN2	28	f	1	2.49	0.89	0.00	0.0189
OSANN2	29	f	1	4.27	0.83	2.56	0.0001
Subtotal OSANN2				3.35	1.72	2.56	
SOBUE	53	m	0	2.74	2.74	0.14	0.0000
SOBUE	54	m	0	2.99	2.81	0.63	0.0000
SOBUE	55	m	0	2.99	2.79	0.64	0.0000
Subtotal SOBUE				2.91	8.35	1.42	
SVENSS	7	f	1	2.27	2.01	0.11	0.0013
SVENSS	12	f	1	3.59	2.20	2.56	0.0000
SVENSS	17	f	1	4.56	0.55	2.34	0.0007
Subtotal SVENSS				3.15	4.77	5.01	
TSUGAN	15	m	0	1.61	0.36	0.29	0.3330
TSUGAN	16	m	0	2.40	0.41	0.01	0.1254
TSUGAN	17	m	0	4.24	0.34	1.02	0.0135
Subtotal TSUGAN				2.70	1.11	1.32	
WAKAI	46	m	1	1.37	1.66	2.14	0.0772
WAKAI	47	m	1	2.34	1.82	0.05	0.0016
WAKAI	48	m	1	3.18	1.76	0.78	0.0000
Subtotal WAKAI				2.32	5.24	2.97	
WU	17	f	2	2.87	0.92	0.12	0.0060
WU	18	f	2	4.55	0.75	3.13	0.0001
Subtotal WU				3.63	1.67	3.25	
WUWILL	14	f	3	1.17	44.55	80.52	0.0000
WUWILL	15	f	3	1.63	15.20	11.92	0.0000
Subtotal WUWILL				1.28	59.75	92.44	
WYNDE2	3	m	0	1.53	2.39	2.31	0.0182
WYNDE2	4	m	0	2.92	2.80	0.48	0.0000
WYNDE2	5	m	0	3.44	2.71	2.35	0.0000
WYNDE2	6	m	0	3.77	2.79	4.43	0.0000
Subtotal WYNDE2				2.96	10.69	9.56	
WYNDE3	4	m	0	1.59	1.96	1.67	0.0265
WYNDE3	5	m	0	2.69	2.70	0.08	0.0000
WYNDE3	6	m	0	3.28	2.70	1.58	0.0000
WYNDE3	7	m	0	4.20	2.50	7.12	0.0000
WYNDE3	63	f	0	-0.22	0.79	5.90	0.8428
WYNDE3	64	f	0	2.11	3.01	0.49	0.0003
WYNDE3	65	f	0	2.50	2.28	0.00	0.0002
WYNDE3	66	f	0	2.72	1.14	0.05	0.0037
Subtotal WYNDE3				2.62	17.07	16.89	
WYNDE4	63	m	2	0.80	4.60	13.52	0.0870
WYNDE4	64	m	2	1.77	6.38	3.52	0.0000
WYNDE4	65	m	2	2.39	7.04	0.10	0.0000
WYNDE4	66	m	2	3.39	6.66	5.09	0.0000
WYNDE4	67	m	2	3.39	6.30	4.82	0.0000
WYNDE4	49	f	2	-0.14	0.90	6.30	0.8951
WYNDE4	50	f	2	1.53	2.64	2.55	0.0130
WYNDE4	51	f	2	2.70	3.07	0.11	0.0000
WYNDE4	52	f	2	3.28	1.11	0.65	0.0006
WYNDE4	53	f	2	3.28	1.11	0.65	0.0006
Subtotal WYNDE4				2.39	39.81	37.31	
WYNDE6	21	m	0	2.57	17.35	0.06	0.0000
WYNDE6	30	m	0	2.98	23.14	5.00	0.0000
WYNDE6	39	m	0	3.39	20.23	15.46	0.0000
WYNDE6	48	m	0	3.99	23.16	50.89	0.0000

Table 2G16 - 2

IESLC - Meta-anal of Ever Smoking (or Curr if Ever not avail) by Amount, Overview, Cigs (or Any Prod if Cigs not avail)
Squamous
Most adjusted

REF	NRR	SEX	AD	Ys	Ws	Qs	Ps
WYNDE6	210	f	0	1.98	16.03	4.47	0.0000
WYNDE6	219	f	0	3.21	26.69	13.04	0.0000
WYNDE6	228	f	0	3.77	17.84	28.12	0.0000
WYNDE6	237	f	0	4.51	20.03	80.09	0.0000
Subtotal WYNDE6				3.34	164.47	197.14	
ZHENG	1	m	0	1.41	2.33	2.81	0.0308
ZHENG	2	m	0	2.19	3.17	0.33	0.0001
ZHENG	3	m	0	2.99	3.51	0.79	0.0000
ZHENG	4	m	0	3.91	3.08	6.06	0.0000
ZHENG	16	f	0	0.75	6.21	19.26	0.0620
ZHENG	17	f	0	2.48	7.48	0.01	0.0000
Subtotal ZHENG				2.17	25.78	29.26	
ZHOU	10	c	0	0.39	3.46	15.57	0.4669
ZHOU	11	c	0	1.01	9.42	21.21	0.0019
ZHOU	12	c	0	1.58	16.68	14.53	0.0000
Subtotal ZHOU				1.26	29.56	51.31	

N	147
NS	36

Table 2G16 - 3

IESLC - Meta-anal of Ever Smoking (or Curr if Ever not avail) by Amount, Overview, Cigs (or Any Prod if Cigs not avail)
Squamous
Most adjusted

	Sex			
	combined	male	female	Total
N	11	84	52	147
NS	4	25	19	48

In this overview table, other than the "N" rows, entries in the "absent" and "Total" columns may be invalid and should be ignored

	Amount smoked (broad categories)				
	absent	<20k5	6-44k20	>20k45	Total
N	39	41	30	37	147
NS	22	30	24	30	106
Wt	516.57	396.78	239.42	227.09	1379.86
Het Chi	272.14	154.02	107.78	143.12	1056.07
Het df	38	40	29	36	146
Het P	***	***	***	***	***
Fixed RR	14.54	6.07	12.36	28.95	12.31
RRl	13.34	5.50	10.89	25.42	11.68
RRu	15.85	6.70	14.03	32.98	12.98
P	+++	+++	+++	+++	+++
Random RR	12.08	4.98	11.86	27.65	11.39
RRl	9.20	3.93	8.92	20.42	9.70
RRu	15.88	6.31	15.76	37.44	13.39
P	+++	+++	+++	+++	+++

	Amount smoked (narrow categories)							
	absent	<10k1	2-19k10	11-29k20	21-39k30	31-98k40	>40k99	Total
N	91	13	9	23	7	1	3	147
NS	36	8	6	19	5	1	2	76
Wt	883.30	96.40	113.42	220.17	56.62	4.12	5.83	1379.86
Het Chi	675.83	57.10	42.50	105.52	8.27	0.00	2.15	1056.07
Het df	90	12	8	22	6	0	2	146
Het P	***	***	***	***	N.S.	N.S.	N.S.	***
Fixed RR	13.42	4.80	8.36	12.35	31.33	10.15	33.86	12.31
RRl	12.57	3.93	6.95	10.82	24.14	3.86	15.04	11.68
RRu	14.34	5.86	10.05	14.09	40.65	26.66	76.22	12.98
P	+++	+++	+++	+++	+++	+++	+++	+++
Random RR	13.07	3.11	6.29	11.88	30.14	10.15	33.54	11.39
RRl	10.59	1.81	3.75	8.52	21.51	3.86	14.41	9.70
RRu	16.13	5.33	10.54	16.58	42.24	26.66	78.06	13.39
P	+++	+++	+++	+++	+++	+++	+++	+++

MALES

	Amount smoked (broad categories)				
	absent	<20k5	6-44k20	>20k45	Total
N	19	23	20	22	84
NS	14	23	20	22	79
Wt	266.93	216.08	156.92	142.77	782.69
Het Chi	37.66	48.34	79.72	62.58	390.54
Het df	18	22	19	21	83
Het P	**	***	***	***	***
Fixed RR	17.28	7.42	12.01	26.76	13.78
RRl	15.32	6.49	10.27	22.71	12.84
RRu	19.48	8.48	14.04	31.52	14.78
P	+++	+++	+++	+++	+++
Random RR	17.31	6.62	11.96	27.70	13.64
RRl	13.46	5.08	8.18	19.71	11.38
RRu	22.25	8.63	17.49	38.93	16.35
P	+++	+++	+++	+++	+++

Table 2G16 - 3

IESLC - Meta-anal of Ever Smoking (or Curr if Ever not avail) by Amount, Overview, Cigs (or Any Prod if Cigs not avail)
Squamous
Most adjusted

MALES

		Amount smoked (narrow categories)						
	absent	<10k1	2-19k10	11-29k20	21-39k30	31-98k40	>40k99	Total
N	49	7	5	15	5	1	2	84
NS	25	7	5	15	5	1	2	59
Wt	466.87	62.26	63.99	143.08	37.67	4.12	4.70	782.69
Het Chi	237.48	12.57	5.35	77.99	5.57	0.00	1.24	390.54
Het df	48	6	4	14	4	0	1	83
Het P	***	(*)	N.S.	***	N.S.	N.S.	N.S.	***
Fixed RR	14.80	7.70	12.85	11.79	27.03	10.15	41.10	13.78
RRl	13.52	6.01	10.06	10.01	19.64	3.86	16.64	12.84
RRu	16.21	9.87	16.42	13.89	37.20	26.66	101.52	14.78
P	+++	+++	+++	+++	+++	+++	+++	+++
Random RR	15.23	5.77	11.33	11.64	26.31	10.15	40.83	13.64
RRl	11.82	3.38	7.66	7.39	17.36	3.86	14.89	11.38
RRu	19.61	9.84	16.76	18.33	39.88	26.66	111.95	16.35
P	+++	+++	+++	+++	+++	+++	+++	+++

FEMALES

	Amount smoked (broad categories)				
	absent	<20k5	6-44k20	>20k45	Total
N	17	15	8	12	52
NS	15	15	8	12	50
Wt	205.78	174.47	78.71	63.00	521.96
Het Chi	174.32	77.04	18.84	71.40	521.04
Het df	16	14	7	11	51
Het P	***	***	**	***	***
Fixed RR	13.17	4.98	13.71	29.26	10.54
RRl	11.49	4.29	10.99	22.86	9.67
RRu	15.10	5.78	17.10	37.45	11.48
P	+++	+++	+++	+++	+++
Random RR	9.58	3.95	13.14	26.18	9.40
RRl	5.66	2.60	8.57	12.49	6.91
RRu	16.21	6.00	20.13	54.88	12.79
P	+++	+++	+++	+++	+++

		Amount smoked (narrow categories)						
	absent	<10k1	2-19k10	11-29k20	21-39k30	31-98k40	>40k99	Total
N	35	5	3	6	2		1	52
NS	19	5	3	6	2		1	36
Wt	357.90	30.67	40.01	73.30	18.94		1.14	521.96
Het Chi	349.86	4.77	6.37	17.57	0.25		0.00	521.04
Het df	34	4	2	5	1		0	51
Het P	***	N.S.	*	**	N.S.		N.S.	***
Fixed RR	11.38	2.10	5.46	14.19	42.01		15.20	10.54
RRl	10.26	1.47	4.00	11.28	26.78		2.42	9.67
RRu	12.62	2.99	7.44	17.84	65.91		95.56	11.48
P	+++	+++	+++	+++	+++		++	+++
Random RR	11.04	1.94	3.97	14.35	42.01		15.20	9.40
RRl	7.55	1.26	1.71	8.73	26.78		2.42	6.91
RRu	16.15	3.01	9.23	23.57	65.91		95.56	12.79
P	+++	++	++	+++	+++		++	+++

Table 2G16 - 4

IESLC - Meta-anal of Ever Smoking (or Curr if Ever not avail) by Amount, Overview, Cigs (or Any Prod if Cigs not avail)
Squamous
Least adjusted

REF	NRR	X	SEX	AGEL	AGEH	RACE	YF	LC TYPE	LOC	START	ST	NLC	R	VB	P	H	AD	SM	PRODUCT	exL	exH	S1	S2	DENOM	De
ALDERS	34		m	0	0	all	-	q+s	Eu:UK	1977	CC	1448	n	V	n	n	1	ev	cig only	1	17	1	0	nev+2	ot
ALDERS	35		m	0	0	all	-	q+s	Eu:UK	1977	CC	1448	n	V	n	n	1	ev	cig only	18	27	2	3	nev+2	ot
ALDERS	36		m	0	0	all	-	q+s	Eu:UK	1977	CC	1448	n	V	n	n	1	ev	cig only	28	99	3	0	nev+2	ot
ALDERS	37		f	0	0	all	-	q+s	Eu:UK	1977	CC	1448	n	V	n	n	1	ev	cig only	1	17	1	0	nev+2	ot
ALDERS	38		f	0	0	all	-	q+s	Eu:UK	1977	CC	1448	n	V	n	n	1	ev	cig only	18	27	2	3	nev+2	ot
ALDERS	39		f	0	0	all	-	q+s	Eu:UK	1977	CC	1448	n	V	n	n	1	ev	cig only	28	99	3	0	nev+2	ot
BARBON	19	x	m	0	0	all	-	q	Eu:wst	1979	CC	755	n	bl	y	y	0	ev	all/unsp	1	9	1	1	nev any	st
BARBON	21	x	m	0	0	all	-	q	Eu:wst	1979	CC	755	n	bl	y	y	0	ev	all/unsp	10	19	0	2	nev any	st
BARBON	23	x	m	0	0	all	-	q	Eu:wst	1979	CC	755	n	bl	y	y	0	ev	all/unsp	20	29	2	3	nev any	st
BARBON	25	x	m	0	0	all	-	q	Eu:wst	1979	CC	755	n	bl	y	y	0	ev	all/unsp	30	39	0	4	nev any	st
BARBON	27	x	m	0	0	all	-	q	Eu:wst	1979	CC	755	n	bl	y	y	0	ev	all/unsp	40	99	3	0	nev any	st
BOUCOT	21	x	m	0	0	all	0	q	NAmer	1951	pr	121	n	bl	n	n	0	cu	cig only	1	20	0	0	nev any	ot
BOUCOT	22	x	m	0	0	all	0	q	NAmer	1951	pr	121	n	bl	n	n	0	cu	cig only	21	99	3	0	nev any	ot
BROWN2	36		m	0	0	wh	-	q	NAmer	1984	CC	14596	n	bl	n	y	2	ev	cig+/-ot	1	19	1	0	nev cigs	or
BROWN2	46		m	0	0	wh	-	q	NAmer	1984	CC	14596	n	bl	n	y	2	ev	cig+/-ot	20	99	0	0	nev cigs	or
BROWN2	35		f	0	0	wh	-	q	NAmer	1984	CC	14596	n	bl	n	y	2	ev	cig+/-ot	1	19	1	0	nev cigs	or
BROWN2	45		f	0	0	wh	-	q	NAmer	1984	CC	14596	n	bl	n	y	2	ev	cig+/-ot	20	99	0	0	nev cigs	or
CHOI	46		m	0	0	all	-	q	As:oth	1985	CC	375	n	bl	n	n	0	ev	cig+/-ot	1	10	1	0	nev cigs	st
CHOI	47		m	0	0	all	-	q	As:oth	1985	CC	375	n	bl	n	n	0	ev	cig+/-ot	11	20	2	3	nev cigs	st
CHOI	48		m	0	0	all	-	q	As:oth	1985	CC	375	n	bl	n	n	0	ev	cig+/-ot	21	30	0	4	nev cigs	st
CHOI	49		m	0	0	all	-	q	As:oth	1985	CC	375	n	bl	n	n	0	ev	cig+/-ot	31	40	0	5	nev cigs	st
CHOI	50		m	0	0	all	-	q	As:oth	1985	CC	375	n	bl	n	n	0	ev	cig+/-ot	41	99	3	6	nev cigs	st
CHOI	56		f	0	0	all	-	q	As:oth	1985	CC	375	n	bl	n	n	0	ev	cig+/-ot	1	10	1	0	nev cigs	st
CHOI	57		f	0	0	all	-	q	As:oth	1985	CC	375	n	bl	n	n	0	ev	cig+/-ot	11	30	2	0	nev cigs	st
CHOI	58		f	0	0	all	-	q	As:oth	1985	CC	375	n	bl	n	n	0	ev	cig+/-ot	31	99	3	0	nev cigs	st
CORREA	47		c	0	0	all	-	q+s	NAmer	1979	CC	1359	n	bl	y	n	1	cu	cig+/-ot	1	20	0	0	nev cigs	or
CORREA	51		c	0	0	all	-	q+s	NAmer	1979	CC	1359	n	bl	y	n	1	cu	cig+/-ot	21	99	3	0	nev cigs	or
DOLL	54	x	m	0	0	all	-	KI	Eu:UK	1948	CC	1465	n	V	n	n	0	ev	all/unsp	1	4	0	1	nev any	st
DOLL	55	x	m	0	0	all	-	KI	Eu:UK	1948	CC	1465	n	V	n	n	0	ev	all/unsp	5	14	1	2	nev any	st
DOLL	56	x	m	0	0	all	-	KI	Eu:UK	1948	CC	1465	n	V	n	n	0	ev	all/unsp	15	24	2	3	nev any	st
DOLL	57	x	m	0	0	all	-	KI	Eu:UK	1948	CC	1465	n	V	n	n	0	ev	all/unsp	25	99	3	0	nev any	st
DOLL	62	x	f	0	0	all	-	KI	Eu:UK	1948	CC	1465	n	V	n	n	0	ev	all/unsp	1	4	0	1	nev any	st
DOLL	63	x	f	0	0	all	-	KI	Eu:UK	1948	CC	1465	n	V	n	n	0	ev	all/unsp	5	14	1	2	nev any	st
DOLL	64	x	f	0	0	all	-	KI	Eu:UK	1948	CC	1465	n	V	n	n	0	ev	all/unsp	15	99	0	0	nev any	st
DORGAN	114		m	0	0	wh	-	q	NAmer	1980	CC	2026	n	bl	y	y	2	ev	cig+/-ot	1	19	1	0	nev any	ot
DORGAN	115		m	0	0	wh	-	q	NAmer	1980	CC	2026	n	bl	y	y	2	ev	cig+/-ot	20	99	0	0	nev any	ot
DORGAN	99		f	0	0	all	-	q	NAmer	1980	CC	2026	n	bl	y	y	3	ev	cig+/-ot	1	19	1	0	nev any	ot
DORGAN	100		f	0	0	all	-	q	NAmer	1980	CC	2026	n	bl	y	y	3	ev	cig+/-ot	20	99	0	0	nev any	ot
DOSEME	7		m	0	0	all	-	q	Eu:bal	1979	CC	1210	n	bl	n	n	2	ev	cig+/-ot	1	10	1	0	nev cigs	or
DOSEME	11		m	0	0	all	-	q	Eu:bal	1979	CC	1210	n	bl	n	n	2	ev	cig+/-ot	11	20	2	3	nev cigs	or
DOSEME	15		m	0	0	all	-	q	Eu:bal	1979	CC	1210	n	bl	n	n	2	ev	cig+/-ot	21	99	3	0	nev cigs	or
ENGELA	57		m	0	0	all	0	q	Eu:Sca	1964	pr	435	n	bl	n	n	7	cu	cig+/-ot	1	4	0	1	nev cigs	or
ENGELA	58		m	0	0	all	0	q	Eu:Sca	1964	pr	435	n	bl	n	n	7	cu	cig+/-ot	5	9	1	0	nev cigs	or
ENGELA	59		m	0	0	all	0	q	Eu:Sca	1964	pr	435	n	bl	n	n	7	cu	cig+/-ot	10	14	0	2	nev cigs	or
ENGELA	60		m	0	0	all	0	q	Eu:Sca	1964	pr	435	n	bl	n	n	7	cu	cig+/-ot	15	19	0	0	nev cigs	or
ENGELA	61		m	0	0	all	0	q	Eu:Sca	1964	pr	435	n	bl	n	n	7	cu	cig+/-ot	20	99	0	0	nev cigs	or
GER	6	x	c	0	0	all	-	q+s	As:oth	1990	CC	141	n	ot	y	n	0	ev	all/unsp	1	10	1	0	nev any	st
GER	7	x	c	0	0	all	-	q+s	As:oth	1990	CC	141	n	ot	y	n	0	ev	all/unsp	11	20	2	3	nev any	st
GER	8	x	c	0	0	all	-	q+s	As:oth	1990	CC	141	n	ot	y	n	0	ev	all/unsp	21	99	3	0	nev any	st
HAENSZ	18		f	0	0	all	-	q+u	NAmer	1955	CC	158	n	bl	n	y	0	cu	cig+/-ot	1	20	0	0	nev any	or
HAENSZ	17		f	0	0	all	-	q+u	NAmer	1955	CC	158	n	bl	n	y	0	cu	cig+/-ot	21	99	3	0	nev any	or
HAMMON	98		m	0	0	wh	0	not a	NAmer	1952	pr	448	n	bl	n	n	1	cu	cig only	1	9	1	1	nev any	ot
HAMMON	99		m	0	0	wh	0	not a	NAmer	1952	pr	448	n	bl	n	n	1	cu	cig only	10	20	2	0	nev any	ot
HAMMON	100		m	0	0	wh	0	not a	NAmer	1952	pr	448	n	bl	n	n	1	cu	cig only	21	39	0	4	nev any	ot
HAMMON	101		m	0	0	wh	0	not a	NAmer	1952	pr	448	n	bl	n	n	1	cu	cig only	40	99	3	0	nev any	ot
JEDRYC	1	x	m	0	0	all	-	q	Eu:est	1980	CC	1630	n	bl	y	n	0	ev	cig+/-ot	1	9	1	1	nev any	st
JEDRYC	2	x	m	0	0	all	-	q	Eu:est	1980	CC	1630	n	bl	y	n	0	ev	cig+/-ot	10	19	0	2	nev any	st
JEDRYC	3	x	m	0	0	all	-	q	Eu:est	1980	CC	1630	n	bl	y	n	0	ev	cig+/-ot	20	29	2	3	nev any	st
JEDRYC	4	x	m	0	0	all	-	q	Eu:est	1980	CC	1630	n	bl	y	n	0	ev	cig+/-ot	30	39	0	4	nev any	st
JEDRYC	5	x	m	0	0	all	-	q	Eu:est	1980	CC	1630	n	bl	y	n	0	ev	cig+/-ot	40	99	3	0	nev any	st
KATSOU	25	x	f	0	0	all	-	KI	Eu:bal	1987	CC	101	n	bl	n	n	0	cu	all/unsp	1	20	0	0	nev any	st
KATSOU	26	x	f	0	0	all	-	KI	Eu:bal	1987	CC	101	n	bl	n	n	0	cu	all/unsp	21	99	3	0	nev any	st
KREYBE	13	x	m	0	0	all	-	KI	Eu:Sca	1948	CC	300	n	bl	n	y	0	ev	all/unsp	1	14	1	0	nev any	st
KREYBE	14	x	m	0	0	all	-	KI	Eu:Sca	1948	CC	300	n	bl	n	y	0	ev	all/unsp	15	24	2	3	nev any	st
KREYBE	15	x	m	0	0	all	-	KI	Eu:Sca	1948	CC	300	n	bl	n	y	0	ev	all/unsp	25	99	3	0	nev any	st
KREYBE	31		f	0	0	all	-	KI	Eu:Sca	1948	CC	300	n	bl	n	y	0	ev	all/unsp	1	14	1	0	nev any	st
KREYBE	32		f	0	0	all	-	KI	Eu:Sca	1948	CC	300	n	bl	n	y	0	ev	all/unsp	15	99	0	0	nev any	st
LAMTH	10		f	0	0	ch	-	q	As:HK	1983	CC	445	n	bl	n	n	0	ev	all/unsp	1	10	1	0	nev any	or
LAMTH	11		f	0	0	ch	-	q	As:HK	1983	CC	445	n	bl	n	n	0	ev	all/unsp	11	20	2	3	nev any	or
LAMTH	12		f	0	0	ch	-	q	As:HK	1983	CC	445	n	bl	n	n	0	ev	all/unsp	21	99	3	0	nev any	st
LUBIN2	149		m	0	0	all	-	q	Eu:mul	1976	CC	7804	n	bl	n	y	0	ev	cig+/-ot	1	9	1	1	nev any	st
LUBIN2	153		m	0	0	all	-	q	Eu:mul	1976	CC	7804	n	bl	n	y	0	ev	cig+/-ot	10	19	0	2	nev any	st

Table 2G16 - 4

IESLC - Meta-anal of Ever Smoking (or Curr if Ever not avail) by Amount, Overview, Cigs (or Any Prod if Cigs not avail)
Squamous
Least adjusted

REF	NRR	X	SEX	AGEL	AGEH	RACE	YF	LC TYPE	LOC	START	ST	NLC	R	VB	P	H	AD	SM	PRODUCT	exL	exH	S1	S2	DENOM	De
LUBIN2	157		m	0	0	all	-	q	Eu:mul	1976	CC	7804	n	bl	n	y	0	ev	cig+/-ot	20	29	2	3	nev any	st
LUBIN2	161		m	0	0	all	-	q	Eu:mul	1976	CC	7804	n	bl	n	y	0	ev	cig+/-ot	30	99	3	0	nev any	st
LUBIN2	169		f	0	0	all	-	q	Eu:mul	1976	CC	7804	n	bl	n	y	0	ev	cig+/-ot	1	9	1	1	nev any	st
LUBIN2	173		f	0	0	all	-	q	Eu:mul	1976	CC	7804	n	bl	n	y	0	ev	cig+/-ot	10	19	0	2	nev any	st
LUBIN2	177		f	0	0	all	-	q	Eu:mul	1976	CC	7804	n	bl	n	y	0	ev	cig+/-ot	20	29	2	3	nev any	st
LUBIN2	181		f	0	0	all	-	q	Eu:mul	1976	CC	7804	n	bl	n	y	0	ev	cig+/-ot	30	99	3	0	nev any	st
LUO	4	x	c	0	0	all	-	q	As:Chi	1990	CC	102	n	ot	n	y	0	ev	cig+/-ot	1	19	1	0	nev cigs	st
LUO	5	x	c	0	0	all	-	q	As:Chi	1990	CC	102	n	ot	n	y	0	ev	cig+/-ot	20	29	2	3	nev cigs	st
LUO	6	x	c	0	0	all	-	q	As:Chi	1990	CC	102	n	ot	n	y	0	ev	cig+/-ot	30	99	3	0	nev cigs	st
MATOS	42	x	m	0	0	all	-	q	SCAmer	1994	CC	200	n	bl	n	n	0	ev	cig+/-ot	1	14	1	0	nev any	st
MATOS	44	x	m	0	0	all	-	q	SCAmer	1994	CC	200	n	bl	n	n	0	ev	cig+/-ot	15	24	2	3	nev any	st
MATOS	46	x	m	0	0	all	-	q	SCAmer	1994	CC	200	n	bl	n	n	0	ev	cig+/-ot	25	99	3	0	nev any	st
MATSUD	4		m	0	0	all	-	q	As:Jap	1965	CC	179	n	bl	n	n	0	ev	cig+/-ot	1	10	1	0	nev cigs	st
MATSUD	5		m	0	0	all	-	q	As:Jap	1965	CC	179	n	bl	n	n	0	ev	cig+/-ot	11	20	2	3	nev cigs	st
MATSUD	6		m	0	0	all	-	q	As:Jap	1965	CC	179	n	bl	n	n	0	ev	cig+/-ot	21	99	3	0	nev cigs	st
ORMOS	5		m	0	0	all	-	q	Eu:est	1947	CC	119	n	bl	y	y	0	ev	cig+/-ot	1	15	1	0	nev any	st
ORMOS	6		m	0	0	all	-	q	Eu:est	1947	CC	119	n	bl	y	y	0	ev	cig+/-ot	16	30	2	0	nev any	st
ORMOS	7		m	0	0	all	-	q	Eu:est	1947	CC	119	n	bl	y	y	0	ev	cig+/-ot	31	99	3	0	nev any	st
OSANN	51		m	0	0	all	-	q	NAmer	1984	CC	1986	n	bl	n	n	2	ev	cig+/-ot	1	39	0	0	nev cigs	or
OSANN	59		m	0	0	all	-	q	NAmer	1984	CC	1986	n	bl	n	n	2	ev	cig+/-ot	40	99	3	0	nev cigs	or
OSANN	52		f	0	0	all	-	q	NAmer	1984	CC	1986	n	bl	n	n	2	ev	cig+/-ot	1	39	0	0	nev cigs	or
OSANN	60		f	0	0	all	-	q	NAmer	1984	CC	1986	n	bl	n	n	2	ev	cig+/-ot	40	99	3	0	nev cigs	or
OSANN2	10	x	f	0	0	all	-	KI	NAmer	1964	ot	217	n	bl	n	y	0	ev	cig+/-ot	1	19	1	0	nev cigs	st
OSANN2	11	x	f	0	0	all	-	KI	NAmer	1964	ot	217	n	bl	n	y	0	ev	cig+/-ot	20	99	0	0	nev cigs	st
SOBUE	53		m	0	0	all	-	q	As:Jap	1986	CC	1376	n	bl	n	y	0	cu	cig+/-ot	1	19	1	0	nev cigs	st
SOBUE	54		m	0	0	all	-	q	As:Jap	1986	CC	1376	n	bl	n	y	0	cu	cig+/-ot	20	29	2	3	nev cigs	st
SOBUE	55		m	0	0	all	-	q	As:Jap	1986	CC	1376	n	bl	n	y	0	cu	cig+/-ot	30	99	3	0	nev cigs	st
SVENSS	27	x	f	0	0	all	-	q	Eu:Sca	1983	CC	210	n	bl	n	n	0	cu	all/unsp	1	10	1	0	nev any	st
SVENSS	32	x	f	0	0	all	-	q	Eu:Sca	1983	CC	210	n	bl	n	n	0	cu	all/unsp	11	20	2	3	nev any	st
SVENSS	37	x	f	0	0	all	-	q	Eu:Sca	1983	CC	210	n	bl	n	n	0	cu	all/unsp	21	99	3	0	nev any	st
TSUGAN	15		m	0	0	all	-	q	As:Jap	1976	CC	134	n	bl	n	y	0	cu	all/unsp	1	15	1	0	nev any	ot
TSUGAN	16		m	0	0	all	-	q	As:Jap	1976	CC	134	n	bl	n	y	0	cu	all/unsp	16	35	2	0	nev any	ot
TSUGAN	17		m	0	0	all	-	q	As:Jap	1976	CC	134	n	bl	n	y	0	cu	all/unsp	36	99	3	0	nev any	ot
WAKAI	43	x	m	0	0	all	-	q	As:Jap	1988	CC	333	n	bl	n	y	0	cu	cig+/-ot	1	19	1	0	nev any	st
WAKAI	44	x	m	0	0	all	-	q	As:Jap	1988	CC	333	n	bl	n	y	0	cu	cig+/-ot	20	29	2	3	nev any	st
WAKAI	45	x	m	0	0	all	-	q	As:Jap	1988	CC	333	n	bl	n	y	0	cu	cig+/-ot	30	99	3	0	nev any	st
WU	12	x	f	0	0	wh	-	q	NAmer	1981	CC	220	n	bl	n	y	0	cu	all/unsp	1	20	0	0	nev any	st
WU	13	x	f	0	0	wh	-	q	NAmer	1981	CC	220	n	bl	n	y	0	cu	all/unsp	21	99	3	0	nev any	st
WUWILL	20	x	f	0	0	all	-	q+s	As:Chi	1985	CC	965	n	ot	n	n	0	ev	cig+/-ot	1	19	1	0	nev cigs	st
WUWILL	21	x	f	0	0	all	-	q+s	As:Chi	1985	CC	965	n	ot	n	n	0	ev	cig+/-ot	20	99	0	0	nev cigs	st
WYNDE2	3		m	0	0	all	-	KI	NAmer	1962	CC	404	n	bl	n	y	0	ev	cig+/-ot	1	10	1	0	nev any	st
WYNDE2	4		m	0	0	all	-	KI	NAmer	1962	CC	404	n	bl	n	y	0	ev	cig+/-ot	11	20	2	3	nev any	st
WYNDE2	5		m	0	0	all	-	KI	NAmer	1962	CC	404	n	bl	n	y	0	ev	cig+/-ot	21	34	0	4	nev any	st
WYNDE2	6		m	0	0	all	-	KI	NAmer	1962	CC	404	n	bl	n	y	0	ev	cig+/-ot	35	99	3	0	nev any	st
WYNDE3	4		m	0	0	all	-	KI	NAmer	1966	CC	350	n	bl	n	y	0	ev	cig+/-ot	1	9	1	1	nev any	st
WYNDE3	5		m	0	0	all	-	KI	NAmer	1966	CC	350	n	bl	n	y	0	ev	cig+/-ot	10	20	2	0	nev any	st
WYNDE3	6		m	0	0	all	-	KI	NAmer	1966	CC	350	n	bl	n	y	0	ev	cig+/-ot	21	40	0	0	nev any	st
WYNDE3	7		m	0	0	all	-	KI	NAmer	1966	CC	350	n	bl	n	y	0	ev	cig+/-ot	41	99	3	6	nev any	st
WYNDE3	63		f	0	0	all	-	KI	NAmer	1966	CC	350	n	bl	n	y	0	ev	cig+/-ot	1	9	1	1	nev any	st
WYNDE3	64		f	0	0	all	-	KI	NAmer	1966	CC	350	n	bl	n	y	0	ev	cig+/-ot	10	20	2	0	nev any	st
WYNDE3	65		f	0	0	all	-	KI	NAmer	1966	CC	350	n	bl	n	y	0	ev	cig+/-ot	21	40	0	0	nev any	st
WYNDE3	66		f	0	0	all	-	KI	NAmer	1966	CC	350	n	bl	n	y	0	ev	cig+/-ot	41	99	3	6	nev any	st
WYNDE4	5	x	m	0	0	all	-	not a	NAmer	1948	CC	684	n	bl	y	n	0	ev	all/unsp	1	9	1	1	nev any	st
WYNDE4	11	x	m	0	0	all	-	not a	NAmer	1948	CC	684	n	bl	y	n	0	ev	all/unsp	10	15	0	2	nev any	st
WYNDE4	17	x	m	0	0	all	-	not a	NAmer	1948	CC	684	n	bl	y	n	0	ev	all/unsp	16	20	2	3	nev any	st
WYNDE4	23	x	m	0	0	all	-	not a	NAmer	1948	CC	684	n	bl	y	n	0	ev	all/unsp	21	34	0	4	nev any	st
WYNDE4	29	x	m	0	0	all	-	not a	NAmer	1948	CC	684	n	bl	y	n	0	ev	all/unsp	35	99	3	0	nev any	st
WYNDE4	49		f	0	0	all	-	not a	NAmer	1948	CC	684	n	bl	y	n	2	ev	all/unsp	1	9	1	1	nev any	ot
WYNDE4	50		f	0	0	all	-	not a	NAmer	1948	CC	684	n	bl	y	n	2	ev	all/unsp	10	15	0	2	nev any	ot
WYNDE4	51		f	0	0	all	-	not a	NAmer	1948	CC	684	n	bl	y	n	2	ev	all/unsp	16	20	2	3	nev any	ot
WYNDE4	52		f	0	0	all	-	not a	NAmer	1948	CC	684	n	bl	y	n	2	ev	all/unsp	21	34	0	4	nev any	ot
WYNDE4	53		f	0	0	all	-	not a	NAmer	1948	CC	684	n	bl	y	n	2	ev	all/unsp	35	99	3	0	nev any	ot
WYNDE6	21		m	0	0	all	-	KI	NAmer	1969	CC	4423	n	bl	n	y	0	cu	cig+/-ot	1	10	1	0	nev any	st
WYNDE6	30		m	0	0	all	-	KI	NAmer	1969	CC	4423	n	bl	n	y	0	cu	cig+/-ot	11	20	2	3	nev any	st
WYNDE6	39		m	0	0	all	-	KI	NAmer	1969	CC	4423	n	bl	n	y	0	cu	cig+/-ot	21	30	0	4	nev any	st
WYNDE6	48		m	0	0	all	-	KI	NAmer	1969	CC	4423	n	bl	n	y	0	cu	cig+/-ot	31	99	3	0	nev any	st
WYNDE6	210		f	0	0	all	-	KI	NAmer	1969	CC	4423	n	bl	n	y	0	cu	cig+/-ot	1	10	1	0	nev cigs	st
WYNDE6	219		f	0	0	all	-	KI	NAmer	1969	CC	4423	n	bl	n	y	0	cu	cig+/-ot	11	20	2	3	nev cigs	st
WYNDE6	228		f	0	0	all	-	KI	NAmer	1969	CC	4423	n	bl	n	y	0	cu	cig+/-ot	21	30	0	4	nev cigs	st
WYNDE6	237		f	0	0	all	-	KI	NAmer	1969	CC	4423	n	bl	n	y	0	cu	cig+/-ot	30	99	3	0	nev cigs	st
ZHENG	1		m	0	0	all	-	q	As:Chi	1982	CC	540	n	ot	*	y	0	ev	cig+/-ot	1	9	1	1	nev cigs	st
ZHENG	2		m	0	0	all	-	q	As:Chi	1982	CC	540	n	ot	*	y	0	ev	cig+/-ot	10	19	0	2	nev cigs	st

Table 2G16 - 4

IESLC - Meta-anal of Ever Smoking (or Curr if Ever not avail) by Amount, Overview, Cigs (or Any Prod if Cigs not avail)
Squamous
Least adjusted

REF	NRR	X	SEX	AGEL	AGEH	RACE	YF	LC TYPE	LOC	START	ST	NLC	R	VB	P	H	AD	SM	PRODUCT	exL	exH	S1	S2	DENOM	De
ZHENG	3		m	0	0	all	-	q	As:Chi	1982	CC	540	n	ot	*	y	0	ev	cig+/-ot	20	29	2	3	nev cigs	st
ZHENG	4		m	0	0	all	-	q	As:Chi	1982	CC	540	n	ot	*	y	0	ev	cig+/-ot	30	99	3	0	nev cigs	st
ZHENG	16		f	0	0	all	-	q	As:Chi	1982	CC	540	n	ot	*	y	0	ev	cig+/-ot	1	9	1	1	nev cigs	st
ZHENG	17		f	0	0	all	-	q	As:Chi	1982	CC	540	n	ot	*	y	0	ev	cig+/-ot	10	99	0	0	nev cigs	st
ZHOU	10		c	0	0	all	-	q	As:Chi	1978	CC	1360	n	ot	n	n	0	ev	all/unsp	1	9	1	1	nev any	st
ZHOU	11		c	0	0	all	-	q	As:Chi	1978	CC	1360	n	ot	n	n	0	ev	all/unsp	10	19	0	2	nev any	st
ZHOU	12		c	0	0	all	-	q	As:Chi	1978	CC	1360	n	ot	n	n	0	ev	all/unsp	20	99	0	0	nev any	st

Cigarette type is all/unspec for all RRs

except for the following:

REF	NRR	CIGTYPE
ALDERS	34	MC only
ALDERS	35	MC only
ALDERS	36	MC only
ALDERS	37	MC only
ALDERS	38	MC only
ALDERS	39	MC only

REF	NRR	Cigarette equivalent
ALDERS	34	-
ALDERS	35	-
ALDERS	36	-
ALDERS	37	-
ALDERS	38	-
ALDERS	39	-
BARBON	19	*
BARBON	21	*
BARBON	23	*
BARBON	25	*
BARBON	27	*
BOUCOT	21	-
BOUCOT	22	-
BROWN2	36	*
BROWN2	46	*
BROWN2	35	*
BROWN2	45	*
CHOI	46	*
CHOI	47	*
CHOI	48	*
CHOI	49	*
CHOI	50	*
CHOI	56	*
CHOI	57	*
CHOI	58	*
CORREA	47	*
CORREA	51	*
DOLL	54	grams
DOLL	55	grams
DOLL	56	grams
DOLL	57	grams
DOLL	62	grams
DOLL	63	grams
DOLL	64	grams
DORGAN	114	*
DORGAN	115	*
DORGAN	99	*
DORGAN	100	*
DOSEME	7	*
DOSEME	11	*
DOSEME	15	*
ENGELA	57	*
ENGELA	58	*
ENGELA	59	*
ENGELA	60	*
ENGELA	61	*
GER	6	*
GER	7	*
GER	8	*

Table 2G16 - 4

IESLC - Meta-anal of Ever Smoking (or Curr if Ever not avail) by Amount, Overview, Cigs (or Any Prod if Cigs not avail)
Squamous
Least adjusted

REF	NRR	Cigarette equivalent
HAENSZ	18	*
HAENSZ	17	*
HAMMON	98	-
HAMMON	99	-
HAMMON	100	-
HAMMON	101	-
JEDRYC	1	*
JEDRYC	2	*
JEDRYC	3	*
JEDRYC	4	*
JEDRYC	5	*
KATSOU	25	*
KATSOU	26	*
KREYBE	13	grams inc 1 cig=1
KREYBE	14	grams inc 1 cig=1
KREYBE	15	grams inc 1 cig=1
KREYBE	31	grams inc 1 cig=1
KREYBE	32	grams inc 1 cig=1
LAMTH	10	*
LAMTH	11	*
LAMTH	12	*
LUBIN2	149	*
LUBIN2	153	*
LUBIN2	157	*
LUBIN2	161	*
LUBIN2	169	*
LUBIN2	173	*
LUBIN2	177	*
LUBIN2	181	*
LUO	4	*
LUO	5	*
LUO	6	*
MATOS	42	*
MATOS	44	*
MATOS	46	*
MATSUD	4	*
MATSUD	5	*
MATSUD	6	*
ORMOS	5	*
ORMOS	6	*
ORMOS	7	*
OSANN	51	*
OSANN	59	*
OSANN	52	*
OSANN	60	*
OSANN2	10	*
OSANN2	11	*
SOBUE	53	*
SOBUE	54	*
SOBUE	55	*
SVENSS	27	*
SVENSS	32	*
SVENSS	37	*
TSUGAN	15	*
TSUGAN	16	*
TSUGAN	17	*
WAKAI	43	*
WAKAI	44	*
WAKAI	45	*
WU	12	*
WU	13	*
WUWILL	20	*
WUWILL	21	*
WYNDE2	3	*
WYNDE2	4	*
WYNDE2	5	*
WYNDE2	6	*
WYNDE3	4	*
WYNDE3	5	*
WYNDE3	6	*
WYNDE3	7	*
WYNDE3	63	*

Table 2G16 - 4

IESLC - Meta-anal of Ever Smoking (or Curr if Ever not avail) by Amount, Overview, Cigs (or Any Prod if Cigs not avail)
Squamous
Least adjusted

REF	NRR	Cigarette equivalent
WYNDE3	64	*
WYNDE3	65	*
WYNDE3	66	*
WYNDE4	5	inc 1 cigar = 5 cigs, 1 pipe = 2.5 cigs
WYNDE4	11	inc 1 cigar = 5 cigs, 1 pipe = 2.5 cigs
WYNDE4	17	inc 1 cigar = 5 cigs, 1 pipe = 2.5 cigs
WYNDE4	23	inc 1 cigar = 5 cigs, 1 pipe = 2.5 cigs
WYNDE4	29	inc 1 cigar = 5 cigs, 1 pipe = 2.5 cigs
WYNDE4	49	inc 1 cigar = 5 cigs, 1 pipe = 2.5 cigs
WYNDE4	50	inc 1 cigar = 5 cigs, 1 pipe = 2.5 cigs
WYNDE4	51	inc 1 cigar = 5 cigs, 1 pipe = 2.5 cigs
WYNDE4	52	inc 1 cigar = 5 cigs, 1 pipe = 2.5 cigs
WYNDE4	53	inc 1 cigar = 5 cigs, 1 pipe = 2.5 cigs
WYNDE6	21	*
WYNDE6	30	*
WYNDE6	39	*
WYNDE6	48	*
WYNDE6	210	*
WYNDE6	219	*
WYNDE6	228	*
WYNDE6	237	*
ZHENG	1	*
ZHENG	2	*
ZHENG	3	*
ZHENG	4	*
ZHENG	16	*
ZHENG	17	*
ZHOU	10	*
ZHOU	11	*
ZHOU	12	*

In this overview table, subtotals and Qs values may be invalid and should be ignored

Table 2G16 - 5

IESLC - Meta-anal of Ever Smoking (or Curr if Ever not avail) by Amount, Overview, Cigs (or Any Prod if Cigs not avail)
Squamous
Least adjusted

REF	NRR	SEX	AD	Number Exposed Case	Number Exposed Cont	Non-exposed Case	Non-exposed Cont	RR	95.00%CI
ALDERS	34	m	1	-	-	-	-	3.79	(1.30- 11.02)
ALDERS	35	m	1	-	-	-	-	7.19	(2.75- 18.79)
ALDERS	36	m	1	-	-	-	-	8.78	(3.46- 22.31)
ALDERS	37	f	1	-	-	-	-	2.55	(1.42- 4.57)
ALDERS	38	f	1	-	-	-	-	9.24	(5.31- 16.09)
ALDERS	39	f	1	-	-	-	-	14.52	(7.93- 26.58)
Subtotal ALDERS								6.82	(5.12- 9.11)
BARBON	19	m	0	11	87	6	188	3.96	(1.42- 11.06)
BARBON	21	m	0	45	111	6	188	12.70	(5.25- 30.73)
BARBON	23	m	0	81	176	6	188	14.42	(6.14- 33.89)
BARBON	25	m	0	46	82	6	188	17.58	(7.22- 42.78)
BARBON	27	m	0	78	111	6	188	22.02	(9.29- 52.18)
Subtotal BARBON								13.16	(8.81- 19.65)
*BOUCOT	21	m	0	21	15208	0	7551	21.35~	(1.29- 352.41)
*BOUCOT	22	m	0	17	6940	0	7551	38.08~	(2.29- 633.12)
Subtotal BOUCOT								28.49	(3.91- 207.42)
BROWN2	36	m	2	-	-	-	-	7.60	(6.20- 9.40)
BROWN2	46	m	2	-	-	-	-	17.20	(14.60- 20.30)
BROWN2	35	f	2	-	-	-	-	11.70	(8.70- 15.80)
BROWN2	45	f	2	-	-	-	-	26.10	(20.70- 32.80)
Subtotal BROWN2								14.51	(13.06- 16.12)
CHOI	46	m	0	12	90	6	95	2.11	(0.76- 5.86)
CHOI	47	m	0	84	281	6	95	4.73	(2.00- 11.19)
CHOI	48	m	0	30	49	6	95	9.69	(3.78- 24.86)
CHOI	49	m	0	25	39	6	95	10.15	(3.86- 26.66)
CHOI	50	m	0	9	6	6	95	23.75	(6.33- 89.09)
CHOI	56	f	0	4	16	10	164	4.10	(1.15- 14.57)
CHOI	57	f	0	5	9	10	164	9.11	(2.57- 32.31)
CHOI	58	f	0	2	1	10	164	32.80	(2.74- 393.20)
Subtotal CHOI								6.86	(4.63- 10.16)
CORREA	47	c	1	-	-	-	-	23.20	(14.60- 37.00)
CORREA	51	c	1	-	-	-	-	54.80	(35.60- 89.20)
Subtotal CORREA								35.84	(25.85- 49.70)
DOLL	54	m	0	29	129	3	61	4.57	(1.34- 15.59)
DOLL	55	m	0	291	570	3	61	10.38	(3.23- 33.37)
DOLL	56	m	0	301	431	3	61	14.20	(4.41- 45.68)
DOLL	57	m	0	208	166	3	61	25.48	(7.85- 82.66)
DOLL	62	f	0	8	25	16	59	1.18	(0.45- 3.11)
DOLL	63	f	0	9	18	16	59	1.84	(0.70- 4.88)
DOLL	64	f	0	15	6	16	59	9.22	(3.08- 27.59)
Subtotal DOLL								5.29	(3.50- 8.02)
DORGAN	114	m	2	-	-	-	-	11.50	(4.10- 32.24)
DORGAN	115	m	2	-	-	-	-	23.29	(8.55- 63.49)
DORGAN	99	f	3	-	-	-	-	7.78	(4.86- 12.44)
DORGAN	100	f	3	-	-	-	-	16.38	(10.22- 26.26)
Subtotal DORGAN								12.06	(8.92- 16.31)
DOSEME	7	m	2	-	-	-	-	2.60	(1.50- 4.60)
DOSEME	11	m	2	-	-	-	-	3.20	(2.20- 4.60)
DOSEME	15	m	2	-	-	-	-	7.00	(4.10- 12.00)
Subtotal DOSEME								3.71	(2.84- 4.84)
*ENGELA	57	m	7	-	-	-	-	4.30	(1.00- 19.00)
*ENGELA	58	m	7	-	-	-	-	7.70	(1.90- 31.00)
*ENGELA	59	m	7	-	-	-	-	15.00	(3.90- 60.00)
*ENGELA	60	m	7	-	-	-	-	30.00	(7.40- 120.00)
*ENGELA	61	m	7	-	-	-	-	24.00	(5.90- 94.00)
Subtotal ENGELA								13.25	(7.08- 24.79)
GER	6	c	0	9	56	11	80	1.17	(0.45- 3.01)
GER	7	c	0	20	87	11	80	1.67	(0.75- 3.71)
GER	8	c	0	19	13	11	80	10.63	(4.13- 27.37)
Subtotal GER								2.59	(1.55- 4.32)
HAENSZ	18	f	0	30	66	44	236	2.44	(1.42- 4.18)
HAENSZ	17	f	0	18	13	44	236	7.43	(3.40- 16.24)
Subtotal HAENSZ								3.49	(2.24- 5.43)
*HAMMON	98	m	1	-	-	-	-	15.12	(4.93- 46.36)
*HAMMON	99	m	1	-	-	-	-	17.44	(6.30- 48.29)
*HAMMON	100	m	1	-	-	-	-	42.32	(15.38- 116.45)
*HAMMON	101	m	1	-	-	-	-	63.91	(22.02- 185.47)
Subtotal HAMMON								29.45	(17.41- 49.82)
JEDRYC	1	m	0	1	67	6	289	0.72	(0.09- 6.07)
JEDRYC	2	m	0	38	199	6	289	9.20	(3.82- 22.17)
JEDRYC	3	m	0	152	434	6	289	16.87	(7.36- 38.66)

Table 2G16 - 5

IESLC - Meta-anal of Ever Smoking (or Curr if Ever not avail) by Amount, Overview, Cigs (or Any Prod if Cigs not avail)
Squamous
Least adjusted

REF	NRR	SEX	AD	Number Exposed Case	Number Exposed Cont	Non-exposed Case	Non-exposed Cont	RR	95.00%CI
JEDRYC	4	m	0	61	118	6	289	24.90	(10.48- 59.17)
JEDRYC	5	m	0	57	82	6	289	33.48	(13.94- 80.42)
Subtotal JEDRYC								16.68	(10.93- 25.44)
KATSOU	25	f	0	9	14	14	67	3.08	(1.11- 8.50)
KATSOU	26	f	0	15	4	14	67	17.95	(5.17- 62.28)
Subtotal KATSOU								6.23	(2.84- 13.69)
KREYBE	13	m	0	123	2341	3	644	11.28	(3.58- 35.57)
KREYBE	14	m	0	49	925	3	644	11.37	(3.53- 36.64)
KREYBE	15	m	0	38	248	3	644	32.89	(10.06- 107.53)
KREYBE	31	f	0	1	286	3	657	0.77	(0.08- 7.39)
KREYBE	32	f	0	1	42	3	657	5.21	(0.53- 51.21)
Subtotal KREYBE								11.71	(6.29- 21.81)
LAMTH	10	f	0	23	11	28	72	5.38	(2.32- 12.46)
LAMTH	11	f	0	28	6	28	72	12.00	(4.49- 32.10)
LAMTH	12	f	0	10	1	28	72	25.71	(3.14- 210.29)
Subtotal LAMTH								8.37	(4.54- 15.43)
LUBIN2	149	m	0	418	2194	54	2616	9.23	(6.91- 12.32)
LUBIN2	153	m	0	1022	3385	54	2616	14.63	(11.07- 19.32)
LUBIN2	157	m	0	1298	3108	54	2616	20.23	(15.33- 26.69)
LUBIN2	161	m	0	849	1746	54	2616	23.56	(17.77- 31.22)
LUBIN2	169	f	0	30	184	72	1180	2.67	(1.70- 4.20)
LUBIN2	173	f	0	91	234	72	1180	6.37	(4.54- 8.95)
LUBIN2	177	f	0	61	110	72	1180	9.09	(6.13- 13.46)
LUBIN2	181	f	0	18	39	72	1180	7.56	(4.12- 13.88)
Subtotal LUBIN2								11.82	(10.51- 13.28)
LUO	4	c	0	3	39	5	51	0.78	(0.18- 3.48)
LUO	5	c	0	22	23	5	51	9.76	(3.28- 28.98)
LUO	6	c	0	9	4	5	51	22.95	(5.15- 102.20)
Subtotal LUO								6.34	(2.97- 13.53)
MATOS	42	m	0	3	88	3	110	1.25	(0.25- 6.35)
MATOS	44	m	0	18	90	3	110	7.33	(2.09- 25.69)
MATOS	46	m	0	26	105	3	110	9.08	(2.67- 30.90)
Subtotal MATOS								5.36	(2.48- 11.58)
MATSUD	4	m	0	21	1237	1	1255	21.31	(2.86- 158.63)
MATSUD	5	m	0	43	1607	1	1255	33.58	(4.62- 244.19)
MATSUD	6	m	0	39	470	1	1255	104.14	(14.27- 760.12)
Subtotal MATSUD								42.26	(13.37- 133.55)
ORMOS	5	m	0	13	329	2	777	15.35	(3.44- 68.41)
ORMOS	6	m	0	10	577	2	777	6.73	(1.47- 30.85)
ORMOS	7	m	0	4	128	2	777	12.14	(2.20- 66.97)
Subtotal ORMOS								10.74	(4.35- 26.54)
OSANN	51	m	2	-	-	-	-	35.30	(17.00- 73.30)
OSANN	59	m	2	-	-	-	-	76.00	(36.80- 157.00)
OSANN	52	f	2	-	-	-	-	24.00	(12.70- 45.50)
OSANN	60	f	2	-	-	-	-	72.30	(36.80- 142.00)
Subtotal OSANN								45.20	(32.03- 63.79)
OSANN2	10	f	0	18	31	7	58	4.81	(1.81- 12.77)
OSANN2	11	f	0	93	29	7	58	26.57	(10.93- 64.58)
Subtotal OSANN2								12.25	(6.35- 23.63)
SOBUE	53	m	0	57	157	3	128	15.49	(4.74- 50.62)
SOBUE	54	m	0	103	222	3	128	19.80	(6.15- 63.68)
SOBUE	55	m	0	87	187	3	128	19.85	(6.14- 64.13)
Subtotal SOBUE								18.28	(9.28- 36.03)
SVENSS	27	f	0	10	30	5	120	8.00	(2.54- 25.16)
SVENSS	32	f	0	28	22	5	120	30.55	(10.64- 87.69)
SVENSS	37	f	0	4	1	5	120	96.00	(9.00-1023.75)
Subtotal SVENSS								19.60	(9.38- 40.97)
TSUGAN	15	m	0	2	5	0	5	5.00~	(0.19- 130.02)
TSUGAN	16	m	0	7	7	0	5	11.00~	(0.51- 236.22)
TSUGAN	17	m	0	9	1	0	5	69.67~	(2.40-2022.74)
Subtotal TSUGAN								14.94	(2.32- 96.11)
WAKAI	43	m	0	13	105	2	65	4.02	(0.88- 18.41)
WAKAI	44	m	0	40	129	2	65	10.08	(2.36- 43.01)
WAKAI	45	m	0	33	48	2	65	22.34	(5.11- 97.69)
Subtotal WAKAI								9.85	(4.19- 23.17)
WU	12	f	0	19	14	2	30	20.36	(4.15- 99.74)
WU	13	f	0	42	9	2	30	70.00	(14.10- 347.48)
Subtotal WU								37.56	(12.15- 116.07)
WUWILL	20	f	0	168	311	117	601	2.77	(2.11- 3.65)
WUWILL	21	f	0	33	40	117	601	4.24	(2.57- 7.00)
Subtotal WUWILL								3.06	(2.41- 3.88)

Table 2G16 - 5

IESLC - Meta-anal of Ever Smoking (or Curr if Ever not avail) by Amount, Overview, Cigs (or Any Prod if Cigs not avail)
Squamous
Least adjusted

REF	NRR	SEX	AD	Number Exposed Case	Number Exposed Cont	Non-exposed Case	Non-exposed Cont	RR	95.00%CI
WYNDE2	3	m	0	15	114	3	105	4.61	(1.30- 16.36)
WYNDE2	4	m	0	108	203	3	105	18.62	(5.77- 60.06)
WYNDE2	5	m	0	74	83	3	105	31.20	(9.50- 102.54)
WYNDE2	6	m	0	139	112	3	105	43.44	(13.42- 140.56)
Subtotal WYNDE2								19.37	(10.64- 35.27)
WYNDE3	4	m	0	7	42	3	88	4.89	(1.20- 19.86)
WYNDE3	5	m	0	57	114	3	88	14.67	(4.44- 48.40)
WYNDE3	6	m	0	74	82	3	88	26.47	(8.03- 87.26)
WYNDE3	7	m	0	59	26	3	88	66.56	(19.27- 229.96)
WYNDE3	63	f	0	1	19	5	76	0.80	(0.09- 7.26)
WYNDE3	64	f	0	13	24	5	76	8.23	(2.66- 25.46)
WYNDE3	65	f	0	8	10	5	76	12.16	(3.32- 44.50)
WYNDE3	66	f	0	3	3	5	76	15.20	(2.42- 95.56)
Subtotal WYNDE3								13.67	(8.51- 21.97)
WYNDE4	5	m	0	14	82	8	115	2.45	(0.98- 6.12)
WYNDE4	11	m	0	61	147	8	115	5.97	(2.74- 12.97)
WYNDE4	17	m	0	213	274	8	115	11.17	(5.34- 23.39)
WYNDE4	23	m	0	186	98	8	115	27.28	(12.79- 58.18)
WYNDE4	29	m	0	123	64	8	115	27.63	(12.69- 60.13)
WYNDE4	49	f	2	-	-	-	-	0.87	(0.11- 6.90)
WYNDE4	50	f	2	-	-	-	-	4.61	(1.38- 15.41)
WYNDE4	51	f	2	-	-	-	-	14.92	(4.88- 45.67)
WYNDE4	52	f	2	-	-	-	-	26.53	(4.12- 171.09)
WYNDE4	53	f	2	-	-	-	-	26.53	(4.12- 171.09)
Subtotal WYNDE4								10.88	(7.98- 14.84)
WYNDE6	21	m	0	75	122	29	617	13.08	(8.17- 20.94)
WYNDE6	30	m	0	270	293	29	617	19.61	(13.04- 29.47)
WYNDE6	39	m	0	179	129	29	617	29.52	(19.09- 45.65)
WYNDE6	48	m	0	502	197	29	617	54.22	(36.08- 81.47)
WYNDE6	210	f	0	37	109	40	856	7.26	(4.45- 11.85)
WYNDE6	219	f	0	191	165	40	856	24.77	(16.95- 36.20)
WYNDE6	228	f	0	101	50	40	856	43.23	(27.18- 68.76)
WYNDE6	237	f	0	221	52	40	856	90.95	(58.70- 140.93)
Subtotal WYNDE6								28.23	(24.23- 32.90)
ZHENG	1	m	0	7	40	4	94	4.11	(1.14- 14.84)
ZHENG	2	m	0	25	66	4	94	8.90	(2.96- 26.78)
ZHENG	3	m	0	75	89	4	94	19.80	(6.95- 56.41)
ZHENG	4	m	0	49	23	4	94	50.07	(16.39- 152.91)
ZHENG	16	f	0	11	29	33	184	2.11	(0.96- 4.64)
ZHENG	17	f	0	32	15	33	184	11.89	(5.81- 24.35)
Subtotal ZHENG								8.76	(5.95- 12.88)
ZHOU	10	c	0	15	5	138	68	1.48	(0.52- 4.24)
ZHOU	11	c	0	78	14	138	68	2.75	(1.45- 5.20)
ZHOU	12	c	0	285	29	138	68	4.84	(3.00- 7.82)
Subtotal ZHOU								3.52	(2.45- 5.04)
Partial Totals				10105	49533	2103	55769		

*prospective study ~ With 0.5 adjustment for zero

REF	NRR	SEX	AD	Ys	Ws	Qs	Ps
ALDERS	34	m	1	1.33	3.36	4.48	0.0145
ALDERS	35	m	1	1.97	4.16	1.10	0.0001
ALDERS	36	m	1	2.17	4.42	0.44	0.0000
ALDERS	37	f	1	0.94	11.25	27.05	0.0017
ALDERS	38	f	1	2.22	12.50	0.87	0.0000
ALDERS	39	f	1	2.68	10.50	0.37	0.0000
Subtotal ALDERS				1.92	46.20	34.32	
BARBON	19	m	0	1.38	3.64	4.49	0.0086
BARBON	21	m	0	2.54	4.92	0.01	0.0000
BARBON	23	m	0	2.67	5.26	0.17	0.0000
BARBON	25	m	0	2.87	4.86	0.70	0.0000
BARBON	27	m	0	3.09	5.16	1.89	0.0000
Subtotal BARBON				2.58	23.84	7.27	
*BOUCOT	21	m	0	3.06	0.49	0.16	0.0324
*BOUCOT	22	m	0	3.64	0.49	0.65	0.0112
Subtotal BOUCOT				3.35	0.97	0.81	
BROWN2	36	m	2	2.03	88.72	18.68	0.0000
BROWN2	46	m	2	2.84	141.44	18.12	0.0000
BROWN2	35	f	2	2.46	43.16	0.03	0.0000
BROWN2	45	f	2	3.26	72.52	43.55	0.0000
Subtotal BROWN2				2.67	345.85	80.38	

Table 2G16 - 5

IESLC - Meta-anal of Ever Smoking (or Curr if Ever not avail) by Amount, Overview, Cigs (or Any Prod if Cigs not avail)
Squamous
Least adjusted

REF	NRR	SEX	AD	Ys	Ws	Qs	Ps
CHOI	46	m	0	0.75	3.68	11.14	0.1517
CHOI	47	m	0	1.55	5.19	4.51	0.0004
CHOI	48	m	0	2.27	4.33	0.20	0.0000
CHOI	49	m	0	2.32	4.12	0.12	0.0000
CHOI	50	m	0	3.17	2.20	1.02	0.0000
CHOI	56	f	0	1.41	2.39	2.77	0.0292
CHOI	57	f	0	2.21	2.40	0.18	0.0006
CHOI	58	f	0	3.49	0.62	0.63	0.0059
Subtotal CHOI				1.93	24.93	20.57	
CORREA	47	c	1	3.14	17.77	7.67	0.0000
CORREA	51	c	1	4.00	18.21	41.89	0.0000
Subtotal CORREA				3.58	35.98	49.57	
DOLL	54	m	0	1.52	2.55	2.39	0.0152
DOLL	55	m	0	2.34	2.82	0.06	0.0001
DOLL	56	m	0	2.65	2.81	0.08	0.0000
DOLL	57	m	0	3.24	2.77	1.56	0.0000
DOLL	62	f	0	0.17	4.09	22.05	0.7378
DOLL	63	f	0	0.61	4.06	14.29	0.2175
DOLL	64	f	0	2.22	3.20	0.23	0.0001
Subtotal DOLL				1.67	22.31	40.65	
DORGAN	114	m	2	2.44	3.61	0.01	0.0000
DORGAN	115	m	2	3.15	3.82	1.67	0.0000
DORGAN	99	f	3	2.05	17.39	3.30	0.0000
DORGAN	100	f	3	2.80	17.25	1.65	0.0000
Subtotal DORGAN				2.49	42.08	6.62	
DOSEME	7	m	2	0.96	12.24	28.70	0.0008
DOSEME	11	m	2	1.16	28.24	49.50	0.0000
DOSEME	15	m	2	1.95	13.32	3.90	0.0000
Subtotal DOSEME				1.31	53.80	82.10	
*ENGELA	57	m	7	1.46	1.77	1.87	0.0522
*ENGELA	58	m	7	2.04	1.97	0.39	0.0042
*ENGELA	59	m	7	2.71	2.06	0.10	0.0001
*ENGELA	60	m	7	3.40	1.98	1.65	0.0000
*ENGELA	61	m	7	3.18	2.01	0.96	0.0000
Subtotal ENGELA				2.58	9.78	4.98	
GER	6	c	0	0.16	4.30	23.38	0.7462
GER	7	c	0	0.51	6.06	23.61	0.2056
GER	8	c	0	2.36	4.29	0.07	0.0000
Subtotal GER				0.95	14.66	47.06	
HAENSZ	18	f	0	0.89	13.25	33.75	0.0012
HAENSZ	17	f	0	2.01	6.27	1.46	0.0000
Subtotal HAENSZ				1.25	19.53	35.21	
*HAMMON	98	m	1	2.72	3.06	0.16	0.0000
*HAMMON	99	m	1	2.86	3.70	0.51	0.0000
*HAMMON	100	m	1	3.75	3.75	5.94	0.0000
*HAMMON	101	m	1	4.16	3.38	9.44	0.0000
Subtotal HAMMON				3.38	13.90	16.05	
JEDRYC	1	m	0	-0.33	0.84	6.70	0.7618
JEDRYC	2	m	0	2.22	4.96	0.36	0.0000
JEDRYC	3	m	0	2.83	5.59	0.64	0.0000
JEDRYC	4	m	0	3.21	5.13	2.72	0.0000
JEDRYC	5	m	0	3.51	5.00	5.25	0.0000
Subtotal JEDRYC				2.81	21.53	15.66	
KATSOU	25	f	0	1.12	3.72	6.91	0.0302
KATSOU	26	f	0	2.89	2.48	0.40	0.0000
Subtotal KATSOU				1.83	6.20	7.31	
KREYBE	13	m	0	2.42	2.91	0.01	0.0000
KREYBE	14	m	0	2.43	2.81	0.01	0.0000
KREYBE	15	m	0	3.49	2.74	2.77	0.0000
KREYBE	31	f	0	-0.27	0.75	5.67	0.8175
KREYBE	32	f	0	1.65	0.74	0.51	0.1566
Subtotal KREYBE				2.46	9.94	8.97	
LAMTH	10	f	0	1.68	5.44	3.52	0.0001
LAMTH	11	f	0	2.48	3.97	0.00	0.0000
LAMTH	12	f	0	3.25	0.87	0.50	0.0025
Subtotal LAMTH				2.12	10.27	4.02	
LUBIN2	149	m	0	2.22	45.98	3.22	0.0000
LUBIN2	153	m	0	2.68	49.57	1.90	0.0000
LUBIN2	157	m	0	3.01	50.02	13.54	0.0000
LUBIN2	161	m	0	3.16	48.42	21.89	0.0000
LUBIN2	169	f	0	0.98	18.69	42.29	0.0000

Table 2G16 - 5

IESLC - Meta-anal of Ever Smoking (or Curr if Ever not avail) by Amount, Overview, Cigs (or Any Prod if Cigs not avail)
Squamous
Least adjusted

REF	NRR	SEX	AD	Ys	Ws	Qs	Ps
LUBIN2	173	f	0	1.85	33.33	13.44	0.0000
LUBIN2	177	f	0	2.21	24.86	1.95	0.0000
LUBIN2	181	f	0	2.02	10.42	2.24	0.0000
Subtotal LUBIN2				2.47	281.30	100.46	
LUO	4	c	0	-0.24	1.73	12.88	0.7498
LUO	5	c	0	2.28	3.24	0.14	0.0000
LUO	6	c	0	3.13	1.72	0.72	0.0000
Subtotal LUO				1.85	6.69	13.74	
MATOS	42	m	0	0.22	1.46	7.46	0.7878
MATOS	44	m	0	1.99	2.44	0.60	0.0018
MATOS	46	m	0	2.21	2.56	0.20	0.0004
Subtotal MATOS				1.68	6.46	8.26	
MATSUD	4	m	0	3.06	0.95	0.31	0.0028
MATSUD	5	m	0	3.51	0.98	1.03	0.0005
MATSUD	6	m	0	4.65	0.97	4.53	0.0000
Subtotal MATSUD				3.74	2.90	5.87	
ORMOS	5	m	0	2.73	1.72	0.10	0.0003
ORMOS	6	m	0	1.91	1.66	0.56	0.0141
ORMOS	7	m	0	2.50	1.32	0.00	0.0042
Subtotal ORMOS				2.37	4.70	0.66	
OSANN	51	m	2	3.56	7.20	8.34	0.0000
OSANN	59	m	2	4.33	7.30	24.82	0.0000
OSANN	52	f	2	3.18	9.44	4.51	0.0000
OSANN	60	f	2	4.28	8.43	27.12	0.0000
Subtotal OSANN				3.81	32.36	64.78	
OSANN2	10	f	0	1.57	4.03	3.39	0.0016
OSANN2	11	f	0	3.28	4.87	3.06	0.0000
Subtotal OSANN2				2.51	8.90	6.45	
SOBUE	53	m	0	2.74	2.74	0.18	0.0000
SOBUE	54	m	0	2.99	2.81	0.70	0.0000
SOBUE	55	m	0	2.99	2.79	0.70	0.0000
Subtotal SOBUE				2.91	8.35	1.58	
SVENSS	27	f	0	2.08	2.93	0.49	0.0004
SVENSS	32	f	0	3.42	3.45	3.00	0.0000
SVENSS	37	f	0	4.56	0.69	2.96	0.0002
Subtotal SVENSS				2.98	7.07	6.45	
TSUGAN	15	m	0	1.61	0.36	0.28	0.3330
TSUGAN	16	m	0	2.40	0.41	0.00	0.1254
TSUGAN	17	m	0	4.24	0.34	1.04	0.0135
Subtotal TSUGAN				2.70	1.11	1.33	
WAKAI	43	m	0	1.39	1.66	1.99	0.0727
WAKAI	44	m	0	2.31	1.82	0.06	0.0018
WAKAI	45	m	0	3.11	1.77	0.68	0.0000
Subtotal WAKAI				2.29	5.25	2.73	
WU	12	f	0	3.01	1.52	0.42	0.0002
WU	13	f	0	4.25	1.50	4.64	0.0000
Subtotal WU				3.63	3.02	5.06	
WUWILL	20	f	0	1.02	51.60	110.97	0.0000
WUWILL	21	f	0	1.44	15.26	16.60	0.0000
Subtotal WUWILL				1.12	66.87	127.57	
WYNDE2	3	m	0	1.53	2.39	2.20	0.0182
WYNDE2	4	m	0	2.92	2.80	0.54	0.0000
WYNDE2	5	m	0	3.44	2.71	2.47	0.0000
WYNDE2	6	m	0	3.77	2.79	4.59	0.0000
Subtotal WYNDE2				2.96	10.69	9.80	
WYNDE3	4	m	0	1.59	1.96	1.58	0.0265
WYNDE3	5	m	0	2.69	2.70	0.11	0.0000
WYNDE3	6	m	0	3.28	2.70	1.68	0.0000
WYNDE3	7	m	0	4.20	2.50	7.32	0.0000
WYNDE3	63	f	0	-0.22	0.79	5.80	0.8428
WYNDE3	64	f	0	2.11	3.01	0.43	0.0003
WYNDE3	65	f	0	2.50	2.28	0.00	0.0002
WYNDE3	66	f	0	2.72	1.14	0.06	0.0037
Subtotal WYNDE3				2.62	17.07	16.99	
WYNDE4	5	m	0	0.90	4.60	11.62	0.0541
WYNDE4	11	m	0	1.79	6.37	3.13	0.0000
WYNDE4	17	m	0	2.41	7.04	0.04	0.0000
WYNDE4	23	m	0	3.31	6.70	4.50	0.0000
WYNDE4	29	m	0	3.32	6.35	4.39	0.0000
WYNDE4	49	f	2	-0.14	0.90	6.19	0.8951
WYNDE4	50	f	2	1.53	2.64	2.43	0.0130

Table 2G16 - 5

IESLC - Meta-anal of Ever Smoking (or Curr if Ever not avail) by Amount, Overview, Cigs (or Any Prod if Cigs not avail)
Squamous
Least adjusted

REF	NRR	SEX	AD	Ys	Ws	Qs	Ps
WYNDE4	51	f	2	2.70	3.07	0.14	0.0000
WYNDE4	52	f	2	3.28	1.11	0.69	0.0006
WYNDE4	53	f	2	3.28	1.11	0.69	0.0006
Subtotal WYNDE4				2.39	39.89	33.82	
WYNDE6	21	m	0	2.57	17.35	0.12	0.0000
WYNDE6	30	m	0	2.98	23.14	5.53	0.0000
WYNDE6	39	m	0	3.39	20.23	16.31	0.0000
WYNDE6	48	m	0	3.99	23.16	52.53	0.0000
WYNDE6	210	f	0	1.98	16.03	4.07	0.0000
WYNDE6	219	f	0	3.21	26.69	13.94	0.0000
WYNDE6	228	f	0	3.77	17.84	29.20	0.0000
WYNDE6	237	f	0	4.51	20.03	82.00	0.0000
Subtotal WYNDE6				3.34	164.47	203.71	
ZHENG	1	m	0	1.41	2.33	2.69	0.0308
ZHENG	2	m	0	2.19	3.17	0.29	0.0001
ZHENG	3	m	0	2.99	3.51	0.87	0.0000
ZHENG	4	m	0	3.91	3.08	6.27	0.0000
ZHENG	16	f	0	0.75	6.21	18.75	0.0620
ZHENG	17	f	0	2.48	7.48	0.00	0.0000
Subtotal ZHENG				2.17	25.78	28.86	
ZHOU	10	c	0	0.39	3.46	15.22	0.4669
ZHOU	11	c	0	1.01	9.42	20.54	0.0019
ZHOU	12	c	0	1.58	16.68	13.80	0.0000
Subtotal ZHOU				1.26	29.56	49.57	

N	151
NS	36

Table 2G16 - 6

IESLC - Meta-anal of Ever Smoking (or Curr if Ever not avail) by Amount, Overview, Cigs (or Any Prod if Cigs not avail)
Squamous
Least adjusted

	Sex combined	male	female	Total
N	11	88	52	151
NS	4	25	19	48

In this overview table, other than the "N" rows, entries in the "absent" and "Total" columns may be invalid and should be ignored

	Amount smoked (broad categories) absent	<20k5	6-44k20	>20k45	Total
N	43	41	30	37	151
NS	24	30	24	30	108
Wt	541.24	405.48	246.36	231.12	1424.21
Het Chi	277.02	181.21	124.35	147.60	1149.23
Het df	42	40	29	36	150
Het P	***	***	***	***	***
Fixed RR	14.54	5.71	11.97	28.64	12.03
RRl	13.37	5.18	10.56	25.17	11.42
RRu	15.82	6.29	13.56	32.58	12.67
P	+++	+++	+++	+++	+++
Random RR	12.37	4.50	11.37	27.18	11.07
RRl	9.62	3.50	8.46	20.11	9.42
RRu	15.92	5.78	15.27	36.76	13.00
P	+++	+++	+++	+++	+++

	absent	Amount smoked (narrow categories) <10k1	2-19k10	11-29k20	21-39k30	31-98k40	>40k99	Total
N	88	15	11	24	9	1	3	151
NS	36	10	8	20	7	1	2	83
Wt	890.93	100.88	123.32	232.48	66.64	4.12	5.83	1424.21
Het Chi	748.13	57.59	42.27	122.56	9.96	0.00	2.15	1149.23
Het df	87	14	10	23	8	0	2	150
Het P	***	***	***	***	N.S.	N.S.	N.S.	***
Fixed RR	13.03	4.74	8.56	12.01	29.28	10.15	33.86	12.03
RRl	12.20	3.90	7.17	10.56	23.03	3.86	15.04	11.42
RRu	13.91	5.76	10.21	13.66	37.22	26.66	76.22	12.67
P	+++	+++	+++	+++	+++	+++	+++	+++
Random RR	12.72	3.07	6.99	11.47	28.21	10.15	33.54	11.07
RRl	10.20	1.88	4.53	8.22	21.18	3.86	14.41	9.42
RRu	15.87	5.01	10.79	16.00	37.56	26.66	78.06	13.00
P	+++	+++	+++	+++	+++	+++	+++	+++

MALES

	Amount smoked (broad categories) absent	<20k5	6-44k20	>20k45	Total
N	23	23	20	22	88
NS	16	23	20	22	81
Wt	286.83	210.37	157.09	142.84	797.13
Het Chi	40.17	53.72	80.09	63.14	402.75
Het df	22	22	19	21	87
Het P	*	***	***	***	***
Fixed RR	17.09	7.28	12.05	26.92	13.82
RRl	15.22	6.36	10.31	22.85	12.89
RRu	19.19	8.34	14.09	31.72	14.81
P	+++	+++	+++	+++	+++
Random RR	16.89	6.17	12.02	28.00	13.58
RRl	13.59	4.63	8.21	19.90	11.37
RRu	20.99	8.23	17.58	39.40	16.22
P	+++	+++	+++	+++	+++

Table 2G16 - 6

IESLC - Meta-anal of Ever Smoking (or Curr if Ever not avail) by Amount, Overview, Cigs (or Any Prod if Cigs not avail)
Squamous
Least adjusted

MALES

		Amount smoked (narrow categories)						
	absent	<10k1	2-19k10	11-29k20	21-39k30	31-98k40	>40k99	Total
N	46	9	7	16	7	1	2	88
NS	25	9	7	16	7	1	2	66
Wt	451.38	66.74	73.87	148.62	47.70	4.12	4.70	797.13
Het Chi	234.71	17.65	5.71	78.91	6.26	0.00	1.24	402.75
Het df	45	8	6	15	6	0	1	87
Het P	***	*	N.S.	***	N.S.	N.S.	N.S.	***
Fixed RR	15.05	7.25	12.57	11.98	25.36	10.15	41.10	13.82
RRl	13.72	5.70	10.01	10.20	19.10	3.86	16.64	12.89
RRu	16.50	9.22	15.79	14.07	33.69	26.66	101.52	14.81
P	+++	+++	+++	+++	+++	+++	+++	+++
Random RR	15.89	5.02	12.57	11.96	25.20	10.15	40.83	13.58
RRl	12.16	3.01	10.01	7.78	18.77	3.86	14.89	11.37
RRu	20.76	8.36	15.79	18.38	33.84	26.66	111.95	16.22
P	+++	+++	+++	+++	+++	+++	+++	+++

FEMALES

	Amount smoked (broad categories)				
	absent	<20k5	6-44k20	>20k45	Total
N	17	15	8	12	52
NS	15	15	8	12	50
Wt	210.55	185.61	79.96	64.06	540.18
Het Chi	177.18	83.83	18.98	71.90	557.15
Het df	16	14	7	11	51
Het P	***	***	**	***	***
Fixed RR	13.20	4.69	13.82	29.39	10.24
RRl	11.53	4.06	11.10	23.01	9.41
RRu	15.10	5.41	17.20	37.55	11.14
P	+++	+++	+++	+++	+++
Random RR	9.67	3.82	13.24	26.24	9.30
RRl	5.79	2.52	8.70	12.70	6.83
RRu	16.16	5.79	20.16	54.22	12.64
P	+++	+++	+++	+++	+++

		Amount smoked (narrow categories)						
	absent	<10k1	2-19k10	11-29k20	21-39k30	31-98k40	>40k99	Total
N	35	5	3	6	2		1	52
NS	19	5	3	6	2		1	36
Wt	374.84	30.67	40.04	74.55	18.94		1.14	540.18
Het Chi	391.89	3.86	5.66	17.67	0.25		0.00	557.15
Het df	34	4	2	5	1		0	51
Het P	***	N.S.	(*)	**	N.S.		N.S.	***
Fixed RR	10.82	2.14	5.50	14.30	42.01		15.20	10.24
RRl	9.78	1.50	4.04	11.39	26.78		2.42	9.41
RRu	11.97	3.05	7.50	17.94	65.91		95.56	11.14
P	+++	+++	+++	+++	+++		++	+++
Random RR	10.72	2.14	4.12	14.43	42.01		15.20	9.30
RRl	7.30	1.50	1.87	8.87	26.78		2.42	6.83
RRu	15.73	3.05	9.08	23.46	65.91		95.56	12.64
P	+++	+++	+++	+++	+++		++	+++

Table 2G16 - 7

IESLC - Meta-anal of Ever Smoking (or Curr if Ever not avail) by Amount, Overview, Cigs (or Any Prod if Cigs not avail)
Squamous
Excluded studies (and stage at which they were excluded)

1	ABELIN ABRAHA AMANDU AMES ANDERS AUSTIN AXELSO BAND BECHER BERRIN BLOHMK BLOT4 BROCKM BROWN1 BYERS1 BYERS2 CARPEN CASCO2 CASCOR CHAN CHEN3 CHIAZZ CHYOU DESTE2 DOCKER DROSTE DU GARCIA GARDIN GENG GODLEY GOODMA GRAHAM GREGOR HEGMAN HEIN HENNEK HINDS HIRAOK HOROWI HORWIT HUANG ISHIMA JAHN JAIN JARVHO JIANG KELLER KIHARA KJUUS KO KOHLME KUBIK LAMWK LAMWK2 LANGE LEI LEMARC LEVIN LIU LOMBA2 LOMBAR MAGNUS MARSH MARSH2 MCDUFF MCLAUG MILLER MILLS NOTANI NOU ODRISC PAWLEG PERSHA POFFIJ QIAO QIAO2 RADZIK REN RONCO ROOTS ROTHSC SAARIK SANKAR SCHWAR SEGI SEOW SHIMIZ SIMARA SIMONA SITAS SOBUE2 STASZE STAYNE STUCKE SUN SUZUK2 SUZUKI TANG TAO TOKARS TOUSEY ULMER VEIERO VUTUC WALD WANG WANG3 WANG4 WICKLU WIGLE WILKIN WU2 WUNSCH WYNDE8 XIANGZ XU XU2 XU4 YONG ZHANG
2	BUELL CHEN MASTRA MZILEN PISANI RESTRE SADOWS
4	BOFFET WYNDE7
5	RIMING TANG2 WYNDE5
6	BLOT1 BLOT2 BLOT3 BOUCHA HIRAY2 JONES LAURIL LICKIN MOLLO MRFIT MURATA SCHWA2 VANDER WARSIN WATSON WYNDER
8	AGUDO AKIBA ARCHER ARMADA AUVINE AXELSS BENSHL BEST BRESLO BRETT BROSS BUFFLE CEDERL CHANG CHATZI CHEN2 CHOW COMSTO COOKSO CPSI CPSII DAMBER DARBY DAVEYS DEAN DEAN2 DEAN3 DEKLER DESTEF DOLL2 DORANT DORN DUNN EBELIN ENSTRO ESAKI FAN GAO GAO2 GARSHI GILLIS GOLLED GSELL HAMMO2 HANSEN HIRAYA HITOSU HOLE HU HU2 HUMBLE JARUP JOLY JUSSAW KAISE2 KAISER KANELL KAUFMA KHUDER KINLEN KNEKT KOO KOULUM KREUZE LAUSSM LETOUR LIAW LIDDEL LIU2 LIU3 LIU4 LIU5 LUBIN MACLEN MARTIS MCCONN MIGRAN MRFITR NAM NOTAN2 PARKIN PASTOR PERNU PERSH2 PETO PEZZO2 PEZZOT PIKE POLEDN PRESCO RACHTA RANDIG SEGI2 SHAW SIEMIA SPEIZE SPITZ STOCKS STOCKW TENKAN TIZZAN TULINI TVERDA WANG2 XU3 YAMAGU YUAN
10	BENHAM

Table 2G16 - 8

Potentially overlapping studies

REF	REFGP	PRINC	.	OVERLAP/LINK
LUBIN2	LUBIN2	1		Lubin-combined
LAMTH	LAMTH	1		KOO/LAMTH/LAMWK
OSANN2	KAISER	2		KAISER/OSANN2
WYNDE6	WYNDE6	1		WYNDE5/6/7/8
MATSUD	MATSUD	1		SOBUE2/MATSUD

Table 2G16 - 9

Most adjusted - insufficient data for metaanalysis

REF	NRR	SEX	AGEL	AGEH	RACE	YF	LC TYPE	LOC	START	ST	NLC	R	VB	P	H	AD	SM	PRODUCT	exL	exH	S1	S2	DENOM	De
CHEN	5	c	0	0	all	-	q	As:oth	1987	CC	323	n	ot	n	y	2	ev	cig+/-ot	1	10	1	0	nev cigs	ot
CHEN	4	c	0	0	all	-	q	As:oth	1987	CC	323	n	ot	n	y	2	ev	cig+/-ot	11	20	2	3	nev cigs	ot
CHEN	3	c	0	0	all	-	q	As:oth	1987	CC	323	n	ot	n	y	2	ev	cig+/-ot	21	30	0	4	nev cigs	ot
CHEN	2	c	0	0	all	-	q	As:oth	1987	CC	323	n	ot	n	y	2	ev	cig+/-ot	31	99	3	0	nev cigs	ot

REF	NRR	RR	SIG	Cigarette equivalent	RRDATA comment
CHEN	5	2.59	n	*	0
CHEN	4	7.05	n	*	0
CHEN	3	7.61	n	*	0
CHEN	2	11.11	y	*	P < 0.001

Table 2G17 -

IESLC - Meta-anal of Ever Smoking (or Current if ev not avail), Amount smoked, “Low”, Cigs (or Any Prod if Cigs not avail)
Squamous

This analysis is restricted to results for:
1) Results by Amount smoked
2) Results complete enough for use in metaanalysis

Within each study, results are then selected (in the following order of preference, within each sex) for:
3) SMKSTA: ever smokers, current smokers
4) PRODUCT: cigarettes regardless of other products, cigarettes only, all/unspec
5) CIGTYPE: all/unspecified, MC regardless of HR, MC only
6) DENOM: never smoked anything, never smoked cigarettes, (never +1 = +long term ex, +2 = +amount unknown,
+3 = never cigs+long term ex)
7) Followup period (YF, prospective studies): whole study (coded as 0) or longest available
8) LCtype: squamous or nearest available, but not adeno. (q = squamous, s = small,
a = adeno, KI = Kreyberg I, u = undifferentiated)
9) Race: all or nearest available, otherwise by race (wh or w = white, bl or b = black, hi = hispanic
ch = chinese, jap = japanese, haw = hawaiian, w+o = white + oriental, sca = scandinavian, as = asian)
10) Amount smoked "low" in key scheme 1 (key value 5, maximum range <20, in numbers of
cigarettes or cigarette equivalents)
11) For overlapping studies: principal rather than subsidiary studies
Finally by Age: whole study (coded as 0) if available, otherwise by widest available age group
and then for single sex results (m, f) in preference to combined sex results (c).

Results adjusted (AD) for the most potential confounders are then chosen in Sections -1 to -3
(and those which actually differ from the adjusted results in Table 2G12 - 1 are marked 'x' in Section -1)
and results adjusted for the least confounders in Sections -4 to -6. (Those least adjusted results which
actually differ from the most adjusted as marked 'x' in column X in Section -4)
(Results adjusted for an unknown number of confounder(s) are coded as 20.)

Section -7 shows excluded studies, together with the stage (as above) at which no qualifying
results were found.

Section -8 lists the potentially overlapping studies which have been included (1=principal, 2=subsidiary).

Section -9 lists any results which would have been included in preference except that they had data not complete
enough for use in meta-analysis, with their significance (yes/no), if known, and any further comment as entered
on the database.

In addition to those mentioned above, the following fields, levels and abbreviations are used:
* or nk = not known, n = no, y = yes, ot = other
ev = ever, cu = current, nev = never
all/unspec = all or unspecified, cig+/-ot = cigarettes irrespective of other products (cigar, pipe etc)
MC = manufactured cigarettes, HR = hand-rolled cigarettes
exL, exH = range of exposure (low and high) in the smoking group, in terms of Amount smoked,
cigarettes or cigarette equivalents
REF: 6-character study reference
NRR: number of the RR on the database within the study
ST : study type (CC = case control, pr or prosp = prospective)
NLC: number of lung cancer cases in whole study
R : risky occupational population (n = no, m = mining, o = other risky)
VB : national cigarette type (V = at least 75% Virginia, bl = at least 75% blended, ot = other)
P : any proxy use
H : full histological confirmation
De : derivation of RR/CI (or = original, st = standard method, ot = other method of estimation)

Table 2G17 - 1

IESLC - Meta-anal of Ever Smoking (or Current if ev not avail), Amount smoked, “Low”, Cigs (or Any Prod if Cigs not avail)
Squamous

Most adjusted

REF	NRR	2G12	SEX	AGEL	AGEH	RACE	YF	LC TYPE	LOC	START	ST	NLC	R	VB	P	H	AD	SM	PRODUCT	exL	exH	DENOM	De
ALDERS	34		m	0	0	all	-	q+s	Eu:UK	1977	CC	1448	n	V	n	n	1	ev	cig only	1	17	nev+2	ot
ALDERS	37		f	0	0	all	-	q+s	Eu:UK	1977	CC	1448	n	V	n	n	1	ev	cig only	1	17	nev+2	ot
BARBON	70		m	0	0	all	-	q	Eu:wst	1979	CC	755	n	bl	y	y	3	ev	all/unsp	1	19	nev any	or
BROWN2	36		m	0	0	wh	-	q	NAmer	1984	CC	14596	n	bl	n	y	2	ev	cig+/-ot	1	19	nev cigs	or
BROWN2	35		f	0	0	wh	-	q	NAmer	1984	CC	14596	n	bl	n	y	2	ev	cig+/-ot	1	19	nev cigs	or
CHOI	46		m	0	0	all	-	q	As:oth	1985	CC	375	n	bl	n	n	0	ev	cig+/-ot	1	10	nev cigs	st
CHOI	56		f	0	0	all	-	q	As:oth	1985	CC	375	n	bl	n	n	0	ev	cig+/-ot	1	10	nev cigs	st
DOLL	69		m	0	0	all	-	KI	Eu:UK	1948	CC	1465	n	V	n	n	1	ev	all/unsp	5	14	nev any	ot
DOLL	77		f	0	0	all	-	KI	Eu:UK	1948	CC	1465	n	V	n	n	1	ev	all/unsp	5	14	nev any	ot
DORGAN	114		m	0	0	wh	-	q	NAmer	1980	CC	2026	n	bl	y	y	2	ev	cig+/-ot	1	19	nev any	ot
DORGAN	99		f	0	0	all	-	q	NAmer	1980	CC	2026	n	bl	y	y	3	ev	cig+/-ot	1	19	nev any	ot
DOSEME	7		m	0	0	all	-	q	Eu:bal	1979	CC	1210	n	bl	n	n	2	ev	cig+/-ot	1	10	nev cigs	or
ENGELA	58		m	0	0	all	0	q	Eu:Sca	1964	pr	435	n	bl	n	n	7	cu	cig+/-ot	5	9	nev cigs	or
GER	14		c	0	0	all	-	q+s	As:oth	1990	CC	141	n	ot	y	n	10	ev	all/unsp	1	10	nev any	ot
HAMMON	98		m	0	0	wh	0	not a	NAmer	1952	pr	448	n	bl	n	n	1	cu	cig only	1	9	nev any	ot
JEDRYC	28		m	0	0	all	-	q	Eu:est	1980	CC	1630	n	bl	y	n	3	ev	cig+/-ot	1	19	nev any	or
KREYBE	1		m	0	0	all	-	KI	Eu:Sca	1948	CC	300	n	bl	n	y	1	ev	all/unsp	1	14	nev any	ot
KREYBE	31		f	0	0	all	-	KI	Eu:Sca	1948	CC	300	n	bl	n	y	0	ev	all/unsp	1	14	nev any	st
LAMTH	10		f	0	0	ch	-	q	As:HK	1983	CC	445	n	bl	n	n	0	ev	all/unsp	1	10	nev any	or
LUBIN2	149		m	0	0	all	-	q	Eu:mul	1976	CC	7804	n	bl	n	y	0	ev	cig+/-ot	1	9	nev any	st
LUBIN2	169		f	0	0	all	-	q	Eu:mul	1976	CC	7804	n	bl	n	y	0	ev	cig+/-ot	1	9	nev any	st
LUO	10		c	0	0	all	-	q	As:Chi	1990	CC	102	n	ot	n	y	20	ev	cig+/-ot	1	19	nev cigs	or
MATOS	43		m	0	0	all	-	q	SCAmer	1994	CC	200	n	bl	n	n	2	ev	cig+/-ot	1	14	nev any	or
MATSUD	4		m	0	0	all	-	q	As:Jap	1965	CC	179	n	bl	n	n	0	ev	cig+/-ot	1	10	nev cigs	st
ORMOS	5		m	0	0	all	-	q	Eu:est	1947	CC	119	n	bl	y	y	0	ev	cig+/-ot	1	15	nev any	st
OSANN2	28		f	0	0	all	-	KI	NAmer	1964	ot	217	n	bl	n	y	1	ev	cig+/-ot	1	19	nev cigs	or
SOBUE	53		m	0	0	all	-	q	As:Jap	1986	CC	1376	n	bl	n	y	0	cu	cig+/-ot	1	19	nev cigs	st
SVENSS	7		f	0	0	all	-	q	Eu:Sca	1983	CC	210	n	bl	n	n	1	cu	all/unsp	1	10	nev any	or
TSUGAN	15		m	0	0	all	-	q	As:Jap	1976	CC	134	n	bl	n	y	0	cu	all/unsp	1	15	nev any	ot
WAKAI	46		m	0	0	all	-	q	As:Jap	1988	CC	333	n	bl	n	y	1	cu	cig+/-ot	1	19	nev any	or
WUWILL	14		f	0	0	all	-	q+s	As:Chi	1985	CC	965	n	ot	n	n	3	ev	cig+/-ot	1	19	nev cigs	ot
WYNDE2	3		m	0	0	all	-	KI	NAmer	1962	CC	404	n	bl	n	y	0	ev	cig+/-ot	1	10	nev any	st
WYNDE3	4		m	0	0	all	-	KI	NAmer	1966	CC	350	n	bl	n	y	0	ev	cig+/-ot	1	9	nev any	st
WYNDE3	63		f	0	0	all	-	KI	NAmer	1966	CC	350	n	bl	n	y	0	ev	cig+/-ot	1	9	nev any	st
WYNDE4	63		m	0	0	all	-	not a	NAmer	1948	CC	684	n	bl	y	n	2	ev	all/unsp	1	9	nev any	ot
WYNDE4	49		f	0	0	all	-	not a	NAmer	1948	CC	684	n	bl	y	n	2	ev	all/unsp	1	9	nev any	ot
WYNDE6	21		m	0	0	all	-	KI	NAmer	1969	CC	4423	n	bl	n	y	0	cu	cig+/-ot	1	10	nev any	st
WYNDE6	210		f	0	0	all	-	KI	NAmer	1969	CC	4423	n	bl	n	y	0	cu	cig+/-ot	1	10	nev cigs	st
ZHENG	1		m	0	0	all	-	q	As:Chi	1982	CC	540	n	ot	*	y	0	ev	cig+/-ot	1	9	nev cigs	st
ZHENG	16		f	0	0	all	-	q	As:Chi	1982	CC	540	n	ot	*	y	0	ev	cig+/-ot	1	9	nev cigs	st
ZHOU	10		c	0	0	all	-	q	As:Chi	1978	CC	1360	n	ot	n	n	0	ev	all/unsp	1	9	nev any	st

Cigarette type is all/unspec for all RRs

except for the following:

REF	NRR	CIGTYPE
ALDERS	34	MC only
ALDERS	37	MC only

Table 2G17 - 2

IESLC - Meta-anal of Ever Smoking (or Current if ev not avail), Amount smoked, “Low”, Cigs (or Any Prod if Cigs not avail)
Squamous
Most adjusted

REF	NRR	SEX	AD	Number Exposed Case	Number Exposed Cont	Non-exposed Case	Non-exposed Cont	RR	95.00%CI
ALDERS	34	m	1	-	-	-	-	3.79	(1.30- 11.02)
ALDERS	37	f	1	-	-	-	-	2.55	(1.42- 4.57)
Subtotal ALDERS								2.79	(1.67- 4.66)
BARBON	70	m	3	-	-	-	-	8.50	(3.60- 20.00)
BROWN2	36	m	2	-	-	-	-	7.60	(6.20- 9.40)
BROWN2	35	f	2	-	-	-	-	11.70	(8.70- 15.80)
Subtotal BROWN2								8.75	(7.38- 10.38)
CHOI	46	m	0	12	90	6	95	2.11	(0.76- 5.86)
CHOI	56	f	0	4	16	10	164	4.10	(1.15- 14.57)
Subtotal CHOI								2.74	(1.24- 6.07)
DOLL	69	m	1	-	-	-	-	10.60	(3.30- 34.07)
DOLL	77	f	1	-	-	-	-	1.70	(0.64- 4.50)
Subtotal DOLL								3.61	(1.71- 7.62)
DORGAN	114	m	2	-	-	-	-	11.50	(4.10- 32.24)
DORGAN	99	f	3	-	-	-	-	7.78	(4.86- 12.44)
Subtotal DORGAN								8.32	(5.43- 12.76)
DOSEME	7	m	2	-	-	-	-	2.60	(1.50- 4.60)
*ENGELA	58	m	7	-	-	-	-	7.70	(1.90- 31.00)
GER	14	c	10	-	-	-	-	1.43	(0.36- 5.61)
*HAMMON	98	m	1	-	-	-	-	15.12	(4.93- 46.36)
JEDRYC	28	m	3	-	-	-	-	7.51	(3.09- 18.27)
KREYBE	1	m	1	-	-	-	-	9.00	(2.85- 28.38)
KREYBE	31	f	0	1	286	3	657	0.77	(0.08- 7.39)
Subtotal KREYBE								5.44	(1.95- 15.16)
LAMTH	10	f	0	23	11	28	72	5.38	(2.32- 12.46)
LUBIN2	149	m	0	418	2194	54	2616	9.23	(6.91- 12.32)
LUBIN2	169	f	0	30	184	72	1180	2.67	(1.70- 4.20)
Subtotal LUBIN2								6.45	(5.06- 8.23)
LUO	10	c	20	-	-	-	-	1.20	(0.10- 10.00)
MATOS	43	m	2	-	-	-	-	1.40	(0.30- 6.90)
MATSUD	4	m	0	21	1237	1	1255	21.31	(2.86- 158.63)
ORMOS	5	m	0	13	329	2	777	15.35	(3.44- 68.41)
OSANN2	28	f	1	-	-	-	-	12.10	(1.50- 96.30)
SOBUE	53	m	0	57	157	3	128	15.49	(4.74- 50.62)
SVENSS	7	f	1	-	-	-	-	9.70	(2.90- 45.90)
TSUGAN	15	m	0	2	5	0	5	5.00~	(0.19- 130.02)
WAKAI	46	m	1	-	-	-	-	3.95	(0.86- 18.10)
WUWILL	14	f	3	-	-	-	-	3.21	(2.39- 4.30)
WYNDE2	3	m	0	15	114	3	105	4.61	(1.30- 16.36)
WYNDE3	4	m	0	7	42	3	88	4.89	(1.20- 19.86)
WYNDE3	63	f	0	1	19	5	76	0.80	(0.09- 7.26)
Subtotal WYNDE3								2.90	(0.89- 9.48)
WYNDE4	63	m	2	-	-	-	-	2.22	(0.89- 5.53)
WYNDE4	49	f	2	-	-	-	-	0.87	(0.11- 6.90)
Subtotal WYNDE4								1.91	(0.83- 4.39)
WYNDE6	21	m	0	75	122	29	617	13.08	(8.17- 20.94)
WYNDE6	210	f	0	37	109	40	856	7.26	(4.45- 11.85)
Subtotal WYNDE6								9.86	(7.02- 13.84)
ZHENG	1	m	0	7	40	4	94	4.11	(1.14- 14.84)
ZHENG	16	f	0	11	29	33	184	2.11	(0.96- 4.64)
Subtotal ZHENG								2.54	(1.30- 4.96)
ZHOU	10	c	0	15	5	138	68	1.48	(0.52- 4.24)
Partial Totals				749	4989	434	9037		

*prospective study ~ With 0.5 adjustment for zero

Table 2G17 - 2

IESLC - Meta-anal of Ever Smoking (or Current if ev not avail), Amount smoked, “Low”, Cigs (or Any Prod if Cigs not avail)
Squamous

Most adjusted

REF	NRR	SEX	AD	Ys	Ws	Qs	Ps
ALDERS	34	m	1	1.33	3.36	0.74	0.0145
ALDERS	37	f	1	0.94	11.25	8.45	0.0017
Subtotal ALDERS				1.03	14.61	9.20	
BARBON	70	m	3	2.14	5.23	0.59	0.0000
BROWN2	36	m	2	2.03	88.72	4.50	0.0000
BROWN2	35	f	2	2.46	43.16	18.61	0.0000
Subtotal BROWN2				2.17	131.88	23.11	
CHOI	46	m	0	0.75	3.68	4.10	0.1517
CHOI	56	f	0	1.41	2.39	0.37	0.0292
Subtotal CHOI				1.01	6.07	4.47	
DOLL	69	m	1	2.36	2.82	0.88	0.0001
DOLL	77	f	1	0.53	4.04	6.54	0.2862
Subtotal DOLL				1.28	6.86	7.42	
DORGAN	114	m	2	2.44	3.61	1.48	0.0000
DORGAN	99	f	3	2.05	17.39	1.07	0.0000
Subtotal DORGAN				2.12	21.01	2.55	
DOSEME	7	m	2	0.96	12.24	8.79	0.0008
*ENGELA	58	m	7	2.04	1.97	0.11	0.0042
GER	14	c	10	0.36	2.04	4.26	0.6097
*HAMMON	98	m	1	2.72	3.06	2.55	0.0000
JEDRYC	28	m	3	2.02	4.87	0.22	0.0000
KREYBE	1	m	1	2.20	2.91	0.45	0.0002
KREYBE	31	f	0	-0.27	0.75	3.20	0.8175
Subtotal KREYBE				1.69	3.66	3.65	
LAMTH	10	f	0	1.68	5.44	0.08	0.0001
LUBIN2	149	m	0	2.22	45.98	8.09	0.0000
LUBIN2	169	f	0	0.98	18.69	12.57	0.0000
Subtotal LUBIN2				1.86	64.67	20.66	
LUO	10	c	20	0.18	0.72	1.90	0.8767
MATOS	43	m	2	0.34	1.56	3.36	0.6740
MATSUD	4	m	0	3.06	0.95	1.50	0.0028
ORMOS	5	m	0	2.73	1.72	1.48	0.0003
OSANN2	28	f	1	2.49	0.89	0.42	0.0189
SOBUE	53	m	0	2.74	2.74	2.41	0.0000
SVENSS	7	f	1	2.27	2.01	0.44	0.0013
TSUGAN	15	m	0	1.61	0.36	0.01	0.3330
WAKAI	46	m	1	1.37	1.66	0.31	0.0772
WUWILL	14	f	3	1.17	44.55	18.06	0.0000
WYNDE2	3	m	0	1.53	2.39	0.18	0.0182
WYNDE3	4	m	0	1.59	1.96	0.09	0.0265
WYNDE3	63	f	0	-0.22	0.79	3.24	0.8428
Subtotal WYNDE3				1.07	2.75	3.33	
WYNDE4	63	m	2	0.80	4.60	4.66	0.0870
WYNDE4	49	f	2	-0.14	0.90	3.38	0.8951
Subtotal WYNDE4				0.64	5.50	8.04	
WYNDE6	21	m	0	2.57	17.35	10.24	0.0000
WYNDE6	210	f	0	1.98	16.03	0.52	0.0000
Subtotal WYNDE6				2.29	33.38	10.76	
ZHENG	1	m	0	1.41	2.33	0.35	0.0308
ZHENG	16	f	0	0.75	6.21	6.89	0.0620
Subtotal ZHENG				0.93	8.54	7.25	
ZHOU	10	c	0	0.39	3.46	6.91	0.4669

Table 2G17 - 2

IESLC - Meta-anal of Ever Smoking (or Current if ev not avail), Amount smoked, “Low”, Cigs (or Any Prod if Cigs not avail)
Squamous

Most adjusted

	N	41
	NS	30
	Wt	396.78
Het	Chi	154.02
Het	df	40
Het	P	***
Fixed	RR	6.07
	RRl	5.50
	RRu	6.70
	P	+++
Random	RR	4.98
	RRl	3.93
	RRu	6.31
	P	+++
Asymm	P	(*)

Table 2G17 - 3

IESLC - Meta-anal of Ever Smoking (or Current if ev not avail), Amount smoked, “Low”, Cigs (or Any Prod if Cigs not avail)
Squamous

Most adjusted

	Sex: combined	male	female	Total
N	3	23	15	41
NS	3	23	15	41
Wt	6.23	216.08	174.47	396.78
Het Chi	0.03	48.34	77.04	154.02
Het df	2	22	14	40
Het P	N.S.	***	***	***
Fixed RR	1.43	7.42	4.98	6.07
RRl	0.65	6.49	4.29	5.50
RRu	3.13	8.48	5.78	6.70
P	N.S.	+++	+++	+++
Random RR	1.43	6.62	3.95	4.98
RRl	0.65	5.08	2.60	3.93
RRu	3.13	8.63	6.00	6.31
P	N.S.	+++	+++	+++
Between Chi				28.63
Between df				2
Between P				***
Btwn(F) P				*
Btwn(R) P				***

Table 2G17 - 4

IESLC - Meta-anal of Ever Smoking (or Current if ev not avail), Amount smoked, “Low”, Cigs (or Any Prod if Cigs not avail)
Squamous
Least adjusted

REF	NRR	X	SEX	AGEL	AGEH	RACE	YF	LC TYPE	LOC	START	ST	NLC	R	VB	P	H	AD	SM	PRODUCT	exL	exH	DENOM	De
ALDERS	34		m	0	0	all	-	q+s	Eu:UK	1977	CC	1448	n	V	n	n	1	ev	cig only	1	17	nev+2	ot
ALDERS	37		f	0	0	all	-	q+s	Eu:UK	1977	CC	1448	n	V	n	n	1	ev	cig only	1	17	nev+2	ot
BARBON	19	x	m	0	0	all	-	q	Eu:wst	1979	CC	755	n	bl	y	y	0	ev	all/unsp	1	9	nev any	st
BROWN2	36		m	0	0	wh	-	q	NAmer	1984	CC	14596	n	bl	n	y	2	ev	cig+/-ot	1	19	nev cigs	or
BROWN2	35		f	0	0	wh	-	q	NAmer	1984	CC	14596	n	bl	n	y	2	ev	cig+/-ot	1	19	nev cigs	or
CHOI	46		m	0	0	all	-	q	As:oth	1985	CC	375	n	bl	n	n	0	ev	cig+/-ot	1	10	nev cigs	st
CHOI	56		f	0	0	all	-	q	As:oth	1985	CC	375	n	bl	n	n	0	ev	cig+/-ot	1	10	nev cigs	st
DOLL	55	x	m	0	0	all	-	KI	Eu:UK	1948	CC	1465	n	V	n	n	0	ev	all/unsp	5	14	nev any	st
DOLL	63	x	f	0	0	all	-	KI	Eu:UK	1948	CC	1465	n	V	n	n	0	ev	all/unsp	5	14	nev any	st
DORGAN	114		m	0	0	wh	-	q	NAmer	1980	CC	2026	n	bl	y	y	2	ev	cig+/-ot	1	19	nev any	ot
DORGAN	99		f	0	0	all	-	q	NAmer	1980	CC	2026	n	bl	y	y	3	ev	cig+/-ot	1	19	nev any	ot
DOSEME	7		m	0	0	all	-	q	Eu:bal	1979	CC	1210	n	bl	n	n	2	ev	cig+/-ot	1	10	nev cigs	or
ENGELA	58		m	0	0	all	0	q	Eu:Sca	1964	pr	435	n	bl	n	n	7	cu	cig+/-ot	5	9	nev cigs	or
GER	6	x	c	0	0	all	-	q+s	As:oth	1990	CC	141	n	ot	y	n	0	ev	all/unsp	1	10	nev any	st
HAMMON	98		m	0	0	wh	0	not a	NAmer	1952	pr	448	n	bl	n	n	1	cu	cig only	1	9	nev any	ot
JEDRYC	1	x	m	0	0	all	-	q	Eu:est	1980	CC	1630	n	bl	y	n	0	ev	cig+/-ot	1	9	nev any	st
KREYBE	13	x	m	0	0	all	-	KI	Eu:Sca	1948	CC	300	n	bl	n	y	0	ev	all/unsp	1	14	nev any	st
KREYBE	31		f	0	0	all	-	KI	Eu:Sca	1948	CC	300	n	bl	n	y	0	ev	all/unsp	1	14	nev any	st
LAMTH	10		f	0	0	ch	-	q	As:HK	1983	CC	445	n	bl	n	n	0	ev	all/unsp	1	10	nev any	or
LUBIN2	149		m	0	0	all	-	q	Eu:mul	1976	CC	7804	n	bl	n	y	0	ev	cig+/-ot	1	9	nev any	st
LUBIN2	169		f	0	0	all	-	q	Eu:mul	1976	CC	7804	n	bl	n	y	0	ev	cig+/-ot	1	9	nev any	st
LUO	4	x	c	0	0	all	-	q	As:Chi	1990	CC	102	n	ot	n	y	0	ev	cig+/-ot	1	19	nev cigs	st
MATOS	42	x	m	0	0	all	-	q	SCAmer	1994	CC	200	n	bl	n	n	0	ev	cig+/-ot	1	14	nev any	st
MATSUD	4		m	0	0	all	-	q	As:Jap	1965	CC	179	n	bl	n	n	0	ev	cig+/-ot	1	10	nev cigs	st
ORMOS	5		m	0	0	all	-	q	Eu:est	1947	CC	119	n	bl	y	y	0	ev	cig+/-ot	1	15	nev any	st
OSANN2	10	x	f	0	0	all	-	KI	NAmer	1964	ot	217	n	bl	n	y	0	ev	cig+/-ot	1	19	nev cigs	st
SOBUE	53		m	0	0	all	-	q	As:Jap	1986	CC	1376	n	bl	n	y	0	cu	cig+/-ot	1	19	nev cigs	st
SVENSS	27	x	f	0	0	all	-	q	Eu:Sca	1983	CC	210	n	bl	n	n	0	cu	all/unsp	1	10	nev any	st
TSUGAN	15		m	0	0	all	-	q	As:Jap	1976	CC	134	n	bl	n	y	0	cu	all/unsp	1	15	nev any	ot
WAKAI	43	x	m	0	0	all	-	q	As:Jap	1988	CC	333	n	bl	n	y	0	cu	cig+/-ot	1	19	nev any	st
WUWILL	20	x	f	0	0	all	-	q+s	As:Chi	1985	CC	965	n	ot	n	n	0	ev	cig+/-ot	1	19	nev cigs	st
WYNDE2	3		m	0	0	all	-	KI	NAmer	1962	CC	404	n	bl	n	y	0	ev	cig+/-ot	1	10	nev any	st
WYNDE3	4		m	0	0	all	-	KI	NAmer	1966	CC	350	n	bl	n	y	0	ev	cig+/-ot	1	9	nev any	st
WYNDE3	63		f	0	0	all	-	KI	NAmer	1966	CC	350	n	bl	n	y	0	ev	cig+/-ot	1	9	nev any	st
WYNDE4	5	x	m	0	0	all	-	not a	NAmer	1948	CC	684	n	bl	y	n	0	ev	all/unsp	1	9	nev any	st
WYNDE4	49		f	0	0	all	-	not a	NAmer	1948	CC	684	n	bl	y	n	2	ev	all/unsp	1	9	nev any	ot
WYNDE6	21		m	0	0	all	-	KI	NAmer	1969	CC	4423	n	bl	n	y	0	cu	cig+/-ot	1	10	nev any	st
WYNDE6	210		f	0	0	all	-	KI	NAmer	1969	CC	4423	n	bl	n	y	0	cu	cig+/-ot	1	10	nev cigs	st
ZHENG	1		m	0	0	all	-	q	As:Chi	1982	CC	540	n	ot	*	y	0	ev	cig+/-ot	1	9	nev cigs	st
ZHENG	16		f	0	0	all	-	q	As:Chi	1982	CC	540	n	ot	*	y	0	ev	cig+/-ot	1	9	nev cigs	st
ZHOU	10		c	0	0	all	-	q	As:Chi	1978	CC	1360	n	ot	n	n	0	ev	all/unsp	1	9	nev any	st

Cigarette type is all/unspec for all RRs

except for the following:

REF	NRR	CIGTYPE
ALDERS	34	MC only
ALDERS	37	MC only

Table 2G17 - 5

IESLC - Meta-anal of Ever Smoking (or Current if ev not avail), Amount smoked, “Low”, Cigs (or Any Prod if Cigs not avail)
Squamous

Least adjusted

REF	NRR	SEX	AD	Number Exposed Case	Cont	Non-exposed Case	Cont	RR	95.00%CI
ALDERS	34	m	1	-	-	-	-	3.79	(1.30- 11.02)
ALDERS	37	f	1	-	-	-	-	2.55	(1.42- 4.57)
Subtotal ALDERS								2.79	(1.67- 4.66)
BARBON	19	m	0	11	87	6	188	3.96	(1.42- 11.06)
BROWN2	36	m	2	-	-	-	-	7.60	(6.20- 9.40)
BROWN2	35	f	2	-	-	-	-	11.70	(8.70- 15.80)
Subtotal BROWN2								8.75	(7.38- 10.38)
CHOI	46	m	0	12	90	6	95	2.11	(0.76- 5.86)
CHOI	56	f	0	4	16	10	164	4.10	(1.15- 14.57)
Subtotal CHOI								2.74	(1.24- 6.07)
DOLL	55	m	0	291	570	3	61	10.38	(3.23- 33.37)
DOLL	63	f	0	9	18	16	59	1.84	(0.70- 4.88)
Subtotal DOLL								3.74	(1.77- 7.90)
DORGAN	114	m	2	-	-	-	-	11.50	(4.10- 32.24)
DORGAN	99	f	3	-	-	-	-	7.78	(4.86- 12.44)
Subtotal DORGAN								8.32	(5.43- 12.76)
DOSEME	7	m	2	-	-	-	-	2.60	(1.50- 4.60)
*ENGELA	58	m	7	-	-	-	-	7.70	(1.90- 31.00)
GER	6	c	0	9	56	11	80	1.17	(0.45- 3.01)
*HAMMON	98	m	1	-	-	-	-	15.12	(4.93- 46.36)
JEDRYC	1	m	0	1	67	6	289	0.72	(0.09- 6.07)
KREYBE	13	m	0	123	2341	3	644	11.28	(3.58- 35.57)
KREYBE	31	f	0	1	286	3	657	0.77	(0.08- 7.39)
Subtotal KREYBE								6.51	(2.34- 18.14)
LAMTH	10	f	0	23	11	28	72	5.38	(2.32- 12.46)
LUBIN2	149	m	0	418	2194	54	2616	9.23	(6.91- 12.32)
LUBIN2	169	f	0	30	184	72	1180	2.67	(1.70- 4.20)
Subtotal LUBIN2								6.45	(5.06- 8.23)
LUO	4	c	0	3	39	5	51	0.78	(0.18- 3.48)
MATOS	42	m	0	3	88	3	110	1.25	(0.25- 6.35)
MATSUD	4	m	0	21	1237	1	1255	21.31	(2.86- 158.63)
ORMOS	5	m	0	13	329	2	777	15.35	(3.44- 68.41)
OSANN2	10	f	0	18	31	7	58	4.81	(1.81- 12.77)
SOBUE	53	m	0	57	157	3	128	15.49	(4.74- 50.62)
SVENSS	27	f	0	10	30	5	120	8.00	(2.54- 25.16)
TSUGAN	15	m	0	2	5	0	5	5.00~	(0.19- 130.02)
WAKAI	43	m	0	13	105	2	65	4.02	(0.88- 18.41)
WUWILL	20	f	0	168	311	117	601	2.77	(2.11- 3.65)
WYNDE2	3	m	0	15	114	3	105	4.61	(1.30- 16.36)
WYNDE3	4	m	0	7	42	3	88	4.89	(1.20- 19.86)
WYNDE3	63	f	0	1	19	5	76	0.80	(0.09- 7.26)
Subtotal WYNDE3								2.90	(0.89- 9.48)
WYNDE4	5	m	0	14	82	8	115	2.45	(0.98- 6.12)
WYNDE4	49	f	2	-	-	-	-	0.87	(0.11- 6.90)
Subtotal WYNDE4								2.07	(0.90- 4.78)
WYNDE6	21	m	0	75	122	29	617	13.08	(8.17- 20.94)
WYNDE6	210	f	0	37	109	40	856	7.26	(4.45- 11.85)
Subtotal WYNDE6								9.86	(7.02- 13.84)
ZHENG	1	m	0	7	40	4	94	4.11	(1.14- 14.84)
ZHENG	16	f	0	11	29	33	184	2.11	(0.96- 4.64)
Subtotal ZHENG								2.54	(1.30- 4.96)
ZHOU	10	c	0	15	5	138	68	1.48	(0.52- 4.24)
Partial Totals				1422	8814	626	11478		

*prospective study ~ With 0.5 adjustment for zero

Table 2G17 - 5

IESLC - Meta-anal of Ever Smoking (or Current if ev not avail), Amount smoked, “Low”, Cigs (or Any Prod if Cigs not avail)
Squamous

Least adjusted

REF	NRR	SEX	AD	Ys	Ws	Qs	Ps
ALDERS	34	m	1	1.33	3.36	0.56	0.0145
ALDERS	37	f	1	0.94	11.25	7.29	0.0017
Subtotal ALDERS				1.03	14.61	7.86	
BARBON	19	m	0	1.38	3.64	0.48	0.0086
BROWN2	36	m	2	2.03	88.72	7.29	0.0000
BROWN2	35	f	2	2.46	43.16	22.26	0.0000
Subtotal BROWN2				2.17	131.88	29.56	
CHOI	46	m	0	0.75	3.68	3.64	0.1517
CHOI	56	f	0	1.41	2.39	0.26	0.0292
Subtotal CHOI				1.01	6.07	3.90	
DOLL	55	m	0	2.34	2.82	1.01	0.0001
DOLL	63	f	0	0.61	4.06	5.18	0.2175
Subtotal DOLL				1.32	6.88	6.19	
DORGAN	114	m	2	2.44	3.61	1.78	0.0000
DORGAN	99	f	3	2.05	17.39	1.67	0.0000
Subtotal DORGAN				2.12	21.01	3.45	
DOSEME	7	m	2	0.96	12.24	7.56	0.0008
*ENGELA	58	m	7	2.04	1.97	0.18	0.0042
GER	6	c	0	0.16	4.30	10.82	0.7462
*HAMMON	98	m	1	2.72	3.06	2.91	0.0000
JEDRYC	1	m	0	-0.33	0.84	3.62	0.7618
KREYBE	13	m	0	2.42	2.91	1.35	0.0000
KREYBE	31	f	0	-0.27	0.75	3.01	0.8175
Subtotal KREYBE				1.87	3.66	4.37	
LAMTH	10	f	0	1.68	5.44	0.02	0.0001
LUBIN2	149	m	0	2.22	45.98	10.64	0.0000
LUBIN2	169	f	0	0.98	18.69	10.75	0.0000
Subtotal LUBIN2				1.86	64.67	21.39	
LUO	4	c	0	-0.24	1.73	6.80	0.7498
MATOS	42	m	0	0.22	1.46	3.35	0.7878
MATSUD	4	m	0	3.06	0.95	1.65	0.0028
ORMOS	5	m	0	2.73	1.72	1.69	0.0003
OSANN2	10	f	0	1.57	4.03	0.12	0.0016
SOBUE	53	m	0	2.74	2.74	2.73	0.0000
SVENSS	27	f	0	2.08	2.93	0.33	0.0004
TSUGAN	15	m	0	1.61	0.36	0.01	0.3330
WAKAI	43	m	0	1.39	1.66	0.20	0.0727
WUWILL	20	f	0	1.02	51.60	26.81	0.0000
WYNDE2	3	m	0	1.53	2.39	0.11	0.0182
WYNDE3	4	m	0	1.59	1.96	0.05	0.0265
WYNDE3	63	f	0	-0.22	0.79	3.05	0.8428
Subtotal WYNDE3				1.07	2.75	3.10	
WYNDE4	5	m	0	0.90	4.60	3.27	0.0541
WYNDE4	49	f	2	-0.14	0.90	3.17	0.8951
Subtotal WYNDE4				0.73	5.50	6.45	
WYNDE6	21	m	0	2.57	17.35	11.94	0.0000
WYNDE6	210	f	0	1.98	16.03	0.94	0.0000
Subtotal WYNDE6				2.29	33.38	12.88	
ZHENG	1	m	0	1.41	2.33	0.25	0.0308
ZHENG	16	f	0	0.75	6.21	6.11	0.0620
Subtotal ZHENG				0.93	8.54	6.36	
ZHOU	10	c	0	0.39	3.46	6.32	0.4669

Table 2G17 - 5

IESLC - Meta-anal of Ever Smoking (or Current if ev not avail), Amount smoked, “Low”, Cigs (or Any Prod if Cigs not avail)
Squamous

Least adjusted

	N	41
	NS	30
	Wt	405.48
Het	Chi	181.21
Het	df	40
Het	P	***
Fixed	RR	5.71
	RRl	5.18
	RRu	6.29
	P	+++
Random	RR	4.50
	RRl	3.50
	RRu	5.78
	P	+++
Asymm	P	*

Table 2G17 - 6

IESLC - Meta-anal of Ever Smoking (or Current if ev not avail), Amount smoked, “Low”, Cigs (or Any Prod if Cigs not avail)
Squamous

Least adjusted

	combined	Sex male	female	Total
N	3	23	15	41
NS	3	23	15	41
Wt	9.50	210.37	185.61	405.48
Het Chi	0.46	53.72	83.83	181.21
Het df	2	22	14	40
Het P	N.S.	***	***	***
Fixed RR	1.18	7.28	4.69	5.71
RRl	0.63	6.36	4.06	5.18
RRu	2.24	8.34	5.41	6.29
P	N.S.	+++	+++	+++
Random RR	1.18	6.17	3.82	4.50
RRl	0.63	4.63	2.52	3.50
RRu	2.24	8.23	5.79	5.78
P	N.S.	+++	+++	+++
Between Chi				43.20
Between df				2
Between P				***
Btwn(F) P				**
Btwn(R) P				***

Table 2G17 - 7

IESLC - Meta-anal of Ever Smoking (or Current if ev not avail), Amount smoked, “Low”, Cigs (or Any Prod if Cigs not avail)
Squamous

Excluded studies (and stage at which they were excluded)

1 ABELIN ABRAHA AMANDU AMES ANDERS AUSTIN AXELSO BAND BECHER BERRIN BLOHMK BLOT4 BROCKM BROWN1 BYERS1 BYERS2
CARPEN CASCO2 CASCOR CHAN CHEN3 CHIAZZ CHYOU DESTE2 DOCKER DROSTE DU GARCIA GARDIN GENG GODLEY GOODMA
GRAHAM GREGOR HEGMAN HEIN HENNEK HINDS HIRAOK HOROWI HORWIT HUANG ISHIMA JAHN JAIN JARVHO JIANG KELLER
KIHARA KJUUS KO KOHLME KUBIK LAMWK LAMWK2 LANGE LEI LEMARC LEVIN LIU LOMBA2 LOMBAR MAGNUS MARSH
MARSH2 MCDUFF MCLAUG MILLER MILLS NOTANI NOU ODRISC PAWLEG PERSHA POFFIJ QIAO QIAO2 RADZIK REN RONCO
ROOTS ROTHSC SAARIK SANKAR SCHWAR SEGI SEOW SHIMIZ SIMARA SIMONA SITAS SOBUE2 STASZE STAYNE STUCKE SUN
SUZUK2 SUZUKI TANG TAO TOKARS TOUSEY ULMER VEIERO VUTUC WALD WANG WANG3 WANG4 WICKLU WIGLE WILKIN
WU2 WUNSCH WYNDE8 XIANGZ XU XU2 XU4 YONG ZHANG

2 BUELL CHEN MASTRA MZILEN PISANI RESTRE SADOWS

4 BOFFET WYNDE7

5 RIMING TANG2 WYNDE5

6 BLOT1 BLOT2 BLOT3 BOUCHA HIRAY2 JONES LAURIL LICKIN MOLLO MRFIT MURATA SCHWA2 VANDER WARSIN WATSON WYNDER

8 AGUDO AKIBA ARCHER ARMADA AUVINE AXELSS BENSHL BEST BRESLO BRETT BROSS BUFFLE CEDERL CHANG CHATZI CHEN2
CHOW COMSTO COOKSO CPSI CPSII DAMBER DARBY DAVEYS DEAN DEAN2 DEAN3 DEKLER DESTEF DOLL2 DORANT DORN
DUNN EBELIN ENSTRO ESAKI FAN GAO GAO2 GARSHI GILLIS GOLLED GSELL HAMMO2 HANSEN HIRAYA HITOSU HOLE
HU HU2 HUMBLE JARUP JOLY JUSSAW KAISE2 KAISER KANELL KAUFMA KHUDER KINLEN KNEKT KOO KOULUM KREUZE
LAUSSM LETOUR LIAW LIDDEL LIU2 LIU3 LIU4 LIU5 LUBIN MACLEN MARTIS MCCONN MIGRAN MRFITR NAM NOTAN2
PARKIN PASTOR PERNU PERSH2 PETO PEZZO2 PEZZOT PIKE POLEDN PRESCO RACHTA RANDIG SEGI2 SHAW SIEMIA SPEIZE
SPITZ STOCKS STOCKW TENKAN TIZZAN TULINI TVERDA WANG2 XU3 YAMAGU YUAN

10 BOUCOT CORREA HAENSZ KATSOU OSANN WU

11 BENHAM

Table 2G17 - 8

Potentially overlapping studies

REF	REFGP	PRINC	.	OVERLAP/LINK
LUBIN2	LUBIN2	1		Lubin-combined
LAMTH	LAMTH	1		KOO/LAMTH/LAMWK
OSANN2	KAISER	2		KAISER/OSANN2
WYNDE6	WYNDE6	1		WYNDE5/6/7/8
MATSUD	MATSUD	1		SOBUE2/MATSUD

Table 2G17 - 9

Most adjusted - insufficient data for metaanalysis

REF	NRR	SEX	AGEL	AGEH	RACE	YF	LC TYPE	LOC	START	ST	NLC	R	VB	P	H	AD	SM	PRODUCT	exL	exH	DENOM	De
CHEN	5	c	0	0	all	-	q	As:oth	1987	CC	323	n	ot	n	y	2	ev	cig+/-ot	1	10	nev cigs	ot

REF	NRR	RR	SIG	RRDATA	comment
CHEN	5	2.59	n	0	

Table 2G18 -

IESLC - Meta-anal of Ever Smoking (or Current if ev not avail), Amount smoked, “Mid”, Cigs (or Any Prod if Cigs not avail)
Squamous

This analysis is restricted to results for:
1) Results by Amount smoked
2) Results complete enough for use in metaanalysis

Within each study, results are then selected (in the following order of preference, within each sex) for:
3) SMKSTA: ever smokers, current smokers
4) PRODUCT: cigarettes regardless of other products, cigarettes only, all/unspec
5) CIGTYPE: all/unspecified, MC regardless of HR, MC only
6) DENOM: never smoked anything, never smoked cigarettes, (never +1 = +long term ex, +2 = +amount unknown, +3 = never cigs+long term ex)
7) Followup period (YF, prospective studies): whole study (coded as 0) or longest available
8) LCtype: squamous or nearest available, but not adeno. (q = squamous, s = small, a = adeno, KI = Kreyberg I, u = undifferentiated)
9) Race: all or nearest available, otherwise by race (wh or w = white, bl or b = black, hi = hispanic ch = chinese, jap = japanese, haw = hawaiian, w+o = white + oriental, sca = scandinavian, as = asian)
10) Amount smoked "mid" in key scheme 1 (key value 20, maximum range 6-44, in numbers of cigarettes or cigarette equivalents)
11) For overlapping studies: principal rather than subsidiary studies
Finally by Age: whole study (coded as 0) if available, otherwise by widest available age group
and then for single sex results (m, f) in preference to combined sex results (c).

Results adjusted (AD) for the most potential confounders are then chosen in Sections -1 to -3
(and those which actually differ from the adjusted results in Table 2G13 - 1 are marked 'x' in Section -1)
and results adjusted for the least confounders in Sections -4 to -6. (Those least adjusted results which
actually differ from the most adjusted as marked 'x' in column X in Section -4)
(Results adjusted for an unknown number of confounder(s) are coded as 20.)

Section -7 shows excluded studies, together with the stage (as above) at which no qualifying
results were found.

Section -8 lists the potentially overlapping studies which have been included (1=principal, 2=subsidiary).

Section -9 lists any results which would have been included in preference except that they had data not complete
enough for use in meta-analysis, with their significance (yes/no), if known, and any further comment as entered
on the database.

In addition to those mentioned above, the following fields, levels and abbreviations are used:
* or nk = not known, n = no, y = yes, ot = other
ev = ever, cu = current, nev = never
all/unspec = all or unspecified, cig+/-ot = cigarettes irrespective of other products (cigar, pipe etc)
MC = manufactured cigarettes, HR = hand-rolled cigarettes
exL, exH = range of exposure (low and high) in the smoking group, in terms of Amount smoked, cigarettes or cigarette equivalents
REF: 6-character study reference
NRR: number of the RR on the database within the study
ST : study type (CC = case control, pr or prosp = prospective)
NLC: number of lung cancer cases in whole study
R : risky occupational population (n = no, m = mining, o = other risky)
VB : national cigarette type (V = at least 75% Virginia, bl = at least 75% blended, ot = other)
P : any proxy use
H : full histological confirmation
De : derivation of RR/CI (or = original, st = standard method, ot = other method of estimation)

Table 2G18 - 1

IESLC - Meta-anal of Ever Smoking (or Current if ev not avail), Amount smoked, “Mid”, Cigs (or Any Prod if Cigs not avail)
Squamous
Most adjusted

REF	NRR	2G13	SEX	AGEL	AGEH	RACE	YF	LC TYPE	LOC	START	ST	NLC	R	VB	P	H	AD	SM	PRODUCT	exL	exH	DENOM	De
ALDERS	35		m	0	0	all	-	q+s	Eu:UK	1977	CC	1448	n	V	n	n	1	ev	cig only	18	27	nev+2	ot
ALDERS	38		f	0	0	all	-	q+s	Eu:UK	1977	CC	1448	n	V	n	n	1	ev	cig only	18	27	nev+2	ot
BARBON	71		m	0	0	all	-	q	Eu:wst	1979	CC	755	n	bl	y	y	3	ev	all/unsp	20	39	nev any	or
CHOI	47		m	0	0	all	-	q	As:oth	1985	CC	375	n	bl	n	n	0	ev	cig+/-ot	11	20	nev cigs	st
CHOI	57		f	0	0	all	-	q	As:oth	1985	CC	375	n	bl	n	n	0	ev	cig+/-ot	11	30	nev cigs	st
DOLL	70		m	0	0	all	-	KI	Eu:UK	1948	CC	1465	n	V	n	n	1	ev	all/unsp	15	24	nev any	ot
DOSEME	11		m	0	0	all	-	q	Eu:bal	1979	CC	1210	n	bl	n	n	2	ev	cig+/-ot	11	20	nev cigs	or
GER	15		c	0	0	all	-	q+s	As:oth	1990	CC	141	n	ot	y	n	10	ev	all/unsp	11	20	nev any	ot
HAMMON	99		m	0	0	wh	0	not a	NAmer	1952	pr	448	n	bl	n	n	1	cu	cig only	10	20	nev any	ot
JEDRYC	29		m	0	0	all	-	q	Eu:est	1980	CC	1630	n	bl	y	n	3	ev	cig+/-ot	20	29	nev any	or
KREYBE	2		m	0	0	all	-	KI	Eu:Sca	1948	CC	300	n	bl	n	y	1	ev	all/unsp	15	24	nev any	ot
LAMTH	11		f	0	0	ch	-	q	As:HK	1983	CC	445	n	bl	n	n	0	ev	all/unsp	11	20	nev any	or
LUBIN2	157		m	0	0	all	-	q	Eu:mul	1976	CC	7804	n	bl	n	y	0	ev	cig+/-ot	20	29	nev any	st
LUBIN2	177		f	0	0	all	-	q	Eu:mul	1976	CC	7804	n	bl	n	y	0	ev	cig+/-ot	20	29	nev any	st
LUO	11		c	0	0	all	-	q	As:Chi	1990	CC	102	n	ot	n	y	20	ev	cig+/-ot	20	29	nev cigs	or
MATOS	45		m	0	0	all	-	q	SCAmer	1994	CC	200	n	bl	n	n	2	ev	cig+/-ot	15	24	nev any	or
MATSUD	5		m	0	0	all	-	q	As:Jap	1965	CC	179	n	bl	n	n	0	ev	cig+/-ot	11	20	nev cigs	st
ORMOS	6		m	0	0	all	-	q	Eu:est	1947	CC	119	n	bl	y	y	0	ev	cig+/-ot	16	30	nev any	st
SOBUE	54		m	0	0	all	-	q	As:Jap	1986	CC	1376	n	bl	n	y	0	cu	cig+/-ot	20	29	nev cigs	st
SVENSS	12		f	0	0	all	-	q	Eu:Sca	1983	CC	210	n	bl	n	n	1	cu	all/unsp	11	20	nev any	or
TSUGAN	16		m	0	0	all	-	q	As:Jap	1976	CC	134	n	bl	n	y	0	cu	all/unsp	16	35	nev any	ot
WAKAI	47		m	0	0	all	-	q	As:Jap	1988	CC	333	n	bl	n	y	1	cu	cig+/-ot	20	29	nev any	or
WYNDE2	4		m	0	0	all	-	KI	NAmer	1962	CC	404	n	bl	n	y	0	ev	cig+/-ot	11	20	nev any	st
WYNDE3	5		m	0	0	all	-	KI	NAmer	1966	CC	350	n	bl	n	y	0	ev	cig+/-ot	10	20	nev any	st
WYNDE3	64		f	0	0	all	-	KI	NAmer	1966	CC	350	n	bl	n	y	0	ev	cig+/-ot	10	20	nev any	st
WYNDE4	65		m	0	0	all	-	not a	NAmer	1948	CC	684	n	bl	y	n	2	ev	all/unsp	16	20	nev any	ot
WYNDE4	51		f	0	0	all	-	not a	NAmer	1948	CC	684	n	bl	y	n	2	ev	all/unsp	16	20	nev any	ot
WYNDE6	30		m	0	0	all	-	KI	NAmer	1969	CC	4423	n	bl	n	y	0	cu	cig+/-ot	11	20	nev any	st
WYNDE6	219		f	0	0	all	-	KI	NAmer	1969	CC	4423	n	bl	n	y	0	cu	cig+/-ot	11	20	nev cigs	st
ZHENG	3		m	0	0	all	-	q	As:Chi	1982	CC	540	n	ot	*	y	0	ev	cig+/-ot	20	29	nev cigs	st

Cigarette type is all/unspec for all RRs

except for the following:

REF	NRR	CIGTYPE
ALDERS	35	MC only
ALDERS	38	MC only

Table 2G18 - 2

IESLC - Meta-anal of Ever Smoking (or Current if ev not avail), Amount smoked, “Mid”, Cigs (or Any Prod if Cigs not avail)
Squamous
Most adjusted

REF	NRR	SEX	AD	Number Exposed Case	Number Exposed Cont	Non-exposed Case	Non-exposed Cont	RR	95.00%CI
ALDERS	35	m	1	-	-	-	-	7.19	(2.75- 18.79)
ALDERS	38	f	1	-	-	-	-	9.24	(5.31- 16.09)
Subtotal ALDERS								8.68	(5.37- 14.03)
BARBON	71	m	3	-	-	-	-	16.30	(7.00- 38.00)
CHOI	47	m	0	84	281	6	95	4.73	(2.00- 11.19)
CHOI	57	f	0	5	9	10	164	9.11	(2.57- 32.31)
Subtotal CHOI								5.82	(2.86- 11.86)
DOLL	70	m	1	-	-	-	-	14.30	(4.45- 46.00)
DOSEME	11	m	2	-	-	-	-	3.20	(2.20- 4.60)
GER	15	c	10	-	-	-	-	2.20	(0.65- 7.48)
*HAMMON	99	m	1	-	-	-	-	17.44	(6.30- 48.29)
JEDRYC	29	m	3	-	-	-	-	13.46	(5.76- 31.47)
KREYBE	2	m	1	-	-	-	-	11.02	(3.42- 35.51)
LAMTH	11	f	0	28	6	28	72	12.00	(4.49- 32.10)
LUBIN2	157	m	0	1298	3108	54	2616	20.23	(15.33- 26.69)
LUBIN2	177	f	0	61	110	72	1180	9.09	(6.13- 13.46)
Subtotal LUBIN2								15.51	(12.37- 19.45)
LUO	11	c	20	-	-	-	-	24.60	(4.20- 145.70)
MATOS	45	m	2	-	-	-	-	7.80	(2.20- 27.40)
MATSUD	5	m	0	43	1607	1	1255	33.58	(4.62- 244.19)
ORMOS	6	m	0	10	577	2	777	6.73	(1.47- 30.85)
SOBUE	54	m	0	103	222	3	128	19.80	(6.15- 63.68)
SVENSS	12	f	1	-	-	-	-	36.20	(12.00- 168.90)
TSUGAN	16	m	0	7	7	0	5	11.00~	(0.51- 236.22)
WAKAI	47	m	1	-	-	-	-	10.40	(2.43- 44.30)
WYNDE2	4	m	0	108	203	3	105	18.62	(5.77- 60.06)
WYNDE3	5	m	0	57	114	3	88	14.67	(4.44- 48.40)
WYNDE3	64	f	0	13	24	5	76	8.23	(2.66- 25.46)
Subtotal WYNDE3								10.81	(4.76- 24.56)
WYNDE4	65	m	2	-	-	-	-	10.92	(5.22- 22.86)
WYNDE4	51	f	2	-	-	-	-	14.92	(4.88- 45.67)
Subtotal WYNDE4								12.01	(6.48- 22.23)
WYNDE6	30	m	0	270	293	29	617	19.61	(13.04- 29.47)
WYNDE6	219	f	0	191	165	40	856	24.77	(16.95- 36.20)
Subtotal WYNDE6								22.22	(16.83- 29.33)
ZHENG	3	m	0	75	89	4	94	19.80	(6.95- 56.41)
Partial Totals				2353	6815	260	8128		

*prospective study
~ With 0.5 adjustment for zero

REF	NRR	SEX	AD	Ys	Ws	Qs	Ps
ALDERS	35	m	1	1.97	4.16	1.22	0.0001
ALDERS	38	f	1	2.22	12.50	1.06	0.0000
Subtotal ALDERS				2.16	16.66	2.28	
BARBON	71	m	3	2.79	5.37	0.41	0.0000
CHOI	47	m	0	1.55	5.19	4.78	0.0004
CHOI	57	f	0	2.21	2.40	0.22	0.0006
Subtotal CHOI				1.76	7.59	5.00	
DOLL	70	m	1	2.66	2.82	0.06	0.0000
DOSEME	11	m	2	1.16	28.24	51.57	0.0000
GER	15	c	10	0.79	2.57	7.67	0.2058
*HAMMON	99	m	1	2.86	3.70	0.44	0.0000
JEDRYC	29	m	3	2.60	5.33	0.04	0.0000
KREYBE	2	m	1	2.40	2.81	0.04	0.0001
LAMTH	11	f	0	2.48	3.97	0.00	0.0000
LUBIN2	157	m	0	3.01	50.02	12.15	0.0000
LUBIN2	177	f	0	2.21	24.86	2.35	0.0000
Subtotal LUBIN2				2.74	74.88	14.50	
LUO	11	c	20	3.20	1.22	0.58	0.0004
MATOS	45	m	2	2.05	2.42	0.51	0.0014
MATSUD	5	m	0	3.51	0.98	0.98	0.0005
ORMOS	6	m	0	1.91	1.66	0.61	0.0141
SOBUE	54	m	0	2.99	2.81	0.62	0.0000
SVENSS	12	f	1	3.59	2.20	2.54	0.0000
TSUGAN	16	m	0	2.40	0.41	0.01	0.1254
WAKAI	47	m	1	2.34	1.82	0.05	0.0016
WYNDE2	4	m	0	2.92	2.80	0.47	0.0000
WYNDE3	5	m	0	2.69	2.70	0.08	0.0000
WYNDE3	64	f	0	2.11	3.01	0.50	0.0003
Subtotal WYNDE3				2.38	5.71	0.58	
WYNDE4	65	m	2	2.39	7.04	0.11	0.0000

Table 2G18 - 2

IESLC - Meta-anal of Ever Smoking (or Current if ev not avail), Amount smoked, “Mid”, Cigs (or Any Prod if Cigs not avail)
Squamous
Most adjusted

REF	NRR	SEX	AD	Ys	Ws	Qs	Ps
WYNDE4	51	f	2	2.70	3.07	0.11	0.0000
Subtotal WYNDE4				2.49	10.12	0.22	
WYNDE6	30	m	0	2.98	23.14	4.93	0.0000
WYNDE6	219	f	0	3.21	26.69	12.91	0.0000
Subtotal WYNDE6				3.10	49.83	17.83	
ZHENG	3	m	0	2.99	3.51	0.78	0.0000

	N	30
	NS	24
	Wt	239.42
Het	Chi	107.78
Het	df	29
Het	P	***
Fixed	RR	12.36
	RRl	10.89
	RRu	14.03
	P	+++
Random	RR	11.86
	RRl	8.92
	RRu	15.76
	P	+++
Asymm	P	N.S.

Table 2G18 - 3

IESLC - Meta-anal of Ever Smoking (or Current if ev not avail), Amount smoked, “Mid”, Cigs (or Any Prod if Cigs not avail)

Squamous

Most adjusted

	Sex combined	male	female	Total
N	2	20	8	30
NS	2	20	8	30
Wt	3.80	156.92	78.71	239.42
Het Chi	4.83	79.72	18.84	107.78
Het df	1	19	7	29
Het P	*	***	**	***
Fixed RR	4.78	12.01	13.71	12.36
RRl	1.75	10.27	10.99	10.89
RRu	13.08	14.04	17.10	14.03
P	++	+++	+++	+++
Random RR	6.73	11.96	13.14	11.86
RRl	0.64	8.18	8.57	8.92
RRu	71.24	17.49	20.13	15.76
P	N.S.	+++	+++	+++
Between Chi				4.39
Between df				2
Between P				N.S.
Btwn(F) P				N.S.
Btwn(R) P				N.S.

Table 2G18 - 4

IESLC - Meta-anal of Ever Smoking (or Current if ev not avail), Amount smoked, “Mid”, Cigs (or Any Prod if Cigs not avail)
Squamous
Least adjusted

REF	NRR	X	SEX	AGEL	AGEH	RACE	YF	LC TYPE	LOC	START	ST	NLC	R	VB	P	H	AD	SM	PRODUCT	exL	exH	DENOM	De
ALDERS	35		m	0	0	all	-	q+s	Eu:UK	1977	CC	1448	n	V	n	n	1	ev	cig only	18	27	nev+2	ot
ALDERS	38		f	0	0	all	-	q+s	Eu:UK	1977	CC	1448	n	V	n	n	1	ev	cig only	18	27	nev+2	ot
BARBON	23	x	m	0	0	all	-	q	Eu:wst	1979	CC	755	n	bl	y	y	0	ev	all/unsp	20	29	nev any	st
CHOI	47		m	0	0	all	-	q	As:oth	1985	CC	375	n	bl	n	n	0	ev	cig+/-ot	11	20	nev cigs	st
CHOI	57		f	0	0	all	-	q	As:oth	1985	CC	375	n	bl	n	n	0	ev	cig+/-ot	11	30	nev cigs	st
DOLL	56	x	m	0	0	all	-	KI	Eu:UK	1948	CC	1465	n	V	n	n	0	ev	all/unsp	15	24	nev any	st
DOSEME	11		m	0	0	all	-	q	Eu:bal	1979	CC	1210	n	bl	n	n	2	ev	cig+/-ot	11	20	nev cigs	or
GER	7	x	c	0	0	all	-	q+s	As:oth	1990	CC	141	n	ot	y	n	0	ev	all/unsp	11	20	nev any	st
HAMMON	99		m	0	0	wh	0	not a	NAmer	1952	pr	448	n	bl	n	n	1	cu	cig only	10	20	nev any	ot
JEDRYC	3	x	m	0	0	all	-	q	Eu:est	1980	CC	1630	n	bl	y	n	0	ev	cig+/-ot	20	29	nev any	st
KREYBE	14	x	m	0	0	all	-	KI	Eu:Sca	1948	CC	300	n	bl	n	y	0	ev	all/unsp	15	24	nev any	st
LAMTH	11		f	0	0	ch	-	q	As:HK	1983	CC	445	n	bl	n	n	0	ev	all/unsp	11	20	nev any	or
LUBIN2	157		m	0	0	all	-	q	Eu:mul	1976	CC	7804	n	bl	n	y	0	ev	cig+/-ot	20	29	nev any	st
LUBIN2	177		f	0	0	all	-	q	Eu:mul	1976	CC	7804	n	bl	n	y	0	ev	cig+/-ot	20	29	nev any	st
LUO	5	x	c	0	0	all	-	q	As:Chi	1990	CC	102	n	ot	n	y	0	ev	cig+/-ot	20	29	nev cigs	st
MATOS	44	x	m	0	0	all	-	q	SCAmer	1994	CC	200	n	bl	n	n	0	ev	cig+/-ot	15	24	nev any	st
MATSUD	5		m	0	0	all	-	q	As:Jap	1965	CC	179	n	bl	n	n	0	ev	cig+/-ot	11	20	nev cigs	st
ORMOS	6		m	0	0	all	-	q	Eu:est	1947	CC	119	n	bl	y	y	0	ev	cig+/-ot	16	30	nev any	st
SOBUE	54		m	0	0	all	-	q	As:Jap	1986	CC	1376	n	bl	n	y	0	cu	cig+/-ot	20	29	nev cigs	st
SVENSS	32	x	f	0	0	all	-	q	Eu:Sca	1983	CC	210	n	bl	n	n	0	cu	all/unsp	11	20	nev any	st
TSUGAN	16		m	0	0	all	-	q	As:Jap	1976	CC	134	n	bl	n	y	0	cu	all/unsp	16	35	nev any	ot
WAKAI	44	x	m	0	0	all	-	q	As:Jap	1988	CC	333	n	bl	n	y	0	cu	cig+/-ot	20	29	nev any	st
WYNDE2	4		m	0	0	all	-	KI	NAmer	1962	CC	404	n	bl	n	y	0	ev	cig+/-ot	11	20	nev any	st
WYNDE3	5		m	0	0	all	-	KI	NAmer	1966	CC	350	n	bl	n	y	0	ev	cig+/-ot	10	20	nev any	st
WYNDE3	64		f	0	0	all	-	KI	NAmer	1966	CC	350	n	bl	n	y	0	ev	cig+/-ot	10	20	nev any	st
WYNDE4	17	x	m	0	0	all	-	not a	NAmer	1948	CC	684	n	bl	y	n	0	ev	all/unsp	16	20	nev any	st
WYNDE4	51		f	0	0	all	-	not a	NAmer	1948	CC	684	n	bl	y	n	2	ev	all/unsp	16	20	nev any	ot
WYNDE6	30		m	0	0	all	-	KI	NAmer	1969	CC	4423	n	bl	n	y	0	cu	cig+/-ot	11	20	nev any	st
WYNDE6	219		f	0	0	all	-	KI	NAmer	1969	CC	4423	n	bl	n	y	0	cu	cig+/-ot	11	20	nev cigs	st
ZHENG	3		m	0	0	all	-	q	As:Chi	1982	CC	540	n	ot	*	y	0	ev	cig+/-ot	20	29	nev cigs	st

Cigarette type is all/unspec for all RRs

except for the following:

REF	NRR	CIGTYPE
ALDERS	35	MC only
ALDERS	38	MC only

Table 2G18 - 5

IESLC - Meta-anal of Ever Smoking (or Current if ev not avail), Amount smoked, “Mid”, Cigs (or Any Prod if Cigs not avail)
Squamous
Least adjusted

REF	NRR	SEX	AD	Number Exposed Case	Number Exposed Cont	Non-exposed Case	Non-exposed Cont	RR	95.00%CI
ALDERS	35	m	1	-	-	-	-	7.19	(2.75- 18.79)
ALDERS	38	f	1	-	-	-	-	9.24	(5.31- 16.09)
Subtotal ALDERS								8.68	(5.37- 14.03)
BARBON	23	m	0	81	176	6	188	14.42	(6.14- 33.89)
CHOI	47	m	0	84	281	6	95	4.73	(2.00- 11.19)
CHOI	57	f	0	5	9	10	164	9.11	(2.57- 32.31)
Subtotal CHOI								5.82	(2.86- 11.86)
DOLL	56	m	0	301	431	3	61	14.20	(4.41- 45.68)
DOSEME	11	m	2	-	-	-	-	3.20	(2.20- 4.60)
GER	7	c	0	20	87	11	80	1.67	(0.75- 3.71)
*HAMMON	99	m	1	-	-	-	-	17.44	(6.30- 48.29)
JEDRYC	3	m	0	152	434	6	289	16.87	(7.36- 38.66)
KREYBE	14	m	0	49	925	3	644	11.37	(3.53- 36.64)
LAMTH	11	f	0	28	6	28	72	12.00	(4.49- 32.10)
LUBIN2	157	m	0	1298	3108	54	2616	20.23	(15.33- 26.69)
LUBIN2	177	f	0	61	110	72	1180	9.09	(6.13- 13.46)
Subtotal LUBIN2								15.51	(12.37- 19.45)
LUO	5	c	0	22	23	5	51	9.76	(3.28- 28.98)
MATOS	44	m	0	18	90	3	110	7.33	(2.09- 25.69)
MATSUD	5	m	0	43	1607	1	1255	33.58	(4.62- 244.19)
ORMOS	6	m	0	10	577	2	777	6.73	(1.47- 30.85)
SOBUE	54	m	0	103	222	3	128	19.80	(6.15- 63.68)
SVENSS	32	f	0	28	22	5	120	30.55	(10.64- 87.69)
TSUGAN	16	m	0	7	7	0	5	11.00~	(0.51- 236.22)
WAKAI	44	m	0	40	129	2	65	10.08	(2.36- 43.01)
WYNDE2	4	m	0	108	203	3	105	18.62	(5.77- 60.06)
WYNDE3	5	m	0	57	114	3	88	14.67	(4.44- 48.40)
WYNDE3	64	f	0	13	24	5	76	8.23	(2.66- 25.46)
Subtotal WYNDE3								10.81	(4.76- 24.56)
WYNDE4	17	m	0	213	274	8	115	11.17	(5.34- 23.39)
WYNDE4	51	f	2	-	-	-	-	14.92	(4.88- 45.67)
Subtotal WYNDE4								12.20	(6.59- 22.60)
WYNDE6	30	m	0	270	293	29	617	19.61	(13.04- 29.47)
WYNDE6	219	f	0	191	165	40	856	24.77	(16.95- 36.20)
Subtotal WYNDE6								22.22	(16.83- 29.33)
ZHENG	3	m	0	75	89	4	94	19.80	(6.95- 56.41)
Partial Totals				3277	9406	312	9851		

*prospective study ~ With 0.5 adjustment for zero

REF	NRR	SEX	AD	Ys	Ws	Qs	Ps
ALDERS	35	m	1	1.97	4.16	1.08	0.0001
ALDERS	38	f	1	2.22	12.50	0.84	0.0000
Subtotal ALDERS				2.16	16.66	1.92	
BARBON	23	m	0	2.67	5.26	0.18	0.0000
CHOI	47	m	0	1.55	5.19	4.47	0.0004
CHOI	57	f	0	2.21	2.40	0.18	0.0006
Subtotal CHOI				1.76	7.59	4.65	
DOLL	56	m	0	2.65	2.81	0.08	0.0000
DOSEME	11	m	2	1.16	28.24	49.14	0.0000
GER	7	c	0	0.51	6.06	23.49	0.2056
*HAMMON	99	m	1	2.86	3.70	0.53	0.0000
JEDRYC	3	m	0	2.83	5.59	0.66	0.0000
KREYBE	14	m	0	2.43	2.81	0.01	0.0000
LAMTH	11	f	0	2.48	3.97	0.00	0.0000
LUBIN2	157	m	0	3.01	50.02	13.79	0.0000
LUBIN2	177	f	0	2.21	24.86	1.88	0.0000
Subtotal LUBIN2				2.74	74.88	15.67	
LUO	5	c	0	2.28	3.24	0.14	0.0000
MATOS	44	m	0	1.99	2.44	0.59	0.0018
MATSUD	5	m	0	3.51	0.98	1.04	0.0005
ORMOS	6	m	0	1.91	1.66	0.55	0.0141
SOBUE	54	m	0	2.99	2.81	0.71	0.0000
SVENSS	32	f	0	3.42	3.45	3.03	0.0000
TSUGAN	16	m	0	2.40	0.41	0.00	0.1254
WAKAI	44	m	0	2.31	1.82	0.05	0.0018
WYNDE2	4	m	0	2.92	2.80	0.55	0.0000
WYNDE3	5	m	0	2.69	2.70	0.11	0.0000
WYNDE3	64	f	0	2.11	3.01	0.42	0.0003
Subtotal WYNDE3				2.38	5.71	0.53	
WYNDE4	17	m	0	2.41	7.04	0.03	0.0000

Table 2G18 - 5

IESLC - Meta-anal of Ever Smoking (or Current if ev not avail), Amount smoked, “Mid”, Cigs (or Any Prod if Cigs not avail)
Squamous
Least adjusted

REF	NRR	SEX	AD	Ys	Ws	Qs	Ps
WYNDE4	51	f	2	2.70	3.07	0.15	0.0000
Subtotal WYNDE4				2.50	10.11	0.18	
WYNDE6	30	m	0	2.98	23.14	5.64	0.0000
WYNDE6	219	f	0	3.21	26.69	14.13	0.0000
Subtotal WYNDE6				3.10	49.83	19.76	
ZHENG	3	m	0	2.99	3.51	0.89	0.0000

	N	30
	NS	24
	Wt	246.36
Het	Chi	124.35
Het	df	29
Het	P	***
Fixed	RR	11.97
	RRl	10.56
	RRu	13.56
	P	+++
Random	RR	11.37
	RRl	8.46
	RRu	15.27
	P	+++
Asymm	P	N.S.

Table 2G18 - 6

IESLC - Meta-anal of Ever Smoking (or Current if ev not avail), Amount smoked, “Mid”, Cigs (or Any Prod if Cigs not avail)

Squamous
Least adjusted

		Sex		
	combined	male	female	Total
N	2	20	8	30
NS	2	20	8	30
Wt	9.31	157.09	79.96	246.36
Het Chi	6.57	80.09	18.98	124.35
Het df	1	19	7	29
Het P	*	***	**	***
Fixed RR	3.09	12.05	13.82	11.97
RRl	1.63	10.31	11.10	10.56
RRu	5.88	14.09	17.20	13.56
P	+++	+++	+++	+++
Random RR	3.88	12.02	13.24	11.37
RRl	0.69	8.21	8.70	8.46
RRu	21.80	17.58	20.16	15.27
P	N.S.	+++	+++	+++
Between Chi				18.71
Between df				2
Between P				***
Btwn(F) P				N.S.
Btwn(R) P				N.S.

Table 2G18 - 7

IESLC - Meta-anal of Ever Smoking (or Current if ev not avail), Amount smoked, “Mid”, Cigs (or Any Prod if Cigs not avail)
Squamous
Excluded studies (and stage at which they were excluded)

1 ABELIN ABRAHA AMANDU AMES ANDERS AUSTIN AXELSO BAND BECHER BERRIN BLOHMK BLOT4 BROCKM BROWN1 BYERS1 BYERS2
CARPEN CASCO2 CASCOR CHAN CHEN3 CHIAZZ CHYOU DESTE2 DOCKER DROSTE DU GARCIA GARDIN GENG GODLEY GOODMA
GRAHAM GREGOR HEGMAN HEIN HENNEK HINDS HIRAOK HOROWI HORWIT HUANG ISHIMA JAHN JAIN JARVHO JIANG KELLER
KIHARA KJUUS KO KOHLME KUBIK LAMWK LAMWK2 LANGE LEI LEMARC LEVIN LIU LOMBA2 LOMBAR MAGNUS MARSH
MARSH2 MCDUFF MCLAUG MILLER MILLS NOTANI NOU ODRISC PAWLEG PERSHA POFFIJ QIAO QIAO2 RADZIK REN RONCO
ROOTS ROTHSC SAARIK SANKAR SCHWAR SEGI SEOW SHIMIZ SIMARA SIMONA SITAS SOBUE2 STASZE STAYNE STUCKE SUN
SUZUK2 SUZUKI TANG TAO TOKARS TOUSEY ULMER VEIERO VUTUC WALD WANG WANG3 WANG4 WICKLU WIGLE WILKIN
WU2 WUNSCH WYNDE8 XIANGZ XU XU2 XU4 YONG ZHANG

2 BUELL CHEN MASTRA MZILEN PISANI RESTRE SADOWS

4 BOFFET WYNDE7

5 RIMING TANG2 WYNDE5

6 BLOT1 BLOT2 BLOT3 BOUCHA HIRAY2 JONES LAURIL LICKIN MOLLO MRFIT MURATA SCHWA2 VANDER WARSIN WATSON WYNDER

8 AGUDO AKIBA ARCHER ARMADA AUVINE AXELSS BENSHL BEST BRESLO BRETT BROSS BUFFLE CEDERL CHANG CHATZI CHEN2
CHOW COMSTO COOKSO CPSI CPSII DAMBER DARBY DAVEYS DEAN DEAN2 DEAN3 DEKLER DESTEF DOLL2 DORANT DORN
DUNN EBELIN ENSTRO ESAKI FAN GAO GAO2 GARSHI GILLIS GOLLED GSELL HAMMO2 HANSEN HIRAYA HITOSU HOLE
HU HU2 HUMBLE JARUP JOLY JUSSAW KAISE2 KAISER KANELL KAUFMA KHUDER KINLEN KNEKT KOO KOULUM KREUZE
LAUSSM LETOUR LIAW LIDDEL LIU2 LIU3 LIU4 LIU5 LUBIN MACLEN MARTIS MCCONN MIGRAN MRFITR NAM NOTAN2
PARKIN PASTOR PERNU PERSH2 PETO PEZZO2 PEZZOT PIKE POLEDN PRESCO RACHTA RANDIG SEGI2 SHAW SIEMIA SPEIZE
SPITZ STOCKS STOCKW TENKAN TIZZAN TULINI TVERDA WANG2 XU3 YAMAGU YUAN

10 BOUCOT BROWN2 CORREA DORGAN ENGELA HAENSZ KATSOU OSANN OSANN2 WU WUWILL ZHOU

11 BENHAM

Table 2G18 - 8

Potentially overlapping studies

REF	REFGP	PRINC	.	OVERLAP/LINK
LUBIN2	LUBIN2	1		Lubin-combined
LAMTH	LAMTH	1		KOO/LAMTH/LAMWK
WYNDE6	WYNDE6	1		WYNDE5/6/7/8
MATSUD	MATSUD	1		SOBUE2/MATSUD

Table 2G18 - 9

Most adjusted - insufficient data for metaanalysis

REF	NRR	SEX	AGEL	AGEH	RACE	YF	LC TYPE	LOC	START	ST	NLC	R	VB	P	H	AD	SM	PRODUCT	exL	exH	DENOM	De
CHEN	4	c	0	0	all	-	q	As:oth	1987	CC	323	n	ot	n	y	2	ev	cig+/-ot	11	20	nev cigs	ot

REF	NRR	RR	SIG	RRDATA comment
CHEN	4	7.05	n	0

Table 2G19 -

IESLC - Meta-anal of Ever Smoking (or Current if ev not avail), Amount smoked, “High”, Cigs (or Any Prod if Cigs not aval)
Squamous

This analysis is restricted to results for:
1) Results by Amount smoked
2) Results complete enough for use in metaanalysis

Within each study, results are then selected (in the following order of preference, within each sex) for:
3) SMKSTA: ever smokers, current smokers
4) PRODUCT: cigarettes regardless of other products, cigarettes only, all/unspec
5) CIGTYPE: all/unspecified, MC regardless of HR, MC only
6) DENOM: never smoked anything, never smoked cigarettes, (never +1 = +long term ex, +2 = +amount unknown, +3 = never cigs+long term ex)
7) Followup period (YF, prospective studies): whole study (coded as 0) or longest available
8) LCtype: squamous or nearest available, but not adeno. (q = squamous, s = small, a = adeno, KI = Kreyberg I, u = undifferentiated)
9) Race: all or nearest available, otherwise by race (wh or w = white, bl or b = black, hi = hispanic ch = chinese, jap = japanese, haw = hawaiian, w+o = white + oriental, sca = scandinavian, as = asian)
10) Amount smoked "high" in key scheme 1 (key value 45, maximum range >20, in numbers of cigarettes or cigarette equivalents)
11) For overlapping studies: principal rather than subsidiary studies
Finally by Age: whole study (coded as 0) if available, otherwise by widest available age group
and then for single sex results (m, f) in preference to combined sex results (c).

Results adjusted (AD) for the most potential confounders are then chosen in Sections -1 to -3
(and those which actually differ from the adjusted results in Table 2G14 - 1 are marked 'x' in Section -1)
and results adjusted for the least confounders in Sections -4 to -6. (Those least adjusted results which
actually differ from the most adjusted as marked 'x' in column X in Section -4)
(Results adjusted for an unknown number of confounder(s) are coded as 20.)

Section -7 shows excluded studies, together with the stage (as above) at which no qualifying
results were found.

Section -8 lists the potentially overlapping studies which have been included (1=principal, 2=subsidiary).

Section -9 lists any results which would have been included in preference except that they had data not complete
enough for use in meta-analysis, with their significance (yes/no), if known, and any further comment as entered
on the database.

In addition to those mentioned above, the following fields, levels and abbreviations are used:
* or nk = not known, n = no, y = yes, ot = other
ev = ever, cu = current, nev = never
all/unspec = all or unspecified, cig+/-ot = cigarettes irrespective of other products (cigar, pipe etc)
MC = manufactured cigarettes, HR = hand-rolled cigarettes
exL, exH = range of exposure (low and high) in the smoking group, in terms of Amount smoked, cigarettes or cigarette equivalents
REF: 6-character study reference
NRR: number of the RR on the database within the study
ST : study type (CC = case control, pr or prosp = prospective)
NLC: number of lung cancer cases in whole study
R : risky occupational population (n = no, m = mining, o = other risky)
VB : national cigarette type (V = at least 75% Virginia, bl = at least 75% blended, ot = other)
P : any proxy use
H : full histological confirmation
De : derivation of RR/CI (or = original, st = standard method, ot = other method of estimation)

Table 2G19 - 1

IESLC - Meta-anal of Ever Smoking (or Current if ev not avail), Amount smoked, “High”, Cigs (or Any Prod if Cigs not aval)
Squamous
Most adjusted

REF	NRR	2G14	SEX	AGEL	AGEH	RACE	YF	LC TYPE	LOC	START	ST	NLC	R	VB	P	H	AD	SM	PRODUCT	exL	exH	DENOM	De
ALDERS	36		m	0	0	all	-	q+s	Eu:UK	1977	CC	1448	n	V	n	n	1	ev	cig only	28	99	nev+2	ot
ALDERS	39		f	0	0	all	-	q+s	Eu:UK	1977	CC	1448	n	V	n	n	1	ev	cig only	28	99	nev+2	ot
BARBON	72		m	0	0	all	-	q	Eu:wst	1979	CC	755	n	bl	y	y	3	ev	all/unsp	40	99	nev any	or
BOUCOT	143		m	0	0	all	0	q	NAmer	1951	pr	121	n	bl	n	n	2	cu	cig only	21	99	nev any	ot
CHOI	50		m	0	0	all	-	q	As:oth	1985	CC	375	n	bl	n	n	0	ev	cig+/-ot	41	99	nev cigs	st
CHOI	58		f	0	0	all	-	q	As:oth	1985	CC	375	n	bl	n	n	0	ev	cig+/-ot	31	99	nev cigs	st
CORREA	51		c	0	0	all	-	q+s	NAmer	1979	CC	1359	n	bl	y	n	1	cu	cig+/-ot	21	99	nev cigs	or
DOLL	71		m	0	0	all	-	KI	Eu:UK	1948	CC	1465	n	V	n	n	1	ev	all/unsp	25	99	nev any	ot
DOSEME	15		m	0	0	all	-	q	Eu:bal	1979	CC	1210	n	bl	n	n	2	ev	cig+/-ot	21	99	nev cigs	or
GER	16		c	0	0	all	-	q+s	As:oth	1990	CC	141	n	ot	y	n	10	ev	all/unsp	21	99	nev any	ot
HAENSZ	17		f	0	0	all	-	q+u	NAmer	1955	CC	158	n	bl	n	y	0	cu	cig+/-ot	21	99	nev any	or
HAMMON	101		m	0	0	wh	0	not a	NAmer	1952	pr	448	n	bl	n	n	1	cu	cig only	40	99	nev any	ot
JEDRYC	30		m	0	0	all	-	q	Eu:est	1980	CC	1630	n	bl	y	n	3	ev	cig+/-ot	30	99	nev any	or
KATSOU	22		f	0	0	all	-	KI	Eu:bal	1987	CC	101	n	bl	n	n	1	cu	all/unsp	21	99	nev any	or
KREYBE	3		m	0	0	all	-	KI	Eu:Sca	1948	CC	300	n	bl	n	y	1	ev	all/unsp	25	99	nev any	ot
LAMTH	12		f	0	0	ch	-	q	As:HK	1983	CC	445	n	bl	n	n	0	ev	all/unsp	21	99	nev any	st
LUBIN2	161		m	0	0	all	-	q	Eu:mul	1976	CC	7804	n	bl	n	y	0	ev	cig+/-ot	30	99	nev any	st
LUBIN2	181		f	0	0	all	-	q	Eu:mul	1976	CC	7804	n	bl	n	y	0	ev	cig+/-ot	30	99	nev any	st
LUO	12		c	0	0	all	-	q	As:Chi	1990	CC	102	n	ot	n	y	20	ev	cig+/-ot	30	99	nev cigs	or
MATOS	47		m	0	0	all	-	q	SCAmer	1994	CC	200	n	bl	n	n	2	ev	cig+/-ot	25	99	nev any	or
MATSUD	6		m	0	0	all	-	q	As:Jap	1965	CC	179	n	bl	n	n	0	ev	cig+/-ot	21	99	nev cigs	st
ORMOS	7		m	0	0	all	-	q	Eu:est	1947	CC	119	n	bl	y	y	0	ev	cig+/-ot	31	99	nev any	st
OSANN	59		m	0	0	all	-	q	NAmer	1984	CC	1986	n	bl	n	n	2	ev	cig+/-ot	40	99	nev cigs	or
OSANN	60		f	0	0	all	-	q	NAmer	1984	CC	1986	n	bl	n	n	2	ev	cig+/-ot	40	99	nev cigs	or
SOBUE	55		m	0	0	all	-	q	As:Jap	1986	CC	1376	n	bl	n	y	0	cu	cig+/-ot	30	99	nev cigs	st
SVENSS	17		f	0	0	all	-	q	Eu:Sca	1983	CC	210	n	bl	n	n	1	cu	all/unsp	21	99	nev any	ot
TSUGAN	17		m	0	0	all	-	q	As:Jap	1976	CC	134	n	bl	n	y	0	cu	all/unsp	36	99	nev any	ot
WAKAI	48		m	0	0	all	-	q	As:Jap	1988	CC	333	n	bl	n	y	1	cu	cig+/-ot	30	99	nev any	or
WU	18		f	0	0	wh	-	q	NAmer	1981	CC	220	n	bl	n	y	2	cu	all/unsp	21	99	nev any	or
WYNDE2	6		m	0	0	all	-	KI	NAmer	1962	CC	404	n	bl	n	y	0	ev	cig+/-ot	35	99	nev any	st
WYNDE3	7		m	0	0	all	-	KI	NAmer	1966	CC	350	n	bl	n	y	0	ev	cig+/-ot	41	99	nev any	st
WYNDE3	66		f	0	0	all	-	KI	NAmer	1966	CC	350	n	bl	n	y	0	ev	cig+/-ot	41	99	nev any	st
WYNDE4	67		m	0	0	all	-	not a	NAmer	1948	CC	684	n	bl	y	n	2	ev	all/unsp	35	99	nev any	ot
WYNDE4	53		f	0	0	all	-	not a	NAmer	1948	CC	684	n	bl	y	n	2	ev	all/unsp	35	99	nev any	ot
WYNDE6	48		m	0	0	all	-	KI	NAmer	1969	CC	4423	n	bl	n	y	0	cu	cig+/-ot	31	99	nev any	st
WYNDE6	237		f	0	0	all	-	KI	NAmer	1969	CC	4423	n	bl	n	y	0	cu	cig+/-ot	30	99	nev cigs	st
ZHENG	4		m	0	0	all	-	q	As:Chi	1982	CC	540	n	ot	*	y	0	ev	cig+/-ot	30	99	nev cigs	st

Cigarette type is all/unspec for all RRs

except for the following:

REF	NRR	CIGTYPE
ALDERS	36	MC only
ALDERS	39	MC only

Table 2G19 - 2

IESLC - Meta-anal of Ever Smoking (or Current if ev not avail), Amount smoked, “High”, Cigs (or Any Prod if Cigs not aval)
Squamous
Most adjusted

REF	NRR	SEX	AD	Number Exposed Case	Cont	Non-exposed Case	Cont	RR	95.00%CI
ALDERS	36	m	1	-	-	-	-	8.78	(3.46- 22.31)
ALDERS	39	f	1	-	-	-	-	14.52	(7.93- 26.58)
Subtotal ALDERS								12.51	(7.53- 20.77)
BARBON	72	m	3	-	-	-	-	28.60	(12.00- 69.00)
*BOUCOT	143	m	2	-	-	-	-	46.64	(2.80- 775.69)
CHOI	50	m	0	9	6	6	95	23.75	(6.33- 89.09)
CHOI	58	f	0	2	1	10	164	32.80	(2.74- 393.20)
Subtotal CHOI								25.50	(7.94- 81.93)
CORREA	51	c	1	-	-	-	-	54.80	(35.60- 89.20)
DOLL	71	m	1	-	-	-	-	25.40	(7.83- 82.40)
DOSEME	15	m	2	-	-	-	-	7.00	(4.10- 12.00)
GER	16	c	10	-	-	-	-	16.04	(4.22- 60.93)
HAENSZ	17	f	0	18	13	44	236	7.43	(3.40- 16.24)
*HAMMON	101	m	1	-	-	-	-	63.91	(22.02- 185.47)
JEDRYC	30	m	3	-	-	-	-	21.42	(9.05- 50.68)
KATSOU	22	f	1	-	-	-	-	19.53	(5.36- 71.11)
KREYBE	3	m	1	-	-	-	-	24.63	(7.54- 80.53)
LAMTH	12	f	0	10	1	28	72	25.71	(3.14- 210.29)
LUBIN2	161	m	0	849	1746	54	2616	23.56	(17.77- 31.22)
LUBIN2	181	f	0	18	39	72	1180	7.56	(4.12- 13.88)
Subtotal LUBIN2								19.26	(14.92- 24.87)
LUO	12	c	20	-	-	-	-	38.70	(5.20- 290.20)
MATOS	47	m	2	-	-	-	-	9.70	(2.80- 33.20)
MATSUD	6	m	0	39	470	1	1255	104.14	(14.27- 760.12)
ORMOS	7	m	0	4	128	2	777	12.14	(2.20- 66.97)
OSANN	59	m	2	-	-	-	-	76.00	(36.80- 157.00)
OSANN	60	f	2	-	-	-	-	72.30	(36.80- 142.00)
Subtotal OSANN								73.99	(45.14- 121.29)
SOBUE	55	m	0	87	187	3	128	19.85	(6.14- 64.13)
SVENSS	17	f	1	-	-	-	-	96.00	(6.90-1335.65)
TSUGAN	17	m	0	9	1	0	5	69.67~	(2.40-2022.74)
WAKAI	48	m	1	-	-	-	-	24.00	(5.46- 105.00)
WU	18	f	2	-	-	-	-	94.40	(9.90- 904.60)
WYNDE2	6	m	0	139	112	3	105	43.44	(13.42- 140.56)
WYNDE3	7	m	0	59	26	3	88	66.56	(19.27- 229.96)
WYNDE3	66	f	0	3	3	5	76	15.20	(2.42- 95.56)
Subtotal WYNDE3								41.95	(15.01- 117.26)
WYNDE4	67	m	2	-	-	-	-	29.54	(13.53- 64.50)
WYNDE4	53	f	2	-	-	-	-	26.53	(4.12- 171.09)
Subtotal WYNDE4								29.07	(14.15- 59.73)
WYNDE6	48	m	0	502	197	29	617	54.22	(36.08- 81.47)
WYNDE6	237	f	0	221	52	40	856	90.95	(58.70- 140.93)
Subtotal WYNDE6								68.92	(51.14- 92.86)
ZHENG	4	m	0	49	23	4	94	50.07	(16.39- 152.91)
Partial Totals				2018	3005	304	8364		

*prospective study ~ With 0.5 adjustment for zero

REF	NRR	SEX	AD	Ys	Ws	Qs	Ps
ALDERS	36	m	1	2.17	4.42	6.30	0.0000
ALDERS	39	f	1	2.68	10.50	5.00	0.0000
Subtotal ALDERS				2.53	14.93	11.30	
BARBON	72	m	3	3.35	5.02	0.00	0.0000
*BOUCOT	143	m	2	3.84	0.49	0.11	0.0074
CHOI	50	m	0	3.17	2.20	0.09	0.0000
CHOI	58	f	0	3.49	0.62	0.01	0.0059
Subtotal CHOI				3.24	2.82	0.10	
CORREA	51	c	1	4.00	18.21	7.41	0.0000
DOLL	71	m	1	3.23	2.77	0.05	0.0000
DOSEME	15	m	2	1.95	13.32	26.86	0.0000
GER	16	c	10	2.78	2.16	0.75	0.0000
HAENSZ	17	f	0	2.01	6.27	11.61	0.0000
*HAMMON	101	m	1	4.16	3.38	2.12	0.0000
JEDRYC	30	m	3	3.06	5.18	0.47	0.0000
KATSOU	22	f	1	2.97	2.30	0.36	0.0000
KREYBE	3	m	1	3.20	2.74	0.07	0.0000
LAMTH	12	f	0	3.25	0.87	0.01	0.0025
LUBIN2	161	m	0	3.16	48.42	2.06	0.0000
LUBIN2	181	f	0	2.02	10.42	18.78	0.0000
Subtotal LUBIN2				2.96	58.85	20.84	
LUO	12	c	20	3.66	0.95	0.08	0.0004

Table 2G19 - 2

IESLC - Meta-anal of Ever Smoking (or Current if ev not avail), Amount smoked, “High”, Cigs (or Any Prod if Cigs not aval)
Squamous
Most adjusted

REF	NRR	SEX	AD	Ys	Ws	Qs	Ps
MATOS	47	m	2	2.27	2.51	3.01	0.0003
MATSUD	6	m	0	4.65	0.97	1.59	0.0000
ORMOS	7	m	0	2.50	1.32	1.00	0.0042
OSANN	59	m	2	4.33	7.30	6.80	0.0000
OSANN	60	f	2	4.28	8.43	7.06	0.0000
Subtotal OSANN				4.30	15.73	13.86	
SOBUE	55	m	0	2.99	2.79	0.40	0.0000
SVENSS	17	f	1	4.56	0.55	0.80	0.0007
TSUGAN	17	m	0	4.24	0.34	0.26	0.0135
WAKAI	48	m	1	3.18	1.76	0.06	0.0000
WU	18	f	2	4.55	0.75	1.05	0.0001
WYNDE2	6	m	0	3.77	2.79	0.46	0.0000
WYNDE3	7	m	0	4.20	2.50	1.73	0.0000
WYNDE3	66	f	0	2.72	1.14	0.47	0.0037
Subtotal WYNDE3				3.74	3.64	2.20	
WYNDE4	67	m	2	3.39	6.30	0.00	0.0000
WYNDE4	53	f	2	3.28	1.11	0.01	0.0006
Subtotal WYNDE4				3.37	7.41	0.01	
WYNDE6	48	m	0	3.99	23.16	9.11	0.0000
WYNDE6	237	f	0	4.51	20.03	26.24	0.0000
Subtotal WYNDE6				4.23	43.19	35.35	
ZHENG	4	m	0	3.91	3.08	0.92	0.0000

	N	37
	NS	30
	Wt	227.09
Het	Chi	143.12
Het	df	36
Het	P	***
Fixed	RR	28.95
	RRl	25.42
	RRu	32.98
	P	+++
Random	RR	27.65
	RRl	20.42
	RRu	37.44
	P	+++
Asymm	P	N.S.

Table 2G19 - 3

IESLC - Meta-anal of Ever Smoking (or Current if ev not avail), Amount smoked, “High”, Cigs (or Any Prod if Cigs not aval)

Squamous

Most adjusted

	Sex combined	male	female	Total
N	3	22	12	37
NS	3	22	12	37
Wt	21.32	142.77	63.00	227.09
Het Chi	2.95	62.58	71.40	143.12
Het df	2	21	11	36
Het P	N.S.	***	***	***
Fixed RR	47.65	26.76	29.26	28.95
RRl	31.17	22.71	22.86	25.42
RRu	72.85	31.52	37.45	32.98
P	+++	+++	+++	+++
Random RR	39.52	27.70	26.18	27.65
RRl	18.61	19.71	12.49	20.42
RRu	83.95	38.93	54.88	37.44
P	+++	+++	+++	+++
Between Chi				6.19
Between df				2
Between P				*
Btwn(F) P				N.S.
Btwn(R) P				N.S.

Table 2G19 - 4

IESLC - Meta-anal of Ever Smoking (or Current if ev not avail), Amount smoked, “High”, Cigs (or Any Prod if Cigs not aval)
Squamous
Least adjusted

REF	NRR	X	SEX	AGEL	AGEH	RACE	YF	LC TYPE	LOC	START	ST	NLC	R	VB	P	H	AD	SM	PRODUCT	exL	exH	DENOM	De
ALDERS	36		m	0	0	all	-	q+s	Eu:UK	1977	CC	1448	n	V	n	n	1	ev	cig only	28	99	nev+2	ot
ALDERS	39		f	0	0	all	-	q+s	Eu:UK	1977	CC	1448	n	V	n	n	1	ev	cig only	28	99	nev+2	ot
BARBON	27	x	m	0	0	all	-	q	Eu:wst	1979	CC	755	n	bl	y	y	0	ev	all/unsp	40	99	nev any	st
BOUCOT	22	x	m	0	0	all	0	q	NAmer	1951	pr	121	n	bl	n	n	0	cu	cig only	21	99	nev any	ot
CHOI	50		m	0	0	all	-	q	As:oth	1985	CC	375	n	bl	n	n	0	ev	cig+/-ot	41	99	nev cigs	st
CHOI	58		f	0	0	all	-	q	As:oth	1985	CC	375	n	bl	n	n	0	ev	cig+/-ot	31	99	nev cigs	st
CORREA	51		c	0	0	all	-	q+s	NAmer	1979	CC	1359	n	bl	y	n	1	cu	cig+/-ot	21	99	nev cigs	or
DOLL	57	x	m	0	0	all	-	KI	Eu:UK	1948	CC	1465	n	V	n	n	0	ev	all/unsp	25	99	nev any	st
DOSEME	15		m	0	0	all	-	q	Eu:bal	1979	CC	1210	n	bl	n	n	2	ev	cig+/-ot	21	99	nev cigs	or
GER	8	x	c	0	0	all	-	q+s	As:oth	1990	CC	141	n	ot	y	n	0	ev	all/unsp	21	99	nev any	st
HAENSZ	17		f	0	0	all	-	q+u	NAmer	1955	CC	158	n	bl	n	y	0	cu	cig+/-ot	21	99	nev any	or
HAMMON	101		m	0	0	wh	0	not a	NAmer	1952	pr	448	n	bl	n	n	1	cu	cig only	40	99	nev any	ot
JEDRYC	5	x	m	0	0	all	-	q	Eu:est	1980	CC	1630	n	bl	y	n	0	ev	cig+/-ot	40	99	nev any	st
KATSOU	26	x	f	0	0	all	-	KI	Eu:bal	1987	CC	101	n	bl	n	n	0	cu	all/unsp	21	99	nev any	st
KREYBE	15	x	m	0	0	all	-	KI	Eu:Sca	1948	CC	300	n	bl	n	y	0	ev	all/unsp	25	99	nev any	st
LAMTH	12		f	0	0	ch	-	q	As:HK	1983	CC	445	n	bl	n	n	0	ev	all/unsp	21	99	nev any	st
LUBIN2	161		m	0	0	all	-	q	Eu:mul	1976	CC	7804	n	bl	n	y	0	ev	cig+/-ot	30	99	nev any	st
LUBIN2	181		f	0	0	all	-	q	Eu:mul	1976	CC	7804	n	bl	n	y	0	ev	cig+/-ot	30	99	nev any	st
LUO	6	x	c	0	0	all	-	q	As:Chi	1990	CC	102	n	ot	n	y	0	ev	cig+/-ot	30	99	nev cigs	st
MATOS	46	x	m	0	0	all	-	q	SCAmer	1994	CC	200	n	bl	n	n	0	ev	cig+/-ot	25	99	nev any	st
MATSUD	6		m	0	0	all	-	q	As:Jap	1965	CC	179	n	bl	n	n	0	ev	cig+/-ot	21	99	nev cigs	st
ORMOS	7		m	0	0	all	-	q	Eu:est	1947	CC	119	n	bl	y	y	0	ev	cig+/-ot	31	99	nev any	st
OSANN	59		m	0	0	all	-	q	NAmer	1984	CC	1986	n	bl	n	n	2	ev	cig+/-ot	40	99	nev cigs	or
OSANN	60		f	0	0	all	-	q	NAmer	1984	CC	1986	n	bl	n	n	2	ev	cig+/-ot	40	99	nev cigs	or
SOBUE	55		m	0	0	all	-	q	As:Jap	1986	CC	1376	n	bl	n	y	0	cu	cig+/-ot	30	99	nev cigs	st
SVENSS	37	x	f	0	0	all	-	q	Eu:Sca	1983	CC	210	n	bl	n	n	0	cu	all/unsp	21	99	nev any	st
TSUGAN	17		m	0	0	all	-	q	As:Jap	1976	CC	134	n	bl	n	y	0	cu	all/unsp	36	99	nev any	ot
WAKAI	45	x	m	0	0	all	-	q	As:Jap	1988	CC	333	n	bl	n	y	0	cu	cig+/-ot	30	99	nev any	st
WU	13	x	f	0	0	wh	-	q	NAmer	1981	CC	220	n	bl	n	y	0	cu	all/unsp	21	99	nev any	st
WYNDE2	6		m	0	0	all	-	KI	NAmer	1962	CC	404	n	bl	n	y	0	ev	cig+/-ot	35	99	nev any	st
WYNDE3	7		m	0	0	all	-	KI	NAmer	1966	CC	350	n	bl	n	y	0	ev	cig+/-ot	41	99	nev any	st
WYNDE3	66		f	0	0	all	-	KI	NAmer	1966	CC	350	n	bl	n	y	0	ev	cig+/-ot	41	99	nev any	st
WYNDE4	29	x	m	0	0	all	-	not a	NAmer	1948	CC	684	n	bl	y	n	0	ev	all/unsp	35	99	nev any	st
WYNDE4	53		f	0	0	all	-	not a	NAmer	1948	CC	684	n	bl	y	n	2	ev	all/unsp	35	99	nev any	ot
WYNDE6	48		m	0	0	all	-	KI	NAmer	1969	CC	4423	n	bl	n	y	0	cu	cig+/-ot	31	99	nev any	st
WYNDE6	237		f	0	0	all	-	KI	NAmer	1969	CC	4423	n	bl	n	y	0	cu	cig+/-ot	30	99	nev cigs	st
ZHENG	4		m	0	0	all	-	q	As:Chi	1982	CC	540	n	ot	*	y	0	ev	cig+/-ot	30	99	nev cigs	st

Cigarette type is all/unspec for all RRs

except for the following:

REF	NRR	CIGTYPE
ALDERS	36	MC only
ALDERS	39	MC only

Table 2G19 - 5

IESLC - Meta-anal of Ever Smoking (or Current if ev not avail), Amount smoked, “High”, Cigs (or Any Prod if Cigs not aval)
Squamous
Least adjusted

REF	NRR	SEX	AD	Number Exposed Case	Cont	Non-exposed Case	Cont	RR	95.00%CI
ALDERS	36	m	1	-	-	-	-	8.78	(3.46- 22.31)
ALDERS	39	f	1	-	-	-	-	14.52	(7.93- 26.58)
Subtotal ALDERS								12.51	(7.53- 20.77)
BARBON	27	m	0	78	111	6	188	22.02	(9.29- 52.18)
*BOUCOT	22	m	0	17	6940	0	7551	38.08~	(2.29- 633.12)
CHOI	50	m	0	9	6	6	95	23.75	(6.33- 89.09)
CHOI	58	f	0	2	1	10	164	32.80	(2.74- 393.20)
Subtotal CHOI								25.50	(7.94- 81.93)
CORREA	51	c	1	-	-	-	-	54.80	(35.60- 89.20)
DOLL	57	m	0	208	166	3	61	25.48	(7.85- 82.66)
DOSEME	15	m	2	-	-	-	-	7.00	(4.10- 12.00)
GER	8	c	0	19	13	11	80	10.63	(4.13- 27.37)
HAENSZ	17	f	0	18	13	44	236	7.43	(3.40- 16.24)
*HAMMON	101	m	1	-	-	-	-	63.91	(22.02- 185.47)
JEDRYC	5	m	0	57	82	6	289	33.48	(13.94- 80.42)
KATSOU	26	f	0	15	4	14	67	17.95	(5.17- 62.28)
KREYBE	15	m	0	38	248	3	644	32.89	(10.06- 107.53)
LAMTH	12	f	0	10	1	28	72	25.71	(3.14- 210.29)
LUBIN2	161	m	0	849	1746	54	2616	23.56	(17.77- 31.22)
LUBIN2	181	f	0	18	39	72	1180	7.56	(4.12- 13.88)
Subtotal LUBIN2								19.26	(14.92- 24.87)
LUO	6	c	0	9	4	5	51	22.95	(5.15- 102.20)
MATOS	46	m	0	26	105	3	110	9.08	(2.67- 30.90)
MATSUD	6	m	0	39	470	1	1255	104.14	(14.27- 760.12)
ORMOS	7	m	0	4	128	2	777	12.14	(2.20- 66.97)
OSANN	59	m	2	-	-	-	-	76.00	(36.80- 157.00)
OSANN	60	f	2	-	-	-	-	72.30	(36.80- 142.00)
Subtotal OSANN								73.99	(45.14- 121.29)
SOBUE	55	m	0	87	187	3	128	19.85	(6.14- 64.13)
SVENSS	37	f	0	4	1	5	120	96.00	(9.00-1023.75)
TSUGAN	17	m	0	9	1	0	5	69.67~	(2.40-2022.74)
WAKAI	45	m	0	33	48	2	65	22.34	(5.11- 97.69)
WU	13	f	0	42	9	2	30	70.00	(14.10- 347.48)
WYNDE2	6	m	0	139	112	3	105	43.44	(13.42- 140.56)
WYNDE3	7	m	0	59	26	3	88	66.56	(19.27- 229.96)
WYNDE3	66	f	0	3	3	5	76	15.20	(2.42- 95.56)
Subtotal WYNDE3								41.95	(15.01- 117.26)
WYNDE4	29	m	0	123	64	8	115	27.63	(12.69- 60.13)
WYNDE4	53	f	2	-	-	-	-	26.53	(4.12- 171.09)
Subtotal WYNDE4								27.46	(13.40- 56.29)
WYNDE6	48	m	0	502	197	29	617	54.22	(36.08- 81.47)
WYNDE6	237	f	0	221	52	40	856	90.95	(58.70- 140.93)
Subtotal WYNDE6								68.92	(51.14- 92.86)
ZHENG	4	m	0	49	23	4	94	50.07	(16.39- 152.91)
Partial Totals				2687	10800	372	17735		

*prospective study ~ With 0.5 adjustment for zero

REF	NRR	SEX	AD	Ys	Ws	Qs	Ps
ALDERS	36	m	1	2.17	4.42	6.18	0.0000
ALDERS	39	f	1	2.68	10.50	4.85	0.0000
Subtotal ALDERS				2.53	14.93	11.03	
BARBON	27	m	0	3.09	5.16	0.36	0.0000
*BOUCOT	22	m	0	3.64	0.49	0.04	0.0112
CHOI	50	m	0	3.17	2.20	0.08	0.0000
CHOI	58	f	0	3.49	0.62	0.01	0.0059
Subtotal CHOI				3.24	2.82	0.09	
CORREA	51	c	1	4.00	18.21	7.67	0.0000
DOLL	57	m	0	3.24	2.77	0.04	0.0000
DOSEME	15	m	2	1.95	13.32	26.45	0.0000
GER	8	c	0	2.36	4.29	4.22	0.0000
HAENSZ	17	f	0	2.01	6.27	11.43	0.0000
*HAMMON	101	m	1	4.16	3.38	2.18	0.0000
JEDRYC	5	m	0	3.51	5.00	0.12	0.0000
KATSOU	26	f	0	2.89	2.48	0.54	0.0000
KREYBE	15	m	0	3.49	2.74	0.05	0.0000
LAMTH	12	f	0	3.25	0.87	0.01	0.0025
LUBIN2	161	m	0	3.16	48.42	1.85	0.0000
LUBIN2	181	f	0	2.02	10.42	18.48	0.0000
Subtotal LUBIN2				2.96	58.85	20.32	
LUO	6	c	0	3.13	1.72	0.08	0.0000

Table 2G19 - 5

IESLC - Meta-anal of Ever Smoking (or Current if ev not avail), Amount smoked, “High”, Cigs (or Any Prod if Cigs not aval)
Squamous
Least adjusted

REF	NRR	SEX	AD	Ys	Ws	Qs	Ps
MATOS	46	m	0	2.21	2.56	3.38	0.0004
MATSUD	6	m	0	4.65	0.97	1.62	0.0000
ORMOS	7	m	0	2.50	1.32	0.97	0.0042
OSANN	59	m	2	4.33	7.30	6.95	0.0000
OSANN	60	f	2	4.28	8.43	7.23	0.0000
Subtotal OSANN				4.30	15.73	14.18	
SOBUE	55	m	0	2.99	2.79	0.38	0.0000
SVENSS	37	f	0	4.56	0.69	1.00	0.0002
TSUGAN	17	m	0	4.24	0.34	0.27	0.0135
WAKAI	45	m	0	3.11	1.77	0.11	0.0000
WU	13	f	0	4.25	1.50	1.20	0.0000
WYNDE2	6	m	0	3.77	2.79	0.48	0.0000
WYNDE3	7	m	0	4.20	2.50	1.78	0.0000
WYNDE3	66	f	0	2.72	1.14	0.46	0.0037
Subtotal WYNDE3				3.74	3.64	2.23	
WYNDE4	29	m	0	3.32	6.35	0.01	0.0000
WYNDE4	53	f	2	3.28	1.11	0.01	0.0006
Subtotal WYNDE4				3.31	7.46	0.01	
WYNDE6	48	m	0	3.99	23.16	9.43	0.0000
WYNDE6	237	f	0	4.51	20.03	26.75	0.0000
Subtotal WYNDE6				4.23	43.19	36.18	
ZHENG	4	m	0	3.91	3.08	0.96	0.0000

	N	37
	NS	30
	Wt	231.12
Het	Chi	147.60
Het	df	36
Het	P	***
Fixed	RR	28.64
	RRl	25.17
	RRu	32.58
	P	+++
Random	RR	27.18
	RRl	20.11
	RRu	36.76
	P	+++
Asymm	P	N.S.

Table 2G19 - 6

IESLC - Meta-anal of Ever Smoking (or Current if ev not avail), Amount smoked, “High”, Cigs (or Any Prod if Cigs not aval)

Squamous

Least adjusted

	Sex<br>combined	male	female	Total
N	3	22	12	37
NS	3	22	12	37
Wt	24.23	142.84	64.06	231.12
Het Chi	9.84	63.14	71.90	147.60
Het df	2	21	11	36
Het P	**	***	***	***
Fixed RR	38.52	26.92	29.39	28.64
RRl	25.87	22.85	23.01	25.17
RRu	57.37	31.72	37.55	32.58
P	+++	+++	+++	+++
Random RR	25.23	28.00	26.24	27.18
RRl	7.86	19.90	12.70	20.11
RRu	80.91	39.40	54.22	36.76
P	+++	+++	+++	+++
Between Chi				2.72
Between df				2
Between P				N.S.
Btwn(F) P				N.S.
Btwn(R) P				N.S.

Table 2G19 - 7

IESLC - Meta-anal of Ever Smoking (or Current if ev not avail), Amount smoked, “High”, Cigs (or Any Prod if Cigs not aval)
Squamous
Excluded studies (and stage at which they were excluded)

1	ABELIN ABRAHA AMANDU AMES ANDERS AUSTIN AXELSO BAND BECHER BERRIN BLOHMK BLOT4 BROCKM BROWN1 BYERS1 BYERS2
	CARPEN CASCO2 CASCOR CHAN CHEN3 CHIAZZ CHYOU DESTE2 DOCKER DROSTE DU GARCIA GARDIN GENG GODLEY GOODMA
	GRAHAM GREGOR HEGMAN HEIN HENNEK HINDS HIRAOK HOROWI HORWIT HUANG ISHIMA JAHN JAIN JARVHO JIANG KELLER
	KIHARA KJUUS KO KOHLME KUBIK LAMWK LAMWK2 LANGE LEI LEMARC LEVIN LIU LOMBA2 LOMBAR MAGNUS MARSH
	MARSH2 MCDUFF MCLAUG MILLER MILLS NOTANI NOU ODRISC PAWLEG PERSHA POFFIJ QIAO QIAO2 RADZIK REN RONCO
	ROOTS ROTHSC SAARIK SANKAR SCHWAR SEGI SEOW SHIMIZ SIMARA SIMONA SITAS SOBUE2 STASZE STAYNE STUCKE SUN
	SUZUK2 SUZUKI TANG TAO TOKARS TOUSEY ULMER VEIERO VUTUC WALD WANG WANG3 WANG4 WICKLU WIGLE WILKIN
	WU2 WUNSCH WYNDE8 XIANGZ XU XU2 XU4 YONG ZHANG
2	BUELL CHEN MASTRA MZILEN PISANI RESTRE SADOWS
4	BOFFET WYNDE7
5	RIMING TANG2 WYNDE5
6	BLOT1 BLOT2 BLOT3 BOUCHA HIRAY2 JONES LAURIL LICKIN MOLLO MRFIT MURATA SCHWA2 VANDER WARSIN WATSON WYNDER
8	AGUDO AKIBA ARCHER ARMADA AUVINE AXELSS BENSHL BEST BRESLO BRETT BROSS BUFFLE CEDERL CHANG CHATZI CHEN2
	CHOW COMSTO COOKSO CPSI CPSII DAMBER DARBY DAVEYS DEAN DEAN2 DEAN3 DEKLER DESTEF DOLL2 DORANT DORN
	DUNN EBELIN ENSTRO ESAKI FAN GAO GAO2 GARSHI GILLIS GOLLED GSELL HAMMO2 HANSEN HIRAYA HITOSU HOLE
	HU HU2 HUMBLE JARUP JOLY JUSSAW KAISE2 KAISER KANELL KAUFMA KHUDER KINLEN KNEKT KOO KOULUM KREUZE
	LAUSSM LETOUR LIAW LIDDEL LIU2 LIU3 LIU4 LIU5 LUBIN MACLEN MARTIS MCCONN MIGRAN MRFITR NAM NOTAN2
	PARKIN PASTOR PERNU PERSH2 PETO PEZZO2 PEZZOT PIKE POLEDN PRESCO RACHTA RANDIG SEGI2 SHAW SIEMIA SPEIZE
	SPITZ STOCKS STOCKW TENKAN TIZZAN TULINI TVERDA WANG2 XU3 YAMAGU YUAN
10	BROWN2 DORGAN ENGELA OSANN2 WUWILL ZHOU
11	BENHAM

Table 2G19 - 8
Potentially overlapping studies

REF	REFGP	PRINC	. OVERLAP/LINK
LUBIN2	LUBIN2	1	Lubin-combined
LAMTH	LAMTH	1	KOO/LAMTH/LAMWK
WYNDE6	WYNDE6	1	WYNDE5/6/7/8
MATSUD	MATSUD	1	SOBUE2/MATSUD

Table 2G19 - 9
Most adjusted - insufficient data for metaanalysis

REF	NRR	SEX	AGEL	AGEH	RACE	YF	LC TYPE	LOC	START	ST	NLC	R	VB	P	H	AD	SM	PRODUCT	exL	exH	DENOM	De
CHEN	2	c	0	0	all	-	q	As:oth	1987	CC	323	n	ot	n	y	2	ev	cig+/-ot	31	99	nev cigs	ot

REF	NRR	RR	SIG	RRDATA comment
CHEN	2	11.11	y	P < 0.001

Table 2G21 -

IESLC - Meta-anal of Ever Smoking (or Curr if Ever not avail) by Amount, Overview, Cigarettes only
Squamous

This analysis is restricted to results for:
1) Results by Amount smoked
   Results by Amount smoked (in numbers of cigarettes) are grouped under 2 schemes (S1, S2).
   Each scheme has a set of "key values". An interval is allocated to the category whose key value
   it includes and intervals which include none or more than one of the key values are excluded.
   (Open-ended intervals are coded as 99.)

S1	key value	maximum range	S2	key value	maximum range
1	5	1-19	1	1	1-9
2	20	6-44	2	10	2-19
3	45	21+	3	20	11-29
			4	30	21-39
			5	40	31-98
			6	99	41+

2) Results complete enough for use in metaanalysis

Within each study, results are then selected (in the following order of preference, within each sex) for:
3) SMKSTA: ever smokers, current smokers
4) PRODUCT: cigarettes only
5) CIGTYPE: all/unspecified, MC regardless of HR, MC only
6) DENOM: never smoked anything, never smoked cigarettes, (never +1 = +long term ex, +2 = +amount unknown,
          +3 = never cigs+long term ex)
7) Followup period (YF, prospective studies): whole study (coded as 0) or longest available
8) LCtype: squamous or nearest available, but not adeno. (q = squamous, s = small,
          a = adeno, KI = Kreyberg I, u = undifferentiated)
9) Race: all or nearest available, otherwise by race (wh or w = white, bl or b = black, hi = hispanic
          ch = chinese, jap = japanese, haw = hawaiian, w+o = white + oriental, sca = scandinavian, as = asian)
10) For overlapping studies: principal rather than subsidiary studies
Finally by Age: whole study (coded as 0) if available, otherwise by widest available age group
and then for single sex results (m, f) in preference to combined sex results (c).

Results adjusted (AD) for the most potential confounders are then chosen in Sections -1 to -3
(and those which actually differ from the adjusted results in Table 2G11 - 1 are marked 'x' in Section -1)
and results adjusted for the least confounders in Sections -4 to -6. (Those least adjusted results which
actually differ from the most adjusted as marked 'x' in column X in Section -4)
(Results adjusted for an unknown number of confounder(s) are coded as 20.)

Section -7 shows excluded studies, together with the stage (as above) at which no qualifying
results were found.

Section -8 lists the potentially overlapping studies which have been included (1=principal, 2=subsidiary).

Section -9 lists any results which would have been included in preference except that they had data not complete
enough for use in meta-analysis, with their significance (yes/no), if known, and any further comment as entered
on the database.

In addition to those mentioned above, the following fields, levels and abbreviations are used:
* or nk = not known, n = no, y = yes, ot = other
ev = ever, cu = current, nev = never
all/unspec = all or unspecified, MC = manufactured cigarettes, HR = hand-rolled cigarettes
exL, exH = range of exposure (low and high) in the smoking group, in terms of Amount smoked,
    cigarettes
REF: 6-character study reference
NRR: number of the RR on the database within the study
ST : study type (CC = case control, pr or prosp = prospective)
NLC: number of lung cancer cases in whole study
R : risky occupational population (n = no, m = mining, o = other risky)
VB : national cigarette type (V = at least 75% Virginia, bl = at least 75% blended, ot = other)
P : any proxy use
H : full histological confirmation
De : derivation of RR/CI (or = original, st = standard method, ot = other method of estimation)

Table 2G21 - 1

IESLC - Meta-anal of Ever Smoking (or Curr if Ever not avail) by Amount, Overview, Cigarettes only
Squamous
Most adjusted

REF	NRR	2G11	SEX	AGEL	AGEH	RACE	YF	LC TYPE	LOC	START	ST	NLC	R	VB	P	H	AD	SM	PRODUCT	exL	exH	S1	S2	DENOM	De
ALDERS	34		m	0	0	all	-	q+s	Eu:UK	1977	CC	1448	n	V	n	n	1	ev	cig only	1	17	1	0	nev+2	ot
ALDERS	35		m	0	0	all	-	q+s	Eu:UK	1977	CC	1448	n	V	n	n	1	ev	cig only	18	27	2	3	nev+2	ot
ALDERS	36		m	0	0	all	-	q+s	Eu:UK	1977	CC	1448	n	V	n	n	1	ev	cig only	28	99	3	0	nev+2	ot
ALDERS	37		f	0	0	all	-	q+s	Eu:UK	1977	CC	1448	n	V	n	n	1	ev	cig only	1	17	1	0	nev+2	ot
ALDERS	38		f	0	0	all	-	q+s	Eu:UK	1977	CC	1448	n	V	n	n	1	ev	cig only	18	27	2	3	nev+2	ot
ALDERS	39		f	0	0	all	-	q+s	Eu:UK	1977	CC	1448	n	V	n	n	1	ev	cig only	28	99	3	0	nev+2	ot
BENHAM	11	x	m	0	0	all	-	KI	Eu:wst	1976	CC	1625	n	bl	n	y	0	ev	cig only	1	9	1	1	nev any	st
BENHAM	12	x	m	0	0	all	-	KI	Eu:wst	1976	CC	1625	n	bl	n	y	0	ev	cig only	10	14	0	2	nev any	st
BENHAM	13	x	m	0	0	all	-	KI	Eu:wst	1976	CC	1625	n	bl	n	y	0	ev	cig only	15	20	2	3	nev any	st
BENHAM	14	x	m	0	0	all	-	KI	Eu:wst	1976	CC	1625	n	bl	n	y	0	ev	cig only	21	39	0	4	nev any	st
BENHAM	15	x	m	0	0	all	-	KI	Eu:wst	1976	CC	1625	n	bl	n	y	0	ev	cig only	40	99	3	0	nev any	st
BOUCOT	142		m	0	0	all	0	q	NAmer	1951	pr	121	n	bl	n	n	2	cu	cig only	1	20	0	0	nev any	ot
BOUCOT	143		m	0	0	all	0	q	NAmer	1951	pr	121	n	bl	n	n	2	cu	cig only	21	99	3	0	nev any	ot
HAMMON	98		m	0	0	wh	0	not a	NAmer	1952	pr	448	n	bl	n	n	1	cu	cig only	1	9	1	1	nev any	ot
HAMMON	99		m	0	0	wh	0	not a	NAmer	1952	pr	448	n	bl	n	n	1	cu	cig only	10	20	2	0	nev any	ot
HAMMON	100		m	0	0	wh	0	not a	NAmer	1952	pr	448	n	bl	n	n	1	cu	cig only	21	39	0	4	nev any	ot
HAMMON	101		m	0	0	wh	0	not a	NAmer	1952	pr	448	n	bl	n	n	1	cu	cig only	40	99	3	0	nev any	ot

Cigarette type is all/unspec for all RRs

except for the following:

REF	NRR	CIGTYPE
ALDERS	34	MC only
ALDERS	35	MC only
ALDERS	36	MC only
ALDERS	37	MC only
ALDERS	38	MC only
ALDERS	39	MC only

In this overview table, subtotals and Qs values may be invalid and should be ignored

Table 2G21 - 2

IESLC - Meta-anal of Ever Smoking (or Curr if Ever not avail) by Amount, Overview, Cigarettes only
Squamous
Most adjusted

REF	NRR	SEX	AD	Number Exposed Case	Cont	Non-exposed Case	Cont	RR	95.00%CI
ALDERS	34	m	1	-	-	-	-	3.79	(1.30- 11.02)
ALDERS	35	m	1	-	-	-	-	7.19	(2.75- 18.79)
ALDERS	36	m	1	-	-	-	-	8.78	(3.46- 22.31)
ALDERS	37	f	1	-	-	-	-	2.55	(1.42- 4.57)
ALDERS	38	f	1	-	-	-	-	9.24	(5.31- 16.09)
ALDERS	39	f	1	-	-	-	-	14.52	(7.93- 26.58)
Subtotal ALDERS								6.82	(5.12- 9.11)
BENHAM	11	m	0	54	182	24	481	5.95	(3.57- 9.90)
BENHAM	12	m	0	125	227	24	481	11.04	(6.94- 17.56)
BENHAM	13	m	0	449	512	24	481	17.58	(11.44- 26.99)
BENHAM	14	m	0	271	206	24	481	26.37	(16.84- 41.27)
BENHAM	15	m	0	175	122	24	481	28.75	(17.96- 46.03)
Subtotal BENHAM								16.08	(13.08- 19.77)
*BOUCOT	142	m	2	-	-	-	-	21.41	(1.40- 385.13)
*BOUCOT	143	m	2	-	-	-	-	46.64	(2.80- 775.69)
Subtotal BOUCOT								31.58	(4.33- 230.41)
*HAMMON	98	m	1	-	-	-	-	15.12	(4.93- 46.36)
*HAMMON	99	m	1	-	-	-	-	17.44	(6.30- 48.29)
*HAMMON	100	m	1	-	-	-	-	42.32	(15.38- 116.45)
*HAMMON	101	m	1	-	-	-	-	63.91	(22.02- 185.47)
Subtotal HAMMON								29.45	(17.41- 49.82)
Partial Totals				1074	1249	120	2405		

*prospective study

REF	NRR	SEX	AD	Ys	Ws	Qs	Ps
ALDERS	34	m	1	1.33	3.36	5.20	0.0145
ALDERS	35	m	1	1.97	4.16	1.51	0.0001
ALDERS	36	m	1	2.17	4.42	0.72	0.0000
ALDERS	37	f	1	0.94	11.25	30.22	0.0017
ALDERS	38	f	1	2.22	12.50	1.55	0.0000
ALDERS	39	f	1	2.68	10.50	0.11	0.0000
Subtotal ALDERS				1.92	46.20	39.30	
BENHAM	11	m	0	1.78	14.76	9.27	0.0000
BENHAM	12	m	0	2.40	17.81	0.54	0.0000
BENHAM	13	m	0	2.87	20.87	1.77	0.0000
BENHAM	14	m	0	3.27	19.12	9.28	0.0000
BENHAM	15	m	0	3.36	17.34	10.64	0.0000
Subtotal BENHAM				2.78	89.90	31.50	
*BOUCOT	142	m	2	3.06	0.49	0.12	0.0325
*BOUCOT	143	m	2	3.84	0.49	0.78	0.0074
Subtotal BOUCOT				3.45	0.97	0.90	
*HAMMON	98	m	1	2.72	3.06	0.06	0.0000
*HAMMON	99	m	1	2.86	3.70	0.30	0.0000
*HAMMON	100	m	1	3.75	3.75	5.13	0.0000
*HAMMON	101	m	1	4.16	3.38	8.47	0.0000
Subtotal HAMMON				3.38	13.90	13.96	

N	17
NS	4

Table 2G21 - 3

IESLC - Meta-anal of Ever Smoking (or Curr if Ever not avail) by Amount, Overview, Cigarettes only
Squamous
Most adjusted

	Sex combined	male	female	Total
N		14	3	17
NS		4	1	5

In this overview table, other than the "N" rows, entries in the "absent" and "Total" columns may be invalid and should be ignored

	Amount smoked (broad categories)				
	absent	<20k5	6-44k20	>20k45	Total
N	4	4	4	5	17
NS	3	3	3	4	13
Wt	41.17	32.43	41.23	36.14	150.97
Het Chi	9.72	9.34	5.12	10.91	85.66
Het df	3	3	3	4	16
Het P	*	*	N.S.	*	***
Fixed RR	18.84	4.62	13.21	22.11	13.14
RRl	13.88	3.27	9.73	15.96	11.20
RRu	25.57	6.52	17.92	30.64	15.41
P	+++	+++	+++	+++	+++
Random RR	21.15	5.02	12.50	22.12	13.18
RRl	10.71	2.57	8.05	11.74	8.81
RRu	41.75	9.81	19.39	41.69	19.74
P	+++	+++	+++	+++	+++

				Amount smoked (narrow categories)				
	absent	<10k1	2-19k10	11-29k20	21-39k30	31-98k40	>40k99	Total
N	9	2	1	3	2			17
NS	4	2	1	2	2			10
Wt	54.94	17.82	17.81	37.53	22.87			150.97
Het Chi	56.43	2.21	0.00	4.81	0.70			85.66
Het df	8	1	0	2	1			16
Het P	***	N.S.	N.S.	(*)	N.S.			***
Fixed RR	12.55	6.98	11.04	12.85	28.49			13.14
RRl	9.63	4.39	6.94	9.33	18.91			11.20
RRu	16.35	11.10	17.56	17.69	42.92			15.41
P	+++	+++	+++	+++	+++			+++
Random RR	13.32	8.25	11.04	11.56	28.49			13.18
RRl	6.02	3.45	6.94	6.71	18.91			8.81
RRu	29.45	19.76	17.56	19.92	42.92			19.74
P	+++	+++	+++	+++	+++			+++

MALES

	Amount smoked (broad categories)				
	absent	<20k5	6-44k20	>20k45	Total
N	4	3	3	4	14
NS	3	3	3	4	13
Wt	41.17	21.18	28.73	25.64	116.72
Het Chi	9.72	3.26	2.84	8.29	49.72
Het df	3	2	2	3	13
Het P	*	N.S.	N.S.	*	***
Fixed RR	18.84	6.33	15.43	26.27	15.83
RRl	13.88	4.14	10.70	17.84	13.21
RRu	25.57	9.70	22.24	38.69	18.98
P	+++	+++	+++	+++	+++
Random RR	21.15	6.59	14.44	26.10	15.59
RRl	10.71	3.51	8.70	11.29	10.44
RRu	41.75	12.37	23.98	60.35	23.28
P	+++	+++	+++	+++	+++

Table 2G21 - 3

IESLC - Meta-anal of Ever Smoking (or Curr if Ever not avail) by Amount, Overview, Cigarettes only
Squamous
Most adjusted

MALES

	Amount smoked (narrow categories) absent	<10k1	2-19k10	11-29k20	21-39k30	31-98k40	>40k99	Total
N	7	2	1	2	2			14
NS	4	2	1	2	2			10
Wt	33.19	17.82	17.81	25.03	22.87			116.72
Het Chi	19.55	2.21	0.00	2.77	0.70			49.72
Het df	6	1	0	1	1			13
Het P	**	N.S.	N.S.	(*)	N.S.			***
Fixed RR	20.57	6.98	11.04	15.15	28.49			15.83
RRl	14.63	4.39	6.94	10.24	18.91			13.21
RRu	28.90	11.10	17.56	22.41	42.92			18.98
P	+++	+++	+++	+++	+++			+++
Random RR	17.76	8.25	11.04	12.52	28.49			15.59
RRl	8.39	3.45	6.94	5.35	18.91			10.44
RRu	37.60	19.76	17.56	29.29	42.92			23.28
P	+++	+++	+++	+++	+++			+++

FEMALES

	Amount smoked (broad categories) absent	<20k5	6-44k20	>20k45	Total
N		1	1	1	3
NS		1	1	1	1
Wt		11.25	12.50	10.50	34.25
Het Chi		0.00	0.00	0.00	18.02
Het df		0	0	0	2
Het P		N.S.	N.S.	N.S.	***
Fixed RR		2.55	9.24	14.52	6.95
RRl		1.42	5.31	7.93	4.98
RRu		4.57	16.08	26.58	9.72
P		++	+++	+++	+++
Random RR		2.55	9.24	14.52	6.99
RRl		1.42	5.31	7.93	2.55
RRu		4.57	16.08	26.58	19.12
P		++	+++	+++	+++

	Amount smoked (narrow categories) absent	<10k1	2-19k10	11-29k20	21-39k30	31-98k40	>40k99	Total
N	2			1				3
NS	1			1				1
Wt	21.75			12.50				34.25
Het Chi	16.43			0.00				18.02
Het df	1			0				2
Het P	***			N.S.				***
Fixed RR	5.91			9.24				6.95
RRl	3.88			5.31				4.98
RRu	8.99			16.08				9.72
P	+++			+++				+++
Random RR	6.07			9.24				6.99
RRl	1.10			5.31				2.55
RRu	33.40			16.08				19.12
P	+			+++				+++

Table 2G21 - 4

IESLC - Meta-anal of Ever Smoking (or Curr if Ever not avail) by Amount, Overview, Cigarettes only
Squamous
Least adjusted

REF	NRR	X	SEX	AGEL	AGEH	RACE	YF	LC TYPE	LOC	START	ST	NLC	R	VB	P	H	AD	SM	PRODUCT	exL	exH	S1	S2	DENOM	De
ALDERS	34		m	0	0	all	-	q+s	Eu:UK	1977	CC	1448	n	V	n	n	1	ev	cig only	1	17	1	0	nev+2	ot
ALDERS	35		m	0	0	all	-	q+s	Eu:UK	1977	CC	1448	n	V	n	n	1	ev	cig only	18	27	2	3	nev+2	ot
ALDERS	36		m	0	0	all	-	q+s	Eu:UK	1977	CC	1448	n	V	n	n	1	ev	cig only	28	99	3	0	nev+2	ot
ALDERS	37		f	0	0	all	-	q+s	Eu:UK	1977	CC	1448	n	V	n	n	1	ev	cig only	1	17	1	0	nev+2	ot
ALDERS	38		f	0	0	all	-	q+s	Eu:UK	1977	CC	1448	n	V	n	n	1	ev	cig only	18	27	2	3	nev+2	ot
ALDERS	39		f	0	0	all	-	q+s	Eu:UK	1977	CC	1448	n	V	n	n	1	ev	cig only	28	99	3	0	nev+2	ot
BENHAM	11		m	0	0	all	-	KI	Eu:wst	1976	CC	1625	n	bl	n	y	0	ev	cig only	1	9	1	1	nev any	st
BENHAM	12		m	0	0	all	-	KI	Eu:wst	1976	CC	1625	n	bl	n	y	0	ev	cig only	10	14	0	2	nev any	st
BENHAM	13		m	0	0	all	-	KI	Eu:wst	1976	CC	1625	n	bl	n	y	0	ev	cig only	15	20	2	3	nev any	st
BENHAM	14		m	0	0	all	-	KI	Eu:wst	1976	CC	1625	n	bl	n	y	0	ev	cig only	21	39	0	4	nev any	st
BENHAM	15		m	0	0	all	-	KI	Eu:wst	1976	CC	1625	n	bl	n	y	0	ev	cig only	40	99	3	0	nev any	st
BOUCOT	21	x	m	0	0	all	0	q	NAmer	1951	pr	121	n	bl	n	n	0	cu	cig only	1	20	0	0	nev any	ot
BOUCOT	22	x	m	0	0	all	0	q	NAmer	1951	pr	121	n	bl	n	n	0	cu	cig only	21	99	3	0	nev any	ot
HAMMON	98		m	0	0	wh	0	not a	NAmer	1952	pr	448	n	bl	n	n	1	cu	cig only	1	9	1	1	nev any	ot
HAMMON	99		m	0	0	wh	0	not a	NAmer	1952	pr	448	n	bl	n	n	1	cu	cig only	10	20	2	0	nev any	ot
HAMMON	100		m	0	0	wh	0	not a	NAmer	1952	pr	448	n	bl	n	n	1	cu	cig only	21	39	0	4	nev any	ot
HAMMON	101		m	0	0	wh	0	not a	NAmer	1952	pr	448	n	bl	n	n	1	cu	cig only	40	99	3	0	nev any	ot

Cigarette type is all/unspec for all RRs

except for the following:

REF	NRR	CIGTYPE
ALDERS	34	MC only
ALDERS	35	MC only
ALDERS	36	MC only
ALDERS	37	MC only
ALDERS	38	MC only
ALDERS	39	MC only

In this overview table, subtotals and Qs values may be invalid and should be ignored

Table 2G21 - 5

IESLC - Meta-anal of Ever Smoking (or Curr if Ever not avail) by Amount, Overview, Cigarettes only
Squamous
Least adjusted

REF	NRR	SEX	AD	Number Case	Exposed Cont	Non-exposed Case	Cont	RR	95.00%CI
ALDERS	34	m	1	-	-	-	-	3.79	(1.30- 11.02)
ALDERS	35	m	1	-	-	-	-	7.19	(2.75- 18.79)
ALDERS	36	m	1	-	-	-	-	8.78	(3.46- 22.31)
ALDERS	37	f	1	-	-	-	-	2.55	(1.42- 4.57)
ALDERS	38	f	1	-	-	-	-	9.24	(5.31- 16.09)
ALDERS	39	f	1	-	-	-	-	14.52	(7.93- 26.58)
Subtotal ALDERS								6.82	(5.12- 9.11)
BENHAM	11	m	0	54	182	24	481	5.95	(3.57- 9.90)
BENHAM	12	m	0	125	227	24	481	11.04	(6.94- 17.56)
BENHAM	13	m	0	449	512	24	481	17.58	(11.44- 26.99)
BENHAM	14	m	0	271	206	24	481	26.37	(16.84- 41.27)
BENHAM	15	m	0	175	122	24	481	28.75	(17.96- 46.03)
Subtotal BENHAM								16.08	(13.08- 19.77)
*BOUCOT	21	m	0	21	15208	0	7551	21.35~	(1.29- 352.41)
*BOUCOT	22	m	0	17	6940	0	7551	38.08~	(2.29- 633.12)
Subtotal BOUCOT								28.49	(3.91- 207.42)
*HAMMON	98	m	1	-	-	-	-	15.12	(4.93- 46.36)
*HAMMON	99	m	1	-	-	-	-	17.44	(6.30- 48.29)
*HAMMON	100	m	1	-	-	-	-	42.32	(15.38- 116.45)
*HAMMON	101	m	1	-	-	-	-	63.91	(22.02- 185.47)
Subtotal HAMMON								29.45	(17.41- 49.82)
Partial Totals				1112	23397	120	17507		

*prospective study ~ With 0.5 adjustment for zero

REF	NRR	SEX	AD	Ys	Ws	Qs	Ps
ALDERS	34	m	1	1.33	3.36	5.19	0.0145
ALDERS	35	m	1	1.97	4.16	1.51	0.0001
ALDERS	36	m	1	2.17	4.42	0.72	0.0000
ALDERS	37	f	1	0.94	11.25	30.20	0.0017
ALDERS	38	f	1	2.22	12.50	1.54	0.0000
ALDERS	39	f	1	2.68	10.50	0.11	0.0000
Subtotal ALDERS				1.92	46.20	39.26	
BENHAM	11	m	0	1.78	14.76	9.26	0.0000
BENHAM	12	m	0	2.40	17.81	0.54	0.0000
BENHAM	13	m	0	2.87	20.87	1.78	0.0000
BENHAM	14	m	0	3.27	19.12	9.30	0.0000
BENHAM	15	m	0	3.36	17.34	10.66	0.0000
Subtotal BENHAM				2.78	89.90	31.52	
*BOUCOT	21	m	0	3.06	0.49	0.12	0.0324
*BOUCOT	22	m	0	3.64	0.49	0.55	0.0112
Subtotal BOUCOT				3.35	0.97	0.67	
*HAMMON	98	m	1	2.72	3.06	0.06	0.0000
*HAMMON	99	m	1	2.86	3.70	0.30	0.0000
*HAMMON	100	m	1	3.75	3.75	5.14	0.0000
*HAMMON	101	m	1	4.16	3.38	8.48	0.0000
Subtotal HAMMON				3.38	13.90	13.97	

N	17
NS	4

Table 2G21 - 6

IESLC - Meta-anal of Ever Smoking (or Curr if Ever not avail) by Amount, Overview, Cigarettes only
Squamous
Least adjusted

	Sex combined	male	female	Total
N		14	3	17
NS		4	1	5

In this overview table, other than the "N" rows, entries in the "absent" and "Total" columns may be invalid and should be ignored

	Amount smoked (broad categories) absent	<20k5	6-44k20	>20k45	Total
N	4	4	4	5	17
NS	3	3	3	4	13
Wt	41.17	32.43	41.23	36.14	150.97
Het Chi	9.72	9.34	5.12	10.78	85.43
Het df	3	3	3	4	16
Het P	*	*	N.S.	*	***
Fixed RR	18.84	4.62	13.21	22.05	13.13
RRl	13.88	3.27	9.73	15.92	11.19
RRu	25.57	6.52	17.92	30.55	15.40
P	+++	+++	+++	+++	+++
Random RR	21.14	5.02	12.50	21.91	13.14
RRl	10.71	2.57	8.05	11.67	8.78
RRu	41.74	9.81	19.39	41.13	19.66
P	+++	+++	+++	+++	+++

	absent	<10k1	2-19k10	11-29k20	21-39k30	31-98k40	>40k99	Total
			Amount smoked (narrow categories)					
N	9	2	1	3	2			17
NS	4	2	1	2	2			10
Wt	54.94	17.82	17.81	37.53	22.87			150.97
Het Chi	56.19	2.21	0.00	4.81	0.70			85.43
Het df	8	1	0	2	1			16
Het P	***	N.S.	N.S.	(*)	N.S.			***
Fixed RR	12.53	6.98	11.04	12.85	28.49			13.13
RRl	9.62	4.39	6.94	9.33	18.91			11.19
RRu	16.32	11.10	17.56	17.69	42.92			15.40
P	+++	+++	+++	+++	+++			+++
Random RR	13.18	8.25	11.04	11.56	28.49			13.14
RRl	5.97	3.45	6.94	6.71	18.91			8.78
RRu	29.08	19.76	17.56	19.92	42.92			19.66
P	+++	+++	+++	+++	+++			+++

MALES

	Amount smoked (broad categories) absent	<20k5	6-44k20	>20k45	Total
N	4	3	3	4	14
NS	3	3	3	4	13
Wt	41.17	21.18	28.73	25.64	116.72
Het Chi	9.72	3.26	2.84	8.20	49.52
Het df	3	2	2	3	13
Het P	*	N.S.	N.S.	*	***
Fixed RR	18.84	6.33	15.43	26.17	15.82
RRl	13.88	4.14	10.70	17.77	13.19
RRu	25.57	9.70	22.24	38.54	18.96
P	+++	+++	+++	+++	+++
Random RR	21.14	6.59	14.44	25.71	15.53
RRl	10.71	3.51	8.70	11.18	10.41
RRu	41.74	12.37	23.98	59.13	23.18
P	+++	+++	+++	+++	+++

Table 2G21 - 6

IESLC - Meta-anal of Ever Smoking (or Curr if Ever not avail) by Amount, Overview, Cigarettes only
Squamous
Least adjusted

MALES

Amount smoked (narrow categories)

	absent	<10k1	2-19k10	11-29k20	21-39k30	31-98k40	>40k99	Total
N	7	2	1	2	2			14
NS	4	2	1	2	2			10
Wt	33.19	17.82	17.81	25.03	22.87			116.72
Het Chi	19.41	2.21	0.00	2.77	0.70			49.52
Het df	6	1	0	1	1			13
Het P	**	N.S.	N.S.	(*)	N.S.			***
Fixed RR	20.50	6.98	11.04	15.15	28.49			15.82
RRl	14.59	4.39	6.94	10.24	18.91			13.19
RRu	28.81	11.10	17.56	22.41	42.92			18.96
P	+++	+++	+++	+++	+++			+++
Random RR	17.56	8.25	11.04	12.52	28.49			15.53
RRl	8.32	3.45	6.94	5.35	18.91			10.41
RRu	37.07	19.76	17.56	29.29	42.92			23.18
P	+++	+++	+++	+++	+++			+++

FEMALES

Amount smoked (broad categories)

	absent	<20k5	6-44k20	>20k45	Total
N		1	1	1	3
NS		1	1	1	1
Wt		11.25	12.50	10.50	34.25
Het Chi		0.00	0.00	0.00	18.02
Het df		0	0	0	2
Het P		N.S.	N.S.	N.S.	***
Fixed RR		2.55	9.24	14.52	6.95
RRl		1.42	5.31	7.93	4.98
RRu		4.57	16.08	26.58	9.72
P		++	+++	+++	+++
Random RR		2.55	9.24	14.52	6.99
RRl		1.42	5.31	7.93	2.55
RRu		4.57	16.08	26.58	19.12
P		++	+++	+++	+++

Amount smoked (narrow categories)

	absent	<10k1	2-19k10	11-29k20	21-39k30	31-98k40	>40k99	Total
N	2			1				3
NS	1			1				1
Wt	21.75			12.50				34.25
Het Chi	16.43			0.00				18.02
Het df	1			0				2
Het P	***			N.S.				***
Fixed RR	5.91			9.24				6.95
RRl	3.88			5.31				4.98
RRu	8.99			16.08				9.72
P	+++			+++				+++
Random RR	6.07			9.24				6.99
RRl	1.10			5.31				2.55
RRu	33.40			16.08				19.12
P	+			+++				+++

Table 2G21 - 7

IESLC - Meta-anal of Ever Smoking (or Curr if Ever not avail) by Amount, Overview, Cigarettes only
Squamous
Excluded studies (and stage at which they were excluded)

1 ABELIN ABRAHA AMANDU AMES ANDERS AUSTIN AXELSO BAND BECHER BERRIN BLOHMK BLOT4 BROCKM BROWN1 BYERS1 BYERS2
CARPEN CASCO2 CASCOR CHAN CHEN3 CHIAZZ CHYOU DESTE2 DOCKER DROSTE DU GARCIA GARDIN GENG GODLEY GOODMA
GRAHAM GREGOR HEGMAN HEIN HENNEK HINDS HIRAOK HOROWI HORWIT HUANG ISHIMA JAHN JAIN JARVHO JIANG KELLER
KIHARA KJUUS KO KOHLME KUBIK LAMWK LAMWK2 LANGE LEI LEMARC LEVIN LIU LOMBA2 LOMBAR MAGNUS MARSH
MARSH2 MCDUFF MCLAUG MILLER MILLS NOTANI NOU ODRISC PAWLEG PERSHA POFFIJ QIAO QIAO2 RADZIK REN RONCO
ROOTS ROTHSC SAARIK SANKAR SCHWAR SEGI SEOW SHIMIZ SIMARA SIMONA SITAS SOBUE2 STASZE STAYNE STUCKE SUN
SUZUK2 SUZUKI TANG TAO TOKARS TOUSEY ULMER VEIERO VUTUC WALD WANG WANG3 WANG4 WICKLU WIGLE WILKIN
WU2 WUNSCH WYNDE8 XIANGZ XU XU2 XU4 YONG ZHANG

2 BUELL CHEN MASTRA MZILEN PISANI RESTRE SADOWS

4 AKIBA ARCHER ARMADA AUVINE AXELSS BARBON BENSHL BLOT1 BLOT2 BLOT3 BOFFET BOUCHA BRESLO BRETT BROSS BROWN2
BUFFLE CHANG CHATZI CHEN2 CHOI CHOW COMSTO COOKSO CORREA DARBY DAVEYS DEKLER DESTEF DOLL DORANT DORGAN
DOSEME DUNN EBELIN ESAKI FAN GAO GAO2 GARSHI GER GILLIS GSELL HAENSZ HAMMO2 HANSEN HIRAYA HITOSU
HOLE HU HU2 HUMBLE JARUP JEDRYC JOLY JONES KAISER KANELL KATSOU KAUFMA KHUDER KINLEN KNEKT KOO
KOULUM KREUZE KREYBE LAMTH LAURIL LAUSSM LETOUR LIAW LICKIN LIDDEL LIU2 LIU3 LIU5 LUBIN2 LUO MACLEN
MARTIS MATOS MATSUD MOLLO MRFIT MRFITR MURATA NAM ORMOS OSANN OSANN2 PARKIN PASTOR PERNU PERSH2 PETO
PEZZO2 PIKE POLEDN PRESCO RACHTA RANDIG SEGI2 SHAW SIEMIA SOBUE SPEIZE SPITZ STOCKS STOCKW SVENSS TENKAN
TSUGAN TULINI VANDER WAKAI WANG2 WARSIN WATSON WU WUWILL WYNDE2 WYNDE3 WYNDE4 WYNDE5 WYNDE6 WYNDE7 WYNDER
XU3 YAMAGU YUAN ZHENG ZHOU

5 RIMING TANG2

6 HIRAY2 SCHWA2

8 AGUDO BEST CEDERL CPSI CPSII DAMBER DEAN DEAN2 DEAN3 DOLL2 DORN ENGELA ENSTRO GOLLED JUSSAW KAISE2
LIU4 LUBIN MCCONN MIGRAN NOTAN2 PEZZOT TIZZAN TVERDA

Table 2G21 - 8

Potentially overlapping studies

REF	REFGP	PRINC	.	OVERLAP/LINK
BENHAM	LUBIN2	2		Subset of Lubin2

Table 2G22 -

IESLC - Meta-anal of Ever Smoking (or Current if ever not avail), Amount smoked, "Low", Cigarettes only
Squamous

This analysis is restricted to results for:
1) Results by Amount smoked
2) Results complete enough for use in metaanalysis

Within each study, results are then selected (in the following order of preference, within each sex) for:
3) SMKSTA: ever smokers, current smokers
4) PRODUCT: cigarettes only
5) CIGTYPE: all/unspecified, MC regardless of HR, MC only
6) DENOM: never smoked anything, never smoked cigarettes, (never +1 = +long term ex, +2 = +amount unknown, +3 = never cigs+long term ex)
7) Followup period (YF, prospective studies): whole study (coded as 0) or longest available
8) LCtype: squamous or nearest available, but not adeno. (q = squamous, s = small, a = adeno, KI = Kreyberg I, u = undifferentiated)
9) Race: all or nearest available, otherwise by race (wh or w = white, bl or b = black, hi = hispanic ch = chinese, jap = japanese, haw = hawaiian, w+o = white + oriental, sca = scandinavian, as = asian)
10) Amount smoked "low" in key scheme 1 (key value 5, maximum range <20, in numbers of cigarettes)
11) For overlapping studies: principal rather than subsidiary studies
Finally by Age: whole study (coded as 0) if available, otherwise by widest available age group
and then for single sex results (m, f) in preference to combined sex results (c).

Results adjusted (AD) for the most potential confounders are then chosen in Sections -1 to -3
(and those which actually differ from the adjusted results in Table 2G12 - 1 are marked 'x' in Section -1)
and results adjusted for the least confounders in Sections -4 to -6. (Those least adjusted results which
actually differ from the most adjusted as marked 'x' in column X in Section -4)
(Results adjusted for an unknown number of confounder(s) are coded as 20.)

Section -7 shows excluded studies, together with the stage (as above) at which no qualifying
results were found.

Section -8 lists the potentially overlapping studies which have been included (1=principal, 2=subsidiary).

Section -9 lists any results which would have been included in preference except that they had data not complete
enough for use in meta-analysis, with their significance (yes/no), if known, and any further comment as entered
on the database.

In addition to those mentioned above, the following fields, levels and abbreviations are used:
* or nk = not known, n = no, y = yes, ot = other
ev = ever, cu = current, nev = never
all/unspec = all or unspecified, MC = manufactured cigarettes, HR = hand-rolled cigarettes
exL, exH = range of exposure (low and high) in the smoking group, in terms of Amount smoked, cigarettes
REF: 6-character study reference
NRR: number of the RR on the database within the study
ST : study type (CC = case control, pr or prosp = prospective)
NLC: number of lung cancer cases in whole study
R : risky occupational population (n = no, m = mining, o = other risky)
VB : national cigarette type (V = at least 75% Virginia, bl = at least 75% blended, ot = other)
P : any proxy use
H : full histological confirmation
De : derivation of RR/CI (or = original, st = standard method, ot = other method of estimation)

Table 2G22 - 1

IESLC - Meta-anal of Ever Smoking (or Current if ever not avail), Amount smoked, "Low", Cigarettes only
Squamous
Most adjusted

REF	NRR	2G12	SEX	AGEL	AGEH	RACE	YF	LC TYPE	LOC	START	ST	NLC	R	VB	P	H	AD	SM	PRODUCT	exL	exH	DENOM	De
ALDERS	34		m	0	0	all	-	q+s	Eu:UK	1977	CC	1448	n	V	n	n	1	ev	cig only	1	17	nev+2	ot
ALDERS	37		f	0	0	all	-	q+s	Eu:UK	1977	CC	1448	n	V	n	n	1	ev	cig only	1	17	nev+2	ot
BENHAM	11	x	m	0	0	all	-	KI	Eu:wst	1976	CC	1625	n	bl	n	y	0	ev	cig only	1	9	nev any	st
HAMMON	98		m	0	0	wh	0	not a	NAmer	1952	pr	448	n	bl	n	n	1	cu	cig only	1	9	nev any	ot

Cigarette type is all/unspec for all RRs

except for the following:

REF	NRR	CIGTYPE
ALDERS	34	MC only
ALDERS	37	MC only

Table 2G22 - 2

IESLC - Meta-anal of Ever Smoking (or Current if ever not avail), Amount smoked, "Low", Cigarettes only
Squamous
Most adjusted

REF	NRR	SEX	AD	Number Exposed Case	Number Exposed Cont	Non-exposed Case	Non-exposed Cont	RR	95.00%CI
ALDERS	34	m	1	-	-	-	-	3.79	(1.30- 11.02)
ALDERS	37	f	1	-	-	-	-	2.55	(1.42- 4.57)
Subtotal ALDERS								2.79	(1.67- 4.66)
BENHAM	11	m	0	54	182	24	481	5.95	(3.57- 9.90)
*HAMMON	98	m	1	-	-	-	-	15.12	(4.93- 46.36)
Partial Totals				54	182	24	481		

*prospective study

REF	NRR	SEX	AD	Ys	Ws	Qs	Ps
ALDERS	34	m	1	1.33	3.36	0.13	0.0145
ALDERS	37	f	1	0.94	11.25	3.97	0.0017
Subtotal ALDERS				1.03	14.61	4.11	
BENHAM	11	m	0	1.78	14.76	0.94	0.0000
*HAMMON	98	m	1	2.72	3.06	4.30	0.0000

	N	4
	NS	3
	Wt	32.43
Het	Chi	9.34
Het	df	3
Het	P	*
Fixed	RR	4.62
	RRl	3.27
	RRu	6.52
	P	+++
Random	RR	5.02
	RRl	2.57
	RRu	9.81
	P	+++
Asymm	P	N.S.

Table 2G22 - 3

IESLC - Meta-anal of Ever Smoking (or Current if ever not avail), Amount smoked, "Low", Cigarettes only
Squamous
Most adjusted

	combined	Sex male	female	Total
N		3	1	4
NS		3	1	4
Wt		21.18	11.25	32.43
Het Chi		3.26	0.00	9.34
Het df		2	0	3
Het P		N.S.	N.S.	*
Fixed RR		6.33	2.55	4.62
RRl		4.14	1.42	3.27
RRu		9.70	4.57	6.52
P		+++	++	+++
Random RR		6.59	2.55	5.02
RRl		3.51	1.42	2.57
RRu		12.37	4.57	9.81
P		+++	++	+++
Between Chi				6.08
Between df				1
Between P				*
Btwn(F) P				N.S.
Btwn(R) P				*

Too few RRs for analysis by factor

Table 2G22 - 4

IESLC - Meta-anal of Ever Smoking (or Current if ever not avail), Amount smoked, "Low", Cigarettes only
Squamous
Least adjusted

REF	NRR	X	SEX	AGEL	AGEH	RACE	YF	LC TYPE	LOC	START	ST	NLC	R	VB	P	H	AD	SM	PRODUCT	exL	exH	DENOM	De
ALDERS	34		m	0	0	all	-	q+s	Eu:UK	1977	CC	1448	n	V	n	n	1	ev	cig only	1	17	nev+2	ot
ALDERS	37		f	0	0	all	-	q+s	Eu:UK	1977	CC	1448	n	V	n	n	1	ev	cig only	1	17	nev+2	ot
BENHAM	11		m	0	0	all	-	KI	Eu:wst	1976	CC	1625	n	bl	n	y	0	ev	cig only	1	9	nev any	st
HAMMON	98		m	0	0	wh	0	not a	NAmer	1952	pr	448	n	bl	n	n	1	cu	cig only	1	9	nev any	ot

Cigarette type is all/unspec for all RRs

except for the following:

REF	NRR	CIGTYPE
ALDERS	34	MC only
ALDERS	37	MC only

Table 2G22 - 5

IESLC - Meta-anal of Ever Smoking (or Current if ever not avail), Amount smoked, "Low", Cigarettes only
Squamous
Least adjusted

REF	NRR	SEX	AD	Number Exposed Case	Cont	Non-exposed Case	Cont	RR	95.00%CI
ALDERS	34	m	1	-	-	-	-	3.79	(1.30- 11.02)
ALDERS	37	f	1	-	-	-	-	2.55	(1.42- 4.57)
Subtotal ALDERS								2.79	(1.67- 4.66)
BENHAM	11	m	0	54	182	24	481	5.95	(3.57- 9.90)
*HAMMON	98	m	1	-	-	-	-	15.12	(4.93- 46.36)
Partial Totals				54	182	24	481		

*prospective study

REF	NRR	SEX	AD	Ys	Ws	Qs	Ps
ALDERS	34	m	1	1.33	3.36	0.13	0.0145
ALDERS	37	f	1	0.94	11.25	3.97	0.0017
Subtotal ALDERS				1.03	14.61	4.11	
BENHAM	11	m	0	1.78	14.76	0.94	0.0000
*HAMMON	98	m	1	2.72	3.06	4.30	0.0000

	N	4
	NS	3
	Wt	32.43
Het	Chi	9.34
Het	df	3
Het	P	*
Fixed	RR	4.62
	RRl	3.27
	RRu	6.52
	P	+++
Random	RR	5.02
	RRl	2.57
	RRu	9.81
	P	+++
Asymm	P	N.S.

Table 2G22 - 6

IESLC - Meta-anal of Ever Smoking (or Current if ever not avail), Amount smoked, "Low", Cigarettes only
Squamous
Least adjusted

	combined	Sex male	female	Total
N		3	1	4
NS		3	1	4
Wt		21.18	11.25	32.43
Het Chi		3.26	0.00	9.34
Het df		2	0	3
Het P		N.S.	N.S.	*
Fixed RR		6.33	2.55	4.62
RRl		4.14	1.42	3.27
RRu		9.70	4.57	6.52
P		+++	++	+++
Random RR		6.59	2.55	5.02
RRl		3.51	1.42	2.57
RRu		12.37	4.57	9.81
P		+++	++	+++
Between Chi				6.08
Between df				1
Between P				*
Btwn(F) P				N.S.
Btwn(R) P				*

Table 2G22 - 7

IESLC - Meta-anal of Ever Smoking (or Current if ever not avail), Amount smoked, "Low", Cigarettes only
Squamous
Excluded studies (and stage at which they were excluded)

1	ABELIN ABRAHA AMANDU AMES ANDERS AUSTIN AXELSO BAND BECHER BERRIN BLOHMK BLOT4 BROCKM BROWN1 BYERS1 BYERS2 CARPEN CASCO2 CASCOR CHAN CHEN3 CHIAZZ CHYOU DESTE2 DOCKER DROSTE DU GARCIA GARDIN GENG GODLEY GOODMA GRAHAM GREGOR HEGMAN HEIN HENNEK HINDS HIRAOK HOROWI HORWIT HUANG ISHIMA JAHN JAIN JARVHO JIANG KELLER KIHARA KJUUS KO KOHLME KUBIK LAMWK LAMWK2 LANGE LEI LEMARC LEVIN LIU LOMBA2 LOMBAR MAGNUS MARSH MARSH2 MCDUFF MCLAUG MILLER MILLS NOTANI NOU ODRISC PAWLEG PERSHA POFFIJ QIAO QIAO2 RADZIK REN RONCO ROOTS ROTHSC SAARIK SANKAR SCHWAR SEGI SEOW SHIMIZ SIMARA SIMONA SITAS SOBUE2 STASZE STAYNE STUCKE SUN SUZUK2 SUZUKI TANG TAO TOKARS TOUSEY ULMER VEIERO VUTUC WALD WANG WANG3 WANG4 WICKLU WIGLE WILKIN WU2 WUNSCH WYNDE8 XIANGZ XU XU2 XU4 YONG ZHANG
2	BUELL CHEN MASTRA MZILEN PISANI RESTRE SADOWS
4	AKIBA ARCHER ARMADA AUVINE AXELSS BARBON BENSHL BLOT1 BLOT2 BLOT3 BOFFET BOUCHA BRESLO BRETT BROSS BROWN2 BUFFLE CHANG CHATZI CHEN2 CHOI CHOW COMSTO COOKSO CORREA DARBY DAVEYS DEKLER DESTEF DOLL DORANT DORGAN DOSEME DUNN EBELIN ESAKI FAN GAO GAO2 GARSHI GER GILLIS GSELL HAENSZ HAMMO2 HANSEN HIRAYA HITOSU HOLE HU HU2 HUMBLE JARUP JEDRYC JOLY JONES KAISER KANELL KATSOU KAUFMA KHUDER KINLEN KNEKT KOO KOULUM KREUZE KREYBE LAMTH LAURIL LAUSSM LETOUR LIAW LICKIN LIDDEL LIU2 LIU3 LIU5 LUBIN2 LUO MACLEN MARTIS MATOS MATSUD MOLLO MRFIT MRFITR MURATA NAM ORMOS OSANN OSANN2 PARKIN PASTOR PERNU PERSH2 PETO PEZZO2 PIKE POLEDN PRESCO RACHTA RANDIG SEGI2 SHAW SIEMIA SOBUE SPEIZE SPITZ STOCKS STOCKW SVENSS TENKAN TSUGAN TULINI VANDER WAKAI WANG2 WARSIN WATSON WU WUWILL WYNDE2 WYNDE3 WYNDE4 WYNDE5 WYNDE6 WYNDE7 WYNDER XU3 YAMAGU YUAN ZHENG ZHOU
5	RIMING TANG2
6	HIRAY2 SCHWA2
8	AGUDO BEST CEDERL CPSI CPSII DAMBER DEAN DEAN2 DEAN3 DOLL2 DORN ENGELA ENSTRO GOLLED JUSSAW KAISE2 LIU4 LUBIN MCCONN MIGRAN NOTAN2 PEZZOT TIZZAN TVERDA
10	BOUCOT

Table 2G22 - 8
Potentially overlapping studies

REF	REFGP	PRINC	. OVERLAP/LINK
BENHAM	LUBIN2	2	Subset of Lubin2

Table 2G23 -

IESLC - Meta-anal of Ever Smoking (or Current if ever not avail), Amount smoked, "Mid", Cigarettes only
Squamous

This analysis is restricted to results for:
1) Results by Amount smoked
2) Results complete enough for use in metaanalysis

Within each study, results are then selected (in the following order of preference, within each sex) for:
3) SMKSTA: ever smokers, current smokers
4) PRODUCT: cigarettes only
5) CIGTYPE: all/unspecified, MC regardless of HR, MC only
6) DENOM: never smoked anything, never smoked cigarettes, (never +1 = +long term ex, +2 = +amount unknown, +3 = never cigs+long term ex)
7) Followup period (YF, prospective studies): whole study (coded as 0) or longest available
8) LCtype: squamous or nearest available, but not adeno. (q = squamous, s = small, a = adeno, KI = Kreyberg I, u = undifferentiated)
9) Race: all or nearest available, otherwise by race (wh or w = white, bl or b = black, hi = hispanic ch = chinese, jap = japanese, haw = hawaiian, w+o = white + oriental, sca = scandinavian, as = asian)
10) Amount smoked "mid" in key scheme 1 (key value 20, maximum range 6-44, in numbers of cigarettes)
11) For overlapping studies: principal rather than subsidiary studies
Finally by Age: whole study (coded as 0) if available, otherwise by widest available age group
and then for single sex results (m, f) in preference to combined sex results (c).

Results adjusted (AD) for the most potential confounders are then chosen in Sections -1 to -3
(and those which actually differ from the adjusted results in Table 2G13 - 1 are marked 'x' in Section -1)
and results adjusted for the least confounders in Sections -4 to -6. (Those least adjusted results which
actually differ from the most adjusted as marked 'x' in column X in Section -4)
(Results adjusted for an unknown number of confounder(s) are coded as 20.)

Section -7 shows excluded studies, together with the stage (as above) at which no qualifying
results were found.

Section -8 lists the potentially overlapping studies which have been included (1=principal, 2=subsidiary).

Section -9 lists any results which would have been included in preference except that they had data not complete
enough for use in meta-analysis, with their significance (yes/no), if known, and any further comment as entered
on the database.

In addition to those mentioned above, the following fields, levels and abbreviations are used:
* or nk = not known, n = no, y = yes, ot = other
ev = ever, cu = current, nev = never
all/unspec = all or unspecified, MC = manufactured cigarettes, HR = hand-rolled cigarettes
exL, exH = range of exposure (low and high) in the smoking group, in terms of Amount smoked, cigarettes
REF: 6-character study reference
NRR: number of the RR on the database within the study
ST : study type (CC = case control, pr or prosp = prospective)
NLC: number of lung cancer cases in whole study
R : risky occupational population (n = no, m = mining, o = other risky)
VB : national cigarette type (V = at least 75% Virginia, bl = at least 75% blended, ot = other)
P : any proxy use
H : full histological confirmation
De : derivation of RR/CI (or = original, st = standard method, ot = other method of estimation)

Table 2G23 - 1

IESLC - Meta-anal of Ever Smoking (or Current if ever not avail), Amount smoked, "Mid", Cigarettes only
Squamous
Most adjusted

REF	NRR	2G13	SEX	AGEL	AGEH	RACE	YF	LC TYPE	LOC	START	ST	NLC	R	VB	P	H	AD	SM	PRODUCT	exL	exH	DENOM	De
ALDERS	35		m	0	0	all	-	q+s	Eu:UK	1977	CC	1448	n	V	n	n	1	ev	cig only	18	27	nev+2	ot
ALDERS	38		f	0	0	all	-	q+s	Eu:UK	1977	CC	1448	n	V	n	n	1	ev	cig only	18	27	nev+2	ot
BENHAM	13	x	m	0	0	all	-	KI	Eu:wst	1976	CC	1625	n	bl	n	y	0	ev	cig only	15	20	nev any	st
HAMMON	99		m	0	0	wh	0	not a	NAmer	1952	pr	448	n	bl	n	n	1	cu	cig only	10	20	nev any	ot

Cigarette type is all/unspec for all RRs

except for the following:

REF	NRR	CIGTYPE
ALDERS	35	MC only
ALDERS	38	MC only

Table 2G23 - 2

IESLC - Meta-anal of Ever Smoking (or Current if ever not avail), Amount smoked, "Mid", Cigarettes only
Squamous
Most adjusted

REF	NRR	SEX	AD	Number Exposed Case	Cont	Non-exposed Case	Cont	RR	95.00%CI
ALDERS	35	m	1	-	-	-	-	7.19	(2.75- 18.79)
ALDERS	38	f	1	-	-	-	-	9.24	(5.31- 16.09)
Subtotal ALDERS								8.68	(5.37- 14.03)
BENHAM	13	m	0	449	512	24	481	17.58	(11.44- 26.99)
*HAMMON	99	m	1	-	-	-	-	17.44	(6.30- 48.29)
Partial Totals				449	512	24	481		

*prospective study

REF	NRR	SEX	AD	Ys	Ws	Qs	Ps
ALDERS	35	m	1	1.97	4.16	1.54	0.0001
ALDERS	38	f	1	2.22	12.50	1.59	0.0000
Subtotal ALDERS				2.16	16.66	3.13	
BENHAM	13	m	0	2.87	20.87	1.70	0.0000
*HAMMON	99	m	1	2.86	3.70	0.29	0.0000

	N	4
	NS	3
	Wt	41.23
Het	Chi	5.12
Het	df	3
Het	P	N.S.
Fixed	RR	13.21
	RRl	9.73
	RRu	17.92
	P	+++
Random	RR	12.50
	RRl	8.05
	RRu	19.39
	P	+++
Asymm	P	N.S.

Table 2G23 - 3

IESLC - Meta-anal of Ever Smoking (or Current if ever not avail), Amount smoked, "Mid", Cigarettes only
Squamous
Most adjusted

	combined	Sex male	female	Total
N		3	1	4
NS		3	1	4
Wt		28.73	12.50	41.23
Het Chi		2.84	0.00	5.12
Het df		2	0	3
Het P		N.S.	N.S.	N.S.
Fixed RR		15.43	9.24	13.21
RRl		10.70	5.31	9.73
RRu		22.24	16.08	17.92
P		+++	+++	+++
Random RR		14.44	9.24	12.50
RRl		8.70	5.31	8.05
RRu		23.98	16.08	19.39
P		+++	+++	+++
Between Chi				2.29
Between df				1
Between P				N.S.
Btwn(F) P				N.S.
Btwn(R) P				N.S.

Too few RRs for analysis by factor

Table 2G23 - 4

IESLC - Meta-anal of Ever Smoking (or Current if ever not avail), Amount smoked, "Mid", Cigarettes only
Squamous
Least adjusted

REF	NRR	X	SEX	AGEL	AGEH	RACE	YF	LC TYPE	LOC	START	ST	NLC	R	VB	P	H	AD	SM	PRODUCT	exL	exH	DENOM	De
ALDERS	35		m	0	0	all	-	q+s	Eu:UK	1977	CC	1448	n	V	n	n	1	ev	cig only	18	27	nev+2	ot
ALDERS	38		f	0	0	all	-	q+s	Eu:UK	1977	CC	1448	n	V	n	n	1	ev	cig only	18	27	nev+2	ot
BENHAM	13		m	0	0	all	-	KI	Eu:wst	1976	CC	1625	n	bl	n	y	0	ev	cig only	15	20	nev any	st
HAMMON	99		m	0	0	wh	0	not a	NAmer	1952	pr	448	n	bl	n	n	1	cu	cig only	10	20	nev any	ot

Cigarette type is all/unspec for all RRs

except for the following:

REF	NRR	CIGTYPE
ALDERS	35	MC only
ALDERS	38	MC only

Table 2G23 - 5

IESLC - Meta-anal of Ever Smoking (or Current if ever not avail), Amount smoked, "Mid", Cigarettes only
Squamous
Least adjusted

REF	NRR	SEX	AD	Number Exposed Case	Number Exposed Cont	Non-exposed Case	Non-exposed Cont	RR	95.00%CI
ALDERS	35	m	1	-	-	-	-	7.19	(2.75- 18.79)
ALDERS	38	f	1	-	-	-	-	9.24	(5.31- 16.09)
Subtotal ALDERS								8.68	(5.37- 14.03)
BENHAM	13	m	0	449	512	24	481	17.58	(11.44- 26.99)
*HAMMON	99	m	1	-	-	-	-	17.44	(6.30- 48.29)
Partial Totals				449	512	24	481		

*prospective study

REF	NRR	SEX	AD	Ys	Ws	Qs	Ps
ALDERS	35	m	1	1.97	4.16	1.54	0.0001
ALDERS	38	f	1	2.22	12.50	1.59	0.0000
Subtotal ALDERS				2.16	16.66	3.13	
BENHAM	13	m	0	2.87	20.87	1.70	0.0000
*HAMMON	99	m	1	2.86	3.70	0.29	0.0000

	N	4
	NS	3
	Wt	41.23
Het	Chi	5.12
Het	df	3
Het	P	N.S.
Fixed	RR	13.21
	RRl	9.73
	RRu	17.92
	P	+++
Random	RR	12.50
	RRl	8.05
	RRu	19.39
	P	+++
Asymm	P	N.S.

Table 2G23 - 6

IESLC - Meta-anal of Ever Smoking (or Current if ever not avail), Amount smoked, "Mid", Cigarettes only
Squamous
Least adjusted

	Sex combined	male	female	Total
N		3	1	4
NS		3	1	4
Wt		28.73	12.50	41.23
Het Chi		2.84	0.00	5.12
Het df		2	0	3
Het P		N.S.	N.S.	N.S.
Fixed RR		15.43	9.24	13.21
RRl		10.70	5.31	9.73
RRu		22.24	16.08	17.92
P		+++	+++	+++
Random RR		14.44	9.24	12.50
RRl		8.70	5.31	8.05
RRu		23.98	16.08	19.39
P		+++	+++	+++
Between Chi				2.29
Between df				1
Between P				N.S.
Btwn(F) P				N.S.
Btwn(R) P				N.S.

Table 2G23 - 7

IESLC - Meta-anal of Ever Smoking (or Current if ever not avail), Amount smoked, "Mid", Cigarettes only
Squamous
Excluded studies (and stage at which they were excluded)

Stage	Studies
1	ABELIN ABRAHA AMANDU AMES ANDERS AUSTIN AXELSO BAND BECHER BERRIN BLOHMK BLOT4 BROCKM BROWN1 BYERS1 BYERS2 CARPEN CASCO2 CASCOR CHAN CHEN3 CHIAZZ CHYOU DESTE2 DOCKER DROSTE DU GARCIA GARDIN GENG GODLEY GOODMA GRAHAM GREGOR HEGMAN HEIN HENNEK HINDS HIRAOK HOROWI HORWIT HUANG ISHIMA JAHN JAIN JARVHO JIANG KELLER KIHARA KJUUS KO KOHLME KUBIK LAMWK LAMWK2 LANGE LEI LEMARC LEVIN LIU LOMBA2 LOMBAR MAGNUS MARSH MARSH2 MCDUFF MCLAUG MILLER MILLS NOTANI NOU ODRISC PAWLEG PERSHA POFFIJ QIAO QIAO2 RADZIK REN RONCO ROOTS ROTHSC SAARIK SANKAR SCHWAR SEGI SEOW SHIMIZ SIMARA SIMONA SITAS SOBUE2 STASZE STAYNE STUCKE SUN SUZUK2 SUZUKI TANG TAO TOKARS TOUSEY ULMER VEIERO VUTUC WALD WANG WANG3 WANG4 WICKLU WIGLE WILKIN WU2 WUNSCH WYNDE8 XIANGZ XU XU2 XU4 YONG ZHANG
2	BUELL CHEN MASTRA MZILEN PISANI RESTRE SADOWS
4	AKIBA ARCHER ARMADA AUVINE AXELSS BARBON BENSHL BLOT1 BLOT2 BLOT3 BOFFET BOUCHA BRESLO BRETT BROSS BROWN2 BUFFLE CHANG CHATZI CHEN2 CHOI CHOW COMSTO COOKSO CORREA DARBY DAVEYS DEKLER DESTEF DOLL DORANT DORGAN DOSEME DUNN EBELIN ESAKI FAN GAO GAO2 GARSHI GER GILLIS GSELL HAENSZ HAMMO2 HANSEN HIRAYA HITOSU HOLE HU HU2 HUMBLE JARUP JEDRYC JOLY JONES KAISER KANELL KATSOU KAUFMA KHUDER KINLEN KNEKT KOO KOULUM KREUZE KREYBE LAMTH LAURIL LAUSSM LETOUR LIAW LICKIN LIDDEL LIU2 LIU3 LIU5 LUBIN2 LUO MACLEN MARTIS MATOS MATSUD MOLLO MRFIT MRFITR MURATA NAM ORMOS OSANN OSANN2 PARKIN PASTOR PERNU PERSH2 PETO PEZZO2 PIKE POLEDN PRESCO RACHTA RANDIG SEGI2 SHAW SIEMIA SOBUE SPEIZE SPITZ STOCKS STOCKW SVENSS TENKAN TSUGAN TULINI VANDER WAKAI WANG2 WARSIN WATSON WU WUWILL WYNDE2 WYNDE3 WYNDE4 WYNDE5 WYNDE6 WYNDE7 WYNDER XU3 YAMAGU YUAN ZHENG ZHOU
5	RIMING TANG2
6	HIRAY2 SCHWA2
8	AGUDO BEST CEDERL CPSI CPSII DAMBER DEAN DEAN2 DEAN3 DOLL2 DORN ENGELA ENSTRO GOLLED JUSSAW KAISE2 LIU4 LUBIN MCCONN MIGRAN NOTAN2 PEZZOT TIZZAN TVERDA
10	BOUCOT

Table 2G23 - 8

Potentially overlapping studies

REF	REFGP	PRINC	.	OVERLAP/LINK
BENHAM	LUBIN2	2		Subset of Lubin2

Table 2G24 -

IESLC - Meta-anal of Ever Smoking (or Current if ever not avail), Amount smoked, "High", Cigarettes only
Squamous

This analysis is restricted to results for:
1) Results by Amount smoked
2) Results complete enough for use in metaanalysis

Within each study, results are then selected (in the following order of preference, within each sex) for:
3) SMKSTA: ever smokers, current smokers
4) PRODUCT: cigarettes only
5) CIGTYPE: all/unspecified, MC regardless of HR, MC only
6) DENOM: never smoked anything, never smoked cigarettes, (never +1 = +long term ex, +2 = +amount unknown, +3 = never cigs+long term ex)
7) Followup period (YF, prospective studies): whole study (coded as 0) or longest available
8) LCtype: squamous or nearest available, but not adeno. (q = squamous, s = small, a = adeno, KI = Kreyberg I, u = undifferentiated)
9) Race: all or nearest available, otherwise by race (wh or w = white, bl or b = black, hi = hispanic ch = chinese, jap = japanese, haw = hawaiian, w+o = white + oriental, sca = scandinavian, as = asian)
10) Amount smoked "high" in key scheme 1 (key value 45, maximum range >20, in numbers of cigarettes)
11) For overlapping studies: principal rather than subsidiary studies
Finally by Age: whole study (coded as 0) if available, otherwise by widest available age group
and then for single sex results (m, f) in preference to combined sex results (c).

Results adjusted (AD) for the most potential confounders are then chosen in Sections -1 to -3
(and those which actually differ from the adjusted results in Table 2G14 - 1 are marked 'x' in Section -1)
and results adjusted for the least confounders in Sections -4 to -6. (Those least adjusted results which
actually differ from the most adjusted as marked 'x' in column X in Section -4)
(Results adjusted for an unknown number of confounder(s) are coded as 20.)

Section -7 shows excluded studies, together with the stage (as above) at which no qualifying
results were found.

Section -8 lists the potentially overlapping studies which have been included (1=principal, 2=subsidiary).

Section -9 lists any results which would have been included in preference except that they had data not complete
enough for use in meta-analysis, with their significance (yes/no), if known, and any further comment as entered
on the database.

In addition to those mentioned above, the following fields, levels and abbreviations are used:
* or nk = not known, n = no, y = yes, ot = other
ev = ever, cu = current, nev = never
all/unspec = all or unspecified, MC = manufactured cigarettes, HR = hand-rolled cigarettes
exL, exH = range of exposure (low and high) in the smoking group, in terms of Amount smoked, cigarettes
REF: 6-character study reference
NRR: number of the RR on the database within the study
ST : study type (CC = case control, pr or prosp = prospective)
NLC: number of lung cancer cases in whole study
R : risky occupational population (n = no, m = mining, o = other risky)
VB : national cigarette type (V = at least 75% Virginia, bl = at least 75% blended, ot = other)
P : any proxy use
H : full histological confirmation
De : derivation of RR/CI (or = original, st = standard method, ot = other method of estimation)

Table 2G24 - 1

IESLC - Meta-anal of Ever Smoking (or Current if ever not avail), Amount smoked, "High", Cigarettes only
Squamous
Most adjusted

REF	NRR	2G14	SEX	AGEL	AGEH	RACE	YF	LC TYPE	LOC	START	ST	NLC	R	VB	P	H	AD	SM	PRODUCT	exL	exH	DENOM	De
ALDERS	36		m	0	0	all	-	q+s	Eu:UK	1977	CC	1448	n	V	n	n	1	ev	cig only	28	99	nev+2	ot
ALDERS	39		f	0	0	all	-	q+s	Eu:UK	1977	CC	1448	n	V	n	n	1	ev	cig only	28	99	nev+2	ot
BENHAM	15	x	m	0	0	all	-	KI	Eu:wst	1976	CC	1625	n	bl	n	y	0	ev	cig only	40	99	nev any	st
BOUCOT	143		m	0	0	all	0	q	NAmer	1951	pr	121	n	bl	n	n	2	cu	cig only	21	99	nev any	ot
HAMMON	101		m	0	0	wh	0	not a	NAmer	1952	pr	448	n	bl	n	n	1	cu	cig only	40	99	nev any	ot

Cigarette type is all/unspec for all RRs

except for the following:

REF	NRR	CIGTYPE
ALDERS	36	MC only
ALDERS	39	MC only

Table 2G24 - 2

IESLC - Meta-anal of Ever Smoking (or Current if ever not avail), Amount smoked, "High", Cigarettes only
Squamous
Most adjusted

REF	NRR	SEX	AD	Number Exposed Case	Number Exposed Cont	Non-exposed Case	Non-exposed Cont	RR	95.00%CI
ALDERS	36	m	1	-	-	-	-	8.78	(3.46- 22.31)
ALDERS	39	f	1	-	-	-	-	14.52	(7.93- 26.58)
Subtotal ALDERS								12.51	(7.53- 20.77)
BENHAM	15	m	0	175	122	24	481	28.75	(17.96- 46.03)
*BOUCOT	143	m	2	-	-	-	-	46.64	(2.80- 775.69)
*HAMMON	101	m	1	-	-	-	-	63.91	(22.02- 185.47)
Partial Totals				175	122	24	481		

*prospective study

REF	NRR	SEX	AD	Ys	Ws	Qs	Ps
ALDERS	36	m	1	2.17	4.42	3.77	0.0000
ALDERS	39	f	1	2.68	10.50	1.86	0.0000
Subtotal ALDERS				2.53	14.93	5.63	
BENHAM	15	m	0	3.36	17.34	1.19	0.0000
*BOUCOT	143	m	2	3.84	0.49	0.27	0.0074
*HAMMON	101	m	1	4.16	3.38	3.81	0.0000

	N	5
	NS	4
	Wt	36.14
Het	Chi	10.91
Het	df	4
Het	P	*
Fixed	RR	22.11
	RRl	15.96
	RRu	30.64
	P	+++
Random	RR	22.12
	RRl	11.74
	RRu	41.69
	P	+++
Asymm	P	N.S.

Table 2G24 - 3

IESLC - Meta-anal of Ever Smoking (or Current if ever not avail), Amount smoked, "High", Cigarettes only
Squamous
Most adjusted

	Sex combined	male	female	Total
N		4	1	5
NS		4	1	5
Wt		25.64	10.50	36.14
Het Chi		8.29	0.00	10.91
Het df		3	0	4
Het P		*	N.S.	*
Fixed RR		26.27	14.52	22.11
RRl		17.84	7.93	15.96
RRu		38.69	26.58	30.64
P		+++	+++	+++
Random RR		26.10	14.52	22.12
RRl		11.29	7.93	11.74
RRu		60.35	26.58	41.69
P		+++	+++	+++
Between Chi				2.62
Between df				1
Between P				N.S.
Btwn(F) P				N.S.
Btwn(R) P				N.S.

Too few RRs for analysis by factor

Table 2G24 - 4

IESLC - Meta-anal of Ever Smoking (or Current if ever not avail), Amount smoked, "High", Cigarettes only
Squamous
Least adjusted

REF	NRR	X	SEX	AGEL	AGEH	RACE	YF	LC TYPE	LOC	START	ST	NLC	R	VB	P	H	AD	SM	PRODUCT	exL	exH	DENOM	De
ALDERS	36		m	0	0	all	-	q+s	Eu:UK	1977	CC	1448	n	V	n	n	1	ev	cig only	28	99	nev+2	ot
ALDERS	39		f	0	0	all	-	q+s	Eu:UK	1977	CC	1448	n	V	n	n	1	ev	cig only	28	99	nev+2	ot
BENHAM	15		m	0	0	all	-	KI	Eu:wst	1976	CC	1625	n	bl	n	y	0	ev	cig only	40	99	nev any	st
BOUCOT	22	x	m	0	0	all	0	q	NAmer	1951	pr	121	n	bl	n	n	0	cu	cig only	21	99	nev any	ot
HAMMON	101		m	0	0	wh	0	not a	NAmer	1952	pr	448	n	bl	n	n	1	cu	cig only	40	99	nev any	ot

Cigarette type is all/unspec for all RRs

except for the following:

REF	NRR	CIGTYPE
ALDERS	36	MC only
ALDERS	39	MC only

Table 2G24 - 5

IESLC - Meta-anal of Ever Smoking (or Current if ever not avail), Amount smoked, "High", Cigarettes only
Squamous
Least adjusted

REF	NRR	SEX	AD	Number Exposed Case	Number Exposed Cont	Non-exposed Case	Non-exposed Cont	RR	95.00%CI
ALDERS	36	m	1	-	-	-	-	8.78	(3.46- 22.31)
ALDERS	39	f	1	-	-	-	-	14.52	(7.93- 26.58)
Subtotal ALDERS								12.51	(7.53- 20.77)
BENHAM	15	m	0	175	122	24	481	28.75	(17.96- 46.03)
*BOUCOT	22	m	0	17	6940	0	7551	38.08~	(2.29- 633.12)
*HAMMON	101	m	1	-	-	-	-	63.91	(22.02- 185.47)
Partial Totals				192	7062	24	8032		

*prospective study ~ With 0.5 adjustment for zero

REF	NRR	SEX	AD	Ys	Ws	Qs	Ps
ALDERS	36	m	1	2.17	4.42	3.75	0.0000
ALDERS	39	f	1	2.68	10.50	1.83	0.0000
Subtotal ALDERS				2.53	14.93	5.59	
BENHAM	15	m	0	3.36	17.34	1.22	0.0000
*BOUCOT	22	m	0	3.64	0.49	0.15	0.0112
*HAMMON	101	m	1	4.16	3.38	3.83	0.0000

	N	5
	NS	4
	Wt	36.14
Het	Chi	10.78
Het	df	4
Het	P	*
Fixed	RR	22.05
	RRl	15.92
	RRu	30.55
	P	+++
Random	RR	21.91
	RRl	11.67
	RRu	41.13
	P	+++
Asymm	P	N.S.

Table 2G24 - 6

IESLC - Meta-anal of Ever Smoking (or Current if ever not avail), Amount smoked, "High", Cigarettes only

Squamous

Least adjusted

	Sex combined	male	female	Total
N		4	1	5
NS		4	1	5
Wt		25.64	10.50	36.14
Het Chi		8.20	0.00	10.78
Het df		3	0	4
Het P		*	N.S.	*
Fixed RR		26.17	14.52	22.05
RRl		17.77	7.93	15.92
RRu		38.54	26.58	30.55
P		+++	+++	+++
Random RR		25.71	14.52	21.91
RRl		11.18	7.93	11.67
RRu		59.13	26.58	41.13
P		+++	+++	+++
Between Chi				2.59
Between df				1
Between P				N.S.
Btwn(F) P				N.S.
Btwn(R) P				N.S.

Table 2G24 - 7

IESLC - Meta-anal of Ever Smoking (or Current if ever not avail), Amount smoked, "High", Cigarettes only
Squamous
Excluded studies (and stage at which they were excluded)

```
1      ABELIN ABRAHA AMANDU   AMES ANDERS AUSTIN AXELSO   BAND BECHER BERRIN BLOHMK  BLOT4 BROCKM BROWN1 BYERS1 BYERS2
       CARPEN CASCO2 CASCOR   CHAN  CHEN3 CHIAZZ  CHYOU DESTE2 DOCKER DROSTE     DU GARCIA GARDIN   GENG GODLEY GOODMA
       GRAHAM GREGOR HEGMAN   HEIN HENNEK  HINDS HIRAOK HOROWI HORWIT  HUANG ISHIMA   JAHN   JAIN JARVHO  JIANG KELLER
       KIHARA  KJUUS     KO KOHLME  KUBIK  LAMWK LAMWK2  LANGE    LEI LEMARC  LEVIN    LIU LOMBA2 LOMBAR MAGNUS  MARSH
       MARSH2 MCDUFF MCLAUG MILLER  MILLS NOTANI    NOU ODRISC PAWLEG PERSHA POFFIJ   QIAO  QIAO2 RADZIK    REN  RONCO
        ROOTS ROTHSC SAARIK SANKAR SCHWAR   SEGI   SEOW SHIMIZ SIMARA SIMONA  SITAS SOBUE2 STASZE STAYNE STUCKE    SUN
       SUZUK2 SUZUKI   TANG    TAO TOKARS TOUSEY  ULMER VEIERO  VUTUC   WALD   WANG  WANG3  WANG4 WICKLU  WIGLE WILKIN
          WU2 WUNSCH WYNDE8 XIANGZ     XU    XU2    XU4   YONG  ZHANG

2       BUELL   CHEN MASTRA MZILEN PISANI RESTRE SADOWS

4       AKIBA ARCHER ARMADA AUVINE AXELSS BARBON BENSHL  BLOT1  BLOT2  BLOT3 BOFFET BOUCHA BRESLO  BRETT  BROSS BROWN2
       BUFFLE  CHANG CHATZI  CHEN2   CHOI   CHOW COMSTO COOKSO CORREA  DARBY DAVEYS DEKLER DESTEF   DOLL DORANT DORGAN
       DOSEME   DUNN EBELIN  ESAKI    FAN    GAO   GAO2 GARSHI    GER GILLIS  GSELL HAENSZ HAMMO2 HANSEN HIRAYA HITOSU
         HOLE     HU    HU2 HUMBLE  JARUP JEDRYC   JOLY  JONES KAISER KANELL KATSOU KAUFMA KHUDER KINLEN  KNEKT    KOO
       KOULUM KREUZE KREYBE  LAMTH LAURIL LAUSSM LETOUR   LIAW LICKIN LIDDEL   LIU2   LIU3   LIU5 LUBIN2    LUO MACLEN
       MARTIS  MATOS MATSUD  MOLLO  MRFIT MRFITR MURATA    NAM  ORMOS  OSANN OSANN2 PARKIN PASTOR  PERNU PERSH2   PETO
       PEZZO2   PIKE POLEDN PRESCO RACHTA RANDIG  SEGI2   SHAW SIEMIA  SOBUE SPEIZE  SPITZ STOCKS STOCKW SVENSS TENKAN
       TSUGAN TULINI VANDER  WAKAI  WANG2 WARSIN WATSON     WU WUWILL WYNDE2 WYNDE3 WYNDE4 WYNDE5 WYNDE6 WYNDE7 WYNDER
          XU3 YAMAGU   YUAN  ZHENG   ZHOU

5      RIMING  TANG2

6      HIRAY2 SCHWA2

8       AGUDO   BEST CEDERL   CPSI  CPSII DAMBER   DEAN  DEAN2  DEAN3  DOLL2   DORN ENGELA ENSTRO GOLLED JUSSAW KAISE2
         LIU4  LUBIN MCCONN MIGRAN NOTAN2 PEZZOT TIZZAN TVERDA
```

Table 2G24 - 8

Potentially overlapping studies

REF	REFGP	PRINC	.	OVERLAP/LINK
BENHAM	LUBIN2	2		Subset of Lubin2

Table 2G26 -

IESLC - Meta-analysis of Ever Smoking by Amount, Overview, Pipe and/or Cigars (not cigs)
Squamous

This analysis is restricted to results for:
1) Results by Amount smoked
Results by Amount smoked are grouped under 2 schemes (S1, S2). Each scheme has a set of "key values". An interval is allocated to the category whose key value it includes and intervals which include none or more than one of the key values are excluded. (Open-ended intervals are coded as 99). Amounts are usually coded as number of cigarette equivalents, defined as shown at the end of Sections -1 and -4.

S1	key value	maximum range	S2	key value	maximum range
1	1	1-98	1	1	1-9
2	99	2-99	2	10	2-98
			3	99	11+

Thus the two levels in scheme S1 correspond to the lowest and highest intervals, irrespective of their values. In scheme C2, the lowest and highest intervals are chosen provided they do not include the value 10, and the interval including the value 10 is also chosen.
2) Smokers of pipe and/or cigars (but not cigarettes)
3) Ever smokers
4) Results complete enough for use in metaanalysis

Within each study, results are then selected (in the following order of preference, within each sex) for:
5) DENOM: never smoked anything, (never +1 = +long term ex)
6) Followup period (prospective studies): whole study (coded as 0) or longest available
7) LCtype: all or nearest available, at least Squamous and Adeno. (q = squamous, s = small, l = large, a = adeno, mix = mixed, alv = alveolar)
8) Race: all or nearest available, otherwise by race (wh or w = white, bl or b = black, hi = hispanic ch = chinese, jap = japanese, haw = hawaiian, w+o = white + oriental, sca = scandinavian, as = asian)
9) For overlapping studies: principal rather than subsidiary studies
Finally by Age: whole study (coded as 0) if available, otherwise by widest available age group and then for single sex results (m, f) in preference to combined sex results (c).

Results adjusted (AD) for the most potential confounders are then chosen in Sections -1 to -3 and results adjusted for the least confounders in Sections -4 to -6. (Those least adjusted results which actually differ from the most adjusted as marked 'x' in column X in Section -4)
(Results adjusted for an unknown number of confounder(s) are coded as 20.)

Section -7 shows excluded studies, together with the stage (as above) at which no qualifying results were found.

Section -8 lists the potentially overlapping studies which have been included (1=principal, 2=subsidiary).

Section -9 lists any results which would have been included in preference except that they had data not complete enough for use in meta-analysis, with their significance (yes/no), if known, and any further comment as entered on the database.

In addition to those mentioned above, the following fields, levels and abbreviations are used:
* or nk = not known, n = no, y = yes, ot = other
nev = never
exL, exH = range of exposure (low and high) in the smoking group, in terms of Amount smoked, cigarette equivalents
REF: 6-character study reference
NRR: number of the RR on the database within the study
ST : study type (CC = case control, pr or prosp = prospective)
NLC: number of lung cancer cases in whole study
R : risky occupational population (n = no, m = mining, o = other risky)
VB : national cigarette type (V = at least 75% Virginia, bl = at least 75% blended, ot = other)
P : any proxy use
H : full histological confirmation
De : derivation of RR/CI (or = original, st = standard method, ot = other method of estimation)

Table 2G26 - 0

No RRs selected for this analysis

Table 2G26 - 7

IESLC - Meta-analysis of Ever Smoking by Amount, Overview, Pipe and/or Cigars (not cigs)
Squamous
Excluded studies (and stage at which they were excluded)

1 ABELIN ABRAHA AMANDU AMES ANDERS AUSTIN AXELSO BAND BECHER BERRIN BLOHMK BLOT4 BROCKM BROWN1 BYERS1 BYERS2 CARPEN
CASCO2 CASCOR CHAN CHEN3 CHIAZZ CHYOU DESTE2 DOCKER DROSTE DU GARCIA GARDIN GENG GODLEY GOODMA GRAHAM GREGOR
HEGMAN HEIN HENNEK HINDS HIRAOK HOROWI HORWIT HUANG ISHIMA JAHN JAIN JARVHO JIANG KELLER KIHARA KJUUS KO
KOHLME KUBIK LAMWK LAMWK2 LANGE LEI LEMARC LEVIN LIU LOMBA2 LOMBAR MAGNUS MARSH MARSH2 MCDUFF MCLAUG MILLER
MILLS NOTANI NOU ODRISC PAWLEG PERSHA POFFIJ QIAO QIAO2 RADZIK REN RONCO ROOTS ROTHSC SAARIK SANKAR SCHWAR
SEGI SEOW SHIMIZ SIMARA SIMONA SITAS SOBUE2 STASZE STAYNE STUCKE SUN SUZUK2 SUZUKI TANG TAO TOKARS TOUSEY
ULMER VEIERO VUTUC WALD WANG WANG3 WANG4 WICKLU WIGLE WILKIN WU2 WUNSCH WYNDE8 XIANGZ XU XU2 XU4
YONG ZHANG

2 AGUDO AKIBA ALDERS ARCHER ARMADA AUVINE AXELSS BARBON BENHAM BENSHL BEST BLOT1 BLOT2 BLOT3 BOFFET BOUCHA BOUCOT
BRESLO BRETT BROSS BROWN2 BUELL BUFFLE CEDERL CHANG CHATZI CHEN CHEN2 CHOI CHOW COMSTO COOKSO CORREA CPSI
CPSII DAMBER DARBY DAVEYS DEAN DEAN2 DEAN3 DEKLER DESTEF DOLL DORANT DORGAN DORN DOSEME DUNN EBELIN ENGELA
ENSTRO ESAKI FAN GAO GAO2 GARSHI GER GILLIS GOLLED GSELL HAENSZ HAMMO2 HAMMON HANSEN HIRAY2 HIRAYA HITOSU
HOLE HU HU2 HUMBLE JARUP JEDRYC JOLY JONES JUSSAW KAISE2 KAISER KANELL KATSOU KAUFMA KHUDER KINLEN KNEKT
KOO KOULUM KREUZE KREYBE LAMTH LAURIL LAUSSM LETOUR LIAW LICKIN LIDDEL LIU2 LIU3 LIU4 LIU5 LUBIN LUBIN2
LUO MACLEN MARTIS MASTRA MATOS MATSUD MCCONN MIGRAN MOLLO MRFIT MRFITR MURATA MZILEN NAM NOTAN2 ORMOS OSANN
OSANN2 PARKIN PASTOR PERNU PERSH2 PETO PEZZO2 PEZZOT PIKE PISANI POLEDN PRESCO RACHTA RANDIG RESTRE RIMING SADOWS
SCHWA2 SEGI2 SHAW SIEMIA SOBUE SPEIZE SPITZ STOCKS STOCKW SVENSS TANG2 TENKAN TIZZAN TSUGAN TULINI TVERDA VANDER
WAKAI WANG2 WARSIN WATSON WU WUWILL WYNDE2 WYNDE3 WYNDE4 WYNDE5 WYNDE6 WYNDER XU3 YAMAGU YUAN ZHENG ZHOU

3 DOLL2

7 WYNDE7

Table 2G28 -

IESLC - Meta-analysis of Current Smoking by Amount, Overview, Pipe and/or Cigars (not cigs)
Squamous

This analysis is restricted to results for:
1) Results by Amount smoked
Results by Amount smoked are grouped under 2 schemes (S1, S2). Each scheme has a set of "key values". An interval is allocated to the category whose key value it includes and intervals which include none or more than one of the key values are excluded. (Open-ended intervals are coded as 99). Amounts are usually coded as number of cigarette equivalents, defined as shown at the end of Sections -1 and -4.

S1	key value	maximum range	S2	key value	maximum range
1	1	1-98	1	1	1-9
2	99	2-99	2	10	2-98
			3	99	11+

Thus the two levels in scheme S1 correspond to the lowest and highest intervals, irrespective of their values. In scheme C2, the lowest and highest intervals are chosen provided they do not include the value 10, and the interval including the value 10 is also chosen.
2) Smokers of pipe and/or cigars (but not cigarettes)
3) Current smokers
4) Results complete enough for use in metaanalysis

Within each study, results are then selected (in the following order of preference, within each sex) for:
5) DENOM: never smoked anything, (never +1 = +long term ex)
6) Followup period (prospective studies): whole study (coded as 0) or longest available
7) LCtype: all or nearest available, at least Squamous and Adeno. (q = squamous, s = small, l = large, a = adeno, mix = mixed, alv = alveolar)
8) Race: all or nearest available, otherwise by race (wh or w = white, bl or b = black, hi = hispanic ch = chinese, jap = japanese, haw = hawaiian, w+o = white + oriental, sca = scandinavian, as = asian)
9) For overlapping studies: principal rather than subsidiary studies
Finally by Age: whole study (coded as 0) if available, otherwise by widest available age group and then for single sex results (m, f) in preference to combined sex results (c).

Results adjusted (AD) for the most potential confounders are then chosen in Sections -1 to -3 and results adjusted for the least confounders in Sections -4 to -6. (Those least adjusted results which actually differ from the most adjusted as marked 'x' in column X in Section -4)
(Results adjusted for an unknown number of confounder(s) are coded as 20.)

Section -7 shows excluded studies, together with the stage (as above) at which no qualifying results were found.

Section -8 lists the potentially overlapping studies which have been included (1=principal, 2=subsidiary).

Section -9 lists any results which would have been included in preference except that they had data not complete enough for use in meta-analysis, with their significance (yes/no), if known, and any further comment as entered on the database.

In addition to those mentioned above, the following fields, levels and abbreviations are used:
* or nk = not known, n = no, y = yes, ot = other
nev = never
exL, exH = range of exposure (low and high) in the smoking group, in terms of Amount smoked, cigarette equivalents
REF: 6-character study reference
NRR: number of the RR on the database within the study
ST : study type (CC = case control, pr or prosp = prospective)
NLC: number of lung cancer cases in whole study
R : risky occupational population (n = no, m = mining, o = other risky)
VB : national cigarette type (V = at least 75% Virginia, bl = at least 75% blended, ot = other)
P : any proxy use
H : full histological confirmation
De : derivation of RR/CI (or = original, st = standard method, ot = other method of estimation)

Table 2G28 - 0

No RRs selected for this analysis

Table 2G28 - 7

IESLC - Meta-analysis of Current Smoking by Amount, Overview, Pipe and/or Cigars (not cigs)
Squamous
Excluded studies (and stage at which they were excluded)

1 ABELIN ABRAHA AMANDU AMES ANDERS AUSTIN AXELSO BAND BECHER BERRIN BLOHMK BLOT4 BROCKM BROWN1 BYERS1 BYERS2 CARPEN
CASCO2 CASCOR CHAN CHEN3 CHIAZZ CHYOU DESTE2 DOCKER DROSTE DU GARCIA GARDIN GENG GODLEY GOODMA GRAHAM GREGOR
HEGMAN HEIN HENNEK HINDS HIRAOK HOROWI HORWIT HUANG ISHIMA JAHN JAIN JARVHO JIANG KELLER KIHARA KJUUS KO
KOHLME KUBIK LAMWK LAMWK2 LANGE LEI LEMARC LEVIN LIU LOMBA2 LOMBAR MAGNUS MARSH MARSH2 MCDUFF MCLAUG MILLER
MILLS NOTANI NOU ODRISC PAWLEG PERSHA POFFIJ QIAO QIAO2 RADZIK REN RONCO ROOTS ROTHSC SAARIK SANKAR SCHWAR
SEGI SEOW SHIMIZ SIMARA SIMONA SITAS SOBUE2 STASZE STAYNE STUCKE SUN SUZUK2 SUZUKI TANG TAO TOKARS TOUSEY
ULMER VEIERO VUTUC WALD WANG WANG3 WANG4 WICKLU WIGLE WILKIN WU2 WUNSCH WYNDE8 XIANGZ XU XU2 XU4
YONG ZHANG

2 AGUDO AKIBA ALDERS ARCHER ARMADA AUVINE AXELSS BARBON BENHAM BENSHL BEST BLOT1 BLOT2 BLOT3 BOFFET BOUCHA BOUCOT
BRESLO BRETT BROSS BROWN2 BUELL BUFFLE CEDERL CHANG CHATZI CHEN CHEN2 CHOI CHOW COMSTO COOKSO CORREA CPSI
CPSII DAMBER DARBY DAVEYS DEAN DEAN2 DEAN3 DEKLER DESTEF DOLL DORANT DORGAN DORN DOSEME DUNN EBELIN ENGELA
ENSTRO ESAKI FAN GAO GAO2 GARSHI GER GILLIS GOLLED GSELL HAENSZ HAMMO2 HAMMON HANSEN HIRAY2 HIRAYA HITOSU
HOLE HU HU2 HUMBLE JARUP JEDRYC JOLY JONES JUSSAW KAISE2 KAISER KANELL KATSOU KAUFMA KHUDER KINLEN KNEKT
KOO KOULUM KREUZE KREYBE LAMTH LAURIL LAUSSM LETOUR LIAW LICKIN LIDDEL LIU2 LIU3 LIU4 LIU5 LUBIN LUBIN2
LUO MACLEN MARTIS MASTRA MATOS MATSUD MCCONN MIGRAN MOLLO MRFIT MRFITR MURATA MZILEN NAM NOTAN2 ORMOS OSANN
OSANN2 PARKIN PASTOR PERNU PERSH2 PETO PEZZO2 PEZZOT PIKE PISANI POLEDN PRESCO RACHTA RANDIG RESTRE RIMING SADOWS
SCHWA2 SEGI2 SHAW SIEMIA SOBUE SPEIZE SPITZ STOCKS STOCKW SVENSS TANG2 TENKAN TIZZAN TSUGAN TULINI TVERDA VANDER
WAKAI WANG2 WARSIN WATSON WU WUWILL WYNDE2 WYNDE3 WYNDE4 WYNDE5 WYNDE6 WYNDER XU3 YAMAGU YUAN ZHENG ZHOU

7 DOLL2 WYNDE7

Table 2G30 -

IESLC - Meta-analysis of Ever smoking (or Current if Ever not avail) by Amount, Overview, Pipe and/or Cigars (not cigs)
Squamous

This analysis is restricted to results for:
1) Results by Amount smoked
Results by Amount smoked are grouped under 2 schemes (S1, S2). Each scheme has a set of "key values". An interval is allocated to the category whose key value it includes and intervals which include none or more than one of the key values are excluded. (Open-ended intervals are coded as 99). Amounts are usually coded as number of cigarette equivalents, defined as shown at the end of Sections -1 and -4.

S1	key value	maximum range	S2	key value	maximum range
1	1	1-98	1	1	1-9
2	99	2-99	2	10	2-98
			3	99	11+

Thus the two levels in scheme S1 correspond to the lowest and highest intervals, irrespective of their values. In scheme C2, the lowest and highest intervals are chosen provided they do not include the value 10, and the interval including the value 10 is also chosen.
2) Smokers of pipe and/or cigars (but not cigarettes)
3) Results complete enough for use in metaanalysis

Within each study, results are then selected (in the following order of preference, within each sex) for:
4) SMKSTA: ever smokers, current smokers
5) DENOM: never smoked anything, (never +1 = +long term ex)
6) Followup period (prospective studies): whole study (coded as 0) or longest available
7) LCtype: all or nearest available, at least Squamous and Adeno. (q = squamous, s = small, l = large, a = adeno, mix = mixed, alv = alveolar)
8) Race: all or nearest available, otherwise by race (wh or w = white, bl or b = black, hi = hispanic ch = chinese, jap = japanese, haw = hawaiian, w+o = white + oriental, sca = scandinavian, as = asian)
9) For overlapping studies: principal rather than subsidiary studies
Finally by Age: whole study (coded as 0) if available, otherwise by widest available age group and then for single sex results (m, f) in preference to combined sex results (c).

Results adjusted (AD) for the most potential confounders are then chosen in Sections -1 to -3 and results adjusted for the least confounders in Sections -4 to -6. (Those least adjusted results which actually differ from the most adjusted as marked 'x' in column X in Section -4)
(Results adjusted for an unknown number of confounder(s) are coded as 20.)

Section -7 shows excluded studies, together with the stage (as above) at which no qualifying results were found.

Section -8 lists the potentially overlapping studies which have been included (1=principal, 2=subsidiary).

Section -9 lists any results which would have been included in preference except that they had data not complete enough for use in meta-analysis, with their significance (yes/no), if known, and any further comment as entered on the database.

In addition to those mentioned above, the following fields, levels and abbreviations are used:
* or nk = not known, n = no, y = yes, ot = other
ev = ever, cu = current, nev = never
exL, exH = range of exposure (low and high) in the smoking group, in terms of Amount smoked, cigarette equivalents
REF: 6-character study reference
NRR: number of the RR on the database within the study
ST : study type (CC = case control, pr or prosp = prospective)
NLC: number of lung cancer cases in whole study
R : risky occupational population (n = no, m = mining, o = other risky)
VB : national cigarette type (V = at least 75% Virginia, bl = at least 75% blended, ot = other)
P : any proxy use
H : full histological confirmation
De : derivation of RR/CI (or = original, st = standard method, ot = other method of estimation)

Table 2G30 - 0

No RRs selected for this analysis

Table 2G30 - 7

IESLC - Meta-analysis of Ever smoking (or Current if Ever not avail) by Amount, Overview, Pipe and/or Cigars (not cigs)
Squamous
Excluded studies (and stage at which they were excluded)

1 ABELIN ABRAHA AMANDU AMES ANDERS AUSTIN AXELSO BAND BECHER BERRIN BLOHMK BLOT4 BROCKM BROWN1 BYERS1 BYERS2 CARPEN
CASCO2 CASCOR CHAN CHEN3 CHIAZZ CHYOU DESTE2 DOCKER DROSTE DU GARCIA GARDIN GENG GODLEY GOODMA GRAHAM GREGOR
HEGMAN HEIN HENNEK HINDS HIRAOK HOROWI HORWIT HUANG ISHIMA JAHN JAIN JARVHO JIANG KELLER KIHARA KJUUS KO
KOHLME KUBIK LAMWK LAMWK2 LANGE LEI LEMARC LEVIN LIU LOMBA2 LOMBAR MAGNUS MARSH MARSH2 MCDUFF MCLAUG MILLER
MILLS NOTANI NOU ODRISC PAWLEG PERSHA POFFIJ QIAO QIAO2 RADZIK REN RONCO ROOTS ROTHSC SAARIK SANKAR SCHWAR
SEGI SEOW SHIMIZ SIMARA SIMONA SITAS SOBUE2 STASZE STAYNE STUCKE SUN SUZUK2 SUZUKI TANG TAO TOKARS TOUSEY
ULMER VEIERO VUTUC WALD WANG WANG3 WANG4 WICKLU WIGLE WILKIN WU2 WUNSCH WYNDE8 XIANGZ XU XU2 XU4
YONG ZHANG

2 AGUDO AKIBA ALDERS ARCHER ARMADA AUVINE AXELSS BARBON BENHAM BENSHL BEST BLOT1 BLOT2 BLOT3 BOFFET BOUCHA BOUCOT
BRESLO BRETT BROSS BROWN2 BUELL BUFFLE CEDERL CHANG CHATZI CHEN CHEN2 CHOI CHOW COMSTO COOKSO CORREA CPSI
CPSII DAMBER DARBY DAVEYS DEAN DEAN2 DEAN3 DEKLER DESTEF DOLL DORANT DORGAN DORN DOSEME DUNN EBELIN ENGELA
ENSTRO ESAKI FAN GAO GAO2 GARSHI GER GILLIS GOLLED GSELL HAENSZ HAMMO2 HAMMON HANSEN HIRAY2 HIRAYA HITOSU
HOLE HU HU2 HUMBLE JARUP JEDRYC JOLY JONES JUSSAW KAISE2 KAISER KANELL KATSOU KAUFMA KHUDER KINLEN KNEKT
KOO KOULUM KREUZE KREYBE LAMTH LAURIL LAUSSM LETOUR LIAW LICKIN LIDDEL LIU2 LIU3 LIU4 LIU5 LUBIN LUBIN2
LUO MACLEN MARTIS MASTRA MATOS MATSUD MCCONN MIGRAN MOLLO MRFIT MRFITR MURATA MZILEN NAM NOTAN2 ORMOS OSANN
OSANN2 PARKIN PASTOR PERNU PERSH2 PETO PEZZO2 PEZZOT PIKE PISANI POLEDN PRESCO RACHTA RANDIG RESTRE RIMING SADOWS
SCHWA2 SEGI2 SHAW SIEMIA SOBUE SPEIZE SPITZ STOCKS STOCKW SVENSS TANG2 TENKAN TIZZAN TSUGAN TULINI TVERDA VANDER
WAKAI WANG2 WARSIN WATSON WU WUWILL WYNDE2 WYNDE3 WYNDE4 WYNDE5 WYNDE6 WYNDER XU3 YAMAGU YUAN ZHENG ZHOU

7 DOLL2 WYNDE7

Table 2G32 -

IESLC - Meta-analysis of Ever Smoking (or Current if ever not available) by Amount, Overview, Pipe only
Squamous

This analysis is restricted to results for:
1) Results by Amount smoked
Results by Amount smoked are grouped under 2 schemes (S1, S2). Each scheme has a set of "key values". An interval is allocated to the category whose key value it includes and intervals which include none or more than one of the key values are excluded. (Open-ended intervals are coded as 99). Amounts are usually coded as number of cigarette equivalents, defined as shown at the end of Sections -1 and -4.

S1	key value	maximum range	S2	key value	maximum range
1	1	1-98	1	1	1-9
2	99	2-99	2	10	2-98
			3	99	11+

Thus the two levels in scheme S1 correspond to the lowest and highest intervals, irrespective of their values. In scheme C2, the lowest and highest intervals are chosen provided they do not include the value 10, and the interval including the value 10 is also chosen.
2) Smokers of pipe only
3) Results complete enough for use in metaanalysis

Within each study, results are then selected (in the following order of preference, within each sex) for:
4) SMKSTA: ever smokers, current smokers
5) DENOM: never smoked anything, (never +1 = +long term ex)
6) Followup period (prospective studies): whole study (coded as 0) or longest available
7) LCtype: all or nearest available, at least Squamous and Adeno. (q = squamous, s = small,
l = large, a = adeno, mix = mixed, alv = alveolar)
8) Race: all or nearest available, otherwise by race (wh or w = white, bl or b = black, hi = hispanic
ch = chinese, jap = japanese, haw = hawaiian, w+o = white + oriental, sca = scandinavian, as = asian)
9) For overlapping studies: principal rather than subsidiary studies
Finally by Age: whole study (coded as 0) if available, otherwise by widest available age group
and then for single sex results (m, f) in preference to combined sex results (c).

Results adjusted (AD) for the most potential confounders are then chosen in Sections -1 to -3
and results adjusted for the least confounders in Sections -4 to -6. (Those least adjusted results which
actually differ from the most adjusted as marked 'x' in column X in Section -4)
(Results adjusted for an unknown number of confounder(s) are coded as 20.)

Section -7 shows excluded studies, together with the stage (as above) at which no qualifying
results were found.

Section -8 lists the potentially overlapping studies which have been included (1=principal, 2=subsidiary).

Section -9 lists any results which would have been included in preference except that they had data not complete
enough for use in meta-analysis, with their significance (yes/no), if known, and any further comment as entered
on the database.

In addition to those mentioned above, the following fields, levels and abbreviations are used:
* or nk = not known, n = no, y = yes, ot = other
ev = ever, cu = current, nev = never
exL, exH = range of exposure (low and high) in the smoking group, in terms of Amount smoked,
cigarette equivalents
REF: 6-character study reference
NRR: number of the RR on the database within the study
ST : study type (CC = case control, pr or prosp = prospective)
NLC: number of lung cancer cases in whole study
R : risky occupational population (n = no, m = mining, o = other risky)
VB : national cigarette type (V = at least 75% Virginia, bl = at least 75% blended, ot = other)
P : any proxy use
H : full histological confirmation
De : derivation of RR/CI (or = original, st = standard method, ot = other method of estimation)

Table 2G32 - 0

No RRs selected for this analysis

Table 2G32 - 7

IESLC - Meta-analysis of Ever Smoking (or Current if ever not available) by Amount, Overview, Pipe only
Squamous
Excluded studies (and stage at which they were excluded)

1 ABELIN ABRAHA AMANDU AMES ANDERS AUSTIN AXELSO BAND BECHER BERRIN BLOHMK BLOT4 BROCKM BROWN1 BYERS1 BYERS2 CARPEN CASCO2 CASCOR CHAN CHEN3 CHIAZZ CHYOU DESTE2 DOCKER DROSTE DU GARCIA GARDIN GENG GODLEY GOODMA GRAHAM GREGOR HEGMAN HEIN HENNEK HINDS HIRAOK HOROWI HORWIT HUANG ISHIMA JAHN JAIN JARVHO JIANG KELLER KIHARA KJUUS KO KOHLME KUBIK LAMWK LAMWK2 LANGE LEI LEMARC LEVIN LIU LOMBA2 LOMBAR MAGNUS MARSH MARSH2 MCDUFF MCLAUG MILLER MILLS NOTANI NOU ODRISC PAWLEG PERSHA POFFIJ QIAO QIAO2 RADZIK REN RONCO ROOTS ROTHSC SAARIK SANKAR SCHWAR SEGI SEOW SHIMIZ SIMARA SIMONA SITAS SOBUE2 STASZE STAYNE STUCKE SUN SUZUK2 SUZUKI TANG TAO TOKARS TOUSEY ULMER VEIERO VUTUC WALD WANG WANG3 WANG4 WICKLU WIGLE WILKIN WU2 WUNSCH WYNDE8 XIANGZ XU XU2 XU4 YONG ZHANG

2 AGUDO AKIBA ALDERS ARCHER ARMADA AUVINE AXELSS BARBON BENHAM BENSHL BLOT1 BLOT2 BLOT3 BOUCHA BOUCOT BRESLO BRETT BROSS BROWN2 BUELL BUFFLE CHANG CHATZI CHEN CHEN2 CHOI CHOW COMSTO COOKSO CORREA CPSI CPSII DARBY DAVEYS DEAN DEAN2 DEKLER DESTEF DOLL2 DORANT DORGAN DOSEME DUNN EBELIN ENGELA ENSTRO ESAKI FAN GAO GAO2 GARSHI GER GILLIS GOLLED GSELL HAENSZ HAMMO2 HAMMON HANSEN HIRAYA HITOSU HOLE HU HU2 HUMBLE JARUP JEDRYC JOLY JONES JUSSAW KAISE2 KAISER KANELL KATSOU KAUFMA KHUDER KINLEN KNEKT KOO KOULUM KREUZE KREYBE LAMTH LAURIL LAUSSM LETOUR LIAW LICKIN LIDDEL LIU2 LIU3 LIU4 LIU5 LUBIN LUO MACLEN MARTIS MASTRA MATOS MATSUD MCCONN MIGRAN MOLLO MRFIT MRFITR MURATA MZILEN NAM NOTAN2 ORMOS OSANN OSANN2 PARKIN PASTOR PERNU PERSH2 PETO PEZZO2 PEZZOT PIKE PISANI POLEDN PRESCO RACHTA RANDIG RESTRE RIMING SCHWA2 SEGI2 SHAW SIEMIA SOBUE SPEIZE SPITZ STOCKS STOCKW SVENSS TANG2 TENKAN TIZZAN TSUGAN TULINI TVERDA VANDER WAKAI WANG2 WARSIN WATSON WU WUWILL WYNDE2 WYNDE3 WYNDE4 WYNDE5 WYNDE6 WYNDER XU3 YAMAGU YUAN ZHENG ZHOU

3 SADOWS

5 HIRAY2

7 BEST BOFFET CEDERL DAMBER DEAN3 DOLL DORN LUBIN2 WYNDE7

Table 2G34 -

IESLC - Meta-analysis of Ever Smoking (or Current if Ever not available) by Amount, Overview, Cigars only
Squamous

This analysis is restricted to results for:
1) Results by Amount smoked
Results by Amount smoked are grouped under 2 schemes (S1, S2). Each scheme has a set of "key values". An interval is allocated to the category whose key value it includes and intervals which include none or more than one of the key values are excluded. (Open-ended intervals are coded as 99). Amounts are usually coded as number of cigarette equivalents, defined as shown at the end of Sections -1 and -4; if originally given as numbers of cigars, we use our own assumption (OA) of 1 cigar = 5 cigarettes.

S1	key value	maximum range	S2	key value	maximum range
1	1	1-98	1	1	1-9
2	99	2-99	2	10	2-98
			3	99	11+

Thus the two levels in scheme S1 correspond to the lowest and highest intervals, irrespective of their values. In scheme C2, the lowest and highest intervals are chosen provided they do not include the value 10, and the interval including the value 10 is also chosen.
2) Smokers of cigars only
3) Results complete enough for use in metaanalysis

Within each study, results are then selected (in the following order of preference, within each sex) for:
4) SMKSTA: ever smokers, current smokers
5) DENOM: never smoked anything, (never +1 = +long term ex)
6) Followup period (prospective studies): whole study (coded as 0) or longest available
7) LCtype: all or nearest available, at least Squamous and Adeno. (q = squamous, s = small, l = large, a = adeno, mix = mixed, alv = alveolar)
8) Race: all or nearest available, otherwise by race (wh or w = white, bl or b = black, hi = hispanic ch = chinese, jap = japanese, haw = hawaiian, w+o = white + oriental, sca = scandinavian, as = asian)
9) For overlapping studies: principal rather than subsidiary studies
Finally by Age: whole study (coded as 0) if available, otherwise by widest available age group and then for single sex results (m, f) in preference to combined sex results (c).

Results adjusted (AD) for the most potential confounders are then chosen in Sections -1 to -3 and results adjusted for the least confounders in Sections -4 to -6. (Those least adjusted results which actually differ from the most adjusted as marked 'x' in column X in Section -4)
(Results adjusted for an unknown number of confounder(s) are coded as 20.)

Section -7 shows excluded studies, together with the stage (as above) at which no qualifying results were found.

Section -8 lists the potentially overlapping studies which have been included (1=principal, 2=subsidiary).

Section -9 lists any results which would have been included in preference except that they had data not complete enough for use in meta-analysis, with their significance (yes/no), if known, and any further comment as entered on the database.

In addition to those mentioned above, the following fields, levels and abbreviations are used:
* or nk = not known, n = no, y = yes, ot = other
ev = ever, cu = current, nev = never
exL, exH = range of exposure (low and high) in the smoking group, in terms of Amount smoked, cigarette equivalents
REF: 6-character study reference
NRR: number of the RR on the database within the study
ST : study type (CC = case control, pr or prosp = prospective)
NLC: number of lung cancer cases in whole study
R : risky occupational population (n = no, m = mining, o = other risky)
VB : national cigarette type (V = at least 75% Virginia, bl = at least 75% blended, ot = other)
P : any proxy use
H : full histological confirmation
De : derivation of RR/CI (or = original, st = standard method, ot = other method of estimation)

Table 2G34 - 0

No RRs selected for this analysis

Table 2G34 - 7

IESLC - Meta-analysis of Ever Smoking (or Current if Ever not available) by Amount, Overview, Cigars only
Squamous
Excluded studies (and stage at which they were excluded)

1	ABELIN	ABRAHA	AMANDU	AMES	ANDERS	AUSTIN	AXELSO	BAND	BECHER	BERRIN	BLOHMK	BLOT4	BROCKM	BROWN1	BYERS1	BYERS2	CARPEN
	CASCO2	CASCOR	CHAN	CHEN3	CHIAZZ	CHYOU	DESTE2	DOCKER	DROSTE	DU	GARCIA	GARDIN	GENG	GODLEY	GOODMA	GRAHAM	GREGOR
	HEGMAN	HEIN	HENNEK	HINDS	HIRAOK	HOROWI	HORWIT	HUANG	ISHIMA	JAHN	JAIN	JARVHO	JIANG	KELLER	KIHARA	KJUUS	KO
	KOHLME	KUBIK	LAMWK	LAMWK2	LANGE	LEI	LEMARC	LEVIN	LIU	LOMBA2	LOMBAR	MAGNUS	MARSH	MARSH2	MCDUFF	MCLAUG	MILLER
	MILLS	NOTANI	NOU	ODRISC	PAWLEG	PERSHA	POFFIJ	QIAO	QIAO2	RADZIK	REN	RONCO	ROOTS	ROTHSC	SAARIK	SANKAR	SCHWAR
	SEGI	SEOW	SHIMIZ	SIMARA	SIMONA	SITAS	SOBUE2	STASZE	STAYNE	STUCKE	SUN	SUZUK2	SUZUKI	TANG	TAO	TOKARS	TOUSEY
	ULMER	VEIERO	VUTUC	WALD	WANG	WANG3	WANG4	WICKLU	WIGLE	WILKIN	WU2	WUNSCH	WYNDE8	XIANGZ	XU	XU2	XU4
	YONG	ZHANG															
2	AGUDO	AKIBA	ALDERS	ARCHER	ARMADA	AUVINE	AXELSS	BARBON	BENHAM	BENSHL	BEST	BLOT1	BLOT2	BLOT3	BOUCHA	BOUCOT	BRESLO
	BRETT	BROSS	BROWN2	BUELL	BUFFLE	CEDERL	CHANG	CHATZI	CHEN	CHEN2	CHOI	CHOW	COMSTO	COOKSO	CORREA	CPSII	DAMBER
	DARBY	DAVEYS	DEAN	DEAN2	DEAN3	DEKLER	DESTEF	DOLL	DOLL2	DORANT	DORGAN	DOSEME	DUNN	EBELIN	ENGELA	ENSTRO	ESAKI
	FAN	GAO	GAO2	GARSHI	GER	GILLIS	GOLLED	GSELL	HAENSZ	HAMMO2	HAMMON	HANSEN	HIRAY2	HIRAYA	HITOSU	HOLE	HU
	HU2	HUMBLE	JARUP	JEDRYC	JONES	JUSSAW	KAISE2	KAISER	KANELL	KATSOU	KAUFMA	KHUDER	KINLEN	KNEKT	KOO	KOULUM	KREUZE
	KREYBE	LAMTH	LAURIL	LAUSSM	LETOUR	LIAW	LICKIN	LIDDEL	LIU2	LIU3	LIU4	LIU5	LUBIN	LUO	MACLEN	MARTIS	MASTRA
	MATOS	MATSUD	MCCONN	MIGRAN	MOLLO	MRFIT	MRFITR	MURATA	MZILEN	NAM	NOTAN2	ORMOS	OSANN	OSANN2	PARKIN	PASTOR	PERNU
	PERSH2	PETO	PEZZO2	PEZZOT	PIKE	PISANI	POLEDN	PRESCO	RACHTA	RANDIG	RESTRE	RIMING	SCHWA2	SEGI2	SHAW	SIEMIA	SOBUE
	SPEIZE	SPITZ	STOCKS	STOCKW	SVENSS	TANG2	TENKAN	TIZZAN	TSUGAN	TULINI	TVERDA	VANDER	WAKAI	WANG2	WARSIN	WATSON	WU
	WUWILL	WYNDE2	WYNDE3	WYNDE4	WYNDE5	WYNDE6	WYNDER	XU3	YAMAGU	YUAN	ZHENG	ZHOU					
3	SADOWS																
7	BOFFET	CPSI	DORN	JOLY	LUBIN2	WYNDE7											

Table 2G36 -

IESLC - Meta-analysis of Ever Smoking (or Current if Ever not available) by Amount, Overview, Mixed smokers
Squamous

This analysis is restricted to results for:
1) Results by Amount smoked
Results by Amount smoked are grouped under 2 schemes (S1, S2). Each scheme has a set of "key values". An interval is allocated to the category whose key value it includes and intervals which include none or more than one of the key values are excluded. (Open-ended intervals are coded as 99). Amounts are usually coded as number of cigarette equivalents, defined as shown at the end of Sections -1 and -4; if originally given as numbers of cigars, we use our own assumption (OA) of 1 cigar = 5 cigarettes.

S1	key value	maximum range	S2	key value	maximum range
1	1	1-98	1	1	1-9
2	99	2-99	2	10	2-98
			3	99	11+

Thus the two levels in scheme S1 correspond to the lowest and highest intervals, irrespective of their values. In scheme C2, the lowest and highest intervals are chosen provided they do not include the value 10, and the interval including the value 10 is also chosen.
2) Mixed smokers (cigarettes and pipe/cigar)
3) Results complete enough for use in metaanalysis

Within each study, results are then selected (in the following order of preference, within each sex) for:
4) SMKSTA: ever smokers, current smokers
5) DENOM: never smoked anything, (never +1 = +long term ex)
6) Followup period (prospective studies): whole study (coded as 0) or longest available
7) LCtype: all or nearest available, at least Squamous and Adeno. (q = squamous, s = small, l = large, a = adeno, mix = mixed, alv = alveolar)
8) Race: all or nearest available, otherwise by race (wh or w = white, bl or b = black, hi = hispanic ch = chinese, jap = japanese, haw = hawaiian, w+o = white + oriental, sca = scandinavian, as = asian)
9) For overlapping studies: principal rather than subsidiary studies
Finally by Age: whole study (coded as 0) if available, otherwise by widest available age group and then for single sex results (m, f) in preference to combined sex results (c).

Results adjusted (AD) for the most potential confounders are then chosen in Sections -1 to -3 and results adjusted for the least confounders in Sections -4 to -6. (Those least adjusted results which actually differ from the most adjusted as marked 'x' in column X in Section -4)
(Results adjusted for an unknown number of confounder(s) are coded as 20.)

Section -7 shows excluded studies, together with the stage (as above) at which no qualifying results were found.

Section -8 lists the potentially overlapping studies which have been included (1=principal, 2=subsidiary).

Section -9 lists any results which would have been included in preference except that they had data not complete enough for use in meta-analysis, with their significance (yes/no), if known, and any further comment as entered on the database.

In addition to those mentioned above, the following fields, levels and abbreviations are used:
* or nk = not known, n = no, y = yes, ot = other
ev = ever, cu = current, nev = never
exL, exH = range of exposure (low and high) in the smoking group, in terms of Amount smoked, cigarette equivalents
REF: 6-character study reference
NRR: number of the RR on the database within the study
ST : study type (CC = case control, pr or prosp = prospective)
NLC: number of lung cancer cases in whole study
R : risky occupational population (n = no, m = mining, o = other risky)
VB : national cigarette type (V = at least 75% Virginia, bl = at least 75% blended, ot = other)
P : any proxy use
H : full histological confirmation
De : derivation of RR/CI (or = original, st = standard method, ot = other method of estimation)

Table 2G36 - 0

No RRs selected for this analysis

Table 2G36 - 7

IESLC - Meta-analysis of Ever Smoking (or Current if Ever not available) by Amount, Overview, Mixed smokers
Squamous
Excluded studies (and stage at which they were excluded)

1 ABELIN ABRAHA AMANDU AMES ANDERS AUSTIN AXELSO BAND BECHER BERRIN BLOHMK BLOT4 BROCKM BROWN1 BYERS1 BYERS2 CARPEN
CASCO2 CASCOR CHAN CHEN3 CHIAZZ CHYOU DESTE2 DOCKER DROSTE DU GARCIA GARDIN GENG GODLEY GOODMA GRAHAM GREGOR
HEGMAN HEIN HENNEK HINDS HIRAOK HOROWI HORWIT HUANG ISHIMA JAHN JAIN JARVHO JIANG KELLER KIHARA KJUUS KO
KOHLME KUBIK LAMWK LAMWK2 LANGE LEI LEMARC LEVIN LIU LOMBA2 LOMBAR MAGNUS MARSH MARSH2 MCDUFF MCLAUG MILLER
MILLS NOTANI NOU ODRISC PAWLEG PERSHA POFFIJ QIAO QIAO2 RADZIK REN RONCO ROOTS ROTHSC SAARIK SANKAR SCHWAR
SEGI SEOW SHIMIZ SIMARA SIMONA SITAS SOBUE2 STASZE STAYNE STUCKE SUN SUZUK2 SUZUKI TANG TAO TOKARS TOUSEY
ULMER VEIERO VUTUC WALD WANG WANG3 WANG4 WICKLU WIGLE WILKIN WU2 WUNSCH WYNDE8 XIANGZ XU XU2 XU4
YONG ZHANG

2 AGUDO AKIBA ALDERS ARCHER ARMADA AUVINE AXELSS BARBON BENHAM BENSHL BEST BLOT1 BLOT2 BLOT3 BOFFET BOUCHA BRESLO
BRETT BROSS BROWN2 BUELL BUFFLE CEDERL CHANG CHATZI CHEN CHEN2 CHOI CHOW COMSTO COOKSO CORREA CPSI CPSII
DAMBER DARBY DAVEYS DEAN DEAN2 DEAN3 DEKLER DESTEF DOLL DOLL2 DORANT DORGAN DOSEME DUNN EBELIN ENGELA ENSTRO
ESAKI FAN GAO GAO2 GARSHI GER GILLIS GOLLED GSELL HAENSZ HAMMO2 HAMMON HANSEN HIRAY2 HIRAYA HITOSU HOLE
HU HU2 HUMBLE JARUP JEDRYC JOLY JONES JUSSAW KAISE2 KAISER KANELL KATSOU KAUFMA KHUDER KINLEN KNEKT KOO
KOULUM KREUZE KREYBE LAMTH LAURIL LAUSSM LETOUR LIAW LICKIN LIDDEL LIU2 LIU3 LIU4 LIU5 LUBIN LUBIN2 LUO
MACLEN MARTIS MASTRA MATOS MATSUD MCCONN MIGRAN MOLLO MRFIT MRFITR MURATA MZILEN NAM NOTAN2 ORMOS OSANN OSANN2
PARKIN PASTOR PERNU PERSH2 PETO PEZZO2 PEZZOT PIKE PISANI POLEDN PRESCO RACHTA RANDIG RESTRE RIMING SADOWS SCHWA2
SEGI2 SHAW SIEMIA SOBUE SPEIZE SPITZ STOCKS STOCKW SVENSS TANG2 TENKAN TIZZAN TSUGAN TULINI TVERDA VANDER WAKAI
WANG2 WARSIN WATSON WU WUWILL WYNDE2 WYNDE3 WYNDE4 WYNDE5 WYNDE6 WYNDE7 WYNDER XU3 YAMAGU YUAN ZHENG ZHOU

7 BOUCOT DORN
