# Supplementary material for: Systematic review with meta-analysis of the epidemiological evidence in the 1900s relating smoking to lung cancer
Source: BMC Cancer. 2012 Sep 3;12:385. doi: 10.1186/1471-2407-12-385 (PMC3505152; doi:10.1186/1471-2407-12-385)
Supplement: Additional file 5 — Detailed Analysis Tables (Individual file names as described in Additional file 1: Methods, Table1). [file 1471-2407-12-385-S5.zip › PDF/2I.pdf]

Table 2I1 -

IESLC - Meta-analysis of Ever Smoking by Duration, Overview
Squamous, Any Product (or Cigarettes if Any not available)

This analysis is restricted to results for:
1) Ever smokers
2) Results by Duration
3) Categorical results by Duration
Results by Duration are grouped under 2 schemes (S1, S2). Each scheme has a set of "key values". An interval is allocated to the category whose key value it includes, and intervals which include none or more than one of the key values are excluded. (Open-ended intervals are coded as 999)

S1	key value	maximum range
1	20	1-34
2	35	21-49
3	50	36+

S2	key value	maximum range
1	5	1-19
2	20	6-29
3	30	21-39
4	40	31-49
5	50	41-998
6	999	51+

4) Squamous (or near equivalent)
5) Results complete enough for use in metaanalysis

Within each study, results are then selected (in the following order of preference, within each sex) for:
6) (not applicable)
7) PRODUCT: all/unspec, cigarettes regardless of other products, cigarettes only
8) CIGTYPE: all/unspecified, MC regardless of HR, MC only
9) (not applicable)
10) DENOM: never smoked anything, never smoked cigarettes, never any + low, never cigs + low
11) Followup period (YF, prospective studies): whole study (coded as 0) or longest available
12) LCtype: squamous or nearest available, but not adeno. (q = squamous, s = small,
a = adeno, KI = Kreyberg I, u = undifferentiated)
13) Race: all or nearest available, otherwise by race (wh or w = white, bl or b = black, hi = hispanic
ch = chinese, jap = japanese, haw = hawaiian, w+o = white + oriental, sca = scandinavian, as = asian)
14) For overlapping studies: principal rather than subsidiary studies
Finally by Age: whole study (coded as 0) if available, otherwise by widest available age group
and then for single sex results (m, f) in preference to results for both sexes combined (c).

Results adjusted (AD) for the most potential confounders are then chosen in Sections -1 to -3
and results adjusted for the least confounders in Sections -4 to -6. (Those least adjusted results which actually differ from the most adjusted are marked 'x' in column X in Section -4)

Section -7 shows excluded studies, together with the stage (as above) at which no qualifying results were found.

Section -8 lists the potentially overlapping studies which have been included (1=principal, 2=subsidiary).

Section -9 lists any results which would have been included in preference except that they had data not complete enough for use in meta-analysis, with their significance (yes/no), if known, and any further comment as entered on the database. It also lists as "gap" any categories for which no data were presented by the original authors.

In addition to those mentioned above, the following fields, levels and abbreviations are used:
* or nk = not known, n = no, y = yes, ot = other
nev = never
all/unspec = all or unspecified, cig+/-ot = cigarettes irrespective of other products (cigar, pipe etc)
MC = manufactured cigarettes, HR = hand-rolled cigarettes
exL, exH = range of exposure (low and high) in the smoking group, in terms of Duration
REF: 6-character study reference
NRR: number of the RR on the database within the study
ST : study type (CC = case control, pr or prosp = prospective)
NLC: number of lung cancer cases in whole study
R : risky occupational population (n = no, m = mining, o = other risky)
VB : national cigarette type (V = at least 75% Virginia, bl = at least 75% blended, ot = other)
P : any proxy use
H : full histological confirmation
De : derivation of RR/CI (or = original, st = standard method, ot = other method of estimation)

Table 2I1 - 1

IESLC - Meta-analysis of Ever Smoking by Duration, Overview
Squamous, Any Product (or Cigarettes if Any not available)
Most adjusted

REF	NRR	SEX	AGEL	AGEH	RACE	YF	LC TYPE	LOC	START	ST	NLC	R	VB	P	H	AD	PRODUCT	exL	exH	S1	S2	DENOM	De
BARBON	562	m	0	0	all	-	q	Eu:wst	1979	CC	755	n	bl	y	y	1	all/unsp	1	29	1	0	nev any	or
BARBON	563	m	0	0	all	-	q	Eu:wst	1979	CC	755	n	bl	y	y	1	all/unsp	30	39	2	3	nev any	or
BARBON	564	m	0	0	all	-	q	Eu:wst	1979	CC	755	n	bl	y	y	1	all/unsp	40	49	0	4	nev any	or
BARBON	565	m	0	0	all	-	q	Eu:wst	1979	CC	755	n	bl	y	y	1	all/unsp	50	999	3	0	nev any	or
BUFFLE	505	m	0	0	wh	-	q	NAmer	1976	CC	943	n	bl	y	n	0	cig+/-ot	1	33	1	0	nev cigs	or
BUFFLE	506	m	0	0	wh	-	q	NAmer	1976	CC	943	n	bl	y	n	0	cig+/-ot	34	43	2	4	nev cigs	or
BUFFLE	507	m	0	0	wh	-	q	NAmer	1976	CC	943	n	bl	y	n	0	cig+/-ot	44	49	0	0	nev cigs	or
BUFFLE	508	m	0	0	wh	-	q	NAmer	1976	CC	943	n	bl	y	n	0	cig+/-ot	50	999	3	0	nev cigs	or
CHOI	559	m	0	0	all	-	q	As:oth	1985	CC	375	n	bl	n	n	0	cig+/-ot	1	29	1	0	nev cigs	st
CHOI	560	m	0	0	all	-	q	As:oth	1985	CC	375	n	bl	n	n	0	cig+/-ot	30	39	2	3	nev cigs	st
CHOI	561	m	0	0	all	-	q	As:oth	1985	CC	375	n	bl	n	n	0	cig+/-ot	40	49	0	4	nev cigs	st
CHOI	562	m	0	0	all	-	q	As:oth	1985	CC	375	n	bl	n	n	0	cig+/-ot	50	999	3	0	nev cigs	st
CHOI	573	f	0	0	all	-	q	As:oth	1985	CC	375	n	bl	n	n	0	cig+/-ot	1	29	1	0	nev cigs	st
CHOI	574	f	0	0	all	-	q	As:oth	1985	CC	375	n	bl	n	n	0	cig+/-ot	30	39	2	3	nev cigs	st
CHOI	575	f	0	0	all	-	q	As:oth	1985	CC	375	n	bl	n	n	0	cig+/-ot	40	999	3	0	nev cigs	st
DAMBER	547	m	0	0	all	-	q	Eu:Sca	1972	CC	579	n	bl	y	n	1	all/unsp	1	30	1	0	nev any	or
DAMBER	548	m	0	0	all	-	q	Eu:Sca	1972	CC	579	n	bl	y	n	1	all/unsp	31	40	2	4	nev any	or
DAMBER	549	m	0	0	all	-	q	Eu:Sca	1972	CC	579	n	bl	y	n	1	all/unsp	41	50	3	5	nev any	or
DAMBER	550	m	0	0	all	-	q	Eu:Sca	1972	CC	579	n	bl	y	n	1	all/unsp	51	999	0	6	nev any	or
DORGAN	572	m	0	0	wh	-	q	NAmer	1980	CC	2026	n	bl	y	y	2	cig+/-ot	1	34	1	0	nev any	ot
DORGAN	573	m	0	0	wh	-	q	NAmer	1980	CC	2026	n	bl	y	y	2	cig+/-ot	35	999	0	0	nev any	ot
DORGAN	564	f	0	0	all	-	q	NAmer	1980	CC	2026	n	bl	y	y	3	cig+/-ot	1	34	1	0	nev any	ot
DORGAN	565	f	0	0	all	-	q	NAmer	1980	CC	2026	n	bl	y	y	3	cig+/-ot	35	999	0	0	nev any	ot
DOSEME	511	m	0	0	all	-	q	Eu:bal	1979	CC	1210	n	bl	n	n	2	cig+/-ot	1	10	0	1	nev cigs	or
DOSEME	512	m	0	0	all	-	q	Eu:bal	1979	CC	1210	n	bl	n	n	2	cig+/-ot	11	20	1	2	nev cigs	or
DOSEME	513	m	0	0	all	-	q	Eu:bal	1979	CC	1210	n	bl	n	n	2	cig+/-ot	21	999	0	0	nev cigs	or
GER	510	c	0	0	all	-	q+s	As:oth	1990	CC	141	n	ot	y	n	5	all/unsp	1	30	1	0	nev any	ot
GER	511	c	0	0	all	-	q+s	As:oth	1990	CC	141	n	ot	y	n	5	all/unsp	31	999	0	0	nev any	ot
HAENSZ	517	f	0	0	all	-	q+u	NAmer	1955	CC	158	n	bl	n	y	0	cig+/-ot	1	14	0	1	nev any	st
HAENSZ	518	f	0	0	all	-	q+u	NAmer	1955	CC	158	n	bl	n	y	0	cig+/-ot	15	999	0	0	nev any	st
JEDRYC	585	m	0	0	all	-	q	Eu:est	1980	CC	1630	n	bl	y	n	3	cig+/-ot	1	19	0	1	nev any	or
JEDRYC	586	m	0	0	all	-	q	Eu:est	1980	CC	1630	n	bl	y	n	3	cig+/-ot	20	39	0	0	nev any	or
JEDRYC	587	m	0	0	all	-	q	Eu:est	1980	CC	1630	n	bl	y	n	3	cig+/-ot	40	999	3	0	nev any	or
JOLY	639	m	0	0	all	-	q	SCAmer	1978	CC	826	n	bl	n	n	0	cig+/-ot	1	29	1	0	nev any	st
JOLY	640	m	0	0	all	-	q	SCAmer	1978	CC	826	n	bl	n	n	0	cig+/-ot	30	39	2	3	nev any	st
JOLY	641	m	0	0	all	-	q	SCAmer	1978	CC	826	n	bl	n	n	0	cig+/-ot	40	49	0	4	nev any	st
JOLY	642	m	0	0	all	-	q	SCAmer	1978	CC	826	n	bl	n	n	0	cig+/-ot	50	999	3	0	nev any	st
JOLY	611	f	0	0	all	-	q	SCAmer	1978	CC	826	n	bl	n	n	0	cig+/-ot	1	29	1	0	nev any	st
JOLY	612	f	0	0	all	-	q	SCAmer	1978	CC	826	n	bl	n	n	0	cig+/-ot	30	39	2	3	nev any	st
JOLY	613	f	0	0	all	-	q	SCAmer	1978	CC	826	n	bl	n	n	0	cig+/-ot	40	49	0	4	nev any	st
JOLY	614	f	0	0	all	-	q	SCAmer	1978	CC	826	n	bl	n	n	0	cig+/-ot	50	999	3	0	nev any	st
LUBIN2	661	m	0	0	all	-	q	Eu:mul	1976	CC	7804	n	bl	n	y	0	cig+/-ot	1	29	1	0	nev any	st
LUBIN2	662	m	0	0	all	-	q	Eu:mul	1976	CC	7804	n	bl	n	y	0	cig+/-ot	30	39	2	3	nev any	st
LUBIN2	663	m	0	0	all	-	q	Eu:mul	1976	CC	7804	n	bl	n	y	0	cig+/-ot	40	49	0	4	nev any	st
LUBIN2	664	m	0	0	all	-	q	Eu:mul	1976	CC	7804	n	bl	n	y	0	cig+/-ot	50	999	3	0	nev any	st
LUBIN2	713	f	0	0	all	-	q	Eu:mul	1976	CC	7804	n	bl	n	y	0	cig+/-ot	1	29	1	0	nev any	st
LUBIN2	714	f	0	0	all	-	q	Eu:mul	1976	CC	7804	n	bl	n	y	0	cig+/-ot	30	39	2	3	nev any	st
LUBIN2	715	f	0	0	all	-	q	Eu:mul	1976	CC	7804	n	bl	n	y	0	cig+/-ot	40	49	0	4	nev any	st
LUBIN2	716	f	0	0	all	-	q	Eu:mul	1976	CC	7804	n	bl	n	y	0	cig+/-ot	50	999	3	0	nev any	st
LUO	504	c	0	0	all	-	q	As:Chi	1990	CC	102	n	ot	n	y	20	cig+/-ot	1	29	1	0	nev cigs	or
LUO	505	c	0	0	all	-	q	As:Chi	1990	CC	102	n	ot	n	y	20	cig+/-ot	30	999	0	0	nev cigs	or
MATOS	606	m	0	0	all	-	q	SCAmer	1994	CC	200	n	bl	n	n	2	cig+/-ot	1	24	1	0	nev any	or
MATOS	607	m	0	0	all	-	q	SCAmer	1994	CC	200	n	bl	n	n	2	cig+/-ot	25	39	2	3	nev any	or
MATOS	608	m	0	0	all	-	q	SCAmer	1994	CC	200	n	bl	n	n	2	cig+/-ot	40	70	3	0	nev any	or
OSANN2	510	f	0	0	all	-	KI	NAmer	1964	ot	217	n	bl	n	y	1	cig+/-ot	1	20	1	0	nev cigs	or
OSANN2	511	f	0	0	all	-	KI	NAmer	1964	ot	217	n	bl	n	y	1	cig+/-ot	21	999	0	0	nev cigs	or
PEZZOT	507	m	0	0	all	-	q	SCAmer	1987	CC	215	n	bl	n	y	0	cig only	1	30	1	0	nev cigs	ot
PEZZOT	508	m	0	0	all	-	q	SCAmer	1987	CC	215	n	bl	n	y	0	cig only	31	40	2	4	nev cigs	ot
PEZZOT	509	m	0	0	all	-	q	SCAmer	1987	CC	215	n	bl	n	y	0	cig only	41	999	3	0	nev cigs	ot
WUWILL	521	f	0	0	all	-	q	As:Chi	1985	CC	965	n	ot	n	n	3	cig+/-ot	1	29	1	0	nev cigs	ot
WUWILL	522	f	0	0	all	-	q	As:Chi	1985	CC	965	n	ot	n	n	3	cig+/-ot	30	39	2	3	nev cigs	ot
WUWILL	523	f	0	0	all	-	q	As:Chi	1985	CC	965	n	ot	n	n	3	cig+/-ot	40	999	3	0	nev cigs	ot
WYNDE2	506	m	0	0	all	-	KI	NAmer	1962	CC	404	n	bl	n	y	0	cig+/-ot	1	29	1	0	nev any	ot
WYNDE2	507	m	0	0	all	-	KI	NAmer	1962	CC	404	n	bl	n	y	0	cig+/-ot	30	40	2	0	nev any	ot
WYNDE2	508	m	0	0	all	-	KI	NAmer	1962	CC	404	n	bl	n	y	0	cig+/-ot	41	999	3	0	nev any	ot
ZHENG	501	m	0	0	all	-	q	As:Chi	1982	CC	540	n	ot	*	y	0	cig+/-ot	1	29	1	0	nev cigs	st
ZHENG	502	m	0	0	all	-	q	As:Chi	1982	CC	540	n	ot	*	y	0	cig+/-ot	30	39	2	3	nev cigs	st
ZHENG	503	m	0	0	all	-	q	As:Chi	1982	CC	540	n	ot	*	y	0	cig+/-ot	40	999	3	0	nev cigs	st
ZHENG	508	f	0	0	all	-	q	As:Chi	1982	CC	540	n	ot	*	y	0	cig+/-ot	1	29	1	0	nev cigs	st
ZHENG	509	f	0	0	all	-	q	As:Chi	1982	CC	540	n	ot	*	y	0	cig+/-ot	30	999	0	0	nev cigs	st
ZHOU	504	c	0	0	all	-	q	As:Chi	1978	CC	1360	n	ot	n	n	0	all/unsp	1	19	0	1	nev any	st
ZHOU	505	c	0	0	all	-	q	As:Chi	1978	CC	1360	n	ot	n	n	0	all/unsp	20	999	0	0	nev any	st

Table 2I1 - 1

IESLC - Meta-analysis of Ever Smoking by Duration, Overview
Squamous, Any Product (or Cigarettes if Any not available)
Most adjusted

Cigarette type is all/unspec for all RRs

In this overview table, subtotals and Qs values may be invalid and should be ignored

Table 2I1 - 2

IESLC - Meta-analysis of Ever Smoking by Duration, Overview
Squamous, Any Product (or Cigarettes if Any not available)
Most adjusted

REF	NRR	SEX	AD	Number Exposed Case	Cont	Non-exposed Case	Cont	RR	95.00%CI
BARBON	562	m	1	7	-	6	-	2.10	(0.70- 6.50)
BARBON	563	m	1	36	-	6	-	9.60	(3.90- 23.90)
BARBON	564	m	1	69	-	6	-	14.60	(6.10- 34.60)
BARBON	565	m	1	149	-	6	-	21.20	(9.10- 49.30)
Subtotal BARBON								10.54	(6.66- 16.67)
BUFFLE	505	m	0	-	-	-	-	9.00	(2.90- 27.90)
BUFFLE	506	m	0	-	-	-	-	14.80	(4.80- 45.30)
BUFFLE	507	m	0	-	-	-	-	12.60	(4.00- 38.80)
BUFFLE	508	m	0	-	-	-	-	22.10	(7.20- 67.70)
Subtotal BUFFLE								13.92	(7.92- 24.46)
CHOI	559	m	0	42	221	6	95	3.01	(1.24- 7.32)
CHOI	560	m	0	73	160	6	95	7.22	(3.03- 17.25)
CHOI	561	m	0	37	64	6	95	9.15	(3.65- 22.95)
CHOI	562	m	0	11	20	6	95	8.71	(2.88- 26.30)
CHOI	573	f	0	6	23	10	164	4.28	(1.42- 12.88)
CHOI	574	f	0	4	2	10	164	32.80	(5.35- 201.12)
CHOI	575	f	0	1	1	10	164	16.40	(0.95- 281.93)
Subtotal CHOI								6.58	(4.35- 9.95)
DAMBER	547	m	1	-	-	14	-	4.40	(1.80- 10.70)
DAMBER	548	m	1	-	-	14	-	8.40	(4.00- 18.30)
DAMBER	549	m	1	-	-	14	-	13.80	(6.80- 29.10)
DAMBER	550	m	1	-	-	14	-	16.70	(8.50- 34.00)
Subtotal DAMBER								10.50	(7.19- 15.34)
DORGAN	572	m	2	-	-	-	-	9.47	(3.39- 26.45)
DORGAN	573	m	2	-	-	-	-	26.21	(9.61- 71.49)
DORGAN	564	f	3	-	-	-	-	4.31	(2.53- 7.35)
DORGAN	565	f	3	-	-	-	-	15.82	(10.05- 24.90)
Subtotal DORGAN								10.17	(7.45- 13.89)
DOSEME	511	m	2	15	-	58	-	1.20	(0.60- 2.50)
DOSEME	512	m	2	70	-	58	-	3.90	(2.30- 6.70)
DOSEME	513	m	2	199	-	58	-	4.90	(3.20- 7.50)
Subtotal DOSEME								3.54	(2.62- 4.79)
GER	510	c	5	6	-	11	-	1.53	(0.40- 5.86)
GER	511	c	5	42	-	11	-	6.41	(2.03- 20.24)
Subtotal GER								3.50	(1.46- 8.37)
HAENSZ	517	f	0	14	26	44	236	2.89	(1.40- 5.96)
HAENSZ	518	f	0	42	77	44	236	2.93	(1.78- 4.80)
Subtotal HAENSZ								2.91	(1.94- 4.38)
JEDRYC	585	m	3	7	-	6	-	5.83	(1.79- 19.04)
JEDRYC	586	m	3	129	-	6	-	12.45	(5.21- 29.74)
JEDRYC	587	m	3	160	-	6	-	13.00	(5.54- 30.48)
Subtotal JEDRYC								10.80	(6.29- 18.57)
JOLY	639	m	0	15	109	2	218	15.00	(3.37- 66.77)
JOLY	640	m	0	24	165	2	218	15.85	(3.69- 68.04)
JOLY	641	m	0	66	182	2	218	39.53	(9.55- 163.60)
JOLY	642	m	0	98	253	2	218	42.22	(10.29- 173.22)
JOLY	611	f	0	5	54	6	283	4.37	(1.29- 14.82)
JOLY	612	f	0	5	24	6	283	9.83	(2.79- 34.57)
JOLY	613	f	0	16	24	6	283	31.44	(11.26- 87.78)
JOLY	614	f	0	22	20	6	283	51.88	(18.89- 142.48)
Subtotal JOLY								21.49	(13.83- 33.39)
LUBIN2	661	m	0	453	2964	54	2616	7.40	(5.56- 9.87)
LUBIN2	662	m	0	1211	3473	54	2616	16.89	(12.80- 22.29)
LUBIN2	663	m	0	1210	2540	54	2616	23.08	(17.48- 30.47)
LUBIN2	664	m	0	746	1460	54	2616	24.75	(18.64- 32.87)
LUBIN2	713	f	0	322	229	72	1180	23.04	(17.21- 30.86)
LUBIN2	714	f	0	767	186	72	1180	67.58	(50.73- 90.03)
LUBIN2	715	f	0	832	118	72	1180	115.56	(85.07- 156.96)
LUBIN2	716	f	0	566	34	72	1180	272.83	(179.26- 415.22)
Subtotal LUBIN2								31.16	(28.05- 34.61)
LUO	504	c	20	6	-	5	-	5.70	(1.00- 32.90)
LUO	505	c	20	28	-	5	-	12.50	(2.80- 55.40)
Subtotal LUO								8.97	(2.89- 27.91)
MATOS	606	m	2	3	-	3	-	1.20	(0.20- 6.20)
MATOS	607	m	2	18	-	3	-	5.80	(1.60- 20.50)
MATOS	608	m	2	26	-	3	-	18.50	(4.90- 69.80)
Subtotal MATOS								6.29	(2.80- 14.15)
OSANN2	510	f	1	11	-	7	-	4.90	(0.50- 44.60)
OSANN2	511	f	1	101	-	7	-	101.10	(8.30-1230.00)
Subtotal OSANN2								18.94	(3.56- 100.64)
PEZZOT	507	m	0	5	134	0	116	9.53~	(0.52- 174.14)

Table 2I1 - 2

IESLC - Meta-analysis of Ever Smoking by Duration, Overview
Squamous, Any Product (or Cigarettes if Any not available)
Most adjusted

REF	NRR	SEX	AD	Number Exposed Case	Cont	Non-exposed Case	Cont	RR	95.00%CI
PEZZOT	508	m	0	35	82	0	116	100.26~	(6.06-1657.79)
PEZZOT	509	m	0	45	101	0	116	104.45~	(6.35-1717.05)
Subtotal PEZZOT								48.16	(9.37- 247.59)
WUWILL	521	f	3	54	-	117	-	2.00	(1.36- 2.94)
WUWILL	522	f	3	66	-	117	-	3.88	(2.64- 5.71)
WUWILL	523	f	3	81	-	117	-	5.57	(3.79- 8.17)
Subtotal WUWILL								3.51	(2.81- 4.39)
WYNDE2	506	m	0	22	55	0	41	33.65~	(1.98- 570.85)
WYNDE2	507	m	0	30	64	0	41	39.25~	(2.34- 659.46)
WYNDE2	508	m	0	94	89	0	41	87.64~	(5.31-1446.06)
Subtotal WYNDE2								48.90	(9.61- 248.90)
ZHENG	501	m	0	13	75	4	94	4.07	(1.28- 13.01)
ZHENG	502	m	0	59	80	4	94	17.33	(6.03- 49.81)
ZHENG	503	m	0	84	63	4	94	31.33	(10.94- 89.77)
ZHENG	508	f	0	8	17	33	184	2.62	(1.05- 6.57)
ZHENG	509	f	0	35	27	33	184	7.23	(3.87- 13.49)
Subtotal ZHENG								7.80	(5.24- 11.62)
ZHOU	504	c	0	60	12	136	68	2.50	(1.26- 4.96)
ZHOU	505	c	0	315	36	136	68	4.38	(2.79- 6.87)
Subtotal ZHOU								3.69	(2.53- 5.38)
Partial Totals				8676	13264	1706	19789		

*prospective study ~ With 0.5 adjustment for zero

REF	NRR	SEX	AD	Ys	Ws	Qs	Ps
BARBON	562	m	1	0.74	3.09	10.82	0.1919
BARBON	563	m	1	2.26	4.68	0.57	0.0000
BARBON	564	m	1	2.68	5.10	0.02	0.0000
BARBON	565	m	1	3.05	5.38	1.05	0.0000
Subtotal BARBON				2.35	18.25	12.47	
BUFFLE	505	m	0	2.20	3.00	0.52	0.0001
BUFFLE	506	m	0	2.69	3.05	0.02	0.0000
BUFFLE	507	m	0	2.53	2.98	0.02	0.0000
BUFFLE	508	m	0	3.10	3.06	0.72	0.0000
Subtotal BUFFLE				2.63	12.08	1.27	
CHOI	559	m	0	1.10	4.87	11.10	0.0151
CHOI	560	m	0	1.98	5.07	2.04	0.0000
CHOI	561	m	0	2.21	4.55	0.72	0.0000
CHOI	562	m	0	2.16	3.14	0.63	0.0001
CHOI	573	f	0	1.45	3.16	4.24	0.0097
CHOI	574	f	0	3.49	1.17	0.90	0.0002
CHOI	575	f	0	2.80	0.47	0.02	0.0539
Subtotal CHOI				1.88	22.44	19.65	
DAMBER	547	m	1	1.48	4.84	6.18	0.0011
DAMBER	548	m	1	2.13	6.65	1.56	0.0000
DAMBER	549	m	1	2.62	7.27	0.00	0.0000
DAMBER	550	m	1	2.82	8.00	0.33	0.0000
Subtotal DAMBER				2.35	26.75	8.07	
DORGAN	572	m	2	2.25	3.64	0.48	0.0000
DORGAN	573	m	2	3.27	3.82	1.63	0.0000
DORGAN	564	f	3	1.46	13.51	17.90	0.0000
DORGAN	565	f	3	2.76	18.67	0.42	0.0000
Subtotal DORGAN				2.32	39.63	20.43	
DOSEME	511	m	2	0.18	7.54	44.54	0.6165
DOSEME	512	m	2	1.36	13.44	21.04	0.0000
DOSEME	513	m	2	1.59	21.18	22.15	0.0000
Subtotal DOSEME				1.26	42.17	87.73	
GER	510	c	5	0.43	2.13	10.20	0.5346
GER	511	c	5	1.86	2.91	1.65	0.0015
Subtotal GER				1.25	5.04	11.85	
HAENSZ	517	f	0	1.06	7.31	17.59	0.0041
HAENSZ	518	f	0	1.07	15.68	37.12	0.0000
Subtotal HAENSZ				1.07	22.99	54.71	
JEDRYC	585	m	3	1.76	2.75	1.98	0.0035
JEDRYC	586	m	3	2.52	5.06	0.04	0.0000
JEDRYC	587	m	3	2.56	5.29	0.01	0.0000
Subtotal JEDRYC				2.38	13.10	2.03	
JOLY	639	m	0	2.71	1.72	0.02	0.0004
JOLY	640	m	0	2.76	1.81	0.04	0.0002
JOLY	641	m	0	3.68	1.90	2.16	0.0000
JOLY	642	m	0	3.74	1.93	2.47	0.0000

Table 2I1 - 2

IESLC - Meta-analysis of Ever Smoking by Duration, Overview
Squamous, Any Product (or Cigarettes if Any not available)
Most adjusted

REF	NRR	SEX	AD	Ys	Ws	Qs	Ps
JOLY	611	f	0	1.47	2.57	3.33	0.0181
JOLY	612	f	0	2.29	2.43	0.26	0.0004
JOLY	613	f	0	3.45	3.64	2.55	0.0000
JOLY	614	f	0	3.95	3.76	6.73	0.0000
Subtotal JOLY				3.07	19.77	17.55	
LUBIN2	661	m	0	2.00	46.63	17.35	0.0000
LUBIN2	662	m	0	2.83	49.96	2.31	0.0000
LUBIN2	663	m	0	3.14	49.70	13.80	0.0000
LUBIN2	664	m	0	3.21	47.79	17.03	0.0000
LUBIN2	713	f	0	3.14	45.03	12.43	0.0000
LUBIN2	714	f	0	4.21	46.69	119.74	0.0000
LUBIN2	715	f	0	4.75	40.96	187.20	0.0000
LUBIN2	716	f	0	5.61	21.78	195.61	0.0000
Subtotal LUBIN2				3.44	348.54	565.46	
LUO	504	c	20	1.74	1.26	0.96	0.0508
LUO	505	c	20	2.53	1.72	0.01	0.0009
Subtotal LUO				2.19	2.98	0.97	
MATOS	606	m	2	0.18	1.30	7.69	0.8351
MATOS	607	m	2	1.76	2.36	1.72	0.0069
MATOS	608	m	2	2.92	2.18	0.20	0.0000
Subtotal MATOS				1.84	5.84	9.62	
OSANN2	510	f	1	1.59	0.76	0.80	0.1654
OSANN2	511	f	1	4.62	0.61	2.47	0.0003
Subtotal OSANN2				2.94	1.38	3.27	
PEZZOT	507	m	0	2.25	0.45	0.06	0.1284
PEZZOT	508	m	0	4.61	0.49	1.94	0.0013
PEZZOT	509	m	0	4.65	0.49	2.03	0.0011
Subtotal PEZZOT				3.87	1.43	4.04	
WUWILL	521	f	3	0.69	25.85	95.19	0.0004
WUWILL	522	f	3	1.36	25.82	40.74	0.0000
WUWILL	523	f	3	1.72	26.04	20.84	0.0000
Subtotal WUWILL				1.26	77.72	156.78	
WYNDE2	506	m	0	3.52	0.48	0.39	0.0149
WYNDE2	507	m	0	3.67	0.48	0.54	0.0108
WYNDE2	508	m	0	4.47	0.49	1.69	0.0018
Subtotal WYNDE2				3.89	1.45	2.62	
ZHENG	501	m	0	1.40	2.85	4.16	0.0177
ZHENG	502	m	0	2.85	3.45	0.20	0.0000
ZHENG	503	m	0	3.44	3.47	2.40	0.0000
ZHENG	508	f	0	0.96	4.55	12.36	0.0395
ZHENG	509	f	0	1.98	9.87	3.97	0.0000
Subtotal ZHENG				2.05	24.19	23.09	
ZHOU	504	c	0	0.92	8.19	23.56	0.0087
ZHOU	505	c	0	1.48	18.86	24.35	0.0000
Subtotal ZHOU				1.31	27.06	47.90	

N	72
NS	19

Table 2I1 - 3

IESLC - Meta-analysis of Ever Smoking by Duration, Overview
Squamous, Any Product (or Cigarettes if Any not available)
Most adjusted

	combined	Sex: male	female	Total
N	6	44	22	72
NS	3	13	8	24

In this overview table, other than the "N" rows, entries in the "absent" and "Total" columns may be invalid and should be ignored

	Duration of smoking (broad categories): absent	1-34k20	21-49k35	36+k50	Total
N	22	21	14	15	72
NS	15	16	11	12	54
Wt	241.01	185.15	154.11	132.54	712.81
Het Chi	388.24	139.16	158.82	196.21	1049.50
Het df	21	20	13	14	71
Het P	***	***	***	***	***
Fixed RR	13.80	6.58	18.37	25.98	13.63
RRl	12.17	5.70	15.69	21.92	12.66
RRu	15.66	7.60	21.51	30.81	14.66
P	+++	+++	+++	+++	+++
Random RR	10.43	4.82	14.19	26.28	10.69
RRl	5.84	3.07	7.30	12.49	7.86
RRu	18.63	7.58	27.62	55.28	14.54
P	+++	+++	+++	+++	+++

	absent	Duration of smoking (narrow categories): 1-19k1	6-29k20	21-39k30	31-49k40	41-998k50	51+k999	Total
N	46	4	1	10	9	1	1	72
NS	19	4	1	7	7	1	1	39
Wt	398.83	25.79	13.44	143.44	116.04	7.27	8.00	712.81
Het Chi	535.92	6.01	0.00	152.77	95.24	0.00	0.00	1049.50
Het df	45	3	0	9	8	0	0	71
Het P	***	N.S.	N.S.	***	***	N.S.	N.S.	***
Fixed RR	10.55	2.30	3.90	18.98	36.83	13.80	16.70	13.63
RRl	9.57	1.56	2.29	16.11	30.70	6.67	8.35	12.66
RRu	11.64	3.38	6.66	22.35	44.18	28.55	33.40	14.66
P	+++	+++	+++	+++	+++	+++	+++	+++
Random RR	9.96	2.42	3.90	13.07	24.11	13.80	16.70	10.69
RRl	6.83	1.38	2.29	5.93	11.16	6.67	8.35	7.86
RRu	14.54	4.24	6.66	28.82	52.09	28.55	33.40	14.54
P	+++	++	+++	+++	+++	+++	+++	+++

Table 2I1 - 3

IESLC - Meta-analysis of Ever Smoking by Duration, Overview
Squamous, Any Product (or Cigarettes if Any not available)
Most adjusted

MALES

Duration of smoking (broad categories)

		absent	1-34k20	21-49k35	36+k50	Total
	N	11	12	10	11	44
	NS	9	12	10	11	42
	Wt	112.58	86.32	78.00	80.48	357.37
Het	Chi	86.09	18.73	10.79	9.86	201.26
Het	df	10	11	9	10	43
Het	P	***	(*)	N.S.	N.S.	***
Fixed	RR	12.23	5.88	14.25	21.98	12.09
	RRl	10.17	4.76	11.41	17.66	10.90
	RRu	14.72	7.26	17.79	27.34	13.41
	P	+++	+++	+++	+++	+++
Random	RR	10.64	5.18	12.90	21.98	10.78
	RRl	5.72	3.61	9.48	17.66	8.29
	RRu	19.80	7.44	17.56	27.34	14.03
	P	+++	+++	+++	+++	+++

Duration of smoking (narrow categories)

		absent	1-19k1	6-29k20	21-39k30	31-49k40	41-998k50	51+k999	Total
	N	26	2	1	6	7	1	1	44
	NS	13	2	1	6	7	1	1	30
	Wt	179.60	10.29	13.44	67.33	71.44	7.27	8.00	357.37
Het	Chi	103.86	5.03	0.00	6.52	11.63	0.00	0.00	201.26
Het	df	25	1	0	5	6	0	0	43
Het	P	***	*	N.S.	N.S.	(*)	N.S.	N.S.	***
Fixed	RR	11.10	1.83	3.90	14.67	19.27	13.80	16.70	12.09
	RRl	9.59	0.99	2.29	11.55	15.28	6.67	8.35	10.90
	RRu	12.85	3.37	6.66	18.63	24.30	28.55	33.40	13.41
	P	+++	(+)	+++	+++	+++	+++	+++	+++
Random	RR	11.06	2.46	3.90	12.82	16.20	13.80	16.70	10.78
	RRl	7.67	0.53	2.29	8.87	10.23	6.67	8.35	8.29
	RRu	15.94	11.50	6.66	18.53	25.63	28.55	33.40	14.03
	P	+++	N.S.	+++	+++	+++	+++	+++	+++

FEMALES

Duration of smoking (broad categories)

		absent	1-34k20	21-49k35	36+k50	Total
	N	7	7	4	4	22
	NS	6	7	4	4	21
	Wt	96.75	95.44	76.11	52.06	320.36
Het	Chi	222.37	112.96	137.83	180.60	759.20
Het	df	6	6	3	3	21
Het	P	***	***	***	***	***
Fixed	RR	23.55	7.55	23.84	33.67	17.83
	RRl	19.29	6.18	19.04	25.66	15.98
	RRu	28.74	9.23	29.84	44.17	19.90
	P	+++	+++	+++	+++	+++
Random	RR	15.27	4.72	16.76	35.26	12.35
	RRl	3.99	1.69	2.48	2.90	6.11
	RRu	58.45	13.17	113.10	429.38	24.96
	P	+++	++	++	++	+++

Table 2I1 - 3

IESLC - Meta-analysis of Ever Smoking by Duration, Overview
Squamous, Any Product (or Cigarettes if Any not available)
Most adjusted

FEMALES

		Duration of smoking (narrow categories)						
	absent	1-19k1	6-29k20	21-39k30	31-49k40	41-998k50	51+k999	Total
N	15	1		4	2			22
NS	8	1		4	2			15
Wt	192.34	7.31		76.11	44.61			320.36
Het Chi	406.82	0.00		137.83	5.67			759.20
Het df	14	0		3	1			21
Het P	***	N.S.		***	*			***
Fixed RR	11.32	2.89		23.84	103.90			17.83
RRl	9.83	1.40		19.04	77.47			15.98
RRu	13.04	5.96		29.84	139.33			19.90
P	+++	++		+++	+++			+++
Random RR	10.00	2.89		16.76	66.35			12.35
RRl	4.37	1.40		2.48	18.79			6.11
RRu	22.90	5.96		113.10	234.28			24.96
P	+++	++		++	+++			+++

Table 2I1 - 4

IESLC - Meta-analysis of Ever Smoking by Duration, Overview
Squamous, Any Product (or Cigarettes if Any not available)
Least adjusted

REF	NRR	X	SEX	AGEL	AGEH	RACE	YF	LC TYPE	LOC	START	ST	NLC	R	VB	P	H	AD	PRODUCT	exL	exH	S1	S2	DENOM	De
BARBON	555	x	m	0	0	all	-	q	Eu:wst	1979	CC	755	n	bl	y	y	0	all/unsp	1	29	1	0	nev any	st
BARBON	556	x	m	0	0	all	-	q	Eu:wst	1979	CC	755	n	bl	y	y	0	all/unsp	30	39	2	3	nev any	st
BARBON	557	x	m	0	0	all	-	q	Eu:wst	1979	CC	755	n	bl	y	y	0	all/unsp	40	49	0	4	nev any	st
BARBON	558	x	m	0	0	all	-	q	Eu:wst	1979	CC	755	n	bl	y	y	0	all/unsp	50	999	3	0	nev any	st
BUFFLE	505		m	0	0	wh	-	q	NAmer	1976	CC	943	n	bl	y	n	0	cig+/-ot	1	33	1	0	nev cigs	or
BUFFLE	506		m	0	0	wh	-	q	NAmer	1976	CC	943	n	bl	y	n	0	cig+/-ot	34	43	2	4	nev cigs	or
BUFFLE	507		m	0	0	wh	-	q	NAmer	1976	CC	943	n	bl	y	n	0	cig+/-ot	44	49	0	0	nev cigs	or
BUFFLE	508		m	0	0	wh	-	q	NAmer	1976	CC	943	n	bl	y	n	0	cig+/-ot	50	999	3	0	nev cigs	or
CHOI	559		m	0	0	all	-	q	As:oth	1985	CC	375	n	bl	n	n	0	cig+/-ot	1	29	1	0	nev cigs	st
CHOI	560		m	0	0	all	-	q	As:oth	1985	CC	375	n	bl	n	n	0	cig+/-ot	30	39	2	3	nev cigs	st
CHOI	561		m	0	0	all	-	q	As:oth	1985	CC	375	n	bl	n	n	0	cig+/-ot	40	49	0	4	nev cigs	st
CHOI	562		m	0	0	all	-	q	As:oth	1985	CC	375	n	bl	n	n	0	cig+/-ot	50	999	3	0	nev cigs	st
CHOI	573		f	0	0	all	-	q	As:oth	1985	CC	375	n	bl	n	n	0	cig+/-ot	1	29	1	0	nev cigs	st
CHOI	574		f	0	0	all	-	q	As:oth	1985	CC	375	n	bl	n	n	0	cig+/-ot	30	39	2	3	nev cigs	st
CHOI	575		f	0	0	all	-	q	As:oth	1985	CC	375	n	bl	n	n	0	cig+/-ot	40	999	3	0	nev cigs	st
DAMBER	547		m	0	0	all	-	q	Eu:Sca	1972	CC	579	n	bl	y	n	1	all/unsp	1	30	1	0	nev any	or
DAMBER	548		m	0	0	all	-	q	Eu:Sca	1972	CC	579	n	bl	y	n	1	all/unsp	31	40	2	4	nev any	or
DAMBER	549		m	0	0	all	-	q	Eu:Sca	1972	CC	579	n	bl	y	n	1	all/unsp	41	50	3	5	nev any	or
DAMBER	550		m	0	0	all	-	q	Eu:Sca	1972	CC	579	n	bl	y	n	1	all/unsp	51	999	0	6	nev any	or
DORGAN	572		m	0	0	wh	-	q	NAmer	1980	CC	2026	n	bl	y	y	2	cig+/-ot	1	34	1	0	nev any	ot
DORGAN	573		m	0	0	wh	-	q	NAmer	1980	CC	2026	n	bl	y	y	2	cig+/-ot	35	999	0	0	nev any	ot
DORGAN	564		f	0	0	all	-	q	NAmer	1980	CC	2026	n	bl	y	y	3	cig+/-ot	1	34	1	0	nev any	ot
DORGAN	565		f	0	0	all	-	q	NAmer	1980	CC	2026	n	bl	y	y	3	cig+/-ot	35	999	0	0	nev any	ot
DOSEME	511		m	0	0	all	-	q	Eu:bal	1979	CC	1210	n	bl	n	n	2	cig+/-ot	1	10	0	1	nev cigs	or
DOSEME	512		m	0	0	all	-	q	Eu:bal	1979	CC	1210	n	bl	n	n	2	cig+/-ot	11	20	1	2	nev cigs	or
DOSEME	513		m	0	0	all	-	q	Eu:bal	1979	CC	1210	n	bl	n	n	2	cig+/-ot	21	999	0	0	nev cigs	or
GER	504	x	c	0	0	all	-	q+s	As:oth	1990	CC	141	n	ot	y	n	0	all/unsp	1	30	1	0	nev any	st
GER	505	x	c	0	0	all	-	q+s	As:oth	1990	CC	141	n	ot	y	n	0	all/unsp	31	999	0	0	nev any	st
HAENSZ	517		f	0	0	all	-	q+u	NAmer	1955	CC	158	n	bl	n	y	0	cig+/-ot	1	14	0	1	nev any	st
HAENSZ	518		f	0	0	all	-	q+u	NAmer	1955	CC	158	n	bl	n	y	0	cig+/-ot	15	999	0	0	nev any	st
JEDRYC	501	x	m	0	0	all	-	q	Eu:est	1980	CC	1630	n	bl	y	n	0	cig+/-ot	1	19	0	1	nev any	st
JEDRYC	502	x	m	0	0	all	-	q	Eu:est	1980	CC	1630	n	bl	y	n	0	cig+/-ot	20	29	1	2	nev any	st
JEDRYC	503	x	m	0	0	all	-	q	Eu:est	1980	CC	1630	n	bl	y	n	0	cig+/-ot	30	39	2	3	nev any	st
JEDRYC	504	x	m	0	0	all	-	q	Eu:est	1980	CC	1630	n	bl	y	n	0	cig+/-ot	40	49	0	4	nev any	st
JEDRYC	505	x	m	0	0	all	-	q	Eu:est	1980	CC	1630	n	bl	y	n	0	cig+/-ot	50	999	3	0	nev any	st
JOLY	639		m	0	0	all	-	q	SCAmer	1978	CC	826	n	bl	n	n	0	cig+/-ot	1	29	1	0	nev any	st
JOLY	640		m	0	0	all	-	q	SCAmer	1978	CC	826	n	bl	n	n	0	cig+/-ot	30	39	2	3	nev any	st
JOLY	641		m	0	0	all	-	q	SCAmer	1978	CC	826	n	bl	n	n	0	cig+/-ot	40	49	0	4	nev any	st
JOLY	642		m	0	0	all	-	q	SCAmer	1978	CC	826	n	bl	n	n	0	cig+/-ot	50	999	3	0	nev any	st
JOLY	611		f	0	0	all	-	q	SCAmer	1978	CC	826	n	bl	n	n	0	cig+/-ot	1	29	1	0	nev any	st
JOLY	612		f	0	0	all	-	q	SCAmer	1978	CC	826	n	bl	n	n	0	cig+/-ot	30	39	2	3	nev any	st
JOLY	613		f	0	0	all	-	q	SCAmer	1978	CC	826	n	bl	n	n	0	cig+/-ot	40	49	0	4	nev any	st
JOLY	614		f	0	0	all	-	q	SCAmer	1978	CC	826	n	bl	n	n	0	cig+/-ot	50	999	3	0	nev any	st
LUBIN2	661		m	0	0	all	-	q	Eu:mul	1976	CC	7804	n	bl	n	y	0	cig+/-ot	1	29	1	0	nev any	st
LUBIN2	662		m	0	0	all	-	q	Eu:mul	1976	CC	7804	n	bl	n	y	0	cig+/-ot	30	39	2	3	nev any	st
LUBIN2	663		m	0	0	all	-	q	Eu:mul	1976	CC	7804	n	bl	n	y	0	cig+/-ot	40	49	0	4	nev any	st
LUBIN2	664		m	0	0	all	-	q	Eu:mul	1976	CC	7804	n	bl	n	y	0	cig+/-ot	50	999	3	0	nev any	st
LUBIN2	713		f	0	0	all	-	q	Eu:mul	1976	CC	7804	n	bl	n	y	0	cig+/-ot	1	29	1	0	nev any	st
LUBIN2	714		f	0	0	all	-	q	Eu:mul	1976	CC	7804	n	bl	n	y	0	cig+/-ot	30	39	2	3	nev any	st
LUBIN2	715		f	0	0	all	-	q	Eu:mul	1976	CC	7804	n	bl	n	y	0	cig+/-ot	40	49	0	4	nev any	st
LUBIN2	716		f	0	0	all	-	q	Eu:mul	1976	CC	7804	n	bl	n	y	0	cig+/-ot	50	999	3	0	nev any	st
LUO	501	x	c	0	0	all	-	q	As:Chi	1990	CC	102	n	ot	n	y	0	cig+/-ot	1	29	1	0	nev cigs	st
LUO	502	x	c	0	0	all	-	q	As:Chi	1990	CC	102	n	ot	n	y	0	cig+/-ot	30	999	0	0	nev cigs	st
MATOS	601	x	m	0	0	all	-	q	SCAmer	1994	CC	200	n	bl	n	n	0	cig+/-ot	1	24	1	0	nev any	st
MATOS	602	x	m	0	0	all	-	q	SCAmer	1994	CC	200	n	bl	n	n	0	cig+/-ot	25	39	2	3	nev any	st
MATOS	603	x	m	0	0	all	-	q	SCAmer	1994	CC	200	n	bl	n	n	0	cig+/-ot	40	70	3	0	nev any	st
OSANN2	507	x	f	0	0	all	-	KI	NAmer	1964	ot	217	n	bl	n	y	0	cig+/-ot	1	20	1	0	nev cigs	st
OSANN2	508	x	f	0	0	all	-	KI	NAmer	1964	ot	217	n	bl	n	y	0	cig+/-ot	21	999	0	0	nev cigs	st
PEZZOT	507		m	0	0	all	-	q	SCAmer	1987	CC	215	n	bl	n	y	0	cig only	1	30	1	0	nev cigs	ot
PEZZOT	508		m	0	0	all	-	q	SCAmer	1987	CC	215	n	bl	n	y	0	cig only	31	40	2	4	nev cigs	ot
PEZZOT	509		m	0	0	all	-	q	SCAmer	1987	CC	215	n	bl	n	y	0	cig only	41	999	3	0	nev cigs	ot
WUWILL	506	x	f	0	0	all	-	q	As:Chi	1985	CC	965	n	ot	n	n	0	cig+/-ot	1	29	1	0	nev cigs	st
WUWILL	507	x	f	0	0	all	-	q	As:Chi	1985	CC	965	n	ot	n	n	0	cig+/-ot	30	39	2	3	nev cigs	st
WUWILL	508	x	f	0	0	all	-	q	As:Chi	1985	CC	965	n	ot	n	n	0	cig+/-ot	40	999	3	0	nev cigs	st
WYNDE2	506		m	0	0	all	-	KI	NAmer	1962	CC	404	n	bl	n	y	0	cig+/-ot	1	29	1	0	nev any	ot
WYNDE2	507		m	0	0	all	-	KI	NAmer	1962	CC	404	n	bl	n	y	0	cig+/-ot	30	40	2	0	nev any	ot
WYNDE2	508		m	0	0	all	-	KI	NAmer	1962	CC	404	n	bl	n	y	0	cig+/-ot	41	999	3	0	nev any	ot
ZHENG	501		m	0	0	all	-	q	As:Chi	1982	CC	540	n	ot	*	y	0	cig+/-ot	1	29	1	0	nev cigs	st
ZHENG	502		m	0	0	all	-	q	As:Chi	1982	CC	540	n	ot	*	y	0	cig+/-ot	30	39	2	3	nev cigs	st
ZHENG	503		m	0	0	all	-	q	As:Chi	1982	CC	540	n	ot	*	y	0	cig+/-ot	40	999	3	0	nev cigs	st
ZHENG	508		f	0	0	all	-	q	As:Chi	1982	CC	540	n	ot	*	y	0	cig+/-ot	1	29	1	0	nev cigs	st
ZHENG	509		f	0	0	all	-	q	As:Chi	1982	CC	540	n	ot	*	y	0	cig+/-ot	30	999	0	0	nev cigs	st

Table 2I1 - 4

IESLC - Meta-analysis of Ever Smoking by Duration, Overview
Squamous, Any Product (or Cigarettes if Any not available)
Least adjusted

REF	NRR	X	SEX	AGEL	AGEH	RACE	YF	LC TYPE	LOC	START	ST	NLC	R	VB	P	H	AD	PRODUCT	exL	exH	S1	S2	DENOM	De
ZHOU	504		c	0	0	all	-	q	As:Chi	1978	CC	1360	n	ot	n	n	0	all/unsp	1	19	0	1	nev any	st
ZHOU	505		c	0	0	all	-	q	As:Chi	1978	CC	1360	n	ot	n	n	0	all/unsp	20	999	0	0	nev any	st

Cigarette type is all/unspec for all RRs

In this overview table, subtotals and Qs values may be invalid and should be ignored

Table 2I1 - 5

IESLC - Meta-analysis of Ever Smoking by Duration, Overview
Squamous, Any Product (or Cigarettes if Any not available)
Least adjusted

REF	NRR	SEX	AD	Number Exposed Case	Cont	Non-exposed Case	Cont	RR	95.00%CI
BARBON	555	m	0	7	91	6	188	2.41	(0.79- 7.38)
BARBON	556	m	0	36	102	6	188	11.06	(4.51- 27.13)
BARBON	557	m	0	69	139	6	188	15.55	(6.57- 36.85)
BARBON	558	m	0	149	235	6	188	19.87	(8.59- 45.94)
Subtotal BARBON								11.23	(7.12- 17.72)
BUFFLE	505	m	0	-	-	-	-	9.00	(2.90- 27.90)
BUFFLE	506	m	0	-	-	-	-	14.80	(4.80- 45.30)
BUFFLE	507	m	0	-	-	-	-	12.60	(4.00- 38.80)
BUFFLE	508	m	0	-	-	-	-	22.10	(7.20- 67.70)
Subtotal BUFFLE								13.92	(7.92- 24.46)
CHOI	559	m	0	42	221	6	95	3.01	(1.24- 7.32)
CHOI	560	m	0	73	160	6	95	7.22	(3.03- 17.25)
CHOI	561	m	0	37	64	6	95	9.15	(3.65- 22.95)
CHOI	562	m	0	11	20	6	95	8.71	(2.88- 26.30)
CHOI	573	f	0	6	23	10	164	4.28	(1.42- 12.88)
CHOI	574	f	0	4	2	10	164	32.80	(5.35- 201.12)
CHOI	575	f	0	1	1	10	164	16.40	(0.95- 281.93)
Subtotal CHOI								6.58	(4.35- 9.95)
DAMBER	547	m	1	-	-	14	-	4.40	(1.80- 10.70)
DAMBER	548	m	1	-	-	14	-	8.40	(4.00- 18.30)
DAMBER	549	m	1	-	-	14	-	13.80	(6.80- 29.10)
DAMBER	550	m	1	-	-	14	-	16.70	(8.50- 34.00)
Subtotal DAMBER								10.50	(7.19- 15.34)
DORGAN	572	m	2	-	-	-	-	9.47	(3.39- 26.45)
DORGAN	573	m	2	-	-	-	-	26.21	(9.61- 71.49)
DORGAN	564	f	3	-	-	-	-	4.31	(2.53- 7.35)
DORGAN	565	f	3	-	-	-	-	15.82	(10.05- 24.90)
Subtotal DORGAN								10.17	(7.45- 13.89)
DOSEME	511	m	2	15	-	58	-	1.20	(0.60- 2.50)
DOSEME	512	m	2	70	-	58	-	3.90	(2.30- 6.70)
DOSEME	513	m	2	199	-	58	-	4.90	(3.20- 7.50)
Subtotal DOSEME								3.54	(2.62- 4.79)
GER	504	c	0	6	37	11	80	1.18	(0.41- 3.43)
GER	505	c	0	42	119	11	80	2.57	(1.25- 5.28)
Subtotal GER								2.01	(1.11- 3.66)
HAENSZ	517	f	0	14	26	44	236	2.89	(1.40- 5.96)
HAENSZ	518	f	0	42	77	44	236	2.93	(1.78- 4.80)
Subtotal HAENSZ								2.91	(1.94- 4.38)
JEDRYC	501	m	0	7	68	6	289	4.96	(1.61- 15.23)
JEDRYC	502	m	0	23	160	6	289	6.92	(2.76- 17.36)
JEDRYC	503	m	0	106	231	6	289	22.10	(9.54- 51.22)
JEDRYC	504	m	0	111	223	6	289	23.98	(10.35- 55.53)
JEDRYC	505	m	0	49	214	6	289	11.03	(4.64- 26.22)
Subtotal JEDRYC								12.77	(8.53- 19.11)
JOLY	639	m	0	15	109	2	218	15.00	(3.37- 66.77)
JOLY	640	m	0	24	165	2	218	15.85	(3.69- 68.04)
JOLY	641	m	0	66	182	2	218	39.53	(9.55- 163.60)
JOLY	642	m	0	98	253	2	218	42.22	(10.29- 173.22)
JOLY	611	f	0	5	54	6	283	4.37	(1.29- 14.82)
JOLY	612	f	0	5	24	6	283	9.83	(2.79- 34.57)
JOLY	613	f	0	16	24	6	283	31.44	(11.26- 87.78)
JOLY	614	f	0	22	20	6	283	51.88	(18.89- 142.48)
Subtotal JOLY								21.49	(13.83- 33.39)
LUBIN2	661	m	0	453	2964	54	2616	7.40	(5.56- 9.87)
LUBIN2	662	m	0	1211	3473	54	2616	16.89	(12.80- 22.29)
LUBIN2	663	m	0	1210	2540	54	2616	23.08	(17.48- 30.47)
LUBIN2	664	m	0	746	1460	54	2616	24.75	(18.64- 32.87)
LUBIN2	713	f	0	322	229	72	1180	23.04	(17.21- 30.86)
LUBIN2	714	f	0	767	186	72	1180	67.58	(50.73- 90.03)
LUBIN2	715	f	0	832	118	72	1180	115.56	(85.07- 156.96)
LUBIN2	716	f	0	566	34	72	1180	272.83	(179.26- 415.22)
Subtotal LUBIN2								31.16	(28.05- 34.61)
LUO	501	c	0	6	21	5	51	2.91	(0.80- 10.60)
LUO	502	c	0	28	45	5	51	6.35	(2.26- 17.82)
Subtotal LUO								4.68	(2.09- 10.49)
MATOS	601	m	0	3	84	3	110	1.31	(0.26- 6.65)
MATOS	602	m	0	18	110	3	110	6.00	(1.72- 20.95)
MATOS	603	m	0	26	89	3	110	10.71	(3.14- 36.55)
Subtotal MATOS								5.35	(2.48- 11.58)
OSANN2	507	f	0	11	26	7	58	3.51	(1.22- 10.06)
OSANN2	508	f	0	101	35	7	58	23.91	(9.98- 57.27)

Table 2I1 - 5

IESLC - Meta-analysis of Ever Smoking by Duration, Overview
Squamous, Any Product (or Cigarettes if Any not available)
Least adjusted

REF	NRR	SEX	AD	Number Exposed Case	Cont	Non-exposed Case	Cont	RR	95.00%CI
Subtotal OSANN2								10.95	(5.59- 21.45)
PEZZOT	507	m	0	5	134	0	116	9.53~	(0.52- 174.14)
PEZZOT	508	m	0	35	82	0	116	100.26~	(6.06-1657.79)
PEZZOT	509	m	0	45	101	0	116	104.45~	(6.35-1717.05)
Subtotal PEZZOT								48.16	(9.37- 247.59)
WUWILL	506	f	0	54	139	117	601	2.00	(1.38- 2.89)
WUWILL	507	f	0	66	98	117	601	3.46	(2.39- 5.01)
WUWILL	508	f	0	81	114	117	601	3.65	(2.58- 5.16)
Subtotal WUWILL								2.96	(2.40- 3.65)
WYNDE2	506	m	0	22	55	0	41	33.65~	(1.98- 570.85)
WYNDE2	507	m	0	30	64	0	41	39.25~	(2.34- 659.46)
WYNDE2	508	m	0	94	89	0	41	87.64~	(5.31-1446.06)
Subtotal WYNDE2								48.90	(9.61- 248.90)
ZHENG	501	m	0	13	75	4	94	4.07	(1.28- 13.01)
ZHENG	502	m	0	59	80	4	94	17.33	(6.03- 49.81)
ZHENG	503	m	0	84	63	4	94	31.33	(10.94- 89.77)
ZHENG	508	f	0	8	17	33	184	2.62	(1.05- 6.57)
ZHENG	509	f	0	35	27	33	184	7.23	(3.87- 13.49)
Subtotal ZHENG								7.80	(5.24- 11.62)
ZHOU	504	c	0	60	12	136	68	2.50	(1.26- 4.96)
ZHOU	505	c	0	315	36	136	68	4.38	(2.79- 6.87)
Subtotal ZHOU								3.69	(2.53- 5.38)
Partial Totals				8676	15644	1718	24497		

*prospective study ~ With 0.5 adjustment for zero

REF	NRR	SEX	AD	Ys	Ws	Qs	Ps
BARBON	555	m	0	0.88	3.07	8.53	0.1233
BARBON	556	m	0	2.40	4.77	0.10	0.0000
BARBON	557	m	0	2.74	5.16	0.20	0.0000
BARBON	558	m	0	2.99	5.47	1.07	0.0000
Subtotal BARBON				2.42	18.47	9.90	
BUFFLE	505	m	0	2.20	3.00	0.37	0.0001
BUFFLE	506	m	0	2.69	3.05	0.07	0.0000
BUFFLE	507	m	0	2.53	2.98	0.00	0.0000
BUFFLE	508	m	0	3.10	3.06	0.92	0.0000
Subtotal BUFFLE				2.63	12.08	1.35	
CHOI	559	m	0	1.10	4.87	10.17	0.0151
CHOI	560	m	0	1.98	5.07	1.65	0.0000
CHOI	561	m	0	2.21	4.55	0.50	0.0000
CHOI	562	m	0	2.16	3.14	0.46	0.0001
CHOI	573	f	0	1.45	3.16	3.78	0.0097
CHOI	574	f	0	3.49	1.17	1.04	0.0002
CHOI	575	f	0	2.80	0.47	0.03	0.0539
Subtotal CHOI				1.88	22.44	17.63	
DAMBER	547	m	1	1.48	4.84	5.49	0.0011
DAMBER	548	m	1	2.13	6.65	1.17	0.0000
DAMBER	549	m	1	2.62	7.27	0.04	0.0000
DAMBER	550	m	1	2.82	8.00	0.58	0.0000
Subtotal DAMBER				2.35	26.75	7.28	
DORGAN	572	m	2	2.25	3.64	0.33	0.0000
DORGAN	573	m	2	3.27	3.82	1.97	0.0000
DORGAN	564	f	3	1.46	13.51	15.94	0.0000
DORGAN	565	f	3	2.76	18.67	0.86	0.0000
Subtotal DORGAN				2.32	39.63	19.09	
DOSEME	511	m	2	0.18	7.54	42.19	0.6165
DOSEME	512	m	2	1.36	13.44	18.91	0.0000
DOSEME	513	m	2	1.59	21.18	19.43	0.0000
Subtotal DOSEME				1.26	42.17	80.53	
GER	504	c	0	0.16	3.37	19.10	0.7621
GER	505	c	0	0.94	7.37	18.98	0.0105
Subtotal GER				0.70	10.74	38.08	
HAENSZ	517	f	0	1.06	7.31	16.15	0.0041
HAENSZ	518	f	0	1.07	15.68	34.05	0.0000
Subtotal HAENSZ				1.07	22.99	50.20	
JEDRYC	501	m	0	1.60	3.05	2.73	0.0052
JEDRYC	502	m	0	1.93	4.55	1.70	0.0000
JEDRYC	503	m	0	3.10	5.44	1.64	0.0000
JEDRYC	504	m	0	3.18	5.45	2.16	0.0000
JEDRYC	505	m	0	2.40	5.12	0.11	0.0000
Subtotal JEDRYC				2.55	23.61	8.34	

Table 2I1 - 5

IESLC - Meta-analysis of Ever Smoking by Duration, Overview
Squamous, Any Product (or Cigarettes if Any not available)
Least adjusted

REF	NRR	SEX	AD	Ys	Ws	Qs	Ps
JOLY	639	m	0	2.71	1.72	0.04	0.0004
JOLY	640	m	0	2.76	1.81	0.08	0.0002
JOLY	641	m	0	3.68	1.90	2.43	0.0000
JOLY	642	m	0	3.74	1.93	2.76	0.0000
JOLY	611	f	0	1.47	2.57	2.96	0.0181
JOLY	612	f	0	2.29	2.43	0.17	0.0004
JOLY	613	f	0	3.45	3.64	2.96	0.0000
JOLY	614	f	0	3.95	3.76	7.40	0.0000
Subtotal JOLY				3.07	19.77	18.80	
LUBIN2	661	m	0	2.00	46.63	13.85	0.0000
LUBIN2	662	m	0	2.83	49.96	3.91	0.0000
LUBIN2	663	m	0	3.14	49.70	17.41	0.0000
LUBIN2	664	m	0	3.21	47.79	20.94	0.0000
LUBIN2	713	f	0	3.14	45.03	15.69	0.0000
LUBIN2	714	f	0	4.21	46.69	129.64	0.0000
LUBIN2	715	f	0	4.75	40.96	198.74	0.0000
LUBIN2	716	f	0	5.61	21.78	204.17	0.0000
Subtotal LUBIN2				3.44	348.54	604.35	
LUO	501	c	0	1.07	2.30	5.03	0.1044
LUO	502	c	0	1.85	3.60	1.76	0.0005
Subtotal LUO				1.54	5.91	6.79	
MATOS	601	m	0	0.27	1.45	7.54	0.7450
MATOS	602	m	0	1.79	2.46	1.40	0.0050
MATOS	603	m	0	2.37	2.55	0.08	0.0002
Subtotal MATOS				1.68	6.46	9.02	
OSANN2	507	f	0	1.25	3.45	5.77	0.0197
OSANN2	508	f	0	3.17	5.04	1.98	0.0000
Subtotal OSANN2				2.39	8.49	7.75	
PEZZOT	507	m	0	2.25	0.45	0.04	0.1284
PEZZOT	508	m	0	4.61	0.49	2.07	0.0013
PEZZOT	509	m	0	4.65	0.49	2.16	0.0011
Subtotal PEZZOT				3.87	1.43	4.28	
WUWILL	506	f	0	0.69	27.84	95.90	0.0003
WUWILL	507	f	0	1.24	28.12	47.95	0.0000
WUWILL	508	f	0	1.29	31.92	50.07	0.0000
Subtotal WUWILL				1.09	87.87	193.92	
WYNDE2	506	m	0	3.52	0.48	0.45	0.0149
WYNDE2	507	m	0	3.67	0.48	0.61	0.0108
WYNDE2	508	m	0	4.47	0.49	1.81	0.0018
Subtotal WYNDE2				3.89	1.45	2.87	
ZHENG	501	m	0	1.40	2.85	3.72	0.0177
ZHENG	502	m	0	2.85	3.45	0.32	0.0000
ZHENG	503	m	0	3.44	3.47	2.79	0.0000
ZHENG	508	f	0	0.96	4.55	11.40	0.0395
ZHENG	509	f	0	1.98	9.87	3.20	0.0000
Subtotal ZHENG				2.05	24.19	21.44	
ZHOU	504	c	0	0.92	8.19	21.79	0.0087
ZHOU	505	c	0	1.48	18.86	21.64	0.0000
Subtotal ZHOU				1.31	27.06	43.43	

N	74
NS	19

Table 2I1 - 6

IESLC - Meta-analysis of Ever Smoking by Duration, Overview
Squamous, Any Product (or Cigarettes if Any not available)
Least adjusted

	Sex			
	combined	male	female	Total
N	6	46	22	74
NS	3	13	8	24

In this overview table, other than the "N" rows, entries in the "absent" and "Total" columns may be invalid and should be ignored

	Duration of smoking (broad categories)				
	absent	1-34k20	21-49k35	36+k50	Total
N	22	22	15	15	74
NS	15	17	12	12	56
Wt	252.53	196.78	162.03	138.71	750.05
Het Chi	411.19	149.08	173.99	255.99	1145.06
Het df	21	21	14	14	73
Het P	***	***	***	***	***
Fixed RR	13.36	6.31	17.79	21.67	12.77
RRl	11.81	5.49	15.25	18.35	11.89
RRu	15.12	7.26	20.75	25.60	13.72
P	+++	+++	+++	+++	+++
Random RR	9.74	4.65	14.72	24.24	10.13
RRl	5.50	3.02	7.72	10.65	7.47
RRu	17.27	7.15	28.07	55.16	13.74
P	+++	+++	+++	+++	+++

		Duration of smoking (narrow categories)						
	absent	1-19k1	6-29k20	21-39k30	31-49k40	41-998k50	51+k999	Total
N	45	4	2	11	10	1	1	74
NS	19	4	2	8	8	1	1	42
Wt	417.78	26.10	17.99	151.37	121.55	7.27	8.00	750.05
Het Chi	590.76	5.43	1.12	168.26	95.68	0.00	0.00	1145.06
Het df	44	3	1	10	9	0	0	73
Het P	***	N.S.	N.S.	***	***	N.S.	N.S.	***
Fixed RR	9.58	2.28	4.51	18.31	36.21	13.80	16.70	12.77
RRl	8.70	1.55	2.84	15.61	30.31	6.67	8.35	11.89
RRu	10.54	3.35	7.16	21.47	43.25	28.55	33.40	13.72
P	+++	+++	+++	+++	+++	+++	+++	+++
Random RR	8.87	2.36	4.58	13.78	24.23	13.80	16.70	10.13
RRl	6.03	1.40	2.76	6.47	12.02	6.67	8.35	7.47
RRu	13.04	4.01	7.58	29.37	48.84	28.55	33.40	13.74
P	+++	++	+++	+++	+++	+++	+++	+++

Table 2I1 - 6

IESLC - Meta-analysis of Ever Smoking by Duration, Overview
Squamous, Any Product (or Cigarettes if Any not available)
Least adjusted

MALES

		Duration of smoking (broad categories)				
		absent	1-34k20	21-49k35	36+k50	Total
	N	11	13	11	11	46
	NS	9	13	11	11	44
	Wt	113.33	90.99	83.63	80.77	368.71
Het	Chi	89.58	17.99	11.24	12.05	206.05
Het	df	10	12	10	10	45
Het	P	***	N.S.	N.S.	N.S.	***
Fixed	RR	12.58	5.95	14.77	21.29	12.17
	RRl	10.47	4.84	11.92	17.12	10.99
	RRu	15.13	7.30	18.30	26.47	13.48
	P	+++	+++	+++	+++	+++
Random	RR	11.22	5.39	14.02	19.79	10.92
	RRl	5.98	3.92	10.72	14.74	8.46
	RRu	21.03	7.41	18.34	26.58	14.08
	P	+++	+++	+++	+++	+++

			Duration of smoking (narrow categories)						
		absent	1-19k1	6-29k20	21-39k30	31-49k40	41-998k50	51+k999	Total
	N	25	2	2	7	8	1	1	46
	NS	13	2	2	7	8	1	1	33
	Wt	174.96	10.60	17.99	72.96	76.94	7.27	8.00	368.71
Het	Chi	101.43	4.37	1.12	6.79	11.71	0.00	0.00	206.05
Het	df	24	1	1	6	7	0	0	45
Het	P	***	*	N.S.	N.S.	N.S.	N.S.	N.S.	***
Fixed	RR	10.93	1.81	4.51	15.26	19.65	13.80	16.70	12.17
	RRl	9.43	0.99	2.84	12.13	15.72	6.67	8.35	10.99
	RRu	12.68	3.30	7.16	19.19	24.57	28.55	33.40	13.48
	P	+++	(+)	+++	+++	+++	+++	+++	+++
Random	RR	10.71	2.28	4.58	14.52	17.32	13.80	16.70	10.92
	RRl	7.35	0.57	2.76	10.85	11.76	6.67	8.35	8.46
	RRu	15.59	9.09	7.58	19.43	25.51	28.55	33.40	14.08
	P	+++	N.S.	+++	+++	+++	+++	+++	+++

FEMALES

		Duration of smoking (broad categories)				
		absent	1-34k20	21-49k35	36+k50	Total
	N	7	7	4	4	22
	NS	6	7	4	4	21
	Wt	101.17	100.12	78.41	57.94	337.63
Het	Chi	221.06	118.26	156.78	243.88	832.26
Het	df	6	6	3	3	21
Het	P	***	***	***	***	***
Fixed	RR	23.36	7.18	21.69	22.22	16.04
	RRl	19.22	5.90	17.39	17.18	14.42
	RRu	28.38	8.73	27.07	28.75	17.85
	P	+++	+++	+++	+++	+++
Random	RR	13.54	4.52	16.27	31.26	11.34
	RRl	3.82	1.68	2.23	1.93	5.59
	RRu	48.00	12.17	118.58	506.39	22.98
	P	+++	++	++	+	+++

Table 2I1 - 6

IESLC - Meta-analysis of Ever Smoking by Duration, Overview
Squamous, Any Product (or Cigarettes if Any not available)
Least adjusted

FEMALES

	Duration of smoking (narrow categories)							
	absent	1-19k1	6-29k20	21-39k30	31-49k40	41-998k50	51+k999	Total
N	15	1		4	2			22
NS	8	1		4	2			15
Wt	207.31	7.31		78.41	44.61			337.63
Het Chi	442.50	0.00		156.78	5.67			832.26
Het df	14	0		3	1			21
Het P	***	N.S.		***	*			***
Fixed RR	10.17	2.89		21.69	103.90			16.04
RRl	8.88	1.40		17.39	77.47			14.42
RRu	11.66	5.96		27.07	139.33			17.85
P	+++	++		+++	+++			+++
Random RR	8.99	2.89		16.27	66.35			11.34
RRl	3.99	1.40		2.23	18.79			5.59
RRu	20.25	5.96		118.58	234.28			22.98
P	+++	++		++	+++			+++

Table 2I1 - 7

IESLC - Meta-analysis of Ever Smoking by Duration, Overview
Squamous, Any Product (or Cigarettes if Any not available)
Excluded studies (and stage at which they were excluded)

1	AKIBA AMANDU AMES BECHER BENSHL BEST BLOT1 BROSS BROWN3 CARPEN CEDERL CHYOU CPSI CPSII DARBY DEAN2 DEAN3 DOLL2 ENGELA GAO2 GARCIA GILLIS GRAHAM GURSEL HAMMO2 HIRAYA HOLE HUMBLE JAHN JAIN KAISE2 KATSOU KAUFMA LAUSSM LIAW MCDUFF MIGRAN MRFITR PEZZO2 PISANI PRESCO QIAO SEGI2 SPEIZE SVENSS TVERDA WAKAI WATSON WIGLE WU WYNDE3 WYNDE8
2	ALDERS BRESLO CHIAZZ DORN GUO HEGMAN KOO KOULUM LIU4 PERNU SOBUE SPITZ SUZUK2 VUTUC YUAN
3	GENG STASZE WU2 ZHANG
4	AGUDO ARMADA AUVINE AXELSS BOFFET BOUCOT CHEN2 CORREA DESTEF DOLL FAN GAO GARSHI HAMMON HU HU2 JUSSAW KREUZE LETOUR LEVIN LIU3 LIU5 MCCONN NOTAN2 QIAO2 RACHTA RESTRE SADOWS TIZZAN WANG2 WYNDE6 WYNDE7
5	CHEN LUBIN XU
10	BOUCHA KHUDER
14	BENHAM

Table 2I1 - 8

Potentially overlapping studies

REF	REFGP	PRINC	OVERLAP/LINK
LUBIN2	LUBIN2	1	Lubin-combined
OSANN2	KAISER	2	KAISER/OSANN2

Table 2I1 - 9

Most adjusted - insufficient data for meta-analysis

REF	NRR	SEX	AGEL	AGEH	RACE	YF	LC TYPE	LOC	START	ST	NLC	R	VB	P	H	AD	PRODUCT	exL	exH	S1	S2	DENOM	De
CHEN	501	c	0	0	all	-	q	As:oth	1987	CC	323	n	ot	n	y	2	cig+/-ot	1	20	1	0	nev cigs	ot
CHEN	502	c	0	0	all	-	q	As:oth	1987	CC	323	n	ot	n	y	2	cig+/-ot	21	30	0	3	nev cigs	ot
CHEN	503	c	0	0	all	-	q	As:oth	1987	CC	323	n	ot	n	y	2	cig+/-ot	31	40	2	4	nev cigs	ot
CHEN	504	c	0	0	all	-	q	As:oth	1987	CC	323	n	ot	n	y	2	cig+/-ot	41	999	3	0	nev cigs	ot
XU	511	m	0	0	all	-	q+s	As:Chi	1985	CC	729	n	ot	n	n	2	all/unsp	1	29	1	0	nev any	or
XU	512	m	0	0	all	-	q+s	As:Chi	1985	CC	729	n	ot	n	n	2	all/unsp	30	39	2	3	nev any	or
XU	513	m	0	0	all	-	q+s	As:Chi	1985	CC	729	n	ot	n	n	2	all/unsp	40	999	3	0	nev any	or

REF	NRR	RR	SIG	RRDATA comment
CHEN	501	1.70	n	0
CHEN	502	2.76	n	0
CHEN	503	6.52	n	0
CHEN	504	8.43	y	p<0.001
XU	511	*		RR for 1-19/day is 2.3(p<0.05), for 20-29/day is 2.6(p<0.05) and for >=30/ day is 7.7(p<0.05)
XU	512	*		RR for 1-19/day is 2.9(p<0.05), for 20-29/day is 3.9(p<0.05) and for >=30/ day is 8.3(p<0.05)
XU	513	*		RR for 1-19/day is 5.0(p<0.05), for 20-29/day is 10.4(p<0.05) and for >=30/ day is 31.2(p<0.05)

Table 2I2 -

IESLC - Meta-analysis of Ever Smoking, Duration, "Low"
Squamous, Any Product (or Cigarettes if Any not available)

This analysis is restricted to results for:
1) Ever smokers
2) Results by Duration
3) Categorical results by Duration
4) Squamous (or near equivalent)
5) Results complete enough for use in metaanalysis

Within each study, results are then selected (in the following order of preference, within each sex) for:
6) (not applicable)
7) PRODUCT: all/unspec, cigarettes regardless of other products, cigarettes only
8) CIGTYPE: all/unspecified, MC regardless of HR, MC only
9) (not applicable)
10) DENOM: never smoked anything, never smoked cigarettes, never any + low, never cigs + low
11) Followup period (YF, prospective studies): whole study (coded as 0) or longest available
12) LCtype: squamous or nearest available, but not adeno. (q = squamous, s = small,
a = adeno, KI = Kreyberg I, u = undifferentiated)
13) Race: all or nearest available, otherwise by race (wh or w = white, bl or b = black, hi = hispanic
ch = chinese, jap = japanese, haw = hawaiian, w+o = white + oriental, sca = scandinavian, as = asian)
14) Duration "low" in key scheme 1 (key value 20, maximum range 1-34)
15) For overlapping studies: principal rather than subsidiary studies
Finally by Age: whole study (coded as 0) if available, otherwise by widest available age group
and then for single sex results (m, f) in preference to results for both sexes combined (c).

Results adjusted (AD) for the most potential confounders are then chosen in Sections -1 to -3
and results adjusted for the least confounders in Sections -4 to -6. (Those least adjusted results which
actually differ from the most adjusted are marked 'x' in column X in Section -4)

Section -7 shows excluded studies, together with the stage (as above) at which no qualifying
results were found.

Section -8 lists the potentially overlapping studies which have been included (1=principal, 2=subsidiary).

Section -9 lists any results which would have been included in preference except that they had data not complete
enough for use in meta-analysis, with their significance (yes/no), if known, and any further comment as entered
on the database. It also lists as "gap" any categories for which no data were presented by the original authors.

In addition to those mentioned above, the following fields, levels and abbreviations are used:
* or nk = not known, n = no, y = yes, ot = other
nev = never
all/unspec = all or unspecified, cig+/-ot = cigarettes irrespective of other products (cigar, pipe etc)
MC = manufactured cigarettes, HR = hand-rolled cigarettes
exL, exH = range of exposure (low and high) in the smoking group, in terms of Duration
REF: 6-character study reference
NRR: number of the RR on the database within the study
ST : study type (CC = case control, pr or prosp = prospective)
NLC: number of lung cancer cases in whole study
R : risky occupational population (n = no, m = mining, o = other risky)
VB : national cigarette type (V = at least 75% Virginia, bl = at least 75% blended, ot = other)
P : any proxy use
H : full histological confirmation
De : derivation of RR/CI (or = original, st = standard method, ot = other method of estimation)

Table 2I2 - 1

IESLC - Meta-analysis of Ever Smoking, Duration, "Low"
Squamous, Any Product (or Cigarettes if Any not available)
Most adjusted

REF	NRR	SEX	AGEL	AGEH	RACE	YF	LC TYPE	LOC	START	ST	NLC	R	VB	P	H	AD	PRODUCT	exL	exH	DENOM	De
BARBON	562	m	0	0	all	-	q	Eu:wst	1979	CC	755	n	bl	y	y	1	all/unsp	1	29	nev any	or
BUFFLE	505	m	0	0	wh	-	q	NAmer	1976	CC	943	n	bl	y	n	0	cig+/-ot	1	33	nev cigs	or
CHOI	559	m	0	0	all	-	q	As:oth	1985	CC	375	n	bl	n	n	0	cig+/-ot	1	29	nev cigs	st
CHOI	573	f	0	0	all	-	q	As:oth	1985	CC	375	n	bl	n	n	0	cig+/-ot	1	29	nev cigs	st
DAMBER	547	m	0	0	all	-	q	Eu:Sca	1972	CC	579	n	bl	y	n	1	all/unsp	1	30	nev any	or
DORGAN	572	m	0	0	wh	-	q	NAmer	1980	CC	2026	n	bl	y	y	2	cig+/-ot	1	34	nev any	ot
DORGAN	564	f	0	0	all	-	q	NAmer	1980	CC	2026	n	bl	y	y	3	cig+/-ot	1	34	nev any	ot
DOSEME	512	m	0	0	all	-	q	Eu:bal	1979	CC	1210	n	bl	n	n	2	cig+/-ot	11	20	nev cigs	or
GER	510	c	0	0	all	-	q+s	As:oth	1990	CC	141	n	ot	y	n	5	all/unsp	1	30	nev any	ot
JEDRYC	502	m	0	0	all	-	q	Eu:est	1980	CC	1630	n	bl	y	n	0	cig+/-ot	20	29	nev any	st
JOLY	639	m	0	0	all	-	q	SCAmer	1978	CC	826	n	bl	n	n	0	cig+/-ot	1	29	nev any	st
JOLY	611	f	0	0	all	-	q	SCAmer	1978	CC	826	n	bl	n	n	0	cig+/-ot	1	29	nev any	st
LUBIN2	661	m	0	0	all	-	q	Eu:mul	1976	CC	7804	n	bl	n	y	0	cig+/-ot	1	29	nev any	st
LUBIN2	713	f	0	0	all	-	q	Eu:mul	1976	CC	7804	n	bl	n	y	0	cig+/-ot	1	29	nev any	st
LUO	504	c	0	0	all	-	q	As:Chi	1990	CC	102	n	ot	n	y	20	cig+/-ot	1	29	nev cigs	or
MATOS	606	m	0	0	all	-	q	SCAmer	1994	CC	200	n	bl	n	n	2	cig+/-ot	1	24	nev any	or
OSANN2	510	f	0	0	all	-	KI	NAmer	1964	ot	217	n	bl	n	y	1	cig+/-ot	1	20	nev cigs	or
PEZZOT	507	m	0	0	all	-	q	SCAmer	1987	CC	215	n	bl	n	y	0	cig only	1	30	nev cigs	ot
WUWILL	521	f	0	0	all	-	q	As:Chi	1985	CC	965	n	ot	n	n	3	cig+/-ot	1	29	nev cigs	ot
WYNDE2	506	m	0	0	all	-	KI	NAmer	1962	CC	404	n	bl	n	y	0	cig+/-ot	1	29	nev any	ot
ZHENG	501	m	0	0	all	-	q	As:Chi	1982	CC	540	n	ot	*	y	0	cig+/-ot	1	29	nev cigs	st
ZHENG	508	f	0	0	all	-	q	As:Chi	1982	CC	540	n	ot	*	y	0	cig+/-ot	1	29	nev cigs	st

Cigarette type is all/unspec for all RRs

Table 2I2 - 2

IESLC - Meta-analysis of Ever Smoking, Duration, "Low"
Squamous, Any Product (or Cigarettes if Any not available)
Most adjusted

REF	NRR	SEX	AD	Number Exposed Case	Cont	Non-exposed Case	Cont	RR	95.00%CI
BARBON	562	m	1	7	-	6	-	2.10	(0.70- 6.50)
BUFFLE	505	m	0	-	-	-	-	9.00	(2.90- 27.90)
CHOI	559	m	0	42	221	6	95	3.01	(1.24- 7.32)
CHOI	573	f	0	6	23	10	164	4.28	(1.42- 12.88)
Subtotal CHOI								3.46	(1.73- 6.90)
DAMBER	547	m	1	-	-	14	-	4.40	(1.80- 10.70)
DORGAN	572	m	2	-	-	-	-	9.47	(3.39- 26.45)
DORGAN	564	f	3	-	-	-	-	4.31	(2.53- 7.35)
Subtotal DORGAN								5.09	(3.17- 8.18)
DOSEME	512	m	2	70	-	58	-	3.90	(2.30- 6.70)
GER	510	c	5	6	-	11	-	1.53	(0.40- 5.86)
JEDRYC	502	m	0	23	160	6	289	6.92	(2.76- 17.36)
JOLY	639	m	0	15	109	2	218	15.00	(3.37- 66.77)
JOLY	611	f	0	5	54	6	283	4.37	(1.29- 14.82)
Subtotal JOLY								7.16	(2.78- 18.44)
LUBIN2	661	m	0	453	2964	54	2616	7.40	(5.56- 9.87)
LUBIN2	713	f	0	322	229	72	1180	23.04	(17.21- 30.86)
Subtotal LUBIN2								12.93	(10.54- 15.87)
LUO	504	c	20	6	-	5	-	5.70	(1.00- 32.90)
MATOS	606	m	2	3	-	3	-	1.20	(0.20- 6.20)
OSANN2	510	f	1	11	-	7	-	4.90	(0.50- 44.60)
PEZZOT	507	m	0	5	134	0	116	9.53~	(0.52- 174.14)
WUWILL	521	f	3	54	-	117	-	2.00	(1.36- 2.94)
WYNDE2	506	m	0	22	55	0	41	33.65~	(1.98- 570.85)
ZHENG	501	m	0	13	75	4	94	4.07	(1.28- 13.01)
ZHENG	508	f	0	8	17	33	184	2.62	(1.05- 6.57)
Subtotal ZHENG								3.11	(1.51- 6.39)
Partial Totals				1071	4041	414	5280		

*prospective study ~ With 0.5 adjustment for zero

REF	NRR	SEX	AD	Ys	Ws	Qs	Ps
BARBON	562	m	1	0.74	3.09	4.05	0.1919
BUFFLE	505	m	0	2.20	3.00	0.29	0.0001
CHOI	559	m	0	1.10	4.87	2.99	0.0151
CHOI	573	f	0	1.45	3.16	0.59	0.0097
Subtotal CHOI				1.24	8.03	3.58	
DAMBER	547	m	1	1.48	4.84	0.79	0.0011
DORGAN	572	m	2	2.25	3.64	0.48	0.0000
DORGAN	564	f	3	1.46	13.51	2.44	0.0000
Subtotal DORGAN				1.63	17.15	2.91	
DOSEME	512	m	2	1.36	13.44	3.70	0.0000
GER	510	c	5	0.43	2.13	4.55	0.5346
JEDRYC	502	m	0	1.93	4.55	0.01	0.0000
JOLY	639	m	0	2.71	1.72	1.17	0.0004
JOLY	611	f	0	1.47	2.57	0.44	0.0181
Subtotal JOLY				1.97	4.30	1.60	
LUBIN2	661	m	0	2.00	46.63	0.63	0.0000
LUBIN2	713	f	0	3.14	45.03	70.57	0.0000
Subtotal LUBIN2				2.56	91.66	71.21	
LUO	504	c	20	1.74	1.26	0.03	0.0508
MATOS	606	m	2	0.18	1.30	3.78	0.8351
OSANN2	510	f	1	1.59	0.76	0.07	0.1654
PEZZOT	507	m	0	2.25	0.45	0.06	0.1284
WUWILL	521	f	3	0.69	25.85	36.76	0.0004
WYNDE2	506	m	0	3.52	0.48	1.27	0.0149
ZHENG	501	m	0	1.40	2.85	0.66	0.0177
ZHENG	508	f	0	0.96	4.55	3.86	0.0395
Subtotal ZHENG				1.13	7.40	4.52	

Table 2I2 - 2

IESLC - Meta-analysis of Ever Smoking, Duration, "Low"
Squamous, Any Product (or Cigarettes if Any not available)
Most adjusted

	N	22
	NS	17
	Wt	189.70
Het	Chi	139.17
Het	df	21
Het	P	***
Fixed	RR	6.59
	RRl	5.72
	RRu	7.60
	P	+++
Random	RR	4.91
	RRl	3.18
	RRu	7.58
	P	+++
Asymm	P	N.S.

Table 2I2 - 3

IESLC - Meta-analysis of Ever Smoking, Duration, "Low"
Squamous, Any Product (or Cigarettes if Any not available)
Most adjusted

	Sex combined	male	female	Total
N	2	13	7	22
NS	2	13	7	22
Wt	3.39	90.86	95.44	189.70
Het Chi	1.37	18.85	112.96	139.17
Het df	1	12	6	21
Het P	N.S.	(*)	***	***
Fixed RR	2.49	5.92	7.55	6.59
RRl	0.86	4.82	6.18	5.72
RRu	7.23	7.28	9.23	7.60
P	(+)	+++	+++	+++
Random RR	2.61	5.33	4.72	4.91
RRl	0.74	3.84	1.69	3.18
RRu	9.25	7.41	13.17	7.58
P	N.S.	+++	++	+++
Between Chi				5.99
Between df				2
Between P				*
Btwn(F) P				N.S.
Btwn(R) P				N.S.

Table 2I2 - 4

IESLC - Meta-analysis of Ever Smoking, Duration, "Low"
Squamous, Any Product (or Cigarettes if Any not available)
Least adjusted

REF	NRR	X	SEX	AGEL	AGEH	RACE	YF	LC TYPE	LOC	START	ST	NLC	R	VB	P	H	AD	PRODUCT	exL	exH	DENOM	De
BARBON	555	x	m	0	0	all	-	q	Eu:wst	1979	CC	755	n	bl	y	y	0	all/unsp	1	29	nev any	st
BUFFLE	505		m	0	0	wh	-	q	NAmer	1976	CC	943	n	bl	y	n	0	cig+/-ot	1	33	nev cigs	or
CHOI	559		m	0	0	all	-	q	As:oth	1985	CC	375	n	bl	n	n	0	cig+/-ot	1	29	nev cigs	st
CHOI	573		f	0	0	all	-	q	As:oth	1985	CC	375	n	bl	n	n	0	cig+/-ot	1	29	nev cigs	st
DAMBER	547		m	0	0	all	-	q	Eu:Sca	1972	CC	579	n	bl	y	n	1	all/unsp	1	30	nev any	or
DORGAN	572		m	0	0	wh	-	q	NAmer	1980	CC	2026	n	bl	y	y	2	cig+/-ot	1	34	nev any	ot
DORGAN	564		f	0	0	all	-	q	NAmer	1980	CC	2026	n	bl	y	y	3	cig+/-ot	1	34	nev any	ot
DOSEME	512		m	0	0	all	-	q	Eu:bal	1979	CC	1210	n	bl	n	n	2	cig+/-ot	11	20	nev cigs	or
GER	504	x	c	0	0	all	-	q+s	As:oth	1990	CC	141	n	ot	y	n	0	all/unsp	1	30	nev any	st
JEDRYC	502		m	0	0	all	-	q	Eu:est	1980	CC	1630	n	bl	y	n	0	cig+/-ot	20	29	nev any	st
JOLY	639		m	0	0	all	-	q	SCAmer	1978	CC	826	n	bl	n	n	0	cig+/-ot	1	29	nev any	st
JOLY	611		f	0	0	all	-	q	SCAmer	1978	CC	826	n	bl	n	n	0	cig+/-ot	1	29	nev any	st
LUBIN2	661		m	0	0	all	-	q	Eu:mul	1976	CC	7804	n	bl	n	y	0	cig+/-ot	1	29	nev any	st
LUBIN2	713		f	0	0	all	-	q	Eu:mul	1976	CC	7804	n	bl	n	y	0	cig+/-ot	1	29	nev any	st
LUO	501	x	c	0	0	all	-	q	As:Chi	1990	CC	102	n	ot	n	y	0	cig+/-ot	1	29	nev cigs	st
MATOS	601	x	m	0	0	all	-	q	SCAmer	1994	CC	200	n	bl	n	n	0	cig+/-ot	1	24	nev any	st
OSANN2	507	x	f	0	0	all	-	KI	NAmer	1964	ot	217	n	bl	n	y	0	cig+/-ot	1	20	nev cigs	st
PEZZOT	507		m	0	0	all	-	q	SCAmer	1987	CC	215	n	bl	n	y	0	cig only	1	30	nev cigs	ot
WUWILL	506	x	f	0	0	all	-	q	As:Chi	1985	CC	965	n	ot	n	n	0	cig+/-ot	1	29	nev cigs	st
WYNDE2	506		m	0	0	all	-	KI	NAmer	1962	CC	404	n	bl	n	y	0	cig+/-ot	1	29	nev any	ot
ZHENG	501		m	0	0	all	-	q	As:Chi	1982	CC	540	n	ot	*	y	0	cig+/-ot	1	29	nev cigs	st
ZHENG	508		f	0	0	all	-	q	As:Chi	1982	CC	540	n	ot	*	y	0	cig+/-ot	1	29	nev cigs	st

Cigarette type is all/unspec for all RRs

Table 2I2 - 5

IESLC - Meta-analysis of Ever Smoking, Duration, "Low"
Squamous, Any Product (or Cigarettes if Any not available)
Least adjusted

REF	NRR	SEX	AD	Number Exposed Case	Exposed Cont	Non-exposed Case	Non-exposed Cont	RR	95.00%CI
BARBON	555	m	0	7	91	6	188	2.41	(0.79- 7.38)
BUFFLE	505	m	0	-	-	-	-	9.00	(2.90- 27.90)
CHOI	559	m	0	42	221	6	95	3.01	(1.24- 7.32)
CHOI	573	f	0	6	23	10	164	4.28	(1.42- 12.88)
Subtotal CHOI								3.46	(1.73- 6.90)
DAMBER	547	m	1	-	-	14	-	4.40	(1.80- 10.70)
DORGAN	572	m	2	-	-	-	-	9.47	(3.39- 26.45)
DORGAN	564	f	3	-	-	-	-	4.31	(2.53- 7.35)
Subtotal DORGAN								5.09	(3.17- 8.18)
DOSEME	512	m	2	70	-	58	-	3.90	(2.30- 6.70)
GER	504	c	0	6	37	11	80	1.18	(0.41- 3.43)
JEDRYC	502	m	0	23	160	6	289	6.92	(2.76- 17.36)
JOLY	639	m	0	15	109	2	218	15.00	(3.37- 66.77)
JOLY	611	f	0	5	54	6	283	4.37	(1.29- 14.82)
Subtotal JOLY								7.16	(2.78- 18.44)
LUBIN2	661	m	0	453	2964	54	2616	7.40	(5.56- 9.87)
LUBIN2	713	f	0	322	229	72	1180	23.04	(17.21- 30.86)
Subtotal LUBIN2								12.93	(10.54- 15.87)
LUO	501	c	0	6	21	5	51	2.91	(0.80- 10.60)
MATOS	601	m	0	3	84	3	110	1.31	(0.26- 6.65)
OSANN2	507	f	0	11	26	7	58	3.51	(1.22- 10.06)
PEZZOT	507	m	0	5	134	0	116	9.53~	(0.52- 174.14)
WUWILL	506	f	0	54	139	117	601	2.00	(1.38- 2.89)
WYNDE2	506	m	0	22	55	0	41	33.65~	(1.98- 570.85)
ZHENG	501	m	0	13	75	4	94	4.07	(1.28- 13.01)
ZHENG	508	f	0	8	17	33	184	2.62	(1.05- 6.57)
Subtotal ZHENG								3.11	(1.51- 6.39)
Partial Totals				1071	4439	414	6368		

*prospective study ~ With 0.5 adjustment for zero

REF	NRR	SEX	AD	Ys	Ws	Qs	Ps
BARBON	555	m	0	0.88	3.07	2.85	0.1233
BUFFLE	505	m	0	2.20	3.00	0.38	0.0001
CHOI	559	m	0	1.10	4.87	2.67	0.0151
CHOI	573	f	0	1.45	3.16	0.48	0.0097
Subtotal CHOI				1.24	8.03	3.15	
DAMBER	547	m	1	1.48	4.84	0.63	0.0011
DORGAN	572	m	2	2.25	3.64	0.60	0.0000
DORGAN	564	f	3	1.46	13.51	1.97	0.0000
Subtotal DORGAN				1.63	17.15	2.57	
DOSEME	512	m	2	1.36	13.44	3.12	0.0000
GER	504	c	0	0.16	3.37	9.47	0.7621
JEDRYC	502	m	0	1.93	4.55	0.04	0.0000
JOLY	639	m	0	2.71	1.72	1.29	0.0004
JOLY	611	f	0	1.47	2.57	0.35	0.0181
Subtotal JOLY				1.97	4.30	1.64	
LUBIN2	661	m	0	2.00	46.63	1.18	0.0000
LUBIN2	713	f	0	3.14	45.03	75.49	0.0000
Subtotal LUBIN2				2.56	91.66	76.67	
LUO	501	c	0	1.07	2.30	1.38	0.1044
MATOS	601	m	0	0.27	1.45	3.60	0.7450
OSANN2	507	f	0	1.25	3.45	1.20	0.0197
PEZZOT	507	m	0	2.25	0.45	0.08	0.1284
WUWILL	506	f	0	0.69	27.84	36.92	0.0003
WYNDE2	506	m	0	3.52	0.48	1.34	0.0149
ZHENG	501	m	0	1.40	2.85	0.55	0.0177
ZHENG	508	f	0	0.96	4.55	3.51	0.0395
Subtotal ZHENG				1.13	7.40	4.06	

Table 2I2 - 5

IESLC - Meta-analysis of Ever Smoking, Duration, "Low"
Squamous, Any Product (or Cigarettes if Any not available)
Least adjusted

	N	22
	NS	17
	Wt	196.78
Het	Chi	149.08
Het	df	21
Het	P	***
Fixed	RR	6.31
	RRl	5.49
	RRu	7.26
	P	+++
Random	RR	4.65
	RRl	3.02
	RRu	7.15
	P	+++
Asymm	P	N.S.

Table 2I2 - 6

IESLC - Meta-analysis of Ever Smoking, Duration, "Low"
Squamous, Any Product (or Cigarettes if Any not available)
Least adjusted

	Sex combined	male	female	Total
N	2	13	7	22
NS	2	13	7	22
Wt	5.67	90.99	100.12	196.78
Het Chi	1.12	17.99	118.26	149.08
Het df	1	12	6	21
Het P	N.S.	N.S.	***	***
Fixed RR	1.70	5.95	7.18	6.31
RRl	0.75	4.84	5.90	5.49
RRu	3.88	7.30	8.73	7.26
P	N.S.	+++	+++	+++
Random RR	1.72	5.39	4.52	4.65
RRl	0.72	3.92	1.68	3.02
RRu	4.12	7.41	12.17	7.15
P	N.S.	+++	++	+++
Between Chi				11.71
Between df				2
Between P				**
Btwn(F) P				N.S.
Btwn(R) P				(*)

Table 2I2 - 7

IESLC - Meta-analysis of Ever Smoking, Duration, "Low"
Squamous, Any Product (or Cigarettes if Any not available)
Excluded studies (and stage at which they were excluded)

1	AKIBA AMANDU AMES BECHER BENSHL BEST BLOT1 BROSS BROWN3 CARPEN CEDERL CHYOU CPSI CPSII DARBY DEAN2 DEAN3 DOLL2 ENGELA GAO2 GARCIA GILLIS GRAHAM GURSEL HAMMO2 HIRAYA HOLE HUMBLE JAHN JAIN KAISE2 KATSOU KAUFMA LAUSSM LIAW MCDUFF MIGRAN MRFITR PEZZO2 PISANI PRESCO QIAO SEGI2 SPEIZE SVENSS TVERDA WAKAI WATSON WIGLE WU WYNDE3 WYNDE8
2	ALDERS BRESLO CHIAZZ DORN GUO HEGMAN KOO KOULUM LIU4 PERNU SOBUE SPITZ SUZUK2 VUTUC YUAN
3	GENG STASZE WU2 ZHANG
4	AGUDO ARMADA AUVINE AXELSS BOFFET BOUCOT CHEN2 CORREA DESTEF DOLL FAN GAO GARSHI HAMMON HU HU2 JUSSAW KREUZE LETOUR LEVIN LIU3 LIU5 MCCONN NOTAN2 QIAO2 RACHTA RESTRE SADOWS TIZZAN WANG2 WYNDE6 WYNDE7
5	CHEN LUBIN XU
10	BOUCHA KHUDER
14	HAENSZ ZHOU
15	BENHAM

Table 2I2 - 8
Potentially overlapping studies

REF	REFGP	PRINC	.	OVERLAP/LINK
LUBIN2	LUBIN2	1		Lubin-combined
OSANN2	KAISER	2		KAISER/OSANN2

Table 2I2 - 9
Most adjusted - insufficient data for meta-analysis

REF	NRR	SEX	AGEL	AGEH	RACE	YF	LC TYPE	LOC	START	ST	NLC	R	VB	P	H	AD	PRODUCT	exL	exH	DENOM	De
CHEN	501	c	0	0	all	-	q	As:oth	1987	CC	323	n	ot	n	y	2	cig+/-ot	1	20	nev cigs	ot
XU	511	m	0	0	all	-	q+s	As:Chi	1985	CC	729	n	ot	n	n	2	all/unsp	1	29	nev any	or

REF	NRR	RR	SIG	RRDATA comment
CHEN	501	1.70	n	0
XU	511	*		RR for 1-19/day is 2.3(p<0.05), for 20-29/day is 2.6(p<0.05) and for >=30/ day is 7.7(p<0.05)

Table 2I3 -

IESLC - Meta-analysis of Ever Smoking, Duration, "Mid"
Squamous, Any Product (or Cigarettes if Any not available)

This analysis is restricted to results for:
1) Ever smokers
2) Results by Duration
3) Categorical results by Duration
4) Squamous (or near equivalent)
5) Results complete enough for use in metaanalysis

Within each study, results are then selected (in the following order of preference, within each sex) for:
6) (not applicable)
7) PRODUCT: all/unspec, cigarettes regardless of other products, cigarettes only
8) CIGTYPE: all/unspecified, MC regardless of HR, MC only
9) (not applicable)
10) DENOM: never smoked anything, never smoked cigarettes, never any + low, never cigs + low
11) Followup period (YF, prospective studies): whole study (coded as 0) or longest available
12) LCtype: squamous or nearest available, but not adeno. (q = squamous, s = small,
a = adeno, KI = Kreyberg I, u = undifferentiated)
13) Race: all or nearest available, otherwise by race (wh or w = white, bl or b = black, hi = hispanic
ch = chinese, jap = japanese, haw = hawaiian, w+o = white + oriental, sca = scandinavian, as = asian)
14) Duration "mid" in key scheme 1 (key value 35, maximum range 21-49)
15) For overlapping studies: principal rather than subsidiary studies
Finally by Age: whole study (coded as 0) if available, otherwise by widest available age group
and then for single sex results (m, f) in preference to results for both sexes combined (c).

Results adjusted (AD) for the most potential confounders are then chosen in Sections -1 to -3
and results adjusted for the least confounders in Sections -4 to -6. (Those least adjusted results which
actually differ from the most adjusted are marked 'x' in column X in Section -4)

Section -7 shows excluded studies, together with the stage (as above) at which no qualifying
results were found.

Section -8 lists the potentially overlapping studies which have been included (1=principal, 2=subsidiary).

Section -9 lists any results which would have been included in preference except that they had data not complete
enough for use in meta-analysis, with their significance (yes/no), if known, and any further comment as entered
on the database. It also lists as "gap" any categories for which no data were presented by the original authors.

In addition to those mentioned above, the following fields, levels and abbreviations are used:
* or nk = not known, n = no, y = yes, ot = other
nev = never
all/unspec = all or unspecified, cig+/-ot = cigarettes irrespective of other products (cigar, pipe etc)
MC = manufactured cigarettes, HR = hand-rolled cigarettes
exL, exH = range of exposure (low and high) in the smoking group, in terms of Duration
REF: 6-character study reference
NRR: number of the RR on the database within the study
ST : study type (CC = case control, pr or prosp = prospective)
NLC: number of lung cancer cases in whole study
R : risky occupational population (n = no, m = mining, o = other risky)
VB : national cigarette type (V = at least 75% Virginia, bl = at least 75% blended, ot = other)
P : any proxy use
H : full histological confirmation
De : derivation of RR/CI (or = original, st = standard method, ot = other method of estimation)

Table 2I3 - 1

IESLC - Meta-analysis of Ever Smoking, Duration, "Mid"
Squamous, Any Product (or Cigarettes if Any not available)
Most adjusted

REF	NRR	SEX	AGEL	AGEH	RACE	YF	LC TYPE	LOC	START	ST	NLC	R	VB	P	H	AD	PRODUCT	exL	exH	DENOM	De
BARBON	563	m	0	0	all	-	q	Eu:wst	1979	CC	755	n	bl	y	y	1	all/unsp	30	39	nev any	or
BUFFLE	506	m	0	0	wh	-	q	NAmer	1976	CC	943	n	bl	y	n	0	cig+/-ot	34	43	nev cigs	or
CHOI	560	m	0	0	all	-	q	As:oth	1985	CC	375	n	bl	n	n	0	cig+/-ot	30	39	nev cigs	st
CHOI	574	f	0	0	all	-	q	As:oth	1985	CC	375	n	bl	n	n	0	cig+/-ot	30	39	nev cigs	st
DAMBER	548	m	0	0	all	-	q	Eu:Sca	1972	CC	579	n	bl	y	n	1	all/unsp	31	40	nev any	or
JEDRYC	503	m	0	0	all	-	q	Eu:est	1980	CC	1630	n	bl	y	n	0	cig+/-ot	30	39	nev any	st
JOLY	640	m	0	0	all	-	q	SCAmer	1978	CC	826	n	bl	n	n	0	cig+/-ot	30	39	nev any	st
JOLY	612	f	0	0	all	-	q	SCAmer	1978	CC	826	n	bl	n	n	0	cig+/-ot	30	39	nev any	st
LUBIN2	662	m	0	0	all	-	q	Eu:mul	1976	CC	7804	n	bl	n	y	0	cig+/-ot	30	39	nev any	st
LUBIN2	714	f	0	0	all	-	q	Eu:mul	1976	CC	7804	n	bl	n	y	0	cig+/-ot	30	39	nev any	st
MATOS	607	m	0	0	all	-	q	SCAmer	1994	CC	200	n	bl	n	n	2	cig+/-ot	25	39	nev any	or
PEZZOT	508	m	0	0	all	-	q	SCAmer	1987	CC	215	n	bl	n	y	0	cig only	31	40	nev cigs	ot
WUWILL	522	f	0	0	all	-	q	As:Chi	1985	CC	965	n	ot	n	n	3	cig+/-ot	30	39	nev cigs	ot
WYNDE2	507	m	0	0	all	-	KI	NAmer	1962	CC	404	n	bl	n	y	0	cig+/-ot	30	40	nev any	ot
ZHENG	502	m	0	0	all	-	q	As:Chi	1982	CC	540	n	ot	*	y	0	cig+/-ot	30	39	nev cigs	st

Cigarette type is all/unspec for all RRs

Table 2I3 - 2

IESLC - Meta-analysis of Ever Smoking, Duration, "Mid"
Squamous, Any Product (or Cigarettes if Any not available)
Most adjusted

REF	NRR	SEX	AD	Number Exposed Case	Cont	Non-exposed Case	Cont	RR	95.00%CI
BARBON	563	m	1	36	-	6	-	9.60	(3.90- 23.90)
BUFFLE	506	m	0	-	-	-	-	14.80	(4.80- 45.30)
CHOI	560	m	0	73	160	6	95	7.22	(3.03- 17.25)
CHOI	574	f	0	4	2	10	164	32.80	(5.35- 201.12)
Subtotal CHOI								9.59	(4.38- 21.01)
DAMBER	548	m	1	-	-	14	-	8.40	(4.00- 18.30)
JEDRYC	503	m	0	106	231	6	289	22.10	(9.54- 51.22)
JOLY	640	m	0	24	165	2	218	15.85	(3.69- 68.04)
JOLY	612	f	0	5	24	6	283	9.83	(2.79- 34.57)
Subtotal JOLY								12.05	(4.65- 31.23)
LUBIN2	662	m	0	1211	3473	54	2616	16.89	(12.80- 22.29)
LUBIN2	714	f	0	767	186	72	1180	67.58	(50.73- 90.03)
Subtotal LUBIN2								33.00	(27.04- 40.29)
MATOS	607	m	2	18	-	3	-	5.80	(1.60- 20.50)
PEZZOT	508	m	0	35	82	0	116	100.26~	(6.06-1657.79)
WUWILL	522	f	3	66	-	117	-	3.88	(2.64- 5.71)
WYNDE2	507	m	0	30	64	0	41	39.25~	(2.34- 659.46)
ZHENG	502	m	0	59	80	4	94	17.33	(6.03- 49.81)
Partial Totals				2434	4467	300	5096		

*prospective study ~ With 0.5 adjustment for zero

REF	NRR	SEX	AD	Ys	Ws	Qs	Ps
BARBON	563	m	1	2.26	4.68	2.01	0.0000
BUFFLE	506	m	0	2.69	3.05	0.15	0.0000
CHOI	560	m	0	1.98	5.07	4.48	0.0000
CHOI	574	f	0	3.49	1.17	0.38	0.0002
Subtotal CHOI				2.26	6.24	4.86	
DAMBER	548	m	1	2.13	6.65	4.14	0.0000
JEDRYC	503	m	0	3.10	5.44	0.17	0.0000
JOLY	640	m	0	2.76	1.81	0.04	0.0002
JOLY	612	f	0	2.29	2.43	0.97	0.0004
Subtotal JOLY				2.49	4.24	1.01	
LUBIN2	662	m	0	2.83	49.96	0.41	0.0000
LUBIN2	714	f	0	4.21	46.69	78.45	0.0000
Subtotal LUBIN2				3.50	96.66	78.86	
MATOS	607	m	2	1.76	2.36	3.17	0.0069
PEZZOT	508	m	0	4.61	0.49	1.40	0.0013
WUWILL	522	f	3	1.36	25.82	62.94	0.0000
WYNDE2	507	m	0	3.67	0.48	0.27	0.0108
ZHENG	502	m	0	2.85	3.45	0.01	0.0000

	N	15
	NS	12
	Wt	159.54
Het	Chi	159.00
Het	df	14
Het	P	***
Fixed	RR	18.49
	RRl	15.83
	RRu	21.59
	P	+++
Random	RR	14.64
	RRl	7.84
	RRu	27.35
	P	+++
Asymm	P	N.S.

Table 2I3 - 3

IESLC - Meta-analysis of Ever Smoking, Duration, "Mid"
Squamous, Any Product (or Cigarettes if Any not available)
Most adjusted

	Sex combined	male	female	Total
N		11	4	15
NS		11	4	15
Wt		83.44	76.11	159.54
Het Chi		11.77	137.83	159.00
Het df		10	3	14
Het P		N.S.	***	***
Fixed RR		14.66	23.84	18.49
RRl		11.83	19.04	15.83
RRu		18.17	29.84	21.59
P		+++	+++	+++
Random RR		13.69	16.76	14.64
RRl		10.30	2.48	7.84
RRu		18.20	113.10	27.35
P		+++	++	+++
Between Chi				9.40
Between df				1
Between P				**
Btwn(F) P				N.S.
Btwn(R) P				N.S.

Table 2I3 - 4

IESLC - Meta-analysis of Ever Smoking, Duration, "Mid"
Squamous, Any Product (or Cigarettes if Any not available)
Least adjusted

REF	NRR	X	SEX	AGEL	AGEH	RACE	YF	LC TYPE	LOC	START	ST	NLC	R	VB	P	H	AD	PRODUCT	exL	exH	DENOM	De
BARBON	556	x	m	0	0	all	-	q	Eu:wst	1979	CC	755	n	bl	y	y	0	all/unsp	30	39	nev any	st
BUFFLE	506		m	0	0	wh	-	q	NAmer	1976	CC	943	n	bl	y	n	0	cig+/-ot	34	43	nev cigs	or
CHOI	560		m	0	0	all	-	q	As:oth	1985	CC	375	n	bl	n	n	0	cig+/-ot	30	39	nev cigs	st
CHOI	574		f	0	0	all	-	q	As:oth	1985	CC	375	n	bl	n	n	0	cig+/-ot	30	39	nev cigs	st
DAMBER	548		m	0	0	all	-	q	Eu:Sca	1972	CC	579	n	bl	y	n	1	all/unsp	31	40	nev any	or
JEDRYC	503		m	0	0	all	-	q	Eu:est	1980	CC	1630	n	bl	y	n	0	cig+/-ot	30	39	nev any	st
JOLY	640		m	0	0	all	-	q	SCAmer	1978	CC	826	n	bl	n	n	0	cig+/-ot	30	39	nev any	st
JOLY	612		f	0	0	all	-	q	SCAmer	1978	CC	826	n	bl	n	n	0	cig+/-ot	30	39	nev any	st
LUBIN2	662		m	0	0	all	-	q	Eu:mul	1976	CC	7804	n	bl	n	y	0	cig+/-ot	30	39	nev any	st
LUBIN2	714		f	0	0	all	-	q	Eu:mul	1976	CC	7804	n	bl	n	y	0	cig+/-ot	30	39	nev any	st
MATOS	602	x	m	0	0	all	-	q	SCAmer	1994	CC	200	n	bl	n	n	0	cig+/-ot	25	39	nev any	st
PEZZOT	508		m	0	0	all	-	q	SCAmer	1987	CC	215	n	bl	n	y	0	cig only	31	40	nev cigs	ot
WUWILL	507	x	f	0	0	all	-	q	As:Chi	1985	CC	965	n	ot	n	n	0	cig+/-ot	30	39	nev cigs	st
WYNDE2	507		m	0	0	all	-	KI	NAmer	1962	CC	404	n	bl	n	y	0	cig+/-ot	30	40	nev any	ot
ZHENG	502		m	0	0	all	-	q	As:Chi	1982	CC	540	n	ot	*	y	0	cig+/-ot	30	39	nev cigs	st

Cigarette type is all/unspec for all RRs

Table 2I3 - 5

IESLC - Meta-analysis of Ever Smoking, Duration, "Mid"
Squamous, Any Product (or Cigarettes if Any not available)
Least adjusted

REF	NRR	SEX	AD	Number Exposed Case	Cont	Non-exposed Case	Cont	RR	95.00%CI
BARBON	556	m	0	36	102	6	188	11.06	(4.51- 27.13)
BUFFLE	506	m	0	-	-	-	-	14.80	(4.80- 45.30)
CHOI	560	m	0	73	160	6	95	7.22	(3.03- 17.25)
CHOI	574	f	0	4	2	10	164	32.80	(5.35- 201.12)
Subtotal CHOI								9.59	(4.38- 21.01)
DAMBER	548	m	1	-	-	14	-	8.40	(4.00- 18.30)
JEDRYC	503	m	0	106	231	6	289	22.10	(9.54- 51.22)
JOLY	640	m	0	24	165	2	218	15.85	(3.69- 68.04)
JOLY	612	f	0	5	24	6	283	9.83	(2.79- 34.57)
Subtotal JOLY								12.05	(4.65- 31.23)
LUBIN2	662	m	0	1211	3473	54	2616	16.89	(12.80- 22.29)
LUBIN2	714	f	0	767	186	72	1180	67.58	(50.73- 90.03)
Subtotal LUBIN2								33.00	(27.04- 40.29)
MATOS	602	m	0	18	110	3	110	6.00	(1.72- 20.95)
PEZZOT	508	m	0	35	82	0	116	100.26~	(6.06-1657.79)
WUWILL	507	f	0	66	98	117	601	3.46	(2.39- 5.01)
WYNDE2	507	m	0	30	64	0	41	39.25~	(2.34- 659.46)
ZHENG	502	m	0	59	80	4	94	17.33	(6.03- 49.81)
Partial Totals				2434	4777	300	5995		

*prospective study ~ With 0.5 adjustment for zero

REF	NRR	SEX	AD	Ys	Ws	Qs	Ps
BARBON	556	m	0	2.40	4.77	1.08	0.0000
BUFFLE	506	m	0	2.69	3.05	0.10	0.0000
CHOI	560	m	0	1.98	5.07	4.12	0.0000
CHOI	574	f	0	3.49	1.17	0.44	0.0002
Subtotal CHOI				2.26	6.24	4.56	
DAMBER	548	m	1	2.13	6.65	3.74	0.0000
JEDRYC	503	m	0	3.10	5.44	0.26	0.0000
JOLY	640	m	0	2.76	1.81	0.02	0.0002
JOLY	612	f	0	2.29	2.43	0.86	0.0004
Subtotal JOLY				2.49	4.24	0.88	
LUBIN2	662	m	0	2.83	49.96	0.13	0.0000
LUBIN2	714	f	0	4.21	46.69	83.17	0.0000
Subtotal LUBIN2				3.50	96.66	83.30	
MATOS	602	m	0	1.79	2.46	2.90	0.0050
PEZZOT	508	m	0	4.61	0.49	1.46	0.0013
WUWILL	507	f	0	1.24	28.12	75.40	0.0000
WYNDE2	507	m	0	3.67	0.48	0.30	0.0108
ZHENG	502	m	0	2.85	3.45	0.00	0.0000

	N	15
	NS	12
	Wt	162.03
Het	Chi	173.99
Het	df	14
Het	P	***
Fixed	RR	17.79
	RRl	15.25
	RRu	20.75
	P	+++
Random	RR	14.72
	RRl	7.72
	RRu	28.07
	P	+++
Asymm	P	N.S.

Table 2I3 - 6

IESLC - Meta-analysis of Ever Smoking, Duration, "Mid"
Squamous, Any Product (or Cigarettes if Any not available)
Least adjusted

	Sex combined	male	female	Total
N		11	4	15
NS		11	4	15
Wt		83.63	78.41	162.03
Het Chi		11.24	156.78	173.99
Het df		10	3	14
Het P		N.S.	***	***
Fixed RR		14.77	21.69	17.79
RRl		11.92	17.39	15.25
RRu		18.30	27.07	20.75
P		+++	+++	+++
Random RR		14.02	16.27	14.72
RRl		10.72	2.23	7.72
RRu		18.34	118.58	28.07
P		+++	++	+++
Between Chi				5.97
Between df				1
Between P				*
Btwn(F) P				N.S.
Btwn(R) P				N.S.

Table 2I3 - 7

IESLC - Meta-analysis of Ever Smoking, Duration, "Mid"
Squamous, Any Product (or Cigarettes if Any not available)
Excluded studies (and stage at which they were excluded)

1	AKIBA AMANDU AMES BECHER BENSHL BEST BLOT1 BROSS BROWN3 CARPEN CEDERL CHYOU CPSI CPSII DARBY DEAN2 DEAN3 DOLL2 ENGELA GAO2 GARCIA GILLIS GRAHAM GURSEL HAMMO2 HIRAYA HOLE HUMBLE JAHN JAIN KAISE2 KATSOU KAUFMA LAUSSM LIAW MCDUFF MIGRAN MRFITR PEZZO2 PISANI PRESCO QIAO SEGI2 SPEIZE SVENSS TVERDA WAKAI WATSON WIGLE WU WYNDE3 WYNDE8
2	ALDERS BRESLO CHIAZZ DORN GUO HEGMAN KOO KOULUM LIU4 PERNU SOBUE SPITZ SUZUK2 VUTUC YUAN
3	GENG STASZE WU2 ZHANG
4	AGUDO ARMADA AUVINE AXELSS BOFFET BOUCOT CHEN2 CORREA DESTEF DOLL FAN GAO GARSHI HAMMON HU HU2 JUSSAW KREUZE LETOUR LEVIN LIU3 LIU5 MCCONN NOTAN2 QIAO2 RACHTA RESTRE SADOWS TIZZAN WANG2 WYNDE6 WYNDE7
5	CHEN LUBIN XU
10	BOUCHA KHUDER
14	DORGAN DOSEME GER HAENSZ LUO OSANN2 ZHOU
15	BENHAM

Table 2I3 - 8
Potentially overlapping studies

REF	REFGP	PRINC	.	OVERLAP/LINK
LUBIN2	LUBIN2	1		Lubin-combined

Table 2I3 - 9
Most adjusted - insufficient data for meta-analysis

REF	NRR	SEX	AGEL	AGEH	RACE	YF	LC TYPE	LOC	START	ST	NLC	R	VB	P	H	AD	PRODUCT	exL	exH	DENOM	De
CHEN	503	c	0	0	all	-	q	As:oth	1987	CC	323	n	ot	n	y	2	cig+/-ot	31	40	nev cigs	ot
XU	512	m	0	0	all	-	q+s	As:Chi	1985	CC	729	n	ot	n	n	2	all/unsp	30	39	nev any	or

REF	NRR	RR	SIG	RRDATA comment
CHEN	503	6.52	n	0
XU	512	*		RR for 1-19/day is 2.9(p<0.05), for 20-29/day is 3.9(p<0.05) and for >=30/ day is 8.3(p<0.05)

Table 2I4 -

IESLC - Meta-analysis of Ever Smoking, Duration, "High"
Squamous, Any Product (or Cigarettes if Any not available)

This analysis is restricted to results for:
1) Ever smokers
2) Results by Duration
3) Categorical results by Duration
4) Squamous (or near equivalent)
5) Results complete enough for use in metaanalysis

Within each study, results are then selected (in the following order of preference, within each sex) for:
6) PRODUCT: all/unspec, cigarettes regardless of other products, cigarettes only
7) CIGTYPE: all/unspecified, MC regardless of HR, MC only
8) (not applicable)
9) DENOM: never smoked anything, never smoked cigarettes, never any + low, never cigs + low
10) Followup period (YF, prospective studies): whole study (coded as 0) or longest available
11) LCtype: squamous or nearest available, but not adeno. (q = squamous, s = small,
a = adeno, KI = Kreyberg I, u = undifferentiated)
12) Race: all or nearest available, otherwise by race (wh or w = white, bl or b = black, hi = hispanic
ch = chinese, jap = japanese, haw = hawaiian, w+o = white + oriental, sca = scandinavian, as = asian)
13) Duration "high" in key scheme 1 (key value 50, maximum range 36+)
14) For overlapping studies: principal rather than subsidiary studies
Finally by Age: whole study (coded as 0) if available, otherwise by widest available age group
and then for single sex results (m, f) in preference to results for both sexes combined (c).

Results adjusted (AD) for the most potential confounders are then chosen in Sections -1 to -3
and results adjusted for the least confounders in Sections -4 to -6. (Those least adjusted results which
actually differ from the most adjusted are marked 'x' in column X in Section -4)

Section -7 shows excluded studies, together with the stage (as above) at which no qualifying
results were found.

Section -8 lists the potentially overlapping studies which have been included (1=principal, 2=subsidiary).

Section -9 lists any results which would have been included in preference except that they had data not complete
enough for use in meta-analysis, with their significance (yes/no), if known, and any further comment as entered
on the database. It also lists as "gap" any categories for which no data were presented by the original authors.

In addition to those mentioned above, the following fields, levels and abbreviations are used:
* or nk = not known, n = no, y = yes, ot = other
nev = never
all/unspec = all or unspecified, cig+/-ot = cigarettes irrespective of other products (cigar, pipe etc)
MC = manufactured cigarettes, HR = hand-rolled cigarettes
exL, exH = range of exposure (low and high) in the smoking group, in terms of Duration
REF: 6-character study reference
NRR: number of the RR on the database within the study
ST : study type (CC = case control, pr or prosp = prospective)
NLC: number of lung cancer cases in whole study
R : risky occupational population (n = no, m = mining, o = other risky)
VB : national cigarette type (V = at least 75% Virginia, bl = at least 75% blended, ot = other)
P : any proxy use
H : full histological confirmation
De : derivation of RR/CI (or = original, st = standard method, ot = other method of estimation)

Table 2I4 - 1

IESLC - Meta-analysis of Ever Smoking, Duration, "High"
Squamous, Any Product (or Cigarettes if Any not available)
Most adjusted

REF	NRR	SEX	AGEL	AGEH	RACE	YF	LC TYPE	LOC	START	ST	NLC	R	VB	P	H	AD	PRODUCT	exL	exH	DENOM	De
BARBON	565	m	0	0	all	-	q	Eu:wst	1979	CC	755	n	bl	y	y	1	all/unsp	50	999	nev any	or
BUFFLE	508	m	0	0	wh	-	q	NAmer	1976	CC	943	n	bl	y	n	0	cig+/-ot	50	999	nev cigs	or
CHOI	562	m	0	0	all	-	q	As:oth	1985	CC	375	n	bl	n	n	0	cig+/-ot	50	999	nev cigs	st
CHOI	575	f	0	0	all	-	q	As:oth	1985	CC	375	n	bl	n	n	0	cig+/-ot	40	999	nev cigs	st
DAMBER	549	m	0	0	all	-	q	Eu:Sca	1972	CC	579	n	bl	y	n	1	all/unsp	41	50	nev any	or
JEDRYC	587	m	0	0	all	-	q	Eu:est	1980	CC	1630	n	bl	y	n	3	cig+/-ot	40	999	nev any	or
JOLY	642	m	0	0	all	-	q	SCAmer	1978	CC	826	n	bl	n	n	0	cig+/-ot	50	999	nev any	st
JOLY	614	f	0	0	all	-	q	SCAmer	1978	CC	826	n	bl	n	n	0	cig+/-ot	50	999	nev any	st
LUBIN2	664	m	0	0	all	-	q	Eu:mul	1976	CC	7804	n	bl	n	y	0	cig+/-ot	50	999	nev any	st
LUBIN2	716	f	0	0	all	-	q	Eu:mul	1976	CC	7804	n	bl	n	y	0	cig+/-ot	50	999	nev any	st
MATOS	608	m	0	0	all	-	q	SCAmer	1994	CC	200	n	bl	n	n	2	cig+/-ot	40	70	nev any	or
PEZZOT	509	m	0	0	all	-	q	SCAmer	1987	CC	215	n	bl	n	y	0	cig only	41	999	nev cigs	ot
WUWILL	523	f	0	0	all	-	q	As:Chi	1985	CC	965	n	ot	n	n	3	cig+/-ot	40	999	nev cigs	ot
WYNDE2	508	m	0	0	all	-	KI	NAmer	1962	CC	404	n	bl	n	y	0	cig+/-ot	41	999	nev any	ot
ZHENG	503	m	0	0	all	-	q	As:Chi	1982	CC	540	n	ot	*	y	0	cig+/-ot	40	999	nev cigs	st

Cigarette type is all/unspec for all RRs

Table 2I4 - 2

IESLC - Meta-analysis of Ever Smoking, Duration, "High"
Squamous, Any Product (or Cigarettes if Any not available)
Most adjusted

REF	NRR	SEX	AD	Number Exposed Case	Cont	Non-exposed Case	Cont	RR	95.00%CI
BARBON	565	m	1	149	-	6	-	21.20	(9.10- 49.30)
BUFFLE	508	m	0	-	-	-	-	22.10	(7.20- 67.70)
CHOI	562	m	0	11	20	6	95	8.71	(2.88- 26.30)
CHOI	575	f	0	1	1	10	164	16.40	(0.95- 281.93)
Subtotal CHOI								9.46	(3.38- 26.51)
DAMBER	549	m	1	-	-	14	-	13.80	(6.80- 29.10)
JEDRYC	587	m	3	160	-	6	-	13.00	(5.54- 30.48)
JOLY	642	m	0	98	253	2	218	42.22	(10.29- 173.22)
JOLY	614	f	0	22	20	6	283	51.88	(18.89- 142.48)
Subtotal JOLY								48.39	(21.28- 110.03)
LUBIN2	664	m	0	746	1460	54	2616	24.75	(18.64- 32.87)
LUBIN2	716	f	0	566	34	72	1180	272.83	(179.26- 415.22)
Subtotal LUBIN2								52.47	(41.48- 66.37)
MATOS	608	m	2	26	-	3	-	18.50	(4.90- 69.80)
PEZZOT	509	m	0	45	101	0	116	104.45~	(6.35-1717.05)
WUWILL	523	f	3	81	-	117	-	5.57	(3.79- 8.17)
WYNDE2	508	m	0	94	89	0	41	87.64~	(5.31-1446.06)
ZHENG	503	m	0	84	63	4	94	31.33	(10.94- 89.77)
Partial Totals				2083	2041	300	4807		

*prospective study ~ With 0.5 adjustment for zero

REF	NRR	SEX	AD	Ys	Ws	Qs	Ps
BARBON	565	m	1	3.05	5.38	0.22	0.0000
BUFFLE	508	m	0	3.10	3.06	0.08	0.0000
CHOI	562	m	0	2.16	3.14	3.76	0.0001
CHOI	575	f	0	2.80	0.47	0.10	0.0539
Subtotal CHOI				2.25	3.62	3.86	
DAMBER	549	m	1	2.62	7.27	2.91	0.0000
JEDRYC	587	m	3	2.56	5.29	2.53	0.0000
JOLY	642	m	0	3.74	1.93	0.45	0.0000
JOLY	614	f	0	3.95	3.76	1.80	0.0000
Subtotal JOLY				3.88	5.69	2.25	
LUBIN2	664	m	0	3.21	47.79	0.11	0.0000
LUBIN2	716	f	0	5.61	21.78	120.42	0.0000
Subtotal LUBIN2				3.96	69.57	120.53	
MATOS	608	m	2	2.92	2.18	0.25	0.0000
PEZZOT	509	m	0	4.65	0.49	0.95	0.0011
WUWILL	523	f	3	1.72	26.04	61.77	0.0000
WYNDE2	508	m	0	4.47	0.49	0.72	0.0018
ZHENG	503	m	0	3.44	3.47	0.12	0.0000

	N	15
	NS	12
	Wt	132.54
Het	Chi	196.21
Het	df	14
Het	P	***
Fixed	RR	25.98
	RRl	21.92
	RRu	30.81
	P	+++
Random	RR	26.28
	RRl	12.49
	RRu	55.28
	P	+++
Asymm	P	N.S.

Table 2I4 - 3

IESLC - Meta-analysis of Ever Smoking, Duration, "High"
Squamous, Any Product (or Cigarettes if Any not available)
Most adjusted

	Sex combined	male	female	Total
N		11	4	15
NS		11	4	15
Wt		80.48	52.06	132.54
Het Chi		9.86	180.60	196.21
Het df		10	3	14
Het P		N.S.	***	***
Fixed RR		21.98	33.67	25.98
RRl		17.66	25.66	21.92
RRu		27.34	44.17	30.81
P		+++	+++	+++
Random RR		21.98	35.26	26.28
RRl		17.66	2.90	12.49
RRu		27.34	429.38	55.28
P		+++	++	+++
Between Chi				5.75
Between df				1
Between P				*
Btwn(F) P				N.S.
Btwn(R) P				N.S.

Table 2I4 - 4

IESLC - Meta-analysis of Ever Smoking, Duration, "High"
Squamous, Any Product (or Cigarettes if Any not available)
Least adjusted

REF	NRR	X	SEX	AGEL	AGEH	RACE	YF	LC TYPE	LOC	START	ST	NLC	R	VB	P	H	AD	PRODUCT	exL	exH	DENOM	De
BARBON	558	x	m	0	0	all	-	q	Eu:wst	1979	CC	755	n	bl	y	y	0	all/unsp	50	999	nev any	st
BUFFLE	508		m	0	0	wh	-	q	NAmer	1976	CC	943	n	bl	y	n	0	cig+/-ot	50	999	nev cigs	or
CHOI	562		m	0	0	all	-	q	As:oth	1985	CC	375	n	bl	n	n	0	cig+/-ot	50	999	nev cigs	st
CHOI	575		f	0	0	all	-	q	As:oth	1985	CC	375	n	bl	n	n	0	cig+/-ot	40	999	nev cigs	st
DAMBER	549		m	0	0	all	-	q	Eu:Sca	1972	CC	579	n	bl	y	n	1	all/unsp	41	50	nev any	or
JEDRYC	505	x	m	0	0	all	-	q	Eu:est	1980	CC	1630	n	bl	y	n	0	cig+/-ot	50	999	nev any	st
JOLY	642		m	0	0	all	-	q	SCAmer	1978	CC	826	n	bl	n	n	0	cig+/-ot	50	999	nev any	st
JOLY	614		f	0	0	all	-	q	SCAmer	1978	CC	826	n	bl	n	n	0	cig+/-ot	50	999	nev any	st
LUBIN2	664		m	0	0	all	-	q	Eu:mul	1976	CC	7804	n	bl	n	y	0	cig+/-ot	50	999	nev any	st
LUBIN2	716		f	0	0	all	-	q	Eu:mul	1976	CC	7804	n	bl	n	y	0	cig+/-ot	50	999	nev any	st
MATOS	603	x	m	0	0	all	-	q	SCAmer	1994	CC	200	n	bl	n	n	0	cig+/-ot	40	70	nev any	st
PEZZOT	509		m	0	0	all	-	q	SCAmer	1987	CC	215	n	bl	n	y	0	cig only	41	999	nev cigs	ot
WUWILL	508	x	f	0	0	all	-	q	As:Chi	1985	CC	965	n	ot	n	n	0	cig+/-ot	40	999	nev cigs	st
WYNDE2	508		m	0	0	all	-	KI	NAmer	1962	CC	404	n	bl	n	y	0	cig+/-ot	41	999	nev any	ot
ZHENG	503		m	0	0	all	-	q	As:Chi	1982	CC	540	n	ot	*	y	0	cig+/-ot	40	999	nev cigs	st

Cigarette type is all/unspec for all RRs

Table 2I4 - 5

IESLC - Meta-analysis of Ever Smoking, Duration, "High"
Squamous, Any Product (or Cigarettes if Any not available)
Least adjusted

REF	NRR	SEX	AD	Number Exposed Case	Cont	Non-exposed Case	Cont	RR	95.00%CI
BARBON	558	m	0	149	235	6	188	19.87	(8.59- 45.94)
BUFFLE	508	m	0	-	-	-	-	22.10	(7.20- 67.70)
CHOI	562	m	0	11	20	6	95	8.71	(2.88- 26.30)
CHOI	575	f	0	1	1	10	164	16.40	(0.95- 281.93)
Subtotal CHOI								9.46	(3.38- 26.51)
DAMBER	549	m	1	-	-	14	-	13.80	(6.80- 29.10)
JEDRYC	505	m	0	49	214	6	289	11.03	(4.64- 26.22)
JOLY	642	m	0	98	253	2	218	42.22	(10.29- 173.22)
JOLY	614	f	0	22	20	6	283	51.88	(18.89- 142.48)
Subtotal JOLY								48.39	(21.28- 110.03)
LUBIN2	664	m	0	746	1460	54	2616	24.75	(18.64- 32.87)
LUBIN2	716	f	0	566	34	72	1180	272.83	(179.26- 415.22)
Subtotal LUBIN2								52.47	(41.48- 66.37)
MATOS	603	m	0	26	89	3	110	10.71	(3.14- 36.55)
PEZZOT	509	m	0	45	101	0	116	104.45~	(6.35-1717.05)
WUWILL	508	f	0	81	114	117	601	3.65	(2.58- 5.16)
WYNDE2	508	m	0	94	89	0	41	87.64~	(5.31-1446.06)
ZHENG	503	m	0	84	63	4	94	31.33	(10.94- 89.77)
Partial Totals				1972	2693	300	5995		

*prospective study ~ With 0.5 adjustment for zero

REF	NRR	SEX	AD	Ys	Ws	Qs	Ps
BARBON	558	m	0	2.99	5.47	0.04	0.0000
BUFFLE	508	m	0	3.10	3.06	0.00	0.0000
CHOI	562	m	0	2.16	3.14	2.61	0.0001
CHOI	575	f	0	2.80	0.47	0.04	0.0539
Subtotal CHOI				2.25	3.62	2.65	
DAMBER	549	m	1	2.62	7.27	1.48	0.0000
JEDRYC	505	m	0	2.40	5.12	2.34	0.0000
JOLY	642	m	0	3.74	1.93	0.86	0.0000
JOLY	614	f	0	3.95	3.76	2.87	0.0000
Subtotal JOLY				3.88	5.69	3.73	
LUBIN2	664	m	0	3.21	47.79	0.84	0.0000
LUBIN2	716	f	0	5.61	21.78	139.71	0.0000
Subtotal LUBIN2				3.96	69.57	140.56	
MATOS	603	m	0	2.37	2.55	1.27	0.0002
PEZZOT	509	m	0	4.65	0.49	1.21	0.0011
WUWILL	508	f	0	1.29	31.92	101.29	0.0000
WYNDE2	508	m	0	4.47	0.49	0.95	0.0018
ZHENG	503	m	0	3.44	3.47	0.47	0.0000

	N	15
	NS	12
	Wt	138.71
Het	Chi	255.99
Het	df	14
Het	P	***
Fixed	RR	21.67
	RRl	18.35
	RRu	25.60
	P	+++
Random	RR	24.24
	RRl	10.65
	RRu	55.16
	P	+++
Asymm	P	N.S.

Table 2I4 - 6

IESLC - Meta-analysis of Ever Smoking, Duration, "High"
Squamous, Any Product (or Cigarettes if Any not available)
Least adjusted

	Sex combined	male	female	Total
N		11	4	15
NS		11	4	15
Wt		80.77	57.94	138.71
Het Chi		12.05	243.88	255.99
Het df		10	3	14
Het P		N.S.	***	***
Fixed RR		21.29	22.22	21.67
RRl		17.12	17.18	18.35
RRu		26.47	28.75	25.60
P		+++	+++	+++
Random RR		19.79	31.26	24.24
RRl		14.74	1.93	10.65
RRu		26.58	506.39	55.16
P		+++	+	+++
Between Chi				0.06
Between df				1
Between P				N.S.
Btwn(F) P				N.S.
Btwn(R) P				N.S.

Table 2I4 - 7

IESLC - Meta-analysis of Ever Smoking, Duration, "High"
Squamous, Any Product (or Cigarettes if Any not available)
Excluded studies (and stage at which they were excluded)

1	AKIBA AMANDU AMES BECHER BENSHL BEST BLOT1 BROSS BROWN3 CARPEN CEDERL CHYOU CPSI CPSII DARBY DEAN2 DEAN3 DOLL2 ENGELA GAO2 GARCIA GILLIS GRAHAM GURSEL HAMMO2 HIRAYA HOLE HUMBLE JAHN JAIN KAISE2 KATSOU KAUFMA LAUSSM LIAW MCDUFF MIGRAN MRFITR PEZZO2 PISANI PRESCO QIAO SEGI2 SPEIZE SVENSS TVERDA WAKAI WATSON WIGLE WU WYNDE3 WYNDE8
2	ALDERS BRESLO CHIAZZ DORN GUO HEGMAN KOO KOULUM LIU4 PERNU SOBUE SPITZ SUZUK2 VUTUC YUAN
3	GENG STASZE WU2 ZHANG
4	AGUDO ARMADA AUVINE AXELSS BOFFET BOUCOT CHEN2 CORREA DESTEF DOLL FAN GAO GARSHI HAMMON HU HU2 JUSSAW KREUZE LETOUR LEVIN LIU3 LIU5 MCCONN NOTAN2 QIAO2 RACHTA RESTRE SADOWS TIZZAN WANG2 WYNDE6 WYNDE7
5	CHEN LUBIN XU
10	BOUCHA KHUDER
14	DORGAN DOSEME GER HAENSZ LUO OSANN2 ZHOU
15	BENHAM

Table 2I4 - 8
Potentially overlapping studies

REF	REFGP	PRINC	.	OVERLAP/LINK
LUBIN2	LUBIN2	1		Lubin-combined

Table 2I4 - 9
Most adjusted - insufficient data for meta-analysis

REF	NRR	SEX	AGEL	AGEH	RACE	YF	LC TYPE	LOC	START	ST	NLC	R	VB	P	H	AD	PRODUCT	exL	exH	DENOM	De
CHEN	504	c	0	0	all	-	q	As:oth	1987	CC	323	n	ot	n	y	2	cig+/-ot	41	999	nev cigs	ot
XU	513	m	0	0	all	-	q+s	As:Chi	1985	CC	729	n	ot	n	n	2	all/unsp	40	999	nev any	or

REF	NRR	RR	SIG	RRDATA comment
CHEN	504	8.43	y	p<0.001
XU	513	*		RR for 1-19/day is 5.0(p<0.05), for 20-29/day is 10.4(p<0.05) and for >=30/ day is 31.2(p<0.05)

Table 2I5 -

IESLC - Meta-analysis of Ever Smoking, Duration, "Highest vs lowest"
Squamous, Any Product (or Cigarettes if Any not available)

This analysis is restricted to results for:
1) Ever smokers
2) Results by Duration
3) Categorical results by Duration
4) Denominator (unexposed) = "low"
5) Squamous (or near equivalent)
6) Results complete enough for use in metaanalysis

Within each study, results are then selected (in the following order of preference, within each sex) for:
7) (not applicable)
8) PRODUCT: all/unspec, cigarettes regardless of other products, cigarettes only
9) CIGTYPE: all/unspecified, MC regardless of HR, MC only
10) Results with least adjustment for other aspects of smoking (ADOS)
11) The highest vs lowest category
12) Followup period (YF, prospective studies): whole study (coded as 0) or longest available
13) LCtype: squamous or nearest available, but not adeno. (q = squamous, s = small,
a = adeno, KI = Kreyberg I, u = undifferentiated)
14) Race: all or nearest available, otherwise by race (wh or w = white, bl or b = black, hi = hispanic
ch = chinese, jap = japanese, haw = hawaiian, w+o = white + oriental, sca = scandinavian, as = asian)
15) For overlapping studies: principal rather than subsidiary studies
Finally by Age: whole study (coded as 0) if available, otherwise by widest available age group
and then for single sex results (m, f) in preference to results for both sexes combined (c).

Results adjusted (AD) for the most potential confounders are then chosen in Sections -1 to -3
and results adjusted for the least confounders in Sections -4 to -6. (Those least adjusted results which
actually differ from the most adjusted are marked 'x' in column X in Section -4)

Section -7 shows excluded studies, together with the stage (as above) at which no qualifying
results were found.

Section -8 lists the potentially overlapping studies which have been included (1=principal, 2=subsidiary).

Section -9 lists any results which would have been included in preference except that they had data not complete
enough for use in meta-analysis, with their significance (yes/no), if known, and any further comment as entered
on the database. It also lists as "gap" any categories for which no data were presented by the original authors.

In addition to those mentioned above, the following fields, levels and abbreviations are used:
* or nk = not known, n = no, y = yes, ot = other
all/unspec = all or unspecified, cig+/-ot = cigarettes irrespective of other products (cigar, pipe etc)
MC = manufactured cigarettes, HR = hand-rolled cigarettes
exL, exH = range of exposure (low and high) in the "highest" group, in terms of Duration
unexL, unexH = range of exposure (low and high) in the "lowest" group, in terms of Duration
REF: 6-character study reference
NRR: number of the RR on the database within the study
ST : study type (CC = case control, pr or prosp = prospective)
NLC: number of lung cancer cases in whole study
R : risky occupational population (n = no, m = mining, o = other risky)
VB : national cigarette type (V = at least 75% Virginia, bl = at least 75% blended, ot = other)
P : any proxy use
H : full histological confirmation
De : derivation of RR/CI (or = original, st = standard method, ot = other method of estimation)

Table 2I5 - 1

IESLC - Meta-analysis of Ever Smoking, Duration, "Highest vs lowest"
Squamous, Any Product (or Cigarettes if Any not available)
Most adjusted

REF	NRR	SEX	AGEL	AGEH	RACE	YF	LC TYPE	LOC	START	ST	NLC	R	VB	P	H	AD	ADOS	PRODUCT	exL	exH	unexL	unexH	De
BARBON	568	m	0	0	all	-	q	Eu:wst	1979	CC	755	n	bl	y	y	1	0	all/unsp	50	999	1	29	ot
BOUCHA	501	c	0	0	wh	-	q+s	Eu:wst	1988	CC	150	n	bl	n	y	0	0	all/unsp	31	999	1	30	st
CHOI	565	m	0	0	all	-	q	As:oth	1985	CC	375	n	bl	n	n	0	0	cig+/-ot	50	999	1	29	st
CHOI	577	f	0	0	all	-	q	As:oth	1985	CC	375	n	bl	n	n	0	0	cig+/-ot	40	999	1	29	st
DAMBER	553	m	0	0	all	-	q	Eu:Sca	1972	CC	579	n	bl	y	n	1	0	all/unsp	51	999	1	30	ot
DORGAN	527	m	0	0	wh	-	q	NAmer	1980	CC	2026	n	bl	y	y	2	0	cig+/-ot	35	999	1	34	ot
DORGAN	523	f	0	0	all	-	q	NAmer	1980	CC	2026	n	bl	y	y	3	0	cig+/-ot	35	999	1	34	ot
DOSEME	515	m	0	0	all	-	q	Eu:bal	1979	CC	1210	n	bl	n	n	2	0	cig+/-ot	21	999	1	10	ot
GER	512	c	0	0	all	-	q+s	As:oth	1990	CC	141	n	ot	y	n	5	0	all/unsp	31	999	1	30	ot
HAENSZ	535	f	0	0	all	-	q+u	NAmer	1955	CC	158	n	bl	n	y	1	0	cig+/-ot	15	999	1	14	ot
JEDRYC	509	m	0	0	all	-	q	Eu:est	1980	CC	1630	n	bl	y	n	0	0	cig+/-ot	50	999	1	19	st
JOLY	645	m	0	0	all	-	q	SCAmer	1978	CC	826	n	bl	n	n	0	0	cig+/-ot	50	999	1	29	st
JOLY	617	f	0	0	all	-	q	SCAmer	1978	CC	826	n	bl	n	n	0	0	cig+/-ot	50	999	1	29	st
KHUDER	530	m	0	0	all	-	q	NAmer	1985	CC	482	n	bl	n	y	5	3#	cig+/-ot	30	999	1	29	or
LUBIN2	667	m	0	0	all	-	q	Eu:mul	1976	CC	7804	n	bl	n	y	0	0	cig+/-ot	50	999	1	29	st
LUBIN2	719	f	0	0	all	-	q	Eu:mul	1976	CC	7804	n	bl	n	y	0	0	cig+/-ot	50	999	1	29	st
LUO	506	c	0	0	all	-	q	As:Chi	1990	CC	102	n	ot	n	y	20	0	cig+/-ot	30	999	1	29	ot
MATOS	610	m	0	0	all	-	q	SCAmer	1994	CC	200	n	bl	n	n	2	0	cig+/-ot	40	70	1	24	ot
OSANN2	512	f	0	0	all	-	KI	NAmer	1964	ot	217	n	bl	n	y	1	0	cig+/-ot	21	999	1	20	ot
PEZZOT	513	m	0	0	all	-	q	SCAmer	1987	CC	215	n	bl	n	y	2	0	cig only	41	999	1	30	ot
WUWILL	510	f	0	0	all	-	q	As:Chi	1985	CC	965	n	ot	n	n	0	0	cig+/-ot	40	999	1	29	st
WYNDE2	513	m	0	0	all	-	KI	NAmer	1962	CC	404	n	bl	n	y	0	0	cig+/-ot	41	999	1	29	st
ZHENG	507	m	0	0	all	-	q	As:Chi	1982	CC	540	n	ot	*	y	1	0	cig+/-ot	40	999	1	29	ot
ZHENG	511	f	0	0	all	-	q	As:Chi	1982	CC	540	n	ot	*	y	1	0	cig+/-ot	30	999	1	29	ot
ZHOU	506	c	0	0	all	-	q	As:Chi	1978	CC	1360	n	ot	n	n	0	0	all/unsp	20	999	1	19	st

Comments on values in listings

KHUDER ADOS Age at starting smoking, No of cigarettes per day, Quitted smoking

Cigarette type is all/unspec for all RRs

Table 2I5 - 2

IESLC - Meta-analysis of Ever Smoking, Duration, "Highest vs lowest"
Squamous, Any Product (or Cigarettes if Any not available)
Most adjusted

REF	NRR	SEX	AD	Number Exposed Case	Cont	Non-exposed Case	Cont	RR	95.00%CI
BARBON	568	m	1	149	-	7	-	10.10	(4.59- 22.21)
BOUCHA	501	c	0	114	92	34	79	2.88	(1.77- 4.69)
CHOI	565	m	0	11	20	42	221	2.89	(1.29- 6.48)
CHOI	577	f	0	1	1	6	23	3.83	(0.21- 70.63)
Subtotal CHOI								2.95	(1.36- 6.42)
DAMBER	553	m	1	-	-	-	-	3.80	(1.82- 7.91)
DORGAN	527	m	2	-	-	-	-	2.77	(2.04- 3.76)
DORGAN	523	f	3	-	-	-	-	3.67	(2.52- 5.34)
Subtotal DORGAN								3.10	(2.44- 3.93)
DOSEME	515	m	2	199	-	15	-	4.08	(2.07- 8.05)
GER	512	c	5	42	-	6	-	4.19	(1.56- 11.28)
HAENSZ	535	f	1	42	-	14	-	1.00	(0.47- 2.12)
JEDRYC	509	m	0	49	214	7	68	2.22	(0.96- 5.14)
JOLY	645	m	0	98	253	15	109	2.81	(1.56- 5.07)
JOLY	617	f	0	22	20	5	54	11.88	(3.96- 35.63)
Subtotal JOLY								3.88	(2.31- 6.51)
KHUDER	530	m	5	-	-	-	-	1.90	(0.90- 3.70)
LUBIN2	667	m	0	746	1460	453	2964	3.34	(2.93- 3.82)
LUBIN2	719	f	0	566	34	322	229	11.84	(8.05- 17.40)
Subtotal LUBIN2								3.82	(3.37- 4.33)
LUO	506	c	20	28	-	6	-	2.19	(0.68- 7.06)
MATOS	610	m	2	26	-	3	-	15.42	(3.96- 60.01)
OSANN2	512	f	1	101	-	11	-	20.63	(3.12- 136.52)
PEZZOT	513	m	2	45	-	5	-	9.90	(3.61- 27.15)
WUWILL	510	f	0	81	114	54	139	1.83	(1.20- 2.80)
WYNDE2	513	m	0	94	89	22	55	2.64	(1.49- 4.68)
ZHENG	507	m	1	84	-	13	-	8.25	(4.21- 16.18)
ZHENG	511	f	1	35	-	8	-	4.42	(1.66- 11.76)
Subtotal ZHENG								6.75	(3.88- 11.76)
ZHOU	506	c	0	315	36	60	12	1.75	(0.86- 3.56)
Partial Totals				2848	2333	1108	3953		

*prospective study

REF	NRR	SEX	AD	Ys	Ws	Qs	Ps
BARBON	568	m	1	2.31	6.18	7.02	0.0000
BOUCHA	501	c	0	1.06	16.20	0.58	0.0000
CHOI	565	m	0	1.06	5.91	0.20	0.0098
CHOI	577	f	0	1.34	0.45	0.00	0.3661
Subtotal CHOI				1.08	6.36	0.20	
DAMBER	553	m	1	1.34	7.12	0.06	0.0004
DORGAN	527	m	2	1.02	41.10	2.13	0.0000
DORGAN	523	f	3	1.30	27.25	0.08	0.0000
Subtotal DORGAN				1.13	68.34	2.21	
DOSEME	515	m	2	1.41	8.33	0.21	0.0000
GER	512	c	5	1.43	3.93	0.14	0.0045
HAENSZ	535	f	1	0.00	6.77	10.52	1.0000
JEDRYC	509	m	0	0.80	5.48	1.10	0.0614
JOLY	645	m	0	1.03	11.11	0.50	0.0006
JOLY	617	f	0	2.47	3.18	4.80	0.0000
Subtotal JOLY				1.36	14.30	5.30	
KHUDER	530	m	5	0.64	7.69	2.81	0.0751
LUBIN2	667	m	0	1.21	218.80	0.35	0.0000
LUBIN2	719	f	0	2.47	25.87	38.81	0.0000
Subtotal LUBIN2				1.34	244.68	39.15	
LUO	506	c	20	0.78	2.81	0.60	0.1891
MATOS	610	m	2	2.74	2.08	4.61	0.0001
OSANN2	512	f	1	3.03	1.08	3.41	0.0017
PEZZOT	513	m	2	2.29	3.77	4.13	0.0000
WUWILL	510	f	0	0.60	21.35	8.83	0.0053
WYNDE2	513	m	0	0.97	11.69	0.89	0.0009
ZHENG	507	m	1	2.11	8.48	6.32	0.0000
ZHENG	511	f	1	1.49	4.01	0.23	0.0029
Subtotal ZHENG				1.91	12.49	6.55	
ZHOU	506	c	0	0.56	7.64	3.61	0.1220

Table 2I5 - 2

IESLC - Meta-analysis of Ever Smoking, Duration, "Highest vs lowest"
Squamous, Any Product (or Cigarettes if Any not available)
Most adjusted

	N	25
	NS	20
	Wt	458.28
Het	Chi	101.93
Het	df	24
Het	P	***
Fixed	RR	3.48
	RRl	3.17
	RRu	3.81
	P	+++
Random	RR	3.74
	RRl	2.94
	RRu	4.76
	P	+++
Asymm	P	N.S.

Table 2I5 - 3

IESLC - Meta-analysis of Ever Smoking, Duration, "Highest vs lowest"
Squamous, Any Product (or Cigarettes if Any not available)
Most adjusted

		Sex			
		combined	male	female	Total
	N	4	13	8	25
	NS	4	13	8	25
	Wt	30.57	337.74	89.97	458.28
Het	Chi	2.34	30.09	63.16	101.93
Het	df	3	12	7	24
Het	P	N.S.	**	***	***
Fixed	RR	2.60	3.39	4.24	3.48
	RRl	1.83	3.05	3.45	3.17
	RRu	3.71	3.77	5.21	3.81
	P	+++	+++	+++	+++
Random	RR	2.60	3.76	4.47	3.74
	RRl	1.83	2.93	2.16	2.94
	RRu	3.71	4.81	9.27	4.76
	P	+++	+++	+++	+++
Between	Chi				6.34
Between	df				2
Between	P				*
Btwn(F)	P				N.S.
Btwn(R)	P				N.S.

		Lung cancer type					
		q	q+s	q+u	KI	not a	Total
	N	20	2	1	2		25
	NS	15	2	1	2		20
	Wt	418.61	20.13	6.77	12.77		458.28
Het	Chi	86.04	0.44	0.00	4.16		101.93
Het	df	19	1	0	1		24
Het	P	***	N.S.	N.S.	*		***
Fixed	RR	3.58	3.10	1.00	3.14		3.48
	RRl	3.25	2.00	0.47	1.81		3.17
	RRu	3.94	4.79	2.12	5.43		3.81
	P	+++	+++	N.S.	+++		+++
Random	RR	4.02	3.10	1.00	6.01		3.74
	RRl	3.07	2.00	0.47	0.83		2.94
	RRu	5.25	4.79	2.12	43.28		4.76
	P	+++	+++	N.S.	(+)		+++
Between	Chi						11.28
Between	df						3
Between	P						*
Btwn(F)	P						N.S.
Btwn(R)	P						**

		Location								
		NAmer	UK	Scand	othEur	China	Japan	othAs	other	Total
	N	6		1	6	5		3	4	25
	NS	5		1	5	4		2	3	20
	Wt	95.57		7.12	280.87	44.28		10.29	20.15	458.28
Het	Chi	14.64		0.00	45.77	16.34		0.33	10.37	101.93
Het	df	5		0	5	4		2	3	24
Het	P	*		N.S.	***	**		N.S.	*	***
Fixed	RR	2.75		3.80	3.81	2.65		3.37	5.33	3.48
	RRl	2.25		1.82	3.39	1.98		1.83	3.45	3.17
	RRu	3.37		7.92	4.28	3.56		6.22	8.25	3.81
	P	+++		+++	+++	+++		+++	+++	+++
Random	RR	2.58		3.80	4.70	3.00		3.37	7.60	3.74
	RRl	1.73		1.82	2.72	1.55		1.83	3.08	2.94
	RRu	3.86		7.92	8.13	5.81		6.22	18.75	4.76
	P	+++		+++	+++	++		+++	+++	+++
Between	Chi									14.49
Between	df									5
Between	P									*
Btwn(F)	P									N.S.
Btwn(R)	P									N.S.

Table 2I5 - 3

IESLC - Meta-analysis of Ever Smoking, Duration, "Highest vs lowest"
Squamous, Any Product (or Cigarettes if Any not available)
Most adjusted

Detailed Country in "other Europe"

	multi	Germany	othWest	East	Balkans	Total
N	2		2	1	1	6
NS	1		2	1	1	5
Wt	244.68		22.39	5.48	8.33	280.87
Het Chi	36.99		7.05	0.00	0.00	45.77
Het df	1		1	0	0	5
Het P	***		**	N.S.	N.S.	***
Fixed RR	3.82		4.07	2.22	4.08	3.81
RRl	3.37		2.69	0.96	2.07	3.39
RRu	4.33		6.16	5.14	8.05	4.28
P	+++		+++	(+)	+++	+++
Random RR	6.21		5.18	2.22	4.08	4.70
RRl	1.80		1.52	0.96	2.07	2.72
RRu	21.43		17.68	5.14	8.05	8.13
P	++		++	(+)	+++	+++
Between Chi						1.73
Between df						3
Between P						N.S.
Btwn(F) P						N.S.
Btwn(R) P						N.S.

Detailed Country in "other Asia"

	India	HongKong	other	Total
N			3	3
NS			2	2
Wt			10.29	10.29
Het Chi			0.33	0.33
Het df			2	2
Het P			N.S.	N.S.
Fixed RR			3.37	3.37
RRl			1.83	1.83
RRu			6.22	6.22
P			+++	+++
Random RR			3.37	3.37
RRl			1.83	1.83
RRu			6.22	6.22
P			+++	+++
Between Chi				
Between df				
Between P				N.S.
Btwn(F) P				N.S.
Btwn(R) P				N.S.

Detailed other continent

	SCAmer	Total
N	4	4
NS	3	3
Wt	20.15	20.15
Het Chi	10.37	10.37
Het df	3	3
Het P	*	*
Fixed RR	5.33	5.33
RRl	3.45	3.45
RRu	8.25	8.25
P	+++	+++
Random RR	7.60	7.60
RRl	3.08	3.08
RRu	18.75	18.75
P	+++	+++
Between Chi		
Between df		
Between P		N.S.
Btwn(F) P		N.S.
Btwn(R) P		N.S.

Table 2I5 - 3

IESLC - Meta-analysis of Ever Smoking, Duration, "Highest vs lowest"
Squamous, Any Product (or Cigarettes if Any not available)
Most adjusted

	Start year of study					
	<1960	1960-69	1970-79	1980-89	1990+	Total
N	1	2	8	11	3	25
NS	1	2	6	8	3	20
Wt	6.77	12.77	288.24	141.69	8.81	458.28
Het Chi	0.00	4.16	52.66	23.41	4.62	101.93
Het df	0	1	7	10	2	24
Het P	N.S.	*	***	**	(*)	***
Fixed RR	1.00	3.14	3.83	3.01	4.63	3.48
RRl	0.47	1.81	3.41	2.55	2.39	3.17
RRu	2.12	5.43	4.30	3.55	8.97	3.81
P	N.S.	+++	+++	+++	+++	+++
Random RR	1.00	6.01	4.80	3.21	4.92	3.74
RRl	0.47	0.83	2.95	2.41	1.77	2.94
RRu	2.12	43.28	7.83	4.27	13.66	4.76
P	N.S.	(+)	+++	+++	++	+++
Between Chi						17.07
Between df						4
Between P						**
Btwn(F) P						N.S.
Btwn(R) P						*

	Study type (1)		
	CC	other	Total
N	24	1	25
NS	19	1	20
Wt	457.20	1.08	458.28
Het Chi	98.51	0.00	101.93
Het df	23	0	24
Het P	***	N.S.	***
Fixed RR	3.46	20.63	3.48
RRl	3.16	3.12	3.17
RRu	3.80	136.46	3.81
P	+++	++	+++
Random RR	3.65	20.63	3.74
RRl	2.87	3.12	2.94
RRu	4.65	136.46	4.76
P	+++	++	+++
Between Chi			3.42
Between df			1
Between P			(*)
Btwn(F) P			N.S.
Btwn(R) P			(*)

	Study type (2)			
	CC	prosp	other	Total
N	24		1	25
NS	19		1	20
Wt	457.20		1.08	458.28
Het Chi	98.51		0.00	101.93
Het df	23		0	24
Het P	***		N.S.	***
Fixed RR	3.46		20.63	3.48
RRl	3.16		3.12	3.17
RRu	3.80		136.46	3.81
P	+++		++	+++
Random RR	3.65		20.63	3.74
RRl	2.87		3.12	2.94
RRu	4.65		136.46	4.76
P	+++		++	+++
Between Chi				3.42
Between df				1
Between P				(*)
Btwn(F) P				N.S.
Btwn(R) P				(*)

Table 2I5 - 3

IESLC - Meta-analysis of Ever Smoking, Duration, "Highest vs lowest"
Squamous, Any Product (or Cigarettes if Any not available)
Most adjusted

	Study size (number of LC cases) 100-249	250-499	500-999	1000+	Total
N	7	4	7	7	25
NS	7	3	5	5	20
Wt	36.64	25.74	61.43	334.46	458.28
Het Chi	23.72	0.82	27.58	46.05	101.93
Het df	6	3	6	6	24
Het P	***	N.S.	***	***	***
Fixed RR	3.20	2.46	3.67	3.57	3.48
RRl	2.31	1.67	2.86	3.21	3.17
RRu	4.42	3.62	4.72	3.97	3.81
P	+++	+++	+++	+++	+++
Random RR	4.27	2.46	4.76	3.66	3.74
RRl	2.07	1.67	2.70	2.46	2.94
RRu	8.81	3.62	8.40	5.43	4.76
P	+++	+++	+++	+++	+++
Between Chi					3.76
Between df					3
Between P					N.S.
Btwn(F) P					N.S.
Btwn(R) P					N.S.

	Risky occupational population no	mining	othRisky	Total
N	25			25
NS	20			20
Wt	458.28			458.28
Het Chi	101.93			101.93
Het df	24			24
Het P	***			***
Fixed RR	3.48			3.48
RRl	3.17			3.17
RRu	3.81			3.81
P	+++			+++
Random RR	3.74			3.74
RRl	2.94			2.94
RRu	4.76			4.76
P	+++			+++
Between Chi				
Between df				
Between P				N.S.
Btwn(F) P				N.S.
Btwn(R) P				N.S.

	National cigarette tobacco type Virginia	blended	other	Total
N		19	6	25
NS		15	5	20
Wt		410.07	48.21	458.28
Het Chi		81.90	17.09	101.93
Het df		18	5	24
Het P		***	**	***
Fixed RR		3.58	2.75	3.48
RRl		3.25	2.08	3.17
RRu		3.94	3.65	3.81
P		+++	+++	+++
Random RR		3.95	3.15	3.74
RRl		3.00	1.77	2.94
RRu		5.20	5.59	4.76
P		+++	+++	+++
Between Chi				2.94
Between df				1
Between P				(*)
Btwn(F) P				N.S.
Btwn(R) P				N.S.

Table 2I5 - 3

IESLC - Meta-analysis of Ever Smoking, Duration, "Highest vs lowest"
Squamous, Any Product (or Cigarettes if Any not available)
Most adjusted

	Any proxy use		
	No/nk	Yes	Total
N	19	6	25
NS	15	5	20
Wt	367.24	91.04	458.28
Het Chi	91.39	10.46	101.93
Het df	18	5	24
Het P	***	(*)	***
Fixed RR	3.50	3.39	3.48
RRl	3.16	2.76	3.17
RRu	3.88	4.16	3.81
P	+++	+++	+++
Random RR	3.77	3.70	3.74
RRl	2.75	2.62	2.94
RRu	5.17	5.22	4.76
P	+++	+++	+++
Between Chi			0.08
Between df			1
Between P			N.S.
Btwn(F) P			N.S.
Btwn(R) P			N.S.

	Full histological confirmation		
	No	Yes	Total
N	11	14	25
NS	9	11	20
Wt	76.58	381.70	458.28
Het Chi	21.06	77.28	101.93
Het df	10	13	24
Het P	*	***	***
Fixed RR	2.86	3.62	3.48
RRl	2.28	3.27	3.17
RRu	3.57	4.00	3.81
P	+++	+++	+++
Random RR	3.29	4.03	3.74
RRl	2.31	2.91	2.94
RRu	4.68	5.58	4.76
P	+++	+++	+++
Between Chi			3.59
Between df			1
Between P			(*)
Btwn(F) P			N.S.
Btwn(R) P			N.S.

	Number of adjustment variables (1)			
	0	1	2+/+nk	Total
N	11	6	8	25
NS	8	5	7	20
Wt	327.70	33.63	96.95	458.28
Het Chi	59.60	25.17	14.48	101.93
Het df	10	5	7	24
Het P	***	***	*	***
Fixed RR	3.43	4.54	3.31	3.48
RRl	3.08	3.24	2.71	3.17
RRu	3.83	6.37	4.04	3.81
P	+++	+++	+++	+++
Random RR	3.34	4.97	3.66	3.74
RRl	2.29	2.25	2.60	2.94
RRu	4.88	11.00	5.16	4.76
P	+++	+++	+++	+++
Between Chi				2.68
Between df				2
Between P				N.S.
Btwn(F) P				N.S.
Btwn(R) P				N.S.

Table 2I5 - 3

IESLC - Meta-analysis of Ever Smoking, Duration, "Highest vs lowest"
Squamous, Any Product (or Cigarettes if Any not available)
Most adjusted

	Number of adjustment variables (2) 0	1	2	3-5	6+/+nk	Total
N	11	6	4	3	1	25
NS	8	5	4	3	1	21
Wt	327.70	33.63	55.28	38.86	2.81	458.28
Het Chi	59.60	25.17	11.07	2.87	0.00	101.93
Het df	10	5	3	2	0	24
Het P	***	***	*	N.S.	N.S.	***
Fixed RR	3.43	4.54	3.42	3.27	2.19	3.48
RRl	3.08	3.24	2.63	2.38	0.68	3.17
RRu	3.83	6.37	4.45	4.47	7.06	3.81
P	+++	+++	+++	+++	N.S.	+++
Random RR	3.34	4.97	5.31	3.13	2.19	3.74
RRl	2.29	2.25	2.61	2.04	0.68	2.94
RRu	4.88	11.00	10.78	4.83	7.06	4.76
P	+++	+++	+++	+++	N.S.	+++
Between Chi						3.22
Between df						4
Between P						N.S.
Btwn(F) P						N.S.
Btwn(R) P						N.S.

	Product all/unsp	cig+/-ot	cig only	Total
N	5	19	1	25
NS	5	14	1	20
Wt	41.07	413.44	3.77	458.28
Het Chi	11.40	86.37	0.00	101.93
Het df	4	18	0	24
Het P	*	***	N.S.	***
Fixed RR	3.45	3.45	9.90	3.48
RRl	2.54	3.13	3.61	3.17
RRu	4.68	3.80	27.15	3.81
P	+++	+++	+++	+++
Random RR	3.69	3.62	9.90	3.74
RRl	2.15	2.73	3.61	2.94
RRu	6.34	4.79	27.15	4.76
P	+++	+++	+++	+++
Between Chi				4.16
Between df				2
Between P				N.S.
Btwn(F) P				N.S.
Btwn(R) P				N.S.

	Derivation of RR/CI Orig	StdCalc	Other	Total
N	1	11	13	25
NS	1	8	11	20
Wt	7.69	327.70	122.89	458.28
Het Chi	0.00	59.60	38.82	101.93
Het df	0	10	12	24
Het P	N.S.	***	***	***
Fixed RR	1.90	3.43	3.74	3.48
RRl	0.94	3.08	3.13	3.17
RRu	3.85	3.83	4.46	3.81
P	(+)	+++	+++	+++
Random RR	1.90	3.34	4.48	3.74
RRl	0.94	2.29	3.11	2.94
RRu	3.85	4.88	6.47	4.76
P	(+)	+++	+++	+++
Between Chi				3.51
Between df				2
Between P				N.S.
Btwn(F) P				N.S.
Btwn(R) P				(*)

Table 2I5 - 4

IESLC - Meta-analysis of Ever Smoking, Duration, "Highest vs lowest"
Squamous, Any Product (or Cigarettes if Any not available)
Least adjusted

REF	NRR	X	SEX	AGEL	AGEH	RACE	YF	LC TYPE	LOC	START	ST	NLC	R	VB	P	H	AD	ADOS	PRODUCT	exL	exH	unexL	unexH	De
BARBON	561	x	m	0	0	all	-	q	Eu:wst	1979	CC	755	n	bl	y	y	0	0	all/unsp	50	999	1	29	st
BOUCHA	501		c	0	0	wh	-	q+s	Eu:wst	1988	CC	150	n	bl	n	y	0	0	all/unsp	31	999	1	30	st
CHOI	565		m	0	0	all	-	q	As:oth	1985	CC	375	n	bl	n	n	0	0	cig+/-ot	50	999	1	29	st
CHOI	577		f	0	0	all	-	q	As:oth	1985	CC	375	n	bl	n	n	0	0	cig+/-ot	40	999	1	29	st
DAMBER	553		m	0	0	all	-	q	Eu:Sca	1972	CC	579	n	bl	y	n	1	0	all/unsp	51	999	1	30	ot
DORGAN	527		m	0	0	wh	-	q	NAmer	1980	CC	2026	n	bl	y	y	2	0	cig+/-ot	35	999	1	34	ot
DORGAN	523		f	0	0	all	-	q	NAmer	1980	CC	2026	n	bl	y	y	3	0	cig+/-ot	35	999	1	34	ot
DOSEME	515		m	0	0	all	-	q	Eu:bal	1979	CC	1210	n	bl	n	n	2	0	cig+/-ot	21	999	1	10	ot
GER	506	x	c	0	0	all	-	q+s	As:oth	1990	CC	141	n	ot	y	n	0	0	all/unsp	31	999	1	30	st
HAENSZ	521	x	f	0	0	all	-	q+u	NAmer	1955	CC	158	n	bl	n	y	0	0	cig+/-ot	15	999	1	14	st
JEDRYC	509		m	0	0	all	-	q	Eu:est	1980	CC	1630	n	bl	y	n	0	0	cig+/-ot	50	999	1	19	st
JOLY	645		m	0	0	all	-	q	SCAmer	1978	CC	826	n	bl	n	n	0	0	cig+/-ot	50	999	1	29	st
JOLY	617		f	0	0	all	-	q	SCAmer	1978	CC	826	n	bl	n	n	0	0	cig+/-ot	50	999	1	29	st
KHUDER	530		m	0	0	all	-	q	NAmer	1985	CC	482	n	bl	n	y	5	3#	cig+/-ot	30	999	1	29	or
LUBIN2	667		m	0	0	all	-	q	Eu:mul	1976	CC	7804	n	bl	n	y	0	0	cig+/-ot	50	999	1	29	st
LUBIN2	719		f	0	0	all	-	q	Eu:mul	1976	CC	7804	n	bl	n	y	0	0	cig+/-ot	50	999	1	29	st
LUO	503	x	c	0	0	all	-	q	As:Chi	1990	CC	102	n	ot	n	y	0	0	cig+/-ot	30	999	1	29	st
MATOS	605	x	m	0	0	all	-	q	SCAmer	1994	CC	200	n	bl	n	n	0	0	cig+/-ot	40	70	1	24	st
OSANN2	509	x	f	0	0	all	-	KI	NAmer	1964	ot	217	n	bl	n	y	0	0	cig+/-ot	21	999	1	20	st
PEZZOT	511	x	m	0	0	all	-	q	SCAmer	1987	CC	215	n	bl	n	y	0	0	cig only	41	999	1	30	st
WUWILL	510		f	0	0	all	-	q	As:Chi	1985	CC	965	n	ot	n	n	0	0	cig+/-ot	40	999	1	29	st
WYNDE2	513		m	0	0	all	-	KI	NAmer	1962	CC	404	n	bl	n	y	0	0	cig+/-ot	41	999	1	29	st
ZHENG	505	x	m	0	0	all	-	q	As:Chi	1982	CC	540	n	ot	*	y	0	0	cig+/-ot	40	999	1	29	st
ZHENG	510	x	f	0	0	all	-	q	As:Chi	1982	CC	540	n	ot	*	y	0	0	cig+/-ot	30	999	1	29	st
ZHOU	506		c	0	0	all	-	q	As:Chi	1978	CC	1360	n	ot	n	n	0	0	all/unsp	20	999	1	19	st

Comments on values in listings

KHUDER ADOS Age at starting smoking, No of cigarettes per day, Quitted smoking

Cigarette type is all/unspec for all RRs

Table 2I5 - 5

IESLC - Meta-analysis of Ever Smoking, Duration, "Highest vs lowest"
Squamous, Any Product (or Cigarettes if Any not available)
Least adjusted

REF	NRR	SEX	AD	Number Exposed Case	Cont	Non-exposed Case	Cont	RR	95.00%CI
BARBON	561	m	0	149	235	7	91	8.24	(3.72- 18.27)
BOUCHA	501	c	0	114	92	34	79	2.88	(1.77- 4.69)
CHOI	565	m	0	11	20	42	221	2.89	(1.29- 6.48)
CHOI	577	f	0	1	1	6	23	3.83	(0.21- 70.63)
Subtotal CHOI								2.95	(1.36- 6.42)
DAMBER	553	m	1	-	-	-	-	3.80	(1.82- 7.91)
DORGAN	527	m	2	-	-	-	-	2.77	(2.04- 3.76)
DORGAN	523	f	3	-	-	-	-	3.67	(2.52- 5.34)
Subtotal DORGAN								3.10	(2.44- 3.93)
DOSEME	515	m	2	199	-	15	-	4.08	(2.07- 8.05)
GER	506	c	0	42	119	6	37	2.18	(0.86- 5.52)
HAENSZ	521	f	0	42	77	14	26	1.01	(0.48- 2.15)
JEDRYC	509	m	0	49	214	7	68	2.22	(0.96- 5.14)
JOLY	645	m	0	98	253	15	109	2.81	(1.56- 5.07)
JOLY	617	f	0	22	20	5	54	11.88	(3.96- 35.63)
Subtotal JOLY								3.88	(2.31- 6.51)
KHUDER	530	m	5	-	-	-	-	1.90	(0.90- 3.70)
LUBIN2	667	m	0	746	1460	453	2964	3.34	(2.93- 3.82)
LUBIN2	719	f	0	566	34	322	229	11.84	(8.05- 17.40)
Subtotal LUBIN2								3.82	(3.37- 4.33)
LUO	503	c	0	28	45	6	21	2.18	(0.78- 6.06)
MATOS	605	m	0	26	89	3	84	8.18	(2.39- 28.03)
OSANN2	509	f	0	101	35	11	26	6.82	(3.06- 15.23)
PEZZOT	511	m	0	45	49	5	45	8.27	(3.01- 22.66)
WUWILL	510	f	0	81	114	54	139	1.83	(1.20- 2.80)
WYNDE2	513	m	0	94	89	22	55	2.64	(1.49- 4.68)
ZHENG	505	m	0	84	63	13	75	7.69	(3.92- 15.08)
ZHENG	510	f	0	35	27	8	17	2.75	(1.04- 7.33)
Subtotal ZHENG								5.53	(3.18- 9.63)
ZHOU	506	c	0	315	36	60	12	1.75	(0.86- 3.56)
Partial Totals				2848	3072	1108	4375		

*prospective study

REF	NRR	SEX	AD	Ys	Ws	Qs	Ps
BARBON	561	m	0	2.11	6.07	4.66	0.0000
BOUCHA	501	c	0	1.06	16.20	0.50	0.0000
CHOI	565	m	0	1.06	5.91	0.17	0.0098
CHOI	577	f	0	1.34	0.45	0.01	0.3661
Subtotal CHOI				1.08	6.36	0.18	
DAMBER	553	m	1	1.34	7.12	0.07	0.0004
DORGAN	527	m	2	1.02	41.10	1.88	0.0000
DORGAN	523	f	3	1.30	27.25	0.12	0.0000
Subtotal DORGAN				1.13	68.34	2.00	
DOSEME	515	m	2	1.41	8.33	0.25	0.0000
GER	506	c	0	0.78	4.43	0.92	0.1018
HAENSZ	521	f	0	0.01	6.82	10.14	0.9731
JEDRYC	509	m	0	0.80	5.48	1.03	0.0614
JOLY	645	m	0	1.03	11.11	0.43	0.0006
JOLY	617	f	0	2.47	3.18	4.92	0.0000
Subtotal JOLY				1.36	14.30	5.35	
KHUDER	530	m	5	0.64	7.69	2.68	0.0751
LUBIN2	667	m	0	1.21	218.80	0.14	0.0000
LUBIN2	719	f	0	2.47	25.87	39.71	0.0000
Subtotal LUBIN2				1.34	244.68	39.85	
LUO	503	c	0	0.78	3.67	0.76	0.1358
MATOS	605	m	0	2.10	2.53	1.91	0.0008
OSANN2	509	f	0	1.92	5.96	2.82	0.0000
PEZZOT	511	m	0	2.11	3.78	2.92	0.0000
WUWILL	510	f	0	0.60	21.35	8.44	0.0053
WYNDE2	513	m	0	0.97	11.69	0.80	0.0009
ZHENG	505	m	0	2.04	8.47	5.53	0.0000
ZHENG	510	f	0	1.01	4.01	0.19	0.0425
Subtotal ZHENG				1.71	12.48	5.72	
ZHOU	506	c	0	0.56	7.64	3.46	0.1220

Table 2I5 - 5

IESLC - Meta-analysis of Ever Smoking, Duration, "Highest vs lowest"
Squamous, Any Product (or Cigarettes if Any not available)
Least adjusted

	N	25
	NS	20
	Wt	464.91
Het	Chi	94.46
Het	df	24
Het	P	***
Fixed	RR	3.43
	RRl	3.13
	RRu	3.76
	P	+++
Random	RR	3.50
	RRl	2.79
	RRu	4.40
	P	+++
Asymm	P	N.S.

Table 2I5 - 6

IESLC - Meta-analysis of Ever Smoking, Duration, "Highest vs lowest"
Squamous, Any Product (or Cigarettes if Any not available)
Least adjusted

	Sex: combined	male	female	Total
N	4	13	8	25
NS	4	13	8	25
Wt	31.94	338.07	94.89	464.91
Het Chi	1.37	22.31	62.39	94.46
Het df	3	12	7	24
Het P	N.S.	*	***	***
Fixed RR	2.38	3.35	4.21	3.43
RRl	1.68	3.01	3.44	3.13
RRu	3.37	3.73	5.14	3.76
P	+++	+++	+++	+++
Random RR	2.38	3.54	3.99	3.50
RRl	1.68	2.86	2.02	2.79
RRu	3.37	4.38	7.87	4.40
P	+++	+++	+++	+++
Between Chi				8.39
Between df				2
Between P				*
Btwn(F) P				N.S.
Btwn(R) P				N.S.

Table 2I5 - 7

IESLC - Meta-analysis of Ever Smoking, Duration, "Highest vs lowest"
Squamous, Any Product (or Cigarettes if Any not available)
Excluded studies (and stage at which they were excluded)

1	AKIBA AMANDU AMES BECHER BENSHL BEST BLOT1 BROSS BROWN3 CARPEN CEDERL CHYOU CPSI CPSII DARBY DEAN2 DEAN3 DOLL2 ENGELA GAO2 GARCIA GILLIS GRAHAM GURSEL HAMMO2 HIRAYA HOLE HUMBLE JAHN JAIN KAISE2 KATSOU KAUFMA LAUSSM LIAW MCDUFF MIGRAN MRFITR PEZZO2 PISANI PRESCO QIAO SEGI2 SPEIZE SVENSS TVERDA WAKAI WATSON WIGLE WU WYNDE3 WYNDE8
2	ALDERS BRESLO CHIAZZ DORN GUO HEGMAN KOO KOULUM LIU4 PERNU SOBUE SPITZ SUZUK2 VUTUC YUAN
3	GENG STASZE WU2 ZHANG
4	GARSHI
5	AGUDO ARMADA AUVINE AXELSS BOFFET BOUCOT CHEN2 CORREA DESTEF DOLL FAN GAO HAMMON HU HU2 JUSSAW KREUZE LETOUR LEVIN LIU3 LIU5 MCCONN NOTAN2 QIAO2 RACHTA RESTRE SADOWS TIZZAN WANG2 WYNDE6 WYNDE7
6	BUFFLE CHEN LUBIN XU
15	BENHAM

Table 2I5 - 8
Potentially overlapping studies

REF	REFGP	PRINC	.	OVERLAP/LINK
LUBIN2	LUBIN2	1		Lubin-combined
OSANN2	KAISER	2		KAISER/OSANN2

Table 2I5 - 9
Most adjusted - insufficient data for meta-analysis

REF	NRR	SEX	AGEL	AGEH	RACE	YF	LC TYPE	LOC	START	ST	NLC	R	VB	P	H	AD	ADOS	PRODUCT	exL	exH	unexL	unexH	De
BUFFLE	551	m	0	0	wh	-	q	NAmer	1976	CC	943	n	bl	y	n	0	0	cig+/-ot	50	999	1	33	st
CHEN	515	c	0	0	all	-	q	As:oth	1987	CC	323	n	ot	n	y	2	0	cig+/-ot	41	999	1	20	st
LUBIN	617	m	0	0	all	-	KI	As:Chi	1984	CC	427	m	ot	y	n	5	1#	cig+/-ot	50	999	1	29	or
XU	515	m	0	0	all	-	q+s	As:Chi	1985	CC	729	n	ot	n	n	2	0	all/unsp	40	999	1	29	st

Comments on values in listings

LUBIN ADOS Duration of pipe use

REF	NRR	RR	SIG	RRDATA comment
BUFFLE	551	2.46		0
CHEN	515	4.96		0
LUBIN	617	4.72		0
XU	515	*		RR for 1-19/day is 2.2, for 20-29/day is 4.0 and for >=30/day is 4.1

Table 2I6 -

IESLC - Meta-analysis of Current Smoking by Duration, Overview
Squamous, Any Product (or Cigarettes if Any not available)

This analysis is restricted to results for:
1) Current smokers
2) Results by Duration
3) Categorical results by Duration
Results by Duration are grouped under 2 schemes (S1, S2). Each scheme has a set of "key values". An interval is allocated to the category whose key value it includes, and intervals which include none or more than one of the key values are excluded. (Open-ended intervals are coded as 999)

S1	key value	maximum range
1	20	1-34
2	35	21-49
3	50	36+

S2	key value	maximum range
1	5	1-19
2	20	6-29
3	30	21-39
4	40	31-49
5	50	41-998
6	999	51+

4) Squamous (or near equivalent)
5) Results complete enough for use in metaanalysis

Within each study, results are then selected (in the following order of preference, within each sex) for:
6) (not applicable)
7) PRODUCT: all/unspec, cigarettes regardless of other products, cigarettes only
8) CIGTYPE: all/unspecified, MC regardless of HR, MC only
9) (not applicable)
10) DENOM: never smoked anything, never smoked cigarettes, never any + low, never cigs + low
11) Followup period (YF, prospective studies): whole study (coded as 0) or longest available
12) LCtype: squamous or nearest available, but not adeno. (q = squamous, s = small,
a = adeno, KI = Kreyberg I, u = undifferentiated)
13) Race: all or nearest available, otherwise by race (wh or w = white, bl or b = black, hi = hispanic
ch = chinese, jap = japanese, haw = hawaiian, w+o = white + oriental, sca = scandinavian, as = asian)
14) For overlapping studies: principal rather than subsidiary studies
Finally by Age: whole study (coded as 0) if available, otherwise by widest available age group
and then for single sex results (m, f) in preference to results for both sexes combined (c).

Results adjusted (AD) for the most potential confounders are then chosen in Sections -1 to -3
and results adjusted for the least confounders in Sections -4 to -6. (Those least adjusted results which actually differ from the most adjusted are marked 'x' in column X in Section -4)

Section -7 shows excluded studies, together with the stage (as above) at which no qualifying results were found.

Section -8 lists the potentially overlapping studies which have been included (1=principal, 2=subsidiary).

Section -9 lists any results which would have been included in preference except that they had data not complete enough for use in meta-analysis, with their significance (yes/no), if known, and any further comment as entered on the database. It also lists as "gap" any categories for which no data were presented by the original authors.

In addition to those mentioned above, the following fields, levels and abbreviations are used:
* or nk = not known, n = no, y = yes, ot = other
nev = never
all/unspec = all or unspecified, cig+/-ot = cigarettes irrespective of other products (cigar, pipe etc)
MC = manufactured cigarettes, HR = hand-rolled cigarettes
exL, exH = range of exposure (low and high) in the smoking group, in terms of Duration
REF: 6-character study reference
NRR: number of the RR on the database within the study
ST : study type (CC = case control, pr or prosp = prospective)
NLC: number of lung cancer cases in whole study
R : risky occupational population (n = no, m = mining, o = other risky)
VB : national cigarette type (V = at least 75% Virginia, bl = at least 75% blended, ot = other)
P : any proxy use
H : full histological confirmation
De : derivation of RR/CI (or = original, st = standard method, ot = other method of estimation)

Table 2I6 - 1

IESLC - Meta-analysis of Current Smoking by Duration, Overview
Squamous, Any Product (or Cigarettes if Any not available)
Most adjusted

REF	NRR	SEX	AGEL	AGEH	RACE	YF	LC TYPE	LOC	START	ST	NLC	R	VB	P	H	AD	PRODUCT	exL	exH	S1	S2	DENOM	De
KATSOU	528	f	0	0	all	-	KI	Eu:bal	1987	CC	101	n	bl	n	n	1	all/unsp	1	29	1	0	nev any	or
KATSOU	529	f	0	0	all	-	KI	Eu:bal	1987	CC	101	n	bl	n	n	1	all/unsp	30	999	0	0	nev any	or
SOBUE	501	m	0	0	all	-	q	As:Jap	1986	CC	1376	n	bl	n	y	0	cig+/-ot	1	29	1	0	nev cigs	st
SOBUE	502	m	0	0	all	-	q	As:Jap	1986	CC	1376	n	bl	n	y	0	cig+/-ot	30	39	2	3	nev cigs	st
SOBUE	503	m	0	0	all	-	q	As:Jap	1986	CC	1376	n	bl	n	y	0	cig+/-ot	40	49	0	4	nev cigs	st
SOBUE	504	m	0	0	all	-	q	As:Jap	1986	CC	1376	n	bl	n	y	0	cig+/-ot	50	999	3	0	nev cigs	st

Cigarette type is all/unspec for all RRs

In this overview table, subtotals and Qs values may be invalid and should be ignored

Table 2I6 - 2

IESLC - Meta-analysis of Current Smoking by Duration, Overview
Squamous, Any Product (or Cigarettes if Any not available)
Most adjusted

REF	NRR	SEX	AD	Number Exposed Case	Cont	Non-exposed Case	Cont	RR	95.00%CI
KATSOU	528	f	1	5	-	14	-	1.77	(0.51- 6.14)
KATSOU	529	f	1	19	-	14	-	14.95	(5.06- 44.20)
Subtotal KATSOU								5.95	(2.63- 13.48)
SOBUE	501	m	0	16	119	3	128	5.74	(1.63- 20.19)
SOBUE	502	m	0	59	200	3	128	12.59	(3.86- 41.00)
SOBUE	503	m	0	95	174	3	128	23.30	(7.22- 75.19)
SOBUE	504	m	0	77	73	3	128	45.00	(13.71- 147.74)
Subtotal SOBUE								17.10	(9.39- 31.14)
Partial Totals				271	566	40	512		

*prospective study

REF	NRR	SEX	AD	Ys	Ws	Qs	Ps
KATSOU	528	f	1	0.57	2.48	8.95	0.3684
KATSOU	529	f	1	2.70	3.27	0.18	0.0000
Subtotal KATSOU				1.78	5.75	9.13	
SOBUE	501	m	0	1.75	2.43	1.27	0.0065
SOBUE	502	m	0	2.53	2.75	0.01	0.0000
SOBUE	503	m	0	3.15	2.80	1.29	0.0000
SOBUE	504	m	0	3.81	2.72	4.86	0.0000
Subtotal SOBUE				2.84	10.70	7.42	

N	6
NS	2

Table 2I6 - 3

IESLC - Meta-analysis of Current Smoking by Duration, Overview
Squamous, Any Product (or Cigarettes if Any not available)
Most adjusted

	Sex combined	male	female	Total
N		4	2	6
NS		1	1	2

In this overview table, other than the "N" rows, entries in the "absent" and "Total" columns may be invalid and should be ignored

	Duration of smoking (broad categories) absent	1-34k20	21-49k35	36+k50	Total
N	2	2	1	1	6
NS	2	2	1	1	4
Wt	6.07	4.91	2.75	2.72	16.45
Het Chi	0.30	1.70	0.00	0.00	16.56
Het df	1	1	0	0	5
Het P	N.S.	N.S.	N.S.	N.S.	**
Fixed RR	18.34	3.17	12.59	45.00	11.83
RRl	8.28	1.31	3.86	13.71	7.29
RRu	40.64	7.67	41.00	147.74	19.17
P	+++	+	+++	+++	+++
Random RR	18.34	3.17	12.59	45.00	11.41
RRl	8.28	1.00	3.86	13.71	4.73
RRu	40.64	10.05	41.00	147.74	27.54
P	+++	+	+++	+++	+++

	absent	Duration of smoking (narrow categories) 1-19k1	6-29k20	21-39k30	31-49k40	41-998k50	51+k999	Total
N	4			1	1			6
NS	2			1	1			2
Wt	10.90			2.75	2.80			16.45
Het Chi	14.87			0.00	0.00			16.56
Het df	3			0	0			5
Het P	**			N.S.	N.S.			**
Fixed RR	9.78			12.59	23.30			11.83
RRl	5.40			3.86	7.22			7.29
RRu	17.71			41.00	75.19			19.17
P	+++			+++	+++			+++
Random RR	9.23			12.59	23.30			11.41
RRl	2.45			3.86	7.22			4.73
RRu	34.74			41.00	75.19			27.54
P	++			+++	+++			+++

Table 2I6 - 3

IESLC - Meta-analysis of Current Smoking by Duration, Overview
Squamous, Any Product (or Cigarettes if Any not available)
Most adjusted

MALES

	Duration of smoking (broad categories) absent	1-34k20	21-49k35	36+k50	Total
N	1	1	1	1	4
NS	1	1	1	1	1
Wt	2.80	2.43	2.75	2.72	10.70
Het Chi	0.00	0.00	0.00	0.00	5.97
Het df	0	0	0	0	3
Het P	N.S.	N.S.	N.S.	N.S.	N.S.
Fixed RR	23.30	5.74	12.59	45.00	17.10
RRl	7.22	1.63	3.86	13.71	9.39
RRu	75.19	20.19	41.00	147.74	31.14
P	+++	++	+++	+++	+++
Random RR	23.30	5.74	12.59	45.00	16.85
RRl	7.22	1.63	3.86	13.71	7.23
RRu	75.19	20.19	41.00	147.74	39.26
P	+++	++	+++	+++	+++

		Duration of smoking (narrow categories)					
	absent	1-19k1	6-29k20	21-39k30	31-49k40	41-998k50	51+k999	Total
N	2			1	1			4
NS	1			1	1			1
Wt	5.15			2.75	2.80			10.70
Het Chi	5.44			0.00	0.00			5.97
Het df	1			0	0			3
Het P	*			N.S.	N.S.			N.S.
Fixed RR	17.03			12.59	23.30			17.10
RRl	7.18			3.86	7.22			9.39
RRu	40.42			41.00	75.19			31.14
P	+++			+++	+++			+++
Random RR	16.24			12.59	23.30			16.85
RRl	2.16			3.86	7.22			7.23
RRu	122.25			41.00	75.19			39.26
P	++			+++	+++			+++

FEMALES

	Duration of smoking (broad categories) absent	1-34k20	21-49k35	36+k50	Total
N	1	1			2
NS	1	1			1
Wt	3.27	2.48			5.75
Het Chi	0.00	0.00			6.42
Het df	0	0			1
Het P	N.S.	N.S.			*
Fixed RR	14.95	1.77			5.95
RRl	5.06	0.51			2.63
RRu	44.19	6.14			13.48
P	+++	N.S.			+++
Random RR	14.95	1.77			5.26
RRl	5.06	0.51			0.65
RRu	44.19	6.14			42.57
P	+++	N.S.			N.S.

Table 2I6 - 3

IESLC - Meta-analysis of Current Smoking by Duration, Overview
Squamous, Any Product (or Cigarettes if Any not available)
Most adjusted

FEMALES

		Duration of smoking (narrow categories)							
		absent	1-19k1	6-29k20	21-39k30	31-49k40	41-998k50	51+k999	Total
	N	2							2
	NS	1							1
	Wt	5.75							5.75
Het	Chi	6.42							6.42
Het	df	1							1
Het	P	*							*
Fixed	RR	5.95							5.95
	RRl	2.63							2.63
	RRu	13.48							13.48
	P	+++							+++
Random	RR	5.26							5.26
	RRl	0.65							0.65
	RRu	42.57							42.57
	P	N.S.							N.S.

Table 2I6 - 4

IESLC - Meta-analysis of Current Smoking by Duration, Overview
Squamous, Any Product (or Cigarettes if Any not available)
Least adjusted

REF	NRR	X	SEX	AGEL	AGEH	RACE	YF	LC TYPE	LOC	START	ST	NLC	R	VB	P	H	AD	PRODUCT	exL	exH	S1	S2	DENOM	De
KATSOU	523	x	f	0	0	all	-	KI	Eu:bal	1987	CC	101	n	bl	n	n	0	all/unsp	1	29	1	0	nev any	st
KATSOU	524	x	f	0	0	all	-	KI	Eu:bal	1987	CC	101	n	bl	n	n	0	all/unsp	30	999	0	0	nev any	st
SOBUE	501		m	0	0	all	-	q	As:Jap	1986	CC	1376	n	bl	n	y	0	cig+/-ot	1	29	1	0	nev cigs	st
SOBUE	502		m	0	0	all	-	q	As:Jap	1986	CC	1376	n	bl	n	y	0	cig+/-ot	30	39	2	3	nev cigs	st
SOBUE	503		m	0	0	all	-	q	As:Jap	1986	CC	1376	n	bl	n	y	0	cig+/-ot	40	49	0	4	nev cigs	st
SOBUE	504		m	0	0	all	-	q	As:Jap	1986	CC	1376	n	bl	n	y	0	cig+/-ot	50	999	3	0	nev cigs	st

Cigarette type is all/unspec for all RRs

In this overview table, subtotals and Qs values may be invalid and should be ignored

Table 2I6 - 5

IESLC - Meta-analysis of Current Smoking by Duration, Overview
Squamous, Any Product (or Cigarettes if Any not available)
Least adjusted

REF	NRR	SEX	AD	Number Exposed Case	Cont	Non-exposed Case	Cont	RR	95.00%CI
KATSOU	523	f	0	5	12	14	67	1.99	(0.61- 6.57)
KATSOU	524	f	0	19	6	14	67	15.15	(5.13- 44.79)
Subtotal KATSOU								6.05	(2.71- 13.49)
SOBUE	501	m	0	16	119	3	128	5.74	(1.63- 20.19)
SOBUE	502	m	0	59	200	3	128	12.59	(3.86- 41.00)
SOBUE	503	m	0	95	174	3	128	23.30	(7.22- 75.19)
SOBUE	504	m	0	77	73	3	128	45.00	(13.71- 147.74)
Subtotal SOBUE								17.10	(9.39- 31.14)
Totals				271	584	40	646		

*prospective study

REF	NRR	SEX	AD	Ys	Ws	Qs	Ps
KATSOU	523	f	0	0.69	2.70	8.54	0.2563
KATSOU	524	f	0	2.72	3.27	0.21	0.0000
Subtotal KATSOU				1.80	5.98	8.75	
SOBUE	501	m	0	1.75	2.43	1.26	0.0065
SOBUE	502	m	0	2.53	2.75	0.01	0.0000
SOBUE	503	m	0	3.15	2.80	1.30	0.0000
SOBUE	504	m	0	3.81	2.72	4.88	0.0000
Subtotal SOBUE				2.84	10.70	7.45	

N	6
NS	2

Table 2I6 - 6

IESLC - Meta-analysis of Current Smoking by Duration, Overview
Squamous, Any Product (or Cigarettes if Any not available)
Least adjusted

	Sex			
	combined	male	female	Total
N		4	2	6
NS		1	1	2

In this overview table, other than the "N" rows, entries in the "absent" and "Total" columns may be invalid and should be ignored

	Duration of smoking (broad categories)				
	absent	1-34k20	21-49k35	36+k50	Total
N	2	2	1	1	6
NS	2	2	1	1	4
Wt	6.07	5.13	2.75	2.72	16.67
Het Chi	0.28	1.43	0.00	0.00	16.20
Het df	1	1	0	0	5
Het P	N.S.	N.S.	N.S.	N.S.	**
Fixed RR	18.48	3.29	12.59	45.00	11.79
RRl	8.34	1.38	3.86	13.71	7.29
RRu	40.94	7.81	41.00	147.74	19.05
P	+++	++	+++	+++	+++
Random RR	18.48	3.32	12.59	45.00	11.57
RRl	8.34	1.18	3.86	13.71	4.87
RRu	40.94	9.33	41.00	147.74	27.49
P	+++	+	+++	+++	+++

	Duration of smoking (narrow categories)							
	absent	1-19k1	6-29k20	21-39k30	31-49k40	41-998k50	51+k999	Total
N	4			1	1			6
NS	2			1	1			2
Wt	11.12			2.75	2.80			16.67
Het Chi	14.49			0.00	0.00			16.20
Het df	3			0	0			5
Het P	**			N.S.	N.S.			**
Fixed RR	9.77			12.59	23.30			11.79
RRl	5.43			3.86	7.22			7.29
RRu	17.58			41.00	75.19			19.05
P	+++			+++	+++			+++
Random RR	9.47			12.59	23.30			11.57
RRl	2.59			3.86	7.22			4.87
RRu	34.58			41.00	75.19			27.49
P	+++			+++	+++			+++

Table 2I6 - 6

IESLC - Meta-analysis of Current Smoking by Duration, Overview
Squamous, Any Product (or Cigarettes if Any not available)
Least adjusted

MALES

Duration of smoking (broad categories)

	absent	1-34k20	21-49k35	36+k50	Total
N	1	1	1	1	4
NS	1	1	1	1	1
Wt	2.80	2.43	2.75	2.72	10.70
Het Chi	0.00	0.00	0.00	0.00	5.97
Het df	0	0	0	0	3
Het P	N.S.	N.S.	N.S.	N.S.	N.S.
Fixed RR	23.30	5.74	12.59	45.00	17.10
RRl	7.22	1.63	3.86	13.71	9.39
RRu	75.19	20.19	41.00	147.74	31.14
P	+++	++	+++	+++	+++
Random RR	23.30	5.74	12.59	45.00	16.85
RRl	7.22	1.63	3.86	13.71	7.23
RRu	75.19	20.19	41.00	147.74	39.26
P	+++	++	+++	+++	+++

Duration of smoking (narrow categories)

	absent	1-19k1	6-29k20	21-39k30	31-49k40	41-998k50	51+k999	Total
N	2			1	1			4
NS	1			1	1			1
Wt	5.15			2.75	2.80			10.70
Het Chi	5.44			0.00	0.00			5.97
Het df	1			0	0			3
Het P	*			N.S.	N.S.			N.S.
Fixed RR	17.03			12.59	23.30			17.10
RRl	7.18			3.86	7.22			9.39
RRu	40.42			41.00	75.19			31.14
P	+++			+++	+++			+++
Random RR	16.24			12.59	23.30			16.85
RRl	2.16			3.86	7.22			7.23
RRu	122.25			41.00	75.19			39.26
P	++			+++	+++			+++

FEMALES

Duration of smoking (broad categories)

	absent	1-34k20	21-49k35	36+k50	Total
N	1	1			2
NS	1	1			1
Wt	3.27	2.70			5.98
Het Chi	0.00	0.00			6.09
Het df	0	0			1
Het P	N.S.	N.S.			*
Fixed RR	15.15	1.99			6.05
RRl	5.13	0.61			2.71
RRu	44.79	6.57			13.49
P	+++	N.S.			+++
Random RR	15.15	1.99			5.58
RRl	5.13	0.61			0.77
RRu	44.79	6.57			40.75
P	+++	N.S.			(+)

Table 2I6 - 6

IESLC - Meta-analysis of Current Smoking by Duration, Overview
Squamous, Any Product (or Cigarettes if Any not available)
Least adjusted

FEMALES

		Duration of smoking (narrow categories)						
	absent	1-19k1	6-29k20	21-39k30	31-49k40	41-998k50	51+k999	Total
N	2							2
NS	1							1
Wt	5.98							5.98
Het Chi	6.09							6.09
Het df	1							1
Het P	*							*
Fixed RR	6.05							6.05
RRl	2.71							2.71
RRu	13.49							13.49
P	+++							+++
Random RR	5.58							5.58
RRl	0.77							0.77
RRu	40.75							40.75
P	(+)							(+)

Table 2I6 - 7

IESLC - Meta-analysis of Current Smoking by Duration, Overview
Squamous, Any Product (or Cigarettes if Any not available)
Excluded studies (and stage at which they were excluded)

Stage																	
1	AGUDO	ALDERS	ARMADA	AUVINE	AXELSS	BARBON	BECHER	BENHAM	BLOT1	BOFFET	BOUCHA	BRESLO	BROWN3	CARPEN	CHEN	CHEN2	
	CHIAZZ	CHOI	CHYOU	CORREA	DAMBER	DARBY	DESTEF	DOLL	DOLL2	DORGAN	DOSEME	FAN	GAO	GARCIA	GARSHI	GENG	
	GER	GRAHAM	GUO	GURSEL	HAENSZ	HAMMO2	HAMMON	HEGMAN	HU	HU2	JAHN	JAIN	JEDRYC	JOLY	JUSSAW	KHUDER	
	KOO	KOULUM	KREUZE	LAUSSM	LETOUR	LEVIN	LIU3	LIU4	LIU5	LUBIN	LUBIN2	LUO	MCCONN	NOTAN2	OSANN2	PERNU	
	PEZZOT	PRESCO	QIAO	QIAO2	RACHTA	RESTRE	SADOWS	STASZE	SUZUK2	TIZZAN	TVERDA	VUTUC	WANG2	WIGLE	WU2	WUWILL	
	WYNDE2	WYNDE3	XU	YUAN	ZHANG	ZHENG	ZHOU										
2	BENSHL	DEAN3	DORN	ENGELA	GAO2	GILLIS	HIRAYA	HOLE	KAUFMA	MIGRAN	MRFITR	SEGI2	SPEIZE	SVENSS	WAKAI	WU	
3	MCDUFF	SPITZ	WYNDE6														
4	AKIBA	AMANDU	AMES	BEST	BOUCOT	BROSS	BUFFLE	CEDERL	CPSI	CPSII	DEAN2	HUMBLE	KAISE2	LIAW	MATOS	PEZZO2	
	PISANI	WATSON	WYNDE7	WYNDE8													

Table 2I7 -

IESLC - Meta-analysis of Current Smoking, Duration, "Low"
Squamous, Any Product (or Cigarettes if Any not available)

This analysis is restricted to results for:
1) Current smokers
2) Results by Duration
3) Categorical results by Duration
4) Squamous (or near equivalent)
5) Results complete enough for use in metaanalysis

Within each study, results are then selected (in the following order of preference, within each sex) for:
6) (not applicable)
7) PRODUCT: all/unspec, cigarettes regardless of other products, cigarettes only
8) CIGTYPE: all/unspecified, MC regardless of HR, MC only
9) (not applicable)
10) DENOM: never smoked anything, never smoked cigarettes, never any + low, never cigs + low
11) Followup period (YF, prospective studies): whole study (coded as 0) or longest available
12) LCtype: squamous or nearest available, but not adeno. (q = squamous, s = small,
a = adeno, KI = Kreyberg I, u = undifferentiated)
13) Race: all or nearest available, otherwise by race (wh or w = white, bl or b = black, hi = hispanic
ch = chinese, jap = japanese, haw = hawaiian, w+o = white + oriental, sca = scandinavian, as = asian)
14) Duration "low" in key scheme 1 (key value 20, maximum range 1-34)
15) For overlapping studies: principal rather than subsidiary studies
Finally by Age: whole study (coded as 0) if available, otherwise by widest available age group
and then for single sex results (m, f) in preference to results for both sexes combined (c).

Results adjusted (AD) for the most potential confounders are then chosen in Sections -1 to -3
and results adjusted for the least confounders in Sections -4 to -6. (Those least adjusted results which
actually differ from the most adjusted are marked 'x' in column X in Section -4)

Section -7 shows excluded studies, together with the stage (as above) at which no qualifying
results were found.

Section -8 lists the potentially overlapping studies which have been included (1=principal, 2=subsidiary).

Section -9 lists any results which would have been included in preference except that they had data not complete
enough for use in meta-analysis, with their significance (yes/no), if known, and any further comment as entered
on the database. It also lists as "gap" any categories for which no data were presented by the original authors.

In addition to those mentioned above, the following fields, levels and abbreviations are used:
* or nk = not known, n = no, y = yes, ot = other
nev = never
all/unspec = all or unspecified, cig+/-ot = cigarettes irrespective of other products (cigar, pipe etc)
MC = manufactured cigarettes, HR = hand-rolled cigarettes
exL, exH = range of exposure (low and high) in the smoking group, in terms of Duration
REF: 6-character study reference
NRR: number of the RR on the database within the study
ST : study type (CC = case control, pr or prosp = prospective)
NLC: number of lung cancer cases in whole study
R : risky occupational population (n = no, m = mining, o = other risky)
VB : national cigarette type (V = at least 75% Virginia, bl = at least 75% blended, ot = other)
P : any proxy use
H : full histological confirmation
De : derivation of RR/CI (or = original, st = standard method, ot = other method of estimation)

Table 2I7 - 1

IESLC - Meta-analysis of Current Smoking, Duration, "Low"
Squamous, Any Product (or Cigarettes if Any not available)
Most adjusted

REF	NRR	SEX	AGEL	AGEH	RACE	YF	LC TYPE	LOC	START	ST	NLC	R	VB	P	H	AD	PRODUCT	exL	exH	DENOM	De
KATSOU	528	f	0	0	all	-	KI	Eu:bal	1987	CC	101	n	bl	n	n	1	all/unsp	1	29	nev any	or
SOBUE	501	m	0	0	all	-	q	As:Jap	1986	CC	1376	n	bl	n	y	0	cig+/-ot	1	29	nev cigs	st

Cigarette type is all/unspec for all RRs

Table 2I7 - 2

IESLC - Meta-analysis of Current Smoking, Duration, "Low"
Squamous, Any Product (or Cigarettes if Any not available)
Most adjusted

REF	NRR	SEX	AD	Number Exposed Case	Cont	Non-exposed Case	Cont	RR	95.00%CI
KATSOU	528	f	1	5	-	14	-	1.77	(0.51- 6.14)
SOBUE	501	m	0	16	119	3	128	5.74	(1.63- 20.19)
Partial Totals				21	119	17	128		

*prospective study

REF	NRR	SEX	AD	Ys	Ws	Qs	Ps
KATSOU	528	f	1	0.57	2.48	0.84	0.3684
SOBUE	501	m	0	1.75	2.43	0.86	0.0065

	N	2
	NS	2
	Wt	4.91
Het	Chi	1.70
Het	df	1
Het	P	N.S.
Fixed	RR	3.17
	RRl	1.31
	RRu	7.67
	P	+
Random	RR	3.17
	RRl	1.00
	RRu	10.05
	P	+
Asymm	P	

Table 2I7 - 3

IESLC - Meta-analysis of Current Smoking, Duration, "Low"
Squamous, Any Product (or Cigarettes if Any not available)
Most adjusted

	combined	Sex male	female	Total
N		1	1	2
NS		1	1	2
Wt		2.43	2.48	4.91
Het Chi		0.00	0.00	1.70
Het df		0	0	1
Het P		N.S.	N.S.	N.S.
Fixed RR		5.74	1.77	3.17
RRl		1.63	0.51	1.31
RRu		20.19	6.14	7.67
P		++	N.S.	+
Random RR		5.74	1.77	3.17
RRl		1.63	0.51	1.00
RRu		20.19	6.14	10.05
P		++	N.S.	+
Between Chi				1.70
Between df				1
Between P				N.S.
Btwn(F) P				N.S.
Btwn(R) P				N.S.

Too few RRs for analysis by factor

Table 2I7 - 4

IESLC - Meta-analysis of Current Smoking, Duration, "Low"
Squamous, Any Product (or Cigarettes if Any not available)
Least adjusted

REF	NRR	X	SEX	AGEL	AGEH	RACE	YF	LC TYPE	LOC	START	ST	NLC	R	VB	P	H	AD	PRODUCT	exL	exH	DENOM	De
KATSOU	523	x	f	0	0	all	-	KI	Eu:bal	1987	CC	101	n	bl	n	n	0	all/unsp	1	29	nev any	st
SOBUE	501		m	0	0	all	-	q	As:Jap	1986	CC	1376	n	bl	n	y	0	cig+/-ot	1	29	nev cigs	st

Cigarette type is all/unspec for all RRs

Table 2I7 - 5

IESLC - Meta-analysis of Current Smoking, Duration, "Low"
Squamous, Any Product (or Cigarettes if Any not available)
Least adjusted

REF	NRR	SEX	AD	Number Exposed Case	Cont	Non-exposed Case	Cont	RR	95.00%CI
KATSOU	523	f	0	5	12	14	67	1.99	(0.61- 6.57)
SOBUE	501	m	0	16	119	3	128	5.74	(1.63- 20.19)
Totals				21	131	17	195		

*prospective study

REF	NRR	SEX	AD	Ys	Ws	Qs	Ps
KATSOU	523	f	0	0.69	2.70	0.68	0.2563
SOBUE	501	m	0	1.75	2.43	0.75	0.0065

	N	2
	NS	2
	Wt	5.13
Het	Chi	1.43
Het	df	1
Het	P	N.S.
Fixed	RR	3.29
	RRl	1.38
	RRu	7.81
	P	++
Random	RR	3.32
	RRl	1.18
	RRu	9.33
	P	+
Asymm	P	

Table 2I7 - 6

IESLC - Meta-analysis of Current Smoking, Duration, "Low"
Squamous, Any Product (or Cigarettes if Any not available)
Least adjusted

	Sex combined	male	female	Total
N		1	1	2
NS		1	1	2
Wt		2.43	2.70	5.13
Het Chi		0.00	0.00	1.43
Het df		0	0	1
Het P		N.S.	N.S.	N.S.
Fixed RR		5.74	1.99	3.29
RRl		1.63	0.61	1.38
RRu		20.19	6.57	7.81
P		++	N.S.	++
Random RR		5.74	1.99	3.32
RRl		1.63	0.61	1.18
RRu		20.19	6.57	9.33
P		++	N.S.	+
Between Chi				1.43
Between df				1
Between P				N.S.
Btwn(F) P				N.S.
Btwn(R) P				N.S.

Table 2I7 - 7

IESLC - Meta-analysis of Current Smoking, Duration, "Low"
Squamous, Any Product (or Cigarettes if Any not available)
Excluded studies (and stage at which they were excluded)

Stage	Excluded studies
1	AGUDO ALDERS ARMADA AUVINE AXELSS BARBON BECHER BENHAM BLOT1 BOFFET BOUCHA BRESLO BROWN3 CARPEN CHEN CHEN2 CHIAZZ CHOI CHYOU CORREA DAMBER DARBY DESTEF DOLL DOLL2 DORGAN DOSEME FAN GAO GARCIA GARSHI GENG GER GRAHAM GUO GURSEL HAENSZ HAMMO2 HAMMON HEGMAN HU HU2 JAHN JAIN JEDRYC JOLY JUSSAW KHUDER KOO KOULUM KREUZE LAUSSM LETOUR LEVIN LIU3 LIU4 LIU5 LUBIN LUBIN2 LUO MCCONN NOTAN2 OSANN2 PERNU PEZZOT PRESCO QIAO QIAO2 RACHTA RESTRE SADOWS STASZE SUZUK2 TIZZAN TVERDA VUTUC WANG2 WIGLE WU2 WUWILL WYNDE2 WYNDE3 XU YUAN ZHANG ZHENG ZHOU
2	BENSHL DEAN3 DORN ENGELA GAO2 GILLIS HIRAYA HOLE KAUFMA MIGRAN MRFITR SEGI2 SPEIZE SVENSS WAKAI WU
3	MCDUFF SPITZ WYNDE6
4	AKIBA AMANDU AMES BEST BOUCOT BROSS BUFFLE CEDERL CPSI CPSII DEAN2 HUMBLE KAISE2 LIAW MATOS PEZZO2 PISANI WATSON WYNDE7 WYNDE8

Table 2I8 -

IESLC - Meta-analysis of Current Smoking, Duration, "Mid"
Squamous, Any Product (or Cigarettes if Any not available)

This analysis is restricted to results for:
1) Current smokers
2) Results by Duration
3) Categorical results by Duration
4) Squamous (or near equivalent)
5) Results complete enough for use in metaanalysis

Within each study, results are then selected (in the following order of preference, within each sex) for:
6) (not applicable)
7) PRODUCT: all/unspec, cigarettes regardless of other products, cigarettes only
8) CIGTYPE: all/unspecified, MC regardless of HR, MC only
9) (not applicable)
10) DENOM: never smoked anything, never smoked cigarettes, never any + low, never cigs + low
11) Followup period (YF, prospective studies): whole study (coded as 0) or longest available
12) LCtype: squamous or nearest available, but not adeno. (q = squamous, s = small,
a = adeno, KI = Kreyberg I, u = undifferentiated)
13) Race: all or nearest available, otherwise by race (wh or w = white, bl or b = black, hi = hispanic
ch = chinese, jap = japanese, haw = hawaiian, w+o = white + oriental, sca = scandinavian, as = asian)
14) Duration "mid" in key scheme 1 (key value 35, maximum range 21-49)
15) For overlapping studies: principal rather than subsidiary studies
Finally by Age: whole study (coded as 0) if available, otherwise by widest available age group
and then for single sex results (m, f) in preference to results for both sexes combined (c).

Results adjusted (AD) for the most potential confounders are then chosen in Sections -1 to -3
and results adjusted for the least confounders in Sections -4 to -6. (Those least adjusted results which
actually differ from the most adjusted are marked 'x' in column X in Section -4)

Section -7 shows excluded studies, together with the stage (as above) at which no qualifying
results were found.

Section -8 lists the potentially overlapping studies which have been included (1=principal, 2=subsidiary).

Section -9 lists any results which would have been included in preference except that they had data not complete
enough for use in meta-analysis, with their significance (yes/no), if known, and any further comment as entered
on the database. It also lists as "gap" any categories for which no data were presented by the original authors.

In addition to those mentioned above, the following fields, levels and abbreviations are used:
* or nk = not known, n = no, y = yes, ot = other
nev = never
all/unspec = all or unspecified, cig+/-ot = cigarettes irrespective of other products (cigar, pipe etc)
MC = manufactured cigarettes, HR = hand-rolled cigarettes
exL, exH = range of exposure (low and high) in the smoking group, in terms of Duration
REF: 6-character study reference
NRR: number of the RR on the database within the study
ST : study type (CC = case control, pr or prosp = prospective)
NLC: number of lung cancer cases in whole study
R : risky occupational population (n = no, m = mining, o = other risky)
VB : national cigarette type (V = at least 75% Virginia, bl = at least 75% blended, ot = other)
P : any proxy use
H : full histological confirmation
De : derivation of RR/CI (or = original, st = standard method, ot = other method of estimation)

Table 2I8 - 1

IESLC - Meta-analysis of Current Smoking, Duration, "Mid"
Squamous, Any Product (or Cigarettes if Any not available)
Most adjusted

REF	NRR	SEX	AGEL	AGEH	RACE	YF	LC TYPE	LOC	START	ST	NLC	R	VB	P	H	AD	PRODUCT	exL	exH	DENOM	De
SOBUE	502	m	0	0	all	-	q	As:Jap	1986	CC	1376	n	bl	n	y	0	cig+/-ot	30	39	nev cigs	st

Cigarette type is all/unspec for all RRs

Table 2I8 - 2

IESLC - Meta-analysis of Current Smoking, Duration, "Mid"
Squamous, Any Product (or Cigarettes if Any not available)
Most adjusted

REF	NRR	SEX	AD	Number Case	Exposed Cont	Non-exposed Case	Cont	RR	95.00%CI
SOBUE	502	m	0	59	200	3	128	12.59	(3.86- 41.00)
Totals				59	200	3	128		

*prospective study

REF	NRR	SEX	AD	Ys	Ws	Qs	Ps
SOBUE	502	m	0	2.53	2.75	0.00	0.0000

	N	1
	NS	1
	Wt	2.75
Het	Chi	0.00
Het	df	0
Het	P	N.S.
Fixed	RR	12.59
	RRl	3.86
	RRu	41.00
	P	+++
Random	RR	12.59
	RRl	3.86
	RRu	41.00
	P	+++
Asymm	P	

Table 2I8 - 3

IESLC - Meta-analysis of Current Smoking, Duration, "Mid"
Squamous, Any Product (or Cigarettes if Any not available)
Most adjusted

	Sex combined	male	female	Total
N		1		1
NS		1		1
Wt		2.75		2.75
Het Chi		0.00		0.00
Het df		0		0
Het P		N.S.		N.S.
Fixed RR		12.59		12.59
RRl		3.86		3.86
RRu		41.00		41.00
P		+++		+++
Random RR		12.59		12.59
RRl		3.86		3.86
RRu		41.00		41.00
P		+++		+++
Between Chi				
Between df				
Between P				N.S.
Btwn(F) P				N.S.
Btwn(R) P				N.S.

Too few RRs for analysis by factor

Table 2I8 - 4

IESLC - Meta-analysis of Current Smoking, Duration, "Mid"
Squamous, Any Product (or Cigarettes if Any not available)
Least adjusted

REF	NRR	X	SEX	AGEL	AGEH	RACE	YF	LC TYPE	LOC	START	ST	NLC	R	VB	P	H	AD	PRODUCT	exL	exH	DENOM	De
SOBUE	502		m	0	0	all	-	q	As:Jap	1986	CC	1376	n	bl	n	y	0	cig+/-ot	30	39	nev cigs	st

Cigarette type is all/unspec for all RRs

Table 2I8 - 5

IESLC - Meta-analysis of Current Smoking, Duration, "Mid"
Squamous, Any Product (or Cigarettes if Any not available)
Least adjusted

REF	NRR	SEX	AD	Number Exposed Case	Cont	Non-exposed Case	Cont	RR	95.00%CI
SOBUE	502	m	0	59	200	3	128	12.59	(3.86- 41.00)
Totals				59	200	3	128		

*prospective study

REF	NRR	SEX	AD	Ys	Ws	Qs	Ps
SOBUE	502	m	0	2.53	2.75	0.00	0.0000

	N	1
	NS	1
	Wt	2.75
Het	Chi	0.00
Het	df	0
Het	P	N.S.
Fixed	RR	12.59
	RRl	3.86
	RRu	41.00
	P	+++
Random	RR	12.59
	RRl	3.86
	RRu	41.00
	P	+++
Asymm	P	

Table 2I8 - 6

IESLC - Meta-analysis of Current Smoking, Duration, "Mid"
Squamous, Any Product (or Cigarettes if Any not available)
Least adjusted

	Sex combined	male	female	Total
N		1		1
NS		1		1
Wt		2.75		2.75
Het Chi		0.00		0.00
Het df		0		0
Het P		N.S.		N.S.
Fixed RR		12.59		12.59
RRl		3.86		3.86
RRu		41.00		41.00
P		+++		+++
Random RR		12.59		12.59
RRl		3.86		3.86
RRu		41.00		41.00
P		+++		+++
Between Chi				
Between df				
Between P				N.S.
Btwn(F) P				N.S.
Btwn(R) P				N.S.

Table 2I8 - 7

IESLC - Meta-analysis of Current Smoking, Duration, "Mid"
Squamous, Any Product (or Cigarettes if Any not available)
Excluded studies (and stage at which they were excluded)

1	AGUDO ALDERS ARMADA AUVINE AXELSS BARBON BECHER BENHAM BLOT1 BOFFET BOUCHA BRESLO BROWN3 CARPEN CHEN CHEN2 CHIAZZ CHOI CHYOU CORREA DAMBER DARBY DESTEF DOLL DOLL2 DORGAN DOSEME FAN GAO GARCIA GARSHI GENG GER GRAHAM GUO GURSEL HAENSZ HAMMO2 HAMMON HEGMAN HU HU2 JAHN JAIN JEDRYC JOLY JUSSAW KHUDER KOO KOULUM KREUZE LAUSSM LETOUR LEVIN LIU3 LIU4 LIU5 LUBIN LUBIN2 LUO MCCONN NOTAN2 OSANN2 PERNU PEZZOT PRESCO QIAO QIAO2 RACHTA RESTRE SADOWS STASZE SUZUK2 TIZZAN TVERDA VUTUC WANG2 WIGLE WU2 WUWILL WYNDE2 WYNDE3 XU YUAN ZHANG ZHENG ZHOU
2	BENSHL DEAN3 DORN ENGELA GAO2 GILLIS HIRAYA HOLE KAUFMA MIGRAN MRFITR SEGI2 SPEIZE SVENSS WAKAI WU
3	MCDUFF SPITZ WYNDE6
4	AKIBA AMANDU AMES BEST BOUCOT BROSS BUFFLE CEDERL CPSI CPSII DEAN2 HUMBLE KAISE2 LIAW MATOS PEZZO2 PISANI WATSON WYNDE7 WYNDE8
14	KATSOU

Table 2I9 -

IESLC - Meta-analysis of Current Smoking, Duration, "High"
Squamous, Any Product (or Cigarettes if Any not available)

This analysis is restricted to results for:
1) Current smokers
2) Results by Duration
3) Categorical results by Duration
4) Squamous (or near equivalent)
5) Results complete enough for use in metaanalysis

Within each study, results are then selected (in the following order of preference, within each sex) for:
6) PRODUCT: all/unspec, cigarettes regardless of other products, cigarettes only
7) CIGTYPE: all/unspecified, MC regardless of HR, MC only
8) (not applicable)
9) DENOM: never smoked anything, never smoked cigarettes, never any + low, never cigs + low
10) Followup period (YF, prospective studies): whole study (coded as 0) or longest available
11) LCtype: squamous or nearest available, but not adeno. (q = squamous, s = small,
a = adeno, KI = Kreyberg I, u = undifferentiated)
12) Race: all or nearest available, otherwise by race (wh or w = white, bl or b = black, hi = hispanic
ch = chinese, jap = japanese, haw = hawaiian, w+o = white + oriental, sca = scandinavian, as = asian)
13) Duration "high" in key scheme 1 (key value 50, maximum range 36+)
14) For overlapping studies: principal rather than subsidiary studies
Finally by Age: whole study (coded as 0) if available, otherwise by widest available age group
and then for single sex results (m, f) in preference to results for both sexes combined (c).

Results adjusted (AD) for the most potential confounders are then chosen in Sections -1 to -3
and results adjusted for the least confounders in Sections -4 to -6. (Those least adjusted results which
actually differ from the most adjusted are marked 'x' in column X in Section -4)

Section -7 shows excluded studies, together with the stage (as above) at which no qualifying
results were found.

Section -8 lists the potentially overlapping studies which have been included (1=principal, 2=subsidiary).

Section -9 lists any results which would have been included in preference except that they had data not complete
enough for use in meta-analysis, with their significance (yes/no), if known, and any further comment as entered
on the database. It also lists as "gap" any categories for which no data were presented by the original authors.

In addition to those mentioned above, the following fields, levels and abbreviations are used:
* or nk = not known, n = no, y = yes, ot = other
nev = never
all/unspec = all or unspecified, cig+/-ot = cigarettes irrespective of other products (cigar, pipe etc)
MC = manufactured cigarettes, HR = hand-rolled cigarettes
exL, exH = range of exposure (low and high) in the smoking group, in terms of Duration
REF: 6-character study reference
NRR: number of the RR on the database within the study
ST : study type (CC = case control, pr or prosp = prospective)
NLC: number of lung cancer cases in whole study
R : risky occupational population (n = no, m = mining, o = other risky)
VB : national cigarette type (V = at least 75% Virginia, bl = at least 75% blended, ot = other)
P : any proxy use
H : full histological confirmation
De : derivation of RR/CI (or = original, st = standard method, ot = other method of estimation)

Table 2I9 - 1

IESLC - Meta-analysis of Current Smoking, Duration, "High"
Squamous, Any Product (or Cigarettes if Any not available)
Most adjusted

REF	NRR	SEX	AGEL	AGEH	RACE	YF	LC TYPE	LOC	START	ST	NLC	R	VB	P	H	AD	PRODUCT	exL	exH	DENOM	De
SOBUE	504	m	0	0	all	-	q	As:Jap	1986	CC	1376	n	bl	n	y	0	cig+/-ot	50	999	nev cigs	st

Cigarette type is all/unspec for all RRs

Table 2I9 - 2

IESLC - Meta-analysis of Current Smoking, Duration, "High"
Squamous, Any Product (or Cigarettes if Any not available)
Most adjusted

REF	NRR	SEX	AD	Number Case	Exposed Cont	Non-exposed Case	Cont	RR	95.00%CI
SOBUE	504	m	0	77	73	3	128	45.00	(13.71- 147.74)
Totals				77	73	3	128		

*prospective study

REF	NRR	SEX	AD	Ys	Ws	Qs	Ps
SOBUE	504	m	0	3.81	2.72	0.00	0.0000

	N	1
	NS	1
	Wt	2.72
Het	Chi	0.00
Het	df	0
Het	P	N.S.
Fixed	RR	45.00
	RRl	13.71
	RRu	147.74
	P	+++
Random	RR	45.00
	RRl	13.71
	RRu	147.74
	P	+++
Asymm	P	

Table 2I9 - 3

IESLC - Meta-analysis of Current Smoking, Duration, "High"
Squamous, Any Product (or Cigarettes if Any not available)
Most adjusted

	Sex<br>combined	male	female	Total
N		1		1
NS		1		1
Wt		2.72		2.72
Het Chi		0.00		0.00
Het df		0		0
Het P		N.S.		N.S.
Fixed RR		45.00		45.00
RRl		13.71		13.71
RRu		147.74		147.74
P		+++		+++
Random RR		45.00		45.00
RRl		13.71		13.71
RRu		147.74		147.74
P		+++		+++
Between Chi				
Between df				
Between P				N.S.
Btwn(F) P				N.S.
Btwn(R) P				N.S.

Too few RRs for analysis by factor

Table 2I9 - 4

IESLC - Meta-analysis of Current Smoking, Duration, "High"
Squamous, Any Product (or Cigarettes if Any not available)
Least adjusted

REF	NRR	X	SEX	AGEL	AGEH	RACE	YF	LC TYPE	LOC	START	ST	NLC	R	VB	P	H	AD	PRODUCT	exL	exH	DENOM	De
SOBUE	504		m	0	0	all	-	q	As:Jap	1986	CC	1376	n	bl	n	y	0	cig+/-ot	50	999	nev cigs	st

Cigarette type is all/unspec for all RRs

Table 2I9 - 5

IESLC - Meta-analysis of Current Smoking, Duration, "High"
Squamous, Any Product (or Cigarettes if Any not available)
Least adjusted

REF	NRR	SEX	AD	Number Case	Exposed Cont	Non-exposed Case	Cont	RR	95.00%CI
SOBUE	504	m	0	77	73	3	128	45.00	(13.71- 147.74)
Totals				77	73	3	128		

*prospective study

REF	NRR	SEX	AD	Ys	Ws	Qs	Ps
SOBUE	504	m	0	3.81	2.72	0.00	0.0000

	N	1
	NS	1
	Wt	2.72
Het	Chi	0.00
Het	df	0
Het	P	N.S.
Fixed	RR	45.00
	RRl	13.71
	RRu	147.74
	P	+++
Random	RR	45.00
	RRl	13.71
	RRu	147.74
	P	+++
Asymm	P	

Table 2I9 - 6

IESLC - Meta-analysis of Current Smoking, Duration, "High"
Squamous, Any Product (or Cigarettes if Any not available)
Least adjusted

	Sex combined	male	female	Total
N		1		1
NS		1		1
Wt		2.72		2.72
Het Chi		0.00		0.00
Het df		0		0
Het P		N.S.		N.S.
Fixed RR		45.00		45.00
RRl		13.71		13.71
RRu		147.74		147.74
P		+++		+++
Random RR		45.00		45.00
RRl		13.71		13.71
RRu		147.74		147.74
P		+++		+++
Between Chi				
Between df				
Between P				N.S.
Btwn(F) P				N.S.
Btwn(R) P				N.S.

Table 2I9 - 7

IESLC - Meta-analysis of Current Smoking, Duration, "High"
Squamous, Any Product (or Cigarettes if Any not available)
Excluded studies (and stage at which they were excluded)

1	AGUDO ALDERS ARMADA AUVINE AXELSS BARBON BECHER BENHAM BLOT1 BOFFET BOUCHA BRESLO BROWN3 CARPEN CHEN CHEN2 CHIAZZ CHOI CHYOU CORREA DAMBER DARBY DESTEF DOLL DOLL2 DORGAN DOSEME FAN GAO GARCIA GARSHI GENG GER GRAHAM GUO GURSEL HAENSZ HAMMO2 HAMMON HEGMAN HU HU2 JAHN JAIN JEDRYC JOLY JUSSAW KHUDER KOO KOULUM KREUZE LAUSSM LETOUR LEVIN LIU3 LIU4 LIU5 LUBIN LUBIN2 LUO MCCONN NOTAN2 OSANN2 PERNU PEZZOT PRESCO QIAO QIAO2 RACHTA RESTRE SADOWS STASZE SUZUK2 TIZZAN TVERDA VUTUC WANG2 WIGLE WU2 WUWILL WYNDE2 WYNDE3 XU YUAN ZHANG ZHENG ZHOU
2	BENSHL DEAN3 DORN ENGELA GAO2 GILLIS HIRAYA HOLE KAUFMA MIGRAN MRFITR SEGI2 SPEIZE SVENSS WAKAI WU
3	MCDUFF SPITZ WYNDE6
4	AKIBA AMANDU AMES BEST BOUCOT BROSS BUFFLE CEDERL CPSI CPSII DEAN2 HUMBLE KAISE2 LIAW MATOS PEZZO2 PISANI WATSON WYNDE7 WYNDE8
14	KATSOU

Table 2I10 -

IESLC - Meta-analysis of Current Smoking, Duration, "Highest vs lowest"
Squamous, Any Product (or Cigarettes if Any not available)

This analysis is restricted to results for:
1) Current smokers
2) Results by Duration
3) Categorical results by Duration
4) Denominator (unexposed) = "low"
5) Squamous (or near equivalent)
6) Results complete enough for use in metaanalysis

Within each study, results are then selected (in the following order of preference, within each sex) for:
7) (not applicable)
8) PRODUCT: all/unspec, cigarettes regardless of other products, cigarettes only
9) CIGTYPE: all/unspecified, MC regardless of HR, MC only
10) Results with least adjustment for other aspects of smoking (ADOS)
11) The highest vs lowest category
12) Followup period (YF, prospective studies): whole study (coded as 0) or longest available
13) LCtype: squamous or nearest available, but not adeno. (q = squamous, s = small,
a = adeno, KI = Kreyberg I, u = undifferentiated)
14) Race: all or nearest available, otherwise by race (wh or w = white, bl or b = black, hi = hispanic
ch = chinese, jap = japanese, haw = hawaiian, w+o = white + oriental, sca = scandinavian, as = asian)
15) For overlapping studies: principal rather than subsidiary studies
Finally by Age: whole study (coded as 0) if available, otherwise by widest available age group
and then for single sex results (m, f) in preference to results for both sexes combined (c).

Results adjusted (AD) for the most potential confounders are then chosen in Sections -1 to -3
and results adjusted for the least confounders in Sections -4 to -6. (Those least adjusted results which
actually differ from the most adjusted are marked 'x' in column X in Section -4)

Section -7 shows excluded studies, together with the stage (as above) at which no qualifying
results were found.

Section -8 lists the potentially overlapping studies which have been included (1=principal, 2=subsidiary).

Section -9 lists any results which would have been included in preference except that they had data not complete
enough for use in meta-analysis, with their significance (yes/no), if known, and any further comment as entered
on the database. It also lists as "gap" any categories for which no data were presented by the original authors.

In addition to those mentioned above, the following fields, levels and abbreviations are used:
* or nk = not known, n = no, y = yes, ot = other
all/unspec = all or unspecified, cig+/-ot = cigarettes irrespective of other products (cigar, pipe etc)
MC = manufactured cigarettes, HR = hand-rolled cigarettes
exL, exH = range of exposure (low and high) in the "highest" group, in terms of Duration
unexL, unexH = range of exposure (low and high) in the "lowest" group, in terms of Duration
REF: 6-character study reference
NRR: number of the RR on the database within the study
ST : study type (CC = case control, pr or prosp = prospective)
NLC: number of lung cancer cases in whole study
R : risky occupational population (n = no, m = mining, o = other risky)
VB : national cigarette type (V = at least 75% Virginia, bl = at least 75% blended, ot = other)
P : any proxy use
H : full histological confirmation
De : derivation of RR/CI (or = original, st = standard method, ot = other method of estimation)

Table 2I10 - 1

IESLC - Meta-analysis of Current Smoking, Duration, "Highest vs lowest"
Squamous, Any Product (or Cigarettes if Any not available)
Most adjusted

REF	NRR	SEX	AGEL	AGEH	RACE	YF	LC TYPE	LOC	START	ST	NLC	R	VB	P	H	AD	ADOS	PRODUCT	exL	exH	unexL	unexH	De
KATSOU	530	f	0	0	all	-	KI	Eu:bal	1987	CC	101	n	bl	n	n	1	0	all/unsp	30	999	1	29	ot
SOBUE	508	m	0	0	all	-	q	As:Jap	1986	CC	1376	n	bl	n	y	0	0	cig+/-ot	50	999	1	29	st

Cigarette type is all/unspec for all RRs

Table 2I10 - 2

IESLC - Meta-analysis of Current Smoking, Duration, "Highest vs lowest"
Squamous, Any Product (or Cigarettes if Any not available)
Most adjusted

REF	NRR	SEX	AD	Number Exposed Case	Cont	Non-exposed Case	Cont	RR	95.00%CI
KATSOU	530	f	1	19	-	5	-	8.45	(2.01- 35.41)
SOBUE	508	m	0	77	73	16	119	7.85	(4.25- 14.47)
Partial Totals				96	73	21	119		

*prospective study

REF	NRR	SEX	AD	Ys	Ws	Qs	Ps
KATSOU	530	f	1	2.13	1.87	0.01	0.0035
SOBUE	508	m	0	2.06	10.25	0.00	0.0000

	N	2
	NS	2
	Wt	12.11
Het	Chi	0.01
Het	df	1
Het	P	N.S.
Fixed	RR	7.94
	RRl	4.52
	RRu	13.94
	P	+++
Random	RR	7.94
	RRl	4.52
	RRu	13.94
	P	+++
Asymm	P	

Table 2I10 - 3

IESLC - Meta-analysis of Current Smoking, Duration, "Highest vs lowest"
Squamous, Any Product (or Cigarettes if Any not available)
Most adjusted

	Sex combined	male	female	Total
N		1	1	2
NS		1	1	2
Wt		10.25	1.87	12.11
Het Chi		0.00	0.00	0.01
Het df		0	0	1
Het P		N.S.	N.S.	N.S.
Fixed RR		7.85	8.45	7.94
RRl		4.25	2.01	4.52
RRu		14.47	35.47	13.94
P		+++	++	+++
Random RR		7.85	8.45	7.94
RRl		4.25	2.01	4.52
RRu		14.47	35.47	13.94
P		+++	++	+++
Between Chi				0.01
Between df				1
Between P				N.S.
Btwn(F) P				N.S.
Btwn(R) P				N.S.

Too few RRs for analysis by factor

Table 2I10 - 4

IESLC - Meta-analysis of Current Smoking, Duration, "Highest vs lowest"
Squamous, Any Product (or Cigarettes if Any not available)
Least adjusted

REF	NRR	X	SEX	AGEL	AGEH	RACE	YF	LC TYPE	LOC	START	ST	NLC	R	VB	P	H	AD	ADOS	PRODUCT	exL	exH	unexL	unexH	De
KATSOU	525	x	f	0	0	all	-	KI	Eu:bal	1987	CC	101	n	bl	n	n	0	0	all/unsp	30	999	1	29	st
SOBUE	508		m	0	0	all	-	q	As:Jap	1986	CC	1376	n	bl	n	y	0	0	cig+/-ot	50	999	1	29	st

Cigarette type is all/unspec for all RRs

Table 2I10 - 5

IESLC - Meta-analysis of Current Smoking, Duration, "Highest vs lowest"
Squamous, Any Product (or Cigarettes if Any not available)
Least adjusted

REF	NRR	SEX	AD	Number Case	Exposed Cont	Non-exposed Case	Cont	RR	95.00%CI
KATSOU	525	f	0	19	6	5	12	7.60	(1.89- 30.50)
SOBUE	508	m	0	77	73	16	119	7.85	(4.25- 14.47)
Totals				96	79	21	131		

*prospective study

REF	NRR	SEX	AD	Ys	Ws	Qs	Ps
KATSOU	525	f	0	2.03	1.99	0.00	0.0042
SOBUE	508	m	0	2.06	10.25	0.00	0.0000

	N	2
	NS	2
	Wt	12.24
Het	Chi	0.00
Het	df	1
Het	P	N.S.
Fixed	RR	7.80
	RRl	4.46
	RRu	13.67
	P	+++
Random	RR	7.80
	RRl	4.46
	RRu	13.67
	P	+++
Asymm	P	

Table 2I10 - 6

IESLC - Meta-analysis of Current Smoking, Duration, "Highest vs lowest"
Squamous, Any Product (or Cigarettes if Any not available)
Least adjusted

	Sex combined	male	female	Total
N		1	1	2
NS		1	1	2
Wt		10.25	1.99	12.24
Het Chi		0.00	0.00	0.00
Het df		0	0	1
Het P		N.S.	N.S.	N.S.
Fixed RR		7.85	7.60	7.80
RRl		4.25	1.89	4.46
RRu		14.47	30.50	13.67
P		+++	++	+++
Random RR		7.85	7.60	7.80
RRl		4.25	1.89	4.46
RRu		14.47	30.50	13.67
P		+++	++	+++
Between Chi				0.00
Between df				1
Between P				N.S.
Btwn(F) P				N.S.
Btwn(R) P				N.S.

Table 2I10 - 7

IESLC - Meta-analysis of Current Smoking, Duration, "Highest vs lowest"
Squamous, Any Product (or Cigarettes if Any not available)
Excluded studies (and stage at which they were excluded)

1	AGUDO ALDERS ARMADA AUVINE AXELSS BARBON BECHER BENHAM BLOT1 BOFFET BOUCHA BRESLO BROWN3 CARPEN CHEN CHEN2 CHIAZZ CHOI CHYOU CORREA DAMBER DARBY DESTEF DOLL DOLL2 DORGAN DOSEME FAN GAO GARCIA GARSHI GENG GER GRAHAM GUO GURSEL HAENSZ HAMMO2 HAMMON HEGMAN HU HU2 JAHN JAIN JEDRYC JOLY JUSSAW KHUDER KOO KOULUM KREUZE LAUSSM LETOUR LEVIN LIU3 LIU4 LIU5 LUBIN LUBIN2 LUO MCCONN NOTAN2 OSANN2 PERNU PEZZOT PRESCO QIAO QIAO2 RACHTA RESTRE SADOWS STASZE SUZUK2 TIZZAN TVERDA VUTUC WANG2 WIGLE WU2 WUWILL WYNDE2 WYNDE3 XU YUAN ZHANG ZHENG ZHOU
2	BENSHL DEAN3 DORN ENGELA GAO2 GILLIS HIRAYA HOLE KAUFMA MIGRAN MRFITR SEGI2 SPEIZE SVENSS WAKAI WU
3	MCDUFF SPITZ WYNDE6
4	AKIBA
5	AMANDU AMES BEST BOUCOT BROSS BUFFLE CEDERL CPSI CPSII DEAN2 HUMBLE KAISE2 LIAW MATOS PEZZO2 PISANI WATSON WYNDE7 WYNDE8

Table 2I11 -

IESLC - Meta-analysis of Ever/current Smoking by Duration, Overview
Squamous, Any Product (or Cigarettes if Any not available)

This analysis is restricted to results for:
1) Ever/current smokers
2) Results by Duration
3) Categorical results by Duration
Results by Duration are grouped under 2 schemes (S1, S2). Each scheme has a set of "key values". An interval is allocated to the category whose key value it includes, and intervals which include none or more than one of the key values are excluded. (Open-ended intervals are coded as 999)

S1	key value	maximum range
1	20	1-34
2	35	21-49
3	50	36+

S2	key value	maximum range
1	5	1-19
2	20	6-29
3	30	21-39
4	40	31-49
5	50	41-998
6	999	51+

4) Squamous (or near equivalent)
5) Results complete enough for use in metaanalysis

Within each study, results are then selected (in the following order of preference, within each sex) for:
6) SMKSTA: ever, current
7) PRODUCT: all/unspec, cigarettes regardless of other products, cigarettes only
8) CIGTYPE: all/unspecified, MC regardless of HR, MC only
9) (not applicable)
10) DENOM: never smoked anything, never smoked cigarettes, never any + low, never cigs + low
11) Followup period (YF, prospective studies): whole study (coded as 0) or longest available
12) LCtype: squamous or nearest available, but not adeno. (q = squamous, s = small, a = adeno, KI = Kreyberg I, u = undifferentiated)
13) Race: all or nearest available, otherwise by race (wh or w = white, bl or b = black, hi = hispanic ch = chinese, jap = japanese, haw = hawaiian, w+o = white + oriental, sca = scandinavian, as = asian)
14) For overlapping studies: principal rather than subsidiary studies
Finally by Age: whole study (coded as 0) if available, otherwise by widest available age group and then for single sex results (m, f) in preference to results for both sexes combined (c).

Results adjusted (AD) for the most potential confounders are then chosen in Sections -1 to -3 and results adjusted for the least confounders in Sections -4 to -6. (Those least adjusted results which actually differ from the most adjusted are marked 'x' in column X in Section -4)

Section -7 shows excluded studies, together with the stage (as above) at which no qualifying results were found.

Section -8 lists the potentially overlapping studies which have been included (1=principal, 2=subsidiary).

Section -9 lists any results which would have been included in preference except that they had data not complete enough for use in meta-analysis, with their significance (yes/no), if known, and any further comment as entered on the database. It also lists as "gap" any categories for which no data were presented by the original authors.

In addition to those mentioned above, the following fields, levels and abbreviations are used:
* or nk = not known, n = no, y = yes, ot = other
ev = ever, cu = current, nev = never
all/unspec = all or unspecified, cig+/-ot = cigarettes irrespective of other products (cigar, pipe etc)
MC = manufactured cigarettes, HR = hand-rolled cigarettes
exL, exH = range of exposure (low and high) in the smoking group, in terms of Duration
REF: 6-character study reference
NRR: number of the RR on the database within the study
ST : study type (CC = case control, pr or prosp = prospective)
NLC: number of lung cancer cases in whole study
R : risky occupational population (n = no, m = mining, o = other risky)
VB : national cigarette type (V = at least 75% Virginia, bl = at least 75% blended, ot = other)
P : any proxy use
H : full histological confirmation
De : derivation of RR/CI (or = original, st = standard method, ot = other method of estimation)

Table 2I11 - 1

IESLC - Meta-analysis of Ever/current Smoking by Duration, Overview
Squamous, Any Product (or Cigarettes if Any not available)
Most adjusted

REF	NRR	SEX	AGEL	AGEH	RACE	YF	LC TYPE	LOC	START	ST	NLC	R	VB	P	H	AD	SM	PRODUCT	exL	exH	S1	S2	DENOM	De
BARBON	562	m	0	0	all	-	q	Eu:wst	1979	CC	755	n	bl	y	y	1	ev	all/unsp	1	29	1	0	nev any	or
BARBON	563	m	0	0	all	-	q	Eu:wst	1979	CC	755	n	bl	y	y	1	ev	all/unsp	30	39	2	3	nev any	or
BARBON	564	m	0	0	all	-	q	Eu:wst	1979	CC	755	n	bl	y	y	1	ev	all/unsp	40	49	0	4	nev any	or
BARBON	565	m	0	0	all	-	q	Eu:wst	1979	CC	755	n	bl	y	y	1	ev	all/unsp	50	999	3	0	nev any	or
BUFFLE	505	m	0	0	wh	-	q	NAmer	1976	CC	943	n	bl	y	n	0	ev	cig+/-ot	1	33	1	0	nev cigs	or
BUFFLE	506	m	0	0	wh	-	q	NAmer	1976	CC	943	n	bl	y	n	0	ev	cig+/-ot	34	43	2	4	nev cigs	or
BUFFLE	507	m	0	0	wh	-	q	NAmer	1976	CC	943	n	bl	y	n	0	ev	cig+/-ot	44	49	0	0	nev cigs	or
BUFFLE	508	m	0	0	wh	-	q	NAmer	1976	CC	943	n	bl	y	n	0	ev	cig+/-ot	50	999	3	0	nev cigs	or
CHOI	559	m	0	0	all	-	q	As:oth	1985	CC	375	n	bl	n	n	0	ev	cig+/-ot	1	29	1	0	nev cigs	st
CHOI	560	m	0	0	all	-	q	As:oth	1985	CC	375	n	bl	n	n	0	ev	cig+/-ot	30	39	2	3	nev cigs	st
CHOI	561	m	0	0	all	-	q	As:oth	1985	CC	375	n	bl	n	n	0	ev	cig+/-ot	40	49	0	4	nev cigs	st
CHOI	562	m	0	0	all	-	q	As:oth	1985	CC	375	n	bl	n	n	0	ev	cig+/-ot	50	999	3	0	nev cigs	st
CHOI	573	f	0	0	all	-	q	As:oth	1985	CC	375	n	bl	n	n	0	ev	cig+/-ot	1	29	1	0	nev cigs	st
CHOI	574	f	0	0	all	-	q	As:oth	1985	CC	375	n	bl	n	n	0	ev	cig+/-ot	30	39	2	3	nev cigs	st
CHOI	575	f	0	0	all	-	q	As:oth	1985	CC	375	n	bl	n	n	0	ev	cig+/-ot	40	999	3	0	nev cigs	st
DAMBER	547	m	0	0	all	-	q	Eu:Sca	1972	CC	579	n	bl	y	n	1	ev	all/unsp	1	30	1	0	nev any	or
DAMBER	548	m	0	0	all	-	q	Eu:Sca	1972	CC	579	n	bl	y	n	1	ev	all/unsp	31	40	2	4	nev any	or
DAMBER	549	m	0	0	all	-	q	Eu:Sca	1972	CC	579	n	bl	y	n	1	ev	all/unsp	41	50	3	5	nev any	or
DAMBER	550	m	0	0	all	-	q	Eu:Sca	1972	CC	579	n	bl	y	n	1	ev	all/unsp	51	999	0	6	nev any	or
DORGAN	572	m	0	0	wh	-	q	NAmer	1980	CC	2026	n	bl	y	y	2	ev	cig+/-ot	1	34	1	0	nev any	ot
DORGAN	573	m	0	0	wh	-	q	NAmer	1980	CC	2026	n	bl	y	y	2	ev	cig+/-ot	35	999	0	0	nev any	ot
DORGAN	564	f	0	0	all	-	q	NAmer	1980	CC	2026	n	bl	y	y	3	ev	cig+/-ot	1	34	1	0	nev any	ot
DORGAN	565	f	0	0	all	-	q	NAmer	1980	CC	2026	n	bl	y	y	3	ev	cig+/-ot	35	999	0	0	nev any	ot
DOSEME	511	m	0	0	all	-	q	Eu:bal	1979	CC	1210	n	bl	n	n	2	ev	cig+/-ot	1	10	0	1	nev cigs	or
DOSEME	512	m	0	0	all	-	q	Eu:bal	1979	CC	1210	n	bl	n	n	2	ev	cig+/-ot	11	20	1	2	nev cigs	or
DOSEME	513	m	0	0	all	-	q	Eu:bal	1979	CC	1210	n	bl	n	n	2	ev	cig+/-ot	21	999	0	0	nev cigs	or
GER	510	c	0	0	all	-	q+s	As:oth	1990	CC	141	n	ot	y	n	5	ev	all/unsp	1	30	1	0	nev any	ot
GER	511	c	0	0	all	-	q+s	As:oth	1990	CC	141	n	ot	y	n	5	ev	all/unsp	31	999	0	0	nev any	ot
HAENSZ	517	f	0	0	all	-	q+u	NAmer	1955	CC	158	n	bl	n	y	0	ev	cig+/-ot	1	14	0	1	nev any	st
HAENSZ	518	f	0	0	all	-	q+u	NAmer	1955	CC	158	n	bl	n	y	0	ev	cig+/-ot	15	999	0	0	nev any	st
JEDRYC	585	m	0	0	all	-	q	Eu:est	1980	CC	1630	n	bl	y	n	3	ev	cig+/-ot	1	19	0	1	nev any	or
JEDRYC	586	m	0	0	all	-	q	Eu:est	1980	CC	1630	n	bl	y	n	3	ev	cig+/-ot	20	39	0	0	nev any	or
JEDRYC	587	m	0	0	all	-	q	Eu:est	1980	CC	1630	n	bl	y	n	3	ev	cig+/-ot	40	999	3	0	nev any	or
JOLY	639	m	0	0	all	-	q	SCAmer	1978	CC	826	n	bl	n	n	0	ev	cig+/-ot	1	29	1	0	nev any	st
JOLY	640	m	0	0	all	-	q	SCAmer	1978	CC	826	n	bl	n	n	0	ev	cig+/-ot	30	39	2	3	nev any	st
JOLY	641	m	0	0	all	-	q	SCAmer	1978	CC	826	n	bl	n	n	0	ev	cig+/-ot	40	49	0	4	nev any	st
JOLY	642	m	0	0	all	-	q	SCAmer	1978	CC	826	n	bl	n	n	0	ev	cig+/-ot	50	999	3	0	nev any	st
JOLY	611	f	0	0	all	-	q	SCAmer	1978	CC	826	n	bl	n	n	0	ev	cig+/-ot	1	29	1	0	nev any	st
JOLY	612	f	0	0	all	-	q	SCAmer	1978	CC	826	n	bl	n	n	0	ev	cig+/-ot	30	39	2	3	nev any	st
JOLY	613	f	0	0	all	-	q	SCAmer	1978	CC	826	n	bl	n	n	0	ev	cig+/-ot	40	49	0	4	nev any	st
JOLY	614	f	0	0	all	-	q	SCAmer	1978	CC	826	n	bl	n	n	0	ev	cig+/-ot	50	999	3	0	nev any	st
KATSOU	528	f	0	0	all	-	KI	Eu:bal	1987	CC	101	n	bl	n	n	1	cu	all/unsp	1	29	1	0	nev any	or
KATSOU	529	f	0	0	all	-	KI	Eu:bal	1987	CC	101	n	bl	n	n	1	cu	all/unsp	30	999	0	0	nev any	or
LUBIN2	661	m	0	0	all	-	q	Eu:mul	1976	CC	7804	n	bl	n	y	0	ev	cig+/-ot	1	29	1	0	nev any	st
LUBIN2	662	m	0	0	all	-	q	Eu:mul	1976	CC	7804	n	bl	n	y	0	ev	cig+/-ot	30	39	2	3	nev any	st
LUBIN2	663	m	0	0	all	-	q	Eu:mul	1976	CC	7804	n	bl	n	y	0	ev	cig+/-ot	40	49	0	4	nev any	st
LUBIN2	664	m	0	0	all	-	q	Eu:mul	1976	CC	7804	n	bl	n	y	0	ev	cig+/-ot	50	999	3	0	nev any	st
LUBIN2	713	f	0	0	all	-	q	Eu:mul	1976	CC	7804	n	bl	n	y	0	ev	cig+/-ot	1	29	1	0	nev any	st
LUBIN2	714	f	0	0	all	-	q	Eu:mul	1976	CC	7804	n	bl	n	y	0	ev	cig+/-ot	30	39	2	3	nev any	st
LUBIN2	715	f	0	0	all	-	q	Eu:mul	1976	CC	7804	n	bl	n	y	0	ev	cig+/-ot	40	49	0	4	nev any	st
LUBIN2	716	f	0	0	all	-	q	Eu:mul	1976	CC	7804	n	bl	n	y	0	ev	cig+/-ot	50	999	3	0	nev any	st
LUO	504	c	0	0	all	-	q	As:Chi	1990	CC	102	n	ot	n	y	20	ev	cig+/-ot	1	29	1	0	nev cigs	or
LUO	505	c	0	0	all	-	q	As:Chi	1990	CC	102	n	ot	n	y	20	ev	cig+/-ot	30	999	0	0	nev cigs	or
MATOS	606	m	0	0	all	-	q	SCAmer	1994	CC	200	n	bl	n	n	2	ev	cig+/-ot	1	24	1	0	nev any	or
MATOS	607	m	0	0	all	-	q	SCAmer	1994	CC	200	n	bl	n	n	2	ev	cig+/-ot	25	39	2	3	nev any	or
MATOS	608	m	0	0	all	-	q	SCAmer	1994	CC	200	n	bl	n	n	2	ev	cig+/-ot	40	70	3	0	nev any	or
OSANN2	510	f	0	0	all	-	KI	NAmer	1964	ot	217	n	bl	n	y	1	ev	cig+/-ot	1	20	1	0	nev cigs	or
OSANN2	511	f	0	0	all	-	KI	NAmer	1964	ot	217	n	bl	n	y	1	ev	cig+/-ot	21	999	0	0	nev cigs	or
PEZZOT	507	m	0	0	all	-	q	SCAmer	1987	CC	215	n	bl	n	y	0	ev	cig only	1	30	1	0	nev cigs	ot
PEZZOT	508	m	0	0	all	-	q	SCAmer	1987	CC	215	n	bl	n	y	0	ev	cig only	31	40	2	4	nev cigs	ot
PEZZOT	509	m	0	0	all	-	q	SCAmer	1987	CC	215	n	bl	n	y	0	ev	cig only	41	999	3	0	nev cigs	ot
SOBUE	501	m	0	0	all	-	q	As:Jap	1986	CC	1376	n	bl	n	y	0	cu	cig+/-ot	1	29	1	0	nev cigs	st
SOBUE	502	m	0	0	all	-	q	As:Jap	1986	CC	1376	n	bl	n	y	0	cu	cig+/-ot	30	39	2	3	nev cigs	st
SOBUE	503	m	0	0	all	-	q	As:Jap	1986	CC	1376	n	bl	n	y	0	cu	cig+/-ot	40	49	0	4	nev cigs	st
SOBUE	504	m	0	0	all	-	q	As:Jap	1986	CC	1376	n	bl	n	y	0	cu	cig+/-ot	50	999	3	0	nev cigs	st
WUWILL	521	f	0	0	all	-	q	As:Chi	1985	CC	965	n	ot	n	n	3	ev	cig+/-ot	1	29	1	0	nev cigs	ot
WUWILL	522	f	0	0	all	-	q	As:Chi	1985	CC	965	n	ot	n	n	3	ev	cig+/-ot	30	39	2	3	nev cigs	ot
WUWILL	523	f	0	0	all	-	q	As:Chi	1985	CC	965	n	ot	n	n	3	ev	cig+/-ot	40	999	3	0	nev cigs	ot
WYNDE2	506	m	0	0	all	-	KI	NAmer	1962	CC	404	n	bl	n	y	0	ev	cig+/-ot	1	29	1	0	nev any	ot
WYNDE2	507	m	0	0	all	-	KI	NAmer	1962	CC	404	n	bl	n	y	0	ev	cig+/-ot	30	40	2	0	nev any	ot
WYNDE2	508	m	0	0	all	-	KI	NAmer	1962	CC	404	n	bl	n	y	0	ev	cig+/-ot	41	999	3	0	nev any	ot
ZHENG	501	m	0	0	all	-	q	As:Chi	1982	CC	540	n	ot	*	y	0	ev	cig+/-ot	1	29	1	0	nev cigs	st

Table 2I11 - 1

IESLC - Meta-analysis of Ever/current Smoking by Duration, Overview
Squamous, Any Product (or Cigarettes if Any not available)
Most adjusted

REF	NRR	SEX	AGEL	AGEH	RACE	YF	LC TYPE	LOC	START	ST	NLC	R	VB	P	H	AD	SM	PRODUCT	exL	exH	S1	S2	DENOM	De
ZHENG	502	m	0	0	all	-	q	As:Chi	1982	CC	540	n	ot	*	y	0	ev	cig+/-ot	30	39	2	3	nev cigs	st
ZHENG	503	m	0	0	all	-	q	As:Chi	1982	CC	540	n	ot	*	y	0	ev	cig+/-ot	40	999	3	0	nev cigs	st
ZHENG	508	f	0	0	all	-	q	As:Chi	1982	CC	540	n	ot	*	y	0	ev	cig+/-ot	1	29	1	0	nev cigs	st
ZHENG	509	f	0	0	all	-	q	As:Chi	1982	CC	540	n	ot	*	y	0	ev	cig+/-ot	30	999	0	0	nev cigs	st
ZHOU	504	c	0	0	all	-	q	As:Chi	1978	CC	1360	n	ot	n	n	0	ev	all/unsp	1	19	0	1	nev any	st
ZHOU	505	c	0	0	all	-	q	As:Chi	1978	CC	1360	n	ot	n	n	0	ev	all/unsp	20	999	0	0	nev any	st

Cigarette type is all/unspec for all RRs

In this overview table, subtotals and Qs values may be invalid and should be ignored

Table 2I11 - 2

IESLC - Meta-analysis of Ever/current Smoking by Duration, Overview
Squamous, Any Product (or Cigarettes if Any not available)
Most adjusted

REF	NRR	SEX	AD	Number Exposed Case	Cont	Non-exposed Case	Cont	RR	95.00%CI
BARBON	562	m	1	7	-	6	-	2.10	(0.70- 6.50)
BARBON	563	m	1	36	-	6	-	9.60	(3.90- 23.90)
BARBON	564	m	1	69	-	6	-	14.60	(6.10- 34.60)
BARBON	565	m	1	149	-	6	-	21.20	(9.10- 49.30)
Subtotal BARBON								10.54	(6.66- 16.67)
BUFFLE	505	m	0	-	-	-	-	9.00	(2.90- 27.90)
BUFFLE	506	m	0	-	-	-	-	14.80	(4.80- 45.30)
BUFFLE	507	m	0	-	-	-	-	12.60	(4.00- 38.80)
BUFFLE	508	m	0	-	-	-	-	22.10	(7.20- 67.70)
Subtotal BUFFLE								13.92	(7.92- 24.46)
CHOI	559	m	0	42	221	6	95	3.01	(1.24- 7.32)
CHOI	560	m	0	73	160	6	95	7.22	(3.03- 17.25)
CHOI	561	m	0	37	64	6	95	9.15	(3.65- 22.95)
CHOI	562	m	0	11	20	6	95	8.71	(2.88- 26.30)
CHOI	573	f	0	6	23	10	164	4.28	(1.42- 12.88)
CHOI	574	f	0	4	2	10	164	32.80	(5.35- 201.12)
CHOI	575	f	0	1	1	10	164	16.40	(0.95- 281.93)
Subtotal CHOI								6.58	(4.35- 9.95)
DAMBER	547	m	1	-	-	14	-	4.40	(1.80- 10.70)
DAMBER	548	m	1	-	-	14	-	8.40	(4.00- 18.30)
DAMBER	549	m	1	-	-	14	-	13.80	(6.80- 29.10)
DAMBER	550	m	1	-	-	14	-	16.70	(8.50- 34.00)
Subtotal DAMBER								10.50	(7.19- 15.34)
DORGAN	572	m	2	-	-	-	-	9.47	(3.39- 26.45)
DORGAN	573	m	2	-	-	-	-	26.21	(9.61- 71.49)
DORGAN	564	f	3	-	-	-	-	4.31	(2.53- 7.35)
DORGAN	565	f	3	-	-	-	-	15.82	(10.05- 24.90)
Subtotal DORGAN								10.17	(7.45- 13.89)
DOSEME	511	m	2	15	-	58	-	1.20	(0.60- 2.50)
DOSEME	512	m	2	70	-	58	-	3.90	(2.30- 6.70)
DOSEME	513	m	2	199	-	58	-	4.90	(3.20- 7.50)
Subtotal DOSEME								3.54	(2.62- 4.79)
GER	510	c	5	6	-	11	-	1.53	(0.40- 5.86)
GER	511	c	5	42	-	11	-	6.41	(2.03- 20.24)
Subtotal GER								3.50	(1.46- 8.37)
HAENSZ	517	f	0	14	26	44	236	2.89	(1.40- 5.96)
HAENSZ	518	f	0	42	77	44	236	2.93	(1.78- 4.80)
Subtotal HAENSZ								2.91	(1.94- 4.38)
JEDRYC	585	m	3	7	-	6	-	5.83	(1.79- 19.04)
JEDRYC	586	m	3	129	-	6	-	12.45	(5.21- 29.74)
JEDRYC	587	m	3	160	-	6	-	13.00	(5.54- 30.48)
Subtotal JEDRYC								10.80	(6.29- 18.57)
JOLY	639	m	0	15	109	2	218	15.00	(3.37- 66.77)
JOLY	640	m	0	24	165	2	218	15.85	(3.69- 68.04)
JOLY	641	m	0	66	182	2	218	39.53	(9.55- 163.60)
JOLY	642	m	0	98	253	2	218	42.22	(10.29- 173.22)
JOLY	611	f	0	5	54	6	283	4.37	(1.29- 14.82)
JOLY	612	f	0	5	24	6	283	9.83	(2.79- 34.57)
JOLY	613	f	0	16	24	6	283	31.44	(11.26- 87.78)
JOLY	614	f	0	22	20	6	283	51.88	(18.89- 142.48)
Subtotal JOLY								21.49	(13.83- 33.39)
KATSOU	528	f	1	5	-	14	-	1.77	(0.51- 6.14)
KATSOU	529	f	1	19	-	14	-	14.95	(5.06- 44.20)
Subtotal KATSOU								5.95	(2.63- 13.48)
LUBIN2	661	m	0	453	2964	54	2616	7.40	(5.56- 9.87)
LUBIN2	662	m	0	1211	3473	54	2616	16.89	(12.80- 22.29)
LUBIN2	663	m	0	1210	2540	54	2616	23.08	(17.48- 30.47)
LUBIN2	664	m	0	746	1460	54	2616	24.75	(18.64- 32.87)
LUBIN2	713	f	0	322	229	72	1180	23.04	(17.21- 30.86)
LUBIN2	714	f	0	767	186	72	1180	67.58	(50.73- 90.03)
LUBIN2	715	f	0	832	118	72	1180	115.56	(85.07- 156.96)
LUBIN2	716	f	0	566	34	72	1180	272.83	(179.26- 415.22)
Subtotal LUBIN2								31.16	(28.05- 34.61)
LUO	504	c	20	6	-	5	-	5.70	(1.00- 32.90)
LUO	505	c	20	28	-	5	-	12.50	(2.80- 55.40)
Subtotal LUO								8.97	(2.89- 27.91)
MATOS	606	m	2	3	-	3	-	1.20	(0.20- 6.20)
MATOS	607	m	2	18	-	3	-	5.80	(1.60- 20.50)
MATOS	608	m	2	26	-	3	-	18.50	(4.90- 69.80)
Subtotal MATOS								6.29	(2.80- 14.15)
OSANN2	510	f	1	11	-	7	-	4.90	(0.50- 44.60)

Table 2I11 - 2

IESLC - Meta-analysis of Ever/current Smoking by Duration, Overview
Squamous, Any Product (or Cigarettes if Any not available)
Most adjusted

REF	NRR	SEX	AD	Number Exposed Case	Cont	Non-exposed Case	Cont	RR	95.00%CI
OSANN2	511	f	1	101	-	7	-	101.10 (	8.30-1230.00)
Subtotal OSANN2								18.94 (	3.56- 100.64)
PEZZOT	507	m	0	5	134	0	116	9.53~(	0.52- 174.14)
PEZZOT	508	m	0	35	82	0	116	100.26~(	6.06-1657.79)
PEZZOT	509	m	0	45	101	0	116	104.45~(	6.35-1717.05)
Subtotal PEZZOT								48.16 (	9.37- 247.59)
SOBUE	501	m	0	16	119	3	128	5.74 (	1.63- 20.19)
SOBUE	502	m	0	59	200	3	128	12.59 (	3.86- 41.00)
SOBUE	503	m	0	95	174	3	128	23.30 (	7.22- 75.19)
SOBUE	504	m	0	77	73	3	128	45.00 (	13.71- 147.74)
Subtotal SOBUE								17.10 (	9.39- 31.14)
WUWILL	521	f	3	54	-	117	-	2.00 (	1.36- 2.94)
WUWILL	522	f	3	66	-	117	-	3.88 (	2.64- 5.71)
WUWILL	523	f	3	81	-	117	-	5.57 (	3.79- 8.17)
Subtotal WUWILL								3.51 (	2.81- 4.39)
WYNDE2	506	m	0	22	55	0	41	33.65~(	1.98- 570.85)
WYNDE2	507	m	0	30	64	0	41	39.25~(	2.34- 659.46)
WYNDE2	508	m	0	94	89	0	41	87.64~(	5.31-1446.06)
Subtotal WYNDE2								48.90 (	9.61- 248.90)
ZHENG	501	m	0	13	75	4	94	4.07 (	1.28- 13.01)
ZHENG	502	m	0	59	80	4	94	17.33 (	6.03- 49.81)
ZHENG	503	m	0	84	63	4	94	31.33 (	10.94- 89.77)
ZHENG	508	f	0	8	17	33	184	2.62 (	1.05- 6.57)
ZHENG	509	f	0	35	27	33	184	7.23 (	3.87- 13.49)
Subtotal ZHENG								7.80 (	5.24- 11.62)
ZHOU	504	c	0	60	12	136	68	2.50 (	1.26- 4.96)
ZHOU	505	c	0	315	36	136	68	4.38 (	2.79- 6.87)
Subtotal ZHOU								3.69 (	2.53- 5.38)
Partial Totals				8947	13830	1746	20301		

*prospective study ~ With 0.5 adjustment for zero

REF	NRR	SEX	AD	Ys	Ws	Qs	Ps
BARBON	562	m	1	0.74	3.09	10.78	0.1919
BARBON	563	m	1	2.26	4.68	0.56	0.0000
BARBON	564	m	1	2.68	5.10	0.03	0.0000
BARBON	565	m	1	3.05	5.38	1.07	0.0000
Subtotal BARBON				2.35	18.25	12.44	
BUFFLE	505	m	0	2.20	3.00	0.51	0.0001
BUFFLE	506	m	0	2.69	3.05	0.02	0.0000
BUFFLE	507	m	0	2.53	2.98	0.02	0.0000
BUFFLE	508	m	0	3.10	3.06	0.73	0.0000
Subtotal BUFFLE				2.63	12.08	1.27	
CHOI	559	m	0	1.10	4.87	11.05	0.0151
CHOI	560	m	0	1.98	5.07	2.02	0.0000
CHOI	561	m	0	2.21	4.55	0.71	0.0000
CHOI	562	m	0	2.16	3.14	0.62	0.0001
CHOI	573	f	0	1.45	3.16	4.22	0.0097
CHOI	574	f	0	3.49	1.17	0.91	0.0002
CHOI	575	f	0	2.80	0.47	0.02	0.0539
Subtotal CHOI				1.88	22.44	19.55	
DAMBER	547	m	1	1.48	4.84	6.14	0.0011
DAMBER	548	m	1	2.13	6.65	1.53	0.0000
DAMBER	549	m	1	2.62	7.27	0.00	0.0000
DAMBER	550	m	1	2.82	8.00	0.34	0.0000
Subtotal DAMBER				2.35	26.75	8.02	
DORGAN	572	m	2	2.25	3.64	0.47	0.0000
DORGAN	573	m	2	3.27	3.82	1.65	0.0000
DORGAN	564	f	3	1.46	13.51	17.80	0.0000
DORGAN	565	f	3	2.76	18.67	0.43	0.0000
Subtotal DORGAN				2.32	39.63	20.36	
DOSEME	511	m	2	0.18	7.54	44.42	0.6165
DOSEME	512	m	2	1.36	13.44	20.93	0.0000
DOSEME	513	m	2	1.59	21.18	22.02	0.0000
Subtotal DOSEME				1.26	42.17	87.36	
GER	510	c	5	0.43	2.13	10.17	0.5346
GER	511	c	5	1.86	2.91	1.64	0.0015
Subtotal GER				1.25	5.04	11.80	
HAENSZ	517	f	0	1.06	7.31	17.51	0.0041
HAENSZ	518	f	0	1.07	15.68	36.97	0.0000
Subtotal HAENSZ				1.07	22.99	54.48	

Table 2I11 - 2

IESLC - Meta-analysis of Ever/current Smoking by Duration, Overview
Squamous, Any Product (or Cigarettes if Any not available)
Most adjusted

REF	NRR	SEX	AD	Ys	Ws	Qs	Ps
JEDRYC	585	m	3	1.76	2.75	1.97	0.0035
JEDRYC	586	m	3	2.52	5.06	0.04	0.0000
JEDRYC	587	m	3	2.56	5.29	0.01	0.0000
Subtotal JEDRYC				2.38	13.10	2.01	
JOLY	639	m	0	2.71	1.72	0.02	0.0004
JOLY	640	m	0	2.76	1.81	0.04	0.0002
JOLY	641	m	0	3.68	1.90	2.17	0.0000
JOLY	642	m	0	3.74	1.93	2.48	0.0000
JOLY	611	f	0	1.47	2.57	3.31	0.0181
JOLY	612	f	0	2.29	2.43	0.25	0.0004
JOLY	613	f	0	3.45	3.64	2.57	0.0000
JOLY	614	f	0	3.95	3.76	6.76	0.0000
Subtotal JOLY				3.07	19.77	17.61	
KATSOU	528	f	1	0.57	2.48	10.31	0.3684
KATSOU	529	f	1	2.70	3.27	0.03	0.0000
Subtotal KATSOU				1.78	5.75	10.34	
LUBIN2	661	m	0	2.00	46.63	17.17	0.0000
LUBIN2	662	m	0	2.83	49.96	2.38	0.0000
LUBIN2	663	m	0	3.14	49.70	13.97	0.0000
LUBIN2	664	m	0	3.21	47.79	17.21	0.0000
LUBIN2	713	f	0	3.14	45.03	12.58	0.0000
LUBIN2	714	f	0	4.21	46.69	120.22	0.0000
LUBIN2	715	f	0	4.75	40.96	187.76	0.0000
LUBIN2	716	f	0	5.61	21.78	196.02	0.0000
Subtotal LUBIN2				3.44	348.54	567.31	
LUO	504	c	20	1.74	1.26	0.95	0.0508
LUO	505	c	20	2.53	1.72	0.01	0.0009
Subtotal LUO				2.19	2.98	0.96	
MATOS	606	m	2	0.18	1.30	7.67	0.8351
MATOS	607	m	2	1.76	2.36	1.71	0.0069
MATOS	608	m	2	2.92	2.18	0.21	0.0000
Subtotal MATOS				1.84	5.84	9.59	
OSANN2	510	f	1	1.59	0.76	0.79	0.1654
OSANN2	511	f	1	4.62	0.61	2.48	0.0003
Subtotal OSANN2				2.94	1.38	3.27	
PEZZOT	507	m	0	2.25	0.45	0.06	0.1284
PEZZOT	508	m	0	4.61	0.49	1.95	0.0013
PEZZOT	509	m	0	4.65	0.49	2.04	0.0011
Subtotal PEZZOT				3.87	1.43	4.05	
SOBUE	501	m	0	1.75	2.43	1.80	0.0065
SOBUE	502	m	0	2.53	2.75	0.02	0.0000
SOBUE	503	m	0	3.15	2.80	0.81	0.0000
SOBUE	504	m	0	3.81	2.72	3.90	0.0000
Subtotal SOBUE				2.84	10.70	6.53	
WUWILL	521	f	3	0.69	25.85	94.88	0.0004
WUWILL	522	f	3	1.36	25.82	40.53	0.0000
WUWILL	523	f	3	1.72	26.04	20.69	0.0000
Subtotal WUWILL				1.26	77.72	156.10	
WYNDE2	506	m	0	3.52	0.48	0.39	0.0149
WYNDE2	507	m	0	3.67	0.48	0.54	0.0108
WYNDE2	508	m	0	4.47	0.49	1.70	0.0018
Subtotal WYNDE2				3.89	1.45	2.64	
ZHENG	501	m	0	1.40	2.85	4.13	0.0177
ZHENG	502	m	0	2.85	3.45	0.20	0.0000
ZHENG	503	m	0	3.44	3.47	2.42	0.0000
ZHENG	508	f	0	0.96	4.55	12.31	0.0395
ZHENG	509	f	0	1.98	9.87	3.93	0.0000
Subtotal ZHENG				2.05	24.19	23.00	
ZHOU	504	c	0	0.92	8.19	23.47	0.0087
ZHOU	505	c	0	1.48	18.86	24.21	0.0000
Subtotal ZHOU				1.31	27.06	47.68	

N	78
NS	21

Table 2I11 - 3

IESLC - Meta-analysis of Ever/current Smoking by Duration, Overview
Squamous, Any Product (or Cigarettes if Any not available)
Most adjusted

	Sex			
	combined	male	female	Total
N	6	48	24	78
NS	3	14	9	26

In this overview table, other than the "N" rows, entries in the "absent" and "Total" columns may be invalid and should be ignored

	Duration of smoking (broad categories)				
	absent	1-34k20	21-49k35	36+k50	Total
N	24	23	15	16	78
NS	17	18	12	13	60
Wt	247.08	190.06	156.86	135.26	729.26
Het Chi	389.01	143.42	159.21	197.01	1066.38
Het df	23	22	14	15	77
Het P	***	***	***	***	***
Fixed RR	13.90	6.46	18.25	26.27	13.58
RRl	12.27	5.60	15.61	22.20	12.63
RRu	15.75	7.45	21.34	31.09	14.60
P	+++	+++	+++	+++	+++
Random RR	10.91	4.66	14.06	27.18	10.74
RRl	6.31	3.03	7.45	13.36	8.00
RRu	18.87	7.16	26.52	55.28	14.41
P	+++	+++	+++	+++	+++

	Duration of smoking (narrow categories)							
	absent	1-19k1	6-29k20	21-39k30	31-49k40	41-998k50	51+k999	Total
N	50	4	1	11	10	1	1	78
NS	21	4	1	8	8	1	1	43
Wt	409.72	25.79	13.44	146.19	118.84	7.27	8.00	729.26
Het Chi	550.85	6.01	0.00	153.23	95.81	0.00	0.00	1066.38
Het df	49	3	0	10	9	0	0	77
Het P	***	N.S.	N.S.	***	***	N.S.	N.S.	***
Fixed RR	10.53	2.30	3.90	18.83	36.43	13.80	16.70	13.58
RRl	9.56	1.56	2.29	16.02	30.44	6.67	8.35	12.63
RRu	11.60	3.38	6.66	22.15	43.61	28.55	33.40	14.60
P	+++	+++	+++	+++	+++	+++	+++	+++
Random RR	9.90	2.42	3.90	13.03	24.01	13.80	16.70	10.74
RRl	6.90	1.38	2.29	6.19	11.71	6.67	8.35	8.00
RRu	14.21	4.24	6.66	27.43	49.23	28.55	33.40	14.41
P	+++	++	+++	+++	+++	+++	+++	+++

Table 2I11 - 3

IESLC - Meta-analysis of Ever/current Smoking by Duration, Overview
Squamous, Any Product (or Cigarettes if Any not available)
Most adjusted

MALES

		Duration of smoking (broad categories)				
		absent	1-34k20	21-49k35	36+k50	Total
	N	12	13	11	12	48
	NS	10	13	11	12	46
	Wt	115.38	88.74	80.75	83.20	368.07
Het	Chi	87.22	18.73	10.83	11.21	208.47
Het	df	11	12	10	11	47
Het	P	***	(*)	N.S.	N.S.	***
Fixed	RR	12.43	5.87	14.19	22.50	12.21
	RRl	10.35	4.77	11.41	18.15	11.03
	RRu	14.91	7.23	17.65	27.89	13.52
	P	+++	+++	+++	+++	+++
Random	RR	11.27	5.23	13.39	22.33	11.16
	RRl	6.26	3.74	10.29	17.81	8.68
	RRu	20.29	7.32	17.41	27.99	14.35
	P	+++	+++	+++	+++	+++

			Duration of smoking (narrow categories)						
		absent	1-19k1	6-29k20	21-39k30	31-49k40	41-998k50	51+k999	Total
	N	28	2	1	7	8	1	1	48
	NS	14	2	1	7	8	1	1	33
	Wt	184.75	10.29	13.44	70.09	74.23	7.27	8.00	368.07
Het	Chi	110.22	5.03	0.00	6.58	11.73	0.00	0.00	208.47
Het	df	27	1	0	6	7	0	0	47
Het	P	***	*	N.S.	N.S.	N.S.	N.S.	N.S.	***
Fixed	RR	11.24	1.83	3.90	14.58	19.41	13.80	16.70	12.21
	RRl	9.73	0.99	2.29	11.54	15.46	6.67	8.35	11.03
	RRu	12.98	3.37	6.66	18.43	24.37	28.55	33.40	13.52
	P	+++	(+)	+++	+++	+++	+++	+++	+++
Random	RR	11.38	2.46	3.90	13.68	16.82	13.80	16.70	11.16
	RRl	7.99	0.53	2.29	10.23	11.20	6.67	8.35	8.68
	RRu	16.22	11.50	6.66	18.28	25.24	28.55	33.40	14.35
	P	+++	N.S.	+++	+++	+++	+++	+++	+++

FEMALES

		Duration of smoking (broad categories)				
		absent	1-34k20	21-49k35	36+k50	Total
	N	8	8	4	4	24
	NS	7	8	4	4	23
	Wt	100.02	97.92	76.11	52.06	326.11
Het	Chi	223.02	118.05	137.83	180.60	772.42
Het	df	7	7	3	3	23
Het	P	***	***	***	***	***
Fixed	RR	23.20	7.28	23.84	33.67	17.49
	RRl	19.07	5.97	19.04	25.66	15.69
	RRu	28.22	8.87	29.84	44.17	19.50
	P	+++	+++	+++	+++	+++
Random	RR	15.18	4.20	16.76	35.26	11.51
	RRl	4.46	1.61	2.48	2.90	5.87
	RRu	51.67	10.97	113.10	429.38	22.55
	P	+++	++	++	++	+++

Table 2I11 - 3

IESLC - Meta-analysis of Ever/current Smoking by Duration, Overview
Squamous, Any Product (or Cigarettes if Any not available)
Most adjusted

FEMALES

		Duration of smoking (narrow categories)						
	absent	1-19k1	6-29k20	21-39k30	31-49k40	41-998k50	51+k999	Total
N	17	1		4	2			24
NS	9	1		4	2			16
Wt	198.09	7.31		76.11	44.61			326.11
Het Chi	415.55	0.00		137.83	5.67			772.42
Het df	16	0		3	1			23
Het P	***	N.S.		***	*			***
Fixed RR	11.11	2.89		23.84	103.90			17.49
RRl	9.67	1.40		19.04	77.47			15.69
RRu	12.77	5.96		29.84	139.33			19.50
P	+++	++		+++	+++			+++
Random RR	9.27	2.89		16.76	66.35			11.51
RRl	4.29	1.40		2.48	18.79			5.87
RRu	20.05	5.96		113.10	234.28			22.55
P	+++	++		++	+++			+++

Table 2I11 - 4

IESLC - Meta-analysis of Ever/current Smoking by Duration, Overview
Squamous, Any Product (or Cigarettes if Any not available)
Least adjusted

REF	NRR	X	SEX	AGEL	AGEH	RACE	YF	LC TYPE	LOC	START	ST	NLC	R	VB	P	H	AD	SM	PRODUCT	exL	exH	S1	S2	DENOM	De
BARBON	555	x	m	0	0	all	-	q	Eu:wst	1979	CC	755	n	bl	y	y	0	ev	all/unsp	1	29	1	0	nev any	st
BARBON	556	x	m	0	0	all	-	q	Eu:wst	1979	CC	755	n	bl	y	y	0	ev	all/unsp	30	39	2	3	nev any	st
BARBON	557	x	m	0	0	all	-	q	Eu:wst	1979	CC	755	n	bl	y	y	0	ev	all/unsp	40	49	0	4	nev any	st
BARBON	558	x	m	0	0	all	-	q	Eu:wst	1979	CC	755	n	bl	y	y	0	ev	all/unsp	50	999	3	0	nev any	st
BUFFLE	505		m	0	0	wh	-	q	NAmer	1976	CC	943	n	bl	y	n	0	ev	cig+/-ot	1	33	1	0	nev cigs	or
BUFFLE	506		m	0	0	wh	-	q	NAmer	1976	CC	943	n	bl	y	n	0	ev	cig+/-ot	34	43	2	4	nev cigs	or
BUFFLE	507		m	0	0	wh	-	q	NAmer	1976	CC	943	n	bl	y	n	0	ev	cig+/-ot	44	49	0	0	nev cigs	or
BUFFLE	508		m	0	0	wh	-	q	NAmer	1976	CC	943	n	bl	y	n	0	ev	cig+/-ot	50	999	3	0	nev cigs	or
CHOI	559		m	0	0	all	-	q	As:oth	1985	CC	375	n	bl	n	n	0	ev	cig+/-ot	1	29	1	0	nev cigs	st
CHOI	560		m	0	0	all	-	q	As:oth	1985	CC	375	n	bl	n	n	0	ev	cig+/-ot	30	39	2	3	nev cigs	st
CHOI	561		m	0	0	all	-	q	As:oth	1985	CC	375	n	bl	n	n	0	ev	cig+/-ot	40	49	0	4	nev cigs	st
CHOI	562		m	0	0	all	-	q	As:oth	1985	CC	375	n	bl	n	n	0	ev	cig+/-ot	50	999	3	0	nev cigs	st
CHOI	573		f	0	0	all	-	q	As:oth	1985	CC	375	n	bl	n	n	0	ev	cig+/-ot	1	29	1	0	nev cigs	st
CHOI	574		f	0	0	all	-	q	As:oth	1985	CC	375	n	bl	n	n	0	ev	cig+/-ot	30	39	2	3	nev cigs	st
CHOI	575		f	0	0	all	-	q	As:oth	1985	CC	375	n	bl	n	n	0	ev	cig+/-ot	40	999	3	0	nev cigs	st
DAMBER	547		m	0	0	all	-	q	Eu:Sca	1972	CC	579	n	bl	y	n	1	ev	all/unsp	1	30	1	0	nev any	or
DAMBER	548		m	0	0	all	-	q	Eu:Sca	1972	CC	579	n	bl	y	n	1	ev	all/unsp	31	40	2	4	nev any	or
DAMBER	549		m	0	0	all	-	q	Eu:Sca	1972	CC	579	n	bl	y	n	1	ev	all/unsp	41	50	3	5	nev any	or
DAMBER	550		m	0	0	all	-	q	Eu:Sca	1972	CC	579	n	bl	y	n	1	ev	all/unsp	51	999	0	6	nev any	or
DORGAN	572		m	0	0	wh	-	q	NAmer	1980	CC	2026	n	bl	y	y	2	ev	cig+/-ot	1	34	1	0	nev any	ot
DORGAN	573		m	0	0	wh	-	q	NAmer	1980	CC	2026	n	bl	y	y	2	ev	cig+/-ot	35	999	0	0	nev any	ot
DORGAN	564		f	0	0	all	-	q	NAmer	1980	CC	2026	n	bl	y	y	3	ev	cig+/-ot	1	34	1	0	nev any	ot
DORGAN	565		f	0	0	all	-	q	NAmer	1980	CC	2026	n	bl	y	y	3	ev	cig+/-ot	35	999	0	0	nev any	ot
DOSEME	511		m	0	0	all	-	q	Eu:bal	1979	CC	1210	n	bl	n	n	2	ev	cig+/-ot	1	10	0	1	nev cigs	or
DOSEME	512		m	0	0	all	-	q	Eu:bal	1979	CC	1210	n	bl	n	n	2	ev	cig+/-ot	11	20	1	2	nev cigs	or
DOSEME	513		m	0	0	all	-	q	Eu:bal	1979	CC	1210	n	bl	n	n	2	ev	cig+/-ot	21	999	0	0	nev cigs	or
GER	504	x	c	0	0	all	-	q+s	As:oth	1990	CC	141	n	ot	y	n	0	ev	all/unsp	1	30	1	0	nev any	st
GER	505	x	c	0	0	all	-	q+s	As:oth	1990	CC	141	n	ot	y	n	0	ev	all/unsp	31	999	0	0	nev any	st
HAENSZ	517		f	0	0	all	-	q+u	NAmer	1955	CC	158	n	bl	n	y	0	ev	cig+/-ot	1	14	0	1	nev any	st
HAENSZ	518		f	0	0	all	-	q+u	NAmer	1955	CC	158	n	bl	n	y	0	ev	cig+/-ot	15	999	0	0	nev any	st
JEDRYC	501	x	m	0	0	all	-	q	Eu:est	1980	CC	1630	n	bl	y	n	0	ev	cig+/-ot	1	19	0	1	nev any	st
JEDRYC	502	x	m	0	0	all	-	q	Eu:est	1980	CC	1630	n	bl	y	n	0	ev	cig+/-ot	20	29	1	2	nev any	st
JEDRYC	503	x	m	0	0	all	-	q	Eu:est	1980	CC	1630	n	bl	y	n	0	ev	cig+/-ot	30	39	2	3	nev any	st
JEDRYC	504	x	m	0	0	all	-	q	Eu:est	1980	CC	1630	n	bl	y	n	0	ev	cig+/-ot	40	49	0	4	nev any	st
JEDRYC	505	x	m	0	0	all	-	q	Eu:est	1980	CC	1630	n	bl	y	n	0	ev	cig+/-ot	50	999	3	0	nev any	st
JOLY	639		m	0	0	all	-	q	SCAmer	1978	CC	826	n	bl	n	n	0	ev	cig+/-ot	1	29	1	0	nev any	st
JOLY	640		m	0	0	all	-	q	SCAmer	1978	CC	826	n	bl	n	n	0	ev	cig+/-ot	30	39	2	3	nev any	st
JOLY	641		m	0	0	all	-	q	SCAmer	1978	CC	826	n	bl	n	n	0	ev	cig+/-ot	40	49	0	4	nev any	st
JOLY	642		m	0	0	all	-	q	SCAmer	1978	CC	826	n	bl	n	n	0	ev	cig+/-ot	50	999	3	0	nev any	st
JOLY	611		f	0	0	all	-	q	SCAmer	1978	CC	826	n	bl	n	n	0	ev	cig+/-ot	1	29	1	0	nev any	st
JOLY	612		f	0	0	all	-	q	SCAmer	1978	CC	826	n	bl	n	n	0	ev	cig+/-ot	30	39	2	3	nev any	st
JOLY	613		f	0	0	all	-	q	SCAmer	1978	CC	826	n	bl	n	n	0	ev	cig+/-ot	40	49	0	4	nev any	st
JOLY	614		f	0	0	all	-	q	SCAmer	1978	CC	826	n	bl	n	n	0	ev	cig+/-ot	50	999	3	0	nev any	st
KATSOU	523	x	f	0	0	all	-	KI	Eu:bal	1987	CC	101	n	bl	n	n	0	cu	all/unsp	1	29	1	0	nev any	st
KATSOU	524	x	f	0	0	all	-	KI	Eu:bal	1987	CC	101	n	bl	n	n	0	cu	all/unsp	30	999	0	0	nev any	st
LUBIN2	661		m	0	0	all	-	q	Eu:mul	1976	CC	7804	n	bl	n	y	0	ev	cig+/-ot	1	29	1	0	nev any	st
LUBIN2	662		m	0	0	all	-	q	Eu:mul	1976	CC	7804	n	bl	n	y	0	ev	cig+/-ot	30	39	2	3	nev any	st
LUBIN2	663		m	0	0	all	-	q	Eu:mul	1976	CC	7804	n	bl	n	y	0	ev	cig+/-ot	40	49	0	4	nev any	st
LUBIN2	664		m	0	0	all	-	q	Eu:mul	1976	CC	7804	n	bl	n	y	0	ev	cig+/-ot	50	999	3	0	nev any	st
LUBIN2	713		f	0	0	all	-	q	Eu:mul	1976	CC	7804	n	bl	n	y	0	ev	cig+/-ot	1	29	1	0	nev any	st
LUBIN2	714		f	0	0	all	-	q	Eu:mul	1976	CC	7804	n	bl	n	y	0	ev	cig+/-ot	30	39	2	3	nev any	st
LUBIN2	715		f	0	0	all	-	q	Eu:mul	1976	CC	7804	n	bl	n	y	0	ev	cig+/-ot	40	49	0	4	nev any	st
LUBIN2	716		f	0	0	all	-	q	Eu:mul	1976	CC	7804	n	bl	n	y	0	ev	cig+/-ot	50	999	3	0	nev any	st
LUO	501	x	c	0	0	all	-	q	As:Chi	1990	CC	102	n	ot	n	y	0	ev	cig+/-ot	1	29	1	0	nev cigs	st
LUO	502	x	c	0	0	all	-	q	As:Chi	1990	CC	102	n	ot	n	y	0	ev	cig+/-ot	30	999	0	0	nev cigs	st
MATOS	601	x	m	0	0	all	-	q	SCAmer	1994	CC	200	n	bl	n	n	0	ev	cig+/-ot	1	24	1	0	nev any	st
MATOS	602	x	m	0	0	all	-	q	SCAmer	1994	CC	200	n	bl	n	n	0	ev	cig+/-ot	25	39	2	3	nev any	st
MATOS	603	x	m	0	0	all	-	q	SCAmer	1994	CC	200	n	bl	n	n	0	ev	cig+/-ot	40	70	3	0	nev any	st
OSANN2	507	x	f	0	0	all	-	KI	NAmer	1964	ot	217	n	bl	n	y	0	ev	cig+/-ot	1	20	1	0	nev cigs	st
OSANN2	508	x	f	0	0	all	-	KI	NAmer	1964	ot	217	n	bl	n	y	0	ev	cig+/-ot	21	999	0	0	nev cigs	st
PEZZOT	507		m	0	0	all	-	q	SCAmer	1987	CC	215	n	bl	n	y	0	ev	cig only	1	30	1	0	nev cigs	ot
PEZZOT	508		m	0	0	all	-	q	SCAmer	1987	CC	215	n	bl	n	y	0	ev	cig only	31	40	2	4	nev cigs	ot
PEZZOT	509		m	0	0	all	-	q	SCAmer	1987	CC	215	n	bl	n	y	0	ev	cig only	41	999	3	0	nev cigs	ot
SOBUE	501		m	0	0	all	-	q	As:Jap	1986	CC	1376	n	bl	n	y	0	cu	cig+/-ot	1	29	1	0	nev cigs	st
SOBUE	502		m	0	0	all	-	q	As:Jap	1986	CC	1376	n	bl	n	y	0	cu	cig+/-ot	30	39	2	3	nev cigs	st
SOBUE	503		m	0	0	all	-	q	As:Jap	1986	CC	1376	n	bl	n	y	0	cu	cig+/-ot	40	49	0	4	nev cigs	st
SOBUE	504		m	0	0	all	-	q	As:Jap	1986	CC	1376	n	bl	n	y	0	cu	cig+/-ot	50	999	3	0	nev cigs	st
WUWILL	506	x	f	0	0	all	-	q	As:Chi	1985	CC	965	n	ot	n	n	0	ev	cig+/-ot	1	29	1	0	nev cigs	st
WUWILL	507	x	f	0	0	all	-	q	As:Chi	1985	CC	965	n	ot	n	n	0	ev	cig+/-ot	30	39	2	3	nev cigs	st
WUWILL	508	x	f	0	0	all	-	q	As:Chi	1985	CC	965	n	ot	n	n	0	ev	cig+/-ot	40	999	3	0	nev cigs	st
WYNDE2	506		m	0	0	all	-	KI	NAmer	1962	CC	404	n	bl	n	y	0	ev	cig+/-ot	1	29	1	0	nev any	ot
WYNDE2	507		m	0	0	all	-	KI	NAmer	1962	CC	404	n	bl	n	y	0	ev	cig+/-ot	30	40	2	0	nev any	ot

Table 2I11 - 4

IESLC - Meta-analysis of Ever/current Smoking by Duration, Overview
Squamous, Any Product (or Cigarettes if Any not available)
Least adjusted

REF	NRR	X	SEX	AGEL	AGEH	RACE	YF	LC TYPE	LOC	START	ST	NLC	R	VB	P	H	AD	SM	PRODUCT	exL	exH	S1	S2	DENOM	De
WYNDE2	508		m	0	0	all	-	KI	NAmer	1962	CC	404	n	bl	n	y	0	ev	cig+/-ot	41	999	3	0	nev any	ot
ZHENG	501		m	0	0	all	-	q	As:Chi	1982	CC	540	n	ot	*	y	0	ev	cig+/-ot	1	29	1	0	nev cigs	st
ZHENG	502		m	0	0	all	-	q	As:Chi	1982	CC	540	n	ot	*	y	0	ev	cig+/-ot	30	39	2	3	nev cigs	st
ZHENG	503		m	0	0	all	-	q	As:Chi	1982	CC	540	n	ot	*	y	0	ev	cig+/-ot	40	999	3	0	nev cigs	st
ZHENG	508		f	0	0	all	-	q	As:Chi	1982	CC	540	n	ot	*	y	0	ev	cig+/-ot	1	29	1	0	nev cigs	st
ZHENG	509		f	0	0	all	-	q	As:Chi	1982	CC	540	n	ot	*	y	0	ev	cig+/-ot	30	999	0	0	nev cigs	st
ZHOU	504		c	0	0	all	-	q	As:Chi	1978	CC	1360	n	ot	n	n	0	ev	all/unsp	1	19	0	1	nev any	st
ZHOU	505		c	0	0	all	-	q	As:Chi	1978	CC	1360	n	ot	n	n	0	ev	all/unsp	20	999	0	0	nev any	st

Cigarette type is all/unspec for all RRs

In this overview table, subtotals and Qs values may be invalid and should be ignored

Table 2I11 - 5

IESLC - Meta-analysis of Ever/current Smoking by Duration, Overview
Squamous, Any Product (or Cigarettes if Any not available)
Least adjusted

REF	NRR	SEX	AD	Number Exposed Case	Cont	Non-exposed Case	Cont	RR	95.00%CI
BARBON	555	m	0	7	91	6	188	2.41	(0.79- 7.38)
BARBON	556	m	0	36	102	6	188	11.06	(4.51- 27.13)
BARBON	557	m	0	69	139	6	188	15.55	(6.57- 36.85)
BARBON	558	m	0	149	235	6	188	19.87	(8.59- 45.94)
Subtotal BARBON								11.23	(7.12- 17.72)
BUFFLE	505	m	0	-	-	-	-	9.00	(2.90- 27.90)
BUFFLE	506	m	0	-	-	-	-	14.80	(4.80- 45.30)
BUFFLE	507	m	0	-	-	-	-	12.60	(4.00- 38.80)
BUFFLE	508	m	0	-	-	-	-	22.10	(7.20- 67.70)
Subtotal BUFFLE								13.92	(7.92- 24.46)
CHOI	559	m	0	42	221	6	95	3.01	(1.24- 7.32)
CHOI	560	m	0	73	160	6	95	7.22	(3.03- 17.25)
CHOI	561	m	0	37	64	6	95	9.15	(3.65- 22.95)
CHOI	562	m	0	11	20	6	95	8.71	(2.88- 26.30)
CHOI	573	f	0	6	23	10	164	4.28	(1.42- 12.88)
CHOI	574	f	0	4	2	10	164	32.80	(5.35- 201.12)
CHOI	575	f	0	1	1	10	164	16.40	(0.95- 281.93)
Subtotal CHOI								6.58	(4.35- 9.95)
DAMBER	547	m	1	-	-	14	-	4.40	(1.80- 10.70)
DAMBER	548	m	1	-	-	14	-	8.40	(4.00- 18.30)
DAMBER	549	m	1	-	-	14	-	13.80	(6.80- 29.10)
DAMBER	550	m	1	-	-	14	-	16.70	(8.50- 34.00)
Subtotal DAMBER								10.50	(7.19- 15.34)
DORGAN	572	m	2	-	-	-	-	9.47	(3.39- 26.45)
DORGAN	573	m	2	-	-	-	-	26.21	(9.61- 71.49)
DORGAN	564	f	3	-	-	-	-	4.31	(2.53- 7.35)
DORGAN	565	f	3	-	-	-	-	15.82	(10.05- 24.90)
Subtotal DORGAN								10.17	(7.45- 13.89)
DOSEME	511	m	2	15	-	58	-	1.20	(0.60- 2.50)
DOSEME	512	m	2	70	-	58	-	3.90	(2.30- 6.70)
DOSEME	513	m	2	199	-	58	-	4.90	(3.20- 7.50)
Subtotal DOSEME								3.54	(2.62- 4.79)
GER	504	c	0	6	37	11	80	1.18	(0.41- 3.43)
GER	505	c	0	42	119	11	80	2.57	(1.25- 5.28)
Subtotal GER								2.01	(1.11- 3.66)
HAENSZ	517	f	0	14	26	44	236	2.89	(1.40- 5.96)
HAENSZ	518	f	0	42	77	44	236	2.93	(1.78- 4.80)
Subtotal HAENSZ								2.91	(1.94- 4.38)
JEDRYC	501	m	0	7	68	6	289	4.96	(1.61- 15.23)
JEDRYC	502	m	0	23	160	6	289	6.92	(2.76- 17.36)
JEDRYC	503	m	0	106	231	6	289	22.10	(9.54- 51.22)
JEDRYC	504	m	0	111	223	6	289	23.98	(10.35- 55.53)
JEDRYC	505	m	0	49	214	6	289	11.03	(4.64- 26.22)
Subtotal JEDRYC								12.77	(8.53- 19.11)
JOLY	639	m	0	15	109	2	218	15.00	(3.37- 66.77)
JOLY	640	m	0	24	165	2	218	15.85	(3.69- 68.04)
JOLY	641	m	0	66	182	2	218	39.53	(9.55- 163.60)
JOLY	642	m	0	98	253	2	218	42.22	(10.29- 173.22)
JOLY	611	f	0	5	54	6	283	4.37	(1.29- 14.82)
JOLY	612	f	0	5	24	6	283	9.83	(2.79- 34.57)
JOLY	613	f	0	16	24	6	283	31.44	(11.26- 87.78)
JOLY	614	f	0	22	20	6	283	51.88	(18.89- 142.48)
Subtotal JOLY								21.49	(13.83- 33.39)
KATSOU	523	f	0	5	12	14	67	1.99	(0.61- 6.57)
KATSOU	524	f	0	19	6	14	67	15.15	(5.13- 44.79)
Subtotal KATSOU								6.05	(2.71- 13.49)
LUBIN2	661	m	0	453	2964	54	2616	7.40	(5.56- 9.87)
LUBIN2	662	m	0	1211	3473	54	2616	16.89	(12.80- 22.29)
LUBIN2	663	m	0	1210	2540	54	2616	23.08	(17.48- 30.47)
LUBIN2	664	m	0	746	1460	54	2616	24.75	(18.64- 32.87)
LUBIN2	713	f	0	322	229	72	1180	23.04	(17.21- 30.86)
LUBIN2	714	f	0	767	186	72	1180	67.58	(50.73- 90.03)
LUBIN2	715	f	0	832	118	72	1180	115.56	(85.07- 156.96)
LUBIN2	716	f	0	566	34	72	1180	272.83	(179.26- 415.22)
Subtotal LUBIN2								31.16	(28.05- 34.61)
LUO	501	c	0	6	21	5	51	2.91	(0.80- 10.60)
LUO	502	c	0	28	45	5	51	6.35	(2.26- 17.82)
Subtotal LUO								4.68	(2.09- 10.49)
MATOS	601	m	0	3	84	3	110	1.31	(0.26- 6.65)
MATOS	602	m	0	18	110	3	110	6.00	(1.72- 20.95)
MATOS	603	m	0	26	89	3	110	10.71	(3.14- 36.55)

Table 2I11 - 5

IESLC - Meta-analysis of Ever/current Smoking by Duration, Overview
Squamous, Any Product (or Cigarettes if Any not available)
Least adjusted

REF	NRR	SEX	AD	Number Exposed Case	Cont	Non-exposed Case	Cont	RR	95.00%CI
Subtotal MATOS								5.35 (	2.48- 11.58)
OSANN2	507	f	0	11	26	7	58	3.51 (	1.22- 10.06)
OSANN2	508	f	0	101	35	7	58	23.91 (	9.98- 57.27)
Subtotal OSANN2								10.95 (	5.59- 21.45)
PEZZOT	507	m	0	5	134	0	116	9.53~(	0.52- 174.14)
PEZZOT	508	m	0	35	82	0	116	100.26~(	6.06-1657.79)
PEZZOT	509	m	0	45	101	0	116	104.45~(	6.35-1717.05)
Subtotal PEZZOT								48.16 (	9.37- 247.59)
SOBUE	501	m	0	16	119	3	128	5.74 (	1.63- 20.19)
SOBUE	502	m	0	59	200	3	128	12.59 (	3.86- 41.00)
SOBUE	503	m	0	95	174	3	128	23.30 (	7.22- 75.19)
SOBUE	504	m	0	77	73	3	128	45.00 (	13.71- 147.74)
Subtotal SOBUE								17.10 (	9.39- 31.14)
WUWILL	506	f	0	54	139	117	601	2.00 (	1.38- 2.89)
WUWILL	507	f	0	66	98	117	601	3.46 (	2.39- 5.01)
WUWILL	508	f	0	81	114	117	601	3.65 (	2.58- 5.16)
Subtotal WUWILL								2.96 (	2.40- 3.65)
WYNDE2	506	m	0	22	55	0	41	33.65~(	1.98- 570.85)
WYNDE2	507	m	0	30	64	0	41	39.25~(	2.34- 659.46)
WYNDE2	508	m	0	94	89	0	41	87.64~(	5.31-1446.06)
Subtotal WYNDE2								48.90 (	9.61- 248.90)
ZHENG	501	m	0	13	75	4	94	4.07 (	1.28- 13.01)
ZHENG	502	m	0	59	80	4	94	17.33 (	6.03- 49.81)
ZHENG	503	m	0	84	63	4	94	31.33 (	10.94- 89.77)
ZHENG	508	f	0	8	17	33	184	2.62 (	1.05- 6.57)
ZHENG	509	f	0	35	27	33	184	7.23 (	3.87- 13.49)
Subtotal ZHENG								7.80 (	5.24- 11.62)
ZHOU	504	c	0	60	12	136	68	2.50 (	1.26- 4.96)
ZHOU	505	c	0	315	36	136	68	4.38 (	2.79- 6.87)
Subtotal ZHOU								3.69 (	2.53- 5.38)
Partial Totals				8947	16228	1758	25143		

*prospective study ~ With 0.5 adjustment for zero

REF	NRR	SEX	AD	Ys	Ws	Qs	Ps
BARBON	555	m	0	0.88	3.07	8.51	0.1233
BARBON	556	m	0	2.40	4.77	0.10	0.0000
BARBON	557	m	0	2.74	5.16	0.20	0.0000
BARBON	558	m	0	2.99	5.47	1.08	0.0000
Subtotal BARBON				2.42	18.47	9.89	
BUFFLE	505	m	0	2.20	3.00	0.36	0.0001
BUFFLE	506	m	0	2.69	3.05	0.07	0.0000
BUFFLE	507	m	0	2.53	2.98	0.00	0.0000
BUFFLE	508	m	0	3.10	3.06	0.93	0.0000
Subtotal BUFFLE				2.63	12.08	1.36	
CHOI	559	m	0	1.10	4.87	10.14	0.0151
CHOI	560	m	0	1.98	5.07	1.64	0.0000
CHOI	561	m	0	2.21	4.55	0.50	0.0000
CHOI	562	m	0	2.16	3.14	0.46	0.0001
CHOI	573	f	0	1.45	3.16	3.77	0.0097
CHOI	574	f	0	3.49	1.17	1.04	0.0002
CHOI	575	f	0	2.80	0.47	0.03	0.0539
Subtotal CHOI				1.88	22.44	17.58	
DAMBER	547	m	1	1.48	4.84	5.47	0.0011
DAMBER	548	m	1	2.13	6.65	1.16	0.0000
DAMBER	549	m	1	2.62	7.27	0.05	0.0000
DAMBER	550	m	1	2.82	8.00	0.58	0.0000
Subtotal DAMBER				2.35	26.75	7.26	
DORGAN	572	m	2	2.25	3.64	0.32	0.0000
DORGAN	573	m	2	3.27	3.82	1.98	0.0000
DORGAN	564	f	3	1.46	13.51	15.89	0.0000
DORGAN	565	f	3	2.76	18.67	0.87	0.0000
Subtotal DORGAN				2.32	39.63	19.06	
DOSEME	511	m	2	0.18	7.54	42.13	0.6165
DOSEME	512	m	2	1.36	13.44	18.85	0.0000
DOSEME	513	m	2	1.59	21.18	19.36	0.0000
Subtotal DOSEME				1.26	42.17	80.34	
GER	504	c	0	0.16	3.37	19.07	0.7621
GER	505	c	0	0.94	7.37	18.94	0.0105
Subtotal GER				0.70	10.74	38.01	
HAENSZ	517	f	0	1.06	7.31	16.11	0.0041

Table 2I11 - 5

IESLC - Meta-analysis of Ever/current Smoking by Duration, Overview
Squamous, Any Product (or Cigarettes if Any not available)
Least adjusted

REF	NRR	SEX	AD	Ys	Ws	Qs	Ps
HAENSZ	518	f	0	1.07	15.68	33.97	0.0000
Subtotal HAENSZ				1.07	22.99	50.08	
JEDRYC	501	m	0	1.60	3.05	2.72	0.0052
JEDRYC	502	m	0	1.93	4.55	1.69	0.0000
JEDRYC	503	m	0	3.10	5.44	1.65	0.0000
JEDRYC	504	m	0	3.18	5.45	2.17	0.0000
JEDRYC	505	m	0	2.40	5.12	0.11	0.0000
Subtotal JEDRYC				2.55	23.61	8.34	
JOLY	639	m	0	2.71	1.72	0.05	0.0004
JOLY	640	m	0	2.76	1.81	0.09	0.0002
JOLY	641	m	0	3.68	1.90	2.44	0.0000
JOLY	642	m	0	3.74	1.93	2.76	0.0000
JOLY	611	f	0	1.47	2.57	2.95	0.0181
JOLY	612	f	0	2.29	2.43	0.16	0.0004
JOLY	613	f	0	3.45	3.64	2.97	0.0000
JOLY	614	f	0	3.95	3.76	7.42	0.0000
Subtotal JOLY				3.07	19.77	18.84	
KATSOU	523	f	0	0.69	2.70	9.31	0.2563
KATSOU	524	f	0	2.72	3.27	0.10	0.0000
Subtotal KATSOU				1.80	5.98	9.41	
LUBIN2	661	m	0	2.00	46.63	13.76	0.0000
LUBIN2	662	m	0	2.83	49.96	3.96	0.0000
LUBIN2	663	m	0	3.14	49.70	17.51	0.0000
LUBIN2	664	m	0	3.21	47.79	21.05	0.0000
LUBIN2	713	f	0	3.14	45.03	15.79	0.0000
LUBIN2	714	f	0	4.21	46.69	129.91	0.0000
LUBIN2	715	f	0	4.75	40.96	199.06	0.0000
LUBIN2	716	f	0	5.61	21.78	204.40	0.0000
Subtotal LUBIN2				3.44	348.54	605.44	
LUO	501	c	0	1.07	2.30	5.02	0.1044
LUO	502	c	0	1.85	3.60	1.75	0.0005
Subtotal LUO				1.54	5.91	6.77	
MATOS	601	m	0	0.27	1.45	7.53	0.7450
MATOS	602	m	0	1.79	2.46	1.39	0.0050
MATOS	603	m	0	2.37	2.55	0.08	0.0002
Subtotal MATOS				1.68	6.46	9.00	
OSANN2	507	f	0	1.25	3.45	5.76	0.0197
OSANN2	508	f	0	3.17	5.04	1.99	0.0000
Subtotal OSANN2				2.39	8.49	7.75	
PEZZOT	507	m	0	2.25	0.45	0.04	0.1284
PEZZOT	508	m	0	4.61	0.49	2.08	0.0013
PEZZOT	509	m	0	4.65	0.49	2.17	0.0011
Subtotal PEZZOT				3.87	1.43	4.28	
SOBUE	501	m	0	1.75	2.43	1.55	0.0065
SOBUE	502	m	0	2.53	2.75	0.00	0.0000
SOBUE	503	m	0	3.15	2.80	1.02	0.0000
SOBUE	504	m	0	3.81	2.72	4.33	0.0000
Subtotal SOBUE				2.84	10.70	6.89	
WUWILL	506	f	0	0.69	27.84	95.72	0.0003
WUWILL	507	f	0	1.24	28.12	47.83	0.0000
WUWILL	508	f	0	1.29	31.92	49.93	0.0000
Subtotal WUWILL				1.09	87.87	193.48	
WYNDE2	506	m	0	3.52	0.48	0.45	0.0149
WYNDE2	507	m	0	3.67	0.48	0.61	0.0108
WYNDE2	508	m	0	4.47	0.49	1.82	0.0018
Subtotal WYNDE2				3.89	1.45	2.88	
ZHENG	501	m	0	1.40	2.85	3.71	0.0177
ZHENG	502	m	0	2.85	3.45	0.33	0.0000
ZHENG	503	m	0	3.44	3.47	2.80	0.0000
ZHENG	508	f	0	0.96	4.55	11.38	0.0395
ZHENG	509	f	0	1.98	9.87	3.18	0.0000
Subtotal ZHENG				2.05	24.19	21.40	
ZHOU	504	c	0	0.92	8.19	21.74	0.0087
ZHOU	505	c	0	1.48	18.86	21.57	0.0000
Subtotal ZHOU				1.31	27.06	43.32	

Table 2I11 - 5

IESLC - Meta-analysis of Ever/current Smoking by Duration, Overview
Squamous, Any Product (or Cigarettes if Any not available)
Least adjusted

N	80
NS	21

Table 2I11 - 6

IESLC - Meta-analysis of Ever/current Smoking by Duration, Overview
Squamous, Any Product (or Cigarettes if Any not available)
Least adjusted

	Sex combined	male	female	Total
N	6	50	24	80
NS	3	14	9	26

In this overview table, other than the "N" rows, entries in the "absent" and "Total" columns may be invalid and should be ignored

Duration of smoking (broad categories)

	absent	1-34k20	21-49k35	36+k50	Total
N	24	24	16	16	80
NS	17	19	13	13	62
Wt	258.59	201.91	164.79	141.43	766.72
Het Chi	412.09	152.64	174.32	257.42	1161.37
Het df	23	23	15	15	79
Het P	***	***	***	***	***
Fixed RR	13.47	6.21	17.69	21.98	12.75
RRl	11.92	5.41	15.18	18.64	11.88
RRu	15.21	7.13	20.61	25.92	13.68
P	+++	+++	+++	+++	+++
Random RR	10.24	4.53	14.56	25.20	10.22
RRl	5.96	3.01	7.85	11.48	7.63
RRu	17.61	6.83	26.99	55.32	13.68
P	+++	+++	+++	+++	+++

Duration of smoking (narrow categories)

	absent	1-19k1	6-29k20	21-39k30	31-49k40	41-998k50	51+k999	Total
N	49	4	2	12	11	1	1	80
NS	21	4	2	9	9	1	1	46
Wt	428.90	26.10	17.99	154.12	124.35	7.27	8.00	766.72
Het Chi	605.26	5.43	1.12	168.64	96.21	0.00	0.00	1161.37
Het df	48	3	1	11	10	0	0	79
Het P	***	N.S.	N.S.	***	***	N.S.	N.S.	***
Fixed RR	9.58	2.28	4.51	18.19	35.85	13.80	16.70	12.75
RRl	8.72	1.55	2.84	15.53	30.07	6.67	8.35	11.88
RRu	10.53	3.35	7.16	21.30	42.74	28.55	33.40	13.68
P	+++	+++	+++	+++	+++	+++	+++	+++
Random RR	8.92	2.36	4.58	13.69	24.13	13.80	16.70	10.22
RRl	6.17	1.40	2.76	6.69	12.50	6.67	8.35	7.63
RRu	12.88	4.01	7.58	28.01	46.60	28.55	33.40	13.68
P	+++	++	+++	+++	+++	+++	+++	+++

Table 2I11 - 6

IESLC - Meta-analysis of Ever/current Smoking by Duration, Overview
Squamous, Any Product (or Cigarettes if Any not available)
Least adjusted

MALES

	Duration of smoking (broad categories) absent	1-34k20	21-49k35	36+k50	Total
N	12	14	12	12	50
NS	10	14	12	12	48
Wt	116.12	93.42	86.38	83.49	379.41
Het Chi	90.62	18.00	11.31	13.53	213.22
Het df	11	13	11	11	49
Het P	***	N.S.	N.S.	N.S.	***
Fixed RR	12.77	5.94	14.70	21.81	12.29
RRl	10.65	4.85	11.90	17.60	11.11
RRu	15.32	7.28	18.15	27.03	13.59
P	+++	+++	+++	+++	+++
Random RR	11.83	5.43	14.45	20.61	11.26
RRl	6.52	4.03	11.52	15.36	8.83
RRu	21.45	7.31	18.13	27.64	14.37
P	+++	+++	+++	+++	+++

	absent	Duration of smoking (narrow categories) 1-19k1	6-29k20	21-39k30	31-49k40	41-998k50	51+k999	Total
N	27	2	2	8	9	1	1	50
NS	14	2	2	8	9	1	1	36
Wt	180.10	10.60	17.99	75.71	79.74	7.27	8.00	379.41
Het Chi	107.86	4.37	1.12	6.89	11.79	0.00	0.00	213.22
Het df	26	1	1	7	8	0	0	49
Het P	***	*	N.S.	N.S.	N.S.	N.S.	N.S.	***
Fixed RR	11.07	1.81	4.51	15.15	19.77	13.80	16.70	12.29
RRl	9.57	0.99	2.84	12.09	15.87	6.67	8.35	11.11
RRu	12.82	3.30	7.16	18.98	24.62	28.55	33.40	13.59
P	+++	(+)	+++	+++	+++	+++	+++	+++
Random RR	11.06	2.28	4.58	15.15	17.82	13.80	16.70	11.26
RRl	7.69	0.57	2.76	12.09	12.60	6.67	8.35	8.83
RRu	15.91	9.09	7.58	18.98	25.22	28.55	33.40	14.37
P	+++	N.S.	+++	+++	+++	+++	+++	+++

FEMALES

	Duration of smoking (broad categories) absent	1-34k20	21-49k35	36+k50	Total
N	8	8	4	4	24
NS	7	8	4	4	23
Wt	104.44	102.82	78.41	57.94	343.61
Het Chi	221.65	122.58	156.78	243.88	843.93
Het df	7	7	3	3	23
Het P	***	***	***	***	***
Fixed RR	23.04	6.94	21.69	22.22	15.77
RRl	19.02	5.72	17.39	17.18	14.19
RRu	27.91	8.42	27.07	28.75	17.53
P	+++	+++	+++	+++	+++
Random RR	13.72	4.11	16.27	31.26	10.70
RRl	4.30	1.63	2.23	1.93	5.45
RRu	43.74	10.37	118.58	506.39	21.02
P	+++	++	++	+	+++

Table 2I11 - 6

IESLC - Meta-analysis of Ever/current Smoking by Duration, Overview
Squamous, Any Product (or Cigarettes if Any not available)
Least adjusted

FEMALES

	absent	1-19k1	6-29k20	21-39k30	31-49k40	41-998k50	51+k999	Total
	Duration of smoking (narrow categories)							
N	17	1		4	2			24
NS	9	1		4	2			16
Wt	213.29	7.31		78.41	44.61			343.61
Het Chi	450.16	0.00		156.78	5.67			843.93
Het df	16	0		3	1			23
Het P	***	N.S.		***	*			***
Fixed RR	10.03	2.89		21.69	103.90			15.77
RRl	8.77	1.40		17.39	77.47			14.19
RRu	11.47	5.96		27.07	139.33			17.53
P	+++	++		+++	+++			+++
Random RR	8.52	2.89		16.27	66.35			10.70
RRl	3.99	1.40		2.23	18.79			5.45
RRu	18.15	5.96		118.58	234.28			21.02
P	+++	++		++	+++			+++

Table 2I11 - 7

IESLC - Meta-analysis of Ever/current Smoking by Duration, Overview
Squamous, Any Product (or Cigarettes if Any not available)
Excluded studies (and stage at which they were excluded)

1	BECHER BLOT1 BROWN3 CARPEN CHYOU DARBY DOLL2 GARCIA GRAHAM GURSEL HAMMO2 JAHN JAIN LAUSSM PRESCO QIAO TVERDA WIGLE WYNDE3
2	ALDERS BENSHL BRESLO CHIAZZ DEAN3 DORN ENGELA GAO2 GILLIS GUO HEGMAN HIRAYA HOLE KAUFMA KOO KOULUM LIU4 MIGRAN MRFITR PERNU SEGI2 SPEIZE SUZUK2 SVENSS VUTUC WAKAI WU YUAN
3	GENG MCDUFF SPITZ STASZE WU2 ZHANG
4	AGUDO AKIBA AMANDU AMES ARMADA AUVINE AXELSS BEST BOFFET BOUCOT BROSS CEDERL CHEN2 CORREA CPSI CPSII DEAN2 DESTEF DOLL FAN GAO GARSHI HAMMON HU HU2 HUMBLE JUSSAW KAISE2 KREUZE LETOUR LEVIN LIAW LIU3 LIU5 MCCONN NOTAN2 PEZZO2 PISANI QIAO2 RACHTA RESTRE SADOWS TIZZAN WANG2 WATSON WYNDE6 WYNDE7 WYNDE8
5	CHEN LUBIN XU
10	BOUCHA KHUDER
14	BENHAM

Table 2I11 - 8

Potentially overlapping studies

REF	REFGP	PRINC	.	OVERLAP/LINK
LUBIN2	LUBIN2	1		Lubin-combined
OSANN2	KAISER	2		KAISER/OSANN2

Table 2I11 - 9

Most adjusted - insufficient data for meta-analysis

REF	NRR	SEX	AGEL	AGEH	RACE	YF	LC TYPE	LOC	START	ST	NLC	R	VB	P	H	AD	SM	PRODUCT	exL	exH	S1	S2	DENOM	De
CHEN	501	c	0	0	all	-	q	As:oth	1987	CC	323	n	ot	n	y	2	ev	cig+/-ot	1	20	1	0	nev cigs	ot
CHEN	502	c	0	0	all	-	q	As:oth	1987	CC	323	n	ot	n	y	2	ev	cig+/-ot	21	30	0	3	nev cigs	ot
CHEN	503	c	0	0	all	-	q	As:oth	1987	CC	323	n	ot	n	y	2	ev	cig+/-ot	31	40	2	4	nev cigs	ot
CHEN	504	c	0	0	all	-	q	As:oth	1987	CC	323	n	ot	n	y	2	ev	cig+/-ot	41	999	3	0	nev cigs	ot
XU	511	m	0	0	all	-	q+s	As:Chi	1985	CC	729	n	ot	n	n	2	ev	all/unsp	1	29	1	0	nev any	or
XU	512	m	0	0	all	-	q+s	As:Chi	1985	CC	729	n	ot	n	n	2	ev	all/unsp	30	39	2	3	nev any	or
XU	513	m	0	0	all	-	q+s	As:Chi	1985	CC	729	n	ot	n	n	2	ev	all/unsp	40	999	3	0	nev any	or

REF	NRR	RR	SIG	RRDATA comment
CHEN	501	1.70	n	0
CHEN	502	2.76	n	0
CHEN	503	6.52	n	0
CHEN	504	8.43	y	p<0.001
XU	511	*		RR for 1-19/day is 2.3(p<0.05), for 20-29/day is 2.6(p<0.05) and for >=30/ day is 7.7(p<0.05)
XU	512	*		RR for 1-19/day is 2.9(p<0.05), for 20-29/day is 3.9(p<0.05) and for >=30/ day is 8.3(p<0.05)
XU	513	*		RR for 1-19/day is 5.0(p<0.05), for 20-29/day is 10.4(p<0.05) and for >=30/ day is 31.2(p<0.05)

Table 2I12 -

IESLC - Meta-analysis of Ever/current Smoking, Duration, "Low"
Squamous, Any Product (or Cigarettes if Any not available)

This analysis is restricted to results for:
1) Ever/current smokers
2) Results by Duration
3) Categorical results by Duration
4) Squamous (or near equivalent)
5) Results complete enough for use in metaanalysis

Within each study, results are then selected (in the following order of preference, within each sex) for:
6) SMKSTA: ever, current
7) PRODUCT: all/unspec, cigarettes regardless of other products, cigarettes only
8) CIGTYPE: all/unspecified, MC regardless of HR, MC only
9) (not applicable)
10) DENOM: never smoked anything, never smoked cigarettes, never any + low, never cigs + low
11) Followup period (YF, prospective studies): whole study (coded as 0) or longest available
12) LCtype: squamous or nearest available, but not adeno. (q = squamous, s = small,
a = adeno, KI = Kreyberg I, u = undifferentiated)
13) Race: all or nearest available, otherwise by race (wh or w = white, bl or b = black, hi = hispanic
ch = chinese, jap = japanese, haw = hawaiian, w+o = white + oriental, sca = scandinavian, as = asian)
14) Duration "low" in key scheme 1 (key value 20, maximum range 1-34)
15) For overlapping studies: principal rather than subsidiary studies
Finally by Age: whole study (coded as 0) if available, otherwise by widest available age group
and then for single sex results (m, f) in preference to results for both sexes combined (c).

Results adjusted (AD) for the most potential confounders are then chosen in Sections -1 to -3
and results adjusted for the least confounders in Sections -4 to -6. (Those least adjusted results which
actually differ from the most adjusted are marked 'x' in column X in Section -4)

Section -7 shows excluded studies, together with the stage (as above) at which no qualifying
results were found.

Section -8 lists the potentially overlapping studies which have been included (1=principal, 2=subsidiary).

Section -9 lists any results which would have been included in preference except that they had data not complete
enough for use in meta-analysis, with their significance (yes/no), if known, and any further comment as entered
on the database. It also lists as "gap" any categories for which no data were presented by the original authors.

In addition to those mentioned above, the following fields, levels and abbreviations are used:
* or nk = not known, n = no, y = yes, ot = other
ev = ever, cu = current, nev = never
all/unspec = all or unspecified, cig+/-ot = cigarettes irrespective of other products (cigar, pipe etc)
MC = manufactured cigarettes, HR = hand-rolled cigarettes
exL, exH = range of exposure (low and high) in the smoking group, in terms of Duration
REF: 6-character study reference
NRR: number of the RR on the database within the study
ST : study type (CC = case control, pr or prosp = prospective)
NLC: number of lung cancer cases in whole study
R : risky occupational population (n = no, m = mining, o = other risky)
VB : national cigarette type (V = at least 75% Virginia, bl = at least 75% blended, ot = other)
P : any proxy use
H : full histological confirmation
De : derivation of RR/CI (or = original, st = standard method, ot = other method of estimation)

Table 2I12 - 1

IESLC - Meta-analysis of Ever/current Smoking, Duration, "Low"
Squamous, Any Product (or Cigarettes if Any not available)
Most adjusted

REF	NRR	SEX	AGEL	AGEH	RACE	YF	LC TYPE	LOC	START	ST	NLC	R	VB	P	H	AD	SM	PRODUCT	exL	exH	DENOM	De
BARBON	562	m	0	0	all	-	q	Eu:wst	1979	CC	755	n	bl	y	y	1	ev	all/unsp	1	29	nev any	or
BUFFLE	505	m	0	0	wh	-	q	NAmer	1976	CC	943	n	bl	y	n	0	ev	cig+/-ot	1	33	nev cigs	or
CHOI	559	m	0	0	all	-	q	As:oth	1985	CC	375	n	bl	n	n	0	ev	cig+/-ot	1	29	nev cigs	st
CHOI	573	f	0	0	all	-	q	As:oth	1985	CC	375	n	bl	n	n	0	ev	cig+/-ot	1	29	nev cigs	st
DAMBER	547	m	0	0	all	-	q	Eu:Sca	1972	CC	579	n	bl	y	n	1	ev	all/unsp	1	30	nev any	or
DORGAN	572	m	0	0	wh	-	q	NAmer	1980	CC	2026	n	bl	y	y	2	ev	cig+/-ot	1	34	nev any	ot
DORGAN	564	f	0	0	all	-	q	NAmer	1980	CC	2026	n	bl	y	y	3	ev	cig+/-ot	1	34	nev any	ot
DOSEME	512	m	0	0	all	-	q	Eu:bal	1979	CC	1210	n	bl	n	n	2	ev	cig+/-ot	11	20	nev cigs	or
GER	510	c	0	0	all	-	q+s	As:oth	1990	CC	141	n	ot	y	n	5	ev	all/unsp	1	30	nev any	ot
JEDRYC	502	m	0	0	all	-	q	Eu:est	1980	CC	1630	n	bl	y	n	0	ev	cig+/-ot	20	29	nev any	st
JOLY	639	m	0	0	all	-	q	SCAmer	1978	CC	826	n	bl	n	n	0	ev	cig+/-ot	1	29	nev any	st
JOLY	611	f	0	0	all	-	q	SCAmer	1978	CC	826	n	bl	n	n	0	ev	cig+/-ot	1	29	nev any	st
KATSOU	528	f	0	0	all	-	KI	Eu:bal	1987	CC	101	n	bl	n	n	1	cu	all/unsp	1	29	nev any	or
LUBIN2	661	m	0	0	all	-	q	Eu:mul	1976	CC	7804	n	bl	n	y	0	ev	cig+/-ot	1	29	nev any	st
LUBIN2	713	f	0	0	all	-	q	Eu:mul	1976	CC	7804	n	bl	n	y	0	ev	cig+/-ot	1	29	nev any	st
LUO	504	c	0	0	all	-	q	As:Chi	1990	CC	102	n	ot	n	y	20	ev	cig+/-ot	1	29	nev cigs	or
MATOS	606	m	0	0	all	-	q	SCAmer	1994	CC	200	n	bl	n	n	2	ev	cig+/-ot	1	24	nev any	or
OSANN2	510	f	0	0	all	-	KI	NAmer	1964	ot	217	n	bl	n	y	1	ev	cig+/-ot	1	20	nev cigs	or
PEZZOT	507	m	0	0	all	-	q	SCAmer	1987	CC	215	n	bl	n	y	0	ev	cig only	1	30	nev cigs	ot
SOBUE	501	m	0	0	all	-	q	As:Jap	1986	CC	1376	n	bl	n	y	0	cu	cig+/-ot	1	29	nev cigs	st
WUWILL	521	f	0	0	all	-	q	As:Chi	1985	CC	965	n	ot	n	n	3	ev	cig+/-ot	1	29	nev cigs	ot
WYNDE2	506	m	0	0	all	-	KI	NAmer	1962	CC	404	n	bl	n	y	0	ev	cig+/-ot	1	29	nev any	ot
ZHENG	501	m	0	0	all	-	q	As:Chi	1982	CC	540	n	ot	*	y	0	ev	cig+/-ot	1	29	nev cigs	st
ZHENG	508	f	0	0	all	-	q	As:Chi	1982	CC	540	n	ot	*	y	0	ev	cig+/-ot	1	29	nev cigs	st

Cigarette type is all/unspec for all RRs

Table 2I12 - 2

IESLC - Meta-analysis of Ever/current Smoking, Duration, "Low"
Squamous, Any Product (or Cigarettes if Any not available)
Most adjusted

REF	NRR	SEX	AD	Number Exposed Case	Cont	Non-exposed Case	Cont	RR	95.00%CI
BARBON	562	m	1	7	-	6	-	2.10	(0.70- 6.50)
BUFFLE	505	m	0	-	-	-	-	9.00	(2.90- 27.90)
CHOI	559	m	0	42	221	6	95	3.01	(1.24- 7.32)
CHOI	573	f	0	6	23	10	164	4.28	(1.42- 12.88)
Subtotal CHOI								3.46	(1.73- 6.90)
DAMBER	547	m	1	-	-	14	-	4.40	(1.80- 10.70)
DORGAN	572	m	2	-	-	-	-	9.47	(3.39- 26.45)
DORGAN	564	f	3	-	-	-	-	4.31	(2.53- 7.35)
Subtotal DORGAN								5.09	(3.17- 8.18)
DOSEME	512	m	2	70	-	58	-	3.90	(2.30- 6.70)
GER	510	c	5	6	-	11	-	1.53	(0.40- 5.86)
JEDRYC	502	m	0	23	160	6	289	6.92	(2.76- 17.36)
JOLY	639	m	0	15	109	2	218	15.00	(3.37- 66.77)
JOLY	611	f	0	5	54	6	283	4.37	(1.29- 14.82)
Subtotal JOLY								7.16	(2.78- 18.44)
KATSOU	528	f	1	5	-	14	-	1.77	(0.51- 6.14)
LUBIN2	661	m	0	453	2964	54	2616	7.40	(5.56- 9.87)
LUBIN2	713	f	0	322	229	72	1180	23.04	(17.21- 30.86)
Subtotal LUBIN2								12.93	(10.54- 15.87)
LUO	504	c	20	6	-	5	-	5.70	(1.00- 32.90)
MATOS	606	m	2	3	-	3	-	1.20	(0.20- 6.20)
OSANN2	510	f	1	11	-	7	-	4.90	(0.50- 44.60)
PEZZOT	507	m	0	5	134	0	116	9.53~	(0.52- 174.14)
SOBUE	501	m	0	16	119	3	128	5.74	(1.63- 20.19)
WUWILL	521	f	3	54	-	117	-	2.00	(1.36- 2.94)
WYNDE2	506	m	0	22	55	0	41	33.65~	(1.98- 570.85)
ZHENG	501	m	0	13	75	4	94	4.07	(1.28- 13.01)
ZHENG	508	f	0	8	17	33	184	2.62	(1.05- 6.57)
Subtotal ZHENG								3.11	(1.51- 6.39)
Partial Totals				1092	4160	431	5408		

*prospective study ~ With 0.5 adjustment for zero

REF	NRR	SEX	AD	Ys	Ws	Qs	Ps
BARBON	562	m	1	0.74	3.09	3.92	0.1919
BUFFLE	505	m	0	2.20	3.00	0.33	0.0001
CHOI	559	m	0	1.10	4.87	2.85	0.0151
CHOI	573	f	0	1.45	3.16	0.54	0.0097
Subtotal CHOI				1.24	8.03	3.39	
DAMBER	547	m	1	1.48	4.84	0.72	0.0011
DORGAN	572	m	2	2.25	3.64	0.53	0.0000
DORGAN	564	f	3	1.46	13.51	2.23	0.0000
Subtotal DORGAN				1.63	17.15	2.76	
DOSEME	512	m	2	1.36	13.44	3.44	0.0000
GER	510	c	5	0.43	2.13	4.43	0.5346
JEDRYC	502	m	0	1.93	4.55	0.02	0.0000
JOLY	639	m	0	2.71	1.72	1.22	0.0004
JOLY	611	f	0	1.47	2.57	0.40	0.0181
Subtotal JOLY				1.97	4.30	1.62	
KATSOU	528	f	1	0.57	2.48	4.17	0.3684
LUBIN2	661	m	0	2.00	46.63	0.85	0.0000
LUBIN2	713	f	0	3.14	45.03	72.67	0.0000
Subtotal LUBIN2				2.56	91.66	73.52	
LUO	504	c	20	1.74	1.26	0.02	0.0508
MATOS	606	m	2	0.18	1.30	3.70	0.8351
OSANN2	510	f	1	1.59	0.76	0.06	0.1654
PEZZOT	507	m	0	2.25	0.45	0.07	0.1284
SOBUE	501	m	0	1.75	2.43	0.04	0.0065
WUWILL	521	f	3	0.69	25.85	35.63	0.0004
WYNDE2	506	m	0	3.52	0.48	1.30	0.0149
ZHENG	501	m	0	1.40	2.85	0.61	0.0177
ZHENG	508	f	0	0.96	4.55	3.71	0.0395
Subtotal ZHENG				1.13	7.40	4.32	

Table 2I12 - 2

IESLC - Meta-analysis of Ever/current Smoking, Duration, "Low"
Squamous, Any Product (or Cigarettes if Any not available)
Most adjusted

	N	24
	NS	19
	Wt	194.61
Het	Chi	143.44
Het	df	23
Het	P	***
Fixed	RR	6.47
	RRl	5.62
	RRu	7.44
	P	+++
Random	RR	4.74
	RRl	3.14
	RRu	7.18
	P	+++
Asymm	P	N.S.

Table 2I12 - 3

IESLC - Meta-analysis of Ever/current Smoking, Duration, "Low"
Squamous, Any Product (or Cigarettes if Any not available)
Most adjusted

	combined	Sex male	female	Total
N	2	14	8	24
NS	2	14	8	24
Wt	3.39	93.29	97.92	194.61
Het Chi	1.37	18.85	118.05	143.44
Het df	1	13	7	23
Het P	N.S.	N.S.	***	***
Fixed RR	2.49	5.92	7.28	6.47
RRl	0.86	4.83	5.97	5.62
RRu	7.23	7.25	8.87	7.44
P	(+)	+++	+++	+++
Random RR	2.61	5.37	4.20	4.74
RRl	0.74	3.95	1.61	3.14
RRu	9.25	7.30	10.97	7.18
P	N.S.	+++	++	+++
Between Chi				5.17
Between df				2
Between P				(*)
Btwn(F) P				N.S.
Btwn(R) P				N.S.

	q	Lung cancer type q+s	q+u	KI	not a	Total
N	20	1		3		24
NS	15	1		3		19
Wt	188.75	2.13		3.72		194.61
Het Chi	133.30	0.00		3.66		143.44
Het df	19	0		2		23
Het P	***	N.S.		N.S.		***
Fixed RR	6.67	1.53		3.18		6.47
RRl	5.78	0.40		1.15		5.62
RRu	7.69	5.86		8.80		7.44
P	+++	N.S.		+		+++
Random RR	5.02	1.53		4.49		4.74
RRl	3.24	0.40		0.93		3.14
RRu	7.78	5.86		21.59		7.18
P	+++	N.S.		(+)		+++
Between Chi						6.47
Between df						2
Between P						*
Btwn(F) P						N.S.
Btwn(R) P						N.S.

	NAmer	UK	Scand	Location othEur	China	Japan	othAs	other	Total
N	5		1	6	4	1	3	4	24
NS	4		1	5	3	1	2	3	19
Wt	21.39		4.84	115.22	34.52	2.43	10.16	6.05	194.61
Het Chi	4.15		0.00	63.11	2.55	0.00	1.36	4.97	143.44
Het df	4		0	5	3	0	2	3	23
Het P	N.S.		N.S.	***	N.S.	N.S.	N.S.	N.S.	***
Fixed RR	5.75		4.40	10.01	2.28	5.74	2.91	4.98	6.47
RRl	3.76		1.80	8.34	1.64	1.63	1.58	2.25	5.62
RRu	8.78		10.73	12.02	3.19	20.19	5.39	11.05	7.44
P	+++		++	+++	+++	++	+++	+++	+++
Random RR	5.88		4.40	5.65	2.28	5.74	2.91	4.96	4.74
RRl	3.76		1.80	2.63	1.64	1.63	1.58	1.68	3.14
RRu	9.19		10.73	12.12	3.19	20.19	5.39	14.63	7.18
P	+++		++	+++	+++	++	+++	++	+++
Between Chi									67.31
Between df									6
Between P									***
Btwn(F) P									(*)
Btwn(R) P									*

Table 2I12 - 3

IESLC - Meta-analysis of Ever/current Smoking, Duration, "Low"
Squamous, Any Product (or Cigarettes if Any not available)
Most adjusted

Detailed Country in "other Europe"

	multi	Germany	othWest	East	Balkans	Total
N	2		1	1	2	6
NS	1		1	1	2	5
Wt	91.66		3.09	4.55	15.92	115.22
Het Chi	29.53		0.00	0.00	1.31	63.11
Het df	1		0	0	1	5
Het P	***		N.S.	N.S.	N.S.	***
Fixed RR	12.93		2.10	6.92	3.45	10.01
RRl	10.54		0.69	2.76	2.11	8.34
RRu	15.87		6.40	17.36	5.64	12.02
P	+++		N.S.	+++	+++	+++
Random RR	13.06		2.10	6.92	3.23	5.65
RRl	4.29		0.69	2.76	1.67	2.63
RRu	39.73		6.40	17.36	6.25	12.12
P	+++		N.S.	+++	+++	+++
Between Chi						32.27
Between df						3
Between P						***
Btwn(F) P						N.S.
Btwn(R) P						(*)

Detailed Country in "other Asia"

	India	HongKong	other	Total
N			3	3
NS			2	2
Wt			10.16	10.16
Het Chi			1.36	1.36
Het df			2	2
Het P			N.S.	N.S.
Fixed RR			2.91	2.91
RRl			1.58	1.58
RRu			5.39	5.39
P			+++	+++
Random RR			2.91	2.91
RRl			1.58	1.58
RRu			5.39	5.39
P			+++	+++
Between Chi				
Between df				
Between P				N.S.
Btwn(F) P				N.S.
Btwn(R) P				N.S.

Detailed other continent

	SCAmer	Total
N	4	4
NS	3	3
Wt	6.05	6.05
Het Chi	4.97	4.97
Het df	3	3
Het P	N.S.	N.S.
Fixed RR	4.98	4.98
RRl	2.25	2.25
RRu	11.05	11.05
P	+++	+++
Random RR	4.96	4.96
RRl	1.68	1.68
RRu	14.63	14.63
P	++	++
Between Chi		
Between df		
Between P		N.S.
Btwn(F) P		N.S.
Btwn(R) P		N.S.

Table 2I12 - 3

IESLC - Meta-analysis of Ever/current Smoking, Duration, "Low"
Squamous, Any Product (or Cigarettes if Any not available)
Most adjusted

	<1960	Start year of study 1960-69	1970-79	1980-89	1990+	Total
N		2	8	11	3	24
NS		2	6	8	3	19
Wt		1.24	120.32	68.35	4.69	194.61
Het Chi		1.09	60.39	16.77	1.87	143.44
Het df		1	7	10	2	23
Het P		N.S.	***	(*)	N.S.	***
Fixed RR		10.31	10.03	3.21	2.04	6.47
RRl		1.78	8.39	2.53	0.82	5.62
RRu		59.89	11.99	4.07	5.03	7.44
P		++	+++	+++	N.S.	+++
Random RR		10.50	6.78	3.69	2.04	4.74
RRl		1.66	3.58	2.60	0.82	3.14
RRu		66.58	12.81	5.24	5.03	7.18
P		+	+++	+++	N.S.	+++
Between Chi						63.31
Between df						3
Between P						***
Btwn(F) P						**
Btwn(R) P						N.S.

	Study type (1) CC	other	Total
N	23	1	24
NS	18	1	19
Wt	193.84	0.76	194.61
Het Chi	143.38	0.00	143.44
Het df	22	0	23
Het P	***	N.S.	***
Fixed RR	6.48	4.90	6.47
RRl	5.63	0.52	5.62
RRu	7.45	46.28	7.44
P	+++	N.S.	+++
Random RR	4.74	4.90	4.74
RRl	3.11	0.52	3.14
RRu	7.22	46.28	7.18
P	+++	N.S.	+++
Between Chi			0.06
Between df			1
Between P			N.S.
Btwn(F) P			N.S.
Btwn(R) P			N.S.

	Study type (2) CC	prosp	other	Total
N	23		1	24
NS	18		1	19
Wt	193.84		0.76	194.61
Het Chi	143.38		0.00	143.44
Het df	22		0	23
Het P	***		N.S.	***
Fixed RR	6.48		4.90	6.47
RRl	5.63		0.52	5.62
RRu	7.45		46.28	7.44
P	+++		N.S.	+++
Random RR	4.74		4.90	4.74
RRl	3.11		0.52	3.14
RRu	7.22		46.28	7.18
P	+++		N.S.	+++
Between Chi				0.06
Between df				1
Between P				N.S.
Btwn(F) P				N.S.
Btwn(R) P				N.S.

Table 2I12 - 3

IESLC - Meta-analysis of Ever/current Smoking, Duration, "Low"
Squamous, Any Product (or Cigarettes if Any not available)
Most adjusted

	Study size (number of LC cases)				
	100-249	250-499	500-999	1000+	Total
N	6	3	8	7	24
NS	6	2	6	5	19
Wt	8.39	8.51	48.48	129.22	194.61
Het Chi	3.47	2.58	14.02	58.35	143.44
Het df	5	2	7	6	23
Het P	N.S.	N.S.	(*)	***	***
Fixed RR	2.30	3.93	2.85	9.72	6.47
RRl	1.17	2.01	2.15	8.18	5.62
RRu	4.52	7.69	3.78	11.55	7.44
P	+	+++	+++	+++	+++
Random RR	2.30	4.19	3.69	7.43	4.74
RRl	1.17	1.84	2.30	4.01	3.14
RRu	4.52	9.52	5.93	13.76	7.18
P	+	+++	+++	+++	+++
Between Chi					65.02
Between df					3
Between P					***
Btwn(F) P					**
Btwn(R) P					(*)

	Risky occupational population			
	no	mining	othRisky	Total
N	24			24
NS	19			19
Wt	194.61			194.61
Het Chi	143.44			143.44
Het df	23			23
Het P	***			***
Fixed RR	6.47			6.47
RRl	5.62			5.62
RRu	7.44			7.44
P	+++			+++
Random RR	4.74			4.74
RRl	3.14			3.14
RRu	7.18			7.18
P	+++			+++
Between Chi				
Between df				
Between P				N.S.
Btwn(F) P				N.S.
Btwn(R) P				N.S.

	National cigarette tobacco type			
	Virginia	blended	other	Total
N		19	5	24
NS		15	4	19
Wt		157.96	36.65	194.61
Het Chi		89.41	2.87	143.44
Het df		18	4	23
Het P		***	N.S.	***
Fixed RR		8.28	2.23	6.47
RRl		7.09	1.61	5.62
RRu		9.68	3.08	7.44
P		+++	+++	+++
Random RR		5.57	2.23	4.74
RRl		3.64	1.61	3.14
RRu		8.53	3.08	7.18
P		+++	+++	+++
Between Chi				51.16
Between df				1
Between P				***
Btwn(F) P				**
Btwn(R) P				***

Table 2I12 - 3

IESLC - Meta-analysis of Ever/current Smoking, Duration, "Low"
Squamous, Any Product (or Cigarettes if Any not available)
Most adjusted

	Any proxy use No/nk	Yes	Total
N	17	7	24
NS	13	6	19
Wt	159.85	34.76	194.61
Het Chi	130.48	8.55	143.44
Het df	16	6	23
Het P	***	N.S.	***
Fixed RR	6.94	4.68	6.47
RRl	5.94	3.36	5.62
RRu	8.10	6.53	7.44
P	+++	+++	+++
Random RR	4.82	4.70	4.74
RRl	2.80	3.09	3.14
RRu	8.30	7.16	7.18
P	+++	+++	+++
Between Chi			4.41
Between df			1
Between P			*
Btwn(F) P			N.S.
Btwn(R) P			N.S.

	Full histological confirmation No	Yes	Total
N	12	12	24
NS	10	9	19
Wt	69.92	124.69	194.61
Het Chi	20.54	65.31	143.44
Het df	11	11	23
Het P	*	***	***
Fixed RR	3.13	9.72	6.47
RRl	2.47	8.16	5.62
RRu	3.95	11.59	7.44
P	+++	+++	+++
Random RR	3.59	6.30	4.74
RRl	2.48	3.58	3.14
RRu	5.19	11.09	7.18
P	+++	+++	+++
Between Chi			57.59
Between df			1
Between P			***
Btwn(F) P			***
Btwn(R) P			N.S.

	Number of adjustment variables (1) 0	1	2+/+nk	Total
N	13	4	7	24
NS	9	4	6	19
Wt	122.29	11.17	61.14	194.61
Het Chi	59.46	1.97	14.34	143.44
Het df	12	3	6	23
Het P	***	N.S.	*	***
Fixed RR	10.18	2.95	3.01	6.47
RRl	8.53	1.64	2.35	5.62
RRu	12.15	5.30	3.87	7.44
P	+++	+++	+++	+++
Random RR	6.79	2.95	3.31	4.74
RRl	4.07	1.64	2.08	3.14
RRu	11.34	5.30	5.28	7.18
P	+++	+++	+++	+++
Between Chi				67.67
Between df				2
Between P				***
Btwn(F) P				**
Btwn(R) P				(*)

Table 2I12 - 3

IESLC - Meta-analysis of Ever/current Smoking, Duration, "Low"
Squamous, Any Product (or Cigarettes if Any not available)
Most adjusted

	Number of adjustment variables (2) 0	1	2	3-5	6+/+nk	Total
N	13	4	3	3	1	24
NS	9	4	3	3	1	20
Wt	122.29	11.17	18.38	41.50	1.26	194.61
Het Chi	59.46	1.97	4.52	5.80	0.00	143.44
Het df	12	3	2	2	0	23
Het P	***	N.S.	N.S.	(*)	N.S.	***
Fixed RR	10.18	2.95	4.28	2.53	5.70	6.47
RRl	8.53	1.64	2.71	1.87	0.99	5.62
RRu	12.15	5.30	6.75	3.43	32.69	7.44
P	+++	+++	+++	+++	(+)	+++
Random RR	6.79	2.95	4.22	2.59	5.70	4.74
RRl	4.07	1.64	1.77	1.39	0.99	3.14
RRu	11.34	5.30	10.06	4.82	32.69	7.18
P	+++	+++	++	++	(+)	+++
Between Chi						71.69
Between df						4
Between P						***
Btwn(F) P						**
Btwn(R) P						N.S.

	Smoking status ever	current	Total
N	22	2	24
NS	17	2	19
Wt	189.70	4.91	194.61
Het Chi	139.17	1.70	143.44
Het df	21	1	23
Het P	***	N.S.	***
Fixed RR	6.59	3.17	6.47
RRl	5.72	1.31	5.62
RRu	7.60	7.67	7.44
P	+++	+	+++
Random RR	4.91	3.17	4.74
RRl	3.18	1.00	3.14
RRu	7.58	10.05	7.18
P	+++	+	+++
Between Chi			2.57
Between df			1
Between P			N.S.
Btwn(F) P			N.S.
Btwn(R) P			N.S.

	Product all/unsp	cig+/-ot	cig only	Total
N	4	19	1	24
NS	4	14	1	19
Wt	12.54	181.61	0.45	194.61
Het Chi	2.44	129.41	0.00	143.44
Het df	3	18	0	23
Het P	N.S.	***	N.S.	***
Fixed RR	2.56	6.89	9.53	6.47
RRl	1.47	5.96	0.52	5.62
RRu	4.45	7.97	174.14	7.44
P	+++	+++	N.S.	+++
Random RR	2.56	5.42	9.53	4.74
RRl	1.47	3.42	0.52	3.14
RRu	4.45	8.60	174.14	7.18
P	+++	+++	N.S.	+++
Between Chi				11.58
Between df				2
Between P				**
Btwn(F) P				N.S.
Btwn(R) P				N.S.

Table 2I12 - 3

IESLC - Meta-analysis of Ever/current Smoking, Duration, "Low"
Squamous, Any Product (or Cigarettes if Any not available)
Most adjusted

	Denominator nev any	nev cigs	Total
N	13	11	24
NS	10	9	19
Wt	131.98	62.63	194.61
Het Chi	79.34	11.85	143.44
Het df	12	10	23
Het P	***	N.S.	***
Fixed RR	9.24	3.05	6.47
RRl	7.79	2.38	5.62
RRu	10.96	3.91	7.44
P	+++	+++	+++
Random RR	5.40	3.33	4.74
RRl	3.13	2.46	3.14
RRu	9.32	4.50	7.18
P	+++	+++	+++
Between Chi			52.25
Between df			1
Between P			***
Btwn(F) P			**
Btwn(R) P			N.S.

	Derivation of RR/CI Orig	StdCalc	Other	Total
N	8	10	6	24
NS	8	6	5	19
Wt	30.18	118.36	46.07	194.61
Het Chi	6.84	58.73	15.18	143.44
Het df	7	9	5	23
Het P	N.S.	***	**	***
Fixed RR	3.69	10.17	2.93	6.47
RRl	2.58	8.49	2.19	5.62
RRu	5.27	12.17	3.91	7.44
P	+++	+++	+++	+++
Random RR	3.69	6.23	3.94	4.74
RRl	2.58	3.52	1.99	3.14
RRu	5.27	11.02	7.82	7.18
P	+++	+++	+++	+++
Between Chi				62.69
Between df				2
Between P				***
Btwn(F) P				**
Btwn(R) P				N.S.

Table 2I12 - 4

IESLC - Meta-analysis of Ever/current Smoking, Duration, "Low"
Squamous, Any Product (or Cigarettes if Any not available)
Least adjusted

REF	NRR	X	SEX	AGEL	AGEH	RACE	YF	LC TYPE	LOC	START	ST	NLC	R	VB	P	H	AD	SM	PRODUCT	exL	exH	DENOM	De
BARBON	555	x	m	0	0	all	-	q	Eu:wst	1979	CC	755	n	bl	y	y	0	ev	all/unsp	1	29	nev any	st
BUFFLE	505		m	0	0	wh	-	q	NAmer	1976	CC	943	n	bl	y	n	0	ev	cig+/-ot	1	33	nev cigs	or
CHOI	559		m	0	0	all	-	q	As:oth	1985	CC	375	n	bl	n	n	0	ev	cig+/-ot	1	29	nev cigs	st
CHOI	573		f	0	0	all	-	q	As:oth	1985	CC	375	n	bl	n	n	0	ev	cig+/-ot	1	29	nev cigs	st
DAMBER	547		m	0	0	all	-	q	Eu:Sca	1972	CC	579	n	bl	y	n	1	ev	all/unsp	1	30	nev any	or
DORGAN	572		m	0	0	wh	-	q	NAmer	1980	CC	2026	n	bl	y	y	2	ev	cig+/-ot	1	34	nev any	ot
DORGAN	564		f	0	0	all	-	q	NAmer	1980	CC	2026	n	bl	y	y	3	ev	cig+/-ot	1	34	nev any	ot
DOSEME	512		m	0	0	all	-	q	Eu:bal	1979	CC	1210	n	bl	n	n	2	ev	cig+/-ot	11	20	nev cigs	or
GER	504	x	c	0	0	all	-	q+s	As:oth	1990	CC	141	n	ot	y	n	0	ev	all/unsp	1	30	nev any	st
JEDRYC	502		m	0	0	all	-	q	Eu:est	1980	CC	1630	n	bl	y	n	0	ev	cig+/-ot	20	29	nev any	st
JOLY	639		m	0	0	all	-	q	SCAmer	1978	CC	826	n	bl	n	n	0	ev	cig+/-ot	1	29	nev any	st
JOLY	611		f	0	0	all	-	q	SCAmer	1978	CC	826	n	bl	n	n	0	ev	cig+/-ot	1	29	nev any	st
KATSOU	523	x	f	0	0	all	-	KI	Eu:bal	1987	CC	101	n	bl	n	n	0	cu	all/unsp	1	29	nev any	st
LUBIN2	661		m	0	0	all	-	q	Eu:mul	1976	CC	7804	n	bl	n	y	0	ev	cig+/-ot	1	29	nev any	st
LUBIN2	713		f	0	0	all	-	q	Eu:mul	1976	CC	7804	n	bl	n	y	0	ev	cig+/-ot	1	29	nev any	st
LUO	501	x	c	0	0	all	-	q	As:Chi	1990	CC	102	n	ot	n	y	0	ev	cig+/-ot	1	29	nev cigs	st
MATOS	601	x	m	0	0	all	-	q	SCAmer	1994	CC	200	n	bl	n	n	0	ev	cig+/-ot	1	24	nev any	st
OSANN2	507	x	f	0	0	all	-	KI	NAmer	1964	ot	217	n	bl	n	y	0	ev	cig+/-ot	1	20	nev cigs	st
PEZZOT	507		m	0	0	all	-	q	SCAmer	1987	CC	215	n	bl	n	y	0	ev	cig only	1	30	nev cigs	ot
SOBUE	501		m	0	0	all	-	q	As:Jap	1986	CC	1376	n	bl	n	y	0	cu	cig+/-ot	1	29	nev cigs	st
WUWILL	506	x	f	0	0	all	-	q	As:Chi	1985	CC	965	n	ot	n	n	0	ev	cig+/-ot	1	29	nev cigs	st
WYNDE2	506		m	0	0	all	-	KI	NAmer	1962	CC	404	n	bl	n	y	0	ev	cig+/-ot	1	29	nev any	ot
ZHENG	501		m	0	0	all	-	q	As:Chi	1982	CC	540	n	ot	*	y	0	ev	cig+/-ot	1	29	nev cigs	st
ZHENG	508		f	0	0	all	-	q	As:Chi	1982	CC	540	n	ot	*	y	0	ev	cig+/-ot	1	29	nev cigs	st

Cigarette type is all/unspec for all RRs

Table 2I12 - 5

IESLC - Meta-analysis of Ever/current Smoking, Duration, "Low"
Squamous, Any Product (or Cigarettes if Any not available)
Least adjusted

REF	NRR	SEX	AD	Number Exposed Case	Exposed Cont	Non-exposed Case	Non-exposed Cont	RR	95.00%CI
BARBON	555	m	0	7	91	6	188	2.41	(0.79- 7.38)
BUFFLE	505	m	0	-	-	-	-	9.00	(2.90- 27.90)
CHOI	559	m	0	42	221	6	95	3.01	(1.24- 7.32)
CHOI	573	f	0	6	23	10	164	4.28	(1.42- 12.88)
Subtotal CHOI								3.46	(1.73- 6.90)
DAMBER	547	m	1	-	-	14	-	4.40	(1.80- 10.70)
DORGAN	572	m	2	-	-	-	-	9.47	(3.39- 26.45)
DORGAN	564	f	3	-	-	-	-	4.31	(2.53- 7.35)
Subtotal DORGAN								5.09	(3.17- 8.18)
DOSEME	512	m	2	70	-	58	-	3.90	(2.30- 6.70)
GER	504	c	0	6	37	11	80	1.18	(0.41- 3.43)
JEDRYC	502	m	0	23	160	6	289	6.92	(2.76- 17.36)
JOLY	639	m	0	15	109	2	218	15.00	(3.37- 66.77)
JOLY	611	f	0	5	54	6	283	4.37	(1.29- 14.82)
Subtotal JOLY								7.16	(2.78- 18.44)
KATSOU	523	f	0	5	12	14	67	1.99	(0.61- 6.57)
LUBIN2	661	m	0	453	2964	54	2616	7.40	(5.56- 9.87)
LUBIN2	713	f	0	322	229	72	1180	23.04	(17.21- 30.86)
Subtotal LUBIN2								12.93	(10.54- 15.87)
LUO	501	c	0	6	21	5	51	2.91	(0.80- 10.60)
MATOS	601	m	0	3	84	3	110	1.31	(0.26- 6.65)
OSANN2	507	f	0	11	26	7	58	3.51	(1.22- 10.06)
PEZZOT	507	m	0	5	134	0	116	9.53~	(0.52- 174.14)
SOBUE	501	m	0	16	119	3	128	5.74	(1.63- 20.19)
WUWILL	506	f	0	54	139	117	601	2.00	(1.38- 2.89)
WYNDE2	506	m	0	22	55	0	41	33.65~	(1.98- 570.85)
ZHENG	501	m	0	13	75	4	94	4.07	(1.28- 13.01)
ZHENG	508	f	0	8	17	33	184	2.62	(1.05- 6.57)
Subtotal ZHENG								3.11	(1.51- 6.39)
Partial Totals				1092	4570	431	6563		

*prospective study ~ With 0.5 adjustment for zero

REF	NRR	SEX	AD	Ys	Ws	Qs	Ps
BARBON	555	m	0	0.88	3.07	2.75	0.1233
BUFFLE	505	m	0	2.20	3.00	0.41	0.0001
CHOI	559	m	0	1.10	4.87	2.55	0.0151
CHOI	573	f	0	1.45	3.16	0.44	0.0097
Subtotal CHOI				1.24	8.03	2.99	
DAMBER	547	m	1	1.48	4.84	0.57	0.0011
DORGAN	572	m	2	2.25	3.64	0.65	0.0000
DORGAN	564	f	3	1.46	13.51	1.80	0.0000
Subtotal DORGAN				1.63	17.15	2.45	
DOSEME	512	m	2	1.36	13.44	2.91	0.0000
GER	504	c	0	0.16	3.37	9.29	0.7621
JEDRYC	502	m	0	1.93	4.55	0.05	0.0000
JOLY	639	m	0	2.71	1.72	1.34	0.0004
JOLY	611	f	0	1.47	2.57	0.32	0.0181
Subtotal JOLY				1.97	4.30	1.66	
KATSOU	523	f	0	0.69	2.70	3.49	0.2563
LUBIN2	661	m	0	2.00	46.63	1.44	0.0000
LUBIN2	713	f	0	3.14	45.03	77.43	0.0000
Subtotal LUBIN2				2.56	91.66	78.88	
LUO	501	c	0	1.07	2.30	1.32	0.1044
MATOS	601	m	0	0.27	1.45	3.52	0.7450
OSANN2	507	f	0	1.25	3.45	1.13	0.0197
PEZZOT	507	m	0	2.25	0.45	0.08	0.1284
SOBUE	501	m	0	1.75	2.43	0.02	0.0065
WUWILL	506	f	0	0.69	27.84	35.87	0.0003
WYNDE2	506	m	0	3.52	0.48	1.37	0.0149
ZHENG	501	m	0	1.40	2.85	0.51	0.0177
ZHENG	508	f	0	0.96	4.55	3.38	0.0395
Subtotal ZHENG				1.13	7.40	3.89	

Table 2I12 - 5

IESLC - Meta-analysis of Ever/current Smoking, Duration, "Low"
Squamous, Any Product (or Cigarettes if Any not available)
Least adjusted

	N	24
	NS	19
	Wt	201.91
Het	Chi	152.64
Het	df	23
Het	P	***
Fixed	RR	6.21
	RRl	5.41
	RRu	7.13
	P	+++
Random	RR	4.53
	RRl	3.01
	RRu	6.83
	P	+++
Asymm	P	N.S.

Table 2I12 - 6

IESLC - Meta-analysis of Ever/current Smoking, Duration, "Low"
Squamous, Any Product (or Cigarettes if Any not available)
Least adjusted

	Sex			
	combined	male	female	Total
N	2	14	8	24
NS	2	14	8	24
Wt	5.67	93.42	102.82	201.91
Het Chi	1.12	18.00	122.58	152.64
Het df	1	13	7	23
Het P	N.S.	N.S.	***	***
Fixed RR	1.70	5.94	6.94	6.21
RRl	0.75	4.85	5.72	5.41
RRu	3.88	7.28	8.42	7.13
P	N.S.	+++	+++	+++
Random RR	1.72	5.43	4.11	4.53
RRl	0.72	4.03	1.63	3.01
RRu	4.12	7.31	10.37	6.83
P	N.S.	+++	++	+++
Between Chi				10.95
Between df				2
Between P				**
Btwn(F) P				N.S.
Btwn(R) P				*

Table 2I12 - 7

IESLC - Meta-analysis of Ever/current Smoking, Duration, "Low"
Squamous, Any Product (or Cigarettes if Any not available)
Excluded studies (and stage at which they were excluded)

1	BECHER BLOT1 BROWN3 CARPEN CHYOU DARBY DOLL2 GARCIA GRAHAM GURSEL HAMMO2 JAHN JAIN LAUSSM PRESCO QIAO TVERDA WIGLE WYNDE3
2	ALDERS BENSHL BRESLO CHIAZZ DEAN3 DORN ENGELA GAO2 GILLIS GUO HEGMAN HIRAYA HOLE KAUFMA KOO KOULUM LIU4 MIGRAN MRFITR PERNU SEGI2 SPEIZE SUZUK2 SVENSS VUTUC WAKAI WU YUAN
3	GENG MCDUFF SPITZ STASZE WU2 ZHANG
4	AGUDO AKIBA AMANDU AMES ARMADA AUVINE AXELSS BEST BOFFET BOUCOT BROSS CEDERL CHEN2 CORREA CPSI CPSII DEAN2 DESTEF DOLL FAN GAO GARSHI HAMMON HU HU2 HUMBLE JUSSAW KAISE2 KREUZE LETOUR LEVIN LIAW LIU3 LIU5 MCCONN NOTAN2 PEZZO2 PISANI QIAO2 RACHTA RESTRE SADOWS TIZZAN WANG2 WATSON WYNDE6 WYNDE7 WYNDE8
5	CHEN LUBIN XU
10	BOUCHA KHUDER
14	HAENSZ ZHOU
15	BENHAM

Table 2I12 - 8

Potentially overlapping studies

REF	REFGP	PRINC	. OVERLAP/LINK
LUBIN2	LUBIN2	1	Lubin-combined
OSANN2	KAISER	2	KAISER/OSANN2

Table 2I12 - 9

Most adjusted - insufficient data for meta-analysis

REF	NRR	SEX	AGEL	AGEH	RACE	YF	LC TYPE	LOC	START	ST	NLC	R	VB	P	H	AD	SM	PRODUCT	exL	exH	DENOM	De
CHEN	501	c	0	0	all	-	q	As:oth	1987	CC	323	n	ot	n	y	2	ev	cig+/-ot	1	20	nev cigs	ot
XU	511	m	0	0	all	-	q+s	As:Chi	1985	CC	729	n	ot	n	n	2	ev	all/unsp	1	29	nev any	or

REF	NRR	RR	SIG	RRDATA comment
CHEN	501	1.70	n	0
XU	511	*		RR for 1-19/day is 2.3(p<0.05), for 20-29/day is 2.6(p<0.05) and for >=30/ day is 7.7(p<0.05)

Table 2I13 -

IESLC - Meta-analysis of Ever/current Smoking, Duration, "Mid"
Squamous, Any Product (or Cigarettes if Any not available)

This analysis is restricted to results for:
1) Ever/current smokers
2) Results by Duration
3) Categorical results by Duration
4) Squamous (or near equivalent)
5) Results complete enough for use in metaanalysis

Within each study, results are then selected (in the following order of preference, within each sex) for:
6) SMKSTA: ever, current
7) PRODUCT: all/unspec, cigarettes regardless of other products, cigarettes only
8) CIGTYPE: all/unspecified, MC regardless of HR, MC only
9) (not applicable)
10) DENOM: never smoked anything, never smoked cigarettes, never any + low, never cigs + low
11) Followup period (YF, prospective studies): whole study (coded as 0) or longest available
12) LCtype: squamous or nearest available, but not adeno. (q = squamous, s = small,
a = adeno, KI = Kreyberg I, u = undifferentiated)
13) Race: all or nearest available, otherwise by race (wh or w = white, bl or b = black, hi = hispanic
ch = chinese, jap = japanese, haw = hawaiian, w+o = white + oriental, sca = scandinavian, as = asian)
14) Duration "mid" in key scheme 1 (key value 35, maximum range 21-49)
15) For overlapping studies: principal rather than subsidiary studies
Finally by Age: whole study (coded as 0) if available, otherwise by widest available age group
and then for single sex results (m, f) in preference to results for both sexes combined (c).

Results adjusted (AD) for the most potential confounders are then chosen in Sections -1 to -3
and results adjusted for the least confounders in Sections -4 to -6. (Those least adjusted results which
actually differ from the most adjusted are marked 'x' in column X in Section -4)

Section -7 shows excluded studies, together with the stage (as above) at which no qualifying
results were found.

Section -8 lists the potentially overlapping studies which have been included (1=principal, 2=subsidiary).

Section -9 lists any results which would have been included in preference except that they had data not complete
enough for use in meta-analysis, with their significance (yes/no), if known, and any further comment as entered
on the database. It also lists as "gap" any categories for which no data were presented by the original authors.

In addition to those mentioned above, the following fields, levels and abbreviations are used:
* or nk = not known, n = no, y = yes, ot = other
ev = ever, cu = current, nev = never
all/unspec = all or unspecified, cig+/-ot = cigarettes irrespective of other products (cigar, pipe etc)
MC = manufactured cigarettes, HR = hand-rolled cigarettes
exL, exH = range of exposure (low and high) in the smoking group, in terms of Duration
REF: 6-character study reference
NRR: number of the RR on the database within the study
ST : study type (CC = case control, pr or prosp = prospective)
NLC: number of lung cancer cases in whole study
R : risky occupational population (n = no, m = mining, o = other risky)
VB : national cigarette type (V = at least 75% Virginia, bl = at least 75% blended, ot = other)
P : any proxy use
H : full histological confirmation
De : derivation of RR/CI (or = original, st = standard method, ot = other method of estimation)

Table 2I13 - 1

IESLC - Meta-analysis of Ever/current Smoking, Duration, "Mid"
Squamous, Any Product (or Cigarettes if Any not available)
Most adjusted

REF	NRR	SEX	AGEL	AGEH	RACE	YF	LC TYPE	LOC	START	ST	NLC	R	VB	P	H	AD	SM	PRODUCT	exL	exH	DENOM	De
BARBON	563	m	0	0	all	-	q	Eu:wst	1979	CC	755	n	bl	y	y	1	ev	all/unsp	30	39	nev any	or
BUFFLE	506	m	0	0	wh	-	q	NAmer	1976	CC	943	n	bl	y	n	0	ev	cig+/-ot	34	43	nev cigs	or
CHOI	560	m	0	0	all	-	q	As:oth	1985	CC	375	n	bl	n	n	0	ev	cig+/-ot	30	39	nev cigs	st
CHOI	574	f	0	0	all	-	q	As:oth	1985	CC	375	n	bl	n	n	0	ev	cig+/-ot	30	39	nev cigs	st
DAMBER	548	m	0	0	all	-	q	Eu:Sca	1972	CC	579	n	bl	y	n	1	ev	all/unsp	31	40	nev any	or
JEDRYC	503	m	0	0	all	-	q	Eu:est	1980	CC	1630	n	bl	y	n	0	ev	cig+/-ot	30	39	nev any	st
JOLY	640	m	0	0	all	-	q	SCAmer	1978	CC	826	n	bl	n	n	0	ev	cig+/-ot	30	39	nev any	st
JOLY	612	f	0	0	all	-	q	SCAmer	1978	CC	826	n	bl	n	n	0	ev	cig+/-ot	30	39	nev any	st
LUBIN2	662	m	0	0	all	-	q	Eu:mul	1976	CC	7804	n	bl	n	y	0	ev	cig+/-ot	30	39	nev any	st
LUBIN2	714	f	0	0	all	-	q	Eu:mul	1976	CC	7804	n	bl	n	y	0	ev	cig+/-ot	30	39	nev any	st
MATOS	607	m	0	0	all	-	q	SCAmer	1994	CC	200	n	bl	n	n	2	ev	cig+/-ot	25	39	nev any	or
PEZZOT	508	m	0	0	all	-	q	SCAmer	1987	CC	215	n	bl	n	y	0	ev	cig only	31	40	nev cigs	ot
SOBUE	502	m	0	0	all	-	q	As:Jap	1986	CC	1376	n	bl	n	y	0	cu	cig+/-ot	30	39	nev cigs	st
WUWILL	522	f	0	0	all	-	q	As:Chi	1985	CC	965	n	ot	n	n	3	ev	cig+/-ot	30	39	nev cigs	ot
WYNDE2	507	m	0	0	all	-	KI	NAmer	1962	CC	404	n	bl	n	y	0	ev	cig+/-ot	30	40	nev any	ot
ZHENG	502	m	0	0	all	-	q	As:Chi	1982	CC	540	n	ot	*	y	0	ev	cig+/-ot	30	39	nev cigs	st

Cigarette type is all/unspec for all RRs

Table 2I13 - 2

IESLC - Meta-analysis of Ever/current Smoking, Duration, "Mid"
Squamous, Any Product (or Cigarettes if Any not available)
Most adjusted

REF	NRR	SEX	AD	Number Exposed Case	Number Exposed Cont	Non-exposed Case	Non-exposed Cont	RR	95.00%CI
BARBON	563	m	1	36	-	6	-	9.60	(3.90- 23.90)
BUFFLE	506	m	0	-	-	-	-	14.80	(4.80- 45.30)
CHOI	560	m	0	73	160	6	95	7.22	(3.03- 17.25)
CHOI	574	f	0	4	2	10	164	32.80	(5.35- 201.12)
Subtotal CHOI								9.59	(4.38- 21.01)
DAMBER	548	m	1	-	-	14	-	8.40	(4.00- 18.30)
JEDRYC	503	m	0	106	231	6	289	22.10	(9.54- 51.22)
JOLY	640	m	0	24	165	2	218	15.85	(3.69- 68.04)
JOLY	612	f	0	5	24	6	283	9.83	(2.79- 34.57)
Subtotal JOLY								12.05	(4.65- 31.23)
LUBIN2	662	m	0	1211	3473	54	2616	16.89	(12.80- 22.29)
LUBIN2	714	f	0	767	186	72	1180	67.58	(50.73- 90.03)
Subtotal LUBIN2								33.00	(27.04- 40.29)
MATOS	607	m	2	18	-	3	-	5.80	(1.60- 20.50)
PEZZOT	508	m	0	35	82	0	116	100.26~	(6.06-1657.79)
SOBUE	502	m	0	59	200	3	128	12.59	(3.86- 41.00)
WUWILL	522	f	3	66	-	117	-	3.88	(2.64- 5.71)
WYNDE2	507	m	0	30	64	0	41	39.25~	(2.34- 659.46)
ZHENG	502	m	0	59	80	4	94	17.33	(6.03- 49.81)
Partial Totals				2493	4667	303	5224		

*prospective study ~ With 0.5 adjustment for zero

REF	NRR	SEX	AD	Ys	Ws	Qs	Ps
BARBON	563	m	1	2.26	4.68	1.97	0.0000
BUFFLE	506	m	0	2.69	3.05	0.14	0.0000
CHOI	560	m	0	1.98	5.07	4.42	0.0000
CHOI	574	f	0	3.49	1.17	0.39	0.0002
Subtotal CHOI				2.26	6.24	4.81	
DAMBER	548	m	1	2.13	6.65	4.07	0.0000
JEDRYC	503	m	0	3.10	5.44	0.19	0.0000
JOLY	640	m	0	2.76	1.81	0.04	0.0002
JOLY	612	f	0	2.29	2.43	0.95	0.0004
Subtotal JOLY				2.49	4.24	0.99	
LUBIN2	662	m	0	2.83	49.96	0.35	0.0000
LUBIN2	714	f	0	4.21	46.69	79.24	0.0000
Subtotal LUBIN2				3.50	96.66	79.59	
MATOS	607	m	2	1.76	2.36	3.14	0.0069
PEZZOT	508	m	0	4.61	0.49	1.41	0.0013
SOBUE	502	m	0	2.53	2.75	0.39	0.0000
WUWILL	522	f	3	1.36	25.82	62.42	0.0000
WYNDE2	507	m	0	3.67	0.48	0.28	0.0108
ZHENG	502	m	0	2.85	3.45	0.01	0.0000

	N	16
	NS	13
	Wt	162.30
Het	Chi	159.40
Het	df	15
Het	P	***
Fixed	RR	18.37
	RRl	15.75
	RRu	21.42
	P	+++
Random	RR	14.48
	RRl	7.97
	RRu	26.33
	P	+++
Asymm	P	N.S.

Table 2I13 - 3

IESLC - Meta-analysis of Ever/current Smoking, Duration, "Mid"
Squamous, Any Product (or Cigarettes if Any not available)
Most adjusted

	Sex			
	combined	male	female	Total
N		12	4	16
NS		12	4	16
Wt		86.19	76.11	162.30
Het Chi		11.83	137.83	159.40
Het df		11	3	15
Het P		N.S.	***	***
Fixed RR		14.59	23.84	18.37
RRl		11.81	19.04	15.75
RRu		18.02	29.84	21.42
P		+++	+++	+++
Random RR		14.01	16.76	14.48
RRl		10.93	2.48	7.97
RRu		17.95	113.10	26.33
P		+++	++	+++
Between Chi				9.74
Between df				1
Between P				**
Btwn(F) P				N.S.
Btwn(R) P				N.S.

	Lung cancer type					
	q	q+s	q+u	KI	not a	Total
N	15			1		16
NS	12			1		13
Wt	161.82			0.48		162.30
Het Chi	159.12			0.00		159.40
Het df	14			0		15
Het P	***			N.S.		***
Fixed RR	18.33			39.25		18.37
RRl	15.71			2.34		15.75
RRu	21.38			659.46		21.42
P	+++			+		+++
Random RR	14.06			39.25		14.48
RRl	7.65			2.34		7.97
RRu	25.86			659.46		26.33
P	+++			+		+++
Between Chi						0.28
Between df						1
Between P						N.S.
Btwn(F) P						N.S.
Btwn(R) P						N.S.

	Location								
	NAmer	UK	Scand	othEur	China	Japan	othAs	other	Total
N	2		1	4	2	1	2	4	16
NS	2		1	3	2	1	1	3	13
Wt	3.53		6.65	106.77	29.27	2.75	6.24	7.09	162.30
Het Chi	0.40		0.00	53.81	6.81	0.00	2.17	3.62	159.40
Het df	1		0	3	1	0	1	3	15
Het P	N.S.		N.S.	***	**	N.S.	N.S.	N.S.	***
Fixed RR	16.91		8.40	30.63	4.63	12.59	9.59	10.93	18.37
RRl	5.96		3.93	25.34	3.22	3.86	4.38	5.23	15.75
RRu	47.98		17.97	37.03	6.65	41.00	21.01	22.82	21.42
P	+++		+++	+++	+++	+++	+++	+++	+++
Random RR	16.91		8.40	23.16	7.54	12.59	12.38	11.36	14.48
RRl	5.96		3.93	9.07	1.76	3.86	2.99	4.97	7.97
RRu	47.98		17.97	59.11	32.38	41.00	51.22	25.96	26.33
P	+++		+++	+++	++	+++	+++	+++	+++
Between Chi									92.58
Between df									6
Between P									***
Btwn(F) P									N.S.
Btwn(R) P									N.S.

Table 2I13 - 3

IESLC - Meta-analysis of Ever/current Smoking, Duration, "Mid"
Squamous, Any Product (or Cigarettes if Any not available)
Most adjusted

Detailed Country in "other Europe"

	multi	Germany	othWest	East	Balkans	Total
N	2		1	1		4
NS	1		1	1		3
Wt	96.66		4.68	5.44		106.77
Het Chi	46.40		0.00	0.00		53.81
Het df	1		0	0		3
Het P	***		N.S.	N.S.		***
Fixed RR	33.00		9.60	22.10		30.63
RRl	27.04		3.88	9.54		25.34
RRu	40.29		23.77	51.22		37.03
P	+++		+++	+++		+++
Random RR	33.77		9.60	22.10		23.16
RRl	8.68		3.88	9.54		9.07
RRu	131.41		23.77	51.22		59.11
P	+++		+++	+++		+++
Between Chi						7.41
Between df						2
Between P						*
Btwn(F) P						N.S.
Btwn(R) P						N.S.

Detailed Country in "other Asia"

	India	HongKong	other	Total
N			2	2
NS			1	1
Wt			6.24	6.24
Het Chi			2.17	2.17
Het df			1	1
Het P			N.S.	N.S.
Fixed RR			9.59	9.59
RRl			4.38	4.38
RRu			21.01	21.01
P			+++	+++
Random RR			12.38	12.38
RRl			2.99	2.99
RRu			51.22	51.22
P			+++	+++
Between Chi				
Between df				
Between P				N.S.
Btwn(F) P				N.S.
Btwn(R) P				N.S.

Detailed other continent

	SCAmer	Total
N	4	4
NS	3	3
Wt	7.09	7.09
Het Chi	3.62	3.62
Het df	3	3
Het P	N.S.	N.S.
Fixed RR	10.93	10.93
RRl	5.23	5.23
RRu	22.82	22.82
P	+++	+++
Random RR	11.36	11.36
RRl	4.97	4.97
RRu	25.96	25.96
P	+++	+++
Between Chi		
Between df		
Between P		N.S.
Btwn(F) P		N.S.
Btwn(R) P		N.S.

Table 2I13 - 3

IESLC - Meta-analysis of Ever/current Smoking, Duration, "Mid"
Squamous, Any Product (or Cigarettes if Any not available)
Most adjusted

	Start year of study <1960	1960-69	1970-79	1980-89	1990+	Total
N		1	7	7	1	16
NS		1	5	6	1	13
Wt		0.48	115.27	44.19	2.36	162.30
Het Chi		0.00	68.43	26.19	0.00	159.40
Het df		0	6	6	0	15
Het P		N.S.	***	***	N.S.	***
Fixed RR		39.25	27.37	6.85	5.80	18.37
RRl		2.34	22.80	5.10	1.62	15.75
RRu		659.46	32.85	9.19	20.76	21.42
P		+	+++	+++	++	+++
Random RR		39.25	16.33	12.91	5.80	14.48
RRl		2.34	7.63	5.92	1.62	7.97
RRu		659.46	34.96	28.16	20.76	26.33
P		+	+++	+++	++	+++
Between Chi						64.78
Between df						3
Between P						***
Btwn(F) P						(*)
Btwn(R) P						N.S.

	Study type (1) CC	other	Total
N	16		16
NS	13		13
Wt	162.30		162.30
Het Chi	159.40		159.40
Het df	15		15
Het P	***		***
Fixed RR	18.37		18.37
RRl	15.75		15.75
RRu	21.42		21.42
P	+++		+++
Random RR	14.48		14.48
RRl	7.97		7.97
RRu	26.33		26.33
P	+++		+++
Between Chi			
Between df			
Between P			N.S.
Btwn(F) P			N.S.
Btwn(R) P			N.S.

	Study type (2) CC	prosp	other	Total
N	16			16
NS	13			13
Wt	162.30			162.30
Het Chi	159.40			159.40
Het df	15			15
Het P	***			***
Fixed RR	18.37			18.37
RRl	15.75			15.75
RRu	21.42			21.42
P	+++			+++
Random RR	14.48			14.48
RRl	7.97			7.97
RRu	26.33			26.33
P	+++			+++
Between Chi				
Between df				
Between P				N.S.
Btwn(F) P				N.S.
Btwn(R) P				N.S.

Table 2I13 - 3

IESLC - Meta-analysis of Ever/current Smoking, Duration, "Mid"
Squamous, Any Product (or Cigarettes if Any not available)
Most adjusted

	Study size (number of LC cases) 100-249	250-499	500-999	1000+	Total
N	2	3	7	4	16
NS	2	2	6	3	13
Wt	2.85	6.72	47.88	104.85	162.30
Het Chi	3.29	3.06	15.22	49.61	159.40
Het df	1	2	6	3	15
Het P	(*)	N.S.	*	***	***
Fixed RR	9.45	10.61	6.33	31.52	18.37
RRl	2.96	4.98	4.77	26.03	15.75
RRu	30.17	22.59	8.40	38.17	21.42
P	+++	+++	+++	+++	+++
Random RR	18.13	13.93	9.09	25.13	14.48
RRl	1.17	4.39	5.31	9.89	7.97
RRu	279.77	44.27	15.57	63.85	26.33
P	+	+++	+++	+++	+++
Between Chi					88.22
Between df					3
Between P					***
Btwn(F) P					*
Btwn(R) P					N.S.

	Risky occupational population no	mining	othRisky	Total
N	16			16
NS	13			13
Wt	162.30			162.30
Het Chi	159.40			159.40
Het df	15			15
Het P	***			***
Fixed RR	18.37			18.37
RRl	15.75			15.75
RRu	21.42			21.42
P	+++			+++
Random RR	14.48			14.48
RRl	7.97			7.97
RRu	26.33			26.33
P	+++			+++
Between Chi				
Between df				
Between P				N.S.
Btwn(F) P				N.S.
Btwn(R) P				N.S.

	National cigarette tobacco type Virginia	blended	other	Total
N		14	2	16
NS		11	2	13
Wt		133.03	29.27	162.30
Het Chi		84.74	6.81	159.40
Het df		13	1	15
Het P		***	**	***
Fixed RR		24.88	4.63	18.37
RRl		20.99	3.22	15.75
RRu		29.48	6.65	21.42
P		+++	+++	+++
Random RR		16.05	7.54	14.48
RRl		9.24	1.76	7.97
RRu		27.87	32.38	26.33
P		+++	++	+++
Between Chi				67.85
Between df				1
Between P				***
Btwn(F) P				**
Btwn(R) P				N.S.

Table 2I13 - 3

IESLC - Meta-analysis of Ever/current Smoking, Duration, "Mid"
Squamous, Any Product (or Cigarettes if Any not available)
Most adjusted

	Any proxy use No/nk	Yes	Total
N	12	4	16
NS	9	4	13
Wt	142.49	19.81	162.30
Het Chi	152.60	3.23	159.40
Het df	11	3	15
Het P	***	N.S.	***
Fixed RR	19.41	12.34	18.37
RRl	16.47	7.94	15.75
RRu	22.88	19.16	21.42
P	+++	+++	+++
Random RR	15.39	12.37	14.48
RRl	7.22	7.82	7.97
RRu	32.82	19.55	26.33
P	+++	+++	+++
Between Chi			3.58
Between df			1
Between P			(*)
Btwn(F) P			N.S.
Btwn(R) P			N.S.

	Full histological confirmation No	Yes	Total
N	9	7	16
NS	7	6	13
Wt	53.79	108.50	162.30
Het Chi	22.42	57.21	159.40
Het df	8	6	15
Het P	**	***	***
Fixed RR	6.79	30.09	18.37
RRl	5.20	24.93	15.75
RRu	8.87	36.32	21.42
P	+++	+++	+++
Random RR	9.66	22.99	14.48
RRl	5.70	10.31	7.97
RRu	16.37	51.27	26.33
P	+++	+++	+++
Between Chi			79.77
Between df			1
Between P			***
Btwn(F) P			**
Btwn(R) P			(*)

	Number of adjustment variables (1) 0	1	2+/+nk	Total
N	12	2	2	16
NS	9	2	2	13
Wt	122.80	11.32	28.18	162.30
Het Chi	66.54	0.05	0.35	159.40
Het df	11	1	1	15
Het P	***	N.S.	N.S.	***
Fixed RR	27.85	8.88	4.01	18.37
RRl	23.33	4.96	2.77	15.75
RRu	33.24	15.89	5.81	21.42
P	+++	+++	+++	+++
Random RR	19.78	8.88	4.01	14.48
RRl	11.09	4.96	2.77	7.97
RRu	35.29	15.89	5.81	26.33
P	+++	+++	+++	+++
Between Chi				92.46
Between df				2
Between P				***
Btwn(F) P				**
Btwn(R) P				***

Table 2I13 - 3

IESLC - Meta-analysis of Ever/current Smoking, Duration, "Mid"
Squamous, Any Product (or Cigarettes if Any not available)
Most adjusted

	Number of adjustment variables (2) 0	1	2	3-5	6+/+nk	Total
N	12	2	1	1		16
NS	9	2	1	1		13
Wt	122.80	11.32	2.36	25.82		162.30
Het Chi	66.54	0.05	0.00	0.00		159.40
Het df	11	1	0	0		15
Het P	***	N.S.	N.S.	N.S.		***
Fixed RR	27.85	8.88	5.80	3.88		18.37
RRl	23.33	4.96	1.62	2.64		15.75
RRu	33.24	15.89	20.76	5.71		21.42
P	+++	+++	++	+++		+++
Random RR	19.78	8.88	5.80	3.88		14.48
RRl	11.09	4.96	1.62	2.64		7.97
RRu	35.29	15.89	20.76	5.71		26.33
P	+++	+++	++	+++		+++
Between Chi						92.81
Between df						3
Between P						***
Btwn(F) P						*
Btwn(R) P						***

	Smoking status ever	current	Total
N	15	1	16
NS	12	1	13
Wt	159.54	2.75	162.30
Het Chi	159.00	0.00	159.40
Het df	14	0	15
Het P	***	N.S.	***
Fixed RR	18.49	12.59	18.37
RRl	15.83	3.86	15.75
RRu	21.59	41.00	21.42
P	+++	+++	+++
Random RR	14.64	12.59	14.48
RRl	7.84	3.86	7.97
RRu	27.35	41.00	26.33
P	+++	+++	+++
Between Chi			0.40
Between df			1
Between P			N.S.
Btwn(F) P			N.S.
Btwn(R) P			N.S.

	Product all/unsp	cig+/-ot	cig only	Total
N	2	13	1	16
NS	2	10	1	13
Wt	11.32	150.49	0.49	162.30
Het Chi	0.05	151.59	0.00	159.40
Het df	1	12	0	15
Het P	N.S.	***	N.S.	***
Fixed RR	8.88	19.29	100.26	18.37
RRl	4.96	16.45	6.06	15.75
RRu	15.89	22.64	1657.79	21.42
P	+++	+++	++	+++
Random RR	8.88	14.70	100.26	14.48
RRl	4.96	7.46	6.06	7.97
RRu	15.89	29.00	1657.79	26.33
P	+++	+++	++	+++
Between Chi				7.76
Between df				2
Between P				*
Btwn(F) P				N.S.
Btwn(R) P				N.S.

Table 2I13 - 3

IESLC - Meta-analysis of Ever/current Smoking, Duration, "Mid"
Squamous, Any Product (or Cigarettes if Any not available)
Most adjusted

	Denominator nev any	nev cigs	Total
N	9	7	16
NS	7	6	13
Wt	120.50	41.80	162.30
Het Chi	73.21	20.15	159.40
Het df	8	6	15
Het P	***	**	***
Fixed RR	26.74	6.22	18.37
RRl	22.37	4.59	15.75
RRu	31.97	8.42	21.42
P	+++	+++	+++
Random RR	16.07	11.56	14.48
RRl	8.09	5.60	7.97
RRu	31.92	23.86	26.33
P	+++	+++	+++
Between Chi			66.04
Between df			1
Between P			***
Btwn(F) P			**
Btwn(R) P			N.S.

	Derivation of RR/CI Orig	StdCalc	Other	Total
N	4	9	3	16
NS	4	6	3	13
Wt	16.73	118.78	26.79	162.30
Het Chi	1.26	64.46	7.47	159.40
Het df	3	8	2	15
Het P	N.S.	***	*	***
Fixed RR	9.17	28.12	4.29	18.37
RRl	5.68	23.49	2.94	15.75
RRu	14.81	33.66	6.27	21.42
P	+++	+++	+++	+++
Random RR	9.17	18.62	17.56	14.48
RRl	5.68	9.74	1.81	7.97
RRu	14.81	35.61	170.36	26.33
P	+++	+++	+	+++
Between Chi				86.22
Between df				2
Between P				***
Btwn(F) P				**
Btwn(R) P				N.S.

Table 2I13 - 4

IESLC - Meta-analysis of Ever/current Smoking, Duration, "Mid"
Squamous, Any Product (or Cigarettes if Any not available)
Least adjusted

REF	NRR	X	SEX	AGEL	AGEH	RACE	YF	LC TYPE	LOC	START	ST	NLC	R	VB	P	H	AD	SM	PRODUCT	exL	exH	DENOM	De
BARBON	556	x	m	0	0	all	-	q	Eu:wst	1979	CC	755	n	bl	y	y	0	ev	all/unsp	30	39	nev any	st
BUFFLE	506		m	0	0	wh	-	q	NAmer	1976	CC	943	n	bl	y	n	0	ev	cig+/-ot	34	43	nev cigs	or
CHOI	560		m	0	0	all	-	q	As:oth	1985	CC	375	n	bl	n	n	0	ev	cig+/-ot	30	39	nev cigs	st
CHOI	574		f	0	0	all	-	q	As:oth	1985	CC	375	n	bl	n	n	0	ev	cig+/-ot	30	39	nev cigs	st
DAMBER	548		m	0	0	all	-	q	Eu:Sca	1972	CC	579	n	bl	y	n	1	ev	all/unsp	31	40	nev any	or
JEDRYC	503		m	0	0	all	-	q	Eu:est	1980	CC	1630	n	bl	y	n	0	ev	cig+/-ot	30	39	nev any	st
JOLY	640		m	0	0	all	-	q	SCAmer	1978	CC	826	n	bl	n	n	0	ev	cig+/-ot	30	39	nev any	st
JOLY	612		f	0	0	all	-	q	SCAmer	1978	CC	826	n	bl	n	n	0	ev	cig+/-ot	30	39	nev any	st
LUBIN2	662		m	0	0	all	-	q	Eu:mul	1976	CC	7804	n	bl	n	y	0	ev	cig+/-ot	30	39	nev any	st
LUBIN2	714		f	0	0	all	-	q	Eu:mul	1976	CC	7804	n	bl	n	y	0	ev	cig+/-ot	30	39	nev any	st
MATOS	602	x	m	0	0	all	-	q	SCAmer	1994	CC	200	n	bl	n	n	0	ev	cig+/-ot	25	39	nev any	st
PEZZOT	508		m	0	0	all	-	q	SCAmer	1987	CC	215	n	bl	n	y	0	ev	cig only	31	40	nev cigs	ot
SOBUE	502		m	0	0	all	-	q	As:Jap	1986	CC	1376	n	bl	n	y	0	cu	cig+/-ot	30	39	nev cigs	st
WUWILL	507	x	f	0	0	all	-	q	As:Chi	1985	CC	965	n	ot	n	n	0	ev	cig+/-ot	30	39	nev cigs	st
WYNDE2	507		m	0	0	all	-	KI	NAmer	1962	CC	404	n	bl	n	y	0	ev	cig+/-ot	30	40	nev any	ot
ZHENG	502		m	0	0	all	-	q	As:Chi	1982	CC	540	n	ot	*	y	0	ev	cig+/-ot	30	39	nev cigs	st

Cigarette type is all/unspec for all RRs

Table 2I13 - 5

IESLC - Meta-analysis of Ever/current Smoking, Duration, "Mid"
Squamous, Any Product (or Cigarettes if Any not available)
Least adjusted

REF	NRR	SEX	AD	Number Exposed Case	Cont	Non-exposed Case	Cont	RR	95.00%CI
BARBON	556	m	0	36	102	6	188	11.06	(4.51- 27.13)
BUFFLE	506	m	0	-	-	-	-	14.80	(4.80- 45.30)
CHOI	560	m	0	73	160	6	95	7.22	(3.03- 17.25)
CHOI	574	f	0	4	2	10	164	32.80	(5.35- 201.12)
Subtotal CHOI								9.59	(4.38- 21.01)
DAMBER	548	m	1	-	-	14	-	8.40	(4.00- 18.30)
JEDRYC	503	m	0	106	231	6	289	22.10	(9.54- 51.22)
JOLY	640	m	0	24	165	2	218	15.85	(3.69- 68.04)
JOLY	612	f	0	5	24	6	283	9.83	(2.79- 34.57)
Subtotal JOLY								12.05	(4.65- 31.23)
LUBIN2	662	m	0	1211	3473	54	2616	16.89	(12.80- 22.29)
LUBIN2	714	f	0	767	186	72	1180	67.58	(50.73- 90.03)
Subtotal LUBIN2								33.00	(27.04- 40.29)
MATOS	602	m	0	18	110	3	110	6.00	(1.72- 20.95)
PEZZOT	508	m	0	35	82	0	116	100.26~	(6.06-1657.79)
SOBUE	502	m	0	59	200	3	128	12.59	(3.86- 41.00)
WUWILL	507	f	0	66	98	117	601	3.46	(2.39- 5.01)
WYNDE2	507	m	0	30	64	0	41	39.25~	(2.34- 659.46)
ZHENG	502	m	0	59	80	4	94	17.33	(6.03- 49.81)
Partial Totals				2493	4977	303	6123		

*prospective study ~ With 0.5 adjustment for zero

REF	NRR	SEX	AD	Ys	Ws	Qs	Ps
BARBON	556	m	0	2.40	4.77	1.05	0.0000
BUFFLE	506	m	0	2.69	3.05	0.10	0.0000
CHOI	560	m	0	1.98	5.07	4.07	0.0000
CHOI	574	f	0	3.49	1.17	0.45	0.0002
Subtotal CHOI				2.26	6.24	4.51	
DAMBER	548	m	1	2.13	6.65	3.69	0.0000
JEDRYC	503	m	0	3.10	5.44	0.27	0.0000
JOLY	640	m	0	2.76	1.81	0.02	0.0002
JOLY	612	f	0	2.29	2.43	0.84	0.0004
Subtotal JOLY				2.49	4.24	0.86	
LUBIN2	662	m	0	2.83	49.96	0.11	0.0000
LUBIN2	714	f	0	4.21	46.69	83.89	0.0000
Subtotal LUBIN2				3.50	96.66	84.00	
MATOS	602	m	0	1.79	2.46	2.87	0.0050
PEZZOT	508	m	0	4.61	0.49	1.47	0.0013
SOBUE	502	m	0	2.53	2.75	0.32	0.0000
WUWILL	507	f	0	1.24	28.12	74.87	0.0000
WYNDE2	507	m	0	3.67	0.48	0.31	0.0108
ZHENG	502	m	0	2.85	3.45	0.00	0.0000

	N	16
	NS	13
	Wt	164.79
Het	Chi	174.32
Het	df	15
Het	P	***
Fixed	RR	17.69
	RRl	15.18
	RRu	20.61
	P	+++
Random	RR	14.56
	RRl	7.85
	RRu	26.99
	P	+++
Asymm	P	N.S.

Table 2I13 - 6

IESLC - Meta-analysis of Ever/current Smoking, Duration, "Mid"
Squamous, Any Product (or Cigarettes if Any not available)
Least adjusted

	Sex combined	male	female	Total
N		12	4	16
NS		12	4	16
Wt		86.38	78.41	164.79
Het Chi		11.31	156.78	174.32
Het df		11	3	15
Het P		N.S.	***	***
Fixed RR		14.70	21.69	17.69
RRl		11.90	17.39	15.18
RRu		18.15	27.07	20.61
P		+++	+++	+++
Random RR		14.45	16.27	14.56
RRl		11.52	2.23	7.85
RRu		18.13	118.58	26.99
P		+++	++	+++
Between Chi				6.23
Between df				1
Between P				*
Btwn(F) P				N.S.
Btwn(R) P				N.S.

Table 2I13 - 7

IESLC - Meta-analysis of Ever/current Smoking, Duration, "Mid"
Squamous, Any Product (or Cigarettes if Any not available)
Excluded studies (and stage at which they were excluded)

Stage	Studies
1	BECHER BLOT1 BROWN3 CARPEN CHYOU DARBY DOLL2 GARCIA GRAHAM GURSEL HAMMO2 JAHN JAIN LAUSSM PRESCO QIAO TVERDA WIGLE WYNDE3
2	ALDERS BENSHL BRESLO CHIAZZ DEAN3 DORN ENGELA GAO2 GILLIS GUO HEGMAN HIRAYA HOLE KAUFMA KOO KOULUM LIU4 MIGRAN MRFITR PERNU SEGI2 SPEIZE SUZUK2 SVENSS VUTUC WAKAI WU YUAN
3	GENG MCDUFF SPITZ STASZE WU2 ZHANG
4	AGUDO AKIBA AMANDU AMES ARMADA AUVINE AXELSS BEST BOFFET BOUCOT BROSS CEDERL CHEN2 CORREA CPSI CPSII DEAN2 DESTEF DOLL FAN GAO GARSHI HAMMON HU HU2 HUMBLE JUSSAW KAISE2 KREUZE LETOUR LEVIN LIAW LIU3 LIU5 MCCONN NOTAN2 PEZZO2 PISANI QIAO2 RACHTA RESTRE SADOWS TIZZAN WANG2 WATSON WYNDE6 WYNDE7 WYNDE8
5	CHEN LUBIN XU
10	BOUCHA KHUDER
14	DORGAN DOSEME GER HAENSZ KATSOU LUO OSANN2 ZHOU
15	BENHAM

Table 2I13 - 8

Potentially overlapping studies

REF	REFGP	PRINC	.	OVERLAP/LINK
LUBIN2	LUBIN2	1		Lubin-combined

Table 2I13 - 9

Most adjusted - insufficient data for meta-analysis

REF	NRR	SEX	AGEL	AGEH	RACE	YF	LC TYPE	LOC	START	ST	NLC	R	VB	P	H	AD	SM	PRODUCT	exL	exH	DENOM	De
CHEN	503	c	0	0	all	-	q	As:oth	1987	CC	323	n	ot	n	y	2	ev	cig+/-ot	31	40	nev cigs	ot
XU	512	m	0	0	all	-	q+s	As:Chi	1985	CC	729	n	ot	n	n	2	ev	all/unsp	30	39	nev any	or

REF	NRR	RR	SIG	RRDATA comment
CHEN	503	6.52	n	0
XU	512	*		RR for 1-19/day is 2.9(p<0.05), for 20-29/day is 3.9(p<0.05) and for >=30/ day is 8.3(p<0.05)

Table 2I14 -

IESLC - Meta-analysis of Ever/current Smoking, Duration, "High"
Squamous, Any Product (or Cigarettes if Any not available)

This analysis is restricted to results for:
1) Ever/current smokers
2) Results by Duration
3) Categorical results by Duration
4) Squamous (or near equivalent)
5) Results complete enough for use in metaanalysis

Within each study, results are then selected (in the following order of preference, within each sex) for:
6) PRODUCT: all/unspec, cigarettes regardless of other products, cigarettes only
7) CIGTYPE: all/unspecified, MC regardless of HR, MC only
8) (not applicable)
9) DENOM: never smoked anything, never smoked cigarettes, never any + low, never cigs + low
10) Followup period (YF, prospective studies): whole study (coded as 0) or longest available
11) LCtype: squamous or nearest available, but not adeno. (q = squamous, s = small,
a = adeno, KI = Kreyberg I, u = undifferentiated)
12) Race: all or nearest available, otherwise by race (wh or w = white, bl or b = black, hi = hispanic
ch = chinese, jap = japanese, haw = hawaiian, w+o = white + oriental, sca = scandinavian, as = asian)
13) Duration "high" in key scheme 1 (key value 50, maximum range 36+)
14) For overlapping studies: principal rather than subsidiary studies
Finally by Age: whole study (coded as 0) if available, otherwise by widest available age group
and then for single sex results (m, f) in preference to results for both sexes combined (c).

Results adjusted (AD) for the most potential confounders are then chosen in Sections -1 to -3
and results adjusted for the least confounders in Sections -4 to -6. (Those least adjusted results which
actually differ from the most adjusted are marked 'x' in column X in Section -4)

Section -7 shows excluded studies, together with the stage (as above) at which no qualifying
results were found.

Section -8 lists the potentially overlapping studies which have been included (1=principal, 2=subsidiary).

Section -9 lists any results which would have been included in preference except that they had data not complete
enough for use in meta-analysis, with their significance (yes/no), if known, and any further comment as entered
on the database. It also lists as "gap" any categories for which no data were presented by the original authors.

In addition to those mentioned above, the following fields, levels and abbreviations are used:
* or nk = not known, n = no, y = yes, ot = other
ev = ever, cu = current, nev = never
all/unspec = all or unspecified, cig+/-ot = cigarettes irrespective of other products (cigar, pipe etc)
MC = manufactured cigarettes, HR = hand-rolled cigarettes
exL, exH = range of exposure (low and high) in the smoking group, in terms of Duration
REF: 6-character study reference
NRR: number of the RR on the database within the study
ST : study type (CC = case control, pr or prosp = prospective)
NLC: number of lung cancer cases in whole study
R : risky occupational population (n = no, m = mining, o = other risky)
VB : national cigarette type (V = at least 75% Virginia, bl = at least 75% blended, ot = other)
P : any proxy use
H : full histological confirmation
De : derivation of RR/CI (or = original, st = standard method, ot = other method of estimation)

Table 2I14 - 1

IESLC - Meta-analysis of Ever/current Smoking, Duration, "High"
Squamous, Any Product (or Cigarettes if Any not available)
Most adjusted

REF	NRR	SEX	AGEL	AGEH	RACE	YF	LC TYPE	LOC	START	ST	NLC	R	VB	P	H	AD	SM	PRODUCT	exL	exH	DENOM	De
BARBON	565	m	0	0	all	-	q	Eu:wst	1979	CC	755	n	bl	y	y	1	ev	all/unsp	50	999	nev any	or
BUFFLE	508	m	0	0	wh	-	q	NAmer	1976	CC	943	n	bl	y	n	0	ev	cig+/-ot	50	999	nev cigs	or
CHOI	562	m	0	0	all	-	q	As:oth	1985	CC	375	n	bl	n	n	0	ev	cig+/-ot	50	999	nev cigs	st
CHOI	575	f	0	0	all	-	q	As:oth	1985	CC	375	n	bl	n	n	0	ev	cig+/-ot	40	999	nev cigs	st
DAMBER	549	m	0	0	all	-	q	Eu:Sca	1972	CC	579	n	bl	y	n	1	ev	all/unsp	41	50	nev any	or
JEDRYC	587	m	0	0	all	-	q	Eu:est	1980	CC	1630	n	bl	y	n	3	ev	cig+/-ot	40	999	nev any	or
JOLY	642	m	0	0	all	-	q	SCAmer	1978	CC	826	n	bl	n	n	0	ev	cig+/-ot	50	999	nev any	st
JOLY	614	f	0	0	all	-	q	SCAmer	1978	CC	826	n	bl	n	n	0	ev	cig+/-ot	50	999	nev any	st
LUBIN2	664	m	0	0	all	-	q	Eu:mul	1976	CC	7804	n	bl	n	y	0	ev	cig+/-ot	50	999	nev any	st
LUBIN2	716	f	0	0	all	-	q	Eu:mul	1976	CC	7804	n	bl	n	y	0	ev	cig+/-ot	50	999	nev any	st
MATOS	608	m	0	0	all	-	q	SCAmer	1994	CC	200	n	bl	n	n	2	ev	cig+/-ot	40	70	nev any	or
PEZZOT	509	m	0	0	all	-	q	SCAmer	1987	CC	215	n	bl	n	y	0	ev	cig only	41	999	nev cigs	ot
SOBUE	504	m	0	0	all	-	q	As:Jap	1986	CC	1376	n	bl	n	y	0	cu	cig+/-ot	50	999	nev cigs	st
WUWILL	523	f	0	0	all	-	q	As:Chi	1985	CC	965	n	ot	n	n	3	ev	cig+/-ot	40	999	nev cigs	ot
WYNDE2	508	m	0	0	all	-	KI	NAmer	1962	CC	404	n	bl	n	y	0	ev	cig+/-ot	41	999	nev any	ot
ZHENG	503	m	0	0	all	-	q	As:Chi	1982	CC	540	n	ot	*	y	0	ev	cig+/-ot	40	999	nev cigs	st

Cigarette type is all/unspec for all RRs

Table 2I14 - 2

IESLC - Meta-analysis of Ever/current Smoking, Duration, "High"
Squamous, Any Product (or Cigarettes if Any not available)
Most adjusted

REF	NRR	SEX	AD	Number Exposed Case	Number Exposed Cont	Non-exposed Case	Non-exposed Cont	RR	95.00%CI
BARBON	565	m	1	149	-	6	-	21.20	(9.10- 49.30)
BUFFLE	508	m	0	-	-	-	-	22.10	(7.20- 67.70)
CHOI	562	m	0	11	20	6	95	8.71	(2.88- 26.30)
CHOI	575	f	0	1	1	10	164	16.40	(0.95- 281.93)
Subtotal CHOI								9.46	(3.38- 26.51)
DAMBER	549	m	1	-	-	14	-	13.80	(6.80- 29.10)
JEDRYC	587	m	3	160	-	6	-	13.00	(5.54- 30.48)
JOLY	642	m	0	98	253	2	218	42.22	(10.29- 173.22)
JOLY	614	f	0	22	20	6	283	51.88	(18.89- 142.48)
Subtotal JOLY								48.39	(21.28- 110.03)
LUBIN2	664	m	0	746	1460	54	2616	24.75	(18.64- 32.87)
LUBIN2	716	f	0	566	34	72	1180	272.83	(179.26- 415.22)
Subtotal LUBIN2								52.47	(41.48- 66.37)
MATOS	608	m	2	26	-	3	-	18.50	(4.90- 69.80)
PEZZOT	509	m	0	45	101	0	116	104.45~	(6.35-1717.05)
SOBUE	504	m	0	77	73	3	128	45.00	(13.71- 147.74)
WUWILL	523	f	3	81	-	117	-	5.57	(3.79- 8.17)
WYNDE2	508	m	0	94	89	0	41	87.64~	(5.31-1446.06)
ZHENG	503	m	0	84	63	4	94	31.33	(10.94- 89.77)
Partial Totals				2160	2114	303	4935		

*prospective study ~ With 0.5 adjustment for zero

REF	NRR	SEX	AD	Ys	Ws	Qs	Ps
BARBON	565	m	1	3.05	5.38	0.25	0.0000
BUFFLE	508	m	0	3.10	3.06	0.09	0.0000
CHOI	562	m	0	2.16	3.14	3.83	0.0001
CHOI	575	f	0	2.80	0.47	0.11	0.0539
Subtotal CHOI				2.25	3.62	3.94	
DAMBER	549	m	1	2.62	7.27	3.01	0.0000
JEDRYC	587	m	3	2.56	5.29	2.62	0.0000
JOLY	642	m	0	3.74	1.93	0.43	0.0000
JOLY	614	f	0	3.95	3.76	1.74	0.0000
Subtotal JOLY				3.88	5.69	2.18	
LUBIN2	664	m	0	3.21	47.79	0.17	0.0000
LUBIN2	716	f	0	5.61	21.78	119.29	0.0000
Subtotal LUBIN2				3.96	69.57	119.46	
MATOS	608	m	2	2.92	2.18	0.27	0.0000
PEZZOT	509	m	0	4.65	0.49	0.93	0.0011
SOBUE	504	m	0	3.81	2.72	0.79	0.0000
WUWILL	523	f	3	1.72	26.04	62.66	0.0000
WYNDE2	508	m	0	4.47	0.49	0.71	0.0018
ZHENG	503	m	0	3.44	3.47	0.11	0.0000

	N	16
	NS	13
	Wt	135.26
Het	Chi	197.01
Het	df	15
Het	P	***
Fixed	RR	26.27
	RRl	22.20
	RRu	31.09
	P	+++
Random	RR	27.18
	RRl	13.36
	RRu	55.28
	P	+++
Asymm	P	N.S.

Table 2I14 - 3

IESLC - Meta-analysis of Ever/current Smoking, Duration, "High"
Squamous, Any Product (or Cigarettes if Any not available)
Most adjusted

	combined	Sex<br>male	female	Total
N		12	4	16
NS		12	4	16
Wt		83.20	52.06	135.26
Het Chi		11.21	180.60	197.01
Het df		11	3	15
Het P		N.S.	***	***
Fixed RR		22.50	33.67	26.27
RRl		18.15	25.66	22.20
RRu		27.89	44.17	31.09
P		+++	+++	+++
Random RR		22.33	35.26	27.18
RRl		17.81	2.90	13.36
RRu		27.99	429.38	55.28
P		+++	++	+++
Between Chi				5.20
Between df				1
Between P				*
Btwn(F) P				N.S.
Btwn(R) P				N.S.

	q	Lung cancer type<br>q+s	q+u	KI	not a	Total
N	15			1		16
NS	12			1		13
Wt	134.77			0.49		135.26
Het Chi	196.30			0.00		197.01
Het df	14			0		15
Het P	***			N.S.		***
Fixed RR	26.16			87.64		26.27
RRl	22.09			5.31		22.20
RRu	30.97			1446.06		31.09
P	+++			++		+++
Random RR	26.04			87.64		27.18
RRl	12.61			5.31		13.36
RRu	53.77			1446.06		55.28
P	+++			++		+++
Between Chi						0.71
Between df						1
Between P						N.S.
Btwn(F) P						N.S.
Btwn(R) P						N.S.

	NAmer	UK	Scand	Location<br>othEur	China	Japan	othAs	other	Total
N	2		1	4	2	1	2	4	16
NS	2		1	3	2	1	1	3	13
Wt	3.55		7.27	80.23	29.51	2.72	3.62	8.36	135.26
Het Chi	0.80		0.00	99.01	9.13	0.00	0.17	2.00	197.01
Het df	1		0	3	1	0	1	3	15
Het P	N.S.		N.S.	***	**	N.S.	N.S.	N.S.	***
Fixed RR	26.72		13.80	45.04	6.82	45.00	9.46	39.40	26.27
RRl	9.44		6.67	36.19	4.76	13.71	3.38	20.01	22.20
RRu	75.63		28.55	56.06	9.79	147.74	26.51	77.62	31.09
P	+++		+++	+++	+++	+++	+++	+++	+++
Random RR	26.72		13.80	37.84	12.29	45.00	9.46	39.40	27.18
RRl	9.44		6.67	8.74	2.27	13.71	3.38	20.01	13.36
RRu	75.63		28.55	163.70	66.38	147.74	26.51	77.62	55.28
P	+++		+++	+++	++	+++	+++	+++	+++
Between Chi									85.91
Between df									6
Between P									***
Btwn(F) P									N.S.
Btwn(R) P									N.S.

Table 2I14 - 3

IESLC - Meta-analysis of Ever/current Smoking, Duration, "High"
Squamous, Any Product (or Cigarettes if Any not available)
Most adjusted

Detailed Country in "other Europe"

	multi	Germany	othWest	East	Balkans	Total
N	2		1	1		4
NS	1		1	1		3
Wt	69.57		5.38	5.29		80.23
Het Chi	86.17		0.00	0.00		99.01
Het df	1		0	0		3
Het P	***		N.S.	N.S.		***
Fixed RR	52.47		21.20	13.00		45.04
RRl	41.48		9.11	5.54		36.19
RRu	66.37		49.34	30.49		56.06
P	+++		+++	+++		+++
Random RR	81.75		21.20	13.00		37.84
RRl	7.78		9.11	5.54		8.74
RRu	858.78		49.34	30.49		163.70
P	+++		+++	+++		+++
Between Chi						12.84
Between df						2
Between P						**
Btwn(F) P						N.S.
Btwn(R) P						N.S.

Detailed Country in "other Asia"

	India	HongKong	other	Total
N			2	2
NS			1	1
Wt			3.62	3.62
Het Chi			0.17	0.17
Het df			1	1
Het P			N.S.	N.S.
Fixed RR			9.46	9.46
RRl			3.38	3.38
RRu			26.51	26.51
P			+++	+++
Random RR			9.46	9.46
RRl			3.38	3.38
RRu			26.51	26.51
P			+++	+++
Between Chi				
Between df				
Between P				N.S.
Btwn(F) P				N.S.
Btwn(R) P				N.S.

Detailed other continent

	SCAmer	Total
N	4	4
NS	3	3
Wt	8.36	8.36
Het Chi	2.00	2.00
Het df	3	3
Het P	N.S.	N.S.
Fixed RR	39.40	39.40
RRl	20.01	20.01
RRu	77.62	77.62
P	+++	+++
Random RR	39.40	39.40
RRl	20.01	20.01
RRu	77.62	77.62
P	+++	+++
Between Chi		
Between df		
Between P		N.S.
Btwn(F) P		N.S.
Btwn(R) P		N.S.

Table 2I14 - 3

IESLC - Meta-analysis of Ever/current Smoking, Duration, "High"
Squamous, Any Product (or Cigarettes if Any not available)
Most adjusted

	<1960	1960-69	1970-79	1980-89	1990+	Total
		Start year of study				
N		1	7	7	1	16
NS		1	5	6	1	13
Wt		0.49	90.97	41.62	2.18	135.26
Het Chi		0.00	102.49	22.26	0.00	197.01
Het df		0	6	6	0	15
Het P		N.S.	***	**	N.S.	***
Fixed RR		87.64	43.20	8.90	18.50	26.27
RRl		5.31	35.17	6.57	4.90	22.20
RRu		1446.06	53.05	12.06	69.82	31.09
P		++	+++	+++	+++	+++
Random RR		87.64	37.59	16.16	18.50	27.18
RRl		5.31	13.82	7.38	4.90	13.36
RRu		1446.06	102.24	35.36	69.82	55.28
P		++	+++	+++	+++	+++
Between Chi						72.25
Between df						3
Between P						***
Btwn(F) P						N.S.
Btwn(R) P						N.S.

	Study type (1)		
	CC	other	Total
N	16		16
NS	13		13
Wt	135.26		135.26
Het Chi	197.01		197.01
Het df	15		15
Het P	***		***
Fixed RR	26.27		26.27
RRl	22.20		22.20
RRu	31.09		31.09
P	+++		+++
Random RR	27.18		27.18
RRl	13.36		13.36
RRu	55.28		55.28
P	+++		+++
Between Chi			
Between df			
Between P			N.S.
Btwn(F) P			N.S.
Btwn(R) P			N.S.

	Study type (2)			
	CC	prosp	other	Total
N	16			16
NS	13			13
Wt	135.26			135.26
Het Chi	197.01			197.01
Het df	15			15
Het P	***			***
Fixed RR	26.27			26.27
RRl	22.20			22.20
RRu	31.09			31.09
P	+++			+++
Random RR	27.18			27.18
RRl	13.36			13.36
RRu	55.28			55.28
P	+++			+++
Between Chi				
Between df				
Between P				N.S.
Btwn(F) P				N.S.
Btwn(R) P				N.S.

Table 2I14 - 3

IESLC - Meta-analysis of Ever/current Smoking, Duration, "High"
Squamous, Any Product (or Cigarettes if Any not available)
Most adjusted

	Study size (number of LC cases)				
	100-249	250-499	500-999	1000+	Total
N	2	3	7	4	16
NS	2	2	6	3	13
Wt	2.67	4.11	50.92	77.57	135.26
Het Chi	1.20	2.30	32.53	95.74	197.01
Het df	1	2	6	3	15
Het P	N.S.	N.S.	***	***	***
Fixed RR	25.43	12.33	11.36	47.46	26.27
RRl	7.66	4.69	8.63	37.99	22.20
RRu	84.42	32.44	14.95	59.29	31.09
P	+++	+++	+++	+++	+++
Random RR	27.84	13.77	20.22	45.35	27.18
RRl	6.59	4.32	9.69	10.00	13.36
RRu	117.60	43.88	42.18	205.64	55.28
P	+++	+++	+++	+++	+++
Between Chi					65.25
Between df					3
Between P					***
Btwn(F) P					N.S.
Btwn(R) P					N.S.

	Risky occupational population			
	no	mining	othRisky	Total
N	16			16
NS	13			13
Wt	135.26			135.26
Het Chi	197.01			197.01
Het df	15			15
Het P	***			***
Fixed RR	26.27			26.27
RRl	22.20			22.20
RRu	31.09			31.09
P	+++			+++
Random RR	27.18			27.18
RRl	13.36			13.36
RRu	55.28			55.28
P	+++			+++
Between Chi				
Between df				
Between P				N.S.
Btwn(F) P				N.S.
Btwn(R) P				N.S.

	National cigarette tobacco type			
	Virginia	blended	other	Total
N		14	2	16
NS		11	2	13
Wt		105.75	29.51	135.26
Het Chi		119.27	9.13	197.01
Het df		13	1	15
Het P		***	**	***
Fixed RR		38.27	6.82	26.27
RRl		31.63	4.76	22.20
RRu		46.31	9.79	31.09
P		+++	+++	+++
Random RR		30.89	12.29	27.18
RRl		15.27	2.27	13.36
RRu		62.48	66.38	55.28
P		+++	++	+++
Between Chi				68.61
Between df				1
Between P				***
Btwn(F) P				*
Btwn(R) P				N.S.

Table 2I14 - 3

IESLC - Meta-analysis of Ever/current Smoking, Duration, "High"
Squamous, Any Product (or Cigarettes if Any not available)
Most adjusted

	Any proxy use No/nk	Yes	Total
N	12	4	16
NS	9	4	13
Wt	114.26	21.00	135.26
Het Chi	190.15	1.13	197.01
Het df	11	3	15
Het P	***	N.S.	***
Fixed RR	28.70	16.25	26.27
RRl	23.89	10.60	22.20
RRu	34.47	24.93	31.09
P	+++	+++	+++
Random RR	32.85	16.25	27.18
RRl	13.02	10.60	13.36
RRu	82.86	24.93	55.28
P	+++	+++	+++
Between Chi			5.73
Between df			1
Between P			*
Btwn(F) P			N.S.
Btwn(R) P			N.S.

	Full histological confirmation No	Yes	Total
N	9	7	16
NS	7	6	13
Wt	53.15	82.11	135.26
Het Chi	27.14	91.42	197.01
Het df	8	6	15
Het P	***	***	***
Fixed RR	10.20	48.48	26.27
RRl	7.79	39.05	22.20
RRu	13.34	60.19	31.09
P	+++	+++	+++
Random RR	15.72	52.78	27.18
RRl	8.70	17.28	13.36
RRu	28.41	161.24	55.28
P	+++	+++	+++
Between Chi			78.45
Between df			1
Between P			***
Btwn(F) P			**
Btwn(R) P			(*)

	Number of adjustment variables (1) 0	1	2+/+nk	Total
N	11	2	3	16
NS	8	2	3	13
Wt	89.10	12.65	33.51	135.26
Het Chi	99.34	0.57	5.43	197.01
Het df	10	1	2	15
Het P	***	N.S.	(*)	***
Fixed RR	46.42	16.57	6.88	26.27
RRl	37.72	9.55	4.91	22.20
RRu	57.13	28.74	9.66	31.09
P	+++	+++	+++	+++
Random RR	40.81	16.57	9.35	27.18
RRl	17.35	9.55	4.37	13.36
RRu	95.97	28.74	20.00	55.28
P	+++	+++	+++	+++
Between Chi				91.67
Between df				2
Between P				***
Btwn(F) P				*
Btwn(R) P				*

Table 2I14 - 3

IESLC - Meta-analysis of Ever/current Smoking, Duration, "High"
Squamous, Any Product (or Cigarettes if Any not available)
Most adjusted

	Number of adjustment variables (2) 0	1	2	3-5	6+/+nk	Total
N	11	2	1	2		16
NS	8	2	1	2		13
Wt	89.10	12.65	2.18	31.33		135.26
Het Chi	99.34	0.57	0.00	3.16		197.01
Het df	10	1	0	1		15
Het P	***	N.S.	N.S.	(*)		***
Fixed RR	46.42	16.57	18.50	6.43		26.27
RRl	37.72	9.55	4.90	4.53		22.20
RRu	57.13	28.74	69.82	9.12		31.09
P	+++	+++	+++	+++		+++
Random RR	40.81	16.57	18.50	7.79		27.18
RRl	17.35	9.55	4.90	3.46		13.36
RRu	95.97	28.74	69.82	17.54		55.28
P	+++	+++	+++	+++		+++
Between Chi						93.95
Between df						3
Between P						***
Btwn(F) P						*
Btwn(R) P						(*)

	Smoking status ever	current	Total
N	15	1	16
NS	12	1	13
Wt	132.54	2.72	135.26
Het Chi	196.21	0.00	197.01
Het df	14	0	15
Het P	***	N.S.	***
Fixed RR	25.98	45.00	26.27
RRl	21.92	13.71	22.20
RRu	30.81	147.74	31.09
P	+++	+++	+++
Random RR	26.28	45.00	27.18
RRl	12.49	13.71	13.36
RRu	55.28	147.74	55.28
P	+++	+++	+++
Between Chi			0.80
Between df			1
Between P			N.S.
Btwn(F) P			N.S.
Btwn(R) P			N.S.

	Product all/unsp	cig+/-ot	cig only	Total
N	2	13	1	16
NS	2	10	1	13
Wt	12.65	122.12	0.49	135.26
Het Chi	0.57	192.60	0.00	197.01
Het df	1	12	0	15
Het P	N.S.	***	N.S.	***
Fixed RR	16.57	27.41	104.45	26.27
RRl	9.55	22.95	6.35	22.20
RRu	28.74	32.72	1717.05	31.09
P	+++	+++	++	+++
Random RR	16.57	27.85	104.45	27.18
RRl	9.55	12.11	6.35	13.36
RRu	28.74	64.04	1717.05	55.28
P	+++	+++	++	+++
Between Chi				3.84
Between df				2
Between P				N.S.
Btwn(F) P				N.S.
Btwn(R) P				N.S.

Table 2I14 - 3

IESLC - Meta-analysis of Ever/current Smoking, Duration, "High"
Squamous, Any Product (or Cigarettes if Any not available)
Most adjusted

		Denominator		
		nev any	nev cigs	Total
	N	9	7	16
	NS	7	6	13
	Wt	95.86	39.40	135.26
Het	Chi	110.20	24.02	197.01
Het	df	8	6	15
Het	P	***	***	***
Fixed	RR	40.66	9.08	26.27
	RRl	33.29	6.64	22.20
	RRu	49.67	12.40	31.09
	P	+++	+++	+++
Random	RR	34.29	18.17	27.18
	RRl	14.02	7.70	13.36
	RRu	83.88	42.87	55.28
	P	+++	+++	+++
Between	Chi			62.79
Between	df			1
Between	P			***
Btwn(F)	P			*
Btwn(R)	P			N.S.

		Derivation of RR/CI			
		Orig	StdCalc	Other	Total
	N	5	8	3	16
	NS	5	5	3	13
	Wt	23.17	85.06	27.02	135.26
Het	Chi	1.16	97.10	7.64	197.01
Het	df	4	7	2	15
Het	P	N.S.	***	*	***
Fixed	RR	16.45	47.28	6.17	26.27
	RRl	10.95	38.23	4.23	22.20
	RRu	24.72	58.47	9.00	31.09
	P	+++	+++	+++	+++
Random	RR	16.45	39.16	26.25	27.18
	RRl	10.95	14.33	2.66	13.36
	RRu	24.72	107.03	258.86	55.28
	P	+++	+++	++	+++
Between	Chi				91.11
Between	df				2
Between	P				***
Btwn(F)	P				*
Btwn(R)	P				N.S.

Table 2I14 - 4

IESLC - Meta-analysis of Ever/current Smoking, Duration, "High"
Squamous, Any Product (or Cigarettes if Any not available)
Least adjusted

REF	NRR	X	SEX	AGEL	AGEH	RACE	YF	LC TYPE	LOC	START	ST	NLC	R	VB	P	H	AD	SM	PRODUCT	exL	exH	DENOM	De
BARBON	558	x	m	0	0	all	-	q	Eu:wst	1979	CC	755	n	bl	y	y	0	ev	all/unsp	50	999	nev any	st
BUFFLE	508		m	0	0	wh	-	q	NAmer	1976	CC	943	n	bl	y	n	0	ev	cig+/-ot	50	999	nev cigs	or
CHOI	562		m	0	0	all	-	q	As:oth	1985	CC	375	n	bl	n	n	0	ev	cig+/-ot	50	999	nev cigs	st
CHOI	575		f	0	0	all	-	q	As:oth	1985	CC	375	n	bl	n	n	0	ev	cig+/-ot	40	999	nev cigs	st
DAMBER	549		m	0	0	all	-	q	Eu:Sca	1972	CC	579	n	bl	y	n	1	ev	all/unsp	41	50	nev any	or
JEDRYC	505	x	m	0	0	all	-	q	Eu:est	1980	CC	1630	n	bl	y	n	0	ev	cig+/-ot	50	999	nev any	st
JOLY	642		m	0	0	all	-	q	SCAmer	1978	CC	826	n	bl	n	n	0	ev	cig+/-ot	50	999	nev any	st
JOLY	614		f	0	0	all	-	q	SCAmer	1978	CC	826	n	bl	n	n	0	ev	cig+/-ot	50	999	nev any	st
LUBIN2	664		m	0	0	all	-	q	Eu:mul	1976	CC	7804	n	bl	n	y	0	ev	cig+/-ot	50	999	nev any	st
LUBIN2	716		f	0	0	all	-	q	Eu:mul	1976	CC	7804	n	bl	n	y	0	ev	cig+/-ot	50	999	nev any	st
MATOS	603	x	m	0	0	all	-	q	SCAmer	1994	CC	200	n	bl	n	n	0	ev	cig+/-ot	40	70	nev any	st
PEZZOT	509		m	0	0	all	-	q	SCAmer	1987	CC	215	n	bl	n	y	0	ev	cig only	41	999	nev cigs	ot
SOBUE	504		m	0	0	all	-	q	As:Jap	1986	CC	1376	n	bl	n	y	0	cu	cig+/-ot	50	999	nev cigs	st
WUWILL	508	x	f	0	0	all	-	q	As:Chi	1985	CC	965	n	ot	n	n	0	ev	cig+/-ot	40	999	nev cigs	st
WYNDE2	508		m	0	0	all	-	KI	NAmer	1962	CC	404	n	bl	n	y	0	ev	cig+/-ot	41	999	nev any	ot
ZHENG	503		m	0	0	all	-	q	As:Chi	1982	CC	540	n	ot	*	y	0	ev	cig+/-ot	40	999	nev cigs	st

Cigarette type is all/unspec for all RRs

Table 2I14 - 5

IESLC - Meta-analysis of Ever/current Smoking, Duration, "High"
Squamous, Any Product (or Cigarettes if Any not available)
Least adjusted

REF	NRR	SEX	AD	Number Case	Exposed Cont	Non-exposed Case	Cont	RR	95.00%CI
BARBON	558	m	0	149	235	6	188	19.87	(8.59- 45.94)
BUFFLE	508	m	0	-	-	-	-	22.10	(7.20- 67.70)
CHOI	562	m	0	11	20	6	95	8.71	(2.88- 26.30)
CHOI	575	f	0	1	1	10	164	16.40	(0.95- 281.93)
Subtotal CHOI								9.46	(3.38- 26.51)
DAMBER	549	m	1	-	-	14	-	13.80	(6.80- 29.10)
JEDRYC	505	m	0	49	214	6	289	11.03	(4.64- 26.22)
JOLY	642	m	0	98	253	2	218	42.22	(10.29- 173.22)
JOLY	614	f	0	22	20	6	283	51.88	(18.89- 142.48)
Subtotal JOLY								48.39	(21.28- 110.03)
LUBIN2	664	m	0	746	1460	54	2616	24.75	(18.64- 32.87)
LUBIN2	716	f	0	566	34	72	1180	272.83	(179.26- 415.22)
Subtotal LUBIN2								52.47	(41.48- 66.37)
MATOS	603	m	0	26	89	3	110	10.71	(3.14- 36.55)
PEZZOT	509	m	0	45	101	0	116	104.45~	(6.35-1717.05)
SOBUE	504	m	0	77	73	3	128	45.00	(13.71- 147.74)
WUWILL	508	f	0	81	114	117	601	3.65	(2.58- 5.16)
WYNDE2	508	m	0	94	89	0	41	87.64~	(5.31-1446.06)
ZHENG	503	m	0	84	63	4	94	31.33	(10.94- 89.77)
Partial Totals				2049	2766	303	6123		

*prospective study ~ With 0.5 adjustment for zero

REF	NRR	SEX	AD	Ys	Ws	Qs	Ps
BARBON	558	m	0	2.99	5.47	0.06	0.0000
BUFFLE	508	m	0	3.10	3.06	0.00	0.0000
CHOI	562	m	0	2.16	3.14	2.69	0.0001
CHOI	575	f	0	2.80	0.47	0.04	0.0539
Subtotal CHOI				2.25	3.62	2.74	
DAMBER	549	m	1	2.62	7.27	1.57	0.0000
JEDRYC	505	m	0	2.40	5.12	2.44	0.0000
JOLY	642	m	0	3.74	1.93	0.82	0.0000
JOLY	614	f	0	3.95	3.76	2.78	0.0000
Subtotal JOLY				3.88	5.69	3.60	
LUBIN2	664	m	0	3.21	47.79	0.67	0.0000
LUBIN2	716	f	0	5.61	21.78	138.17	0.0000
Subtotal LUBIN2				3.96	69.57	138.84	
MATOS	603	m	0	2.37	2.55	1.32	0.0002
PEZZOT	509	m	0	4.65	0.49	1.19	0.0011
SOBUE	504	m	0	3.81	2.72	1.40	0.0000
WUWILL	508	f	0	1.29	31.92	102.90	0.0000
WYNDE2	508	m	0	4.47	0.49	0.94	0.0018
ZHENG	503	m	0	3.44	3.47	0.44	0.0000

	N	16
	NS	13
	Wt	141.43
Het	Chi	257.42
Het	df	15
Het	P	***
Fixed	RR	21.98
	RRl	18.64
	RRu	25.92
	P	+++
Random	RR	25.20
	RRl	11.48
	RRu	55.32
	P	+++
Asymm	P	N.S.

Table 2I14 - 6

IESLC - Meta-analysis of Ever/current Smoking, Duration, "High"
Squamous, Any Product (or Cigarettes if Any not available)
Least adjusted

	Sex combined	male	female	Total
N		12	4	16
NS		12	4	16
Wt		83.49	57.94	141.43
Het Chi		13.53	243.88	257.42
Het df		11	3	15
Het P		N.S.	***	***
Fixed RR		21.81	22.22	21.98
RRl		17.60	17.18	18.64
RRu		27.03	28.75	25.92
P		+++	+++	+++
Random RR		20.61	31.26	25.20
RRl		15.36	1.93	11.48
RRu		27.64	506.39	55.32
P		+++	+	+++
Between Chi				0.01
Between df				1
Between P				N.S.
Btwn(F) P				N.S.
Btwn(R) P				N.S.

Table 2I14 - 7

IESLC - Meta-analysis of Ever/current Smoking, Duration, "High"
Squamous, Any Product (or Cigarettes if Any not available)
Excluded studies (and stage at which they were excluded)

1	BECHER BLOT1 BROWN3 CARPEN CHYOU DARBY DOLL2 GARCIA GRAHAM GURSEL HAMMO2 JAHN JAIN LAUSSM PRESCO QIAO TVERDA WIGLE WYNDE3
2	ALDERS BENSHL BRESLO CHIAZZ DEAN3 DORN ENGELA GAO2 GILLIS GUO HEGMAN HIRAYA HOLE KAUFMA KOO KOULUM LIU4 MIGRAN MRFITR PERNU SEGI2 SPEIZE SUZUK2 SVENSS VUTUC WAKAI WU YUAN
3	GENG MCDUFF SPITZ STASZE WU2 ZHANG
4	AGUDO AKIBA AMANDU AMES ARMADA AUVINE AXELSS BEST BOFFET BOUCOT BROSS CEDERL CHEN2 CORREA CPSI CPSII DEAN2 DESTEF DOLL FAN GAO GARSHI HAMMON HU HU2 HUMBLE JUSSAW KAISE2 KREUZE LETOUR LEVIN LIAW LIU3 LIU5 MCCONN NOTAN2 PEZZO2 PISANI QIAO2 RACHTA RESTRE SADOWS TIZZAN WANG2 WATSON WYNDE6 WYNDE7 WYNDE8
5	CHEN LUBIN XU
10	BOUCHA KHUDER
14	DORGAN DOSEME GER HAENSZ KATSOU LUO OSANN2 ZHOU
15	BENHAM

Table 2I14 - 8
Potentially overlapping studies

REF	REFGP	PRINC	.	OVERLAP/LINK
LUBIN2	LUBIN2	1		Lubin-combined

Table 2I14 - 9
Most adjusted - insufficient data for meta-analysis

REF	NRR	SEX	AGEL	AGEH	RACE	YF	LC TYPE	LOC	START	ST	NLC	R	VB	P	H	AD	SM	PRODUCT	exL	exH	DENOM	De
CHEN	504	c	0	0	all	-	q	As:oth	1987	CC	323	n	ot	n	y	2	ev	cig+/-ot	41	999	nev cigs	ot
XU	513	m	0	0	all	-	q+s	As:Chi	1985	CC	729	n	ot	n	n	2	ev	all/unsp	40	999	nev any	or

REF	NRR	RR	SIG	RRDATA comment
CHEN	504	8.43	y	p<0.001
XU	513	*		RR for 1-19/day is 5.0(p<0.05), for 20-29/day is 10.4(p<0.05) and for >=30/ day is 31.2(p<0.05)

Table 2I15 -

IESLC - Meta-analysis of Ever/current Smoking, Duration, "Highest vs lowest"
Squamous, Any Product (or Cigarettes if Any not available)

This analysis is restricted to results for:
1) Ever/current smokers
2) Results by Duration
3) Categorical results by Duration
4) Denominator (unexposed) = "low"
5) Squamous (or near equivalent)
6) Results complete enough for use in metaanalysis

Within each study, results are then selected (in the following order of preference, within each sex) for:
7) SMKSTA: ever, current
8) PRODUCT: all/unspec, cigarettes regardless of other products, cigarettes only
9) CIGTYPE: all/unspecified, MC regardless of HR, MC only
10) Results with least adjustment for other aspects of smoking (ADOS)
11) The highest vs lowest category
12) Followup period (YF, prospective studies): whole study (coded as 0) or longest available
13) LCtype: squamous or nearest available, but not adeno. (q = squamous, s = small,
a = adeno, KI = Kreyberg I, u = undifferentiated)
14) Race: all or nearest available, otherwise by race (wh or w = white, bl or b = black, hi = hispanic
ch = chinese, jap = japanese, haw = hawaiian, w+o = white + oriental, sca = scandinavian, as = asian)
15) For overlapping studies: principal rather than subsidiary studies
Finally by Age: whole study (coded as 0) if available, otherwise by widest available age group
and then for single sex results (m, f) in preference to results for both sexes combined (c).

Results adjusted (AD) for the most potential confounders are then chosen in Sections -1 to -3
and results adjusted for the least confounders in Sections -4 to -6. (Those least adjusted results which
actually differ from the most adjusted are marked 'x' in column X in Section -4)

Section -7 shows excluded studies, together with the stage (as above) at which no qualifying
results were found.

Section -8 lists the potentially overlapping studies which have been included (1=principal, 2=subsidiary).

Section -9 lists any results which would have been included in preference except that they had data not complete
enough for use in meta-analysis, with their significance (yes/no), if known, and any further comment as entered
on the database. It also lists as "gap" any categories for which no data were presented by the original authors.

In addition to those mentioned above, the following fields, levels and abbreviations are used:
* or nk = not known, n = no, y = yes, ot = other
all/unspec = all or unspecified, cig+/-ot = cigarettes irrespective of other products (cigar, pipe etc)
MC = manufactured cigarettes, HR = hand-rolled cigarettes
exL, exH = range of exposure (low and high) in the "highest" group, in terms of Duration
unexL, unexH = range of exposure (low and high) in the "lowest" group, in terms of Duration
REF: 6-character study reference
NRR: number of the RR on the database within the study
ST : study type (CC = case control, pr or prosp = prospective)
NLC: number of lung cancer cases in whole study
R : risky occupational population (n = no, m = mining, o = other risky)
VB : national cigarette type (V = at least 75% Virginia, bl = at least 75% blended, ot = other)
P : any proxy use
H : full histological confirmation
De : derivation of RR/CI (or = original, st = standard method, ot = other method of estimation)

Table 2I15 - 1

IESLC - Meta-analysis of Ever/current Smoking, Duration, "Highest vs lowest"
Squamous, Any Product (or Cigarettes if Any not available)
Most adjusted

REF	NRR	SEX	AGEL	AGEH	RACE	YF	LC TYPE	LOC	START	ST	NLC	R	VB	P	H	AD	ADOS	SM	PRODUCT	exL	exH	unexL	unexH	De
BARBON	568	m	0	0	all	-	q	Eu:wst	1979	CC	755	n	bl	y	y	1	0	ev	all/unsp	50	999	1	29	ot
BOUCHA	501	c	0	0	wh	-	q+s	Eu:wst	1988	CC	150	n	bl	n	y	0	0	ev	all/unsp	31	999	1	30	st
CHOI	565	m	0	0	all	-	q	As:oth	1985	CC	375	n	bl	n	n	0	0	ev	cig+/-ot	50	999	1	29	st
CHOI	577	f	0	0	all	-	q	As:oth	1985	CC	375	n	bl	n	n	0	0	ev	cig+/-ot	40	999	1	29	st
DAMBER	553	m	0	0	all	-	q	Eu:Sca	1972	CC	579	n	bl	y	n	1	0	ev	all/unsp	51	999	1	30	ot
DORGAN	527	m	0	0	wh	-	q	NAmer	1980	CC	2026	n	bl	y	y	2	0	ev	cig+/-ot	35	999	1	34	ot
DORGAN	523	f	0	0	all	-	q	NAmer	1980	CC	2026	n	bl	y	y	3	0	ev	cig+/-ot	35	999	1	34	ot
DOSEME	515	m	0	0	all	-	q	Eu:bal	1979	CC	1210	n	bl	n	n	2	0	ev	cig+/-ot	21	999	1	10	ot
GER	512	c	0	0	all	-	q+s	As:oth	1990	CC	141	n	ot	y	n	5	0	ev	all/unsp	31	999	1	30	ot
HAENSZ	535	f	0	0	all	-	q+u	NAmer	1955	CC	158	n	bl	n	y	1	0	ev	cig+/-ot	15	999	1	14	ot
JEDRYC	509	m	0	0	all	-	q	Eu:est	1980	CC	1630	n	bl	y	n	0	0	ev	cig+/-ot	50	999	1	19	st
JOLY	645	m	0	0	all	-	q	SCAmer	1978	CC	826	n	bl	n	n	0	0	ev	cig+/-ot	50	999	1	29	st
JOLY	617	f	0	0	all	-	q	SCAmer	1978	CC	826	n	bl	n	n	0	0	ev	cig+/-ot	50	999	1	29	st
KATSOU	530	f	0	0	all	-	KI	Eu:bal	1987	CC	101	n	bl	n	n	1	0	cu	all/unsp	30	999	1	29	ot
KHUDER	530	m	0	0	all	-	q	NAmer	1985	CC	482	n	bl	n	y	5	3#	ev	cig+/-ot	30	999	1	29	or
LUBIN2	667	m	0	0	all	-	q	Eu:mul	1976	CC	7804	n	bl	n	y	0	0	ev	cig+/-ot	50	999	1	29	st
LUBIN2	719	f	0	0	all	-	q	Eu:mul	1976	CC	7804	n	bl	n	y	0	0	ev	cig+/-ot	50	999	1	29	st
LUO	506	c	0	0	all	-	q	As:Chi	1990	CC	102	n	ot	n	y	20	0	ev	cig+/-ot	30	999	1	29	ot
MATOS	610	m	0	0	all	-	q	SCAmer	1994	CC	200	n	bl	n	n	2	0	ev	cig+/-ot	40	70	1	24	ot
OSANN2	512	f	0	0	all	-	KI	NAmer	1964	ot	217	n	bl	n	y	1	0	ev	cig+/-ot	21	999	1	20	ot
PEZZOT	513	m	0	0	all	-	q	SCAmer	1987	CC	215	n	bl	n	y	2	0	ev	cig only	41	999	1	30	ot
SOBUE	508	m	0	0	all	-	q	As:Jap	1986	CC	1376	n	bl	n	y	0	0	cu	cig+/-ot	50	999	1	29	st
WUWILL	510	f	0	0	all	-	q	As:Chi	1985	CC	965	n	ot	n	n	0	0	ev	cig+/-ot	40	999	1	29	st
WYNDE2	513	m	0	0	all	-	KI	NAmer	1962	CC	404	n	bl	n	y	0	0	ev	cig+/-ot	41	999	1	29	st
ZHENG	507	m	0	0	all	-	q	As:Chi	1982	CC	540	n	ot	*	y	1	0	ev	cig+/-ot	40	999	1	29	ot
ZHENG	511	f	0	0	all	-	q	As:Chi	1982	CC	540	n	ot	*	y	1	0	ev	cig+/-ot	30	999	1	29	ot
ZHOU	506	c	0	0	all	-	q	As:Chi	1978	CC	1360	n	ot	n	n	0	0	ev	all/unsp	20	999	1	19	st

Comments on values in listings

KHUDER ADOS Age at starting smoking, No of cigarettes per day, Quitted smoking

Cigarette type is all/unspec for all RRs

Table 2I15 - 2

IESLC - Meta-analysis of Ever/current Smoking, Duration, "Highest vs lowest"
Squamous, Any Product (or Cigarettes if Any not available)
Most adjusted

REF	NRR	SEX	AD	Number Exposed Case	Number Exposed Cont	Non-exposed Case	Non-exposed Cont	RR	95.00%CI
BARBON	568	m	1	149	-	7	-	10.10	(4.59- 22.21)
BOUCHA	501	c	0	114	92	34	79	2.88	(1.77- 4.69)
CHOI	565	m	0	11	20	42	221	2.89	(1.29- 6.48)
CHOI	577	f	0	1	1	6	23	3.83	(0.21- 70.63)
Subtotal CHOI								2.95	(1.36- 6.42)
DAMBER	553	m	1	-	-	-	-	3.80	(1.82- 7.91)
DORGAN	527	m	2	-	-	-	-	2.77	(2.04- 3.76)
DORGAN	523	f	3	-	-	-	-	3.67	(2.52- 5.34)
Subtotal DORGAN								3.10	(2.44- 3.93)
DOSEME	515	m	2	199	-	15	-	4.08	(2.07- 8.05)
GER	512	c	5	42	-	6	-	4.19	(1.56- 11.28)
HAENSZ	535	f	1	42	-	14	-	1.00	(0.47- 2.12)
JEDRYC	509	m	0	49	214	7	68	2.22	(0.96- 5.14)
JOLY	645	m	0	98	253	15	109	2.81	(1.56- 5.07)
JOLY	617	f	0	22	20	5	54	11.88	(3.96- 35.63)
Subtotal JOLY								3.88	(2.31- 6.51)
KATSOU	530	f	1	19	-	5	-	8.45	(2.01- 35.41)
KHUDER	530	m	5	-	-	-	-	1.90	(0.90- 3.70)
LUBIN2	667	m	0	746	1460	453	2964	3.34	(2.93- 3.82)
LUBIN2	719	f	0	566	34	322	229	11.84	(8.05- 17.40)
Subtotal LUBIN2								3.82	(3.37- 4.33)
LUO	506	c	20	28	-	6	-	2.19	(0.68- 7.06)
MATOS	610	m	2	26	-	3	-	15.42	(3.96- 60.01)
OSANN2	512	f	1	101	-	11	-	20.63	(3.12- 136.52)
PEZZOT	513	m	2	45	-	5	-	9.90	(3.61- 27.15)
SOBUE	508	m	0	77	73	16	119	7.85	(4.25- 14.47)
WUWILL	510	f	0	81	114	54	139	1.83	(1.20- 2.80)
WYNDE2	513	m	0	94	89	22	55	2.64	(1.49- 4.68)
ZHENG	507	m	1	84	-	13	-	8.25	(4.21- 16.18)
ZHENG	511	f	1	35	-	8	-	4.42	(1.66- 11.76)
Subtotal ZHENG								6.75	(3.88- 11.76)
ZHOU	506	c	0	315	36	60	12	1.75	(0.86- 3.56)
Partial Totals				2944	2406	1129	4072		

*prospective study

REF	NRR	SEX	AD	Ys	Ws	Qs	Ps
BARBON	568	m	1	2.31	6.18	6.74	0.0000
BOUCHA	501	c	0	1.06	16.20	0.72	0.0000
CHOI	565	m	0	1.06	5.91	0.25	0.0098
CHOI	577	f	0	1.34	0.45	0.00	0.3661
Subtotal CHOI				1.08	6.36	0.25	
DAMBER	553	m	1	1.34	7.12	0.03	0.0004
DORGAN	527	m	2	1.02	41.10	2.55	0.0000
DORGAN	523	f	3	1.30	27.25	0.03	0.0000
Subtotal DORGAN				1.13	68.34	2.58	
DOSEME	515	m	2	1.41	8.33	0.16	0.0000
GER	512	c	5	1.43	3.93	0.11	0.0045
HAENSZ	535	f	1	0.00	6.77	10.89	1.0000
JEDRYC	509	m	0	0.80	5.48	1.20	0.0614
JOLY	645	m	0	1.03	11.11	0.60	0.0006
JOLY	617	f	0	2.47	3.18	4.64	0.0000
Subtotal JOLY				1.36	14.30	5.24	
KATSOU	530	f	1	2.13	1.87	1.40	0.0035
KHUDER	530	m	5	0.64	7.69	3.01	0.0751
LUBIN2	667	m	0	1.21	218.80	0.81	0.0000
LUBIN2	719	f	0	2.47	25.87	37.47	0.0000
Subtotal LUBIN2				1.34	244.68	38.29	
LUO	506	c	20	0.78	2.81	0.66	0.1891
MATOS	610	m	2	2.74	2.08	4.48	0.0001
OSANN2	512	f	1	3.03	1.08	3.33	0.0017
PEZZOT	513	m	2	2.29	3.77	3.96	0.0000
SOBUE	508	m	0	2.06	10.25	6.43	0.0000
WUWILL	510	f	0	0.60	21.35	9.42	0.0053
WYNDE2	513	m	0	0.97	11.69	1.03	0.0009
ZHENG	507	m	1	2.11	8.48	6.01	0.0000
ZHENG	511	f	1	1.49	4.01	0.19	0.0029
Subtotal ZHENG				1.91	12.49	6.20	
ZHOU	506	c	0	0.56	7.64	3.83	0.1220

Table 2I15 - 2

IESLC - Meta-analysis of Ever/current Smoking, Duration, "Highest vs lowest"
Squamous, Any Product (or Cigarettes if Any not available)
Most adjusted

	N	27
	NS	22
	Wt	470.39
Het	Chi	109.96
Het	df	26
Het	P	***
Fixed	RR	3.55
	RRl	3.25
	RRu	3.89
	P	+++
Random	RR	3.93
	RRl	3.10
	RRu	4.97
	P	+++
Asymm	P	N.S.

Table 2I15 - 3

IESLC - Meta-analysis of Ever/current Smoking, Duration, "Highest vs lowest"
Squamous, Any Product (or Cigarettes if Any not available)
Most adjusted

		Sex		
	combined	male	female	Total
N	4	14	9	27
NS	4	14	9	27
Wt	30.57	347.99	91.83	470.39
Het Chi	2.34	37.10	64.03	109.96
Het df	3	13	8	26
Het P	N.S.	***	***	***
Fixed RR	2.60	3.47	4.30	3.55
RRl	1.83	3.13	3.50	3.25
RRu	3.71	3.86	5.28	3.89
P	+++	+++	+++	+++
Random RR	2.60	4.01	4.73	3.93
RRl	1.83	3.12	2.39	3.10
RRu	3.71	5.16	9.39	4.97
P	+++	+++	+++	+++
Between Chi				6.49
Between df				2
Between P				*
Btwn(F) P				N.S.
Btwn(R) P				N.S.

		Lung cancer type				
	q	q+s	q+u	KI	not a	Total
N	21	2	1	3		27
NS	16	2	1	3		22
Wt	428.85	20.13	6.77	14.64		470.39
Het Chi	92.20	0.44	0.00	5.76		109.96
Het df	20	1	0	2		26
Het P	***	N.S.	N.S.	(*)		***
Fixed RR	3.65	3.10	1.00	3.56		3.55
RRl	3.32	2.00	0.47	2.13		3.25
RRu	4.01	4.79	2.12	5.95		3.89
P	+++	+++	N.S.	+++		+++
Random RR	4.17	3.10	1.00	6.05		3.93
RRl	3.21	2.00	0.47	1.78		3.10
RRu	5.42	4.79	2.12	20.62		4.97
P	+++	+++	N.S.	++		+++
Between Chi						11.56
Between df						3
Between P						**
Btwn(F) P						N.S.
Btwn(R) P						**

				Location					
	NAmer	UK	Scand	othEur	China	Japan	othAs	other	Total
N	6		1	7	5	1	3	4	27
NS	5		1	6	4	1	2	3	22
Wt	95.57		7.12	282.73	44.28	10.25	10.29	20.15	470.39
Het Chi	14.64		0.00	46.94	16.34	0.00	0.33	10.37	109.96
Het df	5		0	6	4	0	2	3	26
Het P	*		N.S.	***	**	N.S.	N.S.	*	***
Fixed RR	2.75		3.80	3.83	2.65	7.85	3.37	5.33	3.55
RRl	2.25		1.82	3.41	1.98	4.25	1.83	3.45	3.25
RRu	3.37		7.92	4.30	3.56	14.47	6.22	8.25	3.89
P	+++		+++	+++	+++	+++	+++	+++	+++
Random RR	2.58		3.80	4.92	3.00	7.85	3.37	7.60	3.93
RRl	1.73		1.82	2.92	1.55	4.25	1.83	3.08	3.10
RRu	3.86		7.92	8.29	5.81	14.47	6.22	18.75	4.97
P	+++		+++	+++	++	+++	+++	+++	+++
Between Chi									21.34
Between df									6
Between P									**
Btwn(F) P									N.S.
Btwn(R) P									*

Table 2I15 - 3

IESLC - Meta-analysis of Ever/current Smoking, Duration, "Highest vs lowest"
Squamous, Any Product (or Cigarettes if Any not available)
Most adjusted

Detailed Country in "other Europe"

	multi	Germany	othWest	East	Balkans	Total
N	2		2	1	2	7
NS	1		2	1	2	6
Wt	244.68		22.39	5.48	10.20	282.73
Het Chi	36.99		7.05	0.00	0.81	46.94
Het df	1		1	0	1	6
Het P	***		**	N.S.	N.S.	***
Fixed RR	3.82		4.07	2.22	4.66	3.83
RRl	3.37		2.69	0.96	2.52	3.41
RRu	4.33		6.16	5.14	8.61	4.30
P	+++		+++	(+)	+++	+++
Random RR	6.21		5.18	2.22	4.66	4.92
RRl	1.80		1.52	0.96	2.52	2.92
RRu	21.43		17.68	5.14	8.61	8.29
P	++		++	(+)	+++	+++
Between Chi						2.10
Between df						3
Between P						N.S.
Btwn(F) P						N.S.
Btwn(R) P						N.S.

Detailed Country in "other Asia"

	India	HongKong	other	Total
N			3	3
NS			2	2
Wt			10.29	10.29
Het Chi			0.33	0.33
Het df			2	2
Het P			N.S.	N.S.
Fixed RR			3.37	3.37
RRl			1.83	1.83
RRu			6.22	6.22
P			+++	+++
Random RR			3.37	3.37
RRl			1.83	1.83
RRu			6.22	6.22
P			+++	+++
Between Chi				
Between df				
Between P				N.S.
Btwn(F) P				N.S.
Btwn(R) P				N.S.

Detailed other continent

	SCAmer	Total
N	4	4
NS	3	3
Wt	20.15	20.15
Het Chi	10.37	10.37
Het df	3	3
Het P	*	*
Fixed RR	5.33	5.33
RRl	3.45	3.45
RRu	8.25	8.25
P	+++	+++
Random RR	7.60	7.60
RRl	3.08	3.08
RRu	18.75	18.75
P	+++	+++
Between Chi		
Between df		
Between P		N.S.
Btwn(F) P		N.S.
Btwn(R) P		N.S.

Table 2I15 - 3

IESLC - Meta-analysis of Ever/current Smoking, Duration, "Highest vs lowest"
Squamous, Any Product (or Cigarettes if Any not available)
Most adjusted

	Start year of study <1960	1960-69	1970-79	1980-89	1990+	Total
N	1	2	8	13	3	27
NS	1	2	6	10	3	22
Wt	6.77	12.77	288.24	153.80	8.81	470.39
Het Chi	0.00	4.16	52.66	33.92	4.62	109.96
Het df	0	1	7	12	2	26
Het P	N.S.	*	***	***	(*)	***
Fixed RR	1.00	3.14	3.83	3.25	4.63	3.55
RRl	0.47	1.81	3.41	2.77	2.39	3.25
RRu	2.12	5.43	4.30	3.80	8.97	3.89
P	N.S.	+++	+++	+++	+++	+++
Random RR	1.00	6.01	4.80	3.63	4.92	3.93
RRl	0.47	0.83	2.95	2.68	1.77	3.10
RRu	2.12	43.28	7.83	4.91	13.66	4.97
P	N.S.	(+)	+++	+++	++	+++
Between Chi						14.59
Between df						4
Between P						**
Btwn(F) P						N.S.
Btwn(R) P						*

	Study type (1) CC	other	Total
N	26	1	27
NS	21	1	22
Wt	469.32	1.08	470.39
Het Chi	106.63	0.00	109.96
Het df	25	0	26
Het P	***	N.S.	***
Fixed RR	3.54	20.63	3.55
RRl	3.23	3.12	3.25
RRu	3.87	136.46	3.89
P	+++	++	+++
Random RR	3.84	20.63	3.93
RRl	3.04	3.12	3.10
RRu	4.86	136.46	4.97
P	+++	++	+++
Between Chi			3.34
Between df			1
Between P			(*)
Btwn(F) P			N.S.
Btwn(R) P			(*)

	Study type (2) CC	prosp	other	Total
N	26		1	27
NS	21		1	22
Wt	469.32		1.08	470.39
Het Chi	106.63		0.00	109.96
Het df	25		0	26
Het P	***		N.S.	***
Fixed RR	3.54		20.63	3.55
RRl	3.23		3.12	3.25
RRu	3.87		136.46	3.89
P	+++		++	+++
Random RR	3.84		20.63	3.93
RRl	3.04		3.12	3.10
RRu	4.86		136.46	4.97
P	+++		++	+++
Between Chi				3.34
Between df				1
Between P				(*)
Btwn(F) P				N.S.
Btwn(R) P				(*)

Table 2I15 - 3

IESLC - Meta-analysis of Ever/current Smoking, Duration, "Highest vs lowest"
Squamous, Any Product (or Cigarettes if Any not available)
Most adjusted

	Study size (number of LC cases)				
	100-249	250-499	500-999	1000+	Total
N	8	4	7	8	27
NS	8	3	5	6	22
Wt	38.50	25.74	61.43	344.71	470.39
Het Chi	25.40	0.82	27.58	52.21	109.96
Het df	7	3	6	7	26
Het P	***	N.S.	***	***	***
Fixed RR	3.35	2.46	3.67	3.66	3.55
RRl	2.44	1.67	2.86	3.29	3.25
RRu	4.59	3.62	4.72	4.06	3.89
P	+++	+++	+++	+++	+++
Random RR	4.56	2.46	4.76	3.99	3.93
RRl	2.32	1.67	2.70	2.73	3.10
RRu	8.97	3.62	8.40	5.82	4.97
P	+++	+++	+++	+++	+++
Between Chi					3.96
Between df					3
Between P					N.S.
Btwn(F) P					N.S.
Btwn(R) P					N.S.

	Risky occupational population			
	no	mining	othRisky	Total
N	27			27
NS	22			22
Wt	470.39			470.39
Het Chi	109.96			109.96
Het df	26			26
Het P	***			***
Fixed RR	3.55			3.55
RRl	3.25			3.25
RRu	3.89			3.89
P	+++			+++
Random RR	3.93			3.93
RRl	3.10			3.10
RRu	4.97			4.97
P	+++			+++
Between Chi				
Between df				
Between P				N.S.
Btwn(F) P				N.S.
Btwn(R) P				N.S.

	National cigarette tobacco type			
	Virginia	blended	other	Total
N		21	6	27
NS		17	5	22
Wt		422.18	48.21	470.39
Het Chi		89.39	17.09	109.96
Het df		20	5	26
Het P		***	**	***
Fixed RR		3.66	2.75	3.55
RRl		3.33	2.08	3.25
RRu		4.02	3.65	3.89
P		+++	+++	+++
Random RR		4.18	3.15	3.93
RRl		3.21	1.77	3.10
RRu		5.46	5.59	4.97
P		+++	+++	+++
Between Chi				3.49
Between df				1
Between P				(*)
Btwn(F) P				N.S.
Btwn(R) P				N.S.

Table 2I15 - 3

IESLC - Meta-analysis of Ever/current Smoking, Duration, "Highest vs lowest"
Squamous, Any Product (or Cigarettes if Any not available)
Most adjusted

	Any proxy use No/nk	Yes	Total
N	21	6	27
NS	17	5	22
Wt	379.35	91.04	470.39
Het Chi	99.25	10.46	109.96
Het df	20	5	26
Het P	***	(*)	***
Fixed RR	3.59	3.39	3.55
RRl	3.25	2.76	3.25
RRu	3.97	4.16	3.89
P	+++	+++	+++
Random RR	4.02	3.70	3.93
RRl	2.97	2.62	3.10
RRu	5.44	5.22	4.97
P	+++	+++	+++
Between Chi			0.26
Between df			1
Between P			N.S.
Btwn(F) P			N.S.
Btwn(R) P			N.S.

	Full histological confirmation No	Yes	Total
N	12	15	27
NS	10	12	22
Wt	78.44	391.95	470.39
Het Chi	23.21	83.25	109.96
Het df	11	14	26
Het P	*	***	***
Fixed RR	2.93	3.69	3.55
RRl	2.35	3.35	3.25
RRu	3.66	4.08	3.89
P	+++	+++	+++
Random RR	3.44	4.23	3.93
RRl	2.42	3.08	3.10
RRu	4.90	5.80	4.97
P	+++	+++	+++
Between Chi			3.51
Between df			1
Between P			(*)
Btwn(F) P			N.S.
Btwn(R) P			N.S.

	Number of adjustment variables (1) 0	1	2+/+nk	Total
N	12	7	8	27
NS	9	6	7	22
Wt	337.95	35.50	96.95	470.39
Het Chi	66.38	25.85	14.48	109.96
Het df	11	6	7	26
Het P	***	***	*	***
Fixed RR	3.52	4.69	3.31	3.55
RRl	3.17	3.38	2.71	3.25
RRu	3.92	6.52	4.04	3.89
P	+++	+++	+++	+++
Random RR	3.61	5.25	3.66	3.93
RRl	2.50	2.55	2.60	3.10
RRu	5.20	10.81	5.16	4.97
P	+++	+++	+++	+++
Between Chi				3.25
Between df				2
Between P				N.S.
Btwn(F) P				N.S.
Btwn(R) P				N.S.

Table 2I15 - 3

IESLC - Meta-analysis of Ever/current Smoking, Duration, "Highest vs lowest"
Squamous, Any Product (or Cigarettes if Any not available)
Most adjusted

	Number of adjustment variables (2) 0	1	2	3-5	6+/+nk	Total
N	12	7	4	3	1	27
NS	9	6	4	3	1	23
Wt	337.95	35.50	55.28	38.86	2.81	470.39
Het Chi	66.38	25.85	11.07	2.87	0.00	109.96
Het df	11	6	3	2	0	26
Het P	***	***	*	N.S.	N.S.	***
Fixed RR	3.52	4.69	3.42	3.27	2.19	3.55
RRl	3.17	3.38	2.63	2.38	0.68	3.25
RRu	3.92	6.52	4.45	4.47	7.06	3.89
P	+++	+++	+++	+++	N.S.	+++
Random RR	3.61	5.25	5.31	3.13	2.19	3.93
RRl	2.50	2.55	2.61	2.04	0.68	3.10
RRu	5.20	10.81	10.78	4.83	7.06	4.97
P	+++	+++	+++	+++	N.S.	+++
Between Chi						3.79
Between df						4
Between P						N.S.
Btwn(F) P						N.S.
Btwn(R) P						N.S.

	Smoking status ever	current	Total
N	25	2	27
NS	20	2	22
Wt	458.28	12.11	470.39
Het Chi	101.93	0.01	109.96
Het df	24	1	26
Het P	***	N.S.	***
Fixed RR	3.48	7.94	3.55
RRl	3.17	4.52	3.25
RRu	3.81	13.94	3.89
P	+++	+++	+++
Random RR	3.74	7.94	3.93
RRl	2.94	4.52	3.10
RRu	4.76	13.94	4.97
P	+++	+++	+++
Between Chi			8.03
Between df			1
Between P			**
Btwn(F) P			N.S.
Btwn(R) P			*

	Product all/unsp	cig+/-ot	cig only	Total
N	6	20	1	27
NS	6	15	1	22
Wt	42.93	423.69	3.77	470.39
Het Chi	12.83	93.13	0.00	109.96
Het df	5	19	0	26
Het P	*	***	N.S.	***
Fixed RR	3.59	3.52	9.90	3.55
RRl	2.66	3.20	3.61	3.25
RRu	4.84	3.87	27.15	3.89
P	+++	+++	+++	+++
Random RR	3.97	3.78	9.90	3.93
RRl	2.38	2.87	3.61	3.10
RRu	6.65	4.99	27.15	4.97
P	+++	+++	+++	+++
Between Chi				4.01
Between df				2
Between P				N.S.
Btwn(F) P				N.S.
Btwn(R) P				N.S.

Table 2I15 - 3

IESLC - Meta-analysis of Ever/current Smoking, Duration, "Highest vs lowest"
Squamous, Any Product (or Cigarettes if Any not available)
Most adjusted

		Derivation of RR/CI			
		Orig	StdCalc	Other	Total
	N	1	12	14	27
	NS	1	9	12	22
	Wt	7.69	337.95	124.76	470.39
Het	Chi	0.00	66.38	40.04	109.96
Het	df	0	11	13	26
Het	P	N.S.	***	***	***
Fixed	RR	1.90	3.52	3.79	3.55
	RRl	0.94	3.17	3.18	3.25
	RRu	3.85	3.92	4.51	3.89
	P	(+)	+++	+++	+++
Random	RR	1.90	3.61	4.60	3.93
	RRl	0.94	2.50	3.22	3.10
	RRu	3.85	5.20	6.58	4.97
	P	(+)	+++	+++	+++
Between	Chi				3.54
Between	df				2
Between	P				N.S.
Btwn(F)	P				N.S.
Btwn(R)	P				(*)

Table 2I15 - 4

IESLC - Meta-analysis of Ever/current Smoking, Duration, "Highest vs lowest"
Squamous, Any Product (or Cigarettes if Any not available)
Least adjusted

REF	NRR	X	SEX	AGEL	AGEH	RACE	YF	LC TYPE	LOC	START	ST	NLC	R	VB	P	H	AD	ADOS	SM	PRODUCT	exL	exH	unexL	unexH	De
BARBON	561	x	m	0	0	all	-	q	Eu:wst	1979	CC	755	n	bl	y	y	0	0	ev	all/unsp	50	999	1	29	st
BOUCHA	501		c	0	0	wh	-	q+s	Eu:wst	1988	CC	150	n	bl	n	y	0	0	ev	all/unsp	31	999	1	30	st
CHOI	565		m	0	0	all	-	q	As:oth	1985	CC	375	n	bl	n	n	0	0	ev	cig+/-ot	50	999	1	29	st
CHOI	577		f	0	0	all	-	q	As:oth	1985	CC	375	n	bl	n	n	0	0	ev	cig+/-ot	40	999	1	29	st
DAMBER	553		m	0	0	all	-	q	Eu:Sca	1972	CC	579	n	bl	y	n	1	0	ev	all/unsp	51	999	1	30	ot
DORGAN	527		m	0	0	wh	-	q	NAmer	1980	CC	2026	n	bl	y	y	2	0	ev	cig+/-ot	35	999	1	34	ot
DORGAN	523		f	0	0	all	-	q	NAmer	1980	CC	2026	n	bl	y	y	3	0	ev	cig+/-ot	35	999	1	34	ot
DOSEME	515		m	0	0	all	-	q	Eu:bal	1979	CC	1210	n	bl	n	n	2	0	ev	cig+/-ot	21	999	1	10	ot
GER	506	x	c	0	0	all	-	q+s	As:oth	1990	CC	141	n	ot	y	n	0	0	ev	all/unsp	31	999	1	30	st
HAENSZ	521	x	f	0	0	all	-	q+u	NAmer	1955	CC	158	n	bl	n	y	0	0	ev	cig+/-ot	15	999	1	14	st
JEDRYC	509		m	0	0	all	-	q	Eu:est	1980	CC	1630	n	bl	y	n	0	0	ev	cig+/-ot	50	999	1	19	st
JOLY	645		m	0	0	all	-	q	SCAmer	1978	CC	826	n	bl	n	n	0	0	ev	cig+/-ot	50	999	1	29	st
JOLY	617		f	0	0	all	-	q	SCAmer	1978	CC	826	n	bl	n	n	0	0	ev	cig+/-ot	50	999	1	29	st
KATSOU	525	x	f	0	0	all	-	KI	Eu:bal	1987	CC	101	n	bl	n	n	0	0	cu	all/unsp	30	999	1	29	st
KHUDER	530		m	0	0	all	-	q	NAmer	1985	CC	482	n	bl	n	y	5	3#	ev	cig+/-ot	30	999	1	29	or
LUBIN2	667		m	0	0	all	-	q	Eu:mul	1976	CC	7804	n	bl	n	y	0	0	ev	cig+/-ot	50	999	1	29	st
LUBIN2	719		f	0	0	all	-	q	Eu:mul	1976	CC	7804	n	bl	n	y	0	0	ev	cig+/-ot	50	999	1	29	st
LUO	503	x	c	0	0	all	-	q	As:Chi	1990	CC	102	n	ot	n	y	0	0	ev	cig+/-ot	30	999	1	29	st
MATOS	605	x	m	0	0	all	-	q	SCAmer	1994	CC	200	n	bl	n	n	0	0	ev	cig+/-ot	40	70	1	24	st
OSANN2	509	x	f	0	0	all	-	KI	NAmer	1964	ot	217	n	bl	n	y	0	0	ev	cig+/-ot	21	999	1	20	st
PEZZOT	511	x	m	0	0	all	-	q	SCAmer	1987	CC	215	n	bl	n	y	0	0	ev	cig only	41	999	1	30	st
SOBUE	508		m	0	0	all	-	q	As:Jap	1986	CC	1376	n	bl	n	y	0	0	cu	cig+/-ot	50	999	1	29	st
WUWILL	510		f	0	0	all	-	q	As:Chi	1985	CC	965	n	ot	n	n	0	0	ev	cig+/-ot	40	999	1	29	st
WYNDE2	513		m	0	0	all	-	KI	NAmer	1962	CC	404	n	bl	n	y	0	0	ev	cig+/-ot	41	999	1	29	st
ZHENG	505	x	m	0	0	all	-	q	As:Chi	1982	CC	540	n	ot	*	y	0	0	ev	cig+/-ot	40	999	1	29	st
ZHENG	510	x	f	0	0	all	-	q	As:Chi	1982	CC	540	n	ot	*	y	0	0	ev	cig+/-ot	30	999	1	29	st
ZHOU	506		c	0	0	all	-	q	As:Chi	1978	CC	1360	n	ot	n	n	0	0	ev	all/unsp	20	999	1	19	st

Comments on values in listings

KHUDER ADOS Age at starting smoking, No of cigarettes per day, Quitted smoking

Cigarette type is all/unspec for all RRs

Table 2I15 - 5

IESLC - Meta-analysis of Ever/current Smoking, Duration, "Highest vs lowest"
Squamous, Any Product (or Cigarettes if Any not available)
Least adjusted

				Number Exposed		Non-exposed			
REF	NRR	SEX	AD	Case	Cont	Case	Cont	RR	95.00%CI
BARBON	561	m	0	149	235	7	91	8.24	(3.72- 18.27)
BOUCHA	501	c	0	114	92	34	79	2.88	(1.77- 4.69)
CHOI	565	m	0	11	20	42	221	2.89	(1.29- 6.48)
CHOI	577	f	0	1	1	6	23	3.83	(0.21- 70.63)
Subtotal CHOI								2.95	(1.36- 6.42)
DAMBER	553	m	1	-	-	-	-	3.80	(1.82- 7.91)
DORGAN	527	m	2	-	-	-	-	2.77	(2.04- 3.76)
DORGAN	523	f	3	-	-	-	-	3.67	(2.52- 5.34)
Subtotal DORGAN								3.10	(2.44- 3.93)
DOSEME	515	m	2	199	-	15	-	4.08	(2.07- 8.05)
GER	506	c	0	42	119	6	37	2.18	(0.86- 5.52)
HAENSZ	521	f	0	42	77	14	26	1.01	(0.48- 2.15)
JEDRYC	509	m	0	49	214	7	68	2.22	(0.96- 5.14)
JOLY	645	m	0	98	253	15	109	2.81	(1.56- 5.07)
JOLY	617	f	0	22	20	5	54	11.88	(3.96- 35.63)
Subtotal JOLY								3.88	(2.31- 6.51)
KATSOU	525	f	0	19	6	5	12	7.60	(1.89- 30.50)
KHUDER	530	m	5	-	-	-	-	1.90	(0.90- 3.70)
LUBIN2	667	m	0	746	1460	453	2964	3.34	(2.93- 3.82)
LUBIN2	719	f	0	566	34	322	229	11.84	(8.05- 17.40)
Subtotal LUBIN2								3.82	(3.37- 4.33)
LUO	503	c	0	28	45	6	21	2.18	(0.78- 6.06)
MATOS	605	m	0	26	89	3	84	8.18	(2.39- 28.03)
OSANN2	509	f	0	101	35	11	26	6.82	(3.06- 15.23)
PEZZOT	511	m	0	45	49	5	45	8.27	(3.01- 22.66)
SOBUE	508	m	0	77	73	16	119	7.85	(4.25- 14.47)
WUWILL	510	f	0	81	114	54	139	1.83	(1.20- 2.80)
WYNDE2	513	m	0	94	89	22	55	2.64	(1.49- 4.68)
ZHENG	505	m	0	84	63	13	75	7.69	(3.92- 15.08)
ZHENG	510	f	0	35	27	8	17	2.75	(1.04- 7.33)
Subtotal ZHENG								5.53	(3.18- 9.63)
ZHOU	506	c	0	315	36	60	12	1.75	(0.86- 3.56)
Partial Totals				2944	3151	1129	4506		

*prospective study

REF	NRR	SEX	AD	Ys	Ws	Qs	Ps
BARBON	561	m	0	2.11	6.07	4.44	0.0000
BOUCHA	501	c	0	1.06	16.20	0.62	0.0000
CHOI	565	m	0	1.06	5.91	0.22	0.0098
CHOI	577	f	0	1.34	0.45	0.00	0.3661
Subtotal CHOI				1.08	6.36	0.22	
DAMBER	553	m	1	1.34	7.12	0.05	0.0004
DORGAN	527	m	2	1.02	41.10	2.27	0.0000
DORGAN	523	f	3	1.30	27.25	0.06	0.0000
Subtotal DORGAN				1.13	68.34	2.33	
DOSEME	515	m	2	1.41	8.33	0.19	0.0000
GER	506	c	0	0.78	4.43	1.00	0.1018
HAENSZ	521	f	0	0.01	6.82	10.50	0.9731
JEDRYC	509	m	0	0.80	5.48	1.13	0.0614
JOLY	645	m	0	1.03	11.11	0.53	0.0006
JOLY	617	f	0	2.47	3.18	4.75	0.0000
Subtotal JOLY				1.36	14.30	5.28	
KATSOU	525	f	0	2.03	1.99	1.19	0.0042
KHUDER	530	m	5	0.64	7.69	2.88	0.0751
LUBIN2	667	m	0	1.21	218.80	0.48	0.0000
LUBIN2	719	f	0	2.47	25.87	38.37	0.0000
Subtotal LUBIN2				1.34	244.68	38.84	
LUO	503	c	0	0.78	3.67	0.83	0.1358
MATOS	605	m	0	2.10	2.53	1.82	0.0008
OSANN2	509	f	0	1.92	5.96	2.65	0.0000
PEZZOT	511	m	0	2.11	3.78	2.78	0.0000
SOBUE	508	m	0	2.06	10.25	6.66	0.0000
WUWILL	510	f	0	0.60	21.35	9.02	0.0053
WYNDE2	513	m	0	0.97	11.69	0.93	0.0009
ZHENG	505	m	0	2.04	8.47	5.24	0.0000
ZHENG	510	f	0	1.01	4.01	0.23	0.0425
Subtotal ZHENG				1.71	12.48	5.47	
ZHOU	506	c	0	0.56	7.64	3.68	0.1220

Table 2I15 - 5

IESLC - Meta-analysis of Ever/current Smoking, Duration, "Highest vs lowest"
Squamous, Any Product (or Cigarettes if Any not available)
Least adjusted

	N	27
	NS	22
	Wt	477.15
Het	Chi	102.52
Het	df	26
Het	P	***
Fixed	RR	3.50
	RRl	3.20
	RRu	3.83
	P	+++
Random	RR	3.68
	RRl	2.94
	RRu	4.60
	P	+++
Asymm	P	N.S.

Table 2I15 - 6

IESLC - Meta-analysis of Ever/current Smoking, Duration, "Highest vs lowest"
Squamous, Any Product (or Cigarettes if Any not available)
Least adjusted

		Sex		
	combined	male	female	Total
N	4	14	9	27
NS	4	14	9	27
Wt	31.94	348.32	96.88	477.15
Het Chi	1.37	29.50	63.07	102.52
Het df	3	13	8	26
Het P	N.S.	**	***	***
Fixed RR	2.38	3.44	4.26	3.50
RRl	1.68	3.09	3.49	3.20
RRu	3.37	3.82	5.20	3.83
P	+++	+++	+++	+++
Random RR	2.38	3.81	4.21	3.68
RRl	1.68	3.03	2.22	2.94
RRu	3.37	4.77	7.99	4.60
P	+++	+++	+++	+++
Between Chi				8.58
Between df				2
Between P				*
Btwn(F) P				N.S.
Btwn(R) P				(*)

Table 2I15 - 7

IESLC - Meta-analysis of Ever/current Smoking, Duration, "Highest vs lowest"
Squamous, Any Product (or Cigarettes if Any not available)
Excluded studies (and stage at which they were excluded)

1	BECHER BLOT1 BROWN3 CARPEN CHYOU DARBY DOLL2 GARCIA GRAHAM GURSEL HAMMO2 JAHN JAIN LAUSSM PRESCO QIAO TVERDA WIGLE WYNDE3
2	ALDERS BENSHL BRESLO CHIAZZ DEAN3 DORN ENGELA GAO2 GILLIS GUO HEGMAN HIRAYA HOLE KAUFMA KOO KOULUM LIU4 MIGRAN MRFITR PERNU SEGI2 SPEIZE SUZUK2 SVENSS VUTUC WAKAI WU YUAN
3	GENG MCDUFF SPITZ STASZE WU2 ZHANG
4	AKIBA GARSHI
5	AGUDO AMANDU AMES ARMADA AUVINE AXELSS BEST BOFFET BOUCOT BROSS CEDERL CHEN2 CORREA CPSI CPSII DEAN2 DESTEF DOLL FAN GAO HAMMON HU HU2 HUMBLE JUSSAW KAISE2 KREUZE LETOUR LEVIN LIAW LIU3 LIU5 MCCONN NOTAN2 PEZZO2 PISANI QIAO2 RACHTA RESTRE SADOWS TIZZAN WANG2 WATSON WYNDE6 WYNDE7 WYNDE8
6	BUFFLE CHEN LUBIN XU
15	BENHAM

Table 2I15 - 8

Potentially overlapping studies

REF	REFGP	PRINC	.	OVERLAP/LINK
LUBIN2	LUBIN2	1		Lubin-combined
OSANN2	KAISER	2		KAISER/OSANN2

Table 2I15 - 9

Most adjusted - insufficient data for meta-analysis

REF	NRR	SEX	AGEL	AGEH	RACE	YF	LC TYPE	LOC	START	ST	NLC	R	VB	P	H	AD	ADOS	SM	PRODUCT	exL	exH	unexL	unexH	De
BUFFLE	551	m	0	0	wh	-	q	NAmer	1976	CC	943	n	bl	y	n	0	0	ev	cig+/-ot	50	999	1	33	st
CHEN	515	c	0	0	all	-	q	As:oth	1987	CC	323	n	ot	n	y	2	0	ev	cig+/-ot	41	999	1	20	st
LUBIN	617	m	0	0	all	-	KI	As:Chi	1984	CC	427	m	ot	y	n	5	1#	ev	cig+/-ot	50	999	1	29	or
XU	515	m	0	0	all	-	q+s	As:Chi	1985	CC	729	n	ot	n	n	2	0	ev	all/unsp	40	999	1	29	st

Comments on values in listings

LUBIN ADOS Duration of pipe use

REF	NRR	RR	SIG	RRDATA comment
BUFFLE	551	2.46		0
CHEN	515	4.96		0
LUBIN	617	4.72		0
XU	515	*		RR for 1-19/day is 2.2, for 20-29/day is 4.0 and for >=30/day is 4.1

Table 2I16 -

IESLC - Meta-analysis of Ever/current Smoking by Duration, Overview
Squamous, Cigarettes (or Any Product if Cigarettes not available)

This analysis is restricted to results for:
1) Ever/current smokers
2) Results by Duration
3) Categorical results by Duration
Results by Duration are grouped under 2 schemes (S1, S2). Each scheme has a set of "key values". An interval is allocated to the category whose key value it includes, and intervals which include none or more than one of the key values are excluded. (Open-ended intervals are coded as 999)

S1	key value	maximum range
1	20	1-34
2	35	21-49
3	50	36+

S2	key value	maximum range
1	5	1-19
2	20	6-29
3	30	21-39
4	40	31-49
5	50	41-998
6	999	51+

4) Squamous (or near equivalent)
5) Results complete enough for use in metaanalysis

Within each study, results are then selected (in the following order of preference, within each sex) for:
6) SMKSTA: ever, current
7) PRODUCT: cigarettes regardless of other products, cigarettes only, all/unspec
8) CIGTYPE: all/unspecified, MC regardless of HR, MC only
9) (not applicable)
10) DENOM: never smoked anything, never smoked cigarettes, never any + low, never cigs + low
11) Followup period (YF, prospective studies): whole study (coded as 0) or longest available
12) LCtype: squamous or nearest available, but not adeno. (q = squamous, s = small,
a = adeno, KI = Kreyberg I, u = undifferentiated)
13) Race: all or nearest available, otherwise by race (wh or w = white, bl or b = black, hi = hispanic
ch = chinese, jap = japanese, haw = hawaiian, w+o = white + oriental, sca = scandinavian, as = asian)
14) For overlapping studies: principal rather than subsidiary studies
Finally by Age: whole study (coded as 0) if available, otherwise by widest available age group
and then for single sex results (m, f) in preference to results for both sexes combined (c).

Results adjusted (AD) for the most potential confounders are then chosen in Sections -1 to -3
(and those which actually differ from the adjusted results in Table 2I11 - 1 are marked 'x' in Section -1)
and results adjusted for the least confounders in Sections -4 to -6. (Those least adjusted results which
actually differ from the most adjusted are marked 'x' in column X in Section -4)

Section -7 shows excluded studies, together with the stage (as above) at which no qualifying
results were found.

Section -8 lists the potentially overlapping studies which have been included (1=principal, 2=subsidiary).

Section -9 lists any results which would have been included in preference except that they had data not complete
enough for use in meta-analysis, with their significance (yes/no), if known, and any further comment as entered
on the database. It also lists as "gap" any categories for which no data were presented by the original authors.

In addition to those mentioned above, the following fields, levels and abbreviations are used:
* or nk = not known, n = no, y = yes, ot = other
ev = ever, cu = current, nev = never
all/unspec = all or unspecified, cig+/-ot = cigarettes irrespective of other products (cigar, pipe etc)
MC = manufactured cigarettes, HR = hand-rolled cigarettes
exL, exH = range of exposure (low and high) in the smoking group, in terms of Duration
REF: 6-character study reference
NRR: number of the RR on the database within the study
ST : study type (CC = case control, pr or prosp = prospective)
NLC: number of lung cancer cases in whole study
R : risky occupational population (n = no, m = mining, o = other risky)
VB : national cigarette type (V = at least 75% Virginia, bl = at least 75% blended, ot = other)
P : any proxy use
H : full histological confirmation
De : derivation of RR/CI (or = original, st = standard method, ot = other method of estimation)

Table 2I16 - 1

IESLC - Meta-analysis of Ever/current Smoking by Duration, Overview
Squamous, Cigarettes (or Any Product if Cigarettes not available)
Most adjusted

REF	NRR	2I11	SEX	AGEL	AGEH	RACE	YF	LC TYPE	LOC	START	ST	NLC	R	VB	P	H	AD	SM	PRODUCT	exL	exH	S1	S2	DENOM	De
BARBON	562		m	0	0	all	-	q	Eu:wst	1979	CC	755	n	bl	y	y	1	ev	all/unsp	1	29	1	0	nev any	or
BARBON	563		m	0	0	all	-	q	Eu:wst	1979	CC	755	n	bl	y	y	1	ev	all/unsp	30	39	2	3	nev any	or
BARBON	564		m	0	0	all	-	q	Eu:wst	1979	CC	755	n	bl	y	y	1	ev	all/unsp	40	49	0	4	nev any	or
BARBON	565		m	0	0	all	-	q	Eu:wst	1979	CC	755	n	bl	y	y	1	ev	all/unsp	50	999	3	0	nev any	or
BUFFLE	505		m	0	0	wh	-	q	NAmer	1976	CC	943	n	bl	y	n	0	ev	cig+/-ot	1	33	1	0	nev cigs	or
BUFFLE	506		m	0	0	wh	-	q	NAmer	1976	CC	943	n	bl	y	n	0	ev	cig+/-ot	34	43	2	4	nev cigs	or
BUFFLE	507		m	0	0	wh	-	q	NAmer	1976	CC	943	n	bl	y	n	0	ev	cig+/-ot	44	49	0	0	nev cigs	or
BUFFLE	508		m	0	0	wh	-	q	NAmer	1976	CC	943	n	bl	y	n	0	ev	cig+/-ot	50	999	3	0	nev cigs	or
CHOI	559		m	0	0	all	-	q	As:oth	1985	CC	375	n	bl	n	n	0	ev	cig+/-ot	1	29	1	0	nev cigs	st
CHOI	560		m	0	0	all	-	q	As:oth	1985	CC	375	n	bl	n	n	0	ev	cig+/-ot	30	39	2	3	nev cigs	st
CHOI	561		m	0	0	all	-	q	As:oth	1985	CC	375	n	bl	n	n	0	ev	cig+/-ot	40	49	0	4	nev cigs	st
CHOI	562		m	0	0	all	-	q	As:oth	1985	CC	375	n	bl	n	n	0	ev	cig+/-ot	50	999	3	0	nev cigs	st
CHOI	573		f	0	0	all	-	q	As:oth	1985	CC	375	n	bl	n	n	0	ev	cig+/-ot	1	29	1	0	nev cigs	st
CHOI	574		f	0	0	all	-	q	As:oth	1985	CC	375	n	bl	n	n	0	ev	cig+/-ot	30	39	2	3	nev cigs	st
CHOI	575		f	0	0	all	-	q	As:oth	1985	CC	375	n	bl	n	n	0	ev	cig+/-ot	40	999	3	0	nev cigs	st
DAMBER	547		m	0	0	all	-	q	Eu:Sca	1972	CC	579	n	bl	y	n	1	ev	all/unsp	1	30	1	0	nev any	or
DAMBER	548		m	0	0	all	-	q	Eu:Sca	1972	CC	579	n	bl	y	n	1	ev	all/unsp	31	40	2	4	nev any	or
DAMBER	549		m	0	0	all	-	q	Eu:Sca	1972	CC	579	n	bl	y	n	1	ev	all/unsp	41	50	3	5	nev any	or
DAMBER	550		m	0	0	all	-	q	Eu:Sca	1972	CC	579	n	bl	y	n	1	ev	all/unsp	51	999	0	6	nev any	or
DORGAN	572		m	0	0	wh	-	q	NAmer	1980	CC	2026	n	bl	y	y	2	ev	cig+/-ot	1	34	1	0	nev any	ot
DORGAN	573		m	0	0	wh	-	q	NAmer	1980	CC	2026	n	bl	y	y	2	ev	cig+/-ot	35	999	0	0	nev any	ot
DORGAN	564		f	0	0	all	-	q	NAmer	1980	CC	2026	n	bl	y	y	3	ev	cig+/-ot	1	34	1	0	nev any	ot
DORGAN	565		f	0	0	all	-	q	NAmer	1980	CC	2026	n	bl	y	y	3	ev	cig+/-ot	35	999	0	0	nev any	ot
DOSEME	511		m	0	0	all	-	q	Eu:bal	1979	CC	1210	n	bl	n	n	2	ev	cig+/-ot	1	10	0	1	nev cigs	or
DOSEME	512		m	0	0	all	-	q	Eu:bal	1979	CC	1210	n	bl	n	n	2	ev	cig+/-ot	11	20	1	2	nev cigs	or
DOSEME	513		m	0	0	all	-	q	Eu:bal	1979	CC	1210	n	bl	n	n	2	ev	cig+/-ot	21	999	0	0	nev cigs	or
GER	510		c	0	0	all	-	q+s	As:oth	1990	CC	141	n	ot	y	n	5	ev	all/unsp	1	30	1	0	nev any	ot
GER	511		c	0	0	all	-	q+s	As:oth	1990	CC	141	n	ot	y	n	5	ev	all/unsp	31	999	0	0	nev any	ot
HAENSZ	517		f	0	0	all	-	q+u	NAmer	1955	CC	158	n	bl	n	y	0	ev	cig+/-ot	1	14	0	1	nev any	st
HAENSZ	518		f	0	0	all	-	q+u	NAmer	1955	CC	158	n	bl	n	y	0	ev	cig+/-ot	15	999	0	0	nev any	st
JEDRYC	585		m	0	0	all	-	q	Eu:est	1980	CC	1630	n	bl	y	n	3	ev	cig+/-ot	1	19	0	1	nev any	or
JEDRYC	586		m	0	0	all	-	q	Eu:est	1980	CC	1630	n	bl	y	n	3	ev	cig+/-ot	20	39	0	0	nev any	or
JEDRYC	587		m	0	0	all	-	q	Eu:est	1980	CC	1630	n	bl	y	n	3	ev	cig+/-ot	40	999	3	0	nev any	or
JOLY	639		m	0	0	all	-	q	SCAmer	1978	CC	826	n	bl	n	n	0	ev	cig+/-ot	1	29	1	0	nev any	st
JOLY	640		m	0	0	all	-	q	SCAmer	1978	CC	826	n	bl	n	n	0	ev	cig+/-ot	30	39	2	3	nev any	st
JOLY	641		m	0	0	all	-	q	SCAmer	1978	CC	826	n	bl	n	n	0	ev	cig+/-ot	40	49	0	4	nev any	st
JOLY	642		m	0	0	all	-	q	SCAmer	1978	CC	826	n	bl	n	n	0	ev	cig+/-ot	50	999	3	0	nev any	st
JOLY	611		f	0	0	all	-	q	SCAmer	1978	CC	826	n	bl	n	n	0	ev	cig+/-ot	1	29	1	0	nev any	st
JOLY	612		f	0	0	all	-	q	SCAmer	1978	CC	826	n	bl	n	n	0	ev	cig+/-ot	30	39	2	3	nev any	st
JOLY	613		f	0	0	all	-	q	SCAmer	1978	CC	826	n	bl	n	n	0	ev	cig+/-ot	40	49	0	4	nev any	st
JOLY	614		f	0	0	all	-	q	SCAmer	1978	CC	826	n	bl	n	n	0	ev	cig+/-ot	50	999	3	0	nev any	st
KATSOU	528		f	0	0	all	-	KI	Eu:bal	1987	CC	101	n	bl	n	n	1	cu	all/unsp	1	29	1	0	nev any	or
KATSOU	529		f	0	0	all	-	KI	Eu:bal	1987	CC	101	n	bl	n	n	1	cu	all/unsp	30	999	0	0	nev any	or
LUBIN2	661		m	0	0	all	-	q	Eu:mul	1976	CC	7804	n	bl	n	y	0	ev	cig+/-ot	1	29	1	0	nev any	st
LUBIN2	662		m	0	0	all	-	q	Eu:mul	1976	CC	7804	n	bl	n	y	0	ev	cig+/-ot	30	39	2	3	nev any	st
LUBIN2	663		m	0	0	all	-	q	Eu:mul	1976	CC	7804	n	bl	n	y	0	ev	cig+/-ot	40	49	0	4	nev any	st
LUBIN2	664		m	0	0	all	-	q	Eu:mul	1976	CC	7804	n	bl	n	y	0	ev	cig+/-ot	50	999	3	0	nev any	st
LUBIN2	713		f	0	0	all	-	q	Eu:mul	1976	CC	7804	n	bl	n	y	0	ev	cig+/-ot	1	29	1	0	nev any	st
LUBIN2	714		f	0	0	all	-	q	Eu:mul	1976	CC	7804	n	bl	n	y	0	ev	cig+/-ot	30	39	2	3	nev any	st
LUBIN2	715		f	0	0	all	-	q	Eu:mul	1976	CC	7804	n	bl	n	y	0	ev	cig+/-ot	40	49	0	4	nev any	st
LUBIN2	716		f	0	0	all	-	q	Eu:mul	1976	CC	7804	n	bl	n	y	0	ev	cig+/-ot	50	999	3	0	nev any	st
LUO	504		c	0	0	all	-	q	As:Chi	1990	CC	102	n	ot	n	y	20	ev	cig+/-ot	1	29	1	0	nev cigs	or
LUO	505		c	0	0	all	-	q	As:Chi	1990	CC	102	n	ot	n	y	20	ev	cig+/-ot	30	999	0	0	nev cigs	or
MATOS	606		m	0	0	all	-	q	SCAmer	1994	CC	200	n	bl	n	n	2	ev	cig+/-ot	1	24	1	0	nev any	or
MATOS	607		m	0	0	all	-	q	SCAmer	1994	CC	200	n	bl	n	n	2	ev	cig+/-ot	25	39	2	3	nev any	or
MATOS	608		m	0	0	all	-	q	SCAmer	1994	CC	200	n	bl	n	n	2	ev	cig+/-ot	40	70	3	0	nev any	or
OSANN2	510		f	0	0	all	-	KI	NAmer	1964	ot	217	n	bl	n	y	1	ev	cig+/-ot	1	20	1	0	nev cigs	or
OSANN2	511		f	0	0	all	-	KI	NAmer	1964	ot	217	n	bl	n	y	1	ev	cig+/-ot	21	999	0	0	nev cigs	or
PEZZOT	507		m	0	0	all	-	q	SCAmer	1987	CC	215	n	bl	n	y	0	ev	cig only	1	30	1	0	nev cigs	ot
PEZZOT	508		m	0	0	all	-	q	SCAmer	1987	CC	215	n	bl	n	y	0	ev	cig only	31	40	2	4	nev cigs	ot
PEZZOT	509		m	0	0	all	-	q	SCAmer	1987	CC	215	n	bl	n	y	0	ev	cig only	41	999	3	0	nev cigs	ot
SOBUE	501		m	0	0	all	-	q	As:Jap	1986	CC	1376	n	bl	n	y	0	cu	cig+/-ot	1	29	1	0	nev cigs	st
SOBUE	502		m	0	0	all	-	q	As:Jap	1986	CC	1376	n	bl	n	y	0	cu	cig+/-ot	30	39	2	3	nev cigs	st
SOBUE	503		m	0	0	all	-	q	As:Jap	1986	CC	1376	n	bl	n	y	0	cu	cig+/-ot	40	49	0	4	nev cigs	st
SOBUE	504		m	0	0	all	-	q	As:Jap	1986	CC	1376	n	bl	n	y	0	cu	cig+/-ot	50	999	3	0	nev cigs	st
WUWILL	521		f	0	0	all	-	q	As:Chi	1985	CC	965	n	ot	n	n	3	ev	cig+/-ot	1	29	1	0	nev cigs	ot
WUWILL	522		f	0	0	all	-	q	As:Chi	1985	CC	965	n	ot	n	n	3	ev	cig+/-ot	30	39	2	3	nev cigs	ot
WUWILL	523		f	0	0	all	-	q	As:Chi	1985	CC	965	n	ot	n	n	3	ev	cig+/-ot	40	999	3	0	nev cigs	ot
WYNDE2	506		m	0	0	all	-	KI	NAmer	1962	CC	404	n	bl	n	y	0	ev	cig+/-ot	1	29	1	0	nev any	ot
WYNDE2	507		m	0	0	all	-	KI	NAmer	1962	CC	404	n	bl	n	y	0	ev	cig+/-ot	30	40	2	0	nev any	ot
WYNDE2	508		m	0	0	all	-	KI	NAmer	1962	CC	404	n	bl	n	y	0	ev	cig+/-ot	41	999	3	0	nev any	ot
ZHENG	501		m	0	0	all	-	q	As:Chi	1982	CC	540	n	ot	*	y	0	ev	cig+/-ot	1	29	1	0	nev cigs	st

Table 2I16 - 1

IESLC - Meta-analysis of Ever/current Smoking by Duration, Overview
Squamous, Cigarettes (or Any Product if Cigarettes not available)
Most adjusted

REF	NRR	2I11	SEX	AGEL	AGEH	RACE	YF	LC TYPE	LOC	START	ST	NLC	R	VB	P	H	AD	SM	PRODUCT	exL	exH	S1	S2	DENOM	De
ZHENG	502		m	0	0	all	-	q	As:Chi	1982	CC	540	n	ot	*	y	0	ev	cig+/-ot	30	39	2	3	nev cigs	st
ZHENG	503		m	0	0	all	-	q	As:Chi	1982	CC	540	n	ot	*	y	0	ev	cig+/-ot	40	999	3	0	nev cigs	st
ZHENG	508		f	0	0	all	-	q	As:Chi	1982	CC	540	n	ot	*	y	0	ev	cig+/-ot	1	29	1	0	nev cigs	st
ZHENG	509		f	0	0	all	-	q	As:Chi	1982	CC	540	n	ot	*	y	0	ev	cig+/-ot	30	999	0	0	nev cigs	st
ZHOU	504		c	0	0	all	-	q	As:Chi	1978	CC	1360	n	ot	n	n	0	ev	all/unsp	1	19	0	1	nev any	st
ZHOU	505		c	0	0	all	-	q	As:Chi	1978	CC	1360	n	ot	n	n	0	ev	all/unsp	20	999	0	0	nev any	st

Cigarette type is all/unspec for all RRs

In this overview table, subtotals and Qs values may be invalid and should be ignored

Table 2I16 - 2

IESLC - Meta-analysis of Ever/current Smoking by Duration, Overview
Squamous, Cigarettes (or Any Product if Cigarettes not available)
Most adjusted

REF	NRR	SEX	AD	Number Exposed Case	Cont	Non-exposed Case	Cont	RR	95.00%CI
BARBON	562	m	1	7	-	6	-	2.10	(0.70- 6.50)
BARBON	563	m	1	36	-	6	-	9.60	(3.90- 23.90)
BARBON	564	m	1	69	-	6	-	14.60	(6.10- 34.60)
BARBON	565	m	1	149	-	6	-	21.20	(9.10- 49.30)
Subtotal BARBON								10.54	(6.66- 16.67)
BUFFLE	505	m	0	-	-	-	-	9.00	(2.90- 27.90)
BUFFLE	506	m	0	-	-	-	-	14.80	(4.80- 45.30)
BUFFLE	507	m	0	-	-	-	-	12.60	(4.00- 38.80)
BUFFLE	508	m	0	-	-	-	-	22.10	(7.20- 67.70)
Subtotal BUFFLE								13.92	(7.92- 24.46)
CHOI	559	m	0	42	221	6	95	3.01	(1.24- 7.32)
CHOI	560	m	0	73	160	6	95	7.22	(3.03- 17.25)
CHOI	561	m	0	37	64	6	95	9.15	(3.65- 22.95)
CHOI	562	m	0	11	20	6	95	8.71	(2.88- 26.30)
CHOI	573	f	0	6	23	10	164	4.28	(1.42- 12.88)
CHOI	574	f	0	4	2	10	164	32.80	(5.35- 201.12)
CHOI	575	f	0	1	1	10	164	16.40	(0.95- 281.93)
Subtotal CHOI								6.58	(4.35- 9.95)
DAMBER	547	m	1	-	-	14	-	4.40	(1.80- 10.70)
DAMBER	548	m	1	-	-	14	-	8.40	(4.00- 18.30)
DAMBER	549	m	1	-	-	14	-	13.80	(6.80- 29.10)
DAMBER	550	m	1	-	-	14	-	16.70	(8.50- 34.00)
Subtotal DAMBER								10.50	(7.19- 15.34)
DORGAN	572	m	2	-	-	-	-	9.47	(3.39- 26.45)
DORGAN	573	m	2	-	-	-	-	26.21	(9.61- 71.49)
DORGAN	564	f	3	-	-	-	-	4.31	(2.53- 7.35)
DORGAN	565	f	3	-	-	-	-	15.82	(10.05- 24.90)
Subtotal DORGAN								10.17	(7.45- 13.89)
DOSEME	511	m	2	15	-	58	-	1.20	(0.60- 2.50)
DOSEME	512	m	2	70	-	58	-	3.90	(2.30- 6.70)
DOSEME	513	m	2	199	-	58	-	4.90	(3.20- 7.50)
Subtotal DOSEME								3.54	(2.62- 4.79)
GER	510	c	5	6	-	11	-	1.53	(0.40- 5.86)
GER	511	c	5	42	-	11	-	6.41	(2.03- 20.24)
Subtotal GER								3.50	(1.46- 8.37)
HAENSZ	517	f	0	14	26	44	236	2.89	(1.40- 5.96)
HAENSZ	518	f	0	42	77	44	236	2.93	(1.78- 4.80)
Subtotal HAENSZ								2.91	(1.94- 4.38)
JEDRYC	585	m	3	7	-	6	-	5.83	(1.79- 19.04)
JEDRYC	586	m	3	129	-	6	-	12.45	(5.21- 29.74)
JEDRYC	587	m	3	160	-	6	-	13.00	(5.54- 30.48)
Subtotal JEDRYC								10.80	(6.29- 18.57)
JOLY	639	m	0	15	109	2	218	15.00	(3.37- 66.77)
JOLY	640	m	0	24	165	2	218	15.85	(3.69- 68.04)
JOLY	641	m	0	66	182	2	218	39.53	(9.55- 163.60)
JOLY	642	m	0	98	253	2	218	42.22	(10.29- 173.22)
JOLY	611	f	0	5	54	6	283	4.37	(1.29- 14.82)
JOLY	612	f	0	5	24	6	283	9.83	(2.79- 34.57)
JOLY	613	f	0	16	24	6	283	31.44	(11.26- 87.78)
JOLY	614	f	0	22	20	6	283	51.88	(18.89- 142.48)
Subtotal JOLY								21.49	(13.83- 33.39)
KATSOU	528	f	1	5	-	14	-	1.77	(0.51- 6.14)
KATSOU	529	f	1	19	-	14	-	14.95	(5.06- 44.20)
Subtotal KATSOU								5.95	(2.63- 13.48)
LUBIN2	661	m	0	453	2964	54	2616	7.40	(5.56- 9.87)
LUBIN2	662	m	0	1211	3473	54	2616	16.89	(12.80- 22.29)
LUBIN2	663	m	0	1210	2540	54	2616	23.08	(17.48- 30.47)
LUBIN2	664	m	0	746	1460	54	2616	24.75	(18.64- 32.87)
LUBIN2	713	f	0	322	229	72	1180	23.04	(17.21- 30.86)
LUBIN2	714	f	0	767	186	72	1180	67.58	(50.73- 90.03)
LUBIN2	715	f	0	832	118	72	1180	115.56	(85.07- 156.96)
LUBIN2	716	f	0	566	34	72	1180	272.83	(179.26- 415.22)
Subtotal LUBIN2								31.16	(28.05- 34.61)
LUO	504	c	20	6	-	5	-	5.70	(1.00- 32.90)
LUO	505	c	20	28	-	5	-	12.50	(2.80- 55.40)
Subtotal LUO								8.97	(2.89- 27.91)
MATOS	606	m	2	3	-	3	-	1.20	(0.20- 6.20)
MATOS	607	m	2	18	-	3	-	5.80	(1.60- 20.50)
MATOS	608	m	2	26	-	3	-	18.50	(4.90- 69.80)
Subtotal MATOS								6.29	(2.80- 14.15)
OSANN2	510	f	1	11	-	7	-	4.90	(0.50- 44.60)

Table 2I16 - 2

IESLC - Meta-analysis of Ever/current Smoking by Duration, Overview
Squamous, Cigarettes (or Any Product if Cigarettes not available)
Most adjusted

REF	NRR	SEX	AD	Number Exposed Case	Number Exposed Cont	Non-exposed Case	Non-exposed Cont	RR	95.00%CI
OSANN2	511	f	1	101	-	7	-	101.10	(8.30-1230.00)
Subtotal OSANN2								18.94	(3.56- 100.64)
PEZZOT	507	m	0	5	134	0	116	9.53~	(0.52- 174.14)
PEZZOT	508	m	0	35	82	0	116	100.26~	(6.06-1657.79)
PEZZOT	509	m	0	45	101	0	116	104.45~	(6.35-1717.05)
Subtotal PEZZOT								48.16	(9.37- 247.59)
SOBUE	501	m	0	16	119	3	128	5.74	(1.63- 20.19)
SOBUE	502	m	0	59	200	3	128	12.59	(3.86- 41.00)
SOBUE	503	m	0	95	174	3	128	23.30	(7.22- 75.19)
SOBUE	504	m	0	77	73	3	128	45.00	(13.71- 147.74)
Subtotal SOBUE								17.10	(9.39- 31.14)
WUWILL	521	f	3	54	-	117	-	2.00	(1.36- 2.94)
WUWILL	522	f	3	66	-	117	-	3.88	(2.64- 5.71)
WUWILL	523	f	3	81	-	117	-	5.57	(3.79- 8.17)
Subtotal WUWILL								3.51	(2.81- 4.39)
WYNDE2	506	m	0	22	55	0	41	33.65~	(1.98- 570.85)
WYNDE2	507	m	0	30	64	0	41	39.25~	(2.34- 659.46)
WYNDE2	508	m	0	94	89	0	41	87.64~	(5.31-1446.06)
Subtotal WYNDE2								48.90	(9.61- 248.90)
ZHENG	501	m	0	13	75	4	94	4.07	(1.28- 13.01)
ZHENG	502	m	0	59	80	4	94	17.33	(6.03- 49.81)
ZHENG	503	m	0	84	63	4	94	31.33	(10.94- 89.77)
ZHENG	508	f	0	8	17	33	184	2.62	(1.05- 6.57)
ZHENG	509	f	0	35	27	33	184	7.23	(3.87- 13.49)
Subtotal ZHENG								7.80	(5.24- 11.62)
ZHOU	504	c	0	60	12	136	68	2.50	(1.26- 4.96)
ZHOU	505	c	0	315	36	136	68	4.38	(2.79- 6.87)
Subtotal ZHOU								3.69	(2.53- 5.38)
Partial Totals				8947	13830	1746	20301		

*prospective study ~ With 0.5 adjustment for zero

REF	NRR	SEX	AD	Ys	Ws	Qs	Ps
BARBON	562	m	1	0.74	3.09	10.78	0.1919
BARBON	563	m	1	2.26	4.68	0.56	0.0000
BARBON	564	m	1	2.68	5.10	0.03	0.0000
BARBON	565	m	1	3.05	5.38	1.07	0.0000
Subtotal BARBON				2.35	18.25	12.44	
BUFFLE	505	m	0	2.20	3.00	0.51	0.0001
BUFFLE	506	m	0	2.69	3.05	0.02	0.0000
BUFFLE	507	m	0	2.53	2.98	0.02	0.0000
BUFFLE	508	m	0	3.10	3.06	0.73	0.0000
Subtotal BUFFLE				2.63	12.08	1.27	
CHOI	559	m	0	1.10	4.87	11.05	0.0151
CHOI	560	m	0	1.98	5.07	2.02	0.0000
CHOI	561	m	0	2.21	4.55	0.71	0.0000
CHOI	562	m	0	2.16	3.14	0.62	0.0001
CHOI	573	f	0	1.45	3.16	4.22	0.0097
CHOI	574	f	0	3.49	1.17	0.91	0.0002
CHOI	575	f	0	2.80	0.47	0.02	0.0539
Subtotal CHOI				1.88	22.44	19.55	
DAMBER	547	m	1	1.48	4.84	6.14	0.0011
DAMBER	548	m	1	2.13	6.65	1.53	0.0000
DAMBER	549	m	1	2.62	7.27	0.00	0.0000
DAMBER	550	m	1	2.82	8.00	0.34	0.0000
Subtotal DAMBER				2.35	26.75	8.02	
DORGAN	572	m	2	2.25	3.64	0.47	0.0000
DORGAN	573	m	2	3.27	3.82	1.65	0.0000
DORGAN	564	f	3	1.46	13.51	17.80	0.0000
DORGAN	565	f	3	2.76	18.67	0.43	0.0000
Subtotal DORGAN				2.32	39.63	20.36	
DOSEME	511	m	2	0.18	7.54	44.42	0.6165
DOSEME	512	m	2	1.36	13.44	20.93	0.0000
DOSEME	513	m	2	1.59	21.18	22.02	0.0000
Subtotal DOSEME				1.26	42.17	87.36	
GER	510	c	5	0.43	2.13	10.17	0.5346
GER	511	c	5	1.86	2.91	1.64	0.0015
Subtotal GER				1.25	5.04	11.80	
HAENSZ	517	f	0	1.06	7.31	17.51	0.0041
HAENSZ	518	f	0	1.07	15.68	36.97	0.0000
Subtotal HAENSZ				1.07	22.99	54.48	

Table 2I16 - 2

IESLC - Meta-analysis of Ever/current Smoking by Duration, Overview
Squamous, Cigarettes (or Any Product if Cigarettes not available)
Most adjusted

REF	NRR	SEX	AD	Ys	Ws	Qs	Ps
JEDRYC	585	m	3	1.76	2.75	1.97	0.0035
JEDRYC	586	m	3	2.52	5.06	0.04	0.0000
JEDRYC	587	m	3	2.56	5.29	0.01	0.0000
Subtotal JEDRYC				2.38	13.10	2.01	
JOLY	639	m	0	2.71	1.72	0.02	0.0004
JOLY	640	m	0	2.76	1.81	0.04	0.0002
JOLY	641	m	0	3.68	1.90	2.17	0.0000
JOLY	642	m	0	3.74	1.93	2.48	0.0000
JOLY	611	f	0	1.47	2.57	3.31	0.0181
JOLY	612	f	0	2.29	2.43	0.25	0.0004
JOLY	613	f	0	3.45	3.64	2.57	0.0000
JOLY	614	f	0	3.95	3.76	6.76	0.0000
Subtotal JOLY				3.07	19.77	17.61	
KATSOU	528	f	1	0.57	2.48	10.31	0.3684
KATSOU	529	f	1	2.70	3.27	0.03	0.0000
Subtotal KATSOU				1.78	5.75	10.34	
LUBIN2	661	m	0	2.00	46.63	17.17	0.0000
LUBIN2	662	m	0	2.83	49.96	2.38	0.0000
LUBIN2	663	m	0	3.14	49.70	13.97	0.0000
LUBIN2	664	m	0	3.21	47.79	17.21	0.0000
LUBIN2	713	f	0	3.14	45.03	12.58	0.0000
LUBIN2	714	f	0	4.21	46.69	120.22	0.0000
LUBIN2	715	f	0	4.75	40.96	187.76	0.0000
LUBIN2	716	f	0	5.61	21.78	196.02	0.0000
Subtotal LUBIN2				3.44	348.54	567.31	
LUO	504	c	20	1.74	1.26	0.95	0.0508
LUO	505	c	20	2.53	1.72	0.01	0.0009
Subtotal LUO				2.19	2.98	0.96	
MATOS	606	m	2	0.18	1.30	7.67	0.8351
MATOS	607	m	2	1.76	2.36	1.71	0.0069
MATOS	608	m	2	2.92	2.18	0.21	0.0000
Subtotal MATOS				1.84	5.84	9.59	
OSANN2	510	f	1	1.59	0.76	0.79	0.1654
OSANN2	511	f	1	4.62	0.61	2.48	0.0003
Subtotal OSANN2				2.94	1.38	3.27	
PEZZOT	507	m	0	2.25	0.45	0.06	0.1284
PEZZOT	508	m	0	4.61	0.49	1.95	0.0013
PEZZOT	509	m	0	4.65	0.49	2.04	0.0011
Subtotal PEZZOT				3.87	1.43	4.05	
SOBUE	501	m	0	1.75	2.43	1.80	0.0065
SOBUE	502	m	0	2.53	2.75	0.02	0.0000
SOBUE	503	m	0	3.15	2.80	0.81	0.0000
SOBUE	504	m	0	3.81	2.72	3.90	0.0000
Subtotal SOBUE				2.84	10.70	6.53	
WUWILL	521	f	3	0.69	25.85	94.88	0.0004
WUWILL	522	f	3	1.36	25.82	40.53	0.0000
WUWILL	523	f	3	1.72	26.04	20.69	0.0000
Subtotal WUWILL				1.26	77.72	156.10	
WYNDE2	506	m	0	3.52	0.48	0.39	0.0149
WYNDE2	507	m	0	3.67	0.48	0.54	0.0108
WYNDE2	508	m	0	4.47	0.49	1.70	0.0018
Subtotal WYNDE2				3.89	1.45	2.64	
ZHENG	501	m	0	1.40	2.85	4.13	0.0177
ZHENG	502	m	0	2.85	3.45	0.20	0.0000
ZHENG	503	m	0	3.44	3.47	2.42	0.0000
ZHENG	508	f	0	0.96	4.55	12.31	0.0395
ZHENG	509	f	0	1.98	9.87	3.93	0.0000
Subtotal ZHENG				2.05	24.19	23.00	
ZHOU	504	c	0	0.92	8.19	23.47	0.0087
ZHOU	505	c	0	1.48	18.86	24.21	0.0000
Subtotal ZHOU				1.31	27.06	47.68	

N	78
NS	21

Table 2I16 - 3

IESLC - Meta-analysis of Ever/current Smoking by Duration, Overview
Squamous, Cigarettes (or Any Product if Cigarettes not available)
Most adjusted

	Sex			
	combined	male	female	Total
N	6	48	24	78
NS	3	14	9	26

In this overview table, other than the "N" rows, entries in the "absent" and "Total" columns may be invalid and should be ignored

	Duration of smoking (broad categories)				
	absent	1-34k20	21-49k35	36+k50	Total
N	24	23	15	16	78
NS	17	18	12	13	60
Wt	247.08	190.06	156.86	135.26	729.26
Het Chi	389.01	143.42	159.21	197.01	1066.38
Het df	23	22	14	15	77
Het P	***	***	***	***	***
Fixed RR	13.90	6.46	18.25	26.27	13.58
RRl	12.27	5.60	15.61	22.20	12.63
RRu	15.75	7.45	21.34	31.09	14.60
P	+++	+++	+++	+++	+++
Random RR	10.91	4.66	14.06	27.18	10.74
RRl	6.31	3.03	7.45	13.36	8.00
RRu	18.87	7.16	26.52	55.28	14.41
P	+++	+++	+++	+++	+++

	Duration of smoking (narrow categories)							
	absent	1-19k1	6-29k20	21-39k30	31-49k40	41-998k50	51+k999	Total
N	50	4	1	11	10	1	1	78
NS	21	4	1	8	8	1	1	43
Wt	409.72	25.79	13.44	146.19	118.84	7.27	8.00	729.26
Het Chi	550.85	6.01	0.00	153.23	95.81	0.00	0.00	1066.38
Het df	49	3	0	10	9	0	0	77
Het P	***	N.S.	N.S.	***	***	N.S.	N.S.	***
Fixed RR	10.53	2.30	3.90	18.83	36.43	13.80	16.70	13.58
RRl	9.56	1.56	2.29	16.02	30.44	6.67	8.35	12.63
RRu	11.60	3.38	6.66	22.15	43.61	28.55	33.40	14.60
P	+++	+++	+++	+++	+++	+++	+++	+++
Random RR	9.90	2.42	3.90	13.03	24.01	13.80	16.70	10.74
RRl	6.90	1.38	2.29	6.19	11.71	6.67	8.35	8.00
RRu	14.21	4.24	6.66	27.43	49.23	28.55	33.40	14.41
P	+++	++	+++	+++	+++	+++	+++	+++

Table 2I16 - 3

IESLC - Meta-analysis of Ever/current Smoking by Duration, Overview
Squamous, Cigarettes (or Any Product if Cigarettes not available)
Most adjusted

MALES

		Duration of smoking (broad categories)				
		absent	1-34k20	21-49k35	36+k50	Total
	N	12	13	11	12	48
	NS	10	13	11	12	46
	Wt	115.38	88.74	80.75	83.20	368.07
Het	Chi	87.22	18.73	10.83	11.21	208.47
Het	df	11	12	10	11	47
Het	P	***	(*)	N.S.	N.S.	***
Fixed	RR	12.43	5.87	14.19	22.50	12.21
	RRl	10.35	4.77	11.41	18.15	11.03
	RRu	14.91	7.23	17.65	27.89	13.52
	P	+++	+++	+++	+++	+++
Random	RR	11.27	5.23	13.39	22.33	11.16
	RRl	6.26	3.74	10.29	17.81	8.68
	RRu	20.29	7.32	17.41	27.99	14.35
	P	+++	+++	+++	+++	+++

			Duration of smoking (narrow categories)						
		absent	1-19k1	6-29k20	21-39k30	31-49k40	41-998k50	51+k999	Total
	N	28	2	1	7	8	1	1	48
	NS	14	2	1	7	8	1	1	33
	Wt	184.75	10.29	13.44	70.09	74.23	7.27	8.00	368.07
Het	Chi	110.22	5.03	0.00	6.58	11.73	0.00	0.00	208.47
Het	df	27	1	0	6	7	0	0	47
Het	P	***	*	N.S.	N.S.	N.S.	N.S.	N.S.	***
Fixed	RR	11.24	1.83	3.90	14.58	19.41	13.80	16.70	12.21
	RRl	9.73	0.99	2.29	11.54	15.46	6.67	8.35	11.03
	RRu	12.98	3.37	6.66	18.43	24.37	28.55	33.40	13.52
	P	+++	(+)	+++	+++	+++	+++	+++	+++
Random	RR	11.38	2.46	3.90	13.68	16.82	13.80	16.70	11.16
	RRl	7.99	0.53	2.29	10.23	11.20	6.67	8.35	8.68
	RRu	16.22	11.50	6.66	18.28	25.24	28.55	33.40	14.35
	P	+++	N.S.	+++	+++	+++	+++	+++	+++

FEMALES

		Duration of smoking (broad categories)				
		absent	1-34k20	21-49k35	36+k50	Total
	N	8	8	4	4	24
	NS	7	8	4	4	23
	Wt	100.02	97.92	76.11	52.06	326.11
Het	Chi	223.02	118.05	137.83	180.60	772.42
Het	df	7	7	3	3	23
Het	P	***	***	***	***	***
Fixed	RR	23.20	7.28	23.84	33.67	17.49
	RRl	19.07	5.97	19.04	25.66	15.69
	RRu	28.22	8.87	29.84	44.17	19.50
	P	+++	+++	+++	+++	+++
Random	RR	15.18	4.20	16.76	35.26	11.51
	RRl	4.46	1.61	2.48	2.90	5.87
	RRu	51.67	10.97	113.10	429.38	22.55
	P	+++	++	++	++	+++

Table 2I16 - 3

IESLC - Meta-analysis of Ever/current Smoking by Duration, Overview
Squamous, Cigarettes (or Any Product if Cigarettes not available)
Most adjusted

FEMALES

		Duration of smoking (narrow categories)						
	absent	1-19k1	6-29k20	21-39k30	31-49k40	41-998k50	51+k999	Total
N	17	1		4	2			24
NS	9	1		4	2			16
Wt	198.09	7.31		76.11	44.61			326.11
Het Chi	415.55	0.00		137.83	5.67			772.42
Het df	16	0		3	1			23
Het P	***	N.S.		***	*			***
Fixed RR	11.11	2.89		23.84	103.90			17.49
RRl	9.67	1.40		19.04	77.47			15.69
RRu	12.77	5.96		29.84	139.33			19.50
P	+++	++		+++	+++			+++
Random RR	9.27	2.89		16.76	66.35			11.51
RRl	4.29	1.40		2.48	18.79			5.87
RRu	20.05	5.96		113.10	234.28			22.55
P	+++	++		++	+++			+++

Table 2I16 - 4

IESLC - Meta-analysis of Ever/current Smoking by Duration, Overview
Squamous, Cigarettes (or Any Product if Cigarettes not available)
Least adjusted

REF	NRR	X	SEX	AGEL	AGEH	RACE	YF	LC TYPE	LOC	START	ST	NLC	R	VB	P	H	AD	SM	PRODUCT	exL	exH	S1	S2	DENOM	De
BARBON	555	x	m	0	0	all	-	q	Eu:wst	1979	CC	755	n	bl	y	y	0	ev	all/unsp	1	29	1	0	nev any	st
BARBON	556	x	m	0	0	all	-	q	Eu:wst	1979	CC	755	n	bl	y	y	0	ev	all/unsp	30	39	2	3	nev any	st
BARBON	557	x	m	0	0	all	-	q	Eu:wst	1979	CC	755	n	bl	y	y	0	ev	all/unsp	40	49	0	4	nev any	st
BARBON	558	x	m	0	0	all	-	q	Eu:wst	1979	CC	755	n	bl	y	y	0	ev	all/unsp	50	999	3	0	nev any	st
BUFFLE	505		m	0	0	wh	-	q	NAmer	1976	CC	943	n	bl	y	n	0	ev	cig+/-ot	1	33	1	0	nev cigs	or
BUFFLE	506		m	0	0	wh	-	q	NAmer	1976	CC	943	n	bl	y	n	0	ev	cig+/-ot	34	43	2	4	nev cigs	or
BUFFLE	507		m	0	0	wh	-	q	NAmer	1976	CC	943	n	bl	y	n	0	ev	cig+/-ot	44	49	0	0	nev cigs	or
BUFFLE	508		m	0	0	wh	-	q	NAmer	1976	CC	943	n	bl	y	n	0	ev	cig+/-ot	50	999	3	0	nev cigs	or
CHOI	559		m	0	0	all	-	q	As:oth	1985	CC	375	n	bl	n	n	0	ev	cig+/-ot	1	29	1	0	nev cigs	st
CHOI	560		m	0	0	all	-	q	As:oth	1985	CC	375	n	bl	n	n	0	ev	cig+/-ot	30	39	2	3	nev cigs	st
CHOI	561		m	0	0	all	-	q	As:oth	1985	CC	375	n	bl	n	n	0	ev	cig+/-ot	40	49	0	4	nev cigs	st
CHOI	562		m	0	0	all	-	q	As:oth	1985	CC	375	n	bl	n	n	0	ev	cig+/-ot	50	999	3	0	nev cigs	st
CHOI	573		f	0	0	all	-	q	As:oth	1985	CC	375	n	bl	n	n	0	ev	cig+/-ot	1	29	1	0	nev cigs	st
CHOI	574		f	0	0	all	-	q	As:oth	1985	CC	375	n	bl	n	n	0	ev	cig+/-ot	30	39	2	3	nev cigs	st
CHOI	575		f	0	0	all	-	q	As:oth	1985	CC	375	n	bl	n	n	0	ev	cig+/-ot	40	999	3	0	nev cigs	st
DAMBER	547		m	0	0	all	-	q	Eu:Sca	1972	CC	579	n	bl	y	n	1	ev	all/unsp	1	30	1	0	nev any	or
DAMBER	548		m	0	0	all	-	q	Eu:Sca	1972	CC	579	n	bl	y	n	1	ev	all/unsp	31	40	2	4	nev any	or
DAMBER	549		m	0	0	all	-	q	Eu:Sca	1972	CC	579	n	bl	y	n	1	ev	all/unsp	41	50	3	5	nev any	or
DAMBER	550		m	0	0	all	-	q	Eu:Sca	1972	CC	579	n	bl	y	n	1	ev	all/unsp	51	999	0	6	nev any	or
DORGAN	572		m	0	0	wh	-	q	NAmer	1980	CC	2026	n	bl	y	y	2	ev	cig+/-ot	1	34	1	0	nev any	ot
DORGAN	573		m	0	0	wh	-	q	NAmer	1980	CC	2026	n	bl	y	y	2	ev	cig+/-ot	35	999	0	0	nev any	ot
DORGAN	564		f	0	0	all	-	q	NAmer	1980	CC	2026	n	bl	y	y	3	ev	cig+/-ot	1	34	1	0	nev any	ot
DORGAN	565		f	0	0	all	-	q	NAmer	1980	CC	2026	n	bl	y	y	3	ev	cig+/-ot	35	999	0	0	nev any	ot
DOSEME	511		m	0	0	all	-	q	Eu:bal	1979	CC	1210	n	bl	n	n	2	ev	cig+/-ot	1	10	0	1	nev cigs	or
DOSEME	512		m	0	0	all	-	q	Eu:bal	1979	CC	1210	n	bl	n	n	2	ev	cig+/-ot	11	20	1	2	nev cigs	or
DOSEME	513		m	0	0	all	-	q	Eu:bal	1979	CC	1210	n	bl	n	n	2	ev	cig+/-ot	21	999	0	0	nev cigs	or
GER	504	x	c	0	0	all	-	q+s	As:oth	1990	CC	141	n	ot	y	n	0	ev	all/unsp	1	30	1	0	nev any	st
GER	505	x	c	0	0	all	-	q+s	As:oth	1990	CC	141	n	ot	y	n	0	ev	all/unsp	31	999	0	0	nev any	st
HAENSZ	517		f	0	0	all	-	q+u	NAmer	1955	CC	158	n	bl	n	y	0	ev	cig+/-ot	1	14	0	1	nev any	st
HAENSZ	518		f	0	0	all	-	q+u	NAmer	1955	CC	158	n	bl	n	y	0	ev	cig+/-ot	15	999	0	0	nev any	st
JEDRYC	501	x	m	0	0	all	-	q	Eu:est	1980	CC	1630	n	bl	y	n	0	ev	cig+/-ot	1	19	0	1	nev any	st
JEDRYC	502	x	m	0	0	all	-	q	Eu:est	1980	CC	1630	n	bl	y	n	0	ev	cig+/-ot	20	29	1	2	nev any	st
JEDRYC	503	x	m	0	0	all	-	q	Eu:est	1980	CC	1630	n	bl	y	n	0	ev	cig+/-ot	30	39	2	3	nev any	st
JEDRYC	504	x	m	0	0	all	-	q	Eu:est	1980	CC	1630	n	bl	y	n	0	ev	cig+/-ot	40	49	0	4	nev any	st
JEDRYC	505	x	m	0	0	all	-	q	Eu:est	1980	CC	1630	n	bl	y	n	0	ev	cig+/-ot	50	999	3	0	nev any	st
JOLY	639		m	0	0	all	-	q	SCAmer	1978	CC	826	n	bl	n	n	0	ev	cig+/-ot	1	29	1	0	nev any	st
JOLY	640		m	0	0	all	-	q	SCAmer	1978	CC	826	n	bl	n	n	0	ev	cig+/-ot	30	39	2	3	nev any	st
JOLY	641		m	0	0	all	-	q	SCAmer	1978	CC	826	n	bl	n	n	0	ev	cig+/-ot	40	49	0	4	nev any	st
JOLY	642		m	0	0	all	-	q	SCAmer	1978	CC	826	n	bl	n	n	0	ev	cig+/-ot	50	999	3	0	nev any	st
JOLY	611		f	0	0	all	-	q	SCAmer	1978	CC	826	n	bl	n	n	0	ev	cig+/-ot	1	29	1	0	nev any	st
JOLY	612		f	0	0	all	-	q	SCAmer	1978	CC	826	n	bl	n	n	0	ev	cig+/-ot	30	39	2	3	nev any	st
JOLY	613		f	0	0	all	-	q	SCAmer	1978	CC	826	n	bl	n	n	0	ev	cig+/-ot	40	49	0	4	nev any	st
JOLY	614		f	0	0	all	-	q	SCAmer	1978	CC	826	n	bl	n	n	0	ev	cig+/-ot	50	999	3	0	nev any	st
KATSOU	523	x	f	0	0	all	-	KI	Eu:bal	1987	CC	101	n	bl	n	n	0	cu	all/unsp	1	29	1	0	nev any	st
KATSOU	524	x	f	0	0	all	-	KI	Eu:bal	1987	CC	101	n	bl	n	n	0	cu	all/unsp	30	999	0	0	nev any	st
LUBIN2	661		m	0	0	all	-	q	Eu:mul	1976	CC	7804	n	bl	n	y	0	ev	cig+/-ot	1	29	1	0	nev any	st
LUBIN2	662		m	0	0	all	-	q	Eu:mul	1976	CC	7804	n	bl	n	y	0	ev	cig+/-ot	30	39	2	3	nev any	st
LUBIN2	663		m	0	0	all	-	q	Eu:mul	1976	CC	7804	n	bl	n	y	0	ev	cig+/-ot	40	49	0	4	nev any	st
LUBIN2	664		m	0	0	all	-	q	Eu:mul	1976	CC	7804	n	bl	n	y	0	ev	cig+/-ot	50	999	3	0	nev any	st
LUBIN2	713		f	0	0	all	-	q	Eu:mul	1976	CC	7804	n	bl	n	y	0	ev	cig+/-ot	1	29	1	0	nev any	st
LUBIN2	714		f	0	0	all	-	q	Eu:mul	1976	CC	7804	n	bl	n	y	0	ev	cig+/-ot	30	39	2	3	nev any	st
LUBIN2	715		f	0	0	all	-	q	Eu:mul	1976	CC	7804	n	bl	n	y	0	ev	cig+/-ot	40	49	0	4	nev any	st
LUBIN2	716		f	0	0	all	-	q	Eu:mul	1976	CC	7804	n	bl	n	y	0	ev	cig+/-ot	50	999	3	0	nev any	st
LUO	501	x	c	0	0	all	-	q	As:Chi	1990	CC	102	n	ot	n	y	0	ev	cig+/-ot	1	29	1	0	nev cigs	st
LUO	502	x	c	0	0	all	-	q	As:Chi	1990	CC	102	n	ot	n	y	0	ev	cig+/-ot	30	999	0	0	nev cigs	st
MATOS	601	x	m	0	0	all	-	q	SCAmer	1994	CC	200	n	bl	n	n	0	ev	cig+/-ot	1	24	1	0	nev any	st
MATOS	602	x	m	0	0	all	-	q	SCAmer	1994	CC	200	n	bl	n	n	0	ev	cig+/-ot	25	39	2	3	nev any	st
MATOS	603	x	m	0	0	all	-	q	SCAmer	1994	CC	200	n	bl	n	n	0	ev	cig+/-ot	40	70	3	0	nev any	st
OSANN2	507	x	f	0	0	all	-	KI	NAmer	1964	ot	217	n	bl	n	y	0	ev	cig+/-ot	1	20	1	0	nev cigs	st
OSANN2	508	x	f	0	0	all	-	KI	NAmer	1964	ot	217	n	bl	n	y	0	ev	cig+/-ot	21	999	0	0	nev cigs	st
PEZZOT	507		m	0	0	all	-	q	SCAmer	1987	CC	215	n	bl	n	y	0	ev	cig only	1	30	1	0	nev cigs	ot
PEZZOT	508		m	0	0	all	-	q	SCAmer	1987	CC	215	n	bl	n	y	0	ev	cig only	31	40	2	4	nev cigs	ot
PEZZOT	509		m	0	0	all	-	q	SCAmer	1987	CC	215	n	bl	n	y	0	ev	cig only	41	999	3	0	nev cigs	ot
SOBUE	501		m	0	0	all	-	q	As:Jap	1986	CC	1376	n	bl	n	y	0	cu	cig+/-ot	1	29	1	0	nev cigs	st
SOBUE	502		m	0	0	all	-	q	As:Jap	1986	CC	1376	n	bl	n	y	0	cu	cig+/-ot	30	39	2	3	nev cigs	st
SOBUE	503		m	0	0	all	-	q	As:Jap	1986	CC	1376	n	bl	n	y	0	cu	cig+/-ot	40	49	0	4	nev cigs	st
SOBUE	504		m	0	0	all	-	q	As:Jap	1986	CC	1376	n	bl	n	y	0	cu	cig+/-ot	50	999	3	0	nev cigs	st
WUWILL	506	x	f	0	0	all	-	q	As:Chi	1985	CC	965	n	ot	n	n	0	ev	cig+/-ot	1	29	1	0	nev cigs	st
WUWILL	507	x	f	0	0	all	-	q	As:Chi	1985	CC	965	n	ot	n	n	0	ev	cig+/-ot	30	39	2	3	nev cigs	st
WUWILL	508	x	f	0	0	all	-	q	As:Chi	1985	CC	965	n	ot	n	n	0	ev	cig+/-ot	40	999	3	0	nev cigs	st
WYNDE2	506		m	0	0	all	-	KI	NAmer	1962	CC	404	n	bl	n	y	0	ev	cig+/-ot	1	29	1	0	nev any	ot
WYNDE2	507		m	0	0	all	-	KI	NAmer	1962	CC	404	n	bl	n	y	0	ev	cig+/-ot	30	40	2	0	nev any	ot

Table 2I16 - 4

IESLC - Meta-analysis of Ever/current Smoking by Duration, Overview
Squamous, Cigarettes (or Any Product if Cigarettes not available)
Least adjusted

REF	NRR	X	SEX	AGEL	AGEH	RACE	YF	LC TYPE	LOC	START	ST	NLC	R	VB	P	H	AD	SM	PRODUCT	exL	exH	S1	S2	DENOM	De
WYNDE2	508		m	0	0	all	-	KI	NAmer	1962	CC	404	n	bl	n	y	0	ev	cig+/-ot	41	999	3	0	nev any	ot
ZHENG	501		m	0	0	all	-	q	As:Chi	1982	CC	540	n	ot	*	y	0	ev	cig+/-ot	1	29	1	0	nev cigs	st
ZHENG	502		m	0	0	all	-	q	As:Chi	1982	CC	540	n	ot	*	y	0	ev	cig+/-ot	30	39	2	3	nev cigs	st
ZHENG	503		m	0	0	all	-	q	As:Chi	1982	CC	540	n	ot	*	y	0	ev	cig+/-ot	40	999	3	0	nev cigs	st
ZHENG	508		f	0	0	all	-	q	As:Chi	1982	CC	540	n	ot	*	y	0	ev	cig+/-ot	1	29	1	0	nev cigs	st
ZHENG	509		f	0	0	all	-	q	As:Chi	1982	CC	540	n	ot	*	y	0	ev	cig+/-ot	30	999	0	0	nev cigs	st
ZHOU	504		c	0	0	all	-	q	As:Chi	1978	CC	1360	n	ot	n	n	0	ev	all/unsp	1	19	0	1	nev any	st
ZHOU	505		c	0	0	all	-	q	As:Chi	1978	CC	1360	n	ot	n	n	0	ev	all/unsp	20	999	0	0	nev any	st

Cigarette type is all/unspec for all RRs

In this overview table, subtotals and Qs values may be invalid and should be ignored

Table 2I16 - 5

IESLC - Meta-analysis of Ever/current Smoking by Duration, Overview
Squamous, Cigarettes (or Any Product if Cigarettes not available)
Least adjusted

REF	NRR	SEX	AD	Number Exposed Case	Cont	Non-exposed Case	Cont	RR	95.00%CI
BARBON	555	m	0	7	91	6	188	2.41	(0.79- 7.38)
BARBON	556	m	0	36	102	6	188	11.06	(4.51- 27.13)
BARBON	557	m	0	69	139	6	188	15.55	(6.57- 36.85)
BARBON	558	m	0	149	235	6	188	19.87	(8.59- 45.94)
Subtotal BARBON								11.23	(7.12- 17.72)
BUFFLE	505	m	0	-	-	-	-	9.00	(2.90- 27.90)
BUFFLE	506	m	0	-	-	-	-	14.80	(4.80- 45.30)
BUFFLE	507	m	0	-	-	-	-	12.60	(4.00- 38.80)
BUFFLE	508	m	0	-	-	-	-	22.10	(7.20- 67.70)
Subtotal BUFFLE								13.92	(7.92- 24.46)
CHOI	559	m	0	42	221	6	95	3.01	(1.24- 7.32)
CHOI	560	m	0	73	160	6	95	7.22	(3.03- 17.25)
CHOI	561	m	0	37	64	6	95	9.15	(3.65- 22.95)
CHOI	562	m	0	11	20	6	95	8.71	(2.88- 26.30)
CHOI	573	f	0	6	23	10	164	4.28	(1.42- 12.88)
CHOI	574	f	0	4	2	10	164	32.80	(5.35- 201.12)
CHOI	575	f	0	1	1	10	164	16.40	(0.95- 281.93)
Subtotal CHOI								6.58	(4.35- 9.95)
DAMBER	547	m	1	-	-	14	-	4.40	(1.80- 10.70)
DAMBER	548	m	1	-	-	14	-	8.40	(4.00- 18.30)
DAMBER	549	m	1	-	-	14	-	13.80	(6.80- 29.10)
DAMBER	550	m	1	-	-	14	-	16.70	(8.50- 34.00)
Subtotal DAMBER								10.50	(7.19- 15.34)
DORGAN	572	m	2	-	-	-	-	9.47	(3.39- 26.45)
DORGAN	573	m	2	-	-	-	-	26.21	(9.61- 71.49)
DORGAN	564	f	3	-	-	-	-	4.31	(2.53- 7.35)
DORGAN	565	f	3	-	-	-	-	15.82	(10.05- 24.90)
Subtotal DORGAN								10.17	(7.45- 13.89)
DOSEME	511	m	2	15	-	58	-	1.20	(0.60- 2.50)
DOSEME	512	m	2	70	-	58	-	3.90	(2.30- 6.70)
DOSEME	513	m	2	199	-	58	-	4.90	(3.20- 7.50)
Subtotal DOSEME								3.54	(2.62- 4.79)
GER	504	c	0	6	37	11	80	1.18	(0.41- 3.43)
GER	505	c	0	42	119	11	80	2.57	(1.25- 5.28)
Subtotal GER								2.01	(1.11- 3.66)
HAENSZ	517	f	0	14	26	44	236	2.89	(1.40- 5.96)
HAENSZ	518	f	0	42	77	44	236	2.93	(1.78- 4.80)
Subtotal HAENSZ								2.91	(1.94- 4.38)
JEDRYC	501	m	0	7	68	6	289	4.96	(1.61- 15.23)
JEDRYC	502	m	0	23	160	6	289	6.92	(2.76- 17.36)
JEDRYC	503	m	0	106	231	6	289	22.10	(9.54- 51.22)
JEDRYC	504	m	0	111	223	6	289	23.98	(10.35- 55.53)
JEDRYC	505	m	0	49	214	6	289	11.03	(4.64- 26.22)
Subtotal JEDRYC								12.77	(8.53- 19.11)
JOLY	639	m	0	15	109	2	218	15.00	(3.37- 66.77)
JOLY	640	m	0	24	165	2	218	15.85	(3.69- 68.04)
JOLY	641	m	0	66	182	2	218	39.53	(9.55- 163.60)
JOLY	642	m	0	98	253	2	218	42.22	(10.29- 173.22)
JOLY	611	f	0	5	54	6	283	4.37	(1.29- 14.82)
JOLY	612	f	0	5	24	6	283	9.83	(2.79- 34.57)
JOLY	613	f	0	16	24	6	283	31.44	(11.26- 87.78)
JOLY	614	f	0	22	20	6	283	51.88	(18.89- 142.48)
Subtotal JOLY								21.49	(13.83- 33.39)
KATSOU	523	f	0	5	12	14	67	1.99	(0.61- 6.57)
KATSOU	524	f	0	19	6	14	67	15.15	(5.13- 44.79)
Subtotal KATSOU								6.05	(2.71- 13.49)
LUBIN2	661	m	0	453	2964	54	2616	7.40	(5.56- 9.87)
LUBIN2	662	m	0	1211	3473	54	2616	16.89	(12.80- 22.29)
LUBIN2	663	m	0	1210	2540	54	2616	23.08	(17.48- 30.47)
LUBIN2	664	m	0	746	1460	54	2616	24.75	(18.64- 32.87)
LUBIN2	713	f	0	322	229	72	1180	23.04	(17.21- 30.86)
LUBIN2	714	f	0	767	186	72	1180	67.58	(50.73- 90.03)
LUBIN2	715	f	0	832	118	72	1180	115.56	(85.07- 156.96)
LUBIN2	716	f	0	566	34	72	1180	272.83	(179.26- 415.22)
Subtotal LUBIN2								31.16	(28.05- 34.61)
LUO	501	c	0	6	21	5	51	2.91	(0.80- 10.60)
LUO	502	c	0	28	45	5	51	6.35	(2.26- 17.82)
Subtotal LUO								4.68	(2.09- 10.49)
MATOS	601	m	0	3	84	3	110	1.31	(0.26- 6.65)
MATOS	602	m	0	18	110	3	110	6.00	(1.72- 20.95)
MATOS	603	m	0	26	89	3	110	10.71	(3.14- 36.55)

Table 2I16 - 5

IESLC - Meta-analysis of Ever/current Smoking by Duration, Overview
Squamous, Cigarettes (or Any Product if Cigarettes not available)
Least adjusted

REF	NRR	SEX	AD	Number Exposed Case	Cont	Non-exposed Case	Cont	RR	95.00%CI
Subtotal MATOS								5.35	(2.48- 11.58)
OSANN2	507	f	0	11	26	7	58	3.51	(1.22- 10.06)
OSANN2	508	f	0	101	35	7	58	23.91	(9.98- 57.27)
Subtotal OSANN2								10.95	(5.59- 21.45)
PEZZOT	507	m	0	5	134	0	116	9.53~	(0.52- 174.14)
PEZZOT	508	m	0	35	82	0	116	100.26~	(6.06-1657.79)
PEZZOT	509	m	0	45	101	0	116	104.45~	(6.35-1717.05)
Subtotal PEZZOT								48.16	(9.37- 247.59)
SOBUE	501	m	0	16	119	3	128	5.74	(1.63- 20.19)
SOBUE	502	m	0	59	200	3	128	12.59	(3.86- 41.00)
SOBUE	503	m	0	95	174	3	128	23.30	(7.22- 75.19)
SOBUE	504	m	0	77	73	3	128	45.00	(13.71- 147.74)
Subtotal SOBUE								17.10	(9.39- 31.14)
WUWILL	506	f	0	54	139	117	601	2.00	(1.38- 2.89)
WUWILL	507	f	0	66	98	117	601	3.46	(2.39- 5.01)
WUWILL	508	f	0	81	114	117	601	3.65	(2.58- 5.16)
Subtotal WUWILL								2.96	(2.40- 3.65)
WYNDE2	506	m	0	22	55	0	41	33.65~	(1.98- 570.85)
WYNDE2	507	m	0	30	64	0	41	39.25~	(2.34- 659.46)
WYNDE2	508	m	0	94	89	0	41	87.64~	(5.31-1446.06)
Subtotal WYNDE2								48.90	(9.61- 248.90)
ZHENG	501	m	0	13	75	4	94	4.07	(1.28- 13.01)
ZHENG	502	m	0	59	80	4	94	17.33	(6.03- 49.81)
ZHENG	503	m	0	84	63	4	94	31.33	(10.94- 89.77)
ZHENG	508	f	0	8	17	33	184	2.62	(1.05- 6.57)
ZHENG	509	f	0	35	27	33	184	7.23	(3.87- 13.49)
Subtotal ZHENG								7.80	(5.24- 11.62)
ZHOU	504	c	0	60	12	136	68	2.50	(1.26- 4.96)
ZHOU	505	c	0	315	36	136	68	4.38	(2.79- 6.87)
Subtotal ZHOU								3.69	(2.53- 5.38)
Partial Totals				8947	16228	1758	25143		

*prospective study ~ With 0.5 adjustment for zero

REF	NRR	SEX	AD	Ys	Ws	Qs	Ps
BARBON	555	m	0	0.88	3.07	8.51	0.1233
BARBON	556	m	0	2.40	4.77	0.10	0.0000
BARBON	557	m	0	2.74	5.16	0.20	0.0000
BARBON	558	m	0	2.99	5.47	1.08	0.0000
Subtotal BARBON				2.42	18.47	9.89	
BUFFLE	505	m	0	2.20	3.00	0.36	0.0001
BUFFLE	506	m	0	2.69	3.05	0.07	0.0000
BUFFLE	507	m	0	2.53	2.98	0.00	0.0000
BUFFLE	508	m	0	3.10	3.06	0.93	0.0000
Subtotal BUFFLE				2.63	12.08	1.36	
CHOI	559	m	0	1.10	4.87	10.14	0.0151
CHOI	560	m	0	1.98	5.07	1.64	0.0000
CHOI	561	m	0	2.21	4.55	0.50	0.0000
CHOI	562	m	0	2.16	3.14	0.46	0.0001
CHOI	573	f	0	1.45	3.16	3.77	0.0097
CHOI	574	f	0	3.49	1.17	1.04	0.0002
CHOI	575	f	0	2.80	0.47	0.03	0.0539
Subtotal CHOI				1.88	22.44	17.58	
DAMBER	547	m	1	1.48	4.84	5.47	0.0011
DAMBER	548	m	1	2.13	6.65	1.16	0.0000
DAMBER	549	m	1	2.62	7.27	0.05	0.0000
DAMBER	550	m	1	2.82	8.00	0.58	0.0000
Subtotal DAMBER				2.35	26.75	7.26	
DORGAN	572	m	2	2.25	3.64	0.32	0.0000
DORGAN	573	m	2	3.27	3.82	1.98	0.0000
DORGAN	564	f	3	1.46	13.51	15.89	0.0000
DORGAN	565	f	3	2.76	18.67	0.87	0.0000
Subtotal DORGAN				2.32	39.63	19.06	
DOSEME	511	m	2	0.18	7.54	42.13	0.6165
DOSEME	512	m	2	1.36	13.44	18.85	0.0000
DOSEME	513	m	2	1.59	21.18	19.36	0.0000
Subtotal DOSEME				1.26	42.17	80.34	
GER	504	c	0	0.16	3.37	19.07	0.7621
GER	505	c	0	0.94	7.37	18.94	0.0105
Subtotal GER				0.70	10.74	38.01	
HAENSZ	517	f	0	1.06	7.31	16.11	0.0041

Table 2I16 - 5

IESLC - Meta-analysis of Ever/current Smoking by Duration, Overview
Squamous, Cigarettes (or Any Product if Cigarettes not available)
Least adjusted

REF	NRR	SEX	AD	Ys	Ws	Qs	Ps
HAENSZ	518	f	0	1.07	15.68	33.97	0.0000
Subtotal HAENSZ				1.07	22.99	50.08	
JEDRYC	501	m	0	1.60	3.05	2.72	0.0052
JEDRYC	502	m	0	1.93	4.55	1.69	0.0000
JEDRYC	503	m	0	3.10	5.44	1.65	0.0000
JEDRYC	504	m	0	3.18	5.45	2.17	0.0000
JEDRYC	505	m	0	2.40	5.12	0.11	0.0000
Subtotal JEDRYC				2.55	23.61	8.34	
JOLY	639	m	0	2.71	1.72	0.05	0.0004
JOLY	640	m	0	2.76	1.81	0.09	0.0002
JOLY	641	m	0	3.68	1.90	2.44	0.0000
JOLY	642	m	0	3.74	1.93	2.76	0.0000
JOLY	611	f	0	1.47	2.57	2.95	0.0181
JOLY	612	f	0	2.29	2.43	0.16	0.0004
JOLY	613	f	0	3.45	3.64	2.97	0.0000
JOLY	614	f	0	3.95	3.76	7.42	0.0000
Subtotal JOLY				3.07	19.77	18.84	
KATSOU	523	f	0	0.69	2.70	9.31	0.2563
KATSOU	524	f	0	2.72	3.27	0.10	0.0000
Subtotal KATSOU				1.80	5.98	9.41	
LUBIN2	661	m	0	2.00	46.63	13.76	0.0000
LUBIN2	662	m	0	2.83	49.96	3.96	0.0000
LUBIN2	663	m	0	3.14	49.70	17.51	0.0000
LUBIN2	664	m	0	3.21	47.79	21.05	0.0000
LUBIN2	713	f	0	3.14	45.03	15.79	0.0000
LUBIN2	714	f	0	4.21	46.69	129.91	0.0000
LUBIN2	715	f	0	4.75	40.96	199.06	0.0000
LUBIN2	716	f	0	5.61	21.78	204.40	0.0000
Subtotal LUBIN2				3.44	348.54	605.44	
LUO	501	c	0	1.07	2.30	5.02	0.1044
LUO	502	c	0	1.85	3.60	1.75	0.0005
Subtotal LUO				1.54	5.91	6.77	
MATOS	601	m	0	0.27	1.45	7.53	0.7450
MATOS	602	m	0	1.79	2.46	1.39	0.0050
MATOS	603	m	0	2.37	2.55	0.08	0.0002
Subtotal MATOS				1.68	6.46	9.00	
OSANN2	507	f	0	1.25	3.45	5.76	0.0197
OSANN2	508	f	0	3.17	5.04	1.99	0.0000
Subtotal OSANN2				2.39	8.49	7.75	
PEZZOT	507	m	0	2.25	0.45	0.04	0.1284
PEZZOT	508	m	0	4.61	0.49	2.08	0.0013
PEZZOT	509	m	0	4.65	0.49	2.17	0.0011
Subtotal PEZZOT				3.87	1.43	4.28	
SOBUE	501	m	0	1.75	2.43	1.55	0.0065
SOBUE	502	m	0	2.53	2.75	0.00	0.0000
SOBUE	503	m	0	3.15	2.80	1.02	0.0000
SOBUE	504	m	0	3.81	2.72	4.33	0.0000
Subtotal SOBUE				2.84	10.70	6.89	
WUWILL	506	f	0	0.69	27.84	95.72	0.0003
WUWILL	507	f	0	1.24	28.12	47.83	0.0000
WUWILL	508	f	0	1.29	31.92	49.93	0.0000
Subtotal WUWILL				1.09	87.87	193.48	
WYNDE2	506	m	0	3.52	0.48	0.45	0.0149
WYNDE2	507	m	0	3.67	0.48	0.61	0.0108
WYNDE2	508	m	0	4.47	0.49	1.82	0.0018
Subtotal WYNDE2				3.89	1.45	2.88	
ZHENG	501	m	0	1.40	2.85	3.71	0.0177
ZHENG	502	m	0	2.85	3.45	0.33	0.0000
ZHENG	503	m	0	3.44	3.47	2.80	0.0000
ZHENG	508	f	0	0.96	4.55	11.38	0.0395
ZHENG	509	f	0	1.98	9.87	3.18	0.0000
Subtotal ZHENG				2.05	24.19	21.40	
ZHOU	504	c	0	0.92	8.19	21.74	0.0087
ZHOU	505	c	0	1.48	18.86	21.57	0.0000
Subtotal ZHOU				1.31	27.06	43.32	

Table 2I16 - 5

IESLC - Meta-analysis of Ever/current Smoking by Duration, Overview
Squamous, Cigarettes (or Any Product if Cigarettes not available)
Least adjusted

N	80
NS	21

Table 2I16 - 6

IESLC - Meta-analysis of Ever/current Smoking by Duration, Overview
Squamous, Cigarettes (or Any Product if Cigarettes not available)
Least adjusted

	Sex			
	combined	male	female	Total
N	6	50	24	80
NS	3	14	9	26

In this overview table, other than the "N" rows, entries in the "absent" and "Total" columns may be invalid and should be ignored

	Duration of smoking (broad categories)				
	absent	1-34k20	21-49k35	36+k50	Total
N	24	24	16	16	80
NS	17	19	13	13	62
Wt	258.59	201.91	164.79	141.43	766.72
Het Chi	412.09	152.64	174.32	257.42	1161.37
Het df	23	23	15	15	79
Het P	***	***	***	***	***
Fixed RR	13.47	6.21	17.69	21.98	12.75
RRl	11.92	5.41	15.18	18.64	11.88
RRu	15.21	7.13	20.61	25.92	13.68
P	+++	+++	+++	+++	+++
Random RR	10.24	4.53	14.56	25.20	10.22
RRl	5.96	3.01	7.85	11.48	7.63
RRu	17.61	6.83	26.99	55.32	13.68
P	+++	+++	+++	+++	+++

	Duration of smoking (narrow categories)							
	absent	1-19k1	6-29k20	21-39k30	31-49k40	41-998k50	51+k999	Total
N	49	4	2	12	11	1	1	80
NS	21	4	2	9	9	1	1	46
Wt	428.90	26.10	17.99	154.12	124.35	7.27	8.00	766.72
Het Chi	605.26	5.43	1.12	168.64	96.21	0.00	0.00	1161.37
Het df	48	3	1	11	10	0	0	79
Het P	***	N.S.	N.S.	***	***	N.S.	N.S.	***
Fixed RR	9.58	2.28	4.51	18.19	35.85	13.80	16.70	12.75
RRl	8.72	1.55	2.84	15.53	30.07	6.67	8.35	11.88
RRu	10.53	3.35	7.16	21.30	42.74	28.55	33.40	13.68
P	+++	+++	+++	+++	+++	+++	+++	+++
Random RR	8.92	2.36	4.58	13.69	24.13	13.80	16.70	10.22
RRl	6.17	1.40	2.76	6.69	12.50	6.67	8.35	7.63
RRu	12.88	4.01	7.58	28.01	46.60	28.55	33.40	13.68
P	+++	++	+++	+++	+++	+++	+++	+++

Table 2I16 - 6

IESLC - Meta-analysis of Ever/current Smoking by Duration, Overview
Squamous, Cigarettes (or Any Product if Cigarettes not available)
Least adjusted

MALES

		Duration of smoking (broad categories)				
		absent	1-34k20	21-49k35	36+k50	Total
	N	12	14	12	12	50
	NS	10	14	12	12	48
	Wt	116.12	93.42	86.38	83.49	379.41
Het	Chi	90.62	18.00	11.31	13.53	213.22
Het	df	11	13	11	11	49
Het	P	***	N.S.	N.S.	N.S.	***
Fixed	RR	12.77	5.94	14.70	21.81	12.29
	RRl	10.65	4.85	11.90	17.60	11.11
	RRu	15.32	7.28	18.15	27.03	13.59
	P	+++	+++	+++	+++	+++
Random	RR	11.83	5.43	14.45	20.61	11.26
	RRl	6.52	4.03	11.52	15.36	8.83
	RRu	21.45	7.31	18.13	27.64	14.37
	P	+++	+++	+++	+++	+++

			Duration of smoking (narrow categories)					
		absent	1-19k1	6-29k20	21-39k30	31-49k40	41-998k50	51+k999	Total
	N	27	2	2	8	9	1	1	50
	NS	14	2	2	8	9	1	1	36
	Wt	180.10	10.60	17.99	75.71	79.74	7.27	8.00	379.41
Het	Chi	107.86	4.37	1.12	6.89	11.79	0.00	0.00	213.22
Het	df	26	1	1	7	8	0	0	49
Het	P	***	*	N.S.	N.S.	N.S.	N.S.	N.S.	***
Fixed	RR	11.07	1.81	4.51	15.15	19.77	13.80	16.70	12.29
	RRl	9.57	0.99	2.84	12.09	15.87	6.67	8.35	11.11
	RRu	12.82	3.30	7.16	18.98	24.62	28.55	33.40	13.59
	P	+++	(+)	+++	+++	+++	+++	+++	+++
Random	RR	11.06	2.28	4.58	15.15	17.82	13.80	16.70	11.26
	RRl	7.69	0.57	2.76	12.09	12.60	6.67	8.35	8.83
	RRu	15.91	9.09	7.58	18.98	25.22	28.55	33.40	14.37
	P	+++	N.S.	+++	+++	+++	+++	+++	+++

FEMALES

		Duration of smoking (broad categories)				
		absent	1-34k20	21-49k35	36+k50	Total
	N	8	8	4	4	24
	NS	7	8	4	4	23
	Wt	104.44	102.82	78.41	57.94	343.61
Het	Chi	221.65	122.58	156.78	243.88	843.93
Het	df	7	7	3	3	23
Het	P	***	***	***	***	***
Fixed	RR	23.04	6.94	21.69	22.22	15.77
	RRl	19.02	5.72	17.39	17.18	14.19
	RRu	27.91	8.42	27.07	28.75	17.53
	P	+++	+++	+++	+++	+++
Random	RR	13.72	4.11	16.27	31.26	10.70
	RRl	4.30	1.63	2.23	1.93	5.45
	RRu	43.74	10.37	118.58	506.39	21.02
	P	+++	++	++	+	+++

Table 2I16 - 6

IESLC - Meta-analysis of Ever/current Smoking by Duration, Overview
Squamous, Cigarettes (or Any Product if Cigarettes not available)
Least adjusted

FEMALES

	Duration of smoking (narrow categories)							
	absent	1-19k1	6-29k20	21-39k30	31-49k40	41-998k50	51+k999	Total
N	17	1		4	2			24
NS	9	1		4	2			16
Wt	213.29	7.31		78.41	44.61			343.61
Het Chi	450.16	0.00		156.78	5.67			843.93
Het df	16	0		3	1			23
Het P	***	N.S.		***	*			***
Fixed RR	10.03	2.89		21.69	103.90			15.77
RRl	8.77	1.40		17.39	77.47			14.19
RRu	11.47	5.96		27.07	139.33			17.53
P	+++	++		+++	+++			+++
Random RR	8.52	2.89		16.27	66.35			10.70
RRl	3.99	1.40		2.23	18.79			5.45
RRu	18.15	5.96		118.58	234.28			21.02
P	+++	++		++	+++			+++

Table 2I16 - 7

IESLC - Meta-analysis of Ever/current Smoking by Duration, Overview
Squamous, Cigarettes (or Any Product if Cigarettes not available)
Excluded studies (and stage at which they were excluded)

1	BECHER BLOT1 BROWN3 CARPEN CHYOU DARBY DOLL2 GARCIA GRAHAM GURSEL HAMMO2 JAHN JAIN LAUSSM PRESCO QIAO TVERDA WIGLE WYNDE3
2	ALDERS BENSHL BRESLO CHIAZZ DEAN3 DORN ENGELA GAO2 GILLIS GUO HEGMAN HIRAYA HOLE KAUFMA KOO KOULUM LIU4 MIGRAN MRFITR PERNU SEGI2 SPEIZE SUZUK2 SVENSS VUTUC WAKAI WU YUAN
3	GENG MCDUFF SPITZ STASZE WU2 ZHANG
4	AGUDO AKIBA AMANDU AMES ARMADA AUVINE AXELSS BEST BOFFET BOUCOT BROSS CEDERL CHEN2 CORREA CPSI CPSII DEAN2 DESTEF DOLL FAN GAO GARSHI HAMMON HU HU2 HUMBLE JUSSAW KAISE2 KREUZE LETOUR LEVIN LIAW LIU3 LIU5 MCCONN NOTAN2 PEZZO2 PISANI QIAO2 RACHTA RESTRE SADOWS TIZZAN WANG2 WATSON WYNDE6 WYNDE7 WYNDE8
5	CHEN LUBIN XU
10	BOUCHA KHUDER
14	BENHAM

Table 2I16 - 8

Potentially overlapping studies

REF	REFGP	PRINC	.	OVERLAP/LINK
LUBIN2	LUBIN2	1		Lubin-combined
OSANN2	KAISER	2		KAISER/OSANN2

Table 2I16 - 9

Most adjusted - insufficient data for meta-analysis

REF	NRR	SEX	AGEL	AGEH	RACE	YF	LC TYPE	LOC	START	ST	NLC	R	VB	P	H	AD	SM	PRODUCT	exL	exH	S1	S2	DENOM	De
CHEN	501	c	0	0	all	-	q	As:oth	1987	CC	323	n	ot	n	y	2	ev	cig+/-ot	1	20	1	0	nev cigs	ot
CHEN	502	c	0	0	all	-	q	As:oth	1987	CC	323	n	ot	n	y	2	ev	cig+/-ot	21	30	0	3	nev cigs	ot
CHEN	503	c	0	0	all	-	q	As:oth	1987	CC	323	n	ot	n	y	2	ev	cig+/-ot	31	40	2	4	nev cigs	ot
CHEN	504	c	0	0	all	-	q	As:oth	1987	CC	323	n	ot	n	y	2	ev	cig+/-ot	41	999	3	0	nev cigs	ot
XU	511	m	0	0	all	-	q+s	As:Chi	1985	CC	729	n	ot	n	n	2	ev	all/unsp	1	29	1	0	nev any	or
XU	512	m	0	0	all	-	q+s	As:Chi	1985	CC	729	n	ot	n	n	2	ev	all/unsp	30	39	2	3	nev any	or
XU	513	m	0	0	all	-	q+s	As:Chi	1985	CC	729	n	ot	n	n	2	ev	all/unsp	40	999	3	0	nev any	or

REF	NRR	RR	SIG	RRDATA comment
CHEN	501	1.70	n	0
CHEN	502	2.76	n	0
CHEN	503	6.52	n	0
CHEN	504	8.43	y	p<0.001
XU	511	*		RR for 1-19/day is 2.3(p<0.05), for 20-29/day is 2.6(p<0.05) and for >=30/ day is 7.7(p<0.05)
XU	512	*		RR for 1-19/day is 2.9(p<0.05), for 20-29/day is 3.9(p<0.05) and for >=30/ day is 8.3(p<0.05)
XU	513	*		RR for 1-19/day is 5.0(p<0.05), for 20-29/day is 10.4(p<0.05) and for >=30/ day is 31.2(p<0.05)

Table 2I17 -

IESLC - Meta-analysis of Ever/current Smoking, Duration, "Low"
Squamous, Cigarettes (or Any Product if Cigarettes not available)

This analysis is restricted to results for:
1) Ever/current smokers
2) Results by Duration
3) Categorical results by Duration
4) Squamous (or near equivalent)
5) Results complete enough for use in metaanalysis

Within each study, results are then selected (in the following order of preference, within each sex) for:
6) SMKSTA: ever, current
7) PRODUCT: cigarettes regardless of other products, cigarettes only, all/unspec
8) CIGTYPE: all/unspecified, MC regardless of HR, MC only
9) (not applicable)
10) DENOM: never smoked anything, never smoked cigarettes, never any + low, never cigs + low
11) Followup period (YF, prospective studies): whole study (coded as 0) or longest available
12) LCtype: squamous or nearest available, but not adeno. (q = squamous, s = small,
a = adeno, KI = Kreyberg I, u = undifferentiated)
13) Race: all or nearest available, otherwise by race (wh or w = white, bl or b = black, hi = hispanic
ch = chinese, jap = japanese, haw = hawaiian, w+o = white + oriental, sca = scandinavian, as = asian)
14) Duration "low" in key scheme 1 (key value 20, maximum range 1-34)
15) For overlapping studies: principal rather than subsidiary studies
Finally by Age: whole study (coded as 0) if available, otherwise by widest available age group
and then for single sex results (m, f) in preference to results for both sexes combined (c).

Results adjusted (AD) for the most potential confounders are then chosen in Sections -1 to -3
(and those which actually differ from the adjusted results in Table 2I12 - 1 are marked 'x' in Section -1)
and results adjusted for the least confounders in Sections -4 to -6. (Those least adjusted results which
actually differ from the most adjusted are marked 'x' in column X in Section -4)

Section -7 shows excluded studies, together with the stage (as above) at which no qualifying
results were found.

Section -8 lists the potentially overlapping studies which have been included (1=principal, 2=subsidiary).

Section -9 lists any results which would have been included in preference except that they had data not complete
enough for use in meta-analysis, with their significance (yes/no), if known, and any further comment as entered
on the database. It also lists as "gap" any categories for which no data were presented by the original authors.

In addition to those mentioned above, the following fields, levels and abbreviations are used:
* or nk = not known, n = no, y = yes, ot = other
ev = ever, cu = current, nev = never
all/unspec = all or unspecified, cig+/-ot = cigarettes irrespective of other products (cigar, pipe etc)
MC = manufactured cigarettes, HR = hand-rolled cigarettes
exL, exH = range of exposure (low and high) in the smoking group, in terms of Duration
REF: 6-character study reference
NRR: number of the RR on the database within the study
ST : study type (CC = case control, pr or prosp = prospective)
NLC: number of lung cancer cases in whole study
R : risky occupational population (n = no, m = mining, o = other risky)
VB : national cigarette type (V = at least 75% Virginia, bl = at least 75% blended, ot = other)
P : any proxy use
H : full histological confirmation
De : derivation of RR/CI (or = original, st = standard method, ot = other method of estimation)

Table 2I17 - 1

IESLC - Meta-analysis of Ever/current Smoking, Duration, "Low"
Squamous, Cigarettes (or Any Product if Cigarettes not available)
Most adjusted

REF	NRR	2I12	SEX	AGEL	AGEH	RACE	YF	LC TYPE	LOC	START	ST	NLC	R	VB	P	H	AD	SM	PRODUCT	exL	exH	DENOM	De
BARBON	562		m	0	0	all	-	q	Eu:wst	1979	CC	755	n	bl	y	y	1	ev	all/unsp	1	29	nev any	or
BUFFLE	505		m	0	0	wh	-	q	NAmer	1976	CC	943	n	bl	y	n	0	ev	cig+/-ot	1	33	nev cigs	or
CHOI	559		m	0	0	all	-	q	As:oth	1985	CC	375	n	bl	n	n	0	ev	cig+/-ot	1	29	nev cigs	st
CHOI	573		f	0	0	all	-	q	As:oth	1985	CC	375	n	bl	n	n	0	ev	cig+/-ot	1	29	nev cigs	st
DAMBER	547		m	0	0	all	-	q	Eu:Sca	1972	CC	579	n	bl	y	n	1	ev	all/unsp	1	30	nev any	or
DORGAN	572		m	0	0	wh	-	q	NAmer	1980	CC	2026	n	bl	y	y	2	ev	cig+/-ot	1	34	nev any	ot
DORGAN	564		f	0	0	all	-	q	NAmer	1980	CC	2026	n	bl	y	y	3	ev	cig+/-ot	1	34	nev any	ot
DOSEME	512		m	0	0	all	-	q	Eu:bal	1979	CC	1210	n	bl	n	n	2	ev	cig+/-ot	11	20	nev cigs	or
GER	510		c	0	0	all	-	q+s	As:oth	1990	CC	141	n	ot	y	n	5	ev	all/unsp	1	30	nev any	ot
JEDRYC	502		m	0	0	all	-	q	Eu:est	1980	CC	1630	n	bl	y	n	0	ev	cig+/-ot	20	29	nev any	st
JOLY	639		m	0	0	all	-	q	SCAmer	1978	CC	826	n	bl	n	n	0	ev	cig+/-ot	1	29	nev any	st
JOLY	611		f	0	0	all	-	q	SCAmer	1978	CC	826	n	bl	n	n	0	ev	cig+/-ot	1	29	nev any	st
KATSOU	528		f	0	0	all	-	KI	Eu:bal	1987	CC	101	n	bl	n	n	1	cu	all/unsp	1	29	nev any	or
LUBIN2	661		m	0	0	all	-	q	Eu:mul	1976	CC	7804	n	bl	n	y	0	ev	cig+/-ot	1	29	nev any	st
LUBIN2	713		f	0	0	all	-	q	Eu:mul	1976	CC	7804	n	bl	n	y	0	ev	cig+/-ot	1	29	nev any	st
LUO	504		c	0	0	all	-	q	As:Chi	1990	CC	102	n	ot	n	y	20	ev	cig+/-ot	1	29	nev cigs	or
MATOS	606		m	0	0	all	-	q	SCAmer	1994	CC	200	n	bl	n	n	2	ev	cig+/-ot	1	24	nev any	or
OSANN2	510		f	0	0	all	-	KI	NAmer	1964	ot	217	n	bl	n	y	1	ev	cig+/-ot	1	20	nev cigs	or
PEZZOT	507		m	0	0	all	-	q	SCAmer	1987	CC	215	n	bl	n	y	0	ev	cig only	1	30	nev cigs	ot
SOBUE	501		m	0	0	all	-	q	As:Jap	1986	CC	1376	n	bl	n	y	0	cu	cig+/-ot	1	29	nev cigs	st
WUWILL	521		f	0	0	all	-	q	As:Chi	1985	CC	965	n	ot	n	n	3	ev	cig+/-ot	1	29	nev cigs	ot
WYNDE2	506		m	0	0	all	-	KI	NAmer	1962	CC	404	n	bl	n	y	0	ev	cig+/-ot	1	29	nev any	ot
ZHENG	501		m	0	0	all	-	q	As:Chi	1982	CC	540	n	ot	*	y	0	ev	cig+/-ot	1	29	nev cigs	st
ZHENG	508		f	0	0	all	-	q	As:Chi	1982	CC	540	n	ot	*	y	0	ev	cig+/-ot	1	29	nev cigs	st

Cigarette type is all/unspec for all RRs

Table 2I17 - 2

IESLC - Meta-analysis of Ever/current Smoking, Duration, "Low"
Squamous, Cigarettes (or Any Product if Cigarettes not available)
Most adjusted

REF	NRR	SEX	AD	Number Exposed Case	Number Exposed Cont	Non-exposed Case	Non-exposed Cont	RR	95.00%CI
BARBON	562	m	1	7	-	6	-	2.10	(0.70- 6.50)
BUFFLE	505	m	0	-	-	-	-	9.00	(2.90- 27.90)
CHOI	559	m	0	42	221	6	95	3.01	(1.24- 7.32)
CHOI	573	f	0	6	23	10	164	4.28	(1.42- 12.88)
Subtotal CHOI								3.46	(1.73- 6.90)
DAMBER	547	m	1	-	-	14	-	4.40	(1.80- 10.70)
DORGAN	572	m	2	-	-	-	-	9.47	(3.39- 26.45)
DORGAN	564	f	3	-	-	-	-	4.31	(2.53- 7.35)
Subtotal DORGAN								5.09	(3.17- 8.18)
DOSEME	512	m	2	70	-	58	-	3.90	(2.30- 6.70)
GER	510	c	5	6	-	11	-	1.53	(0.40- 5.86)
JEDRYC	502	m	0	23	160	6	289	6.92	(2.76- 17.36)
JOLY	639	m	0	15	109	2	218	15.00	(3.37- 66.77)
JOLY	611	f	0	5	54	6	283	4.37	(1.29- 14.82)
Subtotal JOLY								7.16	(2.78- 18.44)
KATSOU	528	f	1	5	-	14	-	1.77	(0.51- 6.14)
LUBIN2	661	m	0	453	2964	54	2616	7.40	(5.56- 9.87)
LUBIN2	713	f	0	322	229	72	1180	23.04	(17.21- 30.86)
Subtotal LUBIN2								12.93	(10.54- 15.87)
LUO	504	c	20	6	-	5	-	5.70	(1.00- 32.90)
MATOS	606	m	2	3	-	3	-	1.20	(0.20- 6.20)
OSANN2	510	f	1	11	-	7	-	4.90	(0.50- 44.60)
PEZZOT	507	m	0	5	134	0	116	9.53~	(0.52- 174.14)
SOBUE	501	m	0	16	119	3	128	5.74	(1.63- 20.19)
WUWILL	521	f	3	54	-	117	-	2.00	(1.36- 2.94)
WYNDE2	506	m	0	22	55	0	41	33.65~	(1.98- 570.85)
ZHENG	501	m	0	13	75	4	94	4.07	(1.28- 13.01)
ZHENG	508	f	0	8	17	33	184	2.62	(1.05- 6.57)
Subtotal ZHENG								3.11	(1.51- 6.39)
Partial Totals				1092	4160	431	5408		

*prospective study ~ With 0.5 adjustment for zero

REF	NRR	SEX	AD	Ys	Ws	Qs	Ps
BARBON	562	m	1	0.74	3.09	3.92	0.1919
BUFFLE	505	m	0	2.20	3.00	0.33	0.0001
CHOI	559	m	0	1.10	4.87	2.85	0.0151
CHOI	573	f	0	1.45	3.16	0.54	0.0097
Subtotal CHOI				1.24	8.03	3.39	
DAMBER	547	m	1	1.48	4.84	0.72	0.0011
DORGAN	572	m	2	2.25	3.64	0.53	0.0000
DORGAN	564	f	3	1.46	13.51	2.23	0.0000
Subtotal DORGAN				1.63	17.15	2.76	
DOSEME	512	m	2	1.36	13.44	3.44	0.0000
GER	510	c	5	0.43	2.13	4.43	0.5346
JEDRYC	502	m	0	1.93	4.55	0.02	0.0000
JOLY	639	m	0	2.71	1.72	1.22	0.0004
JOLY	611	f	0	1.47	2.57	0.40	0.0181
Subtotal JOLY				1.97	4.30	1.62	
KATSOU	528	f	1	0.57	2.48	4.17	0.3684
LUBIN2	661	m	0	2.00	46.63	0.85	0.0000
LUBIN2	713	f	0	3.14	45.03	72.67	0.0000
Subtotal LUBIN2				2.56	91.66	73.52	
LUO	504	c	20	1.74	1.26	0.02	0.0508
MATOS	606	m	2	0.18	1.30	3.70	0.8351
OSANN2	510	f	1	1.59	0.76	0.06	0.1654
PEZZOT	507	m	0	2.25	0.45	0.07	0.1284
SOBUE	501	m	0	1.75	2.43	0.04	0.0065
WUWILL	521	f	3	0.69	25.85	35.63	0.0004
WYNDE2	506	m	0	3.52	0.48	1.30	0.0149
ZHENG	501	m	0	1.40	2.85	0.61	0.0177
ZHENG	508	f	0	0.96	4.55	3.71	0.0395
Subtotal ZHENG				1.13	7.40	4.32	

Table 2I17 - 2

IESLC - Meta-analysis of Ever/current Smoking, Duration, "Low"
Squamous, Cigarettes (or Any Product if Cigarettes not available)
Most adjusted

	N	24
	NS	19
	Wt	194.61
Het	Chi	143.44
Het	df	23
Het	P	***
Fixed	RR	6.47
	RRl	5.62
	RRu	7.44
	P	+++
Random	RR	4.74
	RRl	3.14
	RRu	7.18
	P	+++
Asymm	P	N.S.

Table 2I17 - 3

IESLC - Meta-analysis of Ever/current Smoking, Duration, "Low"
Squamous, Cigarettes (or Any Product if Cigarettes not available)
Most adjusted

	Sex combined	male	female	Total
N	2	14	8	24
NS	2	14	8	24
Wt	3.39	93.29	97.92	194.61
Het Chi	1.37	18.85	118.05	143.44
Het df	1	13	7	23
Het P	N.S.	N.S.	***	***
Fixed RR	2.49	5.92	7.28	6.47
RRl	0.86	4.83	5.97	5.62
RRu	7.23	7.25	8.87	7.44
P	(+)	+++	+++	+++
Random RR	2.61	5.37	4.20	4.74
RRl	0.74	3.95	1.61	3.14
RRu	9.25	7.30	10.97	7.18
P	N.S.	+++	++	+++
Between Chi				5.17
Between df				2
Between P				(*)
Btwn(F) P				N.S.
Btwn(R) P				N.S.

Table 2I17 - 4

IESLC - Meta-analysis of Ever/current Smoking, Duration, "Low"
Squamous, Cigarettes (or Any Product if Cigarettes not available)
Least adjusted

REF	NRR	X	SEX	AGEL	AGEH	RACE	YF	LC TYPE	LOC	START	ST	NLC	R	VB	P	H	AD	SM	PRODUCT	exL	exH	DENOM	De
BARBON	555	x	m	0	0	all	-	q	Eu:wst	1979	CC	755	n	bl	y	y	0	ev	all/unsp	1	29	nev any	st
BUFFLE	505		m	0	0	wh	-	q	NAmer	1976	CC	943	n	bl	y	n	0	ev	cig+/-ot	1	33	nev cigs	or
CHOI	559		m	0	0	all	-	q	As:oth	1985	CC	375	n	bl	n	n	0	ev	cig+/-ot	1	29	nev cigs	st
CHOI	573		f	0	0	all	-	q	As:oth	1985	CC	375	n	bl	n	n	0	ev	cig+/-ot	1	29	nev cigs	st
DAMBER	547		m	0	0	all	-	q	Eu:Sca	1972	CC	579	n	bl	y	n	1	ev	all/unsp	1	30	nev any	or
DORGAN	572		m	0	0	wh	-	q	NAmer	1980	CC	2026	n	bl	y	y	2	ev	cig+/-ot	1	34	nev any	ot
DORGAN	564		f	0	0	all	-	q	NAmer	1980	CC	2026	n	bl	y	y	3	ev	cig+/-ot	1	34	nev any	ot
DOSEME	512		m	0	0	all	-	q	Eu:bal	1979	CC	1210	n	bl	n	n	2	ev	cig+/-ot	11	20	nev cigs	or
GER	504	x	c	0	0	all	-	q+s	As:oth	1990	CC	141	n	ot	y	n	0	ev	all/unsp	1	30	nev any	st
JEDRYC	502		m	0	0	all	-	q	Eu:est	1980	CC	1630	n	bl	y	n	0	ev	cig+/-ot	20	29	nev any	st
JOLY	639		m	0	0	all	-	q	SCAmer	1978	CC	826	n	bl	n	n	0	ev	cig+/-ot	1	29	nev any	st
JOLY	611		f	0	0	all	-	q	SCAmer	1978	CC	826	n	bl	n	n	0	ev	cig+/-ot	1	29	nev any	st
KATSOU	523	x	f	0	0	all	-	KI	Eu:bal	1987	CC	101	n	bl	n	n	0	cu	all/unsp	1	29	nev any	st
LUBIN2	661		m	0	0	all	-	q	Eu:mul	1976	CC	7804	n	bl	n	y	0	ev	cig+/-ot	1	29	nev any	st
LUBIN2	713		f	0	0	all	-	q	Eu:mul	1976	CC	7804	n	bl	n	y	0	ev	cig+/-ot	1	29	nev any	st
LUO	501	x	c	0	0	all	-	q	As:Chi	1990	CC	102	n	ot	n	y	0	ev	cig+/-ot	1	29	nev cigs	st
MATOS	601	x	m	0	0	all	-	q	SCAmer	1994	CC	200	n	bl	n	n	0	ev	cig+/-ot	1	24	nev any	st
OSANN2	507	x	f	0	0	all	-	KI	NAmer	1964	ot	217	n	bl	n	y	0	ev	cig+/-ot	1	20	nev cigs	st
PEZZOT	507		m	0	0	all	-	q	SCAmer	1987	CC	215	n	bl	n	y	0	ev	cig only	1	30	nev cigs	ot
SOBUE	501		m	0	0	all	-	q	As:Jap	1986	CC	1376	n	bl	n	y	0	cu	cig+/-ot	1	29	nev cigs	st
WUWILL	506	x	f	0	0	all	-	q	As:Chi	1985	CC	965	n	ot	n	n	0	ev	cig+/-ot	1	29	nev cigs	st
WYNDE2	506		m	0	0	all	-	KI	NAmer	1962	CC	404	n	bl	n	y	0	ev	cig+/-ot	1	29	nev any	ot
ZHENG	501		m	0	0	all	-	q	As:Chi	1982	CC	540	n	ot	*	y	0	ev	cig+/-ot	1	29	nev cigs	st
ZHENG	508		f	0	0	all	-	q	As:Chi	1982	CC	540	n	ot	*	y	0	ev	cig+/-ot	1	29	nev cigs	st

Cigarette type is all/unspec for all RRs

Table 2I17 - 5

IESLC - Meta-analysis of Ever/current Smoking, Duration, "Low"
Squamous, Cigarettes (or Any Product if Cigarettes not available)
Least adjusted

REF	NRR	SEX	AD	Number Exposed Case	Cont	Non-exposed Case	Cont	RR	95.00%CI
BARBON	555	m	0	7	91	6	188	2.41	(0.79- 7.38)
BUFFLE	505	m	0	-	-	-	-	9.00	(2.90- 27.90)
CHOI	559	m	0	42	221	6	95	3.01	(1.24- 7.32)
CHOI	573	f	0	6	23	10	164	4.28	(1.42- 12.88)
Subtotal CHOI								3.46	(1.73- 6.90)
DAMBER	547	m	1	-	-	14	-	4.40	(1.80- 10.70)
DORGAN	572	m	2	-	-	-	-	9.47	(3.39- 26.45)
DORGAN	564	f	3	-	-	-	-	4.31	(2.53- 7.35)
Subtotal DORGAN								5.09	(3.17- 8.18)
DOSEME	512	m	2	70	-	58	-	3.90	(2.30- 6.70)
GER	504	c	0	6	37	11	80	1.18	(0.41- 3.43)
JEDRYC	502	m	0	23	160	6	289	6.92	(2.76- 17.36)
JOLY	639	m	0	15	109	2	218	15.00	(3.37- 66.77)
JOLY	611	f	0	5	54	6	283	4.37	(1.29- 14.82)
Subtotal JOLY								7.16	(2.78- 18.44)
KATSOU	523	f	0	5	12	14	67	1.99	(0.61- 6.57)
LUBIN2	661	m	0	453	2964	54	2616	7.40	(5.56- 9.87)
LUBIN2	713	f	0	322	229	72	1180	23.04	(17.21- 30.86)
Subtotal LUBIN2								12.93	(10.54- 15.87)
LUO	501	c	0	6	21	5	51	2.91	(0.80- 10.60)
MATOS	601	m	0	3	84	3	110	1.31	(0.26- 6.65)
OSANN2	507	f	0	11	26	7	58	3.51	(1.22- 10.06)
PEZZOT	507	m	0	5	134	0	116	9.53~	(0.52- 174.14)
SOBUE	501	m	0	16	119	3	128	5.74	(1.63- 20.19)
WUWILL	506	f	0	54	139	117	601	2.00	(1.38- 2.89)
WYNDE2	506	m	0	22	55	0	41	33.65~	(1.98- 570.85)
ZHENG	501	m	0	13	75	4	94	4.07	(1.28- 13.01)
ZHENG	508	f	0	8	17	33	184	2.62	(1.05- 6.57)
Subtotal ZHENG								3.11	(1.51- 6.39)
Partial Totals				1092	4570	431	6563		

*prospective study ~ With 0.5 adjustment for zero

REF	NRR	SEX	AD	Ys	Ws	Qs	Ps
BARBON	555	m	0	0.88	3.07	2.75	0.1233
BUFFLE	505	m	0	2.20	3.00	0.41	0.0001
CHOI	559	m	0	1.10	4.87	2.55	0.0151
CHOI	573	f	0	1.45	3.16	0.44	0.0097
Subtotal CHOI				1.24	8.03	2.99	
DAMBER	547	m	1	1.48	4.84	0.57	0.0011
DORGAN	572	m	2	2.25	3.64	0.65	0.0000
DORGAN	564	f	3	1.46	13.51	1.80	0.0000
Subtotal DORGAN				1.63	17.15	2.45	
DOSEME	512	m	2	1.36	13.44	2.91	0.0000
GER	504	c	0	0.16	3.37	9.29	0.7621
JEDRYC	502	m	0	1.93	4.55	0.05	0.0000
JOLY	639	m	0	2.71	1.72	1.34	0.0004
JOLY	611	f	0	1.47	2.57	0.32	0.0181
Subtotal JOLY				1.97	4.30	1.66	
KATSOU	523	f	0	0.69	2.70	3.49	0.2563
LUBIN2	661	m	0	2.00	46.63	1.44	0.0000
LUBIN2	713	f	0	3.14	45.03	77.43	0.0000
Subtotal LUBIN2				2.56	91.66	78.88	
LUO	501	c	0	1.07	2.30	1.32	0.1044
MATOS	601	m	0	0.27	1.45	3.52	0.7450
OSANN2	507	f	0	1.25	3.45	1.13	0.0197
PEZZOT	507	m	0	2.25	0.45	0.08	0.1284
SOBUE	501	m	0	1.75	2.43	0.02	0.0065
WUWILL	506	f	0	0.69	27.84	35.87	0.0003
WYNDE2	506	m	0	3.52	0.48	1.37	0.0149
ZHENG	501	m	0	1.40	2.85	0.51	0.0177
ZHENG	508	f	0	0.96	4.55	3.38	0.0395
Subtotal ZHENG				1.13	7.40	3.89	

Table 2I17 - 5

IESLC - Meta-analysis of Ever/current Smoking, Duration, "Low"
Squamous, Cigarettes (or Any Product if Cigarettes not available)
Least adjusted

	N	24
	NS	19
	Wt	201.91
Het	Chi	152.64
Het	df	23
Het	P	***
Fixed	RR	6.21
	RRl	5.41
	RRu	7.13
	P	+++
Random	RR	4.53
	RRl	3.01
	RRu	6.83
	P	+++
Asymm	P	N.S.

Table 2I17 - 6

IESLC - Meta-analysis of Ever/current Smoking, Duration, "Low"
Squamous, Cigarettes (or Any Product if Cigarettes not available)
Least adjusted

	combined	Sex male	female	Total
N	2	14	8	24
NS	2	14	8	24
Wt	5.67	93.42	102.82	201.91
Het Chi	1.12	18.00	122.58	152.64
Het df	1	13	7	23
Het P	N.S.	N.S.	***	***
Fixed RR	1.70	5.94	6.94	6.21
RRl	0.75	4.85	5.72	5.41
RRu	3.88	7.28	8.42	7.13
P	N.S.	+++	+++	+++
Random RR	1.72	5.43	4.11	4.53
RRl	0.72	4.03	1.63	3.01
RRu	4.12	7.31	10.37	6.83
P	N.S.	+++	++	+++
Between Chi				10.95
Between df				2
Between P				**
Btwn(F) P				N.S.
Btwn(R) P				*

Table 2I17 - 7

IESLC - Meta-analysis of Ever/current Smoking, Duration, "Low"
Squamous, Cigarettes (or Any Product if Cigarettes not available)
Excluded studies (and stage at which they were excluded)

1	BECHER BLOT1 BROWN3 CARPEN CHYOU DARBY DOLL2 GARCIA GRAHAM GURSEL HAMMO2 JAHN JAIN LAUSSM PRESCO QIAO TVERDA WIGLE WYNDE3
2	ALDERS BENSHL BRESLO CHIAZZ DEAN3 DORN ENGELA GAO2 GILLIS GUO HEGMAN HIRAYA HOLE KAUFMA KOO KOULUM LIU4 MIGRAN MRFITR PERNU SEGI2 SPEIZE SUZUK2 SVENSS VUTUC WAKAI WU YUAN
3	GENG MCDUFF SPITZ STASZE WU2 ZHANG
4	AGUDO AKIBA AMANDU AMES ARMADA AUVINE AXELSS BEST BOFFET BOUCOT BROSS CEDERL CHEN2 CORREA CPSI CPSII DEAN2 DESTEF DOLL FAN GAO GARSHI HAMMON HU HU2 HUMBLE JUSSAW KAISE2 KREUZE LETOUR LEVIN LIAW LIU3 LIU5 MCCONN NOTAN2 PEZZO2 PISANI QIAO2 RACHTA RESTRE SADOWS TIZZAN WANG2 WATSON WYNDE6 WYNDE7 WYNDE8
5	CHEN LUBIN XU
10	BOUCHA KHUDER
14	HAENSZ ZHOU
15	BENHAM

Table 2I17 - 8
Potentially overlapping studies

REF	REFGP	PRINC	. OVERLAP/LINK
LUBIN2	LUBIN2	1	Lubin-combined
OSANN2	KAISER	2	KAISER/OSANN2

Table 2I17 - 9
Most adjusted - insufficient data for meta-analysis

REF	NRR	SEX	AGEL	AGEH	RACE	YF	LC TYPE	LOC	START	ST	NLC	R	VB	P	H	AD	SM	PRODUCT	exL	exH	DENOM	De
CHEN	501	c	0	0	all	-	q	As:oth	1987	CC	323	n	ot	n	y	2	ev	cig+/-ot	1	20	nev cigs	ot
XU	511	m	0	0	all	-	q+s	As:Chi	1985	CC	729	n	ot	n	n	2	ev	all/unsp	1	29	nev any	or

REF	NRR	RR	SIG	RRDATA comment
CHEN	501	1.70	n	0
XU	511	*		RR for 1-19/day is 2.3(p<0.05), for 20-29/day is 2.6(p<0.05) and for >=30/ day is 7.7(p<0.05)

Table 2I18 -

IESLC - Meta-analysis of Ever/current Smoking, Duration, "Mid"
Squamous, Cigarettes (or Any Product if Cigarettes not available)

This analysis is restricted to results for:
1) Ever/current smokers
2) Results by Duration
3) Categorical results by Duration
4) Squamous (or near equivalent)
5) Results complete enough for use in metaanalysis

Within each study, results are then selected (in the following order of preference, within each sex) for:
6) SMKSTA: ever, current
7) PRODUCT: cigarettes regardless of other products, cigarettes only, all/unspec
8) CIGTYPE: all/unspecified, MC regardless of HR, MC only
9) (not applicable)
10) DENOM: never smoked anything, never smoked cigarettes, never any + low, never cigs + low
11) Followup period (YF, prospective studies): whole study (coded as 0) or longest available
12) LCtype: squamous or nearest available, but not adeno. (q = squamous, s = small,
a = adeno, KI = Kreyberg I, u = undifferentiated)
13) Race: all or nearest available, otherwise by race (wh or w = white, bl or b = black, hi = hispanic
ch = chinese, jap = japanese, haw = hawaiian, w+o = white + oriental, sca = scandinavian, as = asian)
14) Duration "mid" in key scheme 1 (key value 35, maximum range 21-49)
15) For overlapping studies: principal rather than subsidiary studies
Finally by Age: whole study (coded as 0) if available, otherwise by widest available age group
and then for single sex results (m, f) in preference to results for both sexes combined (c).

Results adjusted (AD) for the most potential confounders are then chosen in Sections -1 to -3
(and those which actually differ from the adjusted results in Table 2I13 - 1 are marked 'x' in Section -1)
and results adjusted for the least confounders in Sections -4 to -6. (Those least adjusted results which
actually differ from the most adjusted are marked 'x' in column X in Section -4)

Section -7 shows excluded studies, together with the stage (as above) at which no qualifying
results were found.

Section -8 lists the potentially overlapping studies which have been included (1=principal, 2=subsidiary).

Section -9 lists any results which would have been included in preference except that they had data not complete
enough for use in meta-analysis, with their significance (yes/no), if known, and any further comment as entered
on the database. It also lists as "gap" any categories for which no data were presented by the original authors.

In addition to those mentioned above, the following fields, levels and abbreviations are used:
* or nk = not known, n = no, y = yes, ot = other
ev = ever, cu = current, nev = never
all/unspec = all or unspecified, cig+/-ot = cigarettes irrespective of other products (cigar, pipe etc)
MC = manufactured cigarettes, HR = hand-rolled cigarettes
exL, exH = range of exposure (low and high) in the smoking group, in terms of Duration
REF: 6-character study reference
NRR: number of the RR on the database within the study
ST : study type (CC = case control, pr or prosp = prospective)
NLC: number of lung cancer cases in whole study
R : risky occupational population (n = no, m = mining, o = other risky)
VB : national cigarette type (V = at least 75% Virginia, bl = at least 75% blended, ot = other)
P : any proxy use
H : full histological confirmation
De : derivation of RR/CI (or = original, st = standard method, ot = other method of estimation)

Table 2I18 - 1

IESLC - Meta-analysis of Ever/current Smoking, Duration, "Mid"
Squamous, Cigarettes (or Any Product if Cigarettes not available)
Most adjusted

REF	NRR	2I13	SEX	AGEL	AGEH	RACE	YF	LC TYPE	LOC	START	ST	NLC	R	VB	P	H	AD	SM	PRODUCT	exL	exH	DENOM	De
BARBON	563		m	0	0	all	-	q	Eu:wst	1979	CC	755	n	bl	y	y	1	ev	all/unsp	30	39	nev any	or
BUFFLE	506		m	0	0	wh	-	q	NAmer	1976	CC	943	n	bl	y	n	0	ev	cig+/-ot	34	43	nev cigs	or
CHOI	560		m	0	0	all	-	q	As:oth	1985	CC	375	n	bl	n	n	0	ev	cig+/-ot	30	39	nev cigs	st
CHOI	574		f	0	0	all	-	q	As:oth	1985	CC	375	n	bl	n	n	0	ev	cig+/-ot	30	39	nev cigs	st
DAMBER	548		m	0	0	all	-	q	Eu:Sca	1972	CC	579	n	bl	y	n	1	ev	all/unsp	31	40	nev any	or
JEDRYC	503		m	0	0	all	-	q	Eu:est	1980	CC	1630	n	bl	y	n	0	ev	cig+/-ot	30	39	nev any	st
JOLY	640		m	0	0	all	-	q	SCAmer	1978	CC	826	n	bl	n	n	0	ev	cig+/-ot	30	39	nev any	st
JOLY	612		f	0	0	all	-	q	SCAmer	1978	CC	826	n	bl	n	n	0	ev	cig+/-ot	30	39	nev any	st
LUBIN2	662		m	0	0	all	-	q	Eu:mul	1976	CC	7804	n	bl	n	y	0	ev	cig+/-ot	30	39	nev any	st
LUBIN2	714		f	0	0	all	-	q	Eu:mul	1976	CC	7804	n	bl	n	y	0	ev	cig+/-ot	30	39	nev any	st
MATOS	607		m	0	0	all	-	q	SCAmer	1994	CC	200	n	bl	n	n	2	ev	cig+/-ot	25	39	nev any	or
PEZZOT	508		m	0	0	all	-	q	SCAmer	1987	CC	215	n	bl	n	y	0	ev	cig only	31	40	nev cigs	ot
SOBUE	502		m	0	0	all	-	q	As:Jap	1986	CC	1376	n	bl	n	y	0	cu	cig+/-ot	30	39	nev cigs	st
WUWILL	522		f	0	0	all	-	q	As:Chi	1985	CC	965	n	ot	n	n	3	ev	cig+/-ot	30	39	nev cigs	ot
WYNDE2	507		m	0	0	all	-	KI	NAmer	1962	CC	404	n	bl	n	y	0	ev	cig+/-ot	30	40	nev any	ot
ZHENG	502		m	0	0	all	-	q	As:Chi	1982	CC	540	n	ot	*	y	0	ev	cig+/-ot	30	39	nev cigs	st

Cigarette type is all/unspec for all RRs

Table 2I18 - 2

IESLC - Meta-analysis of Ever/current Smoking, Duration, "Mid"
Squamous, Cigarettes (or Any Product if Cigarettes not available)
Most adjusted

REF	NRR	SEX	AD	Number Exposed Case	Cont	Non-exposed Case	Cont	RR	95.00%CI
BARBON	563	m	1	36	-	6	-	9.60	(3.90- 23.90)
BUFFLE	506	m	0	-	-	-	-	14.80	(4.80- 45.30)
CHOI	560	m	0	73	160	6	95	7.22	(3.03- 17.25)
CHOI	574	f	0	4	2	10	164	32.80	(5.35- 201.12)
Subtotal CHOI								9.59	(4.38- 21.01)
DAMBER	548	m	1	-	-	14	-	8.40	(4.00- 18.30)
JEDRYC	503	m	0	106	231	6	289	22.10	(9.54- 51.22)
JOLY	640	m	0	24	165	2	218	15.85	(3.69- 68.04)
JOLY	612	f	0	5	24	6	283	9.83	(2.79- 34.57)
Subtotal JOLY								12.05	(4.65- 31.23)
LUBIN2	662	m	0	1211	3473	54	2616	16.89	(12.80- 22.29)
LUBIN2	714	f	0	767	186	72	1180	67.58	(50.73- 90.03)
Subtotal LUBIN2								33.00	(27.04- 40.29)
MATOS	607	m	2	18	-	3	-	5.80	(1.60- 20.50)
PEZZOT	508	m	0	35	82	0	116	100.26~	(6.06-1657.79)
SOBUE	502	m	0	59	200	3	128	12.59	(3.86- 41.00)
WUWILL	522	f	3	66	-	117	-	3.88	(2.64- 5.71)
WYNDE2	507	m	0	30	64	0	41	39.25~	(2.34- 659.46)
ZHENG	502	m	0	59	80	4	94	17.33	(6.03- 49.81)
Partial Totals				2493	4667	303	5224		

*prospective study ~ With 0.5 adjustment for zero

REF	NRR	SEX	AD	Ys	Ws	Qs	Ps
BARBON	563	m	1	2.26	4.68	1.97	0.0000
BUFFLE	506	m	0	2.69	3.05	0.14	0.0000
CHOI	560	m	0	1.98	5.07	4.42	0.0000
CHOI	574	f	0	3.49	1.17	0.39	0.0002
Subtotal CHOI				2.26	6.24	4.81	
DAMBER	548	m	1	2.13	6.65	4.07	0.0000
JEDRYC	503	m	0	3.10	5.44	0.19	0.0000
JOLY	640	m	0	2.76	1.81	0.04	0.0002
JOLY	612	f	0	2.29	2.43	0.95	0.0004
Subtotal JOLY				2.49	4.24	0.99	
LUBIN2	662	m	0	2.83	49.96	0.35	0.0000
LUBIN2	714	f	0	4.21	46.69	79.24	0.0000
Subtotal LUBIN2				3.50	96.66	79.59	
MATOS	607	m	2	1.76	2.36	3.14	0.0069
PEZZOT	508	m	0	4.61	0.49	1.41	0.0013
SOBUE	502	m	0	2.53	2.75	0.39	0.0000
WUWILL	522	f	3	1.36	25.82	62.42	0.0000
WYNDE2	507	m	0	3.67	0.48	0.28	0.0108
ZHENG	502	m	0	2.85	3.45	0.01	0.0000

	N	16
	NS	13
	Wt	162.30
Het	Chi	159.40
Het	df	15
Het	P	***
Fixed	RR	18.37
	RRl	15.75
	RRu	21.42
	P	+++
Random	RR	14.48
	RRl	7.97
	RRu	26.33
	P	+++
Asymm	P	N.S.

Table 2I18 - 3

IESLC - Meta-analysis of Ever/current Smoking, Duration, "Mid"
Squamous, Cigarettes (or Any Product if Cigarettes not available)
Most adjusted

	Sex		
combined	male	female	Total
N	12	4	16
NS	12	4	16
Wt	86.19	76.11	162.30
Het Chi	11.83	137.83	159.40
Het df	11	3	15
Het P	N.S.	***	***
Fixed RR	14.59	23.84	18.37
RRl	11.81	19.04	15.75
RRu	18.02	29.84	21.42
P	+++	+++	+++
Random RR	14.01	16.76	14.48
RRl	10.93	2.48	7.97
RRu	17.95	113.10	26.33
P	+++	++	+++
Between Chi			9.74
Between df			1
Between P			**
Btwn(F) P			N.S.
Btwn(R) P			N.S.

Table 2I18 - 4

IESLC - Meta-analysis of Ever/current Smoking, Duration, "Mid"
Squamous, Cigarettes (or Any Product if Cigarettes not available)
Least adjusted

REF	NRR	X	SEX	AGEL	AGEH	RACE	YF	LC TYPE	LOC	START	ST	NLC	R	VB	P	H	AD	SM	PRODUCT	exL	exH	DENOM	De
BARBON	556	x	m	0	0	all	-	q	Eu:wst	1979	CC	755	n	bl	y	y	0	ev	all/unsp	30	39	nev any	st
BUFFLE	506		m	0	0	wh	-	q	NAmer	1976	CC	943	n	bl	y	n	0	ev	cig+/-ot	34	43	nev cigs	or
CHOI	560		m	0	0	all	-	q	As:oth	1985	CC	375	n	bl	n	n	0	ev	cig+/-ot	30	39	nev cigs	st
CHOI	574		f	0	0	all	-	q	As:oth	1985	CC	375	n	bl	n	n	0	ev	cig+/-ot	30	39	nev cigs	st
DAMBER	548		m	0	0	all	-	q	Eu:Sca	1972	CC	579	n	bl	y	n	1	ev	all/unsp	31	40	nev any	or
JEDRYC	503		m	0	0	all	-	q	Eu:est	1980	CC	1630	n	bl	y	n	0	ev	cig+/-ot	30	39	nev any	st
JOLY	640		m	0	0	all	-	q	SCAmer	1978	CC	826	n	bl	n	n	0	ev	cig+/-ot	30	39	nev any	st
JOLY	612		f	0	0	all	-	q	SCAmer	1978	CC	826	n	bl	n	n	0	ev	cig+/-ot	30	39	nev any	st
LUBIN2	662		m	0	0	all	-	q	Eu:mul	1976	CC	7804	n	bl	n	y	0	ev	cig+/-ot	30	39	nev any	st
LUBIN2	714		f	0	0	all	-	q	Eu:mul	1976	CC	7804	n	bl	n	y	0	ev	cig+/-ot	30	39	nev any	st
MATOS	602	x	m	0	0	all	-	q	SCAmer	1994	CC	200	n	bl	n	n	0	ev	cig+/-ot	25	39	nev any	st
PEZZOT	508		m	0	0	all	-	q	SCAmer	1987	CC	215	n	bl	n	y	0	ev	cig only	31	40	nev cigs	ot
SOBUE	502		m	0	0	all	-	q	As:Jap	1986	CC	1376	n	bl	n	y	0	cu	cig+/-ot	30	39	nev cigs	st
WUWILL	507	x	f	0	0	all	-	q	As:Chi	1985	CC	965	n	ot	n	n	0	ev	cig+/-ot	30	39	nev cigs	st
WYNDE2	507		m	0	0	all	-	KI	NAmer	1962	CC	404	n	bl	n	y	0	ev	cig+/-ot	30	40	nev any	ot
ZHENG	502		m	0	0	all	-	q	As:Chi	1982	CC	540	n	ot	*	y	0	ev	cig+/-ot	30	39	nev cigs	st

Cigarette type is all/unspec for all RRs

Table 2I18 - 5

IESLC - Meta-analysis of Ever/current Smoking, Duration, "Mid"
Squamous, Cigarettes (or Any Product if Cigarettes not available)
Least adjusted

REF	NRR	SEX	AD	Number Exposed Case	Number Exposed Cont	Non-exposed Case	Non-exposed Cont	RR	95.00%CI
BARBON	556	m	0	36	102	6	188	11.06	(4.51- 27.13)
BUFFLE	506	m	0	-	-	-	-	14.80	(4.80- 45.30)
CHOI	560	m	0	73	160	6	95	7.22	(3.03- 17.25)
CHOI	574	f	0	4	2	10	164	32.80	(5.35- 201.12)
Subtotal CHOI								9.59	(4.38- 21.01)
DAMBER	548	m	1	-	-	14	-	8.40	(4.00- 18.30)
JEDRYC	503	m	0	106	231	6	289	22.10	(9.54- 51.22)
JOLY	640	m	0	24	165	2	218	15.85	(3.69- 68.04)
JOLY	612	f	0	5	24	6	283	9.83	(2.79- 34.57)
Subtotal JOLY								12.05	(4.65- 31.23)
LUBIN2	662	m	0	1211	3473	54	2616	16.89	(12.80- 22.29)
LUBIN2	714	f	0	767	186	72	1180	67.58	(50.73- 90.03)
Subtotal LUBIN2								33.00	(27.04- 40.29)
MATOS	602	m	0	18	110	3	110	6.00	(1.72- 20.95)
PEZZOT	508	m	0	35	82	0	116	100.26~	(6.06-1657.79)
SOBUE	502	m	0	59	200	3	128	12.59	(3.86- 41.00)
WUWILL	507	f	0	66	98	117	601	3.46	(2.39- 5.01)
WYNDE2	507	m	0	30	64	0	41	39.25~	(2.34- 659.46)
ZHENG	502	m	0	59	80	4	94	17.33	(6.03- 49.81)
Partial Totals				2493	4977	303	6123		

*prospective study ~ With 0.5 adjustment for zero

REF	NRR	SEX	AD	Ys	Ws	Qs	Ps
BARBON	556	m	0	2.40	4.77	1.05	0.0000
BUFFLE	506	m	0	2.69	3.05	0.10	0.0000
CHOI	560	m	0	1.98	5.07	4.07	0.0000
CHOI	574	f	0	3.49	1.17	0.45	0.0002
Subtotal CHOI				2.26	6.24	4.51	
DAMBER	548	m	1	2.13	6.65	3.69	0.0000
JEDRYC	503	m	0	3.10	5.44	0.27	0.0000
JOLY	640	m	0	2.76	1.81	0.02	0.0002
JOLY	612	f	0	2.29	2.43	0.84	0.0004
Subtotal JOLY				2.49	4.24	0.86	
LUBIN2	662	m	0	2.83	49.96	0.11	0.0000
LUBIN2	714	f	0	4.21	46.69	83.89	0.0000
Subtotal LUBIN2				3.50	96.66	84.00	
MATOS	602	m	0	1.79	2.46	2.87	0.0050
PEZZOT	508	m	0	4.61	0.49	1.47	0.0013
SOBUE	502	m	0	2.53	2.75	0.32	0.0000
WUWILL	507	f	0	1.24	28.12	74.87	0.0000
WYNDE2	507	m	0	3.67	0.48	0.31	0.0108
ZHENG	502	m	0	2.85	3.45	0.00	0.0000

	N	16
	NS	13
	Wt	164.79
Het	Chi	174.32
Het	df	15
Het	P	***
Fixed	RR	17.69
	RRl	15.18
	RRu	20.61
	P	+++
Random	RR	14.56
	RRl	7.85
	RRu	26.99
	P	+++
Asymm	P	N.S.

Table 2I18 - 6

IESLC - Meta-analysis of Ever/current Smoking, Duration, "Mid"
Squamous, Cigarettes (or Any Product if Cigarettes not available)
Least adjusted

	Sex combined	male	female	Total
N		12	4	16
NS		12	4	16
Wt		86.38	78.41	164.79
Het Chi		11.31	156.78	174.32
Het df		11	3	15
Het P		N.S.	***	***
Fixed RR		14.70	21.69	17.69
RRl		11.90	17.39	15.18
RRu		18.15	27.07	20.61
P		+++	+++	+++
Random RR		14.45	16.27	14.56
RRl		11.52	2.23	7.85
RRu		18.13	118.58	26.99
P		+++	++	+++
Between Chi				6.23
Between df				1
Between P				*
Btwn(F) P				N.S.
Btwn(R) P				N.S.

Table 2I18 - 7

IESLC - Meta-analysis of Ever/current Smoking, Duration, "Mid"
Squamous, Cigarettes (or Any Product if Cigarettes not available)
Excluded studies (and stage at which they were excluded)

1	BECHER BLOT1 BROWN3 CARPEN CHYOU DARBY DOLL2 GARCIA GRAHAM GURSEL HAMMO2 JAHN JAIN LAUSSM PRESCO QIAO TVERDA WIGLE WYNDE3
2	ALDERS BENSHL BRESLO CHIAZZ DEAN3 DORN ENGELA GAO2 GILLIS GUO HEGMAN HIRAYA HOLE KAUFMA KOO KOULUM LIU4 MIGRAN MRFITR PERNU SEGI2 SPEIZE SUZUK2 SVENSS VUTUC WAKAI WU YUAN
3	GENG MCDUFF SPITZ STASZE WU2 ZHANG
4	AGUDO AKIBA AMANDU AMES ARMADA AUVINE AXELSS BEST BOFFET BOUCOT BROSS CEDERL CHEN2 CORREA CPSI CPSII DEAN2 DESTEF DOLL FAN GAO GARSHI HAMMON HU HU2 HUMBLE JUSSAW KAISE2 KREUZE LETOUR LEVIN LIAW LIU3 LIU5 MCCONN NOTAN2 PEZZO2 PISANI QIAO2 RACHTA RESTRE SADOWS TIZZAN WANG2 WATSON WYNDE6 WYNDE7 WYNDE8
5	CHEN LUBIN XU
10	BOUCHA KHUDER
14	DORGAN DOSEME GER HAENSZ KATSOU LUO OSANN2 ZHOU
15	BENHAM

Table 2I18 - 8

Potentially overlapping studies

REF	REFGP	PRINC .	OVERLAP/LINK
LUBIN2	LUBIN2	1	Lubin-combined

Table 2I18 - 9

Most adjusted - insufficient data for meta-analysis

REF	NRR	SEX	AGEL	AGEH	RACE	YF	LC TYPE	LOC	START	ST	NLC	R	VB	P	H	AD	SM	PRODUCT	exL	exH	DENOM	De
CHEN	503	c	0	0	all	-	q	As:oth	1987	CC	323	n	ot	n	y	2	ev	cig+/-ot	31	40	nev cigs	ot
XU	512	m	0	0	all	-	q+s	As:Chi	1985	CC	729	n	ot	n	n	2	ev	all/unsp	30	39	nev any	or

REF	NRR	RR	SIG	RRDATA comment
CHEN	503	6.52	n	0
XU	512	*		RR for 1-19/day is 2.9(p<0.05), for 20-29/day is 3.9(p<0.05) and for >=30/ day is 8.3(p<0.05)

Table 2I19 -

IESLC - Meta-analysis of Ever/current Smoking, Duration, "High"
Squamous, Cigarettes (or Any Product if Cigarettes not available)

This analysis is restricted to results for:
1) Ever/current smokers
2) Results by Duration
3) Categorical results by Duration
4) Squamous (or near equivalent)
5) Results complete enough for use in metaanalysis

Within each study, results are then selected (in the following order of preference, within each sex) for:
6) PRODUCT: cigarettes regardless of other products, cigarettes only, all/unspec
7) CIGTYPE: all/unspecified, MC regardless of HR, MC only
8) (not applicable)
9) DENOM: never smoked anything, never smoked cigarettes, never any + low, never cigs + low
10) Followup period (YF, prospective studies): whole study (coded as 0) or longest available
11) LCtype: squamous or nearest available, but not adeno. (q = squamous, s = small,
a = adeno, KI = Kreyberg I, u = undifferentiated)
12) Race: all or nearest available, otherwise by race (wh or w = white, bl or b = black, hi = hispanic
ch = chinese, jap = japanese, haw = hawaiian, w+o = white + oriental, sca = scandinavian, as = asian)
13) Duration "high" in key scheme 1 (key value 50, maximum range 36+)
14) For overlapping studies: principal rather than subsidiary studies
Finally by Age: whole study (coded as 0) if available, otherwise by widest available age group
and then for single sex results (m, f) in preference to results for both sexes combined (c).

Results adjusted (AD) for the most potential confounders are then chosen in Sections -1 to -3
(and those which actually differ from the adjusted results in Table 2I14 - 1 are marked 'x' in Section -1)
and results adjusted for the least confounders in Sections -4 to -6. (Those least adjusted results which
actually differ from the most adjusted are marked 'x' in column X in Section -4)

Section -7 shows excluded studies, together with the stage (as above) at which no qualifying
results were found.

Section -8 lists the potentially overlapping studies which have been included (1=principal, 2=subsidiary).

Section -9 lists any results which would have been included in preference except that they had data not complete
enough for use in meta-analysis, with their significance (yes/no), if known, and any further comment as entered
on the database. It also lists as "gap" any categories for which no data were presented by the original authors.

In addition to those mentioned above, the following fields, levels and abbreviations are used:
* or nk = not known, n = no, y = yes, ot = other
ev = ever, cu = current, nev = never
all/unspec = all or unspecified, cig+/-ot = cigarettes irrespective of other products (cigar, pipe etc)
MC = manufactured cigarettes, HR = hand-rolled cigarettes
exL, exH = range of exposure (low and high) in the smoking group, in terms of Duration
REF: 6-character study reference
NRR: number of the RR on the database within the study
ST : study type (CC = case control, pr or prosp = prospective)
NLC: number of lung cancer cases in whole study
R : risky occupational population (n = no, m = mining, o = other risky)
VB : national cigarette type (V = at least 75% Virginia, bl = at least 75% blended, ot = other)
P : any proxy use
H : full histological confirmation
De : derivation of RR/CI (or = original, st = standard method, ot = other method of estimation)

Table 2I19 - 1

IESLC - Meta-analysis of Ever/current Smoking, Duration, "High"
Squamous, Cigarettes (or Any Product if Cigarettes not available)
Most adjusted

REF	NRR	2I14	SEX	AGEL	AGEH	RACE	YF	LC TYPE	LOC	START	ST	NLC	R	VB	P	H	AD	SM	PRODUCT	exL	exH	DENOM	De
BARBON	565		m	0	0	all	-	q	Eu:wst	1979	CC	755	n	bl	y	y	1	ev	all/unsp	50	999	nev any	or
BUFFLE	508		m	0	0	wh	-	q	NAmer	1976	CC	943	n	bl	y	n	0	ev	cig+/-ot	50	999	nev cigs	or
CHOI	562		m	0	0	all	-	q	As:oth	1985	CC	375	n	bl	n	n	0	ev	cig+/-ot	50	999	nev cigs	st
CHOI	575		f	0	0	all	-	q	As:oth	1985	CC	375	n	bl	n	n	0	ev	cig+/-ot	40	999	nev cigs	st
DAMBER	549		m	0	0	all	-	q	Eu:Sca	1972	CC	579	n	bl	y	n	1	ev	all/unsp	41	50	nev any	or
JEDRYC	587		m	0	0	all	-	q	Eu:est	1980	CC	1630	n	bl	y	n	3	ev	cig+/-ot	40	999	nev any	or
JOLY	642		m	0	0	all	-	q	SCAmer	1978	CC	826	n	bl	n	n	0	ev	cig+/-ot	50	999	nev any	st
JOLY	614		f	0	0	all	-	q	SCAmer	1978	CC	826	n	bl	n	n	0	ev	cig+/-ot	50	999	nev any	st
LUBIN2	664		m	0	0	all	-	q	Eu:mul	1976	CC	7804	n	bl	n	y	0	ev	cig+/-ot	50	999	nev any	st
LUBIN2	716		f	0	0	all	-	q	Eu:mul	1976	CC	7804	n	bl	n	y	0	ev	cig+/-ot	50	999	nev any	st
MATOS	608		m	0	0	all	-	q	SCAmer	1994	CC	200	n	bl	n	n	2	ev	cig+/-ot	40	70	nev any	or
PEZZOT	509		m	0	0	all	-	q	SCAmer	1987	CC	215	n	bl	n	y	0	ev	cig only	41	999	nev cigs	ot
SOBUE	504		m	0	0	all	-	q	As:Jap	1986	CC	1376	n	bl	n	y	0	cu	cig+/-ot	50	999	nev cigs	st
WUWILL	523		f	0	0	all	-	q	As:Chi	1985	CC	965	n	ot	n	n	3	ev	cig+/-ot	40	999	nev cigs	ot
WYNDE2	508		m	0	0	all	-	KI	NAmer	1962	CC	404	n	bl	n	y	0	ev	cig+/-ot	41	999	nev any	ot
ZHENG	503		m	0	0	all	-	q	As:Chi	1982	CC	540	n	ot	*	y	0	ev	cig+/-ot	40	999	nev cigs	st

Cigarette type is all/unspec for all RRs

Table 2I19 - 2

IESLC - Meta-analysis of Ever/current Smoking, Duration, "High"
Squamous, Cigarettes (or Any Product if Cigarettes not available)
Most adjusted

REF	NRR	SEX	AD	Number Exposed Case	Number Exposed Cont	Non-exposed Case	Non-exposed Cont	RR	95.00%CI
BARBON	565	m	1	149	-	6	-	21.20	(9.10- 49.30)
BUFFLE	508	m	0	-	-	-	-	22.10	(7.20- 67.70)
CHOI	562	m	0	11	20	6	95	8.71	(2.88- 26.30)
CHOI	575	f	0	1	1	10	164	16.40	(0.95- 281.93)
Subtotal CHOI								9.46	(3.38- 26.51)
DAMBER	549	m	1	-	-	14	-	13.80	(6.80- 29.10)
JEDRYC	587	m	3	160	-	6	-	13.00	(5.54- 30.48)
JOLY	642	m	0	98	253	2	218	42.22	(10.29- 173.22)
JOLY	614	f	0	22	20	6	283	51.88	(18.89- 142.48)
Subtotal JOLY								48.39	(21.28- 110.03)
LUBIN2	664	m	0	746	1460	54	2616	24.75	(18.64- 32.87)
LUBIN2	716	f	0	566	34	72	1180	272.83	(179.26- 415.22)
Subtotal LUBIN2								52.47	(41.48- 66.37)
MATOS	608	m	2	26	-	3	-	18.50	(4.90- 69.80)
PEZZOT	509	m	0	45	101	0	116	104.45~	(6.35-1717.05)
SOBUE	504	m	0	77	73	3	128	45.00	(13.71- 147.74)
WUWILL	523	f	3	81	-	117	-	5.57	(3.79- 8.17)
WYNDE2	508	m	0	94	89	0	41	87.64~	(5.31-1446.06)
ZHENG	503	m	0	84	63	4	94	31.33	(10.94- 89.77)
Partial Totals				2160	2114	303	4935		

*prospective study ~ With 0.5 adjustment for zero

REF	NRR	SEX	AD	Ys	Ws	Qs	Ps
BARBON	565	m	1	3.05	5.38	0.25	0.0000
BUFFLE	508	m	0	3.10	3.06	0.09	0.0000
CHOI	562	m	0	2.16	3.14	3.83	0.0001
CHOI	575	f	0	2.80	0.47	0.11	0.0539
Subtotal CHOI				2.25	3.62	3.94	
DAMBER	549	m	1	2.62	7.27	3.01	0.0000
JEDRYC	587	m	3	2.56	5.29	2.62	0.0000
JOLY	642	m	0	3.74	1.93	0.43	0.0000
JOLY	614	f	0	3.95	3.76	1.74	0.0000
Subtotal JOLY				3.88	5.69	2.18	
LUBIN2	664	m	0	3.21	47.79	0.17	0.0000
LUBIN2	716	f	0	5.61	21.78	119.29	0.0000
Subtotal LUBIN2				3.96	69.57	119.46	
MATOS	608	m	2	2.92	2.18	0.27	0.0000
PEZZOT	509	m	0	4.65	0.49	0.93	0.0011
SOBUE	504	m	0	3.81	2.72	0.79	0.0000
WUWILL	523	f	3	1.72	26.04	62.66	0.0000
WYNDE2	508	m	0	4.47	0.49	0.71	0.0018
ZHENG	503	m	0	3.44	3.47	0.11	0.0000

	N	16
	NS	13
	Wt	135.26
Het	Chi	197.01
Het	df	15
Het	P	***
Fixed	RR	26.27
	RRl	22.20
	RRu	31.09
	P	+++
Random	RR	27.18
	RRl	13.36
	RRu	55.28
	P	+++
Asymm	P	N.S.

Table 2I19 - 3

IESLC - Meta-analysis of Ever/current Smoking, Duration, "High"
Squamous, Cigarettes (or Any Product if Cigarettes not available)
Most adjusted

	combined	Sex male	female	Total
N		12	4	16
NS		12	4	16
Wt		83.20	52.06	135.26
Het Chi		11.21	180.60	197.01
Het df		11	3	15
Het P		N.S.	***	***
Fixed RR		22.50	33.67	26.27
RRl		18.15	25.66	22.20
RRu		27.89	44.17	31.09
P		+++	+++	+++
Random RR		22.33	35.26	27.18
RRl		17.81	2.90	13.36
RRu		27.99	429.38	55.28
P		+++	++	+++
Between Chi				5.20
Between df				1
Between P				*
Btwn(F) P				N.S.
Btwn(R) P				N.S.

Table 2I19 - 4

IESLC - Meta-analysis of Ever/current Smoking, Duration, "High"
Squamous, Cigarettes (or Any Product if Cigarettes not available)
Least adjusted

REF	NRR	X	SEX	AGEL	AGEH	RACE	YF	LC TYPE	LOC	START	ST	NLC	R	VB	P	H	AD	SM	PRODUCT	exL	exH	DENOM	De
BARBON	558	x	m	0	0	all	-	q	Eu:wst	1979	CC	755	n	bl	y	y	0	ev	all/unsp	50	999	nev any	st
BUFFLE	508		m	0	0	wh	-	q	NAmer	1976	CC	943	n	bl	y	n	0	ev	cig+/-ot	50	999	nev cigs	or
CHOI	562		m	0	0	all	-	q	As:oth	1985	CC	375	n	bl	n	n	0	ev	cig+/-ot	50	999	nev cigs	st
CHOI	575		f	0	0	all	-	q	As:oth	1985	CC	375	n	bl	n	n	0	ev	cig+/-ot	40	999	nev cigs	st
DAMBER	549		m	0	0	all	-	q	Eu:Sca	1972	CC	579	n	bl	y	n	1	ev	all/unsp	41	50	nev any	or
JEDRYC	505	x	m	0	0	all	-	q	Eu:est	1980	CC	1630	n	bl	y	n	0	ev	cig+/-ot	50	999	nev any	st
JOLY	642		m	0	0	all	-	q	SCAmer	1978	CC	826	n	bl	n	n	0	ev	cig+/-ot	50	999	nev any	st
JOLY	614		f	0	0	all	-	q	SCAmer	1978	CC	826	n	bl	n	n	0	ev	cig+/-ot	50	999	nev any	st
LUBIN2	664		m	0	0	all	-	q	Eu:mul	1976	CC	7804	n	bl	n	y	0	ev	cig+/-ot	50	999	nev any	st
LUBIN2	716		f	0	0	all	-	q	Eu:mul	1976	CC	7804	n	bl	n	y	0	ev	cig+/-ot	50	999	nev any	st
MATOS	603	x	m	0	0	all	-	q	SCAmer	1994	CC	200	n	bl	n	n	0	ev	cig+/-ot	40	70	nev any	st
PEZZOT	509		m	0	0	all	-	q	SCAmer	1987	CC	215	n	bl	n	y	0	ev	cig only	41	999	nev cigs	ot
SOBUE	504		m	0	0	all	-	q	As:Jap	1986	CC	1376	n	bl	n	y	0	cu	cig+/-ot	50	999	nev cigs	st
WUWILL	508	x	f	0	0	all	-	q	As:Chi	1985	CC	965	n	ot	n	n	0	ev	cig+/-ot	40	999	nev cigs	st
WYNDE2	508		m	0	0	all	-	KI	NAmer	1962	CC	404	n	bl	n	y	0	ev	cig+/-ot	41	999	nev any	ot
ZHENG	503		m	0	0	all	-	q	As:Chi	1982	CC	540	n	ot	*	y	0	ev	cig+/-ot	40	999	nev cigs	st

Cigarette type is all/unspec for all RRs

Table 2I19 - 5

IESLC - Meta-analysis of Ever/current Smoking, Duration, "High"
Squamous, Cigarettes (or Any Product if Cigarettes not available)
Least adjusted

REF	NRR	SEX	AD	Number Exposed Case	Cont	Non-exposed Case	Cont	RR	95.00%CI
BARBON	558	m	0	149	235	6	188	19.87	(8.59- 45.94)
BUFFLE	508	m	0	-	-	-	-	22.10	(7.20- 67.70)
CHOI	562	m	0	11	20	6	95	8.71	(2.88- 26.30)
CHOI	575	f	0	1	1	10	164	16.40	(0.95- 281.93)
Subtotal CHOI								9.46	(3.38- 26.51)
DAMBER	549	m	1	-	-	14	-	13.80	(6.80- 29.10)
JEDRYC	505	m	0	49	214	6	289	11.03	(4.64- 26.22)
JOLY	642	m	0	98	253	2	218	42.22	(10.29- 173.22)
JOLY	614	f	0	22	20	6	283	51.88	(18.89- 142.48)
Subtotal JOLY								48.39	(21.28- 110.03)
LUBIN2	664	m	0	746	1460	54	2616	24.75	(18.64- 32.87)
LUBIN2	716	f	0	566	34	72	1180	272.83	(179.26- 415.22)
Subtotal LUBIN2								52.47	(41.48- 66.37)
MATOS	603	m	0	26	89	3	110	10.71	(3.14- 36.55)
PEZZOT	509	m	0	45	101	0	116	104.45~	(6.35-1717.05)
SOBUE	504	m	0	77	73	3	128	45.00	(13.71- 147.74)
WUWILL	508	f	0	81	114	117	601	3.65	(2.58- 5.16)
WYNDE2	508	m	0	94	89	0	41	87.64~	(5.31-1446.06)
ZHENG	503	m	0	84	63	4	94	31.33	(10.94- 89.77)
Partial Totals				2049	2766	303	6123		

*prospective study ~ With 0.5 adjustment for zero

REF	NRR	SEX	AD	Ys	Ws	Qs	Ps
BARBON	558	m	0	2.99	5.47	0.06	0.0000
BUFFLE	508	m	0	3.10	3.06	0.00	0.0000
CHOI	562	m	0	2.16	3.14	2.69	0.0001
CHOI	575	f	0	2.80	0.47	0.04	0.0539
Subtotal CHOI				2.25	3.62	2.74	
DAMBER	549	m	1	2.62	7.27	1.57	0.0000
JEDRYC	505	m	0	2.40	5.12	2.44	0.0000
JOLY	642	m	0	3.74	1.93	0.82	0.0000
JOLY	614	f	0	3.95	3.76	2.78	0.0000
Subtotal JOLY				3.88	5.69	3.60	
LUBIN2	664	m	0	3.21	47.79	0.67	0.0000
LUBIN2	716	f	0	5.61	21.78	138.17	0.0000
Subtotal LUBIN2				3.96	69.57	138.84	
MATOS	603	m	0	2.37	2.55	1.32	0.0002
PEZZOT	509	m	0	4.65	0.49	1.19	0.0011
SOBUE	504	m	0	3.81	2.72	1.40	0.0000
WUWILL	508	f	0	1.29	31.92	102.90	0.0000
WYNDE2	508	m	0	4.47	0.49	0.94	0.0018
ZHENG	503	m	0	3.44	3.47	0.44	0.0000

	N	16
	NS	13
	Wt	141.43
Het	Chi	257.42
Het	df	15
Het	P	***
Fixed	RR	21.98
	RRl	18.64
	RRu	25.92
	P	+++
Random	RR	25.20
	RRl	11.48
	RRu	55.32
	P	+++
Asymm	P	N.S.

Table 2I19 - 6

IESLC - Meta-analysis of Ever/current Smoking, Duration, "High"
Squamous, Cigarettes (or Any Product if Cigarettes not available)
Least adjusted

	Sex combined	male	female	Total
N		12	4	16
NS		12	4	16
Wt		83.49	57.94	141.43
Het Chi		13.53	243.88	257.42
Het df		11	3	15
Het P		N.S.	***	***
Fixed RR		21.81	22.22	21.98
RRl		17.60	17.18	18.64
RRu		27.03	28.75	25.92
P		+++	+++	+++
Random RR		20.61	31.26	25.20
RRl		15.36	1.93	11.48
RRu		27.64	506.39	55.32
P		+++	+	+++
Between Chi				0.01
Between df				1
Between P				N.S.
Btwn(F) P				N.S.
Btwn(R) P				N.S.

Table 2I19 - 7

IESLC - Meta-analysis of Ever/current Smoking, Duration, "High"
Squamous, Cigarettes (or Any Product if Cigarettes not available)
Excluded studies (and stage at which they were excluded)

1	BECHER BLOT1 BROWN3 CARPEN CHYOU DARBY DOLL2 GARCIA GRAHAM GURSEL HAMMO2 JAHN JAIN LAUSSM PRESCO QIAO TVERDA WIGLE WYNDE3
2	ALDERS BENSHL BRESLO CHIAZZ DEAN3 DORN ENGELA GAO2 GILLIS GUO HEGMAN HIRAYA HOLE KAUFMA KOO KOULUM LIU4 MIGRAN MRFITR PERNU SEGI2 SPEIZE SUZUK2 SVENSS VUTUC WAKAI WU YUAN
3	GENG MCDUFF SPITZ STASZE WU2 ZHANG
4	AGUDO AKIBA AMANDU AMES ARMADA AUVINE AXELSS BEST BOFFET BOUCOT BROSS CEDERL CHEN2 CORREA CPSI CPSII DEAN2 DESTEF DOLL FAN GAO GARSHI HAMMON HU HU2 HUMBLE JUSSAW KAISE2 KREUZE LETOUR LEVIN LIAW LIU3 LIU5 MCCONN NOTAN2 PEZZO2 PISANI QIAO2 RACHTA RESTRE SADOWS TIZZAN WANG2 WATSON WYNDE6 WYNDE7 WYNDE8
5	CHEN LUBIN XU
10	BOUCHA KHUDER
14	DORGAN DOSEME GER HAENSZ KATSOU LUO OSANN2 ZHOU
15	BENHAM

Table 2I19 - 8
Potentially overlapping studies

REF	REFGP	PRINC	.	OVERLAP/LINK
LUBIN2	LUBIN2	1	Lubin-combined

Table 2I19 - 9
Most adjusted - insufficient data for meta-analysis

REF	NRR	SEX	AGEL	AGEH	RACE	YF	LC TYPE	LOC	START	ST	NLC	R	VB	P	H	AD	SM	PRODUCT	exL	exH	DENOM	De
CHEN	504	c	0	0	all	-	q	As:oth	1987	CC	323	n	ot	n	y	2	ev	cig+/-ot	41	999	nev cigs	ot
XU	513	m	0	0	all	-	q+s	As:Chi	1985	CC	729	n	ot	n	n	2	ev	all/unsp	40	999	nev any	or

REF	NRR	RR	SIG	RRDATA comment
CHEN	504	8.43	y	p<0.001
XU	513	*		RR for 1-19/day is 5.0(p<0.05), for 20-29/day is 10.4(p<0.05) and for >=30/ day is 31.2(p<0.05)

Table 2I20 -

IESLC - Meta-analysis of Ever/current Smoking, Duration, "Highest vs lowest"
Squamous, Cigarettes (or Any Product if Cigarettes not available)

This analysis is restricted to results for:
1) Ever/current smokers
2) Results by Duration
3) Categorical results by Duration
4) Denominator (unexposed) = "low"
5) Squamous (or near equivalent)
6) Results complete enough for use in metaanalysis

Within each study, results are then selected (in the following order of preference, within each sex) for:
7) SMKSTA: ever, current
8) PRODUCT: cigarettes regardless of other products, cigarettes only, all/unspec
9) CIGTYPE: all/unspecified, MC regardless of HR, MC only
10) Results with least adjustment for other aspects of smoking (ADOS)
11) The highest vs lowest category
12) Followup period (YF, prospective studies): whole study (coded as 0) or longest available
13) LCtype: squamous or nearest available, but not adeno. (q = squamous, s = small,
a = adeno, KI = Kreyberg I, u = undifferentiated)
14) Race: all or nearest available, otherwise by race (wh or w = white, bl or b = black, hi = hispanic
ch = chinese, jap = japanese, haw = hawaiian, w+o = white + oriental, sca = scandinavian, as = asian)
15) For overlapping studies: principal rather than subsidiary studies
Finally by Age: whole study (coded as 0) if available, otherwise by widest available age group
and then for single sex results (m, f) in preference to results for both sexes combined (c).

Results adjusted (AD) for the most potential confounders are then chosen in Sections -1 to -3
(and those which actually differ from the adjusted results in Table 2I15 - 1 are marked 'x' in Section -1)
and results adjusted for the least confounders in Sections -4 to -6. (Those least adjusted results which
actually differ from the most adjusted are marked 'x' in column X in Section -4)

Section -7 shows excluded studies, together with the stage (as above) at which no qualifying
results were found.

Section -8 lists the potentially overlapping studies which have been included (1=principal, 2=subsidiary).

Section -9 lists any results which would have been included in preference except that they had data not complete
enough for use in meta-analysis, with their significance (yes/no), if known, and any further comment as entered
on the database. It also lists as "gap" any categories for which no data were presented by the original authors.

In addition to those mentioned above, the following fields, levels and abbreviations are used:
* or nk = not known, n = no, y = yes, ot = other
all/unspec = all or unspecified, cig+/-ot = cigarettes irrespective of other products (cigar, pipe etc)
MC = manufactured cigarettes, HR = hand-rolled cigarettes
exL, exH = range of exposure (low and high) in the "highest" group, in terms of Duration
unexL, unexH = range of exposure (low and high) in the "lowest" group, in terms of Duration
REF: 6-character study reference
NRR: number of the RR on the database within the study
ST : study type (CC = case control, pr or prosp = prospective)
NLC: number of lung cancer cases in whole study
R : risky occupational population (n = no, m = mining, o = other risky)
VB : national cigarette type (V = at least 75% Virginia, bl = at least 75% blended, ot = other)
P : any proxy use
H : full histological confirmation
De : derivation of RR/CI (or = original, st = standard method, ot = other method of estimation)

Table 2I20 - 1

IESLC - Meta-analysis of Ever/current Smoking, Duration, "Highest vs lowest"
Squamous, Cigarettes (or Any Product if Cigarettes not available)
Most adjusted

REF	NRR	2I15	SEX	AGEL	AGEH	RACE	YF	LC TYPE	LOC	START	ST	NLC	R	VB	P	H	AD	ADOS	SM	PRODUCT	exL	exH	unexL	unexH	De
BARBON	568		m	0	0	all	-	q	Eu:wst	1979	CC	755	n	bl	y	y	1	0	ev	all/unsp	50	999	1	29	ot
BOUCHA	501		c	0	0	wh	-	q+s	Eu:wst	1988	CC	150	n	bl	n	y	0	0	ev	all/unsp	31	999	1	30	st
CHOI	565		m	0	0	all	-	q	As:oth	1985	CC	375	n	bl	n	n	0	0	ev	cig+/-ot	50	999	1	29	st
CHOI	577		f	0	0	all	-	q	As:oth	1985	CC	375	n	bl	n	n	0	0	ev	cig+/-ot	40	999	1	29	st
DAMBER	553		m	0	0	all	-	q	Eu:Sca	1972	CC	579	n	bl	y	n	1	0	ev	all/unsp	51	999	1	30	ot
DORGAN	527		m	0	0	wh	-	q	NAmer	1980	CC	2026	n	bl	y	y	2	0	ev	cig+/-ot	35	999	1	34	ot
DORGAN	523		f	0	0	all	-	q	NAmer	1980	CC	2026	n	bl	y	y	3	0	ev	cig+/-ot	35	999	1	34	ot
DOSEME	515		m	0	0	all	-	q	Eu:bal	1979	CC	1210	n	bl	n	n	2	0	ev	cig+/-ot	21	999	1	10	ot
GER	512		c	0	0	all	-	q+s	As:oth	1990	CC	141	n	ot	y	n	5	0	ev	all/unsp	31	999	1	30	ot
HAENSZ	535		f	0	0	all	-	q+u	NAmer	1955	CC	158	n	bl	n	y	1	0	ev	cig+/-ot	15	999	1	14	ot
JEDRYC	509		m	0	0	all	-	q	Eu:est	1980	CC	1630	n	bl	y	n	0	0	ev	cig+/-ot	50	999	1	19	st
JOLY	645		m	0	0	all	-	q	SCAmer	1978	CC	826	n	bl	n	n	0	0	ev	cig+/-ot	50	999	1	29	st
JOLY	617		f	0	0	all	-	q	SCAmer	1978	CC	826	n	bl	n	n	0	0	ev	cig+/-ot	50	999	1	29	st
KATSOU	530		f	0	0	all	-	KI	Eu:bal	1987	CC	101	n	bl	n	n	1	0	cu	all/unsp	30	999	1	29	ot
KHUDER	530		m	0	0	all	-	q	NAmer	1985	CC	482	n	bl	n	y	5	3#	ev	cig+/-ot	30	999	1	29	or
LUBIN2	667		m	0	0	all	-	q	Eu:mul	1976	CC	7804	n	bl	n	y	0	0	ev	cig+/-ot	50	999	1	29	st
LUBIN2	719		f	0	0	all	-	q	Eu:mul	1976	CC	7804	n	bl	n	y	0	0	ev	cig+/-ot	50	999	1	29	st
LUO	506		c	0	0	all	-	q	As:Chi	1990	CC	102	n	ot	n	y	20	0	ev	cig+/-ot	30	999	1	29	ot
MATOS	610		m	0	0	all	-	q	SCAmer	1994	CC	200	n	bl	n	n	2	0	ev	cig+/-ot	40	70	1	24	ot
OSANN2	512		f	0	0	all	-	KI	NAmer	1964	ot	217	n	bl	n	y	1	0	ev	cig+/-ot	21	999	1	20	ot
PEZZOT	513		m	0	0	all	-	q	SCAmer	1987	CC	215	n	bl	n	y	2	0	ev	cig only	41	999	1	30	ot
SOBUE	508		m	0	0	all	-	q	As:Jap	1986	CC	1376	n	bl	n	y	0	0	cu	cig+/-ot	50	999	1	29	st
WUWILL	510		f	0	0	all	-	q	As:Chi	1985	CC	965	n	ot	n	n	0	0	ev	cig+/-ot	40	999	1	29	st
WYNDE2	513		m	0	0	all	-	KI	NAmer	1962	CC	404	n	bl	n	y	0	0	ev	cig+/-ot	41	999	1	29	st
ZHENG	507		m	0	0	all	-	q	As:Chi	1982	CC	540	n	ot	*	y	1	0	ev	cig+/-ot	40	999	1	29	ot
ZHENG	511		f	0	0	all	-	q	As:Chi	1982	CC	540	n	ot	*	y	1	0	ev	cig+/-ot	30	999	1	29	ot
ZHOU	506		c	0	0	all	-	q	As:Chi	1978	CC	1360	n	ot	n	n	0	0	ev	all/unsp	20	999	1	19	st

Comments on values in listings

KHUDER ADOS Age at starting smoking, No of cigarettes per day, Quitted smoking

Cigarette type is all/unspec for all RRs

Table 2I20 - 2

IESLC - Meta-analysis of Ever/current Smoking, Duration, "Highest vs lowest"
Squamous, Cigarettes (or Any Product if Cigarettes not available)
Most adjusted

REF	NRR	SEX	AD	Number Exposed Case	Number Exposed Cont	Non-exposed Case	Non-exposed Cont	RR	95.00%CI
BARBON	568	m	1	149	-	7	-	10.10	(4.59- 22.21)
BOUCHA	501	c	0	114	92	34	79	2.88	(1.77- 4.69)
CHOI	565	m	0	11	20	42	221	2.89	(1.29- 6.48)
CHOI	577	f	0	1	1	6	23	3.83	(0.21- 70.63)
Subtotal CHOI								2.95	(1.36- 6.42)
DAMBER	553	m	1	-	-	-	-	3.80	(1.82- 7.91)
DORGAN	527	m	2	-	-	-	-	2.77	(2.04- 3.76)
DORGAN	523	f	3	-	-	-	-	3.67	(2.52- 5.34)
Subtotal DORGAN								3.10	(2.44- 3.93)
DOSEME	515	m	2	199	-	15	-	4.08	(2.07- 8.05)
GER	512	c	5	42	-	6	-	4.19	(1.56- 11.28)
HAENSZ	535	f	1	42	-	14	-	1.00	(0.47- 2.12)
JEDRYC	509	m	0	49	214	7	68	2.22	(0.96- 5.14)
JOLY	645	m	0	98	253	15	109	2.81	(1.56- 5.07)
JOLY	617	f	0	22	20	5	54	11.88	(3.96- 35.63)
Subtotal JOLY								3.88	(2.31- 6.51)
KATSOU	530	f	1	19	-	5	-	8.45	(2.01- 35.41)
KHUDER	530	m	5	-	-	-	-	1.90	(0.90- 3.70)
LUBIN2	667	m	0	746	1460	453	2964	3.34	(2.93- 3.82)
LUBIN2	719	f	0	566	34	322	229	11.84	(8.05- 17.40)
Subtotal LUBIN2								3.82	(3.37- 4.33)
LUO	506	c	20	28	-	6	-	2.19	(0.68- 7.06)
MATOS	610	m	2	26	-	3	-	15.42	(3.96- 60.01)
OSANN2	512	f	1	101	-	11	-	20.63	(3.12- 136.52)
PEZZOT	513	m	2	45	-	5	-	9.90	(3.61- 27.15)
SOBUE	508	m	0	77	73	16	119	7.85	(4.25- 14.47)
WUWILL	510	f	0	81	114	54	139	1.83	(1.20- 2.80)
WYNDE2	513	m	0	94	89	22	55	2.64	(1.49- 4.68)
ZHENG	507	m	1	84	-	13	-	8.25	(4.21- 16.18)
ZHENG	511	f	1	35	-	8	-	4.42	(1.66- 11.76)
Subtotal ZHENG								6.75	(3.88- 11.76)
ZHOU	506	c	0	315	36	60	12	1.75	(0.86- 3.56)
Partial Totals				2944	2406	1129	4072		

*prospective study

REF	NRR	SEX	AD	Ys	Ws	Qs	Ps
BARBON	568	m	1	2.31	6.18	6.74	0.0000
BOUCHA	501	c	0	1.06	16.20	0.72	0.0000
CHOI	565	m	0	1.06	5.91	0.25	0.0098
CHOI	577	f	0	1.34	0.45	0.00	0.3661
Subtotal CHOI				1.08	6.36	0.25	
DAMBER	553	m	1	1.34	7.12	0.03	0.0004
DORGAN	527	m	2	1.02	41.10	2.55	0.0000
DORGAN	523	f	3	1.30	27.25	0.03	0.0000
Subtotal DORGAN				1.13	68.34	2.58	
DOSEME	515	m	2	1.41	8.33	0.16	0.0000
GER	512	c	5	1.43	3.93	0.11	0.0045
HAENSZ	535	f	1	0.00	6.77	10.89	1.0000
JEDRYC	509	m	0	0.80	5.48	1.20	0.0614
JOLY	645	m	0	1.03	11.11	0.60	0.0006
JOLY	617	f	0	2.47	3.18	4.64	0.0000
Subtotal JOLY				1.36	14.30	5.24	
KATSOU	530	f	1	2.13	1.87	1.40	0.0035
KHUDER	530	m	5	0.64	7.69	3.01	0.0751
LUBIN2	667	m	0	1.21	218.80	0.81	0.0000
LUBIN2	719	f	0	2.47	25.87	37.47	0.0000
Subtotal LUBIN2				1.34	244.68	38.29	
LUO	506	c	20	0.78	2.81	0.66	0.1891
MATOS	610	m	2	2.74	2.08	4.48	0.0001
OSANN2	512	f	1	3.03	1.08	3.33	0.0017
PEZZOT	513	m	2	2.29	3.77	3.96	0.0000
SOBUE	508	m	0	2.06	10.25	6.43	0.0000
WUWILL	510	f	0	0.60	21.35	9.42	0.0053
WYNDE2	513	m	0	0.97	11.69	1.03	0.0009
ZHENG	507	m	1	2.11	8.48	6.01	0.0000
ZHENG	511	f	1	1.49	4.01	0.19	0.0029
Subtotal ZHENG				1.91	12.49	6.20	
ZHOU	506	c	0	0.56	7.64	3.83	0.1220

Table 2I20 - 2

IESLC - Meta-analysis of Ever/current Smoking, Duration, "Highest vs lowest"
Squamous, Cigarettes (or Any Product if Cigarettes not available)
Most adjusted

	N	27
	NS	22
	Wt	470.39
Het	Chi	109.96
Het	df	26
Het	P	***
Fixed	RR	3.55
	RRl	3.25
	RRu	3.89
	P	+++
Random	RR	3.93
	RRl	3.10
	RRu	4.97
	P	+++
Asymm	P	N.S.

Table 2I20 - 3

IESLC - Meta-analysis of Ever/current Smoking, Duration, "Highest vs lowest"
Squamous, Cigarettes (or Any Product if Cigarettes not available)
Most adjusted

	Sex			
	combined	male	female	Total
N	4	14	9	27
NS	4	14	9	27
Wt	30.57	347.99	91.83	470.39
Het Chi	2.34	37.10	64.03	109.96
Het df	3	13	8	26
Het P	N.S.	***	***	***
Fixed RR	2.60	3.47	4.30	3.55
RRl	1.83	3.13	3.50	3.25
RRu	3.71	3.86	5.28	3.89
P	+++	+++	+++	+++
Random RR	2.60	4.01	4.73	3.93
RRl	1.83	3.12	2.39	3.10
RRu	3.71	5.16	9.39	4.97
P	+++	+++	+++	+++
Between Chi				6.49
Between df				2
Between P				*
Btwn(F) P				N.S.
Btwn(R) P				N.S.

	Lung cancer type					
	q	q+s	q+u	KI	not a	Total
N	21	2	1	3		27
NS	16	2	1	3		22
Wt	428.85	20.13	6.77	14.64		470.39
Het Chi	92.20	0.44	0.00	5.76		109.96
Het df	20	1	0	2		26
Het P	***	N.S.	N.S.	(*)		***
Fixed RR	3.65	3.10	1.00	3.56		3.55
RRl	3.32	2.00	0.47	2.13		3.25
RRu	4.01	4.79	2.12	5.95		3.89
P	+++	+++	N.S.	+++		+++
Random RR	4.17	3.10	1.00	6.05		3.93
RRl	3.21	2.00	0.47	1.78		3.10
RRu	5.42	4.79	2.12	20.62		4.97
P	+++	+++	N.S.	++		+++
Between Chi						11.56
Between df						3
Between P						**
Btwn(F) P						N.S.
Btwn(R) P						**

	Location								
	NAmer	UK	Scand	othEur	China	Japan	othAs	other	Total
N	6		1	7	5	1	3	4	27
NS	5		1	6	4	1	2	3	22
Wt	95.57		7.12	282.73	44.28	10.25	10.29	20.15	470.39
Het Chi	14.64		0.00	46.94	16.34	0.00	0.33	10.37	109.96
Het df	5		0	6	4	0	2	3	26
Het P	*		N.S.	***	**	N.S.	N.S.	*	***
Fixed RR	2.75		3.80	3.83	2.65	7.85	3.37	5.33	3.55
RRl	2.25		1.82	3.41	1.98	4.25	1.83	3.45	3.25
RRu	3.37		7.92	4.30	3.56	14.47	6.22	8.25	3.89
P	+++		+++	+++	+++	+++	+++	+++	+++
Random RR	2.58		3.80	4.92	3.00	7.85	3.37	7.60	3.93
RRl	1.73		1.82	2.92	1.55	4.25	1.83	3.08	3.10
RRu	3.86		7.92	8.29	5.81	14.47	6.22	18.75	4.97
P	+++		+++	+++	++	+++	+++	+++	+++
Between Chi									21.34
Between df									6
Between P									**
Btwn(F) P									N.S.
Btwn(R) P									*

Table 2I20 - 3

IESLC - Meta-analysis of Ever/current Smoking, Duration, "Highest vs lowest"
Squamous, Cigarettes (or Any Product if Cigarettes not available)
Most adjusted

Detailed Country in "other Europe"

	multi	Germany	othWest	East	Balkans	Total
N	2		2	1	2	7
NS	1		2	1	2	6
Wt	244.68		22.39	5.48	10.20	282.73
Het Chi	36.99		7.05	0.00	0.81	46.94
Het df	1		1	0	1	6
Het P	***		**	N.S.	N.S.	***
Fixed RR	3.82		4.07	2.22	4.66	3.83
RRl	3.37		2.69	0.96	2.52	3.41
RRu	4.33		6.16	5.14	8.61	4.30
P	+++		+++	(+)	+++	+++
Random RR	6.21		5.18	2.22	4.66	4.92
RRl	1.80		1.52	0.96	2.52	2.92
RRu	21.43		17.68	5.14	8.61	8.29
P	++		++	(+)	+++	+++
Between Chi						2.10
Between df						3
Between P						N.S.
Btwn(F) P						N.S.
Btwn(R) P						N.S.

Detailed Country in "other Asia"

	India	HongKong	other	Total
N			3	3
NS			2	2
Wt			10.29	10.29
Het Chi			0.33	0.33
Het df			2	2
Het P			N.S.	N.S.
Fixed RR			3.37	3.37
RRl			1.83	1.83
RRu			6.22	6.22
P			+++	+++
Random RR			3.37	3.37
RRl			1.83	1.83
RRu			6.22	6.22
P			+++	+++
Between Chi				
Between df				
Between P				N.S.
Btwn(F) P				N.S.
Btwn(R) P				N.S.

Detailed other continent

	SCAmer	Total
N	4	4
NS	3	3
Wt	20.15	20.15
Het Chi	10.37	10.37
Het df	3	3
Het P	*	*
Fixed RR	5.33	5.33
RRl	3.45	3.45
RRu	8.25	8.25
P	+++	+++
Random RR	7.60	7.60
RRl	3.08	3.08
RRu	18.75	18.75
P	+++	+++
Between Chi		
Between df		
Between P		N.S.
Btwn(F) P		N.S.
Btwn(R) P		N.S.

Table 2I20 - 3

IESLC - Meta-analysis of Ever/current Smoking, Duration, "Highest vs lowest"
Squamous, Cigarettes (or Any Product if Cigarettes not available)
Most adjusted

	Start year of study					
	<1960	1960-69	1970-79	1980-89	1990+	Total
N	1	2	8	13	3	27
NS	1	2	6	10	3	22
Wt	6.77	12.77	288.24	153.80	8.81	470.39
Het Chi	0.00	4.16	52.66	33.92	4.62	109.96
Het df	0	1	7	12	2	26
Het P	N.S.	*	***	***	(*)	***
Fixed RR	1.00	3.14	3.83	3.25	4.63	3.55
RRl	0.47	1.81	3.41	2.77	2.39	3.25
RRu	2.12	5.43	4.30	3.80	8.97	3.89
P	N.S.	+++	+++	+++	+++	+++
Random RR	1.00	6.01	4.80	3.63	4.92	3.93
RRl	0.47	0.83	2.95	2.68	1.77	3.10
RRu	2.12	43.28	7.83	4.91	13.66	4.97
P	N.S.	(+)	+++	+++	++	+++
Between Chi						14.59
Between df						4
Between P						**
Btwn(F) P						N.S.
Btwn(R) P						*

	Study type (1)		
	CC	other	Total
N	26	1	27
NS	21	1	22
Wt	469.32	1.08	470.39
Het Chi	106.63	0.00	109.96
Het df	25	0	26
Het P	***	N.S.	***
Fixed RR	3.54	20.63	3.55
RRl	3.23	3.12	3.25
RRu	3.87	136.46	3.89
P	+++	++	+++
Random RR	3.84	20.63	3.93
RRl	3.04	3.12	3.10
RRu	4.86	136.46	4.97
P	+++	++	+++
Between Chi			3.34
Between df			1
Between P			(*)
Btwn(F) P			N.S.
Btwn(R) P			(*)

	Study type (2)			
	CC	prosp	other	Total
N	26		1	27
NS	21		1	22
Wt	469.32		1.08	470.39
Het Chi	106.63		0.00	109.96
Het df	25		0	26
Het P	***		N.S.	***
Fixed RR	3.54		20.63	3.55
RRl	3.23		3.12	3.25
RRu	3.87		136.46	3.89
P	+++		++	+++
Random RR	3.84		20.63	3.93
RRl	3.04		3.12	3.10
RRu	4.86		136.46	4.97
P	+++		++	+++
Between Chi				3.34
Between df				1
Between P				(*)
Btwn(F) P				N.S.
Btwn(R) P				(*)

Table 2I20 - 3

IESLC - Meta-analysis of Ever/current Smoking, Duration, "Highest vs lowest"
Squamous, Cigarettes (or Any Product if Cigarettes not available)
Most adjusted

	Study size (number of LC cases)				
	100-249	250-499	500-999	1000+	Total
N	8	4	7	8	27
NS	8	3	5	6	22
Wt	38.50	25.74	61.43	344.71	470.39
Het Chi	25.40	0.82	27.58	52.21	109.96
Het df	7	3	6	7	26
Het P	***	N.S.	***	***	***
Fixed RR	3.35	2.46	3.67	3.66	3.55
RRl	2.44	1.67	2.86	3.29	3.25
RRu	4.59	3.62	4.72	4.06	3.89
P	+++	+++	+++	+++	+++
Random RR	4.56	2.46	4.76	3.99	3.93
RRl	2.32	1.67	2.70	2.73	3.10
RRu	8.97	3.62	8.40	5.82	4.97
P	+++	+++	+++	+++	+++
Between Chi					3.96
Between df					3
Between P					N.S.
Btwn(F) P					N.S.
Btwn(R) P					N.S.

	Risky occupational population			
	no	mining	othRisky	Total
N	27			27
NS	22			22
Wt	470.39			470.39
Het Chi	109.96			109.96
Het df	26			26
Het P	***			***
Fixed RR	3.55			3.55
RRl	3.25			3.25
RRu	3.89			3.89
P	+++			+++
Random RR	3.93			3.93
RRl	3.10			3.10
RRu	4.97			4.97
P	+++			+++
Between Chi				
Between df				
Between P				N.S.
Btwn(F) P				N.S.
Btwn(R) P				N.S.

	National cigarette tobacco type			
	Virginia	blended	other	Total
N		21	6	27
NS		17	5	22
Wt		422.18	48.21	470.39
Het Chi		89.39	17.09	109.96
Het df		20	5	26
Het P		***	**	***
Fixed RR		3.66	2.75	3.55
RRl		3.33	2.08	3.25
RRu		4.02	3.65	3.89
P		+++	+++	+++
Random RR		4.18	3.15	3.93
RRl		3.21	1.77	3.10
RRu		5.46	5.59	4.97
P		+++	+++	+++
Between Chi				3.49
Between df				1
Between P				(*)
Btwn(F) P				N.S.
Btwn(R) P				N.S.

Table 2I20 - 3

IESLC - Meta-analysis of Ever/current Smoking, Duration, "Highest vs lowest"
Squamous, Cigarettes (or Any Product if Cigarettes not available)
Most adjusted

	Any proxy use No/nk	Yes	Total
N	21	6	27
NS	17	5	22
Wt	379.35	91.04	470.39
Het Chi	99.25	10.46	109.96
Het df	20	5	26
Het P	***	(*)	***
Fixed RR	3.59	3.39	3.55
RRl	3.25	2.76	3.25
RRu	3.97	4.16	3.89
P	+++	+++	+++
Random RR	4.02	3.70	3.93
RRl	2.97	2.62	3.10
RRu	5.44	5.22	4.97
P	+++	+++	+++
Between Chi			0.26
Between df			1
Between P			N.S.
Btwn(F) P			N.S.
Btwn(R) P			N.S.

	Full histological confirmation No	Yes	Total
N	12	15	27
NS	10	12	22
Wt	78.44	391.95	470.39
Het Chi	23.21	83.25	109.96
Het df	11	14	26
Het P	*	***	***
Fixed RR	2.93	3.69	3.55
RRl	2.35	3.35	3.25
RRu	3.66	4.08	3.89
P	+++	+++	+++
Random RR	3.44	4.23	3.93
RRl	2.42	3.08	3.10
RRu	4.90	5.80	4.97
P	+++	+++	+++
Between Chi			3.51
Between df			1
Between P			(*)
Btwn(F) P			N.S.
Btwn(R) P			N.S.

	Number of adjustment variables (1) 0	1	2+/+nk	Total
N	12	7	8	27
NS	9	6	7	22
Wt	337.95	35.50	96.95	470.39
Het Chi	66.38	25.85	14.48	109.96
Het df	11	6	7	26
Het P	***	***	*	***
Fixed RR	3.52	4.69	3.31	3.55
RRl	3.17	3.38	2.71	3.25
RRu	3.92	6.52	4.04	3.89
P	+++	+++	+++	+++
Random RR	3.61	5.25	3.66	3.93
RRl	2.50	2.55	2.60	3.10
RRu	5.20	10.81	5.16	4.97
P	+++	+++	+++	+++
Between Chi				3.25
Between df				2
Between P				N.S.
Btwn(F) P				N.S.
Btwn(R) P				N.S.

Table 2I20 - 3

IESLC - Meta-analysis of Ever/current Smoking, Duration, "Highest vs lowest"
Squamous, Cigarettes (or Any Product if Cigarettes not available)
Most adjusted

	Number of adjustment variables (2) 0	1	2	3-5	6+/+nk	Total
N	12	7	4	3	1	27
NS	9	6	4	3	1	23
Wt	337.95	35.50	55.28	38.86	2.81	470.39
Het Chi	66.38	25.85	11.07	2.87	0.00	109.96
Het df	11	6	3	2	0	26
Het P	***	***	*	N.S.	N.S.	***
Fixed RR	3.52	4.69	3.42	3.27	2.19	3.55
RRl	3.17	3.38	2.63	2.38	0.68	3.25
RRu	3.92	6.52	4.45	4.47	7.06	3.89
P	+++	+++	+++	+++	N.S.	+++
Random RR	3.61	5.25	5.31	3.13	2.19	3.93
RRl	2.50	2.55	2.61	2.04	0.68	3.10
RRu	5.20	10.81	10.78	4.83	7.06	4.97
P	+++	+++	+++	+++	N.S.	+++
Between Chi						3.79
Between df						4
Between P						N.S.
Btwn(F) P						N.S.
Btwn(R) P						N.S.

	Smoking status ever	current	Total
N	25	2	27
NS	20	2	22
Wt	458.28	12.11	470.39
Het Chi	101.93	0.01	109.96
Het df	24	1	26
Het P	***	N.S.	***
Fixed RR	3.48	7.94	3.55
RRl	3.17	4.52	3.25
RRu	3.81	13.94	3.89
P	+++	+++	+++
Random RR	3.74	7.94	3.93
RRl	2.94	4.52	3.10
RRu	4.76	13.94	4.97
P	+++	+++	+++
Between Chi			8.03
Between df			1
Between P			**
Btwn(F) P			N.S.
Btwn(R) P			*

	Product all/unsp	cig+/-ot	cig only	Total
N	6	20	1	27
NS	6	15	1	22
Wt	42.93	423.69	3.77	470.39
Het Chi	12.83	93.13	0.00	109.96
Het df	5	19	0	26
Het P	*	***	N.S.	***
Fixed RR	3.59	3.52	9.90	3.55
RRl	2.66	3.20	3.61	3.25
RRu	4.84	3.87	27.15	3.89
P	+++	+++	+++	+++
Random RR	3.97	3.78	9.90	3.93
RRl	2.38	2.87	3.61	3.10
RRu	6.65	4.99	27.15	4.97
P	+++	+++	+++	+++
Between Chi				4.01
Between df				2
Between P				N.S.
Btwn(F) P				N.S.
Btwn(R) P				N.S.

Table 2I20 - 3

IESLC - Meta-analysis of Ever/current Smoking, Duration, "Highest vs lowest"
Squamous, Cigarettes (or Any Product if Cigarettes not available)
Most adjusted

	Derivation of RR/CI			
	Orig	StdCalc	Other	Total
N	1	12	14	27
NS	1	9	12	22
Wt	7.69	337.95	124.76	470.39
Het Chi	0.00	66.38	40.04	109.96
Het df	0	11	13	26
Het P	N.S.	***	***	***
Fixed RR	1.90	3.52	3.79	3.55
RRl	0.94	3.17	3.18	3.25
RRu	3.85	3.92	4.51	3.89
P	(+)	+++	+++	+++
Random RR	1.90	3.61	4.60	3.93
RRl	0.94	2.50	3.22	3.10
RRu	3.85	5.20	6.58	4.97
P	(+)	+++	+++	+++
Between Chi				3.54
Between df				2
Between P				N.S.
Btwn(F) P				N.S.
Btwn(R) P				(*)

Table 2I20 - 4

IESLC - Meta-analysis of Ever/current Smoking, Duration, "Highest vs lowest"
Squamous, Cigarettes (or Any Product if Cigarettes not available)
Least adjusted

REF	NRR	X	SEX	AGEL	AGEH	RACE	YF	LC TYPE	LOC	START	ST	NLC	R	VB	P	H	AD	ADOS	SM	PRODUCT	exL	exH	unexL	unexH	De
BARBON	561	x	m	0	0	all	-	q	Eu:wst	1979	CC	755	n	bl	y	y	0	0	ev	all/unsp	50	999	1	29	st
BOUCHA	501		c	0	0	wh	-	q+s	Eu:wst	1988	CC	150	n	bl	n	y	0	0	ev	all/unsp	31	999	1	30	st
CHOI	565		m	0	0	all	-	q	As:oth	1985	CC	375	n	bl	n	n	0	0	ev	cig+/-ot	50	999	1	29	st
CHOI	577		f	0	0	all	-	q	As:oth	1985	CC	375	n	bl	n	n	0	0	ev	cig+/-ot	40	999	1	29	st
DAMBER	553		m	0	0	all	-	q	Eu:Sca	1972	CC	579	n	bl	y	n	1	0	ev	all/unsp	51	999	1	30	ot
DORGAN	527		m	0	0	wh	-	q	NAmer	1980	CC	2026	n	bl	y	y	2	0	ev	cig+/-ot	35	999	1	34	ot
DORGAN	523		f	0	0	all	-	q	NAmer	1980	CC	2026	n	bl	y	y	3	0	ev	cig+/-ot	35	999	1	34	ot
DOSEME	515		m	0	0	all	-	q	Eu:bal	1979	CC	1210	n	bl	n	n	2	0	ev	cig+/-ot	21	999	1	10	ot
GER	506	x	c	0	0	all	-	q+s	As:oth	1990	CC	141	n	ot	y	n	0	0	ev	all/unsp	31	999	1	30	st
HAENSZ	521	x	f	0	0	all	-	q+u	NAmer	1955	CC	158	n	bl	n	y	0	0	ev	cig+/-ot	15	999	1	14	st
JEDRYC	509		m	0	0	all	-	q	Eu:est	1980	CC	1630	n	bl	y	n	0	0	ev	cig+/-ot	50	999	1	19	st
JOLY	645		m	0	0	all	-	q	SCAmer	1978	CC	826	n	bl	n	n	0	0	ev	cig+/-ot	50	999	1	29	st
JOLY	617		f	0	0	all	-	q	SCAmer	1978	CC	826	n	bl	n	n	0	0	ev	cig+/-ot	50	999	1	29	st
KATSOU	525	x	f	0	0	all	-	KI	Eu:bal	1987	CC	101	n	bl	n	n	0	0	cu	all/unsp	30	999	1	29	st
KHUDER	530		m	0	0	all	-	q	NAmer	1985	CC	482	n	bl	n	y	5	3#	ev	cig+/-ot	30	999	1	29	or
LUBIN2	667		m	0	0	all	-	q	Eu:mul	1976	CC	7804	n	bl	n	y	0	0	ev	cig+/-ot	50	999	1	29	st
LUBIN2	719		f	0	0	all	-	q	Eu:mul	1976	CC	7804	n	bl	n	y	0	0	ev	cig+/-ot	50	999	1	29	st
LUO	503	x	c	0	0	all	-	q	As:Chi	1990	CC	102	n	ot	n	y	0	0	ev	cig+/-ot	30	999	1	29	st
MATOS	605	x	m	0	0	all	-	q	SCAmer	1994	CC	200	n	bl	n	n	0	0	ev	cig+/-ot	40	70	1	24	st
OSANN2	509	x	f	0	0	all	-	KI	NAmer	1964	ot	217	n	bl	n	y	0	0	ev	cig+/-ot	21	999	1	20	st
PEZZOT	511	x	m	0	0	all	-	q	SCAmer	1987	CC	215	n	bl	n	y	0	0	ev	cig only	41	999	1	30	st
SOBUE	508		m	0	0	all	-	q	As:Jap	1986	CC	1376	n	bl	n	y	0	0	cu	cig+/-ot	50	999	1	29	st
WUWILL	510		f	0	0	all	-	q	As:Chi	1985	CC	965	n	ot	n	n	0	0	ev	cig+/-ot	40	999	1	29	st
WYNDE2	513		m	0	0	all	-	KI	NAmer	1962	CC	404	n	bl	n	y	0	0	ev	cig+/-ot	41	999	1	29	st
ZHENG	505	x	m	0	0	all	-	q	As:Chi	1982	CC	540	n	ot	*	y	0	0	ev	cig+/-ot	40	999	1	29	st
ZHENG	510	x	f	0	0	all	-	q	As:Chi	1982	CC	540	n	ot	*	y	0	0	ev	cig+/-ot	30	999	1	29	st
ZHOU	506		c	0	0	all	-	q	As:Chi	1978	CC	1360	n	ot	n	n	0	0	ev	all/unsp	20	999	1	19	st

Comments on values in listings

KHUDER ADOS Age at starting smoking, No of cigarettes per day, Quitted smoking

Cigarette type is all/unspec for all RRs

Table 2I20 - 5

IESLC - Meta-analysis of Ever/current Smoking, Duration, "Highest vs lowest"
Squamous, Cigarettes (or Any Product if Cigarettes not available)
Least adjusted

REF	NRR	SEX	AD	Number Exposed Case	Cont	Non-exposed Case	Cont	RR	95.00%CI
BARBON	561	m	0	149	235	7	91	8.24	(3.72- 18.27)
BOUCHA	501	c	0	114	92	34	79	2.88	(1.77- 4.69)
CHOI	565	m	0	11	20	42	221	2.89	(1.29- 6.48)
CHOI	577	f	0	1	1	6	23	3.83	(0.21- 70.63)
Subtotal CHOI								2.95	(1.36- 6.42)
DAMBER	553	m	1	-	-	-	-	3.80	(1.82- 7.91)
DORGAN	527	m	2	-	-	-	-	2.77	(2.04- 3.76)
DORGAN	523	f	3	-	-	-	-	3.67	(2.52- 5.34)
Subtotal DORGAN								3.10	(2.44- 3.93)
DOSEME	515	m	2	199	-	15	-	4.08	(2.07- 8.05)
GER	506	c	0	42	119	6	37	2.18	(0.86- 5.52)
HAENSZ	521	f	0	42	77	14	26	1.01	(0.48- 2.15)
JEDRYC	509	m	0	49	214	7	68	2.22	(0.96- 5.14)
JOLY	645	m	0	98	253	15	109	2.81	(1.56- 5.07)
JOLY	617	f	0	22	20	5	54	11.88	(3.96- 35.63)
Subtotal JOLY								3.88	(2.31- 6.51)
KATSOU	525	f	0	19	6	5	12	7.60	(1.89- 30.50)
KHUDER	530	m	5	-	-	-	-	1.90	(0.90- 3.70)
LUBIN2	667	m	0	746	1460	453	2964	3.34	(2.93- 3.82)
LUBIN2	719	f	0	566	34	322	229	11.84	(8.05- 17.40)
Subtotal LUBIN2								3.82	(3.37- 4.33)
LUO	503	c	0	28	45	6	21	2.18	(0.78- 6.06)
MATOS	605	m	0	26	89	3	84	8.18	(2.39- 28.03)
OSANN2	509	f	0	101	35	11	26	6.82	(3.06- 15.23)
PEZZOT	511	m	0	45	49	5	45	8.27	(3.01- 22.66)
SOBUE	508	m	0	77	73	16	119	7.85	(4.25- 14.47)
WUWILL	510	f	0	81	114	54	139	1.83	(1.20- 2.80)
WYNDE2	513	m	0	94	89	22	55	2.64	(1.49- 4.68)
ZHENG	505	m	0	84	63	13	75	7.69	(3.92- 15.08)
ZHENG	510	f	0	35	27	8	17	2.75	(1.04- 7.33)
Subtotal ZHENG								5.53	(3.18- 9.63)
ZHOU	506	c	0	315	36	60	12	1.75	(0.86- 3.56)
Partial Totals				2944	3151	1129	4506		

*prospective study

REF	NRR	SEX	AD	Ys	Ws	Qs	Ps
BARBON	561	m	0	2.11	6.07	4.44	0.0000
BOUCHA	501	c	0	1.06	16.20	0.62	0.0000
CHOI	565	m	0	1.06	5.91	0.22	0.0098
CHOI	577	f	0	1.34	0.45	0.00	0.3661
Subtotal CHOI				1.08	6.36	0.22	
DAMBER	553	m	1	1.34	7.12	0.05	0.0004
DORGAN	527	m	2	1.02	41.10	2.27	0.0000
DORGAN	523	f	3	1.30	27.25	0.06	0.0000
Subtotal DORGAN				1.13	68.34	2.33	
DOSEME	515	m	2	1.41	8.33	0.19	0.0000
GER	506	c	0	0.78	4.43	1.00	0.1018
HAENSZ	521	f	0	0.01	6.82	10.50	0.9731
JEDRYC	509	m	0	0.80	5.48	1.13	0.0614
JOLY	645	m	0	1.03	11.11	0.53	0.0006
JOLY	617	f	0	2.47	3.18	4.75	0.0000
Subtotal JOLY				1.36	14.30	5.28	
KATSOU	525	f	0	2.03	1.99	1.19	0.0042
KHUDER	530	m	5	0.64	7.69	2.88	0.0751
LUBIN2	667	m	0	1.21	218.80	0.48	0.0000
LUBIN2	719	f	0	2.47	25.87	38.37	0.0000
Subtotal LUBIN2				1.34	244.68	38.84	
LUO	503	c	0	0.78	3.67	0.83	0.1358
MATOS	605	m	0	2.10	2.53	1.82	0.0008
OSANN2	509	f	0	1.92	5.96	2.65	0.0000
PEZZOT	511	m	0	2.11	3.78	2.78	0.0000
SOBUE	508	m	0	2.06	10.25	6.66	0.0000
WUWILL	510	f	0	0.60	21.35	9.02	0.0053
WYNDE2	513	m	0	0.97	11.69	0.93	0.0009
ZHENG	505	m	0	2.04	8.47	5.24	0.0000
ZHENG	510	f	0	1.01	4.01	0.23	0.0425
Subtotal ZHENG				1.71	12.48	5.47	
ZHOU	506	c	0	0.56	7.64	3.68	0.1220

Table 2I20 - 5

IESLC - Meta-analysis of Ever/current Smoking, Duration, "Highest vs lowest"
Squamous, Cigarettes (or Any Product if Cigarettes not available)
Least adjusted

	N	27
	NS	22
	Wt	477.15
Het	Chi	102.52
Het	df	26
Het	P	***
Fixed	RR	3.50
	RRl	3.20
	RRu	3.83
	P	+++
Random	RR	3.68
	RRl	2.94
	RRu	4.60
	P	+++
Asymm	P	N.S.

Table 2I20 - 6

IESLC - Meta-analysis of Ever/current Smoking, Duration, "Highest vs lowest"
Squamous, Cigarettes (or Any Product if Cigarettes not available)
Least adjusted

	Sex			
	combined	male	female	Total
N	4	14	9	27
NS	4	14	9	27
Wt	31.94	348.32	96.88	477.15
Het Chi	1.37	29.50	63.07	102.52
Het df	3	13	8	26
Het P	N.S.	**	***	***
Fixed RR	2.38	3.44	4.26	3.50
RRl	1.68	3.09	3.49	3.20
RRu	3.37	3.82	5.20	3.83
P	+++	+++	+++	+++
Random RR	2.38	3.81	4.21	3.68
RRl	1.68	3.03	2.22	2.94
RRu	3.37	4.77	7.99	4.60
P	+++	+++	+++	+++
Between Chi				8.58
Between df				2
Between P				*
Btwn(F) P				N.S.
Btwn(R) P				(*)

Table 2I20 - 7

IESLC - Meta-analysis of Ever/current Smoking, Duration, "Highest vs lowest"
Squamous, Cigarettes (or Any Product if Cigarettes not available)
Excluded studies (and stage at which they were excluded)

1	BECHER BLOT1 BROWN3 CARPEN CHYOU DARBY DOLL2 GARCIA GRAHAM GURSEL HAMMO2 JAHN JAIN LAUSSM PRESCO QIAO TVERDA WIGLE WYNDE3
2	ALDERS BENSHL BRESLO CHIAZZ DEAN3 DORN ENGELA GAO2 GILLIS GUO HEGMAN HIRAYA HOLE KAUFMA KOO KOULUM LIU4 MIGRAN MRFITR PERNU SEGI2 SPEIZE SUZUK2 SVENSS VUTUC WAKAI WU YUAN
3	GENG MCDUFF SPITZ STASZE WU2 ZHANG
4	AKIBA GARSHI
5	AGUDO AMANDU AMES ARMADA AUVINE AXELSS BEST BOFFET BOUCOT BROSS CEDERL CHEN2 CORREA CPSI CPSII DEAN2 DESTEF DOLL FAN GAO HAMMON HU HU2 HUMBLE JUSSAW KAISE2 KREUZE LETOUR LEVIN LIAW LIU3 LIU5 MCCONN NOTAN2 PEZZO2 PISANI QIAO2 RACHTA RESTRE SADOWS TIZZAN WANG2 WATSON WYNDE6 WYNDE7 WYNDE8
6	BUFFLE CHEN LUBIN XU
15	BENHAM

Table 2I20 - 8
Potentially overlapping studies

REF	REFGP	PRINC	.	OVERLAP/LINK
LUBIN2	LUBIN2	1		Lubin-combined
OSANN2	KAISER	2		KAISER/OSANN2

Table 2I20 - 9
Most adjusted - insufficient data for meta-analysis

REF	NRR	SEX	AGEL	AGEH	RACE	YF	LC TYPE	LOC	START	ST	NLC	R	VB	P	H	AD	ADOS	SM	PRODUCT	exL	exH	unexL	unexH	De
BUFFLE	551	m	0	0	wh	-	q	NAmer	1976	CC	943	n	bl	y	n	0	0	ev	cig+/-ot	50	999	1	33	st
CHEN	515	c	0	0	all	-	q	As:oth	1987	CC	323	n	ot	n	y	2	0	ev	cig+/-ot	41	999	1	20	st
LUBIN	617	m	0	0	all	-	KI	As:Chi	1984	CC	427	m	ot	y	n	5	1#	ev	cig+/-ot	50	999	1	29	or
XU	515	m	0	0	all	-	q+s	As:Chi	1985	CC	729	n	ot	n	n	2	0	ev	all/unsp	40	999	1	29	st

Comments on values in listings

LUBIN ADOS Duration of pipe use

REF	NRR	RR	SIG	RRDATA comment
BUFFLE	551	2.46		0
CHEN	515	4.96		0
LUBIN	617	4.72		0
XU	515	*		RR for 1-19/day is 2.2, for 20-29/day is 4.0 and for >=30/day is 4.1

Table 2I21 -

IESLC - Meta-analysis of Ever/current Smoking by Duration, Overview
Squamous, Cigarettes only

This analysis is restricted to results for:
1) Ever/current smokers
2) Results by Duration
3) Categorical results by Duration
Results by Duration are grouped under 2 schemes (S1, S2). Each scheme has a set of "key values". An interval is allocated to the category whose key value it includes, and intervals which include none or more than one of the key values are excluded. (Open-ended intervals are coded as 999)

S1	key value	maximum range
1	20	1-34
2	35	21-49
3	50	36+

S2	key value	maximum range
1	5	1-19
2	20	6-29
3	30	21-39
4	40	31-49
5	50	41-998
6	999	51+

4) Squamous (or near equivalent)
5) Results complete enough for use in metaanalysis

Within each study, results are then selected (in the following order of preference, within each sex) for:
6) SMKSTA: ever, current
7) PRODUCT: cigarettes only
8) CIGTYPE: all/unspecified, MC regardless of HR, MC only
9) (not applicable)
10) DENOM: never smoked anything, never smoked cigarettes, never any + low, never cigs + low
11) Followup period (YF, prospective studies): whole study (coded as 0) or longest available
12) LCtype: squamous or nearest available, but not adeno. (q = squamous, s = small,
a = adeno, KI = Kreyberg I, u = undifferentiated)
13) Race: all or nearest available, otherwise by race (wh or w = white, bl or b = black, hi = hispanic
ch = chinese, jap = japanese, haw = hawaiian, w+o = white + oriental, sca = scandinavian, as = asian)
14) For overlapping studies: principal rather than subsidiary studies
Finally by Age: whole study (coded as 0) if available, otherwise by widest available age group
and then for single sex results (m, f) in preference to results for both sexes combined (c).

Results adjusted (AD) for the most potential confounders are then chosen in Sections -1 to -3
(and those which actually differ from the adjusted results in Table 2I11 - 1 are marked 'x' in Section -1)
and results adjusted for the least confounders in Sections -4 to -6. (Those least adjusted results which
actually differ from the most adjusted are marked 'x' in column X in Section -4)

Section -7 shows excluded studies, together with the stage (as above) at which no qualifying
results were found.

Section -8 lists the potentially overlapping studies which have been included (1=principal, 2=subsidiary).

Section -9 lists any results which would have been included in preference except that they had data not complete
enough for use in meta-analysis, with their significance (yes/no), if known, and any further comment as entered
on the database. It also lists as "gap" any categories for which no data were presented by the original authors.

In addition to those mentioned above, the following fields, levels and abbreviations are used:
* or nk = not known, n = no, y = yes, ot = other
ev = ever, cu = current, nev = never
all/unspec = all or unspecified, MC = manufactured cigarettes, HR = hand-rolled cigarettes
exL, exH = range of exposure (low and high) in the smoking group, in terms of Duration
REF: 6-character study reference
NRR: number of the RR on the database within the study
ST : study type (CC = case control, pr or prosp = prospective)
NLC: number of lung cancer cases in whole study
R : risky occupational population (n = no, m = mining, o = other risky)
VB : national cigarette type (V = at least 75% Virginia, bl = at least 75% blended, ot = other)
P : any proxy use
H : full histological confirmation
De : derivation of RR/CI (or = original, st = standard method, ot = other method of estimation)

Table 2I21 - 1

IESLC - Meta-analysis of Ever/current Smoking by Duration, Overview
Squamous, Cigarettes only
Most adjusted

REF	NRR	2I11	SEX	AGEL	AGEH	RACE	YF	LC TYPE	LOC	START	ST	NLC	R	VB	P	H	AD	SM	PRODUCT	exL	exH	S1	S2	DENOM	De
BENHAM	501	x	m	0	0	all	-	KI	Eu:wst	1976	CC	1625	n	bl	n	y	0	ev	cig only	1	25	1	0	nev any	st
BENHAM	502	x	m	0	0	all	-	KI	Eu:wst	1976	CC	1625	n	bl	n	y	0	ev	cig only	26	35	2	3	nev any	st
BENHAM	503	x	m	0	0	all	-	KI	Eu:wst	1976	CC	1625	n	bl	n	y	0	ev	cig only	36	45	0	4	nev any	st
BENHAM	504	x	m	0	0	all	-	KI	Eu:wst	1976	CC	1625	n	bl	n	y	0	ev	cig only	46	999	3	0	nev any	st
PEZZOT	507		m	0	0	all	-	q	SCAmer	1987	CC	215	n	bl	n	y	0	ev	cig only	1	30	1	0	nev cigs	ot
PEZZOT	508		m	0	0	all	-	q	SCAmer	1987	CC	215	n	bl	n	y	0	ev	cig only	31	40	2	4	nev cigs	ot
PEZZOT	509		m	0	0	all	-	q	SCAmer	1987	CC	215	n	bl	n	y	0	ev	cig only	41	999	3	0	nev cigs	ot

Cigarette type is all/unspec for all RRs

In this overview table, subtotals and Qs values may be invalid and should be ignored

Table 2I21 - 2

IESLC - Meta-analysis of Ever/current Smoking by Duration, Overview
Squamous, Cigarettes only
Most adjusted

REF	NRR	SEX	AD	Number Exposed Case	Cont	Non-exposed Case	Cont	RR	95.00%CI
BENHAM	501	m	0	90	283	24	481	6.37	(3.97- 10.23)
BENHAM	502	m	0	345	396	24	481	17.46	(11.31- 26.97)
BENHAM	503	m	0	365	360	24	481	20.32	(13.15- 31.39)
BENHAM	504	m	0	274	210	24	481	26.15	(16.71- 40.91)
Subtotal BENHAM								16.06	(12.84- 20.08)
PEZZOT	507	m	0	5	134	0	116	9.53~	(0.52- 174.14)
PEZZOT	508	m	0	35	82	0	116	100.26~	(6.06-1657.79)
PEZZOT	509	m	0	45	101	0	116	104.45~	(6.35-1717.05)
Subtotal PEZZOT								48.16	(9.37- 247.59)
Totals				1159	1566	96	2272		

*prospective study ~ With 0.5 adjustment for zero

REF	NRR	SEX	AD	Ys	Ws	Qs	Ps
BENHAM	501	m	0	1.85	17.13	15.27	0.0000
BENHAM	502	m	0	2.86	20.34	0.08	0.0000
BENHAM	503	m	0	3.01	20.30	0.94	0.0000
BENHAM	504	m	0	3.26	19.17	4.19	0.0000
Subtotal BENHAM				2.78	76.94	20.48	
PEZZOT	507	m	0	2.25	0.45	0.13	0.1284
PEZZOT	508	m	0	4.61	0.49	1.60	0.0013
PEZZOT	509	m	0	4.65	0.49	1.68	0.0011
Subtotal PEZZOT				3.87	1.43	3.42	

N	7
NS	2

Table 2I21 - 3

IESLC - Meta-analysis of Ever/current Smoking by Duration, Overview
Squamous, Cigarettes only
Most adjusted

	Sex			
	combined	male	female	Total
N		7		7
NS		2		2

In this overview table, other than the "N" rows, entries in the "absent" and "Total" columns may be invalid and should be ignored

	Duration of smoking (broad categories)				
	absent	1-34k20	21-49k35	36+k50	Total
N	1	2	2	2	7
NS	1	2	2	2	6
Wt	20.30	17.58	20.83	19.66	78.37
Het Chi	0.00	0.07	1.46	0.92	23.90
Het df	0	1	1	1	6
Het P	N.S.	N.S.	N.S.	N.S.	***
Fixed RR	20.32	6.44	18.19	27.07	16.38
RRl	13.15	4.04	11.84	17.40	13.13
RRu	31.39	10.28	27.95	42.11	20.45
P	+++	+++	+++	+++	+++
Random RR	20.32	6.44	23.61	27.07	17.51
RRl	13.15	4.04	6.47	17.40	10.08
RRu	31.39	10.28	86.20	42.11	30.45
P	+++	+++	+++	+++	+++

	Duration of smoking (narrow categories)							
	absent	1-19k1	6-29k20	21-39k30	31-49k40	41-998k50	51+k999	Total
N	4			1	2			7
NS	2			1	2			4
Wt	37.24			20.34	20.79			78.37
Het Chi	20.12			0.00	1.21			23.90
Het df	3			0	1			6
Het P	***			N.S.	N.S.			***
Fixed RR	13.74			17.46	21.10			16.38
RRl	9.97			11.31	13.72			13.13
RRu	18.95			26.97	32.43			20.45
P	+++			+++	+++			+++
Random RR	16.11			17.46	24.13			17.51
RRl	5.00			11.31	9.15			10.08
RRu	51.88			26.97	63.60			30.45
P	+++			+++	+++			+++

Table 2I21 - 3

IESLC - Meta-analysis of Ever/current Smoking by Duration, Overview
Squamous, Cigarettes only
Most adjusted

MALES

		Duration of smoking (broad categories)				
		absent	1-34k20	21-49k35	36+k50	Total
	N	1	2	2	2	7
	NS	1	2	2	2	6
	Wt	20.30	17.58	20.83	19.66	78.37
Het	Chi	0.00	0.07	1.46	0.92	23.90
Het	df	0	1	1	1	6
Het	P	N.S.	N.S.	N.S.	N.S.	***
Fixed	RR	20.32	6.44	18.19	27.07	16.38
	RRl	13.15	4.04	11.84	17.40	13.13
	RRu	31.39	10.28	27.95	42.11	20.45
	P	+++	+++	+++	+++	+++
Random	RR	20.32	6.44	23.61	27.07	17.51
	RRl	13.15	4.04	6.47	17.40	10.08
	RRu	31.39	10.28	86.20	42.11	30.45
	P	+++	+++	+++	+++	+++

			Duration of smoking (narrow categories)						
		absent	1-19k1	6-29k20	21-39k30	31-49k40	41-998k50	51+k999	Total
	N	4			1	2			7
	NS	2			1	2			4
	Wt	37.24			20.34	20.79			78.37
Het	Chi	20.12			0.00	1.21			23.90
Het	df	3			0	1			6
Het	P	***			N.S.	N.S.			***
Fixed	RR	13.74			17.46	21.10			16.38
	RRl	9.97			11.31	13.72			13.13
	RRu	18.95			26.97	32.43			20.45
	P	+++			+++	+++			+++
Random	RR	16.11			17.46	24.13			17.51
	RRl	5.00			11.31	9.15			10.08
	RRu	51.88			26.97	63.60			30.45
	P	+++			+++	+++			+++

Table 2I21 - 4

IESLC - Meta-analysis of Ever/current Smoking by Duration, Overview
Squamous, Cigarettes only
Least adjusted

REF	NRR	X	SEX	AGEL	AGEH	RACE	YF	LC TYPE	LOC	START	ST	NLC	R	VB	P	H	AD	SM	PRODUCT	exL	exH	S1	S2	DENOM	De
BENHAM	501		m	0	0	all	-	KI	Eu:wst	1976	CC	1625	n	bl	n	y	0	ev	cig only	1	25	1	0	nev any	st
BENHAM	502		m	0	0	all	-	KI	Eu:wst	1976	CC	1625	n	bl	n	y	0	ev	cig only	26	35	2	3	nev any	st
BENHAM	503		m	0	0	all	-	KI	Eu:wst	1976	CC	1625	n	bl	n	y	0	ev	cig only	36	45	0	4	nev any	st
BENHAM	504		m	0	0	all	-	KI	Eu:wst	1976	CC	1625	n	bl	n	y	0	ev	cig only	46	999	3	0	nev any	st
PEZZOT	507		m	0	0	all	-	q	SCAmer	1987	CC	215	n	bl	n	y	0	ev	cig only	1	30	1	0	nev cigs	ot
PEZZOT	508		m	0	0	all	-	q	SCAmer	1987	CC	215	n	bl	n	y	0	ev	cig only	31	40	2	4	nev cigs	ot
PEZZOT	509		m	0	0	all	-	q	SCAmer	1987	CC	215	n	bl	n	y	0	ev	cig only	41	999	3	0	nev cigs	ot

Cigarette type is all/unspec for all RRs

In this overview table, subtotals and Qs values may be invalid and should be ignored

Table 2I21 - 5

IESLC - Meta-analysis of Ever/current Smoking by Duration, Overview
Squamous, Cigarettes only
Least adjusted

REF	NRR	SEX	AD	Number Exposed Case	Cont	Non-exposed Case	Cont	RR	95.00%CI
BENHAM	501	m	0	90	283	24	481	6.37	(3.97- 10.23)
BENHAM	502	m	0	345	396	24	481	17.46	(11.31- 26.97)
BENHAM	503	m	0	365	360	24	481	20.32	(13.15- 31.39)
BENHAM	504	m	0	274	210	24	481	26.15	(16.71- 40.91)
Subtotal BENHAM								16.06	(12.84- 20.08)
PEZZOT	507	m	0	5	134	0	116	9.53~	(0.52- 174.14)
PEZZOT	508	m	0	35	82	0	116	100.26~	(6.06-1657.79)
PEZZOT	509	m	0	45	101	0	116	104.45~	(6.35-1717.05)
Subtotal PEZZOT								48.16	(9.37- 247.59)
Totals				1159	1566	96	2272		

*prospective study ~ With 0.5 adjustment for zero

REF	NRR	SEX	AD	Ys	Ws	Qs	Ps
BENHAM	501	m	0	1.85	17.13	15.27	0.0000
BENHAM	502	m	0	2.86	20.34	0.08	0.0000
BENHAM	503	m	0	3.01	20.30	0.94	0.0000
BENHAM	504	m	0	3.26	19.17	4.19	0.0000
Subtotal BENHAM				2.78	76.94	20.48	
PEZZOT	507	m	0	2.25	0.45	0.13	0.1284
PEZZOT	508	m	0	4.61	0.49	1.60	0.0013
PEZZOT	509	m	0	4.65	0.49	1.68	0.0011
Subtotal PEZZOT				3.87	1.43	3.42	

N	7
NS	2

Table 2I21 - 6

IESLC - Meta-analysis of Ever/current Smoking by Duration, Overview
Squamous, Cigarettes only
Least adjusted

	Sex combined	male	female	Total
N		7		7
NS		2		2

In this overview table, other than the "N" rows, entries in the "absent" and "Total" columns may be invalid and should be ignored

	Duration of smoking (broad categories) absent	1-34k20	21-49k35	36+k50	Total
N	1	2	2	2	7
NS	1	2	2	2	6
Wt	20.30	17.58	20.83	19.66	78.37
Het Chi	0.00	0.07	1.46	0.92	23.90
Het df	0	1	1	1	6
Het P	N.S.	N.S.	N.S.	N.S.	***
Fixed RR	20.32	6.44	18.19	27.07	16.38
RRl	13.15	4.04	11.84	17.40	13.13
RRu	31.39	10.28	27.95	42.11	20.45
P	+++	+++	+++	+++	+++
Random RR	20.32	6.44	23.61	27.07	17.51
RRl	13.15	4.04	6.47	17.40	10.08
RRu	31.39	10.28	86.20	42.11	30.45
P	+++	+++	+++	+++	+++

	absent	Duration of smoking (narrow categories) 1-19k1	6-29k20	21-39k30	31-49k40	41-998k50	51+k999	Total
N	4			1	2			7
NS	2			1	2			4
Wt	37.24			20.34	20.79			78.37
Het Chi	20.12			0.00	1.21			23.90
Het df	3			0	1			6
Het P	***			N.S.	N.S.			***
Fixed RR	13.74			17.46	21.10			16.38
RRl	9.97			11.31	13.72			13.13
RRu	18.95			26.97	32.43			20.45
P	+++			+++	+++			+++
Random RR	16.11			17.46	24.13			17.51
RRl	5.00			11.31	9.15			10.08
RRu	51.88			26.97	63.60			30.45
P	+++			+++	+++			+++

Table 2I21 - 6

IESLC - Meta-analysis of Ever/current Smoking by Duration, Overview
Squamous, Cigarettes only
Least adjusted

MALES

		Duration of smoking (broad categories)				
		absent	1-34k20	21-49k35	36+k50	Total
	N	1	2	2	2	7
	NS	1	2	2	2	6
	Wt	20.30	17.58	20.83	19.66	78.37
Het	Chi	0.00	0.07	1.46	0.92	23.90
Het	df	0	1	1	1	6
Het	P	N.S.	N.S.	N.S.	N.S.	***
Fixed	RR	20.32	6.44	18.19	27.07	16.38
	RRl	13.15	4.04	11.84	17.40	13.13
	RRu	31.39	10.28	27.95	42.11	20.45
	P	+++	+++	+++	+++	+++
Random	RR	20.32	6.44	23.61	27.07	17.51
	RRl	13.15	4.04	6.47	17.40	10.08
	RRu	31.39	10.28	86.20	42.11	30.45
	P	+++	+++	+++	+++	+++

		Duration of smoking (narrow categories)							
		absent	1-19k1	6-29k20	21-39k30	31-49k40	41-998k50	51+k999	Total
	N	4			1	2			7
	NS	2			1	2			4
	Wt	37.24			20.34	20.79			78.37
Het	Chi	20.12			0.00	1.21			23.90
Het	df	3			0	1			6
Het	P	***			N.S.	N.S.			***
Fixed	RR	13.74			17.46	21.10			16.38
	RRl	9.97			11.31	13.72			13.13
	RRu	18.95			26.97	32.43			20.45
	P	+++			+++	+++			+++
Random	RR	16.11			17.46	24.13			17.51
	RRl	5.00			11.31	9.15			10.08
	RRu	51.88			26.97	63.60			30.45
	P	+++			+++	+++			+++

Table 2I21 - 7

IESLC - Meta-analysis of Ever/current Smoking by Duration, Overview
Squamous, Cigarettes only
Excluded studies (and stage at which they were excluded)

1	BECHER	BLOT1	BROWN3	CARPEN	CHYOU	DARBY	DOLL2	GARCIA	GRAHAM	GURSEL	HAMMO2	JAHN	JAIN	LAUSSM	PRESCO	QIAO
	TVERDA	WIGLE	WYNDE3													
2	ALDERS	BENSHL	BRESLO	CHIAZZ	DEAN3	DORN	ENGELA	GAO2	GILLIS	GUO	HEGMAN	HIRAYA	HOLE	KAUFMA	KOO	KOULUM
	LIU4	MIGRAN	MRFITR	PERNU	SEGI2	SPEIZE	SUZUK2	SVENSS	VUTUC	WAKAI	WU	YUAN				
3	GENG	MCDUFF	SPITZ	STASZE	WU2	ZHANG										
4	AGUDO	AKIBA	AMANDU	AMES	ARMADA	AUVINE	AXELSS	BEST	BOFFET	BOUCOT	BROSS	CEDERL	CHEN2	CORREA	CPSI	CPSII
	DEAN2	DESTEF	DOLL	FAN	GAO	GARSHI	HAMMON	HU	HU2	HUMBLE	JUSSAW	KAISE2	KREUZE	LETOUR	LEVIN	LIAW
	LIU3	LIU5	MCCONN	NOTAN2	PEZZO2	PISANI	QIAO2	RACHTA	RESTRE	SADOWS	TIZZAN	WANG2	WATSON	WYNDE6	WYNDE7	WYNDE8
5	CHEN	LUBIN	XU													
7	BARBON	BOUCHA	BUFFLE	CHOI	DAMBER	DORGAN	DOSEME	GER	HAENSZ	JEDRYC	JOLY	KATSOU	KHUDER	LUBIN2	LUO	MATOS
	OSANN2	SOBUE	WUWILL	WYNDE2	ZHENG	ZHOU										

Table 2I21 - 8

Potentially overlapping studies

REF	REFGP	PRINC	.	OVERLAP/LINK
BENHAM	LUBIN2	2		Subset of Lubin2

Table 2I22 -

IESLC - Meta-analysis of Ever/current Smoking, Duration, "Low"
Squamous, Cigarettes only

This analysis is restricted to results for:
1) Ever/current smokers
2) Results by Duration
3) Categorical results by Duration
4) Squamous (or near equivalent)
5) Results complete enough for use in metaanalysis

Within each study, results are then selected (in the following order of preference, within each sex) for:
6) SMKSTA: ever, current
7) PRODUCT: cigarettes only
8) CIGTYPE: all/unspecified, MC regardless of HR, MC only
9) (not applicable)
10) DENOM: never smoked anything, never smoked cigarettes, never any + low, never cigs + low
11) Followup period (YF, prospective studies): whole study (coded as 0) or longest available
12) LCtype: squamous or nearest available, but not adeno. (q = squamous, s = small,
a = adeno, KI = Kreyberg I, u = undifferentiated)
13) Race: all or nearest available, otherwise by race (wh or w = white, bl or b = black, hi = hispanic
ch = chinese, jap = japanese, haw = hawaiian, w+o = white + oriental, sca = scandinavian, as = asian)
14) Duration "low" in key scheme 1 (key value 20, maximum range 1-34)
15) For overlapping studies: principal rather than subsidiary studies
Finally by Age: whole study (coded as 0) if available, otherwise by widest available age group
and then for single sex results (m, f) in preference to results for both sexes combined (c).

Results adjusted (AD) for the most potential confounders are then chosen in Sections -1 to -3
(and those which actually differ from the adjusted results in Table 2I12 - 1 are marked 'x' in Section -1)
and results adjusted for the least confounders in Sections -4 to -6. (Those least adjusted results which
actually differ from the most adjusted are marked 'x' in column X in Section -4)

Section -7 shows excluded studies, together with the stage (as above) at which no qualifying
results were found.

Section -8 lists the potentially overlapping studies which have been included (1=principal, 2=subsidiary).

Section -9 lists any results which would have been included in preference except that they had data not complete
enough for use in meta-analysis, with their significance (yes/no), if known, and any further comment as entered
on the database. It also lists as "gap" any categories for which no data were presented by the original authors.

In addition to those mentioned above, the following fields, levels and abbreviations are used:
* or nk = not known, n = no, y = yes, ot = other
ev = ever, cu = current, nev = never
all/unspec = all or unspecified, MC = manufactured cigarettes, HR = hand-rolled cigarettes
exL, exH = range of exposure (low and high) in the smoking group, in terms of Duration
REF: 6-character study reference
NRR: number of the RR on the database within the study
ST : study type (CC = case control, pr or prosp = prospective)
NLC: number of lung cancer cases in whole study
R : risky occupational population (n = no, m = mining, o = other risky)
VB : national cigarette type (V = at least 75% Virginia, bl = at least 75% blended, ot = other)
P : any proxy use
H : full histological confirmation
De : derivation of RR/CI (or = original, st = standard method, ot = other method of estimation)

Table 2I22 - 1

IESLC - Meta-analysis of Ever/current Smoking, Duration, "Low"
Squamous, Cigarettes only
Most adjusted

REF	NRR	2I12	SEX	AGEL	AGEH	RACE	YF	LC TYPE	LOC	START	ST	NLC	R	VB	P	H	AD	SM	PRODUCT	exL	exH	DENOM	De
BENHAM	501	x	m	0	0	all	-	KI	Eu:wst	1976	CC	1625	n	bl	n	y	0	ev	cig only	1	25	nev any	st
PEZZOT	507		m	0	0	all	-	q	SCAmer	1987	CC	215	n	bl	n	y	0	ev	cig only	1	30	nev cigs	ot

Cigarette type is all/unspec for all RRs

Table 2I22 - 2

IESLC - Meta-analysis of Ever/current Smoking, Duration, "Low"
Squamous, Cigarettes only
Most adjusted

REF	NRR	SEX	AD	Number Case	Exposed Cont	Non-exposed Case	Cont	RR	95.00%CI
BENHAM	501	m	0	90	283	24	481	6.37	(3.97- 10.23)
PEZZOT	507	m	0	5	134	0	116	9.53~	(0.52- 174.14)
Totals				95	417	24	597		

*prospective study

~ With 0.5 adjustment for zero

REF	NRR	SEX	AD	Ys	Ws	Qs	Ps
BENHAM	501	m	0	1.85	17.13	0.00	0.0000
PEZZOT	507	m	0	2.25	0.45	0.07	0.1284

	N	2
	NS	2
	Wt	17.58
Het	Chi	0.07
Het	df	1
Het	P	N.S.
Fixed	RR	6.44
	RRl	4.04
	RRu	10.28
	P	+++
Random	RR	6.44
	RRl	4.04
	RRu	10.28
	P	+++
Asymm	P	

Table 2I22 - 3

IESLC - Meta-analysis of Ever/current Smoking, Duration, "Low"
Squamous, Cigarettes only
Most adjusted

	Sex combined	male	female	Total
N		2		2
NS		2		2
Wt		17.58		17.58
Het Chi		0.07		0.07
Het df		1		1
Het P		N.S.		N.S.
Fixed RR		6.44		6.44
RRl		4.04		4.04
RRu		10.28		10.28
P		+++		+++
Random RR		6.44		6.44
RRl		4.04		4.04
RRu		10.28		10.28
P		+++		+++
Between Chi				
Between df				
Between P				N.S.
Btwn(F) P				N.S.
Btwn(R) P				N.S.

Too few RRs for analysis by factor

Table 2I22 - 4

IESLC - Meta-analysis of Ever/current Smoking, Duration, "Low"
Squamous, Cigarettes only
Least adjusted

REF	NRR	X	SEX	AGEL	AGEH	RACE	YF	LC TYPE	LOC	START	ST	NLC	R	VB	P	H	AD	SM	PRODUCT	exL	exH	DENOM	De
BENHAM	501		m	0	0	all	-	KI	Eu:wst	1976	CC	1625	n	bl	n	y	0	ev	cig only	1	25	nev any	st
PEZZOT	507		m	0	0	all	-	q	SCAmer	1987	CC	215	n	bl	n	y	0	ev	cig only	1	30	nev cigs	ot

Cigarette type is all/unspec for all RRs

Table 2I22 - 5

IESLC - Meta-analysis of Ever/current Smoking, Duration, "Low"
Squamous, Cigarettes only
Least adjusted

REF	NRR	SEX	AD	Number Exposed Case	Cont	Non-exposed Case	Cont	RR	95.00%CI
BENHAM	501	m	0	90	283	24	481	6.37	(3.97- 10.23)
PEZZOT	507	m	0	5	134	0	116	9.53~	(0.52- 174.14)
Totals				95	417	24	597		

*prospective study

~ With 0.5 adjustment for zero

REF	NRR	SEX	AD	Ys	Ws	Qs	Ps
BENHAM	501	m	0	1.85	17.13	0.00	0.0000
PEZZOT	507	m	0	2.25	0.45	0.07	0.1284

	N	2
	NS	2
	Wt	17.58
Het	Chi	0.07
Het	df	1
Het	P	N.S.
Fixed	RR	6.44
	RRl	4.04
	RRu	10.28
	P	+++
Random	RR	6.44
	RRl	4.04
	RRu	10.28
	P	+++
Asymm	P	

Table 2I22 - 6

IESLC - Meta-analysis of Ever/current Smoking, Duration, "Low"
Squamous, Cigarettes only
Least adjusted

	combined	Sex male	female	Total
N		2		2
NS		2		2
Wt		17.58		17.58
Het Chi		0.07		0.07
Het df		1		1
Het P		N.S.		N.S.
Fixed RR		6.44		6.44
RRl		4.04		4.04
RRu		10.28		10.28
P		+++		+++
Random RR		6.44		6.44
RRl		4.04		4.04
RRu		10.28		10.28
P		+++		+++
Between Chi				
Between df				
Between P				N.S.
Btwn(F) P				N.S.
Btwn(R) P				N.S.

Table 2I22 - 7

IESLC - Meta-analysis of Ever/current Smoking, Duration, "Low"
Squamous, Cigarettes only
Excluded studies (and stage at which they were excluded)

1	BECHER BLOT1 BROWN3 CARPEN CHYOU DARBY DOLL2 GARCIA GRAHAM GURSEL HAMMO2 JAHN JAIN LAUSSM PRESCO QIAO TVERDA WIGLE WYNDE3
2	ALDERS BENSHL BRESLO CHIAZZ DEAN3 DORN ENGELA GAO2 GILLIS GUO HEGMAN HIRAYA HOLE KAUFMA KOO KOULUM LIU4 MIGRAN MRFITR PERNU SEGI2 SPEIZE SUZUK2 SVENSS VUTUC WAKAI WU YUAN
3	GENG MCDUFF SPITZ STASZE WU2 ZHANG
4	AGUDO AKIBA AMANDU AMES ARMADA AUVINE AXELSS BEST BOFFET BOUCOT BROSS CEDERL CHEN2 CORREA CPSI CPSII DEAN2 DESTEF DOLL FAN GAO GARSHI HAMMON HU HU2 HUMBLE JUSSAW KAISE2 KREUZE LETOUR LEVIN LIAW LIU3 LIU5 MCCONN NOTAN2 PEZZO2 PISANI QIAO2 RACHTA RESTRE SADOWS TIZZAN WANG2 WATSON WYNDE6 WYNDE7 WYNDE8
5	CHEN LUBIN XU
7	BARBON BOUCHA BUFFLE CHOI DAMBER DORGAN DOSEME GER HAENSZ JEDRYC JOLY KATSOU KHUDER LUBIN2 LUO MATOS OSANN2 SOBUE WUWILL WYNDE2 ZHENG ZHOU

Table 2I22 - 8

Potentially overlapping studies

REF	REFGP	PRINC	. OVERLAP/LINK
BENHAM	LUBIN2	2	Subset of Lubin2

Table 2I23 -

IESLC - Meta-analysis of Ever/current Smoking, Duration, "Mid"
Squamous, Cigarettes only

This analysis is restricted to results for:
1) Ever/current smokers
2) Results by Duration
3) Categorical results by Duration
4) Squamous (or near equivalent)
5) Results complete enough for use in metaanalysis

Within each study, results are then selected (in the following order of preference, within each sex) for:
6) SMKSTA: ever, current
7) PRODUCT: cigarettes only
8) CIGTYPE: all/unspecified, MC regardless of HR, MC only
9) (not applicable)
10) DENOM: never smoked anything, never smoked cigarettes, never any + low, never cigs + low
11) Followup period (YF, prospective studies): whole study (coded as 0) or longest available
12) LCtype: squamous or nearest available, but not adeno. (q = squamous, s = small,
a = adeno, KI = Kreyberg I, u = undifferentiated)
13) Race: all or nearest available, otherwise by race (wh or w = white, bl or b = black, hi = hispanic
ch = chinese, jap = japanese, haw = hawaiian, w+o = white + oriental, sca = scandinavian, as = asian)
14) Duration "mid" in key scheme 1 (key value 35, maximum range 21-49)
15) For overlapping studies: principal rather than subsidiary studies
Finally by Age: whole study (coded as 0) if available, otherwise by widest available age group
and then for single sex results (m, f) in preference to results for both sexes combined (c).

Results adjusted (AD) for the most potential confounders are then chosen in Sections -1 to -3
(and those which actually differ from the adjusted results in Table 2I13 - 1 are marked 'x' in Section -1)
and results adjusted for the least confounders in Sections -4 to -6. (Those least adjusted results which
actually differ from the most adjusted are marked 'x' in column X in Section -4)

Section -7 shows excluded studies, together with the stage (as above) at which no qualifying
results were found.

Section -8 lists the potentially overlapping studies which have been included (1=principal, 2=subsidiary).

Section -9 lists any results which would have been included in preference except that they had data not complete
enough for use in meta-analysis, with their significance (yes/no), if known, and any further comment as entered
on the database. It also lists as "gap" any categories for which no data were presented by the original authors.

In addition to those mentioned above, the following fields, levels and abbreviations are used:
* or nk = not known, n = no, y = yes, ot = other
ev = ever, cu = current, nev = never
all/unspec = all or unspecified, MC = manufactured cigarettes, HR = hand-rolled cigarettes
exL, exH = range of exposure (low and high) in the smoking group, in terms of Duration
REF: 6-character study reference
NRR: number of the RR on the database within the study
ST : study type (CC = case control, pr or prosp = prospective)
NLC: number of lung cancer cases in whole study
R : risky occupational population (n = no, m = mining, o = other risky)
VB : national cigarette type (V = at least 75% Virginia, bl = at least 75% blended, ot = other)
P : any proxy use
H : full histological confirmation
De : derivation of RR/CI (or = original, st = standard method, ot = other method of estimation)

Table 2I23 - 1

IESLC - Meta-analysis of Ever/current Smoking, Duration, "Mid"
Squamous, Cigarettes only
Most adjusted

REF	NRR	2I13	SEX	AGEL	AGEH	RACE	YF	LC TYPE	LOC	START	ST	NLC	R	VB	P	H	AD	SM	PRODUCT	exL	exH	DENOM	De
BENHAM	502	x	m	0	0	all	-	KI	Eu:wst	1976	CC	1625	n	bl	n	y	0	ev	cig only	26	35	nev any	st
PEZZOT	508		m	0	0	all	-	q	SCAmer	1987	CC	215	n	bl	n	y	0	ev	cig only	31	40	nev cigs	ot

Cigarette type is all/unspec for all RRs

Table 2I23 - 2

IESLC - Meta-analysis of Ever/current Smoking, Duration, "Mid"
Squamous, Cigarettes only
Most adjusted

REF	NRR	SEX	AD	Number Exposed Case	Cont	Non-exposed Case	Cont	RR	95.00%CI
BENHAM	502	m	0	345	396	24	481	17.46	(11.31- 26.97)
PEZZOT	508	m	0	35	82	0	116	100.26~	(6.06-1657.79)
Totals				380	478	24	597		

*prospective study ~ With 0.5 adjustment for zero

REF	NRR	SEX	AD	Ys	Ws	Qs	Ps
BENHAM	502	m	0	2.86	20.34	0.03	0.0000
PEZZOT	508	m	0	4.61	0.49	1.42	0.0013

	N	2
	NS	2
	Wt	20.83
Het	Chi	1.46
Het	df	1
Het	P	N.S.
Fixed	RR	18.19
	RRl	11.84
	RRu	27.95
	P	+++
Random	RR	23.61
	RRl	6.47
	RRu	86.20
	P	+++
Asymm	P	

Table 2I23 - 3

IESLC - Meta-analysis of Ever/current Smoking, Duration, "Mid"
Squamous, Cigarettes only
Most adjusted

	Sex combined	male	female	Total
N		2		2
NS		2		2
Wt		20.83		20.83
Het Chi		1.46		1.46
Het df		1		1
Het P		N.S.		N.S.
Fixed RR		18.19		18.19
RRl		11.84		11.84
RRu		27.95		27.95
P		+++		+++
Random RR		23.61		23.61
RRl		6.47		6.47
RRu		86.20		86.20
P		+++		+++
Between Chi				
Between df				
Between P				N.S.
Btwn(F) P				N.S.
Btwn(R) P				N.S.

Too few RRs for analysis by factor

Table 2I23 - 4

IESLC - Meta-analysis of Ever/current Smoking, Duration, "Mid"
Squamous, Cigarettes only
Least adjusted

REF	NRR	X	SEX	AGEL	AGEH	RACE	YF	LC TYPE	LOC	START	ST	NLC	R	VB	P	H	AD	SM	PRODUCT	exL	exH	DENOM	De
BENHAM	502		m	0	0	all	-	KI	Eu:wst	1976	CC	1625	n	bl	n	y	0	ev	cig only	26	35	nev any	st
PEZZOT	508		m	0	0	all	-	q	SCAmer	1987	CC	215	n	bl	n	y	0	ev	cig only	31	40	nev cigs	ot

Cigarette type is all/unspec for all RRs

Table 2I23 - 5

IESLC - Meta-analysis of Ever/current Smoking, Duration, "Mid"
Squamous, Cigarettes only
Least adjusted

REF	NRR	SEX	AD	Number Exposed Case	Cont	Non-exposed Case	Cont	RR	95.00%CI
BENHAM	502	m	0	345	396	24	481	17.46	(11.31- 26.97)
PEZZOT	508	m	0	35	82	0	116	100.26~	(6.06-1657.79)
Totals				380	478	24	597		

*prospective study ~ With 0.5 adjustment for zero

REF	NRR	SEX	AD	Ys	Ws	Qs	Ps
BENHAM	502	m	0	2.86	20.34	0.03	0.0000
PEZZOT	508	m	0	4.61	0.49	1.42	0.0013

	N	2
	NS	2
	Wt	20.83
Het	Chi	1.46
Het	df	1
Het	P	N.S.
Fixed	RR	18.19
	RRl	11.84
	RRu	27.95
	P	+++
Random	RR	23.61
	RRl	6.47
	RRu	86.20
	P	+++
Asymm	P	

Table 2I23 - 6

IESLC - Meta-analysis of Ever/current Smoking, Duration, "Mid"
Squamous, Cigarettes only
Least adjusted

	Sex combined	male	female	Total
N		2		2
NS		2		2
Wt		20.83		20.83
Het Chi		1.46		1.46
Het df		1		1
Het P		N.S.		N.S.
Fixed RR		18.19		18.19
RRl		11.84		11.84
RRu		27.95		27.95
P		+++		+++
Random RR		23.61		23.61
RRl		6.47		6.47
RRu		86.20		86.20
P		+++		+++
Between Chi				
Between df				
Between P				N.S.
Btwn(F) P				N.S.
Btwn(R) P				N.S.

Table 2I23 - 7

IESLC - Meta-analysis of Ever/current Smoking, Duration, "Mid"
Squamous, Cigarettes only
Excluded studies (and stage at which they were excluded)

1 BECHER BLOT1 BROWN3 CARPEN CHYOU DARBY DOLL2 GARCIA GRAHAM GURSEL HAMMO2 JAHN JAIN LAUSSM PRESCO QIAO TVERDA WIGLE WYNDE3

2 ALDERS BENSHL BRESLO CHIAZZ DEAN3 DORN ENGELA GAO2 GILLIS GUO HEGMAN HIRAYA HOLE KAUFMA KOO KOULUM LIU4 MIGRAN MRFITR PERNU SEGI2 SPEIZE SUZUK2 SVENSS VUTUC WAKAI WU YUAN

3 GENG MCDUFF SPITZ STASZE WU2 ZHANG

4 AGUDO AKIBA AMANDU AMES ARMADA AUVINE AXELSS BEST BOFFET BOUCOT BROSS CEDERL CHEN2 CORREA CPSI CPSII DEAN2 DESTEF DOLL FAN GAO GARSHI HAMMON HU HU2 HUMBLE JUSSAW KAISE2 KREUZE LETOUR LEVIN LIAW LIU3 LIU5 MCCONN NOTAN2 PEZZO2 PISANI QIAO2 RACHTA RESTRE SADOWS TIZZAN WANG2 WATSON WYNDE6 WYNDE7 WYNDE8

5 CHEN LUBIN XU

7 BARBON BOUCHA BUFFLE CHOI DAMBER DORGAN DOSEME GER HAENSZ JEDRYC JOLY KATSOU KHUDER LUBIN2 LUO MATOS OSANN2 SOBUE WUWILL WYNDE2 ZHENG ZHOU

Table 2I23 - 8

Potentially overlapping studies

REF	REFGP	PRINC	. OVERLAP/LINK
BENHAM	LUBIN2	2	Subset of Lubin2

Table 2I24 -

IESLC - Meta-analysis of Ever/current Smoking, Duration, "High"
Squamous, Cigarettes only

This analysis is restricted to results for:
1) Ever/current smokers
2) Results by Duration
3) Categorical results by Duration
4) Squamous (or near equivalent)
5) Results complete enough for use in metaanalysis

Within each study, results are then selected (in the following order of preference, within each sex) for:
6) PRODUCT: cigarettes only
7) CIGTYPE: all/unspecified, MC regardless of HR, MC only
8) (not applicable)
9) DENOM: never smoked anything, never smoked cigarettes, never any + low, never cigs + low
10) Followup period (YF, prospective studies): whole study (coded as 0) or longest available
11) LCtype: squamous or nearest available, but not adeno. (q = squamous, s = small,
a = adeno, KI = Kreyberg I, u = undifferentiated)
12) Race: all or nearest available, otherwise by race (wh or w = white, bl or b = black, hi = hispanic
ch = chinese, jap = japanese, haw = hawaiian, w+o = white + oriental, sca = scandinavian, as = asian)
13) Duration "high" in key scheme 1 (key value 50, maximum range 36+)
14) For overlapping studies: principal rather than subsidiary studies
Finally by Age: whole study (coded as 0) if available, otherwise by widest available age group
and then for single sex results (m, f) in preference to results for both sexes combined (c).

Results adjusted (AD) for the most potential confounders are then chosen in Sections -1 to -3
(and those which actually differ from the adjusted results in Table 2I14 - 1 are marked 'x' in Section -1)
and results adjusted for the least confounders in Sections -4 to -6. (Those least adjusted results which
actually differ from the most adjusted are marked 'x' in column X in Section -4)

Section -7 shows excluded studies, together with the stage (as above) at which no qualifying
results were found.

Section -8 lists the potentially overlapping studies which have been included (1=principal, 2=subsidiary).

Section -9 lists any results which would have been included in preference except that they had data not complete
enough for use in meta-analysis, with their significance (yes/no), if known, and any further comment as entered
on the database. It also lists as "gap" any categories for which no data were presented by the original authors.

In addition to those mentioned above, the following fields, levels and abbreviations are used:
* or nk = not known, n = no, y = yes, ot = other
ev = ever, cu = current, nev = never
all/unspec = all or unspecified, MC = manufactured cigarettes, HR = hand-rolled cigarettes
exL, exH = range of exposure (low and high) in the smoking group, in terms of Duration
REF: 6-character study reference
NRR: number of the RR on the database within the study
ST : study type (CC = case control, pr or prosp = prospective)
NLC: number of lung cancer cases in whole study
R : risky occupational population (n = no, m = mining, o = other risky)
VB : national cigarette type (V = at least 75% Virginia, bl = at least 75% blended, ot = other)
P : any proxy use
H : full histological confirmation
De : derivation of RR/CI (or = original, st = standard method, ot = other method of estimation)

Table 2I24 - 1

IESLC - Meta-analysis of Ever/current Smoking, Duration, "High"
Squamous, Cigarettes only
Most adjusted

REF	NRR	2I14	SEX	AGEL	AGEH	RACE	YF	LC TYPE	LOC	START	ST	NLC	R	VB	P	H	AD	SM	PRODUCT	exL	exH	DENOM	De
BENHAM	504	x	m	0	0	all	-	KI	Eu:wst	1976	CC	1625	n	bl	n	y	0	ev	cig only	46	999	nev any	st
PEZZOT	509		m	0	0	all	-	q	SCAmer	1987	CC	215	n	bl	n	y	0	ev	cig only	41	999	nev cigs	ot

Cigarette type is all/unspec for all RRs

Table 2I24 - 2

IESLC - Meta-analysis of Ever/current Smoking, Duration, "High"
Squamous, Cigarettes only
Most adjusted

REF	NRR	SEX	AD	Number Exposed Case	Cont	Non-exposed Case	Cont	RR	95.00%CI
BENHAM	504	m	0	274	210	24	481	26.15	(16.71- 40.91)
PEZZOT	509	m	0	45	101	0	116	104.45~	(6.35-1717.05)
Totals				319	311	24	597		

*prospective study ~ With 0.5 adjustment for zero

REF	NRR	SEX	AD	Ys	Ws	Qs	Ps
BENHAM	504	m	0	3.26	19.17	0.02	0.0000
PEZZOT	509	m	0	4.65	0.49	0.89	0.0011

	N	2
	NS	2
	Wt	19.66
Het	Chi	0.92
Het	df	1
Het	P	N.S.
Fixed	RR	27.07
	RRl	17.40
	RRu	42.11
	P	+++
Random	RR	27.07
	RRl	17.40
	RRu	42.11
	P	+++
Asymm	P	

Table 2I24 - 3

IESLC - Meta-analysis of Ever/current Smoking, Duration, "High"
Squamous, Cigarettes only
Most adjusted

	Sex			
	combined	male	female	Total
N		2		2
NS		2		2
Wt		19.66		19.66
Het Chi		0.92		0.92
Het df		1		1
Het P		N.S.		N.S.
Fixed RR		27.07		27.07
RRl		17.40		17.40
RRu		42.11		42.11
P		+++		+++
Random RR		27.07		27.07
RRl		17.40		17.40
RRu		42.11		42.11
P		+++		+++
Between Chi				
Between df				
Between P				N.S.
Btwn(F) P				N.S.
Btwn(R) P				N.S.

Too few RRs for analysis by factor

Table 2I24 - 4

IESLC - Meta-analysis of Ever/current Smoking, Duration, "High"
Squamous, Cigarettes only
Least adjusted

REF	NRR	X	SEX	AGEL	AGEH	RACE	YF	LC TYPE	LOC	START	ST	NLC	R	VB	P	H	AD	SM	PRODUCT	exL	exH	DENOM	De
BENHAM	504		m	0	0	all	-	KI	Eu:wst	1976	CC	1625	n	bl	n	y	0	ev	cig only	46	999	nev any	st
PEZZOT	509		m	0	0	all	-	q	SCAmer	1987	CC	215	n	bl	n	y	0	ev	cig only	41	999	nev cigs	ot

Cigarette type is all/unspec for all RRs

Table 2I24 - 5

IESLC - Meta-analysis of Ever/current Smoking, Duration, "High"
Squamous, Cigarettes only
Least adjusted

				Number Exposed		Non-exposed			
REF	NRR	SEX	AD	Case	Cont	Case	Cont	RR	95.00%CI
BENHAM	504	m	0	274	210	24	481	26.15	(16.71- 40.91)
PEZZOT	509	m	0	45	101	0	116	104.45~	(6.35-1717.05)
Totals				319	311	24	597		

*prospective study ~ With 0.5 adjustment for zero

REF	NRR	SEX	AD	Ys	Ws	Qs	Ps
BENHAM	504	m	0	3.26	19.17	0.02	0.0000
PEZZOT	509	m	0	4.65	0.49	0.89	0.0011

	N	2
	NS	2
	Wt	19.66
Het	Chi	0.92
Het	df	1
Het	P	N.S.
Fixed	RR	27.07
	RRl	17.40
	RRu	42.11
	P	+++
Random	RR	27.07
	RRl	17.40
	RRu	42.11
	P	+++
Asymm	P	

Table 2I24 - 6

IESLC - Meta-analysis of Ever/current Smoking, Duration, "High"
Squamous, Cigarettes only
Least adjusted

	combined	Sex male	female	Total
N		2		2
NS		2		2
Wt		19.66		19.66
Het Chi		0.92		0.92
Het df		1		1
Het P		N.S.		N.S.
Fixed RR		27.07		27.07
RRl		17.40		17.40
RRu		42.11		42.11
P		+++		+++
Random RR		27.07		27.07
RRl		17.40		17.40
RRu		42.11		42.11
P		+++		+++
Between Chi				
Between df				
Between P				N.S.
Btwn(F) P				N.S.
Btwn(R) P				N.S.

Table 2I24 - 7

IESLC - Meta-analysis of Ever/current Smoking, Duration, "High"
Squamous, Cigarettes only
Excluded studies (and stage at which they were excluded)

1	BECHER BLOT1 BROWN3 CARPEN CHYOU DARBY DOLL2 GARCIA GRAHAM GURSEL HAMMO2 JAHN JAIN LAUSSM PRESCO QIAO TVERDA WIGLE WYNDE3
2	ALDERS BENSHL BRESLO CHIAZZ DEAN3 DORN ENGELA GAO2 GILLIS GUO HEGMAN HIRAYA HOLE KAUFMA KOO KOULUM LIU4 MIGRAN MRFITR PERNU SEGI2 SPEIZE SUZUK2 SVENSS VUTUC WAKAI WU YUAN
3	GENG MCDUFF SPITZ STASZE WU2 ZHANG
4	AGUDO AKIBA AMANDU AMES ARMADA AUVINE AXELSS BEST BOFFET BOUCOT BROSS CEDERL CHEN2 CORREA CPSI CPSII DEAN2 DESTEF DOLL FAN GAO GARSHI HAMMON HU HU2 HUMBLE JUSSAW KAISE2 KREUZE LETOUR LEVIN LIAW LIU3 LIU5 MCCONN NOTAN2 PEZZO2 PISANI QIAO2 RACHTA RESTRE SADOWS TIZZAN WANG2 WATSON WYNDE6 WYNDE7 WYNDE8
5	CHEN LUBIN XU
7	BARBON BOUCHA BUFFLE CHOI DAMBER DORGAN DOSEME GER HAENSZ JEDRYC JOLY KATSOU KHUDER LUBIN2 LUO MATOS OSANN2 SOBUE WUWILL WYNDE2 ZHENG ZHOU

Table 2I24 - 8

Potentially overlapping studies

REF	REFGP	PRINC	.	OVERLAP/LINK
BENHAM	LUBIN2	2		Subset of Lubin2

Table 2I25 -

IESLC - Meta-analysis of Ever/current Smoking, Duration, "Highest vs lowest"
Squamous, Cigarettes only

This analysis is restricted to results for:
1) Ever/current smokers
2) Results by Duration
3) Categorical results by Duration
4) Denominator (unexposed) = "low"
5) Squamous (or near equivalent)
6) Results complete enough for use in metaanalysis

Within each study, results are then selected (in the following order of preference, within each sex) for:
7) SMKSTA: ever, current
8) PRODUCT: cigarettes only
9) CIGTYPE: all/unspecified, MC regardless of HR, MC only
10) Results with least adjustment for other aspects of smoking (ADOS)
11) The highest vs lowest category
12) Followup period (YF, prospective studies): whole study (coded as 0) or longest available
13) LCtype: squamous or nearest available, but not adeno. (q = squamous, s = small,
a = adeno, KI = Kreyberg I, u = undifferentiated)
14) Race: all or nearest available, otherwise by race (wh or w = white, bl or b = black, hi = hispanic
ch = chinese, jap = japanese, haw = hawaiian, w+o = white + oriental, sca = scandinavian, as = asian)
15) For overlapping studies: principal rather than subsidiary studies
Finally by Age: whole study (coded as 0) if available, otherwise by widest available age group
and then for single sex results (m, f) in preference to results for both sexes combined (c).

Results adjusted (AD) for the most potential confounders are then chosen in Sections -1 to -3
(and those which actually differ from the adjusted results in Table 2I15 - 1 are marked 'x' in Section -1)
and results adjusted for the least confounders in Sections -4 to -6. (Those least adjusted results which
actually differ from the most adjusted are marked 'x' in column X in Section -4)

Section -7 shows excluded studies, together with the stage (as above) at which no qualifying
results were found.

Section -8 lists the potentially overlapping studies which have been included (1=principal, 2=subsidiary).

Section -9 lists any results which would have been included in preference except that they had data not complete
enough for use in meta-analysis, with their significance (yes/no), if known, and any further comment as entered
on the database. It also lists as "gap" any categories for which no data were presented by the original authors.

In addition to those mentioned above, the following fields, levels and abbreviations are used:
* or nk = not known, n = no, y = yes, ot = other
all/unspec = all or unspecified, MC = manufactured cigarettes, HR = hand-rolled cigarettes
exL, exH = range of exposure (low and high) in the "highest" group, in terms of Duration
unexL, unexH = range of exposure (low and high) in the "lowest" group, in terms of Duration
REF: 6-character study reference
NRR: number of the RR on the database within the study
ST : study type (CC = case control, pr or prosp = prospective)
NLC: number of lung cancer cases in whole study
R : risky occupational population (n = no, m = mining, o = other risky)
VB : national cigarette type (V = at least 75% Virginia, bl = at least 75% blended, ot = other)
P : any proxy use
H : full histological confirmation
De : derivation of RR/CI (or = original, st = standard method, ot = other method of estimation)

Table 2I25 - 1

IESLC - Meta-analysis of Ever/current Smoking, Duration, "Highest vs lowest"
Squamous, Cigarettes only
Most adjusted

REF	NRR	2I15	SEX	AGEL	AGEH	RACE	YF	LC TYPE	LOC	START	ST	NLC	R	VB	P	H	AD	ADOS	SM	PRODUCT	exL	exH	unexL	unexH	De
BENHAM	507	x	m	0	0	all	-	KI	Eu:wst	1976	CC	1625	n	bl	n	y	0	0	ev	cig only	46	999	1	25	st
PEZZOT	513		m	0	0	all	-	q	SCAmer	1987	CC	215	n	bl	n	y	2	0	ev	cig only	41	999	1	30	ot

Cigarette type is all/unspec for all RRs

Table 2I25 - 2

IESLC - Meta-analysis of Ever/current Smoking, Duration, "Highest vs lowest"
Squamous, Cigarettes only
Most adjusted

REF	NRR	SEX	AD	Number Exposed Case	Cont	Non-exposed Case	Cont	RR	95.00%CI
BENHAM	507	m	0	274	210	90	283	4.10	(3.05- 5.52)
PEZZOT	513	m	2	45	-	5	-	9.90	(3.61- 27.15)
Partial Totals				319	210	95	283		

*prospective study

REF	NRR	SEX	AD	Ys	Ws	Qs	Ps
BENHAM	507	m	0	1.41	43.37	0.22	0.0000
PEZZOT	513	m	2	2.29	3.77	2.48	0.0000

	N	2
	NS	2
	Wt	47.15
Het	Chi	2.69
Het	df	1
Het	P	N.S.
Fixed	RR	4.40
	RRl	3.31
	RRu	5.86
	P	+++
Random	RR	5.56
	RRl	2.45
	RRu	12.62
	P	+++
Asymm	P	

Table 2I25 - 3

IESLC - Meta-analysis of Ever/current Smoking, Duration, "Highest vs lowest"
Squamous, Cigarettes only
Most adjusted

	Sex			
	combined	male	female	Total
N		2		2
NS		2		2
Wt		47.15		47.15
Het Chi		2.69		2.69
Het df		1		1
Het P		N.S.		N.S.
Fixed RR		4.40		4.40
RRl		3.31		3.31
RRu		5.86		5.86
P		+++		+++
Random RR		5.56		5.56
RRl		2.45		2.45
RRu		12.62		12.62
P		+++		+++
Between Chi				
Between df				
Between P				N.S.
Btwn(F) P				N.S.
Btwn(R) P				N.S.

Too few RRs for analysis by factor

Table 2I25 - 4

IESLC - Meta-analysis of Ever/current Smoking, Duration, "Highest vs lowest"
Squamous, Cigarettes only
Least adjusted

REF	NRR	X	SEX	AGEL	AGEH	RACE	YF	LC TYPE	LOC	START	ST	NLC	R	VB	P	H	AD	ADOS	SM	PRODUCT	exL	exH	unexL	unexH	De
BENHAM	507		m	0	0	all	-	KI	Eu:wst	1976	CC	1625	n	bl	n	y	0	0	ev	cig only	46	999	1	25	st
PEZZOT	511	x	m	0	0	all	-	q	SCAmer	1987	CC	215	n	bl	n	y	0	0	ev	cig only	41	999	1	30	st

Cigarette type is all/unspec for all RRs

Table 2I25 - 5

IESLC - Meta-analysis of Ever/current Smoking, Duration, "Highest vs lowest"
Squamous, Cigarettes only
Least adjusted

				Number Exposed		Non-exposed			
REF	NRR	SEX	AD	Case	Cont	Case	Cont	RR	95.00%CI
BENHAM	507	m	0	274	210	90	283	4.10	(3.05- 5.52)
PEZZOT	511	m	0	45	49	5	45	8.27	(3.01- 22.66)
Totals				319	259	95	328		

*prospective study

REF	NRR	SEX	AD	Ys	Ws	Qs	Ps
BENHAM	507	m	0	1.41	43.37	0.14	0.0000
PEZZOT	511	m	0	2.11	3.78	1.57	0.0000

	N	2
	NS	2
	Wt	47.15
Het	Chi	1.70
Het	df	1
Het	P	N.S.
Fixed	RR	4.34
	RRl	3.26
	RRu	5.77
	P	+++
Random	RR	4.90
	RRl	2.70
	RRu	8.90
	P	+++
Asymm	P	

Table 2I25 - 6

IESLC - Meta-analysis of Ever/current Smoking, Duration, "Highest vs lowest"
Squamous, Cigarettes only
Least adjusted

		Sex		
	combined	male	female	Total
N		2		2
NS		2		2
Wt		47.15		47.15
Het Chi		1.70		1.70
Het df		1		1
Het P		N.S.		N.S.
Fixed RR		4.34		4.34
RRl		3.26		3.26
RRu		5.77		5.77
P		+++		+++
Random RR		4.90		4.90
RRl		2.70		2.70
RRu		8.90		8.90
P		+++		+++
Between Chi				
Between df				
Between P				N.S.
Btwn(F) P				N.S.
Btwn(R) P				N.S.

Table 2I25 - 7

IESLC - Meta-analysis of Ever/current Smoking, Duration, "Highest vs lowest"
Squamous, Cigarettes only
Excluded studies (and stage at which they were excluded)

1	BECHER BLOT1 BROWN3 CARPEN CHYOU DARBY DOLL2 GARCIA GRAHAM GURSEL HAMMO2 JAHN JAIN LAUSSM PRESCO QIAO TVERDA WIGLE WYNDE3
2	ALDERS BENSHL BRESLO CHIAZZ DEAN3 DORN ENGELA GAO2 GILLIS GUO HEGMAN HIRAYA HOLE KAUFMA KOO KOULUM LIU4 MIGRAN MRFITR PERNU SEGI2 SPEIZE SUZUK2 SVENSS VUTUC WAKAI WU YUAN
3	GENG MCDUFF SPITZ STASZE WU2 ZHANG
4	AKIBA GARSHI
5	AGUDO AMANDU AMES ARMADA AUVINE AXELSS BEST BOFFET BOUCOT BROSS CEDERL CHEN2 CORREA CPSI CPSII DEAN2 DESTEF DOLL FAN GAO HAMMON HU HU2 HUMBLE JUSSAW KAISE2 KREUZE LETOUR LEVIN LIAW LIU3 LIU5 MCCONN NOTAN2 PEZZO2 PISANI QIAO2 RACHTA RESTRE SADOWS TIZZAN WANG2 WATSON WYNDE6 WYNDE7 WYNDE8
6	BUFFLE CHEN LUBIN XU
8	BARBON BOUCHA CHOI DAMBER DORGAN DOSEME GER HAENSZ JEDRYC JOLY KATSOU KHUDER LUBIN2 LUO MATOS OSANN2 SOBUE WUWILL WYNDE2 ZHENG ZHOU

Table 2I25 - 8
Potentially overlapping studies

REF	REFGP	PRINC	.	OVERLAP/LINK
BENHAM	LUBIN2	2		Subset of Lubin2
