# Supplementary material for: Systematic review with meta-analysis of the epidemiological evidence in the 1900s relating smoking to lung cancer
Source: BMC Cancer. 2012 Sep 3;12:385. doi: 10.1186/1471-2407-12-385 (PMC3505152; doi:10.1186/1471-2407-12-385)
Supplement: Additional file 5 — Detailed Analysis Tables (Individual file names as described in Additional file 1: Methods, Table1). [file 1471-2407-12-385-S5.zip › PDF/1L.pdf]

Table 1L1 -
IESLC - Meta-analysis of Ever Smoking, Tar, "Highest vs lowest"
All LC types, Cigarettes (or Any Product if Cigarettes not available)

This analysis is restricted to results for:
1) Ever smokers
2) Results by Tar
3) Categorical results by Tar
4) Denominator (unexposed) = "low"
5) All LC types (or near equivalent)
6) Results complete enough for use in metaanalysis

Within each study, results are then selected (in the following order of preference, within each sex) for:
7) (not applicable)
8) PRODUCT: cigarettes regardless of other products, cigarettes only, all/unspec
9) CIGTYPE: all/unspecified, MC regardless of HR, MC only
10) Results with least adjustment for other aspects of smoking (ADOS)
11) The highest vs lowest category
12) Followup period (YF, prospective studies): whole study (coded as 0) or longest available
13) LCtype: all or nearest available, at least Squamous and Adeno. (q = squamous, s = small,
l = large, a = adeno, mix = mixed, alv = alveolar)
14) Race: all or nearest available, otherwise by race (wh or w = white, bl or b = black, hi = hispanic
ch = chinese, jap = japanese, haw = hawaiian, w+o = white + oriental, sca = scandinavian, as = asian)
15) For overlapping studies: principal rather than subsidiary studies
Finally by Age: whole study (coded as 0) if available, otherwise by widest available age group
and then for single sex results (m, f) in preference to results for both sexes combined (c).

Results adjusted (AD) for the most potential confounders are then chosen in Sections -1 to -3
and results adjusted for the least confounders in Sections -4 to -6. (Those least adjusted results which
actually differ from the most adjusted are marked 'x' in column X in Section -4)

Section -7 shows excluded studies, together with the stage (as above) at which no qualifying
results were found.

Section -8 lists the potentially overlapping studies which have been included (1=principal, 2=subsidiary).

Section -9 lists any results which would have been included in preference except that they had data not complete
enough for use in meta-analysis, with their significance (yes/no), if known, and any further comment as entered
on the database. It also lists as "gap" any categories for which no data were presented by the original authors.

In addition to those mentioned above, the following fields, levels and abbreviations are used:
* or nk = not known, n = no, y = yes, ot = other
all/unspec = all or unspecified, cig+/-ot = cigarettes irrespective of other products (cigar, pipe etc)
MC = manufactured cigarettes, HR = hand-rolled cigarettes
exL, exH = range of exposure (low and high) in the "highest" group, in terms of Tar
unexL, unexH = range of exposure (low and high) in the "lowest" group, in terms of Tar
REF: 6-character study reference
NRR: number of the RR on the database within the study
ST : study type (CC = case control, pr or prosp = prospective)
NLC: number of lung cancer cases in whole study
R : risky occupational population (n = no, m = mining, o = other risky)
VB : national cigarette type (V = at least 75% Virginia, bl = at least 75% blended, ot = other)
P : any proxy use
H : full histological confirmation
De : derivation of RR/CI (or = original, st = standard method, ot = other method of estimation)

Table 1L1 - 1

IESLC - Meta-analysis of Ever Smoking, Tar, "Highest vs lowest"
All LC types, Cigarettes (or Any Product if Cigarettes not available)
Most adjusted

REF	NRR	SEX	AGEL	AGEH	RACE	YF	LC TYPE	LOC	START	ST	NLC	R	VB	P	H	AD	ADOS	PRODUCT	exL	exH	unexL	unexH	De
ALDERS	529	m	0	0	all	-	all	Eu:UK	1977	CC	1448	n	V	n	n	2	1#	cig only	17	22	1	16	ot
ALDERS	530	f	0	0	all	-	all	Eu:UK	1977	CC	1448	n	V	n	n	2	1#	cig only	17	22	1	16	ot
DORGAN	540	m	0	0	wh	-	all	NAmer	1980	CC	2026	n	bl	y	y	0	0	cig+/-ot	21	28	1	14	st
LUBIN2	639	m	0	0	all	-	all	Eu:mul	1976	CC	7804	n	bl	n	y	0	0	cig+/-ot	906	906#	901	901	st
LUBIN2	655	f	0	0	all	-	all	Eu:mul	1976	CC	7804	n	bl	n	y	0	0	cig+/-ot	906	906#	901	901	st

Comments on values in listings

ALDERS ADOS Number of cigs/day
ALDERS ADOS Number of cigs/day

exL, exH, unexL, unexH refer to mg tar per cigarette (with “no upper limit” coded as 999) for all RRs except for the following:

LUBIN2 Categories based on percentiles of mean tar level (calculated from current tar levels of brands ever smoked weighted by amounts smoked) with the percentiles calculated within country. Mean tar value for this level is 29.8mg vs a lower level of 15.6mg

Cigarette type is all/unspec for all RRs

except for the following:

REF	NRR	CIGTYPE
ALDERS	529	MC only
ALDERS	530	MC only

Table 1L1 - 2

IESLC - Meta-analysis of Ever Smoking, Tar, "Highest vs lowest"
All LC types, Cigarettes (or Any Product if Cigarettes not available)
Most adjusted

REF	NRR	SEX	AD	Number Exposed Case	Number Exposed Cont	Non-exposed Case	Non-exposed Cont	RR	95.00%CI
ALDERS	529	m	2	156	-	38	-	0.91	(0.54- 1.53)
ALDERS	530	f	2	145	-	85	-	1.04	(0.69- 1.58)
Subtotal ALDERS								0.99	(0.71- 1.37)
DORGAN	540	m	0	139	83	25	28	1.88	(1.03- 3.43)
LUBIN2	639	m	0	133	300	186	428	1.02	(0.78- 1.33)
LUBIN2	655	f	0	9	4	85	151	4.00	(1.20- 13.37)
Subtotal LUBIN2								1.09	(0.84- 1.41)
Partial Totals				582	387	419	607		

*prospective study

REF	NRR	SEX	AD	Ys	Ws	Qs	Ps
ALDERS	529	m	2	-0.09	14.17	0.56	0.7226
ALDERS	530	f	2	0.04	22.39	0.10	0.8528
Subtotal ALDERS				-0.01	36.55	0.66	
DORGAN	540	m	0	0.63	10.53	2.89	0.0412
LUBIN2	639	m	0	0.02	53.87	0.39	0.8836
LUBIN2	655	f	0	1.39	2.64	4.32	0.0245
Subtotal LUBIN2				0.08	56.50	4.71	

	N	5
	NS	3
	Wt	103.58
Het	Chi	8.26
Het	df	4
Het	P	(*)
Fixed	RR	1.11
	RRl	0.92
	RRu	1.35
	P	N.S.
Random	RR	1.20
	RRl	0.88
	RRu	1.66
	P	N.S.
Asymm	P	N.S.

Table 1L1 - 3

IESLC - Meta-analysis of Ever Smoking, Tar, "Highest vs lowest"
All LC types, Cigarettes (or Any Product if Cigarettes not available)
Most adjusted

	Sex			
	combined	male	female	Total
N		3	2	5
NS		3	2	5
Wt		78.56	25.02	103.58
Het Chi		3.80	4.27	8.26
Het df		2	1	4
Het P		N.S.	*	(*)
Fixed RR		1.08	1.20	1.11
RRl		0.87	0.81	0.92
RRu		1.35	1.77	1.35
P		N.S.	N.S.	N.S.
Random RR		1.14	1.80	1.20
RRl		0.80	0.49	0.88
RRu		1.62	6.58	1.66
P		N.S.	N.S.	N.S.
Between Chi				0.19
Between df				1
Between P				N.S.
Btwn(F) P				N.S.
Btwn(R) P				N.S.

Too few RRs for analysis by factor

Table 1L1 - 4

IESLC - Meta-analysis of Ever Smoking, Tar, "Highest vs lowest"
All LC types, Cigarettes (or Any Product if Cigarettes not available)
Least adjusted

REF	NRR	X	SEX	AGEL	AGEH	RACE	YF	LC TYPE	LOC	START	ST	NLC	R	VB	P	H	AD	ADOS	PRODUCT	exL	exH	unexL	unexH	De
ALDERS	529		m	0	0	all	-	all	Eu:UK	1977	CC	1448	n	V	n	n	2	1#	cig only	17	22	1	16	ot
ALDERS	530		f	0	0	all	-	all	Eu:UK	1977	CC	1448	n	V	n	n	2	1#	cig only	17	22	1	16	ot
DORGAN	540		m	0	0	wh	-	all	NAmer	1980	CC	2026	n	bl	y	y	0	0	cig+/-ot	21	28	1	14	st
LUBIN2	639		m	0	0	all	-	all	Eu:mul	1976	CC	7804	n	bl	n	y	0	0	cig+/-ot	906	906#	901	901	st
LUBIN2	655		f	0	0	all	-	all	Eu:mul	1976	CC	7804	n	bl	n	y	0	0	cig+/-ot	906	906#	901	901	st

Comments on values in listings

ALDERS ADOS Number of cigs/day
ALDERS ADOS Number of cigs/day

exL, exH, unexL, unexH refer to mg tar per cigarette (with “no upper limit” coded as 999) for all RRs except for the following:

LUBIN2 Categories based on percentiles of mean tar level (calculated from current tar levels of brands ever smoked weighted by amounts smoked) with the percentiles calculated within country. Mean tar value for this level is 29.8mg vs a lower level of 15.6mg

Cigarette type is all/unspec for all RRs

except for the following:

REF	NRR	CIGTYPE
ALDERS	529	MC only
ALDERS	530	MC only

Table 1L1 - 5

IESLC - Meta-analysis of Ever Smoking, Tar, "Highest vs lowest"
All LC types, Cigarettes (or Any Product if Cigarettes not available)
Least adjusted

REF	NRR	SEX	AD	Number Exposed Case	Cont	Non-exposed Case	Cont	RR	95.00%CI
ALDERS	529	m	2	156	-	38	-	0.91	(0.54- 1.53)
ALDERS	530	f	2	145	-	85	-	1.04	(0.69- 1.58)
Subtotal ALDERS								0.99	(0.71- 1.37)
DORGAN	540	m	0	139	83	25	28	1.88	(1.03- 3.43)
LUBIN2	639	m	0	133	300	186	428	1.02	(0.78- 1.33)
LUBIN2	655	f	0	9	4	85	151	4.00	(1.20- 13.37)
Subtotal LUBIN2								1.09	(0.84- 1.41)
Partial Totals				582	387	419	607		

*prospective study

REF	NRR	SEX	AD	Ys	Ws	Qs	Ps
ALDERS	529	m	2	-0.09	14.17	0.56	0.7226
ALDERS	530	f	2	0.04	22.39	0.10	0.8528
Subtotal ALDERS				-0.01	36.55	0.66	
DORGAN	540	m	0	0.63	10.53	2.89	0.0412
LUBIN2	639	m	0	0.02	53.87	0.39	0.8836
LUBIN2	655	f	0	1.39	2.64	4.32	0.0245
Subtotal LUBIN2				0.08	56.50	4.71	

	N	5
	NS	3
	Wt	103.58
Het	Chi	8.26
Het	df	4
Het	P	(*)
Fixed	RR	1.11
	RRl	0.92
	RRu	1.35
	P	N.S.
Random	RR	1.20
	RRl	0.88
	RRu	1.66
	P	N.S.
Asymm	P	N.S.

Table 1L1 - 6

IESLC - Meta-analysis of Ever Smoking, Tar, "Highest vs lowest"
All LC types, Cigarettes (or Any Product if Cigarettes not available)
Least adjusted

	Sex			
	combined	male	female	Total
N		3	2	5
NS		3	2	5
Wt		78.56	25.02	103.58
Het Chi		3.80	4.27	8.26
Het df		2	1	4
Het P		N.S.	*	(*)
Fixed RR		1.08	1.20	1.11
RRl		0.87	0.81	0.92
RRu		1.35	1.77	1.35
P		N.S.	N.S.	N.S.
Random RR		1.14	1.80	1.20
RRl		0.80	0.49	0.88
RRu		1.62	6.58	1.66
P		N.S.	N.S.	N.S.
Between Chi				0.19
Between df				1
Between P				N.S.
Btwn(F) P				N.S.
Btwn(R) P				N.S.

Table 1L1 - 7

IESLC - Meta-analysis of Ever Smoking, Tar, "Highest vs lowest"
All LC types, Cigarettes (or Any Product if Cigarettes not available)
Excluded studies (and stage at which they were excluded)

1	AKIBA AMANDU AMES BECHER BENSHL BEST BLOT1 BROSS BROWN3 CARPEN CEDERL CHYOU CPSI CPSII DARBY DEAN2 DEAN3 DOLL2 ENGELA GAO2 GARCIA GILLIS GRAHAM GURSEL HAMMO2 HIRAYA HOLE HUMBLE JAHN JAIN KAISE2 KATSOU KAUFMA LAUSSM LIAW MCDUFF MIGRAN MRFITR PEZZO2 PISANI PRESCO QIAO SEGI2 SPEIZE SVENSS TVERDA WAKAI WATSON WIGLE WU WYNDE3 WYNDE8
2	AGUDO ARMADA AUVINE AXELSS BARBON BENHAM BOFFET BOUCHA BOUCOT BRESLO BUFFLE CHEN CHEN2 CHIAZZ CHOI CORREA DAMBER DESTEF DOLL DORN DOSEME FAN GAO GARSHI GENG GER GUO HAENSZ HAMMON HEGMAN HU HU2 JEDRYC JOLY JUSSAW KHUDER KOO KOULUM KREUZE LETOUR LEVIN LIU3 LIU4 LIU5 LUBIN LUO MATOS MCCONN NOTAN2 OSANN2 PERNU PEZZOT QIAO2 RACHTA RESTRE SADOWS SOBUE SPITZ STASZE SUZUK2 TIZZAN WANG2 WU2 WUWILL WYNDE2 WYNDE7 XU YUAN ZHANG ZHENG ZHOU
3	WYNDE6
15	VUTUC

Table 1L1 - 8
Potentially overlapping studies

REF	REFGP	PRINC	.	OVERLAP/LINK
LUBIN2	LUBIN2	1		Lubin-combined

Table 1L2 -

IESLC - Meta-analysis of Current Smoking, Tar, "Highest vs lowest"
All LC types, Cigarettes (or Any Product if Cigarettes not available)

This analysis is restricted to results for:
1) Current smokers
2) Results by Tar
3) Categorical results by Tar
4) Denominator (unexposed) = "low"
5) All LC types (or near equivalent)
6) Results complete enough for use in metaanalysis

Within each study, results are then selected (in the following order of preference, within each sex) for:
7) (not applicable)
8) PRODUCT: cigarettes regardless of other products, cigarettes only, all/unspec
9) CIGTYPE: all/unspecified, MC regardless of HR, MC only
10) Results with least adjustment for other aspects of smoking (ADOS)
11) The highest vs lowest category
12) Followup period (YF, prospective studies): whole study (coded as 0) or longest available
13) LCtype: all or nearest available, at least Squamous and Adeno. (q = squamous, s = small,
l = large, a = adeno, mix = mixed, alv = alveolar)
14) Race: all or nearest available, otherwise by race (wh or w = white, bl or b = black, hi = hispanic
ch = chinese, jap = japanese, haw = hawaiian, w+o = white + oriental, sca = scandinavian, as = asian)
15) For overlapping studies: principal rather than subsidiary studies
Finally by Age: whole study (coded as 0) if available, otherwise by widest available age group
and then for single sex results (m, f) in preference to results for both sexes combined (c).

Results adjusted (AD) for the most potential confounders are then chosen in Sections -1 to -3
and results adjusted for the least confounders in Sections -4 to -6. (Those least adjusted results which
actually differ from the most adjusted are marked 'x' in column X in Section -4)

Section -7 shows excluded studies, together with the stage (as above) at which no qualifying
results were found.

Section -8 lists the potentially overlapping studies which have been included (1=principal, 2=subsidiary).

Section -9 lists any results which would have been included in preference except that they had data not complete
enough for use in meta-analysis, with their significance (yes/no), if known, and any further comment as entered
on the database. It also lists as "gap" any categories for which no data were presented by the original authors.

In addition to those mentioned above, the following fields, levels and abbreviations are used:
* or nk = not known, n = no, y = yes, ot = other
all/unspec = all or unspecified, cig+/-ot = cigarettes irrespective of other products (cigar, pipe etc)
MC = manufactured cigarettes, HR = hand-rolled cigarettes
exL, exH = range of exposure (low and high) in the "highest" group, in terms of Tar
unexL, unexH = range of exposure (low and high) in the "lowest" group, in terms of Tar
REF: 6-character study reference
NRR: number of the RR on the database within the study
ST : study type (CC = case control, pr or prosp = prospective)
NLC: number of lung cancer cases in whole study
R : risky occupational population (n = no, m = mining, o = other risky)
VB : national cigarette type (V = at least 75% Virginia, bl = at least 75% blended, ot = other)
P : any proxy use
H : full histological confirmation
De : derivation of RR/CI (or = original, st = standard method, ot = other method of estimation)

Table 1L2 - 1

IESLC - Meta-analysis of Current Smoking, Tar, "Highest vs lowest"
All LC types, Cigarettes (or Any Product if Cigarettes not available)
Most adjusted

REF	NRR	SEX	AGEL	AGEH	RACE	YF	LC TYPE	LOC	START	ST	NLC	R	VB	P	H	AD	ADOS	PRODUCT	exL	exH	unexL	unexH	De
BENSHL	516	m	40	64	all	10	all	Eu:UK	1967	pr	486	n	V	n	n	2	0	cig+/-ot	33	999	18	23	ot
CPSI	856	m	0	0	wh	6	all	NAmer	1959	pr	5138	n	bl	n	n	1	0	cig+/-ot	26	36	1	18	ot
CPSI	762	f	40	99	all	6	all	NAmer	1959	pr	5138	n	bl	n	n	8	2#	cig only	26	36	1	18	ot
CPSI	767	f	40	99	all	12	all	NAmer	1959	pr	5138	n	bl	n	n	8	2#	cig only	26	36	1	18	ot
GILLIS	507	m	0	0	all	-	all	Eu:UK	1977	CC	656	n	V	n	n	0	0	cig+/-ot	29	999	1	16	st
KAISE2	662	m	0	0	all	9	all	NAmer	1979	pr	318	n	bl	n	n	5	2#	cig only	19	999	1	10	or
KAISE2	664	f	0	0	all	9	all	NAmer	1979	pr	318	n	bl	n	n	5	2#	cig only	19	999	1	10	or
KAUFMA	515	c	0	0	all	-	all	NAmer	1981	CC	881	n	bl	n	n	6	0	cig+/-ot	29	999	1	21	ot
MRFITR	505	m	0	0	all	0	all	NAmer	1973	pr	119	n	bl	n	n	0	0	cig+/-ot	20	999	1	15	st
SPEIZE	544	f	0	0	all	0	all	NAmer	1976	pr	593	n	bl	n	y	2	1#	cig+/-ot	903	903#	901	901	or

Comments on values in listings

CPSI ADOS Number of cigs/day, age started smoking
CPSI ADOS Number of cigs/day, age started smoking
KAISE2 ADOS Cigs/day and years of smoking
KAISE2 ADOS Cigs/day and years of smoking
SPEIZE ADOS Age of start

exL, exH, unexL, unexH refer to mg tar per cigarette (with “no upper limit” coded as 999) for all RRs except for the following:

SPEIZE Risk is that for the highest quartile of tar exposure vs the lowest quartile

Cigarette type is all/unspec for all RRs

Table 1L2 - 2

IESLC - Meta-analysis of Current Smoking, Tar, "Highest vs lowest"
All LC types, Cigarettes (or Any Product if Cigarettes not available)
Most adjusted

REF	NRR	SEX	AD	Number Exposed Case	Cont	Non-exposed Case	Cont	RR	95.00%CI
*BENSHL	516	m	2	38	-	72	-	1.48	(1.00- 2.19)
*CPSI	856	m	1	350	-	93	-	1.47	(1.17- 1.85)
*CPSI	762	f	8	49	-	27	-	1.76	(1.10- 2.82)
*CPSI	767	f	8	58	-	36	-	1.60	(1.06- 2.43)
Subtotal CPSI								1.54	(1.28- 1.85)
GILLIS	507	m	0	23	31	4	5	0.93	(0.22- 3.84)
*KAISE2	662	m	5	29	-	14	-	1.27	(0.67- 2.43)
*KAISE2	664	f	5	13	-	29	-	0.67	(0.34- 1.32)
Subtotal KAISE2								0.94	(0.59- 1.50)
KAUFMA	515	c	6	40	-	35	-	3.27	(1.52- 7.04)
*MRFITR	505	m	0	35	2120	12	992	1.36	(0.71- 2.62)
*SPEIZE	544	f	2	-	-	-	-	2.00	(1.50- 2.80)
Partial Totals				635	2151	322	997		

*prospective study

REF	NRR	SEX	AD	Ys	Ws	Qs	Ps
*BENSHL	516	m	2	0.39	25.01	0.08	0.0499
*CPSI	856	m	1	0.39	73.19	0.28	0.0010
*CPSI	762	f	8	0.57	17.34	0.24	0.0186
*CPSI	767	f	8	0.47	22.33	0.01	0.0264
Subtotal CPSI				0.43	112.86	0.53	
GILLIS	507	m	0	-0.08	1.90	0.52	0.9172
*KAISE2	662	m	5	0.24	9.26	0.40	0.4671
*KAISE2	664	f	5	-0.40	8.35	6.00	0.2471
Subtotal KAISE2				-0.06	17.61	6.40	
KAUFMA	515	c	6	1.18	6.54	3.56	0.0024
*MRFITR	505	m	0	0.31	9.06	0.17	0.3493
*SPEIZE	544	f	2	0.69	39.44	2.39	0.0000

	N	10
	NS	7
	Wt	212.41
Het	Chi	13.64
Het	df	9
Het	P	N.S.
Fixed	RR	1.56
	RRl	1.37
	RRu	1.79
	P	+++
Random	RR	1.55
	RRl	1.29
	RRu	1.86
	P	+++
Asymm	P	N.S.

Table 1L2 - 3

IESLC - Meta-analysis of Current Smoking, Tar, "Highest vs lowest"
All LC types, Cigarettes (or Any Product if Cigarettes not available)
Most adjusted

	Sex: combined	male	female	Total
N	1	5	4	10
NS	1	5	3	9
Wt	6.54	118.42	87.46	212.41
Het Chi	0.00	0.59	8.33	13.64
Het df	0	4	3	9
Het P	N.S.	N.S.	*	N.S.
Fixed RR	3.27	1.44	1.66	1.56
RRl	1.52	1.20	1.35	1.37
RRu	7.04	1.72	2.05	1.79
P	++	+++	+++	+++
Random RR	3.27	1.44	1.51	1.55
RRl	1.52	1.20	1.04	1.29
RRu	7.04	1.72	2.19	1.86
P	++	+++	+	+++
Between Chi				4.72
Between df				2
Between P				(*)
Btwn(F) P				N.S.
Btwn(R) P				N.S.

	Lung cancer type: all	other	Total
N	10		10
NS	7		7
Wt	212.41		212.41
Het Chi	13.64		13.64
Het df	9		9
Het P	N.S.		N.S.
Fixed RR	1.56		1.56
RRl	1.37		1.37
RRu	1.79		1.79
P	+++		+++
Random RR	1.55		1.55
RRl	1.29		1.29
RRu	1.86		1.86
P	+++		+++
Between Chi			
Between df			
Between P			N.S.
Btwn(F) P			N.S.
Btwn(R) P			N.S.

	Location: NAmer	UK	Scand	othEur	China	Japan	othAs	other	Total
N	8	2							10
NS	5	2							7
Wt	185.50	26.91							212.41
Het Chi	13.02	0.39							13.64
Het df	7	1							9
Het P	(*)	N.S.							N.S.
Fixed RR	1.58	1.43							1.56
RRl	1.37	0.98							1.37
RRu	1.83	2.09							1.79
P	+++	(+)							+++
Random RR	1.57	1.43							1.55
RRl	1.26	0.98							1.29
RRu	1.96	2.09							1.86
P	+++	(+)							+++
Between Chi									0.24
Between df									1
Between P									N.S.
Btwn(F) P									N.S.
Btwn(R) P									N.S.

Table 1L2 - 3

IESLC - Meta-analysis of Current Smoking, Tar, "Highest vs lowest"
All LC types, Cigarettes (or Any Product if Cigarettes not available)
Most adjusted

	Detailed Country in "other Europe"					
	multi	Germany	othWest	East	Balkans	Total
N						
NS						
Wt						
Het Chi						
Het df						
Het P						
Fixed RR						
RRl						
RRu						
P						
Random RR						
RRl						
RRu						
P						
Between Chi						
Between df						
Between P						N.S.
Btwn(F) P						N.S.
Btwn(R) P						N.S.

	Detailed Country in "other Asia"			
	India	HongKong	other	Total
N				
NS				
Wt				
Het Chi				
Het df				
Het P				
Fixed RR				
RRl				
RRu				
P				
Random RR				
RRl				
RRu				
P				
Between Chi				
Between df				
Between P				N.S.
Btwn(F) P				N.S.
Btwn(R) P				N.S.

	Detailed other continent	
	SCAmer	Total
N		
NS		
Wt		
Het Chi		
Het df		
Het P		
Fixed RR		
RRl		
RRu		
P		
Random RR		
RRl		
RRu		
P		
Between Chi		
Between df		
Between P		N.S.
Btwn(F) P		N.S.
Btwn(R) P		N.S.

Table 1L2 - 3

IESLC - Meta-analysis of Current Smoking, Tar, "Highest vs lowest"
All LC types, Cigarettes (or Any Product if Cigarettes not available)
Most adjusted

	Start year of study <1960	1960-69	1970-79	1980-89	1990+	Total
N	3	1	5	1		10
NS	1	1	4	1		7
Wt	112.86	25.01	68.01	6.54		212.41
Het Chi	0.50	0.00	9.44	0.00		13.64
Het df	2	0	4	0		9
Het P	N.S.	N.S.	(*)	N.S.		N.S.
Fixed RR	1.54	1.48	1.53	3.27		1.56
RRl	1.28	1.00	1.21	1.52		1.37
RRu	1.85	2.19	1.94	7.04		1.79
P	+++	+	+++	++		+++
Random RR	1.54	1.48	1.28	3.27		1.55
RRl	1.28	1.00	0.83	1.52		1.29
RRu	1.85	2.19	1.98	7.04		1.86
P	+++	+	N.S.	++		+++
Between Chi						3.70
Between df						3
Between P						N.S.
Btwn(F) P						N.S.
Btwn(R) P						N.S.

Study type (1)	CC	other	Total
N	2	8	10
NS	2	5	7
Wt	8.44	203.97	212.41
Het Chi	2.34	9.49	13.64
Het df	1	7	9
Het P	N.S.	N.S.	N.S.
Fixed RR	2.46	1.53	1.56
RRl	1.25	1.34	1.37
RRu	4.83	1.76	1.79
P	++	+++	+++
Random RR	2.02	1.52	1.55
RRl	0.61	1.28	1.29
RRu	6.71	1.80	1.86
P	N.S.	+++	+++
Between Chi			1.81
Between df			1
Between P			N.S.
Btwn(F) P			N.S.
Btwn(R) P			N.S.

	Study type (2) CC	prosp	other	Total
N	2	8		10
NS	2	5		7
Wt	8.44	203.97		212.41
Het Chi	2.34	9.49		13.64
Het df	1	7		9
Het P	N.S.	N.S.		N.S.
Fixed RR	2.46	1.53		1.56
RRl	1.25	1.34		1.37
RRu	4.83	1.76		1.79
P	++	+++		+++
Random RR	2.02	1.52		1.55
RRl	0.61	1.28		1.29
RRu	6.71	1.80		1.86
P	N.S.	+++		+++
Between Chi				1.81
Between df				1
Between P				N.S.
Btwn(F) P				N.S.
Btwn(R) P				N.S.

Table 1L2 - 3

IESLC - Meta-analysis of Current Smoking, Tar, "Highest vs lowest"
All LC types, Cigarettes (or Any Product if Cigarettes not available)
Most adjusted

	Study size (number of LC cases) 100-249	250-499	500-999	1000+	Total
N	1	3	3	3	10
NS	1	2	3	1	7
Wt	9.06	42.61	47.88	112.86	212.41
Het Chi	0.00	3.95	2.64	0.50	13.64
Het df	0	2	2	2	9
Het P	N.S.	N.S.	N.S.	N.S.	N.S.
Fixed RR	1.36	1.23	2.07	1.54	1.56
RRl	0.71	0.91	1.56	1.28	1.37
RRu	2.62	1.65	2.75	1.85	1.79
P	N.S.	N.S.	+++	+++	+++
Random RR	1.36	1.15	2.11	1.54	1.55
RRl	0.71	0.73	1.38	1.28	1.29
RRu	2.62	1.80	3.23	1.85	1.86
P	N.S.	N.S.	+++	+++	+++
Between Chi					6.56
Between df					3
Between P					(*)
Btwn(F) P					N.S.
Btwn(R) P					N.S.

	Risky occupational population no	mining	othRisky	Total
N	10			10
NS	7			7
Wt	212.41			212.41
Het Chi	13.64			13.64
Het df	9			9
Het P	N.S.			N.S.
Fixed RR	1.56			1.56
RRl	1.37			1.37
RRu	1.79			1.79
P	+++			+++
Random RR	1.55			1.55
RRl	1.29			1.29
RRu	1.86			1.86
P	+++			+++
Between Chi				
Between df				
Between P				N.S.
Btwn(F) P				N.S.
Btwn(R) P				N.S.

	National cigarette tobacco type Virginia	blended	other	Total
N	2	8		10
NS	2	5		7
Wt	26.91	185.50		212.41
Het Chi	0.39	13.02		13.64
Het df	1	7		9
Het P	N.S.	(*)		N.S.
Fixed RR	1.43	1.58		1.56
RRl	0.98	1.37		1.37
RRu	2.09	1.83		1.79
P	(+)	+++		+++
Random RR	1.43	1.57		1.55
RRl	0.98	1.26		1.29
RRu	2.09	1.96		1.86
P	(+)	+++		+++
Between Chi				0.24
Between df				1
Between P				N.S.
Btwn(F) P				N.S.
Btwn(R) P				N.S.

Table 1L2 - 3

IESLC - Meta-analysis of Current Smoking, Tar, "Highest vs lowest"
All LC types, Cigarettes (or Any Product if Cigarettes not available)
Most adjusted

	Any proxy use		
	No/nk	Yes	Total
N	10		10
NS	7		7
Wt	212.41		212.41
Het Chi	13.64		13.64
Het df	9		9
Het P	N.S.		N.S.
Fixed RR	1.56		1.56
RRl	1.37		1.37
RRu	1.79		1.79
P	+++		+++
Random RR	1.55		1.55
RRl	1.29		1.29
RRu	1.86		1.86
P	+++		+++
Between Chi			
Between df			
Between P			N.S.
Btwn(F) P			N.S.
Btwn(R) P			N.S.

	Full histological confirmation		
	No	Yes	Total
N	9	1	10
NS	6	1	7
Wt	172.97	39.44	212.41
Het Chi	10.71	0.00	13.64
Het df	8	0	9
Het P	N.S.	N.S.	N.S.
Fixed RR	1.48	2.00	1.56
RRl	1.27	1.46	1.37
RRu	1.72	2.73	1.79
P	+++	+++	+++
Random RR	1.47	2.00	1.55
RRl	1.22	1.46	1.29
RRu	1.78	2.73	1.86
P	+++	+++	+++
Between Chi			2.94
Between df			1
Between P			(*)
Btwn(F) P			N.S.
Btwn(R) P			N.S.

	Number of adjustment variables (1)			
	0	1	2+/+nk	Total
N	2	1	7	10
NS	2	1	5	8
Wt	10.96	73.19	128.26	212.41
Het Chi	0.23	0.00	12.33	13.64
Het df	1	0	6	9
Het P	N.S.	N.S.	(*)	N.S.
Fixed RR	1.28	1.47	1.65	1.56
RRl	0.71	1.17	1.39	1.37
RRu	2.31	1.85	1.96	1.79
P	N.S.	+++	+++	+++
Random RR	1.28	1.47	1.59	1.55
RRl	0.71	1.17	1.22	1.29
RRu	2.31	1.85	2.07	1.86
P	N.S.	+++	+++	+++
Between Chi				1.08
Between df				2
Between P				N.S.
Btwn(F) P				N.S.
Btwn(R) P				N.S.

Table 1L2 - 3

IESLC - Meta-analysis of Current Smoking, Tar, "Highest vs lowest"
All LC types, Cigarettes (or Any Product if Cigarettes not available)
Most adjusted

	Number of adjustment variables (2)					
	0	1	2	3-5	6+/+nk	Total
N	2	1	2	2	3	10
NS	2	1	2	1	2	8
Wt	10.96	73.19	64.45	17.61	46.20	212.41
Het Chi	0.23	0.00	1.39	1.80	2.63	13.64
Het df	1	0	1	1	2	9
Het P	N.S.	N.S.	N.S.	N.S.	N.S.	N.S.
Fixed RR	1.28	1.47	1.78	0.94	1.83	1.56
RRl	0.71	1.17	1.39	0.59	1.38	1.37
RRu	2.31	1.85	2.27	1.50	2.45	1.79
P	N.S.	+++	+++	N.S.	+++	+++
Random RR	1.28	1.47	1.76	0.93	1.88	1.55
RRl	0.71	1.17	1.32	0.50	1.33	1.29
RRu	2.31	1.85	2.36	1.74	2.64	1.86
P	N.S.	+++	+++	N.S.	+++	+++
Between Chi						7.59
Between df						4
Between P						N.S.
Btwn(F) P						N.S.
Btwn(R) P						N.S.

	Product			
	all/unsp	cig+/-ot	cig only	Total
N		6	4	10
NS		6	2	8
Wt		155.14	57.27	212.41
Het Chi		6.73	5.93	13.64
Het df		5	3	9
Het P		N.S.	N.S.	N.S.
Fixed RR		1.63	1.40	1.56
RRl		1.39	1.08	1.37
RRu		1.91	1.81	1.79
P		+++	+	+++
Random RR		1.66	1.33	1.55
RRl		1.35	0.91	1.29
RRu		2.03	1.93	1.86
P		+++	N.S.	+++
Between Chi				0.99
Between df				1
Between P				N.S.
Btwn(F) P				N.S.
Btwn(R) P				N.S.

	Derivation of RR/CI			
	Orig	StdCalc	Other	Total
N	3	2	5	10
NS	2	2	3	7
Wt	57.05	10.96	144.40	212.41
Het Chi	8.78	0.23	4.15	13.64
Het df	2	1	4	9
Het P	*	N.S.	N.S.	N.S.
Fixed RR	1.58	1.28	1.58	1.56
RRl	1.22	0.71	1.34	1.37
RRu	2.05	2.31	1.86	1.79
P	+++	N.S.	+++	+++
Random RR	1.26	1.28	1.59	1.55
RRl	0.66	0.71	1.34	1.29
RRu	2.41	2.31	1.88	1.86
P	N.S.	N.S.	+++	+++
Between Chi				0.48
Between df				2
Between P				N.S.
Btwn(F) P				N.S.
Btwn(R) P				N.S.

Table 1L2 - 4

IESLC - Meta-analysis of Current Smoking, Tar, "Highest vs lowest"
All LC types, Cigarettes (or Any Product if Cigarettes not available)
Least adjusted

REF	NRR	X	SEX	AGEL	AGEH	RACE	YF	LC TYPE	LOC	START	ST	NLC	R	VB	P	H	AD	ADOS	PRODUCT	exL	exH	unexL	unexH	De
BENSHL	516		m	40	64	all	10	all	Eu:UK	1967	pr	486	n	V	n	n	2	0	cig+/-ot	33	999	18	23	ot
CPSI	856		m	0	0	wh	6	all	NAmer	1959	pr	5138	n	bl	n	n	1	0	cig+/-ot	26	36	1	18	ot
CPSI	762		f	40	99	all	6	all	NAmer	1959	pr	5138	n	bl	n	n	8	2#	cig only	26	36	1	18	ot
CPSI	767		f	40	99	all	12	all	NAmer	1959	pr	5138	n	bl	n	n	8	2#	cig only	26	36	1	18	ot
GILLIS	507		m	0	0	all	-	all	Eu:UK	1977	CC	656	n	V	n	n	0	0	cig+/-ot	29	999	1	16	st
KAISE2	662		m	0	0	all	9	all	NAmer	1979	pr	318	n	bl	n	n	5	2#	cig only	19	999	1	10	or
KAISE2	664		f	0	0	all	9	all	NAmer	1979	pr	318	n	bl	n	n	5	2#	cig only	19	999	1	10	or
KAUFMA	505	x	c	0	0	all	-	all	NAmer	1981	CC	881	n	bl	n	n	0	0	cig+/-ot	29	999	1	21	st
MRFITR	505		m	0	0	all	0	all	NAmer	1973	pr	119	n	bl	n	n	0	0	cig+/-ot	20	999	1	15	st
SPEIZE	544		f	0	0	all	0	all	NAmer	1976	pr	593	n	bl	n	y	2	1#	cig+/-ot	903	903#	901	901	or

Comments on values in listings

CPSI ADOS Number of cigs/day, age started smoking
CPSI ADOS Number of cigs/day, age started smoking
KAISE2 ADOS Cigs/day and years of smoking
KAISE2 ADOS Cigs/day and years of smoking
SPEIZE ADOS Age of start

exL, exH, unexL, unexH refer to mg tar per cigarette (with “no upper limit” coded as 999) for all RRs except for the following:

SPEIZE Risk is that for the highest quartile of tar exposure vs the lowest quartile

Cigarette type is all/unspec for all RRs

Table 1L2 - 5

IESLC - Meta-analysis of Current Smoking, Tar, "Highest vs lowest"
All LC types, Cigarettes (or Any Product if Cigarettes not available)
Least adjusted

REF	NRR	SEX	AD	Number Exposed Case	Cont	Non-exposed Case	Cont	RR	95.00%CI
*BENSHL	516	m	2	38	-	72	-	1.48	(1.00- 2.19)
*CPSI	856	m	1	350	-	93	-	1.47	(1.17- 1.85)
*CPSI	762	f	8	49	-	27	-	1.76	(1.10- 2.82)
*CPSI	767	f	8	58	-	36	-	1.60	(1.06- 2.43)
Subtotal CPSI								1.54	(1.28- 1.85)
GILLIS	507	m	0	23	31	4	5	0.93	(0.22- 3.84)
*KAISE2	662	m	5	29	-	14	-	1.27	(0.67- 2.43)
*KAISE2	664	f	5	13	-	29	-	0.67	(0.34- 1.32)
Subtotal KAISE2								0.94	(0.59- 1.50)
KAUFMA	505	c	0	40	23	35	142	7.06	(3.75- 13.28)
*MRFITR	505	m	0	35	2120	12	992	1.36	(0.71- 2.62)
*SPEIZE	544	f	2	-	-	-	-	2.00	(1.50- 2.80)
Partial Totals				635	2174	322	1139		

*prospective study

REF	NRR	SEX	AD	Ys	Ws	Qs	Ps
*BENSHL	516	m	2	0.39	25.01	0.25	0.0499
*CPSI	856	m	1	0.39	73.19	0.83	0.0010
*CPSI	762	f	8	0.57	17.34	0.09	0.0186
*CPSI	767	f	8	0.47	22.33	0.01	0.0264
Subtotal CPSI				0.43	112.86	0.93	
GILLIS	507	m	0	-0.08	1.90	0.61	0.9172
*KAISE2	662	m	5	0.24	9.26	0.59	0.4671
*KAISE2	664	f	5	-0.40	8.35	6.65	0.2471
Subtotal KAISE2				-0.06	17.61	7.24	
KAUFMA	505	c	0	1.95	9.61	20.54	0.0000
*MRFITR	505	m	0	0.31	9.06	0.30	0.3493
*SPEIZE	544	f	2	0.69	39.44	1.60	0.0000

	N	10
	NS	7
	Wt	215.48
Het	Chi	31.47
Het	df	9
Het	P	***
Fixed	RR	1.64
	RRl	1.43
	RRu	1.87
	P	+++
Random	RR	1.64
	RRl	1.24
	RRu	2.17
	P	+++
Asymm	P	N.S.

Table 1L2 - 6

IESLC - Meta-analysis of Current Smoking, Tar, "Highest vs lowest"
All LC types, Cigarettes (or Any Product if Cigarettes not available)
Least adjusted

	Sex			
	combined	male	female	Total
N	1	5	4	10
NS	1	5	3	9
Wt	9.61	118.42	87.46	215.48
Het Chi	0.00	0.59	8.33	31.47
Het df	0	4	3	9
Het P	N.S.	N.S.	*	***
Fixed RR	7.06	1.44	1.66	1.64
RRl	3.75	1.20	1.35	1.43
RRu	13.28	1.72	2.05	1.87
P	+++	+++	+++	+++
Random RR	7.06	1.44	1.51	1.64
RRl	3.75	1.20	1.04	1.24
RRu	13.28	1.72	2.19	2.17
P	+++	+++	+	+++
Between Chi				22.54
Between df				2
Between P				***
Btwn(F) P				*
Btwn(R) P				***

Table 1L2 - 7

IESLC - Meta-analysis of Current Smoking, Tar, "Highest vs lowest"
All LC types, Cigarettes (or Any Product if Cigarettes not available)
Excluded studies (and stage at which they were excluded)

1	AGUDO	ALDERS	ARMADA	AUVINE	AXELSS	BARBON	BECHER	BENHAM	BLOT1	BOFFET	BOUCHA	BRESLO	BROWN3	CARPEN	CHEN	CHEN2
	CHIAZZ	CHOI	CHYOU	CORREA	DAMBER	DARBY	DESTEF	DOLL	DOLL2	DORGAN	DOSEME	FAN	GAO	GARCIA	GARSHI	GENG
	GER	GRAHAM	GUO	GURSEL	HAENSZ	HAMMO2	HAMMON	HEGMAN	HU	HU2	JAHN	JAIN	JEDRYC	JOLY	JUSSAW	KHUDER
	KOO	KOULUM	KREUZE	LAUSSM	LETOUR	LEVIN	LIU3	LIU4	LIU5	LUBIN	LUBIN2	LUO	MCCONN	NOTAN2	OSANN2	PERNU
	PEZZOT	PRESCO	QIAO	QIAO2	RACHTA	RESTRE	SADOWS	STASZE	SUZUK2	TIZZAN	TVERDA	VUTUC	WANG2	WIGLE	WU2	WUWILL
	WYNDE2	WYNDE3	XU	YUAN	ZHANG	ZHENG	ZHOU									
2	AKIBA	AMANDU	AMES	BEST	BOUCOT	BROSS	BUFFLE	CEDERL	DEAN2	DEAN3	DORN	ENGELA	GAO2	HIRAYA	HOLE	HUMBLE
	KATSOU	LIAW	MATOS	MCDUFF	MIGRAN	PEZZO2	PISANI	SEGI2	SOBUE	SPITZ	SVENSS	WAKAI	WATSON	WU	WYNDE7	WYNDE8
3	CPSII															
9	WYNDE6															

Table 1L2 - 8
Potentially overlapping studies

REF	REFGP	PRINC	. OVERLAP/LINK
GILLIS	LUBIN2	2	Subset of Lubin2
BENSHL	TANG2	1	Subset of TANG2
MRFITR	MRFIT	2	Subset of MRFIT
CPSI	CPSI	1	CPSI overall

Table 1L3 -

IESLC - Meta-analysis of Ever/current Smoking, Tar, "Highest vs lowest"
All LC types, Cigarettes (or Any Product if Cigarettes not available)

This analysis is restricted to results for:
1) Ever/current smokers
2) Results by Tar
3) Categorical results by Tar
4) Denominator (unexposed) = "low"
5) All LC types (or near equivalent)
6) Results complete enough for use in metaanalysis

Within each study, results are then selected (in the following order of preference, within each sex) for:
7) SMKSTA: ever, current
8) PRODUCT: cigarettes regardless of other products, cigarettes only, all/unspec
9) CIGTYPE: all/unspecified, MC regardless of HR, MC only
10) Results with least adjustment for other aspects of smoking (ADOS)
11) The highest vs lowest category
12) Followup period (YF, prospective studies): whole study (coded as 0) or longest available
13) LCtype: all or nearest available, at least Squamous and Adeno. (q = squamous, s = small,
l = large, a = adeno, mix = mixed, alv = alveolar)
14) Race: all or nearest available, otherwise by race (wh or w = white, bl or b = black, hi = hispanic
ch = chinese, jap = japanese, haw = hawaiian, w+o = white + oriental, sca = scandinavian, as = asian)
15) For overlapping studies: principal rather than subsidiary studies
Finally by Age: whole study (coded as 0) if available, otherwise by widest available age group
and then for single sex results (m, f) in preference to results for both sexes combined (c).

Results adjusted (AD) for the most potential confounders are then chosen in Sections -1 to -3
and results adjusted for the least confounders in Sections -4 to -6. (Those least adjusted results which
actually differ from the most adjusted are marked 'x' in column X in Section -4)

Section -7 shows excluded studies, together with the stage (as above) at which no qualifying
results were found.

Section -8 lists the potentially overlapping studies which have been included (1=principal, 2=subsidiary).

Section -9 lists any results which would have been included in preference except that they had data not complete
enough for use in meta-analysis, with their significance (yes/no), if known, and any further comment as entered
on the database. It also lists as "gap" any categories for which no data were presented by the original authors.

In addition to those mentioned above, the following fields, levels and abbreviations are used:
* or nk = not known, n = no, y = yes, ot = other
all/unspec = all or unspecified, cig+/-ot = cigarettes irrespective of other products (cigar, pipe etc)
MC = manufactured cigarettes, HR = hand-rolled cigarettes
exL, exH = range of exposure (low and high) in the "highest" group, in terms of Tar
unexL, unexH = range of exposure (low and high) in the "lowest" group, in terms of Tar
REF: 6-character study reference
NRR: number of the RR on the database within the study
ST : study type (CC = case control, pr or prosp = prospective)
NLC: number of lung cancer cases in whole study
R : risky occupational population (n = no, m = mining, o = other risky)
VB : national cigarette type (V = at least 75% Virginia, bl = at least 75% blended, ot = other)
P : any proxy use
H : full histological confirmation
De : derivation of RR/CI (or = original, st = standard method, ot = other method of estimation)

Table 1L3 - 1

IESLC - Meta-analysis of Ever/current Smoking, Tar, "Highest vs lowest"
All LC types, Cigarettes (or Any Product if Cigarettes not available)
Most adjusted

REF	NRR	SEX	AGEL	AGEH	RACE	YF	LC TYPE	LOC	START	ST	NLC	R	VB	P	H	AD	ADOS	SM	PRODUCT	exL	exH	unexL	unexH	De
ALDERS	529	m	0	0	all	-	all	Eu:UK	1977	CC	1448	n	V	n	n	2	1#	ev	cig only	17	22	1	16	ot
ALDERS	530	f	0	0	all	-	all	Eu:UK	1977	CC	1448	n	V	n	n	2	1#	ev	cig only	17	22	1	16	ot
BENSHL	516	m	40	64	all	10	all	Eu:UK	1967	pr	486	n	V	n	n	2	0	cu	cig+/-ot	33	999	18	23	ot
CPSI	856	m	0	0	wh	6	all	NAmer	1959	pr	5138	n	bl	n	n	1	0	cu	cig+/-ot	26	36	1	18	ot
CPSI	762	f	40	99	all	6	all	NAmer	1959	pr	5138	n	bl	n	n	8	2#	cu	cig only	26	36	1	18	ot
CPSI	767	f	40	99	all	12	all	NAmer	1959	pr	5138	n	bl	n	n	8	2#	cu	cig only	26	36	1	18	ot
DORGAN	540	m	0	0	wh	-	all	NAmer	1980	CC	2026	n	bl	y	y	0	0	ev	cig+/-ot	21	28	1	14	st
KAISE2	662	m	0	0	all	9	all	NAmer	1979	pr	318	n	bl	n	n	5	2#	cu	cig only	19	999	1	10	or
KAISE2	664	f	0	0	all	9	all	NAmer	1979	pr	318	n	bl	n	n	5	2#	cu	cig only	19	999	1	10	or
KAUFMA	515	c	0	0	all	-	all	NAmer	1981	CC	881	n	bl	n	n	6	0	cu	cig+/-ot	29	999	1	21	ot
LUBIN2	639	m	0	0	all	-	all	Eu:mul	1976	CC	7804	n	bl	n	y	0	0	ev	cig+/-ot	906	906#	901	901	st
LUBIN2	655	f	0	0	all	-	all	Eu:mul	1976	CC	7804	n	bl	n	y	0	0	ev	cig+/-ot	906	906#	901	901	st
MRFITR	505	m	0	0	all	0	all	NAmer	1973	pr	119	n	bl	n	n	0	0	cu	cig+/-ot	20	999	1	15	st
SPEIZE	544	f	0	0	all	0	all	NAmer	1976	pr	593	n	bl	n	y	2	1#	cu	cig+/-ot	903	903#	901	901	or

Comments on values in listings

ALDERS ADOS Number of cigs/day
ALDERS ADOS Number of cigs/day
CPSI ADOS Number of cigs/day, age started smoking
CPSI ADOS Number of cigs/day, age started smoking
KAISE2 ADOS Cigs/day and years of smoking
KAISE2 ADOS Cigs/day and years of smoking
SPEIZE ADOS Age of start

exL, exH, unexL, unexH refer to mg tar per cigarette (with “no upper limit” coded as 999) for all RRs except for the following:

LUBIN2 Categories based on percentiles of mean tar level (calculated from current tar levels of brands ever smoked weighted by amounts smoked) with the percentiles calculated within country. Mean tar value for this level is 29.8mg vs a lower level of 15.6mg

SPEIZE Risk is that for the highest quartile of tar exposure vs the lowest quartile

Cigarette type is all/unspec for all RRs

except for the following:

REF	NRR	CIGTYPE
ALDERS	529	MC only
ALDERS	530	MC only

Table 1L3 - 2

IESLC - Meta-analysis of Ever/current Smoking, Tar, "Highest vs lowest"
All LC types, Cigarettes (or Any Product if Cigarettes not available)
Most adjusted

REF	NRR	SEX	AD	Number Exposed Case	Number Exposed Cont	Non-exposed Case	Non-exposed Cont	RR	95.00%CI
ALDERS	529	m	2	156	-	38	-	0.91	(0.54- 1.53)
ALDERS	530	f	2	145	-	85	-	1.04	(0.69- 1.58)
Subtotal ALDERS								0.99	(0.71- 1.37)
*BENSHL	516	m	2	38	-	72	-	1.48	(1.00- 2.19)
*CPSI	856	m	1	350	-	93	-	1.47	(1.17- 1.85)
*CPSI	762	f	8	49	-	27	-	1.76	(1.10- 2.82)
*CPSI	767	f	8	58	-	36	-	1.60	(1.06- 2.43)
Subtotal CPSI								1.54	(1.28- 1.85)
DORGAN	540	m	0	139	83	25	28	1.88	(1.03- 3.43)
*KAISE2	662	m	5	29	-	14	-	1.27	(0.67- 2.43)
*KAISE2	664	f	5	13	-	29	-	0.67	(0.34- 1.32)
Subtotal KAISE2								0.94	(0.59- 1.50)
KAUFMA	515	c	6	40	-	35	-	3.27	(1.52- 7.04)
LUBIN2	639	m	0	133	300	186	428	1.02	(0.78- 1.33)
LUBIN2	655	f	0	9	4	85	151	4.00	(1.20- 13.37)
Subtotal LUBIN2								1.09	(0.84- 1.41)
*MRFITR	505	m	0	35	2120	12	992	1.36	(0.71- 2.62)
*SPEIZE	544	f	2	-	-	-	-	2.00	(1.50- 2.80)
Partial Totals				1194	2507	737	1599		

*prospective study

REF	NRR	SEX	AD	Ys	Ws	Qs	Ps
ALDERS	529	m	2	-0.09	14.17	2.64	0.7226
ALDERS	530	f	2	0.04	22.39	1.99	0.8528
Subtotal ALDERS				-0.01	36.55	4.63	
*BENSHL	516	m	2	0.39	25.01	0.07	0.0499
*CPSI	856	m	1	0.39	73.19	0.17	0.0010
*CPSI	762	f	8	0.57	17.34	0.90	0.0186
*CPSI	767	f	8	0.47	22.33	0.39	0.0264
Subtotal CPSI				0.43	112.86	1.46	
DORGAN	540	m	0	0.63	10.53	0.90	0.0412
*KAISE2	662	m	5	0.24	9.26	0.09	0.4671
*KAISE2	664	f	5	-0.40	8.35	4.55	0.2471
Subtotal KAISE2				-0.06	17.61	4.64	
KAUFMA	515	c	6	1.18	6.54	4.70	0.0024
LUBIN2	639	m	0	0.02	53.87	5.43	0.8836
LUBIN2	655	f	0	1.39	2.64	2.90	0.0245
Subtotal LUBIN2				0.08	56.50	8.32	
*MRFITR	505	m	0	0.31	9.06	0.01	0.3493
*SPEIZE	544	f	2	0.69	39.44	4.99	0.0000

	N	14
	NS	9
	Wt	314.09
Het	Chi	29.72
Het	df	13
Het	P	**
Fixed	RR	1.40
	RRl	1.25
	RRu	1.57
	P	+++
Random	RR	1.42
	RRl	1.18
	RRu	1.71
	P	+++
Asymm	P	N.S.

Table 1L3 - 3

IESLC - Meta-analysis of Ever/current Smoking, Tar, "Highest vs lowest"
All LC types, Cigarettes (or Any Product if Cigarettes not available)
Most adjusted

	Sex			
	combined	male	female	Total
N	1	7	6	14
NS	1	7	5	13
Wt	6.54	195.08	112.48	314.09
Het Chi	0.00	7.92	14.67	29.72
Het df	0	6	5	13
Het P	N.S.	N.S.	*	**
Fixed RR	3.27	1.29	1.54	1.40
RRl	1.52	1.12	1.28	1.25
RRu	7.04	1.48	1.86	1.57
P	++	+++	+++	+++
Random RR	3.27	1.29	1.48	1.42
RRl	1.52	1.08	1.05	1.18
RRu	7.04	1.53	2.09	1.71
P	++	++	+	+++
Between Chi				7.13
Between df				2
Between P				*
Btwn(F) P				N.S.
Btwn(R) P				(*)

	Lung cancer type		
	all	other	Total
N	14		14
NS	9		9
Wt	314.09		314.09
Het Chi	29.72		29.72
Het df	13		13
Het P	**		**
Fixed RR	1.40		1.40
RRl	1.25		1.25
RRu	1.57		1.57
P	+++		+++
Random RR	1.42		1.42
RRl	1.18		1.18
RRu	1.71		1.71
P	+++		+++
Between Chi			
Between df			
Between P			N.S.
Btwn(F) P			N.S.
Btwn(R) P			N.S.

	Location								
	NAmer	UK	Scand	othEur	China	Japan	othAs	other	Total
N	9	3		2					14
NS	6	2		1					9
Wt	196.04	61.56		56.50					314.09
Het Chi	13.30	2.59		4.68					29.72
Het df	8	2		1					13
Het P	N.S.	N.S.		*					**
Fixed RR	1.60	1.16		1.09					1.40
RRl	1.39	0.91		0.84					1.25
RRu	1.84	1.49		1.41					1.57
P	+++	N.S.		N.S.					+++
Random RR	1.59	1.15		1.77					1.42
RRl	1.30	0.87		0.48					1.18
RRu	1.95	1.54		6.58					1.71
P	+++	N.S.		N.S.					+++
Between Chi									9.14
Between df									2
Between P									*
Btwn(F) P									N.S.
Btwn(R) P									N.S.

Table 1L3 - 3

IESLC - Meta-analysis of Ever/current Smoking, Tar, "Highest vs lowest"
All LC types, Cigarettes (or Any Product if Cigarettes not available)
Most adjusted

		Detailed Country in "other Europe" multi	Germany	othWest	East	Balkans	Total
	N	2					2
	NS	1					1
	Wt	56.50					56.50
Het	Chi	4.68					4.68
Het	df	1					1
Het	P	*					*
Fixed	RR	1.09					1.09
	RRl	0.84					0.84
	RRu	1.41					1.41
	P	N.S.					N.S.
Random	RR	1.77					1.77
	RRl	0.48					0.48
	RRu	6.58					6.58
	P	N.S.					N.S.
Between	Chi						
Between	df						
Between	P						N.S.
Btwn(F)	P						N.S.
Btwn(R)	P						N.S.

		Detailed Country in "other Asia" India	HongKong	other	Total
	N				
	NS				
	Wt				
Het	Chi				
Het	df				
Het	P				
Fixed	RR				
	RRl				
	RRu				
	P				
Random	RR				
	RRl				
	RRu				
	P				
Between	Chi				
Between	df				
Between	P				N.S.
Btwn(F)	P				N.S.
Btwn(R)	P				N.S.

		Detailed other continent SCAmer	Total
	N		
	NS		
	Wt		
Het	Chi		
Het	df		
Het	P		
Fixed	RR		
	RRl		
	RRu		
	P		
Random	RR		
	RRl		
	RRu		
	P		
Between	Chi		
Between	df		
Between	P		N.S.
Btwn(F)	P		N.S.
Btwn(R)	P		N.S.

Table 1L3 - 3

IESLC - Meta-analysis of Ever/current Smoking, Tar, "Highest vs lowest"
All LC types, Cigarettes (or Any Product if Cigarettes not available)
Most adjusted

	Start year of study <1960	1960-69	1970-79	1980-89	1990+	Total
N	3	1	8	2		14
NS	1	1	5	2		9
Wt	112.86	25.01	159.16	17.07		314.09
Het Chi	0.50	0.00	19.97	1.25		29.72
Het df	2	0	7	1		13
Het P	N.S.	N.S.	**	N.S.		**
Fixed RR	1.54	1.48	1.23	2.32		1.40
RRl	1.28	1.00	1.06	1.44		1.25
RRu	1.85	2.19	1.44	3.73		1.57
P	+++	+	++	+++		+++
Random RR	1.54	1.48	1.22	2.35		1.42
RRl	1.28	1.00	0.91	1.38		1.18
RRu	1.85	2.19	1.64	4.01		1.71
P	+++	+	N.S.	++		+++
Between Chi						8.00
Between df						3
Between P						*
Btwn(F) P						N.S.
Btwn(R) P						N.S.

	Study type (1) CC	other	Total
N	6	8	14
NS	4	5	9
Wt	110.12	203.97	314.09
Het Chi	15.43	9.49	29.72
Het df	5	7	13
Het P	**	N.S.	**
Fixed RR	1.18	1.53	1.40
RRl	0.98	1.34	1.25
RRu	1.43	1.76	1.57
P	(+)	+++	+++
Random RR	1.43	1.52	1.42
RRl	0.97	1.28	1.18
RRu	2.09	1.80	1.71
P	(+)	+++	+++
Between Chi			4.79
Between df			1
Between P			*
Btwn(F) P			N.S.
Btwn(R) P			N.S.

	Study type (2) CC	prosp	other	Total
N	6	8		14
NS	4	5		9
Wt	110.12	203.97		314.09
Het Chi	15.43	9.49		29.72
Het df	5	7		13
Het P	**	N.S.		**
Fixed RR	1.18	1.53		1.40
RRl	0.98	1.34		1.25
RRu	1.43	1.76		1.57
P	(+)	+++		+++
Random RR	1.43	1.52		1.42
RRl	0.97	1.28		1.18
RRu	2.09	1.80		1.71
P	(+)	+++		+++
Between Chi				4.79
Between df				1
Between P				*
Btwn(F) P				N.S.
Btwn(R) P				N.S.

Table 1L3 - 3

IESLC - Meta-analysis of Ever/current Smoking, Tar, "Highest vs lowest"
All LC types, Cigarettes (or Any Product if Cigarettes not available)
Most adjusted

	Study size (number of LC cases)				
	100-249	250-499	500-999	1000+	Total
N	1	3	2	8	14
NS	1	2	2	4	9
Wt	9.06	42.61	45.98	216.44	314.09
Het Chi	0.00	3.95	1.36	14.45	29.72
Het df	0	2	1	7	13
Het P	N.S.	N.S.	N.S.	*	**
Fixed RR	1.36	1.23	2.14	1.32	1.40
RRl	0.71	0.91	1.61	1.15	1.25
RRu	2.62	1.65	2.86	1.50	1.57
P	N.S.	N.S.	+++	+++	+++
Random RR	1.36	1.15	2.25	1.35	1.42
RRl	0.71	0.73	1.49	1.09	1.18
RRu	2.62	1.80	3.38	1.67	1.71
P	N.S.	N.S.	+++	++	+++
Between Chi					9.96
Between df					3
Between P					*
Btwn(F) P					N.S.
Btwn(R) P					N.S.

	Risky occupational population			
	no	mining	othRisky	Total
N	14			14
NS	9			9
Wt	314.09			314.09
Het Chi	29.72			29.72
Het df	13			13
Het P	**			**
Fixed RR	1.40			1.40
RRl	1.25			1.25
RRu	1.57			1.57
P	+++			+++
Random RR	1.42			1.42
RRl	1.18			1.18
RRu	1.71			1.71
P	+++			+++
Between Chi				
Between df				
Between P				N.S.
Btwn(F) P				N.S.
Btwn(R) P				N.S.

	National cigarette tobacco type			
	Virginia	blended	other	Total
N	3	11		14
NS	2	7		9
Wt	61.56	252.54		314.09
Het Chi	2.59	24.49		29.72
Het df	2	10		13
Het P	N.S.	**		**
Fixed RR	1.16	1.47		1.40
RRl	0.91	1.30		1.25
RRu	1.49	1.66		1.57
P	N.S.	+++		+++
Random RR	1.15	1.53		1.42
RRl	0.87	1.23		1.18
RRu	1.54	1.90		1.71
P	N.S.	+++		+++
Between Chi				2.64
Between df				1
Between P				N.S.
Btwn(F) P				N.S.
Btwn(R) P				N.S.

Table 1L3 - 3

IESLC - Meta-analysis of Ever/current Smoking, Tar, "Highest vs lowest"
All LC types, Cigarettes (or Any Product if Cigarettes not available)
Most adjusted

	Any proxy use No/nk	Yes	Total
N	13	1	14
NS	8	1	9
Wt	303.56	10.53	314.09
Het Chi	28.79	0.00	29.72
Het df	12	0	13
Het P	**	N.S.	**
Fixed RR	1.39	1.88	1.40
RRl	1.24	1.03	1.25
RRu	1.55	3.43	1.57
P	+++	+	+++
Random RR	1.40	1.88	1.42
RRl	1.16	1.03	1.18
RRu	1.70	3.43	1.71
P	+++	+	+++
Between Chi			0.93
Between df			1
Between P			N.S.
Btwn(F) P			N.S.
Btwn(R) P			N.S.

	Full histological confirmation No	Yes	Total
N	10	4	14
NS	6	3	9
Wt	207.62	106.47	314.09
Het Chi	15.47	14.14	29.72
Het df	9	3	13
Het P	(*)	**	**
Fixed RR	1.38	1.44	1.40
RRl	1.21	1.19	1.25
RRu	1.58	1.74	1.57
P	+++	+++	+++
Random RR	1.36	1.71	1.42
RRl	1.12	1.04	1.18
RRu	1.65	2.81	1.71
P	++	+	+++
Between Chi			0.11
Between df			1
Between P			N.S.
Btwn(F) P			N.S.
Btwn(R) P			N.S.

	Number of adjustment variables (1) 0	1	2+/+nk	Total
N	4	1	9	14
NS	3	1	6	10
Wt	76.09	73.19	164.81	314.09
Het Chi	7.48	0.00	19.94	29.72
Het df	3	0	8	13
Het P	(*)	N.S.	*	**
Fixed RR	1.20	1.47	1.47	1.40
RRl	0.96	1.17	1.26	1.25
RRu	1.51	1.85	1.71	1.57
P	N.S.	+++	+++	+++
Random RR	1.49	1.47	1.42	1.42
RRl	0.93	1.17	1.10	1.18
RRu	2.38	1.85	1.82	1.71
P	(+)	+++	++	+++
Between Chi				2.29
Between df				2
Between P				N.S.
Btwn(F) P				N.S.
Btwn(R) P				N.S.

Table 1L3 - 3

IESLC - Meta-analysis of Ever/current Smoking, Tar, "Highest vs lowest"
All LC types, Cigarettes (or Any Product if Cigarettes not available)
Most adjusted

	Number of adjustment variables (2)					
	0	1	2	3-5	6+/+nk	Total
N	4	1	4	2	3	14
NS	3	1	3	1	2	10
Wt	76.09	73.19	101.00	17.61	46.20	314.09
Het Chi	7.48	0.00	9.63	1.80	2.63	29.72
Het df	3	0	3	1	2	13
Het P	(*)	N.S.	*	N.S.	N.S.	**
Fixed RR	1.20	1.47	1.44	0.94	1.83	1.40
RRl	0.96	1.17	1.18	0.59	1.38	1.25
RRu	1.51	1.85	1.75	1.50	2.45	1.57
P	N.S.	+++	+++	N.S.	+++	+++
Random RR	1.49	1.47	1.34	0.93	1.88	1.42
RRl	0.93	1.17	0.93	0.50	1.33	1.18
RRu	2.38	1.85	1.92	1.74	2.64	1.71
P	(+)	+++	N.S.	N.S.	+++	+++
Between Chi						8.17
Between df						4
Between P						(*)
Btwn(F) P						N.S.
Btwn(R) P						N.S.

	Smoking status		
	ever	current	Total
N	5	9	14
NS	3	6	9
Wt	103.58	210.51	314.09
Het Chi	8.26	13.12	29.72
Het df	4	8	13
Het P	(*)	N.S.	**
Fixed RR	1.11	1.57	1.40
RRl	0.92	1.37	1.25
RRu	1.35	1.80	1.57
P	N.S.	+++	+++
Random RR	1.20	1.56	1.42
RRl	0.88	1.29	1.18
RRu	1.66	1.89	1.71
P	N.S.	+++	+++
Between Chi			8.33
Between df			1
Between P			**
Btwn(F) P			(*)
Btwn(R) P			N.S.

	Product			
	all/unsp	cig+/-ot	cig only	Total
N		8	6	14
NS		7	3	10
Wt		220.27	93.82	314.09
Het Chi		18.40	8.77	29.72
Het df		7	5	13
Het P		*	N.S.	**
Fixed RR		1.49	1.22	1.40
RRl		1.30	1.00	1.25
RRu		1.70	1.49	1.57
P		+++	(+)	+++
Random RR		1.62	1.19	1.42
RRl		1.27	0.91	1.18
RRu		2.07	1.57	1.71
P		+++	N.S.	+++
Between Chi				2.55
Between df				1
Between P				N.S.
Btwn(F) P				N.S.
Btwn(R) P				N.S.

Table 1L3 - 3

IESLC - Meta-analysis of Ever/current Smoking, Tar, "Highest vs lowest"
All LC types, Cigarettes (or Any Product if Cigarettes not available)
Most adjusted

	Derivation of RR/CI Orig	StdCalc	Other	Total
N	3	4	7	14
NS	2	3	4	9
Wt	57.05	76.09	180.96	314.09
Het Chi	8.78	7.48	10.75	29.72
Het df	2	3	6	13
Het P	*	(*)	(*)	**
Fixed RR	1.58	1.20	1.44	1.40
RRl	1.22	0.96	1.24	1.25
RRu	2.05	1.51	1.66	1.57
P	+++	N.S.	+++	+++
Random RR	1.26	1.49	1.44	1.42
RRl	0.66	0.93	1.17	1.18
RRu	2.41	2.38	1.78	1.71
P	N.S.	(+)	+++	+++
Between Chi				2.70
Between df				2
Between P				N.S.
Btwn(F) P				N.S.
Btwn(R) P				N.S.

Table 1L3 - 4

IESLC - Meta-analysis of Ever/current Smoking, Tar, "Highest vs lowest"
All LC types, Cigarettes (or Any Product if Cigarettes not available)
Least adjusted

REF	NRR	X	SEX	AGEL	AGEH	RACE	YF	LC TYPE	LOC	START	ST	NLC	R	VB	P	H	AD	ADOS	SM	PRODUCT	exL	exH	unexL	unexH	De
ALDERS	529		m	0	0	all	-	all	Eu:UK	1977	CC	1448	n	V	n	n	2	1#	ev	cig only	17	22	1	16	ot
ALDERS	530		f	0	0	all	-	all	Eu:UK	1977	CC	1448	n	V	n	n	2	1#	ev	cig only	17	22	1	16	ot
BENSHL	516		m	40	64	all	10	all	Eu:UK	1967	pr	486	n	V	n	n	2	0	cu	cig+/-ot	33	999	18	23	ot
CPSI	856		m	0	0	wh	6	all	NAmer	1959	pr	5138	n	bl	n	n	1	0	cu	cig+/-ot	26	36	1	18	ot
CPSI	762		f	40	99	all	6	all	NAmer	1959	pr	5138	n	bl	n	n	8	2#	cu	cig only	26	36	1	18	ot
CPSI	767		f	40	99	all	12	all	NAmer	1959	pr	5138	n	bl	n	n	8	2#	cu	cig only	26	36	1	18	ot
DORGAN	540		m	0	0	wh	-	all	NAmer	1980	CC	2026	n	bl	y	y	0	0	ev	cig+/-ot	21	28	1	14	st
KAISE2	662		m	0	0	all	9	all	NAmer	1979	pr	318	n	bl	n	n	5	2#	cu	cig only	19	999	1	10	or
KAISE2	664		f	0	0	all	9	all	NAmer	1979	pr	318	n	bl	n	n	5	2#	cu	cig only	19	999	1	10	or
KAUFMA	505	x	c	0	0	all	-	all	NAmer	1981	CC	881	n	bl	n	n	0	0	cu	cig+/-ot	29	999	1	21	st
LUBIN2	639		m	0	0	all	-	all	Eu:mul	1976	CC	7804	n	bl	n	y	0	0	ev	cig+/-ot	906	906#	901	901	st
LUBIN2	655		f	0	0	all	-	all	Eu:mul	1976	CC	7804	n	bl	n	y	0	0	ev	cig+/-ot	906	906#	901	901	st
MRFITR	505		m	0	0	all	0	all	NAmer	1973	pr	119	n	bl	n	n	0	0	cu	cig+/-ot	20	999	1	15	st
SPEIZE	544		f	0	0	all	0	all	NAmer	1976	pr	593	n	bl	n	y	2	1#	cu	cig+/-ot	903	903#	901	901	or

Comments on values in listings

ALDERS ADOS Number of cigs/day
ALDERS ADOS Number of cigs/day
CPSI ADOS Number of cigs/day, age started smoking
CPSI ADOS Number of cigs/day, age started smoking
KAISE2 ADOS Cigs/day and years of smoking
KAISE2 ADOS Cigs/day and years of smoking
SPEIZE ADOS Age of start

exL, exH, unexL, unexH refer to mg tar per cigarette (with “no upper limit” coded as 999) for all RRs except for the following:

LUBIN2 Categories based on percentiles of mean tar level (calculated from current tar levels of brands ever smoked weighted by amounts smoked) with the percentiles calculated within country. Mean tar value for this level is 29.8mg vs a lower level of 15.6mg

SPEIZE Risk is that for the highest quartile of tar exposure vs the lowest quartile

Cigarette type is all/unspec for all RRs

except for the following:

REF	NRR	CIGTYPE
ALDERS	529	MC only
ALDERS	530	MC only

Table 1L3 - 5

IESLC - Meta-analysis of Ever/current Smoking, Tar, "Highest vs lowest"
All LC types, Cigarettes (or Any Product if Cigarettes not available)
Least adjusted

REF	NRR	SEX	AD	Number Exposed Case	Cont	Non-exposed Case	Cont	RR	95.00%CI
ALDERS	529	m	2	156	-	38	-	0.91	(0.54- 1.53)
ALDERS	530	f	2	145	-	85	-	1.04	(0.69- 1.58)
Subtotal ALDERS								0.99	(0.71- 1.37)
*BENSHL	516	m	2	38	-	72	-	1.48	(1.00- 2.19)
*CPSI	856	m	1	350	-	93	-	1.47	(1.17- 1.85)
*CPSI	762	f	8	49	-	27	-	1.76	(1.10- 2.82)
*CPSI	767	f	8	58	-	36	-	1.60	(1.06- 2.43)
Subtotal CPSI								1.54	(1.28- 1.85)
DORGAN	540	m	0	139	83	25	28	1.88	(1.03- 3.43)
*KAISE2	662	m	5	29	-	14	-	1.27	(0.67- 2.43)
*KAISE2	664	f	5	13	-	29	-	0.67	(0.34- 1.32)
Subtotal KAISE2								0.94	(0.59- 1.50)
KAUFMA	505	c	0	40	23	35	142	7.06	(3.75- 13.28)
LUBIN2	639	m	0	133	300	186	428	1.02	(0.78- 1.33)
LUBIN2	655	f	0	9	4	85	151	4.00	(1.20- 13.37)
Subtotal LUBIN2								1.09	(0.84- 1.41)
*MRFITR	505	m	0	35	2120	12	992	1.36	(0.71- 2.62)
*SPEIZE	544	f	2	-	-	-	-	2.00	(1.50- 2.80)
Partial Totals				1194	2530	737	1741		

*prospective study

REF	NRR	SEX	AD	Ys	Ws	Qs	Ps
ALDERS	529	m	2	-0.09	14.17	3.04	0.7226
ALDERS	530	f	2	0.04	22.39	2.43	0.8528
Subtotal ALDERS				-0.01	36.55	5.47	
*BENSHL	516	m	2	0.39	25.01	0.01	0.0499
*CPSI	856	m	1	0.39	73.19	0.02	0.0010
*CPSI	762	f	8	0.57	17.34	0.67	0.0186
*CPSI	767	f	8	0.47	22.33	0.23	0.0264
Subtotal CPSI				0.43	112.86	0.92	
DORGAN	540	m	0	0.63	10.53	0.71	0.0412
*KAISE2	662	m	5	0.24	9.26	0.16	0.4671
*KAISE2	664	f	5	-0.40	8.35	4.94	0.2471
Subtotal KAISE2				-0.06	17.61	5.10	
KAUFMA	505	c	0	1.95	9.61	24.14	0.0000
LUBIN2	639	m	0	0.02	53.87	6.56	0.8836
LUBIN2	655	f	0	1.39	2.64	2.72	0.0245
Subtotal LUBIN2				0.08	56.50	9.28	
*MRFITR	505	m	0	0.31	9.06	0.03	0.3493
*SPEIZE	544	f	2	0.69	39.44	4.15	0.0000

	N	14
	NS	9
	Wt	317.16
Het	Chi	49.81
Het	df	13
Het	P	***
Fixed	RR	1.45
	RRl	1.30
	RRu	1.61
	P	+++
Random	RR	1.52
	RRl	1.20
	RRu	1.92
	P	+++
Asymm	P	N.S.

Table 1L3 - 6

IESLC - Meta-analysis of Ever/current Smoking, Tar, "Highest vs lowest"
All LC types, Cigarettes (or Any Product if Cigarettes not available)
Least adjusted

	Sex combined	male	female	Total
N	1	7	6	14
NS	1	7	5	13
Wt	9.61	195.08	112.48	317.16
Het Chi	0.00	7.92	14.67	49.81
Het df	0	6	5	13
Het P	N.S.	N.S.	*	***
Fixed RR	7.06	1.29	1.54	1.45
RRl	3.75	1.12	1.28	1.30
RRu	13.28	1.48	1.86	1.61
P	+++	+++	+++	+++
Random RR	7.06	1.29	1.48	1.52
RRl	3.75	1.08	1.05	1.20
RRu	13.28	1.53	2.09	1.92
P	+++	++	+	+++
Between Chi				27.22
Between df				2
Between P				***
Btwn(F) P				*
Btwn(R) P				***

Table 1L3 - 7

IESLC - Meta-analysis of Ever/current Smoking, Tar, "Highest vs lowest"
All LC types, Cigarettes (or Any Product if Cigarettes not available)
Excluded studies (and stage at which they were excluded)

1	BECHER	BLOT1	BROWN3	CARPEN	CHYOU	DARBY	DOLL2	GARCIA	GRAHAM	GURSEL	HAMMO2	JAHN	JAIN	LAUSSM	PRESCO	QIAO
	TVERDA	WIGLE	WYNDE3													
2	AGUDO	AKIBA	AMANDU	AMES	ARMADA	AUVINE	AXELSS	BARBON	BENHAM	BEST	BOFFET	BOUCHA	BOUCOT	BRESLO	BROSS	BUFFLE
	CEDERL	CHEN	CHEN2	CHIAZZ	CHOI	CORREA	DAMBER	DEAN2	DEAN3	DESTEF	DOLL	DORN	DOSEME	ENGELA	FAN	GAO
	GAO2	GARSHI	GENG	GER	GUO	HAENSZ	HAMMON	HEGMAN	HIRAYA	HOLE	HU	HU2	HUMBLE	JEDRYC	JOLY	JUSSAW
	KATSOU	KHUDER	KOO	KOULUM	KREUZE	LETOUR	LEVIN	LIAW	LIU3	LIU4	LIU5	LUBIN	LUO	MATOS	MCCONN	MCDUFF
	MIGRAN	NOTAN2	OSANN2	PERNU	PEZZO2	PEZZOT	PISANI	QIAO2	RACHTA	RESTRE	SADOWS	SEGI2	SOBUE	SPITZ	STASZE	SUZUK2
	SVENSS	TIZZAN	WAKAI	WANG2	WATSON	WU	WU2	WUWILL	WYNDE2	WYNDE7	WYNDE8	XU	YUAN	ZHANG	ZHENG	ZHOU
3	CPSII															
9	WYNDE6															
15	GILLIS	VUTUC														

Table 1L3 - 8
Potentially overlapping studies

REF	REFGP	PRINC	.	OVERLAP/LINK
LUBIN2	LUBIN2	1		Lubin-combined
BENSHL	TANG2	1		Subset of TANG2
MRFITR	MRFIT	2		Subset of MRFIT
CPSI	CPSI	1		CPSI overall

Table 1L4 -

IESLC - Meta-analysis of Ever Smoking, Tar, "Highest vs lowest"
All LC types, Cigarettes only

This analysis is restricted to results for:
1) Ever smokers
2) Results by Tar
3) Categorical results by Tar
4) Denominator (unexposed) = "low"
5) All LC types (or near equivalent)
6) Results complete enough for use in metaanalysis

Within each study, results are then selected (in the following order of preference, within each sex) for:
7) (not applicable)
8) PRODUCT: cigarettes only
9) CIGTYPE: all/unspecified, MC regardless of HR, MC only
10) Results with least adjustment for other aspects of smoking (ADOS)
11) The highest vs lowest category
12) Followup period (YF, prospective studies): whole study (coded as 0) or longest available
13) LCtype: all or nearest available, at least Squamous and Adeno. (q = squamous, s = small,
l = large, a = adeno, mix = mixed, alv = alveolar)
14) Race: all or nearest available, otherwise by race (wh or w = white, bl or b = black, hi = hispanic
ch = chinese, jap = japanese, haw = hawaiian, w+o = white + oriental, sca = scandinavian, as = asian)
15) For overlapping studies: principal rather than subsidiary studies
Finally by Age: whole study (coded as 0) if available, otherwise by widest available age group
and then for single sex results (m, f) in preference to results for both sexes combined (c).

Results adjusted (AD) for the most potential confounders are then chosen in Sections -1 to -3
and results adjusted for the least confounders in Sections -4 to -6. (Those least adjusted results which
actually differ from the most adjusted are marked 'x' in column X in Section -4)

Section -7 shows excluded studies, together with the stage (as above) at which no qualifying
results were found.

Section -8 lists the potentially overlapping studies which have been included (1=principal, 2=subsidiary).

Section -9 lists any results which would have been included in preference except that they had data not complete
enough for use in meta-analysis, with their significance (yes/no), if known, and any further comment as entered
on the database. It also lists as "gap" any categories for which no data were presented by the original authors.

In addition to those mentioned above, the following fields, levels and abbreviations are used:
* or nk = not known, n = no, y = yes, ot = other
all/unspec = all or unspecified, MC = manufactured cigarettes, HR = hand-rolled cigarettes
exL, exH = range of exposure (low and high) in the "highest" group, in terms of Tar
unexL, unexH = range of exposure (low and high) in the "lowest" group, in terms of Tar
REF: 6-character study reference
NRR: number of the RR on the database within the study
ST : study type (CC = case control, pr or prosp = prospective)
NLC: number of lung cancer cases in whole study
R : risky occupational population (n = no, m = mining, o = other risky)
VB : national cigarette type (V = at least 75% Virginia, bl = at least 75% blended, ot = other)
P : any proxy use
H : full histological confirmation
De : derivation of RR/CI (or = original, st = standard method, ot = other method of estimation)

Table 1L4 - 1

IESLC - Meta-analysis of Ever Smoking, Tar, "Highest vs lowest"
All LC types, Cigarettes only
Most adjusted

REF	NRR	SEX	AGEL	AGEH	RACE	YF	LC TYPE	LOC	START	ST	NLC	R	VB	P	H	AD	ADOS	PRODUCT	exL	exH	unexL	unexH	De
ALDERS	529	m	0	0	all	-	all	Eu:UK	1977	CC	1448	n	V	n	n	2		1#cig only	17	22	1	16	ot
ALDERS	530	f	0	0	all	-	all	Eu:UK	1977	CC	1448	n	V	n	n	2		1#cig only	17	22	1	16	ot

Comments on values in listings

ALDERS ADOS Number of cigs/day
ALDERS ADOS Number of cigs/day

Cigarette type is all/unspec for all RRs

except for the following:

REF	NRR	CIGTYPE
ALDERS	529	MC only
ALDERS	530	MC only

Table 1L4 - 2

IESLC - Meta-analysis of Ever Smoking, Tar, "Highest vs lowest"
All LC types, Cigarettes only
Most adjusted

REF	NRR	SEX	AD	Number Exposed Case	Exposed Cont	Non-exposed Case	Non-exposed Cont	RR	95.00%CI
ALDERS	529	m	2	156	-	38	-	0.91	(0.54- 1.53)
ALDERS	530	f	2	145	-	85	-	1.04	(0.69- 1.58)
Subtotal ALDERS								0.99	(0.71- 1.37)
Partial Totals				301	0	123	0		

*prospective study

REF	NRR	SEX	AD	Ys	Ws	Qs	Ps
ALDERS	529	m	2	-0.09	14.17	0.09	0.7226
ALDERS	530	f	2	0.04	22.39	0.06	0.8528
Subtotal ALDERS				-0.01	36.55	0.15	

	N	2
	NS	1
	Wt	36.55
Het	Chi	0.15
Het	df	1
Het	P	N.S.
Fixed	RR	0.99
	RRl	0.71
	RRu	1.37
	P	N.S.
Random	RR	0.99
	RRl	0.71
	RRu	1.37
	P	N.S.
Asymm	P	

Table 1L4 - 3

IESLC - Meta-analysis of Ever Smoking, Tar, "Highest vs lowest"
All LC types, Cigarettes only
Most adjusted

	Sex combined	male	female	Total
N		1	1	2
NS		1	1	1
Wt		14.17	22.39	36.55
Het Chi		0.00	0.00	0.15
Het df		0	0	1
Het P		N.S.	N.S.	N.S.
Fixed RR		0.91	1.04	0.99
RRl		0.54	0.69	0.71
RRu		1.53	1.57	1.37
P		N.S.	N.S.	N.S.
Random RR		0.91	1.04	0.99
RRl		0.54	0.69	0.71
RRu		1.53	1.57	1.37
P		N.S.	N.S.	N.S.
Between Chi				0.15
Between df				1
Between P				N.S.
Btwn(F) P				N.S.
Btwn(R) P				N.S.

Too few RRs for analysis by factor

Table 1L4 - 4

IESLC - Meta-analysis of Ever Smoking, Tar, "Highest vs lowest"
All LC types, Cigarettes only
Least adjusted

REF	NRR	X	SEX	AGEL	AGEH	RACE	YF	LC TYPE	LOC	START	ST	NLC	R	VB	P	H	AD	ADOS	PRODUCT	exL	exH	unexL	unexH	De
ALDERS	529		m	0	0	all	-	all	Eu:UK	1977	CC	1448	n	V	n	n	2		1#cig only	17	22	1	16	ot
ALDERS	530		f	0	0	all	-	all	Eu:UK	1977	CC	1448	n	V	n	n	2		1#cig only	17	22	1	16	ot

Comments on values in listings

ALDERS ADOS Number of cigs/day
ALDERS ADOS Number of cigs/day

Cigarette type is all/unspec for all RRs

except for the following:

REF	NRR	CIGTYPE
ALDERS	529	MC only
ALDERS	530	MC only

Table 1L4 - 5

IESLC - Meta-analysis of Ever Smoking, Tar, "Highest vs lowest"
All LC types, Cigarettes only
Least adjusted

REF	NRR	SEX	AD	Number Exposed Case	Number Exposed Cont	Non-exposed Case	Non-exposed Cont	RR	95.00%CI
ALDERS	529	m	2	156	-	38	-	0.91	(0.54- 1.53)
ALDERS	530	f	2	145	-	85	-	1.04	(0.69- 1.58)
Subtotal ALDERS								0.99	(0.71- 1.37)
Partial Totals				301	0	123	0		

*prospective study

REF	NRR	SEX	AD	Ys	Ws	Qs	Ps
ALDERS	529	m	2	-0.09	14.17	0.09	0.7226
ALDERS	530	f	2	0.04	22.39	0.06	0.8528
Subtotal ALDERS				-0.01	36.55	0.15	

	N	2
	NS	1
	Wt	36.55
Het	Chi	0.15
Het	df	1
Het	P	N.S.
Fixed	RR	0.99
	RRl	0.71
	RRu	1.37
	P	N.S.
Random	RR	0.99
	RRl	0.71
	RRu	1.37
	P	N.S.
Asymm	P	

Table 1L4 - 6

IESLC - Meta-analysis of Ever Smoking, Tar, "Highest vs lowest"
All LC types, Cigarettes only
Least adjusted

	Sex combined	male	female	Total
N		1	1	2
NS		1	1	1
Wt		14.17	22.39	36.55
Het Chi		0.00	0.00	0.15
Het df		0	0	1
Het P		N.S.	N.S.	N.S.
Fixed RR		0.91	1.04	0.99
RRl		0.54	0.69	0.71
RRu		1.53	1.57	1.37
P		N.S.	N.S.	N.S.
Random RR		0.91	1.04	0.99
RRl		0.54	0.69	0.71
RRu		1.53	1.57	1.37
P		N.S.	N.S.	N.S.
Between Chi				0.15
Between df				1
Between P				N.S.
Btwn(F) P				N.S.
Btwn(R) P				N.S.

Table 1L4 - 7

IESLC - Meta-analysis of Ever Smoking, Tar, "Highest vs lowest"
All LC types, Cigarettes only
Excluded studies (and stage at which they were excluded)

1	AKIBA AMANDU AMES BECHER BENSHL BEST BLOT1 BROSS BROWN3 CARPEN CEDERL CHYOU CPSI CPSII DARBY DEAN2 DEAN3 DOLL2 ENGELA GAO2 GARCIA GILLIS GRAHAM GURSEL HAMMO2 HIRAYA HOLE HUMBLE JAHN JAIN KAISE2 KATSOU KAUFMA LAUSSM LIAW MCDUFF MIGRAN MRFITR PEZZO2 PISANI PRESCO QIAO SEGI2 SPEIZE SVENSS TVERDA WAKAI WATSON WIGLE WU WYNDE3 WYNDE8
2	AGUDO ARMADA AUVINE AXELSS BARBON BENHAM BOFFET BOUCHA BOUCOT BRESLO BUFFLE CHEN CHEN2 CHIAZZ CHOI CORREA DAMBER DESTEF DOLL DORN DOSEME FAN GAO GARSHI GENG GER GUO HAENSZ HAMMON HEGMAN HU HU2 JEDRYC JOLY JUSSAW KHUDER KOO KOULUM KREUZE LETOUR LEVIN LIU3 LIU4 LIU5 LUBIN LUO MATOS MCCONN NOTAN2 OSANN2 PERNU PEZZOT QIAO2 RACHTA RESTRE SADOWS SOBUE SPITZ STASZE SUZUK2 TIZZAN WANG2 WU2 WUWILL WYNDE2 WYNDE7 XU YUAN ZHANG ZHENG ZHOU
3	WYNDE6
8	DORGAN LUBIN2 VUTUC

Table 1L5 -

IESLC - Meta-analysis of Current Smoking, Tar, "Highest vs lowest"
All LC types, Cigarettes only

This analysis is restricted to results for:
1) Current smokers
2) Results by Tar
3) Categorical results by Tar
4) Denominator (unexposed) = "low"
5) All LC types (or near equivalent)
6) Results complete enough for use in metaanalysis

Within each study, results are then selected (in the following order of preference, within each sex) for:
7) (not applicable)
8) PRODUCT: cigarettes only
9) CIGTYPE: all/unspecified, MC regardless of HR, MC only
10) Results with least adjustment for other aspects of smoking (ADOS)
11) The highest vs lowest category
12) Followup period (YF, prospective studies): whole study (coded as 0) or longest available
13) LCtype: all or nearest available, at least Squamous and Adeno. (q = squamous, s = small,
l = large, a = adeno, mix = mixed, alv = alveolar)
14) Race: all or nearest available, otherwise by race (wh or w = white, bl or b = black, hi = hispanic
ch = chinese, jap = japanese, haw = hawaiian, w+o = white + oriental, sca = scandinavian, as = asian)
15) For overlapping studies: principal rather than subsidiary studies
Finally by Age: whole study (coded as 0) if available, otherwise by widest available age group
and then for single sex results (m, f) in preference to results for both sexes combined (c).

Results adjusted (AD) for the most potential confounders are then chosen in Sections -1 to -3
and results adjusted for the least confounders in Sections -4 to -6. (Those least adjusted results which
actually differ from the most adjusted are marked 'x' in column X in Section -4)

Section -7 shows excluded studies, together with the stage (as above) at which no qualifying
results were found.

Section -8 lists the potentially overlapping studies which have been included (1=principal, 2=subsidiary).

Section -9 lists any results which would have been included in preference except that they had data not complete
enough for use in meta-analysis, with their significance (yes/no), if known, and any further comment as entered
on the database. It also lists as "gap" any categories for which no data were presented by the original authors.

In addition to those mentioned above, the following fields, levels and abbreviations are used:
* or nk = not known, n = no, y = yes, ot = other
all/unspec = all or unspecified, MC = manufactured cigarettes, HR = hand-rolled cigarettes
exL, exH = range of exposure (low and high) in the "highest" group, in terms of Tar
unexL, unexH = range of exposure (low and high) in the "lowest" group, in terms of Tar
REF: 6-character study reference
NRR: number of the RR on the database within the study
ST : study type (CC = case control, pr or prosp = prospective)
NLC: number of lung cancer cases in whole study
R : risky occupational population (n = no, m = mining, o = other risky)
VB : national cigarette type (V = at least 75% Virginia, bl = at least 75% blended, ot = other)
P : any proxy use
H : full histological confirmation
De : derivation of RR/CI (or = original, st = standard method, ot = other method of estimation)

Table 1L5 - 1

IESLC - Meta-analysis of Current Smoking, Tar, "Highest vs lowest"
All LC types, Cigarettes only
Most adjusted

REF	NRR	SEX	AGEL	AGEH	RACE	YF	LC TYPE	LOC	START	ST	NLC	R	VB	P	H	AD	ADOS	PRODUCT	exL	exH	unexL	unexH	De
CPSI	752	m	40	99	all	6	all	NAmer	1959	pr	5138	n	bl	n	n	9		2#cig only	26	36	1	18	ot
CPSI	757	m	40	99	all	12	all	NAmer	1959	pr	5138	n	bl	n	n	9		2#cig only	26	36	1	18	ot
CPSI	762	f	40	99	all	6	all	NAmer	1959	pr	5138	n	bl	n	n	8		2#cig only	26	36	1	18	ot
CPSI	767	f	40	99	all	12	all	NAmer	1959	pr	5138	n	bl	n	n	8		2#cig only	26	36	1	18	ot
KAISE2	662	m	0	0	all	9	all	NAmer	1979	pr	318	n	bl	n	n	5		2#cig only	19	999	1	10	or
KAISE2	664	f	0	0	all	9	all	NAmer	1979	pr	318	n	bl	n	n	5		2#cig only	19	999	1	10	or

Comments on values in listings

CPSI	ADOS	Number of cigs/day, age started smoking
CPSI	ADOS	Number of cigs/day, age started smoking
CPSI	ADOS	Number of cigs/day, age started smoking
CPSI	ADOS	Number of cigs/day, age started smoking
KAISE2	ADOS	Cigs/day and years of smoking
KAISE2	ADOS	Cigs/day and years of smoking

Cigarette type is all/unspec for all RRs

Table 1L5 - 2

IESLC - Meta-analysis of Current Smoking, Tar, "Highest vs lowest"
All LC types, Cigarettes only
Most adjusted

REF	NRR	SEX	AD	Number Exposed Case	Cont	Non-exposed Case	Cont	RR	95.00%CI
*CPSI	752	m	9	122	-	101	-	1.21	(0.93- 1.58)
*CPSI	757	m	9	90	-	71	-	1.27	(0.93- 1.73)
*CPSI	762	f	8	49	-	27	-	1.76	(1.10- 2.82)
*CPSI	767	f	8	58	-	36	-	1.60	(1.06- 2.43)
Subtotal CPSI								1.35	(1.14- 1.60)
*KAISE2	662	m	5	29	-	14	-	1.27	(0.67- 2.43)
*KAISE2	664	f	5	13	-	29	-	0.67	(0.34- 1.32)
Subtotal KAISE2								0.94	(0.59- 1.50)
Partial Totals				361	0	278	0		

*prospective study

REF	NRR	SEX	AD	Ys	Ws	Qs	Ps
*CPSI	752	m	9	0.19	54.70	0.25	0.1586
*CPSI	757	m	9	0.24	39.88	0.01	0.1312
*CPSI	762	f	8	0.57	17.34	1.64	0.0186
*CPSI	767	f	8	0.47	22.33	1.01	0.0264
Subtotal CPSI				0.30	134.25	2.91	
*KAISE2	662	m	5	0.24	9.26	0.00	0.4671
*KAISE2	664	f	5	-0.40	8.35	3.62	0.2471
Subtotal KAISE2				-0.06	17.61	3.62	

	N	6
	NS	2
	Wt	151.86
Het	Chi	6.53
Het	df	5
Het	P	N.S.
Fixed	RR	1.29
	RRl	1.10
	RRu	1.52
	P	++
Random	RR	1.30
	RRl	1.07
	RRu	1.57
	P	++
Asymm	P	N.S.

Table 1L5 - 3

IESLC - Meta-analysis of Current Smoking, Tar, "Highest vs lowest"
All LC types, Cigarettes only
Most adjusted

	Sex combined	male	female	Total
N		3	3	6
NS		2	2	4
Wt		103.84	48.01	151.86
Het Chi		0.06	5.83	6.53
Het df		2	2	5
Het P		N.S.	(*)	N.S.
Fixed RR		1.24	1.42	1.29
RRl		1.02	1.07	1.10
RRu		1.50	1.89	1.52
P		+	+	++
Random RR		1.24	1.31	1.30
RRl		1.02	0.79	1.07
RRu		1.50	2.17	1.57
P		+	N.S.	++
Between Chi				0.64
Between df				1
Between P				N.S.
Btwn(F) P				N.S.
Btwn(R) P				N.S.

Too few RRs for analysis by factor

Table 1L5 - 4

IESLC - Meta-analysis of Current Smoking, Tar, "Highest vs lowest"
All LC types, Cigarettes only
Least adjusted

REF	NRR	X	SEX	AGEL	AGEH	RACE	YF	LC TYPE	LOC	START	ST	NLC	R	VB	P	H	AD	ADOS	PRODUCT	exL	exH	unexL	unexH	De
CPSI	752		m	40	99	all	6	all	NAmer	1959	pr	5138	n	bl	n	n	9		2#cig only	26	36	1	18	ot
CPSI	757		m	40	99	all	12	all	NAmer	1959	pr	5138	n	bl	n	n	9		2#cig only	26	36	1	18	ot
CPSI	762		f	40	99	all	6	all	NAmer	1959	pr	5138	n	bl	n	n	8		2#cig only	26	36	1	18	ot
CPSI	767		f	40	99	all	12	all	NAmer	1959	pr	5138	n	bl	n	n	8		2#cig only	26	36	1	18	ot
KAISE2	662		m	0	0	all	9	all	NAmer	1979	pr	318	n	bl	n	n	5		2#cig only	19	999	1	10	or
KAISE2	664		f	0	0	all	9	all	NAmer	1979	pr	318	n	bl	n	n	5		2#cig only	19	999	1	10	or

Comments on values in listings

CPSI	ADOS	Number of cigs/day, age started smoking
CPSI	ADOS	Number of cigs/day, age started smoking
CPSI	ADOS	Number of cigs/day, age started smoking
CPSI	ADOS	Number of cigs/day, age started smoking
KAISE2	ADOS	Cigs/day and years of smoking
KAISE2	ADOS	Cigs/day and years of smoking

Cigarette type is all/unspec for all RRs

Table 1L5 - 5

IESLC - Meta-analysis of Current Smoking, Tar, "Highest vs lowest"
All LC types, Cigarettes only
Least adjusted

REF	NRR	SEX	AD	Number Exposed Case	Cont	Non-exposed Case	Cont	RR	95.00%CI
*CPSI	752	m	9	122	-	101	-	1.21	(0.93- 1.58)
*CPSI	757	m	9	90	-	71	-	1.27	(0.93- 1.73)
*CPSI	762	f	8	49	-	27	-	1.76	(1.10- 2.82)
*CPSI	767	f	8	58	-	36	-	1.60	(1.06- 2.43)
Subtotal CPSI								1.35	(1.14- 1.60)
*KAISE2	662	m	5	29	-	14	-	1.27	(0.67- 2.43)
*KAISE2	664	f	5	13	-	29	-	0.67	(0.34- 1.32)
Subtotal KAISE2								0.94	(0.59- 1.50)
Partial Totals				361	0	278	0		

*prospective study

REF	NRR	SEX	AD	Ys	Ws	Qs	Ps
*CPSI	752	m	9	0.19	54.70	0.25	0.1586
*CPSI	757	m	9	0.24	39.88	0.01	0.1312
*CPSI	762	f	8	0.57	17.34	1.64	0.0186
*CPSI	767	f	8	0.47	22.33	1.01	0.0264
Subtotal CPSI				0.30	134.25	2.91	
*KAISE2	662	m	5	0.24	9.26	0.00	0.4671
*KAISE2	664	f	5	-0.40	8.35	3.62	0.2471
Subtotal KAISE2				-0.06	17.61	3.62	

	N	6
	NS	2
	Wt	151.86
Het	Chi	6.53
Het	df	5
Het	P	N.S.
Fixed	RR	1.29
	RRl	1.10
	RRu	1.52
	P	++
Random	RR	1.30
	RRl	1.07
	RRu	1.57
	P	++
Asymm	P	N.S.

Table 1L5 - 6

IESLC - Meta-analysis of Current Smoking, Tar, "Highest vs lowest"
All LC types, Cigarettes only
Least adjusted

	Sex combined	male	female	Total
N		3	3	6
NS		2	2	4
Wt		103.84	48.01	151.86
Het Chi		0.06	5.83	6.53
Het df		2	2	5
Het P		N.S.	(*)	N.S.
Fixed RR		1.24	1.42	1.29
RRl		1.02	1.07	1.10
RRu		1.50	1.89	1.52
P		+	+	++
Random RR		1.24	1.31	1.30
RRl		1.02	0.79	1.07
RRu		1.50	2.17	1.57
P		+	N.S.	++
Between Chi				0.64
Between df				1
Between P				N.S.
Btwn(F) P				N.S.
Btwn(R) P				N.S.

Table 1L5 - 7

IESLC - Meta-analysis of Current Smoking, Tar, "Highest vs lowest"
All LC types, Cigarettes only
Excluded studies (and stage at which they were excluded)

1	AGUDO ALDERS ARMADA AUVINE AXELSS BARBON BECHER BENHAM BLOT1 BOFFET BOUCHA BRESLO BROWN3 CARPEN CHEN CHEN2 CHIAZZ CHOI CHYOU CORREA DAMBER DARBY DESTEF DOLL DOLL2 DORGAN DOSEME FAN GAO GARCIA GARSHI GENG GER GRAHAM GUO GURSEL HAENSZ HAMMO2 HAMMON HEGMAN HU HU2 JAHN JAIN JEDRYC JOLY JUSSAW KHUDER KOO KOULUM KREUZE LAUSSM LETOUR LEVIN LIU3 LIU4 LIU5 LUBIN LUBIN2 LUO MCCONN NOTAN2 OSANN2 PERNU PEZZOT PRESCO QIAO QIAO2 RACHTA RESTRE SADOWS STASZE SUZUK2 TIZZAN TVERDA VUTUC WANG2 WIGLE WU2 WUWILL WYNDE2 WYNDE3 XU YUAN ZHANG ZHENG ZHOU
2	AKIBA AMANDU AMES BEST BOUCOT BROSS BUFFLE CEDERL DEAN2 DEAN3 DORN ENGELA GAO2 HIRAYA HOLE HUMBLE KATSOU LIAW MATOS MCDUFF MIGRAN PEZZO2 PISANI SEGI2 SOBUE SPITZ SVENSS WAKAI WATSON WU WYNDE7 WYNDE8
3	CPSII
8	BENSHL GILLIS KAUFMA MRFITR SPEIZE
9	WYNDE6

Table 1L5 - 8
Potentially overlapping studies

REF	REFGP	PRINC	.	OVERLAP/LINK
CPSI	CPSI	1		CPSI overall

Table 1L6 -

IESLC - Meta-analysis of Ever/current Smoking, Tar, "Highest vs lowest"
All LC types, Cigarettes only

This analysis is restricted to results for:
1) Ever/current smokers
2) Results by Tar
3) Categorical results by Tar
4) Denominator (unexposed) = "low"
5) All LC types (or near equivalent)
6) Results complete enough for use in metaanalysis

Within each study, results are then selected (in the following order of preference, within each sex) for:
7) SMKSTA: ever, current
8) PRODUCT: cigarettes only
9) CIGTYPE: all/unspecified, MC regardless of HR, MC only
10) Results with least adjustment for other aspects of smoking (ADOS)
11) The highest vs lowest category
12) Followup period (YF, prospective studies): whole study (coded as 0) or longest available
13) LCtype: all or nearest available, at least Squamous and Adeno. (q = squamous, s = small,
l = large, a = adeno, mix = mixed, alv = alveolar)
14) Race: all or nearest available, otherwise by race (wh or w = white, bl or b = black, hi = hispanic
ch = chinese, jap = japanese, haw = hawaiian, w+o = white + oriental, sca = scandinavian, as = asian)
15) For overlapping studies: principal rather than subsidiary studies
Finally by Age: whole study (coded as 0) if available, otherwise by widest available age group
and then for single sex results (m, f) in preference to results for both sexes combined (c).

Results adjusted (AD) for the most potential confounders are then chosen in Sections -1 to -3
(and those which actually differ from the adjusted results in Table 1L3 - 1 are marked 'x' in Section -1)
and results adjusted for the least confounders in Sections -4 to -6. (Those least adjusted results which
actually differ from the most adjusted are marked 'x' in column X in Section -4)

Section -7 shows excluded studies, together with the stage (as above) at which no qualifying
results were found.

Section -8 lists the potentially overlapping studies which have been included (1=principal, 2=subsidiary).

Section -9 lists any results which would have been included in preference except that they had data not complete
enough for use in meta-analysis, with their significance (yes/no), if known, and any further comment as entered
on the database. It also lists as "gap" any categories for which no data were presented by the original authors.

In addition to those mentioned above, the following fields, levels and abbreviations are used:
* or nk = not known, n = no, y = yes, ot = other
all/unspec = all or unspecified, MC = manufactured cigarettes, HR = hand-rolled cigarettes
exL, exH = range of exposure (low and high) in the "highest" group, in terms of Tar
unexL, unexH = range of exposure (low and high) in the "lowest" group, in terms of Tar
REF: 6-character study reference
NRR: number of the RR on the database within the study
ST : study type (CC = case control, pr or prosp = prospective)
NLC: number of lung cancer cases in whole study
R : risky occupational population (n = no, m = mining, o = other risky)
VB : national cigarette type (V = at least 75% Virginia, bl = at least 75% blended, ot = other)
P : any proxy use
H : full histological confirmation
De : derivation of RR/CI (or = original, st = standard method, ot = other method of estimation)

Table 1L6 - 1

IESLC - Meta-analysis of Ever/current Smoking, Tar, "Highest vs lowest"
All LC types, Cigarettes only
Most adjusted

REF	NRR	1L3	SEX	AGEL	AGEH	RACE	YF	LC TYPE	LOC	START	ST	NLC	R	VB	P	H	AD	ADOS	SM	PRODUCT	exL	exH	unexL	unexH	De
ALDERS	529		m	0	0	all	-	all	Eu:UK	1977	CC	1448	n	V	n	n	2		1#ev	cig only	17	22	1	16	ot
ALDERS	530		f	0	0	all	-	all	Eu:UK	1977	CC	1448	n	V	n	n	2		1#ev	cig only	17	22	1	16	ot
CPSI	752	x	m	40	99	all	6	all	NAmer	1959	pr	5138	n	bl	n	n	9		2#cu	cig only	26	36	1	18	ot
CPSI	757	x	m	40	99	all	12	all	NAmer	1959	pr	5138	n	bl	n	n	9		2#cu	cig only	26	36	1	18	ot
CPSI	762		f	40	99	all	6	all	NAmer	1959	pr	5138	n	bl	n	n	8		2#cu	cig only	26	36	1	18	ot
CPSI	767		f	40	99	all	12	all	NAmer	1959	pr	5138	n	bl	n	n	8		2#cu	cig only	26	36	1	18	ot
KAISE2	662		m	0	0	all	9	all	NAmer	1979	pr	318	n	bl	n	n	5		2#cu	cig only	19	999	1	10	or
KAISE2	664		f	0	0	all	9	all	NAmer	1979	pr	318	n	bl	n	n	5		2#cu	cig only	19	999	1	10	or

Comments on values in listings

ALDERS	ADOS	Number of cigs/day
ALDERS	ADOS	Number of cigs/day
CPSI	ADOS	Number of cigs/day, age started smoking
CPSI	ADOS	Number of cigs/day, age started smoking
CPSI	ADOS	Number of cigs/day, age started smoking
CPSI	ADOS	Number of cigs/day, age started smoking
KAISE2	ADOS	Cigs/day and years of smoking
KAISE2	ADOS	Cigs/day and years of smoking

Cigarette type is all/unspec for all RRs

except for the following:

REF	NRR	CIGTYPE
ALDERS	529	MC only
ALDERS	530	MC only

Table 1L6 - 2

IESLC - Meta-analysis of Ever/current Smoking, Tar, "Highest vs lowest"
All LC types, Cigarettes only
Most adjusted

REF	NRR	SEX	AD	Number Exposed Case	Cont	Non-exposed Case	Cont	RR	95.00%CI
ALDERS	529	m	2	156	-	38	-	0.91	(0.54- 1.53)
ALDERS	530	f	2	145	-	85	-	1.04	(0.69- 1.58)
Subtotal ALDERS								0.99	(0.71- 1.37)
*CPSI	752	m	9	122	-	101	-	1.21	(0.93- 1.58)
*CPSI	757	m	9	90	-	71	-	1.27	(0.93- 1.73)
*CPSI	762	f	8	49	-	27	-	1.76	(1.10- 2.82)
*CPSI	767	f	8	58	-	36	-	1.60	(1.06- 2.43)
Subtotal CPSI								1.35	(1.14- 1.60)
*KAISE2	662	m	5	29	-	14	-	1.27	(0.67- 2.43)
*KAISE2	664	f	5	13	-	29	-	0.67	(0.34- 1.32)
Subtotal KAISE2								0.94	(0.59- 1.50)
Partial Totals				662	0	401	0		

*prospective study

REF	NRR	SEX	AD	Ys	Ws	Qs	Ps
ALDERS	529	m	2	-0.09	14.17	1.27	0.7226
ALDERS	530	f	2	0.04	22.39	0.62	0.8528
Subtotal ALDERS				-0.01	36.55	1.89	
*CPSI	752	m	9	0.19	54.70	0.01	0.1586
*CPSI	757	m	9	0.24	39.88	0.05	0.1312
*CPSI	762	f	8	0.57	17.34	2.25	0.0186
*CPSI	767	f	8	0.47	22.33	1.57	0.0264
Subtotal CPSI				0.30	134.25	3.87	
*KAISE2	662	m	5	0.24	9.26	0.01	0.4671
*KAISE2	664	f	5	-0.40	8.35	3.06	0.2471
Subtotal KAISE2				-0.06	17.61	3.07	

	N	8
	NS	3
	Wt	188.41
Het	Chi	8.83
Het	df	7
Het	P	N.S.
Fixed	RR	1.23
	RRl	1.06
	RRu	1.42
	P	++
Random	RR	1.22
	RRl	1.03
	RRu	1.44
	P	+
Asymm	P	N.S.

Table 1L6 - 3

IESLC - Meta-analysis of Ever/current Smoking, Tar, "Highest vs lowest"
All LC types, Cigarettes only
Most adjusted

	Sex			
	combined	male	female	Total
N		4	4	8
NS		3	3	6
Wt		118.01	70.40	188.41
Het Chi		1.24	7.33	8.83
Het df		3	3	7
Het P		N.S.	(*)	N.S.
Fixed RR		1.19	1.29	1.23
RRl		1.00	1.02	1.06
RRu		1.43	1.63	1.42
P		(+)	+	++
Random RR		1.19	1.24	1.22
RRl		1.00	0.85	1.03
RRu		1.43	1.81	1.44
P		(+)	N.S.	+
Between Chi				0.26
Between df				1
Between P				N.S.
Btwn(F) P				N.S.
Btwn(R) P				N.S.

Too few RRs for analysis by factor

Table 1L6 - 4

IESLC - Meta-analysis of Ever/current Smoking, Tar, "Highest vs lowest"
All LC types, Cigarettes only
Least adjusted

REF	NRR	X	SEX	AGEL	AGEH	RACE	YF	LC TYPE	LOC	START	ST	NLC	R	VB	P	H	AD	ADOS	SM	PRODUCT	exL	exH	unexL	unexH	De
ALDERS	529		m	0	0	all	-	all	Eu:UK	1977	CC	1448	n	V	n	n	2		1#ev	cig only	17	22	1	16	ot
ALDERS	530		f	0	0	all	-	all	Eu:UK	1977	CC	1448	n	V	n	n	2		1#ev	cig only	17	22	1	16	ot
CPSI	752		m	40	99	all	6	all	NAmer	1959	pr	5138	n	bl	n	n	9		2#cu	cig only	26	36	1	18	ot
CPSI	757		m	40	99	all	12	all	NAmer	1959	pr	5138	n	bl	n	n	9		2#cu	cig only	26	36	1	18	ot
CPSI	762		f	40	99	all	6	all	NAmer	1959	pr	5138	n	bl	n	n	8		2#cu	cig only	26	36	1	18	ot
CPSI	767		f	40	99	all	12	all	NAmer	1959	pr	5138	n	bl	n	n	8		2#cu	cig only	26	36	1	18	ot
KAISE2	662		m	0	0	all	9	all	NAmer	1979	pr	318	n	bl	n	n	5		2#cu	cig only	19	999	1	10	or
KAISE2	664		f	0	0	all	9	all	NAmer	1979	pr	318	n	bl	n	n	5		2#cu	cig only	19	999	1	10	or

Comments on values in listings

ALDERS	ADOS	Number of cigs/day
ALDERS	ADOS	Number of cigs/day
CPSI	ADOS	Number of cigs/day, age started smoking
CPSI	ADOS	Number of cigs/day, age started smoking
CPSI	ADOS	Number of cigs/day, age started smoking
CPSI	ADOS	Number of cigs/day, age started smoking
KAISE2	ADOS	Cigs/day and years of smoking
KAISE2	ADOS	Cigs/day and years of smoking

Cigarette type is all/unspec for all RRs

except for the following:

REF	NRR	CIGTYPE
ALDERS	529	MC only
ALDERS	530	MC only

Table 1L6 - 5

IESLC - Meta-analysis of Ever/current Smoking, Tar, "Highest vs lowest"
All LC types, Cigarettes only
Least adjusted

REF	NRR	SEX	AD	Number Exposed Case	Cont	Non-exposed Case	Cont	RR	95.00%CI
ALDERS	529	m	2	156	-	38	-	0.91	(0.54- 1.53)
ALDERS	530	f	2	145	-	85	-	1.04	(0.69- 1.58)
Subtotal ALDERS								0.99	(0.71- 1.37)
*CPSI	752	m	9	122	-	101	-	1.21	(0.93- 1.58)
*CPSI	757	m	9	90	-	71	-	1.27	(0.93- 1.73)
*CPSI	762	f	8	49	-	27	-	1.76	(1.10- 2.82)
*CPSI	767	f	8	58	-	36	-	1.60	(1.06- 2.43)
Subtotal CPSI								1.35	(1.14- 1.60)
*KAISE2	662	m	5	29	-	14	-	1.27	(0.67- 2.43)
*KAISE2	664	f	5	13	-	29	-	0.67	(0.34- 1.32)
Subtotal KAISE2								0.94	(0.59- 1.50)
Partial Totals				662	0	401	0		

*prospective study

REF	NRR	SEX	AD	Ys	Ws	Qs	Ps
ALDERS	529	m	2	-0.09	14.17	1.27	0.7226
ALDERS	530	f	2	0.04	22.39	0.62	0.8528
Subtotal ALDERS				-0.01	36.55	1.89	
*CPSI	752	m	9	0.19	54.70	0.01	0.1586
*CPSI	757	m	9	0.24	39.88	0.05	0.1312
*CPSI	762	f	8	0.57	17.34	2.25	0.0186
*CPSI	767	f	8	0.47	22.33	1.57	0.0264
Subtotal CPSI				0.30	134.25	3.87	
*KAISE2	662	m	5	0.24	9.26	0.01	0.4671
*KAISE2	664	f	5	-0.40	8.35	3.06	0.2471
Subtotal KAISE2				-0.06	17.61	3.07	

	N	8
	NS	3
	Wt	188.41
Het	Chi	8.83
Het	df	7
Het	P	N.S.
Fixed	RR	1.23
	RRl	1.06
	RRu	1.42
	P	++
Random	RR	1.22
	RRl	1.03
	RRu	1.44
	P	+
Asymm	P	N.S.

Table 1L6 - 6

IESLC - Meta-analysis of Ever/current Smoking, Tar, "Highest vs lowest"
All LC types, Cigarettes only
Least adjusted

	Sex			
	combined	male	female	Total
N		4	4	8
NS		3	3	6
Wt		118.01	70.40	188.41
Het Chi		1.24	7.33	8.83
Het df		3	3	7
Het P		N.S.	(*)	N.S.
Fixed RR		1.19	1.29	1.23
RRl		1.00	1.02	1.06
RRu		1.43	1.63	1.42
P		(+)	+	++
Random RR		1.19	1.24	1.22
RRl		1.00	0.85	1.03
RRu		1.43	1.81	1.44
P		(+)	N.S.	+
Between Chi				0.26
Between df				1
Between P				N.S.
Btwn(F) P				N.S.
Btwn(R) P				N.S.

Table 1L6 - 7

IESLC - Meta-analysis of Ever/current Smoking, Tar, "Highest vs lowest"
All LC types, Cigarettes only
Excluded studies (and stage at which they were excluded)

1	BECHER BLOT1 BROWN3 CARPEN CHYOU DARBY DOLL2 GARCIA GRAHAM GURSEL HAMMO2 JAHN JAIN LAUSSM PRESCO QIAO TVERDA WIGLE WYNDE3
2	AGUDO AKIBA AMANDU AMES ARMADA AUVINE AXELSS BARBON BENHAM BEST BOFFET BOUCHA BOUCOT BRESLO BROSS BUFFLE CEDERL CHEN CHEN2 CHIAZZ CHOI CORREA DAMBER DEAN2 DEAN3 DESTEF DOLL DORN DOSEME ENGELA FAN GAO GAO2 GARSHI GENG GER GUO HAENSZ HAMMON HEGMAN HIRAYA HOLE HU HU2 HUMBLE JEDRYC JOLY JUSSAW KATSOU KHUDER KOO KOULUM KREUZE LETOUR LEVIN LIAW LIU3 LIU4 LIU5 LUBIN LUO MATOS MCCONN MCDUFF MIGRAN NOTAN2 OSANN2 PERNU PEZZO2 PEZZOT PISANI QIAO2 RACHTA RESTRE SADOWS SEGI2 SOBUE SPITZ STASZE SUZUK2 SVENSS TIZZAN WAKAI WANG2 WATSON WU WU2 WUWILL WYNDE2 WYNDE7 WYNDE8 XU YUAN ZHANG ZHENG ZHOU
3	CPSII
8	BENSHL DORGAN GILLIS KAUFMA LUBIN2 MRFITR SPEIZE VUTUC
9	WYNDE6

Table 1L6 - 8
Potentially overlapping studies

REF	REFGP	PRINC	.	OVERLAP/LINK
CPSI	CPSI	1		CPSI overall
