# Supplementary material for: Systematic review with meta-analysis of the epidemiological evidence in the 1900s relating smoking to lung cancer
Source: BMC Cancer. 2012 Sep 3;12:385. doi: 10.1186/1471-2407-12-385 (PMC3505152; doi:10.1186/1471-2407-12-385)
Supplement: Additional file 5 — Detailed Analysis Tables (Individual file names as described in Additional file 1: Methods, Table1). [file 1471-2407-12-385-S5.zip › PDF/2AR.pdf]

Table 2A1R - 1

IESLC - Meta-regression of ever smoking, any product (or cigs if any not available)
Multiple regression of data from Table 2A1
Squamous

Stepwise allowing only characteristics from the fixed model

Log Relative risk
WEIGHTED on Weight

		Deviance	(DF)				
Model 1		522.221	(101)				
		Estimate	S.E.	P	RR	95%CIl	95%CIu
Constant		2.253	0.032	+++	9.517	8.945	10.125

		Deviance	(DF)	Drop Dev	P		
Model 2		419.586	(97)	102.635	***		
		Estimate	S.E.	P	RR	95%CIl	95%CIu
Constant		1.471	0.089	+++	4.354	3.659	5.181
Start year of study							
<1960	14	Aliased			4.354	3.032	6.252
1960-69	14	1.003	0.134	+++	11.866	7.889	17.848
1970-79	26	0.699	0.109	+++	8.761	6.768	11.341
1980-89	40	0.965	0.099	+++	11.424	9.514	13.716
1990+	8	1.046	0.211	+++	12.392	5.667	27.099

		Deviance	(DF)	Drop Dev	P		
Model 3		308.715	(90)	110.870	***		
		Estimate	S.E.	P	RR	95%CIl	95%CIu
Constant		1.743	0.102	+++	5.715	4.679	6.980
Start year of study							
<1960	14	Aliased			4.201	2.994	5.895
1960-69	14	0.813	0.141	+++	9.472	6.492	13.819
1970-79	26	0.953	0.115	+++	10.891	8.243	14.388
1980-89	40	0.937	0.110	+++	10.724	8.942	12.862
1990+	8	1.233	0.238	+++	14.423	6.656	31.254
Location							
NAmer	38	Aliased			12.946	10.846	15.452
UK	4	-0.577	0.248	-	7.274	3.032	17.450
Scand	7	-0.249	0.209	N.S.	10.096	4.868	20.937
othEur	15	-0.612	0.107	---	7.019	5.150	9.566
China	12	-0.969	0.107	---	4.910	3.463	6.962
Japan	8	-0.086	0.193	N.S.	11.878	5.989	23.561
othAs	12	-0.634	0.125	---	6.866	4.536	10.393
other	6	-0.021	0.288	N.S.	12.682	4.591	35.037

Table 2A1R - 1

IESLC - Meta-regression of ever smoking, any product (or cigs if any not available)
Multiple regression of data from Table 2A1
Squamous

Fixed model

Log Relative risk
WEIGHTED on Weight

		Deviance	(DF)				
Model 7		260.432	(82)				
		Estimate	S.E.	P	RR	95%CIl	95%CIu
Constant		1.281	0.174	+++	3.600	2.558	5.068
Sex(RR)							
Male	49	Aliased			9.824	8.323	11.596
Female	42	-0.046	0.072	N.S.	9.381	7.813	11.263
Combined	11	-0.130	0.141	N.S.	8.629	5.496	13.548
Location							
NAmer	38	Aliased			13.105	10.936	15.704
UK	4	-0.861	0.266	--	5.537	2.232	13.738
Scand	7	-0.484	0.241	-	8.079	3.603	18.116
othEur	15	-0.609	0.114	---	7.128	5.149	9.868
China	12	-1.028	0.120	---	4.686	3.242	6.774
Japan	8	-0.562	0.226	-	7.471	3.493	15.976
othAs	12	-0.390	0.158	-	8.869	5.427	14.493
other	6	-0.031	0.301	N.S.	12.707	4.614	34.997
Start year of study							
<1960	14	Aliased			4.184	2.870	6.098
1960-69	14	0.819	0.154	+++	9.492	6.342	14.205
1970-79	26	0.802	0.130	+++	9.328	7.020	12.395
1980-89	40	0.990	0.134	+++	11.256	9.238	13.714
1990+	8	1.788	0.263	+++	25.002	10.486	59.613
Study type (1)							
CC	93	Aliased			9.437	8.438	10.553
other	9	0.395	0.233	(+)	14.008	6.272	31.286
Study size (number of LC cases)							
100-249	22	Aliased			5.466	3.484	8.576
250-499	31	0.251	0.152	N.S.	7.027	4.831	10.222
500-999	18	0.729	0.162	+++	11.336	7.942	16.178
1000+	31	0.655	0.145	+++	10.522	8.979	12.331
Number of adjustment variables (1)							
0	53	Aliased			10.116	8.136	12.578
1	18	0.477	0.147	++	16.300	10.369	25.622
2+/+nk	31	-0.226	0.097	-	8.073	6.610	9.861

Table 2A1R - 2

IESLC - Meta-regression of ever smoking, any product (or cigs if any not available)
Multiple regression of data from Table 2A1
Squamous
Effect of removing characteristics

Log Relative risk
WEIGHTED on Weight

		Deviance	(DF)				
Model 7		260.432	(82)				
		Estimate	S.E.	P	RR	95%CIl	95%CIu
Constant		1.281	0.174	+++	3.600	2.558	5.068
Sex(RR)							
Male	49	Aliased			9.824	8.323	11.596
Female	42	-0.046	0.072	N.S.	9.381	7.813	11.263
Combined	11	-0.130	0.141	N.S.	8.629	5.496	13.548
Location							
NAmer	38	Aliased			13.105	10.936	15.704
UK	4	-0.861	0.266	--	5.537	2.232	13.738
Scand	7	-0.484	0.241	-	8.079	3.603	18.116
othEur	15	-0.609	0.114	---	7.128	5.149	9.868
China	12	-1.028	0.120	---	4.686	3.242	6.774
Japan	8	-0.562	0.226	-	7.471	3.493	15.976
othAs	12	-0.390	0.158	-	8.869	5.427	14.493
other	6	-0.031	0.301	N.S.	12.707	4.614	34.997
Start year of study							
<1960	14	Aliased			4.184	2.870	6.098
1960-69	14	0.819	0.154	+++	9.492	6.342	14.205
1970-79	26	0.802	0.130	+++	9.328	7.020	12.395
1980-89	40	0.990	0.134	+++	11.256	9.238	13.714
1990+	8	1.788	0.263	+++	25.002	10.486	59.613
Study type (1)							
CC	93	Aliased			9.437	8.438	10.553
other	9	0.395	0.233	(+)	14.008	6.272	31.286
Study size (number of LC cases)							
100-249	22	Aliased			5.466	3.484	8.576
250-499	31	0.251	0.152	N.S.	7.027	4.831	10.222
500-999	18	0.729	0.162	+++	11.336	7.942	16.178
1000+	31	0.655	0.145	+++	10.522	8.979	12.331
Number of adjustment variables (1)							
0	53	Aliased			10.116	8.136	12.578
1	18	0.477	0.147	++	16.300	10.369	25.622
2+/+nk	31	-0.226	0.097	-	8.073	6.610	9.861

Omit Sex		Deviance	(DF)	Drop Dev	P		
Model 8		261.503	(84)	-1.070	N.S.		
		Estimate	S.E.	P	RR	95%CIl	95%CIu
Constant		1.213	0.161	+++	3.363	2.455	4.606
Number of adjustment variables (1)							
0	53	Aliased			10.079	8.135	12.489
1	18	0.448	0.143	++	15.774	10.255	24.263
2+/+nk	31	-0.212	0.096	-	8.154	6.719	9.894
Location							
NAmer	38	Aliased			13.095	10.960	15.645
UK	4	-0.836	0.259	--	5.675	2.362	13.634
Scand	7	-0.438	0.236	(-)	8.452	3.863	18.492
othEur	15	-0.616	0.114	---	7.070	5.137	9.729
China	12	-1.033	0.119	---	4.663	3.246	6.698
Japan	8	-0.544	0.224	-	7.603	3.606	16.030
othAs	12	-0.390	0.157	-	8.868	5.466	14.388
other	6	0.008	0.298	N.S.	13.197	4.904	35.516
Start year of study							
<1960	14	Aliased			4.060	2.873	5.738
1960-69	14	0.868	0.143	+++	9.673	6.528	14.334
1970-79	26	0.841	0.120	+++	9.416	7.126	12.443
1980-89	40	1.022	0.125	+++	11.286	9.311	13.682
1990+	8	1.752	0.255	+++	23.404	10.726	51.067
Study type (1)							
CC	93	Aliased			9.436	8.449	10.538
other	9	0.400	0.233	(+)	14.074	6.369	31.103

Table 2A1R - 2

IESLC - Meta-regression of ever smoking, any product (or cigs if any not available)
Multiple regression of data from Table 2A1
Squamous
Effect of removing characteristics

Log Relative risk
WEIGHTED on Weight

		Estimate	S.E.	P	RR	95%CIl	95%CIu
Study size (number of LC cases)							
100-249	22	Aliased			5.454	3.560	8.356
250-499	31	0.253	0.149	(+)	7.023	4.851	10.168
500-999	18	0.724	0.159	+++	11.251	7.924	15.973
1000+	31	0.659	0.139	+++	10.541	9.025	12.312

Omit Location		Deviance	(DF)	Drop Dev	P		
Model 8		361.745	(89)	-101.313	***		
		Estimate	S.E.	P	RR	95%CIl	95%CIu
Constant		0.993	0.156	+++	2.700	1.989	3.664
Number of adjustment variables (1)							
0	53	Aliased			9.036	7.462	10.942
1	18	0.499	0.113	+++	14.888	10.564	20.984
2+/+nk	31	0.002	0.088	N.S.	9.057	7.572	10.833
Sex(RR)							
Male	49	Aliased			10.192	8.708	11.930
Female	42	-0.139	0.071	(-)	8.866	7.461	10.536
Combined	11	-0.107	0.134	N.S.	9.156	6.061	13.832
Start year of study							
<1960	14	Aliased			4.437	3.182	6.188
1960-69	14	1.063	0.146	+++	12.842	8.958	18.411
1970-79	26	0.602	0.121	+++	8.101	6.414	10.231
1980-89	40	0.940	0.118	+++	11.363	9.583	13.474
1990+	8	1.466	0.229	+++	19.222	9.150	40.384
Study type (1)							
CC	93	Aliased			9.416	8.458	10.482
other	9	0.499	0.227	+	15.511	7.306	32.931
Study size (number of LC cases)							
100-249	22	Aliased			5.755	3.833	8.643
250-499	31	0.285	0.146	(+)	7.651	5.531	10.583
500-999	18	0.473	0.151	++	9.233	6.805	12.527
1000+	31	0.618	0.133	+++	10.677	9.287	12.274

Omit start year		Deviance	(DF)	Drop Dev	P		
Model 8		342.591	(86)	-82.159	***		
		Estimate	S.E.	P	RR	95%CIl	95%CIu
Constant		1.921	0.147	+++	6.826	5.113	9.115
Number of adjustment variables (1)							
0	53	Aliased			9.121	7.503	11.088
1	18	0.505	0.143	+++	15.114	9.870	23.146
2+/+nk	31	-0.018	0.084	N.S.	8.957	7.520	10.668
Sex(RR)							
Male	49	Aliased			10.066	8.576	11.814
Female	42	-0.058	0.071	N.S.	9.498	7.981	11.303
Combined	11	-0.331	0.119	--	7.229	4.997	10.457
Location							
NAmer	38	Aliased			12.369	10.533	14.524
UK	4	-1.342	0.250	---	3.233	1.398	7.480
Scand	7	-0.563	0.236	-	7.046	3.229	15.375
othEur	15	-0.668	0.095	---	6.342	4.818	8.347
China	12	-0.735	0.112	---	5.930	4.216	8.342
Japan	8	-0.161	0.214	N.S.	10.535	5.209	21.307
othAs	12	-0.065	0.147	N.S.	11.587	7.332	18.311
other	6	0.595	0.271	+	22.434	9.066	55.513
Study type (1)							
CC	93	Aliased			9.429	8.454	10.517
other	9	0.433	0.233	(+)	14.542	6.639	31.850
Study size (number of LC cases)							
100-249	22	Aliased			5.230	3.493	7.829
250-499	31	0.349	0.148	+	7.416	5.190	10.597
500-999	18	0.571	0.150	+++	9.255	6.616	12.948
1000+	31	0.732	0.131	+++	10.873	9.356	12.635

Table 2A1R - 2

IESLC - Meta-regression of ever smoking, any product (or cigs if any not available)
Multiple regression of data from Table 2A1
Squamous
Effect of removing characteristics

Log Relative risk
WEIGHTED on Weight

Omit study type		Deviance	(DF)	Drop Dev	P		
Model 8		263.310	(83)	-2.878	N.S.		
		Estimate	S.E.	P	RR	95%CIl	95%CIu
Constant		1.303	0.174	+++	3.679	2.616	5.173
Number of adjustment variables (1)							
0	53	Aliased			10.105	8.138	12.548
1	18	0.493	0.146	++	16.552	10.571	25.919
2+/+nk	31	-0.227	0.097	-	8.054	6.603	9.825
Sex(RR)							
Male	49	Aliased			9.821	8.328	11.580
Female	42	-0.043	0.072	N.S.	9.405	7.842	11.279
Combined	11	-0.139	0.141	N.S.	8.546	5.460	13.376
Location							
NAmer	38	Aliased			13.176	11.011	15.767
UK	4	-0.868	0.266	--	5.528	2.241	13.641
Scand	7	-0.499	0.240	-	8.000	3.586	17.847
othEur	15	-0.597	0.114	---	7.256	5.263	10.005
China	12	-1.049	0.120	---	4.617	3.205	6.650
Japan	8	-0.593	0.225	--	7.280	3.426	15.469
othAs	12	-0.437	0.156	--	8.514	5.263	13.774
other	6	-0.065	0.301	N.S.	12.341	4.516	33.723
Start year of study							
<1960	14	Aliased			4.166	2.865	6.057
1960-69	14	0.818	0.154	+++	9.441	6.325	14.093
1970-79	26	0.808	0.130	+++	9.341	7.042	12.390
1980-89	40	0.996	0.134	+++	11.275	9.265	13.721
1990+	8	1.788	0.263	+++	24.911	10.503	59.083
Study size (number of LC cases)							
100-249	22	Aliased			5.540	3.543	8.661
250-499	31	0.285	0.151	(+)	7.366	5.140	10.555
500-999	18	0.734	0.162	+++	11.540	8.118	16.403
1000+	31	0.627	0.144	+++	10.368	8.882	12.104

Omit Study size		Deviance	(DF)	Drop Dev	P		
Model 8		288.497	(85)	-28.064	*		
		Estimate	S.E.	P	RR	95%CIl	95%CIu
Constant		1.782	0.129	+++	5.941	4.614	7.649
Number of adjustment variables (1)							
0	53	Aliased			9.843	7.987	12.130
1	18	0.423	0.142	++	15.025	9.724	23.213
2+/+nk	31	-0.158	0.095	(-)	8.405	6.927	10.199
Sex(RR)							
Male	49	Aliased			10.033	8.538	11.790
Female	42	-0.116	0.070	N.S.	8.933	7.500	10.640
Combined	11	-0.045	0.140	N.S.	9.593	6.202	14.838
Location							
NAmer	38	Aliased			13.248	11.116	15.789
UK	4	-0.712	0.262	--	6.498	2.707	15.599
Scand	7	-0.589	0.230	-	7.348	3.431	15.736
othEur	15	-0.603	0.111	---	7.251	5.366	9.800
China	12	-0.966	0.111	---	5.044	3.607	7.055
Japan	8	-0.507	0.223	-	7.978	3.816	16.683
othAs	12	-0.658	0.143	---	6.861	4.439	10.602
other	6	-0.021	0.293	N.S.	12.969	4.905	34.290
Start year of study							
<1960	14	Aliased			3.932	2.750	5.621
1960-69	14	0.791	0.153	+++	8.674	5.884	12.789
1970-79	26	0.951	0.124	+++	10.179	7.751	13.366
1980-89	40	1.067	0.129	+++	11.428	9.441	13.833
1990+	8	1.391	0.249	+++	15.809	7.112	35.140
Study type (1)							
CC	93	Aliased			9.472	8.488	10.571
other	9	0.219	0.222	N.S.	11.791	5.545	25.075

Table 2A1R - 2

IESLC - Meta-regression of ever smoking, any product (or cigs if any not available)
Multiple regression of data from Table 2A1
Squamous
Effect of removing characteristics

Log Relative risk
WEIGHTED on Weight

Omit	N	adjustment vars Deviance	(DF)	Drop Dev	P		
Model 8		283.106	(84)	-22.674	*		
		Estimate	S.E.	P	RR	95%CIl	95%CIu
Constant		1.259	0.167	+++	3.524	2.542	4.884
Study size (number of LC cases)							
100-249	22	Aliased			5.974	3.850	9.269
250-499	31	0.181	0.148	N.S.	7.156	4.991	10.260
500-999	18	0.588	0.159	+++	10.759	7.586	15.259
1000+	31	0.561	0.144	+++	10.467	8.954	12.236
Sex(RR)							
Male	49	Aliased			9.547	8.115	11.232
Female	42	-0.027	0.072	N.S.	9.297	7.762	11.136
Combined	11	0.082	0.134	N.S.	10.364	6.774	15.857
Location							
NAmer	38	Aliased			12.555	10.572	14.909
UK	4	-0.600	0.256	-	6.890	2.910	16.313
Scand	7	-0.082	0.220	N.S.	11.567	5.610	23.852
othEur	15	-0.625	0.109	---	6.720	4.929	9.160
China	12	-0.967	0.117	---	4.772	3.323	6.851
Japan	8	-0.029	0.196	N.S.	12.195	6.327	23.506
othAs	12	-0.305	0.148	-	9.253	5.820	14.711
other	6	0.081	0.300	N.S.	13.608	5.009	36.972
Start year of study							
<1960	14	Aliased			4.284	2.972	6.175
1960-69	14	0.904	0.152	+++	10.574	7.264	15.393
1970-79	26	0.870	0.128	+++	10.227	7.803	13.405
1980-89	40	0.909	0.128	+++	10.635	8.857	12.770
1990+	8	1.449	0.251	+++	18.250	7.982	41.727
Study type (1)							
CC	93	Aliased			9.422	8.436	10.522
other	9	0.469	0.232	+	15.060	6.822	33.245

Table 2A1R - 3

IESLC - Meta-regression of ever smoking, any product (or cigs if any not available)
Multiple regression of data from Table 2A1
Squamous
Study outliers

Study Ref	NRR	LOGRR	FITVAL	SEFITV	STDRES
STAYNE	3	1.244	2.351	0.425	-2.604
BROWN2	6	2.407	2.700	0.139	-2.111
DOSEME	3	1.281	1.903	0.296	-2.106
SCHWAR	9	0.610	2.926	1.179	-1.964
SOBUE	107	2.168	2.794	0.483	-1.298
LUBIN2	165	1.755	2.083	0.260	-1.262
ZHOU	8	1.144	1.710	0.451	-1.253
GER	13	1.160	2.323	0.952	-1.222
DOLL	88	0.756	1.505	0.613	-1.222
LOMBA2	2	1.445	2.054	0.512	-1.188
ABRAHA	4	1.678	2.552	0.775	-1.128
BRESLO	36	1.306	1.881	0.525	-1.094
KOO	6	1.423	1.834	0.469	-0.876
WUWILL	9	1.435	1.700	0.305	-0.868
KREYBE	25	0.829	1.479	0.834	-0.781
KHUDER	24	2.057	2.522	0.632	-0.735
TIZZAN	18	0.994	1.197	0.277	-0.734
MATOS	67	2.089	2.812	0.989	-0.731
DORGAN	98	2.407	2.654	0.392	-0.629
DAMBER	33	2.468	2.806	0.567	-0.595
CHOI	62	1.695	2.131	0.759	-0.574
DESTE2	16	2.580	3.064	0.895	-0.540
COMSTO	66	2.088	2.729	1.269	-0.505
ZHENG	18	1.695	1.926	0.505	-0.456
WYNDE3	132	1.915	2.305	0.919	-0.425
ZHOU	9	1.338	1.663	0.838	-0.389
KATSOU	37	1.810	2.093	0.729	-0.388
WAKAI	74	2.153	2.437	1.261	-0.225
BUFFLE	62	2.568	2.766	1.065	-0.186
BUFFLE	49	2.641	2.812	0.978	-0.175
CHOI	64	1.937	2.085	0.857	-0.173
ENGELA	62	1.864	2.037	1.016	-0.170
CHAN	15	1.863	1.897	0.564	-0.062
LAMWK2	5	1.870	1.897	0.616	-0.044
LAMWK2	1	1.930	1.944	0.859	-0.016
LAMTH	1	2.092	2.085	0.600	0.011
WYNDE4	54	1.761	1.739	0.742	0.030
XU3	20	1.775	1.719	1.115	0.050
SOBUE	97	2.884	2.841	0.737	0.058
JAIN	48	2.890	2.775	1.355	0.086
NOU	6	1.959	1.804	1.459	0.106
BECHER	11	2.369	2.093	1.317	0.210
HAENSZ	1	1.099	1.009	0.412	0.217
JOLY	52	2.921	2.735	0.761	0.244
TOKARS	10	1.917	1.531	1.486	0.260
DORGAN	113	2.939	2.700	0.904	0.265
ALDERS	55	1.807	1.605	0.719	0.281
SEOW	3	2.862	2.632	0.811	0.284
SVENSS	72	2.535	2.218	1.020	0.311
SCHWAR	10	3.491	2.926	1.800	0.314
SCHWAR	18	3.767	2.879	2.538	0.349
OSANN2	25	3.558	2.926	1.737	0.364
JAIN	43	3.239	2.728	1.344	0.380
LUBIN	33	1.845	1.494	0.912	0.386
FAN	3	2.458	2.162	0.741	0.399
SUZUK2	15	3.434	2.683	1.726	0.435
BYERS1	1	2.115	1.936	0.406	0.441
TSUGAN	13	2.677	1.521	2.608	0.443
ANDERS	10	3.241	2.871	0.804	0.461
COMSTO	78	3.833	2.683	2.490	0.462
SCHWAR	17	4.137	2.879	2.569	0.490
HAMMON	60	2.826	2.404	0.861	0.490

Table 2A1R - 3

IESLC - Meta-regression of ever smoking, any product (or cigs if any not available)
Multiple regression of data from Table 2A1
Squamous
Study outliers

Study Ref	NRR	LOGRR	FITVAL	SEFITV	STDRES
WYNDE6	66	2.923	2.755	0.341	0.491
SIEMIA	7	3.122	2.587	1.074	0.499
WYNDE3	9	2.906	2.351	1.049	0.529
WYNDE6	412	3.477	3.186	0.543	0.536
LUO	8	2.389	1.685	1.302	0.540
JOLY	54	3.441	2.781	1.219	0.541
CHAN	11	2.723	1.944	1.293	0.603
WYNDE2	7	2.981	2.351	1.041	0.605
JEDRYC	54	2.553	2.091	0.753	0.613
LAMWK	2	2.355	1.834	0.830	0.628
WU	32	3.190	1.999	1.776	0.671
CORREA	35	3.343	3.085	0.377	0.684
WAKAI	80	3.228	2.391	1.155	0.725
ISHIMA	6	3.045	1.183	2.358	0.790
PEZZOT	6	4.139	2.240	2.405	0.790
ABRAHA	1	4.529	2.598	2.444	0.790
JAHN	46	3.137	2.317	1.037	0.791
ALDERS	52	2.688	1.651	1.283	0.808
KIHARA	26	3.295	2.628	0.794	0.839
KREYBE	4	2.386	1.526	1.010	0.852
GAO	2	2.128	1.672	0.524	0.872
GAO	12	1.974	1.626	0.398	0.876
STASZE	38	3.480	0.877	2.869	0.907
ZHENG	5	2.822	1.972	0.919	0.925
BARBON	127	2.676	1.978	0.743	0.939
DOLL	86	2.578	1.552	1.022	1.004
HINDS	23	2.781	2.080	0.668	1.049
XU3	24	3.245	1.673	1.464	1.074
OSANN	44	3.273	2.654	0.544	1.139
NOU	1	3.302	1.850	1.249	1.163
WYNDE4	68	2.549	1.785	0.653	1.170
MATSUD	11	3.664	1.538	1.726	1.231
STASZE	12	4.056	0.923	2.494	1.256
ORMOS	8	2.317	0.672	1.302	1.264
HEGMAN	2	3.428	2.392	0.807	1.283
BAND	5	3.623	2.700	0.684	1.349
OSANN	43	3.586	2.700	0.642	1.380
JUSSAW	23	3.236	2.439	0.541	1.473
BROWN2	5	3.001	2.654	0.188	1.849
LUBIN2	145	2.813	2.129	0.247	2.774

Table 2A1R - 4

IESLC - Meta-regression of ever smoking, any product (or cigs if any not available)
Multiple regression of data from Table 2A1
Squamous
Effect of additional characteristics

Log Relative risk
WEIGHTED on Weight

		Deviance	(DF)				
Model 7		260.432	(82)				
		Estimate	S.E.	P	RR	95%CIl	95%CIu
Constant		1.281	0.174	+++	3.600	2.558	5.068
Sex(RR)							
Male	49	Aliased			9.824	8.323	11.596
Female	42	-0.046	0.072	N.S.	9.381	7.813	11.263
Combined	11	-0.130	0.141	N.S.	8.629	5.496	13.548
Location							
NAmer	38	Aliased			13.105	10.936	15.704
UK	4	-0.861	0.266	--	5.537	2.232	13.738
Scand	7	-0.484	0.241	-	8.079	3.603	18.116
othEur	15	-0.609	0.114	---	7.128	5.149	9.868
China	12	-1.028	0.120	---	4.686	3.242	6.774
Japan	8	-0.562	0.226	-	7.471	3.493	15.976
othAs	12	-0.390	0.158	-	8.869	5.427	14.493
other	6	-0.031	0.301	N.S.	12.707	4.614	34.997
Start year of study							
<1960	14	Aliased			4.184	2.870	6.098
1960-69	14	0.819	0.154	+++	9.492	6.342	14.205
1970-79	26	0.802	0.130	+++	9.328	7.020	12.395
1980-89	40	0.990	0.134	+++	11.256	9.238	13.714
1990+	8	1.788	0.263	+++	25.002	10.486	59.613
Study type (1)							
CC	93	Aliased			9.437	8.438	10.553
other	9	0.395	0.233	(+)	14.008	6.272	31.286
Study size (number of LC cases)							
100-249	22	Aliased			5.466	3.484	8.576
250-499	31	0.251	0.152	N.S.	7.027	4.831	10.222
500-999	18	0.729	0.162	+++	11.336	7.942	16.178
1000+	31	0.655	0.145	+++	10.522	8.979	12.331
Number of adjustment variables (1)							
0	53	Aliased			10.116	8.136	12.578
1	18	0.477	0.147	++	16.300	10.369	25.622
2+/+nk	31	-0.226	0.097	-	8.073	6.610	9.861

		Deviance	(DF)	Drop Dev	P		
Model 8		231.324	(78)	29.108	(*)		
		Estimate	S.E.	P	RR	95%CIl	95%CIu
Constant		1.230	0.175	+++	3.421	2.429	4.819
Sex(RR)							
Male	49	Aliased			9.961	8.453	11.739
Female	42	-0.109	0.074	N.S.	8.934	7.441	10.727
Combined	11	-0.002	0.170	N.S.	9.946	5.875	16.835
Location							
NAmer	38	Aliased			11.235	9.702	13.010
UK	4	-0.885	0.267	--	4.635	1.907	11.268
Scand	7	-0.508	0.246	-	6.759	3.056	14.949
othEur	15	Aliased			11.235	9.702	13.010
China	12	-0.930	0.123	---	4.431	3.086	6.361
Japan	8	-0.460	0.229	-	7.090	3.399	14.789
othAs	12	-0.220	0.165	N.S.	9.015	5.460	14.886
other	6	0.093	0.309	N.S.	12.336	4.508	33.755
Start year of study							
<1960	14	Aliased			4.549	3.080	6.719
1960-69	14	0.839	0.156	+++	10.526	7.052	15.711
1970-79	26	0.742	0.150	+++	9.555	7.027	12.992
1980-89	40	0.863	0.140	+++	10.781	8.847	13.136
1990+	8	1.501	0.286	+++	20.410	8.507	48.965
Study type (1)							
CC	93	Aliased			9.460	8.489	10.541
other	9	0.281	0.250	N.S.	12.526	5.445	28.816

Table 2A1R - 4

IESLC - Meta-regression of ever smoking, any product (or cigs if any not available)
Multiple regression of data from Table 2A1
Squamous
Effect of additional characteristics

WEIGHTED on Weight

		Estimate	S.E.	P	RR	95%CIl	95%CIu
Study size (number of LC cases)							
100-249	22	Aliased			5.730	3.676	8.934
250-499	31	0.286	0.153	(+)	7.629	5.281	11.021
500-999	18	0.661	0.164	+++	11.098	7.842	15.705
1000+	31	0.589	0.149	+++	10.325	8.836	12.065
Number of adjustment variables (1)							
0	53	Aliased			8.695	6.878	10.993
1	18	0.645	0.154	+++	16.571	10.569	25.981
2+/+nk	31	0.051	0.112	N.S.	9.150	7.408	11.303
Detailed Country in othEur							
not o E	87	Aliased			10.589	9.251	12.122
multi	2	-0.197	0.174	N.S.	8.698	5.120	14.775
Germany	2	0.186	0.478	N.S.	12.758	2.556	63.673
othWest	2	-0.724	0.216	--	5.133	2.537	10.383
East	7	-0.157	0.287	N.S.	9.055	3.491	23.488
Balkans	2	-1.261	0.192	---	3.002	1.620	5.563

		Deviance	(DF)	Drop Dev	P		
Model 8		248.347	(80)	12.085	N.S.		
		Estimate	S.E.	P	RR	95%CIl	95%CIu
Constant		1.386	0.178	+++	3.998	2.820	5.668
Sex(RR)							
Male	49	Aliased			9.687	8.211	11.427
Female	42	-0.014	0.073	N.S.	9.554	7.964	11.462
Combined	11	-0.124	0.142	N.S.	8.554	5.471	13.376
Location							
NAmer	38	Aliased			12.885	10.944	15.169
UK	4	-0.887	0.266	--	5.307	2.169	12.985
Scand	7	-0.521	0.241	-	7.653	3.430	17.072
othEur	15	-0.647	0.115	---	6.748	4.860	9.370
China	12	-1.042	0.120	---	4.543	3.131	6.594
Japan	8	-0.631	0.227	--	6.855	3.209	14.644
othAs	12	Aliased			12.885	10.944	15.169
other	6	-0.105	0.304	N.S.	11.605	4.192	32.122
Start year of study							
<1960	14	Aliased			4.162	2.867	6.041
1960-69	14	0.656	0.162	+++	8.017	5.180	12.406
1970-79	26	0.840	0.131	+++	9.642	7.218	12.881
1980-89	40	1.010	0.134	+++	11.426	9.393	13.898
1990+	8	1.859	0.280	+++	26.716	10.629	67.151
Study type (1)							
CC	93	Aliased			9.444	8.456	10.548
other	9	0.359	0.233	N.S.	13.518	6.100	29.956
Study size (number of LC cases)							
100-249	22	Aliased			5.954	3.766	9.413
250-499	31	0.263	0.152	(+)	7.743	5.252	11.415
500-999	18	0.544	0.175	++	10.263	7.086	14.865
1000+	31	0.557	0.149	+++	10.395	8.879	12.169
Number of adjustment variables (1)							
0	53	Aliased			10.266	8.273	12.741
1	18	0.459	0.147	++	16.252	10.373	25.463
2+/+nk	31	-0.252	0.098	-	7.977	6.543	9.725
Detailed Country in othAsia							
not o A	90	Aliased			9.905	8.804	11.143
India	1	0.650	0.352	(+)	18.974	5.646	63.762
HongKong	7	-0.610	0.199	--	5.382	2.786	10.399
othAsia	4	-0.888	0.285	--	4.077	1.556	10.686

		Deviance	(DF)	Drop Dev	P		
Model 8		260.197	(81)	0.235	N.S.		
		Estimate	S.E.	P	RR	95%CIl	95%CIu
Constant		1.224	0.211	+++	3.400	2.249	5.139

Table 2A1R - 4

IESLC - Meta-regression of ever smoking, any product (or cigs if any not available)
Multiple regression of data from Table 2A1
Squamous
Effect of additional characteristics

WEIGHTED on Weight

		Estimate	S.E.	P	RR	95%CIl	95%CIu
Sex(RR)							
Male	49	Aliased			9.854	8.328	11.660
Female	42	-0.046	0.072	N.S.	9.407	7.818	11.320
Combined	11	-0.161	0.155	N.S.	8.391	5.107	13.788
Location							
NAmer	38	Aliased			13.108	10.927	15.724
UK	4	-0.865	0.266	--	5.520	2.213	13.771
Scand	7	-0.466	0.243	(-)	8.225	3.614	18.719
othEur	15	-0.607	0.114	---	7.144	5.149	9.912
China	12	-1.029	0.120	---	4.684	3.234	6.785
Japan	8	-0.542	0.229	-	7.620	3.501	16.587
othAs	12	-0.410	0.163	-	8.702	5.212	14.530
other	6	-0.031	0.301	N.S.	12.711	4.588	35.210
Start year of study							
<1960	14	Aliased			4.071	2.655	6.242
1960-69	14	0.819	0.154	+++	9.233	5.874	14.513
1970-79	26	0.832	0.144	+++	9.359	7.025	12.468
1980-89	40	1.026	0.154	+++	11.357	9.214	13.999
1990+	8	1.849	0.292	+++	25.875	10.430	64.192
Study type (1)							
CC	93	Aliased			9.435	8.431	10.558
other	9	0.405	0.234	(+)	14.142	6.284	31.824
Study size (number of LC cases)							
100-249	22	Aliased			5.346	3.306	8.646
250-499	31	0.272	0.158	(+)	7.019	4.814	10.233
500-999	18	0.751	0.168	+++	11.328	7.921	16.201
1000+	31	0.680	0.154	+++	10.556	8.984	12.403
Number of adjustment variables (1)							
0	53	Aliased			10.158	8.143	12.673
1	18	0.455	0.154	++	16.006	9.969	25.700
2+/+nk	31	-0.230	0.098	-	8.075	6.604	9.874
Squamous (or nearest)							
q	74	Aliased			9.373	8.016	10.960
oth	28	0.064	0.132	N.S.	9.993	6.897	14.478

		Deviance	(DF)	Drop Dev	P		
Model 8		248.189	(78)	12.243	N.S.		
		Estimate	S.E.	P	RR	95%CIl	95%CIu
Constant		1.672	0.256	+++	5.322	3.225	8.782
Sex(RR)							
Male	49	Aliased			9.350	7.826	11.169
Female	42	0.007	0.074	N.S.	9.417	7.832	11.322
Combined	11	0.153	0.186	N.S.	10.899	6.110	19.442
Location							
NAmer	38	Aliased			13.093	10.841	15.813
UK	4	-1.070	0.283	---	4.489	1.724	11.691
Scand	7	-0.488	0.253	(-)	8.041	3.467	18.647
othEur	15	-0.590	0.121	---	7.258	5.163	10.202
China	12	-1.035	0.124	---	4.650	3.204	6.750
Japan	8	-0.384	0.235	N.S.	8.918	4.038	19.692
othAs	12	-0.447	0.168	--	8.371	4.900	14.298
other	6	0.034	0.305	N.S.	13.546	4.871	37.668
Start year of study							
<1960	14	Aliased			5.249	3.046	9.044
1960-69	14	0.546	0.188	++	9.063	5.624	14.605
1970-79	26	0.595	0.186	++	9.513	6.894	13.127
1980-89	40	0.721	0.190	+++	10.792	8.686	13.410
1990+	8	1.259	0.340	+++	18.482	7.001	48.793
Study type (1)							
CC	93	Aliased			9.435	8.436	10.553
other	9	0.402	0.236	(+)	14.105	6.251	31.829

Table 2A1R - 4

IESLC - Meta-regression of ever smoking, any product (or cigs if any not available)
Multiple regression of data from Table 2A1
Squamous
Effect of additional characteristics

WEIGHTED on Weight

		Estimate	S.E.	P	RR	95%CIl	95%CIu
Study size (number of LC cases)							
100-249	22	Aliased			6.669	3.916	11.357
250-499	31	0.054	0.172	N.S.	7.042	4.830	10.267
500-999	18	0.493	0.196	+	10.923	7.357	16.217
1000+	31	0.437	0.170	+	10.325	8.762	12.167
Number of adjustment variables (1)							
0	53	Aliased			9.952	7.941	12.473
1	18	0.296	0.163	(+)	13.379	8.039	22.265
2+/+nk	31	-0.154	0.102	N.S.	8.531	6.920	10.518
Squamous (or nearest)							
q	74	Aliased			9.826	8.345	11.570
q+s	7	-0.022	0.197	N.S.	9.613	5.084	18.173
q+u	3	-0.630	0.248	-	5.235	2.404	11.396
KI	14	0.269	0.168	N.S.	12.862	7.544	21.930
not a	4	-0.261	0.290	N.S.	7.572	2.921	19.629

		Deviance	(DF)	Drop Dev	P		
Model 8		260.368	(81)	0.064	N.S.		
		Estimate	S.E.	P	RR	95%CIl	95%CIu
Constant		1.279	0.175	+++	3.593	2.551	5.059
Sex(RR)							
Male	49	Aliased			9.824	8.315	11.608
Female	42	-0.045	0.072	N.S.	9.388	7.808	11.289
Combined	11	-0.134	0.142	N.S.	8.596	5.444	13.574
Location							
NAmer	38	Aliased			13.103	10.922	15.719
UK	4	-0.863	0.266	--	5.529	2.216	13.796
Scand	7	-0.481	0.241	-	8.099	3.592	18.260
othEur	15	-0.608	0.114	---	7.136	5.143	9.901
China	12	-1.029	0.120	---	4.681	3.230	6.784
Japan	8	-0.564	0.226	-	7.457	3.469	16.027
othAs	12	-0.390	0.158	-	8.871	5.412	14.539
other	6	-0.032	0.301	N.S.	12.692	4.579	35.174
Start year of study							
<1960	14	Aliased			4.191	2.867	6.128
1960-69	14	0.815	0.155	+++	9.473	6.309	14.225
1970-79	26	0.798	0.130	+++	9.312	6.989	12.407
1980-89	40	0.988	0.134	+++	11.262	9.230	13.740
1990+	8	1.791	0.263	+++	25.131	10.454	60.411
		Estimate					
Study type (1)							
CC	93	Aliased					
other	9	Aliased					
		Estimate	S.E.	P	RR	95%CIl	95%CIu
Study size (number of LC cases)							
100-249	22	Aliased			5.441	3.444	8.598
250-499	31	0.256	0.153	(+)	7.028	4.820	10.246
500-999	18	0.735	0.164	+++	11.353	7.933	16.249
1000+	31	0.660	0.146	+++	10.525	8.972	12.347
Number of adjustment variables (1)							
0	53	Aliased			10.123	8.129	12.606
1	18	0.478	0.147	++	16.319	10.350	25.730
2+/+nk	31	-0.227	0.098	-	8.066	6.594	9.868
Study type (2)							
CC	93	Aliased			9.437	8.433	10.560
prosp	5	0.365	0.261	N.S.	13.593	5.477	33.741
other	4	0.502	0.481	N.S.	15.587	2.887	84.163

		Deviance	(DF)	Drop Dev	P		
Model 8		260.086	(80)	0.346	N.S.		
		Estimate	S.E.	P	RR	95%CIl	95%CIu
Constant		1.273	0.179	+++	3.571	2.513	5.075

Table 2A1R - 4

IESLC - Meta-regression of ever smoking, any product (or cigs if any not available)
Multiple regression of data from Table 2A1
Squamous
Effect of additional characteristics

WEIGHTED on Weight

		Estimate	S.E.	P	RR	95%CIl	95%CIu
Sex(RR)							
Male	49	Aliased			9.814	8.278	11.636
Female	42	-0.047	0.074	N.S.	9.360	7.759	11.292
Combined	11	-0.114	0.144	N.S.	8.753	5.499	13.932
Location							
NAmer	38	Aliased			13.062	10.836	15.746
UK	4	-0.853	0.266	--	5.565	2.218	13.962
Scand	7	-0.456	0.246	(-)	8.277	3.595	19.057
othEur	15	-0.606	0.118	---	7.126	5.081	9.993
China	12	-1.021	0.123	---	4.703	3.226	6.857
Japan	8	-0.553	0.226	-	7.517	3.480	16.235
othAs	12	-0.378	0.160	-	8.953	5.421	14.787
other	6	-0.049	0.303	N.S.	12.434	4.425	34.945
Start year of study							
<1960	14	Aliased			4.161	2.834	6.108
1960-69	14	0.836	0.159	+++	9.604	6.311	14.615
1970-79	26	0.810	0.130	+++	9.357	7.004	12.501
1980-89	40	0.991	0.135	+++	11.210	9.162	13.716
1990+	8	1.821	0.269	+++	25.712	10.477	63.099
Study type (1)							
CC	93	Aliased			9.431	8.422	10.562
other	9	0.423	0.238	(+)	14.393	6.264	33.070
Study size (number of LC cases)							
100-249	22	Aliased			5.505	3.481	8.704
250-499	31	0.241	0.155	N.S.	7.008	4.771	10.294
500-999	18	0.728	0.167	+++	11.404	7.797	16.680
1000+	31	0.646	0.146	+++	10.507	8.919	12.377
		Estimate					
Number of adjustment variables (1)							
0	53	Aliased					
1	18	Aliased					
2+/+nk	31	Aliased					
		Estimate	S.E.	P	RR	95%CIl	95%CIu
Number of adjustment variables (2)							
0	53	Aliased			10.066	8.048	12.591
1	18	0.474	0.147	++	16.172	10.204	25.630
2	19	-0.207	0.108	(-)	8.182	6.455	10.372
3-5	8	-0.227	0.150	N.S.	8.019	4.997	12.871
6+/+nk	4	-0.417	0.349	N.S.	6.632	1.948	22.582

		Deviance	(DF)	Drop Dev	P		
Model 8		256.693	(81)	3.739	N.S.		
		Estimate	S.E.	P	RR	95%CIl	95%CIu
Constant		1.420	0.189	+++	4.136	2.858	5.985
Sex(RR)							
Male	49	Aliased			9.921	8.399	11.720
Female	42	-0.057	0.073	N.S.	9.371	7.806	11.249
Combined	11	-0.185	0.144	N.S.	8.246	5.216	13.036
Location							
NAmer	38	Aliased			13.306	11.083	15.976
UK	4	-0.923	0.268	---	5.289	2.126	13.160
Scand	7	-0.505	0.241	-	8.034	3.586	18.000
othEur	15	-0.704	0.124	---	6.580	4.611	9.389
China	12	-0.993	0.122	---	4.932	3.375	7.207
Japan	8	-0.501	0.228	-	8.067	3.728	17.454
othAs	12	-0.378	0.158	-	9.115	5.567	14.924
other	6	-0.090	0.303	N.S.	12.165	4.408	33.570
Start year of study							
<1960	14	Aliased			4.409	2.991	6.499
1960-69	14	0.735	0.160	+++	9.197	6.123	13.813
1970-79	26	0.802	0.130	+++	9.831	7.288	13.260
1980-89	40	0.904	0.141	+++	10.889	8.861	13.383
1990+	8	1.730	0.264	+++	24.868	10.439	59.240

Table 2A1R - 4

IESLC - Meta-regression of ever smoking, any product (or cigs if any not available)
Multiple regression of data from Table 2A1
Squamous
Effect of additional characteristics

WEIGHTED on Weight

		Estimate	S.E.	P	RR	95%CIl	95%CIu
Study type (1)							
CC	93	Aliased			9.420	8.424	10.533
other	9	0.480	0.237	+	15.230	6.730	34.467
Study size (number of LC cases)							
100-249	22	Aliased			5.451	3.476	8.549
250-499	31	0.287	0.153	(+)	7.262	4.971	10.609
500-999	18	0.685	0.164	+++	10.811	7.501	15.580
1000+	31	0.660	0.145	+++	10.549	9.002	12.361
Number of adjustment variables (1)							
0	53	Aliased			10.491	8.358	13.169
1	18	0.390	0.153	+	15.496	9.773	24.569
2+/+nk	31	-0.282	0.102	--	7.910	6.456	9.692
RR adjusted for or study matched on age							
Yes	79	Aliased			9.798	8.671	11.073
No	23	-0.264	0.137	(-)	7.524	4.854	11.660

		Deviance	(DF)	Drop Dev	P		
Model 8		251.150	(81)	9.283	(*)		
		Estimate	S.E.	P	RR	95%CIl	95%CIu
Constant		1.556	0.196	+++	4.741	3.226	6.968
Sex(RR)							
Male	49	Aliased			9.787	8.307	11.530
Female	42	-0.050	0.072	N.S.	9.313	7.772	11.160
Combined	11	-0.076	0.142	N.S.	9.074	5.789	14.222
Location							
NAmer	38	Aliased			12.958	10.831	15.501
UK	4	-0.943	0.267	---	5.044	2.043	12.457
Scand	7	-0.358	0.244	N.S.	9.056	4.036	20.321
othEur	15	-0.658	0.115	---	6.711	4.832	9.321
China	12	-0.942	0.123	---	5.051	3.476	7.340
Japan	8	-0.273	0.245	N.S.	9.859	4.368	22.254
othAs	12	-0.386	0.158	-	8.806	5.420	14.308
other	6	0.029	0.302	N.S.	13.345	4.897	36.368
Start year of study							
<1960	14	Aliased			4.776	3.197	7.134
1960-69	14	0.698	0.159	+++	9.594	6.440	14.292
1970-79	26	0.673	0.136	+++	9.358	7.067	12.393
1980-89	40	0.830	0.144	+++	10.950	8.986	13.344
1990+	8	1.471	0.283	+++	20.787	8.591	50.300
Study type (1)							
CC	93	Aliased			9.414	8.429	10.514
other	9	0.508	0.236	+	15.641	7.002	34.940
Study size (number of LC cases)							
100-249	22	Aliased			5.577	3.572	8.709
250-499	31	0.309	0.153	+	7.600	5.193	11.122
500-999	18	0.594	0.168	+++	10.105	6.947	14.700
1000+	31	0.638	0.145	+++	10.553	9.022	12.345
Number of adjustment variables (1)							
0	53	Aliased			10.790	8.596	13.544
1	18	0.346	0.153	+	15.255	9.696	23.999
2+/+nk	31	-0.331	0.103	--	7.748	6.325	9.493
RR adjusted for or matched on factor other than sex, age							
Yes	65	Aliased			10.265	8.935	11.794
No	37	-0.354	0.116	--	7.207	5.164	10.058

		Deviance	(DF)	Drop Dev	P		
Model 8		253.094	(80)	7.338	N.S.		
		Estimate	S.E.	P	RR	95%CIl	95%CIu
Constant		1.264	0.175	+++	3.539	2.512	4.984
Sex(RR)							
Male	49	Aliased			9.755	8.264	11.513
Female	42	-0.031	0.073	N.S.	9.458	7.862	11.377
Combined	11	-0.122	0.144	N.S.	8.630	5.429	13.720

Table 2A1R - 4

IESLC - Meta-regression of ever smoking, any product (or cigs if any not available)
Multiple regression of data from Table 2A1
Squamous
Effect of additional characteristics

WEIGHTED on Weight

		Estimate	S.E.	P	RR	95%CIl	95%CIu
Location							
NAmer	38	Aliased			13.051	10.853	15.693
UK	4	-0.854	0.278	--	5.555	2.171	14.217
Scand	7	-0.477	0.245	(-)	8.096	3.581	18.304
othEur	15	-0.600	0.114	---	7.160	5.173	9.908
China	12	-1.016	0.121	---	4.723	3.268	6.826
Japan	8	-0.551	0.228	-	7.519	3.510	16.106
othAs	12	-0.379	0.164	-	8.931	5.393	14.792
other	6	-0.063	0.302	N.S.	12.251	4.451	33.719
Start year of study							
<1960	14	Aliased			4.247	2.842	6.348
1960-69	14	0.820	0.154	+++	9.640	6.317	14.709
1970-79	26	0.792	0.136	+++	9.377	7.060	12.455
1980-89	40	0.964	0.145	+++	11.141	9.091	13.653
1990+	8	1.785	0.279	+++	25.302	10.418	61.451
Study type (1)							
CC	93	Aliased			9.438	8.441	10.552
other	9	0.391	0.233	(+)	13.950	6.248	31.148
Study size (number of LC cases)							
100-249	22	Aliased			5.450	3.427	8.666
250-499	31	0.263	0.152	(+)	7.091	4.798	10.481
500-999	18	0.738	0.165	+++	11.397	7.988	16.261
1000+	31	0.655	0.152	+++	10.496	8.913	12.361
Number of adjustment variables (1)							
0	53	Aliased			10.116	8.139	12.572
1	18	0.481	0.147	++	16.364	10.403	25.741
2+/+nk	31	-0.226	0.097	-	8.067	6.606	9.850
Product							
all/unsp	54	Aliased			9.345	7.121	12.265
cig+/-ot	46	0.016	0.106	N.S.	9.496	8.068	11.176
cig only	2	1.034	0.390	++	26.294	7.074	97.731

		Deviance	(DF)	Drop Dev	P		
Model 8		254.602	(81)	5.831	N.S.		
		Estimate	S.E.	P	RR	95%CIl	95%CIu
Constant		1.292	0.174	+++	3.638	2.584	5.122
Sex(RR)							
Male	49	Aliased			9.864	8.363	11.634
Female	42	-0.054	0.072	N.S.	9.343	7.788	11.208
Combined	11	-0.137	0.141	N.S.	8.603	5.492	13.477
Location							
NAmer	38	Aliased			13.471	11.203	16.197
UK	4	-1.009	0.273	---	4.911	1.956	12.327
Scand	7	-0.580	0.244	-	7.545	3.358	16.948
othEur	15	-0.692	0.119	---	6.745	4.834	9.412
China	12	-1.023	0.120	---	4.845	3.348	7.011
Japan	8	-0.595	0.226	-	7.431	3.488	15.830
othAs	12	-0.430	0.159	--	8.767	5.377	14.294
other	6	-0.160	0.306	N.S.	11.481	4.147	31.788
Start year of study							
<1960	14	Aliased			3.967	2.705	5.815
1960-69	14	0.830	0.154	+++	9.094	6.061	13.646
1970-79	26	0.841	0.131	+++	9.196	6.926	12.211
1980-89	40	1.069	0.138	+++	11.552	9.457	14.111
1990+	8	1.903	0.267	+++	26.593	11.152	63.410
Study type (1)							
CC	93	Aliased			9.423	8.431	10.532
other	9	0.463	0.235	(+)	14.978	6.695	33.506
Study size (number of LC cases)							
100-249	22	Aliased			5.184	3.291	8.167
250-499	31	0.258	0.152	(+)	6.712	4.597	9.801
500-999	18	0.780	0.164	+++	11.307	7.937	16.109
1000+	31	0.725	0.148	+++	10.699	9.121	12.551

Table 2A1R - 4

IESLC - Meta-regression of ever smoking, any product (or cigs if any not available)
Multiple regression of data from Table 2A1
Squamous
Effect of additional characteristics

WEIGHTED on Weight

		Estimate	S.E.	P	RR	95%CIl	95%CIu
Number of adjustment variables (1)							
0	53	Aliased			9.809	7.862	12.237
1	18	0.539	0.149	+++	16.823	10.703	26.442
2+/+nk	31	-0.175	0.100	(-)	8.233	6.734	10.066
Denominator							
nev any	64	Aliased			10.702	8.749	13.092
nev cigs	38	-0.222	0.092	-	8.570	7.111	10.328

		Deviance	(DF)	Drop Dev	P		
Model 8		239.595	(80)	20.837	*		
		Estimate	S.E.	P	RR	95%CIl	95%CIu
Constant		2.272	0.310	+++	9.694	5.280	17.799
Sex(RR)							
Male	49	Aliased			9.612	8.174	11.303
Female	42	-0.006	0.073	N.S.	9.551	7.983	11.427
Combined	11	-0.076	0.142	N.S.	8.910	5.729	13.857
Location							
NAmer	38	Aliased			11.604	8.517	15.809
UK	4	-1.810	0.361	---	1.899	0.570	6.335
Scand	7	-0.452	0.241	(-)	7.385	3.257	16.741
othEur	15	-0.621	0.114	---	6.233	4.163	9.332
China	12	0.280	0.635	N.S.	15.352	2.307	102.167
Japan	8	-0.589	0.226	-	6.439	2.937	14.114
othAs	12	-0.501	0.168	--	7.030	4.216	11.721
other	6	-0.063	0.304	N.S.	10.895	3.789	31.327
Start year of study							
<1960	14	Aliased			4.210	2.918	6.074
1960-69	14	0.725	0.157	+++	8.689	5.817	12.979
1970-79	26	0.821	0.130	+++	9.570	7.256	12.621
1980-89	40	0.980	0.134	+++	11.219	9.255	13.600
1990+	8	1.901	0.274	+++	28.187	11.709	67.857
Study type (1)							
CC	93	Aliased			9.434	8.463	10.516
other	9	0.409	0.233	(+)	14.207	6.506	31.023
Study size (number of LC cases)							
100-249	22	Aliased			5.901	3.796	9.172
250-499	31	0.220	0.154	N.S.	7.351	5.068	10.661
500-999	18	0.527	0.169	++	9.994	6.947	14.378
1000+	31	0.583	0.146	+++	10.571	9.062	12.333
Number of adjustment variables (1)							
0	53	Aliased			10.150	8.198	12.568
1	18	0.467	0.147	++	16.193	10.437	25.123
2+/+nk	31	-0.230	0.099	-	8.061	6.626	9.806
National cigarette tobacco type							
Virginia	9	Aliased			27.143	12.115	60.813
blended	80	-0.937	0.240	---	10.638	8.054	14.053
other	13	-2.205	0.651	--	2.991	0.460	19.446

		Deviance	(DF)	Drop Dev	P		
Model 8		251.529	(81)	8.903	(*)		
		Estimate	S.E.	P	RR	95%CIl	95%CIu
Constant		1.258	0.175	+++	3.518	2.498	4.953
Sex(RR)							
Male	49	Aliased			9.862	8.370	11.621
Female	42	-0.033	0.073	N.S.	9.544	7.956	11.449
Combined	11	-0.229	0.145	N.S.	7.847	4.956	12.422

Table 2A1R - 4

IESLC - Meta-regression of ever smoking, any product (or cigs if any not available)
Multiple regression of data from Table 2A1
Squamous
Effect of additional characteristics

WEIGHTED on Weight

		Estimate	S.E.	P	RR	95%CIl	95%CIu
Location							
NAmer	38	Aliased			12.549	10.421	15.111
UK	4	-0.782	0.267	--	5.743	2.336	14.120
Scand	7	-0.484	0.241	-	7.737	3.476	17.219
othEur	15	-0.532	0.117	---	7.375	5.334	10.196
China	12	-0.931	0.125	---	4.948	3.419	7.162
Japan	8	-0.424	0.230	(-)	8.211	3.842	17.550
othAs	12	-0.296	0.161	(-)	9.334	5.723	15.224
other	6	0.179	0.309	N.S.	15.014	5.413	41.646
Start year of study							
<1960	14	Aliased			4.424	3.031	6.458
1960-69	14	0.832	0.154	+++	10.171	6.773	15.273
1970-79	26	0.689	0.135	+++	8.813	6.604	11.762
1980-89	40	0.939	0.135	+++	11.319	9.309	13.763
1990+	8	1.635	0.268	+++	22.693	9.541	53.977
Study type (1)							
CC	93	Aliased			9.416	8.431	10.518
other	9	0.496	0.235	+	15.466	6.930	34.515
Study size (number of LC cases)							
100-249	22	Aliased			5.408	3.464	8.444
250-499	31	0.238	0.152	N.S.	6.859	4.730	9.946
500-999	18	0.639	0.165	+++	10.243	7.069	14.841
1000+	31	0.691	0.146	+++	10.794	9.201	12.661
Number of adjustment variables (1)							
0	53	Aliased			10.291	8.289	12.776
1	18	0.394	0.149	+	15.253	9.689	24.012
2+/+nk	31	-0.243	0.098	-	8.068	6.620	9.832
Any proxy use							
No/nk	76	Aliased			9.108	8.074	10.274
Yes	26	0.350	0.117	++	12.929	8.920	18.738

		Deviance	(DF)	Drop Dev	P		
Model 8		257.347	(81)	3.085	N.S.		
		Estimate	S.E.	P	RR	95%CIl	95%CIu
Constant		1.185	0.183	+++	3.269	2.285	4.679
Sex(RR)							
Male	49	Aliased			9.811	8.311	11.581
Female	42	-0.057	0.073	N.S.	9.265	7.704	11.144
Combined	11	-0.068	0.145	N.S.	9.165	5.746	14.618
Location							
NAmer	38	Aliased			12.676	10.455	15.370
UK	4	-0.768	0.271	--	5.881	2.352	14.710
Scand	7	-0.455	0.241	(-)	8.046	3.587	18.044
othEur	15	-0.583	0.115	---	7.073	5.107	9.796
China	12	-0.912	0.137	---	5.093	3.401	7.628
Japan	8	-0.551	0.226	-	7.306	3.411	15.646
othAs	12	-0.265	0.173	N.S.	9.721	5.756	16.417
other	6	0.096	0.310	N.S.	13.952	4.980	39.087
Start year of study							
<1960	14	Aliased			4.342	2.958	6.376
1960-69	14	0.816	0.154	+++	9.818	6.524	14.777
1970-79	26	0.784	0.130	+++	9.513	7.140	12.675
1980-89	40	0.930	0.138	+++	11.011	8.993	13.481
1990+	8	1.674	0.271	+++	23.164	9.586	55.971
Study type (1)							
CC	93	Aliased			9.424	8.426	10.539
other	9	0.459	0.236	(+)	14.912	6.612	33.629
Study size (number of LC cases)							
100-249	22	Aliased			5.521	3.517	8.666
250-499	31	0.273	0.153	(+)	7.257	4.961	10.614
500-999	18	0.719	0.163	+++	11.330	7.938	16.171
1000+	31	0.637	0.145	+++	10.441	8.902	12.245

Table 2A1R - 4

IESLC - Meta-regression of ever smoking, any product (or cigs if any not available)
Multiple regression of data from Table 2A1
Squamous
Effect of additional characteristics

WEIGHTED on Weight

		Estimate	S.E.	P	RR	95%CIl	95%CIu
Number of adjustment variables (1)							
0	53	Aliased			9.878	7.904	12.345
1	18	0.509	0.148	+++	16.431	10.449	25.837
2+/+nk	31	-0.183	0.100	(-)	8.224	6.711	10.080
Full histological confirmation							
No	59	Aliased			8.751	7.163	10.690
Yes	43	0.153	0.087	(+)	10.200	8.548	12.171

		Deviance	(DF)	Drop Dev	P		
Model 8		259.712	(80)	0.721	N.S.		
		Estimate	S.E.	P	RR	95%CIl	95%CIu
Constant		1.275	0.175	+++	3.578	2.539	5.041
Sex(RR)							
Male	49	Aliased			9.806	8.288	11.603
Female	42	-0.040	0.073	N.S.	9.425	7.825	11.352
Combined	11	-0.139	0.144	N.S.	8.531	5.355	13.591
Location							
NAmer	38	Aliased			13.155	10.942	15.816
UK	4	-0.877	0.267	--	5.475	2.181	13.742
Scand	7	-0.484	0.241	-	8.112	3.582	18.370
othEur	15	-0.617	0.115	---	7.099	5.105	9.870
China	12	-1.046	0.122	---	4.624	3.170	6.744
Japan	8	-0.565	0.227	-	7.473	3.459	16.145
othAs	12	-0.386	0.159	-	8.942	5.425	14.739
other	6	-0.042	0.302	N.S.	12.619	4.527	35.179
Start year of study							
<1960	14	Aliased			4.209	2.872	6.171
1960-69	14	0.810	0.157	+++	9.462	6.250	14.326
1970-79	26	0.799	0.130	+++	9.356	7.017	12.474
1980-89	40	0.980	0.135	+++	11.214	9.176	13.704
1990+	8	1.806	0.264	+++	25.612	10.558	62.131
Study type (1)							
CC	93	Aliased			9.441	8.431	10.572
other	9	0.374	0.241	N.S.	13.727	5.916	31.853
Study size (number of LC cases)							
100-249	22	Aliased			5.395	3.404	8.549
250-499	31	0.252	0.154	N.S.	6.940	4.720	10.205
500-999	18	0.748	0.164	+++	11.399	7.950	16.345
1000+	31	0.671	0.147	+++	10.556	8.986	12.400
Number of adjustment variables (1)							
0	53	Aliased			10.118	8.105	12.632
1	18	0.481	0.147	++	16.362	10.339	25.894
2+/+nk	31	-0.227	0.099	-	8.065	6.573	9.896
Risky occupational population							
No	100	Aliased			9.498	8.492	10.623
Mining	1	0.384	0.545	N.S.	13.950	2.046	95.117
Other risky	1	0.441	0.960	N.S.	14.762	0.499	436.457

		Deviance	(DF)	Drop Dev	P		
Model 8		258.129	(79)	2.304	N.S.		
		Estimate	S.E.	P	RR	95%CIl	95%CIu
Constant		1.277	0.176	+++	3.587	2.539	5.067
Sex(RR)							
Male	49	Aliased			9.842	8.316	11.649
Female	42	-0.046	0.072	N.S.	9.395	7.803	11.311
Combined	11	-0.148	0.142	N.S.	8.491	5.352	13.470

Table 2A1R - 4

IESLC - Meta-regression of ever smoking, any product (or cigs if any not available)
Multiple regression of data from Table 2A1
Squamous
Effect of additional characteristics

WEIGHTED on Weight

		Estimate	S.E.	P	RR	95%CIl	95%CIu
Location							
NAmer	38	Aliased			12.925	10.726	15.576
UK	4	-0.823	0.271	--	5.676	2.223	14.494
Scand	7	-0.437	0.244	(-)	8.351	3.641	19.150
othEur	15	-0.608	0.114	---	7.038	5.048	9.812
China	12	-0.946	0.142	---	5.020	3.216	7.837
Japan	8	-0.570	0.227	-	7.309	3.359	15.901
othAs	12	-0.366	0.164	-	8.962	5.312	15.118
other	6	0.001	0.302	N.S.	12.939	4.619	36.249
Start year of study							
<1960	14	Aliased			4.290	2.908	6.328
1960-69	14	0.836	0.155	+++	9.899	6.483	15.116
1970-79	26	0.780	0.131	+++	9.354	6.973	12.549
1980-89	40	0.948	0.139	+++	11.074	8.985	13.648
1990+	8	1.763	0.265	+++	25.018	10.337	60.551
Study type (1)							
CC	93	Aliased			9.471	8.451	10.615
other	9	0.223	0.277	N.S.	11.843	4.509	31.111
Study size (number of LC cases)							
100-249	22	Aliased			5.389	3.407	8.523
250-499	31	0.257	0.155	N.S.	6.970	4.710	10.315
500-999	18	0.745	0.169	+++	11.354	7.713	16.713
1000+	31	0.672	0.146	+++	10.556	8.949	12.451
Number of adjustment variables (1)							
0	53	Aliased			9.975	7.906	12.584
1	18	0.463	0.148	++	15.847	9.959	25.217
2+/+nk	31	-0.194	0.105	(-)	8.219	6.636	10.179
Lowest age in RR							
<25/unlim	72	Aliased			9.590	8.384	10.970
25-39	16	-0.116	0.126	N.S.	8.543	5.742	12.711
40+	12	0.285	0.257	N.S.	12.754	5.262	30.913
unknown	2	-0.033	0.316	N.S.	9.277	3.127	27.522

		Deviance	(DF)	Drop Dev	P		
Model 8		253.750	(78)	6.683	N.S.		
		Estimate	S.E.	P	RR	95%CIl	95%CIu
Constant		0.624	0.494	N.S.	1.867	0.709	4.914
Sex(RR)							
Male	49	Aliased			9.989	8.428	11.840
Female	42	-0.075	0.073	N.S.	9.267	7.694	11.162
Combined	11	-0.178	0.143	N.S.	8.361	5.269	13.269
Location							
NAmer	38	Aliased			14.045	11.346	17.385
UK	4	-1.235	0.346	---	4.084	1.296	12.865
Scand	7	-0.562	0.245	-	8.006	3.506	18.282
othEur	15	-0.690	0.119	---	7.045	5.062	9.804
China	12	-1.285	0.184	---	3.886	2.262	6.673
Japan	8	-0.630	0.229	--	7.481	3.441	16.264
othAs	12	-0.519	0.172	--	8.360	4.916	14.216
other	6	-0.165	0.309	N.S.	11.913	4.206	33.741
Start year of study							
<1960	14	Aliased			4.129	2.809	6.070
1960-69	14	0.767	0.156	+++	8.888	5.834	13.541
1970-79	26	0.831	0.132	+++	9.478	7.028	12.781
1980-89	40	1.005	0.137	+++	11.275	9.173	13.859
1990+	8	1.926	0.270	+++	28.342	11.475	70.001
Study type (1)							
CC	93	Aliased			9.478	8.460	10.617
other	9	0.193	0.263	N.S.	11.492	4.589	28.780

Table 2A1R - 4

IESLC - Meta-regression of ever smoking, any product (or cigs if any not available)
Multiple regression of data from Table 2A1
Squamous
Effect of additional characteristics

WEIGHTED on Weight

		Estimate	S.E.	P	RR	95%CIl	95%CIu
Study size (number of LC cases)							
100-249	22	Aliased			5.625	3.553	8.905
250-499	31	0.228	0.156	N.S.	7.064	4.787	10.425
500-999	18	0.689	0.171	+++	11.201	7.582	16.549
1000+	31	0.624	0.146	+++	10.496	8.906	12.371
Number of adjustment variables (1)							
0	53	Aliased			10.824	8.432	13.893
1	18	0.413	0.150	++	16.363	10.344	25.884
2+/+nk	31	-0.352	0.115	--	7.616	6.071	9.553
Highest age in RR							
<65	5	Aliased			4.150	0.763	22.571
65-74	12	1.160	0.533	+	13.244	6.762	25.940
75-84	13	0.647	0.491	N.S.	7.925	4.786	13.123
85+/unlim	70	0.807	0.480	(+)	9.303	8.023	10.787
unknown	2	0.981	0.573	(+)	11.067	3.558	34.428

		Deviance	(DF)	Drop Dev	P		
Model 8		260.206	(81)	0.226	N.S.		
		Estimate	S.E.	P	RR	95%CIl	95%CIu
Constant		0.864	0.893	N.S.	2.374	0.413	13.656
Sex(RR)							
Male	49	Aliased			9.812	8.302	11.595
Female	42	-0.044	0.073	N.S.	9.391	7.811	11.289
Combined	11	-0.127	0.141	N.S.	8.643	5.489	13.607
Location							
NAmer	38	Aliased			12.997	10.726	15.747
UK	4	-0.794	0.301	-	5.875	2.133	16.181
Scand	7	-0.479	0.241	(-)	8.053	3.574	18.147
othEur	15	-0.596	0.117	---	7.160	5.154	9.947
China	12	-0.996	0.138	---	4.802	3.182	7.246
Japan	8	-0.565	0.226	-	7.384	3.421	15.936
othAs	12	-0.382	0.159	-	8.868	5.412	14.533
other	6	-0.031	0.301	N.S.	12.597	4.538	34.966
Start year of study							
<1960	14	Aliased			4.200	2.872	6.142
1960-69	14	0.825	0.154	+++	9.581	6.350	14.457
1970-79	26	0.795	0.130	+++	9.302	6.984	12.389
1980-89	40	0.984	0.135	+++	11.235	9.207	13.711
1990+	8	1.790	0.263	+++	25.159	10.486	60.360
Study type (1)							
CC	93	Aliased			9.437	8.433	10.560
other	9	0.393	0.233	(+)	13.976	6.228	31.365
Study size (number of LC cases)							
100-249	22	Aliased			5.463	3.473	8.593
250-499	31	0.253	0.152	N.S.	7.035	4.826	10.257
500-999	18	0.737	0.163	+++	11.416	7.952	16.388
1000+	31	0.654	0.145	+++	10.507	8.954	12.329
Number of adjustment variables (1)							
0	53	Aliased			10.084	8.090	12.569
1	18	0.485	0.147	++	16.370	10.376	25.828
2+/+nk	31	-0.221	0.098	-	8.088	6.611	9.894
Midpoint age in RR		0.007	0.015	N.S.	6.379	0.332	122.555

		Deviance	(DF)	Drop Dev	P		
Model 8		238.456	(81)	21.977	**		
		Estimate	S.E.	P	RR	95%CIl	95%CIu
Constant		0.654	0.220	++	1.923	1.250	2.959
Sex(RR)							
Male	49	Aliased			9.331	7.921	10.993
Female	42	0.017	0.074	N.S.	9.494	7.960	11.325
Combined	11	0.130	0.151	N.S.	10.622	6.711	16.813

Table 2A1R - 4

IESLC - Meta-regression of ever smoking, any product (or cigs if any not available)
Multiple regression of data from Table 2A1
Squamous
Effect of additional characteristics

WEIGHTED on Weight

		Estimate	S.E.	P	RR	95%CIl	95%CIu
Location							
NAmer	38	Aliased			12.714	10.667	15.154
UK	4	-0.834	0.266	--	5.521	2.302	13.242
Scand	7	-0.289	0.244	N.S.	9.526	4.339	20.912
othEur	15	-0.541	0.115	---	7.403	5.406	10.136
China	12	-1.022	0.120	---	4.577	3.209	6.528
Japan	8	-0.748	0.229	--	6.020	2.849	12.719
othAs	12	-0.198	0.163	N.S.	10.429	6.409	16.971
other	6	-0.014	0.301	N.S.	12.540	4.728	33.261
Start year of study							
<1960	14	Aliased			3.575	2.445	5.228
1960-69	14	0.974	0.157	+++	9.471	6.424	13.964
1970-79	26	0.936	0.133	+++	9.120	6.933	11.997
1980-89	40	1.204	0.142	+++	11.920	9.812	14.481
1990+	8	1.845	0.263	+++	22.625	9.771	52.389
Study type (1)							
CC	93	Aliased			9.456	8.491	10.531
other	9	0.298	0.234	N.S.	12.735	5.858	27.689
Study size (number of LC cases)							
100-249	22	Aliased			4.707	3.011	7.358
250-499	31	0.325	0.153	+	6.516	4.524	9.385
500-999	18	0.880	0.166	+++	11.348	8.058	15.984
1000+	31	0.839	0.150	+++	10.891	9.330	12.714
Number of adjustment variables (1)							
0	53	Aliased			9.311	7.487	11.579
1	18	0.458	0.147	++	14.723	9.468	22.896
2+/+nk	31	-0.051	0.104	N.S.	8.850	7.220	10.847
Derivation of RR/CI							
Orig/2x2	39	Aliased			8.125	6.955	9.492
Other	63	0.429	0.091	+++	12.476	9.998	15.568

		Deviance	(DF)	Drop Dev	P		
Model 8		254.082	(80)	6.350	N.S.		
		Estimate	S.E.	P	RR	95%CIl	95%CIu
Constant		0.666	0.308	+	1.947	1.066	3.557
Sex(RR)							
Male	49	Aliased			9.722	8.223	11.494
Female	42	-0.049	0.073	N.S.	9.260	7.705	11.128
Combined	11	-0.010	0.153	N.S.	9.622	5.914	15.656
Location							
NAmer	38	Aliased			13.305	11.086	15.968
UK	4	-0.857	0.266	--	5.645	2.272	14.025
Scand	7	-0.438	0.243	(-)	8.583	3.787	19.452
othEur	15	-0.664	0.116	---	6.849	4.916	9.543
China	12	-1.043	0.121	---	4.691	3.240	6.790
Japan	8	-0.735	0.236	--	6.381	2.893	14.073
othAs	12	-0.367	0.160	-	9.217	5.602	15.165
other	6	-0.041	0.303	N.S.	12.770	4.606	35.407
Start year of study							
<1960	14	Aliased			3.626	2.363	5.565
1960-69	14	0.896	0.163	+++	8.885	5.798	13.617
1970-79	26	0.958	0.146	+++	9.449	7.091	12.590
1980-89	40	1.178	0.154	+++	11.774	9.565	14.493
1990+	8	1.901	0.271	+++	24.264	9.916	59.373
Study type (1)							
CC	93	Aliased			9.433	8.435	10.550
other	9	0.412	0.236	(+)	14.243	6.308	32.158
Study size (number of LC cases)							
100-249	22	Aliased			4.999	3.127	7.992
250-499	31	0.305	0.154	(+)	6.781	4.620	9.953
500-999	18	0.808	0.166	+++	11.218	7.812	16.109
1000+	31	0.765	0.153	+++	10.743	9.116	12.660

Table 2A1R - 4

IESLC - Meta-regression of ever smoking, any product (or cigs if any not available)
Multiple regression of data from Table 2A1
Squamous
Effect of additional characteristics

WEIGHTED on Weight

		Estimate	S.E.	P	RR	95%CIl	95%CIu
Number of adjustment variables (1)							
0	53	Aliased			8.548	5.769	12.665
1	18	0.667	0.224	++	16.652	9.656	28.717
2+/+nk	31	0.082	0.187	N.S.	9.275	6.822	12.610
Derivation of RR/CI							
Orig	24	Aliased			7.650	5.417	10.803
StcCalc	42	0.431	0.208	+	11.770	7.688	18.021
Other	36	0.340	0.150	+	10.750	7.152	16.158
