# Supplementary material for: Systematic review with meta-analysis of the epidemiological evidence in the 1900s relating smoking to lung cancer
Source: BMC Cancer. 2012 Sep 3;12:385. doi: 10.1186/1471-2407-12-385 (PMC3505152; doi:10.1186/1471-2407-12-385)
Supplement: Additional file 5 — Detailed Analysis Tables (Individual file names as described in Additional file 1: Methods, Table1). [file 1471-2407-12-385-S5.zip › PDF/2K.pdf]

Table 2K1 -

IESLC - Meta-analysis of Ex Smoking by Years quit (vs current), Overview
Squamous, Any Product (or Cigarettes if Any not available)

This analysis is restricted to results for:
1) Ex smokers
2) Results by Years quit (vs current)
3) Categorical results by Years quit (vs current)
Results by Years quit (vs current) are grouped under 2 schemes (S1, S2). Each scheme has a set of "key values". An interval is allocated to the category whose key value it includes, and intervals which include none or more than one of the key values are excluded. (Open-ended intervals are coded as 999)

S1	key value	maximum range
1	3	1-6
2	7	4-11
3	12	8+

S2	key value	maximum range
1	3	1-11
2	12	4-19
3	20	13+

4) Squamous (or near equivalent)
5) Results complete enough for use in metaanalysis

Within each study, results are then selected (in the following order of preference, within each sex) for:
6) (not applicable)
7) PRODUCT: all/unspec, cigarettes regardless of other products, cigarettes only
8) CIGTYPE: all/unspecified, MC regardless of HR, MC only
9) Results with least adjustment for other aspects of smoking (ADOS)
10) DENOM: current smokers, current + recent smokers (up to number of m=months or y=years, max 2 years)
11) Followup period (YF, prospective studies): whole study (coded as 0) or longest available
12) LCtype: squamous or nearest available, but not adeno. (q = squamous, s = small,
a = adeno, KI = Kreyberg I, u = undifferentiated)
13) Race: all or nearest available, otherwise by race (wh or w = white, bl or b = black, hi = hispanic
ch = chinese, jap = japanese, haw = hawaiian, w+o = white + oriental, sca = scandinavian, as = asian)
14) For overlapping studies: principal rather than subsidiary studies
Finally by Age: whole study (coded as 0) if available, otherwise by widest available age group
and then for single sex results (m, f) in preference to results for both sexes combined (c).

Results adjusted (AD) for the most potential confounders are then chosen in Sections -1 to -3
and results adjusted for the least confounders in Sections -4 to -6. (Those least adjusted results which actually differ from the most adjusted are marked 'x' in column X in Section -4)

Section -7 shows excluded studies, together with the stage (as above) at which no qualifying results were found.

Section -8 lists the potentially overlapping studies which have been included (1=principal, 2=subsidiary).

Section -9 lists any results which would have been included in preference except that they had data not complete enough for use in meta-analysis, with their significance (yes/no), if known, and any further comment as entered on the database. It also lists as "gap" any categories for which no data were presented by the original authors.

In addition to those mentioned above, the following fields, levels and abbreviations are used:
* or nk = not known, n = no, y = yes, ot = other
nev = never
all/unspec = all or unspecified, cig+/-ot = cigarettes irrespective of other products (cigar, pipe etc)
MC = manufactured cigarettes, HR = hand-rolled cigarettes
exL, exH = range of exposure (low and high) in the smoking group, in terms of Years quit (vs current)
REF: 6-character study reference
NRR: number of the RR on the database within the study
ST : study type (CC = case control, pr or prosp = prospective)
NLC: number of lung cancer cases in whole study
R : risky occupational population (n = no, m = mining, o = other risky)
VB : national cigarette type (V = at least 75% Virginia, bl = at least 75% blended, ot = other)
P : any proxy use
H : full histological confirmation
De : derivation of RR/CI (or = original, st = standard method, ot = other method of estimation)

Table 2K1 - 1

IESLC - Meta-analysis of Ex Smoking by Years quit (vs current), Overview
Squamous, Any Product (or Cigarettes if Any not available)
Most adjusted

REF	NRR	SEX	AGEL	AGEH	RACE	YF	LC TYPE	LOC	START	ST	NLC	R	VB	P	H	AD	ADOS	PRODUCT	exL	exH	S1	S2	DENOM	De
BARBON	602	m	0	0	all	-	q	Eu:wst	1979	CC	755	n	bl	y	y	1	0	all/unsp	0.1	4	1	1	current	ot
BARBON	603	m	0	0	all	-	q	Eu:wst	1979	CC	755	n	bl	y	y	1	0	all/unsp	5	14	0	2	current	ot
BARBON	604	m	0	0	all	-	q	Eu:wst	1979	CC	755	n	bl	y	y	1	0	all/unsp	15	24	0	3	current	ot
BARBON	605	m	0	0	all	-	q	Eu:wst	1979	CC	755	n	bl	y	y	1	0	all/unsp	25	999	0	0	current	ot
JAHN	605	m	0	0	all	-	q	Eu:Ger	1988	CC	1004	n	bl	n	n	0	0	cig+/-ot	0.1	0.9	0	0	current	st
JAHN	606	m	0	0	all	-	q	Eu:Ger	1988	CC	1004	n	bl	n	n	0	0	cig+/-ot	1.0	1.9	0	0	current	st
JAHN	607	m	0	0	all	-	q	Eu:Ger	1988	CC	1004	n	bl	n	n	0	0	cig+/-ot	2	5	1	1	current	st
JAHN	608	m	0	0	all	-	q	Eu:Ger	1988	CC	1004	n	bl	n	n	0	0	cig+/-ot	6	10	2	0	current	st
JAHN	609	m	0	0	all	-	q	Eu:Ger	1988	CC	1004	n	bl	n	n	0	0	cig+/-ot	11	20	3	0	current	st
JAHN	610	m	0	0	all	-	q	Eu:Ger	1988	CC	1004	n	bl	n	n	0	0	cig+/-ot	21	999	0	0	current	st
JAIN	546	m	0	0	all	-	q	NAmer	1981	CC	845	n	V	y	n	0	0	cig+/-ot	2	9	0	1	cur+2y	st
JAIN	547	m	0	0	all	-	q	NAmer	1981	CC	845	n	V	y	n	0	0	cig+/-ot	10	999	3	0	cur+2y	st
JAIN	510	f	0	0	all	-	q	NAmer	1981	CC	845	n	V	y	n	0	0	cig+/-ot	2	9	0	1	cur+2y	st
JAIN	511	f	0	0	all	-	q	NAmer	1981	CC	845	n	V	y	n	0	0	cig+/-ot	10	999	3	0	cur+2y	st
LUBIN2	775	m	0	0	all	-	q	Eu:mul	1976	CC	7804	n	bl	n	y	0	0	cig+/-ot	0.1	4	1	1	current	st
LUBIN2	776	m	0	0	all	-	q	Eu:mul	1976	CC	7804	n	bl	n	y	0	0	cig+/-ot	5	9	2	0	current	st
LUBIN2	777	m	0	0	all	-	q	Eu:mul	1976	CC	7804	n	bl	n	y	0	0	cig+/-ot	10	14	3	2	current	st
LUBIN2	778	m	0	0	all	-	q	Eu:mul	1976	CC	7804	n	bl	n	y	0	0	cig+/-ot	15	19	0	0	current	st
LUBIN2	779	m	0	0	all	-	q	Eu:mul	1976	CC	7804	n	bl	n	y	0	0	cig+/-ot	20	999	0	3	current	st
LUBIN2	899	f	0	0	all	-	q	Eu:mul	1976	CC	7804	n	bl	n	y	0	0	cig+/-ot	0.1	9	0	1	current	st
LUBIN2	900	f	0	0	all	-	q	Eu:mul	1976	CC	7804	n	bl	n	y	0	0	cig+/-ot	10	19	3	2	current	st
LUBIN2	901	f	0	0	all	-	q	Eu:mul	1976	CC	7804	n	bl	n	y	0	0	cig+/-ot	20	999	0	3	current	st
MATOS	636	m	0	0	all	-	q	SCAmer	1994	CC	200	n	bl	n	n	2	0	cig+/-ot	1.0	5	1	1	cur+1y	or
MATOS	637	m	0	0	all	-	q	SCAmer	1994	CC	200	n	bl	n	n	2	0	cig+/-ot	6	10	2	0	cur+1y	or
MATOS	638	m	0	0	all	-	q	SCAmer	1994	CC	200	n	bl	n	n	2	0	cig+/-ot	11	999	3	0	cur+1y	or
PEZZOT	582	m	0	0	all	-	q	SCAmer	1987	CC	215	n	bl	n	y	0	0	cig only	1.0	10	0	1	cur+1y	st
PEZZOT	583	m	0	0	all	-	q	SCAmer	1987	CC	215	n	bl	n	y	0	0	cig only	11	999	3	0	cur+1y	st
SOBUE	739	m	0	0	all	-	q	As:Jap	1986	CC	1376	n	bl	n	y	0	0	cig+/-ot	1.0	4	1	1	cur+1y	st
SOBUE	740	m	0	0	all	-	q	As:Jap	1986	CC	1376	n	bl	n	y	0	0	cig+/-ot	5	9	2	0	cur+1y	st
SOBUE	741	m	0	0	all	-	q	As:Jap	1986	CC	1376	n	bl	n	y	0	0	cig+/-ot	10	999	3	0	cur+1y	st
SVENSS	560	f	0	0	all	-	q	Eu:Sca	1983	CC	210	n	bl	n	n	0	0	all/unsp	3	10	0	1	cur+2y	st
SVENSS	561	f	0	0	all	-	q	Eu:Sca	1983	CC	210	n	bl	n	n	0	0	all/unsp	11	999	3	0	cur+2y	st
WYNDE3	511	m	0	0	all	-	KI	NAmer	1966	CC	350	n	bl	n	y	0	0	all/unsp	1.0	3	1	1	cur+1y	st
WYNDE3	512	m	0	0	all	-	KI	NAmer	1966	CC	350	n	bl	n	y	0	0	all/unsp	4	6	0	0	cur+1y	st
WYNDE3	513	m	0	0	all	-	KI	NAmer	1966	CC	350	n	bl	n	y	0	0	all/unsp	7	12	0	2	cur+1y	st
WYNDE3	514	m	0	0	all	-	KI	NAmer	1966	CC	350	n	bl	n	y	0	0	all/unsp	13	999	0	3	cur+1y	st

Cigarette type is all/unspec for all RRs

In this overview table, subtotals and Qs values may be invalid and should be ignored

Table 2K1 - 2

IESLC - Meta-analysis of Ex Smoking by Years quit (vs current), Overview
Squamous, Any Product (or Cigarettes if Any not available)
Most adjusted

REF	NRR	SEX	A	Number Exposed Case	Cont	Non-exposed Case	Cont	RR	95.00%CI
BARBON	602	m	1	11	-	203	-	0.97	(0.45- 2.07)
BARBON	603	m	1	31	-	203	-	0.62	(0.40- 0.96)
BARBON	604	m	1	11	-	203	-	0.42	(0.21- 0.84)
BARBON	605	m	1	4	-	203	-	0.10	(0.03- 0.29)
Subtotal BARBON								0.53	(0.39- 0.74)
JAHN	605	m	0	74	8	153	269	16.26	(7.64- 34.63)
JAHN	606	m	0	25	9	153	269	4.88	(2.22- 10.73)
JAHN	607	m	0	36	46	153	269	1.38	(0.85- 2.22)
JAHN	608	m	0	29	63	153	269	0.81	(0.50- 1.31)
JAHN	609	m	0	18	130	153	269	0.24	(0.14- 0.41)
JAHN	610	m	0	8	146	153	269	0.10	(0.05- 0.20)
Subtotal JAHN								0.92	(0.73- 1.17)
JAIN	546	m	0	24	46	107	118	0.58	(0.33- 1.01)
JAIN	547	m	0	23	113	107	118	0.22	(0.13- 0.38)
JAIN	510	f	0	15	36	81	99	0.51	(0.26- 1.00)
JAIN	511	f	0	7	61	81	99	0.14	(0.06- 0.32)
Subtotal JAIN								0.33	(0.24- 0.45)
LUBIN2	775	m	0	498	1047	2518	6209	1.17	(1.04- 1.32)
LUBIN2	776	m	0	265	882	2518	6209	0.74	(0.64- 0.86)
LUBIN2	777	m	0	146	693	2518	6209	0.52	(0.43- 0.62)
LUBIN2	778	m	0	67	478	2518	6209	0.35	(0.27- 0.45)
LUBIN2	779	m	0	106	1128	2518	6209	0.23	(0.19- 0.28)
LUBIN2	899	f	0	38	95	154	410	1.06	(0.70- 1.62)
LUBIN2	900	f	0	5	33	154	410	0.40	(0.15- 1.05)
LUBIN2	901	f	0	2	29	154	410	0.18	(0.04- 0.78)
Subtotal LUBIN2								0.69	(0.64- 0.74)
MATOS	636	m	2	4	-	33	-	0.70	(0.20- 2.20)
MATOS	637	m	2	5	-	33	-	0.60	(0.20- 1.90)
MATOS	638	m	2	5	-	33	-	0.20	(0.06- 0.50)
Subtotal MATOS								0.42	(0.22- 0.80)
PEZZOT	582	m	0	21	27	56	52	0.72	(0.36- 1.43)
PEZZOT	583	m	0	8	48	56	52	0.15	(0.07- 0.36)
Subtotal PEZZOT								0.39	(0.23- 0.66)
SOBUE	739	m	0	52	116	292	633	0.97	(0.68- 1.39)
SOBUE	740	m	0	32	92	292	633	0.75	(0.49- 1.15)
SOBUE	741	m	0	30	144	292	633	0.45	(0.30- 0.69)
Subtotal SOBUE								0.72	(0.57- 0.90)
SVENSS	560	f	0	5	13	42	53	0.49	(0.16- 1.47)
SVENSS	561	f	0	1	24	42	53	0.05	(0.01- 0.40)
Subtotal SVENSS								0.29	(0.11- 0.77)
WYNDE3	511	m	0	18	22	171	207	0.99	(0.51- 1.91)
WYNDE3	512	m	0	8	17	171	207	0.57	(0.24- 1.35)
WYNDE3	513	m	0	8	31	171	207	0.31	(0.14- 0.70)
WYNDE3	514	m	0	2	55	171	207	0.04	(0.01- 0.18)
Subtotal WYNDE3								0.49	(0.32- 0.74)
Partial Totals				1642	5632	17013	37260		

*prospective study

Table 2K1 - 2

IESLC - Meta-analysis of Ex Smoking by Years quit (vs current), Overview
Squamous, Any Product (or Cigarettes if Any not available)
Most adjusted

REF	NRR	SEX	AD	Ys	Ws	Qs	Ps
BARBON	602	m	1	-0.03	6.60	0.97	0.9376
BARBON	603	m	1	-0.48	20.05	0.08	0.0323
BARBON	604	m	1	-0.87	8.00	1.65	0.0142
BARBON	605	m	1	-2.30	2.99	10.65	0.0001
Subtotal BARBON				-0.63	37.63	13.35	
JAHN	605	m	0	2.79	6.72	68.94	0.0000
JAHN	606	m	0	1.59	6.20	24.78	0.0001
JAHN	607	m	0	0.32	16.73	8.98	0.1917
JAHN	608	m	0	-0.21	16.50	0.67	0.3901
JAHN	609	m	0	-1.41	13.61	13.59	0.0000
JAHN	610	m	0	-2.34	7.04	26.11	0.0000
Subtotal JAHN				-0.08	66.79	143.07	
JAIN	546	m	0	-0.55	12.31	0.24	0.0525
JAIN	547	m	0	-1.49	14.26	16.64	0.0000
JAIN	510	f	0	-0.67	8.55	0.58	0.0484
JAIN	511	f	0	-1.96	5.50	13.23	0.0000
Subtotal JAIN				-1.10	40.63	30.70	
LUBIN2	775	m	0	0.16	283.98	93.26	0.0072
LUBIN2	776	m	0	-0.30	182.96	2.36	0.0000
LUBIN2	777	m	0	-0.65	112.99	6.58	0.0000
LUBIN2	778	m	0	-1.06	56.90	23.95	0.0000
LUBIN2	779	m	0	-1.46	91.92	101.08	0.0000
LUBIN2	899	f	0	0.06	21.85	4.96	0.7687
LUBIN2	900	f	0	-0.91	4.18	1.02	0.0634
LUBIN2	901	f	0	-1.69	1.84	3.02	0.0215
Subtotal LUBIN2				-0.38	756.62	236.23	
MATOS	636	m	2	-0.36	2.67	0.01	0.5598
MATOS	637	m	2	-0.51	3.03	0.03	0.3738
MATOS	638	m	2	-1.61	3.42	4.89	0.0029
Subtotal MATOS				-0.88	9.12	4.93	
PEZZOT	582	m	0	-0.33	8.21	0.06	0.3510
PEZZOT	583	m	0	-1.87	5.47	11.53	0.0000
Subtotal PEZZOT				-0.94	13.68	11.59	
SOBUE	739	m	0	-0.03	30.44	4.51	0.8745
SOBUE	740	m	0	-0.28	21.22	0.37	0.1934
SOBUE	741	m	0	-0.79	22.08	3.21	0.0002
Subtotal SOBUE				-0.33	73.74	8.09	
SVENSS	560	f	0	-0.72	3.13	0.30	0.2010
SVENSS	561	f	0	-2.95	0.92	5.91	0.0047
Subtotal SVENSS				-1.23	4.05	6.21	
WYNDE3	511	m	0	-0.01	8.95	1.46	0.9770
WYNDE3	512	m	0	-0.56	5.14	0.11	0.2020
WYNDE3	513	m	0	-1.16	5.95	3.35	0.0045
WYNDE3	514	m	0	-3.12	1.89	13.88	0.0000
Subtotal WYNDE3				-0.72	21.94	18.81	

N	36
NS	9

Table 2K1 - 3

IESLC - Meta-analysis of Ex Smoking by Years quit (vs current), Overview
Squamous, Any Product (or Cigarettes if Any not available)
Most adjusted

	Sex: combined	male	female	Total
N		29	7	36
NS		8	3	11

In this overview table, other than the "N" rows, entries in the "absent" and "Total" columns may be invalid and should be ignored

Years quit vs current (lower focus)	absent	1-6k3	4-11k7	8+k12	Total
N	17	6	4	9	36
NS	7	6	4	7	24
Wt	268.69	349.37	223.72	182.42	1024.20
Het Chi	220.86	2.56	0.26	32.55	472.97
Het df	16	5	3	8	35
Het P	***	N.S.	N.S.	***	***
Fixed RR	0.41	1.15	0.74	0.40	0.66
RRl	0.36	1.03	0.65	0.35	0.62
RRu	0.46	1.28	0.85	0.47	0.70
P	---	++	---	---	---
Random RR	0.50	1.15	0.74	0.27	0.51
RRl	0.30	1.03	0.65	0.18	0.39
RRu	0.82	1.28	0.85	0.40	0.66
P	--	++	---	---	---

Years quit vs current (higher focus)	absent	1-11k3	4-19k12	13+k20	Total
N	17	11	4	4	36
NS	9	9	3	3	24
Wt	373.95	403.43	143.17	103.65	1024.20
Het Chi	218.40	16.95	2.43	8.13	472.97
Het df	16	10	3	3	35
Het P	***	(*)	N.S.	*	***
Fixed RR	0.57	1.08	0.52	0.23	0.66
RRl	0.51	0.98	0.44	0.19	0.62
RRu	0.63	1.19	0.61	0.28	0.70
P	---	N.S.	---	---	---
Random RR	0.44	0.94	0.52	0.21	0.51
RRl	0.28	0.77	0.44	0.11	0.39
RRu	0.70	1.14	0.61	0.40	0.66
P	---	N.S.	---	---	---

Table 2K1 - 3

IESLC - Meta-analysis of Ex Smoking by Years quit (vs current), Overview
Squamous, Any Product (or Cigarettes if Any not available)
Most adjusted

MALES

		Years quit vs current (lower focus) absent	1-6k3	4-11k7	8+k12	Total
	N	13	6	4	6	29
	NS	6	6	4	6	22
	Wt	233.32	349.37	223.72	171.82	978.22
Het	Chi	196.95	2.56	0.26	22.19	443.88
Het	df	12	5	3	5	28
Het	P	***	N.S.	N.S.	***	***
Fixed	RR	0.37	1.15	0.74	0.42	0.67
	RRl	0.33	1.03	0.65	0.36	0.63
	RRu	0.42	1.28	0.85	0.49	0.71
	P	---	++	---	---	---
Random	RR	0.49	1.15	0.74	0.30	0.55
	RRl	0.27	1.03	0.65	0.20	0.41
	RRu	0.89	1.28	0.85	0.46	0.73
	P	-	++	---	---	---

		Years quit vs current (higher focus) absent	1-11k3	4-19k12	13+k20	Total
	N	15	8	3	3	29
	NS	8	8	3	3	22
	Wt	367.52	369.90	138.99	101.81	978.22
Het	Chi	202.13	9.81	2.17	8.02	443.88
Het	df	14	7	2	2	28
Het	P	***	N.S.	N.S.	*	***
Fixed	RR	0.58	1.11	0.52	0.24	0.67
	RRl	0.53	1.00	0.44	0.19	0.63
	RRu	0.65	1.23	0.62	0.29	0.71
	P	---	+	---	---	---
Random	RR	0.51	1.01	0.52	0.21	0.55
	RRl	0.32	0.84	0.43	0.10	0.41
	RRu	0.81	1.22	0.63	0.46	0.73
	P	--	N.S.	---	---	---

FEMALES

		Years quit vs current (lower focus) absent	1-6k3	4-11k7	8+k12	Total
	N	4			3	7
	NS	3			3	6
	Wt	35.37			10.61	45.98
Het	Chi	8.20			4.39	27.61
Het	df	3			2	6
Het	P	*			N.S.	***
Fixed	RR	0.76			0.20	0.55
	RRl	0.55			0.11	0.42
	RRu	1.05			0.36	0.74
	P	N.S.			---	---
Random	RR	0.57			0.18	0.34
	RRl	0.29			0.07	0.17
	RRu	1.11			0.48	0.70
	P	(-)			---	--

Table 2K1 - 3

IESLC - Meta-analysis of Ex Smoking by Years quit (vs current), Overview
Squamous, Any Product (or Cigarettes if Any not available)
Most adjusted

FEMALES

		Years quit vs current (higher focus)				
		absent	1-11k3	4-19k12	13+k20	Total
	N	2	3	1	1	7
	NS	2	3	1	1	6
	Wt	6.43	33.53	4.18	1.84	45.98
Het	Chi	0.76	4.29	0.00	0.00	27.61
Het	df	1	2	0	0	6
Het	P	N.S.	N.S.	N.S.	N.S.	***
Fixed	RR	0.12	0.82	0.40	0.18	0.55
	RRl	0.06	0.58	0.15	0.04	0.42
	RRu	0.26	1.15	1.05	0.78	0.74
	P	---	N.S.	(-)	-	---
Random	RR	0.12	0.71	0.40	0.18	0.34
	RRl	0.06	0.40	0.15	0.04	0.17
	RRu	0.26	1.27	1.05	0.78	0.70
	P	---	N.S.	(-)	-	--

Table 2K1 - 4

IESLC - Meta-analysis of Ex Smoking by Years quit (vs current), Overview
Squamous, Any Product (or Cigarettes if Any not available)
Least adjusted

REF	NRR	X	SEX	AGEL	AGEH	RACE	YF	LC TYPE	LOC	START	ST	NLC	R	VB	P	H	AD	ADOS	PRODUCT	exL	exH	S1	S2	DENOM	De
BARBON	587	x	m	0	0	all	-	q	Eu:wst	1979	CC	755	n	bl	y	y	0	0	all/unsp	0.1	4	1	1	current	st
BARBON	588	x	m	0	0	all	-	q	Eu:wst	1979	CC	755	n	bl	y	y	0	0	all/unsp	5	14	0	2	current	st
BARBON	589	x	m	0	0	all	-	q	Eu:wst	1979	CC	755	n	bl	y	y	0	0	all/unsp	15	24	0	3	current	st
BARBON	590	x	m	0	0	all	-	q	Eu:wst	1979	CC	755	n	bl	y	y	0	0	all/unsp	25	999	0	0	current	st
JAHN	605		m	0	0	all	-	q	Eu:Ger	1988	CC	1004	n	bl	n	n	0	0	cig+/-ot	0.1	0.9	0	0	current	st
JAHN	606		m	0	0	all	-	q	Eu:Ger	1988	CC	1004	n	bl	n	n	0	0	cig+/-ot	1.0	1.9	0	0	current	st
JAHN	607		m	0	0	all	-	q	Eu:Ger	1988	CC	1004	n	bl	n	n	0	0	cig+/-ot	2	5	1	1	current	st
JAHN	608		m	0	0	all	-	q	Eu:Ger	1988	CC	1004	n	bl	n	n	0	0	cig+/-ot	6	10	2	0	current	st
JAHN	609		m	0	0	all	-	q	Eu:Ger	1988	CC	1004	n	bl	n	n	0	0	cig+/-ot	11	20	3	0	current	st
JAHN	610		m	0	0	all	-	q	Eu:Ger	1988	CC	1004	n	bl	n	n	0	0	cig+/-ot	21	999	0	0	current	st
JAIN	546		m	0	0	all	-	q	NAmer	1981	CC	845	n	V	y	n	0	0	cig+/-ot	2	9	0	1	cur+2y	st
JAIN	547		m	0	0	all	-	q	NAmer	1981	CC	845	n	V	y	n	0	0	cig+/-ot	10	999	3	0	cur+2y	st
JAIN	510		f	0	0	all	-	q	NAmer	1981	CC	845	n	V	y	n	0	0	cig+/-ot	2	9	0	1	cur+2y	st
JAIN	511		f	0	0	all	-	q	NAmer	1981	CC	845	n	V	y	n	0	0	cig+/-ot	10	999	3	0	cur+2y	st
LUBIN2	775		m	0	0	all	-	q	Eu:mul	1976	CC	7804	n	bl	n	y	0	0	cig+/-ot	0.1	4	1	1	current	st
LUBIN2	776		m	0	0	all	-	q	Eu:mul	1976	CC	7804	n	bl	n	y	0	0	cig+/-ot	5	9	2	0	current	st
LUBIN2	777		m	0	0	all	-	q	Eu:mul	1976	CC	7804	n	bl	n	y	0	0	cig+/-ot	10	14	3	2	current	st
LUBIN2	778		m	0	0	all	-	q	Eu:mul	1976	CC	7804	n	bl	n	y	0	0	cig+/-ot	15	19	0	0	current	st
LUBIN2	779		m	0	0	all	-	q	Eu:mul	1976	CC	7804	n	bl	n	y	0	0	cig+/-ot	20	999	0	3	current	st
LUBIN2	899		f	0	0	all	-	q	Eu:mul	1976	CC	7804	n	bl	n	y	0	0	cig+/-ot	0.1	9	0	1	current	st
LUBIN2	900		f	0	0	all	-	q	Eu:mul	1976	CC	7804	n	bl	n	y	0	0	cig+/-ot	10	19	3	2	current	st
LUBIN2	901		f	0	0	all	-	q	Eu:mul	1976	CC	7804	n	bl	n	y	0	0	cig+/-ot	20	999	0	3	current	st
MATOS	626	x	m	0	0	all	-	q	SCAmer	1994	CC	200	n	bl	n	n	0	0	cig+/-ot	1.0	5	1	1	cur+1y	st
MATOS	627	x	m	0	0	all	-	q	SCAmer	1994	CC	200	n	bl	n	n	0	0	cig+/-ot	6	10	2	0	cur+1y	st
MATOS	628	x	m	0	0	all	-	q	SCAmer	1994	CC	200	n	bl	n	n	0	0	cig+/-ot	11	999	3	0	cur+1y	st
PEZZOT	582		m	0	0	all	-	q	SCAmer	1987	CC	215	n	bl	n	y	0	0	cig only	1.0	10	0	1	cur+1y	st
PEZZOT	583		m	0	0	all	-	q	SCAmer	1987	CC	215	n	bl	n	y	0	0	cig only	11	999	3	0	cur+1y	st
SOBUE	739		m	0	0	all	-	q	As:Jap	1986	CC	1376	n	bl	n	y	0	0	cig+/-ot	1.0	4	1	1	cur+1y	st
SOBUE	740		m	0	0	all	-	q	As:Jap	1986	CC	1376	n	bl	n	y	0	0	cig+/-ot	5	9	2	0	cur+1y	st
SOBUE	741		m	0	0	all	-	q	As:Jap	1986	CC	1376	n	bl	n	y	0	0	cig+/-ot	10	999	3	0	cur+1y	st
SVENSS	560		f	0	0	all	-	q	Eu:Sca	1983	CC	210	n	bl	n	n	0	0	all/unsp	3	10	0	1	cur+2y	st
SVENSS	561		f	0	0	all	-	q	Eu:Sca	1983	CC	210	n	bl	n	n	0	0	all/unsp	11	999	3	0	cur+2y	st
WYNDE3	511		m	0	0	all	-	KI	NAmer	1966	CC	350	n	bl	n	y	0	0	all/unsp	1.0	3	1	1	cur+1y	st
WYNDE3	512		m	0	0	all	-	KI	NAmer	1966	CC	350	n	bl	n	y	0	0	all/unsp	4	6	0	0	cur+1y	st
WYNDE3	513		m	0	0	all	-	KI	NAmer	1966	CC	350	n	bl	n	y	0	0	all/unsp	7	12	0	2	cur+1y	st
WYNDE3	514		m	0	0	all	-	KI	NAmer	1966	CC	350	n	bl	n	y	0	0	all/unsp	13	999	0	3	cur+1y	st

Cigarette type is all/unspec for all RRs

In this overview table, subtotals and Qs values may be invalid and should be ignored

Table 2K1 - 5

IESLC - Meta-analysis of Ex Smoking by Years quit (vs current), Overview
Squamous, Any Product (or Cigarettes if Any not available)
Least adjusted

REF	NRR	SEX	A	Number Exposed Case	Cont	Non-exposed Case	Cont	RR	95.00%CI
BARBON	587	m	0	11	20	203	362	0.98	(0.46- 2.09)
BARBON	588	m	0	31	85	203	362	0.65	(0.42- 1.02)
BARBON	589	m	0	11	41	203	362	0.48	(0.24- 0.95)
BARBON	590	m	0	4	59	203	362	0.12	(0.04- 0.34)
Subtotal BARBON								0.56	(0.41- 0.77)
JAHN	605	m	0	74	8	153	269	16.26	(7.64- 34.63)
JAHN	606	m	0	25	9	153	269	4.88	(2.22- 10.73)
JAHN	607	m	0	36	46	153	269	1.38	(0.85- 2.22)
JAHN	608	m	0	29	63	153	269	0.81	(0.50- 1.31)
JAHN	609	m	0	18	130	153	269	0.24	(0.14- 0.41)
JAHN	610	m	0	8	146	153	269	0.10	(0.05- 0.20)
Subtotal JAHN								0.92	(0.73- 1.17)
JAIN	546	m	0	24	46	107	118	0.58	(0.33- 1.01)
JAIN	547	m	0	23	113	107	118	0.22	(0.13- 0.38)
JAIN	510	f	0	15	36	81	99	0.51	(0.26- 1.00)
JAIN	511	f	0	7	61	81	99	0.14	(0.06- 0.32)
Subtotal JAIN								0.33	(0.24- 0.45)
LUBIN2	775	m	0	498	1047	2518	6209	1.17	(1.04- 1.32)
LUBIN2	776	m	0	265	882	2518	6209	0.74	(0.64- 0.86)
LUBIN2	777	m	0	146	693	2518	6209	0.52	(0.43- 0.62)
LUBIN2	778	m	0	67	478	2518	6209	0.35	(0.27- 0.45)
LUBIN2	779	m	0	106	1128	2518	6209	0.23	(0.19- 0.28)
LUBIN2	899	f	0	38	95	154	410	1.06	(0.70- 1.62)
LUBIN2	900	f	0	5	33	154	410	0.40	(0.15- 1.05)
LUBIN2	901	f	0	2	29	154	410	0.18	(0.04- 0.78)
Subtotal LUBIN2								0.69	(0.64- 0.74)
MATOS	626	m	0	4	23	33	132	0.70	(0.23- 2.15)
MATOS	627	m	0	5	27	33	132	0.74	(0.27- 2.07)
MATOS	628	m	0	5	101	33	132	0.20	(0.07- 0.53)
Subtotal MATOS								0.44	(0.24- 0.81)
PEZZOT	582	m	0	21	27	56	52	0.72	(0.36- 1.43)
PEZZOT	583	m	0	8	48	56	52	0.15	(0.07- 0.36)
Subtotal PEZZOT								0.39	(0.23- 0.66)
SOBUE	739	m	0	52	116	292	633	0.97	(0.68- 1.39)
SOBUE	740	m	0	32	92	292	633	0.75	(0.49- 1.15)
SOBUE	741	m	0	30	144	292	633	0.45	(0.30- 0.69)
Subtotal SOBUE								0.72	(0.57- 0.90)
SVENSS	560	f	0	5	13	42	53	0.49	(0.16- 1.47)
SVENSS	561	f	0	1	24	42	53	0.05	(0.01- 0.40)
Subtotal SVENSS								0.29	(0.11- 0.77)
WYNDE3	511	m	0	18	22	171	207	0.99	(0.51- 1.91)
WYNDE3	512	m	0	8	17	171	207	0.57	(0.24- 1.35)
WYNDE3	513	m	0	8	31	171	207	0.31	(0.14- 0.70)
WYNDE3	514	m	0	2	55	171	207	0.04	(0.01- 0.18)
Subtotal WYNDE3								0.49	(0.32- 0.74)
Totals				1642	5988	17013	39104		

*prospective study

Table 2K1 - 5

IESLC - Meta-analysis of Ex Smoking by Years quit (vs current), Overview
Squamous, Any Product (or Cigarettes if Any not available)
Least adjusted

REF	NRR	SEX	AD	Ys	Ws	Qs	Ps
BARBON	587	m	0	-0.02	6.73	1.04	0.9599
BARBON	588	m	0	-0.43	19.34	0.01	0.0585
BARBON	589	m	0	-0.74	8.13	0.86	0.0355
BARBON	590	m	0	-2.11	3.64	10.53	0.0001
Subtotal BARBON				-0.59	37.84	12.43	
JAHN	605	m	0	2.79	6.72	68.88	0.0000
JAHN	606	m	0	1.59	6.20	24.74	0.0001
JAHN	607	m	0	0.32	16.73	8.95	0.1917
JAHN	608	m	0	-0.21	16.50	0.66	0.3901
JAHN	609	m	0	-1.41	13.61	13.62	0.0000
JAHN	610	m	0	-2.34	7.04	26.15	0.0000
Subtotal JAHN				-0.08	66.79	143.01	
JAIN	546	m	0	-0.55	12.31	0.24	0.0525
JAIN	547	m	0	-1.49	14.26	16.69	0.0000
JAIN	510	f	0	-0.67	8.55	0.59	0.0484
JAIN	511	f	0	-1.96	5.50	13.26	0.0000
Subtotal JAIN				-1.10	40.63	30.78	
LUBIN2	775	m	0	0.16	283.98	92.79	0.0072
LUBIN2	776	m	0	-0.30	182.96	2.31	0.0000
LUBIN2	777	m	0	-0.65	112.99	6.66	0.0000
LUBIN2	778	m	0	-1.06	56.90	24.05	0.0000
LUBIN2	779	m	0	-1.46	91.92	101.36	0.0000
LUBIN2	899	f	0	0.06	21.85	4.93	0.7687
LUBIN2	900	f	0	-0.91	4.18	1.03	0.0634
LUBIN2	901	f	0	-1.69	1.84	3.03	0.0215
Subtotal LUBIN2				-0.38	756.62	236.15	
MATOS	626	m	0	-0.36	3.02	0.01	0.5284
MATOS	627	m	0	-0.30	3.64	0.05	0.5671
MATOS	628	m	0	-1.62	4.04	5.88	0.0011
Subtotal MATOS				-0.82	10.69	5.93	
PEZZOT	582	m	0	-0.33	8.21	0.06	0.3510
PEZZOT	583	m	0	-1.87	5.47	11.55	0.0000
Subtotal PEZZOT				-0.94	13.68	11.61	
SOBUE	739	m	0	-0.03	30.44	4.48	0.8745
SOBUE	740	m	0	-0.28	21.22	0.36	0.1934
SOBUE	741	m	0	-0.79	22.08	3.23	0.0002
Subtotal SOBUE				-0.33	73.74	8.07	
SVENSS	560	f	0	-0.72	3.13	0.30	0.2010
SVENSS	561	f	0	-2.95	0.92	5.92	0.0047
Subtotal SVENSS				-1.23	4.05	6.22	
WYNDE3	511	m	0	-0.01	8.95	1.45	0.9770
WYNDE3	512	m	0	-0.56	5.14	0.12	0.2020
WYNDE3	513	m	0	-1.16	5.95	3.36	0.0045
WYNDE3	514	m	0	-3.12	1.89	13.90	0.0000
Subtotal WYNDE3				-0.72	21.94	18.83	

N	36
NS	9

Table 2K1 - 6

IESLC - Meta-analysis of Ex Smoking by Years quit (vs current), Overview
Squamous, Any Product (or Cigarettes if Any not available)
Least adjusted

	Sex combined	male	female	Total
N		29	7	36
NS		8	3	11

In this overview table, other than the "N" rows, entries in the "absent" and "Total" columns may be invalid and should be ignored

	Years quit vs current (lower focus) absent	1-6k3	4-11k7	8+k12	Total
N	17	6	4	9	36
NS	7	6	4	7	24
Wt	268.77	349.85	224.32	183.04	1025.98
Het Chi	221.23	2.64	0.12	32.91	473.03
Het df	16	5	3	8	35
Het P	***	N.S.	N.S.	***	***
Fixed RR	0.41	1.15	0.75	0.40	0.66
RRl	0.36	1.03	0.66	0.35	0.62
RRu	0.46	1.28	0.85	0.47	0.70
P	---	++	---	---	---
Random RR	0.51	1.15	0.75	0.27	0.52
RRl	0.31	1.03	0.66	0.18	0.40
RRu	0.83	1.28	0.85	0.40	0.67
P	--	++	---	---	---

	Years quit vs current (higher focus) absent	1-11k3	4-19k12	13+k20	Total
N	17	11	4	4	36
NS	9	9	3	3	24
Wt	375.83	403.91	142.46	103.78	1025.98
Het Chi	219.11	17.02	2.78	9.54	473.03
Het df	16	10	3	3	35
Het P	***	(*)	N.S.	*	***
Fixed RR	0.57	1.08	0.52	0.24	0.66
RRl	0.51	0.98	0.44	0.20	0.62
RRu	0.63	1.19	0.61	0.29	0.70
P	---	N.S.	---	---	---
Random RR	0.45	0.94	0.52	0.22	0.52
RRl	0.29	0.77	0.44	0.11	0.40
RRu	0.71	1.14	0.61	0.43	0.67
P	---	N.S.	---	---	---

Table 2K1 - 6

IESLC - Meta-analysis of Ex Smoking by Years quit (vs current), Overview
Squamous, Any Product (or Cigarettes if Any not available)
Least adjusted

MALES

Years quit vs current (lower focus)

	absent	1-6k3	4-11k7	8+k12	Total
N	13	6	4	6	29
NS	6	6	4	6	22
Wt	233.40	349.85	224.32	172.43	980.00
Het Chi	197.60	2.64	0.12	22.59	443.91
Het df	12	5	3	5	28
Het P	***	N.S.	N.S.	***	***
Fixed RR	0.37	1.15	0.75	0.42	0.67
RRl	0.33	1.03	0.66	0.36	0.63
RRu	0.42	1.28	0.85	0.49	0.71
P	---	++	---	---	---
Random RR	0.50	1.15	0.75	0.30	0.56
RRl	0.28	1.03	0.66	0.20	0.42
RRu	0.91	1.28	0.85	0.45	0.74
P	-	++	---	---	---

Years quit vs current (higher focus)

	absent	1-11k3	4-19k12	13+k20	Total
N	15	8	3	3	29
NS	8	8	3	3	22
Wt	369.40	370.38	138.28	101.94	980.00
Het Chi	202.86	9.88	2.50	9.42	443.91
Het df	14	7	2	2	28
Het P	***	N.S.	N.S.	**	***
Fixed RR	0.58	1.11	0.52	0.24	0.67
RRl	0.53	1.00	0.44	0.20	0.63
RRu	0.65	1.23	0.62	0.29	0.71
P	---	+	---	---	---
Random RR	0.52	1.01	0.52	0.21	0.56
RRl	0.33	0.84	0.42	0.09	0.42
RRu	0.82	1.22	0.66	0.50	0.74
P	--	N.S.	---	---	---

FEMALES

Years quit vs current (lower focus)

	absent	1-6k3	4-11k7	8+k12	Total
N	4			3	7
NS	3			3	6
Wt	35.37			10.61	45.98
Het Chi	8.20			4.39	27.61
Het df	3			2	6
Het P	*			N.S.	***
Fixed RR	0.76			0.20	0.55
RRl	0.55			0.11	0.42
RRu	1.05			0.36	0.74
P	N.S.			---	---
Random RR	0.57			0.18	0.34
RRl	0.29			0.07	0.17
RRu	1.11			0.48	0.70
P	(-)			---	--

Table 2K1 - 6

IESLC - Meta-analysis of Ex Smoking by Years quit (vs current), Overview
Squamous, Any Product (or Cigarettes if Any not available)
Least adjusted

FEMALES

	Years quit vs current (higher focus) absent	1-11k3	4-19k12	13+k20	Total
N	2	3	1	1	7
NS	2	3	1	1	6
Wt	6.43	33.53	4.18	1.84	45.98
Het Chi	0.76	4.29	0.00	0.00	27.61
Het df	1	2	0	0	6
Het P	N.S.	N.S.	N.S.	N.S.	***
Fixed RR	0.12	0.82	0.40	0.18	0.55
RRl	0.06	0.58	0.15	0.04	0.42
RRu	0.26	1.15	1.05	0.78	0.74
P	---	N.S.	(-)	-	---
Random RR	0.12	0.71	0.40	0.18	0.34
RRl	0.06	0.40	0.15	0.04	0.17
RRu	0.26	1.27	1.05	0.78	0.70
P	---	N.S.	(-)	-	--

Table 2K1 - 7

IESLC - Meta-analysis of Ex Smoking by Years quit (vs current), Overview
Squamous, Any Product (or Cigarettes if Any not available)
Excluded studies (and stage at which they were excluded)

1	AGUDO AKIBA AMANDU AMES AXELSS BEST BOUCHA BOUCOT BRESLO CHEN CHEN2 CHIAZZ DEAN2 DOSEME ENGELA FAN GENG GER GUO HAENSZ HEGMAN HOLE HU HU2 JUSSAW KATSOU KAUFMA KOO KOULUM KREUZE LETOUR LEVIN LIAW LIU3 LIU4 LIU5 MCCONN MIGRAN MRFITR NOTAN2 OSANN2 PERNU QIAO2 RACHTA RESTRE SADOWS SEGI2 STASZE TIZZAN VUTUC WATSON WU WUWILL WYNDE2 WYNDE8 XU YUAN ZHANG ZHENG ZHOU
2	AUVINE BENSHL BLOT1 BROWN3 BUFFLE GURSEL LAUSSM LUO MCDUFF PISANI PRESCO SPITZ WU2 WYNDE7
4	ARMADA BECHER BOFFET BROSS CARPEN CEDERL CHOI CHYOU CORREA CPSI CPSII DAMBER DARBY DEAN3 DESTEF DOLL DOLL2 DORGAN DORN GAO GAO2 GARCIA GARSHI GILLIS GRAHAM HAMMO2 HIRAYA HUMBLE JOLY KAISE2 KHUDER LUBIN PEZZO2 QIAO SPEIZE SUZUK2 TVERDA WANG2 WIGLE
5	ALDERS HAMMON
10	JEDRYC WAKAI WYNDE6
14	BENHAM

Table 2K1 - 8

Potentially overlapping studies

REF	REFGP	PRINC	.	OVERLAP/LINK
LUBIN2	LUBIN2	1		Lubin-combined
JAHN	BOFFET	2		Subset of BOFFET

Table 2K1 - 9

Most adjusted - insufficient data for meta-analysis

REF	NRR	SEX	AGEL	AGEH	RACE	YF	LC TYPE	LOC	START	ST	NLC	R	VB	P	H	AD	ADOS	PRODUCT	exL	exH	S1	S2	DENOM	De
ALDERS	543	m	0	0	all	-	q+s	Eu:UK	1977	CC	1448	n	V	n	n	1	0	cig only	0.1	2	0	0	current	ot
ALDERS	544	m	0	0	all	-	q+s	Eu:UK	1977	CC	1448	n	V	n	n	1	0	cig only	3	9	0	1	current	ot
ALDERS	545	m	0	0	all	-	q+s	Eu:UK	1977	CC	1448	n	V	n	n	1	0	cig only	10	999	3	0	current	ot
ALDERS	554	f	0	0	all	-	q+s	Eu:UK	1977	CC	1448	n	V	n	n	1	0	cig only	0.1	2	0	0	current	ot
ALDERS	555	f	0	0	all	-	q+s	Eu:UK	1977	CC	1448	n	V	n	n	1	0	cig only	3	9	0	1	current	ot
ALDERS	556	f	0	0	all	-	q+s	Eu:UK	1977	CC	1448	n	V	n	n	1	0	cig only	10	999	3	0	current	ot
HAMMON	507	m	0	0	wh	0	not a	NAmer	1952	pr	448	n	bl	n	n	1	0	cig only	0.1	0.9	0	0	current	st
HAMMON	508	m	0	0	wh	0	not a	NAmer	1952	pr	448	n	bl	n	n	1	0	cig only	1.0	9	0	1	current	st
HAMMON	509	m	0	0	wh	0	not a	NAmer	1952	pr	448	n	bl	n	n	1	0	cig only	10	999	3	0	current	st

REF	NRR	RR	SIG	RRDATA comment
ALDERS	543	2.10	y	0.01<p<0.05
ALDERS	544	0.36	y	0.01<p<0.05
ALDERS	545	0.21	y	0.001<p<0.01
ALDERS	554	3.02	y	p<0.001
ALDERS	555	1.51	n	0
ALDERS	556	0.12	y	p<0.001
HAMMON	507	*		RR for <1 pack is 0.97, while that for 1+ packs is 1.26
HAMMON	508	*		RR for <1 pack per day is 0.62, while that for 1+ packs is 0.49
HAMMON	509	*		RR for <1 pack per day is 0.14, while that for 1+ packs is 0.39

Table 2K2 -

IESLC - Meta-analysis of Ex Smoking, Years quit (vs current), "Low"
Squamous, Any Product (or Cigarettes if Any not available)

This analysis is restricted to results for:
1) Ex smokers
2) Results by Years quit (vs current)
3) Categorical results by Years quit (vs current)
4) Squamous (or near equivalent)
5) Results complete enough for use in metaanalysis

Within each study, results are then selected (in the following order of preference, within each sex) for:
6) (not applicable)
7) PRODUCT: all/unspec, cigarettes regardless of other products, cigarettes only
8) CIGTYPE: all/unspecified, MC regardless of HR, MC only
9) Results with least adjustment for other aspects of smoking (ADOS)
10) DENOM: current smokers, current + recent smokers (up to number of m=months or y=years, max 2 years)
11) Followup period (YF, prospective studies): whole study (coded as 0) or longest available
12) LCtype: squamous or nearest available, but not adeno. (q = squamous, s = small,
a = adeno, KI = Kreyberg I, u = undifferentiated)
13) Race: all or nearest available, otherwise by race (wh or w = white, bl or b = black, hi = hispanic
ch = chinese, jap = japanese, haw = hawaiian, w+o = white + oriental, sca = scandinavian, as = asian)
14) Years quit (vs current) "low" in key scheme 1 (key value 3, maximum range 1-6)
15) For overlapping studies: principal rather than subsidiary studies
Finally by Age: whole study (coded as 0) if available, otherwise by widest available age group
and then for single sex results (m, f) in preference to results for both sexes combined (c).

Results adjusted (AD) for the most potential confounders are then chosen in Sections -1 to -3
and results adjusted for the least confounders in Sections -4 to -6. (Those least adjusted results which
actually differ from the most adjusted are marked 'x' in column X in Section -4)

Section -7 shows excluded studies, together with the stage (as above) at which no qualifying
results were found.

Section -8 lists the potentially overlapping studies which have been included (1=principal, 2=subsidiary).

Section -9 lists any results which would have been included in preference except that they had data not complete
enough for use in meta-analysis, with their significance (yes/no), if known, and any further comment as entered
on the database. It also lists as "gap" any categories for which no data were presented by the original authors.

In addition to those mentioned above, the following fields, levels and abbreviations are used:
* or nk = not known, n = no, y = yes, ot = other
nev = never
all/unspec = all or unspecified, cig+/-ot = cigarettes irrespective of other products (cigar, pipe etc)
MC = manufactured cigarettes, HR = hand-rolled cigarettes
exL, exH = range of exposure (low and high) in the smoking group, in terms of Years quit (vs current)
REF: 6-character study reference
NRR: number of the RR on the database within the study
ST : study type (CC = case control, pr or prosp = prospective)
NLC: number of lung cancer cases in whole study
R : risky occupational population (n = no, m = mining, o = other risky)
VB : national cigarette type (V = at least 75% Virginia, bl = at least 75% blended, ot = other)
P : any proxy use
H : full histological confirmation
De : derivation of RR/CI (or = original, st = standard method, ot = other method of estimation)

Table 2K2 - 1

IESLC - Meta-analysis of Ex Smoking, Years quit (vs current), "Low"
Squamous, Any Product (or Cigarettes if Any not available)
Most adjusted

REF	NRR	SEX	AGEL	AGEH	RACE	YF	LC TYPE	LOC	START	ST	NLC	R	VB	P	H	AD	ADOS	PRODUCT	exL	exH	DENOM	De
BARBON	602	m	0	0	all	-	q	Eu:wst	1979	CC	755	n	bl	y	y	1	0	all/unsp	0.1	4	current	ot
JAHN	607	m	0	0	all	-	q	Eu:Ger	1988	CC	1004	n	bl	n	n	0	0	cig+/-ot	2	5	current	st
LUBIN2	775	m	0	0	all	-	q	Eu:mul	1976	CC	7804	n	bl	n	y	0	0	cig+/-ot	0.1	4	current	st
LUBIN2	908	f	0	0	all	-	q	Eu:mul	1976	CC	7804	n	bl	n	y	2	1#	cig+/-ot	0.1	4	current	ot
MATOS	636	m	0	0	all	-	q	SCAmer	1994	CC	200	n	bl	n	n	2	0	cig+/-ot	1.0	5	cur+1y	or
SOBUE	739	m	0	0	all	-	q	As:Jap	1986	CC	1376	n	bl	n	y	0	0	cig+/-ot	1.0	4	cur+1y	st
WYNDE3	511	m	0	0	all	-	KI	NAmer	1966	CC	350	n	bl	n	y	0	0	all/unsp	1.0	3	cur+1y	st

Comments on values in listings

LUBIN2 ADOS Duration of smoking

Cigarette type is all/unspec for all RRs

Table 2K2 - 2

IESLC - Meta-analysis of Ex Smoking, Years quit (vs current), "Low"
Squamous, Any Product (or Cigarettes if Any not available)
Most adjusted

REF	NRR	SEX	A	Number Exposed Case	Cont	Non-exposed Case	Cont	RR	95.00%CI
BARBON	602	m	1	11	-	203	-	0.97	(0.45- 2.07)
JAHN	607	m	0	36	46	153	269	1.38	(0.85- 2.22)
LUBIN2	775	m	0	498	1047	2518	6209	1.17	(1.04- 1.32)
LUBIN2	908	f	2	25	-	154	-	1.10	(0.66- 1.83)
Subtotal LUBIN2								1.17	(1.04- 1.31)
MATOS	636	m	2	4	-	33	-	0.70	(0.20- 2.20)
SOBUE	739	m	0	52	116	292	633	0.97	(0.68- 1.39)
WYNDE3	511	m	0	18	22	171	207	0.99	(0.51- 1.91)
Partial Totals				644	1231	3524	7318		

*prospective study

REF	NRR	SEX	AD	Ys	Ws	Qs	Ps
BARBON	602	m	1	-0.03	6.60	0.19	0.9376
JAHN	607	m	0	0.32	16.73	0.55	0.1917
LUBIN2	775	m	0	0.16	283.98	0.14	0.0072
LUBIN2	908	f	2	0.10	14.77	0.03	0.7141
Subtotal LUBIN2				0.16	298.76	0.17	
MATOS	636	m	2	-0.36	2.67	0.65	0.5598
SOBUE	739	m	0	-0.03	30.44	0.84	0.8745
WYNDE3	511	m	0	-0.01	8.95	0.19	0.9770

	N	7
	NS	6
	Wt	364.15
Het	Chi	2.59
Het	df	6
Het	P	N.S.
Fixed	RR	1.15
	RRl	1.03
	RRu	1.27
	P	++
Random	RR	1.15
	RRl	1.03
	RRu	1.27
	P	++
Asymm	P	N.S.

Table 2K2 - 3

IESLC - Meta-analysis of Ex Smoking, Years quit (vs current), "Low"
Squamous, Any Product (or Cigarettes if Any not available)
Most adjusted

	combined	Sex male	female	Total
N		6	1	7
NS		6	1	7
Wt		349.37	14.77	364.15
Het Chi		2.56	0.00	2.59
Het df		5	0	6
Het P		N.S.	N.S.	N.S.
Fixed RR		1.15	1.10	1.15
RRl		1.03	0.66	1.03
RRu		1.28	1.83	1.27
P		++	N.S.	++
Random RR		1.15	1.10	1.15
RRl		1.03	0.66	1.03
RRu		1.28	1.83	1.27
P		++	N.S.	++
Between Chi				0.03
Between df				1
Between P				N.S.
Btwn(F) P				N.S.
Btwn(R) P				N.S.

Too few RRs for analysis by factor

Table 2K2 - 4

IESLC - Meta-analysis of Ex Smoking, Years quit (vs current), "Low"
Squamous, Any Product (or Cigarettes if Any not available)
Least adjusted

REF	NRR	X	SEX	AGEL	AGEH	RACE	YF	LC TYPE	LOC	START	ST	NLC	R	VB	P	H	AD	ADOS	PRODUCT	exL	exH	DENOM	De
BARBON	587	x	m	0	0	all	-	q	Eu:wst	1979	CC	755	n	bl	y	y	0	0	all/unsp	0.1	4	current	st
JAHN	607		m	0	0	all	-	q	Eu:Ger	1988	CC	1004	n	bl	n	n	0	0	cig+/-ot	2	5	current	st
LUBIN2	775		m	0	0	all	-	q	Eu:mul	1976	CC	7804	n	bl	n	y	0	0	cig+/-ot	0.1	4	current	st
LUBIN2	908		f	0	0	all	-	q	Eu:mul	1976	CC	7804	n	bl	n	y	2	1#	cig+/-ot	0.1	4	current	ot
MATOS	626	x	m	0	0	all	-	q	SCAmer	1994	CC	200	n	bl	n	n	0	0	cig+/-ot	1.0	5	cur+1y	st
SOBUE	739		m	0	0	all	-	q	As:Jap	1986	CC	1376	n	bl	n	y	0	0	cig+/-ot	1.0	4	cur+1y	st
WYNDE3	511		m	0	0	all	-	KI	NAmer	1966	CC	350	n	bl	n	y	0	0	all/unsp	1.0	3	cur+1y	st

Comments on values in listings

LUBIN2 ADOS Duration of smoking

Cigarette type is all/unspec for all RRs

Table 2K2 - 5

IESLC - Meta-analysis of Ex Smoking, Years quit (vs current), "Low"
Squamous, Any Product (or Cigarettes if Any not available)
Least adjusted

REF	NRR	SEX	A	Number Exposed Case	Cont	Non-exposed Case	Cont	RR	95.00%CI
BARBON	587	m	0	11	20	203	362	0.98	(0.46- 2.09)
JAHN	607	m	0	36	46	153	269	1.38	(0.85- 2.22)
LUBIN2	775	m	0	498	1047	2518	6209	1.17	(1.04- 1.32)
LUBIN2	908	f	2	25	-	154	-	1.10	(0.66- 1.83)
Subtotal LUBIN2								1.17	(1.04- 1.31)
MATOS	626	m	0	4	23	33	132	0.70	(0.23- 2.15)
SOBUE	739	m	0	52	116	292	633	0.97	(0.68- 1.39)
WYNDE3	511	m	0	18	22	171	207	0.99	(0.51- 1.91)
Partial Totals				644	1274	3524	7812		

*prospective study

REF	NRR	SEX	AD	Ys	Ws	Qs	Ps
BARBON	587	m	0	-0.02	6.73	0.16	0.9599
JAHN	607	m	0	0.32	16.73	0.56	0.1917
LUBIN2	775	m	0	0.16	283.98	0.15	0.0072
LUBIN2	908	f	2	0.10	14.77	0.03	0.7141
Subtotal LUBIN2				0.16	298.76	0.17	
MATOS	626	m	0	-0.36	3.02	0.75	0.5284
SOBUE	739	m	0	-0.03	30.44	0.83	0.8745
WYNDE3	511	m	0	-0.01	8.95	0.19	0.9770

	N	7
	NS	6
	Wt	364.62
Het	Chi	2.67
Het	df	6
Het	P	N.S.
Fixed	RR	1.15
	RRl	1.03
	RRu	1.27
	P	++
Random	RR	1.15
	RRl	1.03
	RRu	1.27
	P	++
Asymm	P	N.S.

Table 2K2 - 6

IESLC - Meta-analysis of Ex Smoking, Years quit (vs current), "Low"
Squamous, Any Product (or Cigarettes if Any not available)
Least adjusted

	Sex combined male	female	Total
N	6	1	7
NS	6	1	7
Wt	349.85	14.77	364.62
Het Chi	2.64	0.00	2.67
Het df	5	0	6
Het P	N.S.	N.S.	N.S.
Fixed RR	1.15	1.10	1.15
RRl	1.03	0.66	1.03
RRu	1.28	1.83	1.27
P	++	N.S.	++
Random RR	1.15	1.10	1.15
RRl	1.03	0.66	1.03
RRu	1.28	1.83	1.27
P	++	N.S.	++
Between Chi			0.03
Between df			1
Between P			N.S.
Btwn(F) P			N.S.
Btwn(R) P			N.S.

Table 2K2 - 7

IESLC - Meta-analysis of Ex Smoking, Years quit (vs current), "Low"
Squamous, Any Product (or Cigarettes if Any not available)
Excluded studies (and stage at which they were excluded)

1 AGUDO AKIBA AMANDU AMES AXELSS BEST BOUCHA BOUCOT BRESLO CHEN CHEN2 CHIAZZ DEAN2 DOSEME ENGELA FAN GENG GER GUO HAENSZ HEGMAN HOLE HU HU2 JUSSAW KATSOU KAUFMA KOO KOULUM KREUZE LETOUR LEVIN LIAW LIU3 LIU4 LIU5 MCCONN MIGRAN MRFITR NOTAN2 OSANN2 PERNU QIAO2 RACHTA RESTRE SADOWS SEGI2 STASZE TIZZAN VUTUC WATSON WU WUWILL WYNDE2 WYNDE8 XU YUAN ZHANG ZHENG ZHOU

2 AUVINE BENSHL BLOT1 BROWN3 BUFFLE GURSEL LAUSSM LUO MCDUFF PISANI PRESCO SPITZ WU2 WYNDE7

4 ARMADA BECHER BOFFET BROSS CARPEN CEDERL CHOI CHYOU CORREA CPSI CPSII DAMBER DARBY DEAN3 DESTEF DOLL DOLL2 DORGAN DORN GAO GAO2 GARCIA GARSHI GILLIS GRAHAM HAMMO2 HIRAYA HUMBLE JOLY KAISE2 KHUDER LUBIN PEZZO2 QIAO SPEIZE SUZUK2 TVERDA WANG2 WIGLE

5 ALDERS HAMMON

10 JEDRYC WAKAI WYNDE6

14 JAIN PEZZOT SVENSS

15 BENHAM

Table 2K2 - 8
Potentially overlapping studies

REF	REFGP	PRINC	OVERLAP/LINK
LUBIN2	LUBIN2	1	Lubin-combined
JAHN	BOFFET	2	Subset of BOFFET

Table 2K3 -

IESLC - Meta-analysis of Ex Smoking, Years quit (vs current), "Mid"
Squamous, Any Product (or Cigarettes if Any not available)

This analysis is restricted to results for:
1) Ex smokers
2) Results by Years quit (vs current)
3) Categorical results by Years quit (vs current)
4) Squamous (or near equivalent)
5) Results complete enough for use in metaanalysis

Within each study, results are then selected (in the following order of preference, within each sex) for:
6) (not applicable)
7) PRODUCT: all/unspec, cigarettes regardless of other products, cigarettes only
8) CIGTYPE: all/unspecified, MC regardless of HR, MC only
9) Results with least adjustment for other aspects of smoking (ADOS)
10) DENOM: current smokers, current + recent smokers (up to number of m=months or y=years, max 2 years)
11) Followup period (YF, prospective studies): whole study (coded as 0) or longest available
12) LCtype: squamous or nearest available, but not adeno. (q = squamous, s = small,
a = adeno, KI = Kreyberg I, u = undifferentiated)
13) Race: all or nearest available, otherwise by race (wh or w = white, bl or b = black, hi = hispanic
ch = chinese, jap = japanese, haw = hawaiian, w+o = white + oriental, sca = scandinavian, as = asian)
14) Years quit (vs current) "mid" in key scheme 1 (key value 7, maximum range 4-11)
15) For overlapping studies: principal rather than subsidiary studies
Finally by Age: whole study (coded as 0) if available, otherwise by widest available age group
and then for single sex results (m, f) in preference to results for both sexes combined (c).

Results adjusted (AD) for the most potential confounders are then chosen in Sections -1 to -3
and results adjusted for the least confounders in Sections -4 to -6. (Those least adjusted results which
actually differ from the most adjusted are marked 'x' in column X in Section -4)

Section -7 shows excluded studies, together with the stage (as above) at which no qualifying
results were found.

Section -8 lists the potentially overlapping studies which have been included (1=principal, 2=subsidiary).

Section -9 lists any results which would have been included in preference except that they had data not complete
enough for use in meta-analysis, with their significance (yes/no), if known, and any further comment as entered
on the database. It also lists as "gap" any categories for which no data were presented by the original authors.

In addition to those mentioned above, the following fields, levels and abbreviations are used:
* or nk = not known, n = no, y = yes, ot = other
nev = never
all/unspec = all or unspecified, cig+/-ot = cigarettes irrespective of other products (cigar, pipe etc)
MC = manufactured cigarettes, HR = hand-rolled cigarettes
exL, exH = range of exposure (low and high) in the smoking group, in terms of Years quit (vs current)
REF: 6-character study reference
NRR: number of the RR on the database within the study
ST : study type (CC = case control, pr or prosp = prospective)
NLC: number of lung cancer cases in whole study
R : risky occupational population (n = no, m = mining, o = other risky)
VB : national cigarette type (V = at least 75% Virginia, bl = at least 75% blended, ot = other)
P : any proxy use
H : full histological confirmation
De : derivation of RR/CI (or = original, st = standard method, ot = other method of estimation)

Table 2K3 - 1

IESLC - Meta-analysis of Ex Smoking, Years quit (vs current), "Mid"
Squamous, Any Product (or Cigarettes if Any not available)
Most adjusted

REF	NRR	SEX	AGEL	AGEH	RACE	YF	LC TYPE	LOC	START	ST	NLC	R	VB	P	H	AD	ADOS	PRODUCT	exL	exH	DENOM	De
JAHN	608	m	0	0	all	-	q	Eu:Ger	1988	CC	1004	n	bl	n	n	0	0	cig+/-ot	6	10	current	st
LUBIN2	776	m	0	0	all	-	q	Eu:mul	1976	CC	7804	n	bl	n	y	0	0	cig+/-ot	5	9	current	st
LUBIN2	909	f	0	0	all	-	q	Eu:mul	1976	CC	7804	n	bl	n	y	2	1#	cig+/-ot	5	9	current	ot
MATOS	637	m	0	0	all	-	q	SCAmer	1994	CC	200	n	bl	n	n	2	0	cig+/-ot	6	10	cur+1y	or
SOBUE	740	m	0	0	all	-	q	As:Jap	1986	CC	1376	n	bl	n	y	0	0	cig+/-ot	5	9	cur+1y	st

Comments on values in listings

LUBIN2 ADOS Duration of smoking

Cigarette type is all/unspec for all RRs

Table 2K3 - 2

IESLC - Meta-analysis of Ex Smoking, Years quit (vs current), "Mid"
Squamous, Any Product (or Cigarettes if Any not available)
Most adjusted

REF	NRR	SEX	AD	Number Exposed Case	Cont	Non-exposed Case	Cont	RR	95.00%CI
JAHN	608	m	0	29	63	153	269	0.81	(0.50- 1.31)
LUBIN2	776	m	0	265	882	2518	6209	0.74	(0.64- 0.86)
LUBIN2	909	f	2	13	-	154	-	0.90	(0.47- 1.73)
Subtotal LUBIN2								0.75	(0.65- 0.86)
MATOS	637	m	2	5	-	33	-	0.60	(0.20- 1.90)
SOBUE	740	m	0	32	92	292	633	0.75	(0.49- 1.15)
Partial Totals				344	1037	3150	7111		

*prospective study

REF	NRR	SEX	AD	Ys	Ws	Qs	Ps
JAHN	608	m	0	-0.21	16.50	0.09	0.3901
LUBIN2	776	m	0	-0.30	182.96	0.03	0.0000
LUBIN2	909	f	2	-0.11	9.05	0.30	0.7513
Subtotal LUBIN2				-0.29	192.01	0.33	
MATOS	637	m	2	-0.51	3.03	0.15	0.3738
SOBUE	740	m	0	-0.28	21.22	0.00	0.1934

	N	5
	NS	4
	Wt	232.76
Het	Chi	0.58
Het	df	4
Het	P	N.S.
Fixed	RR	0.75
	RRl	0.66
	RRu	0.85
	P	---
Random	RR	0.75
	RRl	0.66
	RRu	0.85
	P	---
Asymm	P	N.S.

Table 2K3 - 3

IESLC - Meta-analysis of Ex Smoking, Years quit (vs current), "Mid"
Squamous, Any Product (or Cigarettes if Any not available)
Most adjusted

	combined	Sex male	female	Total
N		4	1	5
NS		4	1	5
Wt		223.72	9.05	232.76
Het Chi		0.26	0.00	0.58
Het df		3	0	4
Het P		N.S.	N.S.	N.S.
Fixed RR		0.74	0.90	0.75
RRl		0.65	0.47	0.66
RRu		0.85	1.73	0.85
P		---	N.S.	---
Random RR		0.74	0.90	0.75
RRl		0.65	0.47	0.66
RRu		0.85	1.73	0.85
P		---	N.S.	---
Between Chi				0.31
Between df				1
Between P				N.S.
Btwn(F) P				N.S.
Btwn(R) P				N.S.

Too few RRs for analysis by factor

Table 2K3 - 4

IESLC - Meta-analysis of Ex Smoking, Years quit (vs current), "Mid"
Squamous, Any Product (or Cigarettes if Any not available)
Least adjusted

REF	NRR	X	SEX	AGEL	AGEH	RACE	YF	LC TYPE	LOC	START	ST	NLC	R	VB	P	H	AD	ADOS	PRODUCT	exL	exH	DENOM	De
JAHN	608		m	0	0	all	-	q	Eu:Ger	1988	CC	1004	n	bl	n	n	0	0	cig+/-ot	6	10	current	st
LUBIN2	776		m	0	0	all	-	q	Eu:mul	1976	CC	7804	n	bl	n	y	0	0	cig+/-ot	5	9	current	st
LUBIN2	909		f	0	0	all	-	q	Eu:mul	1976	CC	7804	n	bl	n	y	2	1#	cig+/-ot	5	9	current	ot
MATOS	627	x	m	0	0	all	-	q	SCAmer	1994	CC	200	n	bl	n	n	0	0	cig+/-ot	6	10	cur+1y	st
SOBUE	740		m	0	0	all	-	q	As:Jap	1986	CC	1376	n	bl	n	y	0	0	cig+/-ot	5	9	cur+1y	st

Comments on values in listings

LUBIN2 ADOS Duration of smoking

Cigarette type is all/unspec for all RRs

Table 2K3 - 5

IESLC - Meta-analysis of Ex Smoking, Years quit (vs current), "Mid"
Squamous, Any Product (or Cigarettes if Any not available)
Least adjusted

REF	NRR	SEX	A	Number Exposed Case	Cont	Non-exposed Case	Cont	RR	95.00%CI
JAHN	608	m	0	29	63	153	269	0.81	(0.50- 1.31)
LUBIN2	776	m	0	265	882	2518	6209	0.74	(0.64- 0.86)
LUBIN2	909	f	2	13	-	154	-	0.90	(0.47- 1.73)
Subtotal LUBIN2								0.75	(0.65- 0.86)
MATOS	627	m	0	5	27	33	132	0.74	(0.27- 2.07)
SOBUE	740	m	0	32	92	292	633	0.75	(0.49- 1.15)
Partial Totals				344	1064	3150	7243		

*prospective study

REF	NRR	SEX	AD	Ys	Ws	Qs	Ps
JAHN	608	m	0	-0.21	16.50	0.09	0.3901
LUBIN2	776	m	0	-0.30	182.96	0.04	0.0000
LUBIN2	909	f	2	-0.11	9.05	0.29	0.7513
Subtotal LUBIN2				-0.29	192.01	0.33	
MATOS	627	m	0	-0.30	3.64	0.00	0.5671
SOBUE	740	m	0	-0.28	21.22	0.00	0.1934

	N	5
	NS	4
	Wt	233.37
Het	Chi	0.42
Het	df	4
Het	P	N.S.
Fixed	RR	0.75
	RRl	0.66
	RRu	0.86
	P	---
Random	RR	0.75
	RRl	0.66
	RRu	0.86
	P	---
Asymm	P	N.S.

Table 2K3 - 6

IESLC - Meta-analysis of Ex Smoking, Years quit (vs current), "Mid"
Squamous, Any Product (or Cigarettes if Any not available)
Least adjusted

	Sex combined	male	female	Total
N		4	1	5
NS		4	1	5
Wt		224.32	9.05	233.37
Het Chi		0.12	0.00	0.42
Het df		3	0	4
Het P		N.S.	N.S.	N.S.
Fixed RR		0.75	0.90	0.75
RRl		0.66	0.47	0.66
RRu		0.85	1.73	0.86
P		---	N.S.	---
Random RR		0.75	0.90	0.75
RRl		0.66	0.47	0.66
RRu		0.85	1.73	0.86
P		---	N.S.	---
Between Chi				0.30
Between df				1
Between P				N.S.
Btwn(F) P				(*)
Btwn(R) P				N.S.

Table 2K3 - 7

IESLC - Meta-analysis of Ex Smoking, Years quit (vs current), "Mid"
Squamous, Any Product (or Cigarettes if Any not available)
Excluded studies (and stage at which they were excluded)

1	AGUDO AKIBA AMANDU AMES AXELSS BEST BOUCHA BOUCOT BRESLO CHEN CHEN2 CHIAZZ DEAN2 DOSEME ENGELA FAN GENG GER GUO HAENSZ HEGMAN HOLE HU HU2 JUSSAW KATSOU KAUFMA KOO KOULUM KREUZE LETOUR LEVIN LIAW LIU3 LIU4 LIU5 MCCONN MIGRAN MRFITR NOTAN2 OSANN2 PERNU QIAO2 RACHTA RESTRE SADOWS SEGI2 STASZE TIZZAN VUTUC WATSON WU WUWILL WYNDE2 WYNDE8 XU YUAN ZHANG ZHENG ZHOU
2	AUVINE BENSHL BLOT1 BROWN3 BUFFLE GURSEL LAUSSM LUO MCDUFF PISANI PRESCO SPITZ WU2 WYNDE7
4	ARMADA BECHER BOFFET BROSS CARPEN CEDERL CHOI CHYOU CORREA CPSI CPSII DAMBER DARBY DEAN3 DESTEF DOLL DOLL2 DORGAN DORN GAO GAO2 GARCIA GARSHI GILLIS GRAHAM HAMMO2 HIRAYA HUMBLE JOLY KAISE2 KHUDER LUBIN PEZZO2 QIAO SPEIZE SUZUK2 TVERDA WANG2 WIGLE
5	ALDERS HAMMON
10	JEDRYC WAKAI WYNDE6
14	BARBON JAIN PEZZOT SVENSS WYNDE3
15	BENHAM

Table 2K3 - 8
Potentially overlapping studies

REF	REFGP	PRINC	OVERLAP/LINK
LUBIN2	LUBIN2	1	Lubin-combined
JAHN	BOFFET	2	Subset of BOFFET

Table 2K4 -

IESLC - Meta-analysis of Ex Smoking, Years quit (vs current), "High"
Squamous, Any Product (or Cigarettes if Any not available)

This analysis is restricted to results for:
1) Ex smokers
2) Results by Years quit (vs current)
3) Categorical results by Years quit (vs current)
4) Squamous (or near equivalent)
5) Results complete enough for use in metaanalysis

Within each study, results are then selected (in the following order of preference, within each sex) for:
6) PRODUCT: all/unspec, cigarettes regardless of other products, cigarettes only
7) CIGTYPE: all/unspecified, MC regardless of HR, MC only
8) Results with least adjustment for other aspects of smoking (ADOS)
9) DENOM: current smokers, current + recent smokers (up to number of m=months or y=years, max 2 years)
10) Followup period (YF, prospective studies): whole study (coded as 0) or longest available
11) LCtype: squamous or nearest available, but not adeno. (q = squamous, s = small,
a = adeno, KI = Kreyberg I, u = undifferentiated)
12) Race: all or nearest available, otherwise by race (wh or w = white, bl or b = black, hi = hispanic
ch = chinese, jap = japanese, haw = hawaiian, w+o = white + oriental, sca = scandinavian, as = asian)
13) Years quit (vs current) "high" in key scheme 1 (key value 12, maximum range 8+)
14) For overlapping studies: principal rather than subsidiary studies
Finally by Age: whole study (coded as 0) if available, otherwise by widest available age group
and then for single sex results (m, f) in preference to results for both sexes combined (c).

Results adjusted (AD) for the most potential confounders are then chosen in Sections -1 to -3
and results adjusted for the least confounders in Sections -4 to -6. (Those least adjusted results which
actually differ from the most adjusted are marked 'x' in column X in Section -4)

Section -7 shows excluded studies, together with the stage (as above) at which no qualifying
results were found.

Section -8 lists the potentially overlapping studies which have been included (1=principal, 2=subsidiary).

Section -9 lists any results which would have been included in preference except that they had data not complete
enough for use in meta-analysis, with their significance (yes/no), if known, and any further comment as entered
on the database. It also lists as "gap" any categories for which no data were presented by the original authors.

In addition to those mentioned above, the following fields, levels and abbreviations are used:
* or nk = not known, n = no, y = yes, ot = other
nev = never
all/unspec = all or unspecified, cig+/-ot = cigarettes irrespective of other products (cigar, pipe etc)
MC = manufactured cigarettes, HR = hand-rolled cigarettes
exL, exH = range of exposure (low and high) in the smoking group, in terms of Years quit (vs current)
REF: 6-character study reference
NRR: number of the RR on the database within the study
ST : study type (CC = case control, pr or prosp = prospective)
NLC: number of lung cancer cases in whole study
R : risky occupational population (n = no, m = mining, o = other risky)
VB : national cigarette type (V = at least 75% Virginia, bl = at least 75% blended, ot = other)
P : any proxy use
H : full histological confirmation
De : derivation of RR/CI (or = original, st = standard method, ot = other method of estimation)

Table 2K4 - 1

IESLC - Meta-analysis of Ex Smoking, Years quit (vs current), "High"
Squamous, Any Product (or Cigarettes if Any not available)
Most adjusted

REF	NRR	SEX	AGEL	AGEH	RACE	YF	LC TYPE	LOC	START	ST	NLC	R	VB	P	H	AD	ADOS	PRODUCT	exL	exH	DENOM	De
JAHN	609	m	0	0	all	-	q	Eu:Ger	1988	CC	1004	n	bl	n	n	0	0	cig+/-ot	11	20	current	st
JAIN	547	m	0	0	all	-	q	NAmer	1981	CC	845	n	V	y	n	0	0	cig+/-ot	10	999	cur+2y	st
JAIN	511	f	0	0	all	-	q	NAmer	1981	CC	845	n	V	y	n	0	0	cig+/-ot	10	999	cur+2y	st
LUBIN2	777	m	0	0	all	-	q	Eu:mul	1976	CC	7804	n	bl	n	y	0	0	cig+/-ot	10	14	current	st
LUBIN2	900	f	0	0	all	-	q	Eu:mul	1976	CC	7804	n	bl	n	y	0	0	cig+/-ot	10	19	current	st
MATOS	638	m	0	0	all	-	q	SCAmer	1994	CC	200	n	bl	n	n	2	0	cig+/-ot	11	999	cur+1y	or
PEZZOT	583	m	0	0	all	-	q	SCAmer	1987	CC	215	n	bl	n	y	0	0	cig only	11	999	cur+1y	st
SOBUE	741	m	0	0	all	-	q	As:Jap	1986	CC	1376	n	bl	n	y	0	0	cig+/-ot	10	999	cur+1y	st
SVENSS	561	f	0	0	all	-	q	Eu:Sca	1983	CC	210	n	bl	n	n	0	0	all/unsp	11	999	cur+2y	st

Cigarette type is all/unspec for all RRs

Table 2K4 - 2

IESLC - Meta-analysis of Ex Smoking, Years quit (vs current), "High"
Squamous, Any Product (or Cigarettes if Any not available)
Most adjusted

REF	NRR	SEX	A	Number Exposed Case	Cont	Non-exposed Case	Cont	RR	95.00%CI
JAHN	609	m	0	18	130	153	269	0.24	(0.14- 0.41)
JAIN	547	m	0	23	113	107	118	0.22	(0.13- 0.38)
JAIN	511	f	0	7	61	81	99	0.14	(0.06- 0.32)
Subtotal JAIN								0.20	(0.13- 0.31)
LUBIN2	777	m	0	146	693	2518	6209	0.52	(0.43- 0.62)
LUBIN2	900	f	0	5	33	154	410	0.40	(0.15- 1.05)
Subtotal LUBIN2								0.51	(0.43- 0.62)
MATOS	638	m	2	5	-	33	-	0.20	(0.06- 0.50)
PEZZOT	583	m	0	8	48	56	52	0.15	(0.07- 0.36)
SOBUE	741	m	0	30	144	292	633	0.45	(0.30- 0.69)
SVENSS	561	f	0	1	24	42	53	0.05	(0.01- 0.40)
Partial Totals				243	1246	3436	7843		

*prospective study

REF	NRR	SEX	AD	Ys	Ws	Qs	Ps
JAHN	609	m	0	-1.41	13.61	3.51	0.0000
JAIN	547	m	0	-1.49	14.26	4.95	0.0000
JAIN	511	f	0	-1.96	5.50	6.18	0.0000
Subtotal JAIN				-1.63	19.76	11.12	
LUBIN2	777	m	0	-0.65	112.99	7.07	0.0000
LUBIN2	900	f	0	-0.91	4.18	0.00	0.0634
Subtotal LUBIN2				-0.66	117.17	7.07	
MATOS	638	m	2	-1.61	3.42	1.70	0.0029
PEZZOT	583	m	0	-1.87	5.47	5.05	0.0000
SOBUE	741	m	0	-0.79	22.08	0.27	0.0002
SVENSS	561	f	0	-2.95	0.92	3.84	0.0047

	N	9
	NS	7
	Wt	182.42
Het	Chi	32.55
Het	df	8
Het	P	***
Fixed	RR	0.40
	RRl	0.35
	RRu	0.47
	P	---
Random	RR	0.27
	RRl	0.18
	RRu	0.40
	P	---
Asymm	P	**

Table 2K4 - 3

IESLC - Meta-analysis of Ex Smoking, Years quit (vs current), "High"
Squamous, Any Product (or Cigarettes if Any not available)
Most adjusted

	Sex combined	male	female	Total
N		6	3	9
NS		6	3	9
Wt		171.82	10.61	182.42
Het Chi		22.19	4.39	32.55
Het df		5	2	8
Het P		***	N.S.	***
Fixed RR		0.42	0.20	0.40
RRl		0.36	0.11	0.35
RRu		0.49	0.36	0.47
P		---	---	---
Random RR		0.30	0.18	0.27
RRl		0.20	0.07	0.18
RRu		0.46	0.48	0.40
P		---	---	---
Between Chi				5.97
Between df				1
Between P				*
Btwn(F) P				N.S.
Btwn(R) P				N.S.

Too few RRs for analysis by factor

Table 2K4 - 4

IESLC - Meta-analysis of Ex Smoking, Years quit (vs current), "High"
Squamous, Any Product (or Cigarettes if Any not available)
Least adjusted

REF	NRR	X	SEX	AGEL	AGEH	RACE	YF	LC TYPE	LOC	START	ST	NLC	R	VB	P	H	AD	ADOS	PRODUCT	exL	exH	DENOM	De
JAHN	609		m	0	0	all	-	q	Eu:Ger	1988	CC	1004	n	bl	n	n	0	0	cig+/-ot	11	20	current	st
JAIN	547		m	0	0	all	-	q	NAmer	1981	CC	845	n	V	y	n	0	0	cig+/-ot	10	999	cur+2y	st
JAIN	511		f	0	0	all	-	q	NAmer	1981	CC	845	n	V	y	n	0	0	cig+/-ot	10	999	cur+2y	st
LUBIN2	777		m	0	0	all	-	q	Eu:mul	1976	CC	7804	n	bl	n	y	0	0	cig+/-ot	10	14	current	st
LUBIN2	900		f	0	0	all	-	q	Eu:mul	1976	CC	7804	n	bl	n	y	0	0	cig+/-ot	10	19	current	st
MATOS	628	x	m	0	0	all	-	q	SCAmer	1994	CC	200	n	bl	n	n	0	0	cig+/-ot	11	999	cur+1y	st
PEZZOT	583		m	0	0	all	-	q	SCAmer	1987	CC	215	n	bl	n	y	0	0	cig only	11	999	cur+1y	st
SOBUE	741		m	0	0	all	-	q	As:Jap	1986	CC	1376	n	bl	n	y	0	0	cig+/-ot	10	999	cur+1y	st
SVENSS	561		f	0	0	all	-	q	Eu:Sca	1983	CC	210	n	bl	n	n	0	0	all/unsp	11	999	cur+2y	st

Cigarette type is all/unspec for all RRs

Table 2K4 - 5

IESLC - Meta-analysis of Ex Smoking, Years quit (vs current), "High"
Squamous, Any Product (or Cigarettes if Any not available)
Least adjusted

REF	NRR	SEX	A	Number Exposed Case	Cont	Non-exposed Case	Cont	RR	95.00%CI
JAHN	609	m	0	18	130	153	269	0.24	(0.14- 0.41)
JAIN	547	m	0	23	113	107	118	0.22	(0.13- 0.38)
JAIN	511	f	0	7	61	81	99	0.14	(0.06- 0.32)
Subtotal JAIN								0.20	(0.13- 0.31)
LUBIN2	777	m	0	146	693	2518	6209	0.52	(0.43- 0.62)
LUBIN2	900	f	0	5	33	154	410	0.40	(0.15- 1.05)
Subtotal LUBIN2								0.51	(0.43- 0.62)
MATOS	628	m	0	5	101	33	132	0.20	(0.07- 0.53)
PEZZOT	583	m	0	8	48	56	52	0.15	(0.07- 0.36)
SOBUE	741	m	0	30	144	292	633	0.45	(0.30- 0.69)
SVENSS	561	f	0	1	24	42	53	0.05	(0.01- 0.40)
Totals				243	1347	3436	7975		

*prospective study

REF	NRR	SEX	AD	Ys	Ws	Qs	Ps
JAHN	609	m	0	-1.41	13.61	3.47	0.0000
JAIN	547	m	0	-1.49	14.26	4.90	0.0000
JAIN	511	f	0	-1.96	5.50	6.15	0.0000
Subtotal JAIN				-1.63	19.76	11.05	
LUBIN2	777	m	0	-0.65	112.99	7.22	0.0000
LUBIN2	900	f	0	-0.91	4.18	0.00	0.0634
Subtotal LUBIN2				-0.66	117.17	7.22	
MATOS	628	m	0	-1.62	4.04	2.04	0.0011
PEZZOT	583	m	0	-1.87	5.47	5.02	0.0000
SOBUE	741	m	0	-0.79	22.08	0.28	0.0002
SVENSS	561	f	0	-2.95	0.92	3.83	0.0047

	N	9
	NS	7
	Wt	183.04
Het	Chi	32.91
Het	df	8
Het	P	***
Fixed	RR	0.40
	RRl	0.35
	RRu	0.47
	P	---
Random	RR	0.27
	RRl	0.18
	RRu	0.40
	P	---
Asymm	P	**

Table 2K4 - 6

IESLC - Meta-analysis of Ex Smoking, Years quit (vs current), "High"
Squamous, Any Product (or Cigarettes if Any not available)
Least adjusted

Sex combined	male	female	Total
N	6	3	9
NS	6	3	9
Wt	172.43	10.61	183.04
Het Chi	22.59	4.39	32.91
Het df	5	2	8
Het P	***	N.S.	***
Fixed RR	0.42	0.20	0.40
RRl	0.36	0.11	0.35
RRu	0.49	0.36	0.47
P	---	---	---
Random RR	0.30	0.18	0.27
RRl	0.20	0.07	0.18
RRu	0.45	0.48	0.40
P	---	---	---
Between Chi			5.93
Between df			1
Between P			*
Btwn(F) P			N.S.
Btwn(R) P			N.S.

Table 2K4 - 7

IESLC - Meta-analysis of Ex Smoking, Years quit (vs current), "High"
Squamous, Any Product (or Cigarettes if Any not available)
Excluded studies (and stage at which they were excluded)

1	AGUDO AKIBA AMANDU AMES AXELSS BEST BOUCHA BOUCOT BRESLO CHEN CHEN2 CHIAZZ DEAN2 DOSEME ENGELA FAN GENG GER GUO HAENSZ HEGMAN HOLE HU HU2 JUSSAW KATSOU KAUFMA KOO KOULUM KREUZE LETOUR LEVIN LIAW LIU3 LIU4 LIU5 MCCONN MIGRAN MRFITR NOTAN2 OSANN2 PERNU QIAO2 RACHTA RESTRE SADOWS SEGI2 STASZE TIZZAN VUTUC WATSON WU WUWILL WYNDE2 WYNDE8 XU YUAN ZHANG ZHENG ZHOU
2	AUVINE BENSHL BLOT1 BROWN3 BUFFLE GURSEL LAUSSM LUO MCDUFF PISANI PRESCO SPITZ WU2 WYNDE7
4	ARMADA BECHER BOFFET BROSS CARPEN CEDERL CHOI CHYOU CORREA CPSI CPSII DAMBER DARBY DEAN3 DESTEF DOLL DOLL2 DORGAN DORN GAO GAO2 GARCIA GARSHI GILLIS GRAHAM HAMMO2 HIRAYA HUMBLE JOLY KAISE2 KHUDER LUBIN PEZZO2 QIAO SPEIZE SUZUK2 TVERDA WANG2 WIGLE
5	ALDERS HAMMON
10	JEDRYC WAKAI WYNDE6
14	BARBON WYNDE3
15	BENHAM

Table 2K4 - 8
Potentially overlapping studies

REF	REFGP	PRINC	. OVERLAP/LINK
LUBIN2	LUBIN2	1	Lubin-combined
JAHN	BOFFET	2	Subset of BOFFET

Table 2K4 - 9
Most adjusted - insufficient data for meta-analysis

REF	NRR	SEX	AGEL	AGEH	RACE	YF	LC TYPE	LOC	START	ST	NLC	R	VB	P	H	AD	ADOS	PRODUCT	exL	exH	DENOM	De
ALDERS	545	m	0	0	all	-	q+s	Eu:UK	1977	CC	1448	n	V	n	n	1	0	cig only	10	999	current	ot
ALDERS	556	f	0	0	all	-	q+s	Eu:UK	1977	CC	1448	n	V	n	n	1	0	cig only	10	999	current	ot
HAMMON	509	m	0	0	wh	0	not a	NAmer	1952	pr	448	n	bl	n	n	1	0	cig only	10	999	current	st

REF	NRR	RR	SIG	RRDATA comment
ALDERS	545	0.21	y	0.001<p<0.01
ALDERS	556	0.12	y	p<0.001
HAMMON	509	*		RR for <1 pack per day is 0.14, while that for 1+ packs is 0.39

Table 2K5 -

IESLC - Meta-analysis of Ex Smoking, Years quit (vs current), "Highest vs lowest"
Squamous, Any Product (or Cigarettes if Any not available)

This analysis is restricted to results for:
1) Ex smokers
2) Results by Years quit (vs current)
3) Categorical results by Years quit (vs current)
4) Denominator (unexposed) = "low"
5) Squamous (or near equivalent)
6) Results complete enough for use in metaanalysis

Within each study, results are then selected (in the following order of preference, within each sex) for:
7) (not applicable)
8) PRODUCT: all/unspec, cigarettes regardless of other products, cigarettes only
9) CIGTYPE: all/unspecified, MC regardless of HR, MC only
10) Results with least adjustment for other aspects of smoking (ADOS)
11) The highest vs lowest category
12) Followup period (YF, prospective studies): whole study (coded as 0) or longest available
13) LCtype: squamous or nearest available, but not adeno. (q = squamous, s = small,
a = adeno, KI = Kreyberg I, u = undifferentiated)
14) Race: all or nearest available, otherwise by race (wh or w = white, bl or b = black, hi = hispanic
ch = chinese, jap = japanese, haw = hawaiian, w+o = white + oriental, sca = scandinavian, as = asian)
15) For overlapping studies: principal rather than subsidiary studies
Finally by Age: whole study (coded as 0) if available, otherwise by widest available age group
and then for single sex results (m, f) in preference to results for both sexes combined (c).

Results adjusted (AD) for the most potential confounders are then chosen in Sections -1 to -3
and results adjusted for the least confounders in Sections -4 to -6. (Those least adjusted results which
actually differ from the most adjusted are marked 'x' in column X in Section -4)

Section -7 shows excluded studies, together with the stage (as above) at which no qualifying
results were found.

Section -8 lists the potentially overlapping studies which have been included (1=principal, 2=subsidiary).

Section -9 lists any results which would have been included in preference except that they had data not complete
enough for use in meta-analysis, with their significance (yes/no), if known, and any further comment as entered
on the database. It also lists as "gap" any categories for which no data were presented by the original authors.

In addition to those mentioned above, the following fields, levels and abbreviations are used:
* or nk = not known, n = no, y = yes, ot = other
all/unspec = all or unspecified, cig+/-ot = cigarettes irrespective of other products (cigar, pipe etc)
MC = manufactured cigarettes, HR = hand-rolled cigarettes
exL, exH = range of exposure (low and high) in the "highest" group, in terms of Years quit (vs current)
unexL, unexH = range of exposure (low and high) in the "lowest" group, in terms of Years quit (vs current)
REF: 6-character study reference
NRR: number of the RR on the database within the study
ST : study type (CC = case control, pr or prosp = prospective)
NLC: number of lung cancer cases in whole study
R : risky occupational population (n = no, m = mining, o = other risky)
VB : national cigarette type (V = at least 75% Virginia, bl = at least 75% blended, ot = other)
P : any proxy use
H : full histological confirmation
De : derivation of RR/CI (or = original, st = standard method, ot = other method of estimation)

Table 2K5 - 1

IESLC - Meta-analysis of Ex Smoking, Years quit (vs current), "Highest vs lowest"
Squamous, Any Product (or Cigarettes if Any not available)
Most adjusted

REF	NRR	SEX	AGEL	AGEH	RACE	YF	LC TYPE	LOC	START	ST	NLC	R	VB	P	H	AD	ADOS	PRODUCT	exL	exH	unexL	unexH	De
BARBON	608	m	0	0	all	-	q	Eu:wst	1979	CC	755	n	bl	y	y	1	0	all/unsp	25	999	0.1	4	ot
JAHN	615	m	0	0	all	-	q	Eu:Ger	1988	CC	1004	n	bl	n	n	0	0	cig+/-ot	21	999	0.1	0.9	st
JAIN	548	m	0	0	all	-	q	NAmer	1981	CC	845	n	V	y	n	0	0	cig+/-ot	10	999	2	9	st
JAIN	512	f	0	0	all	-	q	NAmer	1981	CC	845	n	V	y	n	0	0	cig+/-ot	10	999	2	9	st
LUBIN2	783	m	0	0	all	-	q	Eu:mul	1976	CC	7804	n	bl	n	y	0	0	cig+/-ot	20	999	0.1	4	st
LUBIN2	903	f	0	0	all	-	q	Eu:mul	1976	CC	7804	n	bl	n	y	0	0	cig+/-ot	20	999	0.1	9	st
MATOS	640	m	0	0	all	-	q	SCAmer	1994	CC	200	n	bl	n	n	2	0	cig+/-ot	11	999	1.0	5	ot
PEZZOT	584	m	0	0	all	-	q	SCAmer	1987	CC	215	n	bl	n	y	0	0	cig only	11	999	1.0	10	st
SOBUE	743	m	0	0	all	-	q	As:Jap	1986	CC	1376	n	bl	n	y	0	0	cig+/-ot	10	999	1.0	4	st
SVENSS	562	f	0	0	all	-	q	Eu:Sca	1983	CC	210	n	bl	n	n	0	0	all/unsp	11	999	3	10	st
WYNDE3	517	m	0	0	all	-	KI	NAmer	1966	CC	350	n	bl	n	y	0	0	all/unsp	13	999	1.0	3	st
WYNDE6	801	m	0	0	all	-	KI	NAmer	1969	CC	4423	n	bl	n	y	2	0	cig+/-ot	16	999	1.0	3	ot

Cigarette type is all/unspec for all RRs

Table 2K5 - 2

IESLC - Meta-analysis of Ex Smoking, Years quit (vs current), "Highest vs lowest"
Squamous, Any Product (or Cigarettes if Any not available)
Most adjusted

REF	NRR	SEX	AD	Number Exposed Case	Number Exposed Cont	Non-exposed Case	Non-exposed Cont	RR	95.00%CI
BARBON	608	m	1	4	-	11	-	0.10	(0.03- 0.37)
JAHN	615	m	0	8	146	74	8	0.01	(0.00- 0.02)
JAIN	548	m	0	23	113	24	46	0.39	(0.20- 0.76)
JAIN	512	f	0	7	61	15	36	0.28	(0.10- 0.74)
Subtotal JAIN								0.35	(0.20- 0.61)
LUBIN2	783	m	0	106	1128	498	1047	0.20	(0.16- 0.25)
LUBIN2	903	f	0	2	29	38	95	0.17	(0.04- 0.76)
Subtotal LUBIN2								0.20	(0.16- 0.25)
MATOS	640	m	2	5	-	4	-	0.29	(0.06- 1.27)
PEZZOT	584	m	0	8	48	21	27	0.21	(0.08- 0.55)
SOBUE	743	m	0	30	144	52	116	0.46	(0.28- 0.78)
SVENSS	562	f	0	1	24	5	13	0.11	(0.01- 1.03)
WYNDE3	517	m	0	2	55	18	22	0.04	(0.01- 0.21)
WYNDE6	801	m	2	19	-	80	-	0.09	(0.05- 0.15)
Partial Totals				215	1748	840	1410		

*prospective study

REF	NRR	SEX	AD	Ys	Ws	Qs	Ps
BARBON	608	m	1	-2.30	2.43	0.96	0.0003
JAHN	615	m	0	-5.13	3.70	44.12	0.0000
JAIN	548	m	0	-0.94	8.64	4.65	0.0057
JAIN	512	f	0	-1.29	3.94	0.59	0.0105
Subtotal JAIN				-1.05	12.58	5.24	
LUBIN2	783	m	0	-1.62	75.28	0.21	0.0000
LUBIN2	903	f	0	-1.76	1.75	0.01	0.0200
Subtotal LUBIN2				-1.62	77.03	0.23	
MATOS	640	m	2	-1.24	1.65	0.32	0.1119
PEZZOT	584	m	0	-1.54	4.34	0.08	0.0013
SOBUE	743	m	0	-0.77	14.68	12.12	0.0033
SVENSS	562	f	0	-2.22	0.76	0.23	0.0529
WYNDE3	517	m	0	-3.11	1.62	3.34	0.0001
WYNDE6	801	m	2	-2.41	12.73	6.84	0.0000

	N	12
	NS	10
	Wt	131.52
Het	Chi	73.47
Het	df	11
Het	P	***
Fixed	RR	0.19
	RRl	0.16
	RRu	0.22
	P	---
Random	RR	0.14
	RRl	0.08
	RRu	0.25
	P	---
Asymm	P	N.S.

Table 2K5 - 3

IESLC - Meta-analysis of Ex Smoking, Years quit (vs current), "Highest vs lowest"
Squamous, Any Product (or Cigarettes if Any not available)
Most adjusted

	Sex			
	combined	male	female	Total
N		9	3	12
NS		9	3	12
Wt		125.07	6.45	131.52
Het Chi		72.63	0.68	73.47
Het df		8	2	11
Het P		***	N.S.	***
Fixed RR		0.19	0.22	0.19
RRl		0.16	0.10	0.16
RRu		0.22	0.47	0.22
P		---	---	---
Random RR		0.13	0.22	0.14
RRl		0.07	0.10	0.08
RRu		0.25	0.47	0.25
P		---	---	---
Between Chi				0.15
Between df				1
Between P				N.S.
Btwn(F) P				N.S.
Btwn(R) P				N.S.

	Lung cancer type					
	q	q+s	q+u	KI	not a	Total
N	10			2		12
NS	8			2		10
Wt	117.17			14.35		131.52
Het Chi	62.13			0.71		73.47
Het df	9			1		11
Het P	***			N.S.		***
Fixed RR	0.21			0.08		0.19
RRl	0.17			0.05		0.16
RRu	0.25			0.14		0.22
P	---			---		---
Random RR	0.16			0.08		0.14
RRl	0.08			0.05		0.08
RRu	0.31			0.14		0.25
P	---			---		---
Between Chi						10.63
Between df						1
Between P						**
Btwn(F) P						N.S.
Btwn(R) P						N.S.

	Location								
	NAmer	UK	Scand	othEur	China	Japan	othAs	other	Total
N	4		1	4		1		2	12
NS	3		1	3		1		2	10
Wt	26.93		0.76	83.16		14.68		5.99	131.52
Het Chi	14.88		0.00	44.00		0.00		0.11	73.47
Het df	3		0	3		0		1	11
Het P	**		N.S.	***		N.S.		N.S.	***
Fixed RR	0.16		0.11	0.17		0.46		0.23	0.19
RRl	0.11		0.01	0.13		0.28		0.10	0.16
RRu	0.24		1.03	0.20		0.78		0.52	0.22
P	---		(-)	---		--		---	---
Random RR	0.16		0.11	0.07		0.46		0.23	0.14
RRl	0.06		0.01	0.01		0.28		0.10	0.08
RRu	0.40		1.03	0.37		0.78		0.52	0.25
P	---		(-)	--		--		---	---
Between Chi									14.48
Between df									4
Between P									**
Btwn(F) P									N.S.
Btwn(R) P									(*)

Table 2K5 - 3

IESLC - Meta-analysis of Ex Smoking, Years quit (vs current), "Highest vs lowest"
Squamous, Any Product (or Cigarettes if Any not available)
Most adjusted

Detailed Country in "other Europe"

	multi	Germany	othWest	East	Balkans	Total
N	2	1	1			4
NS	1	1	1			3
Wt	77.03	3.70	2.43			83.16
Het Chi	0.03	0.00	0.00			44.00
Het df	1	0	0			3
Het P	N.S.	N.S.	N.S.			***
Fixed RR	0.20	0.01	0.10			0.17
RRl	0.16	0.00	0.03			0.13
RRu	0.25	0.02	0.35			0.20
P	---	---	---			---
Random RR	0.20	0.01	0.10			0.07
RRl	0.16	0.00	0.03			0.01
RRu	0.25	0.02	0.35			0.37
P	---	---	---			--
Between Chi						43.97
Between df						2
Between P						***
Btwn(F) P						*
Btwn(R) P						***

Detailed Country in "other Asia"

	India	HongKong	other	Total
N				
NS				
Wt				
Het Chi				
Het df				
Het P				
Fixed RR				
RRl				
RRu				
P				
Random RR				
RRl				
RRu				
P				
Between Chi				
Between df				
Between P				N.S.
Btwn(F) P				N.S.
Btwn(R) P				N.S.

Detailed other continent

	SCAmer	Total
N	2	2
NS	2	2
Wt	5.99	5.99
Het Chi	0.11	0.11
Het df	1	1
Het P	N.S.	N.S.
Fixed RR	0.23	0.23
RRl	0.10	0.10
RRu	0.52	0.52
P	---	---
Random RR	0.23	0.23
RRl	0.10	0.10
RRu	0.52	0.52
P	---	---
Between Chi		
Between df		
Between P		N.S.
Btwn(F) P		N.S.
Btwn(R) P		N.S.

Table 2K5 - 3

IESLC - Meta-analysis of Ex Smoking, Years quit (vs current), "Highest vs lowest"
Squamous, Any Product (or Cigarettes if Any not available)
Most adjusted

	<1960	1960-69	1970-79	1980-89	1990+	Total
	Start year of study					
N		2	3	6	1	12
NS		2	2	5	1	10
Wt		14.35	79.47	36.06	1.65	131.52
Het Chi		0.71	1.12	59.74	0.00	73.47
Het df		1	2	5	0	11
Het P		N.S.	N.S.	***	N.S.	***
Fixed RR		0.08	0.19	0.24	0.29	0.19
RRl		0.05	0.15	0.17	0.06	0.16
RRu		0.14	0.24	0.33	1.33	0.22
P		---	---	---	N.S.	---
Random RR		0.08	0.19	0.14	0.29	0.14
RRl		0.05	0.15	0.04	0.06	0.08
RRu		0.14	0.24	0.50	1.33	0.25
P		---	---	--	N.S.	---
Between Chi						11.90
Between df						3
Between P						**
Btwn(F) P						N.S.
Btwn(R) P						*

	CC	other	Total
	Study type (1)		
N	12		12
NS	10		10
Wt	131.52		131.52
Het Chi	73.47		73.47
Het df	11		11
Het P	***		***
Fixed RR	0.19		0.19
RRl	0.16		0.16
RRu	0.22		0.22
P	---		---
Random RR	0.14		0.14
RRl	0.08		0.08
RRu	0.25		0.25
P	---		---
Between Chi			
Between df			
Between P			N.S.
Btwn(F) P			N.S.
Btwn(R) P			N.S.

	CC	prosp	other	Total
	Study type (2)			
N	12			12
NS	10			10
Wt	131.52			131.52
Het Chi	73.47			73.47
Het df	11			11
Het P	***			***
Fixed RR	0.19			0.19
RRl	0.16			0.16
RRu	0.22			0.22
P	---			---
Random RR	0.14			0.14
RRl	0.08			0.08
RRu	0.25			0.25
P	---			---
Between Chi				
Between df				
Between P				N.S.
Btwn(F) P				N.S.
Btwn(R) P				N.S.

Table 2K5 - 3

IESLC - Meta-analysis of Ex Smoking, Years quit (vs current), "Highest vs lowest"
Squamous, Any Product (or Cigarettes if Any not available)
Most adjusted

	Study size (number of LC cases)				
	100-249	250-499	500-999	1000+	Total
N	3	1	3	5	12
NS	3	1	2	4	10
Wt	6.75	1.62	15.02	108.14	131.52
Het Chi	0.50	0.00	3.53	63.09	73.47
Het df	2	0	2	4	11
Het P	N.S.	N.S.	N.S.	***	***
Fixed RR	0.21	0.04	0.29	0.18	0.19
RRl	0.10	0.01	0.17	0.15	0.16
RRu	0.45	0.21	0.47	0.22	0.22
P	---	---	---	---	---
Random RR	0.21	0.04	0.26	0.10	0.14
RRl	0.10	0.01	0.12	0.04	0.08
RRu	0.45	0.21	0.53	0.28	0.25
P	---	---	---	---	---
Between Chi					6.35
Between df					3
Between P					(*)
Btwn(F) P					N.S.
Btwn(R) P					N.S.

	Risky occupational population			
	no	mining	othRisky	Total
N	12			12
NS	10			10
Wt	131.52			131.52
Het Chi	73.47			73.47
Het df	11			11
Het P	***			***
Fixed RR	0.19			0.19
RRl	0.16			0.16
RRu	0.22			0.22
P	---			---
Random RR	0.14			0.14
RRl	0.08			0.08
RRu	0.25			0.25
P	---			---
Between Chi				
Between df				
Between P				N.S.
Btwn(F) P				N.S.
Btwn(R) P				N.S.

	National cigarette tobacco type			
	Virginia	blended	other	Total
N	2	10		12
NS	1	9		10
Wt	12.58	118.93		131.52
Het Chi	0.33	67.71		73.47
Het df	1	9		11
Het P	N.S.	***		***
Fixed RR	0.35	0.18		0.19
RRl	0.20	0.15		0.16
RRu	0.61	0.21		0.22
P	---	---		---
Random RR	0.35	0.11		0.14
RRl	0.20	0.06		0.08
RRu	0.61	0.23		0.25
P	---	---		---
Between Chi				5.43
Between df				1
Between P				*
Btwn(F) P				N.S.
Btwn(R) P				*

Table 2K5 - 3

IESLC - Meta-analysis of Ex Smoking, Years quit (vs current), "Highest vs lowest"
Squamous, Any Product (or Cigarettes if Any not available)
Most adjusted

	Any proxy use		
	No/nk	Yes	Total
N	9	3	12
NS	8	2	10
Wt	116.50	15.02	131.52
Het Chi	66.93	3.53	73.47
Het df	8	2	11
Het P	***	N.S.	***
Fixed RR	0.18	0.29	0.19
RRl	0.15	0.17	0.16
RRu	0.21	0.47	0.22
P	---	---	---
Random RR	0.12	0.26	0.14
RRl	0.06	0.12	0.08
RRu	0.24	0.53	0.25
P	---	---	---
Between Chi			3.01
Between df			1
Between P			(*)
Btwn(F) P			N.S.
Btwn(R) P			N.S.

	Full histological confirmation		
	No	Yes	Total
N	5	7	12
NS	4	6	10
Wt	18.69	112.83	131.52
Het Chi	48.76	23.38	73.47
Het df	4	6	11
Het P	***	***	***
Fixed RR	0.15	0.20	0.19
RRl	0.09	0.16	0.16
RRu	0.23	0.23	0.22
P	---	---	---
Random RR	0.11	0.17	0.14
RRl	0.02	0.10	0.08
RRu	0.65	0.28	0.25
P	-	---	---
Between Chi			1.33
Between df			1
Between P			N.S.
Btwn(F) P			N.S.
Btwn(R) P			N.S.

	Number of adjustment variables (1)			
	0	1	2+/+nk	Total
N	9	1	2	12
NS	7	1	2	10
Wt	114.70	2.43	14.38	131.52
Het Chi	64.46	0.00	2.00	73.47
Het df	8	0	1	11
Het P	***	N.S.	N.S.	***
Fixed RR	0.20	0.10	0.10	0.19
RRl	0.17	0.03	0.06	0.16
RRu	0.25	0.35	0.17	0.22
P	---	---	---	---
Random RR	0.14	0.10	0.13	0.14
RRl	0.07	0.03	0.04	0.08
RRu	0.29	0.35	0.37	0.25
P	---	---	---	---
Between Chi				7.01
Between df				2
Between P				*
Btwn(F) P				N.S.
Btwn(R) P				N.S.

Table 2K5 - 3

IESLC - Meta-analysis of Ex Smoking, Years quit (vs current), "Highest vs lowest"
Squamous, Any Product (or Cigarettes if Any not available)
Most adjusted

	Number of adjustment variables (2) 0	1	2	3-5	6+/+nk	Total
N	9	1	2			12
NS	7	1	2			10
Wt	114.70	2.43	14.38			131.52
Het Chi	64.46	0.00	2.00			73.47
Het df	8	0	1			11
Het P	***	N.S.	N.S.			***
Fixed RR	0.20	0.10	0.10			0.19
RRl	0.17	0.03	0.06			0.16
RRu	0.25	0.35	0.17			0.22
P	---	---	---			---
Random RR	0.14	0.10	0.13			0.14
RRl	0.07	0.03	0.04			0.08
RRu	0.29	0.35	0.37			0.25
P	---	---	---			---
Between Chi						7.01
Between df						2
Between P						*
Btwn(F) P						N.S.
Btwn(R) P						N.S.

	Product all/unsp	cig+/-ot	cig only	Total
N	3	8	1	12
NS	3	6	1	10
Wt	4.81	122.37	4.34	131.52
Het Chi	0.74	68.75	0.00	73.47
Het df	2	7	0	11
Het P	N.S.	***	N.S.	***
Fixed RR	0.08	0.19	0.21	0.19
RRl	0.03	0.16	0.08	0.16
RRu	0.19	0.23	0.55	0.22
P	---	---	--	---
Random RR	0.08	0.15	0.21	0.14
RRl	0.03	0.08	0.08	0.08
RRu	0.19	0.32	0.55	0.25
P	---	---	--	---
Between Chi				3.98
Between df				2
Between P				N.S.
Btwn(F) P				N.S.
Btwn(R) P				N.S.

	Derivation of RR/CI Orig	StdCalc	Other	Total
N		9	3	12
NS		7	3	10
Wt		114.70	16.81	131.52
Het Chi		64.46	2.00	73.47
Het df		8	2	11
Het P		***	N.S.	***
Fixed RR		0.20	0.10	0.19
RRl		0.17	0.06	0.16
RRu		0.25	0.17	0.22
P		---	---	---
Random RR		0.14	0.10	0.14
RRl		0.07	0.06	0.08
RRu		0.29	0.17	0.25
P		---	---	---
Between Chi				7.01
Between df				1
Between P				**
Btwn(F) P				N.S.
Btwn(R) P				N.S.

Table 2K5 - 4

IESLC - Meta-analysis of Ex Smoking, Years quit (vs current), "Highest vs lowest"
Squamous, Any Product (or Cigarettes if Any not available)
Least adjusted

REF	NRR	X	SEX	AGEL	AGEH	RACE	YF	LC TYPE	LOC	START	ST	NLC	R	VB	P	H	AD	ADOS	PRODUCT	exL	exH	unexL	unexH	De
BARBON	593	x	m	0	0	all	-	q	Eu:wst	1979	CC	755	n	bl	y	y	0	0	all/unsp	25	999	0.1	4	st
JAHN	615		m	0	0	all	-	q	Eu:Ger	1988	CC	1004	n	bl	n	n	0	0	cig+/-ot	21	999	0.1	0.9	st
JAIN	548		m	0	0	all	-	q	NAmer	1981	CC	845	n	V	y	n	0	0	cig+/-ot	10	999	2	9	st
JAIN	512		f	0	0	all	-	q	NAmer	1981	CC	845	n	V	y	n	0	0	cig+/-ot	10	999	2	9	st
LUBIN2	783		m	0	0	all	-	q	Eu:mul	1976	CC	7804	n	bl	n	y	0	0	cig+/-ot	20	999	0.1	4	st
LUBIN2	903		f	0	0	all	-	q	Eu:mul	1976	CC	7804	n	bl	n	y	0	0	cig+/-ot	20	999	0.1	9	st
MATOS	630	x	m	0	0	all	-	q	SCAmer	1994	CC	200	n	bl	n	n	0	0	cig+/-ot	11	999	1.0	5	st
PEZZOT	584		m	0	0	all	-	q	SCAmer	1987	CC	215	n	bl	n	y	0	0	cig only	11	999	1.0	10	st
SOBUE	743		m	0	0	all	-	q	As:Jap	1986	CC	1376	n	bl	n	y	0	0	cig+/-ot	10	999	1.0	4	st
SVENSS	562		f	0	0	all	-	q	Eu:Sca	1983	CC	210	n	bl	n	n	0	0	all/unsp	11	999	3	10	st
WYNDE3	517		m	0	0	all	-	KI	NAmer	1966	CC	350	n	bl	n	y	0	0	all/unsp	13	999	1.0	3	st
WYNDE6	786	x	m	0	0	all	-	KI	NAmer	1969	CC	4423	n	bl	n	y	0	0	cig+/-ot	16	999	1.0	3	st

Cigarette type is all/unspec for all RRs

Table 2K5 - 5

IESLC - Meta-analysis of Ex Smoking, Years quit (vs current), "Highest vs lowest"
Squamous, Any Product (or Cigarettes if Any not available)
Least adjusted

REF	NRR	SEX	AD	Number Exposed Case	Exposed Cont	Non-exposed Case	Non-exposed Cont	RR	95.00%CI
BARBON	593	m	0	4	59	11	20	0.12	(0.04- 0.43)
JAHN	615	m	0	8	146	74	8	0.01	(0.00- 0.02)
JAIN	548	m	0	23	113	24	46	0.39	(0.20- 0.76)
JAIN	512	f	0	7	61	15	36	0.28	(0.10- 0.74)
Subtotal JAIN								0.35	(0.20- 0.61)
LUBIN2	783	m	0	106	1128	498	1047	0.20	(0.16- 0.25)
LUBIN2	903	f	0	2	29	38	95	0.17	(0.04- 0.76)
Subtotal LUBIN2								0.20	(0.16- 0.25)
MATOS	630	m	0	5	101	4	23	0.28	(0.07- 1.14)
PEZZOT	584	m	0	8	48	21	27	0.21	(0.08- 0.55)
SOBUE	743	m	0	30	144	52	116	0.46	(0.28- 0.78)
SVENSS	562	f	0	1	24	5	13	0.11	(0.01- 1.03)
WYNDE3	517	m	0	2	55	18	22	0.04	(0.01- 0.21)
WYNDE6	786	m	0	19	530	80	307	0.14	(0.08- 0.23)
Totals				215	2438	840	1760		

*prospective study

REF	NRR	SEX	AD	Ys	Ws	Qs	Ps
BARBON	593	m	0	-2.09	2.45	0.52	0.0010
JAHN	615	m	0	-5.13	3.70	45.19	0.0000
JAIN	548	m	0	-0.94	8.64	4.14	0.0057
JAIN	512	f	0	-1.29	3.94	0.47	0.0105
Subtotal JAIN				-1.05	12.58	4.60	
LUBIN2	783	m	0	-1.62	75.28	0.01	0.0000
LUBIN2	903	f	0	-1.76	1.75	0.03	0.0200
Subtotal LUBIN2				-1.62	77.03	0.04	
MATOS	630	m	0	-1.26	1.99	0.28	0.0766
PEZZOT	584	m	0	-1.54	4.34	0.04	0.0013
SOBUE	743	m	0	-0.77	14.68	11.04	0.0033
SVENSS	562	f	0	-2.22	0.76	0.26	0.0529
WYNDE3	517	m	0	-3.11	1.62	3.54	0.0001
WYNDE6	786	m	0	-1.98	14.23	1.75	0.0000

	N	12
	NS	10
	Wt	133.37
Het	Chi	67.25
Het	df	11
Het	P	***
Fixed	RR	0.20
	RRl	0.16
	RRu	0.23
	P	---
Random	RR	0.15
	RRl	0.09
	RRu	0.26
	P	---
Asymm	P	N.S.

Table 2K5 - 6

IESLC - Meta-analysis of Ex Smoking, Years quit (vs current), "Highest vs lowest"
Squamous, Any Product (or Cigarettes if Any not available)
Least adjusted

	Sex combined	male	female	Total
N		9	3	12
NS		9	3	12
Wt		126.92	6.45	133.37
Het Chi		66.49	0.68	67.25
Het df		8	2	11
Het P		***	N.S.	***
Fixed RR		0.19	0.22	0.20
RRl		0.16	0.10	0.16
RRu		0.23	0.47	0.23
P		---	---	---
Random RR		0.14	0.22	0.15
RRl		0.07	0.10	0.09
RRu		0.27	0.47	0.26
P		---	---	---
Between Chi				0.08
Between df				1
Between P				N.S.
Btwn(F) P				N.S.
Btwn(R) P				N.S.

Table 2K5 - 7

IESLC - Meta-analysis of Ex Smoking, Years quit (vs current), "Highest vs lowest"
Squamous, Any Product (or Cigarettes if Any not available)
Excluded studies (and stage at which they were excluded)

1	AGUDO AKIBA AMANDU AMES AXELSS BEST BOUCHA BOUCOT BRESLO CHEN CHEN2 CHIAZZ DEAN2 DOSEME ENGELA FAN GENG GER GUO HAENSZ HEGMAN HOLE HU HU2 JUSSAW KATSOU KAUFMA KOO KOULUM KREUZE LETOUR LEVIN LIAW LIU3 LIU4 LIU5 MCCONN MIGRAN MRFITR NOTAN2 OSANN2 PERNU QIAO2 RACHTA RESTRE SADOWS SEGI2 STASZE TIZZAN VUTUC WATSON WU WUWILL WYNDE2 WYNDE8 XU YUAN ZHANG ZHENG ZHOU
2	AUVINE BENSHL BLOT1 BROWN3 BUFFLE GURSEL LAUSSM LUO MCDUFF PISANI PRESCO SPITZ WU2 WYNDE7
4	GARSHI JEDRYC WAKAI
5	ARMADA BECHER BOFFET BROSS CARPEN CEDERL CHOI CHYOU CORREA CPSI CPSII DAMBER DARBY DEAN3 DESTEF DOLL DOLL2 DORGAN DORN GAO GAO2 GARCIA GILLIS GRAHAM HAMMO2 HIRAYA HUMBLE JOLY KAISE2 KHUDER LUBIN PEZZO2 QIAO SPEIZE SUZUK2 TVERDA WANG2 WIGLE
6	ALDERS HAMMON
15	BENHAM

Table 2K5 - 8
Potentially overlapping studies

REF	REFGP	PRINC	OVERLAP/LINK
LUBIN2	LUBIN2	1	Lubin-combined
WYNDE6	WYNDE6	1	WYNDE5/6/7/8
JAHN	BOFFET	2	Subset of BOFFET

Table 2K5 - 9
Most adjusted - insufficient data for meta-analysis

REF	NRR	SEX	AGEL	AGEH	RACE	YF	LC TYPE	LOC	START	ST	NLC	R	VB	P	H	AD	ADOS	PRODUCT	exL	exH	unexL	unexH	De
ALDERS	547	m	0	0	all	-	q+s	Eu:UK	1977	CC	1448	n	V	n	n	1	0	cig only	10	999	0.1	2	st
ALDERS	558	f	0	0	all	-	q+s	Eu:UK	1977	CC	1448	n	V	n	n	1	0	cig only	10	999	0.1	2	st
HAMMON	511	m	0	0	wh	0	not a	NAmer	1952	pr	448	n	bl	n	n	1	0	cig only	10	999	0.1	0.9	st

REF	NRR	RR	SIG	RRDATA comment
ALDERS	547	0.10		0
ALDERS	558	0.04		0
HAMMON	511	*		RR for <1 pack per day is 0.15, while that for 1+ packs is 0.31

Table 2K6 -

IESLC - Meta-analysis of Ex Smoking by Years quit (vs current), Overview
Squamous, Cigarettes (or Any Product if Cigarettes not available)

This analysis is restricted to results for:
1) Ex smokers
2) Results by Years quit (vs current)
3) Categorical results by Years quit (vs current)
Results by Years quit (vs current) are grouped under 2 schemes (S1, S2). Each scheme has a set of "key values". An interval is allocated to the category whose key value it includes, and intervals which include none or more than one of the key values are excluded. (Open-ended intervals are coded as 999)

S1	key value	maximum range
1	3	1-6
2	7	4-11
3	12	8+

S2	key value	maximum range
1	3	1-11
2	12	4-19
3	20	13+

4) Squamous (or near equivalent)
5) Results complete enough for use in metaanalysis

Within each study, results are then selected (in the following order of preference, within each sex) for:
6) (not applicable)
7) PRODUCT: cigarettes regardless of other products, cigarettes only, all/unspec
8) CIGTYPE: all/unspecified, MC regardless of HR, MC only
9) Results with least adjustment for other aspects of smoking (ADOS)
10) DENOM: current smokers, current + recent smokers (up to number of m=months or y=years, max 2 years)
11) Followup period (YF, prospective studies): whole study (coded as 0) or longest available
12) LCtype: squamous or nearest available, but not adeno. (q = squamous, s = small,
a = adeno, KI = Kreyberg I, u = undifferentiated)
13) Race: all or nearest available, otherwise by race (wh or w = white, bl or b = black, hi = hispanic
ch = chinese, jap = japanese, haw = hawaiian, w+o = white + oriental, sca = scandinavian, as = asian)
14) For overlapping studies: principal rather than subsidiary studies
Finally by Age: whole study (coded as 0) if available, otherwise by widest available age group
and then for single sex results (m, f) in preference to results for both sexes combined (c).

Results adjusted (AD) for the most potential confounders are then chosen in Sections -1 to -3
(and those which actually differ from the adjusted results in Table 2K1 - 1 are marked 'x' in Section -1)
and results adjusted for the least confounders in Sections -4 to -6. (Those least adjusted results which
actually differ from the most adjusted are marked 'x' in column X in Section -4)

Section -7 shows excluded studies, together with the stage (as above) at which no qualifying
results were found.

Section -8 lists the potentially overlapping studies which have been included (1=principal, 2=subsidiary).

Section -9 lists any results which would have been included in preference except that they had data not complete
enough for use in meta-analysis, with their significance (yes/no), if known, and any further comment as entered
on the database. It also lists as "gap" any categories for which no data were presented by the original authors.

In addition to those mentioned above, the following fields, levels and abbreviations are used:
* or nk = not known, n = no, y = yes, ot = other
nev = never
all/unspec = all or unspecified, cig+/-ot = cigarettes irrespective of other products (cigar, pipe etc)
MC = manufactured cigarettes, HR = hand-rolled cigarettes
exL, exH = range of exposure (low and high) in the smoking group, in terms of Years quit (vs current)
REF: 6-character study reference
NRR: number of the RR on the database within the study
ST : study type (CC = case control, pr or prosp = prospective)
NLC: number of lung cancer cases in whole study
R : risky occupational population (n = no, m = mining, o = other risky)
VB : national cigarette type (V = at least 75% Virginia, bl = at least 75% blended, ot = other)
P : any proxy use
H : full histological confirmation
De : derivation of RR/CI (or = original, st = standard method, ot = other method of estimation)

Table 2K6 - 1

IESLC - Meta-analysis of Ex Smoking by Years quit (vs current), Overview
Squamous, Cigarettes (or Any Product if Cigarettes not available)
Most adjusted

REF	NRR	2K1	SEX	AGEL	AGEH	RACE	YF	LC TYPE	LOC	START	ST	NLC	R	VB	P	H	AD	ADOS	PRODUCT	exL	exH	S1	S2	DENOM	De
BARBON	602		m	0	0	all	-	q	Eu:wst	1979	CC	755	n	bl	y	y	1	0	all/unsp	0.1	4	1	1	current	ot
BARBON	603		m	0	0	all	-	q	Eu:wst	1979	CC	755	n	bl	y	y	1	0	all/unsp	5	14	0	2	current	ot
BARBON	604		m	0	0	all	-	q	Eu:wst	1979	CC	755	n	bl	y	y	1	0	all/unsp	15	24	0	3	current	ot
BARBON	605		m	0	0	all	-	q	Eu:wst	1979	CC	755	n	bl	y	y	1	0	all/unsp	25	999	0	0	current	ot
JAHN	605		m	0	0	all	-	q	Eu:Ger	1988	CC	1004	n	bl	n	n	0	0	cig+/-ot	0.1	0.9	0	0	current	st
JAHN	606		m	0	0	all	-	q	Eu:Ger	1988	CC	1004	n	bl	n	n	0	0	cig+/-ot	1.0	1.9	0	0	current	st
JAHN	607		m	0	0	all	-	q	Eu:Ger	1988	CC	1004	n	bl	n	n	0	0	cig+/-ot	2	5	1	1	current	st
JAHN	608		m	0	0	all	-	q	Eu:Ger	1988	CC	1004	n	bl	n	n	0	0	cig+/-ot	6	10	2	0	current	st
JAHN	609		m	0	0	all	-	q	Eu:Ger	1988	CC	1004	n	bl	n	n	0	0	cig+/-ot	11	20	3	0	current	st
JAHN	610		m	0	0	all	-	q	Eu:Ger	1988	CC	1004	n	bl	n	n	0	0	cig+/-ot	21	999	0	0	current	st
JAIN	546		m	0	0	all	-	q	NAmer	1981	CC	845	n	V	y	n	0	0	cig+/-ot	2	9	0	1	cur+2y	st
JAIN	547		m	0	0	all	-	q	NAmer	1981	CC	845	n	V	y	n	0	0	cig+/-ot	10	999	3	0	cur+2y	st
JAIN	510		f	0	0	all	-	q	NAmer	1981	CC	845	n	V	y	n	0	0	cig+/-ot	2	9	0	1	cur+2y	st
JAIN	511		f	0	0	all	-	q	NAmer	1981	CC	845	n	V	y	n	0	0	cig+/-ot	10	999	3	0	cur+2y	st
LUBIN2	775		m	0	0	all	-	q	Eu:mul	1976	CC	7804	n	bl	n	y	0	0	cig+/-ot	0.1	4	1	1	current	st
LUBIN2	776		m	0	0	all	-	q	Eu:mul	1976	CC	7804	n	bl	n	y	0	0	cig+/-ot	5	9	2	0	current	st
LUBIN2	777		m	0	0	all	-	q	Eu:mul	1976	CC	7804	n	bl	n	y	0	0	cig+/-ot	10	14	3	2	current	st
LUBIN2	778		m	0	0	all	-	q	Eu:mul	1976	CC	7804	n	bl	n	y	0	0	cig+/-ot	15	19	0	0	current	st
LUBIN2	779		m	0	0	all	-	q	Eu:mul	1976	CC	7804	n	bl	n	y	0	0	cig+/-ot	20	999	0	3	current	st
LUBIN2	899		f	0	0	all	-	q	Eu:mul	1976	CC	7804	n	bl	n	y	0	0	cig+/-ot	0.1	9	0	1	current	st
LUBIN2	900		f	0	0	all	-	q	Eu:mul	1976	CC	7804	n	bl	n	y	0	0	cig+/-ot	10	19	3	2	current	st
LUBIN2	901		f	0	0	all	-	q	Eu:mul	1976	CC	7804	n	bl	n	y	0	0	cig+/-ot	20	999	0	3	current	st
MATOS	636		m	0	0	all	-	q	SCAmer	1994	CC	200	n	bl	n	n	2	0	cig+/-ot	1.0	5	1	1	cur+1y	or
MATOS	637		m	0	0	all	-	q	SCAmer	1994	CC	200	n	bl	n	n	2	0	cig+/-ot	6	10	2	0	cur+1y	or
MATOS	638		m	0	0	all	-	q	SCAmer	1994	CC	200	n	bl	n	n	2	0	cig+/-ot	11	999	3	0	cur+1y	or
PEZZOT	582		m	0	0	all	-	q	SCAmer	1987	CC	215	n	bl	n	y	0	0	cig only	1.0	10	0	1	cur+1y	st
PEZZOT	583		m	0	0	all	-	q	SCAmer	1987	CC	215	n	bl	n	y	0	0	cig only	11	999	3	0	cur+1y	st
SOBUE	739		m	0	0	all	-	q	As:Jap	1986	CC	1376	n	bl	n	y	0	0	cig+/-ot	1.0	4	1	1	cur+1y	st
SOBUE	740		m	0	0	all	-	q	As:Jap	1986	CC	1376	n	bl	n	y	0	0	cig+/-ot	5	9	2	0	cur+1y	st
SOBUE	741		m	0	0	all	-	q	As:Jap	1986	CC	1376	n	bl	n	y	0	0	cig+/-ot	10	999	3	0	cur+1y	st
SVENSS	560		f	0	0	all	-	q	Eu:Sca	1983	CC	210	n	bl	n	n	0	0	all/unsp	3	10	0	1	cur+2y	st
SVENSS	561		f	0	0	all	-	q	Eu:Sca	1983	CC	210	n	bl	n	n	0	0	all/unsp	11	999	3	0	cur+2y	st
WYNDE3	511		m	0	0	all	-	KI	NAmer	1966	CC	350	n	bl	n	y	0	0	all/unsp	1.0	3	1	1	cur+1y	st
WYNDE3	512		m	0	0	all	-	KI	NAmer	1966	CC	350	n	bl	n	y	0	0	all/unsp	4	6	0	0	cur+1y	st
WYNDE3	513		m	0	0	all	-	KI	NAmer	1966	CC	350	n	bl	n	y	0	0	all/unsp	7	12	0	2	cur+1y	st
WYNDE3	514		m	0	0	all	-	KI	NAmer	1966	CC	350	n	bl	n	y	0	0	all/unsp	13	999	0	3	cur+1y	st

Cigarette type is all/unspec for all RRs

In this overview table, subtotals and Qs values may be invalid and should be ignored

Table 2K6 - 2

IESLC - Meta-analysis of Ex Smoking by Years quit (vs current), Overview
Squamous, Cigarettes (or Any Product if Cigarettes not available)
Most adjusted

REF	NRR	SEX	A	Number Exposed Case	Number Exposed Cont	Non-exposed Case	Non-exposed Cont	RR	95.00%CI
BARBON	602	m	1	11	-	203	-	0.97	(0.45- 2.07)
BARBON	603	m	1	31	-	203	-	0.62	(0.40- 0.96)
BARBON	604	m	1	11	-	203	-	0.42	(0.21- 0.84)
BARBON	605	m	1	4	-	203	-	0.10	(0.03- 0.29)
Subtotal BARBON								0.53	(0.39- 0.74)
JAHN	605	m	0	74	8	153	269	16.26	(7.64- 34.63)
JAHN	606	m	0	25	9	153	269	4.88	(2.22- 10.73)
JAHN	607	m	0	36	46	153	269	1.38	(0.85- 2.22)
JAHN	608	m	0	29	63	153	269	0.81	(0.50- 1.31)
JAHN	609	m	0	18	130	153	269	0.24	(0.14- 0.41)
JAHN	610	m	0	8	146	153	269	0.10	(0.05- 0.20)
Subtotal JAHN								0.92	(0.73- 1.17)
JAIN	546	m	0	24	46	107	118	0.58	(0.33- 1.01)
JAIN	547	m	0	23	113	107	118	0.22	(0.13- 0.38)
JAIN	510	f	0	15	36	81	99	0.51	(0.26- 1.00)
JAIN	511	f	0	7	61	81	99	0.14	(0.06- 0.32)
Subtotal JAIN								0.33	(0.24- 0.45)
LUBIN2	775	m	0	498	1047	2518	6209	1.17	(1.04- 1.32)
LUBIN2	776	m	0	265	882	2518	6209	0.74	(0.64- 0.86)
LUBIN2	777	m	0	146	693	2518	6209	0.52	(0.43- 0.62)
LUBIN2	778	m	0	67	478	2518	6209	0.35	(0.27- 0.45)
LUBIN2	779	m	0	106	1128	2518	6209	0.23	(0.19- 0.28)
LUBIN2	899	f	0	38	95	154	410	1.06	(0.70- 1.62)
LUBIN2	900	f	0	5	33	154	410	0.40	(0.15- 1.05)
LUBIN2	901	f	0	2	29	154	410	0.18	(0.04- 0.78)
Subtotal LUBIN2								0.69	(0.64- 0.74)
MATOS	636	m	2	4	-	33	-	0.70	(0.20- 2.20)
MATOS	637	m	2	5	-	33	-	0.60	(0.20- 1.90)
MATOS	638	m	2	5	-	33	-	0.20	(0.06- 0.50)
Subtotal MATOS								0.42	(0.22- 0.80)
PEZZOT	582	m	0	21	27	56	52	0.72	(0.36- 1.43)
PEZZOT	583	m	0	8	48	56	52	0.15	(0.07- 0.36)
Subtotal PEZZOT								0.39	(0.23- 0.66)
SOBUE	739	m	0	52	116	292	633	0.97	(0.68- 1.39)
SOBUE	740	m	0	32	92	292	633	0.75	(0.49- 1.15)
SOBUE	741	m	0	30	144	292	633	0.45	(0.30- 0.69)
Subtotal SOBUE								0.72	(0.57- 0.90)
SVENSS	560	f	0	5	13	42	53	0.49	(0.16- 1.47)
SVENSS	561	f	0	1	24	42	53	0.05	(0.01- 0.40)
Subtotal SVENSS								0.29	(0.11- 0.77)
WYNDE3	511	m	0	18	22	171	207	0.99	(0.51- 1.91)
WYNDE3	512	m	0	8	17	171	207	0.57	(0.24- 1.35)
WYNDE3	513	m	0	8	31	171	207	0.31	(0.14- 0.70)
WYNDE3	514	m	0	2	55	171	207	0.04	(0.01- 0.18)
Subtotal WYNDE3								0.49	(0.32- 0.74)
Partial Totals				1642	5632	17013	37260		

*prospective study

Table 2K6 - 2

IESLC - Meta-analysis of Ex Smoking by Years quit (vs current), Overview
Squamous, Cigarettes (or Any Product if Cigarettes not available)
Most adjusted

REF	NRR	SEX	AD	Ys	Ws	Qs	Ps
BARBON	602	m	1	-0.03	6.60	0.97	0.9376
BARBON	603	m	1	-0.48	20.05	0.08	0.0323
BARBON	604	m	1	-0.87	8.00	1.65	0.0142
BARBON	605	m	1	-2.30	2.99	10.65	0.0001
Subtotal BARBON				-0.63	37.63	13.35	
JAHN	605	m	0	2.79	6.72	68.94	0.0000
JAHN	606	m	0	1.59	6.20	24.78	0.0001
JAHN	607	m	0	0.32	16.73	8.98	0.1917
JAHN	608	m	0	-0.21	16.50	0.67	0.3901
JAHN	609	m	0	-1.41	13.61	13.59	0.0000
JAHN	610	m	0	-2.34	7.04	26.11	0.0000
Subtotal JAHN				-0.08	66.79	143.07	
JAIN	546	m	0	-0.55	12.31	0.24	0.0525
JAIN	547	m	0	-1.49	14.26	16.64	0.0000
JAIN	510	f	0	-0.67	8.55	0.58	0.0484
JAIN	511	f	0	-1.96	5.50	13.23	0.0000
Subtotal JAIN				-1.10	40.63	30.70	
LUBIN2	775	m	0	0.16	283.98	93.26	0.0072
LUBIN2	776	m	0	-0.30	182.96	2.36	0.0000
LUBIN2	777	m	0	-0.65	112.99	6.58	0.0000
LUBIN2	778	m	0	-1.06	56.90	23.95	0.0000
LUBIN2	779	m	0	-1.46	91.92	101.08	0.0000
LUBIN2	899	f	0	0.06	21.85	4.96	0.7687
LUBIN2	900	f	0	-0.91	4.18	1.02	0.0634
LUBIN2	901	f	0	-1.69	1.84	3.02	0.0215
Subtotal LUBIN2				-0.38	756.62	236.23	
MATOS	636	m	2	-0.36	2.67	0.01	0.5598
MATOS	637	m	2	-0.51	3.03	0.03	0.3738
MATOS	638	m	2	-1.61	3.42	4.89	0.0029
Subtotal MATOS				-0.88	9.12	4.93	
PEZZOT	582	m	0	-0.33	8.21	0.06	0.3510
PEZZOT	583	m	0	-1.87	5.47	11.53	0.0000
Subtotal PEZZOT				-0.94	13.68	11.59	
SOBUE	739	m	0	-0.03	30.44	4.51	0.8745
SOBUE	740	m	0	-0.28	21.22	0.37	0.1934
SOBUE	741	m	0	-0.79	22.08	3.21	0.0002
Subtotal SOBUE				-0.33	73.74	8.09	
SVENSS	560	f	0	-0.72	3.13	0.30	0.2010
SVENSS	561	f	0	-2.95	0.92	5.91	0.0047
Subtotal SVENSS				-1.23	4.05	6.21	
WYNDE3	511	m	0	-0.01	8.95	1.46	0.9770
WYNDE3	512	m	0	-0.56	5.14	0.11	0.2020
WYNDE3	513	m	0	-1.16	5.95	3.35	0.0045
WYNDE3	514	m	0	-3.12	1.89	13.88	0.0000
Subtotal WYNDE3				-0.72	21.94	18.81	

N	36
NS	9

Table 2K6 - 3

IESLC - Meta-analysis of Ex Smoking by Years quit (vs current), Overview
Squamous, Cigarettes (or Any Product if Cigarettes not available)
Most adjusted

	Sex combined	male	female	Total
N		29	7	36
NS		8	3	11

In this overview table, other than the "N" rows, entries in the "absent" and "Total" columns may be invalid and should be ignored

Years quit vs current (lower focus)

	absent	1-6k3	4-11k7	8+k12	Total
N	17	6	4	9	36
NS	7	6	4	7	24
Wt	268.69	349.37	223.72	182.42	1024.20
Het Chi	220.86	2.56	0.26	32.55	472.97
Het df	16	5	3	8	35
Het P	***	N.S.	N.S.	***	***
Fixed RR	0.41	1.15	0.74	0.40	0.66
RRl	0.36	1.03	0.65	0.35	0.62
RRu	0.46	1.28	0.85	0.47	0.70
P	---	++	---	---	---
Random RR	0.50	1.15	0.74	0.27	0.51
RRl	0.30	1.03	0.65	0.18	0.39
RRu	0.82	1.28	0.85	0.40	0.66
P	--	++	---	---	---

Years quit vs current (higher focus)

	absent	1-11k3	4-19k12	13+k20	Total
N	17	11	4	4	36
NS	9	9	3	3	24
Wt	373.95	403.43	143.17	103.65	1024.20
Het Chi	218.40	16.95	2.43	8.13	472.97
Het df	16	10	3	3	35
Het P	***	(*)	N.S.	*	***
Fixed RR	0.57	1.08	0.52	0.23	0.66
RRl	0.51	0.98	0.44	0.19	0.62
RRu	0.63	1.19	0.61	0.28	0.70
P	---	N.S.	---	---	---
Random RR	0.44	0.94	0.52	0.21	0.51
RRl	0.28	0.77	0.44	0.11	0.39
RRu	0.70	1.14	0.61	0.40	0.66
P	---	N.S.	---	---	---

Table 2K6 - 3

IESLC - Meta-analysis of Ex Smoking by Years quit (vs current), Overview
Squamous, Cigarettes (or Any Product if Cigarettes not available)
Most adjusted

MALES

Years quit vs current (lower focus)

		absent	1-6k3	4-11k7	8+k12	Total
	N	13	6	4	6	29
	NS	6	6	4	6	22
	Wt	233.32	349.37	223.72	171.82	978.22
Het	Chi	196.95	2.56	0.26	22.19	443.88
Het	df	12	5	3	5	28
Het	P	***	N.S.	N.S.	***	***
Fixed	RR	0.37	1.15	0.74	0.42	0.67
	RRl	0.33	1.03	0.65	0.36	0.63
	RRu	0.42	1.28	0.85	0.49	0.71
	P	---	++	---	---	---
Random	RR	0.49	1.15	0.74	0.30	0.55
	RRl	0.27	1.03	0.65	0.20	0.41
	RRu	0.89	1.28	0.85	0.46	0.73
	P	-	++	---	---	---

Years quit vs current (higher focus)

		absent	1-11k3	4-19k12	13+k20	Total
	N	15	8	3	3	29
	NS	8	8	3	3	22
	Wt	367.52	369.90	138.99	101.81	978.22
Het	Chi	202.13	9.81	2.17	8.02	443.88
Het	df	14	7	2	2	28
Het	P	***	N.S.	N.S.	*	***
Fixed	RR	0.58	1.11	0.52	0.24	0.67
	RRl	0.53	1.00	0.44	0.19	0.63
	RRu	0.65	1.23	0.62	0.29	0.71
	P	---	+	---	---	---
Random	RR	0.51	1.01	0.52	0.21	0.55
	RRl	0.32	0.84	0.43	0.10	0.41
	RRu	0.81	1.22	0.63	0.46	0.73
	P	--	N.S.	---	---	---

FEMALES

Years quit vs current (lower focus)

		absent	1-6k3	4-11k7	8+k12	Total
	N	4			3	7
	NS	3			3	6
	Wt	35.37			10.61	45.98
Het	Chi	8.20			4.39	27.61
Het	df	3			2	6
Het	P	*			N.S.	***
Fixed	RR	0.76			0.20	0.55
	RRl	0.55			0.11	0.42
	RRu	1.05			0.36	0.74
	P	N.S.			---	---
Random	RR	0.57			0.18	0.34
	RRl	0.29			0.07	0.17
	RRu	1.11			0.48	0.70
	P	(-)			---	--

Table 2K6 - 3

IESLC - Meta-analysis of Ex Smoking by Years quit (vs current), Overview
Squamous, Cigarettes (or Any Product if Cigarettes not available)
Most adjusted

FEMALES

	Years quit vs current (higher focus) absent	1-11k3	4-19k12	13+k20	Total
N	2	3	1	1	7
NS	2	3	1	1	6
Wt	6.43	33.53	4.18	1.84	45.98
Het Chi	0.76	4.29	0.00	0.00	27.61
Het df	1	2	0	0	6
Het P	N.S.	N.S.	N.S.	N.S.	***
Fixed RR	0.12	0.82	0.40	0.18	0.55
RRl	0.06	0.58	0.15	0.04	0.42
RRu	0.26	1.15	1.05	0.78	0.74
P	---	N.S.	(-)	-	---
Random RR	0.12	0.71	0.40	0.18	0.34
RRl	0.06	0.40	0.15	0.04	0.17
RRu	0.26	1.27	1.05	0.78	0.70
P	---	N.S.	(-)	-	--

Table 2K6 - 4

IESLC - Meta-analysis of Ex Smoking by Years quit (vs current), Overview
Squamous, Cigarettes (or Any Product if Cigarettes not available)
Least adjusted

REF	NRR	X	SEX	AGEL	AGEH	RACE	YF	LC TYPE	LOC	START	ST	NLC	R	VB	P	H	AD	ADOS	PRODUCT	exL	exH	S1	S2	DENOM	De
BARBON	587	x	m	0	0	all	-	q	Eu:wst	1979	CC	755	n	bl	y	y	0	0	all/unsp	0.1	4	1	1	current	st
BARBON	588	x	m	0	0	all	-	q	Eu:wst	1979	CC	755	n	bl	y	y	0	0	all/unsp	5	14	0	2	current	st
BARBON	589	x	m	0	0	all	-	q	Eu:wst	1979	CC	755	n	bl	y	y	0	0	all/unsp	15	24	0	3	current	st
BARBON	590	x	m	0	0	all	-	q	Eu:wst	1979	CC	755	n	bl	y	y	0	0	all/unsp	25	999	0	0	current	st
JAHN	605		m	0	0	all	-	q	Eu:Ger	1988	CC	1004	n	bl	n	n	0	0	cig+/-ot	0.1	0.9	0	0	current	st
JAHN	606		m	0	0	all	-	q	Eu:Ger	1988	CC	1004	n	bl	n	n	0	0	cig+/-ot	1.0	1.9	0	0	current	st
JAHN	607		m	0	0	all	-	q	Eu:Ger	1988	CC	1004	n	bl	n	n	0	0	cig+/-ot	2	5	1	1	current	st
JAHN	608		m	0	0	all	-	q	Eu:Ger	1988	CC	1004	n	bl	n	n	0	0	cig+/-ot	6	10	2	0	current	st
JAHN	609		m	0	0	all	-	q	Eu:Ger	1988	CC	1004	n	bl	n	n	0	0	cig+/-ot	11	20	3	0	current	st
JAHN	610		m	0	0	all	-	q	Eu:Ger	1988	CC	1004	n	bl	n	n	0	0	cig+/-ot	21	999	0	0	current	st
JAIN	546		m	0	0	all	-	q	NAmer	1981	CC	845	n	V	y	n	0	0	cig+/-ot	2	9	0	1	cur+2y	st
JAIN	547		m	0	0	all	-	q	NAmer	1981	CC	845	n	V	y	n	0	0	cig+/-ot	10	999	3	0	cur+2y	st
JAIN	510		f	0	0	all	-	q	NAmer	1981	CC	845	n	V	y	n	0	0	cig+/-ot	2	9	0	1	cur+2y	st
JAIN	511		f	0	0	all	-	q	NAmer	1981	CC	845	n	V	y	n	0	0	cig+/-ot	10	999	3	0	cur+2y	st
LUBIN2	775		m	0	0	all	-	q	Eu:mul	1976	CC	7804	n	bl	n	y	0	0	cig+/-ot	0.1	4	1	1	current	st
LUBIN2	776		m	0	0	all	-	q	Eu:mul	1976	CC	7804	n	bl	n	y	0	0	cig+/-ot	5	9	2	0	current	st
LUBIN2	777		m	0	0	all	-	q	Eu:mul	1976	CC	7804	n	bl	n	y	0	0	cig+/-ot	10	14	3	2	current	st
LUBIN2	778		m	0	0	all	-	q	Eu:mul	1976	CC	7804	n	bl	n	y	0	0	cig+/-ot	15	19	0	0	current	st
LUBIN2	779		m	0	0	all	-	q	Eu:mul	1976	CC	7804	n	bl	n	y	0	0	cig+/-ot	20	999	0	3	current	st
LUBIN2	899		f	0	0	all	-	q	Eu:mul	1976	CC	7804	n	bl	n	y	0	0	cig+/-ot	0.1	9	0	1	current	st
LUBIN2	900		f	0	0	all	-	q	Eu:mul	1976	CC	7804	n	bl	n	y	0	0	cig+/-ot	10	19	3	2	current	st
LUBIN2	901		f	0	0	all	-	q	Eu:mul	1976	CC	7804	n	bl	n	y	0	0	cig+/-ot	20	999	0	3	current	st
MATOS	626	x	m	0	0	all	-	q	SCAmer	1994	CC	200	n	bl	n	n	0	0	cig+/-ot	1.0	5	1	1	cur+1y	st
MATOS	627	x	m	0	0	all	-	q	SCAmer	1994	CC	200	n	bl	n	n	0	0	cig+/-ot	6	10	2	0	cur+1y	st
MATOS	628	x	m	0	0	all	-	q	SCAmer	1994	CC	200	n	bl	n	n	0	0	cig+/-ot	11	999	3	0	cur+1y	st
PEZZOT	582		m	0	0	all	-	q	SCAmer	1987	CC	215	n	bl	n	y	0	0	cig only	1.0	10	0	1	cur+1y	st
PEZZOT	583		m	0	0	all	-	q	SCAmer	1987	CC	215	n	bl	n	y	0	0	cig only	11	999	3	0	cur+1y	st
SOBUE	739		m	0	0	all	-	q	As:Jap	1986	CC	1376	n	bl	n	y	0	0	cig+/-ot	1.0	4	1	1	cur+1y	st
SOBUE	740		m	0	0	all	-	q	As:Jap	1986	CC	1376	n	bl	n	y	0	0	cig+/-ot	5	9	2	0	cur+1y	st
SOBUE	741		m	0	0	all	-	q	As:Jap	1986	CC	1376	n	bl	n	y	0	0	cig+/-ot	10	999	3	0	cur+1y	st
SVENSS	560		f	0	0	all	-	q	Eu:Sca	1983	CC	210	n	bl	n	n	0	0	all/unsp	3	10	0	1	cur+2y	st
SVENSS	561		f	0	0	all	-	q	Eu:Sca	1983	CC	210	n	bl	n	n	0	0	all/unsp	11	999	3	0	cur+2y	st
WYNDE3	511		m	0	0	all	-	KI	NAmer	1966	CC	350	n	bl	n	y	0	0	all/unsp	1.0	3	1	1	cur+1y	st
WYNDE3	512		m	0	0	all	-	KI	NAmer	1966	CC	350	n	bl	n	y	0	0	all/unsp	4	6	0	0	cur+1y	st
WYNDE3	513		m	0	0	all	-	KI	NAmer	1966	CC	350	n	bl	n	y	0	0	all/unsp	7	12	0	2	cur+1y	st
WYNDE3	514		m	0	0	all	-	KI	NAmer	1966	CC	350	n	bl	n	y	0	0	all/unsp	13	999	0	3	cur+1y	st

Cigarette type is all/unspec for all RRs

In this overview table, subtotals and Qs values may be invalid and should be ignored

Table 2K6 - 5

IESLC - Meta-analysis of Ex Smoking by Years quit (vs current), Overview
Squamous, Cigarettes (or Any Product if Cigarettes not available)
Least adjusted

REF	NRR	SEX	A	Number Exposed Case	Cont	Non-exposed Case	Cont	RR	95.00%CI
BARBON	587	m	0	11	20	203	362	0.98	(0.46- 2.09)
BARBON	588	m	0	31	85	203	362	0.65	(0.42- 1.02)
BARBON	589	m	0	11	41	203	362	0.48	(0.24- 0.95)
BARBON	590	m	0	4	59	203	362	0.12	(0.04- 0.34)
Subtotal BARBON								0.56	(0.41- 0.77)
JAHN	605	m	0	74	8	153	269	16.26	(7.64- 34.63)
JAHN	606	m	0	25	9	153	269	4.88	(2.22- 10.73)
JAHN	607	m	0	36	46	153	269	1.38	(0.85- 2.22)
JAHN	608	m	0	29	63	153	269	0.81	(0.50- 1.31)
JAHN	609	m	0	18	130	153	269	0.24	(0.14- 0.41)
JAHN	610	m	0	8	146	153	269	0.10	(0.05- 0.20)
Subtotal JAHN								0.92	(0.73- 1.17)
JAIN	546	m	0	24	46	107	118	0.58	(0.33- 1.01)
JAIN	547	m	0	23	113	107	118	0.22	(0.13- 0.38)
JAIN	510	f	0	15	36	81	99	0.51	(0.26- 1.00)
JAIN	511	f	0	7	61	81	99	0.14	(0.06- 0.32)
Subtotal JAIN								0.33	(0.24- 0.45)
LUBIN2	775	m	0	498	1047	2518	6209	1.17	(1.04- 1.32)
LUBIN2	776	m	0	265	882	2518	6209	0.74	(0.64- 0.86)
LUBIN2	777	m	0	146	693	2518	6209	0.52	(0.43- 0.62)
LUBIN2	778	m	0	67	478	2518	6209	0.35	(0.27- 0.45)
LUBIN2	779	m	0	106	1128	2518	6209	0.23	(0.19- 0.28)
LUBIN2	899	f	0	38	95	154	410	1.06	(0.70- 1.62)
LUBIN2	900	f	0	5	33	154	410	0.40	(0.15- 1.05)
LUBIN2	901	f	0	2	29	154	410	0.18	(0.04- 0.78)
Subtotal LUBIN2								0.69	(0.64- 0.74)
MATOS	626	m	0	4	23	33	132	0.70	(0.23- 2.15)
MATOS	627	m	0	5	27	33	132	0.74	(0.27- 2.07)
MATOS	628	m	0	5	101	33	132	0.20	(0.07- 0.53)
Subtotal MATOS								0.44	(0.24- 0.81)
PEZZOT	582	m	0	21	27	56	52	0.72	(0.36- 1.43)
PEZZOT	583	m	0	8	48	56	52	0.15	(0.07- 0.36)
Subtotal PEZZOT								0.39	(0.23- 0.66)
SOBUE	739	m	0	52	116	292	633	0.97	(0.68- 1.39)
SOBUE	740	m	0	32	92	292	633	0.75	(0.49- 1.15)
SOBUE	741	m	0	30	144	292	633	0.45	(0.30- 0.69)
Subtotal SOBUE								0.72	(0.57- 0.90)
SVENSS	560	f	0	5	13	42	53	0.49	(0.16- 1.47)
SVENSS	561	f	0	1	24	42	53	0.05	(0.01- 0.40)
Subtotal SVENSS								0.29	(0.11- 0.77)
WYNDE3	511	m	0	18	22	171	207	0.99	(0.51- 1.91)
WYNDE3	512	m	0	8	17	171	207	0.57	(0.24- 1.35)
WYNDE3	513	m	0	8	31	171	207	0.31	(0.14- 0.70)
WYNDE3	514	m	0	2	55	171	207	0.04	(0.01- 0.18)
Subtotal WYNDE3								0.49	(0.32- 0.74)
Totals				1642	5988	17013	39104		

*prospective study

Table 2K6 - 5

IESLC - Meta-analysis of Ex Smoking by Years quit (vs current), Overview
Squamous, Cigarettes (or Any Product if Cigarettes not available)
Least adjusted

REF	NRR	SEX	AD	Ys	Ws	Qs	Ps
BARBON	587	m	0	-0.02	6.73	1.04	0.9599
BARBON	588	m	0	-0.43	19.34	0.01	0.0585
BARBON	589	m	0	-0.74	8.13	0.86	0.0355
BARBON	590	m	0	-2.11	3.64	10.53	0.0001
Subtotal BARBON				-0.59	37.84	12.43	
JAHN	605	m	0	2.79	6.72	68.88	0.0000
JAHN	606	m	0	1.59	6.20	24.74	0.0001
JAHN	607	m	0	0.32	16.73	8.95	0.1917
JAHN	608	m	0	-0.21	16.50	0.66	0.3901
JAHN	609	m	0	-1.41	13.61	13.62	0.0000
JAHN	610	m	0	-2.34	7.04	26.15	0.0000
Subtotal JAHN				-0.08	66.79	143.01	
JAIN	546	m	0	-0.55	12.31	0.24	0.0525
JAIN	547	m	0	-1.49	14.26	16.69	0.0000
JAIN	510	f	0	-0.67	8.55	0.59	0.0484
JAIN	511	f	0	-1.96	5.50	13.26	0.0000
Subtotal JAIN				-1.10	40.63	30.78	
LUBIN2	775	m	0	0.16	283.98	92.79	0.0072
LUBIN2	776	m	0	-0.30	182.96	2.31	0.0000
LUBIN2	777	m	0	-0.65	112.99	6.66	0.0000
LUBIN2	778	m	0	-1.06	56.90	24.05	0.0000
LUBIN2	779	m	0	-1.46	91.92	101.36	0.0000
LUBIN2	899	f	0	0.06	21.85	4.93	0.7687
LUBIN2	900	f	0	-0.91	4.18	1.03	0.0634
LUBIN2	901	f	0	-1.69	1.84	3.03	0.0215
Subtotal LUBIN2				-0.38	756.62	236.15	
MATOS	626	m	0	-0.36	3.02	0.01	0.5284
MATOS	627	m	0	-0.30	3.64	0.05	0.5671
MATOS	628	m	0	-1.62	4.04	5.88	0.0011
Subtotal MATOS				-0.82	10.69	5.93	
PEZZOT	582	m	0	-0.33	8.21	0.06	0.3510
PEZZOT	583	m	0	-1.87	5.47	11.55	0.0000
Subtotal PEZZOT				-0.94	13.68	11.61	
SOBUE	739	m	0	-0.03	30.44	4.48	0.8745
SOBUE	740	m	0	-0.28	21.22	0.36	0.1934
SOBUE	741	m	0	-0.79	22.08	3.23	0.0002
Subtotal SOBUE				-0.33	73.74	8.07	
SVENSS	560	f	0	-0.72	3.13	0.30	0.2010
SVENSS	561	f	0	-2.95	0.92	5.92	0.0047
Subtotal SVENSS				-1.23	4.05	6.22	
WYNDE3	511	m	0	-0.01	8.95	1.45	0.9770
WYNDE3	512	m	0	-0.56	5.14	0.12	0.2020
WYNDE3	513	m	0	-1.16	5.95	3.36	0.0045
WYNDE3	514	m	0	-3.12	1.89	13.90	0.0000
Subtotal WYNDE3				-0.72	21.94	18.83	

N	36
NS	9

Table 2K6 - 6

IESLC - Meta-analysis of Ex Smoking by Years quit (vs current), Overview
Squamous, Cigarettes (or Any Product if Cigarettes not available)
Least adjusted

	Sex combined	male	female	Total
N		29	7	36
NS		8	3	11

In this overview table, other than the "N" rows, entries in the "absent" and "Total" columns may be invalid and should be ignored

Years quit vs current (lower focus)	absent	1-6k3	4-11k7	8+k12	Total
N	17	6	4	9	36
NS	7	6	4	7	24
Wt	268.77	349.85	224.32	183.04	1025.98
Het Chi	221.23	2.64	0.12	32.91	473.03
Het df	16	5	3	8	35
Het P	***	N.S.	N.S.	***	***
Fixed RR	0.41	1.15	0.75	0.40	0.66
RRl	0.36	1.03	0.66	0.35	0.62
RRu	0.46	1.28	0.85	0.47	0.70
P	---	++	---	---	---
Random RR	0.51	1.15	0.75	0.27	0.52
RRl	0.31	1.03	0.66	0.18	0.40
RRu	0.83	1.28	0.85	0.40	0.67
P	--	++	---	---	---

Years quit vs current (higher focus)	absent	1-11k3	4-19k12	13+k20	Total
N	17	11	4	4	36
NS	9	9	3	3	24
Wt	375.83	403.91	142.46	103.78	1025.98
Het Chi	219.11	17.02	2.78	9.54	473.03
Het df	16	10	3	3	35
Het P	***	(*)	N.S.	*	***
Fixed RR	0.57	1.08	0.52	0.24	0.66
RRl	0.51	0.98	0.44	0.20	0.62
RRu	0.63	1.19	0.61	0.29	0.70
P	---	N.S.	---	---	---
Random RR	0.45	0.94	0.52	0.22	0.52
RRl	0.29	0.77	0.44	0.11	0.40
RRu	0.71	1.14	0.61	0.43	0.67
P	---	N.S.	---	---	---

Table 2K6 - 6

IESLC - Meta-analysis of Ex Smoking by Years quit (vs current), Overview
Squamous, Cigarettes (or Any Product if Cigarettes not available)
Least adjusted

MALES

		Years quit vs current (lower focus)				
		absent	1-6k3	4-11k7	8+k12	Total
	N	13	6	4	6	29
	NS	6	6	4	6	22
	Wt	233.40	349.85	224.32	172.43	980.00
Het	Chi	197.60	2.64	0.12	22.59	443.91
Het	df	12	5	3	5	28
Het	P	***	N.S.	N.S.	***	***
Fixed	RR	0.37	1.15	0.75	0.42	0.67
	RRl	0.33	1.03	0.66	0.36	0.63
	RRu	0.42	1.28	0.85	0.49	0.71
	P	---	++	---	---	---
Random	RR	0.50	1.15	0.75	0.30	0.56
	RRl	0.28	1.03	0.66	0.20	0.42
	RRu	0.91	1.28	0.85	0.45	0.74
	P	-	++	---	---	---

		Years quit vs current (higher focus)				
		absent	1-11k3	4-19k12	13+k20	Total
	N	15	8	3	3	29
	NS	8	8	3	3	22
	Wt	369.40	370.38	138.28	101.94	980.00
Het	Chi	202.86	9.88	2.50	9.42	443.91
Het	df	14	7	2	2	28
Het	P	***	N.S.	N.S.	**	***
Fixed	RR	0.58	1.11	0.52	0.24	0.67
	RRl	0.53	1.00	0.44	0.20	0.63
	RRu	0.65	1.23	0.62	0.29	0.71
	P	---	+	---	---	---
Random	RR	0.52	1.01	0.52	0.21	0.56
	RRl	0.33	0.84	0.42	0.09	0.42
	RRu	0.82	1.22	0.66	0.50	0.74
	P	--	N.S.	---	---	---

FEMALES

		Years quit vs current (lower focus)				
		absent	1-6k3	4-11k7	8+k12	Total
	N	4			3	7
	NS	3			3	6
	Wt	35.37			10.61	45.98
Het	Chi	8.20			4.39	27.61
Het	df	3			2	6
Het	P	*			N.S.	***
Fixed	RR	0.76			0.20	0.55
	RRl	0.55			0.11	0.42
	RRu	1.05			0.36	0.74
	P	N.S.			---	---
Random	RR	0.57			0.18	0.34
	RRl	0.29			0.07	0.17
	RRu	1.11			0.48	0.70
	P	(-)			---	--

Table 2K6 - 6

IESLC - Meta-analysis of Ex Smoking by Years quit (vs current), Overview
Squamous, Cigarettes (or Any Product if Cigarettes not available)
Least adjusted

FEMALES

		Years quit vs current (higher focus)				
		absent	1-11k3	4-19k12	13+k20	Total
	N	2	3	1	1	7
	NS	2	3	1	1	6
	Wt	6.43	33.53	4.18	1.84	45.98
Het	Chi	0.76	4.29	0.00	0.00	27.61
Het	df	1	2	0	0	6
Het	P	N.S.	N.S.	N.S.	N.S.	***
Fixed	RR	0.12	0.82	0.40	0.18	0.55
	RRl	0.06	0.58	0.15	0.04	0.42
	RRu	0.26	1.15	1.05	0.78	0.74
	P	---	N.S.	(-)	-	---
Random	RR	0.12	0.71	0.40	0.18	0.34
	RRl	0.06	0.40	0.15	0.04	0.17
	RRu	0.26	1.27	1.05	0.78	0.70
	P	---	N.S.	(-)	-	--

Table 2K6 - 7

IESLC - Meta-analysis of Ex Smoking by Years quit (vs current), Overview
Squamous, Cigarettes (or Any Product if Cigarettes not available)
Excluded studies (and stage at which they were excluded)

1	AGUDO AKIBA AMANDU AMES AXELSS BEST BOUCHA BOUCOT BRESLO CHEN CHEN2 CHIAZZ DEAN2 DOSEME ENGELA FAN GENG GER GUO HAENSZ HEGMAN HOLE HU HU2 JUSSAW KATSOU KAUFMA KOO KOULUM KREUZE LETOUR LEVIN LIAW LIU3 LIU4 LIU5 MCCONN MIGRAN MRFITR NOTAN2 OSANN2 PERNU QIAO2 RACHTA RESTRE SADOWS SEGI2 STASZE TIZZAN VUTUC WATSON WU WUWILL WYNDE2 WYNDE8 XU YUAN ZHANG ZHENG ZHOU
2	AUVINE BENSHL BLOT1 BROWN3 BUFFLE GURSEL LAUSSM LUO MCDUFF PISANI PRESCO SPITZ WU2 WYNDE7
4	ARMADA BECHER BOFFET BROSS CARPEN CEDERL CHOI CHYOU CORREA CPSI CPSII DAMBER DARBY DEAN3 DESTEF DOLL DOLL2 DORGAN DORN GAO GAO2 GARCIA GARSHI GILLIS GRAHAM HAMMO2 HIRAYA HUMBLE JOLY KAISE2 KHUDER LUBIN PEZZO2 QIAO SPEIZE SUZUK2 TVERDA WANG2 WIGLE
5	ALDERS HAMMON
10	JEDRYC WAKAI WYNDE6
14	BENHAM

Table 2K6 - 8

Potentially overlapping studies

REF	REFGP	PRINC	. OVERLAP/LINK
LUBIN2	LUBIN2	1	Lubin-combined
JAHN	BOFFET	2	Subset of BOFFET

Table 2K6 - 9

Most adjusted - insufficient data for meta-analysis

REF	NRR	SEX	AGEL	AGEH	RACE	YF	LC TYPE	LOC	START	ST	NLC	R	VB	P	H	AD	ADOS	PRODUCT	exL	exH	S1	S2	DENOM	De
ALDERS	543	m	0	0	all	-	q+s	Eu:UK	1977	CC	1448	n	V	n	n	1	0	cig only	0.1	2	0	0	current	ot
ALDERS	544	m	0	0	all	-	q+s	Eu:UK	1977	CC	1448	n	V	n	n	1	0	cig only	3	9	0	1	current	ot
ALDERS	545	m	0	0	all	-	q+s	Eu:UK	1977	CC	1448	n	V	n	n	1	0	cig only	10	999	3	0	current	ot
ALDERS	554	f	0	0	all	-	q+s	Eu:UK	1977	CC	1448	n	V	n	n	1	0	cig only	0.1	2	0	0	current	ot
ALDERS	555	f	0	0	all	-	q+s	Eu:UK	1977	CC	1448	n	V	n	n	1	0	cig only	3	9	0	1	current	ot
ALDERS	556	f	0	0	all	-	q+s	Eu:UK	1977	CC	1448	n	V	n	n	1	0	cig only	10	999	3	0	current	ot
HAMMON	507	m	0	0	wh	0	not a	NAmer	1952	pr	448	n	bl	n	n	1	0	cig only	0.1	0.9	0	0	current	st
HAMMON	508	m	0	0	wh	0	not a	NAmer	1952	pr	448	n	bl	n	n	1	0	cig only	1.0	9	0	1	current	st
HAMMON	509	m	0	0	wh	0	not a	NAmer	1952	pr	448	n	bl	n	n	1	0	cig only	10	999	3	0	current	st

REF	NRR	RR	SIG	RRDATA comment
ALDERS	543	2.10	y	0.01<p<0.05
ALDERS	544	0.36	y	0.01<p<0.05
ALDERS	545	0.21	y	0.001<p<0.01
ALDERS	554	3.02	y	p<0.001
ALDERS	555	1.51	n	0
ALDERS	556	0.12	y	p<0.001
HAMMON	507	*		RR for <1 pack is 0.97, while that for 1+ packs is 1.26
HAMMON	508	*		RR for <1 pack per day is 0.62, while that for 1+ packs is 0.49
HAMMON	509	*		RR for <1 pack per day is 0.14, while that for 1+ packs is 0.39

Table 2K7 -

IESLC - Meta-analysis of Ex Smoking, Years quit (vs current), "Low"
Squamous, Cigarettes (or Any Product if Cigarettes not available)

This analysis is restricted to results for:
1) Ex smokers
2) Results by Years quit (vs current)
3) Categorical results by Years quit (vs current)
4) Squamous (or near equivalent)
5) Results complete enough for use in metaanalysis

Within each study, results are then selected (in the following order of preference, within each sex) for:
6) (not applicable)
7) PRODUCT: cigarettes regardless of other products, cigarettes only, all/unspec
8) CIGTYPE: all/unspecified, MC regardless of HR, MC only
9) Results with least adjustment for other aspects of smoking (ADOS)
10) DENOM: current smokers, current + recent smokers (up to number of m=months or y=years, max 2 years)
11) Followup period (YF, prospective studies): whole study (coded as 0) or longest available
12) LCtype: squamous or nearest available, but not adeno. (q = squamous, s = small,
a = adeno, KI = Kreyberg I, u = undifferentiated)
13) Race: all or nearest available, otherwise by race (wh or w = white, bl or b = black, hi = hispanic
ch = chinese, jap = japanese, haw = hawaiian, w+o = white + oriental, sca = scandinavian, as = asian)
14) Years quit (vs current) "low" in key scheme 1 (key value 3, maximum range 1-6)
15) For overlapping studies: principal rather than subsidiary studies
Finally by Age: whole study (coded as 0) if available, otherwise by widest available age group
and then for single sex results (m, f) in preference to results for both sexes combined (c).

Results adjusted (AD) for the most potential confounders are then chosen in Sections -1 to -3
(and those which actually differ from the adjusted results in Table 2K2 - 1 are marked 'x' in Section -1)
and results adjusted for the least confounders in Sections -4 to -6. (Those least adjusted results which
actually differ from the most adjusted are marked 'x' in column X in Section -4)

Section -7 shows excluded studies, together with the stage (as above) at which no qualifying
results were found.

Section -8 lists the potentially overlapping studies which have been included (1=principal, 2=subsidiary).

Section -9 lists any results which would have been included in preference except that they had data not complete
enough for use in meta-analysis, with their significance (yes/no), if known, and any further comment as entered
on the database. It also lists as "gap" any categories for which no data were presented by the original authors.

In addition to those mentioned above, the following fields, levels and abbreviations are used:
* or nk = not known, n = no, y = yes, ot = other
nev = never
all/unspec = all or unspecified, cig+/-ot = cigarettes irrespective of other products (cigar, pipe etc)
MC = manufactured cigarettes, HR = hand-rolled cigarettes
exL, exH = range of exposure (low and high) in the smoking group, in terms of Years quit (vs current)
REF: 6-character study reference
NRR: number of the RR on the database within the study
ST : study type (CC = case control, pr or prosp = prospective)
NLC: number of lung cancer cases in whole study
R : risky occupational population (n = no, m = mining, o = other risky)
VB : national cigarette type (V = at least 75% Virginia, bl = at least 75% blended, ot = other)
P : any proxy use
H : full histological confirmation
De : derivation of RR/CI (or = original, st = standard method, ot = other method of estimation)

Table 2K7 - 1

IESLC - Meta-analysis of Ex Smoking, Years quit (vs current), "Low"
Squamous, Cigarettes (or Any Product if Cigarettes not available)
Most adjusted

REF	NRR	2K2	SEX	AGEL	AGEH	RACE	YF	LC TYPE	LOC	START	ST	NLC	R	VB	P	H	AD	ADOS	PRODUCT	exL	exH	DENOM	De
BARBON	602		m	0	0	all	-	q	Eu:wst	1979	CC	755	n	bl	y	y	1	0	all/unsp	0.1	4	current	ot
JAHN	607		m	0	0	all	-	q	Eu:Ger	1988	CC	1004	n	bl	n	n	0	0	cig+/-ot	2	5	current	st
LUBIN2	775		m	0	0	all	-	q	Eu:mul	1976	CC	7804	n	bl	n	y	0	0	cig+/-ot	0.1	4	current	st
LUBIN2	908		f	0	0	all	-	q	Eu:mul	1976	CC	7804	n	bl	n	y	2	1#	cig+/-ot	0.1	4	current	ot
MATOS	636		m	0	0	all	-	q	SCAmer	1994	CC	200	n	bl	n	n	2	0	cig+/-ot	1.0	5	cur+1y	or
SOBUE	739		m	0	0	all	-	q	As:Jap	1986	CC	1376	n	bl	n	y	0	0	cig+/-ot	1.0	4	cur+1y	st
WYNDE3	511		m	0	0	all	-	KI	NAmer	1966	CC	350	n	bl	n	y	0	0	all/unsp	1.0	3	cur+1y	st

Comments on values in listings

LUBIN2 ADOS Duration of smoking

Cigarette type is all/unspec for all RRs

Table 2K7 - 2

IESLC - Meta-analysis of Ex Smoking, Years quit (vs current), "Low"
Squamous, Cigarettes (or Any Product if Cigarettes not available)
Most adjusted

REF	NRR	SEX	AD	Number Exposed Case	Cont	Non-exposed Case	Cont	RR	95.00%CI
BARBON	602	m	1	11	-	203	-	0.97	(0.45- 2.07)
JAHN	607	m	0	36	46	153	269	1.38	(0.85- 2.22)
LUBIN2	775	m	0	498	1047	2518	6209	1.17	(1.04- 1.32)
LUBIN2	908	f	2	25	-	154	-	1.10	(0.66- 1.83)
Subtotal LUBIN2								1.17	(1.04- 1.31)
MATOS	636	m	2	4	-	33	-	0.70	(0.20- 2.20)
SOBUE	739	m	0	52	116	292	633	0.97	(0.68- 1.39)
WYNDE3	511	m	0	18	22	171	207	0.99	(0.51- 1.91)
Partial Totals				644	1231	3524	7318		

*prospective study

REF	NRR	SEX	AD	Ys	Ws	Qs	Ps
BARBON	602	m	1	-0.03	6.60	0.19	0.9376
JAHN	607	m	0	0.32	16.73	0.55	0.1917
LUBIN2	775	m	0	0.16	283.98	0.14	0.0072
LUBIN2	908	f	2	0.10	14.77	0.03	0.7141
Subtotal LUBIN2				0.16	298.76	0.17	
MATOS	636	m	2	-0.36	2.67	0.65	0.5598
SOBUE	739	m	0	-0.03	30.44	0.84	0.8745
WYNDE3	511	m	0	-0.01	8.95	0.19	0.9770

	N	7
	NS	6
	Wt	364.15
Het	Chi	2.59
Het	df	6
Het	P	N.S.
Fixed	RR	1.15
	RRl	1.03
	RRu	1.27
	P	++
Random	RR	1.15
	RRl	1.03
	RRu	1.27
	P	++
Asymm	P	N.S.

Table 2K7 - 3

IESLC - Meta-analysis of Ex Smoking, Years quit (vs current), "Low"
Squamous, Cigarettes (or Any Product if Cigarettes not available)
Most adjusted

	Sex			
	combined	male	female	Total
N		6	1	7
NS		6	1	7
Wt		349.37	14.77	364.15
Het Chi		2.56	0.00	2.59
Het df		5	0	6
Het P		N.S.	N.S.	N.S.
Fixed RR		1.15	1.10	1.15
RRl		1.03	0.66	1.03
RRu		1.28	1.83	1.27
P		++	N.S.	++
Random RR		1.15	1.10	1.15
RRl		1.03	0.66	1.03
RRu		1.28	1.83	1.27
P		++	N.S.	++
Between Chi				0.03
Between df				1
Between P				N.S.
Btwn(F) P				N.S.
Btwn(R) P				N.S.

Too few RRs for analysis by factor

Table 2K7 - 4

IESLC - Meta-analysis of Ex Smoking, Years quit (vs current), "Low"
Squamous, Cigarettes (or Any Product if Cigarettes not available)
Least adjusted

REF	NRR	X	SEX	AGEL	AGEH	RACE	YF	LC TYPE	LOC	START	ST	NLC	R	VB	P	H	AD	ADOS	PRODUCT	exL	exH	DENOM	De
BARBON	587	x	m	0	0	all	-	q	Eu:wst	1979	CC	755	n	bl	y	y	0	0	all/unsp	0.1	4	current	st
JAHN	607		m	0	0	all	-	q	Eu:Ger	1988	CC	1004	n	bl	n	n	0	0	cig+/-ot	2	5	current	st
LUBIN2	775		m	0	0	all	-	q	Eu:mul	1976	CC	7804	n	bl	n	y	0	0	cig+/-ot	0.1	4	current	st
LUBIN2	908		f	0	0	all	-	q	Eu:mul	1976	CC	7804	n	bl	n	y	2	1#	cig+/-ot	0.1	4	current	ot
MATOS	626	x	m	0	0	all	-	q	SCAmer	1994	CC	200	n	bl	n	n	0	0	cig+/-ot	1.0	5	cur+1y	st
SOBUE	739		m	0	0	all	-	q	As:Jap	1986	CC	1376	n	bl	n	y	0	0	cig+/-ot	1.0	4	cur+1y	st
WYNDE3	511		m	0	0	all	-	KI	NAmer	1966	CC	350	n	bl	n	y	0	0	all/unsp	1.0	3	cur+1y	st

Comments on values in listings

LUBIN2 ADOS Duration of smoking

Cigarette type is all/unspec for all RRs

Table 2K7 - 5

IESLC - Meta-analysis of Ex Smoking, Years quit (vs current), "Low"
Squamous, Cigarettes (or Any Product if Cigarettes not available)
Least adjusted

REF	NRR	SEX	A	Number Exposed Case	Cont	Non-exposed Case	Cont	RR	95.00%CI
BARBON	587	m	0	11	20	203	362	0.98	(0.46- 2.09)
JAHN	607	m	0	36	46	153	269	1.38	(0.85- 2.22)
LUBIN2	775	m	0	498	1047	2518	6209	1.17	(1.04- 1.32)
LUBIN2	908	f	2	25	-	154	-	1.10	(0.66- 1.83)
Subtotal LUBIN2								1.17	(1.04- 1.31)
MATOS	626	m	0	4	23	33	132	0.70	(0.23- 2.15)
SOBUE	739	m	0	52	116	292	633	0.97	(0.68- 1.39)
WYNDE3	511	m	0	18	22	171	207	0.99	(0.51- 1.91)
Partial Totals				644	1274	3524	7812		

*prospective study

REF	NRR	SEX	AD	Ys	Ws	Qs	Ps
BARBON	587	m	0	-0.02	6.73	0.16	0.9599
JAHN	607	m	0	0.32	16.73	0.56	0.1917
LUBIN2	775	m	0	0.16	283.98	0.15	0.0072
LUBIN2	908	f	2	0.10	14.77	0.03	0.7141
Subtotal LUBIN2				0.16	298.76	0.17	
MATOS	626	m	0	-0.36	3.02	0.75	0.5284
SOBUE	739	m	0	-0.03	30.44	0.83	0.8745
WYNDE3	511	m	0	-0.01	8.95	0.19	0.9770

	N	7
	NS	6
	Wt	364.62
Het	Chi	2.67
Het	df	6
Het	P	N.S.
Fixed	RR	1.15
	RRl	1.03
	RRu	1.27
	P	++
Random	RR	1.15
	RRl	1.03
	RRu	1.27
	P	++
Asymm	P	N.S.

Table 2K7 - 6

IESLC - Meta-analysis of Ex Smoking, Years quit (vs current), "Low"
Squamous, Cigarettes (or Any Product if Cigarettes not available)
Least adjusted

	Sex combined	male	female	Total
N		6	1	7
NS		6	1	7
Wt		349.85	14.77	364.62
Het Chi		2.64	0.00	2.67
Het df		5	0	6
Het P		N.S.	N.S.	N.S.
Fixed RR		1.15	1.10	1.15
RRl		1.03	0.66	1.03
RRu		1.28	1.83	1.27
P		++	N.S.	++
Random RR		1.15	1.10	1.15
RRl		1.03	0.66	1.03
RRu		1.28	1.83	1.27
P		++	N.S.	++
Between Chi				0.03
Between df				1
Between P				N.S.
Btwn(F) P				N.S.
Btwn(R) P				N.S.

Table 2K7 - 7

IESLC - Meta-analysis of Ex Smoking, Years quit (vs current), "Low"
Squamous, Cigarettes (or Any Product if Cigarettes not available)
Excluded studies (and stage at which they were excluded)

1	AGUDO AKIBA AMANDU AMES AXELSS BEST BOUCHA BOUCOT BRESLO CHEN CHEN2 CHIAZZ DEAN2 DOSEME ENGELA FAN GENG GER GUO HAENSZ HEGMAN HOLE HU HU2 JUSSAW KATSOU KAUFMA KOO KOULUM KREUZE LETOUR LEVIN LIAW LIU3 LIU4 LIU5 MCCONN MIGRAN MRFITR NOTAN2 OSANN2 PERNU QIAO2 RACHTA RESTRE SADOWS SEGI2 STASZE TIZZAN VUTUC WATSON WU WUWILL WYNDE2 WYNDE8 XU YUAN ZHANG ZHENG ZHOU
2	AUVINE BENSHL BLOT1 BROWN3 BUFFLE GURSEL LAUSSM LUO MCDUFF PISANI PRESCO SPITZ WU2 WYNDE7
4	ARMADA BECHER BOFFET BROSS CARPEN CEDERL CHOI CHYOU CORREA CPSI CPSII DAMBER DARBY DEAN3 DESTEF DOLL DOLL2 DORGAN DORN GAO GAO2 GARCIA GARSHI GILLIS GRAHAM HAMMO2 HIRAYA HUMBLE JOLY KAISE2 KHUDER LUBIN PEZZO2 QIAO SPEIZE SUZUK2 TVERDA WANG2 WIGLE
5	ALDERS HAMMON
10	JEDRYC WAKAI WYNDE6
14	JAIN PEZZOT SVENSS
15	BENHAM

Table 2K7 - 8
Potentially overlapping studies

REF	REFGP	PRINC	OVERLAP/LINK
LUBIN2	LUBIN2	1	Lubin-combined
JAHN	BOFFET	2	Subset of BOFFET

Table 2K8 -

IESLC - Meta-analysis of Ex Smoking, Years quit (vs current), "Mid"
Squamous, Cigarettes (or Any Product if Cigarettes not available)

This analysis is restricted to results for:
1) Ex smokers
2) Results by Years quit (vs current)
3) Categorical results by Years quit (vs current)
4) Squamous (or near equivalent)
5) Results complete enough for use in metaanalysis

Within each study, results are then selected (in the following order of preference, within each sex) for:
6) (not applicable)
7) PRODUCT: cigarettes regardless of other products, cigarettes only, all/unspec
8) CIGTYPE: all/unspecified, MC regardless of HR, MC only
9) Results with least adjustment for other aspects of smoking (ADOS)
10) DENOM: current smokers, current + recent smokers (up to number of m=months or y=years, max 2 years)
11) Followup period (YF, prospective studies): whole study (coded as 0) or longest available
12) LCtype: squamous or nearest available, but not adeno. (q = squamous, s = small,
a = adeno, KI = Kreyberg I, u = undifferentiated)
13) Race: all or nearest available, otherwise by race (wh or w = white, bl or b = black, hi = hispanic
ch = chinese, jap = japanese, haw = hawaiian, w+o = white + oriental, sca = scandinavian, as = asian)
14) Years quit (vs current) "mid" in key scheme 1 (key value 7, maximum range 4-11)
15) For overlapping studies: principal rather than subsidiary studies
Finally by Age: whole study (coded as 0) if available, otherwise by widest available age group
and then for single sex results (m, f) in preference to results for both sexes combined (c).

Results adjusted (AD) for the most potential confounders are then chosen in Sections -1 to -3
(and those which actually differ from the adjusted results in Table 2K3 - 1 are marked 'x' in Section -1)
and results adjusted for the least confounders in Sections -4 to -6. (Those least adjusted results which
actually differ from the most adjusted are marked 'x' in column X in Section -4)

Section -7 shows excluded studies, together with the stage (as above) at which no qualifying
results were found.

Section -8 lists the potentially overlapping studies which have been included (1=principal, 2=subsidiary).

Section -9 lists any results which would have been included in preference except that they had data not complete
enough for use in meta-analysis, with their significance (yes/no), if known, and any further comment as entered
on the database. It also lists as "gap" any categories for which no data were presented by the original authors.

In addition to those mentioned above, the following fields, levels and abbreviations are used:
* or nk = not known, n = no, y = yes, ot = other
nev = never
all/unspec = all or unspecified, cig+/-ot = cigarettes irrespective of other products (cigar, pipe etc)
MC = manufactured cigarettes, HR = hand-rolled cigarettes
exL, exH = range of exposure (low and high) in the smoking group, in terms of Years quit (vs current)
REF: 6-character study reference
NRR: number of the RR on the database within the study
ST : study type (CC = case control, pr or prosp = prospective)
NLC: number of lung cancer cases in whole study
R : risky occupational population (n = no, m = mining, o = other risky)
VB : national cigarette type (V = at least 75% Virginia, bl = at least 75% blended, ot = other)
P : any proxy use
H : full histological confirmation
De : derivation of RR/CI (or = original, st = standard method, ot = other method of estimation)

Table 2K8 - 1

IESLC - Meta-analysis of Ex Smoking, Years quit (vs current), "Mid"
Squamous, Cigarettes (or Any Product if Cigarettes not available)
Most adjusted

REF	NRR	2K3	SEX	AGEL	AGEH	RACE	YF	LC TYPE	LOC	START	ST	NLC	R	VB	P	H	AD	ADOS	PRODUCT	exL	exH	DENOM	De
JAHN	608		m	0	0	all	-	q	Eu:Ger	1988	CC	1004	n	bl	n	n	0	0	cig+/-ot	6	10	current	st
LUBIN2	776		m	0	0	all	-	q	Eu:mul	1976	CC	7804	n	bl	n	y	0	0	cig+/-ot	5	9	current	st
LUBIN2	909		f	0	0	all	-	q	Eu:mul	1976	CC	7804	n	bl	n	y	2	1#	cig+/-ot	5	9	current	ot
MATOS	637		m	0	0	all	-	q	SCAmer	1994	CC	200	n	bl	n	n	2	0	cig+/-ot	6	10	cur+1y	or
SOBUE	740		m	0	0	all	-	q	As:Jap	1986	CC	1376	n	bl	n	y	0	0	cig+/-ot	5	9	cur+1y	st

Comments on values in listings

LUBIN2 ADOS Duration of smoking

Cigarette type is all/unspec for all RRs

Table 2K8 - 2

IESLC - Meta-analysis of Ex Smoking, Years quit (vs current), "Mid"
Squamous, Cigarettes (or Any Product if Cigarettes not available)
Most adjusted

REF	NRR	SEX	A	Number Exposed Case	Cont	Non-exposed Case	Cont	RR	95.00%CI
JAHN	608	m	0	29	63	153	269	0.81	(0.50- 1.31)
LUBIN2	776	m	0	265	882	2518	6209	0.74	(0.64- 0.86)
LUBIN2	909	f	2	13	-	154	-	0.90	(0.47- 1.73)
Subtotal LUBIN2								0.75	(0.65- 0.86)
MATOS	637	m	2	5	-	33	-	0.60	(0.20- 1.90)
SOBUE	740	m	0	32	92	292	633	0.75	(0.49- 1.15)
Partial Totals				344	1037	3150	7111		

*prospective study

REF	NRR	SEX	AD	Ys	Ws	Qs	Ps
JAHN	608	m	0	-0.21	16.50	0.09	0.3901
LUBIN2	776	m	0	-0.30	182.96	0.03	0.0000
LUBIN2	909	f	2	-0.11	9.05	0.30	0.7513
Subtotal LUBIN2				-0.29	192.01	0.33	
MATOS	637	m	2	-0.51	3.03	0.15	0.3738
SOBUE	740	m	0	-0.28	21.22	0.00	0.1934

	N	5
	NS	4
	Wt	232.76
Het	Chi	0.58
Het	df	4
Het	P	N.S.
Fixed	RR	0.75
	RRl	0.66
	RRu	0.85
	P	---
Random	RR	0.75
	RRl	0.66
	RRu	0.85
	P	---
Asymm	P	N.S.

Table 2K8 - 3

IESLC - Meta-analysis of Ex Smoking, Years quit (vs current), "Mid"
Squamous, Cigarettes (or Any Product if Cigarettes not available)
Most adjusted

	combined	Sex male	female	Total
N		4	1	5
NS		4	1	5
Wt		223.72	9.05	232.76
Het Chi		0.26	0.00	0.58
Het df		3	0	4
Het P		N.S.	N.S.	N.S.
Fixed RR		0.74	0.90	0.75
RRl		0.65	0.47	0.66
RRu		0.85	1.73	0.85
P		---	N.S.	---
Random RR		0.74	0.90	0.75
RRl		0.65	0.47	0.66
RRu		0.85	1.73	0.85
P		---	N.S.	---
Between Chi				0.31
Between df				1
Between P				N.S.
Btwn(F) P				N.S.
Btwn(R) P				N.S.

Too few RRs for analysis by factor

Table 2K8 - 4

IESLC - Meta-analysis of Ex Smoking, Years quit (vs current), "Mid"
Squamous, Cigarettes (or Any Product if Cigarettes not available)
Least adjusted

REF	NRR	X	SEX	AGEL	AGEH	RACE	YF	LC TYPE	LOC	START	ST	NLC	R	VB	P	H	AD	ADOS	PRODUCT	exL	exH	DENOM	De
JAHN	608		m	0	0	all	-	q	Eu:Ger	1988	CC	1004	n	bl	n	n	0	0	cig+/-ot	6	10	current	st
LUBIN2	776		m	0	0	all	-	q	Eu:mul	1976	CC	7804	n	bl	n	y	0	0	cig+/-ot	5	9	current	st
LUBIN2	909		f	0	0	all	-	q	Eu:mul	1976	CC	7804	n	bl	n	y	2	1#	cig+/-ot	5	9	current	ot
MATOS	627	x	m	0	0	all	-	q	SCAmer	1994	CC	200	n	bl	n	n	0	0	cig+/-ot	6	10	cur+1y	st
SOBUE	740		m	0	0	all	-	q	As:Jap	1986	CC	1376	n	bl	n	y	0	0	cig+/-ot	5	9	cur+1y	st

Comments on values in listings

LUBIN2 ADOS Duration of smoking

Cigarette type is all/unspec for all RRs

Table 2K8 - 5

IESLC - Meta-analysis of Ex Smoking, Years quit (vs current), "Mid"
Squamous, Cigarettes (or Any Product if Cigarettes not available)
Least adjusted

REF	NRR	SEX	A	Number Exposed Case	Cont	Non-exposed Case	Cont	RR	95.00%CI
JAHN	608	m	0	29	63	153	269	0.81	(0.50- 1.31)
LUBIN2	776	m	0	265	882	2518	6209	0.74	(0.64- 0.86)
LUBIN2	909	f	2	13	-	154	-	0.90	(0.47- 1.73)
Subtotal LUBIN2								0.75	(0.65- 0.86)
MATOS	627	m	0	5	27	33	132	0.74	(0.27- 2.07)
SOBUE	740	m	0	32	92	292	633	0.75	(0.49- 1.15)
Partial Totals				344	1064	3150	7243		

*prospective study

REF	NRR	SEX	AD	Ys	Ws	Qs	Ps
JAHN	608	m	0	-0.21	16.50	0.09	0.3901
LUBIN2	776	m	0	-0.30	182.96	0.04	0.0000
LUBIN2	909	f	2	-0.11	9.05	0.29	0.7513
Subtotal LUBIN2				-0.29	192.01	0.33	
MATOS	627	m	0	-0.30	3.64	0.00	0.5671
SOBUE	740	m	0	-0.28	21.22	0.00	0.1934

	N	5
	NS	4
	Wt	233.37
Het	Chi	0.42
Het	df	4
Het	P	N.S.
Fixed	RR	0.75
	RRl	0.66
	RRu	0.86
	P	---
Random	RR	0.75
	RRl	0.66
	RRu	0.86
	P	---
Asymm	P	N.S.

Table 2K8 - 6

IESLC - Meta-analysis of Ex Smoking, Years quit (vs current), "Mid"
Squamous, Cigarettes (or Any Product if Cigarettes not available)
Least adjusted

	Sex		
combined	male	female	Total
N	4	1	5
NS	4	1	5
Wt	224.32	9.05	233.37
Het Chi	0.12	0.00	0.42
Het df	3	0	4
Het P	N.S.	N.S.	N.S.
Fixed RR	0.75	0.90	0.75
RRl	0.66	0.47	0.66
RRu	0.85	1.73	0.86
P	---	N.S.	---
Random RR	0.75	0.90	0.75
RRl	0.66	0.47	0.66
RRu	0.85	1.73	0.86
P	---	N.S.	---
Between Chi			0.30
Between df			1
Between P			N.S.
Btwn(F) P			(*)
Btwn(R) P			N.S.

Table 2K8 - 7

IESLC - Meta-analysis of Ex Smoking, Years quit (vs current), "Mid"
Squamous, Cigarettes (or Any Product if Cigarettes not available)
Excluded studies (and stage at which they were excluded)

1	AGUDO AKIBA AMANDU AMES AXELSS BEST BOUCHA BOUCOT BRESLO CHEN CHEN2 CHIAZZ DEAN2 DOSEME ENGELA FAN GENG GER GUO HAENSZ HEGMAN HOLE HU HU2 JUSSAW KATSOU KAUFMA KOO KOULUM KREUZE LETOUR LEVIN LIAW LIU3 LIU4 LIU5 MCCONN MIGRAN MRFITR NOTAN2 OSANN2 PERNU QIAO2 RACHTA RESTRE SADOWS SEGI2 STASZE TIZZAN VUTUC WATSON WU WUWILL WYNDE2 WYNDE8 XU YUAN ZHANG ZHENG ZHOU
2	AUVINE BENSHL BLOT1 BROWN3 BUFFLE GURSEL LAUSSM LUO MCDUFF PISANI PRESCO SPITZ WU2 WYNDE7
4	ARMADA BECHER BOFFET BROSS CARPEN CEDERL CHOI CHYOU CORREA CPSI CPSII DAMBER DARBY DEAN3 DESTEF DOLL DOLL2 DORGAN DORN GAO GAO2 GARCIA GARSHI GILLIS GRAHAM HAMMO2 HIRAYA HUMBLE JOLY KAISE2 KHUDER LUBIN PEZZO2 QIAO SPEIZE SUZUK2 TVERDA WANG2 WIGLE
5	ALDERS HAMMON
10	JEDRYC WAKAI WYNDE6
14	BARBON JAIN PEZZOT SVENSS WYNDE3
15	BENHAM

Table 2K8 - 8
Potentially overlapping studies

REF	REFGP	PRINC	OVERLAP/LINK
LUBIN2	LUBIN2	1	Lubin-combined
JAHN	BOFFET	2	Subset of BOFFET

Table 2K9 -

IESLC - Meta-analysis of Ex Smoking, Years quit (vs current), "High"
Squamous, Cigarettes (or Any Product if Cigarettes not available)

This analysis is restricted to results for:
1) Ex smokers
2) Results by Years quit (vs current)
3) Categorical results by Years quit (vs current)
4) Squamous (or near equivalent)
5) Results complete enough for use in metaanalysis

Within each study, results are then selected (in the following order of preference, within each sex) for:
6) PRODUCT: cigarettes regardless of other products, cigarettes only, all/unspec
7) CIGTYPE: all/unspecified, MC regardless of HR, MC only
8) Results with least adjustment for other aspects of smoking (ADOS)
9) DENOM: current smokers, current + recent smokers (up to number of m=months or y=years, max 2 years)
10) Followup period (YF, prospective studies): whole study (coded as 0) or longest available
11) LCtype: squamous or nearest available, but not adeno. (q = squamous, s = small,
a = adeno, KI = Kreyberg I, u = undifferentiated)
12) Race: all or nearest available, otherwise by race (wh or w = white, bl or b = black, hi = hispanic
ch = chinese, jap = japanese, haw = hawaiian, w+o = white + oriental, sca = scandinavian, as = asian)
13) Years quit (vs current) "high" in key scheme 1 (key value 12, maximum range 8+)
14) For overlapping studies: principal rather than subsidiary studies
Finally by Age: whole study (coded as 0) if available, otherwise by widest available age group
and then for single sex results (m, f) in preference to results for both sexes combined (c).

Results adjusted (AD) for the most potential confounders are then chosen in Sections -1 to -3
(and those which actually differ from the adjusted results in Table 2K4 - 1 are marked 'x' in Section -1)
and results adjusted for the least confounders in Sections -4 to -6. (Those least adjusted results which
actually differ from the most adjusted are marked 'x' in column X in Section -4)

Section -7 shows excluded studies, together with the stage (as above) at which no qualifying
results were found.

Section -8 lists the potentially overlapping studies which have been included (1=principal, 2=subsidiary).

Section -9 lists any results which would have been included in preference except that they had data not complete
enough for use in meta-analysis, with their significance (yes/no), if known, and any further comment as entered
on the database. It also lists as "gap" any categories for which no data were presented by the original authors.

In addition to those mentioned above, the following fields, levels and abbreviations are used:
* or nk = not known, n = no, y = yes, ot = other
nev = never
all/unspec = all or unspecified, cig+/-ot = cigarettes irrespective of other products (cigar, pipe etc)
MC = manufactured cigarettes, HR = hand-rolled cigarettes
exL, exH = range of exposure (low and high) in the smoking group, in terms of Years quit (vs current)
REF: 6-character study reference
NRR: number of the RR on the database within the study
ST : study type (CC = case control, pr or prosp = prospective)
NLC: number of lung cancer cases in whole study
R : risky occupational population (n = no, m = mining, o = other risky)
VB : national cigarette type (V = at least 75% Virginia, bl = at least 75% blended, ot = other)
P : any proxy use
H : full histological confirmation
De : derivation of RR/CI (or = original, st = standard method, ot = other method of estimation)

Table 2K9 - 1

IESLC - Meta-analysis of Ex Smoking, Years quit (vs current), "High"
Squamous, Cigarettes (or Any Product if Cigarettes not available)
Most adjusted

REF	NRR	2K4	SEX	AGEL	AGEH	RACE	YF	LC TYPE	LOC	START	ST	NLC	R	VB	P	H	AD	ADOS	PRODUCT	exL	exH	DENOM	De
JAHN	609		m	0	0	all	-	q	Eu:Ger	1988	CC	1004	n	bl	n	n	0	0	cig+/-ot	11	20	current	st
JAIN	547		m	0	0	all	-	q	NAmer	1981	CC	845	n	V	y	n	0	0	cig+/-ot	10	999	cur+2y	st
JAIN	511		f	0	0	all	-	q	NAmer	1981	CC	845	n	V	y	n	0	0	cig+/-ot	10	999	cur+2y	st
LUBIN2	777		m	0	0	all	-	q	Eu:mul	1976	CC	7804	n	bl	n	y	0	0	cig+/-ot	10	14	current	st
LUBIN2	900		f	0	0	all	-	q	Eu:mul	1976	CC	7804	n	bl	n	y	0	0	cig+/-ot	10	19	current	st
MATOS	638		m	0	0	all	-	q	SCAmer	1994	CC	200	n	bl	n	n	2	0	cig+/-ot	11	999	cur+1y	or
PEZZOT	583		m	0	0	all	-	q	SCAmer	1987	CC	215	n	bl	n	y	0	0	cig only	11	999	cur+1y	st
SOBUE	741		m	0	0	all	-	q	As:Jap	1986	CC	1376	n	bl	n	y	0	0	cig+/-ot	10	999	cur+1y	st
SVENSS	561		f	0	0	all	-	q	Eu:Sca	1983	CC	210	n	bl	n	n	0	0	all/unsp	11	999	cur+2y	st

Cigarette type is all/unspec for all RRs

Table 2K9 - 2

IESLC - Meta-analysis of Ex Smoking, Years quit (vs current), "High"
Squamous, Cigarettes (or Any Product if Cigarettes not available)
Most adjusted

REF	NRR	SEX	A	Number Exposed Case	Number Exposed Cont	Non-exposed Case	Non-exposed Cont	RR	95.00%CI
JAHN	609	m	0	18	130	153	269	0.24	(0.14- 0.41)
JAIN	547	m	0	23	113	107	118	0.22	(0.13- 0.38)
JAIN	511	f	0	7	61	81	99	0.14	(0.06- 0.32)
Subtotal JAIN								0.20	(0.13- 0.31)
LUBIN2	777	m	0	146	693	2518	6209	0.52	(0.43- 0.62)
LUBIN2	900	f	0	5	33	154	410	0.40	(0.15- 1.05)
Subtotal LUBIN2								0.51	(0.43- 0.62)
MATOS	638	m	2	5	-	33	-	0.20	(0.06- 0.50)
PEZZOT	583	m	0	8	48	56	52	0.15	(0.07- 0.36)
SOBUE	741	m	0	30	144	292	633	0.45	(0.30- 0.69)
SVENSS	561	f	0	1	24	42	53	0.05	(0.01- 0.40)
Partial Totals				243	1246	3436	7843		

*prospective study

REF	NRR	SEX	AD	Ys	Ws	Qs	Ps
JAHN	609	m	0	-1.41	13.61	3.51	0.0000
JAIN	547	m	0	-1.49	14.26	4.95	0.0000
JAIN	511	f	0	-1.96	5.50	6.18	0.0000
Subtotal JAIN				-1.63	19.76	11.12	
LUBIN2	777	m	0	-0.65	112.99	7.07	0.0000
LUBIN2	900	f	0	-0.91	4.18	0.00	0.0634
Subtotal LUBIN2				-0.66	117.17	7.07	
MATOS	638	m	2	-1.61	3.42	1.70	0.0029
PEZZOT	583	m	0	-1.87	5.47	5.05	0.0000
SOBUE	741	m	0	-0.79	22.08	0.27	0.0002
SVENSS	561	f	0	-2.95	0.92	3.84	0.0047

	N	9
	NS	7
	Wt	182.42
Het	Chi	32.55
Het	df	8
Het	P	***
Fixed	RR	0.40
	RRl	0.35
	RRu	0.47
	P	---
Random	RR	0.27
	RRl	0.18
	RRu	0.40
	P	---
Asymm	P	**

Table 2K9 - 3

IESLC - Meta-analysis of Ex Smoking, Years quit (vs current), "High"
Squamous, Cigarettes (or Any Product if Cigarettes not available)
Most adjusted

	combined	Sex: male	female	Total
N		6	3	9
NS		6	3	9
Wt		171.82	10.61	182.42
Het Chi		22.19	4.39	32.55
Het df		5	2	8
Het P		***	N.S.	***
Fixed RR		0.42	0.20	0.40
RRl		0.36	0.11	0.35
RRu		0.49	0.36	0.47
P		---	---	---
Random RR		0.30	0.18	0.27
RRl		0.20	0.07	0.18
RRu		0.46	0.48	0.40
P		---	---	---
Between Chi				5.97
Between df				1
Between P				*
Btwn(F) P				N.S.
Btwn(R) P				N.S.

Too few RRs for analysis by factor

Table 2K9 - 4

IESLC - Meta-analysis of Ex Smoking, Years quit (vs current), "High"
Squamous, Cigarettes (or Any Product if Cigarettes not available)
Least adjusted

REF	NRR	X	SEX	AGEL	AGEH	RACE	YF	LC TYPE	LOC	START	ST	NLC	R	VB	P	H	AD	ADOS	PRODUCT	exL	exH	DENOM	De
JAHN	609		m	0	0	all	-	q	Eu:Ger	1988	CC	1004	n	bl	n	n	0	0	cig+/-ot	11	20	current	st
JAIN	547		m	0	0	all	-	q	NAmer	1981	CC	845	n	V	y	n	0	0	cig+/-ot	10	999	cur+2y	st
JAIN	511		f	0	0	all	-	q	NAmer	1981	CC	845	n	V	y	n	0	0	cig+/-ot	10	999	cur+2y	st
LUBIN2	777		m	0	0	all	-	q	Eu:mul	1976	CC	7804	n	bl	n	y	0	0	cig+/-ot	10	14	current	st
LUBIN2	900		f	0	0	all	-	q	Eu:mul	1976	CC	7804	n	bl	n	y	0	0	cig+/-ot	10	19	current	st
MATOS	628	x	m	0	0	all	-	q	SCAmer	1994	CC	200	n	bl	n	n	0	0	cig+/-ot	11	999	cur+1y	st
PEZZOT	583		m	0	0	all	-	q	SCAmer	1987	CC	215	n	bl	n	y	0	0	cig only	11	999	cur+1y	st
SOBUE	741		m	0	0	all	-	q	As:Jap	1986	CC	1376	n	bl	n	y	0	0	cig+/-ot	10	999	cur+1y	st
SVENSS	561		f	0	0	all	-	q	Eu:Sca	1983	CC	210	n	bl	n	n	0	0	all/unsp	11	999	cur+2y	st

Cigarette type is all/unspec for all RRs

Table 2K9 - 5

IESLC - Meta-analysis of Ex Smoking, Years quit (vs current), "High"
Squamous, Cigarettes (or Any Product if Cigarettes not available)
Least adjusted

REF	NRR	SEX	A	Number Exposed Case	Cont	Non-exposed Case	Cont	RR	95.00%CI
JAHN	609	m	0	18	130	153	269	0.24	(0.14- 0.41)
JAIN	547	m	0	23	113	107	118	0.22	(0.13- 0.38)
JAIN	511	f	0	7	61	81	99	0.14	(0.06- 0.32)
Subtotal JAIN								0.20	(0.13- 0.31)
LUBIN2	777	m	0	146	693	2518	6209	0.52	(0.43- 0.62)
LUBIN2	900	f	0	5	33	154	410	0.40	(0.15- 1.05)
Subtotal LUBIN2								0.51	(0.43- 0.62)
MATOS	628	m	0	5	101	33	132	0.20	(0.07- 0.53)
PEZZOT	583	m	0	8	48	56	52	0.15	(0.07- 0.36)
SOBUE	741	m	0	30	144	292	633	0.45	(0.30- 0.69)
SVENSS	561	f	0	1	24	42	53	0.05	(0.01- 0.40)
Totals				243	1347	3436	7975		

*prospective study

REF	NRR	SEX	AD	Ys	Ws	Qs	Ps
JAHN	609	m	0	-1.41	13.61	3.47	0.0000
JAIN	547	m	0	-1.49	14.26	4.90	0.0000
JAIN	511	f	0	-1.96	5.50	6.15	0.0000
Subtotal JAIN				-1.63	19.76	11.05	
LUBIN2	777	m	0	-0.65	112.99	7.22	0.0000
LUBIN2	900	f	0	-0.91	4.18	0.00	0.0634
Subtotal LUBIN2				-0.66	117.17	7.22	
MATOS	628	m	0	-1.62	4.04	2.04	0.0011
PEZZOT	583	m	0	-1.87	5.47	5.02	0.0000
SOBUE	741	m	0	-0.79	22.08	0.28	0.0002
SVENSS	561	f	0	-2.95	0.92	3.83	0.0047

	N	9
	NS	7
	Wt	183.04
Het	Chi	32.91
Het	df	8
Het	P	***
Fixed	RR	0.40
	RRl	0.35
	RRu	0.47
	P	---
Random	RR	0.27
	RRl	0.18
	RRu	0.40
	P	---
Asymm	P	**

Table 2K9 - 6

IESLC - Meta-analysis of Ex Smoking, Years quit (vs current), "High"
Squamous, Cigarettes (or Any Product if Cigarettes not available)
Least adjusted

	Sex combined	male	female	Total
N		6	3	9
NS		6	3	9
Wt		172.43	10.61	183.04
Het Chi		22.59	4.39	32.91
Het df		5	2	8
Het P		***	N.S.	***
Fixed RR		0.42	0.20	0.40
RRl		0.36	0.11	0.35
RRu		0.49	0.36	0.47
P		---	---	---
Random RR		0.30	0.18	0.27
RRl		0.20	0.07	0.18
RRu		0.45	0.48	0.40
P		---	---	---
Between Chi				5.93
Between df				1
Between P				*
Btwn(F) P				N.S.
Btwn(R) P				N.S.

Table 2K9 - 7

IESLC - Meta-analysis of Ex Smoking, Years quit (vs current), "High"
Squamous, Cigarettes (or Any Product if Cigarettes not available)
Excluded studies (and stage at which they were excluded)

1	AGUDO AKIBA AMANDU AMES AXELSS BEST BOUCHA BOUCOT BRESLO CHEN CHEN2 CHIAZZ DEAN2 DOSEME ENGELA FAN GENG GER GUO HAENSZ HEGMAN HOLE HU HU2 JUSSAW KATSOU KAUFMA KOO KOULUM KREUZE LETOUR LEVIN LIAW LIU3 LIU4 LIU5 MCCONN MIGRAN MRFITR NOTAN2 OSANN2 PERNU QIAO2 RACHTA RESTRE SADOWS SEGI2 STASZE TIZZAN VUTUC WATSON WU WUWILL WYNDE2 WYNDE8 XU YUAN ZHANG ZHENG ZHOU
2	AUVINE BENSHL BLOT1 BROWN3 BUFFLE GURSEL LAUSSM LUO MCDUFF PISANI PRESCO SPITZ WU2 WYNDE7
4	ARMADA BECHER BOFFET BROSS CARPEN CEDERL CHOI CHYOU CORREA CPSI CPSII DAMBER DARBY DEAN3 DESTEF DOLL DOLL2 DORGAN DORN GAO GAO2 GARCIA GARSHI GILLIS GRAHAM HAMMO2 HIRAYA HUMBLE JOLY KAISE2 KHUDER LUBIN PEZZO2 QIAO SPEIZE SUZUK2 TVERDA WANG2 WIGLE
5	ALDERS HAMMON
10	JEDRYC WAKAI WYNDE6
14	BARBON WYNDE3
15	BENHAM

Table 2K9 - 8

Potentially overlapping studies

REF	REFGP	PRINC	.	OVERLAP/LINK
LUBIN2	LUBIN2	1		Lubin-combined
JAHN	BOFFET	2		Subset of BOFFET

Table 2K9 - 9

Most adjusted - insufficient data for meta-analysis

REF	NRR	SEX	AGEL	AGEH	RACE	YF	LC TYPE	LOC	START	ST	NLC	R	VB	P	H	AD	ADOS	PRODUCT	exL	exH	DENOM	De
ALDERS	545	m	0	0	all	-	q+s	Eu:UK	1977	CC	1448	n	V	n	n	1	0	cig only	10	999	current	ot
ALDERS	556	f	0	0	all	-	q+s	Eu:UK	1977	CC	1448	n	V	n	n	1	0	cig only	10	999	current	ot
HAMMON	509	m	0	0	wh	0	not a	NAmer	1952	pr	448	n	bl	n	n	1	0	cig only	10	999	current	st

REF	NRR	RR	SIG	RRDATA comment
ALDERS	545	0.21	y	0.001<p<0.01
ALDERS	556	0.12	y	p<0.001
HAMMON	509	*		RR for <1 pack per day is 0.14, while that for 1+ packs is 0.39

Table 2K10 -

IESLC - Meta-analysis of Ex Smoking, Years quit (vs current), "Highest vs lowest"
Squamous, Cigarettes (or Any Product if Cigarettes not available)

This analysis is restricted to results for:
1) Ex smokers
2) Results by Years quit (vs current)
3) Categorical results by Years quit (vs current)
4) Denominator (unexposed) = "low"
5) Squamous (or near equivalent)
6) Results complete enough for use in metaanalysis

Within each study, results are then selected (in the following order of preference, within each sex) for:
7) (not applicable)
8) PRODUCT: cigarettes regardless of other products, cigarettes only, all/unspec
9) CIGTYPE: all/unspecified, MC regardless of HR, MC only
10) Results with least adjustment for other aspects of smoking (ADOS)
11) The highest vs lowest category
12) Followup period (YF, prospective studies): whole study (coded as 0) or longest available
13) LCtype: squamous or nearest available, but not adeno. (q = squamous, s = small,
a = adeno, KI = Kreyberg I, u = undifferentiated)
14) Race: all or nearest available, otherwise by race (wh or w = white, bl or b = black, hi = hispanic
ch = chinese, jap = japanese, haw = hawaiian, w+o = white + oriental, sca = scandinavian, as = asian)
15) For overlapping studies: principal rather than subsidiary studies
Finally by Age: whole study (coded as 0) if available, otherwise by widest available age group
and then for single sex results (m, f) in preference to results for both sexes combined (c).

Results adjusted (AD) for the most potential confounders are then chosen in Sections -1 to -3
(and those which actually differ from the adjusted results in Table 2K5 - 1 are marked 'x' in Section -1)
and results adjusted for the least confounders in Sections -4 to -6. (Those least adjusted results which
actually differ from the most adjusted are marked 'x' in column X in Section -4)

Section -7 shows excluded studies, together with the stage (as above) at which no qualifying
results were found.

Section -8 lists the potentially overlapping studies which have been included (1=principal, 2=subsidiary).

Section -9 lists any results which would have been included in preference except that they had data not complete
enough for use in meta-analysis, with their significance (yes/no), if known, and any further comment as entered
on the database. It also lists as "gap" any categories for which no data were presented by the original authors.

In addition to those mentioned above, the following fields, levels and abbreviations are used:
* or nk = not known, n = no, y = yes, ot = other
all/unspec = all or unspecified, cig+/-ot = cigarettes irrespective of other products (cigar, pipe etc)
MC = manufactured cigarettes, HR = hand-rolled cigarettes
exL, exH = range of exposure (low and high) in the "highest" group, in terms of Years quit (vs current)
unexL, unexH = range of exposure (low and high) in the "lowest" group, in terms of Years quit (vs current)
REF: 6-character study reference
NRR: number of the RR on the database within the study
ST : study type (CC = case control, pr or prosp = prospective)
NLC: number of lung cancer cases in whole study
R : risky occupational population (n = no, m = mining, o = other risky)
VB : national cigarette type (V = at least 75% Virginia, bl = at least 75% blended, ot = other)
P : any proxy use
H : full histological confirmation
De : derivation of RR/CI (or = original, st = standard method, ot = other method of estimation)

Table 2K10 - 1

IESLC - Meta-analysis of Ex Smoking, Years quit (vs current), "Highest vs lowest"
Squamous, Cigarettes (or Any Product if Cigarettes not available)
Most adjusted

REF	NRR	2K5	SEX	AGEL	AGEH	RACE	YF	LC TYPE	LOC	START	ST	NLC	R	VB	P	H	AD	ADOS	PRODUCT	exL	exH	unexL	unexH	De
BARBON	608		m	0	0	all	-	q	Eu:wst	1979	CC	755	n	bl	y	y	1	0	all/unsp	25	999	0.1	4	ot
JAHN	615		m	0	0	all	-	q	Eu:Ger	1988	CC	1004	n	bl	n	n	0	0	cig+/-ot	21	999	0.1	0.9	st
JAIN	548		m	0	0	all	-	q	NAmer	1981	CC	845	n	V	y	n	0	0	cig+/-ot	10	999	2	9	st
JAIN	512		f	0	0	all	-	q	NAmer	1981	CC	845	n	V	y	n	0	0	cig+/-ot	10	999	2	9	st
LUBIN2	783		m	0	0	all	-	q	Eu:mul	1976	CC	7804	n	bl	n	y	0	0	cig+/-ot	20	999	0.1	4	st
LUBIN2	903		f	0	0	all	-	q	Eu:mul	1976	CC	7804	n	bl	n	y	0	0	cig+/-ot	20	999	0.1	9	st
MATOS	640		m	0	0	all	-	q	SCAmer	1994	CC	200	n	bl	n	n	2	0	cig+/-ot	11	999	1.0	5	ot
PEZZOT	584		m	0	0	all	-	q	SCAmer	1987	CC	215	n	bl	n	y	0	0	cig only	11	999	1.0	10	st
SOBUE	743		m	0	0	all	-	q	As:Jap	1986	CC	1376	n	bl	n	y	0	0	cig+/-ot	10	999	1.0	4	st
SVENSS	562		f	0	0	all	-	q	Eu:Sca	1983	CC	210	n	bl	n	n	0	0	all/unsp	11	999	3	10	st
WYNDE3	517		m	0	0	all	-	KI	NAmer	1966	CC	350	n	bl	n	y	0	0	all/unsp	13	999	1.0	3	st
WYNDE6	801		m	0	0	all	-	KI	NAmer	1969	CC	4423	n	bl	n	y	2	0	cig+/-ot	16	999	1.0	3	ot

Cigarette type is all/unspec for all RRs

Table 2K10 - 2

IESLC - Meta-analysis of Ex Smoking, Years quit (vs current), "Highest vs lowest"
Squamous, Cigarettes (or Any Product if Cigarettes not available)
Most adjusted

REF	NRR	SEX	AD	Number Exposed Case	Number Exposed Cont	Non-exposed Case	Non-exposed Cont	RR	95.00%CI
BARBON	608	m	1	4	-	11	-	0.10	(0.03- 0.37)
JAHN	615	m	0	8	146	74	8	0.01	(0.00- 0.02)
JAIN	548	m	0	23	113	24	46	0.39	(0.20- 0.76)
JAIN	512	f	0	7	61	15	36	0.28	(0.10- 0.74)
Subtotal JAIN								0.35	(0.20- 0.61)
LUBIN2	783	m	0	106	1128	498	1047	0.20	(0.16- 0.25)
LUBIN2	903	f	0	2	29	38	95	0.17	(0.04- 0.76)
Subtotal LUBIN2								0.20	(0.16- 0.25)
MATOS	640	m	2	5	-	4	-	0.29	(0.06- 1.27)
PEZZOT	584	m	0	8	48	21	27	0.21	(0.08- 0.55)
SOBUE	743	m	0	30	144	52	116	0.46	(0.28- 0.78)
SVENSS	562	f	0	1	24	5	13	0.11	(0.01- 1.03)
WYNDE3	517	m	0	2	55	18	22	0.04	(0.01- 0.21)
WYNDE6	801	m	2	19	-	80	-	0.09	(0.05- 0.15)
Partial Totals				215	1748	840	1410		

*prospective study

REF	NRR	SEX	AD	Ys	Ws	Qs	Ps
BARBON	608	m	1	-2.30	2.43	0.96	0.0003
JAHN	615	m	0	-5.13	3.70	44.12	0.0000
JAIN	548	m	0	-0.94	8.64	4.65	0.0057
JAIN	512	f	0	-1.29	3.94	0.59	0.0105
Subtotal JAIN				-1.05	12.58	5.24	
LUBIN2	783	m	0	-1.62	75.28	0.21	0.0000
LUBIN2	903	f	0	-1.76	1.75	0.01	0.0200
Subtotal LUBIN2				-1.62	77.03	0.23	
MATOS	640	m	2	-1.24	1.65	0.32	0.1119
PEZZOT	584	m	0	-1.54	4.34	0.08	0.0013
SOBUE	743	m	0	-0.77	14.68	12.12	0.0033
SVENSS	562	f	0	-2.22	0.76	0.23	0.0529
WYNDE3	517	m	0	-3.11	1.62	3.34	0.0001
WYNDE6	801	m	2	-2.41	12.73	6.84	0.0000

	N	12
	NS	10
	Wt	131.52
Het	Chi	73.47
Het	df	11
Het	P	***
Fixed	RR	0.19
	RRl	0.16
	RRu	0.22
	P	---
Random	RR	0.14
	RRl	0.08
	RRu	0.25
	P	---
Asymm	P	N.S.

Table 2K10 - 3

IESLC - Meta-analysis of Ex Smoking, Years quit (vs current), "Highest vs lowest"
Squamous, Cigarettes (or Any Product if Cigarettes not available)
Most adjusted

	combined	Sex<br>male	female	Total
N		9	3	12
NS		9	3	12
Wt		125.07	6.45	131.52
Het Chi		72.63	0.68	73.47
Het df		8	2	11
Het P		***	N.S.	***
Fixed RR		0.19	0.22	0.19
RRl		0.16	0.10	0.16
RRu		0.22	0.47	0.22
P		---	---	---
Random RR		0.13	0.22	0.14
RRl		0.07	0.10	0.08
RRu		0.25	0.47	0.25
P		---	---	---
Between Chi				0.15
Between df				1
Between P				N.S.
Btwn(F) P				N.S.
Btwn(R) P				N.S.

	q	Lung cancer type<br>q+s	q+u	KI	not a	Total
N	10			2		12
NS	8			2		10
Wt	117.17			14.35		131.52
Het Chi	62.13			0.71		73.47
Het df	9			1		11
Het P	***			N.S.		***
Fixed RR	0.21			0.08		0.19
RRl	0.17			0.05		0.16
RRu	0.25			0.14		0.22
P	---			---		---
Random RR	0.16			0.08		0.14
RRl	0.08			0.05		0.08
RRu	0.31			0.14		0.25
P	---			---		---
Between Chi						10.63
Between df						1
Between P						**
Btwn(F) P						N.S.
Btwn(R) P						N.S.

	NAmer	UK	Scand	Location<br>othEur	China	Japan	othAs	other	Total
N	4		1	4		1		2	12
NS	3		1	3		1		2	10
Wt	26.93		0.76	83.16		14.68		5.99	131.52
Het Chi	14.88		0.00	44.00		0.00		0.11	73.47
Het df	3		0	3		0		1	11
Het P	**		N.S.	***		N.S.		N.S.	***
Fixed RR	0.16		0.11	0.17		0.46		0.23	0.19
RRl	0.11		0.01	0.13		0.28		0.10	0.16
RRu	0.24		1.03	0.20		0.78		0.52	0.22
P	---		(-)	---		--		---	---
Random RR	0.16		0.11	0.07		0.46		0.23	0.14
RRl	0.06		0.01	0.01		0.28		0.10	0.08
RRu	0.40		1.03	0.37		0.78		0.52	0.25
P	---		(-)	--		--		---	---
Between Chi									14.48
Between df									4
Between P									**
Btwn(F) P									N.S.
Btwn(R) P									(*)

Table 2K10 - 3

IESLC - Meta-analysis of Ex Smoking, Years quit (vs current), "Highest vs lowest"
Squamous, Cigarettes (or Any Product if Cigarettes not available)
Most adjusted

Detailed Country in "other Europe"

	multi	Germany	othWest	East	Balkans	Total
N	2	1	1			4
NS	1	1	1			3
Wt	77.03	3.70	2.43			83.16
Het Chi	0.03	0.00	0.00			44.00
Het df	1	0	0			3
Het P	N.S.	N.S.	N.S.			***
Fixed RR	0.20	0.01	0.10			0.17
RRl	0.16	0.00	0.03			0.13
RRu	0.25	0.02	0.35			0.20
P	---	---	---			---
Random RR	0.20	0.01	0.10			0.07
RRl	0.16	0.00	0.03			0.01
RRu	0.25	0.02	0.35			0.37
P	---	---	---			--
Between Chi						43.97
Between df						2
Between P						***
Btwn(F) P						*
Btwn(R) P						***

Detailed Country in "other Asia"

	India	HongKong	other	Total
N				
NS				
Wt				
Het Chi				
Het df				
Het P				
Fixed RR				
RRl				
RRu				
P				
Random RR				
RRl				
RRu				
P				
Between Chi				
Between df				
Between P				N.S.
Btwn(F) P				N.S.
Btwn(R) P				N.S.

Detailed other continent

	SCAmer	Total
N	2	2
NS	2	2
Wt	5.99	5.99
Het Chi	0.11	0.11
Het df	1	1
Het P	N.S.	N.S.
Fixed RR	0.23	0.23
RRl	0.10	0.10
RRu	0.52	0.52
P	---	---
Random RR	0.23	0.23
RRl	0.10	0.10
RRu	0.52	0.52
P	---	---
Between Chi		
Between df		
Between P		N.S.
Btwn(F) P		N.S.
Btwn(R) P		N.S.

Table 2K10 - 3

IESLC - Meta-analysis of Ex Smoking, Years quit (vs current), "Highest vs lowest"
Squamous, Cigarettes (or Any Product if Cigarettes not available)
Most adjusted

	Start year of study					
	<1960	1960-69	1970-79	1980-89	1990+	Total
N		2	3	6	1	12
NS		2	2	5	1	10
Wt		14.35	79.47	36.06	1.65	131.52
Het Chi		0.71	1.12	59.74	0.00	73.47
Het df		1	2	5	0	11
Het P		N.S.	N.S.	***	N.S.	***
Fixed RR		0.08	0.19	0.24	0.29	0.19
RRl		0.05	0.15	0.17	0.06	0.16
RRu		0.14	0.24	0.33	1.33	0.22
P		---	---	---	N.S.	---
Random RR		0.08	0.19	0.14	0.29	0.14
RRl		0.05	0.15	0.04	0.06	0.08
RRu		0.14	0.24	0.50	1.33	0.25
P		---	---	--	N.S.	---
Between Chi						11.90
Between df						3
Between P						**
Btwn(F) P						N.S.
Btwn(R) P						*

	Study type (1)		
	CC	other	Total
N	12		12
NS	10		10
Wt	131.52		131.52
Het Chi	73.47		73.47
Het df	11		11
Het P	***		***
Fixed RR	0.19		0.19
RRl	0.16		0.16
RRu	0.22		0.22
P	---		---
Random RR	0.14		0.14
RRl	0.08		0.08
RRu	0.25		0.25
P	---		---
Between Chi			
Between df			
Between P			N.S.
Btwn(F) P			N.S.
Btwn(R) P			N.S.

	Study type (2)			
	CC	prosp	other	Total
N	12			12
NS	10			10
Wt	131.52			131.52
Het Chi	73.47			73.47
Het df	11			11
Het P	***			***
Fixed RR	0.19			0.19
RRl	0.16			0.16
RRu	0.22			0.22
P	---			---
Random RR	0.14			0.14
RRl	0.08			0.08
RRu	0.25			0.25
P	---			---
Between Chi				
Between df				
Between P				N.S.
Btwn(F) P				N.S.
Btwn(R) P				N.S.

Table 2K10 - 3

IESLC - Meta-analysis of Ex Smoking, Years quit (vs current), "Highest vs lowest"
Squamous, Cigarettes (or Any Product if Cigarettes not available)
Most adjusted

	Study size (number of LC cases) 100-249	250-499	500-999	1000+	Total
N	3	1	3	5	12
NS	3	1	2	4	10
Wt	6.75	1.62	15.02	108.14	131.52
Het Chi	0.50	0.00	3.53	63.09	73.47
Het df	2	0	2	4	11
Het P	N.S.	N.S.	N.S.	***	***
Fixed RR	0.21	0.04	0.29	0.18	0.19
RRl	0.10	0.01	0.17	0.15	0.16
RRu	0.45	0.21	0.47	0.22	0.22
P	---	---	---	---	---
Random RR	0.21	0.04	0.26	0.10	0.14
RRl	0.10	0.01	0.12	0.04	0.08
RRu	0.45	0.21	0.53	0.28	0.25
P	---	---	---	---	---
Between Chi					6.35
Between df					3
Between P					(*)
Btwn(F) P					N.S.
Btwn(R) P					N.S.

	Risky occupational population no	mining	othRisky	Total
N	12			12
NS	10			10
Wt	131.52			131.52
Het Chi	73.47			73.47
Het df	11			11
Het P	***			***
Fixed RR	0.19			0.19
RRl	0.16			0.16
RRu	0.22			0.22
P	---			---
Random RR	0.14			0.14
RRl	0.08			0.08
RRu	0.25			0.25
P	---			---
Between Chi				
Between df				
Between P				N.S.
Btwn(F) P				N.S.
Btwn(R) P				N.S.

	National cigarette tobacco type Virginia	blended	other	Total
N	2	10		12
NS	1	9		10
Wt	12.58	118.93		131.52
Het Chi	0.33	67.71		73.47
Het df	1	9		11
Het P	N.S.	***		***
Fixed RR	0.35	0.18		0.19
RRl	0.20	0.15		0.16
RRu	0.61	0.21		0.22
P	---	---		---
Random RR	0.35	0.11		0.14
RRl	0.20	0.06		0.08
RRu	0.61	0.23		0.25
P	---	---		---
Between Chi				5.43
Between df				1
Between P				*
Btwn(F) P				N.S.
Btwn(R) P				*

Table 2K10 - 3

IESLC - Meta-analysis of Ex Smoking, Years quit (vs current), "Highest vs lowest"
Squamous, Cigarettes (or Any Product if Cigarettes not available)
Most adjusted

	Any proxy use No/nk	Yes	Total
N	9	3	12
NS	8	2	10
Wt	116.50	15.02	131.52
Het Chi	66.93	3.53	73.47
Het df	8	2	11
Het P	***	N.S.	***
Fixed RR	0.18	0.29	0.19
RRl	0.15	0.17	0.16
RRu	0.21	0.47	0.22
P	---	---	---
Random RR	0.12	0.26	0.14
RRl	0.06	0.12	0.08
RRu	0.24	0.53	0.25
P	---	---	---
Between Chi			3.01
Between df			1
Between P			(*)
Btwn(F) P			N.S.
Btwn(R) P			N.S.

	Full histological confirmation No	Yes	Total
N	5	7	12
NS	4	6	10
Wt	18.69	112.83	131.52
Het Chi	48.76	23.38	73.47
Het df	4	6	11
Het P	***	***	***
Fixed RR	0.15	0.20	0.19
RRl	0.09	0.16	0.16
RRu	0.23	0.23	0.22
P	---	---	---
Random RR	0.11	0.17	0.14
RRl	0.02	0.10	0.08
RRu	0.65	0.28	0.25
P	-	---	---
Between Chi			1.33
Between df			1
Between P			N.S.
Btwn(F) P			N.S.
Btwn(R) P			N.S.

	Number of adjustment variables (1) 0	1	2+/+nk	Total
N	9	1	2	12
NS	7	1	2	10
Wt	114.70	2.43	14.38	131.52
Het Chi	64.46	0.00	2.00	73.47
Het df	8	0	1	11
Het P	***	N.S.	N.S.	***
Fixed RR	0.20	0.10	0.10	0.19
RRl	0.17	0.03	0.06	0.16
RRu	0.25	0.35	0.17	0.22
P	---	---	---	---
Random RR	0.14	0.10	0.13	0.14
RRl	0.07	0.03	0.04	0.08
RRu	0.29	0.35	0.37	0.25
P	---	---	---	---
Between Chi				7.01
Between df				2
Between P				*
Btwn(F) P				N.S.
Btwn(R) P				N.S.

Table 2K10 - 3

IESLC - Meta-analysis of Ex Smoking, Years quit (vs current), "Highest vs lowest"
Squamous, Cigarettes (or Any Product if Cigarettes not available)
Most adjusted

	Number of adjustment variables (2) 0	1	2	3-5	6+/+nk	Total
N	9	1	2			12
NS	7	1	2			10
Wt	114.70	2.43	14.38			131.52
Het Chi	64.46	0.00	2.00			73.47
Het df	8	0	1			11
Het P	***	N.S.	N.S.			***
Fixed RR	0.20	0.10	0.10			0.19
RRl	0.17	0.03	0.06			0.16
RRu	0.25	0.35	0.17			0.22
P	---	---	---			---
Random RR	0.14	0.10	0.13			0.14
RRl	0.07	0.03	0.04			0.08
RRu	0.29	0.35	0.37			0.25
P	---	---	---			---
Between Chi						7.01
Between df						2
Between P						*
Btwn(F) P						N.S.
Btwn(R) P						N.S.

	Product all/unsp	cig+/-ot	cig only	Total
N	3	8	1	12
NS	3	6	1	10
Wt	4.81	122.37	4.34	131.52
Het Chi	0.74	68.75	0.00	73.47
Het df	2	7	0	11
Het P	N.S.	***	N.S.	***
Fixed RR	0.08	0.19	0.21	0.19
RRl	0.03	0.16	0.08	0.16
RRu	0.19	0.23	0.55	0.22
P	---	---	--	---
Random RR	0.08	0.15	0.21	0.14
RRl	0.03	0.08	0.08	0.08
RRu	0.19	0.32	0.55	0.25
P	---	---	--	---
Between Chi				3.98
Between df				2
Between P				N.S.
Btwn(F) P				N.S.
Btwn(R) P				N.S.

	Derivation of RR/CI Orig	StdCalc	Other	Total
N		9	3	12
NS		7	3	10
Wt		114.70	16.81	131.52
Het Chi		64.46	2.00	73.47
Het df		8	2	11
Het P		***	N.S.	***
Fixed RR		0.20	0.10	0.19
RRl		0.17	0.06	0.16
RRu		0.25	0.17	0.22
P		---	---	---
Random RR		0.14	0.10	0.14
RRl		0.07	0.06	0.08
RRu		0.29	0.17	0.25
P		---	---	---
Between Chi				7.01
Between df				1
Between P				**
Btwn(F) P				N.S.
Btwn(R) P				N.S.

Table 2K10 - 4

IESLC - Meta-analysis of Ex Smoking, Years quit (vs current), "Highest vs lowest"
Squamous, Cigarettes (or Any Product if Cigarettes not available)
Least adjusted

REF	NRR	X	SEX	AGEL	AGEH	RACE	YF	LC TYPE	LOC	START	ST	NLC	R	VB	P	H	AD	ADOS	PRODUCT	exL	exH	unexL	unexH	De
BARBON	593	x	m	0	0	all	-	q	Eu:wst	1979	CC	755	n	bl	y	y	0	0	all/unsp	25	999	0.1	4	st
JAHN	615		m	0	0	all	-	q	Eu:Ger	1988	CC	1004	n	bl	n	n	0	0	cig+/-ot	21	999	0.1	0.9	st
JAIN	548		m	0	0	all	-	q	NAmer	1981	CC	845	n	V	y	n	0	0	cig+/-ot	10	999	2	9	st
JAIN	512		f	0	0	all	-	q	NAmer	1981	CC	845	n	V	y	n	0	0	cig+/-ot	10	999	2	9	st
LUBIN2	783		m	0	0	all	-	q	Eu:mul	1976	CC	7804	n	bl	n	y	0	0	cig+/-ot	20	999	0.1	4	st
LUBIN2	903		f	0	0	all	-	q	Eu:mul	1976	CC	7804	n	bl	n	y	0	0	cig+/-ot	20	999	0.1	9	st
MATOS	630	x	m	0	0	all	-	q	SCAmer	1994	CC	200	n	bl	n	n	0	0	cig+/-ot	11	999	1.0	5	st
PEZZOT	584		m	0	0	all	-	q	SCAmer	1987	CC	215	n	bl	n	y	0	0	cig only	11	999	1.0	10	st
SOBUE	743		m	0	0	all	-	q	As:Jap	1986	CC	1376	n	bl	n	y	0	0	cig+/-ot	10	999	1.0	4	st
SVENSS	562		f	0	0	all	-	q	Eu:Sca	1983	CC	210	n	bl	n	n	0	0	all/unsp	11	999	3	10	st
WYNDE3	517		m	0	0	all	-	KI	NAmer	1966	CC	350	n	bl	n	y	0	0	all/unsp	13	999	1.0	3	st
WYNDE6	786	x	m	0	0	all	-	KI	NAmer	1969	CC	4423	n	bl	n	y	0	0	cig+/-ot	16	999	1.0	3	st

Cigarette type is all/unspec for all RRs

Table 2K10 - 5

IESLC - Meta-analysis of Ex Smoking, Years quit (vs current), "Highest vs lowest"
Squamous, Cigarettes (or Any Product if Cigarettes not available)
Least adjusted

REF	NRR	SEX	A	Number Exposed Case	Cont	Non-exposed Case	Cont	RR	95.00%CI
BARBON	593	m	0	4	59	11	20	0.12	(0.04- 0.43)
JAHN	615	m	0	8	146	74	8	0.01	(0.00- 0.02)
JAIN	548	m	0	23	113	24	46	0.39	(0.20- 0.76)
JAIN	512	f	0	7	61	15	36	0.28	(0.10- 0.74)
Subtotal JAIN								0.35	(0.20- 0.61)
LUBIN2	783	m	0	106	1128	498	1047	0.20	(0.16- 0.25)
LUBIN2	903	f	0	2	29	38	95	0.17	(0.04- 0.76)
Subtotal LUBIN2								0.20	(0.16- 0.25)
MATOS	630	m	0	5	101	4	23	0.28	(0.07- 1.14)
PEZZOT	584	m	0	8	48	21	27	0.21	(0.08- 0.55)
SOBUE	743	m	0	30	144	52	116	0.46	(0.28- 0.78)
SVENSS	562	f	0	1	24	5	13	0.11	(0.01- 1.03)
WYNDE3	517	m	0	2	55	18	22	0.04	(0.01- 0.21)
WYNDE6	786	m	0	19	530	80	307	0.14	(0.08- 0.23)
Totals				215	2438	840	1760		

*prospective study

REF	NRR	SEX	AD	Ys	Ws	Qs	Ps
BARBON	593	m	0	-2.09	2.45	0.52	0.0010
JAHN	615	m	0	-5.13	3.70	45.19	0.0000
JAIN	548	m	0	-0.94	8.64	4.14	0.0057
JAIN	512	f	0	-1.29	3.94	0.47	0.0105
Subtotal JAIN				-1.05	12.58	4.60	
LUBIN2	783	m	0	-1.62	75.28	0.01	0.0000
LUBIN2	903	f	0	-1.76	1.75	0.03	0.0200
Subtotal LUBIN2				-1.62	77.03	0.04	
MATOS	630	m	0	-1.26	1.99	0.28	0.0766
PEZZOT	584	m	0	-1.54	4.34	0.04	0.0013
SOBUE	743	m	0	-0.77	14.68	11.04	0.0033
SVENSS	562	f	0	-2.22	0.76	0.26	0.0529
WYNDE3	517	m	0	-3.11	1.62	3.54	0.0001
WYNDE6	786	m	0	-1.98	14.23	1.75	0.0000

	N	12
	NS	10
	Wt	133.37
Het	Chi	67.25
Het	df	11
Het	P	***
Fixed	RR	0.20
	RRl	0.16
	RRu	0.23
	P	---
Random	RR	0.15
	RRl	0.09
	RRu	0.26
	P	---
Asymm	P	N.S.

Table 2K10 - 6

IESLC - Meta-analysis of Ex Smoking, Years quit (vs current), "Highest vs lowest"
Squamous, Cigarettes (or Any Product if Cigarettes not available)
Least adjusted

	Sex combined	male	female	Total
N		9	3	12
NS		9	3	12
Wt		126.92	6.45	133.37
Het Chi		66.49	0.68	67.25
Het df		8	2	11
Het P		***	N.S.	***
Fixed RR		0.19	0.22	0.20
RRl		0.16	0.10	0.16
RRu		0.23	0.47	0.23
P		---	---	---
Random RR		0.14	0.22	0.15
RRl		0.07	0.10	0.09
RRu		0.27	0.47	0.26
P		---	---	---
Between Chi				0.08
Between df				1
Between P				N.S.
Btwn(F) P				N.S.
Btwn(R) P				N.S.

Table 2K10 - 7

IESLC - Meta-analysis of Ex Smoking, Years quit (vs current), "Highest vs lowest"
Squamous, Cigarettes (or Any Product if Cigarettes not available)
Excluded studies (and stage at which they were excluded)

1	AGUDO AKIBA AMANDU AMES AXELSS BEST BOUCHA BOUCOT BRESLO CHEN CHEN2 CHIAZZ DEAN2 DOSEME ENGELA FAN GENG GER GUO HAENSZ HEGMAN HOLE HU HU2 JUSSAW KATSOU KAUFMA KOO KOULUM KREUZE LETOUR LEVIN LIAW LIU3 LIU4 LIU5 MCCONN MIGRAN MRFITR NOTAN2 OSANN2 PERNU QIAO2 RACHTA RESTRE SADOWS SEGI2 STASZE TIZZAN VUTUC WATSON WU WUWILL WYNDE2 WYNDE8 XU YUAN ZHANG ZHENG ZHOU
2	AUVINE BENSHL BLOT1 BROWN3 BUFFLE GURSEL LAUSSM LUO MCDUFF PISANI PRESCO SPITZ WU2 WYNDE7
4	GARSHI JEDRYC WAKAI
5	ARMADA BECHER BOFFET BROSS CARPEN CEDERL CHOI CHYOU CORREA CPSI CPSII DAMBER DARBY DEAN3 DESTEF DOLL DOLL2 DORGAN DORN GAO GAO2 GARCIA GILLIS GRAHAM HAMMO2 HIRAYA HUMBLE JOLY KAISE2 KHUDER LUBIN PEZZO2 QIAO SPEIZE SUZUK2 TVERDA WANG2 WIGLE
6	ALDERS HAMMON
15	BENHAM

Table 2K10 - 8
Potentially overlapping studies

REF	REFGP	PRINC	OVERLAP/LINK
LUBIN2	LUBIN2	1	Lubin-combined
WYNDE6	WYNDE6	1	WYNDE5/6/7/8
JAHN	BOFFET	2	Subset of BOFFET

Table 2K10 - 9
Most adjusted - insufficient data for meta-analysis

REF	NRR	SEX	AGEL	AGEH	RACE	YF	LC TYPE	LOC	START	ST	NLC	R	VB	P	H	AD	ADOS	PRODUCT	exL	exH	unexL	unexH	De
ALDERS	547	m	0	0	all	-	q+s	Eu:UK	1977	CC	1448	n	V	n	n	1	0	cig only	10	999	0.1	2	st
ALDERS	558	f	0	0	all	-	q+s	Eu:UK	1977	CC	1448	n	V	n	n	1	0	cig only	10	999	0.1	2	st
HAMMON	511	m	0	0	wh	0	not a	NAmer	1952	pr	448	n	bl	n	n	1	0	cig only	10	999	0.1	0.9	st

REF	NRR	RR	SIG	RRDATA comment
ALDERS	547	0.10		0
ALDERS	558	0.04		0
HAMMON	511	*		RR for <1 pack per day is 0.15, while that for 1+ packs is 0.31

Table 2K11 -

IESLC - Meta-analysis of Ex Smoking by Years quit (vs current), Overview
Squamous, Cigarettes only

This analysis is restricted to results for:
1) Ex smokers
2) Results by Years quit (vs current)
3) Categorical results by Years quit (vs current)
   Results by Years quit (vs current) are grouped under 2 schemes (S1, S2). Each scheme has a set of "key values". An interval is allocated to the category whose key value it includes, and intervals which include none or more than one of the key values are excluded. (Open-ended intervals are coded as 999)

S1	key value	maximum range
1	3	1-6
2	7	4-11
3	12	8+

S2	key value	maximum range
1	3	1-11
2	12	4-19
3	20	13+

4) Squamous (or near equivalent)
5) Results complete enough for use in metaanalysis

Within each study, results are then selected (in the following order of preference, within each sex) for:
6) (not applicable)
7) PRODUCT: cigarettes only
8) CIGTYPE: all/unspecified, MC regardless of HR, MC only
9) Results with least adjustment for other aspects of smoking (ADOS)
10) DENOM: current smokers, current + recent smokers (up to number of m=months or y=years, max 2 years)
11) Followup period (YF, prospective studies): whole study (coded as 0) or longest available
12) LCtype: squamous or nearest available, but not adeno. (q = squamous, s = small, a = adeno, KI = Kreyberg I, u = undifferentiated)
13) Race: all or nearest available, otherwise by race (wh or w = white, bl or b = black, hi = hispanic ch = chinese, jap = japanese, haw = hawaiian, w+o = white + oriental, sca = scandinavian, as = asian)
14) For overlapping studies: principal rather than subsidiary studies
Finally by Age: whole study (coded as 0) if available, otherwise by widest available age group and then for single sex results (m, f) in preference to results for both sexes combined (c).

Results adjusted (AD) for the most potential confounders are then chosen in Sections -1 to -3 (and those which actually differ from the adjusted results in Table 2K1 - 1 are marked 'x' in Section -1) and results adjusted for the least confounders in Sections -4 to -6. (Those least adjusted results which actually differ from the most adjusted are marked 'x' in column X in Section -4)

Section -7 shows excluded studies, together with the stage (as above) at which no qualifying results were found.

Section -8 lists the potentially overlapping studies which have been included (1=principal, 2=subsidiary).

Section -9 lists any results which would have been included in preference except that they had data not complete enough for use in meta-analysis, with their significance (yes/no), if known, and any further comment as entered on the database. It also lists as "gap" any categories for which no data were presented by the original authors.

In addition to those mentioned above, the following fields, levels and abbreviations are used:
* or nk = not known, n = no, y = yes, ot = other
nev = never
all/unspec = all or unspecified, MC = manufactured cigarettes, HR = hand-rolled cigarettes
exL, exH = range of exposure (low and high) in the smoking group, in terms of Years quit (vs current)
REF: 6-character study reference
NRR: number of the RR on the database within the study
ST : study type (CC = case control, pr or prosp = prospective)
NLC: number of lung cancer cases in whole study
R : risky occupational population (n = no, m = mining, o = other risky)
VB : national cigarette type (V = at least 75% Virginia, bl = at least 75% blended, ot = other)
P : any proxy use
H : full histological confirmation
De : derivation of RR/CI (or = original, st = standard method, ot = other method of estimation)

Table 2K11 - 1

IESLC - Meta-analysis of Ex Smoking by Years quit (vs current), Overview
Squamous, Cigarettes only
Most adjusted

REF	NRR	2K1	SEX	AGEL	AGEH	RACE	YF	LC TYPE	LOC	START	ST	NLC	R	VB	P	H	AD	ADOS	PRODUCT	exL	exH	S1	S2	DENOM	De
BENHAM	539	x	m	0	0	all	-	KI	Eu:wst	1976	CC	1625	n	bl	n	y	0	0	cig only	1.0	3	1	1	cur+1y	st
BENHAM	540	x	m	0	0	all	-	KI	Eu:wst	1976	CC	1625	n	bl	n	y	0	0	cig only	4	6	0	0	cur+1y	st
BENHAM	541	x	m	0	0	all	-	KI	Eu:wst	1976	CC	1625	n	bl	n	y	0	0	cig only	7	10	2	0	cur+1y	st
BENHAM	542	x	m	0	0	all	-	KI	Eu:wst	1976	CC	1625	n	bl	n	y	0	0	cig only	11	19	3	2	cur+1y	st
BENHAM	543	x	m	0	0	all	-	KI	Eu:wst	1976	CC	1625	n	bl	n	y	0	0	cig only	20	999	0	3	cur+1y	st
PEZZOT	582		m	0	0	all	-	q	SCAmer	1987	CC	215	n	bl	n	y	0	0	cig only	1.0	10	0	1	cur+1y	st
PEZZOT	583		m	0	0	all	-	q	SCAmer	1987	CC	215	n	bl	n	y	0	0	cig only	11	999	3	0	cur+1y	st

Cigarette type is all/unspec for all RRs

In this overview table, subtotals and Qs values may be invalid and should be ignored

Table 2K11 - 2

IESLC - Meta-analysis of Ex Smoking by Years quit (vs current), Overview
Squamous, Cigarettes only
Most adjusted

REF	NRR	SEX	A	Number Exposed Case	Cont	Non-exposed Case	Cont	RR	95.00%CI
BENHAM	539	m	0	132	77	789	798	1.73	(1.29- 2.34)
BENHAM	540	m	0	56	92	789	798	0.62	(0.44- 0.87)
BENHAM	541	m	0	41	75	789	798	0.55	(0.37- 0.82)
BENHAM	542	m	0	39	125	789	798	0.32	(0.22- 0.46)
BENHAM	543	m	0	17	82	789	798	0.21	(0.12- 0.36)
Subtotal BENHAM								0.66	(0.56- 0.77)
PEZZOT	582	m	0	21	27	56	52	0.72	(0.36- 1.43)
PEZZOT	583	m	0	8	48	56	52	0.15	(0.07- 0.36)
Subtotal PEZZOT								0.39	(0.23- 0.66)
Totals				314	526	4057	4094		

*prospective study

REF	NRR	SEX	AD	Ys	Ws	Qs	Ps
BENHAM	539	m	0	0.55	43.32	44.83	0.0003
BENHAM	540	m	0	-0.49	32.00	0.01	0.0061
BENHAM	541	m	0	-0.59	24.85	0.39	0.0031
BENHAM	542	m	0	-1.15	27.65	13.03	0.0000
BENHAM	543	m	0	-1.56	13.60	16.31	0.0000
Subtotal BENHAM				-0.42	141.42	74.58	
PEZZOT	582	m	0	-0.33	8.21	0.16	0.3510
PEZZOT	583	m	0	-1.87	5.47	10.70	0.0000
Subtotal PEZZOT				-0.94	13.68	10.86	

N	7
NS	2

Table 2K11 - 3

IESLC - Meta-analysis of Ex Smoking by Years quit (vs current), Overview
Squamous, Cigarettes only
Most adjusted

	Sex			
	combined	male	female	Total
N		7		7
NS		2		2

In this overview table, other than the "N" rows, entries in the "absent" and "Total" columns may be invalid and should be ignored

	Years quit vs current (lower focus)				
	absent	1-6k3	4-11k7	8+k12	Total
N	3	1	1	2	7
NS	2	1	1	2	4
Wt	53.81	43.32	24.85	33.12	155.11
Het Chi	12.68	0.00	0.00	2.32	85.44
Het df	2	0	0	1	6
Het P	**	N.S.	N.S.	N.S.	***
Fixed RR	0.48	1.73	0.55	0.28	0.63
RRl	0.37	1.29	0.37	0.20	0.54
RRu	0.63	2.34	0.82	0.39	0.73
P	---	+++	--	---	---
Random RR	0.45	1.73	0.55	0.24	0.48
RRl	0.22	1.29	0.37	0.13	0.26
RRu	0.95	2.34	0.82	0.48	0.88
P	-	+++	--	---	-

	Years quit vs current (higher focus)				
	absent	1-11k3	4-19k12	13+k20	Total
N	3	2	1	1	7
NS	2	2	1	1	5
Wt	62.32	51.54	27.65	13.60	155.11
Het Chi	9.03	5.30	0.00	0.00	85.44
Het df	2	1	0	0	6
Het P	*	*	N.S.	N.S.	***
Fixed RR	0.52	1.51	0.32	0.21	0.63
RRl	0.41	1.15	0.22	0.12	0.54
RRu	0.67	1.98	0.46	0.36	0.73
P	---	++	---	---	---
Random RR	0.43	1.18	0.32	0.21	0.48
RRl	0.24	0.51	0.22	0.12	0.26
RRu	0.77	2.77	0.46	0.36	0.88
P	--	N.S.	---	---	-

Table 2K11 - 3

IESLC - Meta-analysis of Ex Smoking by Years quit (vs current), Overview
Squamous, Cigarettes only
Most adjusted

MALES

Years quit vs current (lower focus)

		absent	1-6k3	4-11k7	8+k12	Total
	N	3	1	1	2	7
	NS	2	1	1	2	4
	Wt	53.81	43.32	24.85	33.12	155.11
Het	Chi	12.68	0.00	0.00	2.32	85.44
Het	df	2	0	0	1	6
Het	P	**	N.S.	N.S.	N.S.	***
Fixed	RR	0.48	1.73	0.55	0.28	0.63
	RRl	0.37	1.29	0.37	0.20	0.54
	RRu	0.63	2.34	0.82	0.39	0.73
	P	---	+++	--	---	---
Random	RR	0.45	1.73	0.55	0.24	0.48
	RRl	0.22	1.29	0.37	0.13	0.26
	RRu	0.95	2.34	0.82	0.48	0.88
	P	-	+++	--	---	-

Years quit vs current (higher focus)

		absent	1-11k3	4-19k12	13+k20	Total
	N	3	2	1	1	7
	NS	2	2	1	1	5
	Wt	62.32	51.54	27.65	13.60	155.11
Het	Chi	9.03	5.30	0.00	0.00	85.44
Het	df	2	1	0	0	6
Het	P	*	*	N.S.	N.S.	***
Fixed	RR	0.52	1.51	0.32	0.21	0.63
	RRl	0.41	1.15	0.22	0.12	0.54
	RRu	0.67	1.98	0.46	0.36	0.73
	P	---	++	---	---	---
Random	RR	0.43	1.18	0.32	0.21	0.48
	RRl	0.24	0.51	0.22	0.12	0.26
	RRu	0.77	2.77	0.46	0.36	0.88
	P	--	N.S.	---	---	-

Table 2K11 - 4

IESLC - Meta-analysis of Ex Smoking by Years quit (vs current), Overview
Squamous, Cigarettes only
Least adjusted

REF	NRR	X	SEX	AGEL	AGEH	RACE	YF	LC TYPE	LOC	START	ST	NLC	R	VB	P	H	AD	ADOS	PRODUCT	exL	exH	S1	S2	DENOM	De
BENHAM	539		m	0	0	all	-	KI	Eu:wst	1976	CC	1625	n	bl	n	y	0	0	cig only	1.0	3	1	1	cur+1y	st
BENHAM	540		m	0	0	all	-	KI	Eu:wst	1976	CC	1625	n	bl	n	y	0	0	cig only	4	6	0	0	cur+1y	st
BENHAM	541		m	0	0	all	-	KI	Eu:wst	1976	CC	1625	n	bl	n	y	0	0	cig only	7	10	2	0	cur+1y	st
BENHAM	542		m	0	0	all	-	KI	Eu:wst	1976	CC	1625	n	bl	n	y	0	0	cig only	11	19	3	2	cur+1y	st
BENHAM	543		m	0	0	all	-	KI	Eu:wst	1976	CC	1625	n	bl	n	y	0	0	cig only	20	999	0	3	cur+1y	st
PEZZOT	582		m	0	0	all	-	q	SCAmer	1987	CC	215	n	bl	n	y	0	0	cig only	1.0	10	0	1	cur+1y	st
PEZZOT	583		m	0	0	all	-	q	SCAmer	1987	CC	215	n	bl	n	y	0	0	cig only	11	999	3	0	cur+1y	st

Cigarette type is all/unspec for all RRs

In this overview table, subtotals and Qs values may be invalid and should be ignored

Table 2K11 - 5

IESLC - Meta-analysis of Ex Smoking by Years quit (vs current), Overview
Squamous, Cigarettes only
Least adjusted

REF	NRR	SEX	AD	Number Exposed Case	Cont	Non-exposed Case	Cont	RR	95.00%CI
BENHAM	539	m	0	132	77	789	798	1.73	(1.29- 2.34)
BENHAM	540	m	0	56	92	789	798	0.62	(0.44- 0.87)
BENHAM	541	m	0	41	75	789	798	0.55	(0.37- 0.82)
BENHAM	542	m	0	39	125	789	798	0.32	(0.22- 0.46)
BENHAM	543	m	0	17	82	789	798	0.21	(0.12- 0.36)
Subtotal BENHAM								0.66	(0.56- 0.77)
PEZZOT	582	m	0	21	27	56	52	0.72	(0.36- 1.43)
PEZZOT	583	m	0	8	48	56	52	0.15	(0.07- 0.36)
Subtotal PEZZOT								0.39	(0.23- 0.66)
Totals				314	526	4057	4094		

*prospective study

REF	NRR	SEX	AD	Ys	Ws	Qs	Ps
BENHAM	539	m	0	0.55	43.32	44.83	0.0003
BENHAM	540	m	0	-0.49	32.00	0.01	0.0061
BENHAM	541	m	0	-0.59	24.85	0.39	0.0031
BENHAM	542	m	0	-1.15	27.65	13.03	0.0000
BENHAM	543	m	0	-1.56	13.60	16.31	0.0000
Subtotal BENHAM				-0.42	141.42	74.58	
PEZZOT	582	m	0	-0.33	8.21	0.16	0.3510
PEZZOT	583	m	0	-1.87	5.47	10.70	0.0000
Subtotal PEZZOT				-0.94	13.68	10.86	

N	7
NS	2

Table 2K11 - 6

IESLC - Meta-analysis of Ex Smoking by Years quit (vs current), Overview
Squamous, Cigarettes only
Least adjusted

	Sex			
	combined	male	female	Total
N		7		7
NS		2		2

In this overview table, other than the "N" rows, entries in the "absent" and "Total" columns may be invalid and should be ignored

	Years quit vs current (lower focus)				
	absent	1-6k3	4-11k7	8+k12	Total
N	3	1	1	2	7
NS	2	1	1	2	4
Wt	53.81	43.32	24.85	33.12	155.11
Het Chi	12.68	0.00	0.00	2.32	85.44
Het df	2	0	0	1	6
Het P	**	N.S.	N.S.	N.S.	***
Fixed RR	0.48	1.73	0.55	0.28	0.63
RRl	0.37	1.29	0.37	0.20	0.54
RRu	0.63	2.34	0.82	0.39	0.73
P	---	+++	--	---	---
Random RR	0.45	1.73	0.55	0.24	0.48
RRl	0.22	1.29	0.37	0.13	0.26
RRu	0.95	2.34	0.82	0.48	0.88
P	-	+++	--	---	-

	Years quit vs current (higher focus)				
	absent	1-11k3	4-19k12	13+k20	Total
N	3	2	1	1	7
NS	2	2	1	1	5
Wt	62.32	51.54	27.65	13.60	155.11
Het Chi	9.03	5.30	0.00	0.00	85.44
Het df	2	1	0	0	6
Het P	*	*	N.S.	N.S.	***
Fixed RR	0.52	1.51	0.32	0.21	0.63
RRl	0.41	1.15	0.22	0.12	0.54
RRu	0.67	1.98	0.46	0.36	0.73
P	---	++	---	---	---
Random RR	0.43	1.18	0.32	0.21	0.48
RRl	0.24	0.51	0.22	0.12	0.26
RRu	0.77	2.77	0.46	0.36	0.88
P	--	N.S.	---	---	-

Table 2K11 - 6

IESLC - Meta-analysis of Ex Smoking by Years quit (vs current), Overview
Squamous, Cigarettes only
Least adjusted

MALES

	Years quit vs current (lower focus) absent	1-6k3	4-11k7	8+k12	Total
N	3	1	1	2	7
NS	2	1	1	2	4
Wt	53.81	43.32	24.85	33.12	155.11
Het Chi	12.68	0.00	0.00	2.32	85.44
Het df	2	0	0	1	6
Het P	**	N.S.	N.S.	N.S.	***
Fixed RR	0.48	1.73	0.55	0.28	0.63
RRl	0.37	1.29	0.37	0.20	0.54
RRu	0.63	2.34	0.82	0.39	0.73
P	---	+++	--	---	---
Random RR	0.45	1.73	0.55	0.24	0.48
RRl	0.22	1.29	0.37	0.13	0.26
RRu	0.95	2.34	0.82	0.48	0.88
P	-	+++	--	---	-

	Years quit vs current (higher focus) absent	1-11k3	4-19k12	13+k20	Total
N	3	2	1	1	7
NS	2	2	1	1	5
Wt	62.32	51.54	27.65	13.60	155.11
Het Chi	9.03	5.30	0.00	0.00	85.44
Het df	2	1	0	0	6
Het P	*	*	N.S.	N.S.	***
Fixed RR	0.52	1.51	0.32	0.21	0.63
RRl	0.41	1.15	0.22	0.12	0.54
RRu	0.67	1.98	0.46	0.36	0.73
P	---	++	---	---	---
Random RR	0.43	1.18	0.32	0.21	0.48
RRl	0.24	0.51	0.22	0.12	0.26
RRu	0.77	2.77	0.46	0.36	0.88
P	--	N.S.	---	---	-

Table 2K11 - 7

IESLC - Meta-analysis of Ex Smoking by Years quit (vs current), Overview
Squamous, Cigarettes only
Excluded studies (and stage at which they were excluded)

1	AGUDO AKIBA AMANDU AMES AXELSS BEST BOUCHA BOUCOT BRESLO CHEN CHEN2 CHIAZZ DEAN2 DOSEME ENGELA FAN GENG GER GUO HAENSZ HEGMAN HOLE HU HU2 JUSSAW KATSOU KAUFMA KOO KOULUM KREUZE LETOUR LEVIN LIAW LIU3 LIU4 LIU5 MCCONN MIGRAN MRFITR NOTAN2 OSANN2 PERNU QIAO2 RACHTA RESTRE SADOWS SEGI2 STASZE TIZZAN VUTUC WATSON WU WUWILL WYNDE2 WYNDE8 XU YUAN ZHANG ZHENG ZHOU
2	AUVINE BENSHL BLOT1 BROWN3 BUFFLE GURSEL LAUSSM LUO MCDUFF PISANI PRESCO SPITZ WU2 WYNDE7
4	ARMADA BECHER BOFFET BROSS CARPEN CEDERL CHOI CHYOU CORREA CPSI CPSII DAMBER DARBY DEAN3 DESTEF DOLL DOLL2 DORGAN DORN GAO GAO2 GARCIA GARSHI GILLIS GRAHAM HAMMO2 HIRAYA HUMBLE JOLY KAISE2 KHUDER LUBIN PEZZO2 QIAO SPEIZE SUZUK2 TVERDA WANG2 WIGLE
5	ALDERS HAMMON
7	BARBON JAHN JAIN JEDRYC LUBIN2 MATOS SOBUE SVENSS WAKAI WYNDE3 WYNDE6

Table 2K11 - 8

Potentially overlapping studies

REF	REFGP	PRINC	.	OVERLAP/LINK
BENHAM	LUBIN2	2		Subset of Lubin2

Table 2K11 - 9

Most adjusted - insufficient data for meta-analysis

REF	NRR	SEX	AGEL	AGEH	RACE	YF	LC TYPE	LOC	START	ST	NLC	R	VB	P	H	AD	ADOS	PRODUCT	exL	exH	S1	S2	DENOM	De
ALDERS	543	m	0	0	all	-	q+s	Eu:UK	1977	CC	1448	n	V	n	n	1	0	cig only	0.1	2	0	0	current	ot
ALDERS	544	m	0	0	all	-	q+s	Eu:UK	1977	CC	1448	n	V	n	n	1	0	cig only	3	9	0	1	current	ot
ALDERS	545	m	0	0	all	-	q+s	Eu:UK	1977	CC	1448	n	V	n	n	1	0	cig only	10	999	3	0	current	ot
ALDERS	554	f	0	0	all	-	q+s	Eu:UK	1977	CC	1448	n	V	n	n	1	0	cig only	0.1	2	0	0	current	ot
ALDERS	555	f	0	0	all	-	q+s	Eu:UK	1977	CC	1448	n	V	n	n	1	0	cig only	3	9	0	1	current	ot
ALDERS	556	f	0	0	all	-	q+s	Eu:UK	1977	CC	1448	n	V	n	n	1	0	cig only	10	999	3	0	current	ot
HAMMON	507	m	0	0	wh	0	not a	NAmer	1952	pr	448	n	bl	n	n	1	0	cig only	0.1	0.9	0	0	current	st
HAMMON	508	m	0	0	wh	0	not a	NAmer	1952	pr	448	n	bl	n	n	1	0	cig only	1.0	9	0	1	current	st
HAMMON	509	m	0	0	wh	0	not a	NAmer	1952	pr	448	n	bl	n	n	1	0	cig only	10	999	3	0	current	st

REF	NRR	RR	SIG	RRDATA comment
ALDERS	543	2.10	y	0.01<p<0.05
ALDERS	544	0.36	y	0.01<p<0.05
ALDERS	545	0.21	y	0.001<p<0.01
ALDERS	554	3.02	y	p<0.001
ALDERS	555	1.51	n	0
ALDERS	556	0.12	y	p<0.001
HAMMON	507	*		RR for <1 pack is 0.97, while that for 1+ packs is 1.26
HAMMON	508	*		RR for <1 pack per day is 0.62, while that for 1+ packs is 0.49
HAMMON	509	*		RR for <1 pack per day is 0.14, while that for 1+ packs is 0.39

Table 2K12 -

IESLC - Meta-analysis of Ex Smoking, Years quit (vs current), "Low"
Squamous, Cigarettes only

This analysis is restricted to results for:
1) Ex smokers
2) Results by Years quit (vs current)
3) Categorical results by Years quit (vs current)
4) Squamous (or near equivalent)
5) Results complete enough for use in metaanalysis

Within each study, results are then selected (in the following order of preference, within each sex) for:
6) (not applicable)
7) PRODUCT: cigarettes only
8) CIGTYPE: all/unspecified, MC regardless of HR, MC only
9) Results with least adjustment for other aspects of smoking (ADOS)
10) DENOM: current smokers, current + recent smokers (up to number of m=months or y=years, max 2 years)
11) Followup period (YF, prospective studies): whole study (coded as 0) or longest available
12) LCtype: squamous or nearest available, but not adeno. (q = squamous, s = small,
a = adeno, KI = Kreyberg I, u = undifferentiated)
13) Race: all or nearest available, otherwise by race (wh or w = white, bl or b = black, hi = hispanic
ch = chinese, jap = japanese, haw = hawaiian, w+o = white + oriental, sca = scandinavian, as = asian)
14) Years quit (vs current) "low" in key scheme 1 (key value 3, maximum range 1-6)
15) For overlapping studies: principal rather than subsidiary studies
Finally by Age: whole study (coded as 0) if available, otherwise by widest available age group
and then for single sex results (m, f) in preference to results for both sexes combined (c).

Results adjusted (AD) for the most potential confounders are then chosen in Sections -1 to -3
(and those which actually differ from the adjusted results in Table 2K2 - 1 are marked 'x' in Section -1)
and results adjusted for the least confounders in Sections -4 to -6. (Those least adjusted results which
actually differ from the most adjusted are marked 'x' in column X in Section -4)

Section -7 shows excluded studies, together with the stage (as above) at which no qualifying
results were found.

Section -8 lists the potentially overlapping studies which have been included (1=principal, 2=subsidiary).

Section -9 lists any results which would have been included in preference except that they had data not complete
enough for use in meta-analysis, with their significance (yes/no), if known, and any further comment as entered
on the database. It also lists as "gap" any categories for which no data were presented by the original authors.

In addition to those mentioned above, the following fields, levels and abbreviations are used:
* or nk = not known, n = no, y = yes, ot = other
nev = never
all/unspec = all or unspecified, MC = manufactured cigarettes, HR = hand-rolled cigarettes
exL, exH = range of exposure (low and high) in the smoking group, in terms of Years quit (vs current)
REF: 6-character study reference
NRR: number of the RR on the database within the study
ST : study type (CC = case control, pr or prosp = prospective)
NLC: number of lung cancer cases in whole study
R : risky occupational population (n = no, m = mining, o = other risky)
VB : national cigarette type (V = at least 75% Virginia, bl = at least 75% blended, ot = other)
P : any proxy use
H : full histological confirmation
De : derivation of RR/CI (or = original, st = standard method, ot = other method of estimation)

Table 2K12 - 1

IESLC - Meta-analysis of Ex Smoking, Years quit (vs current), "Low"
Squamous, Cigarettes only
Most adjusted

REF	NRR	2K2	SEX	AGEL	AGEH	RACE	YF	LC TYPE	LOC	START	ST	NLC	R	VB	P	H	AD	ADOS	PRODUCT	exL	exH	DENOM	De
BENHAM	539	x	m	0	0	all	-	KI	Eu:wst	1976	CC	1625	n	bl	n	y	0	0	cig only	1.0	3	cur+1y	st

Cigarette type is all/unspec for all RRs

Table 2K12 - 2

IESLC - Meta-analysis of Ex Smoking, Years quit (vs current), "Low"
Squamous, Cigarettes only
Most adjusted

REF	NRR	SEX	AD	Number Exposed Case	Cont	Non-exposed Case	Cont	RR	95.00%CI
BENHAM	539	m	0	132	77	789	798	1.73	(1.29- 2.34)
Totals				132	77	789	798		

*prospective study

REF	NRR	SEX	AD	Ys	Ws	Qs	Ps
BENHAM	539	m	0	0.55	43.32	0.00	0.0003

	N	1
	NS	1
	Wt	43.32
Het	Chi	0.00
Het	df	0
Het	P	N.S.
Fixed	RR	1.73
	RRl	1.29
	RRu	2.34
	P	+++
Random	RR	1.73
	RRl	1.29
	RRu	2.34
	P	+++
Asymm	P	

Table 2K12 - 3

IESLC - Meta-analysis of Ex Smoking, Years quit (vs current), "Low"
Squamous, Cigarettes only
Most adjusted

	combined	Sex male	female	Total
N		1		1
NS		1		1
Wt		43.32		43.32
Het Chi		0.00		0.00
Het df		0		0
Het P		N.S.		N.S.
Fixed RR		1.73		1.73
RRl		1.29		1.29
RRu		2.34		2.34
P		+++		+++
Random RR		1.73		1.73
RRl		1.29		1.29
RRu		2.34		2.34
P		+++		+++
Between Chi				
Between df				
Between P				N.S.
Btwn(F) P				N.S.
Btwn(R) P				N.S.

Too few RRs for analysis by factor

Table 2K12 - 4

IESLC - Meta-analysis of Ex Smoking, Years quit (vs current), "Low"
Squamous, Cigarettes only
Least adjusted

REF	NRR	X	SEX	AGEL	AGEH	RACE	YF	LC TYPE	LOC	START	ST	NLC	R	VB	P	H	AD	ADOS	PRODUCT	exL	exH	DENOM	De
BENHAM	539		m	0	0	all	-	KI	Eu:wst	1976	CC	1625	n	bl	n	y	0	0	cig only	1.0	3	cur+1y	st

Cigarette type is all/unspec for all RRs

Table 2K12 - 5

IESLC - Meta-analysis of Ex Smoking, Years quit (vs current), "Low"
Squamous, Cigarettes only
Least adjusted

REF	NRR	SEX	AD	Number Exposed Case	Cont	Non-exposed Case	Cont	RR	95.00%CI
BENHAM	539	m	0	132	77	789	798	1.73	(1.29- 2.34)
Totals				132	77	789	798		

*prospective study

REF	NRR	SEX	AD	Ys	Ws	Qs	Ps
BENHAM	539	m	0	0.55	43.32	0.00	0.0003

	N	1
	NS	1
	Wt	43.32
Het	Chi	0.00
Het	df	0
Het	P	N.S.
Fixed	RR	1.73
	RRl	1.29
	RRu	2.34
	P	+++
Random	RR	1.73
	RRl	1.29
	RRu	2.34
	P	+++
Asymm	P	

Table 2K12 - 6

IESLC - Meta-analysis of Ex Smoking, Years quit (vs current), "Low"
Squamous, Cigarettes only
Least adjusted

	Sex combined	male	female	Total
N		1		1
NS		1		1
Wt		43.32		43.32
Het Chi		0.00		0.00
Het df		0		0
Het P		N.S.		N.S.
Fixed RR		1.73		1.73
RRl		1.29		1.29
RRu		2.34		2.34
P		+++		+++
Random RR		1.73		1.73
RRl		1.29		1.29
RRu		2.34		2.34
P		+++		+++
Between Chi				
Between df				
Between P				N.S.
Btwn(F) P				N.S.
Btwn(R) P				N.S.

Table 2K12 - 7

IESLC - Meta-analysis of Ex Smoking, Years quit (vs current), "Low"
Squamous, Cigarettes only
Excluded studies (and stage at which they were excluded)

1	AGUDO AKIBA AMANDU AMES AXELSS BEST BOUCHA BOUCOT BRESLO CHEN CHEN2 CHIAZZ DEAN2 DOSEME ENGELA FAN GENG GER GUO HAENSZ HEGMAN HOLE HU HU2 JUSSAW KATSOU KAUFMA KOO KOULUM KREUZE LETOUR LEVIN LIAW LIU3 LIU4 LIU5 MCCONN MIGRAN MRFITR NOTAN2 OSANN2 PERNU QIAO2 RACHTA RESTRE SADOWS SEGI2 STASZE TIZZAN VUTUC WATSON WU WUWILL WYNDE2 WYNDE8 XU YUAN ZHANG ZHENG ZHOU
2	AUVINE BENSHL BLOT1 BROWN3 BUFFLE GURSEL LAUSSM LUO MCDUFF PISANI PRESCO SPITZ WU2 WYNDE7
4	ARMADA BECHER BOFFET BROSS CARPEN CEDERL CHOI CHYOU CORREA CPSI CPSII DAMBER DARBY DEAN3 DESTEF DOLL DOLL2 DORGAN DORN GAO GAO2 GARCIA GARSHI GILLIS GRAHAM HAMMO2 HIRAYA HUMBLE JOLY KAISE2 KHUDER LUBIN PEZZO2 QIAO SPEIZE SUZUK2 TVERDA WANG2 WIGLE
5	ALDERS HAMMON
7	BARBON JAHN JAIN JEDRYC LUBIN2 MATOS SOBUE SVENSS WAKAI WYNDE3 WYNDE6
14	PEZZOT

Table 2K12 - 8
Potentially overlapping studies

REF	REFGP	PRINC	. OVERLAP/LINK
BENHAM	LUBIN2	2	Subset of Lubin2

Table 2K13 -

IESLC - Meta-analysis of Ex Smoking, Years quit (vs current), "Mid"
Squamous, Cigarettes only

This analysis is restricted to results for:
1) Ex smokers
2) Results by Years quit (vs current)
3) Categorical results by Years quit (vs current)
4) Squamous (or near equivalent)
5) Results complete enough for use in metaanalysis

Within each study, results are then selected (in the following order of preference, within each sex) for:
6) (not applicable)
7) PRODUCT: cigarettes only
8) CIGTYPE: all/unspecified, MC regardless of HR, MC only
9) Results with least adjustment for other aspects of smoking (ADOS)
10) DENOM: current smokers, current + recent smokers (up to number of m=months or y=years, max 2 years)
11) Followup period (YF, prospective studies): whole study (coded as 0) or longest available
12) LCtype: squamous or nearest available, but not adeno. (q = squamous, s = small,
a = adeno, KI = Kreyberg I, u = undifferentiated)
13) Race: all or nearest available, otherwise by race (wh or w = white, bl or b = black, hi = hispanic
ch = chinese, jap = japanese, haw = hawaiian, w+o = white + oriental, sca = scandinavian, as = asian)
14) Years quit (vs current) "mid" in key scheme 1 (key value 7, maximum range 4-11)
15) For overlapping studies: principal rather than subsidiary studies
Finally by Age: whole study (coded as 0) if available, otherwise by widest available age group
and then for single sex results (m, f) in preference to results for both sexes combined (c).

Results adjusted (AD) for the most potential confounders are then chosen in Sections -1 to -3
(and those which actually differ from the adjusted results in Table 2K3 - 1 are marked 'x' in Section -1)
and results adjusted for the least confounders in Sections -4 to -6. (Those least adjusted results which
actually differ from the most adjusted are marked 'x' in column X in Section -4)

Section -7 shows excluded studies, together with the stage (as above) at which no qualifying
results were found.

Section -8 lists the potentially overlapping studies which have been included (1=principal, 2=subsidiary).

Section -9 lists any results which would have been included in preference except that they had data not complete
enough for use in meta-analysis, with their significance (yes/no), if known, and any further comment as entered
on the database. It also lists as "gap" any categories for which no data were presented by the original authors.

In addition to those mentioned above, the following fields, levels and abbreviations are used:
* or nk = not known, n = no, y = yes, ot = other
nev = never
all/unspec = all or unspecified, MC = manufactured cigarettes, HR = hand-rolled cigarettes
exL, exH = range of exposure (low and high) in the smoking group, in terms of Years quit (vs current)
REF: 6-character study reference
NRR: number of the RR on the database within the study
ST : study type (CC = case control, pr or prosp = prospective)
NLC: number of lung cancer cases in whole study
R : risky occupational population (n = no, m = mining, o = other risky)
VB : national cigarette type (V = at least 75% Virginia, bl = at least 75% blended, ot = other)
P : any proxy use
H : full histological confirmation
De : derivation of RR/CI (or = original, st = standard method, ot = other method of estimation)

Table 2K13 - 1

IESLC - Meta-analysis of Ex Smoking, Years quit (vs current), "Mid"
Squamous, Cigarettes only
Most adjusted

REF	NRR	2K3	SEX	AGEL	AGEH	RACE	YF	LC TYPE	LOC	START	ST	NLC	R	VB	P	H	AD	ADOS	PRODUCT	exL	exH	DENOM	De
BENHAM	541	x	m	0	0	all	-	KI	Eu:wst	1976	CC	1625	n	bl	n	y	0	0	cig only	7	10	cur+1y	st

Cigarette type is all/unspec for all RRs

Table 2K13 - 2

IESLC - Meta-analysis of Ex Smoking, Years quit (vs current), "Mid"
Squamous, Cigarettes only
Most adjusted

REF	NRR	SEX	AD	Number Exposed Case	Cont	Non-exposed Case	Cont	RR	95.00%CI
BENHAM	541	m	0	41	75	789	798	0.55	(0.37- 0.82)
Totals				41	75	789	798		

*prospective study

REF	NRR	SEX	AD	Ys	Ws	Qs	Ps
BENHAM	541	m	0	-0.59	24.85	0.00	0.0031

	N	1
	NS	1
	Wt	24.85
Het	Chi	0.00
Het	df	0
Het	P	N.S.
Fixed	RR	0.55
	RRl	0.37
	RRu	0.82
	P	--
Random	RR	0.55
	RRl	0.37
	RRu	0.82
	P	--
Asymm	P	

Table 2K13 - 3

IESLC - Meta-analysis of Ex Smoking, Years quit (vs current), "Mid"
Squamous, Cigarettes only
Most adjusted

	Sex combined	male	female	Total
N		1		1
NS		1		1
Wt		24.85		24.85
Het Chi		0.00		0.00
Het df		0		0
Het P		N.S.		N.S.
Fixed RR		0.55		0.55
RRl		0.37		0.37
RRu		0.82		0.82
P		--		--
Random RR		0.55		0.55
RRl		0.37		0.37
RRu		0.82		0.82
P		--		--
Between Chi				
Between df				
Between P				N.S.
Btwn(F) P				N.S.
Btwn(R) P				N.S.

Too few RRs for analysis by factor

Table 2K13 - 4

IESLC - Meta-analysis of Ex Smoking, Years quit (vs current), "Mid"
Squamous, Cigarettes only
Least adjusted

REF	NRR	X	SEX	AGEL	AGEH	RACE	YF	LC TYPE	LOC	START	ST	NLC	R	VB	P	H	AD	ADOS	PRODUCT	exL	exH	DENOM	De
BENHAM	541		m	0	0	all	-	KI	Eu:wst	1976	CC	1625	n	bl	n	y	0	0	cig only	7	10	cur+1y	st

Cigarette type is all/unspec for all RRs

Table 2K13 - 5

IESLC - Meta-analysis of Ex Smoking, Years quit (vs current), "Mid"
Squamous, Cigarettes only
Least adjusted

REF	NRR	SEX	AD	Number Exposed Case	Cont	Non-exposed Case	Cont	RR	95.00%CI
BENHAM	541	m	0	41	75	789	798	0.55	(0.37- 0.82)
Totals				41	75	789	798		

*prospective study

REF	NRR	SEX	AD	Ys	Ws	Qs	Ps
BENHAM	541	m	0	-0.59	24.85	0.00	0.0031

	N	1
	NS	1
	Wt	24.85
Het	Chi	0.00
Het	df	0
Het	P	N.S.
Fixed	RR	0.55
	RRl	0.37
	RRu	0.82
	P	--
Random	RR	0.55
	RRl	0.37
	RRu	0.82
	P	--
Asymm	P	

Table 2K13 - 6

IESLC - Meta-analysis of Ex Smoking, Years quit (vs current), "Mid"
Squamous, Cigarettes only
Least adjusted

	Sex combined	male	female	Total
N		1		1
NS		1		1
Wt		24.85		24.85
Het Chi		0.00		0.00
Het df		0		0
Het P		N.S.		N.S.
Fixed RR		0.55		0.55
RRl		0.37		0.37
RRu		0.82		0.82
P		--		--
Random RR		0.55		0.55
RRl		0.37		0.37
RRu		0.82		0.82
P		--		--
Between Chi				
Between df				
Between P				N.S.
Btwn(F) P				N.S.
Btwn(R) P				N.S.

Table 2K13 - 7

IESLC - Meta-analysis of Ex Smoking, Years quit (vs current), "Mid"
Squamous, Cigarettes only
Excluded studies (and stage at which they were excluded)

1	AGUDO AKIBA AMANDU AMES AXELSS BEST BOUCHA BOUCOT BRESLO CHEN CHEN2 CHIAZZ DEAN2 DOSEME ENGELA FAN GENG GER GUO HAENSZ HEGMAN HOLE HU HU2 JUSSAW KATSOU KAUFMA KOO KOULUM KREUZE LETOUR LEVIN LIAW LIU3 LIU4 LIU5 MCCONN MIGRAN MRFITR NOTAN2 OSANN2 PERNU QIAO2 RACHTA RESTRE SADOWS SEGI2 STASZE TIZZAN VUTUC WATSON WU WUWILL WYNDE2 WYNDE8 XU YUAN ZHANG ZHENG ZHOU
2	AUVINE BENSHL BLOT1 BROWN3 BUFFLE GURSEL LAUSSM LUO MCDUFF PISANI PRESCO SPITZ WU2 WYNDE7
4	ARMADA BECHER BOFFET BROSS CARPEN CEDERL CHOI CHYOU CORREA CPSI CPSII DAMBER DARBY DEAN3 DESTEF DOLL DOLL2 DORGAN DORN GAO GAO2 GARCIA GARSHI GILLIS GRAHAM HAMMO2 HIRAYA HUMBLE JOLY KAISE2 KHUDER LUBIN PEZZO2 QIAO SPEIZE SUZUK2 TVERDA WANG2 WIGLE
5	ALDERS HAMMON
7	BARBON JAHN JAIN JEDRYC LUBIN2 MATOS SOBUE SVENSS WAKAI WYNDE3 WYNDE6
14	PEZZOT

Table 2K13 - 8
Potentially overlapping studies

REF	REFGP	PRINC	.	OVERLAP/LINK
BENHAM	LUBIN2	2		Subset of Lubin2

Table 2K14 -

IESLC - Meta-analysis of Ex Smoking, Years quit (vs current), "High"
Squamous, Cigarettes only

This analysis is restricted to results for:
1) Ex smokers
2) Results by Years quit (vs current)
3) Categorical results by Years quit (vs current)
4) Squamous (or near equivalent)
5) Results complete enough for use in metaanalysis

Within each study, results are then selected (in the following order of preference, within each sex) for:
6) PRODUCT: cigarettes only
7) CIGTYPE: all/unspecified, MC regardless of HR, MC only
8) Results with least adjustment for other aspects of smoking (ADOS)
9) DENOM: current smokers, current + recent smokers (up to number of m=months or y=years, max 2 years)
10) Followup period (YF, prospective studies): whole study (coded as 0) or longest available
11) LCtype: squamous or nearest available, but not adeno. (q = squamous, s = small,
a = adeno, KI = Kreyberg I, u = undifferentiated)
12) Race: all or nearest available, otherwise by race (wh or w = white, bl or b = black, hi = hispanic
ch = chinese, jap = japanese, haw = hawaiian, w+o = white + oriental, sca = scandinavian, as = asian)
13) Years quit (vs current) "high" in key scheme 1 (key value 12, maximum range 8+)
14) For overlapping studies: principal rather than subsidiary studies
Finally by Age: whole study (coded as 0) if available, otherwise by widest available age group
and then for single sex results (m, f) in preference to results for both sexes combined (c).

Results adjusted (AD) for the most potential confounders are then chosen in Sections -1 to -3
(and those which actually differ from the adjusted results in Table 2K4 - 1 are marked 'x' in Section -1)
and results adjusted for the least confounders in Sections -4 to -6. (Those least adjusted results which
actually differ from the most adjusted are marked 'x' in column X in Section -4)

Section -7 shows excluded studies, together with the stage (as above) at which no qualifying
results were found.

Section -8 lists the potentially overlapping studies which have been included (1=principal, 2=subsidiary).

Section -9 lists any results which would have been included in preference except that they had data not complete
enough for use in meta-analysis, with their significance (yes/no), if known, and any further comment as entered
on the database. It also lists as "gap" any categories for which no data were presented by the original authors.

In addition to those mentioned above, the following fields, levels and abbreviations are used:
* or nk = not known, n = no, y = yes, ot = other
nev = never
all/unspec = all or unspecified, MC = manufactured cigarettes, HR = hand-rolled cigarettes
exL, exH = range of exposure (low and high) in the smoking group, in terms of Years quit (vs current)
REF: 6-character study reference
NRR: number of the RR on the database within the study
ST : study type (CC = case control, pr or prosp = prospective)
NLC: number of lung cancer cases in whole study
R : risky occupational population (n = no, m = mining, o = other risky)
VB : national cigarette type (V = at least 75% Virginia, bl = at least 75% blended, ot = other)
P : any proxy use
H : full histological confirmation
De : derivation of RR/CI (or = original, st = standard method, ot = other method of estimation)

Table 2K14 - 1

IESLC - Meta-analysis of Ex Smoking, Years quit (vs current), "High"
Squamous, Cigarettes only
Most adjusted

REF	NRR	2K4	SEX	AGEL	AGEH	RACE	YF	LC TYPE	LOC	START	ST	NLC	R	VB	P	H	AD	ADOS	PRODUCT	exL	exH	DENOM	De
BENHAM	542	x	m	0	0	all	-	KI	Eu:wst	1976	CC	1625	n	bl	n	y	0	0	cig only	11	19	cur+1y	st
PEZZOT	583		m	0	0	all	-	q	SCAmer	1987	CC	215	n	bl	n	y	0	0	cig only	11	999	cur+1y	st

Cigarette type is all/unspec for all RRs

Table 2K14 - 2

IESLC - Meta-analysis of Ex Smoking, Years quit (vs current), "High"
Squamous, Cigarettes only
Most adjusted

REF	NRR	SEX	AD	Number Exposed Case	Number Exposed Cont	Non-exposed Case	Non-exposed Cont	RR	95.00%CI
BENHAM	542	m	0	39	125	789	798	0.32	(0.22- 0.46)
PEZZOT	583	m	0	8	48	56	52	0.15	(0.07- 0.36)
Totals				47	173	845	850		

*prospective study

REF	NRR	SEX	AD	Ys	Ws	Qs	Ps
BENHAM	542	m	0	-1.15	27.65	0.38	0.0000
PEZZOT	583	m	0	-1.87	5.47	1.93	0.0000

	N	2
	NS	2
	Wt	33.12
Het	Chi	2.32
Het	df	1
Het	P	N.S.
Fixed	RR	0.28
	RRl	0.20
	RRu	0.39
	P	---
Random	RR	0.24
	RRl	0.13
	RRu	0.48
	P	---
Asymm	P	

Table 2K14 - 3

IESLC - Meta-analysis of Ex Smoking, Years quit (vs current), "High"
Squamous, Cigarettes only
Most adjusted

	Sex combined	male	female	Total
N		2		2
NS		2		2
Wt		33.12		33.12
Het Chi		2.32		2.32
Het df		1		1
Het P		N.S.		N.S.
Fixed RR		0.28		0.28
RRl		0.20		0.20
RRu		0.39		0.39
P		---		---
Random RR		0.24		0.24
RRl		0.13		0.13
RRu		0.48		0.48
P		---		---
Between Chi				
Between df				
Between P				N.S.
Btwn(F) P				N.S.
Btwn(R) P				N.S.

Too few RRs for analysis by factor

Table 2K14 - 4

IESLC - Meta-analysis of Ex Smoking, Years quit (vs current), "High"
Squamous, Cigarettes only
Least adjusted

REF	NRR	X	SEX	AGEL	AGEH	RACE	YF	LC TYPE	LOC	START	ST	NLC	R	VB	P	H	AD	ADOS	PRODUCT	exL	exH	DENOM	De
BENHAM	542		m	0	0	all	-	KI	Eu:wst	1976	CC	1625	n	bl	n	y	0	0	cig only	11	19	cur+1y	st
PEZZOT	583		m	0	0	all	-	q	SCAmer	1987	CC	215	n	bl	n	y	0	0	cig only	11	999	cur+1y	st

Cigarette type is all/unspec for all RRs

Table 2K14 - 5

IESLC - Meta-analysis of Ex Smoking, Years quit (vs current), "High"
Squamous, Cigarettes only
Least adjusted

REF	NRR	SEX	A	Number Exposed Case	Cont	Non-exposed Case	Cont	RR	95.00%CI
BENHAM	542	m	0	39	125	789	798	0.32	(0.22- 0.46)
PEZZOT	583	m	0	8	48	56	52	0.15	(0.07- 0.36)
Totals				47	173	845	850		

*prospective study

REF	NRR	SEX	AD	Ys	Ws	Qs	Ps
BENHAM	542	m	0	-1.15	27.65	0.38	0.0000
PEZZOT	583	m	0	-1.87	5.47	1.93	0.0000

	N	2
	NS	2
	Wt	33.12
Het	Chi	2.32
Het	df	1
Het	P	N.S.
Fixed	RR	0.28
	RRl	0.20
	RRu	0.39
	P	---
Random	RR	0.24
	RRl	0.13
	RRu	0.48
	P	---
Asymm	P	

Table 2K14 - 6

IESLC - Meta-analysis of Ex Smoking, Years quit (vs current), "High"
Squamous, Cigarettes only
Least adjusted

	Sex combined	male	female	Total
N		2		2
NS		2		2
Wt		33.12		33.12
Het Chi		2.32		2.32
Het df		1		1
Het P		N.S.		N.S.
Fixed RR		0.28		0.28
RRl		0.20		0.20
RRu		0.39		0.39
P		---		---
Random RR		0.24		0.24
RRl		0.13		0.13
RRu		0.48		0.48
P		---		---
Between Chi				
Between df				
Between P				N.S.
Btwn(F) P				N.S.
Btwn(R) P				N.S.

Table 2K14 - 7

IESLC - Meta-analysis of Ex Smoking, Years quit (vs current), "High"
Squamous, Cigarettes only
Excluded studies (and stage at which they were excluded)

1	AGUDO AKIBA AMANDU AMES AXELSS BEST BOUCHA BOUCOT BRESLO CHEN CHEN2 CHIAZZ DEAN2 DOSEME ENGELA FAN GENG GER GUO HAENSZ HEGMAN HOLE HU HU2 JUSSAW KATSOU KAUFMA KOO KOULUM KREUZE LETOUR LEVIN LIAW LIU3 LIU4 LIU5 MCCONN MIGRAN MRFITR NOTAN2 OSANN2 PERNU QIAO2 RACHTA RESTRE SADOWS SEGI2 STASZE TIZZAN VUTUC WATSON WU WUWILL WYNDE2 WYNDE8 XU YUAN ZHANG ZHENG ZHOU
2	AUVINE BENSHL BLOT1 BROWN3 BUFFLE GURSEL LAUSSM LUO MCDUFF PISANI PRESCO SPITZ WU2 WYNDE7
4	ARMADA BECHER BOFFET BROSS CARPEN CEDERL CHOI CHYOU CORREA CPSI CPSII DAMBER DARBY DEAN3 DESTEF DOLL DOLL2 DORGAN DORN GAO GAO2 GARCIA GARSHI GILLIS GRAHAM HAMMO2 HIRAYA HUMBLE JOLY KAISE2 KHUDER LUBIN PEZZO2 QIAO SPEIZE SUZUK2 TVERDA WANG2 WIGLE
5	ALDERS HAMMON
7	BARBON JAHN JAIN JEDRYC LUBIN2 MATOS SOBUE SVENSS WAKAI WYNDE3 WYNDE6

Table 2K14 - 8
Potentially overlapping studies

REF	REFGP	PRINC	. OVERLAP/LINK
BENHAM	LUBIN2	2	Subset of Lubin2

Table 2K14 - 9
Most adjusted - insufficient data for meta-analysis

REF	NRR	SEX	AGEL	AGEH	RACE	YF	LC TYPE	LOC	START	ST	NLC	R	VB	P	H	AD	ADOS	PRODUCT	exL	exH	DENOM	De
ALDERS	545	m	0	0	all	-	q+s	Eu:UK	1977	CC	1448	n	V	n	n	1	0	cig only	10	999	current	ot
ALDERS	556	f	0	0	all	-	q+s	Eu:UK	1977	CC	1448	n	V	n	n	1	0	cig only	10	999	current	ot
HAMMON	509	m	0	0	wh	0	not a	NAmer	1952	pr	448	n	bl	n	n	1	0	cig only	10	999	current	st

REF	NRR	RR	SIG	RRDATA comment
ALDERS	545	0.21	y	0.001<p<0.01
ALDERS	556	0.12	y	p<0.001
HAMMON	509	*		RR for <1 pack per day is 0.14, while that for 1+ packs is 0.39

Table 2K15 -

IESLC - Meta-analysis of Ex Smoking, Years quit (vs current), "Highest vs lowest"
Squamous, Cigarettes only

This analysis is restricted to results for:
1) Ex smokers
2) Results by Years quit (vs current)
3) Categorical results by Years quit (vs current)
4) Denominator (unexposed) = "low"
5) Squamous (or near equivalent)
6) Results complete enough for use in metaanalysis

Within each study, results are then selected (in the following order of preference, within each sex) for:
7) (not applicable)
8) PRODUCT: cigarettes only
9) CIGTYPE: all/unspecified, MC regardless of HR, MC only
10) Results with least adjustment for other aspects of smoking (ADOS)
11) The highest vs lowest category
12) Followup period (YF, prospective studies): whole study (coded as 0) or longest available
13) LCtype: squamous or nearest available, but not adeno. (q = squamous, s = small,
a = adeno, KI = Kreyberg I, u = undifferentiated)
14) Race: all or nearest available, otherwise by race (wh or w = white, bl or b = black, hi = hispanic
ch = chinese, jap = japanese, haw = hawaiian, w+o = white + oriental, sca = scandinavian, as = asian)
15) For overlapping studies: principal rather than subsidiary studies
Finally by Age: whole study (coded as 0) if available, otherwise by widest available age group
and then for single sex results (m, f) in preference to results for both sexes combined (c).

Results adjusted (AD) for the most potential confounders are then chosen in Sections -1 to -3
(and those which actually differ from the adjusted results in Table 2K5 - 1 are marked 'x' in Section -1)
and results adjusted for the least confounders in Sections -4 to -6. (Those least adjusted results which
actually differ from the most adjusted are marked 'x' in column X in Section -4)

Section -7 shows excluded studies, together with the stage (as above) at which no qualifying
results were found.

Section -8 lists the potentially overlapping studies which have been included (1=principal, 2=subsidiary).

Section -9 lists any results which would have been included in preference except that they had data not complete
enough for use in meta-analysis, with their significance (yes/no), if known, and any further comment as entered
on the database. It also lists as "gap" any categories for which no data were presented by the original authors.

In addition to those mentioned above, the following fields, levels and abbreviations are used:
* or nk = not known, n = no, y = yes, ot = other
all/unspec = all or unspecified, MC = manufactured cigarettes, HR = hand-rolled cigarettes
exL, exH = range of exposure (low and high) in the "highest" group, in terms of Years quit (vs current)
unexL, unexH = range of exposure (low and high) in the "lowest" group, in terms of Years quit (vs current)
REF: 6-character study reference
NRR: number of the RR on the database within the study
ST : study type (CC = case control, pr or prosp = prospective)
NLC: number of lung cancer cases in whole study
R : risky occupational population (n = no, m = mining, o = other risky)
VB : national cigarette type (V = at least 75% Virginia, bl = at least 75% blended, ot = other)
P : any proxy use
H : full histological confirmation
De : derivation of RR/CI (or = original, st = standard method, ot = other method of estimation)

Table 2K15 - 1

IESLC - Meta-analysis of Ex Smoking, Years quit (vs current), "Highest vs lowest"
Squamous, Cigarettes only
Most adjusted

REF	NRR	2K5	SEX	AGEL	AGEH	RACE	YF	LC TYPE	LOC	START	ST	NLC	R	VB	P	H	AD	ADOS	PRODUCT	exL	exH	unexL	unexH	De
BENHAM	547	x	m	0	0	all	-	KI	Eu:wst	1976	CC	1625	n	bl	n	y	0	0	cig only	20	999	1.0	3	st
PEZZOT	584		m	0	0	all	-	q	SCAmer	1987	CC	215	n	bl	n	y	0	0	cig only	11	999	1.0	10	st

Cigarette type is all/unspec for all RRs

Table 2K15 - 2

IESLC - Meta-analysis of Ex Smoking, Years quit (vs current), "Highest vs lowest"
Squamous, Cigarettes only
Most adjusted

REF	NRR	SEX	A	Number Exposed Case	Cont	Non-exposed Case	Cont	RR	95.00%CI
BENHAM	547	m	0	17	82	132	77	0.12	(0.07- 0.22)
PEZZOT	584	m	0	8	48	21	27	0.21	(0.08- 0.55)
Totals				25	130	153	104		

*prospective study

REF	NRR	SEX	AD	Ys	Ws	Qs	Ps
BENHAM	547	m	0	-2.11	10.92	0.29	0.0000
PEZZOT	584	m	0	-1.54	4.34	0.73	0.0013

	N	2
	NS	2
	Wt	15.26
Het	Chi	1.02
Het	df	1
Het	P	N.S.
Fixed	RR	0.14
	RRl	0.09
	RRu	0.24
	P	---
Random	RR	0.14
	RRl	0.09
	RRu	0.24
	P	---
Asymm	P	

Table 2K15 - 3

IESLC - Meta-analysis of Ex Smoking, Years quit (vs current), "Highest vs lowest"
Squamous, Cigarettes only
Most adjusted

	Sex combined	male	female	Total
N		2		2
NS		2		2
Wt		15.26		15.26
Het Chi		1.02		1.02
Het df		1		1
Het P		N.S.		N.S.
Fixed RR		0.14		0.14
RRl		0.09		0.09
RRu		0.24		0.24
P		---		---
Random RR		0.14		0.14
RRl		0.09		0.09
RRu		0.24		0.24
P		---		---
Between Chi				
Between df				
Between P				N.S.
Btwn(F) P				N.S.
Btwn(R) P				N.S.

Too few RRs for analysis by factor

Table 2K15 - 4

IESLC - Meta-analysis of Ex Smoking, Years quit (vs current), "Highest vs lowest"
Squamous, Cigarettes only
Least adjusted

REF	NRR	X	SEX	AGEL	AGEH	RACE	YF	LC TYPE	LOC	START	ST	NLC	R	VB	P	H	AD	ADOS	PRODUCT	exL	exH	unexL	unexH	De
BENHAM	547		m	0	0	all	-	KI	Eu:wst	1976	CC	1625	n	bl	n	y	0	0	cig only	20	999	1.0	3	st
PEZZOT	584		m	0	0	all	-	q	SCAmer	1987	CC	215	n	bl	n	y	0	0	cig only	11	999	1.0	10	st

Cigarette type is all/unspec for all RRs

Table 2K15 - 5

IESLC - Meta-analysis of Ex Smoking, Years quit (vs current), "Highest vs lowest"
Squamous, Cigarettes only
Least adjusted

REF	NRR	SEX	AD	Number Exposed Case	Cont	Non-exposed Case	Cont	RR	95.00%CI
BENHAM	547	m	0	17	82	132	77	0.12	(0.07- 0.22)
PEZZOT	584	m	0	8	48	21	27	0.21	(0.08- 0.55)
Totals				25	130	153	104		

*prospective study

REF	NRR	SEX	AD	Ys	Ws	Qs	Ps
BENHAM	547	m	0	-2.11	10.92	0.29	0.0000
PEZZOT	584	m	0	-1.54	4.34	0.73	0.0013

	N	2
	NS	2
	Wt	15.26
Het	Chi	1.02
Het	df	1
Het	P	N.S.
Fixed	RR	0.14
	RRl	0.09
	RRu	0.24
	P	---
Random	RR	0.14
	RRl	0.09
	RRu	0.24
	P	---
Asymm	P	

Table 2K15 - 6

IESLC - Meta-analysis of Ex Smoking, Years quit (vs current), "Highest vs lowest"
Squamous, Cigarettes only
Least adjusted

	Sex combined	male	female	Total
N		2		2
NS		2		2
Wt		15.26		15.26
Het Chi		1.02		1.02
Het df		1		1
Het P		N.S.		N.S.
Fixed RR		0.14		0.14
RRl		0.09		0.09
RRu		0.24		0.24
P		---		---
Random RR		0.14		0.14
RRl		0.09		0.09
RRu		0.24		0.24
P		---		---
Between Chi				
Between df				
Between P				N.S.
Btwn(F) P				N.S.
Btwn(R) P				N.S.

Table 2K15 - 7

IESLC - Meta-analysis of Ex Smoking, Years quit (vs current), "Highest vs lowest"
Squamous, Cigarettes only
Excluded studies (and stage at which they were excluded)

1	AGUDO AKIBA AMANDU AMES AXELSS BEST BOUCHA BOUCOT BRESLO CHEN CHEN2 CHIAZZ DEAN2 DOSEME ENGELA FAN GENG GER GUO HAENSZ HEGMAN HOLE HU HU2 JUSSAW KATSOU KAUFMA KOO KOULUM KREUZE LETOUR LEVIN LIAW LIU3 LIU4 LIU5 MCCONN MIGRAN MRFITR NOTAN2 OSANN2 PERNU QIAO2 RACHTA RESTRE SADOWS SEGI2 STASZE TIZZAN VUTUC WATSON WU WUWILL WYNDE2 WYNDE8 XU YUAN ZHANG ZHENG ZHOU
2	AUVINE BENSHL BLOT1 BROWN3 BUFFLE GURSEL LAUSSM LUO MCDUFF PISANI PRESCO SPITZ WU2 WYNDE7
4	GARSHI JEDRYC WAKAI
5	ARMADA BECHER BOFFET BROSS CARPEN CEDERL CHOI CHYOU CORREA CPSI CPSII DAMBER DARBY DEAN3 DESTEF DOLL DOLL2 DORGAN DORN GAO GAO2 GARCIA GILLIS GRAHAM HAMMO2 HIRAYA HUMBLE JOLY KAISE2 KHUDER LUBIN PEZZO2 QIAO SPEIZE SUZUK2 TVERDA WANG2 WIGLE
6	ALDERS HAMMON
8	BARBON JAHN JAIN LUBIN2 MATOS SOBUE SVENSS WYNDE3 WYNDE6

Table 2K15 - 8

Potentially overlapping studies

REF	REFGP	PRINC	.	OVERLAP/LINK
BENHAM	LUBIN2	2		Subset of Lubin2

Table 2K15 - 9

Most adjusted - insufficient data for meta-analysis

REF	NRR	SEX	AGEL	AGEH	RACE	YF	LC TYPE	LOC	START	ST	NLC	R	VB	P	H	AD	ADOS	PRODUCT	exL	exH	unexL	unexH	De
ALDERS	547	m	0	0	all	-	q+s	Eu:UK	1977	CC	1448	n	V	n	n	1	0	cig only	10	999	0.1	2	st
ALDERS	558	f	0	0	all	-	q+s	Eu:UK	1977	CC	1448	n	V	n	n	1	0	cig only	10	999	0.1	2	st
HAMMON	511	m	0	0	wh	0	not a	NAmer	1952	pr	448	n	bl	n	n	1	0	cig only	10	999	0.1	0.9	st

REF	NRR	RR	SIG	RRDATA comment
ALDERS	547	0.10		0
ALDERS	558	0.04		0
HAMMON	511	*		RR for <1 pack per day is 0.15, while that for 1+ packs is 0.31
