# Supplementary material for: Systematic review with meta-analysis of the epidemiological evidence in the 1900s relating smoking to lung cancer
Source: BMC Cancer. 2012 Sep 3;12:385. doi: 10.1186/1471-2407-12-385 (PMC3505152; doi:10.1186/1471-2407-12-385)
Supplement: Additional file 5 — Detailed Analysis Tables (Individual file names as described in Additional file 1: Methods, Table1). [file 1471-2407-12-385-S5.zip › PDF/1J.pdf]

Table 1J1 -

IESLC - Meta-analysis of Ex Smoking by Years quit (vs never), Overview
All LC types, Any Product (or Cigarettes if Any not available)

This analysis is restricted to results for:
1) Ex smokers
2) Results by Years quit (vs never)
3) Categorical results by Years quit (vs never)
Results by Years quit (vs never) are grouped under 2 schemes (S1, S2). Each scheme has a set of "key values". An interval is allocated to the category whose key value it includes, and intervals which include none or more than one of the key values are excluded. (Open-ended intervals are coded as 999)

S1	key value	maximum range
1	12	8+
2	7	4-11
3	3	1-6

S2	key value	maximum range
1	20	13+
2	12	4-19
3	3	1-11

4) All LC types (or near equivalent)
5) Results complete enough for use in metaanalysis

Within each study, results are then selected (in the following order of preference, within each sex) for:
6) (not applicable)
7) PRODUCT: all/unspec, cigarettes regardless of other products, cigarettes only
8) CIGTYPE: all/unspecified, MC regardless of HR, MC only
9) (not applicable)
10) DENOM: never smoked anything, never smoked cigarettes, never any + low, never cigs + low
11) Followup period (YF, prospective studies): whole study (coded as 0) or longest available
12) LCtype: all or nearest available, at least Squamous and Adeno. (q = squamous, s = small, l = large, a = adeno, mix = mixed, alv = alveolar)
13) Race: all or nearest available, otherwise by race (wh or w = white, bl or b = black, hi = hispanic ch = chinese, jap = japanese, haw = hawaiian, w+o = white + oriental, sca = scandinavian, as = asian)
14) For overlapping studies: principal rather than subsidiary studies
Finally by Age: whole study (coded as 0) if available, otherwise by widest available age group and then for single sex results (m, f) in preference to results for both sexes combined (c).

Results adjusted (AD) for the most potential confounders are then chosen in Sections -1 to -3 and results adjusted for the least confounders in Sections -4 to -6. (Those least adjusted results which actually differ from the most adjusted are marked 'x' in column X in Section -4)

Section -7 shows excluded studies, together with the stage (as above) at which no qualifying results were found.

Section -8 lists the potentially overlapping studies which have been included (1=principal, 2=subsidiary).

Section -9 lists any results which would have been included in preference except that they had data not complete enough for use in meta-analysis, with their significance (yes/no), if known, and any further comment as entered on the database. It also lists as "gap" any categories for which no data were presented by the original authors. This is commonly due to recent quitters having been combined with current smokers

In addition to those mentioned above, the following fields, levels and abbreviations are used:
* or nk = not known, n = no, y = yes, ot = other
nev = never
all/unspec = all or unspecified, cig+/-ot = cigarettes irrespective of other products (cigar, pipe etc)
MC = manufactured cigarettes, HR = hand-rolled cigarettes
exL, exH = range of exposure (low and high) in the smoking group, in terms of Years quit (vs never)
REF: 6-character study reference
NRR: number of the RR on the database within the study
ST : study type (CC = case control, pr or prosp = prospective)
NLC: number of lung cancer cases in whole study
R : risky occupational population (n = no, m = mining, o = other risky)
VB : national cigarette type (V = at least 75% Virginia, bl = at least 75% blended, ot = other)
P : any proxy use
H : full histological confirmation
De : derivation of RR/CI (or = original, st = standard method, ot = other method of estimation)

Table 1J1 - 1

IESLC - Meta-analysis of Ex Smoking by Years quit (vs never), Overview
All LC types, Any Product (or Cigarettes if Any not available)
Most adjusted

REF	NRR	SEX	AGEL	AGEH	RACE	YF	LC TYPE	LOC	START	ST	NLC	R	VB	P	H	AD	PRODUCT	exL	exH	S1	S2	DENOM	De
ALDERS	507	m	0	0	all	-	all	Eu:UK	1977	CC	1448	n	V	n	n	1	cig only	10	999	1	0	nev any	ot
ALDERS	508	m	0	0	all	-	all	Eu:UK	1977	CC	1448	n	V	n	n	1	cig only	3	9	0	3	nev any	ot
ALDERS	509	m	0	0	all	-	all	Eu:UK	1977	CC	1448	n	V	n	n	1	cig only	0.1	2	0	0	nev any	ot
ALDERS	518	f	0	0	all	-	all	Eu:UK	1977	CC	1448	n	V	n	n	1	cig only	10	999	1	0	nev any	ot
ALDERS	519	f	0	0	all	-	all	Eu:UK	1977	CC	1448	n	V	n	n	1	cig only	3	9	0	3	nev any	ot
ALDERS	520	f	0	0	all	-	all	Eu:UK	1977	CC	1448	n	V	n	n	1	cig only	0.1	2	0	0	nev any	ot
ARMADA	515	m	0	0	all	-	all	Eu:wst	1986	CC	325	n	bl	n	y	0	cig+/-ot	6	999	0	0	nev cigs	st
ARMADA	516	m	0	0	all	-	all	Eu:wst	1986	CC	325	n	bl	n	y	0	cig+/-ot	1.0	5	3	3	nev cigs	st
AUVINE	532	c	0	0	all	-	all	Eu:Sca	1986	CC	517	n	bl	y	n	2	cig+/-ot	12	999	1	0	nev cigs	or
BARBON	540	m	0	0	all	-	all	Eu:wst	1979	CC	755	n	bl	y	y	1	all/unsp	25	999	0	0	nev any	or
BARBON	541	m	0	0	all	-	all	Eu:wst	1979	CC	755	n	bl	y	y	1	all/unsp	15	24	0	1	nev any	or
BARBON	542	m	0	0	all	-	all	Eu:wst	1979	CC	755	n	bl	y	y	1	all/unsp	5	14	0	2	nev any	or
BARBON	543	m	0	0	all	-	all	Eu:wst	1979	CC	755	n	bl	y	y	1	all/unsp	0.1	4	3	3	nev any	or
BECHER	501	m	0	0	all	-	all	Eu:Ger	1985	CC	194	n	bl	n	y	0	all/unsp	10	999	1	0	nev any	st
BECHER	502	m	0	0	all	-	all	Eu:Ger	1985	CC	194	n	bl	n	y	0	all/unsp	5	9	2	0	nev any	st
BECHER	503	m	0	0	all	-	all	Eu:Ger	1985	CC	194	n	bl	n	y	0	all/unsp	2	4	3	3	nev any	st
BECHER	511	f	0	0	all	-	all	Eu:Ger	1985	CC	194	n	bl	n	y	0	all/unsp	10	999	1	0	nev any	st
BECHER	512	f	0	0	all	-	all	Eu:Ger	1985	CC	194	n	bl	n	y	0	all/unsp	5	9	2	0	nev any	st
BECHER	513	f	0	0	all	-	all	Eu:Ger	1985	CC	194	n	bl	n	y	0	all/unsp	2	4	3	3	nev any	st
BENSHL	508	m	0	0	all	0	all	Eu:UK	1967	pr	486	n	V	n	n	2	cig+/-ot	30	999	0	0	nev any	or
BENSHL	509	m	0	0	all	0	all	Eu:UK	1967	pr	486	n	V	n	n	2	cig+/-ot	20	29	0	1	nev any	or
BENSHL	510	m	0	0	all	0	all	Eu:UK	1967	pr	486	n	V	n	n	2	cig+/-ot	10	19	1	2	nev any	or
BENSHL	511	m	0	0	all	0	all	Eu:UK	1967	pr	486	n	V	n	n	2	cig+/-ot	1.0	9	0	3	nev any	or
BROSS	515	m	0	0	wh	-	all	NAmer	1960	CC	974	n	bl	n	n	0	cig+/-ot	6	999	0	0	nev any	st
BROSS	516	m	0	0	wh	-	all	NAmer	1960	CC	974	n	bl	n	n	0	cig+/-ot	0.1	5	3	3	nev any	st
BROWN3	503	f	0	0	wh	-	all	NAmer		CC	618		bl	y	n	2	all/unsp	15	999	0	1	nev any	or
CARPEN	501	c	0	0	w+b	-	all	NAmer	1991	CC	356	n	bl	n	n	0	cig+/-ot	15	999	0	1	nev cigs	st
CARPEN	502	c	0	0	w+b	-	all	NAmer	1991	CC	356	n	bl	n	n	0	cig+/-ot	10	14	1	2	nev cigs	st
CARPEN	503	c	0	0	w+b	-	all	NAmer	1991	CC	356	n	bl	n	n	0	cig+/-ot	5	9	2	0	nev cigs	st
CARPEN	504	c	0	0	w+b	-	all	NAmer	1991	CC	356	n	bl	n	n	0	cig+/-ot	0.1	4	3	3	nev cigs	st
CEDERL	528	m	40	69	all	10	all	Eu:Sca	1963	pr	491	n	bl	n	n	1	all/unsp	10	999	1	0	nev any	ot
CEDERL	529	m	40	69	all	10	all	Eu:Sca	1963	pr	491	n	bl	n	n	1	all/unsp	0.1	9	0	3	nev any	ot
CHOI	533	m	0	0	all	-	all	As:oth	1985	CC	375	n	bl	n	n	0	cig+/-ot	15	999	0	1	nev cigs	st
CHOI	534	m	0	0	all	-	all	As:oth	1985	CC	375	n	bl	n	n	0	cig+/-ot	10	14	1	2	nev cigs	st
CHOI	535	m	0	0	all	-	all	As:oth	1985	CC	375	n	bl	n	n	0	cig+/-ot	5	9	2	0	nev cigs	st
CHOI	536	m	0	0	all	-	all	As:oth	1985	CC	375	n	bl	n	n	0	cig+/-ot	0.1	4	3	3	nev cigs	st
CHOI	550	f	0	0	all	-	all	As:oth	1985	CC	375	n	bl	n	n	0	cig+/-ot	5	999	0	0	nev cigs	ot
CHOI	551	f	0	0	all	-	all	As:oth	1985	CC	375	n	bl	n	n	0	cig+/-ot	0.1	4	3	3	nev cigs	st
CHYOU	507	m	0	0	jap	21	all	NAmer	1965	pr	227	n	bl	n	y	2	cig+/-ot	15	999	0	1	nev cigs	or
CHYOU	508	m	0	0	jap	21	all	NAmer	1965	pr	227	n	bl	n	y	2	cig+/-ot	0.1	14	0	0	nev cigs	or
CPSI	807	m	50	74	all	6	all	NAmer	1959	pr	5138	n	bl	n	n	1	cig only	10	999	1	0	nev any	ot
CPSI	808	m	50	74	all	6	all	NAmer	1959	pr	5138	n	bl	n	n	1	cig only	5	9	2	0	nev any	ot
CPSI	809	m	50	74	all	6	all	NAmer	1959	pr	5138	n	bl	n	n	1	cig only	1.0	4	3	3	nev any	ot
CPSI	810	m	50	74	all	6	all	NAmer	1959	pr	5138	n	bl	n	n	1	cig only	0.1	0.9	0	0	nev any	ot
CPSII	652	m	35	99	all	4	all	NAmer	1982	pr	3229	n	bl	n	n	1	cig only	16	999	0	1	nev any	ot
CPSII	653	m	35	99	all	4	all	NAmer	1982	pr	3229	n	bl	n	n	1	cig only	11	15	1	2	nev any	ot
CPSII	654	m	35	99	all	4	all	NAmer	1982	pr	3229	n	bl	n	n	1	cig only	6	10	2	0	nev any	ot
CPSII	655	m	35	99	all	4	all	NAmer	1982	pr	3229	n	bl	n	n	1	cig only	3	5	3	3	nev any	ot
CPSII	656	m	35	99	all	4	all	NAmer	1982	pr	3229	n	bl	n	n	1	cig only	1.0	2	0	0	nev any	ot
CPSII	657	m	35	99	all	4	all	NAmer	1982	pr	3229	n	bl	n	n	1	cig only	0.1	0.9	0	0	nev any	ot
CPSII	633	f	0	0	all	4	all	NAmer	1982	pr	3229	n	bl	n	n	1	cig+/-ot	16	999	0	1	nev cigs	ot
CPSII	634	f	0	0	all	4	all	NAmer	1982	pr	3229	n	bl	n	n	1	cig+/-ot	11	15	1	2	nev cigs	ot
CPSII	635	f	0	0	all	4	all	NAmer	1982	pr	3229	n	bl	n	n	1	cig+/-ot	6	10	2	0	nev cigs	ot
CPSII	636	f	0	0	all	4	all	NAmer	1982	pr	3229	n	bl	n	n	1	cig+/-ot	3	5	3	3	nev cigs	ot
CPSII	637	f	0	0	all	4	all	NAmer	1982	pr	3229	n	bl	n	n	1	cig+/-ot	0.1	2	0	0	nev cigs	ot
DAMBER	523	m	0	0	all	-	all	Eu:Sca	1972	CC	579	n	bl	y	n	1	all/unsp	11	999	1	0	nev any	ot
DAMBER	524	m	0	0	all	-	all	Eu:Sca	1972	CC	579	n	bl	y	n	1	all/unsp	6	10	2	0	nev any	ot
DAMBER	525	m	0	0	all	-	all	Eu:Sca	1972	CC	579	n	bl	y	n	1	all/unsp	0.1	5	3	3	nev any	ot
DARBY	501	m	0	0	wh	-	all	Eu:UK	1988	CC	982	n	V	n	n	0	all/unsp	10	999	1	0	nev any	st
DARBY	502	m	0	0	wh	-	all	Eu:UK	1988	CC	982	n	V	n	n	0	all/unsp	0.1	9	0	3	nev any	st
DARBY	510	f	0	0	wh	-	all	Eu:UK	1988	CC	982	n	V	n	n	0	all/unsp	10	999	1	0	nev any	st
DARBY	511	f	0	0	wh	-	all	Eu:UK	1988	CC	982	n	V	n	n	0	all/unsp	0.1	9	0	3	nev any	st
DEAN3	628	m	0	0	all	-	all	Eu:UK	1969	CC	766	n	V	y	n	1	all/unsp	9	999	1	0	nev any	ot
DEAN3	629	m	0	0	all	-	all	Eu:UK	1969	CC	766	n	V	y	n	1	all/unsp	5	8	2	0	nev any	ot
DEAN3	630	m	0	0	all	-	all	Eu:UK	1969	CC	766	n	V	y	n	1	all/unsp	3	4	3	3	nev any	ot
DEAN3	553	f	0	0	all	-	all	Eu:UK	1969	CC	766	n	V	y	n	1	all/unsp	9	999	1	0	nev any	ot
DEAN3	554	f	0	0	all	-	all	Eu:UK	1969	CC	766	n	V	y	n	1	all/unsp	5	8	2	0	nev any	ot
DEAN3	555	f	0	0	all	-	all	Eu:UK	1969	CC	766	n	V	y	n	1	all/unsp	3	4	3	3	nev any	ot
DESTEF	525	m	0	0	all	-	all	SCAmer	1988	CC	497	n	bl	n	y	4	all/unsp	10	999	1	0	nev any	or
DESTEF	526	m	0	0	all	-	all	SCAmer	1988	CC	497	n	bl	n	y	4	all/unsp	5	9	2	0	nev any	or
DESTEF	527	m	0	0	all	-	all	SCAmer	1988	CC	497	n	bl	n	y	4	all/unsp	0.1	4	3	3	nev any	or
DOLL	529	m	0	0	all	-	all	Eu:UK	1948	CC	1465	n	V	n	n	0	all/unsp	20	999	0	1	nev any	st

Table 1J1 - 1

IESLC - Meta-analysis of Ex Smoking by Years quit (vs never), Overview
All LC types, Any Product (or Cigarettes if Any not available)
Most adjusted

REF	NRR	SEX	AGEL	AGEH	RACE	YF	LC TYPE	LOC	START	ST	NLC	R	VB	P	H	AD	PRODUCT	exL	exH	S1	S2	DENOM	De
DOLL	530	m	0	0	all	-	all	Eu:UK	1948	CC	1465	n	V	n	n	0	all/unsp	10	19	1	2	nev any	st
DOLL	531	m	0	0	all	-	all	Eu:UK	1948	CC	1465	n	V	n	n	0	all/unsp	0.1	9	0	3	nev any	st
DOLL	542	f	0	0	all	-	all	Eu:UK	1948	CC	1465	n	V	n	n	0	all/unsp	10	999	1	0	nev any	st
DOLL	543	f	0	0	all	-	all	Eu:UK	1948	CC	1465	n	V	n	n	0	all/unsp	0.1	9	0	3	nev any	st
DOLL2	501	m	0	0	all	20	all	Eu:UK	1951	pr	920	n	V	n	n	1	cig only	15	999	0	1	nev any	ot
DOLL2	502	m	0	0	all	20	all	Eu:UK	1951	pr	920	n	V	n	n	1	cig only	10	14	1	2	nev any	ot
DOLL2	503	m	0	0	all	20	all	Eu:UK	1951	pr	920	n	V	n	n	1	cig only	5	9	2	0	nev any	ot
DOLL2	504	m	0	0	all	20	all	Eu:UK	1951	pr	920	n	V	n	n	1	cig only	0.1	4	3	3	nev any	ot
DORGAN	501	m	0	0	wh	-	all	NAmer	1980	CC	2026	n	bl	y	y	0	cig+/-ot	10	999	1	0	nev any	st
DORGAN	502	m	0	0	wh	-	all	NAmer	1980	CC	2026	n	bl	y	y	0	cig+/-ot	6	9	2	0	nev any	st
DORGAN	503	m	0	0	wh	-	all	NAmer	1980	CC	2026	n	bl	y	y	0	cig+/-ot	1.1	5	3	3	nev any	st
DORGAN	553	f	0	0	all	-	all	NAmer	1980	CC	2026	n	bl	y	y	0	cig+/-ot	10	999	1	0	nev any	st
DORGAN	554	f	0	0	all	-	all	NAmer	1980	CC	2026	n	bl	y	y	0	cig+/-ot	1.1	9	0	3	nev any	st
DORN	657	m	55	64	wh	8	all	NAmer	1954	pr	5097	n	bl	n	n	0	cig+/-ot	15	999	0	1	nev any	st
DORN	658	m	55	64	wh	8	all	NAmer	1954	pr	5097	n	bl	n	n	0	cig+/-ot	10	14	1	2	nev any	st
DORN	659	m	55	64	wh	8	all	NAmer	1954	pr	5097	n	bl	n	n	0	cig+/-ot	5	9	2	0	nev any	st
DORN	660	m	55	64	wh	8	all	NAmer	1954	pr	5097	n	bl	n	n	0	cig+/-ot	0.1	4	3	3	nev any	st
DORN	680	m	65	74	wh	8	all	NAmer	1954	pr	5097	n	bl	n	n	0	cig+/-ot	15	999	0	1	nev any	st
DORN	681	m	65	74	wh	8	all	NAmer	1954	pr	5097	n	bl	n	n	0	cig+/-ot	10	14	1	2	nev any	st
DORN	682	m	65	74	wh	8	all	NAmer	1954	pr	5097	n	bl	n	n	0	cig+/-ot	5	9	2	0	nev any	st
DORN	683	m	65	74	wh	8	all	NAmer	1954	pr	5097	n	bl	n	n	0	cig+/-ot	0.1	4	3	3	nev any	st
GAO	531	m	0	0	all	-	all	As:Chi	1984	CC	1405	n	ot	n	n	2	cig+/-ot	10	999	1	0	nev cigs	or
GAO	532	m	0	0	all	-	all	As:Chi	1984	CC	1405	n	ot	n	n	2	cig+/-ot	5	9	2	0	nev cigs	or
GAO	533	m	0	0	all	-	all	As:Chi	1984	CC	1405	n	ot	n	n	2	cig+/-ot	0.1	4	3	3	nev cigs	or
GAO	551	f	0	0	all	-	all	As:Chi	1984	CC	1405	n	ot	n	n	2	cig+/-ot	10	999	1	0	nev cigs	or
GAO	552	f	0	0	all	-	all	As:Chi	1984	CC	1405	n	ot	n	n	2	cig+/-ot	5	9	2	0	nev cigs	or
GAO	553	f	0	0	all	-	all	As:Chi	1984	CC	1405	n	ot	n	n	2	cig+/-ot	0.1	4	3	3	nev cigs	or
GAO2	509	m	0	0	all	-	all	As:Jap	1988	CC	282	n	bl	n	n	0	cig+/-ot	20	999	0	1	nev cigs	or
GAO2	510	m	0	0	all	-	all	As:Jap	1988	CC	282	n	bl	n	n	0	cig+/-ot	15	19	0	0	nev cigs	or
GAO2	511	m	0	0	all	-	all	As:Jap	1988	CC	282	n	bl	n	n	0	cig+/-ot	10	14	1	2	nev cigs	or
GAO2	512	m	0	0	all	-	all	As:Jap	1988	CC	282	n	bl	n	n	0	cig+/-ot	5	9	2	0	nev cigs	st
GAO2	513	m	0	0	all	-	all	As:Jap	1988	CC	282	n	bl	n	n	0	cig+/-ot	1.0	4	3	3	nev cigs	or
GARCIA	515	c	0	0	all	-	all	NAmer	1992	CC	416	n	bl	n	y	0	cig+/-ot	30	999	0	0	nev any	st
GARCIA	516	c	0	0	all	-	all	NAmer	1992	CC	416	n	bl	n	y	0	cig+/-ot	15	29	0	1	nev any	st
GARCIA	517	c	0	0	all	-	all	NAmer	1992	CC	416	n	bl	n	y	0	cig+/-ot	5	14	0	2	nev any	st
GARCIA	518	c	0	0	all	-	all	NAmer	1992	CC	416	n	bl	n	y	0	cig+/-ot	1.0	4	3	3	nev any	st
GARSHI	522	m	0	0	all	-	all	NAmer	1981	CC	1081	o	bl	y	n	1	all/unsp	15	999	0	1	nev any	st
GARSHI	523	m	0	0	all	-	all	NAmer	1981	CC	1081	o	bl	y	n	1	all/unsp	5	14	0	2	nev any	st
GRAHAM	535	m	0	0	wh	-	all	NAmer	1956	CC	685	n	bl	n	n	1	cig+/-ot	5	999	0	0	nev any	ot
GRAHAM	536	m	0	0	wh	-	all	NAmer	1956	CC	685	n	bl	n	n	1	cig+/-ot	1.1	5	3	3	nev any	ot
GRAHAM	537	m	0	0	wh	-	all	NAmer	1956	CC	685	n	bl	n	n	1	cig+/-ot	0.1	1.0	0	0	nev any	ot
GURSEL	501	m	0	0	all	-	all	Eu:bal		CC	953		bl	*	n	0	all/unsp	11	999	1	0	nev any	or
HAMMO2	501	m	0	0	all	0	all	NAmer	1967	pr	450	o	bl	n	n	1	cig+/-ot	10	999	1	0	nev any	ot
HAMMO2	502	m	0	0	all	0	all	NAmer	1967	pr	450	o	bl	n	n	1	cig+/-ot	5	9	2	0	nev any	ot
HAMMO2	503	m	0	0	all	0	all	NAmer	1967	pr	450	o	bl	n	n	1	cig+/-ot	0.1	4	3	3	nev any	ot
HIRAYA	507	m	0	0	all	0	all	As:Jap	1965	pr	1917	n	bl	n	n	1	cig+/-ot	10	999	1	0	nev any	st
HIRAYA	508	m	0	0	all	0	all	As:Jap	1965	pr	1917	n	bl	n	n	1	cig+/-ot	5	9	2	0	nev any	st
HIRAYA	509	m	0	0	all	0	all	As:Jap	1965	pr	1917	n	bl	n	n	1	cig+/-ot	0.1	4	3	3	nev any	st
HIRAYA	518	f	0	0	all	0	all	As:Jap	1965	pr	1917	n	bl	n	n	1	cig+/-ot	10	999	1	0	nev any	st
HIRAYA	519	f	0	0	all	0	all	As:Jap	1965	pr	1917	n	bl	n	n	1	cig+/-ot	5	9	2	0	nev any	st
HIRAYA	520	f	0	0	all	0	all	As:Jap	1965	pr	1917	n	bl	n	n	1	cig+/-ot	0.1	4	3	3	nev any	st
JAHN	501	m	0	0	all	-	all	Eu:Ger	1988	CC	1004	n	bl	n	n	0	cig+/-ot	21	999	0	0	nev any	st
JAHN	502	m	0	0	all	-	all	Eu:Ger	1988	CC	1004	n	bl	n	n	0	cig+/-ot	11	20	1	0	nev any	st
JAHN	503	m	0	0	all	-	all	Eu:Ger	1988	CC	1004	n	bl	n	n	0	cig+/-ot	6	10	2	0	nev any	st
JAHN	504	m	0	0	all	-	all	Eu:Ger	1988	CC	1004	n	bl	n	n	0	cig+/-ot	2	5	3	3	nev any	st
JAHN	505	m	0	0	all	-	all	Eu:Ger	1988	CC	1004	n	bl	n	n	0	cig+/-ot	1.0	1.9	0	0	nev any	st
JAHN	506	m	0	0	all	-	all	Eu:Ger	1988	CC	1004	n	bl	n	n	0	cig+/-ot	0.1	0.9	0	0	nev any	st
JAHN	731	f	0	0	all	-	all	Eu:Ger	1988	CC	1004	n	bl	n	n	2	cig+/-ot	21	999	0	0	nev any	or
JAIN	567	m	0	0	all	-	all	NAmer	1981	CC	845	n	V	y	n	0	cig+/-ot	10	999	1	0	nev cigs	st
JAIN	568	m	0	0	all	-	all	NAmer	1981	CC	845	n	V	y	n	0	cig+/-ot	2	9	0	3	nev cigs	st
JAIN	531	f	0	0	all	-	all	NAmer	1981	CC	845	n	V	y	n	0	cig+/-ot	10	999	1	0	nev cigs	st
JAIN	532	f	0	0	all	-	all	NAmer	1981	CC	845	n	V	y	n	0	cig+/-ot	2	9	0	3	nev cigs	st
JEDRYC	611	m	0	0	all	-	all	Eu:est	1980	CC	1630	n	bl	y	n	0	cig+/-ot	10	999	1	0	nev any	st
JEDRYC	612	m	0	0	all	-	all	Eu:est	1980	CC	1630	n	bl	y	n	0	cig+/-ot	5	9	2	0	nev any	st
JOLY	566	m	0	0	all	-	all	SCAmer	1978	CC	826	n	bl	n	n	0	cig+/-ot	5	999	0	0	nev any	st
JOLY	567	m	0	0	all	-	all	SCAmer	1978	CC	826	n	bl	n	n	0	cig+/-ot	1.0	4	3	3	nev any	st
JOLY	553	f	0	0	all	-	all	SCAmer	1978	CC	826	n	bl	n	n	0	cig+/-ot	5	999	0	0	nev any	st
JOLY	554	f	0	0	all	-	all	SCAmer	1978	CC	826	n	bl	n	n	0	cig+/-ot	1.0	4	3	3	nev any	st
KAISE2	646	m	0	0	all	9	all	NAmer	1979	pr	318	n	bl	n	n	1	cig only	21	999	0	0	nev any	st
KAISE2	647	m	0	0	all	9	all	NAmer	1979	pr	318	n	bl	n	n	1	cig only	11	20	1	0	nev any	ot
KAISE2	648	m	0	0	all	9	all	NAmer	1979	pr	318	n	bl	n	n	1	cig only	2	10	0	3	nev any	st
KAISE2	566	f	0	0	all	9	all	NAmer	1979	pr	318	n	bl	n	n	1	cig only	21	999	0	0	nev any	ot

Table 1J1 - 1

IESLC - Meta-analysis of Ex Smoking by Years quit (vs never), Overview
All LC types, Any Product (or Cigarettes if Any not available)
Most adjusted

REF	NRR	SEX	AGEL	AGEH	RACE	YF	LC TYPE	LOC	START	ST	NLC	R	VB	P	H	AD	PRODUCT	exL	exH	S1	S2	DENOM	De
KAISE2	567	f	0	0	all	9	all	NAmer	1979	pr	318	n	bl	n	n	1	cig only	11	20	1	0	nev any	st
KAISE2	568	f	0	0	all	9	all	NAmer	1979	pr	318	n	bl	n	n	1	cig only	2	10	0	3	nev any	st
KHUDER	511	m	0	0	all	-	all	NAmer	1985	CC	482	n	bl	n	y	0	cig+/-ot	15	999	0	1	nev cigs	st
KHUDER	512	m	0	0	all	-	all	NAmer	1985	CC	482	n	bl	n	y	0	cig+/-ot	5	14	0	2	nev cigs	st
KHUDER	513	m	0	0	all	-	all	NAmer	1985	CC	482	n	bl	n	y	0	cig+/-ot	0.1	4	3	3	nev cigs	st
LAUSSM	503	m	0	0	all	-	all	Eu:Ger	1982	CC	432	n	bl	n	n	2	all/unsp	10	999	1	0	nev any	st
LUBIN	585	m	0	0	all	-	all	As:Chi	1984	CC	427	m	ot	y	n	0	cig+/-ot	10	999	1	0	nev any	st
LUBIN	586	m	0	0	all	-	all	As:Chi	1984	CC	427	m	ot	y	n	0	cig+/-ot	5	9	2	0	nev any	st
LUBIN	587	m	0	0	all	-	all	As:Chi	1984	CC	427	m	ot	y	n	0	cig+/-ot	3	4	3	3	nev any	st
LUBIN2	1069	m	0	0	all	-	all	Eu:mul	1976	CC	7804	n	bl	n	y	0	cig+/-ot	25	999	0	0	nev any	st
LUBIN2	1070	m	0	0	all	-	all	Eu:mul	1976	CC	7804	n	bl	n	y	0	cig+/-ot	20	24	0	1	nev any	st
LUBIN2	1071	m	0	0	all	-	all	Eu:mul	1976	CC	7804	n	bl	n	y	0	cig+/-ot	15	19	0	0	nev any	st
LUBIN2	1072	m	0	0	all	-	all	Eu:mul	1976	CC	7804	n	bl	n	y	0	cig+/-ot	10	14	1	2	nev any	st
LUBIN2	1073	m	0	0	all	-	all	Eu:mul	1976	CC	7804	n	bl	n	y	0	cig+/-ot	5	9	2	0	nev any	st
LUBIN2	1074	m	0	0	all	-	all	Eu:mul	1976	CC	7804	n	bl	n	y	0	cig+/-ot	0.1	4	3	3	nev any	st
LUBIN2	1108	f	0	0	all	-	all	Eu:mul	1976	CC	7804	n	bl	n	y	0	cig+/-ot	25	999	0	0	nev any	st
LUBIN2	1109	f	0	0	all	-	all	Eu:mul	1976	CC	7804	n	bl	n	y	0	cig+/-ot	20	24	0	1	nev any	st
LUBIN2	1110	f	0	0	all	-	all	Eu:mul	1976	CC	7804	n	bl	n	y	0	cig+/-ot	15	19	0	0	nev any	st
LUBIN2	1111	f	0	0	all	-	all	Eu:mul	1976	CC	7804	n	bl	n	y	0	cig+/-ot	10	14	1	2	nev any	st
LUBIN2	1112	f	0	0	all	-	all	Eu:mul	1976	CC	7804	n	bl	n	y	0	cig+/-ot	5	9	2	0	nev any	st
LUBIN2	1113	f	0	0	all	-	all	Eu:mul	1976	CC	7804	n	bl	n	y	0	cig+/-ot	0.1	4	3	3	nev any	st
MATOS	591	m	0	0	all	-	all	SCAmer	1994	CC	200	n	bl	n	n	2	cig+/-ot	11	999	1	0	nev any	ot
MATOS	592	m	0	0	all	-	all	SCAmer	1994	CC	200	n	bl	n	n	2	cig+/-ot	6	10	2	0	nev any	ot
MATOS	593	m	0	0	all	-	all	SCAmer	1994	CC	200	n	bl	n	n	2	cig+/-ot	1.0	5	3	3	nev any	ot
PEZZO2	501	m	0	0	all	-	all	SCAmer	1992	CC	367	n	bl	n	y	0	cig+/-ot	11	999	1	0	nev cigs	st
PEZZO2	502	m	0	0	all	-	all	SCAmer	1992	CC	367	n	bl	n	y	0	cig+/-ot	1.0	10	0	3	nev cigs	st
PEZZOT	501	m	0	0	all	-	all	SCAmer	1987	CC	215	n	bl	n	y	0	cig only	11	999	1	0	nev cigs	st
PEZZOT	502	m	0	0	all	-	all	SCAmer	1987	CC	215	n	bl	n	y	0	cig only	1.0	10	0	3	nev cigs	st
SOBUE	717	m	0	0	all	-	all	As:Jap	1986	CC	1376	n	bl	n	y	0	cig+/-ot	25	999	0	0	nev cigs	st
SOBUE	718	m	0	0	all	-	all	As:Jap	1986	CC	1376	n	bl	n	y	0	cig+/-ot	20	24	0	1	nev cigs	st
SOBUE	719	m	0	0	all	-	all	As:Jap	1986	CC	1376	n	bl	n	y	0	cig+/-ot	15	19	0	0	nev cigs	st
SOBUE	720	m	0	0	all	-	all	As:Jap	1986	CC	1376	n	bl	n	y	0	cig+/-ot	10	14	1	2	nev cigs	st
SOBUE	721	m	0	0	all	-	all	As:Jap	1986	CC	1376	n	bl	n	y	0	cig+/-ot	5	9	2	0	nev cigs	st
SOBUE	722	m	0	0	all	-	all	As:Jap	1986	CC	1376	n	bl	n	y	0	cig+/-ot	1.0	4	3	3	nev cigs	st
SPEIZE	501	f	0	0	all	0	all	NAmer	1976	pr	593	n	bl	n	y	0	cig+/-ot	15	999	0	1	nev cigs	st
SPEIZE	502	f	0	0	all	0	all	NAmer	1976	pr	593	n	bl	n	y	0	cig+/-ot	10	15	1	2	nev cigs	st
SPEIZE	503	f	0	0	all	0	all	NAmer	1976	pr	593	n	bl	n	y	0	cig+/-ot	5	10	2	0	nev cigs	st
SPEIZE	504	f	0	0	all	0	all	NAmer	1976	pr	593	n	bl	n	y	0	cig+/-ot	2	5	3	3	nev cigs	st
SPEIZE	505	f	0	0	all	0	all	NAmer	1976	pr	593	n	bl	n	y	0	cig+/-ot	0.1	1.9	0	0	nev cigs	st
SUZUK2	508	c	0	0	all	-	all	SCAmer	1991	CC	123	n	bl	n	y	0	all/unsp	11	999	1	0	nev any	st
SUZUK2	509	c	0	0	all	-	all	SCAmer	1991	CC	123	n	bl	n	y	0	all/unsp	6	10	2	0	nev any	st
SUZUK2	510	c	0	0	all	-	all	SCAmer	1991	CC	123	n	bl	n	y	0	all/unsp	0.1	5	3	3	nev any	st
SVENSS	551	f	0	0	all	-	all	Eu:Sca	1983	CC	210	n	bl	n	n	0	all/unsp	11	999	1	0	nev any	st
SVENSS	552	f	0	0	all	-	all	Eu:Sca	1983	CC	210	n	bl	n	n	0	all/unsp	3	10	0	3	nev any	st
TVERDA	501	m	0	0	all	0	all	Eu:Sca	1972	pr	238	n	bl	n	n	2	cig only	5	999	0	0	nev cigs	ot
TVERDA	502	m	0	0	all	0	all	Eu:Sca	1972	pr	238	n	bl	n	n	2	cig only	1.0	5	3	3	nev cigs	ot
TVERDA	503	m	0	0	all	0	all	Eu:Sca	1972	pr	238	n	bl	n	n	2	cig only	0.1	0.9	0	0	nev cigs	ot
WAKAI	530	m	0	0	all	-	all	As:Jap	1988	CC	333	n	bl	n	y	2	cig+/-ot	20	999	0	1	nev any	or
WAKAI	531	m	0	0	all	-	all	As:Jap	1988	CC	333	n	bl	n	y	2	cig+/-ot	10	19	1	2	nev any	or
WAKAI	532	m	0	0	all	-	all	As:Jap	1988	CC	333	n	bl	n	y	2	cig+/-ot	5	9	2	0	nev any	or
WANG2	510	c	0	0	all	-	all	As:Chi	1980	CC	103	n	ot	n	n	0	cig+/-ot	4	999	0	0	nev cigs	st
WANG2	511	c	0	0	all	-	all	As:Chi	1980	CC	103	n	ot	n	n	0	cig+/-ot	0.1	3	3	3	nev cigs	st
WYNDE3	535	m	0	0	all	-	all	NAmer	1966	CC	350	n	bl	n	y	0	all/unsp	13	999	0	1	nev any	st
WYNDE3	536	m	0	0	all	-	all	NAmer	1966	CC	350	n	bl	n	y	0	all/unsp	7	12	0	2	nev any	st
WYNDE3	537	m	0	0	all	-	all	NAmer	1966	CC	350	n	bl	n	y	0	all/unsp	4	6	0	0	nev any	st
WYNDE3	538	m	0	0	all	-	all	NAmer	1966	CC	350	n	bl	n	y	0	all/unsp	1.0	3	3	3	nev any	st
WYNDE3	587	f	0	0	all	-	all	NAmer	1966	CC	350	n	bl	n	y	0	cig+/-ot	10	999	1	0	nev any	st
WYNDE6	501	m	0	0	all	-	all	NAmer	1969	CC	4423	n	bl	n	y	0	cig only	30	999	0	0	nev any	st
WYNDE6	502	m	0	0	all	-	all	NAmer	1969	CC	4423	n	bl	n	y	0	cig only	20	29	0	1	nev any	st
WYNDE6	503	m	0	0	all	-	all	NAmer	1969	CC	4423	n	bl	n	y	0	cig only	10	19	1	2	nev any	st
WYNDE6	504	m	0	0	all	-	all	NAmer	1969	CC	4423	n	bl	n	y	0	cig only	5	9	2	0	nev any	st
WYNDE6	505	m	0	0	all	-	all	NAmer	1969	CC	4423	n	bl	n	y	0	cig only	1.0	4	3	3	nev any	st
WYNDE6	522	f	0	0	all	-	all	NAmer	1969	CC	4423	n	bl	n	y	0	cig only	30	999	0	0	nev any	st
WYNDE6	523	f	0	0	all	-	all	NAmer	1969	CC	4423	n	bl	n	y	0	cig only	20	29	0	1	nev any	st
WYNDE6	524	f	0	0	all	-	all	NAmer	1969	CC	4423	n	bl	n	y	0	cig only	10	19	1	2	nev any	st
WYNDE6	525	f	0	0	all	-	all	NAmer	1969	CC	4423	n	bl	n	y	0	cig only	5	9	2	0	nev any	st
WYNDE6	526	f	0	0	all	-	all	NAmer	1969	CC	4423	n	bl	n	y	0	cig only	1.0	4	3	3	nev any	st

Cigarette type is all/unspec for all RRs

Table 1J1 - 1

IESLC - Meta-analysis of Ex Smoking by Years quit (vs never), Overview
All LC types, Any Product (or Cigarettes if Any not available)
Most adjusted

except for the following:

REF	NRR	CIGTYPE
ALDERS	507	MC only
ALDERS	508	MC only
ALDERS	509	MC only
ALDERS	518	MC only
ALDERS	519	MC only
ALDERS	520	MC only

In this overview table, subtotals and Qs values may be invalid and should be ignored

Table 1J1 - 2

IESLC - Meta-analysis of Ex Smoking by Years quit (vs never), Overview
All LC types, Any Product (or Cigarettes if Any not available)
Most adjusted

REF	NRR	SEX	AD	Number Exposed Case	Number Exposed Cont	Non-exposed Case	Non-exposed Cont	RR	95.00%CI
ALDERS	507	m	1	29	-	15	-	3.20	(1.61- 6.35)
ALDERS	508	m	1	28	-	15	-	4.30	(2.13- 8.69)
ALDERS	509	m	1	121	-	15	-	18.10	(9.71- 33.74)
ALDERS	518	f	1	26	-	75	-	1.27	(0.76- 2.15)
ALDERS	519	f	1	54	-	75	-	2.95	(1.88- 4.64)
ALDERS	520	f	1	206	-	75	-	9.45	(6.50- 13.74)
Subtotal ALDERS								4.77	(3.86- 5.89)
ARMADA	515	m	0	50	87	8	71	5.10	(2.27- 11.46)
ARMADA	516	m	0	79	45	8	71	15.58	(6.88- 35.29)
Subtotal ARMADA								8.87	(4.99- 15.76)
AUVINE	532	c	2	207	-	44	-	7.50	(4.18- 13.15)
BARBON	540	m	1	15	-	22	-	2.10	(1.00- 4.30)
BARBON	541	m	1	33	-	22	-	6.80	(3.60- 12.80)
BARBON	542	m	1	89	-	22	-	9.10	(5.30- 15.50)
BARBON	543	m	1	32	-	22	-	13.90	(6.80- 28.50)
Subtotal BARBON								6.94	(5.04- 9.55)
BECHER	501	m	0	16	72	3	54	4.00	(1.11- 14.42)
BECHER	502	m	0	16	32	3	54	9.00	(2.43- 33.30)
BECHER	503	m	0	10	12	3	54	15.00	(3.58- 62.92)
BECHER	511	f	0	1	10	10	52	0.52	(0.06- 4.53)
BECHER	512	f	0	2	5	10	52	2.08	(0.35- 12.26)
BECHER	513	f	0	2	3	10	52	3.47	(0.51- 23.48)
Subtotal BECHER								4.76	(2.53- 8.99)
*BENSHL	508	m	2	6	-	10	-	1.00	(0.32- 3.10)
*BENSHL	509	m	2	15	-	10	-	2.59	(1.21- 5.54)
*BENSHL	510	m	2	23	-	10	-	4.08	(2.03- 8.20)
*BENSHL	511	m	2	14	-	10	-	8.68	(4.00- 18.90)
Subtotal BENSHL								3.69	(2.47- 5.51)
BROSS	515	m	0	43	79	38	170	2.44	(1.46- 4.06)
BROSS	516	m	0	169	67	38	170	11.28	(7.19- 17.72)
Subtotal BROSS								5.77	(4.11- 8.09)
BROWN3	503	f	2	186	-	432	-	2.20	(1.80- 2.70)
CARPEN	501	c	0	29	137	8	208	5.50	(2.44- 12.40)
CARPEN	502	c	0	9	51	8	208	4.59	(1.69- 12.48)
CARPEN	503	c	0	25	48	8	208	13.54	(5.75- 31.87)
CARPEN	504	c	0	23	39	8	208	15.33	(6.40- 36.75)
Subtotal CARPEN								8.72	(5.62- 13.52)
*CEDERL	528	m	1	3	-	7	-	1.10	(0.28- 4.25)
*CEDERL	529	m	1	12	-	7	-	6.10	(2.41- 15.46)
Subtotal CEDERL								3.54	(1.64- 7.62)
CHOI	533	m	0	4	19	13	95	1.54	(0.45- 5.23)
CHOI	534	m	0	4	23	13	95	1.27	(0.38- 4.26)
CHOI	535	m	0	5	30	13	95	1.22	(0.40- 3.70)
CHOI	536	m	0	25	64	13	95	2.85	(1.36- 5.99)
CHOI	550	f	0	2	0	76	164	10.75~	(0.51- 226.67)
CHOI	551	f	0	3	2	76	164	3.24	(0.53- 19.77)
Subtotal CHOI								2.04	(1.27- 3.29)
*CHYOU	507	m	2	5	-	8	-	2.80	(0.90- 8.50)
*CHYOU	508	m	2	21	-	8	-	3.80	(1.70- 8.50)
Subtotal CHYOU								3.43	(1.78- 6.59)
*CPSI	807	m	1	15	-	60	-	1.28	(0.73- 2.25)
*CPSI	808	m	1	32	-	60	-	5.15	(3.35- 7.91)
*CPSI	809	m	1	49	-	60	-	8.09	(5.55- 11.80)
*CPSI	810	m	1	37	-	60	-	14.74	(9.78- 22.20)
Subtotal CPSI								6.51	(5.25- 8.07)
*CPSII	652	m	1	256	-	81	-	3.83	(2.98- 4.92)
*CPSII	653	m	1	164	-	81	-	8.61	(6.60- 11.24)
*CPSII	654	m	1	186	-	81	-	11.43	(8.81- 14.84)
*CPSII	655	m	1	178	-	81	-	18.61	(14.31- 24.20)
*CPSII	656	m	1	188	-	81	-	28.07	(21.63- 36.43)
*CPSII	657	m	1	97	-	81	-	38.76	(28.85- 52.07)
*CPSII	633	f	1	50	-	174	-	1.74	(1.27- 2.39)
*CPSII	634	f	1	28	-	174	-	3.86	(2.59- 5.75)
*CPSII	635	f	1	37	-	174	-	4.91	(3.45- 7.01)
*CPSII	636	f	1	56	-	174	-	10.55	(7.81- 14.26)
*CPSII	637	f	1	91	-	174	-	17.02	(13.21- 21.93)
Subtotal CPSII								10.30	(9.46- 11.22)
DAMBER	523	m	1	42	-	42	-	2.60	(1.70- 4.50)
DAMBER	524	m	1	-	-	42	-	4.30	(2.30- 8.10)
DAMBER	525	m	1	-	-	42	-	7.70	(4.50- 13.50)
Subtotal DAMBER								4.22	(3.08- 5.78)

Table 1J1 - 2

IESLC - Meta-analysis of Ex Smoking by Years quit (vs never), Overview
All LC types, Any Product (or Cigarettes if Any not available)
Most adjusted

REF	NRR	SEX	AD	Number Exposed Case	Cont	Non-exposed Case	Cont	RR	95.00%CI
DARBY	501	m	0	139	767	3	384	23.20	(7.34- 73.28)
DARBY	502	m	0	146	339	3	384	55.13	(17.41- 174.53)
DARBY	510	f	0	26	224	23	529	2.67	(1.49- 4.78)
DARBY	511	f	0	68	93	23	529	16.82	(9.98- 28.33)
Subtotal DARBY								9.92	(6.99- 14.09)
DEAN3	628	m	1	32	-	24	-	2.04	(1.18- 3.54)
DEAN3	629	m	1	15	-	24	-	3.29	(1.65- 6.57)
DEAN3	630	m	1	42	-	24	-	4.82	(2.83- 8.20)
DEAN3	553	f	1	2	-	41	-	0.72	(0.17- 3.01)
DEAN3	554	f	1	1	-	41	-	1.09	(0.15- 8.13)
DEAN3	555	f	1	4	-	41	-	1.63	(0.57- 4.63)
Subtotal DEAN3								2.75	(2.02- 3.74)
DESTEF	525	m	4	17	-	27	-	2.80	(1.40- 5.70)
DESTEF	526	m	4	27	-	27	-	6.20	(3.20- 12.20)
DESTEF	527	m	4	64	-	27	-	9.00	(5.20- 15.90)
Subtotal DESTEF								5.86	(4.06- 8.45)
DOLL	529	m	0	8	23	7	61	3.03	(0.99- 9.31)
DOLL	530	m	0	6	26	7	61	2.01	(0.62- 6.56)
DOLL	531	m	0	56	75	7	61	6.51	(2.77- 15.30)
DOLL	542	f	0	1	2	40	59	0.74	(0.06- 8.41)
DOLL	543	f	0	9	6	40	59	2.21	(0.73- 6.70)
Subtotal DOLL								3.24	(1.95- 5.39)
*DOLL2	501	m	1	7	-	7	-	2.00	(0.70- 5.70)
*DOLL2	502	m	1	9	-	7	-	5.30	(1.97- 14.23)
*DOLL2	503	m	1	12	-	7	-	5.90	(2.32- 14.99)
*DOLL2	504	m	1	15	-	7	-	16.00	(6.52- 39.24)
Subtotal DOLL2								6.10	(3.77- 9.87)
DORGAN	501	m	0	134	255	13	140	5.66	(3.09- 10.37)
DORGAN	502	m	0	49	38	13	140	13.89	(6.84- 28.21)
DORGAN	503	m	0	59	51	13	140	12.46	(6.31- 24.61)
DORGAN	553	f	0	34	50	61	213	2.37	(1.41- 4.00)
DORGAN	554	f	0	49	27	61	213	6.34	(3.66- 10.98)
Subtotal DORGAN								5.93	(4.54- 7.76)
*DORN	657	m	0	16	58370	25	213858	2.34	(1.25- 4.39)
*DORN	658	m	0	12	23682	25	213858	4.33	(2.18- 8.63)
*DORN	659	m	0	32	34566	25	213858	7.92	(4.69- 13.36)
*DORN	660	m	0	34	22086	25	213858	13.17	(7.86- 22.07)
*DORN	680	m	0	34	51243	49	171211	2.32	(1.50- 3.59)
*DORN	681	m	0	29	20056	49	171211	5.05	(3.19- 7.99)
*DORN	682	m	0	41	24089	49	171211	5.95	(3.93- 9.00)
*DORN	683	m	0	14	6195	49	171211	7.90	(4.36- 14.29)
Subtotal DORN								5.24	(4.37- 6.27)
GAO	531	m	2	13	-	62	-	1.10	(0.50- 2.20)
GAO	532	m	2	24	-	62	-	3.10	(1.70- 5.90)
GAO	533	m	2	105	-	62	-	6.90	(4.40- 10.80)
GAO	551	f	2	16	-	435	-	2.20	(1.00- 4.60)
GAO	552	f	2	14	-	435	-	3.90	(1.50- 9.90)
GAO	553	f	2	37	-	435	-	7.20	(3.40- 15.10)
Subtotal GAO								3.91	(3.00- 5.11)
GAO2	509	m	0	8	25	13	56	1.38	(0.51- 3.74)
GAO2	510	m	0	7	9	13	56	3.35	(1.05- 10.66)
GAO2	511	m	0	16	18	13	56	3.83	(1.55- 9.46)
GAO2	512	m	0	21	26	13	56	3.48	(1.51- 8.01)
GAO2	513	m	0	31	26	13	56	5.14	(2.31- 11.40)
Subtotal GAO2								3.35	(2.23- 5.04)
GARCIA	515	c	0	10	37	8	80	2.70	(0.99- 7.41)
GARCIA	516	c	0	32	67	8	80	4.78	(2.06- 11.06)
GARCIA	517	c	0	43	36	8	80	11.94	(5.10- 27.97)
GARCIA	518	c	0	33	11	8	80	30.00	(11.07- 81.30)
Subtotal GARCIA								8.14	(5.16- 12.86)
GARSHI	522	m	1	125	-	41	-	3.20	(2.18- 4.69)
GARSHI	523	m	1	166	-	41	-	5.06	(3.47- 7.36)
Subtotal GARSHI								4.04	(3.09- 5.28)
GRAHAM	535	m	1	13	-	18	-	2.59	(1.18- 5.68)
GRAHAM	536	m	1	24	-	18	-	8.50	(4.32- 16.71)
GRAHAM	537	m	1	113	-	18	-	35.79	(20.50- 62.49)
Subtotal GRAHAM								12.49	(8.56- 18.21)
GURSEL	501	m	0	-	-	-	-	2.30	(1.01- 5.22)
*HAMMO2	501	m	1	20	-	5	-	3.45	(1.30- 9.14)
*HAMMO2	502	m	1	11	-	5	-	3.98	(1.39- 11.40)
*HAMMO2	503	m	1	59	-	5	-	10.99	(4.43- 27.26)

Table 1J1 - 2

IESLC - Meta-analysis of Ex Smoking by Years quit (vs never), Overview
All LC types, Any Product (or Cigarettes if Any not available)
Most adjusted

REF	NRR	SEX	AD	Number Case	Exposed Cont	Non-exposed Case	Cont	RR	95.00%CI
Subtotal HAMMO2								5.60	(3.19- 9.82)
*HIRAYA	507	m	1	-	-	-	-	1.38	(0.59- 3.21)
*HIRAYA	508	m	1	-	-	-	-	1.59	(0.66- 3.82)
*HIRAYA	509	m	1	-	-	-	-	2.03	(1.10- 3.75)
*HIRAYA	518	f	1	-	-	-	-	0.97	(0.03- 32.06)
*HIRAYA	519	f	1	-	-	-	-	3.29	(0.56- 19.50)
*HIRAYA	520	f	1	-	-	-	-	3.72	(1.12- 12.37)
Subtotal HIRAYA								1.92	(1.30- 2.85)
JAHN	501	m	0	29	146	18	138	1.52	(0.81- 2.87)
JAHN	502	m	0	64	130	18	138	3.77	(2.12- 6.71)
JAHN	503	m	0	59	63	18	138	7.18	(3.92- 13.16)
JAHN	504	m	0	77	46	18	138	12.83	(6.96- 23.67)
JAHN	505	m	0	60	9	18	138	51.11	(21.72- 120.26)
JAHN	506	m	0	166	8	18	138	159.08	(67.12- 377.03)
JAHN	731	f	2	-	-	-	-	0.30	(0.06- 1.53)
Subtotal JAHN								7.91	(6.06- 10.34)
JAIN	567	m	0	52	113	12	85	3.26	(1.64- 6.48)
JAIN	568	m	0	74	46	12	85	11.39	(5.62- 23.12)
JAIN	531	f	0	19	61	52	214	1.28	(0.71- 2.33)
JAIN	532	f	0	66	36	52	214	7.54	(4.55- 12.52)
Subtotal JAIN								4.36	(3.22- 5.92)
JEDRYC	611	m	0	73	138	49	219	2.36	(1.55- 3.60)
JEDRYC	612	m	0	64	58	49	219	4.93	(3.08- 7.90)
Subtotal JEDRYC								3.27	(2.39- 4.48)
JOLY	566	m	0	63	149	12	218	7.68	(4.00- 14.74)
JOLY	567	m	0	38	36	12	218	19.18	(9.16- 40.14)
JOLY	553	f	0	15	19	52	283	4.30	(2.05- 8.99)
JOLY	554	f	0	19	8	52	283	12.93	(5.38- 31.08)
Subtotal JOLY								9.16	(6.33- 13.26)
*KAISE2	646	m	1	6	-	14	-	1.94	(0.70- 5.40)
*KAISE2	647	m	1	8	-	14	-	3.14	(1.26- 7.82)
*KAISE2	648	m	1	12	-	14	-	8.26	(3.73- 18.28)
*KAISE2	566	f	1	4	-	11	-	6.29	(1.78- 22.20)
*KAISE2	567	f	1	4	-	11	-	4.37	(1.30- 14.72)
*KAISE2	568	f	1	6	-	11	-	7.95	(2.89- 21.86)
Subtotal KAISE2								4.86	(3.24- 7.31)
KHUDER	511	m	0	63	213	23	309	3.97	(2.39- 6.61)
KHUDER	512	m	0	63	133	23	309	6.36	(3.79- 10.69)
KHUDER	513	m	0	88	123	23	309	9.61	(5.80- 15.92)
Subtotal KHUDER								6.25	(4.66- 8.40)
LAUSSM	503	m	2	29	-	63	-	6.54	(3.47- 12.35)
LUBIN	585	m	0	17	73	9	72	1.86	(0.78- 4.45)
LUBIN	586	m	0	20	48	9	72	3.33	(1.40- 7.94)
LUBIN	587	m	0	33	18	9	72	14.67	(5.96- 36.07)
Subtotal LUBIN								4.38	(2.64- 7.28)
LUBIN2	1069	m	0	109	715	190	2616	2.10	(1.64- 2.69)
LUBIN2	1070	m	0	106	413	190	2616	3.53	(2.73- 4.58)
LUBIN2	1071	m	0	130	478	190	2616	3.74	(2.94- 4.78)
LUBIN2	1072	m	0	270	693	190	2616	5.36	(4.38- 6.58)
LUBIN2	1073	m	0	466	822	190	2616	7.81	(6.48- 9.40)
LUBIN2	1074	m	0	866	1047	190	2616	11.39	(9.58- 13.53)
LUBIN2	1108	f	0	4	20	336	1188	0.71	(0.24- 2.08)
LUBIN2	1109	f	0	4	9	336	1188	1.57	(0.48- 5.13)
LUBIN2	1110	f	0	3	7	336	1188	1.52	(0.39- 5.89)
LUBIN2	1111	f	0	10	26	336	1188	1.36	(0.65- 2.85)
LUBIN2	1112	f	0	30	40	336	1188	2.65	(1.63- 4.32)
LUBIN2	1113	f	0	60	55	336	1188	3.86	(2.62- 5.67)
Subtotal LUBIN2								5.29	(4.87- 5.74)
MATOS	591	m	2	27	-	11	-	3.00	(1.43- 6.28)
MATOS	592	m	2	21	-	11	-	9.00	(3.84- 21.08)
MATOS	593	m	2	28	-	11	-	14.00	(6.49- 30.21)
Subtotal MATOS								6.96	(4.43- 10.93)
PEZZO2	501	m	0	43	161	6	117	5.21	(2.15- 12.64)
PEZZO2	502	m	0	85	110	6	117	15.07	(6.33- 35.89)
Subtotal PEZZO2								8.96	(4.82- 16.66)
PEZZOT	501	m	0	20	106	4	116	5.47	(1.81- 16.53)
PEZZOT	502	m	0	46	82	4	116	16.27	(5.64- 46.96)
Subtotal PEZZOT								9.65	(4.49- 20.74)
SOBUE	717	m	0	17	40	29	126	1.85	(0.92- 3.71)
SOBUE	718	m	0	15	23	29	126	2.83	(1.32- 6.09)
SOBUE	719	m	0	24	31	29	126	3.36	(1.72- 6.56)

Table 1J1 - 2

IESLC - Meta-analysis of Ex Smoking by Years quit (vs never), Overview
All LC types, Any Product (or Cigarettes if Any not available)
Most adjusted

REF	NRR	SEX	AD	Number Exposed Case	Cont	Non-exposed Case	Cont	RR	95.00%CI
SOBUE	720	m	0	35	50	29	126	3.04	(1.68- 5.49)
SOBUE	721	m	0	67	92	29	126	3.16	(1.90- 5.28)
SOBUE	722	m	0	128	116	29	126	4.79	(2.98- 7.71)
Subtotal SOBUE								3.27	(2.57- 4.16)
*SPEIZE	501	f	0	28	214271	58	776300	1.75	(1.11- 2.75)
*SPEIZE	502	f	0	17	93933	58	776300	2.42	(1.41- 4.16)
*SPEIZE	503	f	0	41	95585	58	776300	5.74	(3.85- 8.56)
*SPEIZE	504	f	0	34	63060	58	776300	7.22	(4.73- 11.02)
*SPEIZE	505	f	0	24	55232	58	776300	5.82	(3.61- 9.36)
Subtotal SPEIZE								4.24	(3.46- 5.19)
SUZUK2	508	c	0	9	22	11	53	1.97	(0.72- 5.42)
SUZUK2	509	c	0	10	8	11	53	6.02	(1.94- 18.72)
SUZUK2	510	c	0	15	10	11	53	7.23	(2.58- 20.25)
Subtotal SUZUK2								4.28	(2.33- 7.87)
SVENSS	551	f	0	14	24	38	120	1.84	(0.87- 3.91)
SVENSS	552	f	0	16	13	38	120	3.89	(1.72- 8.80)
Subtotal SVENSS								2.60	(1.49- 4.52)
*TVERDA	501	m	2	4	-	4	-	1.34	(0.34- 5.37)
*TVERDA	502	m	2	5	-	4	-	2.83	(0.76- 10.53)
*TVERDA	503	m	2	2	-	4	-	2.77	(0.51- 15.15)
Subtotal TVERDA								2.15	(0.94- 4.93)
WAKAI	530	m	2	7	-	10	-	1.00	(0.35- 2.83)
WAKAI	531	m	2	27	-	10	-	3.63	(1.56- 8.44)
WAKAI	532	m	2	19	-	10	-	2.48	(1.04- 5.92)
Subtotal WAKAI								2.29	(1.35- 3.86)
WANG2	510	c	0	5	11	11	43	1.78	(0.51- 6.19)
WANG2	511	c	0	6	10	11	43	2.35	(0.70- 7.86)
Subtotal WANG2								2.05	(0.86- 4.89)
WYNDE3	535	m	0	5	55	9	88	0.89	(0.28- 2.79)
WYNDE3	536	m	0	11	31	9	88	3.47	(1.31- 9.17)
WYNDE3	537	m	0	11	17	9	88	6.33	(2.28- 17.59)
WYNDE3	538	m	0	21	22	9	88	9.33	(3.76- 23.19)
WYNDE3	587	f	0	1	3	20	76	1.27	(0.12- 12.84)
Subtotal WYNDE3								3.95	(2.42- 6.44)
WYNDE6	501	m	0	21	161	64	918	1.87	(1.11- 3.15)
WYNDE6	502	m	0	55	212	64	918	3.72	(2.52- 5.50)
WYNDE6	503	m	0	159	373	64	918	6.11	(4.47- 8.37)
WYNDE6	504	m	0	98	194	64	918	7.25	(5.10- 10.29)
WYNDE6	505	m	0	201	166	64	918	17.37	(12.53- 24.07)
WYNDE6	522	f	0	10	31	125	991	2.56	(1.22- 5.34)
WYNDE6	523	f	0	16	77	125	991	1.65	(0.93- 2.91)
WYNDE6	524	f	0	36	132	125	991	2.16	(1.43- 3.27)
WYNDE6	525	f	0	51	84	125	991	4.81	(3.24- 7.14)
WYNDE6	526	f	0	82	70	125	991	9.29	(6.42- 13.43)
Subtotal WYNDE6								5.50	(4.84- 6.24)
Partial Totals				10298	773960	11189	5466202		

*prospective study

~ With 0.5 adjustment for zero

Table 1J1 - 2

IESLC - Meta-analysis of Ex Smoking by Years quit (vs never), Overview
All LC types, Any Product (or Cigarettes if Any not available)
Most adjusted

REF	NRR	SEX	AD	Ys	Ws	Qs	Ps
ALDERS	507	m	1	1.16	8.16	2.59	0.0009
ALDERS	508	m	1	1.46	7.77	0.56	0.0000
ALDERS	509	m	1	2.90	9.90	13.55	0.0000
ALDERS	518	f	1	0.24	14.21	31.43	0.3676
ALDERS	519	f	1	1.08	18.83	7.82	0.0000
ALDERS	520	f	1	2.25	27.43	7.41	0.0000
Subtotal ALDERS				1.56	86.30	63.35	
ARMADA	515	m	0	1.63	5.86	0.06	0.0001
ARMADA	516	m	0	2.75	5.75	5.98	0.0000
Subtotal ARMADA				2.18	11.61	6.03	
AUVINE	532	c	2	2.01	11.70	0.97	0.0000
BARBON	540	m	1	0.74	7.22	7.00	0.0462
BARBON	541	m	1	1.92	9.55	0.35	0.0000
BARBON	542	m	1	2.21	13.34	3.10	0.0000
BARBON	543	m	1	2.63	7.48	6.14	0.0000
Subtotal BARBON				1.94	37.60	16.58	
BECHER	501	m	0	1.39	2.34	0.27	0.0341
BECHER	502	m	0	2.20	2.24	0.50	0.0010
BECHER	503	m	0	2.71	1.87	1.80	0.0002
BECHER	511	f	0	-0.65	0.82	4.65	0.5537
BECHER	512	f	0	0.73	1.22	1.21	0.4184
BECHER	513	f	0	1.24	1.05	0.24	0.2027
Subtotal BECHER				1.56	9.54	8.67	
*BENSHL	508	m	2	0.00	2.98	8.88	1.0000
*BENSHL	509	m	2	0.95	6.64	3.98	0.0142
*BENSHL	510	m	2	1.41	7.88	0.81	0.0001
*BENSHL	511	m	2	2.16	6.37	1.20	0.0000
Subtotal BENSHL				1.31	23.87	14.88	
BROSS	515	m	0	0.89	14.68	10.27	0.0006
BROSS	516	m	0	2.42	18.85	9.16	0.0000
Subtotal BROSS				1.75	33.54	19.43	
BROWN3	503	f	2	0.79	93.46	82.20	0.0000
CARPEN	501	c	0	1.71	5.83	0.00	0.0000
CARPEN	502	c	0	1.52	3.84	0.16	0.0028
CARPEN	503	c	0	2.61	5.25	4.06	0.0000
CARPEN	504	c	0	2.73	5.03	5.06	0.0000
Subtotal CARPEN				2.17	19.94	9.28	
*CEDERL	528	m	1	0.10	2.08	5.53	0.8907
*CEDERL	529	m	1	1.81	4.45	0.03	0.0001
Subtotal CEDERL				1.26	6.53	5.56	
CHOI	533	m	0	0.43	2.56	4.30	0.4904
CHOI	534	m	0	0.24	2.63	5.80	0.6977
CHOI	535	m	0	0.20	3.12	7.29	0.7277
CHOI	536	m	0	1.05	6.99	3.21	0.0056
CHOI	550	f	0	2.38	0.41	0.17	0.1268
CHOI	551	f	0	1.17	1.17	0.36	0.2033
Subtotal CHOI				0.71	16.88	21.13	
*CHYOU	507	m	2	1.03	3.05	1.48	0.0723
*CHYOU	508	m	2	1.34	5.93	0.91	0.0011
Subtotal CHYOU				1.23	8.98	2.39	
*CPSI	807	m	1	0.25	12.13	26.54	0.3900
*CPSI	808	m	1	1.64	20.82	0.16	0.0000
*CPSI	809	m	1	2.09	27.01	3.59	0.0000
*CPSI	810	m	1	2.69	22.87	21.26	0.0000
Subtotal CPSI				1.87	82.82	51.55	
*CPSII	652	m	1	1.34	61.12	8.99	0.0000
*CPSII	653	m	1	2.15	54.21	9.87	0.0000
*CPSII	654	m	1	2.44	56.51	28.49	0.0000
*CPSII	655	m	1	2.92	55.67	79.81	0.0000
*CPSII	656	m	1	3.33	56.54	146.27	0.0000
*CPSII	657	m	1	3.66	44.07	164.35	0.0000
*CPSII	633	f	1	0.55	38.44	52.83	0.0006
*CPSII	634	f	1	1.35	24.16	3.41	0.0000
*CPSII	635	f	1	1.59	30.57	0.56	0.0000
*CPSII	636	f	1	2.36	42.39	16.82	0.0000
*CPSII	637	f	1	2.83	59.81	73.44	0.0000
Subtotal CPSII				2.33	523.48	584.82	
DAMBER	523	m	1	0.96	16.22	9.63	0.0001
DAMBER	524	m	1	1.46	9.69	0.69	0.0000
DAMBER	525	m	1	2.04	12.73	1.26	0.0000
Subtotal DAMBER				1.44	38.64	11.59	

Table 1J1 - 2

IESLC - Meta-analysis of Ex Smoking by Years quit (vs never), Overview
All LC types, Any Product (or Cigarettes if Any not available)
Most adjusted

REF	NRR	SEX	AD	Ys	Ws	Qs	Ps
DARBY	501	m	0	3.14	2.90	5.84	0.0000
DARBY	502	m	0	4.01	2.89	15.08	0.0000
DARBY	510	f	0	0.98	11.33	6.27	0.0010
DARBY	511	f	0	2.82	14.12	16.96	0.0000
Subtotal DARBY				2.29	31.24	44.15	
DEAN3	628	m	1	0.71	12.73	13.07	0.0110
DEAN3	629	m	1	1.19	8.05	2.31	0.0007
DEAN3	630	m	1	1.57	13.58	0.32	0.0000
DEAN3	553	f	1	-0.33	1.86	7.85	0.6541
DEAN3	554	f	1	0.09	0.96	2.59	0.9326
DEAN3	555	f	1	0.49	3.50	5.36	0.3606
Subtotal DEAN3				1.01	40.68	31.51	
DESTEF	525	m	4	1.03	7.80	3.78	0.0040
DESTEF	526	m	4	1.82	8.58	0.08	0.0000
DESTEF	527	m	4	2.20	12.30	2.73	0.0000
Subtotal DESTEF				1.77	28.68	6.59	
DOLL	529	m	0	1.11	3.05	1.16	0.0527
DOLL	530	m	0	0.70	2.74	2.90	0.2471
DOLL	531	m	0	1.87	5.25	0.11	0.0000
DOLL	542	f	0	-0.30	0.65	2.67	0.8063
DOLL	543	f	0	0.79	3.13	2.72	0.1602
Subtotal DOLL				1.18	14.82	9.57	
*DOLL2	501	m	1	0.69	3.49	3.73	0.1951
*DOLL2	502	m	1	1.67	3.93	0.01	0.0009
*DOLL2	503	m	1	1.77	4.41	0.01	0.0002
*DOLL2	504	m	1	2.77	4.77	5.22	0.0000
Subtotal DOLL2				1.81	16.61	8.98	
DORGAN	501	m	0	1.73	10.48	0.00	0.0000
DORGAN	502	m	0	2.63	7.65	6.26	0.0000
DORGAN	503	m	0	2.52	8.29	5.25	0.0000
DORGAN	553	f	0	0.86	14.18	10.53	0.0011
DORGAN	554	f	0	1.85	12.73	0.18	0.0000
Subtotal DORGAN				1.78	53.33	22.22	
*DORN	657	m	0	0.85	9.76	7.45	0.0078
*DORN	658	m	0	1.47	8.11	0.55	0.0000
*DORN	659	m	0	2.07	14.04	1.65	0.0000
*DORN	660	m	0	2.58	14.42	10.46	0.0000
*DORN	680	m	0	0.84	20.08	15.74	0.0002
*DORN	681	m	0	1.62	18.24	0.21	0.0000
*DORN	682	m	0	1.78	22.35	0.07	0.0000
*DORN	683	m	0	2.07	10.91	1.26	0.0000
Subtotal DORN				1.66	117.90	37.39	
GAO	531	m	2	0.10	7.00	18.62	0.8009
GAO	532	m	2	1.13	9.92	3.51	0.0004
GAO	533	m	2	1.93	19.06	0.80	0.0000
GAO	551	f	2	0.79	6.60	5.80	0.0428
GAO	552	f	2	1.36	4.31	0.58	0.0047
GAO	553	f	2	1.97	6.91	0.42	0.0000
Subtotal GAO				1.36	53.81	29.74	
GAO2	509	m	0	0.32	3.85	7.60	0.5289
GAO2	510	m	0	1.21	2.87	0.77	0.0406
GAO2	511	m	0	1.34	4.70	0.69	0.0036
GAO2	512	m	0	1.25	5.53	1.27	0.0034
GAO2	513	m	0	1.64	6.04	0.05	0.0001
Subtotal GAO2				1.21	22.99	10.38	
GARCIA	515	c	0	0.99	3.78	2.03	0.0532
GARCIA	516	c	0	1.56	5.44	0.14	0.0003
GARCIA	517	c	0	2.48	5.30	3.02	0.0000
GARCIA	518	c	0	3.40	3.87	10.84	0.0000
Subtotal GARCIA				2.10	18.39	16.03	
GARSHI	522	m	1	1.16	26.18	8.30	0.0000
GARSHI	523	m	1	1.62	27.18	0.30	0.0000
Subtotal GARSHI				1.40	53.36	8.60	
GRAHAM	535	m	1	0.95	6.22	3.73	0.0176
GRAHAM	536	m	1	2.14	8.40	1.44	0.0000
GRAHAM	537	m	1	3.58	12.37	42.40	0.0000
Subtotal GRAHAM				2.52	26.99	47.57	
GURSEL	501	m	0	0.83	5.70	4.55	0.0468
*HAMMO2	501	m	1	1.24	4.04	0.96	0.0128
*HAMMO2	502	m	1	1.38	3.47	0.41	0.0101
*HAMMO2	503	m	1	2.40	4.65	2.09	0.0000

Table 1J1 - 2

IESLC - Meta-analysis of Ex Smoking by Years quit (vs never), Overview
All LC types, Any Product (or Cigarettes if Any not available)
Most adjusted

REF	NRR	SEX	AD	Ys	Ws	Qs	Ps
Subtotal HAMMO2				1.72	12.16	3.47	
*HIRAYA	507	m	1	0.32	5.36	10.56	0.4561
*HIRAYA	508	m	1	0.46	4.98	7.95	0.3005
*HIRAYA	509	m	1	0.71	10.22	10.59	0.0236
*HIRAYA	518	f	1	-0.03	0.32	0.97	0.9863
*HIRAYA	519	f	1	1.19	1.22	0.35	0.1885
*HIRAYA	520	f	1	1.31	2.66	0.45	0.0320
Subtotal HIRAYA				0.65	24.75	30.87	
JAHN	501	m	0	0.42	9.60	16.37	0.1925
JAHN	502	m	0	1.33	11.61	1.84	0.0000
JAHN	503	m	0	1.97	10.46	0.63	0.0000
JAHN	504	m	0	2.55	10.25	6.99	0.0000
JAHN	505	m	0	3.93	5.25	25.57	0.0000
JAHN	506	m	0	5.07	5.16	57.66	0.0000
JAHN	731	f	2	-1.20	1.46	12.58	0.1451
Subtotal JAHN				2.07	53.80	121.65	
JAIN	567	m	0	1.18	8.12	2.41	0.0008
JAIN	568	m	0	2.43	7.67	3.83	0.0000
JAIN	531	f	0	0.25	10.76	23.51	0.4154
JAIN	532	f	0	2.02	14.96	1.30	0.0000
Subtotal JAIN				1.47	41.51	31.05	
JEDRYC	611	m	0	0.86	21.78	16.33	0.0001
JEDRYC	612	m	0	1.60	17.29	0.29	0.0000
Subtotal JEDRYC				1.19	39.07	16.62	
JOLY	566	m	0	2.04	9.05	0.88	0.0000
JOLY	567	m	0	2.95	7.04	10.61	0.0000
JOLY	553	f	0	1.46	7.04	0.51	0.0001
JOLY	554	f	0	2.56	4.99	3.46	0.0000
Subtotal JOLY				2.21	28.12	15.46	
*KAISE2	646	m	1	0.66	3.68	4.16	0.2036
*KAISE2	647	m	1	1.14	4.61	1.56	0.0140
*KAISE2	648	m	1	2.11	6.08	0.90	0.0000
*KAISE2	566	f	1	1.84	2.41	0.03	0.0043
*KAISE2	567	f	1	1.47	2.61	0.17	0.0172
*KAISE2	568	f	1	2.07	3.75	0.45	0.0001
Subtotal KAISE2				1.58	23.15	7.28	
KHUDER	511	m	0	1.38	14.86	1.79	0.0000
KHUDER	512	m	0	1.85	14.26	0.22	0.0000
KHUDER	513	m	0	2.26	15.10	4.35	0.0000
Subtotal KHUDER				1.83	44.23	6.36	
LAUSSM	503	m	2	1.88	9.53	0.22	0.0000
LUBIN	585	m	0	0.62	5.06	6.17	0.1615
LUBIN	586	m	0	1.20	5.11	1.39	0.0065
LUBIN	587	m	0	2.69	4.74	4.36	0.0000
Subtotal LUBIN				1.48	14.91	11.93	
LUBIN2	1069	m	0	0.74	61.66	59.80	0.0000
LUBIN2	1070	m	0	1.26	57.14	12.30	0.0000
LUBIN2	1071	m	0	1.32	64.81	10.68	0.0000
LUBIN2	1072	m	0	1.68	92.66	0.20	0.0000
LUBIN2	1073	m	0	2.05	111.01	11.98	0.0000
LUBIN2	1074	m	0	2.43	128.94	64.33	0.0000
LUBIN2	1108	f	0	-0.35	3.29	14.14	0.5296
LUBIN2	1109	f	0	0.45	2.74	4.45	0.4543
LUBIN2	1110	f	0	0.42	2.08	3.58	0.5486
LUBIN2	1111	f	0	0.31	7.03	14.15	0.4151
LUBIN2	1112	f	0	0.98	16.09	9.08	0.0001
LUBIN2	1113	f	0	1.35	25.86	3.66	0.0000
Subtotal LUBIN2				1.66	573.32	208.35	
MATOS	591	m	2	1.10	7.02	2.76	0.0036
MATOS	592	m	2	2.20	5.30	1.18	0.0000
MATOS	593	m	2	2.64	6.50	5.41	0.0000
Subtotal MATOS				1.94	18.81	9.35	
PEZZO2	501	m	0	1.65	4.89	0.03	0.0003
PEZZO2	502	m	0	2.71	5.10	4.96	0.0000
Subtotal PEZZO2				2.19	9.99	4.99	
PEZZOT	501	m	0	1.70	3.14	0.00	0.0026
PEZZOT	502	m	0	2.79	3.42	3.86	0.0000
Subtotal PEZZOT				2.27	6.56	3.86	
SOBUE	717	m	0	0.61	7.92	9.81	0.0843
SOBUE	718	m	0	1.04	6.55	3.07	0.0077
SOBUE	719	m	0	1.21	8.60	2.26	0.0004

Table 1J1 - 2

IESLC - Meta-analysis of Ex Smoking by Years quit (vs never), Overview
All LC types, Any Product (or Cigarettes if Any not available)
Most adjusted

REF	NRR	SEX	AD	Ys	Ws	Qs	Ps
SOBUE	720	m	0	1.11	10.99	4.14	0.0002
SOBUE	721	m	0	1.15	14.66	4.84	0.0000
SOBUE	722	m	0	1.57	16.99	0.43	0.0000
Subtotal SOBUE				1.18	65.71	24.56	
*SPEIZE	501	f	0	0.56	18.89	25.73	0.0151
*SPEIZE	502	f	0	0.88	13.15	9.31	0.0013
*SPEIZE	503	f	0	1.75	24.03	0.01	0.0000
*SPEIZE	504	f	0	1.98	21.44	1.34	0.0000
*SPEIZE	505	f	0	1.76	16.98	0.02	0.0000
Subtotal SPEIZE				1.44	94.49	36.41	
SUZUK2	508	c	0	0.68	3.75	4.12	0.1886
SUZUK2	509	c	0	1.80	2.99	0.01	0.0019
SUZUK2	510	c	0	1.98	3.62	0.23	0.0002
Subtotal SUZUK2				1.45	10.36	4.36	
SVENSS	551	f	0	0.61	6.77	8.42	0.1120
SVENSS	552	f	0	1.36	5.74	0.78	0.0011
Subtotal SVENSS				0.95	12.51	9.20	
*TVERDA	501	m	2	0.29	2.02	4.15	0.6776
*TVERDA	502	m	2	1.04	2.22	1.05	0.1208
*TVERDA	503	m	2	1.02	1.34	0.67	0.2389
Subtotal TVERDA				0.76	5.58	5.86	
WAKAI	530	m	2	0.00	3.52	10.48	1.0000
WAKAI	531	m	2	1.29	5.39	1.03	0.0028
WAKAI	532	m	2	0.91	5.08	3.40	0.0406
Subtotal WAKAI				0.83	13.99	14.91	
WANG2	510	c	0	0.57	2.47	3.27	0.3664
WANG2	511	c	0	0.85	2.63	2.00	0.1672
Subtotal WANG2				0.72	5.09	5.28	
WYNDE3	535	m	0	-0.12	2.94	9.98	0.8401
WYNDE3	536	m	0	1.24	4.07	0.95	0.0121
WYNDE3	537	m	0	1.84	3.67	0.05	0.0004
WYNDE3	538	m	0	2.23	4.64	1.19	0.0000
WYNDE3	587	f	0	0.24	0.72	1.59	0.8415
Subtotal WYNDE3				1.37	16.04	13.76	
WYNDE6	501	m	0	0.63	14.18	17.15	0.0183
WYNDE6	502	m	0	1.31	25.24	4.29	0.0000
WYNDE6	503	m	0	1.81	38.93	0.28	0.0000
WYNDE6	504	m	0	1.98	31.18	2.01	0.0000
WYNDE6	505	m	0	2.85	36.08	45.94	0.0000
WYNDE6	522	f	0	0.94	7.08	4.39	0.0125
WYNDE6	523	f	0	0.50	11.83	17.82	0.0859
WYNDE6	524	f	0	0.77	22.54	20.57	0.0003
WYNDE6	525	f	0	1.57	24.68	0.59	0.0000
WYNDE6	526	f	0	2.23	28.18	7.11	0.0000
Subtotal WYNDE6				1.70	239.93	120.14	

N	211
NS	51

Table 1J1 - 3

IESLC - Meta-analysis of Ex Smoking by Years quit (vs never), Overview
All LC types, Any Product (or Cigarettes if Any not available)
Most adjusted

	combined	Sex male	female	Total
N	14	141	56	211
NS	5	43	20	68

In this overview table, other than the "N" rows, entries in the "absent" and "Total" columns may be invalid and should be ignored

Years quit vs never (lower focus)

	absent	8+k12	4-11k7	1-6k3	Total
N	82	53	33	43	211
NS	37	39	25	33	134
Wt	1145.36	580.07	492.77	634.80	2852.99
Het Chi	1092.31	204.16	102.48	164.89	1881.67
Het df	81	52	32	42	210
Het P	***	***	***	***	***
Fixed RR	5.02	3.62	6.07	9.69	5.62
RRl	4.74	3.34	5.56	8.96	5.42
RRu	5.32	3.93	6.63	10.47	5.83
P	+++	+++	+++	+++	+++
Random RR	4.51	2.97	5.08	8.60	4.69
RRl	3.60	2.48	4.24	7.22	4.17
RRu	5.65	3.55	6.10	10.23	5.28
P	+++	+++	+++	+++	+++

Years quit vs never (higher focus)

	absent	13+k20	4-19k12	1-11k3	Total
N	106	24	22	59	211
NS	48	20	18	43	129
Wt	1274.41	436.23	385.29	757.07	2852.99
Het Chi	1097.73	59.31	75.84	227.07	1881.67
Het df	105	23	21	58	210
Het P	***	***	***	***	***
Fixed RR	5.56	2.72	4.97	9.24	5.62
RRl	5.27	2.48	4.50	8.60	5.42
RRu	5.88	2.99	5.49	9.92	5.83
P	+++	+++	+++	+++	+++
Random RR	3.96	2.66	4.32	8.35	4.69
RRl	3.27	2.23	3.49	7.14	4.17
RRu	4.79	3.17	5.36	9.76	5.28
P	+++	+++	+++	+++	+++

Table 1J1 - 3

IESLC - Meta-analysis of Ex Smoking by Years quit (vs never), Overview
All LC types, Any Product (or Cigarettes if Any not available)
Most adjusted

MALES

		Years quit vs never (lower focus)				
		absent	8+k12	4-11k7	1-6k3	Total
	N	55	34	23	29	141
	NS	30	33	22	28	113
	Wt	755.91	423.09	381.45	481.50	2041.94
Het	Chi	768.30	124.98	77.66	109.10	1287.64
Het	df	54	33	22	28	140
Het	P	***	***	***	***	***
Fixed	RR	5.48	4.22	6.54	10.48	6.25
	RRl	5.10	3.84	5.91	9.58	5.98
	RRu	5.88	4.64	7.23	11.45	6.53
	P	+++	+++	+++	+++	+++
Random	RR	4.94	3.43	5.25	9.31	5.19
	RRl	3.72	2.79	4.23	7.64	4.52
	RRu	6.56	4.22	6.51	11.35	5.97
	P	+++	+++	+++	+++	+++

		Years quit vs never (higher focus)				
		absent	13+k20	4-19k12	1-11k3	Total
	N	70	17	16	38	141
	NS	40	16	15	37	108
	Wt	942.57	259.59	309.27	530.50	2041.94
Het	Chi	847.11	27.11	31.05	127.63	1287.64
Het	df	69	16	15	37	140
Het	P	***	*	**	***	***
Fixed	RR	5.82	3.25	5.64	10.36	6.25
	RRl	5.46	2.88	5.04	9.52	5.98
	RRu	6.21	3.67	6.30	11.28	6.53
	P	+++	+++	+++	+++	+++
Random	RR	4.50	3.00	5.14	9.42	5.19
	RRl	3.55	2.50	4.25	7.89	4.52
	RRu	5.69	3.60	6.20	11.25	5.97
	P	+++	+++	+++	+++	+++

FEMALES

		Years quit vs never (lower focus)				
		absent	8+k12	4-11k7	1-6k3	Total
	N	22	16	8	10	56
	NS	15	16	8	10	49
	Wt	366.63	137.69	103.08	138.16	745.57
Het	Chi	298.35	23.92	9.12	29.98	465.23
Het	df	21	15	7	9	55
Het	P	***	(*)	N.S.	***	***
Fixed	RR	4.20	2.15	4.43	7.28	4.14
	RRl	3.79	1.82	3.65	6.16	3.85
	RRu	4.65	2.54	5.37	8.60	4.45
	P	+++	+++	+++	+++	+++
Random	RR	3.57	2.03	4.29	6.27	3.32
	RRl	2.33	1.60	3.36	4.36	2.63
	RRu	5.49	2.56	5.47	9.03	4.19
	P	+++	+++	+++	+++	+++

Table 1J1 - 3

IESLC - Meta-analysis of Ex Smoking by Years quit (vs never), Overview
All LC types, Any Product (or Cigarettes if Any not available)
Most adjusted

FEMALES

	Years quit vs never (higher focus) absent	13+k20	4-19k12	1-11k3	Total
N	30	5	4	17	56
NS	19	5	4	17	45
Wt	301.90	165.36	66.88	211.43	745.57
Het Chi	228.38	2.52	7.56	61.38	465.23
Het df	29	4	3	16	55
Het P	***	N.S.	(*)	***	***
Fixed RR	4.84	1.98	2.60	6.83	4.14
RRl	4.33	1.70	2.04	5.97	3.85
RRu	5.42	2.30	3.30	7.82	4.45
P	+++	+++	+++	+++	+++
Random RR	2.74	1.98	2.44	6.03	3.32
RRl	1.90	1.70	1.64	4.49	2.63
RRu	3.95	2.30	3.63	8.11	4.19
P	+++	+++	+++	+++	+++

Table 1J1 - 4

IESLC - Meta-analysis of Ex Smoking by Years quit (vs never), Overview
All LC types, Any Product (or Cigarettes if Any not available)
Least adjusted

REF	NRR	X	SEX	AGEL	AGEH	RACE	YF	LC TYPE	LOC	START	ST	NLC	R	VB	P	H	AD	PRODUCT	exL	exH	S1	S2	DENOM	De
ALDERS	507		m	0	0	all	-	all	Eu:UK	1977	CC	1448	n	V	n	n	1	cig only	10	999	1	0	nev any	ot
ALDERS	508		m	0	0	all	-	all	Eu:UK	1977	CC	1448	n	V	n	n	1	cig only	3	9	0	3	nev any	ot
ALDERS	509		m	0	0	all	-	all	Eu:UK	1977	CC	1448	n	V	n	n	1	cig only	0.1	2	0	0	nev any	ot
ALDERS	518		f	0	0	all	-	all	Eu:UK	1977	CC	1448	n	V	n	n	1	cig only	10	999	1	0	nev any	ot
ALDERS	519		f	0	0	all	-	all	Eu:UK	1977	CC	1448	n	V	n	n	1	cig only	3	9	0	3	nev any	ot
ALDERS	520		f	0	0	all	-	all	Eu:UK	1977	CC	1448	n	V	n	n	1	cig only	0.1	2	0	0	nev any	ot
ARMADA	515		m	0	0	all	-	all	Eu:wst	1986	CC	325	n	bl	n	y	0	cig+/-ot	6	999	0	0	nev cigs	st
ARMADA	516		m	0	0	all	-	all	Eu:wst	1986	CC	325	n	bl	n	y	0	cig+/-ot	1.0	5	3	3	nev cigs	st
AUVINE	530	x	c	0	0	all	-	all	Eu:Sca	1986	CC	517	n	bl	y	n	0	cig+/-ot	12	999	1	0	nev cigs	st
BARBON	525	x	m	0	0	all	-	all	Eu:wst	1979	CC	755	n	bl	y	y	0	all/unsp	25	999	0	0	nev any	st
BARBON	526	x	m	0	0	all	-	all	Eu:wst	1979	CC	755	n	bl	y	y	0	all/unsp	15	24	0	1	nev any	st
BARBON	527	x	m	0	0	all	-	all	Eu:wst	1979	CC	755	n	bl	y	y	0	all/unsp	5	14	0	2	nev any	st
BARBON	528	x	m	0	0	all	-	all	Eu:wst	1979	CC	755	n	bl	y	y	0	all/unsp	0.1	4	3	3	nev any	st
BECHER	501		m	0	0	all	-	all	Eu:Ger	1985	CC	194	n	bl	n	y	0	all/unsp	10	999	1	0	nev any	st
BECHER	502		m	0	0	all	-	all	Eu:Ger	1985	CC	194	n	bl	n	y	0	all/unsp	5	9	2	0	nev any	st
BECHER	503		m	0	0	all	-	all	Eu:Ger	1985	CC	194	n	bl	n	y	0	all/unsp	2	4	3	3	nev any	st
BECHER	511		f	0	0	all	-	all	Eu:Ger	1985	CC	194	n	bl	n	y	0	all/unsp	10	999	1	0	nev any	st
BECHER	512		f	0	0	all	-	all	Eu:Ger	1985	CC	194	n	bl	n	y	0	all/unsp	5	9	2	0	nev any	st
BECHER	513		f	0	0	all	-	all	Eu:Ger	1985	CC	194	n	bl	n	y	0	all/unsp	2	4	3	3	nev any	st
BENSHL	508		m	0	0	all	0	all	Eu:UK	1967	pr	486	n	V	n	n	2	cig+/-ot	30	999	0	0	nev any	or
BENSHL	509		m	0	0	all	0	all	Eu:UK	1967	pr	486	n	V	n	n	2	cig+/-ot	20	29	0	1	nev any	or
BENSHL	510		m	0	0	all	0	all	Eu:UK	1967	pr	486	n	V	n	n	2	cig+/-ot	10	19	1	2	nev any	or
BENSHL	511		m	0	0	all	0	all	Eu:UK	1967	pr	486	n	V	n	n	2	cig+/-ot	1.0	9	0	3	nev any	or
BROSS	515		m	0	0	wh	-	all	NAmer	1960	CC	974	n	bl	n	n	0	cig+/-ot	6	999	0	0	nev any	st
BROSS	516		m	0	0	wh	-	all	NAmer	1960	CC	974	n	bl	n	n	0	cig+/-ot	0.1	5	3	3	nev any	st
BROWN3	501	x	f	0	0	wh	-	all	NAmer		CC	618		bl	y	n	0	all/unsp	15	999	0	1	nev any	st
CARPEN	501		c	0	0	w+b	-	all	NAmer	1991	CC	356	n	bl	n	n	0	cig+/-ot	15	999	0	1	nev cigs	st
CARPEN	502		c	0	0	w+b	-	all	NAmer	1991	CC	356	n	bl	n	n	0	cig+/-ot	10	14	1	2	nev cigs	st
CARPEN	503		c	0	0	w+b	-	all	NAmer	1991	CC	356	n	bl	n	n	0	cig+/-ot	5	9	2	0	nev cigs	st
CARPEN	504		c	0	0	w+b	-	all	NAmer	1991	CC	356	n	bl	n	n	0	cig+/-ot	0.1	4	3	3	nev cigs	st
CEDERL	528		m	40	69	all	10	all	Eu:Sca	1963	pr	491	n	bl	n	n	1	all/unsp	10	999	1	0	nev any	ot
CEDERL	529		m	40	69	all	10	all	Eu:Sca	1963	pr	491	n	bl	n	n	1	all/unsp	0.1	9	0	3	nev any	ot
CHOI	533		m	0	0	all	-	all	As:oth	1985	CC	375	n	bl	n	n	0	cig+/-ot	15	999	0	1	nev cigs	st
CHOI	534		m	0	0	all	-	all	As:oth	1985	CC	375	n	bl	n	n	0	cig+/-ot	10	14	1	2	nev cigs	st
CHOI	535		m	0	0	all	-	all	As:oth	1985	CC	375	n	bl	n	n	0	cig+/-ot	5	9	2	0	nev cigs	st
CHOI	536		m	0	0	all	-	all	As:oth	1985	CC	375	n	bl	n	n	0	cig+/-ot	0.1	4	3	3	nev cigs	st
CHOI	550		f	0	0	all	-	all	As:oth	1985	CC	375	n	bl	n	n	0	cig+/-ot	5	999	0	0	nev cigs	ot
CHOI	551		f	0	0	all	-	all	As:oth	1985	CC	375	n	bl	n	n	0	cig+/-ot	0.1	4	3	3	nev cigs	st
CHYOU	501	x	m	0	0	jap	21	all	NAmer	1965	pr	227	n	bl	n	y	1	cig+/-ot	15	999	0	1	nev cigs	or
CHYOU	502	x	m	0	0	jap	21	all	NAmer	1965	pr	227	n	bl	n	y	1	cig+/-ot	0.1	14	0	0	nev cigs	or
CPSI	807		m	50	74	all	6	all	NAmer	1959	pr	5138	n	bl	n	n	1	cig only	10	999	1	0	nev any	ot
CPSI	808		m	50	74	all	6	all	NAmer	1959	pr	5138	n	bl	n	n	1	cig only	5	9	2	0	nev any	ot
CPSI	809		m	50	74	all	6	all	NAmer	1959	pr	5138	n	bl	n	n	1	cig only	1.0	4	3	3	nev any	ot
CPSI	810		m	50	74	all	6	all	NAmer	1959	pr	5138	n	bl	n	n	1	cig only	0.1	0.9	0	0	nev any	ot
CPSII	652		m	35	99	all	4	all	NAmer	1982	pr	3229	n	bl	n	n	1	cig only	16	999	0	1	nev any	ot
CPSII	653		m	35	99	all	4	all	NAmer	1982	pr	3229	n	bl	n	n	1	cig only	11	15	1	2	nev any	ot
CPSII	654		m	35	99	all	4	all	NAmer	1982	pr	3229	n	bl	n	n	1	cig only	6	10	2	0	nev any	ot
CPSII	655		m	35	99	all	4	all	NAmer	1982	pr	3229	n	bl	n	n	1	cig only	3	5	3	3	nev any	ot
CPSII	656		m	35	99	all	4	all	NAmer	1982	pr	3229	n	bl	n	n	1	cig only	1.0	2	0	0	nev any	ot
CPSII	657		m	35	99	all	4	all	NAmer	1982	pr	3229	n	bl	n	n	1	cig only	0.1	0.9	0	0	nev any	ot
CPSII	633		f	0	0	all	4	all	NAmer	1982	pr	3229	n	bl	n	n	1	cig+/-ot	16	999	0	1	nev cigs	ot
CPSII	634		f	0	0	all	4	all	NAmer	1982	pr	3229	n	bl	n	n	1	cig+/-ot	11	15	1	2	nev cigs	ot
CPSII	635		f	0	0	all	4	all	NAmer	1982	pr	3229	n	bl	n	n	1	cig+/-ot	6	10	2	0	nev cigs	ot
CPSII	636		f	0	0	all	4	all	NAmer	1982	pr	3229	n	bl	n	n	1	cig+/-ot	3	5	3	3	nev cigs	ot
CPSII	637		f	0	0	all	4	all	NAmer	1982	pr	3229	n	bl	n	n	1	cig+/-ot	0.1	2	0	0	nev cigs	ot
DAMBER	523		m	0	0	all	-	all	Eu:Sca	1972	CC	579	n	bl	y	n	1	all/unsp	11	999	1	0	nev any	ot
DAMBER	524		m	0	0	all	-	all	Eu:Sca	1972	CC	579	n	bl	y	n	1	all/unsp	6	10	2	0	nev any	ot
DAMBER	525		m	0	0	all	-	all	Eu:Sca	1972	CC	579	n	bl	y	n	1	all/unsp	0.1	5	3	3	nev any	ot
DARBY	501		m	0	0	wh	-	all	Eu:UK	1988	CC	982	n	V	n	n	0	all/unsp	10	999	1	0	nev any	st
DARBY	502		m	0	0	wh	-	all	Eu:UK	1988	CC	982	n	V	n	n	0	all/unsp	0.1	9	0	3	nev any	st
DARBY	510		f	0	0	wh	-	all	Eu:UK	1988	CC	982	n	V	n	n	0	all/unsp	10	999	1	0	nev any	st
DARBY	511		f	0	0	wh	-	all	Eu:UK	1988	CC	982	n	V	n	n	0	all/unsp	0.1	9	0	3	nev any	st
DEAN3	531	x	m	0	0	all	-	all	Eu:UK	1969	CC	766	n	V	y	n	0	all/unsp	9	999	1	0	nev any	st
DEAN3	532	x	m	0	0	all	-	all	Eu:UK	1969	CC	766	n	V	y	n	0	all/unsp	5	8	2	0	nev any	st
DEAN3	533	x	m	0	0	all	-	all	Eu:UK	1969	CC	766	n	V	y	n	0	all/unsp	3	4	3	3	nev any	st
DEAN3	542	x	f	0	0	all	-	all	Eu:UK	1969	CC	766	n	V	y	n	0	all/unsp	9	999	1	0	nev any	st
DEAN3	543	x	f	0	0	all	-	all	Eu:UK	1969	CC	766	n	V	y	n	0	all/unsp	5	8	2	0	nev any	st
DEAN3	544	x	f	0	0	all	-	all	Eu:UK	1969	CC	766	n	V	y	n	0	all/unsp	3	4	3	3	nev any	st
DESTEF	515	x	m	0	0	all	-	all	SCAmer	1988	CC	497	n	bl	n	y	0	all/unsp	10	999	1	0	nev any	st
DESTEF	516	x	m	0	0	all	-	all	SCAmer	1988	CC	497	n	bl	n	y	0	all/unsp	5	9	2	0	nev any	st
DESTEF	517	x	m	0	0	all	-	all	SCAmer	1988	CC	497	n	bl	n	y	0	all/unsp	0.1	4	3	3	nev any	st
DOLL	529		m	0	0	all	-	all	Eu:UK	1948	CC	1465	n	V	n	n	0	all/unsp	20	999	0	1	nev any	st

Table 1J1 - 4

IESLC - Meta-analysis of Ex Smoking by Years quit (vs never), Overview
All LC types, Any Product (or Cigarettes if Any not available)
Least adjusted

REF	NRR	X	SEX	AGEL	AGEH	RACE	YF	LC TYPE	LOC	START	ST	NLC	R	VB	P	H	AD	PRODUCT	exL	exH	S1	S2	DENOM	De
DOLL	530		m	0	0	all	-	all	Eu:UK	1948	CC	1465	n	V	n	n	0	all/unsp	10	19	1	2	nev any	st
DOLL	531		m	0	0	all	-	all	Eu:UK	1948	CC	1465	n	V	n	n	0	all/unsp	0.1	9	0	3	nev any	st
DOLL	542		f	0	0	all	-	all	Eu:UK	1948	CC	1465	n	V	n	n	0	all/unsp	10	999	1	0	nev any	st
DOLL	543		f	0	0	all	-	all	Eu:UK	1948	CC	1465	n	V	n	n	0	all/unsp	0.1	9	0	3	nev any	st
DOLL2	501		m	0	0	all	20	all	Eu:UK	1951	pr	920	n	V	n	n	1	cig only	15	999	0	1	nev any	st
DOLL2	502		m	0	0	all	20	all	Eu:UK	1951	pr	920	n	V	n	n	1	cig only	10	14	1	2	nev any	ot
DOLL2	503		m	0	0	all	20	all	Eu:UK	1951	pr	920	n	V	n	n	1	cig only	5	9	2	0	nev any	ot
DOLL2	504		m	0	0	all	20	all	Eu:UK	1951	pr	920	n	V	n	n	1	cig only	0.1	4	3	3	nev any	ot
DORGAN	501		m	0	0	wh	-	all	NAmer	1980	CC	2026	n	bl	y	y	0	cig+/-ot	10	999	1	0	nev any	st
DORGAN	502		m	0	0	wh	-	all	NAmer	1980	CC	2026	n	bl	y	y	0	cig+/-ot	6	9	2	0	nev any	st
DORGAN	503		m	0	0	wh	-	all	NAmer	1980	CC	2026	n	bl	y	y	0	cig+/-ot	1.1	5	3	3	nev any	st
DORGAN	553		f	0	0	all	-	all	NAmer	1980	CC	2026	n	bl	y	y	0	cig+/-ot	10	999	1	0	nev any	st
DORGAN	554		f	0	0	all	-	all	NAmer	1980	CC	2026	n	bl	y	y	0	cig+/-ot	1.1	9	0	3	nev any	st
DORN	657		m	55	64	wh	8	all	NAmer	1954	pr	5097	n	bl	n	n	0	cig+/-ot	15	999	0	1	nev any	st
DORN	658		m	55	64	wh	8	all	NAmer	1954	pr	5097	n	bl	n	n	0	cig+/-ot	10	14	1	2	nev any	st
DORN	659		m	55	64	wh	8	all	NAmer	1954	pr	5097	n	bl	n	n	0	cig+/-ot	5	9	2	0	nev any	st
DORN	660		m	55	64	wh	8	all	NAmer	1954	pr	5097	n	bl	n	n	0	cig+/-ot	0.1	4	3	3	nev any	st
DORN	680		m	65	74	wh	8	all	NAmer	1954	pr	5097	n	bl	n	n	0	cig+/-ot	15	999	0	1	nev any	st
DORN	681		m	65	74	wh	8	all	NAmer	1954	pr	5097	n	bl	n	n	0	cig+/-ot	10	14	1	2	nev any	st
DORN	682		m	65	74	wh	8	all	NAmer	1954	pr	5097	n	bl	n	n	0	cig+/-ot	5	9	2	0	nev any	st
DORN	683		m	65	74	wh	8	all	NAmer	1954	pr	5097	n	bl	n	n	0	cig+/-ot	0.1	4	3	3	nev any	st
GAO	521	x	m	0	0	all	-	all	As:Chi	1984	CC	1405	n	ot	n	n	0	cig+/-ot	10	999	1	0	nev cigs	st
GAO	522	x	m	0	0	all	-	all	As:Chi	1984	CC	1405	n	ot	n	n	0	cig+/-ot	5	9	2	0	nev cigs	st
GAO	523	x	m	0	0	all	-	all	As:Chi	1984	CC	1405	n	ot	n	n	0	cig+/-ot	0.1	4	3	3	nev cigs	st
GAO	541	x	f	0	0	all	-	all	As:Chi	1984	CC	1405	n	ot	n	n	0	cig+/-ot	10	999	1	0	nev cigs	st
GAO	542	x	f	0	0	all	-	all	As:Chi	1984	CC	1405	n	ot	n	n	0	cig+/-ot	5	9	2	0	nev cigs	st
GAO	543	x	f	0	0	all	-	all	As:Chi	1984	CC	1405	n	ot	n	n	0	cig+/-ot	0.1	4	3	3	nev cigs	st
GAO2	509		m	0	0	all	-	all	As:Jap	1988	CC	282	n	bl	n	n	0	cig+/-ot	20	999	0	1	nev cigs	or
GAO2	510		m	0	0	all	-	all	As:Jap	1988	CC	282	n	bl	n	n	0	cig+/-ot	15	19	0	0	nev cigs	or
GAO2	511		m	0	0	all	-	all	As:Jap	1988	CC	282	n	bl	n	n	0	cig+/-ot	10	14	1	2	nev cigs	or
GAO2	512		m	0	0	all	-	all	As:Jap	1988	CC	282	n	bl	n	n	0	cig+/-ot	5	9	2	0	nev cigs	st
GAO2	513		m	0	0	all	-	all	As:Jap	1988	CC	282	n	bl	n	n	0	cig+/-ot	1.0	4	3	3	nev cigs	or
GARCIA	515		c	0	0	all	-	all	NAmer	1992	CC	416	n	bl	n	y	0	cig+/-ot	30	999	0	0	nev any	st
GARCIA	516		c	0	0	all	-	all	NAmer	1992	CC	416	n	bl	n	y	0	cig+/-ot	15	29	0	1	nev any	st
GARCIA	517		c	0	0	all	-	all	NAmer	1992	CC	416	n	bl	n	y	0	cig+/-ot	5	14	0	2	nev any	st
GARCIA	518		c	0	0	all	-	all	NAmer	1992	CC	416	n	bl	n	y	0	cig+/-ot	1.0	4	3	3	nev any	st
GARSHI	515	x	m	0	0	all	-	all	NAmer	1981	CC	1081	o	bl	y	n	0	all/unsp	15	999	0	1	nev any	st
GARSHI	516	x	m	0	0	all	-	all	NAmer	1981	CC	1081	o	bl	y	n	0	all/unsp	5	14	0	2	nev any	st
GRAHAM	525	x	m	0	0	wh	-	all	NAmer	1956	CC	685	n	bl	n	n	0	cig+/-ot	5	999	0	0	nev any	st
GRAHAM	526	x	m	0	0	wh	-	all	NAmer	1956	CC	685	n	bl	n	n	0	cig+/-ot	1.1	5	3	3	nev any	st
GRAHAM	527	x	m	0	0	wh	-	all	NAmer	1956	CC	685	n	bl	n	n	0	cig+/-ot	0.1	1.0	0	0	nev any	st
GURSEL	501		m	0	0	all	-	all	Eu:bal		CC	953		bl	*	n	0	all/unsp	11	999	1	0	nev any	or
HAMMO2	501		m	0	0	all	0	all	NAmer	1967	pr	450	o	bl	n	n	1	cig+/-ot	10	999	1	0	nev any	ot
HAMMO2	502		m	0	0	all	0	all	NAmer	1967	pr	450	o	bl	n	n	1	cig+/-ot	5	9	2	0	nev any	ot
HAMMO2	503		m	0	0	all	0	all	NAmer	1967	pr	450	o	bl	n	n	1	cig+/-ot	0.1	4	3	3	nev any	ot
HIRAYA	507		m	0	0	all	0	all	As:Jap	1965	pr	1917	n	bl	n	n	1	cig+/-ot	10	999	1	0	nev any	st
HIRAYA	508		m	0	0	all	0	all	As:Jap	1965	pr	1917	n	bl	n	n	1	cig+/-ot	5	9	2	0	nev any	st
HIRAYA	509		m	0	0	all	0	all	As:Jap	1965	pr	1917	n	bl	n	n	1	cig+/-ot	0.1	4	3	3	nev any	st
HIRAYA	518		f	0	0	all	0	all	As:Jap	1965	pr	1917	n	bl	n	n	1	cig+/-ot	10	999	1	0	nev any	st
HIRAYA	519		f	0	0	all	0	all	As:Jap	1965	pr	1917	n	bl	n	n	1	cig+/-ot	5	9	2	0	nev any	st
HIRAYA	520		f	0	0	all	0	all	As:Jap	1965	pr	1917	n	bl	n	n	1	cig+/-ot	0.1	4	3	3	nev any	st
JAHN	501		m	0	0	all	-	all	Eu:Ger	1988	CC	1004	n	bl	n	n	0	cig+/-ot	21	999	0	0	nev any	st
JAHN	502		m	0	0	all	-	all	Eu:Ger	1988	CC	1004	n	bl	n	n	0	cig+/-ot	11	20	1	0	nev any	st
JAHN	503		m	0	0	all	-	all	Eu:Ger	1988	CC	1004	n	bl	n	n	0	cig+/-ot	6	10	2	0	nev any	st
JAHN	504		m	0	0	all	-	all	Eu:Ger	1988	CC	1004	n	bl	n	n	0	cig+/-ot	2	5	3	3	nev any	st
JAHN	505		m	0	0	all	-	all	Eu:Ger	1988	CC	1004	n	bl	n	n	0	cig+/-ot	1.0	1.9	0	0	nev any	st
JAHN	506		m	0	0	all	-	all	Eu:Ger	1988	CC	1004	n	bl	n	n	0	cig+/-ot	0.1	0.9	0	0	nev any	st
JAHN	731		f	0	0	all	-	all	Eu:Ger	1988	CC	1004	n	bl	n	n	2	cig+/-ot	21	999	0	0	nev any	or
JAIN	567		m	0	0	all	-	all	NAmer	1981	CC	845	n	V	y	n	0	cig+/-ot	10	999	1	0	nev cigs	st
JAIN	568		m	0	0	all	-	all	NAmer	1981	CC	845	n	V	y	n	0	cig+/-ot	2	9	0	3	nev cigs	st
JAIN	531		f	0	0	all	-	all	NAmer	1981	CC	845	n	V	y	n	0	cig+/-ot	10	999	1	0	nev cigs	st
JAIN	532		f	0	0	all	-	all	NAmer	1981	CC	845	n	V	y	n	0	cig+/-ot	2	9	0	3	nev cigs	st
JEDRYC	611		m	0	0	all	-	all	Eu:est	1980	CC	1630	n	bl	y	n	0	cig+/-ot	10	999	1	0	nev any	st
JEDRYC	612		m	0	0	all	-	all	Eu:est	1980	CC	1630	n	bl	y	n	0	cig+/-ot	5	9	2	0	nev any	st
JOLY	566		m	0	0	all	-	all	SCAmer	1978	CC	826	n	bl	n	n	0	cig+/-ot	5	999	0	0	nev any	st
JOLY	567		m	0	0	all	-	all	SCAmer	1978	CC	826	n	bl	n	n	0	cig+/-ot	1.0	4	3	3	nev any	st
JOLY	553		f	0	0	all	-	all	SCAmer	1978	CC	826	n	bl	n	n	0	cig+/-ot	5	999	0	0	nev any	st
JOLY	554		f	0	0	all	-	all	SCAmer	1978	CC	826	n	bl	n	n	0	cig+/-ot	1.0	4	3	3	nev any	st
KAISE2	646		m	0	0	all	9	all	NAmer	1979	pr	318	n	bl	n	n	1	cig only	21	999	0	0	nev any	st
KAISE2	647		m	0	0	all	9	all	NAmer	1979	pr	318	n	bl	n	n	1	cig only	11	20	1	0	nev any	ot
KAISE2	648		m	0	0	all	9	all	NAmer	1979	pr	318	n	bl	n	n	1	cig only	2	10	0	3	nev any	st
KAISE2	566		f	0	0	all	9	all	NAmer	1979	pr	318	n	bl	n	n	1	cig only	21	999	0	0	nev any	ot

Table 1J1 - 4

IESLC - Meta-analysis of Ex Smoking by Years quit (vs never), Overview
All LC types, Any Product (or Cigarettes if Any not available)
Least adjusted

REF	NRR	X	SEX	AGEL	AGEH	RACE	YF	LC TYPE	LOC	START	ST	NLC	R	VB	P	H	AD	PRODUCT	exL	exH	S1	S2	DENOM	De
KAISE2	567		f	0	0	all	9	all	NAmer	1979	pr	318	n	bl	n	n	1	cig only	11	20	1	0	nev any	st
KAISE2	568		f	0	0	all	9	all	NAmer	1979	pr	318	n	bl	n	n	1	cig only	2	10	0	3	nev any	st
KHUDER	511		m	0	0	all	-	all	NAmer	1985	CC	482	n	bl	n	y	0	cig+/-ot	15	999	0	1	nev cigs	st
KHUDER	512		m	0	0	all	-	all	NAmer	1985	CC	482	n	bl	n	y	0	cig+/-ot	5	14	0	2	nev cigs	st
KHUDER	513		m	0	0	all	-	all	NAmer	1985	CC	482	n	bl	n	y	0	cig+/-ot	0.1	4	3	3	nev cigs	st
LAUSSM	501	x	m	0	0	all	-	all	Eu:Ger	1982	CC	432	n	bl	n	n	0	all/unsp	10	999	1	0	nev any	st
LUBIN	585		m	0	0	all	-	all	As:Chi	1984	CC	427	m	ot	y	n	0	cig+/-ot	10	999	1	0	nev any	st
LUBIN	586		m	0	0	all	-	all	As:Chi	1984	CC	427	m	ot	y	n	0	cig+/-ot	5	9	2	0	nev any	st
LUBIN	587		m	0	0	all	-	all	As:Chi	1984	CC	427	m	ot	y	n	0	cig+/-ot	3	4	3	3	nev any	st
LUBIN2	1069		m	0	0	all	-	all	Eu:mul	1976	CC	7804	n	bl	n	y	0	cig+/-ot	25	999	0	0	nev any	st
LUBIN2	1070		m	0	0	all	-	all	Eu:mul	1976	CC	7804	n	bl	n	y	0	cig+/-ot	20	24	0	1	nev any	st
LUBIN2	1071		m	0	0	all	-	all	Eu:mul	1976	CC	7804	n	bl	n	y	0	cig+/-ot	15	19	0	0	nev any	st
LUBIN2	1072		m	0	0	all	-	all	Eu:mul	1976	CC	7804	n	bl	n	y	0	cig+/-ot	10	14	1	2	nev any	st
LUBIN2	1073		m	0	0	all	-	all	Eu:mul	1976	CC	7804	n	bl	n	y	0	cig+/-ot	5	9	2	0	nev any	st
LUBIN2	1074		m	0	0	all	-	all	Eu:mul	1976	CC	7804	n	bl	n	y	0	cig+/-ot	0.1	4	3	3	nev any	st
LUBIN2	1108		f	0	0	all	-	all	Eu:mul	1976	CC	7804	n	bl	n	y	0	cig+/-ot	25	999	0	0	nev any	st
LUBIN2	1109		f	0	0	all	-	all	Eu:mul	1976	CC	7804	n	bl	n	y	0	cig+/-ot	20	24	0	1	nev any	st
LUBIN2	1110		f	0	0	all	-	all	Eu:mul	1976	CC	7804	n	bl	n	y	0	cig+/-ot	15	19	0	0	nev any	st
LUBIN2	1111		f	0	0	all	-	all	Eu:mul	1976	CC	7804	n	bl	n	y	0	cig+/-ot	10	14	1	2	nev any	st
LUBIN2	1112		f	0	0	all	-	all	Eu:mul	1976	CC	7804	n	bl	n	y	0	cig+/-ot	5	9	2	0	nev any	st
LUBIN2	1113		f	0	0	all	-	all	Eu:mul	1976	CC	7804	n	bl	n	y	0	cig+/-ot	0.1	4	3	3	nev any	st
MATOS	581	x	m	0	0	all	-	all	SCAmer	1994	CC	200	n	bl	n	n	0	cig+/-ot	11	999	1	0	nev any	st
MATOS	582	x	m	0	0	all	-	all	SCAmer	1994	CC	200	n	bl	n	n	0	cig+/-ot	6	10	2	0	nev any	st
MATOS	583	x	m	0	0	all	-	all	SCAmer	1994	CC	200	n	bl	n	n	0	cig+/-ot	1.0	5	3	3	nev any	st
PEZZO2	501		m	0	0	all	-	all	SCAmer	1992	CC	367	n	bl	n	y	0	cig+/-ot	11	999	1	0	nev cigs	st
PEZZO2	502		m	0	0	all	-	all	SCAmer	1992	CC	367	n	bl	n	y	0	cig+/-ot	1.0	10	0	3	nev cigs	st
PEZZOT	501		m	0	0	all	-	all	SCAmer	1987	CC	215	n	bl	n	y	0	cig only	11	999	1	0	nev cigs	st
PEZZOT	502		m	0	0	all	-	all	SCAmer	1987	CC	215	n	bl	n	y	0	cig only	1.0	10	0	3	nev cigs	st
SOBUE	717		m	0	0	all	-	all	As:Jap	1986	CC	1376	n	bl	n	y	0	cig+/-ot	25	999	0	0	nev cigs	st
SOBUE	718		m	0	0	all	-	all	As:Jap	1986	CC	1376	n	bl	n	y	0	cig+/-ot	20	24	0	1	nev cigs	st
SOBUE	719		m	0	0	all	-	all	As:Jap	1986	CC	1376	n	bl	n	y	0	cig+/-ot	15	19	0	0	nev cigs	st
SOBUE	720		m	0	0	all	-	all	As:Jap	1986	CC	1376	n	bl	n	y	0	cig+/-ot	10	14	1	2	nev cigs	st
SOBUE	721		m	0	0	all	-	all	As:Jap	1986	CC	1376	n	bl	n	y	0	cig+/-ot	5	9	2	0	nev cigs	st
SOBUE	722		m	0	0	all	-	all	As:Jap	1986	CC	1376	n	bl	n	y	0	cig+/-ot	1.0	4	3	3	nev cigs	st
SPEIZE	501		f	0	0	all	0	all	NAmer	1976	pr	593	n	bl	n	y	0	cig+/-ot	15	999	0	1	nev cigs	st
SPEIZE	502		f	0	0	all	0	all	NAmer	1976	pr	593	n	bl	n	y	0	cig+/-ot	10	15	1	2	nev cigs	st
SPEIZE	503		f	0	0	all	0	all	NAmer	1976	pr	593	n	bl	n	y	0	cig+/-ot	5	10	2	0	nev cigs	st
SPEIZE	504		f	0	0	all	0	all	NAmer	1976	pr	593	n	bl	n	y	0	cig+/-ot	2	5	3	3	nev cigs	st
SPEIZE	505		f	0	0	all	0	all	NAmer	1976	pr	593	n	bl	n	y	0	cig+/-ot	0.1	1.9	0	0	nev cigs	st
SUZUK2	508		c	0	0	all	-	all	SCAmer	1991	CC	123	n	bl	n	y	0	all/unsp	11	999	1	0	nev any	st
SUZUK2	509		c	0	0	all	-	all	SCAmer	1991	CC	123	n	bl	n	y	0	all/unsp	6	10	2	0	nev any	st
SUZUK2	510		c	0	0	all	-	all	SCAmer	1991	CC	123	n	bl	n	y	0	all/unsp	0.1	5	3	3	nev any	st
SVENSS	551		f	0	0	all	-	all	Eu:Sca	1983	CC	210	n	bl	n	n	0	all/unsp	11	999	1	0	nev any	st
SVENSS	552		f	0	0	all	-	all	Eu:Sca	1983	CC	210	n	bl	n	n	0	all/unsp	3	10	0	3	nev any	st
TVERDA	501		m	0	0	all	0	all	Eu:Sca	1972	pr	238	n	bl	n	n	2	cig only	5	999	0	0	nev cigs	ot
TVERDA	502		m	0	0	all	0	all	Eu:Sca	1972	pr	238	n	bl	n	n	2	cig only	1.0	5	3	3	nev cigs	ot
TVERDA	503		m	0	0	all	0	all	Eu:Sca	1972	pr	238	n	bl	n	n	2	cig only	0.1	0.9	0	0	nev cigs	ot
WAKAI	522	x	m	0	0	all	-	all	As:Jap	1988	CC	333	n	bl	n	y	0	cig+/-ot	20	999	0	1	nev any	st
WAKAI	523	x	m	0	0	all	-	all	As:Jap	1988	CC	333	n	bl	n	y	0	cig+/-ot	10	19	1	2	nev any	st
WAKAI	524	x	m	0	0	all	-	all	As:Jap	1988	CC	333	n	bl	n	y	0	cig+/-ot	5	9	2	0	nev any	st
WANG2	510		c	0	0	all	-	all	As:Chi	1980	CC	103	n	ot	n	n	0	cig+/-ot	4	999	0	0	nev cigs	st
WANG2	511		c	0	0	all	-	all	As:Chi	1980	CC	103	n	ot	n	n	0	cig+/-ot	0.1	3	3	3	nev cigs	st
WYNDE3	535		m	0	0	all	-	all	NAmer	1966	CC	350	n	bl	n	y	0	all/unsp	13	999	0	1	nev any	st
WYNDE3	536		m	0	0	all	-	all	NAmer	1966	CC	350	n	bl	n	y	0	all/unsp	7	12	0	2	nev any	st
WYNDE3	537		m	0	0	all	-	all	NAmer	1966	CC	350	n	bl	n	y	0	all/unsp	4	6	0	0	nev any	st
WYNDE3	538		m	0	0	all	-	all	NAmer	1966	CC	350	n	bl	n	y	0	all/unsp	1.0	3	3	3	nev any	st
WYNDE3	587		f	0	0	all	-	all	NAmer	1966	CC	350	n	bl	n	y	0	cig+/-ot	10	999	1	0	nev any	st
WYNDE6	501		m	0	0	all	-	all	NAmer	1969	CC	4423	n	bl	n	y	0	cig only	30	999	0	0	nev any	st
WYNDE6	502		m	0	0	all	-	all	NAmer	1969	CC	4423	n	bl	n	y	0	cig only	20	29	0	1	nev any	st
WYNDE6	503		m	0	0	all	-	all	NAmer	1969	CC	4423	n	bl	n	y	0	cig only	10	19	1	2	nev any	st
WYNDE6	504		m	0	0	all	-	all	NAmer	1969	CC	4423	n	bl	n	y	0	cig only	5	9	2	0	nev any	st
WYNDE6	505		m	0	0	all	-	all	NAmer	1969	CC	4423	n	bl	n	y	0	cig only	1.0	4	3	3	nev any	st
WYNDE6	522		f	0	0	all	-	all	NAmer	1969	CC	4423	n	bl	n	y	0	cig only	30	999	0	0	nev any	st
WYNDE6	523		f	0	0	all	-	all	NAmer	1969	CC	4423	n	bl	n	y	0	cig only	20	29	0	1	nev any	st
WYNDE6	524		f	0	0	all	-	all	NAmer	1969	CC	4423	n	bl	n	y	0	cig only	10	19	1	2	nev any	st
WYNDE6	525		f	0	0	all	-	all	NAmer	1969	CC	4423	n	bl	n	y	0	cig only	5	9	2	0	nev any	st
WYNDE6	526		f	0	0	all	-	all	NAmer	1969	CC	4423	n	bl	n	y	0	cig only	1.0	4	3	3	nev any	st

Cigarette type is all/unspec for all RRs

Table 1J1 - 4

IESLC - Meta-analysis of Ex Smoking by Years quit (vs never), Overview
All LC types, Any Product (or Cigarettes if Any not available)
Least adjusted

except for the following:

REF	NRR	CIGTYPE
ALDERS	507	MC only
ALDERS	508	MC only
ALDERS	509	MC only
ALDERS	518	MC only
ALDERS	519	MC only
ALDERS	520	MC only

In this overview table, subtotals and Qs values may be invalid and should be ignored

Table 1J1 - 5

IESLC - Meta-analysis of Ex Smoking by Years quit (vs never), Overview
All LC types, Any Product (or Cigarettes if Any not available)
Least adjusted

REF	NRR	SEX	AD	Number Exposed Case	Cont	Non-exposed Case	Cont	RR	95.00%CI
ALDERS	507	m	1	29	-	15	-	3.20	(1.61- 6.35)
ALDERS	508	m	1	28	-	15	-	4.30	(2.13- 8.69)
ALDERS	509	m	1	121	-	15	-	18.10	(9.71- 33.74)
ALDERS	518	f	1	26	-	75	-	1.27	(0.76- 2.15)
ALDERS	519	f	1	54	-	75	-	2.95	(1.88- 4.64)
ALDERS	520	f	1	206	-	75	-	9.45	(6.50- 13.74)
Subtotal ALDERS								4.77	(3.86- 5.89)
ARMADA	515	m	0	50	87	8	71	5.10	(2.27- 11.46)
ARMADA	516	m	0	79	45	8	71	15.58	(6.88- 35.29)
Subtotal ARMADA								8.87	(4.99- 15.76)
AUVINE	530	c	0	207	208	44	229	5.18	(3.56- 7.54)
BARBON	525	m	0	15	59	22	188	2.17	(1.06- 4.46)
BARBON	526	m	0	33	41	22	188	6.88	(3.64- 13.00)
BARBON	527	m	0	89	85	22	188	8.95	(5.25- 15.24)
BARBON	528	m	0	32	20	22	188	13.67	(6.71- 27.87)
Subtotal BARBON								6.91	(5.03- 9.50)
BECHER	501	m	0	16	72	3	54	4.00	(1.11- 14.42)
BECHER	502	m	0	16	32	3	54	9.00	(2.43- 33.30)
BECHER	503	m	0	10	12	3	54	15.00	(3.58- 62.92)
BECHER	511	f	0	1	10	10	52	0.52	(0.06- 4.53)
BECHER	512	f	0	2	5	10	52	2.08	(0.35- 12.26)
BECHER	513	f	0	2	3	10	52	3.47	(0.51- 23.48)
Subtotal BECHER								4.76	(2.53- 8.99)
*BENSHL	508	m	2	6	-	10	-	1.00	(0.32- 3.10)
*BENSHL	509	m	2	15	-	10	-	2.59	(1.21- 5.54)
*BENSHL	510	m	2	23	-	10	-	4.08	(2.03- 8.20)
*BENSHL	511	m	2	14	-	10	-	8.68	(4.00- 18.90)
Subtotal BENSHL								3.69	(2.47- 5.51)
BROSS	515	m	0	43	79	38	170	2.44	(1.46- 4.06)
BROSS	516	m	0	169	67	38	170	11.28	(7.19- 17.72)
Subtotal BROSS								5.77	(4.11- 8.09)
BROWN3	501	f	0	186	234	432	1168	2.15	(1.72- 2.68)
CARPEN	501	c	0	29	137	8	208	5.50	(2.44- 12.40)
CARPEN	502	c	0	9	51	8	208	4.59	(1.69- 12.48)
CARPEN	503	c	0	25	48	8	208	13.54	(5.75- 31.87)
CARPEN	504	c	0	23	39	8	208	15.33	(6.40- 36.75)
Subtotal CARPEN								8.72	(5.62- 13.52)
*CEDERL	528	m	1	3	-	7	-	1.10	(0.28- 4.25)
*CEDERL	529	m	1	12	-	7	-	6.10	(2.41- 15.46)
Subtotal CEDERL								3.54	(1.64- 7.62)
CHOI	533	m	0	4	19	13	95	1.54	(0.45- 5.23)
CHOI	534	m	0	4	23	13	95	1.27	(0.38- 4.26)
CHOI	535	m	0	5	30	13	95	1.22	(0.40- 3.70)
CHOI	536	m	0	25	64	13	95	2.85	(1.36- 5.99)
CHOI	550	f	0	2	0	76	164	10.75~	(0.51- 226.67)
CHOI	551	f	0	3	2	76	164	3.24	(0.53- 19.77)
Subtotal CHOI								2.04	(1.27- 3.29)
*CHYOU	501	m	1	5	-	8	-	2.80	(0.90- 8.60)
*CHYOU	502	m	1	21	-	8	-	3.90	(1.70- 8.80)
Subtotal CHYOU								3.48	(1.79- 6.76)
*CPSI	807	m	1	15	-	60	-	1.28	(0.73- 2.25)
*CPSI	808	m	1	32	-	60	-	5.15	(3.35- 7.91)
*CPSI	809	m	1	49	-	60	-	8.09	(5.55- 11.80)
*CPSI	810	m	1	37	-	60	-	14.74	(9.78- 22.20)
Subtotal CPSI								6.51	(5.25- 8.07)
*CPSII	652	m	1	256	-	81	-	3.83	(2.98- 4.92)
*CPSII	653	m	1	164	-	81	-	8.61	(6.60- 11.24)
*CPSII	654	m	1	186	-	81	-	11.43	(8.81- 14.84)
*CPSII	655	m	1	178	-	81	-	18.61	(14.31- 24.20)
*CPSII	656	m	1	188	-	81	-	28.07	(21.63- 36.43)
*CPSII	657	m	1	97	-	81	-	38.76	(28.85- 52.07)
*CPSII	633	f	1	50	-	174	-	1.74	(1.27- 2.39)
*CPSII	634	f	1	28	-	174	-	3.86	(2.59- 5.75)
*CPSII	635	f	1	37	-	174	-	4.91	(3.45- 7.01)
*CPSII	636	f	1	56	-	174	-	10.55	(7.81- 14.26)
*CPSII	637	f	1	91	-	174	-	17.02	(13.21- 21.93)
Subtotal CPSII								10.30	(9.46- 11.22)
DAMBER	523	m	1	42	-	42	-	2.60	(1.70- 4.50)
DAMBER	524	m	1	-	-	42	-	4.30	(2.30- 8.10)
DAMBER	525	m	1	-	-	42	-	7.70	(4.50- 13.50)
Subtotal DAMBER								4.22	(3.08- 5.78)

Table 1J1 - 5

IESLC - Meta-analysis of Ex Smoking by Years quit (vs never), Overview
All LC types, Any Product (or Cigarettes if Any not available)
Least adjusted

REF	NRR	SEX	AD	Number Exposed Case	Cont	Non-exposed Case	Cont	RR	95.00%CI
DARBY	501	m	0	139	767	3	384	23.20	(7.34- 73.28)
DARBY	502	m	0	146	339	3	384	55.13	(17.41- 174.53)
DARBY	510	f	0	26	224	23	529	2.67	(1.49- 4.78)
DARBY	511	f	0	68	93	23	529	16.82	(9.98- 28.33)
Subtotal DARBY								9.92	(6.99- 14.09)
DEAN3	531	m	0	32	204	24	510	3.33	(1.92- 5.80)
DEAN3	532	m	0	15	67	24	510	4.76	(2.38- 9.52)
DEAN3	533	m	0	42	147	24	510	6.07	(3.56- 10.36)
DEAN3	542	f	0	2	114	41	1538	0.66	(0.16- 2.76)
DEAN3	543	f	0	1	38	41	1538	0.99	(0.13- 7.37)
DEAN3	544	f	0	4	110	41	1538	1.36	(0.48- 3.88)
Subtotal DEAN3								3.64	(2.68- 4.96)
DESTEF	515	m	0	17	36	27	163	2.85	(1.41- 5.78)
DESTEF	516	m	0	27	27	27	163	6.04	(3.09- 11.81)
DESTEF	517	m	0	64	45	27	163	8.59	(4.91- 15.00)
Subtotal DESTEF								5.74	(3.98- 8.28)
DOLL	529	m	0	8	23	7	61	3.03	(0.99- 9.31)
DOLL	530	m	0	6	26	7	61	2.01	(0.62- 6.56)
DOLL	531	m	0	56	75	7	61	6.51	(2.77- 15.30)
DOLL	542	f	0	1	2	40	59	0.74	(0.06- 8.41)
DOLL	543	f	0	9	6	40	59	2.21	(0.73- 6.70)
Subtotal DOLL								3.24	(1.95- 5.39)
*DOLL2	501	m	1	7	-	7	-	2.00	(0.70- 5.70)
*DOLL2	502	m	1	9	-	7	-	5.30	(1.97- 14.23)
*DOLL2	503	m	1	12	-	7	-	5.90	(2.32- 14.99)
*DOLL2	504	m	1	15	-	7	-	16.00	(6.52- 39.24)
Subtotal DOLL2								6.10	(3.77- 9.87)
DORGAN	501	m	0	134	255	13	140	5.66	(3.09- 10.37)
DORGAN	502	m	0	49	38	13	140	13.89	(6.84- 28.21)
DORGAN	503	m	0	59	51	13	140	12.46	(6.31- 24.61)
DORGAN	553	f	0	34	50	61	213	2.37	(1.41- 4.00)
DORGAN	554	f	0	49	27	61	213	6.34	(3.66- 10.98)
Subtotal DORGAN								5.93	(4.54- 7.76)
*DORN	657	m	0	16	58370	25	213858	2.34	(1.25- 4.39)
*DORN	658	m	0	12	23682	25	213858	4.33	(2.18- 8.63)
*DORN	659	m	0	32	34566	25	213858	7.92	(4.69- 13.36)
*DORN	660	m	0	34	22086	25	213858	13.17	(7.86- 22.07)
*DORN	680	m	0	34	51243	49	171211	2.32	(1.50- 3.59)
*DORN	681	m	0	29	20056	49	171211	5.05	(3.19- 7.99)
*DORN	682	m	0	41	24089	49	171211	5.95	(3.93- 9.00)
*DORN	683	m	0	14	6195	49	171211	7.90	(4.36- 14.29)
Subtotal DORN								5.24	(4.37- 6.27)
GAO	521	m	0	13	41	62	202	1.03	(0.52- 2.05)
GAO	522	m	0	24	27	62	202	2.90	(1.56- 5.38)
GAO	523	m	0	105	52	62	202	6.58	(4.25- 10.19)
GAO	541	f	0	16	14	435	605	1.59	(0.77- 3.29)
GAO	542	f	0	14	7	435	605	2.78	(1.11- 6.95)
GAO	543	f	0	37	9	435	605	5.72	(2.73- 11.97)
Subtotal GAO								3.35	(2.58- 4.34)
GAO2	509	m	0	8	25	13	56	1.38	(0.51- 3.74)
GAO2	510	m	0	7	9	13	56	3.35	(1.05- 10.66)
GAO2	511	m	0	16	18	13	56	3.83	(1.55- 9.46)
GAO2	512	m	0	21	26	13	56	3.48	(1.51- 8.01)
GAO2	513	m	0	31	26	13	56	5.14	(2.31- 11.40)
Subtotal GAO2								3.35	(2.23- 5.04)
GARCIA	515	c	0	10	37	8	80	2.70	(0.99- 7.41)
GARCIA	516	c	0	32	67	8	80	4.78	(2.06- 11.06)
GARCIA	517	c	0	43	36	8	80	11.94	(5.10- 27.97)
GARCIA	518	c	0	33	11	8	80	30.00	(11.07- 81.30)
Subtotal GARCIA								8.14	(5.16- 12.86)
GARSHI	515	m	0	125	343	41	363	3.23	(2.20- 4.73)
GARSHI	516	m	0	166	290	41	363	5.07	(3.48- 7.37)
Subtotal GARSHI								4.06	(3.11- 5.31)
GRAHAM	525	m	0	13	71	18	346	3.52	(1.65- 7.51)
GRAHAM	526	m	0	24	48	18	346	9.61	(4.86- 19.00)
GRAHAM	527	m	0	113	59	18	346	36.82	(20.84- 65.03)
Subtotal GRAHAM								13.55	(9.28- 19.79)
GURSEL	501	m	0	-	-	-	-	2.30	(1.01- 5.22)
*HAMMO2	501	m	1	20	-	5	-	3.45	(1.30- 9.14)
*HAMMO2	502	m	1	11	-	5	-	3.98	(1.39- 11.40)
*HAMMO2	503	m	1	59	-	5	-	10.99	(4.43- 27.26)

Table 1J1 - 5

IESLC - Meta-analysis of Ex Smoking by Years quit (vs never), Overview
All LC types, Any Product (or Cigarettes if Any not available)
Least adjusted

REF	NRR	SEX	AD	Number Case	Exposed Cont	Non-exposed Case	Cont	RR	95.00%CI
Subtotal HAMMO2								5.60	(3.19- 9.82)
*HIRAYA	507	m	1	-	-	-	-	1.38	(0.59- 3.21)
*HIRAYA	508	m	1	-	-	-	-	1.59	(0.66- 3.82)
*HIRAYA	509	m	1	-	-	-	-	2.03	(1.10- 3.75)
*HIRAYA	518	f	1	-	-	-	-	0.97	(0.03- 32.06)
*HIRAYA	519	f	1	-	-	-	-	3.29	(0.56- 19.50)
*HIRAYA	520	f	1	-	-	-	-	3.72	(1.12- 12.37)
Subtotal HIRAYA								1.92	(1.30- 2.85)
JAHN	501	m	0	29	146	18	138	1.52	(0.81- 2.87)
JAHN	502	m	0	64	130	18	138	3.77	(2.12- 6.71)
JAHN	503	m	0	59	63	18	138	7.18	(3.92- 13.16)
JAHN	504	m	0	77	46	18	138	12.83	(6.96- 23.67)
JAHN	505	m	0	60	9	18	138	51.11	(21.72- 120.26)
JAHN	506	m	0	166	8	18	138	159.08	(67.12- 377.03)
JAHN	731	f	2	-	-	-	-	0.30	(0.06- 1.53)
Subtotal JAHN								7.91	(6.06- 10.34)
JAIN	567	m	0	52	113	12	85	3.26	(1.64- 6.48)
JAIN	568	m	0	74	46	12	85	11.39	(5.62- 23.12)
JAIN	531	f	0	19	61	52	214	1.28	(0.71- 2.33)
JAIN	532	f	0	66	36	52	214	7.54	(4.55- 12.52)
Subtotal JAIN								4.36	(3.22- 5.92)
JEDRYC	611	m	0	73	138	49	219	2.36	(1.55- 3.60)
JEDRYC	612	m	0	64	58	49	219	4.93	(3.08- 7.90)
Subtotal JEDRYC								3.27	(2.39- 4.48)
JOLY	566	m	0	63	149	12	218	7.68	(4.00- 14.74)
JOLY	567	m	0	38	36	12	218	19.18	(9.16- 40.14)
JOLY	553	f	0	15	19	52	283	4.30	(2.05- 8.99)
JOLY	554	f	0	19	8	52	283	12.93	(5.38- 31.08)
Subtotal JOLY								9.16	(6.33- 13.26)
*KAISE2	646	m	1	6	-	14	-	1.94	(0.70- 5.40)
*KAISE2	647	m	1	8	-	14	-	3.14	(1.26- 7.82)
*KAISE2	648	m	1	12	-	14	-	8.26	(3.73- 18.28)
*KAISE2	566	f	1	4	-	11	-	6.29	(1.78- 22.20)
*KAISE2	567	f	1	4	-	11	-	4.37	(1.30- 14.72)
*KAISE2	568	f	1	6	-	11	-	7.95	(2.89- 21.86)
Subtotal KAISE2								4.86	(3.24- 7.31)
KHUDER	511	m	0	63	213	23	309	3.97	(2.39- 6.61)
KHUDER	512	m	0	63	133	23	309	6.36	(3.79- 10.69)
KHUDER	513	m	0	88	123	23	309	9.61	(5.80- 15.92)
Subtotal KHUDER								6.25	(4.66- 8.40)
LAUSSM	501	m	0	29	15	85	226	5.14	(2.63- 10.06)
LUBIN	585	m	0	17	73	9	72	1.86	(0.78- 4.45)
LUBIN	586	m	0	20	48	9	72	3.33	(1.40- 7.94)
LUBIN	587	m	0	33	18	9	72	14.67	(5.96- 36.07)
Subtotal LUBIN								4.38	(2.64- 7.28)
LUBIN2	1069	m	0	109	715	190	2616	2.10	(1.64- 2.69)
LUBIN2	1070	m	0	106	413	190	2616	3.53	(2.73- 4.58)
LUBIN2	1071	m	0	130	478	190	2616	3.74	(2.94- 4.78)
LUBIN2	1072	m	0	270	693	190	2616	5.36	(4.38- 6.58)
LUBIN2	1073	m	0	466	822	190	2616	7.81	(6.48- 9.40)
LUBIN2	1074	m	0	866	1047	190	2616	11.39	(9.58- 13.53)
LUBIN2	1108	f	0	4	20	336	1188	0.71	(0.24- 2.08)
LUBIN2	1109	f	0	4	9	336	1188	1.57	(0.48- 5.13)
LUBIN2	1110	f	0	3	7	336	1188	1.52	(0.39- 5.89)
LUBIN2	1111	f	0	10	26	336	1188	1.36	(0.65- 2.85)
LUBIN2	1112	f	0	30	40	336	1188	2.65	(1.63- 4.32)
LUBIN2	1113	f	0	60	55	336	1188	3.86	(2.62- 5.67)
Subtotal LUBIN2								5.29	(4.87- 5.74)
MATOS	581	m	0	27	101	11	110	2.67	(1.26- 5.67)
MATOS	582	m	0	21	27	11	110	7.78	(3.35- 18.06)
MATOS	583	m	0	28	23	11	110	12.17	(5.31- 27.91)
Subtotal MATOS								5.95	(3.74- 9.47)
PEZZO2	501	m	0	43	161	6	117	5.21	(2.15- 12.64)
PEZZO2	502	m	0	85	110	6	117	15.07	(6.33- 35.89)
Subtotal PEZZO2								8.96	(4.82- 16.66)
PEZZOT	501	m	0	20	106	4	116	5.47	(1.81- 16.53)
PEZZOT	502	m	0	46	82	4	116	16.27	(5.64- 46.96)
Subtotal PEZZOT								9.65	(4.49- 20.74)
SOBUE	717	m	0	17	40	29	126	1.85	(0.92- 3.71)
SOBUE	718	m	0	15	23	29	126	2.83	(1.32- 6.09)
SOBUE	719	m	0	24	31	29	126	3.36	(1.72- 6.56)

Table 1J1 - 5

IESLC - Meta-analysis of Ex Smoking by Years quit (vs never), Overview
All LC types, Any Product (or Cigarettes if Any not available)
Least adjusted

REF	NRR	SEX	AD	Number Exposed Case	Number Exposed Cont	Non-exposed Case	Non-exposed Cont	RR	95.00%CI
SOBUE	720	m	0	35	50	29	126	3.04	(1.68- 5.49)
SOBUE	721	m	0	67	92	29	126	3.16	(1.90- 5.28)
SOBUE	722	m	0	128	116	29	126	4.79	(2.98- 7.71)
Subtotal SOBUE								3.27	(2.57- 4.16)
*SPEIZE	501	f	0	28	214271	58	776300	1.75	(1.11- 2.75)
*SPEIZE	502	f	0	17	93933	58	776300	2.42	(1.41- 4.16)
*SPEIZE	503	f	0	41	95585	58	776300	5.74	(3.85- 8.56)
*SPEIZE	504	f	0	34	63060	58	776300	7.22	(4.73- 11.02)
*SPEIZE	505	f	0	24	55232	58	776300	5.82	(3.61- 9.36)
Subtotal SPEIZE								4.24	(3.46- 5.19)
SUZUK2	508	c	0	9	22	11	53	1.97	(0.72- 5.42)
SUZUK2	509	c	0	10	8	11	53	6.02	(1.94- 18.72)
SUZUK2	510	c	0	15	10	11	53	7.23	(2.58- 20.25)
Subtotal SUZUK2								4.28	(2.33- 7.87)
SVENSS	551	f	0	14	24	38	120	1.84	(0.87- 3.91)
SVENSS	552	f	0	16	13	38	120	3.89	(1.72- 8.80)
Subtotal SVENSS								2.60	(1.49- 4.52)
*TVERDA	501	m	2	4	-	4	-	1.34	(0.34- 5.37)
*TVERDA	502	m	2	5	-	4	-	2.83	(0.76- 10.53)
*TVERDA	503	m	2	2	-	4	-	2.77	(0.51- 15.15)
Subtotal TVERDA								2.15	(0.94- 4.93)
WAKAI	522	m	0	7	47	10	65	0.97	(0.34- 2.73)
WAKAI	523	m	0	27	44	10	65	3.99	(1.76- 9.06)
WAKAI	524	m	0	19	48	10	65	2.57	(1.10- 6.03)
Subtotal WAKAI								2.40	(1.44- 4.02)
WANG2	510	c	0	5	11	11	43	1.78	(0.51- 6.19)
WANG2	511	c	0	6	10	11	43	2.35	(0.70- 7.86)
Subtotal WANG2								2.05	(0.86- 4.89)
WYNDE3	535	m	0	5	55	9	88	0.89	(0.28- 2.79)
WYNDE3	536	m	0	11	31	9	88	3.47	(1.31- 9.17)
WYNDE3	537	m	0	11	17	9	88	6.33	(2.28- 17.59)
WYNDE3	538	m	0	21	22	9	88	9.33	(3.76- 23.19)
WYNDE3	587	f	0	1	3	20	76	1.27	(0.12- 12.84)
Subtotal WYNDE3								3.95	(2.42- 6.44)
WYNDE6	501	m	0	21	161	64	918	1.87	(1.11- 3.15)
WYNDE6	502	m	0	55	212	64	918	3.72	(2.52- 5.50)
WYNDE6	503	m	0	159	373	64	918	6.11	(4.47- 8.37)
WYNDE6	504	m	0	98	194	64	918	7.25	(5.10- 10.29)
WYNDE6	505	m	0	201	166	64	918	17.37	(12.53- 24.07)
WYNDE6	522	f	0	10	31	125	991	2.56	(1.22- 5.34)
WYNDE6	523	f	0	16	77	125	991	1.65	(0.93- 2.91)
WYNDE6	524	f	0	36	132	125	991	2.16	(1.43- 3.27)
WYNDE6	525	f	0	51	84	125	991	4.81	(3.24- 7.14)
WYNDE6	526	f	0	82	70	125	991	9.29	(6.42- 13.43)
Subtotal WYNDE6								5.50	(4.84- 6.24)
Partial Totals				10298	776661	11211	5479920		

*prospective study ~ With 0.5 adjustment for zero

Table 1J1 - 5

IESLC - Meta-analysis of Ex Smoking by Years quit (vs never), Overview
All LC types, Any Product (or Cigarettes if Any not available)
Least adjusted

REF	NRR	SEX	AD	Ys	Ws	Qs	Ps
ALDERS	507	m	1	1.16	8.16	2.61	0.0009
ALDERS	508	m	1	1.46	7.77	0.56	0.0000
ALDERS	509	m	1	2.90	9.90	13.51	0.0000
ALDERS	518	f	1	0.24	14.21	31.51	0.3676
ALDERS	519	f	1	1.08	18.83	7.87	0.0000
ALDERS	520	f	1	2.25	27.43	7.35	0.0000
Subtotal ALDERS				1.56	86.30	63.41	
ARMADA	515	m	0	1.63	5.86	0.06	0.0001
ARMADA	516	m	0	2.75	5.75	5.96	0.0000
Subtotal ARMADA				2.18	11.61	6.01	
AUVINE	530	c	0	1.64	27.22	0.19	0.0000
BARBON	525	m	0	0.78	7.44	6.75	0.0343
BARBON	526	m	0	1.93	9.48	0.38	0.0000
BARBON	527	m	0	2.19	13.55	2.91	0.0000
BARBON	528	m	0	2.62	7.57	5.96	0.0000
Subtotal BARBON				1.93	38.05	16.00	
BECHER	501	m	0	1.39	2.34	0.27	0.0341
BECHER	502	m	0	2.20	2.24	0.49	0.0010
BECHER	503	m	0	2.71	1.87	1.79	0.0002
BECHER	511	f	0	-0.65	0.82	4.65	0.5537
BECHER	512	f	0	0.73	1.22	1.21	0.4184
BECHER	513	f	0	1.24	1.05	0.25	0.2027
Subtotal BECHER				1.56	9.54	8.67	
*BENSHL	508	m	2	0.00	2.98	8.90	1.0000
*BENSHL	509	m	2	0.95	6.64	4.00	0.0142
*BENSHL	510	m	2	1.41	7.88	0.82	0.0001
*BENSHL	511	m	2	2.16	6.37	1.19	0.0000
Subtotal BENSHL				1.31	23.87	14.91	
BROSS	515	m	0	0.89	14.68	10.32	0.0006
BROSS	516	m	0	2.42	18.85	9.11	0.0000
Subtotal BROSS				1.75	33.54	19.43	
BROWN3	501	f	0	0.77	78.00	72.35	0.0000
CARPEN	501	c	0	1.71	5.83	0.00	0.0000
CARPEN	502	c	0	1.52	3.84	0.16	0.0028
CARPEN	503	c	0	2.61	5.25	4.04	0.0000
CARPEN	504	c	0	2.73	5.03	5.05	0.0000
Subtotal CARPEN				2.17	19.94	9.25	
*CEDERL	528	m	1	0.10	2.08	5.54	0.8907
*CEDERL	529	m	1	1.81	4.45	0.03	0.0001
Subtotal CEDERL				1.26	6.53	5.57	
CHOI	533	m	0	0.43	2.56	4.32	0.4904
CHOI	534	m	0	0.24	2.63	5.82	0.6977
CHOI	535	m	0	0.20	3.12	7.31	0.7277
CHOI	536	m	0	1.05	6.99	3.22	0.0056
CHOI	550	f	0	2.38	0.41	0.17	0.1268
CHOI	551	f	0	1.17	1.17	0.36	0.2033
Subtotal CHOI				0.71	16.88	21.20	
*CHYOU	501	m	1	1.03	3.02	1.47	0.0738
*CHYOU	502	m	1	1.36	5.68	0.77	0.0012
Subtotal CHYOU				1.25	8.70	2.24	
*CPSI	807	m	1	0.25	12.13	26.61	0.3900
*CPSI	808	m	1	1.64	20.82	0.17	0.0000
*CPSI	809	m	1	2.09	27.01	3.55	0.0000
*CPSI	810	m	1	2.69	22.87	21.18	0.0000
Subtotal CPSI				1.87	82.82	51.50	
*CPSII	652	m	1	1.34	61.12	9.07	0.0000
*CPSII	653	m	1	2.15	54.21	9.78	0.0000
*CPSII	654	m	1	2.44	56.51	28.33	0.0000
*CPSII	655	m	1	2.92	55.67	79.56	0.0000
*CPSII	656	m	1	3.33	56.54	145.93	0.0000
*CPSII	657	m	1	3.66	44.07	164.03	0.0000
*CPSII	633	f	1	0.55	38.44	53.00	0.0006
*CPSII	634	f	1	1.35	24.16	3.44	0.0000
*CPSII	635	f	1	1.59	30.57	0.57	0.0000
*CPSII	636	f	1	2.36	42.39	16.72	0.0000
*CPSII	637	f	1	2.83	59.81	73.19	0.0000
Subtotal CPSII				2.33	523.48	583.63	
DAMBER	523	m	1	0.96	16.22	9.68	0.0001
DAMBER	524	m	1	1.46	9.69	0.70	0.0000
DAMBER	525	m	1	2.04	12.73	1.25	0.0000
Subtotal DAMBER				1.44	38.64	11.63	

Table 1J1 - 5

IESLC - Meta-analysis of Ex Smoking by Years quit (vs never), Overview
All LC types, Any Product (or Cigarettes if Any not available)
Least adjusted

REF	NRR	SEX	AD	Ys	Ws	Qs	Ps
DARBY	501	m	0	3.14	2.90	5.82	0.0000
DARBY	502	m	0	4.01	2.89	15.06	0.0000
DARBY	510	f	0	0.98	11.33	6.31	0.0010
DARBY	511	f	0	2.82	14.12	16.91	0.0000
Subtotal DARBY				2.29	31.24	44.09	
DEAN3	531	m	0	1.20	12.53	3.44	0.0000
DEAN3	532	m	0	1.56	7.99	0.23	0.0000
DEAN3	533	m	0	1.80	13.47	0.08	0.0000
DEAN3	542	f	0	-0.42	1.87	8.63	0.5669
DEAN3	543	f	0	-0.01	0.95	2.88	0.9899
DEAN3	544	f	0	0.31	3.52	7.07	0.5602
Subtotal DEAN3				1.29	40.33	22.34	
DESTEF	515	m	0	1.05	7.71	3.57	0.0036
DESTEF	516	m	0	1.80	8.53	0.04	0.0000
DESTEF	517	m	0	2.15	12.34	2.20	0.0000
Subtotal DESTEF				1.75	28.58	5.81	
DOLL	529	m	0	1.11	3.05	1.17	0.0527
DOLL	530	m	0	0.70	2.74	2.91	0.2471
DOLL	531	m	0	1.87	5.25	0.11	0.0000
DOLL	542	f	0	-0.30	0.65	2.68	0.8063
DOLL	543	f	0	0.79	3.13	2.73	0.1602
Subtotal DOLL				1.18	14.82	9.60	
*DOLL2	501	m	1	0.69	3.49	3.74	0.1951
*DOLL2	502	m	1	1.67	3.93	0.01	0.0009
*DOLL2	503	m	1	1.77	4.41	0.01	0.0002
*DOLL2	504	m	1	2.77	4.77	5.20	0.0000
Subtotal DOLL2				1.81	16.61	8.97	
DORGAN	501	m	0	1.73	10.48	0.00	0.0000
DORGAN	502	m	0	2.63	7.65	6.23	0.0000
DORGAN	503	m	0	2.52	8.29	5.23	0.0000
DORGAN	553	f	0	0.86	14.18	10.57	0.0011
DORGAN	554	f	0	1.85	12.73	0.18	0.0000
Subtotal DORGAN				1.78	53.33	22.21	
*DORN	657	m	0	0.85	9.76	7.49	0.0078
*DORN	658	m	0	1.47	8.11	0.55	0.0000
*DORN	659	m	0	2.07	14.04	1.63	0.0000
*DORN	660	m	0	2.58	14.42	10.41	0.0000
*DORN	680	m	0	0.84	20.08	15.81	0.0002
*DORN	681	m	0	1.62	18.24	0.21	0.0000
*DORN	682	m	0	1.78	22.35	0.07	0.0000
*DORN	683	m	0	2.07	10.91	1.25	0.0000
Subtotal DORN				1.66	117.90	37.42	
GAO	521	m	0	0.03	8.17	23.49	0.9260
GAO	522	m	0	1.06	10.02	4.43	0.0008
GAO	523	m	0	1.88	20.07	0.49	0.0000
GAO	541	f	0	0.46	7.25	11.60	0.2120
GAO	542	f	0	1.02	4.58	2.28	0.0285
GAO	543	f	0	1.74	7.04	0.00	0.0000
Subtotal GAO				1.21	57.13	42.29	
GAO2	509	m	0	0.32	3.85	7.62	0.5289
GAO2	510	m	0	1.21	2.87	0.77	0.0406
GAO2	511	m	0	1.34	4.70	0.70	0.0036
GAO2	512	m	0	1.25	5.53	1.28	0.0034
GAO2	513	m	0	1.64	6.04	0.05	0.0001
Subtotal GAO2				1.21	22.99	10.43	
GARCIA	515	c	0	0.99	3.78	2.04	0.0532
GARCIA	516	c	0	1.56	5.44	0.15	0.0003
GARCIA	517	c	0	2.48	5.30	3.00	0.0000
GARCIA	518	c	0	3.40	3.87	10.82	0.0000
Subtotal GARCIA				2.10	18.39	16.00	
GARSHI	515	m	0	1.17	26.27	8.14	0.0000
GARSHI	516	m	0	1.62	27.31	0.30	0.0000
Subtotal GARSHI				1.40	53.58	8.45	
GRAHAM	525	m	0	1.26	6.69	1.48	0.0011
GRAHAM	526	m	0	2.26	8.27	2.36	0.0000
GRAHAM	527	m	0	3.61	11.87	41.85	0.0000
Subtotal GRAHAM				2.61	26.83	45.70	
GURSEL	501	m	0	0.83	5.70	4.56	0.0468
*HAMMO2	501	m	1	1.24	4.04	0.97	0.0128
*HAMMO2	502	m	1	1.38	3.47	0.42	0.0101
*HAMMO2	503	m	1	2.40	4.65	2.08	0.0000

Table 1J1 - 5

IESLC - Meta-analysis of Ex Smoking by Years quit (vs never), Overview
All LC types, Any Product (or Cigarettes if Any not available)
Least adjusted

REF	NRR	SEX	AD	Ys	Ws	Qs	Ps
Subtotal HAMMO2				1.72	12.16	3.47	
*HIRAYA	507	m	1	0.32	5.36	10.59	0.4561
*HIRAYA	508	m	1	0.46	4.98	7.97	0.3005
*HIRAYA	509	m	1	0.71	10.22	10.63	0.0236
*HIRAYA	518	f	1	-0.03	0.32	0.98	0.9863
*HIRAYA	519	f	1	1.19	1.22	0.35	0.1885
*HIRAYA	520	f	1	1.31	2.66	0.46	0.0320
Subtotal HIRAYA				0.65	24.75	30.97	
JAHN	501	m	0	0.42	9.60	16.42	0.1925
JAHN	502	m	0	1.33	11.61	1.86	0.0000
JAHN	503	m	0	1.97	10.46	0.62	0.0000
JAHN	504	m	0	2.55	10.25	6.96	0.0000
JAHN	505	m	0	3.93	5.25	25.53	0.0000
JAHN	506	m	0	5.07	5.16	57.60	0.0000
JAHN	731	f	2	-1.20	1.46	12.59	0.1451
Subtotal JAHN				2.07	53.80	121.58	
JAIN	567	m	0	1.18	8.12	2.43	0.0008
JAIN	568	m	0	2.43	7.67	3.81	0.0000
JAIN	531	f	0	0.25	10.76	23.57	0.4154
JAIN	532	f	0	2.02	14.96	1.28	0.0000
Subtotal JAIN				1.47	41.51	31.09	
JEDRYC	611	m	0	0.86	21.78	16.40	0.0001
JEDRYC	612	m	0	1.60	17.29	0.30	0.0000
Subtotal JEDRYC				1.19	39.07	16.70	
JOLY	566	m	0	2.04	9.05	0.87	0.0000
JOLY	567	m	0	2.95	7.04	10.58	0.0000
JOLY	553	f	0	1.46	7.04	0.51	0.0001
JOLY	554	f	0	2.56	4.99	3.45	0.0000
Subtotal JOLY				2.21	28.12	15.41	
*KAISE2	646	m	1	0.66	3.68	4.18	0.2036
*KAISE2	647	m	1	1.14	4.61	1.57	0.0140
*KAISE2	648	m	1	2.11	6.08	0.89	0.0000
*KAISE2	566	f	1	1.84	2.41	0.03	0.0043
*KAISE2	567	f	1	1.47	2.61	0.17	0.0172
*KAISE2	568	f	1	2.07	3.75	0.45	0.0001
Subtotal KAISE2				1.58	23.15	7.29	
KHUDER	511	m	0	1.38	14.86	1.80	0.0000
KHUDER	512	m	0	1.85	14.26	0.21	0.0000
KHUDER	513	m	0	2.26	15.10	4.32	0.0000
Subtotal KHUDER				1.83	44.23	6.34	
LAUSSM	501	m	0	1.64	8.52	0.07	0.0000
LUBIN	585	m	0	0.62	5.06	6.19	0.1615
LUBIN	586	m	0	1.20	5.11	1.40	0.0065
LUBIN	587	m	0	2.69	4.74	4.35	0.0000
Subtotal LUBIN				1.48	14.91	11.94	
LUBIN2	1069	m	0	0.74	61.66	60.03	0.0000
LUBIN2	1070	m	0	1.26	57.14	12.40	0.0000
LUBIN2	1071	m	0	1.32	64.81	10.78	0.0000
LUBIN2	1072	m	0	1.68	92.66	0.22	0.0000
LUBIN2	1073	m	0	2.05	111.01	11.85	0.0000
LUBIN2	1074	m	0	2.43	128.94	63.98	0.0000
LUBIN2	1108	f	0	-0.35	3.29	14.17	0.5296
LUBIN2	1109	f	0	0.45	2.74	4.46	0.4543
LUBIN2	1110	f	0	0.42	2.08	3.59	0.5486
LUBIN2	1111	f	0	0.31	7.03	14.19	0.4151
LUBIN2	1112	f	0	0.98	16.09	9.12	0.0001
LUBIN2	1113	f	0	1.35	25.86	3.70	0.0000
Subtotal LUBIN2				1.66	573.32	208.48	
MATOS	581	m	0	0.98	6.81	3.78	0.0103
MATOS	582	m	0	2.05	5.42	0.57	0.0000
MATOS	583	m	0	2.50	5.58	3.32	0.0000
Subtotal MATOS				1.78	17.80	7.66	
PEZZO2	501	m	0	1.65	4.89	0.03	0.0003
PEZZO2	502	m	0	2.71	5.10	4.94	0.0000
Subtotal PEZZO2				2.19	9.99	4.97	
PEZZOT	501	m	0	1.70	3.14	0.00	0.0026
PEZZOT	502	m	0	2.79	3.42	3.85	0.0000
Subtotal PEZZOT				2.27	6.56	3.85	
SOBUE	717	m	0	0.61	7.92	9.85	0.0843
SOBUE	718	m	0	1.04	6.55	3.09	0.0077
SOBUE	719	m	0	1.21	8.60	2.28	0.0004

Table 1J1 - 5

IESLC - Meta-analysis of Ex Smoking by Years quit (vs never), Overview
All LC types, Any Product (or Cigarettes if Any not available)
Least adjusted

REF	NRR	SEX	AD	Ys	Ws	Qs	Ps
SOBUE	720	m	0	1.11	10.99	4.17	0.0002
SOBUE	721	m	0	1.15	14.66	4.87	0.0000
SOBUE	722	m	0	1.57	16.99	0.44	0.0000
Subtotal SOBUE				1.18	65.71	24.69	
*SPEIZE	501	f	0	0.56	18.89	25.81	0.0151
*SPEIZE	502	f	0	0.88	13.15	9.35	0.0013
*SPEIZE	503	f	0	1.75	24.03	0.01	0.0000
*SPEIZE	504	f	0	1.98	21.44	1.32	0.0000
*SPEIZE	505	f	0	1.76	16.98	0.02	0.0000
Subtotal SPEIZE				1.44	94.49	36.52	
SUZUK2	508	c	0	0.68	3.75	4.14	0.1886
SUZUK2	509	c	0	1.80	2.99	0.01	0.0019
SUZUK2	510	c	0	1.98	3.62	0.23	0.0002
Subtotal SUZUK2				1.45	10.36	4.38	
SVENSS	551	f	0	0.61	6.77	8.45	0.1120
SVENSS	552	f	0	1.36	5.74	0.79	0.0011
Subtotal SVENSS				0.95	12.51	9.24	
*TVERDA	501	m	2	0.29	2.02	4.16	0.6776
*TVERDA	502	m	2	1.04	2.22	1.05	0.1208
*TVERDA	503	m	2	1.02	1.34	0.67	0.2389
Subtotal TVERDA				0.76	5.58	5.88	
WAKAI	522	m	0	-0.03	3.58	11.09	0.9511
WAKAI	523	m	0	1.38	5.71	0.68	0.0009
WAKAI	524	m	0	0.95	5.30	3.25	0.0297
Subtotal WAKAI				0.88	14.58	15.02	
WANG2	510	c	0	0.57	2.47	3.28	0.3664
WANG2	511	c	0	0.85	2.63	2.01	0.1672
Subtotal WANG2				0.72	5.09	5.30	
WYNDE3	535	m	0	-0.12	2.94	10.00	0.8401
WYNDE3	536	m	0	1.24	4.07	0.95	0.0121
WYNDE3	537	m	0	1.84	3.67	0.05	0.0004
WYNDE3	538	m	0	2.23	4.64	1.19	0.0000
WYNDE3	587	f	0	0.24	0.72	1.59	0.8415
Subtotal WYNDE3				1.37	16.04	13.79	
WYNDE6	501	m	0	0.63	14.18	17.21	0.0183
WYNDE6	502	m	0	1.31	25.24	4.33	0.0000
WYNDE6	503	m	0	1.81	38.93	0.26	0.0000
WYNDE6	504	m	0	1.98	31.18	1.98	0.0000
WYNDE6	505	m	0	2.85	36.08	45.79	0.0000
WYNDE6	522	f	0	0.94	7.08	4.41	0.0125
WYNDE6	523	f	0	0.50	11.83	17.88	0.0859
WYNDE6	524	f	0	0.77	22.54	20.65	0.0003
WYNDE6	525	f	0	1.57	24.68	0.61	0.0000
WYNDE6	526	f	0	2.23	28.18	7.06	0.0000
Subtotal WYNDE6				1.70	239.93	120.17	

N	211
NS	51

Table 1J1 - 6

IESLC - Meta-analysis of Ex Smoking by Years quit (vs never), Overview
All LC types, Any Product (or Cigarettes if Any not available)
Least adjusted

	Sex			
	combined	male	female	Total
N	14	141	56	211
NS	5	43	20	68

In this overview table, other than the "N" rows, entries in the "absent" and "Total" columns may be invalid and should be ignored

	Years quit vs never (lower focus)				
	absent	8+k12	4-11k7	1-6k3	Total
N	82	53	33	43	211
NS	37	39	25	33	134
Wt	1130.23	596.24	493.34	634.93	2854.74
Het Chi	1082.06	202.20	102.52	165.08	1868.63
Het df	81	52	32	42	210
Het P	***	***	***	***	***
Fixed RR	5.08	3.62	6.06	9.67	5.63
RRl	4.79	3.34	5.55	8.95	5.43
RRu	5.38	3.92	6.62	10.45	5.84
P	+++	+++	+++	+++	+++
Random RR	4.53	2.94	5.07	8.55	4.67
RRl	3.62	2.46	4.22	7.18	4.16
RRu	5.68	3.51	6.08	10.18	5.25
P	+++	+++	+++	+++	+++

	Years quit vs never (higher focus)				
	absent	13+k20	4-19k12	1-11k3	Total
N	106	24	22	59	211
NS	48	20	18	43	129
Wt	1290.77	420.82	385.95	757.21	2854.74
Het Chi	1095.89	59.93	75.39	227.14	1868.63
Het df	105	23	21	58	210
Het P	***	***	***	***	***
Fixed RR	5.54	2.73	4.97	9.22	5.63
RRl	5.24	2.48	4.50	8.59	5.43
RRu	5.85	3.01	5.50	9.90	5.84
P	+++	+++	+++	+++	+++
Random RR	3.94	2.65	4.34	8.31	4.67
RRl	3.26	2.22	3.50	7.11	4.16
RRu	4.77	3.17	5.37	9.72	5.25
P	+++	+++	+++	+++	+++

Table 1J1 - 6

IESLC - Meta-analysis of Ex Smoking by Years quit (vs never), Overview
All LC types, Any Product (or Cigarettes if Any not available)
Least adjusted

MALES

	Years quit vs never (lower focus) absent	8+k12	4-11k7	1-6k3	Total
N	55	34	23	29	141
NS	30	33	22	28	113
Wt	756.24	423.07	381.77	481.49	2042.57
Het Chi	765.43	121.35	75.27	105.36	1274.62
Het df	54	33	22	28	140
Het P	***	***	***	***	***
Fixed RR	5.49	4.24	6.56	10.50	6.27
RRl	5.11	3.85	5.93	9.60	6.00
RRu	5.89	4.66	7.25	11.48	6.54
P	+++	+++	+++	+++	+++
Random RR	4.97	3.45	5.29	9.35	5.22
RRl	3.75	2.81	4.28	7.70	4.55
RRu	6.60	4.23	6.55	11.37	6.00
P	+++	+++	+++	+++	+++

	Years quit vs never (higher focus) absent	13+k20	4-19k12	1-11k3	Total
N	70	17	16	38	141
NS	40	16	15	37	108
Wt	942.49	259.65	309.93	530.50	2042.57
Het Chi	837.43	27.59	30.51	123.91	1274.62
Het df	69	16	15	37	140
Het P	***	*	*	***	***
Fixed RR	5.85	3.25	5.64	10.39	6.27
RRl	5.49	2.88	5.05	9.54	6.00
RRu	6.23	3.67	6.31	11.31	6.54
P	+++	+++	+++	+++	+++
Random RR	4.54	2.99	5.16	9.45	5.22
RRl	3.59	2.49	4.28	7.93	4.55
RRu	5.74	3.60	6.22	11.26	6.00
P	+++	+++	+++	+++	+++

FEMALES

	Years quit vs never (lower focus) absent	8+k12	4-11k7	1-6k3	Total
N	22	16	8	10	56
NS	15	16	8	10	49
Wt	351.16	138.36	103.34	138.31	731.17
Het Chi	294.10	24.93	10.26	32.38	466.91
Het df	21	15	7	9	55
Het P	***	(*)	N.S.	***	***
Fixed RR	4.30	2.11	4.36	7.16	4.15
RRl	3.87	1.79	3.59	6.06	3.86
RRu	4.77	2.49	5.29	8.46	4.46
P	+++	+++	+++	+++	+++
Random RR	3.57	1.97	4.13	6.00	3.24
RRl	2.32	1.55	3.17	4.11	2.56
RRu	5.49	2.51	5.38	8.75	4.10
P	+++	+++	+++	+++	+++

Table 1J1 - 6

IESLC - Meta-analysis of Ex Smoking by Years quit (vs never), Overview
All LC types, Any Product (or Cigarettes if Any not available)
Least adjusted

FEMALES

		Years quit vs never (higher focus)				
		absent	13+k20	4-19k12	1-11k3	Total
	N	30	5	4	17	56
	NS	19	5	4	17	45
	Wt	302.82	149.90	66.88	211.57	731.17
Het	Chi	235.37	1.91	7.56	63.50	466.91
Het	df	29	4	3	16	55
Het	P	***	N.S.	(*)	***	***
Fixed	RR	4.77	1.93	2.60	6.76	4.15
	RRl	4.26	1.65	2.04	5.91	3.86
	RRu	5.33	2.27	3.30	7.73	4.46
	P	+++	+++	+++	+++	+++
Random	RR	2.65	1.93	2.44	5.89	3.24
	RRl	1.83	1.65	1.64	4.36	2.56
	RRu	3.84	2.27	3.63	7.96	4.10
	P	+++	+++	+++	+++	+++

Table 1J1 - 7

IESLC - Meta-analysis of Ex Smoking by Years quit (vs never), Overview
All LC types, Any Product (or Cigarettes if Any not available)
Excluded studies (and stage at which they were excluded)

1	AGUDO AKIBA AMANDU AMES AXELSS BEST BOUCHA BOUCOT BRESLO CHEN CHEN2 CHIAZZ DEAN2 DOSEME ENGELA FAN GENG GER GUO HAENSZ HEGMAN HOLE HU HU2 JUSSAW KATSOU KAUFMA KOO KOULUM KREUZE LETOUR LEVIN LIAW LIU3 LIU4 LIU5 MCCONN MIGRAN MRFITR NOTAN2 OSANN2 PERNU QIAO2 RACHTA RESTRE SADOWS SEGI2 STASZE TIZZAN VUTUC WATSON WU WUWILL WYNDE2 WYNDE8 XU YUAN ZHANG ZHENG ZHOU
2	BUFFLE HUMBLE PISANI PRESCO WYNDE7
3	MCDUFF SPITZ
4	HAMMON LUO WU2
5	BLOT1 CORREA GILLIS QIAO WIGLE
7	BOFFET
14	BENHAM

Table 1J1 - 8
Potentially overlapping studies

REF	REFGP	PRINC	.	OVERLAP/LINK
LUBIN2	LUBIN2	1		Lubin-combined
TVERDA	TVERDA	1		VEIERO/TVERDAL
BROSS	BYERS1	1		GRAHAM/BROSS/BYERS1
GRAHAM	BYERS1	1		GRAHAM/BROSS/BYERS1
CHYOU	CHYOU	1		GOODMA/CHYOU
BENSHL	TANG2	1		Subset of TANG2
WYNDE6	WYNDE6	1		WYNDE5/6/7/8
CPSI	CPSI	1		CPSI overall
JAHN	BOFFET	2		Subset of BOFFET
LUBIN	XIANGZ	2		LUBIN/XIANGZ/QIAO

Table 1J1 - 9
Most adjusted - insufficient data for meta-analysis

REF	NRR	SEX	AGEL	AGEH	RACE	YF	LC TYPE	LOC	START	ST	NLC	R	VB	P	H	AD	PRODUCT	exL	exH	S1	S2	DENOM	De
ARMADA	524	m	0	0	all	-	all	Eu:wst	1986	CC	325	n	bl	n	y	0	cig+/-ot	0.1	0.9	0	0	nev cigs	ot
AUVINE	533	c	0	0	all	-	all	Eu:Sca	1986	CC	517	n	bl	y	n	2	cig+/-ot	1.0	11	0	3	nev cigs	ot
BECHER	526	m	0	0	all	-	all	Eu:Ger	1985	CC	194	n	bl	n	y	0	all/unsp	1.0	1.0	0	0	nev any	ot
BECHER	528	f	0	0	all	-	all	Eu:Ger	1985	CC	194	n	bl	n	y	0	all/unsp	1.0	1.0	0	0	nev any	ot
BLOT1	501	m	0	0	all	-	all	NAmer	1970	CC	458	n	bl	y	n	0	cig+/-ot	10	999	1	0	nev cigs	or
BLOT1	502	m	0	0	all	-	all	NAmer	1970	CC	458	n	bl	y	n	0	cig+/-ot	1.0	9	0	3	nev cigs	or
BROWN3	504	f	0	0	wh	-	all	NAmer		CC	618		bl	y	n	2	all/unsp	1.0	14	0	0	nev any	ot
CORREA	538	c	0	0	all	-	all	NAmer	1979	CC	1359	n	bl	y	n	2	cig+/-ot	21	999	0	0	nev cigs	or
CORREA	539	c	0	0	all	-	all	NAmer	1979	CC	1359	n	bl	y	n	2	cig+/-ot	6	20	0	0	nev cigs	or
CORREA	540	c	0	0	all	-	all	NAmer	1979	CC	1359	n	bl	y	n	2	cig+/-ot	3	5	3	3	nev cigs	or
CORREA	545	c	0	0	all	-	all	NAmer	1979	CC	1359	n	bl	y	n	2	cig+/-ot	0.1	3	0	0	nev cigs	or
CPSI	718	f	0	0	wh	0	all	NAmer	1959	pr	5138	n	bl	n	n	1	cig only	25	29	0	0	nev cigs	or
CPSI	719	f	0	0	wh	0	all	NAmer	1959	pr	5138	n	bl	n	n	1	cig only	20	24	0	1	nev cigs	or
CPSI	720	f	0	0	wh	0	all	NAmer	1959	pr	5138	n	bl	n	n	1	cig only	15	19	0	0	nev cigs	or
CPSI	721	f	0	0	wh	0	all	NAmer	1959	pr	5138	n	bl	n	n	1	cig only	10	14	1	2	nev cigs	or
CPSI	722	f	0	0	wh	0	all	NAmer	1959	pr	5138	n	bl	n	n	1	cig only	5	9	2	0	nev cigs	or
CPSI	723	f	0	0	wh	0	all	NAmer	1959	pr	5138	n	bl	n	n	1	cig only	2	4	3	3	nev cigs	or
CPSI	935	f	0	0	wh	0	all	NAmer	1959	pr	5138	n	bl	n	n	1	cig only	0.1	1.9	0	0	nev cigs	or
DEAN3	631	m	0	0	all	-	all	Eu:UK	1969	CC	766	n	V	y	n	1	all/unsp	1.0	2	0	0	nev any	ot
DEAN3	626	f	0	0	all	-	all	Eu:UK	1969	CC	766	n	V	y	n	1	all/unsp	1.0	2	0	0	nev any	ot
DORGAN	504	m	0	0	wh	-	all	NAmer	1980	CC	2026	n	bl	y	y	0	cig+/-ot	0.1	1.0	0	0	nev any	ot
DORGAN	555	f	0	0	all	-	all	NAmer	1980	CC	2026	n	bl	y	y	0	cig+/-ot	0.1	1.0	0	0	nev any	ot
GAO2	527	m	0	0	all	-	all	As:Jap	1988	CC	282	n	bl	n	n	0	cig+/-ot	0.1	0.9	0	0	nev cigs	ot
GARCIA	519	c	0	0	all	-	all	NAmer	1992	CC	416	n	bl	n	y	0	cig+/-ot	0.1	0.9	0	0	nev any	ot
GARSHI	524	m	0	0	all	-	all	NAmer	1981	CC	1081	o	bl	y	n	1	all/unsp	1.0	4	3	3	nev any	ot
GURSEL	502	m	0	0	all	-	all	Eu:bal		CC	953		bl	*	n	0	all/unsp	1.0	10	0	3	nev any	ot
JAHN	732	f	0	0	all	-	all	Eu:Ger	1988	CC	1004	n	bl	n	n	2	cig+/-ot	0.1	20	0	0	nev any	ot
JAIN	596	m	0	0	all	-	all	NAmer	1981	CC	845	n	V	y	n	0	cig+/-ot	0.1	1.9	0	0	nev cigs	ot
JAIN	584	f	0	0	all	-	all	NAmer	1981	CC	845	n	V	y	n	0	cig+/-ot	0.1	1.9	0	0	nev cigs	ot
JEDRYC	613	m	0	0	all	-	all	Eu:est	1980	CC	1630	n	bl	y	n	0	cig+/-ot	1.0	4	3	3	nev any	ot
JOLY	568	m	0	0	all	-	all	SCAmer	1978	CC	826	n	bl	n	n	0	cig+/-ot	0.1	0.9	0	0	nev any	ot
JOLY	555	f	0	0	all	-	all	SCAmer	1978	CC	826	n	bl	n	n	0	cig+/-ot	0.1	0.9	0	0	nev any	ot
KAISE2	649	m	0	0	all	9	all	NAmer	1979	pr	318	n	bl	n	n	1	cig only	0.1	1.9	0	0	nev any	ot
KAISE2	569	f	0	0	all	9	all	NAmer	1979	pr	318	n	bl	n	n	1	cig only	0.1	1.9	0	0	nev any	ot
LAUSSM	504	m	0	0	all	-	all	Eu:Ger	1982	CC	432	n	bl	n	n	2	all/unsp	1.0	9	0	3	nev any	ot
LUBIN	588	m	0	0	all	-	all	As:Chi	1984	CC	427	m	ot	y	n	0	cig+/-ot	1.0	2	0	0	nev any	ot

Table 1J1 - 9

IESLC - Meta-analysis of Ex Smoking by Years quit (vs never), Overview
All LC types, Any Product (or Cigarettes if Any not available)
Most adjusted - insufficient data for meta-analysis

REF	NRR	SEX	AGEL	AGEH	RACE	YF	LC TYPE	LOC	START	ST	NLC	R	VB	P	H	AD	PRODUCT	exL	exH	S1	S2	DENOM	De
MATOS	703	m	0	0	all	-	all	SCAmer	1994	CC	200	n	bl	n	n	2	cig+/-ot	0.1	0.9	0	0	nev any	ot
PEZZO2	512	m	0	0	all	-	all	SCAmer	1992	CC	367	n	bl	n	y	0	cig+/-ot	0.1	0.9	0	0	nev cigs	ot
PEZZOT	597	m	0	0	all	-	all	SCAmer	1987	CC	215	n	bl	n	y	0	cig only	0.1	0.9	0	0	nev cigs	ot
SOBUE	778	m	0	0	all	-	all	As:Jap	1986	CC	1376	n	bl	n	y	0	cig+/-ot	0.1	0.9	0	0	nev cigs	ot
SPEIZE	538	f	0	0	all	0	all	NAmer	1976	pr	593	n	bl	n	y	2	cig+/-ot	15	999	0	1	nev cigs	st
SPEIZE	539	f	0	0	all	0	all	NAmer	1976	pr	593	n	bl	n	y	2	cig+/-ot	10	15	1	2	nev cigs	st
SPEIZE	540	f	0	0	all	0	all	NAmer	1976	pr	593	n	bl	n	y	2	cig+/-ot	5	10	2	0	nev cigs	st
SPEIZE	541	f	0	0	all	0	all	NAmer	1976	pr	593	n	bl	n	y	2	cig+/-ot	2	5	3	3	nev cigs	st
SPEIZE	542	f	0	0	all	0	all	NAmer	1976	pr	593	n	bl	n	y	2	cig+/-ot	0.1	1.9	0	0	nev cigs	st
SVENSS	591	f	0	0	all	-	all	Eu:Sca	1983	CC	210	n	bl	n	n	0	all/unsp	1.0	2	0	0	nev any	ot
WAKAI	611	m	0	0	all	-	all	As:Jap	1988	CC	333	n	bl	n	y	2	cig+/-ot	1.0	4	3	3	nev any	ot
WYNDE3	539	m	0	0	all	-	all	NAmer	1966	CC	350	n	bl	n	y	0	all/unsp	0.1	0.9	0	0	nev any	ot
WYNDE3	588	f	0	0	all	-	all	NAmer	1966	CC	350	n	bl	n	y	0	cig+/-ot	1.0	9	0	3	nev any	ot
WYNDE6	506	m	0	0	all	-	all	NAmer	1969	CC	4423	n	bl	n	y	0	cig only	0.1	0.9	0	0	nev any	ot
WYNDE6	527	f	0	0	all	-	all	NAmer	1969	CC	4423	n	bl	n	y	0	cig only	0.1	0.9	0	0	nev any	ot

REF	NRR	RR	SIG	RRDATA comment
ARMADA	524	*	gap	0
AUVINE	533	*	gap	0
BECHER	526	*	gap	0
BECHER	528	*	gap	0
BLOT1	501	1.80		0
BLOT1	502	*	gap	0
BROWN3	504	*	gap	0
CORREA	538	3.90		0
CORREA	539	7.00		0
CORREA	540	7.70		0
CORREA	545	*	gap	0
CPSI	718	2.61		0
CPSI	719	2.52		0
CPSI	720	3.19		0
CPSI	721	0.58		0
CPSI	722	1.51		0
CPSI	723	2.85		0
CPSI	935	*	gap	0
DEAN3	631	*	gap	0
DEAN3	626	*	gap	0
DORGAN	504	*	gap	0
DORGAN	555	*	gap	0
GAO2	527	*	gap	0
GARCIA	519	*	gap	0
GARSHI	524	*	gap	0
GURSEL	502	*	gap	0
JAHN	732	*	gap	0
JAIN	596	*	gap	0
JAIN	584	*	gap	0
JEDRYC	613	*	gap	0
JOLY	568	*	gap	0
JOLY	555	*	gap	0
KAISE2	649	*	gap	0
KAISE2	569	*	gap	0
LAUSSM	504	*	gap	0
LUBIN	588	*	gap	0
MATOS	703	*	gap	0
PEZZO2	512	*	gap	0
PEZZOT	597	*	gap	0
SOBUE	778	*	gap	0
SPEIZE	538	1.00		Insufficient decimals to calculate CIs
SPEIZE	539	2.00		Insufficient decimals to calculate CIs
SPEIZE	540	5.00		Insufficient decimals to calculate CIs
SPEIZE	541	6.00		Insufficient decimals to calculate CIs
SPEIZE	542	6.00		Insufficient decimals to calculate CIs
SVENSS	591	*	gap	0
WAKAI	611	*	gap	0
WYNDE3	539	*	gap	0
WYNDE3	588	*	gap	0
WYNDE6	506	*	gap	0
WYNDE6	527	*	gap	0

Table 1J1 - 9

IESLC - Meta-analysis of Ex Smoking by Years quit (vs never), Overview
All LC types, Any Product (or Cigarettes if Any not available)

Least adjusted - insufficient data for meta-analysis: as for adjusted plus the following

REF	NRR	SEX	AGEL	AGEH	RACE	YF	LC TYPE	LOC	START	ST	NLC	R	VB	P	H	AD	PRODUCT	exL	exH	S1	S2	DENOM	De
AUVINE	531	c	0	0	all	-	all	Eu:Sca	1986	CC	517	n	bl	y	n	0	cig+/-ot	1.0	11	0	3	nev cigs	ot
BROWN3	502	f	0	0	wh	-	all	NAmer		CC	618		bl	y	n	0	all/unsp	1.0	14	0	0	nev any	ot
DEAN3	622	m	0	0	all	-	all	Eu:UK	1969	CC	766	n	V	y	n	0	all/unsp	1.0	2	0	0	nev any	ot
DEAN3	624	f	0	0	all	-	all	Eu:UK	1969	CC	766	n	V	y	n	0	all/unsp	1.0	2	0	0	nev any	ot
GARSHI	517	m	0	0	all	-	all	NAmer	1981	CC	1081	o	bl	y	n	0	all/unsp	1.0	4	3	3	nev any	ot
LAUSSM	502	m	0	0	all	-	all	Eu:Ger	1982	CC	432	n	bl	n	n	0	all/unsp	1.0	9	0	3	nev any	ot
MATOS	701	m	0	0	all	-	all	SCAmer	1994	CC	200	n	bl	n	n	0	cig+/-ot	0.1	0.9	0	0	nev any	ot
WAKAI	609	m	0	0	all	-	all	As:Jap	1988	CC	333	n	bl	n	y	0	cig+/-ot	1.0	4	3	3	nev any	ot

REF	NRR	RR	SIG	RRDATA comment
AUVINE	531	*	gap	0
BROWN3	502	*	gap	0
DEAN3	622	*	gap	0
DEAN3	624	*	gap	0
GARSHI	517	*	gap	0
LAUSSM	502	*	gap	0
MATOS	701	*	gap	0
WAKAI	609	*	gap	0

Table 1J2 -

IESLC - Meta-analysis of Ex Smoking, Years quit (vs never), "Low"
All LC types, Any Product (or Cigarettes if Any not available)

This analysis is restricted to results for:
1) Ex smokers
2) Results by Years quit (vs never)
3) Categorical results by Years quit (vs never)
4) All LC types (or near equivalent)
5) Results complete enough for use in metaanalysis

Within each study, results are then selected (in the following order of preference, within each sex) for:
6) (not applicable)
7) PRODUCT: all/unspec, cigarettes regardless of other products, cigarettes only
8) CIGTYPE: all/unspecified, MC regardless of HR, MC only
9) (not applicable)
10) DENOM: never smoked anything, never smoked cigarettes, never any + low, never cigs + low
11) Followup period (YF, prospective studies): whole study (coded as 0) or longest available
12) LCtype: all or nearest available, at least Squamous and Adeno. (q = squamous, s = small, l = large, a = adeno, mix = mixed, alv = alveolar)
13) Race: all or nearest available, otherwise by race (wh or w = white, bl or b = black, hi = hispanic ch = chinese, jap = japanese, haw = hawaiian, w+o = white + oriental, sca = scandinavian, as = asian)
14) Years quit (vs never) "low" in key scheme 1 (key value 12, maximum range 8+)
15) For overlapping studies: principal rather than subsidiary studies
Finally by Age: whole study (coded as 0) if available, otherwise by widest available age group and then for single sex results (m, f) in preference to results for both sexes combined (c).

Results adjusted (AD) for the most potential confounders are then chosen in Sections -1 to -3 and results adjusted for the least confounders in Sections -4 to -6. (Those least adjusted results which actually differ from the most adjusted are marked 'x' in column X in Section -4)

Section -7 shows excluded studies, together with the stage (as above) at which no qualifying results were found.

Section -8 lists the potentially overlapping studies which have been included (1=principal, 2=subsidiary).

Section -9 lists any results which would have been included in preference except that they had data not complete enough for use in meta-analysis, with their significance (yes/no), if known, and any further comment as entered on the database. It also lists as "gap" any categories for which no data were presented by the original authors. This is commonly due to recent quitters having been combined with current smokers

In addition to those mentioned above, the following fields, levels and abbreviations are used:
* or nk = not known, n = no, y = yes, ot = other
nev = never
all/unspec = all or unspecified, cig+/-ot = cigarettes irrespective of other products (cigar, pipe etc)
MC = manufactured cigarettes, HR = hand-rolled cigarettes
exL, exH = range of exposure (low and high) in the smoking group, in terms of Years quit (vs never)
REF: 6-character study reference
NRR: number of the RR on the database within the study
ST : study type (CC = case control, pr or prosp = prospective)
NLC: number of lung cancer cases in whole study
R : risky occupational population (n = no, m = mining, o = other risky)
VB : national cigarette type (V = at least 75% Virginia, bl = at least 75% blended, ot = other)
P : any proxy use
H : full histological confirmation
De : derivation of RR/CI (or = original, st = standard method, ot = other method of estimation)

Table 1J2 - 1

IESLC - Meta-analysis of Ex Smoking, Years quit (vs never), "Low"
All LC types, Any Product (or Cigarettes if Any not available)
Most adjusted

REF	NRR	SEX	AGEL	AGEH	RACE	YF	LC TYPE	LOC	START	ST	NLC	R	VB	P	H	AD	PRODUCT	exL	exH	DENOM	De
ALDERS	507	m	0	0	all	-	all	Eu:UK	1977	CC	1448	n	V	n	n	1	cig only	10	999	nev any	ot
ALDERS	518	f	0	0	all	-	all	Eu:UK	1977	CC	1448	n	V	n	n	1	cig only	10	999	nev any	ot
AUVINE	532	c	0	0	all	-	all	Eu:Sca	1986	CC	517	n	bl	y	n	2	cig+/-ot	12	999	nev cigs	or
BECHER	501	m	0	0	all	-	all	Eu:Ger	1985	CC	194	n	bl	n	y	0	all/unsp	10	999	nev any	st
BECHER	511	f	0	0	all	-	all	Eu:Ger	1985	CC	194	n	bl	n	y	0	all/unsp	10	999	nev any	st
BENSHL	510	m	0	0	all	0	all	Eu:UK	1967	pr	486	n	V	n	n	2	cig+/-ot	10	19	nev any	or
CARPEN	502	c	0	0	w+b	-	all	NAmer	1991	CC	356	n	bl	n	n	0	cig+/-ot	10	14	nev cigs	st
CEDERL	528	m	40	69	all	10	all	Eu:Sca	1963	pr	491	n	bl	n	n	1	all/unsp	10	999	nev any	ot
CHOI	534	m	0	0	all	-	all	As:oth	1985	CC	375	n	bl	n	n	0	cig+/-ot	10	14	nev cigs	st
CPSI	807	m	50	74	all	6	all	NAmer	1959	pr	5138	n	bl	n	n	1	cig only	10	999	nev any	ot
CPSII	653	m	35	99	all	4	all	NAmer	1982	pr	3229	n	bl	n	n	1	cig only	11	15	nev any	ot
CPSII	634	f	0	0	all	4	all	NAmer	1982	pr	3229	n	bl	n	n	1	cig+/-ot	11	15	nev cigs	ot
DAMBER	523	m	0	0	all	-	all	Eu:Sca	1972	CC	579	n	bl	y	n	1	all/unsp	11	999	nev any	ot
DARBY	501	m	0	0	wh	-	all	Eu:UK	1988	CC	982	n	V	n	n	0	all/unsp	10	999	nev any	st
DARBY	510	f	0	0	wh	-	all	Eu:UK	1988	CC	982	n	V	n	n	0	all/unsp	10	999	nev any	st
DEAN3	628	m	0	0	all	-	all	Eu:UK	1969	CC	766	n	V	y	n	1	all/unsp	9	999	nev any	ot
DEAN3	553	f	0	0	all	-	all	Eu:UK	1969	CC	766	n	V	y	n	1	all/unsp	9	999	nev any	ot
DESTEF	525	m	0	0	all	-	all	SCAmer	1988	CC	497	n	bl	n	y	4	all/unsp	10	999	nev any	or
DOLL	530	m	0	0	all	-	all	Eu:UK	1948	CC	1465	n	V	n	n	0	all/unsp	10	19	nev any	st
DOLL	542	f	0	0	all	-	all	Eu:UK	1948	CC	1465	n	V	n	n	0	all/unsp	10	999	nev any	st
DOLL2	502	m	0	0	all	20	all	Eu:UK	1951	pr	920	n	V	n	n	1	cig only	10	14	nev any	ot
DORGAN	501	m	0	0	wh	-	all	NAmer	1980	CC	2026	n	bl	y	y	0	cig+/-ot	10	999	nev any	st
DORGAN	553	f	0	0	all	-	all	NAmer	1980	CC	2026	n	bl	y	y	0	cig+/-ot	10	999	nev any	st
DORN	658	m	55	64	wh	8	all	NAmer	1954	pr	5097	n	bl	n	n	0	cig+/-ot	10	14	nev any	st
DORN	681	m	65	74	wh	8	all	NAmer	1954	pr	5097	n	bl	n	n	0	cig+/-ot	10	14	nev any	st
GAO	531	m	0	0	all	-	all	As:Chi	1984	CC	1405	n	ot	n	n	2	cig+/-ot	10	999	nev cigs	or
GAO	551	f	0	0	all	-	all	As:Chi	1984	CC	1405	n	ot	n	n	2	cig+/-ot	10	999	nev cigs	or
GAO2	511	m	0	0	all	-	all	As:Jap	1988	CC	282	n	bl	n	n	0	cig+/-ot	10	14	nev cigs	or
GRAHAM	501	m	0	0	wh	-	all	NAmer	1956	CC	685	n	bl	n	n	0	cig only	10	999	nev any	st
GURSEL	501	m	0	0	all	-	all	Eu:bal		CC	953		bl	*	n	0	all/unsp	11	999	nev any	or
HAMMO2	501	m	0	0	all	0	all	NAmer	1967	pr	450	o	bl	n	n	1	cig+/-ot	10	999	nev any	ot
HIRAYA	507	m	0	0	all	0	all	As:Jap	1965	pr	1917	n	bl	n	n	1	cig+/-ot	10	999	nev any	st
HIRAYA	518	f	0	0	all	0	all	As:Jap	1965	pr	1917	n	bl	n	n	1	cig+/-ot	10	999	nev any	st
JAHN	502	m	0	0	all	-	all	Eu:Ger	1988	CC	1004	n	bl	n	n	0	cig+/-ot	11	20	nev any	st
JAIN	567	m	0	0	all	-	all	NAmer	1981	CC	845	n	V	y	n	0	cig+/-ot	10	999	nev cigs	st
JAIN	531	f	0	0	all	-	all	NAmer	1981	CC	845	n	V	y	n	0	cig+/-ot	10	999	nev cigs	st
JEDRYC	611	m	0	0	all	-	all	Eu:est	1980	CC	1630	n	bl	y	n	0	cig+/-ot	10	999	nev any	st
KAISE2	647	m	0	0	all	9	all	NAmer	1979	pr	318	n	bl	n	n	1	cig only	11	20	nev any	ot
KAISE2	567	f	0	0	all	9	all	NAmer	1979	pr	318	n	bl	n	n	1	cig only	11	20	nev any	st
LAUSSM	503	m	0	0	all	-	all	Eu:Ger	1982	CC	432	n	bl	n	n	2	all/unsp	10	999	nev any	st
LUBIN	585	m	0	0	all	-	all	As:Chi	1984	CC	427	m	ot	y	n	0	cig+/-ot	10	999	nev any	st
LUBIN2	1072	m	0	0	all	-	all	Eu:mul	1976	CC	7804	n	bl	n	y	0	cig+/-ot	10	14	nev any	st
LUBIN2	1111	f	0	0	all	-	all	Eu:mul	1976	CC	7804	n	bl	n	y	0	cig+/-ot	10	14	nev any	st
MATOS	591	m	0	0	all	-	all	SCAmer	1994	CC	200	n	bl	n	n	2	cig+/-ot	11	999	nev any	ot
PEZZO2	501	m	0	0	all	-	all	SCAmer	1992	CC	367	n	bl	n	y	0	cig+/-ot	11	999	nev cigs	st
PEZZOT	501	m	0	0	all	-	all	SCAmer	1987	CC	215	n	bl	n	y	0	cig only	11	999	nev cigs	st
SOBUE	720	m	0	0	all	-	all	As:Jap	1986	CC	1376	n	bl	n	y	0	cig+/-ot	10	14	nev cigs	st
SPEIZE	502	f	0	0	all	0	all	NAmer	1976	pr	593	n	bl	n	y	0	cig+/-ot	10	15	nev cigs	st
SUZUK2	508	c	0	0	all	-	all	SCAmer	1991	CC	123	n	bl	n	y	0	all/unsp	11	999	nev any	st
SVENSS	551	f	0	0	all	-	all	Eu:Sca	1983	CC	210	n	bl	n	n	0	all/unsp	11	999	nev any	st
WAKAI	531	m	0	0	all	-	all	As:Jap	1988	CC	333	n	bl	n	y	2	cig+/-ot	10	19	nev any	or
WYNDE3	566	m	0	0	all	-	all	NAmer	1966	CC	350	n	bl	n	y	0	cig+/-ot	10	999	nev any	st
WYNDE3	587	f	0	0	all	-	all	NAmer	1966	CC	350	n	bl	n	y	0	cig+/-ot	10	999	nev any	st
WYNDE6	503	m	0	0	all	-	all	NAmer	1969	CC	4423	n	bl	n	y	0	cig only	10	19	nev any	st
WYNDE6	524	f	0	0	all	-	all	NAmer	1969	CC	4423	n	bl	n	y	0	cig only	10	19	nev any	st

Cigarette type is all/unspec for all RRs
except for the following:

REF	NRR	CIGTYPE
ALDERS	507	MC only
ALDERS	518	MC only

Table 1J2 - 2

IESLC - Meta-analysis of Ex Smoking, Years quit (vs never), "Low"
All LC types, Any Product (or Cigarettes if Any not available)
Most adjusted

REF	NRR	SEX	AD	Number Exposed Case	Cont	Non-exposed Case	Cont	RR	95.00%CI
ALDERS	507	m	1	29	-	15	-	3.20	(1.61- 6.35)
ALDERS	518	f	1	26	-	75	-	1.27	(0.76- 2.15)
Subtotal ALDERS								1.78	(1.18- 2.69)
AUVINE	532	c	2	207	-	44	-	7.50	(4.18- 13.15)
BECHER	501	m	0	16	72	3	54	4.00	(1.11- 14.42)
BECHER	511	f	0	1	10	10	52	0.52	(0.06- 4.53)
Subtotal BECHER								2.35	(0.78- 7.09)
*BENSHL	510	m	2	23	-	10	-	4.08	(2.03- 8.20)
CARPEN	502	c	0	9	51	8	208	4.59	(1.69- 12.48)
*CEDERL	528	m	1	3	-	7	-	1.10	(0.28- 4.25)
CHOI	534	m	0	4	23	13	95	1.27	(0.38- 4.26)
*CPSI	807	m	1	15	-	60	-	1.28	(0.73- 2.25)
*CPSII	653	m	1	164	-	81	-	8.61	(6.60- 11.24)
*CPSII	634	f	1	28	-	174	-	3.86	(2.59- 5.75)
Subtotal CPSII								6.72	(5.39- 8.39)
DAMBER	523	m	1	42	-	42	-	2.60	(1.70- 4.50)
DARBY	501	m	0	139	767	3	384	23.20	(7.34- 73.28)
DARBY	510	f	0	26	224	23	529	2.67	(1.49- 4.78)
Subtotal DARBY								4.15	(2.47- 6.98)
DEAN3	628	m	1	32	-	24	-	2.04	(1.18- 3.54)
DEAN3	553	f	1	2	-	41	-	0.72	(0.17- 3.01)
Subtotal DEAN3								1.79	(1.07- 2.98)
DESTEF	525	m	4	17	-	27	-	2.80	(1.40- 5.70)
DOLL	530	m	0	6	26	7	61	2.01	(0.62- 6.56)
DOLL	542	f	0	1	2	40	59	0.74	(0.06- 8.41)
Subtotal DOLL								1.66	(0.57- 4.81)
*DOLL2	502	m	1	9	-	7	-	5.30	(1.97- 14.23)
DORGAN	501	m	0	134	255	13	140	5.66	(3.09- 10.37)
DORGAN	553	f	0	34	50	61	213	2.37	(1.41- 4.00)
Subtotal DORGAN								3.43	(2.31- 5.10)
*DORN	658	m	0	12	23682	25	213858	4.33	(2.18- 8.63)
*DORN	681	m	0	29	20056	49	171211	5.05	(3.19- 7.99)
Subtotal DORN								4.82	(3.29- 7.06)
GAO	531	m	2	13	-	62	-	1.10	(0.50- 2.20)
GAO	551	f	2	16	-	435	-	2.20	(1.00- 4.60)
Subtotal GAO								1.54	(0.90- 2.62)
GAO2	511	m	0	16	18	13	56	3.83	(1.55- 9.46)
GRAHAM	501	m	0	2	30	18	346	1.28	(0.28- 5.79)
GURSEL	501	m	0	-	-	-	-	2.30	(1.01- 5.22)
*HAMMO2	501	m	1	20	-	5	-	3.45	(1.30- 9.14)
*HIRAYA	507	m	1	-	-	-	-	1.38	(0.59- 3.21)
*HIRAYA	518	f	1	-	-	-	-	0.97	(0.03- 32.06)
Subtotal HIRAYA								1.35	(0.59- 3.08)
JAHN	502	m	0	64	130	18	138	3.77	(2.12- 6.71)
JAIN	567	m	0	52	113	12	85	3.26	(1.64- 6.48)
JAIN	531	f	0	19	61	52	214	1.28	(0.71- 2.33)
Subtotal JAIN								1.91	(1.22- 3.01)
JEDRYC	611	m	0	73	138	49	219	2.36	(1.55- 3.60)
*KAISE2	647	m	1	8	-	14	-	3.14	(1.26- 7.82)
*KAISE2	567	f	1	4	-	11	-	4.37	(1.30- 14.72)
Subtotal KAISE2								3.54	(1.71- 7.34)
LAUSSM	503	m	2	29	-	63	-	6.54	(3.47- 12.35)
LUBIN	585	m	0	17	73	9	72	1.86	(0.78- 4.45)
LUBIN2	1072	m	0	270	693	190	2616	5.36	(4.38- 6.58)
LUBIN2	1111	f	0	10	26	336	1188	1.36	(0.65- 2.85)
Subtotal LUBIN2								4.87	(4.00- 5.93)
MATOS	591	m	2	27	-	11	-	3.00	(1.43- 6.28)
PEZZO2	501	m	0	43	161	6	117	5.21	(2.15- 12.64)
PEZZOT	501	m	0	20	106	4	116	5.47	(1.81- 16.53)
SOBUE	720	m	0	35	50	29	126	3.04	(1.68- 5.49)
*SPEIZE	502	f	0	17	93933	58	776300	2.42	(1.41- 4.16)
SUZUK2	508	c	0	9	22	11	53	1.97	(0.72- 5.42)
SVENSS	551	f	0	14	24	38	120	1.84	(0.87- 3.91)
WAKAI	531	m	2	27	-	10	-	3.63	(1.56- 8.44)
WYNDE3	566	m	0	9	65	9	88	1.35	(0.51- 3.60)
WYNDE3	587	f	0	1	3	20	76	1.27	(0.12- 12.84)
Subtotal WYNDE3								1.34	(0.54- 3.30)
WYNDE6	503	m	0	159	373	64	918	6.11	(4.47- 8.37)
WYNDE6	524	f	0	36	132	125	991	2.16	(1.43- 3.27)
Subtotal WYNDE6								4.18	(3.25- 5.36)
Partial Totals				2018	141369	2534	1170703		

Table 1J2 - 2

IESLC - Meta-analysis of Ex Smoking, Years quit (vs never), "Low"
All LC types, Any Product (or Cigarettes if Any not available)
Most adjusted

REF	NRR	SEX	AD	Number Case	Exposed Cont	Non-exposed Case	Cont	RR	95.00%CI

*prospective study

REF	NRR	SEX	AD	Ys	Ws	Qs	Ps
ALDERS	507	m	1	1.16	8.16	0.11	0.0009
ALDERS	518	f	1	0.24	14.21	15.31	0.3676
Subtotal ALDERS				0.58	22.37	15.41	
AUVINE	532	c	2	2.01	11.70	6.37	0.0000
BECHER	501	m	0	1.39	2.34	0.03	0.0341
BECHER	511	f	0	-0.65	0.82	3.06	0.5537
Subtotal BECHER				0.86	3.16	3.09	
*BENSHL	510	m	2	1.41	7.88	0.13	0.0001
CARPEN	502	c	0	1.52	3.84	0.23	0.0028
*CEDERL	528	m	1	0.10	2.08	2.90	0.8907
CHOI	534	m	0	0.24	2.63	2.82	0.6977
*CPSI	807	m	1	0.25	12.13	12.87	0.3900
*CPSII	653	m	1	2.15	54.21	41.60	0.0000
*CPSII	634	f	1	1.35	24.16	0.13	0.0000
Subtotal CPSII				1.91	78.37	41.74	
DAMBER	523	m	1	0.96	16.22	1.67	0.0001
DARBY	501	m	0	3.14	2.90	10.12	0.0000
DARBY	510	f	0	0.98	11.33	0.99	0.0010
Subtotal DARBY				1.42	14.23	11.11	
DEAN3	628	m	1	0.71	12.73	4.05	0.0110
DEAN3	553	f	1	-0.33	1.86	4.79	0.6541
Subtotal DEAN3				0.58	14.59	8.84	
DESTEF	525	m	4	1.03	7.80	0.48	0.0040
DOLL	530	m	0	0.70	2.74	0.92	0.2471
DOLL	542	f	0	-0.30	0.65	1.62	0.8063
Subtotal DOLL				0.51	3.39	2.54	
*DOLL2	502	m	1	1.67	3.93	0.60	0.0009
DORGAN	501	m	0	1.73	10.48	2.18	0.0000
DORGAN	553	f	0	0.86	14.18	2.41	0.0011
Subtotal DORGAN				1.23	24.66	4.59	
*DORN	658	m	0	1.47	8.11	0.29	0.0000
*DORN	681	m	0	1.62	18.24	2.15	0.0000
Subtotal DORN				1.57	26.35	2.44	
GAO	531	m	2	0.10	7.00	9.77	0.8009
GAO	551	f	2	0.79	6.60	1.57	0.0428
Subtotal GAO				0.43	13.60	11.35	
GAO2	511	m	0	1.34	4.70	0.02	0.0036
GRAHAM	501	m	0	0.25	1.69	1.79	0.7471
GURSEL	501	m	0	0.83	5.70	1.12	0.0468
*HAMMO2	501	m	1	1.24	4.04	0.01	0.0128
*HIRAYA	507	m	1	0.32	5.36	4.88	0.4561
*HIRAYA	518	f	1	-0.03	0.32	0.54	0.9863
Subtotal HIRAYA				0.30	5.67	5.42	
JAHN	502	m	0	1.33	11.61	0.03	0.0000
JAIN	567	m	0	1.18	8.12	0.07	0.0008
JAIN	531	f	0	0.25	10.76	11.38	0.4154
Subtotal JAIN				0.65	18.88	11.46	
JEDRYC	611	m	0	0.86	21.78	3.78	0.0001
*KAISE2	647	m	1	1.14	4.61	0.08	0.0140
*KAISE2	567	f	1	1.47	2.61	0.10	0.0172
Subtotal KAISE2				1.26	7.22	0.18	
LAUSSM	503	m	2	1.88	9.53	3.44	0.0000
LUBIN	585	m	0	0.62	5.06	2.17	0.1615
LUBIN2	1072	m	0	1.68	92.66	15.04	0.0000
LUBIN2	1111	f	0	0.31	7.03	6.61	0.4151
Subtotal LUBIN2				1.58	99.69	21.65	
MATOS	591	m	2	1.10	7.02	0.22	0.0036
PEZZO2	501	m	0	1.65	4.89	0.68	0.0003
PEZZOT	501	m	0	1.70	3.14	0.56	0.0026
SOBUE	720	m	0	1.11	10.99	0.30	0.0002
*SPEIZE	502	f	0	0.88	13.15	2.02	0.0013
SUZUK2	508	c	0	0.68	3.75	1.34	0.1886
SVENSS	551	f	0	0.61	6.77	3.00	0.1120
WAKAI	531	m	2	1.29	5.39	0.00	0.0028
WYNDE3	566	m	0	0.30	4.02	3.81	0.5438
WYNDE3	587	f	0	0.24	0.72	0.78	0.8415
Subtotal WYNDE3				0.29	4.73	4.58	

Table 1J2 - 2

IESLC - Meta-analysis of Ex Smoking, Years quit (vs never), "Low"
All LC types, Any Product (or Cigarettes if Any not available)
Most adjusted

REF	NRR	SEX	AD	Ys	Ws	Qs	Ps
WYNDE6	503	m	0	1.81	38.93	11.09	0.0000
WYNDE6	524	f	0	0.77	22.54	5.77	0.0003
Subtotal WYNDE6				1.43	61.48	16.86	

	N	55
	NS	40
	Wt	585.78
Het	Chi	209.82
Het	df	54
Het	P	***
Fixed	RR	3.59
	RRl	3.31
	RRu	3.89
	P	+++
Random	RR	2.91
	RRl	2.43
	RRu	3.47
	P	+++
Asymm	P	***

Table 1J2 - 3

IESLC - Meta-analysis of Ex Smoking, Years quit (vs never), "Low"
All LC types, Any Product (or Cigarettes if Any not available)
Most adjusted

	Sex			
	combined	male	female	Total
N	3	36	16	55
NS	3	35	16	54
Wt	19.29	428.79	137.69	585.78
Het Chi	5.16	132.47	23.92	209.82
Het df	2	35	15	54
Het P	(*)	***	(*)	***
Fixed RR	5.24	4.15	2.15	3.59
RRl	3.36	3.78	1.82	3.31
RRu	8.19	4.57	2.54	3.89
P	+++	+++	+++	+++
Random RR	4.43	3.31	2.03	2.91
RRl	2.02	2.69	1.60	2.43
RRu	9.74	4.07	2.56	3.47
P	+++	+++	+++	+++
Between Chi				48.26
Between df				2
Between P				***
Btwn(F) P				**
Btwn(R) P				**

	Lung cancer type		
	all	other	Total
N	55		55
NS	40		40
Wt	585.78		585.78
Het Chi	209.82		209.82
Het df	54		54
Het P	***		***
Fixed RR	3.59		3.59
RRl	3.31		3.31
RRu	3.89		3.89
P	+++		+++
Random RR	2.91		2.91
RRl	2.43		2.43
RRu	3.47		3.47
P	+++		+++
Between Chi			
Between df			
Between P			N.S.
Btwn(F) P			N.S.
Btwn(R) P			N.S.

	Location								
	NAmer	UK	Scand	othEur	China	Japan	othAs	other	Total
N	19	10	4	8	3	5	1	5	55
NS	12	6	4	6	2	4	1	5	40
Wt	256.52	66.40	36.76	151.46	18.66	26.75	2.63	26.60	585.78
Het Chi	95.21	30.09	13.61	29.33	1.77	3.93	0.00	3.09	209.82
Het df	18	9	3	7	2	4	0	4	54
Het P	***	***	**	***	N.S.	N.S.	N.S.	N.S.	***
Fixed RR	4.03	2.50	3.26	4.20	1.62	2.76	1.27	3.29	3.59
RRl	3.57	1.97	2.36	3.58	1.03	1.89	0.38	2.25	3.31
RRu	4.56	3.19	4.50	4.92	2.55	4.04	4.26	4.81	3.89
P	+++	+++	+++	+++	+	+++	N.S.	+++	+++
Random RR	3.17	2.76	2.80	3.14	1.62	2.76	1.27	3.29	2.91
RRl	2.32	1.71	1.32	2.03	1.03	1.89	0.38	2.25	2.43
RRu	4.33	4.46	5.95	4.86	2.55	4.04	4.26	4.81	3.47
P	+++	+++	++	+++	+	+++	N.S.	+++	+++
Between Chi									32.80
Between df									7
Between P									***
Btwn(F) P									N.S.
Btwn(R) P									N.S.

Table 1J2 - 3

IESLC - Meta-analysis of Ex Smoking, Years quit (vs never), "Low"
All LC types, Any Product (or Cigarettes if Any not available)
Most adjusted

Detailed Country in "other Europe"

	multi	Germany	othWest	East	Balkans	Total
N	2	4		1	1	8
NS	1	3		1	1	6
Wt	99.69	24.30		21.78	5.70	151.46
Het Chi	12.30	5.53		0.00	0.00	29.33
Het df	1	3		0	0	7
Het P	***	N.S.		N.S.	N.S.	***
Fixed RR	4.87	4.40		2.36	2.30	4.20
RRl	4.00	2.96		1.55	1.01	3.58
RRu	5.93	6.55		3.60	5.23	4.92
P	+++	+++		+++	+	+++
Random RR	2.83	4.04		2.36	2.30	3.14
RRl	0.74	2.16		1.55	1.01	2.03
RRu	10.84	7.53		3.60	5.23	4.86
P	N.S.	+++		+++	+	+++
Between Chi						11.49
Between df						3
Between P						**
Btwn(F) P						N.S.
Btwn(R) P						N.S.

Detailed Country in "other Asia"

	India	HongKong	other	Total
N			1	1
NS			1	1
Wt			2.63	2.63
Het Chi			0.00	0.00
Het df			0	0
Het P			N.S.	N.S.
Fixed RR			1.27	1.27
RRl			0.38	0.38
RRu			4.26	4.26
P			N.S.	N.S.
Random RR			1.27	1.27
RRl			0.38	0.38
RRu			4.26	4.26
P			N.S.	N.S.
Between Chi				
Between df				
Between P				N.S.
Btwn(F) P				N.S.
Btwn(R) P				N.S.

Detailed other continent

	SCAmer	Total
N	5	5
NS	5	5
Wt	26.60	26.60
Het Chi	3.09	3.09
Het df	4	4
Het P	N.S.	N.S.
Fixed RR	3.29	3.29
RRl	2.25	2.25
RRu	4.81	4.81
P	+++	+++
Random RR	3.29	3.29
RRl	2.25	2.25
RRu	4.81	4.81
P	+++	+++
Between Chi		
Between df		
Between P		N.S.
Btwn(F) P		N.S.
Btwn(R) P		N.S.

Table 1J2 - 3

IESLC - Meta-analysis of Ex Smoking, Years quit (vs never), "Low"
All LC types, Any Product (or Cigarettes if Any not available)
Most adjusted

	Start year of study <1960	1960-69	1970-79	1980-89	1990+	Total
N	7	11	8	24	4	54
NS	5	7	5	18	4	39
Wt	47.49	100.47	158.64	253.98	19.50	580.08
Het Chi	19.04	37.18	40.82	104.89	2.45	208.68
Het df	6	10	7	23	3	53
Het P	**	***	***	***	N.S.	***
Fixed RR	3.06	3.16	3.69	3.86	3.45	3.60
RRl	2.30	2.60	3.15	3.42	2.22	3.32
RRu	4.07	3.85	4.31	4.37	5.38	3.91
P	+++	+++	+++	+++	+++	+++
Random RR	2.70	2.23	2.65	3.27	3.45	2.92
RRl	1.50	1.40	1.66	2.47	2.22	2.44
RRu	4.86	3.54	4.22	4.35	5.38	3.50
P	+++	+++	+++	+++	+++	+++
Between Chi						4.31
Between df						4
Between P						N.S.
Btwn(F) P						N.S.
Btwn(R) P						N.S.

	Study type (1) CC	other	Total
N	41	14	55
NS	30	10	40
Wt	424.97	160.81	585.78
Het Chi	138.84	61.22	209.82
Het df	40	13	54
Het P	***	***	***
Fixed RR	3.31	4.42	3.59
RRl	3.01	3.79	3.31
RRu	3.64	5.16	3.89
P	+++	+++	+++
Random RR	2.78	3.30	2.91
RRl	2.28	2.24	2.43
RRu	3.39	4.85	3.47
P	+++	+++	+++
Between Chi			9.76
Between df			1
Between P			**
Btwn(F) P			N.S.
Btwn(R) P			N.S.

	Study type (2) CC	prosp	other	Total
N	41	14		55
NS	30	10		40
Wt	424.97	160.81		585.78
Het Chi	138.84	61.22		209.82
Het df	40	13		54
Het P	***	***		***
Fixed RR	3.31	4.42		3.59
RRl	3.01	3.79		3.31
RRu	3.64	5.16		3.89
P	+++	+++		+++
Random RR	2.78	3.30		2.91
RRl	2.28	2.24		2.43
RRu	3.39	4.85		3.47
P	+++	+++		+++
Between Chi				9.76
Between df				1
Between P				**
Btwn(F) P				N.S.
Btwn(R) P				N.S.

Table 1J2 - 3

IESLC - Meta-analysis of Ex Smoking, Years quit (vs never), "Low"
All LC types, Any Product (or Cigarettes if Any not available)
Most adjusted

	Study size (number of LC cases) 100-249	250-499	500-999	1000+	Total
N	6	15	12	22	55
NS	5	13	9	13	40
Wt	23.84	69.78	100.08	392.08	585.78
Het Chi	5.53	17.23	39.04	135.31	209.82
Het df	5	14	11	21	54
Het P	N.S.	N.S.	***	***	***
Fixed RR	2.56	3.32	2.81	3.95	3.59
RRl	1.72	2.62	2.31	3.58	3.31
RRu	3.83	4.19	3.42	4.36	3.89
P	+++	+++	+++	+++	+++
Random RR	2.57	3.23	2.90	2.88	2.91
RRl	1.67	2.48	1.96	2.17	2.43
RRu	3.95	4.22	4.29	3.80	3.47
P	+++	+++	+++	+++	+++
Between Chi					12.70
Between df					3
Between P					**
Btwn(F) P					N.S.
Btwn(R) P					N.S.

	Risky occupational population no	mining	othRisky	Total
N	52	1	1	54
NS	37	1	1	39
Wt	570.98	5.06	4.04	580.08
Het Chi	206.46	0.00	0.00	208.68
Het df	51	0	0	53
Het P	***	N.S.	N.S.	***
Fixed RR	3.62	1.86	3.45	3.60
RRl	3.34	0.78	1.30	3.32
RRu	3.93	4.45	9.15	3.91
P	+++	N.S.	+	+++
Random RR	2.93	1.86	3.45	2.92
RRl	2.44	0.78	1.30	2.44
RRu	3.53	4.45	9.15	3.50
P	+++	N.S.	+	+++
Between Chi				2.23
Between df				2
Between P				N.S.
Btwn(F) P				N.S.
Btwn(R) P				N.S.

	National cigarette tobacco type Virginia	blended	other	Total
N	12	40	3	55
NS	7	31	2	40
Wt	85.28	481.84	18.66	585.78
Het Chi	35.18	140.93	1.77	209.82
Het df	11	39	2	54
Het P	***	***	N.S.	***
Fixed RR	2.36	3.98	1.62	3.59
RRl	1.91	3.64	1.03	3.31
RRu	2.92	4.35	2.55	3.89
P	+++	+++	+	+++
Random RR	2.59	3.17	1.62	2.91
RRl	1.72	2.61	1.03	2.43
RRu	3.89	3.85	2.55	3.47
P	+++	+++	+	+++
Between Chi				31.95
Between df				2
Between P				***
Btwn(F) P				*
Btwn(R) P				*

Table 1J2 - 3

IESLC - Meta-analysis of Ex Smoking, Years quit (vs never), "Low"
All LC types, Any Product (or Cigarettes if Any not available)
Most adjusted

	Any proxy use No/nk	Yes	Total
N	45	10	55
NS	33	7	40
Wt	472.89	112.89	585.78
Het Chi	168.76	29.80	209.82
Het df	44	9	54
Het P	***	***	***
Fixed RR	3.84	2.70	3.59
RRl	3.51	2.24	3.31
RRu	4.20	3.25	3.89
P	+++	+++	+++
Random RR	2.99	2.63	2.91
RRl	2.44	1.86	2.43
RRu	3.66	3.74	3.47
P	+++	+++	+++
Between Chi			11.26
Between df			1
Between P			***
Btwn(F) P			(*)
Btwn(R) P			N.S.

	Full histological confirmation No	Yes	Total
N	38	17	55
NS	28	12	40
Wt	342.95	242.82	585.78
Het Chi	151.85	53.59	209.82
Het df	37	16	54
Het P	***	***	***
Fixed RR	3.33	3.97	3.59
RRl	3.00	3.50	3.31
RRu	3.71	4.51	3.89
P	+++	+++	+++
Random RR	2.82	3.11	2.91
RRl	2.24	2.35	2.43
RRu	3.56	4.12	3.47
P	+++	+++	+++
Between Chi			4.37
Between df			1
Between P			*
Btwn(F) P			N.S.
Btwn(R) P			N.S.

	Number of adjustment variables (1) 0	1	2+/+nk	Total
N	32	15	8	55
NS	23	10	7	40
Wt	356.25	166.61	62.92	585.78
Het Chi	98.18	89.63	21.99	209.82
Het df	31	14	7	54
Het P	***	***	**	***
Fixed RR	3.59	3.56	3.63	3.59
RRl	3.23	3.06	2.84	3.31
RRu	3.98	4.15	4.65	3.89
P	+++	+++	+++	+++
Random RR	2.97	2.51	3.40	2.91
RRl	2.40	1.62	2.19	2.43
RRu	3.68	3.90	5.29	3.47
P	+++	+++	+++	+++
Between Chi				0.02
Between df				2
Between P				N.S.
Btwn(F) P				N.S.
Btwn(R) P				N.S.

Table 1J2 - 3

IESLC - Meta-analysis of Ex Smoking, Years quit (vs never), "Low"
All LC types, Any Product (or Cigarettes if Any not available)
Most adjusted

	Number of adjustment variables (2) 0	1	2	3-5	6+/+nk	Total
N	32	15	7	1		55
NS	23	10	6	1		40
Wt	356.25	166.61	55.12	7.80		585.78
Het Chi	98.18	89.63	21.39	0.00		209.82
Het df	31	14	6	0		54
Het P	***	***	**	N.S.		***
Fixed RR	3.59	3.56	3.77	2.80		3.59
RRl	3.23	3.06	2.89	1.39		3.31
RRu	3.98	4.15	4.90	5.65		3.89
P	+++	+++	+++	++		+++
Random RR	2.97	2.51	3.49	2.80		2.91
RRl	2.40	1.62	2.11	1.39		2.43
RRu	3.68	3.90	5.77	5.65		3.47
P	+++	+++	+++	++		+++
Between Chi						0.61
Between df						3
Between P						N.S.
Btwn(F) P						N.S.
Btwn(R) P						N.S.

	Product all/unsp	cig+/-ot	cig only	Total
N	15	29	11	55
NS	11	22	8	41
Wt	87.21	332.40	166.16	585.78
Het Chi	32.27	80.34	84.55	209.82
Het df	14	28	10	54
Het P	**	***	***	***
Fixed RR	2.69	3.54	4.29	3.59
RRl	2.18	3.18	3.68	3.31
RRu	3.31	3.94	4.99	3.89
P	+++	+++	+++	+++
Random RR	2.58	2.98	3.20	2.91
RRl	1.82	2.43	1.94	2.43
RRu	3.67	3.66	5.29	3.47
P	+++	+++	+++	+++
Between Chi				12.65
Between df				2
Between P				**
Btwn(F) P				N.S.
Btwn(R) P				N.S.

	Denominator nev any	nev cigs	Total
N	42	13	55
NS	30	11	41
Wt	474.11	111.66	585.78
Het Chi	173.17	32.98	209.82
Het df	41	12	54
Het P	***	***	***
Fixed RR	3.73	3.05	3.59
RRl	3.40	2.53	3.31
RRu	4.08	3.67	3.89
P	+++	+++	+++
Random RR	2.88	2.97	2.91
RRl	2.33	2.15	2.43
RRu	3.55	4.11	3.47
P	+++	+++	+++
Between Chi			3.66
Between df			1
Between P			(*)
Btwn(F) P			N.S.
Btwn(R) P			N.S.

Table 1J2 - 3

IESLC - Meta-analysis of Ex Smoking, Years quit (vs never), "Low"
All LC types, Any Product (or Cigarettes if Any not available)
Most adjusted

	Derivation of RR/CI			
	Orig	StdCalc	Other	Total
N	8	34	13	55
NS	7	24	10	41
Wt	56.76	363.67	165.34	585.78
Het Chi	18.85	105.98	84.29	209.82
Het df	7	33	12	54
Het P	**	***	***	***
Fixed RR	3.23	3.62	3.65	3.59
RRl	2.49	3.26	3.13	3.31
RRu	4.19	4.01	4.25	3.89
P	+++	+++	+++	+++
Random RR	3.06	3.00	2.62	2.91
RRl	1.99	2.42	1.68	2.43
RRu	4.71	3.71	4.09	3.47
P	+++	+++	+++	+++
Between Chi				0.69
Between df				2
Between P				N.S.
Btwn(F) P				N.S.
Btwn(R) P				N.S.

Table 1J2 - 4

IESLC - Meta-analysis of Ex Smoking, Years quit (vs never), "Low"
All LC types, Any Product (or Cigarettes if Any not available)
Least adjusted

REF	NRR	X	SEX	AGEL	AGEH	RACE	YF	LC TYPE	LOC	START	ST	NLC	R	VB	P	H	AD	PRODUCT	exL	exH	DENOM	De
ALDERS	507		m	0	0	all	-	all	Eu:UK	1977	CC	1448	n	V	n	n	1	cig only	10	999	nev any	ot
ALDERS	518		f	0	0	all	-	all	Eu:UK	1977	CC	1448	n	V	n	n	1	cig only	10	999	nev any	ot
AUVINE	530	x	c	0	0	all	-	all	Eu:Sca	1986	CC	517	n	bl	y	n	0	cig+/-ot	12	999	nev cigs	st
BECHER	501		m	0	0	all	-	all	Eu:Ger	1985	CC	194	n	bl	n	y	0	all/unsp	10	999	nev any	st
BECHER	511		f	0	0	all	-	all	Eu:Ger	1985	CC	194	n	bl	n	y	0	all/unsp	10	999	nev any	st
BENSHL	510		m	0	0	all	0	all	Eu:UK	1967	pr	486	n	V	n	n	2	cig+/-ot	10	19	nev any	or
CARPEN	502		c	0	0	w+b	-	all	NAmer	1991	CC	356	n	bl	n	n	0	cig+/-ot	10	14	nev cigs	st
CEDERL	528		m	40	69	all	10	all	Eu:Sca	1963	pr	491	n	bl	n	n	1	all/unsp	10	999	nev any	ot
CHOI	534		m	0	0	all	-	all	As:oth	1985	CC	375	n	bl	n	n	0	cig+/-ot	10	14	nev cigs	st
CPSI	807		m	50	74	all	6	all	NAmer	1959	pr	5138	n	bl	n	n	1	cig only	10	999	nev any	ot
CPSII	653		m	35	99	all	4	all	NAmer	1982	pr	3229	n	bl	n	n	1	cig only	11	15	nev any	ot
CPSII	634		f	0	0	all	4	all	NAmer	1982	pr	3229	n	bl	n	n	1	cig+/-ot	11	15	nev cigs	ot
DAMBER	523		m	0	0	all	-	all	Eu:Sca	1972	CC	579	n	bl	y	n	1	all/unsp	11	999	nev any	ot
DARBY	501		m	0	0	wh	-	all	Eu:UK	1988	CC	982	n	V	n	n	0	all/unsp	10	999	nev any	st
DARBY	510		f	0	0	wh	-	all	Eu:UK	1988	CC	982	n	V	n	n	0	all/unsp	10	999	nev any	st
DEAN3	531	x	m	0	0	all	-	all	Eu:UK	1969	CC	766	n	V	y	n	0	all/unsp	9	999	nev any	st
DEAN3	542	x	f	0	0	all	-	all	Eu:UK	1969	CC	766	n	V	y	n	0	all/unsp	9	999	nev any	st
DESTEF	515	x	m	0	0	all	-	all	SCAmer	1988	CC	497	n	bl	n	y	0	all/unsp	10	999	nev any	st
DOLL	530		m	0	0	all	-	all	Eu:UK	1948	CC	1465	n	V	n	n	0	all/unsp	10	19	nev any	st
DOLL	542		f	0	0	all	-	all	Eu:UK	1948	CC	1465	n	V	n	n	0	all/unsp	10	999	nev any	st
DOLL2	502		m	0	0	all	20	all	Eu:UK	1951	pr	920	n	V	n	n	1	cig only	10	14	nev any	ot
DORGAN	501		m	0	0	wh	-	all	NAmer	1980	CC	2026	n	bl	y	y	0	cig+/-ot	10	999	nev any	st
DORGAN	553		f	0	0	all	-	all	NAmer	1980	CC	2026	n	bl	y	y	0	cig+/-ot	10	999	nev any	st
DORN	658		m	55	64	wh	8	all	NAmer	1954	pr	5097	n	bl	n	n	0	cig+/-ot	10	14	nev any	st
DORN	681		m	65	74	wh	8	all	NAmer	1954	pr	5097	n	bl	n	n	0	cig+/-ot	10	14	nev any	st
GAO	521	x	m	0	0	all	-	all	As:Chi	1984	CC	1405	n	ot	n	n	0	cig+/-ot	10	999	nev cigs	st
GAO	541	x	f	0	0	all	-	all	As:Chi	1984	CC	1405	n	ot	n	n	0	cig+/-ot	10	999	nev cigs	st
GAO2	511		m	0	0	all	-	all	As:Jap	1988	CC	282	n	bl	n	n	0	cig+/-ot	10	14	nev cigs	or
GRAHAM	501		m	0	0	wh	-	all	NAmer	1956	CC	685	n	bl	n	n	0	cig only	10	999	nev any	st
GURSEL	501		m	0	0	all	-	all	Eu:bal		CC	953		bl	*	n	0	all/unsp	11	999	nev any	or
HAMMO2	501		m	0	0	all	0	all	NAmer	1967	pr	450	o	bl	n	n	1	cig+/-ot	10	999	nev any	ot
HIRAYA	507		m	0	0	all	0	all	As:Jap	1965	pr	1917	n	bl	n	n	1	cig+/-ot	10	999	nev any	st
HIRAYA	518		f	0	0	all	0	all	As:Jap	1965	pr	1917	n	bl	n	n	1	cig+/-ot	10	999	nev any	st
JAHN	502		m	0	0	all	-	all	Eu:Ger	1988	CC	1004	n	bl	n	n	0	cig+/-ot	11	20	nev any	st
JAIN	567		m	0	0	all	-	all	NAmer	1981	CC	845	n	V	y	n	0	cig+/-ot	10	999	nev cigs	st
JAIN	531		f	0	0	all	-	all	NAmer	1981	CC	845	n	V	y	n	0	cig+/-ot	10	999	nev cigs	st
JEDRYC	611		m	0	0	all	-	all	Eu:est	1980	CC	1630	n	bl	y	n	0	cig+/-ot	10	999	nev any	st
KAISE2	647		m	0	0	all	9	all	NAmer	1979	pr	318	n	bl	n	n	1	cig only	11	20	nev any	ot
KAISE2	567		f	0	0	all	9	all	NAmer	1979	pr	318	n	bl	n	n	1	cig only	11	20	nev any	st
LAUSSM	501	x	m	0	0	all	-	all	Eu:Ger	1982	CC	432	n	bl	n	n	0	all/unsp	10	999	nev any	st
LUBIN	585		m	0	0	all	-	all	As:Chi	1984	CC	427	m	ot	y	n	0	cig+/-ot	10	999	nev any	st
LUBIN2	1072		m	0	0	all	-	all	Eu:mul	1976	CC	7804	n	bl	n	y	0	cig+/-ot	10	14	nev any	st
LUBIN2	1111		f	0	0	all	-	all	Eu:mul	1976	CC	7804	n	bl	n	y	0	cig+/-ot	10	14	nev any	st
MATOS	581	x	m	0	0	all	-	all	SCAmer	1994	CC	200	n	bl	n	n	0	cig+/-ot	11	999	nev any	st
PEZZO2	501		m	0	0	all	-	all	SCAmer	1992	CC	367	n	bl	n	y	0	cig+/-ot	11	999	nev cigs	st
PEZZOT	501		m	0	0	all	-	all	SCAmer	1987	CC	215	n	bl	n	y	0	cig only	11	999	nev cigs	st
SOBUE	720		m	0	0	all	-	all	As:Jap	1986	CC	1376	n	bl	n	y	0	cig+/-ot	10	14	nev cigs	st
SPEIZE	502		f	0	0	all	0	all	NAmer	1976	pr	593	n	bl	n	y	0	cig+/-ot	10	15	nev cigs	st
SUZUK2	508		c	0	0	all	-	all	SCAmer	1991	CC	123	n	bl	n	y	0	all/unsp	11	999	nev any	st
SVENSS	551		f	0	0	all	-	all	Eu:Sca	1983	CC	210	n	bl	n	n	0	all/unsp	11	999	nev any	st
WAKAI	523	x	m	0	0	all	-	all	As:Jap	1988	CC	333	n	bl	n	y	0	cig+/-ot	10	19	nev any	st
WYNDE3	566		m	0	0	all	-	all	NAmer	1966	CC	350	n	bl	n	y	0	cig+/-ot	10	999	nev any	st
WYNDE3	587		f	0	0	all	-	all	NAmer	1966	CC	350	n	bl	n	y	0	cig+/-ot	10	999	nev any	st
WYNDE6	503		m	0	0	all	-	all	NAmer	1969	CC	4423	n	bl	n	y	0	cig only	10	19	nev any	st
WYNDE6	524		f	0	0	all	-	all	NAmer	1969	CC	4423	n	bl	n	y	0	cig only	10	19	nev any	st

Cigarette type is all/unspec for all RRs
except for the following:

REF	NRR	CIGTYPE
ALDERS	507	MC only
ALDERS	518	MC only

Table 1J2 - 5

IESLC - Meta-analysis of Ex Smoking, Years quit (vs never), "Low"
All LC types, Any Product (or Cigarettes if Any not available)
Least adjusted

REF	NRR	SEX	AD	Number Case	Exposed Cont	Non-exposed Case	Cont	RR	95.00%CI
ALDERS	507	m	1	29	-	15	-	3.20	(1.61- 6.35)
ALDERS	518	f	1	26	-	75	-	1.27	(0.76- 2.15)
Subtotal ALDERS								1.78	(1.18- 2.69)
AUVINE	530	c	0	207	208	44	229	5.18	(3.56- 7.54)
BECHER	501	m	0	16	72	3	54	4.00	(1.11- 14.42)
BECHER	511	f	0	1	10	10	52	0.52	(0.06- 4.53)
Subtotal BECHER								2.35	(0.78- 7.09)
*BENSHL	510	m	2	23	-	10	-	4.08	(2.03- 8.20)
CARPEN	502	c	0	9	51	8	208	4.59	(1.69- 12.48)
*CEDERL	528	m	1	3	-	7	-	1.10	(0.28- 4.25)
CHOI	534	m	0	4	23	13	95	1.27	(0.38- 4.26)
*CPSI	807	m	1	15	-	60	-	1.28	(0.73- 2.25)
*CPSII	653	m	1	164	-	81	-	8.61	(6.60- 11.24)
*CPSII	634	f	1	28	-	174	-	3.86	(2.59- 5.75)
Subtotal CPSII								6.72	(5.39- 8.39)
DAMBER	523	m	1	42	-	42	-	2.60	(1.70- 4.50)
DARBY	501	m	0	139	767	3	384	23.20	(7.34- 73.28)
DARBY	510	f	0	26	224	23	529	2.67	(1.49- 4.78)
Subtotal DARBY								4.15	(2.47- 6.98)
DEAN3	531	m	0	32	204	24	510	3.33	(1.92- 5.80)
DEAN3	542	f	0	2	114	41	1538	0.66	(0.16- 2.76)
Subtotal DEAN3								2.70	(1.61- 4.52)
DESTEF	515	m	0	17	36	27	163	2.85	(1.41- 5.78)
DOLL	530	m	0	6	26	7	61	2.01	(0.62- 6.56)
DOLL	542	f	0	1	2	40	59	0.74	(0.06- 8.41)
Subtotal DOLL								1.66	(0.57- 4.81)
*DOLL2	502	m	1	9	-	7	-	5.30	(1.97- 14.23)
DORGAN	501	m	0	134	255	13	140	5.66	(3.09- 10.37)
DORGAN	553	f	0	34	50	61	213	2.37	(1.41- 4.00)
Subtotal DORGAN								3.43	(2.31- 5.10)
*DORN	658	m	0	12	23682	25	213858	4.33	(2.18- 8.63)
*DORN	681	m	0	29	20056	49	171211	5.05	(3.19- 7.99)
Subtotal DORN								4.82	(3.29- 7.06)
GAO	521	m	0	13	41	62	202	1.03	(0.52- 2.05)
GAO	541	f	0	16	14	435	605	1.59	(0.77- 3.29)
Subtotal GAO								1.27	(0.77- 2.08)
GAO2	511	m	0	16	18	13	56	3.83	(1.55- 9.46)
GRAHAM	501	m	0	2	30	18	346	1.28	(0.28- 5.79)
GURSEL	501	m	0	-	-	-	-	2.30	(1.01- 5.22)
*HAMMO2	501	m	1	20	-	5	-	3.45	(1.30- 9.14)
*HIRAYA	507	m	1	-	-	-	-	1.38	(0.59- 3.21)
*HIRAYA	518	f	1	-	-	-	-	0.97	(0.03- 32.06)
Subtotal HIRAYA								1.35	(0.59- 3.08)
JAHN	502	m	0	64	130	18	138	3.77	(2.12- 6.71)
JAIN	567	m	0	52	113	12	85	3.26	(1.64- 6.48)
JAIN	531	f	0	19	61	52	214	1.28	(0.71- 2.33)
Subtotal JAIN								1.91	(1.22- 3.01)
JEDRYC	611	m	0	73	138	49	219	2.36	(1.55- 3.60)
*KAISE2	647	m	1	8	-	14	-	3.14	(1.26- 7.82)
*KAISE2	567	f	1	4	-	11	-	4.37	(1.30- 14.72)
Subtotal KAISE2								3.54	(1.71- 7.34)
LAUSSM	501	m	0	29	15	85	226	5.14	(2.63- 10.06)
LUBIN	585	m	0	17	73	9	72	1.86	(0.78- 4.45)
LUBIN2	1072	m	0	270	693	190	2616	5.36	(4.38- 6.58)
LUBIN2	1111	f	0	10	26	336	1188	1.36	(0.65- 2.85)
Subtotal LUBIN2								4.87	(4.00- 5.93)
MATOS	581	m	0	27	101	11	110	2.67	(1.26- 5.67)
PEZZO2	501	m	0	43	161	6	117	5.21	(2.15- 12.64)
PEZZOT	501	m	0	20	106	4	116	5.47	(1.81- 16.53)
SOBUE	720	m	0	35	50	29	126	3.04	(1.68- 5.49)
*SPEIZE	502	f	0	17	93933	58	776300	2.42	(1.41- 4.16)
SUZUK2	508	c	0	9	22	11	53	1.97	(0.72- 5.42)
SVENSS	551	f	0	14	24	38	120	1.84	(0.87- 3.91)
WAKAI	523	m	0	27	44	10	65	3.99	(1.76- 9.06)
WYNDE3	566	m	0	9	65	9	88	1.35	(0.51- 3.60)
WYNDE3	587	f	0	1	3	20	76	1.27	(0.12- 12.84)
Subtotal WYNDE3								1.34	(0.54- 3.30)
WYNDE6	503	m	0	159	373	64	918	6.11	(4.47- 8.37)
WYNDE6	524	f	0	36	132	125	991	2.16	(1.43- 3.27)
Subtotal WYNDE6								4.18	(3.25- 5.36)
Partial Totals				2018	142146	2556	1174351		

Table 1J2 - 5

IESLC - Meta-analysis of Ex Smoking, Years quit (vs never), "Low"
All LC types, Any Product (or Cigarettes if Any not available)
Least adjusted

REF	NRR	SEX	AD	Number Exposed Case	Cont	Non-exposed Case	Cont	RR	95.00%CI

*prospective study

REF	NRR	SEX	AD	Ys	Ws	Qs	Ps
ALDERS	507	m	1	1.16	8.16	0.11	0.0009
ALDERS	518	f	1	0.24	14.21	15.31	0.3676
Subtotal ALDERS				0.58	22.37	15.42	
AUVINE	530	c	0	1.64	27.22	3.68	0.0000
BECHER	501	m	0	1.39	2.34	0.03	0.0341
BECHER	511	f	0	-0.65	0.82	3.06	0.5537
Subtotal BECHER				0.86	3.16	3.09	
*BENSHL	510	m	2	1.41	7.88	0.13	0.0001
CARPEN	502	c	0	1.52	3.84	0.23	0.0028
*CEDERL	528	m	1	0.10	2.08	2.90	0.8907
CHOI	534	m	0	0.24	2.63	2.83	0.6977
*CPSI	807	m	1	0.25	12.13	12.87	0.3900
*CPSII	653	m	1	2.15	54.21	41.58	0.0000
*CPSII	634	f	1	1.35	24.16	0.13	0.0000
Subtotal CPSII				1.91	78.37	41.71	
DAMBER	523	m	1	0.96	16.22	1.68	0.0001
DARBY	501	m	0	3.14	2.90	10.12	0.0000
DARBY	510	f	0	0.98	11.33	0.99	0.0010
Subtotal DARBY				1.42	14.23	11.11	
DEAN3	531	m	0	1.20	12.53	0.07	0.0000
DEAN3	542	f	0	-0.42	1.87	5.39	0.5669
Subtotal DEAN3				0.99	14.41	5.45	
DESTEF	515	m	0	1.05	7.71	0.41	0.0036
DOLL	530	m	0	0.70	2.74	0.92	0.2471
DOLL	542	f	0	-0.30	0.65	1.62	0.8063
Subtotal DOLL				0.51	3.39	2.54	
*DOLL2	502	m	1	1.67	3.93	0.60	0.0009
DORGAN	501	m	0	1.73	10.48	2.18	0.0000
DORGAN	553	f	0	0.86	14.18	2.41	0.0011
Subtotal DORGAN				1.23	24.66	4.59	
*DORN	658	m	0	1.47	8.11	0.29	0.0000
*DORN	681	m	0	1.62	18.24	2.14	0.0000
Subtotal DORN				1.57	26.35	2.43	
GAO	521	m	0	0.03	8.17	12.66	0.9260
GAO	541	f	0	0.46	7.25	4.80	0.2120
Subtotal GAO				0.24	15.42	17.46	
GAO2	511	m	0	1.34	4.70	0.02	0.0036
GRAHAM	501	m	0	0.25	1.69	1.79	0.7471
GURSEL	501	m	0	0.83	5.70	1.12	0.0468
*HAMMO2	501	m	1	1.24	4.04	0.01	0.0128
*HIRAYA	507	m	1	0.32	5.36	4.88	0.4561
*HIRAYA	518	f	1	-0.03	0.32	0.54	0.9863
Subtotal HIRAYA				0.30	5.67	5.43	
JAHN	502	m	0	1.33	11.61	0.03	0.0000
JAIN	567	m	0	1.18	8.12	0.07	0.0008
JAIN	531	f	0	0.25	10.76	11.39	0.4154
Subtotal JAIN				0.65	18.88	11.47	
JEDRYC	611	m	0	0.86	21.78	3.78	0.0001
*KAISE2	647	m	1	1.14	4.61	0.08	0.0140
*KAISE2	567	f	1	1.47	2.61	0.10	0.0172
Subtotal KAISE2				1.26	7.22	0.18	
LAUSSM	501	m	0	1.64	8.52	1.10	0.0000
LUBIN	585	m	0	0.62	5.06	2.17	0.1615
LUBIN2	1072	m	0	1.68	92.66	15.02	0.0000
LUBIN2	1111	f	0	0.31	7.03	6.61	0.4151
Subtotal LUBIN2				1.58	99.69	21.63	
MATOS	581	m	0	0.98	6.81	0.59	0.0103
PEZZO2	501	m	0	1.65	4.89	0.68	0.0003
PEZZOT	501	m	0	1.70	3.14	0.56	0.0026
SOBUE	720	m	0	1.11	10.99	0.30	0.0002
*SPEIZE	502	f	0	0.88	13.15	2.02	0.0013
SUZUK2	508	c	0	0.68	3.75	1.35	0.1886
SVENSS	551	f	0	0.61	6.77	3.00	0.1120
WAKAI	523	m	0	1.38	5.71	0.06	0.0009
WYNDE3	566	m	0	0.30	4.02	3.81	0.5438
WYNDE3	587	f	0	0.24	0.72	0.78	0.8415
Subtotal WYNDE3				0.29	4.73	4.59	

Table 1J2 - 5

IESLC - Meta-analysis of Ex Smoking, Years quit (vs never), "Low"
All LC types, Any Product (or Cigarettes if Any not available)
Least adjusted

REF	NRR	SEX	AD	Ys	Ws	Qs	Ps
WYNDE6	503	m	0	1.81	38.93	11.08	0.0000
WYNDE6	524	f	0	0.77	22.54	5.77	0.0003
Subtotal WYNDE6				1.43	61.48	16.85	

	N	55
	NS	40
	Wt	601.95
Het	Chi	207.86
Het	df	54
Het	P	***
Fixed	RR	3.59
	RRl	3.31
	RRu	3.88
	P	+++
Random	RR	2.88
	RRl	2.42
	RRu	3.43
	P	+++
Asymm	P	***

Table 1J2 - 6

IESLC - Meta-analysis of Ex Smoking, Years quit (vs never), "Low"
All LC types, Any Product (or Cigarettes if Any not available)
Least adjusted

		Sex		
	combined	male	female	Total
N	3	36	16	55
NS	3	35	16	54
Wt	34.82	428.77	138.36	601.95
Het Chi	3.08	128.89	24.93	207.86
Het df	2	35	15	54
Het P	N.S.	***	(*)	***
Fixed RR	4.61	4.17	2.11	3.59
RRl	3.30	3.79	1.79	3.31
RRu	6.42	4.59	2.49	3.88
P	+++	+++	+++	+++
Random RR	4.15	3.33	1.97	2.88
RRl	2.45	2.72	1.55	2.42
RRu	7.01	4.09	2.51	3.43
P	+++	+++	+++	+++
Between Chi				50.96
Between df				2
Between P				***
Btwn(F) P				***
Btwn(R) P				**

Table 1J2 - 7

IESLC - Meta-analysis of Ex Smoking, Years quit (vs never), "Low"
All LC types, Any Product (or Cigarettes if Any not available)
Excluded studies (and stage at which they were excluded)

1	AGUDO AKIBA AMANDU AMES AXELSS BEST BOUCHA BOUCOT BRESLO CHEN CHEN2 CHIAZZ DEAN2 DOSEME ENGELA FAN GENG GER GUO HAENSZ HEGMAN HOLE HU HU2 JUSSAW KATSOU KAUFMA KOO KOULUM KREUZE LETOUR LEVIN LIAW LIU3 LIU4 LIU5 MCCONN MIGRAN MRFITR NOTAN2 OSANN2 PERNU QIAO2 RACHTA RESTRE SADOWS SEGI2 STASZE TIZZAN VUTUC WATSON WU WUWILL WYNDE2 WYNDE8 XU YUAN ZHANG ZHENG ZHOU
2	BUFFLE HUMBLE PISANI PRESCO WYNDE7
3	MCDUFF SPITZ
4	HAMMON LUO WU2
5	BLOT1 CORREA GILLIS QIAO WIGLE
7	BOFFET
14	ARMADA BARBON BROSS BROWN3 CHYOU GARCIA GARSHI JOLY KHUDER TVERDA WANG2
15	BENHAM

Table 1J2 - 8
Potentially overlapping studies

REF	REFGP	PRINC	.	OVERLAP/LINK
LUBIN2	LUBIN2	1		Lubin-combined
GRAHAM	BYERS1	1		GRAHAM/BROSS/BYERS1
BENSHL	TANG2	1		Subset of TANG2
WYNDE6	WYNDE6	1		WYNDE5/6/7/8
CPSI	CPSI	1		CPSI overall
JAHN	BOFFET	2		Subset of BOFFET
LUBIN	XIANGZ	2		LUBIN/XIANGZ/QIAO

Table 1J2 - 9
Most adjusted - insufficient data for meta-analysis

REF	NRR	SEX	AGEL	AGEH	RACE	YF	LC TYPE	LOC	START	ST	NLC	R	VB	P	H	AD	PRODUCT	exL	exH	DENOM	De
BLOT1	501	m	0	0	all	-	all	NAmer	1970	CC	458	n	bl	y	n	0	cig+/-ot	10	999	nev cigs	or
CPSI	721	f	0	0	wh	0	all	NAmer	1959	pr	5138	n	bl	n	n	1	cig only	10	14	nev cigs	or
SPEIZE	539	f	0	0	all	0	all	NAmer	1976	pr	593	n	bl	n	y	2	cig+/-ot	10	15	nev cigs	st

REF	NRR	RR	SIG	RRDATA comment
BLOT1	501	1.80		0
CPSI	721	0.58		0
SPEIZE	539	2.00		Insufficient decimals to calculate CIs

Table 1J3 -

IESLC - Meta-analysis of Ex Smoking, Years quit (vs never), "Mid"
All LC types, Any Product (or Cigarettes if Any not available)

This analysis is restricted to results for:
1) Ex smokers
2) Results by Years quit (vs never)
3) Categorical results by Years quit (vs never)
4) All LC types (or near equivalent)
5) Results complete enough for use in metaanalysis

Within each study, results are then selected (in the following order of preference, within each sex) for:
6) (not applicable)
7) PRODUCT: all/unspec, cigarettes regardless of other products, cigarettes only
8) CIGTYPE: all/unspecified, MC regardless of HR, MC only
9) (not applicable)
10) DENOM: never smoked anything, never smoked cigarettes, never any + low, never cigs + low
11) Followup period (YF, prospective studies): whole study (coded as 0) or longest available
12) LCtype: all or nearest available, at least Squamous and Adeno. (q = squamous, s = small,
l = large, a = adeno, mix = mixed, alv = alveolar)
13) Race: all or nearest available, otherwise by race (wh or w = white, bl or b = black, hi = hispanic
ch = chinese, jap = japanese, haw = hawaiian, w+o = white + oriental, sca = scandinavian, as = asian)
14) Years quit (vs never) "mid" in key scheme 1 (key value 7, maximum range 4-11)
15) For overlapping studies: principal rather than subsidiary studies
Finally by Age: whole study (coded as 0) if available, otherwise by widest available age group
and then for single sex results (m, f) in preference to results for both sexes combined (c).

Results adjusted (AD) for the most potential confounders are then chosen in Sections -1 to -3
and results adjusted for the least confounders in Sections -4 to -6. (Those least adjusted results which
actually differ from the most adjusted are marked 'x' in column X in Section -4)

Section -7 shows excluded studies, together with the stage (as above) at which no qualifying
results were found.

Section -8 lists the potentially overlapping studies which have been included (1=principal, 2=subsidiary).

Section -9 lists any results which would have been included in preference except that they had data not complete
enough for use in meta-analysis, with their significance (yes/no), if known, and any further comment as entered
on the database. It also lists as "gap" any categories for which no data were presented by the original authors.
This is commonly due to recent quitters having been combined with current smokers

In addition to those mentioned above, the following fields, levels and abbreviations are used:
* or nk = not known, n = no, y = yes, ot = other
nev = never
all/unspec = all or unspecified, cig+/-ot = cigarettes irrespective of other products (cigar, pipe etc)
MC = manufactured cigarettes, HR = hand-rolled cigarettes
exL, exH = range of exposure (low and high) in the smoking group, in terms of Years quit (vs never)
REF: 6-character study reference
NRR: number of the RR on the database within the study
ST : study type (CC = case control, pr or prosp = prospective)
NLC: number of lung cancer cases in whole study
R : risky occupational population (n = no, m = mining, o = other risky)
VB : national cigarette type (V = at least 75% Virginia, bl = at least 75% blended, ot = other)
P : any proxy use
H : full histological confirmation
De : derivation of RR/CI (or = original, st = standard method, ot = other method of estimation)

Table 1J3 - 1

IESLC - Meta-analysis of Ex Smoking, Years quit (vs never), "Mid"
All LC types, Any Product (or Cigarettes if Any not available)
Most adjusted

REF	NRR	SEX	AGEL	AGEH	RACE	YF	LC TYPE	LOC	START	ST	NLC	R	VB	P	H	AD	PRODUCT	exL	exH	DENOM	De
BECHER	502	m	0	0	all	-	all	Eu:Ger	1985	CC	194	n	bl	n	y	0	all/unsp	5	9	nev any	st
BECHER	512	f	0	0	all	-	all	Eu:Ger	1985	CC	194	n	bl	n	y	0	all/unsp	5	9	nev any	st
CARPEN	503	c	0	0	w+b	-	all	NAmer	1991	CC	356	n	bl	n	n	0	cig+/-ot	5	9	nev cigs	st
CHOI	535	m	0	0	all	-	all	As:oth	1985	CC	375	n	bl	n	n	0	cig+/-ot	5	9	nev cigs	st
CPSI	808	m	50	74	all	6	all	NAmer	1959	pr	5138	n	bl	n	n	1	cig only	5	9	nev any	ot
CPSII	654	m	35	99	all	4	all	NAmer	1982	pr	3229	n	bl	n	n	1	cig only	6	10	nev any	ot
CPSII	635	f	0	0	all	4	all	NAmer	1982	pr	3229	n	bl	n	n	1	cig+/-ot	6	10	nev cigs	ot
DAMBER	524	m	0	0	all	-	all	Eu:Sca	1972	CC	579	n	bl	y	n	1	all/unsp	6	10	nev any	ot
DEAN3	629	m	0	0	all	-	all	Eu:UK	1969	CC	766	n	V	y	n	1	all/unsp	5	8	nev any	ot
DEAN3	554	f	0	0	all	-	all	Eu:UK	1969	CC	766	n	V	y	n	1	all/unsp	5	8	nev any	ot
DESTEF	526	m	0	0	all	-	all	SCAmer	1988	CC	497	n	bl	n	y	4	all/unsp	5	9	nev any	or
DOLL2	503	m	0	0	all	20	all	Eu:UK	1951	pr	920	n	V	n	n	1	cig only	5	9	nev any	ot
DORGAN	502	m	0	0	wh	-	all	NAmer	1980	CC	2026	n	bl	y	y	0	cig+/-ot	6	9	nev any	st
DORN	659	m	55	64	wh	8	all	NAmer	1954	pr	5097	n	bl	n	n	0	cig+/-ot	5	9	nev any	st
DORN	682	m	65	74	wh	8	all	NAmer	1954	pr	5097	n	bl	n	n	0	cig+/-ot	5	9	nev any	st
GAO	532	m	0	0	all	-	all	As:Chi	1984	CC	1405	n	ot	n	n	2	cig+/-ot	5	9	nev cigs	or
GAO	552	f	0	0	all	-	all	As:Chi	1984	CC	1405	n	ot	n	n	2	cig+/-ot	5	9	nev cigs	or
GAO2	512	m	0	0	all	-	all	As:Jap	1988	CC	282	n	bl	n	n	0	cig+/-ot	5	9	nev cigs	st
GRAHAM	502	m	0	0	wh	-	all	NAmer	1956	CC	685	n	bl	n	n	0	cig only	3	10	nev any	st
HAMMO2	502	m	0	0	all	0	all	NAmer	1967	pr	450	o	bl	n	n	1	cig+/-ot	5	9	nev any	ot
HIRAYA	508	m	0	0	all	0	all	As:Jap	1965	pr	1917	n	bl	n	n	1	cig+/-ot	5	9	nev any	st
HIRAYA	519	f	0	0	all	0	all	As:Jap	1965	pr	1917	n	bl	n	n	1	cig+/-ot	5	9	nev any	st
JAHN	503	m	0	0	all	-	all	Eu:Ger	1988	CC	1004	n	bl	n	n	0	cig+/-ot	6	10	nev any	st
JEDRYC	612	m	0	0	all	-	all	Eu:est	1980	CC	1630	n	bl	y	n	0	cig+/-ot	5	9	nev any	st
LUBIN	586	m	0	0	all	-	all	As:Chi	1984	CC	427	m	ot	y	n	0	cig+/-ot	5	9	nev any	st
LUBIN2	1073	m	0	0	all	-	all	Eu:mul	1976	CC	7804	n	bl	n	y	0	cig+/-ot	5	9	nev any	st
LUBIN2	1112	f	0	0	all	-	all	Eu:mul	1976	CC	7804	n	bl	n	y	0	cig+/-ot	5	9	nev any	st
MATOS	592	m	0	0	all	-	all	SCAmer	1994	CC	200	n	bl	n	n	2	cig+/-ot	6	10	nev any	ot
SOBUE	721	m	0	0	all	-	all	As:Jap	1986	CC	1376	n	bl	n	y	0	cig+/-ot	5	9	nev cigs	st
SPEIZE	503	f	0	0	all	0	all	NAmer	1976	pr	593	n	bl	n	y	0	cig+/-ot	5	10	nev cigs	st
SUZUK2	509	c	0	0	all	-	all	SCAmer	1991	CC	123	n	bl	n	y	0	all/unsp	6	10	nev any	st
WAKAI	532	m	0	0	all	-	all	As:Jap	1988	CC	333	n	bl	n	y	2	cig+/-ot	5	9	nev any	or
WYNDE6	504	m	0	0	all	-	all	NAmer	1969	CC	4423	n	bl	n	y	0	cig only	5	9	nev any	st
WYNDE6	525	f	0	0	all	-	all	NAmer	1969	CC	4423	n	bl	n	y	0	cig only	5	9	nev any	st

Cigarette type is all/unspec for all RRs

Table 1J3 - 2

IESLC - Meta-analysis of Ex Smoking, Years quit (vs never), "Mid"
All LC types, Any Product (or Cigarettes if Any not available)
Most adjusted

REF	NRR	SEX	AD	Number Exposed Case	Exposed Cont	Non-exposed Case	Non-exposed Cont	RR	95.00%CI
BECHER	502	m	0	16	32	3	54	9.00	(2.43- 33.30)
BECHER	512	f	0	2	5	10	52	2.08	(0.35- 12.26)
Subtotal BECHER								5.37	(1.87- 15.40)
CARPEN	503	c	0	25	48	8	208	13.54	(5.75- 31.87)
CHOI	535	m	0	5	30	13	95	1.22	(0.40- 3.70)
*CPSI	808	m	1	32	-	60	-	5.15	(3.35- 7.91)
*CPSII	654	m	1	186	-	81	-	11.43	(8.81- 14.84)
*CPSII	635	f	1	37	-	174	-	4.91	(3.45- 7.01)
Subtotal CPSII								8.50	(6.89- 10.48)
DAMBER	524	m	1	-	-	42	-	4.30	(2.30- 8.10)
DEAN3	629	m	1	15	-	24	-	3.29	(1.65- 6.57)
DEAN3	554	f	1	1	-	41	-	1.09	(0.15- 8.13)
Subtotal DEAN3								2.92	(1.52- 5.62)
DESTEF	526	m	4	27	-	27	-	6.20	(3.20- 12.20)
*DOLL2	503	m	1	12	-	7	-	5.90	(2.32- 14.99)
DORGAN	502	m	0	49	38	13	140	13.89	(6.84- 28.21)
*DORN	659	m	0	32	34566	25	213858	7.92	(4.69- 13.36)
*DORN	682	m	0	41	24089	49	171211	5.95	(3.93- 9.00)
Subtotal DORN								6.64	(4.80- 9.19)
GAO	532	m	2	24	-	62	-	3.10	(1.70- 5.90)
GAO	552	f	2	14	-	435	-	3.90	(1.50- 9.90)
Subtotal GAO								3.32	(1.98- 5.59)
GAO2	512	m	0	21	26	13	56	3.48	(1.51- 8.01)
GRAHAM	502	m	0	5	29	18	346	3.31	(1.15- 9.57)
*HAMMO2	502	m	1	11	-	5	-	3.98	(1.39- 11.40)
*HIRAYA	508	m	1	-	-	-	-	1.59	(0.66- 3.82)
*HIRAYA	519	f	1	-	-	-	-	3.29	(0.56- 19.50)
Subtotal HIRAYA								1.83	(0.84- 4.03)
JAHN	503	m	0	59	63	18	138	7.18	(3.92- 13.16)
JEDRYC	612	m	0	64	58	49	219	4.93	(3.08- 7.90)
LUBIN	586	m	0	20	48	9	72	3.33	(1.40- 7.94)
LUBIN2	1073	m	0	466	822	190	2616	7.81	(6.48- 9.40)
LUBIN2	1112	f	0	30	40	336	1188	2.65	(1.63- 4.32)
Subtotal LUBIN2								6.81	(5.72- 8.10)
MATOS	592	m	2	21	-	11	-	9.00	(3.84- 21.08)
SOBUE	721	m	0	67	92	29	126	3.16	(1.90- 5.28)
*SPEIZE	503	f	0	41	95585	58	776300	5.74	(3.85- 8.56)
SUZUK2	509	c	0	10	8	11	53	6.02	(1.94- 18.72)
WAKAI	532	m	2	19	-	10	-	2.48	(1.04- 5.92)
WYNDE6	504	m	0	98	194	64	918	7.25	(5.10- 10.29)
WYNDE6	525	f	0	51	84	125	991	4.81	(3.24- 7.14)
Subtotal WYNDE6								6.05	(4.65- 7.86)
Partial Totals				1501	155857	2020	1168641		

*prospective study

REF	NRR	SEX	AD	Ys	Ws	Qs	Ps
BECHER	502	m	0	2.20	2.24	0.36	0.0010
BECHER	512	f	0	0.73	1.22	1.39	0.4184
Subtotal BECHER				1.68	3.46	1.74	
CARPEN	503	c	0	2.61	5.25	3.41	0.0000
CHOI	535	m	0	0.20	3.12	8.00	0.7277
*CPSI	808	m	1	1.64	20.82	0.53	0.0000
*CPSII	654	m	1	2.44	56.51	22.94	0.0000
*CPSII	635	f	1	1.59	30.57	1.32	0.0000
Subtotal CPSII				2.14	87.08	24.26	
DAMBER	524	m	1	1.46	9.69	1.12	0.0000
DEAN3	629	m	1	1.19	8.05	2.98	0.0007
DEAN3	554	f	1	0.09	0.96	2.83	0.9326
Subtotal DEAN3				1.07	9.01	5.81	
DESTEF	526	m	4	1.82	8.58	0.01	0.0000
*DOLL2	503	m	1	1.77	4.41	0.00	0.0002
DORGAN	502	m	0	2.63	7.65	5.29	0.0000
*DORN	659	m	0	2.07	14.04	1.03	0.0000
*DORN	682	m	0	1.78	22.35	0.01	0.0000
Subtotal DORN				1.89	36.39	1.03	
GAO	532	m	2	1.13	9.92	4.42	0.0004
GAO	552	f	2	1.36	4.31	0.83	0.0047
Subtotal GAO				1.20	14.24	5.25	
GAO2	512	m	0	1.25	5.53	1.69	0.0034
GRAHAM	502	m	0	1.20	3.41	1.23	0.0268

Table 1J3 - 2

IESLC - Meta-analysis of Ex Smoking, Years quit (vs never), "Mid"
All LC types, Any Product (or Cigarettes if Any not available)
Most adjusted

REF	NRR	SEX	AD	Ys	Ws	Qs	Ps
*HAMMO2	502	m	1	1.38	3.47	0.61	0.0101
*HIRAYA	508	m	1	0.46	4.98	8.89	0.3005
*HIRAYA	519	f	1	1.19	1.22	0.45	0.1885
Subtotal HIRAYA				0.61	6.20	9.34	
JAHN	503	m	0	1.97	10.46	0.31	0.0000
JEDRYC	612	m	0	1.60	17.29	0.72	0.0000
LUBIN	586	m	0	1.20	5.11	1.81	0.0065
LUBIN2	1073	m	0	2.05	111.01	7.26	0.0000
LUBIN2	1112	f	0	0.98	16.09	10.92	0.0001
Subtotal LUBIN2				1.92	127.10	18.18	
MATOS	592	m	2	2.20	5.30	0.84	0.0000
SOBUE	721	m	0	1.15	14.66	6.14	0.0000
*SPEIZE	503	f	0	1.75	24.03	0.06	0.0000
SUZUK2	509	c	0	1.80	2.99	0.00	0.0019
WAKAI	532	m	2	0.91	5.08	4.03	0.0406
WYNDE6	504	m	0	1.98	31.18	1.03	0.0000
WYNDE6	525	f	0	1.57	24.68	1.28	0.0000
Subtotal WYNDE6				1.80	55.86	2.30	

	N	34
	NS	26
	Wt	496.18
Het	Chi	103.72
Het	df	33
Het	P	***
Fixed	RR	6.04
	RRl	5.54
	RRu	6.60
	P	+++
Random	RR	5.04
	RRl	4.21
	RRu	6.04
	P	+++
Asymm	P	**

Table 1J3 - 3

IESLC - Meta-analysis of Ex Smoking, Years quit (vs never), "Mid"
All LC types, Any Product (or Cigarettes if Any not available)
Most adjusted

	Sex			
	combined	male	female	Total
N	2	24	8	34
NS	2	23	8	33
Wt	8.23	384.87	103.08	496.18
Het Chi	1.25	79.22	9.12	103.72
Het df	1	23	7	33
Het P	N.S.	***	N.S.	***
Fixed RR	10.09	6.50	4.43	6.04
RRl	5.10	5.88	3.65	5.54
RRu	19.98	7.18	5.37	6.60
P	+++	+++	+++	+++
Random RR	9.87	5.18	4.29	5.04
RRl	4.55	4.19	3.36	4.21
RRu	21.42	6.42	5.47	6.04
P	+++	+++	+++	+++
Between Chi				14.14
Between df				2
Between P				***
Btwn(F) P				N.S.
Btwn(R) P				N.S.

	Lung cancer type		
	all	other	Total
N	34		34
NS	26		26
Wt	496.18		496.18
Het Chi	103.72		103.72
Het df	33		33
Het P	***		***
Fixed RR	6.04		6.04
RRl	5.54		5.54
RRu	6.60		6.60
P	+++		+++
Random RR	5.04		5.04
RRl	4.21		4.21
RRu	6.04		6.04
P	+++		+++
Between Chi			
Between df			
Between P			N.S.
Btwn(F) P			N.S.
Btwn(R) P			N.S.

	Location								
	NAmer	UK	Scand	othEur	China	Japan	othAs	other	Total
N	12	3	1	6	3	5	1	3	34
NS	9	2	1	4	2	4	1	3	26
Wt	243.95	13.43	9.69	158.31	19.35	31.47	3.12	16.87	496.18
Het Chi	33.53	2.51	0.00	19.88	0.16	2.18	0.00	0.53	103.72
Het df	11	2	0	5	2	4	0	2	33
Het P	***	N.S.	N.S.	**	N.S.	N.S.	N.S.	N.S.	***
Fixed RR	7.00	3.68	4.30	6.56	3.33	2.78	1.22	6.93	6.04
RRl	6.17	2.16	2.29	5.62	2.13	1.96	0.40	4.30	5.54
RRu	7.93	6.29	8.07	7.67	5.19	3.94	3.70	11.18	6.60
P	+++	+++	+++	+++	+++	+++	N.S.	+++	+++
Random RR	6.71	3.65	4.30	5.26	3.33	2.78	1.22	6.93	5.04
RRl	5.29	1.92	2.29	3.35	2.13	1.96	0.40	4.30	4.21
RRu	8.52	6.92	8.07	8.28	5.19	3.94	3.70	11.18	6.04
P	+++	+++	+++	+++	+++	+++	N.S.	+++	+++
Between Chi									44.93
Between df									7
Between P									***
Btwn(F) P									*
Btwn(R) P									***

Table 1J3 - 3

IESLC - Meta-analysis of Ex Smoking, Years quit (vs never), "Mid"
All LC types, Any Product (or Cigarettes if Any not available)
Most adjusted

	Detailed Country in "other Europe" multi	Germany	othWest	East	Balkans	Total
N	2	3		1		6
NS	1	2		1		4
Wt	127.10	13.92		17.29		158.31
Het Chi	16.38	1.92		0.00		19.88
Het df	1	2		0		5
Het P	***	N.S.		N.S.		**
Fixed RR	6.81	6.68		4.93		6.56
RRl	5.72	3.95		3.08		5.62
RRu	8.10	11.30		7.90		7.67
P	+++	+++		+++		+++
Random RR	4.66	6.68		4.93		5.26
RRl	1.62	3.95		3.08		3.35
RRu	13.42	11.30		7.90		8.28
P	++	+++		+++		+++
Between Chi						1.59
Between df						2
Between P						N.S.
Btwn(F) P						N.S.
Btwn(R) P						N.S.

	Detailed Country in "other Asia" India	HongKong	other	Total
N			1	1
NS			1	1
Wt			3.12	3.12
Het Chi			0.00	0.00
Het df			0	0
Het P			N.S.	N.S.
Fixed RR			1.22	1.22
RRl			0.40	0.40
RRu			3.70	3.70
P			N.S.	N.S.
Random RR			1.22	1.22
RRl			0.40	0.40
RRu			3.70	3.70
P			N.S.	N.S.
Between Chi				
Between df				
Between P				N.S.
Btwn(F) P				N.S.
Btwn(R) P				N.S.

	Detailed other continent SCAmer	Total
N	3	3
NS	3	3
Wt	16.87	16.87
Het Chi	0.53	0.53
Het df	2	2
Het P	N.S.	N.S.
Fixed RR	6.93	6.93
RRl	4.30	4.30
RRu	11.18	11.18
P	+++	+++
Random RR	6.93	6.93
RRl	4.30	4.30
RRu	11.18	11.18
P	+++	+++
Between Chi		
Between df		
Between P		N.S.
Btwn(F) P		N.S.
Btwn(R) P		N.S.

Table 1J3 - 3

IESLC - Meta-analysis of Ex Smoking, Years quit (vs never), "Mid"
All LC types, Any Product (or Cigarettes if Any not available)
Most adjusted

	Start year of study					
	<1960	1960-69	1970-79	1980-89	1990+	Total
N	5	7	4	15	3	34
NS	4	4	3	12	3	26
Wt	65.03	74.54	160.83	182.25	13.53	496.18
Het Chi	2.73	14.89	18.67	59.25	1.29	103.72
Het df	4	6	3	14	2	33
Het P	N.S.	*	***	***	N.S.	***
Fixed RR	5.86	4.92	6.46	6.06	9.65	6.04
RRl	4.59	3.92	5.53	5.24	5.66	5.54
RRu	7.47	6.17	7.53	7.01	16.44	6.60
P	+++	+++	+++	+++	+++	+++
Random RR	5.86	3.87	4.93	4.73	9.65	5.04
RRl	4.59	2.49	3.02	3.38	5.66	4.21
RRu	7.47	6.02	8.05	6.63	16.44	6.04
P	+++	+++	+++	+++	+++	+++
Between Chi						6.89
Between df						4
Between P						N.S.
Btwn(F) P						N.S.
Btwn(R) P						(*)

	Study type (1)		
	CC	other	Total
N	24	10	34
NS	19	7	26
Wt	313.78	182.40	496.18
Het Chi	66.67	33.74	103.72
Het df	23	9	33
Het P	***	***	***
Fixed RR	5.68	6.73	6.04
RRl	5.08	5.82	5.54
RRu	6.34	7.78	6.60
P	+++	+++	+++
Random RR	4.77	5.62	5.04
RRl	3.81	4.08	4.21
RRu	5.96	7.73	6.04
P	+++	+++	+++
Between Chi			3.32
Between df			1
Between P			(*)
Btwn(F) P			N.S.
Btwn(R) P			N.S.

	Study type (2)			
	CC	prosp	other	Total
N	24	10		34
NS	19	7		26
Wt	313.78	182.40		496.18
Het Chi	66.67	33.74		103.72
Het df	23	9		33
Het P	***	***		***
Fixed RR	5.68	6.73		6.04
RRl	5.08	5.82		5.54
RRu	6.34	7.78		6.60
P	+++	+++		+++
Random RR	4.77	5.62		5.04
RRl	3.81	4.08		4.21
RRu	5.96	7.73		6.04
P	+++	+++		+++
Between Chi				3.32
Between df				1
Between P				(*)
Btwn(F) P				N.S.
Btwn(R) P				N.S.

Table 1J3 - 3

IESLC - Meta-analysis of Ex Smoking, Years quit (vs never), "Mid"
All LC types, Any Product (or Cigarettes if Any not available)
Most adjusted

	Study size (number of LC cases) 100-249	250-499	500-999	1000+	Total
N	4	7	6	17	34
NS	3	7	5	11	26
Wt	11.75	36.13	50.56	397.74	496.18
Het Chi	2.34	15.15	4.76	71.89	103.72
Het df	3	6	5	16	33
Het P	N.S.	*	N.S.	***	***
Fixed RR	6.98	4.26	4.65	6.42	6.04
RRl	3.94	3.08	3.53	5.82	5.54
RRu	12.36	5.91	6.13	7.09	6.60
P	+++	+++	+++	+++	+++
Random RR	6.98	4.00	4.65	5.42	5.04
RRl	3.94	2.36	3.53	4.30	4.21
RRu	12.36	6.77	6.13	6.84	6.04
P	+++	+++	+++	+++	+++
Between Chi					9.58
Between df					3
Between P					*
Btwn(F) P					N.S.
Btwn(R) P					N.S.

	Risky occupational population no	mining	othRisky	Total
N	32	1	1	34
NS	24	1	1	26
Wt	487.61	5.11	3.47	496.18
Het Chi	101.27	0.00	0.00	103.72
Het df	31	0	0	33
Het P	***	N.S.	N.S.	***
Fixed RR	6.10	3.33	3.98	6.04
RRl	5.58	1.40	1.39	5.54
RRu	6.67	7.94	11.40	6.60
P	+++	++	+	+++
Random RR	5.12	3.33	3.98	5.04
RRl	4.25	1.40	1.39	4.21
RRu	6.16	7.94	11.40	6.04
P	+++	++	+	+++
Between Chi				2.46
Between df				2
Between P				N.S.
Btwn(F) P				N.S.
Btwn(R) P				N.S.

	National cigarette tobacco type Virginia	blended	other	Total
N	3	28	3	34
NS	2	22	2	26
Wt	13.43	463.41	19.35	496.18
Het Chi	2.51	90.14	0.16	103.72
Het df	2	27	2	33
Het P	N.S.	***	N.S.	***
Fixed RR	3.68	6.29	3.33	6.04
RRl	2.16	5.74	2.13	5.54
RRu	6.29	6.89	5.19	6.60
P	+++	+++	+++	+++
Random RR	3.65	5.36	3.33	5.04
RRl	1.92	4.43	2.13	4.21
RRu	6.92	6.49	5.19	6.04
P	+++	+++	+++	+++
Between Chi				10.91
Between df				2
Between P				**
Btwn(F) P				N.S.
Btwn(R) P				N.S.

Table 1J3 - 3

IESLC - Meta-analysis of Ex Smoking, Years quit (vs never), "Mid"
All LC types, Any Product (or Cigarettes if Any not available)
Most adjusted

Any proxy use

	No/nk	Yes	Total
N	28	6	34
NS	21	5	26
Wt	447.44	48.75	496.18
Het Chi	88.76	12.67	103.72
Het df	27	5	33
Het P	***	*	***
Fixed RR	6.18	4.92	6.04
RRl	5.63	3.71	5.54
RRu	6.78	6.51	6.60
P	+++	+++	+++
Random RR	5.11	4.70	5.04
RRl	4.20	2.90	4.21
RRu	6.21	7.63	6.04
P	+++	+++	+++
Between Chi			2.29
Between df			1
Between P			N.S.
Btwn(F) P			N.S.
Btwn(R) P			N.S.

Full histological confirmation

	No	Yes	Total
N	22	12	34
NS	17	9	26
Wt	246.78	249.40	496.18
Het Chi	65.81	37.61	103.72
Het df	21	11	33
Het P	***	***	***
Fixed RR	5.90	6.19	6.04
RRl	5.21	5.47	5.54
RRu	6.68	7.01	6.60
P	+++	+++	+++
Random RR	4.80	5.36	5.04
RRl	3.74	4.04	4.21
RRu	6.15	7.10	6.04
P	+++	+++	+++
Between Chi			0.30
Between df			1
Between P			N.S.
Btwn(F) P			N.S.
Btwn(R) P			N.S.

Number of adjustment variables (1)

	0	1	2+/+nk	Total
N	19	10	5	34
NS	15	7	4	26
Wt	322.29	140.69	33.20	496.18
Het Chi	51.89	41.27	6.67	103.72
Het df	18	9	4	33
Het P	***	***	N.S.	***
Fixed RR	6.10	6.38	4.38	6.04
RRl	5.47	5.40	3.11	5.54
RRu	6.81	7.52	6.15	6.60
P	+++	+++	+++	+++
Random RR	5.42	4.42	4.41	5.04
RRl	4.36	2.88	2.82	4.21
RRu	6.74	6.78	6.89	6.04
P	+++	+++	+++	+++
Between Chi				3.89
Between df				2
Between P				N.S.
Btwn(F) P				N.S.
Btwn(R) P				N.S.

Table 1J3 - 3

IESLC - Meta-analysis of Ex Smoking, Years quit (vs never), "Mid"
All LC types, Any Product (or Cigarettes if Any not available)
Most adjusted

Number of adjustment variables (2)

	0	1	2	3-5	6+/+nk	Total
N	19	10	4	1		34
NS	15	7	3	1		26
Wt	322.29	140.69	24.62	8.58		496.18
Het Chi	51.89	41.27	5.27	0.00		103.72
Het df	18	9	3	0		33
Het P	***	***	N.S.	N.S.		***
Fixed RR	6.10	6.38	3.88	6.20		6.04
RRl	5.47	5.40	2.61	3.18		5.54
RRu	6.81	7.52	5.75	12.11		6.60
P	+++	+++	+++	+++		+++
Random RR	5.42	4.42	3.97	6.20		5.04
RRl	4.36	2.88	2.32	3.18		4.21
RRu	6.74	6.78	6.79	12.11		6.04
P	+++	+++	+++	+++		+++
Between Chi						5.29
Between df						3
Between P						N.S.
Btwn(F) P						N.S.
Btwn(R) P						N.S.

Product

	all/unsp	cig+/-ot	cig only	Total
N	7	21	6	34
NS	5	17	5	27
Wt	33.74	321.43	141.01	496.18
Het Chi	5.69	66.72	20.40	103.72
Het df	6	20	5	33
Het P	N.S.	***	**	***
Fixed RR	4.49	5.67	7.51	6.04
RRl	3.20	5.08	6.36	5.54
RRu	6.29	6.33	8.85	6.60
P	+++	+++	+++	+++
Random RR	4.49	4.79	6.37	5.04
RRl	3.20	3.80	4.36	4.21
RRu	6.29	6.03	9.29	6.04
P	+++	+++	+++	+++
Between Chi				10.92
Between df				2
Between P				**
Btwn(F) P				N.S.
Btwn(R) P				N.S.

Denominator

	nev any	nev cigs	Total
N	26	8	34
NS	20	7	27
Wt	398.80	97.39	496.18
Het Chi	75.68	17.00	103.72
Het df	25	7	33
Het P	***	*	***
Fixed RR	6.51	4.47	6.04
RRl	5.90	3.66	5.54
RRu	7.18	5.45	6.60
P	+++	+++	+++
Random RR	5.37	4.22	5.04
RRl	4.39	3.00	4.21
RRu	6.58	5.94	6.04
P	+++	+++	+++
Between Chi			11.05
Between df			1
Between P			***
Btwn(F) P			(*)
Btwn(R) P			N.S.

Table 1J3 - 3

IESLC - Meta-analysis of Ex Smoking, Years quit (vs never), "Mid"
All LC types, Any Product (or Cigarettes if Any not available)
Most adjusted

	Derivation of RR/CI			
	Orig	StdCalc	Other	Total
N	4	21	9	34
NS	3	16	7	26
Wt	27.90	328.49	139.79	496.18
Het Chi	3.40	61.21	31.10	103.72
Het df	3	20	8	33
Het P	N.S.	***	***	***
Fixed RR	3.82	5.97	6.83	6.04
RRl	2.63	5.36	5.78	5.54
RRu	5.53	6.65	8.06	6.60
P	+++	+++	+++	+++
Random RR	3.81	5.12	5.41	5.04
RRl	2.56	4.09	3.67	4.21
RRu	5.67	6.41	7.98	6.04
P	+++	+++	+++	+++
Between Chi				8.02
Between df				2
Between P				*
Btwn(F) P				N.S.
Btwn(R) P				N.S.

Table 1J3 - 4

IESLC - Meta-analysis of Ex Smoking, Years quit (vs never), "Mid"
All LC types, Any Product (or Cigarettes if Any not available)
Least adjusted

REF	NRR	X	SEX	AGEL	AGEH	RACE	YF	LC TYPE	LOC	START	ST	NLC	R	VB	P	H	AD	PRODUCT	exL	exH	DENOM	De
BECHER	502		m	0	0	all	-	all	Eu:Ger	1985	CC	194	n	bl	n	y	0	all/unsp	5	9	nev any	st
BECHER	512		f	0	0	all	-	all	Eu:Ger	1985	CC	194	n	bl	n	y	0	all/unsp	5	9	nev any	st
CARPEN	503		c	0	0	w+b	-	all	NAmer	1991	CC	356	n	bl	n	n	0	cig+/-ot	5	9	nev cigs	st
CHOI	535		m	0	0	all	-	all	As:oth	1985	CC	375	n	bl	n	n	0	cig+/-ot	5	9	nev cigs	st
CPSI	808		m	50	74	all	6	all	NAmer	1959	pr	5138	n	bl	n	n	1	cig only	5	9	nev any	ot
CPSII	654		m	35	99	all	4	all	NAmer	1982	pr	3229	n	bl	n	n	1	cig only	6	10	nev any	ot
CPSII	635		f	0	0	all	4	all	NAmer	1982	pr	3229	n	bl	n	n	1	cig+/-ot	6	10	nev cigs	ot
DAMBER	524		m	0	0	all	-	all	Eu:Sca	1972	CC	579	n	bl	y	n	1	all/unsp	6	10	nev any	ot
DEAN3	532	x	m	0	0	all	-	all	Eu:UK	1969	CC	766	n	V	y	n	0	all/unsp	5	8	nev any	st
DEAN3	543	x	f	0	0	all	-	all	Eu:UK	1969	CC	766	n	V	y	n	0	all/unsp	5	8	nev any	st
DESTEF	516	x	m	0	0	all	-	all	SCAmer	1988	CC	497	n	bl	n	y	0	all/unsp	5	9	nev any	st
DOLL2	503		m	0	0	all	20	all	Eu:UK	1951	pr	920	n	V	n	n	1	cig only	5	9	nev any	ot
DORGAN	502		m	0	0	wh	-	all	NAmer	1980	CC	2026	n	bl	y	y	0	cig+/-ot	6	9	nev any	st
DORN	659		m	55	64	wh	8	all	NAmer	1954	pr	5097	n	bl	n	n	0	cig+/-ot	5	9	nev any	st
DORN	682		m	65	74	wh	8	all	NAmer	1954	pr	5097	n	bl	n	n	0	cig+/-ot	5	9	nev any	st
GAO	522	x	m	0	0	all	-	all	As:Chi	1984	CC	1405	n	ot	n	n	0	cig+/-ot	5	9	nev cigs	st
GAO	542	x	f	0	0	all	-	all	As:Chi	1984	CC	1405	n	ot	n	n	0	cig+/-ot	5	9	nev cigs	st
GAO2	512		m	0	0	all	-	all	As:Jap	1988	CC	282	n	bl	n	n	0	cig+/-ot	5	9	nev cigs	st
GRAHAM	502		m	0	0	wh	-	all	NAmer	1956	CC	685	n	bl	n	n	0	cig only	3	10	nev any	st
HAMMO2	502		m	0	0	all	0	all	NAmer	1967	pr	450	o	bl	n	n	1	cig+/-ot	5	9	nev any	ot
HIRAYA	508		m	0	0	all	0	all	As:Jap	1965	pr	1917	n	bl	n	n	1	cig+/-ot	5	9	nev any	st
HIRAYA	519		f	0	0	all	0	all	As:Jap	1965	pr	1917	n	bl	n	n	1	cig+/-ot	5	9	nev any	st
JAHN	503		m	0	0	all	-	all	Eu:Ger	1988	CC	1004	n	bl	n	n	0	cig+/-ot	6	10	nev any	st
JEDRYC	612		m	0	0	all	-	all	Eu:est	1980	CC	1630	n	bl	y	n	0	cig+/-ot	5	9	nev any	st
LUBIN	586		m	0	0	all	-	all	As:Chi	1984	CC	427	m	ot	y	n	0	cig+/-ot	5	9	nev any	st
LUBIN2	1073		m	0	0	all	-	all	Eu:mul	1976	CC	7804	n	bl	n	y	0	cig+/-ot	5	9	nev any	st
LUBIN2	1112		f	0	0	all	-	all	Eu:mul	1976	CC	7804	n	bl	n	y	0	cig+/-ot	5	9	nev any	st
MATOS	582	x	m	0	0	all	-	all	SCAmer	1994	CC	200	n	bl	n	n	0	cig+/-ot	6	10	nev any	st
SOBUE	721		m	0	0	all	-	all	As:Jap	1986	CC	1376	n	bl	n	y	0	cig+/-ot	5	9	nev cigs	st
SPEIZE	503		f	0	0	all	0	all	NAmer	1976	pr	593	n	bl	n	y	0	cig+/-ot	5	10	nev cigs	st
SUZUK2	509		c	0	0	all	-	all	SCAmer	1991	CC	123	n	bl	n	y	0	all/unsp	6	10	nev any	st
WAKAI	524	x	m	0	0	all	-	all	As:Jap	1988	CC	333	n	bl	n	y	0	cig+/-ot	5	9	nev any	st
WYNDE6	504		m	0	0	all	-	all	NAmer	1969	CC	4423	n	bl	n	y	0	cig only	5	9	nev any	st
WYNDE6	525		f	0	0	all	-	all	NAmer	1969	CC	4423	n	bl	n	y	0	cig only	5	9	nev any	st

Cigarette type is all/unspec for all RRs

Table 1J3 - 5

IESLC - Meta-analysis of Ex Smoking, Years quit (vs never), "Mid"
All LC types, Any Product (or Cigarettes if Any not available)
Least adjusted

REF	NRR	SEX	AD	Number Exposed Case	Number Exposed Cont	Non-exposed Case	Non-exposed Cont	RR	95.00%CI
BECHER	502	m	0	16	32	3	54	9.00	(2.43- 33.30)
BECHER	512	f	0	2	5	10	52	2.08	(0.35- 12.26)
Subtotal BECHER								5.37	(1.87- 15.40)
CARPEN	503	c	0	25	48	8	208	13.54	(5.75- 31.87)
CHOI	535	m	0	5	30	13	95	1.22	(0.40- 3.70)
*CPSI	808	m	1	32	-	60	-	5.15	(3.35- 7.91)
*CPSII	654	m	1	186	-	81	-	11.43	(8.81- 14.84)
*CPSII	635	f	1	37	-	174	-	4.91	(3.45- 7.01)
Subtotal CPSII								8.50	(6.89- 10.48)
DAMBER	524	m	1	-	-	42	-	4.30	(2.30- 8.10)
DEAN3	532	m	0	15	67	24	510	4.76	(2.38- 9.52)
DEAN3	543	f	0	1	38	41	1538	0.99	(0.13- 7.37)
Subtotal DEAN3								4.02	(2.09- 7.75)
DESTEF	516	m	0	27	27	27	163	6.04	(3.09- 11.81)
*DOLL2	503	m	1	12	-	7	-	5.90	(2.32- 14.99)
DORGAN	502	m	0	49	38	13	140	13.89	(6.84- 28.21)
*DORN	659	m	0	32	34566	25	213858	7.92	(4.69- 13.36)
*DORN	682	m	0	41	24089	49	171211	5.95	(3.93- 9.00)
Subtotal DORN								6.64	(4.80- 9.19)
GAO	522	m	0	24	27	62	202	2.90	(1.56- 5.38)
GAO	542	f	0	14	7	435	605	2.78	(1.11- 6.95)
Subtotal GAO								2.86	(1.71- 4.78)
GAO2	512	m	0	21	26	13	56	3.48	(1.51- 8.01)
GRAHAM	502	m	0	5	29	18	346	3.31	(1.15- 9.57)
*HAMMO2	502	m	1	11	-	5	-	3.98	(1.39- 11.40)
*HIRAYA	508	m	1	-	-	-	-	1.59	(0.66- 3.82)
*HIRAYA	519	f	1	-	-	-	-	3.29	(0.56- 19.50)
Subtotal HIRAYA								1.83	(0.84- 4.03)
JAHN	503	m	0	59	63	18	138	7.18	(3.92- 13.16)
JEDRYC	612	m	0	64	58	49	219	4.93	(3.08- 7.90)
LUBIN	586	m	0	20	48	9	72	3.33	(1.40- 7.94)
LUBIN2	1073	m	0	466	822	190	2616	7.81	(6.48- 9.40)
LUBIN2	1112	f	0	30	40	336	1188	2.65	(1.63- 4.32)
Subtotal LUBIN2								6.81	(5.72- 8.10)
MATOS	582	m	0	21	27	11	110	7.78	(3.35- 18.06)
SOBUE	721	m	0	67	92	29	126	3.16	(1.90- 5.28)
*SPEIZE	503	f	0	41	95585	58	776300	5.74	(3.85- 8.56)
SUZUK2	509	c	0	10	8	11	53	6.02	(1.94- 18.72)
WAKAI	524	m	0	19	48	10	65	2.57	(1.10- 6.03)
WYNDE6	504	m	0	98	194	64	918	7.25	(5.10- 10.29)
WYNDE6	525	f	0	51	84	125	991	4.81	(3.24- 7.14)
Subtotal WYNDE6								6.05	(4.65- 7.86)
Partial Totals				1501	156098	2020	1171834		

*prospective study

REF	NRR	SEX	AD	Ys	Ws	Qs	Ps
BECHER	502	m	0	2.20	2.24	0.36	0.0010
BECHER	512	f	0	0.73	1.22	1.39	0.4184
Subtotal BECHER				1.68	3.46	1.74	
CARPEN	503	c	0	2.61	5.25	3.42	0.0000
CHOI	535	m	0	0.20	3.12	7.99	0.7277
*CPSI	808	m	1	1.64	20.82	0.53	0.0000
*CPSII	654	m	1	2.44	56.51	23.01	0.0000
*CPSII	635	f	1	1.59	30.57	1.31	0.0000
Subtotal CPSII				2.14	87.08	24.32	
DAMBER	524	m	1	1.46	9.69	1.12	0.0000
DEAN3	532	m	0	1.56	7.99	0.45	0.0000
DEAN3	543	f	0	-0.01	0.95	3.12	0.9899
Subtotal DEAN3				1.39	8.94	3.57	
DESTEF	516	m	0	1.80	8.53	0.00	0.0000
*DOLL2	503	m	1	1.77	4.41	0.00	0.0002
DORGAN	502	m	0	2.63	7.65	5.30	0.0000
*DORN	659	m	0	2.07	14.04	1.03	0.0000
*DORN	682	m	0	1.78	22.35	0.01	0.0000
Subtotal DORN				1.89	36.39	1.04	
GAO	522	m	0	1.06	10.02	5.41	0.0008
GAO	542	f	0	1.02	4.58	2.75	0.0285
Subtotal GAO				1.05	14.60	8.16	
GAO2	512	m	0	1.25	5.53	1.68	0.0034
GRAHAM	502	m	0	1.20	3.41	1.23	0.0268

Table 1J3 - 5

IESLC - Meta-analysis of Ex Smoking, Years quit (vs never), "Mid"
All LC types, Any Product (or Cigarettes if Any not available)
Least adjusted

REF	NRR	SEX	AD	Ys	Ws	Qs	Ps
*HAMMO2	502	m	1	1.38	3.47	0.60	0.0101
*HIRAYA	508	m	1	0.46	4.98	8.88	0.3005
*HIRAYA	519	f	1	1.19	1.22	0.45	0.1885
Subtotal HIRAYA				0.61	6.20	9.33	
JAHN	503	m	0	1.97	10.46	0.31	0.0000
JEDRYC	612	m	0	1.60	17.29	0.71	0.0000
LUBIN	586	m	0	1.20	5.11	1.80	0.0065
LUBIN2	1073	m	0	2.05	111.01	7.31	0.0000
LUBIN2	1112	f	0	0.98	16.09	10.90	0.0001
Subtotal LUBIN2				1.92	127.10	18.21	
MATOS	582	m	0	2.05	5.42	0.35	0.0000
SOBUE	721	m	0	1.15	14.66	6.12	0.0000
*SPEIZE	503	f	0	1.75	24.03	0.06	0.0000
SUZUK2	509	c	0	1.80	2.99	0.00	0.0019
WAKAI	524	m	0	0.95	5.30	3.85	0.0297
WYNDE6	504	m	0	1.98	31.18	1.04	0.0000
WYNDE6	525	f	0	1.57	24.68	1.27	0.0000
Subtotal WYNDE6				1.80	55.86	2.30	

	N	34
	NS	26
	Wt	496.75
Het	Chi	103.76
Het	df	33
Het	P	***
Fixed	RR	6.04
	RRl	5.53
	RRu	6.59
	P	+++
Random	RR	5.03
	RRl	4.20
	RRu	6.02
	P	+++
Asymm	P	**

Table 1J3 - 6

IESLC - Meta-analysis of Ex Smoking, Years quit (vs never), "Mid"
All LC types, Any Product (or Cigarettes if Any not available)
Least adjusted

		Sex			
		combined	male	female	Total
	N	2	24	8	34
	NS	2	23	8	33
	Wt	8.23	385.18	103.34	496.75
Het	Chi	1.25	76.84	10.26	103.76
Het	df	1	23	7	33
Het	P	N.S.	***	N.S.	***
Fixed	RR	10.09	6.52	4.36	6.04
	RRl	5.10	5.90	3.59	5.53
	RRu	19.98	7.20	5.29	6.59
	P	+++	+++	+++	+++
Random	RR	9.87	5.23	4.13	5.03
	RRl	4.55	4.24	3.17	4.20
	RRu	21.42	6.46	5.38	6.02
	P	+++	+++	+++	+++
Between	Chi				15.41
Between	df				2
Between	P				***
Btwn(F)	P				(*)
Btwn(R)	P				(*)

Table 1J3 - 7

IESLC - Meta-analysis of Ex Smoking, Years quit (vs never), "Mid"
All LC types, Any Product (or Cigarettes if Any not available)
Excluded studies (and stage at which they were excluded)

1	AGUDO AKIBA AMANDU AMES AXELSS BEST BOUCHA BOUCOT BRESLO CHEN CHEN2 CHIAZZ DEAN2 DOSEME ENGELA FAN GENG GER GUO HAENSZ HEGMAN HOLE HU HU2 JUSSAW KATSOU KAUFMA KOO KOULUM KREUZE LETOUR LEVIN LIAW LIU3 LIU4 LIU5 MCCONN MIGRAN MRFITR NOTAN2 OSANN2 PERNU QIAO2 RACHTA RESTRE SADOWS SEGI2 STASZE TIZZAN VUTUC WATSON WU WUWILL WYNDE2 WYNDE8 XU YUAN ZHANG ZHENG ZHOU
2	BUFFLE HUMBLE PISANI PRESCO WYNDE7
3	MCDUFF SPITZ
4	HAMMON LUO WU2
5	BLOT1 CORREA GILLIS QIAO WIGLE
7	BOFFET
14	ALDERS ARMADA AUVINE BARBON BENSHL BROSS BROWN3 CEDERL CHYOU DARBY DOLL GARCIA GARSHI GURSEL JAIN JOLY KAISE2 KHUDER LAUSSM PEZZO2 PEZZOT SVENSS TVERDA WANG2 WYNDE3
15	BENHAM

Table 1J3 - 8
Potentially overlapping studies

REF	REFGP	PRINC	.	OVERLAP/LINK
LUBIN2	LUBIN2	1		Lubin-combined
GRAHAM	BYERS1	1		GRAHAM/BROSS/BYERS1
WYNDE6	WYNDE6	1		WYNDE5/6/7/8
CPSI	CPSI	1		CPSI overall
JAHN	BOFFET	2		Subset of BOFFET
LUBIN	XIANGZ	2		LUBIN/XIANGZ/QIAO

Table 1J3 - 9
Most adjusted - insufficient data for meta-analysis

REF	NRR	SEX	AGEL	AGEH	RACE	YF	LC TYPE	LOC	START	ST	NLC	R	VB	P	H	AD	PRODUCT	exL	exH	DENOM	De
CPSI	722	f	0	0	wh	0	all	NAmer	1959	pr	5138	n	bl	n	n	1	cig only	5	9	nev cigs or	
SPEIZE	540	f	0	0	all	0	all	NAmer	1976	pr	593	n	bl	n	y	2	cig+/-ot	5	10	nev cigs st	

REF	NRR	RR	SIG	RRDATA comment
CPSI	722	1.51		0
SPEIZE	540	5.00		Insufficient decimals to calculate CIs

Table 1J4 -

IESLC - Meta-analysis of Ex Smoking, Years quit (vs never), "High"
All LC types, Any Product (or Cigarettes if Any not available)

This analysis is restricted to results for:
1) Ex smokers
2) Results by Years quit (vs never)
3) Categorical results by Years quit (vs never)
4) All LC types (or near equivalent)
5) Results complete enough for use in metaanalysis

Within each study, results are then selected (in the following order of preference, within each sex) for:
6) PRODUCT: all/unspec, cigarettes regardless of other products, cigarettes only
7) CIGTYPE: all/unspecified, MC regardless of HR, MC only
8) (not applicable)
9) DENOM: never smoked anything, never smoked cigarettes, never any + low, never cigs + low
10) Followup period (YF, prospective studies): whole study (coded as 0) or longest available
11) LCtype: all or nearest available, at least Squamous and Adeno. (q = squamous, s = small,
l = large, a = adeno, mix = mixed, alv = alveolar)
12) Race: all or nearest available, otherwise by race (wh or w = white, bl or b = black, hi = hispanic
ch = chinese, jap = japanese, haw = hawaiian, w+o = white + oriental, sca = scandinavian, as = asian)
13) Years quit (vs never) "high" in key scheme 1 (key value 3, maximum range 1-6)
14) For overlapping studies: principal rather than subsidiary studies
Finally by Age: whole study (coded as 0) if available, otherwise by widest available age group
and then for single sex results (m, f) in preference to results for both sexes combined (c).

Results adjusted (AD) for the most potential confounders are then chosen in Sections -1 to -3
and results adjusted for the least confounders in Sections -4 to -6. (Those least adjusted results which
actually differ from the most adjusted are marked 'x' in column X in Section -4)

Section -7 shows excluded studies, together with the stage (as above) at which no qualifying
results were found.

Section -8 lists the potentially overlapping studies which have been included (1=principal, 2=subsidiary).

Section -9 lists any results which would have been included in preference except that they had data not complete
enough for use in meta-analysis, with their significance (yes/no), if known, and any further comment as entered
on the database. It also lists as "gap" any categories for which no data were presented by the original authors.
This is commonly due to recent quitters having been combined with current smokers

In addition to those mentioned above, the following fields, levels and abbreviations are used:
* or nk = not known, n = no, y = yes, ot = other
nev = never
all/unspec = all or unspecified, cig+/-ot = cigarettes irrespective of other products (cigar, pipe etc)
MC = manufactured cigarettes, HR = hand-rolled cigarettes
exL, exH = range of exposure (low and high) in the smoking group, in terms of Years quit (vs never)
REF: 6-character study reference
NRR: number of the RR on the database within the study
ST : study type (CC = case control, pr or prosp = prospective)
NLC: number of lung cancer cases in whole study
R : risky occupational population (n = no, m = mining, o = other risky)
VB : national cigarette type (V = at least 75% Virginia, bl = at least 75% blended, ot = other)
P : any proxy use
H : full histological confirmation
De : derivation of RR/CI (or = original, st = standard method, ot = other method of estimation)

Table 1J4 - 1

IESLC - Meta-analysis of Ex Smoking, Years quit (vs never), "High"
All LC types, Any Product (or Cigarettes if Any not available)
Most adjusted

REF	NRR	SEX	AGEL	AGEH	RACE	YF	LC TYPE	LOC	START	ST	NLC	R	VB	P	H	AD	PRODUCT	exL	exH	DENOM	De
ARMADA	516	m	0	0	all	-	all	Eu:wst	1986	CC	325	n	bl	n	y	0	cig+/-ot	1.0	5	nev cigs	st
BARBON	543	m	0	0	all	-	all	Eu:wst	1979	CC	755	n	bl	y	y	1	all/unsp	0.1	4	nev any	or
BECHER	503	m	0	0	all	-	all	Eu:Ger	1985	CC	194	n	bl	n	y	0	all/unsp	2	4	nev any	st
BECHER	513	f	0	0	all	-	all	Eu:Ger	1985	CC	194	n	bl	n	y	0	all/unsp	2	4	nev any	st
BROSS	516	m	0	0	wh	-	all	NAmer	1960	CC	974	n	bl	n	n	0	cig+/-ot	0.1	5	nev any	st
CARPEN	504	c	0	0	w+b	-	all	NAmer	1991	CC	356	n	bl	n	n	0	cig+/-ot	0.1	4	nev cigs	st
CHOI	536	m	0	0	all	-	all	As:oth	1985	CC	375	n	bl	n	n	0	cig+/-ot	0.1	4	nev cigs	st
CHOI	551	f	0	0	all	-	all	As:oth	1985	CC	375	n	bl	n	n	0	cig+/-ot	0.1	4	nev cigs	st
CPSI	809	m	50	74	all	6	all	NAmer	1959	pr	5138	n	bl	n	n	1	cig only	1.0	4	nev any	ot
CPSII	655	m	35	99	all	4	all	NAmer	1982	pr	3229	n	bl	n	n	1	cig only	3	5	nev any	ot
CPSII	636	f	0	0	all	4	all	NAmer	1982	pr	3229	n	bl	n	n	1	cig+/-ot	3	5	nev cigs	ot
DAMBER	525	m	0	0	all	-	all	Eu:Sca	1972	CC	579	n	bl	y	n	1	all/unsp	0.1	5	nev any	ot
DEAN3	630	m	0	0	all	-	all	Eu:UK	1969	CC	766	n	V	y	n	1	all/unsp	3	4	nev any	ot
DEAN3	555	f	0	0	all	-	all	Eu:UK	1969	CC	766	n	V	y	n	1	all/unsp	3	4	nev any	ot
DESTEF	527	m	0	0	all	-	all	SCAmer	1988	CC	497	n	bl	n	y	4	all/unsp	0.1	4	nev any	or
DOLL2	504	m	0	0	all	20	all	Eu:UK	1951	pr	920	n	V	n	n	1	cig only	0.1	4	nev any	ot
DORGAN	503	m	0	0	wh	-	all	NAmer	1980	CC	2026	n	bl	y	y	0	cig+/-ot	1.1	5	nev any	st
DORN	660	m	55	64	wh	8	all	NAmer	1954	pr	5097	n	bl	n	n	0	cig+/-ot	0.1	4	nev any	st
DORN	683	m	65	74	wh	8	all	NAmer	1954	pr	5097	n	bl	n	n	0	cig+/-ot	0.1	4	nev any	st
GAO	533	m	0	0	all	-	all	As:Chi	1984	CC	1405	n	ot	n	n	2	cig+/-ot	0.1	4	nev cigs	or
GAO	553	f	0	0	all	-	all	As:Chi	1984	CC	1405	n	ot	n	n	2	cig+/-ot	0.1	4	nev cigs	or
GAO2	513	m	0	0	all	-	all	As:Jap	1988	CC	282	n	bl	n	n	0	cig+/-ot	1.0	4	nev cigs	or
GARCIA	518	c	0	0	all	-	all	NAmer	1992	CC	416	n	bl	n	y	0	cig+/-ot	1.0	4	nev any	st
GRAHAM	536	m	0	0	wh	-	all	NAmer	1956	CC	685	n	bl	n	n	1	cig+/-ot	1.1	5	nev any	ot
HAMMO2	503	m	0	0	all	0	all	NAmer	1967	pr	450	o	bl	n	n	1	cig+/-ot	0.1	4	nev any	ot
HIRAYA	509	m	0	0	all	0	all	As:Jap	1965	pr	1917	n	bl	n	n	1	cig+/-ot	0.1	4	nev any	st
HIRAYA	520	f	0	0	all	0	all	As:Jap	1965	pr	1917	n	bl	n	n	1	cig+/-ot	0.1	4	nev any	st
JAHN	504	m	0	0	all	-	all	Eu:Ger	1988	CC	1004	n	bl	n	n	0	cig+/-ot	2	5	nev any	st
JOLY	567	m	0	0	all	-	all	SCAmer	1978	CC	826	n	bl	n	n	0	cig+/-ot	1.0	4	nev any	st
JOLY	554	f	0	0	all	-	all	SCAmer	1978	CC	826	n	bl	n	n	0	cig+/-ot	1.0	4	nev any	st
KHUDER	513	m	0	0	all	-	all	NAmer	1985	CC	482	n	bl	n	y	0	cig+/-ot	0.1	4	nev cigs	st
LUBIN	587	m	0	0	all	-	all	As:Chi	1984	CC	427	m	ot	y	n	0	cig+/-ot	3	4	nev any	st
LUBIN2	1074	m	0	0	all	-	all	Eu:mul	1976	CC	7804	n	bl	n	y	0	cig+/-ot	0.1	4	nev any	st
LUBIN2	1113	f	0	0	all	-	all	Eu:mul	1976	CC	7804	n	bl	n	y	0	cig+/-ot	0.1	4	nev any	st
MATOS	593	m	0	0	all	-	all	SCAmer	1994	CC	200	n	bl	n	n	2	cig+/-ot	1.0	5	nev any	ot
SOBUE	722	m	0	0	all	-	all	As:Jap	1986	CC	1376	n	bl	n	y	0	cig+/-ot	1.0	4	nev cigs	st
SPEIZE	504	f	0	0	all	0	all	NAmer	1976	pr	593	n	bl	n	y	0	cig+/-ot	2	5	nev cigs	st
SUZUK2	510	c	0	0	all	-	all	SCAmer	1991	CC	123	n	bl	n	y	0	all/unsp	0.1	5	nev any	st
TVERDA	502	m	0	0	all	0	all	Eu:Sca	1972	pr	238	n	bl	n	n	2	cig only	1.0	5	nev cigs	ot
WANG2	511	c	0	0	all	-	all	As:Chi	1980	CC	103	n	ot	n	n	0	cig+/-ot	0.1	3	nev cigs	st
WYNDE3	538	m	0	0	all	-	all	NAmer	1966	CC	350	n	bl	n	y	0	all/unsp	1.0	3	nev any	st
WYNDE6	505	m	0	0	all	-	all	NAmer	1969	CC	4423	n	bl	n	y	0	cig only	1.0	4	nev any	st
WYNDE6	526	f	0	0	all	-	all	NAmer	1969	CC	4423	n	bl	n	y	0	cig only	1.0	4	nev any	st

Cigarette type is all/unspec for all RRs

Table 1J4 - 2

IESLC - Meta-analysis of Ex Smoking, Years quit (vs never), "High"
All LC types, Any Product (or Cigarettes if Any not available)
Most adjusted

REF	NRR	SEX	AD	Number Exposed Case	Cont	Non-exposed Case	Cont	RR	95.00%CI
ARMADA	516	m	0	79	45	8	71	15.58	(6.88- 35.29)
BARBON	543	m	1	32	-	22	-	13.90	(6.80- 28.50)
BECHER	503	m	0	10	12	3	54	15.00	(3.58- 62.92)
BECHER	513	f	0	2	3	10	52	3.47	(0.51- 23.48)
Subtotal BECHER								8.86	(2.81- 27.89)
BROSS	516	m	0	169	67	38	170	11.28	(7.19- 17.72)
CARPEN	504	c	0	23	39	8	208	15.33	(6.40- 36.75)
CHOI	536	m	0	25	64	13	95	2.85	(1.36- 5.99)
CHOI	551	f	0	3	2	76	164	3.24	(0.53- 19.77)
Subtotal CHOI								2.91	(1.46- 5.77)
*CPSI	809	m	1	49	-	60	-	8.09	(5.55- 11.80)
*CPSII	655	m	1	178	-	81	-	18.61	(14.31- 24.20)
*CPSII	636	f	1	56	-	174	-	10.55	(7.81- 14.26)
Subtotal CPSII								14.56	(11.95- 17.75)
DAMBER	525	m	1	-	-	42	-	7.70	(4.50- 13.50)
DEAN3	630	m	1	42	-	24	-	4.82	(2.83- 8.20)
DEAN3	555	f	1	4	-	41	-	1.63	(0.57- 4.63)
Subtotal DEAN3								3.86	(2.40- 6.20)
DESTEF	527	m	4	64	-	27	-	9.00	(5.20- 15.90)
*DOLL2	504	m	1	15	-	7	-	16.00	(6.52- 39.24)
DORGAN	503	m	0	59	51	13	140	12.46	(6.31- 24.61)
*DORN	660	m	0	34	22086	25	213858	13.17	(7.86- 22.07)
*DORN	683	m	0	14	6195	49	171211	7.90	(4.36- 14.29)
Subtotal DORN								10.57	(7.16- 15.60)
GAO	533	m	2	105	-	62	-	6.90	(4.40- 10.80)
GAO	553	f	2	37	-	435	-	7.20	(3.40- 15.10)
Subtotal GAO								6.98	(4.75- 10.25)
GAO2	513	m	0	31	26	13	56	5.14	(2.31- 11.40)
GARCIA	518	c	0	33	11	8	80	30.00	(11.07- 81.30)
GRAHAM	536	m	1	24	-	18	-	8.50	(4.32- 16.71)
*HAMMO2	503	m	1	59	-	5	-	10.99	(4.43- 27.26)
*HIRAYA	509	m	1	-	-	-	-	2.03	(1.10- 3.75)
*HIRAYA	520	f	1	-	-	-	-	3.72	(1.12- 12.37)
Subtotal HIRAYA								2.30	(1.33- 3.97)
JAHN	504	m	0	77	46	18	138	12.83	(6.96- 23.67)
JOLY	567	m	0	38	36	12	218	19.18	(9.16- 40.14)
JOLY	554	f	0	19	8	52	283	12.93	(5.38- 31.08)
Subtotal JOLY								16.28	(9.25- 28.65)
KHUDER	513	m	0	88	123	23	309	9.61	(5.80- 15.92)
LUBIN	587	m	0	33	18	9	72	14.67	(5.96- 36.07)
LUBIN2	1074	m	0	866	1047	190	2616	11.39	(9.58- 13.53)
LUBIN2	1113	f	0	60	55	336	1188	3.86	(2.62- 5.67)
Subtotal LUBIN2								9.50	(8.12- 11.13)
MATOS	593	m	2	28	-	11	-	14.00	(6.49- 30.21)
SOBUE	722	m	0	128	116	29	126	4.79	(2.98- 7.71)
*SPEIZE	504	f	0	34	63060	58	776300	7.22	(4.73- 11.02)
SUZUK2	510	c	0	15	10	11	53	7.23	(2.58- 20.25)
*TVERDA	502	m	2	5	-	4	-	2.83	(0.76- 10.53)
WANG2	511	c	0	6	10	11	43	2.35	(0.70- 7.86)
WYNDE3	538	m	0	21	22	9	88	9.33	(3.76- 23.19)
WYNDE6	505	m	0	201	166	64	918	17.37	(12.53- 24.07)
WYNDE6	526	f	0	82	70	125	991	9.29	(6.42- 13.43)
Subtotal WYNDE6								13.20	(10.34- 16.85)
Partial Totals				2848	93388	2224	1169502		

*prospective study

Table 1J4 - 2

IESLC - Meta-analysis of Ex Smoking, Years quit (vs never), "High"
All LC types, Any Product (or Cigarettes if Any not available)
Most adjusted

REF	NRR	SEX	AD	Ys	Ws	Qs	Ps
ARMADA	516	m	0	2.75	5.75	1.30	0.0000
BARBON	543	m	1	2.63	7.48	0.97	0.0000
BECHER	503	m	0	2.71	1.87	0.36	0.0002
BECHER	513	f	0	1.24	1.05	1.11	0.2027
Subtotal BECHER				2.18	2.92	1.47	
BROSS	516	m	0	2.42	18.85	0.44	0.0000
CARPEN	504	c	0	2.73	5.03	1.06	0.0000
CHOI	536	m	0	1.05	6.99	10.44	0.0056
CHOI	551	f	0	1.17	1.17	1.41	0.2033
Subtotal CHOI				1.07	8.16	11.85	
*CPSI	809	m	1	2.09	27.01	0.88	0.0000
*CPSII	655	m	1	2.92	55.67	23.72	0.0000
*CPSII	636	f	1	2.36	42.39	0.31	0.0000
Subtotal CPSII				2.68	98.06	24.03	
DAMBER	525	m	1	2.04	12.73	0.67	0.0000
DEAN3	630	m	1	1.57	13.58	6.62	0.0000
DEAN3	555	f	1	0.49	3.50	11.13	0.3606
Subtotal DEAN3				1.35	17.08	17.74	
DESTEF	527	m	4	2.20	12.30	0.07	0.0000
*DOLL2	504	m	1	2.77	4.77	1.20	0.0000
DORGAN	503	m	0	2.52	8.29	0.52	0.0000
*DORN	660	m	0	2.58	14.42	1.36	0.0000
*DORN	683	m	0	2.07	10.91	0.46	0.0000
Subtotal DORN				2.36	25.33	1.81	
GAO	533	m	2	1.93	19.06	2.20	0.0000
GAO	553	f	2	1.97	6.91	0.61	0.0000
Subtotal GAO				1.94	25.97	2.80	
GAO2	513	m	0	1.64	6.04	2.43	0.0001
GARCIA	518	c	0	3.40	3.87	4.94	0.0000
GRAHAM	536	m	1	2.14	8.40	0.14	0.0000
*HAMMO2	503	m	1	2.40	4.65	0.07	0.0000
*HIRAYA	509	m	1	0.71	10.22	24.95	0.0236
*HIRAYA	520	f	1	1.31	2.66	2.44	0.0320
Subtotal HIRAYA				0.83	12.88	27.39	
JAHN	504	m	0	2.55	10.25	0.81	0.0000
JOLY	567	m	0	2.95	7.04	3.28	0.0000
JOLY	554	f	0	2.56	4.99	0.41	0.0000
Subtotal JOLY				2.79	12.03	3.70	
KHUDER	513	m	0	2.26	15.10	0.00	0.0000
LUBIN	587	m	0	2.69	4.74	0.82	0.0000
LUBIN2	1074	m	0	2.43	128.94	3.37	0.0000
LUBIN2	1113	f	0	1.35	25.86	21.94	0.0000
Subtotal LUBIN2				2.25	154.81	25.31	
MATOS	593	m	2	2.64	6.50	0.88	0.0000
SOBUE	722	m	0	1.57	16.99	8.41	0.0000
*SPEIZE	504	f	0	1.98	21.44	1.86	0.0000
SUZUK2	510	c	0	1.98	3.62	0.31	0.0002
*TVERDA	502	m	2	1.04	2.22	3.37	0.1208
WANG2	511	c	0	0.85	2.63	5.28	0.1672
WYNDE3	538	m	0	2.23	4.64	0.01	0.0000
WYNDE6	505	m	0	2.85	36.08	12.29	0.0000
WYNDE6	526	f	0	2.23	28.18	0.05	0.0000
Subtotal WYNDE6				2.58	64.26	12.34	

Table 1J4 - 2

IESLC - Meta-analysis of Ex Smoking, Years quit (vs never), "High"
All LC types, Any Product (or Cigarettes if Any not available)
Most adjusted

	N	43
	NS	33
	Wt	634.80
Het	Chi	164.89
Het	df	42
Het	P	***
Fixed	RR	9.69
	RRl	8.96
	RRu	10.47
	P	+++
Random	RR	8.60
	RRl	7.22
	RRu	10.23
	P	+++
Asymm	P	*

Table 1J4 - 3

IESLC - Meta-analysis of Ex Smoking, Years quit (vs never), "High"
All LC types, Any Product (or Cigarettes if Any not available)
Most adjusted

	Sex: combined	male	female	Total
N	4	29	10	43
NS	4	28	10	42
Wt	15.14	481.50	138.16	634.80
Het Chi	11.35	109.10	29.98	164.89
Het df	3	28	9	42
Het P	**	***	***	***
Fixed RR	10.98	10.48	7.28	9.69
RRl	6.63	9.58	6.16	8.96
RRu	18.17	11.45	8.60	10.47
P	+++	+++	+++	+++
Random RR	9.85	9.31	6.27	8.60
RRl	3.66	7.64	4.36	7.22
RRu	26.55	11.35	9.03	10.23
P	+++	+++	+++	+++
Between Chi				14.46
Between df				2
Between P				***
Btwn(F) P				N.S.
Btwn(R) P				N.S.

	Lung cancer type: all	other	Total
N	43		43
NS	33		33
Wt	634.80		634.80
Het Chi	164.89		164.89
Het df	42		42
Het P	***		***
Fixed RR	9.69		9.69
RRl	8.96		8.96
RRu	10.47		10.47
P	+++		+++
Random RR	8.60		8.60
RRl	7.22		7.22
RRu	10.23		10.23
P	+++		+++
Between Chi			
Between df			
Between P			N.S.
Btwn(F) P			N.S.
Btwn(R) P			N.S.

	Location: NAmer	UK	Scand	othEur	China	Japan	othAs	other	Total
N	16	3	2	7	4	4	2	5	43
NS	13	2	2	5	3	3	1	4	33
Wt	304.92	21.85	14.95	181.21	33.34	35.91	8.16	34.45	634.80
Het Chi	35.64	10.81	1.90	29.71	5.73	5.47	0.02	3.66	164.89
Het df	15	2	1	6	3	3	1	4	42
Het P	**	**	N.S.	***	N.S.	N.S.	N.S.	N.S.	***
Fixed RR	11.86	5.26	6.64	9.96	7.12	3.73	2.91	11.76	9.69
RRl	10.60	3.46	4.00	8.61	5.07	2.69	1.46	8.42	8.96
RRu	13.27	8.01	11.01	11.53	9.99	5.17	5.77	16.42	10.47
P	+++	+++	+++	+++	+++	+++	++	+++	+++
Random RR	11.35	5.13	5.62	9.71	7.02	3.67	2.91	11.76	8.60
RRl	9.40	1.73	2.26	5.94	4.13	2.28	1.46	8.42	7.22
RRu	13.70	15.27	13.97	15.87	11.92	5.91	5.77	16.42	10.23
P	+++	++	+++	+++	+++	+++	++	+++	+++
Between Chi									71.94
Between df									7
Between P									***
Btwn(F) P									**
Btwn(R) P									***

Table 1J4 - 3

IESLC - Meta-analysis of Ex Smoking, Years quit (vs never), "High"
All LC types, Any Product (or Cigarettes if Any not available)
Most adjusted

	Detailed Country in "other Europe"					
	multi	Germany	othWest	East	Balkans	Total
N	2	3	2			7
NS	1	2	2			5
Wt	154.81	13.17	13.23			181.21
Het Chi	25.25	1.75	0.04			29.71
Het df	1	2	1			6
Het P	***	N.S.	N.S.			***
Fixed RR	9.50	11.82	14.61			9.96
RRl	8.12	6.89	8.52			8.61
RRu	11.13	20.29	25.04			11.53
P	+++	+++	+++			+++
Random RR	6.72	11.82	14.61			9.71
RRl	2.33	6.89	8.52			5.94
RRu	19.42	20.29	25.04			15.87
P	+++	+++	+++			+++
Between Chi						2.67
Between df						2
Between P						N.S.
Btwn(F) P						N.S.
Btwn(R) P						N.S.

	Detailed Country in "other Asia"			
	India	HongKong	other	Total
N			2	2
NS			1	1
Wt			8.16	8.16
Het Chi			0.02	0.02
Het df			1	1
Het P			N.S.	N.S.
Fixed RR			2.91	2.91
RRl			1.46	1.46
RRu			5.77	5.77
P			++	++
Random RR			2.91	2.91
RRl			1.46	1.46
RRu			5.77	5.77
P			++	++
Between Chi				
Between df				
Between P				N.S.
Btwn(F) P				N.S.
Btwn(R) P				N.S.

	Detailed other continent	
	SCAmer	Total
N	5	5
NS	4	4
Wt	34.45	34.45
Het Chi	3.66	3.66
Het df	4	4
Het P	N.S.	N.S.
Fixed RR	11.76	11.76
RRl	8.42	8.42
RRu	16.42	16.42
P	+++	+++
Random RR	11.76	11.76
RRl	8.42	8.42
RRu	16.42	16.42
P	+++	+++
Between Chi		
Between df		
Between P		N.S.
Btwn(F) P		N.S.
Btwn(R) P		N.S.

Table 1J4 - 3

IESLC - Meta-analysis of Ex Smoking, Years quit (vs never), "High"
All LC types, Any Product (or Cigarettes if Any not available)
Most adjusted

	Start year of study					
	<1960	1960-69	1970-79	1980-89	1990+	Total
N	5	9	8	17	4	43
NS	4	6	6	13	4	33
Wt	65.50	122.36	210.72	217.21	19.01	634.80
Het Chi	4.01	57.00	35.71	59.28	3.81	164.89
Het df	4	8	7	16	3	42
Het P	N.S.	***	***	***	N.S.	***
Fixed RR	9.49	8.85	9.42	10.17	14.77	9.69
RRl	7.45	7.41	8.23	8.90	9.42	8.96
RRu	12.09	10.57	10.78	11.61	23.15	10.47
P	+++	+++	+++	+++	+++	+++
Random RR	9.49	6.46	8.60	8.37	14.76	8.60
RRl	7.44	3.85	5.76	6.25	8.86	7.22
RRu	12.09	10.83	12.82	11.22	24.60	10.23
P	+++	+++	+++	+++	+++	+++
Between Chi						5.08
Between df						4
Between P						N.S.
Btwn(F) P						N.S.
Btwn(R) P						N.S.

	Study type (1)		
	CC	other	Total
N	32	11	43
NS	25	8	33
Wt	438.44	196.36	634.80
Het Chi	103.91	59.80	164.89
Het df	31	10	42
Het P	***	***	***
Fixed RR	9.41	10.33	9.69
RRl	8.57	8.99	8.96
RRu	10.34	11.89	10.47
P	+++	+++	+++
Random RR	8.71	8.17	8.60
RRl	7.15	5.57	7.22
RRu	10.62	11.98	10.23
P	+++	+++	+++
Between Chi			1.19
Between df			1
Between P			N.S.
Btwn(F) P			N.S.
Btwn(R) P			N.S.

	Study type (2)			
	CC	prosp	other	Total
N	32	11		43
NS	25	8		33
Wt	438.44	196.36		634.80
Het Chi	103.91	59.80		164.89
Het df	31	10		42
Het P	***	***		***
Fixed RR	9.41	10.33		9.69
RRl	8.57	8.99		8.96
RRu	10.34	11.89		10.47
P	+++	+++		+++
Random RR	8.71	8.17		8.60
RRl	7.15	5.57		7.22
RRu	10.62	11.98		10.23
P	+++	+++		+++
Between Chi				1.19
Between df				1
Between P				N.S.
Btwn(F) P				N.S.
Btwn(R) P				N.S.

Table 1J4 - 3

IESLC - Meta-analysis of Ex Smoking, Years quit (vs never), "High"
All LC types, Any Product (or Cigarettes if Any not available)
Most adjusted

	Study size (number of LC cases) 100-249	250-499	500-999	1000+	Total
N	6	11	10	16	43
NS	5	10	8	10	33
Wt	17.88	70.29	102.79	443.84	634.80
Het Chi	9.68	22.42	25.36	103.40	164.89
Het df	5	10	9	15	42
Het P	(*)	*	**	***	***
Fixed RR	7.17	9.30	8.63	10.14	9.69
RRl	4.51	7.36	7.12	9.24	8.96
RRu	11.39	11.75	10.48	11.13	10.47
P	+++	+++	+++	+++	+++
Random RR	6.29	9.51	8.80	8.49	8.60
RRl	3.18	6.59	6.26	6.50	7.22
RRu	12.45	13.74	12.37	11.08	10.23
P	+++	+++	+++	+++	+++
Between Chi					4.02
Between df					3
Between P					N.S.
Btwn(F) P					N.S.
Btwn(R) P					N.S.

	Risky occupational population no	mining	othRisky	Total
N	41	1	1	43
NS	31	1	1	33
Wt	625.40	4.74	4.65	634.80
Het Chi	163.99	0.00	0.00	164.89
Het df	40	0	0	42
Het P	***	N.S.	N.S.	***
Fixed RR	9.65	14.67	10.99	9.69
RRl	8.92	5.96	4.43	8.96
RRu	10.44	36.07	27.26	10.47
P	+++	+++	+++	+++
Random RR	8.46	14.67	10.99	8.60
RRl	7.08	5.96	4.43	7.22
RRu	10.12	36.07	27.26	10.23
P	+++	+++	+++	+++
Between Chi				0.90
Between df				2
Between P				N.S.
Btwn(F) P				N.S.
Btwn(R) P				N.S.

	National cigarette tobacco type Virginia	blended	other	Total
N	3	36	4	43
NS	2	28	3	33
Wt	21.85	579.61	33.34	634.80
Het Chi	10.81	136.08	5.73	164.89
Het df	2	35	3	42
Het P	**	***	N.S.	***
Fixed RR	5.26	10.09	7.12	9.69
RRl	3.46	9.30	5.07	8.96
RRu	8.01	10.95	9.99	10.47
P	+++	+++	+++	+++
Random RR	5.13	9.12	7.02	8.60
RRl	1.73	7.60	4.13	7.22
RRu	15.27	10.94	11.92	10.23
P	++	+++	+++	+++
Between Chi				12.26
Between df				2
Between P				**
Btwn(F) P				N.S.
Btwn(R) P				N.S.

Table 1J4 - 3

IESLC - Meta-analysis of Ex Smoking, Years quit (vs never), "High"
All LC types, Any Product (or Cigarettes if Any not available)
Most adjusted

	Any proxy use No/nk	Yes	Total
N	37	6	43
NS	28	5	33
Wt	584.47	50.33	634.80
Het Chi	143.92	17.92	164.89
Het df	36	5	42
Het P	***	**	***
Fixed RR	9.89	7.65	9.69
RRl	9.12	5.80	8.96
RRu	10.72	10.08	10.47
P	+++	+++	+++
Random RR	8.76	7.61	8.60
RRl	7.28	4.42	7.22
RRu	10.54	13.10	10.23
P	+++	+++	+++
Between Chi			3.05
Between df			1
Between P			(*)
Btwn(F) P			N.S.
Btwn(R) P			N.S.

	Full histological confirmation No	Yes	Total
N	27	16	43
NS	20	13	33
Wt	313.33	321.47	634.80
Het Chi	107.18	57.31	164.89
Het df	26	15	42
Het P	***	***	***
Fixed RR	9.44	9.93	9.69
RRl	8.45	8.90	8.96
RRu	10.55	11.08	10.47
P	+++	+++	+++
Random RR	7.91	9.60	8.60
RRl	6.18	7.42	7.22
RRu	10.11	12.42	10.23
P	+++	+++	+++
Between Chi			0.40
Between df			1
Between P			N.S.
Btwn(F) P			N.S.
Btwn(R) P			N.S.

	Number of adjustment variables (1) 0	1	2+/+nk	Total
N	26	12	5	43
NS	20	9	4	33
Wt	394.75	193.06	46.99	634.80
Het Chi	84.53	73.07	5.09	164.89
Het df	25	11	4	42
Het P	***	***	N.S.	***
Fixed RR	9.87	9.81	7.87	9.69
RRl	8.94	8.52	5.91	8.96
RRu	10.89	11.30	10.47	10.47
P	+++	+++	+++	+++
Random RR	9.32	7.44	7.89	8.60
RRl	7.53	4.97	5.63	7.22
RRu	11.54	11.13	11.06	10.23
P	+++	+++	+++	+++
Between Chi				2.20
Between df				2
Between P				N.S.
Btwn(F) P				N.S.
Btwn(R) P				N.S.

Table 1J4 - 3

IESLC - Meta-analysis of Ex Smoking, Years quit (vs never), "High"
All LC types, Any Product (or Cigarettes if Any not available)
Most adjusted

	Number of adjustment variables (2) 0	1	2	3-5	6+/+nk	Total
N	26	12	4	1		43
NS	20	9	3	1		33
Wt	394.75	193.06	34.69	12.30		634.80
Het Chi	84.53	73.07	4.79	0.00		164.89
Het df	25	11	3	0		42
Het P	***	***	N.S.	N.S.		***
Fixed RR	9.87	9.81	7.50	9.00		9.69
RRl	8.94	8.52	5.38	5.15		8.96
RRu	10.89	11.30	10.47	15.74		10.47
P	+++	+++	+++	+++		+++
Random RR	9.32	7.44	7.50	9.00		8.60
RRl	7.53	4.97	4.72	5.15		7.22
RRu	11.54	11.13	11.93	15.74		10.23
P	+++	+++	+++	+++		+++
Between Chi						2.50
Between df						3
Between P						N.S.
Btwn(F) P						N.S.
Btwn(R) P						N.S.

	Product all/unsp	cig+/-ot	cig only	Total
N	9	28	6	43
NS	7	22	5	34
Wt	60.77	420.10	153.93	634.80
Het Chi	15.70	99.60	24.62	164.89
Het df	8	27	5	42
Het P	*	***	***	***
Fixed RR	7.16	8.96	13.49	9.69
RRl	5.57	8.15	11.52	8.96
RRu	9.21	9.86	15.80	10.47
P	+++	+++	+++	+++
Random RR	7.06	8.41	11.75	8.60
RRl	4.82	6.82	7.89	7.22
RRu	10.35	10.38	17.48	10.23
P	+++	+++	+++	+++
Between Chi				24.97
Between df				2
Between P				***
Btwn(F) P				*
Btwn(R) P				N.S.

	Denominator nev any	nev cigs	Total
N	30	13	43
NS	23	11	34
Wt	483.07	151.73	634.80
Het Chi	123.17	28.96	164.89
Het df	29	12	42
Het P	***	**	***
Fixed RR	10.49	7.52	9.69
RRl	9.59	6.42	8.96
RRu	11.47	8.82	10.47
P	+++	+++	+++
Random RR	9.53	6.81	8.60
RRl	7.72	5.16	7.22
RRu	11.75	9.00	10.23
P	+++	+++	+++
Between Chi			12.76
Between df			1
Between P			***
Btwn(F) P			(*)
Btwn(R) P			(*)

Table 1J4 - 3

IESLC - Meta-analysis of Ex Smoking, Years quit (vs never), "High"
All LC types, Any Product (or Cigarettes if Any not available)
Most adjusted

	Derivation of RR/CI			
	Orig	StdCalc	Other	Total
N	5	27	11	43
NS	4	20	9	33
Wt	51.80	401.59	181.42	634.80
Het Chi	4.13	109.49	47.21	164.89
Het df	4	26	10	42
Het P	N.S.	***	***	***
Fixed RR	7.90	9.51	10.70	9.69
RRl	6.02	8.63	9.25	8.96
RRu	10.37	10.49	12.37	10.47
P	+++	+++	+++	+++
Random RR	7.91	8.72	8.50	8.60
RRl	5.99	6.90	5.93	7.22
RRu	10.44	11.02	12.18	10.23
P	+++	+++	+++	+++
Between Chi				4.07
Between df				2
Between P				N.S.
Btwn(F) P				N.S.
Btwn(R) P				N.S.

Table 1J4 - 4

IESLC - Meta-analysis of Ex Smoking, Years quit (vs never), "High"
All LC types, Any Product (or Cigarettes if Any not available)
Least adjusted

REF	NRR	X	SEX	AGEL	AGEH	RACE	YF	LC TYPE	LOC	START	ST	NLC	R	VB	P	H	AD	PRODUCT	exL	exH	DENOM	De
ARMADA	516		m	0	0	all	-	all	Eu:wst	1986	CC	325	n	bl	n	y	0	cig+/-ot	1.0	5	nev cigs	st
BARBON	528	x	m	0	0	all	-	all	Eu:wst	1979	CC	755	n	bl	y	y	0	all/unsp	0.1	4	nev any	st
BECHER	503		m	0	0	all	-	all	Eu:Ger	1985	CC	194	n	bl	n	y	0	all/unsp	2	4	nev any	st
BECHER	513		f	0	0	all	-	all	Eu:Ger	1985	CC	194	n	bl	n	y	0	all/unsp	2	4	nev any	st
BROSS	516		m	0	0	wh	-	all	NAmer	1960	CC	974	n	bl	n	n	0	cig+/-ot	0.1	5	nev any	st
CARPEN	504		c	0	0	w+b	-	all	NAmer	1991	CC	356	n	bl	n	n	0	cig+/-ot	0.1	4	nev cigs	st
CHOI	536		m	0	0	all	-	all	As:oth	1985	CC	375	n	bl	n	n	0	cig+/-ot	0.1	4	nev cigs	st
CHOI	551		f	0	0	all	-	all	As:oth	1985	CC	375	n	bl	n	n	0	cig+/-ot	0.1	4	nev cigs	st
CPSI	809		m	50	74	all	6	all	NAmer	1959	pr	5138	n	bl	n	n	1	cig only	1.0	4	nev any	ot
CPSII	655		m	35	99	all	4	all	NAmer	1982	pr	3229	n	bl	n	n	1	cig only	3	5	nev any	ot
CPSII	636		f	0	0	all	4	all	NAmer	1982	pr	3229	n	bl	n	n	1	cig+/-ot	3	5	nev cigs	ot
DAMBER	525		m	0	0	all	-	all	Eu:Sca	1972	CC	579	n	bl	y	n	1	all/unsp	0.1	5	nev any	ot
DEAN3	533	x	m	0	0	all	-	all	Eu:UK	1969	CC	766	n	V	y	n	0	all/unsp	3	4	nev any	st
DEAN3	544	x	f	0	0	all	-	all	Eu:UK	1969	CC	766	n	V	y	n	0	all/unsp	3	4	nev any	st
DESTEF	517	x	m	0	0	all	-	all	SCAmer	1988	CC	497	n	bl	n	y	0	all/unsp	0.1	4	nev any	st
DOLL2	504		m	0	0	all	20	all	Eu:UK	1951	pr	920	n	V	n	n	1	cig only	0.1	4	nev any	ot
DORGAN	503		m	0	0	wh	-	all	NAmer	1980	CC	2026	n	bl	y	y	0	cig+/-ot	1.1	5	nev any	st
DORN	660		m	55	64	wh	8	all	NAmer	1954	pr	5097	n	bl	n	n	0	cig+/-ot	0.1	4	nev any	st
DORN	683		m	65	74	wh	8	all	NAmer	1954	pr	5097	n	bl	n	n	0	cig+/-ot	0.1	4	nev any	st
GAO	523	x	m	0	0	all	-	all	As:Chi	1984	CC	1405	n	ot	n	n	0	cig+/-ot	0.1	4	nev cigs	st
GAO	543	x	f	0	0	all	-	all	As:Chi	1984	CC	1405	n	ot	n	n	0	cig+/-ot	0.1	4	nev cigs	st
GAO2	513		m	0	0	all	-	all	As:Jap	1988	CC	282	n	bl	n	n	0	cig+/-ot	1.0	4	nev cigs	or
GARCIA	518		c	0	0	all	-	all	NAmer	1992	CC	416	n	bl	n	y	0	cig+/-ot	1.0	4	nev any	st
GRAHAM	526	x	m	0	0	wh	-	all	NAmer	1956	CC	685	n	bl	n	n	0	cig+/-ot	1.1	5	nev any	st
HAMMO2	503		m	0	0	all	0	all	NAmer	1967	pr	450	o	bl	n	n	1	cig+/-ot	0.1	4	nev any	ot
HIRAYA	509		m	0	0	all	0	all	As:Jap	1965	pr	1917	n	bl	n	n	1	cig+/-ot	0.1	4	nev any	st
HIRAYA	520		f	0	0	all	0	all	As:Jap	1965	pr	1917	n	bl	n	n	1	cig+/-ot	0.1	4	nev any	st
JAHN	504		m	0	0	all	-	all	Eu:Ger	1988	CC	1004	n	bl	n	n	0	cig+/-ot	2	5	nev any	st
JOLY	567		m	0	0	all	-	all	SCAmer	1978	CC	826	n	bl	n	n	0	cig+/-ot	1.0	4	nev any	st
JOLY	554		f	0	0	all	-	all	SCAmer	1978	CC	826	n	bl	n	n	0	cig+/-ot	1.0	4	nev any	st
KHUDER	513		m	0	0	all	-	all	NAmer	1985	CC	482	n	bl	n	y	0	cig+/-ot	0.1	4	nev cigs	st
LUBIN	587		m	0	0	all	-	all	As:Chi	1984	CC	427	m	ot	y	n	0	cig+/-ot	3	4	nev any	st
LUBIN2	1074		m	0	0	all	-	all	Eu:mul	1976	CC	7804	n	bl	n	y	0	cig+/-ot	0.1	4	nev any	st
LUBIN2	1113		f	0	0	all	-	all	Eu:mul	1976	CC	7804	n	bl	n	y	0	cig+/-ot	0.1	4	nev any	st
MATOS	583	x	m	0	0	all	-	all	SCAmer	1994	CC	200	n	bl	n	n	0	cig+/-ot	1.0	5	nev any	st
SOBUE	722		m	0	0	all	-	all	As:Jap	1986	CC	1376	n	bl	n	y	0	cig+/-ot	1.0	4	nev cigs	st
SPEIZE	504		f	0	0	all	0	all	NAmer	1976	pr	593	n	bl	n	y	0	cig+/-ot	2	5	nev cigs	st
SUZUK2	510		c	0	0	all	-	all	SCAmer	1991	CC	123	n	bl	n	y	0	all/unsp	0.1	5	nev any	st
TVERDA	502		m	0	0	all	0	all	Eu:Sca	1972	pr	238	n	bl	n	n	2	cig only	1.0	5	nev cigs	ot
WANG2	511		c	0	0	all	-	all	As:Chi	1980	CC	103	n	ot	n	n	0	cig+/-ot	0.1	3	nev cigs	st
WYNDE3	538		m	0	0	all	-	all	NAmer	1966	CC	350	n	bl	n	y	0	all/unsp	1.0	3	nev any	st
WYNDE6	505		m	0	0	all	-	all	NAmer	1969	CC	4423	n	bl	n	y	0	cig only	1.0	4	nev any	st
WYNDE6	526		f	0	0	all	-	all	NAmer	1969	CC	4423	n	bl	n	y	0	cig only	1.0	4	nev any	st

Cigarette type is all/unspec for all RRs

Table 1J4 - 5

IESLC - Meta-analysis of Ex Smoking, Years quit (vs never), "High"
All LC types, Any Product (or Cigarettes if Any not available)
Least adjusted

REF	NRR	SEX	AD	Number Exposed Case	Cont	Non-exposed Case	Cont	RR	95.00%CI
ARMADA	516	m	0	79	45	8	71	15.58	(6.88- 35.29)
BARBON	528	m	0	32	20	22	188	13.67	(6.71- 27.87)
BECHER	503	m	0	10	12	3	54	15.00	(3.58- 62.92)
BECHER	513	f	0	2	3	10	52	3.47	(0.51- 23.48)
Subtotal BECHER								8.86	(2.81- 27.89)
BROSS	516	m	0	169	67	38	170	11.28	(7.19- 17.72)
CARPEN	504	c	0	23	39	8	208	15.33	(6.40- 36.75)
CHOI	536	m	0	25	64	13	95	2.85	(1.36- 5.99)
CHOI	551	f	0	3	2	76	164	3.24	(0.53- 19.77)
Subtotal CHOI								2.91	(1.46- 5.77)
*CPSI	809	m	1	49	-	60	-	8.09	(5.55- 11.80)
*CPSII	655	m	1	178	-	81	-	18.61	(14.31- 24.20)
*CPSII	636	f	1	56	-	174	-	10.55	(7.81- 14.26)
Subtotal CPSII								14.56	(11.95- 17.75)
DAMBER	525	m	1	-	-	42	-	7.70	(4.50- 13.50)
DEAN3	533	m	0	42	147	24	510	6.07	(3.56- 10.36)
DEAN3	544	f	0	4	110	41	1538	1.36	(0.48- 3.88)
Subtotal DEAN3								4.46	(2.77- 7.17)
DESTEF	517	m	0	64	45	27	163	8.59	(4.91- 15.00)
*DOLL2	504	m	1	15	-	7	-	16.00	(6.52- 39.24)
DORGAN	503	m	0	59	51	13	140	12.46	(6.31- 24.61)
*DORN	660	m	0	34	22086	25	213858	13.17	(7.86- 22.07)
*DORN	683	m	0	14	6195	49	171211	7.90	(4.36- 14.29)
Subtotal DORN								10.57	(7.16- 15.60)
GAO	523	m	0	105	52	62	202	6.58	(4.25- 10.19)
GAO	543	f	0	37	9	435	605	5.72	(2.73- 11.97)
Subtotal GAO								6.34	(4.35- 9.24)
GAO2	513	m	0	31	26	13	56	5.14	(2.31- 11.40)
GARCIA	518	c	0	33	11	8	80	30.00	(11.07- 81.30)
GRAHAM	526	m	0	24	48	18	346	9.61	(4.86- 19.00)
*HAMMO2	503	m	1	59	-	5	-	10.99	(4.43- 27.26)
*HIRAYA	509	m	1	-	-	-	-	2.03	(1.10- 3.75)
*HIRAYA	520	f	1	-	-	-	-	3.72	(1.12- 12.37)
Subtotal HIRAYA								2.30	(1.33- 3.97)
JAHN	504	m	0	77	46	18	138	12.83	(6.96- 23.67)
JOLY	567	m	0	38	36	12	218	19.18	(9.16- 40.14)
JOLY	554	f	0	19	8	52	283	12.93	(5.38- 31.08)
Subtotal JOLY								16.28	(9.25- 28.65)
KHUDER	513	m	0	88	123	23	309	9.61	(5.80- 15.92)
LUBIN	587	m	0	33	18	9	72	14.67	(5.96- 36.07)
LUBIN2	1074	m	0	866	1047	190	2616	11.39	(9.58- 13.53)
LUBIN2	1113	f	0	60	55	336	1188	3.86	(2.62- 5.67)
Subtotal LUBIN2								9.50	(8.12- 11.13)
MATOS	583	m	0	28	23	11	110	12.17	(5.31- 27.91)
SOBUE	722	m	0	128	116	29	126	4.79	(2.98- 7.71)
*SPEIZE	504	f	0	34	63060	58	776300	7.22	(4.73- 11.02)
SUZUK2	510	c	0	15	10	11	53	7.23	(2.58- 20.25)
*TVERDA	502	m	2	5	-	4	-	2.83	(0.76- 10.53)
WANG2	511	c	0	6	10	11	43	2.35	(0.70- 7.86)
WYNDE3	538	m	0	21	22	9	88	9.33	(3.76- 23.19)
WYNDE6	505	m	0	201	166	64	918	17.37	(12.53- 24.07)
WYNDE6	526	f	0	82	70	125	991	9.29	(6.42- 13.43)
Subtotal WYNDE6								13.20	(10.34- 16.85)
Partial Totals				2848	93842	2224	1173164		

*prospective study

Table 1J4 - 5

IESLC - Meta-analysis of Ex Smoking, Years quit (vs never), "High"
All LC types, Any Product (or Cigarettes if Any not available)
Least adjusted

REF	NRR	SEX	AD	Ys	Ws	Qs	Ps
ARMADA	516	m	0	2.75	5.75	1.31	0.0000
BARBON	528	m	0	2.62	7.57	0.91	0.0000
BECHER	503	m	0	2.71	1.87	0.36	0.0002
BECHER	513	f	0	1.24	1.05	1.10	0.2027
Subtotal BECHER				2.18	2.92	1.46	
BROSS	516	m	0	2.42	18.85	0.45	0.0000
CARPEN	504	c	0	2.73	5.03	1.07	0.0000
CHOI	536	m	0	1.05	6.99	10.41	0.0056
CHOI	551	f	0	1.17	1.17	1.40	0.2033
Subtotal CHOI				1.07	8.16	11.81	
*CPSI	809	m	1	2.09	27.01	0.86	0.0000
*CPSII	655	m	1	2.92	55.67	23.86	0.0000
*CPSII	636	f	1	2.36	42.39	0.32	0.0000
Subtotal CPSII				2.68	98.06	24.18	
DAMBER	525	m	1	2.04	12.73	0.66	0.0000
DEAN3	533	m	0	1.80	13.47	2.92	0.0000
DEAN3	544	f	0	0.31	3.52	13.50	0.5602
Subtotal DEAN3				1.49	16.99	16.42	
DESTEF	517	m	0	2.15	12.34	0.17	0.0000
*DOLL2	504	m	1	2.77	4.77	1.21	0.0000
DORGAN	503	m	0	2.52	8.29	0.53	0.0000
*DORN	660	m	0	2.58	14.42	1.37	0.0000
*DORN	683	m	0	2.07	10.91	0.45	0.0000
Subtotal DORN				2.36	25.33	1.82	
GAO	523	m	0	1.88	20.07	2.98	0.0000
GAO	543	f	0	1.74	7.04	1.94	0.0000
Subtotal GAO				1.85	27.10	4.92	
GAO2	513	m	0	1.64	6.04	2.42	0.0001
GARCIA	518	c	0	3.40	3.87	4.95	0.0000
GRAHAM	526	m	0	2.26	8.27	0.00	0.0000
*HAMMO2	503	m	1	2.40	4.65	0.08	0.0000
*HIRAYA	509	m	1	0.71	10.22	24.89	0.0236
*HIRAYA	520	f	1	1.31	2.66	2.43	0.0320
Subtotal HIRAYA				0.83	12.88	27.32	
JAHN	504	m	0	2.55	10.25	0.82	0.0000
JOLY	567	m	0	2.95	7.04	3.30	0.0000
JOLY	554	f	0	2.56	4.99	0.42	0.0000
Subtotal JOLY				2.79	12.03	3.72	
KHUDER	513	m	0	2.26	15.10	0.00	0.0000
LUBIN	587	m	0	2.69	4.74	0.82	0.0000
LUBIN2	1074	m	0	2.43	128.94	3.45	0.0000
LUBIN2	1113	f	0	1.35	25.86	21.85	0.0000
Subtotal LUBIN2				2.25	154.81	25.30	
MATOS	583	m	0	2.50	5.58	0.30	0.0000
SOBUE	722	m	0	1.57	16.99	8.36	0.0000
*SPEIZE	504	f	0	1.98	21.44	1.84	0.0000
SUZUK2	510	c	0	1.98	3.62	0.31	0.0002
*TVERDA	502	m	2	1.04	2.22	3.36	0.1208
WANG2	511	c	0	0.85	2.63	5.27	0.1672
WYNDE3	538	m	0	2.23	4.64	0.01	0.0000
WYNDE6	505	m	0	2.85	36.08	12.37	0.0000
WYNDE6	526	f	0	2.23	28.18	0.05	0.0000
Subtotal WYNDE6				2.58	64.26	12.42	

Table 1J4 - 5

IESLC - Meta-analysis of Ex Smoking, Years quit (vs never), "High"
All LC types, Any Product (or Cigarettes if Any not available)
Least adjusted

	N	43
	NS	33
	Wt	634.93
Het	Chi	165.08
Het	df	42
Het	P	***
Fixed	RR	9.67
	RRl	8.95
	RRu	10.45
	P	+++
Random	RR	8.55
	RRl	7.18
	RRu	10.18
	P	+++
Asymm	P	*

Table 1J4 - 6

IESLC - Meta-analysis of Ex Smoking, Years quit (vs never), "High"
All LC types, Any Product (or Cigarettes if Any not available)
Least adjusted

	Sex combined	male	female	Total
N	4	29	10	43
NS	4	28	10	42
Wt	15.14	481.49	138.31	634.93
Het Chi	11.35	105.36	32.38	165.08
Het df	3	28	9	42
Het P	**	***	***	***
Fixed RR	10.98	10.50	7.16	9.67
RRl	6.63	9.60	6.06	8.95
RRu	18.17	11.48	8.46	10.45
P	+++	+++	+++	+++
Random RR	9.85	9.35	6.00	8.55
RRl	3.66	7.70	4.11	7.18
RRu	26.55	11.37	8.75	10.18
P	+++	+++	+++	+++
Between Chi				15.98
Between df				2
Between P				***
Btwn(F) P				N.S.
Btwn(R) P				N.S.

Table 1J4 - 7

IESLC - Meta-analysis of Ex Smoking, Years quit (vs never), "High"
All LC types, Any Product (or Cigarettes if Any not available)
Excluded studies (and stage at which they were excluded)

1	AGUDO AKIBA AMANDU AMES AXELSS BEST BOUCHA BOUCOT BRESLO CHEN CHEN2 CHIAZZ DEAN2 DOSEME ENGELA FAN GENG GER GUO HAENSZ HEGMAN HOLE HU HU2 JUSSAW KATSOU KAUFMA KOO KOULUM KREUZE LETOUR LEVIN LIAW LIU3 LIU4 LIU5 MCCONN MIGRAN MRFITR NOTAN2 OSANN2 PERNU QIAO2 RACHTA RESTRE SADOWS SEGI2 STASZE TIZZAN VUTUC WATSON WU WUWILL WYNDE2 WYNDE8 XU YUAN ZHANG ZHENG ZHOU
2	BUFFLE HUMBLE PISANI PRESCO WYNDE7
3	MCDUFF SPITZ
4	HAMMON LUO WU2
5	BLOT1 CORREA GILLIS QIAO WIGLE
7	BOFFET
14	ALDERS AUVINE BENSHL BROWN3 CEDERL CHYOU DARBY DOLL GARSHI GURSEL JAIN JEDRYC KAISE2 LAUSSM PEZZO2 PEZZOT SVENSS WAKAI
15	BENHAM

Table 1J4 - 8
Potentially overlapping studies

REF	REFGP	PRINC	.	OVERLAP/LINK
LUBIN2	LUBIN2	1		Lubin-combined
TVERDA	TVERDA	1		VEIERO/TVERDAL
BROSS	BYERS1	1		GRAHAM/BROSS/BYERS1
GRAHAM	BYERS1	1		GRAHAM/BROSS/BYERS1
WYNDE6	WYNDE6	1		WYNDE5/6/7/8
CPSI	CPSI	1		CPSI overall
JAHN	BOFFET	2		Subset of BOFFET
LUBIN	XIANGZ	2		LUBIN/XIANGZ/QIAO

Table 1J4 - 9
Most adjusted - insufficient data for meta-analysis

REF	NRR	SEX	AGEL	AGEH	RACE	YF	LC TYPE	LOC	START	ST	NLC	R	VB	P	H	AD	PRODUCT	exL	exH	DENOM	De
CORREA	540	c	0	0	all	-	all	NAmer	1979	CC	1359	n	bl	y	n	2	cig+/-ot	3	5	nev cigs	or
CPSI	723	f	0	0	wh	0	all	NAmer	1959	pr	5138	n	bl	n	n	1	cig only	2	4	nev cigs	or
GARSHI	524	m	0	0	all	-	all	NAmer	1981	CC	1081	o	bl	y	n	1	all/unsp	1.0	4	nev any	ot
JEDRYC	613	m	0	0	all	-	all	Eu:est	1980	CC	1630	n	bl	y	n	0	cig+/-ot	1.0	4	nev any	ot
SPEIZE	541	f	0	0	all	0	all	NAmer	1976	pr	593	n	bl	n	y	2	cig+/-ot	2	5	nev cigs	st
WAKAI	611	m	0	0	all	-	all	As:Jap	1988	CC	333	n	bl	n	y	2	cig+/-ot	1.0	4	nev any	ot

REF	NRR	RR	SIG	RRDATA comment
CORREA	540	7.70		0
CPSI	723	2.85		0
GARSHI	524	*	gap	0
JEDRYC	613	*	gap	0
SPEIZE	541	6.00		Insufficient decimals to calculate CIs
WAKAI	611	*	gap	0

Least adjusted - insufficient data for meta-analysis: as for adjusted plus the following

REF	NRR	SEX	AGEL	AGEH	RACE	YF	LC TYPE	LOC	START	ST	NLC	R	VB	P	H	AD	PRODUCT	exL	exH	DENOM	De
GARSHI	517	m	0	0	all	-	all	NAmer	1981	CC	1081	o	bl	y	n	0	all/unsp	1.0	4	nev any	ot
WAKAI	609	m	0	0	all	-	all	As:Jap	1988	CC	333	n	bl	n	y	0	cig+/-ot	1.0	4	nev any	ot

REF	NRR	RR	SIG	RRDATA comment
GARSHI	517	*	gap	0
WAKAI	609	*	gap	0

Table 1J5 -

IESLC - Meta-analysis of Ex Smoking, Years quit (vs never), "Highest vs lowest"
All LC types, Any Product (or Cigarettes if Any not available)

This analysis is restricted to results for:
1) Ex smokers
2) Results by Years quit (vs never)
3) Categorical results by Years quit (vs never)
4) Denominator (unexposed) = "low"
5) All LC types (or near equivalent)
6) Results complete enough for use in metaanalysis

Within each study, results are then selected (in the following order of preference, within each sex) for:
7) (not applicable)
8) PRODUCT: all/unspec, cigarettes regardless of other products, cigarettes only
9) CIGTYPE: all/unspecified, MC regardless of HR, MC only
10) Results with least adjustment for other aspects of smoking (ADOS)
11) The highest vs lowest category
12) Followup period (YF, prospective studies): whole study (coded as 0) or longest available
13) LCtype: all or nearest available, at least Squamous and Adeno. (q = squamous, s = small,
l = large, a = adeno, mix = mixed, alv = alveolar)
14) Race: all or nearest available, otherwise by race (wh or w = white, bl or b = black, hi = hispanic
ch = chinese, jap = japanese, haw = hawaiian, w+o = white + oriental, sca = scandinavian, as = asian)
15) For overlapping studies: principal rather than subsidiary studies
Finally by Age: whole study (coded as 0) if available, otherwise by widest available age group
and then for single sex results (m, f) in preference to results for both sexes combined (c).

Results adjusted (AD) for the most potential confounders are then chosen in Sections -1 to -3
and results adjusted for the least confounders in Sections -4 to -6. (Those least adjusted results which
actually differ from the most adjusted are marked 'x' in column X in Section -4)

Section -7 shows excluded studies, together with the stage (as above) at which no qualifying
results were found.

Section -8 lists the potentially overlapping studies which have been included (1=principal, 2=subsidiary).

Section -9 lists any results which would have been included in preference except that they had data not complete
enough for use in meta-analysis, with their significance (yes/no), if known, and any further comment as entered
on the database. It also lists as "gap" any categories for which no data were presented by the original authors.
This is commonly due to recent quitters having been combined with current smokers

In addition to those mentioned above, the following fields, levels and abbreviations are used:
* or nk = not known, n = no, y = yes, ot = other
all/unspec = all or unspecified, cig+/-ot = cigarettes irrespective of other products (cigar, pipe etc)
MC = manufactured cigarettes, HR = hand-rolled cigarettes
exL, exH = range of exposure (low and high) in the "highest" group, in terms of Years quit (vs never)
unexL, unexH = range of exposure (low and high) in the "lowest" group, in terms of Years quit (vs never)
REF: 6-character study reference
NRR: number of the RR on the database within the study
ST : study type (CC = case control, pr or prosp = prospective)
NLC: number of lung cancer cases in whole study
R : risky occupational population (n = no, m = mining, o = other risky)
VB : national cigarette type (V = at least 75% Virginia, bl = at least 75% blended, ot = other)
P : any proxy use
H : full histological confirmation
De : derivation of RR/CI (or = original, st = standard method, ot = other method of estimation)

Table 1J5 - 1

IESLC - Meta-analysis of Ex Smoking, Years quit (vs never), "Highest vs lowest"
All LC types, Any Product (or Cigarettes if Any not available)
Most adjusted

REF	NRR	SEX	AGEL	AGEH	RACE	YF	LC TYPE	LOC	START	ST	NLC	R	VB	P	H	AD	ADOS	PRODUCT	exL	exH	unexL	unexH	De
ALDERS	512	m	0	0	all	-	all	Eu:UK	1977	CC	1448	n	V	n	n	1	0	cig only	0.1	2	10	999	ot
ALDERS	523	f	0	0	all	-	all	Eu:UK	1977	CC	1448	n	V	n	n	1	0	cig only	0.1	2	10	999	ot
ARMADA	517	m	0	0	all	-	all	Eu:wst	1986	CC	325	n	bl	n	y	0	0	cig+/-ot	1.0	5	6	999	st
BARBON	547	m	0	0	all	-	all	Eu:wst	1979	CC	755	n	bl	y	y	1	0	all/unsp	0.1	4	25	999	ot
BECHER	505	m	0	0	all	-	all	Eu:Ger	1985	CC	194	n	bl	n	y	0	0	all/unsp	2	4	10	999	st
BECHER	515	f	0	0	all	-	all	Eu:Ger	1985	CC	194	n	bl	n	y	0	0	all/unsp	2	4	10	999	st
BENSHL	514	m	0	0	all	0	all	Eu:UK	1967	pr	486	n	V	n	n	2	0	cig+/-ot	0.1	9	30	999	ot
BROSS	517	m	0	0	wh	-	all	NAmer	1960	CC	974	n	bl	n	n	0	0	cig+/-ot	0.1	5	6	999	st
CARPEN	507	c	0	0	w+b	-	all	NAmer	1991	CC	356	n	bl	n	n	0	0	cig+/-ot	0.1	4	15	999	st
CEDERL	530	m	40	69	all	10	all	Eu:Sca	1963	pr	491	n	bl	n	n	1	0	all/unsp	0.1	9	10	999	ot
CHOI	541	m	0	0	all	-	all	As:oth	1985	CC	375	n	bl	n	n	0	0	cig+/-ot	0.1	4	15	999	st
CHOI	554	f	0	0	all	-	all	As:oth	1985	CC	375	n	bl	n	n	0	0	cig+/-ot	0.1	4	5	999	ot
CHYOU	509	m	0	0	jap	21	all	NAmer	1965	pr	227	n	bl	n	y	2	0	cig+/-ot	0.1	14	15	999	ot
CPSI	814	m	50	74	all	6	all	NAmer	1959	pr	5138	n	bl	n	n	1	0	cig only	0.1	0.9	10	999	ot
CPSII	663	m	35	99	all	4	all	NAmer	1982	pr	3229	n	bl	n	n	1	0	cig only	0.1	0.9	16	999	ot
CPSII	641	f	0	0	all	4	all	NAmer	1982	pr	3229	n	bl	n	n	1	0	cig+/-ot	0.1	2	16	999	ot
DAMBER	527	m	0	0	all	-	all	Eu:Sca	1972	CC	579	n	bl	y	n	1	0	all/unsp	0.1	5	11	999	ot
DARBY	503	m	0	0	wh	-	all	Eu:UK	1988	CC	982	n	V	n	n	0	0	all/unsp	0.1	9	10	999	st
DARBY	512	f	0	0	wh	-	all	Eu:UK	1988	CC	982	n	V	n	n	0	0	all/unsp	0.1	9	10	999	st
DEAN3	634	m	0	0	all	-	all	Eu:UK	1969	CC	766	n	V	y	n	1	0	all/unsp	3	4	9	999	ot
DEAN3	558	f	0	0	all	-	all	Eu:UK	1969	CC	766	n	V	y	n	1	0	all/unsp	3	4	9	999	ot
DESTEF	529	m	0	0	all	-	all	SCAmer	1988	CC	497	n	bl	n	y	4	0	all/unsp	0.1	4	10	999	ot
DOLL	535	m	0	0	all	-	all	Eu:UK	1948	CC	1465	n	V	n	n	0	0	all/unsp	0.1	9	20	999	st
DOLL	546	f	0	0	all	-	all	Eu:UK	1948	CC	1465	n	V	n	n	0	0	all/unsp	0.1	9	10	999	st
DOLL2	508	m	0	0	all	20	all	Eu:UK	1951	pr	920	n	V	n	n	1	0	cig only	0.1	4	15	999	ot
DORGAN	512	m	0	0	wh	-	all	NAmer	1980	CC	2026	n	bl	y	y	0	0	cig+/-ot	1.1	5	10	999	st
DORGAN	557	f	0	0	all	-	all	NAmer	1980	CC	2026	n	bl	y	y	0	0	cig+/-ot	1.1	9	10	999	st
DORN	664	m	55	64	wh	8	all	NAmer	1954	pr	5097	n	bl	n	n	0	0	cig+/-ot	0.1	4	15	999	st
DORN	687	m	65	74	wh	8	all	NAmer	1954	pr	5097	n	bl	n	n	0	0	cig+/-ot	0.1	4	15	999	st
GAO	535	m	0	0	all	-	all	As:Chi	1984	CC	1405	n	ot	n	n	2	0	cig+/-ot	0.1	4	10	999	ot
GAO	555	f	0	0	all	-	all	As:Chi	1984	CC	1405	n	ot	n	n	2	0	cig+/-ot	0.1	4	10	999	ot
GAO2	517	m	0	0	all	-	all	As:Jap	1988	CC	282	n	bl	n	n	0	0	cig+/-ot	1.0	4	20	999	st
GARCIA	507	c	0	0	all	-	all	NAmer	1992	CC	416	n	bl	n	y	0	0	cig+/-ot	1.0	4	30	999	st
GARSHI	525	m	0	0	all	-	all	NAmer	1981	CC	1081	o	bl	y	n	1	0	all/unsp	5	14	15	999	st
GRAHAM	539	m	0	0	wh	-	all	NAmer	1956	CC	685	n	bl	n	n	1	0	cig+/-ot	0.1	1.0	5	999	ot
HAMMO2	509	m	0	0	all	0	all	NAmer	1967	pr	450	o	bl	n	n	1	0	cig+/-ot	0.1	4	10	999	ot
HIRAYA	512	m	0	0	all	0	all	As:Jap	1965	pr	1917	n	bl	n	n	1	0	cig+/-ot	0.1	4	10	999	ot
HIRAYA	523	f	0	0	all	0	all	As:Jap	1965	pr	1917	n	bl	n	n	1	0	cig+/-ot	0.1	4	10	999	ot
JAHN	512	m	0	0	all	-	all	Eu:Ger	1988	CC	1004	n	bl	n	n	0	0	cig+/-ot	0.1	0.9	21	999	st
JAIN	569	m	0	0	all	-	all	NAmer	1981	CC	845	n	V	y	n	0	0	cig+/-ot	2	9	10	999	st
JAIN	533	f	0	0	all	-	all	NAmer	1981	CC	845	n	V	y	n	0	0	cig+/-ot	2	9	10	999	st
JEDRYC	614	m	0	0	all	-	all	Eu:est	1980	CC	1630	n	bl	y	n	0	0	cig+/-ot	5	9	10	999	st
JOLY	569	m	0	0	all	-	all	SCAmer	1978	CC	826	n	bl	n	n	0	0	cig+/-ot	1.0	4	5	999	st
JOLY	556	f	0	0	all	-	all	SCAmer	1978	CC	826	n	bl	n	n	0	0	cig+/-ot	1.0	4	5	999	st
KAISE2	652	m	0	0	all	9	all	NAmer	1979	pr	318	n	bl	n	n	1	0	cig only	2	10	21	999	st
KAISE2	572	f	0	0	all	9	all	NAmer	1979	pr	318	n	bl	n	n	1	0	cig only	2	10	21	999	ot
KHUDER	515	m	0	0	all	-	all	NAmer	1985	CC	482	n	bl	n	y	0	0	cig+/-ot	0.1	4	15	999	st
LUBIN	590	m	0	0	all	-	all	As:Chi	1984	CC	427	m	ot	y	n	0	0	cig+/-ot	3	4	10	999	st
LUBIN2	1080	m	0	0	all	-	all	Eu:mul	1976	CC	7804	n	bl	n	y	0	0	cig+/-ot	0.1	4	25	999	st
LUBIN2	1119	f	0	0	all	-	all	Eu:mul	1976	CC	7804	n	bl	n	y	0	0	cig+/-ot	0.1	4	25	999	st
MATOS	595	m	0	0	all	-	all	SCAmer	1994	CC	200	n	bl	n	n	2	0	cig+/-ot	1.0	5	11	999	ot
PEZZO2	503	m	0	0	all	-	all	SCAmer	1992	CC	367	n	bl	n	y	0	0	cig+/-ot	1.0	10	11	999	st
PEZZOT	503	m	0	0	all	-	all	SCAmer	1987	CC	215	n	bl	n	y	0	0	cig only	1.0	10	11	999	st
SOBUE	727	m	0	0	all	-	all	As:Jap	1986	CC	1376	n	bl	n	y	0	0	cig+/-ot	1.0	4	25	999	st
SPEIZE	510	f	0	0	all	0	all	NAmer	1976	pr	593	n	bl	n	y	2	0	cig+/-ot	0.1	1.9	15	999	ot
SUZUK2	530	c	0	0	all	-	all	SCAmer	1991	CC	123	n	bl	n	y	3	0	all/unsp	0.1	5	11	999	ot
SVENSS	553	f	0	0	all	-	all	Eu:Sca	1983	CC	210	n	bl	n	n	0	0	all/unsp	3	10	11	999	st
TVERDA	505	m	0	0	all	0	all	Eu:Sca	1972	pr	238	n	bl	n	n	2	0	cig only	0.1	0.9	5	999	ot
WAKAI	534	m	0	0	all	-	all	As:Jap	1988	CC	333	n	bl	n	y	2	0	cig+/-ot	5	9	20	999	ot
WANG2	513	c	0	0	all	-	all	As:Chi	1980	CC	103	n	ot	n	n	0	0	cig+/-ot	0.1	3	4	999	st
WYNDE3	543	m	0	0	all	-	all	NAmer	1966	CC	350	n	bl	n	y	0	0	all/unsp	1.0	3	13	999	st
WYNDE6	723	m	0	0	wh	-	all	NAmer	1969	CC	4423	n	bl	n	y	5	1#	cig+/-ot	1.0	10	20	999	ot
WYNDE6	730	m	0	0	bl	-	all	NAmer	1969	CC	4423	n	bl	n	y	5	1#	cig+/-ot	1.0	10	20	999	ot
WYNDE6	735	f	0	0	wh	-	all	NAmer	1969	CC	4423	n	bl	n	y	5	1#	cig+/-ot	1.0	10	11	999	ot
WYNDE6	739	f	0	0	bl	-	all	NAmer	1969	CC	4423	n	bl	n	y	5	1#	cig+/-ot	1.0	10	11	999	ot

Table 1J5 - 1

IESLC - Meta-analysis of Ex Smoking, Years quit (vs never), "Highest vs lowest"
All LC types, Any Product (or Cigarettes if Any not available)
Most adjusted

Comments on values in listings

WYNDE6 ADOS Number of cigs/day
WYNDE6 ADOS Number of cigs/day
WYNDE6 ADOS Number of cigs/day
WYNDE6 ADOS Number of cigs/day

Cigarette type is all/unspec for all RRs
except for the following:

REF	NRR	CIGTYPE
ALDERS	512	MC only
ALDERS	523	MC only

Table 1J5 - 2

IESLC - Meta-analysis of Ex Smoking, Years quit (vs never), "Highest vs lowest"
All LC types, Any Product (or Cigarettes if Any not available)
Most adjusted

REF	NRR	SEX	AD	Number Exposed Case	Cont	Non-exposed Case	Cont	RR	95.00%CI
ALDERS	512	m	1	121	-	29	-	5.66	(3.32- 9.64)
ALDERS	523	f	1	206	-	26	-	7.43	(4.38- 12.59)
Subtotal ALDERS								6.49	(4.46- 9.45)
ARMADA	517	m	0	79	45	50	87	3.05	(1.84- 5.06)
BARBON	547	m	1	32	-	15	-	6.62	(2.95- 14.85)
BECHER	505	m	0	10	12	16	72	3.75	(1.38- 10.18)
BECHER	515	f	0	2	3	1	10	6.67	(0.44- 101.73)
Subtotal BECHER								4.01	(1.57- 10.25)
*BENSHL	514	m	2	14	-	6	-	8.68	(2.96- 25.47)
BROSS	517	m	0	169	67	43	79	4.63	(2.91- 7.39)
CARPEN	507	c	0	28	46	37	158	2.60	(1.44- 4.69)
*CEDERL	530	m	1	12	-	3	-	5.55	(1.56- 19.73)
CHOI	541	m	0	25	64	4	19	1.86	(0.57- 6.00)
CHOI	554	f	0	3	2	2	0	0.28~	(0.01- 8.76)
Subtotal CHOI								1.52	(0.50- 4.63)
*CHYOU	509	m	2	21	-	5	-	1.36	(0.52- 3.57)
*CPSI	814	m	1	37	-	15	-	11.54	(6.33- 21.02)
*CPSII	663	m	1	97	-	256	-	10.12	(8.01- 12.78)
*CPSII	641	f	1	91	-	50	-	9.77	(6.92- 13.79)
Subtotal CPSII								10.01	(8.25- 12.14)
DAMBER	527	m	1	-	-	42	-	2.96	(1.70- 5.16)
DARBY	503	m	0	146	339	139	767	2.38	(1.82- 3.10)
DARBY	512	f	0	68	93	26	224	6.30	(3.77- 10.52)
Subtotal DARBY								2.92	(2.31- 3.69)
DEAN3	634	m	1	42	-	32	-	2.36	(1.42- 3.93)
DEAN3	558	f	1	4	-	2	-	2.26	(0.41- 12.55)
Subtotal DEAN3								2.35	(1.44- 3.83)
DESTEF	529	m	4	64	-	17	-	3.21	(1.64- 6.29)
DOLL	535	m	0	56	75	8	23	2.15	(0.89- 5.15)
DOLL	546	f	0	9	6	1	2	3.00	(0.22- 40.93)
Subtotal DOLL								2.22	(0.97- 5.09)
*DOLL2	508	m	1	15	-	7	-	8.00	(3.26- 19.62)
DORGAN	512	m	0	59	51	134	255	2.20	(1.43- 3.38)
DORGAN	557	f	0	49	27	34	50	2.67	(1.41- 5.06)
Subtotal DORGAN								2.34	(1.64- 3.34)
*DORN	664	m	0	34	22086	16	58370	5.62	(3.10- 10.17)
*DORN	687	m	0	14	6195	34	51243	3.41	(1.83- 6.34)
Subtotal DORN								4.42	(2.88- 6.80)
GAO	535	m	2	105	-	13	-	6.27	(2.91- 13.51)
GAO	555	f	2	37	-	16	-	3.27	(1.14- 9.36)
Subtotal GAO								5.00	(2.69- 9.30)
GAO2	517	m	0	31	26	8	25	3.73	(1.44- 9.65)
GARCIA	507	c	0	33	11	10	37	11.10	(4.18- 29.47)
GARSHI	525	m	1	166	-	125	-	1.59	(1.20- 2.11)
GRAHAM	539	m	1	113	-	13	-	13.80	(6.80- 28.00)
*HAMMO2	509	m	1	59	-	20	-	3.19	(1.94- 5.25)
*HIRAYA	512	m	1	-	-	-	-	1.47	(0.54- 4.02)
*HIRAYA	523	f	1	-	-	-	-	3.84	(0.09- 156.01)
Subtotal HIRAYA								1.57	(0.60- 4.13)
JAHN	512	m	0	166	8	29	146	104.47	(46.30- 235.70)
JAIN	569	m	0	74	46	52	113	3.50	(2.13- 5.72)
JAIN	533	f	0	66	36	19	61	5.89	(3.05- 11.34)
Subtotal JAIN								4.22	(2.85- 6.26)
JEDRYC	614	m	0	64	58	73	138	2.09	(1.32- 3.29)
JOLY	569	m	0	38	36	63	149	2.50	(1.45- 4.30)
JOLY	556	f	0	19	8	15	19	3.01	(1.03- 8.75)
Subtotal JOLY								2.59	(1.60- 4.21)
*KAISE2	652	m	1	12	-	6	-	4.80	(1.62- 14.18)
*KAISE2	572	f	1	6	-	4	-	1.72	(0.45- 6.57)
Subtotal KAISE2								3.20	(1.38- 7.43)
KHUDER	515	m	0	88	123	63	213	2.42	(1.63- 3.58)
LUBIN	590	m	0	33	18	17	73	7.87	(3.61- 17.17)
LUBIN2	1080	m	0	866	1047	109	715	5.43	(4.35- 6.77)
LUBIN2	1119	f	0	60	55	4	20	5.45	(1.75- 16.96)
Subtotal LUBIN2								5.43	(4.37- 6.74)
MATOS	595	m	2	28	-	27	-	4.67	(2.25- 9.67)
PEZZO2	503	m	0	85	110	43	161	2.89	(1.86- 4.49)
PEZZOT	503	m	0	46	82	20	106	2.97	(1.63- 5.41)
SOBUE	727	m	0	128	116	17	40	2.60	(1.40- 4.83)
*SPEIZE	510	f	2	24	-	28	-	6.00	(2.72- 13.21)
SUZUK2	530	c	3	15	-	9	-	6.00	(1.94- 18.51)

Table 1J5 - 2

IESLC - Meta-analysis of Ex Smoking, Years quit (vs never), "Highest vs lowest"
All LC types, Any Product (or Cigarettes if Any not available)
Most adjusted

REF	NRR	SEX	AD	Number Exposed Case	Exposed Cont	Non-exposed Case	Non-exposed Cont	RR	95.00%CI
SVENSS	553	f	0	16	13	14	24	2.11	(0.79- 5.65)
*TVERDA	505	m	2	2	-	4	-	2.07	(0.38- 11.34)
WAKAI	534	m	2	19	-	7	-	2.48	(0.95- 6.47)
WANG2	513	c	0	6	10	5	11	1.32	(0.31- 5.70)
WYNDE3	543	m	0	21	22	5	55	10.50	(3.52- 31.34)
WYNDE6	723	m	5	-	-	-	-	3.00	(2.11- 4.27)
WYNDE6	730	m	5	-	-	-	-	2.33	(0.87- 6.25)
WYNDE6	735	f	5	-	-	-	-	2.50	(1.94- 3.23)
WYNDE6	739	f	5	-	-	-	-	1.50	(0.40- 5.61)
Subtotal WYNDE6								2.61	(2.14- 3.19)
Partial Totals				3933	30936	1888	113465		

*prospective study ~ With 0.5 adjustment for zero

REF	NRR	SEX	AD	Ys	Ws	Qs	Ps
ALDERS	512	m	1	1.73	13.52	1.78	0.0000
ALDERS	523	f	1	2.01	13.78	5.56	0.0000
Subtotal ALDERS				1.87	27.31	7.34	
ARMADA	517	m	0	1.12	15.07	0.97	0.0000
BARBON	547	m	1	1.89	5.88	1.59	0.0000
BECHER	505	m	0	1.32	3.85	0.01	0.0095
BECHER	515	f	0	1.90	0.52	0.14	0.1724
Subtotal BECHER				1.39	4.37	0.15	
*BENSHL	514	m	2	2.16	3.32	2.07	0.0001
BROSS	517	m	0	1.53	17.62	0.47	0.0000
CARPEN	507	c	0	0.96	11.01	1.90	0.0015
*CEDERL	530	m	1	1.71	2.39	0.28	0.0081
CHOI	541	m	0	0.62	2.79	1.58	0.3017
CHOI	554	f	0	-1.27	0.32	2.26	0.4687
Subtotal CHOI				0.42	3.12	3.85	
*CHYOU	509	m	2	0.31	4.14	4.68	0.5315
*CPSI	814	m	1	2.45	10.67	12.33	0.0000
*CPSII	663	m	1	2.31	70.40	62.73	0.0000
*CPSII	641	f	1	2.28	32.32	26.69	0.0000
Subtotal CPSII				2.30	102.72	89.41	
DAMBER	527	m	1	1.09	12.46	1.02	0.0001
DARBY	503	m	0	0.87	54.65	13.94	0.0000
DARBY	512	f	0	1.84	14.62	3.23	0.0000
Subtotal DARBY				1.07	69.28	17.16	
DEAN3	634	m	1	0.86	14.83	3.89	0.0009
DEAN3	558	f	1	0.82	1.31	0.40	0.3502
Subtotal DEAN3				0.86	16.14	4.29	
DESTEF	529	m	4	1.17	8.50	0.35	0.0007
DOLL	535	m	0	0.76	5.01	1.84	0.0873
DOLL	546	f	0	1.10	0.56	0.04	0.4100
Subtotal DOLL				0.80	5.57	1.88	
*DOLL2	508	m	1	2.08	4.77	2.40	0.0000
DORGAN	512	m	0	0.79	20.86	7.05	0.0003
DORGAN	557	f	0	0.98	9.36	1.42	0.0027
Subtotal DORGAN				0.85	30.22	8.47	
*DORN	664	m	0	1.73	10.89	1.37	0.0000
*DORN	687	m	0	1.23	9.93	0.21	0.0001
Subtotal DORN				1.49	20.82	1.58	
GAO	535	m	2	1.84	6.52	1.41	0.0000
GAO	555	f	2	1.18	3.47	0.12	0.0274
Subtotal GAO				1.61	9.99	1.53	
GAO2	517	m	0	1.32	4.24	0.01	0.0067
GARCIA	507	c	0	2.41	4.03	4.33	0.0000
GARSHI	525	m	1	0.46	48.24	39.67	0.0013
GRAHAM	539	m	1	2.62	7.67	12.06	0.0000
*HAMMO2	509	m	1	1.16	15.50	0.69	0.0000
*HIRAYA	512	m	1	0.39	3.81	3.70	0.4519
*HIRAYA	523	f	1	1.35	0.28	0.00	0.4794
Subtotal HIRAYA				0.45	4.09	3.70	
JAHN	512	m	0	4.65	5.80	62.35	0.0000
JAIN	569	m	0	1.25	15.79	0.22	0.0000
JAIN	533	f	0	1.77	8.93	1.44	0.0000
Subtotal JAIN				1.44	24.72	1.67	
JEDRYC	614	m	0	0.74	18.58	7.50	0.0015
JOLY	569	m	0	0.91	13.04	2.71	0.0010
JOLY	556	f	0	1.10	3.37	0.24	0.0433

Table 1J5 - 2

IESLC - Meta-analysis of Ex Smoking, Years quit (vs never), "Highest vs lowest"
All LC types, Any Product (or Cigarettes if Any not available)
Most adjusted

REF	NRR	SEX	AD	Ys	Ws	Qs	Ps
Subtotal JOLY				0.95	16.41	2.95	
*KAISE2	652	m	1	1.57	3.26	0.13	0.0046
*KAISE2	572	f	1	0.54	2.14	1.47	0.4278
Subtotal KAISE2				1.16	5.40	1.59	
KHUDER	515	m	0	0.88	24.96	5.93	0.0000
LUBIN	590	m	0	2.06	6.31	3.03	0.0000
LUBIN2	1080	m	0	1.69	78.85	8.10	0.0000
LUBIN2	1119	f	0	1.70	2.99	0.32	0.0034
Subtotal LUBIN2				1.69	81.83	8.42	
MATOS	595	m	2	1.54	7.23	0.21	0.0000
PEZZO2	503	m	0	1.06	19.87	1.89	0.0000
PEZZOT	503	m	0	1.09	10.71	0.85	0.0004
SOBUE	727	m	0	0.95	9.97	1.73	0.0026
*SPEIZE	510	f	2	1.79	6.15	1.09	0.0000
SUZUK2	530	c	3	1.79	3.02	0.54	0.0018
SVENSS	553	f	0	0.75	3.96	1.54	0.1373
*TVERDA	505	m	2	0.73	1.33	0.55	0.4010
WAKAI	534	m	2	0.91	4.17	0.89	0.0635
WANG2	513	c	0	0.28	1.79	2.14	0.7100
WYNDE3	543	m	0	2.35	3.21	3.09	0.0000
WYNDE6	723	m	5	1.10	30.92	2.29	0.0000
WYNDE6	730	m	5	0.85	3.95	1.09	0.0927
WYNDE6	735	f	5	0.92	59.12	12.20	0.0000
WYNDE6	739	f	5	0.41	2.20	2.05	0.5473
Subtotal WYNDE6				0.96	96.20	17.63	

	N	65
	NS	47
	Wt	820.79
Het	Chi	349.79
Het	df	64
Het	P	***
Fixed	RR	3.94
	RRl	3.68
	RRu	4.22
	P	+++
Random	RR	3.97
	RRl	3.32
	RRu	4.75
	P	+++
Asymm	P	N.S.

Table 1J5 - 3

IESLC - Meta-analysis of Ex Smoking, Years quit (vs never), "Highest vs lowest"
All LC types, Any Product (or Cigarettes if Any not available)
Most adjusted

	Sex			
	combined	male	female	Total
N	4	43	18	65
NS	4	41	17	62
Wt	19.85	635.52	165.41	820.79
Het Chi	8.84	280.47	59.31	349.79
Het df	3	42	17	64
Het P	*	***	***	***
Fixed RR	3.73	3.87	4.24	3.94
RRl	2.40	3.58	3.64	3.68
RRu	5.79	4.18	4.94	4.22
P	+++	+++	+++	+++
Random RR	4.06	3.99	3.96	3.97
RRl	1.74	3.21	2.76	3.32
RRu	9.46	4.95	5.68	4.75
P	++	+++	+++	+++
Between Chi				1.16
Between df				2
Between P				N.S.
Btwn(F) P				N.S.
Btwn(R) P				N.S.

	Lung cancer type		
	all	other	Total
N	65		65
NS	47		47
Wt	820.79		820.79
Het Chi	349.79		349.79
Het df	64		64
Het P	***		***
Fixed RR	3.94		3.94
RRl	3.68		3.68
RRu	4.22		4.22
P	+++		+++
Random RR	3.97		3.97
RRl	3.32		3.32
RRu	4.75		4.75
P	+++		+++
Between Chi			
Between df			
Between P			N.S.
Btwn(F) P			N.S.
Btwn(R) P			N.S.

	Location								
	NAmer	UK	Scand	othEur	China	Japan	othAs	other	Total
N	25	10	4	8	4	5	2	7	65
NS	17	6	4	6	3	4	1	6	47
Wt	433.30	126.38	20.14	131.54	18.09	22.48	3.12	65.74	820.79
Het Chi	206.40	33.89	1.56	72.82	5.43	1.82	1.04	3.26	349.79
Het df	24	9	3	7	3	4	1	6	64
Het P	***	***	N.S.	***	N.S.	N.S.	N.S.	N.S.	***
Fixed RR	4.02	3.56	2.91	5.05	5.13	2.51	1.52	3.12	3.94
RRl	3.66	2.99	1.88	4.26	3.24	1.66	0.50	2.45	3.68
RRu	4.42	4.24	4.51	5.99	8.14	3.80	4.63	3.98	4.22
P	+++	+++	+++	+++	+++	+++	N.S.	+++	+++
Random RR	4.05	4.29	2.91	6.33	4.63	2.51	1.48	3.12	3.97
RRl	3.01	2.85	1.88	3.10	2.42	1.66	0.45	2.45	3.32
RRu	5.46	6.43	4.51	12.91	8.84	3.80	4.92	3.98	4.75
P	+++	+++	+++	+++	+++	+++	N.S.	+++	+++
Between Chi									23.57
Between df									7
Between P									**
Btwn(F) P									N.S.
Btwn(R) P									N.S.

Table 1J5 - 3

IESLC - Meta-analysis of Ex Smoking, Years quit (vs never), "Highest vs lowest"
All LC types, Any Product (or Cigarettes if Any not available)
Most adjusted

Detailed Country in "other Europe"

	multi	Germany	othWest	East	Balkans	Total
N	2	3	2	1		8
NS	1	2	2	1		6
Wt	81.83	10.17	20.95	18.58		131.54
Het Chi	0.00	26.62	2.53	0.00		72.82
Het df	1	2	1	0		7
Het P	N.S.	***	N.S.	N.S.		***
Fixed RR	5.43	25.77	3.80	2.09		5.05
RRl	4.37	13.94	2.47	1.32		4.26
RRu	6.74	47.65	5.82	3.29		5.99
P	+++	+++	+++	++		+++
Random RR	5.43	14.93	4.21	2.09		6.33
RRl	4.37	1.12	1.99	1.32		3.10
RRu	6.74	199.97	8.87	3.29		12.91
P	+++	+	+++	++		+++
Between Chi						43.67
Between df						3
Between P						***
Btwn(F) P						N.S.
Btwn(R) P						**

Detailed Country in "other Asia"

	India	HongKong	other	Total
N			2	2
NS			1	1
Wt			3.12	3.12
Het Chi			1.04	1.04
Het df			1	1
Het P			N.S.	N.S.
Fixed RR			1.52	1.52
RRl			0.50	0.50
RRu			4.63	4.63
P			N.S.	N.S.
Random RR			1.48	1.48
RRl			0.45	0.45
RRu			4.92	4.92
P			N.S.	N.S.
Between Chi				
Between df				
Between P				N.S.
Btwn(F) P				N.S.
Btwn(R) P				N.S.

Detailed other continent

	SCAmer	Total
N	7	7
NS	6	6
Wt	65.74	65.74
Het Chi	3.26	3.26
Het df	6	6
Het P	N.S.	N.S.
Fixed RR	3.12	3.12
RRl	2.45	2.45
RRu	3.98	3.98
P	+++	+++
Random RR	3.12	3.12
RRl	2.45	2.45
RRu	3.98	3.98
P	+++	+++
Between Chi		
Between df		
Between P		N.S.
Btwn(F) P		N.S.
Btwn(R) P		N.S.

Table 1J5 - 3

IESLC - Meta-analysis of Ex Smoking, Years quit (vs never), "Highest vs lowest"
All LC types, Any Product (or Cigarettes if Any not available)
Most adjusted

	Start year of study <1960	1960-69	1970-79	1980-89	1990+	Total
N	7	14	12	27	5	65
NS	5	9	8	20	5	47
Wt	49.50	162.61	156.78	406.73	45.16	820.79
Het Chi	18.91	21.67	17.53	249.20	8.50	349.79
Het df	6	13	11	26	4	64
Het P	**	(*)	(*)	***	(*)	***
Fixed RR	6.36	2.90	4.87	3.91	3.60	3.94
RRl	4.81	2.49	4.16	3.55	2.69	3.68
RRu	8.40	3.38	5.69	4.31	4.82	4.22
P	+++	+++	+++	+++	+++	+++
Random RR	6.07	3.02	4.54	3.81	4.11	3.97
RRl	3.58	2.37	3.55	2.74	2.58	3.32
RRu	10.29	3.84	5.80	5.31	6.54	4.75
P	+++	+++	+++	+++	+++	+++
Between Chi						33.97
Between df						4
Between P						***
Btwn(F) P						N.S.
Btwn(R) P						(*)

	Study type (1) CC	other	Total
N	49	16	65
NS	35	12	47
Wt	639.48	181.30	820.79
Het Chi	211.93	58.74	349.79
Het df	48	15	64
Het P	***	***	***
Fixed RR	3.34	7.05	3.94
RRl	3.09	6.10	3.68
RRu	3.61	8.16	4.22
P	+++	+++	+++
Random RR	3.71	4.99	3.97
RRl	3.09	3.51	3.32
RRu	4.46	7.08	4.75
P	+++	+++	+++
Between Chi			79.12
Between df			1
Between P			***
Btwn(F) P			***
Btwn(R) P			N.S.

	Study type (2) CC	prosp	other	Total
N	49	16		65
NS	35	12		47
Wt	639.48	181.30		820.79
Het Chi	211.93	58.74		349.79
Het df	48	15		64
Het P	***	***		***
Fixed RR	3.34	7.05		3.94
RRl	3.09	6.10		3.68
RRu	3.61	8.16		4.22
P	+++	+++		+++
Random RR	3.71	4.99		3.97
RRl	3.09	3.51		3.32
RRu	4.46	7.08		4.75
P	+++	+++		+++
Between Chi				79.12
Between df				1
Between P				***
Btwn(F) P				***
Btwn(R) P				N.S.

Table 1J5 - 3

IESLC - Meta-analysis of Ex Smoking, Years quit (vs never), "Highest vs lowest"
All LC types, Any Product (or Cigarettes if Any not available)
Most adjusted

	Study size (number of LC cases) 100-249	250-499	500-999	1000+	Total
N	9	17	14	25	65
NS	8	15	10	14	47
Wt	36.55	131.11	181.11	472.01	820.79
Het Chi	7.86	26.91	43.24	258.45	349.79
Het df	8	16	13	24	64
Het P	N.S.	*	***	***	***
Fixed RR	2.99	3.31	3.60	4.37	3.94
RRl	2.16	2.79	3.11	3.99	3.68
RRu	4.13	3.93	4.16	4.78	4.22
P	+++	+++	+++	+++	+++
Random RR	2.99	3.58	4.24	4.27	3.97
RRl	2.16	2.80	3.17	3.07	3.32
RRu	4.13	4.57	5.68	5.94	4.75
P	+++	+++	+++	+++	+++
Between Chi					13.32
Between df					3
Between P					**
Btwn(F) P					N.S.
Btwn(R) P					N.S.

	Risky occupational population no	mining	othRisky	Total
N	62	1	2	65
NS	44	1	2	47
Wt	750.72	6.31	63.75	820.79
Het Chi	303.98	0.00	5.69	349.79
Het df	61	0	1	64
Het P	***	N.S.	*	***
Fixed RR	4.17	7.87	1.88	3.94
RRl	3.88	3.61	1.47	3.68
RRu	4.48	17.17	2.41	4.22
P	+++	+++	+++	+++
Random RR	4.03	7.87	2.18	3.97
RRl	3.37	3.61	1.11	3.32
RRu	4.82	17.17	4.31	4.75
P	+++	+++	+	+++
Between Chi				40.13
Between df				2
Between P				***
Btwn(F) P				*
Btwn(R) P				(*)

	National cigarette tobacco type Virginia	blended	other	Total
N	12	49	4	65
NS	7	37	3	47
Wt	151.10	651.59	18.09	820.79
Het Chi	36.03	306.22	5.43	349.79
Het df	11	48	3	64
Het P	***	***	N.S.	***
Fixed RR	3.66	3.97	5.13	3.94
RRl	3.12	3.68	3.24	3.68
RRu	4.30	4.29	8.14	4.22
P	+++	+++	+++	+++
Random RR	4.30	3.86	4.63	3.97
RRl	3.08	3.10	2.42	3.32
RRu	6.01	4.79	8.84	4.75
P	+++	+++	+++	+++
Between Chi				2.11
Between df				2
Between P				N.S.
Btwn(F) P				N.S.
Btwn(R) P				N.S.

Table 1J5 - 3

IESLC - Meta-analysis of Ex Smoking, Years quit (vs never), "Highest vs lowest"
All LC types, Any Product (or Cigarettes if Any not available)
Most adjusted

	Any proxy use No/nk	Yes	Total
N	54	11	65
NS	39	8	47
Wt	658.22	162.57	820.79
Het Chi	274.22	33.50	349.79
Het df	53	10	64
Het P	***	***	***
Fixed RR	4.41	2.50	3.94
RRl	4.08	2.14	3.68
RRu	4.76	2.91	4.22
P	+++	+++	+++
Random RR	4.22	3.01	3.97
RRl	3.46	2.22	3.32
RRu	5.14	4.09	4.75
P	+++	+++	+++
Between Chi			42.07
Between df			1
Between P			***
Btwn(F) P			**
Btwn(R) P			(*)

	Full histological confirmation No	Yes	Total
N	42	23	65
NS	30	17	47
Wt	488.47	332.32	820.79
Het Chi	281.66	54.45	349.79
Het df	41	22	64
Het P	***	***	***
Fixed RR	4.38	3.37	3.94
RRl	4.01	3.02	3.68
RRu	4.79	3.75	4.22
P	+++	+++	+++
Random RR	4.29	3.33	3.97
RRl	3.31	2.73	3.32
RRu	5.55	4.06	4.75
P	+++	+++	+++
Between Chi			13.68
Between df			1
Between P			***
Btwn(F) P			N.S.
Btwn(R) P			N.S.

	Number of adjustment variables (1) 0	1	2+/+nk	Total
N	33	18	14	65
NS	24	13	10	47
Wt	413.49	263.25	144.05	820.79
Het Chi	140.77	155.55	19.37	349.79
Het df	32	17	13	64
Het P	***	***	N.S.	***
Fixed RR	3.61	5.22	3.02	3.94
RRl	3.28	4.62	2.56	3.68
RRu	3.98	5.89	3.55	4.22
P	+++	+++	+++	+++
Random RR	3.79	4.80	3.28	3.97
RRl	3.02	3.18	2.58	3.32
RRu	4.76	7.27	4.18	4.75
P	+++	+++	+++	+++
Between Chi				34.10
Between df				2
Between P				***
Btwn(F) P				*
Btwn(R) P				N.S.

Table 1J5 - 3

IESLC - Meta-analysis of Ex Smoking, Years quit (vs never), "Highest vs lowest"
All LC types, Any Product (or Cigarettes if Any not available)
Most adjusted

	Number of adjustment variables (2) 0	1	2	3-5	6+/+nk	Total
N	33	18	8	6		65
NS	24	13	7	3		47
Wt	413.49	263.25	36.33	107.72		820.79
Het Chi	140.77	155.55	10.95	3.72		349.79
Het df	32	17	7	5		64
Het P	***	***	N.S.	N.S.		***
Fixed RR	3.61	5.22	4.12	2.72		3.94
RRl	3.28	4.62	2.98	2.25		3.68
RRu	3.98	5.89	5.70	3.28		4.22
P	+++	+++	+++	+++		+++
Random RR	3.79	4.80	3.99	2.72		3.97
RRl	3.02	3.18	2.63	2.25		3.32
RRu	4.76	7.27	6.05	3.28		4.75
P	+++	+++	+++	+++		+++
Between Chi						38.80
Between df						3
Between P						***
Btwn(F) P						(*)
Btwn(R) P						*

	Product all/unsp	cig+/-ot	cig only	Total
N	16	40	9	65
NS	12	29	7	48
Wt	183.03	507.17	130.59	820.79
Het Chi	40.54	190.01	25.80	349.79
Het df	15	39	8	64
Het P	***	***	**	***
Fixed RR	2.61	3.83	7.84	3.94
RRl	2.26	3.51	6.61	3.68
RRu	3.02	4.17	9.31	4.22
P	+++	+++	+++	+++
Random RR	3.28	3.86	6.02	3.97
RRl	2.44	3.12	4.08	3.32
RRu	4.40	4.78	8.87	4.75
P	+++	+++	+++	+++
Between Chi				93.44
Between df				2
Between P				***
Btwn(F) P				***
Btwn(R) P				*

	Derivation of RR/CI Orig	StdCalc	Other	Total
N		34	31	65
NS		26	23	49
Wt		464.67	356.12	820.79
Het Chi		168.31	151.30	349.79
Het df		33	30	64
Het P		***	***	***
Fixed RR		3.33	4.90	3.94
RRl		3.04	4.42	3.68
RRu		3.65	5.44	4.22
P		+++	+++	+++
Random RR		3.72	4.28	3.97
RRl		2.96	3.27	3.32
RRu		4.67	5.59	4.75
P		+++	+++	+++
Between Chi				30.18
Between df				1
Between P				***
Btwn(F) P				*
Btwn(R) P				N.S.

Table 1J5 - 4

IESLC - Meta-analysis of Ex Smoking, Years quit (vs never), "Highest vs lowest"
All LC types, Any Product (or Cigarettes if Any not available)
Least adjusted

REF	NRR	X	SEX	AGEL	AGEH	RACE	YF	LC TYPE	LOC	START	ST	NLC	R	VB	P	H	AD	ADOS	PRODUCT	exL	exH	unexL	unexH	De
ALDERS	512		m	0	0	all	-	all	Eu:UK	1977	CC	1448	n	V	n	n	1	0	cig only	0.1	2	10	999	ot
ALDERS	523		f	0	0	all	-	all	Eu:UK	1977	CC	1448	n	V	n	n	1	0	cig only	0.1	2	10	999	ot
ARMADA	517		m	0	0	all	-	all	Eu:wst	1986	CC	325	n	bl	n	y	0	0	cig+/-ot	1.0	5	6	999	st
BARBON	532	x	m	0	0	all	-	all	Eu:wst	1979	CC	755	n	bl	y	y	0	0	all/unsp	0.1	4	25	999	st
BECHER	505		m	0	0	all	-	all	Eu:Ger	1985	CC	194	n	bl	n	y	0	0	all/unsp	2	4	10	999	st
BECHER	515		f	0	0	all	-	all	Eu:Ger	1985	CC	194	n	bl	n	y	0	0	all/unsp	2	4	10	999	st
BENSHL	514		m	0	0	all	0	all	Eu:UK	1967	pr	486	n	V	n	n	2	0	cig+/-ot	0.1	9	30	999	ot
BROSS	517		m	0	0	wh	-	all	NAmer	1960	CC	974	n	bl	n	n	0	0	cig+/-ot	0.1	5	6	999	st
CARPEN	507		c	0	0	w+b	-	all	NAmer	1991	CC	356	n	bl	n	n	0	0	cig+/-ot	0.1	4	15	999	st
CEDERL	530		m	40	69	all	10	all	Eu:Sca	1963	pr	491	n	bl	n	n	1	0	all/unsp	0.1	9	10	999	ot
CHOI	541		m	0	0	all	-	all	As:oth	1985	CC	375	n	bl	n	n	0	0	cig+/-ot	0.1	4	15	999	st
CHOI	554		f	0	0	all	-	all	As:oth	1985	CC	375	n	bl	n	n	0	0	cig+/-ot	0.1	4	5	999	ot
CHYOU	503	x	m	0	0	jap	21	all	NAmer	1965	pr	227	n	bl	n	y	1	0	cig+/-ot	0.1	14	15	999	ot
CPSI	814		m	50	74	all	6	all	NAmer	1959	pr	5138	n	bl	n	n	1	0	cig only	0.1	0.9	10	999	ot
CPSII	663		m	35	99	all	4	all	NAmer	1982	pr	3229	n	bl	n	n	1	0	cig only	0.1	0.9	16	999	ot
CPSII	641		f	0	0	all	4	all	NAmer	1982	pr	3229	n	bl	n	n	1	0	cig+/-ot	0.1	2	16	999	ot
DAMBER	527		m	0	0	all	-	all	Eu:Sca	1972	CC	579	n	bl	y	n	1	0	all/unsp	0.1	5	11	999	ot
DARBY	503		m	0	0	wh	-	all	Eu:UK	1988	CC	982	n	V	n	n	0	0	all/unsp	0.1	9	10	999	st
DARBY	512		f	0	0	wh	-	all	Eu:UK	1988	CC	982	n	V	n	n	0	0	all/unsp	0.1	9	10	999	st
DEAN3	536	x	m	0	0	all	-	all	Eu:UK	1969	CC	766	n	V	y	n	0	0	all/unsp	3	4	9	999	st
DEAN3	547	x	f	0	0	all	-	all	Eu:UK	1969	CC	766	n	V	y	n	0	0	all/unsp	3	4	9	999	st
DESTEF	519	x	m	0	0	all	-	all	SCAmer	1988	CC	497	n	bl	n	y	0	0	all/unsp	0.1	4	10	999	st
DOLL	535		m	0	0	all	-	all	Eu:UK	1948	CC	1465	n	V	n	n	0	0	all/unsp	0.1	9	20	999	st
DOLL	546		f	0	0	all	-	all	Eu:UK	1948	CC	1465	n	V	n	n	0	0	all/unsp	0.1	9	10	999	st
DOLL2	508		m	0	0	all	20	all	Eu:UK	1951	pr	920	n	V	n	n	1	0	cig only	0.1	4	15	999	ot
DORGAN	512		m	0	0	wh	-	all	NAmer	1980	CC	2026	n	bl	y	y	0	0	cig+/-ot	1.1	5	10	999	st
DORGAN	557		f	0	0	all	-	all	NAmer	1980	CC	2026	n	bl	y	y	0	0	cig+/-ot	1.1	9	10	999	st
DORN	664		m	55	64	wh	8	all	NAmer	1954	pr	5097	n	bl	n	n	0	0	cig+/-ot	0.1	4	15	999	st
DORN	687		m	65	74	wh	8	all	NAmer	1954	pr	5097	n	bl	n	n	0	0	cig+/-ot	0.1	4	15	999	st
GAO	525	x	m	0	0	all	-	all	As:Chi	1984	CC	1405	n	ot	n	n	0	0	cig+/-ot	0.1	4	10	999	st
GAO	545	x	f	0	0	all	-	all	As:Chi	1984	CC	1405	n	ot	n	n	0	0	cig+/-ot	0.1	4	10	999	st
GAO2	517		m	0	0	all	-	all	As:Jap	1988	CC	282	n	bl	n	n	0	0	cig+/-ot	1.0	4	20	999	st
GARCIA	507		c	0	0	all	-	all	NAmer	1992	CC	416	n	bl	n	y	0	0	cig+/-ot	1.0	4	30	999	st
GARSHI	518	x	m	0	0	all	-	all	NAmer	1981	CC	1081	o	bl	y	n	0	0	all/unsp	5	14	15	999	st
GRAHAM	529	x	m	0	0	wh	-	all	NAmer	1956	CC	685	n	bl	n	n	0	0	cig+/-ot	0.1	1.0	5	999	st
HAMMO2	509		m	0	0	all	0	all	NAmer	1967	pr	450	o	bl	n	n	1	0	cig+/-ot	0.1	4	10	999	ot
HIRAYA	512		m	0	0	all	0	all	As:Jap	1965	pr	1917	n	bl	n	n	1	0	cig+/-ot	0.1	4	10	999	ot
HIRAYA	523		f	0	0	all	0	all	As:Jap	1965	pr	1917	n	bl	n	n	1	0	cig+/-ot	0.1	4	10	999	ot
JAHN	512		m	0	0	all	-	all	Eu:Ger	1988	CC	1004	n	bl	n	n	0	0	cig+/-ot	0.1	0.9	21	999	st
JAIN	569		m	0	0	all	-	all	NAmer	1981	CC	845	n	V	y	n	0	0	cig+/-ot	2	9	10	999	st
JAIN	533		f	0	0	all	-	all	NAmer	1981	CC	845	n	V	y	n	0	0	cig+/-ot	2	9	10	999	st
JEDRYC	614		m	0	0	all	-	all	Eu:est	1980	CC	1630	n	bl	y	n	0	0	cig+/-ot	5	9	10	999	st
JOLY	569		m	0	0	all	-	all	SCAmer	1978	CC	826	n	bl	n	n	0	0	cig+/-ot	1.0	4	5	999	st
JOLY	556		f	0	0	all	-	all	SCAmer	1978	CC	826	n	bl	n	n	0	0	cig+/-ot	1.0	4	5	999	st
KAISE2	652		m	0	0	all	9	all	NAmer	1979	pr	318	n	bl	n	n	1	0	cig only	2	10	21	999	st
KAISE2	572		f	0	0	all	9	all	NAmer	1979	pr	318	n	bl	n	n	1	0	cig only	2	10	21	999	ot
KHUDER	515		m	0	0	all	-	all	NAmer	1985	CC	482	n	bl	n	y	0	0	cig+/-ot	0.1	4	15	999	st
LUBIN	590		m	0	0	all	-	all	As:Chi	1984	CC	427	m	ot	y	n	0	0	cig+/-ot	3	4	10	999	st
LUBIN2	1080		m	0	0	all	-	all	Eu:mul	1976	CC	7804	n	bl	n	y	0	0	cig+/-ot	0.1	4	25	999	st
LUBIN2	1119		f	0	0	all	-	all	Eu:mul	1976	CC	7804	n	bl	n	y	0	0	cig+/-ot	0.1	4	25	999	st
MATOS	585	x	m	0	0	all	-	all	SCAmer	1994	CC	200	n	bl	n	n	0	0	cig+/-ot	1.0	5	11	999	st
PEZZO2	503		m	0	0	all	-	all	SCAmer	1992	CC	367	n	bl	n	y	0	0	cig+/-ot	1.0	10	11	999	st
PEZZOT	503		m	0	0	all	-	all	SCAmer	1987	CC	215	n	bl	n	y	0	0	cig only	1.0	10	11	999	st
SOBUE	727		m	0	0	all	-	all	As:Jap	1986	CC	1376	n	bl	n	y	0	0	cig+/-ot	1.0	4	25	999	st
SPEIZE	510		f	0	0	all	0	all	NAmer	1976	pr	593	n	bl	n	y	2	0	cig+/-ot	0.1	1.9	15	999	ot
SUZUK2	512	x	c	0	0	all	-	all	SCAmer	1991	CC	123	n	bl	n	y	0	0	all/unsp	0.1	5	11	999	st
SVENSS	553		f	0	0	all	-	all	Eu:Sca	1983	CC	210	n	bl	n	n	0	0	all/unsp	3	10	11	999	st
TVERDA	505		m	0	0	all	0	all	Eu:Sca	1972	pr	238	n	bl	n	n	2	0	cig only	0.1	0.9	5	999	ot
WAKAI	526	x	m	0	0	all	-	all	As:Jap	1988	CC	333	n	bl	n	y	0	0	cig+/-ot	5	9	20	999	st
WANG2	513		c	0	0	all	-	all	As:Chi	1980	CC	103	n	ot	n	n	0	0	cig+/-ot	0.1	3	4	999	st
WYNDE3	543		m	0	0	all	-	all	NAmer	1966	CC	350	n	bl	n	y	0	0	all/unsp	1.0	3	13	999	st
WYNDE6	723		m	0	0	wh	-	all	NAmer	1969	CC	4423	n	bl	n	y	5	1#	cig+/-ot	1.0	10	20	999	ot
WYNDE6	730		m	0	0	bl	-	all	NAmer	1969	CC	4423	n	bl	n	y	5	1#	cig+/-ot	1.0	10	20	999	ot
WYNDE6	735		f	0	0	wh	-	all	NAmer	1969	CC	4423	n	bl	n	y	5	1#	cig+/-ot	1.0	10	11	999	ot
WYNDE6	739		f	0	0	bl	-	all	NAmer	1969	CC	4423	n	bl	n	y	5	1#	cig+/-ot	1.0	10	11	999	ot

Table 1J5 - 4

IESLC - Meta-analysis of Ex Smoking, Years quit (vs never), "Highest vs lowest"
All LC types, Any Product (or Cigarettes if Any not available)
Least adjusted

Comments on values in listings

WYNDE6 ADOS Number of cigs/day
WYNDE6 ADOS Number of cigs/day
WYNDE6 ADOS Number of cigs/day
WYNDE6 ADOS Number of cigs/day

Cigarette type is all/unspec for all RRs
except for the following:

REF	NRR	CIGTYPE
ALDERS	512	MC only
ALDERS	523	MC only

Table 1J5 - 5

IESLC - Meta-analysis of Ex Smoking, Years quit (vs never), "Highest vs lowest"
All LC types, Any Product (or Cigarettes if Any not available)
Least adjusted

REF	NRR	SEX	AD	Number Exposed Case	Cont	Non-exposed Case	Cont	RR	95.00%CI
ALDERS	512	m	1	121	-	29	-	5.66	(3.32- 9.64)
ALDERS	523	f	1	206	-	26	-	7.43	(4.38- 12.59)
Subtotal ALDERS								6.49	(4.46- 9.45)
ARMADA	517	m	0	79	45	50	87	3.05	(1.84- 5.06)
BARBON	532	m	0	32	20	15	59	6.29	(2.84- 13.95)
BECHER	505	m	0	10	12	16	72	3.75	(1.38- 10.18)
BECHER	515	f	0	2	3	1	10	6.67	(0.44- 101.73)
Subtotal BECHER								4.01	(1.57- 10.25)
*BENSHL	514	m	2	14	-	6	-	8.68	(2.96- 25.47)
BROSS	517	m	0	169	67	43	79	4.63	(2.91- 7.39)
CARPEN	507	c	0	28	46	37	158	2.60	(1.44- 4.69)
*CEDERL	530	m	1	12	-	3	-	5.55	(1.56- 19.73)
CHOI	541	m	0	25	64	4	19	1.86	(0.57- 6.00)
CHOI	554	f	0	3	2	2	0	0.28~	(0.01- 8.76)
Subtotal CHOI								1.52	(0.50- 4.63)
*CHYOU	503	m	1	21	-	5	-	1.39	(0.52- 3.71)
*CPSI	814	m	1	37	-	15	-	11.54	(6.33- 21.02)
*CPSII	663	m	1	97	-	256	-	10.12	(8.01- 12.78)
*CPSII	641	f	1	91	-	50	-	9.77	(6.92- 13.79)
Subtotal CPSII								10.01	(8.25- 12.14)
DAMBER	527	m	1	-	-	42	-	2.96	(1.70- 5.16)
DARBY	503	m	0	146	339	139	767	2.38	(1.82- 3.10)
DARBY	512	f	0	68	93	26	224	6.30	(3.77- 10.52)
Subtotal DARBY								2.92	(2.31- 3.69)
DEAN3	536	m	0	42	147	32	204	1.82	(1.10- 3.02)
DEAN3	547	f	0	4	110	2	114	2.07	(0.37- 11.55)
Subtotal DEAN3								1.84	(1.13- 2.99)
DESTEF	519	m	0	64	45	17	36	3.01	(1.51- 6.01)
DOLL	535	m	0	56	75	8	23	2.15	(0.89- 5.15)
DOLL	546	f	0	9	6	1	2	3.00	(0.22- 40.93)
Subtotal DOLL								2.22	(0.97- 5.09)
*DOLL2	508	m	1	15	-	7	-	8.00	(3.26- 19.62)
DORGAN	512	m	0	59	51	134	255	2.20	(1.43- 3.38)
DORGAN	557	f	0	49	27	34	50	2.67	(1.41- 5.06)
Subtotal DORGAN								2.34	(1.64- 3.34)
*DORN	664	m	0	34	22086	16	58370	5.62	(3.10- 10.17)
*DORN	687	m	0	14	6195	34	51243	3.41	(1.83- 6.34)
Subtotal DORN								4.42	(2.88- 6.80)
GAO	525	m	0	105	52	13	41	6.37	(3.14- 12.91)
GAO	545	f	0	37	9	16	14	3.60	(1.29- 10.00)
Subtotal GAO								5.29	(2.96- 9.47)
GAO2	517	m	0	31	26	8	25	3.73	(1.44- 9.65)
GARCIA	507	c	0	33	11	10	37	11.10	(4.18- 29.47)
GARSHI	518	m	0	166	290	125	343	1.57	(1.19- 2.08)
GRAHAM	529	m	0	113	59	13	71	10.46	(5.35- 20.44)
*HAMMO2	509	m	1	59	-	20	-	3.19	(1.94- 5.25)
*HIRAYA	512	m	1	-	-	-	-	1.47	(0.54- 4.02)
*HIRAYA	523	f	1	-	-	-	-	3.84	(0.09- 156.01)
Subtotal HIRAYA								1.57	(0.60- 4.13)
JAHN	512	m	0	166	8	29	146	104.47	(46.30- 235.70)
JAIN	569	m	0	74	46	52	113	3.50	(2.13- 5.72)
JAIN	533	f	0	66	36	19	61	5.89	(3.05- 11.34)
Subtotal JAIN								4.22	(2.85- 6.26)
JEDRYC	614	m	0	64	58	73	138	2.09	(1.32- 3.29)
JOLY	569	m	0	38	36	63	149	2.50	(1.45- 4.30)
JOLY	556	f	0	19	8	15	19	3.01	(1.03- 8.75)
Subtotal JOLY								2.59	(1.60- 4.21)
*KAISE2	652	m	1	12	-	6	-	4.80	(1.62- 14.18)
*KAISE2	572	f	1	6	-	4	-	1.72	(0.45- 6.57)
Subtotal KAISE2								3.20	(1.38- 7.43)
KHUDER	515	m	0	88	123	63	213	2.42	(1.63- 3.58)
LUBIN	590	m	0	33	18	17	73	7.87	(3.61- 17.17)
LUBIN2	1080	m	0	866	1047	109	715	5.43	(4.35- 6.77)
LUBIN2	1119	f	0	60	55	4	20	5.45	(1.75- 16.96)
Subtotal LUBIN2								5.43	(4.37- 6.74)
MATOS	585	m	0	28	23	27	101	4.55	(2.27- 9.13)
PEZZO2	503	m	0	85	110	43	161	2.89	(1.86- 4.49)
PEZZOT	503	m	0	46	82	20	106	2.97	(1.63- 5.41)
SOBUE	727	m	0	128	116	17	40	2.60	(1.40- 4.83)
*SPEIZE	510	f	2	24	-	28	-	6.00	(2.72- 13.21)
SUZUK2	512	c	0	15	10	9	22	3.67	(1.20- 11.17)

Table 1J5 - 5

IESLC - Meta-analysis of Ex Smoking, Years quit (vs never), "Highest vs lowest"
All LC types, Any Product (or Cigarettes if Any not available)
Least adjusted

REF	NRR	SEX	AD	Number Exposed Case	Cont	Non-exposed Case	Cont	RR	95.00%CI
SVENSS	553	f	0	16	13	14	24	2.11	(0.79- 5.65)
*TVERDA	505	m	2	2	-	4	-	2.07	(0.38- 11.34)
WAKAI	526	m	0	19	48	7	47	2.66	(1.02- 6.91)
WANG2	513	c	0	6	10	5	11	1.32	(0.31- 5.70)
WYNDE3	543	m	0	21	22	5	55	10.50	(3.52- 31.34)
WYNDE6	723	m	5	-	-	-	-	3.00	(2.11- 4.27)
WYNDE6	730	m	5	-	-	-	-	2.33	(0.87- 6.25)
WYNDE6	735	f	5	-	-	-	-	2.50	(1.94- 3.23)
WYNDE6	739	f	5	-	-	-	-	1.50	(0.40- 5.61)
Subtotal WYNDE6								2.61	(2.14- 3.19)
Partial Totals				3933	31749	1888	114517		

*prospective study ~ With 0.5 adjustment for zero

REF	NRR	SEX	AD	Ys	Ws	Qs	Ps
ALDERS	512	m	1	1.73	13.52	1.87	0.0000
ALDERS	523	f	1	2.01	13.78	5.71	0.0000
Subtotal ALDERS				1.87	27.31	7.58	
ARMADA	517	m	0	1.12	15.07	0.90	0.0000
BARBON	532	m	0	1.84	6.07	1.38	0.0000
BECHER	505	m	0	1.32	3.85	0.01	0.0095
BECHER	515	f	0	1.90	0.52	0.15	0.1724
Subtotal BECHER				1.39	4.37	0.15	
*BENSHL	514	m	2	2.16	3.32	2.12	0.0001
BROSS	517	m	0	1.53	17.62	0.52	0.0000
CARPEN	507	c	0	0.96	11.01	1.82	0.0015
*CEDERL	530	m	1	1.71	2.39	0.30	0.0081
CHOI	541	m	0	0.62	2.79	1.54	0.3017
CHOI	554	f	0	-1.27	0.32	2.25	0.4687
Subtotal CHOI				0.42	3.12	3.79	
*CHYOU	503	m	1	0.33	3.98	4.24	0.5112
*CPSI	814	m	1	2.45	10.67	12.54	0.0000
*CPSII	663	m	1	2.31	70.40	63.92	0.0000
*CPSII	641	f	1	2.28	32.32	27.21	0.0000
Subtotal CPSII				2.30	102.72	91.13	
DAMBER	527	m	1	1.09	12.46	0.95	0.0001
DARBY	503	m	0	0.87	54.65	13.45	0.0000
DARBY	512	f	0	1.84	14.62	3.35	0.0000
Subtotal DARBY				1.07	69.28	16.80	
DEAN3	536	m	0	0.60	14.98	8.70	0.0203
DEAN3	547	f	0	0.73	1.30	0.52	0.4055
Subtotal DEAN3				0.61	16.28	9.22	
DESTEF	519	m	0	1.10	8.04	0.54	0.0018
DOLL	535	m	0	0.76	5.01	1.79	0.0873
DOLL	546	f	0	1.10	0.56	0.04	0.4100
Subtotal DOLL				0.80	5.57	1.83	
*DOLL2	508	m	1	2.08	4.77	2.46	0.0000
DORGAN	512	m	0	0.79	20.86	6.84	0.0003
DORGAN	557	f	0	0.98	9.36	1.35	0.0027
Subtotal DORGAN				0.85	30.22	8.19	
*DORN	664	m	0	1.73	10.89	1.44	0.0000
*DORN	687	m	0	1.23	9.93	0.18	0.0001
Subtotal DORN				1.49	20.82	1.63	
GAO	525	m	0	1.85	7.69	1.84	0.0000
GAO	545	f	0	1.28	3.68	0.02	0.0141
Subtotal GAO				1.67	11.36	1.87	
GAO2	517	m	0	1.32	4.24	0.01	0.0067
GARCIA	507	c	0	2.41	4.03	4.40	0.0000
GARSHI	518	m	0	0.45	49.05	40.63	0.0016
GRAHAM	529	m	0	2.35	8.56	8.32	0.0000
*HAMMO2	509	m	1	1.16	15.50	0.63	0.0000
*HIRAYA	512	m	1	0.39	3.81	3.64	0.4519
*HIRAYA	523	f	1	1.35	0.28	0.00	0.4794
Subtotal HIRAYA				0.45	4.09	3.64	
JAHN	512	m	0	4.65	5.80	62.69	0.0000
JAIN	569	m	0	1.25	15.79	0.19	0.0000
JAIN	533	f	0	1.77	8.93	1.51	0.0000
Subtotal JAIN				1.44	24.72	1.70	
JEDRYC	614	m	0	0.74	18.58	7.29	0.0015
JOLY	569	m	0	0.91	13.04	2.60	0.0010
JOLY	556	f	0	1.10	3.37	0.23	0.0433

Table 1J5 - 5

IESLC - Meta-analysis of Ex Smoking, Years quit (vs never), "Highest vs lowest"
All LC types, Any Product (or Cigarettes if Any not available)
Least adjusted

REF	NRR	SEX	AD	Ys	Ws	Qs	Ps
Subtotal JOLY				0.95	16.41	2.83	
*KAISE2	652	m	1	1.57	3.26	0.14	0.0046
*KAISE2	572	f	1	0.54	2.14	1.44	0.4278
Subtotal KAISE2				1.16	5.40	1.57	
KHUDER	515	m	0	0.88	24.96	5.71	0.0000
LUBIN	590	m	0	2.06	6.31	3.11	0.0000
LUBIN2	1080	m	0	1.69	78.85	8.56	0.0000
LUBIN2	1119	f	0	1.70	2.99	0.33	0.0034
Subtotal LUBIN2				1.69	81.83	8.89	
MATOS	585	m	0	1.52	7.93	0.19	0.0000
PEZZO2	503	m	0	1.06	19.87	1.78	0.0000
PEZZOT	503	m	0	1.09	10.71	0.79	0.0004
SOBUE	727	m	0	0.95	9.97	1.66	0.0026
*SPEIZE	510	f	2	1.79	6.15	1.14	0.0000
SUZUK2	512	c	0	1.30	3.09	0.01	0.0223
SVENSS	553	f	0	0.75	3.96	1.50	0.1373
*TVERDA	505	m	2	0.73	1.33	0.54	0.4010
WAKAI	526	m	0	0.98	4.21	0.62	0.0449
WANG2	513	c	0	0.28	1.79	2.11	0.7100
WYNDE3	543	m	0	2.35	3.21	3.15	0.0000
WYNDE6	723	m	5	1.10	30.92	2.14	0.0000
WYNDE6	730	m	5	0.85	3.95	1.05	0.0927
WYNDE6	735	f	5	0.92	59.12	11.73	0.0000
WYNDE6	739	f	5	0.41	2.20	2.01	0.5473
Subtotal WYNDE6				0.96	96.20	16.93	

	N	65
	NS	47
	Wt	824.36
Het	Chi	351.81
Het	df	64
Het	P	***
Fixed	RR	3.90
	RRl	3.65
	RRu	4.18
	P	+++
Random	RR	3.91
	RRl	3.27
	RRu	4.68
	P	+++
Asymm	P	N.S.

Table 1J5 - 6

IESLC - Meta-analysis of Ex Smoking, Years quit (vs never), "Highest vs lowest"
All LC types, Any Product (or Cigarettes if Any not available)
Least adjusted

	Sex combined	male	female	Total
N	4	43	18	65
NS	4	41	17	62
Wt	19.93	638.83	165.61	824.36
Het Chi	8.05	282.76	59.33	351.81
Het df	3	42	17	64
Het P	*	***	***	***
Fixed RR	3.46	3.83	4.25	3.90
RRl	2.23	3.55	3.65	3.65
RRu	5.37	4.14	4.94	4.18
P	+++	+++	+++	+++
Random RR	3.63	3.93	3.97	3.91
RRl	1.62	3.17	2.77	3.27
RRu	8.12	4.89	5.70	4.68
P	++	+++	+++	+++
Between Chi				1.67
Between df				2
Between P				N.S.
Btwn(F) P				N.S.
Btwn(R) P				N.S.

Table 1J5 - 7

IESLC - Meta-analysis of Ex Smoking, Years quit (vs never), "Highest vs lowest"
All LC types, Any Product (or Cigarettes if Any not available)
Excluded studies (and stage at which they were excluded)

1	AGUDO AKIBA AMANDU AMES AXELSS BEST BOUCHA BOUCOT BRESLO CHEN CHEN2 CHIAZZ DEAN2 DOSEME ENGELA FAN GENG GER GUO HAENSZ HEGMAN HOLE HU HU2 JUSSAW KATSOU KAUFMA KOO KOULUM KREUZE LETOUR LEVIN LIAW LIU3 LIU4 LIU5 MCCONN MIGRAN MRFITR NOTAN2 OSANN2 PERNU QIAO2 RACHTA RESTRE SADOWS SEGI2 STASZE TIZZAN VUTUC WATSON WU WUWILL WYNDE2 WYNDE8 XU YUAN ZHANG ZHENG ZHOU
2	BUFFLE HUMBLE PISANI PRESCO WYNDE7
3	MCDUFF SPITZ
4	AUVINE BLOT1 BROWN3 GURSEL LAUSSM LUO WU2
5	HAMMON
6	CORREA GILLIS QIAO WIGLE
8	BOFFET
15	BENHAM

Table 1J5 - 8
Potentially overlapping studies

REF	REFGP	PRINC	OVERLAP/LINK
LUBIN2	LUBIN2	1	Lubin-combined
TVERDA	TVERDA	1	VEIERO/TVERDAL
BROSS	BYERS1	1	GRAHAM/BROSS/BYERS1
GRAHAM	BYERS1	1	GRAHAM/BROSS/BYERS1
CHYOU	CHYOU	1	GOODMA/CHYOU
BENSHL	TANG2	1	Subset of TANG2
WYNDE6	WYNDE6	1	WYNDE5/6/7/8
CPSI	CPSI	1	CPSI overall
JAHN	BOFFET	2	Subset of BOFFET
LUBIN	XIANGZ	2	LUBIN/XIANGZ/QIAO

Table 1J5 - 9
Most adjusted - insufficient data for meta-analysis

REF	NRR	SEX	AGEL	AGEH	RACE	YF	LC TYPE	LOC	START	ST	NLC	R	VB	P	H	AD	ADOS	PRODUCT	exL	exH	unexL	unexH	De
ARMADA	525	m	0	0	all	-	all	Eu:wst	1986	CC	325	n	bl	n	y	0	0	cig+/-ot	0.1	0.9	6	999	ot
BECHER	527	m	0	0	all	-	all	Eu:Ger	1985	CC	194	n	bl	n	y	0	0	all/unsp	1.0	1.0	10	999	ot
BECHER	529	f	0	0	all	-	all	Eu:Ger	1985	CC	194	n	bl	n	y	0	0	all/unsp	1.0	1.0	10	999	ot
CORREA	549	c	0	0	all	-	all	NAmer	1979	CC	1359	n	bl	y	n	2	0	cig+/-ot	0.1	3	21	999	ot
CPSI	710	m	0	0	wh	0	all	NAmer	1959	pr	5138	n	bl	n	n	1	0	cig only	2	4	35	39	st
CPSI	728	f	0	0	wh	0	all	NAmer	1959	pr	5138	n	bl	n	n	1	0	cig only	2	4	25	29	st
DEAN3	635	m	0	0	all	-	all	Eu:UK	1969	CC	766	n	V	y	n	1	0	all/unsp	0.1	2	9	999	ot
DEAN3	627	f	0	0	all	-	all	Eu:UK	1969	CC	766	n	V	y	n	1	0	all/unsp	0.1	2	9	999	ot
DORGAN	513	m	0	0	wh	-	all	NAmer	1980	CC	2026	n	bl	y	y	0	0	cig+/-ot	0.1	1.0	10	999	ot
DORGAN	558	f	0	0	all	-	all	NAmer	1980	CC	2026	n	bl	y	y	0	0	cig+/-ot	0.1	1.0	10	999	ot
GAO2	528	m	0	0	all	-	all	As:Jap	1988	CC	282	n	bl	n	n	0	0	cig+/-ot	0.1	0.9	20	999	ot
GARCIA	521	c	0	0	all	-	all	NAmer	1992	CC	416	n	bl	n	y	0	0	cig+/-ot	0.1	0.9	30	999	ot
GARSHI	526	m	0	0	all	-	all	NAmer	1981	CC	1081	o	bl	y	n	1	0	all/unsp	0.1	4	15	999	ot
JAIN	597	m	0	0	all	-	all	NAmer	1981	CC	845	n	V	y	n	0	0	cig+/-ot	0.1	1.9	10	999	ot
JAIN	585	f	0	0	all	-	all	NAmer	1981	CC	845	n	V	y	n	0	0	cig+/-ot	0.1	1.9	10	999	ot
JEDRYC	615	m	0	0	all	-	all	Eu:est	1980	CC	1630	n	bl	y	n	0	0	cig+/-ot	0.1	4	10	999	ot
JOLY	570	m	0	0	all	-	all	SCAmer	1978	CC	826	n	bl	n	n	0	0	cig+/-ot	0.1	0.9	5	999	ot
JOLY	557	f	0	0	all	-	all	SCAmer	1978	CC	826	n	bl	n	n	0	0	cig+/-ot	0.1	0.9	5	999	ot
KAISE2	653	m	0	0	all	9	all	NAmer	1979	pr	318	n	bl	n	n	1	0	cig only	0.1	1.9	21	999	ot
KAISE2	573	f	0	0	all	9	all	NAmer	1979	pr	318	n	bl	n	n	1	0	cig only	0.1	1.9	21	999	ot
LUBIN	591	m	0	0	all	-	all	As:Chi	1984	CC	427	m	ot	y	n	0	0	cig+/-ot	0.1	2	10	999	ot
MATOS	704	m	0	0	all	-	all	SCAmer	1994	CC	200	n	bl	n	n	2	0	cig+/-ot	0.1	0.9	11	999	ot
PEZZO2	513	m	0	0	all	-	all	SCAmer	1992	CC	367	n	bl	n	y	0	0	cig+/-ot	0.1	0.9	11	999	ot
PEZZOT	598	m	0	0	all	-	all	SCAmer	1987	CC	215	n	bl	n	y	0	0	cig only	0.1	0.9	11	999	ot
SOBUE	779	m	0	0	all	-	all	As:Jap	1986	CC	1376	n	bl	n	y	0	0	cig+/-ot	0.1	0.9	25	999	ot
SVENSS	592	f	0	0	all	-	all	Eu:Sca	1983	CC	210	n	bl	n	n	0	0	all/unsp	0.1	2	11	999	ot
WAKAI	612	m	0	0	all	-	all	As:Jap	1988	CC	333	n	bl	n	y	2	0	cig+/-ot	0.1	4	20	999	ot
WIGLE	515	m	0	0	all	-	all	NAmer	1971	CC	728	n	V	n	n	2	1#	cig+/-ot	0.1	1.0	15	999	st
WIGLE	525	f	0	0	all	-	all	NAmer	1971	CC	728	n	V	n	n	2	1#	cig+/-ot	0.1	1.0	15	999	st
WYNDE3	544	m	0	0	all	-	all	NAmer	1966	CC	350	n	bl	n	y	0	0	all/unsp	0.1	0.9	13	999	ot

Table 1J5 - 9

IESLC - Meta-analysis of Ex Smoking, Years quit (vs never), "Highest vs lowest"
All LC types, Any Product (or Cigarettes if Any not available)
Most adjusted - insufficient data for meta-analysis

Comments on values in listings

WIGLE ADOS Cumulative exposure
WIGLE ADOS Cumulative exposure

REF	NRR	RR	SIG	RRDATA comment
ARMADA	525	*	gap	0
BECHER	527	*	gap	0
BECHER	529	*	gap	0
CORREA	549	*	gap	0
CPSI	710	4.13		0
CPSI	728	1.09		0
DEAN3	635	*	gap	0
DEAN3	627	*	gap	0
DORGAN	513	*	gap	0
DORGAN	558	*	gap	0
GAO2	528	*	gap	0
GARCIA	521	*	gap	0
GARSHI	526	*	gap	0
JAIN	597	*	gap	0
JAIN	585	*	gap	0
JEDRYC	615	*	gap	0
JOLY	570	*	gap	0
JOLY	557	*	gap	0
KAISE2	653	*	gap	0
KAISE2	573	*	gap	0
LUBIN	591	*	gap	0
MATOS	704	*	gap	0
PEZZO2	513	*	gap	0
PEZZOT	598	*	gap	0
SOBUE	779	*	gap	0
SVENSS	592	*	gap	0
WAKAI	612	*	gap	0
WIGLE	515	12.00		0
WIGLE	525	2.25		0
WYNDE3	544	*	gap	0

Least adjusted - insufficient data for meta-analysis: as for adjusted plus the following

REF	NRR	SEX	AGEL	AGEH	RACE	YF	LC TYPE	LOC	START	ST	NLC	R	VB	P	H	AD	ADOS	PRODUCT	exL	exH	unexL	unexH	De
DEAN3	623	m	0	0	all	-	all	Eu:UK	1969	CC	766	n	V	y	n	0	0	all/unsp	0.1	2	9	999	ot
DEAN3	625	f	0	0	all	-	all	Eu:UK	1969	CC	766	n	V	y	n	0	0	all/unsp	0.1	2	9	999	ot
GARSHI	519	m	0	0	all	-	all	NAmer	1981	CC	1081	o	bl	y	n	0	0	all/unsp	0.1	4	15	999	ot
MATOS	702	m	0	0	all	-	all	SCAmer	1994	CC	200	n	bl	n	n	0	0	cig+/-ot	0.1	0.9	11	999	ot
WAKAI	610	m	0	0	all	-	all	As:Jap	1988	CC	333	n	bl	n	y	0	0	cig+/-ot	0.1	4	20	999	ot

REF	NRR	RR	SIG	RRDATA comment
DEAN3	623	*	gap	0
DEAN3	625	*	gap	0
GARSHI	519	*	gap	0
MATOS	702	*	gap	0
WAKAI	610	*	gap	0

Table 1J6 -

IESLC - Meta-analysis of Ex Smoking by Years quit (vs never), Overview
All LC types, Cigarettes (or Any Product if Cigarettes not available)

This analysis is restricted to results for:
1) Ex smokers
2) Results by Years quit (vs never)
3) Categorical results by Years quit (vs never)
Results by Years quit (vs never) are grouped under 2 schemes (S1, S2). Each scheme has a set of "key values". An interval is allocated to the category whose key value it includes, and intervals which include none or more than one of the key values are excluded. (Open-ended intervals are coded as 999)

S1	key value	maximum range
1	12	8+
2	7	4-11
3	3	1-6

S2	key value	maximum range
1	20	13+
2	12	4-19
3	3	1-11

4) All LC types (or near equivalent)
5) Results complete enough for use in metaanalysis

Within each study, results are then selected (in the following order of preference, within each sex) for:
6) (not applicable)
7) PRODUCT: cigarettes regardless of other products, cigarettes only, all/unspec
8) CIGTYPE: all/unspecified, MC regardless of HR, MC only
9) (not applicable)
10) DENOM: never smoked anything, never smoked cigarettes, never any + low, never cigs + low
11) Followup period (YF, prospective studies): whole study (coded as 0) or longest available
12) LCtype: all or nearest available, at least Squamous and Adeno. (q = squamous, s = small, l = large, a = adeno, mix = mixed, alv = alveolar)
13) Race: all or nearest available, otherwise by race (wh or w = white, bl or b = black, hi = hispanic ch = chinese, jap = japanese, haw = hawaiian, w+o = white + oriental, sca = scandinavian, as = asian)
14) For overlapping studies: principal rather than subsidiary studies
Finally by Age: whole study (coded as 0) if available, otherwise by widest available age group and then for single sex results (m, f) in preference to results for both sexes combined (c).

Results adjusted (AD) for the most potential confounders are then chosen in Sections -1 to -3 (and those which actually differ from the adjusted results in Table 1J1 - 1 are marked 'x' in Section -1) and results adjusted for the least confounders in Sections -4 to -6. (Those least adjusted results which actually differ from the most adjusted are marked 'x' in column X in Section -4)

Section -7 shows excluded studies, together with the stage (as above) at which no qualifying results were found.

Section -8 lists the potentially overlapping studies which have been included (1=principal, 2=subsidiary).

Section -9 lists any results which would have been included in preference except that they had data not complete enough for use in meta-analysis, with their significance (yes/no), if known, and any further comment as entered on the database. It also lists as "gap" any categories for which no data were presented by the original authors. This is commonly due to recent quitters having been combined with current smokers

In addition to those mentioned above, the following fields, levels and abbreviations are used:
* or nk = not known, n = no, y = yes, ot = other
nev = never
all/unspec = all or unspecified, cig+/-ot = cigarettes irrespective of other products (cigar, pipe etc)
MC = manufactured cigarettes, HR = hand-rolled cigarettes
exL, exH = range of exposure (low and high) in the smoking group, in terms of Years quit (vs never)
REF: 6-character study reference
NRR: number of the RR on the database within the study
ST : study type (CC = case control, pr or prosp = prospective)
NLC: number of lung cancer cases in whole study
R : risky occupational population (n = no, m = mining, o = other risky)
VB : national cigarette type (V = at least 75% Virginia, bl = at least 75% blended, ot = other)
P : any proxy use
H : full histological confirmation
De : derivation of RR/CI (or = original, st = standard method, ot = other method of estimation)

Table 1J6 - 1

IESLC - Meta-analysis of Ex Smoking by Years quit (vs never), Overview
All LC types, Cigarettes (or Any Product if Cigarettes not available)
Most adjusted

REF	NRR	1J1	SEX	AGEL	AGEH	RACE	YF	LC TYPE	LOC	START	ST	NLC	R	VB	P	H	AD	PRODUCT	exL	exH	S1	S2	DENOM	De
ALDERS	507		m	0	0	all	-	all	Eu:UK	1977	CC	1448	n	V	n	n	1	cig only	10	999	1	0	nev any	ot
ALDERS	508		m	0	0	all	-	all	Eu:UK	1977	CC	1448	n	V	n	n	1	cig only	3	9	0	3	nev any	ot
ALDERS	509		m	0	0	all	-	all	Eu:UK	1977	CC	1448	n	V	n	n	1	cig only	0.1	2	0	0	nev any	ot
ALDERS	518		f	0	0	all	-	all	Eu:UK	1977	CC	1448	n	V	n	n	1	cig only	10	999	1	0	nev any	ot
ALDERS	519		f	0	0	all	-	all	Eu:UK	1977	CC	1448	n	V	n	n	1	cig only	3	9	0	3	nev any	ot
ALDERS	520		f	0	0	all	-	all	Eu:UK	1977	CC	1448	n	V	n	n	1	cig only	0.1	2	0	0	nev any	ot
ARMADA	515		m	0	0	all	-	all	Eu:wst	1986	CC	325	n	bl	n	y	0	cig+/-ot	6	999	0	0	nev cigs	st
ARMADA	516		m	0	0	all	-	all	Eu:wst	1986	CC	325	n	bl	n	y	0	cig+/-ot	1.0	5	3	3	nev cigs	st
AUVINE	532		c	0	0	all	-	all	Eu:Sca	1986	CC	517	n	bl	y	n	2	cig+/-ot	12	999	1	0	nev cigs	or
BARBON	540		m	0	0	all	-	all	Eu:wst	1979	CC	755	n	bl	y	y	1	all/unsp	25	999	0	0	nev any	or
BARBON	541		m	0	0	all	-	all	Eu:wst	1979	CC	755	n	bl	y	y	1	all/unsp	15	24	0	1	nev any	or
BARBON	542		m	0	0	all	-	all	Eu:wst	1979	CC	755	n	bl	y	y	1	all/unsp	5	14	0	2	nev any	or
BARBON	543		m	0	0	all	-	all	Eu:wst	1979	CC	755	n	bl	y	y	1	all/unsp	0.1	4	3	3	nev any	or
BECHER	501		m	0	0	all	-	all	Eu:Ger	1985	CC	194	n	bl	n	y	0	all/unsp	10	999	1	0	nev any	st
BECHER	502		m	0	0	all	-	all	Eu:Ger	1985	CC	194	n	bl	n	y	0	all/unsp	5	9	2	0	nev any	st
BECHER	503		m	0	0	all	-	all	Eu:Ger	1985	CC	194	n	bl	n	y	0	all/unsp	2	4	3	3	nev any	st
BECHER	511		f	0	0	all	-	all	Eu:Ger	1985	CC	194	n	bl	n	y	0	all/unsp	10	999	1	0	nev any	st
BECHER	512		f	0	0	all	-	all	Eu:Ger	1985	CC	194	n	bl	n	y	0	all/unsp	5	9	2	0	nev any	st
BECHER	513		f	0	0	all	-	all	Eu:Ger	1985	CC	194	n	bl	n	y	0	all/unsp	2	4	3	3	nev any	st
BENSHL	508		m	0	0	all	0	all	Eu:UK	1967	pr	486	n	V	n	n	2	cig+/-ot	30	999	0	0	nev any	or
BENSHL	509		m	0	0	all	0	all	Eu:UK	1967	pr	486	n	V	n	n	2	cig+/-ot	20	29	0	1	nev any	or
BENSHL	510		m	0	0	all	0	all	Eu:UK	1967	pr	486	n	V	n	n	2	cig+/-ot	10	19	1	2	nev any	or
BENSHL	511		m	0	0	all	0	all	Eu:UK	1967	pr	486	n	V	n	n	2	cig+/-ot	1.0	9	0	3	nev any	or
BROSS	515		m	0	0	wh	-	all	NAmer	1960	CC	974	n	bl	n	n	0	cig+/-ot	6	999	0	0	nev any	st
BROSS	516		m	0	0	wh	-	all	NAmer	1960	CC	974	n	bl	n	n	0	cig+/-ot	0.1	5	3	3	nev any	st
BROWN3	503		f	0	0	wh	-	all	NAmer		CC	618		bl	y	n	2	all/unsp	15	999	0	1	nev any	or
CARPEN	501		c	0	0	w+b	-	all	NAmer	1991	CC	356	n	bl	n	n	0	cig+/-ot	15	999	0	1	nev cigs	st
CARPEN	502		c	0	0	w+b	-	all	NAmer	1991	CC	356	n	bl	n	n	0	cig+/-ot	10	14	1	2	nev cigs	st
CARPEN	503		c	0	0	w+b	-	all	NAmer	1991	CC	356	n	bl	n	n	0	cig+/-ot	5	9	2	0	nev cigs	st
CARPEN	504		c	0	0	w+b	-	all	NAmer	1991	CC	356	n	bl	n	n	0	cig+/-ot	0.1	4	3	3	nev cigs	st
CEDERL	528		m	40	69	all	10	all	Eu:Sca	1963	pr	491	n	bl	n	n	1	all/unsp	10	999	1	0	nev any	ot
CEDERL	529		m	40	69	all	10	all	Eu:Sca	1963	pr	491	n	bl	n	n	1	all/unsp	0.1	9	0	3	nev any	ot
CHOI	533		m	0	0	all	-	all	As:oth	1985	CC	375	n	bl	n	n	0	cig+/-ot	15	999	0	1	nev cigs	st
CHOI	534		m	0	0	all	-	all	As:oth	1985	CC	375	n	bl	n	n	0	cig+/-ot	10	14	1	2	nev cigs	st
CHOI	535		m	0	0	all	-	all	As:oth	1985	CC	375	n	bl	n	n	0	cig+/-ot	5	9	2	0	nev cigs	st
CHOI	536		m	0	0	all	-	all	As:oth	1985	CC	375	n	bl	n	n	0	cig+/-ot	0.1	4	3	3	nev cigs	st
CHOI	550		f	0	0	all	-	all	As:oth	1985	CC	375	n	bl	n	n	0	cig+/-ot	5	999	0	0	nev cigs	ot
CHOI	551		f	0	0	all	-	all	As:oth	1985	CC	375	n	bl	n	n	0	cig+/-ot	0.1	4	3	3	nev cigs	st
CHYOU	507		m	0	0	jap	21	all	NAmer	1965	pr	227	n	bl	n	y	2	cig+/-ot	15	999	0	1	nev cigs	or
CHYOU	508		m	0	0	jap	21	all	NAmer	1965	pr	227	n	bl	n	y	2	cig+/-ot	0.1	14	0	0	nev cigs	or
CPSI	807		m	50	74	all	6	all	NAmer	1959	pr	5138	n	bl	n	n	1	cig only	10	999	1	0	nev any	ot
CPSI	808		m	50	74	all	6	all	NAmer	1959	pr	5138	n	bl	n	n	1	cig only	5	9	2	0	nev any	ot
CPSI	809		m	50	74	all	6	all	NAmer	1959	pr	5138	n	bl	n	n	1	cig only	1.0	4	3	3	nev any	ot
CPSI	810		m	50	74	all	6	all	NAmer	1959	pr	5138	n	bl	n	n	1	cig only	0.1	0.9	0	0	nev any	ot
CPSII	652		m	35	99	all	4	all	NAmer	1982	pr	3229	n	bl	n	n	1	cig only	16	999	0	1	nev any	ot
CPSII	653		m	35	99	all	4	all	NAmer	1982	pr	3229	n	bl	n	n	1	cig only	11	15	1	2	nev any	ot
CPSII	654		m	35	99	all	4	all	NAmer	1982	pr	3229	n	bl	n	n	1	cig only	6	10	2	0	nev any	ot
CPSII	655		m	35	99	all	4	all	NAmer	1982	pr	3229	n	bl	n	n	1	cig only	3	5	3	3	nev any	ot
CPSII	656		m	35	99	all	4	all	NAmer	1982	pr	3229	n	bl	n	n	1	cig only	1.0	2	0	0	nev any	ot
CPSII	657		m	35	99	all	4	all	NAmer	1982	pr	3229	n	bl	n	n	1	cig only	0.1	0.9	0	0	nev any	ot
CPSII	633		f	0	0	all	4	all	NAmer	1982	pr	3229	n	bl	n	n	1	cig+/-ot	16	999	0	1	nev cigs	ot
CPSII	634		f	0	0	all	4	all	NAmer	1982	pr	3229	n	bl	n	n	1	cig+/-ot	11	15	1	2	nev cigs	ot
CPSII	635		f	0	0	all	4	all	NAmer	1982	pr	3229	n	bl	n	n	1	cig+/-ot	6	10	2	0	nev cigs	ot
CPSII	636		f	0	0	all	4	all	NAmer	1982	pr	3229	n	bl	n	n	1	cig+/-ot	3	5	3	3	nev cigs	ot
CPSII	637		f	0	0	all	4	all	NAmer	1982	pr	3229	n	bl	n	n	1	cig+/-ot	0.1	2	0	0	nev cigs	ot
DAMBER	554	x	m	0	0	all	-	all	Eu:Sca	1972	CC	579	n	bl	y	n	1	cig only	11	999	1	0	nev any	ot
DAMBER	555	x	m	0	0	all	-	all	Eu:Sca	1972	CC	579	n	bl	y	n	1	cig only	0.1	10	0	3	nev any	ot
DARBY	501		m	0	0	wh	-	all	Eu:UK	1988	CC	982	n	V	n	n	0	all/unsp	10	999	1	0	nev any	st
DARBY	502		m	0	0	wh	-	all	Eu:UK	1988	CC	982	n	V	n	n	0	all/unsp	0.1	9	0	3	nev any	st
DARBY	510		f	0	0	wh	-	all	Eu:UK	1988	CC	982	n	V	n	n	0	all/unsp	10	999	1	0	nev any	st
DARBY	511		f	0	0	wh	-	all	Eu:UK	1988	CC	982	n	V	n	n	0	all/unsp	0.1	9	0	3	nev any	st
DEAN3	516	x	m	0	0	all	-	all	Eu:UK	1969	CC	766	n	V	y	n	1	cig only	19	999	0	1	nev any	ot
DEAN3	517	x	m	0	0	all	-	all	Eu:UK	1969	CC	766	n	V	y	n	1	cig only	9	18	1	2	nev any	ot
DEAN3	518	x	m	0	0	all	-	all	Eu:UK	1969	CC	766	n	V	y	n	1	cig only	5	8	2	0	nev any	ot
DEAN3	519	x	m	0	0	all	-	all	Eu:UK	1969	CC	766	n	V	y	n	1	cig only	3	4	3	3	nev any	ot
DEAN3	553		f	0	0	all	-	all	Eu:UK	1969	CC	766	n	V	y	n	1	all/unsp	9	999	1	0	nev any	ot
DEAN3	554		f	0	0	all	-	all	Eu:UK	1969	CC	766	n	V	y	n	1	all/unsp	5	8	2	0	nev any	ot
DEAN3	555		f	0	0	all	-	all	Eu:UK	1969	CC	766	n	V	y	n	1	all/unsp	3	4	3	3	nev any	ot
DESTEF	525		m	0	0	all	-	all	SCAmer	1988	CC	497	n	bl	n	y	4	all/unsp	10	999	1	0	nev any	or
DESTEF	526		m	0	0	all	-	all	SCAmer	1988	CC	497	n	bl	n	y	4	all/unsp	5	9	2	0	nev any	or
DESTEF	527		m	0	0	all	-	all	SCAmer	1988	CC	497	n	bl	n	y	4	all/unsp	0.1	4	3	3	nev any	or
DOLL	529		m	0	0	all	-	all	Eu:UK	1948	CC	1465	n	V	n	n	0	all/unsp	20	999	0	1	nev any	st

Table 1J6 - 1

IESLC - Meta-analysis of Ex Smoking by Years quit (vs never), Overview
All LC types, Cigarettes (or Any Product if Cigarettes not available)
Most adjusted

REF	NRR	1J1	SEX	AGEL	AGEH	RACE	YF	LC TYPE	LOC	START	ST	NLC	R	VB	P	H	AD	PRODUCT	exL	exH	S1	S2	DENOM	De
DOLL	530		m	0	0	all	-	all	Eu:UK	1948	CC	1465	n	V	n	n	0	all/unsp	10	19	1	2	nev any	st
DOLL	531		m	0	0	all	-	all	Eu:UK	1948	CC	1465	n	V	n	n	0	all/unsp	0.1	9	0	3	nev any	st
DOLL	542		f	0	0	all	-	all	Eu:UK	1948	CC	1465	n	V	n	n	0	all/unsp	10	999	1	0	nev any	st
DOLL	543		f	0	0	all	-	all	Eu:UK	1948	CC	1465	n	V	n	n	0	all/unsp	0.1	9	0	3	nev any	st
DOLL2	501		m	0	0	all	20	all	Eu:UK	1951	pr	920	n	V	n	n	1	cig only	15	999	0	1	nev any	ot
DOLL2	502		m	0	0	all	20	all	Eu:UK	1951	pr	920	n	V	n	n	1	cig only	10	14	1	2	nev any	ot
DOLL2	503		m	0	0	all	20	all	Eu:UK	1951	pr	920	n	V	n	n	1	cig only	5	9	2	0	nev any	ot
DOLL2	504		m	0	0	all	20	all	Eu:UK	1951	pr	920	n	V	n	n	1	cig only	0.1	4	3	3	nev any	ot
DORGAN	501		m	0	0	wh	-	all	NAmer	1980	CC	2026	n	bl	y	y	0	cig+/-ot	10	999	1	0	nev any	st
DORGAN	502		m	0	0	wh	-	all	NAmer	1980	CC	2026	n	bl	y	y	0	cig+/-ot	6	9	2	0	nev any	st
DORGAN	503		m	0	0	wh	-	all	NAmer	1980	CC	2026	n	bl	y	y	0	cig+/-ot	1.1	5	3	3	nev any	st
DORGAN	553		f	0	0	all	-	all	NAmer	1980	CC	2026	n	bl	y	y	0	cig+/-ot	10	999	1	0	nev any	st
DORGAN	554		f	0	0	all	-	all	NAmer	1980	CC	2026	n	bl	y	y	0	cig+/-ot	1.1	9	0	3	nev any	st
DORN	657		m	55	64	wh	8	all	NAmer	1954	pr	5097	n	bl	n	n	0	cig+/-ot	15	999	0	1	nev any	st
DORN	658		m	55	64	wh	8	all	NAmer	1954	pr	5097	n	bl	n	n	0	cig+/-ot	10	14	1	2	nev any	st
DORN	659		m	55	64	wh	8	all	NAmer	1954	pr	5097	n	bl	n	n	0	cig+/-ot	5	9	2	0	nev any	st
DORN	660		m	55	64	wh	8	all	NAmer	1954	pr	5097	n	bl	n	n	0	cig+/-ot	0.1	4	3	3	nev any	st
DORN	680		m	65	74	wh	8	all	NAmer	1954	pr	5097	n	bl	n	n	0	cig+/-ot	15	999	0	1	nev any	st
DORN	681		m	65	74	wh	8	all	NAmer	1954	pr	5097	n	bl	n	n	0	cig+/-ot	10	14	1	2	nev any	st
DORN	682		m	65	74	wh	8	all	NAmer	1954	pr	5097	n	bl	n	n	0	cig+/-ot	5	9	2	0	nev any	st
DORN	683		m	65	74	wh	8	all	NAmer	1954	pr	5097	n	bl	n	n	0	cig+/-ot	0.1	4	3	3	nev any	st
GAO	531		m	0	0	all	-	all	As:Chi	1984	CC	1405	n	ot	n	n	2	cig+/-ot	10	999	1	0	nev cigs	or
GAO	532		m	0	0	all	-	all	As:Chi	1984	CC	1405	n	ot	n	n	2	cig+/-ot	5	9	2	0	nev cigs	or
GAO	533		m	0	0	all	-	all	As:Chi	1984	CC	1405	n	ot	n	n	2	cig+/-ot	0.1	4	3	3	nev cigs	or
GAO	551		f	0	0	all	-	all	As:Chi	1984	CC	1405	n	ot	n	n	2	cig+/-ot	10	999	1	0	nev cigs	or
GAO	552		f	0	0	all	-	all	As:Chi	1984	CC	1405	n	ot	n	n	2	cig+/-ot	5	9	2	0	nev cigs	or
GAO	553		f	0	0	all	-	all	As:Chi	1984	CC	1405	n	ot	n	n	2	cig+/-ot	0.1	4	3	3	nev cigs	or
GAO2	509		m	0	0	all	-	all	As:Jap	1988	CC	282	n	bl	n	n	0	cig+/-ot	20	999	0	1	nev cigs	or
GAO2	510		m	0	0	all	-	all	As:Jap	1988	CC	282	n	bl	n	n	0	cig+/-ot	15	19	0	0	nev cigs	or
GAO2	511		m	0	0	all	-	all	As:Jap	1988	CC	282	n	bl	n	n	0	cig+/-ot	10	14	1	2	nev cigs	or
GAO2	512		m	0	0	all	-	all	As:Jap	1988	CC	282	n	bl	n	n	0	cig+/-ot	5	9	2	0	nev cigs	st
GAO2	513		m	0	0	all	-	all	As:Jap	1988	CC	282	n	bl	n	n	0	cig+/-ot	1.0	4	3	3	nev cigs	or
GARCIA	515		c	0	0	all	-	all	NAmer	1992	CC	416	n	bl	n	y	0	cig+/-ot	30	999	0	0	nev any	st
GARCIA	516		c	0	0	all	-	all	NAmer	1992	CC	416	n	bl	n	y	0	cig+/-ot	15	29	0	1	nev any	st
GARCIA	517		c	0	0	all	-	all	NAmer	1992	CC	416	n	bl	n	y	0	cig+/-ot	5	14	0	2	nev any	st
GARCIA	518		c	0	0	all	-	all	NAmer	1992	CC	416	n	bl	n	y	0	cig+/-ot	1.0	4	3	3	nev any	st
GARSHI	522		m	0	0	all	-	all	NAmer	1981	CC	1081	o	bl	y	n	1	all/unsp	15	999	0	1	nev any	st
GARSHI	523		m	0	0	all	-	all	NAmer	1981	CC	1081	o	bl	y	n	1	all/unsp	5	14	0	2	nev any	st
GRAHAM	535		m	0	0	wh	-	all	NAmer	1956	CC	685	n	bl	n	n	1	cig+/-ot	5	999	0	0	nev any	ot
GRAHAM	536		m	0	0	wh	-	all	NAmer	1956	CC	685	n	bl	n	n	1	cig+/-ot	1.1	5	3	3	nev any	ot
GRAHAM	537		m	0	0	wh	-	all	NAmer	1956	CC	685	n	bl	n	n	1	cig+/-ot	0.1	1.0	0	0	nev any	ot
GURSEL	501		m	0	0	all	-	all	Eu:bal		CC	953		bl	*	n	0	all/unsp	11	999	1	0	nev any	or
HAMMO2	501		m	0	0	all	0	all	NAmer	1967	pr	450	o	bl	n	n	1	cig+/-ot	10	999	1	0	nev any	ot
HAMMO2	502		m	0	0	all	0	all	NAmer	1967	pr	450	o	bl	n	n	1	cig+/-ot	5	9	2	0	nev any	ot
HAMMO2	503		m	0	0	all	0	all	NAmer	1967	pr	450	o	bl	n	n	1	cig+/-ot	0.1	4	3	3	nev any	ot
HIRAYA	507		m	0	0	all	0	all	As:Jap	1965	pr	1917	n	bl	n	n	1	cig+/-ot	10	999	1	0	nev any	st
HIRAYA	508		m	0	0	all	0	all	As:Jap	1965	pr	1917	n	bl	n	n	1	cig+/-ot	5	9	2	0	nev any	st
HIRAYA	509		m	0	0	all	0	all	As:Jap	1965	pr	1917	n	bl	n	n	1	cig+/-ot	0.1	4	3	3	nev any	st
HIRAYA	518		f	0	0	all	0	all	As:Jap	1965	pr	1917	n	bl	n	n	1	cig+/-ot	10	999	1	0	nev any	st
HIRAYA	519		f	0	0	all	0	all	As:Jap	1965	pr	1917	n	bl	n	n	1	cig+/-ot	5	9	2	0	nev any	st
HIRAYA	520		f	0	0	all	0	all	As:Jap	1965	pr	1917	n	bl	n	n	1	cig+/-ot	0.1	4	3	3	nev any	st
JAHN	501		m	0	0	all	-	all	Eu:Ger	1988	CC	1004	n	bl	n	n	0	cig+/-ot	21	999	0	0	nev any	st
JAHN	502		m	0	0	all	-	all	Eu:Ger	1988	CC	1004	n	bl	n	n	0	cig+/-ot	11	20	1	0	nev any	st
JAHN	503		m	0	0	all	-	all	Eu:Ger	1988	CC	1004	n	bl	n	n	0	cig+/-ot	6	10	2	0	nev any	st
JAHN	504		m	0	0	all	-	all	Eu:Ger	1988	CC	1004	n	bl	n	n	0	cig+/-ot	2	5	3	3	nev any	st
JAHN	505		m	0	0	all	-	all	Eu:Ger	1988	CC	1004	n	bl	n	n	0	cig+/-ot	1.0	1.9	0	0	nev any	st
JAHN	506		m	0	0	all	-	all	Eu:Ger	1988	CC	1004	n	bl	n	n	0	cig+/-ot	0.1	0.9	0	0	nev any	st
JAHN	731		f	0	0	all	-	all	Eu:Ger	1988	CC	1004	n	bl	n	n	2	cig+/-ot	21	999	0	0	nev any	or
JAIN	567		m	0	0	all	-	all	NAmer	1981	CC	845	n	V	y	n	0	cig+/-ot	10	999	1	0	nev cigs	st
JAIN	568		m	0	0	all	-	all	NAmer	1981	CC	845	n	V	y	n	0	cig+/-ot	2	9	0	3	nev cigs	st
JAIN	531		f	0	0	all	-	all	NAmer	1981	CC	845	n	V	y	n	0	cig+/-ot	10	999	1	0	nev cigs	st
JAIN	532		f	0	0	all	-	all	NAmer	1981	CC	845	n	V	y	n	0	cig+/-ot	2	9	0	3	nev cigs	st
JEDRYC	611		m	0	0	all	-	all	Eu:est	1980	CC	1630	n	bl	y	n	0	cig+/-ot	10	999	1	0	nev any	st
JEDRYC	612		m	0	0	all	-	all	Eu:est	1980	CC	1630	n	bl	y	n	0	cig+/-ot	5	9	2	0	nev any	st
JOLY	566		m	0	0	all	-	all	SCAmer	1978	CC	826	n	bl	n	n	0	cig+/-ot	5	999	0	0	nev any	st
JOLY	567		m	0	0	all	-	all	SCAmer	1978	CC	826	n	bl	n	n	0	cig+/-ot	1.0	4	3	3	nev any	st
JOLY	553		f	0	0	all	-	all	SCAmer	1978	CC	826	n	bl	n	n	0	cig+/-ot	5	999	0	0	nev any	st
JOLY	554		f	0	0	all	-	all	SCAmer	1978	CC	826	n	bl	n	n	0	cig+/-ot	1.0	4	3	3	nev any	st
KAISE2	646		m	0	0	all	9	all	NAmer	1979	pr	318	n	bl	n	n	1	cig only	21	999	0	0	nev any	st
KAISE2	647		m	0	0	all	9	all	NAmer	1979	pr	318	n	bl	n	n	1	cig only	11	20	1	0	nev any	ot
KAISE2	648		m	0	0	all	9	all	NAmer	1979	pr	318	n	bl	n	n	1	cig only	2	10	0	3	nev any	st
KAISE2	566		f	0	0	all	9	all	NAmer	1979	pr	318	n	bl	n	n	1	cig only	21	999	0	0	nev any	ot

Table 1J6 - 1

IESLC - Meta-analysis of Ex Smoking by Years quit (vs never), Overview
All LC types, Cigarettes (or Any Product if Cigarettes not available)
Most adjusted

REF	NRR	1J1	SEX	AGEL	AGEH	RACE	YF	LC TYPE	LOC	START	ST	NLC	R	VB	P	H	AD	PRODUCT	exL	exH	S1	S2	DENOM	De
KAISE2	567		f	0	0	all	9	all	NAmer	1979	pr	318	n	bl	n	n	1	cig only	11	20	1	0	nev any	st
KAISE2	568		f	0	0	all	9	all	NAmer	1979	pr	318	n	bl	n	n	1	cig only	2	10	0	3	nev any	st
KHUDER	511		m	0	0	all	-	all	NAmer	1985	CC	482	n	bl	n	y	0	cig+/-ot	15	999	0	1	nev cigs	st
KHUDER	512		m	0	0	all	-	all	NAmer	1985	CC	482	n	bl	n	y	0	cig+/-ot	5	14	0	2	nev cigs	st
KHUDER	513		m	0	0	all	-	all	NAmer	1985	CC	482	n	bl	n	y	0	cig+/-ot	0.1	4	3	3	nev cigs	st
LAUSSM	503		m	0	0	all	-	all	Eu:Ger	1982	CC	432	n	bl	n	n	2	all/unsp	10	999	1	0	nev any	st
LUBIN	585		m	0	0	all	-	all	As:Chi	1984	CC	427	m	ot	y	n	0	cig+/-ot	10	999	1	0	nev any	st
LUBIN	586		m	0	0	all	-	all	As:Chi	1984	CC	427	m	ot	y	n	0	cig+/-ot	5	9	2	0	nev any	st
LUBIN	587		m	0	0	all	-	all	As:Chi	1984	CC	427	m	ot	y	n	0	cig+/-ot	3	4	3	3	nev any	st
LUBIN2	1069		m	0	0	all	-	all	Eu:mul	1976	CC	7804	n	bl	n	y	0	cig+/-ot	25	999	0	0	nev any	st
LUBIN2	1070		m	0	0	all	-	all	Eu:mul	1976	CC	7804	n	bl	n	y	0	cig+/-ot	20	24	0	1	nev any	st
LUBIN2	1071		m	0	0	all	-	all	Eu:mul	1976	CC	7804	n	bl	n	y	0	cig+/-ot	15	19	0	0	nev any	st
LUBIN2	1072		m	0	0	all	-	all	Eu:mul	1976	CC	7804	n	bl	n	y	0	cig+/-ot	10	14	1	2	nev any	st
LUBIN2	1073		m	0	0	all	-	all	Eu:mul	1976	CC	7804	n	bl	n	y	0	cig+/-ot	5	9	2	0	nev any	st
LUBIN2	1074		m	0	0	all	-	all	Eu:mul	1976	CC	7804	n	bl	n	y	0	cig+/-ot	0.1	4	3	3	nev any	st
LUBIN2	1108		f	0	0	all	-	all	Eu:mul	1976	CC	7804	n	bl	n	y	0	cig+/-ot	25	999	0	0	nev any	st
LUBIN2	1109		f	0	0	all	-	all	Eu:mul	1976	CC	7804	n	bl	n	y	0	cig+/-ot	20	24	0	1	nev any	st
LUBIN2	1110		f	0	0	all	-	all	Eu:mul	1976	CC	7804	n	bl	n	y	0	cig+/-ot	15	19	0	0	nev any	st
LUBIN2	1111		f	0	0	all	-	all	Eu:mul	1976	CC	7804	n	bl	n	y	0	cig+/-ot	10	14	1	2	nev any	st
LUBIN2	1112		f	0	0	all	-	all	Eu:mul	1976	CC	7804	n	bl	n	y	0	cig+/-ot	5	9	2	0	nev any	st
LUBIN2	1113		f	0	0	all	-	all	Eu:mul	1976	CC	7804	n	bl	n	y	0	cig+/-ot	0.1	4	3	3	nev any	st
MATOS	591		m	0	0	all	-	all	SCAmer	1994	CC	200	n	bl	n	n	2	cig+/-ot	11	999	1	0	nev any	ot
MATOS	592		m	0	0	all	-	all	SCAmer	1994	CC	200	n	bl	n	n	2	cig+/-ot	6	10	2	0	nev any	ot
MATOS	593		m	0	0	all	-	all	SCAmer	1994	CC	200	n	bl	n	n	2	cig+/-ot	1.0	5	3	3	nev any	ot
PEZZO2	501		m	0	0	all	-	all	SCAmer	1992	CC	367	n	bl	n	y	0	cig+/-ot	11	999	1	0	nev cigs	st
PEZZO2	502		m	0	0	all	-	all	SCAmer	1992	CC	367	n	bl	n	y	0	cig+/-ot	1.0	10	0	3	nev cigs	st
PEZZOT	501		m	0	0	all	-	all	SCAmer	1987	CC	215	n	bl	n	y	0	cig only	11	999	1	0	nev cigs	st
PEZZOT	502		m	0	0	all	-	all	SCAmer	1987	CC	215	n	bl	n	y	0	cig only	1.0	10	0	3	nev cigs	st
SOBUE	717		m	0	0	all	-	all	As:Jap	1986	CC	1376	n	bl	n	y	0	cig+/-ot	25	999	0	0	nev cigs	st
SOBUE	718		m	0	0	all	-	all	As:Jap	1986	CC	1376	n	bl	n	y	0	cig+/-ot	20	24	0	1	nev cigs	st
SOBUE	719		m	0	0	all	-	all	As:Jap	1986	CC	1376	n	bl	n	y	0	cig+/-ot	15	19	0	0	nev cigs	st
SOBUE	720		m	0	0	all	-	all	As:Jap	1986	CC	1376	n	bl	n	y	0	cig+/-ot	10	14	1	2	nev cigs	st
SOBUE	721		m	0	0	all	-	all	As:Jap	1986	CC	1376	n	bl	n	y	0	cig+/-ot	5	9	2	0	nev cigs	st
SOBUE	722		m	0	0	all	-	all	As:Jap	1986	CC	1376	n	bl	n	y	0	cig+/-ot	1.0	4	3	3	nev cigs	st
SPEIZE	501		f	0	0	all	0	all	NAmer	1976	pr	593	n	bl	n	y	0	cig+/-ot	15	999	0	1	nev cigs	st
SPEIZE	502		f	0	0	all	0	all	NAmer	1976	pr	593	n	bl	n	y	0	cig+/-ot	10	15	1	2	nev cigs	st
SPEIZE	503		f	0	0	all	0	all	NAmer	1976	pr	593	n	bl	n	y	0	cig+/-ot	5	10	2	0	nev cigs	st
SPEIZE	504		f	0	0	all	0	all	NAmer	1976	pr	593	n	bl	n	y	0	cig+/-ot	2	5	3	3	nev cigs	st
SPEIZE	505		f	0	0	all	0	all	NAmer	1976	pr	593	n	bl	n	y	0	cig+/-ot	0.1	1.9	0	0	nev cigs	st
SUZUK2	508		c	0	0	all	-	all	SCAmer	1991	CC	123	n	bl	n	y	0	all/unsp	11	999	1	0	nev any	st
SUZUK2	509		c	0	0	all	-	all	SCAmer	1991	CC	123	n	bl	n	y	0	all/unsp	6	10	2	0	nev any	st
SUZUK2	510		c	0	0	all	-	all	SCAmer	1991	CC	123	n	bl	n	y	0	all/unsp	0.1	5	3	3	nev any	st
SVENSS	551		f	0	0	all	-	all	Eu:Sca	1983	CC	210	n	bl	n	n	0	all/unsp	11	999	1	0	nev any	st
SVENSS	552		f	0	0	all	-	all	Eu:Sca	1983	CC	210	n	bl	n	n	0	all/unsp	3	10	0	3	nev any	st
TVERDA	501		m	0	0	all	0	all	Eu:Sca	1972	pr	238	n	bl	n	n	2	cig only	5	999	0	0	nev cigs	ot
TVERDA	502		m	0	0	all	0	all	Eu:Sca	1972	pr	238	n	bl	n	n	2	cig only	1.0	5	3	3	nev cigs	ot
TVERDA	503		m	0	0	all	0	all	Eu:Sca	1972	pr	238	n	bl	n	n	2	cig only	0.1	0.9	0	0	nev cigs	ot
WAKAI	530		m	0	0	all	-	all	As:Jap	1988	CC	333	n	bl	n	y	2	cig+/-ot	20	999	0	1	nev any	or
WAKAI	531		m	0	0	all	-	all	As:Jap	1988	CC	333	n	bl	n	y	2	cig+/-ot	10	19	1	2	nev any	or
WAKAI	532		m	0	0	all	-	all	As:Jap	1988	CC	333	n	bl	n	y	2	cig+/-ot	5	9	2	0	nev any	or
WANG2	510		c	0	0	all	-	all	As:Chi	1980	CC	103	n	ot	n	n	0	cig+/-ot	4	999	0	0	nev cigs	st
WANG2	511		c	0	0	all	-	all	As:Chi	1980	CC	103	n	ot	n	n	0	cig+/-ot	0.1	3	3	3	nev cigs	st
WYNDE3	566	x	m	0	0	all	-	all	NAmer	1966	CC	350	n	bl	n	y	0	cig+/-ot	10	999	1	0	nev any	st
WYNDE3	587		f	0	0	all	-	all	NAmer	1966	CC	350	n	bl	n	y	0	cig+/-ot	10	999	1	0	nev any	st
WYNDE6	501		m	0	0	all	-	all	NAmer	1969	CC	4423	n	bl	n	y	0	cig only	30	999	0	0	nev any	st
WYNDE6	502		m	0	0	all	-	all	NAmer	1969	CC	4423	n	bl	n	y	0	cig only	20	29	0	1	nev any	st
WYNDE6	503		m	0	0	all	-	all	NAmer	1969	CC	4423	n	bl	n	y	0	cig only	10	19	1	2	nev any	st
WYNDE6	504		m	0	0	all	-	all	NAmer	1969	CC	4423	n	bl	n	y	0	cig only	5	9	2	0	nev any	st
WYNDE6	505		m	0	0	all	-	all	NAmer	1969	CC	4423	n	bl	n	y	0	cig only	1.0	4	3	3	nev any	st
WYNDE6	522		f	0	0	all	-	all	NAmer	1969	CC	4423	n	bl	n	y	0	cig only	30	999	0	0	nev any	st
WYNDE6	523		f	0	0	all	-	all	NAmer	1969	CC	4423	n	bl	n	y	0	cig only	20	29	0	1	nev any	st
WYNDE6	524		f	0	0	all	-	all	NAmer	1969	CC	4423	n	bl	n	y	0	cig only	10	19	1	2	nev any	st
WYNDE6	525		f	0	0	all	-	all	NAmer	1969	CC	4423	n	bl	n	y	0	cig only	5	9	2	0	nev any	st
WYNDE6	526		f	0	0	all	-	all	NAmer	1969	CC	4423	n	bl	n	y	0	cig only	1.0	4	3	3	nev any	st

Cigarette type is all/unspec for all RRs
except for the following:

Table 1J6 - 1

IESLC - Meta-analysis of Ex Smoking by Years quit (vs never), Overview
All LC types, Cigarettes (or Any Product if Cigarettes not available)
Most adjusted

REF	NRR	CIGTYPE
ALDERS	507	MC only
ALDERS	508	MC only
ALDERS	509	MC only
ALDERS	518	MC only
ALDERS	519	MC only
ALDERS	520	MC only
DEAN3	516	MC only
DEAN3	517	MC only
DEAN3	518	MC only
DEAN3	519	MC only

In this overview table, subtotals and Qs values may be invalid and should be ignored

Table 1J6 - 2

IESLC - Meta-analysis of Ex Smoking by Years quit (vs never), Overview
All LC types, Cigarettes (or Any Product if Cigarettes not available)
Most adjusted

REF	NRR	SEX	AD	Number Case	Exposed Cont	Non-exposed Case	Cont	RR	95.00%CI
ALDERS	507	m	1	29	-	15	-	3.20	(1.61- 6.35)
ALDERS	508	m	1	28	-	15	-	4.30	(2.13- 8.69)
ALDERS	509	m	1	121	-	15	-	18.10	(9.71- 33.74)
ALDERS	518	f	1	26	-	75	-	1.27	(0.76- 2.15)
ALDERS	519	f	1	54	-	75	-	2.95	(1.88- 4.64)
ALDERS	520	f	1	206	-	75	-	9.45	(6.50- 13.74)
Subtotal ALDERS								4.77	(3.86- 5.89)
ARMADA	515	m	0	50	87	8	71	5.10	(2.27- 11.46)
ARMADA	516	m	0	79	45	8	71	15.58	(6.88- 35.29)
Subtotal ARMADA								8.87	(4.99- 15.76)
AUVINE	532	c	2	207	-	44	-	7.50	(4.18- 13.15)
BARBON	540	m	1	15	-	22	-	2.10	(1.00- 4.30)
BARBON	541	m	1	33	-	22	-	6.80	(3.60- 12.80)
BARBON	542	m	1	89	-	22	-	9.10	(5.30- 15.50)
BARBON	543	m	1	32	-	22	-	13.90	(6.80- 28.50)
Subtotal BARBON								6.94	(5.04- 9.55)
BECHER	501	m	0	16	72	3	54	4.00	(1.11- 14.42)
BECHER	502	m	0	16	32	3	54	9.00	(2.43- 33.30)
BECHER	503	m	0	10	12	3	54	15.00	(3.58- 62.92)
BECHER	511	f	0	1	10	10	52	0.52	(0.06- 4.53)
BECHER	512	f	0	2	5	10	52	2.08	(0.35- 12.26)
BECHER	513	f	0	2	3	10	52	3.47	(0.51- 23.48)
Subtotal BECHER								4.76	(2.53- 8.99)
*BENSHL	508	m	2	6	-	10	-	1.00	(0.32- 3.10)
*BENSHL	509	m	2	15	-	10	-	2.59	(1.21- 5.54)
*BENSHL	510	m	2	23	-	10	-	4.08	(2.03- 8.20)
*BENSHL	511	m	2	14	-	10	-	8.68	(4.00- 18.90)
Subtotal BENSHL								3.69	(2.47- 5.51)
BROSS	515	m	0	43	79	38	170	2.44	(1.46- 4.06)
BROSS	516	m	0	169	67	38	170	11.28	(7.19- 17.72)
Subtotal BROSS								5.77	(4.11- 8.09)
BROWN3	503	f	2	186	-	432	-	2.20	(1.80- 2.70)
CARPEN	501	c	0	29	137	8	208	5.50	(2.44- 12.40)
CARPEN	502	c	0	9	51	8	208	4.59	(1.69- 12.48)
CARPEN	503	c	0	25	48	8	208	13.54	(5.75- 31.87)
CARPEN	504	c	0	23	39	8	208	15.33	(6.40- 36.75)
Subtotal CARPEN								8.72	(5.62- 13.52)
*CEDERL	528	m	1	3	-	7	-	1.10	(0.28- 4.25)
*CEDERL	529	m	1	12	-	7	-	6.10	(2.41- 15.46)
Subtotal CEDERL								3.54	(1.64- 7.62)
CHOI	533	m	0	4	19	13	95	1.54	(0.45- 5.23)
CHOI	534	m	0	4	23	13	95	1.27	(0.38- 4.26)
CHOI	535	m	0	5	30	13	95	1.22	(0.40- 3.70)
CHOI	536	m	0	25	64	13	95	2.85	(1.36- 5.99)
CHOI	550	f	0	2	0	76	164	10.75~	(0.51- 226.67)
CHOI	551	f	0	3	2	76	164	3.24	(0.53- 19.77)
Subtotal CHOI								2.04	(1.27- 3.29)
*CHYOU	507	m	2	5	-	8	-	2.80	(0.90- 8.50)
*CHYOU	508	m	2	21	-	8	-	3.80	(1.70- 8.50)
Subtotal CHYOU								3.43	(1.78- 6.59)
*CPSI	807	m	1	15	-	60	-	1.28	(0.73- 2.25)
*CPSI	808	m	1	32	-	60	-	5.15	(3.35- 7.91)
*CPSI	809	m	1	49	-	60	-	8.09	(5.55- 11.80)
*CPSI	810	m	1	37	-	60	-	14.74	(9.78- 22.20)
Subtotal CPSI								6.51	(5.25- 8.07)
*CPSII	652	m	1	256	-	81	-	3.83	(2.98- 4.92)
*CPSII	653	m	1	164	-	81	-	8.61	(6.60- 11.24)
*CPSII	654	m	1	186	-	81	-	11.43	(8.81- 14.84)
*CPSII	655	m	1	178	-	81	-	18.61	(14.31- 24.20)
*CPSII	656	m	1	188	-	81	-	28.07	(21.63- 36.43)
*CPSII	657	m	1	97	-	81	-	38.76	(28.85- 52.07)
*CPSII	633	f	1	50	-	174	-	1.74	(1.27- 2.39)
*CPSII	634	f	1	28	-	174	-	3.86	(2.59- 5.75)
*CPSII	635	f	1	37	-	174	-	4.91	(3.45- 7.01)
*CPSII	636	f	1	56	-	174	-	10.55	(7.81- 14.26)
*CPSII	637	f	1	91	-	174	-	17.02	(13.21- 21.93)
Subtotal CPSII								10.30	(9.46- 11.22)
DAMBER	554	m	1	-	-	42	-	1.60	(0.70- 3.40)
DAMBER	555	m	1	-	-	42	-	5.50	(2.80- 11.10)
Subtotal DAMBER								3.23	(1.92- 5.42)
DARBY	501	m	0	139	767	3	384	23.20	(7.34- 73.28)

Table 1J6 - 2

IESLC - Meta-analysis of Ex Smoking by Years quit (vs never), Overview
All LC types, Cigarettes (or Any Product if Cigarettes not available)
Most adjusted

REF	NRR	SEX	AD	Number Exposed Case	Number Exposed Cont	Non-exposed Case	Non-exposed Cont	RR	95.00%CI
DARBY	502	m	0	146	339	3	384	55.13	(17.41- 174.53)
DARBY	510	f	0	26	224	23	529	2.67	(1.49- 4.78)
DARBY	511	f	0	68	93	23	529	16.82	(9.98- 28.33)
Subtotal DARBY								9.92	(6.99- 14.09)
DEAN3	516	m	1	8	-	24	-	1.31	(0.57- 3.04)
DEAN3	517	m	1	15	-	24	-	2.99	(1.51- 5.93)
DEAN3	518	m	1	11	-	24	-	4.16	(1.91- 9.06)
DEAN3	519	m	1	28	-	24	-	4.67	(2.60- 8.38)
DEAN3	553	f	1	2	-	41	-	0.72	(0.17- 3.01)
DEAN3	554	f	1	1	-	41	-	1.09	(0.15- 8.13)
DEAN3	555	f	1	4	-	41	-	1.63	(0.57- 4.63)
Subtotal DEAN3								2.75	(2.00- 3.78)
DESTEF	525	m	4	17	-	27	-	2.80	(1.40- 5.70)
DESTEF	526	m	4	27	-	27	-	6.20	(3.20- 12.20)
DESTEF	527	m	4	64	-	27	-	9.00	(5.20- 15.90)
Subtotal DESTEF								5.86	(4.06- 8.45)
DOLL	529	m	0	8	23	7	61	3.03	(0.99- 9.31)
DOLL	530	m	0	6	26	7	61	2.01	(0.62- 6.56)
DOLL	531	m	0	56	75	7	61	6.51	(2.77- 15.30)
DOLL	542	f	0	1	2	40	59	0.74	(0.06- 8.41)
DOLL	543	f	0	9	6	40	59	2.21	(0.73- 6.70)
Subtotal DOLL								3.24	(1.95- 5.39)
*DOLL2	501	m	1	7	-	7	-	2.00	(0.70- 5.70)
*DOLL2	502	m	1	9	-	7	-	5.30	(1.97- 14.23)
*DOLL2	503	m	1	12	-	7	-	5.90	(2.32- 14.99)
*DOLL2	504	m	1	15	-	7	-	16.00	(6.52- 39.24)
Subtotal DOLL2								6.10	(3.77- 9.87)
DORGAN	501	m	0	134	255	13	140	5.66	(3.09- 10.37)
DORGAN	502	m	0	49	38	13	140	13.89	(6.84- 28.21)
DORGAN	503	m	0	59	51	13	140	12.46	(6.31- 24.61)
DORGAN	553	f	0	34	50	61	213	2.37	(1.41- 4.00)
DORGAN	554	f	0	49	27	61	213	6.34	(3.66- 10.98)
Subtotal DORGAN								5.93	(4.54- 7.76)
*DORN	657	m	0	16	58370	25	213858	2.34	(1.25- 4.39)
*DORN	658	m	0	12	23682	25	213858	4.33	(2.18- 8.63)
*DORN	659	m	0	32	34566	25	213858	7.92	(4.69- 13.36)
*DORN	660	m	0	34	22086	25	213858	13.17	(7.86- 22.07)
*DORN	680	m	0	34	51243	49	171211	2.32	(1.50- 3.59)
*DORN	681	m	0	29	20056	49	171211	5.05	(3.19- 7.99)
*DORN	682	m	0	41	24089	49	171211	5.95	(3.93- 9.00)
*DORN	683	m	0	14	6195	49	171211	7.90	(4.36- 14.29)
Subtotal DORN								5.24	(4.37- 6.27)
GAO	531	m	2	13	-	62	-	1.10	(0.50- 2.20)
GAO	532	m	2	24	-	62	-	3.10	(1.70- 5.90)
GAO	533	m	2	105	-	62	-	6.90	(4.40- 10.80)
GAO	551	f	2	16	-	435	-	2.20	(1.00- 4.60)
GAO	552	f	2	14	-	435	-	3.90	(1.50- 9.90)
GAO	553	f	2	37	-	435	-	7.20	(3.40- 15.10)
Subtotal GAO								3.91	(3.00- 5.11)
GAO2	509	m	0	8	25	13	56	1.38	(0.51- 3.74)
GAO2	510	m	0	7	9	13	56	3.35	(1.05- 10.66)
GAO2	511	m	0	16	18	13	56	3.83	(1.55- 9.46)
GAO2	512	m	0	21	26	13	56	3.48	(1.51- 8.01)
GAO2	513	m	0	31	26	13	56	5.14	(2.31- 11.40)
Subtotal GAO2								3.35	(2.23- 5.04)
GARCIA	515	c	0	10	37	8	80	2.70	(0.99- 7.41)
GARCIA	516	c	0	32	67	8	80	4.78	(2.06- 11.06)
GARCIA	517	c	0	43	36	8	80	11.94	(5.10- 27.97)
GARCIA	518	c	0	33	11	8	80	30.00	(11.07- 81.30)
Subtotal GARCIA								8.14	(5.16- 12.86)
GARSHI	522	m	1	125	-	41	-	3.20	(2.18- 4.69)
GARSHI	523	m	1	166	-	41	-	5.06	(3.47- 7.36)
Subtotal GARSHI								4.04	(3.09- 5.28)
GRAHAM	535	m	1	13	-	18	-	2.59	(1.18- 5.68)
GRAHAM	536	m	1	24	-	18	-	8.50	(4.32- 16.71)
GRAHAM	537	m	1	113	-	18	-	35.79	(20.50- 62.49)
Subtotal GRAHAM								12.49	(8.56- 18.21)
GURSEL	501	m	0	-	-	-	-	2.30	(1.01- 5.22)
*HAMMO2	501	m	1	20	-	5	-	3.45	(1.30- 9.14)
*HAMMO2	502	m	1	11	-	5	-	3.98	(1.39- 11.40)
*HAMMO2	503	m	1	59	-	5	-	10.99	(4.43- 27.26)

Table 1J6 - 2

IESLC - Meta-analysis of Ex Smoking by Years quit (vs never), Overview
All LC types, Cigarettes (or Any Product if Cigarettes not available)
Most adjusted

REF	NRR	SEX	AD	Number Case	Exposed Cont	Non-exposed Case	Cont	RR	95.00%CI
Subtotal HAMMO2								5.60	(3.19- 9.82)
*HIRAYA	507	m	1	-	-	-	-	1.38	(0.59- 3.21)
*HIRAYA	508	m	1	-	-	-	-	1.59	(0.66- 3.82)
*HIRAYA	509	m	1	-	-	-	-	2.03	(1.10- 3.75)
*HIRAYA	518	f	1	-	-	-	-	0.97	(0.03- 32.06)
*HIRAYA	519	f	1	-	-	-	-	3.29	(0.56- 19.50)
*HIRAYA	520	f	1	-	-	-	-	3.72	(1.12- 12.37)
Subtotal HIRAYA								1.92	(1.30- 2.85)
JAHN	501	m	0	29	146	18	138	1.52	(0.81- 2.87)
JAHN	502	m	0	64	130	18	138	3.77	(2.12- 6.71)
JAHN	503	m	0	59	63	18	138	7.18	(3.92- 13.16)
JAHN	504	m	0	77	46	18	138	12.83	(6.96- 23.67)
JAHN	505	m	0	60	9	18	138	51.11	(21.72- 120.26)
JAHN	506	m	0	166	8	18	138	159.08	(67.12- 377.03)
JAHN	731	f	2	-	-	-	-	0.30	(0.06- 1.53)
Subtotal JAHN								7.91	(6.06- 10.34)
JAIN	567	m	0	52	113	12	85	3.26	(1.64- 6.48)
JAIN	568	m	0	74	46	12	85	11.39	(5.62- 23.12)
JAIN	531	f	0	19	61	52	214	1.28	(0.71- 2.33)
JAIN	532	f	0	66	36	52	214	7.54	(4.55- 12.52)
Subtotal JAIN								4.36	(3.22- 5.92)
JEDRYC	611	m	0	73	138	49	219	2.36	(1.55- 3.60)
JEDRYC	612	m	0	64	58	49	219	4.93	(3.08- 7.90)
Subtotal JEDRYC								3.27	(2.39- 4.48)
JOLY	566	m	0	63	149	12	218	7.68	(4.00- 14.74)
JOLY	567	m	0	38	36	12	218	19.18	(9.16- 40.14)
JOLY	553	f	0	15	19	52	283	4.30	(2.05- 8.99)
JOLY	554	f	0	19	8	52	283	12.93	(5.38- 31.08)
Subtotal JOLY								9.16	(6.33- 13.26)
*KAISE2	646	m	1	6	-	14	-	1.94	(0.70- 5.40)
*KAISE2	647	m	1	8	-	14	-	3.14	(1.26- 7.82)
*KAISE2	648	m	1	12	-	14	-	8.26	(3.73- 18.28)
*KAISE2	566	f	1	4	-	11	-	6.29	(1.78- 22.20)
*KAISE2	567	f	1	4	-	11	-	4.37	(1.30- 14.72)
*KAISE2	568	f	1	6	-	11	-	7.95	(2.89- 21.86)
Subtotal KAISE2								4.86	(3.24- 7.31)
KHUDER	511	m	0	63	213	23	309	3.97	(2.39- 6.61)
KHUDER	512	m	0	63	133	23	309	6.36	(3.79- 10.69)
KHUDER	513	m	0	88	123	23	309	9.61	(5.80- 15.92)
Subtotal KHUDER								6.25	(4.66- 8.40)
LAUSSM	503	m	2	29	-	63	-	6.54	(3.47- 12.35)
LUBIN	585	m	0	17	73	9	72	1.86	(0.78- 4.45)
LUBIN	586	m	0	20	48	9	72	3.33	(1.40- 7.94)
LUBIN	587	m	0	33	18	9	72	14.67	(5.96- 36.07)
Subtotal LUBIN								4.38	(2.64- 7.28)
LUBIN2	1069	m	0	109	715	190	2616	2.10	(1.64- 2.69)
LUBIN2	1070	m	0	106	413	190	2616	3.53	(2.73- 4.58)
LUBIN2	1071	m	0	130	478	190	2616	3.74	(2.94- 4.78)
LUBIN2	1072	m	0	270	693	190	2616	5.36	(4.38- 6.58)
LUBIN2	1073	m	0	466	822	190	2616	7.81	(6.48- 9.40)
LUBIN2	1074	m	0	866	1047	190	2616	11.39	(9.58- 13.53)
LUBIN2	1108	f	0	4	20	336	1188	0.71	(0.24- 2.08)
LUBIN2	1109	f	0	4	9	336	1188	1.57	(0.48- 5.13)
LUBIN2	1110	f	0	3	7	336	1188	1.52	(0.39- 5.89)
LUBIN2	1111	f	0	10	26	336	1188	1.36	(0.65- 2.85)
LUBIN2	1112	f	0	30	40	336	1188	2.65	(1.63- 4.32)
LUBIN2	1113	f	0	60	55	336	1188	3.86	(2.62- 5.67)
Subtotal LUBIN2								5.29	(4.87- 5.74)
MATOS	591	m	2	27	-	11	-	3.00	(1.43- 6.28)
MATOS	592	m	2	21	-	11	-	9.00	(3.84- 21.08)
MATOS	593	m	2	28	-	11	-	14.00	(6.49- 30.21)
Subtotal MATOS								6.96	(4.43- 10.93)
PEZZO2	501	m	0	43	161	6	117	5.21	(2.15- 12.64)
PEZZO2	502	m	0	85	110	6	117	15.07	(6.33- 35.89)
Subtotal PEZZO2								8.96	(4.82- 16.66)
PEZZOT	501	m	0	20	106	4	116	5.47	(1.81- 16.53)
PEZZOT	502	m	0	46	82	4	116	16.27	(5.64- 46.96)
Subtotal PEZZOT								9.65	(4.49- 20.74)
SOBUE	717	m	0	17	40	29	126	1.85	(0.92- 3.71)
SOBUE	718	m	0	15	23	29	126	2.83	(1.32- 6.09)
SOBUE	719	m	0	24	31	29	126	3.36	(1.72- 6.56)

Table 1J6 - 2

IESLC - Meta-analysis of Ex Smoking by Years quit (vs never), Overview
All LC types, Cigarettes (or Any Product if Cigarettes not available)
Most adjusted

REF	NRR	SEX	AD	Number Exposed Case	Cont	Non-exposed Case	Cont	RR	95.00%CI
SOBUE	720	m	0	35	50	29	126	3.04	(1.68- 5.49)
SOBUE	721	m	0	67	92	29	126	3.16	(1.90- 5.28)
SOBUE	722	m	0	128	116	29	126	4.79	(2.98- 7.71)
Subtotal SOBUE								3.27	(2.57- 4.16)
*SPEIZE	501	f	0	28	214271	58	776300	1.75	(1.11- 2.75)
*SPEIZE	502	f	0	17	93933	58	776300	2.42	(1.41- 4.16)
*SPEIZE	503	f	0	41	95585	58	776300	5.74	(3.85- 8.56)
*SPEIZE	504	f	0	34	63060	58	776300	7.22	(4.73- 11.02)
*SPEIZE	505	f	0	24	55232	58	776300	5.82	(3.61- 9.36)
Subtotal SPEIZE								4.24	(3.46- 5.19)
SUZUK2	508	c	0	9	22	11	53	1.97	(0.72- 5.42)
SUZUK2	509	c	0	10	8	11	53	6.02	(1.94- 18.72)
SUZUK2	510	c	0	15	10	11	53	7.23	(2.58- 20.25)
Subtotal SUZUK2								4.28	(2.33- 7.87)
SVENSS	551	f	0	14	24	38	120	1.84	(0.87- 3.91)
SVENSS	552	f	0	16	13	38	120	3.89	(1.72- 8.80)
Subtotal SVENSS								2.60	(1.49- 4.52)
*TVERDA	501	m	2	4	-	4	-	1.34	(0.34- 5.37)
*TVERDA	502	m	2	5	-	4	-	2.83	(0.76- 10.53)
*TVERDA	503	m	2	2	-	4	-	2.77	(0.51- 15.15)
Subtotal TVERDA								2.15	(0.94- 4.93)
WAKAI	530	m	2	7	-	10	-	1.00	(0.35- 2.83)
WAKAI	531	m	2	27	-	10	-	3.63	(1.56- 8.44)
WAKAI	532	m	2	19	-	10	-	2.48	(1.04- 5.92)
Subtotal WAKAI								2.29	(1.35- 3.86)
WANG2	510	c	0	5	11	11	43	1.78	(0.51- 6.19)
WANG2	511	c	0	6	10	11	43	2.35	(0.70- 7.86)
Subtotal WANG2								2.05	(0.86- 4.89)
WYNDE3	566	m	0	9	65	9	88	1.35	(0.51- 3.60)
WYNDE3	587	f	0	1	3	20	76	1.27	(0.12- 12.84)
Subtotal WYNDE3								1.34	(0.54- 3.30)
WYNDE6	501	m	0	21	161	64	918	1.87	(1.11- 3.15)
WYNDE6	502	m	0	55	212	64	918	3.72	(2.52- 5.50)
WYNDE6	503	m	0	159	373	64	918	6.11	(4.47- 8.37)
WYNDE6	504	m	0	98	194	64	918	7.25	(5.10- 10.29)
WYNDE6	505	m	0	201	166	64	918	17.37	(12.53- 24.07)
WYNDE6	522	f	0	10	31	125	991	2.56	(1.22- 5.34)
WYNDE6	523	f	0	16	77	125	991	1.65	(0.93- 2.91)
WYNDE6	524	f	0	36	132	125	991	2.16	(1.43- 3.27)
WYNDE6	525	f	0	51	84	125	991	4.81	(3.24- 7.14)
WYNDE6	526	f	0	82	70	125	991	9.29	(6.42- 13.43)
Subtotal WYNDE6								5.50	(4.84- 6.24)
Partial Totals				10190	773900	11144	5465938		

*prospective study ~ With 0.5 adjustment for zero

REF	NRR	SEX	AD	Ys	Ws	Qs	Ps
ALDERS	507	m	1	1.16	8.16	2.60	0.0009
ALDERS	508	m	1	1.46	7.77	0.56	0.0000
ALDERS	509	m	1	2.90	9.90	13.52	0.0000
ALDERS	518	f	1	0.24	14.21	31.49	0.3676
ALDERS	519	f	1	1.08	18.83	7.86	0.0000
ALDERS	520	f	1	2.25	27.43	7.37	0.0000
Subtotal ALDERS				1.56	86.30	63.39	
ARMADA	515	m	0	1.63	5.86	0.06	0.0001
ARMADA	516	m	0	2.75	5.75	5.96	0.0000
Subtotal ARMADA				2.18	11.61	6.02	
AUVINE	532	c	2	2.01	11.70	0.96	0.0000
BARBON	540	m	1	0.74	7.22	7.02	0.0462
BARBON	541	m	1	1.92	9.55	0.34	0.0000
BARBON	542	m	1	2.21	13.34	3.08	0.0000
BARBON	543	m	1	2.63	7.48	6.12	0.0000
Subtotal BARBON				1.94	37.60	16.56	
BECHER	501	m	0	1.39	2.34	0.27	0.0341
BECHER	502	m	0	2.20	2.24	0.49	0.0010
BECHER	503	m	0	2.71	1.87	1.80	0.0002
BECHER	511	f	0	-0.65	0.82	4.65	0.5537
BECHER	512	f	0	0.73	1.22	1.21	0.4184
BECHER	513	f	0	1.24	1.05	0.25	0.2027
Subtotal BECHER				1.56	9.54	8.67	
*BENSHL	508	m	2	0.00	2.98	8.90	1.0000

Table 1J6 - 2

IESLC - Meta-analysis of Ex Smoking by Years quit (vs never), Overview
All LC types, Cigarettes (or Any Product if Cigarettes not available)
Most adjusted

REF	NRR	SEX	AD	Ys	Ws	Qs	Ps
*BENSHL	509	m	2	0.95	6.64	4.00	0.0142
*BENSHL	510	m	2	1.41	7.88	0.82	0.0001
*BENSHL	511	m	2	2.16	6.37	1.20	0.0000
Subtotal BENSHL				1.31	23.87	14.91	
BROSS	515	m	0	0.89	14.68	10.31	0.0006
BROSS	516	m	0	2.42	18.85	9.12	0.0000
Subtotal BROSS				1.75	33.54	19.43	
BROWN3	503	f	2	0.79	93.46	82.46	0.0000
CARPEN	501	c	0	1.71	5.83	0.00	0.0000
CARPEN	502	c	0	1.52	3.84	0.16	0.0028
CARPEN	503	c	0	2.61	5.25	4.04	0.0000
CARPEN	504	c	0	2.73	5.03	5.05	0.0000
Subtotal CARPEN				2.17	19.94	9.26	
*CEDERL	528	m	1	0.10	2.08	5.54	0.8907
*CEDERL	529	m	1	1.81	4.45	0.03	0.0001
Subtotal CEDERL				1.26	6.53	5.56	
CHOI	533	m	0	0.43	2.56	4.31	0.4904
CHOI	534	m	0	0.24	2.63	5.81	0.6977
CHOI	535	m	0	0.20	3.12	7.30	0.7277
CHOI	536	m	0	1.05	6.99	3.22	0.0056
CHOI	550	f	0	2.38	0.41	0.17	0.1268
CHOI	551	f	0	1.17	1.17	0.36	0.2033
Subtotal CHOI				0.71	16.88	21.18	
*CHYOU	507	m	2	1.03	3.05	1.49	0.0723
*CHYOU	508	m	2	1.34	5.93	0.92	0.0011
Subtotal CHYOU				1.23	8.98	2.40	
*CPSI	807	m	1	0.25	12.13	26.60	0.3900
*CPSI	808	m	1	1.64	20.82	0.16	0.0000
*CPSI	809	m	1	2.09	27.01	3.56	0.0000
*CPSI	810	m	1	2.69	22.87	21.20	0.0000
Subtotal CPSI				1.87	82.82	51.51	
*CPSII	652	m	1	1.34	61.12	9.06	0.0000
*CPSII	653	m	1	2.15	54.21	9.80	0.0000
*CPSII	654	m	1	2.44	56.51	28.37	0.0000
*CPSII	655	m	1	2.92	55.67	79.62	0.0000
*CPSII	656	m	1	3.33	56.54	146.00	0.0000
*CPSII	657	m	1	3.66	44.07	164.09	0.0000
*CPSII	633	f	1	0.55	38.44	52.97	0.0006
*CPSII	634	f	1	1.35	24.16	3.44	0.0000
*CPSII	635	f	1	1.59	30.57	0.57	0.0000
*CPSII	636	f	1	2.36	42.39	16.74	0.0000
*CPSII	637	f	1	2.83	59.81	73.24	0.0000
Subtotal CPSII				2.33	523.48	583.88	
DAMBER	554	m	1	0.47	6.15	9.73	0.2437
DAMBER	555	m	1	1.70	8.10	0.00	0.0000
Subtotal DAMBER				1.17	14.25	9.74	
DARBY	501	m	0	3.14	2.90	5.82	0.0000
DARBY	502	m	0	4.01	2.89	15.06	0.0000
DARBY	510	f	0	0.98	11.33	6.30	0.0010
DARBY	511	f	0	2.82	14.12	16.92	0.0000
Subtotal DARBY				2.29	31.24	44.10	
DEAN3	516	m	1	0.27	5.48	11.65	0.5272
DEAN3	517	m	1	1.10	8.21	3.29	0.0017
DEAN3	518	m	1	1.43	6.34	0.58	0.0003
DEAN3	519	m	1	1.54	11.22	0.39	0.0000
DEAN3	553	f	1	-0.33	1.86	7.87	0.6541
DEAN3	554	f	1	0.09	0.96	2.60	0.9326
DEAN3	555	f	1	0.49	3.50	5.38	0.3606
Subtotal DEAN3				1.01	37.58	31.75	
DESTEF	525	m	4	1.03	7.80	3.80	0.0040
DESTEF	526	m	4	1.82	8.58	0.08	0.0000
DESTEF	527	m	4	2.20	12.30	2.71	0.0000
Subtotal DESTEF				1.77	28.68	6.59	
DOLL	529	m	0	1.11	3.05	1.17	0.0527
DOLL	530	m	0	0.70	2.74	2.91	0.2471
DOLL	531	m	0	1.87	5.25	0.11	0.0000
DOLL	542	f	0	-0.30	0.65	2.68	0.8063
DOLL	543	f	0	0.79	3.13	2.73	0.1602
Subtotal DOLL				1.18	14.82	9.59	
*DOLL2	501	m	1	0.69	3.49	3.74	0.1951
*DOLL2	502	m	1	1.67	3.93	0.01	0.0009

Table 1J6 - 2

IESLC - Meta-analysis of Ex Smoking by Years quit (vs never), Overview
All LC types, Cigarettes (or Any Product if Cigarettes not available)
Most adjusted

REF	NRR	SEX	AD	Ys	Ws	Qs	Ps
*DOLL2	503	m	1	1.77	4.41	0.01	0.0002
*DOLL2	504	m	1	2.77	4.77	5.21	0.0000
Subtotal DOLL2				1.81	16.61	8.97	
DORGAN	501	m	0	1.73	10.48	0.00	0.0000
DORGAN	502	m	0	2.63	7.65	6.24	0.0000
DORGAN	503	m	0	2.52	8.29	5.23	0.0000
DORGAN	553	f	0	0.86	14.18	10.56	0.0011
DORGAN	554	f	0	1.85	12.73	0.18	0.0000
Subtotal DORGAN				1.78	53.33	22.22	
*DORN	657	m	0	0.85	9.76	7.48	0.0078
*DORN	658	m	0	1.47	8.11	0.55	0.0000
*DORN	659	m	0	2.07	14.04	1.64	0.0000
*DORN	660	m	0	2.58	14.42	10.42	0.0000
*DORN	680	m	0	0.84	20.08	15.80	0.0002
*DORN	681	m	0	1.62	18.24	0.21	0.0000
*DORN	682	m	0	1.78	22.35	0.07	0.0000
*DORN	683	m	0	2.07	10.91	1.25	0.0000
Subtotal DORN				1.66	117.90	37.42	
GAO	531	m	2	0.10	7.00	18.65	0.8009
GAO	532	m	2	1.13	9.92	3.53	0.0004
GAO	533	m	2	1.93	19.06	0.79	0.0000
GAO	551	f	2	0.79	6.60	5.82	0.0428
GAO	552	f	2	1.36	4.31	0.58	0.0047
GAO	553	f	2	1.97	6.91	0.42	0.0000
Subtotal GAO				1.36	53.81	29.80	
GAO2	509	m	0	0.32	3.85	7.62	0.5289
GAO2	510	m	0	1.21	2.87	0.77	0.0406
GAO2	511	m	0	1.34	4.70	0.70	0.0036
GAO2	512	m	0	1.25	5.53	1.28	0.0034
GAO2	513	m	0	1.64	6.04	0.05	0.0001
Subtotal GAO2				1.21	22.99	10.42	
GARCIA	515	c	0	0.99	3.78	2.03	0.0532
GARCIA	516	c	0	1.56	5.44	0.15	0.0003
GARCIA	517	c	0	2.48	5.30	3.00	0.0000
GARCIA	518	c	0	3.40	3.87	10.82	0.0000
Subtotal GARCIA				2.10	18.39	16.01	
GARSHI	522	m	1	1.16	26.18	8.35	0.0000
GARSHI	523	m	1	1.62	27.18	0.31	0.0000
Subtotal GARSHI				1.40	53.36	8.65	
GRAHAM	535	m	1	0.95	6.22	3.75	0.0176
GRAHAM	536	m	1	2.14	8.40	1.43	0.0000
GRAHAM	537	m	1	3.58	12.37	42.33	0.0000
Subtotal GRAHAM				2.52	26.99	47.50	
GURSEL	501	m	0	0.83	5.70	4.56	0.0468
*HAMMO2	501	m	1	1.24	4.04	0.97	0.0128
*HAMMO2	502	m	1	1.38	3.47	0.42	0.0101
*HAMMO2	503	m	1	2.40	4.65	2.08	0.0000
Subtotal HAMMO2				1.72	12.16	3.47	
*HIRAYA	507	m	1	0.32	5.36	10.58	0.4561
*HIRAYA	508	m	1	0.46	4.98	7.96	0.3005
*HIRAYA	509	m	1	0.71	10.22	10.62	0.0236
*HIRAYA	518	f	1	-0.03	0.32	0.98	0.9863
*HIRAYA	519	f	1	1.19	1.22	0.35	0.1885
*HIRAYA	520	f	1	1.31	2.66	0.46	0.0320
Subtotal HIRAYA				0.65	24.75	30.95	
JAHN	501	m	0	0.42	9.60	16.41	0.1925
JAHN	502	m	0	1.33	11.61	1.85	0.0000
JAHN	503	m	0	1.97	10.46	0.62	0.0000
JAHN	504	m	0	2.55	10.25	6.97	0.0000
JAHN	505	m	0	3.93	5.25	25.54	0.0000
JAHN	506	m	0	5.07	5.16	57.61	0.0000
JAHN	731	f	2	-1.20	1.46	12.59	0.1451
Subtotal JAHN				2.07	53.80	121.59	
JAIN	567	m	0	1.18	8.12	2.42	0.0008
JAIN	568	m	0	2.43	7.67	3.82	0.0000
JAIN	531	f	0	0.25	10.76	23.55	0.4154
JAIN	532	f	0	2.02	14.96	1.29	0.0000
Subtotal JAIN				1.47	41.51	31.08	
JEDRYC	611	m	0	0.86	21.78	16.38	0.0001
JEDRYC	612	m	0	1.60	17.29	0.30	0.0000
Subtotal JEDRYC				1.19	39.07	16.68	

Table 1J6 - 2

IESLC - Meta-analysis of Ex Smoking by Years quit (vs never), Overview
All LC types, Cigarettes (or Any Product if Cigarettes not available)
Most adjusted

REF	NRR	SEX	AD	Ys	Ws	Qs	Ps
JOLY	566	m	0	2.04	9.05	0.88	0.0000
JOLY	567	m	0	2.95	7.04	10.58	0.0000
JOLY	553	f	0	1.46	7.04	0.51	0.0001
JOLY	554	f	0	2.56	4.99	3.45	0.0000
Subtotal JOLY				2.21	28.12	15.42	
*KAISE2	646	m	1	0.66	3.68	4.18	0.2036
*KAISE2	647	m	1	1.14	4.61	1.57	0.0140
*KAISE2	648	m	1	2.11	6.08	0.90	0.0000
*KAISE2	566	f	1	1.84	2.41	0.03	0.0043
*KAISE2	567	f	1	1.47	2.61	0.17	0.0172
*KAISE2	568	f	1	2.07	3.75	0.45	0.0001
Subtotal KAISE2				1.58	23.15	7.29	
KHUDER	511	m	0	1.38	14.86	1.80	0.0000
KHUDER	512	m	0	1.85	14.26	0.22	0.0000
KHUDER	513	m	0	2.26	15.10	4.33	0.0000
Subtotal KHUDER				1.83	44.23	6.34	
LAUSSM	503	m	2	1.88	9.53	0.22	0.0000
LUBIN	585	m	0	0.62	5.06	6.19	0.1615
LUBIN	586	m	0	1.20	5.11	1.40	0.0065
LUBIN	587	m	0	2.69	4.74	4.35	0.0000
Subtotal LUBIN				1.48	14.91	11.94	
LUBIN2	1069	m	0	0.74	61.66	59.98	0.0000
LUBIN2	1070	m	0	1.26	57.14	12.38	0.0000
LUBIN2	1071	m	0	1.32	64.81	10.76	0.0000
LUBIN2	1072	m	0	1.68	92.66	0.21	0.0000
LUBIN2	1073	m	0	2.05	111.01	11.87	0.0000
LUBIN2	1074	m	0	2.43	128.94	64.05	0.0000
LUBIN2	1108	f	0	-0.35	3.29	14.16	0.5296
LUBIN2	1109	f	0	0.45	2.74	4.46	0.4543
LUBIN2	1110	f	0	0.42	2.08	3.59	0.5486
LUBIN2	1111	f	0	0.31	7.03	14.18	0.4151
LUBIN2	1112	f	0	0.98	16.09	9.11	0.0001
LUBIN2	1113	f	0	1.35	25.86	3.69	0.0000
Subtotal LUBIN2				1.66	573.32	208.46	
MATOS	591	m	2	1.10	7.02	2.78	0.0036
MATOS	592	m	2	2.20	5.30	1.17	0.0000
MATOS	593	m	2	2.64	6.50	5.40	0.0000
Subtotal MATOS				1.94	18.81	9.34	
PEZZO2	501	m	0	1.65	4.89	0.03	0.0003
PEZZO2	502	m	0	2.71	5.10	4.95	0.0000
Subtotal PEZZO2				2.19	9.99	4.98	
PEZZOT	501	m	0	1.70	3.14	0.00	0.0026
PEZZOT	502	m	0	2.79	3.42	3.85	0.0000
Subtotal PEZZOT				2.27	6.56	3.85	
SOBUE	717	m	0	0.61	7.92	9.84	0.0843
SOBUE	718	m	0	1.04	6.55	3.09	0.0077
SOBUE	719	m	0	1.21	8.60	2.28	0.0004
SOBUE	720	m	0	1.11	10.99	4.16	0.0002
SOBUE	721	m	0	1.15	14.66	4.86	0.0000
SOBUE	722	m	0	1.57	16.99	0.44	0.0000
Subtotal SOBUE				1.18	65.71	24.66	
*SPEIZE	501	f	0	0.56	18.89	25.80	0.0151
*SPEIZE	502	f	0	0.88	13.15	9.35	0.0013
*SPEIZE	503	f	0	1.75	24.03	0.01	0.0000
*SPEIZE	504	f	0	1.98	21.44	1.33	0.0000
*SPEIZE	505	f	0	1.76	16.98	0.02	0.0000
Subtotal SPEIZE				1.44	94.49	36.49	
SUZUK2	508	c	0	0.68	3.75	4.13	0.1886
SUZUK2	509	c	0	1.80	2.99	0.01	0.0019
SUZUK2	510	c	0	1.98	3.62	0.23	0.0002
Subtotal SUZUK2				1.45	10.36	4.37	
SVENSS	551	f	0	0.61	6.77	8.44	0.1120
SVENSS	552	f	0	1.36	5.74	0.79	0.0011
Subtotal SVENSS				0.95	12.51	9.23	
*TVERDA	501	m	2	0.29	2.02	4.16	0.6776
*TVERDA	502	m	2	1.04	2.22	1.05	0.1208
*TVERDA	503	m	2	1.02	1.34	0.67	0.2389
Subtotal TVERDA				0.76	5.58	5.88	
WAKAI	530	m	2	0.00	3.52	10.50	1.0000
WAKAI	531	m	2	1.29	5.39	1.04	0.0028
WAKAI	532	m	2	0.91	5.08	3.41	0.0406

Table 1J6 - 2

IESLC - Meta-analysis of Ex Smoking by Years quit (vs never), Overview
All LC types, Cigarettes (or Any Product if Cigarettes not available)
Most adjusted

REF	NRR	SEX	AD	Ys	Ws	Qs	Ps
Subtotal WAKAI				0.83	13.99	14.95	
WANG2	510	c	0	0.57	2.47	3.28	0.3664
WANG2	511	c	0	0.85	2.63	2.01	0.1672
Subtotal WANG2				0.72	5.09	5.29	
WYNDE3	566	m	0	0.30	4.02	8.15	0.5438
WYNDE3	587	f	0	0.24	0.72	1.59	0.8415
Subtotal WYNDE3				0.29	4.73	9.75	
WYNDE6	501	m	0	0.63	14.18	17.19	0.0183
WYNDE6	502	m	0	1.31	25.24	4.32	0.0000
WYNDE6	503	m	0	1.81	38.93	0.27	0.0000
WYNDE6	504	m	0	1.98	31.18	1.99	0.0000
WYNDE6	505	m	0	2.85	36.08	45.82	0.0000
WYNDE6	522	f	0	0.94	7.08	4.40	0.0125
WYNDE6	523	f	0	0.50	11.83	17.86	0.0859
WYNDE6	524	f	0	0.77	22.54	20.63	0.0003
WYNDE6	525	f	0	1.57	24.68	0.60	0.0000
WYNDE6	526	f	0	2.23	28.18	7.07	0.0000
Subtotal WYNDE6				1.70	239.93	120.16	

N	208
NS	51

Table 1J6 - 3

IESLC - Meta-analysis of Ex Smoking by Years quit (vs never), Overview
All LC types, Cigarettes (or Any Product if Cigarettes not available)
Most adjusted

	Sex combined	male	female	Total
N	14	138	56	208
NS	5	43	20	68

In this overview table, other than the "N" rows, entries in the "absent" and "Total" columns may be invalid and should be ignored

Years quit vs never (lower focus)	absent	8+k12	4-11k7	1-6k3	Total
N	81	54	32	41	208
NS	38	39	24	31	132
Wt	1148.26	569.50	481.37	615.07	2814.20
Het Chi	1092.72	206.48	99.15	163.54	1875.90
Het df	80	53	31	40	207
Het P	***	***	***	***	***
Fixed RR	5.02	3.63	6.14	9.76	5.63
RRl	4.74	3.35	5.62	9.02	5.42
RRu	5.32	3.95	6.72	10.56	5.84
P	+++	+++	+++	+++	+++
Random RR	4.53	2.93	5.16	8.60	4.65
RRl	3.61	2.45	4.28	7.18	4.13
RRu	5.68	3.51	6.20	10.31	5.24
P	+++	+++	+++	+++	+++

Years quit vs never (higher focus)	absent	13+k20	4-19k12	1-11k3	Total
N	104	24	22	58	208
NS	48	20	18	42	128
Wt	1240.55	438.78	389.43	745.44	2814.20
Het Chi	1090.51	58.56	77.41	228.30	1875.90
Het df	103	23	21	57	207
Het P	***	***	***	***	***
Fixed RR	5.64	2.72	4.94	9.23	5.63
RRl	5.33	2.48	4.47	8.59	5.42
RRu	5.96	2.99	5.45	9.91	5.84
P	+++	+++	+++	+++	+++
Random RR	3.92	2.66	4.28	8.28	4.65
RRl	3.23	2.23	3.45	7.06	4.13
RRu	4.76	3.17	5.31	9.71	5.24
P	+++	+++	+++	+++	+++

Table 1J6 - 3

IESLC - Meta-analysis of Ex Smoking by Years quit (vs never), Overview
All LC types, Cigarettes (or Any Product if Cigarettes not available)
Most adjusted

MALES

		Years quit vs never (lower focus)				
		absent	8+k12	4-11k7	1-6k3	Total
	N	54	35	22	27	138
	NS	31	34	21	26	112
	Wt	758.81	412.52	370.05	461.77	2003.15
Het	Chi	768.89	126.37	73.34	106.89	1280.52
Het	df	53	34	21	26	137
Het	P	***	***	***	***	***
Fixed	RR	5.47	4.26	6.66	10.61	6.28
	RRl	5.10	3.87	6.01	9.69	6.01
	RRu	5.87	4.69	7.37	11.62	6.56
	P	+++	+++	+++	+++	+++
Random	RR	4.98	3.36	5.36	9.38	5.14
	RRl	3.74	2.73	4.30	7.62	4.46
	RRu	6.62	4.14	6.68	11.55	5.93
	P	+++	+++	+++	+++	+++

		Years quit vs never (higher focus)				
		absent	13+k20	4-19k12	1-11k3	Total
	N	68	17	16	37	138
	NS	40	16	15	36	107
	Wt	908.72	262.14	313.41	518.88	2003.15
Het	Chi	838.21	26.70	33.36	128.87	1280.52
Het	df	67	16	15	36	137
Het	P	***	*	**	***	***
Fixed	RR	5.94	3.23	5.58	10.38	6.28
	RRl	5.56	2.87	5.00	9.52	6.01
	RRu	6.34	3.65	6.23	11.31	6.56
	P	+++	+++	+++	+++	+++
Random	RR	4.45	2.99	5.03	9.35	5.14
	RRl	3.49	2.50	4.14	7.79	4.46
	RRu	5.67	3.59	6.10	11.22	5.93
	P	+++	+++	+++	+++	+++

FEMALES

		Years quit vs never (lower focus)				
		absent	8+k12	4-11k7	1-6k3	Total
	N	22	16	8	10	56
	NS	15	16	8	10	49
	Wt	366.63	137.69	103.08	138.16	745.57
Het	Chi	298.35	23.92	9.12	29.98	465.23
Het	df	21	15	7	9	55
Het	P	***	(*)	N.S.	***	***
Fixed	RR	4.20	2.15	4.43	7.28	4.14
	RRl	3.79	1.82	3.65	6.16	3.85
	RRu	4.65	2.54	5.37	8.60	4.45
	P	+++	+++	+++	+++	+++
Random	RR	3.57	2.03	4.29	6.27	3.32
	RRl	2.33	1.60	3.36	4.36	2.63
	RRu	5.49	2.56	5.47	9.03	4.19
	P	+++	+++	+++	+++	+++

Table 1J6 - 3

IESLC - Meta-analysis of Ex Smoking by Years quit (vs never), Overview
All LC types, Cigarettes (or Any Product if Cigarettes not available)
Most adjusted

FEMALES

	Years quit vs never (higher focus) absent	13+k20	4-19k12	1-11k3	Total
N	30	5	4	17	56
NS	19	5	4	17	45
Wt	301.90	165.36	66.88	211.43	745.57
Het Chi	228.38	2.52	7.56	61.38	465.23
Het df	29	4	3	16	55
Het P	***	N.S.	(*)	***	***
Fixed RR	4.84	1.98	2.60	6.83	4.14
RRl	4.33	1.70	2.04	5.97	3.85
RRu	5.42	2.30	3.30	7.82	4.45
P	+++	+++	+++	+++	+++
Random RR	2.74	1.98	2.44	6.03	3.32
RRl	1.90	1.70	1.64	4.49	2.63
RRu	3.95	2.30	3.63	8.11	4.19
P	+++	+++	+++	+++	+++

Table 1J6 - 4

IESLC - Meta-analysis of Ex Smoking by Years quit (vs never), Overview
All LC types, Cigarettes (or Any Product if Cigarettes not available)
Least adjusted

REF	NRR	X	SEX	AGEL	AGEH	RACE	YF	LC TYPE	LOC	START	ST	NLC	R	VB	P	H	AD	PRODUCT	exL	exH	S1	S2	DENOM	De
ALDERS	507		m	0	0	all	-	all	Eu:UK	1977	CC	1448	n	V	n	n	1	cig only	10	999	1	0	nev any	ot
ALDERS	508		m	0	0	all	-	all	Eu:UK	1977	CC	1448	n	V	n	n	1	cig only	3	9	0	3	nev any	ot
ALDERS	509		m	0	0	all	-	all	Eu:UK	1977	CC	1448	n	V	n	n	1	cig only	0.1	2	0	0	nev any	ot
ALDERS	518		f	0	0	all	-	all	Eu:UK	1977	CC	1448	n	V	n	n	1	cig only	10	999	1	0	nev any	ot
ALDERS	519		f	0	0	all	-	all	Eu:UK	1977	CC	1448	n	V	n	n	1	cig only	3	9	0	3	nev any	ot
ALDERS	520		f	0	0	all	-	all	Eu:UK	1977	CC	1448	n	V	n	n	1	cig only	0.1	2	0	0	nev any	ot
ARMADA	515		m	0	0	all	-	all	Eu:wst	1986	CC	325	n	bl	n	y	0	cig+/-ot	6	999	0	0	nev cigs	st
ARMADA	516		m	0	0	all	-	all	Eu:wst	1986	CC	325	n	bl	n	y	0	cig+/-ot	1.0	5	3	3	nev cigs	st
AUVINE	530	x	c	0	0	all	-	all	Eu:Sca	1986	CC	517	n	bl	y	n	0	cig+/-ot	12	999	1	0	nev cigs	st
BARBON	525	x	m	0	0	all	-	all	Eu:wst	1979	CC	755	n	bl	y	y	0	all/unsp	25	999	0	0	nev any	st
BARBON	526	x	m	0	0	all	-	all	Eu:wst	1979	CC	755	n	bl	y	y	0	all/unsp	15	24	0	1	nev any	st
BARBON	527	x	m	0	0	all	-	all	Eu:wst	1979	CC	755	n	bl	y	y	0	all/unsp	5	14	0	2	nev any	st
BARBON	528	x	m	0	0	all	-	all	Eu:wst	1979	CC	755	n	bl	y	y	0	all/unsp	0.1	4	3	3	nev any	st
BECHER	501		m	0	0	all	-	all	Eu:Ger	1985	CC	194	n	bl	n	y	0	all/unsp	10	999	1	0	nev any	st
BECHER	502		m	0	0	all	-	all	Eu:Ger	1985	CC	194	n	bl	n	y	0	all/unsp	5	9	2	0	nev any	st
BECHER	503		m	0	0	all	-	all	Eu:Ger	1985	CC	194	n	bl	n	y	0	all/unsp	2	4	3	3	nev any	st
BECHER	511		f	0	0	all	-	all	Eu:Ger	1985	CC	194	n	bl	n	y	0	all/unsp	10	999	1	0	nev any	st
BECHER	512		f	0	0	all	-	all	Eu:Ger	1985	CC	194	n	bl	n	y	0	all/unsp	5	9	2	0	nev any	st
BECHER	513		f	0	0	all	-	all	Eu:Ger	1985	CC	194	n	bl	n	y	0	all/unsp	2	4	3	3	nev any	st
BENSHL	508		m	0	0	all	0	all	Eu:UK	1967	pr	486	n	V	n	n	2	cig+/-ot	30	999	0	0	nev any	or
BENSHL	509		m	0	0	all	0	all	Eu:UK	1967	pr	486	n	V	n	n	2	cig+/-ot	20	29	0	1	nev any	or
BENSHL	510		m	0	0	all	0	all	Eu:UK	1967	pr	486	n	V	n	n	2	cig+/-ot	10	19	1	2	nev any	or
BENSHL	511		m	0	0	all	0	all	Eu:UK	1967	pr	486	n	V	n	n	2	cig+/-ot	1.0	9	0	3	nev any	or
BROSS	515		m	0	0	wh	-	all	NAmer	1960	CC	974	n	bl	n	n	0	cig+/-ot	6	999	0	0	nev any	st
BROSS	516		m	0	0	wh	-	all	NAmer	1960	CC	974	n	bl	n	n	0	cig+/-ot	0.1	5	3	3	nev any	st
BROWN3	501	x	f	0	0	wh	-	all	NAmer		CC	618		bl	y	n	0	all/unsp	15	999	0	1	nev any	st
CARPEN	501		c	0	0	w+b	-	all	NAmer	1991	CC	356	n	bl	n	n	0	cig+/-ot	15	999	0	1	nev cigs	st
CARPEN	502		c	0	0	w+b	-	all	NAmer	1991	CC	356	n	bl	n	n	0	cig+/-ot	10	14	1	2	nev cigs	st
CARPEN	503		c	0	0	w+b	-	all	NAmer	1991	CC	356	n	bl	n	n	0	cig+/-ot	5	9	2	0	nev cigs	st
CARPEN	504		c	0	0	w+b	-	all	NAmer	1991	CC	356	n	bl	n	n	0	cig+/-ot	0.1	4	3	3	nev cigs	st
CEDERL	528		m	40	69	all	10	all	Eu:Sca	1963	pr	491	n	bl	n	n	1	all/unsp	10	999	1	0	nev any	ot
CEDERL	529		m	40	69	all	10	all	Eu:Sca	1963	pr	491	n	bl	n	n	1	all/unsp	0.1	9	0	3	nev any	ot
CHOI	533		m	0	0	all	-	all	As:oth	1985	CC	375	n	bl	n	n	0	cig+/-ot	15	999	0	1	nev cigs	st
CHOI	534		m	0	0	all	-	all	As:oth	1985	CC	375	n	bl	n	n	0	cig+/-ot	10	14	1	2	nev cigs	st
CHOI	535		m	0	0	all	-	all	As:oth	1985	CC	375	n	bl	n	n	0	cig+/-ot	5	9	2	0	nev cigs	st
CHOI	536		m	0	0	all	-	all	As:oth	1985	CC	375	n	bl	n	n	0	cig+/-ot	0.1	4	3	3	nev cigs	st
CHOI	550		f	0	0	all	-	all	As:oth	1985	CC	375	n	bl	n	n	0	cig+/-ot	5	999	0	0	nev cigs	ot
CHOI	551		f	0	0	all	-	all	As:oth	1985	CC	375	n	bl	n	n	0	cig+/-ot	0.1	4	3	3	nev cigs	st
CHYOU	501	x	m	0	0	jap	21	all	NAmer	1965	pr	227	n	bl	n	y	1	cig+/-ot	15	999	0	1	nev cigs	or
CHYOU	502	x	m	0	0	jap	21	all	NAmer	1965	pr	227	n	bl	n	y	1	cig+/-ot	0.1	14	0	0	nev cigs	or
CPSI	807		m	50	74	all	6	all	NAmer	1959	pr	5138	n	bl	n	n	1	cig only	10	999	1	0	nev any	ot
CPSI	808		m	50	74	all	6	all	NAmer	1959	pr	5138	n	bl	n	n	1	cig only	5	9	2	0	nev any	ot
CPSI	809		m	50	74	all	6	all	NAmer	1959	pr	5138	n	bl	n	n	1	cig only	1.0	4	3	3	nev any	ot
CPSI	810		m	50	74	all	6	all	NAmer	1959	pr	5138	n	bl	n	n	1	cig only	0.1	0.9	0	0	nev any	ot
CPSII	652		m	35	99	all	4	all	NAmer	1982	pr	3229	n	bl	n	n	1	cig only	16	999	0	1	nev any	ot
CPSII	653		m	35	99	all	4	all	NAmer	1982	pr	3229	n	bl	n	n	1	cig only	11	15	1	2	nev any	ot
CPSII	654		m	35	99	all	4	all	NAmer	1982	pr	3229	n	bl	n	n	1	cig only	6	10	2	0	nev any	ot
CPSII	655		m	35	99	all	4	all	NAmer	1982	pr	3229	n	bl	n	n	1	cig only	3	5	3	3	nev any	ot
CPSII	656		m	35	99	all	4	all	NAmer	1982	pr	3229	n	bl	n	n	1	cig only	1.0	2	0	0	nev any	ot
CPSII	657		m	35	99	all	4	all	NAmer	1982	pr	3229	n	bl	n	n	1	cig only	0.1	0.9	0	0	nev any	ot
CPSII	633		f	0	0	all	4	all	NAmer	1982	pr	3229	n	bl	n	n	1	cig+/-ot	16	999	0	1	nev cigs	ot
CPSII	634		f	0	0	all	4	all	NAmer	1982	pr	3229	n	bl	n	n	1	cig+/-ot	11	15	1	2	nev cigs	ot
CPSII	635		f	0	0	all	4	all	NAmer	1982	pr	3229	n	bl	n	n	1	cig+/-ot	6	10	2	0	nev cigs	ot
CPSII	636		f	0	0	all	4	all	NAmer	1982	pr	3229	n	bl	n	n	1	cig+/-ot	3	5	3	3	nev cigs	ot
CPSII	637		f	0	0	all	4	all	NAmer	1982	pr	3229	n	bl	n	n	1	cig+/-ot	0.1	2	0	0	nev cigs	ot
DAMBER	554		m	0	0	all	-	all	Eu:Sca	1972	CC	579	n	bl	y	n	1	cig only	11	999	1	0	nev any	ot
DAMBER	555		m	0	0	all	-	all	Eu:Sca	1972	CC	579	n	bl	y	n	1	cig only	0.1	10	0	3	nev any	ot
DARBY	501		m	0	0	wh	-	all	Eu:UK	1988	CC	982	n	V	n	n	0	all/unsp	10	999	1	0	nev any	st
DARBY	502		m	0	0	wh	-	all	Eu:UK	1988	CC	982	n	V	n	n	0	all/unsp	0.1	9	0	3	nev any	st
DARBY	510		f	0	0	wh	-	all	Eu:UK	1988	CC	982	n	V	n	n	0	all/unsp	10	999	1	0	nev any	st
DARBY	511		f	0	0	wh	-	all	Eu:UK	1988	CC	982	n	V	n	n	0	all/unsp	0.1	9	0	3	nev any	st
DEAN3	501	x	m	0	0	all	-	all	Eu:UK	1969	CC	766	n	V	y	n	0	cig only	19	999	0	1	nev any	st
DEAN3	502	x	m	0	0	all	-	all	Eu:UK	1969	CC	766	n	V	y	n	0	cig only	9	18	1	2	nev any	st
DEAN3	503	x	m	0	0	all	-	all	Eu:UK	1969	CC	766	n	V	y	n	0	cig only	5	8	2	0	nev any	st
DEAN3	504	x	m	0	0	all	-	all	Eu:UK	1969	CC	766	n	V	y	n	0	cig only	3	4	3	3	nev any	st
DEAN3	542	x	f	0	0	all	-	all	Eu:UK	1969	CC	766	n	V	y	n	0	all/unsp	9	999	1	0	nev any	st
DEAN3	543	x	f	0	0	all	-	all	Eu:UK	1969	CC	766	n	V	y	n	0	all/unsp	5	8	2	0	nev any	st
DEAN3	544	x	f	0	0	all	-	all	Eu:UK	1969	CC	766	n	V	y	n	0	all/unsp	3	4	3	3	nev any	st
DESTEF	515	x	m	0	0	all	-	all	SCAmer	1988	CC	497	n	bl	n	y	0	all/unsp	10	999	1	0	nev any	st
DESTEF	516	x	m	0	0	all	-	all	SCAmer	1988	CC	497	n	bl	n	y	0	all/unsp	5	9	2	0	nev any	st
DESTEF	517	x	m	0	0	all	-	all	SCAmer	1988	CC	497	n	bl	n	y	0	all/unsp	0.1	4	3	3	nev any	st
DOLL	529		m	0	0	all	-	all	Eu:UK	1948	CC	1465	n	V	n	n	0	all/unsp	20	999	0	1	nev any	st

Table 1J6 - 4

IESLC - Meta-analysis of Ex Smoking by Years quit (vs never), Overview
All LC types, Cigarettes (or Any Product if Cigarettes not available)
Least adjusted

REF	NRR	X	SEX	AGEL	AGEH	RACE	YF	LC TYPE	LOC	START	ST	NLC	R	VB	P	H	AD	PRODUCT	exL	exH	S1	S2	DENOM	De
DOLL	530		m	0	0	all	-	all	Eu:UK	1948	CC	1465	n	V	n	n	0	all/unsp	10	19	1	2	nev any	st
DOLL	531		m	0	0	all	-	all	Eu:UK	1948	CC	1465	n	V	n	n	0	all/unsp	0.1	9	0	3	nev any	st
DOLL	542		f	0	0	all	-	all	Eu:UK	1948	CC	1465	n	V	n	n	0	all/unsp	10	999	1	0	nev any	st
DOLL	543		f	0	0	all	-	all	Eu:UK	1948	CC	1465	n	V	n	n	0	all/unsp	0.1	9	0	3	nev any	st
DOLL2	501		m	0	0	all	20	all	Eu:UK	1951	pr	920	n	V	n	n	1	cig only	15	999	0	1	nev any	ot
DOLL2	502		m	0	0	all	20	all	Eu:UK	1951	pr	920	n	V	n	n	1	cig only	10	14	1	2	nev any	ot
DOLL2	503		m	0	0	all	20	all	Eu:UK	1951	pr	920	n	V	n	n	1	cig only	5	9	2	0	nev any	ot
DOLL2	504		m	0	0	all	20	all	Eu:UK	1951	pr	920	n	V	n	n	1	cig only	0.1	4	3	3	nev any	ot
DORGAN	501		m	0	0	wh	-	all	NAmer	1980	CC	2026	n	bl	y	y	0	cig+/-ot	10	999	1	0	nev any	st
DORGAN	502		m	0	0	wh	-	all	NAmer	1980	CC	2026	n	bl	y	y	0	cig+/-ot	6	9	2	0	nev any	st
DORGAN	503		m	0	0	wh	-	all	NAmer	1980	CC	2026	n	bl	y	y	0	cig+/-ot	1.1	5	3	3	nev any	st
DORGAN	553		f	0	0	all	-	all	NAmer	1980	CC	2026	n	bl	y	y	0	cig+/-ot	10	999	1	0	nev any	st
DORGAN	554		f	0	0	all	-	all	NAmer	1980	CC	2026	n	bl	y	y	0	cig+/-ot	1.1	9	0	3	nev any	st
DORN	657		m	55	64	wh	8	all	NAmer	1954	pr	5097	n	bl	n	n	0	cig+/-ot	15	999	0	1	nev any	st
DORN	658		m	55	64	wh	8	all	NAmer	1954	pr	5097	n	bl	n	n	0	cig+/-ot	10	14	1	2	nev any	st
DORN	659		m	55	64	wh	8	all	NAmer	1954	pr	5097	n	bl	n	n	0	cig+/-ot	5	9	2	0	nev any	st
DORN	660		m	55	64	wh	8	all	NAmer	1954	pr	5097	n	bl	n	n	0	cig+/-ot	0.1	4	3	3	nev any	st
DORN	680		m	65	74	wh	8	all	NAmer	1954	pr	5097	n	bl	n	n	0	cig+/-ot	15	999	0	1	nev any	st
DORN	681		m	65	74	wh	8	all	NAmer	1954	pr	5097	n	bl	n	n	0	cig+/-ot	10	14	1	2	nev any	st
DORN	682		m	65	74	wh	8	all	NAmer	1954	pr	5097	n	bl	n	n	0	cig+/-ot	5	9	2	0	nev any	st
DORN	683		m	65	74	wh	8	all	NAmer	1954	pr	5097	n	bl	n	n	0	cig+/-ot	0.1	4	3	3	nev any	st
GAO	521	x	m	0	0	all	-	all	As:Chi	1984	CC	1405	n	ot	n	n	0	cig+/-ot	10	999	1	0	nev cigs	st
GAO	522	x	m	0	0	all	-	all	As:Chi	1984	CC	1405	n	ot	n	n	0	cig+/-ot	5	9	2	0	nev cigs	st
GAO	523	x	m	0	0	all	-	all	As:Chi	1984	CC	1405	n	ot	n	n	0	cig+/-ot	0.1	4	3	3	nev cigs	st
GAO	541	x	f	0	0	all	-	all	As:Chi	1984	CC	1405	n	ot	n	n	0	cig+/-ot	10	999	1	0	nev cigs	st
GAO	542	x	f	0	0	all	-	all	As:Chi	1984	CC	1405	n	ot	n	n	0	cig+/-ot	5	9	2	0	nev cigs	st
GAO	543	x	f	0	0	all	-	all	As:Chi	1984	CC	1405	n	ot	n	n	0	cig+/-ot	0.1	4	3	3	nev cigs	st
GAO2	509		m	0	0	all	-	all	As:Jap	1988	CC	282	n	bl	n	n	0	cig+/-ot	20	999	0	1	nev cigs	or
GAO2	510		m	0	0	all	-	all	As:Jap	1988	CC	282	n	bl	n	n	0	cig+/-ot	15	19	0	0	nev cigs	or
GAO2	511		m	0	0	all	-	all	As:Jap	1988	CC	282	n	bl	n	n	0	cig+/-ot	10	14	1	2	nev cigs	or
GAO2	512		m	0	0	all	-	all	As:Jap	1988	CC	282	n	bl	n	n	0	cig+/-ot	5	9	2	0	nev cigs	st
GAO2	513		m	0	0	all	-	all	As:Jap	1988	CC	282	n	bl	n	n	0	cig+/-ot	1.0	4	3	3	nev cigs	or
GARCIA	515		c	0	0	all	-	all	NAmer	1992	CC	416	n	bl	n	y	0	cig+/-ot	30	999	0	0	nev any	st
GARCIA	516		c	0	0	all	-	all	NAmer	1992	CC	416	n	bl	n	y	0	cig+/-ot	15	29	0	1	nev any	st
GARCIA	517		c	0	0	all	-	all	NAmer	1992	CC	416	n	bl	n	y	0	cig+/-ot	5	14	0	2	nev any	st
GARCIA	518		c	0	0	all	-	all	NAmer	1992	CC	416	n	bl	n	y	0	cig+/-ot	1.0	4	3	3	nev any	st
GARSHI	515	x	m	0	0	all	-	all	NAmer	1981	CC	1081	o	bl	y	n	0	all/unsp	15	999	0	1	nev any	st
GARSHI	516	x	m	0	0	all	-	all	NAmer	1981	CC	1081	o	bl	y	n	0	all/unsp	5	14	0	2	nev any	st
GRAHAM	525	x	m	0	0	wh	-	all	NAmer	1956	CC	685	n	bl	n	n	0	cig+/-ot	5	999	0	0	nev any	st
GRAHAM	526	x	m	0	0	wh	-	all	NAmer	1956	CC	685	n	bl	n	n	0	cig+/-ot	1.1	5	3	3	nev any	st
GRAHAM	527	x	m	0	0	wh	-	all	NAmer	1956	CC	685	n	bl	n	n	0	cig+/-ot	0.1	1.0	0	0	nev any	st
GURSEL	501		m	0	0	all	-	all	Eu:bal		CC	953		bl	*	n	0	all/unsp	11	999	1	0	nev any	or
HAMMO2	501		m	0	0	all	0	all	NAmer	1967	pr	450	o	bl	n	n	1	cig+/-ot	10	999	1	0	nev any	ot
HAMMO2	502		m	0	0	all	0	all	NAmer	1967	pr	450	o	bl	n	n	1	cig+/-ot	5	9	2	0	nev any	ot
HAMMO2	503		m	0	0	all	0	all	NAmer	1967	pr	450	o	bl	n	n	1	cig+/-ot	0.1	4	3	3	nev any	ot
HIRAYA	507		m	0	0	all	0	all	As:Jap	1965	pr	1917	n	bl	n	n	1	cig+/-ot	10	999	1	0	nev any	st
HIRAYA	508		m	0	0	all	0	all	As:Jap	1965	pr	1917	n	bl	n	n	1	cig+/-ot	5	9	2	0	nev any	st
HIRAYA	509		m	0	0	all	0	all	As:Jap	1965	pr	1917	n	bl	n	n	1	cig+/-ot	0.1	4	3	3	nev any	st
HIRAYA	518		f	0	0	all	0	all	As:Jap	1965	pr	1917	n	bl	n	n	1	cig+/-ot	10	999	1	0	nev any	st
HIRAYA	519		f	0	0	all	0	all	As:Jap	1965	pr	1917	n	bl	n	n	1	cig+/-ot	5	9	2	0	nev any	st
HIRAYA	520		f	0	0	all	0	all	As:Jap	1965	pr	1917	n	bl	n	n	1	cig+/-ot	0.1	4	3	3	nev any	st
JAHN	501		m	0	0	all	-	all	Eu:Ger	1988	CC	1004	n	bl	n	n	0	cig+/-ot	21	999	0	0	nev any	st
JAHN	502		m	0	0	all	-	all	Eu:Ger	1988	CC	1004	n	bl	n	n	0	cig+/-ot	11	20	1	0	nev any	st
JAHN	503		m	0	0	all	-	all	Eu:Ger	1988	CC	1004	n	bl	n	n	0	cig+/-ot	6	10	2	0	nev any	st
JAHN	504		m	0	0	all	-	all	Eu:Ger	1988	CC	1004	n	bl	n	n	0	cig+/-ot	2	5	3	3	nev any	st
JAHN	505		m	0	0	all	-	all	Eu:Ger	1988	CC	1004	n	bl	n	n	0	cig+/-ot	1.0	1.9	0	0	nev any	st
JAHN	506		m	0	0	all	-	all	Eu:Ger	1988	CC	1004	n	bl	n	n	0	cig+/-ot	0.1	0.9	0	0	nev any	st
JAHN	731		f	0	0	all	-	all	Eu:Ger	1988	CC	1004	n	bl	n	n	2	cig+/-ot	21	999	0	0	nev any	or
JAIN	567		m	0	0	all	-	all	NAmer	1981	CC	845	n	V	y	n	0	cig+/-ot	10	999	1	0	nev cigs	st
JAIN	568		m	0	0	all	-	all	NAmer	1981	CC	845	n	V	y	n	0	cig+/-ot	2	9	0	3	nev cigs	st
JAIN	531		f	0	0	all	-	all	NAmer	1981	CC	845	n	V	y	n	0	cig+/-ot	10	999	1	0	nev cigs	st
JAIN	532		f	0	0	all	-	all	NAmer	1981	CC	845	n	V	y	n	0	cig+/-ot	2	9	0	3	nev cigs	st
JEDRYC	611		m	0	0	all	-	all	Eu:est	1980	CC	1630	n	bl	y	n	0	cig+/-ot	10	999	1	0	nev any	st
JEDRYC	612		m	0	0	all	-	all	Eu:est	1980	CC	1630	n	bl	y	n	0	cig+/-ot	5	9	2	0	nev any	st
JOLY	566		m	0	0	all	-	all	SCAmer	1978	CC	826	n	bl	n	n	0	cig+/-ot	5	999	0	0	nev any	st
JOLY	567		m	0	0	all	-	all	SCAmer	1978	CC	826	n	bl	n	n	0	cig+/-ot	1.0	4	3	3	nev any	st
JOLY	553		f	0	0	all	-	all	SCAmer	1978	CC	826	n	bl	n	n	0	cig+/-ot	5	999	0	0	nev any	st
JOLY	554		f	0	0	all	-	all	SCAmer	1978	CC	826	n	bl	n	n	0	cig+/-ot	1.0	4	3	3	nev any	st
KAISE2	646		m	0	0	all	9	all	NAmer	1979	pr	318	n	bl	n	n	1	cig only	21	999	0	0	nev any	st
KAISE2	647		m	0	0	all	9	all	NAmer	1979	pr	318	n	bl	n	n	1	cig only	11	20	1	0	nev any	ot
KAISE2	648		m	0	0	all	9	all	NAmer	1979	pr	318	n	bl	n	n	1	cig only	2	10	0	3	nev any	st
KAISE2	566		f	0	0	all	9	all	NAmer	1979	pr	318	n	bl	n	n	1	cig only	21	999	0	0	nev any	ot

Table 1J6 - 4

IESLC - Meta-analysis of Ex Smoking by Years quit (vs never), Overview
All LC types, Cigarettes (or Any Product if Cigarettes not available)
Least adjusted

REF	NRR	X	SEX	AGEL	AGEH	RACE	YF	LC TYPE	LOC	START	ST	NLC	R	VB	P	H	AD	PRODUCT	exL	exH	S1	S2	DENOM	De
KAISE2	567		f	0	0	all	9	all	NAmer	1979	pr	318	n	bl	n	n	1	cig only	11	20	1	0	nev any	st
KAISE2	568		f	0	0	all	9	all	NAmer	1979	pr	318	n	bl	n	n	1	cig only	2	10	0	3	nev any	st
KHUDER	511		m	0	0	all	-	all	NAmer	1985	CC	482	n	bl	n	y	0	cig+/-ot	15	999	0	1	nev cigs	st
KHUDER	512		m	0	0	all	-	all	NAmer	1985	CC	482	n	bl	n	y	0	cig+/-ot	5	14	0	2	nev cigs	st
KHUDER	513		m	0	0	all	-	all	NAmer	1985	CC	482	n	bl	n	y	0	cig+/-ot	0.1	4	3	3	nev cigs	st
LAUSSM	501	x	m	0	0	all	-	all	Eu:Ger	1982	CC	432	n	bl	n	n	0	all/unsp	10	999	1	0	nev any	st
LUBIN	585		m	0	0	all	-	all	As:Chi	1984	CC	427	m	ot	y	n	0	cig+/-ot	10	999	1	0	nev any	st
LUBIN	586		m	0	0	all	-	all	As:Chi	1984	CC	427	m	ot	y	n	0	cig+/-ot	5	9	2	0	nev any	st
LUBIN	587		m	0	0	all	-	all	As:Chi	1984	CC	427	m	ot	y	n	0	cig+/-ot	3	4	3	3	nev any	st
LUBIN2	1069		m	0	0	all	-	all	Eu:mul	1976	CC	7804	n	bl	n	y	0	cig+/-ot	25	999	0	0	nev any	st
LUBIN2	1070		m	0	0	all	-	all	Eu:mul	1976	CC	7804	n	bl	n	y	0	cig+/-ot	20	24	0	1	nev any	st
LUBIN2	1071		m	0	0	all	-	all	Eu:mul	1976	CC	7804	n	bl	n	y	0	cig+/-ot	15	19	0	0	nev any	st
LUBIN2	1072		m	0	0	all	-	all	Eu:mul	1976	CC	7804	n	bl	n	y	0	cig+/-ot	10	14	1	2	nev any	st
LUBIN2	1073		m	0	0	all	-	all	Eu:mul	1976	CC	7804	n	bl	n	y	0	cig+/-ot	5	9	2	0	nev any	st
LUBIN2	1074		m	0	0	all	-	all	Eu:mul	1976	CC	7804	n	bl	n	y	0	cig+/-ot	0.1	4	3	3	nev any	st
LUBIN2	1108		f	0	0	all	-	all	Eu:mul	1976	CC	7804	n	bl	n	y	0	cig+/-ot	25	999	0	0	nev any	st
LUBIN2	1109		f	0	0	all	-	all	Eu:mul	1976	CC	7804	n	bl	n	y	0	cig+/-ot	20	24	0	1	nev any	st
LUBIN2	1110		f	0	0	all	-	all	Eu:mul	1976	CC	7804	n	bl	n	y	0	cig+/-ot	15	19	0	0	nev any	st
LUBIN2	1111		f	0	0	all	-	all	Eu:mul	1976	CC	7804	n	bl	n	y	0	cig+/-ot	10	14	1	2	nev any	st
LUBIN2	1112		f	0	0	all	-	all	Eu:mul	1976	CC	7804	n	bl	n	y	0	cig+/-ot	5	9	2	0	nev any	st
LUBIN2	1113		f	0	0	all	-	all	Eu:mul	1976	CC	7804	n	bl	n	y	0	cig+/-ot	0.1	4	3	3	nev any	st
MATOS	581	x	m	0	0	all	-	all	SCAmer	1994	CC	200	n	bl	n	n	0	cig+/-ot	11	999	1	0	nev any	st
MATOS	582	x	m	0	0	all	-	all	SCAmer	1994	CC	200	n	bl	n	n	0	cig+/-ot	6	10	2	0	nev any	st
MATOS	583	x	m	0	0	all	-	all	SCAmer	1994	CC	200	n	bl	n	n	0	cig+/-ot	1.0	5	3	3	nev any	st
PEZZO2	501		m	0	0	all	-	all	SCAmer	1992	CC	367	n	bl	n	y	0	cig+/-ot	11	999	1	0	nev cigs	st
PEZZO2	502		m	0	0	all	-	all	SCAmer	1992	CC	367	n	bl	n	y	0	cig+/-ot	1.0	10	0	3	nev cigs	st
PEZZOT	501		m	0	0	all	-	all	SCAmer	1987	CC	215	n	bl	n	y	0	cig only	11	999	1	0	nev cigs	st
PEZZOT	502		m	0	0	all	-	all	SCAmer	1987	CC	215	n	bl	n	y	0	cig only	1.0	10	0	3	nev cigs	st
SOBUE	717		m	0	0	all	-	all	As:Jap	1986	CC	1376	n	bl	n	y	0	cig+/-ot	25	999	0	0	nev cigs	st
SOBUE	718		m	0	0	all	-	all	As:Jap	1986	CC	1376	n	bl	n	y	0	cig+/-ot	20	24	0	1	nev cigs	st
SOBUE	719		m	0	0	all	-	all	As:Jap	1986	CC	1376	n	bl	n	y	0	cig+/-ot	15	19	0	0	nev cigs	st
SOBUE	720		m	0	0	all	-	all	As:Jap	1986	CC	1376	n	bl	n	y	0	cig+/-ot	10	14	1	2	nev cigs	st
SOBUE	721		m	0	0	all	-	all	As:Jap	1986	CC	1376	n	bl	n	y	0	cig+/-ot	5	9	2	0	nev cigs	st
SOBUE	722		m	0	0	all	-	all	As:Jap	1986	CC	1376	n	bl	n	y	0	cig+/-ot	1.0	4	3	3	nev cigs	st
SPEIZE	501		f	0	0	all	0	all	NAmer	1976	pr	593	n	bl	n	y	0	cig+/-ot	15	999	0	1	nev cigs	st
SPEIZE	502		f	0	0	all	0	all	NAmer	1976	pr	593	n	bl	n	y	0	cig+/-ot	10	15	1	2	nev cigs	st
SPEIZE	503		f	0	0	all	0	all	NAmer	1976	pr	593	n	bl	n	y	0	cig+/-ot	5	10	2	0	nev cigs	st
SPEIZE	504		f	0	0	all	0	all	NAmer	1976	pr	593	n	bl	n	y	0	cig+/-ot	2	5	3	3	nev cigs	st
SPEIZE	505		f	0	0	all	0	all	NAmer	1976	pr	593	n	bl	n	y	0	cig+/-ot	0.1	1.9	0	0	nev cigs	st
SUZUK2	508		c	0	0	all	-	all	SCAmer	1991	CC	123	n	bl	n	y	0	all/unsp	11	999	1	0	nev any	st
SUZUK2	509		c	0	0	all	-	all	SCAmer	1991	CC	123	n	bl	n	y	0	all/unsp	6	10	2	0	nev any	st
SUZUK2	510		c	0	0	all	-	all	SCAmer	1991	CC	123	n	bl	n	y	0	all/unsp	0.1	5	3	3	nev any	st
SVENSS	551		f	0	0	all	-	all	Eu:Sca	1983	CC	210	n	bl	n	n	0	all/unsp	11	999	1	0	nev any	st
SVENSS	552		f	0	0	all	-	all	Eu:Sca	1983	CC	210	n	bl	n	n	0	all/unsp	3	10	0	3	nev any	st
TVERDA	501		m	0	0	all	0	all	Eu:Sca	1972	pr	238	n	bl	n	n	2	cig only	5	999	0	0	nev cigs	ot
TVERDA	502		m	0	0	all	0	all	Eu:Sca	1972	pr	238	n	bl	n	n	2	cig only	1.0	5	3	3	nev cigs	ot
TVERDA	503		m	0	0	all	0	all	Eu:Sca	1972	pr	238	n	bl	n	n	2	cig only	0.1	0.9	0	0	nev cigs	ot
WAKAI	522	x	m	0	0	all	-	all	As:Jap	1988	CC	333	n	bl	n	y	0	cig+/-ot	20	999	0	1	nev any	st
WAKAI	523	x	m	0	0	all	-	all	As:Jap	1988	CC	333	n	bl	n	y	0	cig+/-ot	10	19	1	2	nev any	st
WAKAI	524	x	m	0	0	all	-	all	As:Jap	1988	CC	333	n	bl	n	y	0	cig+/-ot	5	9	2	0	nev any	st
WANG2	510		c	0	0	all	-	all	As:Chi	1980	CC	103	n	ot	n	n	0	cig+/-ot	4	999	0	0	nev cigs	st
WANG2	511		c	0	0	all	-	all	As:Chi	1980	CC	103	n	ot	n	n	0	cig+/-ot	0.1	3	3	3	nev cigs	st
WYNDE3	566		m	0	0	all	-	all	NAmer	1966	CC	350	n	bl	n	y	0	cig+/-ot	10	999	1	0	nev any	st
WYNDE3	587		f	0	0	all	-	all	NAmer	1966	CC	350	n	bl	n	y	0	cig+/-ot	10	999	1	0	nev any	st
WYNDE6	501		m	0	0	all	-	all	NAmer	1969	CC	4423	n	bl	n	y	0	cig only	30	999	0	0	nev any	st
WYNDE6	502		m	0	0	all	-	all	NAmer	1969	CC	4423	n	bl	n	y	0	cig only	20	29	0	1	nev any	st
WYNDE6	503		m	0	0	all	-	all	NAmer	1969	CC	4423	n	bl	n	y	0	cig only	10	19	1	2	nev any	st
WYNDE6	504		m	0	0	all	-	all	NAmer	1969	CC	4423	n	bl	n	y	0	cig only	5	9	2	0	nev any	st
WYNDE6	505		m	0	0	all	-	all	NAmer	1969	CC	4423	n	bl	n	y	0	cig only	1.0	4	3	3	nev any	st
WYNDE6	522		f	0	0	all	-	all	NAmer	1969	CC	4423	n	bl	n	y	0	cig only	30	999	0	0	nev any	st
WYNDE6	523		f	0	0	all	-	all	NAmer	1969	CC	4423	n	bl	n	y	0	cig only	20	29	0	1	nev any	st
WYNDE6	524		f	0	0	all	-	all	NAmer	1969	CC	4423	n	bl	n	y	0	cig only	10	19	1	2	nev any	st
WYNDE6	525		f	0	0	all	-	all	NAmer	1969	CC	4423	n	bl	n	y	0	cig only	5	9	2	0	nev any	st
WYNDE6	526		f	0	0	all	-	all	NAmer	1969	CC	4423	n	bl	n	y	0	cig only	1.0	4	3	3	nev any	st

Cigarette type is all/unspec for all RRs
except for the following:

Table 1J6 - 4

IESLC - Meta-analysis of Ex Smoking by Years quit (vs never), Overview
All LC types, Cigarettes (or Any Product if Cigarettes not available)
Least adjusted

REF	NRR	CIGTYPE
ALDERS	507	MC only
ALDERS	508	MC only
ALDERS	509	MC only
ALDERS	518	MC only
ALDERS	519	MC only
ALDERS	520	MC only
DEAN3	501	MC only
DEAN3	502	MC only
DEAN3	503	MC only
DEAN3	504	MC only

In this overview table, subtotals and Qs values may be invalid and should be ignored

Table 1J6 - 5

IESLC - Meta-analysis of Ex Smoking by Years quit (vs never), Overview
All LC types, Cigarettes (or Any Product if Cigarettes not available)
Least adjusted

REF	NRR	SEX	AD	Number Exposed Case	Cont	Non-exposed Case	Cont	RR	95.00%CI
ALDERS	507	m	1	29	-	15	-	3.20	(1.61- 6.35)
ALDERS	508	m	1	28	-	15	-	4.30	(2.13- 8.69)
ALDERS	509	m	1	121	-	15	-	18.10	(9.71- 33.74)
ALDERS	518	f	1	26	-	75	-	1.27	(0.76- 2.15)
ALDERS	519	f	1	54	-	75	-	2.95	(1.88- 4.64)
ALDERS	520	f	1	206	-	75	-	9.45	(6.50- 13.74)
Subtotal ALDERS								4.77	(3.86- 5.89)
ARMADA	515	m	0	50	87	8	71	5.10	(2.27- 11.46)
ARMADA	516	m	0	79	45	8	71	15.58	(6.88- 35.29)
Subtotal ARMADA								8.87	(4.99- 15.76)
AUVINE	530	c	0	207	208	44	229	5.18	(3.56- 7.54)
BARBON	525	m	0	15	59	22	188	2.17	(1.06- 4.46)
BARBON	526	m	0	33	41	22	188	6.88	(3.64- 13.00)
BARBON	527	m	0	89	85	22	188	8.95	(5.25- 15.24)
BARBON	528	m	0	32	20	22	188	13.67	(6.71- 27.87)
Subtotal BARBON								6.91	(5.03- 9.50)
BECHER	501	m	0	16	72	3	54	4.00	(1.11- 14.42)
BECHER	502	m	0	16	32	3	54	9.00	(2.43- 33.30)
BECHER	503	m	0	10	12	3	54	15.00	(3.58- 62.92)
BECHER	511	f	0	1	10	10	52	0.52	(0.06- 4.53)
BECHER	512	f	0	2	5	10	52	2.08	(0.35- 12.26)
BECHER	513	f	0	2	3	10	52	3.47	(0.51- 23.48)
Subtotal BECHER								4.76	(2.53- 8.99)
*BENSHL	508	m	2	6	-	10	-	1.00	(0.32- 3.10)
*BENSHL	509	m	2	15	-	10	-	2.59	(1.21- 5.54)
*BENSHL	510	m	2	23	-	10	-	4.08	(2.03- 8.20)
*BENSHL	511	m	2	14	-	10	-	8.68	(4.00- 18.90)
Subtotal BENSHL								3.69	(2.47- 5.51)
BROSS	515	m	0	43	79	38	170	2.44	(1.46- 4.06)
BROSS	516	m	0	169	67	38	170	11.28	(7.19- 17.72)
Subtotal BROSS								5.77	(4.11- 8.09)
BROWN3	501	f	0	186	234	432	1168	2.15	(1.72- 2.68)
CARPEN	501	c	0	29	137	8	208	5.50	(2.44- 12.40)
CARPEN	502	c	0	9	51	8	208	4.59	(1.69- 12.48)
CARPEN	503	c	0	25	48	8	208	13.54	(5.75- 31.87)
CARPEN	504	c	0	23	39	8	208	15.33	(6.40- 36.75)
Subtotal CARPEN								8.72	(5.62- 13.52)
*CEDERL	528	m	1	3	-	7	-	1.10	(0.28- 4.25)
*CEDERL	529	m	1	12	-	7	-	6.10	(2.41- 15.46)
Subtotal CEDERL								3.54	(1.64- 7.62)
CHOI	533	m	0	4	19	13	95	1.54	(0.45- 5.23)
CHOI	534	m	0	4	23	13	95	1.27	(0.38- 4.26)
CHOI	535	m	0	5	30	13	95	1.22	(0.40- 3.70)
CHOI	536	m	0	25	64	13	95	2.85	(1.36- 5.99)
CHOI	550	f	0	2	0	76	164	10.75~	(0.51- 226.67)
CHOI	551	f	0	3	2	76	164	3.24	(0.53- 19.77)
Subtotal CHOI								2.04	(1.27- 3.29)
*CHYOU	501	m	1	5	-	8	-	2.80	(0.90- 8.60)
*CHYOU	502	m	1	21	-	8	-	3.90	(1.70- 8.80)
Subtotal CHYOU								3.48	(1.79- 6.76)
*CPSI	807	m	1	15	-	60	-	1.28	(0.73- 2.25)
*CPSI	808	m	1	32	-	60	-	5.15	(3.35- 7.91)
*CPSI	809	m	1	49	-	60	-	8.09	(5.55- 11.80)
*CPSI	810	m	1	37	-	60	-	14.74	(9.78- 22.20)
Subtotal CPSI								6.51	(5.25- 8.07)
*CPSII	652	m	1	256	-	81	-	3.83	(2.98- 4.92)
*CPSII	653	m	1	164	-	81	-	8.61	(6.60- 11.24)
*CPSII	654	m	1	186	-	81	-	11.43	(8.81- 14.84)
*CPSII	655	m	1	178	-	81	-	18.61	(14.31- 24.20)
*CPSII	656	m	1	188	-	81	-	28.07	(21.63- 36.43)
*CPSII	657	m	1	97	-	81	-	38.76	(28.85- 52.07)
*CPSII	633	f	1	50	-	174	-	1.74	(1.27- 2.39)
*CPSII	634	f	1	28	-	174	-	3.86	(2.59- 5.75)
*CPSII	635	f	1	37	-	174	-	4.91	(3.45- 7.01)
*CPSII	636	f	1	56	-	174	-	10.55	(7.81- 14.26)
*CPSII	637	f	1	91	-	174	-	17.02	(13.21- 21.93)
Subtotal CPSII								10.30	(9.46- 11.22)
DAMBER	554	m	1	-	-	42	-	1.60	(0.70- 3.40)
DAMBER	555	m	1	-	-	42	-	5.50	(2.80- 11.10)
Subtotal DAMBER								3.23	(1.92- 5.42)
DARBY	501	m	0	139	767	3	384	23.20	(7.34- 73.28)

Table 1J6 - 5

IESLC - Meta-analysis of Ex Smoking by Years quit (vs never), Overview
All LC types, Cigarettes (or Any Product if Cigarettes not available)
Least adjusted

REF	NRR	SEX	AD	Number Exposed Case	Cont	Non-exposed Case	Cont	RR	95.00%CI
DARBY	502	m	0	146	339	3	384	55.13	(17.41- 174.53)
DARBY	510	f	0	26	224	23	529	2.67	(1.49- 4.78)
DARBY	511	f	0	68	93	23	529	16.82	(9.98- 28.33)
Subtotal DARBY								9.92	(6.99- 14.09)
DEAN3	501	m	0	8	66	24	510	2.58	(1.11- 5.97)
DEAN3	502	m	0	15	86	24	510	3.71	(1.87- 7.35)
DEAN3	503	m	0	11	43	24	510	5.44	(2.50- 11.84)
DEAN3	504	m	0	28	102	24	510	5.83	(3.25- 10.47)
DEAN3	542	f	0	2	114	41	1538	0.66	(0.16- 2.76)
DEAN3	543	f	0	1	38	41	1538	0.99	(0.13- 7.37)
DEAN3	544	f	0	4	110	41	1538	1.36	(0.48- 3.88)
Subtotal DEAN3								3.47	(2.52- 4.78)
DESTEF	515	m	0	17	36	27	163	2.85	(1.41- 5.78)
DESTEF	516	m	0	27	27	27	163	6.04	(3.09- 11.81)
DESTEF	517	m	0	64	45	27	163	8.59	(4.91- 15.00)
Subtotal DESTEF								5.74	(3.98- 8.28)
DOLL	529	m	0	8	23	7	61	3.03	(0.99- 9.31)
DOLL	530	m	0	6	26	7	61	2.01	(0.62- 6.56)
DOLL	531	m	0	56	75	7	61	6.51	(2.77- 15.30)
DOLL	542	f	0	1	2	40	59	0.74	(0.06- 8.41)
DOLL	543	f	0	9	6	40	59	2.21	(0.73- 6.70)
Subtotal DOLL								3.24	(1.95- 5.39)
*DOLL2	501	m	1	7	-	7	-	2.00	(0.70- 5.70)
*DOLL2	502	m	1	9	-	7	-	5.30	(1.97- 14.23)
*DOLL2	503	m	1	12	-	7	-	5.90	(2.32- 14.99)
*DOLL2	504	m	1	15	-	7	-	16.00	(6.52- 39.24)
Subtotal DOLL2								6.10	(3.77- 9.87)
DORGAN	501	m	0	134	255	13	140	5.66	(3.09- 10.37)
DORGAN	502	m	0	49	38	13	140	13.89	(6.84- 28.21)
DORGAN	503	m	0	59	51	13	140	12.46	(6.31- 24.61)
DORGAN	553	f	0	34	50	61	213	2.37	(1.41- 4.00)
DORGAN	554	f	0	49	27	61	213	6.34	(3.66- 10.98)
Subtotal DORGAN								5.93	(4.54- 7.76)
*DORN	657	m	0	16	58370	25	213858	2.34	(1.25- 4.39)
*DORN	658	m	0	12	23682	25	213858	4.33	(2.18- 8.63)
*DORN	659	m	0	32	34566	25	213858	7.92	(4.69- 13.36)
*DORN	660	m	0	34	22086	25	213858	13.17	(7.86- 22.07)
*DORN	680	m	0	34	51243	49	171211	2.32	(1.50- 3.59)
*DORN	681	m	0	29	20056	49	171211	5.05	(3.19- 7.99)
*DORN	682	m	0	41	24089	49	171211	5.95	(3.93- 9.00)
*DORN	683	m	0	14	6195	49	171211	7.90	(4.36- 14.29)
Subtotal DORN								5.24	(4.37- 6.27)
GAO	521	m	0	13	41	62	202	1.03	(0.52- 2.05)
GAO	522	m	0	24	27	62	202	2.90	(1.56- 5.38)
GAO	523	m	0	105	52	62	202	6.58	(4.25- 10.19)
GAO	541	f	0	16	14	435	605	1.59	(0.77- 3.29)
GAO	542	f	0	14	7	435	605	2.78	(1.11- 6.95)
GAO	543	f	0	37	9	435	605	5.72	(2.73- 11.97)
Subtotal GAO								3.35	(2.58- 4.34)
GAO2	509	m	0	8	25	13	56	1.38	(0.51- 3.74)
GAO2	510	m	0	7	9	13	56	3.35	(1.05- 10.66)
GAO2	511	m	0	16	18	13	56	3.83	(1.55- 9.46)
GAO2	512	m	0	21	26	13	56	3.48	(1.51- 8.01)
GAO2	513	m	0	31	26	13	56	5.14	(2.31- 11.40)
Subtotal GAO2								3.35	(2.23- 5.04)
GARCIA	515	c	0	10	37	8	80	2.70	(0.99- 7.41)
GARCIA	516	c	0	32	67	8	80	4.78	(2.06- 11.06)
GARCIA	517	c	0	43	36	8	80	11.94	(5.10- 27.97)
GARCIA	518	c	0	33	11	8	80	30.00	(11.07- 81.30)
Subtotal GARCIA								8.14	(5.16- 12.86)
GARSHI	515	m	0	125	343	41	363	3.23	(2.20- 4.73)
GARSHI	516	m	0	166	290	41	363	5.07	(3.48- 7.37)
Subtotal GARSHI								4.06	(3.11- 5.31)
GRAHAM	525	m	0	13	71	18	346	3.52	(1.65- 7.51)
GRAHAM	526	m	0	24	48	18	346	9.61	(4.86- 19.00)
GRAHAM	527	m	0	113	59	18	346	36.82	(20.84- 65.03)
Subtotal GRAHAM								13.55	(9.28- 19.79)
GURSEL	501	m	0	-	-	-	-	2.30	(1.01- 5.22)
*HAMMO2	501	m	1	20	-	5	-	3.45	(1.30- 9.14)
*HAMMO2	502	m	1	11	-	5	-	3.98	(1.39- 11.40)
*HAMMO2	503	m	1	59	-	5	-	10.99	(4.43- 27.26)

Table 1J6 - 5

IESLC - Meta-analysis of Ex Smoking by Years quit (vs never), Overview
All LC types, Cigarettes (or Any Product if Cigarettes not available)
Least adjusted

REF	NRR	SEX	AD	Number Case	Exposed Cont	Non-exposed Case	Cont	RR	95.00%CI
Subtotal HAMMO2								5.60	(3.19- 9.82)
*HIRAYA	507	m	1	-	-	-	-	1.38	(0.59- 3.21)
*HIRAYA	508	m	1	-	-	-	-	1.59	(0.66- 3.82)
*HIRAYA	509	m	1	-	-	-	-	2.03	(1.10- 3.75)
*HIRAYA	518	f	1	-	-	-	-	0.97	(0.03- 32.06)
*HIRAYA	519	f	1	-	-	-	-	3.29	(0.56- 19.50)
*HIRAYA	520	f	1	-	-	-	-	3.72	(1.12- 12.37)
Subtotal HIRAYA								1.92	(1.30- 2.85)
JAHN	501	m	0	29	146	18	138	1.52	(0.81- 2.87)
JAHN	502	m	0	64	130	18	138	3.77	(2.12- 6.71)
JAHN	503	m	0	59	63	18	138	7.18	(3.92- 13.16)
JAHN	504	m	0	77	46	18	138	12.83	(6.96- 23.67)
JAHN	505	m	0	60	9	18	138	51.11	(21.72- 120.26)
JAHN	506	m	0	166	8	18	138	159.08	(67.12- 377.03)
JAHN	731	f	2	-	-	-	-	0.30	(0.06- 1.53)
Subtotal JAHN								7.91	(6.06- 10.34)
JAIN	567	m	0	52	113	12	85	3.26	(1.64- 6.48)
JAIN	568	m	0	74	46	12	85	11.39	(5.62- 23.12)
JAIN	531	f	0	19	61	52	214	1.28	(0.71- 2.33)
JAIN	532	f	0	66	36	52	214	7.54	(4.55- 12.52)
Subtotal JAIN								4.36	(3.22- 5.92)
JEDRYC	611	m	0	73	138	49	219	2.36	(1.55- 3.60)
JEDRYC	612	m	0	64	58	49	219	4.93	(3.08- 7.90)
Subtotal JEDRYC								3.27	(2.39- 4.48)
JOLY	566	m	0	63	149	12	218	7.68	(4.00- 14.74)
JOLY	567	m	0	38	36	12	218	19.18	(9.16- 40.14)
JOLY	553	f	0	15	19	52	283	4.30	(2.05- 8.99)
JOLY	554	f	0	19	8	52	283	12.93	(5.38- 31.08)
Subtotal JOLY								9.16	(6.33- 13.26)
*KAISE2	646	m	1	6	-	14	-	1.94	(0.70- 5.40)
*KAISE2	647	m	1	8	-	14	-	3.14	(1.26- 7.82)
*KAISE2	648	m	1	12	-	14	-	8.26	(3.73- 18.28)
*KAISE2	566	f	1	4	-	11	-	6.29	(1.78- 22.20)
*KAISE2	567	f	1	4	-	11	-	4.37	(1.30- 14.72)
*KAISE2	568	f	1	6	-	11	-	7.95	(2.89- 21.86)
Subtotal KAISE2								4.86	(3.24- 7.31)
KHUDER	511	m	0	63	213	23	309	3.97	(2.39- 6.61)
KHUDER	512	m	0	63	133	23	309	6.36	(3.79- 10.69)
KHUDER	513	m	0	88	123	23	309	9.61	(5.80- 15.92)
Subtotal KHUDER								6.25	(4.66- 8.40)
LAUSSM	501	m	0	29	15	85	226	5.14	(2.63- 10.06)
LUBIN	585	m	0	17	73	9	72	1.86	(0.78- 4.45)
LUBIN	586	m	0	20	48	9	72	3.33	(1.40- 7.94)
LUBIN	587	m	0	33	18	9	72	14.67	(5.96- 36.07)
Subtotal LUBIN								4.38	(2.64- 7.28)
LUBIN2	1069	m	0	109	715	190	2616	2.10	(1.64- 2.69)
LUBIN2	1070	m	0	106	413	190	2616	3.53	(2.73- 4.58)
LUBIN2	1071	m	0	130	478	190	2616	3.74	(2.94- 4.78)
LUBIN2	1072	m	0	270	693	190	2616	5.36	(4.38- 6.58)
LUBIN2	1073	m	0	466	822	190	2616	7.81	(6.48- 9.40)
LUBIN2	1074	m	0	866	1047	190	2616	11.39	(9.58- 13.53)
LUBIN2	1108	f	0	4	20	336	1188	0.71	(0.24- 2.08)
LUBIN2	1109	f	0	4	9	336	1188	1.57	(0.48- 5.13)
LUBIN2	1110	f	0	3	7	336	1188	1.52	(0.39- 5.89)
LUBIN2	1111	f	0	10	26	336	1188	1.36	(0.65- 2.85)
LUBIN2	1112	f	0	30	40	336	1188	2.65	(1.63- 4.32)
LUBIN2	1113	f	0	60	55	336	1188	3.86	(2.62- 5.67)
Subtotal LUBIN2								5.29	(4.87- 5.74)
MATOS	581	m	0	27	101	11	110	2.67	(1.26- 5.67)
MATOS	582	m	0	21	27	11	110	7.78	(3.35- 18.06)
MATOS	583	m	0	28	23	11	110	12.17	(5.31- 27.91)
Subtotal MATOS								5.95	(3.74- 9.47)
PEZZO2	501	m	0	43	161	6	117	5.21	(2.15- 12.64)
PEZZO2	502	m	0	85	110	6	117	15.07	(6.33- 35.89)
Subtotal PEZZO2								8.96	(4.82- 16.66)
PEZZOT	501	m	0	20	106	4	116	5.47	(1.81- 16.53)
PEZZOT	502	m	0	46	82	4	116	16.27	(5.64- 46.96)
Subtotal PEZZOT								9.65	(4.49- 20.74)
SOBUE	717	m	0	17	40	29	126	1.85	(0.92- 3.71)
SOBUE	718	m	0	15	23	29	126	2.83	(1.32- 6.09)
SOBUE	719	m	0	24	31	29	126	3.36	(1.72- 6.56)

Table 1J6 - 5

IESLC - Meta-analysis of Ex Smoking by Years quit (vs never), Overview
All LC types, Cigarettes (or Any Product if Cigarettes not available)
Least adjusted

REF	NRR	SEX	AD	Number Exposed Case	Cont	Non-exposed Case	Cont	RR	95.00%CI
SOBUE	720	m	0	35	50	29	126	3.04	(1.68- 5.49)
SOBUE	721	m	0	67	92	29	126	3.16	(1.90- 5.28)
SOBUE	722	m	0	128	116	29	126	4.79	(2.98- 7.71)
Subtotal SOBUE								3.27	(2.57- 4.16)
*SPEIZE	501	f	0	28	214271	58	776300	1.75	(1.11- 2.75)
*SPEIZE	502	f	0	17	93933	58	776300	2.42	(1.41- 4.16)
*SPEIZE	503	f	0	41	95585	58	776300	5.74	(3.85- 8.56)
*SPEIZE	504	f	0	34	63060	58	776300	7.22	(4.73- 11.02)
*SPEIZE	505	f	0	24	55232	58	776300	5.82	(3.61- 9.36)
Subtotal SPEIZE								4.24	(3.46- 5.19)
SUZUK2	508	c	0	9	22	11	53	1.97	(0.72- 5.42)
SUZUK2	509	c	0	10	8	11	53	6.02	(1.94- 18.72)
SUZUK2	510	c	0	15	10	11	53	7.23	(2.58- 20.25)
Subtotal SUZUK2								4.28	(2.33- 7.87)
SVENSS	551	f	0	14	24	38	120	1.84	(0.87- 3.91)
SVENSS	552	f	0	16	13	38	120	3.89	(1.72- 8.80)
Subtotal SVENSS								2.60	(1.49- 4.52)
*TVERDA	501	m	2	4	-	4	-	1.34	(0.34- 5.37)
*TVERDA	502	m	2	5	-	4	-	2.83	(0.76- 10.53)
*TVERDA	503	m	2	2	-	4	-	2.77	(0.51- 15.15)
Subtotal TVERDA								2.15	(0.94- 4.93)
WAKAI	522	m	0	7	47	10	65	0.97	(0.34- 2.73)
WAKAI	523	m	0	27	44	10	65	3.99	(1.76- 9.06)
WAKAI	524	m	0	19	48	10	65	2.57	(1.10- 6.03)
Subtotal WAKAI								2.40	(1.44- 4.02)
WANG2	510	c	0	5	11	11	43	1.78	(0.51- 6.19)
WANG2	511	c	0	6	10	11	43	2.35	(0.70- 7.86)
Subtotal WANG2								2.05	(0.86- 4.89)
WYNDE3	566	m	0	9	65	9	88	1.35	(0.51- 3.60)
WYNDE3	587	f	0	1	3	20	76	1.27	(0.12- 12.84)
Subtotal WYNDE3								1.34	(0.54- 3.30)
WYNDE6	501	m	0	21	161	64	918	1.87	(1.11- 3.15)
WYNDE6	502	m	0	55	212	64	918	3.72	(2.52- 5.50)
WYNDE6	503	m	0	159	373	64	918	6.11	(4.47- 8.37)
WYNDE6	504	m	0	98	194	64	918	7.25	(5.10- 10.29)
WYNDE6	505	m	0	201	166	64	918	17.37	(12.53- 24.07)
WYNDE6	522	f	0	10	31	125	991	2.56	(1.22- 5.34)
WYNDE6	523	f	0	16	77	125	991	1.65	(0.93- 2.91)
WYNDE6	524	f	0	36	132	125	991	2.16	(1.43- 3.27)
WYNDE6	525	f	0	51	84	125	991	4.81	(3.24- 7.14)
WYNDE6	526	f	0	82	70	125	991	9.29	(6.42- 13.43)
Subtotal WYNDE6								5.50	(4.84- 6.24)
Partial Totals				10190	776480	11166	5480166		

*prospective study ~ With 0.5 adjustment for zero

REF	NRR	SEX	AD	Ys	Ws	Qs	Ps
ALDERS	507	m	1	1.16	8.16	2.61	0.0009
ALDERS	508	m	1	1.46	7.77	0.57	0.0000
ALDERS	509	m	1	2.90	9.90	13.49	0.0000
ALDERS	518	f	1	0.24	14.21	31.53	0.3676
ALDERS	519	f	1	1.08	18.83	7.88	0.0000
ALDERS	520	f	1	2.25	27.43	7.34	0.0000
Subtotal ALDERS				1.56	86.30	63.42	
ARMADA	515	m	0	1.63	5.86	0.06	0.0001
ARMADA	516	m	0	2.75	5.75	5.95	0.0000
Subtotal ARMADA				2.18	11.61	6.01	
AUVINE	530	c	0	1.64	27.22	0.19	0.0000
BARBON	525	m	0	0.78	7.44	6.76	0.0343
BARBON	526	m	0	1.93	9.48	0.38	0.0000
BARBON	527	m	0	2.19	13.55	2.90	0.0000
BARBON	528	m	0	2.62	7.57	5.96	0.0000
Subtotal BARBON				1.93	38.05	15.99	
BECHER	501	m	0	1.39	2.34	0.27	0.0341
BECHER	502	m	0	2.20	2.24	0.49	0.0010
BECHER	503	m	0	2.71	1.87	1.79	0.0002
BECHER	511	f	0	-0.65	0.82	4.66	0.5537
BECHER	512	f	0	0.73	1.22	1.21	0.4184
BECHER	513	f	0	1.24	1.05	0.25	0.2027
Subtotal BECHER				1.56	9.54	8.67	
*BENSHL	508	m	2	0.00	2.98	8.90	1.0000

Table 1J6 - 5

IESLC - Meta-analysis of Ex Smoking by Years quit (vs never), Overview
All LC types, Cigarettes (or Any Product if Cigarettes not available)
Least adjusted

REF	NRR	SEX	AD	Ys	Ws	Qs	Ps
*BENSHL	509	m	2	0.95	6.64	4.01	0.0142
*BENSHL	510	m	2	1.41	7.88	0.82	0.0001
*BENSHL	511	m	2	2.16	6.37	1.19	0.0000
Subtotal BENSHL				1.31	23.87	14.92	
BROSS	515	m	0	0.89	14.68	10.33	0.0006
BROSS	516	m	0	2.42	18.85	9.10	0.0000
Subtotal BROSS				1.75	33.54	19.43	
BROWN3	501	f	0	0.77	78.00	72.43	0.0000
CARPEN	501	c	0	1.71	5.83	0.00	0.0000
CARPEN	502	c	0	1.52	3.84	0.16	0.0028
CARPEN	503	c	0	2.61	5.25	4.04	0.0000
CARPEN	504	c	0	2.73	5.03	5.04	0.0000
Subtotal CARPEN				2.17	19.94	9.24	
*CEDERL	528	m	1	0.10	2.08	5.54	0.8907
*CEDERL	529	m	1	1.81	4.45	0.03	0.0001
Subtotal CEDERL				1.26	6.53	5.57	
CHOI	533	m	0	0.43	2.56	4.32	0.4904
CHOI	534	m	0	0.24	2.63	5.82	0.6977
CHOI	535	m	0	0.20	3.12	7.31	0.7277
CHOI	536	m	0	1.05	6.99	3.23	0.0056
CHOI	550	f	0	2.38	0.41	0.17	0.1268
CHOI	551	f	0	1.17	1.17	0.36	0.2033
Subtotal CHOI				0.71	16.88	21.21	
*CHYOU	501	m	1	1.03	3.02	1.47	0.0738
*CHYOU	502	m	1	1.36	5.68	0.77	0.0012
Subtotal CHYOU				1.25	8.70	2.24	
*CPSI	807	m	1	0.25	12.13	26.63	0.3900
*CPSI	808	m	1	1.64	20.82	0.17	0.0000
*CPSI	809	m	1	2.09	27.01	3.54	0.0000
*CPSI	810	m	1	2.69	22.87	21.16	0.0000
Subtotal CPSI				1.87	82.82	51.49	
*CPSII	652	m	1	1.34	61.12	9.10	0.0000
*CPSII	653	m	1	2.15	54.21	9.76	0.0000
*CPSII	654	m	1	2.44	56.51	28.29	0.0000
*CPSII	655	m	1	2.92	55.67	79.49	0.0000
*CPSII	656	m	1	3.33	56.54	145.83	0.0000
*CPSII	657	m	1	3.66	44.07	163.93	0.0000
*CPSII	633	f	1	0.55	38.44	53.05	0.0006
*CPSII	634	f	1	1.35	24.16	3.45	0.0000
*CPSII	635	f	1	1.59	30.57	0.58	0.0000
*CPSII	636	f	1	2.36	42.39	16.69	0.0000
*CPSII	637	f	1	2.83	59.81	73.12	0.0000
Subtotal CPSII				2.33	523.48	583.29	
DAMBER	554	m	1	0.47	6.15	9.75	0.2437
DAMBER	555	m	1	1.70	8.10	0.00	0.0000
Subtotal DAMBER				1.17	14.25	9.75	
DARBY	501	m	0	3.14	2.90	5.82	0.0000
DARBY	502	m	0	4.01	2.89	15.05	0.0000
DARBY	510	f	0	0.98	11.33	6.32	0.0010
DARBY	511	f	0	2.82	14.12	16.89	0.0000
Subtotal DARBY				2.29	31.24	44.07	
DEAN3	501	m	0	0.95	5.44	3.33	0.0273
DEAN3	502	m	0	1.31	8.20	1.44	0.0002
DEAN3	503	m	0	1.69	6.34	0.01	0.0000
DEAN3	504	m	0	1.76	11.22	0.01	0.0000
DEAN3	542	f	0	-0.42	1.87	8.64	0.5669
DEAN3	543	f	0	-0.01	0.95	2.89	0.9899
DEAN3	544	f	0	0.31	3.52	7.08	0.5602
Subtotal DEAN3				1.24	37.54	23.39	
DESTEF	515	m	0	1.05	7.71	3.57	0.0036
DESTEF	516	m	0	1.80	8.53	0.04	0.0000
DESTEF	517	m	0	2.15	12.34	2.19	0.0000
Subtotal DESTEF				1.75	28.58	5.81	
DOLL	529	m	0	1.11	3.05	1.17	0.0527
DOLL	530	m	0	0.70	2.74	2.91	0.2471
DOLL	531	m	0	1.87	5.25	0.11	0.0000
DOLL	542	f	0	-0.30	0.65	2.68	0.8063
DOLL	543	f	0	0.79	3.13	2.73	0.1602
Subtotal DOLL				1.18	14.82	9.61	
*DOLL2	501	m	1	0.69	3.49	3.75	0.1951
*DOLL2	502	m	1	1.67	3.93	0.01	0.0009

Table 1J6 - 5

IESLC - Meta-analysis of Ex Smoking by Years quit (vs never), Overview
All LC types, Cigarettes (or Any Product if Cigarettes not available)
Least adjusted

REF	NRR	SEX	AD	Ys	Ws	Qs	Ps
*DOLL2	503	m	1	1.77	4.41	0.01	0.0002
*DOLL2	504	m	1	2.77	4.77	5.20	0.0000
Subtotal DOLL2				1.81	16.61	8.97	
DORGAN	501	m	0	1.73	10.48	0.00	0.0000
DORGAN	502	m	0	2.63	7.65	6.22	0.0000
DORGAN	503	m	0	2.52	8.29	5.22	0.0000
DORGAN	553	f	0	0.86	14.18	10.59	0.0011
DORGAN	554	f	0	1.85	12.73	0.18	0.0000
Subtotal DORGAN				1.78	53.33	22.21	
*DORN	657	m	0	0.85	9.76	7.50	0.0078
*DORN	658	m	0	1.47	8.11	0.56	0.0000
*DORN	659	m	0	2.07	14.04	1.63	0.0000
*DORN	660	m	0	2.58	14.42	10.40	0.0000
*DORN	680	m	0	0.84	20.08	15.83	0.0002
*DORN	681	m	0	1.62	18.24	0.22	0.0000
*DORN	682	m	0	1.78	22.35	0.07	0.0000
*DORN	683	m	0	2.07	10.91	1.24	0.0000
Subtotal DORN				1.66	117.90	37.43	
GAO	521	m	0	0.03	8.17	23.51	0.9260
GAO	522	m	0	1.06	10.02	4.44	0.0008
GAO	523	m	0	1.88	20.07	0.48	0.0000
GAO	541	f	0	0.46	7.25	11.61	0.2120
GAO	542	f	0	1.02	4.58	2.28	0.0285
GAO	543	f	0	1.74	7.04	0.00	0.0000
Subtotal GAO				1.21	57.13	42.32	
GAO2	509	m	0	0.32	3.85	7.63	0.5289
GAO2	510	m	0	1.21	2.87	0.77	0.0406
GAO2	511	m	0	1.34	4.70	0.70	0.0036
GAO2	512	m	0	1.25	5.53	1.28	0.0034
GAO2	513	m	0	1.64	6.04	0.05	0.0001
Subtotal GAO2				1.21	22.99	10.44	
GARCIA	515	c	0	0.99	3.78	2.04	0.0532
GARCIA	516	c	0	1.56	5.44	0.15	0.0003
GARCIA	517	c	0	2.48	5.30	3.00	0.0000
GARCIA	518	c	0	3.40	3.87	10.81	0.0000
Subtotal GARCIA				2.10	18.39	16.00	
GARSHI	515	m	0	1.17	26.27	8.16	0.0000
GARSHI	516	m	0	1.62	27.31	0.31	0.0000
Subtotal GARSHI				1.40	53.58	8.47	
GRAHAM	525	m	0	1.26	6.69	1.48	0.0011
GRAHAM	526	m	0	2.26	8.27	2.36	0.0000
GRAHAM	527	m	0	3.61	11.87	41.83	0.0000
Subtotal GRAHAM				2.61	26.83	45.67	
GURSEL	501	m	0	0.83	5.70	4.57	0.0468
*HAMMO2	501	m	1	1.24	4.04	0.97	0.0128
*HAMMO2	502	m	1	1.38	3.47	0.42	0.0101
*HAMMO2	503	m	1	2.40	4.65	2.08	0.0000
Subtotal HAMMO2				1.72	12.16	3.47	
*HIRAYA	507	m	1	0.32	5.36	10.60	0.4561
*HIRAYA	508	m	1	0.46	4.98	7.98	0.3005
*HIRAYA	509	m	1	0.71	10.22	10.64	0.0236
*HIRAYA	518	f	1	-0.03	0.32	0.98	0.9863
*HIRAYA	519	f	1	1.19	1.22	0.35	0.1885
*HIRAYA	520	f	1	1.31	2.66	0.46	0.0320
Subtotal HIRAYA				0.65	24.75	31.00	
JAHN	501	m	0	0.42	9.60	16.43	0.1925
JAHN	502	m	0	1.33	11.61	1.86	0.0000
JAHN	503	m	0	1.97	10.46	0.62	0.0000
JAHN	504	m	0	2.55	10.25	6.95	0.0000
JAHN	505	m	0	3.93	5.25	25.52	0.0000
JAHN	506	m	0	5.07	5.16	57.58	0.0000
JAHN	731	f	2	-1.20	1.46	12.60	0.1451
Subtotal JAHN				2.07	53.80	121.56	
JAIN	567	m	0	1.18	8.12	2.43	0.0008
JAIN	568	m	0	2.43	7.67	3.81	0.0000
JAIN	531	f	0	0.25	10.76	23.58	0.4154
JAIN	532	f	0	2.02	14.96	1.28	0.0000
Subtotal JAIN				1.47	41.51	31.10	
JEDRYC	611	m	0	0.86	21.78	16.42	0.0001
JEDRYC	612	m	0	1.60	17.29	0.31	0.0000
Subtotal JEDRYC				1.19	39.07	16.72	

Table 1J6 - 5

IESLC - Meta-analysis of Ex Smoking by Years quit (vs never), Overview
All LC types, Cigarettes (or Any Product if Cigarettes not available)
Least adjusted

REF	NRR	SEX	AD	Ys	Ws	Qs	Ps
JOLY	566	m	0	2.04	9.05	0.87	0.0000
JOLY	567	m	0	2.95	7.04	10.57	0.0000
JOLY	553	f	0	1.46	7.04	0.52	0.0001
JOLY	554	f	0	2.56	4.99	3.44	0.0000
Subtotal JOLY				2.21	28.12	15.39	
*KAISE2	646	m	1	0.66	3.68	4.18	0.2036
*KAISE2	647	m	1	1.14	4.61	1.58	0.0140
*KAISE2	648	m	1	2.11	6.08	0.89	0.0000
*KAISE2	566	f	1	1.84	2.41	0.03	0.0043
*KAISE2	567	f	1	1.47	2.61	0.17	0.0172
*KAISE2	568	f	1	2.07	3.75	0.45	0.0001
Subtotal KAISE2				1.58	23.15	7.29	
KHUDER	511	m	0	1.38	14.86	1.81	0.0000
KHUDER	512	m	0	1.85	14.26	0.21	0.0000
KHUDER	513	m	0	2.26	15.10	4.31	0.0000
Subtotal KHUDER				1.83	44.23	6.33	
LAUSSM	501	m	0	1.64	8.52	0.07	0.0000
LUBIN	585	m	0	0.62	5.06	6.20	0.1615
LUBIN	586	m	0	1.20	5.11	1.41	0.0065
LUBIN	587	m	0	2.69	4.74	4.34	0.0000
Subtotal LUBIN				1.48	14.91	11.95	
LUBIN2	1069	m	0	0.74	61.66	60.10	0.0000
LUBIN2	1070	m	0	1.26	57.14	12.43	0.0000
LUBIN2	1071	m	0	1.32	64.81	10.81	0.0000
LUBIN2	1072	m	0	1.68	92.66	0.22	0.0000
LUBIN2	1073	m	0	2.05	111.01	11.81	0.0000
LUBIN2	1074	m	0	2.43	128.94	63.88	0.0000
LUBIN2	1108	f	0	-0.35	3.29	14.17	0.5296
LUBIN2	1109	f	0	0.45	2.74	4.47	0.4543
LUBIN2	1110	f	0	0.42	2.08	3.59	0.5486
LUBIN2	1111	f	0	0.31	7.03	14.20	0.4151
LUBIN2	1112	f	0	0.98	16.09	9.13	0.0001
LUBIN2	1113	f	0	1.35	25.86	3.71	0.0000
Subtotal LUBIN2				1.66	573.32	208.52	
MATOS	581	m	0	0.98	6.81	3.78	0.0103
MATOS	582	m	0	2.05	5.42	0.56	0.0000
MATOS	583	m	0	2.50	5.58	3.31	0.0000
Subtotal MATOS				1.78	17.80	7.66	
PEZZO2	501	m	0	1.65	4.89	0.03	0.0003
PEZZO2	502	m	0	2.71	5.10	4.94	0.0000
Subtotal PEZZO2				2.19	9.99	4.97	
PEZZOT	501	m	0	1.70	3.14	0.00	0.0026
PEZZOT	502	m	0	2.79	3.42	3.84	0.0000
Subtotal PEZZOT				2.27	6.56	3.85	
SOBUE	717	m	0	0.61	7.92	9.85	0.0843
SOBUE	718	m	0	1.04	6.55	3.10	0.0077
SOBUE	719	m	0	1.21	8.60	2.29	0.0004
SOBUE	720	m	0	1.11	10.99	4.18	0.0002
SOBUE	721	m	0	1.15	14.66	4.88	0.0000
SOBUE	722	m	0	1.57	16.99	0.44	0.0000
Subtotal SOBUE				1.18	65.71	24.73	
*SPEIZE	501	f	0	0.56	18.89	25.84	0.0151
*SPEIZE	502	f	0	0.88	13.15	9.37	0.0013
*SPEIZE	503	f	0	1.75	24.03	0.01	0.0000
*SPEIZE	504	f	0	1.98	21.44	1.32	0.0000
*SPEIZE	505	f	0	1.76	16.98	0.02	0.0000
Subtotal SPEIZE				1.44	94.49	36.54	
SUZUK2	508	c	0	0.68	3.75	4.14	0.1886
SUZUK2	509	c	0	1.80	2.99	0.01	0.0019
SUZUK2	510	c	0	1.98	3.62	0.22	0.0002
Subtotal SUZUK2				1.45	10.36	4.38	
SVENSS	551	f	0	0.61	6.77	8.46	0.1120
SVENSS	552	f	0	1.36	5.74	0.79	0.0011
Subtotal SVENSS				0.95	12.51	9.25	
*TVERDA	501	m	2	0.29	2.02	4.16	0.6776
*TVERDA	502	m	2	1.04	2.22	1.05	0.1208
*TVERDA	503	m	2	1.02	1.34	0.67	0.2389
Subtotal TVERDA				0.76	5.58	5.89	
WAKAI	522	m	0	-0.03	3.58	11.10	0.9511
WAKAI	523	m	0	1.38	5.71	0.68	0.0009
WAKAI	524	m	0	0.95	5.30	3.25	0.0297

Table 1J6 - 5

IESLC - Meta-analysis of Ex Smoking by Years quit (vs never), Overview
All LC types, Cigarettes (or Any Product if Cigarettes not available)
Least adjusted

REF	NRR	SEX	AD	Ys	Ws	Qs	Ps
Subtotal WAKAI				0.88	14.58	15.03	
WANG2	510	c	0	0.57	2.47	3.29	0.3664
WANG2	511	c	0	0.85	2.63	2.02	0.1672
Subtotal WANG2				0.72	5.09	5.30	
WYNDE3	566	m	0	0.30	4.02	8.16	0.5438
WYNDE3	587	f	0	0.24	0.72	1.59	0.8415
Subtotal WYNDE3				0.29	4.73	9.76	
WYNDE6	501	m	0	0.63	14.18	17.22	0.0183
WYNDE6	502	m	0	1.31	25.24	4.34	0.0000
WYNDE6	503	m	0	1.81	38.93	0.26	0.0000
WYNDE6	504	m	0	1.98	31.18	1.98	0.0000
WYNDE6	505	m	0	2.85	36.08	45.74	0.0000
WYNDE6	522	f	0	0.94	7.08	4.41	0.0125
WYNDE6	523	f	0	0.50	11.83	17.89	0.0859
WYNDE6	524	f	0	0.77	22.54	20.67	0.0003
WYNDE6	525	f	0	1.57	24.68	0.61	0.0000
WYNDE6	526	f	0	2.23	28.18	7.04	0.0000
Subtotal WYNDE6				1.70	239.93	120.17	

N	208
NS	51

Table 1J6 - 6

IESLC - Meta-analysis of Ex Smoking by Years quit (vs never), Overview
All LC types, Cigarettes (or Any Product if Cigarettes not available)
Least adjusted

	Sex			
	combined	male	female	Total
N	14	138	56	208
NS	5	43	20	68

In this overview table, other than the "N" rows, entries in the "absent" and "Total" columns may be invalid and should be ignored

	Years quit vs never (lower focus)				
	absent	8+k12	4-11k7	1-6k3	Total
N	81	54	32	41	208
NS	38	39	24	31	132
Wt	1133.09	585.86	482.00	615.31	2816.26
Het Chi	1074.93	208.32	100.94	164.34	1863.74
Het df	80	53	31	40	207
Het P	***	***	***	***	***
Fixed RR	5.09	3.61	6.12	9.73	5.63
RRl	4.80	3.33	5.60	8.99	5.43
RRu	5.40	3.91	6.69	10.53	5.85
P	+++	+++	+++	+++	+++
Random RR	4.59	2.88	5.11	8.55	4.64
RRl	3.66	2.41	4.24	7.13	4.12
RRu	5.75	3.44	6.16	10.24	5.22
P	+++	+++	+++	+++	+++

	Years quit vs never (higher focus)				
	absent	13+k20	4-19k12	1-11k3	Total
N	104	24	22	58	208
NS	48	20	18	42	128
Wt	1257.17	423.32	390.08	745.68	2816.26
Het Chi	1099.93	56.23	75.57	228.89	1863.74
Het df	103	23	21	57	207
Het P	***	***	***	***	***
Fixed RR	5.57	2.75	4.96	9.20	5.63
RRl	5.27	2.50	4.49	8.57	5.43
RRu	5.89	3.03	5.48	9.89	5.85
P	+++	+++	+++	+++	+++
Random RR	3.87	2.71	4.34	8.24	4.64
RRl	3.19	2.28	3.51	7.03	4.12
RRu	4.71	3.22	5.36	9.67	5.22
P	+++	+++	+++	+++	+++

Table 1J6 - 6

IESLC - Meta-analysis of Ex Smoking by Years quit (vs never), Overview
All LC types, Cigarettes (or Any Product if Cigarettes not available)
Least adjusted

MALES

Years quit vs never (lower focus)

		absent	8+k12	4-11k7	1-6k3	Total
	N	54	35	22	27	138
	NS	31	34	21	26	112
	Wt	759.10	412.69	370.43	461.86	2004.08
Het	Chi	757.88	127.97	72.86	103.84	1268.95
Het	df	53	34	21	26	137
Het	P	***	***	***	***	***
Fixed	RR	5.51	4.23	6.66	10.62	6.28
	RRl	5.13	3.84	6.01	9.69	6.01
	RRu	5.91	4.66	7.37	11.63	6.57
	P	+++	+++	+++	+++	+++
Random	RR	5.07	3.34	5.37	9.42	5.18
	RRl	3.82	2.71	4.32	7.67	4.50
	RRu	6.73	4.12	6.69	11.56	5.97
	P	+++	+++	+++	+++	+++

Years quit vs never (higher focus)

		absent	13+k20	4-19k12	1-11k3	Total
	N	68	17	16	37	138
	NS	40	16	15	36	107
	Wt	908.89	262.15	314.06	518.97	2004.08
Het	Chi	840.39	22.93	30.99	125.84	1268.95
Het	df	67	16	15	36	137
Het	P	***	N.S.	**	***	***
Fixed	RR	5.91	3.28	5.62	10.38	6.28
	RRl	5.54	2.91	5.03	9.53	6.01
	RRu	6.31	3.70	6.28	11.32	6.57
	P	+++	+++	+++	+++	+++
Random	RR	4.45	3.12	5.13	9.37	5.18
	RRl	3.49	2.64	4.26	7.82	4.50
	RRu	5.67	3.68	6.18	11.23	5.97
	P	+++	+++	+++	+++	+++

FEMALES

Years quit vs never (lower focus)

		absent	8+k12	4-11k7	1-6k3	Total
	N	22	16	8	10	56
	NS	15	16	8	10	49
	Wt	351.16	138.36	103.34	138.31	731.17
Het	Chi	294.10	24.93	10.26	32.38	466.91
Het	df	21	15	7	9	55
Het	P	***	(*)	N.S.	***	***
Fixed	RR	4.30	2.11	4.36	7.16	4.15
	RRl	3.87	1.79	3.59	6.06	3.86
	RRu	4.77	2.49	5.29	8.46	4.46
	P	+++	+++	+++	+++	+++
Random	RR	3.57	1.97	4.13	6.00	3.24
	RRl	2.32	1.55	3.17	4.11	2.56
	RRu	5.49	2.51	5.38	8.75	4.10
	P	+++	+++	+++	+++	+++

Table 1J6 - 6

IESLC - Meta-analysis of Ex Smoking by Years quit (vs never), Overview
All LC types, Cigarettes (or Any Product if Cigarettes not available)
Least adjusted

FEMALES

		Years quit vs never (higher focus)				
		absent	13+k20	4-19k12	1-11k3	Total
	N	30	5	4	17	56
	NS	19	5	4	17	45
	Wt	302.82	149.90	66.88	211.57	731.17
Het	Chi	235.37	1.91	7.56	63.50	466.91
Het	df	29	4	3	16	55
Het	P	***	N.S.	(*)	***	***
Fixed	RR	4.77	1.93	2.60	6.76	4.15
	RRl	4.26	1.65	2.04	5.91	3.86
	RRu	5.33	2.27	3.30	7.73	4.46
	P	+++	+++	+++	+++	+++
Random	RR	2.65	1.93	2.44	5.89	3.24
	RRl	1.83	1.65	1.64	4.36	2.56
	RRu	3.84	2.27	3.63	7.96	4.10
	P	+++	+++	+++	+++	+++

Table 1J6 - 7

IESLC - Meta-analysis of Ex Smoking by Years quit (vs never), Overview
All LC types, Cigarettes (or Any Product if Cigarettes not available)
Excluded studies (and stage at which they were excluded)

1	AGUDO	AKIBA	AMANDU	AMES	AXELSS	BEST	BOUCHA	BOUCOT	BRESLO	CHEN	CHEN2	CHIAZZ	DEAN2	DOSEME	ENGELA	FAN
	GENG	GER	GUO	HAENSZ	HEGMAN	HOLE	HU	HU2	JUSSAW	KATSOU	KAUFMA	KOO	KOULUM	KREUZE	LETOUR	LEVIN
	LIAW	LIU3	LIU4	LIU5	MCCONN	MIGRAN	MRFITR	NOTAN2	OSANN2	PERNU	QIAO2	RACHTA	RESTRE	SADOWS	SEGI2	STASZE
	TIZZAN	VUTUC	WATSON	WU	WUWILL	WYNDE2	WYNDE8	XU	YUAN	ZHANG	ZHENG	ZHOU				
2	BUFFLE	HUMBLE	PISANI	PRESCO	WYNDE7											
3	MCDUFF	SPITZ														
4	HAMMON	LUO	WU2													
5	BLOT1	CORREA	GILLIS	QIAO	WIGLE											
7	BOFFET															
14	BENHAM															

Table 1J6 - 8

Potentially overlapping studies

REF	REFGP	PRINC	. OVERLAP/LINK
LUBIN2	LUBIN2	1	Lubin-combined
TVERDA	TVERDA	1	VEIERO/TVERDAL
BROSS	BYERS1	1	GRAHAM/BROSS/BYERS1
GRAHAM	BYERS1	1	GRAHAM/BROSS/BYERS1
CHYOU	CHYOU	1	GOODMA/CHYOU
BENSHL	TANG2	1	Subset of TANG2
WYNDE6	WYNDE6	1	WYNDE5/6/7/8
CPSI	CPSI	1	CPSI overall
JAHN	BOFFET	2	Subset of BOFFET
LUBIN	XIANGZ	2	LUBIN/XIANGZ/QIAO

Table 1J6 - 9

Most adjusted - insufficient data for meta-analysis

REF	NRR	SEX	AGEL	AGEH	RACE	YF	LC TYPE	LOC	START	ST	NLC	R	VB	P	H	AD	PRODUCT	exL	exH	S1	S2	DENOM	De
ARMADA	524	m	0	0	all	-	all	Eu:wst	1986	CC	325	n	bl	n	y	0	cig+/-ot	0.1	0.9	0	0	nev cigs	ot
AUVINE	533	c	0	0	all	-	all	Eu:Sca	1986	CC	517	n	bl	y	n	2	cig+/-ot	1.0	11	0	3	nev cigs	ot
BECHER	526	m	0	0	all	-	all	Eu:Ger	1985	CC	194	n	bl	n	y	0	all/unsp	1.0	1.0	0	0	nev any	ot
BECHER	528	f	0	0	all	-	all	Eu:Ger	1985	CC	194	n	bl	n	y	0	all/unsp	1.0	1.0	0	0	nev any	ot
BLOT1	501	m	0	0	all	-	all	NAmer	1970	CC	458	n	bl	y	n	0	cig+/-ot	10	999	1	0	nev cigs	or
BLOT1	502	m	0	0	all	-	all	NAmer	1970	CC	458	n	bl	y	n	0	cig+/-ot	1.0	9	0	3	nev cigs	or
BROWN3	504	f	0	0	wh	-	all	NAmer		CC	618		bl	y	n	2	all/unsp	1.0	14	0	0	nev any	ot
CORREA	538	c	0	0	all	-	all	NAmer	1979	CC	1359	n	bl	y	n	2	cig+/-ot	21	999	0	0	nev cigs	or
CORREA	539	c	0	0	all	-	all	NAmer	1979	CC	1359	n	bl	y	n	2	cig+/-ot	6	20	0	0	nev cigs	or
CORREA	540	c	0	0	all	-	all	NAmer	1979	CC	1359	n	bl	y	n	2	cig+/-ot	3	5	3	3	nev cigs	or
CORREA	545	c	0	0	all	-	all	NAmer	1979	CC	1359	n	bl	y	n	2	cig+/-ot	0.1	3	0	0	nev cigs	or
CPSI	718	f	0	0	wh	0	all	NAmer	1959	pr	5138	n	bl	n	n	1	cig only	25	29	0	0	nev cigs	or
CPSI	719	f	0	0	wh	0	all	NAmer	1959	pr	5138	n	bl	n	n	1	cig only	20	24	0	1	nev cigs	or
CPSI	720	f	0	0	wh	0	all	NAmer	1959	pr	5138	n	bl	n	n	1	cig only	15	19	0	0	nev cigs	or
CPSI	721	f	0	0	wh	0	all	NAmer	1959	pr	5138	n	bl	n	n	1	cig only	10	14	1	2	nev cigs	or
CPSI	722	f	0	0	wh	0	all	NAmer	1959	pr	5138	n	bl	n	n	1	cig only	5	9	2	0	nev cigs	or
CPSI	723	f	0	0	wh	0	all	NAmer	1959	pr	5138	n	bl	n	n	1	cig only	2	4	3	3	nev cigs	or
CPSI	935	f	0	0	wh	0	all	NAmer	1959	pr	5138	n	bl	n	n	1	cig only	0.1	1.9	0	0	nev cigs	or
DEAN3	620	m	0	0	all	-	all	Eu:UK	1969	CC	766	n	V	y	n	1	cig only	1.0	2	0	0	nev any	ot
DEAN3	626	f	0	0	all	-	all	Eu:UK	1969	CC	766	n	V	y	n	1	all/unsp	1.0	2	0	0	nev any	ot
DORGAN	504	m	0	0	wh	-	all	NAmer	1980	CC	2026	n	bl	y	y	0	cig+/-ot	0.1	1.0	0	0	nev any	ot
DORGAN	555	f	0	0	all	-	all	NAmer	1980	CC	2026	n	bl	y	y	0	cig+/-ot	0.1	1.0	0	0	nev any	ot
GAO2	527	m	0	0	all	-	all	As:Jap	1988	CC	282	n	bl	n	n	0	cig+/-ot	0.1	0.9	0	0	nev cigs	ot
GARCIA	519	c	0	0	all	-	all	NAmer	1992	CC	416	n	bl	n	y	0	cig+/-ot	0.1	0.9	0	0	nev any	ot
GARSHI	524	m	0	0	all	-	all	NAmer	1981	CC	1081	o	bl	y	n	1	all/unsp	1.0	4	3	3	nev any	ot
GURSEL	502	m	0	0	all	-	all	Eu:bal		CC	953		bl	*	n	0	all/unsp	1.0	10	0	3	nev any	ot
JAHN	732	f	0	0	all	-	all	Eu:Ger	1988	CC	1004	n	bl	n	n	2	cig+/-ot	0.1	20	0	0	nev any	ot
JAIN	596	m	0	0	all	-	all	NAmer	1981	CC	845	n	V	y	n	0	cig+/-ot	0.1	1.9	0	0	nev cigs	ot
JAIN	584	f	0	0	all	-	all	NAmer	1981	CC	845	n	V	y	n	0	cig+/-ot	0.1	1.9	0	0	nev cigs	ot
JEDRYC	613	m	0	0	all	-	all	Eu:est	1980	CC	1630	n	bl	y	n	0	cig+/-ot	1.0	4	3	3	nev any	ot
JOLY	568	m	0	0	all	-	all	SCAmer	1978	CC	826	n	bl	n	n	0	cig+/-ot	0.1	0.9	0	0	nev any	ot
JOLY	555	f	0	0	all	-	all	SCAmer	1978	CC	826	n	bl	n	n	0	cig+/-ot	0.1	0.9	0	0	nev any	ot
KAISE2	649	m	0	0	all	9	all	NAmer	1979	pr	318	n	bl	n	n	1	cig only	0.1	1.9	0	0	nev any	ot
KAISE2	569	f	0	0	all	9	all	NAmer	1979	pr	318	n	bl	n	n	1	cig only	0.1	1.9	0	0	nev any	ot
LAUSSM	504	m	0	0	all	-	all	Eu:Ger	1982	CC	432	n	bl	n	n	2	all/unsp	1.0	9	0	3	nev any	ot
LUBIN	588	m	0	0	all	-	all	As:Chi	1984	CC	427	m	ot	y	n	0	cig+/-ot	1.0	2	0	0	nev any	ot

Table 1J6 - 9

IESLC - Meta-analysis of Ex Smoking by Years quit (vs never), Overview
All LC types, Cigarettes (or Any Product if Cigarettes not available)
Most adjusted - insufficient data for meta-analysis

REF	NRR	SEX	AGEL	AGEH	RACE	YF	LC TYPE	LOC	START	ST	NLC	R	VB	P	H	AD	PRODUCT	exL	exH	S1	S2	DENOM	De
MATOS	703	m	0	0	all	-	all	SCAmer	1994	CC	200	n	bl	n	n	2	cig+/-ot	0.1	0.9	0	0	nev any	ot
PEZZO2	512	m	0	0	all	-	all	SCAmer	1992	CC	367	n	bl	n	y	0	cig+/-ot	0.1	0.9	0	0	nev cigs	ot
PEZZOT	597	m	0	0	all	-	all	SCAmer	1987	CC	215	n	bl	n	y	0	cig only	0.1	0.9	0	0	nev cigs	ot
SOBUE	778	m	0	0	all	-	all	As:Jap	1986	CC	1376	n	bl	n	y	0	cig+/-ot	0.1	0.9	0	0	nev cigs	ot
SPEIZE	538	f	0	0	all	0	all	NAmer	1976	pr	593	n	bl	n	y	2	cig+/-ot	15	999	0	1	nev cigs	st
SPEIZE	539	f	0	0	all	0	all	NAmer	1976	pr	593	n	bl	n	y	2	cig+/-ot	10	15	1	2	nev cigs	st
SPEIZE	540	f	0	0	all	0	all	NAmer	1976	pr	593	n	bl	n	y	2	cig+/-ot	5	10	2	0	nev cigs	st
SPEIZE	541	f	0	0	all	0	all	NAmer	1976	pr	593	n	bl	n	y	2	cig+/-ot	2	5	3	3	nev cigs	st
SPEIZE	542	f	0	0	all	0	all	NAmer	1976	pr	593	n	bl	n	y	2	cig+/-ot	0.1	1.9	0	0	nev cigs	st
SVENSS	591	f	0	0	all	-	all	Eu:Sca	1983	CC	210	n	bl	n	n	0	all/unsp	1.0	2	0	0	nev any	ot
WAKAI	611	m	0	0	all	-	all	As:Jap	1988	CC	333	n	bl	n	y	2	cig+/-ot	1.0	4	3	3	nev any	ot
WYNDE3	567	m	0	0	all	-	all	NAmer	1966	CC	350	n	bl	n	y	0	cig+/-ot	1.0	9	0	3	nev any	ot
WYNDE3	588	f	0	0	all	-	all	NAmer	1966	CC	350	n	bl	n	y	0	cig+/-ot	1.0	9	0	3	nev any	ot
WYNDE6	506	m	0	0	all	-	all	NAmer	1969	CC	4423	n	bl	n	y	0	cig only	0.1	0.9	0	0	nev any	ot
WYNDE6	527	f	0	0	all	-	all	NAmer	1969	CC	4423	n	bl	n	y	0	cig only	0.1	0.9	0	0	nev any	ot

REF	NRR	RR	SIG	RRDATA comment
ARMADA	524	*	gap	0
AUVINE	533	*	gap	0
BECHER	526	*	gap	0
BECHER	528	*	gap	0
BLOT1	501	1.80		0
BLOT1	502	*	gap	0
BROWN3	504	*	gap	0
CORREA	538	3.90		0
CORREA	539	7.00		0
CORREA	540	7.70		0
CORREA	545	*	gap	0
CPSI	718	2.61		0
CPSI	719	2.52		0
CPSI	720	3.19		0
CPSI	721	0.58		0
CPSI	722	1.51		0
CPSI	723	2.85		0
CPSI	935	*	gap	0
DEAN3	620	*	gap	0
DEAN3	626	*	gap	0
DORGAN	504	*	gap	0
DORGAN	555	*	gap	0
GAO2	527	*	gap	0
GARCIA	519	*	gap	0
GARSHI	524	*	gap	0
GURSEL	502	*	gap	0
JAHN	732	*	gap	0
JAIN	596	*	gap	0
JAIN	584	*	gap	0
JEDRYC	613	*	gap	0
JOLY	568	*	gap	0
JOLY	555	*	gap	0
KAISE2	649	*	gap	0
KAISE2	569	*	gap	0
LAUSSM	504	*	gap	0
LUBIN	588	*	gap	0
MATOS	703	*	gap	0
PEZZO2	512	*	gap	0
PEZZOT	597	*	gap	0
SOBUE	778	*	gap	0
SPEIZE	538	1.00		Insufficient decimals to calculate CIs
SPEIZE	539	2.00		Insufficient decimals to calculate CIs
SPEIZE	540	5.00		Insufficient decimals to calculate CIs
SPEIZE	541	6.00		Insufficient decimals to calculate CIs
SPEIZE	542	6.00		Insufficient decimals to calculate CIs
SVENSS	591	*	gap	0
WAKAI	611	*	gap	0
WYNDE3	567	*	gap	0
WYNDE3	588	*	gap	0
WYNDE6	506	*	gap	0
WYNDE6	527	*	gap	0

Table 1J6 - 9

IESLC - Meta-analysis of Ex Smoking by Years quit (vs never), Overview
All LC types, Cigarettes (or Any Product if Cigarettes not available)

Least adjusted - insufficient data for meta-analysis: as for adjusted plus the following

REF	NRR	SEX	AGEL	AGEH	RACE	YF	LC TYPE	LOC	START	ST	NLC	R	VB	P	H	AD	PRODUCT	exL	exH	S1	S2	DENOM	De
AUVINE	531	c	0	0	all	-	all	Eu:Sca	1986	CC	517	n	bl	y	n	0	cig+/-ot	1.0	11	0	3	nev cigs	ot
BROWN3	502	f	0	0	wh	-	all	NAmer		CC	618		bl	y	n	0	all/unsp	1.0	14	0	0	nev any	ot
DEAN3	618	m	0	0	all	-	all	Eu:UK	1969	CC	766	n	V	y	n	0	cig only	1.0	2	0	0	nev any	ot
DEAN3	624	f	0	0	all	-	all	Eu:UK	1969	CC	766	n	V	y	n	0	all/unsp	1.0	2	0	0	nev any	ot
GARSHI	517	m	0	0	all	-	all	NAmer	1981	CC	1081	o	bl	y	n	0	all/unsp	1.0	4	3	3	nev any	ot
LAUSSM	502	m	0	0	all	-	all	Eu:Ger	1982	CC	432	n	bl	n	n	0	all/unsp	1.0	9	0	3	nev any	ot
MATOS	701	m	0	0	all	-	all	SCAmer	1994	CC	200	n	bl	n	n	0	cig+/-ot	0.1	0.9	0	0	nev any	ot
WAKAI	609	m	0	0	all	-	all	As:Jap	1988	CC	333	n	bl	n	y	0	cig+/-ot	1.0	4	3	3	nev any	ot

REF	NRR	RR	SIG	RRDATA comment
AUVINE	531	*	gap	0
BROWN3	502	*	gap	0
DEAN3	618	*	gap	0
DEAN3	624	*	gap	0
GARSHI	517	*	gap	0
LAUSSM	502	*	gap	0
MATOS	701	*	gap	0
WAKAI	609	*	gap	0

Table 1J7 -

IESLC - Meta-analysis of Ex Smoking, Years quit (vs never), "Low"
All LC types, Cigarettes (or Any Product if Cigarettes not available)

This analysis is restricted to results for:
1) Ex smokers
2) Results by Years quit (vs never)
3) Categorical results by Years quit (vs never)
4) All LC types (or near equivalent)
5) Results complete enough for use in metaanalysis

Within each study, results are then selected (in the following order of preference, within each sex) for:
6) (not applicable)
7) PRODUCT: cigarettes regardless of other products, cigarettes only, all/unspec
8) CIGTYPE: all/unspecified, MC regardless of HR, MC only
9) (not applicable)
10) DENOM: never smoked anything, never smoked cigarettes, never any + low, never cigs + low
11) Followup period (YF, prospective studies): whole study (coded as 0) or longest available
12) LCtype: all or nearest available, at least Squamous and Adeno. (q = squamous, s = small,
l = large, a = adeno, mix = mixed, alv = alveolar)
13) Race: all or nearest available, otherwise by race (wh or w = white, bl or b = black, hi = hispanic
ch = chinese, jap = japanese, haw = hawaiian, w+o = white + oriental, sca = scandinavian, as = asian)
14) Years quit (vs never) "low" in key scheme 1 (key value 12, maximum range 8+)
15) For overlapping studies: principal rather than subsidiary studies
Finally by Age: whole study (coded as 0) if available, otherwise by widest available age group
and then for single sex results (m, f) in preference to results for both sexes combined (c).

Results adjusted (AD) for the most potential confounders are then chosen in Sections -1 to -3
(and those which actually differ from the adjusted results in Table 1J2 - 1 are marked 'x' in Section -1)
and results adjusted for the least confounders in Sections -4 to -6. (Those least adjusted results which
actually differ from the most adjusted are marked 'x' in column X in Section -4)

Section -7 shows excluded studies, together with the stage (as above) at which no qualifying
results were found.

Section -8 lists the potentially overlapping studies which have been included (1=principal, 2=subsidiary).

Section -9 lists any results which would have been included in preference except that they had data not complete
enough for use in meta-analysis, with their significance (yes/no), if known, and any further comment as entered
on the database. It also lists as "gap" any categories for which no data were presented by the original authors.
This is commonly due to recent quitters having been combined with current smokers

In addition to those mentioned above, the following fields, levels and abbreviations are used:
* or nk = not known, n = no, y = yes, ot = other
nev = never
all/unspec = all or unspecified, cig+/-ot = cigarettes irrespective of other products (cigar, pipe etc)
MC = manufactured cigarettes, HR = hand-rolled cigarettes
exL, exH = range of exposure (low and high) in the smoking group, in terms of Years quit (vs never)
REF: 6-character study reference
NRR: number of the RR on the database within the study
ST : study type (CC = case control, pr or prosp = prospective)
NLC: number of lung cancer cases in whole study
R : risky occupational population (n = no, m = mining, o = other risky)
VB : national cigarette type (V = at least 75% Virginia, bl = at least 75% blended, ot = other)
P : any proxy use
H : full histological confirmation
De : derivation of RR/CI (or = original, st = standard method, ot = other method of estimation)

Table 1J7 - 1

IESLC - Meta-analysis of Ex Smoking, Years quit (vs never), "Low"
All LC types, Cigarettes (or Any Product if Cigarettes not available)
Most adjusted

REF	NRR	1J2	SEX	AGEL	AGEH	RACE	YF	LC TYPE	LOC	START	ST	NLC	R	VB	P	H	AD	PRODUCT	exL	exH	DENOM	De
ALDERS	507		m	0	0	all	-	all	Eu:UK	1977	CC	1448	n	V	n	n	1	cig only	10	999	nev any	ot
ALDERS	518		f	0	0	all	-	all	Eu:UK	1977	CC	1448	n	V	n	n	1	cig only	10	999	nev any	ot
AUVINE	532		c	0	0	all	-	all	Eu:Sca	1986	CC	517	n	bl	y	n	2	cig+/-ot	12	999	nev cigs	or
BECHER	501		m	0	0	all	-	all	Eu:Ger	1985	CC	194	n	bl	n	y	0	all/unsp	10	999	nev any	st
BECHER	511		f	0	0	all	-	all	Eu:Ger	1985	CC	194	n	bl	n	y	0	all/unsp	10	999	nev any	st
BENSHL	510		m	0	0	all	0	all	Eu:UK	1967	pr	486	n	V	n	n	2	cig+/-ot	10	19	nev any	or
CARPEN	502		c	0	0	w+b	-	all	NAmer	1991	CC	356	n	bl	n	n	0	cig+/-ot	10	14	nev cigs	st
CEDERL	528		m	40	69	all	10	all	Eu:Sca	1963	pr	491	n	bl	n	n	1	all/unsp	10	999	nev any	ot
CHOI	534		m	0	0	all	-	all	As:oth	1985	CC	375	n	bl	n	n	0	cig+/-ot	10	14	nev cigs	st
CPSI	807		m	50	74	all	6	all	NAmer	1959	pr	5138	n	bl	n	n	1	cig only	10	999	nev any	ot
CPSII	653		m	35	99	all	4	all	NAmer	1982	pr	3229	n	bl	n	n	1	cig only	11	15	nev any	ot
CPSII	634		f	0	0	all	4	all	NAmer	1982	pr	3229	n	bl	n	n	1	cig+/-ot	11	15	nev cigs	ot
DAMBER	554	x	m	0	0	all	-	all	Eu:Sca	1972	CC	579	n	bl	y	n	1	cig only	11	999	nev any	ot
DARBY	501		m	0	0	wh	-	all	Eu:UK	1988	CC	982	n	V	n	n	0	all/unsp	10	999	nev any	st
DARBY	510		f	0	0	wh	-	all	Eu:UK	1988	CC	982	n	V	n	n	0	all/unsp	10	999	nev any	st
DEAN3	517	x	m	0	0	all	-	all	Eu:UK	1969	CC	766	n	V	y	n	1	cig only	9	18	nev any	ot
DEAN3	553		f	0	0	all	-	all	Eu:UK	1969	CC	766	n	V	y	n	1	all/unsp	9	999	nev any	ot
DESTEF	525		m	0	0	all	-	all	SCAmer	1988	CC	497	n	bl	n	y	4	all/unsp	10	999	nev any	or
DOLL	530		m	0	0	all	-	all	Eu:UK	1948	CC	1465	n	V	n	n	0	all/unsp	10	19	nev any	st
DOLL	542		f	0	0	all	-	all	Eu:UK	1948	CC	1465	n	V	n	n	0	all/unsp	10	999	nev any	st
DOLL2	502		m	0	0	all	20	all	Eu:UK	1951	pr	920	n	V	n	n	1	cig only	10	14	nev any	ot
DORGAN	501		m	0	0	wh	-	all	NAmer	1980	CC	2026	n	bl	y	y	0	cig+/-ot	10	999	nev any	st
DORGAN	553		f	0	0	all	-	all	NAmer	1980	CC	2026	n	bl	y	y	0	cig+/-ot	10	999	nev any	st
DORN	658		m	55	64	wh	8	all	NAmer	1954	pr	5097	n	bl	n	n	0	cig+/-ot	10	14	nev any	st
DORN	681		m	65	74	wh	8	all	NAmer	1954	pr	5097	n	bl	n	n	0	cig+/-ot	10	14	nev any	st
GAO	531		m	0	0	all	-	all	As:Chi	1984	CC	1405	n	ot	n	n	2	cig+/-ot	10	999	nev cigs	or
GAO	551		f	0	0	all	-	all	As:Chi	1984	CC	1405	n	ot	n	n	2	cig+/-ot	10	999	nev cigs	or
GAO2	511		m	0	0	all	-	all	As:Jap	1988	CC	282	n	bl	n	n	0	cig+/-ot	10	14	nev cigs	or
GRAHAM	501		m	0	0	wh	-	all	NAmer	1956	CC	685	n	bl	n	n	0	cig only	10	999	nev any	st
GURSEL	501		m	0	0	all	-	all	Eu:bal		CC	953		bl	*	n	0	all/unsp	11	999	nev any	or
HAMMO2	501		m	0	0	all	0	all	NAmer	1967	pr	450	o	bl	n	n	1	cig+/-ot	10	999	nev any	ot
HIRAYA	507		m	0	0	all	0	all	As:Jap	1965	pr	1917	n	bl	n	n	1	cig+/-ot	10	999	nev any	st
HIRAYA	518		f	0	0	all	0	all	As:Jap	1965	pr	1917	n	bl	n	n	1	cig+/-ot	10	999	nev any	st
JAHN	502		m	0	0	all	-	all	Eu:Ger	1988	CC	1004	n	bl	n	n	0	cig+/-ot	11	20	nev any	st
JAIN	567		m	0	0	all	-	all	NAmer	1981	CC	845	n	V	y	n	0	cig+/-ot	10	999	nev cigs	st
JAIN	531		f	0	0	all	-	all	NAmer	1981	CC	845	n	V	y	n	0	cig+/-ot	10	999	nev cigs	st
JEDRYC	611		m	0	0	all	-	all	Eu:est	1980	CC	1630	n	bl	y	n	0	cig+/-ot	10	999	nev any	st
KAISE2	647		m	0	0	all	9	all	NAmer	1979	pr	318	n	bl	n	n	1	cig only	11	20	nev any	ot
KAISE2	567		f	0	0	all	9	all	NAmer	1979	pr	318	n	bl	n	n	1	cig only	11	20	nev any	st
LAUSSM	503		m	0	0	all	-	all	Eu:Ger	1982	CC	432	n	bl	n	n	2	all/unsp	10	999	nev any	st
LUBIN	585		m	0	0	all	-	all	As:Chi	1984	CC	427	m	ot	y	n	0	cig+/-ot	10	999	nev any	st
LUBIN2	1072		m	0	0	all	-	all	Eu:mul	1976	CC	7804	n	bl	n	y	0	cig+/-ot	10	14	nev any	st
LUBIN2	1111		f	0	0	all	-	all	Eu:mul	1976	CC	7804	n	bl	n	y	0	cig+/-ot	10	14	nev any	st
MATOS	591		m	0	0	all	-	all	SCAmer	1994	CC	200	n	bl	n	n	2	cig+/-ot	11	999	nev any	ot
PEZZO2	501		m	0	0	all	-	all	SCAmer	1992	CC	367	n	bl	n	y	0	cig+/-ot	11	999	nev cigs	st
PEZZOT	501		m	0	0	all	-	all	SCAmer	1987	CC	215	n	bl	n	y	0	cig only	11	999	nev cigs	st
SOBUE	720		m	0	0	all	-	all	As:Jap	1986	CC	1376	n	bl	n	y	0	cig+/-ot	10	14	nev cigs	st
SPEIZE	502		f	0	0	all	0	all	NAmer	1976	pr	593	n	bl	n	y	0	cig+/-ot	10	15	nev cigs	st
SUZUK2	508		c	0	0	all	-	all	SCAmer	1991	CC	123	n	bl	n	y	0	all/unsp	11	999	nev any	st
SVENSS	551		f	0	0	all	-	all	Eu:Sca	1983	CC	210	n	bl	n	n	0	all/unsp	11	999	nev any	st
WAKAI	531		m	0	0	all	-	all	As:Jap	1988	CC	333	n	bl	n	y	2	cig+/-ot	10	19	nev any	or
WYNDE3	566		m	0	0	all	-	all	NAmer	1966	CC	350	n	bl	n	y	0	cig+/-ot	10	999	nev any	st
WYNDE3	587		f	0	0	all	-	all	NAmer	1966	CC	350	n	bl	n	y	0	cig+/-ot	10	999	nev any	st
WYNDE6	503		m	0	0	all	-	all	NAmer	1969	CC	4423	n	bl	n	y	0	cig only	10	19	nev any	st
WYNDE6	524		f	0	0	all	-	all	NAmer	1969	CC	4423	n	bl	n	y	0	cig only	10	19	nev any	st

Cigarette type is all/unspec for all RRs
except for the following:

REF	NRR	CIGTYPE
ALDERS	507	MC only
ALDERS	518	MC only
DEAN3	517	MC only

Table 1J7 - 2

IESLC - Meta-analysis of Ex Smoking, Years quit (vs never), "Low"
All LC types, Cigarettes (or Any Product if Cigarettes not available)
Most adjusted

REF	NRR	SEX	AD	Number Exposed Case	Cont	Non-exposed Case	Cont	RR	95.00%CI
ALDERS	507	m	1	29	-	15	-	3.20	(1.61- 6.35)
ALDERS	518	f	1	26	-	75	-	1.27	(0.76- 2.15)
Subtotal ALDERS								1.78	(1.18- 2.69)
AUVINE	532	c	2	207	-	44	-	7.50	(4.18- 13.15)
BECHER	501	m	0	16	72	3	54	4.00	(1.11- 14.42)
BECHER	511	f	0	1	10	10	52	0.52	(0.06- 4.53)
Subtotal BECHER								2.35	(0.78- 7.09)
*BENSHL	510	m	2	23	-	10	-	4.08	(2.03- 8.20)
CARPEN	502	c	0	9	51	8	208	4.59	(1.69- 12.48)
*CEDERL	528	m	1	3	-	7	-	1.10	(0.28- 4.25)
CHOI	534	m	0	4	23	13	95	1.27	(0.38- 4.26)
*CPSI	807	m	1	15	-	60	-	1.28	(0.73- 2.25)
*CPSII	653	m	1	164	-	81	-	8.61	(6.60- 11.24)
*CPSII	634	f	1	28	-	174	-	3.86	(2.59- 5.75)
Subtotal CPSII								6.72	(5.39- 8.39)
DAMBER	554	m	1	-	-	42	-	1.60	(0.70- 3.40)
DARBY	501	m	0	139	767	3	384	23.20	(7.34- 73.28)
DARBY	510	f	0	26	224	23	529	2.67	(1.49- 4.78)
Subtotal DARBY								4.15	(2.47- 6.98)
DEAN3	517	m	1	15	-	24	-	2.99	(1.51- 5.93)
DEAN3	553	f	1	2	-	41	-	0.72	(0.17- 3.01)
Subtotal DEAN3								2.30	(1.24- 4.26)
DESTEF	525	m	4	17	-	27	-	2.80	(1.40- 5.70)
DOLL	530	m	0	6	26	7	61	2.01	(0.62- 6.56)
DOLL	542	f	0	1	2	40	59	0.74	(0.06- 8.41)
Subtotal DOLL								1.66	(0.57- 4.81)
*DOLL2	502	m	1	9	-	7	-	5.30	(1.97- 14.23)
DORGAN	501	m	0	134	255	13	140	5.66	(3.09- 10.37)
DORGAN	553	f	0	34	50	61	213	2.37	(1.41- 4.00)
Subtotal DORGAN								3.43	(2.31- 5.10)
*DORN	658	m	0	12	23682	25	213858	4.33	(2.18- 8.63)
*DORN	681	m	0	29	20056	49	171211	5.05	(3.19- 7.99)
Subtotal DORN								4.82	(3.29- 7.06)
GAO	531	m	2	13	-	62	-	1.10	(0.50- 2.20)
GAO	551	f	2	16	-	435	-	2.20	(1.00- 4.60)
Subtotal GAO								1.54	(0.90- 2.62)
GAO2	511	m	0	16	18	13	56	3.83	(1.55- 9.46)
GRAHAM	501	m	0	2	30	18	346	1.28	(0.28- 5.79)
GURSEL	501	m	0	-	-	-	-	2.30	(1.01- 5.22)
*HAMMO2	501	m	1	20	-	5	-	3.45	(1.30- 9.14)
*HIRAYA	507	m	1	-	-	-	-	1.38	(0.59- 3.21)
*HIRAYA	518	f	1	-	-	-	-	0.97	(0.03- 32.06)
Subtotal HIRAYA								1.35	(0.59- 3.08)
JAHN	502	m	0	64	130	18	138	3.77	(2.12- 6.71)
JAIN	567	m	0	52	113	12	85	3.26	(1.64- 6.48)
JAIN	531	f	0	19	61	52	214	1.28	(0.71- 2.33)
Subtotal JAIN								1.91	(1.22- 3.01)
JEDRYC	611	m	0	73	138	49	219	2.36	(1.55- 3.60)
*KAISE2	647	m	1	8	-	14	-	3.14	(1.26- 7.82)
*KAISE2	567	f	1	4	-	11	-	4.37	(1.30- 14.72)
Subtotal KAISE2								3.54	(1.71- 7.34)
LAUSSM	503	m	2	29	-	63	-	6.54	(3.47- 12.35)
LUBIN	585	m	0	17	73	9	72	1.86	(0.78- 4.45)
LUBIN2	1072	m	0	270	693	190	2616	5.36	(4.38- 6.58)
LUBIN2	1111	f	0	10	26	336	1188	1.36	(0.65- 2.85)
Subtotal LUBIN2								4.87	(4.00- 5.93)
MATOS	591	m	2	27	-	11	-	3.00	(1.43- 6.28)
PEZZO2	501	m	0	43	161	6	117	5.21	(2.15- 12.64)
PEZZOT	501	m	0	20	106	4	116	5.47	(1.81- 16.53)
SOBUE	720	m	0	35	50	29	126	3.04	(1.68- 5.49)
*SPEIZE	502	f	0	17	93933	58	776300	2.42	(1.41- 4.16)
SUZUK2	508	c	0	9	22	11	53	1.97	(0.72- 5.42)
SVENSS	551	f	0	14	24	38	120	1.84	(0.87- 3.91)
WAKAI	531	m	2	27	-	10	-	3.63	(1.56- 8.44)
WYNDE3	566	m	0	9	65	9	88	1.35	(0.51- 3.60)
WYNDE3	587	f	0	1	3	20	76	1.27	(0.12- 12.84)
Subtotal WYNDE3								1.34	(0.54- 3.30)
WYNDE6	503	m	0	159	373	64	918	6.11	(4.47- 8.37)
WYNDE6	524	f	0	36	132	125	991	2.16	(1.43- 3.27)
Subtotal WYNDE6								4.18	(3.25- 5.36)
Partial Totals				1959	141369	2534	1170703		

Table 1J7 - 2

IESLC - Meta-analysis of Ex Smoking, Years quit (vs never), "Low"
All LC types, Cigarettes (or Any Product if Cigarettes not available)
Most adjusted

REF	NRR	SEX	AD	Number Case	Exposed Cont	Non-exposed Case	Cont	RR	95.00%CI

*prospective study

REF	NRR	SEX	AD	Ys	Ws	Qs	Ps
ALDERS	507	m	1	1.16	8.16	0.13	0.0009
ALDERS	518	f	1	0.24	14.21	15.61	0.3676
Subtotal ALDERS				0.58	22.37	15.74	
AUVINE	532	c	2	2.01	11.70	6.19	0.0000
BECHER	501	m	0	1.39	2.34	0.02	0.0341
BECHER	511	f	0	-0.65	0.82	3.09	0.5537
Subtotal BECHER				0.86	3.16	3.11	
*BENSHL	510	m	2	1.41	7.88	0.11	0.0001
CARPEN	502	c	0	1.52	3.84	0.21	0.0028
*CEDERL	528	m	1	0.10	2.08	2.95	0.8907
CHOI	534	m	0	0.24	2.63	2.88	0.6977
*CPSI	807	m	1	0.25	12.13	13.13	0.3900
*CPSII	653	m	1	2.15	54.21	40.62	0.0000
*CPSII	634	f	1	1.35	24.16	0.10	0.0000
Subtotal CPSII				1.91	78.37	40.72	
DAMBER	554	m	1	0.47	6.15	4.11	0.2437
DARBY	501	m	0	3.14	2.90	10.01	0.0000
DARBY	510	f	0	0.98	11.33	1.06	0.0010
Subtotal DARBY				1.42	14.23	11.07	
DEAN3	517	m	1	1.10	8.21	0.30	0.0017
DEAN3	553	f	1	-0.33	1.86	4.86	0.6541
Subtotal DEAN3				0.83	10.07	5.16	
DESTEF	525	m	4	1.03	7.80	0.52	0.0040
DOLL	530	m	0	0.70	2.74	0.95	0.2471
DOLL	542	f	0	-0.30	0.65	1.64	0.8063
Subtotal DOLL				0.51	3.39	2.59	
*DOLL2	502	m	1	1.67	3.93	0.57	0.0009
DORGAN	501	m	0	1.73	10.48	2.08	0.0000
DORGAN	553	f	0	0.86	14.18	2.53	0.0011
Subtotal DORGAN				1.23	24.66	4.62	
*DORN	658	m	0	1.47	8.11	0.26	0.0000
*DORN	681	m	0	1.62	18.24	2.02	0.0000
Subtotal DORN				1.57	26.35	2.28	
GAO	531	m	2	0.10	7.00	9.94	0.8009
GAO	551	f	2	0.79	6.60	1.64	0.0428
Subtotal GAO				0.43	13.60	11.59	
GAO2	511	m	0	1.34	4.70	0.01	0.0036
GRAHAM	501	m	0	0.25	1.69	1.83	0.7471
GURSEL	501	m	0	0.83	5.70	1.18	0.0468
*HAMMO2	501	m	1	1.24	4.04	0.01	0.0128
*HIRAYA	507	m	1	0.32	5.36	4.99	0.4561
*HIRAYA	518	f	1	-0.03	0.32	0.55	0.9863
Subtotal HIRAYA				0.30	5.67	5.54	
JAHN	502	m	0	1.33	11.61	0.02	0.0000
JAIN	567	m	0	1.18	8.12	0.09	0.0008
JAIN	531	f	0	0.25	10.76	11.62	0.4154
Subtotal JAIN				0.65	18.88	11.71	
JEDRYC	611	m	0	0.86	21.78	3.97	0.0001
*KAISE2	647	m	1	1.14	4.61	0.09	0.0140
*KAISE2	567	f	1	1.47	2.61	0.09	0.0172
Subtotal KAISE2				1.26	7.22	0.19	
LAUSSM	503	m	2	1.88	9.53	3.33	0.0000
LUBIN	585	m	0	0.62	5.06	2.24	0.1615
LUBIN2	1072	m	0	1.68	92.66	14.28	0.0000
LUBIN2	1111	f	0	0.31	7.03	6.75	0.4151
Subtotal LUBIN2				1.58	99.69	21.02	
MATOS	591	m	2	1.10	7.02	0.25	0.0036
PEZZO2	501	m	0	1.65	4.89	0.64	0.0003
PEZZOT	501	m	0	1.70	3.14	0.53	0.0026
SOBUE	720	m	0	1.11	10.99	0.34	0.0002
*SPEIZE	502	f	0	0.88	13.15	2.13	0.0013
SUZUK2	508	c	0	0.68	3.75	1.39	0.1886
SVENSS	551	f	0	0.61	6.77	3.10	0.1120
WAKAI	531	m	2	1.29	5.39	0.00	0.0028
WYNDE3	566	m	0	0.30	4.02	3.89	0.5438
WYNDE3	587	f	0	0.24	0.72	0.79	0.8415
Subtotal WYNDE3				0.29	4.73	4.68	

Table 1J7 - 2

IESLC - Meta-analysis of Ex Smoking, Years quit (vs never), "Low"
All LC types, Cigarettes (or Any Product if Cigarettes not available)
Most adjusted

REF	NRR	SEX	AD	Ys	Ws	Qs	Ps
WYNDE6	503	m	0	1.81	38.93	10.67	0.0000
WYNDE6	524	f	0	0.77	22.54	6.01	0.0003
Subtotal WYNDE6				1.43	61.48	16.67	

	N	55
	NS	40
	Wt	571.19
Het	Chi	208.31
Het	df	54
Het	P	***
Fixed	RR	3.62
	RRl	3.34
	RRu	3.93
	P	+++
Random	RR	2.91
	RRl	2.43
	RRu	3.48
	P	+++
Asymm	P	***

Table 1J7 - 3

IESLC - Meta-analysis of Ex Smoking, Years quit (vs never), "Low"
All LC types, Cigarettes (or Any Product if Cigarettes not available)
Most adjusted

	Sex			
	combined	male	female	Total
N	3	36	16	55
NS	3	35	16	54
Wt	19.29	414.21	137.69	571.19
Het Chi	5.16	128.80	23.92	208.31
Het df	2	35	15	54
Het P	(*)	***	(*)	***
Fixed RR	5.24	4.24	2.15	3.62
RRl	3.36	3.85	1.82	3.34
RRu	8.19	4.67	2.54	3.93
P	+++	+++	+++	+++
Random RR	4.43	3.32	2.03	2.91
RRl	2.02	2.70	1.60	2.43
RRu	9.74	4.09	2.56	3.48
P	+++	+++	+++	+++
Between Chi				50.43
Between df				2
Between P				***
Btwn(F) P				***
Btwn(R) P				**

	Lung cancer type		
	all	other	Total
N	55		55
NS	40		40
Wt	571.19		571.19
Het Chi	208.31		208.31
Het df	54		54
Het P	***		***
Fixed RR	3.62		3.62
RRl	3.34		3.34
RRu	3.93		3.93
P	+++		+++
Random RR	2.91		2.91
RRl	2.43		2.43
RRu	3.48		3.48
P	+++		+++
Between Chi			
Between df			
Between P			N.S.
Btwn(F) P			N.S.
Btwn(R) P			N.S.

	Location								
	NAmer	UK	Scand	othEur	China	Japan	othAs	other	Total
N	19	10	4	8	3	5	1	5	55
NS	12	6	4	6	2	4	1	5	40
Wt	256.52	61.88	26.69	151.46	18.66	26.75	2.63	26.60	571.19
Het Chi	95.21	29.54	15.87	29.33	1.77	3.93	0.00	3.09	208.31
Het df	18	9	3	7	2	4	0	4	54
Het P	***	***	**	***	N.S.	N.S.	N.S.	N.S.	***
Fixed RR	4.03	2.67	3.17	4.20	1.62	2.76	1.27	3.29	3.62
RRl	3.57	2.08	2.17	3.58	1.03	1.89	0.38	2.25	3.34
RRu	4.56	3.43	4.63	4.92	2.55	4.04	4.26	4.81	3.93
P	+++	+++	+++	+++	+	+++	N.S.	+++	+++
Random RR	3.17	2.90	2.40	3.14	1.62	2.76	1.27	3.29	2.91
RRl	2.32	1.78	0.95	2.03	1.03	1.89	0.38	2.25	2.43
RRu	4.33	4.74	6.10	4.86	2.55	4.04	4.26	4.81	3.48
P	+++	+++	(+)	+++	+	+++	N.S.	+++	+++
Between Chi									29.57
Between df									7
Between P									***
Btwn(F) P									N.S.
Btwn(R) P									N.S.

Table 1J7 - 3

IESLC - Meta-analysis of Ex Smoking, Years quit (vs never), "Low"
All LC types, Cigarettes (or Any Product if Cigarettes not available)
Most adjusted

Detailed Country in "other Europe"

	multi	Germany	othWest	East	Balkans	Total
N	2	4		1	1	8
NS	1	3		1	1	6
Wt	99.69	24.30		21.78	5.70	151.46
Het Chi	12.30	5.53		0.00	0.00	29.33
Het df	1	3		0	0	7
Het P	***	N.S.		N.S.	N.S.	***
Fixed RR	4.87	4.40		2.36	2.30	4.20
RRl	4.00	2.96		1.55	1.01	3.58
RRu	5.93	6.55		3.60	5.23	4.92
P	+++	+++		+++	+	+++
Random RR	2.83	4.04		2.36	2.30	3.14
RRl	0.74	2.16		1.55	1.01	2.03
RRu	10.84	7.53		3.60	5.23	4.86
P	N.S.	+++		+++	+	+++
Between Chi						11.49
Between df						3
Between P						**
Btwn(F) P						N.S.
Btwn(R) P						N.S.

Detailed Country in "other Asia"

	India	HongKong	other	Total
N			1	1
NS			1	1
Wt			2.63	2.63
Het Chi			0.00	0.00
Het df			0	0
Het P			N.S.	N.S.
Fixed RR			1.27	1.27
RRl			0.38	0.38
RRu			4.26	4.26
P			N.S.	N.S.
Random RR			1.27	1.27
RRl			0.38	0.38
RRu			4.26	4.26
P			N.S.	N.S.
Between Chi				
Between df				
Between P				N.S.
Btwn(F) P				N.S.
Btwn(R) P				N.S.

Detailed other continent

	SCAmer	Total
N	5	5
NS	5	5
Wt	26.60	26.60
Het Chi	3.09	3.09
Het df	4	4
Het P	N.S.	N.S.
Fixed RR	3.29	3.29
RRl	2.25	2.25
RRu	4.81	4.81
P	+++	+++
Random RR	3.29	3.29
RRl	2.25	2.25
RRu	4.81	4.81
P	+++	+++
Between Chi		
Between df		
Between P		N.S.
Btwn(F) P		N.S.
Btwn(R) P		N.S.

Table 1J7 - 3

IESLC - Meta-analysis of Ex Smoking, Years quit (vs never), "Low"
All LC types, Cigarettes (or Any Product if Cigarettes not available)
Most adjusted

	Start year of study					
	<1960	1960-69	1970-79	1980-89	1990+	Total
N	7	11	8	24	4	54
NS	5	7	5	18	4	39
Wt	47.49	95.95	148.58	253.98	19.50	565.50
Het Chi	19.04	34.48	43.12	104.89	2.45	207.12
Het df	6	10	7	23	3	53
Het P	**	***	***	***	N.S.	***
Fixed RR	3.06	3.34	3.70	3.86	3.45	3.64
RRl	2.30	2.73	3.15	3.42	2.22	3.35
RRu	4.07	4.08	4.34	4.37	5.38	3.95
P	+++	+++	+++	+++	+++	+++
Random RR	2.70	2.34	2.50	3.27	3.45	2.92
RRl	1.50	1.48	1.50	2.47	2.22	2.43
RRu	4.86	3.71	4.17	4.35	5.38	3.50
P	+++	+++	+++	+++	+++	+++
Between Chi						3.14
Between df						4
Between P						N.S.
Btwn(F) P						N.S.
Btwn(R) P						N.S.

	Study type (1)		
	CC	other	Total
N	41	14	55
NS	30	10	40
Wt	410.38	160.81	571.19
Het Chi	138.18	61.22	208.31
Het df	40	13	54
Het P	***	***	***
Fixed RR	3.35	4.42	3.62
RRl	3.04	3.79	3.34
RRu	3.69	5.16	3.93
P	+++	+++	+++
Random RR	2.78	3.30	2.91
RRl	2.27	2.24	2.43
RRu	3.40	4.85	3.48
P	+++	+++	+++
Between Chi			8.90
Between df			1
Between P			**
Btwn(F) P			N.S.
Btwn(R) P			N.S.

	Study type (2)			
	CC	prosp	other	Total
N	41	14		55
NS	30	10		40
Wt	410.38	160.81		571.19
Het Chi	138.18	61.22		208.31
Het df	40	13		54
Het P	***	***		***
Fixed RR	3.35	4.42		3.62
RRl	3.04	3.79		3.34
RRu	3.69	5.16		3.93
P	+++	+++		+++
Random RR	2.78	3.30		2.91
RRl	2.27	2.24		2.43
RRu	3.40	4.85		3.48
P	+++	+++		+++
Between Chi				8.90
Between df				1
Between P				**
Btwn(F) P				N.S.
Btwn(R) P				N.S.

Table 1J7 - 3

IESLC - Meta-analysis of Ex Smoking, Years quit (vs never), "Low"
All LC types, Cigarettes (or Any Product if Cigarettes not available)
Most adjusted

	Study size (number of LC cases) 100-249	250-499	500-999	1000+	Total
N	6	15	12	22	55
NS	5	13	9	13	40
Wt	23.84	69.78	85.49	392.08	571.19
Het Chi	5.53	17.23	39.56	135.31	208.31
Het df	5	14	11	21	54
Het P	N.S.	N.S.	***	***	***
Fixed RR	2.56	3.32	2.89	3.95	3.62
RRl	1.72	2.62	2.34	3.58	3.34
RRu	3.83	4.19	3.57	4.36	3.93
P	+++	+++	+++	+++	+++
Random RR	2.57	3.23	2.90	2.88	2.91
RRl	1.67	2.48	1.90	2.17	2.43
RRu	3.95	4.22	4.42	3.80	3.48
P	+++	+++	+++	+++	+++
Between Chi					10.68
Between df					3
Between P					*
Btwn(F) P					N.S.
Btwn(R) P					N.S.

	Risky occupational population no	mining	othRisky	Total
N	52	1	1	54
NS	37	1	1	39
Wt	556.40	5.06	4.04	565.50
Het Chi	204.82	0.00	0.00	207.12
Het df	51	0	0	53
Het P	***	N.S.	N.S.	***
Fixed RR	3.66	1.86	3.45	3.64
RRl	3.37	0.78	1.30	3.35
RRu	3.98	4.45	9.15	3.95
P	+++	N.S.	+	+++
Random RR	2.93	1.86	3.45	2.92
RRl	2.43	0.78	1.30	2.43
RRu	3.53	4.45	9.15	3.50
P	+++	N.S.	+	+++
Between Chi				2.31
Between df				2
Between P				N.S.
Btwn(F) P				N.S.
Btwn(R) P				N.S.

	National cigarette tobacco type Virginia	blended	other	Total
N	12	40	3	55
NS	7	31	2	40
Wt	80.76	471.78	18.66	571.19
Het Chi	35.19	143.09	1.77	208.31
Het df	11	39	2	54
Het P	***	***	N.S.	***
Fixed RR	2.47	3.99	1.62	3.62
RRl	1.99	3.65	1.03	3.34
RRu	3.08	4.37	2.55	3.93
P	+++	+++	+	+++
Random RR	2.69	3.13	1.62	2.91
RRl	1.77	2.57	1.03	2.43
RRu	4.09	3.81	2.55	3.48
P	+++	+++	+	+++
Between Chi				28.26
Between df				2
Between P				***
Btwn(F) P				*
Btwn(R) P				*

Table 1J7 - 3

IESLC - Meta-analysis of Ex Smoking, Years quit (vs never), "Low"
All LC types, Cigarettes (or Any Product if Cigarettes not available)
Most adjusted

	Any proxy use No/nk	Yes	Total
N	45	10	55
NS	33	7	40
Wt	472.89	98.30	571.19
Het Chi	168.76	30.51	208.31
Het df	44	9	54
Het P	***	***	***
Fixed RR	3.84	2.75	3.62
RRl	3.51	2.26	3.34
RRu	4.20	3.35	3.93
P	+++	+++	+++
Random RR	2.99	2.62	2.91
RRl	2.44	1.79	2.43
RRu	3.66	3.83	3.48
P	+++	+++	+++
Between Chi			9.04
Between df			1
Between P			**
Btwn(F) P			N.S.
Btwn(R) P			N.S.

	Full histological confirmation No	Yes	Total
N	38	17	55
NS	28	12	40
Wt	328.37	242.82	571.19
Het Chi	151.12	53.59	208.31
Het df	37	16	54
Het P	***	***	***
Fixed RR	3.38	3.97	3.62
RRl	3.04	3.50	3.34
RRu	3.77	4.51	3.93
P	+++	+++	+++
Random RR	2.82	3.11	2.91
RRl	2.22	2.35	2.43
RRu	3.57	4.12	3.48
P	+++	+++	+++
Between Chi			3.60
Between df			1
Between P			(*)
Btwn(F) P			N.S.
Btwn(R) P			N.S.

	Number of adjustment variables (1) 0	1	2+/+nk	Total
N	32	15	8	55
NS	23	10	7	40
Wt	356.25	152.02	62.92	571.19
Het Chi	98.18	88.03	21.99	208.31
Het df	31	14	7	54
Het P	***	***	**	***
Fixed RR	3.59	3.70	3.63	3.62
RRl	3.23	3.16	2.84	3.34
RRu	3.98	4.34	4.65	3.93
P	+++	+++	+++	+++
Random RR	2.97	2.49	3.40	2.91
RRl	2.40	1.58	2.19	2.43
RRu	3.68	3.94	5.29	3.48
P	+++	+++	+++	+++
Between Chi				0.11
Between df				2
Between P				N.S.
Btwn(F) P				N.S.
Btwn(R) P				N.S.

Table 1J7 - 3

IESLC - Meta-analysis of Ex Smoking, Years quit (vs never), "Low"
All LC types, Cigarettes (or Any Product if Cigarettes not available)
Most adjusted

	Number of adjustment variables (2) 0	1	2	3-5	6+/+nk	Total
N	32	15	7	1		55
NS	23	10	6	1		40
Wt	356.25	152.02	55.12	7.80		571.19
Het Chi	98.18	88.03	21.39	0.00		208.31
Het df	31	14	6	0		54
Het P	***	***	**	N.S.		***
Fixed RR	3.59	3.70	3.77	2.80		3.62
RRl	3.23	3.16	2.89	1.39		3.34
RRu	3.98	4.34	4.90	5.65		3.93
P	+++	+++	+++	++		+++
Random RR	2.97	2.49	3.49	2.80		2.91
RRl	2.40	1.58	2.11	1.39		2.43
RRu	3.68	3.94	5.77	5.65		3.48
P	+++	+++	+++	++		+++
Between Chi						0.71
Between df						3
Between P						N.S.
Btwn(F) P						N.S.
Btwn(R) P						N.S.

	Product all/unsp	cig+/-ot	cig only	Total
N	13	29	13	55
NS	10	22	10	42
Wt	58.26	332.40	180.53	571.19
Het Chi	31.01	80.34	91.15	208.31
Het df	12	28	12	54
Het P	**	***	***	***
Fixed RR	2.88	3.54	4.08	3.62
RRl	2.23	3.18	3.53	3.34
RRu	3.72	3.94	4.72	3.93
P	+++	+++	+++	+++
Random RR	2.60	2.98	3.02	2.91
RRl	1.66	2.43	1.93	2.43
RRu	4.07	3.66	4.74	3.48
P	+++	+++	+++	+++
Between Chi				5.81
Between df				2
Between P				(*)
Btwn(F) P				N.S.
Btwn(R) P				N.S.

	Denominator nev any	nev cigs	Total
N	42	13	55
NS	30	11	41
Wt	459.53	111.66	571.19
Het Chi	171.16	32.98	208.31
Het df	41	12	54
Het P	***	***	***
Fixed RR	3.78	3.05	3.62
RRl	3.45	2.53	3.34
RRu	4.14	3.67	3.93
P	+++	+++	+++
Random RR	2.88	2.97	2.91
RRl	2.32	2.15	2.43
RRu	3.56	4.11	3.48
P	+++	+++	+++
Between Chi			4.17
Between df			1
Between P			*
Btwn(F) P			N.S.
Btwn(R) P			N.S.

Table 1J7 - 3

IESLC - Meta-analysis of Ex Smoking, Years quit (vs never), "Low"
All LC types, Cigarettes (or Any Product if Cigarettes not available)
Most adjusted

	Derivation of RR/CI			
	Orig	StdCalc	Other	Total
N	8	34	13	55
NS	7	24	10	41
Wt	56.76	363.67	150.76	571.19
Het Chi	18.85	105.98	82.39	208.31
Het df	7	33	12	54
Het P	**	***	***	***
Fixed RR	3.23	3.62	3.80	3.62
RRl	2.49	3.26	3.24	3.34
RRu	4.19	4.01	4.46	3.93
P	+++	+++	+++	+++
Random RR	3.06	3.00	2.60	2.91
RRl	1.99	2.42	1.63	2.43
RRu	4.71	3.71	4.14	3.48
P	+++	+++	+++	+++
Between Chi				1.09
Between df				2
Between P				N.S.
Btwn(F) P				N.S.
Btwn(R) P				N.S.

Table 1J7 - 4

IESLC - Meta-analysis of Ex Smoking, Years quit (vs never), "Low"
All LC types, Cigarettes (or Any Product if Cigarettes not available)
Least adjusted

REF	NRR	X	SEX	AGEL	AGEH	RACE	YF	LC TYPE	LOC	START	ST	NLC	R	VB	P	H	AD	PRODUCT	exL	exH	DENOM	De
ALDERS	507		m	0	0	all	-	all	Eu:UK	1977	CC	1448	n	V	n	n	1	cig only	10	999	nev any	ot
ALDERS	518		f	0	0	all	-	all	Eu:UK	1977	CC	1448	n	V	n	n	1	cig only	10	999	nev any	ot
AUVINE	530	x	c	0	0	all	-	all	Eu:Sca	1986	CC	517	n	bl	y	n	0	cig+/-ot	12	999	nev cigs	st
BECHER	501		m	0	0	all	-	all	Eu:Ger	1985	CC	194	n	bl	n	y	0	all/unsp	10	999	nev any	st
BECHER	511		f	0	0	all	-	all	Eu:Ger	1985	CC	194	n	bl	n	y	0	all/unsp	10	999	nev any	st
BENSHL	510		m	0	0	all	0	all	Eu:UK	1967	pr	486	n	V	n	n	2	cig+/-ot	10	19	nev any	or
CARPEN	502		c	0	0	w+b	-	all	NAmer	1991	CC	356	n	bl	n	n	0	cig+/-ot	10	14	nev cigs	st
CEDERL	528		m	40	69	all	10	all	Eu:Sca	1963	pr	491	n	bl	n	n	1	all/unsp	10	999	nev any	ot
CHOI	534		m	0	0	all	-	all	As:oth	1985	CC	375	n	bl	n	n	0	cig+/-ot	10	14	nev cigs	st
CPSI	807		m	50	74	all	6	all	NAmer	1959	pr	5138	n	bl	n	n	1	cig only	10	999	nev any	ot
CPSII	653		m	35	99	all	4	all	NAmer	1982	pr	3229	n	bl	n	n	1	cig only	11	15	nev any	ot
CPSII	634		f	0	0	all	4	all	NAmer	1982	pr	3229	n	bl	n	n	1	cig+/-ot	11	15	nev cigs	ot
DAMBER	554		m	0	0	all	-	all	Eu:Sca	1972	CC	579	n	bl	y	n	1	cig only	11	999	nev any	ot
DARBY	501		m	0	0	wh	-	all	Eu:UK	1988	CC	982	n	V	n	n	0	all/unsp	10	999	nev any	st
DARBY	510		f	0	0	wh	-	all	Eu:UK	1988	CC	982	n	V	n	n	0	all/unsp	10	999	nev any	st
DEAN3	502	x	m	0	0	all	-	all	Eu:UK	1969	CC	766	n	V	y	n	0	cig only	9	18	nev any	st
DEAN3	542	x	f	0	0	all	-	all	Eu:UK	1969	CC	766	n	V	y	n	0	all/unsp	9	999	nev any	st
DESTEF	515	x	m	0	0	all	-	all	SCAmer	1988	CC	497	n	bl	n	y	0	all/unsp	10	999	nev any	st
DOLL	530		m	0	0	all	-	all	Eu:UK	1948	CC	1465	n	V	n	n	0	all/unsp	10	19	nev any	st
DOLL	542		f	0	0	all	-	all	Eu:UK	1948	CC	1465	n	V	n	n	0	all/unsp	10	999	nev any	st
DOLL2	502		m	0	0	all	20	all	Eu:UK	1951	pr	920	n	V	n	n	1	cig only	10	14	nev any	ot
DORGAN	501		m	0	0	wh	-	all	NAmer	1980	CC	2026	n	bl	y	y	0	cig+/-ot	10	999	nev any	st
DORGAN	553		f	0	0	all	-	all	NAmer	1980	CC	2026	n	bl	y	y	0	cig+/-ot	10	999	nev any	st
DORN	658		m	55	64	wh	8	all	NAmer	1954	pr	5097	n	bl	n	n	0	cig+/-ot	10	14	nev any	st
DORN	681		m	65	74	wh	8	all	NAmer	1954	pr	5097	n	bl	n	n	0	cig+/-ot	10	14	nev any	st
GAO	521	x	m	0	0	all	-	all	As:Chi	1984	CC	1405	n	ot	n	n	0	cig+/-ot	10	999	nev cigs	st
GAO	541	x	f	0	0	all	-	all	As:Chi	1984	CC	1405	n	ot	n	n	0	cig+/-ot	10	999	nev cigs	st
GAO2	511		m	0	0	all	-	all	As:Jap	1988	CC	282	n	bl	n	n	0	cig+/-ot	10	14	nev cigs	or
GRAHAM	501		m	0	0	wh	-	all	NAmer	1956	CC	685	n	bl	n	n	0	cig only	10	999	nev any	st
GURSEL	501		m	0	0	all	-	all	Eu:bal		CC	953		bl	*	n	0	all/unsp	11	999	nev any	or
HAMMO2	501		m	0	0	all	0	all	NAmer	1967	pr	450	o	bl	n	n	1	cig+/-ot	10	999	nev any	ot
HIRAYA	507		m	0	0	all	0	all	As:Jap	1965	pr	1917	n	bl	n	n	1	cig+/-ot	10	999	nev any	st
HIRAYA	518		f	0	0	all	0	all	As:Jap	1965	pr	1917	n	bl	n	n	1	cig+/-ot	10	999	nev any	st
JAHN	502		m	0	0	all	-	all	Eu:Ger	1988	CC	1004	n	bl	n	n	0	cig+/-ot	11	20	nev any	st
JAIN	567		m	0	0	all	-	all	NAmer	1981	CC	845	n	V	y	n	0	cig+/-ot	10	999	nev cigs	st
JAIN	531		f	0	0	all	-	all	NAmer	1981	CC	845	n	V	y	n	0	cig+/-ot	10	999	nev cigs	st
JEDRYC	611		m	0	0	all	-	all	Eu:est	1980	CC	1630	n	bl	y	n	0	cig+/-ot	10	999	nev any	st
KAISE2	647		m	0	0	all	9	all	NAmer	1979	pr	318	n	bl	n	n	1	cig only	11	20	nev any	ot
KAISE2	567		f	0	0	all	9	all	NAmer	1979	pr	318	n	bl	n	n	1	cig only	11	20	nev any	st
LAUSSM	501	x	m	0	0	all	-	all	Eu:Ger	1982	CC	432	n	bl	n	n	0	all/unsp	10	999	nev any	st
LUBIN	585		m	0	0	all	-	all	As:Chi	1984	CC	427	m	ot	y	n	0	cig+/-ot	10	999	nev any	st
LUBIN2	1072		m	0	0	all	-	all	Eu:mul	1976	CC	7804	n	bl	n	y	0	cig+/-ot	10	14	nev any	st
LUBIN2	1111		f	0	0	all	-	all	Eu:mul	1976	CC	7804	n	bl	n	y	0	cig+/-ot	10	14	nev any	st
MATOS	581	x	m	0	0	all	-	all	SCAmer	1994	CC	200	n	bl	n	n	0	cig+/-ot	11	999	nev any	st
PEZZO2	501		m	0	0	all	-	all	SCAmer	1992	CC	367	n	bl	n	y	0	cig+/-ot	11	999	nev cigs	st
PEZZOT	501		m	0	0	all	-	all	SCAmer	1987	CC	215	n	bl	n	y	0	cig only	11	999	nev cigs	st
SOBUE	720		m	0	0	all	-	all	As:Jap	1986	CC	1376	n	bl	n	y	0	cig+/-ot	10	14	nev cigs	st
SPEIZE	502		f	0	0	all	0	all	NAmer	1976	pr	593	n	bl	n	y	0	cig+/-ot	10	15	nev cigs	st
SUZUK2	508		c	0	0	all	-	all	SCAmer	1991	CC	123	n	bl	n	y	0	all/unsp	11	999	nev any	st
SVENSS	551		f	0	0	all	-	all	Eu:Sca	1983	CC	210	n	bl	n	n	0	all/unsp	11	999	nev any	st
WAKAI	523	x	m	0	0	all	-	all	As:Jap	1988	CC	333	n	bl	n	y	0	cig+/-ot	10	19	nev any	st
WYNDE3	566		m	0	0	all	-	all	NAmer	1966	CC	350	n	bl	n	y	0	cig+/-ot	10	999	nev any	st
WYNDE3	587		f	0	0	all	-	all	NAmer	1966	CC	350	n	bl	n	y	0	cig+/-ot	10	999	nev any	st
WYNDE6	503		m	0	0	all	-	all	NAmer	1969	CC	4423	n	bl	n	y	0	cig only	10	19	nev any	st
WYNDE6	524		f	0	0	all	-	all	NAmer	1969	CC	4423	n	bl	n	y	0	cig only	10	19	nev any	st

Cigarette type is all/unspec for all RRs
except for the following:

REF	NRR	CIGTYPE
ALDERS	507	MC only
ALDERS	518	MC only
DEAN3	502	MC only

Table 1J7 - 5

IESLC - Meta-analysis of Ex Smoking, Years quit (vs never), "Low"
All LC types, Cigarettes (or Any Product if Cigarettes not available)
Least adjusted

REF	NRR	SEX	AD	Number Exposed Case	Cont	Non-exposed Case	Cont	RR	95.00%CI
ALDERS	507	m	1	29	-	15	-	3.20	(1.61- 6.35)
ALDERS	518	f	1	26	-	75	-	1.27	(0.76- 2.15)
Subtotal ALDERS								1.78	(1.18- 2.69)
AUVINE	530	c	0	207	208	44	229	5.18	(3.56- 7.54)
BECHER	501	m	0	16	72	3	54	4.00	(1.11- 14.42)
BECHER	511	f	0	1	10	10	52	0.52	(0.06- 4.53)
Subtotal BECHER								2.35	(0.78- 7.09)
*BENSHL	510	m	2	23	-	10	-	4.08	(2.03- 8.20)
CARPEN	502	c	0	9	51	8	208	4.59	(1.69- 12.48)
*CEDERL	528	m	1	3	-	7	-	1.10	(0.28- 4.25)
CHOI	534	m	0	4	23	13	95	1.27	(0.38- 4.26)
*CPSI	807	m	1	15	-	60	-	1.28	(0.73- 2.25)
*CPSII	653	m	1	164	-	81	-	8.61	(6.60- 11.24)
*CPSII	634	f	1	28	-	174	-	3.86	(2.59- 5.75)
Subtotal CPSII								6.72	(5.39- 8.39)
DAMBER	554	m	1	-	-	42	-	1.60	(0.70- 3.40)
DARBY	501	m	0	139	767	3	384	23.20	(7.34- 73.28)
DARBY	510	f	0	26	224	23	529	2.67	(1.49- 4.78)
Subtotal DARBY								4.15	(2.47- 6.98)
DEAN3	502	m	0	15	86	24	510	3.71	(1.87- 7.35)
DEAN3	542	f	0	2	114	41	1538	0.66	(0.16- 2.76)
Subtotal DEAN3								2.69	(1.45- 4.98)
DESTEF	515	m	0	17	36	27	163	2.85	(1.41- 5.78)
DOLL	530	m	0	6	26	7	61	2.01	(0.62- 6.56)
DOLL	542	f	0	1	2	40	59	0.74	(0.06- 8.41)
Subtotal DOLL								1.66	(0.57- 4.81)
*DOLL2	502	m	1	9	-	7	-	5.30	(1.97- 14.23)
DORGAN	501	m	0	134	255	13	140	5.66	(3.09- 10.37)
DORGAN	553	f	0	34	50	61	213	2.37	(1.41- 4.00)
Subtotal DORGAN								3.43	(2.31- 5.10)
*DORN	658	m	0	12	23682	25	213858	4.33	(2.18- 8.63)
*DORN	681	m	0	29	20056	49	171211	5.05	(3.19- 7.99)
Subtotal DORN								4.82	(3.29- 7.06)
GAO	521	m	0	13	41	62	202	1.03	(0.52- 2.05)
GAO	541	f	0	16	14	435	605	1.59	(0.77- 3.29)
Subtotal GAO								1.27	(0.77- 2.08)
GAO2	511	m	0	16	18	13	56	3.83	(1.55- 9.46)
GRAHAM	501	m	0	2	30	18	346	1.28	(0.28- 5.79)
GURSEL	501	m	0	-	-	-	-	2.30	(1.01- 5.22)
*HAMMO2	501	m	1	20	-	5	-	3.45	(1.30- 9.14)
*HIRAYA	507	m	1	-	-	-	-	1.38	(0.59- 3.21)
*HIRAYA	518	f	1	-	-	-	-	0.97	(0.03- 32.06)
Subtotal HIRAYA								1.35	(0.59- 3.08)
JAHN	502	m	0	64	130	18	138	3.77	(2.12- 6.71)
JAIN	567	m	0	52	113	12	85	3.26	(1.64- 6.48)
JAIN	531	f	0	19	61	52	214	1.28	(0.71- 2.33)
Subtotal JAIN								1.91	(1.22- 3.01)
JEDRYC	611	m	0	73	138	49	219	2.36	(1.55- 3.60)
*KAISE2	647	m	1	8	-	14	-	3.14	(1.26- 7.82)
*KAISE2	567	f	1	4	-	11	-	4.37	(1.30- 14.72)
Subtotal KAISE2								3.54	(1.71- 7.34)
LAUSSM	501	m	0	29	15	85	226	5.14	(2.63- 10.06)
LUBIN	585	m	0	17	73	9	72	1.86	(0.78- 4.45)
LUBIN2	1072	m	0	270	693	190	2616	5.36	(4.38- 6.58)
LUBIN2	1111	f	0	10	26	336	1188	1.36	(0.65- 2.85)
Subtotal LUBIN2								4.87	(4.00- 5.93)
MATOS	581	m	0	27	101	11	110	2.67	(1.26- 5.67)
PEZZO2	501	m	0	43	161	6	117	5.21	(2.15- 12.64)
PEZZOT	501	m	0	20	106	4	116	5.47	(1.81- 16.53)
SOBUE	720	m	0	35	50	29	126	3.04	(1.68- 5.49)
*SPEIZE	502	f	0	17	93933	58	776300	2.42	(1.41- 4.16)
SUZUK2	508	c	0	9	22	11	53	1.97	(0.72- 5.42)
SVENSS	551	f	0	14	24	38	120	1.84	(0.87- 3.91)
WAKAI	523	m	0	27	44	10	65	3.99	(1.76- 9.06)
WYNDE3	566	m	0	9	65	9	88	1.35	(0.51- 3.60)
WYNDE3	587	f	0	1	3	20	76	1.27	(0.12- 12.84)
Subtotal WYNDE3								1.34	(0.54- 3.30)
WYNDE6	503	m	0	159	373	64	918	6.11	(4.47- 8.37)
WYNDE6	524	f	0	36	132	125	991	2.16	(1.43- 3.27)
Subtotal WYNDE6								4.18	(3.25- 5.36)
Partial Totals				1959	142028	2556	1174351		

Table 1J7 - 5

IESLC - Meta-analysis of Ex Smoking, Years quit (vs never), "Low"
All LC types, Cigarettes (or Any Product if Cigarettes not available)
Least adjusted

REF	NRR	SEX	AD	Number Exposed Case	Cont	Non-exposed Case	Cont	RR	95.00%CI
*prospective study									

REF	NRR	SEX	AD	Ys	Ws	Qs	Ps
ALDERS	507	m	1	1.16	8.16	0.11	0.0009
ALDERS	518	f	1	0.24	14.21	15.39	0.3676
Subtotal ALDERS				0.58	22.37	15.50	
AUVINE	530	c	0	1.64	27.22	3.63	0.0000
BECHER	501	m	0	1.39	2.34	0.03	0.0341
BECHER	511	f	0	-0.65	0.82	3.07	0.5537
Subtotal BECHER				0.86	3.16	3.09	
*BENSHL	510	m	2	1.41	7.88	0.13	0.0001
CARPEN	502	c	0	1.52	3.84	0.23	0.0028
*CEDERL	528	m	1	0.10	2.08	2.91	0.8907
CHOI	534	m	0	0.24	2.63	2.84	0.6977
*CPSI	807	m	1	0.25	12.13	12.93	0.3900
*CPSII	653	m	1	2.15	54.21	41.34	0.0000
*CPSII	634	f	1	1.35	24.16	0.12	0.0000
Subtotal CPSII				1.91	78.37	41.47	
DAMBER	554	m	1	0.47	6.15	4.03	0.2437
DARBY	501	m	0	3.14	2.90	10.09	0.0000
DARBY	510	f	0	0.98	11.33	1.00	0.0010
Subtotal DARBY				1.42	14.23	11.10	
DEAN3	502	m	0	1.31	8.20	0.01	0.0002
DEAN3	542	f	0	-0.42	1.87	5.40	0.5669
Subtotal DEAN3				0.99	10.08	5.41	
DESTEF	515	m	0	1.05	7.71	0.41	0.0036
DOLL	530	m	0	0.70	2.74	0.93	0.2471
DOLL	542	f	0	-0.30	0.65	1.63	0.8063
Subtotal DOLL				0.51	3.39	2.55	
*DOLL2	502	m	1	1.67	3.93	0.59	0.0009
DORGAN	501	m	0	1.73	10.48	2.16	0.0000
DORGAN	553	f	0	0.86	14.18	2.44	0.0011
Subtotal DORGAN				1.23	24.66	4.60	
*DORN	658	m	0	1.47	8.11	0.28	0.0000
*DORN	681	m	0	1.62	18.24	2.11	0.0000
Subtotal DORN				1.57	26.35	2.39	
GAO	521	m	0	0.03	8.17	12.71	0.9260
GAO	541	f	0	0.46	7.25	4.83	0.2120
Subtotal GAO				0.24	15.42	17.54	
GAO2	511	m	0	1.34	4.70	0.02	0.0036
GRAHAM	501	m	0	0.25	1.69	1.80	0.7471
GURSEL	501	m	0	0.83	5.70	1.14	0.0468
*HAMMO2	501	m	1	1.24	4.04	0.01	0.0128
*HIRAYA	507	m	1	0.32	5.36	4.91	0.4561
*HIRAYA	518	f	1	-0.03	0.32	0.54	0.9863
Subtotal HIRAYA				0.30	5.67	5.45	
JAHN	502	m	0	1.33	11.61	0.03	0.0000
JAIN	567	m	0	1.18	8.12	0.08	0.0008
JAIN	531	f	0	0.25	10.76	11.45	0.4154
Subtotal JAIN				0.65	18.88	11.52	
JEDRYC	611	m	0	0.86	21.78	3.83	0.0001
*KAISE2	647	m	1	1.14	4.61	0.08	0.0140
*KAISE2	567	f	1	1.47	2.61	0.10	0.0172
Subtotal KAISE2				1.26	7.22	0.18	
LAUSSM	501	m	0	1.64	8.52	1.09	0.0000
LUBIN	585	m	0	0.62	5.06	2.19	0.1615
LUBIN2	1072	m	0	1.68	92.66	14.84	0.0000
LUBIN2	1111	f	0	0.31	7.03	6.64	0.4151
Subtotal LUBIN2				1.58	99.69	21.48	
MATOS	581	m	0	0.98	6.81	0.60	0.0103
PEZZO2	501	m	0	1.65	4.89	0.67	0.0003
PEZZOT	501	m	0	1.70	3.14	0.55	0.0026
SOBUE	720	m	0	1.11	10.99	0.31	0.0002
*SPEIZE	502	f	0	0.88	13.15	2.05	0.0013
SUZUK2	508	c	0	0.68	3.75	1.36	0.1886
SVENSS	551	f	0	0.61	6.77	3.03	0.1120
WAKAI	523	m	0	1.38	5.71	0.06	0.0009
WYNDE3	566	m	0	0.30	4.02	3.83	0.5438
WYNDE3	587	f	0	0.24	0.72	0.78	0.8415
Subtotal WYNDE3				0.29	4.73	4.61	

Table 1J7 - 5

IESLC - Meta-analysis of Ex Smoking, Years quit (vs never), "Low"
All LC types, Cigarettes (or Any Product if Cigarettes not available)
Least adjusted

REF	NRR	SEX	AD	Ys	Ws	Qs	Ps
WYNDE6	503	m	0	1.81	38.93	10.98	0.0000
WYNDE6	524	f	0	0.77	22.54	5.83	0.0003
Subtotal WYNDE6				1.43	61.48	16.81	

	N	55
	NS	40
	Wt	587.55
Het	Chi	210.13
Het	df	54
Het	P	***
Fixed	RR	3.60
	RRl	3.32
	RRu	3.90
	P	+++
Random	RR	2.86
	RRl	2.39
	RRu	3.42
	P	+++
Asymm	P	***

Table 1J7 - 6

IESLC - Meta-analysis of Ex Smoking, Years quit (vs never), "Low"
All LC types, Cigarettes (or Any Product if Cigarettes not available)
Least adjusted

		Sex		
	combined	male	female	Total
N	3	36	16	55
NS	3	35	16	54
Wt	34.82	414.38	138.36	587.55
Het Chi	3.08	130.37	24.93	210.13
Het df	2	35	15	54
Het P	N.S.	***	(*)	***
Fixed RR	4.61	4.21	2.11	3.60
RRl	3.30	3.82	1.79	3.32
RRu	6.42	4.63	2.49	3.90
P	+++	+++	+++	+++
Random RR	4.15	3.30	1.97	2.86
RRl	2.45	2.68	1.55	2.39
RRu	7.01	4.07	2.51	3.42
P	+++	+++	+++	+++
Between Chi				51.75
Between df				2
Between P				***
Btwn(F) P				***
Btwn(R) P				**

Table 1J7 - 7

IESLC - Meta-analysis of Ex Smoking, Years quit (vs never), "Low"
All LC types, Cigarettes (or Any Product if Cigarettes not available)
Excluded studies (and stage at which they were excluded)

1	AGUDO AKIBA AMANDU AMES AXELSS BEST BOUCHA BOUCOT BRESLO CHEN CHEN2 CHIAZZ DEAN2 DOSEME ENGELA FAN GENG GER GUO HAENSZ HEGMAN HOLE HU HU2 JUSSAW KATSOU KAUFMA KOO KOULUM KREUZE LETOUR LEVIN LIAW LIU3 LIU4 LIU5 MCCONN MIGRAN MRFITR NOTAN2 OSANN2 PERNU QIAO2 RACHTA RESTRE SADOWS SEGI2 STASZE TIZZAN VUTUC WATSON WU WUWILL WYNDE2 WYNDE8 XU YUAN ZHANG ZHENG ZHOU
2	BUFFLE HUMBLE PISANI PRESCO WYNDE7
3	MCDUFF SPITZ
4	HAMMON LUO WU2
5	BLOT1 CORREA GILLIS QIAO WIGLE
7	BOFFET
14	ARMADA BARBON BROSS BROWN3 CHYOU GARCIA GARSHI JOLY KHUDER TVERDA WANG2
15	BENHAM

Table 1J7 - 8
Potentially overlapping studies

REF	REFGP	PRINC	. OVERLAP/LINK
LUBIN2	LUBIN2	1	Lubin-combined
GRAHAM	BYERS1	1	GRAHAM/BROSS/BYERS1
BENSHL	TANG2	1	Subset of TANG2
WYNDE6	WYNDE6	1	WYNDE5/6/7/8
CPSI	CPSI	1	CPSI overall
JAHN	BOFFET	2	Subset of BOFFET
LUBIN	XIANGZ	2	LUBIN/XIANGZ/QIAO

Table 1J7 - 9
Most adjusted - insufficient data for meta-analysis

REF	NRR	SEX	AGEL	AGEH	RACE	YF	LC TYPE	LOC	START	ST	NLC	R	VB	P	H	AD	PRODUCT	exL	exH	DENOM	De
BLOT1	501	m	0	0	all	-	all	NAmer	1970	CC	458	n	bl	y	n	0	cig+/-ot	10	999	nev cigs	or
CPSI	721	f	0	0	wh	0	all	NAmer	1959	pr	5138	n	bl	n	n	1	cig only	10	14	nev cigs	or
SPEIZE	539	f	0	0	all	0	all	NAmer	1976	pr	593	n	bl	n	y	2	cig+/-ot	10	15	nev cigs	st

REF	NRR	RR	SIG	RRDATA comment
BLOT1	501	1.80		0
CPSI	721	0.58		0
SPEIZE	539	2.00		Insufficient decimals to calculate CIs

Table 1J8 -

IESLC - Meta-analysis of Ex Smoking, Years quit (vs never), "Mid"
All LC types, Cigarettes (or Any Product if Cigarettes not available)

This analysis is restricted to results for:
1) Ex smokers
2) Results by Years quit (vs never)
3) Categorical results by Years quit (vs never)
4) All LC types (or near equivalent)
5) Results complete enough for use in metaanalysis

Within each study, results are then selected (in the following order of preference, within each sex) for:
6) (not applicable)
7) PRODUCT: cigarettes regardless of other products, cigarettes only, all/unspec
8) CIGTYPE: all/unspecified, MC regardless of HR, MC only
9) (not applicable)
10) DENOM: never smoked anything, never smoked cigarettes, never any + low, never cigs + low
11) Followup period (YF, prospective studies): whole study (coded as 0) or longest available
12) LCtype: all or nearest available, at least Squamous and Adeno. (q = squamous, s = small,
l = large, a = adeno, mix = mixed, alv = alveolar)
13) Race: all or nearest available, otherwise by race (wh or w = white, bl or b = black, hi = hispanic
ch = chinese, jap = japanese, haw = hawaiian, w+o = white + oriental, sca = scandinavian, as = asian)
14) Years quit (vs never) "mid" in key scheme 1 (key value 7, maximum range 4-11)
15) For overlapping studies: principal rather than subsidiary studies
Finally by Age: whole study (coded as 0) if available, otherwise by widest available age group
and then for single sex results (m, f) in preference to results for both sexes combined (c).

Results adjusted (AD) for the most potential confounders are then chosen in Sections -1 to -3
(and those which actually differ from the adjusted results in Table 1J3 - 1 are marked 'x' in Section -1)
and results adjusted for the least confounders in Sections -4 to -6. (Those least adjusted results which
actually differ from the most adjusted are marked 'x' in column X in Section -4)

Section -7 shows excluded studies, together with the stage (as above) at which no qualifying
results were found.

Section -8 lists the potentially overlapping studies which have been included (1=principal, 2=subsidiary).

Section -9 lists any results which would have been included in preference except that they had data not complete
enough for use in meta-analysis, with their significance (yes/no), if known, and any further comment as entered
on the database. It also lists as "gap" any categories for which no data were presented by the original authors.
This is commonly due to recent quitters having been combined with current smokers

In addition to those mentioned above, the following fields, levels and abbreviations are used:
* or nk = not known, n = no, y = yes, ot = other
nev = never
all/unspec = all or unspecified, cig+/-ot = cigarettes irrespective of other products (cigar, pipe etc)
MC = manufactured cigarettes, HR = hand-rolled cigarettes
exL, exH = range of exposure (low and high) in the smoking group, in terms of Years quit (vs never)
REF: 6-character study reference
NRR: number of the RR on the database within the study
ST : study type (CC = case control, pr or prosp = prospective)
NLC: number of lung cancer cases in whole study
R : risky occupational population (n = no, m = mining, o = other risky)
VB : national cigarette type (V = at least 75% Virginia, bl = at least 75% blended, ot = other)
P : any proxy use
H : full histological confirmation
De : derivation of RR/CI (or = original, st = standard method, ot = other method of estimation)

Table 1J8 - 1

IESLC - Meta-analysis of Ex Smoking, Years quit (vs never), "Mid"
All LC types, Cigarettes (or Any Product if Cigarettes not available)
Most adjusted

REF	NRR	1J3	SEX	AGEL	AGEH	RACE	YF	LC TYPE	LOC	START	ST	NLC	R	VB	P	H	AD	PRODUCT	exL	exH	DENOM	De
BECHER	502		m	0	0	all	-	all	Eu:Ger	1985	CC	194	n	bl	n	y	0	all/unsp	5	9	nev any	st
BECHER	512		f	0	0	all	-	all	Eu:Ger	1985	CC	194	n	bl	n	y	0	all/unsp	5	9	nev any	st
CARPEN	503		c	0	0	w+b	-	all	NAmer	1991	CC	356	n	bl	n	n	0	cig+/-ot	5	9	nev cigs	st
CHOI	535		m	0	0	all	-	all	As:oth	1985	CC	375	n	bl	n	n	0	cig+/-ot	5	9	nev cigs	st
CPSI	808		m	50	74	all	6	all	NAmer	1959	pr	5138	n	bl	n	n	1	cig only	5	9	nev any	ot
CPSII	654		m	35	99	all	4	all	NAmer	1982	pr	3229	n	bl	n	n	1	cig only	6	10	nev any	ot
CPSII	635		f	0	0	all	4	all	NAmer	1982	pr	3229	n	bl	n	n	1	cig+/-ot	6	10	nev cigs	ot
DAMBER	524		m	0	0	all	-	all	Eu:Sca	1972	CC	579	n	bl	y	n	1	all/unsp	6	10	nev any	ot
DEAN3	518	x	m	0	0	all	-	all	Eu:UK	1969	CC	766	n	V	y	n	1	cig only	5	8	nev any	ot
DEAN3	554		f	0	0	all	-	all	Eu:UK	1969	CC	766	n	V	y	n	1	all/unsp	5	8	nev any	ot
DESTEF	526		m	0	0	all	-	all	SCAmer	1988	CC	497	n	bl	n	y	4	all/unsp	5	9	nev any	or
DOLL2	503		m	0	0	all	20	all	Eu:UK	1951	pr	920	n	V	n	n	1	cig only	5	9	nev any	ot
DORGAN	502		m	0	0	wh	-	all	NAmer	1980	CC	2026	n	bl	y	y	0	cig+/-ot	6	9	nev any	st
DORN	659		m	55	64	wh	8	all	NAmer	1954	pr	5097	n	bl	n	n	0	cig+/-ot	5	9	nev any	st
DORN	682		m	65	74	wh	8	all	NAmer	1954	pr	5097	n	bl	n	n	0	cig+/-ot	5	9	nev any	st
GAO	532		m	0	0	all	-	all	As:Chi	1984	CC	1405	n	ot	n	n	2	cig+/-ot	5	9	nev cigs	or
GAO	552		f	0	0	all	-	all	As:Chi	1984	CC	1405	n	ot	n	n	2	cig+/-ot	5	9	nev cigs	or
GAO2	512		m	0	0	all	-	all	As:Jap	1988	CC	282	n	bl	n	n	0	cig+/-ot	5	9	nev cigs	st
GRAHAM	502		m	0	0	wh	-	all	NAmer	1956	CC	685	n	bl	n	n	0	cig only	3	10	nev any	st
HAMMO2	502		m	0	0	all	0	all	NAmer	1967	pr	450	o	bl	n	n	1	cig+/-ot	5	9	nev any	ot
HIRAYA	508		m	0	0	all	0	all	As:Jap	1965	pr	1917	n	bl	n	n	1	cig+/-ot	5	9	nev any	st
HIRAYA	519		f	0	0	all	0	all	As:Jap	1965	pr	1917	n	bl	n	n	1	cig+/-ot	5	9	nev any	st
JAHN	503		m	0	0	all	-	all	Eu:Ger	1988	CC	1004	n	bl	n	n	0	cig+/-ot	6	10	nev any	st
JEDRYC	612		m	0	0	all	-	all	Eu:est	1980	CC	1630	n	bl	y	n	0	cig+/-ot	5	9	nev any	st
LUBIN	586		m	0	0	all	-	all	As:Chi	1984	CC	427	m	ot	y	n	0	cig+/-ot	5	9	nev any	st
LUBIN2	1073		m	0	0	all	-	all	Eu:mul	1976	CC	7804	n	bl	n	y	0	cig+/-ot	5	9	nev any	st
LUBIN2	1112		f	0	0	all	-	all	Eu:mul	1976	CC	7804	n	bl	n	y	0	cig+/-ot	5	9	nev any	st
MATOS	592		m	0	0	all	-	all	SCAmer	1994	CC	200	n	bl	n	n	2	cig+/-ot	6	10	nev any	ot
SOBUE	721		m	0	0	all	-	all	As:Jap	1986	CC	1376	n	bl	n	y	0	cig+/-ot	5	9	nev cigs	st
SPEIZE	503		f	0	0	all	0	all	NAmer	1976	pr	593	n	bl	n	y	0	cig+/-ot	5	10	nev cigs	st
SUZUK2	509		c	0	0	all	-	all	SCAmer	1991	CC	123	n	bl	n	y	0	all/unsp	6	10	nev any	st
WAKAI	532		m	0	0	all	-	all	As:Jap	1988	CC	333	n	bl	n	y	2	cig+/-ot	5	9	nev any	or
WYNDE6	504		m	0	0	all	-	all	NAmer	1969	CC	4423	n	bl	n	y	0	cig only	5	9	nev any	st
WYNDE6	525		f	0	0	all	-	all	NAmer	1969	CC	4423	n	bl	n	y	0	cig only	5	9	nev any	st

Cigarette type is all/unspec for all RRs
except for the following:

REF	NRR	CIGTYPE
DEAN3	518	MC only

Table 1J8 - 2

IESLC - Meta-analysis of Ex Smoking, Years quit (vs never), "Mid"
All LC types, Cigarettes (or Any Product if Cigarettes not available)
Most adjusted

REF	NRR	SEX	AD	Number Exposed Case	Exposed Cont	Non-exposed Case	Non-exposed Cont	RR	95.00%CI
BECHER	502	m	0	16	32	3	54	9.00	(2.43- 33.30)
BECHER	512	f	0	2	5	10	52	2.08	(0.35- 12.26)
Subtotal BECHER								5.37	(1.87- 15.40)
CARPEN	503	c	0	25	48	8	208	13.54	(5.75- 31.87)
CHOI	535	m	0	5	30	13	95	1.22	(0.40- 3.70)
*CPSI	808	m	1	32	-	60	-	5.15	(3.35- 7.91)
*CPSII	654	m	1	186	-	81	-	11.43	(8.81- 14.84)
*CPSII	635	f	1	37	-	174	-	4.91	(3.45- 7.01)
Subtotal CPSII								8.50	(6.89- 10.48)
DAMBER	524	m	1	-	-	42	-	4.30	(2.30- 8.10)
DEAN3	518	m	1	11	-	24	-	4.16	(1.91- 9.06)
DEAN3	554	f	1	1	-	41	-	1.09	(0.15- 8.13)
Subtotal DEAN3								3.49	(1.69- 7.20)
DESTEF	526	m	4	27	-	27	-	6.20	(3.20- 12.20)
*DOLL2	503	m	1	12	-	7	-	5.90	(2.32- 14.99)
DORGAN	502	m	0	49	38	13	140	13.89	(6.84- 28.21)
*DORN	659	m	0	32	34566	25	213858	7.92	(4.69- 13.36)
*DORN	682	m	0	41	24089	49	171211	5.95	(3.93- 9.00)
Subtotal DORN								6.64	(4.80- 9.19)
GAO	532	m	2	24	-	62	-	3.10	(1.70- 5.90)
GAO	552	f	2	14	-	435	-	3.90	(1.50- 9.90)
Subtotal GAO								3.32	(1.98- 5.59)
GAO2	512	m	0	21	26	13	56	3.48	(1.51- 8.01)
GRAHAM	502	m	0	5	29	18	346	3.31	(1.15- 9.57)
*HAMMO2	502	m	1	11	-	5	-	3.98	(1.39- 11.40)
*HIRAYA	508	m	1	-	-	-	-	1.59	(0.66- 3.82)
*HIRAYA	519	f	1	-	-	-	-	3.29	(0.56- 19.50)
Subtotal HIRAYA								1.83	(0.84- 4.03)
JAHN	503	m	0	59	63	18	138	7.18	(3.92- 13.16)
JEDRYC	612	m	0	64	58	49	219	4.93	(3.08- 7.90)
LUBIN	586	m	0	20	48	9	72	3.33	(1.40- 7.94)
LUBIN2	1073	m	0	466	822	190	2616	7.81	(6.48- 9.40)
LUBIN2	1112	f	0	30	40	336	1188	2.65	(1.63- 4.32)
Subtotal LUBIN2								6.81	(5.72- 8.10)
MATOS	592	m	2	21	-	11	-	9.00	(3.84- 21.08)
SOBUE	721	m	0	67	92	29	126	3.16	(1.90- 5.28)
*SPEIZE	503	f	0	41	95585	58	776300	5.74	(3.85- 8.56)
SUZUK2	509	c	0	10	8	11	53	6.02	(1.94- 18.72)
WAKAI	532	m	2	19	-	10	-	2.48	(1.04- 5.92)
WYNDE6	504	m	0	98	194	64	918	7.25	(5.10- 10.29)
WYNDE6	525	f	0	51	84	125	991	4.81	(3.24- 7.14)
Subtotal WYNDE6								6.05	(4.65- 7.86)
Partial Totals				1497	155857	2020	1168641		

*prospective study

REF	NRR	SEX	AD	Ys	Ws	Qs	Ps
BECHER	502	m	0	2.20	2.24	0.35	0.0010
BECHER	512	f	0	0.73	1.22	1.40	0.4184
Subtotal BECHER				1.68	3.46	1.75	
CARPEN	503	c	0	2.61	5.25	3.37	0.0000
CHOI	535	m	0	0.20	3.12	8.05	0.7277
*CPSI	808	m	1	1.64	20.82	0.57	0.0000
*CPSII	654	m	1	2.44	56.51	22.58	0.0000
*CPSII	635	f	1	1.59	30.57	1.39	0.0000
Subtotal CPSII				2.14	87.08	23.96	
DAMBER	524	m	1	1.46	9.69	1.16	0.0000
DEAN3	518	m	1	1.43	6.34	0.91	0.0003
DEAN3	554	f	1	0.09	0.96	2.85	0.9326
Subtotal DEAN3				1.25	7.30	3.75	
DESTEF	526	m	4	1.82	8.58	0.00	0.0000
*DOLL2	503	m	1	1.77	4.41	0.00	0.0002
DORGAN	502	m	0	2.63	7.65	5.23	0.0000
*DORN	659	m	0	2.07	14.04	0.99	0.0000
*DORN	682	m	0	1.78	22.35	0.01	0.0000
Subtotal DORN				1.89	36.39	1.00	
GAO	532	m	2	1.13	9.92	4.49	0.0004
GAO	552	f	2	1.36	4.31	0.85	0.0047
Subtotal GAO				1.20	14.24	5.34	
GAO2	512	m	0	1.25	5.53	1.72	0.0034
GRAHAM	502	m	0	1.20	3.41	1.25	0.0268

Table 1J8 - 2

IESLC - Meta-analysis of Ex Smoking, Years quit (vs never), "Mid"
All LC types, Cigarettes (or Any Product if Cigarettes not available)
Most adjusted

REF	NRR	SEX	AD	Ys	Ws	Qs	Ps
*HAMMO2	502	m	1	1.38	3.47	0.62	0.0101
*HIRAYA	508	m	1	0.46	4.98	8.96	0.3005
*HIRAYA	519	f	1	1.19	1.22	0.46	0.1885
Subtotal HIRAYA				0.61	6.20	9.42	
JAHN	503	m	0	1.97	10.46	0.29	0.0000
JEDRYC	612	m	0	1.60	17.29	0.75	0.0000
LUBIN	586	m	0	1.20	5.11	1.84	0.0065
LUBIN2	1073	m	0	2.05	111.01	6.97	0.0000
LUBIN2	1112	f	0	0.98	16.09	11.06	0.0001
Subtotal LUBIN2				1.92	127.10	18.03	
MATOS	592	m	2	2.20	5.30	0.82	0.0000
SOBUE	721	m	0	1.15	14.66	6.24	0.0000
*SPEIZE	503	f	0	1.75	24.03	0.08	0.0000
SUZUK2	509	c	0	1.80	2.99	0.00	0.0019
WAKAI	532	m	2	0.91	5.08	4.08	0.0406
WYNDE6	504	m	0	1.98	31.18	0.97	0.0000
WYNDE6	525	f	0	1.57	24.68	1.34	0.0000
Subtotal WYNDE6				1.80	55.86	2.31	

	N	34
	NS	26
	Wt	494.48
Het	Chi	101.62
Het	df	33
Het	P	***
Fixed	RR	6.08
	RRl	5.56
	RRu	6.63
	P	+++
Random	RR	5.09
	RRl	4.25
	RRu	6.08
	P	+++
Asymm	P	**

Table 1J8 - 3

IESLC - Meta-analysis of Ex Smoking, Years quit (vs never), "Mid"
All LC types, Cigarettes (or Any Product if Cigarettes not available)
Most adjusted

	Sex			
	combined	male	female	Total
N	2	24	8	34
NS	2	23	8	33
Wt	8.23	383.16	103.08	494.48
Het Chi	1.25	76.73	9.12	101.62
Het df	1	23	7	33
Het P	N.S.	***	N.S.	***
Fixed RR	10.09	6.54	4.43	6.08
RRl	5.10	5.92	3.65	5.56
RRu	19.98	7.23	5.37	6.63
P	+++	+++	+++	+++
Random RR	9.87	5.25	4.29	5.09
RRl	4.55	4.25	3.36	4.25
RRu	21.42	6.48	5.47	6.08
P	+++	+++	+++	+++
Between Chi				14.52
Between df				2
Between P				***
Btwn(F) P				(*)
Btwn(R) P				(*)

	Lung cancer type		
	all	other	Total
N	34		34
NS	26		26
Wt	494.48		494.48
Het Chi	101.62		101.62
Het df	33		33
Het P	***		***
Fixed RR	6.08		6.08
RRl	5.56		5.56
RRu	6.63		6.63
P	+++		+++
Random RR	5.09		5.09
RRl	4.25		4.25
RRu	6.08		6.08
P	+++		+++
Between Chi			
Between df			
Between P			N.S.
Btwn(F) P			N.S.
Btwn(R) P			N.S.

	Location								
	NAmer	UK	Scand	othEur	China	Japan	othAs	other	Total
N	12	3	1	6	3	5	1	3	34
NS	9	2	1	4	2	4	1	3	26
Wt	243.95	11.72	9.69	158.31	19.35	31.47	3.12	16.87	494.48
Het Chi	33.53	2.26	0.00	19.88	0.16	2.18	0.00	0.53	101.62
Het df	11	2	0	5	2	4	0	2	33
Het P	***	N.S.	N.S.	**	N.S.	N.S.	N.S.	N.S.	***
Fixed RR	7.00	4.25	4.30	6.56	3.33	2.78	1.22	6.93	6.08
RRl	6.17	2.40	2.29	5.62	2.13	1.96	0.40	4.30	5.56
RRu	7.93	7.53	8.07	7.67	5.19	3.94	3.70	11.18	6.63
P	+++	+++	+++	+++	+++	+++	N.S.	+++	+++
Random RR	6.71	4.19	4.30	5.26	3.33	2.78	1.22	6.93	5.09
RRl	5.29	2.23	2.29	3.35	2.13	1.96	0.40	4.30	4.25
RRu	8.52	7.85	8.07	8.28	5.19	3.94	3.70	11.18	6.08
P	+++	+++	+++	+++	+++	+++	N.S.	+++	+++
Between Chi									43.08
Between df									7
Between P									***
Btwn(F) P									*
Btwn(R) P									***

Table 1J8 - 3

IESLC - Meta-analysis of Ex Smoking, Years quit (vs never), "Mid"
All LC types, Cigarettes (or Any Product if Cigarettes not available)
Most adjusted

Detailed Country in "other Europe"

	multi	Germany	othWest	East	Balkans	Total
N	2	3		1		6
NS	1	2		1		4
Wt	127.10	13.92		17.29		158.31
Het Chi	16.38	1.92		0.00		19.88
Het df	1	2		0		5
Het P	***	N.S.		N.S.		**
Fixed RR	6.81	6.68		4.93		6.56
RRl	5.72	3.95		3.08		5.62
RRu	8.10	11.30		7.90		7.67
P	+++	+++		+++		+++
Random RR	4.66	6.68		4.93		5.26
RRl	1.62	3.95		3.08		3.35
RRu	13.42	11.30		7.90		8.28
P	++	+++		+++		+++
Between Chi						1.59
Between df						2
Between P						N.S.
Btwn(F) P						N.S.
Btwn(R) P						N.S.

Detailed Country in "other Asia"

	India	HongKong	other	Total
N			1	1
NS			1	1
Wt			3.12	3.12
Het Chi			0.00	0.00
Het df			0	0
Het P			N.S.	N.S.
Fixed RR			1.22	1.22
RRl			0.40	0.40
RRu			3.70	3.70
P			N.S.	N.S.
Random RR			1.22	1.22
RRl			0.40	0.40
RRu			3.70	3.70
P			N.S.	N.S.
Between Chi				
Between df				
Between P				N.S.
Btwn(F) P				N.S.
Btwn(R) P				N.S.

Detailed other continent

	SCAmer	Total
N	3	3
NS	3	3
Wt	16.87	16.87
Het Chi	0.53	0.53
Het df	2	2
Het P	N.S.	N.S.
Fixed RR	6.93	6.93
RRl	4.30	4.30
RRu	11.18	11.18
P	+++	+++
Random RR	6.93	6.93
RRl	4.30	4.30
RRu	11.18	11.18
P	+++	+++
Between Chi		
Between df		
Between P		N.S.
Btwn(F) P		N.S.
Btwn(R) P		N.S.

Table 1J8 - 3

IESLC - Meta-analysis of Ex Smoking, Years quit (vs never), "Mid"
All LC types, Cigarettes (or Any Product if Cigarettes not available)
Most adjusted

	Start year of study <1960	1960-69	1970-79	1980-89	1990+	Total
N	5	7	4	15	3	34
NS	4	4	3	12	3	26
Wt	65.03	72.83	160.83	182.25	13.53	494.48
Het Chi	2.73	13.70	18.67	59.25	1.29	101.62
Het df	4	6	3	14	2	33
Het P	N.S.	*	***	***	N.S.	***
Fixed RR	5.86	5.07	6.46	6.06	9.65	6.08
RRl	4.59	4.03	5.53	5.24	5.66	5.56
RRu	7.47	6.38	7.53	7.01	16.44	6.63
P	+++	+++	+++	+++	+++	+++
Random RR	5.86	4.07	4.93	4.73	9.65	5.09
RRl	4.59	2.65	3.02	3.38	5.66	4.25
RRu	7.47	6.27	8.05	6.63	16.44	6.08
P	+++	+++	+++	+++	+++	+++
Between Chi						5.97
Between df						4
Between P						N.S.
Btwn(F) P						N.S.
Btwn(R) P						N.S.

	Study type (1) CC	other	Total
N	24	10	34
NS	19	7	26
Wt	312.07	182.40	494.48
Het Chi	64.86	33.74	101.62
Het df	23	9	33
Het P	***	***	***
Fixed RR	5.72	6.73	6.08
RRl	5.12	5.82	5.56
RRu	6.39	7.78	6.63
P	+++	+++	+++
Random RR	4.83	5.62	5.09
RRl	3.87	4.08	4.25
RRu	6.02	7.73	6.08
P	+++	+++	+++
Between Chi			3.02
Between df			1
Between P			(*)
Btwn(F) P			N.S.
Btwn(R) P			N.S.

	Study type (2) CC	prosp	other	Total
N	24	10		34
NS	19	7		26
Wt	312.07	182.40		494.48
Het Chi	64.86	33.74		101.62
Het df	23	9		33
Het P	***	***		***
Fixed RR	5.72	6.73		6.08
RRl	5.12	5.82		5.56
RRu	6.39	7.78		6.63
P	+++	+++		+++
Random RR	4.83	5.62		5.09
RRl	3.87	4.08		4.25
RRu	6.02	7.73		6.08
P	+++	+++		+++
Between Chi				3.02
Between df				1
Between P				(*)
Btwn(F) P				N.S.
Btwn(R) P				N.S.

Table 1J8 - 3

IESLC - Meta-analysis of Ex Smoking, Years quit (vs never), "Mid"
All LC types, Cigarettes (or Any Product if Cigarettes not available)
Most adjusted

	Study size (number of LC cases) 100-249	250-499	500-999	1000+	Total
N	4	7	6	17	34
NS	3	7	5	11	26
Wt	11.75	36.13	48.85	397.74	494.48
Het Chi	2.34	15.15	3.79	71.89	101.62
Het df	3	6	5	16	33
Het P	N.S.	*	N.S.	***	***
Fixed RR	6.98	4.26	4.85	6.42	6.08
RRl	3.94	3.08	3.67	5.82	5.56
RRu	12.36	5.91	6.42	7.09	6.63
P	+++	+++	+++	+++	+++
Random RR	6.98	4.00	4.85	5.42	5.09
RRl	3.94	2.36	3.67	4.30	4.25
RRu	12.36	6.77	6.42	6.84	6.08
P	+++	+++	+++	+++	+++
Between Chi					8.45
Between df					3
Between P					*
Btwn(F) P					N.S.
Btwn(R) P					N.S.

	Risky occupational population no	mining	othRisky	Total
N	32	1	1	34
NS	24	1	1	26
Wt	485.90	5.11	3.47	494.48
Het Chi	99.12	0.00	0.00	101.62
Het df	31	0	0	33
Het P	***	N.S.	N.S.	***
Fixed RR	6.13	3.33	3.98	6.08
RRl	5.61	1.40	1.39	5.56
RRu	6.70	7.94	11.40	6.63
P	+++	++	+	+++
Random RR	5.16	3.33	3.98	5.09
RRl	4.30	1.40	1.39	4.25
RRu	6.20	7.94	11.40	6.08
P	+++	++	+	+++
Between Chi				2.50
Between df				2
Between P				N.S.
Btwn(F) P				N.S.
Btwn(R) P				N.S.

	National cigarette tobacco type Virginia	blended	other	Total
N	3	28	3	34
NS	2	22	2	26
Wt	11.72	463.41	19.35	494.48
Het Chi	2.26	90.14	0.16	101.62
Het df	2	27	2	33
Het P	N.S.	***	N.S.	***
Fixed RR	4.25	6.29	3.33	6.08
RRl	2.40	5.74	2.13	5.56
RRu	7.53	6.89	5.19	6.63
P	+++	+++	+++	+++
Random RR	4.19	5.36	3.33	5.09
RRl	2.23	4.43	2.13	4.25
RRu	7.85	6.49	5.19	6.08
P	+++	+++	+++	+++
Between Chi				9.06
Between df				2
Between P				*
Btwn(F) P				N.S.
Btwn(R) P				N.S.

Table 1J8 - 3

IESLC - Meta-analysis of Ex Smoking, Years quit (vs never), "Mid"
All LC types, Cigarettes (or Any Product if Cigarettes not available)
Most adjusted

	Any proxy use No/nk	Yes	Total
N	28	6	34
NS	21	5	26
Wt	447.44	47.04	494.48
Het Chi	88.76	11.45	101.62
Het df	27	5	33
Het P	***	*	***
Fixed RR	6.18	5.15	6.08
RRl	5.63	3.87	5.56
RRu	6.78	6.86	6.63
P	+++	+++	+++
Random RR	5.11	4.95	5.09
RRl	4.20	3.10	4.25
RRu	6.21	7.91	6.08
P	+++	+++	+++
Between Chi			1.41
Between df			1
Between P			N.S.
Btwn(F) P			N.S.
Btwn(R) P			N.S.

	Full histological confirmation No	Yes	Total
N	22	12	34
NS	17	9	26
Wt	245.07	249.40	494.48
Het Chi	63.82	37.61	101.62
Het df	21	11	33
Het P	***	***	***
Fixed RR	5.96	6.19	6.08
RRl	5.26	5.47	5.56
RRu	6.75	7.01	6.63
P	+++	+++	+++
Random RR	4.87	5.36	5.09
RRl	3.81	4.04	4.25
RRu	6.22	7.10	6.08
P	+++	+++	+++
Between Chi			0.19
Between df			1
Between P			N.S.
Btwn(F) P			N.S.
Btwn(R) P			N.S.

	Number of adjustment variables (1) 0	1	2+/+nk	Total
N	19	10	5	34
NS	15	7	4	26
Wt	322.29	138.99	33.20	494.48
Het Chi	51.89	38.85	6.67	101.62
Het df	18	9	4	33
Het P	***	***	N.S.	***
Fixed RR	6.10	6.50	4.38	6.08
RRl	5.47	5.50	3.11	5.56
RRu	6.81	7.67	6.15	6.63
P	+++	+++	+++	+++
Random RR	5.42	4.56	4.41	5.09
RRl	4.36	2.99	2.82	4.25
RRu	6.74	6.94	6.89	6.08
P	+++	+++	+++	+++
Between Chi				4.20
Between df				2
Between P				N.S.
Btwn(F) P				N.S.
Btwn(R) P				N.S.

Table 1J8 - 3

IESLC - Meta-analysis of Ex Smoking, Years quit (vs never), "Mid"
All LC types, Cigarettes (or Any Product if Cigarettes not available)
Most adjusted

	Number of adjustment variables (2) 0	1	2	3-5	6+/+nk	Total
N	19	10	4	1		34
NS	15	7	3	1		26
Wt	322.29	138.99	24.62	8.58		494.48
Het Chi	51.89	38.85	5.27	0.00		101.62
Het df	18	9	3	0		33
Het P	***	***	N.S.	N.S.		***
Fixed RR	6.10	6.50	3.88	6.20		6.08
RRl	5.47	5.50	2.61	3.18		5.56
RRu	6.81	7.67	5.75	12.11		6.63
P	+++	+++	+++	+++		+++
Random RR	5.42	4.56	3.97	6.20		5.09
RRl	4.36	2.99	2.32	3.18		4.25
RRu	6.74	6.94	6.79	12.11		6.08
P	+++	+++	+++	+++		+++
Between Chi						5.61
Between df						3
Between P						N.S.
Btwn(F) P						N.S.
Btwn(R) P						N.S.

	Product all/unsp	cig+/-ot	cig only	Total
N	6	21	7	34
NS	5	17	6	28
Wt	25.69	321.43	147.35	494.48
Het Chi	4.67	66.72	22.51	101.62
Het df	5	20	6	33
Het P	N.S.	***	***	***
Fixed RR	4.95	5.67	7.32	6.08
RRl	3.36	5.08	6.23	5.56
RRu	7.28	6.33	8.60	6.63
P	+++	+++	+++	+++
Random RR	4.95	4.79	6.08	5.09
RRl	3.36	3.80	4.26	4.25
RRu	7.28	6.03	8.69	6.08
P	+++	+++	+++	+++
Between Chi				7.72
Between df				2
Between P				*
Btwn(F) P				N.S.
Btwn(R) P				N.S.

	Denominator nev any	nev cigs	Total
N	26	8	34
NS	20	7	27
Wt	397.09	97.39	494.48
Het Chi	73.19	17.00	101.62
Het df	25	7	33
Het P	***	*	***
Fixed RR	6.55	4.47	6.08
RRl	5.94	3.66	5.56
RRu	7.23	5.45	6.63
P	+++	+++	+++
Random RR	5.44	4.22	5.09
RRl	4.45	3.00	4.25
RRu	6.65	5.94	6.08
P	+++	+++	+++
Between Chi			11.44
Between df			1
Between P			***
Btwn(F) P			(*)
Btwn(R) P			N.S.

Table 1J8 - 3

IESLC - Meta-analysis of Ex Smoking, Years quit (vs never), "Mid"
All LC types, Cigarettes (or Any Product if Cigarettes not available)
Most adjusted

	Derivation of RR/CI			
	Orig	StdCalc	Other	Total
N	4	21	9	34
NS	3	16	7	26
Wt	27.90	328.49	138.08	494.48
Het Chi	3.40	61.21	28.31	101.62
Het df	3	20	8	33
Het P	N.S.	***	***	***
Fixed RR	3.82	5.97	6.96	6.08
RRl	2.63	5.36	5.89	5.56
RRu	5.53	6.65	8.23	6.63
P	+++	+++	+++	+++
Random RR	3.81	5.12	5.60	5.09
RRl	2.56	4.09	3.85	4.25
RRu	5.67	6.41	8.16	6.08
P	+++	+++	+++	+++
Between Chi				8.70
Between df				2
Between P				*
Btwn(F) P				N.S.
Btwn(R) P				N.S.

Table 1J8 - 4

IESLC - Meta-analysis of Ex Smoking, Years quit (vs never), "Mid"
All LC types, Cigarettes (or Any Product if Cigarettes not available)
Least adjusted

REF	NRR	X	SEX	AGEL	AGEH	RACE	YF	LC TYPE	LOC	START	ST	NLC	R	VB	P	H	AD	PRODUCT	exL	exH	DENOM	De
BECHER	502		m	0	0	all	-	all	Eu:Ger	1985	CC	194	n	bl	n	y	0	all/unsp	5	9	nev any	st
BECHER	512		f	0	0	all	-	all	Eu:Ger	1985	CC	194	n	bl	n	y	0	all/unsp	5	9	nev any	st
CARPEN	503		c	0	0	w+b	-	all	NAmer	1991	CC	356	n	bl	n	n	0	cig+/-ot	5	9	nev cigs	st
CHOI	535		m	0	0	all	-	all	As:oth	1985	CC	375	n	bl	n	n	0	cig+/-ot	5	9	nev cigs	st
CPSI	808		m	50	74	all	6	all	NAmer	1959	pr	5138	n	bl	n	n	1	cig only	5	9	nev any	ot
CPSII	654		m	35	99	all	4	all	NAmer	1982	pr	3229	n	bl	n	n	1	cig only	6	10	nev any	ot
CPSII	635		f	0	0	all	4	all	NAmer	1982	pr	3229	n	bl	n	n	1	cig+/-ot	6	10	nev cigs	ot
DAMBER	524		m	0	0	all	-	all	Eu:Sca	1972	CC	579	n	bl	y	n	1	all/unsp	6	10	nev any	ot
DEAN3	503	x	m	0	0	all	-	all	Eu:UK	1969	CC	766	n	V	y	n	0	cig only	5	8	nev any	st
DEAN3	543	x	f	0	0	all	-	all	Eu:UK	1969	CC	766	n	V	y	n	0	all/unsp	5	8	nev any	st
DESTEF	516	x	m	0	0	all	-	all	SCAmer	1988	CC	497	n	bl	n	y	0	all/unsp	5	9	nev any	st
DOLL2	503		m	0	0	all	20	all	Eu:UK	1951	pr	920	n	V	n	n	1	cig only	5	9	nev any	ot
DORGAN	502		m	0	0	wh	-	all	NAmer	1980	CC	2026	n	bl	y	y	0	cig+/-ot	6	9	nev any	st
DORN	659		m	55	64	wh	8	all	NAmer	1954	pr	5097	n	bl	n	n	0	cig+/-ot	5	9	nev any	st
DORN	682		m	65	74	wh	8	all	NAmer	1954	pr	5097	n	bl	n	n	0	cig+/-ot	5	9	nev any	st
GAO	522	x	m	0	0	all	-	all	As:Chi	1984	CC	1405	n	ot	n	n	0	cig+/-ot	5	9	nev cigs	st
GAO	542	x	f	0	0	all	-	all	As:Chi	1984	CC	1405	n	ot	n	n	0	cig+/-ot	5	9	nev cigs	st
GAO2	512		m	0	0	all	-	all	As:Jap	1988	CC	282	n	bl	n	n	0	cig+/-ot	5	9	nev cigs	st
GRAHAM	502		m	0	0	wh	-	all	NAmer	1956	CC	685	n	bl	n	n	0	cig only	3	10	nev any	st
HAMMO2	502		m	0	0	all	0	all	NAmer	1967	pr	450	o	bl	n	n	1	cig+/-ot	5	9	nev any	ot
HIRAYA	508		m	0	0	all	0	all	As:Jap	1965	pr	1917	n	bl	n	n	1	cig+/-ot	5	9	nev any	st
HIRAYA	519		f	0	0	all	0	all	As:Jap	1965	pr	1917	n	bl	n	n	1	cig+/-ot	5	9	nev any	st
JAHN	503		m	0	0	all	-	all	Eu:Ger	1988	CC	1004	n	bl	n	n	0	cig+/-ot	6	10	nev any	st
JEDRYC	612		m	0	0	all	-	all	Eu:est	1980	CC	1630	n	bl	y	n	0	cig+/-ot	5	9	nev any	st
LUBIN	586		m	0	0	all	-	all	As:Chi	1984	CC	427	m	ot	y	n	0	cig+/-ot	5	9	nev any	st
LUBIN2	1073		m	0	0	all	-	all	Eu:mul	1976	CC	7804	n	bl	n	y	0	cig+/-ot	5	9	nev any	st
LUBIN2	1112		f	0	0	all	-	all	Eu:mul	1976	CC	7804	n	bl	n	y	0	cig+/-ot	5	9	nev any	st
MATOS	582	x	m	0	0	all	-	all	SCAmer	1994	CC	200	n	bl	n	n	0	cig+/-ot	6	10	nev any	st
SOBUE	721		m	0	0	all	-	all	As:Jap	1986	CC	1376	n	bl	n	y	0	cig+/-ot	5	9	nev cigs	st
SPEIZE	503		f	0	0	all	0	all	NAmer	1976	pr	593	n	bl	n	y	0	cig+/-ot	5	10	nev cigs	st
SUZUK2	509		c	0	0	all	-	all	SCAmer	1991	CC	123	n	bl	n	y	0	all/unsp	6	10	nev any	st
WAKAI	524	x	m	0	0	all	-	all	As:Jap	1988	CC	333	n	bl	n	y	0	cig+/-ot	5	9	nev any	st
WYNDE6	504		m	0	0	all	-	all	NAmer	1969	CC	4423	n	bl	n	y	0	cig only	5	9	nev any	st
WYNDE6	525		f	0	0	all	-	all	NAmer	1969	CC	4423	n	bl	n	y	0	cig only	5	9	nev any	st

Cigarette type is all/unspec for all RRs
except for the following:

REF	NRR	CIGTYPE
DEAN3	503	MC only

Table 1J8 - 5

IESLC - Meta-analysis of Ex Smoking, Years quit (vs never), "Mid"
All LC types, Cigarettes (or Any Product if Cigarettes not available)
Least adjusted

REF	NRR	SEX	AD	Number Exposed Case	Cont	Non-exposed Case	Cont	RR	95.00%CI
BECHER	502	m	0	16	32	3	54	9.00	(2.43- 33.30)
BECHER	512	f	0	2	5	10	52	2.08	(0.35- 12.26)
Subtotal BECHER								5.37	(1.87- 15.40)
CARPEN	503	c	0	25	48	8	208	13.54	(5.75- 31.87)
CHOI	535	m	0	5	30	13	95	1.22	(0.40- 3.70)
*CPSI	808	m	1	32	-	60	-	5.15	(3.35- 7.91)
*CPSII	654	m	1	186	-	81	-	11.43	(8.81- 14.84)
*CPSII	635	f	1	37	-	174	-	4.91	(3.45- 7.01)
Subtotal CPSII								8.50	(6.89- 10.48)
DAMBER	524	m	1	-	-	42	-	4.30	(2.30- 8.10)
DEAN3	503	m	0	11	43	24	510	5.44	(2.50- 11.84)
DEAN3	543	f	0	1	38	41	1538	0.99	(0.13- 7.37)
Subtotal DEAN3								4.35	(2.11- 8.99)
DESTEF	516	m	0	27	27	27	163	6.04	(3.09- 11.81)
*DOLL2	503	m	1	12	-	7	-	5.90	(2.32- 14.99)
DORGAN	502	m	0	49	38	13	140	13.89	(6.84- 28.21)
*DORN	659	m	0	32	34566	25	213858	7.92	(4.69- 13.36)
*DORN	682	m	0	41	24089	49	171211	5.95	(3.93- 9.00)
Subtotal DORN								6.64	(4.80- 9.19)
GAO	522	m	0	24	27	62	202	2.90	(1.56- 5.38)
GAO	542	f	0	14	7	435	605	2.78	(1.11- 6.95)
Subtotal GAO								2.86	(1.71- 4.78)
GAO2	512	m	0	21	26	13	56	3.48	(1.51- 8.01)
GRAHAM	502	m	0	5	29	18	346	3.31	(1.15- 9.57)
*HAMMO2	502	m	1	11	-	5	-	3.98	(1.39- 11.40)
*HIRAYA	508	m	1	-	-	-	-	1.59	(0.66- 3.82)
*HIRAYA	519	f	1	-	-	-	-	3.29	(0.56- 19.50)
Subtotal HIRAYA								1.83	(0.84- 4.03)
JAHN	503	m	0	59	63	18	138	7.18	(3.92- 13.16)
JEDRYC	612	m	0	64	58	49	219	4.93	(3.08- 7.90)
LUBIN	586	m	0	20	48	9	72	3.33	(1.40- 7.94)
LUBIN2	1073	m	0	466	822	190	2616	7.81	(6.48- 9.40)
LUBIN2	1112	f	0	30	40	336	1188	2.65	(1.63- 4.32)
Subtotal LUBIN2								6.81	(5.72- 8.10)
MATOS	582	m	0	21	27	11	110	7.78	(3.35- 18.06)
SOBUE	721	m	0	67	92	29	126	3.16	(1.90- 5.28)
*SPEIZE	503	f	0	41	95585	58	776300	5.74	(3.85- 8.56)
SUZUK2	509	c	0	10	8	11	53	6.02	(1.94- 18.72)
WAKAI	524	m	0	19	48	10	65	2.57	(1.10- 6.03)
WYNDE6	504	m	0	98	194	64	918	7.25	(5.10- 10.29)
WYNDE6	525	f	0	51	84	125	991	4.81	(3.24- 7.14)
Subtotal WYNDE6								6.05	(4.65- 7.86)
Partial Totals				1497	156074	2020	1171834		

*prospective study

REF	NRR	SEX	AD	Ys	Ws	Qs	Ps
BECHER	502	m	0	2.20	2.24	0.35	0.0010
BECHER	512	f	0	0.73	1.22	1.39	0.4184
Subtotal BECHER				1.68	3.46	1.75	
CARPEN	503	c	0	2.61	5.25	3.40	0.0000
CHOI	535	m	0	0.20	3.12	8.02	0.7277
*CPSI	808	m	1	1.64	20.82	0.54	0.0000
*CPSII	654	m	1	2.44	56.51	22.83	0.0000
*CPSII	635	f	1	1.59	30.57	1.34	0.0000
Subtotal CPSII				2.14	87.08	24.17	
DAMBER	524	m	1	1.46	9.69	1.13	0.0000
DEAN3	503	m	0	1.69	6.34	0.07	0.0000
DEAN3	543	f	0	-0.01	0.95	3.13	0.9899
Subtotal DEAN3				1.47	7.29	3.20	
DESTEF	516	m	0	1.80	8.53	0.00	0.0000
*DOLL2	503	m	1	1.77	4.41	0.00	0.0002
DORGAN	502	m	0	2.63	7.65	5.27	0.0000
*DORN	659	m	0	2.07	14.04	1.01	0.0000
*DORN	682	m	0	1.78	22.35	0.01	0.0000
Subtotal DORN				1.89	36.39	1.02	
GAO	522	m	0	1.06	10.02	5.45	0.0008
GAO	542	f	0	1.02	4.58	2.77	0.0285
Subtotal GAO				1.05	14.60	8.22	
GAO2	512	m	0	1.25	5.53	1.70	0.0034
GRAHAM	502	m	0	1.20	3.41	1.24	0.0268

Table 1J8 - 5

IESLC - Meta-analysis of Ex Smoking, Years quit (vs never), "Mid"
All LC types, Cigarettes (or Any Product if Cigarettes not available)
Least adjusted

REF	NRR	SEX	AD	Ys	Ws	Qs	Ps
*HAMMO2	502	m	1	1.38	3.47	0.61	0.0101
*HIRAYA	508	m	1	0.46	4.98	8.91	0.3005
*HIRAYA	519	f	1	1.19	1.22	0.45	0.1885
Subtotal HIRAYA				0.61	6.20	9.36	
JAHN	503	m	0	1.97	10.46	0.30	0.0000
JEDRYC	612	m	0	1.60	17.29	0.73	0.0000
LUBIN	586	m	0	1.20	5.11	1.82	0.0065
LUBIN2	1073	m	0	2.05	111.01	7.17	0.0000
LUBIN2	1112	f	0	0.98	16.09	10.96	0.0001
Subtotal LUBIN2				1.92	127.10	18.13	
MATOS	582	m	0	2.05	5.42	0.34	0.0000
SOBUE	721	m	0	1.15	14.66	6.17	0.0000
*SPEIZE	503	f	0	1.75	24.03	0.07	0.0000
SUZUK2	509	c	0	1.80	2.99	0.00	0.0019
WAKAI	524	m	0	0.95	5.30	3.88	0.0297
WYNDE6	504	m	0	1.98	31.18	1.01	0.0000
WYNDE6	525	f	0	1.57	24.68	1.30	0.0000
Subtotal WYNDE6				1.80	55.86	2.30	

	N	34
	NS	26
	Wt	495.11
Het	Chi	103.37
Het	df	33
Het	P	***
Fixed	RR	6.05
	RRl	5.54
	RRu	6.61
	P	+++
Random	RR	5.05
	RRl	4.21
	RRu	6.04
	P	+++
Asymm	P	**

Table 1J8 - 6

IESLC - Meta-analysis of Ex Smoking, Years quit (vs never), "Mid"
All LC types, Cigarettes (or Any Product if Cigarettes not available)
Least adjusted

	Sex combined	male	female	Total
N	2	24	8	34
NS	2	23	8	33
Wt	8.23	383.53	103.34	495.11
Het Chi	1.25	76.25	10.26	103.37
Het df	1	23	7	33
Het P	N.S.	***	N.S.	***
Fixed RR	10.09	6.54	4.36	6.05
RRl	5.10	5.92	3.59	5.54
RRu	19.98	7.23	5.29	6.61
P	+++	+++	+++	+++
Random RR	9.87	5.26	4.13	5.05
RRl	4.55	4.26	3.17	4.21
RRu	21.42	6.49	5.38	6.04
P	+++	+++	+++	+++
Between Chi				15.61
Between df				2
Between P				***
Btwn(F) P				(*)
Btwn(R) P				(*)

Table 1J8 - 7

IESLC - Meta-analysis of Ex Smoking, Years quit (vs never), "Mid"
All LC types, Cigarettes (or Any Product if Cigarettes not available)
Excluded studies (and stage at which they were excluded)

1	AGUDO AKIBA AMANDU AMES AXELSS BEST BOUCHA BOUCOT BRESLO CHEN CHEN2 CHIAZZ DEAN2 DOSEME ENGELA FAN GENG GER GUO HAENSZ HEGMAN HOLE HU HU2 JUSSAW KATSOU KAUFMA KOO KOULUM KREUZE LETOUR LEVIN LIAW LIU3 LIU4 LIU5 MCCONN MIGRAN MRFITR NOTAN2 OSANN2 PERNU QIAO2 RACHTA RESTRE SADOWS SEGI2 STASZE TIZZAN VUTUC WATSON WU WUWILL WYNDE2 WYNDE8 XU YUAN ZHANG ZHENG ZHOU
2	BUFFLE HUMBLE PISANI PRESCO WYNDE7
3	MCDUFF SPITZ
4	HAMMON LUO WU2
5	BLOT1 CORREA GILLIS QIAO WIGLE
7	BOFFET
14	ALDERS ARMADA AUVINE BARBON BENSHL BROSS BROWN3 CEDERL CHYOU DARBY DOLL GARCIA GARSHI GURSEL JAIN JOLY KAISE2 KHUDER LAUSSM PEZZO2 PEZZOT SVENSS TVERDA WANG2 WYNDE3
15	BENHAM

Table 1J8 - 8
Potentially overlapping studies

REF	REFGP	PRINC	.	OVERLAP/LINK
LUBIN2	LUBIN2	1		Lubin-combined
GRAHAM	BYERS1	1		GRAHAM/BROSS/BYERS1
WYNDE6	WYNDE6	1		WYNDE5/6/7/8
CPSI	CPSI	1		CPSI overall
JAHN	BOFFET	2		Subset of BOFFET
LUBIN	XIANGZ	2		LUBIN/XIANGZ/QIAO

Table 1J8 - 9
Most adjusted - insufficient data for meta-analysis

REF	NRR	SEX	AGEL	AGEH	RACE	YF	LC TYPE	LOC	START	ST	NLC	R	VB	P	H	AD	PRODUCT	exL	exH	DENOM	De
CPSI	722	f	0	0	wh	0	all	NAmer	1959	pr	5138	n	bl	n	n	1	cig only	5	9	nev cigs	or
SPEIZE	540	f	0	0	all	0	all	NAmer	1976	pr	593	n	bl	n	y	2	cig+/-ot	5	10	nev cigs	st

REF	NRR	RR	SIG	RRDATA comment
CPSI	722	1.51		0
SPEIZE	540	5.00		Insufficient decimals to calculate CIs

Table 1J9 -

IESLC - Meta-analysis of Ex Smoking, Years quit (vs never), "High"
All LC types, Cigarettes (or Any Product if Cigarettes not available)

This analysis is restricted to results for:
1) Ex smokers
2) Results by Years quit (vs never)
3) Categorical results by Years quit (vs never)
4) All LC types (or near equivalent)
5) Results complete enough for use in metaanalysis

Within each study, results are then selected (in the following order of preference, within each sex) for:
6) PRODUCT: cigarettes regardless of other products, cigarettes only, all/unspec
7) CIGTYPE: all/unspecified, MC regardless of HR, MC only
8) (not applicable)
9) DENOM: never smoked anything, never smoked cigarettes, never any + low, never cigs + low
10) Followup period (YF, prospective studies): whole study (coded as 0) or longest available
11) LCtype: all or nearest available, at least Squamous and Adeno. (q = squamous, s = small,
l = large, a = adeno, mix = mixed, alv = alveolar)
12) Race: all or nearest available, otherwise by race (wh or w = white, bl or b = black, hi = hispanic
ch = chinese, jap = japanese, haw = hawaiian, w+o = white + oriental, sca = scandinavian, as = asian)
13) Years quit (vs never) "high" in key scheme 1 (key value 3, maximum range 1-6)
14) For overlapping studies: principal rather than subsidiary studies
Finally by Age: whole study (coded as 0) if available, otherwise by widest available age group
and then for single sex results (m, f) in preference to results for both sexes combined (c).

Results adjusted (AD) for the most potential confounders are then chosen in Sections -1 to -3
(and those which actually differ from the adjusted results in Table 1J4 - 1 are marked 'x' in Section -1)
and results adjusted for the least confounders in Sections -4 to -6. (Those least adjusted results which
actually differ from the most adjusted are marked 'x' in column X in Section -4)

Section -7 shows excluded studies, together with the stage (as above) at which no qualifying
results were found.

Section -8 lists the potentially overlapping studies which have been included (1=principal, 2=subsidiary).

Section -9 lists any results which would have been included in preference except that they had data not complete
enough for use in meta-analysis, with their significance (yes/no), if known, and any further comment as entered
on the database. It also lists as "gap" any categories for which no data were presented by the original authors.
This is commonly due to recent quitters having been combined with current smokers

In addition to those mentioned above, the following fields, levels and abbreviations are used:
* or nk = not known, n = no, y = yes, ot = other
nev = never
all/unspec = all or unspecified, cig+/-ot = cigarettes irrespective of other products (cigar, pipe etc)
MC = manufactured cigarettes, HR = hand-rolled cigarettes
exL, exH = range of exposure (low and high) in the smoking group, in terms of Years quit (vs never)
REF: 6-character study reference
NRR: number of the RR on the database within the study
ST : study type (CC = case control, pr or prosp = prospective)
NLC: number of lung cancer cases in whole study
R : risky occupational population (n = no, m = mining, o = other risky)
VB : national cigarette type (V = at least 75% Virginia, bl = at least 75% blended, ot = other)
P : any proxy use
H : full histological confirmation
De : derivation of RR/CI (or = original, st = standard method, ot = other method of estimation)

Table 1J9 - 1

IESLC - Meta-analysis of Ex Smoking, Years quit (vs never), "High"
All LC types, Cigarettes (or Any Product if Cigarettes not available)
Most adjusted

REF	NRR	1J4	SEX	AGEL	AGEH	RACE	YF	LC TYPE	LOC	START	ST	NLC	R	VB	P	H	AD	PRODUCT	exL	exH	DENOM	De
ARMADA	516		m	0	0	all	-	all	Eu:wst	1986	CC	325	n	bl	n	y	0	cig+/-ot	1.0	5	nev cigs	st
BARBON	543		m	0	0	all	-	all	Eu:wst	1979	CC	755	n	bl	y	y	1	all/unsp	0.1	4	nev any	or
BECHER	503		m	0	0	all	-	all	Eu:Ger	1985	CC	194	n	bl	n	y	0	all/unsp	2	4	nev any	st
BECHER	513		f	0	0	all	-	all	Eu:Ger	1985	CC	194	n	bl	n	y	0	all/unsp	2	4	nev any	st
BROSS	516		m	0	0	wh	-	all	NAmer	1960	CC	974	n	bl	n	n	0	cig+/-ot	0.1	5	nev any	st
CARPEN	504		c	0	0	w+b	-	all	NAmer	1991	CC	356	n	bl	n	n	0	cig+/-ot	0.1	4	nev cigs	st
CHOI	536		m	0	0	all	-	all	As:oth	1985	CC	375	n	bl	n	n	0	cig+/-ot	0.1	4	nev cigs	st
CHOI	551		f	0	0	all	-	all	As:oth	1985	CC	375	n	bl	n	n	0	cig+/-ot	0.1	4	nev cigs	st
CPSI	809		m	50	74	all	6	all	NAmer	1959	pr	5138	n	bl	n	n	1	cig only	1.0	4	nev any	ot
CPSII	655		m	35	99	all	4	all	NAmer	1982	pr	3229	n	bl	n	n	1	cig only	3	5	nev any	ot
CPSII	636		f	0	0	all	4	all	NAmer	1982	pr	3229	n	bl	n	n	1	cig+/-ot	3	5	nev cigs	ot
DAMBER	525		m	0	0	all	-	all	Eu:Sca	1972	CC	579	n	bl	y	n	1	all/unsp	0.1	5	nev any	ot
DEAN3	519	x	m	0	0	all	-	all	Eu:UK	1969	CC	766	n	V	y	n	1	cig only	3	4	nev any	ot
DEAN3	555		f	0	0	all	-	all	Eu:UK	1969	CC	766	n	V	y	n	1	all/unsp	3	4	nev any	ot
DESTEF	527		m	0	0	all	-	all	SCAmer	1988	CC	497	n	bl	n	y	4	all/unsp	0.1	4	nev any	or
DOLL2	504		m	0	0	all	20	all	Eu:UK	1951	pr	920	n	V	n	n	1	cig only	0.1	4	nev any	ot
DORGAN	503		m	0	0	wh	-	all	NAmer	1980	CC	2026	n	bl	y	y	0	cig+/-ot	1.1	5	nev any	st
DORN	660		m	55	64	wh	8	all	NAmer	1954	pr	5097	n	bl	n	n	0	cig+/-ot	0.1	4	nev any	st
DORN	683		m	65	74	wh	8	all	NAmer	1954	pr	5097	n	bl	n	n	0	cig+/-ot	0.1	4	nev any	st
GAO	533		m	0	0	all	-	all	As:Chi	1984	CC	1405	n	ot	n	n	2	cig+/-ot	0.1	4	nev cigs	or
GAO	553		f	0	0	all	-	all	As:Chi	1984	CC	1405	n	ot	n	n	2	cig+/-ot	0.1	4	nev cigs	or
GAO2	513		m	0	0	all	-	all	As:Jap	1988	CC	282	n	bl	n	n	0	cig+/-ot	1.0	4	nev cigs	or
GARCIA	518		c	0	0	all	-	all	NAmer	1992	CC	416	n	bl	n	y	0	cig+/-ot	1.0	4	nev any	st
GRAHAM	536		m	0	0	wh	-	all	NAmer	1956	CC	685	n	bl	n	n	1	cig+/-ot	1.1	5	nev any	ot
HAMMO2	503		m	0	0	all	0	all	NAmer	1967	pr	450	o	bl	n	n	1	cig+/-ot	0.1	4	nev any	ot
HIRAYA	509		m	0	0	all	0	all	As:Jap	1965	pr	1917	n	bl	n	n	1	cig+/-ot	0.1	4	nev any	st
HIRAYA	520		f	0	0	all	0	all	As:Jap	1965	pr	1917	n	bl	n	n	1	cig+/-ot	0.1	4	nev any	st
JAHN	504		m	0	0	all	-	all	Eu:Ger	1988	CC	1004	n	bl	n	n	0	cig+/-ot	2	5	nev any	st
JOLY	567		m	0	0	all	-	all	SCAmer	1978	CC	826	n	bl	n	n	0	cig+/-ot	1.0	4	nev any	st
JOLY	554		f	0	0	all	-	all	SCAmer	1978	CC	826	n	bl	n	n	0	cig+/-ot	1.0	4	nev any	st
KHUDER	513		m	0	0	all	-	all	NAmer	1985	CC	482	n	bl	n	y	0	cig+/-ot	0.1	4	nev cigs	st
LUBIN	587		m	0	0	all	-	all	As:Chi	1984	CC	427	m	ot	y	n	0	cig+/-ot	3	4	nev any	st
LUBIN2	1074		m	0	0	all	-	all	Eu:mul	1976	CC	7804	n	bl	n	y	0	cig+/-ot	0.1	4	nev any	st
LUBIN2	1113		f	0	0	all	-	all	Eu:mul	1976	CC	7804	n	bl	n	y	0	cig+/-ot	0.1	4	nev any	st
MATOS	593		m	0	0	all	-	all	SCAmer	1994	CC	200	n	bl	n	n	2	cig+/-ot	1.0	5	nev any	ot
SOBUE	722		m	0	0	all	-	all	As:Jap	1986	CC	1376	n	bl	n	y	0	cig+/-ot	1.0	4	nev cigs	st
SPEIZE	504		f	0	0	all	0	all	NAmer	1976	pr	593	n	bl	n	y	0	cig+/-ot	2	5	nev cigs	st
SUZUK2	510		c	0	0	all	-	all	SCAmer	1991	CC	123	n	bl	n	y	0	all/unsp	0.1	5	nev any	st
TVERDA	502		m	0	0	all	0	all	Eu:Sca	1972	pr	238	n	bl	n	n	2	cig only	1.0	5	nev cigs	ot
WANG2	511		c	0	0	all	-	all	As:Chi	1980	CC	103	n	ot	n	n	0	cig+/-ot	0.1	3	nev cigs	st
WYNDE3	538		m	0	0	all	-	all	NAmer	1966	CC	350	n	bl	n	y	0	all/unsp	1.0	3	nev any	st
WYNDE6	505		m	0	0	all	-	all	NAmer	1969	CC	4423	n	bl	n	y	0	cig only	1.0	4	nev any	st
WYNDE6	526		f	0	0	all	-	all	NAmer	1969	CC	4423	n	bl	n	y	0	cig only	1.0	4	nev any	st

Cigarette type is all/unspec for all RRs
except for the following:

REF	NRR	CIGTYPE
DEAN3	519	MC only

Table 1J9 - 2

IESLC - Meta-analysis of Ex Smoking, Years quit (vs never), "High"
All LC types, Cigarettes (or Any Product if Cigarettes not available)
Most adjusted

REF	NRR	SEX	AD	Number Exposed Case	Number Exposed Cont	Non-exposed Case	Non-exposed Cont	RR	95.00%CI
ARMADA	516	m	0	79	45	8	71	15.58	(6.88- 35.29)
BARBON	543	m	1	32	-	22	-	13.90	(6.80- 28.50)
BECHER	503	m	0	10	12	3	54	15.00	(3.58- 62.92)
BECHER	513	f	0	2	3	10	52	3.47	(0.51- 23.48)
Subtotal BECHER								8.86	(2.81- 27.89)
BROSS	516	m	0	169	67	38	170	11.28	(7.19- 17.72)
CARPEN	504	c	0	23	39	8	208	15.33	(6.40- 36.75)
CHOI	536	m	0	25	64	13	95	2.85	(1.36- 5.99)
CHOI	551	f	0	3	2	76	164	3.24	(0.53- 19.77)
Subtotal CHOI								2.91	(1.46- 5.77)
*CPSI	809	m	1	49	-	60	-	8.09	(5.55- 11.80)
*CPSII	655	m	1	178	-	81	-	18.61	(14.31- 24.20)
*CPSII	636	f	1	56	-	174	-	10.55	(7.81- 14.26)
Subtotal CPSII								14.56	(11.95- 17.75)
DAMBER	525	m	1	-	-	42	-	7.70	(4.50- 13.50)
DEAN3	519	m	1	28	-	24	-	4.67	(2.60- 8.38)
DEAN3	555	f	1	4	-	41	-	1.63	(0.57- 4.63)
Subtotal DEAN3								3.64	(2.18- 6.06)
DESTEF	527	m	4	64	-	27	-	9.00	(5.20- 15.90)
*DOLL2	504	m	1	15	-	7	-	16.00	(6.52- 39.24)
DORGAN	503	m	0	59	51	13	140	12.46	(6.31- 24.61)
*DORN	660	m	0	34	22086	25	213858	13.17	(7.86- 22.07)
*DORN	683	m	0	14	6195	49	171211	7.90	(4.36- 14.29)
Subtotal DORN								10.57	(7.16- 15.60)
GAO	533	m	2	105	-	62	-	6.90	(4.40- 10.80)
GAO	553	f	2	37	-	435	-	7.20	(3.40- 15.10)
Subtotal GAO								6.98	(4.75- 10.25)
GAO2	513	m	0	31	26	13	56	5.14	(2.31- 11.40)
GARCIA	518	c	0	33	11	8	80	30.00	(11.07- 81.30)
GRAHAM	536	m	1	24	-	18	-	8.50	(4.32- 16.71)
*HAMMO2	503	m	1	59	-	5	-	10.99	(4.43- 27.26)
*HIRAYA	509	m	1	-	-	-	-	2.03	(1.10- 3.75)
*HIRAYA	520	f	1	-	-	-	-	3.72	(1.12- 12.37)
Subtotal HIRAYA								2.30	(1.33- 3.97)
JAHN	504	m	0	77	46	18	138	12.83	(6.96- 23.67)
JOLY	567	m	0	38	36	12	218	19.18	(9.16- 40.14)
JOLY	554	f	0	19	8	52	283	12.93	(5.38- 31.08)
Subtotal JOLY								16.28	(9.25- 28.65)
KHUDER	513	m	0	88	123	23	309	9.61	(5.80- 15.92)
LUBIN	587	m	0	33	18	9	72	14.67	(5.96- 36.07)
LUBIN2	1074	m	0	866	1047	190	2616	11.39	(9.58- 13.53)
LUBIN2	1113	f	0	60	55	336	1188	3.86	(2.62- 5.67)
Subtotal LUBIN2								9.50	(8.12- 11.13)
MATOS	593	m	2	28	-	11	-	14.00	(6.49- 30.21)
SOBUE	722	m	0	128	116	29	126	4.79	(2.98- 7.71)
*SPEIZE	504	f	0	34	63060	58	776300	7.22	(4.73- 11.02)
SUZUK2	510	c	0	15	10	11	53	7.23	(2.58- 20.25)
*TVERDA	502	m	2	5	-	4	-	2.83	(0.76- 10.53)
WANG2	511	c	0	6	10	11	43	2.35	(0.70- 7.86)
WYNDE3	538	m	0	21	22	9	88	9.33	(3.76- 23.19)
WYNDE6	505	m	0	201	166	64	918	17.37	(12.53- 24.07)
WYNDE6	526	f	0	82	70	125	991	9.29	(6.42- 13.43)
Subtotal WYNDE6								13.20	(10.34- 16.85)
Partial Totals				2834	93388	2224	1169502		

*prospective study

Table 1J9 - 2

IESLC - Meta-analysis of Ex Smoking, Years quit (vs never), "High"
All LC types, Cigarettes (or Any Product if Cigarettes not available)
Most adjusted

REF	NRR	SEX	AD	Ys	Ws	Qs	Ps
ARMADA	516	m	0	2.75	5.75	1.29	0.0000
BARBON	543	m	1	2.63	7.48	0.96	0.0000
BECHER	503	m	0	2.71	1.87	0.35	0.0002
BECHER	513	f	0	1.24	1.05	1.11	0.2027
Subtotal BECHER				2.18	2.92	1.47	
BROSS	516	m	0	2.42	18.85	0.43	0.0000
CARPEN	504	c	0	2.73	5.03	1.05	0.0000
CHOI	536	m	0	1.05	6.99	10.47	0.0056
CHOI	551	f	0	1.17	1.17	1.42	0.2033
Subtotal CHOI				1.07	8.16	11.89	
*CPSI	809	m	1	2.09	27.01	0.90	0.0000
*CPSII	655	m	1	2.92	55.67	23.57	0.0000
*CPSII	636	f	1	2.36	42.39	0.29	0.0000
Subtotal CPSII				2.68	98.06	23.86	
DAMBER	525	m	1	2.04	12.73	0.68	0.0000
DEAN3	519	m	1	1.54	11.22	6.01	0.0000
DEAN3	555	f	1	0.49	3.50	11.15	0.3606
Subtotal DEAN3				1.29	14.72	17.16	
DESTEF	527	m	4	2.20	12.30	0.07	0.0000
*DOLL2	504	m	1	2.77	4.77	1.19	0.0000
DORGAN	503	m	0	2.52	8.29	0.52	0.0000
*DORN	660	m	0	2.58	14.42	1.34	0.0000
*DORN	683	m	0	2.07	10.91	0.47	0.0000
Subtotal DORN				2.36	25.33	1.81	
GAO	533	m	2	1.93	19.06	2.22	0.0000
GAO	553	f	2	1.97	6.91	0.62	0.0000
Subtotal GAO				1.94	25.97	2.84	
GAO2	513	m	0	1.64	6.04	2.45	0.0001
GARCIA	518	c	0	3.40	3.87	4.92	0.0000
GRAHAM	536	m	1	2.14	8.40	0.15	0.0000
*HAMMO2	503	m	1	2.40	4.65	0.07	0.0000
*HIRAYA	509	m	1	0.71	10.22	25.02	0.0236
*HIRAYA	520	f	1	1.31	2.66	2.45	0.0320
Subtotal HIRAYA				0.83	12.88	27.47	
JAHN	504	m	0	2.55	10.25	0.80	0.0000
JOLY	567	m	0	2.95	7.04	3.26	0.0000
JOLY	554	f	0	2.56	4.99	0.41	0.0000
Subtotal JOLY				2.79	12.03	3.67	
KHUDER	513	m	0	2.26	15.10	0.00	0.0000
LUBIN	587	m	0	2.69	4.74	0.81	0.0000
LUBIN2	1074	m	0	2.43	128.94	3.28	0.0000
LUBIN2	1113	f	0	1.35	25.86	22.04	0.0000
Subtotal LUBIN2				2.25	154.81	25.32	
MATOS	593	m	2	2.64	6.50	0.87	0.0000
SOBUE	722	m	0	1.57	16.99	8.46	0.0000
*SPEIZE	504	f	0	1.98	21.44	1.89	0.0000
SUZUK2	510	c	0	1.98	3.62	0.32	0.0002
*TVERDA	502	m	2	1.04	2.22	3.38	0.1208
WANG2	511	c	0	0.85	2.63	5.30	0.1672
WYNDE3	538	m	0	2.23	4.64	0.01	0.0000
WYNDE6	505	m	0	2.85	36.08	12.21	0.0000
WYNDE6	526	f	0	2.23	28.18	0.06	0.0000
Subtotal WYNDE6				2.58	64.26	12.26	

Table 1J9 - 2

IESLC - Meta-analysis of Ex Smoking, Years quit (vs never), "High"
All LC types, Cigarettes (or Any Product if Cigarettes not available)
Most adjusted

	N	43
	NS	33
	Wt	632.44
Het	Chi	164.24
Het	df	42
Het	P	***
Fixed	RR	9.71
	RRl	8.98
	RRu	10.50
	P	+++
Random	RR	8.60
	RRl	7.22
	RRu	10.23
	P	+++
Asymm	P	*

Table 1J9 - 3

IESLC - Meta-analysis of Ex Smoking, Years quit (vs never), "High"
All LC types, Cigarettes (or Any Product if Cigarettes not available)
Most adjusted

	Sex combined	male	female	Total
N	4	29	10	43
NS	4	28	10	42
Wt	15.14	479.14	138.16	632.44
Het Chi	11.35	108.23	29.98	164.24
Het df	3	28	9	42
Het P	**	***	***	***
Fixed RR	10.98	10.51	7.28	9.71
RRl	6.63	9.61	6.16	8.98
RRu	18.17	11.49	8.60	10.50
P	+++	+++	+++	+++
Random RR	9.85	9.31	6.27	8.60
RRl	3.66	7.64	4.36	7.22
RRu	26.55	11.35	9.03	10.23
P	+++	+++	+++	+++
Between Chi				14.68
Between df				2
Between P				***
Btwn(F) P				N.S.
Btwn(R) P				N.S.

	Lung cancer type all	other	Total
N	43		43
NS	33		33
Wt	632.44		632.44
Het Chi	164.24		164.24
Het df	42		42
Het P	***		***
Fixed RR	9.71		9.71
RRl	8.98		8.98
RRu	10.50		10.50
P	+++		+++
Random RR	8.60		8.60
RRl	7.22		7.22
RRu	10.23		10.23
P	+++		+++
Between Chi			
Between df			
Between P			N.S.
Btwn(F) P			N.S.
Btwn(R) P			N.S.

	NAmer	UK	Scand	Location othEur	China	Japan	othAs	other	Total
N	16	3	2	7	4	4	2	5	43
NS	13	2	2	5	3	3	1	4	33
Wt	304.92	19.49	14.95	181.21	33.34	35.91	8.16	34.45	632.44
Het Chi	35.64	10.87	1.90	29.71	5.73	5.47	0.02	3.66	164.24
Het df	15	2	1	6	3	3	1	4	42
Het P	**	**	N.S.	***	N.S.	N.S.	N.S.	N.S.	***
Fixed RR	11.86	5.22	6.64	9.96	7.12	3.73	2.91	11.76	9.71
RRl	10.60	3.35	4.00	8.61	5.07	2.69	1.46	8.42	8.98
RRu	13.27	8.14	11.01	11.53	9.99	5.17	5.77	16.42	10.50
P	+++	+++	+++	+++	+++	+++	++	+++	+++
Random RR	11.35	5.07	5.62	9.71	7.02	3.67	2.91	11.76	8.60
RRl	9.40	1.66	2.26	5.94	4.13	2.28	1.46	8.42	7.22
RRu	13.70	15.52	13.97	15.87	11.92	5.91	5.77	16.42	10.23
P	+++	++	+++	+++	+++	+++	++	+++	+++
Between Chi									71.24
Between df									7
Between P									***
Btwn(F) P									**
Btwn(R) P									***

Table 1J9 - 3

IESLC - Meta-analysis of Ex Smoking, Years quit (vs never), "High"
All LC types, Cigarettes (or Any Product if Cigarettes not available)
Most adjusted

Detailed Country in "other Europe"

	multi	Germany	othWest	East	Balkans	Total
N	2	3	2			7
NS	1	2	2			5
Wt	154.81	13.17	13.23			181.21
Het Chi	25.25	1.75	0.04			29.71
Het df	1	2	1			6
Het P	***	N.S.	N.S.			***
Fixed RR	9.50	11.82	14.61			9.96
RRl	8.12	6.89	8.52			8.61
RRu	11.13	20.29	25.04			11.53
P	+++	+++	+++			+++
Random RR	6.72	11.82	14.61			9.71
RRl	2.33	6.89	8.52			5.94
RRu	19.42	20.29	25.04			15.87
P	+++	+++	+++			+++
Between Chi						2.67
Between df						2
Between P						N.S.
Btwn(F) P						N.S.
Btwn(R) P						N.S.

Detailed Country in "other Asia"

	India	HongKong	other	Total
N			2	2
NS			1	1
Wt			8.16	8.16
Het Chi			0.02	0.02
Het df			1	1
Het P			N.S.	N.S.
Fixed RR			2.91	2.91
RRl			1.46	1.46
RRu			5.77	5.77
P			++	++
Random RR			2.91	2.91
RRl			1.46	1.46
RRu			5.77	5.77
P			++	++
Between Chi				
Between df				
Between P				N.S.
Btwn(F) P				N.S.
Btwn(R) P				N.S.

Detailed other continent

	SCAmer	Total
N	5	5
NS	4	4
Wt	34.45	34.45
Het Chi	3.66	3.66
Het df	4	4
Het P	N.S.	N.S.
Fixed RR	11.76	11.76
RRl	8.42	8.42
RRu	16.42	16.42
P	+++	+++
Random RR	11.76	11.76
RRl	8.42	8.42
RRu	16.42	16.42
P	+++	+++
Between Chi		
Between df		
Between P		N.S.
Btwn(F) P		N.S.
Btwn(R) P		N.S.

Table 1J9 - 3

IESLC - Meta-analysis of Ex Smoking, Years quit (vs never), "High"
All LC types, Cigarettes (or Any Product if Cigarettes not available)
Most adjusted

	Start year of study					
	<1960	1960-69	1970-79	1980-89	1990+	Total
N	5	9	8	17	4	43
NS	4	6	6	13	4	33
Wt	65.50	120.01	210.72	217.21	19.01	632.44
Het Chi	4.01	56.56	35.71	59.28	3.81	164.24
Het df	4	8	7	16	3	42
Het P	N.S.	***	***	***	N.S.	***
Fixed RR	9.49	8.93	9.42	10.17	14.77	9.71
RRl	7.45	7.47	8.23	8.90	9.42	8.98
RRu	12.09	10.68	10.78	11.61	23.15	10.50
P	+++	+++	+++	+++	+++	+++
Random RR	9.49	6.44	8.60	8.37	14.76	8.60
RRl	7.44	3.82	5.76	6.25	8.86	7.22
RRu	12.09	10.84	12.82	11.22	24.60	10.23
P	+++	+++	+++	+++	+++	+++
Between Chi						4.87
Between df						4
Between P						N.S.
Btwn(F) P						N.S.
Btwn(R) P						N.S.

	Study type (1)		
	CC	other	Total
N	32	11	43
NS	25	8	33
Wt	436.08	196.36	632.44
Het Chi	103.33	59.80	164.24
Het df	31	10	42
Het P	***	***	***
Fixed RR	9.44	10.33	9.71
RRl	8.59	8.99	8.98
RRu	10.37	11.89	10.50
P	+++	+++	+++
Random RR	8.72	8.17	8.60
RRl	7.15	5.57	7.22
RRu	10.62	11.98	10.23
P	+++	+++	+++
Between Chi			1.11
Between df			1
Between P			N.S.
Btwn(F) P			N.S.
Btwn(R) P			N.S.

	Study type (2)			
	CC	prosp	other	Total
N	32	11		43
NS	25	8		33
Wt	436.08	196.36		632.44
Het Chi	103.33	59.80		164.24
Het df	31	10		42
Het P	***	***		***
Fixed RR	9.44	10.33		9.71
RRl	8.59	8.99		8.98
RRu	10.37	11.89		10.50
P	+++	+++		+++
Random RR	8.72	8.17		8.60
RRl	7.15	5.57		7.22
RRu	10.62	11.98		10.23
P	+++	+++		+++
Between Chi				1.11
Between df				1
Between P				N.S.
Btwn(F) P				N.S.
Btwn(R) P				N.S.

Table 1J9 - 3

IESLC - Meta-analysis of Ex Smoking, Years quit (vs never), "High"
All LC types, Cigarettes (or Any Product if Cigarettes not available)
Most adjusted

	Study size (number of LC cases) 100-249	250-499	500-999	1000+	Total
N	6	11	10	16	43
NS	5	10	8	10	33
Wt	17.88	70.29	100.43	443.84	632.44
Het Chi	9.68	22.42	24.98	103.40	164.24
Het df	5	10	9	15	42
Het P	(*)	*	**	***	***
Fixed RR	7.17	9.30	8.72	10.14	9.71
RRl	4.51	7.36	7.17	9.24	8.98
RRu	11.39	11.75	10.61	11.13	10.50
P	+++	+++	+++	+++	+++
Random RR	6.29	9.51	8.80	8.49	8.60
RRl	3.18	6.59	6.25	6.50	7.22
RRu	12.45	13.74	12.39	11.08	10.23
P	+++	+++	+++	+++	+++
Between Chi					3.77
Between df					3
Between P					N.S.
Btwn(F) P					N.S.
Btwn(R) P					N.S.

	Risky occupational population no	mining	othRisky	Total
N	41	1	1	43
NS	31	1	1	33
Wt	623.04	4.74	4.65	632.44
Het Chi	163.35	0.00	0.00	164.24
Het df	40	0	0	42
Het P	***	N.S.	N.S.	***
Fixed RR	9.67	14.67	10.99	9.71
RRl	8.94	5.96	4.43	8.98
RRu	10.46	36.07	27.26	10.50
P	+++	+++	+++	+++
Random RR	8.46	14.67	10.99	8.60
RRl	7.08	5.96	4.43	7.22
RRu	10.12	36.07	27.26	10.23
P	+++	+++	+++	+++
Between Chi				0.89
Between df				2
Between P				N.S.
Btwn(F) P				N.S.
Btwn(R) P				N.S.

	National cigarette tobacco type Virginia	blended	other	Total
N	3	36	4	43
NS	2	28	3	33
Wt	19.49	579.61	33.34	632.44
Het Chi	10.87	136.08	5.73	164.24
Het df	2	35	3	42
Het P	**	***	N.S.	***
Fixed RR	5.22	10.09	7.12	9.71
RRl	3.35	9.30	5.07	8.98
RRu	8.14	10.95	9.99	10.50
P	+++	+++	+++	+++
Random RR	5.07	9.12	7.02	8.60
RRl	1.66	7.60	4.13	7.22
RRu	15.52	10.94	11.92	10.23
P	++	+++	+++	+++
Between Chi				11.56
Between df				2
Between P				**
Btwn(F) P				N.S.
Btwn(R) P				N.S.

Table 1J9 - 3

IESLC - Meta-analysis of Ex Smoking, Years quit (vs never), "High"
All LC types, Cigarettes (or Any Product if Cigarettes not available)
Most adjusted

	Any proxy use No/nk	Yes	Total
N	37	6	43
NS	28	5	33
Wt	584.47	47.97	632.44
Het Chi	143.92	17.74	164.24
Het df	36	5	42
Het P	***	**	***
Fixed RR	9.89	7.77	9.71
RRl	9.12	5.85	8.98
RRu	10.72	10.31	10.50
P	+++	+++	+++
Random RR	8.76	7.59	8.60
RRl	7.28	4.38	7.22
RRu	10.54	13.16	10.23
P	+++	+++	+++
Between Chi			2.58
Between df			1
Between P			N.S.
Btwn(F) P			N.S.
Btwn(R) P			N.S.

	Full histological confirmation No	Yes	Total
N	27	16	43
NS	20	13	33
Wt	310.97	321.47	632.44
Het Chi	106.60	57.31	164.24
Het df	26	15	42
Het P	***	***	***
Fixed RR	9.48	9.93	9.71
RRl	8.48	8.90	8.98
RRu	10.60	11.08	10.50
P	+++	+++	+++
Random RR	7.90	9.60	8.60
RRl	6.18	7.42	7.22
RRu	10.11	12.42	10.23
P	+++	+++	+++
Between Chi			0.34
Between df			1
Between P			N.S.
Btwn(F) P			N.S.
Btwn(R) P			N.S.

	Number of adjustment variables (1) 0	1	2+/+nk	Total
N	26	12	5	43
NS	20	9	4	33
Wt	394.75	190.70	46.99	632.44
Het Chi	84.53	72.39	5.09	164.24
Het df	25	11	4	42
Het P	***	***	N.S.	***
Fixed RR	9.87	9.88	7.87	9.71
RRl	8.94	8.58	5.91	8.98
RRu	10.89	11.39	10.47	10.50
P	+++	+++	+++	+++
Random RR	9.32	7.43	7.89	8.60
RRl	7.53	4.96	5.63	7.22
RRu	11.54	11.12	11.06	10.23
P	+++	+++	+++	+++
Between Chi				2.24
Between df				2
Between P				N.S.
Btwn(F) P				N.S.
Btwn(R) P				N.S.

Table 1J9 - 3

IESLC - Meta-analysis of Ex Smoking, Years quit (vs never), "High"
All LC types, Cigarettes (or Any Product if Cigarettes not available)
Most adjusted

	Number of adjustment variables (2) 0	1	2	3-5	6+/+nk	Total
N	26	12	4	1		43
NS	20	9	3	1		33
Wt	394.75	190.70	34.69	12.30		632.44
Het Chi	84.53	72.39	4.79	0.00		164.24
Het df	25	11	3	0		42
Het P	***	***	N.S.	N.S.		***
Fixed RR	9.87	9.88	7.50	9.00		9.71
RRl	8.94	8.58	5.38	5.15		8.98
RRu	10.89	11.39	10.47	15.74		10.50
P	+++	+++	+++	+++		+++
Random RR	9.32	7.43	7.50	9.00		8.60
RRl	7.53	4.96	4.72	5.15		7.22
RRu	11.54	11.12	11.93	15.74		10.23
P	+++	+++	+++	+++		+++
Between Chi						2.54
Between df						3
Between P						N.S.
Btwn(F) P						N.S.
Btwn(R) P						N.S.

	Product all/unsp	cig+/-ot	cig only	Total
N	8	28	7	43
NS	7	22	6	35
Wt	47.19	420.10	165.14	632.44
Het Chi	12.96	99.60	36.39	164.24
Het df	7	27	6	42
Het P	(*)	***	***	***
Fixed RR	8.03	8.96	12.56	9.71
RRl	6.04	8.15	10.78	8.98
RRu	10.68	9.86	14.62	10.50
P	+++	+++	+++	+++
Random RR	7.63	8.41	10.17	8.60
RRl	5.00	6.82	6.68	7.22
RRu	11.63	10.38	15.47	10.23
P	+++	+++	+++	+++
Between Chi				15.30
Between df				2
Between P				***
Btwn(F) P				N.S.
Btwn(R) P				N.S.

	Denominator nev any	nev cigs	Total
N	30	13	43
NS	23	11	34
Wt	480.71	151.73	632.44
Het Chi	122.30	28.96	164.24
Het df	29	12	42
Het P	***	**	***
Fixed RR	10.52	7.52	9.71
RRl	9.62	6.42	8.98
RRu	11.51	8.82	10.50
P	+++	+++	+++
Random RR	9.53	6.81	8.60
RRl	7.72	5.16	7.22
RRu	11.75	9.00	10.23
P	+++	+++	+++
Between Chi			12.98
Between df			1
Between P			***
Btwn(F) P			(*)
Btwn(R) P			(*)

Table 1J9 - 3

IESLC - Meta-analysis of Ex Smoking, Years quit (vs never), "High"
All LC types, Cigarettes (or Any Product if Cigarettes not available)
Most adjusted

	Derivation of RR/CI Orig	StdCalc	Other	Total
N	5	27	11	43
NS	4	20	9	33
Wt	51.80	401.59	179.06	632.44
Het Chi	4.13	109.49	46.27	164.24
Het df	4	26	10	42
Het P	N.S.	***	***	***
Fixed RR	7.90	9.51	10.79	9.71
RRl	6.02	8.63	9.32	8.98
RRu	10.37	10.49	12.49	10.50
P	+++	+++	+++	+++
Random RR	7.91	8.72	8.50	8.60
RRl	5.99	6.90	5.93	7.22
RRu	10.44	11.02	12.17	10.23
P	+++	+++	+++	+++
Between Chi				4.36
Between df				2
Between P				N.S.
Btwn(F) P				N.S.
Btwn(R) P				N.S.

Table 1J9 - 4

IESLC - Meta-analysis of Ex Smoking, Years quit (vs never), "High"
All LC types, Cigarettes (or Any Product if Cigarettes not available)
Least adjusted

REF	NRR	X	SEX	AGEL	AGEH	RACE	YF	LC TYPE	LOC	START	ST	NLC	R	VB	P	H	AD	PRODUCT	exL	exH	DENOM	De
ARMADA	516		m	0	0	all	-	all	Eu:wst	1986	CC	325	n	bl	n	y	0	cig+/-ot	1.0	5	nev cigs	st
BARBON	528	x	m	0	0	all	-	all	Eu:wst	1979	CC	755	n	bl	y	y	0	all/unsp	0.1	4	nev any	st
BECHER	503		m	0	0	all	-	all	Eu:Ger	1985	CC	194	n	bl	n	y	0	all/unsp	2	4	nev any	st
BECHER	513		f	0	0	all	-	all	Eu:Ger	1985	CC	194	n	bl	n	y	0	all/unsp	2	4	nev any	st
BROSS	516		m	0	0	wh	-	all	NAmer	1960	CC	974	n	bl	n	n	0	cig+/-ot	0.1	5	nev any	st
CARPEN	504		c	0	0	w+b	-	all	NAmer	1991	CC	356	n	bl	n	n	0	cig+/-ot	0.1	4	nev cigs	st
CHOI	536		m	0	0	all	-	all	As:oth	1985	CC	375	n	bl	n	n	0	cig+/-ot	0.1	4	nev cigs	st
CHOI	551		f	0	0	all	-	all	As:oth	1985	CC	375	n	bl	n	n	0	cig+/-ot	0.1	4	nev cigs	st
CPSI	809		m	50	74	all	6	all	NAmer	1959	pr	5138	n	bl	n	n	1	cig only	1.0	4	nev any	ot
CPSII	655		m	35	99	all	4	all	NAmer	1982	pr	3229	n	bl	n	n	1	cig only	3	5	nev any	ot
CPSII	636		f	0	0	all	4	all	NAmer	1982	pr	3229	n	bl	n	n	1	cig+/-ot	3	5	nev cigs	ot
DAMBER	525		m	0	0	all	-	all	Eu:Sca	1972	CC	579	n	bl	y	n	1	all/unsp	0.1	5	nev any	ot
DEAN3	504	x	m	0	0	all	-	all	Eu:UK	1969	CC	766	n	V	y	n	0	cig only	3	4	nev any	st
DEAN3	544	x	f	0	0	all	-	all	Eu:UK	1969	CC	766	n	V	y	n	0	all/unsp	3	4	nev any	st
DESTEF	517	x	m	0	0	all	-	all	SCAmer	1988	CC	497	n	bl	n	y	0	all/unsp	0.1	4	nev any	st
DOLL2	504		m	0	0	all	20	all	Eu:UK	1951	pr	920	n	V	n	n	1	cig only	0.1	4	nev any	ot
DORGAN	503		m	0	0	wh	-	all	NAmer	1980	CC	2026	n	bl	y	y	0	cig+/-ot	1.1	5	nev any	st
DORN	660		m	55	64	wh	8	all	NAmer	1954	pr	5097	n	bl	n	n	0	cig+/-ot	0.1	4	nev any	st
DORN	683		m	65	74	wh	8	all	NAmer	1954	pr	5097	n	bl	n	n	0	cig+/-ot	0.1	4	nev any	st
GAO	523	x	m	0	0	all	-	all	As:Chi	1984	CC	1405	n	ot	n	n	0	cig+/-ot	0.1	4	nev cigs	st
GAO	543	x	f	0	0	all	-	all	As:Chi	1984	CC	1405	n	ot	n	n	0	cig+/-ot	0.1	4	nev cigs	st
GAO2	513		m	0	0	all	-	all	As:Jap	1988	CC	282	n	bl	n	n	0	cig+/-ot	1.0	4	nev cigs	or
GARCIA	518		c	0	0	all	-	all	NAmer	1992	CC	416	n	bl	n	y	0	cig+/-ot	1.0	4	nev any	st
GRAHAM	526	x	m	0	0	wh	-	all	NAmer	1956	CC	685	n	bl	n	n	0	cig+/-ot	1.1	5	nev any	st
HAMMO2	503		m	0	0	all	0	all	NAmer	1967	pr	450	o	bl	n	n	1	cig+/-ot	0.1	4	nev any	ot
HIRAYA	509		m	0	0	all	0	all	As:Jap	1965	pr	1917	n	bl	n	n	1	cig+/-ot	0.1	4	nev any	st
HIRAYA	520		f	0	0	all	0	all	As:Jap	1965	pr	1917	n	bl	n	n	1	cig+/-ot	0.1	4	nev any	st
JAHN	504		m	0	0	all	-	all	Eu:Ger	1988	CC	1004	n	bl	n	n	0	cig+/-ot	2	5	nev any	st
JOLY	567		m	0	0	all	-	all	SCAmer	1978	CC	826	n	bl	n	n	0	cig+/-ot	1.0	4	nev any	st
JOLY	554		f	0	0	all	-	all	SCAmer	1978	CC	826	n	bl	n	n	0	cig+/-ot	1.0	4	nev any	st
KHUDER	513		m	0	0	all	-	all	NAmer	1985	CC	482	n	bl	n	y	0	cig+/-ot	0.1	4	nev cigs	st
LUBIN	587		m	0	0	all	-	all	As:Chi	1984	CC	427	m	ot	y	n	0	cig+/-ot	3	4	nev any	st
LUBIN2	1074		m	0	0	all	-	all	Eu:mul	1976	CC	7804	n	bl	n	y	0	cig+/-ot	0.1	4	nev any	st
LUBIN2	1113		f	0	0	all	-	all	Eu:mul	1976	CC	7804	n	bl	n	y	0	cig+/-ot	0.1	4	nev any	st
MATOS	583	x	m	0	0	all	-	all	SCAmer	1994	CC	200	n	bl	n	n	0	cig+/-ot	1.0	5	nev any	st
SOBUE	722		m	0	0	all	-	all	As:Jap	1986	CC	1376	n	bl	n	y	0	cig+/-ot	1.0	4	nev cigs	st
SPEIZE	504		f	0	0	all	0	all	NAmer	1976	pr	593	n	bl	n	y	0	cig+/-ot	2	5	nev cigs	st
SUZUK2	510		c	0	0	all	-	all	SCAmer	1991	CC	123	n	bl	n	y	0	all/unsp	0.1	5	nev any	st
TVERDA	502		m	0	0	all	0	all	Eu:Sca	1972	pr	238	n	bl	n	n	2	cig only	1.0	5	nev cigs	ot
WANG2	511		c	0	0	all	-	all	As:Chi	1980	CC	103	n	ot	n	n	0	cig+/-ot	0.1	3	nev cigs	st
WYNDE3	538		m	0	0	all	-	all	NAmer	1966	CC	350	n	bl	n	y	0	all/unsp	1.0	3	nev any	st
WYNDE6	505		m	0	0	all	-	all	NAmer	1969	CC	4423	n	bl	n	y	0	cig only	1.0	4	nev any	st
WYNDE6	526		f	0	0	all	-	all	NAmer	1969	CC	4423	n	bl	n	y	0	cig only	1.0	4	nev any	st

Cigarette type is all/unspec for all RRs
except for the following:

REF	NRR	CIGTYPE
DEAN3	504	MC only

Table 1J9 - 5

IESLC - Meta-analysis of Ex Smoking, Years quit (vs never), "High"
All LC types, Cigarettes (or Any Product if Cigarettes not available)
Least adjusted

REF	NRR	SEX	AD	Number Exposed Case	Number Exposed Cont	Non-exposed Case	Non-exposed Cont	RR	95.00%CI
ARMADA	516	m	0	79	45	8	71	15.58	(6.88- 35.29)
BARBON	528	m	0	32	20	22	188	13.67	(6.71- 27.87)
BECHER	503	m	0	10	12	3	54	15.00	(3.58- 62.92)
BECHER	513	f	0	2	3	10	52	3.47	(0.51- 23.48)
Subtotal BECHER								8.86	(2.81- 27.89)
BROSS	516	m	0	169	67	38	170	11.28	(7.19- 17.72)
CARPEN	504	c	0	23	39	8	208	15.33	(6.40- 36.75)
CHOI	536	m	0	25	64	13	95	2.85	(1.36- 5.99)
CHOI	551	f	0	3	2	76	164	3.24	(0.53- 19.77)
Subtotal CHOI								2.91	(1.46- 5.77)
*CPSI	809	m	1	49	-	60	-	8.09	(5.55- 11.80)
*CPSII	655	m	1	178	-	81	-	18.61	(14.31- 24.20)
*CPSII	636	f	1	56	-	174	-	10.55	(7.81- 14.26)
Subtotal CPSII								14.56	(11.95- 17.75)
DAMBER	525	m	1	-	-	42	-	7.70	(4.50- 13.50)
DEAN3	504	m	0	28	102	24	510	5.83	(3.25- 10.47)
DEAN3	544	f	0	4	110	41	1538	1.36	(0.48- 3.88)
Subtotal DEAN3								4.12	(2.47- 6.87)
DESTEF	517	m	0	64	45	27	163	8.59	(4.91- 15.00)
*DOLL2	504	m	1	15	-	7	-	16.00	(6.52- 39.24)
DORGAN	503	m	0	59	51	13	140	12.46	(6.31- 24.61)
*DORN	660	m	0	34	22086	25	213858	13.17	(7.86- 22.07)
*DORN	683	m	0	14	6195	49	171211	7.90	(4.36- 14.29)
Subtotal DORN								10.57	(7.16- 15.60)
GAO	523	m	0	105	52	62	202	6.58	(4.25- 10.19)
GAO	543	f	0	37	9	435	605	5.72	(2.73- 11.97)
Subtotal GAO								6.34	(4.35- 9.24)
GAO2	513	m	0	31	26	13	56	5.14	(2.31- 11.40)
GARCIA	518	c	0	33	11	8	80	30.00	(11.07- 81.30)
GRAHAM	526	m	0	24	48	18	346	9.61	(4.86- 19.00)
*HAMMO2	503	m	1	59	-	5	-	10.99	(4.43- 27.26)
*HIRAYA	509	m	1	-	-	-	-	2.03	(1.10- 3.75)
*HIRAYA	520	f	1	-	-	-	-	3.72	(1.12- 12.37)
Subtotal HIRAYA								2.30	(1.33- 3.97)
JAHN	504	m	0	77	46	18	138	12.83	(6.96- 23.67)
JOLY	567	m	0	38	36	12	218	19.18	(9.16- 40.14)
JOLY	554	f	0	19	8	52	283	12.93	(5.38- 31.08)
Subtotal JOLY								16.28	(9.25- 28.65)
KHUDER	513	m	0	88	123	23	309	9.61	(5.80- 15.92)
LUBIN	587	m	0	33	18	9	72	14.67	(5.96- 36.07)
LUBIN2	1074	m	0	866	1047	190	2616	11.39	(9.58- 13.53)
LUBIN2	1113	f	0	60	55	336	1188	3.86	(2.62- 5.67)
Subtotal LUBIN2								9.50	(8.12- 11.13)
MATOS	583	m	0	28	23	11	110	12.17	(5.31- 27.91)
SOBUE	722	m	0	128	116	29	126	4.79	(2.98- 7.71)
*SPEIZE	504	f	0	34	63060	58	776300	7.22	(4.73- 11.02)
SUZUK2	510	c	0	15	10	11	53	7.23	(2.58- 20.25)
*TVERDA	502	m	2	5	-	4	-	2.83	(0.76- 10.53)
WANG2	511	c	0	6	10	11	43	2.35	(0.70- 7.86)
WYNDE3	538	m	0	21	22	9	88	9.33	(3.76- 23.19)
WYNDE6	505	m	0	201	166	64	918	17.37	(12.53- 24.07)
WYNDE6	526	f	0	82	70	125	991	9.29	(6.42- 13.43)
Subtotal WYNDE6								13.20	(10.34- 16.85)
Partial Totals				2834	93797	2224	1173164		

*prospective study

Table 1J9 - 5

IESLC - Meta-analysis of Ex Smoking, Years quit (vs never), "High"
All LC types, Cigarettes (or Any Product if Cigarettes not available)
Least adjusted

REF	NRR	SEX	AD	Ys	Ws	Qs	Ps
ARMADA	516	m	0	2.75	5.75	1.30	0.0000
BARBON	528	m	0	2.62	7.57	0.90	0.0000
BECHER	503	m	0	2.71	1.87	0.36	0.0002
BECHER	513	f	0	1.24	1.05	1.11	0.2027
Subtotal BECHER				2.18	2.92	1.47	
BROSS	516	m	0	2.42	18.85	0.44	0.0000
CARPEN	504	c	0	2.73	5.03	1.06	0.0000
CHOI	536	m	0	1.05	6.99	10.42	0.0056
CHOI	551	f	0	1.17	1.17	1.41	0.2033
Subtotal CHOI				1.07	8.16	11.83	
*CPSI	809	m	1	2.09	27.01	0.87	0.0000
*CPSII	655	m	1	2.92	55.67	23.79	0.0000
*CPSII	636	f	1	2.36	42.39	0.31	0.0000
Subtotal CPSII				2.68	98.06	24.10	
DAMBER	525	m	1	2.04	12.73	0.67	0.0000
DEAN3	504	m	0	1.76	11.22	2.88	0.0000
DEAN3	544	f	0	0.31	3.52	13.51	0.5602
Subtotal DEAN3				1.42	14.74	16.39	
DESTEF	517	m	0	2.15	12.34	0.18	0.0000
*DOLL2	504	m	1	2.77	4.77	1.20	0.0000
DORGAN	503	m	0	2.52	8.29	0.53	0.0000
*DORN	660	m	0	2.58	14.42	1.37	0.0000
*DORN	683	m	0	2.07	10.91	0.45	0.0000
Subtotal DORN				2.36	25.33	1.82	
GAO	523	m	0	1.88	20.07	2.99	0.0000
GAO	543	f	0	1.74	7.04	1.95	0.0000
Subtotal GAO				1.85	27.10	4.94	
GAO2	513	m	0	1.64	6.04	2.43	0.0001
GARCIA	518	c	0	3.40	3.87	4.95	0.0000
GRAHAM	526	m	0	2.26	8.27	0.00	0.0000
*HAMMO2	503	m	1	2.40	4.65	0.08	0.0000
*HIRAYA	509	m	1	0.71	10.22	24.92	0.0236
*HIRAYA	520	f	1	1.31	2.66	2.44	0.0320
Subtotal HIRAYA				0.83	12.88	27.36	
JAHN	504	m	0	2.55	10.25	0.82	0.0000
JOLY	567	m	0	2.95	7.04	3.29	0.0000
JOLY	554	f	0	2.56	4.99	0.42	0.0000
Subtotal JOLY				2.79	12.03	3.71	
KHUDER	513	m	0	2.26	15.10	0.00	0.0000
LUBIN	587	m	0	2.69	4.74	0.82	0.0000
LUBIN2	1074	m	0	2.43	128.94	3.41	0.0000
LUBIN2	1113	f	0	1.35	25.86	21.89	0.0000
Subtotal LUBIN2				2.25	154.81	25.30	
MATOS	583	m	0	2.50	5.58	0.29	0.0000
SOBUE	722	m	0	1.57	16.99	8.39	0.0000
*SPEIZE	504	f	0	1.98	21.44	1.85	0.0000
SUZUK2	510	c	0	1.98	3.62	0.31	0.0002
*TVERDA	502	m	2	1.04	2.22	3.36	0.1208
WANG2	511	c	0	0.85	2.63	5.28	0.1672
WYNDE3	538	m	0	2.23	4.64	0.01	0.0000
WYNDE6	505	m	0	2.85	36.08	12.33	0.0000
WYNDE6	526	f	0	2.23	28.18	0.05	0.0000
Subtotal WYNDE6				2.58	64.26	12.38	

Table 1J9 - 5

IESLC - Meta-analysis of Ex Smoking, Years quit (vs never), "High"
All LC types, Cigarettes (or Any Product if Cigarettes not available)
Least adjusted

	N	43
	NS	33
	Wt	632.68
Het	Chi	165.02
Het	df	42
Het	P	***
Fixed	RR	9.68
	RRl	8.95
	RRu	10.46
	P	+++
Random	RR	8.54
	RRl	7.18
	RRu	10.17
	P	+++
Asymm	P	*

Table 1J9 - 6

IESLC - Meta-analysis of Ex Smoking, Years quit (vs never), "High"
All LC types, Cigarettes (or Any Product if Cigarettes not available)
Least adjusted

	Sex combined	male	female	Total
N	4	29	10	43
NS	4	28	10	42
Wt	15.14	479.23	138.31	632.68
Het Chi	11.35	105.19	32.38	165.02
Het df	3	28	9	42
Het P	**	***	***	***
Fixed RR	10.98	10.52	7.16	9.68
RRl	6.63	9.62	6.06	8.95
RRu	18.17	11.50	8.46	10.46
P	+++	+++	+++	+++
Random RR	9.85	9.35	6.00	8.54
RRl	3.66	7.69	4.11	7.18
RRu	26.55	11.36	8.75	10.17
P	+++	+++	+++	+++
Between Chi				16.10
Between df				2
Between P				***
Btwn(F) P				N.S.
Btwn(R) P				N.S.

Table 1J9 - 7

IESLC - Meta-analysis of Ex Smoking, Years quit (vs never), "High"
All LC types, Cigarettes (or Any Product if Cigarettes not available)
Excluded studies (and stage at which they were excluded)

1	AGUDO AKIBA AMANDU AMES AXELSS BEST BOUCHA BOUCOT BRESLO CHEN CHEN2 CHIAZZ DEAN2 DOSEME ENGELA FAN GENG GER GUO HAENSZ HEGMAN HOLE HU HU2 JUSSAW KATSOU KAUFMA KOO KOULUM KREUZE LETOUR LEVIN LIAW LIU3 LIU4 LIU5 MCCONN MIGRAN MRFITR NOTAN2 OSANN2 PERNU QIAO2 RACHTA RESTRE SADOWS SEGI2 STASZE TIZZAN VUTUC WATSON WU WUWILL WYNDE2 WYNDE8 XU YUAN ZHANG ZHENG ZHOU
2	BUFFLE HUMBLE PISANI PRESCO WYNDE7
3	MCDUFF SPITZ
4	HAMMON LUO WU2
5	BLOT1 CORREA GILLIS QIAO WIGLE
7	BOFFET
14	ALDERS AUVINE BENSHL BROWN3 CEDERL CHYOU DARBY DOLL GARSHI GURSEL JAIN JEDRYC KAISE2 LAUSSM PEZZO2 PEZZOT SVENSS WAKAI
15	BENHAM

Table 1J9 - 8
Potentially overlapping studies

REF	REFGP	PRINC	.	OVERLAP/LINK
LUBIN2	LUBIN2	1		Lubin-combined
TVERDA	TVERDA	1		VEIERO/TVERDAL
BROSS	BYERS1	1		GRAHAM/BROSS/BYERS1
GRAHAM	BYERS1	1		GRAHAM/BROSS/BYERS1
WYNDE6	WYNDE6	1		WYNDE5/6/7/8
CPSI	CPSI	1		CPSI overall
JAHN	BOFFET	2		Subset of BOFFET
LUBIN	XIANGZ	2		LUBIN/XIANGZ/QIAO

Table 1J9 - 9
Most adjusted - insufficient data for meta-analysis

REF	NRR	SEX	AGEL	AGEH	RACE	YF	LC TYPE	LOC	START	ST	NLC	R	VB	P	H	AD	PRODUCT	exL	exH	DENOM	De
CORREA	540	c	0	0	all	-	all	NAmer	1979	CC	1359	n	bl	y	n	2	cig+/-ot	3	5	nev cigs	or
CPSI	723	f	0	0	wh	0	all	NAmer	1959	pr	5138	n	bl	n	n	1	cig only	2	4	nev cigs	or
GARSHI	524	m	0	0	all	-	all	NAmer	1981	CC	1081	o	bl	y	n	1	all/unsp	1.0	4	nev any	ot
JEDRYC	613	m	0	0	all	-	all	Eu:est	1980	CC	1630	n	bl	y	n	0	cig+/-ot	1.0	4	nev any	ot
SPEIZE	541	f	0	0	all	0	all	NAmer	1976	pr	593	n	bl	n	y	2	cig+/-ot	2	5	nev cigs	st
WAKAI	611	m	0	0	all	-	all	As:Jap	1988	CC	333	n	bl	n	y	2	cig+/-ot	1.0	4	nev any	ot

REF	NRR	RR	SIG	RRDATA comment
CORREA	540	7.70		0
CPSI	723	2.85		0
GARSHI	524	*	gap	0
JEDRYC	613	*	gap	0
SPEIZE	541	6.00		Insufficient decimals to calculate CIs
WAKAI	611	*	gap	0

Least adjusted - insufficient data for meta-analysis: as for adjusted plus the following

REF	NRR	SEX	AGEL	AGEH	RACE	YF	LC TYPE	LOC	START	ST	NLC	R	VB	P	H	AD	PRODUCT	exL	exH	DENOM	De
GARSHI	517	m	0	0	all	-	all	NAmer	1981	CC	1081	o	bl	y	n	0	all/unsp	1.0	4	nev any	ot
WAKAI	609	m	0	0	all	-	all	As:Jap	1988	CC	333	n	bl	n	y	0	cig+/-ot	1.0	4	nev any	ot

REF	NRR	RR	SIG	RRDATA comment
GARSHI	517	*	gap	0
WAKAI	609	*	gap	0

Table 1J10 -

IESLC - Meta-analysis of Ex Smoking, Years quit (vs never), "Highest vs lowest"
All LC types, Cigarettes (or Any Product if Cigarettes not available)

This analysis is restricted to results for:
1) Ex smokers
2) Results by Years quit (vs never)
3) Categorical results by Years quit (vs never)
4) Denominator (unexposed) = "low"
5) All LC types (or near equivalent)
6) Results complete enough for use in metaanalysis

Within each study, results are then selected (in the following order of preference, within each sex) for:
7) (not applicable)
8) PRODUCT: cigarettes regardless of other products, cigarettes only, all/unspec
9) CIGTYPE: all/unspecified, MC regardless of HR, MC only
10) Results with least adjustment for other aspects of smoking (ADOS)
11) The highest vs lowest category
12) Followup period (YF, prospective studies): whole study (coded as 0) or longest available
13) LCtype: all or nearest available, at least Squamous and Adeno. (q = squamous, s = small,
l = large, a = adeno, mix = mixed, alv = alveolar)
14) Race: all or nearest available, otherwise by race (wh or w = white, bl or b = black, hi = hispanic
ch = chinese, jap = japanese, haw = hawaiian, w+o = white + oriental, sca = scandinavian, as = asian)
15) For overlapping studies: principal rather than subsidiary studies
Finally by Age: whole study (coded as 0) if available, otherwise by widest available age group
and then for single sex results (m, f) in preference to results for both sexes combined (c).

Results adjusted (AD) for the most potential confounders are then chosen in Sections -1 to -3
(and those which actually differ from the adjusted results in Table 1J5 - 1 are marked 'x' in Section -1)
and results adjusted for the least confounders in Sections -4 to -6. (Those least adjusted results which
actually differ from the most adjusted are marked 'x' in column X in Section -4)

Section -7 shows excluded studies, together with the stage (as above) at which no qualifying
results were found.

Section -8 lists the potentially overlapping studies which have been included (1=principal, 2=subsidiary).

Section -9 lists any results which would have been included in preference except that they had data not complete
enough for use in meta-analysis, with their significance (yes/no), if known, and any further comment as entered
on the database. It also lists as "gap" any categories for which no data were presented by the original authors.
This is commonly due to recent quitters having been combined with current smokers

In addition to those mentioned above, the following fields, levels and abbreviations are used:
* or nk = not known, n = no, y = yes, ot = other
all/unspec = all or unspecified, cig+/-ot = cigarettes irrespective of other products (cigar, pipe etc)
MC = manufactured cigarettes, HR = hand-rolled cigarettes
exL, exH = range of exposure (low and high) in the "highest" group, in terms of Years quit (vs never)
unexL, unexH = range of exposure (low and high) in the "lowest" group, in terms of Years quit (vs never)
REF: 6-character study reference
NRR: number of the RR on the database within the study
ST : study type (CC = case control, pr or prosp = prospective)
NLC: number of lung cancer cases in whole study
R : risky occupational population (n = no, m = mining, o = other risky)
VB : national cigarette type (V = at least 75% Virginia, bl = at least 75% blended, ot = other)
P : any proxy use
H : full histological confirmation
De : derivation of RR/CI (or = original, st = standard method, ot = other method of estimation)

Table 1J10 - 1

IESLC - Meta-analysis of Ex Smoking, Years quit (vs never), "Highest vs lowest"
All LC types, Cigarettes (or Any Product if Cigarettes not available)
Most adjusted

REF	NRR	1J5	SEX	AGEL	AGEH	RACE	YF	LC TYPE	LOC	START	ST	NLC	R	VB	P	H	AD	ADOS	PRODUCT	exL	exH	unexL	unexH	De
ALDERS	512		m	0	0	all	-	all	Eu:UK	1977	CC	1448	n	V	n	n	1	0	cig only	0.1	2	10	999	ot
ALDERS	523		f	0	0	all	-	all	Eu:UK	1977	CC	1448	n	V	n	n	1	0	cig only	0.1	2	10	999	ot
ARMADA	517		m	0	0	all	-	all	Eu:wst	1986	CC	325	n	bl	n	y	0	0	cig+/-ot	1.0	5	6	999	st
BARBON	547		m	0	0	all	-	all	Eu:wst	1979	CC	755	n	bl	y	y	1	0	all/unsp	0.1	4	25	999	ot
BECHER	505		m	0	0	all	-	all	Eu:Ger	1985	CC	194	n	bl	n	y	0	0	all/unsp	2	4	10	999	st
BECHER	515		f	0	0	all	-	all	Eu:Ger	1985	CC	194	n	bl	n	y	0	0	all/unsp	2	4	10	999	st
BENSHL	514		m	0	0	all	0	all	Eu:UK	1967	pr	486	n	V	n	n	2	0	cig+/-ot	0.1	9	30	999	ot
BROSS	517		m	0	0	wh	-	all	NAmer	1960	CC	974	n	bl	n	n	0	0	cig+/-ot	0.1	5	6	999	st
CARPEN	507		c	0	0	w+b	-	all	NAmer	1991	CC	356	n	bl	n	n	0	0	cig+/-ot	0.1	4	15	999	st
CEDERL	530		m	40	69	all	10	all	Eu:Sca	1963	pr	491	n	bl	n	n	1	0	all/unsp	0.1	9	10	999	ot
CHOI	541		m	0	0	all	-	all	As:oth	1985	CC	375	n	bl	n	n	0	0	cig+/-ot	0.1	4	15	999	st
CHOI	554		f	0	0	all	-	all	As:oth	1985	CC	375	n	bl	n	n	0	0	cig+/-ot	0.1	4	5	999	ot
CHYOU	509		m	0	0	jap	21	all	NAmer	1965	pr	227	n	bl	n	y	2	0	cig+/-ot	0.1	14	15	999	ot
CPSI	814		m	50	74	all	6	all	NAmer	1959	pr	5138	n	bl	n	n	1	0	cig only	0.1	0.9	10	999	ot
CPSII	663		m	35	99	all	4	all	NAmer	1982	pr	3229	n	bl	n	n	1	0	cig only	0.1	0.9	16	999	ot
CPSII	641		f	0	0	all	4	all	NAmer	1982	pr	3229	n	bl	n	n	1	0	cig+/-ot	0.1	2	16	999	ot
DAMBER	556	x	m	0	0	all	-	all	Eu:Sca	1972	CC	579	n	bl	y	n	1	0	cig only	0.1	10	11	999	ot
DARBY	503		m	0	0	wh	-	all	Eu:UK	1988	CC	982	n	V	n	n	0	0	all/unsp	0.1	9	10	999	st
DARBY	512		f	0	0	wh	-	all	Eu:UK	1988	CC	982	n	V	n	n	0	0	all/unsp	0.1	9	10	999	st
DEAN3	523	x	m	0	0	all	-	all	Eu:UK	1969	CC	766	n	V	y	n	1	0	cig only	3	4	19	999	ot
DEAN3	558		f	0	0	all	-	all	Eu:UK	1969	CC	766	n	V	y	n	1	0	all/unsp	3	4	9	999	ot
DESTEF	542	x	m	0	0	all	-	all	SCAmer	1988	CC	497	n	bl	n	y	0	0	cig+/-ot	0.1	4	10	999	st
DOLL	535		m	0	0	all	-	all	Eu:UK	1948	CC	1465	n	V	n	n	0	0	all/unsp	0.1	9	20	999	st
DOLL	546		f	0	0	all	-	all	Eu:UK	1948	CC	1465	n	V	n	n	0	0	all/unsp	0.1	9	10	999	st
DOLL2	508		m	0	0	all	20	all	Eu:UK	1951	pr	920	n	V	n	n	1	0	cig only	0.1	4	15	999	ot
DORGAN	512		m	0	0	wh	-	all	NAmer	1980	CC	2026	n	bl	y	y	0	0	cig+/-ot	1.1	5	10	999	st
DORGAN	557		f	0	0	all	-	all	NAmer	1980	CC	2026	n	bl	y	y	0	0	cig+/-ot	1.1	9	10	999	st
DORN	664		m	55	64	wh	8	all	NAmer	1954	pr	5097	n	bl	n	n	0	0	cig+/-ot	0.1	4	15	999	st
DORN	687		m	65	74	wh	8	all	NAmer	1954	pr	5097	n	bl	n	n	0	0	cig+/-ot	0.1	4	15	999	st
GAO	535		m	0	0	all	-	all	As:Chi	1984	CC	1405	n	ot	n	n	2	0	cig+/-ot	0.1	4	10	999	ot
GAO	555		f	0	0	all	-	all	As:Chi	1984	CC	1405	n	ot	n	n	2	0	cig+/-ot	0.1	4	10	999	ot
GAO2	517		m	0	0	all	-	all	As:Jap	1988	CC	282	n	bl	n	n	0	0	cig+/-ot	1.0	4	20	999	st
GARCIA	507		c	0	0	all	-	all	NAmer	1992	CC	416	n	bl	n	y	0	0	cig+/-ot	1.0	4	30	999	st
GARSHI	525		m	0	0	all	-	all	NAmer	1981	CC	1081	o	bl	y	n	1	0	all/unsp	5	14	15	999	st
GRAHAM	539		m	0	0	wh	-	all	NAmer	1956	CC	685	n	bl	n	n	1	0	cig+/-ot	0.1	1.0	5	999	ot
HAMMO2	509		m	0	0	all	0	all	NAmer	1967	pr	450	o	bl	n	n	1	0	cig+/-ot	0.1	4	10	999	ot
HIRAYA	512		m	0	0	all	0	all	As:Jap	1965	pr	1917	n	bl	n	n	1	0	cig+/-ot	0.1	4	10	999	ot
HIRAYA	523		f	0	0	all	0	all	As:Jap	1965	pr	1917	n	bl	n	n	1	0	cig+/-ot	0.1	4	10	999	ot
JAHN	512		m	0	0	all	-	all	Eu:Ger	1988	CC	1004	n	bl	n	n	0	0	cig+/-ot	0.1	0.9	21	999	st
JAIN	569		m	0	0	all	-	all	NAmer	1981	CC	845	n	V	y	n	0	0	cig+/-ot	2	9	10	999	st
JAIN	533		f	0	0	all	-	all	NAmer	1981	CC	845	n	V	y	n	0	0	cig+/-ot	2	9	10	999	st
JEDRYC	614		m	0	0	all	-	all	Eu:est	1980	CC	1630	n	bl	y	n	0	0	cig+/-ot	5	9	10	999	st
JOLY	569		m	0	0	all	-	all	SCAmer	1978	CC	826	n	bl	n	n	0	0	cig+/-ot	1.0	4	5	999	st
JOLY	556		f	0	0	all	-	all	SCAmer	1978	CC	826	n	bl	n	n	0	0	cig+/-ot	1.0	4	5	999	st
KAISE2	652		m	0	0	all	9	all	NAmer	1979	pr	318	n	bl	n	n	1	0	cig only	2	10	21	999	st
KAISE2	572		f	0	0	all	9	all	NAmer	1979	pr	318	n	bl	n	n	1	0	cig only	2	10	21	999	ot
KHUDER	515		m	0	0	all	-	all	NAmer	1985	CC	482	n	bl	n	y	0	0	cig+/-ot	0.1	4	15	999	st
LUBIN	590		m	0	0	all	-	all	As:Chi	1984	CC	427	m	ot	y	n	0	0	cig+/-ot	3	4	10	999	st
LUBIN2	1080		m	0	0	all	-	all	Eu:mul	1976	CC	7804	n	bl	n	y	0	0	cig+/-ot	0.1	4	25	999	st
LUBIN2	1119		f	0	0	all	-	all	Eu:mul	1976	CC	7804	n	bl	n	y	0	0	cig+/-ot	0.1	4	25	999	st
MATOS	595		m	0	0	all	-	all	SCAmer	1994	CC	200	n	bl	n	n	2	0	cig+/-ot	1.0	5	11	999	ot
PEZZO2	503		m	0	0	all	-	all	SCAmer	1992	CC	367	n	bl	n	y	0	0	cig+/-ot	1.0	10	11	999	st
PEZZOT	503		m	0	0	all	-	all	SCAmer	1987	CC	215	n	bl	n	y	0	0	cig only	1.0	10	11	999	st
SOBUE	727		m	0	0	all	-	all	As:Jap	1986	CC	1376	n	bl	n	y	0	0	cig+/-ot	1.0	4	25	999	st
SPEIZE	510		f	0	0	all	0	all	NAmer	1976	pr	593	n	bl	n	y	2	0	cig+/-ot	0.1	1.9	15	999	ot
SUZUK2	530		c	0	0	all	-	all	SCAmer	1991	CC	123	n	bl	n	y	3	0	all/unsp	0.1	5	11	999	ot
SVENSS	553		f	0	0	all	-	all	Eu:Sca	1983	CC	210	n	bl	n	n	0	0	all/unsp	3	10	11	999	st
TVERDA	505		m	0	0	all	0	all	Eu:Sca	1972	pr	238	n	bl	n	n	2	0	cig only	0.1	0.9	5	999	ot
WAKAI	534		m	0	0	all	-	all	As:Jap	1988	CC	333	n	bl	n	y	2	0	cig+/-ot	5	9	20	999	ot
WANG2	513		c	0	0	all	-	all	As:Chi	1980	CC	103	n	ot	n	n	0	0	cig+/-ot	0.1	3	4	999	st
WYNDE3	543		m	0	0	all	-	all	NAmer	1966	CC	350	n	bl	n	y	0	0	all/unsp	1.0	3	13	999	st
WYNDE6	723		m	0	0	wh	-	all	NAmer	1969	CC	4423	n	bl	n	y	5	1#	cig+/-ot	1.0	10	20	999	ot
WYNDE6	730		m	0	0	bl	-	all	NAmer	1969	CC	4423	n	bl	n	y	5	1#	cig+/-ot	1.0	10	20	999	ot
WYNDE6	735		f	0	0	wh	-	all	NAmer	1969	CC	4423	n	bl	n	y	5	1#	cig+/-ot	1.0	10	11	999	ot
WYNDE6	739		f	0	0	bl	-	all	NAmer	1969	CC	4423	n	bl	n	y	5	1#	cig+/-ot	1.0	10	11	999	ot

Table 1J10 - 1

IESLC - Meta-analysis of Ex Smoking, Years quit (vs never), "Highest vs lowest"
All LC types, Cigarettes (or Any Product if Cigarettes not available)
Most adjusted

Comments on values in listings

WYNDE6	ADOS	Number of cigs/day
WYNDE6	ADOS	Number of cigs/day
WYNDE6	ADOS	Number of cigs/day
WYNDE6	ADOS	Number of cigs/day

Cigarette type is all/unspec for all RRs
except for the following:

REF	NRR	CIGTYPE
ALDERS	512	MC only
ALDERS	523	MC only
DEAN3	523	MC only
DESTEF	542	MC only

Table 1J10 - 2

IESLC - Meta-analysis of Ex Smoking, Years quit (vs never), "Highest vs lowest"
All LC types, Cigarettes (or Any Product if Cigarettes not available)
Most adjusted

REF	NRR	SEX	AD	Number Exposed Case	Number Exposed Cont	Non-exposed Case	Non-exposed Cont	RR	95.00%CI
ALDERS	512	m	1	121	-	29	-	5.66	(3.32- 9.64)
ALDERS	523	f	1	206	-	26	-	7.43	(4.38- 12.59)
Subtotal ALDERS								6.49	(4.46- 9.45)
ARMADA	517	m	0	79	45	50	87	3.05	(1.84- 5.06)
BARBON	547	m	1	32	-	15	-	6.62	(2.95- 14.85)
BECHER	505	m	0	10	12	16	72	3.75	(1.38- 10.18)
BECHER	515	f	0	2	3	1	10	6.67	(0.44- 101.73)
Subtotal BECHER								4.01	(1.57- 10.25)
*BENSHL	514	m	2	14	-	6	-	8.68	(2.96- 25.47)
BROSS	517	m	0	169	67	43	79	4.63	(2.91- 7.39)
CARPEN	507	c	0	28	46	37	158	2.60	(1.44- 4.69)
*CEDERL	530	m	1	12	-	3	-	5.55	(1.56- 19.73)
CHOI	541	m	0	25	64	4	19	1.86	(0.57- 6.00)
CHOI	554	f	0	3	2	2	0	0.28~	(0.01- 8.76)
Subtotal CHOI								1.52	(0.50- 4.63)
*CHYOU	509	m	2	21	-	5	-	1.36	(0.52- 3.57)
*CPSI	814	m	1	37	-	15	-	11.54	(6.33- 21.02)
*CPSII	663	m	1	97	-	256	-	10.12	(8.01- 12.78)
*CPSII	641	f	1	91	-	50	-	9.77	(6.92- 13.79)
Subtotal CPSII								10.01	(8.25- 12.14)
DAMBER	556	m	1	-	-	-	-	3.44	(1.36- 8.68)
DARBY	503	m	0	146	339	139	767	2.38	(1.82- 3.10)
DARBY	512	f	0	68	93	26	224	6.30	(3.77- 10.52)
Subtotal DARBY								2.92	(2.31- 3.69)
DEAN3	523	m	1	28	-	8	-	3.56	(1.52- 8.35)
DEAN3	558	f	1	4	-	2	-	2.26	(0.41- 12.55)
Subtotal DEAN3								3.25	(1.52- 6.97)
DESTEF	542	m	0	10	19	10	41	2.16	(0.77- 6.05)
DOLL	535	m	0	56	75	8	23	2.15	(0.89- 5.15)
DOLL	546	f	0	9	6	1	2	3.00	(0.22- 40.93)
Subtotal DOLL								2.22	(0.97- 5.09)
*DOLL2	508	m	1	15	-	7	-	8.00	(3.26- 19.62)
DORGAN	512	m	0	59	51	134	255	2.20	(1.43- 3.38)
DORGAN	557	f	0	49	27	34	50	2.67	(1.41- 5.06)
Subtotal DORGAN								2.34	(1.64- 3.34)
*DORN	664	m	0	34	22086	16	58370	5.62	(3.10- 10.17)
*DORN	687	m	0	14	6195	34	51243	3.41	(1.83- 6.34)
Subtotal DORN								4.42	(2.88- 6.80)
GAO	535	m	2	105	-	13	-	6.27	(2.91- 13.51)
GAO	555	f	2	37	-	16	-	3.27	(1.14- 9.36)
Subtotal GAO								5.00	(2.69- 9.30)
GAO2	517	m	0	31	26	8	25	3.73	(1.44- 9.65)
GARCIA	507	c	0	33	11	10	37	11.10	(4.18- 29.47)
GARSHI	525	m	1	166	-	125	-	1.59	(1.20- 2.11)
GRAHAM	539	m	1	113	-	13	-	13.80	(6.80- 28.00)
*HAMMO2	509	m	1	59	-	20	-	3.19	(1.94- 5.25)
*HIRAYA	512	m	1	-	-	-	-	1.47	(0.54- 4.02)
*HIRAYA	523	f	1	-	-	-	-	3.84	(0.09- 156.01)
Subtotal HIRAYA								1.57	(0.60- 4.13)
JAHN	512	m	0	166	8	29	146	104.47	(46.30- 235.70)
JAIN	569	m	0	74	46	52	113	3.50	(2.13- 5.72)
JAIN	533	f	0	66	36	19	61	5.89	(3.05- 11.34)
Subtotal JAIN								4.22	(2.85- 6.26)
JEDRYC	614	m	0	64	58	73	138	2.09	(1.32- 3.29)
JOLY	569	m	0	38	36	63	149	2.50	(1.45- 4.30)
JOLY	556	f	0	19	8	15	19	3.01	(1.03- 8.75)
Subtotal JOLY								2.59	(1.60- 4.21)
*KAISE2	652	m	1	12	-	6	-	4.80	(1.62- 14.18)
*KAISE2	572	f	1	6	-	4	-	1.72	(0.45- 6.57)
Subtotal KAISE2								3.20	(1.38- 7.43)
KHUDER	515	m	0	88	123	63	213	2.42	(1.63- 3.58)
LUBIN	590	m	0	33	18	17	73	7.87	(3.61- 17.17)
LUBIN2	1080	m	0	866	1047	109	715	5.43	(4.35- 6.77)
LUBIN2	1119	f	0	60	55	4	20	5.45	(1.75- 16.96)
Subtotal LUBIN2								5.43	(4.37- 6.74)
MATOS	595	m	2	28	-	27	-	4.67	(2.25- 9.67)
PEZZO2	503	m	0	85	110	43	161	2.89	(1.86- 4.49)
PEZZOT	503	m	0	46	82	20	106	2.97	(1.63- 5.41)
SOBUE	727	m	0	128	116	17	40	2.60	(1.40- 4.83)
*SPEIZE	510	f	2	24	-	28	-	6.00	(2.72- 13.21)
SUZUK2	530	c	3	15	-	9	-	6.00	(1.94- 18.51)

Table 1J10 - 2

IESLC - Meta-analysis of Ex Smoking, Years quit (vs never), "Highest vs lowest"
All LC types, Cigarettes (or Any Product if Cigarettes not available)
Most adjusted

REF	NRR	SEX	AD	Number Exposed Case	Cont	Non-exposed Case	Cont	RR	95.00%CI
SVENSS	553	f	0	16	13	14	24	2.11	(0.79- 5.65)
*TVERDA	505	m	2	2	-	4	-	2.07	(0.38- 11.34)
WAKAI	534	m	2	19	-	7	-	2.48	(0.95- 6.47)
WANG2	513	c	0	6	10	5	11	1.32	(0.31- 5.70)
WYNDE3	543	m	0	21	22	5	55	10.50	(3.52- 31.34)
WYNDE6	723	m	5	-	-	-	-	3.00	(2.11- 4.27)
WYNDE6	730	m	5	-	-	-	-	2.33	(0.87- 6.25)
WYNDE6	735	f	5	-	-	-	-	2.50	(1.94- 3.23)
WYNDE6	739	f	5	-	-	-	-	1.50	(0.40- 5.61)
Subtotal WYNDE6								2.61	(2.14- 3.19)
Partial Totals				3865	30955	1815	113506		

*prospective study ~ With 0.5 adjustment for zero

REF	NRR	SEX	AD	Ys	Ws	Qs	Ps
ALDERS	512	m	1	1.73	13.52	1.66	0.0000
ALDERS	523	f	1	2.01	13.78	5.35	0.0000
Subtotal ALDERS				1.87	27.31	7.01	
ARMADA	517	m	0	1.12	15.07	1.07	0.0000
BARBON	547	m	1	1.89	5.88	1.52	0.0000
BECHER	505	m	0	1.32	3.85	0.01	0.0095
BECHER	515	f	0	1.90	0.52	0.14	0.1724
Subtotal BECHER				1.39	4.37	0.15	
*BENSHL	514	m	2	2.16	3.32	2.01	0.0001
BROSS	517	m	0	1.53	17.62	0.40	0.0000
CARPEN	507	c	0	0.96	11.01	2.01	0.0015
*CEDERL	530	m	1	1.71	2.39	0.26	0.0081
CHOI	541	m	0	0.62	2.79	1.63	0.3017
CHOI	554	f	0	-1.27	0.32	2.29	0.4687
Subtotal CHOI				0.42	3.12	3.92	
*CHYOU	509	m	2	0.31	4.14	4.79	0.5315
*CPSI	814	m	1	2.45	10.67	12.06	0.0000
*CPSII	663	m	1	2.31	70.40	61.14	0.0000
*CPSII	641	f	1	2.28	32.32	25.99	0.0000
Subtotal CPSII				2.30	102.72	87.13	
DAMBER	556	m	1	1.24	4.47	0.10	0.0090
DARBY	503	m	0	0.87	54.65	14.61	0.0000
DARBY	512	f	0	1.84	14.62	3.07	0.0000
Subtotal DARBY				1.07	69.28	17.67	
DEAN3	523	m	1	1.27	5.29	0.07	0.0035
DEAN3	558	f	1	0.82	1.31	0.42	0.3502
Subtotal DEAN3				1.18	6.61	0.49	
DESTEF	542	m	0	0.77	3.61	1.36	0.1439
DOLL	535	m	0	0.76	5.01	1.92	0.0873
DOLL	546	f	0	1.10	0.56	0.05	0.4100
Subtotal DOLL				0.80	5.57	1.96	
*DOLL2	508	m	1	2.08	4.77	2.32	0.0000
DORGAN	512	m	0	0.79	20.86	7.35	0.0003
DORGAN	557	f	0	0.98	9.36	1.50	0.0027
Subtotal DORGAN				0.85	30.22	8.85	
*DORN	664	m	0	1.73	10.89	1.28	0.0000
*DORN	687	m	0	1.23	9.93	0.25	0.0001
Subtotal DORN				1.49	20.82	1.53	
GAO	535	m	2	1.84	6.52	1.34	0.0000
GAO	555	f	2	1.18	3.47	0.14	0.0274
Subtotal GAO				1.61	9.99	1.47	
GAO2	517	m	0	1.32	4.24	0.02	0.0067
GARCIA	507	c	0	2.41	4.03	4.23	0.0000
GARSHI	525	m	1	0.46	48.24	40.73	0.0013
GRAHAM	539	m	1	2.62	7.67	11.84	0.0000
*HAMMO2	509	m	1	1.16	15.50	0.77	0.0000
*HIRAYA	512	m	1	0.39	3.81	3.79	0.4519
*HIRAYA	523	f	1	1.35	0.28	0.00	0.4794
Subtotal HIRAYA				0.45	4.09	3.79	
JAHN	512	m	0	4.65	5.80	61.90	0.0000
JAIN	569	m	0	1.25	15.79	0.27	0.0000
JAIN	533	f	0	1.77	8.93	1.36	0.0000
Subtotal JAIN				1.44	24.72	1.63	
JEDRYC	614	m	0	0.74	18.58	7.79	0.0015
JOLY	569	m	0	0.91	13.04	2.85	0.0010
JOLY	556	f	0	1.10	3.37	0.27	0.0433

Table 1J10 - 2

IESLC - Meta-analysis of Ex Smoking, Years quit (vs never), "Highest vs lowest"
All LC types, Cigarettes (or Any Product if Cigarettes not available)
Most adjusted

REF	NRR	SEX	AD	Ys	Ws	Qs	Ps
Subtotal JOLY				0.95	16.41	3.12	
*KAISE2	652	m	1	1.57	3.26	0.11	0.0046
*KAISE2	572	f	1	0.54	2.14	1.51	0.4278
Subtotal KAISE2				1.16	5.40	1.62	
KHUDER	515	m	0	0.88	24.96	6.22	0.0000
LUBIN	590	m	0	2.06	6.31	2.93	0.0000
LUBIN2	1080	m	0	1.69	78.85	7.51	0.0000
LUBIN2	1119	f	0	1.70	2.99	0.29	0.0034
Subtotal LUBIN2				1.69	81.83	7.80	
MATOS	595	m	2	1.54	7.23	0.18	0.0000
PEZZO2	503	m	0	1.06	19.87	2.04	0.0000
PEZZOT	503	m	0	1.09	10.71	0.92	0.0004
SOBUE	727	m	0	0.95	9.97	1.83	0.0026
*SPEIZE	510	f	2	1.79	6.15	1.03	0.0000
SUZUK2	530	c	3	1.79	3.02	0.51	0.0018
SVENSS	553	f	0	0.75	3.96	1.60	0.1373
*TVERDA	505	m	2	0.73	1.33	0.57	0.4010
WAKAI	534	m	2	0.91	4.17	0.94	0.0635
WANG2	513	c	0	0.28	1.79	2.19	0.7100
WYNDE3	543	m	0	2.35	3.21	3.02	0.0000
WYNDE6	723	m	5	1.10	30.92	2.49	0.0000
WYNDE6	730	m	5	0.85	3.95	1.14	0.0927
WYNDE6	735	f	5	0.92	59.12	12.86	0.0000
WYNDE6	739	f	5	0.41	2.20	2.10	0.5473
Subtotal WYNDE6				0.96	96.20	18.59	

	N	65
	NS	47
	Wt	798.37
Het	Chi	345.86
Het	df	64
Het	P	***
Fixed	RR	3.99
	RRl	3.72
	RRu	4.27
	P	+++
Random	RR	4.00
	RRl	3.34
	RRu	4.80
	P	+++
Asymm	P	N.S.

Table 1J10 - 3

IESLC - Meta-analysis of Ex Smoking, Years quit (vs never), "Highest vs lowest"
All LC types, Cigarettes (or Any Product if Cigarettes not available)
Most adjusted

	Sex: combined	male	female	Total
N	4	43	18	65
NS	4	41	17	62
Wt	19.85	613.11	165.41	798.37
Het Chi	8.84	276.84	59.31	345.86
Het df	3	42	17	64
Het P	*	***	***	***
Fixed RR	3.73	3.93	4.24	3.99
RRl	2.40	3.63	3.64	3.72
RRu	5.79	4.25	4.94	4.27
P	+++	+++	+++	+++
Random RR	4.06	4.03	3.96	4.00
RRl	1.74	3.23	2.76	3.34
RRu	9.46	5.02	5.68	4.80
P	++	+++	+++	+++
Between Chi				0.86
Between df				2
Between P				N.S.
Btwn(F) P				N.S.
Btwn(R) P				N.S.

	Lung cancer type: all	other	Total
N	65		65
NS	47		47
Wt	798.37		798.37
Het Chi	345.86		345.86
Het df	64		64
Het P	***		***
Fixed RR	3.99		3.99
RRl	3.72		3.72
RRu	4.27		4.27
P	+++		+++
Random RR	4.00		4.00
RRl	3.34		3.34
RRu	4.80		4.80
P	+++		+++
Between Chi			
Between df			
Between P			N.S.
Btwn(F) P			N.S.
Btwn(R) P			N.S.

	Location: NAmer	UK	Scand	othEur	China	Japan	othAs	other	Total
N	25	10	4	8	4	5	2	7	65
NS	17	6	4	6	3	4	1	6	47
Wt	433.30	116.85	12.15	131.54	18.09	22.48	3.12	60.85	798.37
Het Chi	206.40	31.05	1.66	72.82	5.43	1.82	1.04	3.71	345.86
Het df	24	9	3	7	3	4	1	6	64
Het P	***	***	N.S.	***	N.S.	N.S.	N.S.	N.S.	***
Fixed RR	4.02	3.76	3.05	5.05	5.13	2.51	1.52	3.04	3.99
RRl	3.66	3.13	1.74	4.26	3.24	1.66	0.50	2.37	3.72
RRu	4.42	4.50	5.35	5.99	8.14	3.80	4.63	3.91	4.27
P	+++	+++	+++	+++	+++	+++	N.S.	+++	+++
Random RR	4.05	4.56	3.05	6.33	4.63	2.51	1.48	3.04	4.00
RRl	3.01	3.02	1.74	3.10	2.42	1.66	0.45	2.37	3.34
RRu	5.46	6.90	5.35	12.91	8.84	3.80	4.92	3.91	4.80
P	+++	+++	+++	+++	+++	+++	N.S.	+++	+++
Between Chi									21.94
Between df									7
Between P									**
Btwn(F) P									N.S.
Btwn(R) P									N.S.

Table 1J10 - 3

IESLC - Meta-analysis of Ex Smoking, Years quit (vs never), "Highest vs lowest"
All LC types, Cigarettes (or Any Product if Cigarettes not available)
Most adjusted

Detailed Country in "other Europe"

	multi	Germany	othWest	East	Balkans	Total
N	2	3	2	1		8
NS	1	2	2	1		6
Wt	81.83	10.17	20.95	18.58		131.54
Het Chi	0.00	26.62	2.53	0.00		72.82
Het df	1	2	1	0		7
Het P	N.S.	***	N.S.	N.S.		***
Fixed RR	5.43	25.77	3.80	2.09		5.05
RRl	4.37	13.94	2.47	1.32		4.26
RRu	6.74	47.65	5.82	3.29		5.99
P	+++	+++	+++	++		+++
Random RR	5.43	14.93	4.21	2.09		6.33
RRl	4.37	1.12	1.99	1.32		3.10
RRu	6.74	199.97	8.87	3.29		12.91
P	+++	+	+++	++		+++
Between Chi						43.67
Between df						3
Between P						***
Btwn(F) P						N.S.
Btwn(R) P						**

Detailed Country in "other Asia"

	India	HongKong	other	Total
N			2	2
NS			1	1
Wt			3.12	3.12
Het Chi			1.04	1.04
Het df			1	1
Het P			N.S.	N.S.
Fixed RR			1.52	1.52
RRl			0.50	0.50
RRu			4.63	4.63
P			N.S.	N.S.
Random RR			1.48	1.48
RRl			0.45	0.45
RRu			4.92	4.92
P			N.S.	N.S.
Between Chi				
Between df				
Between P				N.S.
Btwn(F) P				N.S.
Btwn(R) P				N.S.

Detailed other continent

	SCAmer	Total
N	7	7
NS	6	6
Wt	60.85	60.85
Het Chi	3.71	3.71
Het df	6	6
Het P	N.S.	N.S.
Fixed RR	3.04	3.04
RRl	2.37	2.37
RRu	3.91	3.91
P	+++	+++
Random RR	3.04	3.04
RRl	2.37	2.37
RRu	3.91	3.91
P	+++	+++
Between Chi		
Between df		
Between P		N.S.
Btwn(F) P		N.S.
Btwn(R) P		N.S.

Table 1J10 - 3

IESLC - Meta-analysis of Ex Smoking, Years quit (vs never), "Highest vs lowest"
All LC types, Cigarettes (or Any Product if Cigarettes not available)
Most adjusted

	Start year of study <1960	1960-69	1970-79	1980-89	1990+	Total
N	7	14	12	27	5	65
NS	5	9	8	20	5	47
Wt	49.50	153.08	148.79	401.84	45.16	798.37
Het Chi	18.91	21.16	14.85	250.14	8.50	345.86
Het df	6	13	11	26	4	64
Het P	**	(*)	N.S.	***	(*)	***
Fixed RR	6.36	2.98	5.02	3.90	3.60	3.99
RRl	4.81	2.54	4.27	3.54	2.69	3.72
RRu	8.40	3.49	5.89	4.30	4.82	4.27
P	+++	+++	+++	+++	+++	+++
Random RR	6.07	3.14	4.76	3.77	4.11	4.00
RRl	3.58	2.44	3.78	2.69	2.58	3.34
RRu	10.29	4.03	6.00	5.26	6.54	4.80
P	+++	+++	+++	+++	+++	+++
Between Chi						32.30
Between df						4
Between P						***
Btwn(F) P						N.S.
Btwn(R) P						(*)

	Study type (1) CC	other	Total
N	49	16	65
NS	35	12	47
Wt	617.07	181.30	798.37
Het Chi	210.61	58.74	345.86
Het df	48	15	64
Het P	***	***	***
Fixed RR	3.37	7.05	3.99
RRl	3.11	6.10	3.72
RRu	3.65	8.16	4.27
P	+++	+++	+++
Random RR	3.75	4.99	4.00
RRl	3.11	3.51	3.34
RRu	4.52	7.08	4.80
P	+++	+++	+++
Between Chi			76.51
Between df			1
Between P			***
Btwn(F) P			***
Btwn(R) P			N.S.

	Study type (2) CC	prosp	other	Total
N	49	16		65
NS	35	12		47
Wt	617.07	181.30		798.37
Het Chi	210.61	58.74		345.86
Het df	48	15		64
Het P	***	***		***
Fixed RR	3.37	7.05		3.99
RRl	3.11	6.10		3.72
RRu	3.65	8.16		4.27
P	+++	+++		+++
Random RR	3.75	4.99		4.00
RRl	3.11	3.51		3.34
RRu	4.52	7.08		4.80
P	+++	+++		+++
Between Chi				76.51
Between df				1
Between P				***
Btwn(F) P				***
Btwn(R) P				N.S.

Table 1J10 - 3

IESLC - Meta-analysis of Ex Smoking, Years quit (vs never), "Highest vs lowest"
All LC types, Cigarettes (or Any Product if Cigarettes not available)
Most adjusted

	Study size (number of LC cases)				
	100-249	250-499	500-999	1000+	Total
N	9	17	14	25	65
NS	8	15	10	14	47
Wt	36.55	126.22	163.58	472.01	798.37
Het Chi	7.86	27.55	39.70	258.45	345.86
Het df	8	16	13	24	64
Het P	N.S.	*	***	***	***
Fixed RR	2.99	3.28	3.79	4.37	3.99
RRl	2.16	2.75	3.25	3.99	3.72
RRu	4.13	3.90	4.42	4.78	4.27
P	+++	+++	+++	+++	+++
Random RR	2.99	3.54	4.51	4.27	4.00
RRl	2.16	2.75	3.34	3.07	3.34
RRu	4.13	4.57	6.09	5.94	4.80
P	+++	+++	+++	+++	+++
Between Chi					12.30
Between df					3
Between P					**
Btwn(F) P					N.S.
Btwn(R) P					N.S.

	Risky occupational population			
	no	mining	othRisky	Total
N	62	1	2	65
NS	44	1	2	47
Wt	728.31	6.31	63.75	798.37
Het Chi	298.84	0.00	5.69	345.86
Het df	61	0	1	64
Het P	***	N.S.	*	***
Fixed RR	4.23	7.87	1.88	3.99
RRl	3.93	3.61	1.47	3.72
RRu	4.55	17.17	2.41	4.27
P	+++	+++	+++	+++
Random RR	4.07	7.87	2.18	4.00
RRl	3.39	3.61	1.11	3.34
RRu	4.87	17.17	4.31	4.80
P	+++	+++	+	+++
Between Chi				41.33
Between df				2
Between P				***
Btwn(F) P				*
Btwn(R) P				(*)

	National cigarette tobacco type			
	Virginia	blended	other	Total
N	12	49	4	65
NS	7	37	3	47
Wt	141.57	638.70	18.09	798.37
Het Chi	32.88	306.17	5.43	345.86
Het df	11	48	3	64
Het P	***	***	N.S.	***
Fixed RR	3.83	3.99	5.13	3.99
RRl	3.25	3.69	3.24	3.72
RRu	4.52	4.31	8.14	4.27
P	+++	+++	+++	+++
Random RR	4.53	3.85	4.63	4.00
RRl	3.25	3.09	2.42	3.34
RRu	6.33	4.79	8.84	4.80
P	+++	+++	+++	+++
Between Chi				1.38
Between df				2
Between P				N.S.
Btwn(F) P				N.S.
Btwn(R) P				N.S.

Table 1J10 - 3

IESLC - Meta-analysis of Ex Smoking, Years quit (vs never), "Highest vs lowest"
All LC types, Cigarettes (or Any Product if Cigarettes not available)
Most adjusted

	Any proxy use No/nk	Yes	Total
N	54	11	65
NS	39	8	47
Wt	653.33	145.04	798.37
Het Chi	275.21	34.18	345.86
Het df	53	10	64
Het P	***	***	***
Fixed RR	4.41	2.53	3.99
RRl	4.08	2.15	3.72
RRu	4.76	2.98	4.27
P	+++	+++	+++
Random RR	4.20	3.19	4.00
RRl	3.44	2.28	3.34
RRu	5.12	4.47	4.80
P	+++	+++	+++
Between Chi			36.47
Between df			1
Between P			***
Btwn(F) P			**
Btwn(R) P			N.S.

	Full histological confirmation No	Yes	Total
N	42	23	65
NS	30	17	47
Wt	470.94	327.43	798.37
Het Chi	274.27	55.14	345.86
Het df	41	22	64
Het P	***	***	***
Fixed RR	4.49	3.35	3.99
RRl	4.10	3.01	3.72
RRu	4.92	3.74	4.27
P	+++	+++	+++
Random RR	4.37	3.30	4.00
RRl	3.37	2.69	3.34
RRu	5.66	4.04	4.80
P	+++	+++	+++
Between Chi			16.45
Between df			1
Between P			***
Btwn(F) P			(*)
Btwn(R) P			(*)

	Number of adjustment variables (1) 0	1	2+/+nk	Total
N	34	18	13	65
NS	25	13	9	47
Wt	417.10	245.72	135.55	798.37
Het Chi	141.72	142.86	19.34	345.86
Het df	33	17	12	64
Het P	***	***	(*)	***
Fixed RR	3.60	5.54	3.01	3.99
RRl	3.27	4.89	2.54	3.72
RRu	3.96	6.28	3.56	4.27
P	+++	+++	+++	+++
Random RR	3.74	5.02	3.31	4.00
RRl	2.99	3.31	2.54	3.34
RRu	4.68	7.62	4.32	4.80
P	+++	+++	+++	+++
Between Chi				41.95
Between df				2
Between P				***
Btwn(F) P				*
Btwn(R) P				N.S.

Table 1J10 - 3

IESLC - Meta-analysis of Ex Smoking, Years quit (vs never), "Highest vs lowest"
All LC types, Cigarettes (or Any Product if Cigarettes not available)
Most adjusted

	Number of adjustment variables (2) 0	1	2	3-5	6+/+nk	Total
N	34	18	8	5		65
NS	25	13	7	2		47
Wt	417.10	245.72	36.33	99.22		798.37
Het Chi	141.72	142.86	10.95	3.46		345.86
Het df	33	17	7	4		64
Het P	***	***	N.S.	N.S.		***
Fixed RR	3.60	5.54	4.12	2.68		3.99
RRl	3.27	4.89	2.98	2.20		3.72
RRu	3.96	6.28	5.70	3.26		4.27
P	+++	+++	+++	+++		+++
Random RR	3.74	5.02	3.99	2.68		4.00
RRl	2.99	3.31	2.63	2.20		3.34
RRu	4.68	7.62	6.05	3.26		4.80
P	+++	+++	+++	+++		+++
Between Chi						46.87
Between df						3
Between P						***
Btwn(F) P						*
Btwn(R) P						*

	Product all/unsp	cig+/-ot	cig only	Total
N	13	41	11	65
NS	10	30	9	49
Wt	147.23	510.78	140.36	798.37
Het Chi	39.80	191.19	31.70	345.86
Het df	12	40	10	64
Het P	***	***	***	***
Fixed RR	2.58	3.81	7.41	3.99
RRl	2.19	3.49	6.28	3.72
RRu	3.03	4.16	8.75	4.27
P	+++	+++	+++	+++
Random RR	3.56	3.82	5.49	4.00
RRl	2.42	3.10	3.82	3.34
RRu	5.24	4.72	7.89	4.80
P	+++	+++	+++	+++
Between Chi				83.18
Between df				2
Between P				***
Btwn(F) P				***
Btwn(R) P				N.S.

	Derivation of RR/CI Orig	StdCalc	Other	Total
N		35	30	65
NS		27	22	49
Wt		468.28	330.09	798.37
Het Chi		168.98	138.86	345.86
Het df		34	29	64
Het P		***	***	***
Fixed RR		3.32	5.17	3.99
RRl		3.03	4.64	3.72
RRu		3.63	5.76	4.27
P		+++	+++	+++
Random RR		3.67	4.46	4.00
RRl		2.93	3.38	3.34
RRu		4.60	5.87	4.80
P		+++	+++	+++
Between Chi				38.02
Between df				1
Between P				***
Btwn(F) P				**
Btwn(R) P				N.S.

Table 1J10 - 4

IESLC - Meta-analysis of Ex Smoking, Years quit (vs never), "Highest vs lowest"
All LC types, Cigarettes (or Any Product if Cigarettes not available)
Least adjusted

REF	NRR	X	SEX	AGEL	AGEH	RACE	YF	LC TYPE	LOC	START	ST	NLC	R	VB	P	H	AD	ADOS	PRODUCT	exL	exH	unexL	unexH	De
ALDERS	512		m	0	0	all	-	all	Eu:UK	1977	CC	1448	n	V	n	n	1	0	cig only	0.1	2	10	999	ot
ALDERS	523		f	0	0	all	-	all	Eu:UK	1977	CC	1448	n	V	n	n	1	0	cig only	0.1	2	10	999	ot
ARMADA	517		m	0	0	all	-	all	Eu:wst	1986	CC	325	n	bl	n	y	0	0	cig+/-ot	1.0	5	6	999	st
BARBON	532	x	m	0	0	all	-	all	Eu:wst	1979	CC	755	n	bl	y	y	0	0	all/unsp	0.1	4	25	999	st
BECHER	505		m	0	0	all	-	all	Eu:Ger	1985	CC	194	n	bl	n	y	0	0	all/unsp	2	4	10	999	st
BECHER	515		f	0	0	all	-	all	Eu:Ger	1985	CC	194	n	bl	n	y	0	0	all/unsp	2	4	10	999	st
BENSHL	514		m	0	0	all	0	all	Eu:UK	1967	pr	486	n	V	n	n	2	0	cig+/-ot	0.1	9	30	999	ot
BROSS	517		m	0	0	wh	-	all	NAmer	1960	CC	974	n	bl	n	n	0	0	cig+/-ot	0.1	5	6	999	st
CARPEN	507		c	0	0	w+b	-	all	NAmer	1991	CC	356	n	bl	n	n	0	0	cig+/-ot	0.1	4	15	999	st
CEDERL	530		m	40	69	all	10	all	Eu:Sca	1963	pr	491	n	bl	n	n	1	0	all/unsp	0.1	9	10	999	ot
CHOI	541		m	0	0	all	-	all	As:oth	1985	CC	375	n	bl	n	n	0	0	cig+/-ot	0.1	4	15	999	st
CHOI	554		f	0	0	all	-	all	As:oth	1985	CC	375	n	bl	n	n	0	0	cig+/-ot	0.1	4	5	999	ot
CHYOU	503	x	m	0	0	jap	21	all	NAmer	1965	pr	227	n	bl	n	y	1	0	cig+/-ot	0.1	14	15	999	ot
CPSI	814		m	50	74	all	6	all	NAmer	1959	pr	5138	n	bl	n	n	1	0	cig only	0.1	0.9	10	999	ot
CPSII	663		m	35	99	all	4	all	NAmer	1982	pr	3229	n	bl	n	n	1	0	cig only	0.1	0.9	16	999	ot
CPSII	641		f	0	0	all	4	all	NAmer	1982	pr	3229	n	bl	n	n	1	0	cig+/-ot	0.1	2	16	999	ot
DAMBER	556		m	0	0	all	-	all	Eu:Sca	1972	CC	579	n	bl	y	n	1	0	cig only	0.1	10	11	999	ot
DARBY	503		m	0	0	wh	-	all	Eu:UK	1988	CC	982	n	V	n	n	0	0	all/unsp	0.1	9	10	999	st
DARBY	512		f	0	0	wh	-	all	Eu:UK	1988	CC	982	n	V	n	n	0	0	all/unsp	0.1	9	10	999	st
DEAN3	508	x	m	0	0	all	-	all	Eu:UK	1969	CC	766	n	V	y	n	0	0	cig only	3	4	19	999	st
DEAN3	547	x	f	0	0	all	-	all	Eu:UK	1969	CC	766	n	V	y	n	0	0	all/unsp	3	4	9	999	st
DESTEF	542		m	0	0	all	-	all	SCAmer	1988	CC	497	n	bl	n	y	0	0	cig+/-ot	0.1	4	10	999	st
DOLL	535		m	0	0	all	-	all	Eu:UK	1948	CC	1465	n	V	n	n	0	0	all/unsp	0.1	9	20	999	st
DOLL	546		f	0	0	all	-	all	Eu:UK	1948	CC	1465	n	V	n	n	0	0	all/unsp	0.1	9	10	999	st
DOLL2	508		m	0	0	all	20	all	Eu:UK	1951	pr	920	n	V	n	n	1	0	cig only	0.1	4	15	999	ot
DORGAN	512		m	0	0	wh	-	all	NAmer	1980	CC	2026	n	bl	y	y	0	0	cig+/-ot	1.1	5	10	999	st
DORGAN	557		f	0	0	all	-	all	NAmer	1980	CC	2026	n	bl	y	y	0	0	cig+/-ot	1.1	9	10	999	st
DORN	664		m	55	64	wh	8	all	NAmer	1954	pr	5097	n	bl	n	n	0	0	cig+/-ot	0.1	4	15	999	st
DORN	687		m	65	74	wh	8	all	NAmer	1954	pr	5097	n	bl	n	n	0	0	cig+/-ot	0.1	4	15	999	st
GAO	525	x	m	0	0	all	-	all	As:Chi	1984	CC	1405	n	ot	n	n	0	0	cig+/-ot	0.1	4	10	999	st
GAO	545	x	f	0	0	all	-	all	As:Chi	1984	CC	1405	n	ot	n	n	0	0	cig+/-ot	0.1	4	10	999	st
GAO2	517		m	0	0	all	-	all	As:Jap	1988	CC	282	n	bl	n	n	0	0	cig+/-ot	1.0	4	20	999	st
GARCIA	507		c	0	0	all	-	all	NAmer	1992	CC	416	n	bl	n	y	0	0	cig+/-ot	1.0	4	30	999	st
GARSHI	518	x	m	0	0	all	-	all	NAmer	1981	CC	1081	o	bl	y	n	0	0	all/unsp	5	14	15	999	st
GRAHAM	529	x	m	0	0	wh	-	all	NAmer	1956	CC	685	n	bl	n	n	0	0	cig+/-ot	0.1	1.0	5	999	st
HAMMO2	509		m	0	0	all	0	all	NAmer	1967	pr	450	o	bl	n	n	1	0	cig+/-ot	0.1	4	10	999	ot
HIRAYA	512		m	0	0	all	0	all	As:Jap	1965	pr	1917	n	bl	n	n	1	0	cig+/-ot	0.1	4	10	999	ot
HIRAYA	523		f	0	0	all	0	all	As:Jap	1965	pr	1917	n	bl	n	n	1	0	cig+/-ot	0.1	4	10	999	ot
JAHN	512		m	0	0	all	-	all	Eu:Ger	1988	CC	1004	n	bl	n	n	0	0	cig+/-ot	0.1	0.9	21	999	st
JAIN	569		m	0	0	all	-	all	NAmer	1981	CC	845	n	V	y	n	0	0	cig+/-ot	2	9	10	999	st
JAIN	533		f	0	0	all	-	all	NAmer	1981	CC	845	n	V	y	n	0	0	cig+/-ot	2	9	10	999	st
JEDRYC	614		m	0	0	all	-	all	Eu:est	1980	CC	1630	n	bl	y	n	0	0	cig+/-ot	5	9	10	999	st
JOLY	569		m	0	0	all	-	all	SCAmer	1978	CC	826	n	bl	n	n	0	0	cig+/-ot	1.0	4	5	999	st
JOLY	556		f	0	0	all	-	all	SCAmer	1978	CC	826	n	bl	n	n	0	0	cig+/-ot	1.0	4	5	999	st
KAISE2	652		m	0	0	all	9	all	NAmer	1979	pr	318	n	bl	n	n	1	0	cig only	2	10	21	999	st
KAISE2	572		f	0	0	all	9	all	NAmer	1979	pr	318	n	bl	n	n	1	0	cig only	2	10	21	999	ot
KHUDER	515		m	0	0	all	-	all	NAmer	1985	CC	482	n	bl	n	y	0	0	cig+/-ot	0.1	4	15	999	st
LUBIN	590		m	0	0	all	-	all	As:Chi	1984	CC	427	m	ot	y	n	0	0	cig+/-ot	3	4	10	999	st
LUBIN2	1080		m	0	0	all	-	all	Eu:mul	1976	CC	7804	n	bl	n	y	0	0	cig+/-ot	0.1	4	25	999	st
LUBIN2	1119		f	0	0	all	-	all	Eu:mul	1976	CC	7804	n	bl	n	y	0	0	cig+/-ot	0.1	4	25	999	st
MATOS	585	x	m	0	0	all	-	all	SCAmer	1994	CC	200	n	bl	n	n	0	0	cig+/-ot	1.0	5	11	999	st
PEZZO2	503		m	0	0	all	-	all	SCAmer	1992	CC	367	n	bl	n	y	0	0	cig+/-ot	1.0	10	11	999	st
PEZZOT	503		m	0	0	all	-	all	SCAmer	1987	CC	215	n	bl	n	y	0	0	cig only	1.0	10	11	999	st
SOBUE	727		m	0	0	all	-	all	As:Jap	1986	CC	1376	n	bl	n	y	0	0	cig+/-ot	1.0	4	25	999	st
SPEIZE	510		f	0	0	all	0	all	NAmer	1976	pr	593	n	bl	n	y	2	0	cig+/-ot	0.1	1.9	15	999	ot
SUZUK2	512	x	c	0	0	all	-	all	SCAmer	1991	CC	123	n	bl	n	y	0	0	all/unsp	0.1	5	11	999	st
SVENSS	553		f	0	0	all	-	all	Eu:Sca	1983	CC	210	n	bl	n	n	0	0	all/unsp	3	10	11	999	st
TVERDA	505		m	0	0	all	0	all	Eu:Sca	1972	pr	238	n	bl	n	n	2	0	cig only	0.1	0.9	5	999	ot
WAKAI	526	x	m	0	0	all	-	all	As:Jap	1988	CC	333	n	bl	n	y	0	0	cig+/-ot	5	9	20	999	st
WANG2	513		c	0	0	all	-	all	As:Chi	1980	CC	103	n	ot	n	n	0	0	cig+/-ot	0.1	3	4	999	st
WYNDE3	543		m	0	0	all	-	all	NAmer	1966	CC	350	n	bl	n	y	0	0	all/unsp	1.0	3	13	999	st
WYNDE6	723		m	0	0	wh	-	all	NAmer	1969	CC	4423	n	bl	n	y	5	1#	cig+/-ot	1.0	10	20	999	ot
WYNDE6	730		m	0	0	bl	-	all	NAmer	1969	CC	4423	n	bl	n	y	5	1#	cig+/-ot	1.0	10	20	999	ot
WYNDE6	735		f	0	0	wh	-	all	NAmer	1969	CC	4423	n	bl	n	y	5	1#	cig+/-ot	1.0	10	11	999	ot
WYNDE6	739		f	0	0	bl	-	all	NAmer	1969	CC	4423	n	bl	n	y	5	1#	cig+/-ot	1.0	10	11	999	ot

Table 1J10 - 4

IESLC - Meta-analysis of Ex Smoking, Years quit (vs never), "Highest vs lowest"
All LC types, Cigarettes (or Any Product if Cigarettes not available)
Least adjusted

Comments on values in listings

WYNDE6	ADOS	Number of cigs/day
WYNDE6	ADOS	Number of cigs/day
WYNDE6	ADOS	Number of cigs/day
WYNDE6	ADOS	Number of cigs/day

Cigarette type is all/unspec for all RRs
except for the following:

REF	NRR	CIGTYPE
ALDERS	512	MC only
ALDERS	523	MC only
DEAN3	508	MC only
DESTEF	542	MC only

Table 1J10 - 5

IESLC - Meta-analysis of Ex Smoking, Years quit (vs never), "Highest vs lowest"
All LC types, Cigarettes (or Any Product if Cigarettes not available)
Least adjusted

REF	NRR	SEX	AD	Number Exposed Case	Cont	Non-exposed Case	Cont	RR	95.00%CI
ALDERS	512	m	1	121	-	29	-	5.66	(3.32- 9.64)
ALDERS	523	f	1	206	-	26	-	7.43	(4.38- 12.59)
Subtotal ALDERS								6.49	(4.46- 9.45)
ARMADA	517	m	0	79	45	50	87	3.05	(1.84- 5.06)
BARBON	532	m	0	32	20	15	59	6.29	(2.84- 13.95)
BECHER	505	m	0	10	12	16	72	3.75	(1.38- 10.18)
BECHER	515	f	0	2	3	1	10	6.67	(0.44- 101.73)
Subtotal BECHER								4.01	(1.57- 10.25)
*BENSHL	514	m	2	14	-	6	-	8.68	(2.96- 25.47)
BROSS	517	m	0	169	67	43	79	4.63	(2.91- 7.39)
CARPEN	507	c	0	28	46	37	158	2.60	(1.44- 4.69)
*CEDERL	530	m	1	12	-	3	-	5.55	(1.56- 19.73)
CHOI	541	m	0	25	64	4	19	1.86	(0.57- 6.00)
CHOI	554	f	0	3	2	2	0	0.28~	(0.01- 8.76)
Subtotal CHOI								1.52	(0.50- 4.63)
*CHYOU	503	m	1	21	-	5	-	1.39	(0.52- 3.71)
*CPSI	814	m	1	37	-	15	-	11.54	(6.33- 21.02)
*CPSII	663	m	1	97	-	256	-	10.12	(8.01- 12.78)
*CPSII	641	f	1	91	-	50	-	9.77	(6.92- 13.79)
Subtotal CPSII								10.01	(8.25- 12.14)
DAMBER	556	m	1	-	-	-	-	3.44	(1.36- 8.68)
DARBY	503	m	0	146	339	139	767	2.38	(1.82- 3.10)
DARBY	512	f	0	68	93	26	224	6.30	(3.77- 10.52)
Subtotal DARBY								2.92	(2.31- 3.69)
DEAN3	508	m	0	28	102	8	66	2.26	(0.97- 5.27)
DEAN3	547	f	0	4	110	2	114	2.07	(0.37- 11.55)
Subtotal DEAN3								2.23	(1.04- 4.75)
DESTEF	542	m	0	10	19	10	41	2.16	(0.77- 6.05)
DOLL	535	m	0	56	75	8	23	2.15	(0.89- 5.15)
DOLL	546	f	0	9	6	1	2	3.00	(0.22- 40.93)
Subtotal DOLL								2.22	(0.97- 5.09)
*DOLL2	508	m	1	15	-	7	-	8.00	(3.26- 19.62)
DORGAN	512	m	0	59	51	134	255	2.20	(1.43- 3.38)
DORGAN	557	f	0	49	27	34	50	2.67	(1.41- 5.06)
Subtotal DORGAN								2.34	(1.64- 3.34)
*DORN	664	m	0	34	22086	16	58370	5.62	(3.10- 10.17)
*DORN	687	m	0	14	6195	34	51243	3.41	(1.83- 6.34)
Subtotal DORN								4.42	(2.88- 6.80)
GAO	525	m	0	105	52	13	41	6.37	(3.14- 12.91)
GAO	545	f	0	37	9	16	14	3.60	(1.29- 10.00)
Subtotal GAO								5.29	(2.96- 9.47)
GAO2	517	m	0	31	26	8	25	3.73	(1.44- 9.65)
GARCIA	507	c	0	33	11	10	37	11.10	(4.18- 29.47)
GARSHI	518	m	0	166	290	125	343	1.57	(1.19- 2.08)
GRAHAM	529	m	0	113	59	13	71	10.46	(5.35- 20.44)
*HAMMO2	509	m	1	59	-	20	-	3.19	(1.94- 5.25)
*HIRAYA	512	m	1	-	-	-	-	1.47	(0.54- 4.02)
*HIRAYA	523	f	1	-	-	-	-	3.84	(0.09- 156.01)
Subtotal HIRAYA								1.57	(0.60- 4.13)
JAHN	512	m	0	166	8	29	146	104.47	(46.30- 235.70)
JAIN	569	m	0	74	46	52	113	3.50	(2.13- 5.72)
JAIN	533	f	0	66	36	19	61	5.89	(3.05- 11.34)
Subtotal JAIN								4.22	(2.85- 6.26)
JEDRYC	614	m	0	64	58	73	138	2.09	(1.32- 3.29)
JOLY	569	m	0	38	36	63	149	2.50	(1.45- 4.30)
JOLY	556	f	0	19	8	15	19	3.01	(1.03- 8.75)
Subtotal JOLY								2.59	(1.60- 4.21)
*KAISE2	652	m	1	12	-	6	-	4.80	(1.62- 14.18)
*KAISE2	572	f	1	6	-	4	-	1.72	(0.45- 6.57)
Subtotal KAISE2								3.20	(1.38- 7.43)
KHUDER	515	m	0	88	123	63	213	2.42	(1.63- 3.58)
LUBIN	590	m	0	33	18	17	73	7.87	(3.61- 17.17)
LUBIN2	1080	m	0	866	1047	109	715	5.43	(4.35- 6.77)
LUBIN2	1119	f	0	60	55	4	20	5.45	(1.75- 16.96)
Subtotal LUBIN2								5.43	(4.37- 6.74)
MATOS	585	m	0	28	23	27	101	4.55	(2.27- 9.13)
PEZZO2	503	m	0	85	110	43	161	2.89	(1.86- 4.49)
PEZZOT	503	m	0	46	82	20	106	2.97	(1.63- 5.41)
SOBUE	727	m	0	128	116	17	40	2.60	(1.40- 4.83)
*SPEIZE	510	f	2	24	-	28	-	6.00	(2.72- 13.21)
SUZUK2	512	c	0	15	10	9	22	3.67	(1.20- 11.17)

Table 1J10 - 5

IESLC - Meta-analysis of Ex Smoking, Years quit (vs never), "Highest vs lowest"
All LC types, Cigarettes (or Any Product if Cigarettes not available)
Least adjusted

REF	NRR	SEX	AD	Number Exposed Case	Exposed Cont	Non-exposed Case	Non-exposed Cont	RR	95.00%CI
SVENSS	553	f	0	16	13	14	24	2.11	(0.79- 5.65)
*TVERDA	505	m	2	2	-	4	-	2.07	(0.38- 11.34)
WAKAI	526	m	0	19	48	7	47	2.66	(1.02- 6.91)
WANG2	513	c	0	6	10	5	11	1.32	(0.31- 5.70)
WYNDE3	543	m	0	21	22	5	55	10.50	(3.52- 31.34)
WYNDE6	723	m	5	-	-	-	-	3.00	(2.11- 4.27)
WYNDE6	730	m	5	-	-	-	-	2.33	(0.87- 6.25)
WYNDE6	735	f	5	-	-	-	-	2.50	(1.94- 3.23)
WYNDE6	739	f	5	-	-	-	-	1.50	(0.40- 5.61)
Subtotal WYNDE6								2.61	(2.14- 3.19)
Partial Totals				3865	31678	1815	114384		

*prospective study ~ With 0.5 adjustment for zero

REF	NRR	SEX	AD	Ys	Ws	Qs	Ps
ALDERS	512	m	1	1.73	13.52	1.73	0.0000
ALDERS	523	f	1	2.01	13.78	5.47	0.0000
Subtotal ALDERS				1.87	27.31	7.20	
ARMADA	517	m	0	1.12	15.07	1.01	0.0000
BARBON	532	m	0	1.84	6.07	1.30	0.0000
BECHER	505	m	0	1.32	3.85	0.01	0.0095
BECHER	515	f	0	1.90	0.52	0.14	0.1724
Subtotal BECHER				1.39	4.37	0.15	
*BENSHL	514	m	2	2.16	3.32	2.05	0.0001
BROSS	517	m	0	1.53	17.62	0.44	0.0000
CARPEN	507	c	0	0.96	11.01	1.95	0.0015
*CEDERL	530	m	1	1.71	2.39	0.27	0.0081
CHOI	541	m	0	0.62	2.79	1.60	0.3017
CHOI	554	f	0	-1.27	0.32	2.27	0.4687
Subtotal CHOI				0.42	3.12	3.88	
*CHYOU	503	m	1	0.33	3.98	4.36	0.5112
*CPSI	814	m	1	2.45	10.67	12.21	0.0000
*CPSII	663	m	1	2.31	70.40	62.04	0.0000
*CPSII	641	f	1	2.28	32.32	26.38	0.0000
Subtotal CPSII				2.30	102.72	88.42	
DAMBER	556	m	1	1.24	4.47	0.09	0.0090
DARBY	503	m	0	0.87	54.65	14.22	0.0000
DARBY	512	f	0	1.84	14.62	3.16	0.0000
Subtotal DARBY				1.07	69.28	17.38	
DEAN3	508	m	0	0.82	5.39	1.68	0.0578
DEAN3	547	f	0	0.73	1.30	0.55	0.4055
Subtotal DEAN3				0.80	6.69	2.22	
DESTEF	542	m	0	0.77	3.61	1.33	0.1439
DOLL	535	m	0	0.76	5.01	1.87	0.0873
DOLL	546	f	0	1.10	0.56	0.04	0.4100
Subtotal DOLL				0.80	5.57	1.92	
*DOLL2	508	m	1	2.08	4.77	2.36	0.0000
DORGAN	512	m	0	0.79	20.86	7.18	0.0003
DORGAN	557	f	0	0.98	9.36	1.45	0.0027
Subtotal DORGAN				0.85	30.22	8.63	
*DORN	664	m	0	1.73	10.89	1.33	0.0000
*DORN	687	m	0	1.23	9.93	0.22	0.0001
Subtotal DORN				1.49	20.82	1.56	
GAO	525	m	0	1.85	7.69	1.74	0.0000
GAO	545	f	0	1.28	3.68	0.03	0.0141
Subtotal GAO				1.67	11.36	1.77	
GAO2	517	m	0	1.32	4.24	0.02	0.0067
GARCIA	507	c	0	2.41	4.03	4.28	0.0000
GARSHI	518	m	0	0.45	49.05	41.90	0.0016
GRAHAM	529	m	0	2.35	8.56	8.09	0.0000
*HAMMO2	509	m	1	1.16	15.50	0.72	0.0000
*HIRAYA	512	m	1	0.39	3.81	3.74	0.4519
*HIRAYA	523	f	1	1.35	0.28	0.00	0.4794
Subtotal HIRAYA				0.45	4.09	3.74	
JAHN	512	m	0	4.65	5.80	62.16	0.0000
JAIN	569	m	0	1.25	15.79	0.24	0.0000
JAIN	533	f	0	1.77	8.93	1.41	0.0000
Subtotal JAIN				1.44	24.72	1.65	
JEDRYC	614	m	0	0.74	18.58	7.62	0.0015
JOLY	569	m	0	0.91	13.04	2.77	0.0010
JOLY	556	f	0	1.10	3.37	0.25	0.0433

Table 1J10 - 5

IESLC - Meta-analysis of Ex Smoking, Years quit (vs never), "Highest vs lowest"
All LC types, Cigarettes (or Any Product if Cigarettes not available)
Least adjusted

REF	NRR	SEX	AD	Ys	Ws	Qs	Ps
Subtotal JOLY				0.95	16.41	3.02	
*KAISE2	652	m	1	1.57	3.26	0.12	0.0046
*KAISE2	572	f	1	0.54	2.14	1.48	0.4278
Subtotal KAISE2				1.16	5.40	1.61	
KHUDER	515	m	0	0.88	24.96	6.05	0.0000
LUBIN	590	m	0	2.06	6.31	2.99	0.0000
LUBIN2	1080	m	0	1.69	78.85	7.84	0.0000
LUBIN2	1119	f	0	1.70	2.99	0.31	0.0034
Subtotal LUBIN2				1.69	81.83	8.15	
MATOS	585	m	0	1.52	7.93	0.16	0.0000
PEZZO2	503	m	0	1.06	19.87	1.95	0.0000
PEZZOT	503	m	0	1.09	10.71	0.88	0.0004
SOBUE	727	m	0	0.95	9.97	1.77	0.0026
*SPEIZE	510	f	2	1.79	6.15	1.06	0.0000
SUZUK2	512	c	0	1.30	3.09	0.02	0.0223
SVENSS	553	f	0	0.75	3.96	1.57	0.1373
*TVERDA	505	m	2	0.73	1.33	0.56	0.4010
WAKAI	526	m	0	0.98	4.21	0.67	0.0449
WANG2	513	c	0	0.28	1.79	2.16	0.7100
WYNDE3	543	m	0	2.35	3.21	3.06	0.0000
WYNDE6	723	m	5	1.10	30.92	2.38	0.0000
WYNDE6	730	m	5	0.85	3.95	1.11	0.0927
WYNDE6	735	f	5	0.92	59.12	12.48	0.0000
WYNDE6	739	f	5	0.41	2.20	2.07	0.5473
Subtotal WYNDE6				0.96	96.20	18.04	

	N	65
	NS	47
	Wt	802.35
Het	Chi	344.40
Het	df	64
Het	P	***
Fixed	RR	3.96
	RRl	3.69
	RRu	4.24
	P	+++
Random	RR	3.94
	RRl	3.29
	RRu	4.72
	P	+++
Asymm	P	N.S.

Table 1J10 - 6

IESLC - Meta-analysis of Ex Smoking, Years quit (vs never), "Highest vs lowest"
All LC types, Cigarettes (or Any Product if Cigarettes not available)
Least adjusted

		Sex			
		combined	male	female	Total
	N	4	43	18	65
	NS	4	41	17	62
	Wt	19.93	616.82	165.61	802.35
Het	Chi	8.05	275.72	59.33	344.40
Het	df	3	42	17	64
Het	P	*	***	***	***
Fixed	RR	3.46	3.90	4.25	3.96
	RRl	2.23	3.61	3.65	3.69
	RRu	5.37	4.22	4.94	4.24
	P	+++	+++	+++	+++
Random	RR	3.63	3.97	3.97	3.94
	RRl	1.62	3.19	2.77	3.29
	RRu	8.12	4.94	5.70	4.72
	P	++	+++	+++	+++
Between	Chi				1.30
Between	df				2
Between	P				N.S.
Btwn(F)	P				N.S.
Btwn(R)	P				N.S.

Table 1J10 - 7

IESLC - Meta-analysis of Ex Smoking, Years quit (vs never), "Highest vs lowest"
All LC types, Cigarettes (or Any Product if Cigarettes not available)
Excluded studies (and stage at which they were excluded)

1	AGUDO AKIBA AMANDU AMES AXELSS BEST BOUCHA BOUCOT BRESLO CHEN CHEN2 CHIAZZ DEAN2 DOSEME ENGELA FAN GENG GER GUO HAENSZ HEGMAN HOLE HU HU2 JUSSAW KATSOU KAUFMA KOO KOULUM KREUZE LETOUR LEVIN LIAW LIU3 LIU4 LIU5 MCCONN MIGRAN MRFITR NOTAN2 OSANN2 PERNU QIAO2 RACHTA RESTRE SADOWS SEGI2 STASZE TIZZAN VUTUC WATSON WU WUWILL WYNDE2 WYNDE8 XU YUAN ZHANG ZHENG ZHOU
2	BUFFLE HUMBLE PISANI PRESCO WYNDE7
3	MCDUFF SPITZ
4	AUVINE BLOT1 BROWN3 GURSEL LAUSSM LUO WU2
5	HAMMON
6	CORREA GILLIS QIAO WIGLE
8	BOFFET
15	BENHAM

Table 1J10 - 8
Potentially overlapping studies

REF	REFGP	PRINC	. OVERLAP/LINK
LUBIN2	LUBIN2	1	Lubin-combined
TVERDA	TVERDA	1	VEIERO/TVERDAL
BROSS	BYERS1	1	GRAHAM/BROSS/BYERS1
GRAHAM	BYERS1	1	GRAHAM/BROSS/BYERS1
CHYOU	CHYOU	1	GOODMA/CHYOU
BENSHL	TANG2	1	Subset of TANG2
WYNDE6	WYNDE6	1	WYNDE5/6/7/8
CPSI	CPSI	1	CPSI overall
JAHN	BOFFET	2	Subset of BOFFET
LUBIN	XIANGZ	2	LUBIN/XIANGZ/QIAO

Table 1J10 - 9
Most adjusted - insufficient data for meta-analysis

REF	NRR	SEX	AGEL	AGEH	RACE	YF	LC TYPE	LOC	START	ST	NLC	R	VB	P	H	AD	ADOS	PRODUCT	exL	exH	unexL	unexH	De
ARMADA	525	m	0	0	all	-	all	Eu:wst	1986	CC	325	n	bl	n	y	0	0	cig+/-ot	0.1	0.9	6	999	ot
BECHER	527	m	0	0	all	-	all	Eu:Ger	1985	CC	194	n	bl	n	y	0	0	all/unsp	1.0	1.0	10	999	ot
BECHER	529	f	0	0	all	-	all	Eu:Ger	1985	CC	194	n	bl	n	y	0	0	all/unsp	1.0	1.0	10	999	ot
CORREA	549	c	0	0	all	-	all	NAmer	1979	CC	1359	n	bl	y	n	2	0	cig+/-ot	0.1	3	21	999	ot
CPSI	710	m	0	0	wh	0	all	NAmer	1959	pr	5138	n	bl	n	n	1	0	cig only	2	4	35	39	st
CPSI	728	f	0	0	wh	0	all	NAmer	1959	pr	5138	n	bl	n	n	1	0	cig only	2	4	25	29	st
DEAN3	621	m	0	0	all	-	all	Eu:UK	1969	CC	766	n	V	y	n	1	0	cig only	0.1	2	19	999	ot
DEAN3	627	f	0	0	all	-	all	Eu:UK	1969	CC	766	n	V	y	n	1	0	all/unsp	0.1	2	9	999	ot
DORGAN	513	m	0	0	wh	-	all	NAmer	1980	CC	2026	n	bl	y	y	0	0	cig+/-ot	0.1	1.0	10	999	ot
DORGAN	558	f	0	0	all	-	all	NAmer	1980	CC	2026	n	bl	y	y	0	0	cig+/-ot	0.1	1.0	10	999	ot
GAO2	528	m	0	0	all	-	all	As:Jap	1988	CC	282	n	bl	n	n	0	0	cig+/-ot	0.1	0.9	20	999	ot
GARCIA	521	c	0	0	all	-	all	NAmer	1992	CC	416	n	bl	n	y	0	0	cig+/-ot	0.1	0.9	30	999	ot
GARSHI	526	m	0	0	all	-	all	NAmer	1981	CC	1081	o	bl	y	n	1	0	all/unsp	0.1	4	15	999	ot
JAIN	597	m	0	0	all	-	all	NAmer	1981	CC	845	n	V	y	n	0	0	cig+/-ot	0.1	1.9	10	999	ot
JAIN	585	f	0	0	all	-	all	NAmer	1981	CC	845	n	V	y	n	0	0	cig+/-ot	0.1	1.9	10	999	ot
JEDRYC	615	m	0	0	all	-	all	Eu:est	1980	CC	1630	n	bl	y	n	0	0	cig+/-ot	0.1	4	10	999	ot
JOLY	570	m	0	0	all	-	all	SCAmer	1978	CC	826	n	bl	n	n	0	0	cig+/-ot	0.1	0.9	5	999	ot
JOLY	557	f	0	0	all	-	all	SCAmer	1978	CC	826	n	bl	n	n	0	0	cig+/-ot	0.1	0.9	5	999	ot
KAISE2	653	m	0	0	all	9	all	NAmer	1979	pr	318	n	bl	n	n	1	0	cig only	0.1	1.9	21	999	ot
KAISE2	573	f	0	0	all	9	all	NAmer	1979	pr	318	n	bl	n	n	1	0	cig only	0.1	1.9	21	999	ot
LUBIN	591	m	0	0	all	-	all	As:Chi	1984	CC	427	m	ot	y	n	0	0	cig+/-ot	0.1	2	10	999	ot
MATOS	704	m	0	0	all	-	all	SCAmer	1994	CC	200	n	bl	n	n	2	0	cig+/-ot	0.1	0.9	11	999	ot
PEZZO2	513	m	0	0	all	-	all	SCAmer	1992	CC	367	n	bl	n	y	0	0	cig+/-ot	0.1	0.9	11	999	ot
PEZZOT	598	m	0	0	all	-	all	SCAmer	1987	CC	215	n	bl	n	y	0	0	cig only	0.1	0.9	11	999	ot
SOBUE	779	m	0	0	all	-	all	As:Jap	1986	CC	1376	n	bl	n	y	0	0	cig+/-ot	0.1	0.9	25	999	ot
SVENSS	592	f	0	0	all	-	all	Eu:Sca	1983	CC	210	n	bl	n	n	0	0	all/unsp	0.1	2	11	999	ot
WAKAI	612	m	0	0	all	-	all	As:Jap	1988	CC	333	n	bl	n	y	2	0	cig+/-ot	0.1	4	20	999	ot
WIGLE	515	m	0	0	all	-	all	NAmer	1971	CC	728	n	V	n	n	2	1	#cig+/-ot	0.1	1.0	15	999	st
WIGLE	525	f	0	0	all	-	all	NAmer	1971	CC	728	n	V	n	n	2	1	#cig+/-ot	0.1	1.0	15	999	st
WYNDE3	544	m	0	0	all	-	all	NAmer	1966	CC	350	n	bl	n	y	0	0	all/unsp	0.1	0.9	13	999	ot

Table 1J10 - 9

IESLC - Meta-analysis of Ex Smoking, Years quit (vs never), "Highest vs lowest"
All LC types, Cigarettes (or Any Product if Cigarettes not available)
Most adjusted - insufficient data for meta-analysis

Comments on values in listings

WIGLE ADOS Cumulative exposure
WIGLE ADOS Cumulative exposure

REF	NRR	RR	SIG	RRDATA comment
ARMADA	525	*	gap	0
BECHER	527	*	gap	0
BECHER	529	*	gap	0
CORREA	549	*	gap	0
CPSI	710	4.13		0
CPSI	728	1.09		0
DEAN3	621	*	gap	0
DEAN3	627	*	gap	0
DORGAN	513	*	gap	0
DORGAN	558	*	gap	0
GAO2	528	*	gap	0
GARCIA	521	*	gap	0
GARSHI	526	*	gap	0
JAIN	597	*	gap	0
JAIN	585	*	gap	0
JEDRYC	615	*	gap	0
JOLY	570	*	gap	0
JOLY	557	*	gap	0
KAISE2	653	*	gap	0
KAISE2	573	*	gap	0
LUBIN	591	*	gap	0
MATOS	704	*	gap	0
PEZZO2	513	*	gap	0
PEZZOT	598	*	gap	0
SOBUE	779	*	gap	0
SVENSS	592	*	gap	0
WAKAI	612	*	gap	0
WIGLE	515	12.00		0
WIGLE	525	2.25		0
WYNDE3	544	*	gap	0

Least adjusted - insufficient data for meta-analysis: as for adjusted plus the following

REF	NRR	SEX	AGEL	AGEH	RACE	YF	LC TYPE	LOC	START	ST	NLC	R	VB	P	H	AD	ADOS	PRODUCT	exL	exH	unexL	unexH	De
DEAN3	619	m	0	0	all	-	all	Eu:UK	1969	CC	766	n	V	y	n	0	0	cig only	0.1	2	19	999	ot
DEAN3	625	f	0	0	all	-	all	Eu:UK	1969	CC	766	n	V	y	n	0	0	all/unsp	0.1	2	9	999	ot
GARSHI	519	m	0	0	all	-	all	NAmer	1981	CC	1081	o	bl	y	n	0	0	all/unsp	0.1	4	15	999	ot
MATOS	702	m	0	0	all	-	all	SCAmer	1994	CC	200	n	bl	n	n	0	0	cig+/-ot	0.1	0.9	11	999	ot
WAKAI	610	m	0	0	all	-	all	As:Jap	1988	CC	333	n	bl	n	y	0	0	cig+/-ot	0.1	4	20	999	ot

REF	NRR	RR	SIG	RRDATA comment
DEAN3	619	*	gap	0
DEAN3	625	*	gap	0
GARSHI	519	*	gap	0
MATOS	702	*	gap	0
WAKAI	610	*	gap	0

Table 1J11 -

IESLC - Meta-analysis of Ex Smoking by Years quit (vs never), Overview
All LC types, Cigarettes only

This analysis is restricted to results for:
1) Ex smokers
2) Results by Years quit (vs never)
3) Categorical results by Years quit (vs never)
Results by Years quit (vs never) are grouped under 2 schemes (S1, S2). Each scheme has a set of "key values". An interval is allocated to the category whose key value it includes, and intervals which include none or more than one of the key values are excluded. (Open-ended intervals are coded as 999)

S1	key value	maximum range
1	12	8+
2	7	4-11
3	3	1-6

S2	key value	maximum range
1	20	13+
2	12	4-19
3	3	1-11

4) All LC types (or near equivalent)
5) Results complete enough for use in metaanalysis

Within each study, results are then selected (in the following order of preference, within each sex) for:
6) (not applicable)
7) PRODUCT: cigarettes only
8) CIGTYPE: all/unspecified, MC regardless of HR, MC only
9) (not applicable)
10) DENOM: never smoked anything, never smoked cigarettes, never any + low, never cigs + low
11) Followup period (YF, prospective studies): whole study (coded as 0) or longest available
12) LCtype: all or nearest available, at least Squamous and Adeno. (q = squamous, s = small, l = large, a = adeno, mix = mixed, alv = alveolar)
13) Race: all or nearest available, otherwise by race (wh or w = white, bl or b = black, hi = hispanic ch = chinese, jap = japanese, haw = hawaiian, w+o = white + oriental, sca = scandinavian, as = asian)
14) For overlapping studies: principal rather than subsidiary studies
Finally by Age: whole study (coded as 0) if available, otherwise by widest available age group and then for single sex results (m, f) in preference to results for both sexes combined (c).

Results adjusted (AD) for the most potential confounders are then chosen in Sections -1 to -3 (and those which actually differ from the adjusted results in Table 1J1 - 1 are marked 'x' in Section -1) and results adjusted for the least confounders in Sections -4 to -6. (Those least adjusted results which actually differ from the most adjusted are marked 'x' in column X in Section -4)

Section -7 shows excluded studies, together with the stage (as above) at which no qualifying results were found.

Section -8 lists the potentially overlapping studies which have been included (1=principal, 2=subsidiary).

Section -9 lists any results which would have been included in preference except that they had data not complete enough for use in meta-analysis, with their significance (yes/no), if known, and any further comment as entered on the database. It also lists as "gap" any categories for which no data were presented by the original authors. This is commonly due to recent quitters having been combined with current smokers

In addition to those mentioned above, the following fields, levels and abbreviations are used:
* or nk = not known, n = no, y = yes, ot = other
nev = never
all/unspec = all or unspecified, MC = manufactured cigarettes, HR = hand-rolled cigarettes
exL, exH = range of exposure (low and high) in the smoking group, in terms of Years quit (vs never)
REF: 6-character study reference
NRR: number of the RR on the database within the study
ST : study type (CC = case control, pr or prosp = prospective)
NLC: number of lung cancer cases in whole study
R : risky occupational population (n = no, m = mining, o = other risky)
VB : national cigarette type (V = at least 75% Virginia, bl = at least 75% blended, ot = other)
P : any proxy use
H : full histological confirmation
De : derivation of RR/CI (or = original, st = standard method, ot = other method of estimation)

Table 1J11 - 1

IESLC - Meta-analysis of Ex Smoking by Years quit (vs never), Overview
All LC types, Cigarettes only
Most adjusted

REF	NRR	1J1	SEX	AGEL	AGEH	RACE	YF	LC TYPE	LOC	START	ST	NLC	R	VB	P	H	AD	PRODUCT	exL	exH	S1	S2	DENOM	De
ALDERS	507		m	0	0	all	-	all	Eu:UK	1977	CC	1448	n	V	n	n	1	cig only	10	999	1	0	nev any	ot
ALDERS	508		m	0	0	all	-	all	Eu:UK	1977	CC	1448	n	V	n	n	1	cig only	3	9	0	3	nev any	ot
ALDERS	509		m	0	0	all	-	all	Eu:UK	1977	CC	1448	n	V	n	n	1	cig only	0.1	2	0	0	nev any	ot
ALDERS	518		f	0	0	all	-	all	Eu:UK	1977	CC	1448	n	V	n	n	1	cig only	10	999	1	0	nev any	ot
ALDERS	519		f	0	0	all	-	all	Eu:UK	1977	CC	1448	n	V	n	n	1	cig only	3	9	0	3	nev any	ot
ALDERS	520		f	0	0	all	-	all	Eu:UK	1977	CC	1448	n	V	n	n	1	cig only	0.1	2	0	0	nev any	ot
BENHAM	561	x	m	0	0	all	-	not mix	Eu:wst	1976	CC	1625	n	bl	n	y	0	cig only	11	999	1	0	nev any	st
BENHAM	562	x	m	0	0	all	-	not mix	Eu:wst	1976	CC	1625	n	bl	n	y	0	cig only	4	10	2	0	nev any	st
BENHAM	563	x	m	0	0	all	-	not mix	Eu:wst	1976	CC	1625	n	bl	n	y	0	cig only	1.0	3	3	3	nev any	st
CPSI	807		m	50	74	all	6	all	NAmer	1959	pr	5138	n	bl	n	n	1	cig only	10	999	1	0	nev any	ot
CPSI	808		m	50	74	all	6	all	NAmer	1959	pr	5138	n	bl	n	n	1	cig only	5	9	2	0	nev any	ot
CPSI	809		m	50	74	all	6	all	NAmer	1959	pr	5138	n	bl	n	n	1	cig only	1.0	4	3	3	nev any	ot
CPSI	810		m	50	74	all	6	all	NAmer	1959	pr	5138	n	bl	n	n	1	cig only	0.1	0.9	0	0	nev any	ot
CPSII	652		m	35	99	all	4	all	NAmer	1982	pr	3229	n	bl	n	n	1	cig only	16	999	0	1	nev any	ot
CPSII	653		m	35	99	all	4	all	NAmer	1982	pr	3229	n	bl	n	n	1	cig only	11	15	1	2	nev any	ot
CPSII	654		m	35	99	all	4	all	NAmer	1982	pr	3229	n	bl	n	n	1	cig only	6	10	2	0	nev any	ot
CPSII	655		m	35	99	all	4	all	NAmer	1982	pr	3229	n	bl	n	n	1	cig only	3	5	3	3	nev any	ot
CPSII	656		m	35	99	all	4	all	NAmer	1982	pr	3229	n	bl	n	n	1	cig only	1.0	2	0	0	nev any	ot
CPSII	657		m	35	99	all	4	all	NAmer	1982	pr	3229	n	bl	n	n	1	cig only	0.1	0.9	0	0	nev any	ot
DAMBER	554	x	m	0	0	all	-	all	Eu:Sca	1972	CC	579	n	bl	y	n	1	cig only	11	999	1	0	nev any	ot
DAMBER	555	x	m	0	0	all	-	all	Eu:Sca	1972	CC	579	n	bl	y	n	1	cig only	0.1	10	0	3	nev any	ot
DEAN3	516	x	m	0	0	all	-	all	Eu:UK	1969	CC	766	n	V	y	n	1	cig only	19	999	0	1	nev any	ot
DEAN3	517	x	m	0	0	all	-	all	Eu:UK	1969	CC	766	n	V	y	n	1	cig only	9	18	1	2	nev any	ot
DEAN3	518	x	m	0	0	all	-	all	Eu:UK	1969	CC	766	n	V	y	n	1	cig only	5	8	2	0	nev any	ot
DEAN3	519	x	m	0	0	all	-	all	Eu:UK	1969	CC	766	n	V	y	n	1	cig only	3	4	3	3	nev any	ot
DOLL2	501		m	0	0	all	20	all	Eu:UK	1951	pr	920	n	V	n	n	1	cig only	15	999	0	1	nev any	ot
DOLL2	502		m	0	0	all	20	all	Eu:UK	1951	pr	920	n	V	n	n	1	cig only	10	14	1	2	nev any	ot
DOLL2	503		m	0	0	all	20	all	Eu:UK	1951	pr	920	n	V	n	n	1	cig only	5	9	2	0	nev any	ot
DOLL2	504		m	0	0	all	20	all	Eu:UK	1951	pr	920	n	V	n	n	1	cig only	0.1	4	3	3	nev any	ot
DORN	508	x	m	0	0	wh	0	all	NAmer	1954	pr	5097	n	bl	n	n	2	cig only	40	999	0	0	nev any	or
DORN	509	x	m	0	0	wh	0	all	NAmer	1954	pr	5097	n	bl	n	n	2	cig only	30	39	0	0	nev any	or
DORN	510	x	m	0	0	wh	0	all	NAmer	1954	pr	5097	n	bl	n	n	2	cig only	20	29	0	1	nev any	or
DORN	511	x	m	0	0	wh	0	all	NAmer	1954	pr	5097	n	bl	n	n	2	cig only	10	19	1	2	nev any	or
DORN	512	x	m	0	0	wh	0	all	NAmer	1954	pr	5097	n	bl	n	n	2	cig only	5	9	2	0	nev any	or
DORN	513	x	m	0	0	wh	0	all	NAmer	1954	pr	5097	n	bl	n	n	2	cig only	1.0	4	3	3	nev any	or
GRAHAM	501	x	m	0	0	wh	-	all	NAmer	1956	CC	685	n	bl	n	n	0	cig only	10	999	1	0	nev any	st
GRAHAM	502	x	m	0	0	wh	-	all	NAmer	1956	CC	685	n	bl	n	n	0	cig only	3	10	2	0	nev any	st
GRAHAM	503	x	m	0	0	wh	-	all	NAmer	1956	CC	685	n	bl	n	n	0	cig only	1.1	3	3	3	nev any	st
GRAHAM	504	x	m	0	0	wh	-	all	NAmer	1956	CC	685	n	bl	n	n	0	cig only	0.1	1.0	0	0	nev any	st
KAISE2	646		m	0	0	all	9	all	NAmer	1979	pr	318	n	bl	n	n	1	cig only	21	999	0	0	nev any	st
KAISE2	647		m	0	0	all	9	all	NAmer	1979	pr	318	n	bl	n	n	1	cig only	11	20	1	0	nev any	ot
KAISE2	648		m	0	0	all	9	all	NAmer	1979	pr	318	n	bl	n	n	1	cig only	2	10	0	3	nev any	st
KAISE2	566		f	0	0	all	9	all	NAmer	1979	pr	318	n	bl	n	n	1	cig only	21	999	0	0	nev any	ot
KAISE2	567		f	0	0	all	9	all	NAmer	1979	pr	318	n	bl	n	n	1	cig only	11	20	1	0	nev any	st
KAISE2	568		f	0	0	all	9	all	NAmer	1979	pr	318	n	bl	n	n	1	cig only	2	10	0	3	nev any	st
PEZZOT	501		m	0	0	all	-	all	SCAmer	1987	CC	215	n	bl	n	y	0	cig only	11	999	1	0	nev cigs	st
PEZZOT	502		m	0	0	all	-	all	SCAmer	1987	CC	215	n	bl	n	y	0	cig only	1.0	10	0	3	nev cigs	st
TVERDA	501		m	0	0	all	0	all	Eu:Sca	1972	pr	238	n	bl	n	n	2	cig only	5	999	0	0	nev cigs	ot
TVERDA	502		m	0	0	all	0	all	Eu:Sca	1972	pr	238	n	bl	n	n	2	cig only	1.0	5	3	3	nev cigs	ot
TVERDA	503		m	0	0	all	0	all	Eu:Sca	1972	pr	238	n	bl	n	n	2	cig only	0.1	0.9	0	0	nev cigs	ot
WYNDE6	501		m	0	0	all	-	all	NAmer	1969	CC	4423	n	bl	n	y	0	cig only	30	999	0	0	nev any	st
WYNDE6	502		m	0	0	all	-	all	NAmer	1969	CC	4423	n	bl	n	y	0	cig only	20	29	0	1	nev any	st
WYNDE6	503		m	0	0	all	-	all	NAmer	1969	CC	4423	n	bl	n	y	0	cig only	10	19	1	2	nev any	st
WYNDE6	504		m	0	0	all	-	all	NAmer	1969	CC	4423	n	bl	n	y	0	cig only	5	9	2	0	nev any	st
WYNDE6	505		m	0	0	all	-	all	NAmer	1969	CC	4423	n	bl	n	y	0	cig only	1.0	4	3	3	nev any	st
WYNDE6	522		f	0	0	all	-	all	NAmer	1969	CC	4423	n	bl	n	y	0	cig only	30	999	0	0	nev any	st
WYNDE6	523		f	0	0	all	-	all	NAmer	1969	CC	4423	n	bl	n	y	0	cig only	20	29	0	1	nev any	st
WYNDE6	524		f	0	0	all	-	all	NAmer	1969	CC	4423	n	bl	n	y	0	cig only	10	19	1	2	nev any	st
WYNDE6	525		f	0	0	all	-	all	NAmer	1969	CC	4423	n	bl	n	y	0	cig only	5	9	2	0	nev any	st
WYNDE6	526		f	0	0	all	-	all	NAmer	1969	CC	4423	n	bl	n	y	0	cig only	1.0	4	3	3	nev any	st

Cigarette type is all/unspec for all RRs
except for the following:

REF	NRR	CIGTYPE
ALDERS	507	MC only
ALDERS	508	MC only
ALDERS	509	MC only
ALDERS	518	MC only

Table 1J11 - 1

IESLC - Meta-analysis of Ex Smoking by Years quit (vs never), Overview
All LC types, Cigarettes only
Most adjusted

REF	NRR	CIGTYPE
ALDERS	519	MC only
ALDERS	520	MC only
DEAN3	516	MC only
DEAN3	517	MC only
DEAN3	518	MC only
DEAN3	519	MC only

In this overview table, subtotals and Qs values may be invalid and should be ignored

Table 1J11 - 2

IESLC - Meta-analysis of Ex Smoking by Years quit (vs never), Overview
All LC types, Cigarettes only
Most adjusted

REF	NRR	SEX	AD	Number Exposed Case	Cont	Non-exposed Case	Cont	RR	95.00%CI
ALDERS	507	m	1	29	-	15	-	3.20	(1.61- 6.35)
ALDERS	508	m	1	28	-	15	-	4.30	(2.13- 8.69)
ALDERS	509	m	1	121	-	15	-	18.10	(9.71- 33.74)
ALDERS	518	f	1	26	-	75	-	1.27	(0.76- 2.15)
ALDERS	519	f	1	54	-	75	-	2.95	(1.88- 4.64)
ALDERS	520	f	1	206	-	75	-	9.45	(6.50- 13.74)
Subtotal ALDERS								4.77	(3.86- 5.89)
BENHAM	561	m	0	60	228	33	523	4.17	(2.65- 6.56)
BENHAM	562	m	0	105	185	33	523	9.00	(5.88- 13.77)
BENHAM	563	m	0	145	86	33	523	26.72	(17.18- 41.55)
Subtotal BENHAM								10.12	(7.85- 13.04)
*CPSI	807	m	1	15	-	60	-	1.28	(0.73- 2.25)
*CPSI	808	m	1	32	-	60	-	5.15	(3.35- 7.91)
*CPSI	809	m	1	49	-	60	-	8.09	(5.55- 11.80)
*CPSI	810	m	1	37	-	60	-	14.74	(9.78- 22.20)
Subtotal CPSI								6.51	(5.25- 8.07)
*CPSII	652	m	1	256	-	81	-	3.83	(2.98- 4.92)
*CPSII	653	m	1	164	-	81	-	8.61	(6.60- 11.24)
*CPSII	654	m	1	186	-	81	-	11.43	(8.81- 14.84)
*CPSII	655	m	1	178	-	81	-	18.61	(14.31- 24.20)
*CPSII	656	m	1	188	-	81	-	28.07	(21.63- 36.43)
*CPSII	657	m	1	97	-	81	-	38.76	(28.85- 52.07)
Subtotal CPSII								13.29	(11.93- 14.81)
DAMBER	554	m	1	-	-	42	-	1.60	(0.70- 3.40)
DAMBER	555	m	1	-	-	42	-	5.50	(2.80- 11.10)
Subtotal DAMBER								3.23	(1.92- 5.42)
DEAN3	516	m	1	8	-	24	-	1.31	(0.57- 3.04)
DEAN3	517	m	1	15	-	24	-	2.99	(1.51- 5.93)
DEAN3	518	m	1	11	-	24	-	4.16	(1.91- 9.06)
DEAN3	519	m	1	28	-	24	-	4.67	(2.60- 8.38)
Subtotal DEAN3								3.25	(2.29- 4.61)
*DOLL2	501	m	1	7	-	7	-	2.00	(0.70- 5.70)
*DOLL2	502	m	1	9	-	7	-	5.30	(1.97- 14.23)
*DOLL2	503	m	1	12	-	7	-	5.90	(2.32- 14.99)
*DOLL2	504	m	1	15	-	7	-	16.00	(6.52- 39.24)
Subtotal DOLL2								6.10	(3.77- 9.87)
*DORN	508	m	2	49	-	325	-	1.50	(1.10- 2.00)
*DORN	509	m	2	82	-	325	-	2.00	(1.60- 2.60)
*DORN	510	m	2	215	-	325	-	3.30	(2.80- 4.00)
*DORN	511	m	2	261	-	325	-	5.10	(4.20- 6.10)
*DORN	512	m	2	87	-	325	-	7.80	(5.70- 10.50)
*DORN	513	m	2	56	-	325	-	16.10	(10.40- 24.80)
Subtotal DORN								3.73	(3.38- 4.11)
GRAHAM	501	m	0	2	30	18	346	1.28	(0.28- 5.79)
GRAHAM	502	m	0	5	29	18	346	3.31	(1.15- 9.57)
GRAHAM	503	m	0	12	23	18	346	10.03	(4.31- 23.31)
GRAHAM	504	m	0	84	48	18	346	33.64	(18.61- 60.80)
Subtotal GRAHAM								13.27	(8.69- 20.26)
*KAISE2	646	m	1	6	-	14	-	1.94	(0.70- 5.40)
*KAISE2	647	m	1	8	-	14	-	3.14	(1.26- 7.82)
*KAISE2	648	m	1	12	-	14	-	8.26	(3.73- 18.28)
*KAISE2	566	f	1	4	-	11	-	6.29	(1.78- 22.20)
*KAISE2	567	f	1	4	-	11	-	4.37	(1.30- 14.72)
*KAISE2	568	f	1	6	-	11	-	7.95	(2.89- 21.86)
Subtotal KAISE2								4.86	(3.24- 7.31)
PEZZOT	501	m	0	20	106	4	116	5.47	(1.81- 16.53)
PEZZOT	502	m	0	46	82	4	116	16.27	(5.64- 46.96)
Subtotal PEZZOT								9.65	(4.49- 20.74)
*TVERDA	501	m	2	4	-	4	-	1.34	(0.34- 5.37)
*TVERDA	502	m	2	5	-	4	-	2.83	(0.76- 10.53)
*TVERDA	503	m	2	2	-	4	-	2.77	(0.51- 15.15)
Subtotal TVERDA								2.15	(0.94- 4.93)
WYNDE6	501	m	0	21	161	64	918	1.87	(1.11- 3.15)
WYNDE6	502	m	0	55	212	64	918	3.72	(2.52- 5.50)
WYNDE6	503	m	0	159	373	64	918	6.11	(4.47- 8.37)
WYNDE6	504	m	0	98	194	64	918	7.25	(5.10- 10.29)
WYNDE6	505	m	0	201	166	64	918	17.37	(12.53- 24.07)
WYNDE6	522	f	0	10	31	125	991	2.56	(1.22- 5.34)
WYNDE6	523	f	0	16	77	125	991	1.65	(0.93- 2.91)
WYNDE6	524	f	0	36	132	125	991	2.16	(1.43- 3.27)
WYNDE6	525	f	0	51	84	125	991	4.81	(3.24- 7.14)

Table 1J11 - 2

IESLC - Meta-analysis of Ex Smoking by Years quit (vs never), Overview
All LC types, Cigarettes only
Most adjusted

REF	NRR	SEX	AD	Number Exposed Case	Cont	Non-exposed Case	Cont	RR	95.00%CI
WYNDE6	526	f	0	82	70	125	991	9.29	(6.42- 13.43)
Subtotal WYNDE6								5.50	(4.84- 6.24)
Partial Totals				3780	2317	4365	12730		

*prospective study

REF	NRR	SEX	AD	Ys	Ws	Qs	Ps
ALDERS	507	m	1	1.16	8.16	3.62	0.0009
ALDERS	508	m	1	1.46	7.77	1.07	0.0000
ALDERS	509	m	1	2.90	9.90	11.27	0.0000
ALDERS	518	f	1	0.24	14.21	35.94	0.3676
ALDERS	519	f	1	1.08	18.83	10.52	0.0000
ALDERS	520	f	1	2.25	27.43	4.76	0.0000
Subtotal ALDERS				1.56	86.30	67.18	
BENHAM	561	m	0	1.43	18.77	3.02	0.0000
BENHAM	562	m	0	2.20	21.21	2.86	0.0000
BENHAM	563	m	0	3.29	19.71	41.79	0.0000
Subtotal BENHAM				2.31	59.69	47.67	
*CPSI	807	m	1	0.25	12.13	30.37	0.3900
*CPSI	808	m	1	1.64	20.82	0.75	0.0000
*CPSI	809	m	1	2.09	27.01	1.84	0.0000
*CPSI	810	m	1	2.69	22.87	16.96	0.0000
Subtotal CPSI				1.87	82.82	49.93	
*CPSII	652	m	1	1.34	61.12	14.46	0.0000
*CPSII	653	m	1	2.15	54.21	5.68	0.0000
*CPSII	654	m	1	2.44	56.51	20.82	0.0000
*CPSII	655	m	1	2.92	55.67	66.67	0.0000
*CPSII	656	m	1	3.33	56.54	128.13	0.0000
*CPSII	657	m	1	3.66	44.07	147.27	0.0000
Subtotal CPSII				2.59	328.12	383.03	
DAMBER	554	m	1	0.47	6.15	11.37	0.2437
DAMBER	555	m	1	1.70	8.10	0.13	0.0000
Subtotal DAMBER				1.17	14.25	11.49	
DEAN3	516	m	1	0.27	5.48	13.33	0.5272
DEAN3	517	m	1	1.10	8.21	4.42	0.0017
DEAN3	518	m	1	1.43	6.34	1.03	0.0003
DEAN3	519	m	1	1.54	11.22	0.93	0.0000
Subtotal DEAN3				1.18	31.25	19.72	
*DOLL2	501	m	1	0.69	3.49	4.51	0.1951
*DOLL2	502	m	1	1.67	3.93	0.10	0.0009
*DOLL2	503	m	1	1.77	4.41	0.01	0.0002
*DOLL2	504	m	1	2.77	4.77	4.24	0.0000
Subtotal DOLL2				1.81	16.61	8.87	
*DORN	508	m	2	0.41	42.99	87.16	0.0078
*DORN	509	m	2	0.69	65.19	84.15	0.0000
*DORN	510	m	2	1.19	120.78	48.77	0.0000
*DORN	511	m	2	1.63	110.32	4.42	0.0000
*DORN	512	m	2	2.05	41.17	2.08	0.0000
*DORN	513	m	2	2.78	20.35	18.34	0.0000
Subtotal DORN				1.32	400.80	244.92	
GRAHAM	501	m	0	0.25	1.69	4.23	0.7471
GRAHAM	502	m	0	1.20	3.41	1.36	0.0268
GRAHAM	503	m	0	2.31	5.40	1.22	0.0000
GRAHAM	504	m	0	3.52	10.97	31.19	0.0000
Subtotal GRAHAM				2.59	21.47	38.00	
*KAISE2	646	m	1	0.66	3.68	5.01	0.2036
*KAISE2	647	m	1	1.14	4.61	2.16	0.0140
*KAISE2	648	m	1	2.11	6.08	0.48	0.0000
*KAISE2	566	f	1	1.84	2.41	0.00	0.0043
*KAISE2	567	f	1	1.47	2.61	0.33	0.0172
*KAISE2	568	f	1	2.07	3.75	0.22	0.0001
Subtotal KAISE2				1.58	23.15	8.21	
PEZZOT	501	m	0	1.70	3.14	0.05	0.0026
PEZZOT	502	m	0	2.79	3.42	3.15	0.0000
Subtotal PEZZOT				2.27	6.56	3.20	
*TVERDA	501	m	2	0.29	2.02	4.76	0.6776
*TVERDA	502	m	2	1.04	2.22	1.38	0.1208
*TVERDA	503	m	2	1.02	1.34	0.88	0.2389
Subtotal TVERDA				0.76	5.58	7.03	
WYNDE6	501	m	0	0.63	14.18	20.51	0.0183
WYNDE6	502	m	0	1.31	25.24	6.70	0.0000

Table 1J11 - 2

IESLC - Meta-analysis of Ex Smoking by Years quit (vs never), Overview
All LC types, Cigarettes only
Most adjusted

REF	NRR	SEX	AD	Ys	Ws	Qs	Ps
WYNDE6	503	m	0	1.81	38.93	0.01	0.0000
WYNDE6	504	m	0	1.98	31.18	0.71	0.0000
WYNDE6	505	m	0	2.85	36.08	37.93	0.0000
WYNDE6	522	f	0	0.94	7.08	5.61	0.0125
WYNDE6	523	f	0	0.50	11.83	20.94	0.0859
WYNDE6	524	f	0	0.77	22.54	25.24	0.0003
WYNDE6	525	f	0	1.57	24.68	1.64	0.0000
WYNDE6	526	f	0	2.23	28.18	4.49	0.0000
Subtotal WYNDE6				1.70	239.93	123.80	

N	60
NS	13

Table 1J11 - 3

IESLC - Meta-analysis of Ex Smoking by Years quit (vs never), Overview
All LC types, Cigarettes only
Most adjusted

	Sex combined	male	female	Total
N		49	11	60
NS		13	3	16

In this overview table, other than the "N" rows, entries in the "absent" and "Total" columns may be invalid and should be ignored

	Years quit vs never (lower focus) absent	8+k12	4-11k7	1-6k3	Total
N	26	15	9	10	60
NS	12	12	8	9	41
Wt	586.57	309.62	209.74	210.60	1316.53
Het Chi	654.55	94.63	23.35	47.50	1013.04
Het df	25	14	8	9	59
Het P	***	***	**	***	***
Fixed RR	5.24	4.42	7.57	13.72	6.23
RRl	4.84	3.96	6.61	11.99	5.90
RRu	5.69	4.94	8.66	15.71	6.58
P	+++	+++	+++	+++	+++
Random RR	5.03	3.27	6.76	11.96	5.39
RRl	3.23	2.33	5.24	8.52	4.25
RRu	7.83	4.59	8.73	16.79	6.83
P	+++	+++	+++	+++	+++

	Years quit vs never (higher focus) absent	13+k20	4-19k12	1-11k3	Total
N	32	6	6	16	60
NS	13	5	5	13	36
Wt	591.87	227.96	238.15	258.55	1316.53
Het Chi	668.58	12.95	34.50	102.37	1013.04
Het df	31	5	5	15	59
Het P	***	*	***	***	***
Fixed RR	6.55	3.26	5.36	11.31	6.23
RRl	6.04	2.86	4.72	10.02	5.90
RRu	7.09	3.71	6.09	12.78	6.58
P	+++	+++	+++	+++	+++
Random RR	4.86	2.90	4.72	9.29	5.39
RRl	3.28	2.23	3.20	6.58	4.25
RRu	7.21	3.79	6.95	13.12	6.83
P	+++	+++	+++	+++	+++

Table 1J11 - 3

IESLC - Meta-analysis of Ex Smoking by Years quit (vs never), Overview
All LC types, Cigarettes only
Most adjusted

MALES

Years quit vs never (lower focus)

		absent	8+k12	4-11k7	1-6k3	Total
	N	20	12	8	9	49
	NS	12	12	8	9	41
	Wt	515.24	270.26	185.06	182.42	1152.98
Het	Chi	618.31	56.71	17.62	42.54	899.54
Het	df	19	11	7	8	48
Het	P	***	***	*	***	***
Fixed	RR	5.36	5.01	8.04	14.58	6.60
	RRl	4.92	4.45	6.96	12.61	6.23
	RRu	5.85	5.65	9.28	16.85	6.99
	P	+++	+++	+++	+++	+++
Random	RR	5.30	3.75	7.19	12.33	5.81
	RRl	3.12	2.70	5.57	8.51	4.46
	RRu	9.00	5.21	9.30	17.87	7.58
	P	+++	+++	+++	+++	+++

Years quit vs never (higher focus)

		absent	13+k20	4-19k12	1-11k3	Total
	N	26	5	5	13	49
	NS	13	5	5	13	36
	Wt	513.45	216.13	215.61	207.79	1152.98
Het	Chi	616.07	7.15	13.97	61.80	899.54
Het	df	25	4	4	12	48
Het	P	***	N.S.	**	***	***
Fixed	RR	6.92	3.38	5.89	13.21	6.60
	RRl	6.35	2.96	5.16	11.53	6.23
	RRu	7.55	3.86	6.74	15.13	6.99
	P	+++	+++	+++	+++	+++
Random	RR	5.08	3.28	5.75	10.52	5.81
	RRl	3.23	2.63	4.24	7.48	4.46
	RRu	7.99	4.09	7.78	14.80	7.58
	P	+++	+++	+++	+++	+++

FEMALES

Years quit vs never (lower focus)

		absent	8+k12	4-11k7	1-6k3	Total
	N	6	3	1	1	11
	NS	3	3	1	1	6
	Wt	71.33	39.36	24.68	28.18	163.55
Het	Chi	34.15	4.48	0.00	0.00	82.87
Het	df	5	2	0	0	10
Het	P	***	N.S.	N.S.	N.S.	***
Fixed	RR	4.47	1.87	4.81	9.29	4.15
	RRl	3.54	1.37	3.24	6.42	3.56
	RRu	5.63	2.56	7.14	13.43	4.84
	P	+++	+++	+++	+++	+++
Random	RR	4.11	1.95	4.81	9.29	3.80
	RRl	2.11	1.14	3.24	6.42	2.38
	RRu	8.00	3.32	7.14	13.43	6.08
	P	+++	+	+++	+++	+++

Table 1J11 - 3

IESLC - Meta-analysis of Ex Smoking by Years quit (vs never), Overview
All LC types, Cigarettes only
Most adjusted

FEMALES

		Years quit vs never (higher focus)				
		absent	13+k20	4-19k12	1-11k3	Total
	N	6	1	1	3	11
	NS	3	1	1	3	6
	Wt	78.41	11.83	22.54	50.76	163.55
Het	Chi	40.47	0.00	0.00	15.16	82.87
Het	df	5	0	0	2	10
Het	P	***	N.S.	N.S.	***	***
Fixed	RR	4.54	1.65	2.16	6.00	4.15
	RRl	3.64	0.93	1.43	4.56	3.56
	RRu	5.67	2.91	3.27	7.90	4.84
	P	+++	(+)	+++	+++	+++
Random	RR	3.93	1.65	2.16	5.88	3.80
	RRl	1.95	0.93	1.43	2.49	2.38
	RRu	7.93	2.91	3.27	13.85	6.08
	P	+++	(+)	+++	+++	+++

Table 1J11 - 4

IESLC - Meta-analysis of Ex Smoking by Years quit (vs never), Overview
All LC types, Cigarettes only
Least adjusted

REF	NRR	X	SEX	AGEL	AGEH	RACE	YF	LC TYPE	LOC	START	ST	NLC	R	VB	P	H	AD	PRODUCT	exL	exH	S1	S2	DENOM	De
ALDERS	507		m	0	0	all	-	all	Eu:UK	1977	CC	1448	n	V	n	n	1	cig only	10	999	1	0	nev any	ot
ALDERS	508		m	0	0	all	-	all	Eu:UK	1977	CC	1448	n	V	n	n	1	cig only	3	9	0	3	nev any	ot
ALDERS	509		m	0	0	all	-	all	Eu:UK	1977	CC	1448	n	V	n	n	1	cig only	0.1	2	0	0	nev any	ot
ALDERS	518		f	0	0	all	-	all	Eu:UK	1977	CC	1448	n	V	n	n	1	cig only	10	999	1	0	nev any	ot
ALDERS	519		f	0	0	all	-	all	Eu:UK	1977	CC	1448	n	V	n	n	1	cig only	3	9	0	3	nev any	ot
ALDERS	520		f	0	0	all	-	all	Eu:UK	1977	CC	1448	n	V	n	n	1	cig only	0.1	2	0	0	nev any	ot
BENHAM	561		m	0	0	all	-	not mix	Eu:wst	1976	CC	1625	n	bl	n	y	0	cig only	11	999	1	0	nev any	st
BENHAM	562		m	0	0	all	-	not mix	Eu:wst	1976	CC	1625	n	bl	n	y	0	cig only	4	10	2	0	nev any	st
BENHAM	563		m	0	0	all	-	not mix	Eu:wst	1976	CC	1625	n	bl	n	y	0	cig only	1.0	3	3	3	nev any	st
CPSI	807		m	50	74	all	6	all	NAmer	1959	pr	5138	n	bl	n	n	1	cig only	10	999	1	0	nev any	ot
CPSI	808		m	50	74	all	6	all	NAmer	1959	pr	5138	n	bl	n	n	1	cig only	5	9	2	0	nev any	ot
CPSI	809		m	50	74	all	6	all	NAmer	1959	pr	5138	n	bl	n	n	1	cig only	1.0	4	3	3	nev any	ot
CPSI	810		m	50	74	all	6	all	NAmer	1959	pr	5138	n	bl	n	n	1	cig only	0.1	0.9	0	0	nev any	ot
CPSII	652		m	35	99	all	4	all	NAmer	1982	pr	3229	n	bl	n	n	1	cig only	16	999	0	1	nev any	ot
CPSII	653		m	35	99	all	4	all	NAmer	1982	pr	3229	n	bl	n	n	1	cig only	11	15	1	2	nev any	ot
CPSII	654		m	35	99	all	4	all	NAmer	1982	pr	3229	n	bl	n	n	1	cig only	6	10	2	0	nev any	ot
CPSII	655		m	35	99	all	4	all	NAmer	1982	pr	3229	n	bl	n	n	1	cig only	3	5	3	3	nev any	ot
CPSII	656		m	35	99	all	4	all	NAmer	1982	pr	3229	n	bl	n	n	1	cig only	1.0	2	0	0	nev any	ot
CPSII	657		m	35	99	all	4	all	NAmer	1982	pr	3229	n	bl	n	n	1	cig only	0.1	0.9	0	0	nev any	ot
DAMBER	554		m	0	0	all	-	all	Eu:Sca	1972	CC	579	n	bl	y	n	1	cig only	11	999	1	0	nev any	ot
DAMBER	555		m	0	0	all	-	all	Eu:Sca	1972	CC	579	n	bl	y	n	1	cig only	0.1	10	0	3	nev any	ot
DEAN3	501	x	m	0	0	all	-	all	Eu:UK	1969	CC	766	n	V	y	n	0	cig only	19	999	0	1	nev any	st
DEAN3	502	x	m	0	0	all	-	all	Eu:UK	1969	CC	766	n	V	y	n	0	cig only	9	18	1	2	nev any	st
DEAN3	503	x	m	0	0	all	-	all	Eu:UK	1969	CC	766	n	V	y	n	0	cig only	5	8	2	0	nev any	st
DEAN3	504	x	m	0	0	all	-	all	Eu:UK	1969	CC	766	n	V	y	n	0	cig only	3	4	3	3	nev any	st
DOLL2	501		m	0	0	all	20	all	Eu:UK	1951	pr	920	n	V	n	n	1	cig only	15	999	0	1	nev any	ot
DOLL2	502		m	0	0	all	20	all	Eu:UK	1951	pr	920	n	V	n	n	1	cig only	10	14	1	2	nev any	ot
DOLL2	503		m	0	0	all	20	all	Eu:UK	1951	pr	920	n	V	n	n	1	cig only	5	9	2	0	nev any	ot
DOLL2	504		m	0	0	all	20	all	Eu:UK	1951	pr	920	n	V	n	n	1	cig only	0.1	4	3	3	nev any	ot
DORN	508		m	0	0	wh	0	all	NAmer	1954	pr	5097	n	bl	n	n	2	cig only	40	999	0	0	nev any	or
DORN	509		m	0	0	wh	0	all	NAmer	1954	pr	5097	n	bl	n	n	2	cig only	30	39	0	0	nev any	or
DORN	510		m	0	0	wh	0	all	NAmer	1954	pr	5097	n	bl	n	n	2	cig only	20	29	0	1	nev any	or
DORN	511		m	0	0	wh	0	all	NAmer	1954	pr	5097	n	bl	n	n	2	cig only	10	19	1	2	nev any	or
DORN	512		m	0	0	wh	0	all	NAmer	1954	pr	5097	n	bl	n	n	2	cig only	5	9	2	0	nev any	or
DORN	513		m	0	0	wh	0	all	NAmer	1954	pr	5097	n	bl	n	n	2	cig only	1.0	4	3	3	nev any	or
GRAHAM	501		m	0	0	wh	-	all	NAmer	1956	CC	685	n	bl	n	n	0	cig only	10	999	1	0	nev any	st
GRAHAM	502		m	0	0	wh	-	all	NAmer	1956	CC	685	n	bl	n	n	0	cig only	3	10	2	0	nev any	st
GRAHAM	503		m	0	0	wh	-	all	NAmer	1956	CC	685	n	bl	n	n	0	cig only	1.1	3	3	3	nev any	st
GRAHAM	504		m	0	0	wh	-	all	NAmer	1956	CC	685	n	bl	n	n	0	cig only	0.1	1.0	0	0	nev any	st
KAISE2	646		m	0	0	all	9	all	NAmer	1979	pr	318	n	bl	n	n	1	cig only	21	999	0	0	nev any	st
KAISE2	647		m	0	0	all	9	all	NAmer	1979	pr	318	n	bl	n	n	1	cig only	11	20	1	0	nev any	ot
KAISE2	648		m	0	0	all	9	all	NAmer	1979	pr	318	n	bl	n	n	1	cig only	2	10	0	3	nev any	st
KAISE2	566		f	0	0	all	9	all	NAmer	1979	pr	318	n	bl	n	n	1	cig only	21	999	0	0	nev any	ot
KAISE2	567		f	0	0	all	9	all	NAmer	1979	pr	318	n	bl	n	n	1	cig only	11	20	1	0	nev any	st
KAISE2	568		f	0	0	all	9	all	NAmer	1979	pr	318	n	bl	n	n	1	cig only	2	10	0	3	nev any	st
PEZZOT	501		m	0	0	all	-	all	SCAmer	1987	CC	215	n	bl	n	y	0	cig only	11	999	1	0	nev cigs	st
PEZZOT	502		m	0	0	all	-	all	SCAmer	1987	CC	215	n	bl	n	y	0	cig only	1.0	10	0	3	nev cigs	st
TVERDA	501		m	0	0	all	0	all	Eu:Sca	1972	pr	238	n	bl	n	n	2	cig only	5	999	0	0	nev cigs	ot
TVERDA	502		m	0	0	all	0	all	Eu:Sca	1972	pr	238	n	bl	n	n	2	cig only	1.0	5	3	3	nev cigs	ot
TVERDA	503		m	0	0	all	0	all	Eu:Sca	1972	pr	238	n	bl	n	n	2	cig only	0.1	0.9	0	0	nev cigs	ot
WYNDE6	501		m	0	0	all	-	all	NAmer	1969	CC	4423	n	bl	n	y	0	cig only	30	999	0	0	nev any	st
WYNDE6	502		m	0	0	all	-	all	NAmer	1969	CC	4423	n	bl	n	y	0	cig only	20	29	0	1	nev any	st
WYNDE6	503		m	0	0	all	-	all	NAmer	1969	CC	4423	n	bl	n	y	0	cig only	10	19	1	2	nev any	st
WYNDE6	504		m	0	0	all	-	all	NAmer	1969	CC	4423	n	bl	n	y	0	cig only	5	9	2	0	nev any	st
WYNDE6	505		m	0	0	all	-	all	NAmer	1969	CC	4423	n	bl	n	y	0	cig only	1.0	4	3	3	nev any	st
WYNDE6	522		f	0	0	all	-	all	NAmer	1969	CC	4423	n	bl	n	y	0	cig only	30	999	0	0	nev any	st
WYNDE6	523		f	0	0	all	-	all	NAmer	1969	CC	4423	n	bl	n	y	0	cig only	20	29	0	1	nev any	st
WYNDE6	524		f	0	0	all	-	all	NAmer	1969	CC	4423	n	bl	n	y	0	cig only	10	19	1	2	nev any	st
WYNDE6	525		f	0	0	all	-	all	NAmer	1969	CC	4423	n	bl	n	y	0	cig only	5	9	2	0	nev any	st
WYNDE6	526		f	0	0	all	-	all	NAmer	1969	CC	4423	n	bl	n	y	0	cig only	1.0	4	3	3	nev any	st

Cigarette type is all/unspec for all RRs
except for the following:

REF	NRR	CIGTYPE
ALDERS	507	MC only
ALDERS	508	MC only
ALDERS	509	MC only
ALDERS	518	MC only

Table 1J11 - 4

IESLC - Meta-analysis of Ex Smoking by Years quit (vs never), Overview
All LC types, Cigarettes only
Least adjusted

REF	NRR	CIGTYPE
ALDERS	519	MC only
ALDERS	520	MC only
DEAN3	501	MC only
DEAN3	502	MC only
DEAN3	503	MC only
DEAN3	504	MC only

In this overview table, subtotals and Qs values may be invalid and should be ignored

Table 1J11 - 5

IESLC - Meta-analysis of Ex Smoking by Years quit (vs never), Overview
All LC types, Cigarettes only
Least adjusted

REF	NRR	SEX	AD	Number Exposed Case	Number Exposed Cont	Non-exposed Case	Non-exposed Cont	RR	95.00%CI
ALDERS	507	m	1	29	-	15	-	3.20	(1.61- 6.35)
ALDERS	508	m	1	28	-	15	-	4.30	(2.13- 8.69)
ALDERS	509	m	1	121	-	15	-	18.10	(9.71- 33.74)
ALDERS	518	f	1	26	-	75	-	1.27	(0.76- 2.15)
ALDERS	519	f	1	54	-	75	-	2.95	(1.88- 4.64)
ALDERS	520	f	1	206	-	75	-	9.45	(6.50- 13.74)
Subtotal ALDERS								4.77	(3.86- 5.89)
BENHAM	561	m	0	60	228	33	523	4.17	(2.65- 6.56)
BENHAM	562	m	0	105	185	33	523	9.00	(5.88- 13.77)
BENHAM	563	m	0	145	86	33	523	26.72	(17.18- 41.55)
Subtotal BENHAM								10.12	(7.85- 13.04)
*CPSI	807	m	1	15	-	60	-	1.28	(0.73- 2.25)
*CPSI	808	m	1	32	-	60	-	5.15	(3.35- 7.91)
*CPSI	809	m	1	49	-	60	-	8.09	(5.55- 11.80)
*CPSI	810	m	1	37	-	60	-	14.74	(9.78- 22.20)
Subtotal CPSI								6.51	(5.25- 8.07)
*CPSII	652	m	1	256	-	81	-	3.83	(2.98- 4.92)
*CPSII	653	m	1	164	-	81	-	8.61	(6.60- 11.24)
*CPSII	654	m	1	186	-	81	-	11.43	(8.81- 14.84)
*CPSII	655	m	1	178	-	81	-	18.61	(14.31- 24.20)
*CPSII	656	m	1	188	-	81	-	28.07	(21.63- 36.43)
*CPSII	657	m	1	97	-	81	-	38.76	(28.85- 52.07)
Subtotal CPSII								13.29	(11.93- 14.81)
DAMBER	554	m	1	-	-	42	-	1.60	(0.70- 3.40)
DAMBER	555	m	1	-	-	42	-	5.50	(2.80- 11.10)
Subtotal DAMBER								3.23	(1.92- 5.42)
DEAN3	501	m	0	8	66	24	510	2.58	(1.11- 5.97)
DEAN3	502	m	0	15	86	24	510	3.71	(1.87- 7.35)
DEAN3	503	m	0	11	43	24	510	5.44	(2.50- 11.84)
DEAN3	504	m	0	28	102	24	510	5.83	(3.25- 10.47)
Subtotal DEAN3								4.43	(3.12- 6.29)
*DOLL2	501	m	1	7	-	7	-	2.00	(0.70- 5.70)
*DOLL2	502	m	1	9	-	7	-	5.30	(1.97- 14.23)
*DOLL2	503	m	1	12	-	7	-	5.90	(2.32- 14.99)
*DOLL2	504	m	1	15	-	7	-	16.00	(6.52- 39.24)
Subtotal DOLL2								6.10	(3.77- 9.87)
*DORN	508	m	2	49	-	325	-	1.50	(1.10- 2.00)
*DORN	509	m	2	82	-	325	-	2.00	(1.60- 2.60)
*DORN	510	m	2	215	-	325	-	3.30	(2.80- 4.00)
*DORN	511	m	2	261	-	325	-	5.10	(4.20- 6.10)
*DORN	512	m	2	87	-	325	-	7.80	(5.70- 10.50)
*DORN	513	m	2	56	-	325	-	16.10	(10.40- 24.80)
Subtotal DORN								3.73	(3.38- 4.11)
GRAHAM	501	m	0	2	30	18	346	1.28	(0.28- 5.79)
GRAHAM	502	m	0	5	29	18	346	3.31	(1.15- 9.57)
GRAHAM	503	m	0	12	23	18	346	10.03	(4.31- 23.31)
GRAHAM	504	m	0	84	48	18	346	33.64	(18.61- 60.80)
Subtotal GRAHAM								13.27	(8.69- 20.26)
*KAISE2	646	m	1	6	-	14	-	1.94	(0.70- 5.40)
*KAISE2	647	m	1	8	-	14	-	3.14	(1.26- 7.82)
*KAISE2	648	m	1	12	-	14	-	8.26	(3.73- 18.28)
*KAISE2	566	f	1	4	-	11	-	6.29	(1.78- 22.20)
*KAISE2	567	f	1	4	-	11	-	4.37	(1.30- 14.72)
*KAISE2	568	f	1	6	-	11	-	7.95	(2.89- 21.86)
Subtotal KAISE2								4.86	(3.24- 7.31)
PEZZOT	501	m	0	20	106	4	116	5.47	(1.81- 16.53)
PEZZOT	502	m	0	46	82	4	116	16.27	(5.64- 46.96)
Subtotal PEZZOT								9.65	(4.49- 20.74)
*TVERDA	501	m	2	4	-	4	-	1.34	(0.34- 5.37)
*TVERDA	502	m	2	5	-	4	-	2.83	(0.76- 10.53)
*TVERDA	503	m	2	2	-	4	-	2.77	(0.51- 15.15)
Subtotal TVERDA								2.15	(0.94- 4.93)
WYNDE6	501	m	0	21	161	64	918	1.87	(1.11- 3.15)
WYNDE6	502	m	0	55	212	64	918	3.72	(2.52- 5.50)
WYNDE6	503	m	0	159	373	64	918	6.11	(4.47- 8.37)
WYNDE6	504	m	0	98	194	64	918	7.25	(5.10- 10.29)
WYNDE6	505	m	0	201	166	64	918	17.37	(12.53- 24.07)
WYNDE6	522	f	0	10	31	125	991	2.56	(1.22- 5.34)
WYNDE6	523	f	0	16	77	125	991	1.65	(0.93- 2.91)
WYNDE6	524	f	0	36	132	125	991	2.16	(1.43- 3.27)
WYNDE6	525	f	0	51	84	125	991	4.81	(3.24- 7.14)

Table 1J11 - 5

IESLC - Meta-analysis of Ex Smoking by Years quit (vs never), Overview
All LC types, Cigarettes only
Least adjusted

REF	NRR	SEX	AD	Number Exposed Case	Number Exposed Cont	Non-exposed Case	Non-exposed Cont	RR	95.00%CI
WYNDE6	526	f	0	82	70	125	991	9.29	(6.42- 13.43)
Subtotal WYNDE6								5.50	(4.84- 6.24)
Partial Totals				3780	2614	4365	14770		

*prospective study

REF	NRR	SEX	AD	Ys	Ws	Qs	Ps
ALDERS	507	m	1	1.16	8.16	3.70	0.0009
ALDERS	508	m	1	1.46	7.77	1.11	0.0000
ALDERS	509	m	1	2.90	9.90	11.11	0.0000
ALDERS	518	f	1	0.24	14.21	36.27	0.3676
ALDERS	519	f	1	1.08	18.83	10.73	0.0000
ALDERS	520	f	1	2.25	27.43	4.59	0.0000
Subtotal ALDERS				1.56	86.30	67.52	
BENHAM	561	m	0	1.43	18.77	3.13	0.0000
BENHAM	562	m	0	2.20	21.21	2.75	0.0000
BENHAM	563	m	0	3.29	19.71	41.37	0.0000
Subtotal BENHAM				2.31	59.69	47.25	
*CPSI	807	m	1	0.25	12.13	30.65	0.3900
*CPSI	808	m	1	1.64	20.82	0.81	0.0000
*CPSI	809	m	1	2.09	27.01	1.74	0.0000
*CPSI	810	m	1	2.69	22.87	16.67	0.0000
Subtotal CPSI				1.87	82.82	49.88	
*CPSII	652	m	1	1.34	61.12	14.91	0.0000
*CPSII	653	m	1	2.15	54.21	5.42	0.0000
*CPSII	654	m	1	2.44	56.51	20.31	0.0000
*CPSII	655	m	1	2.92	55.67	65.77	0.0000
*CPSII	656	m	1	3.33	56.54	126.88	0.0000
*CPSII	657	m	1	3.66	44.07	146.09	0.0000
Subtotal CPSII				2.59	328.12	379.38	
DAMBER	554	m	1	0.47	6.15	11.49	0.2437
DAMBER	555	m	1	1.70	8.10	0.14	0.0000
Subtotal DAMBER				1.17	14.25	11.63	
DEAN3	501	m	0	0.95	5.44	4.32	0.0273
DEAN3	502	m	0	1.31	8.20	2.27	0.0002
DEAN3	503	m	0	1.69	6.34	0.13	0.0000
DEAN3	504	m	0	1.76	11.22	0.06	0.0000
Subtotal DEAN3				1.49	31.20	6.78	
*DOLL2	501	m	1	0.69	3.49	4.57	0.1951
*DOLL2	502	m	1	1.67	3.93	0.11	0.0009
*DOLL2	503	m	1	1.77	4.41	0.02	0.0002
*DOLL2	504	m	1	2.77	4.77	4.18	0.0000
Subtotal DOLL2				1.81	16.61	8.88	
*DORN	508	m	2	0.41	42.99	88.07	0.0078
*DORN	509	m	2	0.69	65.19	85.25	0.0000
*DORN	510	m	2	1.19	120.78	49.90	0.0000
*DORN	511	m	2	1.63	110.32	4.75	0.0000
*DORN	512	m	2	2.05	41.17	1.95	0.0000
*DORN	513	m	2	2.78	20.35	18.06	0.0000
Subtotal DORN				1.32	400.80	247.97	
GRAHAM	501	m	0	0.25	1.69	4.26	0.7471
GRAHAM	502	m	0	1.20	3.41	1.39	0.0268
GRAHAM	503	m	0	2.31	5.40	1.19	0.0000
GRAHAM	504	m	0	3.52	10.97	30.92	0.0000
Subtotal GRAHAM				2.59	21.47	37.76	
*KAISE2	646	m	1	0.66	3.68	5.07	0.2036
*KAISE2	647	m	1	1.14	4.61	2.21	0.0140
*KAISE2	648	m	1	2.11	6.08	0.46	0.0000
*KAISE2	566	f	1	1.84	2.41	0.00	0.0043
*KAISE2	567	f	1	1.47	2.61	0.34	0.0172
*KAISE2	568	f	1	2.07	3.75	0.21	0.0001
Subtotal KAISE2				1.58	23.15	8.30	
PEZZOT	501	m	0	1.70	3.14	0.06	0.0026
PEZZOT	502	m	0	2.79	3.42	3.10	0.0000
Subtotal PEZZOT				2.27	6.56	3.16	
*TVERDA	501	m	2	0.29	2.02	4.81	0.6776
*TVERDA	502	m	2	1.04	2.22	1.41	0.1208
*TVERDA	503	m	2	1.02	1.34	0.89	0.2389
Subtotal TVERDA				0.76	5.58	7.11	
WYNDE6	501	m	0	0.63	14.18	20.76	0.0183
WYNDE6	502	m	0	1.31	25.24	6.90	0.0000

Table 1J11 - 5

IESLC - Meta-analysis of Ex Smoking by Years quit (vs never), Overview
All LC types, Cigarettes only
Least adjusted

REF	NRR	SEX	AD	Ys	Ws	Qs	Ps
WYNDE6	503	m	0	1.81	38.93	0.03	0.0000
WYNDE6	504	m	0	1.98	31.18	0.64	0.0000
WYNDE6	505	m	0	2.85	36.08	37.39	0.0000
WYNDE6	522	f	0	0.94	7.08	5.70	0.0125
WYNDE6	523	f	0	0.50	11.83	21.17	0.0859
WYNDE6	524	f	0	0.77	22.54	25.60	0.0003
WYNDE6	525	f	0	1.57	24.68	1.74	0.0000
WYNDE6	526	f	0	2.23	28.18	4.33	0.0000
Subtotal WYNDE6				1.70	239.93	124.26	

N	60
NS	13

Table 1J11 - 6

IESLC - Meta-analysis of Ex Smoking by Years quit (vs never), Overview
All LC types, Cigarettes only
Least adjusted

	Sex: combined	male	female	Total
N		49	11	60
NS		13	3	16

In this overview table, other than the "N" rows, entries in the "absent" and "Total" columns may be invalid and should be ignored

	Years quit vs never (lower focus): absent	8+k12	4-11k7	1-6k3	Total
N	26	15	9	10	60
NS	12	12	8	9	41
Wt	586.53	309.61	209.73	210.60	1316.47
Het Chi	646.73	93.62	21.76	42.64	999.87
Het df	25	14	8	9	59
Het P	***	***	**	***	***
Fixed RR	5.28	4.45	7.63	13.89	6.28
RRl	4.87	3.98	6.66	12.13	5.95
RRu	5.72	4.97	8.73	15.89	6.62
P	+++	+++	+++	+++	+++
Random RR	5.16	3.32	6.92	12.29	5.51
RRl	3.32	2.37	5.41	8.91	4.35
RRu	8.01	4.65	8.85	16.97	6.97
P	+++	+++	+++	+++	+++

	Years quit vs never (higher focus): absent	13+k20	4-19k12	1-11k3	Total
N	32	6	6	16	60
NS	13	5	5	13	36
Wt	591.87	227.92	238.14	258.55	1316.47
Het Chi	667.49	8.64	32.81	98.49	999.87
Het df	31	5	5	15	59
Het P	***	N.S.	***	***	***
Fixed RR	6.56	3.31	5.40	11.42	6.28
RRl	6.06	2.91	4.76	10.11	5.95
RRu	7.11	3.77	6.13	12.90	6.62
P	+++	+++	+++	+++	+++
Random RR	4.90	3.16	4.86	9.44	5.51
RRl	3.31	2.56	3.32	6.73	4.35
RRu	7.27	3.90	7.10	13.25	6.97
P	+++	+++	+++	+++	+++

Table 1J11 - 6

IESLC - Meta-analysis of Ex Smoking by Years quit (vs never), Overview
All LC types, Cigarettes only
Least adjusted

MALES

		Years quit vs never (lower focus)				
		absent	8+k12	4-11k7	1-6k3	Total
	N	20	12	8	9	49
	NS	12	12	8	9	41
	Wt	515.20	270.25	185.06	182.42	1152.93
Het	Chi	610.31	55.26	15.83	37.38	885.24
Het	df	19	11	7	8	48
Het	P	***	***	*	***	***
Fixed	RR	5.40	5.05	8.11	14.78	6.65
	RRl	4.95	4.48	7.02	12.78	6.28
	RRu	5.89	5.69	9.37	17.09	7.05
	P	+++	+++	+++	+++	+++
Random	RR	5.48	3.82	7.40	12.75	5.98
	RRl	3.24	2.76	5.81	8.99	4.59
	RRu	9.28	5.30	9.43	18.07	7.78
	P	+++	+++	+++	+++	+++

		Years quit vs never (higher focus)				
		absent	13+k20	4-19k12	1-11k3	Total
	N	26	5	5	13	49
	NS	13	5	5	13	36
	Wt	513.45	216.09	215.60	207.79	1152.93
Het	Chi	614.79	2.55	11.94	57.14	885.24
Het	df	25	4	4	12	48
Het	P	***	N.S.	*	***	***
Fixed	RR	6.94	3.44	5.94	13.37	6.65
	RRl	6.37	3.01	5.20	11.67	6.28
	RRu	7.57	3.93	6.79	15.32	7.05
	P	+++	+++	+++	+++	+++
Random	RR	5.13	3.44	5.93	10.76	5.98
	RRl	3.26	3.01	4.48	7.74	4.59
	RRu	8.06	3.93	7.85	14.96	7.78
	P	+++	+++	+++	+++	+++

FEMALES

		Years quit vs never (lower focus)				
		absent	8+k12	4-11k7	1-6k3	Total
	N	6	3	1	1	11
	NS	3	3	1	1	6
	Wt	71.33	39.36	24.68	28.18	163.55
Het	Chi	34.15	4.48	0.00	0.00	82.87
Het	df	5	2	0	0	10
Het	P	***	N.S.	N.S.	N.S.	***
Fixed	RR	4.47	1.87	4.81	9.29	4.15
	RRl	3.54	1.37	3.24	6.42	3.56
	RRu	5.63	2.56	7.14	13.43	4.84
	P	+++	+++	+++	+++	+++
Random	RR	4.11	1.95	4.81	9.29	3.80
	RRl	2.11	1.14	3.24	6.42	2.38
	RRu	8.00	3.32	7.14	13.43	6.08
	P	+++	+	+++	+++	+++

Table 1J11 - 6

IESLC - Meta-analysis of Ex Smoking by Years quit (vs never), Overview
All LC types, Cigarettes only
Least adjusted

FEMALES

	Years quit vs never (higher focus) absent	13+k20	4-19k12	1-11k3	Total
N	6	1	1	3	11
NS	3	1	1	3	6
Wt	78.41	11.83	22.54	50.76	163.55
Het Chi	40.47	0.00	0.00	15.16	82.87
Het df	5	0	0	2	10
Het P	***	N.S.	N.S.	***	***
Fixed RR	4.54	1.65	2.16	6.00	4.15
RRl	3.64	0.93	1.43	4.56	3.56
RRu	5.67	2.91	3.27	7.90	4.84
P	+++	(+)	+++	+++	+++
Random RR	3.93	1.65	2.16	5.88	3.80
RRl	1.95	0.93	1.43	2.49	2.38
RRu	7.93	2.91	3.27	13.85	6.08
P	+++	(+)	+++	+++	+++

Table 1J11 - 7

IESLC - Meta-analysis of Ex Smoking by Years quit (vs never), Overview
All LC types, Cigarettes only
Excluded studies (and stage at which they were excluded)

1	AGUDO AKIBA AMANDU AMES AXELSS BEST BOUCHA BOUCOT BRESLO CHEN CHEN2 CHIAZZ DEAN2 DOSEME ENGELA FAN GENG GER GUO HAENSZ HEGMAN HOLE HU HU2 JUSSAW KATSOU KAUFMA KOO KOULUM KREUZE LETOUR LEVIN LIAW LIU3 LIU4 LIU5 MCCONN MIGRAN MRFITR NOTAN2 OSANN2 PERNU QIAO2 RACHTA RESTRE SADOWS SEGI2 STASZE TIZZAN VUTUC WATSON WU WUWILL WYNDE2 WYNDE8 XU YUAN ZHANG ZHENG ZHOU
2	BUFFLE HUMBLE PISANI PRESCO WYNDE7
3	MCDUFF SPITZ
4	HAMMON LUO WU2
5	BLOT1 CORREA GILLIS QIAO WIGLE
7	ARMADA AUVINE BARBON BECHER BENSHL BOFFET BROSS BROWN3 CARPEN CEDERL CHOI CHYOU DARBY DESTEF DOLL DORGAN GAO GAO2 GARCIA GARSHI GURSEL HAMMO2 HIRAYA JAHN JAIN JEDRYC JOLY KHUDER LAUSSM LUBIN LUBIN2 MATOS PEZZO2 SOBUE SPEIZE SUZUK2 SVENSS WAKAI WANG2 WYNDE3

Table 1J11 - 8
Potentially overlapping studies

REF	REFGP	PRINC	. OVERLAP/LINK
BENHAM	LUBIN2	2	Subset of Lubin2
TVERDA	TVERDA	1	VEIERO/TVERDAL
GRAHAM	BYERS1	1	GRAHAM/BROSS/BYERS1
WYNDE6	WYNDE6	1	WYNDE5/6/7/8
CPSI	CPSI	1	CPSI overall

Table 1J11 - 9
Most adjusted - insufficient data for meta-analysis

REF	NRR	SEX	AGEL	AGEH	RACE	YF	LC TYPE	LOC	START	ST	NLC	R	VB	P	H	AD	PRODUCT	exL	exH	S1	S2	DENOM	De
BENHAM	564	m	0	0	all	-	not mix	Eu:wst	1976	CC	1625	n	bl	n	y	0	cig only	0.1	0.9	0	0	nev any	ot
CPSI	718	f	0	0	wh	0	all	NAmer	1959	pr	5138	n	bl	n	n	1	cig only	25	29	0	0	nev cigs	or
CPSI	719	f	0	0	wh	0	all	NAmer	1959	pr	5138	n	bl	n	n	1	cig only	20	24	0	1	nev cigs	or
CPSI	720	f	0	0	wh	0	all	NAmer	1959	pr	5138	n	bl	n	n	1	cig only	15	19	0	0	nev cigs	or
CPSI	721	f	0	0	wh	0	all	NAmer	1959	pr	5138	n	bl	n	n	1	cig only	10	14	1	2	nev cigs	or
CPSI	722	f	0	0	wh	0	all	NAmer	1959	pr	5138	n	bl	n	n	1	cig only	5	9	2	0	nev cigs	or
CPSI	723	f	0	0	wh	0	all	NAmer	1959	pr	5138	n	bl	n	n	1	cig only	2	4	3	3	nev cigs	or
CPSI	935	f	0	0	wh	0	all	NAmer	1959	pr	5138	n	bl	n	n	1	cig only	0.1	1.9	0	0	nev cigs	ot
DEAN3	620	m	0	0	all	-	all	Eu:UK	1969	CC	766	n	V	y	n	1	cig only	1.0	2	0	0	nev any	ot
KAISE2	649	m	0	0	all	9	all	NAmer	1979	pr	318	n	bl	n	n	1	cig only	0.1	1.9	0	0	nev any	ot
KAISE2	569	f	0	0	all	9	all	NAmer	1979	pr	318	n	bl	n	n	1	cig only	0.1	1.9	0	0	nev any	ot
PEZZOT	597	m	0	0	all	-	all	SCAmer	1987	CC	215	n	bl	n	y	0	cig only	0.1	0.9	0	0	nev cigs	ot
WYNDE6	506	m	0	0	all	-	all	NAmer	1969	CC	4423	n	bl	n	y	0	cig only	0.1	0.9	0	0	nev any	ot
WYNDE6	527	f	0	0	all	-	all	NAmer	1969	CC	4423	n	bl	n	y	0	cig only	0.1	0.9	0	0	nev any	ot

REF	NRR	RR	SIG	RRDATA comment
BENHAM	564	*	gap	0
CPSI	718	2.61		0
CPSI	719	2.52		0
CPSI	720	3.19		0
CPSI	721	0.58		0
CPSI	722	1.51		0
CPSI	723	2.85		0
CPSI	935	*	gap	0
DEAN3	620	*	gap	0
KAISE2	649	*	gap	0
KAISE2	569	*	gap	0
PEZZOT	597	*	gap	0
WYNDE6	506	*	gap	0
WYNDE6	527	*	gap	0

Least adjusted - insufficient data for meta-analysis: as for adjusted plus the following

REF	NRR	SEX	AGEL	AGEH	RACE	YF	LC TYPE	LOC	START	ST	NLC	R	VB	P	H	AD	PRODUCT	exL	exH	S1	S2	DENOM	De
DEAN3	618	m	0	0	all	-	all	Eu:UK	1969	CC	766	n	V	y	n	0	cig only	1.0	2	0	0	nev any	ot

Table 1J11 - 9

IESLC - Meta-analysis of Ex Smoking by Years quit (vs never), Overview
All LC types, Cigarettes only
Least adjusted - insufficient data for meta-analysis: as for adjusted plus the following

REF	NRR	RR	SIG	RRDATA comment
DEAN3	618	*	gap	0

Table 1J12 -

IESLC - Meta-analysis of Ex Smoking, Years quit (vs never), "Low"
All LC types, Cigarettes only

This analysis is restricted to results for:
1) Ex smokers
2) Results by Years quit (vs never)
3) Categorical results by Years quit (vs never)
4) All LC types (or near equivalent)
5) Results complete enough for use in metaanalysis

Within each study, results are then selected (in the following order of preference, within each sex) for:
6) (not applicable)
7) PRODUCT: cigarettes only
8) CIGTYPE: all/unspecified, MC regardless of HR, MC only
9) (not applicable)
10) DENOM: never smoked anything, never smoked cigarettes, never any + low, never cigs + low
11) Followup period (YF, prospective studies): whole study (coded as 0) or longest available
12) LCtype: all or nearest available, at least Squamous and Adeno. (q = squamous, s = small,
l = large, a = adeno, mix = mixed, alv = alveolar)
13) Race: all or nearest available, otherwise by race (wh or w = white, bl or b = black, hi = hispanic
ch = chinese, jap = japanese, haw = hawaiian, w+o = white + oriental, sca = scandinavian, as = asian)
14) Years quit (vs never) "low" in key scheme 1 (key value 12, maximum range 8+)
15) For overlapping studies: principal rather than subsidiary studies
Finally by Age: whole study (coded as 0) if available, otherwise by widest available age group
and then for single sex results (m, f) in preference to results for both sexes combined (c).

Results adjusted (AD) for the most potential confounders are then chosen in Sections -1 to -3
(and those which actually differ from the adjusted results in Table 1J2 - 1 are marked 'x' in Section -1)
and results adjusted for the least confounders in Sections -4 to -6. (Those least adjusted results which
actually differ from the most adjusted are marked 'x' in column X in Section -4)

Section -7 shows excluded studies, together with the stage (as above) at which no qualifying
results were found.

Section -8 lists the potentially overlapping studies which have been included (1=principal, 2=subsidiary).

Section -9 lists any results which would have been included in preference except that they had data not complete
enough for use in meta-analysis, with their significance (yes/no), if known, and any further comment as entered
on the database. It also lists as "gap" any categories for which no data were presented by the original authors.
This is commonly due to recent quitters having been combined with current smokers

In addition to those mentioned above, the following fields, levels and abbreviations are used:
* or nk = not known, n = no, y = yes, ot = other
nev = never
all/unspec = all or unspecified, MC = manufactured cigarettes, HR = hand-rolled cigarettes
exL, exH = range of exposure (low and high) in the smoking group, in terms of Years quit (vs never)
REF: 6-character study reference
NRR: number of the RR on the database within the study
ST : study type (CC = case control, pr or prosp = prospective)
NLC: number of lung cancer cases in whole study
R : risky occupational population (n = no, m = mining, o = other risky)
VB : national cigarette type (V = at least 75% Virginia, bl = at least 75% blended, ot = other)
P : any proxy use
H : full histological confirmation
De : derivation of RR/CI (or = original, st = standard method, ot = other method of estimation)

Table 1J12 - 1

IESLC - Meta-analysis of Ex Smoking, Years quit (vs never), "Low"
All LC types, Cigarettes only
Most adjusted

REF	NRR	1J2	SEX	AGEL	AGEH	RACE	YF	LC TYPE	LOC	START	ST	NLC	R	VB	P	H	AD	PRODUCT	exL	exH	DENOM	De
ALDERS	507		m	0	0	all	-	all	Eu:UK	1977	CC	1448	n	V	n	n	1	cig only	10	999	nev any	ot
ALDERS	518		f	0	0	all	-	all	Eu:UK	1977	CC	1448	n	V	n	n	1	cig only	10	999	nev any	ot
BENHAM	561	x	m	0	0	all	-	not mix	Eu:wst	1976	CC	1625	n	bl	n	y	0	cig only	11	999	nev any	st
CPSI	807		m	50	74	all	6	all	NAmer	1959	pr	5138	n	bl	n	n	1	cig only	10	999	nev any	ot
CPSII	653		m	35	99	all	4	all	NAmer	1982	pr	3229	n	bl	n	n	1	cig only	11	15	nev any	ot
DAMBER	554	x	m	0	0	all	-	all	Eu:Sca	1972	CC	579	n	bl	y	n	1	cig only	11	999	nev any	ot
DEAN3	517	x	m	0	0	all	-	all	Eu:UK	1969	CC	766	n	V	y	n	1	cig only	9	18	nev any	ot
DOLL2	502		m	0	0	all	20	all	Eu:UK	1951	pr	920	n	V	n	n	1	cig only	10	14	nev any	ot
DORN	511	x	m	0	0	wh	0	all	NAmer	1954	pr	5097	n	bl	n	n	2	cig only	10	19	nev any	or
GRAHAM	501		m	0	0	wh	-	all	NAmer	1956	CC	685	n	bl	n	n	0	cig only	10	999	nev any	st
KAISE2	647		m	0	0	all	9	all	NAmer	1979	pr	318	n	bl	n	n	1	cig only	11	20	nev any	ot
KAISE2	567		f	0	0	all	9	all	NAmer	1979	pr	318	n	bl	n	n	1	cig only	11	20	nev any	st
PEZZOT	501		m	0	0	all	-	all	SCAmer	1987	CC	215	n	bl	n	y	0	cig only	11	999	nev cigs	st
WYNDE6	503		m	0	0	all	-	all	NAmer	1969	CC	4423	n	bl	n	y	0	cig only	10	19	nev any	st
WYNDE6	524		f	0	0	all	-	all	NAmer	1969	CC	4423	n	bl	n	y	0	cig only	10	19	nev any	st

Cigarette type is all/unspec for all RRs
except for the following:

REF	NRR	CIGTYPE
ALDERS	507	MC only
ALDERS	518	MC only
DEAN3	517	MC only

Table 1J12 - 2

IESLC - Meta-analysis of Ex Smoking, Years quit (vs never), "Low"
All LC types, Cigarettes only
Most adjusted

REF	NRR	SEX	AD	Number Exposed Case	Cont	Non-exposed Case	Cont	RR	95.00%CI
ALDERS	507	m	1	29	-	15	-	3.20	(1.61- 6.35)
ALDERS	518	f	1	26	-	75	-	1.27	(0.76- 2.15)
Subtotal ALDERS								1.78	(1.18- 2.69)
BENHAM	561	m	0	60	228	33	523	4.17	(2.65- 6.56)
*CPSI	807	m	1	15	-	60	-	1.28	(0.73- 2.25)
*CPSII	653	m	1	164	-	81	-	8.61	(6.60- 11.24)
DAMBER	554	m	1	-	-	42	-	1.60	(0.70- 3.40)
DEAN3	517	m	1	15	-	24	-	2.99	(1.51- 5.93)
*DOLL2	502	m	1	9	-	7	-	5.30	(1.97- 14.23)
*DORN	511	m	2	261	-	325	-	5.10	(4.20- 6.10)
GRAHAM	501	m	0	2	30	18	346	1.28	(0.28- 5.79)
*KAISE2	647	m	1	8	-	14	-	3.14	(1.26- 7.82)
*KAISE2	567	f	1	4	-	11	-	4.37	(1.30- 14.72)
Subtotal KAISE2								3.54	(1.71- 7.34)
PEZZOT	501	m	0	20	106	4	116	5.47	(1.81- 16.53)
WYNDE6	503	m	0	159	373	64	918	6.11	(4.47- 8.37)
WYNDE6	524	f	0	36	132	125	991	2.16	(1.43- 3.27)
Subtotal WYNDE6								4.18	(3.25- 5.36)
Partial Totals				808	869	898	2894		

*prospective study

REF	NRR	SEX	AD	Ys	Ws	Qs	Ps
ALDERS	507	m	1	1.16	8.16	0.85	0.0009
ALDERS	518	f	1	0.24	14.21	22.12	0.3676
Subtotal ALDERS				0.58	22.37	22.98	
BENHAM	561	m	0	1.43	18.77	0.06	0.0000
*CPSI	807	m	1	0.25	12.13	18.64	0.3900
*CPSII	653	m	1	2.15	54.21	24.05	0.0000
DAMBER	554	m	1	0.47	6.15	6.36	0.2437
DEAN3	517	m	1	1.10	8.21	1.26	0.0017
*DOLL2	502	m	1	1.67	3.93	0.13	0.0009
*DORN	511	m	2	1.63	110.32	2.24	0.0000
GRAHAM	501	m	0	0.25	1.69	2.59	0.7471
*KAISE2	647	m	1	1.14	4.61	0.54	0.0140
*KAISE2	567	f	1	1.47	2.61	0.00	0.0172
Subtotal KAISE2				1.26	7.22	0.54	
PEZZOT	501	m	0	1.70	3.14	0.14	0.0026
WYNDE6	503	m	0	1.81	38.93	4.08	0.0000
WYNDE6	524	f	0	0.77	22.54	11.55	0.0003
Subtotal WYNDE6				1.43	61.48	15.63	

	N	15
	NS	12
	Wt	309.62
Het	Chi	94.63
Het	df	14
Het	P	***
Fixed	RR	4.42
	RRl	3.96
	RRu	4.94
	P	+++
Random	RR	3.27
	RRl	2.33
	RRu	4.59
	P	+++
Asymm	P	*

Table 1J12 - 3

IESLC - Meta-analysis of Ex Smoking, Years quit (vs never), "Low"
All LC types, Cigarettes only
Most adjusted

	Sex			
	combined	male	female	Total
N		12	3	15
NS		12	3	15
Wt		270.26	39.36	309.62
Het Chi		56.71	4.48	94.63
Het df		11	2	14
Het P		***	N.S.	***
Fixed RR		5.01	1.87	4.42
RRl		4.45	1.37	3.96
RRu		5.65	2.56	4.94
P		+++	+++	+++
Random RR		3.75	1.95	3.27
RRl		2.70	1.14	2.33
RRu		5.21	3.32	4.59
P		+++	+	+++
Between Chi				33.44
Between df				1
Between P				***
Btwn(F) P				*
Btwn(R) P				*

	Lung cancer type		
	all	other	Total
N	14	1	15
NS	11	1	12
Wt	290.85	18.77	309.62
Het Chi	94.56	0.00	94.63
Het df	13	0	14
Het P	***	N.S.	***
Fixed RR	4.44	4.17	4.42
RRl	3.96	2.65	3.96
RRu	4.98	6.56	4.94
P	+++	+++	+++
Random RR	3.19	4.17	3.27
RRl	2.21	2.65	2.33
RRu	4.61	6.56	4.59
P	+++	+++	+++
Between Chi			0.07
Between df			1
Between P			N.S.
Btwn(F) P			N.S.
Btwn(R) P			N.S.

	Location								
	NAmer	UK	Scand	othEur	China	Japan	othAs	other	Total
N	8	4	1	1				1	15
NS	6	3	1	1				1	12
Wt	247.04	34.51	6.15	18.77				3.14	309.62
Het Chi	60.16	9.20	0.00	0.00				0.00	94.63
Het df	7	3	0	0				0	14
Het P	***	*	N.S.	N.S.				N.S.	***
Fixed RR	4.99	2.28	1.60	4.17				5.47	4.42
RRl	4.40	1.63	0.73	2.65				1.81	3.96
RRu	5.65	3.18	3.53	6.56				16.53	4.94
P	+++	+++	N.S.	+++				++	+++
Random RR	3.63	2.63	1.60	4.17				5.47	3.27
RRl	2.33	1.43	0.73	2.65				1.81	2.33
RRu	5.66	4.84	3.53	6.56				16.53	4.59
P	+++	++	N.S.	+++				++	+++
Between Chi									25.27
Between df									4
Between P									***
Btwn(F) P									N.S.
Btwn(R) P									N.S.

Table 1J12 - 3

IESLC - Meta-analysis of Ex Smoking, Years quit (vs never), "Low"
All LC types, Cigarettes only
Most adjusted

Detailed Country in "other Europe"

	multi	Germany	othWest	East	Balkans	Total
N			1			1
NS			1			1
Wt			18.77			18.77
Het Chi			0.00			0.00
Het df			0			0
Het P			N.S.			N.S.
Fixed RR			4.17			4.17
RRl			2.65			2.65
RRu			6.56			6.56
P			+++			+++
Random RR			4.17			4.17
RRl			2.65			2.65
RRu			6.56			6.56
P			+++			+++
Between Chi						
Between df						
Between P						N.S.
Btwn(F) P						N.S.
Btwn(R) P						N.S.

Detailed Country in "other Asia"

	India	HongKong	other	Total
N				
NS				
Wt				
Het Chi				
Het df				
Het P				
Fixed RR				
RRl				
RRu				
P				
Random RR				
RRl				
RRu				
P				
Between Chi				
Between df				
Between P				N.S.
Btwn(F) P				N.S.
Btwn(R) P				N.S.

Detailed other continent

	SCAmer	Total
N	1	1
NS	1	1
Wt	3.14	3.14
Het Chi	0.00	0.00
Het df	0	0
Het P	N.S.	N.S.
Fixed RR	5.47	5.47
RRl	1.81	1.81
RRu	16.53	16.53
P	++	++
Random RR	5.47	5.47
RRl	1.81	1.81
RRu	16.53	16.53
P	++	++
Between Chi		
Between df		
Between P		N.S.
Btwn(F) P		N.S.
Btwn(R) P		N.S.

Table 1J12 - 3

IESLC - Meta-analysis of Ex Smoking, Years quit (vs never), "Low"
All LC types, Cigarettes only
Most adjusted

	Start year of study <1960	1960-69	1970-79	1980-89	1990+	Total
N	4	3	6	2		15
NS	4	2	4	2		12
Wt	128.07	69.69	54.51	57.35		309.62
Het Chi	23.60	16.24	14.15	0.61		94.63
Het df	3	2	5	1		14
Het P	***	***	*	N.S.		***
Fixed RR	4.40	4.02	2.58	8.40		4.42
RRl	3.70	3.17	1.98	6.48		3.96
RRu	5.23	5.08	3.37	10.88		4.94
P	+++	+++	+++	+++		+++
Random RR	2.75	3.47	2.58	8.40		3.27
RRl	1.12	1.67	1.60	6.48		2.33
RRu	6.73	7.21	4.17	10.88		4.59
P	+	+++	+++	+++		+++
Between Chi						40.03
Between df						3
Between P						***
Btwn(F) P						(*)
Btwn(R) P						***

	Study type (1) CC	other	Total
N	9	6	15
NS	7	5	12
Wt	121.82	187.81	309.62
Het Chi	38.45	38.75	94.63
Het df	8	5	14
Het P	***	***	***
Fixed RR	3.29	5.35	4.42
RRl	2.76	4.64	3.96
RRu	3.93	6.18	4.94
P	+++	+++	+++
Random RR	2.81	4.10	3.27
RRl	1.83	2.41	2.33
RRu	4.32	6.98	4.59
P	+++	+++	+++
Between Chi			17.43
Between df			1
Between P			***
Btwn(F) P			N.S.
Btwn(R) P			N.S.

	Study type (2) CC	prosp	other	Total
N	9	6		15
NS	7	5		12
Wt	121.82	187.81		309.62
Het Chi	38.45	38.75		94.63
Het df	8	5		14
Het P	***	***		***
Fixed RR	3.29	5.35		4.42
RRl	2.76	4.64		3.96
RRu	3.93	6.18		4.94
P	+++	+++		+++
Random RR	2.81	4.10		3.27
RRl	1.83	2.41		2.33
RRu	4.32	6.98		4.59
P	+++	+++		+++
Between Chi				17.43
Between df				1
Between P				***
Btwn(F) P				N.S.
Btwn(R) P				N.S.

Table 1J12 - 3

IESLC - Meta-analysis of Ex Smoking, Years quit (vs never), "Low"
All LC types, Cigarettes only
Most adjusted

	Study size (number of LC cases)				
	100-249	250-499	500-999	1000+	Total
N	1	2	4	8	15
NS	1	1	4	6	12
Wt	3.14	7.22	19.98	279.28	309.62
Het Chi	0.00	0.18	4.45	83.11	94.63
Het df	0	1	3	7	14
Het P	N.S.	N.S.	N.S.	***	***
Fixed RR	5.47	3.54	2.57	4.61	4.42
RRl	1.81	1.71	1.66	4.10	3.96
RRu	16.53	7.34	3.98	5.19	4.94
P	++	+++	+++	+++	+++
Random RR	5.47	3.54	2.55	3.37	3.27
RRl	1.81	1.71	1.46	2.17	2.33
RRu	16.53	7.34	4.45	5.24	4.59
P	++	+++	+++	+++	+++
Between Chi					6.89
Between df					3
Between P					(*)
Btwn(F) P					N.S.
Btwn(R) P					N.S.

	Risky occupational population			
	no	mining	othRisky	Total
N	15			15
NS	12			12
Wt	309.62			309.62
Het Chi	94.63			94.63
Het df	14			14
Het P	***			***
Fixed RR	4.42			4.42
RRl	3.96			3.96
RRu	4.94			4.94
P	+++			+++
Random RR	3.27			3.27
RRl	2.33			2.33
RRu	4.59			4.59
P	+++			+++
Between Chi				
Between df				
Between P				N.S.
Btwn(F) P				N.S.
Btwn(R) P				N.S.

	National cigarette tobacco type			
	Virginia	blended	other	Total
N	4	11		15
NS	3	9		12
Wt	34.51	275.11		309.62
Het Chi	9.20	68.37		94.63
Het df	3	10		14
Het P	*	***		***
Fixed RR	2.28	4.81		4.42
RRl	1.63	4.27		3.96
RRu	3.18	5.41		4.94
P	+++	+++		+++
Random RR	2.63	3.55		3.27
RRl	1.43	2.45		2.33
RRu	4.84	5.15		4.59
P	++	+++		+++
Between Chi				17.06
Between df				1
Between P				***
Btwn(F) P				N.S.
Btwn(R) P				N.S.

Table 1J12 - 3

IESLC - Meta-analysis of Ex Smoking, Years quit (vs never), "Low"
All LC types, Cigarettes only
Most adjusted

	Any proxy use No/nk	Yes	Total
N	13	2	15
NS	10	2	12
Wt	295.26	14.36	309.62
Het Chi	86.71	1.38	94.63
Het df	12	1	14
Het P	***	N.S.	***
Fixed RR	4.57	2.29	4.42
RRl	4.07	1.36	3.96
RRu	5.12	3.84	4.94
P	+++	++	+++
Random RR	3.46	2.26	3.27
RRl	2.41	1.23	2.33
RRu	4.97	4.16	4.59
P	+++	++	+++
Between Chi			6.55
Between df			1
Between P			*
Btwn(F) P			N.S.
Btwn(R) P			N.S.

	Full histological confirmation No	Yes	Total
N	11	4	15
NS	9	3	12
Wt	226.23	83.39	309.62
Het Chi	78.73	15.65	94.63
Het df	10	3	14
Het P	***	**	***
Fixed RR	4.50	4.22	4.42
RRl	3.95	3.40	3.96
RRu	5.13	5.23	4.94
P	+++	+++	+++
Random RR	2.96	4.04	3.27
RRl	1.88	2.34	2.33
RRu	4.66	6.98	4.59
P	+++	+++	+++
Between Chi			0.26
Between df			1
Between P			N.S.
Btwn(F) P			N.S.
Btwn(R) P			N.S.

	Number of adjustment variables (1) 0	1	2+/+nk	Total
N	5	9	1	15
NS	4	7	1	12
Wt	85.08	114.22	110.32	309.62
Het Chi	18.00	73.15	0.00	94.63
Het df	4	8	0	14
Het P	**	***	N.S.	***
Fixed RR	4.12	4.06	5.10	4.42
RRl	3.33	3.38	4.23	3.96
RRu	5.09	4.88	6.15	4.94
P	+++	+++	+++	+++
Random RR	3.65	2.89	5.10	3.27
RRl	2.14	1.54	4.23	2.33
RRu	6.23	5.43	6.15	4.59
P	+++	+++	+++	+++
Between Chi				3.49
Between df				2
Between P				N.S.
Btwn(F) P				N.S.
Btwn(R) P				N.S.

Table 1J12 - 3

IESLC - Meta-analysis of Ex Smoking, Years quit (vs never), "Low"
All LC types, Cigarettes only
Most adjusted

	Number of adjustment variables (2) 0	1	2	3-5	6+/+nk	Total
N	5	9	1			15
NS	4	7	1			12
Wt	85.08	114.22	110.32			309.62
Het Chi	18.00	73.15	0.00			94.63
Het df	4	8	0			14
Het P	**	***	N.S.			***
Fixed RR	4.12	4.06	5.10			4.42
RRl	3.33	3.38	4.23			3.96
RRu	5.09	4.88	6.15			4.94
P	+++	+++	+++			+++
Random RR	3.65	2.89	5.10			3.27
RRl	2.14	1.54	4.23			2.33
RRu	6.23	5.43	6.15			4.59
P	+++	+++	+++			+++
Between Chi						3.49
Between df						2
Between P						N.S.
Btwn(F) P						N.S.
Btwn(R) P						N.S.

	Denominator nev any	nev cigs	Total
N	14	1	15
NS	11	1	12
Wt	306.48	3.14	309.62
Het Chi	94.49	0.00	94.63
Het df	13	0	14
Het P	***	N.S.	***
Fixed RR	4.41	5.47	4.42
RRl	3.95	1.81	3.96
RRu	4.94	16.53	4.94
P	+++	++	+++
Random RR	3.19	5.47	3.27
RRl	2.25	1.81	2.33
RRu	4.52	16.53	4.59
P	+++	++	+++
Between Chi			0.14
Between df			1
Between P			N.S.
Btwn(F) P			N.S.
Btwn(R) P			N.S.

	Derivation of RR/CI Orig	StdCalc	Other	Total
N	1	6	8	15
NS	1	5	7	13
Wt	110.32	87.69	111.61	309.62
Het Chi	0.00	18.01	73.13	94.63
Het df	0	5	7	14
Het P	N.S.	**	***	***
Fixed RR	5.10	4.13	4.06	4.42
RRl	4.23	3.35	3.37	3.96
RRu	6.15	5.09	4.88	4.94
P	+++	+++	+++	+++
Random RR	5.10	3.74	2.78	3.27
RRl	4.23	2.31	1.41	2.33
RRu	6.15	6.05	5.46	4.59
P	+++	+++	++	+++
Between Chi				3.49
Between df				2
Between P				N.S.
Btwn(F) P				N.S.
Btwn(R) P				N.S.

Table 1J12 - 4

IESLC - Meta-analysis of Ex Smoking, Years quit (vs never), "Low"
All LC types, Cigarettes only
Least adjusted

REF	NRR	X	SEX	AGEL	AGEH	RACE	YF	LC TYPE	LOC	START	ST	NLC	R	VB	P	H	AD	PRODUCT	exL	exH	DENOM	De
ALDERS	507		m	0	0	all	-	all	Eu:UK	1977	CC	1448	n	V	n	n	1	cig only	10	999	nev any	ot
ALDERS	518		f	0	0	all	-	all	Eu:UK	1977	CC	1448	n	V	n	n	1	cig only	10	999	nev any	ot
BENHAM	561		m	0	0	all	-	not mix	Eu:wst	1976	CC	1625	n	bl	n	y	0	cig only	11	999	nev any	st
CPSI	807		m	50	74	all	6	all	NAmer	1959	pr	5138	n	bl	n	n	1	cig only	10	999	nev any	ot
CPSII	653		m	35	99	all	4	all	NAmer	1982	pr	3229	n	bl	n	n	1	cig only	11	15	nev any	ot
DAMBER	554		m	0	0	all	-	all	Eu:Sca	1972	CC	579	n	bl	y	n	1	cig only	11	999	nev any	ot
DEAN3	502	x	m	0	0	all	-	all	Eu:UK	1969	CC	766	n	V	y	n	0	cig only	9	18	nev any	st
DOLL2	502		m	0	0	all	20	all	Eu:UK	1951	pr	920	n	V	n	n	1	cig only	10	14	nev any	ot
DORN	511		m	0	0	wh	0	all	NAmer	1954	pr	5097	n	bl	n	n	2	cig only	10	19	nev any	or
GRAHAM	501		m	0	0	wh	-	all	NAmer	1956	CC	685	n	bl	n	n	0	cig only	10	999	nev any	st
KAISE2	647		m	0	0	all	9	all	NAmer	1979	pr	318	n	bl	n	n	1	cig only	11	20	nev any	ot
KAISE2	567		f	0	0	all	9	all	NAmer	1979	pr	318	n	bl	n	n	1	cig only	11	20	nev any	st
PEZZOT	501		m	0	0	all	-	all	SCAmer	1987	CC	215	n	bl	n	y	0	cig only	11	999	nev cigs	st
WYNDE6	503		m	0	0	all	-	all	NAmer	1969	CC	4423	n	bl	n	y	0	cig only	10	19	nev any	st
WYNDE6	524		f	0	0	all	-	all	NAmer	1969	CC	4423	n	bl	n	y	0	cig only	10	19	nev any	st

Cigarette type is all/unspec for all RRs
except for the following:

REF	NRR	CIGTYPE
ALDERS	507	MC only
ALDERS	518	MC only
DEAN3	502	MC only

Table 1J12 - 5

IESLC - Meta-analysis of Ex Smoking, Years quit (vs never), "Low"
All LC types, Cigarettes only
Least adjusted

REF	NRR	SEX	AD	Number Exposed Case	Exposed Cont	Non-exposed Case	Non-exposed Cont	RR	95.00%CI
ALDERS	507	m	1	29	-	15	-	3.20	(1.61- 6.35)
ALDERS	518	f	1	26	-	75	-	1.27	(0.76- 2.15)
Subtotal ALDERS								1.78	(1.18- 2.69)
BENHAM	561	m	0	60	228	33	523	4.17	(2.65- 6.56)
*CPSI	807	m	1	15	-	60	-	1.28	(0.73- 2.25)
*CPSII	653	m	1	164	-	81	-	8.61	(6.60- 11.24)
DAMBER	554	m	1	-	-	42	-	1.60	(0.70- 3.40)
DEAN3	502	m	0	15	86	24	510	3.71	(1.87- 7.35)
*DOLL2	502	m	1	9	-	7	-	5.30	(1.97- 14.23)
*DORN	511	m	2	261	-	325	-	5.10	(4.20- 6.10)
GRAHAM	501	m	0	2	30	18	346	1.28	(0.28- 5.79)
*KAISE2	647	m	1	8	-	14	-	3.14	(1.26- 7.82)
*KAISE2	567	f	1	4	-	11	-	4.37	(1.30- 14.72)
Subtotal KAISE2								3.54	(1.71- 7.34)
PEZZOT	501	m	0	20	106	4	116	5.47	(1.81- 16.53)
WYNDE6	503	m	0	159	373	64	918	6.11	(4.47- 8.37)
WYNDE6	524	f	0	36	132	125	991	2.16	(1.43- 3.27)
Subtotal WYNDE6								4.18	(3.25- 5.36)
Partial Totals				808	955	898	3404		

*prospective study

REF	NRR	SEX	AD	Ys	Ws	Qs	Ps
ALDERS	507	m	1	1.16	8.16	0.89	0.0009
ALDERS	518	f	1	0.24	14.21	22.33	0.3676
Subtotal ALDERS				0.58	22.37	23.21	
BENHAM	561	m	0	1.43	18.77	0.08	0.0000
*CPSI	807	m	1	0.25	12.13	18.82	0.3900
*CPSII	653	m	1	2.15	54.21	23.64	0.0000
DAMBER	554	m	1	0.47	6.15	6.43	0.2437
DEAN3	502	m	0	1.31	8.20	0.27	0.0002
*DOLL2	502	m	1	1.67	3.93	0.12	0.0009
*DORN	511	m	2	1.63	110.32	2.06	0.0000
GRAHAM	501	m	0	0.25	1.69	2.62	0.7471
*KAISE2	647	m	1	1.14	4.61	0.56	0.0140
*KAISE2	567	f	1	1.47	2.61	0.00	0.0172
Subtotal KAISE2				1.26	7.22	0.56	
PEZZOT	501	m	0	1.70	3.14	0.13	0.0026
WYNDE6	503	m	0	1.81	38.93	3.94	0.0000
WYNDE6	524	f	0	0.77	22.54	11.73	0.0003
Subtotal WYNDE6				1.43	61.48	15.67	

	N	15
	NS	12
	Wt	309.61
Het	Chi	93.62
Het	df	14
Het	P	***
Fixed	RR	4.45
	RRl	3.98
	RRu	4.97
	P	+++
Random	RR	3.32
	RRl	2.37
	RRu	4.65
	P	+++
Asymm	P	(*)

Table 1J12 - 6

IESLC - Meta-analysis of Ex Smoking, Years quit (vs never), "Low"
All LC types, Cigarettes only
Least adjusted

Sex combined	male	female	Total
N	12	3	15
NS	12	3	15
Wt	270.25	39.36	309.61
Het Chi	55.26	4.48	93.62
Het df	11	2	14
Het P	***	N.S.	***
Fixed RR	5.05	1.87	4.45
RRl	4.48	1.37	3.98
RRu	5.69	2.56	4.97
P	+++	+++	+++
Random RR	3.82	1.95	3.32
RRl	2.76	1.14	2.37
RRu	5.30	3.32	4.65
P	+++	+	+++
Between Chi			33.88
Between df			1
Between P			***
Btwn(F) P			*
Btwn(R) P			*

Table 1J12 - 7

IESLC - Meta-analysis of Ex Smoking, Years quit (vs never), "Low"
All LC types, Cigarettes only
Excluded studies (and stage at which they were excluded)

1	AGUDO AKIBA AMANDU AMES AXELSS BEST BOUCHA BOUCOT BRESLO CHEN CHEN2 CHIAZZ DEAN2 DOSEME ENGELA FAN GENG GER GUO HAENSZ HEGMAN HOLE HU HU2 JUSSAW KATSOU KAUFMA KOO KOULUM KREUZE LETOUR LEVIN LIAW LIU3 LIU4 LIU5 MCCONN MIGRAN MRFITR NOTAN2 OSANN2 PERNU QIAO2 RACHTA RESTRE SADOWS SEGI2 STASZE TIZZAN VUTUC WATSON WU WUWILL WYNDE2 WYNDE8 XU YUAN ZHANG ZHENG ZHOU
2	BUFFLE HUMBLE PISANI PRESCO WYNDE7
3	MCDUFF SPITZ
4	HAMMON LUO WU2
5	BLOT1 CORREA GILLIS QIAO WIGLE
7	ARMADA AUVINE BARBON BECHER BENSHL BOFFET BROSS BROWN3 CARPEN CEDERL CHOI CHYOU DARBY DESTEF DOLL DORGAN GAO GAO2 GARCIA GARSHI GURSEL HAMMO2 HIRAYA JAHN JAIN JEDRYC JOLY KHUDER LAUSSM LUBIN LUBIN2 MATOS PEZZO2 SOBUE SPEIZE SUZUK2 SVENSS WAKAI WANG2 WYNDE3
14	TVERDA

Table 1J12 - 8
Potentially overlapping studies

REF	REFGP	PRINC	. OVERLAP/LINK
BENHAM	LUBIN2	2	Subset of Lubin2
GRAHAM	BYERS1	1	GRAHAM/BROSS/BYERS1
WYNDE6	WYNDE6	1	WYNDE5/6/7/8
CPSI	CPSI	1	CPSI overall

Table 1J12 - 9
Most adjusted - insufficient data for meta-analysis

REF	NRR	SEX	AGEL	AGEH	RACE	YF	LC TYPE	LOC	START	ST	NLC	R	VB	P	H	AD	PRODUCT	exL	exH	DENOM	De
CPSI	721	f	0	0	wh	0	all	NAmer	1959	pr	5138	n	bl	n	n	1	cig only	10	14	nev cigs	or

REF	NRR	RR	SIG	RRDATA comment
CPSI	721	0.58		0

Table 1J13 -

IESLC - Meta-analysis of Ex Smoking, Years quit (vs never), "Mid"
All LC types, Cigarettes only

This analysis is restricted to results for:
1) Ex smokers
2) Results by Years quit (vs never)
3) Categorical results by Years quit (vs never)
4) All LC types (or near equivalent)
5) Results complete enough for use in metaanalysis

Within each study, results are then selected (in the following order of preference, within each sex) for:
6) (not applicable)
7) PRODUCT: cigarettes only
8) CIGTYPE: all/unspecified, MC regardless of HR, MC only
9) (not applicable)
10) DENOM: never smoked anything, never smoked cigarettes, never any + low, never cigs + low
11) Followup period (YF, prospective studies): whole study (coded as 0) or longest available
12) LCtype: all or nearest available, at least Squamous and Adeno. (q = squamous, s = small,
l = large, a = adeno, mix = mixed, alv = alveolar)
13) Race: all or nearest available, otherwise by race (wh or w = white, bl or b = black, hi = hispanic
ch = chinese, jap = japanese, haw = hawaiian, w+o = white + oriental, sca = scandinavian, as = asian)
14) Years quit (vs never) "mid" in key scheme 1 (key value 7, maximum range 4-11)
15) For overlapping studies: principal rather than subsidiary studies
Finally by Age: whole study (coded as 0) if available, otherwise by widest available age group
and then for single sex results (m, f) in preference to results for both sexes combined (c).

Results adjusted (AD) for the most potential confounders are then chosen in Sections -1 to -3
(and those which actually differ from the adjusted results in Table 1J3 - 1 are marked 'x' in Section -1)
and results adjusted for the least confounders in Sections -4 to -6. (Those least adjusted results which
actually differ from the most adjusted are marked 'x' in column X in Section -4)

Section -7 shows excluded studies, together with the stage (as above) at which no qualifying
results were found.

Section -8 lists the potentially overlapping studies which have been included (1=principal, 2=subsidiary).

Section -9 lists any results which would have been included in preference except that they had data not complete
enough for use in meta-analysis, with their significance (yes/no), if known, and any further comment as entered
on the database. It also lists as "gap" any categories for which no data were presented by the original authors.
This is commonly due to recent quitters having been combined with current smokers

In addition to those mentioned above, the following fields, levels and abbreviations are used:
* or nk = not known, n = no, y = yes, ot = other
nev = never
all/unspec = all or unspecified, MC = manufactured cigarettes, HR = hand-rolled cigarettes
exL, exH = range of exposure (low and high) in the smoking group, in terms of Years quit (vs never)
REF: 6-character study reference
NRR: number of the RR on the database within the study
ST : study type (CC = case control, pr or prosp = prospective)
NLC: number of lung cancer cases in whole study
R : risky occupational population (n = no, m = mining, o = other risky)
VB : national cigarette type (V = at least 75% Virginia, bl = at least 75% blended, ot = other)
P : any proxy use
H : full histological confirmation
De : derivation of RR/CI (or = original, st = standard method, ot = other method of estimation)

Table 1J13 - 1

IESLC - Meta-analysis of Ex Smoking, Years quit (vs never), "Mid"
All LC types, Cigarettes only
Most adjusted

REF	NRR	1J3	SEX	AGEL	AGEH	RACE	YF	LC TYPE	LOC	START	ST	NLC	R	VB	P	H	AD	PRODUCT	exL	exH	DENOM	De
BENHAM	562	x	m	0	0	all	-	not mix	Eu:wst	1976	CC	1625	n	bl	n	y	0	cig only	4	10	nev any	st
CPSI	808		m	50	74	all	6	all	NAmer	1959	pr	5138	n	bl	n	n	1	cig only	5	9	nev any	ot
CPSII	654		m	35	99	all	4	all	NAmer	1982	pr	3229	n	bl	n	n	1	cig only	6	10	nev any	ot
DEAN3	518	x	m	0	0	all	-	all	Eu:UK	1969	CC	766	n	V	y	n	1	cig only	5	8	nev any	ot
DOLL2	503		m	0	0	all	20	all	Eu:UK	1951	pr	920	n	V	n	n	1	cig only	5	9	nev any	ot
DORN	512	x	m	0	0	wh	0	all	NAmer	1954	pr	5097	n	bl	n	n	2	cig only	5	9	nev any	or
GRAHAM	502		m	0	0	wh	-	all	NAmer	1956	CC	685	n	bl	n	n	0	cig only	3	10	nev any	st
WYNDE6	504		m	0	0	all	-	all	NAmer	1969	CC	4423	n	bl	n	y	0	cig only	5	9	nev any	st
WYNDE6	525		f	0	0	all	-	all	NAmer	1969	CC	4423	n	bl	n	y	0	cig only	5	9	nev any	st

Cigarette type is all/unspec for all RRs
except for the following:

REF	NRR	CIGTYPE
DEAN3	518	MC only

Table 1J13 - 2

IESLC - Meta-analysis of Ex Smoking, Years quit (vs never), "Mid"
All LC types, Cigarettes only
Most adjusted

REF	NRR	SEX	AD	Number Exposed Case	Cont	Non-exposed Case	Cont	RR	95.00%CI
BENHAM	562	m	0	105	185	33	523	9.00	(5.88- 13.77)
*CPSI	808	m	1	32	-	60	-	5.15	(3.35- 7.91)
*CPSII	654	m	1	186	-	81	-	11.43	(8.81- 14.84)
DEAN3	518	m	1	11	-	24	-	4.16	(1.91- 9.06)
*DOLL2	503	m	1	12	-	7	-	5.90	(2.32- 14.99)
*DORN	512	m	2	87	-	325	-	7.80	(5.70- 10.50)
GRAHAM	502	m	0	5	29	18	346	3.31	(1.15- 9.57)
WYNDE6	504	m	0	98	194	64	918	7.25	(5.10- 10.29)
WYNDE6	525	f	0	51	84	125	991	4.81	(3.24- 7.14)
Subtotal WYNDE6								6.05	(4.65- 7.86)
Partial Totals				587	492	737	2778		

*prospective study

REF	NRR	SEX	AD	Ys	Ws	Qs	Ps
BENHAM	562	m	0	2.20	21.21	0.63	0.0000
*CPSI	808	m	1	1.64	20.82	3.08	0.0000
*CPSII	654	m	1	2.44	56.51	9.62	0.0000
DEAN3	518	m	1	1.43	6.34	2.27	0.0003
*DOLL2	503	m	1	1.77	4.41	0.27	0.0002
*DORN	512	m	2	2.05	41.17	0.04	0.0000
GRAHAM	502	m	0	1.20	3.41	2.33	0.0268
WYNDE6	504	m	0	1.98	31.18	0.06	0.0000
WYNDE6	525	f	0	1.57	24.68	5.05	0.0000
Subtotal WYNDE6				1.80	55.86	5.11	

	N	9
	NS	8
	Wt	209.74
Het	Chi	23.35
Het	df	8
Het	P	**
Fixed	RR	7.57
	RRl	6.61
	RRu	8.66
	P	+++
Random	RR	6.76
	RRl	5.24
	RRu	8.73
	P	+++
Asymm	P	(*)

Table 1J13 - 3

IESLC - Meta-analysis of Ex Smoking, Years quit (vs never), "Mid"
All LC types, Cigarettes only
Most adjusted

	Sex combined	male	female	Total
N		8	1	9
NS		8	1	8
Wt		185.06	24.68	209.74
Het Chi		17.62	0.00	23.35
Het df		7	0	8
Het P		*	N.S.	**
Fixed RR		8.04	4.81	7.57
RRl		6.96	3.24	6.61
RRu		9.28	7.14	8.66
P		+++	+++	+++
Random RR		7.19	4.81	6.76
RRl		5.57	3.24	5.24
RRu		9.30	7.14	8.73
P		+++	+++	+++
Between Chi				5.72
Between df				1
Between P				*
Btwn(F) P				N.S.
Btwn(R) P				(*)

Too few RRs for analysis by factor

Table 1J13 - 4

IESLC - Meta-analysis of Ex Smoking, Years quit (vs never), "Mid"
All LC types, Cigarettes only
Least adjusted

REF	NRR	X	SEX	AGEL	AGEH	RACE	YF	LC TYPE	LOC	START	ST	NLC	R	VB	P	H	AD	PRODUCT	exL	exH	DENOM	De
BENHAM	562		m	0	0	all	-	not mix	Eu:wst	1976	CC	1625	n	bl	n	y	0	cig only	4	10	nev any	st
CPSI	808		m	50	74	all	6	all	NAmer	1959	pr	5138	n	bl	n	n	1	cig only	5	9	nev any	ot
CPSII	654		m	35	99	all	4	all	NAmer	1982	pr	3229	n	bl	n	n	1	cig only	6	10	nev any	ot
DEAN3	503	x	m	0	0	all	-	all	Eu:UK	1969	CC	766	n	V	y	n	0	cig only	5	8	nev any	st
DOLL2	503		m	0	0	all	20	all	Eu:UK	1951	pr	920	n	V	n	n	1	cig only	5	9	nev any	ot
DORN	512		m	0	0	wh	0	all	NAmer	1954	pr	5097	n	bl	n	n	2	cig only	5	9	nev any	or
GRAHAM	502		m	0	0	wh	-	all	NAmer	1956	CC	685	n	bl	n	n	0	cig only	3	10	nev any	st
WYNDE6	504		m	0	0	all	-	all	NAmer	1969	CC	4423	n	bl	n	y	0	cig only	5	9	nev any	st
WYNDE6	525		f	0	0	all	-	all	NAmer	1969	CC	4423	n	bl	n	y	0	cig only	5	9	nev any	st

Cigarette type is all/unspec for all RRs
except for the following:

REF	NRR	CIGTYPE
DEAN3	503	MC only

Table 1J13 - 5

IESLC - Meta-analysis of Ex Smoking, Years quit (vs never), "Mid"
All LC types, Cigarettes only
Least adjusted

REF	NRR	SEX	AD	Number Exposed Case	Cont	Non-exposed Case	Cont	RR	95.00%CI
BENHAM	562	m	0	105	185	33	523	9.00	(5.88- 13.77)
*CPSI	808	m	1	32	-	60	-	5.15	(3.35- 7.91)
*CPSII	654	m	1	186	-	81	-	11.43	(8.81- 14.84)
DEAN3	503	m	0	11	43	24	510	5.44	(2.50- 11.84)
*DOLL2	503	m	1	12	-	7	-	5.90	(2.32- 14.99)
*DORN	512	m	2	87	-	325	-	7.80	(5.70- 10.50)
GRAHAM	502	m	0	5	29	18	346	3.31	(1.15- 9.57)
WYNDE6	504	m	0	98	194	64	918	7.25	(5.10- 10.29)
WYNDE6	525	f	0	51	84	125	991	4.81	(3.24- 7.14)
Subtotal WYNDE6								6.05	(4.65- 7.86)
Partial Totals				587	535	737	3288		

*prospective study

REF	NRR	SEX	AD	Ys	Ws	Qs	Ps
BENHAM	562	m	0	2.20	21.21	0.58	0.0000
*CPSI	808	m	1	1.64	20.82	3.21	0.0000
*CPSII	654	m	1	2.44	56.51	9.24	0.0000
DEAN3	503	m	0	1.69	6.34	0.73	0.0000
*DOLL2	503	m	1	1.77	4.41	0.29	0.0002
*DORN	512	m	2	2.05	41.17	0.02	0.0000
GRAHAM	502	m	0	1.20	3.41	2.37	0.0268
WYNDE6	504	m	0	1.98	31.18	0.08	0.0000
WYNDE6	525	f	0	1.57	24.68	5.23	0.0000
Subtotal WYNDE6				1.80	55.86	5.31	

	N	9
	NS	8
	Wt	209.73
Het	Chi	21.76
Het	df	8
Het	P	**
Fixed	RR	7.63
	RRl	6.66
	RRu	8.73
	P	+++
Random	RR	6.92
	RRl	5.41
	RRu	8.85
	P	+++
Asymm	P	(*)

Table 1J13 - 6

IESLC - Meta-analysis of Ex Smoking, Years quit (vs never), "Mid"
All LC types, Cigarettes only
Least adjusted

	Sex combined male	female	Total
N	8	1	9
NS	8	1	8
Wt	185.06	24.68	209.73
Het Chi	15.83	0.00	21.76
Het df	7	0	8
Het P	*	N.S.	**
Fixed RR	8.11	4.81	7.63
RRl	7.02	3.24	6.66
RRu	9.37	7.14	8.73
P	+++	+++	+++
Random RR	7.40	4.81	6.92
RRl	5.81	3.24	5.41
RRu	9.43	7.14	8.85
P	+++	+++	+++
Between Chi			5.93
Between df			1
Between P			*
Btwn(F) P			N.S.
Btwn(R) P			(*)

Table 1J13 - 7

IESLC - Meta-analysis of Ex Smoking, Years quit (vs never), "Mid"
All LC types, Cigarettes only
Excluded studies (and stage at which they were excluded)

1	AGUDO AKIBA AMANDU AMES AXELSS BEST BOUCHA BOUCOT BRESLO CHEN CHEN2 CHIAZZ DEAN2 DOSEME ENGELA FAN GENG GER GUO HAENSZ HEGMAN HOLE HU HU2 JUSSAW KATSOU KAUFMA KOO KOULUM KREUZE LETOUR LEVIN LIAW LIU3 LIU4 LIU5 MCCONN MIGRAN MRFITR NOTAN2 OSANN2 PERNU QIAO2 RACHTA RESTRE SADOWS SEGI2 STASZE TIZZAN VUTUC WATSON WU WUWILL WYNDE2 WYNDE8 XU YUAN ZHANG ZHENG ZHOU
2	BUFFLE HUMBLE PISANI PRESCO WYNDE7
3	MCDUFF SPITZ
4	HAMMON LUO WU2
5	BLOT1 CORREA GILLIS QIAO WIGLE
7	ARMADA AUVINE BARBON BECHER BENSHL BOFFET BROSS BROWN3 CARPEN CEDERL CHOI CHYOU DARBY DESTEF DOLL DORGAN GAO GAO2 GARCIA GARSHI GURSEL HAMMO2 HIRAYA JAHN JAIN JEDRYC JOLY KHUDER LAUSSM LUBIN LUBIN2 MATOS PEZZO2 SOBUE SPEIZE SUZUK2 SVENSS WAKAI WANG2 WYNDE3
14	ALDERS DAMBER KAISE2 PEZZOT TVERDA

Table 1J13 - 8
Potentially overlapping studies

REF	REFGP	PRINC	.	OVERLAP/LINK
BENHAM	LUBIN2	2		Subset of Lubin2
GRAHAM	BYERS1	1		GRAHAM/BROSS/BYERS1
WYNDE6	WYNDE6	1		WYNDE5/6/7/8
CPSI	CPSI	1		CPSI overall

Table 1J13 - 9
Most adjusted - insufficient data for meta-analysis

REF	NRR	SEX	AGEL	AGEH	RACE	YF	LC TYPE	LOC	START	ST	NLC	R	VB	P	H	AD	PRODUCT	exL	exH	DENOM	De
CPSI	722	f	0	0	wh	0	all	NAmer	1959	pr	5138	n	bl	n	n	1	cig only	5	9	nev cigs or	

REF	NRR	RR	SIG	RRDATA comment
CPSI	722	1.51		0

Table 1J14 -

IESLC - Meta-analysis of Ex Smoking, Years quit (vs never), "High"
All LC types, Cigarettes only

This analysis is restricted to results for:
1) Ex smokers
2) Results by Years quit (vs never)
3) Categorical results by Years quit (vs never)
4) All LC types (or near equivalent)
5) Results complete enough for use in metaanalysis

Within each study, results are then selected (in the following order of preference, within each sex) for:
6) PRODUCT: cigarettes only
7) CIGTYPE: all/unspecified, MC regardless of HR, MC only
8) (not applicable)
9) DENOM: never smoked anything, never smoked cigarettes, never any + low, never cigs + low
10) Followup period (YF, prospective studies): whole study (coded as 0) or longest available
11) LCtype: all or nearest available, at least Squamous and Adeno. (q = squamous, s = small,
l = large, a = adeno, mix = mixed, alv = alveolar)
12) Race: all or nearest available, otherwise by race (wh or w = white, bl or b = black, hi = hispanic
ch = chinese, jap = japanese, haw = hawaiian, w+o = white + oriental, sca = scandinavian, as = asian)
13) Years quit (vs never) "high" in key scheme 1 (key value 3, maximum range 1-6)
14) For overlapping studies: principal rather than subsidiary studies
Finally by Age: whole study (coded as 0) if available, otherwise by widest available age group
and then for single sex results (m, f) in preference to results for both sexes combined (c).

Results adjusted (AD) for the most potential confounders are then chosen in Sections -1 to -3
(and those which actually differ from the adjusted results in Table 1J4 - 1 are marked 'x' in Section -1)
and results adjusted for the least confounders in Sections -4 to -6. (Those least adjusted results which
actually differ from the most adjusted are marked 'x' in column X in Section -4)

Section -7 shows excluded studies, together with the stage (as above) at which no qualifying
results were found.

Section -8 lists the potentially overlapping studies which have been included (1=principal, 2=subsidiary).

Section -9 lists any results which would have been included in preference except that they had data not complete
enough for use in meta-analysis, with their significance (yes/no), if known, and any further comment as entered
on the database. It also lists as "gap" any categories for which no data were presented by the original authors.
This is commonly due to recent quitters having been combined with current smokers

In addition to those mentioned above, the following fields, levels and abbreviations are used:
* or nk = not known, n = no, y = yes, ot = other
nev = never
all/unspec = all or unspecified, MC = manufactured cigarettes, HR = hand-rolled cigarettes
exL, exH = range of exposure (low and high) in the smoking group, in terms of Years quit (vs never)
REF: 6-character study reference
NRR: number of the RR on the database within the study
ST : study type (CC = case control, pr or prosp = prospective)
NLC: number of lung cancer cases in whole study
R : risky occupational population (n = no, m = mining, o = other risky)
VB : national cigarette type (V = at least 75% Virginia, bl = at least 75% blended, ot = other)
P : any proxy use
H : full histological confirmation
De : derivation of RR/CI (or = original, st = standard method, ot = other method of estimation)

Table 1J14 - 1

IESLC - Meta-analysis of Ex Smoking, Years quit (vs never), "High"
All LC types, Cigarettes only
Most adjusted

REF	NRR	1J4	SEX	AGEL	AGEH	RACE	YF	LC TYPE	LOC	START	ST	NLC	R	VB	P	H	AD	PRODUCT	exL	exH	DENOM	De
BENHAM	563	x	m	0	0	all	-	not mix	Eu:wst	1976	CC	1625	n	bl	n	y	0	cig only	1.0	3	nev any	st
CPSI	809		m	50	74	all	6	all	NAmer	1959	pr	5138	n	bl	n	n	1	cig only	1.0	4	nev any	ot
CPSII	655		m	35	99	all	4	all	NAmer	1982	pr	3229	n	bl	n	n	1	cig only	3	5	nev any	ot
DEAN3	519	x	m	0	0	all	-	all	Eu:UK	1969	CC	766	n	V	y	n	1	cig only	3	4	nev any	ot
DOLL2	504		m	0	0	all	20	all	Eu:UK	1951	pr	920	n	V	n	n	1	cig only	0.1	4	nev any	ot
DORN	513	x	m	0	0	wh	0	all	NAmer	1954	pr	5097	n	bl	n	n	2	cig only	1.0	4	nev any	or
GRAHAM	503	x	m	0	0	wh	-	all	NAmer	1956	CC	685	n	bl	n	n	0	cig only	1.1	3	nev any	st
TVERDA	502		m	0	0	all	0	all	Eu:Sca	1972	pr	238	n	bl	n	n	2	cig only	1.0	5	nev cigs	ot
WYNDE6	505		m	0	0	all	-	all	NAmer	1969	CC	4423	n	bl	n	y	0	cig only	1.0	4	nev any	st
WYNDE6	526		f	0	0	all	-	all	NAmer	1969	CC	4423	n	bl	n	y	0	cig only	1.0	4	nev any	st

Cigarette type is all/unspec for all RRs
except for the following:

REF	NRR	CIGTYPE
DEAN3	519	MC only

Table 1J14 - 2

IESLC - Meta-analysis of Ex Smoking, Years quit (vs never), "High"
All LC types, Cigarettes only
Most adjusted

REF	NRR	SEX	AD	Number Exposed Case	Exposed Cont	Non-exposed Case	Non-exposed Cont	RR	95.00%CI
BENHAM	563	m	0	145	86	33	523	26.72	(17.18- 41.55)
*CPSI	809	m	1	49	-	60	-	8.09	(5.55- 11.80)
*CPSII	655	m	1	178	-	81	-	18.61	(14.31- 24.20)
DEAN3	519	m	1	28	-	24	-	4.67	(2.60- 8.38)
*DOLL2	504	m	1	15	-	7	-	16.00	(6.52- 39.24)
*DORN	513	m	2	56	-	325	-	16.10	(10.40- 24.80)
GRAHAM	503	m	0	12	23	18	346	10.03	(4.31- 23.31)
*TVERDA	502	m	2	5	-	4	-	2.83	(0.76- 10.53)
WYNDE6	505	m	0	201	166	64	918	17.37	(12.53- 24.07)
WYNDE6	526	f	0	82	70	125	991	9.29	(6.42- 13.43)
Subtotal WYNDE6								13.20	(10.34- 16.85)
Partial Totals				771	345	741	2778		

*prospective study

REF	NRR	SEX	AD	Ys	Ws	Qs	Ps
BENHAM	563	m	0	3.29	19.71	8.75	0.0000
*CPSI	809	m	1	2.09	27.01	7.54	0.0000
*CPSII	655	m	1	2.92	55.67	5.16	0.0000
DEAN3	519	m	1	1.54	11.22	13.04	0.0000
*DOLL2	504	m	1	2.77	4.77	0.11	0.0000
*DORN	513	m	2	2.78	20.35	0.52	0.0000
GRAHAM	503	m	0	2.31	5.40	0.53	0.0000
*TVERDA	502	m	2	1.04	2.22	5.54	0.1208
WYNDE6	505	m	0	2.85	36.08	2.00	0.0000
WYNDE6	526	f	0	2.23	28.18	4.30	0.0000
Subtotal WYNDE6				2.58	64.26	6.30	

	N	10
	NS	9
	Wt	210.60
Het	Chi	47.50
Het	df	9
Het	P	***
Fixed	RR	13.72
	RRl	11.99
	RRu	15.71
	P	+++
Random	RR	11.96
	RRl	8.52
	RRu	16.79
	P	+++
Asymm	P	N.S.

Table 1J14 - 3

IESLC - Meta-analysis of Ex Smoking, Years quit (vs never), "High"
All LC types, Cigarettes only
Most adjusted

	Sex combined	male	female	Total
N		9	1	10
NS		9	1	9
Wt		182.42	28.18	210.60
Het Chi		42.54	0.00	47.50
Het df		8	0	9
Het P		***	N.S.	***
Fixed RR		14.58	9.29	13.72
RRl		12.61	6.42	11.99
RRu		16.85	13.43	15.71
P		+++	+++	+++
Random RR		12.33	9.29	11.96
RRl		8.51	6.42	8.52
RRu		17.87	13.43	16.79
P		+++	+++	+++
Between Chi				4.96
Between df				1
Between P				*
Btwn(F) P				N.S.
Btwn(R) P				N.S.

	Lung cancer type all	other	Total
N	9	1	10
NS	8	1	9
Wt	190.89	19.71	210.60
Het Chi	37.84	0.00	47.50
Het df	8	0	9
Het P	***	N.S.	***
Fixed RR	12.81	26.72	13.72
RRl	11.12	17.18	11.99
RRu	14.76	41.55	15.71
P	+++	+++	+++
Random RR	10.87	26.72	11.96
RRl	7.72	17.18	8.52
RRu	15.32	41.55	16.79
P	+++	+++	+++
Between Chi			9.66
Between df			1
Between P			**
Btwn(F) P			N.S.
Btwn(R) P			**

	Location								
	NAmer	UK	Scand	othEur	China	Japan	othAs	other	Total
N	6	2	1	1					10
NS	5	2	1	1					9
Wt	172.68	15.99	2.22	19.71					210.60
Het Chi	20.04	5.08	0.00	0.00					47.50
Het df	5	1	0	0					9
Het P	**	*	N.S.	N.S.					***
Fixed RR	13.86	6.74	2.83	26.72					13.72
RRl	11.94	4.13	0.76	17.18					11.99
RRu	16.09	11.01	10.53	41.55					15.71
P	+++	+++	N.S.	+++					+++
Random RR	12.94	8.23	2.83	26.72					11.96
RRl	9.43	2.47	0.76	17.18					8.52
RRu	17.77	27.41	10.53	41.55					16.79
P	+++	+++	N.S.	+++					+++
Between Chi									22.38
Between df									3
Between P									***
Btwn(F) P									N.S.
Btwn(R) P									**

Table 1J14 - 3

IESLC - Meta-analysis of Ex Smoking, Years quit (vs never), "High"
All LC types, Cigarettes only
Most adjusted

Detailed Country in "other Europe"

	multi	Germany	othWest	East	Balkans	Total
N			1			1
NS			1			1
Wt			19.71			19.71
Het Chi			0.00			0.00
Het df			0			0
Het P			N.S.			N.S.
Fixed RR			26.72			26.72
RRl			17.18			17.18
RRu			41.55			41.55
P			+++			+++
Random RR			26.72			26.72
RRl			17.18			17.18
RRu			41.55			41.55
P			+++			+++
Between Chi						
Between df						
Between P						N.S.
Btwn(F) P						N.S.
Btwn(R) P						N.S.

Detailed Country in "other Asia"

	India	HongKong	other	Total
N				
NS				
Wt				
Het Chi				
Het df				
Het P				
Fixed RR				
RRl				
RRu				
P				
Random RR				
RRl				
RRu				
P				
Between Chi				
Between df				
Between P				N.S.
Btwn(F) P				N.S.
Btwn(R) P				N.S.

Detailed other continent

	SCAmer	Total
N		
NS		
Wt		
Het Chi		
Het df		
Het P		
Fixed RR		
RRl		
RRu		
P		
Random RR		
RRl		
RRu		
P		
Between Chi		
Between df		
Between P		N.S.
Btwn(F) P		N.S.
Btwn(R) P		N.S.

Table 1J14 - 3

IESLC - Meta-analysis of Ex Smoking, Years quit (vs never), "High"
All LC types, Cigarettes only
Most adjusted

	Start year of study <1960	1960-69	1970-79	1980-89	1990+	Total
N	4	3	2	1		10
NS	4	2	2	1		9
Wt	57.52	75.48	21.93	55.67		210.60
Het Chi	6.21	16.51	10.07	0.00		47.50
Het df	3	2	1	0		9
Het P	N.S.	***	**	N.S.		***
Fixed RR	11.14	11.31	21.28	18.61		13.72
RRl	8.60	9.03	14.00	14.31		11.99
RRu	14.43	14.17	32.34	24.20		15.71
P	+++	+++	+++	+++		+++
Random RR	11.63	9.43	9.50	18.61		11.96
RRl	7.69	4.76	1.06	14.31		8.52
RRu	17.59	18.66	85.20	24.20		16.79
P	+++	+++	+	+++		+++
Between Chi						14.71
Between df						3
Between P						**
Btwn(F) P						N.S.
Btwn(R) P						N.S.

	Study type (1) CC	other	Total
N	5	5	10
NS	4	5	9
Wt	100.59	110.01	210.60
Het Chi	28.52	18.79	47.50
Het df	4	4	9
Het P	***	***	***
Fixed RR	13.30	14.12	13.72
RRl	10.94	11.72	11.99
RRu	16.17	17.02	15.71
P	+++	+++	+++
Random RR	11.84	11.98	11.96
RRl	6.80	7.37	8.52
RRu	20.62	19.45	16.79
P	+++	+++	+++
Between Chi			0.19
Between df			1
Between P			N.S.
Btwn(F) P			N.S.
Btwn(R) P			N.S.

	Study type (2) CC	prosp	other	Total
N	5	5		10
NS	4	5		9
Wt	100.59	110.01		210.60
Het Chi	28.52	18.79		47.50
Het df	4	4		9
Het P	***	***		***
Fixed RR	13.30	14.12		13.72
RRl	10.94	11.72		11.99
RRu	16.17	17.02		15.71
P	+++	+++		+++
Random RR	11.84	11.98		11.96
RRl	6.80	7.37		8.52
RRu	20.62	19.45		16.79
P	+++	+++		+++
Between Chi				0.19
Between df				1
Between P				N.S.
Btwn(F) P				N.S.
Btwn(R) P				N.S.

Table 1J14 - 3

IESLC - Meta-analysis of Ex Smoking, Years quit (vs never), "High"
All LC types, Cigarettes only
Most adjusted

	Study size (number of LC cases) 100-249	250-499	500-999	1000+	Total
N	1		3	6	10
NS	1		3	5	9
Wt	2.22		21.39	186.99	210.60
Het Chi	0.00		5.71	26.81	47.50
Het df	0		2	5	9
Het P	N.S.		(*)	***	***
Fixed RR	2.83		7.45	14.99	13.72
RRl	0.76		4.88	12.99	11.99
RRu	10.53		11.39	17.31	15.71
P	N.S.		+++	+++	+++
Random RR	2.83		8.54	14.75	11.96
RRl	0.76		4.03	10.50	8.52
RRu	10.53		18.06	20.71	16.79
P	N.S.		+++	+++	+++
Between Chi					14.98
Between df					2
Between P					***
Btwn(F) P					N.S.
Btwn(R) P					*

	Risky occupational population no	mining	othRisky	Total
N	10			10
NS	9			9
Wt	210.60			210.60
Het Chi	47.50			47.50
Het df	9			9
Het P	***			***
Fixed RR	13.72			13.72
RRl	11.99			11.99
RRu	15.71			15.71
P	+++			+++
Random RR	11.96			11.96
RRl	8.52			8.52
RRu	16.79			16.79
P	+++			+++
Between Chi				
Between df				
Between P				N.S.
Btwn(F) P				N.S.
Btwn(R) P				N.S.

	National cigarette tobacco type Virginia	blended	other	Total
N	2	8		10
NS	2	7		9
Wt	15.99	194.61		210.60
Het Chi	5.08	33.69		47.50
Het df	1	7		9
Het P	*	***		***
Fixed RR	6.74	14.55		13.72
RRl	4.13	12.64		11.99
RRu	11.01	16.74		15.71
P	+++	+++		+++
Random RR	8.23	13.20		11.96
RRl	2.47	9.44		8.52
RRu	27.41	18.47		16.79
P	+++	+++		+++
Between Chi				8.74
Between df				1
Between P				**
Btwn(F) P				N.S.
Btwn(R) P				N.S.

Table 1J14 - 3

IESLC - Meta-analysis of Ex Smoking, Years quit (vs never), "High"
All LC types, Cigarettes only
Most adjusted

Any proxy use

	No/nk	Yes	Total
N	9	1	10
NS	8	1	9
Wt	199.38	11.22	210.60
Het Chi	33.73	0.00	47.50
Het df	8	0	9
Het P	***	N.S.	***
Fixed RR	14.58	4.67	13.72
RRl	12.69	2.60	11.99
RRu	16.75	8.38	15.71
P	+++	+++	+++
Random RR	13.43	4.67	11.96
RRl	9.80	2.60	8.52
RRu	18.40	8.38	16.79
P	+++	+++	+++
Between Chi			13.77
Between df			1
Between P			***
Btwn(F) P			N.S.
Btwn(R) P			**

Full histological confirmation

	No	Yes	Total
N	7	3	10
NS	7	2	9
Wt	126.63	83.97	210.60
Het Chi	31.56	13.70	47.50
Het df	6	2	9
Het P	***	**	***
Fixed RR	12.62	15.58	13.72
RRl	10.60	12.58	11.99
RRu	15.02	19.29	15.71
P	+++	+++	+++
Random RR	10.02	16.13	11.96
RRl	6.31	9.13	8.52
RRu	15.93	28.49	16.79
P	+++	+++	+++
Between Chi			2.24
Between df			1
Between P			N.S.
Btwn(F) P			N.S.
Btwn(R) P			N.S.

Number of adjustment variables (1)

	0	1	2+/+nk	Total
N	4	4	2	10
NS	3	4	2	9
Wt	89.37	98.66	22.57	210.60
Het Chi	14.69	25.09	6.06	47.50
Het df	3	3	1	9
Het P	**	***	*	***
Fixed RR	15.17	12.57	13.57	13.72
RRl	12.33	10.32	8.98	11.99
RRu	18.66	15.31	20.49	15.71
P	+++	+++	+++	+++
Random RR	14.88	10.26	7.57	11.96
RRl	9.09	5.29	1.40	8.52
RRu	24.37	19.90	41.00	16.79
P	+++	+++	+	+++
Between Chi				1.66
Between df				2
Between P				N.S.
Btwn(F) P				N.S.
Btwn(R) P				N.S.

Table 1J14 - 3

IESLC - Meta-analysis of Ex Smoking, Years quit (vs never), "High"
All LC types, Cigarettes only
Most adjusted

	Number of adjustment variables (2) 0	1	2	3-5	6+/+nk	Total
N	4	4	2			10
NS	3	4	2			9
Wt	89.37	98.66	22.57			210.60
Het Chi	14.69	25.09	6.06			47.50
Het df	3	3	1			9
Het P	**	***	*			***
Fixed RR	15.17	12.57	13.57			13.72
RRl	12.33	10.32	8.98			11.99
RRu	18.66	15.31	20.49			15.71
P	+++	+++	+++			+++
Random RR	14.88	10.26	7.57			11.96
RRl	9.09	5.29	1.40			8.52
RRu	24.37	19.90	41.00			16.79
P	+++	+++	+			+++
Between Chi						1.66
Between df						2
Between P						N.S.
Btwn(F) P						N.S.
Btwn(R) P						N.S.

	Denominator nev any	nev cigs	Total
N	9	1	10
NS	8	1	9
Wt	208.37	2.22	210.60
Het Chi	41.89	0.00	47.50
Het df	8	0	9
Het P	***	N.S.	***
Fixed RR	13.96	2.83	13.72
RRl	12.18	0.76	11.99
RRu	15.99	10.53	15.71
P	+++	N.S.	+++
Random RR	12.82	2.83	11.96
RRl	9.20	0.76	8.52
RRu	17.87	10.53	16.79
P	+++	N.S.	+++
Between Chi			5.60
Between df			1
Between P			*
Btwn(F) P			N.S.
Btwn(R) P			*

	Derivation of RR/CI Orig	StdCalc	Other	Total
N	1	4	5	10
NS	1	3	5	9
Wt	20.35	89.37	100.88	210.60
Het Chi	0.00	14.69	29.93	47.50
Het df	0	3	4	9
Het P	N.S.	**	***	***
Fixed RR	16.10	15.17	12.16	13.72
RRl	10.43	12.33	10.01	11.99
RRu	24.86	18.66	14.78	15.71
P	+++	+++	+++	+++
Random RR	16.10	14.88	8.73	11.96
RRl	10.43	9.09	4.56	8.52
RRu	24.86	24.37	16.69	16.79
P	+++	+++	+++	+++
Between Chi				2.89
Between df				2
Between P				N.S.
Btwn(F) P				N.S.
Btwn(R) P				N.S.

Table 1J14 - 4

IESLC - Meta-analysis of Ex Smoking, Years quit (vs never), "High"
All LC types, Cigarettes only
Least adjusted

REF	NRR	X	SEX	AGEL	AGEH	RACE	YF	LC TYPE	LOC	START	ST	NLC	R	VB	P	H	AD	PRODUCT	exL	exH	DENOM	De
BENHAM	563		m	0	0	all	-	not mix	Eu:wst	1976	CC	1625	n	bl	n	y	0	cig only	1.0	3	nev any	st
CPSI	809		m	50	74	all	6	all	NAmer	1959	pr	5138	n	bl	n	n	1	cig only	1.0	4	nev any	ot
CPSII	655		m	35	99	all	4	all	NAmer	1982	pr	3229	n	bl	n	n	1	cig only	3	5	nev any	ot
DEAN3	504	x	m	0	0	all	-	all	Eu:UK	1969	CC	766	n	V	y	n	0	cig only	3	4	nev any	st
DOLL2	504		m	0	0	all	20	all	Eu:UK	1951	pr	920	n	V	n	n	1	cig only	0.1	4	nev any	ot
DORN	513		m	0	0	wh	0	all	NAmer	1954	pr	5097	n	bl	n	n	2	cig only	1.0	4	nev any	or
GRAHAM	503		m	0	0	wh	-	all	NAmer	1956	CC	685	n	bl	n	n	0	cig only	1.1	3	nev any	st
TVERDA	502		m	0	0	all	0	all	Eu:Sca	1972	pr	238	n	bl	n	n	2	cig only	1.0	5	nev cigs	ot
WYNDE6	505		m	0	0	all	-	all	NAmer	1969	CC	4423	n	bl	n	y	0	cig only	1.0	4	nev any	st
WYNDE6	526		f	0	0	all	-	all	NAmer	1969	CC	4423	n	bl	n	y	0	cig only	1.0	4	nev any	st

Cigarette type is all/unspec for all RRs
except for the following:

REF	NRR	CIGTYPE
DEAN3	504	MC only

Table 1J14 - 5

IESLC - Meta-analysis of Ex Smoking, Years quit (vs never), "High"
All LC types, Cigarettes only
Least adjusted

REF	NRR	SEX	AD	Number Case	Exposed Cont	Non-exposed Case	Cont	RR	95.00%CI
BENHAM	563	m	0	145	86	33	523	26.72	(17.18- 41.55)
*CPSI	809	m	1	49	-	60	-	8.09	(5.55- 11.80)
*CPSII	655	m	1	178	-	81	-	18.61	(14.31- 24.20)
DEAN3	504	m	0	28	102	24	510	5.83	(3.25- 10.47)
*DOLL2	504	m	1	15	-	7	-	16.00	(6.52- 39.24)
*DORN	513	m	2	56	-	325	-	16.10	(10.40- 24.80)
GRAHAM	503	m	0	12	23	18	346	10.03	(4.31- 23.31)
*TVERDA	502	m	2	5	-	4	-	2.83	(0.76- 10.53)
WYNDE6	505	m	0	201	166	64	918	17.37	(12.53- 24.07)
WYNDE6	526	f	0	82	70	125	991	9.29	(6.42- 13.43)
Subtotal WYNDE6								13.20	(10.34- 16.85)
Partial Totals				771	447	741	3288		

*prospective study

REF	NRR	SEX	AD	Ys	Ws	Qs	Ps
BENHAM	563	m	0	3.29	19.71	8.44	0.0000
*CPSI	809	m	1	2.09	27.01	7.88	0.0000
*CPSII	655	m	1	2.92	55.67	4.77	0.0000
DEAN3	504	m	0	1.76	11.22	8.44	0.0000
*DOLL2	504	m	1	2.77	4.77	0.10	0.0000
*DORN	513	m	2	2.78	20.35	0.44	0.0000
GRAHAM	503	m	0	2.31	5.40	0.57	0.0000
*TVERDA	502	m	2	1.04	2.22	5.63	0.1208
WYNDE6	505	m	0	2.85	36.08	1.81	0.0000
WYNDE6	526	f	0	2.23	28.18	4.56	0.0000
Subtotal WYNDE6				2.58	64.26	6.37	

	N	10
	NS	9
	Wt	210.60
Het	Chi	42.64
Het	df	9
Het	P	***
Fixed	RR	13.89
	RRl	12.13
	RRu	15.89
	P	+++
Random	RR	12.29
	RRl	8.91
	RRu	16.97
	P	+++
Asymm	P	N.S.

Table 1J14 - 6

IESLC - Meta-analysis of Ex Smoking, Years quit (vs never), "High"
All LC types, Cigarettes only
Least adjusted

	Sex combined male	female	Total
N	9	1	10
NS	9	1	9
Wt	182.42	28.18	210.60
Het Chi	37.38	0.00	42.64
Het df	8	0	9
Het P	***	N.S.	***
Fixed RR	14.78	9.29	13.89
RRl	12.78	6.42	12.13
RRu	17.09	13.43	15.89
P	+++	+++	+++
Random RR	12.75	9.29	12.29
RRl	8.99	6.42	8.91
RRu	18.07	13.43	16.97
P	+++	+++	+++
Between Chi			5.27
Between df			1
Between P			*
Btwn(F) P			N.S.
Btwn(R) P			N.S.

Table 1J14 - 7

IESLC - Meta-analysis of Ex Smoking, Years quit (vs never), "High"
All LC types, Cigarettes only
Excluded studies (and stage at which they were excluded)

1	AGUDO AKIBA AMANDU AMES AXELSS BEST BOUCHA BOUCOT BRESLO CHEN CHEN2 CHIAZZ DEAN2 DOSEME ENGELA FAN GENG GER GUO HAENSZ HEGMAN HOLE HU HU2 JUSSAW KATSOU KAUFMA KOO KOULUM KREUZE LETOUR LEVIN LIAW LIU3 LIU4 LIU5 MCCONN MIGRAN MRFITR NOTAN2 OSANN2 PERNU QIAO2 RACHTA RESTRE SADOWS SEGI2 STASZE TIZZAN VUTUC WATSON WU WUWILL WYNDE2 WYNDE8 XU YUAN ZHANG ZHENG ZHOU
2	BUFFLE HUMBLE PISANI PRESCO WYNDE7
3	MCDUFF SPITZ
4	HAMMON LUO WU2
5	BLOT1 CORREA GILLIS QIAO WIGLE
7	ARMADA AUVINE BARBON BECHER BENSHL BOFFET BROSS BROWN3 CARPEN CEDERL CHOI CHYOU DARBY DESTEF DOLL DORGAN GAO GAO2 GARCIA GARSHI GURSEL HAMMO2 HIRAYA JAHN JAIN JEDRYC JOLY KHUDER LAUSSM LUBIN LUBIN2 MATOS PEZZO2 SOBUE SPEIZE SUZUK2 SVENSS WAKAI WANG2 WYNDE3
14	ALDERS DAMBER KAISE2 PEZZOT

Table 1J14 - 8
Potentially overlapping studies

REF	REFGP	PRINC	. OVERLAP/LINK
BENHAM	LUBIN2	2	Subset of Lubin2
TVERDA	TVERDA	1	VEIERO/TVERDAL
GRAHAM	BYERS1	1	GRAHAM/BROSS/BYERS1
WYNDE6	WYNDE6	1	WYNDE5/6/7/8
CPSI	CPSI	1	CPSI overall

Table 1J14 - 9
Most adjusted - insufficient data for meta-analysis

REF	NRR	SEX	AGEL	AGEH	RACE	YF	LC TYPE	LOC	START	ST	NLC	R	VB	P	H	AD	PRODUCT	exL	exH	DENOM	De
CPSI	723	f	0	0	wh	0	all	NAmer	1959	pr	5138	n	bl	n	n	1	cig only	2	4	nev cigs	or

REF	NRR	RR	SIG	RRDATA comment
CPSI	723	2.85		0

Table 1J15 -

IESLC - Meta-analysis of Ex Smoking, Years quit (vs never), "Highest vs lowest"
All LC types, Cigarettes only

This analysis is restricted to results for:
1) Ex smokers
2) Results by Years quit (vs never)
3) Categorical results by Years quit (vs never)
4) Denominator (unexposed) = "low"
5) All LC types (or near equivalent)
6) Results complete enough for use in metaanalysis

Within each study, results are then selected (in the following order of preference, within each sex) for:
7) (not applicable)
8) PRODUCT: cigarettes only
9) CIGTYPE: all/unspecified, MC regardless of HR, MC only
10) Results with least adjustment for other aspects of smoking (ADOS)
11) The highest vs lowest category
12) Followup period (YF, prospective studies): whole study (coded as 0) or longest available
13) LCtype: all or nearest available, at least Squamous and Adeno. (q = squamous, s = small,
l = large, a = adeno, mix = mixed, alv = alveolar)
14) Race: all or nearest available, otherwise by race (wh or w = white, bl or b = black, hi = hispanic
ch = chinese, jap = japanese, haw = hawaiian, w+o = white + oriental, sca = scandinavian, as = asian)
15) For overlapping studies: principal rather than subsidiary studies
Finally by Age: whole study (coded as 0) if available, otherwise by widest available age group
and then for single sex results (m, f) in preference to results for both sexes combined (c).

Results adjusted (AD) for the most potential confounders are then chosen in Sections -1 to -3
(and those which actually differ from the adjusted results in Table 1J5 - 1 are marked 'x' in Section -1)
and results adjusted for the least confounders in Sections -4 to -6. (Those least adjusted results which
actually differ from the most adjusted are marked 'x' in column X in Section -4)

Section -7 shows excluded studies, together with the stage (as above) at which no qualifying
results were found.

Section -8 lists the potentially overlapping studies which have been included (1=principal, 2=subsidiary).

Section -9 lists any results which would have been included in preference except that they had data not complete
enough for use in meta-analysis, with their significance (yes/no), if known, and any further comment as entered
on the database. It also lists as "gap" any categories for which no data were presented by the original authors.
This is commonly due to recent quitters having been combined with current smokers

In addition to those mentioned above, the following fields, levels and abbreviations are used:
* or nk = not known, n = no, y = yes, ot = other
all/unspec = all or unspecified, MC = manufactured cigarettes, HR = hand-rolled cigarettes
exL, exH = range of exposure (low and high) in the "highest" group, in terms of Years quit (vs never)
unexL, unexH = range of exposure (low and high) in the "lowest" group, in terms of Years quit (vs never)
REF: 6-character study reference
NRR: number of the RR on the database within the study
ST : study type (CC = case control, pr or prosp = prospective)
NLC: number of lung cancer cases in whole study
R : risky occupational population (n = no, m = mining, o = other risky)
VB : national cigarette type (V = at least 75% Virginia, bl = at least 75% blended, ot = other)
P : any proxy use
H : full histological confirmation
De : derivation of RR/CI (or = original, st = standard method, ot = other method of estimation)

Table 1J15 - 1

IESLC - Meta-analysis of Ex Smoking, Years quit (vs never), "Highest vs lowest"
All LC types, Cigarettes only
Most adjusted

REF	NRR	1J5	SEX	AGEL	AGEH	RACE	YF	LC TYPE	LOC	START	ST	NLC	R	VB	P	H	AD	ADOS	PRODUCT	exL	exH	unexL	unexH	De
ALDERS	512		m	0	0	all	-	all	Eu:UK	1977	CC	1448	n	V	n	n	1	0	cig only	0.1	2	10	999	ot
ALDERS	523		f	0	0	all	-	all	Eu:UK	1977	CC	1448	n	V	n	n	1	0	cig only	0.1	2	10	999	ot
BENHAM	638	x	m	0	0	all	-	all	Eu:wst	1976	CC	1625	n	bl	n	y	0	0	cig only	1.0	4	20	999	st
CPSI	814		m	50	74	all	6	all	NAmer	1959	pr	5138	n	bl	n	n	1	0	cig only	0.1	0.9	10	999	ot
CPSII	663		m	35	99	all	4	all	NAmer	1982	pr	3229	n	bl	n	n	1	0	cig only	0.1	0.9	16	999	ot
DAMBER	556	x	m	0	0	all	-	all	Eu:Sca	1972	CC	579	n	bl	y	n	1	0	cig only	0.1	10	11	999	ot
DEAN3	523	x	m	0	0	all	-	all	Eu:UK	1969	CC	766	n	V	y	n	1	0	cig only	3	4	19	999	ot
DOLL2	508		m	0	0	all	20	all	Eu:UK	1951	pr	920	n	V	n	n	1	0	cig only	0.1	4	15	999	ot
DORN	518	x	m	0	0	wh	0	all	NAmer	1954	pr	5097	n	bl	n	n	2	0	cig only	0.1	4	40	999	ot
GRAHAM	507	x	m	0	0	wh	-	all	NAmer	1956	CC	685	n	bl	n	n	0	0	cig only	0.1	1.0	10	999	st
KAISE2	652		m	0	0	all	9	all	NAmer	1979	pr	318	n	bl	n	n	1	0	cig only	2	10	21	999	st
KAISE2	572		f	0	0	all	9	all	NAmer	1979	pr	318	n	bl	n	n	1	0	cig only	2	10	21	999	ot
PEZZOT	503		m	0	0	all	-	all	SCAmer	1987	CC	215	n	bl	n	y	0	0	cig only	1.0	10	11	999	st
TVERDA	505		m	0	0	all	0	all	Eu:Sca	1972	pr	238	n	bl	n	n	2	0	cig only	0.1	0.9	5	999	ot
WYNDE6	511	x	m	0	0	all	-	all	NAmer	1969	CC	4423	n	bl	n	y	0	0	cig only	1.0	4	30	999	st
WYNDE6	532	x	f	0	0	all	-	all	NAmer	1969	CC	4423	n	bl	n	y	0	0	cig only	1.0	4	30	999	st

Cigarette type is all/unspec for all RRs
except for the following:

REF	NRR	CIGTYPE
ALDERS	512	MC only
ALDERS	523	MC only
DEAN3	523	MC only

Table 1J15 - 2

IESLC - Meta-analysis of Ex Smoking, Years quit (vs never), "Highest vs lowest"
All LC types, Cigarettes only
Most adjusted

REF	NRR	SEX	AD	Number Exposed Case	Exposed Cont	Non-exposed Case	Non-exposed Cont	RR	95.00%CI
ALDERS	512	m	1	121	-	29	-	5.66	(3.32- 9.64)
ALDERS	523	f	1	206	-	26	-	7.43	(4.38- 12.59)
Subtotal ALDERS								6.49	(4.46- 9.45)
BENHAM	638	m	0	154	138	19	129	7.58	(4.44- 12.92)
*CPSI	814	m	1	37	-	15	-	11.54	(6.33- 21.02)
*CPSII	663	m	1	97	-	256	-	10.12	(8.01- 12.78)
DAMBER	556	m	1	-	-	-	-	3.44	(1.36- 8.68)
DEAN3	523	m	1	28	-	8	-	3.56	(1.52- 8.35)
*DOLL2	508	m	1	15	-	7	-	8.00	(3.26- 19.62)
*DORN	518	m	2	56	-	49	-	10.73	(6.60- 17.45)
GRAHAM	507	m	0	84	48	2	30	26.25	(6.01- 114.70)
*KAISE2	652	m	1	12	-	6	-	4.80	(1.62- 14.18)
*KAISE2	572	f	1	6	-	4	-	1.72	(0.45- 6.57)
Subtotal KAISE2								3.20	(1.38- 7.43)
PEZZOT	503	m	0	46	82	20	106	2.97	(1.63- 5.41)
*TVERDA	505	m	2	2	-	4	-	2.07	(0.38- 11.34)
WYNDE6	511	m	0	201	166	21	161	9.28	(5.64- 15.29)
WYNDE6	532	f	0	82	70	10	31	3.63	(1.66- 7.93)
Subtotal WYNDE6								7.07	(4.64- 10.77)
Partial Totals				1147	504	476	457		

*prospective study

REF	NRR	SEX	AD	Ys	Ws	Qs	Ps
ALDERS	512	m	1	1.73	13.52	1.29	0.0000
ALDERS	523	f	1	2.01	13.78	0.02	0.0000
Subtotal ALDERS				1.87	27.31	1.31	
BENHAM	638	m	0	2.03	13.49	0.00	0.0000
*CPSI	814	m	1	2.45	10.67	1.74	0.0000
*CPSII	663	m	1	2.31	70.40	5.22	0.0000
DAMBER	556	m	1	1.24	4.47	2.91	0.0090
DEAN3	523	m	1	1.27	5.29	3.16	0.0035
*DOLL2	508	m	1	2.08	4.77	0.01	0.0000
*DORN	518	m	2	2.37	16.25	1.78	0.0000
GRAHAM	507	m	0	3.27	1.77	2.65	0.0000
*KAISE2	652	m	1	1.57	3.26	0.73	0.0046
*KAISE2	572	f	1	0.54	2.14	4.81	0.4278
Subtotal KAISE2				1.16	5.40	5.54	
PEZZOT	503	m	0	1.09	10.71	9.72	0.0004
*TVERDA	505	m	2	0.73	1.33	2.30	0.4010
WYNDE6	511	m	0	2.23	15.43	0.53	0.0000
WYNDE6	532	f	0	1.29	6.30	3.57	0.0012
Subtotal WYNDE6				1.96	21.72	4.10	

	N	16
	NS	13
	Wt	193.59
Het	Chi	40.44
Het	df	15
Het	P	***
Fixed	RR	7.71
	RRl	6.70
	RRu	8.87
	P	+++
Random	RR	6.44
	RRl	4.92
	RRu	8.42
	P	+++
Asymm	P	*

Table 1J15 - 3

IESLC - Meta-analysis of Ex Smoking, Years quit (vs never), "Highest vs lowest"
All LC types, Cigarettes only
Most adjusted

	combined	Sex male	female	Total
N		13	3	16
NS		13	3	16
Wt		171.37	22.22	193.59
Het Chi		31.63	5.18	40.44
Het df		12	2	15
Het P		**	(*)	***
Fixed RR		8.10	5.27	7.71
RRl		6.97	3.48	6.70
RRu		9.41	7.99	8.87
P		+++	+++	+++
Random RR		6.96	4.27	6.44
RRl		5.22	1.99	4.92
RRu		9.27	9.17	8.42
P		+++	+++	+++
Between Chi				3.63
Between df				1
Between P				(*)
Btwn(F) P				N.S.
Btwn(R) P				N.S.

	Lung cancer type all	other	Total
N	16		16
NS	13		13
Wt	193.59		193.59
Het Chi	40.44		40.44
Het df	15		15
Het P	***		***
Fixed RR	7.71		7.71
RRl	6.70		6.70
RRu	8.87		8.87
P	+++		+++
Random RR	6.44		6.44
RRl	4.92		4.92
RRu	8.42		8.42
P	+++		+++
Between Chi			
Between df			
Between P			N.S.
Btwn(F) P			N.S.
Btwn(R) P			N.S.

	Location NAmer	UK	Scand	othEur	China	Japan	othAs	other	Total
N	8	4	2	1				1	16
NS	6	3	2	1				1	13
Wt	126.22	37.37	5.80	13.49				10.71	193.59
Het Chi	16.32	2.50	0.26	0.00				0.00	40.44
Het df	7	3	1	0				0	15
Het P	*	N.S.	N.S.	N.S.				N.S.	***
Fixed RR	9.35	6.12	3.06	7.58				2.97	7.71
RRl	7.85	4.44	1.36	4.44				1.63	6.70
RRu	11.13	8.44	6.91	12.92				5.41	8.87
P	+++	+++	++	+++				+++	+++
Random RR	8.27	6.12	3.06	7.58				2.97	6.44
RRl	5.88	4.44	1.36	4.44				1.63	4.92
RRu	11.62	8.44	6.91	12.92				5.41	8.42
P	+++	+++	++	+++				+++	+++
Between Chi									21.36
Between df									4
Between P									***
Btwn(F) P									(*)
Btwn(R) P									*

Table 1J15 - 3

IESLC - Meta-analysis of Ex Smoking, Years quit (vs never), "Highest vs lowest"
All LC types, Cigarettes only
Most adjusted

Detailed Country in "other Europe"

	multi	Germany	othWest	East	Balkans	Total
N			1			1
NS			1			1
Wt			13.49			13.49
Het Chi			0.00			0.00
Het df			0			0
Het P			N.S.			N.S.
Fixed RR			7.58			7.58
RRl			4.44			4.44
RRu			12.92			12.92
P			+++			+++
Random RR			7.58			7.58
RRl			4.44			4.44
RRu			12.92			12.92
P			+++			+++
Between Chi						
Between df						
Between P						N.S.
Btwn(F) P						N.S.
Btwn(R) P						N.S.

Detailed Country in "other Asia"

	India	HongKong	other	Total
N				
NS				
Wt				
Het Chi				
Het df				
Het P				
Fixed RR				
RRl				
RRu				
P				
Random RR				
RRl				
RRu				
P				
Between Chi				
Between df				
Between P				N.S.
Btwn(F) P				N.S.
Btwn(R) P				N.S.

Detailed other continent

	SCAmer	Total
N	1	1
NS	1	1
Wt	10.71	10.71
Het Chi	0.00	0.00
Het df	0	0
Het P	N.S.	N.S.
Fixed RR	2.97	2.97
RRl	1.63	1.63
RRu	5.41	5.41
P	+++	+++
Random RR	2.97	2.97
RRl	1.63	1.63
RRu	5.41	5.41
P	+++	+++
Between Chi		
Between df		
Between P		N.S.
Btwn(F) P		N.S.
Btwn(R) P		N.S.

Table 1J15 - 3

IESLC - Meta-analysis of Ex Smoking, Years quit (vs never), "Highest vs lowest"
All LC types, Cigarettes only
Most adjusted

	Start year of study					
	<1960	1960-69	1970-79	1980-89	1990+	Total
N	4	3	7	2		16
NS	4	2	5	2		13
Wt	33.46	27.02	52.00	81.11		193.59
Het Chi	1.85	5.95	7.73	13.95		40.44
Het df	3	2	6	1		15
Het P	N.S.	(*)	N.S.	***		***
Fixed RR	11.04	6.18	5.77	8.61		7.71
RRl	7.87	4.24	4.40	6.93		6.70
RRu	15.49	9.01	7.58	10.70		8.87
P	+++	+++	+++	+++		+++
Random RR	11.04	5.26	5.49	5.67		6.44
RRl	7.87	2.62	3.96	1.71		4.92
RRu	15.49	10.58	7.62	18.79		8.42
P	+++	+++	+++	++		+++
Between Chi						10.97
Between df						3
Between P						*
Btwn(F) P						N.S.
Btwn(R) P						*

	Study type (1)		
	CC	other	Total
N	9	7	16
NS	7	6	13
Wt	84.77	108.83	193.59
Het Chi	17.87	11.93	40.44
Het df	8	6	15
Het P	*	(*)	***
Fixed RR	5.91	9.48	7.71
RRl	4.78	7.86	6.70
RRu	7.31	11.44	8.87
P	+++	+++	+++
Random RR	5.70	8.06	6.44
RRl	4.08	5.60	4.92
RRu	7.95	11.62	8.42
P	+++	+++	+++
Between Chi			10.65
Between df			1
Between P			**
Btwn(F) P			*
Btwn(R) P			N.S.

	Study type (2)			
	CC	prosp	other	Total
N	9	7		16
NS	7	6		13
Wt	84.77	108.83		193.59
Het Chi	17.87	11.93		40.44
Het df	8	6		15
Het P	*	(*)		***
Fixed RR	5.91	9.48		7.71
RRl	4.78	7.86		6.70
RRu	7.31	11.44		8.87
P	+++	+++		+++
Random RR	5.70	8.06		6.44
RRl	4.08	5.60		4.92
RRu	7.95	11.62		8.42
P	+++	+++		+++
Between Chi				10.65
Between df				1
Between P				**
Btwn(F) P				*
Btwn(R) P				N.S.

Table 1J15 - 3

IESLC - Meta-analysis of Ex Smoking, Years quit (vs never), "Highest vs lowest"
All LC types, Cigarettes only
Most adjusted

	Study size (number of LC cases)				
	100-249	250-499	500-999	1000+	Total
N	2	2	4	8	16
NS	2	1	4	6	13
Wt	12.04	5.40	16.30	159.84	193.59
Het Chi	0.16	1.36	6.97	11.10	40.44
Het df	1	1	3	7	15
Het P	N.S.	N.S.	(*)	N.S.	***
Fixed RR	2.86	3.20	5.55	8.85	7.71
RRl	1.62	1.38	3.42	7.58	6.70
RRu	5.02	7.43	9.02	10.33	8.87
P	+++	++	+++	+++	+++
Random RR	2.86	3.11	6.16	8.38	6.44
RRl	1.62	1.15	2.88	6.75	4.92
RRu	5.02	8.40	13.20	10.40	8.42
P	+++	+	+++	+++	+++
Between Chi					20.85
Between df					3
Between P					***
Btwn(F) P					*
Btwn(R) P					**

	Risky occupational population			
	no	mining	othRisky	Total
N	16			16
NS	13			13
Wt	193.59			193.59
Het Chi	40.44			40.44
Het df	15			15
Het P	***			***
Fixed RR	7.71			7.71
RRl	6.70			6.70
RRu	8.87			8.87
P	+++			+++
Random RR	6.44			6.44
RRl	4.92			4.92
RRu	8.42			8.42
P	+++			+++
Between Chi				
Between df				
Between P				N.S.
Btwn(F) P				N.S.
Btwn(R) P				N.S.

	National cigarette tobacco type			
	Virginia	blended	other	Total
N	4	12		16
NS	3	10		13
Wt	37.37	156.22		193.59
Het Chi	2.50	35.50		40.44
Het df	3	11		15
Het P	N.S.	***		***
Fixed RR	6.12	8.14		7.71
RRl	4.44	6.96		6.70
RRu	8.44	9.53		8.87
P	+++	+++		+++
Random RR	6.12	6.50		6.44
RRl	4.44	4.62		4.92
RRu	8.44	9.15		8.42
P	+++	+++		+++
Between Chi				2.45
Between df				1
Between P				N.S.
Btwn(F) P				N.S.
Btwn(R) P				N.S.

Table 1J15 - 3

IESLC - Meta-analysis of Ex Smoking, Years quit (vs never), "Highest vs lowest"
All LC types, Cigarettes only
Most adjusted

	Any proxy use No/nk	Yes	Total
N	14	2	16
NS	11	2	13
Wt	183.83	9.77	193.59
Het Chi	34.05	0.00	40.44
Het df	13	1	15
Het P	**	N.S.	***
Fixed RR	8.04	3.50	7.71
RRl	6.96	1.87	6.70
RRu	9.29	6.56	8.87
P	+++	+++	+++
Random RR	6.94	3.50	6.44
RRl	5.28	1.87	4.92
RRu	9.12	6.56	8.42
P	+++	+++	+++
Between Chi			6.39
Between df			1
Between P			*
Btwn(F) P			N.S.
Btwn(R) P			(*)

	Full histological confirmation No	Yes	Total
N	12	4	16
NS	10	3	13
Wt	147.67	45.93	193.59
Het Chi	25.59	10.53	40.44
Het df	11	3	15
Het P	**	*	***
Fixed RR	8.38	5.90	7.71
RRl	7.13	4.42	6.70
RRu	9.84	7.87	8.87
P	+++	+++	+++
Random RR	6.96	5.44	6.44
RRl	5.12	3.13	4.92
RRu	9.45	9.46	8.42
P	+++	+++	+++
Between Chi			4.32
Between df			1
Between P			*
Btwn(F) P			N.S.
Btwn(R) P			N.S.

	Number of adjustment variables (1) 0	1	2+/+nk	Total
N	5	9	2	16
NS	4	7	2	13
Wt	47.69	128.31	17.59	193.59
Het Chi	14.32	19.55	3.33	40.44
Het df	4	8	1	15
Het P	**	*	(*)	***
Fixed RR	6.23	8.11	9.47	7.71
RRl	4.69	6.82	5.94	6.70
RRu	8.28	9.64	15.12	8.87
P	+++	+++	+++	+++
Random RR	6.34	6.41	5.81	6.44
RRl	3.57	4.56	1.22	4.92
RRu	11.29	9.00	27.64	8.42
P	+++	+++	+	+++
Between Chi				3.24
Between df				2
Between P				N.S.
Btwn(F) P				N.S.
Btwn(R) P				N.S.

Table 1J15 - 3

IESLC - Meta-analysis of Ex Smoking, Years quit (vs never), "Highest vs lowest"
All LC types, Cigarettes only
Most adjusted

	Number of adjustment variables (2) 0	1	2	3-5	6+/+nk	Total
N	5	9	2			16
NS	4	7	2			13
Wt	47.69	128.31	17.59			193.59
Het Chi	14.32	19.55	3.33			40.44
Het df	4	8	1			15
Het P	**	*	(*)			***
Fixed RR	6.23	8.11	9.47			7.71
RRl	4.69	6.82	5.94			6.70
RRu	8.28	9.64	15.12			8.87
P	+++	+++	+++			+++
Random RR	6.34	6.41	5.81			6.44
RRl	3.57	4.56	1.22			4.92
RRu	11.29	9.00	27.64			8.42
P	+++	+++	+			+++
Between Chi						3.24
Between df						2
Between P						N.S.
Btwn(F) P						N.S.
Btwn(R) P						N.S.

	Derivation of RR/CI Orig	StdCalc	Other	Total
N		6	10	16
NS		5	9	14
Wt		50.96	142.64	193.59
Het Chi		14.53	22.28	40.44
Het df		5	9	15
Het P		*	**	***
Fixed RR		6.13	8.37	7.71
RRl		4.66	7.10	6.70
RRu		8.06	9.86	8.87
P		+++	+++	+++
Random RR		6.09	6.78	6.44
RRl		3.67	4.94	4.92
RRu		10.11	9.32	8.42
P		+++	+++	+++
Between Chi				3.64
Between df				1
Between P				(*)
Btwn(F) P				N.S.
Btwn(R) P				N.S.

Table 1J15 - 4

IESLC - Meta-analysis of Ex Smoking, Years quit (vs never), "Highest vs lowest"
All LC types, Cigarettes only
Least adjusted

REF	NRR	X	SEX	AGEL	AGEH	RACE	YF	LC TYPE	LOC	START	ST	NLC	R	VB	P	H	AD	ADOS	PRODUCT	exL	exH	unexL	unexH	De
ALDERS	512		m	0	0	all	-	all	Eu:UK	1977	CC	1448	n	V	n	n	1	0	cig only	0.1	2	10	999	ot
ALDERS	523		f	0	0	all	-	all	Eu:UK	1977	CC	1448	n	V	n	n	1	0	cig only	0.1	2	10	999	ot
BENHAM	638		m	0	0	all	-	all	Eu:wst	1976	CC	1625	n	bl	n	y	0	0	cig only	1.0	4	20	999	st
CPSI	814		m	50	74	all	6	all	NAmer	1959	pr	5138	n	bl	n	n	1	0	cig only	0.1	0.9	10	999	ot
CPSII	663		m	35	99	all	4	all	NAmer	1982	pr	3229	n	bl	n	n	1	0	cig only	0.1	0.9	16	999	ot
DAMBER	556		m	0	0	all	-	all	Eu:Sca	1972	CC	579	n	bl	y	n	1	0	cig only	0.1	10	11	999	ot
DEAN3	508	x	m	0	0	all	-	all	Eu:UK	1969	CC	766	n	V	y	n	0	0	cig only	3	4	19	999	st
DOLL2	508		m	0	0	all	20	all	Eu:UK	1951	pr	920	n	V	n	n	1	0	cig only	0.1	4	15	999	ot
DORN	518		m	0	0	wh	0	all	NAmer	1954	pr	5097	n	bl	n	n	2	0	cig only	0.1	4	40	999	ot
GRAHAM	507		m	0	0	wh	-	all	NAmer	1956	CC	685	n	bl	n	n	0	0	cig only	0.1	1.0	10	999	st
KAISE2	652		m	0	0	all	9	all	NAmer	1979	pr	318	n	bl	n	n	1	0	cig only	2	10	21	999	st
KAISE2	572		f	0	0	all	9	all	NAmer	1979	pr	318	n	bl	n	n	1	0	cig only	2	10	21	999	ot
PEZZOT	503		m	0	0	all	-	all	SCAmer	1987	CC	215	n	bl	n	y	0	0	cig only	1.0	10	11	999	st
TVERDA	505		m	0	0	all	0	all	Eu:Sca	1972	pr	238	n	bl	n	n	2	0	cig only	0.1	0.9	5	999	ot
WYNDE6	511		m	0	0	all	-	all	NAmer	1969	CC	4423	n	bl	n	y	0	0	cig only	1.0	4	30	999	st
WYNDE6	532		f	0	0	all	-	all	NAmer	1969	CC	4423	n	bl	n	y	0	0	cig only	1.0	4	30	999	st

Cigarette type is all/unspec for all RRs
except for the following:

REF	NRR	CIGTYPE
ALDERS	512	MC only
ALDERS	523	MC only
DEAN3	508	MC only

Table 1J15 - 5

IESLC - Meta-analysis of Ex Smoking, Years quit (vs never), "Highest vs lowest"
All LC types, Cigarettes only
Least adjusted

REF	NRR	SEX	AD	Number Exposed Case	Exposed Cont	Non-exposed Case	Non-exposed Cont	RR	95.00%CI
ALDERS	512	m	1	121	-	29	-	5.66	(3.32- 9.64)
ALDERS	523	f	1	206	-	26	-	7.43	(4.38- 12.59)
Subtotal ALDERS								6.49	(4.46- 9.45)
BENHAM	638	m	0	154	138	19	129	7.58	(4.44- 12.92)
*CPSI	814	m	1	37	-	15	-	11.54	(6.33- 21.02)
*CPSII	663	m	1	97	-	256	-	10.12	(8.01- 12.78)
DAMBER	556	m	1	-	-	-	-	3.44	(1.36- 8.68)
DEAN3	508	m	0	28	102	8	66	2.26	(0.97- 5.27)
*DOLL2	508	m	1	15	-	7	-	8.00	(3.26- 19.62)
*DORN	518	m	2	56	-	49	-	10.73	(6.60- 17.45)
GRAHAM	507	m	0	84	48	2	30	26.25	(6.01- 114.70)
*KAISE2	652	m	1	12	-	6	-	4.80	(1.62- 14.18)
*KAISE2	572	f	1	6	-	4	-	1.72	(0.45- 6.57)
Subtotal KAISE2								3.20	(1.38- 7.43)
PEZZOT	503	m	0	46	82	20	106	2.97	(1.63- 5.41)
*TVERDA	505	m	2	2	-	4	-	2.07	(0.38- 11.34)
WYNDE6	511	m	0	201	166	21	161	9.28	(5.64- 15.29)
WYNDE6	532	f	0	82	70	10	31	3.63	(1.66- 7.93)
Subtotal WYNDE6								7.07	(4.64- 10.77)
Partial Totals				1147	606	476	523		

*prospective study

REF	NRR	SEX	AD	Ys	Ws	Qs	Ps
ALDERS	512	m	1	1.73	13.52	1.18	0.0000
ALDERS	523	f	1	2.01	13.78	0.01	0.0000
Subtotal ALDERS				1.87	27.31	1.19	
BENHAM	638	m	0	2.03	13.49	0.00	0.0000
*CPSI	814	m	1	2.45	10.67	1.85	0.0000
*CPSII	663	m	1	2.31	70.40	5.73	0.0000
DAMBER	556	m	1	1.24	4.47	2.82	0.0090
DEAN3	508	m	0	0.82	5.39	7.91	0.0578
*DOLL2	508	m	1	2.08	4.77	0.01	0.0000
*DORN	518	m	2	2.37	16.25	1.92	0.0000
GRAHAM	507	m	0	3.27	1.77	2.71	0.0000
*KAISE2	652	m	1	1.57	3.26	0.69	0.0046
*KAISE2	572	f	1	0.54	2.14	4.73	0.4278
Subtotal KAISE2				1.16	5.40	5.42	
PEZZOT	503	m	0	1.09	10.71	9.46	0.0004
*TVERDA	505	m	2	0.73	1.33	2.26	0.4010
WYNDE6	511	m	0	2.23	15.43	0.61	0.0000
WYNDE6	532	f	0	1.29	6.30	3.45	0.0012
Subtotal WYNDE6				1.96	21.72	4.06	

	N	16
	NS	13
	Wt	193.68
Het	Chi	45.33
Het	df	15
Het	P	***
Fixed	RR	7.61
	RRl	6.61
	RRu	8.76
	P	+++
Random	RR	6.22
	RRl	4.69
	RRu	8.26
	P	+++
Asymm	P	*

Table 1J15 - 6

IESLC - Meta-analysis of Ex Smoking, Years quit (vs never), "Highest vs lowest"
All LC types, Cigarettes only
Least adjusted

	Sex combined	male	female	Total
N		13	3	16
NS		13	3	16
Wt		171.46	22.22	193.68
Het Chi		36.76	5.18	45.33
Het df		12	2	15
Het P		***	(*)	***
Fixed RR		7.98	5.27	7.61
RRl		6.87	3.48	6.61
RRu		9.27	7.99	8.76
P		+++	+++	+++
Random RR		6.69	4.27	6.22
RRl		4.92	1.99	4.69
RRu		9.10	9.17	8.26
P		+++	+++	+++
Between Chi				3.39
Between df				1
Between P				(*)
Btwn(F) P				N.S.
Btwn(R) P				N.S.

Table 1J15 - 7

IESLC - Meta-analysis of Ex Smoking, Years quit (vs never), "Highest vs lowest"
All LC types, Cigarettes only
Excluded studies (and stage at which they were excluded)

Stage	Studies
1	AGUDO AKIBA AMANDU AMES AXELSS BEST BOUCHA BOUCOT BRESLO CHEN CHEN2 CHIAZZ DEAN2 DOSEME ENGELA FAN GENG GER GUO HAENSZ HEGMAN HOLE HU HU2 JUSSAW KATSOU KAUFMA KOO KOULUM KREUZE LETOUR LEVIN LIAW LIU3 LIU4 LIU5 MCCONN MIGRAN MRFITR NOTAN2 OSANN2 PERNU QIAO2 RACHTA RESTRE SADOWS SEGI2 STASZE TIZZAN VUTUC WATSON WU WUWILL WYNDE2 WYNDE8 XU YUAN ZHANG ZHENG ZHOU
2	BUFFLE HUMBLE PISANI PRESCO WYNDE7
3	MCDUFF SPITZ
4	AUVINE BLOT1 BROWN3 GURSEL LAUSSM LUO WU2
5	HAMMON
6	CORREA GILLIS QIAO WIGLE
8	ARMADA BARBON BECHER BENSHL BOFFET BROSS CARPEN CEDERL CHOI CHYOU DARBY DESTEF DOLL DORGAN GAO GAO2 GARCIA GARSHI HAMMO2 HIRAYA JAHN JAIN JEDRYC JOLY KHUDER LUBIN LUBIN2 MATOS PEZZO2 SOBUE SPEIZE SUZUK2 SVENSS WAKAI WANG2 WYNDE3

Table 1J15 - 8
Potentially overlapping studies

REF	REFGP	PRINC	OVERLAP/LINK
BENHAM	LUBIN2	2	Subset of Lubin2
TVERDA	TVERDA	1	VEIERO/TVERDAL
GRAHAM	BYERS1	1	GRAHAM/BROSS/BYERS1
WYNDE6	WYNDE6	1	WYNDE5/6/7/8
CPSI	CPSI	1	CPSI overall

Table 1J15 - 9
Most adjusted - insufficient data for meta-analysis

REF	NRR	SEX	AGEL	AGEH	RACE	YF	LC TYPE	LOC	START	ST	NLC	R	VB	P	H	AD	ADOS	PRODUCT	exL	exH	unexL	unexH	De
CPSI	710	m	0	0	wh	0	all	NAmer	1959	pr	5138	n	bl	n	n	1	0	cig only	2	4	35	39	st
CPSI	728	f	0	0	wh	0	all	NAmer	1959	pr	5138	n	bl	n	n	1	0	cig only	2	4	25	29	st
DEAN3	621	m	0	0	all	-	all	Eu:UK	1969	CC	766	n	V	y	n	1	0	cig only	0.1	2	19	999	ot
KAISE2	653	m	0	0	all	9	all	NAmer	1979	pr	318	n	bl	n	n	1	0	cig only	0.1	1.9	21	999	ot
KAISE2	573	f	0	0	all	9	all	NAmer	1979	pr	318	n	bl	n	n	1	0	cig only	0.1	1.9	21	999	ot
PEZZOT	598	m	0	0	all	-	all	SCAmer	1987	CC	215	n	bl	n	y	0	0	cig only	0.1	0.9	11	999	ot
WYNDE6	512	m	0	0	all	-	all	NAmer	1969	CC	4423	n	bl	n	y	0	0	cig only	0.1	0.9	30	999	ot
WYNDE6	533	f	0	0	all	-	all	NAmer	1969	CC	4423	n	bl	n	y	0	0	cig only	0.1	0.9	30	999	ot

REF	NRR	RR	SIG	RRDATA comment
CPSI	710	4.13		0
CPSI	728	1.09		0
DEAN3	621	*	gap	0
KAISE2	653	*	gap	0
KAISE2	573	*	gap	0
PEZZOT	598	*	gap	0
WYNDE6	512	*	gap	0
WYNDE6	533	*	gap	0

Least adjusted - insufficient data for meta-analysis: as for adjusted plus the following

REF	NRR	SEX	AGEL	AGEH	RACE	YF	LC TYPE	LOC	START	ST	NLC	R	VB	P	H	AD	ADOS	PRODUCT	exL	exH	unexL	unexH	De
DEAN3	619	m	0	0	all	-	all	Eu:UK	1969	CC	766	n	V	y	n	0	0	cig only	0.1	2	19	999	ot

REF	NRR	RR	SIG	RRDATA comment
DEAN3	619	*	gap	0

Table 1J16 -

IESLC - Meta-analysis of Ex Smoking by Years quit (vs never), Overview
All LC types, Pipes and/or cigars (not cigs)

This analysis is restricted to results for:
1) Ex smokers
2) Results by Years quit (vs never)
3) Categorical results by Years quit (vs never)
Results by Years quit (vs never) are grouped under 2 schemes (S1, S2). Each scheme has a set of "key values". An interval is allocated to the category whose key value it includes, and intervals which include none or more than one of the key values are excluded. (Open-ended intervals are coded as 999)

S1	key value	maximum range
1	12	8+
2	7	4-11
3	3	1-6

S2	key value	maximum range
1	20	13+
2	12	4-19
3	3	1-11

4) All LC types (or near equivalent)
5) Results complete enough for use in metaanalysis
6) pipes and/or cigars (not cigs)

Within each study, results are then selected (in the following order of preference, within each sex) for:
7) (not applicable)
8) DENOM: never smoked anything, never any + low
9) Followup period (YF, prospective studies): whole study (coded as 0) or longest available
10) LCtype: all or nearest available, at least Squamous and Adeno. (q = squamous, s = small, l = large, a = adeno, mix = mixed, alv = alveolar)
11) Race: all or nearest available, otherwise by race (wh or w = white, bl or b = black, hi = hispanic ch = chinese, jap = japanese, haw = hawaiian, w+o = white + oriental, sca = scandinavian, as = asian)
12) For overlapping studies: principal rather than subsidiary studies
Finally by Age: whole study (coded as 0) if available, otherwise by widest available age group and then for single sex results (m, f) in preference to results for both sexes combined (c).

Results adjusted (AD) for the most potential confounders are then chosen in Sections -1 to -3 and results adjusted for the least confounders in Sections -4 to -6. (Those least adjusted results which actually differ from the most adjusted are marked 'x' in column X in Section -4)

Section -7 shows excluded studies, together with the stage (as above) at which no qualifying results were found.

Section -8 lists the potentially overlapping studies which have been included (1=principal, 2=subsidiary).

Section -9 lists any results which would have been included in preference except that they had data not complete enough for use in meta-analysis, with their significance (yes/no), if known, and any further comment as entered on the database. It also lists as "gap" any categories for which no data were presented by the original authors. This is commonly due to recent quitters having been combined with current smokers

In addition to those mentioned above, the following fields, levels and abbreviations are used:
* or nk = not known, n = no, y = yes, ot = other
nev = never
all/unspec = all or unspecified, cig+/-ot = cigarettes irrespective of other products (cigar, pipe etc)
MC = manufactured cigarettes, HR = hand-rolled cigarettes
exL, exH = range of exposure (low and high) in the smoking group, in terms of Years quit (vs never)
REF: 6-character study reference
NRR: number of the RR on the database within the study
ST : study type (CC = case control, pr or prosp = prospective)
NLC: number of lung cancer cases in whole study
R : risky occupational population (n = no, m = mining, o = other risky)
VB : national cigarette type (V = at least 75% Virginia, bl = at least 75% blended, ot = other)
P : any proxy use
H : full histological confirmation
De : derivation of RR/CI (or = original, st = standard method, ot = other method of estimation)

Table 1J16 - 1

IESLC - Meta-analysis of Ex Smoking by Years quit (vs never), Overview
All LC types, Pipes and/or cigars (not cigs)
Most adjusted

REF	NRR	SEX	AGEL	AGEH	RACE	YF	LC TYPE	LOC	START	ST	NLC	R	VB	P	H	AD	exL	exH	S1	S2	DENOM	De
WYNDE3	602	m	0	0	all	-	all	NAmer	1966	CC	350	n	bl	n	y	0	10	999	1	0	nev	any st

In this overview table, subtotals and Qs values may be invalid and should be ignored

Table 1J16 - 2

IESLC - Meta-analysis of Ex Smoking by Years quit (vs never), Overview
All LC types, Pipes and/or cigars (not cigs)
Most adjusted

REF	NRR	SEX	AD	Number Case	Exposed Cont	Non-exposed Case	Cont	RR	95.00%CI
WYNDE3	602	m	0	1	4	9	88	2.44	(0.25- 24.29)
Totals				1	4	9	88		

*prospective study

REF	NRR	SEX	AD	Ys	Ws	Qs	Ps
WYNDE3	602	m	0	0.89	0.73	0.00	0.4455

N	1
NS	1

Table 1J16 - 3

IESLC - Meta-analysis of Ex Smoking by Years quit (vs never), Overview
All LC types, Pipes and/or cigars (not cigs)
Most adjusted

	Sex: combined	male	female	Total
N		1		1
NS		1		1

In this overview table, other than the "N" rows, entries in the "absent" and "Total" columns may be invalid and should be ignored

Years quit vs never (lower focus)	absent	8+k12	4-11k7	1-6k3	Total
N		1			1
NS		1			1
Wt		0.73			0.73
Het Chi		0.00			0.00
Het df		0			0
Het P		N.S.			N.S.
Fixed RR		2.44			2.44
RRl		0.25			0.25
RRu		24.29			24.29
P		N.S.			N.S.
Random RR		2.44			2.44
RRl		0.25			0.25
RRu		24.29			24.29
P		N.S.			N.S.

Years quit vs never (higher focus)	absent	13+k20	4-19k12	1-11k3	Total
N	1				1
NS	1				1
Wt	0.73				0.73
Het Chi	0.00				0.00
Het df	0				0
Het P	N.S.				N.S.
Fixed RR	2.44				2.44
RRl	0.25				0.25
RRu	24.29				24.29
P	N.S.				N.S.
Random RR	2.44				2.44
RRl	0.25				0.25
RRu	24.29				24.29
P	N.S.				N.S.

Table 1J16 - 4

IESLC - Meta-analysis of Ex Smoking by Years quit (vs never), Overview
All LC types, Pipes and/or cigars (not cigs)
Least adjusted

REF	NRR	X	SEX	AGEL	AGEH	RACE	YF	LC TYPE	LOC	START	ST	NLC	R	VB	P	H	AD	exL	exH	S1	S2	DENOM	De
WYNDE3	602		m	0	0	all	-	all	NAmer	1966	CC	350	n	bl	n	y	0	10	999	1	0	nev any	st

In this overview table, subtotals and Qs values may be invalid and should be ignored

Table 1J16 - 5

IESLC - Meta-analysis of Ex Smoking by Years quit (vs never), Overview
All LC types, Pipes and/or cigars (not cigs)
Least adjusted

REF	NRR	SEX	AD	Number Case	Exposed Cont	Non-exposed Case	Cont	RR	95.00%CI
WYNDE3	602	m	0	1	4	9	88	2.44	(0.25- 24.29)
Totals				1	4	9	88		

*prospective study

REF	NRR	SEX	AD	Ys	Ws	Qs	Ps
WYNDE3	602	m	0	0.89	0.73	0.00	0.4455

N	1
NS	1

Table 1J16 - 6

IESLC - Meta-analysis of Ex Smoking by Years quit (vs never), Overview
All LC types, Pipes and/or cigars (not cigs)
Least adjusted

	Sex			
	combined	male	female	Total
N		1		1
NS		1		1

In this overview table, other than the "N" rows, entries in the "absent" and "Total" columns may be invalid and should be ignored

	Years quit vs never (lower focus)				
	absent	8+k12	4-11k7	1-6k3	Total
N		1			1
NS		1			1
Wt		0.73			0.73
Het Chi		0.00			0.00
Het df		0			0
Het P		N.S.			N.S.
Fixed RR		2.44			2.44
RRl		0.25			0.25
RRu		24.29			24.29
P		N.S.			N.S.
Random RR		2.44			2.44
RRl		0.25			0.25
RRu		24.29			24.29
P		N.S.			N.S.

	Years quit vs never (higher focus)				
	absent	13+k20	4-19k12	1-11k3	Total
N	1				1
NS	1				1
Wt	0.73				0.73
Het Chi	0.00				0.00
Het df	0				0
Het P	N.S.				N.S.
Fixed RR	2.44				2.44
RRl	0.25				0.25
RRu	24.29				24.29
P	N.S.				N.S.
Random RR	2.44				2.44
RRl	0.25				0.25
RRu	24.29				24.29
P	N.S.				N.S.

Table 1J16 - 7

IESLC - Meta-analysis of Ex Smoking by Years quit (vs never), Overview
All LC types, Pipes and/or cigars (not cigs)
Excluded studies (and stage at which they were excluded)

1	AGUDO AKIBA AMANDU AMES AXELSS BEST BOUCHA BOUCOT BRESLO CHEN CHEN2 CHIAZZ DEAN2 DOSEME ENGELA FAN GENG GER GUO HAENSZ HEGMAN HOLE HU HU2 JUSSAW KATSOU KAUFMA KOO KOULUM KREUZE LETOUR LEVIN LIAW LIU3 LIU4 LIU5 MCCONN MIGRAN MRFITR NOTAN2 OSANN2 PERNU QIAO2 RACHTA RESTRE SADOWS SEGI2 STASZE TIZZAN VUTUC WATSON WU WUWILL WYNDE2 WYNDE8 XU YUAN ZHANG ZHENG ZHOU
2	BUFFLE HUMBLE PISANI PRESCO WYNDE7
3	MCDUFF SPITZ
4	HAMMON LUO WU2
5	BLOT1 CORREA GILLIS QIAO WIGLE
7	ALDERS ARMADA AUVINE BARBON BECHER BENHAM BENSHL BOFFET BROSS BROWN3 CARPEN CEDERL CHOI CHYOU CPSI CPSII DAMBER DARBY DEAN3 DESTEF DOLL DOLL2 DORGAN DORN GAO GAO2 GARCIA GARSHI GRAHAM GURSEL HAMMO2 HIRAYA JAHN JAIN JEDRYC JOLY KAISE2 KHUDER LAUSSM LUBIN LUBIN2 MATOS PEZZO2 PEZZOT SOBUE SPEIZE SUZUK2 SVENSS TVERDA WAKAI WANG2 WYNDE6

Table 1J16 - 8

Table 1J16 - 9
Most adjusted - insufficient data for meta-analysis

REF	NRR	SEX	AGEL	AGEH	RACE	YF	LC TYPE	LOC	START	ST	NLC	R	VB	P	H	AD	exL	exH	S1	S2	DENOM	De
WYNDE3	603	m	0	0	all	-	all	NAmer	1966	CC	350	n	bl	n	y	0	1.0	9	0	3	nev	any ot

REF	NRR	RR	SIG	RRDATA comment
WYNDE3	603	*	gap	0

Table 1J17 -

IESLC - Meta-analysis of Ex Smoking, Years quit (vs never), "Highest vs lowest"
All LC types, Pipes and/or cigars (not cigs)

This analysis is restricted to results for:
1) Ex smokers
2) Results by Years quit (vs never)
3) Categorical results by Years quit (vs never)
4) Denominator (unexposed) = "low"
5) All LC types (or near equivalent)
6) Results complete enough for use in metaanalysis
7) (not applicable)
8) PRODUCT: pipes and/or cigars (not cigs)

Within each study, results are then selected (in the following order of preference, within each sex) for:
9) Results with least adjustment for other aspects of smoking (ADOS)
10) The highest vs lowest category
11) Followup period (YF, prospective studies): whole study (coded as 0) or longest available
12) LCtype: all or nearest available, at least Squamous and Adeno. (q = squamous, s = small,
l = large, a = adeno, mix = mixed, alv = alveolar)
13) Race: all or nearest available, otherwise by race (wh or w = white, bl or b = black, hi = hispanic
ch = chinese, jap = japanese, haw = hawaiian, w+o = white + oriental, sca = scandinavian, as = asian)
14) For overlapping studies: principal rather than subsidiary studies
Finally by Age: whole study (coded as 0) if available, otherwise by widest available age group
and then for single sex results (m, f) in preference to results for both sexes combined (c).

Results adjusted (AD) for the most potential confounders are then chosen in Sections -1 to -3
and results adjusted for the least confounders in Sections -4 to -6. (Those least adjusted results which
actually differ from the most adjusted are marked 'x' in column X in Section -4)

Section -7 shows excluded studies, together with the stage (as above) at which no qualifying
results were found.

Section -8 lists the potentially overlapping studies which have been included (1=principal, 2=subsidiary).

Section -9 lists any results which would have been included in preference except that they had data not complete
enough for use in meta-analysis, with their significance (yes/no), if known, and any further comment as entered
on the database. It also lists as "gap" any categories for which no data were presented by the original authors.
This is commonly due to recent quitters having been combined with current smokers

In addition to those mentioned above, the following fields, levels and abbreviations are used:
* or nk = not known, n = no, y = yes, ot = other
all/unspec = all or unspecified, cig+/-ot = cigarettes irrespective of other products (cigar, pipe etc)
MC = manufactured cigarettes, HR = hand-rolled cigarettes
exL, exH = range of exposure (low and high) in the "highest" group, in terms of Years quit (vs never)
unexL, unexH = range of exposure (low and high) in the "lowest" group, in terms of Years quit (vs never)
REF: 6-character study reference
NRR: number of the RR on the database within the study
ST : study type (CC = case control, pr or prosp = prospective)
NLC: number of lung cancer cases in whole study
R : risky occupational population (n = no, m = mining, o = other risky)
VB : national cigarette type (V = at least 75% Virginia, bl = at least 75% blended, ot = other)
P : any proxy use
H : full histological confirmation
De : derivation of RR/CI (or = original, st = standard method, ot = other method of estimation)

Table 1J17 - 0

No RRs selected for this analysis

Table 1J17 - 7

IESLC - Meta-analysis of Ex Smoking, Years quit (vs never), "Highest vs lowest"
All LC types, Pipes and/or cigars (not cigs)
Excluded studies (and stage at which they were excluded)

1 AGUDO AKIBA AMANDU AMES AXELSS BEST BOUCHA BOUCOT BRESLO CHEN CHEN2 CHIAZZ DEAN2 DOSEME ENGELA FAN GENG GER GUO HAENSZ HEGMAN HOLE HU HU2 JUSSAW KATSOU KAUFMA KOO KOULUM KREUZE LETOUR LEVIN LIAW LIU3 LIU4 LIU5 MCCONN MIGRAN MRFITR NOTAN2 OSANN2 PERNU QIAO2 RACHTA RESTRE SADOWS SEGI2 STASZE TIZZAN VUTUC WATSON WU WUWILL WYNDE2 WYNDE8 XU YUAN ZHANG ZHENG ZHOU

2 BUFFLE HUMBLE PISANI PRESCO WYNDE7

3 MCDUFF SPITZ

4 AUVINE BLOT1 BROWN3 GURSEL LAUSSM LUO WU2

5 HAMMON

6 CORREA GILLIS QIAO WIGLE

8 ALDERS ARMADA BARBON BECHER BENHAM BENSHL BOFFET BROSS CARPEN CEDERL CHOI CHYOU CPSI CPSII DAMBER DARBY DEAN3 DESTEF DOLL DOLL2 DORGAN DORN GAO GAO2 GARCIA GARSHI GRAHAM HAMMO2 HIRAYA JAHN JAIN JEDRYC JOLY KAISE2 KHUDER LUBIN LUBIN2 MATOS PEZZO2 PEZZOT SOBUE SPEIZE SUZUK2 SVENSS TVERDA WAKAI WANG2 WYNDE3 WYNDE6

Table 1J18 -

IESLC - Meta-analysis of Ex Smoking by Years quit (vs never), Overview
All LC types, Pipes only

This analysis is restricted to results for:
1) Ex smokers
2) Results by Years quit (vs never)
3) Categorical results by Years quit (vs never)
Results by Years quit (vs never) are grouped under 2 schemes (S1, S2). Each scheme has a set of "key values". An interval is allocated to the category whose key value it includes, and intervals which include none or more than one of the key values are excluded. (Open-ended intervals are coded as 999)

S1	key value	maximum range
1	12	8+
2	7	4-11
3	3	1-6

S2	key value	maximum range
1	20	13+
2	12	4-19
3	3	1-11

4) All LC types (or near equivalent)
5) Results complete enough for use in metaanalysis
6) pipes only

Within each study, results are then selected (in the following order of preference, within each sex) for:
7) (not applicable)
8) DENOM: never smoked anything, never any + low
9) Followup period (YF, prospective studies): whole study (coded as 0) or longest available
10) LCtype: all or nearest available, at least Squamous and Adeno. (q = squamous, s = small, l = large, a = adeno, mix = mixed, alv = alveolar)
11) Race: all or nearest available, otherwise by race (wh or w = white, bl or b = black, hi = hispanic ch = chinese, jap = japanese, haw = hawaiian, w+o = white + oriental, sca = scandinavian, as = asian)
12) For overlapping studies: principal rather than subsidiary studies
Finally by Age: whole study (coded as 0) if available, otherwise by widest available age group and then for single sex results (m, f) in preference to results for both sexes combined (c).

Results adjusted (AD) for the most potential confounders are then chosen in Sections -1 to -3 and results adjusted for the least confounders in Sections -4 to -6. (Those least adjusted results which actually differ from the most adjusted are marked 'x' in column X in Section -4)

Section -7 shows excluded studies, together with the stage (as above) at which no qualifying results were found.

Section -8 lists the potentially overlapping studies which have been included (1=principal, 2=subsidiary).

Section -9 lists any results which would have been included in preference except that they had data not complete enough for use in meta-analysis, with their significance (yes/no), if known, and any further comment as entered on the database. It also lists as "gap" any categories for which no data were presented by the original authors. This is commonly due to recent quitters having been combined with current smokers

In addition to those mentioned above, the following fields, levels and abbreviations are used:
* or nk = not known, n = no, y = yes, ot = other
nev = never
all/unspec = all or unspecified, cig+/-ot = cigarettes irrespective of other products (cigar, pipe etc)
MC = manufactured cigarettes, HR = hand-rolled cigarettes
exL, exH = range of exposure (low and high) in the smoking group, in terms of Years quit (vs never)
REF: 6-character study reference
NRR: number of the RR on the database within the study
ST : study type (CC = case control, pr or prosp = prospective)
NLC: number of lung cancer cases in whole study
R : risky occupational population (n = no, m = mining, o = other risky)
VB : national cigarette type (V = at least 75% Virginia, bl = at least 75% blended, ot = other)
P : any proxy use
H : full histological confirmation
De : derivation of RR/CI (or = original, st = standard method, ot = other method of estimation)

Table 1J18 - 1

IESLC - Meta-analysis of Ex Smoking by Years quit (vs never), Overview
All LC types, Pipes only
Most adjusted

REF	NRR	SEX	AGEL	AGEH	RACE	YF	LC TYPE	LOC	START	ST	NLC	R	VB	P	H	AD	exL	exH	S1	S2	DENOM	De
BOFFET	549	m	0	0	all	-	all	Eu:mul	1988	CC	5621	n	bl	y	n	2	15	999	0	1	nev any	or
BOFFET	550	m	0	0	all	-	all	Eu:mul	1988	CC	5621	n	bl	y	n	2	0.1	14	0	0	nev any	or
DAMBER	560	m	0	0	all	-	all	Eu:Sca	1972	CC	579	n	bl	y	n	1	11	999	1	0	nev any	ot
DAMBER	561	m	0	0	all	-	all	Eu:Sca	1972	CC	579	n	bl	y	n	1	0.1	10	0	3	nev any	ot

In this overview table, subtotals and Qs values may be invalid and should be ignored

Table 1J18 - 2

IESLC - Meta-analysis of Ex Smoking by Years quit (vs never), Overview
All LC types, Pipes only
Most adjusted

REF	NRR	SEX	AD	Number Exposed Case	Cont	Non-exposed Case	Cont	RR	95.00%CI
BOFFET	549	m	2	-	-	117	-	1.40	(0.50- 4.00)
BOFFET	550	m	2	-	-	117	-	10.30	(5.10- 20.50)
Subtotal BOFFET								5.56	(3.12- 9.91)
DAMBER	560	m	1	-	-	42	-	4.50	(2.30- 9.30)
DAMBER	561	m	1	-	-	42	-	5.00	(2.60- 9.20)
Subtotal DAMBER								4.77	(2.98- 7.62)
Partial Totals				0	0	318	0		

*prospective study

REF	NRR	SEX	AD	Ys	Ws	Qs	Ps
BOFFET	549	m	2	0.34	3.55	5.88	0.5259
BOFFET	550	m	2	2.33	7.94	4.00	0.0000
Subtotal BOFFET				1.72	11.49	9.87	
DAMBER	560	m	1	1.50	7.87	0.11	0.0000
DAMBER	561	m	1	1.61	9.62	0.00	0.0000
Subtotal DAMBER				1.56	17.49	0.11	

N	4
NS	2

Table 1J18 - 3

IESLC - Meta-analysis of Ex Smoking by Years quit (vs never), Overview
All LC types, Pipes only
Most adjusted

	Sex			
	combined	male	female	Total
N		4		4
NS		2		2

In this overview table, other than the "N" rows, entries in the "absent" and "Total" columns may be invalid and should be ignored

MALES

	Years quit vs never (lower focus)				
	absent	8+k12	4-11k7	1-6k3	Total
N	3	1			4
NS	2	1			2
Wt	21.12	7.87			28.99
Het Chi	9.84	0.00			9.99
Het df	2	0			3
Het P	**	N.S.			*
Fixed RR	5.30	4.50			5.07
RRl	3.46	2.24			3.52
RRu	8.11	9.05			7.29
P	+++	+++			+++
Random RR	4.46	4.50			4.58
RRl	1.67	2.24			2.32
RRu	11.96	9.05			9.05
P	++	+++			+++

	Years quit vs never (higher focus)				
	absent	13+k20	4-19k12	1-11k3	Total
N	2	1		1	4
NS	2	1		1	4
Wt	15.81	3.55		9.62	28.99
Het Chi	2.71	0.00		0.00	9.99
Het df	1	0		0	3
Het P	(*)	N.S.		N.S.	*
Fixed RR	6.82	1.40		5.00	5.07
RRl	4.17	0.49		2.66	3.52
RRu	11.16	3.96		9.41	7.29
P	+++	N.S.		+++	+++
Random RR	6.81	1.40		5.00	4.58
RRl	3.03	0.49		2.66	2.32
RRu	15.34	3.96		9.41	9.05
P	+++	N.S.		+++	+++

Table 1J18 - 4

IESLC - Meta-analysis of Ex Smoking by Years quit (vs never), Overview
All LC types, Pipes only
Least adjusted

REF	NRR	X	SEX	AGEL	AGEH	RACE	YF	LC TYPE	LOC	START	ST	NLC	R	VB	P	H	AD	exL	exH	S1	S2	DENOM	De
BOFFET	549		m	0	0	all	-	all	Eu:mul	1988	CC	5621	n	bl	y	n	2	15	999	0	1	nev any	or
BOFFET	550		m	0	0	all	-	all	Eu:mul	1988	CC	5621	n	bl	y	n	2	0.1	14	0	0	nev any	or
DAMBER	560		m	0	0	all	-	all	Eu:Sca	1972	CC	579	n	bl	y	n	1	11	999	1	0	nev any	ot
DAMBER	561		m	0	0	all	-	all	Eu:Sca	1972	CC	579	n	bl	y	n	1	0.1	10	0	3	nev any	ot

In this overview table, subtotals and Qs values may be invalid and should be ignored

Table 1J18 - 5

IESLC - Meta-analysis of Ex Smoking by Years quit (vs never), Overview
All LC types, Pipes only
Least adjusted

REF	NRR	SEX	AD	Number Exposed Case	Cont	Non-exposed Case	Cont	RR	95.00%CI
BOFFET	549	m	2	-	-	117	-	1.40	(0.50- 4.00)
BOFFET	550	m	2	-	-	117	-	10.30	(5.10- 20.50)
Subtotal BOFFET								5.56	(3.12- 9.91)
DAMBER	560	m	1	-	-	42	-	4.50	(2.30- 9.30)
DAMBER	561	m	1	-	-	42	-	5.00	(2.60- 9.20)
Subtotal DAMBER								4.77	(2.98- 7.62)
Partial Totals				0	0	318	0		

*prospective study

REF	NRR	SEX	AD	Ys	Ws	Qs	Ps
BOFFET	549	m	2	0.34	3.55	5.88	0.5259
BOFFET	550	m	2	2.33	7.94	4.00	0.0000
Subtotal BOFFET				1.72	11.49	9.87	
DAMBER	560	m	1	1.50	7.87	0.11	0.0000
DAMBER	561	m	1	1.61	9.62	0.00	0.0000
Subtotal DAMBER				1.56	17.49	0.11	

N	4
NS	2

Table 1J18 - 6

IESLC - Meta-analysis of Ex Smoking by Years quit (vs never), Overview
All LC types, Pipes only
Least adjusted

	Sex			
	combined	male	female	Total
N		4		4
NS		2		2

In this overview table, other than the "N" rows, entries in the "absent" and "Total" columns may be invalid and should be ignored

MALES

	Years quit vs never (lower focus)				
	absent	8+k12	4-11k7	1-6k3	Total
N	3	1			4
NS	2	1			2
Wt	21.12	7.87			28.99
Het Chi	9.84	0.00			9.99
Het df	2	0			3
Het P	**	N.S.			*
Fixed RR	5.30	4.50			5.07
RRl	3.46	2.24			3.52
RRu	8.11	9.05			7.29
P	+++	+++			+++
Random RR	4.46	4.50			4.58
RRl	1.67	2.24			2.32
RRu	11.96	9.05			9.05
P	++	+++			+++

	Years quit vs never (higher focus)				
	absent	13+k20	4-19k12	1-11k3	Total
N	2	1		1	4
NS	2	1		1	4
Wt	15.81	3.55		9.62	28.99
Het Chi	2.71	0.00		0.00	9.99
Het df	1	0		0	3
Het P	(*)	N.S.		N.S.	*
Fixed RR	6.82	1.40		5.00	5.07
RRl	4.17	0.49		2.66	3.52
RRu	11.16	3.96		9.41	7.29
P	+++	N.S.		+++	+++
Random RR	6.81	1.40		5.00	4.58
RRl	3.03	0.49		2.66	2.32
RRu	15.34	3.96		9.41	9.05
P	+++	N.S.		+++	+++

Table 1J18 - 7

IESLC - Meta-analysis of Ex Smoking by Years quit (vs never), Overview
All LC types, Pipes only
Excluded studies (and stage at which they were excluded)

1	AGUDO AKIBA AMANDU AMES AXELSS BEST BOUCHA BOUCOT BRESLO CHEN CHEN2 CHIAZZ DEAN2 DOSEME ENGELA FAN GENG GER GUO HAENSZ HEGMAN HOLE HU HU2 JUSSAW KATSOU KAUFMA KOO KOULUM KREUZE LETOUR LEVIN LIAW LIU3 LIU4 LIU5 MCCONN MIGRAN MRFITR NOTAN2 OSANN2 PERNU QIAO2 RACHTA RESTRE SADOWS SEGI2 STASZE TIZZAN VUTUC WATSON WU WUWILL WYNDE2 WYNDE8 XU YUAN ZHANG ZHENG ZHOU
2	BUFFLE HUMBLE PISANI PRESCO WYNDE7
3	MCDUFF SPITZ
4	HAMMON LUO WU2
5	BLOT1 CORREA GILLIS QIAO WIGLE
7	ALDERS ARMADA AUVINE BARBON BECHER BENHAM BENSHL BROSS BROWN3 CARPEN CEDERL CHOI CHYOU CPSI CPSII DARBY DEAN3 DESTEF DOLL DOLL2 DORGAN DORN GAO GAO2 GARCIA GARSHI GRAHAM GURSEL HAMMO2 HIRAYA JAHN JAIN JEDRYC JOLY KAISE2 KHUDER LAUSSM LUBIN LUBIN2 MATOS PEZZO2 PEZZOT SOBUE SPEIZE SUZUK2 SVENSS TVERDA WAKAI WANG2 WYNDE3 WYNDE6

Table 1J18 - 8

Potentially overlapping studies

REF	REFGP	PRINC	.	OVERLAP/LINK
BOFFET	BOFFET	1		BOFFET-combined

Table 1J19 -

IESLC - Meta-analysis of Ex Smoking, Years quit (vs never), "Highest vs lowest"
All LC types, Pipes only

This analysis is restricted to results for:
1) Ex smokers
2) Results by Years quit (vs never)
3) Categorical results by Years quit (vs never)
4) Denominator (unexposed) = "low"
5) All LC types (or near equivalent)
6) Results complete enough for use in metaanalysis
7) (not applicable)
8) PRODUCT: pipes only

Within each study, results are then selected (in the following order of preference, within each sex) for:
9) Results with least adjustment for other aspects of smoking (ADOS)
10) The highest vs lowest category
11) Followup period (YF, prospective studies): whole study (coded as 0) or longest available
12) LCtype: all or nearest available, at least Squamous and Adeno. (q = squamous, s = small,
l = large, a = adeno, mix = mixed, alv = alveolar)
13) Race: all or nearest available, otherwise by race (wh or w = white, bl or b = black, hi = hispanic
ch = chinese, jap = japanese, haw = hawaiian, w+o = white + oriental, sca = scandinavian, as = asian)
14) For overlapping studies: principal rather than subsidiary studies
Finally by Age: whole study (coded as 0) if available, otherwise by widest available age group
and then for single sex results (m, f) in preference to results for both sexes combined (c).

Results adjusted (AD) for the most potential confounders are then chosen in Sections -1 to -3
and results adjusted for the least confounders in Sections -4 to -6. (Those least adjusted results which
actually differ from the most adjusted are marked 'x' in column X in Section -4)

Section -7 shows excluded studies, together with the stage (as above) at which no qualifying
results were found.

Section -8 lists the potentially overlapping studies which have been included (1=principal, 2=subsidiary).

Section -9 lists any results which would have been included in preference except that they had data not complete
enough for use in meta-analysis, with their significance (yes/no), if known, and any further comment as entered
on the database. It also lists as "gap" any categories for which no data were presented by the original authors.
This is commonly due to recent quitters having been combined with current smokers

In addition to those mentioned above, the following fields, levels and abbreviations are used:
* or nk = not known, n = no, y = yes, ot = other
all/unspec = all or unspecified, cig+/-ot = cigarettes irrespective of other products (cigar, pipe etc)
MC = manufactured cigarettes, HR = hand-rolled cigarettes
exL, exH = range of exposure (low and high) in the "highest" group, in terms of Years quit (vs never)
unexL, unexH = range of exposure (low and high) in the "lowest" group, in terms of Years quit (vs never)
REF: 6-character study reference
NRR: number of the RR on the database within the study
ST : study type (CC = case control, pr or prosp = prospective)
NLC: number of lung cancer cases in whole study
R : risky occupational population (n = no, m = mining, o = other risky)
VB : national cigarette type (V = at least 75% Virginia, bl = at least 75% blended, ot = other)
P : any proxy use
H : full histological confirmation
De : derivation of RR/CI (or = original, st = standard method, ot = other method of estimation)

Table 1J19 - 1

IESLC - Meta-analysis of Ex Smoking, Years quit (vs never), "Highest vs lowest"
All LC types, Pipes only
Most adjusted

REF	NRR	SEX	AGEL	AGEH	RACE	YF	LC TYPE	LOC	START	ST	NLC	R	VB	P	H	AD	ADOS	exL	exH	unexL	unexH	De
BOFFET	551	m	0	0	all	-	all	Eu:mul	1988	CC	5621	n	bl	y	n	2	0	0.1	14	15	999	ot
DAMBER	562	m	0	0	all	-	all	Eu:Sca	1972	CC	579	n	bl	y	n	1	0	0.1	10	11	999	ot

Table 1J19 - 2

IESLC - Meta-analysis of Ex Smoking, Years quit (vs never), "Highest vs lowest"
All LC types, Pipes only
Most adjusted

REF	NRR	SEX	AD	Number Exposed Case	Cont	Non-exposed Case	Cont	RR	95.00%CI
BOFFET	551	m	2	-	-	-	-	7.36	(2.18- 24.85)
DAMBER	562	m	1	-	-	-	-	1.11	(0.50- 2.47)
Partial Totals				0	0	0	0		

*prospective study

REF	NRR	SEX	AD	Ys	Ws	Qs	Ps
BOFFET	551	m	2	2.00	2.59	4.54	0.0013
DAMBER	562	m	1	0.10	6.02	1.95	0.7979

	N	2
	NS	2
	Wt	8.62
Het	Chi	6.49
Het	df	1
Het	P	*
Fixed	RR	1.96
	RRl	1.01
	RRu	3.83
	P	+
Random	RR	2.70
	RRl	0.42
	RRu	17.16
	P	N.S.
Asymm	P	

Table 1J19 - 3

IESLC - Meta-analysis of Ex Smoking, Years quit (vs never), "Highest vs lowest"
All LC types, Pipes only
Most adjusted

	combined	Sex male	female	Total
N		2		2
NS		2		2
Wt		8.62		8.62
Het Chi		6.49		6.49
Het df		1		1
Het P		*		*
Fixed RR		1.96		1.96
RRl		1.01		1.01
RRu		3.83		3.83
P		+		+
Random RR		2.70		2.70
RRl		0.42		0.42
RRu		17.16		17.16
P		N.S.		N.S.
Between Chi				
Between df				
Between P				N.S.
Btwn(F) P				N.S.
Btwn(R) P				N.S.

MALES

Too few RRs for analysis by factor

Table 1J19 - 4

IESLC - Meta-analysis of Ex Smoking, Years quit (vs never), "Highest vs lowest"
All LC types, Pipes only
Least adjusted

REF	NRR	X	SEX	AGEL	AGEH	RACE	YF	LC TYPE	LOC	START	ST	NLC	R	VB	P	H	AD	ADOS	exL	exH	unexL	unexH	De
BOFFET	551		m	0	0	all	-	all	Eu:mul	1988	CC	5621	n	bl	y	n	2	0	0.1	14	15	999	ot
DAMBER	562		m	0	0	all	-	all	Eu:Sca	1972	CC	579	n	bl	y	n	1	0	0.1	10	11	999	ot

Table 1J19 - 5

IESLC - Meta-analysis of Ex Smoking, Years quit (vs never), "Highest vs lowest"
All LC types, Pipes only
Least adjusted

REF	NRR	SEX	AD	Number Exposed Case	Number Exposed Cont	Non-exposed Case	Non-exposed Cont	RR	95.00%CI
BOFFET	551	m	2	-	-	-	-	7.36	(2.18- 24.85)
DAMBER	562	m	1	-	-	-	-	1.11	(0.50- 2.47)
Partial Totals				0	0	0	0		

*prospective study

REF	NRR	SEX	AD	Ys	Ws	Qs	Ps
BOFFET	551	m	2	2.00	2.59	4.54	0.0013
DAMBER	562	m	1	0.10	6.02	1.95	0.7979

	N	2
	NS	2
	Wt	8.62
Het	Chi	6.49
Het	df	1
Het	P	*
Fixed	RR	1.96
	RRl	1.01
	RRu	3.83
	P	+
Random	RR	2.70
	RRl	0.42
	RRu	17.16
	P	N.S.
Asymm	P	

Table 1J19 - 6

IESLC - Meta-analysis of Ex Smoking, Years quit (vs never), "Highest vs lowest"
All LC types, Pipes only
Least adjusted

	Sex combined	male	female	Total
N		2		2
NS		2		2
Wt		8.62		8.62
Het Chi		6.49		6.49
Het df		1		1
Het P		*		*
Fixed RR		1.96		1.96
RRl		1.01		1.01
RRu		3.83		3.83
P		+		+
Random RR		2.70		2.70
RRl		0.42		0.42
RRu		17.16		17.16
P		N.S.		N.S.
Between Chi				
Between df				
Between P				N.S.
Btwn(F) P				N.S.
Btwn(R) P				N.S.

Table 1J19 - 7

IESLC - Meta-analysis of Ex Smoking, Years quit (vs never), "Highest vs lowest"
All LC types, Pipes only
Excluded studies (and stage at which they were excluded)

1	AGUDO AKIBA AMANDU AMES AXELSS BEST BOUCHA BOUCOT BRESLO CHEN CHEN2 CHIAZZ DEAN2 DOSEME ENGELA FAN GENG GER GUO HAENSZ HEGMAN HOLE HU HU2 JUSSAW KATSOU KAUFMA KOO KOULUM KREUZE LETOUR LEVIN LIAW LIU3 LIU4 LIU5 MCCONN MIGRAN MRFITR NOTAN2 OSANN2 PERNU QIAO2 RACHTA RESTRE SADOWS SEGI2 STASZE TIZZAN VUTUC WATSON WU WUWILL WYNDE2 WYNDE8 XU YUAN ZHANG ZHENG ZHOU
2	BUFFLE HUMBLE PISANI PRESCO WYNDE7
3	MCDUFF SPITZ
4	AUVINE BLOT1 BROWN3 GURSEL LAUSSM LUO WU2
5	HAMMON
6	CORREA GILLIS QIAO WIGLE
8	ALDERS ARMADA BARBON BECHER BENHAM BENSHL BROSS CARPEN CEDERL CHOI CHYOU CPSI CPSII DARBY DEAN3 DESTEF DOLL DOLL2 DORGAN DORN GAO GAO2 GARCIA GARSHI GRAHAM HAMMO2 HIRAYA JAHN JAIN JEDRYC JOLY KAISE2 KHUDER LUBIN LUBIN2 MATOS PEZZO2 PEZZOT SOBUE SPEIZE SUZUK2 SVENSS TVERDA WAKAI WANG2 WYNDE3 WYNDE6

Table 1J19 - 8
Potentially overlapping studies

REF	REFGP	PRINC	.	OVERLAP/LINK
BOFFET	BOFFET	1		BOFFET-combined

Table 1J19 - 9
Most adjusted - insufficient data for meta-analysis

REF	NRR	SEX	AGEL	AGEH	RACE	YF	LC TYPE	LOC	START	ST	NLC	R	VB	P	H	AD	ADOS	exL	exH	unexL	unexH	De
LUBIN2	1068	m	0	0	all	-	all	Eu:mul	1976	CC	7804	n	bl	n	y	2	1#0.1	4	5	999	st	

Comments on values in listings

LUBIN2 ADOS Duration of pipe smoking

REF	NRR	RR	SIG	RRDATA comment
LUBIN2	1068	2.24		0

Table 1J20 -

IESLC - Meta-analysis of Ex Smoking by Years quit (vs never), Overview
All LC types, Cigars only

This analysis is restricted to results for:
1) Ex smokers
2) Results by Years quit (vs never)
3) Categorical results by Years quit (vs never)
Results by Years quit (vs never) are grouped under 2 schemes (S1, S2). Each scheme has a set of "key values". An interval is allocated to the category whose key value it includes, and intervals which include none or more than one of the key values are excluded. (Open-ended intervals are coded as 999)

S1	key value	maximum range
1	12	8+
2	7	4-11
3	3	1-6

S2	key value	maximum range
1	20	13+
2	12	4-19
3	3	1-11

4) All LC types (or near equivalent)
5) Results complete enough for use in metaanalysis
6) cigars only

Within each study, results are then selected (in the following order of preference, within each sex) for:
7) (not applicable)
8) DENOM: never smoked anything, never any + low
9) Followup period (YF, prospective studies): whole study (coded as 0) or longest available
10) LCtype: all or nearest available, at least Squamous and Adeno. (q = squamous, s = small, l = large, a = adeno, mix = mixed, alv = alveolar)
11) Race: all or nearest available, otherwise by race (wh or w = white, bl or b = black, hi = hispanic ch = chinese, jap = japanese, haw = hawaiian, w+o = white + oriental, sca = scandinavian, as = asian)
12) For overlapping studies: principal rather than subsidiary studies
Finally by Age: whole study (coded as 0) if available, otherwise by widest available age group and then for single sex results (m, f) in preference to results for both sexes combined (c).

Results adjusted (AD) for the most potential confounders are then chosen in Sections -1 to -3 and results adjusted for the least confounders in Sections -4 to -6. (Those least adjusted results which actually differ from the most adjusted are marked 'x' in column X in Section -4)

Section -7 shows excluded studies, together with the stage (as above) at which no qualifying results were found.

Section -8 lists the potentially overlapping studies which have been included (1=principal, 2=subsidiary).

Section -9 lists any results which would have been included in preference except that they had data not complete enough for use in meta-analysis, with their significance (yes/no), if known, and any further comment as entered on the database. It also lists as "gap" any categories for which no data were presented by the original authors. This is commonly due to recent quitters having been combined with current smokers

In addition to those mentioned above, the following fields, levels and abbreviations are used:
* or nk = not known, n = no, y = yes, ot = other
nev = never
all/unspec = all or unspecified, MC = manufactured cigarettes, HR = hand-rolled cigarettes
exL, exH = range of exposure (low and high) in the smoking group, in terms of Years quit (vs never)
REF: 6-character study reference
NRR: number of the RR on the database within the study
ST : study type (CC = case control, pr or prosp = prospective)
NLC: number of lung cancer cases in whole study
R : risky occupational population (n = no, m = mining, o = other risky)
VB : national cigarette type (V = at least 75% Virginia, bl = at least 75% blended, ot = other)
P : any proxy use
H : full histological confirmation
De : derivation of RR/CI (or = original, st = standard method, ot = other method of estimation)

Table 1J20 - 1

IESLC - Meta-analysis of Ex Smoking by Years quit (vs never), Overview
All LC types, Cigars only
Most adjusted

REF	NRR	SEX	AGEL	AGEH	RACE	YF	LC TYPE	LOC	START	ST	NLC	R	VB	P	H	AD	exL	exH	S1	S2	DENOM	De
BOFFET	555	m	0	0	all	-	all	Eu:mul	1988	CC	5621	n	bl	y	n	2	15	999	0	1	nev any	or
BOFFET	556	m	0	0	all	-	all	Eu:mul	1988	CC	5621	n	bl	y	n	2	0.1	14	0	0	nev any	or
JOLY	684	m	0	0	all	-	all	SCAmer	1978	CC	826	n	bl	n	n	0	5	999	0	0	nev any	st
JOLY	685	m	0	0	all	-	all	SCAmer	1978	CC	826	n	bl	n	n	0	1.0	4	3	3	nev any	st

In this overview table, subtotals and Qs values may be invalid and should be ignored

Table 1J20 - 2

IESLC - Meta-analysis of Ex Smoking by Years quit (vs never), Overview
All LC types, Cigars only
Most adjusted

REF	NRR	SEX	AD	Number Exposed Case	Cont	Non-exposed Case	Cont	RR	95.00%CI
BOFFET	555	m	2	-	-	117	-	6.90	(3.10- 15.10)
BOFFET	556	m	2	-	-	117	-	8.80	(4.00- 19.50)
Subtotal BOFFET								7.79	(4.45- 13.64)
JOLY	684	m	0	5	25	12	218	3.63	(1.18- 11.16)
JOLY	685	m	0	2	13	12	218	2.79	(0.57- 13.82)
Subtotal JOLY								3.33	(1.33- 8.35)
Partial Totals				7	38	258	436		

*prospective study

REF	NRR	SEX	AD	Ys	Ws	Qs	Ps
BOFFET	555	m	2	1.93	6.13	0.07	0.0000
BOFFET	556	m	2	2.17	6.12	0.76	0.0000
Subtotal BOFFET				2.05	12.25	0.83	
JOLY	684	m	0	1.29	3.05	0.87	0.0243
JOLY	685	m	0	1.03	1.50	0.95	0.2075
Subtotal JOLY				1.20	4.55	1.82	

N	4
NS	2

Table 1J20 - 3

IESLC - Meta-analysis of Ex Smoking by Years quit (vs never), Overview
All LC types, Cigars only
Most adjusted

	combined	Sex male	female	Total
N		4		4
NS		2		2

In this overview table, other than the "N" rows, entries in the "absent" and "Total" columns may be invalid and should be ignored

MALES

	Years quit vs never (lower focus) absent	8+k12	4-11k7	1-6k3	Total
N	3			1	4
NS	2			1	2
Wt	15.30			1.50	16.81
Het Chi	1.60			0.00	2.65
Het df	2			0	3
Het P	N.S.			N.S.	N.S.
Fixed RR	6.69			2.79	6.19
RRl	4.06			0.57	3.84
RRu	11.05			13.82	9.98
P	+++			N.S.	+++
Random RR	6.69			2.79	6.19
RRl	4.06			0.57	3.84
RRu	11.05			13.82	9.98
P	+++			N.S.	+++

	Years quit vs never (higher focus) absent	13+k20	4-19k12	1-11k3	Total
N	2	1		1	4
NS	2	1		1	4
Wt	9.17	6.13		1.50	16.81
Het Chi	1.59	0.00		0.00	2.65
Het df	1	0		0	3
Het P	N.S.	N.S.		N.S.	N.S.
Fixed RR	6.56	6.90		2.79	6.19
RRl	3.43	3.13		0.57	3.84
RRu	12.53	15.23		13.82	9.98
P	+++	+++		N.S.	+++
Random RR	6.21	6.90		2.79	6.19
RRl	2.66	3.13		0.57	3.84
RRu	14.48	15.23		13.82	9.98
P	+++	+++		N.S.	+++

Table 1J20 - 4

IESLC - Meta-analysis of Ex Smoking by Years quit (vs never), Overview
All LC types, Cigars only
Least adjusted

REF	NRR	X	SEX	AGEL	AGEH	RACE	YF	LC TYPE	LOC	START	ST	NLC	R	VB	P	H	AD	exL	exH	S1	S2	DENOM	De
BOFFET	555		m	0	0	all	-	all	Eu:mul	1988	CC	5621	n	bl	y	n	2	15	999	0	1	nev any	or
BOFFET	556		m	0	0	all	-	all	Eu:mul	1988	CC	5621	n	bl	y	n	2	0.1	14	0	0	nev any	or
JOLY	684		m	0	0	all	-	all	SCAmer	1978	CC	826	n	bl	n	n	0	5	999	0	0	nev any	st
JOLY	685		m	0	0	all	-	all	SCAmer	1978	CC	826	n	bl	n	n	0	1.0	4	3	3	nev any	st

In this overview table, subtotals and Qs values may be invalid and should be ignored

Table 1J20 - 5

IESLC - Meta-analysis of Ex Smoking by Years quit (vs never), Overview
All LC types, Cigars only
Least adjusted

REF	NRR	SEX	AD	Number Exposed Case	Cont	Non-exposed Case	Cont	RR	95.00%CI
BOFFET	555	m	2	-	-	117	-	6.90	(3.10- 15.10)
BOFFET	556	m	2	-	-	117	-	8.80	(4.00- 19.50)
Subtotal BOFFET								7.79	(4.45- 13.64)
JOLY	684	m	0	5	25	12	218	3.63	(1.18- 11.16)
JOLY	685	m	0	2	13	12	218	2.79	(0.57- 13.82)
Subtotal JOLY								3.33	(1.33- 8.35)
Partial Totals				7	38	258	436		

*prospective study

REF	NRR	SEX	AD	Ys	Ws	Qs	Ps
BOFFET	555	m	2	1.93	6.13	0.07	0.0000
BOFFET	556	m	2	2.17	6.12	0.76	0.0000
Subtotal BOFFET				2.05	12.25	0.83	
JOLY	684	m	0	1.29	3.05	0.87	0.0243
JOLY	685	m	0	1.03	1.50	0.95	0.2075
Subtotal JOLY				1.20	4.55	1.82	

N	4
NS	2

Table 1J20 - 6

IESLC - Meta-analysis of Ex Smoking by Years quit (vs never), Overview
All LC types, Cigars only
Least adjusted

	Sex combined	male	female	Total
N		4		4
NS		2		2

In this overview table, other than the "N" rows, entries in the "absent" and "Total" columns may be invalid and should be ignored

MALES

Years quit vs never (lower focus)	absent	8+k12	4-11k7	1-6k3	Total
N	3			1	4
NS	2			1	2
Wt	15.30			1.50	16.81
Het Chi	1.60			0.00	2.65
Het df	2			0	3
Het P	N.S.			N.S.	N.S.
Fixed RR	6.69			2.79	6.19
RRl	4.06			0.57	3.84
RRu	11.05			13.82	9.98
P	+++			N.S.	+++
Random RR	6.69			2.79	6.19
RRl	4.06			0.57	3.84
RRu	11.05			13.82	9.98
P	+++			N.S.	+++

Years quit vs never (higher focus)	absent	13+k20	4-19k12	1-11k3	Total
N	2	1		1	4
NS	2	1		1	4
Wt	9.17	6.13		1.50	16.81
Het Chi	1.59	0.00		0.00	2.65
Het df	1	0		0	3
Het P	N.S.	N.S.		N.S.	N.S.
Fixed RR	6.56	6.90		2.79	6.19
RRl	3.43	3.13		0.57	3.84
RRu	12.53	15.23		13.82	9.98
P	+++	+++		N.S.	+++
Random RR	6.21	6.90		2.79	6.19
RRl	2.66	3.13		0.57	3.84
RRu	14.48	15.23		13.82	9.98
P	+++	+++		N.S.	+++

Table 1J20 - 7

IESLC - Meta-analysis of Ex Smoking by Years quit (vs never), Overview
All LC types, Cigars only
Excluded studies (and stage at which they were excluded)

1 AGUDO AKIBA AMANDU AMES AXELSS BEST BOUCHA BOUCOT BRESLO CHEN CHEN2 CHIAZZ DEAN2 DOSEME ENGELA FAN
GENG GER GUO HAENSZ HEGMAN HOLE HU HU2 JUSSAW KATSOU KAUFMA KOO KOULUM KREUZE LETOUR LEVIN
LIAW LIU3 LIU4 LIU5 MCCONN MIGRAN MRFITR NOTAN2 OSANN2 PERNU QIAO2 RACHTA RESTRE SADOWS SEGI2 STASZE
TIZZAN VUTUC WATSON WU WUWILL WYNDE2 WYNDE8 XU YUAN ZHANG ZHENG ZHOU

2 BUFFLE HUMBLE PISANI PRESCO WYNDE7

3 MCDUFF SPITZ

4 HAMMON LUO WU2

5 BLOT1 CORREA GILLIS QIAO WIGLE

7 ALDERS ARMADA AUVINE BARBON BECHER BENHAM BENSHL BROSS BROWN3 CARPEN CEDERL CHOI CHYOU CPSI CPSII DAMBER
DARBY DEAN3 DESTEF DOLL DOLL2 DORGAN DORN GAO GAO2 GARCIA GARSHI GRAHAM GURSEL HAMMO2 HIRAYA JAHN
JAIN JEDRYC KAISE2 KHUDER LAUSSM LUBIN LUBIN2 MATOS PEZZO2 PEZZOT SOBUE SPEIZE SUZUK2 SVENSS TVERDA WAKAI
WANG2 WYNDE3 WYNDE6

Table 1J20 - 8

Potentially overlapping studies

REF	REFGP	PRINC	.	OVERLAP/LINK
BOFFET	BOFFET	1		BOFFET-combined

Table 1J20 - 9

Most adjusted - insufficient data for meta-analysis

REF	NRR	SEX	AGEL	AGEH	RACE	YF	LC TYPE	LOC	START	ST	NLC	R	VB	P	H	AD	exL	exH	S1	S2	DENOM	De
JOLY	686	m	0	0	all	-	all	SCAmer	1978	CC	826	n	bl	n	n	0	0.1	0.9	0	0	nev any	ot

REF	NRR	RR	SIG	RRDATA comment
JOLY	686	*	gap	0

Table 1J21 -

IESLC - Meta-analysis of Ex Smoking, Years quit (vs never), "Highest vs lowest"
All LC types, Cigars only

This analysis is restricted to results for:
1) Ex smokers
2) Results by Years quit (vs never)
3) Categorical results by Years quit (vs never)
4) Denominator (unexposed) = "low"
5) All LC types (or near equivalent)
6) Results complete enough for use in metaanalysis
7) (not applicable)
8) PRODUCT: cigars only

Within each study, results are then selected (in the following order of preference, within each sex) for:
9) Results with least adjustment for other aspects of smoking (ADOS)
10) The highest vs lowest category
11) Followup period (YF, prospective studies): whole study (coded as 0) or longest available
12) LCtype: all or nearest available, at least Squamous and Adeno. (q = squamous, s = small,
l = large, a = adeno, mix = mixed, alv = alveolar)
13) Race: all or nearest available, otherwise by race (wh or w = white, bl or b = black, hi = hispanic
ch = chinese, jap = japanese, haw = hawaiian, w+o = white + oriental, sca = scandinavian, as = asian)
14) For overlapping studies: principal rather than subsidiary studies
Finally by Age: whole study (coded as 0) if available, otherwise by widest available age group
and then for single sex results (m, f) in preference to results for both sexes combined (c).

Results adjusted (AD) for the most potential confounders are then chosen in Sections -1 to -3
and results adjusted for the least confounders in Sections -4 to -6. (Those least adjusted results which
actually differ from the most adjusted are marked 'x' in column X in Section -4)

Section -7 shows excluded studies, together with the stage (as above) at which no qualifying
results were found.

Section -8 lists the potentially overlapping studies which have been included (1=principal, 2=subsidiary).

Section -9 lists any results which would have been included in preference except that they had data not complete
enough for use in meta-analysis, with their significance (yes/no), if known, and any further comment as entered
on the database. It also lists as "gap" any categories for which no data were presented by the original authors.
This is commonly due to recent quitters having been combined with current smokers

In addition to those mentioned above, the following fields, levels and abbreviations are used:
* or nk = not known, n = no, y = yes, ot = other
all/unspec = all or unspecified, MC = manufactured cigarettes, HR = hand-rolled cigarettes
exL, exH = range of exposure (low and high) in the "highest" group, in terms of Years quit (vs never)
unexL, unexH = range of exposure (low and high) in the "lowest" group, in terms of Years quit (vs never)
REF: 6-character study reference
NRR: number of the RR on the database within the study
ST : study type (CC = case control, pr or prosp = prospective)
NLC: number of lung cancer cases in whole study
R : risky occupational population (n = no, m = mining, o = other risky)
VB : national cigarette type (V = at least 75% Virginia, bl = at least 75% blended, ot = other)
P : any proxy use
H : full histological confirmation
De : derivation of RR/CI (or = original, st = standard method, ot = other method of estimation)

Table 1J21 - 1

IESLC - Meta-analysis of Ex Smoking, Years quit (vs never), "Highest vs lowest"
All LC types, Cigars only
Most adjusted

REF	NRR	SEX	AGEL	AGEH	RACE	YF	LC TYPE	LOC	START	ST	NLC	R	VB	P	H	AD	ADOS	exL	exH	unexL	unexH	De
BOFFET	557	m	0	0	all	-	all	Eu:mul	1988	CC	5621	n	bl	y	n	2	0	0.1	14	15	999	ot
JOLY	687	m	0	0	all	-	all	SCAmer	1978	CC	826	n	bl	n	n	0	0	1.0	4	5	999	st

Table 1J21 - 2

IESLC - Meta-analysis of Ex Smoking, Years quit (vs never), "Highest vs lowest"
All LC types, Cigars only
Most adjusted

REF	NRR	SEX	AD	Number Exposed Case	Cont	Non-exposed Case	Cont	RR	95.00%CI
BOFFET	557	m	2	-	-	-	-	1.28	(0.43- 3.79)
JOLY	687	m	0	2	13	5	25	0.77	(0.13- 4.52)
Partial Totals				2	13	5	25		

*prospective study

REF	NRR	SEX	AD	Ys	Ws	Qs	Ps
BOFFET	557	m	2	0.25	3.24	0.06	0.6566
JOLY	687	m	0	-0.26	1.22	0.17	0.7716

	N	2
	NS	2
	Wt	4.47
Het	Chi	0.23
Het	df	1
Het	P	N.S.
Fixed	RR	1.11
	RRl	0.44
	RRu	2.81
	P	N.S.
Random	RR	1.11
	RRl	0.44
	RRu	2.81
	P	N.S.
Asymm	P	

Table 1J21 - 3

IESLC - Meta-analysis of Ex Smoking, Years quit (vs never), "Highest vs lowest"
All LC types, Cigars only
Most adjusted

	Sex combined	male	female	Total
N		2		2
NS		2		2
Wt		4.47		4.47
Het Chi		0.23		0.23
Het df		1		1
Het P		N.S.		N.S.
Fixed RR		1.11		1.11
RRl		0.44		0.44
RRu		2.81		2.81
P		N.S.		N.S.
Random RR		1.11		1.11
RRl		0.44		0.44
RRu		2.81		2.81
P		N.S.		N.S.
Between Chi				
Between df				
Between P				N.S.
Btwn(F) P				N.S.
Btwn(R) P				N.S.

MALES

Too few RRs for analysis by factor

Table 1J21 - 4

IESLC - Meta-analysis of Ex Smoking, Years quit (vs never), "Highest vs lowest"
All LC types, Cigars only
Least adjusted

REF	NRR	X	SEX	AGEL	AGEH	RACE	YF	LC TYPE	LOC	START	ST	NLC	R	VB	P	H	AD	ADOS	exL	exH	unexL	unexH	De
BOFFET	557		m	0	0	all	-	all	Eu:mul	1988	CC	5621	n	bl	y	n	2	0	0.1	14	15	999	ot
JOLY	687		m	0	0	all	-	all	SCAmer	1978	CC	826	n	bl	n	n	0	0	1.0	4	5	999	st

Table 1J21 - 5

IESLC - Meta-analysis of Ex Smoking, Years quit (vs never), "Highest vs lowest"
All LC types, Cigars only
Least adjusted

REF	NRR	SEX	AD	Number Exposed Case	Cont	Non-exposed Case	Cont	RR	95.00%CI
BOFFET	557	m	2	-	-	-	-	1.28	(0.43- 3.79)
JOLY	687	m	0	2	13	5	25	0.77	(0.13- 4.52)
Partial Totals				2	13	5	25		

*prospective study

REF	NRR	SEX	AD	Ys	Ws	Qs	Ps
BOFFET	557	m	2	0.25	3.24	0.06	0.6566
JOLY	687	m	0	-0.26	1.22	0.17	0.7716

	N	2
	NS	2
	Wt	4.47
Het	Chi	0.23
Het	df	1
Het	P	N.S.
Fixed	RR	1.11
	RRl	0.44
	RRu	2.81
	P	N.S.
Random	RR	1.11
	RRl	0.44
	RRu	2.81
	P	N.S.
Asymm	P	

Table 1J21 - 6

IESLC - Meta-analysis of Ex Smoking, Years quit (vs never), "Highest vs lowest"
All LC types, Cigars only
Least adjusted

	Sex combined	male	female	Total
N		2		2
NS		2		2
Wt		4.47		4.47
Het Chi		0.23		0.23
Het df		1		1
Het P		N.S.		N.S.
Fixed RR		1.11		1.11
RRl		0.44		0.44
RRu		2.81		2.81
P		N.S.		N.S.
Random RR		1.11		1.11
RRl		0.44		0.44
RRu		2.81		2.81
P		N.S.		N.S.
Between Chi				
Between df				
Between P				N.S.
Btwn(F) P				N.S.
Btwn(R) P				N.S.

Table 1J21 - 7

IESLC - Meta-analysis of Ex Smoking, Years quit (vs never), "Highest vs lowest"
All LC types, Cigars only
Excluded studies (and stage at which they were excluded)

1	AGUDO AKIBA AMANDU AMES AXELSS BEST BOUCHA BOUCOT BRESLO CHEN CHEN2 CHIAZZ DEAN2 DOSEME ENGELA FAN GENG GER GUO HAENSZ HEGMAN HOLE HU HU2 JUSSAW KATSOU KAUFMA KOO KOULUM KREUZE LETOUR LEVIN LIAW LIU3 LIU4 LIU5 MCCONN MIGRAN MRFITR NOTAN2 OSANN2 PERNU QIAO2 RACHTA RESTRE SADOWS SEGI2 STASZE TIZZAN VUTUC WATSON WU WUWILL WYNDE2 WYNDE8 XU YUAN ZHANG ZHENG ZHOU
2	BUFFLE HUMBLE PISANI PRESCO WYNDE7
3	MCDUFF SPITZ
4	AUVINE BLOT1 BROWN3 GURSEL LAUSSM LUO WU2
5	HAMMON
6	CORREA GILLIS QIAO WIGLE
8	ALDERS ARMADA BARBON BECHER BENHAM BENSHL BROSS CARPEN CEDERL CHOI CHYOU CPSI CPSII DAMBER DARBY DEAN3 DESTEF DOLL DOLL2 DORGAN DORN GAO GAO2 GARCIA GARSHI GRAHAM HAMMO2 HIRAYA JAHN JAIN JEDRYC KAISE2 KHUDER LUBIN LUBIN2 MATOS PEZZO2 PEZZOT SOBUE SPEIZE SUZUK2 SVENSS TVERDA WAKAI WANG2 WYNDE3 WYNDE6

Table 1J21 - 8
Potentially overlapping studies

REF	REFGP	PRINC	.	OVERLAP/LINK
BOFFET	BOFFET	1	BOFFET-combined

Table 1J21 - 9
Most adjusted - insufficient data for meta-analysis

REF	NRR	SEX	AGEL	AGEH	RACE	YF	LC TYPE	LOC	START	ST	NLC	R	VB	P	H	AD	ADOS	exL	exH	unexL	unexH	De
JOLY	688	m	0	0	all	-	all	SCAmer	1978	CC	826	n	bl	n	n	0	0	0.1	0.9	5	999	ot
LUBIN2	1064	m	0	0	all	-	all	Eu:mul	1976	CC	7804	n	bl	n	y	2	1#	0.1	4	5	999	st

Comments on values in listings

LUBIN2 ADOS Duration of cigar smoking

REF	NRR	RR	SIG	RRDATA comment
JOLY	688	*	gap	0
LUBIN2	1064	0.84		0

Table 1J22 -

IESLC - Meta-analysis of Ex Smoking by Years quit (vs never), Overview
All LC types, Mixed smokers

This analysis is restricted to results for:
1) Ex smokers
2) Results by Years quit (vs never)
3) Categorical results by Years quit (vs never)
Results by Years quit (vs never) are grouped under 2 schemes (S1, S2). Each scheme has a set of "key values". An interval is allocated to the category whose key value it includes, and intervals which include none or more than one of the key values are excluded. (Open-ended intervals are coded as 999)

S1	key value	maximum range
1	12	8+
2	7	4-11
3	3	1-6

S2	key value	maximum range
1	20	13+
2	12	4-19
3	3	1-11

4) All LC types (or near equivalent)
5) Results complete enough for use in metaanalysis
6) mixed smokers (cigarettes and pipe/cigar)

Within each study, results are then selected (in the following order of preference, within each sex) for:
7) (not applicable)
8) DENOM: never smoked anything, never any + low
9) Followup period (YF, prospective studies): whole study (coded as 0) or longest available
10) LCtype: all or nearest available, at least Squamous and Adeno. (q = squamous, s = small, l = large, a = adeno, mix = mixed, alv = alveolar)
11) Race: all or nearest available, otherwise by race (wh or w = white, bl or b = black, hi = hispanic ch = chinese, jap = japanese, haw = hawaiian, w+o = white + oriental, sca = scandinavian, as = asian)
12) For overlapping studies: principal rather than subsidiary studies
Finally by Age: whole study (coded as 0) if available, otherwise by widest available age group and then for single sex results (m, f) in preference to results for both sexes combined (c).

Results adjusted (AD) for the most potential confounders are then chosen in Sections -1 to -3 and results adjusted for the least confounders in Sections -4 to -6. (Those least adjusted results which actually differ from the most adjusted are marked 'x' in column X in Section -4)

Section -7 shows excluded studies, together with the stage (as above) at which no qualifying results were found.

Section -8 lists the potentially overlapping studies which have been included (1=principal, 2=subsidiary).

Section -9 lists any results which would have been included in preference except that they had data not complete enough for use in meta-analysis, with their significance (yes/no), if known, and any further comment as entered on the database. It also lists as "gap" any categories for which no data were presented by the original authors. This is commonly due to recent quitters having been combined with current smokers

In addition to those mentioned above, the following fields, levels and abbreviations are used:
* or nk = not known, n = no, y = yes, ot = other
nev = never
exL, exH = range of exposure (low and high) in the smoking group, in terms of Years quit (vs never)
REF: 6-character study reference
NRR: number of the RR on the database within the study
ST : study type (CC = case control, pr or prosp = prospective)
NLC: number of lung cancer cases in whole study
R : risky occupational population (n = no, m = mining, o = other risky)
VB : national cigarette type (V = at least 75% Virginia, bl = at least 75% blended, ot = other)
P : any proxy use
H : full histological confirmation
De : derivation of RR/CI (or = original, st = standard method, ot = other method of estimation)

Table 1J22 - 1

IESLC - Meta-analysis of Ex Smoking by Years quit (vs never), Overview
All LC types, Mixed smokers
Most adjusted

REF	NRR	SEX	AGEL	AGEH	RACE	YF	LC TYPE	LOC	START	ST	NLC	R	VB	P	H	AD	exL	exH	S1	S2	DENOM	De
GRAHAM	515	m	0	0	wh	-	all	NAmer	1956	CC	685	n	bl	n	n	0	5	999	0	0	nev any	st
GRAHAM	516	m	0	0	wh	-	all	NAmer	1956	CC	685	n	bl	n	n	0	1.1	5	3	3	nev any	st
GRAHAM	517	m	0	0	wh	-	all	NAmer	1956	CC	685	n	bl	n	n	0	0.1	1.0	0	0	nev any	st

In this overview table, subtotals and Qs values may be invalid and should be ignored

Table 1J22 - 2

IESLC - Meta-analysis of Ex Smoking by Years quit (vs never), Overview
All LC types, Mixed smokers
Most adjusted

				Number Exposed		Non-exposed			
REF	NRR	SEX	AD	Case	Cont	Case	Cont	RR	95.00%CI
GRAHAM	515	m	0	6	28	18	346	4.12	(1.51- 11.21)
GRAHAM	516	m	0	12	9	18	346	25.63	(9.57- 68.67)
GRAHAM	517	m	0	29	11	18	346	50.68	(21.87- 117.43)
Subtotal GRAHAM								19.97	(11.65- 34.23)
Totals				47	48	54	1038		

*prospective study

REF	NRR	SEX	AD	Ys	Ws	Qs	Ps
GRAHAM	515	m	0	1.42	3.83	9.55	0.0056
GRAHAM	516	m	0	3.24	3.95	0.25	0.0000
GRAHAM	517	m	0	3.93	5.44	4.72	0.0000
Subtotal GRAHAM				2.99	13.23	14.52	

N	3
NS	1

Table 1J22 - 3

IESLC - Meta-analysis of Ex Smoking by Years quit (vs never), Overview
All LC types, Mixed smokers
Most adjusted

	Sex: combined	male	female	Total
N		3		3
NS		1		1

In this overview table, other than the "N" rows, entries in the "absent" and "Total" columns may be invalid and should be ignored

MALES

Years quit vs never (lower focus)	absent	8+k12	4-11k7	1-6k3	Total
N	2			1	3
NS	1			1	1
Wt	9.27			3.95	13.23
Het Chi	14.17			0.00	14.52
Het df	1			0	2
Het P	***			N.S.	***
Fixed RR	17.95			25.63	19.97
RRl	9.43			9.57	11.65
RRu	34.17			68.67	34.23
P	+++			+++	+++
Random RR	14.67			25.63	17.79
RRl	1.25			9.57	4.12
RRu	171.62			68.67	76.82
P	+			+++	+++

Years quit vs never (higher focus)	absent	13+k20	4-19k12	1-11k3	Total
N	2			1	3
NS	1			1	1
Wt	9.27			3.95	13.23
Het Chi	14.17			0.00	14.52
Het df	1			0	2
Het P	***			N.S.	***
Fixed RR	17.95			25.63	19.97
RRl	9.43			9.57	11.65
RRu	34.17			68.67	34.23
P	+++			+++	+++
Random RR	14.67			25.63	17.79
RRl	1.25			9.57	4.12
RRu	171.62			68.67	76.82
P	+			+++	+++

Table 1J22 - 4

IESLC - Meta-analysis of Ex Smoking by Years quit (vs never), Overview
All LC types, Mixed smokers
Least adjusted

REF	NRR	X	SEX	AGEL	AGEH	RACE	YF	LC TYPE	LOC	START	ST	NLC	R	VB	P	H	AD	exL	exH	S1	S2	DENOM	De
GRAHAM	515		m	0	0	wh	-	all	NAmer	1956	CC	685	n	bl	n	n	0	5	999	0	0	nev any	st
GRAHAM	516		m	0	0	wh	-	all	NAmer	1956	CC	685	n	bl	n	n	0	1.1	5	3	3	nev any	st
GRAHAM	517		m	0	0	wh	-	all	NAmer	1956	CC	685	n	bl	n	n	0	0.1	1.0	0	0	nev any	st

In this overview table, subtotals and Qs values may be invalid and should be ignored

Table 1J22 - 5

IESLC - Meta-analysis of Ex Smoking by Years quit (vs never), Overview
All LC types, Mixed smokers
Least adjusted

REF	NRR	SEX	AD	Number Exposed Case	Cont	Non-exposed Case	Cont	RR	95.00%CI
GRAHAM	515	m	0	6	28	18	346	4.12	(1.51- 11.21)
GRAHAM	516	m	0	12	9	18	346	25.63	(9.57- 68.67)
GRAHAM	517	m	0	29	11	18	346	50.68	(21.87- 117.43)
Subtotal GRAHAM								19.97	(11.65- 34.23)
Totals				47	48	54	1038		

*prospective study

REF	NRR	SEX	AD	Ys	Ws	Qs	Ps
GRAHAM	515	m	0	1.42	3.83	9.55	0.0056
GRAHAM	516	m	0	3.24	3.95	0.25	0.0000
GRAHAM	517	m	0	3.93	5.44	4.72	0.0000
Subtotal GRAHAM				2.99	13.23	14.52	

N	3
NS	1

Table 1J22 - 6

IESLC - Meta-analysis of Ex Smoking by Years quit (vs never), Overview
All LC types, Mixed smokers
Least adjusted

	Sex combined	male	female	Total
N		3		3
NS		1		1

In this overview table, other than the "N" rows, entries in the "absent" and "Total" columns may be invalid and should be ignored

MALES

Years quit vs never (lower focus)	absent	8+k12	4-11k7	1-6k3	Total
N	2			1	3
NS	1			1	1
Wt	9.27			3.95	13.23
Het Chi	14.17			0.00	14.52
Het df	1			0	2
Het P	***			N.S.	***
Fixed RR	17.95			25.63	19.97
RRl	9.43			9.57	11.65
RRu	34.17			68.67	34.23
P	+++			+++	+++
Random RR	14.67			25.63	17.79
RRl	1.25			9.57	4.12
RRu	171.62			68.67	76.82
P	+			+++	+++

Years quit vs never (higher focus)	absent	13+k20	4-19k12	1-11k3	Total
N	2			1	3
NS	1			1	1
Wt	9.27			3.95	13.23
Het Chi	14.17			0.00	14.52
Het df	1			0	2
Het P	***			N.S.	***
Fixed RR	17.95			25.63	19.97
RRl	9.43			9.57	11.65
RRu	34.17			68.67	34.23
P	+++			+++	+++
Random RR	14.67			25.63	17.79
RRl	1.25			9.57	4.12
RRu	171.62			68.67	76.82
P	+			+++	+++

Table 1J22 - 7

IESLC - Meta-analysis of Ex Smoking by Years quit (vs never), Overview
All LC types, Mixed smokers
Excluded studies (and stage at which they were excluded)

1	AGUDO AKIBA AMANDU AMES AXELSS BEST BOUCHA BOUCOT BRESLO CHEN CHEN2 CHIAZZ DEAN2 DOSEME ENGELA FAN GENG GER GUO HAENSZ HEGMAN HOLE HU HU2 JUSSAW KATSOU KAUFMA KOO KOULUM KREUZE LETOUR LEVIN LIAW LIU3 LIU4 LIU5 MCCONN MIGRAN MRFITR NOTAN2 OSANN2 PERNU QIAO2 RACHTA RESTRE SADOWS SEGI2 STASZE TIZZAN VUTUC WATSON WU WUWILL WYNDE2 WYNDE8 XU YUAN ZHANG ZHENG ZHOU
2	BUFFLE HUMBLE PISANI PRESCO WYNDE7
3	MCDUFF SPITZ
4	HAMMON LUO WU2
5	BLOT1 CORREA GILLIS QIAO WIGLE
7	ALDERS ARMADA AUVINE BARBON BECHER BENHAM BENSHL BOFFET BROSS BROWN3 CARPEN CEDERL CHOI CHYOU CPSI CPSII DAMBER DARBY DEAN3 DESTEF DOLL DOLL2 DORGAN DORN GAO GAO2 GARCIA GARSHI GURSEL HAMMO2 HIRAYA JAHN JAIN JEDRYC JOLY KAISE2 KHUDER LAUSSM LUBIN LUBIN2 MATOS PEZZO2 PEZZOT SOBUE SPEIZE SUZUK2 SVENSS TVERDA WAKAI WANG2 WYNDE3 WYNDE6

Table 1J22 - 8

Potentially overlapping studies

REF	REFGP	PRINC	.	OVERLAP/LINK
GRAHAM	BYERS1		1	GRAHAM/BROSS/BYERS1

Table 1J23 -

IESLC - Meta-analysis of Ex Smoking, Years quit (vs never), "Highest vs lowest"
All LC types, Mixed smokers

This analysis is restricted to results for:
1) Ex smokers
2) Results by Years quit (vs never)
3) Categorical results by Years quit (vs never)
4) Denominator (unexposed) = "low"
5) All LC types (or near equivalent)
6) Results complete enough for use in metaanalysis
7) (not applicable)
8) PRODUCT: mixed smokers (cigarettes and pipe/cigar)

Within each study, results are then selected (in the following order of preference, within each sex) for:
9) Results with least adjustment for other aspects of smoking (ADOS)
10) The highest vs lowest category
11) Followup period (YF, prospective studies): whole study (coded as 0) or longest available
12) LCtype: all or nearest available, at least Squamous and Adeno. (q = squamous, s = small,
l = large, a = adeno, mix = mixed, alv = alveolar)
13) Race: all or nearest available, otherwise by race (wh or w = white, bl or b = black, hi = hispanic
ch = chinese, jap = japanese, haw = hawaiian, w+o = white + oriental, sca = scandinavian, as = asian)
14) For overlapping studies: principal rather than subsidiary studies
Finally by Age: whole study (coded as 0) if available, otherwise by widest available age group
and then for single sex results (m, f) in preference to results for both sexes combined (c).

Results adjusted (AD) for the most potential confounders are then chosen in Sections -1 to -3
and results adjusted for the least confounders in Sections -4 to -6. (Those least adjusted results which
actually differ from the most adjusted are marked 'x' in column X in Section -4)

Section -7 shows excluded studies, together with the stage (as above) at which no qualifying
results were found.

Section -8 lists the potentially overlapping studies which have been included (1=principal, 2=subsidiary).

Section -9 lists any results which would have been included in preference except that they had data not complete
enough for use in meta-analysis, with their significance (yes/no), if known, and any further comment as entered
on the database. It also lists as "gap" any categories for which no data were presented by the original authors.
This is commonly due to recent quitters having been combined with current smokers

In addition to those mentioned above, the following fields, levels and abbreviations are used:
* or nk = not known, n = no, y = yes, ot = other
exL, exH = range of exposure (low and high) in the "highest" group, in terms of Years quit (vs never)
unexL, unexH = range of exposure (low and high) in the "lowest" group, in terms of Years quit (vs never)
REF: 6-character study reference
NRR: number of the RR on the database within the study
ST : study type (CC = case control, pr or prosp = prospective)
NLC: number of lung cancer cases in whole study
R : risky occupational population (n = no, m = mining, o = other risky)
VB : national cigarette type (V = at least 75% Virginia, bl = at least 75% blended, ot = other)
P : any proxy use
H : full histological confirmation
De : derivation of RR/CI (or = original, st = standard method, ot = other method of estimation)

Table 1J23 - 1

IESLC - Meta-analysis of Ex Smoking, Years quit (vs never), "Highest vs lowest"
All LC types, Mixed smokers
Most adjusted

REF	NRR	SEX	AGEL	AGEH	RACE	YF	LC TYPE	LOC	START	ST	NLC	R	VB	P	H	AD	ADOS	exL	exH	unexL	unexH	De
GRAHAM	519	m	0	0	wh	-	all	NAmer	1956	CC	685	n	bl	n	n	0	0	0.1	1.0	5	999	st

Table 1J23 - 2

IESLC - Meta-analysis of Ex Smoking, Years quit (vs never), "Highest vs lowest"
All LC types, Mixed smokers
Most adjusted

REF	NRR	SEX	AD	Number Exposed Case	Cont	Non-exposed Case	Cont	RR	95.00%CI
GRAHAM	519	m	0	29	11	6	28	12.30	(4.01- 37.79)
Totals				29	11	6	28		

*prospective study

REF	NRR	SEX	AD	Ys	Ws	Qs	Ps
GRAHAM	519	m	0	2.51	3.05	0.00	0.0000

	N	1
	NS	1
	Wt	3.05
Het	Chi	0.00
Het	df	0
Het	P	N.S.
Fixed	RR	12.30
	RRl	4.01
	RRu	37.79
	P	+++
Random	RR	12.30
	RRl	4.01
	RRu	37.79
	P	+++
Asymm	P	

Table 1J23 - 3

IESLC - Meta-analysis of Ex Smoking, Years quit (vs never), "Highest vs lowest"
All LC types, Mixed smokers
Most adjusted

	Sex combined	male	female	Total
N		1		1
NS		1		1
Wt		3.05		3.05
Het Chi		0.00		0.00
Het df		0		0
Het P		N.S.		N.S.
Fixed RR		12.30		12.30
RRl		4.01		4.01
RRu		37.79		37.79
P		+++		+++
Random RR		12.30		12.30
RRl		4.01		4.01
RRu		37.79		37.79
P		+++		+++
Between Chi				
Between df				
Between P				N.S.
Btwn(F) P				N.S.
Btwn(R) P				N.S.

MALES

Too few RRs for analysis by factor

Table 1J23 - 4

IESLC - Meta-analysis of Ex Smoking, Years quit (vs never), "Highest vs lowest"
All LC types, Mixed smokers
Least adjusted

REF	NRR	X	SEX	AGEL	AGEH	RACE	YF	LC TYPE	LOC	START	ST	NLC	R	VB	P	H	AD	ADOS	exL	exH	unexL	unexH	De
GRAHAM	519		m	0	0	wh	-	all	NAmer	1956	CC	685	n	bl	n	n	0	0	0.1	1.0	5	999	st

Table 1J23 - 5

IESLC - Meta-analysis of Ex Smoking, Years quit (vs never), "Highest vs lowest"
All LC types, Mixed smokers
Least adjusted

REF	NRR	SEX	AD	Number Case	Exposed Cont	Non-exposed Case	Cont	RR	95.00%CI
GRAHAM	519	m	0	29	11	6	28	12.30	(4.01- 37.79)
Totals				29	11	6	28		

*prospective study

REF	NRR	SEX	AD	Ys	Ws	Qs	Ps
GRAHAM	519	m	0	2.51	3.05	0.00	0.0000

	N	1
	NS	1
	Wt	3.05
Het	Chi	0.00
Het	df	0
Het	P	N.S.
Fixed	RR	12.30
	RRl	4.01
	RRu	37.79
	P	+++
Random	RR	12.30
	RRl	4.01
	RRu	37.79
	P	+++
Asymm	P	

Table 1J23 - 6

IESLC - Meta-analysis of Ex Smoking, Years quit (vs never), "Highest vs lowest"
All LC types, Mixed smokers
Least adjusted

	combined	Sex: male	female	Total
N		1		1
NS		1		1
Wt		3.05		3.05
Het Chi		0.00		0.00
Het df		0		0
Het P		N.S.		N.S.
Fixed RR		12.30		12.30
RRl		4.01		4.01
RRu		37.79		37.79
P		+++		+++
Random RR		12.30		12.30
RRl		4.01		4.01
RRu		37.79		37.79
P		+++		+++
Between Chi				
Between df				
Between P				N.S.
Btwn(F) P				N.S.
Btwn(R) P				N.S.

Table 1J23 - 7

IESLC - Meta-analysis of Ex Smoking, Years quit (vs never), "Highest vs lowest"
All LC types, Mixed smokers
Excluded studies (and stage at which they were excluded)

1	AGUDO AKIBA AMANDU AMES AXELSS BEST BOUCHA BOUCOT BRESLO CHEN CHEN2 CHIAZZ DEAN2 DOSEME ENGELA FAN GENG GER GUO HAENSZ HEGMAN HOLE HU HU2 JUSSAW KATSOU KAUFMA KOO KOULUM KREUZE LETOUR LEVIN LIAW LIU3 LIU4 LIU5 MCCONN MIGRAN MRFITR NOTAN2 OSANN2 PERNU QIAO2 RACHTA RESTRE SADOWS SEGI2 STASZE TIZZAN VUTUC WATSON WU WUWILL WYNDE2 WYNDE8 XU YUAN ZHANG ZHENG ZHOU
2	BUFFLE HUMBLE PISANI PRESCO WYNDE7
3	MCDUFF SPITZ
4	AUVINE BLOT1 BROWN3 GURSEL LAUSSM LUO WU2
5	HAMMON
6	CORREA GILLIS QIAO WIGLE
8	ALDERS ARMADA BARBON BECHER BENHAM BENSHL BOFFET BROSS CARPEN CEDERL CHOI CHYOU CPSI CPSII DAMBER DARBY DEAN3 DESTEF DOLL DOLL2 DORGAN DORN GAO GAO2 GARCIA GARSHI HAMMO2 HIRAYA JAHN JAIN JEDRYC JOLY KAISE2 KHUDER LUBIN LUBIN2 MATOS PEZZO2 PEZZOT SOBUE SPEIZE SUZUK2 SVENSS TVERDA WAKAI WANG2 WYNDE3 WYNDE6

Table 1J23 - 8
Potentially overlapping studies

REF	REFGP	PRINC	.	OVERLAP/LINK
GRAHAM	BYERS1	1	GRAHAM/BROSS/BYERS1
