# Supplementary material for: Systematic review with meta-analysis of the epidemiological evidence in the 1900s relating smoking to lung cancer
Source: BMC Cancer. 2012 Sep 3;12:385. doi: 10.1186/1471-2407-12-385 (PMC3505152; doi:10.1186/1471-2407-12-385)
Supplement: Additional file 5 — Detailed Analysis Tables (Individual file names as described in Additional file 1: Methods, Table1). [file 1471-2407-12-385-S5.zip › PDF/2J.pdf]

Table 2J1 -

IESLC - Meta-analysis of Ex Smoking by Years quit (vs never), Overview
Squamous, Any Product (or Cigarettes if Any not available)

This analysis is restricted to results for:
1) Ex smokers
2) Results by Years quit (vs never)
3) Categorical results by Years quit (vs never)
Results by Years quit (vs never) are grouped under 2 schemes (S1, S2). Each scheme has a set of "key values". An interv is allocated to the category whose key value it includes, and intervals which include none or more than one of the key values are excluded. (Open-ended intervals are coded as 999)

| S1 | key value | maximum range |
|---|---|---|
| 1 | 12 | 8+ |
| 2 | 7 | 4-11 |
| 3 | 3 | 1-6 |

| S2 | key value | maximum range |
|---|---|---|
| 1 | 20 | 13+ |
| 2 | 12 | 4-19 |
| 3 | 3 | 1-11 |

4) Squamous (or near equivalent)
5) Results complete enough for use in metaanalysis

Within each study, results are then selected (in the following order of preference, within each sex) for:
6) (not applicable)
7) PRODUCT: all/unspec, cigarettes regardless of other products, cigarettes only
8) CIGTYPE: all/unspecified, MC regardless of HR, MC only
9) (not applicable)
10) DENOM: never smoked anything, never smoked cigarettes, never any + low, never cigs + low
11) Followup period (YF, prospective studies): whole study (coded as 0) or longest available
12) LCtype: squamous or nearest available, but not adeno. (q = squamous, s = small, a = adeno, KI = Kreyberg I, u = undifferentiated)
13) Race: all or nearest available, otherwise by race (wh or w = white, bl or b = black, hi = hispanic ch = chinese, jap = japanese, haw = hawaiian, w+o = white + oriental, sca = scandinavian, as = asian)
14) For overlapping studies: principal rather than subsidiary studies
Finally by Age: whole study (coded as 0) if available, otherwise by widest available age group and then for single sex results (m, f) in preference to results for both sexes combined (c).

Results adjusted (AD) for the most potential confounders are then chosen in Sections -1 to -3 and results adjusted for the least confounders in Sections -4 to -6. (Those least adjusted results which actually differ from the most adjusted are marked 'x' in column X in Section -4)

Section -7 shows excluded studies, together with the stage (as above) at which no qualifying results were found.

Section -8 lists the potentially overlapping studies which have been included (1=principal, 2=subsidiary).

Section -9 lists any results which would have been included in preference except that they had data not complete enough for use in meta-analysis, with their significance (yes/no), if known, and any further comment as entered on the database. It also lists as "gap" any categories for which no data were presented by the original authors. This is commonly due to recent quitters having been combined with current smokers

In addition to those mentioned above, the following fields, levels and abbreviations are used:
* or nk = not known, n = no, y = yes, ot = other
nev = never
all/unspec = all or unspecified, cig+/-ot = cigarettes irrespective of other products (cigar, pipe etc)
MC = manufactured cigarettes, HR = hand-rolled cigarettes
exL, exH = range of exposure (low and high) in the smoking group, in terms of Years quit (vs never)
REF: 6-character study reference
NRR: number of the RR on the database within the study
ST : study type (CC = case control, pr or prosp = prospective)
NLC: number of lung cancer cases in whole study
R : risky occupational population (n = no, m = mining, o = other risky)
VB : national cigarette type (V = at least 75% Virginia, bl = at least 75% blended, ot = other)
P : any proxy use
H : full histological confirmation
De : derivation of RR/CI (or = original, st = standard method, ot = other method of estimation)

Table 2J1 - 1

IESLC - Meta-analysis of Ex Smoking by Years quit (vs never), Overview
Squamous, Any Product (or Cigarettes if Any not available)
Most adjusted

| REF | NRR | SEX | AGEL | AGEH | RACE | YF | LC TYPE | LOC | START | ST | NLC | R | VB | P | H | AD | PRODUCT | exL | exH | S1 | S2 | DENOM | De |
|---|---|---|---|---|---|---|---|---|---|---|---|---|---|---|---|---|---|---|---|---|---|---|---|
| BARBON | 594 | m | 0 | 0 | all | - | q | Eu:wst | 1979 | CC | 755 | n | bl | y | y | 1 | all/unsp | 25 | 999 | 0 | 0 | nev any | or |
| BARBON | 595 | m | 0 | 0 | all | - | q | Eu:wst | 1979 | CC | 755 | n | bl | y | y | 1 | all/unsp | 15 | 24 | 0 | 1 | nev any | or |
| BARBON | 596 | m | 0 | 0 | all | - | q | Eu:wst | 1979 | CC | 755 | n | bl | y | y | 1 | all/unsp | 5 | 14 | 0 | 2 | nev any | or |
| BARBON | 597 | m | 0 | 0 | all | - | q | Eu:wst | 1979 | CC | 755 | n | bl | y | y | 1 | all/unsp | 0.1 | 4 | 3 | 3 | nev any | or |
| BROWN3 | 507 | f | 0 | 0 | wh | - | q | NAmer | | CC | 618 | | bl | y | n | 0 | all/unsp | 15 | 999 | 0 | 1 | nev any | st |
| JAHN | 593 | m | 0 | 0 | all | - | q | Eu:Ger | 1988 | CC | 1004 | n | bl | n | n | 0 | cig+/-ot | 21 | 999 | 0 | 0 | nev any | st |
| JAHN | 594 | m | 0 | 0 | all | - | q | Eu:Ger | 1988 | CC | 1004 | n | bl | n | n | 0 | cig+/-ot | 11 | 20 | 1 | 0 | nev any | st |
| JAHN | 595 | m | 0 | 0 | all | - | q | Eu:Ger | 1988 | CC | 1004 | n | bl | n | n | 0 | cig+/-ot | 6 | 10 | 2 | 0 | nev any | st |
| JAHN | 596 | m | 0 | 0 | all | - | q | Eu:Ger | 1988 | CC | 1004 | n | bl | n | n | 0 | cig+/-ot | 2 | 5 | 3 | 3 | nev any | st |
| JAHN | 597 | m | 0 | 0 | all | - | q | Eu:Ger | 1988 | CC | 1004 | n | bl | n | n | 0 | cig+/-ot | 1.0 | 1.9 | 0 | 0 | nev any | st |
| JAHN | 598 | m | 0 | 0 | all | - | q | Eu:Ger | 1988 | CC | 1004 | n | bl | n | n | 0 | cig+/-ot | 0.1 | 0.9 | 0 | 0 | nev any | st |
| JAIN | 543 | m | 0 | 0 | all | - | q | NAmer | 1981 | CC | 845 | n | V | y | n | 0 | cig+/-ot | 10 | 999 | 1 | 0 | nev cigs | st |
| JAIN | 544 | m | 0 | 0 | all | - | q | NAmer | 1981 | CC | 845 | n | V | y | n | 0 | cig+/-ot | 2 | 9 | 0 | 3 | nev cigs | st |
| JAIN | 507 | f | 0 | 0 | all | - | q | NAmer | 1981 | CC | 845 | n | V | y | n | 0 | cig+/-ot | 10 | 999 | 1 | 0 | nev cigs | st |
| JAIN | 508 | f | 0 | 0 | all | - | q | NAmer | 1981 | CC | 845 | n | V | y | n | 0 | cig+/-ot | 2 | 9 | 0 | 3 | nev cigs | st |
| JEDRYC | 543 | m | 0 | 0 | all | - | q | Eu:est | 1980 | CC | 1630 | n | bl | y | n | 0 | cig+/-ot | 10 | 999 | 1 | 0 | nev any | st |
| JEDRYC | 544 | m | 0 | 0 | all | - | q | Eu:est | 1980 | CC | 1630 | n | bl | y | n | 0 | cig+/-ot | 5 | 9 | 2 | 0 | nev any | st |
| LUBIN2 | 765 | m | 0 | 0 | all | - | q | Eu:mul | 1976 | CC | 7804 | n | bl | n | y | 0 | cig+/-ot | 20 | 999 | 0 | 1 | nev any | st |
| LUBIN2 | 766 | m | 0 | 0 | all | - | q | Eu:mul | 1976 | CC | 7804 | n | bl | n | y | 0 | cig+/-ot | 15 | 19 | 0 | 0 | nev any | st |
| LUBIN2 | 767 | m | 0 | 0 | all | - | q | Eu:mul | 1976 | CC | 7804 | n | bl | n | y | 0 | cig+/-ot | 10 | 14 | 1 | 2 | nev any | st |
| LUBIN2 | 768 | m | 0 | 0 | all | - | q | Eu:mul | 1976 | CC | 7804 | n | bl | n | y | 0 | cig+/-ot | 5 | 9 | 2 | 0 | nev any | st |
| LUBIN2 | 769 | m | 0 | 0 | all | - | q | Eu:mul | 1976 | CC | 7804 | n | bl | n | y | 0 | cig+/-ot | 0.1 | 4 | 3 | 3 | nev any | st |
| LUBIN2 | 893 | f | 0 | 0 | all | - | q | Eu:mul | 1976 | CC | 7804 | n | bl | n | y | 0 | cig+/-ot | 20 | 999 | 0 | 1 | nev any | st |
| LUBIN2 | 894 | f | 0 | 0 | all | - | q | Eu:mul | 1976 | CC | 7804 | n | bl | n | y | 0 | cig+/-ot | 10 | 19 | 1 | 2 | nev any | st |
| LUBIN2 | 895 | f | 0 | 0 | all | - | q | Eu:mul | 1976 | CC | 7804 | n | bl | n | y | 0 | cig+/-ot | 0.1 | 9 | 0 | 3 | nev any | st |
| LUO | 522 | c | 0 | 0 | all | - | q | As:Chi | 1990 | CC | 102 | n | ot | n | y | 20 | cig+/-ot | 10 | 999 | 1 | 0 | nev cigs | or |
| MATOS | 631 | m | 0 | 0 | all | - | q | SCAmer | 1994 | CC | 200 | n | bl | n | n | 2 | cig+/-ot | 11 | 999 | 1 | 0 | nev any | ot |
| MATOS | 632 | m | 0 | 0 | all | - | q | SCAmer | 1994 | CC | 200 | n | bl | n | n | 2 | cig+/-ot | 6 | 10 | 2 | 0 | nev any | ot |
| MATOS | 633 | m | 0 | 0 | all | - | q | SCAmer | 1994 | CC | 200 | n | bl | n | n | 2 | cig+/-ot | 1.0 | 5 | 3 | 3 | nev any | ot |
| PEZZOT | 579 | m | 0 | 0 | all | - | q | SCAmer | 1987 | CC | 215 | n | bl | n | y | 0 | cig only | 11 | 999 | 1 | 0 | nev cigs | ot |
| PEZZOT | 580 | m | 0 | 0 | all | - | q | SCAmer | 1987 | CC | 215 | n | bl | n | y | 0 | cig only | 1.0 | 10 | 0 | 3 | nev cigs | ot |
| SVENSS | 557 | f | 0 | 0 | all | - | q | Eu:Sca | 1983 | CC | 210 | n | bl | n | n | 0 | all/unsp | 11 | 999 | 1 | 0 | nev any | st |
| SVENSS | 558 | f | 0 | 0 | all | - | q | Eu:Sca | 1983 | CC | 210 | n | bl | n | n | 0 | all/unsp | 3 | 10 | 0 | 3 | nev any | st |
| WAKAI | 546 | m | 0 | 0 | all | - | q | As:Jap | 1988 | CC | 333 | n | bl | n | y | 1 | cig+/-ot | 20 | 999 | 0 | 1 | nev any | or |
| WAKAI | 547 | m | 0 | 0 | all | - | q | As:Jap | 1988 | CC | 333 | n | bl | n | y | 1 | cig+/-ot | 10 | 19 | 1 | 2 | nev any | or |
| WAKAI | 548 | m | 0 | 0 | all | - | q | As:Jap | 1988 | CC | 333 | n | bl | n | y | 1 | cig+/-ot | 5 | 9 | 2 | 0 | nev any | or |
| WYNDE3 | 501 | m | 0 | 0 | all | - | KI | NAmer | 1966 | CC | 350 | n | bl | n | y | 0 | all/unsp | 13 | 999 | 0 | 1 | nev any | st |
| WYNDE3 | 502 | m | 0 | 0 | all | - | KI | NAmer | 1966 | CC | 350 | n | bl | n | y | 0 | all/unsp | 7 | 12 | 0 | 2 | nev any | st |
| WYNDE3 | 503 | m | 0 | 0 | all | - | KI | NAmer | 1966 | CC | 350 | n | bl | n | y | 0 | all/unsp | 4 | 6 | 0 | 0 | nev any | st |
| WYNDE3 | 504 | m | 0 | 0 | all | - | KI | NAmer | 1966 | CC | 350 | n | bl | n | y | 0 | all/unsp | 1.0 | 3 | 3 | 3 | nev any | st |
| WYNDE3 | 573 | f | 0 | 0 | all | - | KI | NAmer | 1966 | CC | 350 | n | bl | n | y | 0 | cig+/-ot | 10 | 999 | 1 | 0 | nev any | st |
| WYNDE6 | 787 | m | 0 | 0 | all | - | KI | NAmer | 1969 | CC | 4423 | n | bl | n | y | 2 | cig+/-ot | 16 | 999 | 0 | 1 | nev any | ot |
| WYNDE6 | 788 | m | 0 | 0 | all | - | KI | NAmer | 1969 | CC | 4423 | n | bl | n | y | 2 | cig+/-ot | 11 | 15 | 1 | 2 | nev any | ot |
| WYNDE6 | 789 | m | 0 | 0 | all | - | KI | NAmer | 1969 | CC | 4423 | n | bl | n | y | 2 | cig+/-ot | 7 | 10 | 2 | 0 | nev any | ot |
| WYNDE6 | 790 | m | 0 | 0 | all | - | KI | NAmer | 1969 | CC | 4423 | n | bl | n | y | 2 | cig+/-ot | 4 | 6 | 0 | 0 | nev any | ot |
| WYNDE6 | 791 | m | 0 | 0 | all | - | KI | NAmer | 1969 | CC | 4423 | n | bl | n | y | 2 | cig+/-ot | 1.0 | 3 | 3 | 3 | nev any | ot |

Cigarette type is all/unspec for all RRs

In this overview table, subtotals and Qs values may be invalid and should be ignored

Table 2J1 - 2

IESLC - Meta-analysis of Ex Smoking by Years quit (vs never), Overview
Squamous, Any Product (or Cigarettes if Any not available)
Most adjusted

| REF | NRR | SEX | AD | Number Exposed Case | Cont | Non-exposed Case | Cont | RR | 95.00%CI |
|---|---|---|---|---|---|---|---|---|---|
| BARBON | 594 | m | 1 | 4 | - | 6 | - | 1.90 | ( 0.50- 7.20) |
| BARBON | 595 | m | 1 | 11 | - | 6 | - | 8.10 | ( 2.80- 23.20) |
| BARBON | 596 | m | 1 | 31 | - | 6 | - | 11.90 | ( 4.80- 29.80) |
| BARBON | 597 | m | 1 | 11 | - | 6 | - | 18.70 | ( 6.20- 56.30) |
| Subtotal BARBON | | | | | | | | 8.92 | ( 5.22- 15.24) |
| BROWN3 | 507 | f | 0 | 17 | 10 | 432 | 1168 | 4.60 | ( 2.09- 10.12) |
| JAHN | 593 | m | 0 | 8 | 146 | 3 | 138 | 2.52 | ( 0.66- 9.70) |
| JAHN | 594 | m | 0 | 18 | 130 | 3 | 138 | 6.37 | ( 1.83- 22.13) |
| JAHN | 595 | m | 0 | 29 | 63 | 3 | 138 | 21.17 | ( 6.22- 72.12) |
| JAHN | 596 | m | 0 | 36 | 46 | 3 | 138 | 36.00 | ( 10.58- 122.45) |
| JAHN | 597 | m | 0 | 25 | 9 | 3 | 138 | 127.78 | ( 32.33- 505.04) |
| JAHN | 598 | m | 0 | 74 | 8 | 3 | 138 | 425.50 | (109.58-1652.23) |
| Subtotal JAHN | | | | | | | | 27.87 | ( 16.45- 47.20) |
| JAIN | 543 | m | 0 | 23 | 113 | 2 | 85 | 8.65 | ( 1.98- 37.70) |
| JAIN | 544 | m | 0 | 24 | 46 | 2 | 85 | 22.17 | ( 5.02- 98.04) |
| JAIN | 507 | f | 0 | 7 | 61 | 6 | 214 | 4.09 | ( 1.33- 12.63) |
| JAIN | 508 | f | 0 | 15 | 36 | 6 | 214 | 14.86 | ( 5.41- 40.82) |
| Subtotal JAIN | | | | | | | | 9.92 | ( 5.39- 18.27) |
| JEDRYC | 543 | m | 0 | 23 | 230 | 6 | 289 | 4.82 | ( 1.93- 12.03) |
| JEDRYC | 544 | m | 0 | 22 | 82 | 6 | 289 | 12.92 | ( 5.07- 32.93) |
| Subtotal JEDRYC | | | | | | | | 7.80 | ( 4.06- 15.01) |
| LUBIN2 | 765 | m | 0 | 106 | 1128 | 54 | 2616 | 4.55 | ( 3.26- 6.36) |
| LUBIN2 | 766 | m | 0 | 67 | 478 | 54 | 2616 | 6.79 | ( 4.68- 9.84) |
| LUBIN2 | 767 | m | 0 | 146 | 693 | 54 | 2616 | 10.21 | ( 7.39- 14.10) |
| LUBIN2 | 768 | m | 0 | 265 | 882 | 54 | 2616 | 14.56 | ( 10.76- 19.70) |
| LUBIN2 | 769 | m | 0 | 498 | 1047 | 54 | 2616 | 23.04 | ( 17.25- 30.79) |
| LUBIN2 | 893 | f | 0 | 2 | 29 | 72 | 1180 | 1.13 | ( 0.26- 4.83) |
| LUBIN2 | 894 | f | 0 | 5 | 33 | 72 | 1180 | 2.48 | ( 0.94- 6.55) |
| LUBIN2 | 895 | f | 0 | 38 | 95 | 72 | 1180 | 6.56 | ( 4.20- 10.23) |
| Subtotal LUBIN2 | | | | | | | | 9.96 | ( 8.71- 11.40) |
| LUO | 522 | c | 20 | 1 | - | 5 | - | 2.00 | ( 0.20- 23.10) |
| MATOS | 631 | m | 2 | 5 | - | 3 | - | 2.00 | ( 0.41- 9.65) |
| MATOS | 632 | m | 2 | 5 | - | 3 | - | 6.00 | ( 1.19- 30.28) |
| MATOS | 633 | m | 2 | 4 | - | 3 | - | 7.00 | ( 1.32- 37.20) |
| Subtotal MATOS | | | | | | | | 4.28 | ( 1.68- 10.92) |
| PEZZOT | 579 | m | 0 | 8 | 106 | 0 | 116 | 18.60~ | ( 1.06- 326.10) |
| PEZZOT | 580 | m | 0 | 21 | 82 | 0 | 116 | 60.72~ | ( 3.63-1016.67) |
| Subtotal PEZZOT | | | | | | | | 33.93 | ( 4.55- 252.91) |
| SVENSS | 557 | f | 0 | 1 | 24 | 5 | 120 | 1.00 | ( 0.11- 8.95) |
| SVENSS | 558 | f | 0 | 5 | 13 | 5 | 120 | 9.23 | ( 2.36- 36.16) |
| Subtotal SVENSS | | | | | | | | 4.96 | ( 1.56- 15.80) |
| WAKAI | 546 | m | 1 | 3 | - | 2 | - | 2.05 | ( 0.33- 12.80) |
| WAKAI | 547 | m | 1 | 12 | - | 2 | - | 8.95 | ( 1.91- 42.00) |
| WAKAI | 548 | m | 1 | 11 | - | 2 | - | 7.47 | ( 1.58- 35.30) |
| Subtotal WAKAI | | | | | | | | 5.68 | ( 2.22- 14.53) |
| WYNDE3 | 501 | m | 0 | 2 | 55 | 3 | 88 | 1.07 | ( 0.17- 6.59) |
| WYNDE3 | 502 | m | 0 | 8 | 31 | 3 | 88 | 7.57 | ( 1.89- 30.35) |
| WYNDE3 | 503 | m | 0 | 8 | 17 | 3 | 88 | 13.80 | ( 3.32- 57.39) |
| WYNDE3 | 504 | m | 0 | 18 | 22 | 3 | 88 | 24.00 | ( 6.49- 88.81) |
| WYNDE3 | 573 | f | 0 | 1 | 3 | 5 | 76 | 5.07 | ( 0.44- 57.98) |
| Subtotal WYNDE3 | | | | | | | | 8.80 | ( 4.39- 17.65) |
| WYNDE6 | 787 | m | 2 | 19 | - | 8 | - | 5.00 | ( 2.18- 11.49) |
| WYNDE6 | 788 | m | 2 | 22 | - | 8 | - | 13.70 | ( 6.04- 31.10) |
| WYNDE6 | 789 | m | 2 | 36 | - | 8 | - | 17.20 | ( 7.92- 37.33) |
| WYNDE6 | 790 | m | 2 | 44 | - | 8 | - | 24.90 | ( 11.61- 53.39) |
| WYNDE6 | 791 | m | 2 | 80 | - | 8 | - | 53.80 | ( 25.75- 112.41) |
| Subtotal WYNDE6 | | | | | | | | 18.53 | ( 13.06- 26.30) |
| Partial Totals | | | | 1819 | 5718 | 1075 | 20692 | | |

*prospective study ~ With 0.5 adjustment for zero

Table 2J1 - 2

IESLC - Meta-analysis of Ex Smoking by Years quit (vs never), Overview
Squamous, Any Product (or Cigarettes if Any not available)
Most adjusted

| REF | NRR | SEX | AD | Ys | Ws | Qs | Ps |
|---|---|---|---|---|---|---|---|
| BARBON | 594 | m | 1 | 0.64 | 2.16 | 6.30 | 0.3455 |
| BARBON | 595 | m | 1 | 2.09 | 3.44 | 0.23 | 0.0001 |
| BARBON | 596 | m | 1 | 2.48 | 4.61 | 0.07 | 0.0000 |
| BARBON | 597 | m | 1 | 2.93 | 3.16 | 1.06 | 0.0000 |
| Subtotal BARBON | | | | 2.19 | 13.36 | 7.66 | |
| BROWN3 | 507 | f | 0 | 1.53 | 6.17 | 4.19 | 0.0002 |
| JAHN | 593 | m | 0 | 0.92 | 2.12 | 4.30 | 0.1786 |
| JAHN | 594 | m | 0 | 1.85 | 2.48 | 0.61 | 0.0036 |
| JAHN | 595 | m | 0 | 3.05 | 2.56 | 1.27 | 0.0000 |
| JAHN | 596 | m | 0 | 3.58 | 2.56 | 3.90 | 0.0000 |
| JAHN | 597 | m | 0 | 4.85 | 2.03 | 12.72 | 0.0000 |
| JAHN | 598 | m | 0 | 6.05 | 2.09 | 28.63 | 0.0000 |
| Subtotal JAHN | | | | 3.33 | 13.84 | 51.43 | |
| JAIN | 543 | m | 0 | 2.16 | 1.77 | 0.07 | 0.0041 |
| JAIN | 544 | m | 0 | 3.10 | 1.74 | 0.98 | 0.0000 |
| JAIN | 507 | f | 0 | 1.41 | 3.02 | 2.67 | 0.0142 |
| JAIN | 508 | f | 0 | 2.70 | 3.76 | 0.46 | 0.0000 |
| Subtotal JAIN | | | | 2.29 | 10.30 | 4.17 | |
| JEDRYC | 543 | m | 0 | 1.57 | 4.59 | 2.77 | 0.0008 |
| JEDRYC | 544 | m | 0 | 2.56 | 4.39 | 0.19 | 0.0000 |
| Subtotal JEDRYC | | | | 2.05 | 8.98 | 2.97 | |
| LUBIN2 | 765 | m | 0 | 1.52 | 34.22 | 23.80 | 0.0000 |
| LUBIN2 | 766 | m | 0 | 1.92 | 27.84 | 5.25 | 0.0000 |
| LUBIN2 | 767 | m | 0 | 2.32 | 36.77 | 0.03 | 0.0000 |
| LUBIN2 | 768 | m | 0 | 2.68 | 42.00 | 4.53 | 0.0000 |
| LUBIN2 | 769 | m | 0 | 3.14 | 45.74 | 28.39 | 0.0000 |
| LUBIN2 | 893 | f | 0 | 0.12 | 1.82 | 9.03 | 0.8688 |
| LUBIN2 | 894 | f | 0 | 0.91 | 4.08 | 8.46 | 0.0662 |
| LUBIN2 | 895 | f | 0 | 1.88 | 19.39 | 4.27 | 0.0000 |
| Subtotal LUBIN2 | | | | 2.30 | 211.87 | 83.75 | |
| LUO | 522 | c | 20 | 0.69 | 0.68 | 1.87 | 0.5673 |
| MATOS | 631 | m | 2 | 0.69 | 1.54 | 4.23 | 0.3897 |
| MATOS | 632 | m | 2 | 1.79 | 1.47 | 0.46 | 0.0300 |
| MATOS | 633 | m | 2 | 1.95 | 1.38 | 0.22 | 0.0223 |
| Subtotal MATOS | | | | 1.45 | 4.39 | 4.91 | |
| PEZZOT | 579 | m | 0 | 2.92 | 0.47 | 0.15 | 0.0455 |
| PEZZOT | 580 | m | 0 | 4.11 | 0.48 | 1.49 | 0.0043 |
| Subtotal PEZZOT | | | | 3.52 | 0.95 | 1.65 | |
| SVENSS | 557 | f | 0 | 0.00 | 0.80 | 4.42 | 1.0000 |
| SVENSS | 558 | f | 0 | 2.22 | 2.06 | 0.03 | 0.0014 |
| Subtotal SVENSS | | | | 1.60 | 2.86 | 4.45 | |
| WAKAI | 546 | m | 1 | 0.72 | 1.15 | 3.06 | 0.4418 |
| WAKAI | 547 | m | 1 | 2.19 | 1.61 | 0.04 | 0.0054 |
| WAKAI | 548 | m | 1 | 2.01 | 1.59 | 0.18 | 0.0112 |
| Subtotal WAKAI | | | | 1.74 | 4.35 | 3.28 | |
| WYNDE3 | 501 | m | 0 | 0.06 | 1.16 | 6.05 | 0.9446 |
| WYNDE3 | 502 | m | 0 | 2.02 | 1.99 | 0.21 | 0.0043 |
| WYNDE3 | 503 | m | 0 | 2.62 | 1.89 | 0.14 | 0.0003 |
| WYNDE3 | 504 | m | 0 | 3.18 | 2.24 | 1.54 | 0.0000 |
| WYNDE3 | 573 | f | 0 | 1.62 | 0.65 | 0.34 | 0.1919 |
| Subtotal WYNDE3 | | | | 2.17 | 7.93 | 8.29 | |
| WYNDE6 | 787 | m | 2 | 1.61 | 5.56 | 3.05 | 0.0001 |
| WYNDE6 | 788 | m | 2 | 2.62 | 5.72 | 0.41 | 0.0000 |
| WYNDE6 | 789 | m | 2 | 2.84 | 6.39 | 1.57 | 0.0000 |
| WYNDE6 | 790 | m | 2 | 3.21 | 6.60 | 4.94 | 0.0000 |
| WYNDE6 | 791 | m | 2 | 3.99 | 7.08 | 18.93 | 0.0000 |
| Subtotal WYNDE6 | | | | 2.92 | 31.35 | 28.90 | |

| | |
|---|---|
| N | 46 |
| NS | 13 |

Table 2J1 - 3

IESLC - Meta-analysis of Ex Smoking by Years quit (vs never), Overview
Squamous, Any Product (or Cigarettes if Any not available)
Most adjusted

| | Sex combined | male | female | Total |
|---|---|---|---|---|
| N | 1 | 36 | 9 | 46 |
| NS | 1 | 10 | 5 | 16 |

In this overview table, other than the "N" rows, entries in the "absent" and "Total" columns may be invalid and should be ignored

| Years quit vs never (lower focus) | absent | 8+k12 | 4-11k7 | 1-6k3 | Total |
|---|---|---|---|---|---|
| N | 21 | 13 | 6 | 6 | 46 |
| NS | 10 | 11 | 6 | 6 | 33 |
| Wt | 132.29 | 64.18 | 58.40 | 62.16 | 317.03 |
| Het Chi | 98.05 | 20.26 | 2.45 | 7.30 | 207.51 |
| Het df | 20 | 12 | 5 | 5 | 45 |
| Het P | *** | (*) | N.S. | N.S. | *** |
| Fixed RR | 7.03 | 7.76 | 14.34 | 24.95 | 10.48 |
| RRl | 5.93 | 6.07 | 11.10 | 19.45 | 9.39 |
| RRu | 8.33 | 9.91 | 18.54 | 31.99 | 11.70 |
| P | +++ | +++ | +++ | +++ | +++ |
| Random RR | 8.75 | 5.89 | 14.34 | 26.22 | 9.56 |
| RRl | 5.65 | 3.85 | 11.10 | 17.19 | 7.24 |
| RRu | 13.57 | 9.00 | 18.54 | 39.98 | 12.63 |
| P | +++ | +++ | +++ | +++ | +++ |

| Years quit vs never (higher focus) | absent | 13+k20 | 4-19k12 | 1-11k3 | Total |
|---|---|---|---|---|---|
| N | 22 | 7 | 6 | 11 | 46 |
| NS | 12 | 6 | 5 | 9 | 32 |
| Wt | 119.13 | 53.52 | 54.79 | 89.59 | 317.03 |
| Het Chi | 87.41 | 7.77 | 8.64 | 35.55 | 207.51 |
| Het df | 21 | 6 | 5 | 10 | 45 |
| Het P | *** | N.S. | N.S. | *** | *** |
| Fixed RR | 10.92 | 4.34 | 9.46 | 17.91 | 10.48 |
| RRl | 9.12 | 3.32 | 7.26 | 14.56 | 9.39 |
| RRu | 13.06 | 5.67 | 12.32 | 22.03 | 11.70 |
| P | +++ | +++ | +++ | +++ | +++ |
| Random RR | 9.83 | 4.12 | 8.65 | 18.57 | 9.56 |
| RRl | 6.29 | 2.82 | 5.47 | 11.16 | 7.24 |
| RRu | 15.37 | 6.03 | 13.68 | 30.93 | 12.63 |
| P | +++ | +++ | +++ | +++ | +++ |

Table 2J1 - 3

IESLC - Meta-analysis of Ex Smoking by Years quit (vs never), Overview
Squamous, Any Product (or Cigarettes if Any not available)
Most adjusted

MALES

| | Years quit vs never (lower focus) absent | 8+k12 | 4-11k7 | 1-6k3 | Total |
|---|---|---|---|---|---|
| N | 16 | 8 | 6 | 6 | 36 |
| NS | 8 | 8 | 6 | 6 | 28 |
| Wt | 99.08 | 54.95 | 58.40 | 62.16 | 274.59 |
| Het Chi | 88.34 | 7.39 | 2.45 | 7.30 | 168.61 |
| Het df | 15 | 7 | 5 | 5 | 35 |
| Het P | *** | N.S. | N.S. | N.S. | *** |
| Fixed RR | 7.31 | 9.21 | 14.34 | 24.95 | 11.66 |
| RRl | 6.00 | 7.07 | 11.10 | 19.45 | 10.36 |
| RRu | 8.90 | 11.99 | 18.54 | 31.99 | 13.13 |
| P | +++ | +++ | +++ | +++ | +++ |
| Random RR | 10.24 | 8.96 | 14.34 | 26.22 | 11.71 |
| RRl | 5.80 | 6.62 | 11.10 | 17.19 | 8.61 |
| RRu | 18.11 | 12.12 | 18.54 | 39.98 | 15.94 |
| P | +++ | +++ | +++ | +++ | +++ |

| | Years quit vs never (higher focus) absent | 13+k20 | 4-19k12 | 1-11k3 | Total |
|---|---|---|---|---|---|
| N | 18 | 5 | 5 | 8 | 36 |
| NS | 10 | 5 | 5 | 8 | 28 |
| Wt | 113.98 | 45.53 | 50.71 | 64.38 | 274.59 |
| Het Chi | 77.22 | 4.36 | 0.76 | 7.71 | 168.61 |
| Het df | 17 | 4 | 4 | 7 | 35 |
| Het P | *** | N.S. | N.S. | N.S. | *** |
| Fixed RR | 11.56 | 4.54 | 10.53 | 25.03 | 11.66 |
| RRl | 9.62 | 3.40 | 8.00 | 19.61 | 10.36 |
| RRu | 13.89 | 6.07 | 13.87 | 31.96 | 13.13 |
| P | +++ | +++ | +++ | +++ | +++ |
| Random RR | 11.80 | 4.52 | 10.53 | 25.85 | 11.71 |
| RRl | 7.36 | 3.18 | 8.00 | 18.91 | 8.61 |
| RRu | 18.93 | 6.42 | 13.87 | 35.34 | 15.94 |
| P | +++ | +++ | +++ | +++ | +++ |

FEMALES

| | Years quit vs never (lower focus) absent | 8+k12 | 4-11k7 | 1-6k3 | Total |
|---|---|---|---|---|---|
| N | 5 | 4 | | | 9 |
| NS | 4 | 4 | | | 8 |
| Wt | 33.20 | 8.55 | | | 41.76 |
| Het Chi | 9.09 | 1.56 | | | 14.76 |
| Het df | 4 | 3 | | | 8 |
| Het P | (*) | N.S. | | | (*) |
| Fixed RR | 6.25 | 2.87 | | | 5.33 |
| RRl | 4.44 | 1.47 | | | 3.93 |
| RRu | 8.78 | 5.61 | | | 7.21 |
| P | +++ | ++ | | | +++ |
| Random RR | 5.94 | 2.87 | | | 4.70 |
| RRl | 3.22 | 1.47 | | | 2.89 |
| RRu | 10.97 | 5.61 | | | 7.64 |
| P | +++ | ++ | | | +++ |

Table 2J1 - 3

IESLC - Meta-analysis of Ex Smoking by Years quit (vs never), Overview
Squamous, Any Product (or Cigarettes if Any not available)
Most adjusted

FEMALES

| | Years quit vs never (higher focus) absent | 13+k20 | 4-19k12 | 1-11k3 | Total |
|---|---|---|---|---|---|
| N | 3 | 2 | 1 | 3 | 9 |
| NS | 3 | 2 | 1 | 3 | 8 |
| Wt | 4.47 | 7.99 | 4.08 | 25.21 | 41.76 |
| Het Chi | 1.40 | 2.77 | 0.00 | 2.19 | 14.76 |
| Het df | 2 | 1 | 0 | 2 | 8 |
| Het P | N.S. | (*) | N.S. | N.S. | (*) |
| Fixed RR | 3.28 | 3.34 | 2.48 | 7.62 | 5.33 |
| RRl | 1.30 | 1.67 | 0.94 | 5.16 | 3.93 |
| RRu | 8.29 | 6.68 | 6.55 | 11.25 | 7.21 |
| P | + | +++ | (+) | +++ | +++ |
| Random RR | 3.28 | 2.62 | 2.48 | 7.87 | 4.70 |
| RRl | 1.30 | 0.68 | 0.94 | 5.04 | 2.89 |
| RRu | 8.29 | 10.07 | 6.55 | 12.28 | 7.64 |
| P | + | N.S. | (+) | +++ | +++ |

Table 2J1 - 4

IESLC - Meta-analysis of Ex Smoking by Years quit (vs never), Overview
Squamous, Any Product (or Cigarettes if Any not available)
Least adjusted

| REF | NRR | X | SEX | AGEL | AGEH | RACE | YF | LC TYPE | LOC | START | ST | NLC | R | VB | P | H | AD | PRODUCT | exL | exH | S1 | S2 | DENOM | De |
|---|---|---|---|---|---|---|---|---|---|---|---|---|---|---|---|---|---|---|---|---|---|---|---|---|
| BARBON | 579 | x | m | 0 | 0 | all | - | q | Eu:wst | 1979 | CC | 755 | n | bl | y | y | 0 | all/unsp | 25 | 999 | 0 | 0 | nev any | st |
| BARBON | 580 | x | m | 0 | 0 | all | - | q | Eu:wst | 1979 | CC | 755 | n | bl | y | y | 0 | all/unsp | 15 | 24 | 0 | 1 | nev any | st |
| BARBON | 581 | x | m | 0 | 0 | all | - | q | Eu:wst | 1979 | CC | 755 | n | bl | y | y | 0 | all/unsp | 5 | 14 | 0 | 2 | nev any | st |
| BARBON | 582 | x | m | 0 | 0 | all | - | q | Eu:wst | 1979 | CC | 755 | n | bl | y | y | 0 | all/unsp | 0.1 | 4 | 3 | 3 | nev any | st |
| BROWN3 | 507 | | f | 0 | 0 | wh | - | q | NAmer | | CC | 618 | | bl | y | n | 0 | all/unsp | 15 | 999 | 0 | 1 | nev any | st |
| JAHN | 593 | | m | 0 | 0 | all | - | q | Eu:Ger | 1988 | CC | 1004 | n | bl | n | n | 0 | cig+/-ot | 21 | 999 | 0 | 0 | nev any | st |
| JAHN | 594 | | m | 0 | 0 | all | - | q | Eu:Ger | 1988 | CC | 1004 | n | bl | n | n | 0 | cig+/-ot | 11 | 20 | 1 | 0 | nev any | st |
| JAHN | 595 | | m | 0 | 0 | all | - | q | Eu:Ger | 1988 | CC | 1004 | n | bl | n | n | 0 | cig+/-ot | 6 | 10 | 2 | 0 | nev any | st |
| JAHN | 596 | | m | 0 | 0 | all | - | q | Eu:Ger | 1988 | CC | 1004 | n | bl | n | n | 0 | cig+/-ot | 2 | 5 | 3 | 3 | nev any | st |
| JAHN | 597 | | m | 0 | 0 | all | - | q | Eu:Ger | 1988 | CC | 1004 | n | bl | n | n | 0 | cig+/-ot | 1.0 | 1.9 | 0 | 0 | nev any | st |
| JAHN | 598 | | m | 0 | 0 | all | - | q | Eu:Ger | 1988 | CC | 1004 | n | bl | n | n | 0 | cig+/-ot | 0.1 | 0.9 | 0 | 0 | nev any | st |
| JAIN | 543 | | m | 0 | 0 | all | - | q | NAmer | 1981 | CC | 845 | n | V | y | n | 0 | cig+/-ot | 10 | 999 | 1 | 0 | nev cigs | st |
| JAIN | 544 | | m | 0 | 0 | all | - | q | NAmer | 1981 | CC | 845 | n | V | y | n | 0 | cig+/-ot | 2 | 9 | 0 | 3 | nev cigs | st |
| JAIN | 507 | | f | 0 | 0 | all | - | q | NAmer | 1981 | CC | 845 | n | V | y | n | 0 | cig+/-ot | 10 | 999 | 1 | 0 | nev cigs | st |
| JAIN | 508 | | f | 0 | 0 | all | - | q | NAmer | 1981 | CC | 845 | n | V | y | n | 0 | cig+/-ot | 2 | 9 | 0 | 3 | nev cigs | st |
| JEDRYC | 543 | | m | 0 | 0 | all | - | q | Eu:est | 1980 | CC | 1630 | n | bl | y | n | 0 | cig+/-ot | 10 | 999 | 1 | 0 | nev any | st |
| JEDRYC | 544 | | m | 0 | 0 | all | - | q | Eu:est | 1980 | CC | 1630 | n | bl | y | n | 0 | cig+/-ot | 5 | 9 | 2 | 0 | nev any | st |
| LUBIN2 | 765 | | m | 0 | 0 | all | - | q | Eu:mul | 1976 | CC | 7804 | n | bl | n | y | 0 | cig+/-ot | 20 | 999 | 0 | 1 | nev any | st |
| LUBIN2 | 766 | | m | 0 | 0 | all | - | q | Eu:mul | 1976 | CC | 7804 | n | bl | n | y | 0 | cig+/-ot | 15 | 19 | 0 | 0 | nev any | st |
| LUBIN2 | 767 | | m | 0 | 0 | all | - | q | Eu:mul | 1976 | CC | 7804 | n | bl | n | y | 0 | cig+/-ot | 10 | 14 | 1 | 2 | nev any | st |
| LUBIN2 | 768 | | m | 0 | 0 | all | - | q | Eu:mul | 1976 | CC | 7804 | n | bl | n | y | 0 | cig+/-ot | 5 | 9 | 2 | 0 | nev any | st |
| LUBIN2 | 769 | | m | 0 | 0 | all | - | q | Eu:mul | 1976 | CC | 7804 | n | bl | n | y | 0 | cig+/-ot | 0.1 | 4 | 3 | 3 | nev any | st |
| LUBIN2 | 893 | | f | 0 | 0 | all | - | q | Eu:mul | 1976 | CC | 7804 | n | bl | n | y | 0 | cig+/-ot | 20 | 999 | 0 | 1 | nev any | st |
| LUBIN2 | 894 | | f | 0 | 0 | all | - | q | Eu:mul | 1976 | CC | 7804 | n | bl | n | y | 0 | cig+/-ot | 10 | 19 | 1 | 2 | nev any | st |
| LUBIN2 | 895 | | f | 0 | 0 | all | - | q | Eu:mul | 1976 | CC | 7804 | n | bl | n | y | 0 | cig+/-ot | 0.1 | 9 | 0 | 3 | nev any | st |
| LUO | 519 | x | c | 0 | 0 | all | - | q | As:Chi | 1990 | CC | 102 | n | ot | n | y | 0 | cig+/-ot | 10 | 999 | 1 | 0 | nev cigs | st |
| MATOS | 621 | x | m | 0 | 0 | all | - | q | SCAmer | 1994 | CC | 200 | n | bl | n | n | 0 | cig+/-ot | 11 | 999 | 1 | 0 | nev any | st |
| MATOS | 622 | x | m | 0 | 0 | all | - | q | SCAmer | 1994 | CC | 200 | n | bl | n | n | 0 | cig+/-ot | 6 | 10 | 2 | 0 | nev any | st |
| MATOS | 623 | x | m | 0 | 0 | all | - | q | SCAmer | 1994 | CC | 200 | n | bl | n | n | 0 | cig+/-ot | 1.0 | 5 | 3 | 3 | nev any | st |
| PEZZOT | 579 | | m | 0 | 0 | all | - | q | SCAmer | 1987 | CC | 215 | n | bl | n | y | 0 | cig only | 11 | 999 | 1 | 0 | nev cigs | ot |
| PEZZOT | 580 | | m | 0 | 0 | all | - | q | SCAmer | 1987 | CC | 215 | n | bl | n | y | 0 | cig only | 1.0 | 10 | 0 | 3 | nev cigs | ot |
| SVENSS | 557 | | f | 0 | 0 | all | - | q | Eu:Sca | 1983 | CC | 210 | n | bl | n | n | 0 | all/unsp | 11 | 999 | 1 | 0 | nev any | st |
| SVENSS | 558 | | f | 0 | 0 | all | - | q | Eu:Sca | 1983 | CC | 210 | n | bl | n | n | 0 | all/unsp | 3 | 10 | 0 | 3 | nev any | st |
| WAKAI | 538 | x | m | 0 | 0 | all | - | q | As:Jap | 1988 | CC | 333 | n | bl | n | y | 0 | cig+/-ot | 20 | 999 | 0 | 1 | nev any | st |
| WAKAI | 539 | x | m | 0 | 0 | all | - | q | As:Jap | 1988 | CC | 333 | n | bl | n | y | 0 | cig+/-ot | 10 | 19 | 1 | 2 | nev any | st |
| WAKAI | 540 | x | m | 0 | 0 | all | - | q | As:Jap | 1988 | CC | 333 | n | bl | n | y | 0 | cig+/-ot | 5 | 9 | 2 | 0 | nev any | st |
| WYNDE3 | 501 | | m | 0 | 0 | all | - | KI | NAmer | 1966 | CC | 350 | n | bl | n | y | 0 | all/unsp | 13 | 999 | 0 | 1 | nev any | st |
| WYNDE3 | 502 | | m | 0 | 0 | all | - | KI | NAmer | 1966 | CC | 350 | n | bl | n | y | 0 | all/unsp | 7 | 12 | 0 | 2 | nev any | st |
| WYNDE3 | 503 | | m | 0 | 0 | all | - | KI | NAmer | 1966 | CC | 350 | n | bl | n | y | 0 | all/unsp | 4 | 6 | 0 | 0 | nev any | st |
| WYNDE3 | 504 | | m | 0 | 0 | all | - | KI | NAmer | 1966 | CC | 350 | n | bl | n | y | 0 | all/unsp | 1.0 | 3 | 3 | 3 | nev any | st |
| WYNDE3 | 573 | | f | 0 | 0 | all | - | KI | NAmer | 1966 | CC | 350 | n | bl | n | y | 0 | cig+/-ot | 10 | 999 | 1 | 0 | nev any | st |
| WYNDE6 | 772 | x | m | 0 | 0 | all | - | KI | NAmer | 1969 | CC | 4423 | n | bl | n | y | 0 | cig+/-ot | 16 | 999 | 0 | 1 | nev any | st |
| WYNDE6 | 773 | x | m | 0 | 0 | all | - | KI | NAmer | 1969 | CC | 4423 | n | bl | n | y | 0 | cig+/-ot | 11 | 15 | 1 | 2 | nev any | st |
| WYNDE6 | 774 | x | m | 0 | 0 | all | - | KI | NAmer | 1969 | CC | 4423 | n | bl | n | y | 0 | cig+/-ot | 7 | 10 | 2 | 0 | nev any | st |
| WYNDE6 | 775 | x | m | 0 | 0 | all | - | KI | NAmer | 1969 | CC | 4423 | n | bl | n | y | 0 | cig+/-ot | 4 | 6 | 0 | 0 | nev any | st |
| WYNDE6 | 776 | x | m | 0 | 0 | all | - | KI | NAmer | 1969 | CC | 4423 | n | bl | n | y | 0 | cig+/-ot | 1.0 | 3 | 3 | 3 | nev any | st |

Cigarette type is all/unspec for all RRs

In this overview table, subtotals and Qs values may be invalid and should be ignored

Table 2J1 - 5

IESLC - Meta-analysis of Ex Smoking by Years quit (vs never), Overview
Squamous, Any Product (or Cigarettes if Any not available)
Least adjusted

| REF | NRR | SEX | AD | Number Exposed Case | Number Exposed Cont | Non-exposed Case | Non-exposed Cont | RR | 95.00%CI |
|---|---|---|---|---|---|---|---|---|---|
| BARBON | 579 | m | 0 | 4 | 59 | 6 | 188 | 2.12 | ( 0.58- 7.78) |
| BARBON | 580 | m | 0 | 11 | 41 | 6 | 188 | 8.41 | ( 2.94- 24.04) |
| BARBON | 581 | m | 0 | 31 | 85 | 6 | 188 | 11.43 | ( 4.60- 28.42) |
| BARBON | 582 | m | 0 | 11 | 20 | 6 | 188 | 17.23 | ( 5.76- 51.58) |
| Subtotal BARBON | | | | | | | | 8.77 | ( 5.16- 14.93) |
| BROWN3 | 507 | f | 0 | 17 | 10 | 432 | 1168 | 4.60 | ( 2.09- 10.12) |
| JAHN | 593 | m | 0 | 8 | 146 | 3 | 138 | 2.52 | ( 0.66- 9.70) |
| JAHN | 594 | m | 0 | 18 | 130 | 3 | 138 | 6.37 | ( 1.83- 22.13) |
| JAHN | 595 | m | 0 | 29 | 63 | 3 | 138 | 21.17 | ( 6.22- 72.12) |
| JAHN | 596 | m | 0 | 36 | 46 | 3 | 138 | 36.00 | ( 10.58- 122.45) |
| JAHN | 597 | m | 0 | 25 | 9 | 3 | 138 | 127.78 | ( 32.33- 505.04) |
| JAHN | 598 | m | 0 | 74 | 8 | 3 | 138 | 425.50 | (109.58-1652.23) |
| Subtotal JAHN | | | | | | | | 27.87 | ( 16.45- 47.20) |
| JAIN | 543 | m | 0 | 23 | 113 | 2 | 85 | 8.65 | ( 1.98- 37.70) |
| JAIN | 544 | m | 0 | 24 | 46 | 2 | 85 | 22.17 | ( 5.02- 98.04) |
| JAIN | 507 | f | 0 | 7 | 61 | 6 | 214 | 4.09 | ( 1.33- 12.63) |
| JAIN | 508 | f | 0 | 15 | 36 | 6 | 214 | 14.86 | ( 5.41- 40.82) |
| Subtotal JAIN | | | | | | | | 9.92 | ( 5.39- 18.27) |
| JEDRYC | 543 | m | 0 | 23 | 230 | 6 | 289 | 4.82 | ( 1.93- 12.03) |
| JEDRYC | 544 | m | 0 | 22 | 82 | 6 | 289 | 12.92 | ( 5.07- 32.93) |
| Subtotal JEDRYC | | | | | | | | 7.80 | ( 4.06- 15.01) |
| LUBIN2 | 765 | m | 0 | 106 | 1128 | 54 | 2616 | 4.55 | ( 3.26- 6.36) |
| LUBIN2 | 766 | m | 0 | 67 | 478 | 54 | 2616 | 6.79 | ( 4.68- 9.84) |
| LUBIN2 | 767 | m | 0 | 146 | 693 | 54 | 2616 | 10.21 | ( 7.39- 14.10) |
| LUBIN2 | 768 | m | 0 | 265 | 882 | 54 | 2616 | 14.56 | ( 10.76- 19.70) |
| LUBIN2 | 769 | m | 0 | 498 | 1047 | 54 | 2616 | 23.04 | ( 17.25- 30.79) |
| LUBIN2 | 893 | f | 0 | 2 | 29 | 72 | 1180 | 1.13 | ( 0.26- 4.83) |
| LUBIN2 | 894 | f | 0 | 5 | 33 | 72 | 1180 | 2.48 | ( 0.94- 6.55) |
| LUBIN2 | 895 | f | 0 | 38 | 95 | 72 | 1180 | 6.56 | ( 4.20- 10.23) |
| Subtotal LUBIN2 | | | | | | | | 9.96 | ( 8.71- 11.40) |
| LUO | 519 | c | 0 | 1 | 10 | 5 | 51 | 1.02 | ( 0.11- 9.69) |
| MATOS | 621 | m | 0 | 5 | 101 | 3 | 110 | 1.82 | ( 0.42- 7.79) |
| MATOS | 622 | m | 0 | 5 | 27 | 3 | 110 | 6.79 | ( 1.53- 30.19) |
| MATOS | 623 | m | 0 | 4 | 23 | 3 | 110 | 6.38 | ( 1.34- 30.44) |
| Subtotal MATOS | | | | | | | | 4.17 | ( 1.75- 9.93) |
| PEZZOT | 579 | m | 0 | 8 | 106 | 0 | 116 | 18.60~ | ( 1.06- 326.10) |
| PEZZOT | 580 | m | 0 | 21 | 82 | 0 | 116 | 60.72~ | ( 3.63-1016.67) |
| Subtotal PEZZOT | | | | | | | | 33.93 | ( 4.55- 252.91) |
| SVENSS | 557 | f | 0 | 1 | 24 | 5 | 120 | 1.00 | ( 0.11- 8.95) |
| SVENSS | 558 | f | 0 | 5 | 13 | 5 | 120 | 9.23 | ( 2.36- 36.16) |
| Subtotal SVENSS | | | | | | | | 4.96 | ( 1.56- 15.80) |
| WAKAI | 538 | m | 0 | 3 | 47 | 2 | 65 | 2.07 | ( 0.33- 12.91) |
| WAKAI | 539 | m | 0 | 12 | 44 | 2 | 65 | 8.86 | ( 1.89- 41.56) |
| WAKAI | 540 | m | 0 | 11 | 48 | 2 | 65 | 7.45 | ( 1.58- 35.17) |
| Subtotal WAKAI | | | | | | | | 5.67 | ( 2.22- 14.50) |
| WYNDE3 | 501 | m | 0 | 2 | 55 | 3 | 88 | 1.07 | ( 0.17- 6.59) |
| WYNDE3 | 502 | m | 0 | 8 | 31 | 3 | 88 | 7.57 | ( 1.89- 30.35) |
| WYNDE3 | 503 | m | 0 | 8 | 17 | 3 | 88 | 13.80 | ( 3.32- 57.39) |
| WYNDE3 | 504 | m | 0 | 18 | 22 | 3 | 88 | 24.00 | ( 6.49- 88.81) |
| WYNDE3 | 573 | f | 0 | 1 | 3 | 5 | 76 | 5.07 | ( 0.44- 57.98) |
| Subtotal WYNDE3 | | | | | | | | 8.80 | ( 4.39- 17.65) |
| WYNDE6 | 772 | m | 0 | 19 | 530 | 8 | 1667 | 7.47 | ( 3.25- 17.16) |
| WYNDE6 | 773 | m | 0 | 22 | 259 | 8 | 1667 | 17.70 | ( 7.80- 40.18) |
| WYNDE6 | 774 | m | 0 | 36 | 340 | 8 | 1667 | 22.06 | ( 10.17- 47.89) |
| WYNDE6 | 775 | m | 0 | 44 | 321 | 8 | 1667 | 28.56 | ( 13.32- 61.24) |
| WYNDE6 | 776 | m | 0 | 80 | 307 | 8 | 1667 | 54.30 | ( 25.99- 113.46) |
| Subtotal WYNDE6 | | | | | | | | 22.64 | ( 15.95- 32.12) |
| Totals | | | | 1819 | 7980 | 1075 | 30355 | | |

*prospective study ~ With 0.5 adjustment for zero

Table 2J1 - 5

IESLC - Meta-analysis of Ex Smoking by Years quit (vs never), Overview
Squamous, Any Product (or Cigarettes if Any not available)
Least adjusted

| REF | NRR | SEX | AD | Ys | Ws | Qs | Ps |
|---|---|---|---|---|---|---|---|
| BARBON | 579 | m | 0 | 0.75 | 2.28 | 5.91 | 0.2554 |
| BARBON | 580 | m | 0 | 2.13 | 3.48 | 0.19 | 0.0001 |
| BARBON | 581 | m | 0 | 2.44 | 4.63 | 0.02 | 0.0000 |
| BARBON | 582 | m | 0 | 2.85 | 3.20 | 0.75 | 0.0000 |
| Subtotal BARBON | | | | 2.17 | 13.58 | 6.87 | |
| BROWN3 | 507 | f | 0 | 1.53 | 6.17 | 4.34 | 0.0002 |
| JAHN | 593 | m | 0 | 0.92 | 2.12 | 4.39 | 0.1786 |
| JAHN | 594 | m | 0 | 1.85 | 2.48 | 0.65 | 0.0036 |
| JAHN | 595 | m | 0 | 3.05 | 2.56 | 1.21 | 0.0000 |
| JAHN | 596 | m | 0 | 3.58 | 2.56 | 3.81 | 0.0000 |
| JAHN | 597 | m | 0 | 4.85 | 2.03 | 12.57 | 0.0000 |
| JAHN | 598 | m | 0 | 6.05 | 2.09 | 28.41 | 0.0000 |
| Subtotal JAHN | | | | 3.33 | 13.84 | 51.05 | |
| JAIN | 543 | m | 0 | 2.16 | 1.77 | 0.08 | 0.0041 |
| JAIN | 544 | m | 0 | 3.10 | 1.74 | 0.94 | 0.0000 |
| JAIN | 507 | f | 0 | 1.41 | 3.02 | 2.76 | 0.0142 |
| JAIN | 508 | f | 0 | 2.70 | 3.76 | 0.42 | 0.0000 |
| Subtotal JAIN | | | | 2.29 | 10.30 | 4.19 | |
| JEDRYC | 543 | m | 0 | 1.57 | 4.59 | 2.88 | 0.0008 |
| JEDRYC | 544 | m | 0 | 2.56 | 4.39 | 0.17 | 0.0000 |
| Subtotal JEDRYC | | | | 2.05 | 8.98 | 3.04 | |
| LUBIN2 | 765 | m | 0 | 1.52 | 34.22 | 24.63 | 0.0000 |
| LUBIN2 | 766 | m | 0 | 1.92 | 27.84 | 5.60 | 0.0000 |
| LUBIN2 | 767 | m | 0 | 2.32 | 36.77 | 0.06 | 0.0000 |
| LUBIN2 | 768 | m | 0 | 2.68 | 42.00 | 4.14 | 0.0000 |
| LUBIN2 | 769 | m | 0 | 3.14 | 45.74 | 27.36 | 0.0000 |
| LUBIN2 | 893 | f | 0 | 0.12 | 1.82 | 9.15 | 0.8688 |
| LUBIN2 | 894 | f | 0 | 0.91 | 4.08 | 8.63 | 0.0662 |
| LUBIN2 | 895 | f | 0 | 1.88 | 19.39 | 4.54 | 0.0000 |
| Subtotal LUBIN2 | | | | 2.30 | 211.87 | 84.10 | |
| LUO | 519 | c | 0 | 0.02 | 0.76 | 4.16 | 0.9862 |
| MATOS | 621 | m | 0 | 0.60 | 1.81 | 5.66 | 0.4224 |
| MATOS | 622 | m | 0 | 1.92 | 1.73 | 0.35 | 0.0119 |
| MATOS | 623 | m | 0 | 1.85 | 1.57 | 0.41 | 0.0202 |
| Subtotal MATOS | | | | 1.43 | 5.11 | 6.42 | |
| PEZZOT | 579 | m | 0 | 2.92 | 0.47 | 0.15 | 0.0455 |
| PEZZOT | 580 | m | 0 | 4.11 | 0.48 | 1.47 | 0.0043 |
| Subtotal PEZZOT | | | | 3.52 | 0.95 | 1.61 | |
| SVENSS | 557 | f | 0 | 0.00 | 0.80 | 4.47 | 1.0000 |
| SVENSS | 558 | f | 0 | 2.22 | 2.06 | 0.04 | 0.0014 |
| Subtotal SVENSS | | | | 1.60 | 2.86 | 4.51 | |
| WAKAI | 538 | m | 0 | 0.73 | 1.15 | 3.07 | 0.4340 |
| WAKAI | 539 | m | 0 | 2.18 | 1.61 | 0.05 | 0.0056 |
| WAKAI | 540 | m | 0 | 2.01 | 1.59 | 0.20 | 0.0112 |
| Subtotal WAKAI | | | | 1.73 | 4.35 | 3.33 | |
| WYNDE3 | 501 | m | 0 | 0.06 | 1.16 | 6.13 | 0.9446 |
| WYNDE3 | 502 | m | 0 | 2.02 | 1.99 | 0.23 | 0.0043 |
| WYNDE3 | 503 | m | 0 | 2.62 | 1.89 | 0.13 | 0.0003 |
| WYNDE3 | 504 | m | 0 | 3.18 | 2.24 | 1.49 | 0.0000 |
| WYNDE3 | 573 | f | 0 | 1.62 | 0.65 | 0.36 | 0.1919 |
| Subtotal WYNDE3 | | | | 2.17 | 7.93 | 8.33 | |
| WYNDE6 | 772 | m | 0 | 2.01 | 5.55 | 0.69 | 0.0000 |
| WYNDE6 | 773 | m | 0 | 2.87 | 5.72 | 1.48 | 0.0000 |
| WYNDE6 | 774 | m | 0 | 3.09 | 6.40 | 3.41 | 0.0000 |
| WYNDE6 | 775 | m | 0 | 3.35 | 6.60 | 6.45 | 0.0000 |
| WYNDE6 | 776 | m | 0 | 3.99 | 7.07 | 18.81 | 0.0000 |
| Subtotal WYNDE6 | | | | 3.12 | 31.34 | 30.84 | |

| | |
|---|---|
| N | 46 |
| NS | 13 |

Table 2J1 - 6

IESLC - Meta-analysis of Ex Smoking by Years quit (vs never), Overview
Squamous, Any Product (or Cigarettes if Any not available)
Least adjusted

| | Sex | | | |
|---|---|---|---|---|
| | combined | male | female | Total |
| N | 1 | 36 | 9 | 46 |
| NS | 1 | 10 | 5 | 16 |

In this overview table, other than the "N" rows, entries in the "absent" and "Total" columns may be invalid and should be ignored

| | Years quit vs never (lower focus) | | | | |
|---|---|---|---|---|---|
| | absent | 8+k12 | 4-11k7 | 1-6k3 | Total |
| N | 21 | 13 | 6 | 6 | 46 |
| NS | 10 | 11 | 6 | 6 | 33 |
| Wt | 132.46 | 64.53 | 58.67 | 62.39 | 318.05 |
| Het Chi | 99.16 | 25.15 | 3.24 | 8.27 | 212.80 |
| Het df | 20 | 12 | 5 | 5 | 45 |
| Het P | *** | * | N.S. | N.S. | *** |
| Fixed RR | 7.20 | 7.79 | 14.73 | 24.71 | 10.63 |
| RRl | 6.07 | 6.10 | 11.41 | 19.28 | 9.53 |
| RRu | 8.54 | 9.94 | 19.03 | 31.66 | 11.87 |
| P | +++ | +++ | +++ | +++ | +++ |
| Random RR | 9.06 | 5.62 | 14.73 | 25.52 | 9.70 |
| RRl | 5.84 | 3.50 | 11.41 | 16.15 | 7.33 |
| RRu | 14.07 | 9.01 | 19.03 | 40.31 | 12.84 |
| P | +++ | +++ | +++ | +++ | +++ |

| | Years quit vs never (higher focus) | | | | |
|---|---|---|---|---|---|
| | absent | 13+k20 | 4-19k12 | 1-11k3 | Total |
| N | 22 | 7 | 6 | 11 | 46 |
| NS | 12 | 6 | 5 | 9 | 32 |
| Wt | 119.87 | 53.56 | 54.80 | 89.82 | 318.05 |
| Het Chi | 93.92 | 9.36 | 10.00 | 36.15 | 212.80 |
| Het df | 21 | 6 | 5 | 10 | 45 |
| Het P | *** | N.S. | (*) | *** | *** |
| Fixed RR | 11.04 | 4.54 | 9.68 | 17.81 | 10.63 |
| RRl | 9.23 | 3.47 | 7.43 | 14.48 | 9.53 |
| RRu | 13.20 | 5.93 | 12.61 | 21.90 | 11.87 |
| P | +++ | +++ | +++ | +++ | +++ |
| Random RR | 9.81 | 4.31 | 8.90 | 18.26 | 9.70 |
| RRl | 6.20 | 2.79 | 5.41 | 10.95 | 7.33 |
| RRu | 15.50 | 6.66 | 14.64 | 30.46 | 12.84 |
| P | +++ | +++ | +++ | +++ | +++ |

Table 2J1 - 6

IESLC - Meta-analysis of Ex Smoking by Years quit (vs never), Overview
Squamous, Any Product (or Cigarettes if Any not available)
Least adjusted

MALES

| | | Years quit vs never (lower focus) | | | | |
|---|---|---|---|---|---|---|
| | | absent | 8+k12 | 4-11k7 | 1-6k3 | Total |
| | N | 16 | 8 | 6 | 6 | 36 |
| | NS | 8 | 8 | 6 | 6 | 28 |
| | Wt | 99.26 | 55.22 | 58.67 | 62.39 | 275.53 |
| Het | Chi | 89.18 | 10.10 | 3.24 | 8.27 | 170.52 |
| Het | df | 15 | 7 | 5 | 5 | 35 |
| Het | P | *** | N.S. | N.S. | N.S. | *** |
| Fixed | RR | 7.55 | 9.35 | 14.73 | 24.71 | 11.88 |
| | RRl | 6.20 | 7.18 | 11.41 | 19.28 | 10.56 |
| | RRu | 9.19 | 12.17 | 19.03 | 31.66 | 13.37 |
| | P | +++ | +++ | +++ | +++ | +++ |
| Random | RR | 10.73 | 8.40 | 14.73 | 25.52 | 12.06 |
| | RRl | 6.06 | 5.48 | 11.41 | 16.15 | 8.86 |
| | RRu | 18.99 | 12.89 | 19.03 | 40.31 | 16.41 |
| | P | +++ | +++ | +++ | +++ | +++ |

| | | Years quit vs never (higher focus) | | | | |
|---|---|---|---|---|---|---|
| | | absent | 13+k20 | 4-19k12 | 1-11k3 | Total |
| | N | 18 | 5 | 5 | 8 | 36 |
| | NS | 10 | 5 | 5 | 8 | 28 |
| | Wt | 114.64 | 45.56 | 50.72 | 64.61 | 275.53 |
| Het | Chi | 81.19 | 5.71 | 1.84 | 8.68 | 170.52 |
| Het | df | 17 | 4 | 4 | 7 | 35 |
| Het | P | *** | N.S. | N.S. | N.S. | *** |
| Fixed | RR | 11.76 | 4.79 | 10.80 | 24.80 | 11.88 |
| | RRl | 9.79 | 3.58 | 8.20 | 19.43 | 10.56 |
| | RRu | 14.12 | 6.40 | 14.22 | 31.65 | 13.37 |
| | P | +++ | +++ | +++ | +++ | +++ |
| Random | RR | 12.08 | 4.86 | 10.80 | 25.77 | 12.06 |
| | RRl | 7.48 | 3.02 | 8.20 | 17.88 | 8.86 |
| | RRu | 19.52 | 7.85 | 14.22 | 37.14 | 16.41 |
| | P | +++ | +++ | +++ | +++ | +++ |

FEMALES

| | | Years quit vs never (lower focus) | | | | |
|---|---|---|---|---|---|---|
| | | absent | 8+k12 | 4-11k7 | 1-6k3 | Total |
| | N | 5 | 4 | | | 9 |
| | NS | 4 | 4 | | | 8 |
| | Wt | 33.20 | 8.55 | | | 41.76 |
| Het | Chi | 9.09 | 1.56 | | | 14.76 |
| Het | df | 4 | 3 | | | 8 |
| Het | P | (*) | N.S. | | | (*) |
| Fixed | RR | 6.25 | 2.87 | | | 5.33 |
| | RRl | 4.44 | 1.47 | | | 3.93 |
| | RRu | 8.78 | 5.61 | | | 7.21 |
| | P | +++ | ++ | | | +++ |
| Random | RR | 5.94 | 2.87 | | | 4.70 |
| | RRl | 3.22 | 1.47 | | | 2.89 |
| | RRu | 10.97 | 5.61 | | | 7.64 |
| | P | +++ | ++ | | | +++ |

Table 2J1 - 6

IESLC - Meta-analysis of Ex Smoking by Years quit (vs never), Overview
Squamous, Any Product (or Cigarettes if Any not available)
Least adjusted

FEMALES

| | Years quit vs never (higher focus) absent | 13+k20 | 4-19k12 | 1-11k3 | Total |
|---|---|---|---|---|---|
| N | 3 | 2 | 1 | 3 | 9 |
| NS | 3 | 2 | 1 | 3 | 8 |
| Wt | 4.47 | 7.99 | 4.08 | 25.21 | 41.76 |
| Het Chi | 1.40 | 2.77 | 0.00 | 2.19 | 14.76 |
| Het df | 2 | 1 | 0 | 2 | 8 |
| Het P | N.S. | (*) | N.S. | N.S. | (*) |
| Fixed RR | 3.28 | 3.34 | 2.48 | 7.62 | 5.33 |
| RRl | 1.30 | 1.67 | 0.94 | 5.16 | 3.93 |
| RRu | 8.29 | 6.68 | 6.55 | 11.25 | 7.21 |
| P | + | +++ | (+) | +++ | +++ |
| Random RR | 3.28 | 2.62 | 2.48 | 7.87 | 4.70 |
| RRl | 1.30 | 0.68 | 0.94 | 5.04 | 2.89 |
| RRu | 8.29 | 10.07 | 6.55 | 12.28 | 7.64 |
| P | + | N.S. | (+) | +++ | +++ |

Table 2J1 - 7

IESLC - Meta-analysis of Ex Smoking by Years quit (vs never), Overview
Squamous, Any Product (or Cigarettes if Any not available)
Excluded studies (and stage at which they were excluded)

| | |
|---|---|
| 1 | AGUDO AKIBA AMANDU AMES AXELSS BEST BOUCHA BOUCOT BRESLO CHEN CHEN2 CHIAZZ DEAN2 DOSEME ENGELA FAN GENG GER GUO HAENSZ HEGMAN HOLE HU HU2 JUSSAW KATSOU KAUFMA KOO KOULUM KREUZE LETOUR LEVIN LIAW LIU3 LIU4 LIU5 MCCONN MIGRAN MRFITR NOTAN2 OSANN2 PERNU QIAO2 RACHTA RESTRE SADOWS SEGI2 STASZE TIZZAN VUTUC WATSON WU WUWILL WYNDE2 WYNDE8 XU YUAN ZHANG ZHENG ZHOU |
| 2 | BUFFLE HUMBLE PISANI PRESCO WYNDE7 |
| 3 | MCDUFF SPITZ |
| 4 | ARMADA AUVINE BECHER BENSHL BLOT1 BOFFET BROSS CARPEN CEDERL CHOI CHYOU CORREA CPSI CPSII DAMBER DARBY DEAN3 DESTEF DOLL DOLL2 DORGAN DORN GAO GAO2 GARCIA GARSHI GILLIS GRAHAM GURSEL HAMMO2 HIRAYA JOLY KAISE2 KHUDER LAUSSM LUBIN PEZZO2 QIAO SPEIZE SUZUK2 TVERDA WANG2 WIGLE WU2 |
| 5 | ALDERS HAMMON |
| 10 | SOBUE |
| 14 | BENHAM |

Table 2J1 - 8
Potentially overlapping studies

| REF | REFGP | PRINC | . OVERLAP/LINK |
|---|---|---|---|
| LUBIN2 | LUBIN2 | 1 | Lubin-combined |
| WYNDE6 | WYNDE6 | 1 | WYNDE5/6/7/8 |
| JAHN | BOFFET | 2 | Subset of BOFFET |

Table 2J1 - 9
Most adjusted - insufficient data for meta-analysis

| REF | NRR | SEX | AGEL | AGEH | RACE | YF | LC TYPE | LOC | START | ST | NLC | R | VB | P | H | AD | PRODUCT | exL | exH | S1 | S2 | DENOM | De |
|---|---|---|---|---|---|---|---|---|---|---|---|---|---|---|---|---|---|---|---|---|---|---|---|
| ALDERS | 537 | m | 0 | 0 | all | - | q+s | Eu:UK | 1977 | CC | 1448 | n | V | n | n | 1 | cig only | 10 | 999 | 1 | 0 | nev any | st |
| ALDERS | 538 | m | 0 | 0 | all | - | q+s | Eu:UK | 1977 | CC | 1448 | n | V | n | n | 1 | cig only | 3 | 9 | 0 | 3 | nev any | st |
| ALDERS | 539 | m | 0 | 0 | all | - | q+s | Eu:UK | 1977 | CC | 1448 | n | V | n | n | 1 | cig only | 0.1 | 2 | 0 | 0 | nev any | st |
| ALDERS | 548 | f | 0 | 0 | all | - | q+s | Eu:UK | 1977 | CC | 1448 | n | V | n | n | 1 | cig only | 10 | 999 | 1 | 0 | nev any | st |
| ALDERS | 549 | f | 0 | 0 | all | - | q+s | Eu:UK | 1977 | CC | 1448 | n | V | n | n | 1 | cig only | 3 | 9 | 0 | 3 | nev any | st |
| ALDERS | 550 | f | 0 | 0 | all | - | q+s | Eu:UK | 1977 | CC | 1448 | n | V | n | n | 1 | cig only | 0.1 | 2 | 0 | 0 | nev any | st |
| BROWN3 | 508 | f | 0 | 0 | wh | - | q | NAmer | | CC | 618 | | bl | y | n | 0 | all/unsp | 1.0 | 14 | 0 | 0 | nev any | ot |
| HAMMON | 501 | m | 0 | 0 | wh | 0 | not a | NAmer | 1952 | pr | 448 | n | bl | n | n | 1 | cig only | 10 | 999 | 1 | 0 | nev any | st |
| HAMMON | 502 | m | 0 | 0 | wh | 0 | not a | NAmer | 1952 | pr | 448 | n | bl | n | n | 1 | cig only | 1.0 | 9 | 0 | 3 | nev any | st |
| HAMMON | 503 | m | 0 | 0 | wh | 0 | not a | NAmer | 1952 | pr | 448 | n | bl | n | n | 1 | cig only | 0.1 | 0.9 | 0 | 0 | nev any | st |
| JAIN | 588 | m | 0 | 0 | all | - | q | NAmer | 1981 | CC | 845 | n | V | y | n | 0 | cig+/-ot | 0.1 | 1.9 | 0 | 0 | nev cigs | ot |
| JAIN | 576 | f | 0 | 0 | all | - | q | NAmer | 1981 | CC | 845 | n | V | y | n | 0 | cig+/-ot | 0.1 | 1.9 | 0 | 0 | nev cigs | ot |
| JEDRYC | 545 | m | 0 | 0 | all | - | q | Eu:est | 1980 | CC | 1630 | n | bl | y | n | 0 | cig+/-ot | 1.0 | 4 | 3 | 3 | nev any | ot |
| LUO | 523 | c | 0 | 0 | all | - | q | As:Chi | 1990 | CC | 102 | n | ot | n | y | 20 | cig+/-ot | 1.0 | 9 | 0 | 3 | nev cigs | ot |
| MATOS | 707 | m | 0 | 0 | all | - | q | SCAmer | 1994 | CC | 200 | n | bl | n | n | 2 | cig+/-ot | 0.1 | 0.9 | 0 | 0 | nev any | ot |
| PEZZOT | 599 | m | 0 | 0 | all | - | q | SCAmer | 1987 | CC | 215 | n | bl | n | y | 0 | cig only | 0.1 | 0.9 | 0 | 0 | nev cigs | ot |
| SVENSS | 593 | f | 0 | 0 | all | - | q | Eu:Sca | 1983 | CC | 210 | n | bl | n | n | 0 | all/unsp | 1.0 | 2 | 0 | 0 | nev any | ot |
| WAKAI | 615 | m | 0 | 0 | all | - | q | As:Jap | 1988 | CC | 333 | n | bl | n | y | 1 | cig+/-ot | 1.0 | 4 | 3 | 3 | nev any | ot |
| WYNDE3 | 505 | m | 0 | 0 | all | - | KI | NAmer | 1966 | CC | 350 | n | bl | n | y | 0 | all/unsp | 0.1 | 0.9 | 0 | 0 | nev any | ot |
| WYNDE3 | 574 | f | 0 | 0 | all | - | KI | NAmer | 1966 | CC | 350 | n | bl | n | y | 0 | cig+/-ot | 1.0 | 9 | 0 | 3 | nev any | ot |
| WYNDE6 | 792 | m | 0 | 0 | all | - | KI | NAmer | 1969 | CC | 4423 | n | bl | n | y | 2 | cig+/-ot | 0.1 | 0.9 | 0 | 0 | nev any | ot |

| REF | NRR | RR | SIG | RRDATA | comment |
|---|---|---|---|---|---|
| ALDERS | 537 | 2.33 | | | 0 |
| ALDERS | 538 | 4.00 | | | 0 |
| ALDERS | 539 | 23.33 | | | 0 |
| ALDERS | 548 | 0.67 | | | 0 |
| ALDERS | 549 | 8.39 | | | 0 |
| ALDERS | 550 | 16.78 | | | 0 |
| BROWN3 | 508 | * | gap | | 0 |
| HAMMON | 501 | * | | RR for <1 pack per day is 2.44, while that for 1+ packs is 17.79 | |
| HAMMON | 502 | * | | RR for <1 pack per day is 10.44, while that for 1+ packs is 22.82 | |
| HAMMON | 503 | * | | RR for <1 pack per day is 16.50, while that for 1+ packs is 58.24 | |
| JAIN | 588 | * | gap | | 0 |
| JAIN | 576 | * | gap | | 0 |
| JEDRYC | 545 | * | gap | | 0 |

Table 2J1 - 9

IESLC - Meta-analysis of Ex Smoking by Years quit (vs never), Overview
Squamous, Any Product (or Cigarettes if Any not available)
Most adjusted - insufficient data for meta-analysis

| REF | NRR | RR | SIG | RRDATA comment |
|---|---|---|---|---|
| LUO | 523 | * | gap | 0 |
| MATOS | 707 | * | gap | 0 |
| PEZZOT | 599 | * | gap | 0 |
| SVENSS | 593 | * | gap | 0 |
| WAKAI | 615 | * | gap | 0 |
| WYNDE3 | 505 | * | gap | 0 |
| WYNDE3 | 574 | * | gap | 0 |
| WYNDE6 | 792 | * | gap | 0 |

Least adjusted - insufficient data for meta-analysis: as for adjusted plus the following

| REF | NRR | SEX | AGEL | AGEH | RACE | YF | LC TYPE | LOC | START | ST | NLC | R | VB | P | H | AD | PRODUCT | exL | exH | S1 | S2 | DENOM | De |
|---|---|---|---|---|---|---|---|---|---|---|---|---|---|---|---|---|---|---|---|---|---|---|---|
| LUO | 520 | c | 0 | 0 | all | - | q | As:Chi | 1990 | CC | 102 | n | ot | n | y | 0 | cig+/-ot | 1.0 | 9 | 0 | 3 | nev cigs | ot |
| MATOS | 705 | m | 0 | 0 | all | - | q | SCAmer | 1994 | CC | 200 | n | bl | n | n | 0 | cig+/-ot | 0.1 | 0.9 | 0 | 0 | nev any | ot |
| WAKAI | 613 | m | 0 | 0 | all | - | q | As:Jap | 1988 | CC | 333 | n | bl | n | y | 0 | cig+/-ot | 1.0 | 4 | 3 | 3 | nev any | ot |
| WYNDE6 | 777 | m | 0 | 0 | all | - | KI | NAmer | 1969 | CC | 4423 | n | bl | n | y | 0 | cig+/-ot | 0.1 | 0.9 | 0 | 0 | nev any | ot |

| REF | NRR | RR | SIG | RRDATA comment |
|---|---|---|---|---|
| LUO | 520 | * | gap | 0 |
| MATOS | 705 | * | gap | 0 |
| WAKAI | 613 | * | gap | 0 |
| WYNDE6 | 777 | * | gap | 0 |

Table 2J2 -

IESLC - Meta-analysis of Ex Smoking, Years quit (vs never), "Low"
Squamous, Any Product (or Cigarettes if Any not available)

This analysis is restricted to results for:
1) Ex smokers
2) Results by Years quit (vs never)
3) Categorical results by Years quit (vs never)
4) Squamous (or near equivalent)
5) Results complete enough for use in metaanalysis

Within each study, results are then selected (in the following order of preference, within each sex) for:
6) (not applicable)
7) PRODUCT: all/unspec, cigarettes regardless of other products, cigarettes only
8) CIGTYPE: all/unspecified, MC regardless of HR, MC only
9) (not applicable)
10) DENOM: never smoked anything, never smoked cigarettes, never any + low, never cigs + low
11) Followup period (YF, prospective studies): whole study (coded as 0) or longest available
12) LCtype: squamous or nearest available, but not adeno. (q = squamous, s = small,
a = adeno, KI = Kreyberg I, u = undifferentiated)
13) Race: all or nearest available, otherwise by race (wh or w = white, bl or b = black, hi = hispanic
ch = chinese, jap = japanese, haw = hawaiian, w+o = white + oriental, sca = scandinavian, as = asian)
14) Years quit (vs never) "low" in key scheme 1 (key value 12, maximum range 8+)
15) For overlapping studies: principal rather than subsidiary studies
Finally by Age: whole study (coded as 0) if available, otherwise by widest available age group
and then for single sex results (m, f) in preference to results for both sexes combined (c).

Results adjusted (AD) for the most potential confounders are then chosen in Sections -1 to -3
and results adjusted for the least confounders in Sections -4 to -6. (Those least adjusted results which
actually differ from the most adjusted are marked 'x' in column X in Section -4)

Section -7 shows excluded studies, together with the stage (as above) at which no qualifying
results were found.

Section -8 lists the potentially overlapping studies which have been included (1=principal, 2=subsidiary).

Section -9 lists any results which would have been included in preference except that they had data not complete
enough for use in meta-analysis, with their significance (yes/no), if known, and any further comment as entered
on the database. It also lists as "gap" any categories for which no data were presented by the original authors.
This is commonly due to recent quitters having been combined with current smokers

In addition to those mentioned above, the following fields, levels and abbreviations are used:
* or nk = not known, n = no, y = yes, ot = other
nev = never
all/unspec = all or unspecified, cig+/-ot = cigarettes irrespective of other products (cigar, pipe etc)
MC = manufactured cigarettes, HR = hand-rolled cigarettes
exL, exH = range of exposure (low and high) in the smoking group, in terms of Years quit (vs never)
REF: 6-character study reference
NRR: number of the RR on the database within the study
ST : study type (CC = case control, pr or prosp = prospective)
NLC: number of lung cancer cases in whole study
R : risky occupational population (n = no, m = mining, o = other risky)
VB : national cigarette type (V = at least 75% Virginia, bl = at least 75% blended, ot = other)
P : any proxy use
H : full histological confirmation
De : derivation of RR/CI (or = original, st = standard method, ot = other method of estimation)

Table 2J2 - 1

IESLC - Meta-analysis of Ex Smoking, Years quit (vs never), "Low"
Squamous, Any Product (or Cigarettes if Any not available)
Most adjusted

| REF | NRR | SEX | AGEL | AGEH | RACE | YF | LC TYPE | LOC | START | ST | NLC | R | VB | P | H | AD | PRODUCT | exL | exH | DENOM | De |
|---|---|---|---|---|---|---|---|---|---|---|---|---|---|---|---|---|---|---|---|---|---|
| JAHN | 594 | m | 0 | 0 | all | - | q | Eu:Ger | 1988 | CC | 1004 | n | bl | n | n | 0 | cig+/-ot | 11 | 20 | nev any | st |
| JAIN | 543 | m | 0 | 0 | all | - | q | NAmer | 1981 | CC | 845 | n | V | y | n | 0 | cig+/-ot | 10 | 999 | nev cigs | st |
| JAIN | 507 | f | 0 | 0 | all | - | q | NAmer | 1981 | CC | 845 | n | V | y | n | 0 | cig+/-ot | 10 | 999 | nev cigs | st |
| JEDRYC | 543 | m | 0 | 0 | all | - | q | Eu:est | 1980 | CC | 1630 | n | bl | y | n | 0 | cig+/-ot | 10 | 999 | nev any | st |
| LUBIN2 | 767 | m | 0 | 0 | all | - | q | Eu:mul | 1976 | CC | 7804 | n | bl | n | y | 0 | cig+/-ot | 10 | 14 | nev any | st |
| LUBIN2 | 894 | f | 0 | 0 | all | - | q | Eu:mul | 1976 | CC | 7804 | n | bl | n | y | 0 | cig+/-ot | 10 | 19 | nev any | st |
| LUO | 522 | c | 0 | 0 | all | - | q | As:Chi | 1990 | CC | 102 | n | ot | n | y | 20 | cig+/-ot | 10 | 999 | nev cigs | or |
| MATOS | 631 | m | 0 | 0 | all | - | q | SCAmer | 1994 | CC | 200 | n | bl | n | n | 2 | cig+/-ot | 11 | 999 | nev any | ot |
| PEZZOT | 579 | m | 0 | 0 | all | - | q | SCAmer | 1987 | CC | 215 | n | bl | n | y | 0 | cig only | 11 | 999 | nev cigs | ot |
| SVENSS | 557 | f | 0 | 0 | all | - | q | Eu:Sca | 1983 | CC | 210 | n | bl | n | n | 0 | all/unsp | 11 | 999 | nev any | st |
| WAKAI | 547 | m | 0 | 0 | all | - | q | As:Jap | 1988 | CC | 333 | n | bl | n | y | 1 | cig+/-ot | 10 | 19 | nev any | or |
| WYNDE3 | 552 | m | 0 | 0 | all | - | KI | NAmer | 1966 | CC | 350 | n | bl | n | y | 0 | cig+/-ot | 10 | 999 | nev any | st |
| WYNDE3 | 573 | f | 0 | 0 | all | - | KI | NAmer | 1966 | CC | 350 | n | bl | n | y | 0 | cig+/-ot | 10 | 999 | nev any | st |
| WYNDE6 | 788 | m | 0 | 0 | all | - | KI | NAmer | 1969 | CC | 4423 | n | bl | n | y | 2 | cig+/-ot | 11 | 15 | nev any | ot |

Cigarette type is all/unspec for all RRs

Table 2J2 - 2

IESLC - Meta-analysis of Ex Smoking, Years quit (vs never), "Low"
Squamous, Any Product (or Cigarettes if Any not available)
Most adjusted

| REF | NRR | SEX | AD | Number Exposed Case | Cont | Non-exposed Case | Cont | RR | 95.00%CI |
|---|---|---|---|---|---|---|---|---|---|
| JAHN | 594 | m | 0 | 18 | 130 | 3 | 138 | 6.37 | ( 1.83- 22.13) |
| JAIN | 543 | m | 0 | 23 | 113 | 2 | 85 | 8.65 | ( 1.98- 37.70) |
| JAIN | 507 | f | 0 | 7 | 61 | 6 | 214 | 4.09 | ( 1.33- 12.63) |
| Subtotal JAIN | | | | | | | | 5.40 | ( 2.21- 13.20) |
| JEDRYC | 543 | m | 0 | 23 | 230 | 6 | 289 | 4.82 | ( 1.93- 12.03) |
| LUBIN2 | 767 | m | 0 | 146 | 693 | 54 | 2616 | 10.21 | ( 7.39- 14.10) |
| LUBIN2 | 894 | f | 0 | 5 | 33 | 72 | 1180 | 2.48 | ( 0.94- 6.55) |
| Subtotal LUBIN2 | | | | | | | | 8.86 | ( 6.52- 12.04) |
| LUO | 522 | c | 20 | 1 | - | 5 | - | 2.00 | ( 0.20- 23.10) |
| MATOS | 631 | m | 2 | 5 | - | 3 | - | 2.00 | ( 0.41- 9.65) |
| PEZZOT | 579 | m | 0 | 8 | 106 | 0 | 116 | 18.60~ | ( 1.06- 326.10) |
| SVENSS | 557 | f | 0 | 1 | 24 | 5 | 120 | 1.00 | ( 0.11- 8.95) |
| WAKAI | 547 | m | 1 | 12 | - | 2 | - | 8.95 | ( 1.91- 42.00) |
| WYNDE3 | 552 | m | 0 | 6 | 65 | 3 | 88 | 2.71 | ( 0.65- 11.23) |
| WYNDE3 | 573 | f | 0 | 1 | 3 | 5 | 76 | 5.07 | ( 0.44- 57.98) |
| Subtotal WYNDE3 | | | | | | | | 3.17 | ( 0.93- 10.85) |
| WYNDE6 | 788 | m | 2 | 22 | - | 8 | - | 13.70 | ( 6.04- 31.10) |
| Partial Totals | | | | 278 | 1458 | 174 | 4922 | | |

*prospective study ~ With 0.5 adjustment for zero

| REF | NRR | SEX | AD | Ys | Ws | Qs | Ps |
|---|---|---|---|---|---|---|---|
| JAHN | 594 | m | 0 | 1.85 | 2.48 | 0.07 | 0.0036 |
| JAIN | 543 | m | 0 | 2.16 | 1.77 | 0.03 | 0.0041 |
| JAIN | 507 | f | 0 | 1.41 | 3.02 | 1.12 | 0.0142 |
| Subtotal JAIN | | | | 1.69 | 4.80 | 1.16 | |
| JEDRYC | 543 | m | 0 | 1.57 | 4.59 | 0.91 | 0.0008 |
| LUBIN2 | 767 | m | 0 | 2.32 | 36.77 | 3.41 | 0.0000 |
| LUBIN2 | 894 | f | 0 | 0.91 | 4.08 | 5.02 | 0.0662 |
| Subtotal LUBIN2 | | | | 2.18 | 40.86 | 8.43 | |
| LUO | 522 | c | 20 | 0.69 | 0.68 | 1.20 | 0.5673 |
| MATOS | 631 | m | 2 | 0.69 | 1.54 | 2.70 | 0.3897 |
| PEZZOT | 579 | m | 0 | 2.92 | 0.47 | 0.38 | 0.0455 |
| SVENSS | 557 | f | 0 | 0.00 | 0.80 | 3.26 | 1.0000 |
| WAKAI | 547 | m | 1 | 2.19 | 1.61 | 0.05 | 0.0054 |
| WYNDE3 | 552 | m | 0 | 1.00 | 1.90 | 1.98 | 0.1699 |
| WYNDE3 | 573 | f | 0 | 1.62 | 0.65 | 0.10 | 0.1919 |
| Subtotal WYNDE3 | | | | 1.16 | 2.55 | 2.08 | |
| WYNDE6 | 788 | m | 2 | 2.62 | 5.72 | 2.05 | 0.0000 |

| | | |
|---|---|---|
| | N | 14 |
| | NS | 11 |
| | Wt | 66.08 |
| Het | Chi | 22.30 |
| Het | df | 13 |
| Het | P | (*) |
| Fixed | RR | 7.53 |
| | RRl | 5.91 |
| | RRu | 9.58 |
| | P | +++ |
| Random | RR | 5.59 |
| | RRl | 3.68 |
| | RRu | 8.48 |
| | P | +++ |
| Asymm | P | * |

Table 2J2 - 3

IESLC - Meta-analysis of Ex Smoking, Years quit (vs never), "Low"
Squamous, Any Product (or Cigarettes if Any not available)
Most adjusted

| | Sex | | | |
|---|---|---|---|---|
| | combined | male | female | Total |
| N | 1 | 9 | 4 | 14 |
| NS | 1 | 9 | 4 | 14 |
| Wt | 0.68 | 56.85 | 8.55 | 66.08 |
| Het Chi | 0.00 | 10.13 | 1.56 | 22.30 |
| Het df | 0 | 8 | 3 | 13 |
| Het P | N.S. | N.S. | N.S. | (*) |
| Fixed RR | 2.00 | 8.84 | 2.87 | 7.53 |
| RRl | 0.19 | 6.82 | 1.47 | 5.91 |
| RRu | 21.49 | 11.46 | 5.61 | 9.58 |
| P | N.S. | +++ | ++ | +++ |
| Random RR | 2.00 | 7.84 | 2.87 | 5.59 |
| RRl | 0.19 | 5.37 | 1.47 | 3.68 |
| RRu | 21.49 | 11.44 | 5.61 | 8.48 |
| P | N.S. | +++ | ++ | +++ |
| Between Chi | | | | 10.60 |
| Between df | | | | 2 |
| Between P | | | | ** |
| Btwn(F) P | | | | * |
| Btwn(R) P | | | | * |

| | Lung cancer type | | | | | |
|---|---|---|---|---|---|---|
| | q | q+s | q+u | KI | not a | Total |
| N | 11 | | | 3 | | 14 |
| NS | 9 | | | 2 | | 11 |
| Wt | 57.82 | | | 8.27 | | 66.08 |
| Het Chi | 18.14 | | | 3.95 | | 22.30 |
| Het df | 10 | | | 2 | | 13 |
| Het P | (*) | | | N.S. | | (*) |
| Fixed RR | 7.37 | | | 8.73 | | 7.53 |
| RRl | 5.69 | | | 4.42 | | 5.91 |
| RRu | 9.53 | | | 17.27 | | 9.58 |
| P | +++ | | | +++ | | +++ |
| Random RR | 5.13 | | | 6.78 | | 5.59 |
| RRl | 3.16 | | | 2.15 | | 3.68 |
| RRu | 8.32 | | | 21.40 | | 8.48 |
| P | +++ | | | ++ | | +++ |
| Between Chi | | | | | | 0.21 |
| Between df | | | | | | 1 |
| Between P | | | | | | N.S. |
| Btwn(F) P | | | | | | N.S. |
| Btwn(R) P | | | | | | N.S. |

| | Location | | | | | | | | |
|---|---|---|---|---|---|---|---|---|---|
| | NAmer | UK | Scand | othEur | China | Japan | othAs | other | Total |
| N | 5 | | 1 | 4 | 1 | 1 | | 2 | 14 |
| NS | 3 | | 1 | 3 | 1 | 1 | | 2 | 11 |
| Wt | 13.06 | | 0.80 | 47.92 | 0.68 | 1.61 | | 2.01 | 66.08 |
| Het Chi | 5.28 | | 0.00 | 9.04 | 0.00 | 0.00 | | 1.79 | 22.30 |
| Het df | 4 | | 0 | 3 | 0 | 0 | | 1 | 13 |
| Het P | N.S. | | N.S. | * | N.S. | N.S. | | N.S. | (*) |
| Fixed RR | 7.32 | | 1.00 | 8.22 | 2.00 | 8.95 | | 3.36 | 7.53 |
| RRl | 4.26 | | 0.11 | 6.19 | 0.19 | 1.91 | | 0.84 | 5.91 |
| RRu | 12.59 | | 8.95 | 10.91 | 21.49 | 41.97 | | 13.41 | 9.58 |
| P | +++ | | N.S. | +++ | N.S. | ++ | | (+) | +++ |
| Random RR | 6.75 | | 1.00 | 5.73 | 2.00 | 8.95 | | 4.37 | 5.59 |
| RRl | 3.49 | | 0.11 | 2.85 | 0.19 | 1.91 | | 0.54 | 3.68 |
| RRu | 13.05 | | 8.95 | 11.53 | 21.49 | 41.97 | | 35.16 | 8.48 |
| P | +++ | | N.S. | +++ | N.S. | ++ | | N.S. | +++ |
| Between Chi | | | | | | | | | 6.19 |
| Between df | | | | | | | | | 5 |
| Between P | | | | | | | | | N.S. |
| Btwn(F) P | | | | | | | | | N.S. |
| Btwn(R) P | | | | | | | | | N.S. |

Table 2J2 - 3

IESLC - Meta-analysis of Ex Smoking, Years quit (vs never), "Low"
Squamous, Any Product (or Cigarettes if Any not available)
Most adjusted

Detailed Country in "other Europe"

| | multi | Germany | othWest | East | Balkans | Total |
|---|---|---|---|---|---|---|
| N | 2 | 1 | | 1 | | 4 |
| NS | 1 | 1 | | 1 | | 3 |
| Wt | 40.86 | 2.48 | | 4.59 | | 47.92 |
| Het Chi | 7.34 | 0.00 | | 0.00 | | 9.04 |
| Het df | 1 | 0 | | 0 | | 3 |
| Het P | ** | N.S. | | N.S. | | * |
| Fixed RR | 8.86 | 6.37 | | 4.82 | | 8.22 |
| RRl | 6.52 | 1.83 | | 1.93 | | 6.19 |
| RRu | 12.04 | 22.13 | | 12.03 | | 10.91 |
| P | +++ | ++ | | +++ | | +++ |
| Random RR | 5.44 | 6.37 | | 4.82 | | 5.73 |
| RRl | 1.37 | 1.83 | | 1.93 | | 2.85 |
| RRu | 21.55 | 22.13 | | 12.03 | | 11.53 |
| P | + | ++ | | +++ | | +++ |
| Between Chi | | | | | | 1.70 |
| Between df | | | | | | 2 |
| Between P | | | | | | N.S. |
| Btwn(F) P | | | | | | N.S. |
| Btwn(R) P | | | | | | N.S. |

Detailed Country in "other Asia"

| | India | HongKong | other | Total |
|---|---|---|---|---|
| N | | | | |
| NS | | | | |
| Wt | | | | |
| Het Chi | | | | |
| Het df | | | | |
| Het P | | | | N.S. |
| Fixed RR | | | | |
| RRl | | | | |
| RRu | | | | |
| P | | | | +++ |
| Random RR | | | | |
| RRl | | | | |
| RRu | | | | |
| P | | | | +++ |
| Between Chi | | | | |
| Between df | | | | |
| Between P | | | | N.S. |
| Btwn(F) P | | | | N.S. |
| Btwn(R) P | | | | N.S. |

Detailed other continent

| | SCAmer | Total |
|---|---|---|
| N | 2 | 2 |
| NS | 2 | 2 |
| Wt | 2.01 | 2.01 |
| Het Chi | 1.79 | 1.79 |
| Het df | 1 | 1 |
| Het P | N.S. | N.S. |
| Fixed RR | 3.36 | 3.36 |
| RRl | 0.84 | 0.84 |
| RRu | 13.41 | 13.41 |
| P | (+) | (+) |
| Random RR | 4.37 | 4.37 |
| RRl | 0.54 | 0.54 |
| RRu | 35.16 | 35.16 |
| P | N.S. | N.S. |
| Between Chi | | |
| Between df | | |
| Between P | | N.S. |
| Btwn(F) P | | N.S. |
| Btwn(R) P | | N.S. |

Table 2J2 - 3

IESLC - Meta-analysis of Ex Smoking, Years quit (vs never), "Low"
Squamous, Any Product (or Cigarettes if Any not available)
Most adjusted

| | <1960 | Start year of study 1960-69 | 1970-79 | 1980-89 | 1990+ | Total |
|---|---|---|---|---|---|---|
| N | | 3 | 2 | 7 | 2 | 14 |
| NS | | 2 | 1 | 6 | 2 | 11 |
| Wt | | 8.27 | 40.86 | 14.74 | 2.22 | 66.08 |
| Het Chi | | 3.95 | 7.34 | 4.15 | 0.00 | 22.30 |
| Het df | | 2 | 1 | 6 | 1 | 13 |
| Het P | | N.S. | ** | N.S. | N.S. | (*) |
| Fixed RR | | 8.73 | 8.86 | 5.37 | 2.00 | 7.53 |
| RRl | | 4.42 | 6.52 | 3.22 | 0.54 | 5.91 |
| RRu | | 17.27 | 12.04 | 8.95 | 7.45 | 9.58 |
| P | | +++ | +++ | +++ | N.S. | +++ |
| Random RR | | 6.78 | 5.44 | 5.37 | 2.00 | 5.59 |
| RRl | | 2.15 | 1.37 | 3.22 | 0.54 | 3.68 |
| RRu | | 21.40 | 21.55 | 8.95 | 7.45 | 8.48 |
| P | | ++ | + | +++ | N.S. | +++ |
| Between Chi | | | | | | 6.85 |
| Between df | | | | | | 3 |
| Between P | | | | | | (*) |
| Btwn(F) P | | | | | | N.S. |
| Btwn(R) P | | | | | | N.S. |

| | Study type (1) CC | other | Total |
|---|---|---|---|
| N | 14 | | 14 |
| NS | 11 | | 11 |
| Wt | 66.08 | | 66.08 |
| Het Chi | 22.30 | | 22.30 |
| Het df | 13 | | 13 |
| Het P | (*) | | (*) |
| Fixed RR | 7.53 | | 7.53 |
| RRl | 5.91 | | 5.91 |
| RRu | 9.58 | | 9.58 |
| P | +++ | | +++ |
| Random RR | 5.59 | | 5.59 |
| RRl | 3.68 | | 3.68 |
| RRu | 8.48 | | 8.48 |
| P | +++ | | +++ |
| Between Chi | | | |
| Between df | | | |
| Between P | | | N.S. |
| Btwn(F) P | | | N.S. |
| Btwn(R) P | | | N.S. |

| | Study type (2) CC | prosp | other | Total |
|---|---|---|---|---|
| N | 14 | | | 14 |
| NS | 11 | | | 11 |
| Wt | 66.08 | | | 66.08 |
| Het Chi | 22.30 | | | 22.30 |
| Het df | 13 | | | 13 |
| Het P | (*) | | | (*) |
| Fixed RR | 7.53 | | | 7.53 |
| RRl | 5.91 | | | 5.91 |
| RRu | 9.58 | | | 9.58 |
| P | +++ | | | +++ |
| Random RR | 5.59 | | | 5.59 |
| RRl | 3.68 | | | 3.68 |
| RRu | 8.48 | | | 8.48 |
| P | +++ | | | +++ |
| Between Chi | | | | |
| Between df | | | | |
| Between P | | | | N.S. |
| Btwn(F) P | | | | N.S. |
| Btwn(R) P | | | | N.S. |

Table 2J2 - 3

IESLC - Meta-analysis of Ex Smoking, Years quit (vs never), "Low"
Squamous, Any Product (or Cigarettes if Any not available)
Most adjusted

| | Study size (number of LC cases) 100-249 | 250-499 | 500-999 | 1000+ | Total |
|---|---|---|---|---|---|
| N | 4 | 3 | 2 | 5 | 14 |
| NS | 4 | 2 | 1 | 4 | 11 |
| Wt | 3.49 | 4.15 | 4.80 | 53.64 | 66.08 |
| Het Chi | 2.64 | 1.25 | 0.63 | 10.38 | 22.30 |
| Het df | 3 | 2 | 1 | 4 | 13 |
| Het P | N.S. | N.S. | N.S. | * | (*) |
| Fixed RR | 2.30 | 4.74 | 5.40 | 8.68 | 7.53 |
| RRl | 0.81 | 1.81 | 2.21 | 6.64 | 5.91 |
| RRu | 6.57 | 12.41 | 13.20 | 11.34 | 9.58 |
| P | N.S. | ++ | +++ | +++ | +++ |
| Random RR | 2.30 | 4.74 | 5.40 | 6.97 | 5.59 |
| RRl | 0.81 | 1.81 | 2.21 | 3.96 | 3.68 |
| RRu | 6.57 | 12.41 | 13.20 | 12.25 | 8.48 |
| P | N.S. | ++ | +++ | +++ | +++ |
| Between Chi | | | | | 7.41 |
| Between df | | | | | 3 |
| Between P | | | | | (*) |
| Btwn(F) P | | | | | N.S. |
| Btwn(R) P | | | | | N.S. |

| | Risky occupational population no | mining | othRisky | Total |
|---|---|---|---|---|
| N | 14 | | | 14 |
| NS | 11 | | | 11 |
| Wt | 66.08 | | | 66.08 |
| Het Chi | 22.30 | | | 22.30 |
| Het df | 13 | | | 13 |
| Het P | (*) | | | (*) |
| Fixed RR | 7.53 | | | 7.53 |
| RRl | 5.91 | | | 5.91 |
| RRu | 9.58 | | | 9.58 |
| P | +++ | | | +++ |
| Random RR | 5.59 | | | 5.59 |
| RRl | 3.68 | | | 3.68 |
| RRu | 8.48 | | | 8.48 |
| P | +++ | | | +++ |
| Between Chi | | | | |
| Between df | | | | |
| Between P | | | | N.S. |
| Btwn(F) P | | | | N.S. |
| Btwn(R) P | | | | N.S. |

| | National cigarette tobacco type Virginia | blended | other | Total |
|---|---|---|---|---|
| N | 2 | 11 | 1 | 14 |
| NS | 1 | 9 | 1 | 11 |
| Wt | 4.80 | 60.60 | 0.68 | 66.08 |
| Het Chi | 0.63 | 19.84 | 0.00 | 22.30 |
| Het df | 1 | 10 | 0 | 13 |
| Het P | N.S. | * | N.S. | (*) |
| Fixed RR | 5.40 | 7.84 | 2.00 | 7.53 |
| RRl | 2.21 | 6.10 | 0.19 | 5.91 |
| RRu | 13.20 | 10.09 | 21.49 | 9.58 |
| P | +++ | +++ | N.S. | +++ |
| Random RR | 5.40 | 5.65 | 2.00 | 5.59 |
| RRl | 2.21 | 3.48 | 0.19 | 3.68 |
| RRu | 13.20 | 9.19 | 21.49 | 8.48 |
| P | +++ | +++ | N.S. | +++ |
| Between Chi | | | | 1.83 |
| Between df | | | | 2 |
| Between P | | | | N.S. |
| Btwn(F) P | | | | N.S. |
| Btwn(R) P | | | | N.S. |

Table 2J2 - 3

IESLC - Meta-analysis of Ex Smoking, Years quit (vs never), "Low"
Squamous, Any Product (or Cigarettes if Any not available)
Most adjusted

| | Any proxy use No/nk | Yes | Total |
|---|---|---|---|
| N | 11 | 3 | 14 |
| NS | 9 | 2 | 11 |
| Wt | 56.70 | 9.39 | 66.08 |
| Het Chi | 20.00 | 0.66 | 22.30 |
| Het df | 10 | 2 | 13 |
| Het P | * | N.S. | (*) |
| Fixed RR | 8.02 | 5.10 | 7.53 |
| RRl | 6.19 | 2.69 | 5.91 |
| RRu | 10.41 | 9.68 | 9.58 |
| P | +++ | +++ | +++ |
| Random RR | 5.45 | 5.10 | 5.59 |
| RRl | 3.20 | 2.69 | 3.68 |
| RRu | 9.28 | 9.68 | 8.48 |
| P | +++ | +++ | +++ |
| Between Chi | | | 1.65 |
| Between df | | | 1 |
| Between P | | | N.S. |
| Btwn(F) P | | | N.S. |
| Btwn(R) P | | | N.S. |

| | Full histological confirmation No | Yes | Total |
|---|---|---|---|
| N | 6 | 8 | 14 |
| NS | 5 | 6 | 11 |
| Wt | 14.20 | 51.88 | 66.08 |
| Het Chi | 3.92 | 13.05 | 22.30 |
| Het df | 5 | 7 | 13 |
| Het P | N.S. | (*) | (*) |
| Fixed RR | 4.37 | 8.73 | 7.53 |
| RRl | 2.60 | 6.65 | 5.91 |
| RRu | 7.36 | 11.46 | 9.58 |
| P | +++ | +++ | +++ |
| Random RR | 4.37 | 6.67 | 5.59 |
| RRl | 2.60 | 3.80 | 3.68 |
| RRu | 7.36 | 11.72 | 8.48 |
| P | +++ | +++ | +++ |
| Between Chi | | | 5.33 |
| Between df | | | 1 |
| Between P | | | * |
| Btwn(F) P | | | (*) |
| Btwn(R) P | | | N.S. |

| | Number of adjustment variables (1) 0 | 1 | 2+/+nk | Total |
|---|---|---|---|---|
| N | 10 | 1 | 3 | 14 |
| NS | 7 | 1 | 3 | 11 |
| Wt | 56.53 | 1.61 | 7.94 | 66.08 |
| Het Chi | 16.29 | 0.00 | 5.92 | 22.30 |
| Het df | 9 | 0 | 2 | 13 |
| Het P | (*) | N.S. | (*) | (*) |
| Fixed RR | 7.42 | 8.95 | 8.00 | 7.53 |
| RRl | 5.72 | 1.91 | 3.99 | 5.91 |
| RRu | 9.64 | 41.97 | 16.03 | 9.58 |
| P | +++ | ++ | +++ | +++ |
| Random RR | 5.19 | 8.95 | 4.73 | 5.59 |
| RRl | 3.17 | 1.91 | 1.06 | 3.68 |
| RRu | 8.50 | 41.97 | 21.16 | 8.48 |
| P | +++ | ++ | + | +++ |
| Between Chi | | | | 0.09 |
| Between df | | | | 2 |
| Between P | | | | N.S. |
| Btwn(F) P | | | | N.S. |
| Btwn(R) P | | | | N.S. |

Table 2J2 - 3

IESLC - Meta-analysis of Ex Smoking, Years quit (vs never), "Low"
Squamous, Any Product (or Cigarettes if Any not available)
Most adjusted

| | Number of adjustment variables (2) | | | | | |
|---|---|---|---|---|---|---|
| | 0 | 1 | 2 | 3-5 | 6+/+nk | Total |
| N | 10 | 1 | 2 | | 1 | 14 |
| NS | 7 | 1 | 2 | | 1 | 11 |
| Wt | 56.53 | 1.61 | 7.26 | | 0.68 | 66.08 |
| Het Chi | 16.29 | 0.00 | 4.49 | | 0.00 | 22.30 |
| Het df | 9 | 0 | 1 | | 0 | 13 |
| Het P | (*) | N.S. | * | | N.S. | (*) |
| Fixed RR | 7.42 | 8.95 | 9.11 | | 2.00 | 7.53 |
| RRl | 5.72 | 1.91 | 4.40 | | 0.19 | 5.91 |
| RRu | 9.64 | 41.97 | 18.85 | | 21.49 | 9.58 |
| P | +++ | ++ | +++ | | N.S. | +++ |
| Random RR | 5.19 | 8.95 | 5.92 | | 2.00 | 5.59 |
| RRl | 3.17 | 1.91 | 0.91 | | 0.19 | 3.68 |
| RRu | 8.50 | 41.97 | 38.43 | | 21.49 | 8.48 |
| P | +++ | ++ | (+) | | N.S. | +++ |
| Between Chi | | | | | | 1.52 |
| Between df | | | | | | 3 |
| Between P | | | | | | N.S. |
| Btwn(F) P | | | | | | N.S. |
| Btwn(R) P | | | | | | N.S. |

| | Product | | | |
|---|---|---|---|---|
| | all/unsp | cig+/-ot | cig only | Total |
| N | 1 | 12 | 1 | 14 |
| NS | 1 | 9 | 1 | 11 |
| Wt | 0.80 | 64.81 | 0.47 | 66.08 |
| Het Chi | 0.00 | 18.64 | 0.00 | 22.30 |
| Het df | 0 | 11 | 0 | 13 |
| Het P | N.S. | (*) | N.S. | (*) |
| Fixed RR | 1.00 | 7.67 | 18.60 | 7.53 |
| RRl | 0.11 | 6.01 | 1.06 | 5.91 |
| RRu | 8.95 | 9.78 | 326.10 | 9.58 |
| P | N.S. | +++ | + | +++ |
| Random RR | 1.00 | 5.84 | 18.60 | 5.59 |
| RRl | 0.11 | 3.87 | 1.06 | 3.68 |
| RRu | 8.95 | 8.82 | 326.10 | 8.48 |
| P | N.S. | +++ | + | +++ |
| Between Chi | | | | 3.66 |
| Between df | | | | 2 |
| Between P | | | | N.S. |
| Btwn(F) P | | | | N.S. |
| Btwn(R) P | | | | N.S. |

| | Denominator | | |
|---|---|---|---|
| | nev any | nev cigs | Total |
| N | 10 | 4 | 14 |
| NS | 8 | 3 | 11 |
| Wt | 60.13 | 5.95 | 66.08 |
| Het Chi | 19.49 | 2.01 | 22.30 |
| Het df | 9 | 3 | 13 |
| Het P | * | N.S. | (*) |
| Fixed RR | 7.79 | 5.31 | 7.53 |
| RRl | 6.05 | 2.38 | 5.91 |
| RRu | 10.03 | 11.86 | 9.58 |
| P | +++ | +++ | +++ |
| Random RR | 5.43 | 5.31 | 5.59 |
| RRl | 3.29 | 2.38 | 3.68 |
| RRu | 8.99 | 11.86 | 8.48 |
| P | +++ | +++ | +++ |
| Between Chi | | | 0.80 |
| Between df | | | 1 |
| Between P | | | N.S. |
| Btwn(F) P | | | N.S. |
| Btwn(R) P | | | N.S. |

Table 2J2 - 3

IESLC - Meta-analysis of Ex Smoking, Years quit (vs never), "Low"
Squamous, Any Product (or Cigarettes if Any not available)
Most adjusted

| | Derivation of RR/CI Orig | StdCalc | Other | Total |
|---|---|---|---|---|
| N | 2 | 9 | 3 | 14 |
| NS | 2 | 6 | 3 | 11 |
| Wt | 2.29 | 56.06 | 7.73 | 66.08 |
| Het Chi | 1.07 | 15.89 | 4.72 | 22.30 |
| Het df | 1 | 8 | 2 | 13 |
| Het P | N.S. | * | (*) | (*) |
| Fixed RR | 5.73 | 7.37 | 9.51 | 7.53 |
| RRl | 1.57 | 5.67 | 4.70 | 5.91 |
| RRu | 20.93 | 9.57 | 19.25 | 9.58 |
| P | ++ | +++ | +++ | +++ |
| Random RR | 5.61 | 4.96 | 7.54 | 5.59 |
| RRl | 1.44 | 2.97 | 1.86 | 3.68 |
| RRu | 21.87 | 8.29 | 30.53 | 8.48 |
| P | + | +++ | ++ | +++ |
| Between Chi | | | | 0.62 |
| Between df | | | | 2 |
| Between P | | | | N.S. |
| Btwn(F) P | | | | N.S. |
| Btwn(R) P | | | | N.S. |

Table 2J2 - 4

IESLC - Meta-analysis of Ex Smoking, Years quit (vs never), "Low"
Squamous, Any Product (or Cigarettes if Any not available)
Least adjusted

| REF | NRR | X | SEX | AGEL | AGEH | RACE | YF | LC TYPE | LOC | START | ST | NLC | R | VB | P | H | AD | PRODUCT | exL | exH | DENOM | De |
|---|---|---|---|---|---|---|---|---|---|---|---|---|---|---|---|---|---|---|---|---|---|---|
| JAHN | 594 | | m | 0 | 0 | all | - | q | Eu:Ger | 1988 | CC | 1004 | n | bl | n | n | 0 | cig+/-ot | 11 | 20 | nev any | st |
| JAIN | 543 | | m | 0 | 0 | all | - | q | NAmer | 1981 | CC | 845 | n | V | y | n | 0 | cig+/-ot | 10 | 999 | nev cigs | st |
| JAIN | 507 | | f | 0 | 0 | all | - | q | NAmer | 1981 | CC | 845 | n | V | y | n | 0 | cig+/-ot | 10 | 999 | nev cigs | st |
| JEDRYC | 543 | | m | 0 | 0 | all | - | q | Eu:est | 1980 | CC | 1630 | n | bl | y | n | 0 | cig+/-ot | 10 | 999 | nev any | st |
| LUBIN2 | 767 | | m | 0 | 0 | all | - | q | Eu:mul | 1976 | CC | 7804 | n | bl | n | y | 0 | cig+/-ot | 10 | 14 | nev any | st |
| LUBIN2 | 894 | | f | 0 | 0 | all | - | q | Eu:mul | 1976 | CC | 7804 | n | bl | n | y | 0 | cig+/-ot | 10 | 19 | nev any | st |
| LUO | 519 | x | c | 0 | 0 | all | - | q | As:Chi | 1990 | CC | 102 | n | ot | n | y | 0 | cig+/-ot | 10 | 999 | nev cigs | st |
| MATOS | 621 | x | m | 0 | 0 | all | - | q | SCAmer | 1994 | CC | 200 | n | bl | n | n | 0 | cig+/-ot | 11 | 999 | nev any | st |
| PEZZOT | 579 | | m | 0 | 0 | all | - | q | SCAmer | 1987 | CC | 215 | n | bl | n | y | 0 | cig only | 11 | 999 | nev cigs | ot |
| SVENSS | 557 | | f | 0 | 0 | all | - | q | Eu:Sca | 1983 | CC | 210 | n | bl | n | n | 0 | all/unsp | 11 | 999 | nev any | st |
| WAKAI | 539 | x | m | 0 | 0 | all | - | q | As:Jap | 1988 | CC | 333 | n | bl | n | y | 0 | cig+/-ot | 10 | 19 | nev any | st |
| WYNDE3 | 552 | | m | 0 | 0 | all | - | KI | NAmer | 1966 | CC | 350 | n | bl | n | y | 0 | cig+/-ot | 10 | 999 | nev any | st |
| WYNDE3 | 573 | | f | 0 | 0 | all | - | KI | NAmer | 1966 | CC | 350 | n | bl | n | y | 0 | cig+/-ot | 10 | 999 | nev any | st |
| WYNDE6 | 773 | x | m | 0 | 0 | all | - | KI | NAmer | 1969 | CC | 4423 | n | bl | n | y | 0 | cig+/-ot | 11 | 15 | nev any | st |

Cigarette type is all/unspec for all RRs

Table 2J2 - 5

IESLC - Meta-analysis of Ex Smoking, Years quit (vs never), "Low"
Squamous, Any Product (or Cigarettes if Any not available)
Least adjusted

| REF | NRR | SEX | AD | Number Exposed Case | Exposed Cont | Non-exposed Case | Non-exposed Cont | RR | 95.00%CI |
|---|---|---|---|---|---|---|---|---|---|
| JAHN | 594 | m | 0 | 18 | 130 | 3 | 138 | 6.37 | ( 1.83- 22.13) |
| JAIN | 543 | m | 0 | 23 | 113 | 2 | 85 | 8.65 | ( 1.98- 37.70) |
| JAIN | 507 | f | 0 | 7 | 61 | 6 | 214 | 4.09 | ( 1.33- 12.63) |
| Subtotal JAIN | | | | | | | | 5.40 | ( 2.21- 13.20) |
| JEDRYC | 543 | m | 0 | 23 | 230 | 6 | 289 | 4.82 | ( 1.93- 12.03) |
| LUBIN2 | 767 | m | 0 | 146 | 693 | 54 | 2616 | 10.21 | ( 7.39- 14.10) |
| LUBIN2 | 894 | f | 0 | 5 | 33 | 72 | 1180 | 2.48 | ( 0.94- 6.55) |
| Subtotal LUBIN2 | | | | | | | | 8.86 | ( 6.52- 12.04) |
| LUO | 519 | c | 0 | 1 | 10 | 5 | 51 | 1.02 | ( 0.11- 9.69) |
| MATOS | 621 | m | 0 | 5 | 101 | 3 | 110 | 1.82 | ( 0.42- 7.79) |
| PEZZOT | 579 | m | 0 | 8 | 106 | 0 | 116 | 18.60~ | ( 1.06- 326.10) |
| SVENSS | 557 | f | 0 | 1 | 24 | 5 | 120 | 1.00 | ( 0.11- 8.95) |
| WAKAI | 539 | m | 0 | 12 | 44 | 2 | 65 | 8.86 | ( 1.89- 41.56) |
| WYNDE3 | 552 | m | 0 | 6 | 65 | 3 | 88 | 2.71 | ( 0.65- 11.23) |
| WYNDE3 | 573 | f | 0 | 1 | 3 | 5 | 76 | 5.07 | ( 0.44- 57.98) |
| Subtotal WYNDE3 | | | | | | | | 3.17 | ( 0.93- 10.85) |
| WYNDE6 | 773 | m | 0 | 22 | 259 | 8 | 1667 | 17.70 | ( 7.80- 40.18) |
| Totals | | | | 278 | 1872 | 174 | 6815 | | |

*prospective study ~ With 0.5 adjustment for zero

| REF | NRR | SEX | AD | Ys | Ws | Qs | Ps |
|---|---|---|---|---|---|---|---|
| JAHN | 594 | m | 0 | 1.85 | 2.48 | 0.07 | 0.0036 |
| JAIN | 543 | m | 0 | 2.16 | 1.77 | 0.03 | 0.0041 |
| JAIN | 507 | f | 0 | 1.41 | 3.02 | 1.14 | 0.0142 |
| Subtotal JAIN | | | | 1.69 | 4.80 | 1.17 | |
| JEDRYC | 543 | m | 0 | 1.57 | 4.59 | 0.93 | 0.0008 |
| LUBIN2 | 767 | m | 0 | 2.32 | 36.77 | 3.31 | 0.0000 |
| LUBIN2 | 894 | f | 0 | 0.91 | 4.08 | 5.06 | 0.0662 |
| Subtotal LUBIN2 | | | | 2.18 | 40.86 | 8.37 | |
| LUO | 519 | c | 0 | 0.02 | 0.76 | 3.04 | 0.9862 |
| MATOS | 621 | m | 0 | 0.60 | 1.81 | 3.68 | 0.4224 |
| PEZZOT | 579 | m | 0 | 2.92 | 0.47 | 0.38 | 0.0455 |
| SVENSS | 557 | f | 0 | 0.00 | 0.80 | 3.27 | 1.0000 |
| WAKAI | 539 | m | 0 | 2.18 | 1.61 | 0.04 | 0.0056 |
| WYNDE3 | 552 | m | 0 | 1.00 | 1.90 | 2.00 | 0.1699 |
| WYNDE3 | 573 | f | 0 | 1.62 | 0.65 | 0.10 | 0.1919 |
| Subtotal WYNDE3 | | | | 1.16 | 2.55 | 2.10 | |
| WYNDE6 | 773 | m | 0 | 2.87 | 5.72 | 4.14 | 0.0000 |

| | | |
|---|---|---|
| | N | 14 |
| | NS | 11 |
| | Wt | 66.42 |
| Het | Chi | 27.21 |
| Het | df | 13 |
| Het | P | * |
| Fixed | RR | 7.56 |
| | RRl | 5.94 |
| | RRu | 9.61 |
| | P | +++ |
| Random | RR | 5.35 |
| | RRl | 3.38 |
| | RRu | 8.47 |
| | P | +++ |
| Asymm | P | * |

Table 2J2 - 6

IESLC - Meta-analysis of Ex Smoking, Years quit (vs never), "Low"
Squamous, Any Product (or Cigarettes if Any not available)
Least adjusted

| | Sex | | | |
|---|---|---|---|---|
| | combined | male | female | Total |
| N | 1 | 9 | 4 | 14 |
| NS | 1 | 9 | 4 | 14 |
| Wt | 0.76 | 57.11 | 8.55 | 66.42 |
| Het Chi | 0.00 | 12.92 | 1.56 | 27.21 |
| Het df | 0 | 8 | 3 | 13 |
| Het P | N.S. | N.S. | N.S. | * |
| Fixed RR | 1.02 | 8.97 | 2.87 | 7.56 |
| RRl | 0.11 | 6.92 | 1.47 | 5.94 |
| RRu | 9.69 | 11.63 | 5.61 | 9.61 |
| P | N.S. | +++ | ++ | +++ |
| Random RR | 1.02 | 7.56 | 2.87 | 5.35 |
| RRl | 0.11 | 4.83 | 1.47 | 3.38 |
| RRu | 9.69 | 11.83 | 5.61 | 8.47 |
| P | N.S. | +++ | ++ | +++ |
| Between Chi | | | | 12.73 |
| Between df | | | | 2 |
| Between P | | | | ** |
| Btwn(F) P | | | | * |
| Btwn(R) P | | | | * |

Table 2J2 - 7

IESLC - Meta-analysis of Ex Smoking, Years quit (vs never), "Low"
Squamous, Any Product (or Cigarettes if Any not available)
Excluded studies (and stage at which they were excluded)

| | |
|---|---|
| 1 | AGUDO AKIBA AMANDU AMES AXELSS BEST BOUCHA BOUCOT BRESLO CHEN CHEN2 CHIAZZ DEAN2 DOSEME ENGELA FAN GENG GER GUO HAENSZ HEGMAN HOLE HU HU2 JUSSAW KATSOU KAUFMA KOO KOULUM KREUZE LETOUR LEVIN LIAW LIU3 LIU4 LIU5 MCCONN MIGRAN MRFITR NOTAN2 OSANN2 PERNU QIAO2 RACHTA RESTRE SADOWS SEGI2 STASZE TIZZAN VUTUC WATSON WU WUWILL WYNDE2 WYNDE8 XU YUAN ZHANG ZHENG ZHOU |
| 2 | BUFFLE HUMBLE PISANI PRESCO WYNDE7 |
| 3 | MCDUFF SPITZ |
| 4 | ARMADA AUVINE BECHER BENSHL BLOT1 BOFFET BROSS CARPEN CEDERL CHOI CHYOU CORREA CPSI CPSII DAMBER DARBY DEAN3 DESTEF DOLL DOLL2 DORGAN DORN GAO GAO2 GARCIA GARSHI GILLIS GRAHAM GURSEL HAMMO2 HIRAYA JOLY KAISE2 KHUDER LAUSSM LUBIN PEZZO2 QIAO SPEIZE SUZUK2 TVERDA WANG2 WIGLE WU2 |
| 5 | ALDERS HAMMON |
| 10 | SOBUE |
| 14 | BARBON BROWN3 |
| 15 | BENHAM |

Table 2J2 - 8

Potentially overlapping studies

| REF | REFGP | PRINC | . OVERLAP/LINK |
|---|---|---|---|
| LUBIN2 | LUBIN2 | 1 | Lubin-combined |
| WYNDE6 | WYNDE6 | 1 | WYNDE5/6/7/8 |
| JAHN | BOFFET | 2 | Subset of BOFFET |

Table 2J2 - 9

Most adjusted - insufficient data for meta-analysis

| REF | NRR | SEX | AGEL | AGEH | RACE | YF | LC TYPE | LOC | START | ST | NLC | R | VB | P | H | AD | PRODUCT | exL | exH | DENOM | De |
|---|---|---|---|---|---|---|---|---|---|---|---|---|---|---|---|---|---|---|---|---|---|
| ALDERS | 537 | m | 0 | 0 | all | - | q+s | Eu:UK | 1977 | CC | 1448 | n | V | n | n | 1 | cig only | 10 | 999 | nev any st | |
| ALDERS | 548 | f | 0 | 0 | all | - | q+s | Eu:UK | 1977 | CC | 1448 | n | V | n | n | 1 | cig only | 10 | 999 | nev any st | |
| HAMMON | 501 | m | 0 | 0 | wh | 0 | not a | NAmer | 1952 | pr | 448 | n | bl | n | n | 1 | cig only | 10 | 999 | nev any st | |

| REF | NRR | RR | SIG | RRDATA comment |
|---|---|---|---|---|
| ALDERS | 537 | 2.33 | | 0 |
| ALDERS | 548 | 0.67 | | 0 |
| HAMMON | 501 | * | | RR for <1 pack per day is 2.44, while that for 1+ packs is 17.79 |

Table 2J3 -

IESLC - Meta-analysis of Ex Smoking, Years quit (vs never), "Mid"
Squamous, Any Product (or Cigarettes if Any not available)

This analysis is restricted to results for:
1) Ex smokers
2) Results by Years quit (vs never)
3) Categorical results by Years quit (vs never)
4) Squamous (or near equivalent)
5) Results complete enough for use in metaanalysis

Within each study, results are then selected (in the following order of preference, within each sex) for:
6) (not applicable)
7) PRODUCT: all/unspec, cigarettes regardless of other products, cigarettes only
8) CIGTYPE: all/unspecified, MC regardless of HR, MC only
9) (not applicable)
10) DENOM: never smoked anything, never smoked cigarettes, never any + low, never cigs + low
11) Followup period (YF, prospective studies): whole study (coded as 0) or longest available
12) LCtype: squamous or nearest available, but not adeno. (q = squamous, s = small,
a = adeno, KI = Kreyberg I, u = undifferentiated)
13) Race: all or nearest available, otherwise by race (wh or w = white, bl or b = black, hi = hispanic
ch = chinese, jap = japanese, haw = hawaiian, w+o = white + oriental, sca = scandinavian, as = asian)
14) Years quit (vs never) "mid" in key scheme 1 (key value 7, maximum range 4-11)
15) For overlapping studies: principal rather than subsidiary studies
Finally by Age: whole study (coded as 0) if available, otherwise by widest available age group
and then for single sex results (m, f) in preference to results for both sexes combined (c).

Results adjusted (AD) for the most potential confounders are then chosen in Sections -1 to -3
and results adjusted for the least confounders in Sections -4 to -6. (Those least adjusted results which
actually differ from the most adjusted are marked 'x' in column X in Section -4)

Section -7 shows excluded studies, together with the stage (as above) at which no qualifying
results were found.

Section -8 lists the potentially overlapping studies which have been included (1=principal, 2=subsidiary).

Section -9 lists any results which would have been included in preference except that they had data not complete
enough for use in meta-analysis, with their significance (yes/no), if known, and any further comment as entered
on the database. It also lists as "gap" any categories for which no data were presented by the original authors.
This is commonly due to recent quitters having been combined with current smokers

In addition to those mentioned above, the following fields, levels and abbreviations are used:
* or nk = not known, n = no, y = yes, ot = other
nev = never
all/unspec = all or unspecified, cig+/-ot = cigarettes irrespective of other products (cigar, pipe etc)
MC = manufactured cigarettes, HR = hand-rolled cigarettes
exL, exH = range of exposure (low and high) in the smoking group, in terms of Years quit (vs never)
REF: 6-character study reference
NRR: number of the RR on the database within the study
ST : study type (CC = case control, pr or prosp = prospective)
NLC: number of lung cancer cases in whole study
R : risky occupational population (n = no, m = mining, o = other risky)
VB : national cigarette type (V = at least 75% Virginia, bl = at least 75% blended, ot = other)
P : any proxy use
H : full histological confirmation
De : derivation of RR/CI (or = original, st = standard method, ot = other method of estimation)

Table 2J3 - 1

IESLC - Meta-analysis of Ex Smoking, Years quit (vs never), "Mid"
Squamous, Any Product (or Cigarettes if Any not available)
Most adjusted

| REF | NRR | SEX | AGEL | AGEH | RACE | YF | LC TYPE | LOC | START | ST | NLC | R | VB | P | H | AD | PRODUCT | exL | exH | DENOM | De |
|---|---|---|---|---|---|---|---|---|---|---|---|---|---|---|---|---|---|---|---|---|---|
| JAHN | 595 | m | 0 | 0 | all | - | q | Eu:Ger | 1988 | CC | 1004 | n | bl | n | n | 0 | cig+/-ot | 6 | 10 | nev any | st |
| JEDRYC | 544 | m | 0 | 0 | all | - | q | Eu:est | 1980 | CC | 1630 | n | bl | y | n | 0 | cig+/-ot | 5 | 9 | nev any | st |
| LUBIN2 | 768 | m | 0 | 0 | all | - | q | Eu:mul | 1976 | CC | 7804 | n | bl | n | y | 0 | cig+/-ot | 5 | 9 | nev any | st |
| MATOS | 632 | m | 0 | 0 | all | - | q | SCAmer | 1994 | CC | 200 | n | bl | n | n | 2 | cig+/-ot | 6 | 10 | nev any | ot |
| WAKAI | 548 | m | 0 | 0 | all | - | q | As:Jap | 1988 | CC | 333 | n | bl | n | y | 1 | cig+/-ot | 5 | 9 | nev any | or |
| WYNDE6 | 789 | m | 0 | 0 | all | - | KI | NAmer | 1969 | CC | 4423 | n | bl | n | y | 2 | cig+/-ot | 7 | 10 | nev any | ot |

Cigarette type is all/unspec for all RRs

Table 2J3 - 2

IESLC - Meta-analysis of Ex Smoking, Years quit (vs never), "Mid"
Squamous, Any Product (or Cigarettes if Any not available)
Most adjusted

| REF | NRR | SEX | AD | Number Exposed Case | Cont | Non-exposed Case | Cont | RR | 95.00%CI |
|---|---|---|---|---|---|---|---|---|---|
| JAHN | 595 | m | 0 | 29 | 63 | 3 | 138 | 21.17 | ( 6.22- 72.12) |
| JEDRYC | 544 | m | 0 | 22 | 82 | 6 | 289 | 12.92 | ( 5.07- 32.93) |
| LUBIN2 | 768 | m | 0 | 265 | 882 | 54 | 2616 | 14.56 | ( 10.76- 19.70) |
| MATOS | 632 | m | 2 | 5 | - | 3 | - | 6.00 | ( 1.19- 30.28) |
| WAKAI | 548 | m | 1 | 11 | - | 2 | - | 7.47 | ( 1.58- 35.30) |
| WYNDE6 | 789 | m | 2 | 36 | - | 8 | - | 17.20 | ( 7.92- 37.33) |
| Partial Totals | | | | 368 | 1027 | 76 | 3043 | | |

*prospective study

| REF | NRR | SEX | AD | Ys | Ws | Qs | Ps |
|---|---|---|---|---|---|---|---|
| JAHN | 595 | m | 0 | 3.05 | 2.56 | 0.39 | 0.0000 |
| JEDRYC | 544 | m | 0 | 2.56 | 4.39 | 0.05 | 0.0000 |
| LUBIN2 | 768 | m | 0 | 2.68 | 42.00 | 0.01 | 0.0000 |
| MATOS | 632 | m | 2 | 1.79 | 1.47 | 1.11 | 0.0300 |
| WAKAI | 548 | m | 1 | 2.01 | 1.59 | 0.68 | 0.0112 |
| WYNDE6 | 789 | m | 2 | 2.84 | 6.39 | 0.21 | 0.0000 |

| | | |
|---|---|---|
| | N | 6 |
| | NS | 6 |
| | Wt | 58.40 |
| Het | Chi | 2.45 |
| Het | df | 5 |
| Het | P | N.S. |
| Fixed | RR | 14.34 |
| | RRl | 11.10 |
| | RRu | 18.54 |
| | P | +++ |
| Random | RR | 14.34 |
| | RRl | 11.10 |
| | RRu | 18.54 |
| | P | +++ |
| Asymm | P | N.S. |

Table 2J3 - 3

IESLC - Meta-analysis of Ex Smoking, Years quit (vs never), "Mid"
Squamous, Any Product (or Cigarettes if Any not available)
Most adjusted

| | Sex combined | male | female | Total |
|---|---|---|---|---|
| N | | 6 | | 6 |
| NS | | 6 | | 6 |
| Wt | | 58.40 | | 58.40 |
| Het Chi | | 2.45 | | 2.45 |
| Het df | | 5 | | 5 |
| Het P | | N.S. | | N.S. |
| Fixed RR | | 14.34 | | 14.34 |
| RRl | | 11.10 | | 11.10 |
| RRu | | 18.54 | | 18.54 |
| P | | +++ | | +++ |
| Random RR | | 14.34 | | 14.34 |
| RRl | | 11.10 | | 11.10 |
| RRu | | 18.54 | | 18.54 |
| P | | +++ | | +++ |
| Between Chi | | | | |
| Between df | | | | |
| Between P | | | | N.S. |
| Btwn(F) P | | | | N.S. |
| Btwn(R) P | | | | N.S. |

Too few RRs for analysis by factor

Table 2J3 - 4

IESLC - Meta-analysis of Ex Smoking, Years quit (vs never), "Mid"
Squamous, Any Product (or Cigarettes if Any not available)
Least adjusted

| REF | NRR | X | SEX | AGEL | AGEH | RACE | YF | LC TYPE | LOC | START | ST | NLC | R | VB | P | H | AD | PRODUCT | exL | exH | DENOM | De |
|---|---|---|---|---|---|---|---|---|---|---|---|---|---|---|---|---|---|---|---|---|---|---|
| JAHN | 595 | | m | 0 | 0 | all | - | q | Eu:Ger | 1988 | CC | 1004 | n | bl | n | n | 0 | cig+/-ot | 6 | 10 | nev any | st |
| JEDRYC | 544 | | m | 0 | 0 | all | - | q | Eu:est | 1980 | CC | 1630 | n | bl | y | n | 0 | cig+/-ot | 5 | 9 | nev any | st |
| LUBIN2 | 768 | | m | 0 | 0 | all | - | q | Eu:mul | 1976 | CC | 7804 | n | bl | n | y | 0 | cig+/-ot | 5 | 9 | nev any | st |
| MATOS | 622 | x | m | 0 | 0 | all | - | q | SCAmer | 1994 | CC | 200 | n | bl | n | n | 0 | cig+/-ot | 6 | 10 | nev any | st |
| WAKAI | 540 | x | m | 0 | 0 | all | - | q | As:Jap | 1988 | CC | 333 | n | bl | n | y | 0 | cig+/-ot | 5 | 9 | nev any | st |
| WYNDE6 | 774 | x | m | 0 | 0 | all | - | KI | NAmer | 1969 | CC | 4423 | n | bl | n | y | 0 | cig+/-ot | 7 | 10 | nev any | st |

Cigarette type is all/unspec for all RRs

Table 2J3 - 5

IESLC - Meta-analysis of Ex Smoking, Years quit (vs never), "Mid"
Squamous, Any Product (or Cigarettes if Any not available)
Least adjusted

| REF | NRR | SEX | AD | Number Exposed Case | Cont | Non-exposed Case | Cont | RR | 95.00%CI |
|---|---|---|---|---|---|---|---|---|---|
| JAHN | 595 | m | 0 | 29 | 63 | 3 | 138 | 21.17 | ( 6.22- 72.12) |
| JEDRYC | 544 | m | 0 | 22 | 82 | 6 | 289 | 12.92 | ( 5.07- 32.93) |
| LUBIN2 | 768 | m | 0 | 265 | 882 | 54 | 2616 | 14.56 | ( 10.76- 19.70) |
| MATOS | 622 | m | 0 | 5 | 27 | 3 | 110 | 6.79 | ( 1.53- 30.19) |
| WAKAI | 540 | m | 0 | 11 | 48 | 2 | 65 | 7.45 | ( 1.58- 35.17) |
| WYNDE6 | 774 | m | 0 | 36 | 340 | 8 | 1667 | 22.06 | ( 10.17- 47.89) |
| Totals | | | | 368 | 1442 | 76 | 4885 | | |

*prospective study

| REF | NRR | SEX | AD | Ys | Ws | Qs | Ps |
|---|---|---|---|---|---|---|---|
| JAHN | 595 | m | 0 | 3.05 | 2.56 | 0.34 | 0.0000 |
| JEDRYC | 544 | m | 0 | 2.56 | 4.39 | 0.08 | 0.0000 |
| LUBIN2 | 768 | m | 0 | 2.68 | 42.00 | 0.01 | 0.0000 |
| MATOS | 622 | m | 0 | 1.92 | 1.73 | 1.04 | 0.0119 |
| WAKAI | 540 | m | 0 | 2.01 | 1.59 | 0.74 | 0.0112 |
| WYNDE6 | 774 | m | 0 | 3.09 | 6.40 | 1.04 | 0.0000 |

| | | |
|---|---|---|
| | N | 6 |
| | NS | 6 |
| | Wt | 58.67 |
| Het | Chi | 3.24 |
| Het | df | 5 |
| Het | P | N.S. |
| Fixed | RR | 14.73 |
| | RRl | 11.41 |
| | RRu | 19.03 |
| | P | +++ |
| Random | RR | 14.73 |
| | RRl | 11.41 |
| | RRu | 19.03 |
| | P | +++ |
| Asymm | P | N.S. |

Table 2J3 - 6

IESLC - Meta-analysis of Ex Smoking, Years quit (vs never), "Mid"
Squamous, Any Product (or Cigarettes if Any not available)
Least adjusted

| | Sex combined | male | female | Total |
|---|---|---|---|---|
| N | | 6 | | 6 |
| NS | | 6 | | 6 |
| Wt | | 58.67 | | 58.67 |
| Het Chi | | 3.24 | | 3.24 |
| Het df | | 5 | | 5 |
| Het P | | N.S. | | N.S. |
| Fixed RR | | 14.73 | | 14.73 |
| RRl | | 11.41 | | 11.41 |
| RRu | | 19.03 | | 19.03 |
| P | | +++ | | +++ |
| Random RR | | 14.73 | | 14.73 |
| RRl | | 11.41 | | 11.41 |
| RRu | | 19.03 | | 19.03 |
| P | | +++ | | +++ |
| Between Chi | | | | |
| Between df | | | | |
| Between P | | | | N.S. |
| Btwn(F) P | | | | N.S. |
| Btwn(R) P | | | | N.S. |

Table 2J3 - 7

IESLC - Meta-analysis of Ex Smoking, Years quit (vs never), "Mid"
Squamous, Any Product (or Cigarettes if Any not available)
Excluded studies (and stage at which they were excluded)

| | |
|---|---|
| 1 | AGUDO AKIBA AMANDU AMES AXELSS BEST BOUCHA BOUCOT BRESLO CHEN CHEN2 CHIAZZ DEAN2 DOSEME ENGELA FAN GENG GER GUO HAENSZ HEGMAN HOLE HU HU2 JUSSAW KATSOU KAUFMA KOO KOULUM KREUZE LETOUR LEVIN LIAW LIU3 LIU4 LIU5 MCCONN MIGRAN MRFITR NOTAN2 OSANN2 PERNU QIAO2 RACHTA RESTRE SADOWS SEGI2 STASZE TIZZAN VUTUC WATSON WU WUWILL WYNDE2 WYNDE8 XU YUAN ZHANG ZHENG ZHOU |
| 2 | BUFFLE HUMBLE PISANI PRESCO WYNDE7 |
| 3 | MCDUFF SPITZ |
| 4 | ARMADA AUVINE BECHER BENSHL BLOT1 BOFFET BROSS CARPEN CEDERL CHOI CHYOU CORREA CPSI CPSII DAMBER DARBY DEAN3 DESTEF DOLL DOLL2 DORGAN DORN GAO GAO2 GARCIA GARSHI GILLIS GRAHAM GURSEL HAMMO2 HIRAYA JOLY KAISE2 KHUDER LAUSSM LUBIN PEZZO2 QIAO SPEIZE SUZUK2 TVERDA WANG2 WIGLE WU2 |
| 5 | ALDERS HAMMON |
| 10 | SOBUE |
| 14 | BARBON BROWN3 JAIN LUO PEZZOT SVENSS WYNDE3 |
| 15 | BENHAM |

Table 2J3 - 8
Potentially overlapping studies

| REF | REFGP | PRINC | OVERLAP/LINK |
|---|---|---|---|
| LUBIN2 | LUBIN2 | 1 | Lubin-combined |
| WYNDE6 | WYNDE6 | 1 | WYNDE5/6/7/8 |
| JAHN | BOFFET | 2 | Subset of BOFFET |

Table 2J4 -

IESLC - Meta-analysis of Ex Smoking, Years quit (vs never), "High"
Squamous, Any Product (or Cigarettes if Any not available)

This analysis is restricted to results for:
1) Ex smokers
2) Results by Years quit (vs never)
3) Categorical results by Years quit (vs never)
4) Squamous (or near equivalent)
5) Results complete enough for use in metaanalysis

Within each study, results are then selected (in the following order of preference, within each sex) for:
6) PRODUCT: all/unspec, cigarettes regardless of other products, cigarettes only
7) CIGTYPE: all/unspecified, MC regardless of HR, MC only
8) (not applicable)
9) DENOM: never smoked anything, never smoked cigarettes, never any + low, never cigs + low
10) Followup period (YF, prospective studies): whole study (coded as 0) or longest available
11) LCtype: squamous or nearest available, but not adeno. (q = squamous, s = small,
a = adeno, KI = Kreyberg I, u = undifferentiated)
12) Race: all or nearest available, otherwise by race (wh or w = white, bl or b = black, hi = hispanic
ch = chinese, jap = japanese, haw = hawaiian, w+o = white + oriental, sca = scandinavian, as = asian)
13) Years quit (vs never) "high" in key scheme 1 (key value 3, maximum range 1-6)
14) For overlapping studies: principal rather than subsidiary studies
Finally by Age: whole study (coded as 0) if available, otherwise by widest available age group
and then for single sex results (m, f) in preference to results for both sexes combined (c).

Results adjusted (AD) for the most potential confounders are then chosen in Sections -1 to -3
and results adjusted for the least confounders in Sections -4 to -6. (Those least adjusted results which
actually differ from the most adjusted are marked 'x' in column X in Section -4)

Section -7 shows excluded studies, together with the stage (as above) at which no qualifying
results were found.

Section -8 lists the potentially overlapping studies which have been included (1=principal, 2=subsidiary).

Section -9 lists any results which would have been included in preference except that they had data not complete
enough for use in meta-analysis, with their significance (yes/no), if known, and any further comment as entered
on the database. It also lists as "gap" any categories for which no data were presented by the original authors.
This is commonly due to recent quitters having been combined with current smokers

In addition to those mentioned above, the following fields, levels and abbreviations are used:
* or nk = not known, n = no, y = yes, ot = other
nev = never
all/unspec = all or unspecified, cig+/-ot = cigarettes irrespective of other products (cigar, pipe etc)
MC = manufactured cigarettes, HR = hand-rolled cigarettes
exL, exH = range of exposure (low and high) in the smoking group, in terms of Years quit (vs never)
REF: 6-character study reference
NRR: number of the RR on the database within the study
ST : study type (CC = case control, pr or prosp = prospective)
NLC: number of lung cancer cases in whole study
R : risky occupational population (n = no, m = mining, o = other risky)
VB : national cigarette type (V = at least 75% Virginia, bl = at least 75% blended, ot = other)
P : any proxy use
H : full histological confirmation
De : derivation of RR/CI (or = original, st = standard method, ot = other method of estimation)

Table 2J4 - 1

IESLC - Meta-analysis of Ex Smoking, Years quit (vs never), "High"
Squamous, Any Product (or Cigarettes if Any not available)
Most adjusted

| REF | NRR | SEX | AGEL | AGEH | RACE | YF | LC TYPE | LOC | START | ST | NLC | R | VB | P | H | AD | PRODUCT | exL | exH | DENOM | De |
|---|---|---|---|---|---|---|---|---|---|---|---|---|---|---|---|---|---|---|---|---|---|
| BARBON | 597 | m | 0 | 0 | all | - | q | Eu:wst | 1979 | CC | 755 | n | bl | y | y | 1 | all/unsp | 0.1 | 4 | nev any | or |
| JAHN | 596 | m | 0 | 0 | all | - | q | Eu:Ger | 1988 | CC | 1004 | n | bl | n | n | 0 | cig+/-ot | 2 | 5 | nev any | st |
| LUBIN2 | 769 | m | 0 | 0 | all | - | q | Eu:mul | 1976 | CC | 7804 | n | bl | n | y | 0 | cig+/-ot | 0.1 | 4 | nev any | st |
| MATOS | 633 | m | 0 | 0 | all | - | q | SCAmer | 1994 | CC | 200 | n | bl | n | n | 2 | cig+/-ot | 1.0 | 5 | nev any | ot |
| WYNDE3 | 504 | m | 0 | 0 | all | - | KI | NAmer | 1966 | CC | 350 | n | bl | n | y | 0 | all/unsp | 1.0 | 3 | nev any | st |
| WYNDE6 | 791 | m | 0 | 0 | all | - | KI | NAmer | 1969 | CC | 4423 | n | bl | n | y | 2 | cig+/-ot | 1.0 | 3 | nev any | ot |

Cigarette type is all/unspec for all RRs

Table 2J4 - 2

IESLC - Meta-analysis of Ex Smoking, Years quit (vs never), "High"
Squamous, Any Product (or Cigarettes if Any not available)
Most adjusted

| REF | NRR | SEX | AD | Number Exposed Case | Exposed Cont | Non-exposed Case | Non-exposed Cont | RR | 95.00%CI |
|---|---|---|---|---|---|---|---|---|---|
| BARBON | 597 | m | 1 | 11 | - | 6 | - | 18.70 | ( 6.20- 56.30) |
| JAHN | 596 | m | 0 | 36 | 46 | 3 | 138 | 36.00 | ( 10.58- 122.45) |
| LUBIN2 | 769 | m | 0 | 498 | 1047 | 54 | 2616 | 23.04 | ( 17.25- 30.79) |
| MATOS | 633 | m | 2 | 4 | - | 3 | - | 7.00 | ( 1.32- 37.20) |
| WYNDE3 | 504 | m | 0 | 18 | 22 | 3 | 88 | 24.00 | ( 6.49- 88.81) |
| WYNDE6 | 791 | m | 2 | 80 | - | 8 | - | 53.80 | ( 25.75- 112.41) |
| Partial Totals | | | | 647 | 1115 | 77 | 2842 | | |

*prospective study

| REF | NRR | SEX | AD | Ys | Ws | Qs | Ps |
|---|---|---|---|---|---|---|---|
| BARBON | 597 | m | 1 | 2.93 | 3.16 | 0.26 | 0.0000 |
| JAHN | 596 | m | 0 | 3.58 | 2.56 | 0.34 | 0.0000 |
| LUBIN2 | 769 | m | 0 | 3.14 | 45.74 | 0.29 | 0.0000 |
| MATOS | 633 | m | 2 | 1.95 | 1.38 | 2.23 | 0.0223 |
| WYNDE3 | 504 | m | 0 | 3.18 | 2.24 | 0.00 | 0.0000 |
| WYNDE6 | 791 | m | 2 | 3.99 | 7.08 | 4.18 | 0.0000 |

| | | |
|---|---|---|
| | N | 6 |
| | NS | 6 |
| | Wt | 62.16 |
| Het | Chi | 7.30 |
| Het | df | 5 |
| Het | P | N.S. |
| Fixed | RR | 24.95 |
| | RRl | 19.45 |
| | RRu | 31.99 |
| | P | +++ |
| Random | RR | 26.22 |
| | RRl | 17.19 |
| | RRu | 39.98 |
| | P | +++ |
| Asymm | P | N.S. |

Table 2J4 - 3

IESLC - Meta-analysis of Ex Smoking, Years quit (vs never), "High"
Squamous, Any Product (or Cigarettes if Any not available)
Most adjusted

| | Sex combined | male | female | Total |
|---|---|---|---|---|
| N | | 6 | | 6 |
| NS | | 6 | | 6 |
| Wt | | 62.16 | | 62.16 |
| Het Chi | | 7.30 | | 7.30 |
| Het df | | 5 | | 5 |
| Het P | | N.S. | | N.S. |
| Fixed RR | | 24.95 | | 24.95 |
| RRl | | 19.45 | | 19.45 |
| RRu | | 31.99 | | 31.99 |
| P | | +++ | | +++ |
| Random RR | | 26.22 | | 26.22 |
| RRl | | 17.19 | | 17.19 |
| RRu | | 39.98 | | 39.98 |
| P | | +++ | | +++ |
| Between Chi | | | | |
| Between df | | | | |
| Between P | | | | N.S. |
| Btwn(F) P | | | | N.S. |
| Btwn(R) P | | | | N.S. |

Too few RRs for analysis by factor

Table 2J4 - 4

IESLC - Meta-analysis of Ex Smoking, Years quit (vs never), "High"
Squamous, Any Product (or Cigarettes if Any not available)
Least adjusted

| REF | NRR | X | SEX | AGEL | AGEH | RACE | YF | LC TYPE | LOC | START | ST | NLC | R | VB | P | H | AD | PRODUCT | exL | exH | DENOM | De |
|---|---|---|---|---|---|---|---|---|---|---|---|---|---|---|---|---|---|---|---|---|---|---|
| BARBON | 582 | x | m | 0 | 0 | all | - | q | Eu:wst | 1979 | CC | 755 | n | bl | y | y | 0 | all/unsp | 0.1 | 4 | nev any | st |
| JAHN | 596 | | m | 0 | 0 | all | - | q | Eu:Ger | 1988 | CC | 1004 | n | bl | n | n | 0 | cig+/-ot | 2 | 5 | nev any | st |
| LUBIN2 | 769 | | m | 0 | 0 | all | - | q | Eu:mul | 1976 | CC | 7804 | n | bl | n | y | 0 | cig+/-ot | 0.1 | 4 | nev any | st |
| MATOS | 623 | x | m | 0 | 0 | all | - | q | SCAmer | 1994 | CC | 200 | n | bl | n | n | 0 | cig+/-ot | 1.0 | 5 | nev any | st |
| WYNDE3 | 504 | | m | 0 | 0 | all | - | KI | NAmer | 1966 | CC | 350 | n | bl | n | y | 0 | all/unsp | 1.0 | 3 | nev any | st |
| WYNDE6 | 776 | x | m | 0 | 0 | all | - | KI | NAmer | 1969 | CC | 4423 | n | bl | n | y | 0 | cig+/-ot | 1.0 | 3 | nev any | st |

Cigarette type is all/unspec for all RRs

Table 2J4 - 5

IESLC - Meta-analysis of Ex Smoking, Years quit (vs never), "High"
Squamous, Any Product (or Cigarettes if Any not available)
Least adjusted

| REF | NRR | SEX | AD | Number Exposed Case | Exposed Cont | Non-exposed Case | Non-exposed Cont | RR | 95.00%CI |
|---|---|---|---|---|---|---|---|---|---|
| BARBON | 582 | m | 0 | 11 | 20 | 6 | 188 | 17.23 | ( 5.76- 51.58) |
| JAHN | 596 | m | 0 | 36 | 46 | 3 | 138 | 36.00 | ( 10.58- 122.45) |
| LUBIN2 | 769 | m | 0 | 498 | 1047 | 54 | 2616 | 23.04 | ( 17.25- 30.79) |
| MATOS | 623 | m | 0 | 4 | 23 | 3 | 110 | 6.38 | ( 1.34- 30.44) |
| WYNDE3 | 504 | m | 0 | 18 | 22 | 3 | 88 | 24.00 | ( 6.49- 88.81) |
| WYNDE6 | 776 | m | 0 | 80 | 307 | 8 | 1667 | 54.30 | ( 25.99- 113.46) |
| Totals | | | | 647 | 1465 | 77 | 4807 | | |

*prospective study

| REF | NRR | SEX | AD | Ys | Ws | Qs | Ps |
|---|---|---|---|---|---|---|---|
| BARBON | 582 | m | 0 | 2.85 | 3.20 | 0.41 | 0.0000 |
| JAHN | 596 | m | 0 | 3.58 | 2.56 | 0.36 | 0.0000 |
| LUBIN2 | 769 | m | 0 | 3.14 | 45.74 | 0.22 | 0.0000 |
| MATOS | 623 | m | 0 | 1.85 | 1.57 | 2.88 | 0.0202 |
| WYNDE3 | 504 | m | 0 | 3.18 | 2.24 | 0.00 | 0.0000 |
| WYNDE6 | 776 | m | 0 | 3.99 | 7.07 | 4.39 | 0.0000 |

| | | |
|---|---|---|
| | N | 6 |
| | NS | 6 |
| | Wt | 62.39 |
| Het | Chi | 8.27 |
| Het | df | 5 |
| Het | P | N.S. |
| Fixed | RR | 24.71 |
| | RRl | 19.28 |
| | RRu | 31.66 |
| | P | +++ |
| Random | RR | 25.52 |
| | RRl | 16.15 |
| | RRu | 40.31 |
| | P | +++ |
| Asymm | P | N.S. |

Table 2J4 - 6

IESLC - Meta-analysis of Ex Smoking, Years quit (vs never), "High"
Squamous, Any Product (or Cigarettes if Any not available)
Least adjusted

| | Sex combined | male | female | Total |
|---|---|---|---|---|
| N | | 6 | | 6 |
| NS | | 6 | | 6 |
| Wt | | 62.39 | | 62.39 |
| Het Chi | | 8.27 | | 8.27 |
| Het df | | 5 | | 5 |
| Het P | | N.S. | | N.S. |
| Fixed RR | | 24.71 | | 24.71 |
| RRl | | 19.28 | | 19.28 |
| RRu | | 31.66 | | 31.66 |
| P | | +++ | | +++ |
| Random RR | | 25.52 | | 25.52 |
| RRl | | 16.15 | | 16.15 |
| RRu | | 40.31 | | 40.31 |
| P | | +++ | | +++ |
| Between Chi | | | | |
| Between df | | | | |
| Between P | | | | N.S. |
| Btwn(F) P | | | | N.S. |
| Btwn(R) P | | | | N.S. |

Table 2J4 - 7

IESLC - Meta-analysis of Ex Smoking, Years quit (vs never), "High"
Squamous, Any Product (or Cigarettes if Any not available)
Excluded studies (and stage at which they were excluded)

| | |
|---|---|
| 1 | AGUDO AKIBA AMANDU AMES AXELSS BEST BOUCHA BOUCOT BRESLO CHEN CHEN2 CHIAZZ DEAN2 DOSEME ENGELA FAN GENG GER GUO HAENSZ HEGMAN HOLE HU HU2 JUSSAW KATSOU KAUFMA KOO KOULUM KREUZE LETOUR LEVIN LIAW LIU3 LIU4 LIU5 MCCONN MIGRAN MRFITR NOTAN2 OSANN2 PERNU QIAO2 RACHTA RESTRE SADOWS SEGI2 STASZE TIZZAN VUTUC WATSON WU WUWILL WYNDE2 WYNDE8 XU YUAN ZHANG ZHENG ZHOU |
| 2 | BUFFLE HUMBLE PISANI PRESCO WYNDE7 |
| 3 | MCDUFF SPITZ |
| 4 | ARMADA AUVINE BECHER BENSHL BLOT1 BOFFET BROSS CARPEN CEDERL CHOI CHYOU CORREA CPSI CPSII DAMBER DARBY DEAN3 DESTEF DOLL DOLL2 DORGAN DORN GAO GAO2 GARCIA GARSHI GILLIS GRAHAM GURSEL HAMMO2 HIRAYA JOLY KAISE2 KHUDER LAUSSM LUBIN PEZZO2 QIAO SPEIZE SUZUK2 TVERDA WANG2 WIGLE WU2 |
| 5 | ALDERS HAMMON |
| 10 | SOBUE |
| 14 | BROWN3 JAIN JEDRYC LUO PEZZOT SVENSS WAKAI |
| 15 | BENHAM |

Table 2J4 - 8
Potentially overlapping studies

| REF | REFGP | PRINC | . | OVERLAP/LINK |
|---|---|---|---|---|
| LUBIN2 | LUBIN2 | 1 | | Lubin-combined |
| WYNDE6 | WYNDE6 | 1 | | WYNDE5/6/7/8 |
| JAHN | BOFFET | 2 | | Subset of BOFFET |

Table 2J4 - 9
Most adjusted - insufficient data for meta-analysis

| REF | NRR | SEX | AGEL | AGEH | RACE | YF | LC TYPE | LOC | START | ST | NLC | R | VB | P | H | AD | PRODUCT | exL | exH | DENOM | De |
|---|---|---|---|---|---|---|---|---|---|---|---|---|---|---|---|---|---|---|---|---|---|
| JEDRYC | 545 | m | 0 | 0 | all | - | q | Eu:est | 1980 | CC | 1630 | n | bl | y | n | 0 | cig+/-ot | 1.0 | 4 | nev any | ot |
| WAKAI | 615 | m | 0 | 0 | all | - | q | As:Jap | 1988 | CC | 333 | n | bl | n | y | 1 | cig+/-ot | 1.0 | 4 | nev any | ot |

| REF | NRR | RR | SIG | RRDATA comment |
|---|---|---|---|---|
| JEDRYC | 545 | * | gap | 0 |
| WAKAI | 615 | * | gap | 0 |

Least adjusted - insufficient data for meta-analysis: as for adjusted plus the following

| REF | NRR | SEX | AGEL | AGEH | RACE | YF | LC TYPE | LOC | START | ST | NLC | R | VB | P | H | AD | PRODUCT | exL | exH | DENOM | De |
|---|---|---|---|---|---|---|---|---|---|---|---|---|---|---|---|---|---|---|---|---|---|
| WAKAI | 613 | m | 0 | 0 | all | - | q | As:Jap | 1988 | CC | 333 | n | bl | n | y | 0 | cig+/-ot | 1.0 | 4 | nev any | ot |

| REF | NRR | RR | SIG | RRDATA comment |
|---|---|---|---|---|
| WAKAI | 613 | * | gap | 0 |

Table 2J5 -

IESLC - Meta-analysis of Ex Smoking, Years quit (vs never), "Highest vs lowest"
Squamous, Any Product (or Cigarettes if Any not available)

This analysis is restricted to results for:
1) Ex smokers
2) Results by Years quit (vs never)
3) Categorical results by Years quit (vs never)
4) Denominator (unexposed) = "low"
5) Squamous (or near equivalent)
6) Results complete enough for use in metaanalysis

Within each study, results are then selected (in the following order of preference, within each sex) for:
7) (not applicable)
8) PRODUCT: all/unspec, cigarettes regardless of other products, cigarettes only
9) CIGTYPE: all/unspecified, MC regardless of HR, MC only
10) Results with least adjustment for other aspects of smoking (ADOS)
11) The highest vs lowest category
12) Followup period (YF, prospective studies): whole study (coded as 0) or longest available
13) LCtype: squamous or nearest available, but not adeno. (q = squamous, s = small,
a = adeno, KI = Kreyberg I, u = undifferentiated)
14) Race: all or nearest available, otherwise by race (wh or w = white, bl or b = black, hi = hispanic
ch = chinese, jap = japanese, haw = hawaiian, w+o = white + oriental, sca = scandinavian, as = asian)
15) For overlapping studies: principal rather than subsidiary studies
Finally by Age: whole study (coded as 0) if available, otherwise by widest available age group
and then for single sex results (m, f) in preference to results for both sexes combined (c).

Results adjusted (AD) for the most potential confounders are then chosen in Sections -1 to -3
and results adjusted for the least confounders in Sections -4 to -6. (Those least adjusted results which
actually differ from the most adjusted are marked 'x' in column X in Section -4)

Section -7 shows excluded studies, together with the stage (as above) at which no qualifying
results were found.

Section -8 lists the potentially overlapping studies which have been included (1=principal, 2=subsidiary).

Section -9 lists any results which would have been included in preference except that they had data not complete
enough for use in meta-analysis, with their significance (yes/no), if known, and any further comment as entered
on the database. It also lists as "gap" any categories for which no data were presented by the original authors.
This is commonly due to recent quitters having been combined with current smokers

In addition to those mentioned above, the following fields, levels and abbreviations are used:
* or nk = not known, n = no, y = yes, ot = other
all/unspec = all or unspecified, cig+/-ot = cigarettes irrespective of other products (cigar, pipe etc)
MC = manufactured cigarettes, HR = hand-rolled cigarettes
exL, exH = range of exposure (low and high) in the "highest" group, in terms of Years quit (vs never)
unexL, unexH = range of exposure (low and high) in the "lowest" group, in terms of Years quit (vs never)
REF: 6-character study reference
NRR: number of the RR on the database within the study
ST : study type (CC = case control, pr or prosp = prospective)
NLC: number of lung cancer cases in whole study
R : risky occupational population (n = no, m = mining, o = other risky)
VB : national cigarette type (V = at least 75% Virginia, bl = at least 75% blended, ot = other)
P : any proxy use
H : full histological confirmation
De : derivation of RR/CI (or = original, st = standard method, ot = other method of estimation)

Table 2J5 - 1

IESLC - Meta-analysis of Ex Smoking, Years quit (vs never), "Highest vs lowest"
Squamous, Any Product (or Cigarettes if Any not available)
Most adjusted

| REF | NRR | SEX | AGEL | AGEH | RACE | YF | LC TYPE | LOC | START | ST | NLC | R | VB | P | H | AD | ADOS | PRODUCT | exL | exH | unexL | unexH | De |
|---|---|---|---|---|---|---|---|---|---|---|---|---|---|---|---|---|---|---|---|---|---|---|---|
| BARBON | 601 | m | 0 | 0 | all | - | q | Eu:wst | 1979 | CC | 755 | n | bl | y | y | 1 | 0 | all/unsp | 0.1 | 4 | 25 | 999 | ot |
| JAHN | 604 | m | 0 | 0 | all | - | q | Eu:Ger | 1988 | CC | 1004 | n | bl | n | n | 0 | 0 | cig+/-ot | 0.1 | 0.9 | 21 | 999 | st |
| JAIN | 545 | m | 0 | 0 | all | - | q | NAmer | 1981 | CC | 845 | n | V | y | n | 0 | 0 | cig+/-ot | 2 | 9 | 10 | 999 | st |
| JAIN | 509 | f | 0 | 0 | all | - | q | NAmer | 1981 | CC | 845 | n | V | y | n | 0 | 0 | cig+/-ot | 2 | 9 | 10 | 999 | st |
| JEDRYC | 547 | m | 0 | 0 | all | - | q | Eu:est | 1980 | CC | 1630 | n | bl | y | n | 0 | 0 | cig+/-ot | 5 | 9 | 10 | 999 | st |
| LUBIN2 | 774 | m | 0 | 0 | all | - | q | Eu:mul | 1976 | CC | 7804 | n | bl | n | y | 0 | 0 | cig+/-ot | 0.1 | 4 | 20 | 999 | st |
| LUBIN2 | 898 | f | 0 | 0 | all | - | q | Eu:mul | 1976 | CC | 7804 | n | bl | n | y | 0 | 0 | cig+/-ot | 0.1 | 9 | 20 | 999 | st |
| MATOS | 635 | m | 0 | 0 | all | - | q | SCAmer | 1994 | CC | 200 | n | bl | n | n | 2 | 0 | cig+/-ot | 1.0 | 5 | 11 | 999 | ot |
| PEZZOT | 581 | m | 0 | 0 | all | - | q | SCAmer | 1987 | CC | 215 | n | bl | n | y | 0 | 0 | cig only | 1.0 | 10 | 11 | 999 | st |
| SOBUE | 782 | m | 0 | 0 | all | - | q | As:Jap | 1986 | CC | 1376 | n | bl | n | y | 0 | 0 | cig+/-ot | 1.0 | 4 | 10 | 999 | st |
| SVENSS | 559 | f | 0 | 0 | all | - | q | Eu:Sca | 1983 | CC | 210 | n | bl | n | n | 0 | 0 | all/unsp | 3 | 10 | 11 | 999 | st |
| WAKAI | 550 | m | 0 | 0 | all | - | q | As:Jap | 1988 | CC | 333 | n | bl | n | y | 1 | 0 | cig+/-ot | 5 | 9 | 20 | 999 | ot |
| WYNDE3 | 509 | m | 0 | 0 | all | - | KI | NAmer | 1966 | CC | 350 | n | bl | n | y | 0 | 0 | all/unsp | 1.0 | 3 | 13 | 999 | st |
| WYNDE6 | 796 | m | 0 | 0 | all | - | KI | NAmer | 1969 | CC | 4423 | n | bl | n | y | 2 | 0 | cig+/-ot | 1.0 | 3 | 16 | 999 | ot |

Cigarette type is all/unspec for all RRs

Table 2J5 - 2

IESLC - Meta-analysis of Ex Smoking, Years quit (vs never), "Highest vs lowest"
Squamous, Any Product (or Cigarettes if Any not available)
Most adjusted

| REF | NRR | SEX | AD | Number Case | Exposed Cont | Non-exposed Case | Cont | RR | 95.00%CI |
|---|---|---|---|---|---|---|---|---|---|
| BARBON | 601 | m | 1 | 11 | - | 4 | - | 9.84 | ( 2.71- 35.70) |
| JAHN | 604 | m | 0 | 74 | 8 | 8 | 146 | 168.81 | ( 60.93- 467.73) |
| JAIN | 545 | m | 0 | 24 | 46 | 23 | 113 | 2.56 | ( 1.32- 4.99) |
| JAIN | 509 | f | 0 | 15 | 36 | 7 | 61 | 3.63 | ( 1.35- 9.74) |
| Subtotal JAIN | | | | | | | | 2.86 | ( 1.65- 4.97) |
| JEDRYC | 547 | m | 0 | 22 | 82 | 23 | 230 | 2.68 | ( 1.42- 5.07) |
| LUBIN2 | 774 | m | 0 | 498 | 1047 | 106 | 1128 | 5.06 | ( 4.04- 6.34) |
| LUBIN2 | 898 | f | 0 | 38 | 95 | 2 | 29 | 5.80 | ( 1.32- 25.52) |
| Subtotal LUBIN2 | | | | | | | | 5.08 | ( 4.06- 6.35) |
| MATOS | 635 | m | 2 | 4 | - | 5 | - | 3.50 | ( 0.78- 15.62) |
| PEZZOT | 581 | m | 0 | 21 | 27 | 8 | 48 | 4.67 | ( 1.82- 11.96) |
| SOBUE | 782 | m | 0 | 52 | 116 | 30 | 144 | 2.15 | ( 1.29- 3.59) |
| SVENSS | 559 | f | 0 | 5 | 13 | 1 | 24 | 9.23 | ( 0.97- 87.63) |
| WAKAI | 550 | m | 1 | 11 | - | 3 | - | 3.64 | ( 0.96- 13.87) |
| WYNDE3 | 509 | m | 0 | 18 | 22 | 2 | 55 | 22.50 | ( 4.81- 105.19) |
| WYNDE6 | 796 | m | 2 | 80 | - | 19 | - | 10.76 | ( 6.40- 18.09) |
| Partial Totals | | | | 873 | 1492 | 241 | 1978 | | |

*prospective study

| REF | NRR | SEX | AD | Ys | Ws | Qs | Ps |
|---|---|---|---|---|---|---|---|
| BARBON | 601 | m | 1 | 2.29 | 2.31 | 1.00 | 0.0005 |
| JAHN | 604 | m | 0 | 5.13 | 3.70 | 45.33 | 0.0000 |
| JAIN | 545 | m | 0 | 0.94 | 8.64 | 4.07 | 0.0057 |
| JAIN | 509 | f | 0 | 1.29 | 3.94 | 0.45 | 0.0105 |
| Subtotal JAIN | | | | 1.05 | 12.58 | 4.52 | |
| JEDRYC | 547 | m | 0 | 0.99 | 9.48 | 3.89 | 0.0024 |
| LUBIN2 | 774 | m | 0 | 1.62 | 75.28 | 0.00 | 0.0000 |
| LUBIN2 | 898 | f | 0 | 1.76 | 1.75 | 0.03 | 0.0200 |
| Subtotal LUBIN2 | | | | 1.62 | 77.03 | 0.03 | |
| MATOS | 635 | m | 2 | 1.25 | 1.71 | 0.24 | 0.1013 |
| PEZZOT | 581 | m | 0 | 1.54 | 4.34 | 0.03 | 0.0013 |
| SOBUE | 782 | m | 0 | 0.77 | 14.68 | 10.90 | 0.0033 |
| SVENSS | 559 | f | 0 | 2.22 | 0.76 | 0.27 | 0.0529 |
| WAKAI | 550 | m | 1 | 1.29 | 2.15 | 0.24 | 0.0579 |
| WYNDE3 | 509 | m | 0 | 3.11 | 1.62 | 3.56 | 0.0001 |
| WYNDE6 | 796 | m | 2 | 2.38 | 14.23 | 7.96 | 0.0000 |

| | | |
|---|---|---|
| | N | 14 |
| | NS | 12 |
| | Wt | 144.59 |
| Het | Chi | 78.00 |
| Het | df | 13 |
| Het | P | *** |
| Fixed | RR | 5.09 |
| | RRl | 4.33 |
| | RRu | 5.99 |
| | P | +++ |
| Random | RR | 6.22 |
| | RRl | 3.75 |
| | RRu | 10.30 |
| | P | +++ |
| Asymm | P | N.S. |

Table 2J5 - 3

IESLC - Meta-analysis of Ex Smoking, Years quit (vs never), "Highest vs lowest"
Squamous, Any Product (or Cigarettes if Any not available)
Most adjusted

| | Sex | | | |
|---|---|---|---|---|
| | combined | male | female | Total |
| N | | 11 | 3 | 14 |
| NS | | 11 | 3 | 14 |
| Wt | | 138.14 | 6.45 | 144.59 |
| Het Chi | | 77.24 | 0.68 | 78.00 |
| Het df | | 10 | 2 | 13 |
| Het P | | *** | N.S. | *** |
| Fixed RR | | 5.12 | 4.60 | 5.09 |
| RRl | | 4.33 | 2.13 | 4.33 |
| RRu | | 6.05 | 9.95 | 5.99 |
| P | | +++ | +++ | +++ |
| Random RR | | 6.49 | 4.60 | 6.22 |
| RRl | | 3.64 | 2.13 | 3.75 |
| RRu | | 11.56 | 9.95 | 10.30 |
| P | | +++ | +++ | +++ |
| Between Chi | | | | 0.07 |
| Between df | | | | 1 |
| Between P | | | | N.S. |
| Btwn(F) P | | | | N.S. |
| Btwn(R) P | | | | N.S. |

| | Lung cancer type | | | | | |
|---|---|---|---|---|---|---|
| | q | q+s | q+u | KI | not a | Total |
| N | 12 | | | 2 | | 14 |
| NS | 10 | | | 2 | | 12 |
| Wt | 128.74 | | | 15.85 | | 144.59 |
| Het Chi | 65.15 | | | 0.79 | | 78.00 |
| Het df | 11 | | | 1 | | 13 |
| Het P | *** | | | N.S. | | *** |
| Fixed RR | 4.60 | | | 11.60 | | 5.09 |
| RRl | 3.87 | | | 7.09 | | 4.33 |
| RRu | 5.47 | | | 18.98 | | 5.99 |
| P | +++ | | | +++ | | +++ |
| Random RR | 5.42 | | | 11.60 | | 6.22 |
| RRl | 3.12 | | | 7.09 | | 3.75 |
| RRu | 9.42 | | | 18.98 | | 10.30 |
| P | +++ | | | +++ | | +++ |
| Between Chi | | | | | | 12.06 |
| Between df | | | | | | 1 |
| Between P | | | | | | *** |
| Btwn(F) P | | | | | | N.S. |
| Btwn(R) P | | | | | | * |

| | Location | | | | | | | | |
|---|---|---|---|---|---|---|---|---|---|
| | NAmer | UK | Scand | othEur | China | Japan | othAs | other | Total |
| N | 4 | | 1 | 5 | | 2 | | 2 | 14 |
| NS | 3 | | 1 | 4 | | 2 | | 2 | 12 |
| Wt | 28.43 | | 0.76 | 92.52 | | 16.83 | | 6.05 | 144.59 |
| Het Chi | 14.88 | | 0.00 | 49.54 | | 0.52 | | 0.10 | 78.00 |
| Het df | 3 | | 0 | 4 | | 1 | | 1 | 13 |
| Het P | ** | | N.S. | *** | | N.S. | | N.S. | *** |
| Fixed RR | 6.24 | | 9.23 | 5.56 | | 2.30 | | 4.30 | 5.09 |
| RRl | 4.32 | | 0.97 | 4.54 | | 1.43 | | 1.94 | 4.33 |
| RRu | 9.01 | | 87.63 | 6.82 | | 3.71 | | 9.54 | 5.99 |
| P | +++ | | (+) | +++ | | +++ | | +++ | +++ |
| Random RR | 6.25 | | 9.23 | 10.20 | | 2.30 | | 4.30 | 6.22 |
| RRl | 2.49 | | 0.97 | 3.18 | | 1.43 | | 1.94 | 3.75 |
| RRu | 15.68 | | 87.63 | 32.75 | | 3.71 | | 9.54 | 10.30 |
| P | +++ | | (+) | +++ | | +++ | | +++ | +++ |
| Between Chi | | | | | | | | | 12.95 |
| Between df | | | | | | | | | 4 |
| Between P | | | | | | | | | * |
| Btwn(F) P | | | | | | | | | N.S. |
| Btwn(R) P | | | | | | | | | (*) |

Table 2J5 - 3

IESLC - Meta-analysis of Ex Smoking, Years quit (vs never), "Highest vs lowest"
Squamous, Any Product (or Cigarettes if Any not available)
Most adjusted

Detailed Country in "other Europe"

| | multi | Germany | othWest | East | Balkans | Total |
|---|---|---|---|---|---|---|
| N | 2 | 1 | 1 | 1 | | 5 |
| NS | 1 | 1 | 1 | 1 | | 4 |
| Wt | 77.03 | 3.70 | 2.31 | 9.48 | | 92.52 |
| Het Chi | 0.03 | 0.00 | 0.00 | 0.00 | | 49.54 |
| Het df | 1 | 0 | 0 | 0 | | 4 |
| Het P | N.S. | N.S. | N.S. | N.S. | | *** |
| Fixed RR | 5.08 | 168.81 | 9.84 | 2.68 | | 5.56 |
| RRl | 4.06 | 60.93 | 2.71 | 1.42 | | 4.54 |
| RRu | 6.35 | 467.73 | 35.71 | 5.07 | | 6.82 |
| P | +++ | +++ | +++ | ++ | | +++ |
| Random RR | 5.08 | 168.81 | 9.84 | 2.68 | | 10.20 |
| RRl | 4.06 | 60.93 | 2.71 | 1.42 | | 3.18 |
| RRu | 6.35 | 467.73 | 35.71 | 5.07 | | 32.75 |
| P | +++ | +++ | +++ | ++ | | +++ |
| Between Chi | | | | | | 49.51 |
| Between df | | | | | | 3 |
| Between P | | | | | | *** |
| Btwn(F) P | | | | | | * |
| Btwn(R) P | | | | | | *** |

Detailed Country in "other Asia"

| | India | HongKong | other | Total |
|---|---|---|---|---|
| N | | | | |
| NS | | | | |
| Wt | | | | |
| Het Chi | | | | |
| Het df | | | | |
| Het P | | | | N.S. |
| Fixed RR | | | | |
| RRl | | | | |
| RRu | | | | |
| P | | | | ++ |
| Random RR | | | | |
| RRl | | | | |
| RRu | | | | |
| P | | | | ++ |
| Between Chi | | | | |
| Between df | | | | |
| Between P | | | | N.S. |
| Btwn(F) P | | | | N.S. |
| Btwn(R) P | | | | N.S. |

Detailed other continent

| | SCAmer | Total |
|---|---|---|
| N | 2 | 2 |
| NS | 2 | 2 |
| Wt | 6.05 | 6.05 |
| Het Chi | 0.10 | 0.10 |
| Het df | 1 | 1 |
| Het P | N.S. | N.S. |
| Fixed RR | 4.30 | 4.30 |
| RRl | 1.94 | 1.94 |
| RRu | 9.54 | 9.54 |
| P | +++ | +++ |
| Random RR | 4.30 | 4.30 |
| RRl | 1.94 | 1.94 |
| RRu | 9.54 | 9.54 |
| P | +++ | +++ |
| Between Chi | | |
| Between df | | |
| Between P | | N.S. |
| Btwn(F) P | | N.S. |
| Btwn(R) P | | N.S. |

Table 2J5 - 3

IESLC - Meta-analysis of Ex Smoking, Years quit (vs never), "Highest vs lowest"
Squamous, Any Product (or Cigarettes if Any not available)
Most adjusted

| | Start year of study | | | | | |
|---|---|---|---|---|---|---|
| | <1960 | 1960-69 | 1970-79 | 1980-89 | 1990+ | Total |
| N | | 2 | 3 | 8 | 1 | 14 |
| NS | | 2 | 2 | 7 | 1 | 12 |
| Wt | | 15.85 | 79.34 | 47.69 | 1.71 | 144.59 |
| Het Chi | | 0.79 | 1.01 | 61.26 | 0.00 | 78.00 |
| Het df | | 1 | 2 | 7 | 0 | 13 |
| Het P | | N.S. | N.S. | *** | N.S. | *** |
| Fixed RR | | 11.60 | 5.18 | 3.82 | 3.50 | 5.09 |
| RRl | | 7.09 | 4.15 | 2.88 | 0.78 | 4.33 |
| RRu | | 18.98 | 6.45 | 5.08 | 15.66 | 5.99 |
| P | | +++ | +++ | +++ | N.S. | +++ |
| Random RR | | 11.60 | 5.18 | 5.56 | 3.50 | 6.22 |
| RRl | | 7.09 | 4.15 | 2.25 | 0.78 | 3.75 |
| RRu | | 18.98 | 6.45 | 13.71 | 15.66 | 10.30 |
| P | | +++ | +++ | +++ | N.S. | +++ |
| Between Chi | | | | | | 14.93 |
| Between df | | | | | | 3 |
| Between P | | | | | | ** |
| Btwn(F) P | | | | | | N.S. |
| Btwn(R) P | | | | | | * |

| | Study type (1) | | |
|---|---|---|---|
| | CC | other | Total |
| N | 14 | | 14 |
| NS | 12 | | 12 |
| Wt | 144.59 | | 144.59 |
| Het Chi | 78.00 | | 78.00 |
| Het df | 13 | | 13 |
| Het P | *** | | *** |
| Fixed RR | 5.09 | | 5.09 |
| RRl | 4.33 | | 4.33 |
| RRu | 5.99 | | 5.99 |
| P | +++ | | +++ |
| Random RR | 6.22 | | 6.22 |
| RRl | 3.75 | | 3.75 |
| RRu | 10.30 | | 10.30 |
| P | +++ | | +++ |
| Between Chi | | | |
| Between df | | | |
| Between P | | | N.S. |
| Btwn(F) P | | | N.S. |
| Btwn(R) P | | | N.S. |

| | Study type (2) | | | |
|---|---|---|---|---|
| | CC | prosp | other | Total |
| N | 14 | | | 14 |
| NS | 12 | | | 12 |
| Wt | 144.59 | | | 144.59 |
| Het Chi | 78.00 | | | 78.00 |
| Het df | 13 | | | 13 |
| Het P | *** | | | *** |
| Fixed RR | 5.09 | | | 5.09 |
| RRl | 4.33 | | | 4.33 |
| RRu | 5.99 | | | 5.99 |
| P | +++ | | | +++ |
| Random RR | 6.22 | | | 6.22 |
| RRl | 3.75 | | | 3.75 |
| RRu | 10.30 | | | 10.30 |
| P | +++ | | | +++ |
| Between Chi | | | | |
| Between df | | | | |
| Between P | | | | N.S. |
| Btwn(F) P | | | | N.S. |
| Btwn(R) P | | | | N.S. |

Table 2J5 - 3

IESLC - Meta-analysis of Ex Smoking, Years quit (vs never), "Highest vs lowest"
Squamous, Any Product (or Cigarettes if Any not available)
Most adjusted

| | Study size (number of LC cases) 100-249 | 250-499 | 500-999 | 1000+ | Total |
|---|---|---|---|---|---|
| N | 3 | 2 | 3 | 6 | 14 |
| NS | 3 | 2 | 2 | 5 | 12 |
| Wt | 6.81 | 3.77 | 14.89 | 119.12 | 144.59 |
| Het Chi | 0.49 | 3.06 | 3.31 | 67.94 | 78.00 |
| Het df | 2 | 1 | 2 | 5 | 13 |
| Het P | N.S. | (*) | N.S. | *** | *** |
| Fixed RR | 4.68 | 7.94 | 3.46 | 5.29 | 5.09 |
| RRl | 2.21 | 2.89 | 2.08 | 4.42 | 4.33 |
| RRu | 9.93 | 21.80 | 5.76 | 6.34 | 5.99 |
| P | +++ | +++ | +++ | +++ | +++ |
| Random RR | 4.68 | 8.67 | 3.83 | 7.67 | 6.22 |
| RRl | 2.21 | 1.46 | 1.90 | 3.32 | 3.75 |
| RRu | 9.93 | 51.59 | 7.70 | 17.71 | 10.30 |
| P | +++ | + | +++ | +++ | +++ |
| Between Chi | | | | | 3.19 |
| Between df | | | | | 3 |
| Between P | | | | | N.S. |
| Btwn(F) P | | | | | N.S. |
| Btwn(R) P | | | | | N.S. |

| | Risky occupational population no | mining | othRisky | Total |
|---|---|---|---|---|
| N | 14 | | | 14 |
| NS | 12 | | | 12 |
| Wt | 144.59 | | | 144.59 |
| Het Chi | 78.00 | | | 78.00 |
| Het df | 13 | | | 13 |
| Het P | *** | | | *** |
| Fixed RR | 5.09 | | | 5.09 |
| RRl | 4.33 | | | 4.33 |
| RRu | 5.99 | | | 5.99 |
| P | +++ | | | +++ |
| Random RR | 6.22 | | | 6.22 |
| RRl | 3.75 | | | 3.75 |
| RRu | 10.30 | | | 10.30 |
| P | +++ | | | +++ |
| Between Chi | | | | |
| Between df | | | | |
| Between P | | | | N.S. |
| Btwn(F) P | | | | N.S. |
| Btwn(R) P | | | | N.S. |

| | National cigarette tobacco type Virginia | blended | other | Total |
|---|---|---|---|---|
| N | 2 | 12 | | 14 |
| NS | 1 | 11 | | 12 |
| Wt | 12.58 | 132.01 | | 144.59 |
| Het Chi | 0.33 | 73.07 | | 78.00 |
| Het df | 1 | 11 | | 13 |
| Het P | N.S. | *** | | *** |
| Fixed RR | 2.86 | 5.38 | | 5.09 |
| RRl | 1.65 | 4.54 | | 4.33 |
| RRu | 4.97 | 6.38 | | 5.99 |
| P | +++ | +++ | | +++ |
| Random RR | 2.86 | 7.19 | | 6.22 |
| RRl | 1.65 | 4.04 | | 3.75 |
| RRu | 4.97 | 12.81 | | 10.30 |
| P | +++ | +++ | | +++ |
| Between Chi | | | | 4.60 |
| Between df | | | | 1 |
| Between P | | | | * |
| Btwn(F) P | | | | N.S. |
| Btwn(R) P | | | | * |

Table 2J5 - 3

IESLC - Meta-analysis of Ex Smoking, Years quit (vs never), "Highest vs lowest"
Squamous, Any Product (or Cigarettes if Any not available)
Most adjusted

| | Any proxy use No/nk | Yes | Total |
|---|---|---|---|
| N | 10 | 4 | 14 |
| NS | 9 | 3 | 12 |
| Wt | 120.22 | 24.37 | 144.59 |
| Het Chi | 67.41 | 3.69 | 78.00 |
| Het df | 9 | 3 | 13 |
| Het P | *** | N.S. | *** |
| Fixed RR | 5.62 | 3.14 | 5.09 |
| RRl | 4.70 | 2.11 | 4.33 |
| RRu | 6.72 | 4.66 | 5.99 |
| P | +++ | +++ | +++ |
| Random RR | 7.95 | 3.23 | 6.22 |
| RRl | 4.06 | 2.05 | 3.75 |
| RRu | 15.58 | 5.08 | 10.30 |
| P | +++ | +++ | +++ |
| Between Chi | | | 6.89 |
| Between df | | | 1 |
| Between P | | | ** |
| Btwn(F) P | | | N.S. |
| Btwn(R) P | | | * |

| | Full histological confirmation No | Yes | Total |
|---|---|---|---|
| N | 6 | 8 | 14 |
| NS | 5 | 7 | 12 |
| Wt | 28.23 | 116.36 | 144.59 |
| Het Chi | 54.25 | 23.73 | 78.00 |
| Het df | 5 | 7 | 13 |
| Het P | *** | ** | *** |
| Fixed RR | 4.99 | 5.12 | 5.09 |
| RRl | 3.45 | 4.27 | 4.33 |
| RRu | 7.21 | 6.14 | 5.99 |
| P | +++ | +++ | +++ |
| Random RR | 7.01 | 5.66 | 6.22 |
| RRl | 1.88 | 3.51 | 3.75 |
| RRu | 26.14 | 9.12 | 10.30 |
| P | ++ | +++ | +++ |
| Between Chi | | | 0.01 |
| Between df | | | 1 |
| Between P | | | N.S. |
| Btwn(F) P | | | N.S. |
| Btwn(R) P | | | N.S. |

| | Number of adjustment variables (1) 0 | 1 | 2+/+nk | Total |
|---|---|---|---|---|
| N | 10 | 2 | 2 | 14 |
| NS | 8 | 2 | 2 | 12 |
| Wt | 124.18 | 4.47 | 15.94 | 144.59 |
| Het Chi | 67.61 | 1.10 | 1.93 | 78.00 |
| Het df | 9 | 1 | 1 | 13 |
| Het P | *** | N.S. | N.S. | *** |
| Fixed RR | 4.67 | 6.09 | 9.54 | 5.09 |
| RRl | 3.92 | 2.41 | 5.84 | 4.33 |
| RRu | 5.57 | 15.40 | 15.58 | 5.99 |
| P | +++ | +++ | +++ | +++ |
| Random RR | 6.18 | 6.08 | 7.72 | 6.22 |
| RRl | 3.28 | 2.30 | 2.82 | 3.75 |
| RRu | 11.67 | 16.11 | 21.08 | 10.30 |
| P | +++ | +++ | +++ | +++ |
| Between Chi | | | | 7.36 |
| Between df | | | | 2 |
| Between P | | | | * |
| Btwn(F) P | | | | N.S. |
| Btwn(R) P | | | | N.S. |

Table 2J5 - 3

IESLC - Meta-analysis of Ex Smoking, Years quit (vs never), "Highest vs lowest"
Squamous, Any Product (or Cigarettes if Any not available)
Most adjusted

| | Number of adjustment variables (2) 0 | 1 | 2 | 3-5 | 6+/+nk | Total |
|---|---|---|---|---|---|---|
| N | 10 | 2 | 2 | | | 14 |
| NS | 8 | 2 | 2 | | | 12 |
| Wt | 124.18 | 4.47 | 15.94 | | | 144.59 |
| Het Chi | 67.61 | 1.10 | 1.93 | | | 78.00 |
| Het df | 9 | 1 | 1 | | | 13 |
| Het P | *** | N.S. | N.S. | | | *** |
| Fixed RR | 4.67 | 6.09 | 9.54 | | | 5.09 |
| RRl | 3.92 | 2.41 | 5.84 | | | 4.33 |
| RRu | 5.57 | 15.40 | 15.58 | | | 5.99 |
| P | +++ | +++ | +++ | | | +++ |
| Random RR | 6.18 | 6.08 | 7.72 | | | 6.22 |
| RRl | 3.28 | 2.30 | 2.82 | | | 3.75 |
| RRu | 11.67 | 16.11 | 21.08 | | | 10.30 |
| P | +++ | +++ | +++ | | | +++ |
| Between Chi | | | | | | 7.36 |
| Between df | | | | | | 2 |
| Between P | | | | | | * |
| Btwn(F) P | | | | | | N.S. |
| Btwn(R) P | | | | | | N.S. |

| | Product all/unsp | cig+/-ot | cig only | Total |
|---|---|---|---|---|
| N | 3 | 10 | 1 | 14 |
| NS | 3 | 8 | 1 | 12 |
| Wt | 4.69 | 135.57 | 4.34 | 144.59 |
| Het Chi | 0.75 | 73.01 | 0.00 | 78.00 |
| Het df | 2 | 9 | 0 | 13 |
| Het P | N.S. | *** | N.S. | *** |
| Fixed RR | 12.95 | 4.94 | 4.67 | 5.09 |
| RRl | 5.24 | 4.18 | 1.82 | 4.33 |
| RRu | 32.03 | 5.85 | 11.96 | 5.99 |
| P | +++ | +++ | ++ | +++ |
| Random RR | 12.95 | 5.57 | 4.67 | 6.22 |
| RRl | 5.24 | 3.06 | 1.82 | 3.75 |
| RRu | 32.03 | 10.13 | 11.96 | 10.30 |
| P | +++ | +++ | ++ | +++ |
| Between Chi | | | | 4.23 |
| Between df | | | | 2 |
| Between P | | | | N.S. |
| Btwn(F) P | | | | N.S. |
| Btwn(R) P | | | | N.S. |

| | Derivation of RR/CI Orig | StdCalc | Other | Total |
|---|---|---|---|---|
| N | | 10 | 4 | 14 |
| NS | | 8 | 4 | 12 |
| Wt | | 124.18 | 20.41 | 144.59 |
| Het Chi | | 67.61 | 3.73 | 78.00 |
| Het df | | 9 | 3 | 13 |
| Het P | | *** | N.S. | *** |
| Fixed RR | | 4.67 | 8.65 | 5.09 |
| RRl | | 3.92 | 5.60 | 4.33 |
| RRu | | 5.57 | 13.34 | 5.99 |
| P | | +++ | +++ | +++ |
| Random RR | | 6.18 | 7.83 | 6.22 |
| RRl | | 3.28 | 4.48 | 3.75 |
| RRu | | 11.67 | 13.71 | 10.30 |
| P | | +++ | +++ | +++ |
| Between Chi | | | | 6.66 |
| Between df | | | | 1 |
| Between P | | | | ** |
| Btwn(F) P | | | | N.S. |
| Btwn(R) P | | | | N.S. |

Table 2J5 - 4

IESLC - Meta-analysis of Ex Smoking, Years quit (vs never), "Highest vs lowest"
Squamous, Any Product (or Cigarettes if Any not available)
Least adjusted

| REF | NRR | X | SEX | AGEL | AGEH | RACE | YF | LC TYPE | LOC | START | ST | NLC | R | VB | P | H | AD | ADOS | PRODUCT | exL | exH | unexL | unexH | De |
|---|---|---|---|---|---|---|---|---|---|---|---|---|---|---|---|---|---|---|---|---|---|---|---|---|
| BARBON | 586 | x | m | 0 | 0 | all | - | q | Eu:wst | 1979 | CC | 755 | n | bl | y | y | 0 | 0 | all/unsp | 0.1 | 4 | 25 | 999 | st |
| JAHN | 604 | | m | 0 | 0 | all | - | q | Eu:Ger | 1988 | CC | 1004 | n | bl | n | n | 0 | 0 | cig+/-ot | 0.1 | 0.9 | 21 | 999 | st |
| JAIN | 545 | | m | 0 | 0 | all | - | q | NAmer | 1981 | CC | 845 | n | V | y | n | 0 | 0 | cig+/-ot | 2 | 9 | 10 | 999 | st |
| JAIN | 509 | | f | 0 | 0 | all | - | q | NAmer | 1981 | CC | 845 | n | V | y | n | 0 | 0 | cig+/-ot | 2 | 9 | 10 | 999 | st |
| JEDRYC | 547 | | m | 0 | 0 | all | - | q | Eu:est | 1980 | CC | 1630 | n | bl | y | n | 0 | 0 | cig+/-ot | 5 | 9 | 10 | 999 | st |
| LUBIN2 | 774 | | m | 0 | 0 | all | - | q | Eu:mul | 1976 | CC | 7804 | n | bl | n | y | 0 | 0 | cig+/-ot | 0.1 | 4 | 20 | 999 | st |
| LUBIN2 | 898 | | f | 0 | 0 | all | - | q | Eu:mul | 1976 | CC | 7804 | n | bl | n | y | 0 | 0 | cig+/-ot | 0.1 | 9 | 20 | 999 | st |
| MATOS | 625 | x | m | 0 | 0 | all | - | q | SCAmer | 1994 | CC | 200 | n | bl | n | n | 0 | 0 | cig+/-ot | 1.0 | 5 | 11 | 999 | st |
| PEZZOT | 581 | | m | 0 | 0 | all | - | q | SCAmer | 1987 | CC | 215 | n | bl | n | y | 0 | 0 | cig only | 1.0 | 10 | 11 | 999 | st |
| SOBUE | 782 | | m | 0 | 0 | all | - | q | As:Jap | 1986 | CC | 1376 | n | bl | n | y | 0 | 0 | cig+/-ot | 1.0 | 4 | 10 | 999 | st |
| SVENSS | 559 | | f | 0 | 0 | all | - | q | Eu:Sca | 1983 | CC | 210 | n | bl | n | n | 0 | 0 | all/unsp | 3 | 10 | 11 | 999 | st |
| WAKAI | 542 | x | m | 0 | 0 | all | - | q | As:Jap | 1988 | CC | 333 | n | bl | n | y | 0 | 0 | cig+/-ot | 5 | 9 | 20 | 999 | st |
| WYNDE3 | 509 | | m | 0 | 0 | all | - | KI | NAmer | 1966 | CC | 350 | n | bl | n | y | 0 | 0 | all/unsp | 1.0 | 3 | 13 | 999 | st |
| WYNDE6 | 781 | x | m | 0 | 0 | all | - | KI | NAmer | 1969 | CC | 4423 | n | bl | n | y | 0 | 0 | cig+/-ot | 1.0 | 3 | 16 | 999 | st |

Cigarette type is all/unspec for all RRs

Table 2J5 - 5

IESLC - Meta-analysis of Ex Smoking, Years quit (vs never), "Highest vs lowest"
Squamous, Any Product (or Cigarettes if Any not available)
Least adjusted

| REF | NRR | SEX | AD | Number Exposed Case | Cont | Non-exposed Case | Cont | RR | 95.00%CI |
|---|---|---|---|---|---|---|---|---|---|
| BARBON | 586 | m | 0 | 11 | 20 | 4 | 59 | 8.11 | ( 2.32- 28.36) |
| JAHN | 604 | m | 0 | 74 | 8 | 8 | 146 | 168.81 | ( 60.93- 467.73) |
| JAIN | 545 | m | 0 | 24 | 46 | 23 | 113 | 2.56 | ( 1.32- 4.99) |
| JAIN | 509 | f | 0 | 15 | 36 | 7 | 61 | 3.63 | ( 1.35- 9.74) |
| Subtotal JAIN | | | | | | | | 2.86 | ( 1.65- 4.97) |
| JEDRYC | 547 | m | 0 | 22 | 82 | 23 | 230 | 2.68 | ( 1.42- 5.07) |
| LUBIN2 | 774 | m | 0 | 498 | 1047 | 106 | 1128 | 5.06 | ( 4.04- 6.34) |
| LUBIN2 | 898 | f | 0 | 38 | 95 | 2 | 29 | 5.80 | ( 1.32- 25.52) |
| Subtotal LUBIN2 | | | | | | | | 5.08 | ( 4.06- 6.35) |
| MATOS | 625 | m | 0 | 4 | 23 | 5 | 101 | 3.51 | ( 0.87- 14.11) |
| PEZZOT | 581 | m | 0 | 21 | 27 | 8 | 48 | 4.67 | ( 1.82- 11.96) |
| SOBUE | 782 | m | 0 | 52 | 116 | 30 | 144 | 2.15 | ( 1.29- 3.59) |
| SVENSS | 559 | f | 0 | 5 | 13 | 1 | 24 | 9.23 | ( 0.97- 87.63) |
| WAKAI | 542 | m | 0 | 11 | 48 | 3 | 47 | 3.59 | ( 0.94- 13.69) |
| WYNDE3 | 509 | m | 0 | 18 | 22 | 2 | 55 | 22.50 | ( 4.81- 105.19) |
| WYNDE6 | 781 | m | 0 | 80 | 307 | 19 | 530 | 7.27 | ( 4.32- 12.22) |
| Totals | | | | 873 | 1890 | 241 | 2715 | | |

*prospective study

| REF | NRR | SEX | AD | Ys | Ws | Qs | Ps |
|---|---|---|---|---|---|---|---|
| BARBON | 586 | m | 0 | 2.09 | 2.45 | 0.63 | 0.0010 |
| JAHN | 604 | m | 0 | 5.13 | 3.70 | 46.43 | 0.0000 |
| JAIN | 545 | m | 0 | 0.94 | 8.64 | 3.59 | 0.0057 |
| JAIN | 509 | f | 0 | 1.29 | 3.94 | 0.35 | 0.0105 |
| Subtotal JAIN | | | | 1.05 | 12.58 | 3.94 | |
| JEDRYC | 547 | m | 0 | 0.99 | 9.48 | 3.40 | 0.0024 |
| LUBIN2 | 774 | m | 0 | 1.62 | 75.28 | 0.10 | 0.0000 |
| LUBIN2 | 898 | f | 0 | 1.76 | 1.75 | 0.05 | 0.0200 |
| Subtotal LUBIN2 | | | | 1.62 | 77.03 | 0.15 | |
| MATOS | 625 | m | 0 | 1.26 | 1.99 | 0.22 | 0.0766 |
| PEZZOT | 581 | m | 0 | 1.54 | 4.34 | 0.01 | 0.0013 |
| SOBUE | 782 | m | 0 | 0.77 | 14.68 | 9.86 | 0.0033 |
| SVENSS | 559 | f | 0 | 2.22 | 0.76 | 0.31 | 0.0529 |
| WAKAI | 542 | m | 0 | 1.28 | 2.14 | 0.20 | 0.0612 |
| WYNDE3 | 509 | m | 0 | 3.11 | 1.62 | 3.77 | 0.0001 |
| WYNDE6 | 781 | m | 0 | 1.98 | 14.23 | 2.25 | 0.0000 |

| | | |
|---|---|---|
| | N | 14 |
| | NS | 12 |
| | Wt | 145.00 |
| Het | Chi | 71.16 |
| Het | df | 13 |
| Het | P | *** |
| Fixed | RR | 4.88 |
| | RRl | 4.15 |
| | RRu | 5.75 |
| | P | +++ |
| Random | RR | 5.87 |
| | RRl | 3.63 |
| | RRu | 9.51 |
| | P | +++ |
| Asymm | P | N.S. |

Table 2J5 - 6

IESLC - Meta-analysis of Ex Smoking, Years quit (vs never), "Highest vs lowest"
Squamous, Any Product (or Cigarettes if Any not available)
Least adjusted

| | Sex | | |
|---|---|---|---|
| combined | male | female | Total |
| N | 11 | 3 | 14 |
| NS | 11 | 3 | 14 |
| Wt | 138.54 | 6.45 | 145.00 |
| Het Chi | 70.45 | 0.68 | 71.16 |
| Het df | 10 | 2 | 13 |
| Het P | *** | N.S. | *** |
| Fixed RR | 4.90 | 4.60 | 4.88 |
| RRl | 4.15 | 2.13 | 4.15 |
| RRu | 5.78 | 9.95 | 5.75 |
| P | +++ | +++ | +++ |
| Random RR | 6.07 | 4.60 | 5.87 |
| RRl | 3.50 | 2.13 | 3.63 |
| RRu | 10.53 | 9.95 | 9.51 |
| P | +++ | +++ | +++ |
| Between Chi | | | 0.02 |
| Between df | | | 1 |
| Between P | | | N.S. |
| Btwn(F) P | | | N.S. |
| Btwn(R) P | | | N.S. |

Table 2J5 - 7

IESLC - Meta-analysis of Ex Smoking, Years quit (vs never), "Highest vs lowest"
Squamous, Any Product (or Cigarettes if Any not available)
Excluded studies (and stage at which they were excluded)

| | |
|---|---|
| 1 | AGUDO AKIBA AMANDU AMES AXELSS BEST BOUCHA BOUCOT BRESLO CHEN CHEN2 CHIAZZ DEAN2 DOSEME ENGELA FAN GENG GER GUO HAENSZ HEGMAN HOLE HU HU2 JUSSAW KATSOU KAUFMA KOO KOULUM KREUZE LETOUR LEVIN LIAW LIU3 LIU4 LIU5 MCCONN MIGRAN MRFITR NOTAN2 OSANN2 PERNU QIAO2 RACHTA RESTRE SADOWS SEGI2 STASZE TIZZAN VUTUC WATSON WU WUWILL WYNDE2 WYNDE8 XU YUAN ZHANG ZHENG ZHOU |
| 2 | BUFFLE HUMBLE PISANI PRESCO WYNDE7 |
| 3 | MCDUFF SPITZ |
| 4 | AUVINE BLOT1 BROWN3 GURSEL LAUSSM LUO WU2 |
| 5 | ARMADA BECHER BENSHL BOFFET BROSS CARPEN CEDERL CHOI CHYOU CORREA CPSI CPSII DAMBER DARBY DEAN3 DESTEF DOLL DOLL2 DORGAN DORN GAO GAO2 GARCIA GARSHI GILLIS GRAHAM HAMMO2 HIRAYA JOLY KAISE2 KHUDER LUBIN PEZZO2 QIAO SPEIZE SUZUK2 TVERDA WANG2 WIGLE |
| 6 | ALDERS HAMMON |
| 15 | BENHAM |

Table 2J5 - 8

Potentially overlapping studies

| REF | REFGP | PRINC | . OVERLAP/LINK |
|---|---|---|---|
| LUBIN2 | LUBIN2 | 1 | Lubin-combined |
| WYNDE6 | WYNDE6 | 1 | WYNDE5/6/7/8 |
| JAHN | BOFFET | 2 | Subset of BOFFET |

Table 2J5 - 9

Most adjusted - insufficient data for meta-analysis

| REF | NRR | SEX | AGEL | AGEH | RACE | YF | LC TYPE | LOC | START | ST | NLC | R | VB | P | H | AD | ADOS | PRODUCT | exL | exH | unexL | unexH | De |
|---|---|---|---|---|---|---|---|---|---|---|---|---|---|---|---|---|---|---|---|---|---|---|---|
| ALDERS | 542 | m | 0 | 0 | all | - | q+s | Eu:UK | 1977 | CC | 1448 | n | V | n | n | 1 | 0 | cig only | 0.1 | 2 | 10 | 999 | st |
| ALDERS | 553 | f | 0 | 0 | all | - | q+s | Eu:UK | 1977 | CC | 1448 | n | V | n | n | 1 | 0 | cig only | 0.1 | 2 | 10 | 999 | st |
| HAMMON | 506 | m | 0 | 0 | wh | 0 | not a | NAmer | 1952 | pr | 448 | n | bl | n | n | 1 | 0 | cig only | 0.1 | 0.9 | 10 | 999 | st |
| JAIN | 589 | m | 0 | 0 | all | - | q | NAmer | 1981 | CC | 845 | n | V | y | n | 0 | 0 | cig+/-ot | 0.1 | 1.9 | 10 | 999 | ot |
| JAIN | 577 | f | 0 | 0 | all | - | q | NAmer | 1981 | CC | 845 | n | V | y | n | 0 | 0 | cig+/-ot | 0.1 | 1.9 | 10 | 999 | ot |
| JEDRYC | 548 | m | 0 | 0 | all | - | q | Eu:est | 1980 | CC | 1630 | n | bl | y | n | 0 | 0 | cig+/-ot | 0.1 | 4 | 10 | 999 | ot |
| MATOS | 708 | m | 0 | 0 | all | - | q | SCAmer | 1994 | CC | 200 | n | bl | n | n | 2 | 0 | cig+/-ot | 0.1 | 0.9 | 11 | 999 | ot |
| PEZZOT | 600 | m | 0 | 0 | all | - | q | SCAmer | 1987 | CC | 215 | n | bl | n | y | 0 | 0 | cig only | 0.1 | 0.9 | 11 | 999 | ot |
| SOBUE | 783 | m | 0 | 0 | all | - | q | As:Jap | 1986 | CC | 1376 | n | bl | n | y | 0 | 0 | cig+/-ot | 0.1 | 0.9 | 10 | 999 | ot |
| SVENSS | 594 | f | 0 | 0 | all | - | q | Eu:Sca | 1983 | CC | 210 | n | bl | n | n | 0 | 0 | all/unsp | 0.1 | 2 | 11 | 999 | ot |
| WAKAI | 616 | m | 0 | 0 | all | - | q | As:Jap | 1988 | CC | 333 | n | bl | n | y | 1 | 0 | cig+/-ot | 0.1 | 4 | 20 | 999 | ot |
| WYNDE3 | 510 | m | 0 | 0 | all | - | KI | NAmer | 1966 | CC | 350 | n | bl | n | y | 0 | 0 | all/unsp | 0.1 | 0.9 | 13 | 999 | ot |
| WYNDE6 | 797 | m | 0 | 0 | all | - | KI | NAmer | 1969 | CC | 4423 | n | bl | n | y | 2 | 0 | cig+/-ot | 0.1 | 0.9 | 16 | 999 | ot |

| REF | NRR | RR | SIG | RRDATA comment |
|---|---|---|---|---|
| ALDERS | 542 | 10.00 | | 0 |
| ALDERS | 553 | 25.17 | | 0 |
| HAMMON | 506 | * | | RR for <1 pack per day is 6.76, while that for 1+ packs is 3.27 |
| JAIN | 589 | * | gap | 0 |
| JAIN | 577 | * | gap | 0 |
| JEDRYC | 548 | * | gap | 0 |
| MATOS | 708 | * | gap | 0 |
| PEZZOT | 600 | * | gap | 0 |
| SOBUE | 783 | * | gap | 0 |
| SVENSS | 594 | * | gap | 0 |
| WAKAI | 616 | * | gap | 0 |
| WYNDE3 | 510 | * | gap | 0 |
| WYNDE6 | 797 | * | gap | 0 |

Least adjusted - insufficient data for meta-analysis: as for adjusted plus the following

| REF | NRR | SEX | AGEL | AGEH | RACE | YF | LC TYPE | LOC | START | ST | NLC | R | VB | P | H | AD | ADOS | PRODUCT | exL | exH | unexL | unexH | De |
|---|---|---|---|---|---|---|---|---|---|---|---|---|---|---|---|---|---|---|---|---|---|---|---|
| MATOS | 706 | m | 0 | 0 | all | - | q | SCAmer | 1994 | CC | 200 | n | bl | n | n | 0 | 0 | cig+/-ot | 0.1 | 0.9 | 11 | 999 | ot |
| WAKAI | 614 | m | 0 | 0 | all | - | q | As:Jap | 1988 | CC | 333 | n | bl | n | y | 0 | 0 | cig+/-ot | 0.1 | 4 | 20 | 999 | ot |
| WYNDE6 | 782 | m | 0 | 0 | all | - | KI | NAmer | 1969 | CC | 4423 | n | bl | n | y | 0 | 0 | cig+/-ot | 0.1 | 0.9 | 16 | 999 | ot |

Table 2J5 - 9

IESLC - Meta-analysis of Ex Smoking, Years quit (vs never), "Highest vs lowest"
Squamous, Any Product (or Cigarettes if Any not available)
Least adjusted - insufficient data for meta-analysis: as for adjusted plus the following

| REF | NRR | RR | SIG | RRDATA comment |
|---|---|---|---|---|
| MATOS | 706 | * | gap | 0 |
| WAKAI | 614 | * | gap | 0 |
| WYNDE6 | 782 | * | gap | 0 |

Table 2J6 -

IESLC - Meta-analysis of Ex Smoking by Years quit (vs never), Overview
Squamous, Cigarettes (or Any Product if Cigarettes not available)

This analysis is restricted to results for:
1) Ex smokers
2) Results by Years quit (vs never)
3) Categorical results by Years quit (vs never)
Results by Years quit (vs never) are grouped under 2 schemes (S1, S2). Each scheme has a set of "key values". An interv is allocated to the category whose key value it includes, and intervals which include none or more than one of the key values are excluded. (Open-ended intervals are coded as 999)

| S1 | key value | maximum range |
|---|---|---|
| 1 | 12 | 8+ |
| 2 | 7 | 4-11 |
| 3 | 3 | 1-6 |

| S2 | key value | maximum range |
|---|---|---|
| 1 | 20 | 13+ |
| 2 | 12 | 4-19 |
| 3 | 3 | 1-11 |

4) Squamous (or near equivalent)
5) Results complete enough for use in metaanalysis

Within each study, results are then selected (in the following order of preference, within each sex) for:
6) (not applicable)
7) PRODUCT: cigarettes regardless of other products, cigarettes only, all/unspec
8) CIGTYPE: all/unspecified, MC regardless of HR, MC only
9) (not applicable)
10) DENOM: never smoked anything, never smoked cigarettes, never any + low, never cigs + low
11) Followup period (YF, prospective studies): whole study (coded as 0) or longest available
12) LCtype: squamous or nearest available, but not adeno. (q = squamous, s = small,
a = adeno, KI = Kreyberg I, u = undifferentiated)
13) Race: all or nearest available, otherwise by race (wh or w = white, bl or b = black, hi = hispanic
ch = chinese, jap = japanese, haw = hawaiian, w+o = white + oriental, sca = scandinavian, as = asian)
14) For overlapping studies: principal rather than subsidiary studies
Finally by Age: whole study (coded as 0) if available, otherwise by widest available age group
and then for single sex results (m, f) in preference to results for both sexes combined (c).

Results adjusted (AD) for the most potential confounders are then chosen in Sections -1 to -3
(and those which actually differ from the adjusted results in Table 2J1 - 1 are marked 'x' in Section -1)
and results adjusted for the least confounders in Sections -4 to -6. (Those least adjusted results which
actually differ from the most adjusted are marked 'x' in column X in Section -4)

Section -7 shows excluded studies, together with the stage (as above) at which no qualifying
results were found.

Section -8 lists the potentially overlapping studies which have been included (1=principal, 2=subsidiary).

Section -9 lists any results which would have been included in preference except that they had data not complete
enough for use in meta-analysis, with their significance (yes/no), if known, and any further comment as entered
on the database. It also lists as "gap" any categories for which no data were presented by the original authors.
This is commonly due to recent quitters having been combined with current smokers

In addition to those mentioned above, the following fields, levels and abbreviations are used:
* or nk = not known, n = no, y = yes, ot = other
nev = never
all/unspec = all or unspecified, cig+/-ot = cigarettes irrespective of other products (cigar, pipe etc)
MC = manufactured cigarettes, HR = hand-rolled cigarettes
exL, exH = range of exposure (low and high) in the smoking group, in terms of Years quit (vs never)
REF: 6-character study reference
NRR: number of the RR on the database within the study
ST : study type (CC = case control, pr or prosp = prospective)
NLC: number of lung cancer cases in whole study
R : risky occupational population (n = no, m = mining, o = other risky)
VB : national cigarette type (V = at least 75% Virginia, bl = at least 75% blended, ot = other)
P : any proxy use
H : full histological confirmation
De : derivation of RR/CI (or = original, st = standard method, ot = other method of estimation)

Table 2J6 - 1

IESLC - Meta-analysis of Ex Smoking by Years quit (vs never), Overview
Squamous, Cigarettes (or Any Product if Cigarettes not available)
Most adjusted

| REF | NRR | 2J1 | SEX | AGEL | AGEH | RACE | YF | LC TYPE | LOC | START | ST | NLC | R | VB | P | H | AD | PRODUCT | exL | exH | S1 | S2 | DENOM | De |
|---|---|---|---|---|---|---|---|---|---|---|---|---|---|---|---|---|---|---|---|---|---|---|---|---|
| BARBON | 594 | | m | 0 | 0 | all | - | q | Eu:wst | 1979 | CC | 755 | n | bl | y | y | 1 | all/unsp | 25 | 999 | 0 | 0 | nev any | or |
| BARBON | 595 | | m | 0 | 0 | all | - | q | Eu:wst | 1979 | CC | 755 | n | bl | y | y | 1 | all/unsp | 15 | 24 | 0 | 1 | nev any | or |
| BARBON | 596 | | m | 0 | 0 | all | - | q | Eu:wst | 1979 | CC | 755 | n | bl | y | y | 1 | all/unsp | 5 | 14 | 0 | 2 | nev any | or |
| BARBON | 597 | | m | 0 | 0 | all | - | q | Eu:wst | 1979 | CC | 755 | n | bl | y | y | 1 | all/unsp | 0.1 | 4 | 3 | 3 | nev any | or |
| BROWN3 | 507 | | f | 0 | 0 | wh | - | q | NAmer | | CC | 618 | | bl | y | n | 0 | all/unsp | 15 | 999 | 0 | 1 | nev any | st |
| JAHN | 593 | | m | 0 | 0 | all | - | q | Eu:Ger | 1988 | CC | 1004 | n | bl | n | n | 0 | cig+/-ot | 21 | 999 | 0 | 0 | nev any | st |
| JAHN | 594 | | m | 0 | 0 | all | - | q | Eu:Ger | 1988 | CC | 1004 | n | bl | n | n | 0 | cig+/-ot | 11 | 20 | 1 | 0 | nev any | st |
| JAHN | 595 | | m | 0 | 0 | all | - | q | Eu:Ger | 1988 | CC | 1004 | n | bl | n | n | 0 | cig+/-ot | 6 | 10 | 2 | 0 | nev any | st |
| JAHN | 596 | | m | 0 | 0 | all | - | q | Eu:Ger | 1988 | CC | 1004 | n | bl | n | n | 0 | cig+/-ot | 2 | 5 | 3 | 3 | nev any | st |
| JAHN | 597 | | m | 0 | 0 | all | - | q | Eu:Ger | 1988 | CC | 1004 | n | bl | n | n | 0 | cig+/-ot | 1.0 | 1.9 | 0 | 0 | nev any | st |
| JAHN | 598 | | m | 0 | 0 | all | - | q | Eu:Ger | 1988 | CC | 1004 | n | bl | n | n | 0 | cig+/-ot | 0.1 | 0.9 | 0 | 0 | nev any | st |
| JAIN | 543 | | m | 0 | 0 | all | - | q | NAmer | 1981 | CC | 845 | n | V | y | n | 0 | cig+/-ot | 10 | 999 | 1 | 0 | nev cigs | st |
| JAIN | 544 | | m | 0 | 0 | all | - | q | NAmer | 1981 | CC | 845 | n | V | y | n | 0 | cig+/-ot | 2 | 9 | 0 | 3 | nev cigs | st |
| JAIN | 507 | | f | 0 | 0 | all | - | q | NAmer | 1981 | CC | 845 | n | V | y | n | 0 | cig+/-ot | 10 | 999 | 1 | 0 | nev cigs | st |
| JAIN | 508 | | f | 0 | 0 | all | - | q | NAmer | 1981 | CC | 845 | n | V | y | n | 0 | cig+/-ot | 2 | 9 | 0 | 3 | nev cigs | st |
| JEDRYC | 543 | | m | 0 | 0 | all | - | q | Eu:est | 1980 | CC | 1630 | n | bl | y | n | 0 | cig+/-ot | 10 | 999 | 1 | 0 | nev any | st |
| JEDRYC | 544 | | m | 0 | 0 | all | - | q | Eu:est | 1980 | CC | 1630 | n | bl | y | n | 0 | cig+/-ot | 5 | 9 | 2 | 0 | nev any | st |
| LUBIN2 | 765 | | m | 0 | 0 | all | - | q | Eu:mul | 1976 | CC | 7804 | n | bl | n | y | 0 | cig+/-ot | 20 | 999 | 0 | 1 | nev any | st |
| LUBIN2 | 766 | | m | 0 | 0 | all | - | q | Eu:mul | 1976 | CC | 7804 | n | bl | n | y | 0 | cig+/-ot | 15 | 19 | 0 | 0 | nev any | st |
| LUBIN2 | 767 | | m | 0 | 0 | all | - | q | Eu:mul | 1976 | CC | 7804 | n | bl | n | y | 0 | cig+/-ot | 10 | 14 | 1 | 2 | nev any | st |
| LUBIN2 | 768 | | m | 0 | 0 | all | - | q | Eu:mul | 1976 | CC | 7804 | n | bl | n | y | 0 | cig+/-ot | 5 | 9 | 2 | 0 | nev any | st |
| LUBIN2 | 769 | | m | 0 | 0 | all | - | q | Eu:mul | 1976 | CC | 7804 | n | bl | n | y | 0 | cig+/-ot | 0.1 | 4 | 3 | 3 | nev any | st |
| LUBIN2 | 893 | | f | 0 | 0 | all | - | q | Eu:mul | 1976 | CC | 7804 | n | bl | n | y | 0 | cig+/-ot | 20 | 999 | 0 | 1 | nev any | st |
| LUBIN2 | 894 | | f | 0 | 0 | all | - | q | Eu:mul | 1976 | CC | 7804 | n | bl | n | y | 0 | cig+/-ot | 10 | 19 | 1 | 2 | nev any | st |
| LUBIN2 | 895 | | f | 0 | 0 | all | - | q | Eu:mul | 1976 | CC | 7804 | n | bl | n | y | 0 | cig+/-ot | 0.1 | 9 | 0 | 3 | nev any | st |
| LUO | 522 | | c | 0 | 0 | all | - | q | As:Chi | 1990 | CC | 102 | n | ot | n | y | 20 | cig+/-ot | 10 | 999 | 1 | 0 | nev cigs | or |
| MATOS | 631 | | m | 0 | 0 | all | - | q | SCAmer | 1994 | CC | 200 | n | bl | n | n | 2 | cig+/-ot | 11 | 999 | 1 | 0 | nev any | ot |
| MATOS | 632 | | m | 0 | 0 | all | - | q | SCAmer | 1994 | CC | 200 | n | bl | n | n | 2 | cig+/-ot | 6 | 10 | 2 | 0 | nev any | ot |
| MATOS | 633 | | m | 0 | 0 | all | - | q | SCAmer | 1994 | CC | 200 | n | bl | n | n | 2 | cig+/-ot | 1.0 | 5 | 3 | 3 | nev any | ot |
| PEZZOT | 579 | | m | 0 | 0 | all | - | q | SCAmer | 1987 | CC | 215 | n | bl | n | y | 0 | cig only | 11 | 999 | 1 | 0 | nev cigs | ot |
| PEZZOT | 580 | | m | 0 | 0 | all | - | q | SCAmer | 1987 | CC | 215 | n | bl | n | y | 0 | cig only | 1.0 | 10 | 0 | 3 | nev cigs | ot |
| SVENSS | 557 | | f | 0 | 0 | all | - | q | Eu:Sca | 1983 | CC | 210 | n | bl | n | n | 0 | all/unsp | 11 | 999 | 1 | 0 | nev any | st |
| SVENSS | 558 | | f | 0 | 0 | all | - | q | Eu:Sca | 1983 | CC | 210 | n | bl | n | n | 0 | all/unsp | 3 | 10 | 0 | 3 | nev any | st |
| WAKAI | 546 | | m | 0 | 0 | all | - | q | As:Jap | 1988 | CC | 333 | n | bl | n | y | 1 | cig+/-ot | 20 | 999 | 0 | 1 | nev any | or |
| WAKAI | 547 | | m | 0 | 0 | all | - | q | As:Jap | 1988 | CC | 333 | n | bl | n | y | 1 | cig+/-ot | 10 | 19 | 1 | 2 | nev any | or |
| WAKAI | 548 | | m | 0 | 0 | all | - | q | As:Jap | 1988 | CC | 333 | n | bl | n | y | 1 | cig+/-ot | 5 | 9 | 2 | 0 | nev any | or |
| WYNDE3 | 552 | x | m | 0 | 0 | all | - | KI | NAmer | 1966 | CC | 350 | n | bl | n | y | 0 | cig+/-ot | 10 | 999 | 1 | 0 | nev any | st |
| WYNDE3 | 573 | | f | 0 | 0 | all | - | KI | NAmer | 1966 | CC | 350 | n | bl | n | y | 0 | cig+/-ot | 10 | 999 | 1 | 0 | nev any | st |
| WYNDE6 | 787 | | m | 0 | 0 | all | - | KI | NAmer | 1969 | CC | 4423 | n | bl | n | y | 2 | cig+/-ot | 16 | 999 | 0 | 1 | nev any | ot |
| WYNDE6 | 788 | | m | 0 | 0 | all | - | KI | NAmer | 1969 | CC | 4423 | n | bl | n | y | 2 | cig+/-ot | 11 | 15 | 1 | 2 | nev any | ot |
| WYNDE6 | 789 | | m | 0 | 0 | all | - | KI | NAmer | 1969 | CC | 4423 | n | bl | n | y | 2 | cig+/-ot | 7 | 10 | 2 | 0 | nev any | ot |
| WYNDE6 | 790 | | m | 0 | 0 | all | - | KI | NAmer | 1969 | CC | 4423 | n | bl | n | y | 2 | cig+/-ot | 4 | 6 | 0 | 0 | nev any | ot |
| WYNDE6 | 791 | | m | 0 | 0 | all | - | KI | NAmer | 1969 | CC | 4423 | n | bl | n | y | 2 | cig+/-ot | 1.0 | 3 | 3 | 3 | nev any | ot |

Cigarette type is all/unspec for all RRs

In this overview table, subtotals and Qs values may be invalid and should be ignored

Table 2J6 - 2

IESLC - Meta-analysis of Ex Smoking by Years quit (vs never), Overview
Squamous, Cigarettes (or Any Product if Cigarettes not available)
Most adjusted

| REF | NRR | SEX | AD | Number Exposed Case | Cont | Non-exposed Case | Cont | RR | 95.00%CI |
|---|---|---|---|---|---|---|---|---|---|
| BARBON | 594 | m | 1 | 4 | - | 6 | - | 1.90 | ( 0.50- 7.20) |
| BARBON | 595 | m | 1 | 11 | - | 6 | - | 8.10 | ( 2.80- 23.20) |
| BARBON | 596 | m | 1 | 31 | - | 6 | - | 11.90 | ( 4.80- 29.80) |
| BARBON | 597 | m | 1 | 11 | - | 6 | - | 18.70 | ( 6.20- 56.30) |
| Subtotal BARBON | | | | | | | | 8.92 | ( 5.22- 15.24) |
| BROWN3 | 507 | f | 0 | 17 | 10 | 432 | 1168 | 4.60 | ( 2.09- 10.12) |
| JAHN | 593 | m | 0 | 8 | 146 | 3 | 138 | 2.52 | ( 0.66- 9.70) |
| JAHN | 594 | m | 0 | 18 | 130 | 3 | 138 | 6.37 | ( 1.83- 22.13) |
| JAHN | 595 | m | 0 | 29 | 63 | 3 | 138 | 21.17 | ( 6.22- 72.12) |
| JAHN | 596 | m | 0 | 36 | 46 | 3 | 138 | 36.00 | ( 10.58- 122.45) |
| JAHN | 597 | m | 0 | 25 | 9 | 3 | 138 | 127.78 | ( 32.33- 505.04) |
| JAHN | 598 | m | 0 | 74 | 8 | 3 | 138 | 425.50 | (109.58-1652.23) |
| Subtotal JAHN | | | | | | | | 27.87 | ( 16.45- 47.20) |
| JAIN | 543 | m | 0 | 23 | 113 | 2 | 85 | 8.65 | ( 1.98- 37.70) |
| JAIN | 544 | m | 0 | 24 | 46 | 2 | 85 | 22.17 | ( 5.02- 98.04) |
| JAIN | 507 | f | 0 | 7 | 61 | 6 | 214 | 4.09 | ( 1.33- 12.63) |
| JAIN | 508 | f | 0 | 15 | 36 | 6 | 214 | 14.86 | ( 5.41- 40.82) |
| Subtotal JAIN | | | | | | | | 9.92 | ( 5.39- 18.27) |
| JEDRYC | 543 | m | 0 | 23 | 230 | 6 | 289 | 4.82 | ( 1.93- 12.03) |
| JEDRYC | 544 | m | 0 | 22 | 82 | 6 | 289 | 12.92 | ( 5.07- 32.93) |
| Subtotal JEDRYC | | | | | | | | 7.80 | ( 4.06- 15.01) |
| LUBIN2 | 765 | m | 0 | 106 | 1128 | 54 | 2616 | 4.55 | ( 3.26- 6.36) |
| LUBIN2 | 766 | m | 0 | 67 | 478 | 54 | 2616 | 6.79 | ( 4.68- 9.84) |
| LUBIN2 | 767 | m | 0 | 146 | 693 | 54 | 2616 | 10.21 | ( 7.39- 14.10) |
| LUBIN2 | 768 | m | 0 | 265 | 882 | 54 | 2616 | 14.56 | ( 10.76- 19.70) |
| LUBIN2 | 769 | m | 0 | 498 | 1047 | 54 | 2616 | 23.04 | ( 17.25- 30.79) |
| LUBIN2 | 893 | f | 0 | 2 | 29 | 72 | 1180 | 1.13 | ( 0.26- 4.83) |
| LUBIN2 | 894 | f | 0 | 5 | 33 | 72 | 1180 | 2.48 | ( 0.94- 6.55) |
| LUBIN2 | 895 | f | 0 | 38 | 95 | 72 | 1180 | 6.56 | ( 4.20- 10.23) |
| Subtotal LUBIN2 | | | | | | | | 9.96 | ( 8.71- 11.40) |
| LUO | 522 | c | 20 | 1 | - | 5 | - | 2.00 | ( 0.20- 23.10) |
| MATOS | 631 | m | 2 | 5 | - | 3 | - | 2.00 | ( 0.41- 9.65) |
| MATOS | 632 | m | 2 | 5 | - | 3 | - | 6.00 | ( 1.19- 30.28) |
| MATOS | 633 | m | 2 | 4 | - | 3 | - | 7.00 | ( 1.32- 37.20) |
| Subtotal MATOS | | | | | | | | 4.28 | ( 1.68- 10.92) |
| PEZZOT | 579 | m | 0 | 8 | 106 | 0 | 116 | 18.60~ | ( 1.06- 326.10) |
| PEZZOT | 580 | m | 0 | 21 | 82 | 0 | 116 | 60.72~ | ( 3.63-1016.67) |
| Subtotal PEZZOT | | | | | | | | 33.93 | ( 4.55- 252.91) |
| SVENSS | 557 | f | 0 | 1 | 24 | 5 | 120 | 1.00 | ( 0.11- 8.95) |
| SVENSS | 558 | f | 0 | 5 | 13 | 5 | 120 | 9.23 | ( 2.36- 36.16) |
| Subtotal SVENSS | | | | | | | | 4.96 | ( 1.56- 15.80) |
| WAKAI | 546 | m | 1 | 3 | - | 2 | - | 2.05 | ( 0.33- 12.80) |
| WAKAI | 547 | m | 1 | 12 | - | 2 | - | 8.95 | ( 1.91- 42.00) |
| WAKAI | 548 | m | 1 | 11 | - | 2 | - | 7.47 | ( 1.58- 35.30) |
| Subtotal WAKAI | | | | | | | | 5.68 | ( 2.22- 14.53) |
| WYNDE3 | 552 | m | 0 | 6 | 65 | 3 | 88 | 2.71 | ( 0.65- 11.23) |
| WYNDE3 | 573 | f | 0 | 1 | 3 | 5 | 76 | 5.07 | ( 0.44- 57.98) |
| Subtotal WYNDE3 | | | | | | | | 3.17 | ( 0.93- 10.85) |
| WYNDE6 | 787 | m | 2 | 19 | - | 8 | - | 5.00 | ( 2.18- 11.49) |
| WYNDE6 | 788 | m | 2 | 22 | - | 8 | - | 13.70 | ( 6.04- 31.10) |
| WYNDE6 | 789 | m | 2 | 36 | - | 8 | - | 17.20 | ( 7.92- 37.33) |
| WYNDE6 | 790 | m | 2 | 44 | - | 8 | - | 24.90 | ( 11.61- 53.39) |
| WYNDE6 | 791 | m | 2 | 80 | - | 8 | - | 53.80 | ( 25.75- 112.41) |
| Subtotal WYNDE6 | | | | | | | | 18.53 | ( 13.06- 26.30) |
| Partial Totals | | | | 1789 | 5658 | 1066 | 20428 | | |

*prospective study ~ With 0.5 adjustment for zero

Table 2J6 - 2

IESLC - Meta-analysis of Ex Smoking by Years quit (vs never), Overview
Squamous, Cigarettes (or Any Product if Cigarettes not available)
Most adjusted

| REF | NRR | SEX | AD | Ys | Ws | Qs | Ps |
|---|---|---|---|---|---|---|---|
| BARBON | 594 | m | 1 | 0.64 | 2.16 | 6.26 | 0.3455 |
| BARBON | 595 | m | 1 | 2.09 | 3.44 | 0.22 | 0.0001 |
| BARBON | 596 | m | 1 | 2.48 | 4.61 | 0.08 | 0.0000 |
| BARBON | 597 | m | 1 | 2.93 | 3.16 | 1.08 | 0.0000 |
| Subtotal BARBON | | | | 2.19 | 13.36 | 7.64 | |
| BROWN3 | 507 | f | 0 | 1.53 | 6.17 | 4.14 | 0.0002 |
| JAHN | 593 | m | 0 | 0.92 | 2.12 | 4.27 | 0.1786 |
| JAHN | 594 | m | 0 | 1.85 | 2.48 | 0.60 | 0.0036 |
| JAHN | 595 | m | 0 | 3.05 | 2.56 | 1.28 | 0.0000 |
| JAHN | 596 | m | 0 | 3.58 | 2.56 | 3.94 | 0.0000 |
| JAHN | 597 | m | 0 | 4.85 | 2.03 | 12.77 | 0.0000 |
| JAHN | 598 | m | 0 | 6.05 | 2.09 | 28.71 | 0.0000 |
| Subtotal JAHN | | | | 3.33 | 13.84 | 51.58 | |
| JAIN | 543 | m | 0 | 2.16 | 1.77 | 0.06 | 0.0041 |
| JAIN | 544 | m | 0 | 3.10 | 1.74 | 0.99 | 0.0000 |
| JAIN | 507 | f | 0 | 1.41 | 3.02 | 2.64 | 0.0142 |
| JAIN | 508 | f | 0 | 2.70 | 3.76 | 0.47 | 0.0000 |
| Subtotal JAIN | | | | 2.29 | 10.30 | 4.17 | |
| JEDRYC | 543 | m | 0 | 1.57 | 4.59 | 2.74 | 0.0008 |
| JEDRYC | 544 | m | 0 | 2.56 | 4.39 | 0.20 | 0.0000 |
| Subtotal JEDRYC | | | | 2.05 | 8.98 | 2.94 | |
| LUBIN2 | 765 | m | 0 | 1.52 | 34.22 | 23.49 | 0.0000 |
| LUBIN2 | 766 | m | 0 | 1.92 | 27.84 | 5.12 | 0.0000 |
| LUBIN2 | 767 | m | 0 | 2.32 | 36.77 | 0.02 | 0.0000 |
| LUBIN2 | 768 | m | 0 | 2.68 | 42.00 | 4.68 | 0.0000 |
| LUBIN2 | 769 | m | 0 | 3.14 | 45.74 | 28.77 | 0.0000 |
| LUBIN2 | 893 | f | 0 | 0.12 | 1.82 | 8.99 | 0.8688 |
| LUBIN2 | 894 | f | 0 | 0.91 | 4.08 | 8.40 | 0.0662 |
| LUBIN2 | 895 | f | 0 | 1.88 | 19.39 | 4.17 | 0.0000 |
| Subtotal LUBIN2 | | | | 2.30 | 211.87 | 83.64 | |
| LUO | 522 | c | 20 | 0.69 | 0.68 | 1.86 | 0.5673 |
| MATOS | 631 | m | 2 | 0.69 | 1.54 | 4.20 | 0.3897 |
| MATOS | 632 | m | 2 | 1.79 | 1.47 | 0.45 | 0.0300 |
| MATOS | 633 | m | 2 | 1.95 | 1.38 | 0.22 | 0.0223 |
| Subtotal MATOS | | | | 1.45 | 4.39 | 4.87 | |
| PEZZOT | 579 | m | 0 | 2.92 | 0.47 | 0.16 | 0.0455 |
| PEZZOT | 580 | m | 0 | 4.11 | 0.48 | 1.50 | 0.0043 |
| Subtotal PEZZOT | | | | 3.52 | 0.95 | 1.66 | |
| SVENSS | 557 | f | 0 | 0.00 | 0.80 | 4.40 | 1.0000 |
| SVENSS | 558 | f | 0 | 2.22 | 2.06 | 0.03 | 0.0014 |
| Subtotal SVENSS | | | | 1.60 | 2.86 | 4.43 | |
| WAKAI | 546 | m | 1 | 0.72 | 1.15 | 3.04 | 0.4418 |
| WAKAI | 547 | m | 1 | 2.19 | 1.61 | 0.04 | 0.0054 |
| WAKAI | 548 | m | 1 | 2.01 | 1.59 | 0.18 | 0.0112 |
| Subtotal WAKAI | | | | 1.74 | 4.35 | 3.25 | |
| WYNDE3 | 552 | m | 0 | 1.00 | 1.90 | 3.45 | 0.1699 |
| WYNDE3 | 573 | f | 0 | 1.62 | 0.65 | 0.34 | 0.1919 |
| Subtotal WYNDE3 | | | | 1.16 | 2.55 | 3.79 | |
| WYNDE6 | 787 | m | 2 | 1.61 | 5.56 | 3.00 | 0.0001 |
| WYNDE6 | 788 | m | 2 | 2.62 | 5.72 | 0.43 | 0.0000 |
| WYNDE6 | 789 | m | 2 | 2.84 | 6.39 | 1.60 | 0.0000 |
| WYNDE6 | 790 | m | 2 | 3.21 | 6.60 | 5.00 | 0.0000 |
| WYNDE6 | 791 | m | 2 | 3.99 | 7.08 | 19.05 | 0.0000 |
| Subtotal WYNDE6 | | | | 2.92 | 31.35 | 29.09 | |

| | |
|---|---|
| N | 43 |
| NS | 13 |

Table 2J6 - 3

IESLC - Meta-analysis of Ex Smoking by Years quit (vs never), Overview
Squamous, Cigarettes (or Any Product if Cigarettes not available)
Most adjusted

| | Sex | | | |
|---|---|---|---|---|
| | combined | male | female | Total |
| N | 1 | 33 | 9 | 43 |
| NS | 1 | 10 | 5 | 16 |

In this overview table, other than the "N" rows, entries in the "absent" and "Total" columns may be invalid and should be ignored

| | Years quit vs never (lower focus) | | | | |
|---|---|---|---|---|---|
| | absent | 8+k12 | 4-11k7 | 1-6k3 | Total |
| N | 18 | 14 | 6 | 5 | 43 |
| NS | 9 | 11 | 6 | 5 | 31 |
| Wt | 127.24 | 66.08 | 58.40 | 59.91 | 311.64 |
| Het Chi | 93.05 | 22.30 | 2.45 | 7.30 | 203.03 |
| Het df | 17 | 13 | 5 | 4 | 42 |
| Het P | *** | (*) | N.S. | N.S. | *** |
| Fixed RR | 7.07 | 7.53 | 14.34 | 24.98 | 10.43 |
| RRl | 5.94 | 5.91 | 11.10 | 19.39 | 9.33 |
| RRu | 8.41 | 9.58 | 18.54 | 32.18 | 11.65 |
| P | +++ | +++ | +++ | +++ | +++ |
| Random RR | 9.33 | 5.59 | 14.34 | 26.38 | 9.43 |
| RRl | 5.83 | 3.68 | 11.10 | 15.92 | 7.08 |
| RRu | 14.93 | 8.48 | 18.54 | 43.71 | 12.56 |
| P | +++ | +++ | +++ | +++ | +++ |

| | Years quit vs never (higher focus) | | | | |
|---|---|---|---|---|---|
| | absent | 13+k20 | 4-19k12 | 1-11k3 | Total |
| N | 22 | 6 | 5 | 10 | 43 |
| NS | 12 | 5 | 4 | 8 | 29 |
| Wt | 119.14 | 52.36 | 52.79 | 87.35 | 311.64 |
| Het Chi | 90.92 | 5.44 | 8.54 | 35.35 | 203.03 |
| Het df | 21 | 5 | 4 | 9 | 42 |
| Het P | *** | N.S. | (*) | *** | *** |
| Fixed RR | 10.64 | 4.48 | 9.54 | 17.78 | 10.43 |
| RRl | 8.89 | 3.41 | 7.28 | 14.41 | 9.33 |
| RRu | 12.73 | 5.87 | 12.49 | 21.92 | 11.65 |
| P | +++ | +++ | +++ | +++ | +++ |
| Random RR | 9.15 | 4.44 | 8.66 | 18.19 | 9.43 |
| RRl | 5.80 | 3.25 | 5.12 | 10.54 | 7.08 |
| RRu | 14.41 | 6.07 | 14.64 | 31.39 | 12.56 |
| P | +++ | +++ | +++ | +++ | +++ |

Table 2J6 - 3

IESLC - Meta-analysis of Ex Smoking by Years quit (vs never), Overview
Squamous, Cigarettes (or Any Product if Cigarettes not available)
Most adjusted

MALES

| | | Years quit vs never (lower focus) | | | | |
|---|---|---|---|---|---|---|
| | | absent | 8+k12 | 4-11k7 | 1-6k3 | Total |
| | N | 13 | 9 | 6 | 5 | 33 |
| | NS | 7 | 9 | 6 | 5 | 27 |
| | Wt | 94.04 | 56.85 | 58.40 | 59.91 | 269.20 |
| Het | Chi | 83.27 | 10.13 | 2.45 | 7.30 | 164.43 |
| Het | df | 12 | 8 | 5 | 4 | 32 |
| Het | P | *** | N.S. | N.S. | N.S. | *** |
| Fixed | RR | 7.38 | 8.84 | 14.34 | 24.98 | 11.62 |
| | RRl | 6.03 | 6.82 | 11.10 | 19.39 | 10.31 |
| | RRu | 9.04 | 11.46 | 18.54 | 32.18 | 13.09 |
| | P | +++ | +++ | +++ | +++ | +++ |
| Random | RR | 11.67 | 7.84 | 14.34 | 26.38 | 11.67 |
| | RRl | 6.18 | 5.37 | 11.10 | 15.92 | 8.47 |
| | RRu | 22.02 | 11.44 | 18.54 | 43.71 | 16.09 |
| | P | +++ | +++ | +++ | +++ | +++ |

| | | Years quit vs never (higher focus) | | | | |
|---|---|---|---|---|---|---|
| | | absent | 13+k20 | 4-19k12 | 1-11k3 | Total |
| | N | 18 | 4 | 4 | 7 | 33 |
| | NS | 10 | 4 | 4 | 7 | 25 |
| | Wt | 113.99 | 44.37 | 48.71 | 62.13 | 269.20 |
| Het | Chi | 81.07 | 1.86 | 0.53 | 7.71 | 164.43 |
| Het | df | 17 | 3 | 3 | 6 | 32 |
| Het | P | *** | N.S. | N.S. | N.S. | *** |
| Fixed | RR | 11.25 | 4.72 | 10.67 | 25.07 | 11.62 |
| | RRl | 9.36 | 3.52 | 8.06 | 19.55 | 10.31 |
| | RRu | 13.52 | 6.33 | 14.13 | 32.15 | 13.09 |
| | P | +++ | +++ | +++ | +++ | +++ |
| Random | RR | 10.89 | 4.72 | 10.67 | 26.49 | 11.67 |
| | RRl | 6.72 | 3.52 | 8.06 | 17.89 | 8.47 |
| | RRu | 17.65 | 6.33 | 14.13 | 39.23 | 16.09 |
| | P | +++ | +++ | +++ | +++ | +++ |

FEMALES

| | | Years quit vs never (lower focus) | | | | |
|---|---|---|---|---|---|---|
| | | absent | 8+k12 | 4-11k7 | 1-6k3 | Total |
| | N | 5 | 4 | | | 9 |
| | NS | 4 | 4 | | | 8 |
| | Wt | 33.20 | 8.55 | | | 41.76 |
| Het | Chi | 9.09 | 1.56 | | | 14.76 |
| Het | df | 4 | 3 | | | 8 |
| Het | P | (*) | N.S. | | | (*) |
| Fixed | RR | 6.25 | 2.87 | | | 5.33 |
| | RRl | 4.44 | 1.47 | | | 3.93 |
| | RRu | 8.78 | 5.61 | | | 7.21 |
| | P | +++ | ++ | | | +++ |
| Random | RR | 5.94 | 2.87 | | | 4.70 |
| | RRl | 3.22 | 1.47 | | | 2.89 |
| | RRu | 10.97 | 5.61 | | | 7.64 |
| | P | +++ | ++ | | | +++ |

Table 2J6 - 3

IESLC - Meta-analysis of Ex Smoking by Years quit (vs never), Overview
Squamous, Cigarettes (or Any Product if Cigarettes not available)
Most adjusted

FEMALES

| | Years quit vs never (higher focus) absent | 13+k20 | 4-19k12 | 1-11k3 | Total |
|---|---|---|---|---|---|
| N | 3 | 2 | 1 | 3 | 9 |
| NS | 3 | 2 | 1 | 3 | 8 |
| Wt | 4.47 | 7.99 | 4.08 | 25.21 | 41.76 |
| Het Chi | 1.40 | 2.77 | 0.00 | 2.19 | 14.76 |
| Het df | 2 | 1 | 0 | 2 | 8 |
| Het P | N.S. | (*) | N.S. | N.S. | (*) |
| Fixed RR | 3.28 | 3.34 | 2.48 | 7.62 | 5.33 |
| RRl | 1.30 | 1.67 | 0.94 | 5.16 | 3.93 |
| RRu | 8.29 | 6.68 | 6.55 | 11.25 | 7.21 |
| P | + | +++ | (+) | +++ | +++ |
| Random RR | 3.28 | 2.62 | 2.48 | 7.87 | 4.70 |
| RRl | 1.30 | 0.68 | 0.94 | 5.04 | 2.89 |
| RRu | 8.29 | 10.07 | 6.55 | 12.28 | 7.64 |
| P | + | N.S. | (+) | +++ | +++ |

Table 2J6 - 4

IESLC - Meta-analysis of Ex Smoking by Years quit (vs never), Overview
Squamous, Cigarettes (or Any Product if Cigarettes not available)
Least adjusted

| REF | NRR | X | SEX | AGEL | AGEH | RACE | YF | LC TYPE | LOC | START | ST | NLC | R | VB | P | H | AD | PRODUCT | exL | exH | S1 | S2 | DENOM | De |
|---|---|---|---|---|---|---|---|---|---|---|---|---|---|---|---|---|---|---|---|---|---|---|---|---|
| BARBON | 579 | x | m | 0 | 0 | all | - | q | Eu:wst | 1979 | CC | 755 | n | bl | y | y | 0 | all/unsp | 25 | 999 | 0 | 0 | nev any | st |
| BARBON | 580 | x | m | 0 | 0 | all | - | q | Eu:wst | 1979 | CC | 755 | n | bl | y | y | 0 | all/unsp | 15 | 24 | 0 | 1 | nev any | st |
| BARBON | 581 | x | m | 0 | 0 | all | - | q | Eu:wst | 1979 | CC | 755 | n | bl | y | y | 0 | all/unsp | 5 | 14 | 0 | 2 | nev any | st |
| BARBON | 582 | x | m | 0 | 0 | all | - | q | Eu:wst | 1979 | CC | 755 | n | bl | y | y | 0 | all/unsp | 0.1 | 4 | 3 | 3 | nev any | st |
| BROWN3 | 507 | | f | 0 | 0 | wh | - | q | NAmer | | CC | 618 | | bl | y | n | 0 | all/unsp | 15 | 999 | 0 | 1 | nev any | st |
| JAHN | 593 | | m | 0 | 0 | all | - | q | Eu:Ger | 1988 | CC | 1004 | n | bl | n | n | 0 | cig+/-ot | 21 | 999 | 0 | 0 | nev any | st |
| JAHN | 594 | | m | 0 | 0 | all | - | q | Eu:Ger | 1988 | CC | 1004 | n | bl | n | n | 0 | cig+/-ot | 11 | 20 | 1 | 0 | nev any | st |
| JAHN | 595 | | m | 0 | 0 | all | - | q | Eu:Ger | 1988 | CC | 1004 | n | bl | n | n | 0 | cig+/-ot | 6 | 10 | 2 | 0 | nev any | st |
| JAHN | 596 | | m | 0 | 0 | all | - | q | Eu:Ger | 1988 | CC | 1004 | n | bl | n | n | 0 | cig+/-ot | 2 | 5 | 3 | 3 | nev any | st |
| JAHN | 597 | | m | 0 | 0 | all | - | q | Eu:Ger | 1988 | CC | 1004 | n | bl | n | n | 0 | cig+/-ot | 1.0 | 1.9 | 0 | 0 | nev any | st |
| JAHN | 598 | | m | 0 | 0 | all | - | q | Eu:Ger | 1988 | CC | 1004 | n | bl | n | n | 0 | cig+/-ot | 0.1 | 0.9 | 0 | 0 | nev any | st |
| JAIN | 543 | | m | 0 | 0 | all | - | q | NAmer | 1981 | CC | 845 | n | V | y | n | 0 | cig+/-ot | 10 | 999 | 1 | 0 | nev cigs | st |
| JAIN | 544 | | m | 0 | 0 | all | - | q | NAmer | 1981 | CC | 845 | n | V | y | n | 0 | cig+/-ot | 2 | 9 | 0 | 3 | nev cigs | st |
| JAIN | 507 | | f | 0 | 0 | all | - | q | NAmer | 1981 | CC | 845 | n | V | y | n | 0 | cig+/-ot | 10 | 999 | 1 | 0 | nev cigs | st |
| JAIN | 508 | | f | 0 | 0 | all | - | q | NAmer | 1981 | CC | 845 | n | V | y | n | 0 | cig+/-ot | 2 | 9 | 0 | 3 | nev cigs | st |
| JEDRYC | 543 | | m | 0 | 0 | all | - | q | Eu:est | 1980 | CC | 1630 | n | bl | y | n | 0 | cig+/-ot | 10 | 999 | 1 | 0 | nev any | st |
| JEDRYC | 544 | | m | 0 | 0 | all | - | q | Eu:est | 1980 | CC | 1630 | n | bl | y | n | 0 | cig+/-ot | 5 | 9 | 2 | 0 | nev any | st |
| LUBIN2 | 765 | | m | 0 | 0 | all | - | q | Eu:mul | 1976 | CC | 7804 | n | bl | n | y | 0 | cig+/-ot | 20 | 999 | 0 | 1 | nev any | st |
| LUBIN2 | 766 | | m | 0 | 0 | all | - | q | Eu:mul | 1976 | CC | 7804 | n | bl | n | y | 0 | cig+/-ot | 15 | 19 | 0 | 0 | nev any | st |
| LUBIN2 | 767 | | m | 0 | 0 | all | - | q | Eu:mul | 1976 | CC | 7804 | n | bl | n | y | 0 | cig+/-ot | 10 | 14 | 1 | 2 | nev any | st |
| LUBIN2 | 768 | | m | 0 | 0 | all | - | q | Eu:mul | 1976 | CC | 7804 | n | bl | n | y | 0 | cig+/-ot | 5 | 9 | 2 | 0 | nev any | st |
| LUBIN2 | 769 | | m | 0 | 0 | all | - | q | Eu:mul | 1976 | CC | 7804 | n | bl | n | y | 0 | cig+/-ot | 0.1 | 4 | 3 | 3 | nev any | st |
| LUBIN2 | 893 | | f | 0 | 0 | all | - | q | Eu:mul | 1976 | CC | 7804 | n | bl | n | y | 0 | cig+/-ot | 20 | 999 | 0 | 1 | nev any | st |
| LUBIN2 | 894 | | f | 0 | 0 | all | - | q | Eu:mul | 1976 | CC | 7804 | n | bl | n | y | 0 | cig+/-ot | 10 | 19 | 1 | 2 | nev any | st |
| LUBIN2 | 895 | | f | 0 | 0 | all | - | q | Eu:mul | 1976 | CC | 7804 | n | bl | n | y | 0 | cig+/-ot | 0.1 | 9 | 0 | 3 | nev any | st |
| LUO | 519 | x | c | 0 | 0 | all | - | q | As:Chi | 1990 | CC | 102 | n | ot | n | y | 0 | cig+/-ot | 10 | 999 | 1 | 0 | nev cigs | st |
| MATOS | 621 | x | m | 0 | 0 | all | - | q | SCAmer | 1994 | CC | 200 | n | bl | n | n | 0 | cig+/-ot | 11 | 999 | 1 | 0 | nev any | st |
| MATOS | 622 | x | m | 0 | 0 | all | - | q | SCAmer | 1994 | CC | 200 | n | bl | n | n | 0 | cig+/-ot | 6 | 10 | 2 | 0 | nev any | st |
| MATOS | 623 | x | m | 0 | 0 | all | - | q | SCAmer | 1994 | CC | 200 | n | bl | n | n | 0 | cig+/-ot | 1.0 | 5 | 3 | 3 | nev any | st |
| PEZZOT | 579 | | m | 0 | 0 | all | - | q | SCAmer | 1987 | CC | 215 | n | bl | n | y | 0 | cig only | 11 | 999 | 1 | 0 | nev cigs | ot |
| PEZZOT | 580 | | m | 0 | 0 | all | - | q | SCAmer | 1987 | CC | 215 | n | bl | n | y | 0 | cig only | 1.0 | 10 | 0 | 3 | nev cigs | ot |
| SVENSS | 557 | | f | 0 | 0 | all | - | q | Eu:Sca | 1983 | CC | 210 | n | bl | n | n | 0 | all/unsp | 11 | 999 | 1 | 0 | nev any | st |
| SVENSS | 558 | | f | 0 | 0 | all | - | q | Eu:Sca | 1983 | CC | 210 | n | bl | n | n | 0 | all/unsp | 3 | 10 | 0 | 3 | nev any | st |
| WAKAI | 538 | x | m | 0 | 0 | all | - | q | As:Jap | 1988 | CC | 333 | n | bl | n | y | 0 | cig+/-ot | 20 | 999 | 0 | 1 | nev any | st |
| WAKAI | 539 | x | m | 0 | 0 | all | - | q | As:Jap | 1988 | CC | 333 | n | bl | n | y | 0 | cig+/-ot | 10 | 19 | 1 | 2 | nev any | st |
| WAKAI | 540 | x | m | 0 | 0 | all | - | q | As:Jap | 1988 | CC | 333 | n | bl | n | y | 0 | cig+/-ot | 5 | 9 | 2 | 0 | nev any | st |
| WYNDE3 | 552 | | m | 0 | 0 | all | - | KI | NAmer | 1966 | CC | 350 | n | bl | n | y | 0 | cig+/-ot | 10 | 999 | 1 | 0 | nev any | st |
| WYNDE3 | 573 | | f | 0 | 0 | all | - | KI | NAmer | 1966 | CC | 350 | n | bl | n | y | 0 | cig+/-ot | 10 | 999 | 1 | 0 | nev any | st |
| WYNDE6 | 772 | x | m | 0 | 0 | all | - | KI | NAmer | 1969 | CC | 4423 | n | bl | n | y | 0 | cig+/-ot | 16 | 999 | 0 | 1 | nev any | st |
| WYNDE6 | 773 | x | m | 0 | 0 | all | - | KI | NAmer | 1969 | CC | 4423 | n | bl | n | y | 0 | cig+/-ot | 11 | 15 | 1 | 2 | nev any | st |
| WYNDE6 | 774 | x | m | 0 | 0 | all | - | KI | NAmer | 1969 | CC | 4423 | n | bl | n | y | 0 | cig+/-ot | 7 | 10 | 2 | 0 | nev any | st |
| WYNDE6 | 775 | x | m | 0 | 0 | all | - | KI | NAmer | 1969 | CC | 4423 | n | bl | n | y | 0 | cig+/-ot | 4 | 6 | 0 | 0 | nev any | st |
| WYNDE6 | 776 | x | m | 0 | 0 | all | - | KI | NAmer | 1969 | CC | 4423 | n | bl | n | y | 0 | cig+/-ot | 1.0 | 3 | 3 | 3 | nev any | st |

Cigarette type is all/unspec for all RRs

In this overview table, subtotals and Qs values may be invalid and should be ignored

Table 2J6 - 5

IESLC - Meta-analysis of Ex Smoking by Years quit (vs never), Overview
Squamous, Cigarettes (or Any Product if Cigarettes not available)
Least adjusted

| REF | NRR | SEX | AD | Number Exposed Case | Cont | Non-exposed Case | Cont | RR | 95.00%CI |
|---|---|---|---|---|---|---|---|---|---|
| BARBON | 579 | m | 0 | 4 | 59 | 6 | 188 | 2.12 | ( 0.58- 7.78) |
| BARBON | 580 | m | 0 | 11 | 41 | 6 | 188 | 8.41 | ( 2.94- 24.04) |
| BARBON | 581 | m | 0 | 31 | 85 | 6 | 188 | 11.43 | ( 4.60- 28.42) |
| BARBON | 582 | m | 0 | 11 | 20 | 6 | 188 | 17.23 | ( 5.76- 51.58) |
| Subtotal BARBON | | | | | | | | 8.77 | ( 5.16- 14.93) |
| BROWN3 | 507 | f | 0 | 17 | 10 | 432 | 1168 | 4.60 | ( 2.09- 10.12) |
| JAHN | 593 | m | 0 | 8 | 146 | 3 | 138 | 2.52 | ( 0.66- 9.70) |
| JAHN | 594 | m | 0 | 18 | 130 | 3 | 138 | 6.37 | ( 1.83- 22.13) |
| JAHN | 595 | m | 0 | 29 | 63 | 3 | 138 | 21.17 | ( 6.22- 72.12) |
| JAHN | 596 | m | 0 | 36 | 46 | 3 | 138 | 36.00 | ( 10.58- 122.45) |
| JAHN | 597 | m | 0 | 25 | 9 | 3 | 138 | 127.78 | ( 32.33- 505.04) |
| JAHN | 598 | m | 0 | 74 | 8 | 3 | 138 | 425.50 | (109.58-1652.23) |
| Subtotal JAHN | | | | | | | | 27.87 | ( 16.45- 47.20) |
| JAIN | 543 | m | 0 | 23 | 113 | 2 | 85 | 8.65 | ( 1.98- 37.70) |
| JAIN | 544 | m | 0 | 24 | 46 | 2 | 85 | 22.17 | ( 5.02- 98.04) |
| JAIN | 507 | f | 0 | 7 | 61 | 6 | 214 | 4.09 | ( 1.33- 12.63) |
| JAIN | 508 | f | 0 | 15 | 36 | 6 | 214 | 14.86 | ( 5.41- 40.82) |
| Subtotal JAIN | | | | | | | | 9.92 | ( 5.39- 18.27) |
| JEDRYC | 543 | m | 0 | 23 | 230 | 6 | 289 | 4.82 | ( 1.93- 12.03) |
| JEDRYC | 544 | m | 0 | 22 | 82 | 6 | 289 | 12.92 | ( 5.07- 32.93) |
| Subtotal JEDRYC | | | | | | | | 7.80 | ( 4.06- 15.01) |
| LUBIN2 | 765 | m | 0 | 106 | 1128 | 54 | 2616 | 4.55 | ( 3.26- 6.36) |
| LUBIN2 | 766 | m | 0 | 67 | 478 | 54 | 2616 | 6.79 | ( 4.68- 9.84) |
| LUBIN2 | 767 | m | 0 | 146 | 693 | 54 | 2616 | 10.21 | ( 7.39- 14.10) |
| LUBIN2 | 768 | m | 0 | 265 | 882 | 54 | 2616 | 14.56 | ( 10.76- 19.70) |
| LUBIN2 | 769 | m | 0 | 498 | 1047 | 54 | 2616 | 23.04 | ( 17.25- 30.79) |
| LUBIN2 | 893 | f | 0 | 2 | 29 | 72 | 1180 | 1.13 | ( 0.26- 4.83) |
| LUBIN2 | 894 | f | 0 | 5 | 33 | 72 | 1180 | 2.48 | ( 0.94- 6.55) |
| LUBIN2 | 895 | f | 0 | 38 | 95 | 72 | 1180 | 6.56 | ( 4.20- 10.23) |
| Subtotal LUBIN2 | | | | | | | | 9.96 | ( 8.71- 11.40) |
| LUO | 519 | c | 0 | 1 | 10 | 5 | 51 | 1.02 | ( 0.11- 9.69) |
| MATOS | 621 | m | 0 | 5 | 101 | 3 | 110 | 1.82 | ( 0.42- 7.79) |
| MATOS | 622 | m | 0 | 5 | 27 | 3 | 110 | 6.79 | ( 1.53- 30.19) |
| MATOS | 623 | m | 0 | 4 | 23 | 3 | 110 | 6.38 | ( 1.34- 30.44) |
| Subtotal MATOS | | | | | | | | 4.17 | ( 1.75- 9.93) |
| PEZZOT | 579 | m | 0 | 8 | 106 | 0 | 116 | 18.60~ | ( 1.06- 326.10) |
| PEZZOT | 580 | m | 0 | 21 | 82 | 0 | 116 | 60.72~ | ( 3.63-1016.67) |
| Subtotal PEZZOT | | | | | | | | 33.93 | ( 4.55- 252.91) |
| SVENSS | 557 | f | 0 | 1 | 24 | 5 | 120 | 1.00 | ( 0.11- 8.95) |
| SVENSS | 558 | f | 0 | 5 | 13 | 5 | 120 | 9.23 | ( 2.36- 36.16) |
| Subtotal SVENSS | | | | | | | | 4.96 | ( 1.56- 15.80) |
| WAKAI | 538 | m | 0 | 3 | 47 | 2 | 65 | 2.07 | ( 0.33- 12.91) |
| WAKAI | 539 | m | 0 | 12 | 44 | 2 | 65 | 8.86 | ( 1.89- 41.56) |
| WAKAI | 540 | m | 0 | 11 | 48 | 2 | 65 | 7.45 | ( 1.58- 35.17) |
| Subtotal WAKAI | | | | | | | | 5.67 | ( 2.22- 14.50) |
| WYNDE3 | 552 | m | 0 | 6 | 65 | 3 | 88 | 2.71 | ( 0.65- 11.23) |
| WYNDE3 | 573 | f | 0 | 1 | 3 | 5 | 76 | 5.07 | ( 0.44- 57.98) |
| Subtotal WYNDE3 | | | | | | | | 3.17 | ( 0.93- 10.85) |
| WYNDE6 | 772 | m | 0 | 19 | 530 | 8 | 1667 | 7.47 | ( 3.25- 17.16) |
| WYNDE6 | 773 | m | 0 | 22 | 259 | 8 | 1667 | 17.70 | ( 7.80- 40.18) |
| WYNDE6 | 774 | m | 0 | 36 | 340 | 8 | 1667 | 22.06 | ( 10.17- 47.89) |
| WYNDE6 | 775 | m | 0 | 44 | 321 | 8 | 1667 | 28.56 | ( 13.32- 61.24) |
| WYNDE6 | 776 | m | 0 | 80 | 307 | 8 | 1667 | 54.30 | ( 25.99- 113.46) |
| Subtotal WYNDE6 | | | | | | | | 22.64 | ( 15.95- 32.12) |
| Totals | | | | 1789 | 7920 | 1066 | 30091 | | |

*prospective study ~ With 0.5 adjustment for zero

Table 2J6 - 5

IESLC - Meta-analysis of Ex Smoking by Years quit (vs never), Overview
Squamous, Cigarettes (or Any Product if Cigarettes not available)
Least adjusted

| REF | NRR | SEX | AD | Ys | Ws | Qs | Ps |
|---|---|---|---|---|---|---|---|
| BARBON | 579 | m | 0 | 0.75 | 2.28 | 5.87 | 0.2554 |
| BARBON | 580 | m | 0 | 2.13 | 3.48 | 0.18 | 0.0001 |
| BARBON | 581 | m | 0 | 2.44 | 4.63 | 0.03 | 0.0000 |
| BARBON | 582 | m | 0 | 2.85 | 3.20 | 0.76 | 0.0000 |
| Subtotal BARBON | | | | 2.17 | 13.58 | 6.84 | |
| BROWN3 | 507 | f | 0 | 1.53 | 6.17 | 4.29 | 0.0002 |
| JAHN | 593 | m | 0 | 0.92 | 2.12 | 4.36 | 0.1786 |
| JAHN | 594 | m | 0 | 1.85 | 2.48 | 0.64 | 0.0036 |
| JAHN | 595 | m | 0 | 3.05 | 2.56 | 1.23 | 0.0000 |
| JAHN | 596 | m | 0 | 3.58 | 2.56 | 3.84 | 0.0000 |
| JAHN | 597 | m | 0 | 4.85 | 2.03 | 12.62 | 0.0000 |
| JAHN | 598 | m | 0 | 6.05 | 2.09 | 28.49 | 0.0000 |
| Subtotal JAHN | | | | 3.33 | 13.84 | 51.18 | |
| JAIN | 543 | m | 0 | 2.16 | 1.77 | 0.07 | 0.0041 |
| JAIN | 544 | m | 0 | 3.10 | 1.74 | 0.95 | 0.0000 |
| JAIN | 507 | f | 0 | 1.41 | 3.02 | 2.73 | 0.0142 |
| JAIN | 508 | f | 0 | 2.70 | 3.76 | 0.43 | 0.0000 |
| Subtotal JAIN | | | | 2.29 | 10.30 | 4.19 | |
| JEDRYC | 543 | m | 0 | 1.57 | 4.59 | 2.84 | 0.0008 |
| JEDRYC | 544 | m | 0 | 2.56 | 4.39 | 0.18 | 0.0000 |
| Subtotal JEDRYC | | | | 2.05 | 8.98 | 3.02 | |
| LUBIN2 | 765 | m | 0 | 1.52 | 34.22 | 24.34 | 0.0000 |
| LUBIN2 | 766 | m | 0 | 1.92 | 27.84 | 5.47 | 0.0000 |
| LUBIN2 | 767 | m | 0 | 2.32 | 36.77 | 0.05 | 0.0000 |
| LUBIN2 | 768 | m | 0 | 2.68 | 42.00 | 4.27 | 0.0000 |
| LUBIN2 | 769 | m | 0 | 3.14 | 45.74 | 27.71 | 0.0000 |
| LUBIN2 | 893 | f | 0 | 0.12 | 1.82 | 9.11 | 0.8688 |
| LUBIN2 | 894 | f | 0 | 0.91 | 4.08 | 8.57 | 0.0662 |
| LUBIN2 | 895 | f | 0 | 1.88 | 19.39 | 4.44 | 0.0000 |
| Subtotal LUBIN2 | | | | 2.30 | 211.87 | 83.97 | |
| LUO | 519 | c | 0 | 0.02 | 0.76 | 4.15 | 0.9862 |
| MATOS | 621 | m | 0 | 0.60 | 1.81 | 5.63 | 0.4224 |
| MATOS | 622 | m | 0 | 1.92 | 1.73 | 0.34 | 0.0119 |
| MATOS | 623 | m | 0 | 1.85 | 1.57 | 0.40 | 0.0202 |
| Subtotal MATOS | | | | 1.43 | 5.11 | 6.37 | |
| PEZZOT | 579 | m | 0 | 2.92 | 0.47 | 0.15 | 0.0455 |
| PEZZOT | 580 | m | 0 | 4.11 | 0.48 | 1.48 | 0.0043 |
| Subtotal PEZZOT | | | | 3.52 | 0.95 | 1.63 | |
| SVENSS | 557 | f | 0 | 0.00 | 0.80 | 4.45 | 1.0000 |
| SVENSS | 558 | f | 0 | 2.22 | 2.06 | 0.04 | 0.0014 |
| Subtotal SVENSS | | | | 1.60 | 2.86 | 4.49 | |
| WAKAI | 538 | m | 0 | 0.73 | 1.15 | 3.05 | 0.4340 |
| WAKAI | 539 | m | 0 | 2.18 | 1.61 | 0.05 | 0.0056 |
| WAKAI | 540 | m | 0 | 2.01 | 1.59 | 0.20 | 0.0112 |
| Subtotal WAKAI | | | | 1.73 | 4.35 | 3.30 | |
| WYNDE3 | 552 | m | 0 | 1.00 | 1.90 | 3.53 | 0.1699 |
| WYNDE3 | 573 | f | 0 | 1.62 | 0.65 | 0.35 | 0.1919 |
| Subtotal WYNDE3 | | | | 1.16 | 2.55 | 3.88 | |
| WYNDE6 | 772 | m | 0 | 2.01 | 5.55 | 0.67 | 0.0000 |
| WYNDE6 | 773 | m | 0 | 2.87 | 5.72 | 1.51 | 0.0000 |
| WYNDE6 | 774 | m | 0 | 3.09 | 6.40 | 3.46 | 0.0000 |
| WYNDE6 | 775 | m | 0 | 3.35 | 6.60 | 6.51 | 0.0000 |
| WYNDE6 | 776 | m | 0 | 3.99 | 7.07 | 18.92 | 0.0000 |
| Subtotal WYNDE6 | | | | 3.12 | 31.34 | 31.08 | |

| | |
|---|---|
| N | 43 |
| NS | 13 |

Table 2J6 - 6

IESLC - Meta-analysis of Ex Smoking by Years quit (vs never), Overview
Squamous, Cigarettes (or Any Product if Cigarettes not available)
Least adjusted

| | Sex | | | |
|---|---|---|---|---|
| | combined | male | female | Total |
| N | 1 | 33 | 9 | 43 |
| NS | 1 | 10 | 5 | 16 |

In this overview table, other than the "N" rows, entries in the "absent" and "Total" columns may be invalid and should be ignored

| | Years quit vs never (lower focus) | | | | |
|---|---|---|---|---|---|
| | absent | 8+k12 | 4-11k7 | 1-6k3 | Total |
| N | 18 | 14 | 6 | 5 | 43 |
| NS | 9 | 11 | 6 | 5 | 31 |
| Wt | 127.42 | 66.42 | 58.67 | 60.14 | 312.66 |
| Het Chi | 94.12 | 27.21 | 3.24 | 8.27 | 208.37 |
| Het df | 17 | 13 | 5 | 4 | 42 |
| Het P | *** | * | N.S. | (*) | *** |
| Fixed RR | 7.25 | 7.56 | 14.73 | 24.73 | 10.58 |
| RRl | 6.09 | 5.94 | 11.41 | 19.21 | 9.47 |
| RRu | 8.62 | 9.61 | 19.03 | 31.84 | 11.82 |
| P | +++ | +++ | +++ | +++ | +++ |
| Random RR | 9.70 | 5.35 | 14.73 | 25.44 | 9.58 |
| RRl | 6.05 | 3.38 | 11.41 | 14.84 | 7.17 |
| RRu | 15.56 | 8.47 | 19.03 | 43.61 | 12.78 |
| P | +++ | +++ | +++ | +++ | +++ |

| | Years quit vs never (higher focus) | | | | |
|---|---|---|---|---|---|
| | absent | 13+k20 | 4-19k12 | 1-11k3 | Total |
| N | 22 | 6 | 5 | 10 | 43 |
| NS | 12 | 5 | 4 | 8 | 29 |
| Wt | 119.87 | 52.40 | 52.81 | 87.58 | 312.66 |
| Het Chi | 97.49 | 6.87 | 9.88 | 35.94 | 208.37 |
| Het df | 21 | 5 | 4 | 9 | 42 |
| Het P | *** | N.S. | * | *** | *** |
| Fixed RR | 10.76 | 4.68 | 9.77 | 17.67 | 10.58 |
| RRl | 8.99 | 3.57 | 7.46 | 14.33 | 9.47 |
| RRu | 12.86 | 6.14 | 12.79 | 21.79 | 11.82 |
| P | +++ | +++ | +++ | +++ | +++ |
| Random RR | 9.12 | 4.68 | 8.98 | 17.86 | 9.58 |
| RRl | 5.73 | 3.15 | 5.09 | 10.34 | 7.17 |
| RRu | 14.53 | 6.96 | 15.82 | 30.86 | 12.78 |
| P | +++ | +++ | +++ | +++ | +++ |

Table 2J6 - 6

IESLC - Meta-analysis of Ex Smoking by Years quit (vs never), Overview
Squamous, Cigarettes (or Any Product if Cigarettes not available)
Least adjusted

MALES

Years quit vs never (lower focus)

| | | absent | 8+k12 | 4-11k7 | 1-6k3 | Total |
|---|---|---|---|---|---|---|
| | N | 13 | 9 | 6 | 5 | 33 |
| | NS | 7 | 9 | 6 | 5 | 27 |
| | Wt | 94.22 | 57.11 | 58.67 | 60.14 | 270.14 |
| Het | Chi | 84.04 | 12.92 | 3.24 | 8.27 | 166.38 |
| Het | df | 12 | 8 | 5 | 4 | 32 |
| Het | P | *** | N.S. | N.S. | (*) | *** |
| Fixed | RR | 7.64 | 8.97 | 14.73 | 24.73 | 11.84 |
| | RRl | 6.24 | 6.92 | 11.41 | 19.21 | 10.51 |
| | RRu | 9.35 | 11.63 | 19.03 | 31.84 | 13.34 |
| | P | +++ | +++ | +++ | +++ | +++ |
| Random | RR | 12.33 | 7.56 | 14.73 | 25.44 | 12.04 |
| | RRl | 6.52 | 4.83 | 11.41 | 14.84 | 8.74 |
| | RRu | 23.30 | 11.83 | 19.03 | 43.61 | 16.59 |
| | P | +++ | +++ | +++ | +++ | +++ |

Years quit vs never (higher focus)

| | | absent | 13+k20 | 4-19k12 | 1-11k3 | Total |
|---|---|---|---|---|---|---|
| | N | 18 | 4 | 4 | 7 | 33 |
| | NS | 10 | 4 | 4 | 7 | 25 |
| | Wt | 114.64 | 44.40 | 48.73 | 62.37 | 270.14 |
| Het | Chi | 85.15 | 3.02 | 1.58 | 8.68 | 166.38 |
| Het | df | 17 | 3 | 3 | 6 | 32 |
| Het | P | *** | N.S. | N.S. | N.S. | *** |
| Fixed | RR | 11.44 | 4.98 | 10.95 | 24.83 | 11.84 |
| | RRl | 9.53 | 3.71 | 8.27 | 19.37 | 10.51 |
| | RRu | 13.74 | 6.68 | 14.50 | 31.83 | 13.34 |
| | P | +++ | +++ | +++ | +++ | +++ |
| Random | RR | 11.15 | 4.99 | 10.95 | 25.97 | 12.04 |
| | RRl | 6.83 | 3.70 | 8.27 | 16.83 | 8.74 |
| | RRu | 18.20 | 6.74 | 14.50 | 40.07 | 16.59 |
| | P | +++ | +++ | +++ | +++ | +++ |

FEMALES

Years quit vs never (lower focus)

| | | absent | 8+k12 | 4-11k7 | 1-6k3 | Total |
|---|---|---|---|---|---|---|
| | N | 5 | 4 | | | 9 |
| | NS | 4 | 4 | | | 8 |
| | Wt | 33.20 | 8.55 | | | 41.76 |
| Het | Chi | 9.09 | 1.56 | | | 14.76 |
| Het | df | 4 | 3 | | | 8 |
| Het | P | (*) | N.S. | | | (*) |
| Fixed | RR | 6.25 | 2.87 | | | 5.33 |
| | RRl | 4.44 | 1.47 | | | 3.93 |
| | RRu | 8.78 | 5.61 | | | 7.21 |
| | P | +++ | ++ | | | +++ |
| Random | RR | 5.94 | 2.87 | | | 4.70 |
| | RRl | 3.22 | 1.47 | | | 2.89 |
| | RRu | 10.97 | 5.61 | | | 7.64 |
| | P | +++ | ++ | | | +++ |

Table 2J6 - 6

IESLC - Meta-analysis of Ex Smoking by Years quit (vs never), Overview
Squamous, Cigarettes (or Any Product if Cigarettes not available)
Least adjusted

FEMALES

| | Years quit vs never (higher focus) absent | 13+k20 | 4-19k12 | 1-11k3 | Total |
|---|---|---|---|---|---|
| N | 3 | 2 | 1 | 3 | 9 |
| NS | 3 | 2 | 1 | 3 | 8 |
| Wt | 4.47 | 7.99 | 4.08 | 25.21 | 41.76 |
| Het Chi | 1.40 | 2.77 | 0.00 | 2.19 | 14.76 |
| Het df | 2 | 1 | 0 | 2 | 8 |
| Het P | N.S. | (*) | N.S. | N.S. | (*) |
| Fixed RR | 3.28 | 3.34 | 2.48 | 7.62 | 5.33 |
| RRl | 1.30 | 1.67 | 0.94 | 5.16 | 3.93 |
| RRu | 8.29 | 6.68 | 6.55 | 11.25 | 7.21 |
| P | + | +++ | (+) | +++ | +++ |
| Random RR | 3.28 | 2.62 | 2.48 | 7.87 | 4.70 |
| RRl | 1.30 | 0.68 | 0.94 | 5.04 | 2.89 |
| RRu | 8.29 | 10.07 | 6.55 | 12.28 | 7.64 |
| P | + | N.S. | (+) | +++ | +++ |

Table 2J6 - 7

IESLC - Meta-analysis of Ex Smoking by Years quit (vs never), Overview
Squamous, Cigarettes (or Any Product if Cigarettes not available)
Excluded studies (and stage at which they were excluded)

1 AGUDO AKIBA AMANDU AMES AXELSS BEST BOUCHA BOUCOT BRESLO CHEN CHEN2 CHIAZZ DEAN2 DOSEME ENGELA FAN
GENG GER GUO HAENSZ HEGMAN HOLE HU HU2 JUSSAW KATSOU KAUFMA KOO KOULUM KREUZE LETOUR LEVIN
LIAW LIU3 LIU4 LIU5 MCCONN MIGRAN MRFITR NOTAN2 OSANN2 PERNU QIAO2 RACHTA RESTRE SADOWS SEGI2 STASZE
TIZZAN VUTUC WATSON WU WUWILL WYNDE2 WYNDE8 XU YUAN ZHANG ZHENG ZHOU

2 BUFFLE HUMBLE PISANI PRESCO WYNDE7

3 MCDUFF SPITZ

4 ARMADA AUVINE BECHER BENSHL BLOT1 BOFFET BROSS CARPEN CEDERL CHOI CHYOU CORREA CPSI CPSII DAMBER DARBY
DEAN3 DESTEF DOLL DOLL2 DORGAN DORN GAO GAO2 GARCIA GARSHI GILLIS GRAHAM GURSEL HAMMO2 HIRAYA JOLY
KAISE2 KHUDER LAUSSM LUBIN PEZZO2 QIAO SPEIZE SUZUK2 TVERDA WANG2 WIGLE WU2

5 ALDERS HAMMON

10 SOBUE

14 BENHAM

Table 2J6 - 8

Potentially overlapping studies

| REF | REFGP | PRINC | . OVERLAP/LINK |
|---|---|---|---|
| LUBIN2 | LUBIN2 | 1 | Lubin-combined |
| WYNDE6 | WYNDE6 | 1 | WYNDE5/6/7/8 |
| JAHN | BOFFET | 2 | Subset of BOFFET |

Table 2J6 - 9

Most adjusted - insufficient data for meta-analysis

| REF | NRR | SEX | AGEL | AGEH | RACE | YF | LC TYPE | LOC | START | ST | NLC | R | VB | P | H | AD | PRODUCT | exL | exH | S1 | S2 | DENOM | De |
|---|---|---|---|---|---|---|---|---|---|---|---|---|---|---|---|---|---|---|---|---|---|---|---|
| ALDERS | 537 | m | 0 | 0 | all | - | q+s | Eu:UK | 1977 | CC | 1448 | n | V | n | n | 1 | cig only | 10 | 999 | 1 | 0 | nev any | st |
| ALDERS | 538 | m | 0 | 0 | all | - | q+s | Eu:UK | 1977 | CC | 1448 | n | V | n | n | 1 | cig only | 3 | 9 | 0 | 3 | nev any | st |
| ALDERS | 539 | m | 0 | 0 | all | - | q+s | Eu:UK | 1977 | CC | 1448 | n | V | n | n | 1 | cig only | 0.1 | 2 | 0 | 0 | nev any | st |
| ALDERS | 548 | f | 0 | 0 | all | - | q+s | Eu:UK | 1977 | CC | 1448 | n | V | n | n | 1 | cig only | 10 | 999 | 1 | 0 | nev any | st |
| ALDERS | 549 | f | 0 | 0 | all | - | q+s | Eu:UK | 1977 | CC | 1448 | n | V | n | n | 1 | cig only | 3 | 9 | 0 | 3 | nev any | st |
| ALDERS | 550 | f | 0 | 0 | all | - | q+s | Eu:UK | 1977 | CC | 1448 | n | V | n | n | 1 | cig only | 0.1 | 2 | 0 | 0 | nev any | st |
| BROWN3 | 508 | f | 0 | 0 | wh | - | q | NAmer | | CC | 618 | | bl | y | n | 0 | all/unsp | 1.0 | 14 | 0 | 0 | nev any | ot |
| HAMMON | 501 | m | 0 | 0 | wh | 0 | not a | NAmer | 1952 | pr | 448 | n | bl | n | n | 1 | cig only | 10 | 999 | 1 | 0 | nev any | st |
| HAMMON | 502 | m | 0 | 0 | wh | 0 | not a | NAmer | 1952 | pr | 448 | n | bl | n | n | 1 | cig only | 1.0 | 9 | 0 | 3 | nev any | st |
| HAMMON | 503 | m | 0 | 0 | wh | 0 | not a | NAmer | 1952 | pr | 448 | n | bl | n | n | 1 | cig only | 0.1 | 0.9 | 0 | 0 | nev any | st |
| JAIN | 588 | m | 0 | 0 | all | - | q | NAmer | 1981 | CC | 845 | n | V | y | n | 0 | cig+/-ot | 0.1 | 1.9 | 0 | 0 | nev cigs | ot |
| JAIN | 576 | f | 0 | 0 | all | - | q | NAmer | 1981 | CC | 845 | n | V | y | n | 0 | cig+/-ot | 0.1 | 1.9 | 0 | 0 | nev cigs | ot |
| JEDRYC | 545 | m | 0 | 0 | all | - | q | Eu:est | 1980 | CC | 1630 | n | bl | y | n | 0 | cig+/-ot | 1.0 | 4 | 3 | 3 | nev any | ot |
| LUO | 523 | c | 0 | 0 | all | - | q | As:Chi | 1990 | CC | 102 | n | ot | n | y | 20 | cig+/-ot | 1.0 | 9 | 0 | 3 | nev cigs | ot |
| MATOS | 707 | m | 0 | 0 | all | - | q | SCAmer | 1994 | CC | 200 | n | bl | n | n | 2 | cig+/-ot | 0.1 | 0.9 | 0 | 0 | nev any | ot |
| PEZZOT | 599 | m | 0 | 0 | all | - | q | SCAmer | 1987 | CC | 215 | n | bl | n | y | 0 | cig only | 0.1 | 0.9 | 0 | 0 | nev cigs | ot |
| SVENSS | 593 | f | 0 | 0 | all | - | q | Eu:Sca | 1983 | CC | 210 | n | bl | n | n | 0 | all/unsp | 1.0 | 2 | 0 | 0 | nev any | ot |
| WAKAI | 615 | m | 0 | 0 | all | - | q | As:Jap | 1988 | CC | 333 | n | bl | n | y | 1 | cig+/-ot | 1.0 | 4 | 3 | 3 | nev any | ot |
| WYNDE3 | 553 | m | 0 | 0 | all | - | KI | NAmer | 1966 | CC | 350 | n | bl | n | y | 0 | cig+/-ot | 1.0 | 9 | 0 | 3 | nev any | ot |
| WYNDE3 | 574 | f | 0 | 0 | all | - | KI | NAmer | 1966 | CC | 350 | n | bl | n | y | 0 | cig+/-ot | 1.0 | 9 | 0 | 3 | nev any | ot |
| WYNDE6 | 792 | m | 0 | 0 | all | - | KI | NAmer | 1969 | CC | 4423 | n | bl | n | y | 2 | cig+/-ot | 0.1 | 0.9 | 0 | 0 | nev any | ot |

| REF | NRR | RR | SIG | RRDATA comment |
|---|---|---|---|---|
| ALDERS | 537 | 2.33 | | 0 |
| ALDERS | 538 | 4.00 | | 0 |
| ALDERS | 539 | 23.33 | | 0 |
| ALDERS | 548 | 0.67 | | 0 |
| ALDERS | 549 | 8.39 | | 0 |
| ALDERS | 550 | 16.78 | | 0 |
| BROWN3 | 508 | * | gap | 0 |
| HAMMON | 501 | * | | RR for <1 pack per day is 2.44, while that for 1+ packs is 17.79 |
| HAMMON | 502 | * | | RR for <1 pack per day is 10.44, while that for 1+ packs is 22.82 |
| HAMMON | 503 | * | | RR for <1 pack per day is 16.50, while that for 1+ packs is 58.24 |
| JAIN | 588 | * | gap | 0 |
| JAIN | 576 | * | gap | 0 |
| JEDRYC | 545 | * | gap | 0 |

Table 2J6 - 9

IESLC - Meta-analysis of Ex Smoking by Years quit (vs never), Overview
Squamous, Cigarettes (or Any Product if Cigarettes not available)
Most adjusted - insufficient data for meta-analysis

| REF | NRR | RR | SIG | RRDATA comment |
|---|---|---|---|---|
| LUO | 523 | * | gap | 0 |
| MATOS | 707 | * | gap | 0 |
| PEZZOT | 599 | * | gap | 0 |
| SVENSS | 593 | * | gap | 0 |
| WAKAI | 615 | * | gap | 0 |
| WYNDE3 | 553 | * | gap | 0 |
| WYNDE3 | 574 | * | gap | 0 |
| WYNDE6 | 792 | * | gap | 0 |

Least adjusted - insufficient data for meta-analysis: as for adjusted plus the following

| REF | NRR | SEX | AGEL | AGEH | RACE | YF | LC TYPE | LOC | START | ST | NLC | R | VB | P | H | AD | PRODUCT | exL | exH | S1 | S2 | DENOM | De |
|---|---|---|---|---|---|---|---|---|---|---|---|---|---|---|---|---|---|---|---|---|---|---|---|
| LUO | 520 | c | 0 | 0 | all | - | q | As:Chi | 1990 | CC | 102 | n | ot | n | y | 0 | cig+/-ot | 1.0 | 9 | 0 | 3 | nev cigs | ot |
| MATOS | 705 | m | 0 | 0 | all | - | q | SCAmer | 1994 | CC | 200 | n | bl | n | n | 0 | cig+/-ot | 0.1 | 0.9 | 0 | 0 | nev any | ot |
| WAKAI | 613 | m | 0 | 0 | all | - | q | As:Jap | 1988 | CC | 333 | n | bl | n | y | 0 | cig+/-ot | 1.0 | 4 | 3 | 3 | nev any | ot |
| WYNDE6 | 777 | m | 0 | 0 | all | - | KI | NAmer | 1969 | CC | 4423 | n | bl | n | y | 0 | cig+/-ot | 0.1 | 0.9 | 0 | 0 | nev any | ot |

| REF | NRR | RR | SIG | RRDATA comment |
|---|---|---|---|---|
| LUO | 520 | * | gap | 0 |
| MATOS | 705 | * | gap | 0 |
| WAKAI | 613 | * | gap | 0 |
| WYNDE6 | 777 | * | gap | 0 |

Table 2J7 -

IESLC - Meta-analysis of Ex Smoking, Years quit (vs never), "Low"
Squamous, Cigarettes (or Any Product if Cigarettes not available)

This analysis is restricted to results for:
1) Ex smokers
2) Results by Years quit (vs never)
3) Categorical results by Years quit (vs never)
4) Squamous (or near equivalent)
5) Results complete enough for use in metaanalysis

Within each study, results are then selected (in the following order of preference, within each sex) for:
6) (not applicable)
7) PRODUCT: cigarettes regardless of other products, cigarettes only, all/unspec
8) CIGTYPE: all/unspecified, MC regardless of HR, MC only
9) (not applicable)
10) DENOM: never smoked anything, never smoked cigarettes, never any + low, never cigs + low
11) Followup period (YF, prospective studies): whole study (coded as 0) or longest available
12) LCtype: squamous or nearest available, but not adeno. (q = squamous, s = small,
a = adeno, KI = Kreyberg I, u = undifferentiated)
13) Race: all or nearest available, otherwise by race (wh or w = white, bl or b = black, hi = hispanic
ch = chinese, jap = japanese, haw = hawaiian, w+o = white + oriental, sca = scandinavian, as = asian)
14) Years quit (vs never) "low" in key scheme 1 (key value 12, maximum range 8+)
15) For overlapping studies: principal rather than subsidiary studies
Finally by Age: whole study (coded as 0) if available, otherwise by widest available age group
and then for single sex results (m, f) in preference to results for both sexes combined (c).

Results adjusted (AD) for the most potential confounders are then chosen in Sections -1 to -3
(and those which actually differ from the adjusted results in Table 2J2 - 1 are marked 'x' in Section -1)
and results adjusted for the least confounders in Sections -4 to -6. (Those least adjusted results which
actually differ from the most adjusted are marked 'x' in column X in Section -4)

Section -7 shows excluded studies, together with the stage (as above) at which no qualifying
results were found.

Section -8 lists the potentially overlapping studies which have been included (1=principal, 2=subsidiary).

Section -9 lists any results which would have been included in preference except that they had data not complete
enough for use in meta-analysis, with their significance (yes/no), if known, and any further comment as entered
on the database. It also lists as "gap" any categories for which no data were presented by the original authors.
This is commonly due to recent quitters having been combined with current smokers

In addition to those mentioned above, the following fields, levels and abbreviations are used:
* or nk = not known, n = no, y = yes, ot = other
nev = never
all/unspec = all or unspecified, cig+/-ot = cigarettes irrespective of other products (cigar, pipe etc)
MC = manufactured cigarettes, HR = hand-rolled cigarettes
exL, exH = range of exposure (low and high) in the smoking group, in terms of Years quit (vs never)
REF: 6-character study reference
NRR: number of the RR on the database within the study
ST : study type (CC = case control, pr or prosp = prospective)
NLC: number of lung cancer cases in whole study
R : risky occupational population (n = no, m = mining, o = other risky)
VB : national cigarette type (V = at least 75% Virginia, bl = at least 75% blended, ot = other)
P : any proxy use
H : full histological confirmation
De : derivation of RR/CI (or = original, st = standard method, ot = other method of estimation)

Table 2J7 - 1

IESLC - Meta-analysis of Ex Smoking, Years quit (vs never), "Low"
Squamous, Cigarettes (or Any Product if Cigarettes not available)
Most adjusted

| REF | NRR | 2J2 | SEX | AGEL | AGEH | RACE | YF | LC TYPE | LOC | START | ST | NLC | R | VB | P | H | AD | PRODUCT | exL | exH | DENOM | De |
|---|---|---|---|---|---|---|---|---|---|---|---|---|---|---|---|---|---|---|---|---|---|---|
| JAHN | 594 | | m | 0 | 0 | all | - | q | Eu:Ger | 1988 | CC | 1004 | n | bl | n | n | 0 | cig+/-ot | 11 | 20 | nev any | st |
| JAIN | 543 | | m | 0 | 0 | all | - | q | NAmer | 1981 | CC | 845 | n | V | y | n | 0 | cig+/-ot | 10 | 999 | nev cigs | st |
| JAIN | 507 | | f | 0 | 0 | all | - | q | NAmer | 1981 | CC | 845 | n | V | y | n | 0 | cig+/-ot | 10 | 999 | nev cigs | st |
| JEDRYC | 543 | | m | 0 | 0 | all | - | q | Eu:est | 1980 | CC | 1630 | n | bl | y | n | 0 | cig+/-ot | 10 | 999 | nev any | st |
| LUBIN2 | 767 | | m | 0 | 0 | all | - | q | Eu:mul | 1976 | CC | 7804 | n | bl | n | y | 0 | cig+/-ot | 10 | 14 | nev any | st |
| LUBIN2 | 894 | | f | 0 | 0 | all | - | q | Eu:mul | 1976 | CC | 7804 | n | bl | n | y | 0 | cig+/-ot | 10 | 19 | nev any | st |
| LUO | 522 | | c | 0 | 0 | all | - | q | As:Chi | 1990 | CC | 102 | n | ot | n | y | 20 | cig+/-ot | 10 | 999 | nev cigs | or |
| MATOS | 631 | | m | 0 | 0 | all | - | q | SCAmer | 1994 | CC | 200 | n | bl | n | n | 2 | cig+/-ot | 11 | 999 | nev any | ot |
| PEZZOT | 579 | | m | 0 | 0 | all | - | q | SCAmer | 1987 | CC | 215 | n | bl | n | y | 0 | cig only | 11 | 999 | nev cigs | ot |
| SVENSS | 557 | | f | 0 | 0 | all | - | q | Eu:Sca | 1983 | CC | 210 | n | bl | n | n | 0 | all/unsp | 11 | 999 | nev any | st |
| WAKAI | 547 | | m | 0 | 0 | all | - | q | As:Jap | 1988 | CC | 333 | n | bl | n | y | 1 | cig+/-ot | 10 | 19 | nev any | or |
| WYNDE3 | 552 | | m | 0 | 0 | all | - | KI | NAmer | 1966 | CC | 350 | n | bl | n | y | 0 | cig+/-ot | 10 | 999 | nev any | st |
| WYNDE3 | 573 | | f | 0 | 0 | all | - | KI | NAmer | 1966 | CC | 350 | n | bl | n | y | 0 | cig+/-ot | 10 | 999 | nev any | st |
| WYNDE6 | 788 | | m | 0 | 0 | all | - | KI | NAmer | 1969 | CC | 4423 | n | bl | n | y | 2 | cig+/-ot | 11 | 15 | nev any | ot |

Cigarette type is all/unspec for all RRs

Table 2J7 - 2

IESLC - Meta-analysis of Ex Smoking, Years quit (vs never), "Low"
Squamous, Cigarettes (or Any Product if Cigarettes not available)
Most adjusted

| REF | NRR | SEX | AD | Number Exposed Case | Number Exposed Cont | Non-exposed Case | Non-exposed Cont | RR | 95.00%CI |
|---|---|---|---|---|---|---|---|---|---|
| JAHN | 594 | m | 0 | 18 | 130 | 3 | 138 | 6.37 | ( 1.83- 22.13) |
| JAIN | 543 | m | 0 | 23 | 113 | 2 | 85 | 8.65 | ( 1.98- 37.70) |
| JAIN | 507 | f | 0 | 7 | 61 | 6 | 214 | 4.09 | ( 1.33- 12.63) |
| Subtotal JAIN | | | | | | | | 5.40 | ( 2.21- 13.20) |
| JEDRYC | 543 | m | 0 | 23 | 230 | 6 | 289 | 4.82 | ( 1.93- 12.03) |
| LUBIN2 | 767 | m | 0 | 146 | 693 | 54 | 2616 | 10.21 | ( 7.39- 14.10) |
| LUBIN2 | 894 | f | 0 | 5 | 33 | 72 | 1180 | 2.48 | ( 0.94- 6.55) |
| Subtotal LUBIN2 | | | | | | | | 8.86 | ( 6.52- 12.04) |
| LUO | 522 | c | 20 | 1 | - | 5 | - | 2.00 | ( 0.20- 23.10) |
| MATOS | 631 | m | 2 | 5 | - | 3 | - | 2.00 | ( 0.41- 9.65) |
| PEZZOT | 579 | m | 0 | 8 | 106 | 0 | 116 | 18.60~ | ( 1.06- 326.10) |
| SVENSS | 557 | f | 0 | 1 | 24 | 5 | 120 | 1.00 | ( 0.11- 8.95) |
| WAKAI | 547 | m | 1 | 12 | - | 2 | - | 8.95 | ( 1.91- 42.00) |
| WYNDE3 | 552 | m | 0 | 6 | 65 | 3 | 88 | 2.71 | ( 0.65- 11.23) |
| WYNDE3 | 573 | f | 0 | 1 | 3 | 5 | 76 | 5.07 | ( 0.44- 57.98) |
| Subtotal WYNDE3 | | | | | | | | 3.17 | ( 0.93- 10.85) |
| WYNDE6 | 788 | m | 2 | 22 | - | 8 | - | 13.70 | ( 6.04- 31.10) |
| Partial Totals | | | | 278 | 1458 | 174 | 4922 | | |

*prospective study ~ With 0.5 adjustment for zero

| REF | NRR | SEX | AD | Ys | Ws | Qs | Ps |
|---|---|---|---|---|---|---|---|
| JAHN | 594 | m | 0 | 1.85 | 2.48 | 0.07 | 0.0036 |
| JAIN | 543 | m | 0 | 2.16 | 1.77 | 0.03 | 0.0041 |
| JAIN | 507 | f | 0 | 1.41 | 3.02 | 1.12 | 0.0142 |
| Subtotal JAIN | | | | 1.69 | 4.80 | 1.16 | |
| JEDRYC | 543 | m | 0 | 1.57 | 4.59 | 0.91 | 0.0008 |
| LUBIN2 | 767 | m | 0 | 2.32 | 36.77 | 3.41 | 0.0000 |
| LUBIN2 | 894 | f | 0 | 0.91 | 4.08 | 5.02 | 0.0662 |
| Subtotal LUBIN2 | | | | 2.18 | 40.86 | 8.43 | |
| LUO | 522 | c | 20 | 0.69 | 0.68 | 1.20 | 0.5673 |
| MATOS | 631 | m | 2 | 0.69 | 1.54 | 2.70 | 0.3897 |
| PEZZOT | 579 | m | 0 | 2.92 | 0.47 | 0.38 | 0.0455 |
| SVENSS | 557 | f | 0 | 0.00 | 0.80 | 3.26 | 1.0000 |
| WAKAI | 547 | m | 1 | 2.19 | 1.61 | 0.05 | 0.0054 |
| WYNDE3 | 552 | m | 0 | 1.00 | 1.90 | 1.98 | 0.1699 |
| WYNDE3 | 573 | f | 0 | 1.62 | 0.65 | 0.10 | 0.1919 |
| Subtotal WYNDE3 | | | | 1.16 | 2.55 | 2.08 | |
| WYNDE6 | 788 | m | 2 | 2.62 | 5.72 | 2.05 | 0.0000 |

| | | |
|---|---|---|
| | N | 14 |
| | NS | 11 |
| | Wt | 66.08 |
| Het | Chi | 22.30 |
| Het | df | 13 |
| Het | P | (*) |
| Fixed | RR | 7.53 |
| | RRl | 5.91 |
| | RRu | 9.58 |
| | P | +++ |
| Random | RR | 5.59 |
| | RRl | 3.68 |
| | RRu | 8.48 |
| | P | +++ |
| Asymm | P | * |

Table 2J7 - 3

IESLC - Meta-analysis of Ex Smoking, Years quit (vs never), "Low"
Squamous, Cigarettes (or Any Product if Cigarettes not available)
Most adjusted

| | combined | Sex male | female | Total |
|---|---|---|---|---|
| N | 1 | 9 | 4 | 14 |
| NS | 1 | 9 | 4 | 14 |
| Wt | 0.68 | 56.85 | 8.55 | 66.08 |
| Het Chi | 0.00 | 10.13 | 1.56 | 22.30 |
| Het df | 0 | 8 | 3 | 13 |
| Het P | N.S. | N.S. | N.S. | (*) |
| Fixed RR | 2.00 | 8.84 | 2.87 | 7.53 |
| RRl | 0.19 | 6.82 | 1.47 | 5.91 |
| RRu | 21.49 | 11.46 | 5.61 | 9.58 |
| P | N.S. | +++ | ++ | +++ |
| Random RR | 2.00 | 7.84 | 2.87 | 5.59 |
| RRl | 0.19 | 5.37 | 1.47 | 3.68 |
| RRu | 21.49 | 11.44 | 5.61 | 8.48 |
| P | N.S. | +++ | ++ | +++ |
| Between Chi | | | | 10.60 |
| Between df | | | | 2 |
| Between P | | | | ** |
| Btwn(F) P | | | | * |
| Btwn(R) P | | | | * |

| | q | Lung cancer type q+s | q+u | KI | not a | Total |
|---|---|---|---|---|---|---|
| N | 11 | | | 3 | | 14 |
| NS | 9 | | | 2 | | 11 |
| Wt | 57.82 | | | 8.27 | | 66.08 |
| Het Chi | 18.14 | | | 3.95 | | 22.30 |
| Het df | 10 | | | 2 | | 13 |
| Het P | (*) | | | N.S. | | (*) |
| Fixed RR | 7.37 | | | 8.73 | | 7.53 |
| RRl | 5.69 | | | 4.42 | | 5.91 |
| RRu | 9.53 | | | 17.27 | | 9.58 |
| P | +++ | | | +++ | | +++ |
| Random RR | 5.13 | | | 6.78 | | 5.59 |
| RRl | 3.16 | | | 2.15 | | 3.68 |
| RRu | 8.32 | | | 21.40 | | 8.48 |
| P | +++ | | | ++ | | +++ |
| Between Chi | | | | | | 0.21 |
| Between df | | | | | | 1 |
| Between P | | | | | | N.S. |
| Btwn(F) P | | | | | | N.S. |
| Btwn(R) P | | | | | | N.S. |

| | NAmer | UK | Scand | Location othEur | China | Japan | othAs | other | Total |
|---|---|---|---|---|---|---|---|---|---|
| N | 5 | | 1 | 4 | 1 | 1 | | 2 | 14 |
| NS | 3 | | 1 | 3 | 1 | 1 | | 2 | 11 |
| Wt | 13.06 | | 0.80 | 47.92 | 0.68 | 1.61 | | 2.01 | 66.08 |
| Het Chi | 5.28 | | 0.00 | 9.04 | 0.00 | 0.00 | | 1.79 | 22.30 |
| Het df | 4 | | 0 | 3 | 0 | 0 | | 1 | 13 |
| Het P | N.S. | | N.S. | * | N.S. | N.S. | | N.S. | (*) |
| Fixed RR | 7.32 | | 1.00 | 8.22 | 2.00 | 8.95 | | 3.36 | 7.53 |
| RRl | 4.26 | | 0.11 | 6.19 | 0.19 | 1.91 | | 0.84 | 5.91 |
| RRu | 12.59 | | 8.95 | 10.91 | 21.49 | 41.97 | | 13.41 | 9.58 |
| P | +++ | | N.S. | +++ | N.S. | ++ | | (+) | +++ |
| Random RR | 6.75 | | 1.00 | 5.73 | 2.00 | 8.95 | | 4.37 | 5.59 |
| RRl | 3.49 | | 0.11 | 2.85 | 0.19 | 1.91 | | 0.54 | 3.68 |
| RRu | 13.05 | | 8.95 | 11.53 | 21.49 | 41.97 | | 35.16 | 8.48 |
| P | +++ | | N.S. | +++ | N.S. | ++ | | N.S. | +++ |
| Between Chi | | | | | | | | | 6.19 |
| Between df | | | | | | | | | 5 |
| Between P | | | | | | | | | N.S. |
| Btwn(F) P | | | | | | | | | N.S. |
| Btwn(R) P | | | | | | | | | N.S. |

Table 2J7 - 3

IESLC - Meta-analysis of Ex Smoking, Years quit (vs never), "Low"
Squamous, Cigarettes (or Any Product if Cigarettes not available)
Most adjusted

Detailed Country in "other Europe"

| | multi | Germany | othWest | East | Balkans | Total |
|---|---|---|---|---|---|---|
| N | 2 | 1 | | 1 | | 4 |
| NS | 1 | 1 | | 1 | | 3 |
| Wt | 40.86 | 2.48 | | 4.59 | | 47.92 |
| Het Chi | 7.34 | 0.00 | | 0.00 | | 9.04 |
| Het df | 1 | 0 | | 0 | | 3 |
| Het P | ** | N.S. | | N.S. | | * |
| Fixed RR | 8.86 | 6.37 | | 4.82 | | 8.22 |
| RRl | 6.52 | 1.83 | | 1.93 | | 6.19 |
| RRu | 12.04 | 22.13 | | 12.03 | | 10.91 |
| P | +++ | ++ | | +++ | | +++ |
| Random RR | 5.44 | 6.37 | | 4.82 | | 5.73 |
| RRl | 1.37 | 1.83 | | 1.93 | | 2.85 |
| RRu | 21.55 | 22.13 | | 12.03 | | 11.53 |
| P | + | ++ | | +++ | | +++ |
| Between Chi | | | | | | 1.70 |
| Between df | | | | | | 2 |
| Between P | | | | | | N.S. |
| Btwn(F) P | | | | | | N.S. |
| Btwn(R) P | | | | | | N.S. |

Detailed Country in "other Asia"

| | India | HongKong | other | Total |
|---|---|---|---|---|
| N | | | | |
| NS | | | | |
| Wt | | | | |
| Het Chi | | | | |
| Het df | | | | |
| Het P | | | | N.S. |
| Fixed RR | | | | |
| RRl | | | | |
| RRu | | | | |
| P | | | | +++ |
| Random RR | | | | |
| RRl | | | | |
| RRu | | | | |
| P | | | | +++ |
| Between Chi | | | | |
| Between df | | | | |
| Between P | | | | N.S. |
| Btwn(F) P | | | | N.S. |
| Btwn(R) P | | | | N.S. |

Detailed other continent

| | SCAmer | Total |
|---|---|---|
| N | 2 | 2 |
| NS | 2 | 2 |
| Wt | 2.01 | 2.01 |
| Het Chi | 1.79 | 1.79 |
| Het df | 1 | 1 |
| Het P | N.S. | N.S. |
| Fixed RR | 3.36 | 3.36 |
| RRl | 0.84 | 0.84 |
| RRu | 13.41 | 13.41 |
| P | (+) | (+) |
| Random RR | 4.37 | 4.37 |
| RRl | 0.54 | 0.54 |
| RRu | 35.16 | 35.16 |
| P | N.S. | N.S. |
| Between Chi | | |
| Between df | | |
| Between P | | N.S. |
| Btwn(F) P | | N.S. |
| Btwn(R) P | | N.S. |

Table 2J7 - 3

IESLC - Meta-analysis of Ex Smoking, Years quit (vs never), "Low"
Squamous, Cigarettes (or Any Product if Cigarettes not available)
Most adjusted

| | <1960 | Start year of study 1960-69 | 1970-79 | 1980-89 | 1990+ | Total |
|---|---|---|---|---|---|---|
| N | | 3 | 2 | 7 | 2 | 14 |
| NS | | 2 | 1 | 6 | 2 | 11 |
| Wt | | 8.27 | 40.86 | 14.74 | 2.22 | 66.08 |
| Het Chi | | 3.95 | 7.34 | 4.15 | 0.00 | 22.30 |
| Het df | | 2 | 1 | 6 | 1 | 13 |
| Het P | | N.S. | ** | N.S. | N.S. | (*) |
| Fixed RR | | 8.73 | 8.86 | 5.37 | 2.00 | 7.53 |
| RRl | | 4.42 | 6.52 | 3.22 | 0.54 | 5.91 |
| RRu | | 17.27 | 12.04 | 8.95 | 7.45 | 9.58 |
| P | | +++ | +++ | +++ | N.S. | +++ |
| Random RR | | 6.78 | 5.44 | 5.37 | 2.00 | 5.59 |
| RRl | | 2.15 | 1.37 | 3.22 | 0.54 | 3.68 |
| RRu | | 21.40 | 21.55 | 8.95 | 7.45 | 8.48 |
| P | | ++ | + | +++ | N.S. | +++ |
| Between Chi | | | | | | 6.85 |
| Between df | | | | | | 3 |
| Between P | | | | | | (*) |
| Btwn(F) P | | | | | | N.S. |
| Btwn(R) P | | | | | | N.S. |

| | Study type (1) CC | other | Total |
|---|---|---|---|
| N | 14 | | 14 |
| NS | 11 | | 11 |
| Wt | 66.08 | | 66.08 |
| Het Chi | 22.30 | | 22.30 |
| Het df | 13 | | 13 |
| Het P | (*) | | (*) |
| Fixed RR | 7.53 | | 7.53 |
| RRl | 5.91 | | 5.91 |
| RRu | 9.58 | | 9.58 |
| P | +++ | | +++ |
| Random RR | 5.59 | | 5.59 |
| RRl | 3.68 | | 3.68 |
| RRu | 8.48 | | 8.48 |
| P | +++ | | +++ |
| Between Chi | | | |
| Between df | | | |
| Between P | | | N.S. |
| Btwn(F) P | | | N.S. |
| Btwn(R) P | | | N.S. |

| | Study type (2) CC | prosp | other | Total |
|---|---|---|---|---|
| N | 14 | | | 14 |
| NS | 11 | | | 11 |
| Wt | 66.08 | | | 66.08 |
| Het Chi | 22.30 | | | 22.30 |
| Het df | 13 | | | 13 |
| Het P | (*) | | | (*) |
| Fixed RR | 7.53 | | | 7.53 |
| RRl | 5.91 | | | 5.91 |
| RRu | 9.58 | | | 9.58 |
| P | +++ | | | +++ |
| Random RR | 5.59 | | | 5.59 |
| RRl | 3.68 | | | 3.68 |
| RRu | 8.48 | | | 8.48 |
| P | +++ | | | +++ |
| Between Chi | | | | |
| Between df | | | | |
| Between P | | | | N.S. |
| Btwn(F) P | | | | N.S. |
| Btwn(R) P | | | | N.S. |

Table 2J7 - 3

IESLC - Meta-analysis of Ex Smoking, Years quit (vs never), "Low"
Squamous, Cigarettes (or Any Product if Cigarettes not available)
Most adjusted

| | Study size (number of LC cases) | | | | |
|---|---|---|---|---|---|
| | 100-249 | 250-499 | 500-999 | 1000+ | Total |
| N | 4 | 3 | 2 | 5 | 14 |
| NS | 4 | 2 | 1 | 4 | 11 |
| Wt | 3.49 | 4.15 | 4.80 | 53.64 | 66.08 |
| Het Chi | 2.64 | 1.25 | 0.63 | 10.38 | 22.30 |
| Het df | 3 | 2 | 1 | 4 | 13 |
| Het P | N.S. | N.S. | N.S. | * | (*) |
| Fixed RR | 2.30 | 4.74 | 5.40 | 8.68 | 7.53 |
| RRl | 0.81 | 1.81 | 2.21 | 6.64 | 5.91 |
| RRu | 6.57 | 12.41 | 13.20 | 11.34 | 9.58 |
| P | N.S. | ++ | +++ | +++ | +++ |
| Random RR | 2.30 | 4.74 | 5.40 | 6.97 | 5.59 |
| RRl | 0.81 | 1.81 | 2.21 | 3.96 | 3.68 |
| RRu | 6.57 | 12.41 | 13.20 | 12.25 | 8.48 |
| P | N.S. | ++ | +++ | +++ | +++ |
| Between Chi | | | | | 7.41 |
| Between df | | | | | 3 |
| Between P | | | | | (*) |
| Btwn(F) P | | | | | N.S. |
| Btwn(R) P | | | | | N.S. |

| | Risky occupational population | | | |
|---|---|---|---|---|
| | no | mining | othRisky | Total |
| N | 14 | | | 14 |
| NS | 11 | | | 11 |
| Wt | 66.08 | | | 66.08 |
| Het Chi | 22.30 | | | 22.30 |
| Het df | 13 | | | 13 |
| Het P | (*) | | | (*) |
| Fixed RR | 7.53 | | | 7.53 |
| RRl | 5.91 | | | 5.91 |
| RRu | 9.58 | | | 9.58 |
| P | +++ | | | +++ |
| Random RR | 5.59 | | | 5.59 |
| RRl | 3.68 | | | 3.68 |
| RRu | 8.48 | | | 8.48 |
| P | +++ | | | +++ |
| Between Chi | | | | |
| Between df | | | | |
| Between P | | | | N.S. |
| Btwn(F) P | | | | N.S. |
| Btwn(R) P | | | | N.S. |

| | National cigarette tobacco type | | | |
|---|---|---|---|---|
| | Virginia | blended | other | Total |
| N | 2 | 11 | 1 | 14 |
| NS | 1 | 9 | 1 | 11 |
| Wt | 4.80 | 60.60 | 0.68 | 66.08 |
| Het Chi | 0.63 | 19.84 | 0.00 | 22.30 |
| Het df | 1 | 10 | 0 | 13 |
| Het P | N.S. | * | N.S. | (*) |
| Fixed RR | 5.40 | 7.84 | 2.00 | 7.53 |
| RRl | 2.21 | 6.10 | 0.19 | 5.91 |
| RRu | 13.20 | 10.09 | 21.49 | 9.58 |
| P | +++ | +++ | N.S. | +++ |
| Random RR | 5.40 | 5.65 | 2.00 | 5.59 |
| RRl | 2.21 | 3.48 | 0.19 | 3.68 |
| RRu | 13.20 | 9.19 | 21.49 | 8.48 |
| P | +++ | +++ | N.S. | +++ |
| Between Chi | | | | 1.83 |
| Between df | | | | 2 |
| Between P | | | | N.S. |
| Btwn(F) P | | | | N.S. |
| Btwn(R) P | | | | N.S. |

Table 2J7 - 3

IESLC - Meta-analysis of Ex Smoking, Years quit (vs never), "Low"
Squamous, Cigarettes (or Any Product if Cigarettes not available)
Most adjusted

| | Any proxy use No/nk | Yes | Total |
|---|---|---|---|
| N | 11 | 3 | 14 |
| NS | 9 | 2 | 11 |
| Wt | 56.70 | 9.39 | 66.08 |
| Het Chi | 20.00 | 0.66 | 22.30 |
| Het df | 10 | 2 | 13 |
| Het P | * | N.S. | (*) |
| Fixed RR | 8.02 | 5.10 | 7.53 |
| RRl | 6.19 | 2.69 | 5.91 |
| RRu | 10.41 | 9.68 | 9.58 |
| P | +++ | +++ | +++ |
| Random RR | 5.45 | 5.10 | 5.59 |
| RRl | 3.20 | 2.69 | 3.68 |
| RRu | 9.28 | 9.68 | 8.48 |
| P | +++ | +++ | +++ |
| Between Chi | | | 1.65 |
| Between df | | | 1 |
| Between P | | | N.S. |
| Btwn(F) P | | | N.S. |
| Btwn(R) P | | | N.S. |

| | Full histological confirmation No | Yes | Total |
|---|---|---|---|
| N | 6 | 8 | 14 |
| NS | 5 | 6 | 11 |
| Wt | 14.20 | 51.88 | 66.08 |
| Het Chi | 3.92 | 13.05 | 22.30 |
| Het df | 5 | 7 | 13 |
| Het P | N.S. | (*) | (*) |
| Fixed RR | 4.37 | 8.73 | 7.53 |
| RRl | 2.60 | 6.65 | 5.91 |
| RRu | 7.36 | 11.46 | 9.58 |
| P | +++ | +++ | +++ |
| Random RR | 4.37 | 6.67 | 5.59 |
| RRl | 2.60 | 3.80 | 3.68 |
| RRu | 7.36 | 11.72 | 8.48 |
| P | +++ | +++ | +++ |
| Between Chi | | | 5.33 |
| Between df | | | 1 |
| Between P | | | * |
| Btwn(F) P | | | (*) |
| Btwn(R) P | | | N.S. |

| | Number of adjustment variables (1) 0 | 1 | 2+/+nk | Total |
|---|---|---|---|---|
| N | 10 | 1 | 3 | 14 |
| NS | 7 | 1 | 3 | 11 |
| Wt | 56.53 | 1.61 | 7.94 | 66.08 |
| Het Chi | 16.29 | 0.00 | 5.92 | 22.30 |
| Het df | 9 | 0 | 2 | 13 |
| Het P | (*) | N.S. | (*) | (*) |
| Fixed RR | 7.42 | 8.95 | 8.00 | 7.53 |
| RRl | 5.72 | 1.91 | 3.99 | 5.91 |
| RRu | 9.64 | 41.97 | 16.03 | 9.58 |
| P | +++ | ++ | +++ | +++ |
| Random RR | 5.19 | 8.95 | 4.73 | 5.59 |
| RRl | 3.17 | 1.91 | 1.06 | 3.68 |
| RRu | 8.50 | 41.97 | 21.16 | 8.48 |
| P | +++ | ++ | + | +++ |
| Between Chi | | | | 0.09 |
| Between df | | | | 2 |
| Between P | | | | N.S. |
| Btwn(F) P | | | | N.S. |
| Btwn(R) P | | | | N.S. |

Table 2J7 - 3

IESLC - Meta-analysis of Ex Smoking, Years quit (vs never), "Low"
Squamous, Cigarettes (or Any Product if Cigarettes not available)
Most adjusted

| | Number of adjustment variables (2) 0 | 1 | 2 | 3-5 | 6+/+nk | Total |
|---|---|---|---|---|---|---|
| N | 10 | 1 | 2 | | 1 | 14 |
| NS | 7 | 1 | 2 | | 1 | 11 |
| Wt | 56.53 | 1.61 | 7.26 | | 0.68 | 66.08 |
| Het Chi | 16.29 | 0.00 | 4.49 | | 0.00 | 22.30 |
| Het df | 9 | 0 | 1 | | 0 | 13 |
| Het P | (*) | N.S. | * | | N.S. | (*) |
| Fixed RR | 7.42 | 8.95 | 9.11 | | 2.00 | 7.53 |
| RRl | 5.72 | 1.91 | 4.40 | | 0.19 | 5.91 |
| RRu | 9.64 | 41.97 | 18.85 | | 21.49 | 9.58 |
| P | +++ | ++ | +++ | | N.S. | +++ |
| Random RR | 5.19 | 8.95 | 5.92 | | 2.00 | 5.59 |
| RRl | 3.17 | 1.91 | 0.91 | | 0.19 | 3.68 |
| RRu | 8.50 | 41.97 | 38.43 | | 21.49 | 8.48 |
| P | +++ | ++ | (+) | | N.S. | +++ |
| Between Chi | | | | | | 1.52 |
| Between df | | | | | | 3 |
| Between P | | | | | | N.S. |
| Btwn(F) P | | | | | | N.S. |
| Btwn(R) P | | | | | | N.S. |

| | Product all/unsp | cig+/-ot | cig only | Total |
|---|---|---|---|---|
| N | 1 | 12 | 1 | 14 |
| NS | 1 | 9 | 1 | 11 |
| Wt | 0.80 | 64.81 | 0.47 | 66.08 |
| Het Chi | 0.00 | 18.64 | 0.00 | 22.30 |
| Het df | 0 | 11 | 0 | 13 |
| Het P | N.S. | (*) | N.S. | (*) |
| Fixed RR | 1.00 | 7.67 | 18.60 | 7.53 |
| RRl | 0.11 | 6.01 | 1.06 | 5.91 |
| RRu | 8.95 | 9.78 | 326.10 | 9.58 |
| P | N.S. | +++ | + | +++ |
| Random RR | 1.00 | 5.84 | 18.60 | 5.59 |
| RRl | 0.11 | 3.87 | 1.06 | 3.68 |
| RRu | 8.95 | 8.82 | 326.10 | 8.48 |
| P | N.S. | +++ | + | +++ |
| Between Chi | | | | 3.66 |
| Between df | | | | 2 |
| Between P | | | | N.S. |
| Btwn(F) P | | | | N.S. |
| Btwn(R) P | | | | N.S. |

| | Denominator nev any | nev cigs | Total |
|---|---|---|---|
| N | 10 | 4 | 14 |
| NS | 8 | 3 | 11 |
| Wt | 60.13 | 5.95 | 66.08 |
| Het Chi | 19.49 | 2.01 | 22.30 |
| Het df | 9 | 3 | 13 |
| Het P | * | N.S. | (*) |
| Fixed RR | 7.79 | 5.31 | 7.53 |
| RRl | 6.05 | 2.38 | 5.91 |
| RRu | 10.03 | 11.86 | 9.58 |
| P | +++ | +++ | +++ |
| Random RR | 5.43 | 5.31 | 5.59 |
| RRl | 3.29 | 2.38 | 3.68 |
| RRu | 8.99 | 11.86 | 8.48 |
| P | +++ | +++ | +++ |
| Between Chi | | | 0.80 |
| Between df | | | 1 |
| Between P | | | N.S. |
| Btwn(F) P | | | N.S. |
| Btwn(R) P | | | N.S. |

Table 2J7 - 3

IESLC - Meta-analysis of Ex Smoking, Years quit (vs never), "Low"
Squamous, Cigarettes (or Any Product if Cigarettes not available)
Most adjusted

| | | Derivation of RR/CI | | | |
|---|---|---|---|---|---|
| | | Orig | StdCalc | Other | Total |
| | N | 2 | 9 | 3 | 14 |
| | NS | 2 | 6 | 3 | 11 |
| | Wt | 2.29 | 56.06 | 7.73 | 66.08 |
| Het | Chi | 1.07 | 15.89 | 4.72 | 22.30 |
| Het | df | 1 | 8 | 2 | 13 |
| Het | P | N.S. | * | (*) | (*) |
| Fixed | RR | 5.73 | 7.37 | 9.51 | 7.53 |
| | RRl | 1.57 | 5.67 | 4.70 | 5.91 |
| | RRu | 20.93 | 9.57 | 19.25 | 9.58 |
| | P | ++ | +++ | +++ | +++ |
| Random | RR | 5.61 | 4.96 | 7.54 | 5.59 |
| | RRl | 1.44 | 2.97 | 1.86 | 3.68 |
| | RRu | 21.87 | 8.29 | 30.53 | 8.48 |
| | P | + | +++ | ++ | +++ |
| Between | Chi | | | | 0.62 |
| Between | df | | | | 2 |
| Between | P | | | | N.S. |
| Btwn(F) | P | | | | N.S. |
| Btwn(R) | P | | | | N.S. |

Table 2J7 - 4

IESLC - Meta-analysis of Ex Smoking, Years quit (vs never), "Low"
Squamous, Cigarettes (or Any Product if Cigarettes not available)
Least adjusted

| REF | NRR | X | SEX | AGEL | AGEH | RACE | YF | LC TYPE | LOC | START | ST | NLC | R | VB | P | H | AD | PRODUCT | exL | exH | DENOM | De |
|---|---|---|---|---|---|---|---|---|---|---|---|---|---|---|---|---|---|---|---|---|---|---|
| JAHN | 594 | | m | 0 | 0 | all | - | q | Eu:Ger | 1988 | CC | 1004 | n | bl | n | n | 0 | cig+/-ot | 11 | 20 | nev any | st |
| JAIN | 543 | | m | 0 | 0 | all | - | q | NAmer | 1981 | CC | 845 | n | V | y | n | 0 | cig+/-ot | 10 | 999 | nev cigs | st |
| JAIN | 507 | | f | 0 | 0 | all | - | q | NAmer | 1981 | CC | 845 | n | V | y | n | 0 | cig+/-ot | 10 | 999 | nev cigs | st |
| JEDRYC | 543 | | m | 0 | 0 | all | - | q | Eu:est | 1980 | CC | 1630 | n | bl | y | n | 0 | cig+/-ot | 10 | 999 | nev any | st |
| LUBIN2 | 767 | | m | 0 | 0 | all | - | q | Eu:mul | 1976 | CC | 7804 | n | bl | n | y | 0 | cig+/-ot | 10 | 14 | nev any | st |
| LUBIN2 | 894 | | f | 0 | 0 | all | - | q | Eu:mul | 1976 | CC | 7804 | n | bl | n | y | 0 | cig+/-ot | 10 | 19 | nev any | st |
| LUO | 519 | x | c | 0 | 0 | all | - | q | As:Chi | 1990 | CC | 102 | n | ot | n | y | 0 | cig+/-ot | 10 | 999 | nev cigs | st |
| MATOS | 621 | x | m | 0 | 0 | all | - | q | SCAmer | 1994 | CC | 200 | n | bl | n | n | 0 | cig+/-ot | 11 | 999 | nev any | st |
| PEZZOT | 579 | | m | 0 | 0 | all | - | q | SCAmer | 1987 | CC | 215 | n | bl | n | y | 0 | cig only | 11 | 999 | nev cigs | ot |
| SVENSS | 557 | | f | 0 | 0 | all | - | q | Eu:Sca | 1983 | CC | 210 | n | bl | n | n | 0 | all/unsp | 11 | 999 | nev any | st |
| WAKAI | 539 | x | m | 0 | 0 | all | - | q | As:Jap | 1988 | CC | 333 | n | bl | n | y | 0 | cig+/-ot | 10 | 19 | nev any | st |
| WYNDE3 | 552 | | m | 0 | 0 | all | - | KI | NAmer | 1966 | CC | 350 | n | bl | n | y | 0 | cig+/-ot | 10 | 999 | nev any | st |
| WYNDE3 | 573 | | f | 0 | 0 | all | - | KI | NAmer | 1966 | CC | 350 | n | bl | n | y | 0 | cig+/-ot | 10 | 999 | nev any | st |
| WYNDE6 | 773 | x | m | 0 | 0 | all | - | KI | NAmer | 1969 | CC | 4423 | n | bl | n | y | 0 | cig+/-ot | 11 | 15 | nev any | st |

Cigarette type is all/unspec for all RRs

Table 2J7 - 5

IESLC - Meta-analysis of Ex Smoking, Years quit (vs never), "Low"
Squamous, Cigarettes (or Any Product if Cigarettes not available)
Least adjusted

| REF | NRR | SEX | AD | Number Exposed Case | Cont | Non-exposed Case | Cont | RR | 95.00%CI |
|---|---|---|---|---|---|---|---|---|---|
| JAHN | 594 | m | 0 | 18 | 130 | 3 | 138 | 6.37 | ( 1.83- 22.13) |
| JAIN | 543 | m | 0 | 23 | 113 | 2 | 85 | 8.65 | ( 1.98- 37.70) |
| JAIN | 507 | f | 0 | 7 | 61 | 6 | 214 | 4.09 | ( 1.33- 12.63) |
| Subtotal JAIN | | | | | | | | 5.40 | ( 2.21- 13.20) |
| JEDRYC | 543 | m | 0 | 23 | 230 | 6 | 289 | 4.82 | ( 1.93- 12.03) |
| LUBIN2 | 767 | m | 0 | 146 | 693 | 54 | 2616 | 10.21 | ( 7.39- 14.10) |
| LUBIN2 | 894 | f | 0 | 5 | 33 | 72 | 1180 | 2.48 | ( 0.94- 6.55) |
| Subtotal LUBIN2 | | | | | | | | 8.86 | ( 6.52- 12.04) |
| LUO | 519 | c | 0 | 1 | 10 | 5 | 51 | 1.02 | ( 0.11- 9.69) |
| MATOS | 621 | m | 0 | 5 | 101 | 3 | 110 | 1.82 | ( 0.42- 7.79) |
| PEZZOT | 579 | m | 0 | 8 | 106 | 0 | 116 | 18.60~ | ( 1.06- 326.10) |
| SVENSS | 557 | f | 0 | 1 | 24 | 5 | 120 | 1.00 | ( 0.11- 8.95) |
| WAKAI | 539 | m | 0 | 12 | 44 | 2 | 65 | 8.86 | ( 1.89- 41.56) |
| WYNDE3 | 552 | m | 0 | 6 | 65 | 3 | 88 | 2.71 | ( 0.65- 11.23) |
| WYNDE3 | 573 | f | 0 | 1 | 3 | 5 | 76 | 5.07 | ( 0.44- 57.98) |
| Subtotal WYNDE3 | | | | | | | | 3.17 | ( 0.93- 10.85) |
| WYNDE6 | 773 | m | 0 | 22 | 259 | 8 | 1667 | 17.70 | ( 7.80- 40.18) |
| Totals | | | | 278 | 1872 | 174 | 6815 | | |

*prospective study ~ With 0.5 adjustment for zero

| REF | NRR | SEX | AD | Ys | Ws | Qs | Ps |
|---|---|---|---|---|---|---|---|
| JAHN | 594 | m | 0 | 1.85 | 2.48 | 0.07 | 0.0036 |
| JAIN | 543 | m | 0 | 2.16 | 1.77 | 0.03 | 0.0041 |
| JAIN | 507 | f | 0 | 1.41 | 3.02 | 1.14 | 0.0142 |
| Subtotal JAIN | | | | 1.69 | 4.80 | 1.17 | |
| JEDRYC | 543 | m | 0 | 1.57 | 4.59 | 0.93 | 0.0008 |
| LUBIN2 | 767 | m | 0 | 2.32 | 36.77 | 3.31 | 0.0000 |
| LUBIN2 | 894 | f | 0 | 0.91 | 4.08 | 5.06 | 0.0662 |
| Subtotal LUBIN2 | | | | 2.18 | 40.86 | 8.37 | |
| LUO | 519 | c | 0 | 0.02 | 0.76 | 3.04 | 0.9862 |
| MATOS | 621 | m | 0 | 0.60 | 1.81 | 3.68 | 0.4224 |
| PEZZOT | 579 | m | 0 | 2.92 | 0.47 | 0.38 | 0.0455 |
| SVENSS | 557 | f | 0 | 0.00 | 0.80 | 3.27 | 1.0000 |
| WAKAI | 539 | m | 0 | 2.18 | 1.61 | 0.04 | 0.0056 |
| WYNDE3 | 552 | m | 0 | 1.00 | 1.90 | 2.00 | 0.1699 |
| WYNDE3 | 573 | f | 0 | 1.62 | 0.65 | 0.10 | 0.1919 |
| Subtotal WYNDE3 | | | | 1.16 | 2.55 | 2.10 | |
| WYNDE6 | 773 | m | 0 | 2.87 | 5.72 | 4.14 | 0.0000 |

| | | |
|---|---|---|
| | N | 14 |
| | NS | 11 |
| | Wt | 66.42 |
| Het | Chi | 27.21 |
| Het | df | 13 |
| Het | P | * |
| Fixed | RR | 7.56 |
| | RRl | 5.94 |
| | RRu | 9.61 |
| | P | +++ |
| Random | RR | 5.35 |
| | RRl | 3.38 |
| | RRu | 8.47 |
| | P | +++ |
| Asymm | P | * |

Table 2J7 - 6

IESLC - Meta-analysis of Ex Smoking, Years quit (vs never), "Low"
Squamous, Cigarettes (or Any Product if Cigarettes not available)
Least adjusted

| | Sex combined | male | female | Total |
|---|---|---|---|---|
| N | 1 | 9 | 4 | 14 |
| NS | 1 | 9 | 4 | 14 |
| Wt | 0.76 | 57.11 | 8.55 | 66.42 |
| Het Chi | 0.00 | 12.92 | 1.56 | 27.21 |
| Het df | 0 | 8 | 3 | 13 |
| Het P | N.S. | N.S. | N.S. | * |
| Fixed RR | 1.02 | 8.97 | 2.87 | 7.56 |
| RRl | 0.11 | 6.92 | 1.47 | 5.94 |
| RRu | 9.69 | 11.63 | 5.61 | 9.61 |
| P | N.S. | +++ | ++ | +++ |
| Random RR | 1.02 | 7.56 | 2.87 | 5.35 |
| RRl | 0.11 | 4.83 | 1.47 | 3.38 |
| RRu | 9.69 | 11.83 | 5.61 | 8.47 |
| P | N.S. | +++ | ++ | +++ |
| Between Chi | | | | 12.73 |
| Between df | | | | 2 |
| Between P | | | | ** |
| Btwn(F) P | | | | * |
| Btwn(R) P | | | | * |

Table 2J7 - 7

IESLC - Meta-analysis of Ex Smoking, Years quit (vs never), "Low"
Squamous, Cigarettes (or Any Product if Cigarettes not available)
Excluded studies (and stage at which they were excluded)

| | |
|---|---|
| 1 | AGUDO AKIBA AMANDU AMES AXELSS BEST BOUCHA BOUCOT BRESLO CHEN CHEN2 CHIAZZ DEAN2 DOSEME ENGELA FAN GENG GER GUO HAENSZ HEGMAN HOLE HU HU2 JUSSAW KATSOU KAUFMA KOO KOULUM KREUZE LETOUR LEVIN LIAW LIU3 LIU4 LIU5 MCCONN MIGRAN MRFITR NOTAN2 OSANN2 PERNU QIAO2 RACHTA RESTRE SADOWS SEGI2 STASZE TIZZAN VUTUC WATSON WU WUWILL WYNDE2 WYNDE8 XU YUAN ZHANG ZHENG ZHOU |
| 2 | BUFFLE HUMBLE PISANI PRESCO WYNDE7 |
| 3 | MCDUFF SPITZ |
| 4 | ARMADA AUVINE BECHER BENSHL BLOT1 BOFFET BROSS CARPEN CEDERL CHOI CHYOU CORREA CPSI CPSII DAMBER DARBY DEAN3 DESTEF DOLL DOLL2 DORGAN DORN GAO GAO2 GARCIA GARSHI GILLIS GRAHAM GURSEL HAMMO2 HIRAYA JOLY KAISE2 KHUDER LAUSSM LUBIN PEZZO2 QIAO SPEIZE SUZUK2 TVERDA WANG2 WIGLE WU2 |
| 5 | ALDERS HAMMON |
| 10 | SOBUE |
| 14 | BARBON BROWN3 |
| 15 | BENHAM |

Table 2J7 - 8
Potentially overlapping studies

| REF | REFGP | PRINC | . | OVERLAP/LINK |
|---|---|---|---|---|
| LUBIN2 | LUBIN2 | 1 | | Lubin-combined |
| WYNDE6 | WYNDE6 | 1 | | WYNDE5/6/7/8 |
| JAHN | BOFFET | 2 | | Subset of BOFFET |

Table 2J7 - 9
Most adjusted - insufficient data for meta-analysis

| REF | NRR | SEX | AGEL | AGEH | RACE | YF | LC TYPE | LOC | START | ST | NLC | R | VB | P | H | AD | PRODUCT | exL | exH | DENOM | De |
|---|---|---|---|---|---|---|---|---|---|---|---|---|---|---|---|---|---|---|---|---|---|
| ALDERS | 537 | m | 0 | 0 | all | - | q+s | Eu:UK | 1977 | CC | 1448 | n | V | n | n | 1 | cig only | 10 | 999 | nev any | st |
| ALDERS | 548 | f | 0 | 0 | all | - | q+s | Eu:UK | 1977 | CC | 1448 | n | V | n | n | 1 | cig only | 10 | 999 | nev any | st |
| HAMMON | 501 | m | 0 | 0 | wh | 0 | not a | NAmer | 1952 | pr | 448 | n | bl | n | n | 1 | cig only | 10 | 999 | nev any | st |

| REF | NRR | RR | SIG | RRDATA comment |
|---|---|---|---|---|
| ALDERS | 537 | 2.33 | | 0 |
| ALDERS | 548 | 0.67 | | 0 |
| HAMMON | 501 | * | | RR for <1 pack per day is 2.44, while that for 1+ packs is 17.79 |

Table 2J8 -

IESLC - Meta-analysis of Ex Smoking, Years quit (vs never), "Mid"
Squamous, Cigarettes (or Any Product if Cigarettes not available)

This analysis is restricted to results for:
1) Ex smokers
2) Results by Years quit (vs never)
3) Categorical results by Years quit (vs never)
4) Squamous (or near equivalent)
5) Results complete enough for use in metaanalysis

Within each study, results are then selected (in the following order of preference, within each sex) for:
6) (not applicable)
7) PRODUCT: cigarettes regardless of other products, cigarettes only, all/unspec
8) CIGTYPE: all/unspecified, MC regardless of HR, MC only
9) (not applicable)
10) DENOM: never smoked anything, never smoked cigarettes, never any + low, never cigs + low
11) Followup period (YF, prospective studies): whole study (coded as 0) or longest available
12) LCtype: squamous or nearest available, but not adeno. (q = squamous, s = small,
a = adeno, KI = Kreyberg I, u = undifferentiated)
13) Race: all or nearest available, otherwise by race (wh or w = white, bl or b = black, hi = hispanic
ch = chinese, jap = japanese, haw = hawaiian, w+o = white + oriental, sca = scandinavian, as = asian)
14) Years quit (vs never) "mid" in key scheme 1 (key value 7, maximum range 4-11)
15) For overlapping studies: principal rather than subsidiary studies
Finally by Age: whole study (coded as 0) if available, otherwise by widest available age group
and then for single sex results (m, f) in preference to results for both sexes combined (c).

Results adjusted (AD) for the most potential confounders are then chosen in Sections -1 to -3
(and those which actually differ from the adjusted results in Table 2J3 - 1 are marked 'x' in Section -1)
and results adjusted for the least confounders in Sections -4 to -6. (Those least adjusted results which
actually differ from the most adjusted are marked 'x' in column X in Section -4)

Section -7 shows excluded studies, together with the stage (as above) at which no qualifying
results were found.

Section -8 lists the potentially overlapping studies which have been included (1=principal, 2=subsidiary).

Section -9 lists any results which would have been included in preference except that they had data not complete
enough for use in meta-analysis, with their significance (yes/no), if known, and any further comment as entered
on the database. It also lists as "gap" any categories for which no data were presented by the original authors.
This is commonly due to recent quitters having been combined with current smokers

In addition to those mentioned above, the following fields, levels and abbreviations are used:
* or nk = not known, n = no, y = yes, ot = other
nev = never
all/unspec = all or unspecified, cig+/-ot = cigarettes irrespective of other products (cigar, pipe etc)
MC = manufactured cigarettes, HR = hand-rolled cigarettes
exL, exH = range of exposure (low and high) in the smoking group, in terms of Years quit (vs never)
REF: 6-character study reference
NRR: number of the RR on the database within the study
ST : study type (CC = case control, pr or prosp = prospective)
NLC: number of lung cancer cases in whole study
R : risky occupational population (n = no, m = mining, o = other risky)
VB : national cigarette type (V = at least 75% Virginia, bl = at least 75% blended, ot = other)
P : any proxy use
H : full histological confirmation
De : derivation of RR/CI (or = original, st = standard method, ot = other method of estimation)

Table 2J8 - 1

IESLC - Meta-analysis of Ex Smoking, Years quit (vs never), "Mid"
Squamous, Cigarettes (or Any Product if Cigarettes not available)
Most adjusted

| REF | NRR | 2J3 | SEX | AGEL | AGEH | RACE | YF | LC TYPE | LOC | START | ST | NLC | R | VB | P | H | AD | PRODUCT | exL | exH | DENOM | De |
|---|---|---|---|---|---|---|---|---|---|---|---|---|---|---|---|---|---|---|---|---|---|---|
| JAHN | 595 | | m | 0 | 0 | all | - | q | Eu:Ger | 1988 | CC | 1004 | n | bl | n | n | 0 | cig+/-ot | 6 | 10 | nev any | st |
| JEDRYC | 544 | | m | 0 | 0 | all | - | q | Eu:est | 1980 | CC | 1630 | n | bl | y | n | 0 | cig+/-ot | 5 | 9 | nev any | st |
| LUBIN2 | 768 | | m | 0 | 0 | all | - | q | Eu:mul | 1976 | CC | 7804 | n | bl | n | y | 0 | cig+/-ot | 5 | 9 | nev any | st |
| MATOS | 632 | | m | 0 | 0 | all | - | q | SCAmer | 1994 | CC | 200 | n | bl | n | n | 2 | cig+/-ot | 6 | 10 | nev any | ot |
| WAKAI | 548 | | m | 0 | 0 | all | - | q | As:Jap | 1988 | CC | 333 | n | bl | n | y | 1 | cig+/-ot | 5 | 9 | nev any | or |
| WYNDE6 | 789 | | m | 0 | 0 | all | - | KI | NAmer | 1969 | CC | 4423 | n | bl | n | y | 2 | cig+/-ot | 7 | 10 | nev any | ot |

Cigarette type is all/unspec for all RRs

Table 2J8 - 2

IESLC - Meta-analysis of Ex Smoking, Years quit (vs never), "Mid"
Squamous, Cigarettes (or Any Product if Cigarettes not available)
Most adjusted

| REF | NRR | SEX | AD | Number Exposed Case | Exposed Cont | Non-exposed Case | Non-exposed Cont | RR | 95.00%CI |
|---|---|---|---|---|---|---|---|---|---|
| JAHN | 595 | m | 0 | 29 | 63 | 3 | 138 | 21.17 | ( 6.22- 72.12) |
| JEDRYC | 544 | m | 0 | 22 | 82 | 6 | 289 | 12.92 | ( 5.07- 32.93) |
| LUBIN2 | 768 | m | 0 | 265 | 882 | 54 | 2616 | 14.56 | ( 10.76- 19.70) |
| MATOS | 632 | m | 2 | 5 | - | 3 | - | 6.00 | ( 1.19- 30.28) |
| WAKAI | 548 | m | 1 | 11 | - | 2 | - | 7.47 | ( 1.58- 35.30) |
| WYNDE6 | 789 | m | 2 | 36 | - | 8 | - | 17.20 | ( 7.92- 37.33) |
| Partial Totals | | | | 368 | 1027 | 76 | 3043 | | |

*prospective study

| REF | NRR | SEX | AD | Ys | Ws | Qs | Ps |
|---|---|---|---|---|---|---|---|
| JAHN | 595 | m | 0 | 3.05 | 2.56 | 0.39 | 0.0000 |
| JEDRYC | 544 | m | 0 | 2.56 | 4.39 | 0.05 | 0.0000 |
| LUBIN2 | 768 | m | 0 | 2.68 | 42.00 | 0.01 | 0.0000 |
| MATOS | 632 | m | 2 | 1.79 | 1.47 | 1.11 | 0.0300 |
| WAKAI | 548 | m | 1 | 2.01 | 1.59 | 0.68 | 0.0112 |
| WYNDE6 | 789 | m | 2 | 2.84 | 6.39 | 0.21 | 0.0000 |

| | | |
|---|---|---|
| | N | 6 |
| | NS | 6 |
| | Wt | 58.40 |
| Het | Chi | 2.45 |
| Het | df | 5 |
| Het | P | N.S. |
| Fixed | RR | 14.34 |
| | RRl | 11.10 |
| | RRu | 18.54 |
| | P | +++ |
| Random | RR | 14.34 |
| | RRl | 11.10 |
| | RRu | 18.54 |
| | P | +++ |
| Asymm | P | N.S. |

Table 2J8 - 3

IESLC - Meta-analysis of Ex Smoking, Years quit (vs never), "Mid"
Squamous, Cigarettes (or Any Product if Cigarettes not available)
Most adjusted

| | Sex combined | male | female | Total |
|---|---|---|---|---|
| N | | 6 | | 6 |
| NS | | 6 | | 6 |
| Wt | | 58.40 | | 58.40 |
| Het Chi | | 2.45 | | 2.45 |
| Het df | | 5 | | 5 |
| Het P | | N.S. | | N.S. |
| Fixed RR | | 14.34 | | 14.34 |
| RRl | | 11.10 | | 11.10 |
| RRu | | 18.54 | | 18.54 |
| P | | +++ | | +++ |
| Random RR | | 14.34 | | 14.34 |
| RRl | | 11.10 | | 11.10 |
| RRu | | 18.54 | | 18.54 |
| P | | +++ | | +++ |
| Between Chi | | | | |
| Between df | | | | |
| Between P | | | | N.S. |
| Btwn(F) P | | | | N.S. |
| Btwn(R) P | | | | N.S. |

Too few RRs for analysis by factor

Table 2J8 - 4

IESLC - Meta-analysis of Ex Smoking, Years quit (vs never), "Mid"
Squamous, Cigarettes (or Any Product if Cigarettes not available)
Least adjusted

| REF | NRR | X | SEX | AGEL | AGEH | RACE | YF | LC TYPE | LOC | START | ST | NLC | R | VB | P | H | AD | PRODUCT | exL | exH | DENOM | De |
|---|---|---|---|---|---|---|---|---|---|---|---|---|---|---|---|---|---|---|---|---|---|---|
| JAHN | 595 | | m | 0 | 0 | all | - | q | Eu:Ger | 1988 | CC | 1004 | n | bl | n | n | 0 | cig+/-ot | 6 | 10 | nev any | st |
| JEDRYC | 544 | | m | 0 | 0 | all | - | q | Eu:est | 1980 | CC | 1630 | n | bl | y | n | 0 | cig+/-ot | 5 | 9 | nev any | st |
| LUBIN2 | 768 | | m | 0 | 0 | all | - | q | Eu:mul | 1976 | CC | 7804 | n | bl | n | y | 0 | cig+/-ot | 5 | 9 | nev any | st |
| MATOS | 622 | x | m | 0 | 0 | all | - | q | SCAmer | 1994 | CC | 200 | n | bl | n | n | 0 | cig+/-ot | 6 | 10 | nev any | st |
| WAKAI | 540 | x | m | 0 | 0 | all | - | q | As:Jap | 1988 | CC | 333 | n | bl | n | y | 0 | cig+/-ot | 5 | 9 | nev any | st |
| WYNDE6 | 774 | x | m | 0 | 0 | all | - | KI | NAmer | 1969 | CC | 4423 | n | bl | n | y | 0 | cig+/-ot | 7 | 10 | nev any | st |

Cigarette type is all/unspec for all RRs

Table 2J8 - 5

IESLC - Meta-analysis of Ex Smoking, Years quit (vs never), "Mid"
Squamous, Cigarettes (or Any Product if Cigarettes not available)
Least adjusted

| REF | NRR | SEX | AD | Number Exposed Case | Cont | Non-exposed Case | Cont | RR | 95.00%CI |
|---|---|---|---|---|---|---|---|---|---|
| JAHN | 595 | m | 0 | 29 | 63 | 3 | 138 | 21.17 | ( 6.22- 72.12) |
| JEDRYC | 544 | m | 0 | 22 | 82 | 6 | 289 | 12.92 | ( 5.07- 32.93) |
| LUBIN2 | 768 | m | 0 | 265 | 882 | 54 | 2616 | 14.56 | ( 10.76- 19.70) |
| MATOS | 622 | m | 0 | 5 | 27 | 3 | 110 | 6.79 | ( 1.53- 30.19) |
| WAKAI | 540 | m | 0 | 11 | 48 | 2 | 65 | 7.45 | ( 1.58- 35.17) |
| WYNDE6 | 774 | m | 0 | 36 | 340 | 8 | 1667 | 22.06 | ( 10.17- 47.89) |
| Totals | | | | 368 | 1442 | 76 | 4885 | | |

*prospective study

| REF | NRR | SEX | AD | Ys | Ws | Qs | Ps |
|---|---|---|---|---|---|---|---|
| JAHN | 595 | m | 0 | 3.05 | 2.56 | 0.34 | 0.0000 |
| JEDRYC | 544 | m | 0 | 2.56 | 4.39 | 0.08 | 0.0000 |
| LUBIN2 | 768 | m | 0 | 2.68 | 42.00 | 0.01 | 0.0000 |
| MATOS | 622 | m | 0 | 1.92 | 1.73 | 1.04 | 0.0119 |
| WAKAI | 540 | m | 0 | 2.01 | 1.59 | 0.74 | 0.0112 |
| WYNDE6 | 774 | m | 0 | 3.09 | 6.40 | 1.04 | 0.0000 |

| | | |
|---|---|---|
| | N | 6 |
| | NS | 6 |
| | Wt | 58.67 |
| Het | Chi | 3.24 |
| Het | df | 5 |
| Het | P | N.S. |
| Fixed | RR | 14.73 |
| | RRl | 11.41 |
| | RRu | 19.03 |
| | P | +++ |
| Random | RR | 14.73 |
| | RRl | 11.41 |
| | RRu | 19.03 |
| | P | +++ |
| Asymm | P | N.S. |

Table 2J8 - 6

IESLC - Meta-analysis of Ex Smoking, Years quit (vs never), "Mid"
Squamous, Cigarettes (or Any Product if Cigarettes not available)
Least adjusted

| | Sex combined | male | female | Total |
|---|---|---|---|---|
| N | | 6 | | 6 |
| NS | | 6 | | 6 |
| Wt | | 58.67 | | 58.67 |
| Het Chi | | 3.24 | | 3.24 |
| Het df | | 5 | | 5 |
| Het P | | N.S. | | N.S. |
| Fixed RR | | 14.73 | | 14.73 |
| RRl | | 11.41 | | 11.41 |
| RRu | | 19.03 | | 19.03 |
| P | | +++ | | +++ |
| Random RR | | 14.73 | | 14.73 |
| RRl | | 11.41 | | 11.41 |
| RRu | | 19.03 | | 19.03 |
| P | | +++ | | +++ |
| Between Chi | | | | |
| Between df | | | | |
| Between P | | | | N.S. |
| Btwn(F) P | | | | N.S. |
| Btwn(R) P | | | | N.S. |

Table 2J8 - 7

IESLC - Meta-analysis of Ex Smoking, Years quit (vs never), "Mid"
Squamous, Cigarettes (or Any Product if Cigarettes not available)
Excluded studies (and stage at which they were excluded)

| | |
|---|---|
| 1 | AGUDO AKIBA AMANDU AMES AXELSS BEST BOUCHA BOUCOT BRESLO CHEN CHEN2 CHIAZZ DEAN2 DOSEME ENGELA FAN GENG GER GUO HAENSZ HEGMAN HOLE HU HU2 JUSSAW KATSOU KAUFMA KOO KOULUM KREUZE LETOUR LEVIN LIAW LIU3 LIU4 LIU5 MCCONN MIGRAN MRFITR NOTAN2 OSANN2 PERNU QIAO2 RACHTA RESTRE SADOWS SEGI2 STASZE TIZZAN VUTUC WATSON WU WUWILL WYNDE2 WYNDE8 XU YUAN ZHANG ZHENG ZHOU |
| 2 | BUFFLE HUMBLE PISANI PRESCO WYNDE7 |
| 3 | MCDUFF SPITZ |
| 4 | ARMADA AUVINE BECHER BENSHL BLOT1 BOFFET BROSS CARPEN CEDERL CHOI CHYOU CORREA CPSI CPSII DAMBER DARBY DEAN3 DESTEF DOLL DOLL2 DORGAN DORN GAO GAO2 GARCIA GARSHI GILLIS GRAHAM GURSEL HAMMO2 HIRAYA JOLY KAISE2 KHUDER LAUSSM LUBIN PEZZO2 QIAO SPEIZE SUZUK2 TVERDA WANG2 WIGLE WU2 |
| 5 | ALDERS HAMMON |
| 10 | SOBUE |
| 14 | BARBON BROWN3 JAIN LUO PEZZOT SVENSS WYNDE3 |
| 15 | BENHAM |

Table 2J8 - 8
Potentially overlapping studies

| REF | REFGP | PRINC | OVERLAP/LINK |
|---|---|---|---|
| LUBIN2 | LUBIN2 | 1 | Lubin-combined |
| WYNDE6 | WYNDE6 | 1 | WYNDE5/6/7/8 |
| JAHN | BOFFET | 2 | Subset of BOFFET |

Table 2J9 -

IESLC - Meta-analysis of Ex Smoking, Years quit (vs never), "High"
Squamous, Cigarettes (or Any Product if Cigarettes not available)

This analysis is restricted to results for:
1) Ex smokers
2) Results by Years quit (vs never)
3) Categorical results by Years quit (vs never)
4) Squamous (or near equivalent)
5) Results complete enough for use in metaanalysis

Within each study, results are then selected (in the following order of preference, within each sex) for:
6) PRODUCT: cigarettes regardless of other products, cigarettes only, all/unspec
7) CIGTYPE: all/unspecified, MC regardless of HR, MC only
8) (not applicable)
9) DENOM: never smoked anything, never smoked cigarettes, never any + low, never cigs + low
10) Followup period (YF, prospective studies): whole study (coded as 0) or longest available
11) LCtype: squamous or nearest available, but not adeno. (q = squamous, s = small,
a = adeno, KI = Kreyberg I, u = undifferentiated)
12) Race: all or nearest available, otherwise by race (wh or w = white, bl or b = black, hi = hispanic
ch = chinese, jap = japanese, haw = hawaiian, w+o = white + oriental, sca = scandinavian, as = asian)
13) Years quit (vs never) "high" in key scheme 1 (key value 3, maximum range 1-6)
14) For overlapping studies: principal rather than subsidiary studies
Finally by Age: whole study (coded as 0) if available, otherwise by widest available age group
and then for single sex results (m, f) in preference to results for both sexes combined (c).

Results adjusted (AD) for the most potential confounders are then chosen in Sections -1 to -3
(and those which actually differ from the adjusted results in Table 2J4 - 1 are marked 'x' in Section -1)
and results adjusted for the least confounders in Sections -4 to -6. (Those least adjusted results which
actually differ from the most adjusted are marked 'x' in column X in Section -4)

Section -7 shows excluded studies, together with the stage (as above) at which no qualifying
results were found.

Section -8 lists the potentially overlapping studies which have been included (1=principal, 2=subsidiary).

Section -9 lists any results which would have been included in preference except that they had data not complete
enough for use in meta-analysis, with their significance (yes/no), if known, and any further comment as entered
on the database. It also lists as "gap" any categories for which no data were presented by the original authors.
This is commonly due to recent quitters having been combined with current smokers

In addition to those mentioned above, the following fields, levels and abbreviations are used:
* or nk = not known, n = no, y = yes, ot = other
nev = never
all/unspec = all or unspecified, cig+/-ot = cigarettes irrespective of other products (cigar, pipe etc)
MC = manufactured cigarettes, HR = hand-rolled cigarettes
exL, exH = range of exposure (low and high) in the smoking group, in terms of Years quit (vs never)
REF: 6-character study reference
NRR: number of the RR on the database within the study
ST : study type (CC = case control, pr or prosp = prospective)
NLC: number of lung cancer cases in whole study
R : risky occupational population (n = no, m = mining, o = other risky)
VB : national cigarette type (V = at least 75% Virginia, bl = at least 75% blended, ot = other)
P : any proxy use
H : full histological confirmation
De : derivation of RR/CI (or = original, st = standard method, ot = other method of estimation)

Table 2J9 - 1

IESLC - Meta-analysis of Ex Smoking, Years quit (vs never), "High"
Squamous, Cigarettes (or Any Product if Cigarettes not available)
Most adjusted

| REF | NRR | 2J4 | SEX | AGEL | AGEH | RACE | YF | LC TYPE | LOC | START | ST | NLC | R | VB | P | H | AD | PRODUCT | exL | exH | DENOM | De |
|---|---|---|---|---|---|---|---|---|---|---|---|---|---|---|---|---|---|---|---|---|---|---|
| BARBON | 597 | | m | 0 | 0 | all | - | q | Eu:wst | 1979 | CC | 755 | n | bl | y | y | 1 | all/unsp | 0.1 | 4 | nev any | or |
| JAHN | 596 | | m | 0 | 0 | all | - | q | Eu:Ger | 1988 | CC | 1004 | n | bl | n | n | 0 | cig+/-ot | 2 | 5 | nev any | st |
| LUBIN2 | 769 | | m | 0 | 0 | all | - | q | Eu:mul | 1976 | CC | 7804 | n | bl | n | y | 0 | cig+/-ot | 0.1 | 4 | nev any | st |
| MATOS | 633 | | m | 0 | 0 | all | - | q | SCAmer | 1994 | CC | 200 | n | bl | n | n | 2 | cig+/-ot | 1.0 | 5 | nev any | ot |
| WYNDE3 | 504 | | m | 0 | 0 | all | - | KI | NAmer | 1966 | CC | 350 | n | bl | n | y | 0 | all/unsp | 1.0 | 3 | nev any | st |
| WYNDE6 | 791 | | m | 0 | 0 | all | - | KI | NAmer | 1969 | CC | 4423 | n | bl | n | y | 2 | cig+/-ot | 1.0 | 3 | nev any | ot |

Cigarette type is all/unspec for all RRs

Table 2J9 - 2

IESLC - Meta-analysis of Ex Smoking, Years quit (vs never), "High"
Squamous, Cigarettes (or Any Product if Cigarettes not available)
Most adjusted

| REF | NRR | SEX | AD | Number Exposed Case | Exposed Cont | Non-exposed Case | Non-exposed Cont | RR | 95.00%CI |
|---|---|---|---|---|---|---|---|---|---|
| BARBON | 597 | m | 1 | 11 | - | 6 | - | 18.70 | ( 6.20- 56.30) |
| JAHN | 596 | m | 0 | 36 | 46 | 3 | 138 | 36.00 | ( 10.58- 122.45) |
| LUBIN2 | 769 | m | 0 | 498 | 1047 | 54 | 2616 | 23.04 | ( 17.25- 30.79) |
| MATOS | 633 | m | 2 | 4 | - | 3 | - | 7.00 | ( 1.32- 37.20) |
| WYNDE3 | 504 | m | 0 | 18 | 22 | 3 | 88 | 24.00 | ( 6.49- 88.81) |
| WYNDE6 | 791 | m | 2 | 80 | - | 8 | - | 53.80 | ( 25.75- 112.41) |
| Partial Totals | | | | 647 | 1115 | 77 | 2842 | | |

*prospective study

| REF | NRR | SEX | AD | Ys | Ws | Qs | Ps |
|---|---|---|---|---|---|---|---|
| BARBON | 597 | m | 1 | 2.93 | 3.16 | 0.26 | 0.0000 |
| JAHN | 596 | m | 0 | 3.58 | 2.56 | 0.34 | 0.0000 |
| LUBIN2 | 769 | m | 0 | 3.14 | 45.74 | 0.29 | 0.0000 |
| MATOS | 633 | m | 2 | 1.95 | 1.38 | 2.23 | 0.0223 |
| WYNDE3 | 504 | m | 0 | 3.18 | 2.24 | 0.00 | 0.0000 |
| WYNDE6 | 791 | m | 2 | 3.99 | 7.08 | 4.18 | 0.0000 |

| | | |
|---|---|---|
| | N | 6 |
| | NS | 6 |
| | Wt | 62.16 |
| Het | Chi | 7.30 |
| Het | df | 5 |
| Het | P | N.S. |
| Fixed | RR | 24.95 |
| | RRl | 19.45 |
| | RRu | 31.99 |
| | P | +++ |
| Random | RR | 26.22 |
| | RRl | 17.19 |
| | RRu | 39.98 |
| | P | +++ |
| Asymm | P | N.S. |

Table 2J9 - 3

IESLC - Meta-analysis of Ex Smoking, Years quit (vs never), "High"
Squamous, Cigarettes (or Any Product if Cigarettes not available)
Most adjusted

| | Sex combined | male | female | Total |
|---|---|---|---|---|
| N | | 6 | | 6 |
| NS | | 6 | | 6 |
| Wt | | 62.16 | | 62.16 |
| Het Chi | | 7.30 | | 7.30 |
| Het df | | 5 | | 5 |
| Het P | | N.S. | | N.S. |
| Fixed RR | | 24.95 | | 24.95 |
| RRl | | 19.45 | | 19.45 |
| RRu | | 31.99 | | 31.99 |
| P | | +++ | | +++ |
| Random RR | | 26.22 | | 26.22 |
| RRl | | 17.19 | | 17.19 |
| RRu | | 39.98 | | 39.98 |
| P | | +++ | | +++ |
| Between Chi | | | | |
| Between df | | | | |
| Between P | | | | N.S. |
| Btwn(F) P | | | | N.S. |
| Btwn(R) P | | | | N.S. |

Too few RRs for analysis by factor

Table 2J9 - 4

IESLC - Meta-analysis of Ex Smoking, Years quit (vs never), "High"
Squamous, Cigarettes (or Any Product if Cigarettes not available)
Least adjusted

| REF | NRR | X | SEX | AGEL | AGEH | RACE | YF | LC TYPE | LOC | START | ST | NLC | R | VB | P | H | AD | PRODUCT | exL | exH | DENOM | De |
|---|---|---|---|---|---|---|---|---|---|---|---|---|---|---|---|---|---|---|---|---|---|---|
| BARBON | 582 | x | m | 0 | 0 | all | - | q | Eu:wst | 1979 | CC | 755 | n | bl | y | y | 0 | all/unsp | 0.1 | 4 | nev any | st |
| JAHN | 596 | | m | 0 | 0 | all | - | q | Eu:Ger | 1988 | CC | 1004 | n | bl | n | n | 0 | cig+/-ot | 2 | 5 | nev any | st |
| LUBIN2 | 769 | | m | 0 | 0 | all | - | q | Eu:mul | 1976 | CC | 7804 | n | bl | n | y | 0 | cig+/-ot | 0.1 | 4 | nev any | st |
| MATOS | 623 | x | m | 0 | 0 | all | - | q | SCAmer | 1994 | CC | 200 | n | bl | n | n | 0 | cig+/-ot | 1.0 | 5 | nev any | st |
| WYNDE3 | 504 | | m | 0 | 0 | all | - | KI | NAmer | 1966 | CC | 350 | n | bl | n | y | 0 | all/unsp | 1.0 | 3 | nev any | st |
| WYNDE6 | 776 | x | m | 0 | 0 | all | - | KI | NAmer | 1969 | CC | 4423 | n | bl | n | y | 0 | cig+/-ot | 1.0 | 3 | nev any | st |

Cigarette type is all/unspec for all RRs

Table 2J9 - 5

IESLC - Meta-analysis of Ex Smoking, Years quit (vs never), "High"
Squamous, Cigarettes (or Any Product if Cigarettes not available)
Least adjusted

| REF | NRR | SEX | AD | Number Exposed Case | Exposed Cont | Non-exposed Case | Non-exposed Cont | RR | 95.00%CI |
|---|---|---|---|---|---|---|---|---|---|
| BARBON | 582 | m | 0 | 11 | 20 | 6 | 188 | 17.23 | ( 5.76- 51.58) |
| JAHN | 596 | m | 0 | 36 | 46 | 3 | 138 | 36.00 | ( 10.58- 122.45) |
| LUBIN2 | 769 | m | 0 | 498 | 1047 | 54 | 2616 | 23.04 | ( 17.25- 30.79) |
| MATOS | 623 | m | 0 | 4 | 23 | 3 | 110 | 6.38 | ( 1.34- 30.44) |
| WYNDE3 | 504 | m | 0 | 18 | 22 | 3 | 88 | 24.00 | ( 6.49- 88.81) |
| WYNDE6 | 776 | m | 0 | 80 | 307 | 8 | 1667 | 54.30 | ( 25.99- 113.46) |
| Totals | | | | 647 | 1465 | 77 | 4807 | | |

*prospective study

| REF | NRR | SEX | AD | Ys | Ws | Qs | Ps |
|---|---|---|---|---|---|---|---|
| BARBON | 582 | m | 0 | 2.85 | 3.20 | 0.41 | 0.0000 |
| JAHN | 596 | m | 0 | 3.58 | 2.56 | 0.36 | 0.0000 |
| LUBIN2 | 769 | m | 0 | 3.14 | 45.74 | 0.22 | 0.0000 |
| MATOS | 623 | m | 0 | 1.85 | 1.57 | 2.88 | 0.0202 |
| WYNDE3 | 504 | m | 0 | 3.18 | 2.24 | 0.00 | 0.0000 |
| WYNDE6 | 776 | m | 0 | 3.99 | 7.07 | 4.39 | 0.0000 |

| | | |
|---|---|---|
| | N | 6 |
| | NS | 6 |
| | Wt | 62.39 |
| Het | Chi | 8.27 |
| Het | df | 5 |
| Het | P | N.S. |
| Fixed | RR | 24.71 |
| | RRl | 19.28 |
| | RRu | 31.66 |
| | P | +++ |
| Random | RR | 25.52 |
| | RRl | 16.15 |
| | RRu | 40.31 |
| | P | +++ |
| Asymm | P | N.S. |

Table 2J9 - 6

IESLC - Meta-analysis of Ex Smoking, Years quit (vs never), "High"
Squamous, Cigarettes (or Any Product if Cigarettes not available)
Least adjusted

| | Sex combined | male | female | Total |
|---|---|---|---|---|
| N | | 6 | | 6 |
| NS | | 6 | | 6 |
| Wt | | 62.39 | | 62.39 |
| Het Chi | | 8.27 | | 8.27 |
| Het df | | 5 | | 5 |
| Het P | | N.S. | | N.S. |
| Fixed RR | | 24.71 | | 24.71 |
| RRl | | 19.28 | | 19.28 |
| RRu | | 31.66 | | 31.66 |
| P | | +++ | | +++ |
| Random RR | | 25.52 | | 25.52 |
| RRl | | 16.15 | | 16.15 |
| RRu | | 40.31 | | 40.31 |
| P | | +++ | | +++ |
| Between Chi | | | | |
| Between df | | | | |
| Between P | | | | N.S. |
| Btwn(F) P | | | | N.S. |
| Btwn(R) P | | | | N.S. |

Table 2J9 - 7

IESLC - Meta-analysis of Ex Smoking, Years quit (vs never), "High"
Squamous, Cigarettes (or Any Product if Cigarettes not available)
Excluded studies (and stage at which they were excluded)

| | |
|---|---|
| 1 | AGUDO AKIBA AMANDU AMES AXELSS BEST BOUCHA BOUCOT BRESLO CHEN CHEN2 CHIAZZ DEAN2 DOSEME ENGELA FAN GENG GER GUO HAENSZ HEGMAN HOLE HU HU2 JUSSAW KATSOU KAUFMA KOO KOULUM KREUZE LETOUR LEVIN LIAW LIU3 LIU4 LIU5 MCCONN MIGRAN MRFITR NOTAN2 OSANN2 PERNU QIAO2 RACHTA RESTRE SADOWS SEGI2 STASZE TIZZAN VUTUC WATSON WU WUWILL WYNDE2 WYNDE8 XU YUAN ZHANG ZHENG ZHOU |
| 2 | BUFFLE HUMBLE PISANI PRESCO WYNDE7 |
| 3 | MCDUFF SPITZ |
| 4 | ARMADA AUVINE BECHER BENSHL BLOT1 BOFFET BROSS CARPEN CEDERL CHOI CHYOU CORREA CPSI CPSII DAMBER DARBY DEAN3 DESTEF DOLL DOLL2 DORGAN DORN GAO GAO2 GARCIA GARSHI GILLIS GRAHAM GURSEL HAMMO2 HIRAYA JOLY KAISE2 KHUDER LAUSSM LUBIN PEZZO2 QIAO SPEIZE SUZUK2 TVERDA WANG2 WIGLE WU2 |
| 5 | ALDERS HAMMON |
| 10 | SOBUE |
| 14 | BROWN3 JAIN JEDRYC LUO PEZZOT SVENSS WAKAI |
| 15 | BENHAM |

Table 2J9 - 8
Potentially overlapping studies

| REF | REFGP | PRINC | . OVERLAP/LINK |
|---|---|---|---|
| LUBIN2 | LUBIN2 | 1 | Lubin-combined |
| WYNDE6 | WYNDE6 | 1 | WYNDE5/6/7/8 |
| JAHN | BOFFET | 2 | Subset of BOFFET |

Table 2J9 - 9
Most adjusted - insufficient data for meta-analysis

| REF | NRR | SEX | AGEL | AGEH | RACE | YF | LC TYPE | LOC | START | ST | NLC | R | VB | P | H | AD | PRODUCT | exL | exH | DENOM | De |
|---|---|---|---|---|---|---|---|---|---|---|---|---|---|---|---|---|---|---|---|---|---|
| JEDRYC | 545 | m | 0 | 0 | all | - | q | Eu:est | 1980 | CC | 1630 | n | bl | y | n | 0 | cig+/-ot | 1.0 | 4 | nev any | ot |
| WAKAI | 615 | m | 0 | 0 | all | - | q | As:Jap | 1988 | CC | 333 | n | bl | n | y | 1 | cig+/-ot | 1.0 | 4 | nev any | ot |

| REF | NRR | RR | SIG | RRDATA comment |
|---|---|---|---|---|
| JEDRYC | 545 | * | gap | 0 |
| WAKAI | 615 | * | gap | 0 |

Least adjusted - insufficient data for meta-analysis: as for adjusted plus the following

| REF | NRR | SEX | AGEL | AGEH | RACE | YF | LC TYPE | LOC | START | ST | NLC | R | VB | P | H | AD | PRODUCT | exL | exH | DENOM | De |
|---|---|---|---|---|---|---|---|---|---|---|---|---|---|---|---|---|---|---|---|---|---|
| WAKAI | 613 | m | 0 | 0 | all | - | q | As:Jap | 1988 | CC | 333 | n | bl | n | y | 0 | cig+/-ot | 1.0 | 4 | nev any | ot |

| REF | NRR | RR | SIG | RRDATA comment |
|---|---|---|---|---|
| WAKAI | 613 | * | gap | 0 |

Table 2J10 -

IESLC - Meta-analysis of Ex Smoking, Years quit (vs never), "Highest vs lowest"
Squamous, Cigarettes (or Any Product if Cigarettes not available)

This analysis is restricted to results for:
1) Ex smokers
2) Results by Years quit (vs never)
3) Categorical results by Years quit (vs never)
4) Denominator (unexposed) = "low"
5) Squamous (or near equivalent)
6) Results complete enough for use in metaanalysis

Within each study, results are then selected (in the following order of preference, within each sex) for:
7) (not applicable)
8) PRODUCT: cigarettes regardless of other products, cigarettes only, all/unspec
9) CIGTYPE: all/unspecified, MC regardless of HR, MC only
10) Results with least adjustment for other aspects of smoking (ADOS)
11) The highest vs lowest category
12) Followup period (YF, prospective studies): whole study (coded as 0) or longest available
13) LCtype: squamous or nearest available, but not adeno. (q = squamous, s = small,
a = adeno, KI = Kreyberg I, u = undifferentiated)
14) Race: all or nearest available, otherwise by race (wh or w = white, bl or b = black, hi = hispanic
ch = chinese, jap = japanese, haw = hawaiian, w+o = white + oriental, sca = scandinavian, as = asian)
15) For overlapping studies: principal rather than subsidiary studies
Finally by Age: whole study (coded as 0) if available, otherwise by widest available age group
and then for single sex results (m, f) in preference to results for both sexes combined (c).

Results adjusted (AD) for the most potential confounders are then chosen in Sections -1 to -3
(and those which actually differ from the adjusted results in Table 2J5 - 1 are marked 'x' in Section -1)
and results adjusted for the least confounders in Sections -4 to -6. (Those least adjusted results which
actually differ from the most adjusted are marked 'x' in column X in Section -4)

Section -7 shows excluded studies, together with the stage (as above) at which no qualifying
results were found.

Section -8 lists the potentially overlapping studies which have been included (1=principal, 2=subsidiary).

Section -9 lists any results which would have been included in preference except that they had data not complete
enough for use in meta-analysis, with their significance (yes/no), if known, and any further comment as entered
on the database. It also lists as "gap" any categories for which no data were presented by the original authors.
This is commonly due to recent quitters having been combined with current smokers

In addition to those mentioned above, the following fields, levels and abbreviations are used:
* or nk = not known, n = no, y = yes, ot = other
all/unspec = all or unspecified, cig+/-ot = cigarettes irrespective of other products (cigar, pipe etc)
MC = manufactured cigarettes, HR = hand-rolled cigarettes
exL, exH = range of exposure (low and high) in the "highest" group, in terms of Years quit (vs never)
unexL, unexH = range of exposure (low and high) in the "lowest" group, in terms of Years quit (vs never)
REF: 6-character study reference
NRR: number of the RR on the database within the study
ST : study type (CC = case control, pr or prosp = prospective)
NLC: number of lung cancer cases in whole study
R : risky occupational population (n = no, m = mining, o = other risky)
VB : national cigarette type (V = at least 75% Virginia, bl = at least 75% blended, ot = other)
P : any proxy use
H : full histological confirmation
De : derivation of RR/CI (or = original, st = standard method, ot = other method of estimation)

Table 2J10 - 1

IESLC - Meta-analysis of Ex Smoking, Years quit (vs never), "Highest vs lowest"
Squamous, Cigarettes (or Any Product if Cigarettes not available)
Most adjusted

| REF | NRR | 2J5 | SEX | AGEL | AGEH | RACE | YF | LC TYPE | LOC | START | ST | NLC | R | VB | P | H | AD | ADOS | PRODUCT | exL | exH | unexL | unexH | De |
|---|---|---|---|---|---|---|---|---|---|---|---|---|---|---|---|---|---|---|---|---|---|---|---|---|
| BARBON | 601 | | m | 0 | 0 | all | - | q | Eu:wst | 1979 | CC | 755 | n | bl | y | y | 1 | 0 | all/unsp | 0.1 | 4 | 25 | 999 | ot |
| JAHN | 604 | | m | 0 | 0 | all | - | q | Eu:Ger | 1988 | CC | 1004 | n | bl | n | n | 0 | 0 | cig+/-ot | 0.1 | 0.9 | 21 | 999 | st |
| JAIN | 545 | | m | 0 | 0 | all | - | q | NAmer | 1981 | CC | 845 | n | V | y | n | 0 | 0 | cig+/-ot | 2 | 9 | 10 | 999 | st |
| JAIN | 509 | | f | 0 | 0 | all | - | q | NAmer | 1981 | CC | 845 | n | V | y | n | 0 | 0 | cig+/-ot | 2 | 9 | 10 | 999 | st |
| JEDRYC | 547 | | m | 0 | 0 | all | - | q | Eu:est | 1980 | CC | 1630 | n | bl | y | n | 0 | 0 | cig+/-ot | 5 | 9 | 10 | 999 | st |
| LUBIN2 | 774 | | m | 0 | 0 | all | - | q | Eu:mul | 1976 | CC | 7804 | n | bl | n | y | 0 | 0 | cig+/-ot | 0.1 | 4 | 20 | 999 | st |
| LUBIN2 | 898 | | f | 0 | 0 | all | - | q | Eu:mul | 1976 | CC | 7804 | n | bl | n | y | 0 | 0 | cig+/-ot | 0.1 | 9 | 20 | 999 | st |
| MATOS | 635 | | m | 0 | 0 | all | - | q | SCAmer | 1994 | CC | 200 | n | bl | n | n | 2 | 0 | cig+/-ot | 1.0 | 5 | 11 | 999 | ot |
| PEZZOT | 581 | | m | 0 | 0 | all | - | q | SCAmer | 1987 | CC | 215 | n | bl | n | y | 0 | 0 | cig only | 1.0 | 10 | 11 | 999 | st |
| SOBUE | 782 | | m | 0 | 0 | all | - | q | As:Jap | 1986 | CC | 1376 | n | bl | n | y | 0 | 0 | cig+/-ot | 1.0 | 4 | 10 | 999 | st |
| SVENSS | 559 | | f | 0 | 0 | all | - | q | Eu:Sca | 1983 | CC | 210 | n | bl | n | n | 0 | 0 | all/unsp | 3 | 10 | 11 | 999 | st |
| WAKAI | 550 | | m | 0 | 0 | all | - | q | As:Jap | 1988 | CC | 333 | n | bl | n | y | 1 | 0 | cig+/-ot | 5 | 9 | 20 | 999 | ot |
| WYNDE3 | 509 | | m | 0 | 0 | all | - | KI | NAmer | 1966 | CC | 350 | n | bl | n | y | 0 | 0 | all/unsp | 1.0 | 3 | 13 | 999 | st |
| WYNDE6 | 796 | | m | 0 | 0 | all | - | KI | NAmer | 1969 | CC | 4423 | n | bl | n | y | 2 | 0 | cig+/-ot | 1.0 | 3 | 16 | 999 | ot |

Cigarette type is all/unspec for all RRs

Table 2J10 - 2

IESLC - Meta-analysis of Ex Smoking, Years quit (vs never), "Highest vs lowest"
Squamous, Cigarettes (or Any Product if Cigarettes not available)
Most adjusted

| REF | NRR | SEX | AD | Number Exposed Case | Cont | Non-exposed Case | Cont | RR | 95.00%CI |
|---|---|---|---|---|---|---|---|---|---|
| BARBON | 601 | m | 1 | 11 | - | 4 | - | 9.84 | ( 2.71- 35.70) |
| JAHN | 604 | m | 0 | 74 | 8 | 8 | 146 | 168.81 | ( 60.93- 467.73) |
| JAIN | 545 | m | 0 | 24 | 46 | 23 | 113 | 2.56 | ( 1.32- 4.99) |
| JAIN | 509 | f | 0 | 15 | 36 | 7 | 61 | 3.63 | ( 1.35- 9.74) |
| Subtotal JAIN | | | | | | | | 2.86 | ( 1.65- 4.97) |
| JEDRYC | 547 | m | 0 | 22 | 82 | 23 | 230 | 2.68 | ( 1.42- 5.07) |
| LUBIN2 | 774 | m | 0 | 498 | 1047 | 106 | 1128 | 5.06 | ( 4.04- 6.34) |
| LUBIN2 | 898 | f | 0 | 38 | 95 | 2 | 29 | 5.80 | ( 1.32- 25.52) |
| Subtotal LUBIN2 | | | | | | | | 5.08 | ( 4.06- 6.35) |
| MATOS | 635 | m | 2 | 4 | - | 5 | - | 3.50 | ( 0.78- 15.62) |
| PEZZOT | 581 | m | 0 | 21 | 27 | 8 | 48 | 4.67 | ( 1.82- 11.96) |
| SOBUE | 782 | m | 0 | 52 | 116 | 30 | 144 | 2.15 | ( 1.29- 3.59) |
| SVENSS | 559 | f | 0 | 5 | 13 | 1 | 24 | 9.23 | ( 0.97- 87.63) |
| WAKAI | 550 | m | 1 | 11 | - | 3 | - | 3.64 | ( 0.96- 13.87) |
| WYNDE3 | 509 | m | 0 | 18 | 22 | 2 | 55 | 22.50 | ( 4.81- 105.19) |
| WYNDE6 | 796 | m | 2 | 80 | - | 19 | - | 10.76 | ( 6.40- 18.09) |
| Partial Totals | | | | 873 | 1492 | 241 | 1978 | | |

*prospective study

| REF | NRR | SEX | AD | Ys | Ws | Qs | Ps |
|---|---|---|---|---|---|---|---|
| BARBON | 601 | m | 1 | 2.29 | 2.31 | 1.00 | 0.0005 |
| JAHN | 604 | m | 0 | 5.13 | 3.70 | 45.33 | 0.0000 |
| JAIN | 545 | m | 0 | 0.94 | 8.64 | 4.07 | 0.0057 |
| JAIN | 509 | f | 0 | 1.29 | 3.94 | 0.45 | 0.0105 |
| Subtotal JAIN | | | | 1.05 | 12.58 | 4.52 | |
| JEDRYC | 547 | m | 0 | 0.99 | 9.48 | 3.89 | 0.0024 |
| LUBIN2 | 774 | m | 0 | 1.62 | 75.28 | 0.00 | 0.0000 |
| LUBIN2 | 898 | f | 0 | 1.76 | 1.75 | 0.03 | 0.0200 |
| Subtotal LUBIN2 | | | | 1.62 | 77.03 | 0.03 | |
| MATOS | 635 | m | 2 | 1.25 | 1.71 | 0.24 | 0.1013 |
| PEZZOT | 581 | m | 0 | 1.54 | 4.34 | 0.03 | 0.0013 |
| SOBUE | 782 | m | 0 | 0.77 | 14.68 | 10.90 | 0.0033 |
| SVENSS | 559 | f | 0 | 2.22 | 0.76 | 0.27 | 0.0529 |
| WAKAI | 550 | m | 1 | 1.29 | 2.15 | 0.24 | 0.0579 |
| WYNDE3 | 509 | m | 0 | 3.11 | 1.62 | 3.56 | 0.0001 |
| WYNDE6 | 796 | m | 2 | 2.38 | 14.23 | 7.96 | 0.0000 |

| | | |
|---|---|---|
| | N | 14 |
| | NS | 12 |
| | Wt | 144.59 |
| Het | Chi | 78.00 |
| Het | df | 13 |
| Het | P | *** |
| Fixed | RR | 5.09 |
| | RRl | 4.33 |
| | RRu | 5.99 |
| | P | +++ |
| Random | RR | 6.22 |
| | RRl | 3.75 |
| | RRu | 10.30 |
| | P | +++ |
| Asymm | P | N.S. |

Table 2J10 - 3

IESLC - Meta-analysis of Ex Smoking, Years quit (vs never), "Highest vs lowest"
Squamous, Cigarettes (or Any Product if Cigarettes not available)
Most adjusted

| | Sex combined | male | female | Total |
|---|---|---|---|---|
| N | | 11 | 3 | 14 |
| NS | | 11 | 3 | 14 |
| Wt | | 138.14 | 6.45 | 144.59 |
| Het Chi | | 77.24 | 0.68 | 78.00 |
| Het df | | 10 | 2 | 13 |
| Het P | | *** | N.S. | *** |
| Fixed RR | | 5.12 | 4.60 | 5.09 |
| RRl | | 4.33 | 2.13 | 4.33 |
| RRu | | 6.05 | 9.95 | 5.99 |
| P | | +++ | +++ | +++ |
| Random RR | | 6.49 | 4.60 | 6.22 |
| RRl | | 3.64 | 2.13 | 3.75 |
| RRu | | 11.56 | 9.95 | 10.30 |
| P | | +++ | +++ | +++ |
| Between Chi | | | | 0.07 |
| Between df | | | | 1 |
| Between P | | | | N.S. |
| Btwn(F) P | | | | N.S. |
| Btwn(R) P | | | | N.S. |

| | Lung cancer type q | q+s | q+u | KI | not a | Total |
|---|---|---|---|---|---|---|
| N | 12 | | | 2 | | 14 |
| NS | 10 | | | 2 | | 12 |
| Wt | 128.74 | | | 15.85 | | 144.59 |
| Het Chi | 65.15 | | | 0.79 | | 78.00 |
| Het df | 11 | | | 1 | | 13 |
| Het P | *** | | | N.S. | | *** |
| Fixed RR | 4.60 | | | 11.60 | | 5.09 |
| RRl | 3.87 | | | 7.09 | | 4.33 |
| RRu | 5.47 | | | 18.98 | | 5.99 |
| P | +++ | | | +++ | | +++ |
| Random RR | 5.42 | | | 11.60 | | 6.22 |
| RRl | 3.12 | | | 7.09 | | 3.75 |
| RRu | 9.42 | | | 18.98 | | 10.30 |
| P | +++ | | | +++ | | +++ |
| Between Chi | | | | | | 12.06 |
| Between df | | | | | | 1 |
| Between P | | | | | | *** |
| Btwn(F) P | | | | | | N.S. |
| Btwn(R) P | | | | | | * |

| | Location NAmer | UK | Scand | othEur | China | Japan | othAs | other | Total |
|---|---|---|---|---|---|---|---|---|---|
| N | 4 | | 1 | 5 | | 2 | | 2 | 14 |
| NS | 3 | | 1 | 4 | | 2 | | 2 | 12 |
| Wt | 28.43 | | 0.76 | 92.52 | | 16.83 | | 6.05 | 144.59 |
| Het Chi | 14.88 | | 0.00 | 49.54 | | 0.52 | | 0.10 | 78.00 |
| Het df | 3 | | 0 | 4 | | 1 | | 1 | 13 |
| Het P | ** | | N.S. | *** | | N.S. | | N.S. | *** |
| Fixed RR | 6.24 | | 9.23 | 5.56 | | 2.30 | | 4.30 | 5.09 |
| RRl | 4.32 | | 0.97 | 4.54 | | 1.43 | | 1.94 | 4.33 |
| RRu | 9.01 | | 87.63 | 6.82 | | 3.71 | | 9.54 | 5.99 |
| P | +++ | | (+) | +++ | | +++ | | +++ | +++ |
| Random RR | 6.25 | | 9.23 | 10.20 | | 2.30 | | 4.30 | 6.22 |
| RRl | 2.49 | | 0.97 | 3.18 | | 1.43 | | 1.94 | 3.75 |
| RRu | 15.68 | | 87.63 | 32.75 | | 3.71 | | 9.54 | 10.30 |
| P | +++ | | (+) | +++ | | +++ | | +++ | +++ |
| Between Chi | | | | | | | | | 12.95 |
| Between df | | | | | | | | | 4 |
| Between P | | | | | | | | | * |
| Btwn(F) P | | | | | | | | | N.S. |
| Btwn(R) P | | | | | | | | | (*) |

Table 2J10 - 3

IESLC - Meta-analysis of Ex Smoking, Years quit (vs never), "Highest vs lowest"
Squamous, Cigarettes (or Any Product if Cigarettes not available)
Most adjusted

Detailed Country in "other Europe"

| | multi | Germany | othWest | East | Balkans | Total |
|---|---|---|---|---|---|---|
| N | 2 | 1 | 1 | 1 | | 5 |
| NS | 1 | 1 | 1 | 1 | | 4 |
| Wt | 77.03 | 3.70 | 2.31 | 9.48 | | 92.52 |
| Het Chi | 0.03 | 0.00 | 0.00 | 0.00 | | 49.54 |
| Het df | 1 | 0 | 0 | 0 | | 4 |
| Het P | N.S. | N.S. | N.S. | N.S. | | *** |
| Fixed RR | 5.08 | 168.81 | 9.84 | 2.68 | | 5.56 |
| RRl | 4.06 | 60.93 | 2.71 | 1.42 | | 4.54 |
| RRu | 6.35 | 467.73 | 35.71 | 5.07 | | 6.82 |
| P | +++ | +++ | +++ | ++ | | +++ |
| Random RR | 5.08 | 168.81 | 9.84 | 2.68 | | 10.20 |
| RRl | 4.06 | 60.93 | 2.71 | 1.42 | | 3.18 |
| RRu | 6.35 | 467.73 | 35.71 | 5.07 | | 32.75 |
| P | +++ | +++ | +++ | ++ | | +++ |
| Between Chi | | | | | | 49.51 |
| Between df | | | | | | 3 |
| Between P | | | | | | *** |
| Btwn(F) P | | | | | | * |
| Btwn(R) P | | | | | | *** |

Detailed Country in "other Asia"

| | India | HongKong | other | Total |
|---|---|---|---|---|
| N | | | | |
| NS | | | | |
| Wt | | | | |
| Het Chi | | | | |
| Het df | | | | |
| Het P | | | | N.S. |
| Fixed RR | | | | |
| RRl | | | | |
| RRu | | | | |
| P | | | | ++ |
| Random RR | | | | |
| RRl | | | | |
| RRu | | | | |
| P | | | | ++ |
| Between Chi | | | | |
| Between df | | | | |
| Between P | | | | N.S. |
| Btwn(F) P | | | | N.S. |
| Btwn(R) P | | | | N.S. |

Detailed other continent

| | SCAmer | Total |
|---|---|---|
| N | 2 | 2 |
| NS | 2 | 2 |
| Wt | 6.05 | 6.05 |
| Het Chi | 0.10 | 0.10 |
| Het df | 1 | 1 |
| Het P | N.S. | N.S. |
| Fixed RR | 4.30 | 4.30 |
| RRl | 1.94 | 1.94 |
| RRu | 9.54 | 9.54 |
| P | +++ | +++ |
| Random RR | 4.30 | 4.30 |
| RRl | 1.94 | 1.94 |
| RRu | 9.54 | 9.54 |
| P | +++ | +++ |
| Between Chi | | |
| Between df | | |
| Between P | | N.S. |
| Btwn(F) P | | N.S. |
| Btwn(R) P | | N.S. |

Table 2J10 - 3

IESLC - Meta-analysis of Ex Smoking, Years quit (vs never), "Highest vs lowest"
Squamous, Cigarettes (or Any Product if Cigarettes not available)
Most adjusted

| | <1960 | 1960-69 | 1970-79 | 1980-89 | 1990+ | Total |
|---|---|---|---|---|---|---|
| | | Start year of study | | | | |
| N | | 2 | 3 | 8 | 1 | 14 |
| NS | | 2 | 2 | 7 | 1 | 12 |
| Wt | | 15.85 | 79.34 | 47.69 | 1.71 | 144.59 |
| Het Chi | | 0.79 | 1.01 | 61.26 | 0.00 | 78.00 |
| Het df | | 1 | 2 | 7 | 0 | 13 |
| Het P | | N.S. | N.S. | *** | N.S. | *** |
| Fixed RR | | 11.60 | 5.18 | 3.82 | 3.50 | 5.09 |
| RRl | | 7.09 | 4.15 | 2.88 | 0.78 | 4.33 |
| RRu | | 18.98 | 6.45 | 5.08 | 15.66 | 5.99 |
| P | | +++ | +++ | +++ | N.S. | +++ |
| Random RR | | 11.60 | 5.18 | 5.56 | 3.50 | 6.22 |
| RRl | | 7.09 | 4.15 | 2.25 | 0.78 | 3.75 |
| RRu | | 18.98 | 6.45 | 13.71 | 15.66 | 10.30 |
| P | | +++ | +++ | +++ | N.S. | +++ |
| Between Chi | | | | | | 14.93 |
| Between df | | | | | | 3 |
| Between P | | | | | | ** |
| Btwn(F) P | | | | | | N.S. |
| Btwn(R) P | | | | | | * |

| | CC | other | Total |
|---|---|---|---|
| | Study type (1) | | |
| N | 14 | | 14 |
| NS | 12 | | 12 |
| Wt | 144.59 | | 144.59 |
| Het Chi | 78.00 | | 78.00 |
| Het df | 13 | | 13 |
| Het P | *** | | *** |
| Fixed RR | 5.09 | | 5.09 |
| RRl | 4.33 | | 4.33 |
| RRu | 5.99 | | 5.99 |
| P | +++ | | +++ |
| Random RR | 6.22 | | 6.22 |
| RRl | 3.75 | | 3.75 |
| RRu | 10.30 | | 10.30 |
| P | +++ | | +++ |
| Between Chi | | | |
| Between df | | | |
| Between P | | | N.S. |
| Btwn(F) P | | | N.S. |
| Btwn(R) P | | | N.S. |

| | CC | prosp | other | Total |
|---|---|---|---|---|
| | Study type (2) | | | |
| N | 14 | | | 14 |
| NS | 12 | | | 12 |
| Wt | 144.59 | | | 144.59 |
| Het Chi | 78.00 | | | 78.00 |
| Het df | 13 | | | 13 |
| Het P | *** | | | *** |
| Fixed RR | 5.09 | | | 5.09 |
| RRl | 4.33 | | | 4.33 |
| RRu | 5.99 | | | 5.99 |
| P | +++ | | | +++ |
| Random RR | 6.22 | | | 6.22 |
| RRl | 3.75 | | | 3.75 |
| RRu | 10.30 | | | 10.30 |
| P | +++ | | | +++ |
| Between Chi | | | | |
| Between df | | | | |
| Between P | | | | N.S. |
| Btwn(F) P | | | | N.S. |
| Btwn(R) P | | | | N.S. |

Table 2J10 - 3

IESLC - Meta-analysis of Ex Smoking, Years quit (vs never), "Highest vs lowest"
Squamous, Cigarettes (or Any Product if Cigarettes not available)
Most adjusted

| | Study size (number of LC cases) 100-249 | 250-499 | 500-999 | 1000+ | Total |
|---|---|---|---|---|---|
| N | 3 | 2 | 3 | 6 | 14 |
| NS | 3 | 2 | 2 | 5 | 12 |
| Wt | 6.81 | 3.77 | 14.89 | 119.12 | 144.59 |
| Het Chi | 0.49 | 3.06 | 3.31 | 67.94 | 78.00 |
| Het df | 2 | 1 | 2 | 5 | 13 |
| Het P | N.S. | (*) | N.S. | *** | *** |
| Fixed RR | 4.68 | 7.94 | 3.46 | 5.29 | 5.09 |
| RRl | 2.21 | 2.89 | 2.08 | 4.42 | 4.33 |
| RRu | 9.93 | 21.80 | 5.76 | 6.34 | 5.99 |
| P | +++ | +++ | +++ | +++ | +++ |
| Random RR | 4.68 | 8.67 | 3.83 | 7.67 | 6.22 |
| RRl | 2.21 | 1.46 | 1.90 | 3.32 | 3.75 |
| RRu | 9.93 | 51.59 | 7.70 | 17.71 | 10.30 |
| P | +++ | + | +++ | +++ | +++ |
| Between Chi | | | | | 3.19 |
| Between df | | | | | 3 |
| Between P | | | | | N.S. |
| Btwn(F) P | | | | | N.S. |
| Btwn(R) P | | | | | N.S. |

| | Risky occupational population no | mining | othRisky | Total |
|---|---|---|---|---|
| N | 14 | | | 14 |
| NS | 12 | | | 12 |
| Wt | 144.59 | | | 144.59 |
| Het Chi | 78.00 | | | 78.00 |
| Het df | 13 | | | 13 |
| Het P | *** | | | *** |
| Fixed RR | 5.09 | | | 5.09 |
| RRl | 4.33 | | | 4.33 |
| RRu | 5.99 | | | 5.99 |
| P | +++ | | | +++ |
| Random RR | 6.22 | | | 6.22 |
| RRl | 3.75 | | | 3.75 |
| RRu | 10.30 | | | 10.30 |
| P | +++ | | | +++ |
| Between Chi | | | | |
| Between df | | | | |
| Between P | | | | N.S. |
| Btwn(F) P | | | | N.S. |
| Btwn(R) P | | | | N.S. |

| | National cigarette tobacco type Virginia | blended | other | Total |
|---|---|---|---|---|
| N | 2 | 12 | | 14 |
| NS | 1 | 11 | | 12 |
| Wt | 12.58 | 132.01 | | 144.59 |
| Het Chi | 0.33 | 73.07 | | 78.00 |
| Het df | 1 | 11 | | 13 |
| Het P | N.S. | *** | | *** |
| Fixed RR | 2.86 | 5.38 | | 5.09 |
| RRl | 1.65 | 4.54 | | 4.33 |
| RRu | 4.97 | 6.38 | | 5.99 |
| P | +++ | +++ | | +++ |
| Random RR | 2.86 | 7.19 | | 6.22 |
| RRl | 1.65 | 4.04 | | 3.75 |
| RRu | 4.97 | 12.81 | | 10.30 |
| P | +++ | +++ | | +++ |
| Between Chi | | | | 4.60 |
| Between df | | | | 1 |
| Between P | | | | * |
| Btwn(F) P | | | | N.S. |
| Btwn(R) P | | | | * |

Table 2J10 - 3

IESLC - Meta-analysis of Ex Smoking, Years quit (vs never), "Highest vs lowest"
Squamous, Cigarettes (or Any Product if Cigarettes not available)
Most adjusted

| | Any proxy use No/nk | Yes | Total |
|---|---|---|---|
| N | 10 | 4 | 14 |
| NS | 9 | 3 | 12 |
| Wt | 120.22 | 24.37 | 144.59 |
| Het Chi | 67.41 | 3.69 | 78.00 |
| Het df | 9 | 3 | 13 |
| Het P | *** | N.S. | *** |
| Fixed RR | 5.62 | 3.14 | 5.09 |
| RRl | 4.70 | 2.11 | 4.33 |
| RRu | 6.72 | 4.66 | 5.99 |
| P | +++ | +++ | +++ |
| Random RR | 7.95 | 3.23 | 6.22 |
| RRl | 4.06 | 2.05 | 3.75 |
| RRu | 15.58 | 5.08 | 10.30 |
| P | +++ | +++ | +++ |
| Between Chi | | | 6.89 |
| Between df | | | 1 |
| Between P | | | ** |
| Btwn(F) P | | | N.S. |
| Btwn(R) P | | | * |

| | Full histological confirmation No | Yes | Total |
|---|---|---|---|
| N | 6 | 8 | 14 |
| NS | 5 | 7 | 12 |
| Wt | 28.23 | 116.36 | 144.59 |
| Het Chi | 54.25 | 23.73 | 78.00 |
| Het df | 5 | 7 | 13 |
| Het P | *** | ** | *** |
| Fixed RR | 4.99 | 5.12 | 5.09 |
| RRl | 3.45 | 4.27 | 4.33 |
| RRu | 7.21 | 6.14 | 5.99 |
| P | +++ | +++ | +++ |
| Random RR | 7.01 | 5.66 | 6.22 |
| RRl | 1.88 | 3.51 | 3.75 |
| RRu | 26.14 | 9.12 | 10.30 |
| P | ++ | +++ | +++ |
| Between Chi | | | 0.01 |
| Between df | | | 1 |
| Between P | | | N.S. |
| Btwn(F) P | | | N.S. |
| Btwn(R) P | | | N.S. |

| | Number of adjustment variables (1) 0 | 1 | 2+/+nk | Total |
|---|---|---|---|---|
| N | 10 | 2 | 2 | 14 |
| NS | 8 | 2 | 2 | 12 |
| Wt | 124.18 | 4.47 | 15.94 | 144.59 |
| Het Chi | 67.61 | 1.10 | 1.93 | 78.00 |
| Het df | 9 | 1 | 1 | 13 |
| Het P | *** | N.S. | N.S. | *** |
| Fixed RR | 4.67 | 6.09 | 9.54 | 5.09 |
| RRl | 3.92 | 2.41 | 5.84 | 4.33 |
| RRu | 5.57 | 15.40 | 15.58 | 5.99 |
| P | +++ | +++ | +++ | +++ |
| Random RR | 6.18 | 6.08 | 7.72 | 6.22 |
| RRl | 3.28 | 2.30 | 2.82 | 3.75 |
| RRu | 11.67 | 16.11 | 21.08 | 10.30 |
| P | +++ | +++ | +++ | +++ |
| Between Chi | | | | 7.36 |
| Between df | | | | 2 |
| Between P | | | | * |
| Btwn(F) P | | | | N.S. |
| Btwn(R) P | | | | N.S. |

Table 2J10 - 3

IESLC - Meta-analysis of Ex Smoking, Years quit (vs never), "Highest vs lowest"
Squamous, Cigarettes (or Any Product if Cigarettes not available)
Most adjusted

| | Number of adjustment variables (2) 0 | 1 | 2 | 3-5 | 6+/+nk | Total |
|---|---|---|---|---|---|---|
| N | 10 | 2 | 2 | | | 14 |
| NS | 8 | 2 | 2 | | | 12 |
| Wt | 124.18 | 4.47 | 15.94 | | | 144.59 |
| Het Chi | 67.61 | 1.10 | 1.93 | | | 78.00 |
| Het df | 9 | 1 | 1 | | | 13 |
| Het P | *** | N.S. | N.S. | | | *** |
| Fixed RR | 4.67 | 6.09 | 9.54 | | | 5.09 |
| RRl | 3.92 | 2.41 | 5.84 | | | 4.33 |
| RRu | 5.57 | 15.40 | 15.58 | | | 5.99 |
| P | +++ | +++ | +++ | | | +++ |
| Random RR | 6.18 | 6.08 | 7.72 | | | 6.22 |
| RRl | 3.28 | 2.30 | 2.82 | | | 3.75 |
| RRu | 11.67 | 16.11 | 21.08 | | | 10.30 |
| P | +++ | +++ | +++ | | | +++ |
| Between Chi | | | | | | 7.36 |
| Between df | | | | | | 2 |
| Between P | | | | | | * |
| Btwn(F) P | | | | | | N.S. |
| Btwn(R) P | | | | | | N.S. |

| | Product all/unsp | cig+/-ot | cig only | Total |
|---|---|---|---|---|
| N | 3 | 10 | 1 | 14 |
| NS | 3 | 8 | 1 | 12 |
| Wt | 4.69 | 135.57 | 4.34 | 144.59 |
| Het Chi | 0.75 | 73.01 | 0.00 | 78.00 |
| Het df | 2 | 9 | 0 | 13 |
| Het P | N.S. | *** | N.S. | *** |
| Fixed RR | 12.95 | 4.94 | 4.67 | 5.09 |
| RRl | 5.24 | 4.18 | 1.82 | 4.33 |
| RRu | 32.03 | 5.85 | 11.96 | 5.99 |
| P | +++ | +++ | ++ | +++ |
| Random RR | 12.95 | 5.57 | 4.67 | 6.22 |
| RRl | 5.24 | 3.06 | 1.82 | 3.75 |
| RRu | 32.03 | 10.13 | 11.96 | 10.30 |
| P | +++ | +++ | ++ | +++ |
| Between Chi | | | | 4.23 |
| Between df | | | | 2 |
| Between P | | | | N.S. |
| Btwn(F) P | | | | N.S. |
| Btwn(R) P | | | | N.S. |

| | Derivation of RR/CI Orig | StdCalc | Other | Total |
|---|---|---|---|---|
| N | | 10 | 4 | 14 |
| NS | | 8 | 4 | 12 |
| Wt | | 124.18 | 20.41 | 144.59 |
| Het Chi | | 67.61 | 3.73 | 78.00 |
| Het df | | 9 | 3 | 13 |
| Het P | | *** | N.S. | *** |
| Fixed RR | | 4.67 | 8.65 | 5.09 |
| RRl | | 3.92 | 5.60 | 4.33 |
| RRu | | 5.57 | 13.34 | 5.99 |
| P | | +++ | +++ | +++ |
| Random RR | | 6.18 | 7.83 | 6.22 |
| RRl | | 3.28 | 4.48 | 3.75 |
| RRu | | 11.67 | 13.71 | 10.30 |
| P | | +++ | +++ | +++ |
| Between Chi | | | | 6.66 |
| Between df | | | | 1 |
| Between P | | | | ** |
| Btwn(F) P | | | | N.S. |
| Btwn(R) P | | | | N.S. |

Table 2J10 - 4

IESLC - Meta-analysis of Ex Smoking, Years quit (vs never), "Highest vs lowest"
Squamous, Cigarettes (or Any Product if Cigarettes not available)
Least adjusted

| REF | NRR | X | SEX | AGEL | AGEH | RACE | YF | LC TYPE | LOC | START | ST | NLC | R | VB | P | H | AD | ADOS | PRODUCT | exL | exH | unexL | unexH | De |
|---|---|---|---|---|---|---|---|---|---|---|---|---|---|---|---|---|---|---|---|---|---|---|---|---|
| BARBON | 586 | x | m | 0 | 0 | all | - | q | Eu:wst | 1979 | CC | 755 | n | bl | y | y | 0 | 0 | all/unsp | 0.1 | 4 | 25 | 999 | st |
| JAHN | 604 | | m | 0 | 0 | all | - | q | Eu:Ger | 1988 | CC | 1004 | n | bl | n | n | 0 | 0 | cig+/-ot | 0.1 | 0.9 | 21 | 999 | st |
| JAIN | 545 | | m | 0 | 0 | all | - | q | NAmer | 1981 | CC | 845 | n | V | y | n | 0 | 0 | cig+/-ot | 2 | 9 | 10 | 999 | st |
| JAIN | 509 | | f | 0 | 0 | all | - | q | NAmer | 1981 | CC | 845 | n | V | y | n | 0 | 0 | cig+/-ot | 2 | 9 | 10 | 999 | st |
| JEDRYC | 547 | | m | 0 | 0 | all | - | q | Eu:est | 1980 | CC | 1630 | n | bl | y | n | 0 | 0 | cig+/-ot | 5 | 9 | 10 | 999 | st |
| LUBIN2 | 774 | | m | 0 | 0 | all | - | q | Eu:mul | 1976 | CC | 7804 | n | bl | n | y | 0 | 0 | cig+/-ot | 0.1 | 4 | 20 | 999 | st |
| LUBIN2 | 898 | | f | 0 | 0 | all | - | q | Eu:mul | 1976 | CC | 7804 | n | bl | n | y | 0 | 0 | cig+/-ot | 0.1 | 9 | 20 | 999 | st |
| MATOS | 625 | x | m | 0 | 0 | all | - | q | SCAmer | 1994 | CC | 200 | n | bl | n | n | 0 | 0 | cig+/-ot | 1.0 | 5 | 11 | 999 | st |
| PEZZOT | 581 | | m | 0 | 0 | all | - | q | SCAmer | 1987 | CC | 215 | n | bl | n | y | 0 | 0 | cig only | 1.0 | 10 | 11 | 999 | st |
| SOBUE | 782 | | m | 0 | 0 | all | - | q | As:Jap | 1986 | CC | 1376 | n | bl | n | y | 0 | 0 | cig+/-ot | 1.0 | 4 | 10 | 999 | st |
| SVENSS | 559 | | f | 0 | 0 | all | - | q | Eu:Sca | 1983 | CC | 210 | n | bl | n | n | 0 | 0 | all/unsp | 3 | 10 | 11 | 999 | st |
| WAKAI | 542 | x | m | 0 | 0 | all | - | q | As:Jap | 1988 | CC | 333 | n | bl | n | y | 0 | 0 | cig+/-ot | 5 | 9 | 20 | 999 | st |
| WYNDE3 | 509 | | m | 0 | 0 | all | - | KI | NAmer | 1966 | CC | 350 | n | bl | n | y | 0 | 0 | all/unsp | 1.0 | 3 | 13 | 999 | st |
| WYNDE6 | 781 | x | m | 0 | 0 | all | - | KI | NAmer | 1969 | CC | 4423 | n | bl | n | y | 0 | 0 | cig+/-ot | 1.0 | 3 | 16 | 999 | st |

Cigarette type is all/unspec for all RRs

Table 2J10 - 5

IESLC - Meta-analysis of Ex Smoking, Years quit (vs never), "Highest vs lowest"
Squamous, Cigarettes (or Any Product if Cigarettes not available)
Least adjusted

| REF | NRR | SEX | AD | Number Exposed Case | Cont | Non-exposed Case | Cont | RR | 95.00%CI |
|---|---|---|---|---|---|---|---|---|---|
| BARBON | 586 | m | 0 | 11 | 20 | 4 | 59 | 8.11 | ( 2.32- 28.36) |
| JAHN | 604 | m | 0 | 74 | 8 | 8 | 146 | 168.81 | ( 60.93- 467.73) |
| JAIN | 545 | m | 0 | 24 | 46 | 23 | 113 | 2.56 | ( 1.32- 4.99) |
| JAIN | 509 | f | 0 | 15 | 36 | 7 | 61 | 3.63 | ( 1.35- 9.74) |
| Subtotal JAIN | | | | | | | | 2.86 | ( 1.65- 4.97) |
| JEDRYC | 547 | m | 0 | 22 | 82 | 23 | 230 | 2.68 | ( 1.42- 5.07) |
| LUBIN2 | 774 | m | 0 | 498 | 1047 | 106 | 1128 | 5.06 | ( 4.04- 6.34) |
| LUBIN2 | 898 | f | 0 | 38 | 95 | 2 | 29 | 5.80 | ( 1.32- 25.52) |
| Subtotal LUBIN2 | | | | | | | | 5.08 | ( 4.06- 6.35) |
| MATOS | 625 | m | 0 | 4 | 23 | 5 | 101 | 3.51 | ( 0.87- 14.11) |
| PEZZOT | 581 | m | 0 | 21 | 27 | 8 | 48 | 4.67 | ( 1.82- 11.96) |
| SOBUE | 782 | m | 0 | 52 | 116 | 30 | 144 | 2.15 | ( 1.29- 3.59) |
| SVENSS | 559 | f | 0 | 5 | 13 | 1 | 24 | 9.23 | ( 0.97- 87.63) |
| WAKAI | 542 | m | 0 | 11 | 48 | 3 | 47 | 3.59 | ( 0.94- 13.69) |
| WYNDE3 | 509 | m | 0 | 18 | 22 | 2 | 55 | 22.50 | ( 4.81- 105.19) |
| WYNDE6 | 781 | m | 0 | 80 | 307 | 19 | 530 | 7.27 | ( 4.32- 12.22) |
| Totals | | | | 873 | 1890 | 241 | 2715 | | |

*prospective study

| REF | NRR | SEX | AD | Ys | Ws | Qs | Ps |
|---|---|---|---|---|---|---|---|
| BARBON | 586 | m | 0 | 2.09 | 2.45 | 0.63 | 0.0010 |
| JAHN | 604 | m | 0 | 5.13 | 3.70 | 46.43 | 0.0000 |
| JAIN | 545 | m | 0 | 0.94 | 8.64 | 3.59 | 0.0057 |
| JAIN | 509 | f | 0 | 1.29 | 3.94 | 0.35 | 0.0105 |
| Subtotal JAIN | | | | 1.05 | 12.58 | 3.94 | |
| JEDRYC | 547 | m | 0 | 0.99 | 9.48 | 3.40 | 0.0024 |
| LUBIN2 | 774 | m | 0 | 1.62 | 75.28 | 0.10 | 0.0000 |
| LUBIN2 | 898 | f | 0 | 1.76 | 1.75 | 0.05 | 0.0200 |
| Subtotal LUBIN2 | | | | 1.62 | 77.03 | 0.15 | |
| MATOS | 625 | m | 0 | 1.26 | 1.99 | 0.22 | 0.0766 |
| PEZZOT | 581 | m | 0 | 1.54 | 4.34 | 0.01 | 0.0013 |
| SOBUE | 782 | m | 0 | 0.77 | 14.68 | 9.86 | 0.0033 |
| SVENSS | 559 | f | 0 | 2.22 | 0.76 | 0.31 | 0.0529 |
| WAKAI | 542 | m | 0 | 1.28 | 2.14 | 0.20 | 0.0612 |
| WYNDE3 | 509 | m | 0 | 3.11 | 1.62 | 3.77 | 0.0001 |
| WYNDE6 | 781 | m | 0 | 1.98 | 14.23 | 2.25 | 0.0000 |

| | | |
|---|---|---|
| | N | 14 |
| | NS | 12 |
| | Wt | 145.00 |
| Het | Chi | 71.16 |
| Het | df | 13 |
| Het | P | *** |
| Fixed | RR | 4.88 |
| | RRl | 4.15 |
| | RRu | 5.75 |
| | P | +++ |
| Random | RR | 5.87 |
| | RRl | 3.63 |
| | RRu | 9.51 |
| | P | +++ |
| Asymm | P | N.S. |

Table 2J10 - 6

IESLC - Meta-analysis of Ex Smoking, Years quit (vs never), "Highest vs lowest"
Squamous, Cigarettes (or Any Product if Cigarettes not available)
Least adjusted

| | Sex combined | male | female | Total |
|---|---|---|---|---|
| N | | 11 | 3 | 14 |
| NS | | 11 | 3 | 14 |
| Wt | | 138.54 | 6.45 | 145.00 |
| Het Chi | | 70.45 | 0.68 | 71.16 |
| Het df | | 10 | 2 | 13 |
| Het P | | *** | N.S. | *** |
| Fixed RR | | 4.90 | 4.60 | 4.88 |
| RRl | | 4.15 | 2.13 | 4.15 |
| RRu | | 5.78 | 9.95 | 5.75 |
| P | | +++ | +++ | +++ |
| Random RR | | 6.07 | 4.60 | 5.87 |
| RRl | | 3.50 | 2.13 | 3.63 |
| RRu | | 10.53 | 9.95 | 9.51 |
| P | | +++ | +++ | +++ |
| Between Chi | | | | 0.02 |
| Between df | | | | 1 |
| Between P | | | | N.S. |
| Btwn(F) P | | | | N.S. |
| Btwn(R) P | | | | N.S. |

## Table 2J10 - 7

IESLC - Meta-analysis of Ex Smoking, Years quit (vs never), "Highest vs lowest"
Squamous, Cigarettes (or Any Product if Cigarettes not available)
Excluded studies (and stage at which they were excluded)

| | |
|---|---|
| 1 | AGUDO AKIBA AMANDU AMES AXELSS BEST BOUCHA BOUCOT BRESLO CHEN CHEN2 CHIAZZ DEAN2 DOSEME ENGELA FAN GENG GER GUO HAENSZ HEGMAN HOLE HU HU2 JUSSAW KATSOU KAUFMA KOO KOULUM KREUZE LETOUR LEVIN LIAW LIU3 LIU4 LIU5 MCCONN MIGRAN MRFITR NOTAN2 OSANN2 PERNU QIAO2 RACHTA RESTRE SADOWS SEGI2 STASZE TIZZAN VUTUC WATSON WU WUWILL WYNDE2 WYNDE8 XU YUAN ZHANG ZHENG ZHOU |
| 2 | BUFFLE HUMBLE PISANI PRESCO WYNDE7 |
| 3 | MCDUFF SPITZ |
| 4 | AUVINE BLOT1 BROWN3 GURSEL LAUSSM LUO WU2 |
| 5 | ARMADA BECHER BENSHL BOFFET BROSS CARPEN CEDERL CHOI CHYOU CORREA CPSI CPSII DAMBER DARBY DEAN3 DESTEF DOLL DOLL2 DORGAN DORN GAO GAO2 GARCIA GARSHI GILLIS GRAHAM HAMMO2 HIRAYA JOLY KAISE2 KHUDER LUBIN PEZZO2 QIAO SPEIZE SUZUK2 TVERDA WANG2 WIGLE |
| 6 | ALDERS HAMMON |
| 15 | BENHAM |

## Table 2J10 - 8

Potentially overlapping studies

| REF | REFGP | PRINC | . OVERLAP/LINK |
|---|---|---|---|
| LUBIN2 | LUBIN2 | 1 | Lubin-combined |
| WYNDE6 | WYNDE6 | 1 | WYNDE5/6/7/8 |
| JAHN | BOFFET | 2 | Subset of BOFFET |

## Table 2J10 - 9

Most adjusted - insufficient data for meta-analysis

| REF | NRR | SEX | AGEL | AGEH | RACE | YF | LC TYPE | LOC | START | ST | NLC | R | VB | P | H | AD | ADOS | PRODUCT | exL | exH | unexL | unexH | De |
|---|---|---|---|---|---|---|---|---|---|---|---|---|---|---|---|---|---|---|---|---|---|---|---|
| ALDERS | 542 | m | 0 | 0 | all | - | q+s | Eu:UK | 1977 | CC | 1448 | n | V | n | n | 1 | 0 | cig only | 0.1 | 2 | 10 | 999 | st |
| ALDERS | 553 | f | 0 | 0 | all | - | q+s | Eu:UK | 1977 | CC | 1448 | n | V | n | n | 1 | 0 | cig only | 0.1 | 2 | 10 | 999 | st |
| HAMMON | 506 | m | 0 | 0 | wh | 0 | not a | NAmer | 1952 | pr | 448 | n | bl | n | n | 1 | 0 | cig only | 0.1 | 0.9 | 10 | 999 | st |
| JAIN | 589 | m | 0 | 0 | all | - | q | NAmer | 1981 | CC | 845 | n | V | y | n | 0 | 0 | cig+/-ot | 0.1 | 1.9 | 10 | 999 | ot |
| JAIN | 577 | f | 0 | 0 | all | - | q | NAmer | 1981 | CC | 845 | n | V | y | n | 0 | 0 | cig+/-ot | 0.1 | 1.9 | 10 | 999 | ot |
| JEDRYC | 548 | m | 0 | 0 | all | - | q | Eu:est | 1980 | CC | 1630 | n | bl | y | n | 0 | 0 | cig+/-ot | 0.1 | 4 | 10 | 999 | ot |
| MATOS | 708 | m | 0 | 0 | all | - | q | SCAmer | 1994 | CC | 200 | n | bl | n | n | 2 | 0 | cig+/-ot | 0.1 | 0.9 | 11 | 999 | ot |
| PEZZOT | 600 | m | 0 | 0 | all | - | q | SCAmer | 1987 | CC | 215 | n | bl | n | y | 0 | 0 | cig only | 0.1 | 0.9 | 11 | 999 | ot |
| SOBUE | 783 | m | 0 | 0 | all | - | q | As:Jap | 1986 | CC | 1376 | n | bl | n | y | 0 | 0 | cig+/-ot | 0.1 | 0.9 | 10 | 999 | ot |
| SVENSS | 594 | f | 0 | 0 | all | - | q | Eu:Sca | 1983 | CC | 210 | n | bl | n | n | 0 | 0 | all/unsp | 0.1 | 2 | 11 | 999 | ot |
| WAKAI | 616 | m | 0 | 0 | all | - | q | As:Jap | 1988 | CC | 333 | n | bl | n | y | 1 | 0 | cig+/-ot | 0.1 | 4 | 20 | 999 | ot |
| WYNDE3 | 510 | m | 0 | 0 | all | - | KI | NAmer | 1966 | CC | 350 | n | bl | n | y | 0 | 0 | all/unsp | 0.1 | 0.9 | 13 | 999 | ot |
| WYNDE6 | 797 | m | 0 | 0 | all | - | KI | NAmer | 1969 | CC | 4423 | n | bl | n | y | 2 | 0 | cig+/-ot | 0.1 | 0.9 | 16 | 999 | ot |

| REF | NRR | RR | SIG | RRDATA comment |
|---|---|---|---|---|
| ALDERS | 542 | 10.00 | | 0 |
| ALDERS | 553 | 25.17 | | 0 |
| HAMMON | 506 | * | | RR for <1 pack per day is 6.76, while that for 1+ packs is 3.27 |
| JAIN | 589 | * | gap | 0 |
| JAIN | 577 | * | gap | 0 |
| JEDRYC | 548 | * | gap | 0 |
| MATOS | 708 | * | gap | 0 |
| PEZZOT | 600 | * | gap | 0 |
| SOBUE | 783 | * | gap | 0 |
| SVENSS | 594 | * | gap | 0 |
| WAKAI | 616 | * | gap | 0 |
| WYNDE3 | 510 | * | gap | 0 |
| WYNDE6 | 797 | * | gap | 0 |

Least adjusted - insufficient data for meta-analysis: as for adjusted plus the following

| REF | NRR | SEX | AGEL | AGEH | RACE | YF | LC TYPE | LOC | START | ST | NLC | R | VB | P | H | AD | ADOS | PRODUCT | exL | exH | unexL | unexH | De |
|---|---|---|---|---|---|---|---|---|---|---|---|---|---|---|---|---|---|---|---|---|---|---|---|
| MATOS | 706 | m | 0 | 0 | all | - | q | SCAmer | 1994 | CC | 200 | n | bl | n | n | 0 | 0 | cig+/-ot | 0.1 | 0.9 | 11 | 999 | ot |
| WAKAI | 614 | m | 0 | 0 | all | - | q | As:Jap | 1988 | CC | 333 | n | bl | n | y | 0 | 0 | cig+/-ot | 0.1 | 4 | 20 | 999 | ot |
| WYNDE6 | 782 | m | 0 | 0 | all | - | KI | NAmer | 1969 | CC | 4423 | n | bl | n | y | 0 | 0 | cig+/-ot | 0.1 | 0.9 | 16 | 999 | ot |

Table 2J10 - 9

IESLC - Meta-analysis of Ex Smoking, Years quit (vs never), "Highest vs lowest"
Squamous, Cigarettes (or Any Product if Cigarettes not available)
Least adjusted - insufficient data for meta-analysis: as for adjusted plus the following

| REF | NRR | RR | SIG | RRDATA comment |
|---|---|---|---|---|
| MATOS | 706 | * | gap | 0 |
| WAKAI | 614 | * | gap | 0 |
| WYNDE6 | 782 | * | gap | 0 |

Table 2J11 -

IESLC - Meta-analysis of Ex Smoking by Years quit (vs never), Overview
Squamous, Cigarettes only

This analysis is restricted to results for:
1) Ex smokers
2) Results by Years quit (vs never)
3) Categorical results by Years quit (vs never)
   Results by Years quit (vs never) are grouped under 2 schemes (S1, S2). Each scheme has a set of "key values". An interv
   is allocated to the category whose key value it includes, and intervals which include none or more than
   one of the key values are excluded. (Open-ended intervals are coded as 999)

| S1 | key value | maximum range |
|---|---|---|
| 1 | 12 | 8+ |
| 2 | 7 | 4-11 |
| 3 | 3 | 1-6 |

| S2 | key value | maximum range |
|---|---|---|
| 1 | 20 | 13+ |
| 2 | 12 | 4-19 |
| 3 | 3 | 1-11 |

4) Squamous (or near equivalent)
5) Results complete enough for use in metaanalysis

Within each study, results are then selected (in the following order of preference, within each sex) for:
6) (not applicable)
7) PRODUCT: cigarettes only
8) CIGTYPE: all/unspecified, MC regardless of HR, MC only
9) (not applicable)
10) DENOM: never smoked anything, never smoked cigarettes, never any + low, never cigs + low
11) Followup period (YF, prospective studies): whole study (coded as 0) or longest available
12) LCtype: squamous or nearest available, but not adeno. (q = squamous, s = small,
    a = adeno, KI = Kreyberg I, u = undifferentiated)
13) Race: all or nearest available, otherwise by race (wh or w = white, bl or b = black, hi = hispanic
    ch = chinese, jap = japanese, haw = hawaiian, w+o = white + oriental, sca = scandinavian, as = asian)
14) For overlapping studies: principal rather than subsidiary studies
Finally by Age: whole study (coded as 0) if available, otherwise by widest available age group
and then for single sex results (m, f) in preference to results for both sexes combined (c).

Results adjusted (AD) for the most potential confounders are then chosen in Sections -1 to -3
(and those which actually differ from the adjusted results in Table 2J1 - 1 are marked 'x' in Section -1)
and results adjusted for the least confounders in Sections -4 to -6. (Those least adjusted results which
actually differ from the most adjusted are marked 'x' in column X in Section -4)

Section -7 shows excluded studies, together with the stage (as above) at which no qualifying
results were found.

Section -8 lists the potentially overlapping studies which have been included (1=principal, 2=subsidiary).

Section -9 lists any results which would have been included in preference except that they had data not complete
enough for use in meta-analysis, with their significance (yes/no), if known, and any further comment as entered
on the database. It also lists as "gap" any categories for which no data were presented by the original authors.
This is commonly due to recent quitters having been combined with current smokers

In addition to those mentioned above, the following fields, levels and abbreviations are used:
* or nk = not known, n = no, y = yes, ot = other
nev = never
all/unspec = all or unspecified, MC = manufactured cigarettes, HR = hand-rolled cigarettes
exL, exH = range of exposure (low and high) in the smoking group, in terms of Years quit (vs never)
REF: 6-character study reference
NRR: number of the RR on the database within the study
ST : study type (CC = case control, pr or prosp = prospective)
NLC: number of lung cancer cases in whole study
R  : risky occupational population (n = no, m = mining, o = other risky)
VB : national cigarette type (V = at least 75% Virginia, bl = at least 75% blended, ot = other)
P  : any proxy use
H  : full histological confirmation
De : derivation of RR/CI (or = original, st = standard method, ot = other method of estimation)

Table 2J11 - 1

IESLC - Meta-analysis of Ex Smoking by Years quit (vs never), Overview
Squamous, Cigarettes only
Most adjusted

| REF | NRR | 2J1 | SEX | AGEL | AGEH | RACE | YF | LC TYPE | LOC | START | ST | NLC | R | VB | P | H | AD | PRODUCT | exL | exH | S1 | S2 | DENOM | De |
|---|---|---|---|---|---|---|---|---|---|---|---|---|---|---|---|---|---|---|---|---|---|---|---|---|
| BENHAM | 527 | x | m | 0 | 0 | all | - | KI | Eu:wst | 1976 | CC | 1625 | n | bl | n | y | 0 | cig only | 20 | 999 | 0 | 1 | nev any | st |
| BENHAM | 528 | x | m | 0 | 0 | all | - | KI | Eu:wst | 1976 | CC | 1625 | n | bl | n | y | 0 | cig only | 11 | 19 | 1 | 2 | nev any | st |
| BENHAM | 529 | x | m | 0 | 0 | all | - | KI | Eu:wst | 1976 | CC | 1625 | n | bl | n | y | 0 | cig only | 7 | 10 | 2 | 0 | nev any | st |
| BENHAM | 530 | x | m | 0 | 0 | all | - | KI | Eu:wst | 1976 | CC | 1625 | n | bl | n | y | 0 | cig only | 4 | 6 | 0 | 0 | nev any | st |
| BENHAM | 531 | x | m | 0 | 0 | all | - | KI | Eu:wst | 1976 | CC | 1625 | n | bl | n | y | 0 | cig only | 1.0 | 3 | 3 | 3 | nev any | st |
| PEZZOT | 579 | | m | 0 | 0 | all | - | q | SCAmer | 1987 | CC | 215 | n | bl | n | y | 0 | cig only | 11 | 999 | 1 | 0 | nev cigs | ot |
| PEZZOT | 580 | | m | 0 | 0 | all | - | q | SCAmer | 1987 | CC | 215 | n | bl | n | y | 0 | cig only | 1.0 | 10 | 0 | 3 | nev cigs | ot |

Cigarette type is all/unspec for all RRs

In this overview table, subtotals and Qs values may be invalid and should be ignored

Table 2J11 - 2

IESLC - Meta-analysis of Ex Smoking by Years quit (vs never), Overview
Squamous, Cigarettes only
Most adjusted

| REF | NRR | SEX | AD | Number Exposed Case | Cont | Non-exposed Case | Cont | RR | 95.00%CI |
|---|---|---|---|---|---|---|---|---|---|
| BENHAM | 527 | m | 0 | 17 | 82 | 24 | 481 | 4.15 | ( 2.14- 8.07) |
| BENHAM | 528 | m | 0 | 39 | 125 | 24 | 481 | 6.25 | ( 3.62- 10.79) |
| BENHAM | 529 | m | 0 | 41 | 75 | 24 | 481 | 10.96 | ( 6.26- 19.17) |
| BENHAM | 530 | m | 0 | 56 | 92 | 24 | 481 | 12.20 | ( 7.20- 20.68) |
| BENHAM | 531 | m | 0 | 132 | 77 | 24 | 481 | 34.36 | ( 20.90- 56.48) |
| Subtotal BENHAM | | | | | | | | 11.59 | ( 9.06- 14.83) |
| PEZZOT | 579 | m | 0 | 8 | 106 | 0 | 116 | 18.60~ | ( 1.06- 326.10) |
| PEZZOT | 580 | m | 0 | 21 | 82 | 0 | 116 | 60.72~ | ( 3.63-1016.67) |
| Subtotal PEZZOT | | | | | | | | 33.93 | ( 4.55- 252.91) |
| Totals | | | | 314 | 639 | 120 | 2637 | | |

*prospective study ~ With 0.5 adjustment for zero

| REF | NRR | SEX | AD | Ys | Ws | Qs | Ps |
|---|---|---|---|---|---|---|---|
| BENHAM | 527 | m | 0 | 1.42 | 8.71 | 9.46 | 0.0000 |
| BENHAM | 528 | m | 0 | 1.83 | 12.92 | 5.18 | 0.0000 |
| BENHAM | 529 | m | 0 | 2.39 | 12.27 | 0.06 | 0.0000 |
| BENHAM | 530 | m | 0 | 2.50 | 13.80 | 0.02 | 0.0000 |
| BENHAM | 531 | m | 0 | 3.54 | 15.55 | 17.83 | 0.0000 |
| Subtotal BENHAM | | | | 2.45 | 63.26 | 32.54 | |
| PEZZOT | 579 | m | 0 | 2.92 | 0.47 | 0.10 | 0.0455 |
| PEZZOT | 580 | m | 0 | 4.11 | 0.48 | 1.30 | 0.0043 |
| Subtotal PEZZOT | | | | 3.52 | 0.95 | 1.40 | |

| | |
|---|---|
| N | 7 |
| NS | 2 |

Table 2J11 - 3

IESLC - Meta-analysis of Ex Smoking by Years quit (vs never), Overview
Squamous, Cigarettes only
Most adjusted

| | Sex | | | |
|---|---|---|---|---|
| | combined | male | female | Total |
| N | | 7 | | 7 |
| NS | | 2 | | 2 |

In this overview table, other than the "N" rows, entries in the "absent" and "Total" columns may be invalid and should be ignored

| | Years quit vs never (lower focus) | | | | |
|---|---|---|---|---|---|
| | absent | 8+k12 | 4-11k7 | 1-6k3 | Total |
| N | 3 | 2 | 1 | 1 | 7 |
| NS | 2 | 2 | 1 | 1 | 5 |
| Wt | 23.00 | 13.39 | 12.27 | 15.55 | 64.21 |
| Het Chi | 8.13 | 0.54 | 0.00 | 0.00 | 33.94 |
| Het df | 2 | 1 | 0 | 0 | 6 |
| Het P | * | N.S. | N.S. | N.S. | *** |
| Fixed RR | 8.39 | 6.50 | 10.96 | 34.36 | 11.78 |
| RRl | 5.58 | 3.80 | 6.26 | 20.90 | 9.22 |
| RRu | 12.63 | 11.10 | 19.17 | 56.48 | 15.04 |
| P | +++ | +++ | +++ | +++ | +++ |
| Random RR | 9.12 | 6.50 | 10.96 | 34.36 | 11.64 |
| RRl | 3.23 | 3.80 | 6.26 | 20.90 | 6.00 |
| RRu | 25.78 | 11.10 | 19.17 | 56.48 | 22.59 |
| P | +++ | +++ | +++ | +++ | +++ |

| | Years quit vs never (higher focus) | | | | |
|---|---|---|---|---|---|
| | absent | 13+k20 | 4-19k12 | 1-11k3 | Total |
| N | 3 | 1 | 1 | 2 | 7 |
| NS | 2 | 1 | 1 | 2 | 4 |
| Wt | 26.54 | 8.71 | 12.92 | 16.03 | 64.21 |
| Het Chi | 0.18 | 0.00 | 0.00 | 0.15 | 33.94 |
| Het df | 2 | 0 | 0 | 1 | 6 |
| Het P | N.S. | N.S. | N.S. | N.S. | *** |
| Fixed RR | 11.69 | 4.15 | 6.25 | 34.95 | 11.78 |
| RRl | 7.99 | 2.14 | 3.62 | 21.42 | 9.22 |
| RRu | 17.11 | 8.07 | 10.79 | 57.02 | 15.04 |
| P | +++ | +++ | +++ | +++ | +++ |
| Random RR | 11.69 | 4.15 | 6.25 | 34.95 | 11.64 |
| RRl | 7.99 | 2.14 | 3.62 | 21.42 | 6.00 |
| RRu | 17.11 | 8.07 | 10.79 | 57.02 | 22.59 |
| P | +++ | +++ | +++ | +++ | +++ |

Table 2J11 - 3

IESLC - Meta-analysis of Ex Smoking by Years quit (vs never), Overview
Squamous, Cigarettes only
Most adjusted

MALES

| Years quit vs never (lower focus) | absent | 8+k12 | 4-11k7 | 1-6k3 | Total |
|---|---|---|---|---|---|
| N | 3 | 2 | 1 | 1 | 7 |
| NS | 2 | 2 | 1 | 1 | 5 |
| Wt | 23.00 | 13.39 | 12.27 | 15.55 | 64.21 |
| Het Chi | 8.13 | 0.54 | 0.00 | 0.00 | 33.94 |
| Het df | 2 | 1 | 0 | 0 | 6 |
| Het P | * | N.S. | N.S. | N.S. | *** |
| Fixed RR | 8.39 | 6.50 | 10.96 | 34.36 | 11.78 |
| RRl | 5.58 | 3.80 | 6.26 | 20.90 | 9.22 |
| RRu | 12.63 | 11.10 | 19.17 | 56.48 | 15.04 |
| P | +++ | +++ | +++ | +++ | +++ |
| Random RR | 9.12 | 6.50 | 10.96 | 34.36 | 11.64 |
| RRl | 3.23 | 3.80 | 6.26 | 20.90 | 6.00 |
| RRu | 25.78 | 11.10 | 19.17 | 56.48 | 22.59 |
| P | +++ | +++ | +++ | +++ | +++ |

| Years quit vs never (higher focus) | absent | 13+k20 | 4-19k12 | 1-11k3 | Total |
|---|---|---|---|---|---|
| N | 3 | 1 | 1 | 2 | 7 |
| NS | 2 | 1 | 1 | 2 | 4 |
| Wt | 26.54 | 8.71 | 12.92 | 16.03 | 64.21 |
| Het Chi | 0.18 | 0.00 | 0.00 | 0.15 | 33.94 |
| Het df | 2 | 0 | 0 | 1 | 6 |
| Het P | N.S. | N.S. | N.S. | N.S. | *** |
| Fixed RR | 11.69 | 4.15 | 6.25 | 34.95 | 11.78 |
| RRl | 7.99 | 2.14 | 3.62 | 21.42 | 9.22 |
| RRu | 17.11 | 8.07 | 10.79 | 57.02 | 15.04 |
| P | +++ | +++ | +++ | +++ | +++ |
| Random RR | 11.69 | 4.15 | 6.25 | 34.95 | 11.64 |
| RRl | 7.99 | 2.14 | 3.62 | 21.42 | 6.00 |
| RRu | 17.11 | 8.07 | 10.79 | 57.02 | 22.59 |
| P | +++ | +++ | +++ | +++ | +++ |

Table 2J11 - 4

IESLC - Meta-analysis of Ex Smoking by Years quit (vs never), Overview
Squamous, Cigarettes only
Least adjusted

| REF | NRR | X | SEX | AGEL | AGEH | RACE | YF | LC TYPE | LOC | START | ST | NLC | R | VB | P | H | AD | PRODUCT | exL | exH | S1 | S2 | DENOM | De |
|---|---|---|---|---|---|---|---|---|---|---|---|---|---|---|---|---|---|---|---|---|---|---|---|---|
| BENHAM | 527 | | m | 0 | 0 | all | - | KI | Eu:wst | 1976 | CC | 1625 | n | bl | n | y | 0 | cig only | 20 | 999 | 0 | 1 | nev any | st |
| BENHAM | 528 | | m | 0 | 0 | all | - | KI | Eu:wst | 1976 | CC | 1625 | n | bl | n | y | 0 | cig only | 11 | 19 | 1 | 2 | nev any | st |
| BENHAM | 529 | | m | 0 | 0 | all | - | KI | Eu:wst | 1976 | CC | 1625 | n | bl | n | y | 0 | cig only | 7 | 10 | 2 | 0 | nev any | st |
| BENHAM | 530 | | m | 0 | 0 | all | - | KI | Eu:wst | 1976 | CC | 1625 | n | bl | n | y | 0 | cig only | 4 | 6 | 0 | 0 | nev any | st |
| BENHAM | 531 | | m | 0 | 0 | all | - | KI | Eu:wst | 1976 | CC | 1625 | n | bl | n | y | 0 | cig only | 1.0 | 3 | 3 | 3 | nev any | st |
| PEZZOT | 579 | | m | 0 | 0 | all | - | q | SCAmer | 1987 | CC | 215 | n | bl | n | y | 0 | cig only | 11 | 999 | 1 | 0 | nev cigs | ot |
| PEZZOT | 580 | | m | 0 | 0 | all | - | q | SCAmer | 1987 | CC | 215 | n | bl | n | y | 0 | cig only | 1.0 | 10 | 0 | 3 | nev cigs | ot |

Cigarette type is all/unspec for all RRs

In this overview table, subtotals and Qs values may be invalid and should be ignored

Table 2J11 - 5

IESLC - Meta-analysis of Ex Smoking by Years quit (vs never), Overview
Squamous, Cigarettes only
Least adjusted

| REF | NRR | SEX | AD | Number Exposed Case | Cont | Non-exposed Case | Cont | RR | 95.00%CI |
|---|---|---|---|---|---|---|---|---|---|
| BENHAM | 527 | m | 0 | 17 | 82 | 24 | 481 | 4.15 | ( 2.14- 8.07) |
| BENHAM | 528 | m | 0 | 39 | 125 | 24 | 481 | 6.25 | ( 3.62- 10.79) |
| BENHAM | 529 | m | 0 | 41 | 75 | 24 | 481 | 10.96 | ( 6.26- 19.17) |
| BENHAM | 530 | m | 0 | 56 | 92 | 24 | 481 | 12.20 | ( 7.20- 20.68) |
| BENHAM | 531 | m | 0 | 132 | 77 | 24 | 481 | 34.36 | ( 20.90- 56.48) |
| Subtotal BENHAM | | | | | | | | 11.59 | ( 9.06- 14.83) |
| PEZZOT | 579 | m | 0 | 8 | 106 | 0 | 116 | 18.60~ | ( 1.06- 326.10) |
| PEZZOT | 580 | m | 0 | 21 | 82 | 0 | 116 | 60.72~ | ( 3.63-1016.67) |
| Subtotal PEZZOT | | | | | | | | 33.93 | ( 4.55- 252.91) |
| Totals | | | | 314 | 639 | 120 | 2637 | | |

*prospective study ~ With 0.5 adjustment for zero

| REF | NRR | SEX | AD | Ys | Ws | Qs | Ps |
|---|---|---|---|---|---|---|---|
| BENHAM | 527 | m | 0 | 1.42 | 8.71 | 9.46 | 0.0000 |
| BENHAM | 528 | m | 0 | 1.83 | 12.92 | 5.18 | 0.0000 |
| BENHAM | 529 | m | 0 | 2.39 | 12.27 | 0.06 | 0.0000 |
| BENHAM | 530 | m | 0 | 2.50 | 13.80 | 0.02 | 0.0000 |
| BENHAM | 531 | m | 0 | 3.54 | 15.55 | 17.83 | 0.0000 |
| Subtotal BENHAM | | | | 2.45 | 63.26 | 32.54 | |
| PEZZOT | 579 | m | 0 | 2.92 | 0.47 | 0.10 | 0.0455 |
| PEZZOT | 580 | m | 0 | 4.11 | 0.48 | 1.30 | 0.0043 |
| Subtotal PEZZOT | | | | 3.52 | 0.95 | 1.40 | |

| | |
|---|---|
| N | 7 |
| NS | 2 |

Table 2J11 - 6

IESLC - Meta-analysis of Ex Smoking by Years quit (vs never), Overview
Squamous, Cigarettes only
Least adjusted

| | Sex | | | |
|---|---|---|---|---|
| | combined | male | female | Total |
| N | | 7 | | 7 |
| NS | | 2 | | 2 |

In this overview table, other than the "N" rows, entries in the "absent" and "Total" columns may be invalid and should be ignored

| | Years quit vs never (lower focus) | | | | |
|---|---|---|---|---|---|
| | absent | 8+k12 | 4-11k7 | 1-6k3 | Total |
| N | 3 | 2 | 1 | 1 | 7 |
| NS | 2 | 2 | 1 | 1 | 5 |
| Wt | 23.00 | 13.39 | 12.27 | 15.55 | 64.21 |
| Het Chi | 8.13 | 0.54 | 0.00 | 0.00 | 33.94 |
| Het df | 2 | 1 | 0 | 0 | 6 |
| Het P | * | N.S. | N.S. | N.S. | *** |
| Fixed RR | 8.39 | 6.50 | 10.96 | 34.36 | 11.78 |
| RRl | 5.58 | 3.80 | 6.26 | 20.90 | 9.22 |
| RRu | 12.63 | 11.10 | 19.17 | 56.48 | 15.04 |
| P | +++ | +++ | +++ | +++ | +++ |
| Random RR | 9.12 | 6.50 | 10.96 | 34.36 | 11.64 |
| RRl | 3.23 | 3.80 | 6.26 | 20.90 | 6.00 |
| RRu | 25.78 | 11.10 | 19.17 | 56.48 | 22.59 |
| P | +++ | +++ | +++ | +++ | +++ |

| | Years quit vs never (higher focus) | | | | |
|---|---|---|---|---|---|
| | absent | 13+k20 | 4-19k12 | 1-11k3 | Total |
| N | 3 | 1 | 1 | 2 | 7 |
| NS | 2 | 1 | 1 | 2 | 4 |
| Wt | 26.54 | 8.71 | 12.92 | 16.03 | 64.21 |
| Het Chi | 0.18 | 0.00 | 0.00 | 0.15 | 33.94 |
| Het df | 2 | 0 | 0 | 1 | 6 |
| Het P | N.S. | N.S. | N.S. | N.S. | *** |
| Fixed RR | 11.69 | 4.15 | 6.25 | 34.95 | 11.78 |
| RRl | 7.99 | 2.14 | 3.62 | 21.42 | 9.22 |
| RRu | 17.11 | 8.07 | 10.79 | 57.02 | 15.04 |
| P | +++ | +++ | +++ | +++ | +++ |
| Random RR | 11.69 | 4.15 | 6.25 | 34.95 | 11.64 |
| RRl | 7.99 | 2.14 | 3.62 | 21.42 | 6.00 |
| RRu | 17.11 | 8.07 | 10.79 | 57.02 | 22.59 |
| P | +++ | +++ | +++ | +++ | +++ |

Table 2J11 - 6

IESLC - Meta-analysis of Ex Smoking by Years quit (vs never), Overview
Squamous, Cigarettes only
Least adjusted

MALES

| | Years quit vs never (lower focus) absent | 8+k12 | 4-11k7 | 1-6k3 | Total |
|---|---|---|---|---|---|
| N | 3 | 2 | 1 | 1 | 7 |
| NS | 2 | 2 | 1 | 1 | 5 |
| Wt | 23.00 | 13.39 | 12.27 | 15.55 | 64.21 |
| Het Chi | 8.13 | 0.54 | 0.00 | 0.00 | 33.94 |
| Het df | 2 | 1 | 0 | 0 | 6 |
| Het P | * | N.S. | N.S. | N.S. | *** |
| Fixed RR | 8.39 | 6.50 | 10.96 | 34.36 | 11.78 |
| RRl | 5.58 | 3.80 | 6.26 | 20.90 | 9.22 |
| RRu | 12.63 | 11.10 | 19.17 | 56.48 | 15.04 |
| P | +++ | +++ | +++ | +++ | +++ |
| Random RR | 9.12 | 6.50 | 10.96 | 34.36 | 11.64 |
| RRl | 3.23 | 3.80 | 6.26 | 20.90 | 6.00 |
| RRu | 25.78 | 11.10 | 19.17 | 56.48 | 22.59 |
| P | +++ | +++ | +++ | +++ | +++ |

| | Years quit vs never (higher focus) absent | 13+k20 | 4-19k12 | 1-11k3 | Total |
|---|---|---|---|---|---|
| N | 3 | 1 | 1 | 2 | 7 |
| NS | 2 | 1 | 1 | 2 | 4 |
| Wt | 26.54 | 8.71 | 12.92 | 16.03 | 64.21 |
| Het Chi | 0.18 | 0.00 | 0.00 | 0.15 | 33.94 |
| Het df | 2 | 0 | 0 | 1 | 6 |
| Het P | N.S. | N.S. | N.S. | N.S. | *** |
| Fixed RR | 11.69 | 4.15 | 6.25 | 34.95 | 11.78 |
| RRl | 7.99 | 2.14 | 3.62 | 21.42 | 9.22 |
| RRu | 17.11 | 8.07 | 10.79 | 57.02 | 15.04 |
| P | +++ | +++ | +++ | +++ | +++ |
| Random RR | 11.69 | 4.15 | 6.25 | 34.95 | 11.64 |
| RRl | 7.99 | 2.14 | 3.62 | 21.42 | 6.00 |
| RRu | 17.11 | 8.07 | 10.79 | 57.02 | 22.59 |
| P | +++ | +++ | +++ | +++ | +++ |

Table 2J11 - 7

IESLC - Meta-analysis of Ex Smoking by Years quit (vs never), Overview
Squamous, Cigarettes only
Excluded studies (and stage at which they were excluded)

| | |
|---|---|
| 1 | AGUDO AKIBA AMANDU AMES AXELSS BEST BOUCHA BOUCOT BRESLO CHEN CHEN2 CHIAZZ DEAN2 DOSEME ENGELA FAN GENG GER GUO HAENSZ HEGMAN HOLE HU HU2 JUSSAW KATSOU KAUFMA KOO KOULUM KREUZE LETOUR LEVIN LIAW LIU3 LIU4 LIU5 MCCONN MIGRAN MRFITR NOTAN2 OSANN2 PERNU QIAO2 RACHTA RESTRE SADOWS SEGI2 STASZE TIZZAN VUTUC WATSON WU WUWILL WYNDE2 WYNDE8 XU YUAN ZHANG ZHENG ZHOU |
| 2 | BUFFLE HUMBLE PISANI PRESCO WYNDE7 |
| 3 | MCDUFF SPITZ |
| 4 | ARMADA AUVINE BECHER BENSHL BLOT1 BOFFET BROSS CARPEN CEDERL CHOI CHYOU CORREA CPSI CPSII DAMBER DARBY DEAN3 DESTEF DOLL DOLL2 DORGAN DORN GAO GAO2 GARCIA GARSHI GILLIS GRAHAM GURSEL HAMMO2 HIRAYA JOLY KAISE2 KHUDER LAUSSM LUBIN PEZZO2 QIAO SPEIZE SUZUK2 TVERDA WANG2 WIGLE WU2 |
| 5 | ALDERS HAMMON |
| 7 | BARBON BROWN3 JAHN JAIN JEDRYC LUBIN2 LUO MATOS SOBUE SVENSS WAKAI WYNDE3 WYNDE6 |

Table 2J11 - 8
Potentially overlapping studies

| REF | REFGP | PRINC | . | OVERLAP/LINK |
|---|---|---|---|---|
| BENHAM | LUBIN2 | 2 | | Subset of Lubin2 |

Table 2J11 - 9
Most adjusted - insufficient data for meta-analysis

| REF | NRR | SEX | AGEL | AGEH | RACE | YF | LC TYPE | LOC | START | ST | NLC | R | VB | P | H | AD | PRODUCT | exL | exH | S1 | S2 | DENOM | De |
|---|---|---|---|---|---|---|---|---|---|---|---|---|---|---|---|---|---|---|---|---|---|---|---|
| ALDERS | 537 | m | 0 | 0 | all | - | q+s | Eu:UK | 1977 | CC | 1448 | n | V | n | n | 1 | cig only | 10 | 999 | 1 | 0 | nev any | st |
| ALDERS | 538 | m | 0 | 0 | all | - | q+s | Eu:UK | 1977 | CC | 1448 | n | V | n | n | 1 | cig only | 3 | 9 | 0 | 3 | nev any | st |
| ALDERS | 539 | m | 0 | 0 | all | - | q+s | Eu:UK | 1977 | CC | 1448 | n | V | n | n | 1 | cig only | 0.1 | 2 | 0 | 0 | nev any | st |
| ALDERS | 548 | f | 0 | 0 | all | - | q+s | Eu:UK | 1977 | CC | 1448 | n | V | n | n | 1 | cig only | 10 | 999 | 1 | 0 | nev any | st |
| ALDERS | 549 | f | 0 | 0 | all | - | q+s | Eu:UK | 1977 | CC | 1448 | n | V | n | n | 1 | cig only | 3 | 9 | 0 | 3 | nev any | st |
| ALDERS | 550 | f | 0 | 0 | all | - | q+s | Eu:UK | 1977 | CC | 1448 | n | V | n | n | 1 | cig only | 0.1 | 2 | 0 | 0 | nev any | st |
| BENHAM | 532 | m | 0 | 0 | all | - | KI | Eu:wst | 1976 | CC | 1625 | n | bl | n | y | 0 | cig only | 0.1 | 0.9 | 0 | 0 | nev any | ot |
| HAMMON | 501 | m | 0 | 0 | wh | 0 | not a | NAmer | 1952 | pr | 448 | n | bl | n | n | 1 | cig only | 10 | 999 | 1 | 0 | nev any | st |
| HAMMON | 502 | m | 0 | 0 | wh | 0 | not a | NAmer | 1952 | pr | 448 | n | bl | n | n | 1 | cig only | 1.0 | 9 | 0 | 3 | nev any | st |
| HAMMON | 503 | m | 0 | 0 | wh | 0 | not a | NAmer | 1952 | pr | 448 | n | bl | n | n | 1 | cig only | 0.1 | 0.9 | 0 | 0 | nev any | st |
| PEZZOT | 599 | m | 0 | 0 | all | - | q | SCAmer | 1987 | CC | 215 | n | bl | n | y | 0 | cig only | 0.1 | 0.9 | 0 | 0 | nev cigs | ot |

| REF | NRR | RR | SIG | RRDATA comment |
|---|---|---|---|---|
| ALDERS | 537 | 2.33 | | 0 |
| ALDERS | 538 | 4.00 | | 0 |
| ALDERS | 539 | 23.33 | | 0 |
| ALDERS | 548 | 0.67 | | 0 |
| ALDERS | 549 | 8.39 | | 0 |
| ALDERS | 550 | 16.78 | | 0 |
| BENHAM | 532 | * | gap | 0 |
| HAMMON | 501 | * | | RR for <1 pack per day is 2.44, while that for 1+ packs is 17.79 |
| HAMMON | 502 | * | | RR for <1 pack per day is 10.44, while that for 1+ packs is 22.82 |
| HAMMON | 503 | * | | RR for <1 pack per day is 16.50, while that for 1+ packs is 58.24 |
| PEZZOT | 599 | * | gap | 0 |

Table 2J12 -

IESLC - Meta-analysis of Ex Smoking, Years quit (vs never), "Low"
Squamous, Cigarettes only

This analysis is restricted to results for:
1) Ex smokers
2) Results by Years quit (vs never)
3) Categorical results by Years quit (vs never)
4) Squamous (or near equivalent)
5) Results complete enough for use in metaanalysis

Within each study, results are then selected (in the following order of preference, within each sex) for:
6) (not applicable)
7) PRODUCT: cigarettes only
8) CIGTYPE: all/unspecified, MC regardless of HR, MC only
9) (not applicable)
10) DENOM: never smoked anything, never smoked cigarettes, never any + low, never cigs + low
11) Followup period (YF, prospective studies): whole study (coded as 0) or longest available
12) LCtype: squamous or nearest available, but not adeno. (q = squamous, s = small,
a = adeno, KI = Kreyberg I, u = undifferentiated)
13) Race: all or nearest available, otherwise by race (wh or w = white, bl or b = black, hi = hispanic
ch = chinese, jap = japanese, haw = hawaiian, w+o = white + oriental, sca = scandinavian, as = asian)
14) Years quit (vs never) "low" in key scheme 1 (key value 12, maximum range 8+)
15) For overlapping studies: principal rather than subsidiary studies
Finally by Age: whole study (coded as 0) if available, otherwise by widest available age group
and then for single sex results (m, f) in preference to results for both sexes combined (c).

Results adjusted (AD) for the most potential confounders are then chosen in Sections -1 to -3
(and those which actually differ from the adjusted results in Table 2J2 - 1 are marked 'x' in Section -1)
and results adjusted for the least confounders in Sections -4 to -6. (Those least adjusted results which
actually differ from the most adjusted are marked 'x' in column X in Section -4)

Section -7 shows excluded studies, together with the stage (as above) at which no qualifying
results were found.

Section -8 lists the potentially overlapping studies which have been included (1=principal, 2=subsidiary).

Section -9 lists any results which would have been included in preference except that they had data not complete
enough for use in meta-analysis, with their significance (yes/no), if known, and any further comment as entered
on the database. It also lists as "gap" any categories for which no data were presented by the original authors.
This is commonly due to recent quitters having been combined with current smokers

In addition to those mentioned above, the following fields, levels and abbreviations are used:
* or nk = not known, n = no, y = yes, ot = other
nev = never
all/unspec = all or unspecified, MC = manufactured cigarettes, HR = hand-rolled cigarettes
exL, exH = range of exposure (low and high) in the smoking group, in terms of Years quit (vs never)
REF: 6-character study reference
NRR: number of the RR on the database within the study
ST : study type (CC = case control, pr or prosp = prospective)
NLC: number of lung cancer cases in whole study
R  : risky occupational population (n = no, m = mining, o = other risky)
VB : national cigarette type (V = at least 75% Virginia, bl = at least 75% blended, ot = other)
P  : any proxy use
H  : full histological confirmation
De : derivation of RR/CI (or = original, st = standard method, ot = other method of estimation)

Table 2J12 - 1

IESLC - Meta-analysis of Ex Smoking, Years quit (vs never), "Low"
Squamous, Cigarettes only
Most adjusted

| REF | NRR | 2J2 | SEX | AGEL | AGEH | RACE | YF | LC TYPE | LOC | START | ST | NLC | R | VB | P | H | AD | PRODUCT | exL | exH | DENOM | De |
|---|---|---|---|---|---|---|---|---|---|---|---|---|---|---|---|---|---|---|---|---|---|---|
| BENHAM | 528 | x | m | 0 | 0 | all | - | KI | Eu:wst | 1976 | CC | 1625 | n | bl | n | y | 0 | cig only | 11 | 19 | nev any | st |
| PEZZOT | 579 | | m | 0 | 0 | all | - | q | SCAmer | 1987 | CC | 215 | n | bl | n | y | 0 | cig only | 11 | 999 | nev cigs | ot |

Cigarette type is all/unspec for all RRs

Table 2J12 - 2

IESLC - Meta-analysis of Ex Smoking, Years quit (vs never), "Low"
Squamous, Cigarettes only
Most adjusted

| REF | NRR | SEX | AD | Number Case | Exposed Cont | Non-exposed Case | Cont | RR | 95.00%CI |
|---|---|---|---|---|---|---|---|---|---|
| BENHAM | 528 | m | 0 | 39 | 125 | 24 | 481 | 6.25 | ( 3.62- 10.79) |
| PEZZOT | 579 | m | 0 | 8 | 106 | 0 | 116 | 18.60~ | ( 1.06- 326.10) |
| Totals | | | | 47 | 231 | 24 | 597 | | |

*prospective study ~ With 0.5 adjustment for zero

| REF | NRR | SEX | AD | Ys | Ws | Qs | Ps |
|---|---|---|---|---|---|---|---|
| BENHAM | 528 | m | 0 | 1.83 | 12.92 | 0.02 | 0.0000 |
| PEZZOT | 579 | m | 0 | 2.92 | 0.47 | 0.52 | 0.0455 |

| | | |
|---|---|---|
| | N | 2 |
| | NS | 2 |
| | Wt | 13.39 |
| Het | Chi | 0.54 |
| Het | df | 1 |
| Het | P | N.S. |
| Fixed | RR | 6.50 |
| | RRl | 3.80 |
| | RRu | 11.10 |
| | P | +++ |
| Random | RR | 6.50 |
| | RRl | 3.80 |
| | RRu | 11.10 |
| | P | +++ |
| Asymm | P | |

Table 2J12 - 3

IESLC - Meta-analysis of Ex Smoking, Years quit (vs never), "Low"
Squamous, Cigarettes only
Most adjusted

| | Sex combined | male | female | Total |
|---|---|---|---|---|
| N | | 2 | | 2 |
| NS | | 2 | | 2 |
| Wt | | 13.39 | | 13.39 |
| Het Chi | | 0.54 | | 0.54 |
| Het df | | 1 | | 1 |
| Het P | | N.S. | | N.S. |
| Fixed RR | | 6.50 | | 6.50 |
| RRl | | 3.80 | | 3.80 |
| RRu | | 11.10 | | 11.10 |
| P | | +++ | | +++ |
| Random RR | | 6.50 | | 6.50 |
| RRl | | 3.80 | | 3.80 |
| RRu | | 11.10 | | 11.10 |
| P | | +++ | | +++ |
| Between Chi | | | | |
| Between df | | | | |
| Between P | | | | N.S. |
| Btwn(F) P | | | | N.S. |
| Btwn(R) P | | | | N.S. |

Too few RRs for analysis by factor

Table 2J12 - 4

IESLC - Meta-analysis of Ex Smoking, Years quit (vs never), "Low"
Squamous, Cigarettes only
Least adjusted

| REF | NRR | X | SEX | AGEL | AGEH | RACE | YF | LC TYPE | LOC | START | ST | NLC | R | VB | P | H | AD | PRODUCT | exL | exH | DENOM | De |
|---|---|---|---|---|---|---|---|---|---|---|---|---|---|---|---|---|---|---|---|---|---|---|
| BENHAM | 528 | | m | 0 | 0 | all | - | KI | Eu:wst | 1976 | CC | 1625 | n | bl | n | y | 0 | cig only | 11 | 19 | nev any | st |
| PEZZOT | 579 | | m | 0 | 0 | all | - | q | SCAmer | 1987 | CC | 215 | n | bl | n | y | 0 | cig only | 11 | 999 | nev cigs | ot |

Cigarette type is all/unspec for all RRs

Table 2J12 - 5

IESLC - Meta-analysis of Ex Smoking, Years quit (vs never), "Low"
Squamous, Cigarettes only
Least adjusted

| REF | NRR | SEX | AD | Number Case | Exposed Cont | Non-exposed Case | Cont | RR | 95.00%CI |
|---|---|---|---|---|---|---|---|---|---|
| BENHAM | 528 | m | 0 | 39 | 125 | 24 | 481 | 6.25 | ( 3.62- 10.79) |
| PEZZOT | 579 | m | 0 | 8 | 106 | 0 | 116 | 18.60~ | ( 1.06- 326.10) |
| Totals | | | | 47 | 231 | 24 | 597 | | |

*prospective study ~ With 0.5 adjustment for zero

| REF | NRR | SEX | AD | Ys | Ws | Qs | Ps |
|---|---|---|---|---|---|---|---|
| BENHAM | 528 | m | 0 | 1.83 | 12.92 | 0.02 | 0.0000 |
| PEZZOT | 579 | m | 0 | 2.92 | 0.47 | 0.52 | 0.0455 |

| | | |
|---|---|---|
| | N | 2 |
| | NS | 2 |
| | Wt | 13.39 |
| Het | Chi | 0.54 |
| Het | df | 1 |
| Het | P | N.S. |
| Fixed | RR | 6.50 |
| | RRl | 3.80 |
| | RRu | 11.10 |
| | P | +++ |
| Random | RR | 6.50 |
| | RRl | 3.80 |
| | RRu | 11.10 |
| | P | +++ |
| Asymm | P | |

Table 2J12 - 6

IESLC - Meta-analysis of Ex Smoking, Years quit (vs never), "Low"
Squamous, Cigarettes only
Least adjusted

| | Sex combined | male | female | Total |
|---|---|---|---|---|
| N | | 2 | | 2 |
| NS | | 2 | | 2 |
| Wt | | 13.39 | | 13.39 |
| Het Chi | | 0.54 | | 0.54 |
| Het df | | 1 | | 1 |
| Het P | | N.S. | | N.S. |
| Fixed RR | | 6.50 | | 6.50 |
| RRl | | 3.80 | | 3.80 |
| RRu | | 11.10 | | 11.10 |
| P | | +++ | | +++ |
| Random RR | | 6.50 | | 6.50 |
| RRl | | 3.80 | | 3.80 |
| RRu | | 11.10 | | 11.10 |
| P | | +++ | | +++ |
| Between Chi | | | | |
| Between df | | | | |
| Between P | | | | N.S. |
| Btwn(F) P | | | | N.S. |
| Btwn(R) P | | | | N.S. |

Table 2J12 - 7

IESLC - Meta-analysis of Ex Smoking, Years quit (vs never), "Low"
Squamous, Cigarettes only
Excluded studies (and stage at which they were excluded)

| | |
|---|---|
| 1 | AGUDO AKIBA AMANDU AMES AXELSS BEST BOUCHA BOUCOT BRESLO CHEN CHEN2 CHIAZZ DEAN2 DOSEME ENGELA FAN GENG GER GUO HAENSZ HEGMAN HOLE HU HU2 JUSSAW KATSOU KAUFMA KOO KOULUM KREUZE LETOUR LEVIN LIAW LIU3 LIU4 LIU5 MCCONN MIGRAN MRFITR NOTAN2 OSANN2 PERNU QIAO2 RACHTA RESTRE SADOWS SEGI2 STASZE TIZZAN VUTUC WATSON WU WUWILL WYNDE2 WYNDE8 XU YUAN ZHANG ZHENG ZHOU |
| 2 | BUFFLE HUMBLE PISANI PRESCO WYNDE7 |
| 3 | MCDUFF SPITZ |
| 4 | ARMADA AUVINE BECHER BENSHL BLOT1 BOFFET BROSS CARPEN CEDERL CHOI CHYOU CORREA CPSI CPSII DAMBER DARBY DEAN3 DESTEF DOLL DOLL2 DORGAN DORN GAO GAO2 GARCIA GARSHI GILLIS GRAHAM GURSEL HAMMO2 HIRAYA JOLY KAISE2 KHUDER LAUSSM LUBIN PEZZO2 QIAO SPEIZE SUZUK2 TVERDA WANG2 WIGLE WU2 |
| 5 | ALDERS HAMMON |
| 7 | BARBON BROWN3 JAHN JAIN JEDRYC LUBIN2 LUO MATOS SOBUE SVENSS WAKAI WYNDE3 WYNDE6 |

Table 2J12 - 8

Potentially overlapping studies

| REF | REFGP | PRINC | . | OVERLAP/LINK |
|---|---|---|---|---|
| BENHAM | LUBIN2 | 2 | | Subset of Lubin2 |

Table 2J12 - 9

Most adjusted - insufficient data for meta-analysis

| REF | NRR | SEX | AGEL | AGEH | RACE | YF | LC TYPE | LOC | START | ST | NLC | R | VB | P | H | AD | PRODUCT | exL | exH | DENOM | De |
|---|---|---|---|---|---|---|---|---|---|---|---|---|---|---|---|---|---|---|---|---|---|
| ALDERS | 537 | m | 0 | 0 | all | - | q+s | Eu:UK | 1977 | CC | 1448 | n | V | n | n | 1 | cig only | 10 | 999 | nev | any st |
| ALDERS | 548 | f | 0 | 0 | all | - | q+s | Eu:UK | 1977 | CC | 1448 | n | V | n | n | 1 | cig only | 10 | 999 | nev | any st |
| HAMMON | 501 | m | 0 | 0 | wh | 0 | not a | NAmer | 1952 | pr | 448 | n | bl | n | n | 1 | cig only | 10 | 999 | nev | any st |

| REF | NRR | RR | SIG | RRDATA comment |
|---|---|---|---|---|
| ALDERS | 537 | 2.33 | | 0 |
| ALDERS | 548 | 0.67 | | 0 |
| HAMMON | 501 | * | | RR for <1 pack per day is 2.44, while that for 1+ packs is 17.79 |

Table 2J13 -

IESLC - Meta-analysis of Ex Smoking, Years quit (vs never), "Mid"
Squamous, Cigarettes only

This analysis is restricted to results for:
1) Ex smokers
2) Results by Years quit (vs never)
3) Categorical results by Years quit (vs never)
4) Squamous (or near equivalent)
5) Results complete enough for use in metaanalysis

Within each study, results are then selected (in the following order of preference, within each sex) for:
6) (not applicable)
7) PRODUCT: cigarettes only
8) CIGTYPE: all/unspecified, MC regardless of HR, MC only
9) (not applicable)
10) DENOM: never smoked anything, never smoked cigarettes, never any + low, never cigs + low
11) Followup period (YF, prospective studies): whole study (coded as 0) or longest available
12) LCtype: squamous or nearest available, but not adeno. (q = squamous, s = small,
a = adeno, KI = Kreyberg I, u = undifferentiated)
13) Race: all or nearest available, otherwise by race (wh or w = white, bl or b = black, hi = hispanic
ch = chinese, jap = japanese, haw = hawaiian, w+o = white + oriental, sca = scandinavian, as = asian)
14) Years quit (vs never) "mid" in key scheme 1 (key value 7, maximum range 4-11)
15) For overlapping studies: principal rather than subsidiary studies
Finally by Age: whole study (coded as 0) if available, otherwise by widest available age group
and then for single sex results (m, f) in preference to results for both sexes combined (c).

Results adjusted (AD) for the most potential confounders are then chosen in Sections -1 to -3
(and those which actually differ from the adjusted results in Table 2J3 - 1 are marked 'x' in Section -1)
and results adjusted for the least confounders in Sections -4 to -6. (Those least adjusted results which
actually differ from the most adjusted are marked 'x' in column X in Section -4)

Section -7 shows excluded studies, together with the stage (as above) at which no qualifying
results were found.

Section -8 lists the potentially overlapping studies which have been included (1=principal, 2=subsidiary).

Section -9 lists any results which would have been included in preference except that they had data not complete
enough for use in meta-analysis, with their significance (yes/no), if known, and any further comment as entered
on the database. It also lists as "gap" any categories for which no data were presented by the original authors.
This is commonly due to recent quitters having been combined with current smokers

In addition to those mentioned above, the following fields, levels and abbreviations are used:
* or nk = not known, n = no, y = yes, ot = other
nev = never
all/unspec = all or unspecified, MC = manufactured cigarettes, HR = hand-rolled cigarettes
exL, exH = range of exposure (low and high) in the smoking group, in terms of Years quit (vs never)
REF: 6-character study reference
NRR: number of the RR on the database within the study
ST : study type (CC = case control, pr or prosp = prospective)
NLC: number of lung cancer cases in whole study
R  : risky occupational population (n = no, m = mining, o = other risky)
VB : national cigarette type (V = at least 75% Virginia, bl = at least 75% blended, ot = other)
P  : any proxy use
H  : full histological confirmation
De : derivation of RR/CI (or = original, st = standard method, ot = other method of estimation)

Table 2J13 - 1

IESLC - Meta-analysis of Ex Smoking, Years quit (vs never), "Mid"
Squamous, Cigarettes only
Most adjusted

| REF | NRR | 2J3 | SEX | AGEL | AGEH | RACE | YF | LC TYPE | LOC | START | ST | NLC | R | VB | P | H | AD | PRODUCT | exL | exH | DENOM | De |
|---|---|---|---|---|---|---|---|---|---|---|---|---|---|---|---|---|---|---|---|---|---|---|
| BENHAM | 529 | x | m | 0 | 0 | all | - | KI | Eu:wst | 1976 | CC | 1625 | n | bl | n | y | 0 | cig only | 7 | 10 | nev any | st |

Cigarette type is all/unspec for all RRs

Table 2J13 - 2

IESLC - Meta-analysis of Ex Smoking, Years quit (vs never), "Mid"
Squamous, Cigarettes only
Most adjusted

| REF | NRR | SEX | AD | Number Exposed Case | Number Exposed Cont | Non-exposed Case | Non-exposed Cont | RR | 95.00%CI |
|---|---|---|---|---|---|---|---|---|---|
| BENHAM | 529 | m | 0 | 41 | 75 | 24 | 481 | 10.96 | ( 6.26- 19.17) |
| Totals | | | | 41 | 75 | 24 | 481 | | |

*prospective study

| REF | NRR | SEX | AD | Ys | Ws | Qs | Ps |
|---|---|---|---|---|---|---|---|
| BENHAM | 529 | m | 0 | 2.39 | 12.27 | 0.00 | 0.0000 |

| | | |
|---|---|---|
| | N | 1 |
| | NS | 1 |
| | Wt | 12.27 |
| Het | Chi | 0.00 |
| Het | df | 0 |
| Het | P | N.S. |
| Fixed | RR | 10.96 |
| | RRl | 6.26 |
| | RRu | 19.17 |
| | P | +++ |
| Random | RR | 10.96 |
| | RRl | 6.26 |
| | RRu | 19.17 |
| | P | +++ |
| Asymm | P | |

Table 2J13 - 3

IESLC - Meta-analysis of Ex Smoking, Years quit (vs never), "Mid"
Squamous, Cigarettes only
Most adjusted

| | Sex combined | male | female | Total |
|---|---|---|---|---|
| N | | 1 | | 1 |
| NS | | 1 | | 1 |
| Wt | | 12.27 | | 12.27 |
| Het Chi | | 0.00 | | 0.00 |
| Het df | | 0 | | 0 |
| Het P | | N.S. | | N.S. |
| Fixed RR | | 10.96 | | 10.96 |
| RRl | | 6.26 | | 6.26 |
| RRu | | 19.17 | | 19.17 |
| P | | +++ | | +++ |
| Random RR | | 10.96 | | 10.96 |
| RRl | | 6.26 | | 6.26 |
| RRu | | 19.17 | | 19.17 |
| P | | +++ | | +++ |
| Between Chi | | | | |
| Between df | | | | |
| Between P | | | | N.S. |
| Btwn(F) P | | | | N.S. |
| Btwn(R) P | | | | N.S. |

Too few RRs for analysis by factor

Table 2J13 - 4

IESLC - Meta-analysis of Ex Smoking, Years quit (vs never), "Mid"
Squamous, Cigarettes only
Least adjusted

| REF | NRR | X | SEX | AGEL | AGEH | RACE | YF | LC TYPE | LOC | START | ST | NLC | R | VB | P | H | AD | PRODUCT | exL | exH | DENOM | De |
|---|---|---|---|---|---|---|---|---|---|---|---|---|---|---|---|---|---|---|---|---|---|---|
| BENHAM | 529 | | m | 0 | 0 | all | - | KI | Eu:wst | 1976 | CC | 1625 | n | bl | n | y | 0 | cig only | 7 | 10 | nev any | st |

Cigarette type is all/unspec for all RRs

Table 2J13 - 5

IESLC - Meta-analysis of Ex Smoking, Years quit (vs never), "Mid"
Squamous, Cigarettes only
Least adjusted

| REF | NRR | SEX | AD | Number Exposed Case | Number Exposed Cont | Non-exposed Case | Non-exposed Cont | RR | 95.00%CI |
|---|---|---|---|---|---|---|---|---|---|
| BENHAM | 529 | m | 0 | 41 | 75 | 24 | 481 | 10.96 | ( 6.26- 19.17) |
| Totals | | | | 41 | 75 | 24 | 481 | | |

*prospective study

| REF | NRR | SEX | AD | Ys | Ws | Qs | Ps |
|---|---|---|---|---|---|---|---|
| BENHAM | 529 | m | 0 | 2.39 | 12.27 | 0.00 | 0.0000 |

| | | |
|---|---|---|
| | N | 1 |
| | NS | 1 |
| | Wt | 12.27 |
| Het | Chi | 0.00 |
| Het | df | 0 |
| Het | P | N.S. |
| Fixed | RR | 10.96 |
| | RRl | 6.26 |
| | RRu | 19.17 |
| | P | +++ |
| Random | RR | 10.96 |
| | RRl | 6.26 |
| | RRu | 19.17 |
| | P | +++ |
| Asymm | P | |

Table 2J13 - 6

IESLC - Meta-analysis of Ex Smoking, Years quit (vs never), "Mid"
Squamous, Cigarettes only
Least adjusted

| | Sex combined | male | female | Total |
|---|---|---|---|---|
| N | | 1 | | 1 |
| NS | | 1 | | 1 |
| Wt | | 12.27 | | 12.27 |
| Het Chi | | 0.00 | | 0.00 |
| Het df | | 0 | | 0 |
| Het P | | N.S. | | N.S. |
| Fixed RR | | 10.96 | | 10.96 |
| RRl | | 6.26 | | 6.26 |
| RRu | | 19.17 | | 19.17 |
| P | | +++ | | +++ |
| Random RR | | 10.96 | | 10.96 |
| RRl | | 6.26 | | 6.26 |
| RRu | | 19.17 | | 19.17 |
| P | | +++ | | +++ |
| Between Chi | | | | |
| Between df | | | | |
| Between P | | | | N.S. |
| Btwn(F) P | | | | N.S. |
| Btwn(R) P | | | | N.S. |

Table 2J13 - 7

IESLC - Meta-analysis of Ex Smoking, Years quit (vs never), "Mid"
Squamous, Cigarettes only
Excluded studies (and stage at which they were excluded)

| | |
|---|---|
| 1 | AGUDO AKIBA AMANDU AMES AXELSS BEST BOUCHA BOUCOT BRESLO CHEN CHEN2 CHIAZZ DEAN2 DOSEME ENGELA FAN GENG GER GUO HAENSZ HEGMAN HOLE HU HU2 JUSSAW KATSOU KAUFMA KOO KOULUM KREUZE LETOUR LEVIN LIAW LIU3 LIU4 LIU5 MCCONN MIGRAN MRFITR NOTAN2 OSANN2 PERNU QIAO2 RACHTA RESTRE SADOWS SEGI2 STASZE TIZZAN VUTUC WATSON WU WUWILL WYNDE2 WYNDE8 XU YUAN ZHANG ZHENG ZHOU |
| 2 | BUFFLE HUMBLE PISANI PRESCO WYNDE7 |
| 3 | MCDUFF SPITZ |
| 4 | ARMADA AUVINE BECHER BENSHL BLOT1 BOFFET BROSS CARPEN CEDERL CHOI CHYOU CORREA CPSI CPSII DAMBER DARBY DEAN3 DESTEF DOLL DOLL2 DORGAN DORN GAO GAO2 GARCIA GARSHI GILLIS GRAHAM GURSEL HAMMO2 HIRAYA JOLY KAISE2 KHUDER LAUSSM LUBIN PEZZO2 QIAO SPEIZE SUZUK2 TVERDA WANG2 WIGLE WU2 |
| 5 | ALDERS HAMMON |
| 7 | BARBON BROWN3 JAHN JAIN JEDRYC LUBIN2 LUO MATOS SOBUE SVENSS WAKAI WYNDE3 WYNDE6 |
| 14 | PEZZOT |

Table 2J13 - 8
Potentially overlapping studies

| REF | REFGP | PRINC | . OVERLAP/LINK |
|---|---|---|---|
| BENHAM | LUBIN2 | 2 | Subset of Lubin2 |

Table 2J14 -

IESLC - Meta-analysis of Ex Smoking, Years quit (vs never), "High"
Squamous, Cigarettes only

This analysis is restricted to results for:
1) Ex smokers
2) Results by Years quit (vs never)
3) Categorical results by Years quit (vs never)
4) Squamous (or near equivalent)
5) Results complete enough for use in metaanalysis

Within each study, results are then selected (in the following order of preference, within each sex) for:
6) PRODUCT: cigarettes only
7) CIGTYPE: all/unspecified, MC regardless of HR, MC only
8) (not applicable)
9) DENOM: never smoked anything, never smoked cigarettes, never any + low, never cigs + low
10) Followup period (YF, prospective studies): whole study (coded as 0) or longest available
11) LCtype: squamous or nearest available, but not adeno. (q = squamous, s = small,
a = adeno, KI = Kreyberg I, u = undifferentiated)
12) Race: all or nearest available, otherwise by race (wh or w = white, bl or b = black, hi = hispanic
ch = chinese, jap = japanese, haw = hawaiian, w+o = white + oriental, sca = scandinavian, as = asian)
13) Years quit (vs never) "high" in key scheme 1 (key value 3, maximum range 1-6)
14) For overlapping studies: principal rather than subsidiary studies
Finally by Age: whole study (coded as 0) if available, otherwise by widest available age group
and then for single sex results (m, f) in preference to results for both sexes combined (c).

Results adjusted (AD) for the most potential confounders are then chosen in Sections -1 to -3
(and those which actually differ from the adjusted results in Table 2J4 - 1 are marked 'x' in Section -1)
and results adjusted for the least confounders in Sections -4 to -6. (Those least adjusted results which
actually differ from the most adjusted are marked 'x' in column X in Section -4)

Section -7 shows excluded studies, together with the stage (as above) at which no qualifying
results were found.

Section -8 lists the potentially overlapping studies which have been included (1=principal, 2=subsidiary).

Section -9 lists any results which would have been included in preference except that they had data not complete
enough for use in meta-analysis, with their significance (yes/no), if known, and any further comment as entered
on the database. It also lists as "gap" any categories for which no data were presented by the original authors.
This is commonly due to recent quitters having been combined with current smokers

In addition to those mentioned above, the following fields, levels and abbreviations are used:
* or nk = not known, n = no, y = yes, ot = other
nev = never
all/unspec = all or unspecified, MC = manufactured cigarettes, HR = hand-rolled cigarettes
exL, exH = range of exposure (low and high) in the smoking group, in terms of Years quit (vs never)
REF: 6-character study reference
NRR: number of the RR on the database within the study
ST : study type (CC = case control, pr or prosp = prospective)
NLC: number of lung cancer cases in whole study
R : risky occupational population (n = no, m = mining, o = other risky)
VB : national cigarette type (V = at least 75% Virginia, bl = at least 75% blended, ot = other)
P : any proxy use
H : full histological confirmation
De : derivation of RR/CI (or = original, st = standard method, ot = other method of estimation)

Table 2J14 - 1

IESLC - Meta-analysis of Ex Smoking, Years quit (vs never), "High"
Squamous, Cigarettes only
Most adjusted

| REF | NRR | 2J4 | SEX | AGEL | AGEH | RACE | YF | LC TYPE | LOC | START | ST | NLC | R | VB | P | H | AD | PRODUCT | exL | exH | DENOM | De |
|---|---|---|---|---|---|---|---|---|---|---|---|---|---|---|---|---|---|---|---|---|---|---|
| BENHAM | 531 | x | m | 0 | 0 | all | - | KI | Eu:wst | 1976 | CC | 1625 | n | bl | n | y | 0 | cig only | 1.0 | 3 | nev any | st |

Cigarette type is all/unspec for all RRs

Table 2J14 - 2

IESLC - Meta-analysis of Ex Smoking, Years quit (vs never), "High"
Squamous, Cigarettes only
Most adjusted

| REF | NRR | SEX | AD | Number Case | Exposed Cont | Non-exposed Case | Cont | RR | 95.00%CI |
|---|---|---|---|---|---|---|---|---|---|
| BENHAM | 531 | m | 0 | 132 | 77 | 24 | 481 | 34.36 | ( 20.90- 56.48) |
| Totals | | | | 132 | 77 | 24 | 481 | | |

*prospective study

| REF | NRR | SEX | AD | Ys | Ws | Qs | Ps |
|---|---|---|---|---|---|---|---|
| BENHAM | 531 | m | 0 | 3.54 | 15.55 | 0.00 | 0.0000 |

| | | |
|---|---|---|
| | N | 1 |
| | NS | 1 |
| | Wt | 15.55 |
| Het | Chi | 0.00 |
| Het | df | 0 |
| Het | P | N.S. |
| Fixed | RR | 34.36 |
| | RRl | 20.90 |
| | RRu | 56.48 |
| | P | +++ |
| Random | RR | 34.36 |
| | RRl | 20.90 |
| | RRu | 56.48 |
| | P | +++ |
| Asymm | P | |

Table 2J14 - 3

IESLC - Meta-analysis of Ex Smoking, Years quit (vs never), "High"
Squamous, Cigarettes only
Most adjusted

| | Sex combined | male | female | Total |
|---|---|---|---|---|
| N | | 1 | | 1 |
| NS | | 1 | | 1 |
| Wt | | 15.55 | | 15.55 |
| Het Chi | | 0.00 | | 0.00 |
| Het df | | 0 | | 0 |
| Het P | | N.S. | | N.S. |
| Fixed RR | | 34.36 | | 34.36 |
| RRl | | 20.90 | | 20.90 |
| RRu | | 56.48 | | 56.48 |
| P | | +++ | | +++ |
| Random RR | | 34.36 | | 34.36 |
| RRl | | 20.90 | | 20.90 |
| RRu | | 56.48 | | 56.48 |
| P | | +++ | | +++ |
| Between Chi | | | | |
| Between df | | | | |
| Between P | | | | N.S. |
| Btwn(F) P | | | | N.S. |
| Btwn(R) P | | | | N.S. |

Too few RRs for analysis by factor

Table 2J14 - 4

IESLC - Meta-analysis of Ex Smoking, Years quit (vs never), "High"
Squamous, Cigarettes only
Least adjusted

| REF | NRR | X | SEX | AGEL | AGEH | RACE | YF | LC TYPE | LOC | START | ST | NLC | R | VB | P | H | AD | PRODUCT | exL | exH | DENOM | De |
|---|---|---|---|---|---|---|---|---|---|---|---|---|---|---|---|---|---|---|---|---|---|---|
| BENHAM | 531 | | m | 0 | 0 | all | - | KI | Eu:wst | 1976 | CC | 1625 | n | bl | n | y | 0 | cig only | 1.0 | 3 | nev any | st |

Cigarette type is all/unspec for all RRs

Table 2J14 - 5

IESLC - Meta-analysis of Ex Smoking, Years quit (vs never), "High"
Squamous, Cigarettes only
Least adjusted

| REF | NRR | SEX | AD | Number Case | Exposed Cont | Non-exposed Case | Cont | RR | 95.00%CI |
|---|---|---|---|---|---|---|---|---|---|
| BENHAM | 531 | m | 0 | 132 | 77 | 24 | 481 | 34.36 | ( 20.90- 56.48) |
| Totals | | | | 132 | 77 | 24 | 481 | | |

*prospective study

| REF | NRR | SEX | AD | Ys | Ws | Qs | Ps |
|---|---|---|---|---|---|---|---|
| BENHAM | 531 | m | 0 | 3.54 | 15.55 | 0.00 | 0.0000 |

| | | |
|---|---|---|
| | N | 1 |
| | NS | 1 |
| | Wt | 15.55 |
| Het | Chi | 0.00 |
| Het | df | 0 |
| Het | P | N.S. |
| Fixed | RR | 34.36 |
| | RRl | 20.90 |
| | RRu | 56.48 |
| | P | +++ |
| Random | RR | 34.36 |
| | RRl | 20.90 |
| | RRu | 56.48 |
| | P | +++ |
| Asymm | P | |

Table 2J14 - 6

IESLC - Meta-analysis of Ex Smoking, Years quit (vs never), "High"
Squamous, Cigarettes only
Least adjusted

| | Sex combined | male | female | Total |
|---|---|---|---|---|
| N | | 1 | | 1 |
| NS | | 1 | | 1 |
| Wt | | 15.55 | | 15.55 |
| Het Chi | | 0.00 | | 0.00 |
| Het df | | 0 | | 0 |
| Het P | | N.S. | | N.S. |
| Fixed RR | | 34.36 | | 34.36 |
| RRl | | 20.90 | | 20.90 |
| RRu | | 56.48 | | 56.48 |
| P | | +++ | | +++ |
| Random RR | | 34.36 | | 34.36 |
| RRl | | 20.90 | | 20.90 |
| RRu | | 56.48 | | 56.48 |
| P | | +++ | | +++ |
| Between Chi | | | | |
| Between df | | | | |
| Between P | | | | N.S. |
| Btwn(F) P | | | | N.S. |
| Btwn(R) P | | | | N.S. |

Table 2J14 - 7

IESLC - Meta-analysis of Ex Smoking, Years quit (vs never), "High"
Squamous, Cigarettes only
Excluded studies (and stage at which they were excluded)

| Stage | Studies |
|---|---|
| 1 | AGUDO AKIBA AMANDU AMES AXELSS BEST BOUCHA BOUCOT BRESLO CHEN CHEN2 CHIAZZ DEAN2 DOSEME ENGELA FAN GENG GER GUO HAENSZ HEGMAN HOLE HU HU2 JUSSAW KATSOU KAUFMA KOO KOULUM KREUZE LETOUR LEVIN LIAW LIU3 LIU4 LIU5 MCCONN MIGRAN MRFITR NOTAN2 OSANN2 PERNU QIAO2 RACHTA RESTRE SADOWS SEGI2 STASZE TIZZAN VUTUC WATSON WU WUWILL WYNDE2 WYNDE8 XU YUAN ZHANG ZHENG ZHOU |
| 2 | BUFFLE HUMBLE PISANI PRESCO WYNDE7 |
| 3 | MCDUFF SPITZ |
| 4 | ARMADA AUVINE BECHER BENSHL BLOT1 BOFFET BROSS CARPEN CEDERL CHOI CHYOU CORREA CPSI CPSII DAMBER DARBY DEAN3 DESTEF DOLL DOLL2 DORGAN DORN GAO GAO2 GARCIA GARSHI GILLIS GRAHAM GURSEL HAMMO2 HIRAYA JOLY KAISE2 KHUDER LAUSSM LUBIN PEZZO2 QIAO SPEIZE SUZUK2 TVERDA WANG2 WIGLE WU2 |
| 5 | ALDERS HAMMON |
| 7 | BARBON BROWN3 JAHN JAIN JEDRYC LUBIN2 LUO MATOS SOBUE SVENSS WAKAI WYNDE3 WYNDE6 |
| 14 | PEZZOT |

Table 2J14 - 8
Potentially overlapping studies

| REF | REFGP | PRINC | . | OVERLAP/LINK |
|---|---|---|---|---|
| BENHAM | LUBIN2 | 2 | | Subset of Lubin2 |

Table 2J15 -

IESLC - Meta-analysis of Ex Smoking, Years quit (vs never), "Highest vs lowest"
Squamous, Cigarettes only

This analysis is restricted to results for:
1) Ex smokers
2) Results by Years quit (vs never)
3) Categorical results by Years quit (vs never)
4) Denominator (unexposed) = "low"
5) Squamous (or near equivalent)
6) Results complete enough for use in metaanalysis

Within each study, results are then selected (in the following order of preference, within each sex) for:
7) (not applicable)
8) PRODUCT: cigarettes only
9) CIGTYPE: all/unspecified, MC regardless of HR, MC only
10) Results with least adjustment for other aspects of smoking (ADOS)
11) The highest vs lowest category
12) Followup period (YF, prospective studies): whole study (coded as 0) or longest available
13) LCtype: squamous or nearest available, but not adeno. (q = squamous, s = small,
a = adeno, KI = Kreyberg I, u = undifferentiated)
14) Race: all or nearest available, otherwise by race (wh or w = white, bl or b = black, hi = hispanic
ch = chinese, jap = japanese, haw = hawaiian, w+o = white + oriental, sca = scandinavian, as = asian)
15) For overlapping studies: principal rather than subsidiary studies
Finally by Age: whole study (coded as 0) if available, otherwise by widest available age group
and then for single sex results (m, f) in preference to results for both sexes combined (c).

Results adjusted (AD) for the most potential confounders are then chosen in Sections -1 to -3
(and those which actually differ from the adjusted results in Table 2J5 - 1 are marked 'x' in Section -1)
and results adjusted for the least confounders in Sections -4 to -6. (Those least adjusted results which
actually differ from the most adjusted are marked 'x' in column X in Section -4)

Section -7 shows excluded studies, together with the stage (as above) at which no qualifying
results were found.

Section -8 lists the potentially overlapping studies which have been included (1=principal, 2=subsidiary).

Section -9 lists any results which would have been included in preference except that they had data not complete
enough for use in meta-analysis, with their significance (yes/no), if known, and any further comment as entered
on the database. It also lists as "gap" any categories for which no data were presented by the original authors.
This is commonly due to recent quitters having been combined with current smokers

In addition to those mentioned above, the following fields, levels and abbreviations are used:
* or nk = not known, n = no, y = yes, ot = other
all/unspec = all or unspecified, MC = manufactured cigarettes, HR = hand-rolled cigarettes
exL, exH = range of exposure (low and high) in the "highest" group, in terms of Years quit (vs never)
unexL, unexH = range of exposure (low and high) in the "lowest" group, in terms of Years quit (vs never)
REF: 6-character study reference
NRR: number of the RR on the database within the study
ST : study type (CC = case control, pr or prosp = prospective)
NLC: number of lung cancer cases in whole study
R : risky occupational population (n = no, m = mining, o = other risky)
VB : national cigarette type (V = at least 75% Virginia, bl = at least 75% blended, ot = other)
P : any proxy use
H : full histological confirmation
De : derivation of RR/CI (or = original, st = standard method, ot = other method of estimation)

Table 2J15 - 1

IESLC - Meta-analysis of Ex Smoking, Years quit (vs never), "Highest vs lowest"
Squamous, Cigarettes only
Most adjusted

| REF | NRR | 2J5 | SEX | AGEL | AGEH | RACE | YF | LC TYPE | LOC | START | ST | NLC | R | VB | P | H | AD | ADOS | PRODUCT | exL | exH | unexL | unexH | De |
|---|---|---|---|---|---|---|---|---|---|---|---|---|---|---|---|---|---|---|---|---|---|---|---|---|
| BENHAM | 537 | x | m | 0 | 0 | all | - | KI | Eu:wst | 1976 | CC | 1625 | n | bl | n | y | 0 | 0 | cig only | 1.0 | 3 | 20 | 999 | st |
| PEZZOT | 581 | | m | 0 | 0 | all | - | q | SCAmer | 1987 | CC | 215 | n | bl | n | y | 0 | 0 | cig only | 1.0 | 10 | 11 | 999 | st |

Cigarette type is all/unspec for all RRs

Table 2J15 - 2

IESLC - Meta-analysis of Ex Smoking, Years quit (vs never), "Highest vs lowest"
Squamous, Cigarettes only
Most adjusted

| REF | NRR | SEX | AD | Number Exposed Case | Cont | Non-exposed Case | Cont | RR | 95.00%CI |
|---|---|---|---|---|---|---|---|---|---|
| BENHAM | 537 | m | 0 | 132 | 77 | 17 | 82 | 8.27 | ( 4.57- 14.96) |
| PEZZOT | 581 | m | 0 | 21 | 27 | 8 | 48 | 4.67 | ( 1.82- 11.96) |
| Totals | | | | 153 | 104 | 25 | 130 | | |

*prospective study

| REF | NRR | SEX | AD | Ys | Ws | Qs | Ps |
|---|---|---|---|---|---|---|---|
| BENHAM | 537 | m | 0 | 2.11 | 10.92 | 0.29 | 0.0000 |
| PEZZOT | 581 | m | 0 | 1.54 | 4.34 | 0.73 | 0.0013 |

| | | |
|---|---|---|
| | N | 2 |
| | NS | 2 |
| | Wt | 15.26 |
| Het | Chi | 1.02 |
| Het | df | 1 |
| Het | P | N.S. |
| Fixed | RR | 7.03 |
| | RRl | 4.25 |
| | RRu | 11.61 |
| | P | +++ |
| Random | RR | 7.01 |
| | RRl | 4.22 |
| | RRu | 11.65 |
| | P | +++ |
| Asymm | P | |

Table 2J15 - 3

IESLC - Meta-analysis of Ex Smoking, Years quit (vs never), "Highest vs lowest"
Squamous, Cigarettes only
Most adjusted

| | Sex combined | male | female | Total |
|---|---|---|---|---|
| N | | 2 | | 2 |
| NS | | 2 | | 2 |
| Wt | | 15.26 | | 15.26 |
| Het Chi | | 1.02 | | 1.02 |
| Het df | | 1 | | 1 |
| Het P | | N.S. | | N.S. |
| Fixed RR | | 7.03 | | 7.03 |
| RRl | | 4.25 | | 4.25 |
| RRu | | 11.61 | | 11.61 |
| P | | +++ | | +++ |
| Random RR | | 7.01 | | 7.01 |
| RRl | | 4.22 | | 4.22 |
| RRu | | 11.65 | | 11.65 |
| P | | +++ | | +++ |
| Between Chi | | | | |
| Between df | | | | |
| Between P | | | | N.S. |
| Btwn(F) P | | | | N.S. |
| Btwn(R) P | | | | N.S. |

Too few RRs for analysis by factor

Table 2J15 - 4

IESLC - Meta-analysis of Ex Smoking, Years quit (vs never), "Highest vs lowest"
Squamous, Cigarettes only
Least adjusted

| REF | NRR | X | SEX | AGEL | AGEH | RACE | YF | LC TYPE | LOC | START | ST | NLC | R | VB | P | H | AD | ADOS | PRODUCT | exL | exH | unexL | unexH | De |
|---|---|---|---|---|---|---|---|---|---|---|---|---|---|---|---|---|---|---|---|---|---|---|---|---|
| BENHAM | 537 | | m | 0 | 0 | all | - | KI | Eu:wst | 1976 | CC | 1625 | n | bl | n | y | 0 | 0 | cig only | 1.0 | 3 | 20 | 999 | st |
| PEZZOT | 581 | | m | 0 | 0 | all | - | q | SCAmer | 1987 | CC | 215 | n | bl | n | y | 0 | 0 | cig only | 1.0 | 10 | 11 | 999 | st |

Cigarette type is all/unspec for all RRs

Table 2J15 - 5

IESLC - Meta-analysis of Ex Smoking, Years quit (vs never), "Highest vs lowest"
Squamous, Cigarettes only
Least adjusted

| REF | NRR | SEX | AD | Number Exposed Case | Cont | Non-exposed Case | Cont | RR | 95.00%CI |
|---|---|---|---|---|---|---|---|---|---|
| BENHAM | 537 | m | 0 | 132 | 77 | 17 | 82 | 8.27 | ( 4.57- 14.96) |
| PEZZOT | 581 | m | 0 | 21 | 27 | 8 | 48 | 4.67 | ( 1.82- 11.96) |
| Totals | | | | 153 | 104 | 25 | 130 | | |

*prospective study

| REF | NRR | SEX | AD | Ys | Ws | Qs | Ps |
|---|---|---|---|---|---|---|---|
| BENHAM | 537 | m | 0 | 2.11 | 10.92 | 0.29 | 0.0000 |
| PEZZOT | 581 | m | 0 | 1.54 | 4.34 | 0.73 | 0.0013 |

| | | |
|---|---|---|
| | N | 2 |
| | NS | 2 |
| | Wt | 15.26 |
| Het | Chi | 1.02 |
| Het | df | 1 |
| Het | P | N.S. |
| Fixed | RR | 7.03 |
| | RRl | 4.25 |
| | RRu | 11.61 |
| | P | +++ |
| Random | RR | 7.01 |
| | RRl | 4.22 |
| | RRu | 11.65 |
| | P | +++ |
| Asymm | P | |

Table 2J15 - 6

IESLC - Meta-analysis of Ex Smoking, Years quit (vs never), "Highest vs lowest"
Squamous, Cigarettes only
Least adjusted

| | Sex combined | male | female | Total |
|---|---|---|---|---|
| N | | 2 | | 2 |
| NS | | 2 | | 2 |
| Wt | | 15.26 | | 15.26 |
| Het Chi | | 1.02 | | 1.02 |
| Het df | | 1 | | 1 |
| Het P | | N.S. | | N.S. |
| Fixed RR | | 7.03 | | 7.03 |
| RRl | | 4.25 | | 4.25 |
| RRu | | 11.61 | | 11.61 |
| P | | +++ | | +++ |
| Random RR | | 7.01 | | 7.01 |
| RRl | | 4.22 | | 4.22 |
| RRu | | 11.65 | | 11.65 |
| P | | +++ | | +++ |
| Between Chi | | | | |
| Between df | | | | |
| Between P | | | | N.S. |
| Btwn(F) P | | | | N.S. |
| Btwn(R) P | | | | N.S. |

Table 2J15 - 7

IESLC - Meta-analysis of Ex Smoking, Years quit (vs never), "Highest vs lowest"
Squamous, Cigarettes only
Excluded studies (and stage at which they were excluded)

| Stage | Studies |
|---|---|
| 1 | AGUDO AKIBA AMANDU AMES AXELSS BEST BOUCHA BOUCOT BRESLO CHEN CHEN2 CHIAZZ DEAN2 DOSEME ENGELA FAN GENG GER GUO HAENSZ HEGMAN HOLE HU HU2 JUSSAW KATSOU KAUFMA KOO KOULUM KREUZE LETOUR LEVIN LIAW LIU3 LIU4 LIU5 MCCONN MIGRAN MRFITR NOTAN2 OSANN2 PERNU QIAO2 RACHTA RESTRE SADOWS SEGI2 STASZE TIZZAN VUTUC WATSON WU WUWILL WYNDE2 WYNDE8 XU YUAN ZHANG ZHENG ZHOU |
| 2 | BUFFLE HUMBLE PISANI PRESCO WYNDE7 |
| 3 | MCDUFF SPITZ |
| 4 | AUVINE BLOT1 BROWN3 GURSEL LAUSSM LUO WU2 |
| 5 | ARMADA BECHER BENSHL BOFFET BROSS CARPEN CEDERL CHOI CHYOU CORREA CPSI CPSII DAMBER DARBY DEAN3 DESTEF DOLL DOLL2 DORGAN DORN GAO GAO2 GARCIA GARSHI GILLIS GRAHAM HAMMO2 HIRAYA JOLY KAISE2 KHUDER LUBIN PEZZO2 QIAO SPEIZE SUZUK2 TVERDA WANG2 WIGLE |
| 6 | ALDERS HAMMON |
| 8 | BARBON JAHN JAIN JEDRYC LUBIN2 MATOS SOBUE SVENSS WAKAI WYNDE3 WYNDE6 |

Table 2J15 - 8

Potentially overlapping studies

| REF | REFGP | PRINC | . | OVERLAP/LINK |
|---|---|---|---|---|
| BENHAM | LUBIN2 | 2 | | Subset of Lubin2 |

Table 2J15 - 9

Most adjusted - insufficient data for meta-analysis

| REF | NRR | SEX | AGEL | AGEH | RACE | YF | LC TYPE | LOC | START | ST | NLC | R | VB | P | H | AD | ADOS | PRODUCT | exL | exH | unexL | unexH | De |
|---|---|---|---|---|---|---|---|---|---|---|---|---|---|---|---|---|---|---|---|---|---|---|---|
| ALDERS | 542 | m | 0 | 0 | all | - | q+s | Eu:UK | 1977 | CC | 1448 | n | V | n | n | 1 | 0 | cig only | 0.1 | 2 | 10 | 999 | st |
| ALDERS | 553 | f | 0 | 0 | all | - | q+s | Eu:UK | 1977 | CC | 1448 | n | V | n | n | 1 | 0 | cig only | 0.1 | 2 | 10 | 999 | st |
| BENHAM | 538 | m | 0 | 0 | all | - | KI | Eu:wst | 1976 | CC | 1625 | n | bl | n | y | 0 | 0 | cig only | 0.1 | 0.9 | 20 | 999 | ot |
| HAMMON | 506 | m | 0 | 0 | wh | 0 | not a | NAmer | 1952 | pr | 448 | n | bl | n | n | 1 | 0 | cig only | 0.1 | 0.9 | 10 | 999 | st |
| PEZZOT | 600 | m | 0 | 0 | all | - | q | SCAmer | 1987 | CC | 215 | n | bl | n | y | 0 | 0 | cig only | 0.1 | 0.9 | 11 | 999 | ot |

| REF | NRR | RR | SIG | RRDATA comment |
|---|---|---|---|---|
| ALDERS | 542 | 10.00 | | 0 |
| ALDERS | 553 | 25.17 | | 0 |
| BENHAM | 538 | * | gap | 0 |
| HAMMON | 506 | * | | RR for <1 pack per day is 6.76, while that for 1+ packs is 3.27 |
| PEZZOT | 600 | * | gap | 0 |
