# Supplementary material for: Systematic review with meta-analysis of the epidemiological evidence in the 1900s relating smoking to lung cancer
Source: BMC Cancer. 2012 Sep 3;12:385. doi: 10.1186/1471-2407-12-385 (PMC3505152; doi:10.1186/1471-2407-12-385)
Supplement: Additional file 5 — Detailed Analysis Tables (Individual file names as described in Additional file 1: Methods, Table1). [file 1471-2407-12-385-S5.zip › PDF/1E.pdf]

Table 1E1 -

IESLC - Meta-analysis of Ever Smoking, Pipe and/or Cigars (not cigs)
All LC types

This analysis is restricted to results for:
1) Non-dose-response data
2) Smokers of pipe and/or cigars (but not cigarettes)
3) Ever smokers
4) Results complete enough for use in metaanalysis

Within each study, results are then selected (in the following order of preference, within each sex) for:
5) DENOM: never smoked anything, (never +1 = +long term ex)
6) Followup period (prospective studies): whole study (coded as 0) or longest available
7) LCtype: all or nearest available, at least Squamous and Adeno. (q = squamous, s = small,
l = large, a = adeno, mix = mixed, alv = alveolar)
8) Race: all or nearest available, otherwise by race (wh or w = white, bl or b = black, hi = hispanic
ch = chinese, jap = japanese, haw = hawaiian, w+o = white + oriental, sca = scandinavian, as = asian)
9) For overlapping studies: principal rather than subsidiary studies
Finally by Age: whole study (coded as 0) if available, otherwise by widest available age group
and then for single sex results (m, f) in preference to combined sex results (c).

Results adjusted (AD) for the most potential confounders are then chosen in Sections -1 to -3
and results adjusted for the least confounders in Sections -4 to -6. (Those least adjusted results which
actually differ from the most adjusted as marked 'x' in column X in Section -4)
(Results adjusted for an unknown number of confounder(s) are coded as 20.)

Section -7 shows excluded studies, together with the stage (as above) at which no qualifying
results were found.

Section -8 lists the potentially overlapping studies which have been included (1=principal, 2=subsidiary).

Section -9 lists any results which would have been included in preference except that they had data not complete
enough for use in meta-analysis, with their significance (yes/no), if known, and any further comment as entered
on the database.

In addition to those mentioned above, the following fields, levels and abbreviations are used:
* or nk = not known, n = no, y = yes, ot = other
nev = never
REF: 6-character study reference
NRR: number of the RR on the database within the study
ST : study type (CC = case control, pr or prosp = prospective)
NLC: number of lung cancer cases in whole study
R : risky occupational population (n = no, m = mining, o = other risky)
VB : national cigarette type (V = at least 75% Virginia, bl = at least 75% blended, ot = other)
P : any proxy use
H : full histological confirmation
De : derivation of RR/CI (or = original, st = standard method, ot = other method of estimation)

Table 1E1 - 1

IESLC - Meta-analysis of Ever Smoking, Pipe and/or Cigars (not cigs)
All LC types
Most adjusted

REF	NRR	SEX	AGEL	AGEH	RACE	YF	LC TYPE	LOC	START	ST	NLC	R	VB	P	H	AD	DENOM	De
ABELIN	46	m	0	0	all	-	all	Eu:wst	1941	CC	118	n	bl	y	n	1	nev any	st
ALDERS	1	m	0	0	all	-	all	Eu:UK	1977	CC	1448	n	V	n	n	1	nev any	ot
ARMADA	30	m	0	0	all	-	all	Eu:wst	1986	CC	325	n	bl	n	y	0	nev any	st
BECHER	23	m	0	0	all	-	all	Eu:Ger	1985	CC	194	n	bl	n	y	2	nev any	or
BEST	19	m	55	79	all	3	all	NAmer	1955	pr	381	n	V	n	n	0	nev any	st
BOFFET	30	m	0	0	all	-	all	Eu:mul	1988	CC	5621	n	bl	y	n	0	nev any	st
BOUCOT	117	m	0	0	all	0	all	NAmer	1951	pr	121	n	bl	n	n	2	nev any	ot
BRESLO	33	m	0	0	all	-	all	NAmer	1949	CC	518	n	bl	n	y	0	nev+1	st
CHOW	8	m	0	0	wh	0	all	NAmer	1966	pr	219	n	bl	n	n	2	nev any	ot
COMSTO	1	m	0	0	all	-	all	NAmer	1975	ot	258	n	bl	n	n	0	nev any	st
CPSI	186	m	35	84	all	6	all	NAmer	1959	pr	5138	n	bl	n	n	1	nev any	ot
DAMBER	28	m	0	0	all	-	all	Eu:Sca	1972	CC	579	n	bl	y	n	0	nev any	st
DEAN	11	m	0	0	wh	-	all	Africa	1947	CC	603	n	V	y	n	0	nev any	st
DEAN2	11	m	0	0	all	-	all	Eu:UK	1960	CC	954	n	V	y	n	0	nev any	st
DOLL2	33	m	0	0	all	10	all	Eu:UK	1951	pr	920	n	V	n	n	1	nev any	ot
DORGAN	1	m	0	0	wh	-	all	NAmer	1980	CC	2026	n	bl	y	y	0	nev any	st
DORGAN	25	m	0	0	bl	-	all	NAmer	1980	CC	2026	n	bl	y	y	0	nev any	st
DORN	35	m	0	0	wh	2	all	NAmer	1954	pr	5097	n	bl	n	n	1	nev any	ot
GOLLED	5	m	35	99	all	-	all	Eu:UK	1952	CC	443	n	V	y	n	1	nev any	ot
GRAHAM	11	m	0	0	wh	-	all	NAmer	1956	CC	685	n	bl	n	n	1	nev any	ot
HAMMO2	14	m	0	0	all	0	all	NAmer	1967	pr	450	o	bl	n	n	1	nev any	ot
HAMMON	118	m	0	0	wh	0	all	NAmer	1952	pr	448	n	bl	n	n	1	nev any	ot
KJUUS	4	m	0	0	all	-	all	Eu:Sca	1979	CC	176	n	bl	n	n	0	nev any	st
LEVIN	31	m	0	0	all	-	all	NAmer	1938	CC	475	n	bl	n	n	1	nev any	st
LOMBAR	1	m	0	0	all	-	all	NAmer	1951	CC	1040	n	bl	n	n	0	nev any	st
LUBIN2	50	m	0	0	all	-	all	Eu:mul	1976	CC	7804	n	bl	n	y	2	nev any	ot
MARSH	5	m	0	0	all	-	all	NAmer	1979	CC	150	n	bl	y	n	0	nev any	ot
MARSH2	3	c	0	0	all	-	all	NAmer	1979	CC	114	n	bl	y	n	0	nev any	ot
SADOWS	70	m	0	0	wh	-	all	NAmer	1938	CC	477	n	bl	n	n	0	nev any	st
STASZE	3	m	0	0	all	-	all	Eu:est	1954	CC	281	n	bl	n	y	0	nev any	st
STOCKW	5	c	0	0	all	-	all	NAmer	1981	CC	22161	n	bl	n	n	0	nev any	st
TOUSEY	9	m	0	0	all	-	all	NAmer	1993	CC	507	n	bl	y	y	3	nev any	or
TOUSEY	5	f	0	0	all	-	all	NAmer	1993	CC	507	n	bl	y	y	0	nev any	st
WIGLE	14	m	0	0	all	-	all	NAmer	1971	CC	728	n	V	n	n	0	nev any	st
WIGLE	17	f	0	0	all	-	all	NAmer	1971	CC	728	n	V	n	n	0	nev any	ot
WYNDE2	15	m	0	0	all	-	all	NAmer	1962	CC	404	n	bl	n	y	0	nev any	st
WYNDE3	152	m	0	0	all	-	all	NAmer	1966	CC	350	n	bl	n	y	0	nev any	st
WYNDE6	63	m	0	0	all	-	all	NAmer	1969	CC	4423	n	bl	n	y	0	nev any	st

Table 1E1 - 2

IESLC - Meta-analysis of Ever Smoking, Pipe and/or Cigars (not cigs)
All LC types
Most adjusted

REF	NRR	SEX	AD	Number Exposed Case	Cont	Non-exposed Case	Cont	RR	95.00%CI
ABELIN	46	m	1	-	-	-	-	30.49 (	7.32- 127.00)
ALDERS	1	m	1	-	-	-	-	3.82 (	1.74- 8.40)
ARMADA	30	m	0	4	7	4	64	9.14 (	1.86- 44.85)
BECHER	23	m	2	-	-	-	-	4.62 (	1.15- 18.56)
*BEST	19	m	0	8	2972	1	2854	7.68 (	0.96- 61.38)
BOFFET	30	m	0	118	266	117	1750	6.64 (	4.98- 8.84)
*BOUCOT	117	m	2	-	-	-	-	6.80 (	0.37- 126.25)
BRESLO	33	m	0	15	68	7	42	1.32 (	0.50- 3.51)
*CHOW	8	m	2	-	-	-	-	3.95 (	1.18- 13.26)
COMSTO	1	m	0	2	15	4	69	2.30 (	0.39- 13.73)
*CPSI	186	m	1	-	-	-	-	1.94 (	1.44- 2.62)
DAMBER	28	m	0	205	149	42	208	6.81 (	4.60- 10.09)
DEAN	11	m	0	51	74	12	61	3.50 (	1.71- 7.16)
DEAN2	11	m	0	81	128	33	112	2.15 (	1.33- 3.46)
*DOLL2	33	m	1	-	-	-	-	6.14 (	1.85- 20.40)
DORGAN	1	m	0	22	55	15	93	2.48 (	1.19- 5.18)
DORGAN	25	m	0	3	19	3	35	1.84 (	0.34- 10.03)
Subtotal DORGAN								2.37 (	1.20- 4.65)
*DORN	35	m	1	-	-	-	-	1.60 (	0.81- 3.16)
GOLLED	5	m	1	-	-	-	-	3.13 (	1.73- 5.67)
GRAHAM	11	m	1	-	-	-	-	2.55 (	1.49- 4.38)
*HAMMO2	14	m	1	-	-	-	-	0.88 (	0.21- 3.67)
*HAMMON	118	m	1	-	-	-	-	1.55 (	0.83- 2.90)
KJUUS	4	m	0	23	25	2	24	11.04 (	2.34- 52.00)
LEVIN	31	m	1	-	-	-	-	1.31 (	0.81- 2.12)
LOMBAR	1	m	0	48	146	14	112	2.63 (	1.38- 5.01)
LUBIN2	50	m	2	-	-	-	-	3.14 (	2.41- 4.08)
MARSH	5	m	0	0	5	2	31	1.15~(	0.05- 27.23)
MARSH2	3	c	0	0	4	12	56	0.50~(	0.03- 9.94)
SADOWS	70	m	0	38	88	18	81	1.94 (	1.03- 3.67)
STASZE	3	m	0	4	101	5	158	1.25 (	0.33- 4.77)
STOCKW	5	c	0	715	655	2791	10641	4.16 (	3.71- 4.66)
TOUSEY	9	m	3	-	-	-	-	2.80 (	0.70- 11.60)
TOUSEY	5	f	0	1	2	13	226	8.69 (	0.74- 102.22)
Subtotal TOUSEY								3.70 (	1.09- 12.52)
WIGLE	14	m	0	30	113	15	204	3.61 (	1.86- 6.99)
WIGLE	17	f	0	1	0	36	439	36.12~(	1.45- 902.62)
Subtotal WIGLE								3.96 (	2.07- 7.57)
WYNDE2	15	m	0	14	104	8	105	1.77 (	0.71- 4.39)
WYNDE3	152	m	0	14	68	9	88	2.01 (	0.82- 4.93)
WYNDE6	63	m	0	58	199	87	617	2.07 (	1.43- 2.99)
Partial Totals				1455	5263	3250	18070		

*prospective study ~ With 0.5 adjustment for zero

REF	NRR	SEX	AD	Ys	Ws	Qs	Ps
ABELIN	46	m	1	3.42	1.89	8.94	0.0000
ALDERS	1	m	1	1.34	6.20	0.06	0.0008
ARMADA	30	m	0	2.21	1.52	1.44	0.0064
BECHER	23	m	2	1.53	1.99	0.17	0.0310
*BEST	19	m	0	2.04	0.89	0.57	0.0545
BOFFET	30	m	0	1.89	46.83	19.91	0.0000
*BOUCOT	117	m	2	1.92	0.45	0.21	0.1976
BRESLO	33	m	0	0.28	4.03	3.72	0.5736
*CHOW	8	m	2	1.37	2.63	0.05	0.0260
COMSTO	1	m	0	0.83	1.20	0.20	0.3609
*CPSI	186	m	1	0.66	42.89	14.32	0.0000
DAMBER	28	m	0	1.92	24.87	11.45	0.0000
DEAN	11	m	0	1.25	7.53	0.00	0.0006
DEAN2	11	m	0	0.76	16.84	3.82	0.0017
*DOLL2	33	m	1	1.81	2.67	0.88	0.0030
DORGAN	1	m	0	0.91	7.09	0.78	0.0156
DORGAN	25	m	0	0.61	1.34	0.53	0.4799
Subtotal DORGAN				0.86	8.43	1.31	
*DORN	35	m	1	0.47	8.29	4.92	0.1759
GOLLED	5	m	1	1.14	10.90	0.11	0.0002
GRAHAM	11	m	1	0.94	13.22	1.22	0.0007
*HAMMO2	14	m	1	-0.13	1.88	3.51	0.8610
*HAMMON	118	m	1	0.44	9.82	6.32	0.1697
KJUUS	4	m	0	2.40	1.60	2.16	0.0024
LEVIN	31	m	1	0.27	16.60	15.63	0.2713

Table 1E1 - 2

IESLC - Meta-analysis of Ever Smoking, Pipe and/or Cigars (not cigs)
All LC types
Most adjusted

REF	NRR	SEX	AD	Ys	Ws	Qs	Ps
LOMBAR	1	m	0	0.97	9.26	0.69	0.0033
LUBIN2	50	m	2	1.14	55.44	0.51	0.0000
MARSH	5	m	0	0.14	0.38	0.47	0.9331
MARSH2	3	c	0	-0.69	0.43	1.60	0.6511
SADOWS	70	m	0	0.66	9.47	3.14	0.0409
STASZE	3	m	0	0.22	2.14	2.21	0.7425
STOCKW	5	c	0	1.43	296.07	10.19	0.0000
TOUSEY	9	m	3	1.03	1.95	0.09	0.1506
TOUSEY	5	f	0	2.16	0.63	0.54	0.0855
Subtotal TOUSEY				1.31	2.58	0.62	
WIGLE	14	m	0	1.28	8.79	0.02	0.0001
WIGLE	17	f	0	3.59	0.37	2.04	0.0289
Subtotal WIGLE				1.38	9.16	2.06	
WYNDE2	15	m	0	0.57	4.64	2.09	0.2202
WYNDE3	152	m	0	0.70	4.79	1.40	0.1256
WYNDE6	63	m	0	0.73	28.26	7.48	0.0001

	N	38
	NS	35
	Wt	655.79
Het	Chi	133.38
Het	df	37
Het	P	***
Fixed	RR	3.46
	RRl	3.20
	RRu	3.73
	P	+++
Random	RR	2.92
	RRl	2.38
	RRu	3.57
	P	+++
Asymm	P	N.S.

Table 1E1 - 3

IESLC - Meta-analysis of Ever Smoking, Pipe and/or Cigars (not cigs)
All LC types
Most adjusted

	Sex: combined	male	female	Total
N	2	34	2	38
NS	2	33	2	37
Wt	296.50	358.28	1.00	655.79
Het Chi	1.92	110.39	0.47	133.38
Het df	1	33	1	37
Het P	N.S.	***	N.S.	***
Fixed RR	4.15	2.96	14.72	3.46
RRl	3.70	2.67	2.08	3.20
RRu	4.65	3.28	104.15	3.73
P	+++	+++	++	+++
Random RR	2.50	2.84	14.72	2.92
RRl	0.42	2.27	2.08	2.38
RRu	14.72	3.55	104.15	3.57
P	N.S.	+++	++	+++
Between Chi				20.59
Between df				2
Between P				***
Btwn(F) P				(*)
Btwn(R) P				N.S.

	All LC (or nearest): all	other	Total
N	38		38
NS	35		35
Wt	655.79		655.79
Het Chi	133.38		133.38
Het df	37		37
Het P	***		***
Fixed RR	3.46		3.46
RRl	3.20		3.20
RRu	3.73		3.73
P	+++		+++
Random RR	2.92		2.92
RRl	2.38		2.38
RRu	3.57		3.57
P	+++		+++
Between Chi			
Between df			
Between P			N.S.
Btwn(F) P			N.S.
Btwn(R) P			N.S.

	Location: NAmer	UK	Scand	othEur	China	Japan	othAs	other	Total
N	25	4	2	6				1	38
NS	22	4	2	6				1	35
Wt	475.37	36.61	26.47	109.81				7.53	655.79
Het Chi	78.18	3.55	0.35	25.43				0.00	133.38
Het df	24	3	1	5				0	37
Het P	***	N.S.	N.S.	***				N.S.	***
Fixed RR	3.17	2.86	7.02	4.51				3.50	3.46
RRl	2.90	2.07	4.79	3.74				1.71	3.20
RRu	3.47	3.95	10.27	5.44				7.16	3.73
P	+++	+++	+++	+++				+++	+++
Random RR	2.25	2.94	7.02	5.17				3.50	2.92
RRl	1.74	2.04	4.79	2.81				1.71	2.38
RRu	2.91	4.23	10.27	9.52				7.16	3.57
P	+++	+++	+++	+++				+++	+++
Between Chi									25.88
Between df									4
Between P									***
Btwn(F) P									N.S.
Btwn(R) P									***

Table 1E1 - 3

IESLC - Meta-analysis of Ever Smoking, Pipe and/or Cigars (not cigs)
All LC types
Most adjusted

	Detailed Country in "other Europe"					
	multi	Germany	othWest	East	Balkans	Total
N	2	1	2	1		6
NS	2	1	2	1		6
Wt	102.27	1.99	3.41	2.14		109.81
Het Chi	14.21	0.00	1.22	0.00		25.43
Het df	1	0	1	0		5
Het P	***	N.S.	N.S.	N.S.		***
Fixed RR	4.42	4.62	17.82	1.25		4.51
RRl	3.64	1.15	6.16	0.33		3.74
RRu	5.37	18.56	51.54	4.77		5.44
P	+++	+	+++	N.S.		+++
Random RR	4.55	4.62	17.61	1.25		5.17
RRl	2.19	1.15	5.44	0.33		2.81
RRu	9.48	18.56	57.07	4.77		9.52
P	+++	+	+++	N.S.		+++
Between Chi						9.99
Between df						3
Between P						*
Btwn(F) P						N.S.
Btwn(R) P						*

	Detailed Country in "other Asia"			
	India	HongKong	other	Total
N				
NS				
Wt				
Het Chi				
Het df				
Het P				N.S.
Fixed RR				
RRl				
RRu				
P				N.S.
Random RR				
RRl				
RRu				
P				N.S.
Between Chi				
Between df				
Between P				N.S.
Btwn(F) P				N.S.
Btwn(R) P				N.S.

	Detailed other continent			
	SCAmer	Auslia	Africa	Total
N			1	1
NS			1	1
Wt			7.53	7.53
Het Chi			0.00	0.00
Het df			0	0
Het P			N.S.	N.S.
Fixed RR			3.50	3.50
RRl			1.71	1.71
RRu			7.16	7.16
P			+++	+++
Random RR			3.50	3.50
RRl			1.71	1.71
RRu			7.16	7.16
P			+++	+++
Between Chi				
Between df				
Between P				N.S.
Btwn(F) P				N.S.
Btwn(R) P				N.S.

Table 1E1 - 3

IESLC - Meta-analysis of Ever Smoking, Pipe and/or Cigars (not cigs)
All LC types
Most adjusted

	Start year of study <1960	1960-69	1970-79	1980-89	1990+	Total
N	15	6	9	6	2	38
NS	15	6	8	5	1	35
Wt	140.05	59.04	99.29	354.83	2.58	655.79
Het Chi	30.31	2.61	16.67	12.98	0.61	133.38
Het df	14	5	8	5	1	37
Het P	**	N.S.	*	*	N.S.	***
Fixed RR	2.13	2.06	3.96	4.38	3.70	3.46
RRl	1.81	1.60	3.25	3.95	1.09	3.20
RRu	2.52	2.66	4.82	4.87	12.52	3.73
P	+++	+++	+++	+++	+	+++
Random RR	2.35	2.06	4.20	4.52	3.70	2.92
RRl	1.78	1.60	2.77	3.18	1.09	2.38
RRu	3.10	2.66	6.36	6.43	12.52	3.57
P	+++	+++	+++	+++	+	+++
Between Chi						70.20
Between df						4
Between P						***
Btwn(F) P						***
Btwn(R) P						**

	Study type (1) CC	other	Total
N	29	9	38
NS	26	9	35
Wt	585.07	70.72	655.79
Het Chi	99.78	9.23	133.38
Het df	28	8	37
Het P	***	N.S.	***
Fixed RR	3.70	1.99	3.46
RRl	3.41	1.57	3.20
RRu	4.01	2.51	3.73
P	+++	+++	+++
Random RR	3.11	2.04	2.92
RRl	2.51	1.51	2.38
RRu	3.87	2.76	3.57
P	+++	+++	+++
Between Chi			24.36
Between df			1
Between P			***
Btwn(F) P			**
Btwn(R) P			*

	Study type (2) CC	prosp	other	Total
N	29	8	1	38
NS	26	8	1	35
Wt	585.07	69.51	1.20	655.79
Het Chi	99.78	9.21	0.00	133.38
Het df	28	7	0	37
Het P	***	N.S.	N.S.	***
Fixed RR	3.70	1.98	2.30	3.46
RRl	3.41	1.57	0.39	3.20
RRu	4.01	2.51	13.73	3.73
P	+++	+++	N.S.	+++
Random RR	3.11	2.07	2.30	2.92
RRl	2.51	1.47	0.39	2.38
RRu	3.87	2.92	13.73	3.57
P	+++	+++	N.S.	+++
Between Chi				24.39
Between df				2
Between P				***
Btwn(F) P				*
Btwn(R) P				N.S.

Table 1E1 - 3

IESLC - Meta-analysis of Ever Smoking, Pipe and/or Cigars (not cigs)
All LC types
Most adjusted

	Study size (number of LC cases)				
	100-249	250-499	500-999	1000+	Total
N	7	11	10	10	38
NS	7	11	8	9	35
Wt	9.36	63.86	80.89	501.67	655.79
Het Chi	9.82	12.46	23.57	57.68	133.38
Het df	6	10	9	9	37
Het P	N.S.	N.S.	**	***	***
Fixed RR	6.52	1.87	3.63	3.67	3.46
RRl	3.44	1.46	2.92	3.36	3.20
RRu	12.37	2.39	4.52	4.00	3.73
P	+++	+++	+++	+++	+++
Random RR	6.02	1.91	3.46	2.95	2.92
RRl	2.50	1.43	2.29	2.19	2.38
RRu	14.46	2.56	5.22	3.97	3.57
P	+++	+++	+++	+++	+++
Between Chi					29.85
Between df					3
Between P					***
Btwn(F) P					*
Btwn(R) P					*

	Risky occupational population			
	no	mining	othRisky	Total
N	37		1	38
NS	34		1	35
Wt	653.91		1.88	655.79
Het Chi	129.85		0.00	133.38
Het df	36		0	37
Het P	***		N.S.	***
Fixed RR	3.47		0.88	3.46
RRl	3.21		0.21	3.20
RRu	3.75		3.68	3.73
P	+++		N.S.	+++
Random RR	2.97		0.88	2.92
RRl	2.42		0.21	2.38
RRu	3.63		3.68	3.57
P	+++		N.S.	+++
Between Chi				3.52
Between df				1
Between P				(*)
Btwn(F) P				N.S.
Btwn(R) P				(*)

	National cigarette tobacco type			
	Virginia	blended	other	Total
N	8	30		38
NS	7	28		35
Wt	54.19	601.60		655.79
Het Chi	7.05	125.85		133.38
Het df	7	29		37
Het P	N.S.	***		***
Fixed RR	3.16	3.49		3.46
RRl	2.42	3.22		3.20
RRu	4.12	3.78		3.73
P	+++	+++		+++
Random RR	3.16	2.76		2.92
RRl	2.42	2.18		2.38
RRu	4.14	3.50		3.57
P	+++	+++		+++
Between Chi				0.48
Between df				1
Between P				N.S.
Btwn(F) P				N.S.
Btwn(R) P				N.S.

Table 1E1 - 3

IESLC - Meta-analysis of Ever Smoking, Pipe and/or Cigars (not cigs)
All LC types
Most adjusted

	Any proxy use No/nk	Yes	Total
N	26	12	38
NS	25	10	35
Wt	535.10	120.68	655.79
Het Chi	82.41	36.11	133.38
Het df	25	11	37
Het P	***	***	***
Fixed RR	3.22	4.75	3.46
RRl	2.96	3.97	3.20
RRu	3.50	5.67	3.73
P	+++	+++	+++
Random RR	2.54	3.98	2.92
RRl	2.02	2.63	2.38
RRu	3.19	6.04	3.57
P	+++	+++	+++
Between Chi			14.85
Between df			1
Between P			***
Btwn(F) P			*
Btwn(R) P			(*)

	Full histological confirmation No	Yes	Total
N	26	12	38
NS	25	10	35
Wt	541.96	113.82	655.79
Het Chi	110.46	11.62	133.38
Het df	25	11	37
Het P	***	N.S.	***
Fixed RR	3.67	2.60	3.46
RRl	3.37	2.16	3.20
RRu	3.99	3.12	3.73
P	+++	+++	+++
Random RR	3.14	2.54	2.92
RRl	2.44	2.07	2.38
RRu	4.06	3.12	3.57
P	+++	+++	+++
Between Chi			11.30
Between df			1
Between P			***
Btwn(F) P			(*)
Btwn(R) P			N.S.

	Number of adjustment variables (1) 0	1	2+/+nk	Total
N	23	10	5	38
NS	21	10	5	36
Wt	478.98	114.35	62.45	655.79
Het Chi	68.65	27.93	0.69	133.38
Het df	22	9	4	37
Het P	***	***	N.S.	***
Fixed RR	3.93	2.11	3.22	3.46
RRl	3.59	1.75	2.51	3.20
RRu	4.30	2.53	4.12	3.73
P	+++	+++	+++	+++
Random RR	3.15	2.42	3.22	2.92
RRl	2.45	1.67	2.51	2.38
RRu	4.05	3.50	4.12	3.57
P	+++	+++	+++	+++
Between Chi				36.10
Between df				2
Between P				***
Btwn(F) P				**
Btwn(R) P				N.S.

Table 1E1 - 3

IESLC - Meta-analysis of Ever Smoking, Pipe and/or Cigars (not cigs)
All LC types
Most adjusted

	Number of adjustment variables (2) 0	1	2	3-5	6+/+nk	Total
N	23	10	4	1		38
NS	21	10	4	1		36
Wt	478.98	114.35	60.50	1.95		655.79
Het Chi	68.65	27.93	0.66	0.00		133.38
Het df	22	9	3	0		37
Het P	***	***	N.S.	N.S.		***
Fixed RR	3.93	2.11	3.23	2.80		3.46
RRl	3.59	1.75	2.51	0.69		3.20
RRu	4.30	2.53	4.16	11.40		3.73
P	+++	+++	+++	N.S.		+++
Random RR	3.15	2.42	3.23	2.80		2.92
RRl	2.45	1.67	2.51	0.69		2.38
RRu	4.05	3.50	4.16	11.40		3.57
P	+++	+++	+++	N.S.		+++
Between Chi						36.14
Between df						3
Between P						***
Btwn(F) P						*
Btwn(R) P						N.S.

	Derivation of RR/CI Orig	StdCalc	Other	Total
N	2	22	14	38
NS	2	21	14	37
Wt	3.94	496.29	155.57	655.79
Het Chi	0.25	91.97	20.39	133.38
Het df	1	21	13	37
Het P	N.S.	***	(*)	***
Fixed RR	3.61	3.82	2.51	3.46
RRl	1.34	3.50	2.15	3.20
RRu	9.68	4.17	2.94	3.73
P	+	+++	+++	+++
Random RR	3.61	3.13	2.49	2.92
RRl	1.34	2.39	1.94	2.38
RRu	9.68	4.11	3.19	3.57
P	+	+++	+++	+++
Between Chi				20.77
Between df				2
Between P				***
Btwn(F) P				(*)
Btwn(R) P				N.S.

Table 1E1 - 4

IESLC - Meta-analysis of Ever Smoking, Pipe and/or Cigars (not cigs)
All LC types
Least adjusted

REF	NRR	X	SEX	AGEL	AGEH	RACE	YF	LC TYPE	LOC	START	ST	NLC	R	VB	P	H	AD	DENOM	De
ABELIN	3	x	m	0	0	all	-	all	Eu:wst	1941	CC	118	n	bl	y	n	0	nev any	st
ALDERS	7	x	m	0	0	all	-	all	Eu:UK	1977	CC	1448	n	V	n	n	0	nev any	st
ARMADA	30		m	0	0	all	-	all	Eu:wst	1986	CC	325	n	bl	n	y	0	nev any	st
BECHER	3	x	m	0	0	all	-	all	Eu:Ger	1985	CC	194	n	bl	n	y	0	nev any	st
BEST	19		m	55	79	all	3	all	NAmer	1955	pr	381	n	V	n	n	0	nev any	st
BOFFET	30		m	0	0	all	-	all	Eu:mul	1988	CC	5621	n	bl	y	n	0	nev any	st
BOUCOT	5	x	m	0	0	all	0	all	NAmer	1951	pr	121	n	bl	n	n	0	nev any	ot
BRESLO	33		m	0	0	all	-	all	NAmer	1949	CC	518	n	bl	n	y	0	nev+1	st
CHOW	1	x	m	0	0	wh	0	all	NAmer	1966	pr	219	n	bl	n	n	0	nev any	st
COMSTO	1		m	0	0	all	-	all	NAmer	1975	ot	258	n	bl	n	n	0	nev any	st
CPSI	186		m	35	84	all	6	all	NAmer	1959	pr	5138	n	bl	n	n	1	nev any	ot
DAMBER	28		m	0	0	all	-	all	Eu:Sca	1972	CC	579	n	bl	y	n	0	nev any	st
DEAN	11		m	0	0	wh	-	all	Africa	1947	CC	603	n	V	y	n	0	nev any	st
DEAN2	11		m	0	0	all	-	all	Eu:UK	1960	CC	954	n	V	y	n	0	nev any	st
DOLL2	33		m	0	0	all	10	all	Eu:UK	1951	pr	920	n	V	n	n	1	nev any	ot
DORGAN	1		m	0	0	wh	-	all	NAmer	1980	CC	2026	n	bl	y	y	0	nev any	st
DORGAN	25		m	0	0	bl	-	all	NAmer	1980	CC	2026	n	bl	y	y	0	nev any	st
DORN	44	x	m	0	0	wh	2	all	NAmer	1954	pr	5097	n	bl	n	n	0	nev any	st
GOLLED	19	x	m	35	99	all	-	all	Eu:UK	1952	CC	443	n	V	y	n	0	nev any	st
GRAHAM	6	x	m	0	0	wh	-	all	NAmer	1956	CC	685	n	bl	n	n	0	nev any	st
HAMMO2	28	x	m	0	0	all	0	all	NAmer	1967	pr	450	o	bl	n	n	0	nev any	st
HAMMON	130	x	m	0	0	wh	0	all	NAmer	1952	pr	448	n	bl	n	n	0	nev any	st
KJUUS	4		m	0	0	all	-	all	Eu:Sca	1979	CC	176	n	bl	n	n	0	nev any	st
LEVIN	31		m	0	0	all	-	all	NAmer	1938	CC	475	n	bl	n	n	1	nev any	st
LOMBAR	1		m	0	0	all	-	all	NAmer	1951	CC	1040	n	bl	n	n	0	nev any	st
LUBIN2	49	x	m	0	0	all	-	all	Eu:mul	1976	CC	7804	n	bl	n	y	0	nev any	st
MARSH	5		m	0	0	all	-	all	NAmer	1979	CC	150	n	bl	y	n	0	nev any	ot
MARSH2	3		c	0	0	all	-	all	NAmer	1979	CC	114	n	bl	y	n	0	nev any	ot
SADOWS	70		m	0	0	wh	-	all	NAmer	1938	CC	477	n	bl	n	n	0	nev any	st
STASZE	3		m	0	0	all	-	all	Eu:est	1954	CC	281	n	bl	n	y	0	nev any	st
STOCKW	5		c	0	0	all	-	all	NAmer	1981	CC	22161	n	bl	n	n	0	nev any	st
TOUSEY	1	x	m	0	0	all	-	all	NAmer	1993	CC	507	n	bl	y	y	0	nev any	st
TOUSEY	5		f	0	0	all	-	all	NAmer	1993	CC	507	n	bl	y	y	0	nev any	st
WIGLE	14		m	0	0	all	-	all	NAmer	1971	CC	728	n	V	n	n	0	nev any	st
WIGLE	17		f	0	0	all	-	all	NAmer	1971	CC	728	n	V	n	n	0	nev any	ot
WYNDE2	15		m	0	0	all	-	all	NAmer	1962	CC	404	n	bl	n	y	0	nev any	st
WYNDE3	152		m	0	0	all	-	all	NAmer	1966	CC	350	n	bl	n	y	0	nev any	st
WYNDE6	63		m	0	0	all	-	all	NAmer	1969	CC	4423	n	bl	n	y	0	nev any	st

Table 1E1 - 5

IESLC - Meta-analysis of Ever Smoking, Pipe and/or Cigars (not cigs)
All LC types
Least adjusted

REF	NRR	SEX	AD	Number Exposed Case	Cont	Non-exposed Case	Cont	RR	95.00%CI
ABELIN	3	m	0	69	187	2	183	33.76	(8.16- 139.75)
ALDERS	7	m	0	17	35	15	133	4.31	(1.96- 9.47)
ARMADA	30	m	0	4	7	4	64	9.14	(1.86- 44.85)
BECHER	3	m	0	6	21	3	54	5.14	(1.18- 22.47)
*BEST	19	m	0	8	2972	1	2854	7.68	(0.96- 61.38)
BOFFET	30	m	0	118	266	117	1750	6.64	(4.98- 8.84)
*BOUCOT	5	m	0	4	8926	0	7551	7.61~	(0.41- 141.39)
BRESLO	33	m	0	15	68	7	42	1.32	(0.50- 3.51)
*CHOW	1	m	0	5	13677	6	62913	3.83	(1.17- 12.56)
COMSTO	1	m	0	2	15	4	69	2.30	(0.39- 13.73)
*CPSI	186	m	1	-	-	-	-	1.94	(1.44- 2.62)
DAMBER	28	m	0	205	149	42	208	6.81	(4.60- 10.09)
DEAN	11	m	0	51	74	12	61	3.50	(1.71- 7.16)
DEAN2	11	m	0	81	128	33	112	2.15	(1.33- 3.46)
*DOLL2	33	m	1	-	-	-	-	6.14	(1.85- 20.40)
DORGAN	1	m	0	22	55	15	93	2.48	(1.19- 5.18)
DORGAN	25	m	0	3	19	3	35	1.84	(0.34- 10.03)
Subtotal DORGAN								2.37	(1.20- 4.65)
*DORN	44	m	0	16	68146	17	117918	1.63	(0.82- 3.22)
GOLLED	19	m	0	48	331	15	490	4.74	(2.61- 8.60)
GRAHAM	6	m	0	49	367	18	346	2.57	(1.47- 4.49)
*HAMMO2	28	m	0	3	488	5	891	1.10	(0.26- 4.56)
*HAMMON	130	m	0	28	127770	15	115884	1.69	(0.90- 3.17)
KJUUS	4	m	0	23	25	2	24	11.04	(2.34- 52.00)
LEVIN	31	m	1	-	-	-	-	1.31	(0.81- 2.12)
LOMBAR	1	m	0	48	146	14	112	2.63	(1.38- 5.01)
LUBIN2	49	m	0	99	406	190	2617	3.36	(2.58- 4.38)
MARSH	5	m	0	0	5	2	31	1.15~	(0.05- 27.23)
MARSH2	3	c	0	0	4	12	56	0.50~	(0.03- 9.94)
SADOWS	70	m	0	38	88	18	81	1.94	(1.03- 3.67)
STASZE	3	m	0	4	101	5	158	1.25	(0.33- 4.77)
STOCKW	5	c	0	715	655	2791	10641	4.16	(3.71- 4.66)
TOUSEY	1	m	0	4	48	4	130	2.71	(0.65- 11.26)
TOUSEY	5	f	0	1	2	13	226	8.69	(0.74- 102.22)
Subtotal TOUSEY								3.63	(1.06- 12.45)
WIGLE	14	m	0	30	113	15	204	3.61	(1.86- 6.99)
WIGLE	17	f	0	1	0	36	439	36.12~	(1.45- 902.62)
Subtotal WIGLE								3.96	(2.07- 7.57)
WYNDE2	15	m	0	14	104	8	105	1.77	(0.71- 4.39)
WYNDE3	152	m	0	14	68	9	88	2.01	(0.82- 4.93)
WYNDE6	63	m	0	58	199	87	617	2.07	(1.43- 2.99)
Partial Totals				1803	225665	3540	327180		

*prospective study ~ With 0.5 adjustment for zero

REF	NRR	SEX	AD	Ys	Ws	Qs	Ps
ABELIN	3	m	0	3.52	1.90	9.74	0.0000
ALDERS	7	m	0	1.46	6.19	0.25	0.0003
ARMADA	30	m	0	2.21	1.52	1.39	0.0064
BECHER	3	m	0	1.64	1.77	0.26	0.0295
*BEST	19	m	0	2.04	0.89	0.54	0.0545
BOFFET	30	m	0	1.89	46.83	18.88	0.0000
*BOUCOT	5	m	0	2.03	0.45	0.27	0.1733
BRESLO	33	m	0	0.28	4.03	3.85	0.5736
*CHOW	1	m	0	1.34	2.73	0.02	0.0265
COMSTO	1	m	0	0.83	1.20	0.22	0.3609
*CPSI	186	m	1	0.66	42.89	15.17	0.0000
DAMBER	28	m	0	1.92	24.87	10.88	0.0000
DEAN	11	m	0	1.25	7.53	0.00	0.0006
DEAN2	11	m	0	0.76	16.84	4.09	0.0017
*DOLL2	33	m	1	1.81	2.67	0.83	0.0030
DORGAN	1	m	0	0.91	7.09	0.86	0.0156
DORGAN	25	m	0	0.61	1.34	0.56	0.4799
Subtotal DORGAN				0.86	8.43	1.42	
*DORN	44	m	0	0.49	8.24	4.88	0.1614
GOLLED	19	m	0	1.56	10.80	0.96	0.0000
GRAHAM	6	m	0	0.94	12.26	1.22	0.0010
*HAMMO2	28	m	0	0.09	1.89	2.57	0.9003
*HAMMON	130	m	0	0.53	9.77	5.22	0.0998
KJUUS	4	m	0	2.40	1.60	2.09	0.0024
LEVIN	31	m	1	0.27	16.60	16.18	0.2713

Table 1E1 - 5

IESLC - Meta-analysis of Ever Smoking, Pipe and/or Cigars (not cigs)
All LC types
Least adjusted

REF	NRR	SEX	AD	Ys	Ws	Qs	Ps
LOMBAR	1	m	0	0.97	9.26	0.78	0.0033
LUBIN2	49	m	0	1.21	54.92	0.12	0.0000
MARSH	5	m	0	0.14	0.38	0.48	0.9331
MARSH2	3	c	0	-0.69	0.43	1.63	0.6511
SADOWS	70	m	0	0.66	9.47	3.33	0.0409
STASZE	3	m	0	0.22	2.14	2.29	0.7425
STOCKW	5	c	0	1.43	296.07	8.41	0.0000
TOUSEY	1	m	0	1.00	1.89	0.13	0.1705
TOUSEY	5	f	0	2.16	0.63	0.52	0.0855
Subtotal TOUSEY				1.29	2.52	0.65	
WIGLE	14	m	0	1.28	8.79	0.01	0.0001
WIGLE	17	f	0	3.59	0.37	2.01	0.0289
Subtotal WIGLE				1.38	9.16	2.02	
WYNDE2	15	m	0	0.57	4.64	2.20	0.2202
WYNDE3	152	m	0	0.70	4.79	1.49	0.1256
WYNDE6	63	m	0	0.73	28.26	7.98	0.0001

	N	38
	NS	35
	Wt	653.95
Het	Chi	132.31
Het	df	37
Het	P	***
Fixed	RR	3.52
	RRl	3.26
	RRu	3.80
	P	+++
Random	RR	3.01
	RRl	2.46
	RRu	3.68
	P	+++
Asymm	P	N.S.

Table 1E1 - 6

IESLC - Meta-analysis of Ever Smoking, Pipe and/or Cigars (not cigs)
All LC types
Least adjusted

		Sex			
		combined	male	female	Total
	N	2	34	2	38
	NS	2	33	2	37
	Wt	296.50	356.45	1.00	653.95
Het	Chi	1.92	112.59	0.47	132.31
Het	df	1	33	1	37
Het	P	N.S.	***	N.S.	***
Fixed	RR	4.15	3.05	14.72	3.52
	RRl	3.70	2.75	2.08	3.26
	RRu	4.65	3.39	104.15	3.80
	P	+++	+++	++	+++
Random	RR	2.50	2.94	14.72	3.01
	RRl	0.42	2.35	2.08	2.46
	RRu	14.72	3.69	104.15	3.68
	P	N.S.	+++	++	+++
Between	Chi				17.33
Between	df				2
Between	P				***
Btwn(F)	P				(*)
Btwn(R)	P				N.S.

Table 1E1 - 7

IESLC - Meta-analysis of Ever Smoking, Pipe and/or Cigars (not cigs)
All LC types
Excluded studies (and stage at which they were excluded)

1	BOUCHA BUELL LAURIL MZILEN
2	ABRAHA AGUDO AKIBA AMANDU AMES ANDERS ARCHER AUSTIN AUVINE AXELSO AXELSS BAND BARBON BERRIN BLOHMK BLOT1 BLOT2 BLOT3 BLOT4 BRETT BROCKM BROWN1 BROWN2 BUFFLE BYERS1 BYERS2 CARPEN CASCO2 CASCOR CEDERL CHAN CHANG CHATZI CHEN CHEN2 CHEN3 CHIAZZ CHOI CHYOU COOKSO CORREA CPSII DAVEYS DEAN3 DESTE2 DESTEF DOCKER DOLL DORANT DOSEME DROSTE DU DUNN EBELIN ENSTRO ESAKI FAN GAO GAO2 GARCIA GARDIN GARSHI GENG GER GILLIS GODLEY GOODMA GREGOR GSELL HAENSZ HANSEN HEGMAN HEIN HENNEK HINDS HIRAOK HIRAY2 HIRAYA HITOSU HOROWI HORWIT HU HU2 HUANG HUMBLE ISHIMA JAIN JARUP JARVHO JEDRYC JIANG JOLY JONES JUSSAW KAISE2 KAISER KANELL KATSOU KAUFMA KELLER KHUDER KIHARA KO KOHLME KOO KOULUM KREYBE KUBIK LAMTH LAMWK LAMWK2 LANGE LAUSSM LEI LEMARC LETOUR LIAW LICKIN LIDDEL LIU LIU2 LIU3 LIU4 LIU5 LOMBA2 LUBIN LUO MACLEN MAGNUS MARTIS MASTRA MATSUD MCCONN MCDUFF MCLAUG MILLER MILLS MOLLO MRFIT MRFITR MURATA NAM NOTAN2 NOTANI NOU ODRISC ORMOS OSANN OSANN2 PARKIN PASTOR PAWLEG PERNU PERSH2 PERSHA PETO PEZZO2 PEZZOT PIKE PISANI POFFIJ POLEDN PRESCO QIAO QIAO2 RACHTA RADZIK RANDIG REN RESTRE RIMING RONCO ROOTS ROTHSC SAARIK SANKAR SCHWA2 SCHWAR SEGI SEGI2 SEOW SHAW SHIMIZ SIEMIA SIMARA SIMONA SITAS SOBUE SOBUE2 SPEIZE SPITZ STAYNE STOCKS STUCKE SUN SUZUK2 SUZUKI SVENSS TANG TANG2 TAO TENKAN TIZZAN TOKARS TSUGAN TVERDA ULMER VANDER VEIERO VUTUC WAKAI WANG WANG2 WANG3 WANG4 WARSIN WATSON WICKLU WILKIN WU WU2 WUNSCH WUWILL WYNDE4 WYNDE5 WYNDE8 WYNDER XIANGZ XU XU2 XU3 XU4 YAMAGU YONG YUAN ZHANG ZHENG ZHOU
3	BENSHL BROSS DARBY DEKLER ENGELA HOLE KINLEN KNEKT KREUZE MATOS MIGRAN TULINI WALD WYNDE7
9	BENHAM JAHN

Table 1E1 - 8

Potentially overlapping studies

REF	REFGP	PRINC	.	OVERLAP/LINK
LUBIN2	LUBIN2	1		Lubin-combined
GRAHAM	BYERS1	1		GRAHAM/BROSS/BYERS1
WYNDE6	WYNDE6	1		WYNDE5/6/7/8
CPSI	CPSI	1		CPSI overall
BOFFET	BOFFET	1		BOFFET-combined

Table 1E1 - 9

Most adjusted - insufficient data for metaanalysis

REF	NRR	SEX	AGEL	AGEH	RACE	YF	LC TYPE	LOC	START	ST	NLC	R	VB	P	H	AD	DENOM	De
BECHER	4	f	0	0	all	-	all	Eu:Ger	1985	CC	194	n	bl	n	y	0	nev any	st
BRESLO	34	f	0	0	all	-	all	NAmer	1949	CC	518	n	bl	n	y	0	nev+1	st

REF	NRR	RR	SIG	RRDATA comment
BECHER	4		*	No relevant smokers
BRESLO	34		*	No relevant smokers

Table 1E2 -

IESLC - Meta-analysis of Current Smoking, Pipe and/or Cigars (not cigs)
All LC types

This analysis is restricted to results for:
1) Non-dose-response data
2) Smokers of pipe and/or cigars (but not cigarettes)
3) Current smokers
4) Results complete enough for use in metaanalysis

Within each study, results are then selected (in the following order of preference, within each sex) for:
5) DENOM: never smoked anything, (never +1 = +long term ex)
6) Followup period (prospective studies): whole study (coded as 0) or longest available
7) LCtype: all or nearest available, at least Squamous and Adeno. (q = squamous, s = small,
l = large, a = adeno, mix = mixed, alv = alveolar)
8) Race: all or nearest available, otherwise by race (wh or w = white, bl or b = black, hi = hispanic
ch = chinese, jap = japanese, haw = hawaiian, w+o = white + oriental, sca = scandinavian, as = asian)
9) For overlapping studies: principal rather than subsidiary studies
Finally by Age: whole study (coded as 0) if available, otherwise by widest available age group
and then for single sex results (m, f) in preference to combined sex results (c).

Results adjusted (AD) for the most potential confounders are then chosen in Sections -1 to -3
and results adjusted for the least confounders in Sections -4 to -6. (Those least adjusted results which
actually differ from the most adjusted as marked 'x' in column X in Section -4)
(Results adjusted for an unknown number of confounder(s) are coded as 20.)

Section -7 shows excluded studies, together with the stage (as above) at which no qualifying
results were found.

Section -8 lists the potentially overlapping studies which have been included (1=principal, 2=subsidiary).

Section -9 lists any results which would have been included in preference except that they had data not complete
enough for use in meta-analysis, with their significance (yes/no), if known, and any further comment as entered
on the database.

In addition to those mentioned above, the following fields, levels and abbreviations are used:
* or nk = not known, n = no, y = yes, ot = other
nev = never
REF: 6-character study reference
NRR: number of the RR on the database within the study
ST : study type (CC = case control, pr or prosp = prospective)
NLC: number of lung cancer cases in whole study
R : risky occupational population (n = no, m = mining, o = other risky)
VB : national cigarette type (V = at least 75% Virginia, bl = at least 75% blended, ot = other)
P : any proxy use
H : full histological confirmation
De : derivation of RR/CI (or = original, st = standard method, ot = other method of estimation)

Table 1E2 - 1

IESLC - Meta-analysis of Current Smoking, Pipe and/or Cigars (not cigs)
All LC types
Most adjusted

REF	NRR	SEX	AGEL	AGEH	RACE	YF	LC TYPE	LOC	START	ST	NLC	R	VB	P	H	AD	DENOM	De
BENSHL	10	m	40	64	all	10	all	Eu:UK	1967	pr	486	n	V	n	n	1	nev any	ot
BROSS	5	m	0	0	wh	-	all	NAmer	1960	CC	974	n	bl	n	n	0	nev any	st
CHOW	13	m	0	0	wh	0	all	NAmer	1966	pr	219	n	bl	n	n	0	nev any	st
DARBY	6	m	0	0	wh	-	all	Eu:UK	1988	CC	982	n	V	n	n	0	nev any	st
DARBY	13	f	0	0	wh	-	all	Eu:UK	1988	CC	982	n	V	n	n	0	nev any	st
DEKLER	5	m	0	0	all	0	all	Auslia	1961	pr	138	m	V	n	n	2	nev any	or
DOLL2	20	m	0	0	all	20	all	Eu:UK	1951	pr	920	n	V	n	n	1	nev any	ot
DORN	90	m	35	84	wh	8	all	NAmer	1954	pr	5097	n	bl	n	n	1	nev any	ot
ENGELA	167	m	0	0	all	12	all	Eu:Sca	1964	pr	435	n	bl	n	n	1	nev any	ot
GRAHAM	24	m	0	0	wh	-	all	NAmer	1956	CC	685	n	bl	n	n	1	nev any	ot
HOLE	6	m	0	0	all	0	all	Eu:UK	1972	pr	225	n	V	n	n	1	nev any	ot
KINLEN	13	m	0	0	all	0	all	Eu:UK	1967	pr	718	n	V	n	n	2	nev any	ot
KNEKT	28	m	20	69	all	21	all	Eu:Sca	1966	pr	515	n	bl	n	n	1	nev any	or
KREUZE	9	m	1	45	all	-	all	Eu:Ger	1990	CC	2260	n	bl	n	n	0	nev any	ot
KREUZE	11	m	55	69	all	-	all	Eu:Ger	1990	CC	2260	n	bl	n	n	0	nev any	st
KREUZE	10	f	1	45	all	-	all	Eu:Ger	1990	CC	2260	n	bl	n	n	0	nev any	ot
KREUZE	12	f	55	69	all	-	all	Eu:Ger	1990	CC	2260	n	bl	n	n	0	nev any	ot
LUBIN2	30	m	0	0	all	-	all	Eu:mul	1976	CC	7804	n	bl	n	y	2	nev any	ot
MATOS	1	m	0	0	all	-	all	SCAmer	1994	CC	200	n	bl	n	n	0	nev any	st
MIGRAN	16	m	0	0	all	0	all	Eu:UK	1964	pr	259	n	V	n	n	2	nev any	ot
TULINI	35	m	0	0	all	0	all	Eu:Sca	1967	pr	472	n	bl	n	n	3	nev any	ot
TULINI	31	f	0	0	all	0	all	Eu:Sca	1967	pr	472	n	bl	n	n	3	nev any	or
WALD	3	m	0	0	all	0	all	Eu:UK	1975	pr	102	n	V	n	n	1	nev any	or
WIGLE	29	m	0	0	all	-	all	NAmer	1971	CC	728	n	V	n	n	1	nev any	ot
WIGLE	5	f	0	0	all	-	all	NAmer	1971	CC	728	n	V	n	n	0	nev any	ot
WYNDE7	46	m	0	0	all	-	all	NAmer	1977	CC	2085	n	bl	n	y	0	nev any	st

Table 1E2 - 2

IESLC - Meta-analysis of Current Smoking, Pipe and/or Cigars (not cigs)
All LC types
Most adjusted

REF	NRR	SEX	AD	Number Exposed Case	Number Exposed Cont	Non-exposed Case	Non-exposed Cont	RR	95.00%CI
*BENSHL	10	m	1	-	-	-	-	2.33	(0.58- 9.33)
BROSS	5	m	0	125	211	38	170	2.65	(1.75- 4.02)
*CHOW	13	m	0	37	47084	6	62913	8.24	(3.48- 19.52)
DARBY	6	m	0	57	165	3	384	44.22	(13.65- 143.22)
DARBY	13	f	0	3	14	23	529	4.93	(1.32- 18.36)
Subtotal DARBY								16.69	(6.95- 40.09)
*DEKLER	5	m	2	-	-	-	-	9.10	(0.82- 101.10)
*DOLL2	20	m	1	-	-	-	-	5.80	(2.65- 12.72)
*DORN	90	m	1	-	-	-	-	1.70	(1.21- 2.37)
*ENGELA	167	m	1	-	-	-	-	2.60	(1.02- 6.60)
GRAHAM	24	m	1	-	-	-	-	2.88	(1.70- 4.89)
*HOLE	6	m	1	-	-	-	-	2.29	(0.48- 11.04)
*KINLEN	13	m	2	-	-	-	-	6.51	(2.95- 14.35)
*KNEKT	28	m	1	-	-	-	-	6.10	(2.10- 18.20)
KREUZE	9	m	0	0	6	6	54	0.64~	(0.03- 12.82)
KREUZE	11	m	0	26	15	23	403	30.37	(14.18- 65.06)
KREUZE	10	f	0	0	1	6	38	1.97~	(0.07- 53.89)
KREUZE	12	f	0	3	0	95	177	13.01~	(0.67- 254.52)
Subtotal KREUZE								20.75	(10.30- 41.79)
LUBIN2	30	m	2	-	-	-	-	4.05	(3.18- 5.16)
MATOS	1	m	0	1	4	11	110	2.50	(0.26- 24.38)
*MIGRAN	16	m	2	-	-	-	-	3.81	(1.19- 12.23)
*TULINI	35	m	3	-	-	-	-	7.57	(4.02- 14.26)
*TULINI	31	f	3	-	-	-	-	14.70	(6.35- 34.10)
Subtotal TULINI								9.63	(5.81- 15.96)
*WALD	3	m	1	-	-	-	-	3.19	(1.07- 9.50)
WIGLE	29	m	1	-	-	-	-	2.10	(1.05- 4.20)
WIGLE	5	f	0	1	0	36	439	36.12~	(1.45- 902.62)
Subtotal WIGLE								2.38	(1.21- 4.69)
WYNDE7	46	m	0	31	171	64	918	2.60	(1.64- 4.11)
Partial Totals				284	47671	311	66135		

*prospective study ~ With 0.5 adjustment for zero

REF	NRR	SEX	AD	Ys	Ws	Qs	Ps
*BENSHL	10	m	1	0.85	1.99	0.45	0.2326
BROSS	5	m	0	0.97	22.25	2.65	0.0000
*CHOW	13	m	0	2.11	5.16	3.21	0.0000
DARBY	6	m	0	3.79	2.78	16.96	0.0000
DARBY	13	f	0	1.60	2.22	0.17	0.0174
Subtotal DARBY				2.81	5.00	17.12	
*DEKLER	5	m	2	2.21	0.66	0.52	0.0722
*DOLL2	20	m	1	1.76	6.24	1.20	0.0000
*DORN	90	m	1	0.53	34.00	21.19	0.0020
*ENGELA	167	m	1	0.96	4.41	0.59	0.0449
GRAHAM	24	m	1	1.06	13.76	0.95	0.0001
*HOLE	6	m	1	0.83	1.56	0.38	0.3003
*KINLEN	13	m	2	1.87	6.14	1.88	0.0000
*KNEKT	28	m	1	1.81	3.30	0.79	0.0010
KREUZE	9	m	0	-0.44	0.43	1.33	0.7737
KREUZE	11	m	0	3.41	6.62	29.01	0.0000
KREUZE	10	f	0	0.68	0.35	0.14	0.6868
KREUZE	12	f	0	2.57	0.43	0.67	0.0908
Subtotal KREUZE				3.03	7.83	31.15	
LUBIN2	30	m	2	1.40	65.58	0.41	0.0000
MATOS	1	m	0	0.92	0.74	0.12	0.4303
*MIGRAN	16	m	2	1.34	2.83	0.00	0.0244
*TULINI	35	m	3	2.02	9.58	4.75	0.0000
*TULINI	31	f	3	2.69	5.44	10.18	0.0000
Subtotal TULINI				2.26	15.02	14.93	
*WALD	3	m	1	1.16	3.22	0.08	0.0373
WIGLE	29	m	1	0.74	8.00	2.67	0.0359
WIGLE	5	f	0	3.59	0.37	1.91	0.0289
Subtotal WIGLE				0.87	8.37	4.58	
WYNDE7	46	m	0	0.96	18.24	2.42	0.0000

Table 1E2 - 2

IESLC - Meta-analysis of Current Smoking, Pipe and/or Cigars (not cigs)
All LC types
Most adjusted

	N	26
	NS	20
	Wt	226.33
Het	Chi	104.61
Het	df	25
Het	P	***
Fixed	RR	3.74
	RRl	3.29
	RRu	4.26
	P	+++
Random	RR	4.76
	RRl	3.44
	RRu	6.59
	P	+++
Asymm	P	N.S.

Table 1E2 - 3

IESLC - Meta-analysis of Current Smoking, Pipe and/or Cigars (not cigs)
All LC types
Most adjusted

	Sex			
	combined	male	female	Total
N		21	5	26
NS		20	4	24
Wt		217.51	8.82	226.33
Het Chi		91.16	3.45	104.61
Het df		20	4	25
Het P		***	N.S.	***
Fixed RR		3.59	10.64	3.74
RRl		3.14	5.50	3.29
RRu		4.10	20.58	4.26
P		+++	+++	+++
Random RR		4.38	10.64	4.76
RRl		3.13	5.50	3.44
RRu		6.13	20.58	6.59
P		+++	+++	+++
Between Chi				10.00
Between df				1
Between P				**
Btwn(F) P				N.S.
Btwn(R) P				*

	All LC (or nearest)		
	all	other	Total
N	26		26
NS	20		20
Wt	226.33		226.33
Het Chi	104.61		104.61
Het df	25		25
Het P	***		***
Fixed RR	3.74		3.74
RRl	3.29		3.29
RRu	4.26		4.26
P	+++		+++
Random RR	4.76		4.76
RRl	3.44		3.44
RRu	6.59		6.59
P	+++		+++
Between Chi			
Between df			
Between P			N.S.
Btwn(F) P			N.S.
Btwn(R) P			N.S.

	Location								
	NAmer	UK	Scand	othEur	China	Japan	othAs	other	Total
N	7	8	4	5				2	26
NS	6	7	3	2				2	20
Wt	101.79	26.99	22.73	73.41				1.40	226.33
Het Chi	15.56	16.27	7.44	26.86				0.58	104.61
Het df	6	7	3	4				1	25
Het P	*	*	(*)	***				N.S.	***
Fixed RR	2.42	5.72	6.99	4.82				4.60	3.74
RRl	1.99	3.92	4.63	3.84				0.88	3.29
RRu	2.94	8.34	10.54	6.06				24.07	4.26
P	+++	+++	+++	+++				(+)	+++
Random RR	2.73	5.48	6.69	6.20				4.60	4.76
RRl	1.92	3.01	3.42	1.51				0.88	3.44
RRu	3.88	9.96	13.10	25.49				24.07	6.59
P	+++	+++	+++	+				(+)	+++
Between Chi									37.91
Between df									4
Between P									***
Btwn(F) P									*
Btwn(R) P									(*)

Table 1E2 - 3

IESLC - Meta-analysis of Current Smoking, Pipe and/or Cigars (not cigs)
All LC types
Most adjusted

Detailed Country in "other Europe"

	multi	Germany	othWest	East	Balkans	Total
N	1	4				5
NS	1	1				2
Wt	65.58	7.83				73.41
Het Chi	0.00	8.18				26.86
Het df	0	3				4
Het P	N.S.	*				***
Fixed RR	4.05	20.75				4.82
RRl	3.18	10.30				3.84
RRu	5.16	41.79				6.06
P	+++	+++				+++
Random RR	4.05	6.75				6.20
RRl	3.18	0.96				1.51
RRu	5.16	47.32				25.49
P	+++	(+)				+
Between Chi						18.68
Between df						1
Between P						***
Btwn(F) P						(*)
Btwn(R) P						N.S.

Detailed Country in "other Asia"

	India	HongKong	other	Total
N				
NS				
Wt				
Het Chi				
Het df				
Het P				
Fixed RR				
RRl				
RRu				
P				
Random RR				
RRl				
RRu				
P				
Between Chi				
Between df				
Between P				N.S.
Btwn(F) P				N.S.
Btwn(R) P				N.S.

Detailed other continent

	SCAmer	Auslia	Africa	Total
N	1	1		2
NS	1	1		2
Wt	0.74	0.66		1.40
Het Chi	0.00	0.00		0.58
Het df	0	0		1
Het P	N.S.	N.S.		N.S.
Fixed RR	2.50	9.10		4.60
RRl	0.26	0.82		0.88
RRu	24.38	101.04		24.07
P	N.S.	(+)		(+)
Random RR	2.50	9.10		4.60
RRl	0.26	0.82		0.88
RRu	24.38	101.04		24.07
P	N.S.	(+)		(+)
Between Chi				0.58
Between df				1
Between P				N.S.
Btwn(F) P				N.S.
Btwn(R) P				N.S.

Table 1E2 - 3

IESLC - Meta-analysis of Current Smoking, Pipe and/or Cigars (not cigs)
All LC types
Most adjusted

	Start year of study					
	<1960	1960-69	1970-79	1980-89	1990+	Total
N	3	10	6	2	5	26
NS	3	9	5	1	2	20
Wt	54.01	61.77	96.97	5.00	8.58	226.33
Het Chi	9.11	21.99	7.42	5.95	11.21	104.61
Het df	2	9	5	1	4	25
Het P	*	**	N.S.	*	*	***
Fixed RR	2.24	4.67	3.50	16.69	17.28	3.74
RRl	1.72	3.64	2.87	6.95	8.85	3.29
RRu	2.93	5.99	4.27	40.09	33.75	4.26
P	+++	+++	+++	+++	+++	+++
Random RR	2.82	5.28	3.16	15.07	5.61	4.76
RRl	1.47	3.42	2.27	1.76	1.05	3.44
RRu	5.41	8.17	4.40	129.35	29.98	6.59
P	++	+++	+++	+	+	+++
Between Chi						48.93
Between df						4
Between P						***
Btwn(F) P						**
Btwn(R) P						N.S.

	Study type (1)		
	CC	other	Total
N	13	13	26
NS	8	12	20
Wt	141.78	84.54	226.33
Het Chi	59.29	45.02	104.61
Het df	12	12	25
Het P	***	***	***
Fixed RR	3.85	3.57	3.74
RRl	3.27	2.88	3.29
RRu	4.54	4.42	4.26
P	+++	+++	+++
Random RR	4.90	4.68	4.76
RRl	2.98	2.92	3.44
RRu	8.05	7.52	6.59
P	+++	+++	+++
Between Chi			0.31
Between df			1
Between P			N.S.
Btwn(F) P			N.S.
Btwn(R) P			N.S.

	Study type (2)			
	CC	prosp	other	Total
N	13	13		26
NS	8	12		20
Wt	141.78	84.54		226.33
Het Chi	59.29	45.02		104.61
Het df	12	12		25
Het P	***	***		***
Fixed RR	3.85	3.57		3.74
RRl	3.27	2.88		3.29
RRu	4.54	4.42		4.26
P	+++	+++		+++
Random RR	4.90	4.68		4.76
RRl	2.98	2.92		3.44
RRu	8.05	7.52		6.59
P	+++	+++		+++
Between Chi				0.31
Between df				1
Between P				N.S.
Btwn(F) P				N.S.
Btwn(R) P				N.S.

Table 1E2 - 3

IESLC - Meta-analysis of Current Smoking, Pipe and/or Cigars (not cigs)
All LC types
Most adjusted

	Study size (number of LC cases) 100-249	250-499	500-999	1000+	Total
N	5	5	9	7	26
NS	5	4	7	4	20
Wt	11.35	24.25	65.07	125.65	226.33
Het Chi	3.48	10.33	29.17	53.40	104.61
Het df	4	4	8	6	25
Het P	N.S.	*	***	***	***
Fixed RR	4.91	6.06	3.75	3.32	3.74
RRl	2.74	4.07	2.94	2.79	3.29
RRu	8.78	9.02	4.78	3.96	4.26
P	+++	+++	+++	+++	+++
Random RR	4.91	5.32	5.10	4.16	4.76
RRl	2.74	2.70	3.02	1.99	3.44
RRu	8.78	10.47	8.63	8.70	6.59
P	+++	+++	+++	+++	+++
Between Chi					8.24
Between df					3
Between P					*
Btwn(F) P					N.S.
Btwn(R) P					N.S.

	Risky occupational population no	mining	othRisky	Total
N	25	1		26
NS	19	1		20
Wt	225.66	0.66		226.33
Het Chi	104.09	0.00		104.61
Het df	24	0		25
Het P	***	N.S.		***
Fixed RR	3.73	9.10		3.74
RRl	3.28	0.82		3.29
RRu	4.25	101.04		4.26
P	+++	(+)		+++
Random RR	4.72	9.10		4.76
RRl	3.40	0.82		3.44
RRu	6.55	101.04		6.59
P	+++	(+)		+++
Between Chi				0.52
Between df				1
Between P				N.S.
Btwn(F) P				N.S.
Btwn(R) P				N.S.

	National cigarette tobacco type Virginia	blended	other	Total
N	11	15		26
NS	9	11		20
Wt	36.02	190.30		226.33
Het Chi	24.33	78.05		104.61
Het df	10	14		25
Het P	**	***		***
Fixed RR	4.71	3.58		3.74
RRl	3.39	3.11		3.29
RRu	6.52	4.13		4.26
P	+++	+++		+++
Random RR	5.14	4.57		4.76
RRl	2.95	3.02		3.44
RRu	8.93	6.90		6.59
P	+++	+++		+++
Between Chi				2.24
Between df				1
Between P				N.S.
Btwn(F) P				N.S.
Btwn(R) P				N.S.

Table 1E2 - 3

IESLC - Meta-analysis of Current Smoking, Pipe and/or Cigars (not cigs)
All LC types
Most adjusted

	Any proxy use No/nk	Yes	Total
N	26		26
NS	20		20
Wt	226.33		226.33
Het Chi	104.61		104.61
Het df	25		25
Het P	***		***
Fixed RR	3.74		3.74
RRl	3.29		3.29
RRu	4.26		4.26
P	+++		+++
Random RR	4.76		4.76
RRl	3.44		3.44
RRu	6.59		6.59
P	+++		+++
Between Chi			
Between df			
Between P			N.S.
Btwn(F) P			N.S.
Btwn(R) P			N.S.

	Full histological confirmation No	Yes	Total
N	24	2	26
NS	18	2	20
Wt	142.51	83.82	226.33
Het Chi	101.77	2.80	104.61
Het df	23	1	25
Het P	***	(*)	***
Fixed RR	3.78	3.68	3.74
RRl	3.21	2.97	3.29
RRu	4.46	4.56	4.26
P	+++	+++	+++
Random RR	5.06	3.39	4.76
RRl	3.39	2.22	3.44
RRu	7.57	5.19	6.59
P	+++	+++	+++
Between Chi			0.04
Between df			1
Between P			N.S.
Btwn(F) P			N.S.
Btwn(R) P			N.S.

	Number of adjustment variables (1) 0	1	2+/+nk	Total
N	11	9	6	26
NS	7	9	5	21
Wt	59.61	76.48	90.24	226.33
Het Chi	56.50	12.66	11.68	104.61
Het df	10	8	5	25
Het P	***	N.S.	*	***
Fixed RR	4.52	2.38	4.85	3.74
RRl	3.50	1.90	3.95	3.29
RRu	5.82	2.98	5.96	4.26
P	+++	+++	+++	+++
Random RR	6.79	2.71	6.30	4.76
RRl	3.12	1.95	3.96	3.44
RRu	14.76	3.76	10.03	6.59
P	+++	+++	+++	+++
Between Chi				23.77
Between df				2
Between P				***
Btwn(F) P				(*)
Btwn(R) P				**

Table 1E2 - 3

IESLC - Meta-analysis of Current Smoking, Pipe and/or Cigars (not cigs)
All LC types
Most adjusted

	Number of adjustment variables (2)					
	0	1	2	3-5	6+/+nk	Total
N	11	9	4	2		26
NS	7	9	4	1		21
Wt	59.61	76.48	75.21	15.02		226.33
Het Chi	56.50	12.66	1.69	1.53		104.61
Het df	10	8	3	1		25
Het P	***	N.S.	N.S.	N.S.		***
Fixed RR	4.52	2.38	4.23	9.63		3.74
RRl	3.50	1.90	3.37	5.81		3.29
RRu	5.82	2.98	5.30	15.96		4.26
P	+++	+++	+++	+++		+++
Random RR	6.79	2.71	4.23	9.94		4.76
RRl	3.12	1.95	3.37	5.24		3.44
RRu	14.76	3.76	5.30	18.84		6.59
P	+++	+++	+++	+++		+++
Between Chi						32.24
Between df						3
Between P						***
Btwn(F) P						*
Btwn(R) P						**

	Derivation of RR/CI			
	Orig	StdCalc	Other	Total
N	4	7	15	26
NS	4	6	12	22
Wt	12.62	58.02	155.69	226.33
Het Chi	4.97	52.54	35.96	104.61
Het df	3	6	14	25
Het P	N.S.	***	**	***
Fixed RR	7.71	4.51	3.29	3.74
RRl	4.44	3.48	2.82	3.29
RRu	13.39	5.83	3.85	4.26
P	+++	+++	+++	+++
Random RR	7.24	7.30	3.47	4.76
RRl	3.40	3.05	2.48	3.44
RRu	15.44	17.45	4.85	6.59
P	+++	+++	+++	+++
Between Chi				11.14
Between df				2
Between P				**
Btwn(F) P				N.S.
Btwn(R) P				(*)

Table 1E2 - 4

IESLC - Meta-analysis of Current Smoking, Pipe and/or Cigars (not cigs)
All LC types
Least adjusted

REF	NRR	X	SEX	AGEL	AGEH	RACE	YF	LC TYPE	LOC	START	ST	NLC	R	VB	P	H	AD	DENOM	De
BENSHL	10		m	40	64	all	10	all	Eu:UK	1967	pr	486	n	V	n	n	1	nev any	ot
BROSS	5		m	0	0	wh	-	all	NAmer	1960	CC	974	n	bl	n	n	0	nev any	st
CHOW	13		m	0	0	wh	0	all	NAmer	1966	pr	219	n	bl	n	n	0	nev any	st
DARBY	6		m	0	0	wh	-	all	Eu:UK	1988	CC	982	n	V	n	n	0	nev any	st
DARBY	13		f	0	0	wh	-	all	Eu:UK	1988	CC	982	n	V	n	n	0	nev any	st
DEKLER	5		m	0	0	all	0	all	Auslia	1961	pr	138	m	V	n	n	2	nev any	or
DOLL2	20		m	0	0	all	20	all	Eu:UK	1951	pr	920	n	V	n	n	1	nev any	ot
DORN	90		m	35	84	wh	8	all	NAmer	1954	pr	5097	n	bl	n	n	1	nev any	ot
ENGELA	167		m	0	0	all	12	all	Eu:Sca	1964	pr	435	n	bl	n	n	1	nev any	ot
GRAHAM	16	x	m	0	0	wh	-	all	NAmer	1956	CC	685	n	bl	n	n	0	nev any	st
HOLE	13	x	m	0	0	all	0	all	Eu:UK	1972	pr	225	n	V	n	n	0	nev any	st
KINLEN	2	x	m	0	0	all	0	all	Eu:UK	1967	pr	718	n	V	n	n	0	nev any	st
KNEKT	24	x	m	20	69	all	21	all	Eu:Sca	1966	pr	515	n	bl	n	n	0	nev any	st
KREUZE	9		m	1	45	all	-	all	Eu:Ger	1990	CC	2260	n	bl	n	n	0	nev any	ot
KREUZE	11		m	55	69	all	-	all	Eu:Ger	1990	CC	2260	n	bl	n	n	0	nev any	st
KREUZE	10		f	1	45	all	-	all	Eu:Ger	1990	CC	2260	n	bl	n	n	0	nev any	ot
KREUZE	12		f	55	69	all	-	all	Eu:Ger	1990	CC	2260	n	bl	n	n	0	nev any	ot
LUBIN2	29	x	m	0	0	all	-	all	Eu:mul	1976	CC	7804	n	bl	n	y	0	nev any	st
MATOS	1		m	0	0	all	-	all	SCAmer	1994	CC	200	n	bl	n	n	0	nev any	st
MIGRAN	15	x	m	0	0	all	0	all	Eu:UK	1964	pr	259	n	V	n	n	0	nev any	st
TULINI	12	x	m	0	0	all	0	all	Eu:Sca	1967	pr	472	n	bl	n	n	1	nev any	ot
TULINI	8	x	f	0	0	all	0	all	Eu:Sca	1967	pr	472	n	bl	n	n	1	nev any	or
WALD	1	x	m	0	0	all	0	all	Eu:UK	1975	pr	102	n	V	n	n	0	nev any	st
WIGLE	2	x	m	0	0	all	-	all	NAmer	1971	CC	728	n	V	n	n	0	nev any	st
WIGLE	5		f	0	0	all	-	all	NAmer	1971	CC	728	n	V	n	n	0	nev any	ot
WYNDE7	46		m	0	0	all	-	all	NAmer	1977	CC	2085	n	bl	n	y	0	nev any	st

Table 1E2 - 5

IESLC - Meta-analysis of Current Smoking, Pipe and/or Cigars (not cigs)
All LC types
Least adjusted

REF	NRR	SEX	AD	Number Exposed Case	Number Exposed Cont	Non-exposed Case	Non-exposed Cont	RR	95.00%CI
*BENSHL	10	m	1	-	-	-	-	2.33	(0.58- 9.33)
BROSS	5	m	0	125	211	38	170	2.65	(1.75- 4.02)
*CHOW	13	m	0	37	47084	6	62913	8.24	(3.48- 19.52)
DARBY	6	m	0	57	165	3	384	44.22	(13.65- 143.22)
DARBY	13	f	0	3	14	23	529	4.93	(1.32- 18.36)
Subtotal DARBY								16.69	(6.95- 40.09)
*DEKLER	5	m	2	-	-	-	-	9.10	(0.82- 101.10)
*DOLL2	20	m	1	-	-	-	-	5.80	(2.65- 12.72)
*DORN	90	m	1	-	-	-	-	1.70	(1.21- 2.37)
*ENGELA	167	m	1	-	-	-	-	2.60	(1.02- 6.60)
GRAHAM	16	m	0	64	398	18	346	3.09	(1.80- 5.32)
*HOLE	13	m	0	2	141	7	1189	2.41	(0.51- 11.49)
*KINLEN	2	m	0	47	1367	7	1333	6.55	(2.97- 14.43)
*KNEKT	24	m	0	7	2822	6	17814	7.36	(2.48- 21.90)
KREUZE	9	m	0	0	6	6	54	0.64~	(0.03- 12.82)
KREUZE	11	m	0	26	15	23	403	30.37	(14.18- 65.06)
KREUZE	10	f	0	0	1	6	38	1.97~	(0.07- 53.89)
KREUZE	12	f	0	3	0	95	177	13.01~	(0.67- 254.52)
Subtotal KREUZE								20.75	(10.30- 41.79)
LUBIN2	29	m	0	142	465	190	2617	4.21	(3.31- 5.34)
MATOS	1	m	0	1	4	11	110	2.50	(0.26- 24.38)
*MIGRAN	15	m	0	16	575	4	867	6.03	(2.03- 17.95)
*TULINI	12	m	1	-	-	-	-	7.92	(4.20- 14.90)
*TULINI	8	f	1	-	-	-	-	15.70	(6.78- 36.30)
Subtotal TULINI								10.15	(6.12- 16.83)
*WALD	1	m	0	6	1309	7	6539	4.28	(1.44- 12.72)
WIGLE	2	m	0	24	86	15	204	3.80	(1.90- 7.59)
WIGLE	5	f	0	1	0	36	439	36.12~	(1.45- 902.62)
Subtotal WIGLE								4.19	(2.13- 8.25)
WYNDE7	46	m	0	31	171	64	918	2.60	(1.64- 4.11)
Partial Totals				592	54834	565	97044		

*prospective study ~ With 0.5 adjustment for zero

REF	NRR	SEX	AD	Ys	Ws	Qs	Ps
*BENSHL	10	m	1	0.85	1.99	0.56	0.2326
BROSS	5	m	0	0.97	22.25	3.56	0.0000
*CHOW	13	m	0	2.11	5.16	2.78	0.0000
DARBY	6	m	0	3.79	2.78	16.21	0.0000
DARBY	13	f	0	1.60	2.22	0.11	0.0174
Subtotal DARBY				2.81	5.00	16.32	
*DEKLER	5	m	2	2.21	0.66	0.46	0.0722
*DOLL2	20	m	1	1.76	6.24	0.92	0.0000
*DORN	90	m	1	0.53	34.00	24.23	0.0020
*ENGELA	167	m	1	0.96	4.41	0.77	0.0449
GRAHAM	16	m	0	1.13	13.06	0.79	0.0000
*HOLE	13	m	0	0.88	1.57	0.39	0.2698
*KINLEN	2	m	0	1.88	6.15	1.56	0.0000
*KNEKT	24	m	0	2.00	3.24	1.25	0.0003
KREUZE	9	m	0	-0.44	0.43	1.41	0.7737
KREUZE	11	m	0	3.41	6.62	27.51	0.0000
KREUZE	10	f	0	0.68	0.35	0.17	0.6868
KREUZE	12	f	0	2.57	0.43	0.62	0.0908
Subtotal KREUZE				3.03	7.83	29.71	
LUBIN2	29	m	0	1.44	67.39	0.26	0.0000
MATOS	1	m	0	0.92	0.74	0.16	0.4303
*MIGRAN	15	m	0	1.80	3.23	0.58	0.0012
*TULINI	12	m	1	2.07	9.58	4.62	0.0000
*TULINI	8	f	1	2.75	5.46	10.38	0.0000
Subtotal TULINI				2.32	15.04	15.00	
*WALD	1	m	0	1.45	3.24	0.02	0.0088
WIGLE	2	m	0	1.33	8.01	0.01	0.0002
WIGLE	5	f	0	3.59	0.37	1.81	0.0289
Subtotal WIGLE				1.43	8.38	1.83	
WYNDE7	46	m	0	0.96	18.24	3.21	0.0000

Table 1E2 - 5

IESLC - Meta-analysis of Current Smoking, Pipe and/or Cigars (not cigs)
All LC types
Least adjusted

	N	26
	NS	20
	Wt	227.84
Het	Chi	104.35
Het	df	25
Het	P	***
Fixed	RR	3.95
	RRl	3.47
	RRu	4.50
	P	+++
Random	RR	5.16
	RRl	3.73
	RRu	7.13
	P	+++
Asymm	P	(*)

Table 1E2 - 6

IESLC - Meta-analysis of Current Smoking, Pipe and/or Cigars (not cigs)
All LC types
Least adjusted

	Sex		
combined	male	female	Total
N	21	5	26
NS	20	4	24
Wt	219.01	8.84	227.84
Het Chi	90.88	3.70	104.35
Het df	20	4	25
Het P	***	N.S.	***
Fixed RR	3.79	11.09	3.95
RRl	3.32	5.73	3.47
RRu	4.33	21.43	4.50
P	+++	+++	+++
Random RR	4.77	11.09	5.16
RRl	3.41	5.73	3.73
RRu	6.67	21.43	7.13
P	+++	+++	+++
Between Chi			9.77
Between df			1
Between P			**
Btwn(F) P			N.S.
Btwn(R) P			*

Table 1E2 - 7

IESLC - Meta-analysis of Current Smoking, Pipe and/or Cigars (not cigs)
All LC types
Excluded studies (and stage at which they were excluded)

1	BOUCHA BUELL LAURIL MZILEN
2	ABRAHA AGUDO AKIBA AMANDU AMES ANDERS ARCHER AUSTIN AUVINE AXELSO AXELSS BAND BARBON BERRIN BLOHMK BLOT1 BLOT2 BLOT3 BLOT4 BRETT BROCKM BROWN1 BROWN2 BUFFLE BYERS1 BYERS2 CARPEN CASCO2 CASCOR CEDERL CHAN CHANG CHATZI CHEN CHEN2 CHEN3 CHIAZZ CHOI CHYOU COOKSO CORREA CPSII DAVEYS DEAN3 DESTE2 DESTEF DOCKER DOLL DORANT DOSEME DROSTE DU DUNN EBELIN ENSTRO ESAKI FAN GAO GAO2 GARCIA GARDIN GARSHI GENG GER GILLIS GODLEY GOODMA GREGOR GSELL HAENSZ HANSEN HEGMAN HEIN HENNEK HINDS HIRAOK HIRAY2 HIRAYA HITOSU HOROWI HORWIT HU HU2 HUANG HUMBLE ISHIMA JAIN JARUP JARVHO JEDRYC JIANG JOLY JONES JUSSAW KAISE2 KAISER KANELL KATSOU KAUFMA KELLER KHUDER KIHARA KO KOHLME KOO KOULUM KREYBE KUBIK LAMTH LAMWK LAMWK2 LANGE LAUSSM LEI LEMARC LETOUR LIAW LICKIN LIDDEL LIU LIU2 LIU3 LIU4 LIU5 LOMBA2 LUBIN LUO MACLEN MAGNUS MARTIS MASTRA MATSUD MCCONN MCDUFF MCLAUG MILLER MILLS MOLLO MRFIT MRFITR MURATA NAM NOTAN2 NOTANI NOU ODRISC ORMOS OSANN OSANN2 PARKIN PASTOR PAWLEG PERNU PERSH2 PERSHA PETO PEZZO2 PEZZOT PIKE PISANI POFFIJ POLEDN PRESCO QIAO QIAO2 RACHTA RADZIK RANDIG REN RESTRE RIMING RONCO ROOTS ROTHSC SAARIK SANKAR SCHWA2 SCHWAR SEGI SEGI2 SEOW SHAW SHIMIZ SIEMIA SIMARA SIMONA SITAS SOBUE SOBUE2 SPEIZE SPITZ STAYNE STOCKS STUCKE SUN SUZUK2 SUZUKI SVENSS TANG TANG2 TAO TENKAN TIZZAN TOKARS TSUGAN TVERDA ULMER VANDER VEIERO VUTUC WAKAI WANG WANG2 WANG3 WANG4 WARSIN WATSON WICKLU WILKIN WU WU2 WUNSCH WUWILL WYNDE4 WYNDE5 WYNDE8 WYNDER XIANGZ XU XU2 XU3 XU4 YAMAGU YONG YUAN ZHANG ZHENG ZHOU
3	ABELIN ALDERS ARMADA BECHER BENHAM BEST BOFFET BOUCOT BRESLO COMSTO CPSI DAMBER DEAN DEAN2 DORGAN GOLLED HAMMO2 HAMMON JAHN KJUUS LEVIN LOMBAR MARSH MARSH2 SADOWS STASZE STOCKW TOUSEY WYNDE2 WYNDE3 WYNDE6

Table 1E2 - 8

Potentially overlapping studies

REF	REFGP	PRINC	OVERLAP/LINK
LUBIN2	LUBIN2	1	Lubin-combined
BROSS	BYERS1	1	GRAHAM/BROSS/BYERS1
GRAHAM	BYERS1	1	GRAHAM/BROSS/BYERS1
HOLE	TANG2	1	Subset of TANG2
BENSHL	TANG2	1	Subset of TANG2
WALD	TANG2	1	Subset of TANG2
WYNDE7	WYNDE6	2	WYNDE5/6/7/8

Table 1E3 -

IESLC - Meta-analysis of Ever Smoking (or Current if Ever not available), Pipe and/or Cigars (not cigs)
All LC types

This analysis is restricted to results for:
1) Non-dose-response data
2) Smokers of pipe and/or cigars (but not cigarettes)
3) Results complete enough for use in metaanalysis

Within each study, results are then selected (in the following order of preference, within each sex) for:
4) SMKSTA: ever smokers, current smokers
5) DENOM: never smoked anything, (never +1 = +long term ex)
6) Followup period (prospective studies): whole study (coded as 0) or longest available
7) LCtype: all or nearest available, at least Squamous and Adeno. (q = squamous, s = small,
          l = large, a = adeno, mix = mixed, alv = alveolar)
8) Race: all or nearest available, otherwise by race (wh or w = white, bl or b = black, hi = hispanic
          ch = chinese, jap = japanese, haw = hawaiian, w+o = white + oriental, sca = scandinavian, as = asian)
9) For overlapping studies: principal rather than subsidiary studies
Finally by Age: whole study (coded as 0) if available, otherwise by widest available age group
and then for single sex results (m, f) in preference to combined sex results (c).

Results adjusted (AD) for the most potential confounders are then chosen in Sections -1 to -3
and results adjusted for the least confounders in Sections -4 to -6. (Those least adjusted results which
actually differ from the most adjusted as marked 'x' in column X in Section -4)
(Results adjusted for an unknown number of confounder(s) are coded as 20.)

Section -7 shows excluded studies, together with the stage (as above) at which no qualifying
results were found.

Section -8 lists the potentially overlapping studies which have been included (1=principal, 2=subsidiary).

Section -9 lists any results which would have been included in preference except that they had data not complete
enough for use in meta-analysis, with their significance (yes/no), if known, and any further comment as entered
on the database.

In addition to those mentioned above, the following fields, levels and abbreviations are used:
* or nk = not known, n = no, y = yes, ot = other
ev = ever, cu = current, nev = never
REF: 6-character study reference
NRR: number of the RR on the database within the study
ST : study type (CC = case control, pr or prosp = prospective)
NLC: number of lung cancer cases in whole study
R : risky occupational population (n = no, m = mining, o = other risky)
VB : national cigarette type (V = at least 75% Virginia, bl = at least 75% blended, ot = other)
P : any proxy use
H : full histological confirmation
De : derivation of RR/CI (or = original, st = standard method, ot = other method of estimation)

Table 1E3 - 1

IESLC - Meta-analysis of Ever Smoking (or Current if Ever not available), Pipe and/or Cigars (not cigs)
All LC types
Most adjusted

REF	NRR	SEX	AGEL	AGEH	RACE	YF	LC TYPE	LOC	START	ST	NLC	R	VB	P	H	AD	SM	DENOM	De
ABELIN	46	m	0	0	all	-	all	Eu:wst	1941	CC	118	n	bl	y	n	1	ev	nev any	st
ALDERS	1	m	0	0	all	-	all	Eu:UK	1977	CC	1448	n	V	n	n	1	ev	nev any	ot
ARMADA	30	m	0	0	all	-	all	Eu:wst	1986	CC	325	n	bl	n	y	0	ev	nev any	st
BECHER	23	m	0	0	all	-	all	Eu:Ger	1985	CC	194	n	bl	n	y	2	ev	nev any	or
BENSHL	10	m	40	64	all	10	all	Eu:UK	1967	pr	486	n	V	n	n	1	cu	nev any	ot
BEST	19	m	55	79	all	3	all	NAmer	1955	pr	381	n	V	n	n	0	ev	nev any	st
BOFFET	30	m	0	0	all	-	all	Eu:mul	1988	CC	5621	n	bl	y	n	0	ev	nev any	st
BOUCOT	117	m	0	0	all	0	all	NAmer	1951	pr	121	n	bl	n	n	2	ev	nev any	ot
BRESLO	33	m	0	0	all	-	all	NAmer	1949	CC	518	n	bl	n	y	0	ev	nev+1	st
BROSS	5	m	0	0	wh	-	all	NAmer	1960	CC	974	n	bl	n	n	0	cu	nev any	st
CHOW	8	m	0	0	wh	0	all	NAmer	1966	pr	219	n	bl	n	n	2	ev	nev any	ot
COMSTO	1	m	0	0	all	-	all	NAmer	1975	ot	258	n	bl	n	n	0	ev	nev any	st
CPSI	186	m	35	84	all	6	all	NAmer	1959	pr	5138	n	bl	n	n	1	ev	nev any	ot
DAMBER	28	m	0	0	all	-	all	Eu:Sca	1972	CC	579	n	bl	y	n	0	ev	nev any	st
DARBY	6	m	0	0	wh	-	all	Eu:UK	1988	CC	982	n	V	n	n	0	cu	nev any	st
DARBY	13	f	0	0	wh	-	all	Eu:UK	1988	CC	982	n	V	n	n	0	cu	nev any	st
DEAN	11	m	0	0	wh	-	all	Africa	1947	CC	603	n	V	y	n	0	ev	nev any	st
DEAN2	11	m	0	0	all	-	all	Eu:UK	1960	CC	954	n	V	y	n	0	ev	nev any	st
DEKLER	5	m	0	0	all	0	all	Auslia	1961	pr	138	m	V	n	n	2	cu	nev any	or
DOLL2	33	m	0	0	all	10	all	Eu:UK	1951	pr	920	n	V	n	n	1	ev	nev any	ot
DORGAN	1	m	0	0	wh	-	all	NAmer	1980	CC	2026	n	bl	y	y	0	ev	nev any	st
DORGAN	25	m	0	0	bl	-	all	NAmer	1980	CC	2026	n	bl	y	y	0	ev	nev any	st
DORN	35	m	0	0	wh	2	all	NAmer	1954	pr	5097	n	bl	n	n	1	ev	nev any	ot
ENGELA	167	m	0	0	all	12	all	Eu:Sca	1964	pr	435	n	bl	n	n	1	cu	nev any	ot
GOLLED	5	m	35	99	all	-	all	Eu:UK	1952	CC	443	n	V	y	n	1	ev	nev any	ot
GRAHAM	11	m	0	0	wh	-	all	NAmer	1956	CC	685	n	bl	n	n	1	ev	nev any	ot
HAMMO2	14	m	0	0	all	0	all	NAmer	1967	pr	450	o	bl	n	n	1	ev	nev any	ot
HAMMON	118	m	0	0	wh	0	all	NAmer	1952	pr	448	n	bl	n	n	1	ev	nev any	ot
HOLE	6	m	0	0	all	0	all	Eu:UK	1972	pr	225	n	V	n	n	1	cu	nev any	ot
KINLEN	13	m	0	0	all	0	all	Eu:UK	1967	pr	718	n	V	n	n	2	cu	nev any	ot
KJUUS	4	m	0	0	all	-	all	Eu:Sca	1979	CC	176	n	bl	n	n	0	ev	nev any	st
KNEKT	28	m	20	69	all	21	all	Eu:Sca	1966	pr	515	n	bl	n	n	1	cu	nev any	or
KREUZE	10	f	1	45	all	-	all	Eu:Ger	1990	CC	2260	n	bl	n	n	0	cu	nev any	ot
KREUZE	12	f	55	69	all	-	all	Eu:Ger	1990	CC	2260	n	bl	n	n	0	cu	nev any	ot
LEVIN	31	m	0	0	all	-	all	NAmer	1938	CC	475	n	bl	n	n	1	ev	nev any	st
LOMBAR	1	m	0	0	all	-	all	NAmer	1951	CC	1040	n	bl	n	n	0	ev	nev any	st
LUBIN2	50	m	0	0	all	-	all	Eu:mul	1976	CC	7804	n	bl	n	y	2	ev	nev any	ot
MARSH	5	m	0	0	all	-	all	NAmer	1979	CC	150	n	bl	y	n	0	ev	nev any	ot
MARSH2	3	c	0	0	all	-	all	NAmer	1979	CC	114	n	bl	y	n	0	ev	nev any	ot
MATOS	1	m	0	0	all	-	all	SCAmer	1994	CC	200	n	bl	n	n	0	cu	nev any	st
MIGRAN	16	m	0	0	all	0	all	Eu:UK	1964	pr	259	n	V	n	n	2	cu	nev any	ot
SADOWS	70	m	0	0	wh	-	all	NAmer	1938	CC	477	n	bl	n	n	0	ev	nev any	st
STASZE	3	m	0	0	all	-	all	Eu:est	1954	CC	281	n	bl	n	y	0	ev	nev any	st
STOCKW	5	c	0	0	all	-	all	NAmer	1981	CC	22161	n	bl	n	n	0	ev	nev any	st
TOUSEY	9	m	0	0	all	-	all	NAmer	1993	CC	507	n	bl	y	y	3	ev	nev any	or
TOUSEY	5	f	0	0	all	-	all	NAmer	1993	CC	507	n	bl	y	y	0	ev	nev any	st
TULINI	35	m	0	0	all	0	all	Eu:Sca	1967	pr	472	n	bl	n	n	3	cu	nev any	ot
TULINI	31	f	0	0	all	0	all	Eu:Sca	1967	pr	472	n	bl	n	n	3	cu	nev any	or
WALD	3	m	0	0	all	0	all	Eu:UK	1975	pr	102	n	V	n	n	1	cu	nev any	or
WIGLE	14	m	0	0	all	-	all	NAmer	1971	CC	728	n	V	n	n	0	ev	nev any	st
WIGLE	17	f	0	0	all	-	all	NAmer	1971	CC	728	n	V	n	n	0	ev	nev any	ot
WYNDE2	15	m	0	0	all	-	all	NAmer	1962	CC	404	n	bl	n	y	0	ev	nev any	st
WYNDE3	152	m	0	0	all	-	all	NAmer	1966	CC	350	n	bl	n	y	0	ev	nev any	st
WYNDE6	63	m	0	0	all	-	all	NAmer	1969	CC	4423	n	bl	n	y	0	ev	nev any	st

Table 1E3 - 2

IESLC - Meta-analysis of Ever Smoking (or Current if Ever not available), Pipe and/or Cigars (not cigs)
All LC types
Most adjusted

REF	NRR	SEX	AD	Number Exposed Case	Number Exposed Cont	Non-exposed Case	Non-exposed Cont	RR	95.00%CI
ABELIN	46	m	1	-	-	-	-	30.49	(7.32- 127.00)
ALDERS	1	m	1	-	-	-	-	3.82	(1.74- 8.40)
ARMADA	30	m	0	4	7	4	64	9.14	(1.86- 44.85)
BECHER	23	m	2	-	-	-	-	4.62	(1.15- 18.56)
*BENSHL	10	m	1	-	-	-	-	2.33	(0.58- 9.33)
*BEST	19	m	0	8	2972	1	2854	7.68	(0.96- 61.38)
BOFFET	30	m	0	118	266	117	1750	6.64	(4.98- 8.84)
*BOUCOT	117	m	2	-	-	-	-	6.80	(0.37- 126.25)
BRESLO	33	m	0	15	68	7	42	1.32	(0.50- 3.51)
BROSS	5	m	0	125	211	38	170	2.65	(1.75- 4.02)
*CHOW	8	m	2	-	-	-	-	3.95	(1.18- 13.26)
COMSTO	1	m	0	2	15	4	69	2.30	(0.39- 13.73)
*CPSI	186	m	1	-	-	-	-	1.94	(1.44- 2.62)
DAMBER	28	m	0	205	149	42	208	6.81	(4.60- 10.09)
DARBY	6	m	0	57	165	3	384	44.22	(13.65- 143.22)
DARBY	13	f	0	3	14	23	529	4.93	(1.32- 18.36)
Subtotal DARBY								16.69	(6.95- 40.09)
DEAN	11	m	0	51	74	12	61	3.50	(1.71- 7.16)
DEAN2	11	m	0	81	128	33	112	2.15	(1.33- 3.46)
*DEKLER	5	m	2	-	-	-	-	9.10	(0.82- 101.10)
*DOLL2	33	m	1	-	-	-	-	6.14	(1.85- 20.40)
DORGAN	1	m	0	22	55	15	93	2.48	(1.19- 5.18)
DORGAN	25	m	0	3	19	3	35	1.84	(0.34- 10.03)
Subtotal DORGAN								2.37	(1.20- 4.65)
*DORN	35	m	1	-	-	-	-	1.60	(0.81- 3.16)
*ENGELA	167	m	1	-	-	-	-	2.60	(1.02- 6.60)
GOLLED	5	m	1	-	-	-	-	3.13	(1.73- 5.67)
GRAHAM	11	m	1	-	-	-	-	2.55	(1.49- 4.38)
*HAMMO2	14	m	1	-	-	-	-	0.88	(0.21- 3.67)
*HAMMON	118	m	1	-	-	-	-	1.55	(0.83- 2.90)
*HOLE	6	m	1	-	-	-	-	2.29	(0.48- 11.04)
*KINLEN	13	m	2	-	-	-	-	6.51	(2.95- 14.35)
KJUUS	4	m	0	23	25	2	24	11.04	(2.34- 52.00)
*KNEKT	28	m	1	-	-	-	-	6.10	(2.10- 18.20)
KREUZE	10	f	0	0	1	6	38	1.97~	(0.07- 53.89)
KREUZE	12	f	0	3	0	95	177	13.01~	(0.67- 254.52)
Subtotal KREUZE								5.60	(0.61- 51.10)
LEVIN	31	m	1	-	-	-	-	1.31	(0.81- 2.12)
LOMBAR	1	m	0	48	146	14	112	2.63	(1.38- 5.01)
LUBIN2	50	m	2	-	-	-	-	3.14	(2.41- 4.08)
MARSH	5	m	0	0	5	2	31	1.15~	(0.05- 27.23)
MARSH2	3	c	0	0	4	12	56	0.50~	(0.03- 9.94)
MATOS	1	m	0	1	4	11	110	2.50	(0.26- 24.38)
*MIGRAN	16	m	2	-	-	-	-	3.81	(1.19- 12.23)
SADOWS	70	m	0	38	88	18	81	1.94	(1.03- 3.67)
STASZE	3	m	0	4	101	5	158	1.25	(0.33- 4.77)
STOCKW	5	c	0	715	655	2791	10641	4.16	(3.71- 4.66)
TOUSEY	9	m	3	-	-	-	-	2.80	(0.70- 11.60)
TOUSEY	5	f	0	1	2	13	226	8.69	(0.74- 102.22)
Subtotal TOUSEY								3.70	(1.09- 12.52)
*TULINI	35	m	3	-	-	-	-	7.57	(4.02- 14.26)
*TULINI	31	f	3	-	-	-	-	14.70	(6.35- 34.10)
Subtotal TULINI								9.63	(5.81- 15.96)
*WALD	3	m	1	-	-	-	-	3.19	(1.07- 9.50)
WIGLE	14	m	0	30	113	15	204	3.61	(1.86- 6.99)
WIGLE	17	f	0	1	0	36	439	36.12~	(1.45- 902.62)
Subtotal WIGLE								3.96	(2.07- 7.57)
WYNDE2	15	m	0	14	104	8	105	1.77	(0.71- 4.39)
WYNDE3	152	m	0	14	68	9	88	2.01	(0.82- 4.93)
WYNDE6	63	m	0	58	199	87	617	2.07	(1.43- 2.99)
Partial Totals				1644	5658	3426	19478		

*prospective study ~ With 0.5 adjustment for zero

Table 1E3 - 2

IESLC - Meta-analysis of Ever Smoking (or Current if Ever not available), Pipe and/or Cigars (not cigs)
All LC types
Most adjusted

REF	NRR	SEX	AD	Ys	Ws	Qs	Ps
ABELIN	46	m	1	3.42	1.89	8.70	0.0000
ALDERS	1	m	1	1.34	6.20	0.03	0.0008
ARMADA	30	m	0	2.21	1.52	1.35	0.0064
BECHER	23	m	2	1.53	1.99	0.13	0.0310
*BENSHL	10	m	1	0.85	1.99	0.36	0.2326
*BEST	19	m	0	2.04	0.89	0.53	0.0545
BOFFET	30	m	0	1.89	46.83	18.16	0.0000
*BOUCOT	117	m	2	1.92	0.45	0.19	0.1976
BRESLO	33	m	0	0.28	4.03	3.95	0.5736
BROSS	5	m	0	0.97	22.25	1.94	0.0000
*CHOW	8	m	2	1.37	2.63	0.03	0.0260
COMSTO	1	m	0	0.83	1.20	0.23	0.3609
*CPSI	186	m	1	0.66	42.89	15.81	0.0000
DAMBER	28	m	0	1.92	24.87	10.48	0.0000
DARBY	6	m	0	3.79	2.78	17.65	0.0000
DARBY	13	f	0	1.60	2.22	0.24	0.0174
Subtotal DARBY				2.81	5.00	17.89	
DEAN	11	m	0	1.25	7.53	0.00	0.0006
DEAN2	11	m	0	0.76	16.84	4.30	0.0017
*DEKLER	5	m	2	2.21	0.66	0.58	0.0722
*DOLL2	33	m	1	1.81	2.67	0.79	0.0030
DORGAN	1	m	0	0.91	7.09	0.93	0.0156
DORGAN	25	m	0	0.61	1.34	0.58	0.4799
Subtotal DORGAN				0.86	8.43	1.51	
*DORN	35	m	1	0.47	8.29	5.30	0.1759
*ENGELA	167	m	1	0.96	4.41	0.44	0.0449
GOLLED	5	m	1	1.14	10.90	0.18	0.0002
GRAHAM	11	m	1	0.94	13.22	1.47	0.0007
*HAMMO2	14	m	1	-0.13	1.88	3.67	0.8610
*HAMMON	118	m	1	0.44	9.82	6.79	0.1697
*HOLE	6	m	1	0.83	1.56	0.30	0.3003
*KINLEN	13	m	2	1.87	6.14	2.24	0.0000
KJUUS	4	m	0	2.40	1.60	2.05	0.0024
*KNEKT	28	m	1	1.81	3.30	0.96	0.0010
KREUZE	10	f	0	0.68	0.35	0.12	0.6868
KREUZE	12	f	0	2.57	0.43	0.73	0.0908
Subtotal KREUZE				1.72	0.79	0.85	
LEVIN	31	m	1	0.27	16.60	16.59	0.2713
LOMBAR	1	m	0	0.97	9.26	0.85	0.0033
LUBIN2	50	m	2	1.14	55.44	0.87	0.0000
MARSH	5	m	0	0.14	0.38	0.49	0.9331
MARSH2	3	c	0	-0.69	0.43	1.65	0.6511
MATOS	1	m	0	0.92	0.74	0.09	0.4303
*MIGRAN	16	m	2	1.34	2.83	0.01	0.0244
SADOWS	70	m	0	0.66	9.47	3.47	0.0409
STASZE	3	m	0	0.22	2.14	2.34	0.7425
STOCKW	5	c	0	1.43	296.07	7.23	0.0000
TOUSEY	9	m	3	1.03	1.95	0.11	0.1506
TOUSEY	5	f	0	2.16	0.63	0.50	0.0855
Subtotal TOUSEY				1.31	2.58	0.62	
*TULINI	35	m	3	2.02	9.58	5.46	0.0000
*TULINI	31	f	3	2.69	5.44	10.94	0.0000
Subtotal TULINI				2.26	15.02	16.39	
*WALD	3	m	1	1.16	3.22	0.04	0.0373
WIGLE	14	m	0	1.28	8.79	0.00	0.0001
WIGLE	17	f	0	3.59	0.37	1.99	0.0289
Subtotal WIGLE				1.38	9.16	1.99	
WYNDE2	15	m	0	0.57	4.64	2.28	0.2202
WYNDE3	152	m	0	0.70	4.79	1.56	0.1256
WYNDE6	63	m	0	0.73	28.26	8.35	0.0001

Table 1E3 - 2

IESLC - Meta-analysis of Ever Smoking (or Current if Ever not available), Pipe and/or Cigars (not cigs)
All LC types
Most adjusted

	N	54
	NS	48
	Wt	723.71
Het	Chi	176.03
Het	df	53
Het	P	***
Fixed	RR	3.56
	RRl	3.31
	RRu	3.83
	P	+++
Random	RR	3.37
	RRl	2.81
	RRu	4.04
	P	+++
Asymm	P	N.S.

Table 1E3 - 3

IESLC - Meta-analysis of Ever Smoking (or Current if Ever not available), Pipe and/or Cigars (not cigs)
All LC types
Most adjusted

	Sex			
	combined	male	female	Total
N	2	46	6	54
NS	2	45	5	52
Wt	296.50	417.76	9.45	723.71
Het Chi	1.92	145.23	3.48	176.03
Het df	1	45	5	53
Het P	N.S.	***	N.S.	***
Fixed RR	4.15	3.12	10.49	3.56
RRl	3.70	2.83	5.55	3.31
RRu	4.65	3.43	19.85	3.83
P	+++	+++	+++	+++
Random RR	2.50	3.19	10.49	3.37
RRl	0.42	2.61	5.55	2.81
RRu	14.72	3.91	19.85	4.04
P	N.S.	+++	+++	+++
Between Chi				25.40
Between df				2
Between P				***
Btwn(F) P				*
Btwn(R) P				**

	All LC (or nearest)		
	all	other	Total
N	54		54
NS	48		48
Wt	723.71		723.71
Het Chi	176.03		176.03
Het df	53		53
Het P	***		***
Fixed RR	3.56		3.56
RRl	3.31		3.31
RRu	3.83		3.83
P	+++		+++
Random RR	3.37		3.37
RRl	2.81		2.81
RRu	4.04		4.04
P	+++		+++
Between Chi			
Between df			
Between P			N.S.
Btwn(F) P			N.S.
Btwn(R) P			N.S.

	Location								
	NAmer	UK	Scand	othEur	China	Japan	othAs	other	Total
N	26	11	6	8				3	54
NS	23	10	5	7				3	48
Wt	497.63	57.36	49.20	110.59				8.93	723.71
Het Chi	78.86	26.09	7.79	26.15				0.67	176.03
Het df	25	10	5	7				2	53
Het P	***	**	N.S.	***				N.S.	***
Fixed RR	3.15	3.67	7.00	4.52				3.66	3.56
RRl	2.88	2.83	5.30	3.75				1.90	3.31
RRu	3.43	4.75	9.26	5.44				7.05	3.83
P	+++	+++	+++	+++				+++	+++
Random RR	2.27	4.32	7.02	5.17				3.66	3.37
RRl	1.79	2.73	4.72	2.91				1.90	2.81
RRu	2.89	6.84	10.44	9.19				7.05	4.04
P	+++	+++	+++	+++				+++	+++
Between Chi									36.46
Between df									4
Between P									***
Btwn(F) P									*
Btwn(R) P									***

Table 1E3 - 3

IESLC - Meta-analysis of Ever Smoking (or Current if Ever not available), Pipe and/or Cigars (not cigs)
All LC types
Most adjusted

	Detailed Country in "other Europe"					
	multi	Germany	othWest	East	Balkans	Total
N	2	3	2	1		8
NS	2	2	2	1		7
Wt	102.27	2.77	3.41	2.14		110.59
Het Chi	14.21	0.71	1.22	0.00		26.15
Het df	1	2	1	0		7
Het P	***	N.S.	N.S.	N.S.		***
Fixed RR	4.42	4.88	17.82	1.25		4.52
RRl	3.64	1.50	6.16	0.33		3.75
RRu	5.37	15.83	51.54	4.77		5.44
P	+++	++	+++	N.S.		+++
Random RR	4.55	4.88	17.61	1.25		5.17
RRl	2.19	1.50	5.44	0.33		2.91
RRu	9.48	15.83	57.07	4.77		9.19
P	+++	++	+++	N.S.		+++
Between Chi						10.01
Between df						3
Between P						*
Btwn(F) P						N.S.
Btwn(R) P						*

	Detailed Country in "other Asia"			
	India	HongKong	other	Total
N				
NS				
Wt				
Het Chi				
Het df				
Het P				N.S.
Fixed RR				
RRl				
RRu				
P				N.S.
Random RR				
RRl				
RRu				
P				N.S.
Between Chi				
Between df				
Between P				N.S.
Btwn(F) P				N.S.
Btwn(R) P				N.S.

	Detailed other continent			
	SCAmer	Auslia	Africa	Total
N	1	1	1	3
NS	1	1	1	3
Wt	0.74	0.66	7.53	8.93
Het Chi	0.00	0.00	0.00	0.67
Het df	0	0	0	2
Het P	N.S.	N.S.	N.S.	N.S.
Fixed RR	2.50	9.10	3.50	3.66
RRl	0.26	0.82	1.71	1.90
RRu	24.38	101.04	7.16	7.05
P	N.S.	(+)	+++	+++
Random RR	2.50	9.10	3.50	3.66
RRl	0.26	0.82	1.71	1.90
RRu	24.38	101.04	7.16	7.05
P	N.S.	(+)	+++	+++
Between Chi				0.67
Between df				2
Between P				N.S.
Btwn(F) P				N.S.
Btwn(R) P				N.S.

Table 1E3 - 3

IESLC - Meta-analysis of Ever Smoking (or Current if Ever not available), Pipe and/or Cigars (not cigs)
All LC types
Most adjusted

	Start year of study					
	<1960	1960-69	1970-79	1980-89	1990+	Total
N	15	15	11	8	5	54
NS	15	14	10	6	3	48
Wt	140.05	115.64	104.07	359.84	4.11	723.71
Het Chi	30.31	39.72	17.27	27.74	1.55	176.03
Het df	14	14	10	7	4	53
Het P	**	***	(*)	***	N.S.	***
Fixed RR	2.13	3.00	3.90	4.47	3.73	3.56
RRl	1.81	2.50	3.22	4.03	1.42	3.31
RRu	2.52	3.60	4.73	4.95	9.81	3.83
P	+++	+++	+++	+++	++	+++
Random RR	2.35	3.38	4.00	5.46	3.73	3.37
RRl	1.78	2.39	2.80	3.52	1.42	2.81
RRu	3.10	4.79	5.72	8.45	9.81	4.04
P	+++	+++	+++	+++	++	+++
Between Chi						59.45
Between df						4
Between P						***
Btwn(F) P						***
Btwn(R) P						*

	Study type (1)		
	CC	other	Total
N	35	19	54
NS	30	18	48
Wt	613.85	109.85	723.71
Het Chi	120.50	49.70	176.03
Het df	34	18	53
Het P	***	***	***
Fixed RR	3.70	2.88	3.56
RRl	3.42	2.39	3.31
RRu	4.00	3.47	3.83
P	+++	+++	+++
Random RR	3.31	3.54	3.37
RRl	2.67	2.44	2.81
RRu	4.09	5.14	4.04
P	+++	+++	+++
Between Chi			5.83
Between df			1
Between P			*
Btwn(F) P			N.S.
Btwn(R) P			N.S.

	Study type (2)			
	CC	prosp	other	Total
N	35	18	1	54
NS	30	17	1	48
Wt	613.85	108.65	1.20	723.71
Het Chi	120.50	49.64	0.00	176.03
Het df	34	17	0	53
Het P	***	***	N.S.	***
Fixed RR	3.70	2.89	2.30	3.56
RRl	3.42	2.39	0.39	3.31
RRu	4.00	3.48	13.73	3.83
P	+++	+++	N.S.	+++
Random RR	3.31	3.59	2.30	3.37
RRl	2.67	2.44	0.39	2.81
RRu	4.09	5.27	13.73	4.04
P	+++	+++	N.S.	+++
Between Chi				5.89
Between df				2
Between P				(*)
Btwn(F) P				N.S.
Btwn(R) P				N.S.

Table 1E3 - 3

IESLC - Meta-analysis of Ever Smoking (or Current if Ever not available), Pipe and/or Cigars (not cigs)
All LC types
Most adjusted

	Study size (number of LC cases) 100-249	250-499	500-999	1000+	Total
N	11	16	15	12	54
NS	11	15	12	10	48
Wt	15.55	88.11	117.59	502.46	723.71
Het Chi	12.67	47.13	46.04	58.51	176.03
Het df	10	15	14	11	53
Het P	N.S.	***	***	***	***
Fixed RR	4.91	2.58	3.82	3.67	3.56
RRl	2.98	2.10	3.19	3.36	3.31
RRu	8.06	3.18	4.58	4.00	3.83
P	+++	+++	+++	+++	+++
Random RR	4.87	2.77	4.29	2.98	3.37
RRl	2.72	1.85	2.95	2.23	2.81
RRu	8.72	4.15	6.24	3.99	4.04
P	+++	+++	+++	+++	+++
Between Chi					11.68
Between df					3
Between P					**
Btwn(F) P					N.S.
Btwn(R) P					N.S.

	Risky occupational population no	mining	othRisky	Total
N	52	1	1	54
NS	46	1	1	48
Wt	721.16	0.66	1.88	723.71
Het Chi	171.77	0.00	0.00	176.03
Het df	51	0	0	53
Het P	***	N.S.	N.S.	***
Fixed RR	3.57	9.10	0.88	3.56
RRl	3.32	0.82	0.21	3.31
RRu	3.84	101.04	3.68	3.83
P	+++	(+)	N.S.	+++
Random RR	3.40	9.10	0.88	3.37
RRl	2.84	0.82	0.21	2.81
RRu	4.08	101.04	3.68	4.04
P	+++	(+)	N.S.	+++
Between Chi				4.26
Between df				2
Between P				N.S.
Btwn(F) P				N.S.
Btwn(R) P				N.S.

	National cigarette tobacco type Virginia	blended	other	Total
N	16	38		54
NS	14	34		48
Wt	75.60	648.10		723.71
Het Chi	29.05	146.76		176.03
Het df	15	37		53
Het P	*	***		***
Fixed RR	3.75	3.54		3.56
RRl	2.99	3.28		3.31
RRu	4.70	3.82		3.83
P	+++	+++		+++
Random RR	4.32	3.08		3.37
RRl	3.04	2.48		2.81
RRu	6.13	3.82		4.04
P	+++	+++		+++
Between Chi				0.22
Between df				1
Between P				N.S.
Btwn(F) P				N.S.
Btwn(R) P				N.S.

Table 1E3 - 3

IESLC - Meta-analysis of Ever Smoking (or Current if Ever not available), Pipe and/or Cigars (not cigs)
All LC types
Most adjusted

	Any proxy use No/nk	Yes	Total
N	42	12	54
NS	38	10	48
Wt	603.02	120.68	723.71
Het Chi	127.94	36.11	176.03
Het df	41	11	53
Het P	***	***	***
Fixed RR	3.36	4.75	3.56
RRl	3.10	3.97	3.31
RRu	3.64	5.67	3.83
P	+++	+++	+++
Random RR	3.19	3.98	3.37
RRl	2.61	2.63	2.81
RRu	3.91	6.04	4.04
P	+++	+++	+++
Between Chi			11.97
Between df			1
Between P			***
Btwn(F) P			(*)
Btwn(R) P			N.S.

	Full histological confirmation No	Yes	Total
N	42	12	54
NS	38	10	48
Wt	609.88	113.82	723.71
Het Chi	150.95	11.62	176.03
Het df	41	11	53
Het P	***	N.S.	***
Fixed RR	3.78	2.60	3.56
RRl	3.49	2.16	3.31
RRu	4.09	3.12	3.83
P	+++	+++	+++
Random RR	3.70	2.54	3.37
RRl	2.99	2.07	2.81
RRu	4.59	3.12	4.04
P	+++	+++	+++
Between Chi			13.46
Between df			1
Between P			***
Btwn(F) P			*
Btwn(R) P			*

	Number of adjustment variables (1) 0	1	2+/+nk	Total
N	29	15	10	54
NS	25	15	9	49
Wt	507.77	128.83	87.11	723.71
Het Chi	89.45	32.14	18.59	176.03
Het df	28	14	9	53
Het P	***	**	*	***
Fixed RR	3.92	2.21	4.14	3.56
RRl	3.59	1.86	3.35	3.31
RRu	4.27	2.63	5.11	3.83
P	+++	+++	+++	+++
Random RR	3.39	2.55	5.35	3.37
RRl	2.66	1.88	3.49	2.81
RRu	4.34	3.45	8.21	4.04
P	+++	+++	+++	+++
Between Chi				35.85
Between df				2
Between P				***
Btwn(F) P				**
Btwn(R) P				*

Table 1E3 - 3

IESLC - Meta-analysis of Ever Smoking (or Current if Ever not available), Pipe and/or Cigars (not cigs)
All LC types
Most adjusted

	Number of adjustment variables (2) 0	1	2	3-5	6+/+nk	Total
N	29	15	7	3		54
NS	25	15	7	2		49
Wt	507.77	128.83	70.14	16.97		723.71
Het Chi	89.45	32.14	4.03	4.16		176.03
Het df	28	14	6	2		53
Het P	***	**	N.S.	N.S.		***
Fixed RR	3.92	2.21	3.49	8.35		3.56
RRl	3.59	1.86	2.76	5.19		3.31
RRu	4.27	2.63	4.41	13.44		3.83
P	+++	+++	+++	+++		+++
Random RR	3.39	2.55	3.49	7.88		3.37
RRl	2.66	1.88	2.76	3.72		2.81
RRu	4.34	3.45	4.41	16.68		4.04
P	+++	+++	+++	+++		+++
Between Chi						46.25
Between df						3
Between P						***
Btwn(F) P						**
Btwn(R) P						*

	Derivation of RR/CI Orig	StdCalc	Other	Total
N	6	26	22	54
NS	6	24	21	51
Wt	16.55	524.28	182.87	723.71
Het Chi	6.96	111.90	37.54	176.03
Het df	5	25	21	53
Het P	N.S.	***	*	***
Fixed RR	6.44	3.81	2.77	3.56
RRl	3.98	3.50	2.40	3.31
RRu	10.42	4.15	3.21	3.83
P	+++	+++	+++	+++
Random RR	6.03	3.38	2.94	3.37
RRl	3.34	2.61	2.30	2.81
RRu	10.88	4.40	3.76	4.04
P	+++	+++	+++	+++
Between Chi				19.64
Between df				2
Between P				***
Btwn(F) P				*
Btwn(R) P				(*)

	Smoking status ever	current	Total
N	38	16	54
NS	35	13	48
Wt	655.79	67.92	723.71
Het Chi	133.38	36.65	176.03
Het df	37	15	53
Het P	***	**	***
Fixed RR	3.46	4.72	3.56
RRl	3.20	3.72	3.31
RRu	3.73	5.99	3.83
P	+++	+++	+++
Random RR	2.92	5.31	3.37
RRl	2.38	3.45	2.81
RRu	3.57	8.19	4.04
P	+++	+++	+++
Between Chi			6.01
Between df			1
Between P			*
Btwn(F) P			N.S.
Btwn(R) P			*

Table 1E3 - 4

IESLC - Meta-analysis of Ever Smoking (or Current if Ever not available), Pipe and/or Cigars (not cigs)
All LC types
Least adjusted

REF	NRR	X	SEX	AGEL	AGEH	RACE	YF	LC TYPE	LOC	START	ST	NLC	R	VB	P	H	AD	SM	DENOM	De
ABELIN	3	x	m	0	0	all	-	all	Eu:wst	1941	CC	118	n	bl	y	n	0	ev	nev any	st
ALDERS	7	x	m	0	0	all	-	all	Eu:UK	1977	CC	1448	n	V	n	n	0	ev	nev any	st
ARMADA	30		m	0	0	all	-	all	Eu:wst	1986	CC	325	n	bl	n	y	0	ev	nev any	st
BECHER	3	x	m	0	0	all	-	all	Eu:Ger	1985	CC	194	n	bl	n	y	0	ev	nev any	st
BENSHL	10		m	40	64	all	10	all	Eu:UK	1967	pr	486	n	V	n	n	1	cu	nev any	ot
BEST	19		m	55	79	all	3	all	NAmer	1955	pr	381	n	V	n	n	0	ev	nev any	st
BOFFET	30		m	0	0	all	-	all	Eu:mul	1988	CC	5621	n	bl	y	n	0	ev	nev any	st
BOUCOT	5	x	m	0	0	all	0	all	NAmer	1951	pr	121	n	bl	n	n	0	ev	nev any	ot
BRESLO	33		m	0	0	all	-	all	NAmer	1949	CC	518	n	bl	n	y	0	ev	nev+1	st
BROSS	5		m	0	0	wh	-	all	NAmer	1960	CC	974	n	bl	n	n	0	cu	nev any	st
CHOW	1	x	m	0	0	wh	0	all	NAmer	1966	pr	219	n	bl	n	n	0	ev	nev any	st
COMSTO	1		m	0	0	all	-	all	NAmer	1975	ot	258	n	bl	n	n	0	ev	nev any	st
CPSI	186		m	35	84	all	6	all	NAmer	1959	pr	5138	n	bl	n	n	1	ev	nev any	ot
DAMBER	28		m	0	0	all	-	all	Eu:Sca	1972	CC	579	n	bl	y	n	0	ev	nev any	st
DARBY	6		m	0	0	wh	-	all	Eu:UK	1988	CC	982	n	V	n	n	0	cu	nev any	st
DARBY	13		f	0	0	wh	-	all	Eu:UK	1988	CC	982	n	V	n	n	0	cu	nev any	st
DEAN	11		m	0	0	wh	-	all	Africa	1947	CC	603	n	V	y	n	0	ev	nev any	st
DEAN2	11		m	0	0	all	-	all	Eu:UK	1960	CC	954	n	V	y	n	0	ev	nev any	st
DEKLER	5		m	0	0	all	0	all	Auslia	1961	pr	138	m	V	n	n	2	cu	nev any	or
DOLL2	33		m	0	0	all	10	all	Eu:UK	1951	pr	920	n	V	n	n	1	ev	nev any	ot
DORGAN	1		m	0	0	wh	-	all	NAmer	1980	CC	2026	n	bl	y	y	0	ev	nev any	st
DORGAN	25		m	0	0	bl	-	all	NAmer	1980	CC	2026	n	bl	y	y	0	ev	nev any	st
DORN	44	x	m	0	0	wh	2	all	NAmer	1954	pr	5097	n	bl	n	n	0	ev	nev any	st
ENGELA	167		m	0	0	all	12	all	Eu:Sca	1964	pr	435	n	bl	n	n	1	cu	nev any	ot
GOLLED	19	x	m	35	99	all	-	all	Eu:UK	1952	CC	443	n	V	y	n	0	ev	nev any	st
GRAHAM	6	x	m	0	0	wh	-	all	NAmer	1956	CC	685	n	bl	n	n	0	ev	nev any	st
HAMMO2	28	x	m	0	0	all	0	all	NAmer	1967	pr	450	o	bl	n	n	0	ev	nev any	st
HAMMON	130	x	m	0	0	wh	0	all	NAmer	1952	pr	448	n	bl	n	n	0	ev	nev any	st
HOLE	13	x	m	0	0	all	0	all	Eu:UK	1972	pr	225	n	V	n	n	0	cu	nev any	st
KINLEN	2	x	m	0	0	all	0	all	Eu:UK	1967	pr	718	n	V	n	n	0	cu	nev any	st
KJUUS	4		m	0	0	all	-	all	Eu:Sca	1979	CC	176	n	bl	n	n	0	ev	nev any	st
KNEKT	24	x	m	20	69	all	21	all	Eu:Sca	1966	pr	515	n	bl	n	n	0	cu	nev any	st
KREUZE	10		f	1	45	all	-	all	Eu:Ger	1990	CC	2260	n	bl	n	n	0	cu	nev any	ot
KREUZE	12		f	55	69	all	-	all	Eu:Ger	1990	CC	2260	n	bl	n	n	0	cu	nev any	ot
LEVIN	31		m	0	0	all	-	all	NAmer	1938	CC	475	n	bl	n	n	1	ev	nev any	st
LOMBAR	1		m	0	0	all	-	all	NAmer	1951	CC	1040	n	bl	n	n	0	ev	nev any	st
LUBIN2	49	x	m	0	0	all	-	all	Eu:mul	1976	CC	7804	n	bl	n	y	0	ev	nev any	st
MARSH	5		m	0	0	all	-	all	NAmer	1979	CC	150	n	bl	y	n	0	ev	nev any	ot
MARSH2	3		c	0	0	all	-	all	NAmer	1979	CC	114	n	bl	y	n	0	ev	nev any	ot
MATOS	1		m	0	0	all	-	all	SCAmer	1994	CC	200	n	bl	n	n	0	cu	nev any	st
MIGRAN	15	x	m	0	0	all	0	all	Eu:UK	1964	pr	259	n	V	n	n	0	cu	nev any	st
SADOWS	70		m	0	0	wh	-	all	NAmer	1938	CC	477	n	bl	n	n	0	ev	nev any	st
STASZE	3		m	0	0	all	-	all	Eu:est	1954	CC	281	n	bl	n	y	0	ev	nev any	st
STOCKW	5		c	0	0	all	-	all	NAmer	1981	CC	22161	n	bl	n	n	0	ev	nev any	st
TOUSEY	1	x	m	0	0	all	-	all	NAmer	1993	CC	507	n	bl	y	y	0	ev	nev any	st
TOUSEY	5		f	0	0	all	-	all	NAmer	1993	CC	507	n	bl	y	y	0	ev	nev any	st
TULINI	12	x	m	0	0	all	0	all	Eu:Sca	1967	pr	472	n	bl	n	n	1	cu	nev any	ot
TULINI	8	x	f	0	0	all	0	all	Eu:Sca	1967	pr	472	n	bl	n	n	1	cu	nev any	or
WALD	1	x	m	0	0	all	0	all	Eu:UK	1975	pr	102	n	V	n	n	0	cu	nev any	st
WIGLE	14		m	0	0	all	-	all	NAmer	1971	CC	728	n	V	n	n	0	ev	nev any	st
WIGLE	17		f	0	0	all	-	all	NAmer	1971	CC	728	n	V	n	n	0	ev	nev any	ot
WYNDE2	15		m	0	0	all	-	all	NAmer	1962	CC	404	n	bl	n	y	0	ev	nev any	st
WYNDE3	152		m	0	0	all	-	all	NAmer	1966	CC	350	n	bl	n	y	0	ev	nev any	st
WYNDE6	63		m	0	0	all	-	all	NAmer	1969	CC	4423	n	bl	n	y	0	ev	nev any	st

Table 1E3 - 5

IESLC - Meta-analysis of Ever Smoking (or Current if Ever not available), Pipe and/or Cigars (not cigs)
All LC types
Least adjusted

REF	NRR	SEX	AD	Number Exposed Case	Number Exposed Cont	Non-exposed Case	Non-exposed Cont	RR	95.00%CI
ABELIN	3	m	0	69	187	2	183	33.76	(8.16- 139.75)
ALDERS	7	m	0	17	35	15	133	4.31	(1.96- 9.47)
ARMADA	30	m	0	4	7	4	64	9.14	(1.86- 44.85)
BECHER	3	m	0	6	21	3	54	5.14	(1.18- 22.47)
*BENSHL	10	m	1	-	-	-	-	2.33	(0.58- 9.33)
*BEST	19	m	0	8	2972	1	2854	7.68	(0.96- 61.38)
BOFFET	30	m	0	118	266	117	1750	6.64	(4.98- 8.84)
*BOUCOT	5	m	0	4	8926	0	7551	7.61~	(0.41- 141.39)
BRESLO	33	m	0	15	68	7	42	1.32	(0.50- 3.51)
BROSS	5	m	0	125	211	38	170	2.65	(1.75- 4.02)
*CHOW	1	m	0	5	13677	6	62913	3.83	(1.17- 12.56)
COMSTO	1	m	0	2	15	4	69	2.30	(0.39- 13.73)
*CPSI	186	m	1	-	-	-	-	1.94	(1.44- 2.62)
DAMBER	28	m	0	205	149	42	208	6.81	(4.60- 10.09)
DARBY	6	m	0	57	165	3	384	44.22	(13.65- 143.22)
DARBY	13	f	0	3	14	23	529	4.93	(1.32- 18.36)
Subtotal DARBY								16.69	(6.95- 40.09)
DEAN	11	m	0	51	74	12	61	3.50	(1.71- 7.16)
DEAN2	11	m	0	81	128	33	112	2.15	(1.33- 3.46)
*DEKLER	5	m	2	-	-	-	-	9.10	(0.82- 101.10)
*DOLL2	33	m	1	-	-	-	-	6.14	(1.85- 20.40)
DORGAN	1	m	0	22	55	15	93	2.48	(1.19- 5.18)
DORGAN	25	m	0	3	19	3	35	1.84	(0.34- 10.03)
Subtotal DORGAN								2.37	(1.20- 4.65)
*DORN	44	m	0	16	68146	17	117918	1.63	(0.82- 3.22)
*ENGELA	167	m	1	-	-	-	-	2.60	(1.02- 6.60)
GOLLED	19	m	0	48	331	15	490	4.74	(2.61- 8.60)
GRAHAM	6	m	0	49	367	18	346	2.57	(1.47- 4.49)
*HAMMO2	28	m	0	3	488	5	891	1.10	(0.26- 4.56)
*HAMMON	130	m	0	28	127770	15	115884	1.69	(0.90- 3.17)
*HOLE	13	m	0	2	141	7	1189	2.41	(0.51- 11.49)
*KINLEN	2	m	0	47	1367	7	1333	6.55	(2.97- 14.43)
KJUUS	4	m	0	23	25	2	24	11.04	(2.34- 52.00)
*KNEKT	24	m	0	7	2822	6	17814	7.36	(2.48- 21.90)
KREUZE	10	f	0	0	1	6	38	1.97~	(0.07- 53.89)
KREUZE	12	f	0	3	0	95	177	13.01~	(0.67- 254.52)
Subtotal KREUZE								5.60	(0.61- 51.10)
LEVIN	31	m	1	-	-	-	-	1.31	(0.81- 2.12)
LOMBAR	1	m	0	48	146	14	112	2.63	(1.38- 5.01)
LUBIN2	49	m	0	99	406	190	2617	3.36	(2.58- 4.38)
MARSH	5	m	0	0	5	2	31	1.15~	(0.05- 27.23)
MARSH2	3	c	0	0	4	12	56	0.50~	(0.03- 9.94)
MATOS	1	m	0	1	4	11	110	2.50	(0.26- 24.38)
*MIGRAN	15	m	0	16	575	4	867	6.03	(2.03- 17.95)
SADOWS	70	m	0	38	88	18	81	1.94	(1.03- 3.67)
STASZE	3	m	0	4	101	5	158	1.25	(0.33- 4.77)
STOCKW	5	c	0	715	655	2791	10641	4.16	(3.71- 4.66)
TOUSEY	1	m	0	4	48	4	130	2.71	(0.65- 11.26)
TOUSEY	5	f	0	1	2	13	226	8.69	(0.74- 102.22)
Subtotal TOUSEY								3.63	(1.06- 12.45)
*TULINI	12	m	1	-	-	-	-	7.92	(4.20- 14.90)
*TULINI	8	f	1	-	-	-	-	15.70	(6.78- 36.30)
Subtotal TULINI								10.15	(6.12- 16.83)
*WALD	1	m	0	6	1309	7	6539	4.28	(1.44- 12.72)
WIGLE	14	m	0	30	113	15	204	3.61	(1.86- 6.99)
WIGLE	17	f	0	1	0	36	439	36.12~	(1.45- 902.62)
Subtotal WIGLE								3.96	(2.07- 7.57)
WYNDE2	15	m	0	14	104	8	105	1.77	(0.71- 4.39)
WYNDE3	152	m	0	14	68	9	88	2.01	(0.82- 4.93)
WYNDE6	63	m	0	58	199	87	617	2.07	(1.43- 2.99)
Partial Totals				2070	232274	3747	356330		

*prospective study

~ With 0.5 adjustment for zero

Table 1E3 - 5

IESLC - Meta-analysis of Ever Smoking (or Current if Ever not available), Pipe and/or Cigars (not cigs)
All LC types
Least adjusted

REF	NRR	SEX	AD	Ys	Ws	Qs	Ps
ABELIN	3	m	0	3.52	1.90	9.46	0.0000
ALDERS	7	m	0	1.46	6.19	0.18	0.0003
ARMADA	30	m	0	2.21	1.52	1.29	0.0064
BECHER	3	m	0	1.64	1.77	0.21	0.0295
*BENSHL	10	m	1	0.85	1.99	0.39	0.2326
*BEST	19	m	0	2.04	0.89	0.50	0.0545
BOFFET	30	m	0	1.89	46.83	16.95	0.0000
*BOUCOT	5	m	0	2.03	0.45	0.25	0.1733
BRESLO	33	m	0	0.28	4.03	4.12	0.5736
BROSS	5	m	0	0.97	22.25	2.22	0.0000
*CHOW	1	m	0	1.34	2.73	0.01	0.0265
COMSTO	1	m	0	0.83	1.20	0.25	0.3609
*CPSI	186	m	1	0.66	42.89	16.92	0.0000
DAMBER	28	m	0	1.92	24.87	9.82	0.0000
DARBY	6	m	0	3.79	2.78	17.36	0.0000
DARBY	13	f	0	1.60	2.22	0.21	0.0174
Subtotal DARBY				2.81	5.00	17.57	
DEAN	11	m	0	1.25	7.53	0.01	0.0006
DEAN2	11	m	0	0.76	16.84	4.66	0.0017
*DEKLER	5	m	2	2.21	0.66	0.56	0.0722
*DOLL2	33	m	1	1.81	2.67	0.73	0.0030
DORGAN	1	m	0	0.91	7.09	1.04	0.0156
DORGAN	25	m	0	0.61	1.34	0.62	0.4799
Subtotal DORGAN				0.86	8.43	1.65	
*DORN	44	m	0	0.49	8.24	5.32	0.1614
*ENGELA	167	m	1	0.96	4.41	0.50	0.0449
GOLLED	19	m	0	1.56	10.80	0.76	0.0000
GRAHAM	6	m	0	0.94	12.26	1.49	0.0010
*HAMMO2	28	m	0	0.09	1.89	2.71	0.9003
*HAMMON	130	m	0	0.53	9.77	5.71	0.0998
*HOLE	13	m	0	0.88	1.57	0.27	0.2698
*KINLEN	2	m	0	1.88	6.15	2.13	0.0000
KJUUS	4	m	0	2.40	1.60	1.97	0.0024
*KNEKT	24	m	0	2.00	3.24	1.61	0.0003
KREUZE	10	f	0	0.68	0.35	0.13	0.6868
KREUZE	12	f	0	2.57	0.43	0.71	0.0908
Subtotal KREUZE				1.72	0.79	0.84	
LEVIN	31	m	1	0.27	16.60	17.29	0.2713
LOMBAR	1	m	0	0.97	9.26	0.97	0.0033
LUBIN2	49	m	0	1.21	54.92	0.34	0.0000
MARSH	5	m	0	0.14	0.38	0.51	0.9331
MARSH2	3	c	0	-0.69	0.43	1.69	0.6511
MATOS	1	m	0	0.92	0.74	0.10	0.4303
*MIGRAN	15	m	0	1.80	3.23	0.83	0.0012
SADOWS	70	m	0	0.66	9.47	3.72	0.0409
STASZE	3	m	0	0.22	2.14	2.44	0.7425
STOCKW	5	c	0	1.43	296.07	5.42	0.0000
TOUSEY	1	m	0	1.00	1.89	0.16	0.1705
TOUSEY	5	f	0	2.16	0.63	0.48	0.0855
Subtotal TOUSEY				1.29	2.52	0.64	
*TULINI	12	m	1	2.07	9.58	5.81	0.0000
*TULINI	8	f	1	2.75	5.46	11.68	0.0000
Subtotal TULINI				2.32	15.04	17.49	
*WALD	1	m	0	1.45	3.24	0.09	0.0088
WIGLE	14	m	0	1.28	8.79	0.00	0.0001
WIGLE	17	f	0	3.59	0.37	1.96	0.0289
Subtotal WIGLE				1.38	9.16	1.96	
WYNDE2	15	m	0	0.57	4.64	2.42	0.2202
WYNDE3	152	m	0	0.70	4.79	1.67	0.1256
WYNDE6	63	m	0	0.73	28.26	9.01	0.0001

Table 1E3 - 5

IESLC - Meta-analysis of Ever Smoking (or Current if Ever not available), Pipe and/or Cigars (not cigs)
All LC types
Least adjusted

	N	54
	NS	48
	Wt	722.26
Het	Chi	177.63
Het	df	53
Het	P	***
Fixed	RR	3.64
	RRl	3.38
	RRu	3.91
	P	+++
Random	RR	3.52
	RRl	2.93
	RRu	4.22
	P	+++
Asymm	P	N.S.

Table 1E3 - 6

IESLC - Meta-analysis of Ever Smoking (or Current if Ever not available), Pipe and/or Cigars (not cigs)
All LC types
Least adjusted

	Sex combined	male	female	Total
N	2	46	6	54
NS	2	45	5	52
Wt	296.50	416.29	9.47	722.26
Het Chi	1.92	149.45	3.73	177.63
Het df	1	45	5	53
Het P	N.S.	***	N.S.	***
Fixed RR	4.15	3.23	10.91	3.64
RRl	3.70	2.93	5.77	3.38
RRu	4.65	3.55	20.62	3.91
P	+++	+++	+++	+++
Random RR	2.50	3.35	10.91	3.52
RRl	0.42	2.73	5.77	2.93
RRu	14.72	4.12	20.62	4.22
P	N.S.	+++	+++	+++
Between Chi				22.52
Between df				2
Between P				***
Btwn(F) P				*
Btwn(R) P				**

Table 1E3 - 7

IESLC - Meta-analysis of Ever Smoking (or Current if Ever not available), Pipe and/or Cigars (not cigs)
All LC types
Excluded studies (and stage at which they were excluded)

1 BOUCHA BUELL LAURIL MZILEN

2 ABRAHA AGUDO AKIBA AMANDU AMES ANDERS ARCHER AUSTIN AUVINE AXELSO AXELSS BAND BARBON BERRIN BLOHMK BLOT1
BLOT2 BLOT3 BLOT4 BRETT BROCKM BROWN1 BROWN2 BUFFLE BYERS1 BYERS2 CARPEN CASCO2 CASCOR CEDERL CHAN CHANG
CHATZI CHEN CHEN2 CHEN3 CHIAZZ CHOI CHYOU COOKSO CORREA CPSII DAVEYS DEAN3 DESTE2 DESTEF DOCKER DOLL
DORANT DOSEME DROSTE DU DUNN EBELIN ENSTRO ESAKI FAN GAO GAO2 GARCIA GARDIN GARSHI GENG GER
GILLIS GODLEY GOODMA GREGOR GSELL HAENSZ HANSEN HEGMAN HEIN HENNEK HINDS HIRAOK HIRAY2 HIRAYA HITOSU HOROWI
HORWIT HU HU2 HUANG HUMBLE ISHIMA JAIN JARUP JARVHO JEDRYC JIANG JOLY JONES JUSSAW KAISE2 KAISER
KANELL KATSOU KAUFMA KELLER KHUDER KIHARA KO KOHLME KOO KOULUM KREYBE KUBIK LAMTH LAMWK LAMWK2 LANGE
LAUSSM LEI LEMARC LETOUR LIAW LICKIN LIDDEL LIU LIU2 LIU3 LIU4 LIU5 LOMBA2 LUBIN LUO MACLEN
MAGNUS MARTIS MASTRA MATSUD MCCONN MCDUFF MCLAUG MILLER MILLS MOLLO MRFIT MRFITR MURATA NAM NOTAN2 NOTANI
NOU ODRISC ORMOS OSANN OSANN2 PARKIN PASTOR PAWLEG PERNU PERSH2 PERSHA PETO PEZZO2 PEZZOT PIKE PISANI
POFFIJ POLEDN PRESCO QIAO QIAO2 RACHTA RADZIK RANDIG REN RESTRE RIMING RONCO ROOTS ROTHSC SAARIK SANKAR
SCHWA2 SCHWAR SEGI SEGI2 SEOW SHAW SHIMIZ SIEMIA SIMARA SIMONA SITAS SOBUE SOBUE2 SPEIZE SPITZ STAYNE
STOCKS STUCKE SUN SUZUK2 SUZUKI SVENSS TANG TANG2 TAO TENKAN TIZZAN TOKARS TSUGAN TVERDA ULMER VANDER
VEIERO VUTUC WAKAI WANG WANG2 WANG3 WANG4 WARSIN WATSON WICKLU WILKIN WU WU2 WUNSCH WUWILL WYNDE4
WYNDE5 WYNDE8 WYNDER XIANGZ XU XU2 XU3 XU4 YAMAGU YONG YUAN ZHANG ZHENG ZHOU

9 BENHAM JAHN WYNDE7

Table 1E3 - 8
Potentially overlapping studies

REF	REFGP	PRINC	.	OVERLAP/LINK
LUBIN2	LUBIN2	1		Lubin-combined
BROSS	BYERS1	1		GRAHAM/BROSS/BYERS1
GRAHAM	BYERS1	1		GRAHAM/BROSS/BYERS1
HOLE	TANG2	1		Subset of TANG2
BENSHL	TANG2	1		Subset of TANG2
WALD	TANG2	1		Subset of TANG2
WYNDE6	WYNDE6	1		WYNDE5/6/7/8
CPSI	CPSI	1		CPSI overall
BOFFET	BOFFET	1		BOFFET-combined

Table 1E3 - 9
Most adjusted - insufficient data for metaanalysis

REF	NRR	SEX	AGEL	AGEH	RACE	YF	LC TYPE	LOC	START	ST	NLC	R	VB	P	H	AD	SM	DENOM	De
BECHER	4	f	0	0	all	-	all	Eu:Ger	1985	CC	194	n	bl	n	y	0	ev	nev	any st
BRESLO	34	f	0	0	all	-	all	NAmer	1949	CC	518	n	bl	n	y	0	ev	nev+1	st

REF	NRR	RR	SIG	RRDATA comment
BECHER	4		*	No relevant smokers
BRESLO	34		*	No relevant smokers

Table 1E4 -

IESLC - Meta-analysis of Current Smoking (or Ever if Current not available), Pipe and/or Cigars (not cigs)
All LC types

This analysis is restricted to results for:
1) Non-dose-response data
2) Smokers of pipe and/or cigars (but not cigarettes)
3) Results complete enough for use in metaanalysis

Within each study, results are then selected (in the following order of preference, within each sex) for:
4) SMKSTA: current smokers, ever smokers
5) DENOM: never smoked anything, (never +1 = +long term ex)
6) Followup period (prospective studies): whole study (coded as 0) or longest available
7) LCtype: all or nearest available, at least Squamous and Adeno. (q = squamous, s = small,
l = large, a = adeno, mix = mixed, alv = alveolar)
8) Race: all or nearest available, otherwise by race (wh or w = white, bl or b = black, hi = hispanic
ch = chinese, jap = japanese, haw = hawaiian, w+o = white + oriental, sca = scandinavian, as = asian)
9) For overlapping studies: principal rather than subsidiary studies
Finally by Age: whole study (coded as 0) if available, otherwise by widest available age group
and then for single sex results (m, f) in preference to combined sex results (c).

Results adjusted (AD) for the most potential confounders are then chosen in Sections -1 to -3
(and those which actually differ from the adjusted results in Table 1E3 - 1 are marked 'x' in Section -1)
and results adjusted for the least confounders in Sections -4 to -6. (Those least adjusted results which
actually differ from the most adjusted as marked 'x' in column X in Section -4)
(Results adjusted for an unknown number of confounder(s) are coded as 20.)

Section -7 shows excluded studies, together with the stage (as above) at which no qualifying
results were found.

Section -8 lists the potentially overlapping studies which have been included (1=principal, 2=subsidiary).

Section -9 lists any results which would have been included in preference except that they had data not complete
enough for use in meta-analysis, with their significance (yes/no), if known, and any further comment as entered
on the database.

In addition to those mentioned above, the following fields, levels and abbreviations are used:
* or nk = not known, n = no, y = yes, ot = other
ev = ever, cu = current, nev = never
REF: 6-character study reference
NRR: number of the RR on the database within the study
ST : study type (CC = case control, pr or prosp = prospective)
NLC: number of lung cancer cases in whole study
R : risky occupational population (n = no, m = mining, o = other risky)
VB : national cigarette type (V = at least 75% Virginia, bl = at least 75% blended, ot = other)
P : any proxy use
H : full histological confirmation
De : derivation of RR/CI (or = original, st = standard method, ot = other method of estimation)

Table 1E4 - 1

IESLC - Meta-analysis of Current Smoking (or Ever if Current not available), Pipe and/or Cigars (not cigs)
All LC types
Most adjusted

REF	NRR	1E3	SEX	AGEL	AGEH	RACE	YF	LC TYPE	LOC	START	ST	NLC	R	VB	P	H	AD	SM	DENOM	De
ABELIN	46		m	0	0	all	-	all	Eu:wst	1941	CC	118	n	bl	y	n	1	ev	nev any	st
ALDERS	1		m	0	0	all	-	all	Eu:UK	1977	CC	1448	n	V	n	n	1	ev	nev any	ot
ARMADA	30		m	0	0	all	-	all	Eu:wst	1986	CC	325	n	bl	n	y	0	ev	nev any	st
BECHER	23		m	0	0	all	-	all	Eu:Ger	1985	CC	194	n	bl	n	y	2	ev	nev any	or
BENSHL	10		m	40	64	all	10	all	Eu:UK	1967	pr	486	n	V	n	n	1	cu	nev any	ot
BEST	19		m	55	79	all	3	all	NAmer	1955	pr	381	n	V	n	n	0	ev	nev any	st
BOFFET	30		m	0	0	all	-	all	Eu:mul	1988	CC	5621	n	bl	y	n	0	ev	nev any	st
BOUCOT	117		m	0	0	all	0	all	NAmer	1951	pr	121	n	bl	n	n	2	ev	nev any	ot
BRESLO	33		m	0	0	all	-	all	NAmer	1949	CC	518	n	bl	n	y	0	ev	nev+1	st
BROSS	5		m	0	0	wh	-	all	NAmer	1960	CC	974	n	bl	n	n	0	cu	nev any	st
CHOW	13	x	m	0	0	wh	0	all	NAmer	1966	pr	219	n	bl	n	n	0	cu	nev any	st
COMSTO	1		m	0	0	all	-	all	NAmer	1975	ot	258	n	bl	n	n	0	ev	nev any	st
CPSI	186		m	35	84	all	6	all	NAmer	1959	pr	5138	n	bl	n	n	1	ev	nev any	ot
DAMBER	28		m	0	0	all	-	all	Eu:Sca	1972	CC	579	n	bl	y	n	0	ev	nev any	st
DARBY	6		m	0	0	wh	-	all	Eu:UK	1988	CC	982	n	V	n	n	0	cu	nev any	st
DARBY	13		f	0	0	wh	-	all	Eu:UK	1988	CC	982	n	V	n	n	0	cu	nev any	st
DEAN	11		m	0	0	wh	-	all	Africa	1947	CC	603	n	V	y	n	0	ev	nev any	st
DEAN2	11		m	0	0	all	-	all	Eu:UK	1960	CC	954	n	V	y	n	0	ev	nev any	st
DEKLER	5		m	0	0	all	0	all	Auslia	1961	pr	138	m	V	n	n	2	cu	nev any	or
DOLL2	20	x	m	0	0	all	20	all	Eu:UK	1951	pr	920	n	V	n	n	1	cu	nev any	ot
DORGAN	1		m	0	0	wh	-	all	NAmer	1980	CC	2026	n	bl	y	y	0	ev	nev any	st
DORGAN	25		m	0	0	bl	-	all	NAmer	1980	CC	2026	n	bl	y	y	0	ev	nev any	st
DORN	90	x	m	35	84	wh	8	all	NAmer	1954	pr	5097	n	bl	n	n	1	cu	nev any	ot
ENGELA	167		m	0	0	all	12	all	Eu:Sca	1964	pr	435	n	bl	n	n	1	cu	nev any	ot
GOLLED	5		m	35	99	all	-	all	Eu:UK	1952	CC	443	n	V	y	n	1	ev	nev any	ot
GRAHAM	24	x	m	0	0	wh	-	all	NAmer	1956	CC	685	n	bl	n	n	1	cu	nev any	ot
HAMMO2	14		m	0	0	all	0	all	NAmer	1967	pr	450	o	bl	n	n	1	ev	nev any	ot
HAMMON	118		m	0	0	wh	0	all	NAmer	1952	pr	448	n	bl	n	n	1	ev	nev any	ot
HOLE	6		m	0	0	all	0	all	Eu:UK	1972	pr	225	n	V	n	n	1	cu	nev any	ot
KINLEN	13		m	0	0	all	0	all	Eu:UK	1967	pr	718	n	V	n	n	2	cu	nev any	ot
KJUUS	4		m	0	0	all	-	all	Eu:Sca	1979	CC	176	n	bl	n	n	0	ev	nev any	st
KNEKT	28		m	20	69	all	21	all	Eu:Sca	1966	pr	515	n	bl	n	n	1	cu	nev any	or
KREUZE	10		f	1	45	all	-	all	Eu:Ger	1990	CC	2260	n	bl	n	n	0	cu	nev any	ot
KREUZE	12		f	55	69	all	-	all	Eu:Ger	1990	CC	2260	n	bl	n	n	0	cu	nev any	ot
LEVIN	31		m	0	0	all	-	all	NAmer	1938	CC	475	n	bl	n	n	1	ev	nev any	st
LOMBAR	1		m	0	0	all	-	all	NAmer	1951	CC	1040	n	bl	n	n	0	ev	nev any	st
LUBIN2	30	x	m	0	0	all	-	all	Eu:mul	1976	CC	7804	n	bl	n	y	2	cu	nev any	ot
MARSH	5		m	0	0	all	-	all	NAmer	1979	CC	150	n	bl	y	n	0	ev	nev any	ot
MARSH2	3		c	0	0	all	-	all	NAmer	1979	CC	114	n	bl	y	n	0	ev	nev any	ot
MATOS	1		m	0	0	all	-	all	SCAmer	1994	CC	200	n	bl	n	n	0	cu	nev any	st
MIGRAN	16		m	0	0	all	0	all	Eu:UK	1964	pr	259	n	V	n	n	2	cu	nev any	ot
SADOWS	70		m	0	0	wh	-	all	NAmer	1938	CC	477	n	bl	n	n	0	ev	nev any	st
STASZE	3		m	0	0	all	-	all	Eu:est	1954	CC	281	n	bl	n	y	0	ev	nev any	st
STOCKW	5		c	0	0	all	-	all	NAmer	1981	CC	22161	n	bl	n	n	0	ev	nev any	st
TOUSEY	9		m	0	0	all	-	all	NAmer	1993	CC	507	n	bl	y	y	3	ev	nev any	or
TOUSEY	5		f	0	0	all	-	all	NAmer	1993	CC	507	n	bl	y	y	0	ev	nev any	st
TULINI	35		m	0	0	all	0	all	Eu:Sca	1967	pr	472	n	bl	n	n	3	cu	nev any	ot
TULINI	31		f	0	0	all	0	all	Eu:Sca	1967	pr	472	n	bl	n	n	3	cu	nev any	or
WALD	3		m	0	0	all	0	all	Eu:UK	1975	pr	102	n	V	n	n	1	cu	nev any	or
WIGLE	29	x	m	0	0	all	-	all	NAmer	1971	CC	728	n	V	n	n	1	cu	nev any	ot
WIGLE	5	x	f	0	0	all	-	all	NAmer	1971	CC	728	n	V	n	n	0	cu	nev any	ot
WYNDE2	15		m	0	0	all	-	all	NAmer	1962	CC	404	n	bl	n	y	0	ev	nev any	st
WYNDE3	152		m	0	0	all	-	all	NAmer	1966	CC	350	n	bl	n	y	0	ev	nev any	st
WYNDE6	63		m	0	0	all	-	all	NAmer	1969	CC	4423	n	bl	n	y	0	ev	nev any	st

Table 1E4 - 2

IESLC - Meta-analysis of Current Smoking (or Ever if Current not available), Pipe and/or Cigars (not cigs)
All LC types
Most adjusted

				Number Exposed		Non-exposed			
REF	NRR	SEX	AD	Case	Cont	Case	Cont	RR	95.00%CI
ABELIN	46	m	1	-	-	-	-	30.49	(7.32- 127.00)
ALDERS	1	m	1	-	-	-	-	3.82	(1.74- 8.40)
ARMADA	30	m	0	4	7	4	64	9.14	(1.86- 44.85)
BECHER	23	m	2	-	-	-	-	4.62	(1.15- 18.56)
*BENSHL	10	m	1	-	-	-	-	2.33	(0.58- 9.33)
*BEST	19	m	0	8	2972	1	2854	7.68	(0.96- 61.38)
BOFFET	30	m	0	118	266	117	1750	6.64	(4.98- 8.84)
*BOUCOT	117	m	2	-	-	-	-	6.80	(0.37- 126.25)
BRESLO	33	m	0	15	68	7	42	1.32	(0.50- 3.51)
BROSS	5	m	0	125	211	38	170	2.65	(1.75- 4.02)
*CHOW	13	m	0	37	47084	6	62913	8.24	(3.48- 19.52)
COMSTO	1	m	0	2	15	4	69	2.30	(0.39- 13.73)
*CPSI	186	m	1	-	-	-	-	1.94	(1.44- 2.62)
DAMBER	28	m	0	205	149	42	208	6.81	(4.60- 10.09)
DARBY	6	m	0	57	165	3	384	44.22	(13.65- 143.22)
DARBY	13	f	0	3	14	23	529	4.93	(1.32- 18.36)
Subtotal DARBY								16.69	(6.95- 40.09)
DEAN	11	m	0	51	74	12	61	3.50	(1.71- 7.16)
DEAN2	11	m	0	81	128	33	112	2.15	(1.33- 3.46)
*DEKLER	5	m	2	-	-	-	-	9.10	(0.82- 101.10)
*DOLL2	20	m	1	-	-	-	-	5.80	(2.65- 12.72)
DORGAN	1	m	0	22	55	15	93	2.48	(1.19- 5.18)
DORGAN	25	m	0	3	19	3	35	1.84	(0.34- 10.03)
Subtotal DORGAN								2.37	(1.20- 4.65)
*DORN	90	m	1	-	-	-	-	1.70	(1.21- 2.37)
*ENGELA	167	m	1	-	-	-	-	2.60	(1.02- 6.60)
GOLLED	5	m	1	-	-	-	-	3.13	(1.73- 5.67)
GRAHAM	24	m	1	-	-	-	-	2.88	(1.70- 4.89)
*HAMMO2	14	m	1	-	-	-	-	0.88	(0.21- 3.67)
*HAMMON	118	m	1	-	-	-	-	1.55	(0.83- 2.90)
*HOLE	6	m	1	-	-	-	-	2.29	(0.48- 11.04)
*KINLEN	13	m	2	-	-	-	-	6.51	(2.95- 14.35)
KJUUS	4	m	0	23	25	2	24	11.04	(2.34- 52.00)
*KNEKT	28	m	1	-	-	-	-	6.10	(2.10- 18.20)
KREUZE	10	f	0	0	1	6	38	1.97~	(0.07- 53.89)
KREUZE	12	f	0	3	0	95	177	13.01~	(0.67- 254.52)
Subtotal KREUZE								5.60	(0.61- 51.10)
LEVIN	31	m	1	-	-	-	-	1.31	(0.81- 2.12)
LOMBAR	1	m	0	48	146	14	112	2.63	(1.38- 5.01)
LUBIN2	30	m	2	-	-	-	-	4.05	(3.18- 5.16)
MARSH	5	m	0	0	5	2	31	1.15~	(0.05- 27.23)
MARSH2	3	c	0	0	4	12	56	0.50~	(0.03- 9.94)
MATOS	1	m	0	1	4	11	110	2.50	(0.26- 24.38)
*MIGRAN	16	m	2	-	-	-	-	3.81	(1.19- 12.23)
SADOWS	70	m	0	38	88	18	81	1.94	(1.03- 3.67)
STASZE	3	m	0	4	101	5	158	1.25	(0.33- 4.77)
STOCKW	5	c	0	715	655	2791	10641	4.16	(3.71- 4.66)
TOUSEY	9	m	3	-	-	-	-	2.80	(0.70- 11.60)
TOUSEY	5	f	0	1	2	13	226	8.69	(0.74- 102.22)
Subtotal TOUSEY								3.70	(1.09- 12.52)
*TULINI	35	m	3	-	-	-	-	7.57	(4.02- 14.26)
*TULINI	31	f	3	-	-	-	-	14.70	(6.35- 34.10)
Subtotal TULINI								9.63	(5.81- 15.96)
*WALD	3	m	1	-	-	-	-	3.19	(1.07- 9.50)
WIGLE	29	m	1	-	-	-	-	2.10	(1.05- 4.20)
WIGLE	5	f	0	1	0	36	439	36.12~	(1.45- 902.62)
Subtotal WIGLE								2.38	(1.21- 4.69)
WYNDE2	15	m	0	14	104	8	105	1.77	(0.71- 4.39)
WYNDE3	152	m	0	14	68	9	88	2.01	(0.82- 4.93)
WYNDE6	63	m	0	58	199	87	617	2.07	(1.43- 2.99)
Partial Totals				1651	52629	3417	82187		

*prospective study ~ With 0.5 adjustment for zero

Table 1E4 - 2

IESLC - Meta-analysis of Current Smoking (or Ever if Current not available), Pipe and/or Cigars (not cigs)
All LC types
Most adjusted

REF	NRR	SEX	AD	Ys	Ws	Qs	Ps
ABELIN	46	m	1	3.42	1.89	8.71	0.0000
ALDERS	1	m	1	1.34	6.20	0.03	0.0008
ARMADA	30	m	0	2.21	1.52	1.35	0.0064
BECHER	23	m	2	1.53	1.99	0.14	0.0310
*BENSHL	10	m	1	0.85	1.99	0.36	0.2326
*BEST	19	m	0	2.04	0.89	0.53	0.0545
BOFFET	30	m	0	1.89	46.83	18.18	0.0000
*BOUCOT	117	m	2	1.92	0.45	0.19	0.1976
BRESLO	33	m	0	0.28	4.03	3.94	0.5736
BROSS	5	m	0	0.97	22.25	1.93	0.0000
*CHOW	13	m	0	2.11	5.16	3.64	0.0000
COMSTO	1	m	0	0.83	1.20	0.23	0.3609
*CPSI	186	m	1	0.66	42.89	15.79	0.0000
DAMBER	28	m	0	1.92	24.87	10.49	0.0000
DARBY	6	m	0	3.79	2.78	17.66	0.0000
DARBY	13	f	0	1.60	2.22	0.24	0.0174
Subtotal DARBY				2.81	5.00	17.89	
DEAN	11	m	0	1.25	7.53	0.00	0.0006
DEAN2	11	m	0	0.76	16.84	4.29	0.0017
*DEKLER	5	m	2	2.21	0.66	0.58	0.0722
*DOLL2	20	m	1	1.76	6.24	1.49	0.0000
DORGAN	1	m	0	0.91	7.09	0.92	0.0156
DORGAN	25	m	0	0.61	1.34	0.58	0.4799
Subtotal DORGAN				0.86	8.43	1.50	
*DORN	90	m	1	0.53	34.00	18.56	0.0020
*ENGELA	167	m	1	0.96	4.41	0.43	0.0449
GOLLED	5	m	1	1.14	10.90	0.18	0.0002
GRAHAM	24	m	1	1.06	13.76	0.62	0.0001
*HAMMO2	14	m	1	-0.13	1.88	3.67	0.8610
*HAMMON	118	m	1	0.44	9.82	6.78	0.1697
*HOLE	6	m	1	0.83	1.56	0.30	0.3003
*KINLEN	13	m	2	1.87	6.14	2.24	0.0000
KJUUS	4	m	0	2.40	1.60	2.05	0.0024
*KNEKT	28	m	1	1.81	3.30	0.96	0.0010
KREUZE	10	f	0	0.68	0.35	0.12	0.6868
KREUZE	12	f	0	2.57	0.43	0.73	0.0908
Subtotal KREUZE				1.72	0.79	0.85	
LEVIN	31	m	1	0.27	16.60	16.58	0.2713
LOMBAR	1	m	0	0.97	9.26	0.85	0.0033
LUBIN2	30	m	2	1.40	65.58	1.10	0.0000
MARSH	5	m	0	0.14	0.38	0.49	0.9331
MARSH2	3	c	0	-0.69	0.43	1.65	0.6511
MATOS	1	m	0	0.92	0.74	0.09	0.4303
*MIGRAN	16	m	2	1.34	2.83	0.01	0.0244
SADOWS	70	m	0	0.66	9.47	3.47	0.0409
STASZE	3	m	0	0.22	2.14	2.34	0.7425
STOCKW	5	c	0	1.43	296.07	7.26	0.0000
TOUSEY	9	m	3	1.03	1.95	0.11	0.1506
TOUSEY	5	f	0	2.16	0.63	0.50	0.0855
Subtotal TOUSEY				1.31	2.58	0.62	
*TULINI	35	m	3	2.02	9.58	5.46	0.0000
*TULINI	31	f	3	2.69	5.44	10.94	0.0000
Subtotal TULINI				2.26	15.02	16.40	
*WALD	3	m	1	1.16	3.22	0.04	0.0373
WIGLE	29	m	1	0.74	8.00	2.22	0.0359
WIGLE	5	f	0	3.59	0.37	1.99	0.0289
Subtotal WIGLE				0.87	8.37	4.22	
WYNDE2	15	m	0	0.57	4.64	2.27	0.2202
WYNDE3	152	m	0	0.70	4.79	1.56	0.1256
WYNDE6	63	m	0	0.73	28.26	8.34	0.0001

Table 1E4 - 2

IESLC - Meta-analysis of Current Smoking (or Ever if Current not available), Pipe and/or Cigars (not cigs)
All LC types
Most adjusted

	N	54
	NS	48
	Wt	765.42
Het	Chi	195.19
Het	df	53
Het	P	***
Fixed	RR	3.56
	RRl	3.32
	RRu	3.82
	P	+++
Random	RR	3.42
	RRl	2.85
	RRu	4.11
	P	+++
Asymm	P	N.S.

Table 1E4 - 3

IESLC - Meta-analysis of Current Smoking (or Ever if Current not available), Pipe and/or Cigars (not cigs)
All LC types
Most adjusted

		Sex			
		combined	male	female	Total
	N	2	46	6	54
	NS	2	45	5	52
	Wt	296.50	459.47	9.45	765.42
Het	Chi	1.92	165.00	3.48	195.19
Het	df	1	45	5	53
Het	P	N.S.	***	N.S.	***
Fixed	RR	4.15	3.15	10.49	3.56
	RRl	3.70	2.88	5.55	3.32
	RRu	4.65	3.45	19.85	3.82
	P	+++	+++	+++	+++
Random	RR	2.50	3.26	10.49	3.42
	RRl	0.42	2.65	5.55	2.85
	RRu	14.72	4.00	19.85	4.11
	P	N.S.	+++	+++	+++
Between	Chi				24.80
Between	df				2
Between	P				***
Btwn(F)	P				*
Btwn(R)	P				**

		Smoking status		
		ever	current	Total
	N	31	23	54
	NS	29	19	48
	Wt	564.39	201.04	765.42
Het	Chi	123.45	71.65	195.19
Het	df	30	22	53
Het	P	***	***	***
Fixed	RR	3.54	3.63	3.56
	RRl	3.26	3.16	3.32
	RRu	3.84	4.16	3.82
	P	+++	+++	+++
Random	RR	2.87	4.50	3.42
	RRl	2.25	3.30	2.85
	RRu	3.65	6.15	4.11
	P	+++	+++	+++
Between	Chi			0.09
Between	df			1
Between	P			N.S.
Btwn(F)	P			N.S.
Btwn(R)	P			*

Table 1E4 - 4

IESLC - Meta-analysis of Current Smoking (or Ever if Current not available), Pipe and/or Cigars (not cigs)
All LC types
Least adjusted

REF	NRR	X	SEX	AGEL	AGEH	RACE	YF	LC TYPE	LOC	START	ST	NLC	R	VB	P	H	AD	SM	DENOM	De
ABELIN	3	x	m	0	0	all	-	all	Eu:wst	1941	CC	118	n	bl	y	n	0	ev	nev any	st
ALDERS	7	x	m	0	0	all	-	all	Eu:UK	1977	CC	1448	n	V	n	n	0	ev	nev any	st
ARMADA	30		m	0	0	all	-	all	Eu:wst	1986	CC	325	n	bl	n	y	0	ev	nev any	st
BECHER	3	x	m	0	0	all	-	all	Eu:Ger	1985	CC	194	n	bl	n	y	0	ev	nev any	st
BENSHL	10		m	40	64	all	10	all	Eu:UK	1967	pr	486	n	V	n	n	1	cu	nev any	ot
BEST	19		m	55	79	all	3	all	NAmer	1955	pr	381	n	V	n	n	0	ev	nev any	st
BOFFET	30		m	0	0	all	-	all	Eu:mul	1988	CC	5621	n	bl	y	n	0	ev	nev any	st
BOUCOT	5	x	m	0	0	all	0	all	NAmer	1951	pr	121	n	bl	n	n	0	ev	nev any	ot
BRESLO	33		m	0	0	all	-	all	NAmer	1949	CC	518	n	bl	n	y	0	ev	nev+1	st
BROSS	5		m	0	0	wh	-	all	NAmer	1960	CC	974	n	bl	n	n	0	cu	nev any	st
CHOW	13		m	0	0	wh	0	all	NAmer	1966	pr	219	n	bl	n	n	0	cu	nev any	st
COMSTO	1		m	0	0	all	-	all	NAmer	1975	ot	258	n	bl	n	n	0	ev	nev any	st
CPSI	186		m	35	84	all	6	all	NAmer	1959	pr	5138	n	bl	n	n	1	ev	nev any	ot
DAMBER	28		m	0	0	all	-	all	Eu:Sca	1972	CC	579	n	bl	y	n	0	ev	nev any	st
DARBY	6		m	0	0	wh	-	all	Eu:UK	1988	CC	982	n	V	n	n	0	cu	nev any	st
DARBY	13		f	0	0	wh	-	all	Eu:UK	1988	CC	982	n	V	n	n	0	cu	nev any	st
DEAN	11		m	0	0	wh	-	all	Africa	1947	CC	603	n	V	y	n	0	ev	nev any	st
DEAN2	11		m	0	0	all	-	all	Eu:UK	1960	CC	954	n	V	y	n	0	ev	nev any	st
DEKLER	5		m	0	0	all	0	all	Auslia	1961	pr	138	m	V	n	n	2	cu	nev any	or
DOLL2	20		m	0	0	all	20	all	Eu:UK	1951	pr	920	n	V	n	n	1	cu	nev any	ot
DORGAN	1		m	0	0	wh	-	all	NAmer	1980	CC	2026	n	bl	y	y	0	ev	nev any	st
DORGAN	25		m	0	0	bl	-	all	NAmer	1980	CC	2026	n	bl	y	y	0	ev	nev any	st
DORN	90		m	35	84	wh	8	all	NAmer	1954	pr	5097	n	bl	n	n	1	cu	nev any	ot
ENGELA	167		m	0	0	all	12	all	Eu:Sca	1964	pr	435	n	bl	n	n	1	cu	nev any	ot
GOLLED	19	x	m	35	99	all	-	all	Eu:UK	1952	CC	443	n	V	y	n	0	ev	nev any	st
GRAHAM	16	x	m	0	0	wh	-	all	NAmer	1956	CC	685	n	bl	n	n	0	cu	nev any	st
HAMMO2	28	x	m	0	0	all	0	all	NAmer	1967	pr	450	o	bl	n	n	0	ev	nev any	st
HAMMON	130	x	m	0	0	wh	0	all	NAmer	1952	pr	448	n	bl	n	n	0	ev	nev any	st
HOLE	13	x	m	0	0	all	0	all	Eu:UK	1972	pr	225	n	V	n	n	0	cu	nev any	st
KINLEN	2	x	m	0	0	all	0	all	Eu:UK	1967	pr	718	n	V	n	n	0	cu	nev any	st
KJUUS	4		m	0	0	all	-	all	Eu:Sca	1979	CC	176	n	bl	n	n	0	ev	nev any	st
KNEKT	24	x	m	20	69	all	21	all	Eu:Sca	1966	pr	515	n	bl	n	n	0	cu	nev any	st
KREUZE	10		f	1	45	all	-	all	Eu:Ger	1990	CC	2260	n	bl	n	n	0	cu	nev any	ot
KREUZE	12		f	55	69	all	-	all	Eu:Ger	1990	CC	2260	n	bl	n	n	0	cu	nev any	ot
LEVIN	31		m	0	0	all	-	all	NAmer	1938	CC	475	n	bl	n	n	1	ev	nev any	st
LOMBAR	1		m	0	0	all	-	all	NAmer	1951	CC	1040	n	bl	n	n	0	ev	nev any	st
LUBIN2	29	x	m	0	0	all	-	all	Eu:mul	1976	CC	7804	n	bl	n	y	0	cu	nev any	st
MARSH	5		m	0	0	all	-	all	NAmer	1979	CC	150	n	bl	y	n	0	ev	nev any	ot
MARSH2	3		c	0	0	all	-	all	NAmer	1979	CC	114	n	bl	y	n	0	ev	nev any	ot
MATOS	1		m	0	0	all	-	all	SCAmer	1994	CC	200	n	bl	n	n	0	cu	nev any	st
MIGRAN	15	x	m	0	0	all	0	all	Eu:UK	1964	pr	259	n	V	n	n	0	cu	nev any	st
SADOWS	70		m	0	0	wh	-	all	NAmer	1938	CC	477	n	bl	n	n	0	ev	nev any	st
STASZE	3		m	0	0	all	-	all	Eu:est	1954	CC	281	n	bl	n	y	0	ev	nev any	st
STOCKW	5		c	0	0	all	-	all	NAmer	1981	CC	22161	n	bl	n	n	0	ev	nev any	st
TOUSEY	1	x	m	0	0	all	-	all	NAmer	1993	CC	507	n	bl	y	y	0	ev	nev any	st
TOUSEY	5		f	0	0	all	-	all	NAmer	1993	CC	507	n	bl	y	y	0	ev	nev any	st
TULINI	12	x	m	0	0	all	0	all	Eu:Sca	1967	pr	472	n	bl	n	n	1	cu	nev any	ot
TULINI	8	x	f	0	0	all	0	all	Eu:Sca	1967	pr	472	n	bl	n	n	1	cu	nev any	or
WALD	1	x	m	0	0	all	0	all	Eu:UK	1975	pr	102	n	V	n	n	0	cu	nev any	st
WIGLE	2	x	m	0	0	all	-	all	NAmer	1971	CC	728	n	V	n	n	0	cu	nev any	st
WIGLE	5		f	0	0	all	-	all	NAmer	1971	CC	728	n	V	n	n	0	cu	nev any	ot
WYNDE2	15		m	0	0	all	-	all	NAmer	1962	CC	404	n	bl	n	y	0	ev	nev any	st
WYNDE3	152		m	0	0	all	-	all	NAmer	1966	CC	350	n	bl	n	y	0	ev	nev any	st
WYNDE6	63		m	0	0	all	-	all	NAmer	1969	CC	4423	n	bl	n	y	0	ev	nev any	st

Table 1E4 - 5

IESLC - Meta-analysis of Current Smoking (or Ever if Current not available), Pipe and/or Cigars (not cigs)
All LC types
Least adjusted

REF	NRR	SEX	AD	Number Exposed Case	Number Exposed Cont	Non-exposed Case	Non-exposed Cont	RR	95.00%CI
ABELIN	3	m	0	69	187	2	183	33.76	(8.16- 139.75)
ALDERS	7	m	0	17	35	15	133	4.31	(1.96- 9.47)
ARMADA	30	m	0	4	7	4	64	9.14	(1.86- 44.85)
BECHER	3	m	0	6	21	3	54	5.14	(1.18- 22.47)
*BENSHL	10	m	1	-	-	-	-	2.33	(0.58- 9.33)
*BEST	19	m	0	8	2972	1	2854	7.68	(0.96- 61.38)
BOFFET	30	m	0	118	266	117	1750	6.64	(4.98- 8.84)
*BOUCOT	5	m	0	4	8926	0	7551	7.61~	(0.41- 141.39)
BRESLO	33	m	0	15	68	7	42	1.32	(0.50- 3.51)
BROSS	5	m	0	125	211	38	170	2.65	(1.75- 4.02)
*CHOW	13	m	0	37	47084	6	62913	8.24	(3.48- 19.52)
COMSTO	1	m	0	2	15	4	69	2.30	(0.39- 13.73)
*CPSI	186	m	1	-	-	-	-	1.94	(1.44- 2.62)
DAMBER	28	m	0	205	149	42	208	6.81	(4.60- 10.09)
DARBY	6	m	0	57	165	3	384	44.22	(13.65- 143.22)
DARBY	13	f	0	3	14	23	529	4.93	(1.32- 18.36)
Subtotal DARBY								16.69	(6.95- 40.09)
DEAN	11	m	0	51	74	12	61	3.50	(1.71- 7.16)
DEAN2	11	m	0	81	128	33	112	2.15	(1.33- 3.46)
*DEKLER	5	m	2	-	-	-	-	9.10	(0.82- 101.10)
*DOLL2	20	m	1	-	-	-	-	5.80	(2.65- 12.72)
DORGAN	1	m	0	22	55	15	93	2.48	(1.19- 5.18)
DORGAN	25	m	0	3	19	3	35	1.84	(0.34- 10.03)
Subtotal DORGAN								2.37	(1.20- 4.65)
*DORN	90	m	1	-	-	-	-	1.70	(1.21- 2.37)
*ENGELA	167	m	1	-	-	-	-	2.60	(1.02- 6.60)
GOLLED	19	m	0	48	331	15	490	4.74	(2.61- 8.60)
GRAHAM	16	m	0	64	398	18	346	3.09	(1.80- 5.32)
*HAMMO2	28	m	0	3	488	5	891	1.10	(0.26- 4.56)
*HAMMON	130	m	0	28	127770	15	115884	1.69	(0.90- 3.17)
*HOLE	13	m	0	2	141	7	1189	2.41	(0.51- 11.49)
*KINLEN	2	m	0	47	1367	7	1333	6.55	(2.97- 14.43)
KJUUS	4	m	0	23	25	2	24	11.04	(2.34- 52.00)
*KNEKT	24	m	0	7	2822	6	17814	7.36	(2.48- 21.90)
KREUZE	10	f	0	0	1	6	38	1.97~	(0.07- 53.89)
KREUZE	12	f	0	3	0	95	177	13.01~	(0.67- 254.52)
Subtotal KREUZE								5.60	(0.61- 51.10)
LEVIN	31	m	1	-	-	-	-	1.31	(0.81- 2.12)
LOMBAR	1	m	0	48	146	14	112	2.63	(1.38- 5.01)
LUBIN2	29	m	0	142	465	190	2617	4.21	(3.31- 5.34)
MARSH	5	m	0	0	5	2	31	1.15~	(0.05- 27.23)
MARSH2	3	c	0	0	4	12	56	0.50~	(0.03- 9.94)
MATOS	1	m	0	1	4	11	110	2.50	(0.26- 24.38)
*MIGRAN	15	m	0	16	575	4	867	6.03	(2.03- 17.95)
SADOWS	70	m	0	38	88	18	81	1.94	(1.03- 3.67)
STASZE	3	m	0	4	101	5	158	1.25	(0.33- 4.77)
STOCKW	5	c	0	715	655	2791	10641	4.16	(3.71- 4.66)
TOUSEY	1	m	0	4	48	4	130	2.71	(0.65- 11.26)
TOUSEY	5	f	0	1	2	13	226	8.69	(0.74- 102.22)
Subtotal TOUSEY								3.63	(1.06- 12.45)
*TULINI	12	m	1	-	-	-	-	7.92	(4.20- 14.90)
*TULINI	8	f	1	-	-	-	-	15.70	(6.78- 36.30)
Subtotal TULINI								10.15	(6.12- 16.83)
*WALD	1	m	0	6	1309	7	6539	4.28	(1.44- 12.72)
WIGLE	2	m	0	24	86	15	204	3.80	(1.90- 7.59)
WIGLE	5	f	0	1	0	36	439	36.12~	(1.45- 902.62)
Subtotal WIGLE								4.19	(2.13- 8.25)
WYNDE2	15	m	0	14	104	8	105	1.77	(0.71- 4.39)
WYNDE3	152	m	0	14	68	9	88	2.01	(0.82- 4.93)
WYNDE6	63	m	0	58	199	87	617	2.07	(1.43- 2.99)
Partial Totals				2138	197598	3730	238412		

*prospective study ~ With 0.5 adjustment for zero

Table 1E4 - 5

IESLC - Meta-analysis of Current Smoking (or Ever if Current not available), Pipe and/or Cigars (not cigs)
All LC types
Least adjusted

REF	NRR	SEX	AD	Ys	Ws	Qs	Ps
ABELIN	3	m	0	3.52	1.90	9.42	0.0000
ALDERS	7	m	0	1.46	6.19	0.17	0.0003
ARMADA	30	m	0	2.21	1.52	1.28	0.0064
BECHER	3	m	0	1.64	1.77	0.21	0.0295
*BENSHL	10	m	1	0.85	1.99	0.40	0.2326
*BEST	19	m	0	2.04	0.89	0.49	0.0545
BOFFET	30	m	0	1.89	46.83	16.73	0.0000
*BOUCOT	5	m	0	2.03	0.45	0.24	0.1733
BRESLO	33	m	0	0.28	4.03	4.15	0.5736
BROSS	5	m	0	0.97	22.25	2.28	0.0000
*CHOW	13	m	0	2.11	5.16	3.42	0.0000
COMSTO	1	m	0	0.83	1.20	0.26	0.3609
*CPSI	186	m	1	0.66	42.89	17.14	0.0000
DAMBER	28	m	0	1.92	24.87	9.69	0.0000
DARBY	6	m	0	3.79	2.78	17.30	0.0000
DARBY	13	f	0	1.60	2.22	0.20	0.0174
Subtotal DARBY				2.81	5.00	17.51	
DEAN	11	m	0	1.25	7.53	0.01	0.0006
DEAN2	11	m	0	0.76	16.84	4.74	0.0017
*DEKLER	5	m	2	2.21	0.66	0.55	0.0722
*DOLL2	20	m	1	1.76	6.24	1.34	0.0000
DORGAN	1	m	0	0.91	7.09	1.06	0.0156
DORGAN	25	m	0	0.61	1.34	0.63	0.4799
Subtotal DORGAN				0.86	8.43	1.68	
*DORN	90	m	1	0.53	34.00	19.85	0.0020
*ENGELA	167	m	1	0.96	4.41	0.51	0.0449
GOLLED	19	m	0	1.56	10.80	0.73	0.0000
GRAHAM	16	m	0	1.13	13.06	0.36	0.0000
*HAMMO2	28	m	0	0.09	1.89	2.73	0.9003
*HAMMON	130	m	0	0.53	9.77	5.77	0.0998
*HOLE	13	m	0	0.88	1.57	0.27	0.2698
*KINLEN	2	m	0	1.88	6.15	2.10	0.0000
KJUUS	4	m	0	2.40	1.60	1.96	0.0024
*KNEKT	24	m	0	2.00	3.24	1.59	0.0003
KREUZE	10	f	0	0.68	0.35	0.13	0.6868
KREUZE	12	f	0	2.57	0.43	0.70	0.0908
Subtotal KREUZE				1.72	0.79	0.83	
LEVIN	31	m	1	0.27	16.60	17.43	0.2713
LOMBAR	1	m	0	0.97	9.26	0.99	0.0033
LUBIN2	29	m	0	1.44	67.39	1.35	0.0000
MARSH	5	m	0	0.14	0.38	0.51	0.9331
MARSH2	3	c	0	-0.69	0.43	1.70	0.6511
MATOS	1	m	0	0.92	0.74	0.11	0.4303
*MIGRAN	15	m	0	1.80	3.23	0.81	0.0012
SADOWS	70	m	0	0.66	9.47	3.76	0.0409
STASZE	3	m	0	0.22	2.14	2.46	0.7425
STOCKW	5	c	0	1.43	296.07	5.10	0.0000
TOUSEY	1	m	0	1.00	1.89	0.17	0.1705
TOUSEY	5	f	0	2.16	0.63	0.48	0.0855
Subtotal TOUSEY				1.29	2.52	0.64	
*TULINI	12	m	1	2.07	9.58	5.75	0.0000
*TULINI	8	f	1	2.75	5.46	11.62	0.0000
Subtotal TULINI				2.32	15.04	17.37	
*WALD	1	m	0	1.45	3.24	0.08	0.0088
WIGLE	2	m	0	1.33	8.01	0.01	0.0002
WIGLE	5	f	0	3.59	0.37	1.95	0.0289
Subtotal WIGLE				1.43	8.38	1.96	
WYNDE2	15	m	0	0.57	4.64	2.44	0.2202
WYNDE3	152	m	0	0.70	4.79	1.70	0.1256
WYNDE6	63	m	0	0.73	28.26	9.14	0.0001

Table 1E4 - 5

IESLC - Meta-analysis of Current Smoking (or Ever if Current not available), Pipe and/or Cigars (not cigs)
All LC types
Least adjusted

	N	54
	NS	48
	Wt	766.53
Het	Chi	195.98
Het	df	53
Het	P	***
Fixed	RR	3.65
	RRl	3.40
	RRu	3.92
	P	+++
Random	RR	3.63
	RRl	3.02
	RRu	4.36
	P	+++
Asymm	P	N.S.

Table 1E4 - 6

IESLC - Meta-analysis of Current Smoking (or Ever if Current not available), Pipe and/or Cigars (not cigs)
All LC types
Least adjusted

	Sex: combined	male	female	Total
N	2	46	6	54
NS	2	45	5	52
Wt	296.50	460.56	9.47	766.53
Het Chi	1.92	169.04	3.73	195.98
Het df	1	45	5	53
Het P	N.S.	***	N.S.	***
Fixed RR	4.15	3.29	10.91	3.65
RRl	3.70	3.00	5.77	3.40
RRu	4.65	3.60	20.62	3.92
P	+++	+++	+++	+++
Random RR	2.50	3.47	10.91	3.63
RRl	0.42	2.82	5.77	3.02
RRu	14.72	4.28	20.62	4.36
P	N.S.	+++	+++	+++
Between Chi				21.29
Between df				2
Between P				***
Btwn(F) P				(*)
Btwn(R) P				**

Table 1E4 - 7

IESLC - Meta-analysis of Current Smoking (or Ever if Current not available), Pipe and/or Cigars (not cigs)
All LC types
Excluded studies (and stage at which they were excluded)

1 BOUCHA BUELL LAURIL MZILEN

2 ABRAHA AGUDO AKIBA AMANDU AMES ANDERS ARCHER AUSTIN AUVINE AXELSO AXELSS BAND BARBON BERRIN BLOHMK BLOT1
BLOT2 BLOT3 BLOT4 BRETT BROCKM BROWN1 BROWN2 BUFFLE BYERS1 BYERS2 CARPEN CASCO2 CASCOR CEDERL CHAN CHANG
CHATZI CHEN CHEN2 CHEN3 CHIAZZ CHOI CHYOU COOKSO CORREA CPSII DAVEYS DEAN3 DESTE2 DESTEF DOCKER DOLL
DORANT DOSEME DROSTE DU DUNN EBELIN ENSTRO ESAKI FAN GAO GAO2 GARCIA GARDIN GARSHI GENG GER
GILLIS GODLEY GOODMA GREGOR GSELL HAENSZ HANSEN HEGMAN HEIN HENNEK HINDS HIRAOK HIRAY2 HIRAYA HITOSU HOROWI
HORWIT HU HU2 HUANG HUMBLE ISHIMA JAIN JARUP JARVHO JEDRYC JIANG JOLY JONES JUSSAW KAISE2 KAISER
KANELL KATSOU KAUFMA KELLER KHUDER KIHARA KO KOHLME KOO KOULUM KREYBE KUBIK LAMTH LAMWK LAMWK2 LANGE
LAUSSM LEI LEMARC LETOUR LIAW LICKIN LIDDEL LIU LIU2 LIU3 LIU4 LIU5 LOMBA2 LUBIN LUO MACLEN
MAGNUS MARTIS MASTRA MATSUD MCCONN MCDUFF MCLAUG MILLER MILLS MOLLO MRFIT MRFITR MURATA NAM NOTAN2 NOTANI
NOU ODRISC ORMOS OSANN OSANN2 PARKIN PASTOR PAWLEG PERNU PERSH2 PERSHA PETO PEZZO2 PEZZOT PIKE PISANI
POFFIJ POLEDN PRESCO QIAO QIAO2 RACHTA RADZIK RANDIG REN RESTRE RIMING RONCO ROOTS ROTHSC SAARIK SANKAR
SCHWA2 SCHWAR SEGI SEGI2 SEOW SHAW SHIMIZ SIEMIA SIMARA SIMONA SITAS SOBUE SOBUE2 SPEIZE SPITZ STAYNE
STOCKS STUCKE SUN SUZUK2 SUZUKI SVENSS TANG TANG2 TAO TENKAN TIZZAN TOKARS TSUGAN TVERDA ULMER VANDER
VEIERO VUTUC WAKAI WANG WANG2 WANG3 WANG4 WARSIN WATSON WICKLU WILKIN WU WU2 WUNSCH WUWILL WYNDE4
WYNDE5 WYNDE8 WYNDER XIANGZ XU XU2 XU3 XU4 YAMAGU YONG YUAN ZHANG ZHENG ZHOU

9 BENHAM JAHN WYNDE7

Table 1E4 - 8

Potentially overlapping studies

REF	REFGP	PRINC	.	OVERLAP/LINK
LUBIN2	LUBIN2	1		Lubin-combined
BROSS	BYERS1	1		GRAHAM/BROSS/BYERS1
GRAHAM	BYERS1	1		GRAHAM/BROSS/BYERS1
HOLE	TANG2	1		Subset of TANG2
BENSHL	TANG2	1		Subset of TANG2
WALD	TANG2	1		Subset of TANG2
WYNDE6	WYNDE6	1		WYNDE5/6/7/8
CPSI	CPSI	1		CPSI overall
BOFFET	BOFFET	1		BOFFET-combined

Table 1E4 - 9

Most adjusted - insufficient data for metaanalysis

REF	NRR	SEX	AGEL	AGEH	RACE	YF	LC TYPE	LOC	START	ST	NLC	R	VB	P	H	AD	SM	DENOM	De
BECHER	4	f	0	0	all	-	all	Eu:Ger	1985	CC	194	n	bl	n	y	0	ev	nev any	st
BRESLO	34	f	0	0	all	-	all	NAmer	1949	CC	518	n	bl	n	y	0	ev	nev+1	st

REF	NRR	RR	SIG	RRDATA comment
BECHER	4		*	No relevant smokers
BRESLO	34		*	No relevant smokers

Table 1E5 -

IESLC - Meta-analysis of Ex Smoking, Pipe and/or Cigars (not cigs)
All LC types

This analysis is restricted to results for:
1) Non-dose-response data
2) Smokers of pipe and/or cigars (but not cigarettes)
3) Ex smokers
4) Results complete enough for use in metaanalysis

Within each study, results are then selected (in the following order of preference, within each sex) for:
5) DENOM: never smoked anything, (never +1 = +long term ex)
6) Followup period (prospective studies): whole study (coded as 0) or longest available
7) LCtype: all or nearest available, at least Squamous and Adeno. (q = squamous, s = small,
l = large, a = adeno, mix = mixed, alv = alveolar)
8) Race: all or nearest available, otherwise by race (wh or w = white, bl or b = black, hi = hispanic
ch = chinese, jap = japanese, haw = hawaiian, w+o = white + oriental, sca = scandinavian, as = asian)
9) For overlapping studies: principal rather than subsidiary studies
Finally by Age: whole study (coded as 0) if available, otherwise by widest available age group
and then for single sex results (m, f) in preference to combined sex results (c).

Results adjusted (AD) for the most potential confounders are then chosen in Sections -1 to -3
and results adjusted for the least confounders in Sections -4 to -6. (Those least adjusted results which
actually differ from the most adjusted as marked 'x' in column X in Section -4)
(Results adjusted for an unknown number of confounder(s) are coded as 20.)

Section -7 shows excluded studies, together with the stage (as above) at which no qualifying
results were found.

Section -8 lists the potentially overlapping studies which have been included (1=principal, 2=subsidiary).

Section -9 lists any results which would have been included in preference except that they had data not complete
enough for use in meta-analysis, with their significance (yes/no), if known, and any further comment as entered
on the database.

In addition to those mentioned above, the following fields, levels and abbreviations are used:
* or nk = not known, n = no, y = yes, ot = other
nev = never
REF: 6-character study reference
NRR: number of the RR on the database within the study
ST : study type (CC = case control, pr or prosp = prospective)
NLC: number of lung cancer cases in whole study
R : risky occupational population (n = no, m = mining, o = other risky)
VB : national cigarette type (V = at least 75% Virginia, bl = at least 75% blended, ot = other)
P : any proxy use
H : full histological confirmation
De : derivation of RR/CI (or = original, st = standard method, ot = other method of estimation)

Table 1E5 - 1

IESLC - Meta-analysis of Ex Smoking, Pipe and/or Cigars (not cigs)
All LC types
Most adjusted

REF	NRR	SEX	AGEL	AGEH	RACE	YF	LC TYPE	LOC	START	ST	NLC	R	VB	P	H	AD	DENOM	De
CHOW	14	m	0	0	wh	0	all	NAmer	1966	pr	219	n	bl	n	n	0	nev any	st
DOLL2	42	m	0	0	all	10	all	Eu:UK	1951	pr	920	n	V	n	n	1	nev any	ot
DORN	135	m	35	84	wh	8	all	NAmer	1954	pr	5097	n	bl	n	n	1	nev any	ot
LUBIN2	40	m	0	0	all	-	all	Eu:mul	1976	CC	7804	n	bl	n	y	2	nev any	ot
MIGRAN	23	m	0	0	all	0	all	Eu:UK	1964	pr	259	n	V	n	n	0	nev any	st
WIGLE	8	m	0	0	all	-	all	NAmer	1971	CC	728	n	V	n	n	0	nev any	st
WYNDE7	50	m	0	0	all	-	all	NAmer	1977	CC	2085	n	bl	n	y	0	nev any	st

Table 1E5 - 2

IESLC - Meta-analysis of Ex Smoking, Pipe and/or Cigars (not cigs)
All LC types
Most adjusted

REF	NRR	SEX	AD	Number Exposed Case	Number Exposed Cont	Non-exposed Case	Non-exposed Cont	RR	95.00%CI
*CHOW	14	m	0	1	5494	6	62913	1.91	(0.23- 15.85)
*DOLL2	42	m	1	-	-	-	-	3.29	(0.66- 16.28)
*DORN	135	m	1	-	-	-	-	1.46	(0.85- 2.51)
LUBIN2	40	m	2	-	-	-	-	2.37	(1.43- 3.94)
*MIGRAN	23	m	0	1	103	4	867	2.10	(0.24- 18.65)
WIGLE	8	m	0	6	27	15	204	3.02	(1.08- 8.45)
WYNDE7	50	m	0	18	137	64	918	1.88	(1.08- 3.28)
Partial Totals				26	5761	89	64902		

*prospective study

REF	NRR	SEX	AD	Ys	Ws	Qs	Ps
*CHOW	14	m	0	0.65	0.86	0.00	0.5495
*DOLL2	42	m	1	1.19	1.50	0.37	0.1453
*DORN	135	m	1	0.38	13.11	1.28	0.1707
LUBIN2	40	m	2	0.86	14.96	0.44	0.0008
*MIGRAN	23	m	0	0.74	0.81	0.00	0.5039
WIGLE	8	m	0	1.11	3.63	0.62	0.0350
WYNDE7	50	m	0	0.63	12.57	0.04	0.0247

	N	7
	NS	7
	Wt	47.42
Het	Chi	2.77
Het	df	6
Het	P	N.S.
Fixed	RR	2.00
	RRl	1.50
	RRu	2.65
	P	+++
Random	RR	2.00
	RRl	1.50
	RRu	2.65
	P	+++
Asymm	P	N.S.

Table 1E5 - 3

IESLC - Meta-analysis of Ex Smoking, Pipe and/or Cigars (not cigs)
All LC types
Most adjusted

	Sex: combined	male	female	Total
N		7		7
NS		7		7
Wt		47.42		47.42
Het Chi		2.77		2.77
Het df		6		6
Het P		N.S.		N.S.
Fixed RR		2.00		2.00
RRl		1.50		1.50
RRu		2.65		2.65
P		+++		+++
Random RR		2.00		2.00
RRl		1.50		1.50
RRu		2.65		2.65
P		+++		+++
Between Chi				
Between df				
Between P				N.S.
Btwn(F) P				N.S.
Btwn(R) P				N.S.

Too few RRs for analysis by factor

Table 1E5 - 4

IESLC - Meta-analysis of Ex Smoking, Pipe and/or Cigars (not cigs)
All LC types
Least adjusted

REF	NRR	X	SEX	AGEL	AGEH	RACE	YF	LC TYPE	LOC	START	ST	NLC	R	VB	P	H	AD	DENOM	De
CHOW	14		m	0	0	wh	0	all	NAmer	1966	pr	219	n	bl	n	n	0	nev any	st
DOLL2	42		m	0	0	all	10	all	Eu:UK	1951	pr	920	n	V	n	n	1	nev any	ot
DORN	135		m	35	84	wh	8	all	NAmer	1954	pr	5097	n	bl	n	n	1	nev any	ot
LUBIN2	39	x	m	0	0	all	-	all	Eu:mul	1976	CC	7804	n	bl	n	y	0	nev any	st
MIGRAN	23		m	0	0	all	0	all	Eu:UK	1964	pr	259	n	V	n	n	0	nev any	st
WIGLE	8		m	0	0	all	-	all	NAmer	1971	CC	728	n	V	n	n	0	nev any	st
WYNDE7	50		m	0	0	all	-	all	NAmer	1977	CC	2085	n	bl	n	y	0	nev any	st

Table 1E5 - 5

IESLC - Meta-analysis of Ex Smoking, Pipe and/or Cigars (not cigs)
All LC types
Least adjusted

REF	NRR	SEX	AD	Number Exposed Case	Exposed Cont	Non-exposed Case	Non-exposed Cont	RR	95.00%CI
*CHOW	14	m	0	1	5494	6	62913	1.91	(0.23- 15.85)
*DOLL2	42	m	1	-	-	-	-	3.29	(0.66- 16.28)
*DORN	135	m	1	-	-	-	-	1.46	(0.85- 2.51)
LUBIN2	39	m	0	19	95	190	2617	2.75	(1.65- 4.61)
*MIGRAN	23	m	0	1	103	4	867	2.10	(0.24- 18.65)
WIGLE	8	m	0	6	27	15	204	3.02	(1.08- 8.45)
WYNDE7	50	m	0	18	137	64	918	1.88	(1.08- 3.28)
Partial Totals				45	5856	279	67519		

*prospective study

REF	NRR	SEX	AD	Ys	Ws	Qs	Ps
*CHOW	14	m	0	0.65	0.86	0.01	0.5495
*DOLL2	42	m	1	1.19	1.50	0.31	0.1453
*DORN	135	m	1	0.38	13.11	1.68	0.1707
LUBIN2	39	m	0	1.01	14.53	1.12	0.0001
*MIGRAN	23	m	0	0.74	0.81	0.00	0.5039
WIGLE	8	m	0	1.11	3.63	0.50	0.0350
WYNDE7	50	m	0	0.63	12.57	0.13	0.0247

	N	7
	NS	7
	Wt	47.00
Het	Chi	3.74
Het	df	6
Het	P	N.S.
Fixed	RR	2.09
	RRl	1.57
	RRu	2.78
	P	+++
Random	RR	2.09
	RRl	1.57
	RRu	2.78
	P	+++
Asymm	P	N.S.

Table 1E5 - 6

IESLC - Meta-analysis of Ex Smoking, Pipe and/or Cigars (not cigs)
All LC types
Least adjusted

	Sex			
	combined	male	female	Total
N		7		7
NS		7		7
Wt		47.00		47.00
Het Chi		3.74		3.74
Het df		6		6
Het P		N.S.		N.S.
Fixed RR		2.09		2.09
RRl		1.57		1.57
RRu		2.78		2.78
P		+++		+++
Random RR		2.09		2.09
RRl		1.57		1.57
RRu		2.78		2.78
P		+++		+++
Between Chi				
Between df				
Between P				N.S.
Btwn(F) P				N.S.
Btwn(R) P				N.S.

Table 1E5 - 7

IESLC - Meta-analysis of Ex Smoking, Pipe and/or Cigars (not cigs)
All LC types
Excluded studies (and stage at which they were excluded)

1 BOUCHA BUELL LAURIL MZILEN

2 ABRAHA AGUDO AKIBA AMANDU AMES ANDERS ARCHER AUSTIN AUVINE AXELSO AXELSS BAND BARBON BERRIN BLOHMK BLOT1 BLOT2 BLOT3 BLOT4 BRETT BROCKM BROWN1 BROWN2 BUFFLE BYERS1 BYERS2 CARPEN CASCO2 CASCOR CEDERL CHAN CHANG CHATZI CHEN CHEN2 CHEN3 CHIAZZ CHOI CHYOU COOKSO CORREA CPSII DAVEYS DEAN3 DESTE2 DESTEF DOCKER DOLL DORANT DOSEME DROSTE DU DUNN EBELIN ENSTRO ESAKI FAN GAO GAO2 GARCIA GARDIN GARSHI GENG GER GILLIS GODLEY GOODMA GREGOR GSELL HAENSZ HANSEN HEGMAN HEIN HENNEK HINDS HIRAOK HIRAY2 HIRAYA HITOSU HOROWI HORWIT HU HU2 HUANG HUMBLE ISHIMA JAIN JARUP JARVHO JEDRYC JIANG JOLY JONES JUSSAW KAISE2 KAISER KANELL KATSOU KAUFMA KELLER KHUDER KIHARA KO KOHLME KOO KOULUM KREYBE KUBIK LAMTH LAMWK LAMWK2 LANGE LAUSSM LEI LEMARC LETOUR LIAW LICKIN LIDDEL LIU LIU2 LIU3 LIU4 LIU5 LOMBA2 LUBIN LUO MACLEN MAGNUS MARTIS MASTRA MATSUD MCCONN MCDUFF MCLAUG MILLER MILLS MOLLO MRFIT MRFITR MURATA NAM NOTAN2 NOTANI NOU ODRISC ORMOS OSANN OSANN2 PARKIN PASTOR PAWLEG PERNU PERSH2 PERSHA PETO PEZZO2 PEZZOT PIKE PISANI POFFIJ POLEDN PRESCO QIAO QIAO2 RACHTA RADZIK RANDIG REN RESTRE RIMING RONCO ROOTS ROTHSC SAARIK SANKAR SCHWA2 SCHWAR SEGI SEGI2 SEOW SHAW SHIMIZ SIEMIA SIMARA SIMONA SITAS SOBUE SOBUE2 SPEIZE SPITZ STAYNE STOCKS STUCKE SUN SUZUK2 SUZUKI SVENSS TANG TANG2 TAO TENKAN TIZZAN TOKARS TSUGAN TVERDA ULMER VANDER VEIERO VUTUC WAKAI WANG WANG2 WANG3 WANG4 WARSIN WATSON WICKLU WILKIN WU WU2 WUNSCH WUWILL WYNDE4 WYNDE5 WYNDE8 WYNDER XIANGZ XU XU2 XU3 XU4 YAMAGU YONG YUAN ZHANG ZHENG ZHOU

3 ABELIN ALDERS ARMADA BECHER BENHAM BENSHL BEST BOFFET BOUCOT BRESLO BROSS COMSTO CPSI DAMBER DARBY DEAN DEAN2 DEKLER DORGAN ENGELA GOLLED GRAHAM HAMMO2 HAMMON HOLE JAHN KINLEN KJUUS KNEKT KREUZE LEVIN LOMBAR MARSH MARSH2 MATOS SADOWS STASZE STOCKW TOUSEY TULINI WALD WYNDE2 WYNDE3 WYNDE6

Table 1E5 - 8

Potentially overlapping studies

REF	REFGP	PRINC	.	OVERLAP/LINK
LUBIN2	LUBIN2	1		Lubin-combined
WYNDE7	WYNDE6	2		WYNDE5/6/7/8

Table 1E6 -

IESLC - Meta-analysis of Ever Smoking, Pipe only
All LC types

This analysis is restricted to results for:
1) Non-dose-response data
2) Smokers of pipe only
3) Ever smokers
4) Results complete enough for use in metaanalysis

Within each study, results are then selected (in the following order of preference, within each sex) for:
5) DENOM: never smoked anything, (never +1 = +long term ex)
6) Followup period (prospective studies): whole study (coded as 0) or longest available
7) LCtype: all or nearest available, at least Squamous and Adeno. (q = squamous, s = small,
l = large, a = adeno, mix = mixed, alv = alveolar)
8) Race: all or nearest available, otherwise by race (wh or w = white, bl or b = black, hi = hispanic
ch = chinese, jap = japanese, haw = hawaiian, w+o = white + oriental, sca = scandinavian, as = asian)
9) For overlapping studies: principal rather than subsidiary studies
Finally by Age: whole study (coded as 0) if available, otherwise by widest available age group
and then for single sex results (m, f) in preference to combined sex results (c).

Results adjusted (AD) for the most potential confounders are then chosen in Sections -1 to -3
and results adjusted for the least confounders in Sections -4 to -6. (Those least adjusted results which
actually differ from the most adjusted as marked 'x' in column X in Section -4)
(Results adjusted for an unknown number of confounder(s) are coded as 20.)

Section -7 shows excluded studies, together with the stage (as above) at which no qualifying
results were found.

Section -8 lists the potentially overlapping studies which have been included (1=principal, 2=subsidiary).

Section -9 lists any results which would have been included in preference except that they had data not complete
enough for use in meta-analysis, with their significance (yes/no), if known, and any further comment as entered
on the database.

In addition to those mentioned above, the following fields, levels and abbreviations are used:
* or nk = not known, n = no, y = yes, ot = other
nev = never
REF: 6-character study reference
NRR: number of the RR on the database within the study
ST : study type (CC = case control, pr or prosp = prospective)
NLC: number of lung cancer cases in whole study
R : risky occupational population (n = no, m = mining, o = other risky)
VB : national cigarette type (V = at least 75% Virginia, bl = at least 75% blended, ot = other)
P : any proxy use
H : full histological confirmation
De : derivation of RR/CI (or = original, st = standard method, ot = other method of estimation)

Table 1E6 - 1

IESLC - Meta-analysis of Ever Smoking, Pipe only
All LC types
Most adjusted

REF	NRR	SEX	AGEL	AGEH	RACE	YF	LC TYPE	LOC	START	ST	NLC	R	VB	P	H	AD	DENOM	De
ABELIN	50	m	0	0	all	-	all	Eu:wst	1941	CC	118	n	bl	y	n	1	nev any	st
ARMADA	2	m	0	0	all	-	all	Eu:wst	1986	CC	325	n	bl	n	y	0	nev any	st
BEST	17	m	0	0	all	0	all	NAmer	1955	pr	381	n	V	n	n	1	nev any	ot
BOFFET	2	m	0	0	all	-	all	Eu:mul	1988	CC	5621	n	bl	y	n	2	nev any	or
BOUCOT	118	m	0	0	all	0	all	NAmer	1951	pr	121	n	bl	n	n	2	nev any	ot
COOKSO	3	c	0	0	bl	-	all	Africa	1961	CC	234	n	V	n	y	0	nev any	st
CPSI	181	m	35	84	all	6	all	NAmer	1959	pr	5138	n	bl	n	n	1	nev any	ot
DAMBER	2	m	0	0	all	-	all	Eu:Sca	1972	CC	579	n	bl	y	n	0	nev any	st
DEAN	5	m	0	0	wh	-	all	Africa	1947	CC	603	n	V	y	n	0	nev any	st
DOLL	18	m	0	0	all	-	all	Eu:UK	1948	CC	1465	n	V	n	n	0	nev any	st
DORN	34	m	0	0	wh	2	all	NAmer	1954	pr	5097	n	bl	n	n	1	nev any	ot
HAMMON	112	m	0	0	wh	0	all	NAmer	1952	pr	448	n	bl	n	n	1	nev any	ot
KOULUM	3	m	0	0	all	-	all	Eu:Sca	1936	CC	812	n	bl	n	n	0	nev any	st
LEVIN	28	m	0	0	all	-	all	NAmer	1938	CC	475	n	bl	n	n	1	nev any	st
LUBIN2	22	m	0	0	all	-	all	Eu:mul	1976	CC	7804	n	bl	n	y	2	nev any	ot
MCCONN	17	c	0	0	all	-	all	Eu:UK	1946	CC	100	n	V	n	y	0	nev any	st
PERNU	9	m	0	0	all	-	all	Eu:Sca	1944	CC	1606	n	bl	n	n	0	nev any	st
PERNU	5	f	0	0	all	-	all	Eu:Sca	1944	CC	1606	n	bl	n	n	0	nev any	st
SADOWS	30	m	0	0	wh	-	all	NAmer	1938	CC	477	n	bl	n	n	1	nev any	ot
STOCKS	40	m	0	0	all	-	all	Eu:UK	1952	CC	2932	n	V	y	n	2	nev any	st
TIZZAN	4	m	0	0	all	-	all	Eu:wst	1959	CC	1358	n	bl	n	n	0	nev any	st
WYNDE7	41	m	0	0	all	-	all	NAmer	1977	CC	2085	n	bl	n	y	0	nev any	st
XIANGZ	10	m	0	0	all	0	all	As:Chi	1976	pr	983	m	ot	n	n	2	nev any	ot

Table 1E6 - 2

IESLC - Meta-analysis of Ever Smoking, Pipe only
All LC types
Most adjusted

REF	NRR	SEX	AD	Number Exposed Case	Number Exposed Cont	Non-exposed Case	Non-exposed Cont	RR	95.00%CI
ABELIN	50	m	1	-	-	-	-	23.54	(4.73- 117.28)
ARMADA	2	m	0	1	1	4	64	16.00	(0.84- 305.74)
*BEST	17	m	1	-	-	-	-	4.35	(1.82- 10.41)
BOFFET	2	m	2	-	-	-	-	7.90	(5.30- 11.80)
*BOUCOT	118	m	2	-	-	-	-	9.06	(0.37- 222.47)
COOKSO	3	c	0	5	1	45	61	6.78	(0.77- 60.04)
*CPSI	181	m	1	-	-	-	-	2.54	(1.68- 3.83)
DAMBER	2	m	0	198	142	42	208	6.91	(4.65- 10.25)
DEAN	5	m	0	49	71	12	61	3.51	(1.71- 7.19)
DOLL	18	m	0	53	93	7	61	4.97	(2.12- 11.64)
*DORN	34	m	1	-	-	-	-	1.33	(0.45- 3.95)
*HAMMON	112	m	1	-	-	-	-	3.01	(1.52- 5.97)
KOULUM	3	m	0	15	17	5	54	9.53	(3.02- 30.08)
LEVIN	28	m	1	-	-	-	-	1.30	(0.74- 2.30)
LUBIN2	22	m	2	-	-	-	-	2.67	(1.85- 3.84)
MCCONN	17	c	0	8	7	9	23	2.92	(0.82- 10.44)
PERNU	9	m	0	55	37	97	275	4.21	(2.62- 6.79)
PERNU	5	f	0	1	1	110	971	8.83	(0.55- 142.12)
Subtotal PERNU								4.30	(2.69- 6.89)
SADOWS	30	m	1	-	-	-	-	1.17	(0.36- 3.82)
STOCKS	40	m	2	-	-	-	-	2.61	(1.79- 3.82)
TIZZAN	4	m	0	16	18	180	305	1.51	(0.75- 3.03)
WYNDE7	41	m	0	9	97	64	918	1.33	(0.64- 2.76)
*XIANGZ	10	m	2	-	-	-	-	3.00	(2.02- 4.46)
Partial Totals				410	485	575	3001		

*prospective study

REF	NRR	SEX	AD	Ys	Ws	Qs	Ps
ABELIN	50	m	1	3.16	1.49	5.66	0.0001
ARMADA	2	m	0	2.77	0.44	1.08	0.0655
*BEST	17	m	1	1.47	5.05	0.34	0.0010
BOFFET	2	m	2	2.07	23.99	17.57	0.0000
*BOUCOT	118	m	2	2.20	0.38	0.37	0.1770
COOKSO	3	c	0	1.91	0.81	0.40	0.0855
*CPSI	181	m	1	0.93	22.63	1.76	0.0000
DAMBER	2	m	0	1.93	24.56	12.78	0.0000
DEAN	5	m	0	1.26	7.45	0.01	0.0006
DOLL	18	m	0	1.60	5.29	0.81	0.0002
*DORN	34	m	1	0.29	3.26	2.79	0.6068
*HAMMON	112	m	1	1.10	8.21	0.10	0.0016
KOULUM	3	m	0	2.25	2.91	3.17	0.0001
LEVIN	28	m	1	0.26	11.95	10.75	0.3645
LUBIN2	22	m	2	0.98	28.81	1.51	0.0000
MCCONN	17	c	0	1.07	2.37	0.05	0.0991
PERNU	9	m	0	1.44	16.90	0.88	0.0000
PERNU	5	f	0	2.18	0.50	0.47	0.1245
Subtotal PERNU				1.46	17.40	1.34	
SADOWS	30	m	1	0.16	2.75	3.06	0.7944
STOCKS	40	m	2	0.96	26.74	1.69	0.0000
TIZZAN	4	m	0	0.41	7.88	5.06	0.2502
WYNDE7	41	m	0	0.29	7.24	6.19	0.4419
*XIANGZ	10	m	2	1.10	24.49	0.31	0.0000

Table 1E6 - 2

IESLC - Meta-analysis of Ever Smoking, Pipe only
All LC types
Most adjusted

	N	23
	NS	22
	Wt	236.10
Het	Chi	76.80
Het	df	22
Het	P	***
Fixed	RR	3.36
	RRl	2.95
	RRu	3.81
	P	+++
Random	RR	3.31
	RRl	2.51
	RRu	4.35
	P	+++
Asymm	P	N.S.

Table 1E6 - 3

IESLC - Meta-analysis of Ever Smoking, Pipe only
All LC types
Most adjusted

	Sex			
	combined	male	female	Total
N	2	20	1	23
NS	2	20	1	23
Wt	3.17	232.43	0.50	236.10
Het Chi	0.43	75.89	0.00	76.80
Het df	1	19	0	22
Het P	N.S.	***	N.S.	***
Fixed RR	3.62	3.35	8.83	3.36
RRl	1.20	2.94	0.55	2.95
RRu	10.87	3.81	142.12	3.81
P	+	+++	N.S.	+++
Random RR	3.62	3.26	8.83	3.31
RRl	1.20	2.45	0.55	2.51
RRu	10.87	4.35	142.12	4.35
P	+	+++	N.S.	+++
Between Chi				0.49
Between df				2
Between P				N.S.
Btwn(F) P				N.S.
Btwn(R) P				N.S.

	All LC (or nearest)		
	all	other	Total
N	23		23
NS	22		22
Wt	236.10		236.10
Het Chi	76.80		76.80
Het df	22		22
Het P	***		***
Fixed RR	3.36		3.36
RRl	2.95		2.95
RRu	3.81		3.81
P	+++		+++
Random RR	3.31		3.31
RRl	2.51		2.51
RRu	4.35		4.35
P	+++		+++
Between Chi			
Between df			
Between P			N.S.
Btwn(F) P			N.S.
Btwn(R) P			N.S.

	Location								
	NAmer	UK	Scand	othEur	China	Japan	othAs	other	Total
N	8	3	4	5	1			2	23
NS	8	3	3	5	1			2	22
Wt	61.46	34.40	44.87	62.61	24.49			8.26	236.10
Het Chi	11.23	1.83	3.27	28.87	0.00			0.32	76.80
Het df	7	2	3	4	0			1	22
Het P	N.S.	N.S.	N.S.	***	N.S.			N.S.	***
Fixed RR	2.08	2.90	5.87	4.01	3.00			3.74	3.36
RRl	1.62	2.08	4.38	3.13	2.02			1.89	2.95
RRu	2.67	4.06	7.87	5.14	4.46			7.40	3.81
P	+++	+++	+++	+++	+++			+++	+++
Random RR	2.03	2.90	5.87	4.78	3.00			3.74	3.31
RRl	1.43	2.08	4.25	2.05	2.02			1.89	2.51
RRu	2.89	4.06	8.10	11.11	4.46			7.40	4.35
P	+++	+++	+++	+++	+++			+++	+++
Between Chi									31.28
Between df									5
Between P									***
Btwn(F) P									(*)
Btwn(R) P									***

Table 1E6 - 3

IESLC - Meta-analysis of Ever Smoking, Pipe only
All LC types
Most adjusted

Detailed Country in "other Europe"

	multi	Germany	othWest	East	Balkans	Total
N	2		3			5
NS	2		3			5
Wt	52.80		9.81			62.61
Het Chi	15.40		11.04			28.87
Het df	1		2			4
Het P	***		**			***
Fixed RR	4.37		2.54			4.01
RRl	3.34		1.36			3.13
RRu	5.72		4.75			5.14
P	+++		++			+++
Random RR	4.58		6.89			4.78
RRl	1.58		0.80			2.05
RRu	13.25		59.32			11.11
P	++		(+)			+++
Between Chi						2.43
Between df						1
Between P						N.S.
Btwn(F) P						N.S.
Btwn(R) P						N.S.

Detailed Country in "other Asia"

	India	HongKong	other	Total
N				
NS				
Wt				
Het Chi				
Het df				
Het P				
Fixed RR				
RRl				
RRu				
P				
Random RR				
RRl				
RRu				
P				
Between Chi				
Between df				
Between P				N.S.
Btwn(F) P				N.S.
Btwn(R) P				N.S.

Detailed other continent

	SCAmer	Auslia	Africa	Total
N			2	2
NS			2	2
Wt			8.26	8.26
Het Chi			0.32	0.32
Het df			1	1
Het P			N.S.	N.S.
Fixed RR			3.74	3.74
RRl			1.89	1.89
RRu			7.40	7.40
P			+++	+++
Random RR			3.74	3.74
RRl			1.89	1.89
RRu			7.40	7.40
P			+++	+++
Between Chi				
Between df				
Between P				N.S.
Btwn(F) P				N.S.
Btwn(R) P				N.S.

Table 1E6 - 3

IESLC - Meta-analysis of Ever Smoking, Pipe only
All LC types
Most adjusted

	Start year of study <1960	1960-69	1970-79	1980-89	1990+	Total
N	16	1	4	2		23
NS	15	1	4	2		22
Wt	125.76	0.81	85.11	24.43		236.10
Het Chi	32.57	0.00	20.76	0.22		76.80
Het df	15	0	3	1		22
Het P	**	N.S.	***	N.S.		***
Fixed RR	2.79	6.78	3.42	8.00		3.36
RRl	2.34	0.77	2.77	5.38		2.95
RRu	3.32	60.04	4.23	11.90		3.81
P	+++	(+)	+++	+++		+++
Random RR	2.95	6.78	3.07	8.00		3.31
RRl	2.20	0.77	1.72	5.38		2.51
RRu	3.96	60.04	5.49	11.90		4.35
P	+++	(+)	+++	+++		+++
Between Chi						23.25
Between df						3
Between P						***
Btwn(F) P						(*)
Btwn(R) P						***

	Study type (1) CC	other	Total
N	17	6	23
NS	16	6	22
Wt	172.09	64.01	236.10
Het Chi	70.39	3.68	76.80
Het df	16	5	22
Het P	***	N.S.	***
Fixed RR	3.58	2.81	3.36
RRl	3.09	2.20	2.95
RRu	4.16	3.60	3.81
P	+++	+++	+++
Random RR	3.55	2.81	3.31
RRl	2.47	2.20	2.51
RRu	5.09	3.60	4.35
P	+++	+++	+++
Between Chi			2.73
Between df			1
Between P			(*)
Btwn(F) P			N.S.
Btwn(R) P			N.S.

	Study type (2) CC	prosp	other	Total
N	17	6		23
NS	16	6		22
Wt	172.09	64.01		236.10
Het Chi	70.39	3.68		76.80
Het df	16	5		22
Het P	***	N.S.		***
Fixed RR	3.58	2.81		3.36
RRl	3.09	2.20		2.95
RRu	4.16	3.60		3.81
P	+++	+++		+++
Random RR	3.55	2.81		3.31
RRl	2.47	2.20		2.51
RRu	5.09	3.60		4.35
P	+++	+++		+++
Between Chi				2.73
Between df				1
Between P				(*)
Btwn(F) P				N.S.
Btwn(R) P				N.S.

Table 1E6 - 3

IESLC - Meta-analysis of Ever Smoking, Pipe only
All LC types
Most adjusted

	Study size (number of LC cases)				
	100-249	250-499	500-999	1000+	Total
N	4	5	4	10	23
NS	4	5	4	9	22
Wt	5.04	28.41	59.41	143.24	236.10
Het Chi	4.02	9.25	10.62	38.20	76.80
Het df	3	4	3	9	22
Het P	N.S.	(*)	*	***	***
Fixed RR	6.74	2.11	4.57	3.16	3.36
RRl	2.82	1.46	3.54	2.68	2.95
RRu	16.14	3.05	5.89	3.72	3.81
P	+++	+++	+++	+++	+++
Random RR	7.23	2.33	4.76	2.91	3.31
RRl	2.49	1.24	2.77	2.01	2.51
RRu	20.99	4.39	8.17	4.23	4.35
P	+++	++	+++	+++	+++
Between Chi					14.71
Between df					3
Between P					**
Btwn(F) P					N.S.
Btwn(R) P					N.S.

	Risky occupational population			
	no	mining	othRisky	Total
N	22	1		23
NS	21	1		22
Wt	211.61	24.49		236.10
Het Chi	76.45	0.00		76.80
Het df	21	0		22
Het P	***	N.S.		***
Fixed RR	3.40	3.00		3.36
RRl	2.97	2.02		2.95
RRu	3.89	4.46		3.81
P	+++	+++		+++
Random RR	3.34	3.00		3.31
RRl	2.48	2.02		2.51
RRu	4.50	4.46		4.35
P	+++	+++		+++
Between Chi				0.34
Between df				1
Between P				N.S.
Btwn(F) P				N.S.
Btwn(R) P				N.S.

	National cigarette tobacco type			
	Virginia	blended	other	Total
N	6	16	1	23
NS	6	15	1	22
Wt	47.71	163.89	24.49	236.10
Het Chi	3.14	73.00	0.00	76.80
Het df	5	15	0	22
Het P	N.S.	***	N.S.	***
Fixed RR	3.17	3.47	3.00	3.36
RRl	2.38	2.98	2.02	2.95
RRu	4.21	4.05	4.46	3.81
P	+++	+++	+++	+++
Random RR	3.17	3.27	3.00	3.31
RRl	2.38	2.21	2.02	2.51
RRu	4.21	4.84	4.46	4.35
P	+++	+++	+++	+++
Between Chi				0.66
Between df				2
Between P				N.S.
Btwn(F) P				N.S.
Btwn(R) P				N.S.

Table 1E6 - 3

IESLC - Meta-analysis of Ever Smoking, Pipe only
All LC types
Most adjusted

	Any proxy use No/nk	Yes	Total
N	18	5	23
NS	17	5	22
Wt	151.87	84.23	236.10
Het Chi	31.12	23.38	76.80
Het df	17	4	22
Het P	*	***	***
Fixed RR	2.67	5.07	3.36
RRl	2.28	4.10	2.95
RRu	3.13	6.28	3.81
P	+++	+++	+++
Random RR	2.69	5.51	3.31
RRl	2.09	3.10	2.51
RRu	3.44	9.79	4.35
P	+++	+++	+++
Between Chi			22.30
Between df			1
Between P			***
Btwn(F) P			**
Btwn(R) P			*

	Full histological confirmation No	Yes	Total
N	18	5	23
NS	17	5	22
Wt	196.43	39.67	236.10
Het Chi	66.80	5.37	76.80
Het df	17	4	22
Het P	***	N.S.	***
Fixed RR	3.57	2.46	3.36
RRl	3.11	1.80	2.95
RRu	4.11	3.35	3.81
P	+++	+++	+++
Random RR	3.48	2.43	3.31
RRl	2.55	1.50	2.51
RRu	4.76	3.94	4.35
P	+++	+++	+++
Between Chi			4.63
Between df			1
Between P			*
Btwn(F) P			N.S.
Btwn(R) P			N.S.

	Number of adjustment variables (1) 0	1	2+/+nk	Total
N	11	7	5	23
NS	10	7	5	22
Wt	76.35	55.34	104.41	236.10
Het Chi	27.79	17.05	21.23	76.80
Het df	10	6	4	22
Het P	**	**	***	***
Fixed RR	4.11	2.33	3.52	3.36
RRl	3.28	1.79	2.90	2.95
RRu	5.14	3.03	4.26	3.81
P	+++	+++	+++	+++
Random RR	3.84	2.49	3.65	3.31
RRl	2.47	1.51	2.23	2.51
RRu	5.97	4.12	5.97	4.35
P	+++	+++	+++	+++
Between Chi				10.73
Between df				2
Between P				**
Btwn(F) P				N.S.
Btwn(R) P				N.S.

Table 1E6 - 3

IESLC - Meta-analysis of Ever Smoking, Pipe only
All LC types
Most adjusted

	Number of adjustment variables (2) 0	1	2	3-5	6+/+nk	Total
N	11	7	5			23
NS	10	7	5			22
Wt	76.35	55.34	104.41			236.10
Het Chi	27.79	17.05	21.23			76.80
Het df	10	6	4			22
Het P	**	**	***			***
Fixed RR	4.11	2.33	3.52			3.36
RRl	3.28	1.79	2.90			2.95
RRu	5.14	3.03	4.26			3.81
P	+++	+++	+++			+++
Random RR	3.84	2.49	3.65			3.31
RRl	2.47	1.51	2.23			2.51
RRu	5.97	4.12	5.97			4.35
P	+++	+++	+++			+++
Between Chi						10.73
Between df						2
Between P						**
Btwn(F) P						N.S.
Btwn(R) P						N.S.

	Derivation of RR/CI Orig	StdCalc	Other	Total
N	1	14	8	23
NS	1	13	8	22
Wt	23.99	116.53	95.58	236.10
Het Chi	0.00	48.99	5.71	76.80
Het df	0	13	7	22
Het P	N.S.	***	N.S.	***
Fixed RR	7.90	3.36	2.70	3.36
RRl	5.29	2.81	2.21	2.95
RRu	11.79	4.03	3.30	3.81
P	+++	+++	+++	+++
Random RR	7.90	3.59	2.70	3.31
RRl	5.29	2.38	2.21	2.51
RRu	11.79	5.42	3.30	4.35
P	+++	+++	+++	+++
Between Chi				22.09
Between df				2
Between P				***
Btwn(F) P				*
Btwn(R) P				***

Table 1E6 - 4

IESLC - Meta-analysis of Ever Smoking, Pipe only
All LC types
Least adjusted

REF	NRR	X	SEX	AGEL	AGEH	RACE	YF	LC TYPE	LOC	START	ST	NLC	R	VB	P	H	AD	DENOM	De
ABELIN	7	x	m	0	0	all	-	all	Eu:wst	1941	CC	118	n	bl	y	n	0	nev any	st
ARMADA	2		m	0	0	all	-	all	Eu:wst	1986	CC	325	n	bl	n	y	0	nev any	st
BEST	17		m	0	0	all	0	all	NAmer	1955	pr	381	n	V	n	n	1	nev any	ot
BOFFET	5	x	m	0	0	all	-	all	Eu:mul	1988	CC	5621	n	bl	y	n	0	nev any	st
BOUCOT	6	x	m	0	0	all	0	all	NAmer	1951	pr	121	n	bl	n	n	0	nev any	ot
COOKSO	3		c	0	0	bl	-	all	Africa	1961	CC	234	n	V	n	y	0	nev any	st
CPSI	181		m	35	84	all	6	all	NAmer	1959	pr	5138	n	bl	n	n	1	nev any	ot
DAMBER	2		m	0	0	all	-	all	Eu:Sca	1972	CC	579	n	bl	y	n	0	nev any	st
DEAN	5		m	0	0	wh	-	all	Africa	1947	CC	603	n	V	y	n	0	nev any	st
DOLL	18		m	0	0	all	-	all	Eu:UK	1948	CC	1465	n	V	n	n	0	nev any	st
DORN	43	x	m	0	0	wh	2	all	NAmer	1954	pr	5097	n	bl	n	n	0	nev any	st
HAMMON	124	x	m	0	0	wh	0	all	NAmer	1952	pr	448	n	bl	n	n	0	nev any	st
KOULUM	3		m	0	0	all	-	all	Eu:Sca	1936	CC	812	n	bl	n	n	0	nev any	st
LEVIN	28		m	0	0	all	-	all	NAmer	1938	CC	475	n	bl	n	n	1	nev any	st
LUBIN2	21	x	m	0	0	all	-	all	Eu:mul	1976	CC	7804	n	bl	n	y	0	nev any	st
MCCONN	17		c	0	0	all	-	all	Eu:UK	1946	CC	100	n	V	n	y	0	nev any	st
PERNU	9		m	0	0	all	-	all	Eu:Sca	1944	CC	1606	n	bl	n	n	0	nev any	st
PERNU	5		f	0	0	all	-	all	Eu:Sca	1944	CC	1606	n	bl	n	n	0	nev any	st
SADOWS	3	x	m	0	0	wh	-	all	NAmer	1938	CC	477	n	bl	n	n	0	nev any	st
STOCKS	24	x	m	0	0	all	-	all	Eu:UK	1952	CC	2932	n	V	y	n	0	nev any	st
TIZZAN	4		m	0	0	all	-	all	Eu:wst	1959	CC	1358	n	bl	n	n	0	nev any	st
WYNDE7	41		m	0	0	all	-	all	NAmer	1977	CC	2085	n	bl	n	y	0	nev any	st
XIANGZ	3	x	m	0	0	all	0	all	As:Chi	1976	pr	983	m	ot	n	n	0	nev any	st

Table 1E6 - 5

IESLC - Meta-analysis of Ever Smoking, Pipe only
All LC types
Least adjusted

REF	NRR	SEX	AD	Number Exposed Case	Exposed Cont	Non-exposed Case	Non-exposed Cont	RR	95.00%CI
ABELIN	7	m	0	8	34	2	183	21.53	(4.38- 105.80)
ARMADA	2	m	0	1	1	4	64	16.00	(0.84- 305.74)
*BEST	17	m	1	-	-	-	-	4.35	(1.82- 10.41)
BOFFET	5	m	0	61	129	117	1750	7.07	(4.95- 10.11)
*BOUCOT	6	m	0	1	2389	0	7551	9.48~	(0.39- 232.65)
COOKSO	3	c	0	5	1	45	61	6.78	(0.77- 60.04)
*CPSI	181	m	1	-	-	-	-	2.54	(1.68- 3.83)
DAMBER	2	m	0	198	142	42	208	6.91	(4.65- 10.25)
DEAN	5	m	0	49	71	12	61	3.51	(1.71- 7.19)
DOLL	18	m	0	53	93	7	61	4.97	(2.12- 11.64)
*DORN	43	m	0	4	17780	17	117918	1.56	(0.53- 4.64)
*HAMMON	124	m	0	18	43041	15	115884	3.23	(1.63- 6.41)
KOULUM	3	m	0	15	17	5	54	9.53	(3.02- 30.08)
LEVIN	28	m	1	-	-	-	-	1.30	(0.74- 2.30)
LUBIN2	21	m	0	39	197	190	2617	2.73	(1.88- 3.96)
MCCONN	17	c	0	8	7	9	23	2.92	(0.82- 10.44)
PERNU	9	m	0	55	37	97	275	4.21	(2.62- 6.79)
PERNU	5	f	0	1	1	110	971	8.83	(0.55- 142.12)
Subtotal PERNU								4.30	(2.69- 6.89)
SADOWS	3	m	0	13	43	18	81	1.36	(0.61- 3.04)
STOCKS	24	m	0	211	994	45	638	3.01	(2.15- 4.21)
TIZZAN	4	m	0	16	18	180	305	1.51	(0.75- 3.03)
WYNDE7	41	m	0	9	97	64	918	1.33	(0.64- 2.76)
*XIANGZ	3	m	0	356	1483	25	974	9.35	(6.29- 13.92)
Partial Totals				1121	66575	1004	250597		

*prospective study ~ With 0.5 adjustment for zero

REF	NRR	SEX	AD	Ys	Ws	Qs	Ps
ABELIN	7	m	0	3.07	1.52	4.54	0.0002
ARMADA	2	m	0	2.77	0.44	0.91	0.0655
*BEST	17	m	1	1.47	5.05	0.09	0.0010
BOFFET	5	m	0	1.96	30.06	11.47	0.0000
*BOUCOT	6	m	0	2.25	0.38	0.31	0.1683
COOKSO	3	c	0	1.91	0.81	0.27	0.0855
*CPSI	181	m	1	0.93	22.63	3.74	0.0000
DAMBER	2	m	0	1.93	24.56	8.66	0.0000
DEAN	5	m	0	1.26	7.45	0.05	0.0006
DOLL	18	m	0	1.60	5.29	0.37	0.0002
*DORN	43	m	0	0.44	3.24	2.59	0.4232
*HAMMON	124	m	0	1.17	8.18	0.22	0.0008
KOULUM	3	m	0	2.25	2.91	2.44	0.0001
LEVIN	28	m	1	0.26	11.95	13.84	0.3645
LUBIN2	21	m	0	1.00	27.50	3.09	0.0000
MCCONN	17	c	0	1.07	2.37	0.17	0.0991
PERNU	9	m	0	1.44	16.90	0.17	0.0000
PERNU	5	f	0	2.18	0.50	0.35	0.1245
Subtotal PERNU				1.46	17.40	0.52	
SADOWS	3	m	0	0.31	5.95	6.32	0.4527
STOCKS	24	m	0	1.10	33.86	1.90	0.0000
TIZZAN	4	m	0	0.41	7.88	6.80	0.2502
WYNDE7	41	m	0	0.29	7.24	8.02	0.4419
*XIANGZ	3	m	0	2.24	24.33	19.58	0.0000

Table 1E6 - 5

IESLC - Meta-analysis of Ever Smoking, Pipe only
All LC types
Least adjusted

	N	23
	NS	22
	Wt	250.99
Het	Chi	95.89
Het	df	22
Het	P	***
Fixed	RR	3.81
	RRl	3.37
	RRu	4.32
	P	+++
Random	RR	3.60
	RRl	2.68
	RRu	4.84
	P	+++
Asymm	P	N.S.

Table 1E6 - 6

IESLC - Meta-analysis of Ever Smoking, Pipe only
All LC types
Least adjusted

		Sex			
		combined	male	female	Total
	N	2	20	1	23
	NS	2	20	1	23
	Wt	3.17	247.32	0.50	250.99
Het	Chi	0.43	95.11	0.00	95.89
Het	df	1	19	0	22
Het	P	N.S.	***	N.S.	***
Fixed	RR	3.62	3.81	8.83	3.81
	RRl	1.20	3.36	0.55	3.37
	RRu	10.87	4.32	142.12	4.32
	P	+	+++	N.S.	+++
Random	RR	3.62	3.56	8.83	3.60
	RRl	1.20	2.61	0.55	2.68
	RRu	10.87	4.85	142.12	4.84
	P	+	+++	N.S.	+++
Between	Chi				0.36
Between	df				2
Between	P				N.S.
Btwn(F)	P				N.S.
Btwn(R)	P				N.S.

Table 1E6 - 7

IESLC - Meta-analysis of Ever Smoking, Pipe only
All LC types
Excluded studies (and stage at which they were excluded)

1	BOUCHA BUELL LAURIL MZILEN
2	ABRAHA AGUDO AKIBA ALDERS AMANDU AMES ANDERS ARCHER AUSTIN AUVINE AXELSO AXELSS BAND BARBON BECHER BERRIN BLOHMK BLOT1 BLOT2 BLOT3 BLOT4 BRESLO BRETT BROCKM BROSS BROWN1 BROWN2 BUFFLE BYERS1 BYERS2 CARPEN CASCO2 CASCOR CHAN CHANG CHATZI CHEN CHEN2 CHEN3 CHIAZZ CHOI CHOW CHYOU COMSTO CORREA CPSII DARBY DAVEYS DEAN2 DEKLER DESTE2 DESTEF DOCKER DORANT DORGAN DOSEME DROSTE DU DUNN EBELIN ENGELA ENSTRO ESAKI FAN GAO GAO2 GARCIA GARSHI GENG GER GILLIS GODLEY GOLLED GOODMA GRAHAM GREGOR GSELL HAENSZ HAMMO2 HANSEN HEGMAN HENNEK HINDS HIRAOK HIRAYA HITOSU HOLE HOROWI HORWIT HU HU2 HUANG HUMBLE ISHIMA JAHN JAIN JARUP JARVHO JEDRYC JIANG JOLY JONES JUSSAW KAISE2 KAISER KANELL KATSOU KAUFMA KELLER KHUDER KIHARA KINLEN KJUUS KNEKT KO KOHLME KOO KREUZE KREYBE KUBIK LAMTH LAMWK LAMWK2 LAUSSM LEI LEMARC LETOUR LIAW LICKIN LIDDEL LIU LIU2 LIU3 LIU4 LIU5 LOMBA2 LOMBAR LUO MACLEN MAGNUS MARSH MARSH2 MARTIS MASTRA MATOS MATSUD MCDUFF MCLAUG MIGRAN MILLER MILLS MOLLO MRFIT MRFITR MURATA NAM NOTAN2 NOTANI NOU ODRISC ORMOS OSANN OSANN2 PARKIN PASTOR PAWLEG PERSH2 PERSHA PETO PEZZO2 PEZZOT PIKE PISANI POFFIJ POLEDN PRESCO QIAO2 RACHTA RADZIK RANDIG REN RESTRE RIMING RONCO ROOTS ROTHSC SAARIK SANKAR SCHWAR SEGI SEGI2 SEOW SHAW SHIMIZ SIEMIA SIMARA SIMONA SITAS SOBUE SOBUE2 SPEIZE SPITZ STASZE STAYNE STOCKW STUCKE SUN SUZUK2 SUZUKI SVENSS TANG TANG2 TAO TENKAN TOKARS TOUSEY TSUGAN ULMER VANDER VEIERO VUTUC WAKAI WALD WANG WANG2 WANG3 WANG4 WARSIN WATSON WICKLU WIGLE WILKIN WU WU2 WUNSCH WUWILL WYNDE2 WYNDE3 WYNDE4 WYNDE5 WYNDE6 WYNDE8 WYNDER XU XU2 XU3 XU4 YAMAGU YONG YUAN ZHANG ZHENG ZHOU
3	BENSHL CEDERL DEAN3 GARDIN HEIN HIRAY2 LANGE TULINI TVERDA
4	DOLL2
5	SCHWA2
9	BENHAM LUBIN QIAO

Table 1E6 - 8
Potentially overlapping studies

REF	REFGP	PRINC	OVERLAP/LINK
LUBIN2	LUBIN2	1	Lubin-combined
WYNDE7	WYNDE6	2	WYNDE5/6/7/8
CPSI	CPSI	1	CPSI overall
BOFFET	BOFFET	1	BOFFET-combined
XIANGZ	XIANGZ	1	LUBIN/XIANGZ/QIAO

Table 1E6 - 9
Most adjusted - insufficient data for metaanalysis

REF	NRR	SEX	AGEL	AGEH	RACE	YF	LC TYPE	LOC	START	ST	NLC	R	VB	P	H	AD	DENOM	De
DOLL2	49	m	35	99	all	5	all	Eu:UK	1951	pr	920	n	V	n	n	1	nev any st	

REF	NRR	RR	SIG	RRDATA comment
DOLL2	49	5.43		0

Table 1E7 -

IESLC - Meta-analysis of Current Smoking, Pipe only
All LC types

This analysis is restricted to results for:
1) Non-dose-response data
2) Smokers of pipe only
3) Current smokers
4) Results complete enough for use in metaanalysis

Within each study, results are then selected (in the following order of preference, within each sex) for:
5) DENOM: never smoked anything, (never +1 = +long term ex)
6) Followup period (prospective studies): whole study (coded as 0) or longest available
7) LCtype: all or nearest available, at least Squamous and Adeno. (q = squamous, s = small,
l = large, a = adeno, mix = mixed, alv = alveolar)
8) Race: all or nearest available, otherwise by race (wh or w = white, bl or b = black, hi = hispanic
ch = chinese, jap = japanese, haw = hawaiian, w+o = white + oriental, sca = scandinavian, as = asian)
9) For overlapping studies: principal rather than subsidiary studies
Finally by Age: whole study (coded as 0) if available, otherwise by widest available age group
and then for single sex results (m, f) in preference to combined sex results (c).

Results adjusted (AD) for the most potential confounders are then chosen in Sections -1 to -3
and results adjusted for the least confounders in Sections -4 to -6. (Those least adjusted results which
actually differ from the most adjusted as marked 'x' in column X in Section -4)
(Results adjusted for an unknown number of confounder(s) are coded as 20.)

Section -7 shows excluded studies, together with the stage (as above) at which no qualifying
results were found.

Section -8 lists the potentially overlapping studies which have been included (1=principal, 2=subsidiary).

Section -9 lists any results which would have been included in preference except that they had data not complete
enough for use in meta-analysis, with their significance (yes/no), if known, and any further comment as entered
on the database.

In addition to those mentioned above, the following fields, levels and abbreviations are used:
* or nk = not known, n = no, y = yes, ot = other
nev = never
REF: 6-character study reference
NRR: number of the RR on the database within the study
ST : study type (CC = case control, pr or prosp = prospective)
NLC: number of lung cancer cases in whole study
R : risky occupational population (n = no, m = mining, o = other risky)
VB : national cigarette type (V = at least 75% Virginia, bl = at least 75% blended, ot = other)
P : any proxy use
H : full histological confirmation
De : derivation of RR/CI (or = original, st = standard method, ot = other method of estimation)

Table 1E7 - 1

IESLC - Meta-analysis of Current Smoking, Pipe only
All LC types
Most adjusted

REF	NRR	SEX	AGEL	AGEH	RACE	YF	LC TYPE	LOC	START	ST	NLC	R	VB	P	H	AD	DENOM	De
BENSHL	2	m	0	0	all	0	all	Eu:UK	1967	pr	486	n	V	n	n	1	nev any	ot
BOFFET	24	m	0	0	all	-	all	Eu:mul	1988	CC	5621	n	bl	y	n	2	nev any	or
CEDERL	84	m	0	0	all	16	all	Eu:Sca	1963	pr	491	n	bl	n	n	2	nev any	ot
DAMBER	18	m	0	0	all	-	all	Eu:Sca	1972	CC	579	n	bl	y	n	1	nev any	ot
DEAN3	178	m	0	0	all	-	all	Eu:UK	1969	CC	766	n	V	y	n	1	nev any	ot
DORN	23	m	0	0	wh	15	all	NAmer	1954	pr	5097	n	bl	n	n	2	nev any	ot
GARDIN	3	c	0	0	all	-	all	Eu:UK	1988	CC	143	n	V	y	n	0	nev any	st
HEIN	3	m	0	0	all	0	all	Eu:Sca	1970	pr	144	n	bl	n	n	0	nev any	st
LANGE	18	m	0	0	all	0	all	Eu:Sca	1976	pr	268	n	bl	n	n	1	nev any	or
LUBIN2	6	m	0	0	all	-	all	Eu:mul	1976	CC	7804	n	bl	n	y	2	nev any	ot
TULINI	25	m	0	0	all	0	all	Eu:Sca	1967	pr	472	n	bl	n	n	3	nev any	or
WYNDE7	3	m	0	0	all	-	all	NAmer	1977	CC	2085	n	bl	n	y	0	nev any	st

Table 1E7 - 2

IESLC - Meta-analysis of Current Smoking, Pipe only
All LC types
Most adjusted

REF	NRR	SEX	AD	Number Exposed Case	Number Exposed Cont	Non-exposed Case	Non-exposed Cont	RR	95.00%CI
*BENSHL	2	m	1	-	-	-	-	4.52	(2.22- 9.19)
BOFFET	24	m	2	-	-	-	-	12.50	(7.70- 20.20)
*CEDERL	84	m	2	-	-	-	-	7.20	(4.45- 11.66)
DAMBER	18	m	1	-	-	-	-	8.30	(5.30- 13.10)
DEAN3	178	m	1	-	-	-	-	2.15	(1.17- 3.94)
*DORN	23	m	2	-	-	-	-	2.14	(1.46- 3.13)
GARDIN	3	c	0	3	2	5	41	12.30	(1.64- 92.33)
*HEIN	3	m	0	10	499	1	457	9.16	(1.18- 71.26)
*LANGE	18	m	1	-	-	-	-	4.10	(1.40- 13.00)
LUBIN2	6	m	2	-	-	-	-	5.85	(4.52- 7.58)
*TULINI	25	m	3	-	-	-	-	10.20	(5.38- 19.50)
WYNDE7	3	m	0	7	54	64	918	1.86	(0.81- 4.25)
Partial Totals				20	555	70	1416		

*prospective study

REF	NRR	SEX	AD	Ys	Ws	Qs	Ps
*BENSHL	2	m	1	1.51	7.61	0.19	0.0000
BOFFET	24	m	2	2.53	16.52	12.25	0.0000
*CEDERL	84	m	2	1.97	16.56	1.59	0.0000
DAMBER	18	m	1	2.12	18.77	3.83	0.0000
DEAN3	178	m	1	0.77	10.42	8.42	0.0135
*DORN	23	m	2	0.76	26.42	21.58	0.0001
GARDIN	3	c	0	2.51	0.95	0.68	0.0147
*HEIN	3	m	0	2.21	0.91	0.28	0.0344
*LANGE	18	m	1	1.41	3.09	0.20	0.0131
LUBIN2	6	m	2	1.77	57.49	0.60	0.0000
*TULINI	25	m	3	2.32	9.27	4.01	0.0000
WYNDE7	3	m	0	0.62	5.62	6.12	0.1416

	N	12
	NS	12
	Wt	173.62
Het	Chi	59.74
Het	df	11
Het	P	***
Fixed	RR	5.28
	RRl	4.55
	RRu	6.13
	P	+++
Random	RR	5.20
	RRl	3.50
	RRu	7.73
	P	+++
Asymm	P	N.S.

Table 1E7 - 3

IESLC - Meta-analysis of Current Smoking, Pipe only
All LC types
Most adjusted

		Sex		
	combined	male	female	Total
N	1	11		12
NS	1	11		12
Wt	0.95	172.68		173.62
Het Chi	0.00	59.06		59.74
Het df	0	10		11
Het P	N.S.	***		***
Fixed RR	12.30	5.26		5.28
RRl	1.64	4.53		4.55
RRu	92.33	6.10		6.13
P	+	+++		+++
Random RR	12.30	5.07		5.20
RRl	1.64	3.39		3.50
RRu	92.33	7.59		7.73
P	+	+++		+++
Between Chi				0.68
Between df				1
Between P				N.S.
Btwn(F) P				N.S.
Btwn(R) P				N.S.

	All LC (or nearest)		
	all	other	Total
N	12		12
NS	12		12
Wt	173.62		173.62
Het Chi	59.74		59.74
Het df	11		11
Het P	***		***
Fixed RR	5.28		5.28
RRl	4.55		4.55
RRu	6.13		6.13
P	+++		+++
Random RR	5.20		5.20
RRl	3.50		3.50
RRu	7.73		7.73
P	+++		+++
Between Chi			
Between df			
Between P			N.S.
Btwn(F) P			N.S.
Btwn(R) P			N.S.

				Location					
	NAmer	UK	Scand	othEur	China	Japan	othAs	other	Total
N	2	3	5	2					12
NS	2	3	5	2					12
Wt	32.04	18.98	48.60	74.01					173.62
Het Chi	0.09	4.26	2.14	7.40					59.74
Het df	1	2	4	1					11
Het P	N.S.	N.S.	N.S.	**					***
Fixed RR	2.09	3.16	7.88	6.93					5.28
RRl	1.48	2.01	5.95	5.52					4.55
RRu	2.95	4.95	10.44	8.70					6.13
P	+++	+++	+++	+++					+++
Random RR	2.09	3.59	7.88	8.31					5.20
RRl	1.48	1.69	5.95	3.96					3.50
RRu	2.95	7.64	10.44	17.46					7.73
P	+++	+++	+++	+++					+++
Between Chi									45.84
Between df									3
Between P									***
Btwn(F) P									**
Btwn(R) P									***

Table 1E7 - 3

IESLC - Meta-analysis of Current Smoking, Pipe only
All LC types
Most adjusted

	Detailed Country in "other Europe"					
	multi	Germany	othWest	East	Balkans	Total
N	2					2
NS	2					2
Wt	74.01					74.01
Het Chi	7.40					7.40
Het df	1					1
Het P	**					**
Fixed RR	6.93					6.93
RRl	5.52					5.52
RRu	8.70					8.70
P	+++					+++
Random RR	8.31					8.31
RRl	3.96					3.96
RRu	17.46					17.46
P	+++					+++
Between Chi						
Between df						
Between P						N.S.
Btwn(F) P						N.S.
Btwn(R) P						N.S.

	Detailed Country in "other Asia"			
	India	HongKong	other	Total
N				
NS				
Wt				
Het Chi				
Het df				
Het P				
Fixed RR				
RRl				
RRu				
P				
Random RR				
RRl				
RRu				
P				
Between Chi				
Between df				
Between P				N.S.
Btwn(F) P				N.S.
Btwn(R) P				N.S.

	Detailed other continent			
	SCAmer	Auslia	Africa	Total
N				
NS				
Wt				
Het Chi				
Het df				
Het P				
Fixed RR				
RRl				
RRu				
P				
Random RR				
RRl				
RRu				
P				
Between Chi				
Between df				
Between P				N.S.
Btwn(F) P				N.S.
Btwn(R) P				N.S.

Table 1E7 - 3

IESLC - Meta-analysis of Current Smoking, Pipe only
All LC types
Most adjusted

	<1960	1960-69	1970-79	1980-89	1990+	Total
		Start year of study				
N	1	4	5	2		12
NS	1	4	5	2		12
Wt	26.42	43.86	85.87	17.46		173.62
Het Chi	0.00	14.20	10.24	0.00		59.74
Het df	0	3	4	1		11
Het P	N.S.	**	*	N.S.		***
Fixed RR	2.14	5.36	5.81	12.49		5.28
RRl	1.46	3.99	4.70	7.81		4.55
RRu	3.13	7.21	7.18	19.96		6.13
P	+++	+++	+++	+++		+++
Random RR	2.14	5.19	5.15	12.49		5.20
RRl	1.46	2.69	3.21	7.81		3.50
RRu	3.13	10.02	8.27	19.96		7.73
P	+++	+++	+++	+++		+++
Between Chi						35.30
Between df						3
Between P						***
Btwn(F) P						(*)
Btwn(R) P						***

	Study type (1) CC	other	Total
N	6	6	12
NS	6	6	12
Wt	109.76	63.87	173.62
Het Chi	30.08	24.71	59.74
Het df	5	5	11
Het P	***	***	***
Fixed RR	6.01	4.23	5.28
RRl	4.98	3.31	4.55
RRu	7.25	5.41	6.13
P	+++	+++	+++
Random RR	5.33	5.11	5.20
RRl	3.07	2.74	3.50
RRu	9.25	9.53	7.73
P	+++	+++	+++
Between Chi			4.94
Between df			1
Between P			*
Btwn(F) P			N.S.
Btwn(R) P			N.S.

	Study type (2) CC	prosp	other	Total
N	6	6		12
NS	6	6		12
Wt	109.76	63.87		173.62
Het Chi	30.08	24.71		59.74
Het df	5	5		11
Het P	***	***		***
Fixed RR	6.01	4.23		5.28
RRl	4.98	3.31		4.55
RRu	7.25	5.41		6.13
P	+++	+++		+++
Random RR	5.33	5.11		5.20
RRl	3.07	2.74		3.50
RRu	9.25	9.53		7.73
P	+++	+++		+++
Between Chi				4.94
Between df				1
Between P				*
Btwn(F) P				N.S.
Btwn(R) P				N.S.

Table 1E7 - 3

IESLC - Meta-analysis of Current Smoking, Pipe only
All LC types
Most adjusted

	Study size (number of LC cases) 100-249	250-499	500-999	1000+	Total
N	2	4	2	4	12
NS	2	4	2	4	12
Wt	1.86	36.53	29.19	106.04	173.62
Het Chi	0.04	3.64	12.23	39.67	59.74
Het df	1	3	1	3	11
Het P	N.S.	N.S.	***	***	***
Fixed RR	10.64	6.80	5.12	4.82	5.28
RRl	2.53	4.92	3.56	3.99	4.55
RRu	44.82	9.41	7.36	5.83	6.13
P	++	+++	+++	+++	+++
Random RR	10.64	6.71	4.29	4.27	5.20
RRl	2.53	4.64	1.14	1.96	3.50
RRu	44.82	9.71	16.12	9.28	7.73
P	++	+++	+	+++	+++
Between Chi					4.16
Between df					3
Between P					N.S.
Btwn(F) P					N.S.
Btwn(R) P					N.S.

	Risky occupational population no	mining	othRisky	Total
N	12			12
NS	12			12
Wt	173.62			173.62
Het Chi	59.74			59.74
Het df	11			11
Het P	***			***
Fixed RR	5.28			5.28
RRl	4.55			4.55
RRu	6.13			6.13
P	+++			+++
Random RR	5.20			5.20
RRl	3.50			3.50
RRu	7.73			7.73
P	+++			+++
Between Chi				
Between df				
Between P				N.S.
Btwn(F) P				N.S.
Btwn(R) P				N.S.

	National cigarette tobacco type Virginia	blended	other	Total
N	3	9		12
NS	3	9		12
Wt	18.98	154.64		173.62
Het Chi	4.26	49.84		59.74
Het df	2	8		11
Het P	N.S.	***		***
Fixed RR	3.16	5.63		5.28
RRl	2.01	4.81		4.55
RRu	4.95	6.59		6.13
P	+++	+++		+++
Random RR	3.59	5.68		5.20
RRl	1.69	3.64		3.50
RRu	7.64	8.89		7.73
P	+++	+++		+++
Between Chi				5.64
Between df				1
Between P				*
Btwn(F) P				N.S.
Btwn(R) P				N.S.

Table 1E7 - 3

IESLC - Meta-analysis of Current Smoking, Pipe only
All LC types
Most adjusted

	Any proxy use No/nk	Yes	Total
N	8	4	12
NS	8	4	12
Wt	126.97	46.65	173.62
Het Chi	32.99	20.90	59.74
Het df	7	3	11
Het P	***	***	***
Fixed RR	4.73	7.15	5.28
RRl	3.97	5.37	4.55
RRu	5.62	9.53	6.13
P	+++	+++	+++
Random RR	4.61	6.70	5.20
RRl	2.94	2.85	3.50
RRu	7.24	15.78	7.73
P	+++	+++	+++
Between Chi			5.85
Between df			1
Between P			*
Btwn(F) P			N.S.
Btwn(R) P			N.S.

	Full histological confirmation No	Yes	Total
N	10	2	12
NS	10	2	12
Wt	110.52	63.10	173.62
Het Chi	53.02	6.72	59.74
Het df	9	1	11
Het P	***	**	***
Fixed RR	5.28	5.28	5.28
RRl	4.38	4.13	4.55
RRu	6.37	6.76	6.13
P	+++	+++	+++
Random RR	5.70	3.54	5.20
RRl	3.46	1.16	3.50
RRu	9.40	10.79	7.73
P	+++	+	+++
Between Chi			
Between df			1
Between P			N.S.
Btwn(F) P			N.S.
Btwn(R) P			N.S.

	Number of adjustment variables (1) 0	1	2+/+nk	Total
N	3	4	5	12
NS	3	4	5	12
Wt	7.47	39.90	126.26	173.62
Het Chi	4.29	12.43	39.59	59.74
Het df	2	3	4	11
Het P	N.S.	**	***	***
Fixed RR	2.87	4.92	5.60	5.28
RRl	1.40	3.60	4.71	4.55
RRu	5.88	6.70	6.67	6.13
P	++	+++	+++	+++
Random RR	4.48	4.36	6.35	5.20
RRl	1.18	2.20	3.52	3.50
RRu	17.02	8.63	11.44	7.73
P	+	+++	+++	+++
Between Chi				3.43
Between df				2
Between P				N.S.
Btwn(F) P				N.S.
Btwn(R) P				N.S.

Table 1E7 - 3

IESLC - Meta-analysis of Current Smoking, Pipe only
All LC types
Most adjusted

	Number of adjustment variables (2) 0	1	2	3-5	6+/+nk	Total
N	3	4	4	1		12
NS	3	4	4	1		12
Wt	7.47	39.90	116.99	9.27		173.62
Het Chi	4.29	12.43	36.00	0.00		59.74
Het df	2	3	3	0		11
Het P	N.S.	**	***	N.S.		***
Fixed RR	2.87	4.92	5.34	10.20		5.28
RRl	1.40	3.60	4.46	5.36		4.55
RRu	5.88	6.70	6.41	19.42		6.13
P	++	+++	+++	+++		+++
Random RR	4.48	4.36	5.73	10.20		5.20
RRl	1.18	2.20	2.93	5.36		3.50
RRu	17.02	8.63	11.18	19.42		7.73
P	+	+++	+++	+++		+++
Between Chi						7.02
Between df						3
Between P						(*)
Btwn(F) P						N.S.
Btwn(R) P						N.S.

	Derivation of RR/CI Orig	StdCalc	Other	Total
N	3	3	6	12
NS	3	3	6	12
Wt	28.88	7.47	137.27	173.62
Het Chi	3.24	4.29	34.57	59.74
Het df	2	2	5	11
Het P	N.S.	N.S.	***	***
Fixed RR	10.39	2.87	4.74	5.28
RRl	7.22	1.40	4.01	4.55
RRu	14.97	5.88	5.60	6.13
P	+++	++	+++	+++
Random RR	9.68	4.48	4.45	5.20
RRl	5.85	1.18	2.77	3.50
RRu	16.01	17.02	7.15	7.73
P	+++	+	+++	+++
Between Chi				17.64
Between df				2
Between P				***
Btwn(F) P				N.S.
Btwn(R) P				(*)

Table 1E7 - 4

IESLC - Meta-analysis of Current Smoking, Pipe only
All LC types
Least adjusted

REF	NRR	X	SEX	AGEL	AGEH	RACE	YF	LC TYPE	LOC	START	ST	NLC	R	VB	P	H	AD	DENOM	De
BENSHL	2		m	0	0	all	0	all	Eu:UK	1967	pr	486	n	V	n	n	1	nev any	ot
BOFFET	24		m	0	0	all	-	all	Eu:mul	1988	CC	5621	n	bl	y	n	2	nev any	or
CEDERL	84		m	0	0	all	16	all	Eu:Sca	1963	pr	491	n	bl	n	n	2	nev any	ot
DAMBER	18		m	0	0	all	-	all	Eu:Sca	1972	CC	579	n	bl	y	n	1	nev any	ot
DEAN3	177	x	m	0	0	all	-	all	Eu:UK	1969	CC	766	n	V	y	n	0	nev any	st
DORN	23		m	0	0	wh	15	all	NAmer	1954	pr	5097	n	bl	n	n	2	nev any	ot
GARDIN	3		c	0	0	all	-	all	Eu:UK	1988	CC	143	n	V	y	n	0	nev any	st
HEIN	3		m	0	0	all	0	all	Eu:Sca	1970	pr	144	n	bl	n	n	0	nev any	st
LANGE	9	x	m	0	0	all	0	all	Eu:Sca	1976	pr	268	n	bl	n	n	0	nev any	st
LUBIN2	5	x	m	0	0	all	-	all	Eu:mul	1976	CC	7804	n	bl	n	y	0	nev any	st
TULINI	2	x	m	0	0	all	0	all	Eu:Sca	1967	pr	472	n	bl	n	n	1	nev any	or
WYNDE7	3		m	0	0	all	-	all	NAmer	1977	CC	2085	n	bl	n	y	0	nev any	st

Table 1E7 - 5

IESLC - Meta-analysis of Current Smoking, Pipe only
All LC types
Least adjusted

REF	NRR	SEX	AD	Number Exposed Case	Cont	Non-exposed Case	Cont	RR	95.00%CI
*BENSHL	2	m	1	-	-	-	-	4.52	(2.22- 9.19)
BOFFET	24	m	2	-	-	-	-	12.50	(7.70- 20.20)
*CEDERL	84	m	2	-	-	-	-	7.20	(4.45- 11.66)
DAMBER	18	m	1	-	-	-	-	8.30	(5.30- 13.10)
DEAN3	177	m	0	23	116	24	510	4.21	(2.30- 7.73)
*DORN	23	m	2	-	-	-	-	2.14	(1.46- 3.13)
GARDIN	3	c	0	3	2	5	41	12.30	(1.64- 92.33)
*HEIN	3	m	0	10	499	1	457	9.16	(1.18- 71.26)
*LANGE	9	m	0	16	433	5	721	5.33	(1.97- 14.44)
LUBIN2	5	m	0	68	232	190	2617	4.04	(2.97- 5.49)
*TULINI	2	m	1	-	-	-	-	11.10	(5.86- 21.20)
WYNDE7	3	m	0	7	54	64	918	1.86	(0.81- 4.25)
Partial Totals				127	1336	289	5264		

*prospective study

REF	NRR	SEX	AD	Ys	Ws	Qs	Ps
*BENSHL	2	m	1	1.51	7.61	0.08	0.0000
BOFFET	24	m	2	2.53	16.52	13.77	0.0000
*CEDERL	84	m	2	1.97	16.56	2.16	0.0000
DAMBER	18	m	1	2.12	18.77	4.76	0.0000
DEAN3	177	m	0	1.44	10.45	0.32	0.0000
*DORN	23	m	2	0.76	26.42	19.18	0.0001
GARDIN	3	c	0	2.51	0.95	0.76	0.0147
*HEIN	3	m	0	2.21	0.91	0.33	0.0344
*LANGE	9	m	0	1.67	3.86	0.01	0.0010
LUBIN2	5	m	0	1.40	40.55	1.91	0.0000
*TULINI	2	m	1	2.41	9.29	5.86	0.0000
WYNDE7	3	m	0	0.62	5.62	5.53	0.1416

	N	12
	NS	12
	Wt	157.51
Het	Chi	54.68
Het	df	11
Het	P	***
Fixed	RR	5.02
	RRl	4.29
	RRu	5.86
	P	+++
Random	RR	5.44
	RRl	3.69
	RRu	8.02
	P	+++
Asymm	P	N.S.

Table 1E7 - 6

IESLC - Meta-analysis of Current Smoking, Pipe only
All LC types
Least adjusted

	Sex combined	male	female	Total
N	1	11		12
NS	1	11		12
Wt	0.95	156.56		157.51
Het Chi	0.00	53.91		54.68
Het df	0	10		11
Het P	N.S.	***		***
Fixed RR	12.30	4.99		5.02
RRl	1.64	4.27		4.29
RRu	92.33	5.84		5.86
P	+	+++		+++
Random RR	12.30	5.31		5.44
RRl	1.64	3.58		3.69
RRu	92.33	7.89		8.02
P	+	+++		+++
Between Chi				0.76
Between df				1
Between P				N.S.
Btwn(F) P				N.S.
Btwn(R) P				N.S.

Table 1E7 - 7

IESLC - Meta-analysis of Current Smoking, Pipe only
All LC types
Excluded studies (and stage at which they were excluded)

1	BOUCHA BUELL LAURIL MZILEN
2	ABRAHA AGUDO AKIBA ALDERS AMANDU AMES ANDERS ARCHER AUSTIN AUVINE AXELSO AXELSS BAND BARBON BECHER BERRIN BLOHMK BLOT1 BLOT2 BLOT3 BLOT4 BRESLO BRETT BROCKM BROSS BROWN1 BROWN2 BUFFLE BYERS1 BYERS2 CARPEN CASCO2 CASCOR CHAN CHANG CHATZI CHEN CHEN2 CHEN3 CHIAZZ CHOI CHOW CHYOU COMSTO CORREA CPSII DARBY DAVEYS DEAN2 DEKLER DESTE2 DESTEF DOCKER DORANT DORGAN DOSEME DROSTE DU DUNN EBELIN ENGELA ENSTRO ESAKI FAN GAO GAO2 GARCIA GARSHI GENG GER GILLIS GODLEY GOLLED GOODMA GRAHAM GREGOR GSELL HAENSZ HAMMO2 HANSEN HEGMAN HENNEK HINDS HIRAOK HIRAYA HITOSU HOLE HOROWI HORWIT HU HU2 HUANG HUMBLE ISHIMA JAHN JAIN JARUP JARVHO JEDRYC JIANG JOLY JONES JUSSAW KAISE2 KAISER KANELL KATSOU KAUFMA KELLER KHUDER KIHARA KINLEN KJUUS KNEKT KO KOHLME KOO KREUZE KREYBE KUBIK LAMTH LAMWK LAMWK2 LAUSSM LEI LEMARC LETOUR LIAW LICKIN LIDDEL LIU LIU2 LIU3 LIU4 LIU5 LOMBA2 LOMBAR LUO MACLEN MAGNUS MARSH MARSH2 MARTIS MASTRA MATOS MATSUD MCDUFF MCLAUG MIGRAN MILLER MILLS MOLLO MRFIT MRFITR MURATA NAM NOTAN2 NOTANI NOU ODRISC ORMOS OSANN OSANN2 PARKIN PASTOR PAWLEG PERSH2 PERSHA PETO PEZZO2 PEZZOT PIKE PISANI POFFIJ POLEDN PRESCO QIAO2 RACHTA RADZIK RANDIG REN RESTRE RIMING RONCO ROOTS ROTHSC SAARIK SANKAR SCHWAR SEGI SEGI2 SEOW SHAW SHIMIZ SIEMIA SIMARA SIMONA SITAS SOBUE SOBUE2 SPEIZE SPITZ STASZE STAYNE STOCKW STUCKE SUN SUZUK2 SUZUKI SVENSS TANG TANG2 TAO TENKAN TOKARS TOUSEY TSUGAN ULMER VANDER VEIERO VUTUC WAKAI WALD WANG WANG2 WANG3 WANG4 WARSIN WATSON WICKLU WIGLE WILKIN WU WU2 WUNSCH WUWILL WYNDE2 WYNDE3 WYNDE4 WYNDE5 WYNDE6 WYNDE8 WYNDER XU XU2 XU3 XU4 YAMAGU YONG YUAN ZHANG ZHENG ZHOU
3	ABELIN ARMADA BENHAM BEST BOUCOT COOKSO CPSI DEAN DOLL DOLL2 HAMMON KOULUM LEVIN LUBIN MCCONN PERNU QIAO SADOWS SCHWA2 STOCKS TIZZAN XIANGZ
5	HIRAY2 TVERDA

Table 1E7 - 8
Potentially overlapping studies

REF	REFGP	PRINC	OVERLAP/LINK
LUBIN2	LUBIN2	1	Lubin-combined
HEIN	PRESCO	1	Subset of PRESCO
LANGE	PRESCO	1	Subset of PRESCO
BENSHL	TANG2	1	Subset of TANG2
WYNDE7	WYNDE6	2	WYNDE5/6/7/8
BOFFET	BOFFET	1	BOFFET-combined

Table 1E8 -

IESLC - Meta-analysis of Ever Smoking (or Current if Ever not available), Pipe only
All LC types

This analysis is restricted to results for:
1) Non-dose-response data
2) Smokers of pipe only
3) Results complete enough for use in metaanalysis

Within each study, results are then selected (in the following order of preference, within each sex) for:
4) SMKSTA: ever smokers, current smokers
5) DENOM: never smoked anything, (never +1 = +long term ex)
6) Followup period (prospective studies): whole study (coded as 0) or longest available
7) LCtype: all or nearest available, at least Squamous and Adeno. (q = squamous, s = small,
l = large, a = adeno, mix = mixed, alv = alveolar)
8) Race: all or nearest available, otherwise by race (wh or w = white, bl or b = black, hi = hispanic
ch = chinese, jap = japanese, haw = hawaiian, w+o = white + oriental, sca = scandinavian, as = asian)
9) For overlapping studies: principal rather than subsidiary studies
Finally by Age: whole study (coded as 0) if available, otherwise by widest available age group
and then for single sex results (m, f) in preference to combined sex results (c).

Results adjusted (AD) for the most potential confounders are then chosen in Sections -1 to -3
and results adjusted for the least confounders in Sections -4 to -6. (Those least adjusted results which
actually differ from the most adjusted as marked 'x' in column X in Section -4)
(Results adjusted for an unknown number of confounder(s) are coded as 20.)

Section -7 shows excluded studies, together with the stage (as above) at which no qualifying
results were found.

Section -8 lists the potentially overlapping studies which have been included (1=principal, 2=subsidiary).

Section -9 lists any results which would have been included in preference except that they had data not complete
enough for use in meta-analysis, with their significance (yes/no), if known, and any further comment as entered
on the database.

In addition to those mentioned above, the following fields, levels and abbreviations are used:
* or nk = not known, n = no, y = yes, ot = other
ev = ever, cu = current, nev = never
REF: 6-character study reference
NRR: number of the RR on the database within the study
ST : study type (CC = case control, pr or prosp = prospective)
NLC: number of lung cancer cases in whole study
R : risky occupational population (n = no, m = mining, o = other risky)
VB : national cigarette type (V = at least 75% Virginia, bl = at least 75% blended, ot = other)
P : any proxy use
H : full histological confirmation
De : derivation of RR/CI (or = original, st = standard method, ot = other method of estimation)

Table 1E8 - 1

IESLC - Meta-analysis of Ever Smoking (or Current if Ever not available), Pipe only
All LC types
Most adjusted

REF	NRR	SEX	AGEL	AGEH	RACE	YF	LC TYPE	LOC	START	ST	NLC	R	VB	P	H	AD	SM	DENOM	De
ABELIN	50	m	0	0	all	-	all	Eu:wst	1941	CC	118	n	bl	y	n	1	ev	nev any	st
ARMADA	2	m	0	0	all	-	all	Eu:wst	1986	CC	325	n	bl	n	y	0	ev	nev any	st
BENSHL	2	m	0	0	all	0	all	Eu:UK	1967	pr	486	n	V	n	n	1	cu	nev any	ot
BEST	17	m	0	0	all	0	all	NAmer	1955	pr	381	n	V	n	n	1	ev	nev any	ot
BOFFET	2	m	0	0	all	-	all	Eu:mul	1988	CC	5621	n	bl	y	n	2	ev	nev any	or
BOUCOT	118	m	0	0	all	0	all	NAmer	1951	pr	121	n	bl	n	n	2	ev	nev any	ot
CEDERL	84	m	0	0	all	16	all	Eu:Sca	1963	pr	491	n	bl	n	n	2	cu	nev any	ot
COOKSO	3	c	0	0	bl	-	all	Africa	1961	CC	234	n	V	n	y	0	ev	nev any	st
CPSI	181	m	35	84	all	6	all	NAmer	1959	pr	5138	n	bl	n	n	1	ev	nev any	ot
DAMBER	2	m	0	0	all	-	all	Eu:Sca	1972	CC	579	n	bl	y	n	0	ev	nev any	st
DEAN	5	m	0	0	wh	-	all	Africa	1947	CC	603	n	V	y	n	0	ev	nev any	st
DEAN3	178	m	0	0	all	-	all	Eu:UK	1969	CC	766	n	V	y	n	1	cu	nev any	ot
DOLL	18	m	0	0	all	-	all	Eu:UK	1948	CC	1465	n	V	n	n	0	ev	nev any	st
DORN	34	m	0	0	wh	2	all	NAmer	1954	pr	5097	n	bl	n	n	1	ev	nev any	ot
GARDIN	3	c	0	0	all	-	all	Eu:UK	1988	CC	143	n	V	y	n	0	cu	nev any	st
HAMMON	112	m	0	0	wh	0	all	NAmer	1952	pr	448	n	bl	n	n	1	ev	nev any	ot
HEIN	3	m	0	0	all	0	all	Eu:Sca	1970	pr	144	n	bl	n	n	0	cu	nev any	st
KOULUM	3	m	0	0	all	-	all	Eu:Sca	1936	CC	812	n	bl	n	n	0	ev	nev any	st
LANGE	18	m	0	0	all	0	all	Eu:Sca	1976	pr	268	n	bl	n	n	1	cu	nev any	or
LEVIN	28	m	0	0	all	-	all	NAmer	1938	CC	475	n	bl	n	n	1	ev	nev any	st
LUBIN2	22	m	0	0	all	-	all	Eu:mul	1976	CC	7804	n	bl	n	y	2	ev	nev any	ot
MCCONN	17	c	0	0	all	-	all	Eu:UK	1946	CC	100	n	V	n	y	0	ev	nev any	st
PERNU	9	m	0	0	all	-	all	Eu:Sca	1944	CC	1606	n	bl	n	n	0	ev	nev any	st
PERNU	5	f	0	0	all	-	all	Eu:Sca	1944	CC	1606	n	bl	n	n	0	ev	nev any	st
SADOWS	30	m	0	0	wh	-	all	NAmer	1938	CC	477	n	bl	n	n	1	ev	nev any	ot
STOCKS	40	m	0	0	all	-	all	Eu:UK	1952	CC	2932	n	V	y	n	2	ev	nev any	st
TIZZAN	4	m	0	0	all	-	all	Eu:wst	1959	CC	1358	n	bl	n	n	0	ev	nev any	st
TULINI	25	m	0	0	all	0	all	Eu:Sca	1967	pr	472	n	bl	n	n	3	cu	nev any	or
WYNDE7	41	m	0	0	all	-	all	NAmer	1977	CC	2085	n	bl	n	y	0	ev	nev any	st
XIANGZ	10	m	0	0	all	0	all	As:Chi	1976	pr	983	m	ot	n	n	2	ev	nev any	ot

Table 1E8 - 2

IESLC - Meta-analysis of Ever Smoking (or Current if Ever not available), Pipe only
All LC types
Most adjusted

REF	NRR	SEX	AD	Number Exposed Case	Exposed Cont	Non-exposed Case	Non-exposed Cont	RR	95.00%CI
ABELIN	50	m	1	-	-	-	-	23.54	(4.73- 117.28)
ARMADA	2	m	0	1	1	4	64	16.00	(0.84- 305.74)
*BENSHL	2	m	1	-	-	-	-	4.52	(2.22- 9.19)
*BEST	17	m	1	-	-	-	-	4.35	(1.82- 10.41)
BOFFET	2	m	2	-	-	-	-	7.90	(5.30- 11.80)
*BOUCOT	118	m	2	-	-	-	-	9.06	(0.37- 222.47)
*CEDERL	84	m	2	-	-	-	-	7.20	(4.45- 11.66)
COOKSO	3	c	0	5	1	45	61	6.78	(0.77- 60.04)
*CPSI	181	m	1	-	-	-	-	2.54	(1.68- 3.83)
DAMBER	2	m	0	198	142	42	208	6.91	(4.65- 10.25)
DEAN	5	m	0	49	71	12	61	3.51	(1.71- 7.19)
DEAN3	178	m	1	-	-	-	-	2.15	(1.17- 3.94)
DOLL	18	m	0	53	93	7	61	4.97	(2.12- 11.64)
*DORN	34	m	1	-	-	-	-	1.33	(0.45- 3.95)
GARDIN	3	c	0	3	2	5	41	12.30	(1.64- 92.33)
*HAMMON	112	m	1	-	-	-	-	3.01	(1.52- 5.97)
*HEIN	3	m	0	10	499	1	457	9.16	(1.18- 71.26)
KOULUM	3	m	0	15	17	5	54	9.53	(3.02- 30.08)
*LANGE	18	m	1	-	-	-	-	4.10	(1.40- 13.00)
LEVIN	28	m	1	-	-	-	-	1.30	(0.74- 2.30)
LUBIN2	22	m	2	-	-	-	-	2.67	(1.85- 3.84)
MCCONN	17	c	0	8	7	9	23	2.92	(0.82- 10.44)
PERNU	9	m	0	55	37	97	275	4.21	(2.62- 6.79)
PERNU	5	f	0	1	1	110	971	8.83	(0.55- 142.12)
Subtotal PERNU								4.30	(2.69- 6.89)
SADOWS	30	m	1	-	-	-	-	1.17	(0.36- 3.82)
STOCKS	40	m	2	-	-	-	-	2.61	(1.79- 3.82)
TIZZAN	4	m	0	16	18	180	305	1.51	(0.75- 3.03)
*TULINI	25	m	3	-	-	-	-	10.20	(5.38- 19.50)
WYNDE7	41	m	0	9	97	64	918	1.33	(0.64- 2.76)
*XIANGZ	10	m	2	-	-	-	-	3.00	(2.02- 4.46)
Partial Totals				423	986	581	3499		

*prospective study

REF	NRR	SEX	AD	Ys	Ws	Qs	Ps
ABELIN	50	m	1	3.16	1.49	5.19	0.0001
ARMADA	2	m	0	2.77	0.44	0.97	0.0655
*BENSHL	2	m	1	1.51	7.61	0.35	0.0000
*BEST	17	m	1	1.47	5.05	0.16	0.0010
BOFFET	2	m	2	2.07	23.99	14.37	0.0000
*BOUCOT	118	m	2	2.20	0.38	0.31	0.1770
*CEDERL	84	m	2	1.97	16.56	7.69	0.0000
COOKSO	3	c	0	1.91	0.81	0.31	0.0855
*CPSI	181	m	1	0.93	22.63	2.94	0.0000
DAMBER	2	m	0	1.93	24.56	10.05	0.0000
DEAN	5	m	0	1.26	7.45	0.01	0.0006
DEAN3	178	m	1	0.77	10.42	2.90	0.0135
DOLL	18	m	0	1.60	5.29	0.51	0.0002
*DORN	34	m	1	0.29	3.26	3.31	0.6068
GARDIN	3	c	0	2.51	0.95	1.40	0.0147
*HAMMON	112	m	1	1.10	8.21	0.30	0.0016
*HEIN	3	m	0	2.21	0.91	0.78	0.0344
KOULUM	3	m	0	2.25	2.91	2.69	0.0001
*LANGE	18	m	1	1.41	3.09	0.04	0.0131
LEVIN	28	m	1	0.26	11.95	12.69	0.3645
LUBIN2	22	m	2	0.98	28.81	2.78	0.0000
MCCONN	17	c	0	1.07	2.37	0.12	0.0991
PERNU	9	m	0	1.44	16.90	0.36	0.0000
PERNU	5	f	0	2.18	0.50	0.39	0.1245
Subtotal PERNU				1.46	17.40	0.75	
SADOWS	30	m	1	0.16	2.75	3.55	0.7944
STOCKS	40	m	2	0.96	26.74	2.97	0.0000
TIZZAN	4	m	0	0.41	7.88	6.15	0.2502
*TULINI	25	m	3	2.32	9.27	9.82	0.0000
WYNDE7	41	m	0	0.29	7.24	7.34	0.4419
*XIANGZ	10	m	2	1.10	24.49	0.92	0.0000

Table 1E8 - 2

IESLC - Meta-analysis of Ever Smoking (or Current if Ever not available), Pipe only
All LC types
Most adjusted

	N	30
	NS	29
	Wt	284.92
Het	Chi	101.36
Het	df	29
Het	P	***
Fixed	RR	3.64
	RRl	3.24
	RRu	4.09
	P	+++
Random	RR	3.70
	RRl	2.89
	RRu	4.74
	P	+++
Asymm	P	N.S.

Table 1E8 - 3

IESLC - Meta-analysis of Ever Smoking (or Current if Ever not available), Pipe only
All LC types
Most adjusted

	Sex			
	combined	male	female	Total
N	3	26	1	30
NS	3	26	1	30
Wt	4.12	280.30	0.50	284.92
Het Chi	1.52	99.13	0.00	101.36
Het df	2	25	0	29
Het P	N.S.	***	N.S.	***
Fixed RR	4.79	3.62	8.83	3.64
RRl	1.82	3.22	0.55	3.24
RRu	12.58	4.07	142.12	4.09
P	++	+++	N.S.	+++
Random RR	4.79	3.62	8.83	3.70
RRl	1.82	2.80	0.55	2.89
RRu	12.58	4.69	142.12	4.74
P	++	+++	N.S.	+++
Between Chi				0.71
Between df				2
Between P				N.S.
Btwn(F) P				N.S.
Btwn(R) P				N.S.

	All LC (or nearest)		
	all	other	Total
N	30		30
NS	29		29
Wt	284.92		284.92
Het Chi	101.36		101.36
Het df	29		29
Het P	***		***
Fixed RR	3.64		3.64
RRl	3.24		3.24
RRu	4.09		4.09
P	+++		+++
Random RR	3.70		3.70
RRl	2.89		2.89
RRu	4.74		4.74
P	+++		+++
Between Chi			
Between df			
Between P			N.S.
Btwn(F) P			N.S.
Btwn(R) P			N.S.

	Location								
	NAmer	UK	Scand	othEur	China	Japan	othAs	other	Total
N	8	6	8	5	1			2	30
NS	8	6	7	5	1			2	29
Wt	61.46	53.39	74.71	62.61	24.49			8.26	284.92
Het Chi	11.23	6.18	6.55	28.87	0.00			0.32	101.36
Het df	7	5	7	4	0			1	29
Het P	N.S.	N.S.	N.S.	***	N.S.			N.S.	***
Fixed RR	2.08	2.99	6.52	4.01	3.00			3.74	3.64
RRl	1.62	2.29	5.19	3.13	2.02			1.89	3.24
RRu	2.67	3.91	8.17	5.14	4.46			7.40	4.09
P	+++	+++	+++	+++	+++			+++	+++
Random RR	2.03	3.12	6.52	4.78	3.00			3.74	3.70
RRl	1.43	2.25	5.19	2.05	2.02			1.89	2.89
RRu	2.89	4.33	8.17	11.11	4.46			7.40	4.74
P	+++	+++	+++	+++	+++			+++	+++
Between Chi									48.21
Between df									5
Between P									***
Btwn(F) P									**
Btwn(R) P									***

Table 1E8 - 3

IESLC - Meta-analysis of Ever Smoking (or Current if Ever not available), Pipe only
All LC types
Most adjusted

Detailed Country in "other Europe"

	multi	Germany	othWest	East	Balkans	Total
N	2		3			5
NS	2		3			5
Wt	52.80		9.81			62.61
Het Chi	15.40		11.04			28.87
Het df	1		2			4
Het P	***		**			***
Fixed RR	4.37		2.54			4.01
RRl	3.34		1.36			3.13
RRu	5.72		4.75			5.14
P	+++		++			+++
Random RR	4.58		6.89			4.78
RRl	1.58		0.80			2.05
RRu	13.25		59.32			11.11
P	++		(+)			+++
Between Chi						2.43
Between df						1
Between P						N.S.
Btwn(F) P						N.S.
Btwn(R) P						N.S.

Detailed Country in "other Asia"

	India	HongKong	other	Total
N				
NS				
Wt				
Het Chi				
Het df				
Het P				
Fixed RR				
RRl				
RRu				
P				
Random RR				
RRl				
RRu				
P				
Between Chi				
Between df				
Between P				N.S.
Btwn(F) P				N.S.
Btwn(R) P				N.S.

Detailed other continent

	SCAmer	Auslia	Africa	Total
N			2	2
NS			2	2
Wt			8.26	8.26
Het Chi			0.32	0.32
Het df			1	1
Het P			N.S.	N.S.
Fixed RR			3.74	3.74
RRl			1.89	1.89
RRu			7.40	7.40
P			+++	+++
Random RR			3.74	3.74
RRl			1.89	1.89
RRu			7.40	7.40
P			+++	+++
Between Chi				
Between df				
Between P				N.S.
Btwn(F) P				N.S.
Btwn(R) P				N.S.

Table 1E8 - 3

IESLC - Meta-analysis of Ever Smoking (or Current if Ever not available), Pipe only
All LC types
Most adjusted

	Start year of study <1960	1960-69	1970-79	1980-89	1990+	Total
N	16	5	6	3		30
NS	15	5	6	3		29
Wt	125.76	44.67	89.12	25.37		284.92
Het Chi	32.57	14.24	21.72	0.38		101.36
Het df	15	4	5	2		29
Het P	**	**	***	N.S.		***
Fixed RR	2.79	5.39	3.48	8.13		3.64
RRl	2.34	4.02	2.83	5.51		3.24
RRu	3.32	7.22	4.28	12.00		4.09
P	+++	+++	+++	+++		+++
Random RR	2.95	5.28	3.36	8.13		3.70
RRl	2.20	2.88	2.03	5.51		2.89
RRu	3.96	9.69	5.55	12.00		4.74
P	+++	+++	+++	+++		+++
Between Chi						32.44
Between df						3
Between P						***
Btwn(F) P						*
Btwn(R) P						***

	Study type (1) CC	other	Total
N	19	11	30
NS	18	11	29
Wt	183.45	101.46	284.92
Het Chi	74.46	26.12	101.36
Het df	18	10	29
Het P	***	**	***
Fixed RR	3.50	3.91	3.64
RRl	3.03	3.22	3.24
RRu	4.05	4.75	4.09
P	+++	+++	+++
Random RR	3.51	4.10	3.70
RRl	2.50	2.87	2.89
RRu	4.94	5.86	4.74
P	+++	+++	+++
Between Chi			0.78
Between df			1
Between P			N.S.
Btwn(F) P			N.S.
Btwn(R) P			N.S.

	Study type (2) CC	prosp	other	Total
N	19	11		30
NS	18	11		29
Wt	183.45	101.46		284.92
Het Chi	74.46	26.12		101.36
Het df	18	10		29
Het P	***	**		***
Fixed RR	3.50	3.91		3.64
RRl	3.03	3.22		3.24
RRu	4.05	4.75		4.09
P	+++	+++		+++
Random RR	3.51	4.10		3.70
RRl	2.50	2.87		2.89
RRu	4.94	5.86		4.74
P	+++	+++		+++
Between Chi				0.78
Between df				1
Between P				N.S.
Btwn(F) P				N.S.
Btwn(R) P				N.S.

Table 1E8 - 3

IESLC - Meta-analysis of Ever Smoking (or Current if Ever not available), Pipe only
All LC types
Most adjusted

	Study size (number of LC cases) 100-249	250-499	500-999	1000+	Total
N	6	9	5	10	30
NS	6	9	5	9	29
Wt	6.90	64.94	69.84	143.24	284.92
Het Chi	4.34	34.73	15.66	38.20	101.36
Het df	5	8	4	9	29
Het P	N.S.	***	**	***	***
Fixed RR	7.62	4.08	4.08	3.16	3.64
RRl	3.61	3.20	3.23	2.68	3.24
RRu	16.08	5.20	5.16	3.72	4.09
P	+++	+++	+++	+++	+++
Random RR	7.62	3.88	4.06	2.91	3.70
RRl	3.61	2.24	2.44	2.01	2.89
RRu	16.08	6.73	6.75	4.23	4.74
P	+++	+++	+++	+++	+++
Between Chi					8.43
Between df					3
Between P					*
Btwn(F) P					N.S.
Btwn(R) P					N.S.

	Risky occupational population no	mining	othRisky	Total
N	29	1		30
NS	28	1		29
Wt	260.42	24.49		284.92
Het Chi	100.35	0.00		101.36
Het df	28	0		29
Het P	***	N.S.		***
Fixed RR	3.71	3.00		3.64
RRl	3.29	2.02		3.24
RRu	4.19	4.46		4.09
P	+++	+++		+++
Random RR	3.76	3.00		3.70
RRl	2.89	2.02		2.89
RRu	4.88	4.46		4.74
P	+++	+++		+++
Between Chi				1.01
Between df				1
Between P				N.S.
Btwn(F) P				N.S.
Btwn(R) P				N.S.

	National cigarette tobacco type Virginia	blended	other	Total
N	9	20	1	30
NS	9	19	1	29
Wt	66.70	193.73	24.49	284.92
Het Chi	7.41	90.68	0.00	101.36
Het df	8	19	0	29
Het P	N.S.	***	N.S.	***
Fixed RR	3.16	3.92	3.00	3.64
RRl	2.49	3.40	2.02	3.24
RRu	4.02	4.51	4.46	4.09
P	+++	+++	+++	+++
Random RR	3.16	3.83	3.00	3.70
RRl	2.49	2.69	2.02	2.89
RRu	4.02	5.46	4.46	4.74
P	+++	+++	+++	+++
Between Chi				3.28
Between df				2
Between P				N.S.
Btwn(F) P				N.S.
Btwn(R) P				N.S.

Table 1E8 - 3

IESLC - Meta-analysis of Ever Smoking (or Current if Ever not available), Pipe only
All LC types
Most adjusted

	Any proxy use No/nk	Yes	Total
N	23	7	30
NS	22	7	29
Wt	189.32	95.60	284.92
Het Chi	61.55	31.11	101.36
Het df	22	6	29
Het P	***	***	***
Fixed RR	3.22	4.66	3.64
RRl	2.79	3.81	3.24
RRu	3.71	5.69	4.09
P	+++	+++	+++
Random RR	3.34	4.93	3.70
RRl	2.54	2.92	2.89
RRu	4.39	8.33	4.74
P	+++	+++	+++
Between Chi			8.71
Between df			1
Between P			**
Btwn(F) P			N.S.
Btwn(R) P			N.S.

	Full histological confirmation No	Yes	Total
N	25	5	30
NS	24	5	29
Wt	245.25	39.67	284.92
Het Chi	88.85	5.37	101.36
Het df	24	4	29
Het P	***	N.S.	***
Fixed RR	3.88	2.46	3.64
RRl	3.43	1.80	3.24
RRu	4.40	3.35	4.09
P	+++	+++	+++
Random RR	3.91	2.43	3.70
RRl	2.99	1.50	2.89
RRu	5.13	3.94	4.74
P	+++	+++	+++
Between Chi			7.13
Between df			1
Between P			**
Btwn(F) P			N.S.
Btwn(R) P			(*)

	Number of adjustment variables (1) 0	1	2+/+nk	Total
N	13	10	7	30
NS	12	10	7	29
Wt	78.21	76.47	130.23	284.92
Het Chi	29.47	20.98	36.62	101.36
Het df	12	9	6	29
Het P	**	*	***	***
Fixed RR	4.20	2.52	4.15	3.64
RRl	3.37	2.01	3.50	3.24
RRu	5.24	3.15	4.93	4.09
P	+++	+++	+++	+++
Random RR	4.11	2.68	4.71	3.70
RRl	2.70	1.85	2.97	2.89
RRu	6.25	3.90	7.48	4.74
P	+++	+++	+++	+++
Between Chi				14.28
Between df				2
Between P				***
Btwn(F) P				N.S.
Btwn(R) P				N.S.

Table 1E8 - 3

IESLC - Meta-analysis of Ever Smoking (or Current if Ever not available), Pipe only
All LC types
Most adjusted

	Number of adjustment variables (2) 0	1	2	3-5	6+/+nk	Total
N	13	10	6	1		30
NS	12	10	6	1		29
Wt	78.21	76.47	120.97	9.27		284.92
Het Chi	29.47	20.98	28.57	0.00		101.36
Het df	12	9	5	0		29
Het P	**	*	***	N.S.		***
Fixed RR	4.20	2.52	3.88	10.20		3.64
RRl	3.37	2.01	3.25	5.36		3.24
RRu	5.24	3.15	4.63	19.42		4.09
P	+++	+++	+++	+++		+++
Random RR	4.11	2.68	4.14	10.20		3.70
RRl	2.70	1.85	2.60	5.36		2.89
RRu	6.25	3.90	6.60	19.42		4.74
P	+++	+++	+++	+++		+++
Between Chi						22.33
Between df						3
Between P						***
Btwn(F) P						(*)
Btwn(R) P						**

	Derivation of RR/CI Orig	StdCalc	Other	Total
N	3	16	11	30
NS	3	15	11	29
Wt	36.35	118.39	130.18	284.92
Het Chi	1.93	51.46	21.77	101.36
Het df	2	15	10	29
Het P	N.S.	***	*	***
Fixed RR	7.97	3.42	3.10	3.64
RRl	5.76	2.86	2.61	3.24
RRu	11.04	4.10	3.68	4.09
P	+++	+++	+++	+++
Random RR	7.97	3.82	3.07	3.70
RRl	5.76	2.56	2.31	2.89
RRu	11.04	5.69	4.06	4.74
P	+++	+++	+++	+++
Between Chi				26.20
Between df				2
Between P				***
Btwn(F) P				*
Btwn(R) P				***

	Smoking status ever	current	Total
N	23	7	30
NS	22	7	29
Wt	236.10	48.82	284.92
Het Chi	76.80	15.33	101.36
Het df	22	6	29
Het P	***	*	***
Fixed RR	3.36	5.41	3.64
RRl	2.95	4.09	3.24
RRu	3.81	7.16	4.09
P	+++	+++	+++
Random RR	3.31	5.44	3.70
RRl	2.51	3.27	2.89
RRu	4.35	9.04	4.74
P	+++	+++	+++
Between Chi			9.23
Between df			1
Between P			**
Btwn(F) P			N.S.
Btwn(R) P			(*)

Table 1E8 - 4

IESLC - Meta-analysis of Ever Smoking (or Current if Ever not available), Pipe only
All LC types
Least adjusted

REF	NRR	X	SEX	AGEL	AGEH	RACE	YF	LC TYPE	LOC	START	ST	NLC	R	VB	P	H	AD	SM	DENOM	De
ABELIN	7	x	m	0	0	all	-	all	Eu:wst	1941	CC	118	n	bl	y	n	0	ev	nev any	st
ARMADA	2		m	0	0	all	-	all	Eu:wst	1986	CC	325	n	bl	n	y	0	ev	nev any	st
BENSHL	2		m	0	0	all	0	all	Eu:UK	1967	pr	486	n	V	n	n	1	cu	nev any	ot
BEST	17		m	0	0	all	0	all	NAmer	1955	pr	381	n	V	n	n	1	ev	nev any	ot
BOFFET	5	x	m	0	0	all	-	all	Eu:mul	1988	CC	5621	n	bl	y	n	0	ev	nev any	st
BOUCOT	6	x	m	0	0	all	0	all	NAmer	1951	pr	121	n	bl	n	n	0	ev	nev any	ot
CEDERL	84		m	0	0	all	16	all	Eu:Sca	1963	pr	491	n	bl	n	n	2	cu	nev any	ot
COOKSO	3		c	0	0	bl	-	all	Africa	1961	CC	234	n	V	n	y	0	ev	nev any	st
CPSI	181		m	35	84	all	6	all	NAmer	1959	pr	5138	n	bl	n	n	1	ev	nev any	ot
DAMBER	2		m	0	0	all	-	all	Eu:Sca	1972	CC	579	n	bl	y	n	0	ev	nev any	st
DEAN	5		m	0	0	wh	-	all	Africa	1947	CC	603	n	V	y	n	0	ev	nev any	st
DEAN3	177	x	m	0	0	all	-	all	Eu:UK	1969	CC	766	n	V	y	n	0	cu	nev any	st
DOLL	18		m	0	0	all	-	all	Eu:UK	1948	CC	1465	n	V	n	n	0	ev	nev any	st
DORN	43	x	m	0	0	wh	2	all	NAmer	1954	pr	5097	n	bl	n	n	0	ev	nev any	st
GARDIN	3		c	0	0	all	-	all	Eu:UK	1988	CC	143	n	V	y	n	0	cu	nev any	st
HAMMON	124	x	m	0	0	wh	0	all	NAmer	1952	pr	448	n	bl	n	n	0	ev	nev any	st
HEIN	3		m	0	0	all	0	all	Eu:Sca	1970	pr	144	n	bl	n	n	0	cu	nev any	st
KOULUM	3		m	0	0	all	-	all	Eu:Sca	1936	CC	812	n	bl	n	n	0	ev	nev any	st
LANGE	9	x	m	0	0	all	0	all	Eu:Sca	1976	pr	268	n	bl	n	n	0	cu	nev any	st
LEVIN	28		m	0	0	all	-	all	NAmer	1938	CC	475	n	bl	n	n	1	ev	nev any	st
LUBIN2	21	x	m	0	0	all	-	all	Eu:mul	1976	CC	7804	n	bl	n	y	0	ev	nev any	st
MCCONN	17		c	0	0	all	-	all	Eu:UK	1946	CC	100	n	V	n	y	0	ev	nev any	st
PERNU	9		m	0	0	all	-	all	Eu:Sca	1944	CC	1606	n	bl	n	n	0	ev	nev any	st
PERNU	5		f	0	0	all	-	all	Eu:Sca	1944	CC	1606	n	bl	n	n	0	ev	nev any	st
SADOWS	3	x	m	0	0	wh	-	all	NAmer	1938	CC	477	n	bl	n	n	0	ev	nev any	st
STOCKS	24	x	m	0	0	all	-	all	Eu:UK	1952	CC	2932	n	V	y	n	0	ev	nev any	st
TIZZAN	4		m	0	0	all	-	all	Eu:wst	1959	CC	1358	n	bl	n	n	0	ev	nev any	st
TULINI	2	x	m	0	0	all	0	all	Eu:Sca	1967	pr	472	n	bl	n	n	1	cu	nev any	or
WYNDE7	41		m	0	0	all	-	all	NAmer	1977	CC	2085	n	bl	n	y	0	ev	nev any	st
XIANGZ	3	x	m	0	0	all	0	all	As:Chi	1976	pr	983	m	ot	n	n	0	ev	nev any	st

Table 1E8 - 5

IESLC - Meta-analysis of Ever Smoking (or Current if Ever not available), Pipe only
All LC types
Least adjusted

REF	NRR	SEX	AD	Number Exposed Case	Number Exposed Cont	Non-exposed Case	Non-exposed Cont	RR	95.00%CI
ABELIN	7	m	0	8	34	2	183	21.53	(4.38- 105.80)
ARMADA	2	m	0	1	1	4	64	16.00	(0.84- 305.74)
*BENSHL	2	m	1	-	-	-	-	4.52	(2.22- 9.19)
*BEST	17	m	1	-	-	-	-	4.35	(1.82- 10.41)
BOFFET	5	m	0	61	129	117	1750	7.07	(4.95- 10.11)
*BOUCOT	6	m	0	1	2389	0	7551	9.48~	(0.39- 232.65)
*CEDERL	84	m	2	-	-	-	-	7.20	(4.45- 11.66)
COOKSO	3	c	0	5	1	45	61	6.78	(0.77- 60.04)
*CPSI	181	m	1	-	-	-	-	2.54	(1.68- 3.83)
DAMBER	2	m	0	198	142	42	208	6.91	(4.65- 10.25)
DEAN	5	m	0	49	71	12	61	3.51	(1.71- 7.19)
DEAN3	177	m	0	23	116	24	510	4.21	(2.30- 7.73)
DOLL	18	m	0	53	93	7	61	4.97	(2.12- 11.64)
*DORN	43	m	0	4	17780	17	117918	1.56	(0.53- 4.64)
GARDIN	3	c	0	3	2	5	41	12.30	(1.64- 92.33)
*HAMMON	124	m	0	18	43041	15	115884	3.23	(1.63- 6.41)
*HEIN	3	m	0	10	499	1	457	9.16	(1.18- 71.26)
KOULUM	3	m	0	15	17	5	54	9.53	(3.02- 30.08)
*LANGE	9	m	0	16	433	5	721	5.33	(1.97- 14.44)
LEVIN	28	m	1	-	-	-	-	1.30	(0.74- 2.30)
LUBIN2	21	m	0	39	197	190	2617	2.73	(1.88- 3.96)
MCCONN	17	c	0	8	7	9	23	2.92	(0.82- 10.44)
PERNU	9	m	0	55	37	97	275	4.21	(2.62- 6.79)
PERNU	5	f	0	1	1	110	971	8.83	(0.55- 142.12)
Subtotal PERNU								4.30	(2.69- 6.89)
SADOWS	3	m	0	13	43	18	81	1.36	(0.61- 3.04)
STOCKS	24	m	0	211	994	45	638	3.01	(2.15- 4.21)
TIZZAN	4	m	0	16	18	180	305	1.51	(0.75- 3.03)
*TULINI	2	m	1	-	-	-	-	11.10	(5.86- 21.20)
WYNDE7	41	m	0	9	97	64	918	1.33	(0.64- 2.76)
*XIANGZ	3	m	0	356	1483	25	974	9.35	(6.29- 13.92)
Partial Totals				1173	67625	1039	252326		

*prospective study ~ With 0.5 adjustment for zero

REF	NRR	SEX	AD	Ys	Ws	Qs	Ps
ABELIN	7	m	0	3.07	1.52	4.10	0.0002
ARMADA	2	m	0	2.77	0.44	0.80	0.0655
*BENSHL	2	m	1	1.51	7.61	0.05	0.0000
*BEST	17	m	1	1.47	5.05	0.01	0.0010
BOFFET	5	m	0	1.96	30.06	8.49	0.0000
*BOUCOT	6	m	0	2.25	0.38	0.25	0.1683
*CEDERL	84	m	2	1.97	16.56	4.99	0.0000
COOKSO	3	c	0	1.91	0.81	0.19	0.0855
*CPSI	181	m	1	0.93	22.63	5.50	0.0000
DAMBER	2	m	0	1.93	24.56	6.32	0.0000
DEAN	5	m	0	1.26	7.45	0.21	0.0006
DEAN3	177	m	0	1.44	10.45	0.00	0.0000
DOLL	18	m	0	1.60	5.29	0.17	0.0002
*DORN	43	m	0	0.44	3.24	3.11	0.4232
GARDIN	3	c	0	2.51	0.95	1.11	0.0147
*HAMMON	124	m	0	1.17	8.18	0.52	0.0008
*HEIN	3	m	0	2.21	0.91	0.57	0.0344
KOULUM	3	m	0	2.25	2.91	2.00	0.0001
*LANGE	9	m	0	1.67	3.86	0.24	0.0010
LEVIN	28	m	1	0.26	11.95	16.15	0.3645
LUBIN2	21	m	0	1.00	27.50	4.89	0.0000
MCCONN	17	c	0	1.07	2.37	0.30	0.0991
PERNU	9	m	0	1.44	16.90	0.00	0.0000
PERNU	5	f	0	2.18	0.50	0.28	0.1245
Subtotal PERNU				1.46	17.40	0.29	
SADOWS	3	m	0	0.31	5.95	7.43	0.4527
STOCKS	24	m	0	1.10	33.86	3.54	0.0000
TIZZAN	4	m	0	0.41	7.88	8.13	0.2502
*TULINI	2	m	1	2.41	9.29	8.96	0.0000
WYNDE7	41	m	0	0.29	7.24	9.39	0.4419
*XIANGZ	3	m	0	2.24	24.33	15.99	0.0000

Table 1E8 - 5

IESLC - Meta-analysis of Ever Smoking (or Current if Ever not available), Pipe only
All LC types
Least adjusted

	N	30
	NS	29
	Wt	300.63
Het	Chi	113.69
Het	df	29
Het	P	***
Fixed	RR	4.16
	RRl	3.71
	RRu	4.66
	P	+++
Random	RR	4.10
	RRl	3.18
	RRu	5.29
	P	+++
Asymm	P	N.S.

Table 1E8 - 6

IESLC - Meta-analysis of Ever Smoking (or Current if Ever not available), Pipe only
All LC types
Least adjusted

		Sex		
	combined	male	female	Total
N	3	26	1	30
NS	3	26	1	30
Wt	4.12	296.01	0.50	300.63
Het Chi	1.52	111.81	0.00	113.69
Het df	2	25	0	29
Het P	N.S.	***	N.S.	***
Fixed RR	4.79	4.14	8.83	4.16
RRl	1.82	3.70	0.55	3.71
RRu	12.58	4.64	142.12	4.66
P	++	+++	N.S.	+++
Random RR	4.79	4.03	8.83	4.10
RRl	1.82	3.09	0.55	3.18
RRu	12.58	5.26	142.12	5.29
P	++	+++	N.S.	+++
Between Chi				0.37
Between df				2
Between P				N.S.
Btwn(F) P				N.S.
Btwn(R) P				N.S.

Table 1E8 - 7

IESLC - Meta-analysis of Ever Smoking (or Current if Ever not available), Pipe only
All LC types
Excluded studies (and stage at which they were excluded)

1 BOUCHA BUELL LAURIL MZILEN

2 ABRAHA AGUDO AKIBA ALDERS AMANDU AMES ANDERS ARCHER AUSTIN AUVINE AXELSO AXELSS BAND BARBON BECHER BERRIN
BLOHMK BLOT1 BLOT2 BLOT3 BLOT4 BRESLO BRETT BROCKM BROSS BROWN1 BROWN2 BUFFLE BYERS1 BYERS2 CARPEN CASCO2
CASCOR CHAN CHANG CHATZI CHEN CHEN2 CHEN3 CHIAZZ CHOI CHOW CHYOU COMSTO CORREA CPSII DARBY DAVEYS
DEAN2 DEKLER DESTE2 DESTEF DOCKER DORANT DORGAN DOSEME DROSTE DU DUNN EBELIN ENGELA ENSTRO ESAKI FAN
GAO GAO2 GARCIA GARSHI GENG GER GILLIS GODLEY GOLLED GOODMA GRAHAM GREGOR GSELL HAENSZ HAMMO2 HANSEN
HEGMAN HENNEK HINDS HIRAOK HIRAYA HITOSU HOLE HOROWI HORWIT HU HU2 HUANG HUMBLE ISHIMA JAHN JAIN
JARUP JARVHO JEDRYC JIANG JOLY JONES JUSSAW KAISE2 KAISER KANELL KATSOU KAUFMA KELLER KHUDER KIHARA KINLEN
KJUUS KNEKT KO KOHLME KOO KREUZE KREYBE KUBIK LAMTH LAMWK LAMWK2 LAUSSM LEI LEMARC LETOUR LIAW
LICKIN LIDDEL LIU LIU2 LIU3 LIU4 LIU5 LOMBA2 LOMBAR LUO MACLEN MAGNUS MARSH MARSH2 MARTIS MASTRA
MATOS MATSUD MCDUFF MCLAUG MIGRAN MILLER MILLS MOLLO MRFIT MRFITR MURATA NAM NOTAN2 NOTANI NOU ODRISC
ORMOS OSANN OSANN2 PARKIN PASTOR PAWLEG PERSH2 PERSHA PETO PEZZO2 PEZZOT PIKE PISANI POFFIJ POLEDN PRESCO
QIAO2 RACHTA RADZIK RANDIG REN RESTRE RIMING RONCO ROOTS ROTHSC SAARIK SANKAR SCHWAR SEGI SEGI2 SEOW
SHAW SHIMIZ SIEMIA SIMARA SIMONA SITAS SOBUE SOBUE2 SPEIZE SPITZ STASZE STAYNE STOCKW STUCKE SUN SUZUK2
SUZUKI SVENSS TANG TANG2 TAO TENKAN TOKARS TOUSEY TSUGAN ULMER VANDER VEIERO VUTUC WAKAI WALD WANG
WANG2 WANG3 WANG4 WARSIN WATSON WICKLU WIGLE WILKIN WU WU2 WUNSCH WUWILL WYNDE2 WYNDE3 WYNDE4 WYNDE5
WYNDE6 WYNDE8 WYNDER XU XU2 XU3 XU4 YAMAGU YONG YUAN ZHANG ZHENG ZHOU

3 DOLL2

5 HIRAY2 SCHWA2 TVERDA

9 BENHAM LUBIN QIAO

Table 1E8 - 8

Potentially overlapping studies

REF	REFGP	PRINC	.	OVERLAP/LINK
LUBIN2	LUBIN2	1		Lubin-combined
HEIN	PRESCO	1		Subset of PRESCO
LANGE	PRESCO	1		Subset of PRESCO
BENSHL	TANG2	1		Subset of TANG2
WYNDE7	WYNDE6	2		WYNDE5/6/7/8
CPSI	CPSI	1		CPSI overall
BOFFET	BOFFET	1		BOFFET-combined
XIANGZ	XIANGZ	1		LUBIN/XIANGZ/QIAO

Table 1E8 - 9

Most adjusted - insufficient data for metaanalysis

REF	NRR	SEX	AGEL	AGEH	RACE	YF	LC TYPE	LOC	START	ST	NLC	R	VB	P	H	AD	SM	DENOM	De
DOLL2	49	m	35	99	all	5	all	Eu:UK	1951	pr	920	n	V	n	n	1	ev	nev any	st

REF	NRR	RR	SIG	RRDATA	comment
DOLL2	49	5.43			0

Table 1E9 -

IESLC - Meta-analysis of Current Smoking (or Ever if Current not available), Pipe only
All LC types

This analysis is restricted to results for:
1) Non-dose-response data
2) Smokers of pipe only
3) Results complete enough for use in metaanalysis

Within each study, results are then selected (in the following order of preference, within each sex) for:
4) SMKSTA: current smokers, ever smokers
5) DENOM: never smoked anything, (never +1 = +long term ex)
6) Followup period (prospective studies): whole study (coded as 0) or longest available
7) LCtype: all or nearest available, at least Squamous and Adeno. (q = squamous, s = small,
l = large, a = adeno, mix = mixed, alv = alveolar)
8) Race: all or nearest available, otherwise by race (wh or w = white, bl or b = black, hi = hispanic
ch = chinese, jap = japanese, haw = hawaiian, w+o = white + oriental, sca = scandinavian, as = asian)
9) For overlapping studies: principal rather than subsidiary studies
Finally by Age: whole study (coded as 0) if available, otherwise by widest available age group
and then for single sex results (m, f) in preference to combined sex results (c).

Results adjusted (AD) for the most potential confounders are then chosen in Sections -1 to -3
(and those which actually differ from the adjusted results in Table 1E8 - 1 are marked 'x' in Section -1)
and results adjusted for the least confounders in Sections -4 to -6. (Those least adjusted results which
actually differ from the most adjusted as marked 'x' in column X in Section -4)
(Results adjusted for an unknown number of confounder(s) are coded as 20.)

Section -7 shows excluded studies, together with the stage (as above) at which no qualifying
results were found.

Section -8 lists the potentially overlapping studies which have been included (1=principal, 2=subsidiary).

Section -9 lists any results which would have been included in preference except that they had data not complete
enough for use in meta-analysis, with their significance (yes/no), if known, and any further comment as entered
on the database.

In addition to those mentioned above, the following fields, levels and abbreviations are used:
* or nk = not known, n = no, y = yes, ot = other
ev = ever, cu = current, nev = never
REF: 6-character study reference
NRR: number of the RR on the database within the study
ST : study type (CC = case control, pr or prosp = prospective)
NLC: number of lung cancer cases in whole study
R : risky occupational population (n = no, m = mining, o = other risky)
VB : national cigarette type (V = at least 75% Virginia, bl = at least 75% blended, ot = other)
P : any proxy use
H : full histological confirmation
De : derivation of RR/CI (or = original, st = standard method, ot = other method of estimation)

Table 1E9 - 1

IESLC - Meta-analysis of Current Smoking (or Ever if Current not available), Pipe only
All LC types
Most adjusted

REF	NRR	1E8	SEX	AGEL	AGEH	RACE	YF	LC TYPE	LOC	START	ST	NLC	R	VB	P	H	AD	SM	DENOM	De
ABELIN	50		m	0	0	all	-	all	Eu:wst	1941	CC	118	n	bl	y	n	1	ev	nev any	st
ARMADA	2		m	0	0	all	-	all	Eu:wst	1986	CC	325	n	bl	n	y	0	ev	nev any	st
BENSHL	2		m	0	0	all	0	all	Eu:UK	1967	pr	486	n	V	n	n	1	cu	nev any	ot
BEST	17		m	0	0	all	0	all	NAmer	1955	pr	381	n	V	n	n	1	ev	nev any	ot
BOFFET	24	x	m	0	0	all	-	all	Eu:mul	1988	CC	5621	n	bl	y	n	2	cu	nev any	or
BOUCOT	118		m	0	0	all	0	all	NAmer	1951	pr	121	n	bl	n	n	2	ev	nev any	ot
CEDERL	84		m	0	0	all	16	all	Eu:Sca	1963	pr	491	n	bl	n	n	2	cu	nev any	ot
COOKSO	3		c	0	0	bl	-	all	Africa	1961	CC	234	n	V	n	y	0	ev	nev any	st
CPSI	181		m	35	84	all	6	all	NAmer	1959	pr	5138	n	bl	n	n	1	ev	nev any	ot
DAMBER	18	x	m	0	0	all	-	all	Eu:Sca	1972	CC	579	n	bl	y	n	1	cu	nev any	ot
DEAN	5		m	0	0	wh	-	all	Africa	1947	CC	603	n	V	y	n	0	ev	nev any	st
DEAN3	178		m	0	0	all	-	all	Eu:UK	1969	CC	766	n	V	y	n	1	cu	nev any	ot
DOLL	18		m	0	0	all	-	all	Eu:UK	1948	CC	1465	n	V	n	n	0	ev	nev any	st
DORN	23	x	m	0	0	wh	15	all	NAmer	1954	pr	5097	n	bl	n	n	2	cu	nev any	ot
GARDIN	3		c	0	0	all	-	all	Eu:UK	1988	CC	143	n	V	y	n	0	cu	nev any	st
HAMMON	112		m	0	0	wh	0	all	NAmer	1952	pr	448	n	bl	n	n	1	ev	nev any	ot
HEIN	3		m	0	0	all	0	all	Eu:Sca	1970	pr	144	n	bl	n	n	0	cu	nev any	st
KOULUM	3		m	0	0	all	-	all	Eu:Sca	1936	CC	812	n	bl	n	n	0	ev	nev any	st
LANGE	18		m	0	0	all	0	all	Eu:Sca	1976	pr	268	n	bl	n	n	1	cu	nev any	or
LEVIN	28		m	0	0	all	-	all	NAmer	1938	CC	475	n	bl	n	n	1	ev	nev any	st
LUBIN2	6	x	m	0	0	all	-	all	Eu:mul	1976	CC	7804	n	bl	n	y	2	cu	nev any	ot
MCCONN	17		c	0	0	all	-	all	Eu:UK	1946	CC	100	n	V	n	y	0	ev	nev any	st
PERNU	9		m	0	0	all	-	all	Eu:Sca	1944	CC	1606	n	bl	n	n	0	ev	nev any	st
PERNU	5		f	0	0	all	-	all	Eu:Sca	1944	CC	1606	n	bl	n	n	0	ev	nev any	st
SADOWS	30		m	0	0	wh	-	all	NAmer	1938	CC	477	n	bl	n	n	1	ev	nev any	ot
STOCKS	40		m	0	0	all	-	all	Eu:UK	1952	CC	2932	n	V	y	n	2	ev	nev any	st
TIZZAN	4		m	0	0	all	-	all	Eu:wst	1959	CC	1358	n	bl	n	n	0	ev	nev any	st
TULINI	25		m	0	0	all	0	all	Eu:Sca	1967	pr	472	n	bl	n	n	3	cu	nev any	or
WYNDE7	3	x	m	0	0	all	-	all	NAmer	1977	CC	2085	n	bl	n	y	0	cu	nev any	st
XIANGZ	10		m	0	0	all	0	all	As:Chi	1976	pr	983	m	ot	n	n	2	ev	nev any	ot

Table 1E9 - 2

IESLC - Meta-analysis of Current Smoking (or Ever if Current not available), Pipe only
All LC types
Most adjusted

REF	NRR	SEX	AD	Number Exposed Case	Number Exposed Cont	Non-exposed Case	Non-exposed Cont	RR	95.00%CI
ABELIN	50	m	1	-	-	-	-	23.54	(4.73- 117.28)
ARMADA	2	m	0	1	1	4	64	16.00	(0.84- 305.74)
*BENSHL	2	m	1	-	-	-	-	4.52	(2.22- 9.19)
*BEST	17	m	1	-	-	-	-	4.35	(1.82- 10.41)
BOFFET	24	m	2	-	-	-	-	12.50	(7.70- 20.20)
*BOUCOT	118	m	2	-	-	-	-	9.06	(0.37- 222.47)
*CEDERL	84	m	2	-	-	-	-	7.20	(4.45- 11.66)
COOKSO	3	c	0	5	1	45	61	6.78	(0.77- 60.04)
*CPSI	181	m	1	-	-	-	-	2.54	(1.68- 3.83)
DAMBER	18	m	1	-	-	-	-	8.30	(5.30- 13.10)
DEAN	5	m	0	49	71	12	61	3.51	(1.71- 7.19)
DEAN3	178	m	1	-	-	-	-	2.15	(1.17- 3.94)
DOLL	18	m	0	53	93	7	61	4.97	(2.12- 11.64)
*DORN	23	m	2	-	-	-	-	2.14	(1.46- 3.13)
GARDIN	3	c	0	3	2	5	41	12.30	(1.64- 92.33)
*HAMMON	112	m	1	-	-	-	-	3.01	(1.52- 5.97)
*HEIN	3	m	0	10	499	1	457	9.16	(1.18- 71.26)
KOULUM	3	m	0	15	17	5	54	9.53	(3.02- 30.08)
*LANGE	18	m	1	-	-	-	-	4.10	(1.40- 13.00)
LEVIN	28	m	1	-	-	-	-	1.30	(0.74- 2.30)
LUBIN2	6	m	2	-	-	-	-	5.85	(4.52- 7.58)
MCCONN	17	c	0	8	7	9	23	2.92	(0.82- 10.44)
PERNU	9	m	0	55	37	97	275	4.21	(2.62- 6.79)
PERNU	5	f	0	1	1	110	971	8.83	(0.55- 142.12)
Subtotal PERNU								4.30	(2.69- 6.89)
SADOWS	30	m	1	-	-	-	-	1.17	(0.36- 3.82)
STOCKS	40	m	2	-	-	-	-	2.61	(1.79- 3.82)
TIZZAN	4	m	0	16	18	180	305	1.51	(0.75- 3.03)
*TULINI	25	m	3	-	-	-	-	10.20	(5.38- 19.50)
WYNDE7	3	m	0	7	54	64	918	1.86	(0.81- 4.25)
*XIANGZ	10	m	2	-	-	-	-	3.00	(2.02- 4.46)
Partial Totals				223	801	539	3291		

*prospective study

REF	NRR	SEX	AD	Ys	Ws	Qs	Ps
ABELIN	50	m	1	3.16	1.49	4.68	0.0001
ARMADA	2	m	0	2.77	0.44	0.85	0.0655
*BENSHL	2	m	1	1.51	7.61	0.11	0.0000
*BEST	17	m	1	1.47	5.05	0.03	0.0010
BOFFET	24	m	2	2.53	16.52	21.41	0.0000
*BOUCOT	118	m	2	2.20	0.38	0.25	0.1770
*CEDERL	84	m	2	1.97	16.56	5.70	0.0000
COOKSO	3	c	0	1.91	0.81	0.22	0.0855
*CPSI	181	m	1	0.93	22.63	4.69	0.0000
DAMBER	18	m	1	2.12	18.77	9.97	0.0000
DEAN	5	m	0	1.26	7.45	0.13	0.0006
DEAN3	178	m	1	0.77	10.42	4.03	0.0135
DOLL	18	m	0	1.60	5.29	0.25	0.0002
*DORN	23	m	2	0.76	26.42	10.37	0.0001
GARDIN	3	c	0	2.51	0.95	1.19	0.0147
*HAMMON	112	m	1	1.10	8.21	0.67	0.0016
*HEIN	3	m	0	2.21	0.91	0.62	0.0344
KOULUM	3	m	0	2.25	2.91	2.19	0.0001
*LANGE	18	m	1	1.41	3.09	0.00	0.0131
LEVIN	28	m	1	0.26	11.95	15.12	0.3645
LUBIN2	6	m	2	1.77	57.49	8.26	0.0000
MCCONN	17	c	0	1.07	2.37	0.24	0.0991
PERNU	9	m	0	1.44	16.90	0.04	0.0000
PERNU	5	f	0	2.18	0.50	0.31	0.1245
Subtotal PERNU				1.46	17.40	0.35	
SADOWS	30	m	1	0.16	2.75	4.17	0.7944
STOCKS	40	m	2	0.96	26.74	4.90	0.0000
TIZZAN	4	m	0	0.41	7.88	7.53	0.2502
*TULINI	25	m	3	2.32	9.27	8.10	0.0000
WYNDE7	3	m	0	0.62	5.62	3.30	0.1416
*XIANGZ	10	m	2	1.10	24.49	2.04	0.0000

Table 1E9 - 2

IESLC - Meta-analysis of Current Smoking (or Ever if Current not available), Pipe only
All LC types
Most adjusted

	N	30
	NS	29
	Wt	321.87
Het	Chi	121.38
Het	df	29
Het	P	***
Fixed	RR	4.00
	RRl	3.59
	RRu	4.47
	P	+++
Random	RR	4.04
	RRl	3.13
	RRu	5.23
	P	+++
Asymm	P	N.S.

Table 1E9 - 3

IESLC - Meta-analysis of Current Smoking (or Ever if Current not available), Pipe only
All LC types
Most adjusted

		Sex			
		combined	male	female	Total
	N	3	26	1	30
	NS	3	26	1	30
	Wt	4.12	317.25	0.50	321.87
Het	Chi	1.52	119.42	0.00	121.38
Het	df	2	25	0	29
Het	P	N.S.	***	N.S.	***
Fixed	RR	4.79	3.99	8.83	4.00
	RRl	1.82	3.57	0.55	3.59
	RRu	12.58	4.45	142.12	4.47
	P	++	+++	N.S.	+++
Random	RR	4.79	3.97	8.83	4.04
	RRl	1.82	3.04	0.55	3.13
	RRu	12.58	5.19	142.12	5.23
	P	++	+++	N.S.	+++
Between	Chi				0.45
Between	df				2
Between	P				N.S.
Btwn(F)	P				N.S.
Btwn(R)	P				N.S.

		Smoking status		
		ever	current	Total
	N	18	12	30
	NS	17	12	29
	Wt	148.24	173.62	321.87
Het	Chi	32.70	59.74	121.38
Het	df	17	11	29
Het	P	*	***	***
Fixed	RR	2.89	5.28	4.00
	RRl	2.46	4.55	3.59
	RRu	3.40	6.13	4.47
	P	+++	+++	+++
Random	RR	3.10	5.20	4.04
	RRl	2.39	3.50	3.13
	RRu	4.02	7.73	5.23
	P	+++	+++	+++
Between	Chi			28.95
Between	df			1
Between	P			***
Btwn(F)	P			**
Btwn(R)	P			*

Table 1E9 - 4

IESLC - Meta-analysis of Current Smoking (or Ever if Current not available), Pipe only
All LC types
Least adjusted

REF	NRR	X	SEX	AGEL	AGEH	RACE	YF	LC TYPE	LOC	START	ST	NLC	R	VB	P	H	AD	SM	DENOM	De
ABELIN	7	x	m	0	0	all	-	all	Eu:wst	1941	CC	118	n	bl	y	n	0	ev	nev any	st
ARMADA	2		m	0	0	all	-	all	Eu:wst	1986	CC	325	n	bl	n	y	0	ev	nev any	st
BENSHL	2		m	0	0	all	0	all	Eu:UK	1967	pr	486	n	V	n	n	1	cu	nev any	ot
BEST	17		m	0	0	all	0	all	NAmer	1955	pr	381	n	V	n	n	1	ev	nev any	ot
BOFFET	24		m	0	0	all	-	all	Eu:mul	1988	CC	5621	n	bl	y	n	2	cu	nev any	or
BOUCOT	6	x	m	0	0	all	0	all	NAmer	1951	pr	121	n	bl	n	n	0	ev	nev any	ot
CEDERL	84		m	0	0	all	16	all	Eu:Sca	1963	pr	491	n	bl	n	n	2	cu	nev any	ot
COOKSO	3		c	0	0	bl	-	all	Africa	1961	CC	234	n	V	n	y	0	ev	nev any	st
CPSI	181		m	35	84	all	6	all	NAmer	1959	pr	5138	n	bl	n	n	1	ev	nev any	ot
DAMBER	18		m	0	0	all	-	all	Eu:Sca	1972	CC	579	n	bl	y	n	1	cu	nev any	ot
DEAN	5		m	0	0	wh	-	all	Africa	1947	CC	603	n	V	y	n	0	ev	nev any	st
DEAN3	177	x	m	0	0	all	-	all	Eu:UK	1969	CC	766	n	V	y	n	0	cu	nev any	st
DOLL	18		m	0	0	all	-	all	Eu:UK	1948	CC	1465	n	V	n	n	0	ev	nev any	st
DORN	23		m	0	0	wh	15	all	NAmer	1954	pr	5097	n	bl	n	n	2	cu	nev any	ot
GARDIN	3		c	0	0	all	-	all	Eu:UK	1988	CC	143	n	V	y	n	0	cu	nev any	st
HAMMON	124	x	m	0	0	wh	0	all	NAmer	1952	pr	448	n	bl	n	n	0	ev	nev any	st
HEIN	3		m	0	0	all	0	all	Eu:Sca	1970	pr	144	n	bl	n	n	0	cu	nev any	st
KOULUM	3		m	0	0	all	-	all	Eu:Sca	1936	CC	812	n	bl	n	n	0	ev	nev any	st
LANGE	9	x	m	0	0	all	0	all	Eu:Sca	1976	pr	268	n	bl	n	n	0	cu	nev any	st
LEVIN	28		m	0	0	all	-	all	NAmer	1938	CC	475	n	bl	n	n	1	ev	nev any	st
LUBIN2	5	x	m	0	0	all	-	all	Eu:mul	1976	CC	7804	n	bl	n	y	0	cu	nev any	st
MCCONN	17		c	0	0	all	-	all	Eu:UK	1946	CC	100	n	V	n	y	0	ev	nev any	st
PERNU	9		m	0	0	all	-	all	Eu:Sca	1944	CC	1606	n	bl	n	n	0	ev	nev any	st
PERNU	5		f	0	0	all	-	all	Eu:Sca	1944	CC	1606	n	bl	n	n	0	ev	nev any	st
SADOWS	3	x	m	0	0	wh	-	all	NAmer	1938	CC	477	n	bl	n	n	0	ev	nev any	st
STOCKS	24	x	m	0	0	all	-	all	Eu:UK	1952	CC	2932	n	V	y	n	0	ev	nev any	st
TIZZAN	4		m	0	0	all	-	all	Eu:wst	1959	CC	1358	n	bl	n	n	0	ev	nev any	st
TULINI	2	x	m	0	0	all	0	all	Eu:Sca	1967	pr	472	n	bl	n	n	1	cu	nev any	or
WYNDE7	3		m	0	0	all	-	all	NAmer	1977	CC	2085	n	bl	n	y	0	cu	nev any	st
XIANGZ	3	x	m	0	0	all	0	all	As:Chi	1976	pr	983	m	ot	n	n	0	ev	nev any	st

Table 1E9 - 5

IESLC - Meta-analysis of Current Smoking (or Ever if Current not available), Pipe only
All LC types
Least adjusted

REF	NRR	SEX	AD	Number Exposed Case	Number Exposed Cont	Non-exposed Case	Non-exposed Cont	RR	95.00%CI
ABELIN	7	m	0	8	34	2	183	21.53	(4.38- 105.80)
ARMADA	2	m	0	1	1	4	64	16.00	(0.84- 305.74)
*BENSHL	2	m	1	-	-	-	-	4.52	(2.22- 9.19)
*BEST	17	m	1	-	-	-	-	4.35	(1.82- 10.41)
BOFFET	24	m	2	-	-	-	-	12.50	(7.70- 20.20)
*BOUCOT	6	m	0	1	2389	0	7551	9.48~	(0.39- 232.65)
*CEDERL	84	m	2	-	-	-	-	7.20	(4.45- 11.66)
COOKSO	3	c	0	5	1	45	61	6.78	(0.77- 60.04)
*CPSI	181	m	1	-	-	-	-	2.54	(1.68- 3.83)
DAMBER	18	m	1	-	-	-	-	8.30	(5.30- 13.10)
DEAN	5	m	0	49	71	12	61	3.51	(1.71- 7.19)
DEAN3	177	m	0	23	116	24	510	4.21	(2.30- 7.73)
DOLL	18	m	0	53	93	7	61	4.97	(2.12- 11.64)
*DORN	23	m	2	-	-	-	-	2.14	(1.46- 3.13)
GARDIN	3	c	0	3	2	5	41	12.30	(1.64- 92.33)
*HAMMON	124	m	0	18	43041	15	115884	3.23	(1.63- 6.41)
*HEIN	3	m	0	10	499	1	457	9.16	(1.18- 71.26)
KOULUM	3	m	0	15	17	5	54	9.53	(3.02- 30.08)
*LANGE	9	m	0	16	433	5	721	5.33	(1.97- 14.44)
LEVIN	28	m	1	-	-	-	-	1.30	(0.74- 2.30)
LUBIN2	5	m	0	68	232	190	2617	4.04	(2.97- 5.49)
MCCONN	17	c	0	8	7	9	23	2.92	(0.82- 10.44)
PERNU	9	m	0	55	37	97	275	4.21	(2.62- 6.79)
PERNU	5	f	0	1	1	110	971	8.83	(0.55- 142.12)
Subtotal PERNU								4.30	(2.69- 6.89)
SADOWS	3	m	0	13	43	18	81	1.36	(0.61- 3.04)
STOCKS	24	m	0	211	994	45	638	3.01	(2.15- 4.21)
TIZZAN	4	m	0	16	18	180	305	1.51	(0.75- 3.03)
*TULINI	2	m	1	-	-	-	-	11.10	(5.86- 21.20)
WYNDE7	3	m	0	7	54	64	918	1.86	(0.81- 4.25)
*XIANGZ	3	m	0	356	1483	25	974	9.35	(6.29- 13.92)
Partial Totals				937	49566	863	132450		

*prospective study ~ With 0.5 adjustment for zero

REF	NRR	SEX	AD	Ys	Ws	Qs	Ps
ABELIN	7	m	0	3.07	1.52	4.06	0.0002
ARMADA	2	m	0	2.77	0.44	0.79	0.0655
*BENSHL	2	m	1	1.51	7.61	0.04	0.0000
*BEST	17	m	1	1.47	5.05	0.01	0.0010
BOFFET	24	m	2	2.53	16.52	19.73	0.0000
*BOUCOT	6	m	0	2.25	0.38	0.25	0.1683
*CEDERL	84	m	2	1.97	16.56	4.85	0.0000
COOKSO	3	c	0	1.91	0.81	0.19	0.0855
*CPSI	181	m	1	0.93	22.63	5.67	0.0000
DAMBER	18	m	1	2.12	18.77	8.76	0.0000
DEAN	5	m	0	1.26	7.45	0.24	0.0006
DEAN3	177	m	0	1.44	10.45	0.00	0.0000
DOLL	18	m	0	1.60	5.29	0.15	0.0002
*DORN	23	m	2	0.76	26.42	11.93	0.0001
GARDIN	3	c	0	2.51	0.95	1.10	0.0147
*HAMMON	124	m	0	1.17	8.18	0.55	0.0008
*HEIN	3	m	0	2.21	0.91	0.56	0.0344
KOULUM	3	m	0	2.25	2.91	1.96	0.0001
*LANGE	9	m	0	1.67	3.86	0.22	0.0010
LEVIN	28	m	1	0.26	11.95	16.37	0.3645
LUBIN2	5	m	0	1.40	40.55	0.06	0.0000
MCCONN	17	c	0	1.07	2.37	0.31	0.0991
PERNU	9	m	0	1.44	16.90	0.00	0.0000
PERNU	5	f	0	2.18	0.50	0.28	0.1245
Subtotal PERNU				1.46	17.40	0.28	
SADOWS	3	m	0	0.31	5.95	7.53	0.4527
STOCKS	24	m	0	1.10	33.86	3.71	0.0000
TIZZAN	4	m	0	0.41	7.88	8.25	0.2502
*TULINI	2	m	1	2.41	9.29	8.82	0.0000
WYNDE7	3	m	0	0.62	5.62	3.71	0.1416
*XIANGZ	3	m	0	2.24	24.33	15.68	0.0000

Table 1E9 - 5

IESLC - Meta-analysis of Current Smoking (or Ever if Current not available), Pipe only
All LC types
Least adjusted

	N	30
	NS	29
	Wt	315.89
Het	Chi	125.78
Het	df	29
Het	P	***
Fixed	RR	4.19
	RRl	3.75
	RRu	4.68
	P	+++
Random	RR	4.37
	RRl	3.37
	RRu	5.67
	P	+++
Asymm	P	N.S.

Table 1E9 - 6

IESLC - Meta-analysis of Current Smoking (or Ever if Current not available), Pipe only
All LC types
Least adjusted

		combined	Sex<br>male	female	Total
	N	3	26	1	30
	NS	3	26	1	30
	Wt	4.12	311.28	0.50	315.89
Het	Chi	1.52	123.91	0.00	125.78
Het	df	2	25	0	29
Het	P	N.S.	***	N.S.	***
Fixed	RR	4.79	4.18	8.83	4.19
	RRl	1.82	3.74	0.55	3.75
	RRu	12.58	4.67	142.12	4.68
	P	++	+++	N.S.	+++
Random	RR	4.79	4.31	8.83	4.37
	RRl	1.82	3.29	0.55	3.37
	RRu	12.58	5.66	142.12	5.67
	P	++	+++	N.S.	+++
Between	Chi				0.35
Between	df				2
Between	P				N.S.
Btwn(F)	P				N.S.
Btwn(R)	P				N.S.

Table 1E9 - 7

IESLC - Meta-analysis of Current Smoking (or Ever if Current not available), Pipe only
All LC types
Excluded studies (and stage at which they were excluded)

1 BOUCHA BUELL LAURIL MZILEN

2 ABRAHA AGUDO AKIBA ALDERS AMANDU AMES ANDERS ARCHER AUSTIN AUVINE AXELSO AXELSS BAND BARBON BECHER BERRIN
BLOHMK BLOT1 BLOT2 BLOT3 BLOT4 BRESLO BRETT BROCKM BROSS BROWN1 BROWN2 BUFFLE BYERS1 BYERS2 CARPEN CASCO2
CASCOR CHAN CHANG CHATZI CHEN CHEN2 CHEN3 CHIAZZ CHOI CHOW CHYOU COMSTO CORREA CPSII DARBY DAVEYS
DEAN2 DEKLER DESTE2 DESTEF DOCKER DORANT DORGAN DOSEME DROSTE DU DUNN EBELIN ENGELA ENSTRO ESAKI FAN
GAO GAO2 GARCIA GARSHI GENG GER GILLIS GODLEY GOLLED GOODMA GRAHAM GREGOR GSELL HAENSZ HAMMO2 HANSEN
HEGMAN HENNEK HINDS HIRAOK HIRAYA HITOSU HOLE HOROWI HORWIT HU HU2 HUANG HUMBLE ISHIMA JAHN JAIN
JARUP JARVHO JEDRYC JIANG JOLY JONES JUSSAW KAISE2 KAISER KANELL KATSOU KAUFMA KELLER KHUDER KIHARA KINLEN
KJUUS KNEKT KO KOHLME KOO KREUZE KREYBE KUBIK LAMTH LAMWK LAMWK2 LAUSSM LEI LEMARC LETOUR LIAW
LICKIN LIDDEL LIU LIU2 LIU3 LIU4 LIU5 LOMBA2 LOMBAR LUO MACLEN MAGNUS MARSH MARSH2 MARTIS MASTRA
MATOS MATSUD MCDUFF MCLAUG MIGRAN MILLER MILLS MOLLO MRFIT MRFITR MURATA NAM NOTAN2 NOTANI NOU ODRISC
ORMOS OSANN OSANN2 PARKIN PASTOR PAWLEG PERSH2 PERSHA PETO PEZZO2 PEZZOT PIKE PISANI POFFIJ POLEDN PRESCO
QIAO2 RACHTA RADZIK RANDIG REN RESTRE RIMING RONCO ROOTS ROTHSC SAARIK SANKAR SCHWAR SEGI SEGI2 SEOW
SHAW SHIMIZ SIEMIA SIMARA SIMONA SITAS SOBUE SOBUE2 SPEIZE SPITZ STASZE STAYNE STOCKW STUCKE SUN SUZUK2
SUZUKI SVENSS TANG TANG2 TAO TENKAN TOKARS TOUSEY TSUGAN ULMER VANDER VEIERO VUTUC WAKAI WALD WANG
WANG2 WANG3 WANG4 WARSIN WATSON WICKLU WIGLE WILKIN WU WU2 WUNSCH WUWILL WYNDE2 WYNDE3 WYNDE4 WYNDE5
WYNDE6 WYNDE8 WYNDER XU XU2 XU3 XU4 YAMAGU YONG YUAN ZHANG ZHENG ZHOU

3 DOLL2

5 HIRAY2 SCHWA2 TVERDA

9 BENHAM LUBIN QIAO

Table 1E9 - 8

Potentially overlapping studies

REF	REFGP	PRINC	.	OVERLAP/LINK
LUBIN2	LUBIN2	1		Lubin-combined
HEIN	PRESCO	1		Subset of PRESCO
LANGE	PRESCO	1		Subset of PRESCO
BENSHL	TANG2	1		Subset of TANG2
WYNDE7	WYNDE6	2		WYNDE5/6/7/8
CPSI	CPSI	1		CPSI overall
BOFFET	BOFFET	1		BOFFET-combined
XIANGZ	XIANGZ	1		LUBIN/XIANGZ/QIAO

Table 1E9 - 9

Most adjusted - insufficient data for metaanalysis

REF	NRR	SEX	AGEL	AGEH	RACE	YF	LC TYPE	LOC	START	ST	NLC	R	VB	P	H	AD	SM	DENOM	De
DOLL2	49	m	35	99	all	5	all	Eu:UK	1951	pr	920	n	V	n	n	1	ev	nev any	st

REF	NRR	RR	SIG	RRDATA comment
DOLL2	49	5.43		0

Table 1E10 -

IESLC - Meta-analysis of Ex Smoking, Pipe only
All LC types

This analysis is restricted to results for:
1) Non-dose-response data
2) Smokers of pipe only
3) Ex smokers
4) Results complete enough for use in metaanalysis

Within each study, results are then selected (in the following order of preference, within each sex) for:
5) DENOM: never smoked anything, (never +1 = +long term ex)
6) Followup period (prospective studies): whole study (coded as 0) or longest available
7) LCtype: all or nearest available, at least Squamous and Adeno. (q = squamous, s = small,
l = large, a = adeno, mix = mixed, alv = alveolar)
8) Race: all or nearest available, otherwise by race (wh or w = white, bl or b = black, hi = hispanic
ch = chinese, jap = japanese, haw = hawaiian, w+o = white + oriental, sca = scandinavian, as = asian)
9) For overlapping studies: principal rather than subsidiary studies
Finally by Age: whole study (coded as 0) if available, otherwise by widest available age group
and then for single sex results (m, f) in preference to combined sex results (c).

Results adjusted (AD) for the most potential confounders are then chosen in Sections -1 to -3
and results adjusted for the least confounders in Sections -4 to -6. (Those least adjusted results which
actually differ from the most adjusted as marked 'x' in column X in Section -4)
(Results adjusted for an unknown number of confounder(s) are coded as 20.)

Section -7 shows excluded studies, together with the stage (as above) at which no qualifying
results were found.

Section -8 lists the potentially overlapping studies which have been included (1=principal, 2=subsidiary).

Section -9 lists any results which would have been included in preference except that they had data not complete
enough for use in meta-analysis, with their significance (yes/no), if known, and any further comment as entered
on the database.

In addition to those mentioned above, the following fields, levels and abbreviations are used:
* or nk = not known, n = no, y = yes, ot = other
nev = never
REF: 6-character study reference
NRR: number of the RR on the database within the study
ST : study type (CC = case control, pr or prosp = prospective)
NLC: number of lung cancer cases in whole study
R : risky occupational population (n = no, m = mining, o = other risky)
VB : national cigarette type (V = at least 75% Virginia, bl = at least 75% blended, ot = other)
P : any proxy use
H : full histological confirmation
De : derivation of RR/CI (or = original, st = standard method, ot = other method of estimation)

Table 1E10 - 1

IESLC - Meta-analysis of Ex Smoking, Pipe only
All LC types
Most adjusted

REF	NRR	SEX	AGEL	AGEH	RACE	YF	LC TYPE	LOC	START	ST	NLC	R	VB	P	H	AD	DENOM	De
BOFFET	34	m	0	0	all	-	all	Eu:mul	1988	CC	5621	n	bl	y	n	2	nev any	ot
DAMBER	19	m	0	0	all	-	all	Eu:Sca	1972	CC	579	n	bl	y	n	1	nev any	ot
DORN	140	m	35	84	wh	8	all	NAmer	1954	pr	5097	n	bl	n	n	1	nev any	ot
LUBIN2	14	m	0	0	all	-	all	Eu:mul	1976	CC	7804	n	bl	n	y	2	nev any	or
WYNDE7	8	m	0	0	all	-	all	NAmer	1977	CC	2085	n	bl	n	y	0	nev any	st

Table 1E10 - 2

IESLC - Meta-analysis of Ex Smoking, Pipe only
All LC types
Most adjusted

REF	NRR	SEX	AD	Number Exposed Case	Number Exposed Cont	Non-exposed Case	Non-exposed Cont	RR	95.00%CI
BOFFET	34	m	2	-	-	-	-	4.32	(2.48- 7.53)
DAMBER	19	m	1	-	-	-	-	4.78	(2.82- 8.10)
*DORN	140	m	1	-	-	-	-	2.26	(1.04- 4.91)
LUBIN2	14	m	2	-	-	-	-	1.54	(0.60- 4.50)
WYNDE7	8	m	0	2	43	64	918	0.67	(0.16- 2.82)
Partial Totals				2	43	64	918		

*prospective study

REF	NRR	SEX	AD	Ys	Ws	Qs	Ps
BOFFET	34	m	2	1.46	12.46	0.87	0.0000
DAMBER	19	m	1	1.56	13.80	1.84	0.0000
*DORN	140	m	1	0.82	6.38	0.94	0.0395
LUBIN2	14	m	2	0.43	3.78	2.23	0.4009
WYNDE7	8	m	0	-0.40	1.85	4.77	0.5818

	N	5
	NS	5
	Wt	38.27
Het	Chi	10.64
Het	df	4
Het	P	*
Fixed	RR	3.32
	RRl	2.42
	RRu	4.55
	P	+++
Random	RR	2.69
	RRl	1.53
	RRu	4.72
	P	+++
Asymm	P	**

Table 1E10 - 3

IESLC - Meta-analysis of Ex Smoking, Pipe only
All LC types
Most adjusted

	Sex combined	male	female	Total
N		5		5
NS		5		5
Wt		38.27		38.27
Het Chi		10.64		10.64
Het df		4		4
Het P		*		*
Fixed RR		3.32		3.32
RRl		2.42		2.42
RRu		4.55		4.55
P		+++		+++
Random RR		2.69		2.69
RRl		1.53		1.53
RRu		4.72		4.72
P		+++		+++
Between Chi				
Between df				
Between P				N.S.
Btwn(F) P				N.S.
Btwn(R) P				N.S.

Too few RRs for analysis by factor

Table 1E10 - 4

IESLC - Meta-analysis of Ex Smoking, Pipe only
All LC types
Least adjusted

REF	NRR	X	SEX	AGEL	AGEH	RACE	YF	LC TYPE	LOC	START	ST	NLC	R	VB	P	H	AD	DENOM	De
BOFFET	34		m	0	0	all	-	all	Eu:mul	1988	CC	5621	n	bl	y	n	2	nev any	ot
DAMBER	19		m	0	0	all	-	all	Eu:Sca	1972	CC	579	n	bl	y	n	1	nev any	ot
DORN	140		m	35	84	wh	8	all	NAmer	1954	pr	5097	n	bl	n	n	1	nev any	ot
LUBIN2	13	x	m	0	0	all	-	all	Eu:mul	1976	CC	7804	n	bl	n	y	0	nev any	st
WYNDE7	8		m	0	0	all	-	all	NAmer	1977	CC	2085	n	bl	n	y	0	nev any	st

Table 1E10 - 5

IESLC - Meta-analysis of Ex Smoking, Pipe only
All LC types
Least adjusted

REF	NRR	SEX	AD	Number Exposed Case	Number Exposed Cont	Non-exposed Case	Non-exposed Cont	RR	95.00%CI
BOFFET	34	m	2	-	-	-	-	4.32	(2.48- 7.53)
DAMBER	19	m	1	-	-	-	-	4.78	(2.82- 8.10)
*DORN	140	m	1	-	-	-	-	2.26	(1.04- 4.91)
LUBIN2	13	m	0	4	32	190	2617	1.72	(0.60- 4.92)
WYNDE7	8	m	0	2	43	64	918	0.67	(0.16- 2.82)
Partial Totals				6	75	254	3535		

*prospective study

REF	NRR	SEX	AD	Ys	Ws	Qs	Ps
BOFFET	34	m	2	1.46	12.46	0.76	0.0000
DAMBER	19	m	1	1.56	13.80	1.68	0.0000
*DORN	140	m	1	0.82	6.38	1.02	0.0395
LUBIN2	13	m	0	0.54	3.49	1.58	0.3104
WYNDE7	8	m	0	-0.40	1.85	4.86	0.5818

	N	5
	NS	5
	Wt	37.98
Het	Chi	9.90
Het	df	4
Het	P	*
Fixed	RR	3.37
	RRl	2.45
	RRu	4.64
	P	+++
Random	RR	2.77
	RRl	1.61
	RRu	4.79
	P	+++
Asymm	P	**

Table 1E10 - 6

IESLC - Meta-analysis of Ex Smoking, Pipe only
All LC types
Least adjusted

	Sex combined	male	female	Total
N		5		5
NS		5		5
Wt		37.98		37.98
Het Chi		9.90		9.90
Het df		4		4
Het P		*		*
Fixed RR		3.37		3.37
RRl		2.45		2.45
RRu		4.64		4.64
P		+++		+++
Random RR		2.77		2.77
RRl		1.61		1.61
RRu		4.79		4.79
P		+++		+++
Between Chi				
Between df				
Between P				N.S.
Btwn(F) P				N.S.
Btwn(R) P				N.S.

Table 1E10 - 7

IESLC - Meta-analysis of Ex Smoking, Pipe only
All LC types
Excluded studies (and stage at which they were excluded)

```
1      BOUCHA  BUELL LAURIL MZILEN

2      ABRAHA  AGUDO  AKIBA ALDERS AMANDU   AMES ANDERS ARCHER AUSTIN AUVINE AXELSO AXELSS   BAND BARBON BECHER BERRIN
       BLOHMK  BLOT1  BLOT2  BLOT3  BLOT4 BRESLO  BRETT BROCKM  BROSS BROWN1 BROWN2 BUFFLE BYERS1 BYERS2 CARPEN CASCO2
       CASCOR   CHAN  CHANG CHATZI   CHEN  CHEN2  CHEN3 CHIAZZ   CHOI   CHOW  CHYOU COMSTO CORREA  CPSII  DARBY DAVEYS
        DEAN2 DEKLER DESTE2 DESTEF DOCKER DORANT DORGAN DOSEME DROSTE     DU   DUNN EBELIN ENGELA ENSTRO  ESAKI    FAN
          GAO   GAO2 GARCIA GARSHI   GENG    GER GILLIS GODLEY GOLLED GOODMA GRAHAM GREGOR  GSELL HAENSZ HAMMO2 HANSEN
       HEGMAN HENNEK  HINDS HIRAOK HIRAYA HITOSU   HOLE HOROWI HORWIT     HU    HU2  HUANG HUMBLE ISHIMA   JAHN   JAIN
        JARUP JARVHO JEDRYC  JIANG   JOLY  JONES JUSSAW KAISE2 KAISER KANELL KATSOU KAUFMA KELLER KHUDER KIHARA KINLEN
        KJUUS  KNEKT     KO KOHLME    KOO KREUZE KREYBE  KUBIK  LAMTH  LAMWK LAMWK2 LAUSSM    LEI LEMARC LETOUR   LIAW
       LICKIN LIDDEL    LIU   LIU2   LIU3   LIU4   LIU5 LOMBA2 LOMBAR    LUO MACLEN MAGNUS  MARSH MARSH2 MARTIS MASTRA
        MATOS MATSUD MCDUFF MCLAUG MIGRAN MILLER  MILLS  MOLLO  MRFIT MRFITR MURATA    NAM NOTAN2 NOTANI    NOU ODRISC
        ORMOS  OSANN OSANN2 PARKIN PASTOR PAWLEG PERSH2 PERSHA   PETO PEZZO2 PEZZOT   PIKE PISANI POFFIJ POLEDN PRESCO
        QIAO2 RACHTA RADZIK RANDIG    REN RESTRE RIMING  RONCO  ROOTS ROTHSC SAARIK SANKAR SCHWAR   SEGI  SEGI2   SEOW
         SHAW SHIMIZ SIEMIA SIMARA SIMONA  SITAS  SOBUE SOBUE2 SPEIZE  SPITZ STASZE STAYNE STOCKW STUCKE    SUN SUZUK2
       SUZUKI SVENSS   TANG  TANG2    TAO TENKAN TOKARS TOUSEY TSUGAN  ULMER VANDER VEIERO  VUTUC  WAKAI   WALD   WANG
        WANG2  WANG3  WANG4 WARSIN WATSON WICKLU  WIGLE WILKIN     WU    WU2 WUNSCH WUWILL WYNDE2 WYNDE3 WYNDE4 WYNDE5
       WYNDE6 WYNDE8 WYNDER     XU    XU2    XU3    XU4 YAMAGU   YONG   YUAN  ZHANG  ZHENG   ZHOU

3      ABELIN ARMADA BENHAM BENSHL   BEST BOUCOT CEDERL COOKSO   CPSI   DEAN  DEAN3   DOLL  DOLL2 GARDIN HAMMON   HEIN
       HIRAY2 KOULUM  LANGE  LEVIN  LUBIN MCCONN  PERNU   QIAO SADOWS SCHWA2 STOCKS TIZZAN TULINI TVERDA XIANGZ
```

Table 1E10 - 8

Potentially overlapping studies

REF	REFGP	PRINC	. OVERLAP/LINK
LUBIN2	LUBIN2	1	Lubin-combined
WYNDE7	WYNDE6	2	WYNDE5/6/7/8
BOFFET	BOFFET	1	BOFFET-combined

Table 1E11 -

IESLC - Meta-analysis of Ever Smoking, Cigars only
All LC types

This analysis is restricted to results for:
1) Non-dose-response data
2) Smokers of cigars only
3) Ever smokers
4) Results complete enough for use in metaanalysis

Within each study, results are then selected (in the following order of preference, within each sex) for:
5) DENOM: never smoked anything, (never +1 = +long term ex)
6) Followup period (prospective studies): whole study (coded as 0) or longest available
7) LCtype: all or nearest available, at least Squamous and Adeno. (q = squamous, s = small,
l = large, a = adeno, mix = mixed, alv = alveolar)
8) Race: all or nearest available, otherwise by race (wh or w = white, bl or b = black, hi = hispanic
ch = chinese, jap = japanese, haw = hawaiian, w+o = white + oriental, sca = scandinavian, as = asian)
9) For overlapping studies: principal rather than subsidiary studies
Finally by Age: whole study (coded as 0) if available, otherwise by widest available age group
and then for single sex results (m, f) in preference to combined sex results (c).

Results adjusted (AD) for the most potential confounders are then chosen in Sections -1 to -3
and results adjusted for the least confounders in Sections -4 to -6. (Those least adjusted results which
actually differ from the most adjusted as marked 'x' in column X in Section -4)
(Results adjusted for an unknown number of confounder(s) are coded as 20.)

Section -7 shows excluded studies, together with the stage (as above) at which no qualifying
results were found.

Section -8 lists the potentially overlapping studies which have been included (1=principal, 2=subsidiary).

Section -9 lists any results which would have been included in preference except that they had data not complete
enough for use in meta-analysis, with their significance (yes/no), if known, and any further comment as entered
on the database.

In addition to those mentioned above, the following fields, levels and abbreviations are used:
* or nk = not known, n = no, y = yes, ot = other
nev = never
REF: 6-character study reference
NRR: number of the RR on the database within the study
ST : study type (CC = case control, pr or prosp = prospective)
NLC: number of lung cancer cases in whole study
R : risky occupational population (n = no, m = mining, o = other risky)
VB : national cigarette type (V = at least 75% Virginia, bl = at least 75% blended, ot = other)
P : any proxy use
H : full histological confirmation
De : derivation of RR/CI (or = original, st = standard method, ot = other method of estimation)

Table 1E11 - 1

IESLC - Meta-analysis of Ever Smoking, Cigars only
All LC types
Most adjusted

REF	NRR	SEX	AGEL	AGEH	RACE	YF	LC TYPE	LOC	START	ST	NLC	R	VB	P	H	AD	DENOM	De
ABELIN	51	m	0	0	all	-	all	Eu:wst	1941	CC	118	n	bl	y	n	1	nev any	st
ARMADA	3	m	0	0	all	-	all	Eu:wst	1986	CC	325	n	bl	n	y	0	nev any	st
BEST	16	m	0	0	all	0	all	NAmer	1955	pr	381	n	V	n	n	1	nev any	ot
BOFFET	1	m	0	0	all	-	all	Eu:mul	1988	CC	5621	n	bl	y	n	2	nev any	or
BOUCOT	119	m	0	0	all	0	all	NAmer	1951	pr	121	n	bl	n	n	2	nev any	ot
CPSI	183	m	35	84	all	6	all	NAmer	1959	pr	5138	n	bl	n	n	1	nev any	ot
DAMBER	4	m	0	0	all	-	all	Eu:Sca	1972	CC	579	n	bl	y	n	0	nev any	st
DORN	32	m	0	0	wh	2	all	NAmer	1954	pr	5097	n	bl	n	n	1	nev any	ot
HAMMON	111	m	0	0	wh	0	all	NAmer	1952	pr	448	n	bl	n	n	1	nev any	ot
JOLY	13	m	0	0	all	-	all	SCAmer	1978	CC	826	n	bl	n	n	0	nev any	st
LEVIN	29	m	0	0	all	-	all	NAmer	1938	CC	475	n	bl	n	n	1	nev any	st
LUBIN2	20	m	0	0	all	-	all	Eu:mul	1976	CC	7804	n	bl	n	y	2	nev any	ot
SADOWS	29	m	0	0	wh	-	all	NAmer	1938	CC	477	n	bl	n	n	1	nev any	ot
TIZZAN	3	m	0	0	all	-	all	Eu:wst	1959	CC	1358	n	bl	n	n	0	nev any	st
WYNDE7	40	m	0	0	all	-	all	NAmer	1977	CC	2085	n	bl	n	y	0	nev any	st

Table 1E11 - 2

IESLC - Meta-analysis of Ever Smoking, Cigars only
All LC types
Most adjusted

REF	NRR	SEX	AD	Number Exposed Case	Number Exposed Cont	Non-exposed Case	Non-exposed Cont	RR	95.00%CI
ABELIN	51	m	1	-	-	-	-	25.09	(5.77- 109.07)
ARMADA	3	m	0	3	6	4	64	8.00	(1.44- 44.45)
*BEST	16	m	1	-	-	-	-	2.94	(0.61- 14.15)
BOFFET	1	m	2	-	-	-	-	9.00	(5.80- 14.10)
*BOUCOT	119	m	2	-	-	-	-	8.81	(0.45- 170.58)
*CPSI	183	m	1	-	-	-	-	2.11	(1.45- 3.07)
DAMBER	4	m	0	7	7	42	208	4.95	(1.65- 14.86)
*DORN	32	m	1	-	-	-	-	1.50	(0.59- 3.80)
*HAMMON	111	m	1	-	-	-	-	1.02	(0.42- 2.51)
JOLY	13	m	0	43	179	12	218	4.36	(2.23- 8.53)
LEVIN	29	m	1	-	-	-	-	1.41	(0.76- 2.60)
LUBIN2	20	m	2	-	-	-	-	3.26	(2.21- 4.79)
SADOWS	29	m	1	-	-	-	-	2.98	(1.06- 8.33)
TIZZAN	3	m	0	26	57	180	305	0.77	(0.47- 1.27)
WYNDE7	40	m	0	30	152	64	918	2.83	(1.78- 4.51)
Partial Totals				109	401	302	1713		

*prospective study

REF	NRR	SEX	AD	Ys	Ws	Qs	Ps
ABELIN	51	m	1	3.22	1.78	8.75	0.0000
ARMADA	3	m	0	2.08	1.31	1.51	0.0175
*BEST	16	m	1	1.08	1.55	0.01	0.1788
BOFFET	1	m	2	2.20	19.47	27.70	0.0000
*BOUCOT	119	m	2	2.18	0.44	0.60	0.1509
*CPSI	183	m	1	0.75	27.31	1.82	0.0001
DAMBER	4	m	0	1.60	3.18	1.13	0.0043
*DORN	32	m	1	0.41	4.43	1.59	0.3935
*HAMMON	111	m	1	0.02	4.81	4.66	0.9654
JOLY	13	m	0	1.47	8.56	1.88	0.0000
LEVIN	29	m	1	0.34	10.16	4.44	0.2735
LUBIN2	20	m	2	1.18	25.68	0.81	0.0000
SADOWS	29	m	1	1.09	3.62	0.03	0.0379
TIZZAN	3	m	0	-0.26	15.42	24.57	0.3117
WYNDE7	40	m	0	1.04	17.66	0.02	0.0000

	N	15
	NS	15
	Wt	145.37
Het	Chi	79.50
Het	df	14
Het	P	***
Fixed	RR	2.73
	RRl	2.32
	RRu	3.21
	P	+++
Random	RR	2.95
	RRl	1.91
	RRu	4.56
	P	+++
Asymm	P	N.S.

Table 1E11 - 3

IESLC - Meta-analysis of Ever Smoking, Cigars only
All LC types
Most adjusted

	Sex			
	combined	male	female	Total
N		15		15
NS		15		15
Wt		145.37		145.37
Het Chi		79.50		79.50
Het df		14		14
Het P		***		***
Fixed RR		2.73		2.73
RRl		2.32		2.32
RRu		3.21		3.21
P		+++		+++
Random RR		2.95		2.95
RRl		1.91		1.91
RRu		4.56		4.56
P		+++		+++
Between Chi				
Between df				
Between P				N.S.
Btwn(F) P				N.S.
Btwn(R) P				N.S.

	All LC (or nearest)		
	all	other	Total
N	15		15
NS	15		15
Wt	145.37		145.37
Het Chi	79.50		79.50
Het df	14		14
Het P	***		***
Fixed RR	2.73		2.73
RRl	2.32		2.32
RRu	3.21		3.21
P	+++		+++
Random RR	2.95		2.95
RRl	1.91		1.91
RRu	4.56		4.56
P	+++		+++
Between Chi			
Between df			
Between P			N.S.
Btwn(F) P			N.S.
Btwn(R) P			N.S.

	Location								
	NAmer	UK	Scand	othEur	China	Japan	othAs	other	Total
N	8		1	5				1	15
NS	8		1	5				1	15
Wt	69.97		3.18	63.66				8.56	145.37
Het Chi	7.69		0.00	60.40				0.00	79.50
Het df	7		0	4				0	14
Het P	N.S.		N.S.	***				N.S.	***
Fixed RR	2.06		4.95	3.38				4.36	2.73
RRl	1.63		1.65	2.65				2.23	2.32
RRu	2.61		14.86	4.33				8.53	3.21
P	+++		++	+++				+++	+++
Random RR	2.05		4.95	4.76				4.36	2.95
RRl	1.58		1.65	1.60				2.23	1.91
RRu	2.65		14.86	14.12				8.53	4.56
P	+++		++	++				+++	+++
Between Chi									11.41
Between df									3
Between P									**
Btwn(F) P									N.S.
Btwn(R) P									(*)

Table 1E11 - 3

IESLC - Meta-analysis of Ever Smoking, Cigars only
All LC types
Most adjusted

Detailed Country in "other Europe"

	multi	Germany	othWest	East	Balkans	Total
N	2		3			5
NS	2		3			5
Wt	45.15		18.51			63.66
Het Chi	11.42		24.06			60.40
Het df	1		2			4
Het P	***		***			***
Fixed RR	5.05		1.27			3.38
RRl	3.77		0.81			2.65
RRu	6.76		2.01			4.33
P	+++		N.S.			+++
Random RR	5.38		4.96			4.76
RRl	1.99		0.45			1.60
RRu	14.56		55.17			14.12
P	+++		N.S.			++
Between Chi						24.92
Between df						1
Between P						***
Btwn(F) P						N.S.
Btwn(R) P						N.S.

Detailed Country in "other Asia"

	India	HongKong	other	Total
N				
NS				
Wt				
Het Chi				
Het df				
Het P				
Fixed RR				
RRl				
RRu				
P				
Random RR				
RRl				
RRu				
P				
Between Chi				
Between df				
Between P				N.S.
Btwn(F) P				N.S.
Btwn(R) P				N.S.

Detailed other continent

	SCAmer	Auslia	Africa	Total
N	1			1
NS	1			1
Wt	8.56			8.56
Het Chi	0.00			0.00
Het df	0			0
Het P	N.S.			N.S.
Fixed RR	4.36			4.36
RRl	2.23			2.23
RRu	8.53			8.53
P	+++			+++
Random RR	4.36			4.36
RRl	2.23			2.23
RRu	8.53			8.53
P	+++			+++
Between Chi				
Between df				
Between P				N.S.
Btwn(F) P				N.S.
Btwn(R) P				N.S.

Table 1E11 - 3

IESLC - Meta-analysis of Ever Smoking, Cigars only
All LC types
Most adjusted

	Start year of study					
	<1960	1960-69	1970-79	1980-89	1990+	Total
N	9		4	2		15
NS	9		4	2		15
Wt	69.51		55.09	20.78		145.37
Het Chi	28.10		1.60	0.02		79.50
Het df	8		3	1		14
Het P	***		N.S.	N.S.		***
Fixed RR	1.63		3.34	8.93		2.73
RRl	1.29		2.56	5.81		2.32
RRu	2.07		4.35	13.73		3.21
P	+++		+++	+++		+++
Random RR	1.98		3.34	8.93		2.95
RRl	1.17		2.56	5.81		1.91
RRu	3.34		4.35	13.73		4.56
P	+		+++	+++		+++
Between Chi						49.79
Between df						2
Between P						***
Btwn(F) P						**
Btwn(R) P						***

	Study type (1)		
	CC	other	Total
N	10	5	15
NS	10	5	15
Wt	106.84	38.54	145.37
Het Chi	69.05	3.73	79.50
Het df	9	4	14
Het P	***	N.S.	***
Fixed RR	3.11	1.91	2.73
RRl	2.57	1.39	2.32
RRu	3.76	2.62	3.21
P	+++	+++	+++
Random RR	3.59	1.91	2.95
RRl	2.03	1.39	1.91
RRu	6.37	2.62	4.56
P	+++	+++	+++
Between Chi			6.73
Between df			1
Between P			**
Btwn(F) P			N.S.
Btwn(R) P			(*)

	Study type (2)			
	CC	prosp	other	Total
N	10	5		15
NS	10	5		15
Wt	106.84	38.54		145.37
Het Chi	69.05	3.73		79.50
Het df	9	4		14
Het P	***	N.S.		***
Fixed RR	3.11	1.91		2.73
RRl	2.57	1.39		2.32
RRu	3.76	2.62		3.21
P	+++	+++		+++
Random RR	3.59	1.91		2.95
RRl	2.03	1.39		1.91
RRu	6.37	2.62		4.56
P	+++	+++		+++
Between Chi				6.73
Between df				1
Between P				**
Btwn(F) P				N.S.
Btwn(R) P				(*)

Table 1E11 - 3

IESLC - Meta-analysis of Ever Smoking, Cigars only
All LC types
Most adjusted

	Study size (number of LC cases) 100-249	250-499	500-999	1000+	Total
N	2	5	2	6	15
NS	2	5	2	6	15
Wt	2.21	21.44	11.75	109.97	145.37
Het Chi	0.38	6.33	0.04	56.50	79.50
Het df	1	4	1	5	14
Het P	N.S.	N.S.	N.S.	***	***
Fixed RR	20.42	1.74	4.52	2.71	2.73
RRl	5.47	1.14	2.55	2.25	2.32
RRu	76.22	2.66	8.00	3.27	3.21
P	+++	+	+++	+++	+++
Random RR	20.42	1.95	4.52	2.48	2.95
RRl	5.47	1.09	2.55	1.30	1.91
RRu	76.22	3.49	8.00	4.74	4.56
P	+++	+	+++	++	+++
Between Chi					16.25
Between df					3
Between P					**
Btwn(F) P					N.S.
Btwn(R) P					**

	Risky occupational population no	mining	othRisky	Total
N	15			15
NS	15			15
Wt	145.37			145.37
Het Chi	79.50			79.50
Het df	14			14
Het P	***			***
Fixed RR	2.73			2.73
RRl	2.32			2.32
RRu	3.21			3.21
P	+++			+++
Random RR	2.95			2.95
RRl	1.91			1.91
RRu	4.56			4.56
P	+++			+++
Between Chi				
Between df				
Between P				N.S.
Btwn(F) P				N.S.
Btwn(R) P				N.S.

	National cigarette tobacco type Virginia	blended	other	Total
N	1	14		15
NS	1	14		15
Wt	1.55	143.82		145.37
Het Chi	0.00	79.50		79.50
Het df	0	13		14
Het P	N.S.	***		***
Fixed RR	2.94	2.73		2.73
RRl	0.61	2.32		2.32
RRu	14.16	3.21		3.21
P	N.S.	+++		+++
Random RR	2.94	2.95		2.95
RRl	0.61	1.88		1.91
RRu	14.16	4.63		4.56
P	N.S.	+++		+++
Between Chi				0.01
Between df				1
Between P				N.S.
Btwn(F) P				N.S.
Btwn(R) P				N.S.

Table 1E11 - 3

IESLC - Meta-analysis of Ever Smoking, Cigars only
All LC types
Most adjusted

	Any proxy use No/nk	Yes	Total
N	12	3	15
NS	12	3	15
Wt	120.94	24.43	145.37
Het Chi	34.95	3.00	79.50
Het df	11	2	14
Het P	***	N.S.	***
Fixed RR	2.15	8.97	2.73
RRl	1.80	6.03	2.32
RRu	2.57	13.34	3.21
P	+++	+++	+++
Random RR	2.16	9.12	2.95
RRl	1.50	4.85	1.91
RRu	3.11	17.17	4.56
P	+++	+++	+++
Between Chi			41.55
Between df			1
Between P			***
Btwn(F) P			**
Btwn(R) P			***

	Full histological confirmation No	Yes	Total
N	12	3	15
NS	12	3	15
Wt	100.73	44.65	145.37
Het Chi	76.73	1.37	79.50
Het df	11	2	14
Het P	***	N.S.	***
Fixed RR	2.56	3.17	2.73
RRl	2.10	2.36	2.32
RRu	3.11	4.24	3.21
P	+++	+++	+++
Random RR	2.85	3.17	2.95
RRl	1.60	2.36	1.91
RRu	5.08	4.24	4.56
P	+++	+++	+++
Between Chi			1.40
Between df			1
Between P			N.S.
Btwn(F) P			N.S.
Btwn(R) P			N.S.

	Number of adjustment variables (1) 0	1	2+/+nk	Total
N	5	7	3	15
NS	5	7	3	15
Wt	46.13	53.65	45.59	145.37
Het Chi	26.24	16.05	11.55	79.50
Het df	4	6	2	14
Het P	***	*	**	***
Fixed RR	2.13	2.00	5.08	2.73
RRl	1.59	1.53	3.80	2.32
RRu	2.84	2.61	6.79	3.21
P	+++	+++	+++	+++
Random RR	2.87	2.21	5.59	2.95
RRl	1.26	1.31	2.26	1.91
RRu	6.58	3.73	13.84	4.56
P	+	++	+++	+++
Between Chi				25.66
Between df				2
Between P				***
Btwn(F) P				(*)
Btwn(R) P				N.S.

Table 1E11 - 3

IESLC - Meta-analysis of Ever Smoking, Cigars only
All LC types
Most adjusted

	Number of adjustment variables (2) 0	1	2	3-5	6+/+nk	Total
N	5	7	3			15
NS	5	7	3			15
Wt	46.13	53.65	45.59			145.37
Het Chi	26.24	16.05	11.55			79.50
Het df	4	6	2			14
Het P	***	*	**			***
Fixed RR	2.13	2.00	5.08			2.73
RRl	1.59	1.53	3.80			2.32
RRu	2.84	2.61	6.79			3.21
P	+++	+++	+++			+++
Random RR	2.87	2.21	5.59			2.95
RRl	1.26	1.31	2.26			1.91
RRu	6.58	3.73	13.84			4.56
P	+	++	+++			+++
Between Chi						25.66
Between df						2
Between P						***
Btwn(F) P						(*)
Btwn(R) P						N.S.

	Derivation of RR/CI Orig	StdCalc	Other	Total
N	1	7	7	15
NS	1	7	7	15
Wt	19.47	58.07	67.83	145.37
Het Chi	0.00	38.78	8.33	79.50
Het df	0	6	6	14
Het P	N.S.	***	N.S.	***
Fixed RR	9.00	2.14	2.39	2.73
RRl	5.77	1.65	1.89	2.32
RRu	14.03	2.76	3.04	3.21
P	+++	+++	+++	+++
Random RR	9.00	3.22	2.30	2.95
RRl	5.77	1.57	1.65	1.91
RRu	14.03	6.63	3.20	4.56
P	+++	++	+++	+++
Between Chi				32.39
Between df				2
Between P				***
Btwn(F) P				*
Btwn(R) P				***

Table 1E11 - 4

IESLC - Meta-analysis of Ever Smoking, Cigars only
All LC types
Least adjusted

REF	NRR	X	SEX	AGEL	AGEH	RACE	YF	LC TYPE	LOC	START	ST	NLC	R	VB	P	H	AD	DENOM	De
ABELIN	8	x	m	0	0	all	-	all	Eu:wst	1941	CC	118	n	bl	y	n	0	nev any	st
ARMADA	3		m	0	0	all	-	all	Eu:wst	1986	CC	325	n	bl	n	y	0	nev any	st
BEST	16		m	0	0	all	0	all	NAmer	1955	pr	381	n	V	n	n	1	nev any	ot
BOFFET	4	x	m	0	0	all	-	all	Eu:mul	1988	CC	5621	n	bl	y	n	0	nev any	st
BOUCOT	7	x	m	0	0	all	0	all	NAmer	1951	pr	121	n	bl	n	n	0	nev any	ot
CPSI	183		m	35	84	all	6	all	NAmer	1959	pr	5138	n	bl	n	n	1	nev any	ot
DAMBER	4		m	0	0	all	-	all	Eu:Sca	1972	CC	579	n	bl	y	n	0	nev any	st
DORN	41	x	m	0	0	wh	2	all	NAmer	1954	pr	5097	n	bl	n	n	0	nev any	st
HAMMON	123	x	m	0	0	wh	0	all	NAmer	1952	pr	448	n	bl	n	n	0	nev any	st
JOLY	13		m	0	0	all	-	all	SCAmer	1978	CC	826	n	bl	n	n	0	nev any	st
LEVIN	29		m	0	0	all	-	all	NAmer	1938	CC	475	n	bl	n	n	1	nev any	st
LUBIN2	19	x	m	0	0	all	-	all	Eu:mul	1976	CC	7804	n	bl	n	y	0	nev any	st
SADOWS	2	x	m	0	0	wh	-	all	NAmer	1938	CC	477	n	bl	n	n	0	nev any	st
TIZZAN	3		m	0	0	all	-	all	Eu:wst	1959	CC	1358	n	bl	n	n	0	nev any	st
WYNDE7	40		m	0	0	all	-	all	NAmer	1977	CC	2085	n	bl	n	y	0	nev any	st

Table 1E11 - 5

IESLC - Meta-analysis of Ever Smoking, Cigars only
All LC types
Least adjusted

REF	NRR	SEX	AD	Number Exposed Case	Number Exposed Cont	Non-exposed Case	Non-exposed Cont	RR	95.00%CI
ABELIN	8	m	0	33	99	2	183	30.50	(7.17- 129.78)
ARMADA	3	m	0	3	6	4	64	8.00	(1.44- 44.45)
*BEST	16	m	1	-	-	-	-	2.94	(0.61- 14.15)
BOFFET	4	m	0	43	77	117	1750	8.35	(5.50- 12.68)
*BOUCOT	7	m	0	3	5084	0	7551	10.40~	(0.54- 201.23)
*CPSI	183	m	1	-	-	-	-	2.11	(1.45- 3.07)
DAMBER	4	m	0	7	7	42	208	4.95	(1.65- 14.86)
*DORN	41	m	0	6	28422	17	117918	1.46	(0.58- 3.71)
*HAMMON	123	m	0	7	51480	15	115884	1.05	(0.43- 2.58)
JOLY	13	m	0	43	179	12	218	4.36	(2.23- 8.53)
LEVIN	29	m	1	-	-	-	-	1.41	(0.76- 2.60)
LUBIN2	19	m	0	37	144	190	2617	3.54	(2.40- 5.23)
SADOWS	2	m	0	11	21	18	81	2.36	(0.97- 5.74)
TIZZAN	3	m	0	26	57	180	305	0.77	(0.47- 1.27)
WYNDE7	40	m	0	30	152	64	918	2.83	(1.78- 4.51)
Partial Totals				249	85728	661	247697		

*prospective study ~ With 0.5 adjustment for zero

REF	NRR	SEX	AD	Ys	Ws	Qs	Ps
ABELIN	8	m	0	3.42	1.83	10.49	0.0000
ARMADA	3	m	0	2.08	1.31	1.45	0.0175
*BEST	16	m	1	1.08	1.55	0.00	0.1788
BOFFET	4	m	0	2.12	22.05	26.57	0.0000
*BOUCOT	7	m	0	2.34	0.44	0.76	0.1214
*CPSI	183	m	1	0.75	27.31	2.11	0.0001
DAMBER	4	m	0	1.60	3.18	1.05	0.0043
*DORN	41	m	0	0.38	4.44	1.84	0.4219
*HAMMON	123	m	0	0.05	4.77	4.54	0.9143
JOLY	13	m	0	1.47	8.56	1.72	0.0000
LEVIN	29	m	1	0.34	10.16	4.71	0.2735
LUBIN2	19	m	0	1.26	25.24	1.44	0.0000
SADOWS	2	m	0	0.86	4.84	0.14	0.0591
TIZZAN	3	m	0	-0.26	15.42	25.36	0.3117
WYNDE7	40	m	0	1.04	17.66	0.00	0.0000

	N	15
	NS	15
	Wt	148.76
Het	Chi	82.20
Het	df	14
Het	P	***
Fixed	RR	2.79
	RRl	2.37
	RRu	3.27
	P	+++
Random	RR	2.95
	RRl	1.91
	RRu	4.56
	P	+++
Asymm	P	N.S.

Table 1E11 - 6

IESLC - Meta-analysis of Ever Smoking, Cigars only
All LC types
Least adjusted

	Sex combined	male	female	Total
N		15		15
NS		15		15
Wt		148.76		148.76
Het Chi		82.20		82.20
Het df		14		14
Het P		***		***
Fixed RR		2.79		2.79
RRl		2.37		2.37
RRu		3.27		3.27
P		+++		+++
Random RR		2.95		2.95
RRl		1.91		1.91
RRu		4.56		4.56
P		+++		+++
Between Chi				
Between df				
Between P				N.S.
Btwn(F) P				N.S.
Btwn(R) P				N.S.

Table 1E11 - 7

IESLC - Meta-analysis of Ever Smoking, Cigars only
All LC types
Excluded studies (and stage at which they were excluded)

1	BOUCHA BUELL LAURIL MZILEN
2	ABRAHA AGUDO AKIBA ALDERS AMANDU AMES ANDERS ARCHER AUSTIN AUVINE AXELSO AXELSS BAND BARBON BECHER BERRIN BLOHMK BLOT1 BLOT2 BLOT3 BLOT4 BRESLO BRETT BROCKM BROSS BROWN1 BROWN2 BUFFLE BYERS1 BYERS2 CARPEN CASCO2 CASCOR CHAN CHANG CHATZI CHEN CHEN2 CHEN3 CHIAZZ CHOI CHOW CHYOU COMSTO COOKSO CORREA DARBY DAVEYS DEAN DEAN2 DEKLER DESTE2 DESTEF DOCKER DOLL DOLL2 DORANT DORGAN DOSEME DROSTE DU DUNN EBELIN ENGELA ENSTRO ESAKI FAN GAO GAO2 GARCIA GARSHI GENG GER GILLIS GODLEY GOLLED GOODMA GRAHAM GREGOR GSELL HAENSZ HAMMO2 HANSEN HEGMAN HENNEK HINDS HIRAOK HIRAY2 HIRAYA HITOSU HOLE HOROWI HORWIT HU HU2 HUANG HUMBLE ISHIMA JAHN JAIN JARUP JARVHO JEDRYC JIANG JONES JUSSAW KAISE2 KAISER KANELL KATSOU KAUFMA KELLER KHUDER KIHARA KINLEN KJUUS KNEKT KO KOHLME KOO KOULUM KREUZE KREYBE KUBIK LAMTH LAMWK LAMWK2 LAUSSM LEI LEMARC LETOUR LIAW LICKIN LIDDEL LIU LIU2 LIU3 LIU4 LIU5 LOMBA2 LOMBAR LUBIN LUO MACLEN MAGNUS MARSH MARSH2 MARTIS MASTRA MATOS MATSUD MCCONN MCDUFF MCLAUG MIGRAN MILLER MILLS MOLLO MRFIT MRFITR MURATA NAM NOTAN2 NOTANI NOU ODRISC ORMOS OSANN OSANN2 PARKIN PASTOR PAWLEG PERNU PERSH2 PERSHA PETO PEZZO2 PEZZOT PIKE PISANI POFFIJ POLEDN PRESCO QIAO QIAO2 RACHTA RADZIK RANDIG REN RESTRE RIMING RONCO ROOTS ROTHSC SAARIK SANKAR SCHWA2 SCHWAR SEGI SEGI2 SEOW SHAW SHIMIZ SIEMIA SIMARA SIMONA SITAS SOBUE SOBUE2 SPEIZE SPITZ STASZE STAYNE STOCKS STOCKW STUCKE SUN SUZUK2 SUZUKI SVENSS TANG TANG2 TAO TENKAN TOKARS TOUSEY TSUGAN TVERDA ULMER VANDER VEIERO VUTUC WAKAI WALD WANG WANG2 WANG3 WANG4 WARSIN WATSON WICKLU WIGLE WILKIN WU WU2 WUNSCH WUWILL WYNDE2 WYNDE3 WYNDE4 WYNDE5 WYNDE6 WYNDE8 XIANGZ XU XU2 XU3 XU4 YAMAGU YONG YUAN ZHANG ZHENG ZHOU
3	BENSHL CEDERL CPSII DEAN3 GARDIN HEIN LANGE TULINI WYNDER
9	BENHAM

Table 1E11 - 8

Potentially overlapping studies

REF	REFGP	PRINC	. OVERLAP/LINK
LUBIN2	LUBIN2	1	Lubin-combined
WYNDE7	WYNDE6	2	WYNDE5/6/7/8
CPSI	CPSI	1	CPSI overall
BOFFET	BOFFET	1	BOFFET-combined

Table 1E12 -

IESLC - Meta-analysis of Current Smoking, Cigars only
All LC types

This analysis is restricted to results for:
1) Non-dose-response data
2) Smokers of cigars only
3) Current smokers
4) Results complete enough for use in metaanalysis

Within each study, results are then selected (in the following order of preference, within each sex) for:
5) DENOM: never smoked anything, (never +1 = +long term ex)
6) Followup period (prospective studies): whole study (coded as 0) or longest available
7) LCtype: all or nearest available, at least Squamous and Adeno. (q = squamous, s = small,
l = large, a = adeno, mix = mixed, alv = alveolar)
8) Race: all or nearest available, otherwise by race (wh or w = white, bl or b = black, hi = hispanic
ch = chinese, jap = japanese, haw = hawaiian, w+o = white + oriental, sca = scandinavian, as = asian)
9) For overlapping studies: principal rather than subsidiary studies
Finally by Age: whole study (coded as 0) if available, otherwise by widest available age group
and then for single sex results (m, f) in preference to combined sex results (c).

Results adjusted (AD) for the most potential confounders are then chosen in Sections -1 to -3
and results adjusted for the least confounders in Sections -4 to -6. (Those least adjusted results which
actually differ from the most adjusted as marked 'x' in column X in Section -4)
(Results adjusted for an unknown number of confounder(s) are coded as 20.)

Section -7 shows excluded studies, together with the stage (as above) at which no qualifying
results were found.

Section -8 lists the potentially overlapping studies which have been included (1=principal, 2=subsidiary).

Section -9 lists any results which would have been included in preference except that they had data not complete
enough for use in meta-analysis, with their significance (yes/no), if known, and any further comment as entered
on the database.

In addition to those mentioned above, the following fields, levels and abbreviations are used:
* or nk = not known, n = no, y = yes, ot = other
nev = never
REF: 6-character study reference
NRR: number of the RR on the database within the study
ST : study type (CC = case control, pr or prosp = prospective)
NLC: number of lung cancer cases in whole study
R : risky occupational population (n = no, m = mining, o = other risky)
VB : national cigarette type (V = at least 75% Virginia, bl = at least 75% blended, ot = other)
P : any proxy use
H : full histological confirmation
De : derivation of RR/CI (or = original, st = standard method, ot = other method of estimation)

Table 1E12 - 1

IESLC - Meta-analysis of Current Smoking, Cigars only
All LC types
Most adjusted

REF	NRR	SEX	AGEL	AGEH	RACE	YF	LC TYPE	LOC	START	ST	NLC	R	VB	P	H	AD	DENOM	De
BENSHL	3	m	0	0	all	0	all	Eu:UK	1967	pr	486	n	V	n	n	1	nev any	ot
BOFFET	25	m	0	0	all	-	all	Eu:mul	1988	CC	5621	n	bl	y	n	2	nev any	or
CEDERL	85	m	0	0	all	16	all	Eu:Sca	1963	pr	491	n	bl	n	n	2	nev any	ot
CPSI	153	m	35	99	wh	0	all	NAmer	1959	pr	5138	n	bl	n	n	1	nev any	ot
CPSII	1	m	0	0	all	0	all	NAmer	1982	pr	3229	n	bl	n	n	2	nev any	or
DEAN3	179	m	0	0	all	-	all	Eu:UK	1969	CC	766	n	V	y	n	0	nev any	ot
DORN	22	m	0	0	wh	15	all	NAmer	1954	pr	5097	n	bl	n	n	2	nev any	ot
GARDIN	4	c	0	0	all	-	all	Eu:UK	1988	CC	143	n	V	y	n	0	nev any	st
HEIN	2	m	0	0	all	0	all	Eu:Sca	1970	pr	144	n	bl	n	n	0	nev any	st
JOLY	17	m	0	0	all	-	all	SCAmer	1978	CC	826	n	bl	n	n	0	nev any	st
LANGE	17	m	0	0	all	0	all	Eu:Sca	1976	pr	268	n	bl	n	n	1	nev any	or
LANGE	13	f	0	0	all	0	all	Eu:Sca	1976	pr	268	n	bl	n	n	1	nev any	or
LUBIN2	4	m	0	0	all	-	all	Eu:mul	1976	CC	7804	n	bl	n	y	2	nev any	ot
TULINI	26	m	0	0	all	0	all	Eu:Sca	1967	pr	472	n	bl	n	n	3	nev any	or
WYNDE7	2	m	0	0	all	-	all	NAmer	1977	CC	2085	n	bl	n	y	0	nev any	st

Table 1E12 - 2

IESLC - Meta-analysis of Current Smoking, Cigars only
All LC types
Most adjusted

REF	NRR	SEX	AD	Number Exposed Case	Number Exposed Cont	Non-exposed Case	Non-exposed Cont	RR	95.00%CI
*BENSHL	3	m	1	-	-	-	-	6.62	(3.12- 14.05)
BOFFET	25	m	2	-	-	-	-	10.60	(5.90- 19.10)
*CEDERL	85	m	2	-	-	-	-	7.60	(3.70- 15.59)
*CPSI	153	m	1	-	-	-	-	3.30	(2.68- 4.06)
*CPSII	1	m	2	-	-	-	-	5.20	(4.10- 6.60)
DEAN3	179	m	0	0	26	24	510	0.39~	(0.02- 6.64)
*DORN	22	m	2	-	-	-	-	1.66	(1.18- 2.34)
GARDIN	4	c	0	3	2	5	41	12.30	(1.64- 92.33)
*HEIN	2	m	0	23	420	1	457	25.03	(3.39- 184.50)
JOLY	17	m	0	36	141	12	218	4.64	(2.33- 9.22)
*LANGE	17	m	1	-	-	-	-	6.00	(2.20- 17.00)
*LANGE	13	f	1	-	-	-	-	4.90	(3.00- 12.00)
Subtotal LANGE								5.22	(2.94- 9.27)
LUBIN2	4	m	2	-	-	-	-	5.18	(3.72- 7.22)
*TULINI	26	m	3	-	-	-	-	4.16	(2.00- 8.63)
WYNDE7	2	m	0	18	82	64	918	3.15	(1.78- 5.57)
Partial Totals				80	671	106	2144		

*prospective study ~ With 0.5 adjustment for zero

REF	NRR	SEX	AD	Ys	Ws	Qs	Ps
*BENSHL	3	m	1	1.89	6.79	1.64	0.0000
BOFFET	25	m	2	2.36	11.13	10.33	0.0000
*CEDERL	85	m	2	2.03	7.43	2.95	0.0000
*CPSI	153	m	1	1.19	89.06	3.70	0.0000
*CPSII	1	m	2	1.65	67.79	4.26	0.0000
DEAN3	179	m	0	-0.93	0.48	2.61	0.5175
*DORN	22	m	2	0.51	32.78	26.03	0.0037
GARDIN	4	c	0	2.51	0.95	1.17	0.0147
*HEIN	2	m	0	3.22	0.96	3.20	0.0016
JOLY	17	m	0	1.53	8.14	0.15	0.0000
*LANGE	17	m	1	1.79	3.68	0.57	0.0006
*LANGE	13	f	1	1.59	8.00	0.29	0.0000
Subtotal LANGE				1.65	11.67	0.86	
LUBIN2	4	m	2	1.64	34.94	2.13	0.0000
*TULINI	26	m	3	1.43	7.19	0.01	0.0001
WYNDE7	2	m	0	1.15	11.84	0.75	0.0001

	N	15
	NS	14
	Wt	291.16
Het	Chi	59.79
Het	df	14
Het	P	***
Fixed	RR	4.05
	RRl	3.61
	RRu	4.54
	P	+++
Random	RR	4.67
	RRl	3.49
	RRu	6.25
	P	+++
Asymm	P	N.S.

Table 1E12 - 3

IESLC - Meta-analysis of Current Smoking, Cigars only
All LC types
Most adjusted

	combined	Sex: male	female	Total
N	1	13	1	15
NS	1	13	1	15
Wt	0.95	282.22	8.00	291.16
Het Chi	0.00	58.30	0.00	59.79
Het df	0	12	0	14
Het P	N.S.	***	N.S.	***
Fixed RR	12.30	4.01	4.90	4.05
RRl	1.64	3.57	2.45	3.61
RRu	92.33	4.51	9.80	4.54
P	+	+++	+++	+++
Random RR	12.30	4.58	4.90	4.67
RRl	1.64	3.36	2.45	3.49
RRu	92.33	6.24	9.80	6.25
P	+	+++	+++	+++
Between Chi				1.48
Between df				2
Between P				N.S.
Btwn(F) P				N.S.
Btwn(R) P				N.S.

	All LC (or nearest): all	other	Total
N	15		15
NS	14		14
Wt	291.16		291.16
Het Chi	59.79		59.79
Het df	14		14
Het P	***		***
Fixed RR	4.05		4.05
RRl	3.61		3.61
RRu	4.54		4.54
P	+++		+++
Random RR	4.67		4.67
RRl	3.49		3.49
RRu	6.25		6.25
P	+++		+++
Between Chi			
Between df			
Between P			N.S.
Btwn(F) P			N.S.
Btwn(R) P			N.S.

	Location: NAmer	UK	Scand	othEur	China	Japan	othAs	other	Total
N	4	3	5	2				1	15
NS	4	3	4	2				1	14
Wt	201.48	8.21	27.25	46.08				8.14	291.16
Het Chi	29.22	4.12	3.62	4.33				0.00	59.79
Het df	3	2	4	1				0	14
Het P	***	N.S.	N.S.	*				N.S.	***
Fixed RR	3.43	6.02	5.76	6.16				4.64	4.05
RRl	2.99	3.04	3.96	4.61				2.33	3.61
RRu	3.94	11.94	8.38	8.22				9.22	4.54
P	+++	+++	+++	+++				+++	+++
Random RR	3.11	4.69	5.76	7.10				4.64	4.67
RRl	1.95	1.11	3.96	3.54				2.33	3.49
RRu	4.97	19.80	8.38	14.25				9.22	6.25
P	+++	+	+++	+++				+++	+++
Between Chi									18.49
Between df									4
Between P									***
Btwn(F) P									N.S.
Btwn(R) P									N.S.

Table 1E12 - 3

IESLC - Meta-analysis of Current Smoking, Cigars only
All LC types
Most adjusted

	Detailed Country in "other Europe" multi	Germany	othWest	East	Balkans	Total
N	2					2
NS	2					2
Wt	46.08					46.08
Het Chi	4.33					4.33
Het df	1					1
Het P	*					*
Fixed RR	6.16					6.16
RRl	4.61					4.61
RRu	8.22					8.22
P	+++					+++
Random RR	7.10					7.10
RRl	3.54					3.54
RRu	14.25					14.25
P	+++					+++
Between Chi						
Between df						
Between P						N.S.
Btwn(F) P						N.S.
Btwn(R) P						N.S.

	Detailed Country in "other Asia" India	HongKong	other	Total
N				
NS				
Wt				
Het Chi				
Het df				
Het P				
Fixed RR				
RRl				
RRu				
P				
Random RR				
RRl				
RRu				
P				
Between Chi				
Between df				
Between P				N.S.
Btwn(F) P				N.S.
Btwn(R) P				N.S.

	Detailed other continent SCAmer	Auslia	Africa	Total
N	1			1
NS	1			1
Wt	8.14			8.14
Het Chi	0.00			0.00
Het df	0			0
Het P	N.S.			N.S.
Fixed RR	4.64			4.64
RRl	2.33			2.33
RRu	9.22			9.22
P	+++			+++
Random RR	4.64			4.64
RRl	2.33			2.33
RRu	9.22			9.22
P	+++			+++
Between Chi				
Between df				
Between P				N.S.
Btwn(F) P				N.S.
Btwn(R) P				N.S.

Table 1E12 - 3

IESLC - Meta-analysis of Current Smoking, Cigars only
All LC types
Most adjusted

	Start year of study <1960	1960-69	1970-79	1980-89	1990+	Total
N	2	4	6	3		15
NS	2	4	5	3		14
Wt	121.84	21.88	67.56	79.87		291.16
Het Chi	11.31	4.91	5.13	5.39		59.79
Het df	1	3	5	2		14
Het P	***	N.S.	N.S.	(*)		***
Fixed RR	2.74	5.60	4.80	5.80		4.05
RRl	2.30	3.68	3.78	4.66		3.61
RRu	3.28	8.51	6.09	7.22		4.54
P	+++	+++	+++	+++		+++
Random RR	2.37	5.34	4.79	7.30		4.67
RRl	1.21	3.01	3.75	3.97		3.49
RRu	4.65	9.49	6.13	13.40		6.25
P	+	+++	+++	+++		+++
Between Chi						33.05
Between df						3
Between P						***
Btwn(F) P						*
Btwn(R) P						N.S.

	Study type (1) CC	other	Total
N	6	9	15
NS	6	8	14
Wt	67.49	223.67	291.16
Het Chi	12.64	41.30	59.79
Het df	5	8	14
Het P	*	***	***
Fixed RR	5.24	3.74	4.05
RRl	4.13	3.28	3.61
RRu	6.65	4.27	4.54
P	+++	+++	+++
Random RR	5.17	4.44	4.67
RRl	3.23	3.07	3.49
RRu	8.28	6.44	6.25
P	+++	+++	+++
Between Chi			5.85
Between df			1
Between P			*
Btwn(F) P			N.S.
Btwn(R) P			N.S.

	Study type (2) CC	prosp	other	Total
N	6	9		15
NS	6	8		14
Wt	67.49	223.67		291.16
Het Chi	12.64	41.30		59.79
Het df	5	8		14
Het P	*	***		***
Fixed RR	5.24	3.74		4.05
RRl	4.13	3.28		3.61
RRu	6.65	4.27		4.54
P	+++	+++		+++
Random RR	5.17	4.44		4.67
RRl	3.23	3.07		3.49
RRu	8.28	6.44		6.25
P	+++	+++		+++
Between Chi				5.85
Between df				1
Between P				*
Btwn(F) P				N.S.
Btwn(R) P				N.S.

Table 1E12 - 3

IESLC - Meta-analysis of Current Smoking, Cigars only
All LC types
Most adjusted

	Study size (number of LC cases) 100-249	250-499	500-999	1000+	Total
N	2	5	2	6	15
NS	2	4	2	6	14
Wt	1.91	33.07	8.62	247.55	291.16
Het Chi	0.24	1.67	2.76	46.41	59.79
Het df	1	4	1	5	14
Het P	N.S.	N.S.	(*)	***	***
Fixed RR	17.60	5.68	4.04	3.82	4.05
RRl	4.26	4.04	2.07	3.38	3.61
RRu	72.74	7.98	7.88	4.33	4.54
P	+++	+++	+++	+++	+++
Random RR	17.60	5.68	2.01	4.03	4.67
RRl	4.26	4.04	0.20	2.68	3.49
RRu	72.74	7.98	19.83	6.07	6.25
P	+++	+++	N.S.	+++	+++
Between Chi					8.70
Between df					3
Between P					*
Btwn(F) P					N.S.
Btwn(R) P					N.S.

	Risky occupational population no	mining	othRisky	Total
N	15			15
NS	14			14
Wt	291.16			291.16
Het Chi	59.79			59.79
Het df	14			14
Het P	***			***
Fixed RR	4.05			4.05
RRl	3.61			3.61
RRu	4.54			4.54
P	+++			+++
Random RR	4.67			4.67
RRl	3.49			3.49
RRu	6.25			6.25
P	+++			+++
Between Chi				
Between df				
Between P				N.S.
Btwn(F) P				N.S.
Btwn(R) P				N.S.

	National cigarette tobacco type Virginia	blended	other	Total
N	3	12		15
NS	3	11		14
Wt	8.21	282.95		291.16
Het Chi	4.12	54.33		59.79
Het df	2	11		14
Het P	N.S.	***		***
Fixed RR	6.02	4.00		4.05
RRl	3.04	3.56		3.61
RRu	11.94	4.49		4.54
P	+++	+++		+++
Random RR	4.69	4.59		4.67
RRl	1.11	3.39		3.49
RRu	19.80	6.21		6.25
P	+	+++		+++
Between Chi				1.34
Between df				1
Between P				N.S.
Btwn(F) P				N.S.
Btwn(R) P				N.S.

Table 1E12 - 3

IESLC - Meta-analysis of Current Smoking, Cigars only
All LC types
Most adjusted

	Any proxy use No/nk	Yes	Total
N	12	3	15
NS	11	3	14
Wt	278.60	12.56	291.16
Het Chi	45.27	5.07	59.79
Het df	11	2	14
Het P	***	(*)	***
Fixed RR	3.89	9.45	4.05
RRl	3.46	5.44	3.61
RRu	4.38	16.43	4.54
P	+++	+++	+++
Random RR	4.33	5.74	4.67
RRl	3.26	1.18	3.49
RRu	5.75	27.87	6.25
P	+++	+	+++
Between Chi			9.44
Between df			1
Between P			**
Btwn(F) P			N.S.
Btwn(R) P			N.S.

	Full histological confirmation No	Yes	Total
N	13	2	15
NS	12	2	14
Wt	244.38	46.78	291.16
Het Chi	56.78	2.19	59.79
Het df	12	1	14
Het P	***	N.S.	***
Fixed RR	3.95	4.57	4.05
RRl	3.49	3.43	3.61
RRu	4.48	6.08	4.54
P	+++	+++	+++
Random RR	4.85	4.27	4.67
RRl	3.43	2.66	3.49
RRu	6.85	6.87	6.25
P	+++	+++	+++
Between Chi			0.82
Between df			1
Between P			N.S.
Btwn(F) P			N.S.
Btwn(R) P			N.S.

	Number of adjustment variables (1) 0	1	2+/+nk	Total
N	5	4	6	15
NS	5	3	6	14
Wt	22.37	107.52	161.27	291.16
Het Chi	7.87	4.90	44.81	59.79
Het df	4	3	5	14
Het P	(*)	N.S.	***	***
Fixed RR	4.02	3.62	4.36	4.05
RRl	2.65	3.00	3.74	3.61
RRu	6.08	4.38	5.09	4.54
P	+++	+++	+++	+++
Random RR	4.59	4.30	4.83	4.67
RRl	2.18	2.96	2.90	3.49
RRu	9.67	6.24	8.04	6.25
P	+++	+++	+++	+++
Between Chi				2.20
Between df				2
Between P				N.S.
Btwn(F) P				N.S.
Btwn(R) P				N.S.

Table 1E12 - 3

IESLC - Meta-analysis of Current Smoking, Cigars only
All LC types
Most adjusted

	Number of adjustment variables (2) 0	1	2	3-5	6+/+nk	Total
N	5	4	5	1		15
NS	5	3	5	1		14
Wt	22.37	107.52	154.08	7.19		291.16
Het Chi	7.87	4.90	44.79	0.00		59.79
Het df	4	3	4	0		14
Het P	(*)	N.S.	***	N.S.		***
Fixed RR	4.02	3.62	4.37	4.16		4.05
RRl	2.65	3.00	3.73	2.00		3.61
RRu	6.08	4.38	5.12	8.64		4.54
P	+++	+++	+++	+++		+++
Random RR	4.59	4.30	4.96	4.16		4.67
RRl	2.18	2.96	2.79	2.00		3.49
RRu	9.67	6.24	8.83	8.64		6.25
P	+++	+++	+++	+++		+++
Between Chi						2.22
Between df						3
Between P						N.S.
Btwn(F) P						N.S.
Btwn(R) P						N.S.

	Derivation of RR/CI Orig	StdCalc	Other	Total
N	5	4	6	15
NS	4	4	6	14
Wt	97.79	21.89	171.48	291.16
Het Chi	5.69	5.22	33.15	59.79
Het df	4	3	5	14
Het P	N.S.	N.S.	***	***
Fixed RR	5.55	4.23	3.36	4.05
RRl	4.55	2.78	2.89	3.61
RRu	6.77	6.42	3.90	4.54
P	+++	+++	+++	+++
Random RR	5.77	5.03	3.76	4.67
RRl	4.28	2.59	2.32	3.49
RRu	7.77	9.76	6.11	6.25
P	+++	+++	+++	+++
Between Chi				15.72
Between df				2
Between P				***
Btwn(F) P				N.S.
Btwn(R) P				N.S.

Table 1E12 - 4

IESLC - Meta-analysis of Current Smoking, Cigars only
All LC types
Least adjusted

REF	NRR	X	SEX	AGEL	AGEH	RACE	YF	LC TYPE	LOC	START	ST	NLC	R	VB	P	H	AD	DENOM	De
BENSHL	3		m	0	0	all	0	all	Eu:UK	1967	pr	486	n	V	n	n	1	nev any	ot
BOFFET	25		m	0	0	all	-	all	Eu:mul	1988	CC	5621	n	bl	y	n	2	nev any	or
CEDERL	85		m	0	0	all	16	all	Eu:Sca	1963	pr	491	n	bl	n	n	2	nev any	ot
CPSI	153		m	35	99	wh	0	all	NAmer	1959	pr	5138	n	bl	n	n	1	nev any	ot
CPSII	1		m	0	0	all	0	all	NAmer	1982	pr	3229	n	bl	n	n	2	nev any	or
DEAN3	179		m	0	0	all	-	all	Eu:UK	1969	CC	766	n	V	y	n	0	nev any	ot
DORN	22		m	0	0	wh	15	all	NAmer	1954	pr	5097	n	bl	n	n	2	nev any	ot
GARDIN	4		c	0	0	all	-	all	Eu:UK	1988	CC	143	n	V	y	n	0	nev any	st
HEIN	2		m	0	0	all	0	all	Eu:Sca	1970	pr	144	n	bl	n	n	0	nev any	st
JOLY	17		m	0	0	all	-	all	SCAmer	1978	CC	826	n	bl	n	n	0	nev any	st
LANGE	8	x	m	0	0	all	0	all	Eu:Sca	1976	pr	268	n	bl	n	n	0	nev any	st
LANGE	4	x	f	0	0	all	0	all	Eu:Sca	1976	pr	268	n	bl	n	n	0	nev any	st
LUBIN2	3	x	m	0	0	all	-	all	Eu:mul	1976	CC	7804	n	bl	n	y	0	nev any	st
TULINI	3	x	m	0	0	all	0	all	Eu:Sca	1967	pr	472	n	bl	n	n	1	nev any	or
WYNDE7	2		m	0	0	all	-	all	NAmer	1977	CC	2085	n	bl	n	y	0	nev any	st

Table 1E12 - 5

IESLC - Meta-analysis of Current Smoking, Cigars only
All LC types
Least adjusted

REF	NRR	SEX	AD	Number Exposed Case	Number Exposed Cont	Non-exposed Case	Non-exposed Cont	RR	95.00%CI
*BENSHL	3	m	1	-	-	-	-	6.62	(3.12- 14.05)
BOFFET	25	m	2	-	-	-	-	10.60	(5.90- 19.10)
*CEDERL	85	m	2	-	-	-	-	7.60	(3.70- 15.59)
*CPSI	153	m	1	-	-	-	-	3.30	(2.68- 4.06)
*CPSII	1	m	2	-	-	-	-	5.20	(4.10- 6.60)
DEAN3	179	m	0	0	26	24	510	0.39~	(0.02- 6.64)
*DORN	22	m	2	-	-	-	-	1.66	(1.18- 2.34)
GARDIN	4	c	0	3	2	5	41	12.30	(1.64- 92.33)
*HEIN	2	m	0	23	420	1	457	25.03	(3.39- 184.50)
JOLY	17	m	0	36	141	12	218	4.64	(2.33- 9.22)
*LANGE	8	m	0	47	808	5	721	8.39	(3.35- 20.97)
*LANGE	4	f	0	14	770	7	2159	5.61	(2.27- 13.84)
Subtotal LANGE								6.84	(3.59- 13.01)
LUBIN2	3	m	0	57	185	190	2617	4.24	(3.05- 5.91)
*TULINI	3	m	1	-	-	-	-	4.05	(1.95- 8.40)
WYNDE7	2	m	0	18	82	64	918	3.15	(1.78- 5.57)
Partial Totals				198	2434	308	7641		

*prospective study ~ With 0.5 adjustment for zero

REF	NRR	SEX	AD	Ys	Ws	Qs	Ps
*BENSHL	3	m	1	1.89	6.79	1.77	0.0000
BOFFET	25	m	2	2.36	11.13	10.72	0.0000
*CEDERL	85	m	2	2.03	7.43	3.12	0.0000
*CPSI	153	m	1	1.19	89.06	3.07	0.0000
*CPSII	1	m	2	1.65	67.79	4.91	0.0000
DEAN3	179	m	0	-0.93	0.48	2.57	0.5175
*DORN	22	m	2	0.51	32.78	24.97	0.0037
GARDIN	4	c	0	2.51	0.95	1.21	0.0147
*HEIN	2	m	0	3.22	0.96	3.26	0.0016
JOLY	17	m	0	1.53	8.14	0.19	0.0000
*LANGE	8	m	0	2.13	4.57	2.55	0.0000
*LANGE	4	f	0	1.72	4.71	0.56	0.0002
Subtotal LANGE				1.92	9.28	3.11	
LUBIN2	3	m	0	1.45	34.97	0.15	0.0000
*TULINI	3	m	1	1.40	7.20	0.00	0.0002
WYNDE7	2	m	0	1.15	11.84	0.64	0.0001

	N	15
	NS	14
	Wt	288.81
Het	Chi	59.70
Het	df	14
Het	P	***
Fixed	RR	3.97
	RRl	3.54
	RRu	4.46
	P	+++
Random	RR	4.69
	RRl	3.50
	RRu	6.29
	P	+++
Asymm	P	N.S.

Table 1E12 - 6

IESLC - Meta-analysis of Current Smoking, Cigars only
All LC types
Least adjusted

		combined	Sex male	female	Total
	N	1	13	1	15
	NS	1	13	1	15
	Wt	0.95	283.16	4.71	288.81
Het	Chi	0.00	57.91	0.00	59.70
Het	df	0	12	0	14
Het	P	N.S.	***	N.S.	***
Fixed	RR	12.30	3.94	5.61	3.97
	RRl	1.64	3.50	2.27	3.54
	RRu	92.33	4.42	13.84	4.46
	P	+	+++	+++	+++
Random	RR	12.30	4.57	5.61	4.69
	RRl	1.64	3.36	2.27	3.50
	RRu	92.33	6.21	13.84	6.29
	P	+	+++	+++	+++
Between	Chi				1.79
Between	df				2
Between	P				N.S.
Btwn(F)	P				N.S.
Btwn(R)	P				N.S.

Table 1E12 - 7

IESLC - Meta-analysis of Current Smoking, Cigars only
All LC types
Excluded studies (and stage at which they were excluded)

1	BOUCHA BUELL LAURIL MZILEN
2	ABRAHA AGUDO AKIBA ALDERS AMANDU AMES ANDERS ARCHER AUSTIN AUVINE AXELSO AXELSS BAND BARBON BECHER BERRIN BLOHMK BLOT1 BLOT2 BLOT3 BLOT4 BRESLO BRETT BROCKM BROSS BROWN1 BROWN2 BUFFLE BYERS1 BYERS2 CARPEN CASCO2 CASCOR CHAN CHANG CHATZI CHEN CHEN2 CHEN3 CHIAZZ CHOI CHOW CHYOU COMSTO COOKSO CORREA DARBY DAVEYS DEAN DEAN2 DEKLER DESTE2 DESTEF DOCKER DOLL DOLL2 DORANT DORGAN DOSEME DROSTE DU DUNN EBELIN ENGELA ENSTRO ESAKI FAN GAO GAO2 GARCIA GARSHI GENG GER GILLIS GODLEY GOLLED GOODMA GRAHAM GREGOR GSELL HAENSZ HAMMO2 HANSEN HEGMAN HENNEK HINDS HIRAOK HIRAY2 HIRAYA HITOSU HOLE HOROWI HORWIT HU HU2 HUANG HUMBLE ISHIMA JAHN JAIN JARUP JARVHO JEDRYC JIANG JONES JUSSAW KAISE2 KAISER KANELL KATSOU KAUFMA KELLER KHUDER KIHARA KINLEN KJUUS KNEKT KO KOHLME KOO KOULUM KREUZE KREYBE KUBIK LAMTH LAMWK LAMWK2 LAUSSM LEI LEMARC LETOUR LIAW LICKIN LIDDEL LIU LIU2 LIU3 LIU4 LIU5 LOMBA2 LOMBAR LUBIN LUO MACLEN MAGNUS MARSH MARSH2 MARTIS MASTRA MATOS MATSUD MCCONN MCDUFF MCLAUG MIGRAN MILLER MILLS MOLLO MRFIT MRFITR MURATA NAM NOTAN2 NOTANI NOU ODRISC ORMOS OSANN OSANN2 PARKIN PASTOR PAWLEG PERNU PERSH2 PERSHA PETO PEZZO2 PEZZOT PIKE PISANI POFFIJ POLEDN PRESCO QIAO QIAO2 RACHTA RADZIK RANDIG REN RESTRE RIMING RONCO ROOTS ROTHSC SAARIK SANKAR SCHWA2 SCHWAR SEGI SEGI2 SEOW SHAW SHIMIZ SIEMIA SIMARA SIMONA SITAS SOBUE SOBUE2 SPEIZE SPITZ STASZE STAYNE STOCKS STOCKW STUCKE SUN SUZUK2 SUZUKI SVENSS TANG TANG2 TAO TENKAN TOKARS TOUSEY TSUGAN TVERDA ULMER VANDER VEIERO VUTUC WAKAI WALD WANG WANG2 WANG3 WANG4 WARSIN WATSON WICKLU WIGLE WILKIN WU WU2 WUNSCH WUWILL WYNDE2 WYNDE3 WYNDE4 WYNDE5 WYNDE6 WYNDE8 XIANGZ XU XU2 XU3 XU4 YAMAGU YONG YUAN ZHANG ZHENG ZHOU
3	ABELIN ARMADA BENHAM BEST BOUCOT DAMBER HAMMON LEVIN SADOWS TIZZAN
5	WYNDER

Table 1E12 - 8
Potentially overlapping studies

REF	REFGP	PRINC	OVERLAP/LINK
LUBIN2	LUBIN2	1	Lubin-combined
HEIN	PRESCO	1	Subset of PRESCO
LANGE	PRESCO	1	Subset of PRESCO
BENSHL	TANG2	1	Subset of TANG2
WYNDE7	WYNDE6	2	WYNDE5/6/7/8
CPSI	CPSI	1	CPSI overall
BOFFET	BOFFET	1	BOFFET-combined

Table 1E13 -

IESLC - Meta-analysis of Ever Smoking (or Current if Ever not available), Cigars only
All LC types

This analysis is restricted to results for:
1) Non-dose-response data
2) Smokers of cigars only
3) Results complete enough for use in metaanalysis

Within each study, results are then selected (in the following order of preference, within each sex) for:
4) SMKSTA: ever smokers, current smokers
5) DENOM: never smoked anything, (never +1 = +long term ex)
6) Followup period (prospective studies): whole study (coded as 0) or longest available
7) LCtype: all or nearest available, at least Squamous and Adeno. (q = squamous, s = small,
l = large, a = adeno, mix = mixed, alv = alveolar)
8) Race: all or nearest available, otherwise by race (wh or w = white, bl or b = black, hi = hispanic
ch = chinese, jap = japanese, haw = hawaiian, w+o = white + oriental, sca = scandinavian, as = asian)
9) For overlapping studies: principal rather than subsidiary studies
Finally by Age: whole study (coded as 0) if available, otherwise by widest available age group
and then for single sex results (m, f) in preference to combined sex results (c).

Results adjusted (AD) for the most potential confounders are then chosen in Sections -1 to -3
and results adjusted for the least confounders in Sections -4 to -6. (Those least adjusted results which
actually differ from the most adjusted as marked 'x' in column X in Section -4)
(Results adjusted for an unknown number of confounder(s) are coded as 20.)

Section -7 shows excluded studies, together with the stage (as above) at which no qualifying
results were found.

Section -8 lists the potentially overlapping studies which have been included (1=principal, 2=subsidiary).

Section -9 lists any results which would have been included in preference except that they had data not complete
enough for use in meta-analysis, with their significance (yes/no), if known, and any further comment as entered
on the database.

In addition to those mentioned above, the following fields, levels and abbreviations are used:
* or nk = not known, n = no, y = yes, ot = other
ev = ever, cu = current, nev = never
REF: 6-character study reference
NRR: number of the RR on the database within the study
ST : study type (CC = case control, pr or prosp = prospective)
NLC: number of lung cancer cases in whole study
R : risky occupational population (n = no, m = mining, o = other risky)
VB : national cigarette type (V = at least 75% Virginia, bl = at least 75% blended, ot = other)
P : any proxy use
H : full histological confirmation
De : derivation of RR/CI (or = original, st = standard method, ot = other method of estimation)

Table 1E13 - 1

IESLC - Meta-analysis of Ever Smoking (or Current if Ever not available), Cigars only
All LC types
Most adjusted

REF	NRR	SEX	AGEL	AGEH	RACE	YF	LC TYPE	LOC	START	ST	NLC	R	VB	P	H	AD	SM	DENOM	De
ABELIN	51	m	0	0	all	-	all	Eu:wst	1941	CC	118	n	bl	y	n	1	ev	nev any	st
ARMADA	3	m	0	0	all	-	all	Eu:wst	1986	CC	325	n	bl	n	y	0	ev	nev any	st
BENSHL	3	m	0	0	all	0	all	Eu:UK	1967	pr	486	n	V	n	n	1	cu	nev any	ot
BEST	16	m	0	0	all	0	all	NAmer	1955	pr	381	n	V	n	n	1	ev	nev any	ot
BOFFET	1	m	0	0	all	-	all	Eu:mul	1988	CC	5621	n	bl	y	n	2	ev	nev any	or
BOUCOT	119	m	0	0	all	0	all	NAmer	1951	pr	121	n	bl	n	n	2	ev	nev any	ot
CEDERL	85	m	0	0	all	16	all	Eu:Sca	1963	pr	491	n	bl	n	n	2	cu	nev any	ot
CPSI	183	m	35	84	all	6	all	NAmer	1959	pr	5138	n	bl	n	n	1	ev	nev any	ot
CPSII	1	m	0	0	all	0	all	NAmer	1982	pr	3229	n	bl	n	n	2	cu	nev any	or
DAMBER	4	m	0	0	all	-	all	Eu:Sca	1972	CC	579	n	bl	y	n	0	ev	nev any	st
DEAN3	179	m	0	0	all	-	all	Eu:UK	1969	CC	766	n	V	y	n	0	cu	nev any	ot
DORN	32	m	0	0	wh	2	all	NAmer	1954	pr	5097	n	bl	n	n	1	ev	nev any	ot
GARDIN	4	c	0	0	all	-	all	Eu:UK	1988	CC	143	n	V	y	n	0	cu	nev any	st
HAMMON	111	m	0	0	wh	0	all	NAmer	1952	pr	448	n	bl	n	n	1	ev	nev any	ot
HEIN	2	m	0	0	all	0	all	Eu:Sca	1970	pr	144	n	bl	n	n	0	cu	nev any	st
JOLY	13	m	0	0	all	-	all	SCAmer	1978	CC	826	n	bl	n	n	0	ev	nev any	st
LANGE	17	m	0	0	all	0	all	Eu:Sca	1976	pr	268	n	bl	n	n	1	cu	nev any	or
LANGE	13	f	0	0	all	0	all	Eu:Sca	1976	pr	268	n	bl	n	n	1	cu	nev any	or
LEVIN	29	m	0	0	all	-	all	NAmer	1938	CC	475	n	bl	n	n	1	ev	nev any	st
LUBIN2	20	m	0	0	all	-	all	Eu:mul	1976	CC	7804	n	bl	n	y	2	ev	nev any	ot
SADOWS	29	m	0	0	wh	-	all	NAmer	1938	CC	477	n	bl	n	n	1	ev	nev any	ot
TIZZAN	3	m	0	0	all	-	all	Eu:wst	1959	CC	1358	n	bl	n	n	0	ev	nev any	st
TULINI	26	m	0	0	all	0	all	Eu:Sca	1967	pr	472	n	bl	n	n	3	cu	nev any	or
WYNDE7	40	m	0	0	all	-	all	NAmer	1977	CC	2085	n	bl	n	y	0	ev	nev any	st

Table 1E13 - 2

IESLC - Meta-analysis of Ever Smoking (or Current if Ever not available), Cigars only
All LC types
Most adjusted

REF	NRR	SEX	AD	Number Exposed Case	Number Exposed Cont	Non-exposed Case	Non-exposed Cont	RR	95.00%CI
ABELIN	51	m	1	-	-	-	-	25.09	(5.77- 109.07)
ARMADA	3	m	0	3	6	4	64	8.00	(1.44- 44.45)
*BENSHL	3	m	1	-	-	-	-	6.62	(3.12- 14.05)
*BEST	16	m	1	-	-	-	-	2.94	(0.61- 14.15)
BOFFET	1	m	2	-	-	-	-	9.00	(5.80- 14.10)
*BOUCOT	119	m	2	-	-	-	-	8.81	(0.45- 170.58)
*CEDERL	85	m	2	-	-	-	-	7.60	(3.70- 15.59)
*CPSI	183	m	1	-	-	-	-	2.11	(1.45- 3.07)
*CPSII	1	m	2	-	-	-	-	5.20	(4.10- 6.60)
DAMBER	4	m	0	7	7	42	208	4.95	(1.65- 14.86)
DEAN3	179	m	0	0	26	24	510	0.39~	(0.02- 6.64)
*DORN	32	m	1	-	-	-	-	1.50	(0.59- 3.80)
GARDIN	4	c	0	3	2	5	41	12.30	(1.64- 92.33)
*HAMMON	111	m	1	-	-	-	-	1.02	(0.42- 2.51)
*HEIN	2	m	0	23	420	1	457	25.03	(3.39- 184.50)
JOLY	13	m	0	43	179	12	218	4.36	(2.23- 8.53)
*LANGE	17	m	1	-	-	-	-	6.00	(2.20- 17.00)
*LANGE	13	f	1	-	-	-	-	4.90	(3.00- 12.00)
Subtotal LANGE								5.22	(2.94- 9.27)
LEVIN	29	m	1	-	-	-	-	1.41	(0.76- 2.60)
LUBIN2	20	m	2	-	-	-	-	3.26	(2.21- 4.79)
SADOWS	29	m	1	-	-	-	-	2.98	(1.06- 8.33)
TIZZAN	3	m	0	26	57	180	305	0.77	(0.47- 1.27)
*TULINI	26	m	3	-	-	-	-	4.16	(2.00- 8.63)
WYNDE7	40	m	0	30	152	64	918	2.83	(1.78- 4.51)
Partial Totals				135	849	332	2721		

*prospective study ~ With 0.5 adjustment for zero

REF	NRR	SEX	AD	Ys	Ws	Qs	Ps
ABELIN	51	m	1	3.22	1.78	6.65	0.0000
ARMADA	3	m	0	2.08	1.31	0.82	0.0175
*BENSHL	3	m	1	1.89	6.79	2.46	0.0000
*BEST	16	m	1	1.08	1.55	0.07	0.1788
BOFFET	1	m	2	2.20	19.47	16.09	0.0000
*BOUCOT	119	m	2	2.18	0.44	0.34	0.1509
*CEDERL	85	m	2	2.03	7.43	4.07	0.0000
*CPSI	183	m	1	0.75	27.31	8.00	0.0001
*CPSII	1	m	2	1.65	67.79	8.81	0.0000
DAMBER	4	m	0	1.60	3.18	0.31	0.0043
DEAN3	179	m	0	-0.93	0.48	2.37	0.5175
*DORN	32	m	1	0.41	4.43	3.45	0.3935
GARDIN	4	c	0	2.51	0.95	1.41	0.0147
*HAMMON	111	m	1	0.02	4.81	7.73	0.9654
*HEIN	2	m	0	3.22	0.96	3.59	0.0016
JOLY	13	m	0	1.47	8.56	0.29	0.0000
*LANGE	17	m	1	1.79	3.68	0.93	0.0006
*LANGE	13	f	1	1.59	8.00	0.73	0.0000
Subtotal LANGE				1.65	11.67	1.66	
LEVIN	29	m	1	0.34	10.16	9.06	0.2735
LUBIN2	20	m	2	1.18	25.68	0.29	0.0000
SADOWS	29	m	1	1.09	3.62	0.14	0.0379
TIZZAN	3	m	0	-0.26	15.42	36.85	0.3117
*TULINI	26	m	3	1.43	7.19	0.14	0.0001
WYNDE7	40	m	0	1.04	17.66	1.08	0.0000

Table 1E13 - 2

IESLC - Meta-analysis of Ever Smoking (or Current if Ever not available), Cigars only
All LC types
Most adjusted

	N	24
	NS	23
	Wt	248.63
Het	Chi	115.70
Het	df	23
Het	P	***
Fixed	RR	3.63
	RRl	3.20
	RRu	4.11
	P	+++
Random	RR	3.71
	RRl	2.68
	RRu	5.15
	P	+++
Asymm	P	N.S.

Table 1E13 - 3

IESLC - Meta-analysis of Ever Smoking (or Current if Ever not available), Cigars only
All LC types
Most adjusted

	Sex combined	male	female	Total
N	1	22	1	24
NS	1	22	1	24
Wt	0.95	239.69	8.00	248.63
Het Chi	0.00	113.51	0.00	115.70
Het df	0	21	0	23
Het P	N.S.	***	N.S.	***
Fixed RR	12.30	3.57	4.90	3.63
RRl	1.64	3.15	2.45	3.20
RRu	92.33	4.05	9.80	4.11
P	+	+++	+++	+++
Random RR	12.30	3.58	4.90	3.71
RRl	1.64	2.54	2.45	2.68
RRu	92.33	5.05	9.80	5.15
P	+	+++	+++	+++
Between Chi				2.19
Between df				2
Between P				N.S.
Btwn(F) P				N.S.
Btwn(R) P				N.S.

	All LC (or nearest) all	other	Total
N	24		24
NS	23		23
Wt	248.63		248.63
Het Chi	115.70		115.70
Het df	23		23
Het P	***		***
Fixed RR	3.63		3.63
RRl	3.20		3.20
RRu	4.11		4.11
P	+++		+++
Random RR	3.71		3.71
RRl	2.68		2.68
RRu	5.15		5.15
P	+++		+++
Between Chi			
Between df			
Between P			N.S.
Btwn(F) P			N.S.
Btwn(R) P			N.S.

	Location NAmer	UK	Scand	othEur	China	Japan	othAs	other	Total
N	9	3	6	5				1	24
NS	9	3	5	5				1	23
Wt	137.76	8.21	30.43	63.66				8.56	248.63
Het Chi	37.07	4.12	3.69	60.40				0.00	115.70
Het df	8	2	5	4				0	23
Het P	***	N.S.	N.S.	***				N.S.	***
Fixed RR	3.25	6.02	5.67	3.38				4.36	3.63
RRl	2.75	3.04	3.97	2.65				2.23	3.20
RRu	3.84	11.94	8.09	4.33				8.53	4.11
P	+++	+++	+++	+++				+++	+++
Random RR	2.36	4.69	5.67	4.76				4.36	3.71
RRl	1.49	1.11	3.97	1.60				2.23	2.68
RRu	3.73	19.80	8.09	14.12				8.53	5.15
P	+++	+	+++	++				+++	+++
Between Chi									10.41
Between df									4
Between P									*
Btwn(F) P									N.S.
Btwn(R) P									(*)

Table 1E13 - 3

IESLC - Meta-analysis of Ever Smoking (or Current if Ever not available), Cigars only
All LC types
Most adjusted

Detailed Country in "other Europe"

	multi	Germany	othWest	East	Balkans	Total
N	2		3			5
NS	2		3			5
Wt	45.15		18.51			63.66
Het Chi	11.42		24.06			60.40
Het df	1		2			4
Het P	***		***			***
Fixed RR	5.05		1.27			3.38
RRl	3.77		0.81			2.65
RRu	6.76		2.01			4.33
P	+++		N.S.			+++
Random RR	5.38		4.96			4.76
RRl	1.99		0.45			1.60
RRu	14.56		55.17			14.12
P	+++		N.S.			++
Between Chi						24.92
Between df						1
Between P						***
Btwn(F) P						N.S.
Btwn(R) P						N.S.

Detailed Country in "other Asia"

	India	HongKong	other	Total
N				
NS				
Wt				
Het Chi				
Het df				
Het P				
Fixed RR				
RRl				
RRu				
P				
Random RR				
RRl				
RRu				
P				
Between Chi				
Between df				
Between P				N.S.
Btwn(F) P				N.S.
Btwn(R) P				N.S.

Detailed other continent

	SCAmer	Auslia	Africa	Total
N	1			1
NS	1			1
Wt	8.56			8.56
Het Chi	0.00			0.00
Het df	0			0
Het P	N.S.			N.S.
Fixed RR	4.36			4.36
RRl	2.23			2.23
RRu	8.53			8.53
P	+++			+++
Random RR	4.36			4.36
RRl	2.23			2.23
RRu	8.53			8.53
P	+++			+++
Between Chi				
Between df				
Between P				N.S.
Btwn(F) P				N.S.
Btwn(R) P				N.S.

Table 1E13 - 3

IESLC - Meta-analysis of Ever Smoking (or Current if Ever not available), Cigars only
All LC types
Most adjusted

	Start year of study					
	<1960	1960-69	1970-79	1980-89	1990+	Total
N	9	4	7	4		24
NS	9	4	6	4		23
Wt	69.51	21.88	67.72	89.52		248.63
Het Chi	28.10	4.91	7.19	5.18		115.70
Het df	8	3	6	3		23
Het P	***	N.S.	N.S.	N.S.		***
Fixed RR	1.63	5.60	3.71	5.95		3.63
RRl	1.29	3.68	2.93	4.84		3.20
RRu	2.07	8.51	4.71	7.32		4.11
P	+++	+++	+++	+++		+++
Random RR	1.98	5.34	3.85	6.73		3.71
RRl	1.17	3.01	2.92	4.49		2.68
RRu	3.34	9.49	5.08	10.09		5.15
P	+	+++	+++	+++		+++
Between Chi						70.33
Between df						3
Between P						***
Btwn(F) P						***
Btwn(R) P						**

	Study type (1)		
	CC	other	Total
N	12	12	24
NS	12	11	23
Wt	108.26	140.36	248.63
Het Chi	72.89	38.41	115.70
Het df	11	11	23
Het P	***	***	***
Fixed RR	3.12	4.08	3.63
RRl	2.58	3.45	3.20
RRu	3.76	4.81	4.11
P	+++	+++	+++
Random RR	3.57	3.92	3.71
RRl	2.05	2.64	2.68
RRu	6.21	5.82	5.15
P	+++	+++	+++
Between Chi			4.40
Between df			1
Between P			*
Btwn(F) P			N.S.
Btwn(R) P			N.S.

	Study type (2)			
	CC	prosp	other	Total
N	12	12		24
NS	12	11		23
Wt	108.26	140.36		248.63
Het Chi	72.89	38.41		115.70
Het df	11	11		23
Het P	***	***		***
Fixed RR	3.12	4.08		3.63
RRl	2.58	3.45		3.20
RRu	3.76	4.81		4.11
P	+++	+++		+++
Random RR	3.57	3.92		3.71
RRl	2.05	2.64		2.68
RRu	6.21	5.82		5.15
P	+++	+++		+++
Between Chi				4.40
Between df				1
Between P				*
Btwn(F) P				N.S.
Btwn(R) P				N.S.

Table 1E13 - 3

IESLC - Meta-analysis of Ever Smoking (or Current if Ever not available), Cigars only
All LC types
Most adjusted

	Study size (number of LC cases) 100-249	250-499	500-999	1000+	Total
N	4	10	3	7	24
NS	4	9	3	7	23
Wt	4.12	54.51	12.23	177.77	248.63
Het Chi	0.65	26.12	2.79	74.27	115.70
Het df	3	9	2	6	23
Het P	N.S.	**	N.S.	***	***
Fixed RR	19.06	3.57	4.10	3.48	3.63
RRl	7.26	2.74	2.34	3.00	3.20
RRu	50.05	4.65	7.19	4.03	4.11
P	+++	+++	+++	+++	+++
Random RR	19.06	3.68	3.85	2.79	3.71
RRl	7.26	2.29	1.78	1.61	2.68
RRu	50.05	5.92	8.34	4.85	5.15
P	+++	+++	+++	+++	+++
Between Chi					11.87
Between df					3
Between P					**
Btwn(F) P					N.S.
Btwn(R) P					**

	Risky occupational population no	mining	othRisky	Total
N	24			24
NS	23			23
Wt	248.63			248.63
Het Chi	115.70			115.70
Het df	23			23
Het P	***			***
Fixed RR	3.63			3.63
RRl	3.20			3.20
RRu	4.11			4.11
P	+++			+++
Random RR	3.71			3.71
RRl	2.68			2.68
RRu	5.15			5.15
P	+++			+++
Between Chi				
Between df				
Between P				N.S.
Btwn(F) P				N.S.
Btwn(R) P				N.S.

	National cigarette tobacco type Virginia	blended	other	Total
N	4	20		24
NS	4	19		23
Wt	9.77	238.86		248.63
Het Chi	4.80	109.33		115.70
Het df	3	19		23
Het P	N.S.	***		***
Fixed RR	5.37	3.57		3.63
RRl	2.87	3.14		3.20
RRu	10.06	4.05		4.11
P	+++	+++		+++
Random RR	4.52	3.64		3.71
RRl	1.69	2.57		2.68
RRu	12.11	5.16		5.15
P	++	+++		+++
Between Chi				1.57
Between df				1
Between P				N.S.
Btwn(F) P				N.S.
Btwn(R) P				N.S.

Table 1E13 - 3

IESLC - Meta-analysis of Ever Smoking (or Current if Ever not available), Cigars only
All LC types
Most adjusted

	Any proxy use No/nk	Yes	Total
N	19	5	24
NS	18	5	23
Wt	222.77	25.86	248.63
Het Chi	86.64	7.74	115.70
Het df	18	4	23
Het P	***	N.S.	***
Fixed RR	3.28	8.56	3.63
RRl	2.88	5.82	3.20
RRu	3.74	12.59	4.11
P	+++	+++	+++
Random RR	3.22	7.86	3.71
RRl	2.31	3.58	2.68
RRu	4.50	17.27	5.15
P	+++	+++	+++
Between Chi			21.31
Between df			1
Between P			***
Btwn(F) P			*
Btwn(R) P			*

	Full histological confirmation No	Yes	Total
N	21	3	24
NS	20	3	23
Wt	203.98	44.65	248.63
Het Chi	113.33	1.37	115.70
Het df	20	2	23
Het P	***	N.S.	***
Fixed RR	3.74	3.17	3.63
RRl	3.26	2.36	3.20
RRu	4.28	4.24	4.11
P	+++	+++	+++
Random RR	3.78	3.17	3.71
RRl	2.57	2.36	2.68
RRu	5.55	4.24	5.15
P	+++	+++	+++
Between Chi			1.01
Between df			1
Between P			N.S.
Btwn(F) P			N.S.
Btwn(R) P			N.S.

	Number of adjustment variables (1) 0	1	2+/+nk	Total
N	8	10	6	24
NS	8	9	6	23
Wt	48.52	72.11	128.00	248.63
Het Chi	36.15	31.48	13.01	115.70
Het df	7	9	5	23
Het P	***	***	*	***
Fixed RR	2.27	2.61	5.21	3.63
RRl	1.72	2.07	4.38	3.20
RRu	3.01	3.29	6.19	4.11
P	+++	+++	+++	+++
Random RR	3.55	3.07	5.39	3.71
RRl	1.62	1.89	3.82	2.68
RRu	7.78	4.97	7.62	5.15
P	++	+++	+++	+++
Between Chi				35.06
Between df				2
Between P				***
Btwn(F) P				*
Btwn(R) P				N.S.

Table 1E13 - 3

IESLC - Meta-analysis of Ever Smoking (or Current if Ever not available), Cigars only
All LC types
Most adjusted

	Number of adjustment variables (2) 0	1	2	3-5	6+/+nk	Total
N	8	10	5	1		24
NS	8	9	5	1		23
Wt	48.52	72.11	120.81	7.19		248.63
Het Chi	36.15	31.48	12.62	0.00		115.70
Het df	7	9	4	0		23
Het P	***	***	*	N.S.		***
Fixed RR	2.27	2.61	5.28	4.16		3.63
RRl	1.72	2.07	4.41	2.00		3.20
RRu	3.01	3.29	6.31	8.64		4.11
P	+++	+++	+++	+++		+++
Random RR	3.55	3.07	5.64	4.16		3.71
RRl	1.62	1.89	3.79	2.00		2.68
RRu	7.78	4.97	8.40	8.64		5.15
P	++	+++	+++	+++		+++
Between Chi						35.44
Between df						3
Between P						***
Btwn(F) P						(*)
Btwn(R) P						N.S.

	Derivation of RR/CI Orig	StdCalc	Other	Total
N	5	9	10	24
NS	4	9	10	23
Wt	106.12	59.98	82.53	248.63
Het Chi	5.54	47.24	24.24	115.70
Het df	4	8	9	23
Het P	N.S.	***	**	***
Fixed RR	5.67	2.28	2.86	3.63
RRl	4.69	1.77	2.30	3.20
RRu	6.85	2.94	3.55	4.11
P	+++	+++	+++	+++
Random RR	5.80	4.13	2.88	3.71
RRl	4.43	2.04	1.87	2.68
RRu	7.59	8.37	4.43	5.15
P	+++	+++	+++	+++
Between Chi				38.67
Between df				2
Between P				***
Btwn(F) P				*
Btwn(R) P				*

	Smoking status ever	current	Total
N	15	9	24
NS	15	8	23
Wt	145.37	103.25	248.63
Het Chi	79.50	8.05	115.70
Het df	14	8	23
Het P	***	N.S.	***
Fixed RR	2.73	5.40	3.63
RRl	2.32	4.46	3.20
RRu	3.21	6.55	4.11
P	+++	+++	+++
Random RR	2.95	5.41	3.71
RRl	1.91	4.44	2.68
RRu	4.56	6.59	5.15
P	+++	+++	+++
Between Chi			28.14
Between df			1
Between P			***
Btwn(F) P			*
Btwn(R) P			*

Table 1E13 - 4

IESLC - Meta-analysis of Ever Smoking (or Current if Ever not available), Cigars only
All LC types
Least adjusted

REF	NRR	X	SEX	AGEL	AGEH	RACE	YF	LC TYPE	LOC	START	ST	NLC	R	VB	P	H	AD	SM	DENOM	De
ABELIN	8	x	m	0	0	all	-	all	Eu:wst	1941	CC	118	n	bl	y	n	0	ev	nev any	st
ARMADA	3		m	0	0	all	-	all	Eu:wst	1986	CC	325	n	bl	n	y	0	ev	nev any	st
BENSHL	3		m	0	0	all	0	all	Eu:UK	1967	pr	486	n	V	n	n	1	cu	nev any	ot
BEST	16		m	0	0	all	0	all	NAmer	1955	pr	381	n	V	n	n	1	ev	nev any	ot
BOFFET	4	x	m	0	0	all	-	all	Eu:mul	1988	CC	5621	n	bl	y	n	0	ev	nev any	st
BOUCOT	7	x	m	0	0	all	0	all	NAmer	1951	pr	121	n	bl	n	n	0	ev	nev any	ot
CEDERL	85		m	0	0	all	16	all	Eu:Sca	1963	pr	491	n	bl	n	n	2	cu	nev any	ot
CPSI	183		m	35	84	all	6	all	NAmer	1959	pr	5138	n	bl	n	n	1	ev	nev any	ot
CPSII	1		m	0	0	all	0	all	NAmer	1982	pr	3229	n	bl	n	n	2	cu	nev any	or
DAMBER	4		m	0	0	all	-	all	Eu:Sca	1972	CC	579	n	bl	y	n	0	ev	nev any	st
DEAN3	179		m	0	0	all	-	all	Eu:UK	1969	CC	766	n	V	y	n	0	cu	nev any	ot
DORN	41	x	m	0	0	wh	2	all	NAmer	1954	pr	5097	n	bl	n	n	0	ev	nev any	st
GARDIN	4		c	0	0	all	-	all	Eu:UK	1988	CC	143	n	V	y	n	0	cu	nev any	st
HAMMON	123	x	m	0	0	wh	0	all	NAmer	1952	pr	448	n	bl	n	n	0	ev	nev any	st
HEIN	2		m	0	0	all	0	all	Eu:Sca	1970	pr	144	n	bl	n	n	0	cu	nev any	st
JOLY	13		m	0	0	all	-	all	SCAmer	1978	CC	826	n	bl	n	n	0	ev	nev any	st
LANGE	8	x	m	0	0	all	0	all	Eu:Sca	1976	pr	268	n	bl	n	n	0	cu	nev any	st
LANGE	4	x	f	0	0	all	0	all	Eu:Sca	1976	pr	268	n	bl	n	n	0	cu	nev any	st
LEVIN	29		m	0	0	all	-	all	NAmer	1938	CC	475	n	bl	n	n	1	ev	nev any	st
LUBIN2	19	x	m	0	0	all	-	all	Eu:mul	1976	CC	7804	n	bl	n	y	0	ev	nev any	st
SADOWS	2	x	m	0	0	wh	-	all	NAmer	1938	CC	477	n	bl	n	n	0	ev	nev any	st
TIZZAN	3		m	0	0	all	-	all	Eu:wst	1959	CC	1358	n	bl	n	n	0	ev	nev any	st
TULINI	3	x	m	0	0	all	0	all	Eu:Sca	1967	pr	472	n	bl	n	n	1	cu	nev any	or
WYNDE7	40		m	0	0	all	-	all	NAmer	1977	CC	2085	n	bl	n	y	0	ev	nev any	st

Table 1E13 - 5

IESLC - Meta-analysis of Ever Smoking (or Current if Ever not available), Cigars only
All LC types
Least adjusted

REF	NRR	SEX	AD	Number Exposed Case	Exposed Cont	Non-exposed Case	Non-exposed Cont	RR	95.00%CI
ABELIN	8	m	0	33	99	2	183	30.50	(7.17- 129.78)
ARMADA	3	m	0	3	6	4	64	8.00	(1.44- 44.45)
*BENSHL	3	m	1	-	-	-	-	6.62	(3.12- 14.05)
*BEST	16	m	1	-	-	-	-	2.94	(0.61- 14.15)
BOFFET	4	m	0	43	77	117	1750	8.35	(5.50- 12.68)
*BOUCOT	7	m	0	3	5084	0	7551	10.40~	(0.54- 201.23)
*CEDERL	85	m	2	-	-	-	-	7.60	(3.70- 15.59)
*CPSI	183	m	1	-	-	-	-	2.11	(1.45- 3.07)
*CPSII	1	m	2	-	-	-	-	5.20	(4.10- 6.60)
DAMBER	4	m	0	7	7	42	208	4.95	(1.65- 14.86)
DEAN3	179	m	0	0	26	24	510	0.39~	(0.02- 6.64)
*DORN	41	m	0	6	28422	17	117918	1.46	(0.58- 3.71)
GARDIN	4	c	0	3	2	5	41	12.30	(1.64- 92.33)
*HAMMON	123	m	0	7	51480	15	115884	1.05	(0.43- 2.58)
*HEIN	2	m	0	23	420	1	457	25.03	(3.39- 184.50)
JOLY	13	m	0	43	179	12	218	4.36	(2.23- 8.53)
*LANGE	8	m	0	47	808	5	721	8.39	(3.35- 20.97)
*LANGE	4	f	0	14	770	7	2159	5.61	(2.27- 13.84)
Subtotal LANGE								6.84	(3.59- 13.01)
LEVIN	29	m	1	-	-	-	-	1.41	(0.76- 2.60)
LUBIN2	19	m	0	37	144	190	2617	3.54	(2.40- 5.23)
SADOWS	2	m	0	11	21	18	81	2.36	(0.97- 5.74)
TIZZAN	3	m	0	26	57	180	305	0.77	(0.47- 1.27)
*TULINI	3	m	1	-	-	-	-	4.05	(1.95- 8.40)
WYNDE7	40	m	0	30	152	64	918	2.83	(1.78- 4.51)
Partial Totals				336	87754	703	251585		

*prospective study ~ With 0.5 adjustment for zero

REF	NRR	SEX	AD	Ys	Ws	Qs	Ps
ABELIN	8	m	0	3.42	1.83	8.20	0.0000
ARMADA	3	m	0	2.08	1.31	0.79	0.0175
*BENSHL	3	m	1	1.89	6.79	2.34	0.0000
*BEST	16	m	1	1.08	1.55	0.08	0.1788
BOFFET	4	m	0	2.12	22.05	14.84	0.0000
*BOUCOT	7	m	0	2.34	0.44	0.47	0.1214
*CEDERL	85	m	2	2.03	7.43	3.92	0.0000
*CPSI	183	m	1	0.75	27.31	8.42	0.0001
*CPSII	1	m	2	1.65	67.79	8.15	0.0000
DAMBER	4	m	0	1.60	3.18	0.28	0.0043
DEAN3	179	m	0	-0.93	0.48	2.40	0.5175
*DORN	41	m	0	0.38	4.44	3.76	0.4219
GARDIN	4	c	0	2.51	0.95	1.38	0.0147
*HAMMON	123	m	0	0.05	4.77	7.49	0.9143
*HEIN	2	m	0	3.22	0.96	3.54	0.0016
JOLY	13	m	0	1.47	8.56	0.25	0.0000
*LANGE	8	m	0	2.13	4.57	3.11	0.0000
*LANGE	4	f	0	1.72	4.71	0.84	0.0002
Subtotal LANGE				1.92	9.28	3.95	
LEVIN	29	m	1	0.34	10.16	9.33	0.2735
LUBIN2	19	m	0	1.26	25.24	0.04	0.0000
SADOWS	2	m	0	0.86	4.84	0.96	0.0591
TIZZAN	3	m	0	-0.26	15.42	37.51	0.3117
*TULINI	3	m	1	1.40	7.20	0.07	0.0002
WYNDE7	40	m	0	1.04	17.66	1.21	0.0000

Table 1E13 - 5

IESLC - Meta-analysis of Ever Smoking (or Current if Ever not available), Cigars only
All LC types
Least adjusted

	N	24
	NS	23
	Wt	249.64
Het	Chi	119.38
Het	df	23
Het	P	***
Fixed	RR	3.68
	RRl	3.25
	RRu	4.16
	P	+++
Random	RR	3.79
	RRl	2.72
	RRu	5.27
	P	+++
Asymm	P	N.S.

Table 1E13 - 6

IESLC - Meta-analysis of Ever Smoking (or Current if Ever not available), Cigars only
All LC types
Least adjusted

		Sex			
		combined	male	female	Total
N		1	22	1	24
NS		1	22	1	24
Wt		0.95	243.99	4.71	249.64
Het	Chi	0.00	117.12	0.00	119.38
Het	df	0	21	0	23
Het	P	N.S.	***	N.S.	***
Fixed	RR	12.30	3.63	5.61	3.68
	RRl	1.64	3.20	2.27	3.25
	RRu	92.33	4.12	13.84	4.16
	P	+	+++	+++	+++
Random	RR	12.30	3.63	5.61	3.79
	RRl	1.64	2.57	2.27	2.72
	RRu	92.33	5.13	13.84	5.27
	P	+	+++	+++	+++
Between	Chi				2.26
Between	df				2
Between	P				N.S.
Btwn(F)	P				N.S.
Btwn(R)	P				N.S.

Table 1E13 - 7

IESLC - Meta-analysis of Ever Smoking (or Current if Ever not available), Cigars only
All LC types
Excluded studies (and stage at which they were excluded)

1 BOUCHA BUELL LAURIL MZILEN

2 ABRAHA AGUDO AKIBA ALDERS AMANDU AMES ANDERS ARCHER AUSTIN AUVINE AXELSO AXELSS BAND BARBON BECHER BERRIN
BLOHMK BLOT1 BLOT2 BLOT3 BLOT4 BRESLO BRETT BROCKM BROSS BROWN1 BROWN2 BUFFLE BYERS1 BYERS2 CARPEN CASCO2
CASCOR CHAN CHANG CHATZI CHEN CHEN2 CHEN3 CHIAZZ CHOI CHOW CHYOU COMSTO COOKSO CORREA DARBY DAVEYS
DEAN DEAN2 DEKLER DESTE2 DESTEF DOCKER DOLL DOLL2 DORANT DORGAN DOSEME DROSTE DU DUNN EBELIN ENGELA
ENSTRO ESAKI FAN GAO GAO2 GARCIA GARSHI GENG GER GILLIS GODLEY GOLLED GOODMA GRAHAM GREGOR GSELL
HAENSZ HAMMO2 HANSEN HEGMAN HENNEK HINDS HIRAOK HIRAY2 HIRAYA HITOSU HOLE HOROWI HORWIT HU HU2 HUANG
HUMBLE ISHIMA JAHN JAIN JARUP JARVHO JEDRYC JIANG JONES JUSSAW KAISE2 KAISER KANELL KATSOU KAUFMA KELLER
KHUDER KIHARA KINLEN KJUUS KNEKT KO KOHLME KOO KOULUM KREUZE KREYBE KUBIK LAMTH LAMWK LAMWK2 LAUSSM
LEI LEMARC LETOUR LIAW LICKIN LIDDEL LIU LIU2 LIU3 LIU4 LIU5 LOMBA2 LOMBAR LUBIN LUO MACLEN
MAGNUS MARSH MARSH2 MARTIS MASTRA MATOS MATSUD MCCONN MCDUFF MCLAUG MIGRAN MILLER MILLS MOLLO MRFIT MRFITR
MURATA NAM NOTAN2 NOTANI NOU ODRISC ORMOS OSANN OSANN2 PARKIN PASTOR PAWLEG PERNU PERSH2 PERSHA PETO
PEZZO2 PEZZOT PIKE PISANI POFFIJ POLEDN PRESCO QIAO QIAO2 RACHTA RADZIK RANDIG REN RESTRE RIMING RONCO
ROOTS ROTHSC SAARIK SANKAR SCHWA2 SCHWAR SEGI SEGI2 SEOW SHAW SHIMIZ SIEMIA SIMARA SIMONA SITAS SOBUE
SOBUE2 SPEIZE SPITZ STASZE STAYNE STOCKS STOCKW STUCKE SUN SUZUK2 SUZUKI SVENSS TANG TANG2 TAO TENKAN
TOKARS TOUSEY TSUGAN TVERDA ULMER VANDER VEIERO VUTUC WAKAI WALD WANG WANG2 WANG3 WANG4 WARSIN WATSON
WICKLU WIGLE WILKIN WU WU2 WUNSCH WUWILL WYNDE2 WYNDE3 WYNDE4 WYNDE5 WYNDE6 WYNDE8 XIANGZ XU XU2
XU3 XU4 YAMAGU YONG YUAN ZHANG ZHENG ZHOU

5 WYNDER

9 BENHAM

Table 1E13 - 8
Potentially overlapping studies

REF	REFGP	PRINC	OVERLAP/LINK
LUBIN2	LUBIN2	1	Lubin-combined
HEIN	PRESCO	1	Subset of PRESCO
LANGE	PRESCO	1	Subset of PRESCO
BENSHL	TANG2	1	Subset of TANG2
WYNDE7	WYNDE6	2	WYNDE5/6/7/8
CPSI	CPSI	1	CPSI overall
BOFFET	BOFFET	1	BOFFET-combined

Table 1E14 -

IESLC - Meta-analysis of Current Smoking (or Ever if Current not available), Cigars only
All LC types

This analysis is restricted to results for:
1) Non-dose-response data
2) Smokers of cigars only
3) Results complete enough for use in metaanalysis

Within each study, results are then selected (in the following order of preference, within each sex) for:
4) SMKSTA: current smokers, ever smokers
5) DENOM: never smoked anything, (never +1 = +long term ex)
6) Followup period (prospective studies): whole study (coded as 0) or longest available
7) LCtype: all or nearest available, at least Squamous and Adeno. (q = squamous, s = small,
l = large, a = adeno, mix = mixed, alv = alveolar)
8) Race: all or nearest available, otherwise by race (wh or w = white, bl or b = black, hi = hispanic
ch = chinese, jap = japanese, haw = hawaiian, w+o = white + oriental, sca = scandinavian, as = asian)
9) For overlapping studies: principal rather than subsidiary studies
Finally by Age: whole study (coded as 0) if available, otherwise by widest available age group
and then for single sex results (m, f) in preference to combined sex results (c).

Results adjusted (AD) for the most potential confounders are then chosen in Sections -1 to -3
(and those which actually differ from the adjusted results in Table 1E13 - 1 are marked 'x' in Section -1)
and results adjusted for the least confounders in Sections -4 to -6. (Those least adjusted results which
actually differ from the most adjusted as marked 'x' in column X in Section -4)
(Results adjusted for an unknown number of confounder(s) are coded as 20.)

Section -7 shows excluded studies, together with the stage (as above) at which no qualifying
results were found.

Section -8 lists the potentially overlapping studies which have been included (1=principal, 2=subsidiary).

Section -9 lists any results which would have been included in preference except that they had data not complete
enough for use in meta-analysis, with their significance (yes/no), if known, and any further comment as entered
on the database.

In addition to those mentioned above, the following fields, levels and abbreviations are used:
* or nk = not known, n = no, y = yes, ot = other
ev = ever, cu = current, nev = never
REF: 6-character study reference
NRR: number of the RR on the database within the study
ST : study type (CC = case control, pr or prosp = prospective)
NLC: number of lung cancer cases in whole study
R : risky occupational population (n = no, m = mining, o = other risky)
VB : national cigarette type (V = at least 75% Virginia, bl = at least 75% blended, ot = other)
P : any proxy use
H : full histological confirmation
De : derivation of RR/CI (or = original, st = standard method, ot = other method of estimation)

Table 1E14 - 1

IESLC - Meta-analysis of Current Smoking (or Ever if Current not available), Cigars only
All LC types
Most adjusted

REF	NRR	1E13	SEX	AGEL	AGEH	RACE	YF	LC TYPE	LOC	START	ST	NLC	R	VB	P	H	AD	SM	DENOM	De
ABELIN	51		m	0	0	all	-	all	Eu:wst	1941	CC	118	n	bl	y	n	1	ev	nev any	st
ARMADA	3		m	0	0	all	-	all	Eu:wst	1986	CC	325	n	bl	n	y	0	ev	nev any	st
BENSHL	3		m	0	0	all	0	all	Eu:UK	1967	pr	486	n	V	n	n	1	cu	nev any	ot
BEST	16		m	0	0	all	0	all	NAmer	1955	pr	381	n	V	n	n	1	ev	nev any	ot
BOFFET	25	x	m	0	0	all	-	all	Eu:mul	1988	CC	5621	n	bl	y	n	2	cu	nev any	or
BOUCOT	119		m	0	0	all	0	all	NAmer	1951	pr	121	n	bl	n	n	2	ev	nev any	ot
CEDERL	85		m	0	0	all	16	all	Eu:Sca	1963	pr	491	n	bl	n	n	2	cu	nev any	ot
CPSI	153	x	m	35	99	wh	0	all	NAmer	1959	pr	5138	n	bl	n	n	1	cu	nev any	ot
CPSII	1		m	0	0	all	0	all	NAmer	1982	pr	3229	n	bl	n	n	2	cu	nev any	or
DAMBER	4		m	0	0	all	-	all	Eu:Sca	1972	CC	579	n	bl	y	n	0	ev	nev any	st
DEAN3	179		m	0	0	all	-	all	Eu:UK	1969	CC	766	n	V	y	n	0	cu	nev any	ot
DORN	22	x	m	0	0	wh	15	all	NAmer	1954	pr	5097	n	bl	n	n	2	cu	nev any	ot
GARDIN	4		c	0	0	all	-	all	Eu:UK	1988	CC	143	n	V	y	n	0	cu	nev any	st
HAMMON	111		m	0	0	wh	0	all	NAmer	1952	pr	448	n	bl	n	n	1	ev	nev any	ot
HEIN	2		m	0	0	all	0	all	Eu:Sca	1970	pr	144	n	bl	n	n	0	cu	nev any	st
JOLY	17	x	m	0	0	all	-	all	SCAmer	1978	CC	826	n	bl	n	n	0	cu	nev any	st
LANGE	17		m	0	0	all	0	all	Eu:Sca	1976	pr	268	n	bl	n	n	1	cu	nev any	or
LANGE	13		f	0	0	all	0	all	Eu:Sca	1976	pr	268	n	bl	n	n	1	cu	nev any	or
LEVIN	29		m	0	0	all	-	all	NAmer	1938	CC	475	n	bl	n	n	1	ev	nev any	st
LUBIN2	4	x	m	0	0	all	-	all	Eu:mul	1976	CC	7804	n	bl	n	y	2	cu	nev any	ot
SADOWS	29		m	0	0	wh	-	all	NAmer	1938	CC	477	n	bl	n	n	1	ev	nev any	ot
TIZZAN	3		m	0	0	all	-	all	Eu:wst	1959	CC	1358	n	bl	n	n	0	ev	nev any	st
TULINI	26		m	0	0	all	0	all	Eu:Sca	1967	pr	472	n	bl	n	n	3	cu	nev any	or
WYNDE7	2	x	m	0	0	all	-	all	NAmer	1977	CC	2085	n	bl	n	y	0	cu	nev any	st

Table 1E14 - 2

IESLC - Meta-analysis of Current Smoking (or Ever if Current not available), Cigars only
All LC types
Most adjusted

REF	NRR	SEX	AD	Number Exposed Case	Number Exposed Cont	Non-exposed Case	Non-exposed Cont	RR	95.00%CI
ABELIN	51	m	1	-	-	-	-	25.09	(5.77- 109.07)
ARMADA	3	m	0	3	6	4	64	8.00	(1.44- 44.45)
*BENSHL	3	m	1	-	-	-	-	6.62	(3.12- 14.05)
*BEST	16	m	1	-	-	-	-	2.94	(0.61- 14.15)
BOFFET	25	m	2	-	-	-	-	10.60	(5.90- 19.10)
*BOUCOT	119	m	2	-	-	-	-	8.81	(0.45- 170.58)
*CEDERL	85	m	2	-	-	-	-	7.60	(3.70- 15.59)
*CPSI	153	m	1	-	-	-	-	3.30	(2.68- 4.06)
*CPSII	1	m	2	-	-	-	-	5.20	(4.10- 6.60)
DAMBER	4	m	0	7	7	42	208	4.95	(1.65- 14.86)
DEAN3	179	m	0	0	26	24	510	0.39~	(0.02- 6.64)
*DORN	22	m	2	-	-	-	-	1.66	(1.18- 2.34)
GARDIN	4	c	0	3	2	5	41	12.30	(1.64- 92.33)
*HAMMON	111	m	1	-	-	-	-	1.02	(0.42- 2.51)
*HEIN	2	m	0	23	420	1	457	25.03	(3.39- 184.50)
JOLY	17	m	0	36	141	12	218	4.64	(2.33- 9.22)
*LANGE	17	m	1	-	-	-	-	6.00	(2.20- 17.00)
*LANGE	13	f	1	-	-	-	-	4.90	(3.00- 12.00)
Subtotal LANGE								5.22	(2.94- 9.27)
LEVIN	29	m	1	-	-	-	-	1.41	(0.76- 2.60)
LUBIN2	4	m	2	-	-	-	-	5.18	(3.72- 7.22)
SADOWS	29	m	1	-	-	-	-	2.98	(1.06- 8.33)
TIZZAN	3	m	0	26	57	180	305	0.77	(0.47- 1.27)
*TULINI	26	m	3	-	-	-	-	4.16	(2.00- 8.63)
WYNDE7	2	m	0	18	82	64	918	3.15	(1.78- 5.57)
Partial Totals				116	741	332	2721		

*prospective study ~ With 0.5 adjustment for zero

REF	NRR	SEX	AD	Ys	Ws	Qs	Ps
ABELIN	51	m	1	3.22	1.78	6.71	0.0000
ARMADA	3	m	0	2.08	1.31	0.84	0.0175
*BENSHL	3	m	1	1.89	6.79	2.52	0.0000
*BEST	16	m	1	1.08	1.55	0.06	0.1788
BOFFET	25	m	2	2.36	11.13	13.01	0.0000
*BOUCOT	119	m	2	2.18	0.44	0.35	0.1509
*CEDERL	85	m	2	2.03	7.43	4.16	0.0000
*CPSI	153	m	1	1.19	89.06	0.66	0.0000
*CPSII	1	m	2	1.65	67.79	9.22	0.0000
DAMBER	4	m	0	1.60	3.18	0.33	0.0043
DEAN3	179	m	0	-0.93	0.48	2.35	0.5175
*DORN	22	m	2	0.51	32.78	19.59	0.0037
GARDIN	4	c	0	2.51	0.95	1.43	0.0147
*HAMMON	111	m	1	0.02	4.81	7.63	0.9654
*HEIN	2	m	0	3.22	0.96	3.62	0.0016
JOLY	17	m	0	1.53	8.14	0.53	0.0000
*LANGE	17	m	1	1.79	3.68	0.96	0.0006
*LANGE	13	f	1	1.59	8.00	0.77	0.0000
Subtotal LANGE				1.65	11.67	1.73	
LEVIN	29	m	1	0.34	10.16	8.90	0.2735
LUBIN2	4	m	2	1.64	34.94	4.65	0.0000
SADOWS	29	m	1	1.09	3.62	0.13	0.0379
TIZZAN	3	m	0	-0.26	15.42	36.46	0.3117
*TULINI	26	m	3	1.43	7.19	0.15	0.0001
WYNDE7	2	m	0	1.15	11.84	0.21	0.0001

Table 1E14 - 2

IESLC - Meta-analysis of Current Smoking (or Ever if Current not available), Cigars only
All LC types
Most adjusted

	N	24
	NS	23
	Wt	333.42
Het	Chi	125.25
Het	df	23
Het	P	***
Fixed	RR	3.60
	RRl	3.23
	RRu	4.00
	P	+++
Random	RR	3.90
	RRl	2.88
	RRu	5.29
	P	+++
Asymm	P	N.S.

Table 1E14 - 3

IESLC - Meta-analysis of Current Smoking (or Ever if Current not available), Cigars only
All LC types
Most adjusted

	Sex combined	male	female	Total
N	1	22	1	24
NS	1	22	1	24
Wt	0.95	324.48	8.00	333.42
Het Chi	0.00	123.01	0.00	125.25
Het df	0	21	0	23
Het P	N.S.	***	N.S.	***
Fixed RR	12.30	3.56	4.90	3.60
RRl	1.64	3.19	2.45	3.23
RRu	92.33	3.96	9.80	4.00
P	+	+++	+++	+++
Random RR	12.30	3.78	4.90	3.90
RRl	1.64	2.75	2.45	2.88
RRu	92.33	5.20	9.80	5.29
P	+	+++	+++	+++
Between Chi				2.24
Between df				2
Between P				N.S.
Btwn(F) P				N.S.
Btwn(R) P				N.S.

	Smoking status ever	current	Total
N	9	15	24
NS	9	14	23
Wt	42.26	291.16	333.42
Het Chi	33.46	59.79	125.25
Het df	8	14	23
Het P	***	***	***
Fixed RR	1.59	4.05	3.60
RRl	1.18	3.61	3.23
RRu	2.16	4.54	4.00
P	++	+++	+++
Random RR	2.79	4.67	3.90
RRl	1.37	3.49	2.88
RRu	5.66	6.25	5.29
P	++	+++	+++
Between Chi			32.00
Between df			1
Between P			***
Btwn(F) P			*
Btwn(R) P			N.S.

Table 1E14 - 4

IESLC - Meta-analysis of Current Smoking (or Ever if Current not available), Cigars only
All LC types
Least adjusted

REF	NRR	X	SEX	AGEL	AGEH	RACE	YF	LC TYPE	LOC	START	ST	NLC	R	VB	P	H	AD	SM	DENOM	De
ABELIN	8	x	m	0	0	all	-	all	Eu:wst	1941	CC	118	n	bl	y	n	0	ev	nev any	st
ARMADA	3		m	0	0	all	-	all	Eu:wst	1986	CC	325	n	bl	n	y	0	ev	nev any	st
BENSHL	3		m	0	0	all	0	all	Eu:UK	1967	pr	486	n	V	n	n	1	cu	nev any	ot
BEST	16		m	0	0	all	0	all	NAmer	1955	pr	381	n	V	n	n	1	ev	nev any	ot
BOFFET	25		m	0	0	all	-	all	Eu:mul	1988	CC	5621	n	bl	y	n	2	cu	nev any	or
BOUCOT	7	x	m	0	0	all	0	all	NAmer	1951	pr	121	n	bl	n	n	0	ev	nev any	ot
CEDERL	85		m	0	0	all	16	all	Eu:Sca	1963	pr	491	n	bl	n	n	2	cu	nev any	ot
CPSI	153		m	35	99	wh	0	all	NAmer	1959	pr	5138	n	bl	n	n	1	cu	nev any	ot
CPSII	1		m	0	0	all	0	all	NAmer	1982	pr	3229	n	bl	n	n	2	cu	nev any	or
DAMBER	4		m	0	0	all	-	all	Eu:Sca	1972	CC	579	n	bl	y	n	0	ev	nev any	st
DEAN3	179		m	0	0	all	-	all	Eu:UK	1969	CC	766	n	V	y	n	0	cu	nev any	ot
DORN	22		m	0	0	wh	15	all	NAmer	1954	pr	5097	n	bl	n	n	2	cu	nev any	ot
GARDIN	4		c	0	0	all	-	all	Eu:UK	1988	CC	143	n	V	y	n	0	cu	nev any	st
HAMMON	123	x	m	0	0	wh	0	all	NAmer	1952	pr	448	n	bl	n	n	0	ev	nev any	st
HEIN	2		m	0	0	all	0	all	Eu:Sca	1970	pr	144	n	bl	n	n	0	cu	nev any	st
JOLY	17		m	0	0	all	-	all	SCAmer	1978	CC	826	n	bl	n	n	0	cu	nev any	st
LANGE	8	x	m	0	0	all	0	all	Eu:Sca	1976	pr	268	n	bl	n	n	0	cu	nev any	st
LANGE	4	x	f	0	0	all	0	all	Eu:Sca	1976	pr	268	n	bl	n	n	0	cu	nev any	st
LEVIN	29		m	0	0	all	-	all	NAmer	1938	CC	475	n	bl	n	n	1	ev	nev any	st
LUBIN2	3	x	m	0	0	all	-	all	Eu:mul	1976	CC	7804	n	bl	n	y	0	cu	nev any	st
SADOWS	2	x	m	0	0	wh	-	all	NAmer	1938	CC	477	n	bl	n	n	0	ev	nev any	st
TIZZAN	3		m	0	0	all	-	all	Eu:wst	1959	CC	1358	n	bl	n	n	0	ev	nev any	st
TULINI	3	x	m	0	0	all	0	all	Eu:Sca	1967	pr	472	n	bl	n	n	1	cu	nev any	or
WYNDE7	2		m	0	0	all	-	all	NAmer	1977	CC	2085	n	bl	n	y	0	cu	nev any	st

Table 1E14 - 5

IESLC - Meta-analysis of Current Smoking (or Ever if Current not available), Cigars only
All LC types
Least adjusted

REF	NRR	SEX	AD	Number Exposed Case	Number Exposed Cont	Non-exposed Case	Non-exposed Cont	RR	95.00%CI
ABELIN	8	m	0	33	99	2	183	30.50	(7.17- 129.78)
ARMADA	3	m	0	3	6	4	64	8.00	(1.44- 44.45)
*BENSHL	3	m	1	-	-	-	-	6.62	(3.12- 14.05)
*BEST	16	m	1	-	-	-	-	2.94	(0.61- 14.15)
BOFFET	25	m	2	-	-	-	-	10.60	(5.90- 19.10)
*BOUCOT	7	m	0	3	5084	0	7551	10.40~	(0.54- 201.23)
*CEDERL	85	m	2	-	-	-	-	7.60	(3.70- 15.59)
*CPSI	153	m	1	-	-	-	-	3.30	(2.68- 4.06)
*CPSII	1	m	2	-	-	-	-	5.20	(4.10- 6.60)
DAMBER	4	m	0	7	7	42	208	4.95	(1.65- 14.86)
DEAN3	179	m	0	0	26	24	510	0.39~	(0.02- 6.64)
*DORN	22	m	2	-	-	-	-	1.66	(1.18- 2.34)
GARDIN	4	c	0	3	2	5	41	12.30	(1.64- 92.33)
*HAMMON	123	m	0	7	51480	15	115884	1.05	(0.43- 2.58)
*HEIN	2	m	0	23	420	1	457	25.03	(3.39- 184.50)
JOLY	17	m	0	36	141	12	218	4.64	(2.33- 9.22)
*LANGE	8	m	0	47	808	5	721	8.39	(3.35- 20.97)
*LANGE	4	f	0	14	770	7	2159	5.61	(2.27- 13.84)
Subtotal LANGE								6.84	(3.59- 13.01)
LEVIN	29	m	1	-	-	-	-	1.41	(0.76- 2.60)
LUBIN2	3	m	0	57	185	190	2617	4.24	(3.05- 5.91)
SADOWS	2	m	0	11	21	18	81	2.36	(0.97- 5.74)
TIZZAN	3	m	0	26	57	180	305	0.77	(0.47- 1.27)
*TULINI	3	m	1	-	-	-	-	4.05	(1.95- 8.40)
WYNDE7	2	m	0	18	82	64	918	3.15	(1.78- 5.57)
Partial Totals				288	59188	569	131917		

*prospective study ~ With 0.5 adjustment for zero

REF	NRR	SEX	AD	Ys	Ws	Qs	Ps
ABELIN	8	m	0	3.42	1.83	8.52	0.0000
ARMADA	3	m	0	2.08	1.31	0.87	0.0175
*BENSHL	3	m	1	1.89	6.79	2.68	0.0000
*BEST	16	m	1	1.08	1.55	0.05	0.1788
BOFFET	25	m	2	2.36	11.13	13.46	0.0000
*BOUCOT	7	m	0	2.34	0.44	0.51	0.1214
*CEDERL	85	m	2	2.03	7.43	4.37	0.0000
*CPSI	153	m	1	1.19	89.06	0.40	0.0000
*CPSII	1	m	2	1.65	67.79	10.18	0.0000
DAMBER	4	m	0	1.60	3.18	0.36	0.0043
DEAN3	179	m	0	-0.93	0.48	2.32	0.5175
*DORN	22	m	2	0.51	32.78	18.66	0.0037
GARDIN	4	c	0	2.51	0.95	1.47	0.0147
*HAMMON	123	m	0	0.05	4.77	7.01	0.9143
*HEIN	2	m	0	3.22	0.96	3.69	0.0016
JOLY	17	m	0	1.53	8.14	0.61	0.0000
*LANGE	8	m	0	2.13	4.57	3.43	0.0000
*LANGE	4	f	0	1.72	4.71	1.01	0.0002
Subtotal LANGE				1.92	9.28	4.43	
LEVIN	29	m	1	0.34	10.16	8.55	0.2735
LUBIN2	3	m	0	1.45	34.97	1.19	0.0000
SADOWS	2	m	0	0.86	4.84	0.79	0.0591
TIZZAN	3	m	0	-0.26	15.42	35.58	0.3117
*TULINI	3	m	1	1.40	7.20	0.14	0.0002
WYNDE7	2	m	0	1.15	11.84	0.15	0.0001

Table 1E14 - 5

IESLC - Meta-analysis of Current Smoking (or Ever if Current not available), Cigars only
All LC types
Least adjusted

	N	24
	NS	23
	Wt	332.32
Het	Chi	126.00
Het	df	23
Het	P	***
Fixed	RR	3.53
	RRl	3.17
	RRu	3.93
	P	+++
Random	RR	3.92
	RRl	2.89
	RRu	5.32
	P	+++
Asymm	P	N.S.

Table 1E14 - 6

IESLC - Meta-analysis of Current Smoking (or Ever if Current not available), Cigars only
All LC types
Least adjusted

		combined	Sex male	female	Total
	N	1	22	1	24
	NS	1	22	1	24
	Wt	0.95	326.67	4.71	332.32
Het	Chi	0.00	123.49	0.00	126.00
Het	df	0	21	0	23
Het	P	N.S.	***	N.S.	***
Fixed	RR	12.30	3.49	5.61	3.53
	RRl	1.64	3.13	2.27	3.17
	RRu	92.33	3.89	13.84	3.93
	P	+	+++	+++	+++
Random	RR	12.30	3.78	5.61	3.92
	RRl	1.64	2.75	2.27	2.89
	RRu	92.33	5.19	13.84	5.32
	P	+	+++	+++	+++
Between	Chi				2.52
Between	df				2
Between	P				N.S.
Btwn(F)	P				N.S.
Btwn(R)	P				N.S.

Table 1E14 - 7

IESLC - Meta-analysis of Current Smoking (or Ever if Current not available), Cigars only
All LC types
Excluded studies (and stage at which they were excluded)

1	BOUCHA	BUELL	LAURIL	MZILEN												
2	ABRAHA	AGUDO	AKIBA	ALDERS	AMANDU	AMES	ANDERS	ARCHER	AUSTIN	AUVINE	AXELSO	AXELSS	BAND	BARBON	BECHER	BERRIN
	BLOHMK	BLOT1	BLOT2	BLOT3	BLOT4	BRESLO	BRETT	BROCKM	BROSS	BROWN1	BROWN2	BUFFLE	BYERS1	BYERS2	CARPEN	CASCO2
	CASCOR	CHAN	CHANG	CHATZI	CHEN	CHEN2	CHEN3	CHIAZZ	CHOI	CHOW	CHYOU	COMSTO	COOKSO	CORREA	DARBY	DAVEYS
	DEAN	DEAN2	DEKLER	DESTE2	DESTEF	DOCKER	DOLL	DOLL2	DORANT	DORGAN	DOSEME	DROSTE	DU	DUNN	EBELIN	ENGELA
	ENSTRO	ESAKI	FAN	GAO	GAO2	GARCIA	GARSHI	GENG	GER	GILLIS	GODLEY	GOLLED	GOODMA	GRAHAM	GREGOR	GSELL
	HAENSZ	HAMMO2	HANSEN	HEGMAN	HENNEK	HINDS	HIRAOK	HIRAY2	HIRAYA	HITOSU	HOLE	HOROWI	HORWIT	HU	HU2	HUANG
	HUMBLE	ISHIMA	JAHN	JAIN	JARUP	JARVHO	JEDRYC	JIANG	JONES	JUSSAW	KAISE2	KAISER	KANELL	KATSOU	KAUFMA	KELLER
	KHUDER	KIHARA	KINLEN	KJUUS	KNEKT	KO	KOHLME	KOO	KOULUM	KREUZE	KREYBE	KUBIK	LAMTH	LAMWK	LAMWK2	LAUSSM
	LEI	LEMARC	LETOUR	LIAW	LICKIN	LIDDEL	LIU	LIU2	LIU3	LIU4	LIU5	LOMBA2	LOMBAR	LUBIN	LUO	MACLEN
	MAGNUS	MARSH	MARSH2	MARTIS	MASTRA	MATOS	MATSUD	MCCONN	MCDUFF	MCLAUG	MIGRAN	MILLER	MILLS	MOLLO	MRFIT	MRFITR
	MURATA	NAM	NOTAN2	NOTANI	NOU	ODRISC	ORMOS	OSANN	OSANN2	PARKIN	PASTOR	PAWLEG	PERNU	PERSH2	PERSHA	PETO
	PEZZO2	PEZZOT	PIKE	PISANI	POFFIJ	POLEDN	PRESCO	QIAO	QIAO2	RACHTA	RADZIK	RANDIG	REN	RESTRE	RIMING	RONCO
	ROOTS	ROTHSC	SAARIK	SANKAR	SCHWA2	SCHWAR	SEGI	SEGI2	SEOW	SHAW	SHIMIZ	SIEMIA	SIMARA	SIMONA	SITAS	SOBUE
	SOBUE2	SPEIZE	SPITZ	STASZE	STAYNE	STOCKS	STOCKW	STUCKE	SUN	SUZUK2	SUZUKI	SVENSS	TANG	TANG2	TAO	TENKAN
	TOKARS	TOUSEY	TSUGAN	TVERDA	ULMER	VANDER	VEIERO	VUTUC	WAKAI	WALD	WANG	WANG2	WANG3	WANG4	WARSIN	WATSON
	WICKLU	WIGLE	WILKIN	WU	WU2	WUNSCH	WUWILL	WYNDE2	WYNDE3	WYNDE4	WYNDE5	WYNDE6	WYNDE8	XIANGZ	XU	XU2
	XU3	XU4	YAMAGU	YONG	YUAN	ZHANG	ZHENG	ZHOU								
5	WYNDER															
9	BENHAM															

Table 1E14 - 8
Potentially overlapping studies

REF	REFGP	PRINC	OVERLAP/LINK
LUBIN2	LUBIN2	1	Lubin-combined
HEIN	PRESCO	1	Subset of PRESCO
LANGE	PRESCO	1	Subset of PRESCO
BENSHL	TANG2	1	Subset of TANG2
WYNDE7	WYNDE6	2	WYNDE5/6/7/8
CPSI	CPSI	1	CPSI overall
BOFFET	BOFFET	1	BOFFET-combined

Table 1E15 -

IESLC - Meta-analysis of Ex Smoking, Cigars only
All LC types

This analysis is restricted to results for:
1) Non-dose-response data
2) Smokers of cigars only
3) Ex smokers
4) Results complete enough for use in metaanalysis

Within each study, results are then selected (in the following order of preference, within each sex) for:
5) DENOM: never smoked anything, (never +1 = +long term ex)
6) Followup period (prospective studies): whole study (coded as 0) or longest available
7) LCtype: all or nearest available, at least Squamous and Adeno. (q = squamous, s = small,
l = large, a = adeno, mix = mixed, alv = alveolar)
8) Race: all or nearest available, otherwise by race (wh or w = white, bl or b = black, hi = hispanic
ch = chinese, jap = japanese, haw = hawaiian, w+o = white + oriental, sca = scandinavian, as = asian)
9) For overlapping studies: principal rather than subsidiary studies
Finally by Age: whole study (coded as 0) if available, otherwise by widest available age group
and then for single sex results (m, f) in preference to combined sex results (c).

Results adjusted (AD) for the most potential confounders are then chosen in Sections -1 to -3
and results adjusted for the least confounders in Sections -4 to -6. (Those least adjusted results which
actually differ from the most adjusted as marked 'x' in column X in Section -4)
(Results adjusted for an unknown number of confounder(s) are coded as 20.)

Section -7 shows excluded studies, together with the stage (as above) at which no qualifying
results were found.

Section -8 lists the potentially overlapping studies which have been included (1=principal, 2=subsidiary).

Section -9 lists any results which would have been included in preference except that they had data not complete
enough for use in meta-analysis, with their significance (yes/no), if known, and any further comment as entered
on the database.

In addition to those mentioned above, the following fields, levels and abbreviations are used:
* or nk = not known, n = no, y = yes, ot = other
nev = never
REF: 6-character study reference
NRR: number of the RR on the database within the study
ST : study type (CC = case control, pr or prosp = prospective)
NLC: number of lung cancer cases in whole study
R : risky occupational population (n = no, m = mining, o = other risky)
VB : national cigarette type (V = at least 75% Virginia, bl = at least 75% blended, ot = other)
P : any proxy use
H : full histological confirmation
De : derivation of RR/CI (or = original, st = standard method, ot = other method of estimation)

Table 1E15 - 1

IESLC - Meta-analysis of Ex Smoking, Cigars only
All LC types
Most adjusted

REF	NRR	SEX	AGEL	AGEH	RACE	YF	LC TYPE	LOC	START	ST	NLC	R	VB	P	H	AD	DENOM		De
BOFFET	35	m	0	0	all	-	all	Eu:mul	1988	CC	5621	n	bl	y	n	2	nev	any	ot
DORN	138	m	35	84	wh	8	all	NAmer	1954	pr	5097	n	bl	n	n	1	nev	any	ot
JOLY	21	m	0	0	all	-	all	SCAmer	1978	CC	826	n	bl	n	n	0	nev	any	st
LUBIN2	12	m	0	0	all	-	all	Eu:mul	1976	CC	7804	n	bl	n	y	2	nev	any	or
WYNDE7	7	m	0	0	all	-	all	NAmer	1977	CC	2085	n	bl	n	y	0	nev	any	st

Table 1E15 - 2

IESLC - Meta-analysis of Ex Smoking, Cigars only
All LC types
Most adjusted

REF	NRR	SEX	AD	Number Exposed Case	Number Exposed Cont	Non-exposed Case	Non-exposed Cont	RR	95.00%CI
BOFFET	35	m	2	-	-	-	-	7.77	(4.38- 13.81)
*DORN	138	m	1	-	-	-	-	1.02	(0.41- 2.54)
JOLY	21	m	0	7	38	12	218	3.35	(1.24- 9.04)
LUBIN2	12	m	2	-	-	-	-	2.40	(1.20- 5.00)
WYNDE7	7	m	0	12	70	64	918	2.46	(1.27- 4.77)
Partial Totals				19	108	76	1136		

*prospective study

REF	NRR	SEX	AD	Ys	Ws	Qs	Ps
BOFFET	35	m	2	2.05	11.65	8.75	0.0000
*DORN	138	m	1	0.02	4.62	6.26	0.9660
JOLY	21	m	0	1.21	3.89	0.00	0.0172
LUBIN2	12	m	2	0.88	7.54	0.72	0.0162
WYNDE7	7	m	0	0.90	8.75	0.71	0.0078

	N	5
	NS	5
	Wt	36.45
Het	Chi	16.43
Het	df	4
Het	P	**
Fixed	RR	3.27
	RRl	2.36
	RRu	4.52
	P	+++
Random	RR	2.85
	RRl	1.45
	RRu	5.61
	P	++
Asymm	P	N.S.

Table 1E15 - 3

IESLC - Meta-analysis of Ex Smoking, Cigars only
All LC types
Most adjusted

	Sex			
	combined	male	female	Total
N		5		5
NS		5		5
Wt		36.45		36.45
Het Chi		16.43		16.43
Het df		4		4
Het P		**		**
Fixed RR		3.27		3.27
RRl		2.36		2.36
RRu		4.52		4.52
P		+++		+++
Random RR		2.85		2.85
RRl		1.45		1.45
RRu		5.61		5.61
P		++		++
Between Chi				
Between df				
Between P				N.S.
Btwn(F) P				N.S.
Btwn(R) P				N.S.

Too few RRs for analysis by factor

Table 1E15 - 4

IESLC - Meta-analysis of Ex Smoking, Cigars only
All LC types
Least adjusted

REF	NRR	X	SEX	AGEL	AGEH	RACE	YF	LC TYPE	LOC	START	ST	NLC	R	VB	P	H	AD	DENOM	De
BOFFET	35		m	0	0	all	-	all	Eu:mul	1988	CC	5621	n	bl	y	n	2	nev any	ot
DORN	138		m	35	84	wh	8	all	NAmer	1954	pr	5097	n	bl	n	n	1	nev any	ot
JOLY	21		m	0	0	all	-	all	SCAmer	1978	CC	826	n	bl	n	n	0	nev any	st
LUBIN2	11	x	m	0	0	all	-	all	Eu:mul	1976	CC	7804	n	bl	n	y	0	nev any	st
WYNDE7	7		m	0	0	all	-	all	NAmer	1977	CC	2085	n	bl	n	y	0	nev any	st

Table 1E15 - 5

IESLC - Meta-analysis of Ex Smoking, Cigars only
All LC types
Least adjusted

REF	NRR	SEX	AD	Number Exposed Case	Cont	Non-exposed Case	Cont	RR	95.00%CI
BOFFET	35	m	2	-	-	-	-	7.77	(4.38- 13.81)
*DORN	138	m	1	-	-	-	-	1.02	(0.41- 2.54)
JOLY	21	m	0	7	38	12	218	3.35	(1.24- 9.04)
LUBIN2	11	m	0	9	46	190	2617	2.69	(1.30- 5.59)
WYNDE7	7	m	0	12	70	64	918	2.46	(1.27- 4.77)
Partial Totals				28	154	266	3753		

*prospective study

REF	NRR	SEX	AD	Ys	Ws	Qs	Ps
BOFFET	35	m	2	2.05	11.65	8.23	0.0000
*DORN	138	m	1	0.02	4.62	6.54	0.9660
JOLY	21	m	0	1.21	3.89	0.00	0.0172
LUBIN2	11	m	0	0.99	7.22	0.34	0.0077
WYNDE7	7	m	0	0.90	8.75	0.84	0.0078

	N	5
	NS	5
	Wt	36.13
Het	Chi	15.96
Het	df	4
Het	P	**
Fixed	RR	3.35
	RRl	2.42
	RRu	4.65
	P	+++
Random	RR	2.92
	RRl	1.49
	RRu	5.72
	P	++
Asymm	P	N.S.

Table 1E15 - 6

IESLC - Meta-analysis of Ex Smoking, Cigars only
All LC types
Least adjusted

	combined	Sex male	female	Total
N		5		5
NS		5		5
Wt		36.13		36.13
Het Chi		15.96		15.96
Het df		4		4
Het P		**		**
Fixed RR		3.35		3.35
RRl		2.42		2.42
RRu		4.65		4.65
P		+++		+++
Random RR		2.92		2.92
RRl		1.49		1.49
RRu		5.72		5.72
P		++		++
Between Chi				
Between df				
Between P				N.S.
Btwn(F) P				N.S.
Btwn(R) P				N.S.

Table 1E15 - 7

IESLC - Meta-analysis of Ex Smoking, Cigars only
All LC types
Excluded studies (and stage at which they were excluded)

1 BOUCHA BUELL LAURIL MZILEN

2 ABRAHA AGUDO AKIBA ALDERS AMANDU AMES ANDERS ARCHER AUSTIN AUVINE AXELSO AXELSS BAND BARBON BECHER BERRIN
BLOHMK BLOT1 BLOT2 BLOT3 BLOT4 BRESLO BRETT BROCKM BROSS BROWN1 BROWN2 BUFFLE BYERS1 BYERS2 CARPEN CASCO2
CASCOR CHAN CHANG CHATZI CHEN CHEN2 CHEN3 CHIAZZ CHOI CHOW CHYOU COMSTO COOKSO CORREA DARBY DAVEYS
DEAN DEAN2 DEKLER DESTE2 DESTEF DOCKER DOLL DOLL2 DORANT DORGAN DOSEME DROSTE DU DUNN EBELIN ENGELA
ENSTRO ESAKI FAN GAO GAO2 GARCIA GARSHI GENG GER GILLIS GODLEY GOLLED GOODMA GRAHAM GREGOR GSELL
HAENSZ HAMMO2 HANSEN HEGMAN HENNEK HINDS HIRAOK HIRAY2 HIRAYA HITOSU HOLE HOROWI HORWIT HU HU2 HUANG
HUMBLE ISHIMA JAHN JAIN JARUP JARVHO JEDRYC JIANG JONES JUSSAW KAISE2 KAISER KANELL KATSOU KAUFMA KELLER
KHUDER KIHARA KINLEN KJUUS KNEKT KO KOHLME KOO KOULUM KREUZE KREYBE KUBIK LAMTH LAMWK LAMWK2 LAUSSM
LEI LEMARC LETOUR LIAW LICKIN LIDDEL LIU LIU2 LIU3 LIU4 LIU5 LOMBA2 LOMBAR LUBIN LUO MACLEN
MAGNUS MARSH MARSH2 MARTIS MASTRA MATOS MATSUD MCCONN MCDUFF MCLAUG MIGRAN MILLER MILLS MOLLO MRFIT MRFITR
MURATA NAM NOTAN2 NOTANI NOU ODRISC ORMOS OSANN OSANN2 PARKIN PASTOR PAWLEG PERNU PERSH2 PERSHA PETO
PEZZO2 PEZZOT PIKE PISANI POFFIJ POLEDN PRESCO QIAO QIAO2 RACHTA RADZIK RANDIG REN RESTRE RIMING RONCO
ROOTS ROTHSC SAARIK SANKAR SCHWA2 SCHWAR SEGI SEGI2 SEOW SHAW SHIMIZ SIEMIA SIMARA SIMONA SITAS SOBUE
SOBUE2 SPEIZE SPITZ STASZE STAYNE STOCKS STOCKW STUCKE SUN SUZUK2 SUZUKI SVENSS TANG TANG2 TAO TENKAN
TOKARS TOUSEY TSUGAN TVERDA ULMER VANDER VEIERO VUTUC WAKAI WALD WANG WANG2 WANG3 WANG4 WARSIN WATSON
WICKLU WIGLE WILKIN WU WU2 WUNSCH WUWILL WYNDE2 WYNDE3 WYNDE4 WYNDE5 WYNDE6 WYNDE8 XIANGZ XU XU2
XU3 XU4 YAMAGU YONG YUAN ZHANG ZHENG ZHOU

3 ABELIN ARMADA BENHAM BENSHL BEST BOUCOT CEDERL CPSI CPSII DAMBER DEAN3 GARDIN HAMMON HEIN LANGE LEVIN
SADOWS TIZZAN TULINI WYNDER

Table 1E15 - 8
Potentially overlapping studies

REF	REFGP	PRINC	OVERLAP/LINK
LUBIN2	LUBIN2	1	Lubin-combined
WYNDE7	WYNDE6	2	WYNDE5/6/7/8
BOFFET	BOFFET	1	BOFFET-combined

Table 1E16 -

IESLC - Meta-analysis of Ever Smoking, Mixed smokers
All LC types

This analysis is restricted to results for:
1) Non-dose-response data
2) Mixed smokers (cigarettes and pipe/cigar)
3) Ever smokers
4) Results complete enough for use in metaanalysis

Within each study, results are then selected (in the following order of preference, within each sex) for:
5) DENOM: never smoked anything, (never +1 = +long term ex)
6) Followup period (prospective studies): whole study (coded as 0) or longest available
7) LCtype: all or nearest available, at least Squamous and Adeno. (q = squamous, s = small,
l = large, a = adeno, mix = mixed, alv = alveolar)
8) Race: all or nearest available, otherwise by race (wh or w = white, bl or b = black, hi = hispanic
ch = chinese, jap = japanese, haw = hawaiian, w+o = white + oriental, sca = scandinavian, as = asian)
9) For overlapping studies: principal rather than subsidiary studies
Finally by Age: whole study (coded as 0) if available, otherwise by widest available age group
and then for single sex results (m, f) in preference to combined sex results (c).

Results adjusted (AD) for the most potential confounders are then chosen in Sections -1 to -3
and results adjusted for the least confounders in Sections -4 to -6. (Those least adjusted results which
actually differ from the most adjusted as marked 'x' in column X in Section -4)
(Results adjusted for an unknown number of confounder(s) are coded as 20.)

Section -7 shows excluded studies, together with the stage (as above) at which no qualifying
results were found.

Section -8 lists the potentially overlapping studies which have been included (1=principal, 2=subsidiary).

Section -9 lists any results which would have been included in preference except that they had data not complete
enough for use in meta-analysis, with their significance (yes/no), if known, and any further comment as entered
on the database.

In addition to those mentioned above, the following fields, levels and abbreviations are used:
* or nk = not known, n = no, y = yes, ot = other
nev = never
REF: 6-character study reference
NRR: number of the RR on the database within the study
ST : study type (CC = case control, pr or prosp = prospective)
NLC: number of lung cancer cases in whole study
R : risky occupational population (n = no, m = mining, o = other risky)
VB : national cigarette type (V = at least 75% Virginia, bl = at least 75% blended, ot = other)
P : any proxy use
H : full histological confirmation
De : derivation of RR/CI (or = original, st = standard method, ot = other method of estimation)

Table 1E16 - 1

IESLC - Meta-analysis of Ever Smoking, Mixed smokers
All LC types
Most adjusted

REF	NRR	SEX	AGEL	AGEH	RACE	YF	LC TYPE	LOC	START	ST	NLC	R	VB	P	H	AD	DENOM	De
ABELIN	48	m	0	0	all	-	all	Eu:wst	1941	CC	118	n	bl	y	n	1	nev any	st
ALDERS	2	m	0	0	all	-	all	Eu:UK	1977	CC	1448	n	V	n	n	1	nev any	ot
ARMADA	31	m	0	0	all	-	all	Eu:wst	1986	CC	325	n	bl	n	y	0	nev any	st
BEST	20	m	55	79	all	3	all	NAmer	1955	pr	381	n	V	n	n	0	nev any	st
BOFFET	26	m	0	0	all	-	all	Eu:mul	1988	CC	5621	n	bl	y	n	2	nev any	or
BOUCOT	116	m	0	0	all	0	all	NAmer	1951	pr	121	n	bl	n	n	2	nev any	ot
BRESLO	19	m	0	0	all	-	all	NAmer	1949	CC	518	n	bl	n	y	0	nev+1	st
CHOW	22	m	0	0	wh	0	all	NAmer	1966	pr	219	n	bl	n	n	0	nev any	st
CPSI	184	m	35	84	all	6	all	NAmer	1959	pr	5138	n	bl	n	n	1	nev any	ot
DAMBER	3	m	0	0	all	-	all	Eu:Sca	1972	CC	579	n	bl	y	n	0	nev any	st
DEAN	6	m	0	0	wh	-	all	Africa	1947	CC	603	n	V	y	n	0	nev any	st
DEAN2	10	m	0	0	all	-	all	Eu:UK	1960	CC	954	n	V	y	n	0	nev any	st
DEAN2	18	f	0	0	all	-	all	Eu:UK	1960	CC	954	n	V	y	n	0	nev any	ot
DOLL2	85	m	0	0	all	10	all	Eu:UK	1951	pr	920	n	V	n	n	1	nev any	ot
DORN	31	m	0	0	wh	2	all	NAmer	1954	pr	5097	n	bl	n	n	1	nev any	ot
GOLLED	4	m	35	99	all	-	all	Eu:UK	1952	CC	443	n	V	y	n	1	nev any	ot
GRAHAM	19	m	0	0	wh	-	all	NAmer	1956	CC	685	n	bl	n	n	0	nev any	st
HAMMON	114	m	0	0	wh	0	all	NAmer	1952	pr	448	n	bl	n	n	1	nev any	ot
JOLY	35	m	0	0	all	-	all	SCAmer	1978	CC	826	n	bl	n	n	0	nev any	st
LOMBAR	11	m	0	0	all	-	all	NAmer	1951	CC	1040	n	bl	n	n	0	nev any	st
LUBIN2	52	m	0	0	all	-	all	Eu:mul	1976	CC	7804	n	bl	n	y	2	nev any	ot
LUBIN2	100	f	0	0	all	-	all	Eu:mul	1976	CC	7804	n	bl	n	y	1	nev any	ot
MCCONN	18	c	0	0	all	-	all	Eu:UK	1946	CC	100	n	V	n	y	0	nev any	st
SADOWS	69	m	0	0	wh	-	all	NAmer	1938	CC	477	n	bl	n	n	0	nev any	st
STASZE	6	m	0	0	all	-	all	Eu:est	1954	CC	281	n	bl	n	y	0	nev any	st
WYNDE7	43	m	0	0	all	-	all	NAmer	1977	CC	2085	n	bl	n	y	0	nev any	st
XIANGZ	11	m	0	0	all	0	all	As:Chi	1976	pr	983	m	ot	n	n	2	nev any	ot

Table 1E16 - 2

IESLC - Meta-analysis of Ever Smoking, Mixed smokers
All LC types
Most adjusted

REF	NRR	SEX	AD	Number Exposed Case	Number Exposed Cont	Non-exposed Case	Non-exposed Cont	RR	95.00%CI
ABELIN	48	m	1	-	-	-	-	29.10	(6.25- 135.39)
ALDERS	2	m	1	-	-	-	-	9.09	(5.14- 16.08)
ARMADA	31	m	0	72	57	4	64	20.21	(6.94- 58.82)
*BEST	20	m	0	115	13479	1	2854	24.35	(3.40- 174.26)
BOFFET	26	m	2	-	-	-	-	12.70	(10.30- 15.60)
*BOUCOT	116	m	2	-	-	-	-	47.79	(2.91- 786.03)
BRESLO	19	m	0	155	154	7	42	6.04	(2.63- 13.86)
*CHOW	22	m	0	132	122272	6	62913	11.32	(5.00- 25.65)
*CPSI	184	m	1	-	-	-	-	8.04	(6.38- 10.12)
DAMBER	3	m	0	134	75	42	208	8.85	(5.72- 13.68)
DEAN	6	m	0	137	115	12	61	6.06	(3.11- 11.80)
DEAN2	10	m	0	57	48	33	112	4.03	(2.33- 6.96)
DEAN2	18	f	0	1	0	88	121	4.12~	(0.17- 102.29)
Subtotal DEAN2								4.03	(2.35- 6.91)
*DOLL2	85	m	1	-	-	-	-	7.57	(2.35- 24.43)
*DORN	31	m	1	-	-	-	-	6.40	(3.82- 10.72)
GOLLED	4	m	1	-	-	-	-	6.46	(3.46- 12.07)
GRAHAM	19	m	0	144	333	18	346	8.31	(4.98- 13.88)
*HAMMON	114	m	1	-	-	-	-	7.63	(4.49- 12.98)
JOLY	35	m	0	173	210	12	218	14.97	(8.09- 27.68)
LOMBAR	11	m	0	492	480	14	112	8.20	(4.64- 14.49)
LUBIN2	52	m	2	-	-	-	-	7.77	(6.45- 9.36)
LUBIN2	100	f	1	-	-	-	-	3.30	(0.30- 36.51)
Subtotal LUBIN2								7.73	(6.42- 9.31)
MCCONN	18	c	0	15	32	9	23	1.20	(0.45- 3.21)
SADOWS	69	m	0	148	118	18	81	5.64	(3.21- 9.93)
STASZE	6	m	0	33	101	5	158	10.32	(3.90- 27.32)
WYNDE7	43	m	0	327	614	64	918	7.64	(5.73- 10.18)
*XIANGZ	11	m	2	-	-	-	-	1.90	(1.27- 2.85)
Partial Totals				2135	138088	333	68231		

*prospective study ~ With 0.5 adjustment for zero

REF	NRR	SEX	AD	Ys	Ws	Qs	Ps
ABELIN	48	m	1	3.37	1.62	2.78	0.0000
ALDERS	2	m	1	2.21	11.81	0.24	0.0000
ARMADA	31	m	0	3.01	3.37	2.99	0.0000
*BEST	20	m	0	3.19	0.99	1.26	0.0015
BOFFET	26	m	2	2.54	89.16	20.38	0.0000
*BOUCOT	116	m	2	3.87	0.49	1.59	0.0068
BRESLO	19	m	0	1.80	5.57	0.39	0.0000
*CHOW	22	m	0	2.43	5.74	0.76	0.0000
*CPSI	184	m	1	2.08	72.19	0.03	0.0000
DAMBER	3	m	0	2.18	20.24	0.28	0.0000
DEAN	6	m	0	1.80	8.64	0.60	0.0000
DEAN2	10	m	0	1.39	12.89	5.78	0.0000
DEAN2	18	f	0	1.42	0.37	0.16	0.3878
Subtotal DEAN2				1.39	13.26	5.94	
*DOLL2	85	m	1	2.02	2.80	0.00	0.0007
*DORN	31	m	1	1.86	14.43	0.62	0.0000
GOLLED	4	m	1	1.87	9.84	0.39	0.0000
GRAHAM	19	m	0	2.12	14.62	0.04	0.0000
*HAMMON	114	m	1	2.03	13.64	0.01	0.0000
JOLY	35	m	0	2.71	10.16	4.19	0.0000
LOMBAR	11	m	0	2.10	11.84	0.02	0.0000
LUBIN2	52	m	2	2.05	110.82	0.02	0.0000
LUBIN2	100	f	1	1.19	0.67	0.50	0.3297
Subtotal LUBIN2				2.05	111.49	0.52	
MCCONN	18	c	0	0.18	3.96	14.04	0.7193
SADOWS	69	m	0	1.73	12.03	1.33	0.0000
STASZE	6	m	0	2.33	4.06	0.30	0.0000
WYNDE7	43	m	0	2.03	46.73	0.04	0.0000
*XIANGZ	11	m	2	0.64	23.52	47.54	0.0019

Table 1E16 - 2

IESLC - Meta-analysis of Ever Smoking, Mixed smokers
All LC types
Most adjusted

	N	27
	NS	25
	Wt	512.19
Het	Chi	106.28
Het	df	26
Het	P	***
Fixed	RR	7.87
	RRl	7.22
	RRu	8.59
	P	+++
Random	RR	7.37
	RRl	5.97
	RRu	9.11
	P	+++
Asymm	P	N.S.

Table 1E16 - 3

IESLC - Meta-analysis of Ever Smoking, Mixed smokers
All LC types
Most adjusted

	Sex			
	combined	male	female	Total
N	1	24	2	27
NS	1	24	2	27
Wt	3.96	507.19	1.04	512.19
Het Chi	0.00	91.44	0.01	106.28
Het df	0	23	1	26
Het P	N.S.	***	N.S.	***
Fixed RR	1.20	8.00	3.57	7.87
RRl	0.45	7.34	0.52	7.22
RRu	3.21	8.73	24.44	8.59
P	N.S.	+++	N.S.	+++
Random RR	1.20	7.79	3.57	7.37
RRl	0.45	6.35	0.52	5.97
RRu	3.21	9.56	24.44	9.11
P	N.S.	+++	N.S.	+++
Between Chi				14.83
Between df				2
Between P				***
Btwn(F) P				N.S.
Btwn(R) P				**

	All LC (or nearest)		
	all	other	Total
N	27		27
NS	25		25
Wt	512.19		512.19
Het Chi	106.28		106.28
Het df	26		26
Het P	***		***
Fixed RR	7.87		7.87
RRl	7.22		7.22
RRu	8.59		8.59
P	+++		+++
Random RR	7.37		7.37
RRl	5.97		5.97
RRu	9.11		9.11
P	+++		+++
Between Chi			
Between df			
Between P			N.S.
Btwn(F) P			N.S.
Btwn(R) P			N.S.

	Location								
	NAmer	UK	Scand	othEur	China	Japan	othAs	other	Total
N	11	6	1	6	1			2	27
NS	11	5	1	5	1			2	25
Wt	198.27	41.68	20.24	209.70	23.52			18.80	512.19
Het Chi	6.05	13.93	0.00	16.44	0.00			3.82	106.28
Het df	10	5	0	5	0			1	26
Het P	N.S.	*	N.S.	**	N.S.			(*)	***
Fixed RR	7.74	5.28	8.85	9.85	1.90			9.87	7.87
RRl	6.73	3.89	5.72	8.60	1.27			6.28	7.22
RRu	8.89	7.15	13.68	11.28	2.85			15.52	8.59
P	+++	+++	+++	+++	++			+++	+++
Random RR	7.74	4.84	8.85	11.03	1.90			9.61	7.37
RRl	6.73	2.74	5.72	7.48	1.27			3.96	5.97
RRu	8.89	8.55	13.68	16.28	2.85			23.32	9.11
P	+++	+++	+++	+++	++			+++	+++
Between Chi									66.04
Between df									5
Between P									***
Btwn(F) P									***
Btwn(R) P									***

Table 1E16 - 3

IESLC - Meta-analysis of Ever Smoking, Mixed smokers
All LC types
Most adjusted

Detailed Country in "other Europe"

	multi	Germany	othWest	East	Balkans	Total
N	3		2	1		6
NS	2		2	1		5
Wt	200.65		4.99	4.06		209.70
Het Chi	12.70		0.15	0.00		16.44
Het df	2		1	0		5
Het P	**		N.S.	N.S.		**
Fixed RR	9.64		22.76	10.32		9.85
RRl	8.39		9.46	3.90		8.60
RRu	11.07		54.72	27.32		11.28
P	+++		+++	+++		+++
Random RR	9.53		22.76	10.32		11.03
RRl	5.98		9.46	3.90		7.48
RRu	15.20		54.72	27.32		16.28
P	+++		+++	+++		+++
Between Chi						3.60
Between df						2
Between P						N.S.
Btwn(F) P						N.S.
Btwn(R) P						N.S.

Detailed Country in "other Asia"

	India	HongKong	other	Total
N				
NS				
Wt				
Het Chi				
Het df				
Het P				N.S.
Fixed RR				
RRl				
RRu				
P				+++
Random RR				
RRl				
RRu				
P				+++
Between Chi				
Between df				
Between P				N.S.
Btwn(F) P				N.S.
Btwn(R) P				N.S.

Detailed other continent

	SCAmer	Auslia	Africa	Total
N	1		1	2
NS	1		1	2
Wt	10.16		8.64	18.80
Het Chi	0.00		0.00	3.82
Het df	0		0	1
Het P	N.S.		N.S.	(*)
Fixed RR	14.97		6.06	9.87
RRl	8.09		3.11	6.28
RRu	27.68		11.80	15.52
P	+++		+++	+++
Random RR	14.97		6.06	9.61
RRl	8.09		3.11	3.96
RRu	27.68		11.80	23.32
P	+++		+++	+++
Between Chi				3.82
Between df				1
Between P				(*)
Btwn(F) P				N.S.
Btwn(R) P				(*)

Table 1E16 - 3

IESLC - Meta-analysis of Ever Smoking, Mixed smokers
All LC types
Most adjusted

	Start year of study					
	<1960	1960-69	1970-79	1980-89	1990+	Total
N	15	3	7	2		27
NS	15	2	6	2		25
Wt	176.73	19.00	223.94	92.53		512.19
Het Chi	22.50	4.27	49.72	0.70		106.28
Het df	14	2	6	1		26
Het P	(*)	N.S.	***	N.S.		***
Fixed RR	7.33	5.51	7.00	12.92		7.87
RRl	6.32	3.51	6.14	10.54		7.22
RRu	8.49	8.64	7.98	15.84		8.59
P	+++	+++	+++	+++		+++
Random RR	7.07	6.17	6.86	12.92		7.37
RRl	5.67	2.68	4.36	10.54		5.97
RRu	8.83	14.22	10.80	15.84		9.11
P	+++	+++	+++	+++		+++
Between Chi						29.10
Between df						3
Between P						***
Btwn(F) P						(*)
Btwn(R) P						***

	Study type (1)		
	CC	other	Total
N	19	8	27
NS	17	8	25
Wt	378.39	133.80	512.19
Het Chi	51.86	44.46	106.28
Het df	18	7	26
Het P	***	***	***
Fixed RR	8.55	6.23	7.87
RRl	7.73	5.26	7.22
RRu	9.46	7.38	8.59
P	+++	+++	+++
Random RR	7.81	7.04	7.37
RRl	6.32	4.04	5.97
RRu	9.65	12.28	9.11
P	+++	+++	+++
Between Chi			9.96
Between df			1
Between P			**
Btwn(F) P			N.S.
Btwn(R) P			N.S.

	Study type (2)			
	CC	prosp	other	Total
N	19	8		27
NS	17	8		25
Wt	378.39	133.80		512.19
Het Chi	51.86	44.46		106.28
Het df	18	7		26
Het P	***	***		***
Fixed RR	8.55	6.23		7.87
RRl	7.73	5.26		7.22
RRu	9.46	7.38		8.59
P	+++	+++		+++
Random RR	7.81	7.04		7.37
RRl	6.32	4.04		5.97
RRu	9.65	12.28		9.11
P	+++	+++		+++
Between Chi				9.96
Between df				1
Between P				**
Btwn(F) P				N.S.
Btwn(R) P				N.S.

Table 1E16 - 3

IESLC - Meta-analysis of Ever Smoking, Mixed smokers
All LC types
Most adjusted

	Study size (number of LC cases) 100-249	250-499	500-999	1000+	Total
N	4	6	9	8	27
NS	4	6	8	7	25
Wt	11.81	43.92	98.80	357.65	512.19
Het Chi	18.69	6.26	45.50	17.42	106.28
Het df	3	5	8	7	26
Het P	***	N.S.	***	*	***
Fixed RR	6.44	7.70	5.44	8.80	7.87
RRl	3.64	5.73	4.47	7.94	7.22
RRu	11.40	10.35	6.63	9.76	8.59
P	+++	+++	+++	+++	+++
Random RR	9.50	7.97	6.07	8.53	7.37
RRl	1.89	5.63	3.63	7.05	5.97
RRu	47.79	11.28	10.15	10.33	9.11
P	++	+++	+++	+++	+++
Between Chi					18.41
Between df					3
Between P					***
Btwn(F) P					N.S.
Btwn(R) P					N.S.

	Risky occupational population no	mining	othRisky	Total
N	26	1		27
NS	24	1		25
Wt	488.68	23.52		512.19
Het Chi	56.46	0.00		106.28
Het df	25	0		26
Het P	***	N.S.		***
Fixed RR	8.43	1.90		7.87
RRl	7.72	1.27		7.22
RRu	9.21	2.85		8.59
P	+++	++		+++
Random RR	7.98	1.90		7.37
RRl	6.76	1.27		5.97
RRu	9.41	2.85		9.11
P	+++	++		+++
Between Chi				49.83
Between df				1
Between P				***
Btwn(F) P				***
Btwn(R) P				***

	National cigarette tobacco type Virginia	blended	other	Total
N	8	18	1	27
NS	7	17	1	25
Wt	51.31	437.37	23.52	512.19
Het Chi	16.27	30.26	0.00	106.28
Het df	7	17	0	26
Het P	*	*	N.S.	***
Fixed RR	5.56	8.85	1.90	7.87
RRl	4.23	8.06	1.27	7.22
RRu	7.31	9.72	2.85	8.59
P	+++	+++	++	+++
Random RR	5.45	8.80	1.90	7.37
RRl	3.41	7.55	1.27	5.97
RRu	8.69	10.26	2.85	9.11
P	+++	+++	++	+++
Between Chi				59.75
Between df				2
Between P				***
Btwn(F) P				***
Btwn(R) P				***

Table 1E16 - 3

IESLC - Meta-analysis of Ever Smoking, Mixed smokers
All LC types
Most adjusted

	Any proxy use No/nk	Yes	Total
N	20	7	27
NS	19	6	25
Wt	369.43	142.77	512.19
Het Chi	72.81	22.24	106.28
Het df	19	6	26
Het P	***	**	***
Fixed RR	7.18	9.99	7.87
RRl	6.49	8.48	7.22
RRu	7.95	11.77	8.59
P	+++	+++	+++
Random RR	7.18	7.96	7.37
RRl	5.62	5.16	5.97
RRu	9.16	12.26	9.11
P	+++	+++	+++
Between Chi			11.24
Between df			1
Between P			***
Btwn(F) P			(*)
Btwn(R) P			N.S.

	Full histological confirmation No	Yes	Total
N	20	7	27
NS	19	6	25
Wt	337.03	175.16	512.19
Het Chi	87.80	17.91	106.28
Het df	19	6	26
Het P	***	**	***
Fixed RR	8.07	7.52	7.87
RRl	7.25	6.48	7.22
RRu	8.98	8.72	8.59
P	+++	+++	+++
Random RR	7.66	6.81	7.37
RRl	5.85	4.61	5.97
RRu	10.03	10.05	9.11
P	+++	+++	+++
Between Chi			0.57
Between df			1
Between P			N.S.
Btwn(F) P			N.S.
Btwn(R) P			N.S.

	Number of adjustment variables (1) 0	1	2+/+nk	Total
N	15	8	4	27
NS	14	8	4	26
Wt	161.19	127.01	223.99	512.19
Het Chi	31.79	4.57	69.20	106.28
Het df	14	7	3	26
Het P	**	N.S.	***	***
Fixed RR	7.50	7.83	8.18	7.87
RRl	6.43	6.58	7.18	7.22
RRu	8.75	9.32	9.33	8.59
P	+++	+++	+++	+++
Random RR	7.45	7.83	6.70	7.37
RRl	5.73	6.58	3.03	5.97
RRu	9.69	9.32	14.80	9.11
P	+++	+++	+++	+++
Between Chi				0.72
Between df				2
Between P				N.S.
Btwn(F) P				N.S.
Btwn(R) P				N.S.

Table 1E16 - 3

IESLC - Meta-analysis of Ever Smoking, Mixed smokers
All LC types
Most adjusted

	Number of adjustment variables (2) 0	1	2	3-5	6+/+nk	Total
N	15	8	4			27
NS	14	8	4			26
Wt	161.19	127.01	223.99			512.19
Het Chi	31.79	4.57	69.20			106.28
Het df	14	7	3			26
Het P	**	N.S.	***			***
Fixed RR	7.50	7.83	8.18			7.87
RRl	6.43	6.58	7.18			7.22
RRu	8.75	9.32	9.33			8.59
P	+++	+++	+++			+++
Random RR	7.45	7.83	6.70			7.37
RRl	5.73	6.58	3.03			5.97
RRu	9.69	9.32	14.80			9.11
P	+++	+++	+++			+++
Between Chi						0.72
Between df						2
Between P						N.S.
Btwn(F) P						N.S.
Btwn(R) P						N.S.

	Derivation of RR/CI Orig	StdCalc	Other	Total
N	1	15	11	27
NS	1	15	10	26
Wt	89.16	162.44	260.59	512.19
Het Chi	0.00	34.61	45.82	106.28
Het df	0	14	10	26
Het P	N.S.	**	***	***
Fixed RR	12.70	7.61	6.83	7.87
RRl	10.32	6.53	6.05	7.22
RRu	15.63	8.88	7.71	8.59
P	+++	+++	+++	+++
Random RR	12.70	7.74	6.30	7.37
RRl	10.32	5.91	4.46	5.97
RRu	15.63	10.15	8.90	9.11
P	+++	+++	+++	+++
Between Chi				25.85
Between df				2
Between P				***
Btwn(F) P				*
Btwn(R) P				***

Table 1E16 - 4

IESLC - Meta-analysis of Ever Smoking, Mixed smokers
All LC types
Least adjusted

REF	NRR	X	SEX	AGEL	AGEH	RACE	YF	LC TYPE	LOC	START	ST	NLC	R	VB	P	H	AD	DENOM	De
ABELIN	5	x	m	0	0	all	-	all	Eu:wst	1941	CC	118	n	bl	y	n	0	nev any	st
ALDERS	8	x	m	0	0	all	-	all	Eu:UK	1977	CC	1448	n	V	n	n	0	nev any	st
ARMADA	31		m	0	0	all	-	all	Eu:wst	1986	CC	325	n	bl	n	y	0	nev any	st
BEST	20		m	55	79	all	3	all	NAmer	1955	pr	381	n	V	n	n	0	nev any	st
BOFFET	28	x	m	0	0	all	-	all	Eu:mul	1988	CC	5621	n	bl	y	n	0	nev any	st
BOUCOT	4	x	m	0	0	all	0	all	NAmer	1951	pr	121	n	bl	n	n	0	nev any	ot
BRESLO	19		m	0	0	all	-	all	NAmer	1949	CC	518	n	bl	n	y	0	nev+1	st
CHOW	22		m	0	0	wh	0	all	NAmer	1966	pr	219	n	bl	n	n	0	nev any	st
CPSI	184		m	35	84	all	6	all	NAmer	1959	pr	5138	n	bl	n	n	1	nev any	ot
DAMBER	3		m	0	0	all	-	all	Eu:Sca	1972	CC	579	n	bl	y	n	0	nev any	st
DEAN	6		m	0	0	wh	-	all	Africa	1947	CC	603	n	V	y	n	0	nev any	st
DEAN2	10		m	0	0	all	-	all	Eu:UK	1960	CC	954	n	V	y	n	0	nev any	st
DEAN2	18		f	0	0	all	-	all	Eu:UK	1960	CC	954	n	V	y	n	0	nev any	ot
DOLL2	85		m	0	0	all	10	all	Eu:UK	1951	pr	920	n	V	n	n	1	nev any	ot
DORN	40	x	m	0	0	wh	2	all	NAmer	1954	pr	5097	n	bl	n	n	0	nev any	st
GOLLED	18	x	m	35	99	all	-	all	Eu:UK	1952	CC	443	n	V	y	n	0	nev any	st
GRAHAM	19		m	0	0	wh	-	all	NAmer	1956	CC	685	n	bl	n	n	0	nev any	st
HAMMON	126	x	m	0	0	wh	0	all	NAmer	1952	pr	448	n	bl	n	n	0	nev any	st
JOLY	35		m	0	0	all	-	all	SCAmer	1978	CC	826	n	bl	n	n	0	nev any	st
LOMBAR	11		m	0	0	all	-	all	NAmer	1951	CC	1040	n	bl	n	n	0	nev any	st
LUBIN2	51	x	m	0	0	all	-	all	Eu:mul	1976	CC	7804	n	bl	n	y	0	nev any	st
LUBIN2	99	x	f	0	0	all	-	all	Eu:mul	1976	CC	7804	n	bl	n	y	0	nev any	st
MCCONN	18		c	0	0	all	-	all	Eu:UK	1946	CC	100	n	V	n	y	0	nev any	st
SADOWS	69		m	0	0	wh	-	all	NAmer	1938	CC	477	n	bl	n	n	0	nev any	st
STASZE	6		m	0	0	all	-	all	Eu:est	1954	CC	281	n	bl	n	y	0	nev any	st
WYNDE7	43		m	0	0	all	-	all	NAmer	1977	CC	2085	n	bl	n	y	0	nev any	st
XIANGZ	4	x	m	0	0	all	0	all	As:Chi	1976	pr	983	m	ot	n	n	0	nev any	st

Table 1E16 - 5

IESLC - Meta-analysis of Ever Smoking, Mixed smokers
All LC types
Least adjusted

REF	NRR	SEX	AD	Number Exposed Case	Number Exposed Cont	Non-exposed Case	Non-exposed Cont	RR	95.00%CI
ABELIN	5	m	0	17	64	2	183	24.30	(5.46- 108.12)
ALDERS	8	m	0	206	179	15	133	10.20	(5.77- 18.05)
ARMADA	31	m	0	72	57	4	64	20.21	(6.94- 58.82)
*BEST	20	m	0	115	13479	1	2854	24.35	(3.40- 174.26)
BOFFET	28	m	0	1182	1309	117	1750	13.51	(11.02- 16.55)
*BOUCOT	4	m	0	24	7607	0	7551	48.64~	(2.96- 799.67)
BRESLO	19	m	0	155	154	7	42	6.04	(2.63- 13.86)
*CHOW	22	m	0	132	122272	6	62913	11.32	(5.00- 25.65)
*CPSI	184	m	1	-	-	-	-	8.04	(6.38- 10.12)
DAMBER	3	m	0	134	75	42	208	8.85	(5.72- 13.68)
DEAN	6	m	0	137	115	12	61	6.06	(3.11- 11.80)
DEAN2	10	m	0	57	48	33	112	4.03	(2.33- 6.96)
DEAN2	18	f	0	1	0	88	121	4.12~	(0.17- 102.29)
Subtotal DEAN2								4.03	(2.35- 6.91)
*DOLL2	85	m	1	-	-	-	-	7.57	(2.35- 24.43)
*DORN	40	m	0	96	110585	17	117918	6.02	(3.60- 10.08)
GOLLED	18	m	0	36	175	15	490	6.72	(3.59- 12.57)
GRAHAM	19	m	0	144	333	18	346	8.31	(4.98- 13.88)
*HAMMON	126	m	0	148	156773	15	115884	7.29	(4.29- 12.40)
JOLY	35	m	0	173	210	12	218	14.97	(8.09- 27.68)
LOMBAR	11	m	0	492	480	14	112	8.20	(4.64- 14.49)
LUBIN2	51	m	0	588	1092	190	2617	7.42	(6.21- 8.86)
LUBIN2	99	f	0	1	2	336	1188	1.77	(0.16- 19.56)
Subtotal LUBIN2								7.36	(6.16- 8.79)
MCCONN	18	c	0	15	32	9	23	1.20	(0.45- 3.21)
SADOWS	69	m	0	148	118	18	81	5.64	(3.21- 9.93)
STASZE	6	m	0	33	101	5	158	10.32	(3.90- 27.32)
WYNDE7	43	m	0	327	614	64	918	7.64	(5.73- 10.18)
*XIANGZ	4	m	0	237	2569	25	974	3.59	(2.40- 5.39)
Partial Totals				4670	418443	1065	316919		

*prospective study ~ With 0.5 adjustment for zero

REF	NRR	SEX	AD	Ys	Ws	Qs	Ps
ABELIN	5	m	0	3.19	1.72	2.07	0.0000
ALDERS	8	m	0	2.32	11.82	0.61	0.0000
ARMADA	31	m	0	3.01	3.37	2.79	0.0000
*BEST	20	m	0	3.19	0.99	1.19	0.0015
BOFFET	28	m	0	2.60	93.21	24.04	0.0000
*BOUCOT	4	m	0	3.88	0.49	1.57	0.0065
BRESLO	19	m	0	1.80	5.57	0.49	0.0000
*CHOW	22	m	0	2.43	5.74	0.63	0.0000
*CPSI	184	m	1	2.08	72.19	0.01	0.0000
DAMBER	3	m	0	2.18	20.24	0.15	0.0000
DEAN	6	m	0	1.80	8.64	0.75	0.0000
DEAN2	10	m	0	1.39	12.89	6.34	0.0000
DEAN2	18	f	0	1.42	0.37	0.17	0.3878
Subtotal DEAN2				1.39	13.26	6.51	
*DOLL2	85	m	1	2.02	2.80	0.01	0.0007
*DORN	40	m	0	1.80	14.45	1.30	0.0000
GOLLED	18	m	0	1.91	9.78	0.35	0.0000
GRAHAM	19	m	0	2.12	14.62	0.01	0.0000
*HAMMON	126	m	0	1.99	13.62	0.16	0.0000
JOLY	35	m	0	2.71	10.16	3.79	0.0000
LOMBAR	11	m	0	2.10	11.84	0.00	0.0000
LUBIN2	51	m	0	2.00	121.04	1.01	0.0000
LUBIN2	99	f	0	0.57	0.66	1.55	0.6422
Subtotal LUBIN2				2.00	121.71	2.56	
MCCONN	18	c	0	0.18	3.96	14.52	0.7193
SADOWS	69	m	0	1.73	12.03	1.60	0.0000
STASZE	6	m	0	2.33	4.06	0.23	0.0000
WYNDE7	43	m	0	2.03	46.73	0.18	0.0000
*XIANGZ	4	m	0	1.28	23.36	15.55	0.0000

Table 1E16 - 5

IESLC - Meta-analysis of Ever Smoking, Mixed smokers
All LC types
Least adjusted

	N	27
	NS	25
	Wt	526.35
Het	Chi	81.08
Het	df	26
Het	P	***
Fixed	RR	8.13
	RRl	7.46
	RRu	8.85
	P	+++
Random	RR	7.60
	RRl	6.31
	RRu	9.14
	P	+++
Asymm	P	N.S.

Table 1E16 - 6

IESLC - Meta-analysis of Ever Smoking, Mixed smokers
All LC types
Least adjusted

	combined	Sex male	female	Total
N	1	24	2	27
NS	1	24	2	27
Wt	3.96	521.35	1.04	526.35
Het Chi	0.00	64.69	0.17	81.08
Het df	0	23	1	26
Het P	N.S.	***	N.S.	***
Fixed RR	1.20	8.27	2.39	8.13
RRl	0.45	7.59	0.35	7.46
RRu	3.21	9.01	16.41	8.85
P	N.S.	+++	N.S.	+++
Random RR	1.20	8.00	2.39	7.60
RRl	0.45	6.73	0.35	6.31
RRu	3.21	9.51	16.41	9.14
P	N.S.	+++	N.S.	+++
Between Chi				16.22
Between df				2
Between P				***
Btwn(F) P				(*)
Btwn(R) P				***

Table 1E16 - 7

IESLC - Meta-analysis of Ever Smoking, Mixed smokers
All LC types
Excluded studies (and stage at which they were excluded)

```
1      BOUCHA  BUELL LAURIL MZILEN

2      ABRAHA  AGUDO  AKIBA AMANDU   AMES ANDERS ARCHER AUSTIN AUVINE AXELSO AXELSS   BAND BARBON BECHER BENSHL BERRIN
       BLOHMK  BLOT1  BLOT2  BLOT3  BLOT4  BRETT BROCKM  BROSS BROWN1 BROWN2 BUFFLE BYERS1 BYERS2 CARPEN CASCO2 CASCOR
         CHAN  CHANG CHATZI   CHEN  CHEN2  CHEN3 CHIAZZ   CHOI  CHYOU COMSTO COOKSO CORREA  CPSII  DARBY DAVEYS  DEAN3
       DEKLER DESTE2 DESTEF DOCKER   DOLL DORANT DORGAN DOSEME DROSTE     DU   DUNN EBELIN ENGELA ENSTRO  ESAKI    FAN
          GAO   GAO2 GARCIA GARDIN GARSHI   GENG    GER GILLIS GODLEY GOODMA GREGOR  GSELL HAENSZ HAMMO2 HANSEN HEGMAN
         HEIN HENNEK  HINDS HIRAOK HIRAYA HITOSU   HOLE HOROWI HORWIT     HU    HU2  HUANG HUMBLE ISHIMA   JAHN   JAIN
        JARUP JARVHO JEDRYC  JIANG  JONES JUSSAW KAISE2 KAISER KANELL KATSOU KAUFMA KELLER KHUDER KIHARA KINLEN  KJUUS
        KNEKT     KO KOHLME    KOO KOULUM KREUZE KREYBE  KUBIK  LAMTH  LAMWK LAMWK2  LANGE LAUSSM    LEI LEMARC LETOUR
        LEVIN   LIAW LICKIN LIDDEL    LIU   LIU2   LIU3   LIU4   LIU5 LOMBA2    LUO MACLEN MAGNUS  MARSH MARSH2 MARTIS
       MASTRA  MATOS MATSUD MCDUFF MCLAUG MILLER  MILLS  MOLLO  MRFIT MRFITR MURATA    NAM NOTAN2 NOTANI    NOU ODRISC
        ORMOS  OSANN OSANN2 PARKIN PASTOR PAWLEG  PERNU PERSH2 PERSHA   PETO PEZZO2 PEZZOT   PIKE PISANI POFFIJ POLEDN
       PRESCO  QIAO2 RACHTA RADZIK RANDIG    REN RESTRE RIMING  RONCO  ROOTS ROTHSC SAARIK SANKAR SCHWAR   SEGI  SEGI2
         SEOW   SHAW SHIMIZ SIEMIA SIMARA SIMONA  SITAS  SOBUE SOBUE2 SPEIZE  SPITZ STAYNE STOCKS STOCKW STUCKE    SUN
       SUZUK2 SUZUKI SVENSS   TANG  TANG2    TAO TENKAN TIZZAN TOKARS TOUSEY TSUGAN TULINI  ULMER VANDER VEIERO  VUTUC
        WAKAI   WALD   WANG  WANG2  WANG3  WANG4 WARSIN WATSON WICKLU  WIGLE WILKIN     WU    WU2 WUNSCH WUWILL WYNDE2
       WYNDE3 WYNDE4 WYNDE5 WYNDE6 WYNDE8     XU    XU2    XU3    XU4 YAMAGU   YONG   YUAN  ZHANG  ZHENG   ZHOU

3      CEDERL HIRAY2 MIGRAN TVERDA WYNDER

5      SCHWA2

9      BENHAM  LUBIN   QIAO
```

Table 1E16 - 8

Potentially overlapping studies

REF	REFGP	PRINC	.	OVERLAP/LINK
LUBIN2	LUBIN2	1		Lubin-combined
GRAHAM	BYERS1	1		GRAHAM/BROSS/BYERS1
WYNDE7	WYNDE6	2		WYNDE5/6/7/8
CPSI	CPSI	1		CPSI overall
BOFFET	BOFFET	1		BOFFET-combined
XIANGZ	XIANGZ	1		LUBIN/XIANGZ/QIAO

Table 1E16 - 9

Most adjusted - insufficient data for metaanalysis

REF	NRR	SEX	AGEL	AGEH	RACE	YF	LC TYPE	LOC	START	ST	NLC	R	VB	P	H	AD	DENOM	De
BRESLO	25	f	0	0	all	-	all	NAmer	1949	CC	518	n	bl	n	y	0	nev+1	st

REF	NRR	RR	SIG	RRDATA comment
BRESLO	25		*	0

Table 1E17 -

IESLC - Meta-analysis of Current Smoking, Mixed smokers
All LC types

This analysis is restricted to results for:
1) Non-dose-response data
2) Mixed smokers (cigarettes and pipe/cigar)
3) Current smokers
4) Results complete enough for use in metaanalysis

Within each study, results are then selected (in the following order of preference, within each sex) for:
5) DENOM: never smoked anything, (never +1 = +long term ex)
6) Followup period (prospective studies): whole study (coded as 0) or longest available
7) LCtype: all or nearest available, at least Squamous and Adeno. (q = squamous, s = small,
l = large, a = adeno, mix = mixed, alv = alveolar)
8) Race: all or nearest available, otherwise by race (wh or w = white, bl or b = black, hi = hispanic
ch = chinese, jap = japanese, haw = hawaiian, w+o = white + oriental, sca = scandinavian, as = asian)
9) For overlapping studies: principal rather than subsidiary studies
Finally by Age: whole study (coded as 0) if available, otherwise by widest available age group
and then for single sex results (m, f) in preference to combined sex results (c).

Results adjusted (AD) for the most potential confounders are then chosen in Sections -1 to -3
and results adjusted for the least confounders in Sections -4 to -6. (Those least adjusted results which
actually differ from the most adjusted as marked 'x' in column X in Section -4)
(Results adjusted for an unknown number of confounder(s) are coded as 20.)

Section -7 shows excluded studies, together with the stage (as above) at which no qualifying
results were found.

Section -8 lists the potentially overlapping studies which have been included (1=principal, 2=subsidiary).

Section -9 lists any results which would have been included in preference except that they had data not complete
enough for use in meta-analysis, with their significance (yes/no), if known, and any further comment as entered
on the database.

In addition to those mentioned above, the following fields, levels and abbreviations are used:
* or nk = not known, n = no, y = yes, ot = other
nev = never
REF: 6-character study reference
NRR: number of the RR on the database within the study
ST : study type (CC = case control, pr or prosp = prospective)
NLC: number of lung cancer cases in whole study
R : risky occupational population (n = no, m = mining, o = other risky)
VB : national cigarette type (V = at least 75% Virginia, bl = at least 75% blended, ot = other)
P : any proxy use
H : full histological confirmation
De : derivation of RR/CI (or = original, st = standard method, ot = other method of estimation)

Table 1E17 - 1

IESLC - Meta-analysis of Current Smoking, Mixed smokers
All LC types
Most adjusted

REF	NRR	SEX	AGEL	AGEH	RACE	YF	LC TYPE	LOC	START	ST	NLC	R	VB	P	H	AD	DENOM	De
CEDERL	28	m	0	0	all	10	all	Eu:Sca	1963	pr	491	n	bl	n	n	1	nev any	ot
CHOW	23	m	0	0	wh	0	all	NAmer	1966	pr	219	n	bl	n	n	0	nev any	st
DOLL2	21	m	0	0	all	20	all	Eu:UK	1951	pr	920	n	V	n	n	1	nev any	ot
DORN	146	m	35	84	wh	8	all	NAmer	1954	pr	5097	n	bl	n	n	1	nev any	ot
GRAHAM	5	m	0	0	wh	-	all	NAmer	1956	CC	685	n	bl	n	n	0	nev any	st
LOMBAR	7	m	0	0	all	-	all	NAmer	1951	CC	1040	n	bl	n	n	0	nev any	st
LUBIN2	32	m	0	0	all	-	all	Eu:mul	1976	CC	7804	n	bl	n	y	2	nev any	ot
MIGRAN	14	m	0	0	all	0	all	Eu:UK	1964	pr	259	n	V	n	n	2	nev any	ot
WYNDE7	5	m	0	0	all	-	all	NAmer	1977	CC	2085	n	bl	n	y	0	nev any	st

Table 1E17 - 2

IESLC - Meta-analysis of Current Smoking, Mixed smokers
All LC types
Most adjusted

REF	NRR	SEX	AD	Number Exposed Case	Number Exposed Cont	Non-exposed Case	Non-exposed Cont	RR	95.00%CI
*CEDERL	28	m	1	-	-	-	-	10.90	(4.75- 25.01)
*CHOW	23	m	0	59	36605	6	62913	16.90	(7.30- 39.14)
*DOLL2	21	m	1	-	-	-	-	8.20	(3.75- 17.92)
*DORN	146	m	1	-	-	-	-	8.85	(6.93- 11.30)
GRAHAM	5	m	0	82	254	18	346	6.21	(3.63- 10.60)
LOMBAR	7	m	0	420	361	14	112	9.31	(5.25- 16.51)
LUBIN2	32	m	2	-	-	-	-	10.61	(8.52- 13.20)
*MIGRAN	14	m	2	-	-	-	-	3.87	(1.18- 12.71)
WYNDE7	5	m	0	211	287	64	918	10.55	(7.74- 14.37)
Partial Totals				772	37507	102	64289		

*prospective study

REF	NRR	SEX	AD	Ys	Ws	Qs	Ps
*CEDERL	28	m	1	2.39	5.57	0.09	0.0000
*CHOW	23	m	0	2.83	5.45	1.72	0.0000
*DOLL2	21	m	1	2.10	6.28	0.16	0.0000
*DORN	146	m	1	2.18	64.27	0.46	0.0000
GRAHAM	5	m	0	1.83	13.41	2.59	0.0000
LOMBAR	7	m	0	2.23	11.69	0.01	0.0000
LUBIN2	32	m	2	2.36	80.17	0.76	0.0000
*MIGRAN	14	m	2	1.35	2.72	2.26	0.0256
WYNDE7	5	m	0	2.36	40.10	0.33	0.0000

	N	9
	NS	9
	Wt	229.66
Het	Chi	8.38
Het	df	8
Het	P	N.S.
Fixed	RR	9.63
	RRl	8.46
	RRu	10.96
	P	+++
Random	RR	9.60
	RRl	8.37
	RRu	11.00
	P	+++
Asymm	P	N.S.

Table 1E17 - 3

IESLC - Meta-analysis of Current Smoking, Mixed smokers
All LC types
Most adjusted

	Sex combined	male	female	Total
N		9		9
NS		9		9
Wt		229.66		229.66
Het Chi		8.38		8.38
Het df		8		8
Het P		N.S.		N.S.
Fixed RR		9.63		9.63
RRl		8.46		8.46
RRu		10.96		10.96
P		+++		+++
Random RR		9.60		9.60
RRl		8.37		8.37
RRu		11.00		11.00
P		+++		+++
Between Chi				
Between df				
Between P				N.S.
Btwn(F) P				N.S.
Btwn(R) P				N.S.

Too few RRs for analysis by factor

Table 1E17 - 4

IESLC - Meta-analysis of Current Smoking, Mixed smokers
All LC types
Least adjusted

REF	NRR	X	SEX	AGEL	AGEH	RACE	YF	LC TYPE	LOC	START	ST	NLC	R	VB	P	H	AD	DENOM	De
CEDERL	4	x	m	0	0	all	10	all	Eu:Sca	1963	pr	491	n	bl	n	n	0	nev any	st
CHOW	23		m	0	0	wh	0	all	NAmer	1966	pr	219	n	bl	n	n	0	nev any	st
DOLL2	21		m	0	0	all	20	all	Eu:UK	1951	pr	920	n	V	n	n	1	nev any	ot
DORN	146		m	35	84	wh	8	all	NAmer	1954	pr	5097	n	bl	n	n	1	nev any	ot
GRAHAM	5		m	0	0	wh	-	all	NAmer	1956	CC	685	n	bl	n	n	0	nev any	st
LOMBAR	7		m	0	0	all	-	all	NAmer	1951	CC	1040	n	bl	n	n	0	nev any	st
LUBIN2	31	x	m	0	0	all	-	all	Eu:mul	1976	CC	7804	n	bl	n	y	0	nev any	st
MIGRAN	13	x	m	0	0	all	0	all	Eu:UK	1964	pr	259	n	V	n	n	0	nev any	st
WYNDE7	5		m	0	0	all	-	all	NAmer	1977	CC	2085	n	bl	n	y	0	nev any	st

Table 1E17 - 5

IESLC - Meta-analysis of Current Smoking, Mixed smokers
All LC types
Least adjusted

REF	NRR	SEX	AD	Number Exposed Case	Number Exposed Cont	Non-exposed Case	Non-exposed Cont	RR	95.00%CI
*CEDERL	4	m	0	27	3762	7	6352	6.51	(2.84- 14.94)
*CHOW	23	m	0	59	36605	6	62913	16.90	(7.30- 39.14)
*DOLL2	21	m	1	-	-	-	-	8.20	(3.75- 17.92)
*DORN	146	m	1	-	-	-	-	8.85	(6.93- 11.30)
GRAHAM	5	m	0	82	254	18	346	6.21	(3.63- 10.60)
LOMBAR	7	m	0	420	361	14	112	9.31	(5.25- 16.51)
LUBIN2	31	m	0	314	444	190	2617	9.74	(7.92- 11.97)
*MIGRAN	13	m	0	9	353	4	867	5.53	(1.71- 17.83)
WYNDE7	5	m	0	211	287	64	918	10.55	(7.74- 14.37)
Partial Totals				1122	42066	303	74125		

*prospective study

REF	NRR	SEX	AD	Ys	Ws	Qs	Ps
*CEDERL	4	m	0	1.87	5.57	0.70	0.0000
*CHOW	23	m	0	2.83	5.45	1.95	0.0000
*DOLL2	21	m	1	2.10	6.28	0.10	0.0000
*DORN	146	m	1	2.18	64.27	0.15	0.0000
GRAHAM	5	m	0	1.83	13.41	2.18	0.0000
LOMBAR	7	m	0	2.23	11.69	0.00	0.0000
LUBIN2	31	m	0	2.28	90.23	0.21	0.0000
*MIGRAN	13	m	0	1.71	2.80	0.75	0.0042
WYNDE7	5	m	0	2.36	40.10	0.65	0.0000

	N	9
	NS	9
	Wt	239.81
Het	Chi	6.69
Het	df	8
Het	P	N.S.
Fixed	RR	9.29
	RRl	8.18
	RRu	10.54
	P	+++
Random	RR	9.29
	RRl	8.18
	RRu	10.54
	P	+++
Asymm	P	N.S.

Table 1E17 - 6

IESLC - Meta-analysis of Current Smoking, Mixed smokers
All LC types
Least adjusted

	Sex combined	male	female	Total
N		9		9
NS		9		9
Wt		239.81		239.81
Het Chi		6.69		6.69
Het df		8		8
Het P		N.S.		N.S.
Fixed RR		9.29		9.29
RRl		8.18		8.18
RRu		10.54		10.54
P		+++		+++
Random RR		9.29		9.29
RRl		8.18		8.18
RRu		10.54		10.54
P		+++		+++
Between Chi				
Between df				
Between P				N.S.
Btwn(F) P				N.S.
Btwn(R) P				N.S.

Table 1E17 - 7

IESLC - Meta-analysis of Current Smoking, Mixed smokers
All LC types
Excluded studies (and stage at which they were excluded)

Stage	Studies
1	BOUCHA BUELL LAURIL MZILEN
2	ABRAHA AGUDO AKIBA AMANDU AMES ANDERS ARCHER AUSTIN AUVINE AXELSO AXELSS BAND BARBON BECHER BENSHL BERRIN BLOHMK BLOT1 BLOT2 BLOT3 BLOT4 BRETT BROCKM BROSS BROWN1 BROWN2 BUFFLE BYERS1 BYERS2 CARPEN CASCO2 CASCOR CHAN CHANG CHATZI CHEN CHEN2 CHEN3 CHIAZZ CHOI CHYOU COMSTO COOKSO CORREA CPSII DARBY DAVEYS DEAN3 DEKLER DESTE2 DESTEF DOCKER DOLL DORANT DORGAN DOSEME DROSTE DU DUNN EBELIN ENGELA ENSTRO ESAKI FAN GAO GAO2 GARCIA GARDIN GARSHI GENG GER GILLIS GODLEY GOODMA GREGOR GSELL HAENSZ HAMMO2 HANSEN HEGMAN HEIN HENNEK HINDS HIRAOK HIRAYA HITOSU HOLE HOROWI HORWIT HU HU2 HUANG HUMBLE ISHIMA JAHN JAIN JARUP JARVHO JEDRYC JIANG JONES JUSSAW KAISE2 KAISER KANELL KATSOU KAUFMA KELLER KHUDER KIHARA KINLEN KJUUS KNEKT KO KOHLME KOO KOULUM KREUZE KREYBE KUBIK LAMTH LAMWK LAMWK2 LANGE LAUSSM LEI LEMARC LETOUR LEVIN LIAW LICKIN LIDDEL LIU LIU2 LIU3 LIU4 LIU5 LOMBA2 LUO MACLEN MAGNUS MARSH MARSH2 MARTIS MASTRA MATOS MATSUD MCDUFF MCLAUG MILLER MILLS MOLLO MRFIT MRFITR MURATA NAM NOTAN2 NOTANI NOU ODRISC ORMOS OSANN OSANN2 PARKIN PASTOR PAWLEG PERNU PERSH2 PERSHA PETO PEZZO2 PEZZOT PIKE PISANI POFFIJ POLEDN PRESCO QIAO2 RACHTA RADZIK RANDIG REN RESTRE RIMING RONCO ROOTS ROTHSC SAARIK SANKAR SCHWAR SEGI SEGI2 SEOW SHAW SHIMIZ SIEMIA SIMARA SIMONA SITAS SOBUE SOBUE2 SPEIZE SPITZ STAYNE STOCKS STOCKW STUCKE SUN SUZUK2 SUZUKI SVENSS TANG TANG2 TAO TENKAN TIZZAN TOKARS TOUSEY TSUGAN TULINI ULMER VANDER VEIERO VUTUC WAKAI WALD WANG WANG2 WANG3 WANG4 WARSIN WATSON WICKLU WIGLE WILKIN WU WU2 WUNSCH WUWILL WYNDE2 WYNDE3 WYNDE4 WYNDE5 WYNDE6 WYNDE8 XU XU2 XU3 XU4 YAMAGU YONG YUAN ZHANG ZHENG ZHOU
3	ABELIN ALDERS ARMADA BENHAM BEST BOFFET BOUCOT BRESLO CPSI DAMBER DEAN DEAN2 GOLLED HAMMON JOLY LUBIN MCCONN QIAO SADOWS SCHWA2 STASZE XIANGZ
5	HIRAY2 TVERDA WYNDER

Table 1E17 - 8

Potentially overlapping studies

REF	REFGP	PRINC	.	OVERLAP/LINK
LUBIN2	LUBIN2	1		Lubin-combined
GRAHAM	BYERS1	1		GRAHAM/BROSS/BYERS1
WYNDE7	WYNDE6	2		WYNDE5/6/7/8

Table 1E18 -

IESLC - Meta-analysis of Ever Smoking (or Current if Ever not available), Mixed smokers
All LC types

This analysis is restricted to results for:
1) Non-dose-response data
2) Mixed smokers (cigarettes and pipe/cigar)
3) Results complete enough for use in metaanalysis

Within each study, results are then selected (in the following order of preference, within each sex) for:
4) SMKSTA: ever smokers, current smokers
5) DENOM: never smoked anything, (never +1 = +long term ex)
6) Followup period (prospective studies): whole study (coded as 0) or longest available
7) LCtype: all or nearest available, at least Squamous and Adeno. (q = squamous, s = small,
l = large, a = adeno, mix = mixed, alv = alveolar)
8) Race: all or nearest available, otherwise by race (wh or w = white, bl or b = black, hi = hispanic
ch = chinese, jap = japanese, haw = hawaiian, w+o = white + oriental, sca = scandinavian, as = asian)
9) For overlapping studies: principal rather than subsidiary studies
Finally by Age: whole study (coded as 0) if available, otherwise by widest available age group
and then for single sex results (m, f) in preference to combined sex results (c).

Results adjusted (AD) for the most potential confounders are then chosen in Sections -1 to -3
and results adjusted for the least confounders in Sections -4 to -6. (Those least adjusted results which
actually differ from the most adjusted as marked 'x' in column X in Section -4)
(Results adjusted for an unknown number of confounder(s) are coded as 20.)

Section -7 shows excluded studies, together with the stage (as above) at which no qualifying
results were found.

Section -8 lists the potentially overlapping studies which have been included (1=principal, 2=subsidiary).

Section -9 lists any results which would have been included in preference except that they had data not complete
enough for use in meta-analysis, with their significance (yes/no), if known, and any further comment as entered
on the database.

In addition to those mentioned above, the following fields, levels and abbreviations are used:
* or nk = not known, n = no, y = yes, ot = other
ev = ever, cu = current, nev = never
REF: 6-character study reference
NRR: number of the RR on the database within the study
ST : study type (CC = case control, pr or prosp = prospective)
NLC: number of lung cancer cases in whole study
R : risky occupational population (n = no, m = mining, o = other risky)
VB : national cigarette type (V = at least 75% Virginia, bl = at least 75% blended, ot = other)
P : any proxy use
H : full histological confirmation
De : derivation of RR/CI (or = original, st = standard method, ot = other method of estimation)

Table 1E18 - 1

IESLC - Meta-analysis of Ever Smoking (or Current if Ever not available), Mixed smokers
All LC types
Most adjusted

REF	NRR	SEX	AGEL	AGEH	RACE	YF	LC TYPE	LOC	START	ST	NLC	R	VB	P	H	AD	SM	DENOM	De
ABELIN	48	m	0	0	all	-	all	Eu:wst	1941	CC	118	n	bl	y	n	1	ev	nev any	st
ALDERS	2	m	0	0	all	-	all	Eu:UK	1977	CC	1448	n	V	n	n	1	ev	nev any	ot
ARMADA	31	m	0	0	all	-	all	Eu:wst	1986	CC	325	n	bl	n	y	0	ev	nev any	st
BEST	20	m	55	79	all	3	all	NAmer	1955	pr	381	n	V	n	n	0	ev	nev any	st
BOFFET	26	m	0	0	all	-	all	Eu:mul	1988	CC	5621	n	bl	y	n	2	ev	nev any	or
BOUCOT	116	m	0	0	all	0	all	NAmer	1951	pr	121	n	bl	n	n	2	ev	nev any	ot
BRESLO	19	m	0	0	all	-	all	NAmer	1949	CC	518	n	bl	n	y	0	ev	nev+1	st
CEDERL	28	m	0	0	all	10	all	Eu:Sca	1963	pr	491	n	bl	n	n	1	cu	nev any	ot
CHOW	22	m	0	0	wh	0	all	NAmer	1966	pr	219	n	bl	n	n	0	ev	nev any	st
CPSI	184	m	35	84	all	6	all	NAmer	1959	pr	5138	n	bl	n	n	1	ev	nev any	ot
DAMBER	3	m	0	0	all	-	all	Eu:Sca	1972	CC	579	n	bl	y	n	0	ev	nev any	st
DEAN	6	m	0	0	wh	-	all	Africa	1947	CC	603	n	V	y	n	0	ev	nev any	st
DEAN2	10	m	0	0	all	-	all	Eu:UK	1960	CC	954	n	V	y	n	0	ev	nev any	st
DEAN2	18	f	0	0	all	-	all	Eu:UK	1960	CC	954	n	V	y	n	0	ev	nev any	ot
DOLL2	85	m	0	0	all	10	all	Eu:UK	1951	pr	920	n	V	n	n	1	ev	nev any	ot
DORN	31	m	0	0	wh	2	all	NAmer	1954	pr	5097	n	bl	n	n	1	ev	nev any	ot
GOLLED	4	m	35	99	all	-	all	Eu:UK	1952	CC	443	n	V	y	n	1	ev	nev any	ot
GRAHAM	19	m	0	0	wh	-	all	NAmer	1956	CC	685	n	bl	n	n	0	ev	nev any	st
HAMMON	114	m	0	0	wh	0	all	NAmer	1952	pr	448	n	bl	n	n	1	ev	nev any	ot
JOLY	35	m	0	0	all	-	all	SCAmer	1978	CC	826	n	bl	n	n	0	ev	nev any	st
LOMBAR	11	m	0	0	all	-	all	NAmer	1951	CC	1040	n	bl	n	n	0	ev	nev any	st
LUBIN2	52	m	0	0	all	-	all	Eu:mul	1976	CC	7804	n	bl	n	y	2	ev	nev any	ot
LUBIN2	100	f	0	0	all	-	all	Eu:mul	1976	CC	7804	n	bl	n	y	1	ev	nev any	ot
MCCONN	18	c	0	0	all	-	all	Eu:UK	1946	CC	100	n	V	n	y	0	ev	nev any	st
MIGRAN	14	m	0	0	all	0	all	Eu:UK	1964	pr	259	n	V	n	n	2	cu	nev any	ot
SADOWS	69	m	0	0	wh	-	all	NAmer	1938	CC	477	n	bl	n	n	0	ev	nev any	st
STASZE	6	m	0	0	all	-	all	Eu:est	1954	CC	281	n	bl	n	y	0	ev	nev any	st
WYNDE7	43	m	0	0	all	-	all	NAmer	1977	CC	2085	n	bl	n	y	0	ev	nev any	st
XIANGZ	11	m	0	0	all	0	all	As:Chi	1976	pr	983	m	ot	n	n	2	ev	nev any	ot

Table 1E18 - 2

IESLC - Meta-analysis of Ever Smoking (or Current if Ever not available), Mixed smokers
All LC types
Most adjusted

REF	NRR	SEX	AD	Number Exposed Case	Cont	Non-exposed Case	Cont	RR	95.00%CI
ABELIN	48	m	1	-	-	-	-	29.10	(6.25- 135.39)
ALDERS	2	m	1	-	-	-	-	9.09	(5.14- 16.08)
ARMADA	31	m	0	72	57	4	64	20.21	(6.94- 58.82)
*BEST	20	m	0	115	13479	1	2854	24.35	(3.40- 174.26)
BOFFET	26	m	2	-	-	-	-	12.70	(10.30- 15.60)
*BOUCOT	116	m	2	-	-	-	-	47.79	(2.91- 786.03)
BRESLO	19	m	0	155	154	7	42	6.04	(2.63- 13.86)
*CEDERL	28	m	1	-	-	-	-	10.90	(4.75- 25.01)
*CHOW	22	m	0	132	122272	6	62913	11.32	(5.00- 25.65)
*CPSI	184	m	1	-	-	-	-	8.04	(6.38- 10.12)
DAMBER	3	m	0	134	75	42	208	8.85	(5.72- 13.68)
DEAN	6	m	0	137	115	12	61	6.06	(3.11- 11.80)
DEAN2	10	m	0	57	48	33	112	4.03	(2.33- 6.96)
DEAN2	18	f	0	1	0	88	121	4.12~	(0.17- 102.29)
Subtotal DEAN2								4.03	(2.35- 6.91)
*DOLL2	85	m	1	-	-	-	-	7.57	(2.35- 24.43)
*DORN	31	m	1	-	-	-	-	6.40	(3.82- 10.72)
GOLLED	4	m	1	-	-	-	-	6.46	(3.46- 12.07)
GRAHAM	19	m	0	144	333	18	346	8.31	(4.98- 13.88)
*HAMMON	114	m	1	-	-	-	-	7.63	(4.49- 12.98)
JOLY	35	m	0	173	210	12	218	14.97	(8.09- 27.68)
LOMBAR	11	m	0	492	480	14	112	8.20	(4.64- 14.49)
LUBIN2	52	m	2	-	-	-	-	7.77	(6.45- 9.36)
LUBIN2	100	f	1	-	-	-	-	3.30	(0.30- 36.51)
Subtotal LUBIN2								7.73	(6.42- 9.31)
MCCONN	18	c	0	15	32	9	23	1.20	(0.45- 3.21)
*MIGRAN	14	m	2	-	-	-	-	3.87	(1.18- 12.71)
SADOWS	69	m	0	148	118	18	81	5.64	(3.21- 9.93)
STASZE	6	m	0	33	101	5	158	10.32	(3.90- 27.32)
WYNDE7	43	m	0	327	614	64	918	7.64	(5.73- 10.18)
*XIANGZ	11	m	2	-	-	-	-	1.90	(1.27- 2.85)
Partial Totals				2135	138088	333	68231		

*prospective study ~ With 0.5 adjustment for zero

REF	NRR	SEX	AD	Ys	Ws	Qs	Ps
ABELIN	48	m	1	3.37	1.62	2.78	0.0000
ALDERS	2	m	1	2.21	11.81	0.24	0.0000
ARMADA	31	m	0	3.01	3.37	2.99	0.0000
*BEST	20	m	0	3.19	0.99	1.26	0.0015
BOFFET	26	m	2	2.54	89.16	20.40	0.0000
*BOUCOT	116	m	2	3.87	0.49	1.59	0.0068
BRESLO	19	m	0	1.80	5.57	0.39	0.0000
*CEDERL	28	m	1	2.39	5.57	0.59	0.0000
*CHOW	22	m	0	2.43	5.74	0.76	0.0000
*CPSI	184	m	1	2.08	72.19	0.03	0.0000
DAMBER	3	m	0	2.18	20.24	0.28	0.0000
DEAN	6	m	0	1.80	8.64	0.59	0.0000
DEAN2	10	m	0	1.39	12.89	5.78	0.0000
DEAN2	18	f	0	1.42	0.37	0.16	0.3878
Subtotal DEAN2				1.39	13.26	5.93	
*DOLL2	85	m	1	2.02	2.80	0.00	0.0007
*DORN	31	m	1	1.86	14.43	0.62	0.0000
GOLLED	4	m	1	1.87	9.84	0.38	0.0000
GRAHAM	19	m	0	2.12	14.62	0.04	0.0000
*HAMMON	114	m	1	2.03	13.64	0.01	0.0000
JOLY	35	m	0	2.71	10.16	4.19	0.0000
LOMBAR	11	m	0	2.10	11.84	0.02	0.0000
LUBIN2	52	m	2	2.05	110.82	0.02	0.0000
LUBIN2	100	f	1	1.19	0.67	0.50	0.3297
Subtotal LUBIN2				2.05	111.49	0.52	
MCCONN	18	c	0	0.18	3.96	14.04	0.7193
*MIGRAN	14	m	2	1.35	2.72	1.37	0.0256
SADOWS	69	m	0	1.73	12.03	1.33	0.0000
STASZE	6	m	0	2.33	4.06	0.30	0.0000
WYNDE7	43	m	0	2.03	46.73	0.04	0.0000
*XIANGZ	11	m	2	0.64	23.52	47.52	0.0019

Table 1E18 - 2

IESLC - Meta-analysis of Ever Smoking (or Current if Ever not available), Mixed smokers
All LC types
Most adjusted

	N	29
	NS	27
	Wt	520.48
Het	Chi	108.24
Het	df	28
Het	P	***
Fixed	RR	7.87
	RRl	7.22
	RRu	8.58
	P	+++
Random	RR	7.37
	RRl	6.00
	RRu	9.04
	P	+++
Asymm	P	N.S.

Table 1E18 - 3

IESLC - Meta-analysis of Ever Smoking (or Current if Ever not available), Mixed smokers
All LC types
Most adjusted

		Sex		
	combined	male	female	Total
N	1	26	2	29
NS	1	26	2	29
Wt	3.96	515.48	1.04	520.48
Het Chi	0.00	93.41	0.01	108.24
Het df	0	25	1	28
Het P	N.S.	***	N.S.	***
Fixed RR	1.20	8.00	3.57	7.87
RRl	0.45	7.34	0.52	7.22
RRu	3.21	8.72	24.44	8.58
P	N.S.	+++	N.S.	+++
Random RR	1.20	7.76	3.57	7.37
RRl	0.45	6.36	0.52	6.00
RRu	3.21	9.46	24.44	9.04
P	N.S.	+++	N.S.	+++
Between Chi				14.82
Between df				2
Between P				***
Btwn(F) P				N.S.
Btwn(R) P				**

	All LC (or nearest)		
	all	other	Total
N	29		29
NS	27		27
Wt	520.48		520.48
Het Chi	108.24		108.24
Het df	28		28
Het P	***		***
Fixed RR	7.87		7.87
RRl	7.22		7.22
RRu	8.58		8.58
P	+++		+++
Random RR	7.37		7.37
RRl	6.00		6.00
RRu	9.04		9.04
P	+++		+++
Between Chi			
Between df			
Between P			N.S.
Btwn(F) P			N.S.
Btwn(R) P			N.S.

				Location					
	NAmer	UK	Scand	othEur	China	Japan	othAs	other	Total
N	11	7	2	6	1			2	29
NS	11	6	2	5	1			2	27
Wt	198.27	44.40	25.81	209.70	23.52			18.80	520.48
Het Chi	6.05	14.17	0.19	16.44	0.00			3.82	108.24
Het df	10	6	1	5	0			1	28
Het P	N.S.	*	N.S.	**	N.S.			(*)	***
Fixed RR	7.74	5.18	9.26	9.85	1.90			9.87	7.87
RRl	6.73	3.86	6.29	8.60	1.27			6.28	7.22
RRu	8.89	6.95	13.61	11.28	2.85			15.52	8.58
P	+++	+++	+++	+++	++			+++	+++
Random RR	7.74	4.75	9.26	11.03	1.90			9.61	7.37
RRl	6.73	2.86	6.29	7.48	1.27			3.96	6.00
RRu	8.89	7.87	13.61	16.28	2.85			23.32	9.04
P	+++	+++	+++	+++	++			+++	+++
Between Chi									67.56
Between df									5
Between P									***
Btwn(F) P									***
Btwn(R) P									***

Table 1E18 - 3

IESLC - Meta-analysis of Ever Smoking (or Current if Ever not available), Mixed smokers
All LC types
Most adjusted

	Detailed Country in "other Europe"					
	multi	Germany	othWest	East	Balkans	Total
N	3		2	1		6
NS	2		2	1		5
Wt	200.65		4.99	4.06		209.70
Het Chi	12.70		0.15	0.00		16.44
Het df	2		1	0		5
Het P	**		N.S.	N.S.		**
Fixed RR	9.64		22.76	10.32		9.85
RRl	8.39		9.46	3.90		8.60
RRu	11.07		54.72	27.32		11.28
P	+++		+++	+++		+++
Random RR	9.53		22.76	10.32		11.03
RRl	5.98		9.46	3.90		7.48
RRu	15.20		54.72	27.32		16.28
P	+++		+++	+++		+++
Between Chi						3.60
Between df						2
Between P						N.S.
Btwn(F) P						N.S.
Btwn(R) P						N.S.

	Detailed Country in "other Asia"			
	India	HongKong	other	Total
N				
NS				
Wt				
Het Chi				
Het df				
Het P				N.S.
Fixed RR				
RRl				
RRu				
P				+++
Random RR				
RRl				
RRu				
P				+++
Between Chi				
Between df				
Between P				N.S.
Btwn(F) P				N.S.
Btwn(R) P				N.S.

	Detailed other continent			
	SCAmer	Auslia	Africa	Total
N	1		1	2
NS	1		1	2
Wt	10.16		8.64	18.80
Het Chi	0.00		0.00	3.82
Het df	0		0	1
Het P	N.S.		N.S.	(*)
Fixed RR	14.97		6.06	9.87
RRl	8.09		3.11	6.28
RRu	27.68		11.80	15.52
P	+++		+++	+++
Random RR	14.97		6.06	9.61
RRl	8.09		3.11	3.96
RRu	27.68		11.80	23.32
P	+++		+++	+++
Between Chi				3.82
Between df				1
Between P				(*)
Btwn(F) P				N.S.
Btwn(R) P				(*)

Table 1E18 - 3

IESLC - Meta-analysis of Ever Smoking (or Current if Ever not available), Mixed smokers
All LC types
Most adjusted

	Start year of study <1960	1960-69	1970-79	1980-89	1990+	Total
N	15	5	7	2		29
NS	15	4	6	2		27
Wt	176.73	27.29	223.94	92.53		520.48
Het Chi	22.50	6.90	49.72	0.70		108.24
Het df	14	4	6	1		28
Het P	(*)	N.S.	***	N.S.		***
Fixed RR	7.33	6.11	7.00	12.92		7.87
RRl	6.32	4.20	6.14	10.54		7.22
RRu	8.49	8.90	7.98	15.84		8.58
P	+++	+++	+++	+++		+++
Random RR	7.07	6.51	6.86	12.92		7.37
RRl	5.67	3.74	4.36	10.54		6.00
RRu	8.83	11.33	10.80	15.84		9.04
P	+++	+++	+++	+++		+++
Between Chi						28.42
Between df						3
Between P						***
Btwn(F) P						(*)
Btwn(R) P						***

	Study type (1) CC	other	Total
N	19	10	29
NS	17	10	27
Wt	378.39	142.09	520.48
Het Chi	51.86	46.80	108.24
Het df	18	9	28
Het P	***	***	***
Fixed RR	8.55	6.31	7.87
RRl	7.73	5.35	7.22
RRu	9.46	7.44	8.58
P	+++	+++	+++
Random RR	7.81	6.97	7.37
RRl	6.32	4.31	6.00
RRu	9.65	11.28	9.04
P	+++	+++	+++
Between Chi			9.58
Between df			1
Between P			**
Btwn(F) P			N.S.
Btwn(R) P			N.S.

	Study type (2) CC	prosp	other	Total
N	19	10		29
NS	17	10		27
Wt	378.39	142.09		520.48
Het Chi	51.86	46.80		108.24
Het df	18	9		28
Het P	***	***		***
Fixed RR	8.55	6.31		7.87
RRl	7.73	5.35		7.22
RRu	9.46	7.44		8.58
P	+++	+++		+++
Random RR	7.81	6.97		7.37
RRl	6.32	4.31		6.00
RRu	9.65	11.28		9.04
P	+++	+++		+++
Between Chi				9.58
Between df				1
Between P				**
Btwn(F) P				N.S.
Btwn(R) P				N.S.

Table 1E18 - 3

IESLC - Meta-analysis of Ever Smoking (or Current if Ever not available), Mixed smokers
All LC types
Most adjusted

	Study size (number of LC cases) 100-249	250-499	500-999	1000+	Total
N	4	8	9	8	29
NS	4	8	8	7	27
Wt	11.81	52.21	98.80	357.65	520.48
Het Chi	18.69	8.22	45.50	17.42	108.24
Het df	3	7	8	7	28
Het P	***	N.S.	***	*	***
Fixed RR	6.44	7.71	5.44	8.80	7.87
RRl	3.64	5.88	4.47	7.94	7.22
RRu	11.40	10.11	6.63	9.76	8.58
P	+++	+++	+++	+++	+++
Random RR	9.50	7.86	6.07	8.53	7.37
RRl	1.89	5.80	3.63	7.05	6.00
RRu	47.79	10.66	10.15	10.33	9.04
P	++	+++	+++	+++	+++
Between Chi					18.41
Between df					3
Between P					***
Btwn(F) P					N.S.
Btwn(R) P					N.S.

	Risky occupational population no	mining	othRisky	Total
N	28	1		29
NS	26	1		27
Wt	496.96	23.52		520.48
Het Chi	58.47	0.00		108.24
Het df	27	0		28
Het P	***	N.S.		***
Fixed RR	8.42	1.90		7.87
RRl	7.71	1.27		7.22
RRu	9.19	2.85		8.58
P	+++	++		+++
Random RR	7.96	1.90		7.37
RRl	6.77	1.27		6.00
RRu	9.35	2.85		9.04
P	+++	++		+++
Between Chi				49.77
Between df				1
Between P				***
Btwn(F) P				***
Btwn(R) P				***

	National cigarette tobacco type Virginia	blended	other	Total
N	9	19	1	29
NS	8	18	1	27
Wt	54.03	442.93	23.52	520.48
Het Chi	16.61	30.50	0.00	108.24
Het df	8	18	0	28
Het P	*	*	N.S.	***
Fixed RR	5.46	8.88	1.90	7.87
RRl	4.18	8.09	1.27	7.22
RRu	7.13	9.74	2.85	8.58
P	+++	+++	++	+++
Random RR	5.29	8.85	1.90	7.37
RRl	3.45	7.63	1.27	6.00
RRu	8.12	10.26	2.85	9.04
P	+++	+++	++	+++
Between Chi				61.13
Between df				2
Between P				***
Btwn(F) P				***
Btwn(R) P				***

Table 1E18 - 3

IESLC - Meta-analysis of Ever Smoking (or Current if Ever not available), Mixed smokers
All LC types
Most adjusted

	Any proxy use No/nk	Yes	Total
N	22	7	29
NS	21	6	27
Wt	377.71	142.77	520.48
Het Chi	74.81	22.24	108.24
Het df	21	6	28
Het P	***	**	***
Fixed RR	7.19	9.99	7.87
RRl	6.50	8.48	7.22
RRu	7.96	11.77	8.58
P	+++	+++	+++
Random RR	7.18	7.96	7.37
RRl	5.69	5.16	6.00
RRu	9.06	12.26	9.04
P	+++	+++	+++
Between Chi			11.19
Between df			1
Between P			***
Btwn(F) P			(*)
Btwn(R) P			N.S.

	Full histological confirmation No	Yes	Total
N	22	7	29
NS	21	6	27
Wt	345.32	175.16	520.48
Het Chi	89.77	17.91	108.24
Het df	21	6	28
Het P	***	**	***
Fixed RR	8.06	7.52	7.87
RRl	7.25	6.48	7.22
RRu	8.96	8.72	8.58
P	+++	+++	+++
Random RR	7.62	6.81	7.37
RRl	5.89	4.61	6.00
RRu	9.84	10.05	9.04
P	+++	+++	+++
Between Chi			0.56
Between df			1
Between P			N.S.
Btwn(F) P			N.S.
Btwn(R) P			N.S.

	Number of adjustment variables (1) 0	1	2+/+nk	Total
N	15	9	5	29
NS	14	9	5	28
Wt	161.19	132.58	226.71	520.48
Het Chi	31.79	5.16	70.70	108.24
Het df	14	8	4	28
Het P	**	N.S.	***	***
Fixed RR	7.50	7.94	8.11	7.87
RRl	6.43	6.70	7.12	7.22
RRu	8.75	9.41	9.24	8.58
P	+++	+++	+++	+++
Random RR	7.45	7.94	6.14	7.37
RRl	5.73	6.70	2.98	6.00
RRu	9.69	9.41	12.62	9.04
P	+++	+++	+++	+++
Between Chi				0.59
Between df				2
Between P				N.S.
Btwn(F) P				N.S.
Btwn(R) P				N.S.

Table 1E18 - 3

IESLC - Meta-analysis of Ever Smoking (or Current if Ever not available), Mixed smokers
All LC types
Most adjusted

	Number of adjustment variables (2) 0	1	2	3-5	6+/+nk	Total
N	15	9	5			29
NS	14	9	5			28
Wt	161.19	132.58	226.71			520.48
Het Chi	31.79	5.16	70.70			108.24
Het df	14	8	4			28
Het P	**	N.S.	***			***
Fixed RR	7.50	7.94	8.11			7.87
RRl	6.43	6.70	7.12			7.22
RRu	8.75	9.41	9.24			8.58
P	+++	+++	+++			+++
Random RR	7.45	7.94	6.14			7.37
RRl	5.73	6.70	2.98			6.00
RRu	9.69	9.41	12.62			9.04
P	+++	+++	+++			+++
Between Chi						0.59
Between df						2
Between P						N.S.
Btwn(F) P						N.S.
Btwn(R) P						N.S.

	Derivation of RR/CI Orig	StdCalc	Other	Total
N	1	15	13	29
NS	1	15	12	28
Wt	89.16	162.44	268.88	520.48
Het Chi	0.00	34.61	47.91	108.24
Het df	0	14	12	28
Het P	N.S.	**	***	***
Fixed RR	12.70	7.61	6.86	7.87
RRl	10.32	6.53	6.08	7.22
RRu	15.63	8.88	7.73	8.58
P	+++	+++	+++	+++
Random RR	12.70	7.74	6.41	7.37
RRl	10.32	5.91	4.66	6.00
RRu	15.63	10.15	8.80	9.04
P	+++	+++	+++	+++
Between Chi				25.72
Between df				2
Between P				***
Btwn(F) P				*
Btwn(R) P				***

	Smoking status ever	current	Total
N	27	2	29
NS	25	2	27
Wt	512.19	8.29	520.48
Het Chi	106.28	1.96	108.24
Het df	26	1	28
Het P	***	N.S.	***
Fixed RR	7.87	7.76	7.87
RRl	7.22	3.93	7.22
RRu	8.59	15.33	8.58
P	+++	+++	+++
Random RR	7.37	7.11	7.37
RRl	5.97	2.62	6.00
RRu	9.11	19.31	9.04
P	+++	+++	+++
Between Chi			0.00
Between df			1
Between P			N.S.
Btwn(F) P			N.S.
Btwn(R) P			N.S.

Table 1E18 - 4

IESLC - Meta-analysis of Ever Smoking (or Current if Ever not available), Mixed smokers
All LC types
Least adjusted

REF	NRR	X	SEX	AGEL	AGEH	RACE	YF	LC TYPE	LOC	START	ST	NLC	R	VB	P	H	AD	SM	DENOM	De
ABELIN	5	x	m	0	0	all	-	all	Eu:wst	1941	CC	118	n	bl	y	n	0	ev	nev any	st
ALDERS	8	x	m	0	0	all	-	all	Eu:UK	1977	CC	1448	n	V	n	n	0	ev	nev any	st
ARMADA	31		m	0	0	all	-	all	Eu:wst	1986	CC	325	n	bl	n	y	0	ev	nev any	st
BEST	20		m	55	79	all	3	all	NAmer	1955	pr	381	n	V	n	n	0	ev	nev any	st
BOFFET	28	x	m	0	0	all	-	all	Eu:mul	1988	CC	5621	n	bl	y	n	0	ev	nev any	st
BOUCOT	4	x	m	0	0	all	0	all	NAmer	1951	pr	121	n	bl	n	n	0	ev	nev any	ot
BRESLO	19		m	0	0	all	-	all	NAmer	1949	CC	518	n	bl	n	y	0	ev	nev+1	st
CEDERL	4	x	m	0	0	all	10	all	Eu:Sca	1963	pr	491	n	bl	n	n	0	cu	nev any	st
CHOW	22		m	0	0	wh	0	all	NAmer	1966	pr	219	n	bl	n	n	0	ev	nev any	st
CPSI	184		m	35	84	all	6	all	NAmer	1959	pr	5138	n	bl	n	n	1	ev	nev any	ot
DAMBER	3		m	0	0	all	-	all	Eu:Sca	1972	CC	579	n	bl	y	n	0	ev	nev any	st
DEAN	6		m	0	0	wh	-	all	Africa	1947	CC	603	n	V	y	n	0	ev	nev any	st
DEAN2	10		m	0	0	all	-	all	Eu:UK	1960	CC	954	n	V	y	n	0	ev	nev any	st
DEAN2	18		f	0	0	all	-	all	Eu:UK	1960	CC	954	n	V	y	n	0	ev	nev any	ot
DOLL2	85		m	0	0	all	10	all	Eu:UK	1951	pr	920	n	V	n	n	1	ev	nev any	ot
DORN	40	x	m	0	0	wh	2	all	NAmer	1954	pr	5097	n	bl	n	n	0	ev	nev any	st
GOLLED	18	x	m	35	99	all	-	all	Eu:UK	1952	CC	443	n	V	y	n	0	ev	nev any	st
GRAHAM	19		m	0	0	wh	-	all	NAmer	1956	CC	685	n	bl	n	n	0	ev	nev any	st
HAMMON	126	x	m	0	0	wh	0	all	NAmer	1952	pr	448	n	bl	n	n	0	ev	nev any	st
JOLY	35		m	0	0	all	-	all	SCAmer	1978	CC	826	n	bl	n	n	0	ev	nev any	st
LOMBAR	11		m	0	0	all	-	all	NAmer	1951	CC	1040	n	bl	n	n	0	ev	nev any	st
LUBIN2	51	x	m	0	0	all	-	all	Eu:mul	1976	CC	7804	n	bl	n	y	0	ev	nev any	st
LUBIN2	99	x	f	0	0	all	-	all	Eu:mul	1976	CC	7804	n	bl	n	y	0	ev	nev any	st
MCCONN	18		c	0	0	all	-	all	Eu:UK	1946	CC	100	n	V	n	y	0	ev	nev any	st
MIGRAN	13	x	m	0	0	all	0	all	Eu:UK	1964	pr	259	n	V	n	n	0	cu	nev any	st
SADOWS	69		m	0	0	wh	-	all	NAmer	1938	CC	477	n	bl	n	n	0	ev	nev any	st
STASZE	6		m	0	0	all	-	all	Eu:est	1954	CC	281	n	bl	n	y	0	ev	nev any	st
WYNDE7	43		m	0	0	all	-	all	NAmer	1977	CC	2085	n	bl	n	y	0	ev	nev any	st
XIANGZ	4	x	m	0	0	all	0	all	As:Chi	1976	pr	983	m	ot	n	n	0	ev	nev any	st

Table 1E18 - 5

IESLC - Meta-analysis of Ever Smoking (or Current if Ever not available), Mixed smokers
All LC types
Least adjusted

REF	NRR	SEX	AD	Number Exposed Case	Exposed Cont	Non-exposed Case	Non-exposed Cont	RR	95.00%CI
ABELIN	5	m	0	17	64	2	183	24.30	(5.46- 108.12)
ALDERS	8	m	0	206	179	15	133	10.20	(5.77- 18.05)
ARMADA	31	m	0	72	57	4	64	20.21	(6.94- 58.82)
*BEST	20	m	0	115	13479	1	2854	24.35	(3.40- 174.26)
BOFFET	28	m	0	1182	1309	117	1750	13.51	(11.02- 16.55)
*BOUCOT	4	m	0	24	7607	0	7551	48.64~	(2.96- 799.67)
BRESLO	19	m	0	155	154	7	42	6.04	(2.63- 13.86)
*CEDERL	4	m	0	27	3762	7	6352	6.51	(2.84- 14.94)
*CHOW	22	m	0	132	122272	6	62913	11.32	(5.00- 25.65)
*CPSI	184	m	1	-	-	-	-	8.04	(6.38- 10.12)
DAMBER	3	m	0	134	75	42	208	8.85	(5.72- 13.68)
DEAN	6	m	0	137	115	12	61	6.06	(3.11- 11.80)
DEAN2	10	m	0	57	48	33	112	4.03	(2.33- 6.96)
DEAN2	18	f	0	1	0	88	121	4.12~	(0.17- 102.29)
Subtotal DEAN2								4.03	(2.35- 6.91)
*DOLL2	85	m	1	-	-	-	-	7.57	(2.35- 24.43)
*DORN	40	m	0	96	110585	17	117918	6.02	(3.60- 10.08)
GOLLED	18	m	0	36	175	15	490	6.72	(3.59- 12.57)
GRAHAM	19	m	0	144	333	18	346	8.31	(4.98- 13.88)
*HAMMON	126	m	0	148	156773	15	115884	7.29	(4.29- 12.40)
JOLY	35	m	0	173	210	12	218	14.97	(8.09- 27.68)
LOMBAR	11	m	0	492	480	14	112	8.20	(4.64- 14.49)
LUBIN2	51	m	0	588	1092	190	2617	7.42	(6.21- 8.86)
LUBIN2	99	f	0	1	2	336	1188	1.77	(0.16- 19.56)
Subtotal LUBIN2								7.36	(6.16- 8.79)
MCCONN	18	c	0	15	32	9	23	1.20	(0.45- 3.21)
*MIGRAN	13	m	0	9	353	4	867	5.53	(1.71- 17.83)
SADOWS	69	m	0	148	118	18	81	5.64	(3.21- 9.93)
STASZE	6	m	0	33	101	5	158	10.32	(3.90- 27.32)
WYNDE7	43	m	0	327	614	64	918	7.64	(5.73- 10.18)
*XIANGZ	4	m	0	237	2569	25	974	3.59	(2.40- 5.39)
Partial Totals				4706	422558	1076	324138		

*prospective study ~ With 0.5 adjustment for zero

REF	NRR	SEX	AD	Ys	Ws	Qs	Ps
ABELIN	5	m	0	3.19	1.72	2.09	0.0000
ALDERS	8	m	0	2.32	11.82	0.64	0.0000
ARMADA	31	m	0	3.01	3.37	2.82	0.0000
*BEST	20	m	0	3.19	0.99	1.20	0.0015
BOFFET	28	m	0	2.60	93.21	24.45	0.0000
*BOUCOT	4	m	0	3.88	0.49	1.58	0.0065
BRESLO	19	m	0	1.80	5.57	0.48	0.0000
*CEDERL	4	m	0	1.87	5.57	0.26	0.0000
*CHOW	22	m	0	2.43	5.74	0.65	0.0000
*CPSI	184	m	1	2.08	72.19	0.00	0.0000
DAMBER	3	m	0	2.18	20.24	0.16	0.0000
DEAN	6	m	0	1.80	8.64	0.73	0.0000
DEAN2	10	m	0	1.39	12.89	6.26	0.0000
DEAN2	18	f	0	1.42	0.37	0.17	0.3878
Subtotal DEAN2				1.39	13.26	6.43	
*DOLL2	85	m	1	2.02	2.80	0.01	0.0007
*DORN	40	m	0	1.80	14.45	1.26	0.0000
GOLLED	18	m	0	1.91	9.78	0.34	0.0000
GRAHAM	19	m	0	2.12	14.62	0.01	0.0000
*HAMMON	126	m	0	1.99	13.62	0.15	0.0000
JOLY	35	m	0	2.71	10.16	3.84	0.0000
LOMBAR	11	m	0	2.10	11.84	0.00	0.0000
LUBIN2	51	m	0	2.00	121.04	0.92	0.0000
LUBIN2	99	f	0	0.57	0.66	1.54	0.6422
Subtotal LUBIN2				2.00	121.71	2.46	
MCCONN	18	c	0	0.18	3.96	14.45	0.7193
*MIGRAN	13	m	0	1.71	2.80	0.41	0.0042
SADOWS	69	m	0	1.73	12.03	1.56	0.0000
STASZE	6	m	0	2.33	4.06	0.24	0.0000
WYNDE7	43	m	0	2.03	46.73	0.16	0.0000
*XIANGZ	4	m	0	1.28	23.36	15.39	0.0000

Table 1E18 - 5

IESLC - Meta-analysis of Ever Smoking (or Current if Ever not available), Mixed smokers
All LC types
Least adjusted

	N	29
	NS	27
	Wt	534.72
Het	Chi	81.76
Het	df	28
Het	P	***
Fixed	RR	8.09
	RRl	7.44
	RRu	8.81
	P	+++
Random	RR	7.52
	RRl	6.29
	RRu	8.99
	P	+++
Asymm	P	N.S.

Table 1E18 - 6

IESLC - Meta-analysis of Ever Smoking (or Current if Ever not available), Mixed smokers
All LC types
Least adjusted

		combined	Sex male	female	Total
	N	1	26	2	29
	NS	1	26	2	29
	Wt	3.96	529.72	1.04	534.72
Het	Chi	0.00	65.45	0.17	81.76
Het	df	0	25	1	28
Het	P	N.S.	***	N.S.	***
Fixed	RR	1.20	8.23	2.39	8.09
	RRl	0.45	7.56	0.35	7.44
	RRu	3.21	8.96	16.41	8.81
	P	N.S.	+++	N.S.	+++
Random	RR	1.20	7.90	2.39	7.52
	RRl	0.45	6.69	0.35	6.29
	RRu	3.21	9.34	16.41	8.99
	P	N.S.	+++	N.S.	+++
Between	Chi				16.14
Between	df				2
Between	P				***
Btwn(F)	P				(*)
Btwn(R)	P				***

Table 1E18 - 7

IESLC - Meta-analysis of Ever Smoking (or Current if Ever not available), Mixed smokers
All LC types
Excluded studies (and stage at which they were excluded)

1 BOUCHA BUELL LAURIL MZILEN

2 ABRAHA AGUDO AKIBA AMANDU AMES ANDERS ARCHER AUSTIN AUVINE AXELSO AXELSS BAND BARBON BECHER BENSHL BERRIN
BLOHMK BLOT1 BLOT2 BLOT3 BLOT4 BRETT BROCKM BROSS BROWN1 BROWN2 BUFFLE BYERS1 BYERS2 CARPEN CASCO2 CASCOR
CHAN CHANG CHATZI CHEN CHEN2 CHEN3 CHIAZZ CHOI CHYOU COMSTO COOKSO CORREA CPSII DARBY DAVEYS DEAN3
DEKLER DESTE2 DESTEF DOCKER DOLL DORANT DORGAN DOSEME DROSTE DU DUNN EBELIN ENGELA ENSTRO ESAKI FAN
GAO GAO2 GARCIA GARDIN GARSHI GENG GER GILLIS GODLEY GOODMA GREGOR GSELL HAENSZ HAMMO2 HANSEN HEGMAN
HEIN HENNEK HINDS HIRAOK HIRAYA HITOSU HOLE HOROWI HORWIT HU HU2 HUANG HUMBLE ISHIMA JAHN JAIN
JARUP JARVHO JEDRYC JIANG JONES JUSSAW KAISE2 KAISER KANELL KATSOU KAUFMA KELLER KHUDER KIHARA KINLEN KJUUS
KNEKT KO KOHLME KOO KOULUM KREUZE KREYBE KUBIK LAMTH LAMWK LAMWK2 LANGE LAUSSM LEI LEMARC LETOUR
LEVIN LIAW LICKIN LIDDEL LIU LIU2 LIU3 LIU4 LIU5 LOMBA2 LUO MACLEN MAGNUS MARSH MARSH2 MARTIS
MASTRA MATOS MATSUD MCDUFF MCLAUG MILLER MILLS MOLLO MRFIT MRFITR MURATA NAM NOTAN2 NOTANI NOU ODRISC
ORMOS OSANN OSANN2 PARKIN PASTOR PAWLEG PERNU PERSH2 PERSHA PETO PEZZO2 PEZZOT PIKE PISANI POFFIJ POLEDN
PRESCO QIAO2 RACHTA RADZIK RANDIG REN RESTRE RIMING RONCO ROOTS ROTHSC SAARIK SANKAR SCHWAR SEGI SEGI2
SEOW SHAW SHIMIZ SIEMIA SIMARA SIMONA SITAS SOBUE SOBUE2 SPEIZE SPITZ STAYNE STOCKS STOCKW STUCKE SUN
SUZUK2 SUZUKI SVENSS TANG TANG2 TAO TENKAN TIZZAN TOKARS TOUSEY TSUGAN TULINI ULMER VANDER VEIERO VUTUC
WAKAI WALD WANG WANG2 WANG3 WANG4 WARSIN WATSON WICKLU WIGLE WILKIN WU WU2 WUNSCH WUWILL WYNDE2
WYNDE3 WYNDE4 WYNDE5 WYNDE6 WYNDE8 XU XU2 XU3 XU4 YAMAGU YONG YUAN ZHANG ZHENG ZHOU

5 HIRAY2 SCHWA2 TVERDA WYNDER

9 BENHAM LUBIN QIAO

Table 1E18 - 8
Potentially overlapping studies

REF	REFGP	PRINC	.	OVERLAP/LINK
LUBIN2	LUBIN2	1		Lubin-combined
GRAHAM	BYERS1	1		GRAHAM/BROSS/BYERS1
WYNDE7	WYNDE6	2		WYNDE5/6/7/8
CPSI	CPSI	1		CPSI overall
BOFFET	BOFFET	1		BOFFET-combined
XIANGZ	XIANGZ	1		LUBIN/XIANGZ/QIAO

Table 1E18 - 9
Most adjusted - insufficient data for metaanalysis

REF	NRR	SEX	AGEL	AGEH	RACE	YF	LC TYPE	LOC	START	ST	NLC	R	VB	P	H	AD	SM	DENOM	De
BRESLO	25	f	0	0	all	-	all	NAmer	1949	CC	518	n	bl	n	y	0	ev	nev+1	st

REF	NRR	RR	SIG	RRDATA comment
BRESLO	25		*	0

Table 1E19 -

IESLC - Meta-analysis of Current Smoking (or Ever if Current not available), Mixed smokers
All LC types

This analysis is restricted to results for:
1) Non-dose-response data
2) Mixed smokers (cigarettes and pipe/cigar)
3) Results complete enough for use in metaanalysis

Within each study, results are then selected (in the following order of preference, within each sex) for:
4) SMKSTA: current smokers, ever smokers
5) DENOM: never smoked anything, (never +1 = +long term ex)
6) Followup period (prospective studies): whole study (coded as 0) or longest available
7) LCtype: all or nearest available, at least Squamous and Adeno. (q = squamous, s = small,
l = large, a = adeno, mix = mixed, alv = alveolar)
8) Race: all or nearest available, otherwise by race (wh or w = white, bl or b = black, hi = hispanic
ch = chinese, jap = japanese, haw = hawaiian, w+o = white + oriental, sca = scandinavian, as = asian)
9) For overlapping studies: principal rather than subsidiary studies
Finally by Age: whole study (coded as 0) if available, otherwise by widest available age group
and then for single sex results (m, f) in preference to combined sex results (c).

Results adjusted (AD) for the most potential confounders are then chosen in Sections -1 to -3
(and those which actually differ from the adjusted results in Table 1E13 - 1 are marked 'x' in Section -1)
and results adjusted for the least confounders in Sections -4 to -6. (Those least adjusted results which
actually differ from the most adjusted as marked 'x' in column X in Section -4)
(Results adjusted for an unknown number of confounder(s) are coded as 20.)

Section -7 shows excluded studies, together with the stage (as above) at which no qualifying
results were found.

Section -8 lists the potentially overlapping studies which have been included (1=principal, 2=subsidiary).

Section -9 lists any results which would have been included in preference except that they had data not complete
enough for use in meta-analysis, with their significance (yes/no), if known, and any further comment as entered
on the database.

In addition to those mentioned above, the following fields, levels and abbreviations are used:
* or nk = not known, n = no, y = yes, ot = other
ev = ever, cu = current, nev = never
REF: 6-character study reference
NRR: number of the RR on the database within the study
ST : study type (CC = case control, pr or prosp = prospective)
NLC: number of lung cancer cases in whole study
R : risky occupational population (n = no, m = mining, o = other risky)
VB : national cigarette type (V = at least 75% Virginia, bl = at least 75% blended, ot = other)
P : any proxy use
H : full histological confirmation
De : derivation of RR/CI (or = original, st = standard method, ot = other method of estimation)

Table 1E19 - 1

IESLC - Meta-analysis of Current Smoking (or Ever if Current not available), Mixed smokers
All LC types
Most adjusted

REF	NRR	1E13	SEX	AGEL	AGEH	RACE	YF	LC TYPE	LOC	START	ST	NLC	R	VB	P	H	AD	SM	DENOM	De
ABELIN	48		m	0	0	all	-	all	Eu:wst	1941	CC	118	n	bl	y	n	1	ev	nev any	st
ALDERS	2		m	0	0	all	-	all	Eu:UK	1977	CC	1448	n	V	n	n	1	ev	nev any	ot
ARMADA	31		m	0	0	all	-	all	Eu:wst	1986	CC	325	n	bl	n	y	0	ev	nev any	st
BEST	20		m	55	79	all	3	all	NAmer	1955	pr	381	n	V	n	n	0	ev	nev any	st
BOFFET	26		m	0	0	all	-	all	Eu:mul	1988	CC	5621	n	bl	y	n	2	ev	nev any	or
BOUCOT	116		m	0	0	all	0	all	NAmer	1951	pr	121	n	bl	n	n	2	ev	nev any	ot
BRESLO	19		m	0	0	all	-	all	NAmer	1949	CC	518	n	bl	n	y	0	ev	nev+1	st
CEDERL	28		m	0	0	all	10	all	Eu:Sca	1963	pr	491	n	bl	n	n	1	cu	nev any	ot
CHOW	23	x	m	0	0	wh	0	all	NAmer	1966	pr	219	n	bl	n	n	0	cu	nev any	st
CPSI	184		m	35	84	all	6	all	NAmer	1959	pr	5138	n	bl	n	n	1	ev	nev any	ot
DAMBER	3		m	0	0	all	-	all	Eu:Sca	1972	CC	579	n	bl	y	n	0	ev	nev any	st
DEAN	6		m	0	0	wh	-	all	Africa	1947	CC	603	n	V	y	n	0	ev	nev any	st
DEAN2	10		m	0	0	all	-	all	Eu:UK	1960	CC	954	n	V	y	n	0	ev	nev any	st
DEAN2	18		f	0	0	all	-	all	Eu:UK	1960	CC	954	n	V	y	n	0	ev	nev any	ot
DOLL2	21	x	m	0	0	all	20	all	Eu:UK	1951	pr	920	n	V	n	n	1	cu	nev any	ot
DORN	146	x	m	35	84	wh	8	all	NAmer	1954	pr	5097	n	bl	n	n	1	cu	nev any	ot
GOLLED	4		m	35	99	all	-	all	Eu:UK	1952	CC	443	n	V	y	n	1	ev	nev any	ot
GRAHAM	5	x	m	0	0	wh	-	all	NAmer	1956	CC	685	n	bl	n	n	0	cu	nev any	st
HAMMON	114		m	0	0	wh	0	all	NAmer	1952	pr	448	n	bl	n	n	1	ev	nev any	ot
JOLY	35		m	0	0	all	-	all	SCAmer	1978	CC	826	n	bl	n	n	0	ev	nev any	st
LOMBAR	7	x	m	0	0	all	-	all	NAmer	1951	CC	1040	n	bl	n	n	0	cu	nev any	st
LUBIN2	32	x	m	0	0	all	-	all	Eu:mul	1976	CC	7804	n	bl	n	y	2	cu	nev any	ot
LUBIN2	100		f	0	0	all	-	all	Eu:mul	1976	CC	7804	n	bl	n	y	1	ev	nev any	ot
MCCONN	18		c	0	0	all	-	all	Eu:UK	1946	CC	100	n	V	n	y	0	ev	nev any	st
MIGRAN	14		m	0	0	all	0	all	Eu:UK	1964	pr	259	n	V	n	n	2	cu	nev any	ot
SADOWS	69		m	0	0	wh	-	all	NAmer	1938	CC	477	n	bl	n	n	0	ev	nev any	st
STASZE	6		m	0	0	all	-	all	Eu:est	1954	CC	281	n	bl	n	y	0	ev	nev any	st
WYNDE7	5	x	m	0	0	all	-	all	NAmer	1977	CC	2085	n	bl	n	y	0	cu	nev any	st
XIANGZ	11		m	0	0	all	0	all	As:Chi	1976	pr	983	m	ot	n	n	2	ev	nev any	ot

Table 1E19 - 2

IESLC - Meta-analysis of Current Smoking (or Ever if Current not available), Mixed smokers
All LC types
Most adjusted

REF	NRR	SEX	AD	Number Case	Exposed Cont	Non-exposed Case	Cont	RR	95.00%CI
ABELIN	48	m	1	-	-	-	-	29.10	(6.25- 135.39)
ALDERS	2	m	1	-	-	-	-	9.09	(5.14- 16.08)
ARMADA	31	m	0	72	57	4	64	20.21	(6.94- 58.82)
*BEST	20	m	0	115	13479	1	2854	24.35	(3.40- 174.26)
BOFFET	26	m	2	-	-	-	-	12.70	(10.30- 15.60)
*BOUCOT	116	m	2	-	-	-	-	47.79	(2.91- 786.03)
BRESLO	19	m	0	155	154	7	42	6.04	(2.63- 13.86)
*CEDERL	28	m	1	-	-	-	-	10.90	(4.75- 25.01)
*CHOW	23	m	0	59	36605	6	62913	16.90	(7.30- 39.14)
*CPSI	184	m	1	-	-	-	-	8.04	(6.38- 10.12)
DAMBER	3	m	0	134	75	42	208	8.85	(5.72- 13.68)
DEAN	6	m	0	137	115	12	61	6.06	(3.11- 11.80)
DEAN2	10	m	0	57	48	33	112	4.03	(2.33- 6.96)
DEAN2	18	f	0	1	0	88	121	4.12~	(0.17- 102.29)
Subtotal DEAN2								4.03	(2.35- 6.91)
*DOLL2	21	m	1	-	-	-	-	8.20	(3.75- 17.92)
*DORN	146	m	1	-	-	-	-	8.85	(6.93- 11.30)
GOLLED	4	m	1	-	-	-	-	6.46	(3.46- 12.07)
GRAHAM	5	m	0	82	254	18	346	6.21	(3.63- 10.60)
*HAMMON	114	m	1	-	-	-	-	7.63	(4.49- 12.98)
JOLY	35	m	0	173	210	12	218	14.97	(8.09- 27.68)
LOMBAR	7	m	0	420	361	14	112	9.31	(5.25- 16.51)
LUBIN2	32	m	2	-	-	-	-	10.61	(8.52- 13.20)
LUBIN2	100	f	1	-	-	-	-	3.30	(0.30- 36.51)
Subtotal LUBIN2								10.51	(8.45- 13.07)
MCCONN	18	c	0	15	32	9	23	1.20	(0.45- 3.21)
*MIGRAN	14	m	2	-	-	-	-	3.87	(1.18- 12.71)
SADOWS	69	m	0	148	118	18	81	5.64	(3.21- 9.93)
STASZE	6	m	0	33	101	5	158	10.32	(3.90- 27.32)
WYNDE7	5	m	0	211	287	64	918	10.55	(7.74- 14.37)
*XIANGZ	11	m	2	-	-	-	-	1.90	(1.27- 2.85)
Partial Totals				1812	51896	333	68231		

*prospective study ~ With 0.5 adjustment for zero

REF	NRR	SEX	AD	Ys	Ws	Qs	Ps
ABELIN	48	m	1	3.37	1.62	2.40	0.0000
ALDERS	2	m	1	2.21	11.81	0.03	0.0000
ARMADA	31	m	0	3.01	3.37	2.44	0.0000
*BEST	20	m	0	3.19	0.99	1.07	0.0015
BOFFET	26	m	2	2.54	89.16	13.34	0.0000
*BOUCOT	116	m	2	3.87	0.49	1.44	0.0068
BRESLO	19	m	0	1.80	5.57	0.71	0.0000
*CEDERL	28	m	1	2.39	5.57	0.30	0.0000
*CHOW	23	m	0	2.83	5.45	2.46	0.0000
*CPSI	184	m	1	2.08	72.19	0.36	0.0000
DAMBER	3	m	0	2.18	20.24	0.01	0.0000
DEAN	6	m	0	1.80	8.64	1.08	0.0000
DEAN2	10	m	0	1.39	12.89	7.46	0.0000
DEAN2	18	f	0	1.42	0.37	0.20	0.3878
Subtotal DEAN2				1.39	13.26	7.67	
*DOLL2	21	m	1	2.10	6.28	0.02	0.0000
*DORN	146	m	1	2.18	64.27	0.04	0.0000
GOLLED	4	m	1	1.87	9.84	0.82	0.0000
GRAHAM	5	m	0	1.83	13.41	1.45	0.0000
*HAMMON	114	m	1	2.03	13.64	0.21	0.0000
JOLY	35	m	0	2.71	10.16	3.08	0.0000
LOMBAR	7	m	0	2.23	11.69	0.07	0.0000
LUBIN2	32	m	2	2.36	80.17	3.43	0.0000
LUBIN2	100	f	1	1.19	0.67	0.62	0.3297
Subtotal LUBIN2				2.35	80.83	4.05	
MCCONN	18	c	0	0.18	3.96	15.44	0.7193
*MIGRAN	14	m	2	1.35	2.72	1.75	0.0256
SADOWS	69	m	0	1.73	12.03	2.17	0.0000
STASZE	6	m	0	2.33	4.06	0.13	0.0000
WYNDE7	5	m	0	2.36	40.10	1.62	0.0000
*XIANGZ	11	m	2	0.64	23.52	53.84	0.0019

Table 1E19 - 2

IESLC - Meta-analysis of Current Smoking (or Ever if Current not available), Mixed smokers
All LC types
Most adjusted

	N	29
	NS	27
	Wt	534.88
Het	Chi	117.99
Het	df	28
Het	P	***
Fixed	RR	8.63
	RRl	7.93
	RRu	9.39
	P	+++
Random	RR	7.81
	RRl	6.35
	RRu	9.61
	P	+++
Asymm	P	N.S.

Table 1E19 - 3

IESLC - Meta-analysis of Current Smoking (or Ever if Current not available), Mixed smokers
All LC types
Most adjusted

	Sex combined	male	female	Total
N	1	26	2	29
NS	1	26	2	29
Wt	3.96	529.88	1.04	534.88
Het Chi	0.00	101.59	0.01	117.99
Het df	0	25	1	28
Het P	N.S.	***	N.S.	***
Fixed RR	1.20	8.77	3.57	8.63
RRl	0.45	8.05	0.52	7.93
RRu	3.21	9.55	24.44	9.39
P	N.S.	+++	N.S.	+++
Random RR	1.20	8.25	3.57	7.81
RRl	0.45	6.75	0.52	6.35
RRu	3.21	10.09	24.44	9.61
P	N.S.	+++	N.S.	+++
Between Chi				16.39
Between df				2
Between P				***
Btwn(F) P				N.S.
Btwn(R) P				***

	Smoking status ever	current	Total
N	20	9	29
NS	19	9	28
Wt	305.21	229.66	534.88
Het Chi	104.75	8.38	117.99
Het df	19	8	28
Het P	***	N.S.	***
Fixed RR	7.94	9.63	8.63
RRl	7.10	8.46	7.93
RRu	8.89	10.96	9.39
P	+++	+++	+++
Random RR	7.23	9.60	7.81
RRl	5.25	8.37	6.35
RRu	9.96	11.00	9.61
P	+++	+++	+++
Between Chi			4.86
Between df			1
Between P			*
Btwn(F) P			N.S.
Btwn(R) P			N.S.

Table 1E19 - 4

IESLC - Meta-analysis of Current Smoking (or Ever if Current not available), Mixed smokers
All LC types
Least adjusted

REF	NRR	X	SEX	AGEL	AGEH	RACE	YF	LC TYPE	LOC	START	ST	NLC	R	VB	P	H	AD	SM	DENOM	De
ABELIN	5	x	m	0	0	all	-	all	Eu:wst	1941	CC	118	n	bl	y	n	0	ev	nev any	st
ALDERS	8	x	m	0	0	all	-	all	Eu:UK	1977	CC	1448	n	V	n	n	0	ev	nev any	st
ARMADA	31		m	0	0	all	-	all	Eu:wst	1986	CC	325	n	bl	n	y	0	ev	nev any	st
BEST	20		m	55	79	all	3	all	NAmer	1955	pr	381	n	V	n	n	0	ev	nev any	st
BOFFET	28	x	m	0	0	all	-	all	Eu:mul	1988	CC	5621	n	bl	y	n	0	ev	nev any	st
BOUCOT	4	x	m	0	0	all	0	all	NAmer	1951	pr	121	n	bl	n	n	0	ev	nev any	ot
BRESLO	19		m	0	0	all	-	all	NAmer	1949	CC	518	n	bl	n	y	0	ev	nev+1	st
CEDERL	4	x	m	0	0	all	10	all	Eu:Sca	1963	pr	491	n	bl	n	n	0	cu	nev any	st
CHOW	23		m	0	0	wh	0	all	NAmer	1966	pr	219	n	bl	n	n	0	cu	nev any	st
CPSI	184		m	35	84	all	6	all	NAmer	1959	pr	5138	n	bl	n	n	1	ev	nev any	ot
DAMBER	3		m	0	0	all	-	all	Eu:Sca	1972	CC	579	n	bl	y	n	0	ev	nev any	st
DEAN	6		m	0	0	wh	-	all	Africa	1947	CC	603	n	V	y	n	0	ev	nev any	st
DEAN2	10		m	0	0	all	-	all	Eu:UK	1960	CC	954	n	V	y	n	0	ev	nev any	st
DEAN2	18		f	0	0	all	-	all	Eu:UK	1960	CC	954	n	V	y	n	0	ev	nev any	ot
DOLL2	21		m	0	0	all	20	all	Eu:UK	1951	pr	920	n	V	n	n	1	cu	nev any	ot
DORN	146		m	35	84	wh	8	all	NAmer	1954	pr	5097	n	bl	n	n	1	cu	nev any	ot
GOLLED	18	x	m	35	99	all	-	all	Eu:UK	1952	CC	443	n	V	y	n	0	ev	nev any	st
GRAHAM	5		m	0	0	wh	-	all	NAmer	1956	CC	685	n	bl	n	n	0	cu	nev any	st
HAMMON	126	x	m	0	0	wh	0	all	NAmer	1952	pr	448	n	bl	n	n	0	ev	nev any	st
JOLY	35		m	0	0	all	-	all	SCAmer	1978	CC	826	n	bl	n	n	0	ev	nev any	st
LOMBAR	7		m	0	0	all	-	all	NAmer	1951	CC	1040	n	bl	n	n	0	cu	nev any	st
LUBIN2	31	x	m	0	0	all	-	all	Eu:mul	1976	CC	7804	n	bl	n	y	0	cu	nev any	st
LUBIN2	99	x	f	0	0	all	-	all	Eu:mul	1976	CC	7804	n	bl	n	y	0	ev	nev any	st
MCCONN	18		c	0	0	all	-	all	Eu:UK	1946	CC	100	n	V	n	y	0	ev	nev any	st
MIGRAN	13	x	m	0	0	all	0	all	Eu:UK	1964	pr	259	n	V	n	n	0	cu	nev any	st
SADOWS	69		m	0	0	wh	-	all	NAmer	1938	CC	477	n	bl	n	n	0	ev	nev any	st
STASZE	6		m	0	0	all	-	all	Eu:est	1954	CC	281	n	bl	n	y	0	ev	nev any	st
WYNDE7	5		m	0	0	all	-	all	NAmer	1977	CC	2085	n	bl	n	y	0	cu	nev any	st
XIANGZ	4	x	m	0	0	all	0	all	As:Chi	1976	pr	983	m	ot	n	n	0	ev	nev any	st

Table 1E19 - 5

IESLC - Meta-analysis of Current Smoking (or Ever if Current not available), Mixed smokers
All LC types
Least adjusted

REF	NRR	SEX	AD	Number Exposed Case	Number Exposed Cont	Non-exposed Case	Non-exposed Cont	RR	95.00%CI
ABELIN	5	m	0	17	64	2	183	24.30	(5.46- 108.12)
ALDERS	8	m	0	206	179	15	133	10.20	(5.77- 18.05)
ARMADA	31	m	0	72	57	4	64	20.21	(6.94- 58.82)
*BEST	20	m	0	115	13479	1	2854	24.35	(3.40- 174.26)
BOFFET	28	m	0	1182	1309	117	1750	13.51	(11.02- 16.55)
*BOUCOT	4	m	0	24	7607	0	7551	48.64~	(2.96- 799.67)
BRESLO	19	m	0	155	154	7	42	6.04	(2.63- 13.86)
*CEDERL	4	m	0	27	3762	7	6352	6.51	(2.84- 14.94)
*CHOW	23	m	0	59	36605	6	62913	16.90	(7.30- 39.14)
*CPSI	184	m	1	-	-	-	-	8.04	(6.38- 10.12)
DAMBER	3	m	0	134	75	42	208	8.85	(5.72- 13.68)
DEAN	6	m	0	137	115	12	61	6.06	(3.11- 11.80)
DEAN2	10	m	0	57	48	33	112	4.03	(2.33- 6.96)
DEAN2	18	f	0	1	0	88	121	4.12~	(0.17- 102.29)
Subtotal DEAN2								4.03	(2.35- 6.91)
*DOLL2	21	m	1	-	-	-	-	8.20	(3.75- 17.92)
*DORN	146	m	1	-	-	-	-	8.85	(6.93- 11.30)
GOLLED	18	m	0	36	175	15	490	6.72	(3.59- 12.57)
GRAHAM	5	m	0	82	254	18	346	6.21	(3.63- 10.60)
*HAMMON	126	m	0	148	156773	15	115884	7.29	(4.29- 12.40)
JOLY	35	m	0	173	210	12	218	14.97	(8.09- 27.68)
LOMBAR	7	m	0	420	361	14	112	9.31	(5.25- 16.51)
LUBIN2	31	m	0	314	444	190	2617	9.74	(7.92- 11.97)
LUBIN2	99	f	0	1	2	336	1188	1.77	(0.16- 19.56)
Subtotal LUBIN2								9.62	(7.83- 11.82)
MCCONN	18	c	0	15	32	9	23	1.20	(0.45- 3.21)
*MIGRAN	13	m	0	9	353	4	867	5.53	(1.71- 17.83)
SADOWS	69	m	0	148	118	18	81	5.64	(3.21- 9.93)
STASZE	6	m	0	33	101	5	158	10.32	(3.90- 27.32)
WYNDE7	5	m	0	211	287	64	918	10.55	(7.74- 14.37)
*XIANGZ	4	m	0	237	2569	25	974	3.59	(2.40- 5.39)
Partial Totals				4013	225133	1059	206220		

*prospective study ~ With 0.5 adjustment for zero

REF	NRR	SEX	AD	Ys	Ws	Qs	Ps
ABELIN	5	m	0	3.19	1.72	1.75	0.0000
ALDERS	8	m	0	2.32	11.82	0.23	0.0000
ARMADA	31	m	0	3.01	3.37	2.28	0.0000
*BEST	20	m	0	3.19	0.99	1.01	0.0015
BOFFET	28	m	0	2.60	93.21	16.46	0.0000
*BOUCOT	4	m	0	3.88	0.49	1.42	0.0065
BRESLO	19	m	0	1.80	5.57	0.82	0.0000
*CEDERL	4	m	0	1.87	5.57	0.53	0.0000
*CHOW	23	m	0	2.83	5.45	2.26	0.0000
*CPSI	184	m	1	2.08	72.19	0.70	0.0000
DAMBER	3	m	0	2.18	20.24	0.00	0.0000
DEAN	6	m	0	1.80	8.64	1.26	0.0000
DEAN2	10	m	0	1.39	12.89	8.02	0.0000
DEAN2	18	f	0	1.42	0.37	0.22	0.3878
Subtotal DEAN2				1.39	13.26	8.24	
*DOLL2	21	m	1	2.10	6.28	0.04	0.0000
*DORN	146	m	1	2.18	64.27	0.00	0.0000
GOLLED	18	m	0	1.91	9.78	0.76	0.0000
GRAHAM	5	m	0	1.83	13.41	1.71	0.0000
*HAMMON	126	m	0	1.99	13.62	0.52	0.0000
JOLY	35	m	0	2.71	10.16	2.78	0.0000
LOMBAR	7	m	0	2.23	11.69	0.03	0.0000
LUBIN2	31	m	0	2.28	90.23	0.79	0.0000
LUBIN2	99	f	0	0.57	0.66	1.73	0.6422
Subtotal LUBIN2				2.26	90.90	2.52	
MCCONN	18	c	0	0.18	3.96	15.88	0.7193
*MIGRAN	13	m	0	1.71	2.80	0.63	0.0042
SADOWS	69	m	0	1.73	12.03	2.46	0.0000
STASZE	6	m	0	2.33	4.06	0.09	0.0000
WYNDE7	5	m	0	2.36	40.10	1.20	0.0000
*XIANGZ	4	m	0	1.28	23.36	19.08	0.0000

Table 1E19 - 5

IESLC - Meta-analysis of Current Smoking (or Ever if Current not available), Mixed smokers
All LC types
Least adjusted

	N	29
	NS	27
	Wt	548.95
Het	Chi	84.66
Het	df	28
Het	P	***
Fixed	RR	8.87
	RRl	8.16
	RRu	9.65
	P	+++
Random	RR	8.00
	RRl	6.71
	RRu	9.55
	P	+++
Asymm	P	N.S.

Table 1E19 - 6

IESLC - Meta-analysis of Current Smoking (or Ever if Current not available), Mixed smokers
All LC types
Least adjusted

	Sex combined	male	female	Total
N	1	26	2	29
NS	1	26	2	29
Wt	3.96	543.95	1.04	548.95
Het Chi	0.00	66.67	0.17	84.66
Het df	0	25	1	28
Het P	N.S.	***	N.S.	***
Fixed RR	1.20	9.03	2.39	8.87
RRl	0.45	8.30	0.35	8.16
RRu	3.21	9.82	16.41	9.65
P	N.S.	+++	N.S.	+++
Random RR	1.20	8.43	2.39	8.00
RRl	0.45	7.15	0.35	6.71
RRu	3.21	9.93	16.41	9.55
P	N.S.	+++	N.S.	+++
Between Chi				17.82
Between df				2
Between P				***
Btwn(F) P				*
Btwn(R) P				***

Table 1E19 - 7

IESLC - Meta-analysis of Current Smoking (or Ever if Current not available), Mixed smokers
All LC types
Excluded studies (and stage at which they were excluded)

1	BOUCHA BUELL LAURIL MZILEN
2	ABRAHA AGUDO AKIBA AMANDU AMES ANDERS ARCHER AUSTIN AUVINE AXELSO AXELSS BAND BARBON BECHER BENSHL BERRIN BLOHMK BLOT1 BLOT2 BLOT3 BLOT4 BRETT BROCKM BROSS BROWN1 BROWN2 BUFFLE BYERS1 BYERS2 CARPEN CASCO2 CASCOR CHAN CHANG CHATZI CHEN CHEN2 CHEN3 CHIAZZ CHOI CHYOU COMSTO COOKSO CORREA CPSII DARBY DAVEYS DEAN3 DEKLER DESTE2 DESTEF DOCKER DOLL DORANT DORGAN DOSEME DROSTE DU DUNN EBELIN ENGELA ENSTRO ESAKI FAN GAO GAO2 GARCIA GARDIN GARSHI GENG GER GILLIS GODLEY GOODMA GREGOR GSELL HAENSZ HAMMO2 HANSEN HEGMAN HEIN HENNEK HINDS HIRAOK HIRAYA HITOSU HOLE HOROWI HORWIT HU HU2 HUANG HUMBLE ISHIMA JAHN JAIN JARUP JARVHO JEDRYC JIANG JONES JUSSAW KAISE2 KAISER KANELL KATSOU KAUFMA KELLER KHUDER KIHARA KINLEN KJUUS KNEKT KO KOHLME KOO KOULUM KREUZE KREYBE KUBIK LAMTH LAMWK LAMWK2 LANGE LAUSSM LEI LEMARC LETOUR LEVIN LIAW LICKIN LIDDEL LIU LIU2 LIU3 LIU4 LIU5 LOMBA2 LUO MACLEN MAGNUS MARSH MARSH2 MARTIS MASTRA MATOS MATSUD MCDUFF MCLAUG MILLER MILLS MOLLO MRFIT MRFITR MURATA NAM NOTAN2 NOTANI NOU ODRISC ORMOS OSANN OSANN2 PARKIN PASTOR PAWLEG PERNU PERSH2 PERSHA PETO PEZZO2 PEZZOT PIKE PISANI POFFIJ POLEDN PRESCO QIAO2 RACHTA RADZIK RANDIG REN RESTRE RIMING RONCO ROOTS ROTHSC SAARIK SANKAR SCHWAR SEGI SEGI2 SEOW SHAW SHIMIZ SIEMIA SIMARA SIMONA SITAS SOBUE SOBUE2 SPEIZE SPITZ STAYNE STOCKS STOCKW STUCKE SUN SUZUK2 SUZUKI SVENSS TANG TANG2 TAO TENKAN TIZZAN TOKARS TOUSEY TSUGAN TULINI ULMER VANDER VEIERO VUTUC WAKAI WALD WANG WANG2 WANG3 WANG4 WARSIN WATSON WICKLU WIGLE WILKIN WU WU2 WUNSCH WUWILL WYNDE2 WYNDE3 WYNDE4 WYNDE5 WYNDE6 WYNDE8 XU XU2 XU3 XU4 YAMAGU YONG YUAN ZHANG ZHENG ZHOU
5	HIRAY2 SCHWA2 TVERDA WYNDER
9	BENHAM LUBIN QIAO

Table 1E19 - 8
Potentially overlapping studies

REF	REFGP	PRINC	. OVERLAP/LINK
LUBIN2	LUBIN2	1	Lubin-combined
GRAHAM	BYERS1	1	GRAHAM/BROSS/BYERS1
WYNDE7	WYNDE6	2	WYNDE5/6/7/8
CPSI	CPSI	1	CPSI overall
BOFFET	BOFFET	1	BOFFET-combined
XIANGZ	XIANGZ	1	LUBIN/XIANGZ/QIAO

Table 1E19 - 9
Most adjusted - insufficient data for metaanalysis

REF	NRR	SEX	AGEL	AGEH	RACE	YF	LC TYPE	LOC	START	ST	NLC	R	VB	P	H	AD	SM	DENOM	De
BRESLO	25	f	0	0	all	-	all	NAmer	1949	CC	518	n	bl	n	y	0	ev	nev+1	st

REF	NRR	RR	SIG	RRDATA comment
BRESLO	25		*	0

Table 1E20 -

IESLC - Meta-analysis of Ex Smoking, Mixed smokers
All LC types

This analysis is restricted to results for:
1) Non-dose-response data
2) Mixed smokers (cigarettes and pipe/cigar)
3) Ex smokers
4) Results complete enough for use in metaanalysis

Within each study, results are then selected (in the following order of preference, within each sex) for:
5) DENOM: never smoked anything, (never +1 = +long term ex)
6) Followup period (prospective studies): whole study (coded as 0) or longest available
7) LCtype: all or nearest available, at least Squamous and Adeno. (q = squamous, s = small,
l = large, a = adeno, mix = mixed, alv = alveolar)
8) Race: all or nearest available, otherwise by race (wh or w = white, bl or b = black, hi = hispanic
ch = chinese, jap = japanese, haw = hawaiian, w+o = white + oriental, sca = scandinavian, as = asian)
9) For overlapping studies: principal rather than subsidiary studies
Finally by Age: whole study (coded as 0) if available, otherwise by widest available age group
and then for single sex results (m, f) in preference to combined sex results (c).

Results adjusted (AD) for the most potential confounders are then chosen in Sections -1 to -3
and results adjusted for the least confounders in Sections -4 to -6. (Those least adjusted results which
actually differ from the most adjusted as marked 'x' in column X in Section -4)
(Results adjusted for an unknown number of confounder(s) are coded as 20.)

Section -7 shows excluded studies, together with the stage (as above) at which no qualifying
results were found.

Section -8 lists the potentially overlapping studies which have been included (1=principal, 2=subsidiary).

Section -9 lists any results which would have been included in preference except that they had data not complete
enough for use in meta-analysis, with their significance (yes/no), if known, and any further comment as entered
on the database.

In addition to those mentioned above, the following fields, levels and abbreviations are used:
* or nk = not known, n = no, y = yes, ot = other
nev = never
REF: 6-character study reference
NRR: number of the RR on the database within the study
ST : study type (CC = case control, pr or prosp = prospective)
NLC: number of lung cancer cases in whole study
R : risky occupational population (n = no, m = mining, o = other risky)
VB : national cigarette type (V = at least 75% Virginia, bl = at least 75% blended, ot = other)
P : any proxy use
H : full histological confirmation
De : derivation of RR/CI (or = original, st = standard method, ot = other method of estimation)

Table 1E20 - 1

IESLC - Meta-analysis of Ex Smoking, Mixed smokers
All LC types
Most adjusted

REF	NRR	SEX	AGEL	AGEH	RACE	YF	LC TYPE	LOC	START	ST	NLC	R	VB	P	H	AD	DENOM	De
CHOW	24	m	0	0	wh	0	all	NAmer	1966	pr	219	n	bl	n	n	0	nev any	st
DOLL2	87	m	0	0	all	10	all	Eu:UK	1951	pr	920	n	V	n	n	1	nev any	ot
DORN	181	m	35	84	wh	8	all	NAmer	1954	pr	5097	n	bl	n	n	1	nev any	ot
GRAHAM	4	m	0	0	wh	-	all	NAmer	1956	CC	685	n	bl	n	n	0	nev any	st
LOMBAR	6	m	0	0	all	-	all	NAmer	1951	CC	1040	n	bl	n	n	0	nev any	st
LUBIN2	42	m	0	0	all	-	all	Eu:mul	1976	CC	7804	n	bl	n	y	2	nev any	ot
WYNDE7	10	m	0	0	all	-	all	NAmer	1977	CC	2085	n	bl	n	y	0	nev any	st

Table 1E20 - 2

IESLC - Meta-analysis of Ex Smoking, Mixed smokers
All LC types
Most adjusted

REF	NRR	SEX	AD	Number Exposed Case	Number Exposed Cont	Non-exposed Case	Non-exposed Cont	RR	95.00%CI
*CHOW	24	m	0	14	34648	6	62913	4.24	(1.63- 11.02)
*DOLL2	87	m	1	-	-	-	-	6.86	(1.71- 27.42)
*DORN	181	m	1	-	-	-	-	4.43	(3.31- 5.93)
GRAHAM	4	m	0	47	48	18	346	18.82	(10.11- 35.04)
LOMBAR	6	m	0	72	119	14	112	4.84	(2.58- 9.07)
LUBIN2	42	m	2	-	-	-	-	3.77	(2.87- 4.94)
WYNDE7	10	m	0	116	327	64	918	5.09	(3.66- 7.08)
Partial Totals				249	35142	102	64289		

*prospective study

REF	NRR	SEX	AD	Ys	Ws	Qs	Ps
*CHOW	24	m	0	1.44	4.20	0.06	0.0031
*DOLL2	87	m	1	1.93	2.00	0.26	0.0065
*DORN	181	m	1	1.49	45.20	0.28	0.0000
GRAHAM	4	m	0	2.94	9.94	18.62	0.0000
LOMBAR	6	m	0	1.58	9.74	0.00	0.0000
LUBIN2	42	m	2	1.33	52.10	2.99	0.0000
WYNDE7	10	m	0	1.63	35.22	0.13	0.0000

	N	7
	NS	7
	Wt	158.40
Het	Chi	22.34
Het	df	6
Het	P	**
Fixed	RR	4.79
	RRl	4.10
	RRu	5.60
	P	+++
Random	RR	5.51
	RRl	3.88
	RRu	7.82
	P	+++
Asymm	P	N.S.

Table 1E20 - 3

IESLC - Meta-analysis of Ex Smoking, Mixed smokers
All LC types
Most adjusted

	Sex: combined	male	female	Total
N		7		7
NS		7		7
Wt		158.40		158.40
Het Chi		22.34		22.34
Het df		6		6
Het P		**		**
Fixed RR		4.79		4.79
RRl		4.10		4.10
RRu		5.60		5.60
P		+++		+++
Random RR		5.51		5.51
RRl		3.88		3.88
RRu		7.82		7.82
P		+++		+++
Between Chi				
Between df				
Between P				N.S.
Btwn(F) P				N.S.
Btwn(R) P				N.S.

Too few RRs for analysis by factor

Table 1E20 - 4

IESLC - Meta-analysis of Ex Smoking, Mixed smokers
All LC types
Least adjusted

REF	NRR	X	SEX	AGEL	AGEH	RACE	YF	LC TYPE	LOC	START	ST	NLC	R	VB	P	H	AD	DENOM	De
CHOW	24		m	0	0	wh	0	all	NAmer	1966	pr	219	n	bl	n	n	0	nev any	st
DOLL2	87		m	0	0	all	10	all	Eu:UK	1951	pr	920	n	V	n	n	1	nev any	ot
DORN	181		m	35	84	wh	8	all	NAmer	1954	pr	5097	n	bl	n	n	1	nev any	ot
GRAHAM	4		m	0	0	wh	-	all	NAmer	1956	CC	685	n	bl	n	n	0	nev any	st
LOMBAR	6		m	0	0	all	-	all	NAmer	1951	CC	1040	n	bl	n	n	0	nev any	st
LUBIN2	41	x	m	0	0	all	-	all	Eu:mul	1976	CC	7804	n	bl	n	y	0	nev any	st
WYNDE7	10		m	0	0	all	-	all	NAmer	1977	CC	2085	n	bl	n	y	0	nev any	st

Table 1E20 - 5

IESLC - Meta-analysis of Ex Smoking, Mixed smokers
All LC types
Least adjusted

REF	NRR	SEX	AD	Number Exposed Case	Cont	Non-exposed Case	Cont	RR	95.00%CI
*CHOW	24	m	0	14	34648	6	62913	4.24	(1.63- 11.02)
*DOLL2	87	m	1	-	-	-	-	6.86	(1.71- 27.42)
*DORN	181	m	1	-	-	-	-	4.43	(3.31- 5.93)
GRAHAM	4	m	0	47	48	18	346	18.82	(10.11- 35.04)
LOMBAR	6	m	0	72	119	14	112	4.84	(2.58- 9.07)
LUBIN2	41	m	0	99	340	190	2617	4.01	(3.07- 5.24)
WYNDE7	10	m	0	116	327	64	918	5.09	(3.66- 7.08)
Partial Totals				348	35482	292	66906		

*prospective study

REF	NRR	SEX	AD	Ys	Ws	Qs	Ps
*CHOW	24	m	0	1.44	4.20	0.08	0.0031
*DOLL2	87	m	1	1.93	2.00	0.23	0.0065
*DORN	181	m	1	1.49	45.20	0.42	0.0000
GRAHAM	4	m	0	2.94	9.94	18.12	0.0000
LOMBAR	6	m	0	1.58	9.74	0.00	0.0000
LUBIN2	41	m	0	1.39	53.51	2.06	0.0000
WYNDE7	10	m	0	1.63	35.22	0.06	0.0000

	N	7
	NS	7
	Wt	159.81
Het	Chi	20.98
Het	df	6
Het	P	**
Fixed	RR	4.88
	RRl	4.18
	RRu	5.70
	P	+++
Random	RR	5.56
	RRl	3.96
	RRu	7.80
	P	+++
Asymm	P	N.S.

Table 1E20 - 6

IESLC - Meta-analysis of Ex Smoking, Mixed smokers
All LC types
Least adjusted

	combined	Sex male	female	Total
N		7		7
NS		7		7
Wt		159.81		159.81
Het Chi		20.98		20.98
Het df		6		6
Het P		**		**
Fixed RR		4.88		4.88
RRl		4.18		4.18
RRu		5.70		5.70
P		+++		+++
Random RR		5.56		5.56
RRl		3.96		3.96
RRu		7.80		7.80
P		+++		+++
Between Chi				
Between df				
Between P				N.S.
Btwn(F) P				N.S.
Btwn(R) P				N.S.

Table 1E20 - 7

IESLC - Meta-analysis of Ex Smoking, Mixed smokers
All LC types
Excluded studies (and stage at which they were excluded)

1	BOUCHA BUELL LAURIL MZILEN
2	ABRAHA AGUDO AKIBA AMANDU AMES ANDERS ARCHER AUSTIN AUVINE AXELSO AXELSS BAND BARBON BECHER BENSHL BERRIN BLOHMK BLOT1 BLOT2 BLOT3 BLOT4 BRETT BROCKM BROSS BROWN1 BROWN2 BUFFLE BYERS1 BYERS2 CARPEN CASCO2 CASCOR CHAN CHANG CHATZI CHEN CHEN2 CHEN3 CHIAZZ CHOI CHYOU COMSTO COOKSO CORREA CPSII DARBY DAVEYS DEAN3 DEKLER DESTE2 DESTEF DOCKER DOLL DORANT DORGAN DOSEME DROSTE DU DUNN EBELIN ENGELA ENSTRO ESAKI FAN GAO GAO2 GARCIA GARDIN GARSHI GENG GER GILLIS GODLEY GOODMA GREGOR GSELL HAENSZ HAMMO2 HANSEN HEGMAN HEIN HENNEK HINDS HIRAOK HIRAYA HITOSU HOLE HOROWI HORWIT HU HU2 HUANG HUMBLE ISHIMA JAHN JAIN JARUP JARVHO JEDRYC JIANG JONES JUSSAW KAISE2 KAISER KANELL KATSOU KAUFMA KELLER KHUDER KIHARA KINLEN KJUUS KNEKT KO KOHLME KOO KOULUM KREUZE KREYBE KUBIK LAMTH LAMWK LAMWK2 LANGE LAUSSM LEI LEMARC LETOUR LEVIN LIAW LICKIN LIDDEL LIU LIU2 LIU3 LIU4 LIU5 LOMBA2 LUO MACLEN MAGNUS MARSH MARSH2 MARTIS MASTRA MATOS MATSUD MCDUFF MCLAUG MILLER MILLS MOLLO MRFIT MRFITR MURATA NAM NOTAN2 NOTANI NOU ODRISC ORMOS OSANN OSANN2 PARKIN PASTOR PAWLEG PERNU PERSH2 PERSHA PETO PEZZO2 PEZZOT PIKE PISANI POFFIJ POLEDN PRESCO QIAO2 RACHTA RADZIK RANDIG REN RESTRE RIMING RONCO ROOTS ROTHSC SAARIK SANKAR SCHWAR SEGI SEGI2 SEOW SHAW SHIMIZ SIEMIA SIMARA SIMONA SITAS SOBUE SOBUE2 SPEIZE SPITZ STAYNE STOCKS STOCKW STUCKE SUN SUZUK2 SUZUKI SVENSS TANG TANG2 TAO TENKAN TIZZAN TOKARS TOUSEY TSUGAN TULINI ULMER VANDER VEIERO VUTUC WAKAI WALD WANG WANG2 WANG3 WANG4 WARSIN WATSON WICKLU WIGLE WILKIN WU WU2 WUNSCH WUWILL WYNDE2 WYNDE3 WYNDE4 WYNDE5 WYNDE6 WYNDE8 XU XU2 XU3 XU4 YAMAGU YONG YUAN ZHANG ZHENG ZHOU
3	ABELIN ALDERS ARMADA BENHAM BEST BOFFET BOUCOT BRESLO CEDERL CPSI DAMBER DEAN DEAN2 GOLLED HAMMON HIRAY2 JOLY LUBIN MCCONN MIGRAN QIAO SADOWS SCHWA2 STASZE TVERDA WYNDER XIANGZ

Table 1E20 - 8

Potentially overlapping studies

REF	REFGP	PRINC	.	OVERLAP/LINK
LUBIN2	LUBIN2	1		Lubin-combined
GRAHAM	BYERS1	1		GRAHAM/BROSS/BYERS1
WYNDE7	WYNDE6	2		WYNDE5/6/7/8
