# Supplementary material for: Systematic review with meta-analysis of the epidemiological evidence in the 1900s relating smoking to lung cancer
Source: BMC Cancer. 2012 Sep 3;12:385. doi: 10.1186/1471-2407-12-385 (PMC3505152; doi:10.1186/1471-2407-12-385)
Supplement: Additional file 5 — Detailed Analysis Tables (Individual file names as described in Additional file 1: Methods, Table1). [file 1471-2407-12-385-S5.zip › PDF/3E.pdf]

Table 3E1 -

IESLC - Meta-analysis of Ever Smoking, Pipe and/or Cigars (not cigs)
Adenocarcinoma

This analysis is restricted to results for:
1) Non-dose-response data
2) Smokers of pipe and/or cigars (but not cigarettes)
3) Ever smokers
4) Results complete enough for use in metaanalysis

Within each study, results are then selected (in the following order of preference, within each sex) for:
5) DENOM: never smoked anything, (never +1 = +long term ex)
6) Followup period (prospective studies): whole study (coded as 0) or longest available
7) LCtype: all or nearest available, at least Squamous and Adeno. (q = squamous, s = small,
l = large, a = adeno, mix = mixed, alv = alveolar)
8) Race: all or nearest available, otherwise by race (wh or w = white, bl or b = black, hi = hispanic
ch = chinese, jap = japanese, haw = hawaiian, w+o = white + oriental, sca = scandinavian, as = asian)
9) For overlapping studies: principal rather than subsidiary studies
Finally by Age: whole study (coded as 0) if available, otherwise by widest available age group
and then for single sex results (m, f) in preference to combined sex results (c).

Results adjusted (AD) for the most potential confounders are then chosen in Sections -1 to -3
and results adjusted for the least confounders in Sections -4 to -6. (Those least adjusted results which
actually differ from the most adjusted as marked 'x' in column X in Section -4)
(Results adjusted for an unknown number of confounder(s) are coded as 20.)

Section -7 shows excluded studies, together with the stage (as above) at which no qualifying
results were found.

Section -8 lists the potentially overlapping studies which have been included (1=principal, 2=subsidiary).

Section -9 lists any results which would have been included in preference except that they had data not complete
enough for use in meta-analysis, with their significance (yes/no), if known, and any further comment as entered
on the database.

In addition to those mentioned above, the following fields, levels and abbreviations are used:
* or nk = not known, n = no, y = yes, ot = other
nev = never
REF: 6-character study reference
NRR: number of the RR on the database within the study
ST : study type (CC = case control, pr or prosp = prospective)
NLC: number of lung cancer cases in whole study
R : risky occupational population (n = no, m = mining, o = other risky)
VB : national cigarette type (V = at least 75% Virginia, bl = at least 75% blended, ot = other)
P : any proxy use
H : full histological confirmation
De : derivation of RR/CI (or = original, st = standard method, ot = other method of estimation)

Table 3E1 - 1

IESLC - Meta-analysis of Ever Smoking, Pipe and/or Cigars (not cigs)
Adenocarcinoma
Most adjusted

REF	NRR	SEX	AGEL	AGEH	RACE	YF	LC TYPE	LOC	START	ST	NLC	R	VB	P	H	AD	DENOM	De
ALDERS	40	m	0	0	all	-	not q+s	Eu:UK	1977	CC	1448	n	V	n	n	1	nev any	ot
BRESLO	31	c	0	0	all	-	a	NAmer	1949	CC	518	n	bl	n	y	0	nev+1	st
HAMMON	93	m	0	0	wh	0	a	NAmer	1952	pr	448	n	bl	n	n	0	nev any	st
JAHN	18	m	0	0	all	-	a	Eu:Ger	1988	CC	1004	n	bl	n	n	0	nev any	st
WYNDE2	8	m	0	0	all	-	KII	NAmer	1962	CC	404	n	bl	n	y	0	nev any	st
WYNDE3	151	m	0	0	all	-	KII	NAmer	1966	CC	350	n	bl	n	y	0	nev any	st
WYNDE6	60	m	0	0	all	-	KII	NAmer	1969	CC	4423	n	bl	n	y	0	nev any	st

Table 3E1 - 2

IESLC - Meta-analysis of Ever Smoking, Pipe and/or Cigars (not cigs)
Adenocarcinoma
Most adjusted

REF	NRR	SEX	AD	Number Exposed Case	Number Exposed Cont	Non-exposed Case	Non-exposed Cont	RR	95.00%CI
ALDERS	40	m	1	-	-	-	-	0.56	(0.11- 2.90)
BRESLO	31	c	0	2	68	4	56	0.41	(0.07- 2.33)
*HAMMON	93	m	0	3	127770	2	115884	1.36	(0.23- 8.14)
JAHN	18	m	0	2	30	8	138	1.15	(0.23- 5.69)
WYNDE2	8	m	0	3	104	5	105	0.61	(0.14- 2.60)
WYNDE3	151	m	0	4	68	6	88	0.86	(0.23- 3.18)
WYNDE6	60	m	0	20	199	58	617	1.07	(0.63- 1.82)
Partial Totals				34	128239	83	116888		

*prospective study

REF	NRR	SEX	AD	Ys	Ws	Qs	Ps
ALDERS	40	m	1	-0.58	1.44	0.37	0.4873
BRESLO	31	c	0	-0.89	1.28	0.84	0.3158
*HAMMON	93	m	0	0.31	1.20	0.18	0.7360
JAHN	18	m	0	0.14	1.50	0.07	0.8640
WYNDE2	8	m	0	-0.50	1.81	0.33	0.5001
WYNDE3	151	m	0	-0.15	2.26	0.01	0.8244
WYNDE6	60	m	0	0.07	13.53	0.27	0.8057

	N	7
	NS	7
	Wt	23.02
Het	Chi	2.07
Het	df	6
Het	P	N.S.
Fixed	RR	0.93
	RRl	0.62
	RRu	1.40
	P	N.S.
Random	RR	0.93
	RRl	0.62
	RRu	1.40
	P	N.S.
Asymm	P	N.S.

Table 3E1 - 3

IESLC - Meta-analysis of Ever Smoking, Pipe and/or Cigars (not cigs)
Adenocarcinoma
Most adjusted

	Sex			
	combined	male	female	Total
N	1	6		7
NS	1	6		7
Wt	1.28	21.74		23.02
Het Chi	0.00	1.17		2.07
Het df	0	5		6
Het P	N.S.	N.S.		N.S.
Fixed RR	0.41	0.97		0.93
RRl	0.07	0.64		0.62
RRu	2.33	1.48		1.40
P	N.S.	N.S.		N.S.
Random RR	0.41	0.97		0.93
RRl	0.07	0.64		0.62
RRu	2.33	1.48		1.40
P	N.S.	N.S.		N.S.
Between Chi				0.89
Between df				1
Between P				N.S.
Btwn(F) P				N.S.
Btwn(R) P				N.S.

Too few RRs for analysis by factor

Table 3E1 - 4

IESLC - Meta-analysis of Ever Smoking, Pipe and/or Cigars (not cigs)
Adenocarcinoma
Least adjusted

REF	NRR	X	SEX	AGEL	AGEH	RACE	YF	LC TYPE	LOC	START	ST	NLC	R	VB	P	H	AD	DENOM	De
ALDERS	99	x	m	0	0	all	-	not q+s	Eu:UK	1977	CC	1448	n	V	n	n	0	nev any	st
BRESLO	31		c	0	0	all	-	a	NAmer	1949	CC	518	n	bl	n	y	0	nev+1	st
HAMMON	93		m	0	0	wh	0	a	NAmer	1952	pr	448	n	bl	n	n	0	nev any	st
JAHN	18		m	0	0	all	-	a	Eu:Ger	1988	CC	1004	n	bl	n	n	0	nev any	st
WYNDE2	8		m	0	0	all	-	KII	NAmer	1962	CC	404	n	bl	n	y	0	nev any	st
WYNDE3	151		m	0	0	all	-	KII	NAmer	1966	CC	350	n	bl	n	y	0	nev any	st
WYNDE6	60		m	0	0	all	-	KII	NAmer	1969	CC	4423	n	bl	n	y	0	nev any	st

Table 3E1 - 5

IESLC - Meta-analysis of Ever Smoking, Pipe and/or Cigars (not cigs)
Adenocarcinoma
Least adjusted

REF	NRR	SEX	AD	Number Exposed Case	Cont	Non-exposed Case	Cont	RR	95.00%CI
ALDERS	99	m	0	2	35	6	133	1.27	(0.24- 6.55)
BRESLO	31	c	0	2	68	4	56	0.41	(0.07- 2.33)
*HAMMON	93	m	0	3	127770	2	115884	1.36	(0.23- 8.14)
JAHN	18	m	0	2	30	8	138	1.15	(0.23- 5.69)
WYNDE2	8	m	0	3	104	5	105	0.61	(0.14- 2.60)
WYNDE3	151	m	0	4	68	6	88	0.86	(0.23- 3.18)
WYNDE6	60	m	0	20	199	58	617	1.07	(0.63- 1.82)
Totals				36	128274	89	117021		

*prospective study

REF	NRR	SEX	AD	Ys	Ws	Qs	Ps
ALDERS	99	m	0	0.24	1.42	0.10	0.7780
BRESLO	31	c	0	-0.89	1.28	0.95	0.3158
*HAMMON	93	m	0	0.31	1.20	0.13	0.7360
JAHN	18	m	0	0.14	1.50	0.04	0.8640
WYNDE2	8	m	0	-0.50	1.81	0.41	0.5001
WYNDE3	151	m	0	-0.15	2.26	0.03	0.8244
WYNDE6	60	m	0	0.07	13.53	0.11	0.8057

	N	7
	NS	7
	Wt	23.01
Het	Chi	1.78
Het	df	6
Het	P	N.S.
Fixed	RR	0.98
	RRl	0.65
	RRu	1.47
	P	N.S.
Random	RR	0.98
	RRl	0.65
	RRu	1.47
	P	N.S.
Asymm	P	N.S.

Table 3E1 - 6

IESLC - Meta-analysis of Ever Smoking, Pipe and/or Cigars (not cigs)
Adenocarcinoma
Least adjusted

		Sex		
	combined	male	female	Total
N	1	6		7
NS	1	6		7
Wt	1.28	21.73		23.01
Het Chi	0.00	0.77		1.78
Het df	0	5		6
Het P	N.S.	N.S.		N.S.
Fixed RR	0.41	1.03		0.98
RRl	0.07	0.67		0.65
RRu	2.33	1.56		1.47
P	N.S.	N.S.		N.S.
Random RR	0.41	1.03		0.98
RRl	0.07	0.67		0.65
RRu	2.33	1.56		1.47
P	N.S.	N.S.		N.S.
Between Chi				1.01
Between df				1
Between P				N.S.
Btwn(F) P				(*)
Btwn(R) P				N.S.

Table 3E1 - 7

IESLC - Meta-analysis of Ever Smoking, Pipe and/or Cigars (not cigs)
Adenocarcinoma
Excluded studies (and stage at which they were excluded)

1 BOUCHA BUELL LAURIL MZILEN

2 ABRAHA AGUDO AKIBA AMANDU AMES ANDERS ARCHER AUSTIN AUVINE AXELSO AXELSS BAND BARBON BERRIN BLOHMK BLOT1
BLOT2 BLOT3 BLOT4 BRETT BROCKM BROWN1 BROWN2 BUFFLE BYERS1 BYERS2 CARPEN CASCO2 CASCOR CEDERL CHAN CHANG
CHATZI CHEN CHEN2 CHEN3 CHIAZZ CHOI CHYOU COOKSO CORREA CPSII DAVEYS DEAN3 DESTE2 DESTEF DOCKER DOLL
DORANT DOSEME DROSTE DU DUNN EBELIN ENSTRO ESAKI FAN GAO GAO2 GARCIA GARDIN GARSHI GENG GER
GILLIS GODLEY GOODMA GREGOR GSELL HAENSZ HANSEN HEGMAN HEIN HENNEK HINDS HIRAOK HIRAY2 HIRAYA HITOSU HOROWI
HORWIT HU HU2 HUANG HUMBLE ISHIMA JAIN JARUP JARVHO JEDRYC JIANG JOLY JONES JUSSAW KAISE2 KAISER
KANELL KATSOU KAUFMA KELLER KHUDER KIHARA KO KOHLME KOO KOULUM KREYBE KUBIK LAMTH LAMWK LAMWK2 LANGE
LAUSSM LEI LEMARC LETOUR LIAW LICKIN LIDDEL LIU LIU2 LIU3 LIU4 LIU5 LOMBA2 LUBIN LUO MACLEN
MAGNUS MARTIS MASTRA MATSUD MCCONN MCDUFF MCLAUG MILLER MILLS MOLLO MRFIT MRFITR MURATA NAM NOTAN2 NOTANI
NOU ODRISC ORMOS OSANN OSANN2 PARKIN PASTOR PAWLEG PERNU PERSH2 PERSHA PETO PEZZO2 PEZZOT PIKE PISANI
POFFIJ POLEDN PRESCO QIAO QIAO2 RACHTA RADZIK RANDIG REN RESTRE RIMING RONCO ROOTS ROTHSC SAARIK SANKAR
SCHWA2 SCHWAR SEGI SEGI2 SEOW SHAW SHIMIZ SIEMIA SIMARA SIMONA SITAS SOBUE SOBUE2 SPEIZE SPITZ STAYNE
STOCKS STUCKE SUN SUZUK2 SUZUKI SVENSS TANG TANG2 TAO TENKAN TIZZAN TOKARS TSUGAN TVERDA ULMER VANDER
VEIERO VUTUC WAKAI WANG WANG2 WANG3 WANG4 WARSIN WATSON WICKLU WILKIN WU WU2 WUNSCH WUWILL WYNDE4
WYNDE5 WYNDE8 WYNDER XIANGZ XU XU2 XU3 XU4 YAMAGU YONG YUAN ZHANG ZHENG ZHOU

3 BENSHL BROSS DARBY DEKLER ENGELA HOLE KINLEN KNEKT KREUZE MATOS MIGRAN TULINI WALD WYNDE7

7 ABELIN ARMADA BECHER BENHAM BEST BOFFET BOUCOT CHOW COMSTO CPSI DAMBER DEAN DEAN2 DOLL2 DORGAN DORN
GOLLED GRAHAM HAMMO2 KJUUS LEVIN LOMBAR LUBIN2 MARSH MARSH2 SADOWS STASZE STOCKW TOUSEY WIGLE

Table 3E1 - 8
Potentially overlapping studies

REF	REFGP	PRINC	.	OVERLAP/LINK
WYNDE6	WYNDE6	1		WYNDE5/6/7/8
JAHN	BOFFET	2		Subset of BOFFET

Table 3E2 -

IESLC - Meta-analysis of Current Smoking, Pipe and/or Cigars (not cigs)
Adenocarcinoma

This analysis is restricted to results for:
1) Non-dose-response data
2) Smokers of pipe and/or cigars (but not cigarettes)
3) Current smokers
4) Results complete enough for use in metaanalysis

Within each study, results are then selected (in the following order of preference, within each sex) for:
5) DENOM: never smoked anything, (never +1 = +long term ex)
6) Followup period (prospective studies): whole study (coded as 0) or longest available
7) LCtype: all or nearest available, at least Squamous and Adeno. (q = squamous, s = small,
l = large, a = adeno, mix = mixed, alv = alveolar)
8) Race: all or nearest available, otherwise by race (wh or w = white, bl or b = black, hi = hispanic
ch = chinese, jap = japanese, haw = hawaiian, w+o = white + oriental, sca = scandinavian, as = asian)
9) For overlapping studies: principal rather than subsidiary studies
Finally by Age: whole study (coded as 0) if available, otherwise by widest available age group
and then for single sex results (m, f) in preference to combined sex results (c).

Results adjusted (AD) for the most potential confounders are then chosen in Sections -1 to -3
and results adjusted for the least confounders in Sections -4 to -6. (Those least adjusted results which
actually differ from the most adjusted as marked 'x' in column X in Section -4)
(Results adjusted for an unknown number of confounder(s) are coded as 20.)

Section -7 shows excluded studies, together with the stage (as above) at which no qualifying
results were found.

Section -8 lists the potentially overlapping studies which have been included (1=principal, 2=subsidiary).

Section -9 lists any results which would have been included in preference except that they had data not complete
enough for use in meta-analysis, with their significance (yes/no), if known, and any further comment as entered
on the database.

In addition to those mentioned above, the following fields, levels and abbreviations are used:
* or nk = not known, n = no, y = yes, ot = other
nev = never
REF: 6-character study reference
NRR: number of the RR on the database within the study
ST : study type (CC = case control, pr or prosp = prospective)
NLC: number of lung cancer cases in whole study
R : risky occupational population (n = no, m = mining, o = other risky)
VB : national cigarette type (V = at least 75% Virginia, bl = at least 75% blended, ot = other)
P : any proxy use
H : full histological confirmation
De : derivation of RR/CI (or = original, st = standard method, ot = other method of estimation)

Table 3E2 - 1

IESLC - Meta-analysis of Current Smoking, Pipe and/or Cigars (not cigs)
Adenocarcinoma
Most adjusted

REF	NRR	SEX	AGEL	AGEH	RACE	YF	LC TYPE	LOC	START	ST	NLC	R	VB	P	H	AD	DENOM	De
WYNDE7	79	m	0	0	all	-	KII	NAmer	1977	CC	2085	n	bl	n	y	0	nev any	st

Table 3E2 - 2

IESLC - Meta-analysis of Current Smoking, Pipe and/or Cigars (not cigs)
Adenocarcinoma
Most adjusted

REF	NRR	SEX	AD	Number Exposed Case	Cont	Non-exposed Case	Cont	RR	95.00%CI
WYNDE7	79	m	0	7	171	42	918	0.89	(0.40- 2.02)
Totals				7	171	42	918		

*prospective study

REF	NRR	SEX	AD	Ys	Ws	Qs	Ps
WYNDE7	79	m	0	-0.11	5.76	0.00	0.7895

	N	1
	NS	1
	Wt	5.76
Het	Chi	0.00
Het	df	0
Het	P	N.S.
Fixed	RR	0.89
	RRl	0.40
	RRu	2.02
	P	N.S.
Random	RR	0.89
	RRl	0.40
	RRu	2.02
	P	N.S.
Asymm	P	

Table 3E2 - 3

IESLC - Meta-analysis of Current Smoking, Pipe and/or Cigars (not cigs)
Adenocarcinoma
Most adjusted

	Sex combined	male	female	Total
N		1		1
NS		1		1
Wt		5.76		5.76
Het Chi		0.00		0.00
Het df		0		0
Het P		N.S.		N.S.
Fixed RR		0.89		0.89
RRl		0.40		0.40
RRu		2.02		2.02
P		N.S.		N.S.
Random RR		0.89		0.89
RRl		0.40		0.40
RRu		2.02		2.02
P		N.S.		N.S.
Between Chi				
Between df				
Between P				N.S.
Btwn(F) P				N.S.
Btwn(R) P				N.S.

Too few RRs for analysis by factor

Table 3E2 - 4

IESLC - Meta-analysis of Current Smoking, Pipe and/or Cigars (not cigs)
Adenocarcinoma
Least adjusted

REF	NRR	X	SEX	AGEL	AGEH	RACE	YF	LC TYPE	LOC	START	ST	NLC	R	VB	P	H	AD	DENOM	De
WYNDE7	79		m	0	0	all	-	KII	NAmer	1977	CC	2085	n	bl	n	y	0	nev any	st

Table 3E2 - 5

IESLC - Meta-analysis of Current Smoking, Pipe and/or Cigars (not cigs)
Adenocarcinoma
Least adjusted

REF	NRR	SEX	AD	Number Exposed Case	Cont	Non-exposed Case	Cont	RR	95.00%CI
WYNDE7	79	m	0	7	171	42	918	0.89	(0.40- 2.02)
Totals				7	171	42	918		

*prospective study

REF	NRR	SEX	AD	Ys	Ws	Qs	Ps
WYNDE7	79	m	0	-0.11	5.76	0.00	0.7895

	N	1
	NS	1
	Wt	5.76
Het	Chi	0.00
Het	df	0
Het	P	N.S.
Fixed	RR	0.89
	RRl	0.40
	RRu	2.02
	P	N.S.
Random	RR	0.89
	RRl	0.40
	RRu	2.02
	P	N.S.
Asymm	P	

Table 3E2 - 6

IESLC - Meta-analysis of Current Smoking, Pipe and/or Cigars (not cigs)
Adenocarcinoma
Least adjusted

	Sex combined	male	female	Total
N		1		1
NS		1		1
Wt		5.76		5.76
Het Chi		0.00		0.00
Het df		0		0
Het P		N.S.		N.S.
Fixed RR		0.89		0.89
RRl		0.40		0.40
RRu		2.02		2.02
P		N.S.		N.S.
Random RR		0.89		0.89
RRl		0.40		0.40
RRu		2.02		2.02
P		N.S.		N.S.
Between Chi				
Between df				
Between P				N.S.
Btwn(F) P				N.S.
Btwn(R) P				N.S.

Table 3E2 - 7

IESLC - Meta-analysis of Current Smoking, Pipe and/or Cigars (not cigs)
Adenocarcinoma
Excluded studies (and stage at which they were excluded)

1	BOUCHA BUELL LAURIL MZILEN
2	ABRAHA AGUDO AKIBA AMANDU AMES ANDERS ARCHER AUSTIN AUVINE AXELSO AXELSS BAND BARBON BERRIN BLOHMK BLOT1 BLOT2 BLOT3 BLOT4 BRETT BROCKM BROWN1 BROWN2 BUFFLE BYERS1 BYERS2 CARPEN CASCO2 CASCOR CEDERL CHAN CHANG CHATZI CHEN CHEN2 CHEN3 CHIAZZ CHOI CHYOU COOKSO CORREA CPSII DAVEYS DEAN3 DESTE2 DESTEF DOCKER DOLL DORANT DOSEME DROSTE DU DUNN EBELIN ENSTRO ESAKI FAN GAO GAO2 GARCIA GARDIN GARSHI GENG GER GILLIS GODLEY GOODMA GREGOR GSELL HAENSZ HANSEN HEGMAN HEIN HENNEK HINDS HIRAOK HIRAY2 HIRAYA HITOSU HOROWI HORWIT HU HU2 HUANG HUMBLE ISHIMA JAIN JARUP JARVHO JEDRYC JIANG JOLY JONES JUSSAW KAISE2 KAISER KANELL KATSOU KAUFMA KELLER KHUDER KIHARA KO KOHLME KOO KOULUM KREYBE KUBIK LAMTH LAMWK LAMWK2 LANGE LAUSSM LEI LEMARC LETOUR LIAW LICKIN LIDDEL LIU LIU2 LIU3 LIU4 LIU5 LOMBA2 LUBIN LUO MACLEN MAGNUS MARTIS MASTRA MATSUD MCCONN MCDUFF MCLAUG MILLER MILLS MOLLO MRFIT MRFITR MURATA NAM NOTAN2 NOTANI NOU ODRISC ORMOS OSANN OSANN2 PARKIN PASTOR PAWLEG PERNU PERSH2 PERSHA PETO PEZZO2 PEZZOT PIKE PISANI POFFIJ POLEDN PRESCO QIAO QIAO2 RACHTA RADZIK RANDIG REN RESTRE RIMING RONCO ROOTS ROTHSC SAARIK SANKAR SCHWA2 SCHWAR SEGI SEGI2 SEOW SHAW SHIMIZ SIEMIA SIMARA SIMONA SITAS SOBUE SOBUE2 SPEIZE SPITZ STAYNE STOCKS STUCKE SUN SUZUK2 SUZUKI SVENSS TANG TANG2 TAO TENKAN TIZZAN TOKARS TSUGAN TVERDA ULMER VANDER VEIERO VUTUC WAKAI WANG WANG2 WANG3 WANG4 WARSIN WATSON WICKLU WILKIN WU WU2 WUNSCH WUWILL WYNDE4 WYNDE5 WYNDE8 WYNDER XIANGZ XU XU2 XU3 XU4 YAMAGU YONG YUAN ZHANG ZHENG ZHOU
3	ABELIN ALDERS ARMADA BECHER BENHAM BEST BOFFET BOUCOT BRESLO COMSTO CPSI DAMBER DEAN DEAN2 DORGAN GOLLED HAMMO2 HAMMON JAHN KJUUS LEVIN LOMBAR MARSH MARSH2 SADOWS STASZE STOCKW TOUSEY WYNDE2 WYNDE3 WYNDE6
7	BENSHL BROSS CHOW DARBY DEKLER DOLL2 DORN ENGELA GRAHAM HOLE KINLEN KNEKT KREUZE LUBIN2 MATOS MIGRAN TULINI WALD WIGLE

Table 3E2 - 8
Potentially overlapping studies

REF	REFGP	PRINC	.	OVERLAP/LINK
WYNDE7	WYNDE6	2		WYNDE5/6/7/8

Table 3E3 -

IESLC - Meta-analysis of Ever Smoking (or Current if Ever not available), Pipe and/or Cigars (not cigs)
Adenocarcinoma

This analysis is restricted to results for:
1) Non-dose-response data
2) Smokers of pipe and/or cigars (but not cigarettes)
3) Results complete enough for use in metaanalysis

Within each study, results are then selected (in the following order of preference, within each sex) for:
4) SMKSTA: ever smokers, current smokers
5) DENOM: never smoked anything, (never +1 = +long term ex)
6) Followup period (prospective studies): whole study (coded as 0) or longest available
7) LCtype: all or nearest available, at least Squamous and Adeno. (q = squamous, s = small,
l = large, a = adeno, mix = mixed, alv = alveolar)
8) Race: all or nearest available, otherwise by race (wh or w = white, bl or b = black, hi = hispanic
ch = chinese, jap = japanese, haw = hawaiian, w+o = white + oriental, sca = scandinavian, as = asian)
9) For overlapping studies: principal rather than subsidiary studies
Finally by Age: whole study (coded as 0) if available, otherwise by widest available age group
and then for single sex results (m, f) in preference to combined sex results (c).

Results adjusted (AD) for the most potential confounders are then chosen in Sections -1 to -3
and results adjusted for the least confounders in Sections -4 to -6. (Those least adjusted results which
actually differ from the most adjusted as marked 'x' in column X in Section -4)
(Results adjusted for an unknown number of confounder(s) are coded as 20.)

Section -7 shows excluded studies, together with the stage (as above) at which no qualifying
results were found.

Section -8 lists the potentially overlapping studies which have been included (1=principal, 2=subsidiary).

Section -9 lists any results which would have been included in preference except that they had data not complete
enough for use in meta-analysis, with their significance (yes/no), if known, and any further comment as entered
on the database.

In addition to those mentioned above, the following fields, levels and abbreviations are used:
* or nk = not known, n = no, y = yes, ot = other
ev = ever, cu = current, nev = never
REF: 6-character study reference
NRR: number of the RR on the database within the study
ST : study type (CC = case control, pr or prosp = prospective)
NLC: number of lung cancer cases in whole study
R : risky occupational population (n = no, m = mining, o = other risky)
VB : national cigarette type (V = at least 75% Virginia, bl = at least 75% blended, ot = other)
P : any proxy use
H : full histological confirmation
De : derivation of RR/CI (or = original, st = standard method, ot = other method of estimation)

Table 3E3 - 1

IESLC - Meta-analysis of Ever Smoking (or Current if Ever not available), Pipe and/or Cigars (not cigs)
Adenocarcinoma
Most adjusted

REF	NRR	SEX	AGEL	AGEH	RACE	YF	LC TYPE	LOC	START	ST	NLC	R	VB	P	H	AD	SM	DENOM	De
ALDERS	40	m	0	0	all	-	not q+s	Eu:UK	1977	CC	1448	n	V	n	n	1	ev	nev any	ot
BRESLO	31	c	0	0	all	-	a	NAmer	1949	CC	518	n	bl	n	y	0	ev	nev+1	st
HAMMON	93	m	0	0	wh	0	a	NAmer	1952	pr	448	n	bl	n	n	0	ev	nev any	st
JAHN	18	m	0	0	all	-	a	Eu:Ger	1988	CC	1004	n	bl	n	n	0	ev	nev any	st
WYNDE2	8	m	0	0	all	-	KII	NAmer	1962	CC	404	n	bl	n	y	0	ev	nev any	st
WYNDE3	151	m	0	0	all	-	KII	NAmer	1966	CC	350	n	bl	n	y	0	ev	nev any	st
WYNDE6	60	m	0	0	all	-	KII	NAmer	1969	CC	4423	n	bl	n	y	0	ev	nev any	st

Table 3E3 - 2

IESLC - Meta-analysis of Ever Smoking (or Current if Ever not available), Pipe and/or Cigars (not cigs)
Adenocarcinoma
Most adjusted

REF	NRR	SEX	AD	Number Exposed Case	Number Exposed Cont	Non-exposed Case	Non-exposed Cont	RR	95.00%CI
ALDERS	40	m	1	-	-	-	-	0.56	(0.11- 2.90)
BRESLO	31	c	0	2	68	4	56	0.41	(0.07- 2.33)
*HAMMON	93	m	0	3	127770	2	115884	1.36	(0.23- 8.14)
JAHN	18	m	0	2	30	8	138	1.15	(0.23- 5.69)
WYNDE2	8	m	0	3	104	5	105	0.61	(0.14- 2.60)
WYNDE3	151	m	0	4	68	6	88	0.86	(0.23- 3.18)
WYNDE6	60	m	0	20	199	58	617	1.07	(0.63- 1.82)
Partial Totals				34	128239	83	116888		

*prospective study

REF	NRR	SEX	AD	Ys	Ws	Qs	Ps
ALDERS	40	m	1	-0.58	1.44	0.37	0.4873
BRESLO	31	c	0	-0.89	1.28	0.84	0.3158
*HAMMON	93	m	0	0.31	1.20	0.18	0.7360
JAHN	18	m	0	0.14	1.50	0.07	0.8640
WYNDE2	8	m	0	-0.50	1.81	0.33	0.5001
WYNDE3	151	m	0	-0.15	2.26	0.01	0.8244
WYNDE6	60	m	0	0.07	13.53	0.27	0.8057

	N	7
	NS	7
	Wt	23.02
Het	Chi	2.07
Het	df	6
Het	P	N.S.
Fixed	RR	0.93
	RRl	0.62
	RRu	1.40
	P	N.S.
Random	RR	0.93
	RRl	0.62
	RRu	1.40
	P	N.S.
Asymm	P	N.S.

Table 3E3 - 3

IESLC - Meta-analysis of Ever Smoking (or Current if Ever not available), Pipe and/or Cigars (not cigs)
Adenocarcinoma
Most adjusted

		Sex		
	combined	male	female	Total
N	1	6		7
NS	1	6		7
Wt	1.28	21.74		23.02
Het Chi	0.00	1.17		2.07
Het df	0	5		6
Het P	N.S.	N.S.		N.S.
Fixed RR	0.41	0.97		0.93
RRl	0.07	0.64		0.62
RRu	2.33	1.48		1.40
P	N.S.	N.S.		N.S.
Random RR	0.41	0.97		0.93
RRl	0.07	0.64		0.62
RRu	2.33	1.48		1.40
P	N.S.	N.S.		N.S.
Between Chi				0.89
Between df				1
Between P				N.S.
Btwn(F) P				N.S.
Btwn(R) P				N.S.

Too few RRs for analysis by factor

Table 3E3 - 4

IESLC - Meta-analysis of Ever Smoking (or Current if Ever not available), Pipe and/or Cigars (not cigs)
Adenocarcinoma
Least adjusted

REF	NRR	X	SEX	AGEL	AGEH	RACE	YF	LC TYPE	LOC	START	ST	NLC	R	VB	P	H	AD	SM	DENOM	De
ALDERS	99	x	m	0	0	all	-	not q+s	Eu:UK	1977	CC	1448	n	V	n	n	0	ev	nev any	st
BRESLO	31		c	0	0	all	-	a	NAmer	1949	CC	518	n	bl	n	y	0	ev	nev+1	st
HAMMON	93		m	0	0	wh	0	a	NAmer	1952	pr	448	n	bl	n	n	0	ev	nev any	st
JAHN	18		m	0	0	all	-	a	Eu:Ger	1988	CC	1004	n	bl	n	n	0	ev	nev any	st
WYNDE2	8		m	0	0	all	-	KII	NAmer	1962	CC	404	n	bl	n	y	0	ev	nev any	st
WYNDE3	151		m	0	0	all	-	KII	NAmer	1966	CC	350	n	bl	n	y	0	ev	nev any	st
WYNDE6	60		m	0	0	all	-	KII	NAmer	1969	CC	4423	n	bl	n	y	0	ev	nev any	st

Table 3E3 - 5

IESLC - Meta-analysis of Ever Smoking (or Current if Ever not available), Pipe and/or Cigars (not cigs)
Adenocarcinoma
Least adjusted

REF	NRR	SEX	AD	Number Exposed Case	Cont	Non-exposed Case	Cont	RR	95.00%CI
ALDERS	99	m	0	2	35	6	133	1.27	(0.24- 6.55)
BRESLO	31	c	0	2	68	4	56	0.41	(0.07- 2.33)
*HAMMON	93	m	0	3	127770	2	115884	1.36	(0.23- 8.14)
JAHN	18	m	0	2	30	8	138	1.15	(0.23- 5.69)
WYNDE2	8	m	0	3	104	5	105	0.61	(0.14- 2.60)
WYNDE3	151	m	0	4	68	6	88	0.86	(0.23- 3.18)
WYNDE6	60	m	0	20	199	58	617	1.07	(0.63- 1.82)
Totals				36	128274	89	117021		

*prospective study

REF	NRR	SEX	AD	Ys	Ws	Qs	Ps
ALDERS	99	m	0	0.24	1.42	0.10	0.7780
BRESLO	31	c	0	-0.89	1.28	0.95	0.3158
*HAMMON	93	m	0	0.31	1.20	0.13	0.7360
JAHN	18	m	0	0.14	1.50	0.04	0.8640
WYNDE2	8	m	0	-0.50	1.81	0.41	0.5001
WYNDE3	151	m	0	-0.15	2.26	0.03	0.8244
WYNDE6	60	m	0	0.07	13.53	0.11	0.8057

	N	7
	NS	7
	Wt	23.01
Het	Chi	1.78
Het	df	6
Het	P	N.S.
Fixed	RR	0.98
	RRl	0.65
	RRu	1.47
	P	N.S.
Random	RR	0.98
	RRl	0.65
	RRu	1.47
	P	N.S.
Asymm	P	N.S.

Table 3E3 - 6

IESLC - Meta-analysis of Ever Smoking (or Current if Ever not available), Pipe and/or Cigars (not cigs)
Adenocarcinoma
Least adjusted

		combined	Sex: male	female	Total
	N	1	6		7
	NS	1	6		7
	Wt	1.28	21.73		23.01
Het	Chi	0.00	0.77		1.78
Het	df	0	5		6
Het	P	N.S.	N.S.		N.S.
Fixed	RR	0.41	1.03		0.98
	RRl	0.07	0.67		0.65
	RRu	2.33	1.56		1.47
	P	N.S.	N.S.		N.S.
Random	RR	0.41	1.03		0.98
	RRl	0.07	0.67		0.65
	RRu	2.33	1.56		1.47
	P	N.S.	N.S.		N.S.
Between	Chi				1.01
Between	df				1
Between	P				N.S.
Btwn(F)	P				(*)
Btwn(R)	P				N.S.

Table 3E3 - 7

IESLC - Meta-analysis of Ever Smoking (or Current if Ever not available), Pipe and/or Cigars (not cigs)
Adenocarcinoma
Excluded studies (and stage at which they were excluded)

1	BOUCHA BUELL LAURIL MZILEN
2	ABRAHA AGUDO AKIBA AMANDU AMES ANDERS ARCHER AUSTIN AUVINE AXELSO AXELSS BAND BARBON BERRIN BLOHMK BLOT1 BLOT2 BLOT3 BLOT4 BRETT BROCKM BROWN1 BROWN2 BUFFLE BYERS1 BYERS2 CARPEN CASCO2 CASCOR CEDERL CHAN CHANG CHATZI CHEN CHEN2 CHEN3 CHIAZZ CHOI CHYOU COOKSO CORREA CPSII DAVEYS DEAN3 DESTE2 DESTEF DOCKER DOLL DORANT DOSEME DROSTE DU DUNN EBELIN ENSTRO ESAKI FAN GAO GAO2 GARCIA GARDIN GARSHI GENG GER GILLIS GODLEY GOODMA GREGOR GSELL HAENSZ HANSEN HEGMAN HEIN HENNEK HINDS HIRAOK HIRAY2 HIRAYA HITOSU HOROWI HORWIT HU HU2 HUANG HUMBLE ISHIMA JAIN JARUP JARVHO JEDRYC JIANG JOLY JONES JUSSAW KAISE2 KAISER KANELL KATSOU KAUFMA KELLER KHUDER KIHARA KO KOHLME KOO KOULUM KREYBE KUBIK LAMTH LAMWK LAMWK2 LANGE LAUSSM LEI LEMARC LETOUR LIAW LICKIN LIDDEL LIU LIU2 LIU3 LIU4 LIU5 LOMBA2 LUBIN LUO MACLEN MAGNUS MARTIS MASTRA MATSUD MCCONN MCDUFF MCLAUG MILLER MILLS MOLLO MRFIT MRFITR MURATA NAM NOTAN2 NOTANI NOU ODRISC ORMOS OSANN OSANN2 PARKIN PASTOR PAWLEG PERNU PERSH2 PERSHA PETO PEZZO2 PEZZOT PIKE PISANI POFFIJ POLEDN PRESCO QIAO QIAO2 RACHTA RADZIK RANDIG REN RESTRE RIMING RONCO ROOTS ROTHSC SAARIK SANKAR SCHWA2 SCHWAR SEGI SEGI2 SEOW SHAW SHIMIZ SIEMIA SIMARA SIMONA SITAS SOBUE SOBUE2 SPEIZE SPITZ STAYNE STOCKS STUCKE SUN SUZUK2 SUZUKI SVENSS TANG TANG2 TAO TENKAN TIZZAN TOKARS TSUGAN TVERDA ULMER VANDER VEIERO VUTUC WAKAI WANG WANG2 WANG3 WANG4 WARSIN WATSON WICKLU WILKIN WU WU2 WUNSCH WUWILL WYNDE4 WYNDE5 WYNDE8 WYNDER XIANGZ XU XU2 XU3 XU4 YAMAGU YONG YUAN ZHANG ZHENG ZHOU
7	ABELIN ARMADA BECHER BENHAM BENSHL BEST BOFFET BOUCOT BROSS CHOW COMSTO CPSI DAMBER DARBY DEAN DEAN2 DEKLER DOLL2 DORGAN DORN ENGELA GOLLED GRAHAM HAMMO2 HOLE KINLEN KJUUS KNEKT KREUZE LEVIN LOMBAR LUBIN2 MARSH MARSH2 MATOS MIGRAN SADOWS STASZE STOCKW TOUSEY TULINI WALD WIGLE
9	WYNDE7

Table 3E3 - 8
Potentially overlapping studies

REF	REFGP	PRINC	OVERLAP/LINK
WYNDE6	WYNDE6	1	WYNDE5/6/7/8
JAHN	BOFFET	2	Subset of BOFFET

Table 3E4 -

IESLC - Meta-analysis of Current Smoking (or Ever if Current not available), Pipe and/or Cigars (not cigs)
Adenocarcinoma

This analysis is restricted to results for:
1) Non-dose-response data
2) Smokers of pipe and/or cigars (but not cigarettes)
3) Results complete enough for use in metaanalysis

Within each study, results are then selected (in the following order of preference, within each sex) for:
4) SMKSTA: current smokers, ever smokers
5) DENOM: never smoked anything, (never +1 = +long term ex)
6) Followup period (prospective studies): whole study (coded as 0) or longest available
7) LCtype: all or nearest available, at least Squamous and Adeno. (q = squamous, s = small,
l = large, a = adeno, mix = mixed, alv = alveolar)
8) Race: all or nearest available, otherwise by race (wh or w = white, bl or b = black, hi = hispanic
ch = chinese, jap = japanese, haw = hawaiian, w+o = white + oriental, sca = scandinavian, as = asian)
9) For overlapping studies: principal rather than subsidiary studies
Finally by Age: whole study (coded as 0) if available, otherwise by widest available age group
and then for single sex results (m, f) in preference to combined sex results (c).

Results adjusted (AD) for the most potential confounders are then chosen in Sections -1 to -3
(and those which actually differ from the adjusted results in Table 3E3 - 1 are marked 'x' in Section -1)
and results adjusted for the least confounders in Sections -4 to -6. (Those least adjusted results which
actually differ from the most adjusted as marked 'x' in column X in Section -4)
(Results adjusted for an unknown number of confounder(s) are coded as 20.)

Section -7 shows excluded studies, together with the stage (as above) at which no qualifying
results were found.

Section -8 lists the potentially overlapping studies which have been included (1=principal, 2=subsidiary).

Section -9 lists any results which would have been included in preference except that they had data not complete
enough for use in meta-analysis, with their significance (yes/no), if known, and any further comment as entered
on the database.

In addition to those mentioned above, the following fields, levels and abbreviations are used:
* or nk = not known, n = no, y = yes, ot = other
ev = ever, cu = current, nev = never
REF: 6-character study reference
NRR: number of the RR on the database within the study
ST : study type (CC = case control, pr or prosp = prospective)
NLC: number of lung cancer cases in whole study
R : risky occupational population (n = no, m = mining, o = other risky)
VB : national cigarette type (V = at least 75% Virginia, bl = at least 75% blended, ot = other)
P : any proxy use
H : full histological confirmation
De : derivation of RR/CI (or = original, st = standard method, ot = other method of estimation)

Table 3E4 - 1

IESLC - Meta-analysis of Current Smoking (or Ever if Current not available), Pipe and/or Cigars (not cigs)
Adenocarcinoma
Most adjusted

REF	NRR	3E3	SEX	AGEL	AGEH	RACE	YF	LC TYPE	LOC	START	ST	NLC	R	VB	P	H	AD	SM	DENOM	De
ALDERS	40		m	0	0	all	-	not q+s	Eu:UK	1977	CC	1448	n	V	n	n	1	ev	nev any	ot
BRESLO	31		c	0	0	all	-	a	NAmer	1949	CC	518	n	bl	n	y	0	ev	nev+1	st
HAMMON	93		m	0	0	wh	0	a	NAmer	1952	pr	448	n	bl	n	n	0	ev	nev any	st
JAHN	18		m	0	0	all	-	a	Eu:Ger	1988	CC	1004	n	bl	n	n	0	ev	nev any	st
WYNDE2	8		m	0	0	all	-	KII	NAmer	1962	CC	404	n	bl	n	y	0	ev	nev any	st
WYNDE3	151		m	0	0	all	-	KII	NAmer	1966	CC	350	n	bl	n	y	0	ev	nev any	st
WYNDE6	60		m	0	0	all	-	KII	NAmer	1969	CC	4423	n	bl	n	y	0	ev	nev any	st

Table 3E4 - 2

IESLC - Meta-analysis of Current Smoking (or Ever if Current not available), Pipe and/or Cigars (not cigs)
Adenocarcinoma
Most adjusted

REF	NRR	SEX	AD	Number Exposed Case	Number Exposed Cont	Non-exposed Case	Non-exposed Cont	RR	95.00%CI
ALDERS	40	m	1	-	-	-	-	0.56	(0.11- 2.90)
BRESLO	31	c	0	2	68	4	56	0.41	(0.07- 2.33)
*HAMMON	93	m	0	3	127770	2	115884	1.36	(0.23- 8.14)
JAHN	18	m	0	2	30	8	138	1.15	(0.23- 5.69)
WYNDE2	8	m	0	3	104	5	105	0.61	(0.14- 2.60)
WYNDE3	151	m	0	4	68	6	88	0.86	(0.23- 3.18)
WYNDE6	60	m	0	20	199	58	617	1.07	(0.63- 1.82)
Partial Totals				34	128239	83	116888		

*prospective study

REF	NRR	SEX	AD	Ys	Ws	Qs	Ps
ALDERS	40	m	1	-0.58	1.44	0.37	0.4873
BRESLO	31	c	0	-0.89	1.28	0.84	0.3158
*HAMMON	93	m	0	0.31	1.20	0.18	0.7360
JAHN	18	m	0	0.14	1.50	0.07	0.8640
WYNDE2	8	m	0	-0.50	1.81	0.33	0.5001
WYNDE3	151	m	0	-0.15	2.26	0.01	0.8244
WYNDE6	60	m	0	0.07	13.53	0.27	0.8057

	N	7
	NS	7
	Wt	23.02
Het	Chi	2.07
Het	df	6
Het	P	N.S.
Fixed	RR	0.93
	RRl	0.62
	RRu	1.40
	P	N.S.
Random	RR	0.93
	RRl	0.62
	RRu	1.40
	P	N.S.
Asymm	P	N.S.

Table 3E4 - 3

IESLC - Meta-analysis of Current Smoking (or Ever if Current not available), Pipe and/or Cigars (not cigs)
Adenocarcinoma
Most adjusted

	Sex: combined	male	female	Total
N	1	6		7
NS	1	6		7
Wt	1.28	21.74		23.02
Het Chi	0.00	1.17		2.07
Het df	0	5		6
Het P	N.S.	N.S.		N.S.
Fixed RR	0.41	0.97		0.93
RRl	0.07	0.64		0.62
RRu	2.33	1.48		1.40
P	N.S.	N.S.		N.S.
Random RR	0.41	0.97		0.93
RRl	0.07	0.64		0.62
RRu	2.33	1.48		1.40
P	N.S.	N.S.		N.S.
Between Chi				0.89
Between df				1
Between P				N.S.
Btwn(F) P				N.S.
Btwn(R) P				N.S.

Too few RRs for analysis by factor

Table 3E4 - 4

IESLC - Meta-analysis of Current Smoking (or Ever if Current not available), Pipe and/or Cigars (not cigs)
Adenocarcinoma
Least adjusted

REF	NRR	X	SEX	AGEL	AGEH	RACE	YF	LC TYPE	LOC	START	ST	NLC	R	VB	P	H	AD	SM	DENOM	De
ALDERS	99	x	m	0	0	all	-	not q+s	Eu:UK	1977	CC	1448	n	V	n	n	0	ev	nev any	st
BRESLO	31		c	0	0	all	-	a	NAmer	1949	CC	518	n	bl	n	y	0	ev	nev+1	st
HAMMON	93		m	0	0	wh	0	a	NAmer	1952	pr	448	n	bl	n	n	0	ev	nev any	st
JAHN	18		m	0	0	all	-	a	Eu:Ger	1988	CC	1004	n	bl	n	n	0	ev	nev any	st
WYNDE2	8		m	0	0	all	-	KII	NAmer	1962	CC	404	n	bl	n	y	0	ev	nev any	st
WYNDE3	151		m	0	0	all	-	KII	NAmer	1966	CC	350	n	bl	n	y	0	ev	nev any	st
WYNDE6	60		m	0	0	all	-	KII	NAmer	1969	CC	4423	n	bl	n	y	0	ev	nev any	st

Table 3E4 - 5

IESLC - Meta-analysis of Current Smoking (or Ever if Current not available), Pipe and/or Cigars (not cigs)
Adenocarcinoma
Least adjusted

REF	NRR	SEX	AD	Number Exposed Case	Cont	Non-exposed Case	Cont	RR	95.00%CI
ALDERS	99	m	0	2	35	6	133	1.27	(0.24- 6.55)
BRESLO	31	c	0	2	68	4	56	0.41	(0.07- 2.33)
*HAMMON	93	m	0	3	127770	2	115884	1.36	(0.23- 8.14)
JAHN	18	m	0	2	30	8	138	1.15	(0.23- 5.69)
WYNDE2	8	m	0	3	104	5	105	0.61	(0.14- 2.60)
WYNDE3	151	m	0	4	68	6	88	0.86	(0.23- 3.18)
WYNDE6	60	m	0	20	199	58	617	1.07	(0.63- 1.82)
Totals				36	128274	89	117021		

*prospective study

REF	NRR	SEX	AD	Ys	Ws	Qs	Ps
ALDERS	99	m	0	0.24	1.42	0.10	0.7780
BRESLO	31	c	0	-0.89	1.28	0.95	0.3158
*HAMMON	93	m	0	0.31	1.20	0.13	0.7360
JAHN	18	m	0	0.14	1.50	0.04	0.8640
WYNDE2	8	m	0	-0.50	1.81	0.41	0.5001
WYNDE3	151	m	0	-0.15	2.26	0.03	0.8244
WYNDE6	60	m	0	0.07	13.53	0.11	0.8057

	N	7
	NS	7
	Wt	23.01
Het	Chi	1.78
Het	df	6
Het	P	N.S.
Fixed	RR	0.98
	RRl	0.65
	RRu	1.47
	P	N.S.
Random	RR	0.98
	RRl	0.65
	RRu	1.47
	P	N.S.
Asymm	P	N.S.

Table 3E4 - 6

IESLC - Meta-analysis of Current Smoking (or Ever if Current not available), Pipe and/or Cigars (not cigs)
Adenocarcinoma
Least adjusted

	combined	Sex: male	female	Total
N	1	6		7
NS	1	6		7
Wt	1.28	21.73		23.01
Het Chi	0.00	0.77		1.78
Het df	0	5		6
Het P	N.S.	N.S.		N.S.
Fixed RR	0.41	1.03		0.98
RRl	0.07	0.67		0.65
RRu	2.33	1.56		1.47
P	N.S.	N.S.		N.S.
Random RR	0.41	1.03		0.98
RRl	0.07	0.67		0.65
RRu	2.33	1.56		1.47
P	N.S.	N.S.		N.S.
Between Chi				1.01
Between df				1
Between P				N.S.
Btwn(F) P				(*)
Btwn(R) P				N.S.

Table 3E4 - 7

IESLC - Meta-analysis of Current Smoking (or Ever if Current not available), Pipe and/or Cigars (not cigs)
Adenocarcinoma
Excluded studies (and stage at which they were excluded)

1 BOUCHA BUELL LAURIL MZILEN

2 ABRAHA AGUDO AKIBA AMANDU AMES ANDERS ARCHER AUSTIN AUVINE AXELSO AXELSS BAND BARBON BERRIN BLOHMK BLOT1
BLOT2 BLOT3 BLOT4 BRETT BROCKM BROWN1 BROWN2 BUFFLE BYERS1 BYERS2 CARPEN CASCO2 CASCOR CEDERL CHAN CHANG
CHATZI CHEN CHEN2 CHEN3 CHIAZZ CHOI CHYOU COOKSO CORREA CPSII DAVEYS DEAN3 DESTE2 DESTEF DOCKER DOLL
DORANT DOSEME DROSTE DU DUNN EBELIN ENSTRO ESAKI FAN GAO GAO2 GARCIA GARDIN GARSHI GENG GER
GILLIS GODLEY GOODMA GREGOR GSELL HAENSZ HANSEN HEGMAN HEIN HENNEK HINDS HIRAOK HIRAY2 HIRAYA HITOSU HOROWI
HORWIT HU HU2 HUANG HUMBLE ISHIMA JAIN JARUP JARVHO JEDRYC JIANG JOLY JONES JUSSAW KAISE2 KAISER
KANELL KATSOU KAUFMA KELLER KHUDER KIHARA KO KOHLME KOO KOULUM KREYBE KUBIK LAMTH LAMWK LAMWK2 LANGE
LAUSSM LEI LEMARC LETOUR LIAW LICKIN LIDDEL LIU LIU2 LIU3 LIU4 LIU5 LOMBA2 LUBIN LUO MACLEN
MAGNUS MARTIS MASTRA MATSUD MCCONN MCDUFF MCLAUG MILLER MILLS MOLLO MRFIT MRFITR MURATA NAM NOTAN2 NOTANI
NOU ODRISC ORMOS OSANN OSANN2 PARKIN PASTOR PAWLEG PERNU PERSH2 PERSHA PETO PEZZO2 PEZZOT PIKE PISANI
POFFIJ POLEDN PRESCO QIAO QIAO2 RACHTA RADZIK RANDIG REN RESTRE RIMING RONCO ROOTS ROTHSC SAARIK SANKAR
SCHWA2 SCHWAR SEGI SEGI2 SEOW SHAW SHIMIZ SIEMIA SIMARA SIMONA SITAS SOBUE SOBUE2 SPEIZE SPITZ STAYNE
STOCKS STUCKE SUN SUZUK2 SUZUKI SVENSS TANG TANG2 TAO TENKAN TIZZAN TOKARS TSUGAN TVERDA ULMER VANDER
VEIERO VUTUC WAKAI WANG WANG2 WANG3 WANG4 WARSIN WATSON WICKLU WILKIN WU WU2 WUNSCH WUWILL WYNDE4
WYNDE5 WYNDE8 WYNDER XIANGZ XU XU2 XU3 XU4 YAMAGU YONG YUAN ZHANG ZHENG ZHOU

7 ABELIN ARMADA BECHER BENHAM BENSHL BEST BOFFET BOUCOT BROSS CHOW COMSTO CPSI DAMBER DARBY DEAN DEAN2
DEKLER DOLL2 DORGAN DORN ENGELA GOLLED GRAHAM HAMMO2 HOLE KINLEN KJUUS KNEKT KREUZE LEVIN LOMBAR LUBIN2
MARSH MARSH2 MATOS MIGRAN SADOWS STASZE STOCKW TOUSEY TULINI WALD WIGLE

9 WYNDE7

Table 3E4 - 8

Potentially overlapping studies

REF	REFGP	PRINC	.	OVERLAP/LINK
WYNDE6	WYNDE6	1		WYNDE5/6/7/8
JAHN	BOFFET	2		Subset of BOFFET

Table 3E5 -

IESLC - Meta-analysis of Ex Smoking, Pipe and/or Cigars (not cigs)
Adenocarcinoma

This analysis is restricted to results for:
1) Non-dose-response data
2) Smokers of pipe and/or cigars (but not cigarettes)
3) Ex smokers
4) Results complete enough for use in metaanalysis

Within each study, results are then selected (in the following order of preference, within each sex) for:
5) DENOM: never smoked anything, (never +1 = +long term ex)
6) Followup period (prospective studies): whole study (coded as 0) or longest available
7) LCtype: all or nearest available, at least Squamous and Adeno. (q = squamous, s = small,
l = large, a = adeno, mix = mixed, alv = alveolar)
8) Race: all or nearest available, otherwise by race (wh or w = white, bl or b = black, hi = hispanic
ch = chinese, jap = japanese, haw = hawaiian, w+o = white + oriental, sca = scandinavian, as = asian)
9) For overlapping studies: principal rather than subsidiary studies
Finally by Age: whole study (coded as 0) if available, otherwise by widest available age group
and then for single sex results (m, f) in preference to combined sex results (c).

Results adjusted (AD) for the most potential confounders are then chosen in Sections -1 to -3
and results adjusted for the least confounders in Sections -4 to -6. (Those least adjusted results which
actually differ from the most adjusted as marked 'x' in column X in Section -4)
(Results adjusted for an unknown number of confounder(s) are coded as 20.)

Section -7 shows excluded studies, together with the stage (as above) at which no qualifying
results were found.

Section -8 lists the potentially overlapping studies which have been included (1=principal, 2=subsidiary).

Section -9 lists any results which would have been included in preference except that they had data not complete
enough for use in meta-analysis, with their significance (yes/no), if known, and any further comment as entered
on the database.

In addition to those mentioned above, the following fields, levels and abbreviations are used:
* or nk = not known, n = no, y = yes, ot = other
nev = never
REF: 6-character study reference
NRR: number of the RR on the database within the study
ST : study type (CC = case control, pr or prosp = prospective)
NLC: number of lung cancer cases in whole study
R : risky occupational population (n = no, m = mining, o = other risky)
VB : national cigarette type (V = at least 75% Virginia, bl = at least 75% blended, ot = other)
P : any proxy use
H : full histological confirmation
De : derivation of RR/CI (or = original, st = standard method, ot = other method of estimation)

Table 3E5 - 1

IESLC - Meta-analysis of Ex Smoking, Pipe and/or Cigars (not cigs)
Adenocarcinoma
Most adjusted

REF	NRR	SEX	AGEL	AGEH	RACE	YF	LC TYPE	LOC	START	ST	NLC	R	VB	P	H	AD	DENOM	De
WYNDE7	83	m	0	0	all	-	KII	NAmer	1977	CC	2085	n	bl	n	y	0	nev any	st

Table 3E5 - 2

IESLC - Meta-analysis of Ex Smoking, Pipe and/or Cigars (not cigs)
Adenocarcinoma
Most adjusted

REF	NRR	SEX	AD	Number Exposed Case	Cont	Non-exposed Case	Cont	RR	95.00%CI
WYNDE7	83	m	0	9	137	42	918	1.44	(0.68- 3.02)
Totals				9	137	42	918		

*prospective study

REF	NRR	SEX	AD	Ys	Ws	Qs	Ps
WYNDE7	83	m	0	0.36	6.98	0.00	0.3393

	N	1
	NS	1
	Wt	6.98
Het	Chi	0.00
Het	df	0
Het	P	N.S.
Fixed	RR	1.44
	RRl	0.68
	RRu	3.02
	P	N.S.
Random	RR	1.44
	RRl	0.68
	RRu	3.02
	P	N.S.
Asymm	P	

Table 3E5 - 3

IESLC - Meta-analysis of Ex Smoking, Pipe and/or Cigars (not cigs)
Adenocarcinoma
Most adjusted

	Sex			
	combined	male	female	Total
N		1		1
NS		1		1
Wt		6.98		6.98
Het Chi		0.00		0.00
Het df		0		0
Het P		N.S.		N.S.
Fixed RR		1.44		1.44
RRl		0.68		0.68
RRu		3.02		3.02
P		N.S.		N.S.
Random RR		1.44		1.44
RRl		0.68		0.68
RRu		3.02		3.02
P		N.S.		N.S.
Between Chi				
Between df				
Between P				N.S.
Btwn(F) P				N.S.
Btwn(R) P				N.S.

Too few RRs for analysis by factor

Table 3E5 - 4

IESLC - Meta-analysis of Ex Smoking, Pipe and/or Cigars (not cigs)
Adenocarcinoma
Least adjusted

REF	NRR	X	SEX	AGEL	AGEH	RACE	YF	LC TYPE	LOC	START	ST	NLC	R	VB	P	H	AD	DENOM	De
WYNDE7	83		m	0	0	all	-	KII	NAmer	1977	CC	2085	n	bl	n	y	0	nev any	st

Table 3E5 - 5

IESLC - Meta-analysis of Ex Smoking, Pipe and/or Cigars (not cigs)
Adenocarcinoma
Least adjusted

REF	NRR	SEX	AD	Number Exposed Case	Cont	Non-exposed Case	Cont	RR	95.00%CI
WYNDE7	83	m	0	9	137	42	918	1.44	(0.68- 3.02)
Totals				9	137	42	918		

*prospective study

REF	NRR	SEX	AD	Ys	Ws	Qs	Ps
WYNDE7	83	m	0	0.36	6.98	0.00	0.3393

	N	1
	NS	1
	Wt	6.98
Het	Chi	0.00
Het	df	0
Het	P	N.S.
Fixed	RR	1.44
	RRl	0.68
	RRu	3.02
	P	N.S.
Random	RR	1.44
	RRl	0.68
	RRu	3.02
	P	N.S.
Asymm	P	

Table 3E5 - 6

IESLC - Meta-analysis of Ex Smoking, Pipe and/or Cigars (not cigs)
Adenocarcinoma
Least adjusted

	Sex combined	male	female	Total
N		1		1
NS		1		1
Wt		6.98		6.98
Het Chi		0.00		0.00
Het df		0		0
Het P		N.S.		N.S.
Fixed RR		1.44		1.44
RRl		0.68		0.68
RRu		3.02		3.02
P		N.S.		N.S.
Random RR		1.44		1.44
RRl		0.68		0.68
RRu		3.02		3.02
P		N.S.		N.S.
Between Chi				
Between df				
Between P				N.S.
Btwn(F) P				N.S.
Btwn(R) P				N.S.

Table 3E5 - 7

IESLC - Meta-analysis of Ex Smoking, Pipe and/or Cigars (not cigs)
Adenocarcinoma
Excluded studies (and stage at which they were excluded)

```
1      BOUCHA  BUELL LAURIL MZILEN

2      ABRAHA  AGUDO  AKIBA AMANDU   AMES ANDERS ARCHER AUSTIN AUVINE AXELSO AXELSS   BAND BARBON BERRIN BLOHMK  BLOT1
        BLOT2  BLOT3  BLOT4  BRETT BROCKM BROWN1 BROWN2 BUFFLE BYERS1 BYERS2 CARPEN CASCO2 CASCOR CEDERL   CHAN  CHANG
       CHATZI   CHEN  CHEN2  CHEN3 CHIAZZ   CHOI  CHYOU COOKSO CORREA  CPSII DAVEYS  DEAN3 DESTE2 DESTEF DOCKER   DOLL
       DORANT DOSEME DROSTE     DU   DUNN EBELIN ENSTRO  ESAKI    FAN    GAO   GAO2 GARCIA GARDIN GARSHI   GENG    GER
       GILLIS GODLEY GOODMA GREGOR  GSELL HAENSZ HANSEN HEGMAN   HEIN HENNEK  HINDS HIRAOK HIRAY2 HIRAYA HITOSU HOROWI
       HORWIT     HU    HU2  HUANG HUMBLE ISHIMA   JAIN  JARUP JARVHO JEDRYC  JIANG   JOLY  JONES JUSSAW KAISE2 KAISER
       KANELL KATSOU KAUFMA KELLER KHUDER KIHARA     KO KOHLME    KOO KOULUM KREYBE  KUBIK  LAMTH  LAMWK LAMWK2  LANGE
       LAUSSM    LEI LEMARC LETOUR   LIAW LICKIN LIDDEL    LIU   LIU2   LIU3   LIU4   LIU5 LOMBA2  LUBIN    LUO MACLEN
       MAGNUS MARTIS MASTRA MATSUD MCCONN MCDUFF MCLAUG MILLER  MILLS  MOLLO  MRFIT MRFITR MURATA    NAM NOTAN2 NOTANI
          NOU ODRISC  ORMOS  OSANN OSANN2 PARKIN PASTOR PAWLEG  PERNU PERSH2 PERSHA   PETO PEZZO2 PEZZOT   PIKE PISANI
       POFFIJ POLEDN PRESCO   QIAO  QIAO2 RACHTA RADZIK RANDIG    REN RESTRE RIMING  RONCO  ROOTS ROTHSC SAARIK SANKAR
       SCHWA2 SCHWAR   SEGI  SEGI2   SEOW   SHAW SHIMIZ SIEMIA SIMARA SIMONA  SITAS  SOBUE SOBUE2 SPEIZE  SPITZ STAYNE
       STOCKS STUCKE    SUN SUZUK2 SUZUKI SVENSS   TANG  TANG2    TAO TENKAN TIZZAN TOKARS TSUGAN TVERDA  ULMER VANDER
       VEIERO  VUTUC  WAKAI   WANG  WANG2  WANG3  WANG4 WARSIN WATSON WICKLU WILKIN     WU    WU2 WUNSCH WUWILL WYNDE4
       WYNDE5 WYNDE8 WYNDER XIANGZ     XU    XU2    XU3    XU4 YAMAGU   YONG   YUAN  ZHANG  ZHENG   ZHOU

3      ABELIN ALDERS ARMADA BECHER BENHAM BENSHL   BEST BOFFET BOUCOT BRESLO  BROSS COMSTO   CPSI DAMBER  DARBY   DEAN
        DEAN2 DEKLER DORGAN ENGELA GOLLED GRAHAM HAMMO2 HAMMON   HOLE   JAHN KINLEN  KJUUS  KNEKT KREUZE  LEVIN LOMBAR
        MARSH MARSH2  MATOS SADOWS STASZE STOCKW TOUSEY TULINI   WALD WYNDE2 WYNDE3 WYNDE6

7        CHOW  DOLL2   DORN LUBIN2 MIGRAN  WIGLE
```

Table 3E5 - 8
Potentially overlapping studies

REF	REFGP	PRINC	.	OVERLAP/LINK
WYNDE7	WYNDE6	2		WYNDE5/6/7/8

Table 3E6 -

IESLC - Meta-analysis of Ever Smoking, Pipe only
Adenocarcinoma

This analysis is restricted to results for:
1) Non-dose-response data
2) Smokers of pipe only
3) Ever smokers
4) Results complete enough for use in metaanalysis

Within each study, results are then selected (in the following order of preference, within each sex) for:
5) DENOM: never smoked anything, (never +1 = +long term ex)
6) Followup period (prospective studies): whole study (coded as 0) or longest available
7) LCtype: all or nearest available, at least Squamous and Adeno. (q = squamous, s = small,
 l = large, a = adeno, mix = mixed, alv = alveolar)
8) Race: all or nearest available, otherwise by race (wh or w = white, bl or b = black, hi = hispanic
 ch = chinese, jap = japanese, haw = hawaiian, w+o = white + oriental, sca = scandinavian, as = asian)
9) For overlapping studies: principal rather than subsidiary studies
Finally by Age: whole study (coded as 0) if available, otherwise by widest available age group
and then for single sex results (m, f) in preference to combined sex results (c).

Results adjusted (AD) for the most potential confounders are then chosen in Sections -1 to -3
and results adjusted for the least confounders in Sections -4 to -6. (Those least adjusted results which
actually differ from the most adjusted as marked 'x' in column X in Section -4)
(Results adjusted for an unknown number of confounder(s) are coded as 20.)

Section -7 shows excluded studies, together with the stage (as above) at which no qualifying
results were found.

Section -8 lists the potentially overlapping studies which have been included (1=principal, 2=subsidiary).

Section -9 lists any results which would have been included in preference except that they had data not complete
enough for use in meta-analysis, with their significance (yes/no), if known, and any further comment as entered
on the database.

In addition to those mentioned above, the following fields, levels and abbreviations are used:
* or nk = not known, n = no, y = yes, ot = other
nev = never
REF: 6-character study reference
NRR: number of the RR on the database within the study
ST : study type (CC = case control, pr or prosp = prospective)
NLC: number of lung cancer cases in whole study
R : risky occupational population (n = no, m = mining, o = other risky)
VB : national cigarette type (V = at least 75% Virginia, bl = at least 75% blended, ot = other)
P : any proxy use
H : full histological confirmation
De : derivation of RR/CI (or = original, st = standard method, ot = other method of estimation)

Table 3E6 - 1

IESLC - Meta-analysis of Ever Smoking, Pipe only
Adenocarcinoma
Most adjusted

REF	NRR	SEX	AGEL	AGEH	RACE	YF	LC TYPE	LOC	START	ST	NLC	R	VB	P	H	AD	DENOM	De
HAMMON	81	m	0	0	wh	0	a	NAmer	1952	pr	448	n	bl	n	n	1	nev any	ot
LUBIN	19	m	0	0	all	-	KII	As:Chi	1984	CC	427	m	ot	y	n	0	nev any	st
LUBIN2	140	m	0	0	all	-	a	Eu:mul	1976	CC	7804	n	bl	n	y	0	nev any	st
WYNDE7	62	m	0	0	all	-	KII	NAmer	1977	CC	2085	n	bl	n	y	0	nev any	st

Table 3E6 - 2

IESLC - Meta-analysis of Ever Smoking, Pipe only
Adenocarcinoma
Most adjusted

REF	NRR	SEX	AD	Number Exposed Case	Number Exposed Cont	Non-exposed Case	Non-exposed Cont	RR	95.00%CI
*HAMMON	81	m	1	-	-	-	-	2.44	(0.34- 17.35)
LUBIN	19	m	0	5	151	4	72	0.60	(0.16- 2.29)
LUBIN2	140	m	0	4	197	195	2616	0.27	(0.10- 0.74)
WYNDE7	62	m	0	2	97	42	918	0.45	(0.11- 1.89)
Partial Totals				11	445	241	3606		

*prospective study

REF	NRR	SEX	AD	Ys	Ws	Qs	Ps
*HAMMON	81	m	1	0.89	0.99	2.71	0.3739
LUBIN	19	m	0	-0.52	2.13	0.12	0.4506
LUBIN2	140	m	0	-1.30	3.84	1.13	0.0108
WYNDE7	62	m	0	-0.80	1.87	0.00	0.2759

N	4
NS	4
Wt	8.82
Het Chi	3.96
Het df	3
Het P	N.S.
Fixed RR	0.47
RRl	0.24
RRu	0.91
P	-
Random RR	0.50
RRl	0.23
RRu	1.10
P	(-)
Asymm P	*

Table 3E6 - 3

IESLC - Meta-analysis of Ever Smoking, Pipe only
Adenocarcinoma
Most adjusted

	Sex combined	male	female	Total
N		4		4
NS		4		4
Wt		8.82		8.82
Het Chi		3.96		3.96
Het df		3		3
Het P		N.S.		N.S.
Fixed RR		0.47		0.47
RRl		0.24		0.24
RRu		0.91		0.91
P		-		-
Random RR		0.50		0.50
RRl		0.23		0.23
RRu		1.10		1.10
P		(-)		(-)
Between Chi				
Between df				
Between P				N.S.
Btwn(F) P				N.S.
Btwn(R) P				N.S.

Too few RRs for analysis by factor

Table 3E6 - 4

IESLC - Meta-analysis of Ever Smoking, Pipe only
Adenocarcinoma
Least adjusted

REF	NRR	X	SEX	AGEL	AGEH	RACE	YF	LC TYPE	LOC	START	ST	NLC	R	VB	P	H	AD	DENOM	De
HAMMON	87	x	m	0	0	wh	0	a	NAmer	1952	pr	448	n	bl	n	n	0	nev any	st
LUBIN	19		m	0	0	all	-	KII	As:Chi	1984	CC	427	m	ot	y	n	0	nev any	st
LUBIN2	140		m	0	0	all	-	a	Eu:mul	1976	CC	7804	n	bl	n	y	0	nev any	st
WYNDE7	62		m	0	0	all	-	KII	NAmer	1977	CC	2085	n	bl	n	y	0	nev any	st

Table 3E6 - 5

IESLC - Meta-analysis of Ever Smoking, Pipe only
Adenocarcinoma
Least adjusted

REF	NRR	SEX	AD	Number Exposed Case	Cont	Non-exposed Case	Cont	RR	95.00%CI
*HAMMON	87	m	0	2	43041	2	115884	2.69	(0.38- 19.11)
LUBIN	19	m	0	5	151	4	72	0.60	(0.16- 2.29)
LUBIN2	140	m	0	4	197	195	2616	0.27	(0.10- 0.74)
WYNDE7	62	m	0	2	97	42	918	0.45	(0.11- 1.89)
Totals				13	43486	243	119490		

*prospective study

REF	NRR	SEX	AD	Ys	Ws	Qs	Ps
*HAMMON	87	m	0	0.99	1.00	3.02	0.3220
LUBIN	19	m	0	-0.52	2.13	0.11	0.4506
LUBIN2	140	m	0	-1.30	3.84	1.18	0.0108
WYNDE7	62	m	0	-0.80	1.87	0.00	0.2759

	N	4
	NS	4
	Wt	8.83
Het	Chi	4.31
Het	df	3
Het	P	N.S.
Fixed	RR	0.47
	RRl	0.25
	RRu	0.92
	P	-
Random	RR	0.52
	RRl	0.23
	RRu	1.18
	P	N.S.
Asymm	P	*

Table 3E6 - 6

IESLC - Meta-analysis of Ever Smoking, Pipe only
Adenocarcinoma
Least adjusted

	Sex combined	male	female	Total
N		4		4
NS		4		4
Wt		8.83		8.83
Het Chi		4.31		4.31
Het df		3		3
Het P		N.S.		N.S.
Fixed RR		0.47		0.47
RRl		0.25		0.25
RRu		0.92		0.92
P		-		-
Random RR		0.52		0.52
RRl		0.23		0.23
RRu		1.18		1.18
P		N.S.		N.S.
Between Chi				
Between df				
Between P				N.S.
Btwn(F) P				N.S.
Btwn(R) P				N.S.

Table 3E6 - 7

IESLC - Meta-analysis of Ever Smoking, Pipe only
Adenocarcinoma
Excluded studies (and stage at which they were excluded)

1	BOUCHA BUELL LAURIL MZILEN
2	ABRAHA AGUDO AKIBA ALDERS AMANDU AMES ANDERS ARCHER AUSTIN AUVINE AXELSO AXELSS BAND BARBON BECHER BERRIN BLOHMK BLOT1 BLOT2 BLOT3 BLOT4 BRESLO BRETT BROCKM BROSS BROWN1 BROWN2 BUFFLE BYERS1 BYERS2 CARPEN CASCO2 CASCOR CHAN CHANG CHATZI CHEN CHEN2 CHEN3 CHIAZZ CHOI CHOW CHYOU COMSTO CORREA CPSII DARBY DAVEYS DEAN2 DEKLER DESTE2 DESTEF DOCKER DORANT DORGAN DOSEME DROSTE DU DUNN EBELIN ENGELA ENSTRO ESAKI FAN GAO GAO2 GARCIA GARSHI GENG GER GILLIS GODLEY GOLLED GOODMA GRAHAM GREGOR GSELL HAENSZ HAMMO2 HANSEN HEGMAN HENNEK HINDS HIRAOK HIRAYA HITOSU HOLE HOROWI HORWIT HU HU2 HUANG HUMBLE ISHIMA JAHN JAIN JARUP JARVHO JEDRYC JIANG JOLY JONES JUSSAW KAISE2 KAISER KANELL KATSOU KAUFMA KELLER KHUDER KIHARA KINLEN KJUUS KNEKT KO KOHLME KOO KREUZE KREYBE KUBIK LAMTH LAMWK LAMWK2 LAUSSM LEI LEMARC LETOUR LIAW LICKIN LIDDEL LIU LIU2 LIU3 LIU4 LIU5 LOMBA2 LOMBAR LUO MACLEN MAGNUS MARSH MARSH2 MARTIS MASTRA MATOS MATSUD MCDUFF MCLAUG MIGRAN MILLER MILLS MOLLO MRFIT MRFITR MURATA NAM NOTAN2 NOTANI NOU ODRISC ORMOS OSANN OSANN2 PARKIN PASTOR PAWLEG PERSH2 PERSHA PETO PEZZO2 PEZZOT PIKE PISANI POFFIJ POLEDN PRESCO QIAO2 RACHTA RADZIK RANDIG REN RESTRE RIMING RONCO ROOTS ROTHSC SAARIK SANKAR SCHWAR SEGI SEGI2 SEOW SHAW SHIMIZ SIEMIA SIMARA SIMONA SITAS SOBUE SOBUE2 SPEIZE SPITZ STASZE STAYNE STOCKW STUCKE SUN SUZUK2 SUZUKI SVENSS TANG TANG2 TAO TENKAN TOKARS TOUSEY TSUGAN ULMER VANDER VEIERO VUTUC WAKAI WALD WANG WANG2 WANG3 WANG4 WARSIN WATSON WICKLU WIGLE WILKIN WU WU2 WUNSCH WUWILL WYNDE2 WYNDE3 WYNDE4 WYNDE5 WYNDE6 WYNDE8 WYNDER XU XU2 XU3 XU4 YAMAGU YONG YUAN ZHANG ZHENG ZHOU
3	BENSHL CEDERL DEAN3 GARDIN HEIN HIRAY2 LANGE TULINI TVERDA
4	DOLL2
5	SCHWA2
7	ABELIN ARMADA BENHAM BEST BOFFET BOUCOT COOKSO CPSI DAMBER DEAN DOLL DORN KOULUM LEVIN MCCONN PERNU QIAO SADOWS STOCKS TIZZAN XIANGZ

Table 3E6 - 8
Potentially overlapping studies

REF	REFGP	PRINC	. OVERLAP/LINK
LUBIN2	LUBIN2	1	Lubin-combined
WYNDE7	WYNDE6	2	WYNDE5/6/7/8
LUBIN	XIANGZ	2	LUBIN/XIANGZ/QIAO

Table 3E7 -

IESLC - Meta-analysis of Current Smoking, Pipe only
Adenocarcinoma

This analysis is restricted to results for:
1) Non-dose-response data
2) Smokers of pipe only
3) Current smokers
4) Results complete enough for use in metaanalysis

Within each study, results are then selected (in the following order of preference, within each sex) for:
5) DENOM: never smoked anything, (never +1 = +long term ex)
6) Followup period (prospective studies): whole study (coded as 0) or longest available
7) LCtype: all or nearest available, at least Squamous and Adeno. (q = squamous, s = small,
l = large, a = adeno, mix = mixed, alv = alveolar)
8) Race: all or nearest available, otherwise by race (wh or w = white, bl or b = black, hi = hispanic
ch = chinese, jap = japanese, haw = hawaiian, w+o = white + oriental, sca = scandinavian, as = asian)
9) For overlapping studies: principal rather than subsidiary studies
Finally by Age: whole study (coded as 0) if available, otherwise by widest available age group
and then for single sex results (m, f) in preference to combined sex results (c).

Results adjusted (AD) for the most potential confounders are then chosen in Sections -1 to -3
and results adjusted for the least confounders in Sections -4 to -6. (Those least adjusted results which
actually differ from the most adjusted as marked 'x' in column X in Section -4)
(Results adjusted for an unknown number of confounder(s) are coded as 20.)

Section -7 shows excluded studies, together with the stage (as above) at which no qualifying
results were found.

Section -8 lists the potentially overlapping studies which have been included (1=principal, 2=subsidiary).

Section -9 lists any results which would have been included in preference except that they had data not complete
enough for use in meta-analysis, with their significance (yes/no), if known, and any further comment as entered
on the database.

In addition to those mentioned above, the following fields, levels and abbreviations are used:
* or nk = not known, n = no, y = yes, ot = other
nev = never
REF: 6-character study reference
NRR: number of the RR on the database within the study
ST : study type (CC = case control, pr or prosp = prospective)
NLC: number of lung cancer cases in whole study
R : risky occupational population (n = no, m = mining, o = other risky)
VB : national cigarette type (V = at least 75% Virginia, bl = at least 75% blended, ot = other)
P : any proxy use
H : full histological confirmation
De : derivation of RR/CI (or = original, st = standard method, ot = other method of estimation)

Table 3E7 - 1

IESLC - Meta-analysis of Current Smoking, Pipe only
Adenocarcinoma
Most adjusted

REF	NRR	SEX	AGEL	AGEH	RACE	YF	LC TYPE	LOC	START	ST	NLC	R	VB	P	H	AD	DENOM	De
WYNDE7	30	m	0	0	all	-	KII	NAmer	1977	CC	2085	n	bl	n	y	0	nev any	st

Table 3E7 - 2

IESLC - Meta-analysis of Current Smoking, Pipe only
Adenocarcinoma
Most adjusted

REF	NRR	SEX	AD	Number Exposed Case	Cont	Non-exposed Case	Cont	RR	95.00%CI
WYNDE7	30	m	0	1	54	42	918	0.40	(0.05- 3.00)
Totals				1	54	42	918		

*prospective study

REF	NRR	SEX	AD	Ys	Ws	Qs	Ps
WYNDE7	30	m	0	-0.90	0.96	0.00	0.3759

	N	1
	NS	1
	Wt	0.96
Het	Chi	0.00
Het	df	0
Het	P	N.S.
Fixed	RR	0.40
	RRl	0.05
	RRu	3.00
	P	N.S.
Random	RR	0.40
	RRl	0.05
	RRu	3.00
	P	N.S.
Asymm	P	

Table 3E7 - 3

IESLC - Meta-analysis of Current Smoking, Pipe only
Adenocarcinoma
Most adjusted

	Sex: combined	male	female	Total
N		1		1
NS		1		1
Wt		0.96		0.96
Het Chi		0.00		0.00
Het df		0		0
Het P		N.S.		N.S.
Fixed RR		0.40		0.40
RRl		0.05		0.05
RRu		3.00		3.00
P		N.S.		N.S.
Random RR		0.40		0.40
RRl		0.05		0.05
RRu		3.00		3.00
P		N.S.		N.S.
Between Chi				
Between df				
Between P				N.S.
Btwn(F) P				N.S.
Btwn(R) P				N.S.

Too few RRs for analysis by factor

Table 3E7 - 4

IESLC - Meta-analysis of Current Smoking, Pipe only
Adenocarcinoma
Least adjusted

REF	NRR	X	SEX	AGEL	AGEH	RACE	YF	LC TYPE	LOC	START	ST	NLC	R	VB	P	H	AD	DENOM	De
WYNDE7	30		m	0	0	all	-	KII	NAmer	1977	CC	2085	n	bl	n	y	0	nev any	st

Table 3E7 - 5

IESLC - Meta-analysis of Current Smoking, Pipe only
Adenocarcinoma
Least adjusted

REF	NRR	SEX	AD	Number Exposed Case	Cont	Non-exposed Case	Cont	RR	95.00%CI
WYNDE7	30	m	0	1	54	42	918	0.40	(0.05- 3.00)
Totals				1	54	42	918		

*prospective study

REF	NRR	SEX	AD	Ys	Ws	Qs	Ps
WYNDE7	30	m	0	-0.90	0.96	0.00	0.3759

	N	1
	NS	1
	Wt	0.96
Het	Chi	0.00
Het	df	0
Het	P	N.S.
Fixed	RR	0.40
	RRl	0.05
	RRu	3.00
	P	N.S.
Random	RR	0.40
	RRl	0.05
	RRu	3.00
	P	N.S.
Asymm	P	

Table 3E7 - 6

IESLC - Meta-analysis of Current Smoking, Pipe only
Adenocarcinoma
Least adjusted

	Sex combined	male	female	Total
N		1		1
NS		1		1
Wt		0.96		0.96
Het Chi		0.00		0.00
Het df		0		0
Het P		N.S.		N.S.
Fixed RR		0.40		0.40
RRl		0.05		0.05
RRu		3.00		3.00
P		N.S.		N.S.
Random RR		0.40		0.40
RRl		0.05		0.05
RRu		3.00		3.00
P		N.S.		N.S.
Between Chi				
Between df				
Between P				N.S.
Btwn(F) P				N.S.
Btwn(R) P				N.S.

Table 3E7 - 7

IESLC - Meta-analysis of Current Smoking, Pipe only
Adenocarcinoma
Excluded studies (and stage at which they were excluded)

1	BOUCHA BUELL LAURIL MZILEN
2	ABRAHA AGUDO AKIBA ALDERS AMANDU AMES ANDERS ARCHER AUSTIN AUVINE AXELSO AXELSS BAND BARBON BECHER BERRIN BLOHMK BLOT1 BLOT2 BLOT3 BLOT4 BRESLO BRETT BROCKM BROSS BROWN1 BROWN2 BUFFLE BYERS1 BYERS2 CARPEN CASCO2 CASCOR CHAN CHANG CHATZI CHEN CHEN2 CHEN3 CHIAZZ CHOI CHOW CHYOU COMSTO CORREA CPSII DARBY DAVEYS DEAN2 DEKLER DESTE2 DESTEF DOCKER DORANT DORGAN DOSEME DROSTE DU DUNN EBELIN ENGELA ENSTRO ESAKI FAN GAO GAO2 GARCIA GARSHI GENG GER GILLIS GODLEY GOLLED GOODMA GRAHAM GREGOR GSELL HAENSZ HAMMO2 HANSEN HEGMAN HENNEK HINDS HIRAOK HIRAYA HITOSU HOLE HOROWI HORWIT HU HU2 HUANG HUMBLE ISHIMA JAHN JAIN JARUP JARVHO JEDRYC JIANG JOLY JONES JUSSAW KAISE2 KAISER KANELL KATSOU KAUFMA KELLER KHUDER KIHARA KINLEN KJUUS KNEKT KO KOHLME KOO KREUZE KREYBE KUBIK LAMTH LAMWK LAMWK2 LAUSSM LEI LEMARC LETOUR LIAW LICKIN LIDDEL LIU LIU2 LIU3 LIU4 LIU5 LOMBA2 LOMBAR LUO MACLEN MAGNUS MARSH MARSH2 MARTIS MASTRA MATOS MATSUD MCDUFF MCLAUG MIGRAN MILLER MILLS MOLLO MRFIT MRFITR MURATA NAM NOTAN2 NOTANI NOU ODRISC ORMOS OSANN OSANN2 PARKIN PASTOR PAWLEG PERSH2 PERSHA PETO PEZZO2 PEZZOT PIKE PISANI POFFIJ POLEDN PRESCO QIAO2 RACHTA RADZIK RANDIG REN RESTRE RIMING RONCO ROOTS ROTHSC SAARIK SANKAR SCHWAR SEGI SEGI2 SEOW SHAW SHIMIZ SIEMIA SIMARA SIMONA SITAS SOBUE SOBUE2 SPEIZE SPITZ STASZE STAYNE STOCKW STUCKE SUN SUZUK2 SUZUKI SVENSS TANG TANG2 TAO TENKAN TOKARS TOUSEY TSUGAN ULMER VANDER VEIERO VUTUC WAKAI WALD WANG WANG2 WANG3 WANG4 WARSIN WATSON WICKLU WIGLE WILKIN WU WU2 WUNSCH WUWILL WYNDE2 WYNDE3 WYNDE4 WYNDE5 WYNDE6 WYNDE8 WYNDER XU XU2 XU3 XU4 YAMAGU YONG YUAN ZHANG ZHENG ZHOU
3	ABELIN ARMADA BENHAM BEST BOUCOT COOKSO CPSI DEAN DOLL DOLL2 HAMMON KOULUM LEVIN LUBIN MCCONN PERNU QIAO SADOWS SCHWA2 STOCKS TIZZAN XIANGZ
5	HIRAY2 TVERDA
7	BENSHL BOFFET CEDERL DAMBER DEAN3 DORN GARDIN HEIN LANGE LUBIN2 TULINI

Table 3E7 - 8

Potentially overlapping studies

REF	REFGP	PRINC	.	OVERLAP/LINK
WYNDE7	WYNDE6	2		WYNDE5/6/7/8

Table 3E8 -

IESLC - Meta-analysis of Ever Smoking (or Current if Ever not available), Pipe only
Adenocarcinoma

This analysis is restricted to results for:
1) Non-dose-response data
2) Smokers of pipe only
3) Results complete enough for use in metaanalysis

Within each study, results are then selected (in the following order of preference, within each sex) for:
4) SMKSTA: ever smokers, current smokers
5) DENOM: never smoked anything, (never +1 = +long term ex)
6) Followup period (prospective studies): whole study (coded as 0) or longest available
7) LCtype: all or nearest available, at least Squamous and Adeno. (q = squamous, s = small,
l = large, a = adeno, mix = mixed, alv = alveolar)
8) Race: all or nearest available, otherwise by race (wh or w = white, bl or b = black, hi = hispanic
ch = chinese, jap = japanese, haw = hawaiian, w+o = white + oriental, sca = scandinavian, as = asian)
9) For overlapping studies: principal rather than subsidiary studies
Finally by Age: whole study (coded as 0) if available, otherwise by widest available age group
and then for single sex results (m, f) in preference to combined sex results (c).

Results adjusted (AD) for the most potential confounders are then chosen in Sections -1 to -3
and results adjusted for the least confounders in Sections -4 to -6. (Those least adjusted results which
actually differ from the most adjusted as marked 'x' in column X in Section -4)
(Results adjusted for an unknown number of confounder(s) are coded as 20.)

Section -7 shows excluded studies, together with the stage (as above) at which no qualifying
results were found.

Section -8 lists the potentially overlapping studies which have been included (1=principal, 2=subsidiary).

Section -9 lists any results which would have been included in preference except that they had data not complete
enough for use in meta-analysis, with their significance (yes/no), if known, and any further comment as entered
on the database.

In addition to those mentioned above, the following fields, levels and abbreviations are used:
* or nk = not known, n = no, y = yes, ot = other
ev = ever, cu = current, nev = never
REF: 6-character study reference
NRR: number of the RR on the database within the study
ST : study type (CC = case control, pr or prosp = prospective)
NLC: number of lung cancer cases in whole study
R : risky occupational population (n = no, m = mining, o = other risky)
VB : national cigarette type (V = at least 75% Virginia, bl = at least 75% blended, ot = other)
P : any proxy use
H : full histological confirmation
De : derivation of RR/CI (or = original, st = standard method, ot = other method of estimation)

Table 3E8 - 1

IESLC - Meta-analysis of Ever Smoking (or Current if Ever not available), Pipe only
Adenocarcinoma
Most adjusted

REF	NRR	SEX	AGEL	AGEH	RACE	YF	LC TYPE	LOC	START	ST	NLC	R	VB	P	H	AD	SM	DENOM	De
HAMMON	81	m	0	0	wh	0	a	NAmer	1952	pr	448	n	bl	n	n	1	ev	nev any	ot
LUBIN	19	m	0	0	all	-	KII	As:Chi	1984	CC	427	m	ot	y	n	0	ev	nev any	st
LUBIN2	140	m	0	0	all	-	a	Eu:mul	1976	CC	7804	n	bl	n	y	0	ev	nev any	st
WYNDE7	62	m	0	0	all	-	KII	NAmer	1977	CC	2085	n	bl	n	y	0	ev	nev any	st

Table 3E8 - 2

IESLC - Meta-analysis of Ever Smoking (or Current if Ever not available), Pipe only
Adenocarcinoma
Most adjusted

REF	NRR	SEX	AD	Number Exposed Case	Exposed Cont	Non-exposed Case	Non-exposed Cont	RR	95.00%CI
*HAMMON	81	m	1	-	-	-	-	2.44	(0.34- 17.35)
LUBIN	19	m	0	5	151	4	72	0.60	(0.16- 2.29)
LUBIN2	140	m	0	4	197	195	2616	0.27	(0.10- 0.74)
WYNDE7	62	m	0	2	97	42	918	0.45	(0.11- 1.89)
Partial Totals				11	445	241	3606		

*prospective study

REF	NRR	SEX	AD	Ys	Ws	Qs	Ps
*HAMMON	81	m	1	0.89	0.99	2.71	0.3739
LUBIN	19	m	0	-0.52	2.13	0.12	0.4506
LUBIN2	140	m	0	-1.30	3.84	1.13	0.0108
WYNDE7	62	m	0	-0.80	1.87	0.00	0.2759

N	4
NS	4
Wt	8.82
Het Chi	3.96
Het df	3
Het P	N.S.
Fixed RR	0.47
RRl	0.24
RRu	0.91
P	-
Random RR	0.50
RRl	0.23
RRu	1.10
P	(-)
Asymm P	*

Table 3E8 - 3

IESLC - Meta-analysis of Ever Smoking (or Current if Ever not available), Pipe only
Adenocarcinoma
Most adjusted

	Sex combined	male	female	Total
N		4		4
NS		4		4
Wt		8.82		8.82
Het Chi		3.96		3.96
Het df		3		3
Het P		N.S.		N.S.
Fixed RR		0.47		0.47
RRl		0.24		0.24
RRu		0.91		0.91
P		-		-
Random RR		0.50		0.50
RRl		0.23		0.23
RRu		1.10		1.10
P		(-)		(-)
Between Chi				
Between df				
Between P				N.S.
Btwn(F) P				N.S.
Btwn(R) P				N.S.

Too few RRs for analysis by factor

Table 3E8 - 4

IESLC - Meta-analysis of Ever Smoking (or Current if Ever not available), Pipe only
Adenocarcinoma
Least adjusted

REF	NRR	X	SEX	AGEL	AGEH	RACE	YF	LC TYPE	LOC	START	ST	NLC	R	VB	P	H	AD	SM	DENOM	De
HAMMON	87	x	m	0	0	wh	0	a	NAmer	1952	pr	448	n	bl	n	n	0	ev	nev any	st
LUBIN	19		m	0	0	all	-	KII	As:Chi	1984	CC	427	m	ot	y	n	0	ev	nev any	st
LUBIN2	140		m	0	0	all	-	a	Eu:mul	1976	CC	7804	n	bl	n	y	0	ev	nev any	st
WYNDE7	62		m	0	0	all	-	KII	NAmer	1977	CC	2085	n	bl	n	y	0	ev	nev any	st

Table 3E8 - 5

IESLC - Meta-analysis of Ever Smoking (or Current if Ever not available), Pipe only
Adenocarcinoma
Least adjusted

REF	NRR	SEX	AD	Number Exposed Case	Exposed Cont	Non-exposed Case	Non-exposed Cont	RR	95.00%CI
*HAMMON	87	m	0	2	43041	2	115884	2.69	(0.38- 19.11)
LUBIN	19	m	0	5	151	4	72	0.60	(0.16- 2.29)
LUBIN2	140	m	0	4	197	195	2616	0.27	(0.10- 0.74)
WYNDE7	62	m	0	2	97	42	918	0.45	(0.11- 1.89)
Totals				13	43486	243	119490		

*prospective study

REF	NRR	SEX	AD	Ys	Ws	Qs	Ps
*HAMMON	87	m	0	0.99	1.00	3.02	0.3220
LUBIN	19	m	0	-0.52	2.13	0.11	0.4506
LUBIN2	140	m	0	-1.30	3.84	1.18	0.0108
WYNDE7	62	m	0	-0.80	1.87	0.00	0.2759

	N	4
	NS	4
	Wt	8.83
Het	Chi	4.31
Het	df	3
Het	P	N.S.
Fixed	RR	0.47
	RRl	0.25
	RRu	0.92
	P	-
Random	RR	0.52
	RRl	0.23
	RRu	1.18
	P	N.S.
Asymm	P	*

Table 3E8 - 6

IESLC - Meta-analysis of Ever Smoking (or Current if Ever not available), Pipe only
Adenocarcinoma
Least adjusted

	Sex combined	male	female	Total
N		4		4
NS		4		4
Wt		8.83		8.83
Het Chi		4.31		4.31
Het df		3		3
Het P		N.S.		N.S.
Fixed RR		0.47		0.47
RRl		0.25		0.25
RRu		0.92		0.92
P		-		-
Random RR		0.52		0.52
RRl		0.23		0.23
RRu		1.18		1.18
P		N.S.		N.S.
Between Chi				
Between df				
Between P				N.S.
Btwn(F) P				N.S.
Btwn(R) P				N.S.

Table 3E8 - 7

IESLC - Meta-analysis of Ever Smoking (or Current if Ever not available), Pipe only
Adenocarcinoma
Excluded studies (and stage at which they were excluded)

1	BOUCHA BUELL LAURIL MZILEN
2	ABRAHA AGUDO AKIBA ALDERS AMANDU AMES ANDERS ARCHER AUSTIN AUVINE AXELSO AXELSS BAND BARBON BECHER BERRIN BLOHMK BLOT1 BLOT2 BLOT3 BLOT4 BRESLO BRETT BROCKM BROSS BROWN1 BROWN2 BUFFLE BYERS1 BYERS2 CARPEN CASCO2 CASCOR CHAN CHANG CHATZI CHEN CHEN2 CHEN3 CHIAZZ CHOI CHOW CHYOU COMSTO CORREA CPSII DARBY DAVEYS DEAN2 DEKLER DESTE2 DESTEF DOCKER DORANT DORGAN DOSEME DROSTE DU DUNN EBELIN ENGELA ENSTRO ESAKI FAN GAO GAO2 GARCIA GARSHI GENG GER GILLIS GODLEY GOLLED GOODMA GRAHAM GREGOR GSELL HAENSZ HAMMO2 HANSEN HEGMAN HENNEK HINDS HIRAOK HIRAYA HITOSU HOLE HOROWI HORWIT HU HU2 HUANG HUMBLE ISHIMA JAHN JAIN JARUP JARVHO JEDRYC JIANG JOLY JONES JUSSAW KAISE2 KAISER KANELL KATSOU KAUFMA KELLER KHUDER KIHARA KINLEN KJUUS KNEKT KO KOHLME KOO KREUZE KREYBE KUBIK LAMTH LAMWK LAMWK2 LAUSSM LEI LEMARC LETOUR LIAW LICKIN LIDDEL LIU LIU2 LIU3 LIU4 LIU5 LOMBA2 LOMBAR LUO MACLEN MAGNUS MARSH MARSH2 MARTIS MASTRA MATOS MATSUD MCDUFF MCLAUG MIGRAN MILLER MILLS MOLLO MRFIT MRFITR MURATA NAM NOTAN2 NOTANI NOU ODRISC ORMOS OSANN OSANN2 PARKIN PASTOR PAWLEG PERSH2 PERSHA PETO PEZZO2 PEZZOT PIKE PISANI POFFIJ POLEDN PRESCO QIAO2 RACHTA RADZIK RANDIG REN RESTRE RIMING RONCO ROOTS ROTHSC SAARIK SANKAR SCHWAR SEGI SEGI2 SEOW SHAW SHIMIZ SIEMIA SIMARA SIMONA SITAS SOBUE SOBUE2 SPEIZE SPITZ STASZE STAYNE STOCKW STUCKE SUN SUZUK2 SUZUKI SVENSS TANG TANG2 TAO TENKAN TOKARS TOUSEY TSUGAN ULMER VANDER VEIERO VUTUC WAKAI WALD WANG WANG2 WANG3 WANG4 WARSIN WATSON WICKLU WIGLE WILKIN WU WU2 WUNSCH WUWILL WYNDE2 WYNDE3 WYNDE4 WYNDE5 WYNDE6 WYNDE8 WYNDER XU XU2 XU3 XU4 YAMAGU YONG YUAN ZHANG ZHENG ZHOU
3	DOLL2
5	HIRAY2 SCHWA2 TVERDA
7	ABELIN ARMADA BENHAM BENSHL BEST BOFFET BOUCOT CEDERL COOKSO CPSI DAMBER DEAN DEAN3 DOLL DORN GARDIN HEIN KOULUM LANGE LEVIN MCCONN PERNU QIAO SADOWS STOCKS TIZZAN TULINI XIANGZ

Table 3E8 - 8

Potentially overlapping studies

REF	REFGP	PRINC	. OVERLAP/LINK
LUBIN2	LUBIN2	1	Lubin-combined
WYNDE7	WYNDE6	2	WYNDE5/6/7/8
LUBIN	XIANGZ	2	LUBIN/XIANGZ/QIAO

Table 3E9 -

IESLC - Meta-analysis of Current Smoking (or Ever if Current not available), Pipe only
Adenocarcinoma

This analysis is restricted to results for:
1) Non-dose-response data
2) Smokers of pipe only
3) Results complete enough for use in metaanalysis

Within each study, results are then selected (in the following order of preference, within each sex) for:
4) SMKSTA: current smokers, ever smokers
5) DENOM: never smoked anything, (never +1 = +long term ex)
6) Followup period (prospective studies): whole study (coded as 0) or longest available
7) LCtype: all or nearest available, at least Squamous and Adeno. (q = squamous, s = small,
l = large, a = adeno, mix = mixed, alv = alveolar)
8) Race: all or nearest available, otherwise by race (wh or w = white, bl or b = black, hi = hispanic
ch = chinese, jap = japanese, haw = hawaiian, w+o = white + oriental, sca = scandinavian, as = asian)
9) For overlapping studies: principal rather than subsidiary studies
Finally by Age: whole study (coded as 0) if available, otherwise by widest available age group
and then for single sex results (m, f) in preference to combined sex results (c).

Results adjusted (AD) for the most potential confounders are then chosen in Sections -1 to -3
(and those which actually differ from the adjusted results in Table 3E8 - 1 are marked 'x' in Section -1)
and results adjusted for the least confounders in Sections -4 to -6. (Those least adjusted results which
actually differ from the most adjusted as marked 'x' in column X in Section -4)
(Results adjusted for an unknown number of confounder(s) are coded as 20.)

Section -7 shows excluded studies, together with the stage (as above) at which no qualifying
results were found.

Section -8 lists the potentially overlapping studies which have been included (1=principal, 2=subsidiary).

Section -9 lists any results which would have been included in preference except that they had data not complete
enough for use in meta-analysis, with their significance (yes/no), if known, and any further comment as entered
on the database.

In addition to those mentioned above, the following fields, levels and abbreviations are used:
* or nk = not known, n = no, y = yes, ot = other
ev = ever, cu = current, nev = never
REF: 6-character study reference
NRR: number of the RR on the database within the study
ST : study type (CC = case control, pr or prosp = prospective)
NLC: number of lung cancer cases in whole study
R : risky occupational population (n = no, m = mining, o = other risky)
VB : national cigarette type (V = at least 75% Virginia, bl = at least 75% blended, ot = other)
P : any proxy use
H : full histological confirmation
De : derivation of RR/CI (or = original, st = standard method, ot = other method of estimation)

Table 3E9 - 1

IESLC - Meta-analysis of Current Smoking (or Ever if Current not available), Pipe only
Adenocarcinoma
Most adjusted

REF	NRR	3E8	SEX	AGEL	AGEH	RACE	YF	LC TYPE	LOC	START	ST	NLC	R	VB	P	H	AD	SM	DENOM	De
HAMMON	81		m	0	0	wh	0	a	NAmer	1952	pr	448	n	bl	n	n	1	ev	nev any	ot
LUBIN	19		m	0	0	all	-	KII	As:Chi	1984	CC	427	m	ot	y	n	0	ev	nev any	st
LUBIN2	140		m	0	0	all	-	a	Eu:mul	1976	CC	7804	n	bl	n	y	0	ev	nev any	st
WYNDE7	30	x	m	0	0	all	-	KII	NAmer	1977	CC	2085	n	bl	n	y	0	cu	nev any	st

Table 3E9 - 2

IESLC - Meta-analysis of Current Smoking (or Ever if Current not available), Pipe only
Adenocarcinoma
Most adjusted

REF	NRR	SEX	AD	Number Exposed Case	Number Exposed Cont	Non-exposed Case	Non-exposed Cont	RR	95.00%CI
*HAMMON	81	m	1	-	-	-	-	2.44	(0.34- 17.35)
LUBIN	19	m	0	5	151	4	72	0.60	(0.16- 2.29)
LUBIN2	140	m	0	4	197	195	2616	0.27	(0.10- 0.74)
WYNDE7	30	m	0	1	54	42	918	0.40	(0.05- 3.00)
Partial Totals				10	402	241	3606		

*prospective study

REF	NRR	SEX	AD	Ys	Ws	Qs	Ps
*HAMMON	81	m	1	0.89	0.99	2.73	0.3739
LUBIN	19	m	0	-0.52	2.13	0.13	0.4506
LUBIN2	140	m	0	-1.30	3.84	1.09	0.0108
WYNDE7	30	m	0	-0.90	0.96	0.02	0.3759

	N	4
	NS	4
	Wt	7.91
Het	Chi	3.98
Het	df	3
Het	P	N.S.
Fixed	RR	0.46
	RRl	0.23
	RRu	0.93
	P	-
Random	RR	0.51
	RRl	0.22
	RRu	1.18
	P	N.S.
Asymm	P	N.S.

Table 3E9 - 3

IESLC - Meta-analysis of Current Smoking (or Ever if Current not available), Pipe only
Adenocarcinoma
Most adjusted

	Sex combined	male	female	Total
N		4		4
NS		4		4
Wt		7.91		7.91
Het Chi		3.98		3.98
Het df		3		3
Het P		N.S.		N.S.
Fixed RR		0.46		0.46
RRl		0.23		0.23
RRu		0.93		0.93
P		-		-
Random RR		0.51		0.51
RRl		0.22		0.22
RRu		1.18		1.18
P		N.S.		N.S.
Between Chi				
Between df				
Between P				N.S.
Btwn(F) P				N.S.
Btwn(R) P				N.S.

Too few RRs for analysis by factor

Table 3E9 - 4

IESLC - Meta-analysis of Current Smoking (or Ever if Current not available), Pipe only
Adenocarcinoma
Least adjusted

REF	NRR	X	SEX	AGEL	AGEH	RACE	YF	LC TYPE	LOC	START	ST	NLC	R	VB	P	H	AD	SM	DENOM	De
HAMMON	87	x	m	0	0	wh	0	a	NAmer	1952	pr	448	n	bl	n	n	0	ev	nev any	st
LUBIN	19		m	0	0	all	-	KII	As:Chi	1984	CC	427	m	ot	y	n	0	ev	nev any	st
LUBIN2	140		m	0	0	all	-	a	Eu:mul	1976	CC	7804	n	bl	n	y	0	ev	nev any	st
WYNDE7	30		m	0	0	all	-	KII	NAmer	1977	CC	2085	n	bl	n	y	0	cu	nev any	st

Table 3E9 - 5

IESLC - Meta-analysis of Current Smoking (or Ever if Current not available), Pipe only
Adenocarcinoma
Least adjusted

REF	NRR	SEX	AD	Number Exposed Case	Exposed Cont	Non-exposed Case	Non-exposed Cont	RR	95.00%CI
*HAMMON	87	m	0	2	43041	2	115884	2.69	(0.38- 19.11)
LUBIN	19	m	0	5	151	4	72	0.60	(0.16- 2.29)
LUBIN2	140	m	0	4	197	195	2616	0.27	(0.10- 0.74)
WYNDE7	30	m	0	1	54	42	918	0.40	(0.05- 3.00)
Totals				12	43443	243	119490		

*prospective study

REF	NRR	SEX	AD	Ys	Ws	Qs	Ps
*HAMMON	87	m	0	0.99	1.00	3.04	0.3220
LUBIN	19	m	0	-0.52	2.13	0.12	0.4506
LUBIN2	140	m	0	-1.30	3.84	1.15	0.0108
WYNDE7	30	m	0	-0.90	0.96	0.02	0.3759

N	4
NS	4
Wt	7.92
Het Chi	4.33
Het df	3
Het P	N.S.
Fixed RR	0.47
RRl	0.23
RRu	0.94
P	-
Random RR	0.53
RRl	0.22
RRu	1.27
P	N.S.
Asymm P	N.S.

Table 3E9 - 6

IESLC - Meta-analysis of Current Smoking (or Ever if Current not available), Pipe only
Adenocarcinoma
Least adjusted

	Sex combined	male	female	Total
N		4		4
NS		4		4
Wt		7.92		7.92
Het Chi		4.33		4.33
Het df		3		3
Het P		N.S.		N.S.
Fixed RR		0.47		0.47
RRl		0.23		0.23
RRu		0.94		0.94
P		-		-
Random RR		0.53		0.53
RRl		0.22		0.22
RRu		1.27		1.27
P		N.S.		N.S.
Between Chi				
Between df				
Between P				N.S.
Btwn(F) P				N.S.
Btwn(R) P				N.S.

Table 3E9 - 7

IESLC - Meta-analysis of Current Smoking (or Ever if Current not available), Pipe only
Adenocarcinoma
Excluded studies (and stage at which they were excluded)

1 BOUCHA BUELL LAURIL MZILEN

2 ABRAHA AGUDO AKIBA ALDERS AMANDU AMES ANDERS ARCHER AUSTIN AUVINE AXELSO AXELSS BAND BARBON BECHER BERRIN
BLOHMK BLOT1 BLOT2 BLOT3 BLOT4 BRESLO BRETT BROCKM BROSS BROWN1 BROWN2 BUFFLE BYERS1 BYERS2 CARPEN CASCO2
CASCOR CHAN CHANG CHATZI CHEN CHEN2 CHEN3 CHIAZZ CHOI CHOW CHYOU COMSTO CORREA CPSII DARBY DAVEYS
DEAN2 DEKLER DESTE2 DESTEF DOCKER DORANT DORGAN DOSEME DROSTE DU DUNN EBELIN ENGELA ENSTRO ESAKI FAN
GAO GAO2 GARCIA GARSHI GENG GER GILLIS GODLEY GOLLED GOODMA GRAHAM GREGOR GSELL HAENSZ HAMMO2 HANSEN
HEGMAN HENNEK HINDS HIRAOK HIRAYA HITOSU HOLE HOROWI HORWIT HU HU2 HUANG HUMBLE ISHIMA JAHN JAIN
JARUP JARVHO JEDRYC JIANG JOLY JONES JUSSAW KAISE2 KAISER KANELL KATSOU KAUFMA KELLER KHUDER KIHARA KINLEN
KJUUS KNEKT KO KOHLME KOO KREUZE KREYBE KUBIK LAMTH LAMWK LAMWK2 LAUSSM LEI LEMARC LETOUR LIAW
LICKIN LIDDEL LIU LIU2 LIU3 LIU4 LIU5 LOMBA2 LOMBAR LUO MACLEN MAGNUS MARSH MARSH2 MARTIS MASTRA
MATOS MATSUD MCDUFF MCLAUG MIGRAN MILLER MILLS MOLLO MRFIT MRFITR MURATA NAM NOTAN2 NOTANI NOU ODRISC
ORMOS OSANN OSANN2 PARKIN PASTOR PAWLEG PERSH2 PERSHA PETO PEZZO2 PEZZOT PIKE PISANI POFFIJ POLEDN PRESCO
QIAO2 RACHTA RADZIK RANDIG REN RESTRE RIMING RONCO ROOTS ROTHSC SAARIK SANKAR SCHWAR SEGI SEGI2 SEOW
SHAW SHIMIZ SIEMIA SIMARA SIMONA SITAS SOBUE SOBUE2 SPEIZE SPITZ STASZE STAYNE STOCKW STUCKE SUN SUZUK2
SUZUKI SVENSS TANG TANG2 TAO TENKAN TOKARS TOUSEY TSUGAN ULMER VANDER VEIERO VUTUC WAKAI WALD WANG
WANG2 WANG3 WANG4 WARSIN WATSON WICKLU WIGLE WILKIN WU WU2 WUNSCH WUWILL WYNDE2 WYNDE3 WYNDE4 WYNDE5
WYNDE6 WYNDE8 WYNDER XU XU2 XU3 XU4 YAMAGU YONG YUAN ZHANG ZHENG ZHOU

3 DOLL2

5 HIRAY2 SCHWA2 TVERDA

7 ABELIN ARMADA BENHAM BENSHL BEST BOFFET BOUCOT CEDERL COOKSO CPSI DAMBER DEAN DEAN3 DOLL DORN GARDIN
HEIN KOULUM LANGE LEVIN MCCONN PERNU QIAO SADOWS STOCKS TIZZAN TULINI XIANGZ

Table 3E9 - 8

Potentially overlapping studies

REF	REFGP	PRINC	OVERLAP/LINK
LUBIN2	LUBIN2	1	Lubin-combined
WYNDE7	WYNDE6	2	WYNDE5/6/7/8
LUBIN	XIANGZ	2	LUBIN/XIANGZ/QIAO

Table 3E10 -

IESLC - Meta-analysis of Ex Smoking, Pipe only
Adenocarcinoma

This analysis is restricted to results for:
1) Non-dose-response data
2) Smokers of pipe only
3) Ex smokers
4) Results complete enough for use in metaanalysis

Within each study, results are then selected (in the following order of preference, within each sex) for:
5) DENOM: never smoked anything, (never +1 = +long term ex)
6) Followup period (prospective studies): whole study (coded as 0) or longest available
7) LCtype: all or nearest available, at least Squamous and Adeno. (q = squamous, s = small,
l = large, a = adeno, mix = mixed, alv = alveolar)
8) Race: all or nearest available, otherwise by race (wh or w = white, bl or b = black, hi = hispanic
ch = chinese, jap = japanese, haw = hawaiian, w+o = white + oriental, sca = scandinavian, as = asian)
9) For overlapping studies: principal rather than subsidiary studies
Finally by Age: whole study (coded as 0) if available, otherwise by widest available age group
and then for single sex results (m, f) in preference to combined sex results (c).

Results adjusted (AD) for the most potential confounders are then chosen in Sections -1 to -3
and results adjusted for the least confounders in Sections -4 to -6. (Those least adjusted results which
actually differ from the most adjusted as marked 'x' in column X in Section -4)
(Results adjusted for an unknown number of confounder(s) are coded as 20.)

Section -7 shows excluded studies, together with the stage (as above) at which no qualifying
results were found.

Section -8 lists the potentially overlapping studies which have been included (1=principal, 2=subsidiary).

Section -9 lists any results which would have been included in preference except that they had data not complete
enough for use in meta-analysis, with their significance (yes/no), if known, and any further comment as entered
on the database.

In addition to those mentioned above, the following fields, levels and abbreviations are used:
* or nk = not known, n = no, y = yes, ot = other
nev = never
REF: 6-character study reference
NRR: number of the RR on the database within the study
ST : study type (CC = case control, pr or prosp = prospective)
NLC: number of lung cancer cases in whole study
R : risky occupational population (n = no, m = mining, o = other risky)
VB : national cigarette type (V = at least 75% Virginia, bl = at least 75% blended, ot = other)
P : any proxy use
H : full histological confirmation
De : derivation of RR/CI (or = original, st = standard method, ot = other method of estimation)

Table 3E10 - 1

IESLC - Meta-analysis of Ex Smoking, Pipe only
Adenocarcinoma
Most adjusted

REF	NRR	SEX	AGEL	AGEH	RACE	YF	LC TYPE	LOC	START	ST	NLC	R	VB	P	H	AD	DENOM	De
WYNDE7	35	m	0	0	all	-	KII	NAmer	1977	CC	2085	n	bl	n	y	0	nev any	st

Table 3E10 - 2

IESLC - Meta-analysis of Ex Smoking, Pipe only
Adenocarcinoma
Most adjusted

REF	NRR	SEX	AD	Number Exposed Case	Cont	Non-exposed Case	Cont	RR	95.00%CI
WYNDE7	35	m	0	1	43	42	918	0.51	(0.07- 3.78)
Totals				1	43	42	918		

*prospective study

REF	NRR	SEX	AD	Ys	Ws	Qs	Ps
WYNDE7	35	m	0	-0.68	0.95	0.00	0.5086

	N	1
	NS	1
	Wt	0.95
Het	Chi	0.00
Het	df	0
Het	P	N.S.
Fixed	RR	0.51
	RRl	0.07
	RRu	3.78
	P	N.S.
Random	RR	0.51
	RRl	0.07
	RRu	3.78
	P	N.S.
Asymm	P	

Table 3E10 - 3

IESLC - Meta-analysis of Ex Smoking, Pipe only
Adenocarcinoma
Most adjusted

	Sex combined	male	female	Total
N		1		1
NS		1		1
Wt		0.95		0.95
Het Chi		0.00		0.00
Het df		0		0
Het P		N.S.		N.S.
Fixed RR		0.51		0.51
RRl		0.07		0.07
RRu		3.78		3.78
P		N.S.		N.S.
Random RR		0.51		0.51
RRl		0.07		0.07
RRu		3.78		3.78
P		N.S.		N.S.
Between Chi				
Between df				
Between P				N.S.
Btwn(F) P				N.S.
Btwn(R) P				N.S.

Too few RRs for analysis by factor

Table 3E10 - 4

IESLC - Meta-analysis of Ex Smoking, Pipe only
Adenocarcinoma
Least adjusted

REF	NRR	X	SEX	AGEL	AGEH	RACE	YF	LC TYPE	LOC	START	ST	NLC	R	VB	P	H	AD	DENOM	De
WYNDE7	35		m	0	0	all	-	KII	NAmer	1977	CC	2085	n	bl	n	y	0	nev any	st

Table 3E10 - 5

IESLC - Meta-analysis of Ex Smoking, Pipe only
Adenocarcinoma
Least adjusted

REF	NRR	SEX	AD	Number Exposed Case	Exposed Cont	Non-exposed Case	Non-exposed Cont	RR	95.00%CI
WYNDE7	35	m	0	1	43	42	918	0.51	(0.07- 3.78)
Totals				1	43	42	918		

*prospective study

REF	NRR	SEX	AD	Ys	Ws	Qs	Ps
WYNDE7	35	m	0	-0.68	0.95	0.00	0.5086

	N	1
	NS	1
	Wt	0.95
Het	Chi	0.00
Het	df	0
Het	P	N.S.
Fixed	RR	0.51
	RRl	0.07
	RRu	3.78
	P	N.S.
Random	RR	0.51
	RRl	0.07
	RRu	3.78
	P	N.S.
Asymm	P	

Table 3E10 - 6

IESLC - Meta-analysis of Ex Smoking, Pipe only
Adenocarcinoma
Least adjusted

	Sex combined	male	female	Total
N		1		1
NS		1		1
Wt		0.95		0.95
Het Chi		0.00		0.00
Het df		0		0
Het P		N.S.		N.S.
Fixed RR		0.51		0.51
RRl		0.07		0.07
RRu		3.78		3.78
P		N.S.		N.S.
Random RR		0.51		0.51
RRl		0.07		0.07
RRu		3.78		3.78
P		N.S.		N.S.
Between Chi				
Between df				
Between P				N.S.
Btwn(F) P				N.S.
Btwn(R) P				N.S.

Table 3E10 - 7

IESLC - Meta-analysis of Ex Smoking, Pipe only
Adenocarcinoma
Excluded studies (and stage at which they were excluded)

1	BOUCHA BUELL LAURIL MZILEN
2	ABRAHA AGUDO AKIBA ALDERS AMANDU AMES ANDERS ARCHER AUSTIN AUVINE AXELSO AXELSS BAND BARBON BECHER BERRIN BLOHMK BLOT1 BLOT2 BLOT3 BLOT4 BRESLO BRETT BROCKM BROSS BROWN1 BROWN2 BUFFLE BYERS1 BYERS2 CARPEN CASCO2 CASCOR CHAN CHANG CHATZI CHEN CHEN2 CHEN3 CHIAZZ CHOI CHOW CHYOU COMSTO CORREA CPSII DARBY DAVEYS DEAN2 DEKLER DESTE2 DESTEF DOCKER DORANT DORGAN DOSEME DROSTE DU DUNN EBELIN ENGELA ENSTRO ESAKI FAN GAO GAO2 GARCIA GARSHI GENG GER GILLIS GODLEY GOLLED GOODMA GRAHAM GREGOR GSELL HAENSZ HAMMO2 HANSEN HEGMAN HENNEK HINDS HIRAOK HIRAYA HITOSU HOLE HOROWI HORWIT HU HU2 HUANG HUMBLE ISHIMA JAHN JAIN JARUP JARVHO JEDRYC JIANG JOLY JONES JUSSAW KAISE2 KAISER KANELL KATSOU KAUFMA KELLER KHUDER KIHARA KINLEN KJUUS KNEKT KO KOHLME KOO KREUZE KREYBE KUBIK LAMTH LAMWK LAMWK2 LAUSSM LEI LEMARC LETOUR LIAW LICKIN LIDDEL LIU LIU2 LIU3 LIU4 LIU5 LOMBA2 LOMBAR LUO MACLEN MAGNUS MARSH MARSH2 MARTIS MASTRA MATOS MATSUD MCDUFF MCLAUG MIGRAN MILLER MILLS MOLLO MRFIT MRFITR MURATA NAM NOTAN2 NOTANI NOU ODRISC ORMOS OSANN OSANN2 PARKIN PASTOR PAWLEG PERSH2 PERSHA PETO PEZZO2 PEZZOT PIKE PISANI POFFIJ POLEDN PRESCO QIAO2 RACHTA RADZIK RANDIG REN RESTRE RIMING RONCO ROOTS ROTHSC SAARIK SANKAR SCHWAR SEGI SEGI2 SEOW SHAW SHIMIZ SIEMIA SIMARA SIMONA SITAS SOBUE SOBUE2 SPEIZE SPITZ STASZE STAYNE STOCKW STUCKE SUN SUZUK2 SUZUKI SVENSS TANG TANG2 TAO TENKAN TOKARS TOUSEY TSUGAN ULMER VANDER VEIERO VUTUC WAKAI WALD WANG WANG2 WANG3 WANG4 WARSIN WATSON WICKLU WIGLE WILKIN WU WU2 WUNSCH WUWILL WYNDE2 WYNDE3 WYNDE4 WYNDE5 WYNDE6 WYNDE8 WYNDER XU XU2 XU3 XU4 YAMAGU YONG YUAN ZHANG ZHENG ZHOU
3	ABELIN ARMADA BENHAM BENSHL BEST BOUCOT CEDERL COOKSO CPSI DEAN DEAN3 DOLL DOLL2 GARDIN HAMMON HEIN HIRAY2 KOULUM LANGE LEVIN LUBIN MCCONN PERNU QIAO SADOWS SCHWA2 STOCKS TIZZAN TULINI TVERDA XIANGZ
7	BOFFET DAMBER DORN LUBIN2

Table 3E10 - 8
Potentially overlapping studies

REF	REFGP	PRINC	.	OVERLAP/LINK
WYNDE7	WYNDE6	2		WYNDE5/6/7/8

Table 3E11 -

IESLC - Meta-analysis of Ever Smoking, Cigars only
Adenocarcinoma

This analysis is restricted to results for:
1) Non-dose-response data
2) Smokers of cigars only
3) Ever smokers
4) Results complete enough for use in metaanalysis

Within each study, results are then selected (in the following order of preference, within each sex) for:
5) DENOM: never smoked anything, (never +1 = +long term ex)
6) Followup period (prospective studies): whole study (coded as 0) or longest available
7) LCtype: all or nearest available, at least Squamous and Adeno. (q = squamous, s = small,
l = large, a = adeno, mix = mixed, alv = alveolar)
8) Race: all or nearest available, otherwise by race (wh or w = white, bl or b = black, hi = hispanic
ch = chinese, jap = japanese, haw = hawaiian, w+o = white + oriental, sca = scandinavian, as = asian)
9) For overlapping studies: principal rather than subsidiary studies
Finally by Age: whole study (coded as 0) if available, otherwise by widest available age group
and then for single sex results (m, f) in preference to combined sex results (c).

Results adjusted (AD) for the most potential confounders are then chosen in Sections -1 to -3
and results adjusted for the least confounders in Sections -4 to -6. (Those least adjusted results which
actually differ from the most adjusted as marked 'x' in column X in Section -4)
(Results adjusted for an unknown number of confounder(s) are coded as 20.)

Section -7 shows excluded studies, together with the stage (as above) at which no qualifying
results were found.

Section -8 lists the potentially overlapping studies which have been included (1=principal, 2=subsidiary).

Section -9 lists any results which would have been included in preference except that they had data not complete
enough for use in meta-analysis, with their significance (yes/no), if known, and any further comment as entered
on the database.

In addition to those mentioned above, the following fields, levels and abbreviations are used:
* or nk = not known, n = no, y = yes, ot = other
nev = never
REF: 6-character study reference
NRR: number of the RR on the database within the study
ST : study type (CC = case control, pr or prosp = prospective)
NLC: number of lung cancer cases in whole study
R : risky occupational population (n = no, m = mining, o = other risky)
VB : national cigarette type (V = at least 75% Virginia, bl = at least 75% blended, ot = other)
P : any proxy use
H : full histological confirmation
De : derivation of RR/CI (or = original, st = standard method, ot = other method of estimation)

Table 3E11 - 1

IESLC - Meta-analysis of Ever Smoking, Cigars only
Adenocarcinoma
Most adjusted

REF	NRR	SEX	AGEL	AGEH	RACE	YF	LC TYPE	LOC	START	ST	NLC	R	VB	P	H	AD	DENOM	De
HAMMON	80	m	0	0	wh	0	a	NAmer	1952	pr	448	n	bl	n	n	1	nev any	ot
LUBIN2	136	m	0	0	all	-	a	Eu:mul	1976	CC	7804	n	bl	n	y	0	nev any	st
WYNDE7	61	m	0	0	all	-	KII	NAmer	1977	CC	2085	n	bl	n	y	0	nev any	st

Table 3E11 - 2

IESLC - Meta-analysis of Ever Smoking, Cigars only
Adenocarcinoma
Most adjusted

REF	NRR	SEX	AD	Number Exposed Case	Cont	Non-exposed Case	Cont	RR	95.00%CI
*HAMMON	80	m	1	-	-	-	-	0.94	(0.09- 10.42)
LUBIN2	136	m	0	1	145	195	2616	0.09	(0.01- 0.66)
WYNDE7	61	m	0	9	152	42	918	1.29	(0.62- 2.71)
Partial Totals				10	297	237	3534		

*prospective study

REF	NRR	SEX	AD	Ys	Ws	Qs	Ps
*HAMMON	80	m	1	-0.06	0.68	0.00	0.9593
LUBIN2	136	m	0	-2.38	0.99	5.28	0.0180
WYNDE7	61	m	0	0.26	7.01	0.74	0.4947

	N	3
	NS	3
	Wt	8.68
Het	Chi	6.03
Het	df	2
Het	P	*
Fixed	RR	0.93
	RRl	0.48
	RRu	1.82
	P	N.S.
Random	RR	0.55
	RRl	0.11
	RRu	2.88
	P	N.S.
Asymm	P	N.S.

Table 3E11 - 3

IESLC - Meta-analysis of Ever Smoking, Cigars only
Adenocarcinoma
Most adjusted

	Sex combined	male	female	Total
N		3		3
NS		3		3
Wt		8.68		8.68
Het Chi		6.03		6.03
Het df		2		2
Het P		*		*
Fixed RR		0.93		0.93
RRl		0.48		0.48
RRu		1.82		1.82
P		N.S.		N.S.
Random RR		0.55		0.55
RRl		0.11		0.11
RRu		2.88		2.88
P		N.S.		N.S.
Between Chi				
Between df				
Between P				N.S.
Btwn(F) P				N.S.
Btwn(R) P				N.S.

Too few RRs for analysis by factor

Table 3E11 - 4

IESLC - Meta-analysis of Ever Smoking, Cigars only
Adenocarcinoma
Least adjusted

REF	NRR	X	SEX	AGEL	AGEH	RACE	YF	LC TYPE	LOC	START	ST	NLC	R	VB	P	H	AD	DENOM	De
HAMMON	86	x	m	0	0	wh	0	a	NAmer	1952	pr	448	n	bl	n	n	0	nev any	st
LUBIN2	136		m	0	0	all	-	a	Eu:mul	1976	CC	7804	n	bl	n	y	0	nev any	st
WYNDE7	61		m	0	0	all	-	KII	NAmer	1977	CC	2085	n	bl	n	y	0	nev any	st

Table 3E11 - 5

IESLC - Meta-analysis of Ever Smoking, Cigars only
Adenocarcinoma
Least adjusted

REF	NRR	SEX	AD	Number Case	Exposed Cont	Non-exposed Case	Cont	RR	95.00%CI
*HAMMON	86	m	0	1	51480	2	115884	1.13	(0.10- 12.41)
LUBIN2	136	m	0	1	145	195	2616	0.09	(0.01- 0.66)
WYNDE7	61	m	0	9	152	42	918	1.29	(0.62- 2.71)
Totals				11	51777	239	119418		

*prospective study

REF	NRR	SEX	AD	Ys	Ws	Qs	Ps
*HAMMON	86	m	0	0.12	0.67	0.02	0.9231
LUBIN2	136	m	0	-2.38	0.99	5.35	0.0180
WYNDE7	61	m	0	0.26	7.01	0.68	0.4947

	N	3
	NS	3
	Wt	8.67
Het	Chi	6.05
Het	df	2
Het	P	*
Fixed	RR	0.95
	RRl	0.49
	RRu	1.84
	P	N.S.
Random	RR	0.57
	RRl	0.11
	RRu	3.03
	P	N.S.
Asymm	P	N.S.

Table 3E11 - 6

IESLC - Meta-analysis of Ever Smoking, Cigars only
Adenocarcinoma
Least adjusted

	Sex combined	male	female	Total
N		3		3
NS		3		3
Wt		8.67		8.67
Het Chi		6.05		6.05
Het df		2		2
Het P		*		*
Fixed RR		0.95		0.95
RRl		0.49		0.49
RRu		1.84		1.84
P		N.S.		N.S.
Random RR		0.57		0.57
RRl		0.11		0.11
RRu		3.03		3.03
P		N.S.		N.S.
Between Chi				
Between df				
Between P				N.S.
Btwn(F) P				N.S.
Btwn(R) P				N.S.

Table 3E11 - 7

IESLC - Meta-analysis of Ever Smoking, Cigars only
Adenocarcinoma
Excluded studies (and stage at which they were excluded)

1	BOUCHA BUELL LAURIL MZILEN
2	ABRAHA AGUDO AKIBA ALDERS AMANDU AMES ANDERS ARCHER AUSTIN AUVINE AXELSO AXELSS BAND BARBON BECHER BERRIN BLOHMK BLOT1 BLOT2 BLOT3 BLOT4 BRESLO BRETT BROCKM BROSS BROWN1 BROWN2 BUFFLE BYERS1 BYERS2 CARPEN CASCO2 CASCOR CHAN CHANG CHATZI CHEN CHEN2 CHEN3 CHIAZZ CHOI CHOW CHYOU COMSTO COOKSO CORREA DARBY DAVEYS DEAN DEAN2 DEKLER DESTE2 DESTEF DOCKER DOLL DOLL2 DORANT DORGAN DOSEME DROSTE DU DUNN EBELIN ENGELA ENSTRO ESAKI FAN GAO GAO2 GARCIA GARSHI GENG GER GILLIS GODLEY GOLLED GOODMA GRAHAM GREGOR GSELL HAENSZ HAMMO2 HANSEN HEGMAN HENNEK HINDS HIRAOK HIRAY2 HIRAYA HITOSU HOLE HOROWI HORWIT HU HU2 HUANG HUMBLE ISHIMA JAHN JAIN JARUP JARVHO JEDRYC JIANG JONES JUSSAW KAISE2 KAISER KANELL KATSOU KAUFMA KELLER KHUDER KIHARA KINLEN KJUUS KNEKT KO KOHLME KOO KOULUM KREUZE KREYBE KUBIK LAMTH LAMWK LAMWK2 LAUSSM LEI LEMARC LETOUR LIAW LICKIN LIDDEL LIU LIU2 LIU3 LIU4 LIU5 LOMBA2 LOMBAR LUBIN LUO MACLEN MAGNUS MARSH MARSH2 MARTIS MASTRA MATOS MATSUD MCCONN MCDUFF MCLAUG MIGRAN MILLER MILLS MOLLO MRFIT MRFITR MURATA NAM NOTAN2 NOTANI NOU ODRISC ORMOS OSANN OSANN2 PARKIN PASTOR PAWLEG PERNU PERSH2 PERSHA PETO PEZZO2 PEZZOT PIKE PISANI POFFIJ POLEDN PRESCO QIAO QIAO2 RACHTA RADZIK RANDIG REN RESTRE RIMING RONCO ROOTS ROTHSC SAARIK SANKAR SCHWA2 SCHWAR SEGI SEGI2 SEOW SHAW SHIMIZ SIEMIA SIMARA SIMONA SITAS SOBUE SOBUE2 SPEIZE SPITZ STASZE STAYNE STOCKS STOCKW STUCKE SUN SUZUK2 SUZUKI SVENSS TANG TANG2 TAO TENKAN TOKARS TOUSEY TSUGAN TVERDA ULMER VANDER VEIERO VUTUC WAKAI WALD WANG WANG2 WANG3 WANG4 WARSIN WATSON WICKLU WIGLE WILKIN WU WU2 WUNSCH WUWILL WYNDE2 WYNDE3 WYNDE4 WYNDE5 WYNDE6 WYNDE8 XIANGZ XU XU2 XU3 XU4 YAMAGU YONG YUAN ZHANG ZHENG ZHOU
3	BENSHL CEDERL CPSII DEAN3 GARDIN HEIN LANGE TULINI WYNDER
7	ABELIN ARMADA BENHAM BEST BOFFET BOUCOT CPSI DAMBER DORN JOLY LEVIN SADOWS TIZZAN

Table 3E11 - 8

Potentially overlapping studies

REF	REFGP	PRINC	.	OVERLAP/LINK
LUBIN2	LUBIN2	1		Lubin-combined
WYNDE7	WYNDE6	2		WYNDE5/6/7/8

Table 3E12 -

IESLC - Meta-analysis of Current Smoking, Cigars only
Adenocarcinoma

This analysis is restricted to results for:
1) Non-dose-response data
2) Smokers of cigars only
3) Current smokers
4) Results complete enough for use in metaanalysis

Within each study, results are then selected (in the following order of preference, within each sex) for:
5) DENOM: never smoked anything, (never +1 = +long term ex)
6) Followup period (prospective studies): whole study (coded as 0) or longest available
7) LCtype: all or nearest available, at least Squamous and Adeno. (q = squamous, s = small,
l = large, a = adeno, mix = mixed, alv = alveolar)
8) Race: all or nearest available, otherwise by race (wh or w = white, bl or b = black, hi = hispanic
ch = chinese, jap = japanese, haw = hawaiian, w+o = white + oriental, sca = scandinavian, as = asian)
9) For overlapping studies: principal rather than subsidiary studies
Finally by Age: whole study (coded as 0) if available, otherwise by widest available age group
and then for single sex results (m, f) in preference to combined sex results (c).

Results adjusted (AD) for the most potential confounders are then chosen in Sections -1 to -3
and results adjusted for the least confounders in Sections -4 to -6. (Those least adjusted results which
actually differ from the most adjusted as marked 'x' in column X in Section -4)
(Results adjusted for an unknown number of confounder(s) are coded as 20.)

Section -7 shows excluded studies, together with the stage (as above) at which no qualifying
results were found.

Section -8 lists the potentially overlapping studies which have been included (1=principal, 2=subsidiary).

Section -9 lists any results which would have been included in preference except that they had data not complete
enough for use in meta-analysis, with their significance (yes/no), if known, and any further comment as entered
on the database.

In addition to those mentioned above, the following fields, levels and abbreviations are used:
* or nk = not known, n = no, y = yes, ot = other
nev = never
REF: 6-character study reference
NRR: number of the RR on the database within the study
ST : study type (CC = case control, pr or prosp = prospective)
NLC: number of lung cancer cases in whole study
R : risky occupational population (n = no, m = mining, o = other risky)
VB : national cigarette type (V = at least 75% Virginia, bl = at least 75% blended, ot = other)
P : any proxy use
H : full histological confirmation
De : derivation of RR/CI (or = original, st = standard method, ot = other method of estimation)

Table 3E12 - 1

IESLC - Meta-analysis of Current Smoking, Cigars only
Adenocarcinoma
Most adjusted

REF	NRR	SEX	AGEL	AGEH	RACE	YF	LC TYPE	LOC	START	ST	NLC	R	VB	P	H	AD	DENOM	De
WYNDE7	29	m	0	0	all	-	KII	NAmer	1977	CC	2085	n	bl	n	y	0	nev any	st

Table 3E12 - 2

IESLC - Meta-analysis of Current Smoking, Cigars only
Adenocarcinoma
Most adjusted

REF	NRR	SEX	AD	Number Exposed Case	Cont	Non-exposed Case	Cont	RR	95.00%CI
WYNDE7	29	m	0	3	82	42	918	0.80	(0.24- 2.64)
Totals				3	82	42	918		

*prospective study

REF	NRR	SEX	AD	Ys	Ws	Qs	Ps
WYNDE7	29	m	0	-0.22	2.70	0.00	0.7134

	N	1
	NS	1
	Wt	2.70
Het	Chi	0.00
Het	df	0
Het	P	N.S.
Fixed	RR	0.80
	RRl	0.24
	RRu	2.64
	P	N.S.
Random	RR	0.80
	RRl	0.24
	RRu	2.64
	P	N.S.
Asymm	P	

Table 3E12 - 3

IESLC - Meta-analysis of Current Smoking, Cigars only
Adenocarcinoma
Most adjusted

	Sex combined	male	female	Total
N		1		1
NS		1		1
Wt		2.70		2.70
Het Chi		0.00		0.00
Het df		0		0
Het P		N.S.		N.S.
Fixed RR		0.80		0.80
RRl		0.24		0.24
RRu		2.64		2.64
P		N.S.		N.S.
Random RR		0.80		0.80
RRl		0.24		0.24
RRu		2.64		2.64
P		N.S.		N.S.
Between Chi				
Between df				
Between P				N.S.
Btwn(F) P				N.S.
Btwn(R) P				N.S.

Too few RRs for analysis by factor

Table 3E12 - 4

IESLC - Meta-analysis of Current Smoking, Cigars only
Adenocarcinoma
Least adjusted

REF	NRR	X	SEX	AGEL	AGEH	RACE	YF	LC TYPE	LOC	START	ST	NLC	R	VB	P	H	AD	DENOM	De
WYNDE7	29		m	0	0	all	-	KII	NAmer	1977	CC	2085	n	bl	n	y	0	nev any	st

Table 3E12 - 5

IESLC - Meta-analysis of Current Smoking, Cigars only
Adenocarcinoma
Least adjusted

REF	NRR	SEX	AD	Number Exposed Case	Number Exposed Cont	Non-exposed Case	Non-exposed Cont	RR	95.00%CI
WYNDE7	29	m	0	3	82	42	918	0.80	(0.24- 2.64)
Totals				3	82	42	918		

*prospective study

REF	NRR	SEX	AD	Ys	Ws	Qs	Ps
WYNDE7	29	m	0	-0.22	2.70	0.00	0.7134

	N	1
	NS	1
	Wt	2.70
Het	Chi	0.00
Het	df	0
Het	P	N.S.
Fixed	RR	0.80
	RRl	0.24
	RRu	2.64
	P	N.S.
Random	RR	0.80
	RRl	0.24
	RRu	2.64
	P	N.S.
Asymm	P	

Table 3E12 - 6

IESLC - Meta-analysis of Current Smoking, Cigars only
Adenocarcinoma
Least adjusted

	Sex combined	male	female	Total
N		1		1
NS		1		1
Wt		2.70		2.70
Het Chi		0.00		0.00
Het df		0		0
Het P		N.S.		N.S.
Fixed RR		0.80		0.80
RRl		0.24		0.24
RRu		2.64		2.64
P		N.S.		N.S.
Random RR		0.80		0.80
RRl		0.24		0.24
RRu		2.64		2.64
P		N.S.		N.S.
Between Chi				
Between df				
Between P				N.S.
Btwn(F) P				N.S.
Btwn(R) P				N.S.

Table 3E12 - 7

IESLC - Meta-analysis of Current Smoking, Cigars only
Adenocarcinoma
Excluded studies (and stage at which they were excluded)

1	BOUCHA BUELL LAURIL MZILEN
2	ABRAHA AGUDO AKIBA ALDERS AMANDU AMES ANDERS ARCHER AUSTIN AUVINE AXELSO AXELSS BAND BARBON BECHER BERRIN BLOHMK BLOT1 BLOT2 BLOT3 BLOT4 BRESLO BRETT BROCKM BROSS BROWN1 BROWN2 BUFFLE BYERS1 BYERS2 CARPEN CASCO2 CASCOR CHAN CHANG CHATZI CHEN CHEN2 CHEN3 CHIAZZ CHOI CHOW CHYOU COMSTO COOKSO CORREA DARBY DAVEYS DEAN DEAN2 DEKLER DESTE2 DESTEF DOCKER DOLL DOLL2 DORANT DORGAN DOSEME DROSTE DU DUNN EBELIN ENGELA ENSTRO ESAKI FAN GAO GAO2 GARCIA GARSHI GENG GER GILLIS GODLEY GOLLED GOODMA GRAHAM GREGOR GSELL HAENSZ HAMMO2 HANSEN HEGMAN HENNEK HINDS HIRAOK HIRAY2 HIRAYA HITOSU HOLE HOROWI HORWIT HU HU2 HUANG HUMBLE ISHIMA JAHN JAIN JARUP JARVHO JEDRYC JIANG JONES JUSSAW KAISE2 KAISER KANELL KATSOU KAUFMA KELLER KHUDER KIHARA KINLEN KJUUS KNEKT KO KOHLME KOO KOULUM KREUZE KREYBE KUBIK LAMTH LAMWK LAMWK2 LAUSSM LEI LEMARC LETOUR LIAW LICKIN LIDDEL LIU LIU2 LIU3 LIU4 LIU5 LOMBA2 LOMBAR LUBIN LUO MACLEN MAGNUS MARSH MARSH2 MARTIS MASTRA MATOS MATSUD MCCONN MCDUFF MCLAUG MIGRAN MILLER MILLS MOLLO MRFIT MRFITR MURATA NAM NOTAN2 NOTANI NOU ODRISC ORMOS OSANN OSANN2 PARKIN PASTOR PAWLEG PERNU PERSH2 PERSHA PETO PEZZO2 PEZZOT PIKE PISANI POFFIJ POLEDN PRESCO QIAO QIAO2 RACHTA RADZIK RANDIG REN RESTRE RIMING RONCO ROOTS ROTHSC SAARIK SANKAR SCHWA2 SCHWAR SEGI SEGI2 SEOW SHAW SHIMIZ SIEMIA SIMARA SIMONA SITAS SOBUE SOBUE2 SPEIZE SPITZ STASZE STAYNE STOCKS STOCKW STUCKE SUN SUZUK2 SUZUKI SVENSS TANG TANG2 TAO TENKAN TOKARS TOUSEY TSUGAN TVERDA ULMER VANDER VEIERO VUTUC WAKAI WALD WANG WANG2 WANG3 WANG4 WARSIN WATSON WICKLU WIGLE WILKIN WU WU2 WUNSCH WUWILL WYNDE2 WYNDE3 WYNDE4 WYNDE5 WYNDE6 WYNDE8 XIANGZ XU XU2 XU3 XU4 YAMAGU YONG YUAN ZHANG ZHENG ZHOU
3	ABELIN ARMADA BENHAM BEST BOUCOT DAMBER HAMMON LEVIN SADOWS TIZZAN
5	WYNDER
7	BENSHL BOFFET CEDERL CPSI CPSII DEAN3 DORN GARDIN HEIN JOLY LANGE LUBIN2 TULINI

Table 3E12 - 8
Potentially overlapping studies

REF	REFGP	PRINC	.	OVERLAP/LINK
WYNDE7	WYNDE6	2		WYNDE5/6/7/8

Table 3E13 -

IESLC - Meta-analysis of Ever Smoking (or Current if Ever not available), Cigars only
Adenocarcinoma

This analysis is restricted to results for:
1) Non-dose-response data
2) Smokers of cigars only
3) Results complete enough for use in metaanalysis

Within each study, results are then selected (in the following order of preference, within each sex) for:
4) SMKSTA: ever smokers, current smokers
5) DENOM: never smoked anything, (never +1 = +long term ex)
6) Followup period (prospective studies): whole study (coded as 0) or longest available
7) LCtype: all or nearest available, at least Squamous and Adeno. (q = squamous, s = small,
l = large, a = adeno, mix = mixed, alv = alveolar)
8) Race: all or nearest available, otherwise by race (wh or w = white, bl or b = black, hi = hispanic
ch = chinese, jap = japanese, haw = hawaiian, w+o = white + oriental, sca = scandinavian, as = asian)
9) For overlapping studies: principal rather than subsidiary studies
Finally by Age: whole study (coded as 0) if available, otherwise by widest available age group
and then for single sex results (m, f) in preference to combined sex results (c).

Results adjusted (AD) for the most potential confounders are then chosen in Sections -1 to -3
and results adjusted for the least confounders in Sections -4 to -6. (Those least adjusted results which
actually differ from the most adjusted as marked 'x' in column X in Section -4)
(Results adjusted for an unknown number of confounder(s) are coded as 20.)

Section -7 shows excluded studies, together with the stage (as above) at which no qualifying
results were found.

Section -8 lists the potentially overlapping studies which have been included (1=principal, 2=subsidiary).

Section -9 lists any results which would have been included in preference except that they had data not complete
enough for use in meta-analysis, with their significance (yes/no), if known, and any further comment as entered
on the database.

In addition to those mentioned above, the following fields, levels and abbreviations are used:
* or nk = not known, n = no, y = yes, ot = other
ev = ever, cu = current, nev = never
REF: 6-character study reference
NRR: number of the RR on the database within the study
ST : study type (CC = case control, pr or prosp = prospective)
NLC: number of lung cancer cases in whole study
R : risky occupational population (n = no, m = mining, o = other risky)
VB : national cigarette type (V = at least 75% Virginia, bl = at least 75% blended, ot = other)
P : any proxy use
H : full histological confirmation
De : derivation of RR/CI (or = original, st = standard method, ot = other method of estimation)

Table 3E13 - 1

IESLC - Meta-analysis of Ever Smoking (or Current if Ever not available), Cigars only
Adenocarcinoma
Most adjusted

REF	NRR	SEX	AGEL	AGEH	RACE	YF	LC TYPE	LOC	START	ST	NLC	R	VB	P	H	AD	SM	DENOM		De
HAMMON	80	m	0	0	wh	0	a	NAmer	1952	pr	448	n	bl	n	n	1	ev	nev	any	ot
LUBIN2	136	m	0	0	all	-	a	Eu:mul	1976	CC	7804	n	bl	n	y	0	ev	nev	any	st
WYNDE7	61	m	0	0	all	-	KII	NAmer	1977	CC	2085	n	bl	n	y	0	ev	nev	any	st

Table 3E13 - 2

IESLC - Meta-analysis of Ever Smoking (or Current if Ever not available), Cigars only
Adenocarcinoma
Most adjusted

REF	NRR	SEX	AD	Number Exposed Case	Cont	Non-exposed Case	Cont	RR	95.00%CI
*HAMMON	80	m	1	-	-	-	-	0.94	(0.09- 10.42)
LUBIN2	136	m	0	1	145	195	2616	0.09	(0.01- 0.66)
WYNDE7	61	m	0	9	152	42	918	1.29	(0.62- 2.71)
Partial Totals				10	297	237	3534		

*prospective study

REF	NRR	SEX	AD	Ys	Ws	Qs	Ps
*HAMMON	80	m	1	-0.06	0.68	0.00	0.9593
LUBIN2	136	m	0	-2.38	0.99	5.28	0.0180
WYNDE7	61	m	0	0.26	7.01	0.74	0.4947

	N	3
	NS	3
	Wt	8.68
Het	Chi	6.03
Het	df	2
Het	P	*
Fixed	RR	0.93
	RRl	0.48
	RRu	1.82
	P	N.S.
Random	RR	0.55
	RRl	0.11
	RRu	2.88
	P	N.S.
Asymm	P	N.S.

Table 3E13 - 3

IESLC - Meta-analysis of Ever Smoking (or Current if Ever not available), Cigars only
Adenocarcinoma
Most adjusted

	Sex combined	male	female	Total
N		3		3
NS		3		3
Wt		8.68		8.68
Het Chi		6.03		6.03
Het df		2		2
Het P		*		*
Fixed RR		0.93		0.93
RRl		0.48		0.48
RRu		1.82		1.82
P		N.S.		N.S.
Random RR		0.55		0.55
RRl		0.11		0.11
RRu		2.88		2.88
P		N.S.		N.S.
Between Chi				
Between df				
Between P				N.S.
Btwn(F) P				N.S.
Btwn(R) P				N.S.

Too few RRs for analysis by factor

Table 3E13 - 4

IESLC - Meta-analysis of Ever Smoking (or Current if Ever not available), Cigars only
Adenocarcinoma
Least adjusted

REF	NRR	X	SEX	AGEL	AGEH	RACE	YF	LC TYPE	LOC	START	ST	NLC	R	VB	P	H	AD	SM	DENOM	De
HAMMON	86	x	m	0	0	wh	0	a	NAmer	1952	pr	448	n	bl	n	n	0	ev	nev any	st
LUBIN2	136		m	0	0	all	-	a	Eu:mul	1976	CC	7804	n	bl	n	y	0	ev	nev any	st
WYNDE7	61		m	0	0	all	-	KII	NAmer	1977	CC	2085	n	bl	n	y	0	ev	nev any	st

Table 3E13 - 5

IESLC - Meta-analysis of Ever Smoking (or Current if Ever not available), Cigars only
Adenocarcinoma
Least adjusted

REF	NRR	SEX	AD	Number Exposed Case	Cont	Non-exposed Case	Cont	RR	95.00%CI
*HAMMON	86	m	0	1	51480	2	115884	1.13	(0.10- 12.41)
LUBIN2	136	m	0	1	145	195	2616	0.09	(0.01- 0.66)
WYNDE7	61	m	0	9	152	42	918	1.29	(0.62- 2.71)
Totals				11	51777	239	119418		

*prospective study

REF	NRR	SEX	AD	Ys	Ws	Qs	Ps
*HAMMON	86	m	0	0.12	0.67	0.02	0.9231
LUBIN2	136	m	0	-2.38	0.99	5.35	0.0180
WYNDE7	61	m	0	0.26	7.01	0.68	0.4947

	N	3
	NS	3
	Wt	8.67
Het	Chi	6.05
Het	df	2
Het	P	*
Fixed	RR	0.95
	RRl	0.49
	RRu	1.84
	P	N.S.
Random	RR	0.57
	RRl	0.11
	RRu	3.03
	P	N.S.
Asymm	P	N.S.

Table 3E13 - 6

IESLC - Meta-analysis of Ever Smoking (or Current if Ever not available), Cigars only
Adenocarcinoma
Least adjusted

	Sex combined	male	female	Total
N		3		3
NS		3		3
Wt		8.67		8.67
Het Chi		6.05		6.05
Het df		2		2
Het P		*		*
Fixed RR		0.95		0.95
RRl		0.49		0.49
RRu		1.84		1.84
P		N.S.		N.S.
Random RR		0.57		0.57
RRl		0.11		0.11
RRu		3.03		3.03
P		N.S.		N.S.
Between Chi				
Between df				
Between P				N.S.
Btwn(F) P				N.S.
Btwn(R) P				N.S.

Table 3E13 - 7

IESLC - Meta-analysis of Ever Smoking (or Current if Ever not available), Cigars only
Adenocarcinoma
Excluded studies (and stage at which they were excluded)

1 BOUCHA BUELL LAURIL MZILEN

2 ABRAHA AGUDO AKIBA ALDERS AMANDU AMES ANDERS ARCHER AUSTIN AUVINE AXELSO AXELSS BAND BARBON BECHER BERRIN
BLOHMK BLOT1 BLOT2 BLOT3 BLOT4 BRESLO BRETT BROCKM BROSS BROWN1 BROWN2 BUFFLE BYERS1 BYERS2 CARPEN CASCO2
CASCOR CHAN CHANG CHATZI CHEN CHEN2 CHEN3 CHIAZZ CHOI CHOW CHYOU COMSTO COOKSO CORREA DARBY DAVEYS
DEAN DEAN2 DEKLER DESTE2 DESTEF DOCKER DOLL DOLL2 DORANT DORGAN DOSEME DROSTE DU DUNN EBELIN ENGELA
ENSTRO ESAKI FAN GAO GAO2 GARCIA GARSHI GENG GER GILLIS GODLEY GOLLED GOODMA GRAHAM GREGOR GSELL
HAENSZ HAMMO2 HANSEN HEGMAN HENNEK HINDS HIRAOK HIRAY2 HIRAYA HITOSU HOLE HOROWI HORWIT HU HU2 HUANG
HUMBLE ISHIMA JAHN JAIN JARUP JARVHO JEDRYC JIANG JONES JUSSAW KAISE2 KAISER KANELL KATSOU KAUFMA KELLER
KHUDER KIHARA KINLEN KJUUS KNEKT KO KOHLME KOO KOULUM KREUZE KREYBE KUBIK LAMTH LAMWK LAMWK2 LAUSSM
LEI LEMARC LETOUR LIAW LICKIN LIDDEL LIU LIU2 LIU3 LIU4 LIU5 LOMBA2 LOMBAR LUBIN LUO MACLEN
MAGNUS MARSH MARSH2 MARTIS MASTRA MATOS MATSUD MCCONN MCDUFF MCLAUG MIGRAN MILLER MILLS MOLLO MRFIT MRFITR
MURATA NAM NOTAN2 NOTANI NOU ODRISC ORMOS OSANN OSANN2 PARKIN PASTOR PAWLEG PERNU PERSH2 PERSHA PETO
PEZZO2 PEZZOT PIKE PISANI POFFIJ POLEDN PRESCO QIAO QIAO2 RACHTA RADZIK RANDIG REN RESTRE RIMING RONCO
ROOTS ROTHSC SAARIK SANKAR SCHWA2 SCHWAR SEGI SEGI2 SEOW SHAW SHIMIZ SIEMIA SIMARA SIMONA SITAS SOBUE
SOBUE2 SPEIZE SPITZ STASZE STAYNE STOCKS STOCKW STUCKE SUN SUZUK2 SUZUKI SVENSS TANG TANG2 TAO TENKAN
TOKARS TOUSEY TSUGAN TVERDA ULMER VANDER VEIERO VUTUC WAKAI WALD WANG WANG2 WANG3 WANG4 WARSIN WATSON
WICKLU WIGLE WILKIN WU WU2 WUNSCH WUWILL WYNDE2 WYNDE3 WYNDE4 WYNDE5 WYNDE6 WYNDE8 XIANGZ XU XU2
XU3 XU4 YAMAGU YONG YUAN ZHANG ZHENG ZHOU

5 WYNDER

7 ABELIN ARMADA BENHAM BENSHL BEST BOFFET BOUCOT CEDERL CPSI CPSII DAMBER DEAN3 DORN GARDIN HEIN JOLY
LANGE LEVIN SADOWS TIZZAN TULINI

Table 3E13 - 8

Potentially overlapping studies

REF	REFGP	PRINC	. OVERLAP/LINK
LUBIN2	LUBIN2	1	Lubin-combined
WYNDE7	WYNDE6	2	WYNDE5/6/7/8

Table 3E14 -

IESLC - Meta-analysis of Current Smoking (or Ever if Current not available), Cigars only
Adenocarcinoma

This analysis is restricted to results for:
1) Non-dose-response data
2) Smokers of cigars only
3) Results complete enough for use in metaanalysis

Within each study, results are then selected (in the following order of preference, within each sex) for:
4) SMKSTA: current smokers, ever smokers
5) DENOM: never smoked anything, (never +1 = +long term ex)
6) Followup period (prospective studies): whole study (coded as 0) or longest available
7) LCtype: all or nearest available, at least Squamous and Adeno. (q = squamous, s = small,
l = large, a = adeno, mix = mixed, alv = alveolar)
8) Race: all or nearest available, otherwise by race (wh or w = white, bl or b = black, hi = hispanic
ch = chinese, jap = japanese, haw = hawaiian, w+o = white + oriental, sca = scandinavian, as = asian)
9) For overlapping studies: principal rather than subsidiary studies
Finally by Age: whole study (coded as 0) if available, otherwise by widest available age group
and then for single sex results (m, f) in preference to combined sex results (c).

Results adjusted (AD) for the most potential confounders are then chosen in Sections -1 to -3
(and those which actually differ from the adjusted results in Table 3E13 - 1 are marked 'x' in Section -1)
and results adjusted for the least confounders in Sections -4 to -6. (Those least adjusted results which
actually differ from the most adjusted as marked 'x' in column X in Section -4)
(Results adjusted for an unknown number of confounder(s) are coded as 20.)

Section -7 shows excluded studies, together with the stage (as above) at which no qualifying
results were found.

Section -8 lists the potentially overlapping studies which have been included (1=principal, 2=subsidiary).

Section -9 lists any results which would have been included in preference except that they had data not complete
enough for use in meta-analysis, with their significance (yes/no), if known, and any further comment as entered
on the database.

In addition to those mentioned above, the following fields, levels and abbreviations are used:
* or nk = not known, n = no, y = yes, ot = other
ev = ever, cu = current, nev = never
REF: 6-character study reference
NRR: number of the RR on the database within the study
ST : study type (CC = case control, pr or prosp = prospective)
NLC: number of lung cancer cases in whole study
R : risky occupational population (n = no, m = mining, o = other risky)
VB : national cigarette type (V = at least 75% Virginia, bl = at least 75% blended, ot = other)
P : any proxy use
H : full histological confirmation
De : derivation of RR/CI (or = original, st = standard method, ot = other method of estimation)

Table 3E14 - 1

IESLC - Meta-analysis of Current Smoking (or Ever if Current not available), Cigars only
Adenocarcinoma
Most adjusted

REF	NRR	3E13	SEX	AGEL	AGEH	RACE	YF	LC TYPE	LOC	START	ST	NLC	R	VB	P	H	AD	SM	DENOM	De
HAMMON	80		m	0	0	wh	0	a	NAmer	1952	pr	448	n	bl	n	n	1	ev	nev any	ot
LUBIN2	136		m	0	0	all	-	a	Eu:mul	1976	CC	7804	n	bl	n	y	0	ev	nev any	st
WYNDE7	29	x	m	0	0	all	-	KII	NAmer	1977	CC	2085	n	bl	n	y	0	cu	nev any	st

Table 3E14 - 2

IESLC - Meta-analysis of Current Smoking (or Ever if Current not available), Cigars only
Adenocarcinoma
Most adjusted

REF	NRR	SEX	AD	Number Exposed Case	Number Exposed Cont	Non-exposed Case	Non-exposed Cont	RR	95.00%CI
*HAMMON	80	m	1	-	-	-	-	0.94	(0.09- 10.42)
LUBIN2	136	m	0	1	145	195	2616	0.09	(0.01- 0.66)
WYNDE7	29	m	0	3	82	42	918	0.80	(0.24- 2.64)
Partial Totals				4	227	237	3534		

*prospective study

REF	NRR	SEX	AD	Ys	Ws	Qs	Ps
*HAMMON	80	m	1	-0.06	0.68	0.27	0.9593
LUBIN2	136	m	0	-2.38	0.99	2.84	0.0180
WYNDE7	29	m	0	-0.22	2.70	0.58	0.7134

	N	3
	NS	3
	Wt	4.37
Het	Chi	3.68
Het	df	2
Het	P	N.S.
Fixed	RR	0.50
	RRl	0.20
	RRu	1.29
	P	N.S.
Random	RR	0.44
	RRl	0.11
	RRu	1.78
	P	N.S.
Asymm	P	N.S.

Table 3E14 - 3

IESLC - Meta-analysis of Current Smoking (or Ever if Current not available), Cigars only
Adenocarcinoma
Most adjusted

	Sex combined	male	female	Total
N		3		3
NS		3		3
Wt		4.37		4.37
Het Chi		3.68		3.68
Het df		2		2
Het P		N.S.		N.S.
Fixed RR		0.50		0.50
RRl		0.20		0.20
RRu		1.29		1.29
P		N.S.		N.S.
Random RR		0.44		0.44
RRl		0.11		0.11
RRu		1.78		1.78
P		N.S.		N.S.
Between Chi				
Between df				
Between P				N.S.
Btwn(F) P				N.S.
Btwn(R) P				N.S.

Too few RRs for analysis by factor

Table 3E14 - 4

IESLC - Meta-analysis of Current Smoking (or Ever if Current not available), Cigars only
Adenocarcinoma
Least adjusted

REF	NRR	X	SEX	AGEL	AGEH	RACE	YF	LC TYPE	LOC	START	ST	NLC	R	VB	P	H	AD	SM	DENOM	De
HAMMON	86	x	m	0	0	wh	0	a	NAmer	1952	pr	448	n	bl	n	n	0	ev	nev any	st
LUBIN2	136		m	0	0	all	-	a	Eu:mul	1976	CC	7804	n	bl	n	y	0	ev	nev any	st
WYNDE7	29		m	0	0	all	-	KII	NAmer	1977	CC	2085	n	bl	n	y	0	cu	nev any	st

Table 3E14 - 5

IESLC - Meta-analysis of Current Smoking (or Ever if Current not available), Cigars only
Adenocarcinoma
Least adjusted

REF	NRR	SEX	AD	Number Exposed Case	Cont	Non-exposed Case	Cont	RR	95.00%CI
*HAMMON	86	m	0	1	51480	2	115884	1.13	(0.10- 12.41)
LUBIN2	136	m	0	1	145	195	2616	0.09	(0.01- 0.66)
WYNDE7	29	m	0	3	82	42	918	0.80	(0.24- 2.64)
Totals				5	51707	239	119418		

*prospective study

REF	NRR	SEX	AD	Ys	Ws	Qs	Ps
*HAMMON	86	m	0	0.12	0.67	0.40	0.9231
LUBIN2	136	m	0	-2.38	0.99	2.92	0.0180
WYNDE7	29	m	0	-0.22	2.70	0.52	0.7134

	N	3
	NS	3
	Wt	4.35
Het	Chi	3.84
Het	df	2
Het	P	N.S.
Fixed	RR	0.52
	RRl	0.20
	RRu	1.32
	P	N.S.
Random	RR	0.45
	RRl	0.11
	RRu	1.91
	P	N.S.
Asymm	P	N.S.

Table 3E14 - 6

IESLC - Meta-analysis of Current Smoking (or Ever if Current not available), Cigars only
Adenocarcinoma
Least adjusted

	Sex combined	male	female	Total
N		3		3
NS		3		3
Wt		4.35		4.35
Het Chi		3.84		3.84
Het df		2		2
Het P		N.S.		N.S.
Fixed RR		0.52		0.52
RRl		0.20		0.20
RRu		1.32		1.32
P		N.S.		N.S.
Random RR		0.45		0.45
RRl		0.11		0.11
RRu		1.91		1.91
P		N.S.		N.S.
Between Chi				
Between df				
Between P				N.S.
Btwn(F) P				N.S.
Btwn(R) P				N.S.

Table 3E14 - 7

IESLC - Meta-analysis of Current Smoking (or Ever if Current not available), Cigars only
Adenocarcinoma
Excluded studies (and stage at which they were excluded)

1 BOUCHA BUELL LAURIL MZILEN

2 ABRAHA AGUDO AKIBA ALDERS AMANDU AMES ANDERS ARCHER AUSTIN AUVINE AXELSO AXELSS BAND BARBON BECHER BERRIN
BLOHMK BLOT1 BLOT2 BLOT3 BLOT4 BRESLO BRETT BROCKM BROSS BROWN1 BROWN2 BUFFLE BYERS1 BYERS2 CARPEN CASCO2
CASCOR CHAN CHANG CHATZI CHEN CHEN2 CHEN3 CHIAZZ CHOI CHOW CHYOU COMSTO COOKSO CORREA DARBY DAVEYS
DEAN DEAN2 DEKLER DESTE2 DESTEF DOCKER DOLL DOLL2 DORANT DORGAN DOSEME DROSTE DU DUNN EBELIN ENGELA
ENSTRO ESAKI FAN GAO GAO2 GARCIA GARSHI GENG GER GILLIS GODLEY GOLLED GOODMA GRAHAM GREGOR GSELL
HAENSZ HAMMO2 HANSEN HEGMAN HENNEK HINDS HIRAOK HIRAY2 HIRAYA HITOSU HOLE HOROWI HORWIT HU HU2 HUANG
HUMBLE ISHIMA JAHN JAIN JARUP JARVHO JEDRYC JIANG JONES JUSSAW KAISE2 KAISER KANELL KATSOU KAUFMA KELLER
KHUDER KIHARA KINLEN KJUUS KNEKT KO KOHLME KOO KOULUM KREUZE KREYBE KUBIK LAMTH LAMWK LAMWK2 LAUSSM
LEI LEMARC LETOUR LIAW LICKIN LIDDEL LIU LIU2 LIU3 LIU4 LIU5 LOMBA2 LOMBAR LUBIN LUO MACLEN
MAGNUS MARSH MARSH2 MARTIS MASTRA MATOS MATSUD MCCONN MCDUFF MCLAUG MIGRAN MILLER MILLS MOLLO MRFIT MRFITR
MURATA NAM NOTAN2 NOTANI NOU ODRISC ORMOS OSANN OSANN2 PARKIN PASTOR PAWLEG PERNU PERSH2 PERSHA PETO
PEZZO2 PEZZOT PIKE PISANI POFFIJ POLEDN PRESCO QIAO QIAO2 RACHTA RADZIK RANDIG REN RESTRE RIMING RONCO
ROOTS ROTHSC SAARIK SANKAR SCHWA2 SCHWAR SEGI SEGI2 SEOW SHAW SHIMIZ SIEMIA SIMARA SIMONA SITAS SOBUE
SOBUE2 SPEIZE SPITZ STASZE STAYNE STOCKS STOCKW STUCKE SUN SUZUK2 SUZUKI SVENSS TANG TANG2 TAO TENKAN
TOKARS TOUSEY TSUGAN TVERDA ULMER VANDER VEIERO VUTUC WAKAI WALD WANG WANG2 WANG3 WANG4 WARSIN WATSON
WICKLU WIGLE WILKIN WU WU2 WUNSCH WUWILL WYNDE2 WYNDE3 WYNDE4 WYNDE5 WYNDE6 WYNDE8 XIANGZ XU XU2
XU3 XU4 YAMAGU YONG YUAN ZHANG ZHENG ZHOU

5 WYNDER

7 ABELIN ARMADA BENHAM BENSHL BEST BOFFET BOUCOT CEDERL CPSI CPSII DAMBER DEAN3 DORN GARDIN HEIN JOLY
LANGE LEVIN SADOWS TIZZAN TULINI

Table 3E14 - 8

Potentially overlapping studies

REF	REFGP	PRINC	. OVERLAP/LINK
LUBIN2	LUBIN2	1	Lubin-combined
WYNDE7	WYNDE6	2	WYNDE5/6/7/8

Table 3E15 -

IESLC - Meta-analysis of Ex Smoking, Cigars only
Adenocarcinoma

This analysis is restricted to results for:
1) Non-dose-response data
2) Smokers of cigars only
3) Ex smokers
4) Results complete enough for use in metaanalysis

Within each study, results are then selected (in the following order of preference, within each sex) for:
5) DENOM: never smoked anything, (never +1 = +long term ex)
6) Followup period (prospective studies): whole study (coded as 0) or longest available
7) LCtype: all or nearest available, at least Squamous and Adeno. (q = squamous, s = small,
l = large, a = adeno, mix = mixed, alv = alveolar)
8) Race: all or nearest available, otherwise by race (wh or w = white, bl or b = black, hi = hispanic
ch = chinese, jap = japanese, haw = hawaiian, w+o = white + oriental, sca = scandinavian, as = asian)
9) For overlapping studies: principal rather than subsidiary studies
Finally by Age: whole study (coded as 0) if available, otherwise by widest available age group
and then for single sex results (m, f) in preference to combined sex results (c).

Results adjusted (AD) for the most potential confounders are then chosen in Sections -1 to -3
and results adjusted for the least confounders in Sections -4 to -6. (Those least adjusted results which
actually differ from the most adjusted as marked 'x' in column X in Section -4)
(Results adjusted for an unknown number of confounder(s) are coded as 20.)

Section -7 shows excluded studies, together with the stage (as above) at which no qualifying
results were found.

Section -8 lists the potentially overlapping studies which have been included (1=principal, 2=subsidiary).

Section -9 lists any results which would have been included in preference except that they had data not complete
enough for use in meta-analysis, with their significance (yes/no), if known, and any further comment as entered
on the database.

In addition to those mentioned above, the following fields, levels and abbreviations are used:
* or nk = not known, n = no, y = yes, ot = other
nev = never
REF: 6-character study reference
NRR: number of the RR on the database within the study
ST : study type (CC = case control, pr or prosp = prospective)
NLC: number of lung cancer cases in whole study
R : risky occupational population (n = no, m = mining, o = other risky)
VB : national cigarette type (V = at least 75% Virginia, bl = at least 75% blended, ot = other)
P : any proxy use
H : full histological confirmation
De : derivation of RR/CI (or = original, st = standard method, ot = other method of estimation)

Table 3E15 - 1

IESLC - Meta-analysis of Ex Smoking, Cigars only
Adenocarcinoma
Most adjusted

REF	NRR	SEX	AGEL	AGEH	RACE	YF	LC TYPE	LOC	START	ST	NLC	R	VB	P	H	AD	DENOM	De
WYNDE7	34	m	0	0	all	-	KII	NAmer	1977	CC	2085	n	bl	n	y	0	nev any	st

Table 3E15 - 2

IESLC - Meta-analysis of Ex Smoking, Cigars only
Adenocarcinoma
Most adjusted

REF	NRR	SEX	AD	Number Case	Exposed Cont	Non-exposed Case	Cont	RR	95.00%CI
WYNDE7	34	m	0	6	70	42	918	1.87	(0.77- 4.56)
Totals				6	70	42	918		

*prospective study

REF	NRR	SEX	AD	Ys	Ws	Qs	Ps
WYNDE7	34	m	0	0.63	4.86	0.00	0.1665

	N	1
	NS	1
	Wt	4.86
Het	Chi	0.00
Het	df	0
Het	P	N.S.
Fixed	RR	1.87
	RRl	0.77
	RRu	4.56
	P	N.S.
Random	RR	1.87
	RRl	0.77
	RRu	4.56
	P	N.S.
Asymm	P	

Table 3E15 - 3

IESLC - Meta-analysis of Ex Smoking, Cigars only
Adenocarcinoma
Most adjusted

	Sex combined	male	female	Total
N		1		1
NS		1		1
Wt		4.86		4.86
Het Chi		0.00		0.00
Het df		0		0
Het P		N.S.		N.S.
Fixed RR		1.87		1.87
RRl		0.77		0.77
RRu		4.56		4.56
P		N.S.		N.S.
Random RR		1.87		1.87
RRl		0.77		0.77
RRu		4.56		4.56
P		N.S.		N.S.
Between Chi				
Between df				
Between P				N.S.
Btwn(F) P				N.S.
Btwn(R) P				N.S.

Too few RRs for analysis by factor

Table 3E15 - 4

IESLC - Meta-analysis of Ex Smoking, Cigars only
Adenocarcinoma
Least adjusted

REF	NRR	X	SEX	AGEL	AGEH	RACE	YF	LC TYPE	LOC	START	ST	NLC	R	VB	P	H	AD	DENOM	De
WYNDE7	34		m	0	0	all	-	KII	NAmer	1977	CC	2085	n	bl	n	y	0	nev any	st

Table 3E15 - 5

IESLC - Meta-analysis of Ex Smoking, Cigars only
Adenocarcinoma
Least adjusted

REF	NRR	SEX	AD	Number Exposed Case	Cont	Non-exposed Case	Cont	RR	95.00%CI
WYNDE7	34	m	0	6	70	42	918	1.87	(0.77- 4.56)
Totals				6	70	42	918		

*prospective study

REF	NRR	SEX	AD	Ys	Ws	Qs	Ps
WYNDE7	34	m	0	0.63	4.86	0.00	0.1665

	N	1
	NS	1
	Wt	4.86
Het	Chi	0.00
Het	df	0
Het	P	N.S.
Fixed	RR	1.87
	RRl	0.77
	RRu	4.56
	P	N.S.
Random	RR	1.87
	RRl	0.77
	RRu	4.56
	P	N.S.
Asymm	P	

Table 3E15 - 6

IESLC - Meta-analysis of Ex Smoking, Cigars only
Adenocarcinoma
Least adjusted

	Sex combined	male	female	Total
N		1		1
NS		1		1
Wt		4.86		4.86
Het Chi		0.00		0.00
Het df		0		0
Het P		N.S.		N.S.
Fixed RR		1.87		1.87
RRl		0.77		0.77
RRu		4.56		4.56
P		N.S.		N.S.
Random RR		1.87		1.87
RRl		0.77		0.77
RRu		4.56		4.56
P		N.S.		N.S.
Between Chi				
Between df				
Between P				N.S.
Btwn(F) P				N.S.
Btwn(R) P				N.S.

Table 3E15 - 7

IESLC - Meta-analysis of Ex Smoking, Cigars only
Adenocarcinoma
Excluded studies (and stage at which they were excluded)

1	BOUCHA BUELL LAURIL MZILEN
2	ABRAHA AGUDO AKIBA ALDERS AMANDU AMES ANDERS ARCHER AUSTIN AUVINE AXELSO AXELSS BAND BARBON BECHER BERRIN BLOHMK BLOT1 BLOT2 BLOT3 BLOT4 BRESLO BRETT BROCKM BROSS BROWN1 BROWN2 BUFFLE BYERS1 BYERS2 CARPEN CASCO2 CASCOR CHAN CHANG CHATZI CHEN CHEN2 CHEN3 CHIAZZ CHOI CHOW CHYOU COMSTO COOKSO CORREA DARBY DAVEYS DEAN DEAN2 DEKLER DESTE2 DESTEF DOCKER DOLL DOLL2 DORANT DORGAN DOSEME DROSTE DU DUNN EBELIN ENGELA ENSTRO ESAKI FAN GAO GAO2 GARCIA GARSHI GENG GER GILLIS GODLEY GOLLED GOODMA GRAHAM GREGOR GSELL HAENSZ HAMMO2 HANSEN HEGMAN HENNEK HINDS HIRAOK HIRAY2 HIRAYA HITOSU HOLE HOROWI HORWIT HU HU2 HUANG HUMBLE ISHIMA JAHN JAIN JARUP JARVHO JEDRYC JIANG JONES JUSSAW KAISE2 KAISER KANELL KATSOU KAUFMA KELLER KHUDER KIHARA KINLEN KJUUS KNEKT KO KOHLME KOO KOULUM KREUZE KREYBE KUBIK LAMTH LAMWK LAMWK2 LAUSSM LEI LEMARC LETOUR LIAW LICKIN LIDDEL LIU LIU2 LIU3 LIU4 LIU5 LOMBA2 LOMBAR LUBIN LUO MACLEN MAGNUS MARSH MARSH2 MARTIS MASTRA MATOS MATSUD MCCONN MCDUFF MCLAUG MIGRAN MILLER MILLS MOLLO MRFIT MRFITR MURATA NAM NOTAN2 NOTANI NOU ODRISC ORMOS OSANN OSANN2 PARKIN PASTOR PAWLEG PERNU PERSH2 PERSHA PETO PEZZO2 PEZZOT PIKE PISANI POFFIJ POLEDN PRESCO QIAO QIAO2 RACHTA RADZIK RANDIG REN RESTRE RIMING RONCO ROOTS ROTHSC SAARIK SANKAR SCHWA2 SCHWAR SEGI SEGI2 SEOW SHAW SHIMIZ SIEMIA SIMARA SIMONA SITAS SOBUE SOBUE2 SPEIZE SPITZ STASZE STAYNE STOCKS STOCKW STUCKE SUN SUZUK2 SUZUKI SVENSS TANG TANG2 TAO TENKAN TOKARS TOUSEY TSUGAN TVERDA ULMER VANDER VEIERO VUTUC WAKAI WALD WANG WANG2 WANG3 WANG4 WARSIN WATSON WICKLU WIGLE WILKIN WU WU2 WUNSCH WUWILL WYNDE2 WYNDE3 WYNDE4 WYNDE5 WYNDE6 WYNDE8 XIANGZ XU XU2 XU3 XU4 YAMAGU YONG YUAN ZHANG ZHENG ZHOU
3	ABELIN ARMADA BENHAM BENSHL BEST BOUCOT CEDERL CPSI CPSII DAMBER DEAN3 GARDIN HAMMON HEIN LANGE LEVIN SADOWS TIZZAN TULINI WYNDER
7	BOFFET DORN JOLY LUBIN2

Table 3E15 - 8
Potentially overlapping studies

REF	REFGP	PRINC	.	OVERLAP/LINK
WYNDE7	WYNDE6	2		WYNDE5/6/7/8

Table 3E16 -

IESLC - Meta-analysis of Ever Smoking, Mixed smokers
Adenocarcinoma

This analysis is restricted to results for:
1) Non-dose-response data
2) Mixed smokers (cigarettes and pipe/cigar)
3) Ever smokers
4) Results complete enough for use in metaanalysis

Within each study, results are then selected (in the following order of preference, within each sex) for:
5) DENOM: never smoked anything, (never +1 = +long term ex)
6) Followup period (prospective studies): whole study (coded as 0) or longest available
7) LCtype: all or nearest available, at least Squamous and Adeno. (q = squamous, s = small,
l = large, a = adeno, mix = mixed, alv = alveolar)
8) Race: all or nearest available, otherwise by race (wh or w = white, bl or b = black, hi = hispanic
ch = chinese, jap = japanese, haw = hawaiian, w+o = white + oriental, sca = scandinavian, as = asian)
9) For overlapping studies: principal rather than subsidiary studies
Finally by Age: whole study (coded as 0) if available, otherwise by widest available age group
and then for single sex results (m, f) in preference to combined sex results (c).

Results adjusted (AD) for the most potential confounders are then chosen in Sections -1 to -3
and results adjusted for the least confounders in Sections -4 to -6. (Those least adjusted results which
actually differ from the most adjusted as marked 'x' in column X in Section -4)
(Results adjusted for an unknown number of confounder(s) are coded as 20.)

Section -7 shows excluded studies, together with the stage (as above) at which no qualifying
results were found.

Section -8 lists the potentially overlapping studies which have been included (1=principal, 2=subsidiary).

Section -9 lists any results which would have been included in preference except that they had data not complete
enough for use in meta-analysis, with their significance (yes/no), if known, and any further comment as entered
on the database.

In addition to those mentioned above, the following fields, levels and abbreviations are used:
* or nk = not known, n = no, y = yes, ot = other
nev = never
REF: 6-character study reference
NRR: number of the RR on the database within the study
ST : study type (CC = case control, pr or prosp = prospective)
NLC: number of lung cancer cases in whole study
R : risky occupational population (n = no, m = mining, o = other risky)
VB : national cigarette type (V = at least 75% Virginia, bl = at least 75% blended, ot = other)
P : any proxy use
H : full histological confirmation
De : derivation of RR/CI (or = original, st = standard method, ot = other method of estimation)

Table 3E16 - 1

IESLC - Meta-analysis of Ever Smoking, Mixed smokers
Adenocarcinoma
Most adjusted

REF	NRR	SEX	AGEL	AGEH	RACE	YF	LC TYPE	LOC	START	ST	NLC	R	VB	P	H	AD	DENOM	De
ALDERS	41	m	0	0	all	-	not q+s	Eu:UK	1977	CC	1448	n	V	n	n	1	nev any	ot
BRESLO	3	c	0	0	all	-	a	NAmer	1949	CC	518	n	bl	n	y	0	nev+1	st
HAMMON	82	m	0	0	wh	0	a	NAmer	1952	pr	448	n	bl	n	n	1	nev any	ot
LUBIN	20	m	0	0	all	-	KII	As:Chi	1984	CC	427	m	ot	y	n	0	nev any	st
STASZE	23	m	0	0	all	-	a	Eu:est	1954	CC	281	n	bl	n	y	0	nev any	ot
WYNDE7	65	m	0	0	all	-	KII	NAmer	1977	CC	2085	n	bl	n	y	0	nev any	st

Table 3E16 - 2

IESLC - Meta-analysis of Ever Smoking, Mixed smokers
Adenocarcinoma
Most adjusted

REF	NRR	SEX	AD	Number Exposed Case	Number Exposed Cont	Non-exposed Case	Non-exposed Cont	RR	95.00%CI
ALDERS	41	m	1	-	-	-	-	4.13	(1.70- 10.04)
BRESLO	3	c	0	9	154	4	56	0.82	(0.24- 2.76)
*HAMMON	82	m	1	-	-	-	-	4.06	(0.91- 18.12)
LUBIN	20	m	0	27	597	4	72	0.81	(0.28- 2.39)
STASZE	23	m	0	5	101	0	158	17.18~	(0.94- 313.99)
WYNDE7	65	m	0	104	614	42	918	3.70	(2.55- 5.37)
Partial Totals				145	1466	50	1204		

*prospective study ~ With 0.5 adjustment for zero

REF	NRR	SEX	AD	Ys	Ws	Qs	Ps
ALDERS	41	m	1	1.42	4.87	0.42	0.0017
BRESLO	3	c	0	-0.20	2.59	4.55	0.7465
*HAMMON	82	m	1	1.40	1.72	0.13	0.0663
LUBIN	20	m	0	-0.21	3.30	5.84	0.7084
STASZE	23	m	0	2.84	0.45	1.35	0.0551
WYNDE7	65	m	0	1.31	27.67	0.95	0.0000

	N	6
	NS	6
	Wt	40.61
Het	Chi	13.24
Het	df	5
Het	P	*
Fixed	RR	3.08
	RRl	2.26
	RRu	4.18
	P	+++
Random	RR	2.48
	RRl	1.25
	RRu	4.95
	P	++
Asymm	P	N.S.

Table 3E16 - 3

IESLC - Meta-analysis of Ever Smoking, Mixed smokers
Adenocarcinoma
Most adjusted

		Sex		
	combined	male	female	Total
N	1	5		6
NS	1	5		6
Wt	2.59	38.02		40.61
Het Chi	0.00	8.38		13.24
Het df	0	4		5
Het P	N.S.	(*)		*
Fixed RR	0.82	3.37		3.08
RRl	0.24	2.45		2.26
RRu	2.76	4.63		4.18
P	N.S.	+++		+++
Random RR	0.82	3.05		2.48
RRl	0.24	1.58		1.25
RRu	2.76	5.88		4.95
P	N.S.	+++		++
Between Chi				4.86
Between df				1
Between P				*
Btwn(F) P				N.S.
Btwn(R) P				(*)

Too few RRs for analysis by factor

Table 3E16 - 4

IESLC - Meta-analysis of Ever Smoking, Mixed smokers
Adenocarcinoma
Least adjusted

REF	NRR	X	SEX	AGEL	AGEH	RACE	YF	LC TYPE	LOC	START	ST	NLC	R	VB	P	H	AD	DENOM	De
ALDERS	100	x	m	0	0	all	-	not q+s	Eu:UK	1977	CC	1448	n	V	n	n	0	nev any	st
BRESLO	3		c	0	0	all	-	a	NAmer	1949	CC	518	n	bl	n	y	0	nev+1	st
HAMMON	89	x	m	0	0	wh	0	a	NAmer	1952	pr	448	n	bl	n	n	0	nev any	st
LUBIN	20		m	0	0	all	-	KII	As:Chi	1984	CC	427	m	ot	y	n	0	nev any	st
STASZE	23		m	0	0	all	-	a	Eu:est	1954	CC	281	n	bl	n	y	0	nev any	ot
WYNDE7	65		m	0	0	all	-	KII	NAmer	1977	CC	2085	n	bl	n	y	0	nev any	st

Table 3E16 - 5

IESLC - Meta-analysis of Ever Smoking, Mixed smokers
Adenocarcinoma
Least adjusted

REF	NRR	SEX	AD	Number Exposed Case	Cont	Non-exposed Case	Cont	RR	95.00%CI
ALDERS	100	m	0	39	179	6	133	4.83	(1.99- 11.74)
BRESLO	3	c	0	9	154	4	56	0.82	(0.24- 2.76)
*HAMMON	89	m	0	12	156773	2	115884	4.44	(0.99- 19.82)
LUBIN	20	m	0	27	597	4	72	0.81	(0.28- 2.39)
STASZE	23	m	0	5	101	0	158	17.18~	(0.94- 313.99)
WYNDE7	65	m	0	104	614	42	918	3.70	(2.55- 5.37)
Totals				196	158418	58	117221		

*prospective study ~ With 0.5 adjustment for zero

REF	NRR	SEX	AD	Ys	Ws	Qs	Ps
ALDERS	100	m	0	1.57	4.87	0.90	0.0005
BRESLO	3	c	0	-0.20	2.59	4.70	0.7465
*HAMMON	89	m	0	1.49	1.71	0.20	0.0511
LUBIN	20	m	0	-0.21	3.30	6.04	0.7084
STASZE	23	m	0	2.84	0.45	1.31	0.0551
WYNDE7	65	m	0	1.31	27.67	0.74	0.0000

	N	6
	NS	6
	Wt	40.60
Het	Chi	13.89
Het	df	5
Het	P	*
Fixed	RR	3.15
	RRl	2.31
	RRu	4.28
	P	+++
Random	RR	2.59
	RRl	1.28
	RRu	5.26
	P	++
Asymm	P	N.S.

Table 3E16 - 6

IESLC - Meta-analysis of Ever Smoking, Mixed smokers
Adenocarcinoma
Least adjusted

	Sex			
	combined	male	female	Total
N	1	5		6
NS	1	5		6
Wt	2.59	38.01		40.60
Het Chi	0.00	8.86		13.89
Het df	0	4		5
Het P	N.S.	(*)		*
Fixed RR	0.82	3.45		3.15
RRl	0.24	2.51		2.31
RRu	2.76	4.74		4.28
P	N.S.	+++		+++
Random RR	0.82	3.21		2.59
RRl	0.24	1.63		1.28
RRu	2.76	6.32		5.26
P	N.S.	+++		++
Between Chi				5.03
Between df				1
Between P				*
Btwn(F) P				N.S.
Btwn(R) P				(*)

Table 3E16 - 7

IESLC - Meta-analysis of Ever Smoking, Mixed smokers
Adenocarcinoma
Excluded studies (and stage at which they were excluded)

1	BOUCHA BUELL LAURIL MZILEN
2	ABRAHA AGUDO AKIBA AMANDU AMES ANDERS ARCHER AUSTIN AUVINE AXELSO AXELSS BAND BARBON BECHER BENSHL BERRIN BLOHMK BLOT1 BLOT2 BLOT3 BLOT4 BRETT BROCKM BROSS BROWN1 BROWN2 BUFFLE BYERS1 BYERS2 CARPEN CASCO2 CASCOR CHAN CHANG CHATZI CHEN CHEN2 CHEN3 CHIAZZ CHOI CHYOU COMSTO COOKSO CORREA CPSII DARBY DAVEYS DEAN3 DEKLER DESTE2 DESTEF DOCKER DOLL DORANT DORGAN DOSEME DROSTE DU DUNN EBELIN ENGELA ENSTRO ESAKI FAN GAO GAO2 GARCIA GARDIN GARSHI GENG GER GILLIS GODLEY GOODMA GREGOR GSELL HAENSZ HAMMO2 HANSEN HEGMAN HEIN HENNEK HINDS HIRAOK HIRAYA HITOSU HOLE HOROWI HORWIT HU HU2 HUANG HUMBLE ISHIMA JAHN JAIN JARUP JARVHO JEDRYC JIANG JONES JUSSAW KAISE2 KAISER KANELL KATSOU KAUFMA KELLER KHUDER KIHARA KINLEN KJUUS KNEKT KO KOHLME KOO KOULUM KREUZE KREYBE KUBIK LAMTH LAMWK LAMWK2 LANGE LAUSSM LEI LEMARC LETOUR LEVIN LIAW LICKIN LIDDEL LIU LIU2 LIU3 LIU4 LIU5 LOMBA2 LUO MACLEN MAGNUS MARSH MARSH2 MARTIS MASTRA MATOS MATSUD MCDUFF MCLAUG MILLER MILLS MOLLO MRFIT MRFITR MURATA NAM NOTAN2 NOTANI NOU ODRISC ORMOS OSANN OSANN2 PARKIN PASTOR PAWLEG PERNU PERSH2 PERSHA PETO PEZZO2 PEZZOT PIKE PISANI POFFIJ POLEDN PRESCO QIAO2 RACHTA RADZIK RANDIG REN RESTRE RIMING RONCO ROOTS ROTHSC SAARIK SANKAR SCHWAR SEGI SEGI2 SEOW SHAW SHIMIZ SIEMIA SIMARA SIMONA SITAS SOBUE SOBUE2 SPEIZE SPITZ STAYNE STOCKS STOCKW STUCKE SUN SUZUK2 SUZUKI SVENSS TANG TANG2 TAO TENKAN TIZZAN TOKARS TOUSEY TSUGAN TULINI ULMER VANDER VEIERO VUTUC WAKAI WALD WANG WANG2 WANG3 WANG4 WARSIN WATSON WICKLU WIGLE WILKIN WU WU2 WUNSCH WUWILL WYNDE2 WYNDE3 WYNDE4 WYNDE5 WYNDE6 WYNDE8 XU XU2 XU3 XU4 YAMAGU YONG YUAN ZHANG ZHENG ZHOU
3	CEDERL HIRAY2 MIGRAN TVERDA WYNDER
5	SCHWA2
7	ABELIN ARMADA BENHAM BEST BOFFET BOUCOT CHOW CPSI DAMBER DEAN DEAN2 DOLL2 DORN GOLLED GRAHAM JOLY LOMBAR LUBIN2 MCCONN QIAO SADOWS XIANGZ

Table 3E16 - 8
Potentially overlapping studies

REF	REFGP	PRINC	.	OVERLAP/LINK
WYNDE7	WYNDE6	2		WYNDE5/6/7/8
LUBIN	XIANGZ	2		LUBIN/XIANGZ/QIAO

Table 3E17 -

IESLC - Meta-analysis of Current Smoking, Mixed smokers
Adenocarcinoma

This analysis is restricted to results for:
1) Non-dose-response data
2) Mixed smokers (cigarettes and pipe/cigar)
3) Current smokers
4) Results complete enough for use in metaanalysis

Within each study, results are then selected (in the following order of preference, within each sex) for:
5) DENOM: never smoked anything, (never +1 = +long term ex)
6) Followup period (prospective studies): whole study (coded as 0) or longest available
7) LCtype: all or nearest available, at least Squamous and Adeno. (q = squamous, s = small,
l = large, a = adeno, mix = mixed, alv = alveolar)
8) Race: all or nearest available, otherwise by race (wh or w = white, bl or b = black, hi = hispanic
ch = chinese, jap = japanese, haw = hawaiian, w+o = white + oriental, sca = scandinavian, as = asian)
9) For overlapping studies: principal rather than subsidiary studies
Finally by Age: whole study (coded as 0) if available, otherwise by widest available age group
and then for single sex results (m, f) in preference to combined sex results (c).

Results adjusted (AD) for the most potential confounders are then chosen in Sections -1 to -3
and results adjusted for the least confounders in Sections -4 to -6. (Those least adjusted results which
actually differ from the most adjusted as marked 'x' in column X in Section -4)
(Results adjusted for an unknown number of confounder(s) are coded as 20.)

Section -7 shows excluded studies, together with the stage (as above) at which no qualifying
results were found.

Section -8 lists the potentially overlapping studies which have been included (1=principal, 2=subsidiary).

Section -9 lists any results which would have been included in preference except that they had data not complete
enough for use in meta-analysis, with their significance (yes/no), if known, and any further comment as entered
on the database.

In addition to those mentioned above, the following fields, levels and abbreviations are used:
* or nk = not known, n = no, y = yes, ot = other
nev = never
REF: 6-character study reference
NRR: number of the RR on the database within the study
ST : study type (CC = case control, pr or prosp = prospective)
NLC: number of lung cancer cases in whole study
R : risky occupational population (n = no, m = mining, o = other risky)
VB : national cigarette type (V = at least 75% Virginia, bl = at least 75% blended, ot = other)
P : any proxy use
H : full histological confirmation
De : derivation of RR/CI (or = original, st = standard method, ot = other method of estimation)

Table 3E17 - 1

IESLC - Meta-analysis of Current Smoking, Mixed smokers
Adenocarcinoma
Most adjusted

REF	NRR	SEX	AGEL	AGEH	RACE	YF	LC TYPE	LOC	START	ST	NLC	R	VB	P	H	AD	DENOM	De
WYNDE7	33	m	0	0	all	-	KII	NAmer	1977	CC	2085	n	bl	n	y	0	nev any	st

Table 3E17 - 2

IESLC - Meta-analysis of Current Smoking, Mixed smokers
Adenocarcinoma
Most adjusted

REF	NRR	SEX	AD	Number Exposed Case	Number Exposed Cont	Non-exposed Case	Non-exposed Cont	RR	95.00%CI
WYNDE7	33	m	0	59	287	42	918	4.49	(2.96- 6.82)
Totals				59	287	42	918		

*prospective study

REF	NRR	SEX	AD	Ys	Ws	Qs	Ps
WYNDE7	33	m	0	1.50	22.06	0.00	0.0000

	N	1
	NS	1
	Wt	22.06
Het	Chi	0.00
Het	df	0
Het	P	N.S.
Fixed	RR	4.49
	RRl	2.96
	RRu	6.82
	P	+++
Random	RR	4.49
	RRl	2.96
	RRu	6.82
	P	+++
Asymm	P	

Table 3E17 - 3

IESLC - Meta-analysis of Current Smoking, Mixed smokers
Adenocarcinoma
Most adjusted

	Sex combined	male	female	Total
N		1		1
NS		1		1
Wt		22.06		22.06
Het Chi		0.00		0.00
Het df		0		0
Het P		N.S.		N.S.
Fixed RR		4.49		4.49
RRl		2.96		2.96
RRu		6.82		6.82
P		+++		+++
Random RR		4.49		4.49
RRl		2.96		2.96
RRu		6.82		6.82
P		+++		+++
Between Chi				
Between df				
Between P				N.S.
Btwn(F) P				N.S.
Btwn(R) P				N.S.

Too few RRs for analysis by factor

Table 3E17 - 4

IESLC - Meta-analysis of Current Smoking, Mixed smokers
Adenocarcinoma
Least adjusted

REF	NRR	X	SEX	AGEL	AGEH	RACE	YF	LC TYPE	LOC	START	ST	NLC	R	VB	P	H	AD	DENOM	De
WYNDE7	33		m	0	0	all	-	KII	NAmer	1977	CC	2085	n	bl	n	y	0	nev any	st

Table 3E17 - 5

IESLC - Meta-analysis of Current Smoking, Mixed smokers
Adenocarcinoma
Least adjusted

REF	NRR	SEX	AD	Number Exposed Case	Number Exposed Cont	Non-exposed Case	Non-exposed Cont	RR	95.00%CI
WYNDE7	33	m	0	59	287	42	918	4.49	(2.96- 6.82)
Totals				59	287	42	918		

*prospective study

REF	NRR	SEX	AD	Ys	Ws	Qs	Ps
WYNDE7	33	m	0	1.50	22.06	0.00	0.0000

	N	1
	NS	1
	Wt	22.06
Het	Chi	0.00
Het	df	0
Het	P	N.S.
Fixed	RR	4.49
	RRl	2.96
	RRu	6.82
	P	+++
Random	RR	4.49
	RRl	2.96
	RRu	6.82
	P	+++
Asymm	P	

Table 3E17 - 6

IESLC - Meta-analysis of Current Smoking, Mixed smokers
Adenocarcinoma
Least adjusted

	Sex combined	male	female	Total
N		1		1
NS		1		1
Wt		22.06		22.06
Het Chi		0.00		0.00
Het df		0		0
Het P		N.S.		N.S.
Fixed RR		4.49		4.49
RRl		2.96		2.96
RRu		6.82		6.82
P		+++		+++
Random RR		4.49		4.49
RRl		2.96		2.96
RRu		6.82		6.82
P		+++		+++
Between Chi				
Between df				
Between P				N.S.
Btwn(F) P				N.S.
Btwn(R) P				N.S.

Table 3E17 - 7

IESLC - Meta-analysis of Current Smoking, Mixed smokers
Adenocarcinoma
Excluded studies (and stage at which they were excluded)

1 BOUCHA BUELL LAURIL MZILEN

2 ABRAHA AGUDO AKIBA AMANDU AMES ANDERS ARCHER AUSTIN AUVINE AXELSO AXELSS BAND BARBON BECHER BENSHL BERRIN
BLOHMK BLOT1 BLOT2 BLOT3 BLOT4 BRETT BROCKM BROSS BROWN1 BROWN2 BUFFLE BYERS1 BYERS2 CARPEN CASCO2 CASCOR
CHAN CHANG CHATZI CHEN CHEN2 CHEN3 CHIAZZ CHOI CHYOU COMSTO COOKSO CORREA CPSII DARBY DAVEYS DEAN3
DEKLER DESTE2 DESTEF DOCKER DOLL DORANT DORGAN DOSEME DROSTE DU DUNN EBELIN ENGELA ENSTRO ESAKI FAN
GAO GAO2 GARCIA GARDIN GARSHI GENG GER GILLIS GODLEY GOODMA GREGOR GSELL HAENSZ HAMMO2 HANSEN HEGMAN
HEIN HENNEK HINDS HIRAOK HIRAYA HITOSU HOLE HOROWI HORWIT HU HU2 HUANG HUMBLE ISHIMA JAHN JAIN
JARUP JARVHO JEDRYC JIANG JONES JUSSAW KAISE2 KAISER KANELL KATSOU KAUFMA KELLER KHUDER KIHARA KINLEN KJUUS
KNEKT KO KOHLME KOO KOULUM KREUZE KREYBE KUBIK LAMTH LAMWK LAMWK2 LANGE LAUSSM LEI LEMARC LETOUR
LEVIN LIAW LICKIN LIDDEL LIU LIU2 LIU3 LIU4 LIU5 LOMBA2 LUO MACLEN MAGNUS MARSH MARSH2 MARTIS
MASTRA MATOS MATSUD MCDUFF MCLAUG MILLER MILLS MOLLO MRFIT MRFITR MURATA NAM NOTAN2 NOTANI NOU ODRISC
ORMOS OSANN OSANN2 PARKIN PASTOR PAWLEG PERNU PERSH2 PERSHA PETO PEZZO2 PEZZOT PIKE PISANI POFFIJ POLEDN
PRESCO QIAO2 RACHTA RADZIK RANDIG REN RESTRE RIMING RONCO ROOTS ROTHSC SAARIK SANKAR SCHWAR SEGI SEGI2
SEOW SHAW SHIMIZ SIEMIA SIMARA SIMONA SITAS SOBUE SOBUE2 SPEIZE SPITZ STAYNE STOCKS STOCKW STUCKE SUN
SUZUK2 SUZUKI SVENSS TANG TANG2 TAO TENKAN TIZZAN TOKARS TOUSEY TSUGAN TULINI ULMER VANDER VEIERO VUTUC
WAKAI WALD WANG WANG2 WANG3 WANG4 WARSIN WATSON WICKLU WIGLE WILKIN WU WU2 WUNSCH WUWILL WYNDE2
WYNDE3 WYNDE4 WYNDE5 WYNDE6 WYNDE8 XU XU2 XU3 XU4 YAMAGU YONG YUAN ZHANG ZHENG ZHOU

3 ABELIN ALDERS ARMADA BENHAM BEST BOFFET BOUCOT BRESLO CPSI DAMBER DEAN DEAN2 GOLLED HAMMON JOLY LUBIN
MCCONN QIAO SADOWS SCHWA2 STASZE XIANGZ

5 HIRAY2 TVERDA WYNDER

7 CEDERL CHOW DOLL2 DORN GRAHAM LOMBAR LUBIN2 MIGRAN

Table 3E17 - 8

Potentially overlapping studies

REF	REFGP	PRINC	.	OVERLAP/LINK
WYNDE7	WYNDE6	2		WYNDE5/6/7/8

Table 3E18 -

IESLC - Meta-analysis of Ever Smoking (or Current if Ever not available), Mixed smokers
Adenocarcinoma

This analysis is restricted to results for:
1) Non-dose-response data
2) Mixed smokers (cigarettes and pipe/cigar)
3) Results complete enough for use in metaanalysis

Within each study, results are then selected (in the following order of preference, within each sex) for:
4) SMKSTA: ever smokers, current smokers
5) DENOM: never smoked anything, (never +1 = +long term ex)
6) Followup period (prospective studies): whole study (coded as 0) or longest available
7) LCtype: all or nearest available, at least Squamous and Adeno. (q = squamous, s = small,
l = large, a = adeno, mix = mixed, alv = alveolar)
8) Race: all or nearest available, otherwise by race (wh or w = white, bl or b = black, hi = hispanic
ch = chinese, jap = japanese, haw = hawaiian, w+o = white + oriental, sca = scandinavian, as = asian)
9) For overlapping studies: principal rather than subsidiary studies
Finally by Age: whole study (coded as 0) if available, otherwise by widest available age group
and then for single sex results (m, f) in preference to combined sex results (c).

Results adjusted (AD) for the most potential confounders are then chosen in Sections -1 to -3
and results adjusted for the least confounders in Sections -4 to -6. (Those least adjusted results which
actually differ from the most adjusted as marked 'x' in column X in Section -4)
(Results adjusted for an unknown number of confounder(s) are coded as 20.)

Section -7 shows excluded studies, together with the stage (as above) at which no qualifying
results were found.

Section -8 lists the potentially overlapping studies which have been included (1=principal, 2=subsidiary).

Section -9 lists any results which would have been included in preference except that they had data not complete
enough for use in meta-analysis, with their significance (yes/no), if known, and any further comment as entered
on the database.

In addition to those mentioned above, the following fields, levels and abbreviations are used:
* or nk = not known, n = no, y = yes, ot = other
ev = ever, cu = current, nev = never
REF: 6-character study reference
NRR: number of the RR on the database within the study
ST : study type (CC = case control, pr or prosp = prospective)
NLC: number of lung cancer cases in whole study
R : risky occupational population (n = no, m = mining, o = other risky)
VB : national cigarette type (V = at least 75% Virginia, bl = at least 75% blended, ot = other)
P : any proxy use
H : full histological confirmation
De : derivation of RR/CI (or = original, st = standard method, ot = other method of estimation)

Table 3E18 - 1

IESLC - Meta-analysis of Ever Smoking (or Current if Ever not available), Mixed smokers
Adenocarcinoma
Most adjusted

REF	NRR	SEX	AGEL	AGEH	RACE	YF	LC TYPE	LOC	START	ST	NLC	R	VB	P	H	AD	SM	DENOM	De
ALDERS	41	m	0	0	all	-	not q+s	Eu:UK	1977	CC	1448	n	V	n	n	1	ev	nev any	ot
BRESLO	3	c	0	0	all	-	a	NAmer	1949	CC	518	n	bl	n	y	0	ev	nev+1	st
HAMMON	82	m	0	0	wh	0	a	NAmer	1952	pr	448	n	bl	n	n	1	ev	nev any	ot
LUBIN	20	m	0	0	all	-	KII	As:Chi	1984	CC	427	m	ot	y	n	0	ev	nev any	st
STASZE	23	m	0	0	all	-	a	Eu:est	1954	CC	281	n	bl	n	y	0	ev	nev any	ot
WYNDE7	65	m	0	0	all	-	KII	NAmer	1977	CC	2085	n	bl	n	y	0	ev	nev any	st

Table 3E18 - 2

IESLC - Meta-analysis of Ever Smoking (or Current if Ever not available), Mixed smokers
Adenocarcinoma
Most adjusted

REF	NRR	SEX	AD	Number Exposed Case	Exposed Cont	Non-exposed Case	Non-exposed Cont	RR	95.00%CI
ALDERS	41	m	1	-	-	-	-	4.13	(1.70- 10.04)
BRESLO	3	c	0	9	154	4	56	0.82	(0.24- 2.76)
*HAMMON	82	m	1	-	-	-	-	4.06	(0.91- 18.12)
LUBIN	20	m	0	27	597	4	72	0.81	(0.28- 2.39)
STASZE	23	m	0	5	101	0	158	17.18~	(0.94- 313.99)
WYNDE7	65	m	0	104	614	42	918	3.70	(2.55- 5.37)
Partial Totals				145	1466	50	1204		

*prospective study ~ With 0.5 adjustment for zero

REF	NRR	SEX	AD	Ys	Ws	Qs	Ps
ALDERS	41	m	1	1.42	4.87	0.42	0.0017
BRESLO	3	c	0	-0.20	2.59	4.55	0.7465
*HAMMON	82	m	1	1.40	1.72	0.13	0.0663
LUBIN	20	m	0	-0.21	3.30	5.84	0.7084
STASZE	23	m	0	2.84	0.45	1.35	0.0551
WYNDE7	65	m	0	1.31	27.67	0.95	0.0000

	N	6
	NS	6
	Wt	40.61
Het	Chi	13.24
Het	df	5
Het	P	*
Fixed	RR	3.08
	RRl	2.26
	RRu	4.18
	P	+++
Random	RR	2.48
	RRl	1.25
	RRu	4.95
	P	++
Asymm	P	N.S.

Table 3E18 - 3

IESLC - Meta-analysis of Ever Smoking (or Current if Ever not available), Mixed smokers
Adenocarcinoma
Most adjusted

	Sex combined	male	female	Total
N	1	5		6
NS	1	5		6
Wt	2.59	38.02		40.61
Het Chi	0.00	8.38		13.24
Het df	0	4		5
Het P	N.S.	(*)		*
Fixed RR	0.82	3.37		3.08
RRl	0.24	2.45		2.26
RRu	2.76	4.63		4.18
P	N.S.	+++		+++
Random RR	0.82	3.05		2.48
RRl	0.24	1.58		1.25
RRu	2.76	5.88		4.95
P	N.S.	+++		++
Between Chi				4.86
Between df				1
Between P				*
Btwn(F) P				N.S.
Btwn(R) P				(*)

Too few RRs for analysis by factor

Table 3E18 - 4

IESLC - Meta-analysis of Ever Smoking (or Current if Ever not available), Mixed smokers
Adenocarcinoma
Least adjusted

REF	NRR	X	SEX	AGEL	AGEH	RACE	YF	LC TYPE	LOC	START	ST	NLC	R	VB	P	H	AD	SM	DENOM	De
ALDERS	100	x	m	0	0	all	-	not q+s	Eu:UK	1977	CC	1448	n	V	n	n	0	ev	nev any	st
BRESLO	3		c	0	0	all	-	a	NAmer	1949	CC	518	n	bl	n	y	0	ev	nev+1	st
HAMMON	89	x	m	0	0	wh	0	a	NAmer	1952	pr	448	n	bl	n	n	0	ev	nev any	st
LUBIN	20		m	0	0	all	-	KII	As:Chi	1984	CC	427	m	ot	y	n	0	ev	nev any	st
STASZE	23		m	0	0	all	-	a	Eu:est	1954	CC	281	n	bl	n	y	0	ev	nev any	ot
WYNDE7	65		m	0	0	all	-	KII	NAmer	1977	CC	2085	n	bl	n	y	0	ev	nev any	st

Table 3E18 - 5

IESLC - Meta-analysis of Ever Smoking (or Current if Ever not available), Mixed smokers
Adenocarcinoma
Least adjusted

REF	NRR	SEX	AD	Number Exposed Case	Cont	Non-exposed Case	Cont	RR	95.00%CI
ALDERS	100	m	0	39	179	6	133	4.83	(1.99- 11.74)
BRESLO	3	c	0	9	154	4	56	0.82	(0.24- 2.76)
*HAMMON	89	m	0	12	156773	2	115884	4.44	(0.99- 19.82)
LUBIN	20	m	0	27	597	4	72	0.81	(0.28- 2.39)
STASZE	23	m	0	5	101	0	158	17.18~	(0.94- 313.99)
WYNDE7	65	m	0	104	614	42	918	3.70	(2.55- 5.37)
Totals				196	158418	58	117221		

*prospective study ~ With 0.5 adjustment for zero

REF	NRR	SEX	AD	Ys	Ws	Qs	Ps
ALDERS	100	m	0	1.57	4.87	0.90	0.0005
BRESLO	3	c	0	-0.20	2.59	4.70	0.7465
*HAMMON	89	m	0	1.49	1.71	0.20	0.0511
LUBIN	20	m	0	-0.21	3.30	6.04	0.7084
STASZE	23	m	0	2.84	0.45	1.31	0.0551
WYNDE7	65	m	0	1.31	27.67	0.74	0.0000

	N	6
	NS	6
	Wt	40.60
Het	Chi	13.89
Het	df	5
Het	P	*
Fixed	RR	3.15
	RRl	2.31
	RRu	4.28
	P	+++
Random	RR	2.59
	RRl	1.28
	RRu	5.26
	P	++
Asymm	P	N.S.

Table 3E18 - 6

IESLC - Meta-analysis of Ever Smoking (or Current if Ever not available), Mixed smokers
Adenocarcinoma
Least adjusted

	combined	Sex male	female	Total
N	1	5		6
NS	1	5		6
Wt	2.59	38.01		40.60
Het Chi	0.00	8.86		13.89
Het df	0	4		5
Het P	N.S.	(*)		*
Fixed RR	0.82	3.45		3.15
RRl	0.24	2.51		2.31
RRu	2.76	4.74		4.28
P	N.S.	+++		+++
Random RR	0.82	3.21		2.59
RRl	0.24	1.63		1.28
RRu	2.76	6.32		5.26
P	N.S.	+++		++
Between Chi				5.03
Between df				1
Between P				*
Btwn(F) P				N.S.
Btwn(R) P				(*)

Table 3E18 - 7

IESLC - Meta-analysis of Ever Smoking (or Current if Ever not available), Mixed smokers
Adenocarcinoma
Excluded studies (and stage at which they were excluded)

1	BOUCHA BUELL LAURIL MZILEN
2	ABRAHA AGUDO AKIBA AMANDU AMES ANDERS ARCHER AUSTIN AUVINE AXELSO AXELSS BAND BARBON BECHER BENSHL BERRIN BLOHMK BLOT1 BLOT2 BLOT3 BLOT4 BRETT BROCKM BROSS BROWN1 BROWN2 BUFFLE BYERS1 BYERS2 CARPEN CASCO2 CASCOR CHAN CHANG CHATZI CHEN CHEN2 CHEN3 CHIAZZ CHOI CHYOU COMSTO COOKSO CORREA CPSII DARBY DAVEYS DEAN3 DEKLER DESTE2 DESTEF DOCKER DOLL DORANT DORGAN DOSEME DROSTE DU DUNN EBELIN ENGELA ENSTRO ESAKI FAN GAO GAO2 GARCIA GARDIN GARSHI GENG GER GILLIS GODLEY GOODMA GREGOR GSELL HAENSZ HAMMO2 HANSEN HEGMAN HEIN HENNEK HINDS HIRAOK HIRAYA HITOSU HOLE HOROWI HORWIT HU HU2 HUANG HUMBLE ISHIMA JAHN JAIN JARUP JARVHO JEDRYC JIANG JONES JUSSAW KAISE2 KAISER KANELL KATSOU KAUFMA KELLER KHUDER KIHARA KINLEN KJUUS KNEKT KO KOHLME KOO KOULUM KREUZE KREYBE KUBIK LAMTH LAMWK LAMWK2 LANGE LAUSSM LEI LEMARC LETOUR LEVIN LIAW LICKIN LIDDEL LIU LIU2 LIU3 LIU4 LIU5 LOMBA2 LUO MACLEN MAGNUS MARSH MARSH2 MARTIS MASTRA MATOS MATSUD MCDUFF MCLAUG MILLER MILLS MOLLO MRFIT MRFITR MURATA NAM NOTAN2 NOTANI NOU ODRISC ORMOS OSANN OSANN2 PARKIN PASTOR PAWLEG PERNU PERSH2 PERSHA PETO PEZZO2 PEZZOT PIKE PISANI POFFIJ POLEDN PRESCO QIAO2 RACHTA RADZIK RANDIG REN RESTRE RIMING RONCO ROOTS ROTHSC SAARIK SANKAR SCHWAR SEGI SEGI2 SEOW SHAW SHIMIZ SIEMIA SIMARA SIMONA SITAS SOBUE SOBUE2 SPEIZE SPITZ STAYNE STOCKS STOCKW STUCKE SUN SUZUK2 SUZUKI SVENSS TANG TANG2 TAO TENKAN TIZZAN TOKARS TOUSEY TSUGAN TULINI ULMER VANDER VEIERO VUTUC WAKAI WALD WANG WANG2 WANG3 WANG4 WARSIN WATSON WICKLU WIGLE WILKIN WU WU2 WUNSCH WUWILL WYNDE2 WYNDE3 WYNDE4 WYNDE5 WYNDE6 WYNDE8 XU XU2 XU3 XU4 YAMAGU YONG YUAN ZHANG ZHENG ZHOU
5	HIRAY2 SCHWA2 TVERDA WYNDER
7	ABELIN ARMADA BENHAM BEST BOFFET BOUCOT CEDERL CHOW CPSI DAMBER DEAN DEAN2 DOLL2 DORN GOLLED GRAHAM JOLY LOMBAR LUBIN2 MCCONN MIGRAN QIAO SADOWS XIANGZ

Table 3E18 - 8

Potentially overlapping studies

REF	REFGP	PRINC	.	OVERLAP/LINK
WYNDE7	WYNDE6	2		WYNDE5/6/7/8
LUBIN	XIANGZ	2		LUBIN/XIANGZ/QIAO

Table 3E19 -

IESLC - Meta-analysis of Current Smoking (or Ever if Current not available), Mixed smokers
Adenocarcinoma

This analysis is restricted to results for:
1) Non-dose-response data
2) Mixed smokers (cigarettes and pipe/cigar)
3) Results complete enough for use in metaanalysis

Within each study, results are then selected (in the following order of preference, within each sex) for:
4) SMKSTA: current smokers, ever smokers
5) DENOM: never smoked anything, (never +1 = +long term ex)
6) Followup period (prospective studies): whole study (coded as 0) or longest available
7) LCtype: all or nearest available, at least Squamous and Adeno. (q = squamous, s = small,
l = large, a = adeno, mix = mixed, alv = alveolar)
8) Race: all or nearest available, otherwise by race (wh or w = white, bl or b = black, hi = hispanic
ch = chinese, jap = japanese, haw = hawaiian, w+o = white + oriental, sca = scandinavian, as = asian)
9) For overlapping studies: principal rather than subsidiary studies
Finally by Age: whole study (coded as 0) if available, otherwise by widest available age group
and then for single sex results (m, f) in preference to combined sex results (c).

Results adjusted (AD) for the most potential confounders are then chosen in Sections -1 to -3
(and those which actually differ from the adjusted results in Table 3E18 - 1 are marked 'x' in Section -1)
and results adjusted for the least confounders in Sections -4 to -6. (Those least adjusted results which
actually differ from the most adjusted as marked 'x' in column X in Section -4)
(Results adjusted for an unknown number of confounder(s) are coded as 20.)

Section -7 shows excluded studies, together with the stage (as above) at which no qualifying
results were found.

Section -8 lists the potentially overlapping studies which have been included (1=principal, 2=subsidiary).

Section -9 lists any results which would have been included in preference except that they had data not complete
enough for use in meta-analysis, with their significance (yes/no), if known, and any further comment as entered
on the database.

In addition to those mentioned above, the following fields, levels and abbreviations are used:
* or nk = not known, n = no, y = yes, ot = other
ev = ever, cu = current, nev = never
REF: 6-character study reference
NRR: number of the RR on the database within the study
ST : study type (CC = case control, pr or prosp = prospective)
NLC: number of lung cancer cases in whole study
R : risky occupational population (n = no, m = mining, o = other risky)
VB : national cigarette type (V = at least 75% Virginia, bl = at least 75% blended, ot = other)
P : any proxy use
H : full histological confirmation
De : derivation of RR/CI (or = original, st = standard method, ot = other method of estimation)

Table 3E19 - 1

IESLC - Meta-analysis of Current Smoking (or Ever if Current not available), Mixed smokers
Adenocarcinoma
Most adjusted

REF	NRR	3E18	SEX	AGEL	AGEH	RACE	YF	LC TYPE	LOC	START	ST	NLC	R	VB	P	H	AD	SM	DENOM	De
ALDERS	41		m	0	0	all	-	not q+s	Eu:UK	1977	CC	1448	n	V	n	n	1	ev	nev any	ot
BRESLO	3		c	0	0	all	-	a	NAmer	1949	CC	518	n	bl	n	y	0	ev	nev+1	st
HAMMON	82		m	0	0	wh	0	a	NAmer	1952	pr	448	n	bl	n	n	1	ev	nev any	ot
LUBIN	20		m	0	0	all	-	KII	As:Chi	1984	CC	427	m	ot	y	n	0	ev	nev any	st
STASZE	23		m	0	0	all	-	a	Eu:est	1954	CC	281	n	bl	n	y	0	ev	nev any	ot
WYNDE7	33	x	m	0	0	all	-	KII	NAmer	1977	CC	2085	n	bl	n	y	0	cu	nev any	st

Table 3E19 - 2

IESLC - Meta-analysis of Current Smoking (or Ever if Current not available), Mixed smokers
Adenocarcinoma
Most adjusted

REF	NRR	SEX	AD	Number Exposed Case	Number Exposed Cont	Non-exposed Case	Non-exposed Cont	RR	95.00%CI
ALDERS	41	m	1	-	-	-	-	4.13	(1.70- 10.04)
BRESLO	3	c	0	9	154	4	56	0.82	(0.24- 2.76)
*HAMMON	82	m	1	-	-	-	-	4.06	(0.91- 18.12)
LUBIN	20	m	0	27	597	4	72	0.81	(0.28- 2.39)
STASZE	23	m	0	5	101	0	158	17.18~	(0.94- 313.99)
WYNDE7	33	m	0	59	287	42	918	4.49	(2.96- 6.82)
Partial Totals				100	1139	50	1204		

*prospective study

~ With 0.5 adjustment for zero

REF	NRR	SEX	AD	Ys	Ws	Qs	Ps
ALDERS	41	m	1	1.42	4.87	0.20	0.0017
BRESLO	3	c	0	-0.20	2.59	5.20	0.7465
*HAMMON	82	m	1	1.40	1.72	0.06	0.0663
LUBIN	20	m	0	-0.21	3.30	6.68	0.7084
STASZE	23	m	0	2.84	0.45	1.21	0.0551
WYNDE7	33	m	0	1.50	22.06	1.81	0.0000

	N	6
	NS	6
	Wt	35.00
Het	Chi	15.16
Het	df	5
Het	P	**
Fixed	RR	3.37
	RRl	2.42
	RRu	4.70
	P	+++
Random	RR	2.61
	RRl	1.23
	RRu	5.52
	P	+
Asymm	P	N.S.

Table 3E19 - 3

IESLC - Meta-analysis of Current Smoking (or Ever if Current not available), Mixed smokers
Adenocarcinoma
Most adjusted

		Sex		
	combined	male	female	Total
N	1	5		6
NS	1	5		6
Wt	2.59	32.41		35.00
Het Chi	0.00	9.54		15.16
Het df	0	4		5
Het P	N.S.	*		**
Fixed RR	0.82	3.78		3.37
RRl	0.24	2.68		2.42
RRu	2.76	5.33		4.70
P	N.S.	+++		+++
Random RR	0.82	3.26		2.61
RRl	0.24	1.59		1.23
RRu	2.76	6.69		5.52
P	N.S.	++		+
Between Chi				5.62
Between df				1
Between P				*
Btwn(F) P				N.S.
Btwn(R) P				(*)

Too few RRs for analysis by factor

Table 3E19 - 4

IESLC - Meta-analysis of Current Smoking (or Ever if Current not available), Mixed smokers
Adenocarcinoma
Least adjusted

REF	NRR	X	SEX	AGEL	AGEH	RACE	YF	LC TYPE	LOC	START	ST	NLC	R	VB	P	H	AD	SM	DENOM	De
ALDERS	100	x	m	0	0	all	-	not q+s	Eu:UK	1977	CC	1448	n	V	n	n	0	ev	nev any	st
BRESLO	3		c	0	0	all	-	a	NAmer	1949	CC	518	n	bl	n	y	0	ev	nev+1	st
HAMMON	89	x	m	0	0	wh	0	a	NAmer	1952	pr	448	n	bl	n	n	0	ev	nev any	st
LUBIN	20		m	0	0	all	-	KII	As:Chi	1984	CC	427	m	ot	y	n	0	ev	nev any	st
STASZE	23		m	0	0	all	-	a	Eu:est	1954	CC	281	n	bl	n	y	0	ev	nev any	ot
WYNDE7	33		m	0	0	all	-	KII	NAmer	1977	CC	2085	n	bl	n	y	0	cu	nev any	st

Table 3E19 - 5

IESLC - Meta-analysis of Current Smoking (or Ever if Current not available), Mixed smokers
Adenocarcinoma
Least adjusted

REF	NRR	SEX	AD	Number Exposed Case	Cont	Non-exposed Case	Cont	RR	95.00%CI
ALDERS	100	m	0	39	179	6	133	4.83	(1.99- 11.74)
BRESLO	3	c	0	9	154	4	56	0.82	(0.24- 2.76)
*HAMMON	89	m	0	12	156773	2	115884	4.44	(0.99- 19.82)
LUBIN	20	m	0	27	597	4	72	0.81	(0.28- 2.39)
STASZE	23	m	0	5	101	0	158	17.18~	(0.94- 313.99)
WYNDE7	33	m	0	59	287	42	918	4.49	(2.96- 6.82)
Totals				151	158091	58	117221		

*prospective study ~ With 0.5 adjustment for zero

REF	NRR	SEX	AD	Ys	Ws	Qs	Ps
ALDERS	100	m	0	1.57	4.87	0.54	0.0005
BRESLO	3	c	0	-0.20	2.59	5.40	0.7465
*HAMMON	89	m	0	1.49	1.71	0.11	0.0511
LUBIN	20	m	0	-0.21	3.30	6.92	0.7084
STASZE	23	m	0	2.84	0.45	1.17	0.0551
WYNDE7	33	m	0	1.50	22.06	1.50	0.0000

	N	6
	NS	6
	Wt	35.00
Het	Chi	15.63
Het	df	5
Het	P	**
Fixed	RR	3.46
	RRl	2.49
	RRu	4.82
	P	+++
Random	RR	2.72
	RRl	1.27
	RRu	5.84
	P	+
Asymm	P	N.S.

Table 3E19 - 6

IESLC - Meta-analysis of Current Smoking (or Ever if Current not available), Mixed smokers
Adenocarcinoma
Least adjusted

	combined	Sex: male	female	Total
N	1	5		6
NS	1	5		6
Wt	2.59	32.40		35.00
Het Chi	0.00	9.80		15.63
Het df	0	4		5
Het P	N.S.	*		**
Fixed RR	0.82	3.89		3.46
RRl	0.24	2.75		2.49
RRu	2.76	5.48		4.82
P	N.S.	+++		+++
Random RR	0.82	3.43		2.72
RRl	0.24	1.65		1.27
RRu	2.76	7.12		5.84
P	N.S.	+++		+
Between Chi				5.83
Between df				1
Between P				*
Btwn(F) P				N.S.
Btwn(R) P				*

Table 3E19 - 7

IESLC - Meta-analysis of Current Smoking (or Ever if Current not available), Mixed smokers
Adenocarcinoma
Excluded studies (and stage at which they were excluded)

Stage	Excluded studies
1	BOUCHA BUELL LAURIL MZILEN
2	ABRAHA AGUDO AKIBA AMANDU AMES ANDERS ARCHER AUSTIN AUVINE AXELSO AXELSS BAND BARBON BECHER BENSHL BERRIN BLOHMK BLOT1 BLOT2 BLOT3 BLOT4 BRETT BROCKM BROSS BROWN1 BROWN2 BUFFLE BYERS1 BYERS2 CARPEN CASCO2 CASCOR CHAN CHANG CHATZI CHEN CHEN2 CHEN3 CHIAZZ CHOI CHYOU COMSTO COOKSO CORREA CPSII DARBY DAVEYS DEAN3 DEKLER DESTE2 DESTEF DOCKER DOLL DORANT DORGAN DOSEME DROSTE DU DUNN EBELIN ENGELA ENSTRO ESAKI FAN GAO GAO2 GARCIA GARDIN GARSHI GENG GER GILLIS GODLEY GOODMA GREGOR GSELL HAENSZ HAMMO2 HANSEN HEGMAN HEIN HENNEK HINDS HIRAOK HIRAYA HITOSU HOLE HOROWI HORWIT HU HU2 HUANG HUMBLE ISHIMA JAHN JAIN JARUP JARVHO JEDRYC JIANG JONES JUSSAW KAISE2 KAISER KANELL KATSOU KAUFMA KELLER KHUDER KIHARA KINLEN KJUUS KNEKT KO KOHLME KOO KOULUM KREUZE KREYBE KUBIK LAMTH LAMWK LAMWK2 LANGE LAUSSM LEI LEMARC LETOUR LEVIN LIAW LICKIN LIDDEL LIU LIU2 LIU3 LIU4 LIU5 LOMBA2 LUO MACLEN MAGNUS MARSH MARSH2 MARTIS MASTRA MATOS MATSUD MCDUFF MCLAUG MILLER MILLS MOLLO MRFIT MRFITR MURATA NAM NOTAN2 NOTANI NOU ODRISC ORMOS OSANN OSANN2 PARKIN PASTOR PAWLEG PERNU PERSH2 PERSHA PETO PEZZO2 PEZZOT PIKE PISANI POFFIJ POLEDN PRESCO QIAO2 RACHTA RADZIK RANDIG REN RESTRE RIMING RONCO ROOTS ROTHSC SAARIK SANKAR SCHWAR SEGI SEGI2 SEOW SHAW SHIMIZ SIEMIA SIMARA SIMONA SITAS SOBUE SOBUE2 SPEIZE SPITZ STAYNE STOCKS STOCKW STUCKE SUN SUZUK2 SUZUKI SVENSS TANG TANG2 TAO TENKAN TIZZAN TOKARS TOUSEY TSUGAN TULINI ULMER VANDER VEIERO VUTUC WAKAI WALD WANG WANG2 WANG3 WANG4 WARSIN WATSON WICKLU WIGLE WILKIN WU WU2 WUNSCH WUWILL WYNDE2 WYNDE3 WYNDE4 WYNDE5 WYNDE6 WYNDE8 XU XU2 XU3 XU4 YAMAGU YONG YUAN ZHANG ZHENG ZHOU
5	HIRAY2 SCHWA2 TVERDA WYNDER
7	ABELIN ARMADA BENHAM BEST BOFFET BOUCOT CEDERL CHOW CPSI DAMBER DEAN DEAN2 DOLL2 DORN GOLLED GRAHAM JOLY LOMBAR LUBIN2 MCCONN MIGRAN QIAO SADOWS XIANGZ

Table 3E19 - 8
Potentially overlapping studies

REF	REFGP	PRINC	.	OVERLAP/LINK
WYNDE7	WYNDE6	2		WYNDE5/6/7/8
LUBIN	XIANGZ	2		LUBIN/XIANGZ/QIAO

Table 3E20 -

IESLC - Meta-analysis of Ex Smoking, Mixed smokers
Adenocarcinoma

This analysis is restricted to results for:
1) Non-dose-response data
2) Mixed smokers (cigarettes and pipe/cigar)
3) Ex smokers
4) Results complete enough for use in metaanalysis

Within each study, results are then selected (in the following order of preference, within each sex) for:
5) DENOM: never smoked anything, (never +1 = +long term ex)
6) Followup period (prospective studies): whole study (coded as 0) or longest available
7) LCtype: all or nearest available, at least Squamous and Adeno. (q = squamous, s = small,
l = large, a = adeno, mix = mixed, alv = alveolar)
8) Race: all or nearest available, otherwise by race (wh or w = white, bl or b = black, hi = hispanic
ch = chinese, jap = japanese, haw = hawaiian, w+o = white + oriental, sca = scandinavian, as = asian)
9) For overlapping studies: principal rather than subsidiary studies
Finally by Age: whole study (coded as 0) if available, otherwise by widest available age group
and then for single sex results (m, f) in preference to combined sex results (c).

Results adjusted (AD) for the most potential confounders are then chosen in Sections -1 to -3
and results adjusted for the least confounders in Sections -4 to -6. (Those least adjusted results which
actually differ from the most adjusted as marked 'x' in column X in Section -4)
(Results adjusted for an unknown number of confounder(s) are coded as 20.)

Section -7 shows excluded studies, together with the stage (as above) at which no qualifying
results were found.

Section -8 lists the potentially overlapping studies which have been included (1=principal, 2=subsidiary).

Section -9 lists any results which would have been included in preference except that they had data not complete
enough for use in meta-analysis, with their significance (yes/no), if known, and any further comment as entered
on the database.

In addition to those mentioned above, the following fields, levels and abbreviations are used:
* or nk = not known, n = no, y = yes, ot = other
nev = never
REF: 6-character study reference
NRR: number of the RR on the database within the study
ST : study type (CC = case control, pr or prosp = prospective)
NLC: number of lung cancer cases in whole study
R : risky occupational population (n = no, m = mining, o = other risky)
VB : national cigarette type (V = at least 75% Virginia, bl = at least 75% blended, ot = other)
P : any proxy use
H : full histological confirmation
De : derivation of RR/CI (or = original, st = standard method, ot = other method of estimation)

Table 3E20 - 1

IESLC - Meta-analysis of Ex Smoking, Mixed smokers
Adenocarcinoma
Most adjusted

REF	NRR	SEX	AGEL	AGEH	RACE	YF	LC TYPE	LOC	START	ST	NLC	R	VB	P	H	AD	DENOM	De
WYNDE7	38	m	0	0	all	-	KII	NAmer	1977	CC	2085	n	bl	n	y	0	nev any	st

Table 3E20 - 2

IESLC - Meta-analysis of Ex Smoking, Mixed smokers
Adenocarcinoma
Most adjusted

REF	NRR	SEX	AD	Number Exposed Case	Number Exposed Cont	Non-exposed Case	Non-exposed Cont	RR	95.00%CI
WYNDE7	38	m	0	45	327	42	918	3.01	(1.94- 4.67)
Totals				45	327	42	918		

*prospective study

REF	NRR	SEX	AD	Ys	Ws	Qs	Ps
WYNDE7	38	m	0	1.10	19.93	0.00	0.0000

	N	1
	NS	1
	Wt	19.93
Het	Chi	0.00
Het	df	0
Het	P	N.S.
Fixed	RR	3.01
	RRl	1.94
	RRu	4.67
	P	+++
Random	RR	3.01
	RRl	1.94
	RRu	4.67
	P	+++
Asymm	P	

Table 3E20 - 3

IESLC - Meta-analysis of Ex Smoking, Mixed smokers
Adenocarcinoma
Most adjusted

	Sex combined	male	female	Total
N		1		1
NS		1		1
Wt		19.93		19.93
Het Chi		0.00		0.00
Het df		0		0
Het P		N.S.		N.S.
Fixed RR		3.01		3.01
RRl		1.94		1.94
RRu		4.67		4.67
P		+++		+++
Random RR		3.01		3.01
RRl		1.94		1.94
RRu		4.67		4.67
P		+++		+++
Between Chi				
Between df				
Between P				N.S.
Btwn(F) P				N.S.
Btwn(R) P				N.S.

Too few RRs for analysis by factor

Table 3E20 - 4

IESLC - Meta-analysis of Ex Smoking, Mixed smokers
Adenocarcinoma
Least adjusted

REF	NRR	X	SEX	AGEL	AGEH	RACE	YF	LC TYPE	LOC	START	ST	NLC	R	VB	P	H	AD	DENOM	De
WYNDE7	38		m	0	0	all	-	KII	NAmer	1977	CC	2085	n	bl	n	y	0	nev any	st

Table 3E20 - 5

IESLC - Meta-analysis of Ex Smoking, Mixed smokers
Adenocarcinoma
Least adjusted

REF	NRR	SEX	AD	Number Exposed Case	Cont	Non-exposed Case	Cont	RR	95.00%CI
WYNDE7	38	m	0	45	327	42	918	3.01	(1.94- 4.67)
Totals				45	327	42	918		

*prospective study

REF	NRR	SEX	AD	Ys	Ws	Qs	Ps
WYNDE7	38	m	0	1.10	19.93	0.00	0.0000

	N	1
	NS	1
	Wt	19.93
Het	Chi	0.00
Het	df	0
Het	P	N.S.
Fixed	RR	3.01
	RRl	1.94
	RRu	4.67
	P	+++
Random	RR	3.01
	RRl	1.94
	RRu	4.67
	P	+++
Asymm	P	

Table 3E20 - 6

IESLC - Meta-analysis of Ex Smoking, Mixed smokers
Adenocarcinoma
Least adjusted

	Sex			
	combined	male	female	Total
N		1		1
NS		1		1
Wt		19.93		19.93
Het Chi		0.00		0.00
Het df		0		0
Het P		N.S.		N.S.
Fixed RR		3.01		3.01
RRl		1.94		1.94
RRu		4.67		4.67
P		+++		+++
Random RR		3.01		3.01
RRl		1.94		1.94
RRu		4.67		4.67
P		+++		+++
Between Chi				
Between df				
Between P				N.S.
Btwn(F) P				N.S.
Btwn(R) P				N.S.

Table 3E20 - 7

IESLC - Meta-analysis of Ex Smoking, Mixed smokers
Adenocarcinoma
Excluded studies (and stage at which they were excluded)

1 BOUCHA BUELL LAURIL MZILEN

2 ABRAHA AGUDO AKIBA AMANDU AMES ANDERS ARCHER AUSTIN AUVINE AXELSO AXELSS BAND BARBON BECHER BENSHL BERRIN
BLOHMK BLOT1 BLOT2 BLOT3 BLOT4 BRETT BROCKM BROSS BROWN1 BROWN2 BUFFLE BYERS1 BYERS2 CARPEN CASCO2 CASCOR
CHAN CHANG CHATZI CHEN CHEN2 CHEN3 CHIAZZ CHOI CHYOU COMSTO COOKSO CORREA CPSII DARBY DAVEYS DEAN3
DEKLER DESTE2 DESTEF DOCKER DOLL DORANT DORGAN DOSEME DROSTE DU DUNN EBELIN ENGELA ENSTRO ESAKI FAN
GAO GAO2 GARCIA GARDIN GARSHI GENG GER GILLIS GODLEY GOODMA GREGOR GSELL HAENSZ HAMMO2 HANSEN HEGMAN
HEIN HENNEK HINDS HIRAOK HIRAYA HITOSU HOLE HOROWI HORWIT HU HU2 HUANG HUMBLE ISHIMA JAHN JAIN
JARUP JARVHO JEDRYC JIANG JONES JUSSAW KAISE2 KAISER KANELL KATSOU KAUFMA KELLER KHUDER KIHARA KINLEN KJUUS
KNEKT KO KOHLME KOO KOULUM KREUZE KREYBE KUBIK LAMTH LAMWK LAMWK2 LANGE LAUSSM LEI LEMARC LETOUR
LEVIN LIAW LICKIN LIDDEL LIU LIU2 LIU3 LIU4 LIU5 LOMBA2 LUO MACLEN MAGNUS MARSH MARSH2 MARTIS
MASTRA MATOS MATSUD MCDUFF MCLAUG MILLER MILLS MOLLO MRFIT MRFITR MURATA NAM NOTAN2 NOTANI NOU ODRISC
ORMOS OSANN OSANN2 PARKIN PASTOR PAWLEG PERNU PERSH2 PERSHA PETO PEZZO2 PEZZOT PIKE PISANI POFFIJ POLEDN
PRESCO QIAO2 RACHTA RADZIK RANDIG REN RESTRE RIMING RONCO ROOTS ROTHSC SAARIK SANKAR SCHWAR SEGI SEGI2
SEOW SHAW SHIMIZ SIEMIA SIMARA SIMONA SITAS SOBUE SOBUE2 SPEIZE SPITZ STAYNE STOCKS STOCKW STUCKE SUN
SUZUK2 SUZUKI SVENSS TANG TANG2 TAO TENKAN TIZZAN TOKARS TOUSEY TSUGAN TULINI ULMER VANDER VEIERO VUTUC
WAKAI WALD WANG WANG2 WANG3 WANG4 WARSIN WATSON WICKLU WIGLE WILKIN WU WU2 WUNSCH WUWILL WYNDE2
WYNDE3 WYNDE4 WYNDE5 WYNDE6 WYNDE8 XU XU2 XU3 XU4 YAMAGU YONG YUAN ZHANG ZHENG ZHOU

3 ABELIN ALDERS ARMADA BENHAM BEST BOFFET BOUCOT BRESLO CEDERL CPSI DAMBER DEAN DEAN2 GOLLED HAMMON HIRAY2
JOLY LUBIN MCCONN MIGRAN QIAO SADOWS SCHWA2 STASZE TVERDA WYNDER XIANGZ

7 CHOW DOLL2 DORN GRAHAM LOMBAR LUBIN2

Table 3E20 - 8

Potentially overlapping studies

REF	REFGP	PRINC	.	OVERLAP/LINK
WYNDE7	WYNDE6	2		WYNDE5/6/7/8
