# Supplementary material for: Systematic review with meta-analysis of the epidemiological evidence in the 1900s relating smoking to lung cancer
Source: BMC Cancer. 2012 Sep 3;12:385. doi: 10.1186/1471-2407-12-385 (PMC3505152; doi:10.1186/1471-2407-12-385)
Supplement: Additional file 5 — Detailed Analysis Tables (Individual file names as described in Additional file 1: Methods, Table1). [file 1471-2407-12-385-S5.zip › PDF/1A.pdf]

Table 1A1 -

IESLC - Meta-analysis of Ever Smoking, Any product (or Cigarettes if Any not available)
All LC types

This analysis is restricted to results for:
1) Non-dose-response data
2) Ever smokers
3) Results complete enough for use in metaanalysis

Within each study, results are then selected (in the following order of preference, within each sex) for:
4) PRODUCT: all/unspec, cigarettes regardless of other products, cigarettes only
5) CIGTYPE: all/unspecified, MC regardless of HR, MC only
6) DENOM: never smoked anything, never smoked cigarettes, (never +1 = +long term ex, +2 = +amount unknown, +3 = never cigs+long term ex)
7) Followup period (YF, prospective studies): whole study (coded as 0) or longest available
8) LCtype: all or nearest available, at least Squamous and Adeno. (q = squamous, s = small, l = large, a = adeno, mix = mixed, alv = alveolar)
9) Race: all or nearest available, otherwise by race (wh or w = white, bl or b = black, hi = hispanic ch = chinese, jap = japanese, haw = hawaiian, w+o = white + oriental, sca = scandinavian, as = asian)
10) For overlapping studies: principal rather than subsidiary studies
Finally by Age: whole study (coded as 0) if available, otherwise by widest available age group and then for single sex results (m, f) in preference to combined sex results (c).

Results adjusted (AD) for the most potential confounders are then chosen in Sections -1 to -3 and results adjusted for the least confounders in Sections -4 to -6. (Those least adjusted results which actually differ from the most adjusted as marked 'x' in column X in Section -4)
(Results adjusted for an unknown number of confounder(s) are coded as 20.)

Section -7 shows excluded studies, together with the stage (as above) at which no qualifying results were found.

Section -8 lists the potentially overlapping studies which have been included (1=principal, 2=subsidiary).

Section -9 lists any results which would have been included in preference except that they had data not complete enough for use in meta-analysis, with their significance (yes/no), if known, and any further comment as entered on the database.

In addition to those mentioned above, the following fields, levels and abbreviations are used:
* or nk = not known, n = no, y = yes, ot = other
nev = never
all/unspec = all or unspecified, cig+/-ot = cigarettes irrespective of other products (cigar, pipe etc)
MC = manufactured cigarettes, HR = hand-rolled cigarettes
REF: 6-character study reference
NRR: number of the RR on the database within the study
ST : study type (CC = case control, pr or prosp = prospective)
NLC: number of lung cancer cases in whole study
R : risky occupational population (n = no, m = mining, o = other risky)
VB : national cigarette type (V = at least 75% Virginia, bl = at least 75% blended, ot = other)
P : any proxy use
H : full histological confirmation
De : derivation of RR/CI (or = original, st = standard method, ot = other method of estimation)

Table 1A1 - 1

IESLC - Meta-analysis of Ever Smoking, Any product (or Cigarettes if Any not available)
All LC types
Most adjusted

REF	NRR	SEX	AGEL	AGEH	RACE	YF	LC TYPE	LOC	START	ST	NLC	R	VB	P	H	AD	PRODUCT	DENOM	De
ABELIN	44	m	0	0	all	-	all	Eu:wst	1941	CC	118	n	bl	y	n	1	all/unsp	nev any	st
ABRAHA	7	m	0	0	all	0	q+s+a	Eu:est	1975	pr	571	n	bl	n	n	0	all/unsp	nev any	ot
ABRAHA	8	f	0	0	all	0	q+s+a	Eu:est	1975	pr	571	n	bl	n	n	0	all/unsp	nev any	ot
AGUDO	1	f	0	0	all	-	all	Eu:wst	1989	CC	103	n	bl	n	n	3	cig only	nev any	or
AKIBA	11	m	0	0	all	0	all	As:Jap	1963	pr	610	n	bl	n	n	5	cig+/-ot	nev cigs	ot
AKIBA	15	f	0	0	all	0	all	As:Jap	1963	pr	610	n	bl	n	n	5	cig+/-ot	nev cigs	ot
ALDERS	69	m	0	0	all	-	all	Eu:UK	1977	CC	1448	n	V	n	n	1	all/unsp	nev any	ot
ALDERS	6	f	0	0	all	-	all	Eu:UK	1977	CC	1448	n	V	n	n	1	cig only	nev any	ot
AMANDU	7	m	0	0	wh	0	all	NAmer	1959	pr	132	m	bl	n	n	2	cig+/-ot	nev cigs	ot
AMES	4	m	0	0	wh	-	all	NAmer	1959	ot	317	m	bl	n	n	0	all/unsp	nev any	st
ANDERS	3	f	0	0	all	0	all	NAmer	1986	pr	343	n	bl	n	n	0	cig+/-ot	nev cigs	st
ARCHER	6	m	0	0	wh	0	all	NAmer	1950	pr	146	m	bl	n	n	0	cig+/-ot	nev cigs	st
ARMADA	29	m	0	0	all	-	all	Eu:wst	1986	CC	325	n	bl	n	y	0	all/unsp	nev any	st
AUSTIN	7	c	0	0	all	-	all	NAmer	1970	CC	166	o	bl	y	n	3	cig+/-ot	nev cigs	ot
AUVINE	19	c	0	0	all	-	all	Eu:Sca	1986	CC	517	n	bl	y	n	2	cig+/-ot	nev cigs	ot
AXELSO	1	c	0	0	all	-	all	Eu:Sca	1960	CC	152	n	bl	y	n	0	all/unsp	nev any	st
AXELSS	8	m	0	0	sca	-	all	Eu:Sca	1989	CC	436	n	bl	n	n	6	all/unsp	nev any	ot
AXELSS	11	f	0	0	sca	-	all	Eu:Sca	1989	CC	436	n	bl	n	n	0	all/unsp	nev any	st
BAND	1	m	0	0	all	-	all	NAmer	1983	CC	2831	n	V	y	y	2	cig only	nev any	ot
BARBON	131	m	0	0	all	-	all	Eu:wst	1979	CC	755	n	bl	y	y	3	all/unsp	nev any	ot
BECHER	1	m	0	0	all	-	all	Eu:Ger	1985	CC	194	n	bl	n	y	0	all/unsp	nev any	st
BECHER	24	f	0	0	all	-	all	Eu:Ger	1985	CC	194	n	bl	n	y	2	all/unsp	nev any	ot
BENSHL	18	m	40	64	all	10	all	Eu:UK	1967	pr	486	n	V	n	n	1	all/unsp	nev any	ot
BEST	22	m	55	79	all	3	all	NAmer	1955	pr	381	n	V	n	n	0	all/unsp	nev any	st
BEST	18	f	0	0	all	0	all	NAmer	1955	pr	381	n	V	n	n	1	cig only	nev any	ot
BLOHMK	3	m	0	0	all	-	all	Eu:Ger	1978	CC	888	n	bl	n	y	0	all/unsp	nev any	st
BLOT4	1	m	0	0	wh	-	all	NAmer	1974	CC	335	n	bl	y	n	0	cig+/-ot	nev cigs	st
BOFFET	33	m	0	0	all	-	all	Eu:mul	1988	CC	5621	n	bl	y	n	2	all/unsp	nev any	or
BOUCOT	121	m	0	0	all	0	all	NAmer	1951	pr	121	n	bl	n	n	2	all/unsp	nev any	ot
BRESLO	37	m	0	0	all	-	all	NAmer	1949	CC	518	n	bl	n	y	0	all/unsp	nev+1	st
BRESLO	38	f	0	0	all	-	all	NAmer	1949	CC	518	n	bl	n	y	0	all/unsp	nev+1	st
BRETT	10	m	0	0	all	0	all	Eu:UK	1960	pr	150	n	V	n	n	0	cig+/-ot	nev cigs	st
BROCKM	1	m	0	0	wh	-	all	Eu:Ger	1990	CC	117	n	bl	n	y	0	cig+/-ot	nev cigs	st
BROCKM	2	f	0	0	wh	-	all	Eu:Ger	1990	CC	117	n	bl	n	y	0	cig+/-ot	nev cigs	st
BROSS	12	m	0	0	wh	-	all	NAmer	1960	CC	974	n	bl	n	n	0	all/unsp	nev any	st
BROWN2	2	m	0	0	wh	-	all	NAmer	1984	CC	14596	n	bl	n	y	2	cig+/-ot	nev cigs	or
BROWN2	1	f	0	0	wh	-	all	NAmer	1984	CC	14596	n	bl	n	y	2	cig+/-ot	nev cigs	or
BUFFLE	1	m	0	0	wh	-	all	NAmer	1976	CC	943	n	bl	y	n	0	all/unsp	nev any	st
BUFFLE	5	f	0	0	wh	-	all	NAmer	1976	CC	943	n	bl	y	n	0	all/unsp	nev any	st
CARPEN	12	c	0	0	w+b	-	all	NAmer	1991	CC	356	n	bl	n	n	3	cig+/-ot	nev cigs	ot
CASCO2	1	c	0	0	wh	-	all	Eu:Ger	1991	CC	155	n	bl	n	n	0	all/unsp	nev any	st
CASCOR	1	c	0	0	wh	-	all	Eu:Ger	1985	CC	389	n	bl	n	y	0	all/unsp	nev any	st
CEDERL	107	m	0	0	all	16	all	Eu:Sca	1963	pr	491	n	bl	n	n	2	all/unsp	nev any	ot
CEDERL	112	f	0	0	all	0	all	Eu:Sca	1963	pr	491	n	bl	n	n	2	all/unsp	nev any	ot
CHAN	9	m	0	0	all	-	all	As:HK	1976	CC	397	n	bl	n	n	0	all/unsp	nev any	st
CHAN	10	f	0	0	all	-	all	As:HK	1976	CC	397	n	bl	n	n	0	all/unsp	nev any	st
CHANG	6	m	0	0	all	0	all	NAmer	1972	pr	136	n	bl	n	n	0	cig+/-ot	nev cigs	st
CHANG	12	f	0	0	all	0	all	NAmer	1972	pr	136	n	bl	n	n	0	cig+/-ot	nev cigs	st
CHATZI	4	c	0	0	all	-	all	Eu:bal	1987	CC	282	n	bl	n	y	0	all/unsp	nev any	st
CHEN2	1	m	0	0	all	-	all	As:Chi	1983	CC	193	n	ot	y	n	0	all/unsp	nev any	st
CHEN2	2	f	0	0	all	-	all	As:Chi	1983	CC	193	n	ot	y	n	0	all/unsp	nev any	st
CHEN3	1	c	0	0	all	-	all	As:Chi	1981	CC	254	n	ot	y	n	0	all/unsp	nev any	st
CHIAZZ	3	m	0	0	all	-	all	NAmer	1940	CC	144	o	bl	y	n	11	cig+/-ot	nev cigs	or
CHOI	1	m	0	0	all	-	all	As:oth	1985	CC	375	n	bl	n	n	0	cig+/-ot	nev cigs	st
CHOI	5	f	0	0	all	-	all	As:oth	1985	CC	375	n	bl	n	n	0	cig+/-ot	nev cigs	st
CHOW	55	m	0	0	wh	0	all	NAmer	1966	pr	219	n	bl	n	n	2	all/unsp	nev any	ot
CHYOU	7	m	0	0	jap	0	all	NAmer	1965	pr	227	n	bl	n	y	1	cig+/-ot	nev cigs	ot
COMSTO	34	m	0	0	all	-	all	NAmer	1975	ot	258	n	bl	n	n	0	all/unsp	nev any	st
COMSTO	46	f	0	0	all	-	all	NAmer	1975	ot	258	n	bl	n	n	0	all/unsp	nev any	st
COOKSO	5	c	0	0	bl	-	all	Africa	1961	CC	234	n	V	n	y	0	all/unsp	nev any	st
CORREA	34	c	0	0	all	-	all	NAmer	1979	CC	1359	n	bl	y	n	1	cig+/-ot	nev cigs	or
CPSI	187	m	35	84	all	6	all	NAmer	1959	pr	5138	n	bl	n	n	1	cig+/-ot	nev any	ot
CPSI	274	f	40	74	all	6	all	NAmer	1959	pr	5138	n	bl	n	n	1	cig+/-ot	nev cigs	ot
CPSII	104	m	35	99	all	4	all	NAmer	1982	pr	3229	n	bl	n	n	1	cig only	nev any	ot
CPSII	79	f	0	0	all	4	all	NAmer	1982	pr	3229	n	bl	n	n	1	cig+/-ot	nev cigs	ot
DAMBER	25	m	0	0	all	-	all	Eu:Sca	1972	CC	579	n	bl	y	n	1	all/unsp	nev any	st
DARBY	15	m	0	0	wh	-	all	Eu:UK	1988	CC	982	n	V	n	n	0	all/unsp	nev any	st
DARBY	16	f	0	0	wh	-	all	Eu:UK	1988	CC	982	n	V	n	n	0	all/unsp	nev any	st
DAVEYS	5	m	0	0	all	-	all	Eu:Ger	1930	CC	109	n	bl	y	n	0	all/unsp	nev any	st
DAVEYS	6	f	0	0	all	-	all	Eu:Ger	1930	CC	109	n	bl	y	n	0	all/unsp	nev any	ot
DEAN	7	m	0	0	wh	-	all	Africa	1947	CC	603	n	V	y	n	0	all/unsp	nev any	st
DEAN2	3	m	0	0	all	-	all	Eu:UK	1960	CC	954	n	V	y	n	0	all/unsp	nev any	st

Table 1A1 - 1

IESLC - Meta-analysis of Ever Smoking, Any product (or Cigarettes if Any not available)
All LC types
Most adjusted

REF	NRR	SEX	AGEL	AGEH	RACE	YF	LC TYPE	LOC	START	ST	NLC	R	VB	P	H	AD	PRODUCT	DENOM	De
DEAN2	7	f	0	0	all	-	all	Eu:UK	1960	CC	954	n	V	y	n	0	all/unsp	nev any	st
DEAN3	49	m	0	0	all	-	all	Eu:UK	1969	CC	766	n	V	y	n	3	all/unsp	nev any	ot
DEAN3	126	f	0	0	all	-	all	Eu:UK	1969	CC	766	n	V	y	n	3	cig only	nev any	ot
DEKLER	6	m	0	0	all	0	all	Auslia	1961	pr	138	m	V	n	n	2	all/unsp	nev any	ot
DESTE2	14	c	0	0	all	-	all	SCAmer	1993	CC	463	n	bl	n	n	7	all/unsp	nev any	st
DESTEF	48	m	0	0	all	-	all	SCAmer	1988	CC	497	n	bl	n	y	4	all/unsp	nev any	ot
DOCKER	3	c	0	0	wh	0	all	NAmer	1974	pr	120	n	bl	n	n	4	cig+/-ot	nev cigs	ot
DOLL	6	m	0	0	all	-	all	Eu:UK	1948	CC	1465	n	V	n	n	0	all/unsp	nev any	st
DOLL	12	f	0	0	all	-	all	Eu:UK	1948	CC	1465	n	V	n	n	0	all/unsp	nev any	st
DOLL2	56	m	0	0	all	0	all	Eu:UK	1951	pr	920	n	V	n	n	1	all/unsp	nev any	ot
DORANT	10	c	0	0	all	0	all	Eu:wst	1986	ot	550	n	bl	n	y	0	all/unsp	nev any	st
DORGAN	6	m	0	0	wh	-	all	NAmer	1980	CC	2026	n	bl	y	y	0	all/unsp	nev any	st
DORGAN	30	m	0	0	bl	-	all	NAmer	1980	CC	2026	n	bl	y	y	0	all/unsp	nev any	st
DORGAN	53	f	0	0	wh	-	all	NAmer	1980	CC	2026	n	bl	y	y	0	all/unsp	nev any	st
DORGAN	76	f	0	0	bl	-	all	NAmer	1980	CC	2026	n	bl	y	y	0	all/unsp	nev any	st
DORN	196	m	35	84	wh	8	all	NAmer	1954	pr	5097	n	bl	n	n	1	all/unsp	nev any	ot
DOSEME	1	m	0	0	all	-	all	Eu:bal	1979	CC	1210	n	bl	n	n	2	cig+/-ot	nev cigs	or
DROSTE	7	m	0	0	all	-	all	Eu:wst	1995	CC	478	n	bl	n	y	4	all/unsp	nev any	ot
DU	1	m	0	0	all	-	all	As:Chi	1985	CC	849	n	ot	y	n	0	all/unsp	nev any	or
DU	2	f	0	0	all	-	all	As:Chi	1985	CC	849	n	ot	y	n	0	all/unsp	nev any	or
DUNN	6	m	0	0	all	0	all	NAmer	1954	pr	139	o	bl	n	n	0	cig+/-ot	nev cigs	st
EBELIN	1	m	0	0	all	-	all	Eu:Ger	1980	CC	130	n	bl	n	n	0	all/unsp	nev any	st
ENGELA	159	m	0	0	all	12	all	Eu:Sca	1964	pr	435	n	bl	n	n	1	all/unsp	nev any	ot
ENGELA	165	f	0	0	all	12	all	Eu:Sca	1964	pr	435	n	bl	n	n	1	all/unsp	nev any	ot
ESAKI	4	m	0	0	all	-	all	As:Jap	1961	CC	245	n	bl	y	n	0	cig+/-ot	nev cigs	st
ESAKI	5	f	0	0	all	-	all	As:Jap	1961	CC	245	n	bl	y	n	0	cig+/-ot	nev cigs	st
FAN	1	m	0	0	all	-	all	As:Chi	1990	CC	403	n	ot	y	n	0	cig+/-ot	nev cigs	st
FAN	2	f	0	0	all	-	all	As:Chi	1990	CC	403	n	ot	y	n	0	cig+/-ot	nev cigs	st
GAO	1	m	0	0	all	-	all	As:Chi	1984	CC	1405	n	ot	n	n	2	cig+/-ot	nev cigs	or
GAO	11	f	0	0	all	-	all	As:Chi	1984	CC	1405	n	ot	n	n	2	cig+/-ot	nev cigs	or
GAO2	10	m	0	0	all	-	all	As:Jap	1988	CC	282	n	bl	n	n	1	cig+/-ot	nev cigs	ot
GARCIA	3	c	0	0	all	-	all	NAmer	1992	CC	416	n	bl	n	y	0	cig+/-ot	nev cigs	st
GARDIN	7	c	0	0	all	-	all	Eu:UK	1988	CC	143	n	V	y	n	0	all/unsp	nev any	st
GARSHI	25	m	0	0	all	-	all	NAmer	1981	CC	1081	o	bl	y	n	1	all/unsp	nev any	st
GENG	1	m	0	0	all	-	all	As:Chi	1985	CC	292	n	ot	*	n	0	cig+/-ot	nev any	st
GENG	2	f	0	0	all	-	all	As:Chi	1985	CC	292	n	ot	*	n	0	cig+/-ot	nev any	st
GER	21	c	0	0	all	-	all	As:oth	1990	CC	141	n	ot	y	n	14	all/unsp	nev any	ot
GODLEY	5	m	0	0	all	-	all	NAmer	1966	CC	1986	n	bl	y	n	1	cig+/-ot	nev cigs	ot
GODLEY	6	f	0	0	all	-	all	NAmer	1966	CC	1986	n	bl	y	n	1	cig+/-ot	nev cigs	ot
GOLLED	7	m	35	99	all	-	all	Eu:UK	1952	CC	443	n	V	y	n	1	cig+/-ot	nev any	ot
GOODMA	3	m	0	0	w+o	-	all	NAmer	1983	CC	326	n	bl	y	y	0	cig+/-ot	nev any	st
GOODMA	7	f	0	0	w+o	-	all	NAmer	1983	CC	326	n	bl	y	y	0	cig+/-ot	nev any	st
GRAHAM	27	m	0	0	wh	-	all	NAmer	1956	CC	685	n	bl	n	n	1	all/unsp	nev any	ot
GREGOR	3	m	0	0	all	-	all	Eu:UK	1976	CC	104	n	V	n	y	0	cig+/-ot	nev cigs	st
GREGOR	7	f	0	0	all	-	all	Eu:UK	1976	CC	104	n	V	n	y	0	cig+/-ot	nev cigs	st
GSELL	8	m	0	0	all	-	all	Eu:wst	1937	CC	150	n	bl	n	y	0	all/unsp	nev any	st
HAENSZ	11	f	0	0	all	-	not alv	NAmer	1955	CC	158	n	bl	n	y	2	all/unsp	nev any	ot
HAMMO2	4	m	0	0	all	6	all	NAmer	1967	pr	450	o	bl	n	n	1	all/unsp	nev any	ot
HAMMON	117	m	0	0	wh	0	all	NAmer	1952	pr	448	n	bl	n	n	1	all/unsp	nev any	ot
HANSEN	3	m	0	0	all	0	all	Eu:Sca	1968	pr	105	o	bl	y	n	2	all/unsp	nev any	ot
HEGMAN	1	c	0	0	all	-	all	NAmer	1989	CC	282	n	bl	y	y	0	all/unsp	nev any	st
HEIN	7	m	0	0	all	0	all	Eu:Sca	1970	pr	144	n	bl	n	n	0	all/unsp	nev any	st
HENNEK	3	m	0	0	all	0	all	NAmer	1982	pr	169	n	bl	n	n	0	all/unsp	nev any	st
HINDS	22	f	0	0	o	-	all	NAmer	1968	CC	292	n	bl	n	n	3	all/unsp	nev any	st
HIRAYA	147	m	0	0	all	0	all	As:Jap	1965	pr	1917	n	bl	n	n	1	cig+/-ot	nev any	ot
HIRAYA	150	f	0	0	all	0	all	As:Jap	1965	pr	1917	n	bl	n	n	1	cig+/-ot	nev any	ot
HITOSU	38	m	0	0	all	-	all	As:Jap	1960	CC	216	n	bl	y	n	1	all/unsp	nev any	st
HITOSU	62	f	0	0	all	-	all	As:Jap	1960	CC	216	n	bl	y	n	1	all/unsp	nev any	st
HOLE	8	m	0	0	all	0	all	Eu:UK	1972	pr	225	n	V	n	n	1	all/unsp	nev any	ot
HOROWI	1	m	0	0	all	-	all	NAmer	1956	CC	236	n	V	n	n	0	cig+/-ot	nev any	st
HOROWI	2	f	0	0	all	-	all	NAmer	1956	CC	236	n	V	n	n	0	cig+/-ot	nev any	st
HORWIT	1	f	0	0	all	-	all	NAmer	1977	CC	112	n	bl	n	n	0	cig+/-ot	nev cigs	st
HU	15	m	0	0	all	-	all	As:Chi	1985	CC	227	n	ot	n	y	0	cig+/-ot	nev any	st
HU	16	f	0	0	all	-	all	As:Chi	1985	CC	227	n	ot	n	y	0	cig+/-ot	nev any	st
HU2	9	m	0	0	all	-	all	As:Chi	1977	CC	523	n	ot	y	n	0	cig+/-ot	nev cigs	st
HU2	10	f	0	0	all	-	all	As:Chi	1977	CC	523	n	ot	y	n	0	cig+/-ot	nev cigs	st
HUANG	1	c	0	0	all	-	all	As:Chi	1990	CC	135	n	ot	y	n	0	all/unsp	nev any	st
HUMBLE	14	m	0	0	w-hi	-	all	NAmer	1980	CC	521	n	bl	y	n	1	cig+/-ot	nev cigs	ot
HUMBLE	16	m	0	0	hi	-	all	NAmer	1980	CC	521	n	bl	y	n	1	cig+/-ot	nev cigs	ot
HUMBLE	18	f	0	0	w-hi	-	all	NAmer	1980	CC	521	n	bl	y	n	1	cig+/-ot	nev cigs	ot
HUMBLE	20	f	0	0	hi	-	all	NAmer	1980	CC	521	n	bl	y	n	1	cig+/-ot	nev cigs	ot
JAHN	22	f	0	0	all	-	all	Eu:Ger	1988	CC	1004	n	bl	n	n	2	cig+/-ot	nev any	ot

Table 1A1 - 1

IESLC - Meta-analysis of Ever Smoking, Any product (or Cigarettes if Any not available)
All LC types
Most adjusted

REF	NRR	SEX	AGEL	AGEH	RACE	YF	LC TYPE	LOC	START	ST	NLC	R	VB	P	H	AD	PRODUCT	DENOM	De
JAIN	46	m	0	0	all	-	all	NAmer	1981	CC	845	n	V	y	n	2	cig+/-ot	nev cigs	or
JAIN	41	f	0	0	all	-	all	NAmer	1981	CC	845	n	V	y	n	2	cig+/-ot	nev cigs	or
JARUP	6	m	0	0	all	-	all	Eu:Sca	1928	CC	102	o	bl	y	n	2	all/unsp	nev any	ot
JARVHO	3	m	0	0	all	-	all	Eu:Sca	1983	CC	147	n	bl	n	n	0	all/unsp	nev any	st
JARVHO	7	f	0	0	all	-	all	Eu:Sca	1983	CC	147	n	bl	n	n	0	all/unsp	nev any	st
JEDRYC	58	m	0	0	all	-	all	Eu:est	1980	CC	1630	n	bl	y	n	4	cig+/-ot	nev any	ot
JEDRYC	59	f	0	0	all	-	all	Eu:est	1980	CC	1630	n	bl	y	n	4	cig+/-ot	nev any	ot
JIANG	1	m	0	0	all	-	all	As:Chi	1984	CC	125	n	ot	n	n	0	all/unsp	nev any	st
JIANG	2	f	0	0	all	-	all	As:Chi	1984	CC	125	n	ot	n	n	0	all/unsp	nev any	st
JOLY	14	m	0	0	all	-	all	SCAmer	1978	CC	826	n	bl	n	n	0	all/unsp	nev any	st
JOLY	1	f	0	0	all	-	all	SCAmer	1978	CC	826	n	bl	n	n	0	cig+/-ot	nev any	st
JUSSAW	29	m	0	0	all	-	all	As:Ind	1964	CC	792	n	V	n	n	2	all/unsp	nev any	st
KAISE2	72	m	35	99	all	9	all	NAmer	1979	pr	318	n	bl	n	n	1	cig only	nev any	st
KAISE2	64	f	35	99	all	9	all	NAmer	1979	pr	318	n	bl	n	n	1	cig only	nev any	st
KAISER	13	m	0	0	all	0	all	NAmer	1964	pr	714	n	bl	n	n	2	cig+/-ot	nev cigs	ot
KAISER	10	f	0	0	all	0	all	NAmer	1964	pr	714	n	bl	n	n	2	cig+/-ot	nev cigs	ot
KATSOU	29	f	0	0	all	-	all	Eu:bal	1987	CC	101	n	bl	n	n	1	all/unsp	nev any	ot
KAUFMA	17	c	0	0	all	-	all	NAmer	1981	CC	881	n	bl	n	n	6	cig+/-ot	nev cigs	ot
KELLER	3	m	0	0	wh	-	all	NAmer	1985	CC	15038	n	bl	n	n	0	all/unsp	nev any	st
KELLER	11	m	0	0	nonw	-	all	NAmer	1985	CC	15038	n	bl	n	n	0	all/unsp	nev any	st
KELLER	7	f	0	0	wh	-	all	NAmer	1985	CC	15038	n	bl	n	n	0	all/unsp	nev any	st
KELLER	15	f	0	0	nonw	-	all	NAmer	1985	CC	15038	n	bl	n	n	0	all/unsp	nev any	st
KHUDER	4	m	0	0	all	-	all	NAmer	1985	CC	482	n	bl	n	y	0	cig+/-ot	nev cigs	st
KIHARA	31	c	0	0	jap	-	all	As:Jap	1991	CC	440	n	bl	n	n	0	all/unsp	nev any	st
KINLEN	17	m	0	0	all	0	all	Eu:UK	1967	pr	718	n	V	n	n	2	all/unsp	nev any	ot
KJUUS	10	m	0	0	all	-	all	Eu:Sca	1979	CC	176	n	bl	n	n	0	all/unsp	nev any	st
KNEKT	87	m	20	69	all	21	all	Eu:Sca	1966	pr	515	n	bl	n	n	1	all/unsp	nev any	ot
KO	1	f	0	0	all	-	all	As:oth	1992	CC	117	n	ot	n	y	3	cig+/-ot	nev cigs	or
KOHLME	2	c	0	0	all	-	all	Eu:Ger	1990	CC	239	n	bl	n	n	4	all/unsp	nev any	or
KOO	1	f	0	0	all	-	all	As:HK	1981	CC	200	n	bl	n	n	0	all/unsp	nev any	st
KOULUM	1	m	0	0	all	-	all	Eu:Sca	1936	CC	812	n	bl	n	n	0	all/unsp	nev any	st
KREUZE	14	f	1	45	all	-	all	Eu:Ger	1990	CC	2260	n	bl	n	n	0	all/unsp	nev any	st
KREUZE	16	f	55	69	all	-	all	Eu:Ger	1990	CC	2260	n	bl	n	n	0	all/unsp	nev any	st
KREYBE	12	m	0	0	all	-	all	Eu:Sca	1948	CC	300	n	bl	n	y	1	all/unsp	nev any	ot
KREYBE	30	f	0	0	all	-	all	Eu:Sca	1948	CC	300	n	bl	n	y	1	all/unsp	nev any	ot
KUBIK	28	m	0	0	all	0	all	Eu:est	1965	pr	108	n	bl	n	n	0	all/unsp	nev any	st
LAMTH	6	f	0	0	ch	-	all	As:HK	1983	CC	445	n	bl	n	n	0	all/unsp	nev any	or
LAMWK	1	f	0	0	ch	-	all	As:HK	1981	CC	163	n	bl	n	n	0	all/unsp	nev any	st
LAMWK2	9	m	0	0	all	-	q+s+l+a	As:HK	1976	CC	480	n	bl	n	n	0	all/unsp	nev any	st
LAMWK2	10	f	0	0	all	-	q+s+l+a	As:HK	1976	CC	480	n	bl	n	n	0	all/unsp	nev any	st
LANGE	40	m	0	0	all	0	all	Eu:Sca	1976	pr	268	n	bl	n	n	1	all/unsp	nev any	ot
LANGE	37	f	0	0	all	0	all	Eu:Sca	1976	pr	268	n	bl	n	n	1	all/unsp	nev any	ot
LAUSSM	11	m	0	0	all	-	all	Eu:Ger	1982	CC	432	n	bl	n	n	3	all/unsp	nev any	or
LEI	1	m	0	0	all	-	all	As:Chi	1986	CC	792	n	ot	y	n	0	all/unsp	nev any	st
LEI	2	f	0	0	all	-	all	As:Chi	1986	CC	792	n	ot	y	n	0	all/unsp	nev any	st
LEMARC	3	c	0	0	w+o	-	all	NAmer	1992	CC	341	n	bl	n	y	0	all/unsp	nev any	st
LETOUR	1	c	0	0	all	-	all	NAmer	1983	CC	738	n	V	y	y	0	cig+/-ot	nev cigs	st
LEVIN	32	m	0	0	all	-	all	NAmer	1938	CC	475	n	bl	n	n	1	all/unsp	nev any	st
LIDDEL	5	m	0	0	all	18	all	NAmer	1970	pr	304	m	V	n	n	1	cig+/-ot	nev cigs	ot
LIU	2	c	0	0	all	-	all	As:Chi	1980	CC	229	n	ot	*	n	2	all/unsp	nev any	or
LIU2	2	m	0	0	all	-	all	As:Chi	1983	CC	316	n	ot	n	n	3	all/unsp	nev any	ot
LIU2	4	f	0	0	all	-	all	As:Chi	1983	CC	316	n	ot	n	n	3	all/unsp	nev any	ot
LIU3	2	m	0	0	all	-	all	As:Chi	1985	CC	110	n	ot	n	n	2	all/unsp	nev any	or
LIU4	11	m	0	0	all	-	all	As:Chi	1986	CC	1000-00	n	ot	y	n	2	all/unsp	nev any	ot
LIU4	12	f	0	0	all	-	all	As:Chi	1986	CC	1000-00	n	ot	y	n	2	all/unsp	nev any	ot
LIU5	1	c	0	0	all	-	all	As:Chi	1978	CC	111	n	ot	y	n	0	all/unsp	nev any	st
LOMBA2	1	f	0	0	all	-	all	NAmer	1960	CC	225	n	bl	n	n	0	cig+/-ot	nev cigs	st
LOMBAR	12	m	0	0	all	-	all	NAmer	1951	CC	1040	n	bl	n	n	0	all/unsp	nev any	st
LUBIN2	46	m	0	0	all	-	all	Eu:mul	1976	CC	7804	n	bl	n	y	2	all/unsp	nev any	ot
LUBIN2	102	f	0	0	all	-	all	Eu:mul	1976	CC	7804	n	bl	n	y	1	all/unsp	nev any	ot
LUO	7	c	0	0	all	-	all	As:Chi	1990	CC	102	n	ot	n	y	20	cig+/-ot	nev cigs	or
MACLEN	73	c	0	0	ch	-	all	As:oth	1972	CC	233	n	bl	n	n	2	cig+/-ot	nev cigs	ot
MAGNUS	5	m	0	0	all	0	all	Eu:Sca	1953	pr	203	o	bl	y	n	3	all/unsp	nev any	ot
MARSH	7	c	0	0	all	-	all	NAmer	1979	CC	150	n	bl	y	n	2	all/unsp	nev any	or
MARSH2	5	m	0	0	all	-	all	NAmer	1979	CC	114	n	bl	y	n	1	all/unsp	nev any	or
MARSH2	6	f	0	0	all	-	all	NAmer	1979	CC	114	n	bl	y	n	1	all/unsp	nev any	ot
MARTIS	4	m	0	0	all	-	all	Eu:UK	1972	CC	201	n	V	n	n	0	cig+/-ot	nev cigs	st
MASTRA	2	m	0	0	all	-	all	Eu:wst	1973	CC	309	n	bl	n	n	2	all/unsp	nev any	st
MATOS	27	m	0	0	all	-	all	SCAmer	1994	CC	200	n	bl	n	n	2	cig+/-ot	nev any	or
MATSUD	10	m	0	0	all	-	all	As:Jap	1965	CC	179	n	bl	n	n	0	cig+/-ot	nev cigs	st

Table 1A1 - 1

IESLC - Meta-analysis of Ever Smoking, Any product (or Cigarettes if Any not available)
All LC types
Most adjusted

REF	NRR	SEX	AGEL	AGEH	RACE	YF	LC TYPE	LOC	START	ST	NLC	R	VB	P	H	AD	PRODUCT	DENOM	De
MCCONN	1	m	0	0	all	-	all	Eu:UK	1946	CC	100	n	V	n	y	0	all/unsp	nev any	st
MCCONN	2	f	0	0	all	-	all	Eu:UK	1946	CC	100	n	V	n	y	0	all/unsp	nev any	st
MCDUFF	1	m	0	0	all	-	all	NAmer	1979	CC	165	n	V	y	n	0	cig+/-ot	nev cigs	st
MCLAUG	1	m	0	0	all	-	all	As:Chi	1972	CC	316	o	ot	y	n	0	all/unsp	nev any	st
MIGRAN	27	m	0	0	all	0	all	Eu:UK	1964	pr	259	n	V	n	n	2	all/unsp	nev any	ot
MIGRAN	42	f	0	0	all	0	all	Eu:UK	1964	pr	259	n	V	n	n	2	all/unsp	nev any	ot
MILLER	2	f	0	0	all	-	all	NAmer	1972	CC	168	n	bl	y	n	1	cig+/-ot	nev any	ot
MILLS	3	m	0	0	wh	-	all	NAmer	1940	CC	444	n	bl	y	n	1	all/unsp	nev any	ot
MRFITR	6	m	0	0	all	0	all	NAmer	1973	pr	119	n	bl	n	n	0	cig+/-ot	nev cigs	ot
NAM	77	m	0	0	all	-	all	NAmer	1986	CC	1199	n	bl	y	n	1	cig+/-ot	nev cigs	ot
NAM	93	f	0	0	all	-	all	NAmer	1986	CC	1199	n	bl	y	n	1	cig+/-ot	nev cigs	ot
NOTAN2	15	m	0	0	all	-	all	As:Ind	1963	CC	683	n	V	n	n	2	all/unsp	nev any	ot
NOU	11	m	30	64	all	-	all	Eu:Sca	1971	CC	273	n	bl	y	n	0	all/unsp	nev any	st
NOU	12	f	30	64	all	-	all	Eu:Sca	1971	CC	273	n	bl	y	n	0	all/unsp	nev any	st
ODRISC	3	c	0	0	all	-	all	Eu:UK	1992	CC	446	n	V	n	n	0	all/unsp	nev any	st
ORMOS	4	m	0	0	all	-	all	Eu:est	1947	CC	119	n	bl	y	y	0	cig+/-ot	nev any	st
ORMOS	26	f	0	0	all	-	all	Eu:est	1947	CC	119	n	bl	y	y	0	cig+/-ot	nev any	st
OSANN	41	m	0	0	all	-	all	NAmer	1984	CC	1986	n	bl	n	n	2	cig+/-ot	nev cigs	or
OSANN	42	f	0	0	all	-	all	NAmer	1984	CC	1986	n	bl	n	n	2	cig+/-ot	nev cigs	or
PARKIN	28	m	0	0	bl	-	all	Africa	1963	CC	877	n	V	y	n	6	all/unsp	nev any	ot
PASTOR	10	m	0	0	all	-	all	Eu:wst	1976	CC	204	n	bl	y	n	1	all/unsp	nev any	or
PAWLEG	2	m	0	0	all	-	all	Eu:est	1992	CC	176	n	bl	n	y	6	all/unsp	nev any	ot
PERNU	2	m	0	0	all	-	all	Eu:Sca	1944	CC	1606	n	bl	n	n	0	all/unsp	nev any	st
PERNU	1	f	0	0	all	-	all	Eu:Sca	1944	CC	1606	n	bl	n	n	0	all/unsp	nev any	st
PERSH2	11	c	0	0	all	-	all	Eu:Sca	1980	CC	1022	n	bl	y	n	4	all/unsp	nev any	ot
PETO	5	m	0	0	all	0	all	Eu:UK	1954	pr	103	n	V	n	n	0	all/unsp	nev any	st
PEZZO2	10	m	0	0	all	-	all	SCAmer	1992	CC	367	n	bl	n	y	0	cig+/-ot	nev cigs	st
PEZZOT	25	m	0	0	all	-	all	SCAmer	1987	CC	215	n	bl	n	y	0	cig only	nev cigs	st
PIKE	4	m	0	0	w-hi	-	all	NAmer	1972	CC	731	n	bl	y	n	0	all/unsp	nev any	st
PIKE	8	f	0	0	w-hi	-	all	NAmer	1972	CC	731	n	bl	y	n	0	all/unsp	nev any	st
POFFIJ	1	c	0	0	all	-	all	Eu:mul	1990	CC	971	n	bl	n	n	0	all/unsp	nev any	st
POLEDN	1	c	0	0	all	-	all	NAmer	1978	CC	209	n	bl	y	n	1	cig+/-ot	nev cigs	or
QIAO2	15	m	0	0	all	0	all	As:Chi	1992	pr	241	m	ot	n	n	1	all/unsp	nev any	ot
RACHTA	15	f	0	0	all	-	all	Eu:est	1991	CC	118	n	bl	n	y	4	cig+/-ot	nev cigs	ot
RADZIK	1	c	0	0	all	-	all	Eu:est	1986	CC	189	n	bl	n	n	0	all/unsp	nev any	st
RANDIG	23	m	0	0	all	-	all	Eu:Ger	1951	CC	448	n	bl	n	n	0	all/unsp	nev any	st
RANDIG	24	f	0	0	all	-	all	Eu:Ger	1951	CC	448	n	bl	n	n	0	all/unsp	nev any	st
REN	1	m	0	0	all	-	all	As:Chi	1980	CC	244	n	ot	*	n	0	all/unsp	nev any	st
REN	2	f	0	0	all	-	all	As:Chi	1980	CC	244	n	ot	*	n	0	all/unsp	nev any	st
RONCO	1	m	0	0	all	-	all	Eu:wst	1976	CC	126	n	bl	y	n	0	all/unsp	nev any	st
ROTHSC	2	c	0	0	all	-	all	NAmer	1971	CC	284	n	bl	y	n	1	all/unsp	nev any	st
SADOWS	31	m	0	0	wh	-	all	NAmer	1938	CC	477	n	bl	n	n	1	all/unsp	nev any	ot
SANKAR	2	m	0	0	all	-	all	As:Ind	1990	CC	281	n	V	n	n	3	all/unsp	nev any	ot
SCHWAR	1	m	0	0	wh	-	all	NAmer	1984	CC	5588	n	bl	y	y	0	cig+/-ot	nev cigs	st
SCHWAR	2	m	0	0	bl	-	all	NAmer	1984	CC	5588	n	bl	y	y	0	cig+/-ot	nev cigs	st
SCHWAR	3	f	0	0	wh	-	all	NAmer	1984	CC	5588	n	bl	y	y	0	cig+/-ot	nev cigs	st
SCHWAR	4	f	0	0	bl	-	all	NAmer	1984	CC	5588	n	bl	y	y	0	cig+/-ot	nev cigs	st
SEGI	1	m	0	0	all	-	all	As:Jap	1948	CC	159	n	bl	n	n	0	all/unsp	nev any	ot
SEOW	6	f	0	0	ch	-	q+s+l+a	As:oth	1997	CC	153	n	bl	n	y	1	cig+/-ot	nev cigs	st
SHAW	12	c	0	0	wh	-	all	NAmer	1988	CC	335	n	V	n	y	0	all/unsp	nev any	st
SIEMIA	5	m	0	0	all	-	all	NAmer	1979	CC	857	n	V	y	y	7	cig+/-ot	nev cigs	or
SIMARA	3	m	0	0	all	-	all	As:oth	1971	CC	115	n	bl	n	n	6	cig+/-ot	nev cigs	ot
SIMARA	4	f	0	0	all	-	all	As:oth	1971	CC	115	n	bl	n	n	6	cig+/-ot	nev cigs	ot
SOBUE	105	m	0	0	all	-	q+s+l+a	As:Jap	1986	CC	1376	n	bl	n	y	1	cig+/-ot	nev cigs	ot
SOBUE	115	f	0	0	all	-	q+s+l+a	As:Jap	1986	CC	1376	n	bl	n	y	1	cig+/-ot	nev cigs	ot
SPEIZE	8	f	0	0	all	0	all	NAmer	1976	pr	593	n	bl	n	y	0	cig+/-ot	nev cigs	st
SPITZ	3	c	0	0	b+hi	-	all	NAmer	1992	CC	177	n	bl	n	y	0	cig+/-ot	nev cigs	st
STASZE	1	m	0	0	all	-	all	Eu:est	1954	CC	281	n	bl	n	y	0	all/unsp	nev any	st
STASZE	5	f	0	0	all	-	all	Eu:est	1954	CC	281	n	bl	n	y	0	all/unsp	nev any	st
STAYNE	1	m	0	0	all	-	all	NAmer	1969	CC	420	n	bl	n	n	0	all/unsp	nev any	st
STOCKS	47	m	0	0	all	-	all	Eu:UK	1952	CC	2932	n	V	y	n	2	all/unsp	nev any	st
STOCKS	50	f	0	0	all	-	all	Eu:UK	1952	CC	2932	n	V	y	n	1	cig+/-ot	nev any	ot
STOCKW	6	c	0	0	all	-	all	NAmer	1981	CC	22161	n	bl	n	n	0	all/unsp	nev any	st
STUCKE	3	m	0	0	all	-	all	Eu:wst	1989	CC	247	n	bl	n	y	0	all/unsp	nev any	ot
SUN	1	c	0	0	all	-	all	As:Chi	1992	CC	207	n	ot	n	y	0	all/unsp	nev any	st
SUZUK2	20	c	0	0	all	-	all	SCAmer	1991	CC	123	n	bl	n	y	3	all/unsp	nev any	ot
SVENSS	71	f	0	0	all	-	all	Eu:Sca	1983	CC	210	n	bl	n	n	1	all/unsp	nev any	ot
TANG	3	c	0	0	all	-	not s	NAmer	1992	CC	119	n	bl	n	y	0	cig+/-ot	nev cigs	st
TENKAN	22	m	0	0	all	17	all	Eu:Sca	1962	pr	242	n	bl	n	n	1	all/unsp	nev any	ot
TIZZAN	1	m	0	0	all	-	all	Eu:wst	1959	CC	1358	n	bl	n	n	0	all/unsp	nev any	st
TIZZAN	12	f	0	0	all	-	all	Eu:wst	1959	CC	1358	n	bl	n	n	0	all/unsp	nev any	st
TOKARS	6	c	0	0	all	-	all	Eu:est	1966	ot	162	o	bl	n	y	3	all/unsp	nev any	or

Table 1A1 - 1

IESLC - Meta-analysis of Ever Smoking, Any product (or Cigarettes if Any not available)
All LC types
Most adjusted

REF	NRR	SEX	AGEL	AGEH	RACE	YF	LC TYPE	LOC	START	ST	NLC	R	VB	P	H	AD	PRODUCT	DENOM	De
TOUSEY	21	m	0	0	all	-	all	NAmer	1993	CC	507	n	bl	y	y	3	all/unsp	nev any	ot
TOUSEY	26	f	0	0	all	-	all	NAmer	1993	CC	507	n	bl	y	y	0	all/unsp	nev any	st
TSUGAN	27	m	0	0	all	-	q+a	As:Jap	1976	CC	134	n	bl	n	y	0	all/unsp	nev any	st
TULINI	38	m	0	0	all	0	all	Eu:Sca	1967	pr	472	n	bl	n	n	3	all/unsp	nev any	ot
TULINI	44	f	0	0	all	0	all	Eu:Sca	1967	pr	472	n	bl	n	n	3	all/unsp	nev any	ot
TVERDA	22	m	0	0	all	0	all	Eu:Sca	1972	pr	238	n	bl	n	n	2	cig+/-ot	nev cigs	ot
WAKAI	72	m	0	0	all	-	all	As:Jap	1988	CC	333	n	bl	n	y	2	all/unsp	nev any	ot
WAKAI	78	f	0	0	all	-	all	As:Jap	1988	CC	333	n	bl	n	y	2	all/unsp	nev any	ot
WANG	5	c	0	0	all	-	all	As:Chi	1990	CC	390	n	ot	*	y	6	all/unsp	nev any	or
WANG2	16	c	0	0	all	-	all	As:Chi	1980	CC	103	n	ot	n	n	4	cig+/-ot	nev cigs	ot
WANG3	1	c	0	0	all	-	all	As:Chi	1981	CC	293	n	ot	*	n	0	all/unsp	nev any	st
WANG4	2	m	0	0	all	-	all	As:Chi	1976	CC	1170	n	ot	y	n	2	all/unsp	nev any	st
WICKLU	1	m	0	0	wh	-	all	NAmer	1968	CC	155	n	bl	y	n	0	cig+/-ot	nev+3	or
WIGLE	27	m	0	0	all	-	all	NAmer	1971	CC	728	n	V	n	n	1	all/unsp	nev any	ot
WIGLE	32	f	0	0	all	-	all	NAmer	1971	CC	728	n	V	n	n	1	all/unsp	nev any	ot
WILKIN	3	c	0	0	all	-	all	Eu:UK	1992	CC	271	n	V	n	n	4	cig+/-ot	nev cigs	ot
WU	45	f	0	0	wh	-	q+a	NAmer	1981	CC	220	n	bl	n	y	2	all/unsp	nev any	ot
WUNSCH	4	m	0	0	all	-	all	SCAmer	1990	CC	398	n	bl	y	n	1	cig+/-ot	nev any	or
WUNSCH	10	f	0	0	all	-	all	SCAmer	1990	CC	398	n	bl	y	n	1	cig+/-ot	nev any	or
WUWILL	8	f	0	0	all	-	all	As:Chi	1985	CC	965	n	ot	n	n	3	cig+/-ot	nev cigs	or
WYNDE2	21	m	0	0	all	-	all	NAmer	1962	CC	404	n	bl	n	y	0	all/unsp	nev any	st
WYNDE3	49	m	0	0	all	-	all	NAmer	1966	CC	350	n	bl	n	y	0	all/unsp	nev any	st
WYNDE3	138	f	0	0	all	-	all	NAmer	1966	CC	350	n	bl	n	y	0	all/unsp	nev any	st
WYNDE4	48	m	0	0	all	-	all	NAmer	1948	CC	684	n	bl	y	n	0	all/unsp	nev any	st
WYNDE4	62	f	0	0	all	-	all	NAmer	1948	CC	684	n	bl	y	n	2	all/unsp	nev any	ot
WYNDE6	72	m	0	0	all	-	all	NAmer	1969	CC	4423	n	bl	n	y	0	all/unsp	nev any	st
WYNDE6	252	f	0	0	all	-	all	NAmer	1969	CC	4423	n	bl	n	y	0	cig+/-ot	nev cigs	st
XIANGZ	13	m	0	0	all	0	all	As:Chi	1976	pr	983	m	ot	n	n	2	all/unsp	nev any	ot
XU	2	m	0	0	all	-	all	As:Chi	1985	CC	729	n	ot	n	n	2	all/unsp	nev any	or
XU2	2	c	0	0	all	-	all	As:Chi	1987	CC	610	o	ot	y	n	7	all/unsp	nev any	ot
XU3	2	m	0	0	all	-	all	As:Chi	1981	CC	135	n	ot	n	n	1	all/unsp	nev any	or
XU3	4	f	0	0	all	-	all	As:Chi	1981	CC	135	n	ot	n	n	1	all/unsp	nev any	or
XU4	1	c	0	0	all	-	all	As:Chi	1981	CC	206	n	ot	*	n	0	all/unsp	nev any	st
YAMAGU	11	c	0	0	all	-	all	As:Jap	1989	CC	144	n	bl	n	y	1	all/unsp	nev any	ot
YONG	2	c	0	0	all	0	all	NAmer	1971	pr	216	n	bl	n	n	1	cig+/-ot	nev cigs	ot
YUAN	1	m	0	0	all	0	all	As:Chi	1986	pr	142	n	ot	n	n	2	cig+/-ot	nev cigs	ot
ZHANG	2	m	0	0	all	-	all	As:Chi	1988	CC	100	n	ot	n	n	7	all/unsp	nev any	or
ZHANG	3	f	0	0	all	-	all	As:Chi	1988	CC	100	n	ot	n	n	7	all/unsp	nev any	or
ZHENG	15	m	0	0	all	-	all	As:Chi	1982	CC	540	n	ot	*	y	0	cig+/-ot	nev cigs	st
ZHENG	24	f	0	0	all	-	all	As:Chi	1982	CC	540	n	ot	*	y	0	cig+/-ot	nev cigs	st
ZHOU	2	m	0	0	all	-	all	As:Chi	1978	CC	1360	n	ot	n	n	0	all/unsp	nev any	st
ZHOU	3	f	0	0	all	-	all	As:Chi	1978	CC	1360	n	ot	n	n	0	all/unsp	nev any	st

Cigarette type is all/unspec for all RRs
except for the following:

REF	NRR	CIGTYPE
ALDERS	6	MC only
DEAN3	126	MC only

Table 1A1 - 2

IESLC - Meta-analysis of Ever Smoking, Any product (or Cigarettes if Any not available)
All LC types
Most adjusted

REF	NRR	SEX	AD	Number Exposed Case	Number Exposed Cont	Non-exposed Case	Non-exposed Cont	RR	95.00%CI
ABELIN	44	m	1	-	-	-	-	35.38	(8.62- 145.24)
*ABRAHA	7	m	0	269	10351	10	3365	8.74	(4.66- 16.42)
*ABRAHA	8	f	0	62	5256	28	11589	4.88	(3.13- 7.62)
Subtotal ABRAHA								5.93	(4.12- 8.53)
AGUDO	1	f	3	-	-	-	-	3.10	(1.42- 6.75)
*AKIBA	11	m	5	-	-	-	-	4.75	(3.07- 7.34)
*AKIBA	15	f	5	-	-	-	-	3.16	(2.37- 4.21)
Subtotal AKIBA								3.58	(2.81- 4.54)
ALDERS	69	m	1	-	-	-	-	9.65	(5.61- 16.61)
ALDERS	6	f	1	-	-	-	-	4.75	(3.55- 6.35)
Subtotal ALDERS								5.56	(4.31- 7.19)
*AMANDU	7	m	2	-	-	-	-	5.89	(2.27- 15.28)
AMES	4	m	0	297	251	15	62	4.89	(2.72- 8.81)
*ANDERS	3	f	0	297	96164	46	195158	13.10	(9.61- 17.87)
*ARCHER	6	m	0	140	36269	6	9842	6.33	(2.80- 14.33)
ARMADA	29	m	0	321	261	4	64	19.68	(7.07- 54.75)
AUSTIN	7	c	3	-	-	-	-	11.12	(3.95- 31.28)
AUVINE	19	c	2	-	-	-	-	13.84	(7.90- 24.25)
AXELSO	1	c	0	90	86	62	371	6.26	(4.20- 9.34)
AXELSS	8	m	6	-	-	-	-	8.02	(4.62- 13.94)
AXELSS	11	f	0	110	109	18	154	8.63	(4.95- 15.05)
Subtotal AXELSS								8.32	(5.62- 12.31)
BAND	1	m	2	-	-	-	-	9.96	(7.38- 13.44)
BARBON	131	m	3	-	-	-	-	11.13	(7.02- 17.64)
BECHER	1	m	0	143	238	3	54	10.82	(3.32- 35.23)
BECHER	24	f	2	-	-	-	-	11.81	(3.20- 43.56)
Subtotal BECHER								11.25	(4.69- 27.01)
*BENSHL	18	m	1	-	-	-	-	5.90	(2.62- 13.31)
*BEST	22	m	0	221	24776	1	2854	25.46	(3.57- 181.46)
*BEST	18	f	1	-	-	-	-	2.24	(0.59- 8.44)
Subtotal BEST								4.81	(1.60- 14.47)
BLOHMK	3	m	0	762	587	126	301	3.10	(2.45- 3.92)
BLOT4	1	m	0	327	245	8	87	14.51	(6.91- 30.51)
BOFFET	33	m	2	-	-	-	-	14.20	(11.70- 17.20)
*BOUCOT	121	m	2	-	-	-	-	40.95	(2.55- 658.45)
BRESLO	37	m	0	486	451	7	42	6.47	(2.88- 14.54)
BRESLO	38	f	0	13	11	12	14	1.38	(0.45- 4.20)
Subtotal BRESLO								3.79	(1.97- 7.29)
*BRETT	10	m	0	144	47930	6	6530	3.27	(1.45- 7.40)
BROCKM	1	m	0	87	81	2	2	1.07	(0.15- 7.80)
BROCKM	2	f	0	24	54	4	18	2.00	(0.61- 6.54)
Subtotal BROCKM								1.70	(0.61- 4.70)
BROSS	12	m	0	902	784	38	170	5.15	(3.57- 7.41)
BROWN2	2	m	2	-	-	-	-	9.10	(8.30- 10.00)
BROWN2	1	f	2	-	-	-	-	12.70	(11.50- 13.90)
Subtotal BROWN2								10.72	(10.03- 11.46)
BUFFLE	1	m	0	470	419	5	47	10.54	(4.15- 26.76)
BUFFLE	5	f	0	419	284	41	198	7.12	(4.93- 10.30)
Subtotal BUFFLE								7.51	(5.33- 10.58)
CARPEN	12	c	3	-	-	-	-	14.88	(8.46- 26.18)
CASCO2	1	c	0	149	212	6	98	11.48	(4.90- 26.87)
CASCOR	1	c	0	365	362	22	295	13.52	(8.56- 21.35)
*CEDERL	107	m	2	-	-	-	-	5.92	(3.85- 9.12)
*CEDERL	112	f	2	-	-	-	-	4.18	(2.94- 5.93)
Subtotal CEDERL								4.80	(3.66- 6.30)
CHAN	9	m	0	206	161	2	43	27.51	(6.57- 115.26)
CHAN	10	f	0	105	50	84	139	3.48	(2.26- 5.35)
Subtotal CHAN								4.13	(2.73- 6.25)
*CHANG	6	m	0	78	1506	5	502	5.20	(2.12- 12.77)
*CHANG	12	f	0	42	1183	11	1139	3.68	(1.90- 7.10)
Subtotal CHANG								4.15	(2.44- 7.06)
CHATZI	4	c	0	255	365	27	129	3.34	(2.14- 5.21)
CHEN2	1	m	0	121	97	9	33	4.57	(2.09- 10.02)
CHEN2	2	f	0	38	30	25	33	1.67	(0.82- 3.39)
Subtotal CHEN2								2.62	(1.55- 4.44)
CHEN3	1	c	0	182	156	72	98	1.59	(1.09- 2.30)
CHIAZZ	3	m	11	-	-	-	-	26.17	(3.32- 206.50)
CHOI	1	m	0	267	465	13	95	4.20	(2.31- 7.64)
CHOI	5	f	0	19	26	76	164	1.58	(0.82- 3.02)
Subtotal CHOI								2.68	(1.72- 4.16)
*CHOW	55	m	2	-	-	-	-	11.08	(4.87- 25.21)

Table 1A1 - 2

IESLC - Meta-analysis of Ever Smoking, Any product (or Cigarettes if Any not available)
All LC types
Most adjusted

REF	NRR	SEX	AD	Number Exposed Case	Number Exposed Cont	Non-exposed Case	Non-exposed Cont	RR	95.00%CI
*CHYOU	7	m	1	-	-	-	-	8.35	(4.76- 14.64)
COMSTO	34	m	0	153	244	4	69	10.82	(3.87- 30.24)
COMSTO	46	f	0	88	87	13	115	8.95	(4.69- 17.06)
Subtotal COMSTO								9.44	(5.46- 16.31)
COOKSO	5	c	0	189	39	45	61	6.57	(3.92- 11.02)
CORREA	34	c	1	-	-	-	-	11.40	(8.70- 15.00)
*CPSI	187	m	1	-	-	-	-	9.18	(7.36- 11.45)
*CPSI	274	f	1	-	-	-	-	2.79	(2.22- 3.51)
Subtotal CPSI								5.17	(4.41- 6.06)
*CPSII	104	m	1	-	-	-	-	12.83	(10.28- 16.01)
*CPSII	79	f	1	-	-	-	-	8.16	(6.93- 9.62)
Subtotal CPSII								9.58	(8.40- 10.93)
DAMBER	25	m	1	-	-	-	-	7.15	(4.98- 10.26)
DARBY	15	m	0	664	1724	3	384	49.30	(15.77- 154.07)
DARBY	16	f	0	292	548	23	529	12.26	(7.89- 19.05)
Subtotal DARBY								14.69	(9.74- 22.16)
DAVEYS	5	m	0	90	144	3	23	4.79	(1.40- 16.42)
DAVEYS	6	f	0	0	3	16	83	0.72~	(0.04- 14.66)
Subtotal DAVEYS								3.65	(1.17- 11.42)
DEAN	7	m	0	591	574	12	61	5.23	(2.79- 9.82)
DEAN2	3	m	0	769	688	33	112	3.79	(2.54- 5.67)
DEAN2	7	f	0	64	30	88	121	2.93	(1.76- 4.90)
Subtotal DEAN2								3.44	(2.51- 4.72)
DEAN3	49	m	3	-	-	-	-	6.14	(3.92- 9.61)
DEAN3	126	f	3	-	-	-	-	4.63	(3.03- 7.09)
Subtotal DEAN3								5.29	(3.89- 7.20)
*DEKLER	6	m	2	-	-	-	-	20.29	(2.84- 145.18)
DESTE2	14	c	7	-	-	-	-	8.70	(5.10- 14.90)
DESTEF	48	m	4	-	-	-	-	9.11	(5.86- 14.15)
*DOCKER	3	c	4	-	-	-	-	4.29	(1.66- 11.06)
DOLL	6	m	0	1350	1296	7	61	9.08	(4.14- 19.92)
DOLL	12	f	0	68	49	40	59	2.05	(1.19- 3.53)
Subtotal DOLL								3.32	(2.12- 5.19)
*DOLL2	56	m	1	-	-	-	-	7.66	(4.86- 12.07)
DORANT	10	c	0	470	2033	14	1090	18.00	(10.52- 30.78)
DORGAN	6	m	0	721	455	15	93	9.82	(5.62- 17.16)
DORGAN	30	m	0	266	135	3	35	22.99	(6.94- 76.10)
DORGAN	53	f	0	757	229	103	244	7.83	(5.96- 10.30)
DORGAN	76	f	0	79	27	7	20	8.36	(3.18- 21.95)
Subtotal DORGAN								8.52	(6.75- 10.76)
*DORN	196	m	1	-	-	-	-	7.04	(5.60- 8.84)
DOSEME	1	m	2	-	-	-	-	3.30	(2.60- 4.40)
DROSTE	7	m	4	-	-	-	-	8.62	(3.80- 19.56)
DU	1	m	0	538	-	28	-	3.53	(2.44- 5.11)
DU	2	f	0	191	-	92	-	1.93	(1.30- 2.87)
Subtotal DU								2.66	(2.03- 3.49)
*DUNN	6	m	0	137	52634	2	14160	18.43	(4.56- 74.42)
EBELIN	1	m	0	101	142	12	117	6.93	(3.63- 13.24)
*ENGELA	159	m	1	-	-	-	-	6.34	(2.95- 13.60)
*ENGELA	165	f	1	-	-	-	-	4.74	(2.20- 10.22)
Subtotal ENGELA								5.49	(3.19- 9.43)
ESAKI	4	m	0	155	143	16	28	1.90	(0.99- 3.65)
ESAKI	5	f	0	34	19	40	55	2.46	(1.23- 4.92)
Subtotal ESAKI								2.14	(1.33- 3.45)
FAN	1	m	0	216	498	36	236	2.84	(1.93- 4.18)
FAN	2	f	0	82	97	69	320	3.92	(2.65- 5.81)
Subtotal FAN								3.33	(2.53- 4.38)
GAO	1	m	2	-	-	-	-	3.90	(2.90- 5.40)
GAO	11	f	2	-	-	-	-	3.30	(2.50- 4.20)
Subtotal GAO								3.53	(2.90- 4.31)
GAO2	10	m	1	-	-	-	-	5.17	(2.76- 9.69)
GARCIA	3	c	0	395	307	21	139	8.52	(5.26- 13.80)
GARDIN	7	c	0	138	102	5	41	11.09	(4.23- 29.06)
GARSHI	25	m	1	-	-	-	-	5.81	(4.17- 8.10)
GENG	1	m	0	92	68	7	31	5.99	(2.49- 14.42)
GENG	2	f	0	126	75	67	118	2.96	(1.96- 4.48)
Subtotal GENG								3.36	(2.31- 4.89)
GER	21	c	14	-	-	-	-	1.84	(1.06- 3.20)
GODLEY	5	m	1	-	-	-	-	6.84	(5.60- 8.35)
GODLEY	6	f	1	-	-	-	-	5.54	(4.29- 7.15)
Subtotal GODLEY								6.31	(5.39- 7.39)

Table 1A1 - 2

IESLC - Meta-analysis of Ever Smoking, Any product (or Cigarettes if Any not available)
All LC types
Most adjusted

REF	NRR	SEX	AD	Number Exposed Case	Number Exposed Cont	Non-exposed Case	Non-exposed Cont	RR	95.00%CI
GOLLED	7	m	1	-	-	-	-	7.51	(4.44- 12.71)
GOODMA	3	m	0	216	398	10	199	10.80	(5.60- 20.82)
GOODMA	7	f	0	81	91	19	177	8.29	(4.74- 14.52)
Subtotal GOODMA								9.27	(6.05- 14.19)
GRAHAM	27	m	1	-	-	-	-	7.01	(4.39- 11.20)
GREGOR	3	m	0	72	98	10	14	1.03	(0.43- 2.45)
GREGOR	7	f	0	21	42	1	22	11.00	(1.39- 87.29)
Subtotal GREGOR								1.46	(0.66- 3.26)
GSELL	8	m	0	148	121	2	29	17.74	(4.15- 75.83)
HAENSZ	11	f	2	-	-	-	-	2.19	(1.48- 3.24)
*HAMMO2	4	m	1	-	-	-	-	22.82	(3.20- 162.75)
*HAMMON	117	m	1	-	-	-	-	6.80	(4.06- 11.37)
*HANSEN	3	m	2	-	-	-	-	1.53	(0.71- 3.91)
HEGMAN	1	c	0	255	1202	27	2080	16.34	(10.92- 24.45)
*HEIN	7	m	0	143	4471	1	457	14.62	(2.05- 104.23)
*HENNEK	3	m	0	146	11112	23	10919	6.24	(4.02- 9.67)
HINDS	22	f	3	-	-	-	-	5.65	(4.14- 7.72)
*HIRAYA	147	m	1	-	-	-	-	4.36	(3.53- 5.39)
*HIRAYA	150	f	1	-	-	-	-	2.36	(1.90- 2.94)
Subtotal HIRAYA								3.24	(2.78- 3.77)
HITOSU	38	m	1	-	-	-	-	2.91	(1.34- 6.34)
HITOSU	62	f	1	-	-	-	-	3.40	(2.05- 5.64)
Subtotal HITOSU								3.25	(2.12- 4.96)
*HOLE	8	m	1	-	-	-	-	6.44	(3.03- 13.69)
HOROWI	1	m	0	182	525	19	196	3.58	(2.17- 5.90)
HOROWI	2	f	0	21	382	14	463	1.82	(0.91- 3.62)
Subtotal HOROWI								2.83	(1.89- 4.25)
HORWIT	1	f	0	97	92	11	118	11.31	(5.73- 22.34)
HU	15	m	0	120	94	41	67	2.09	(1.30- 3.35)
HU	16	f	0	26	18	40	48	1.73	(0.83- 3.61)
Subtotal HU								1.98	(1.33- 2.94)
HU2	9	m	0	294	228	49	115	3.03	(2.08- 4.41)
HU2	10	f	0	108	80	72	100	1.88	(1.23- 2.85)
Subtotal HU2								2.44	(1.85- 3.23)
HUANG	1	c	0	98	77	37	58	2.00	(1.20- 3.32)
HUMBLE	14	m	1	-	-	-	-	12.10	(5.12- 28.60)
HUMBLE	16	m	1	-	-	-	-	11.88	(2.65- 53.30)
HUMBLE	18	f	1	-	-	-	-	11.36	(5.32- 24.23)
HUMBLE	20	f	1	-	-	-	-	15.40	(4.87- 48.74)
Subtotal HUMBLE								12.28	(7.58- 19.90)
JAHN	22	f	2	-	-	-	-	3.30	(1.99- 5.49)
JAIN	46	m	2	-	-	-	-	8.30	(4.53- 17.00)
JAIN	41	f	2	-	-	-	-	9.20	(5.95- 15.10)
Subtotal JAIN								8.89	(6.08- 13.01)
JARUP	6	m	2	-	-	-	-	7.54	(2.80- 20.33)
JARVHO	3	m	0	99	57	1	16	27.79	(3.59- 215.09)
JARVHO	7	f	0	41	15	6	21	9.57	(3.24- 28.26)
Subtotal JARVHO								12.08	(4.64- 31.46)
JEDRYC	58	m	4	-	-	-	-	5.46	(3.85- 7.73)
JEDRYC	59	f	4	-	-	-	-	4.54	(2.56- 8.05)
Subtotal JEDRYC								5.19	(3.86- 7.00)
JIANG	1	m	0	93	83	7	17	2.72	(1.08- 6.89)
JIANG	2	f	0	11	6	14	19	2.49	(0.74- 8.35)
Subtotal JIANG								2.63	(1.26- 5.50)
JOLY	14	m	0	595	888	12	218	12.17	(6.75- 21.97)
JOLY	1	f	0	166	123	52	283	7.34	(5.04- 10.70)
Subtotal JOLY								8.50	(6.19- 11.68)
JUSSAW	29	m	2	-	-	-	-	16.83	(11.65- 25.21)
*KAISE2	72	m	1	-	-	-	-	5.40	(3.05- 9.57)
*KAISE2	64	f	1	-	-	-	-	10.09	(5.29- 19.27)
Subtotal KAISE2								7.11	(4.63- 10.90)
*KAISER	13	m	2	-	-	-	-	17.63	(11.98- 25.96)
*KAISER	10	f	2	-	-	-	-	5.63	(3.89- 8.14)
Subtotal KAISER								9.70	(7.43- 12.67)
KATSOU	29	f	1	-	-	-	-	3.30	(1.77- 6.15)
KAUFMA	17	c	6	-	-	-	-	12.38	(8.59- 17.85)
KELLER	3	m	0	8066	2517	323	1017	10.09	(8.83- 11.52)
KELLER	11	m	0	1493	340	38	117	13.52	(9.20- 19.86)
KELLER	7	f	0	3998	1269	469	1860	12.49	(11.09- 14.08)
KELLER	15	f	0	584	214	67	232	9.45	(6.91- 12.93)
Subtotal KELLER								11.30	(10.40- 12.29)

Table 1A1 - 2

IESLC - Meta-analysis of Ever Smoking, Any product (or Cigarettes if Any not available)
All LC types
Most adjusted

REF	NRR	SEX	AD	Number Exposed Case	Number Exposed Cont	Non-exposed Case	Non-exposed Cont	RR	95.00%CI
KHUDER	4	m	0	459	785	23	309	7.86	(5.06- 12.19)
KIHARA	31	c	0	338	232	102	237	3.39	(2.54- 4.51)
*KINLEN	17	m	2	-	-	-	-	10.99	(5.24- 23.06)
KJUUS	10	m	0	174	152	2	24	13.74	(3.19- 59.08)
*KNEKT	87	m	1	-	-	-	-	6.42	(2.82- 14.62)
KO	1	f	3	-	-	-	-	4.20	(1.10- 15.60)
KOHLME	2	c	4	-	-	-	-	16.40	(6.90- 38.42)
KOO	1	f	0	112	63	88	137	2.77	(1.84- 4.16)
KOULUM	1	m	0	807	246	5	54	35.43	(14.02- 89.55)
KREUZE	14	f	0	62	42	6	38	9.35	(3.63- 24.08)
KREUZE	16	f	0	205	101	95	177	3.78	(2.68- 5.34)
Subtotal KREUZE								4.21	(3.04- 5.81)
KREYBE	12	m	1	-	-	-	-	6.61	(2.93- 14.92)
KREYBE	30	f	1	-	-	-	-	1.43	(0.71- 2.86)
Subtotal KREYBE								2.73	(1.61- 4.64)
*KUBIK	28	m	0	106	8051	2	4271	28.12	(6.94- 113.84)
LAMTH	6	f	0	242	106	202	337	3.81	(2.86- 5.08)
LAMWK	1	f	0	88	41	75	144	4.12	(2.59- 6.55)
LAMWK2	9	m	0	244	161	23	43	2.83	(1.64- 4.88)
LAMWK2	10	f	0	75	50	65	139	3.21	(2.02- 5.10)
Subtotal LAMWK2								3.04	(2.14- 4.33)
*LANGE	40	m	1	-	-	-	-	4.74	(1.77- 12.67)
*LANGE	37	f	1	-	-	-	-	4.93	(2.48- 9.81)
Subtotal LANGE								4.87	(2.77- 8.55)
LAUSSM	11	m	3	-	-	-	-	5.70	(4.10- 7.80)
LEI	1	m	0	443	361	41	123	3.68	(2.52- 5.38)
LEI	2	f	0	123	61	85	147	3.49	(2.32- 5.24)
Subtotal LEI								3.59	(2.72- 4.74)
LEMARC	3	c	0	309	288	32	168	5.63	(3.74- 8.49)
LETOUR	1	c	0	714	514	24	224	12.96	(8.38- 20.05)
LEVIN	32	m	1	-	-	-	-	4.86	(3.41- 6.92)
*LIDDEL	5	m	1	-	-	-	-	3.61	(2.27- 5.73)
LIU	2	c	2	-	-	-	-	1.92	(1.40- 2.64)
LIU2	2	m	3	-	-	-	-	5.19	(2.03- 13.25)
LIU2	4	f	3	-	-	-	-	4.65	(2.18- 9.93)
Subtotal LIU2								4.86	(2.69- 8.76)
LIU3	2	m	2	-	-	-	-	1.26	(0.30- 5.26)
LIU4	11	m	2	-	-	-	-	2.76	(2.69- 2.83)
LIU4	12	f	2	-	-	-	-	2.86	(2.77- 2.95)
Subtotal LIU4								2.80	(2.74- 2.85)
LIU5	1	c	0	85	70	26	41	1.91	(1.07- 3.44)
LOMBA2	1	f	0	149	353	76	239	1.33	(0.96- 1.83)
LOMBAR	12	m	0	1026	928	14	112	8.84	(5.04- 15.53)
LUBIN2	46	m	2	-	-	-	-	8.50	(7.29- 9.91)
LUBIN2	102	f	1	-	-	-	-	3.90	(3.29- 4.62)
Subtotal LUBIN2								5.99	(5.34- 6.71)
LUO	7	c	20	-	-	-	-	2.70	(1.50- 5.00)
MACLEN	73	c	2	-	-	-	-	2.67	(1.66- 4.29)
*MAGNUS	5	m	3	-	-	-	-	4.13	(1.94- 8.77)
MARSH	7	c	2	-	-	-	-	6.80	(3.30- 13.99)
MARSH2	5	m	1	-	-	-	-	1.89	(0.70- 5.14)
MARSH2	6	f	1	-	-	-	-	5.28	(1.89- 14.72)
Subtotal MARSH2								3.11	(1.52- 6.36)
MARTIS	4	m	0	197	176	4	25	7.00	(2.39- 20.49)
MASTRA	2	m	2	-	-	-	-	8.14	(3.32- 20.00)
MATOS	27	m	2	-	-	-	-	6.80	(3.50- 13.10)
MATSUD	10	m	0	170	3314	3	1255	21.46	(6.84- 67.33)
MCCONN	1	m	0	88	174	5	12	1.21	(0.41- 3.55)
MCCONN	2	f	0	3	3	4	11	2.75	(0.38- 19.67)
Subtotal MCCONN								1.46	(0.57- 3.76)
MCDUFF	1	m	0	159	134	6	31	6.13	(2.48- 15.14)
MCLAUG	1	m	0	294	1082	22	270	3.33	(2.12- 5.25)
*MIGRAN	27	m	2	-	-	-	-	3.61	(1.34- 9.72)
*MIGRAN	42	f	2	-	-	-	-	4.62	(1.63- 13.09)
Subtotal MIGRAN								4.06	(1.98- 8.32)
MILLER	2	f	1	-	-	-	-	4.99	(2.06- 12.10)
MILLS	3	m	1	-	-	-	-	1.33	(1.09- 1.63)
*MRFITR	6	m	0	119	11007	0	1859	40.37~	(2.51- 648.95)
NAM	77	m	1	-	-	-	-	8.71	(5.87- 12.93)
NAM	93	f	1	-	-	-	-	8.88	(6.35- 12.40)
Subtotal NAM								8.81	(6.82- 11.37)

Table 1A1 - 2

IESLC - Meta-analysis of Ever Smoking, Any product (or Cigarettes if Any not available)
All LC types
Most adjusted

REF	NRR	SEX	AD	Number Exposed Case	Number Exposed Cont	Non-exposed Case	Non-exposed Cont	RR	95.00%CI
NOTAN2	15	m	2	-	-	-	-	2.99	(2.40- 3.72)
NOU	11	m	0	74	247	6	122	6.09	(2.58- 14.39)
NOU	12	f	0	10	92	4	261	7.09	(2.17- 23.17)
Subtotal NOU								6.42	(3.20- 12.87)
ODRISC	3	c	0	440	996	6	664	48.89	(21.71- 110.07)
ORMOS	4	m	0	87	1034	7	777	9.34	(4.30- 20.28)
ORMOS	26	f	0	1	234	23	1044	0.19	(0.03- 1.44)
Subtotal ORMOS								5.65	(2.74- 11.64)
OSANN	41	m	2	-	-	-	-	19.70	(14.40- 26.80)
OSANN	42	f	2	-	-	-	-	15.00	(11.80- 19.10)
Subtotal OSANN								16.62	(13.74- 20.10)
PARKIN	28	m	6	-	-	-	-	4.03	(3.15- 5.15)
PASTOR	10	m	1	-	-	-	-	6.81	(3.38- 13.70)
PAWLEG	2	m	6	-	-	-	-	12.26	(4.07- 36.95)
PERNU	2	m	0	1380	438	97	275	8.93	(6.92- 11.53)
PERNU	1	f	0	19	89	110	971	1.88	(1.11- 3.21)
Subtotal PERNU								6.68	(5.31- 8.41)
PERSH2	11	c	4	-	-	-	-	6.55	(5.46- 7.86)
*PETO	5	m	0	101	2423	2	295	6.15	(1.52- 24.79)
PEZZO2	10	m	0	361	469	6	117	15.01	(6.53- 34.48)
PEZZOT	25	m	0	211	317	4	116	19.30	(7.02- 53.10)
PIKE	4	m	0	514	375	18	69	5.25	(3.08- 8.98)
PIKE	8	f	0	163	90	36	96	4.83	(3.04- 7.66)
Subtotal PIKE								5.01	(3.53- 7.10)
POFFIJ	1	c	0	913	918	58	452	7.75	(5.81- 10.34)
POLEDN	1	c	1	-	-	-	-	9.24	(5.23- 16.33)
*QIAO2	15	m	1	-	-	-	-	1.53	(0.81- 2.89)
RACHTA	15	f	4	-	-	-	-	8.21	(3.96- 17.05)
RADZIK	1	c	0	180	198	9	13	1.31	(0.55- 3.15)
RANDIG	23	m	0	410	359	5	22	5.03	(1.88- 13.41)
RANDIG	24	f	0	16	39	17	92	2.22	(1.02- 4.84)
Subtotal RANDIG								3.04	(1.65- 5.60)
REN	1	m	0	106	84	12	34	3.58	(1.74- 7.33)
REN	2	f	0	78	20	48	50	4.06	(2.16- 7.64)
Subtotal REN								3.84	(2.39- 6.17)
RONCO	1	m	0	120	306	6	78	5.10	(2.16- 12.01)
ROTHSC	2	c	1	-	-	-	-	5.55	(2.97- 10.37)
SADOWS	31	m	1	-	-	-	-	3.63	(1.81- 7.30)
SANKAR	2	m	3	-	-	-	-	13.62	(9.00- 20.62)
SCHWAR	1	m	0	2648	1019	119	376	8.21	(6.60- 10.22)
SCHWAR	2	m	0	863	275	50	104	6.53	(4.54- 9.39)
SCHWAR	3	f	0	1351	637	182	855	9.96	(8.28- 12.00)
SCHWAR	4	f	0	335	179	40	247	11.56	(7.90- 16.90)
Subtotal SCHWAR								9.05	(7.99- 10.25)
SEGI	1	m	0	140	1742	18	382	1.71	(1.03- 2.82)
SEOW	6	f	1	-	-	-	-	5.25	(2.80- 9.84)
SHAW	12	c	0	324	266	11	107	11.85	(6.24- 22.50)
SIEMIA	5	m	7	-	-	-	-	12.10	(6.60- 22.30)
SIMARA	3	m	6	-	-	-	-	1.65	(0.97- 2.81)
SIMARA	4	f	6	-	-	-	-	1.63	(0.87- 3.06)
Subtotal SIMARA								1.64	(1.09- 2.46)
SOBUE	105	m	1	-	-	-	-	3.72	(2.57- 5.38)
SOBUE	115	f	1	-	-	-	-	2.51	(1.89- 3.33)
Subtotal SOBUE								2.90	(2.32- 3.64)
*SPEIZE	8	f	0	535	1012074	58	776300	7.08	(5.40- 9.28)
SPITZ	3	c	0	170	169	7	128	18.39	(8.35- 40.53)
STASZE	1	m	0	255	754	5	158	10.69	(4.34- 26.33)
STASZE	5	f	0	6	153	15	1660	4.34	(1.66- 11.35)
Subtotal STASZE								7.01	(3.63- 13.53)
STAYNE	1	m	0	362	567	58	333	3.67	(2.69- 4.99)
STOCKS	47	m	2	-	-	-	-	5.95	(4.23- 8.36)
STOCKS	50	f	1	-	-	-	-	3.04	(2.35- 3.93)
Subtotal STOCKS								3.88	(3.16- 4.76)
STOCKW	6	c	0	19370	7069	2791	10641	10.45	(9.94- 10.98)
STUCKE	3	m	0	247	203	0	51	125.27~	(7.68-2042.38)
SUN	1	c	0	140	173	67	191	2.31	(1.62- 3.30)
SUZUK2	20	c	3	-	-	-	-	14.02	(4.36- 45.04)
SVENSS	71	f	1	-	-	-	-	6.18	(3.79- 10.07)
TANG	3	c	0	110	59	9	39	8.08	(3.66- 17.82)
*TENKAN	22	m	1	-	-	-	-	14.64	(6.29- 34.07)
TIZZAN	1	m	0	1036	911	180	305	1.93	(1.57- 2.36)

Table 1A1 - 2

IESLC - Meta-analysis of Ever Smoking, Any product (or Cigarettes if Any not available)
All LC types
Most adjusted

REF	NRR	SEX	AD	Number Exposed Case	Number Exposed Cont	Non-exposed Case	Non-exposed Cont	RR	95.00%CI
TIZZAN	12	f	0	25	28	25	114	4.07	(2.04- 8.13)
Subtotal TIZZAN								2.05	(1.68- 2.49)
TOKARS	6	c	3	-	-	-	-	6.60	(3.20- 13.70)
TOUSEY	21	m	3	-	-	-	-	19.63	(7.18- 53.68)
TOUSEY	26	f	0	193	214	13	226	15.68	(8.67- 28.34)
Subtotal TOUSEY								16.61	(9.97- 27.67)
TSUGAN	27	m	0	73	71	18	22	1.26	(0.62- 2.54)
*TULINI	38	m	3	-	-	-	-	7.71	(4.19- 14.18)
*TULINI	44	f	3	-	-	-	-	13.01	(7.24- 23.40)
Subtotal TULINI								10.12	(6.63- 15.44)
*TVERDA	22	m	2	-	-	-	-	4.58	(2.97- 7.06)
WAKAI	72	m	2	-	-	-	-	3.67	(1.84- 7.32)
WAKAI	78	f	2	-	-	-	-	4.49	(2.35- 8.59)
Subtotal WAKAI								4.09	(2.55- 6.55)
WANG	5	c	6	-	-	-	-	2.88	(1.74- 4.77)
WANG2	16	c	4	-	-	-	-	2.29	(1.12- 4.70)
WANG3	1	c	0	235	172	58	121	2.85	(1.97- 4.13)
WANG4	2	m	2	-	-	-	-	1.16	(0.96- 1.42)
WICKLU	1	m	0	-	-	-	-	4.60	(2.80- 7.60)
WIGLE	27	m	1	-	-	-	-	8.80	(5.15- 15.02)
WIGLE	32	f	1	-	-	-	-	4.40	(2.88- 6.73)
Subtotal WIGLE								5.75	(4.12- 8.02)
WILKIN	3	c	4	-	-	-	-	7.83	(4.45- 13.78)
WU	45	f	2	-	-	-	-	3.03	(1.81- 5.07)
WUNSCH	4	m	1	-	-	-	-	4.75	(2.66- 8.50)
WUNSCH	10	f	1	-	-	-	-	4.43	(2.62- 7.47)
Subtotal WUNSCH								4.57	(3.10- 6.74)
WUWILL	8	f	3	-	-	-	-	2.30	(1.90- 2.80)
WYNDE2	21	m	0	396	616	8	105	8.44	(4.07- 17.51)
WYNDE3	49	m	0	275	332	9	88	8.10	(4.00- 16.38)
WYNDE3	138	f	0	46	56	20	76	3.12	(1.67- 5.85)
Subtotal WYNDE3								4.76	(2.98- 7.61)
WYNDE4	48	m	0	632	665	12	115	9.11	(4.98- 16.67)
WYNDE4	62	f	2	-	-	-	-	2.87	(1.48- 5.55)
Subtotal WYNDE4								5.38	(3.45- 8.41)
WYNDE6	72	m	0	2823	1996	87	617	10.03	(7.96- 12.65)
WYNDE6	252	f	0	1354	701	159	856	10.40	(8.58- 12.60)
Subtotal WYNDE6								10.25	(8.84- 11.88)
*XIANGZ	13	m	2	-	-	-	-	2.16	(1.46- 3.18)
XU	2	m	2	-	-	-	-	2.70	(2.10- 3.50)
XU2	2	c	7	-	-	-	-	3.80	(2.84- 5.07)
XU3	2	m	1	-	-	-	-	5.99	(2.65- 13.50)
XU3	4	f	1	-	-	-	-	3.86	(1.39- 10.70)
Subtotal XU3								5.05	(2.67- 9.54)
XU4	1	c	0	161	113	45	93	2.94	(1.92- 4.52)
YAMAGU	11	c	1	-	-	-	-	3.97	(2.12- 7.40)
*YONG	2	c	1	-	-	-	-	6.74	(4.47- 10.18)
*YUAN	1	m	2	-	-	-	-	6.50	(3.64- 11.60)
ZHANG	2	m	7	-	-	-	-	4.00	(1.61- 9.91)
ZHANG	3	f	7	-	-	-	-	3.75	(1.80- 10.76)
Subtotal ZHANG								3.87	(2.05- 7.32)
ZHENG	15	m	0	279	218	33	94	3.65	(2.36- 5.63)
ZHENG	24	f	0	76	44	152	184	2.09	(1.36- 3.21)
Subtotal ZHENG								2.75	(2.03- 3.73)
ZHOU	2	m	0	740	41	275	36	2.36	(1.48- 3.77)
ZHOU	3	f	0	112	7	231	32	2.22	(0.95- 5.18)
Subtotal ZHOU								2.33	(1.54- 3.51)
Partial Totals				80688	1384900	9242	1081588		

*prospective study ~ With 0.5 adjustment for zero

Table 1A1 - 2

IESLC - Meta-analysis of Ever Smoking, Any product (or Cigarettes if Any not available)
All LC types
Most adjusted

REF	NRR	SEX	AD	Ys	Ws	Qs	Ps
ABELIN	44	m	1	3.57	1.93	8.70	0.0000
*ABRAHA	7	m	0	2.17	9.68	5.13	0.0000
*ABRAHA	8	f	0	1.59	19.39	0.41	0.0000
Subtotal ABRAHA				1.78	29.07	5.54	
AGUDO	1	f	3	1.13	6.32	0.60	0.0044
*AKIBA	11	m	5	1.56	20.22	0.28	0.0000
*AKIBA	15	f	5	1.15	46.54	3.91	0.0000
Subtotal AKIBA				1.27	66.77	4.19	
ALDERS	69	m	1	2.27	13.04	8.91	0.0000
ALDERS	6	f	1	1.56	45.44	0.63	0.0000
Subtotal ALDERS				1.72	58.48	9.54	
*AMANDU	7	m	2	1.77	4.23	0.47	0.0003
AMES	4	m	0	1.59	11.09	0.24	0.0000
*ANDERS	3	f	0	2.57	39.86	51.10	0.0000
*ARCHER	6	m	0	1.85	5.76	0.94	0.0000
ARMADA	29	m	0	2.98	3.67	8.69	0.0000
AUSTIN	7	c	3	2.41	3.59	3.36	0.0000
AUVINE	19	c	2	2.63	12.22	17.21	0.0000
AXELSO	1	c	0	1.83	24.06	3.74	0.0000
AXELSS	8	m	6	2.08	12.60	5.18	0.0000
AXELSS	11	f	0	2.16	12.45	6.37	0.0000
Subtotal AXELSS				2.12	25.05	11.55	
BAND	1	m	2	2.30	42.76	31.48	0.0000
BARBON	131	m	3	2.41	18.10	17.00	0.0000
BECHER	1	m	0	2.38	2.75	2.44	0.0001
BECHER	24	f	2	2.47	2.25	2.38	0.0002
Subtotal BECHER				2.42	5.01	4.82	
*BENSHL	18	m	1	1.77	5.82	0.65	0.0000
*BEST	22	m	0	3.24	1.00	3.21	0.0012
*BEST	18	f	1	0.81	2.17	0.87	0.2348
Subtotal BEST				1.57	3.17	4.09	
BLOHMK	3	m	0	1.13	70.05	6.68	0.0000
BLOT4	1	m	0	2.68	6.96	10.61	0.0000
BOFFET	33	m	2	2.65	103.49	152.21	0.0000
*BOUCOT	121	m	2	3.71	0.50	2.57	0.0088
BRESLO	37	m	0	1.87	5.85	1.06	0.0000
BRESLO	38	f	0	0.32	3.10	3.88	0.5717
Subtotal BRESLO				1.33	8.95	4.95	
*BRETT	10	m	0	1.18	5.77	0.38	0.0044
BROCKM	1	m	0	0.07	0.98	1.83	0.9437
BROCKM	2	f	0	0.69	2.73	1.53	0.2517
Subtotal BROCKM				0.53	3.71	3.36	
BROSS	12	m	0	1.64	28.92	1.13	0.0000
BROWN2	2	m	2	2.21	442.58	260.89	0.0000
BROWN2	1	f	2	2.54	427.71	518.57	0.0000
Subtotal BROWN2				2.37	870.29	779.46	
BUFFLE	1	m	0	2.36	4.43	3.71	0.0000
BUFFLE	5	f	0	1.96	28.29	7.74	0.0000
Subtotal BUFFLE				2.02	32.72	11.45	
CARPEN	12	c	3	2.70	12.04	19.10	0.0000
CASCO2	1	c	0	2.44	5.31	5.31	0.0000
CASCOR	1	c	0	2.60	18.40	24.92	0.0000
*CEDERL	107	m	2	1.78	20.66	2.36	0.0000
*CEDERL	112	f	2	1.43	31.21	0.00	0.0000
Subtotal CEDERL				1.57	51.88	2.36	
CHAN	9	m	0	3.31	1.87	6.57	0.0000
CHAN	10	f	0	1.25	20.57	0.78	0.0000
Subtotal CHAN				1.42	22.44	7.35	
*CHANG	6	m	0	1.65	4.76	0.21	0.0003
*CHANG	12	f	0	1.30	8.85	0.17	0.0001
Subtotal CHANG				1.42	13.61	0.38	
CHATZI	4	c	0	1.21	19.44	1.07	0.0000
CHEN2	1	m	0	1.52	6.25	0.04	0.0001
CHEN2	2	f	0	0.51	7.70	6.61	0.1539
Subtotal CHEN2				0.97	13.95	6.65	
CHEN3	1	c	0	0.46	27.78	26.57	0.0148
CHIAZZ	3	m	11	3.26	0.90	3.00	0.0019
CHOI	1	m	0	1.43	10.71	0.00	0.0000
CHOI	5	f	0	0.46	9.06	8.79	0.1703
Subtotal CHOI				0.99	19.78	8.79	
*CHOW	55	m	2	2.41	5.68	5.29	0.0000

Table 1A1 - 2

IESLC - Meta-analysis of Ever Smoking, Any product (or Cigarettes if Any not available)
All LC types
Most adjusted

REF	NRR	SEX	AD	Ys	Ws	Qs	Ps
*CHYOU	7	m	1	2.12	12.17	5.66	0.0000
COMSTO	34	m	0	2.38	3.63	3.22	0.0000
COMSTO	46	f	0	2.19	9.22	5.20	0.0000
Subtotal COMSTO				2.25	12.85	8.41	
COOKSO	5	c	0	1.88	14.38	2.81	0.0000
CORREA	34	c	1	2.43	51.78	51.07	0.0000
*CPSI	187	m	1	2.22	78.68	47.44	0.0000
*CPSI	274	f	1	1.03	73.22	12.58	0.0000
Subtotal CPSI				1.64	151.90	60.02	
*CPSII	104	m	1	2.55	78.29	96.69	0.0000
*CPSII	79	f	1	2.10	142.84	61.99	0.0000
Subtotal CPSII				2.26	221.13	158.67	
DAMBER	25	m	1	1.97	29.41	8.16	0.0000
DARBY	15	m	0	3.90	2.96	17.87	0.0000
DARBY	16	f	0	2.51	19.76	22.43	0.0000
Subtotal DARBY				2.69	22.71	40.29	
DAVEYS	5	m	0	1.57	2.53	0.04	0.0126
DAVEYS	6	f	0	-0.32	0.42	1.32	0.8327
Subtotal DAVEYS				1.30	2.96	1.36	
DEAN	7	m	0	1.66	9.69	0.45	0.0000
DEAN2	3	m	0	1.33	23.82	0.27	0.0000
DEAN2	7	f	0	1.08	14.58	1.94	0.0000
Subtotal DEAN2				1.24	38.40	2.21	
DEAN3	49	m	3	1.81	19.11	2.68	0.0000
DEAN3	126	f	3	1.53	21.26	0.18	0.0000
Subtotal DEAN3				1.67	40.37	2.86	
*DEKLER	6	m	2	3.01	0.99	2.45	0.0027
DESTE2	14	c	7	2.16	13.37	6.98	0.0000
DESTEF	48	m	4	2.21	19.77	11.69	0.0000
*DOCKER	3	c	4	1.46	4.27	0.00	0.0026
DOLL	6	m	0	2.21	6.22	3.64	0.0000
DOLL	12	f	0	0.72	12.98	6.80	0.0099
Subtotal DOLL				1.20	19.20	10.45	
*DOLL2	56	m	1	2.04	18.57	6.59	0.0000
DORANT	10	c	0	2.89	13.34	28.04	0.0000
DORGAN	6	m	0	2.28	12.35	8.80	0.0000
DORGAN	30	m	0	3.13	2.68	7.70	0.0000
DORGAN	53	f	0	2.06	51.30	19.56	0.0000
DORGAN	76	f	0	2.12	4.12	1.92	0.0000
Subtotal DORGAN				2.14	70.44	37.99	
*DORN	196	m	1	1.95	73.73	19.26	0.0000
DOSEME	1	m	2	1.19	55.52	3.38	0.0000
DROSTE	7	m	4	2.15	5.72	2.91	0.0000
DU	1	m	0	1.26	28.12	0.90	0.0000
DU	2	f	0	0.66	24.50	15.02	0.0011
Subtotal DU				0.98	52.62	15.92	
*DUNN	6	m	0	2.91	1.97	4.28	0.0000
EBELIN	1	m	0	1.94	9.19	2.26	0.0000
*ENGELA	159	m	1	1.85	6.58	1.09	0.0000
*ENGELA	165	f	1	1.56	6.51	0.09	0.0001
Subtotal ENGELA				1.70	13.09	1.17	
ESAKI	4	m	0	0.64	8.96	5.74	0.0554
ESAKI	5	f	0	0.90	7.99	2.33	0.0109
Subtotal ESAKI				0.76	16.94	8.07	
FAN	1	m	0	1.04	25.87	4.05	0.0000
FAN	2	f	0	1.37	24.92	0.14	0.0000
Subtotal FAN				1.20	50.80	4.18	
GAO	1	m	2	1.36	39.76	0.25	0.0000
GAO	11	f	2	1.19	57.09	3.47	0.0000
Subtotal GAO				1.26	96.85	3.72	
GAO2	10	m	1	1.64	9.74	0.40	0.0000
GARCIA	3	c	0	2.14	16.50	8.12	0.0000
GARDIN	7	c	0	2.41	4.14	3.86	0.0000
GARSHI	25	m	1	1.76	34.86	3.55	0.0000
GENG	1	m	0	1.79	4.98	0.61	0.0001
GENG	2	f	0	1.08	22.39	2.83	0.0000
Subtotal GENG				1.21	27.37	3.44	
GER	21	c	14	0.61	12.59	8.69	0.0305
GODLEY	5	m	1	1.92	96.28	22.40	0.0000
GODLEY	6	f	1	1.71	58.89	4.34	0.0000
Subtotal GODLEY				1.84	155.17	26.74	

Table 1A1 - 2

IESLC - Meta-analysis of Ever Smoking, Any product (or Cigarettes if Any not available)
All LC types
Most adjusted

REF	NRR	SEX	AD	Ys	Ws	Qs	Ps
GOLLED	7	m	1	2.02	13.89	4.60	0.0000
GOODMA	3	m	0	2.38	8.92	7.86	0.0000
GOODMA	7	f	0	2.12	12.25	5.58	0.0000
Subtotal GOODMA				2.23	21.17	13.44	
GRAHAM	27	m	1	1.95	17.52	4.50	0.0000
GREGOR	3	m	0	0.03	5.11	10.20	0.9492
GREGOR	7	f	0	2.40	0.90	0.82	0.0233
Subtotal GREGOR				0.38	6.01	11.02	
GSELL	8	m	0	2.88	1.82	3.75	0.0001
HAENSZ	11	f	2	0.78	25.03	10.79	0.0001
*HAMMO2	4	m	1	3.13	1.00	2.83	0.0018
*HAMMON	117	m	1	1.92	14.49	3.29	0.0000
*HANSEN	3	m	2	0.43	5.28	5.44	0.3285
HEGMAN	1	c	0	2.79	23.66	43.33	0.0000
*HEIN	7	m	0	2.68	1.00	1.53	0.0074
*HENNEK	3	m	0	1.83	19.94	3.03	0.0000
HINDS	22	f	3	1.73	39.57	3.35	0.0000
*HIRAYA	147	m	1	1.47	85.78	0.09	0.0000
*HIRAYA	150	f	1	0.86	80.63	27.29	0.0000
Subtotal HIRAYA				1.18	166.40	27.38	
HITOSU	38	m	1	1.07	6.36	0.88	0.0071
HITOSU	62	f	1	1.22	15.00	0.70	0.0000
Subtotal HITOSU				1.18	21.36	1.59	
*HOLE	8	m	1	1.86	6.76	1.20	0.0000
HOROWI	1	m	0	1.27	15.35	0.42	0.0000
HOROWI	2	f	0	0.60	8.08	5.74	0.0894
Subtotal HOROWI				1.04	23.43	6.16	
HORWIT	1	f	0	2.43	8.29	8.05	0.0000
HU	15	m	0	0.74	17.16	8.53	0.0023
HU	16	f	0	0.55	7.15	5.67	0.1413
Subtotal HU				0.68	24.31	14.20	
HU2	9	m	0	1.11	27.11	3.01	0.0000
HU2	10	f	0	0.63	21.91	14.44	0.0033
Subtotal HU2				0.89	49.01	17.45	
HUANG	1	c	0	0.69	14.82	8.33	0.0078
HUMBLE	14	m	1	2.49	5.19	5.75	0.0000
HUMBLE	16	m	1	2.47	1.71	1.82	0.0012
HUMBLE	18	f	1	2.43	6.68	6.55	0.0000
HUMBLE	20	f	1	2.73	2.90	4.85	0.0000
Subtotal HUMBLE				2.51	16.48	18.97	
JAHN	22	f	2	1.19	14.92	0.91	0.0000
JAIN	46	m	2	2.12	8.79	4.01	0.0000
JAIN	41	f	2	2.22	17.72	10.74	0.0000
Subtotal JAIN				2.19	26.50	14.75	
JARUP	6	m	2	2.02	3.91	1.31	0.0001
JARVHO	3	m	0	3.32	0.92	3.26	0.0015
JARVHO	7	f	0	2.26	3.28	2.19	0.0000
Subtotal JARVHO				2.49	4.19	5.45	
JEDRYC	58	m	4	1.70	31.63	2.09	0.0000
JEDRYC	59	f	4	1.51	11.71	0.06	0.0000
Subtotal JEDRYC				1.65	43.33	2.15	
JIANG	1	m	0	1.00	4.45	0.86	0.0346
JIANG	2	f	0	0.91	2.62	0.73	0.1401
Subtotal JIANG				0.97	7.08	1.59	
JOLY	14	m	0	2.50	11.02	12.35	0.0000
JOLY	1	f	0	1.99	27.09	8.30	0.0000
Subtotal JOLY				2.14	38.11	20.65	
JUSSAW	29	m	2	2.82	25.79	49.30	0.0000
*KAISE2	72	m	1	1.69	11.75	0.71	0.0000
*KAISE2	64	f	1	2.31	9.19	6.98	0.0000
Subtotal KAISE2				1.96	20.95	7.69	
*KAISER	13	m	2	2.87	25.69	52.48	0.0000
*KAISER	10	f	2	1.73	28.18	2.33	0.0000
Subtotal KAISER				2.27	53.88	54.81	
KATSOU	29	f	1	1.19	9.91	0.60	0.0002
KAUFMA	17	c	6	2.52	28.72	33.23	0.0000
KELLER	3	m	0	2.31	217.37	164.92	0.0000
KELLER	11	m	0	2.60	25.99	35.20	0.0000
KELLER	7	f	0	2.53	269.69	317.37	0.0000
KELLER	15	f	0	2.25	39.03	25.32	0.0000
Subtotal KELLER				2.43	552.08	542.81	

Table 1A1 - 2

IESLC - Meta-analysis of Ever Smoking, Any product (or Cigarettes if Any not available)
All LC types
Most adjusted

REF	NRR	SEX	AD	Ys	Ws	Qs	Ps
KHUDER	4	m	0	2.06	19.93	7.68	0.0000
KIHARA	31	c	0	1.22	46.97	2.30	0.0000
*KINLEN	17	m	2	2.40	7.00	6.40	0.0000
KJUUS	10	m	0	2.62	1.81	2.51	0.0004
*KNEKT	87	m	1	1.86	5.67	1.00	0.0000
KO	1	f	3	1.44	2.18	0.00	0.0339
KOHLME	2	c	4	2.80	5.21	9.59	0.0000
KOO	1	f	0	1.02	23.01	4.11	0.0000
KOULUM	1	m	0	3.57	4.47	20.21	0.0000
KREUZE	14	f	0	2.24	4.29	2.71	0.0000
KREUZE	16	f	0	1.33	32.30	0.39	0.0000
Subtotal KREUZE				1.44	36.60	3.11	
KREYBE	12	m	1	1.89	5.80	1.16	0.0000
KREYBE	30	f	1	0.36	7.92	9.28	0.3143
Subtotal KREYBE				1.01	13.71	10.45	
*KUBIK	28	m	0	3.34	1.96	7.06	0.0000
LAMTH	6	f	0	1.34	46.55	0.50	0.0000
LAMWK	1	f	0	1.42	17.85	0.01	0.0000
LAMWK2	9	m	0	1.04	12.98	2.07	0.0002
LAMWK2	10	f	0	1.17	17.89	1.35	0.0000
Subtotal LAMWK2				1.11	30.86	3.42	
*LANGE	40	m	1	1.56	3.97	0.05	0.0019
*LANGE	37	f	1	1.60	8.13	0.19	0.0000
Subtotal LANGE				1.58	12.09	0.25	
LAUSSM	11	m	3	1.74	37.15	3.34	0.0000
LEI	1	m	0	1.30	26.63	0.50	0.0000
LEI	2	f	0	1.25	23.21	0.85	0.0000
Subtotal LEI				1.28	49.84	1.35	
LEMARC	3	c	0	1.73	22.77	1.89	0.0000
LETOUR	1	c	0	2.56	20.21	25.43	0.0000
LEVIN	32	m	1	1.58	30.68	0.61	0.0000
*LIDDEL	5	m	1	1.28	17.92	0.44	0.0000
LIU	2	c	2	0.65	38.19	23.72	0.0001
LIU2	2	m	3	1.65	4.37	0.19	0.0006
LIU2	4	f	3	1.54	6.68	0.06	0.0001
Subtotal LIU2				1.58	11.05	0.25	
LIU3	2	m	2	0.23	1.87	2.74	0.7518
LIU4	11	m	2	1.02	5969.41	1079.57	0.0000
LIU4	12	f	2	1.05	3876.64	588.65	0.0000
Subtotal LIU4				1.03	9846.05	1668.22	
LIU5	1	c	0	0.65	11.25	7.04	0.0293
LOMBA2	1	f	0	0.28	37.19	49.81	0.0841
LOMBAR	12	m	0	2.18	12.13	6.63	0.0000
LUBIN2	46	m	2	2.14	162.99	79.77	0.0000
LUBIN2	102	f	1	1.36	133.31	0.84	0.0000
Subtotal LUBIN2				1.79	296.30	80.61	
LUO	7	c	20	0.99	10.60	2.12	0.0012
MACLEN	73	c	2	0.98	17.04	3.58	0.0001
*MAGNUS	5	m	3	1.42	6.75	0.00	0.0002
MARSH	7	c	2	1.92	7.36	1.67	0.0000
MARSH2	5	m	1	0.64	3.87	2.50	0.2107
MARSH2	6	f	1	1.66	3.65	0.18	0.0015
Subtotal MARSH2				1.14	7.51	2.68	
MARTIS	4	m	0	1.95	3.32	0.85	0.0004
MASTRA	2	m	2	2.10	4.76	2.05	0.0000
MATOS	27	m	2	1.92	8.82	2.00	0.0000
MATSUD	10	m	0	3.07	2.94	7.77	0.0000
MCCONN	1	m	0	0.19	3.33	5.17	0.7237
MCCONN	2	f	0	1.01	0.99	0.18	0.3136
Subtotal MCCONN				0.38	4.32	5.36	
MCDUFF	1	m	0	1.81	4.70	0.65	0.0001
MCLAUG	1	m	0	1.20	18.70	1.04	0.0000
*MIGRAN	27	m	2	1.28	3.91	0.10	0.0111
*MIGRAN	42	f	2	1.53	3.54	0.03	0.0040
Subtotal MIGRAN				1.40	7.45	0.12	
MILLER	2	f	1	1.61	4.90	0.14	0.0004
MILLS	3	m	1	0.29	96.62	128.22	0.0046
*MRFITR	6	m	0	3.70	0.50	2.54	0.0091
NAM	77	m	1	2.16	24.64	12.91	0.0000
NAM	93	f	1	2.18	34.31	18.96	0.0000
Subtotal NAM				2.18	58.95	31.87	

Table 1A1 - 2

IESLC - Meta-analysis of Ever Smoking, Any product (or Cigarettes if Any not available)
All LC types
Most adjusted

REF	NRR	SEX	AD	Ys	Ws	Qs	Ps
NOTAN2	15	m	2	1.10	80.00	9.53	0.0000
NOU	11	m	0	1.81	5.20	0.70	0.0000
NOU	12	f	0	1.96	2.74	0.74	0.0012
Subtotal NOU				1.86	7.94	1.44	
ODRISC	3	c	0	3.89	5.83	34.98	0.0000
ORMOS	4	m	0	2.23	6.39	4.02	0.0000
ORMOS	26	f	0	-1.64	0.95	9.05	0.1093
Subtotal ORMOS				1.73	7.34	13.07	
OSANN	41	m	2	2.98	39.82	94.46	0.0000
OSANN	42	f	2	2.71	66.25	106.45	0.0000
Subtotal OSANN				2.81	106.08	200.91	
PARKIN	28	m	6	1.39	63.58	0.14	0.0000
PASTOR	10	m	1	1.92	7.85	1.79	0.0000
PAWLEG	2	m	6	2.51	3.16	3.59	0.0000
PERNU	2	m	0	2.19	58.99	33.11	0.0000
PERNU	1	f	0	0.63	13.52	8.80	0.0198
Subtotal PERNU				1.90	72.50	41.91	
PERSH2	11	c	4	1.88	115.76	22.31	0.0000
*PETO	5	m	0	1.82	1.98	0.28	0.0107
PEZZO2	10	m	0	2.71	5.55	8.93	0.0000
PEZZOT	25	m	0	2.96	3.75	8.67	0.0000
PIKE	4	m	0	1.66	13.39	0.64	0.0000
PIKE	8	f	0	1.57	18.04	0.33	0.0000
Subtotal PIKE				1.61	31.43	0.96	
POFFIJ	1	c	0	2.05	46.21	17.04	0.0000
POLEDN	1	c	1	2.22	11.85	7.27	0.0000
*QIAO2	15	m	1	0.43	9.50	9.79	0.1900
RACHTA	15	f	4	2.11	7.21	3.19	0.0000
RADZIK	1	c	0	0.27	5.03	6.87	0.5411
RANDIG	23	m	0	1.61	3.99	0.12	0.0013
RANDIG	24	f	0	0.80	6.34	2.62	0.0447
Subtotal RANDIG				1.11	10.32	2.74	
REN	1	m	0	1.27	7.46	0.21	0.0005
REN	2	f	0	1.40	9.65	0.01	0.0000
Subtotal REN				1.35	17.11	0.22	
RONCO	1	m	0	1.63	5.23	0.19	0.0002
ROTHSC	2	c	1	1.71	9.83	0.73	0.0000
SADOWS	31	m	1	1.29	7.90	0.18	0.0003
SANKAR	2	m	3	2.61	22.36	30.66	0.0000
SCHWAR	1	m	0	2.11	80.50	35.60	0.0000
SCHWAR	2	m	0	1.88	29.06	5.51	0.0000
SCHWAR	3	f	0	2.30	111.43	82.11	0.0000
SCHWAR	4	f	0	2.45	26.58	26.94	0.0000
Subtotal SCHWAR				2.20	247.58	150.16	
SEGI	1	m	0	0.53	15.18	12.47	0.0375
SEOW	6	f	1	1.66	9.73	0.46	0.0000
SHAW	12	c	0	2.47	9.34	9.94	0.0000
SIEMIA	5	m	7	2.49	10.37	11.49	0.0000
SIMARA	3	m	6	0.50	13.58	11.99	0.0650
SIMARA	4	f	6	0.49	9.71	8.80	0.1278
Subtotal SIMARA				0.50	23.30	20.80	
SOBUE	105	m	1	1.31	28.15	0.45	0.0000
SOBUE	115	f	1	0.92	47.90	12.96	0.0000
Subtotal SOBUE				1.07	76.05	13.41	
*SPEIZE	8	f	0	1.96	52.33	13.94	0.0000
SPITZ	3	c	0	2.91	6.16	13.33	0.0000
STASZE	1	m	0	2.37	4.73	4.07	0.0000
STASZE	5	f	0	1.47	4.16	0.00	0.0028
Subtotal STASZE				1.95	8.88	4.08	
STAYNE	1	m	0	1.30	40.37	0.81	0.0000
STOCKS	47	m	2	1.78	33.11	3.89	0.0000
STOCKS	50	f	1	1.11	58.11	6.28	0.0000
Subtotal STOCKS				1.36	91.22	10.17	
STOCKW	6	c	0	2.35	1549.52	1271.41	0.0000
STUCKE	3	m	0	4.83	0.49	5.67	0.0007
SUN	1	c	0	0.84	30.23	11.05	0.0000
SUZUK2	20	c	3	2.64	2.82	4.06	0.0000
SVENSS	71	f	1	1.82	16.09	2.33	0.0000
TANG	3	c	0	2.09	6.14	2.59	0.0000
*TENKAN	22	m	1	2.68	5.38	8.32	0.0000
TIZZAN	1	m	0	0.66	91.77	56.49	0.0000

Table 1A1 - 2

IESLC - Meta-analysis of Ever Smoking, Any product (or Cigarettes if Any not available)
All LC types
Most adjusted

REF	NRR	SEX	AD	Ys	Ws	Qs	Ps
TIZZAN	12	f	0	1.40	8.03	0.01	0.0001
Subtotal TIZZAN				0.72	99.80	56.50	
TOKARS	6	c	3	1.89	7.27	1.45	0.0000
TOUSEY	21	m	3	2.98	3.80	8.96	0.0000
TOUSEY	26	f	0	2.75	10.96	18.87	0.0000
Subtotal TOUSEY				2.81	14.76	27.83	
TSUGAN	27	m	0	0.23	7.76	11.41	0.5244
*TULINI	38	m	3	2.04	10.34	3.75	0.0000
*TULINI	44	f	3	2.57	11.17	14.14	0.0000
Subtotal TULINI				2.31	21.50	17.88	
*TVERDA	22	m	2	1.52	20.49	0.14	0.0000
WAKAI	72	m	2	1.30	8.06	0.16	0.0002
WAKAI	78	f	2	1.50	9.15	0.03	0.0000
Subtotal WAKAI				1.41	17.20	0.19	
WANG	5	c	6	1.06	15.11	2.21	0.0000
WANG2	16	c	4	0.83	7.47	2.80	0.0235
WANG3	1	c	0	1.05	28.11	4.34	0.0000
WANG4	2	m	2	0.15	100.26	167.38	0.1372
WICKLU	1	m	0	1.53	15.41	0.11	0.0000
WIGLE	27	m	1	2.17	13.41	7.23	0.0000
WIGLE	32	f	1	1.48	21.33	0.04	0.0000
Subtotal WIGLE				1.75	34.74	7.27	
WILKIN	3	c	4	2.06	12.03	4.59	0.0000
WU	45	f	2	1.11	14.48	1.60	0.0000
WUNSCH	4	m	1	1.56	11.39	0.16	0.0000
WUNSCH	10	f	1	1.49	14.00	0.03	0.0000
Subtotal WUNSCH				1.52	25.38	0.19	
WUWILL	8	f	3	0.83	102.19	37.73	0.0000
WYNDE2	21	m	0	2.13	7.21	3.46	0.0000
WYNDE3	49	m	0	2.09	7.74	3.28	0.0000
WYNDE3	138	f	0	1.14	9.73	0.89	0.0004
Subtotal WYNDE3				1.56	17.48	4.17	
WYNDE4	48	m	0	2.21	10.51	6.21	0.0000
WYNDE4	62	f	2	1.05	8.80	1.31	0.0018
Subtotal WYNDE4				1.68	19.31	7.52	
WYNDE6	72	m	0	2.31	71.58	53.57	0.0000
WYNDE6	252	f	0	2.34	103.92	84.40	0.0000
Subtotal WYNDE6				2.33	175.50	137.97	
*XIANGZ	13	m	2	0.77	25.36	11.40	0.0001
XU	2	m	2	0.99	58.89	11.78	0.0000
XU2	2	c	7	1.34	45.75	0.51	0.0000
XU3	2	m	1	1.79	5.80	0.71	0.0000
XU3	4	f	1	1.35	3.69	0.03	0.0095
Subtotal XU3				1.62	9.49	0.74	
XU4	1	c	0	1.08	20.82	2.71	0.0000
YAMAGU	11	c	1	1.38	9.83	0.04	0.0000
*YONG	2	c	1	1.91	22.68	4.96	0.0000
*YUAN	1	m	2	1.87	11.44	2.13	0.0000
ZHANG	2	m	7	1.39	4.65	0.01	0.0028
ZHANG	3	f	7	1.32	4.81	0.07	0.0038
Subtotal ZHANG				1.35	9.46	0.08	
ZHENG	15	m	0	1.29	20.36	0.44	0.0000
ZHENG	24	f	0	0.74	20.88	10.31	0.0008
Subtotal ZHENG				1.01	41.24	10.75	
ZHOU	2	m	0	0.86	17.50	5.90	0.0003
ZHOU	3	f	0	0.80	5.34	2.22	0.0660
Subtotal ZHOU				0.84	22.83	8.12	

Table 1A1 - 2

IESLC - Meta-analysis of Ever Smoking, Any product (or Cigarettes if Any not available)
All LC types
Most adjusted

	N	328
	NS	236
	Wt	19346.08
Het	Chi	7470.28
Het	df	327
Het	P	***
Fixed	RR	4.22
	RRl	4.16
	RRu	4.28
	P	+++
Random	RR	5.50
	RRl	5.07
	RRu	5.96
	P	+++
Asymm	P	***

Table 1A1 - 3

IESLC - Meta-analysis of Ever Smoking, Any product (or Cigarettes if Any not available)
All LC types
Most adjusted

	Sex			
	combined	male	female	Total
N	49	171	108	328
NS	49	167	103	319
Wt	2467.81	9857.19	7021.09	19346.08
Het Chi	685.94	3146.98	2350.06	7470.28
Het df	48	170	107	327
Het P	***	***	***	***
Fixed RR	8.26	3.74	3.95	4.22
RRl	7.94	3.67	3.86	4.16
RRu	8.60	3.82	4.05	4.28
P	+++	+++	+++	+++
Random RR	6.09	6.18	4.43	5.50
RRl	4.98	5.49	3.84	5.07
RRu	7.44	6.95	5.10	5.96
P	+++	+++	+++	+++
Between Chi				1287.30
Between df				2
Between P				***
Btwn(F) P				***
Btwn(R) P				**

	Lung cancer type		
	all	other	Total
N	317	11	328
NS	228	8	236
Wt	19146.95	199.13	19346.08
Het Chi	7420.92	34.96	7470.28
Het df	316	10	327
Het P	***	***	***
Fixed RR	4.23	3.23	4.22
RRl	4.17	2.81	4.16
RRu	4.29	3.71	4.28
P	+++	+++	+++
Random RR	5.59	3.48	5.50
RRl	5.15	2.65	5.07
RRu	6.07	4.57	5.96
P	+++	+++	+++
Between Chi			14.40
Between df			1
Between P			***
Btwn(F) P			N.S.
Btwn(R) P			**

	Location								
	NAmer	UK	Scand	othEur	China	Japan	othAs	other	Total
N	116	29	32	50	51	18	18	14	328
NS	81	20	23	39	35	12	14	12	236
Wt	5542.36	373.26	474.25	1032.00	10907.37	457.15	353.46	206.22	19346.08
Het Chi	1176.08	139.56	102.83	429.48	166.47	52.89	143.00	37.28	7470.28
Het df	115	28	31	49	50	17	17	13	327
Het P	***	***	***	***	***	***	***	***	***
Fixed RR	8.86	5.22	6.43	5.55	2.77	3.19	3.79	6.25	4.22
RRl	8.63	4.72	5.88	5.22	2.72	2.91	3.41	5.45	4.16
RRu	9.10	5.78	7.04	5.90	2.83	3.49	4.20	7.16	4.28
P	+++	+++	+++	+++	+++	+++	+++	+++	+++
Random RR	7.49	5.83	6.39	6.09	2.69	3.21	3.76	7.41	5.50
RRl	6.78	4.54	5.29	4.95	2.50	2.68	2.72	5.72	5.07
RRu	8.27	7.49	7.71	7.51	2.88	3.85	5.18	9.60	5.96
P	+++	+++	+++	+++	+++	+++	+++	+++	+++
Between Chi									5222.67
Between df									7
Between P									***
Btwn(F) P									***
Btwn(R) P									***

Table 1A1 - 3

IESLC - Meta-analysis of Ever Smoking, Any product (or Cigarettes if Any not available)
All LC types
Most adjusted

	Detailed Country in "other Europe"					
	multi	Germany	othWest	East	Balkans	Total
N	4	17	13	13	3	50
NS	3	12	12	9	3	39
Wt	446.01	218.84	169.04	113.26	84.86	1032.00
Het Chi	101.78	66.23	135.12	36.35	0.00	429.48
Het df	3	16	12	12	2	49
Het P	***	***	***	***	N.S.	***
Fixed RR	7.51	4.66	3.95	5.74	3.31	5.55
RRl	6.85	4.09	3.40	4.77	2.67	5.22
RRu	8.24	5.32	4.59	6.90	4.09	5.90
P	+++	+++	+++	+++	+++	+++
Random RR	7.76	5.47	8.78	5.85	3.31	6.09
RRl	4.45	3.94	4.70	4.10	2.67	4.95
RRu	13.54	7.57	16.40	8.34	4.09	7.51
P	+++	+++	+++	+++	+++	+++
Between Chi						89.99
Between df						4
Between P						***
Btwn(F) P						*
Btwn(R) P						***

	Detailed Country in "other Asia"			
	India	HongKong	other	Total
N	3	7	8	18
NS	3	5	6	14
Wt	128.15	140.70	84.62	353.46
Het Chi	80.36	10.74	16.02	143.00
Het df	2	6	7	17
Het P	***	(*)	*	***
Fixed RR	5.52	3.52	2.42	3.79
RRl	4.64	2.99	1.95	3.41
RRu	6.56	4.15	2.99	4.20
P	+++	+++	+++	+++
Random RR	8.74	3.56	2.46	3.76
RRl	2.58	2.81	1.76	2.72
RRu	29.65	4.50	3.43	5.18
P	+++	+++	+++	+++
Between Chi				35.88
Between df				2
Between P				***
Btwn(F) P				N.S.
Btwn(R) P				(*)

	Detailed other continent			
	SCAmer	Auslia	Africa	Total
N	10	1	3	14
NS	8	1	3	12
Wt	117.57	0.99	87.66	206.22
Het Chi	16.77	0.00	3.05	37.28
Het df	9	0	2	13
Het P	(*)	N.S.	N.S.	***
Fixed RR	7.91	20.29	4.49	6.25
RRl	6.61	2.84	3.65	5.45
RRu	9.48	145.07	5.54	7.16
P	+++	++	+++	+++
Random RR	8.23	20.29	4.78	7.41
RRl	6.34	2.84	3.52	5.72
RRu	10.69	145.07	6.50	9.60
P	+++	++	+++	+++
Between Chi				17.46
Between df				2
Between P				***
Btwn(F) P				*
Btwn(R) P				*

Table 1A1 - 3

IESLC - Meta-analysis of Ever Smoking, Any product (or Cigarettes if Any not available)
All LC types
Most adjusted

	Start year of study <1960	1960-69	1970-79	1980-89	1990+	Total
N	54	52	71	114	37	328
NS	39	38	52	75	32	236
Wt	917.74	1289.61	1372.29	15276.26	490.19	19346.08
Het Chi	478.45	418.91	580.44	5654.47	238.00	7470.28
Het df	53	51	70	113	36	327
Het P	***	***	***	***	***	***
Fixed RR	3.96	5.16	4.58	4.11	5.28	4.22
RRl	3.71	4.88	4.34	4.04	4.83	4.16
RRu	4.23	5.45	4.82	4.17	5.77	4.28
P	+++	+++	+++	+++	+++	+++
Random RR	4.67	5.45	5.05	5.95	6.22	5.50
RRl	3.75	4.60	4.27	5.18	4.89	5.07
RRu	5.82	6.46	5.96	6.83	7.92	5.96
P	+++	+++	+++	+++	+++	+++
Between Chi						99.99
Between df						4
Between P						***
Btwn(F) P						N.S.
Btwn(R) P						N.S.

	Study type (1) CC	other	Total
N	262	66	328
NS	185	51	236
Wt	18051.91	1294.18	19346.08
Het Chi	6890.71	407.55	7470.28
Het df	261	65	327
Het P	***	***	***
Fixed RR	4.12	6.01	4.22
RRl	4.06	5.69	4.16
RRu	4.18	6.34	4.28
P	+++	+++	+++
Random RR	5.32	6.24	5.50
RRl	4.87	5.34	5.07
RRu	5.82	7.28	5.96
P	+++	+++	+++
Between Chi			172.01
Between df			1
Between P			***
Btwn(F) P			**
Btwn(R) P			(*)

	Study type (2) CC	prosp	other	Total
N	262	61	5	328
NS	185	47	4	236
Wt	18051.91	1249.63	44.55	19346.08
Het Chi	6890.71	387.94	11.34	7470.28
Het df	261	60	4	327
Het P	***	***	*	***
Fixed RR	4.12	5.92	9.17	4.22
RRl	4.06	5.60	6.84	4.16
RRu	4.18	6.25	12.30	4.28
P	+++	+++	+++	+++
Random RR	5.32	6.05	8.92	5.50
RRl	4.87	5.16	5.36	5.07
RRu	5.82	7.10	14.86	5.96
P	+++	+++	+++	+++
Between Chi				180.28
Between df				2
Between P				***
Btwn(F) P				*
Btwn(R) P				(*)

Table 1A1 - 3

IESLC - Meta-analysis of Ever Smoking, Any product (or Cigarettes if Any not available)
All LC types
Most adjusted

	Study size (number of LC cases)				
	100-249	250-499	500-999	1000+	Total
N	115	86	64	63	328
NS	96	63	43	34	236
Wt	934.48	1260.88	1461.27	15689.46	19346.08
Het Chi	459.45	573.04	573.94	5786.26	7470.28
Het df	114	85	63	62	327
Het P	***	***	***	***	***
Fixed RR	3.74	4.85	4.93	4.15	4.22
RRl	3.51	4.59	4.68	4.08	4.16
RRu	3.99	5.12	5.19	4.21	4.28
P	+++	+++	+++	+++	+++
Random RR	4.43	5.75	6.17	6.14	5.50
RRl	3.86	4.95	5.25	5.15	5.07
RRu	5.09	6.69	7.25	7.32	5.96
P	+++	+++	+++	+++	+++
Between Chi					77.58
Between df					3
Between P					***
Btwn(F) P					N.S.
Btwn(R) P					**

	Risky occupational population			
	no	mining	othRisky	Total
N	310	7	11	328
NS	218	7	11	236
Wt	19141.27	74.85	129.97	19346.08
Het Chi	7417.77	18.94	25.75	7470.28
Het df	309	6	10	327
Het P	***	**	**	***
Fixed RR	4.23	3.13	4.62	4.22
RRl	4.17	2.49	3.89	4.16
RRu	4.29	3.92	5.48	4.28
P	+++	+++	+++	+++
Random RR	5.53	3.66	5.25	5.50
RRl	5.09	2.34	3.74	5.07
RRu	6.01	5.73	7.38	5.96
P	+++	+++	+++	+++
Between Chi				7.82
Between df				2
Between P				*
Btwn(F) P				N.S.
Btwn(R) P				N.S.

	National cigarette tobacco type			
	Virginia	blended	other	Total
N	50	225	53	328
NS	37	162	37	236
Wt	783.19	7640.75	10922.14	19346.08
Het Chi	301.14	2503.36	168.96	7470.28
Het df	49	224	52	327
Het P	***	***	***	***
Fixed RR	5.60	7.48	2.77	4.22
RRl	5.22	7.32	2.72	4.16
RRu	6.00	7.65	2.83	4.28
P	+++	+++	+++	+++
Random RR	6.24	6.30	2.68	5.50
RRl	5.16	5.79	2.49	5.07
RRu	7.54	6.87	2.87	5.96
P	+++	+++	+++	+++
Between Chi				4496.81
Between df				2
Between P				***
Btwn(F) P				***
Btwn(R) P				***

Table 1A1 - 3

IESLC - Meta-analysis of Ever Smoking, Any product (or Cigarettes if Any not available)
All LC types
Most adjusted

	Any proxy use No/nk	Yes	Total
N	227	101	328
NS	169	67	236
Wt	7284.35	12061.73	19346.08
Het Chi	2850.32	1845.32	7470.28
Het df	226	100	327
Het P	***	***	***
Fixed RR	6.87	3.15	4.22
RRl	6.72	3.09	4.16
RRu	7.03	3.20	4.28
P	+++	+++	+++
Random RR	5.51	5.39	5.50
RRl	5.02	4.84	5.07
RRu	6.04	6.02	5.96
P	+++	+++	+++
Between Chi			2774.63
Between df			1
Between P			***
Btwn(F) P			***
Btwn(R) P			N.S.

	Full histological confirmation No	Yes	Total
N	245	83	328
NS	177	59	236
Wt	16826.38	2519.71	19346.08
Het Chi	5647.29	789.61	7470.28
Het df	244	82	327
Het P	***	***	***
Fixed RR	3.86	7.67	4.22
RRl	3.80	7.38	4.16
RRu	3.92	7.98	4.28
P	+++	+++	+++
Random RR	5.25	6.30	5.50
RRl	4.80	5.47	5.07
RRu	5.74	7.25	5.96
P	+++	+++	+++
Between Chi			1033.37
Between df			1
Between P			***
Btwn(F) P			***
Btwn(R) P			*

	Number of adjustment variables (1) 0	1	2+/+nk	Total
N	164	69	95	328
NS	114	50	79	243
Wt	4667.87	1824.48	12853.73	19346.08
Het Chi	1870.84	604.35	3045.73	7470.28
Het df	163	68	94	327
Het P	***	***	***	***
Fixed RR	7.12	5.13	3.40	4.22
RRl	6.92	4.90	3.34	4.16
RRu	7.33	5.37	3.46	4.28
P	+++	+++	+++	+++
Random RR	5.44	5.48	5.56	5.50
RRl	4.86	4.72	4.87	5.07
RRu	6.09	6.37	6.34	5.96
P	+++	+++	+++	+++
Between Chi				1949.36
Between df				2
Between P				***
Btwn(F) P				***
Btwn(R) P				N.S.

Table 1A1 - 3

IESLC - Meta-analysis of Ever Smoking, Any product (or Cigarettes if Any not available)
All LC types
Most adjusted

	Number of adjustment variables (2) 0	1	2	3-5	6+/+nk	Total
N	164	69	49	31	15	328
NS	114	50	40	26	13	243
Wt	4667.87	1824.48	11979.62	624.61	249.50	19346.08
Het Chi	1870.84	604.35	2613.40	180.15	97.34	7470.28
Het df	163	68	48	30	14	327
Het P	***	***	***	***	***	***
Fixed RR	7.12	5.13	3.30	5.33	4.45	4.22
RRl	6.92	4.90	3.24	4.93	3.93	4.16
RRu	7.33	5.37	3.36	5.77	5.04	4.28
P	+++	+++	+++	+++	+++	+++
Random RR	5.44	5.48	5.39	6.41	4.55	5.50
RRl	4.86	4.72	4.50	5.18	3.18	5.07
RRu	6.09	6.37	6.46	7.93	6.51	5.96
P	+++	+++	+++	+++	+++	+++
Between Chi						2104.19
Between df						4
Between P						***
Btwn(F) P						***
Btwn(R) P						N.S.

	Product all/unsp	cig+/-ot	cig only	Total
N	205	114	9	328
NS	154	81	8	243
Wt	15543.15	3581.99	220.95	19346.08
Het Chi	5010.61	1453.31	52.61	7470.28
Het df	204	113	8	327
Het P	***	***	***	***
Fixed RR	3.79	6.52	8.11	4.22
RRl	3.73	6.31	7.11	4.16
RRu	3.85	6.73	9.26	4.28
P	+++	+++	+++	+++
Random RR	5.41	5.54	6.83	5.50
RRl	4.90	4.87	4.63	5.07
RRu	5.97	6.30	10.08	5.96
P	+++	+++	+++	+++
Between Chi				953.75
Between df				2
Between P				***
Btwn(F) P				***
Btwn(R) P				N.S.

	Denominator nev any	nev cigs	Total
N	236	92	328
NS	171	68	239
Wt	16305.48	3040.60	19346.08
Het Chi	5338.21	1204.65	7470.28
Het df	235	91	327
Het P	***	***	***
Fixed RR	3.84	7.01	4.22
RRl	3.78	6.77	4.16
RRu	3.90	7.26	4.28
P	+++	+++	+++
Random RR	5.31	5.96	5.50
RRl	4.84	5.17	5.07
RRu	5.81	6.88	5.96
P	+++	+++	+++
Between Chi			927.41
Between df			1
Between P			***
Btwn(F) P			***
Btwn(R) P			N.S.

Table 1A1 - 3

IESLC - Meta-analysis of Ever Smoking, Any product (or Cigarettes if Any not available)
All LC types
Most adjusted

		Derivation of RR/CI			
		Orig	StdCalc	Other	Total
	N	39	170	119	328
	NS	31	121	92	244
	Wt	1805.41	4883.24	12657.44	19346.08
Het	Chi	777.03	2130.89	1807.64	7470.28
Het	df	38	169	118	327
Het	P	***	***	***	***
Fixed	RR	7.41	6.98	3.21	4.22
	RRl	7.08	6.79	3.16	4.16
	RRu	7.76	7.18	3.27	4.28
	P	+++	+++	+++	+++
Random	RR	5.36	5.57	5.34	5.50
	RRl	4.23	4.97	4.85	5.07
	RRu	6.79	6.24	5.89	5.96
	P	+++	+++	+++	+++
Between	Chi				2754.71
Between	df				2
Between	P				***
Btwn(F)	P				***
Btwn(R)	P				N.S.

		Study LIU4		
		LIU4	others	Total
	N	2	326	328
	NS	1	235	236
	Wt	9846.05	9500.03	19346.08
Het	Chi	2.98	4076.16	7470.28
Het	df	1	325	327
Het	P	(*)	***	***
Fixed	RR	2.80	6.47	4.22
	RRl	2.74	6.34	4.16
	RRu	2.85	6.60	4.28
	P	+++	+++	+++
Random	RR	2.81	5.52	5.50
	RRl	2.71	5.10	5.07
	RRu	2.91	5.97	5.96
	P	+++	+++	+++
Between	Chi			3391.14
Between	df			1
Between	P			***
Btwn(F)	P			***
Btwn(R)	P			***

Table 1A1 - 4

IESLC - Meta-analysis of Ever Smoking, Any product (or Cigarettes if Any not available)
All LC types
Least adjusted

REF	NRR	X	SEX	AGEL	AGEH	RACE	YF	LC TYPE	LOC	START	ST	NLC	R	VB	P	H	AD	PRODUCT	DENOM	De
ABELIN	1	x	m	0	0	all	-	all	Eu:wst	1941	CC	118	n	bl	y	n	0	all/unsp	nev any	st
ABRAHA	7		m	0	0	all	0	q+s+a	Eu:est	1975	pr	571	n	bl	n	n	0	all/unsp	nev any	ot
ABRAHA	8		f	0	0	all	0	q+s+a	Eu:est	1975	pr	571	n	bl	n	n	0	all/unsp	nev any	ot
AGUDO	8	x	f	0	0	all	-	all	Eu:wst	1989	CC	103	n	bl	n	n	0	cig only	nev any	st
AKIBA	3	x	m	0	0	all	0	all	As:Jap	1963	pr	610	n	bl	n	n	0	cig+/-ot	nev cigs	st
AKIBA	7	x	f	0	0	all	0	all	As:Jap	1963	pr	610	n	bl	n	n	0	cig+/-ot	nev cigs	st
ALDERS	62	x	m	0	0	all	-	all	Eu:UK	1977	CC	1448	n	V	n	n	0	all/unsp	nev any	st
ALDERS	12	x	f	0	0	all	-	all	Eu:UK	1977	CC	1448	n	V	n	n	0	cig only	nev any	st
AMANDU	3	x	m	0	0	wh	0	all	NAmer	1959	pr	132	m	bl	n	n	0	cig+/-ot	nev cigs	st
AMES	4		m	0	0	wh	-	all	NAmer	1959	ot	317	m	bl	n	n	0	all/unsp	nev any	st
ANDERS	3		f	0	0	all	0	all	NAmer	1986	pr	343	n	bl	n	n	0	cig+/-ot	nev cigs	st
ARCHER	6		m	0	0	wh	0	all	NAmer	1950	pr	146	m	bl	n	n	0	cig+/-ot	nev cigs	st
ARMADA	29		m	0	0	all	-	all	Eu:wst	1986	CC	325	n	bl	n	y	0	all/unsp	nev any	st
AUSTIN	3	x	c	0	0	all	-	all	NAmer	1970	CC	166	o	bl	y	n	0	cig+/-ot	nev cigs	st
AUVINE	1	x	c	0	0	all	-	all	Eu:Sca	1986	CC	517	n	bl	y	n	0	cig+/-ot	nev cigs	st
AXELSO	1		c	0	0	all	-	all	Eu:Sca	1960	CC	152	n	bl	y	n	0	all/unsp	nev any	st
AXELSS	1	x	m	0	0	sca	-	all	Eu:Sca	1989	CC	436	n	bl	n	n	0	all/unsp	nev any	st
AXELSS	11		f	0	0	sca	-	all	Eu:Sca	1989	CC	436	n	bl	n	n	0	all/unsp	nev any	st
BAND	1		m	0	0	all	-	all	NAmer	1983	CC	2831	n	V	y	y	2	cig only	nev any	ot
BARBON	106	x	m	0	0	all	-	all	Eu:wst	1979	CC	755	n	bl	y	y	0	all/unsp	nev any	st
BECHER	1		m	0	0	all	-	all	Eu:Ger	1985	CC	194	n	bl	n	y	0	all/unsp	nev any	st
BECHER	2	x	f	0	0	all	-	all	Eu:Ger	1985	CC	194	n	bl	n	y	0	all/unsp	nev any	st
BENSHL	18		m	40	64	all	10	all	Eu:UK	1967	pr	486	n	V	n	n	1	all/unsp	nev any	ot
BEST	22		m	55	79	all	3	all	NAmer	1955	pr	381	n	V	n	n	0	all/unsp	nev any	st
BEST	18		f	0	0	all	0	all	NAmer	1955	pr	381	n	V	n	n	1	cig only	nev any	ot
BLOHMK	3		m	0	0	all	-	all	Eu:Ger	1978	CC	888	n	bl	n	y	0	all/unsp	nev any	st
BLOT4	1		m	0	0	wh	-	all	NAmer	1974	CC	335	n	bl	y	n	0	cig+/-ot	nev cigs	st
BOFFET	32	x	m	0	0	all	-	all	Eu:mul	1988	CC	5621	n	bl	y	n	0	all/unsp	nev any	st
BOUCOT	9	x	m	0	0	all	0	all	NAmer	1951	pr	121	n	bl	n	n	0	all/unsp	nev any	ot
BRESLO	37		m	0	0	all	-	all	NAmer	1949	CC	518	n	bl	n	y	0	all/unsp	nev+1	st
BRESLO	38		f	0	0	all	-	all	NAmer	1949	CC	518	n	bl	n	y	0	all/unsp	nev+1	st
BRETT	10		m	0	0	all	0	all	Eu:UK	1960	pr	150	n	V	n	n	0	cig+/-ot	nev cigs	st
BROCKM	1		m	0	0	wh	-	all	Eu:Ger	1990	CC	117	n	bl	n	y	0	cig+/-ot	nev cigs	st
BROCKM	2		f	0	0	wh	-	all	Eu:Ger	1990	CC	117	n	bl	n	y	0	cig+/-ot	nev cigs	st
BROSS	12		m	0	0	wh	-	all	NAmer	1960	CC	974	n	bl	n	n	0	all/unsp	nev any	st
BROWN2	2		m	0	0	wh	-	all	NAmer	1984	CC	14596	n	bl	n	y	2	cig+/-ot	nev cigs	or
BROWN2	1		f	0	0	wh	-	all	NAmer	1984	CC	14596	n	bl	n	y	2	cig+/-ot	nev cigs	or
BUFFLE	1		m	0	0	wh	-	all	NAmer	1976	CC	943	n	bl	y	n	0	all/unsp	nev any	st
BUFFLE	5		f	0	0	wh	-	all	NAmer	1976	CC	943	n	bl	y	n	0	all/unsp	nev any	st
CARPEN	7	x	c	0	0	w+b	-	all	NAmer	1991	CC	356	n	bl	n	n	0	cig+/-ot	nev cigs	st
CASCO2	1		c	0	0	wh	-	all	Eu:Ger	1991	CC	155	n	bl	n	n	0	all/unsp	nev any	st
CASCOR	1		c	0	0	wh	-	all	Eu:Ger	1985	CC	389	n	bl	n	y	0	all/unsp	nev any	st
CEDERL	107		m	0	0	all	16	all	Eu:Sca	1963	pr	491	n	bl	n	n	2	all/unsp	nev any	ot
CEDERL	112		f	0	0	all	0	all	Eu:Sca	1963	pr	491	n	bl	n	n	2	all/unsp	nev any	ot
CHAN	9		m	0	0	all	-	all	As:HK	1976	CC	397	n	bl	n	n	0	all/unsp	nev any	st
CHAN	10		f	0	0	all	-	all	As:HK	1976	CC	397	n	bl	n	n	0	all/unsp	nev any	st
CHANG	6		m	0	0	all	0	all	NAmer	1972	pr	136	n	bl	n	n	0	cig+/-ot	nev cigs	st
CHANG	12		f	0	0	all	0	all	NAmer	1972	pr	136	n	bl	n	n	0	cig+/-ot	nev cigs	st
CHATZI	4		c	0	0	all	-	all	Eu:bal	1987	CC	282	n	bl	n	y	0	all/unsp	nev any	st
CHEN2	1		m	0	0	all	-	all	As:Chi	1983	CC	193	n	ot	y	n	0	all/unsp	nev any	st
CHEN2	2		f	0	0	all	-	all	As:Chi	1983	CC	193	n	ot	y	n	0	all/unsp	nev any	st
CHEN3	1		c	0	0	all	-	all	As:Chi	1981	CC	254	n	ot	y	n	0	all/unsp	nev any	st
CHIAZZ	2	x	m	0	0	all	-	all	NAmer	1940	CC	144	o	bl	y	n	0	cig+/-ot	nev cigs	st
CHOI	1		m	0	0	all	-	all	As:oth	1985	CC	375	n	bl	n	n	0	cig+/-ot	nev cigs	st
CHOI	5		f	0	0	all	-	all	As:oth	1985	CC	375	n	bl	n	n	0	cig+/-ot	nev cigs	st
CHOW	7	x	m	0	0	wh	0	all	NAmer	1966	pr	219	n	bl	n	n	0	all/unsp	nev any	st
CHYOU	9	x	m	0	0	jap	0	all	NAmer	1965	pr	227	n	bl	n	y	0	cig+/-ot	nev cigs	st
COMSTO	34		m	0	0	all	-	all	NAmer	1975	ot	258	n	bl	n	n	0	all/unsp	nev any	st
COMSTO	46		f	0	0	all	-	all	NAmer	1975	ot	258	n	bl	n	n	0	all/unsp	nev any	st
COOKSO	5		c	0	0	bl	-	all	Africa	1961	CC	234	n	V	n	y	0	all/unsp	nev any	st
CORREA	33	x	c	0	0	all	-	all	NAmer	1979	CC	1359	n	bl	y	n	0	cig+/-ot	nev cigs	st
CPSI	187		m	35	84	all	6	all	NAmer	1959	pr	5138	n	bl	n	n	1	cig+/-ot	nev any	ot
CPSI	274		f	40	74	all	6	all	NAmer	1959	pr	5138	n	bl	n	n	1	cig+/-ot	nev cigs	ot
CPSII	104		m	35	99	all	4	all	NAmer	1982	pr	3229	n	bl	n	n	1	cig only	nev any	ot
CPSII	79		f	0	0	all	4	all	NAmer	1982	pr	3229	n	bl	n	n	1	cig+/-ot	nev cigs	ot
DAMBER	5	x	m	0	0	all	-	all	Eu:Sca	1972	CC	579	n	bl	y	n	0	all/unsp	nev any	st
DARBY	15		m	0	0	wh	-	all	Eu:UK	1988	CC	982	n	V	n	n	0	all/unsp	nev any	st
DARBY	16		f	0	0	wh	-	all	Eu:UK	1988	CC	982	n	V	n	n	0	all/unsp	nev any	st
DAVEYS	5		m	0	0	all	-	all	Eu:Ger	1930	CC	109	n	bl	y	n	0	all/unsp	nev any	st
DAVEYS	6		f	0	0	all	-	all	Eu:Ger	1930	CC	109	n	bl	y	n	0	all/unsp	nev any	ot
DEAN	7		m	0	0	wh	-	all	Africa	1947	CC	603	n	V	y	n	0	all/unsp	nev any	st
DEAN2	3		m	0	0	all	-	all	Eu:UK	1960	CC	954	n	V	y	n	0	all/unsp	nev any	st

Table 1A1 - 4

IESLC - Meta-analysis of Ever Smoking, Any product (or Cigarettes if Any not available)
All LC types
Least adjusted

REF	NRR	X	SEX	AGEL	AGEH	RACE	YF	LC TYPE	LOC	START	ST	NLC	R	VB	P	H	AD	PRODUCT	DENOM	De
DEAN2	7		f	0	0	all	-	all	Eu:UK	1960	CC	954	n	V	y	n	0	all/unsp	nev any	st
DEAN3	47	x	m	0	0	all	-	all	Eu:UK	1969	CC	766	n	V	y	n	0	all/unsp	nev any	st
DEAN3	124	x	f	0	0	all	-	all	Eu:UK	1969	CC	766	n	V	y	n	0	cig only	nev any	st
DEKLER	6		m	0	0	all	0	all	Auslia	1961	pr	138	m	V	n	n	2	all/unsp	nev any	ot
DESTE2	13	x	c	0	0	all	-	all	SCAmer	1993	CC	463	n	bl	n	n	0	all/unsp	nev any	st
DESTEF	5	x	m	0	0	all	-	all	SCAmer	1988	CC	497	n	bl	n	y	0	all/unsp	nev any	st
DOCKER	3		c	0	0	wh	0	all	NAmer	1974	pr	120	n	bl	n	n	4	cig+/-ot	nev cigs	ot
DOLL	6		m	0	0	all	-	all	Eu:UK	1948	CC	1465	n	V	n	n	0	all/unsp	nev any	st
DOLL	12		f	0	0	all	-	all	Eu:UK	1948	CC	1465	n	V	n	n	0	all/unsp	nev any	st
DOLL2	56		m	0	0	all	0	all	Eu:UK	1951	pr	920	n	V	n	n	1	all/unsp	nev any	ot
DORANT	10		c	0	0	all	0	all	Eu:wst	1986	ot	550	n	bl	n	y	0	all/unsp	nev any	st
DORGAN	6		m	0	0	wh	-	all	NAmer	1980	CC	2026	n	bl	y	y	0	all/unsp	nev any	st
DORGAN	30		m	0	0	bl	-	all	NAmer	1980	CC	2026	n	bl	y	y	0	all/unsp	nev any	st
DORGAN	53		f	0	0	wh	-	all	NAmer	1980	CC	2026	n	bl	y	y	0	all/unsp	nev any	st
DORGAN	76		f	0	0	bl	-	all	NAmer	1980	CC	2026	n	bl	y	y	0	all/unsp	nev any	st
DORN	196		m	35	84	wh	8	all	NAmer	1954	pr	5097	n	bl	n	n	1	all/unsp	nev any	ot
DOSEME	17	x	m	0	0	all	-	all	Eu:bal	1979	CC	1210	n	bl	n	n	0	cig+/-ot	nev cigs	st
DROSTE	3	x	m	0	0	all	-	all	Eu:wst	1995	CC	478	n	bl	n	y	0	all/unsp	nev any	st
DU	1		m	0	0	all	-	all	As:Chi	1985	CC	849	n	ot	y	n	0	all/unsp	nev any	or
DU	2		f	0	0	all	-	all	As:Chi	1985	CC	849	n	ot	y	n	0	all/unsp	nev any	or
DUNN	6		m	0	0	all	0	all	NAmer	1954	pr	139	o	bl	n	n	0	cig+/-ot	nev cigs	st
EBELIN	1		m	0	0	all	-	all	Eu:Ger	1980	CC	130	n	bl	n	n	0	all/unsp	nev any	st
ENGELA	155	x	m	0	0	all	12	all	Eu:Sca	1964	pr	435	n	bl	n	n	0	all/unsp	nev any	st
ENGELA	162	x	f	0	0	all	12	all	Eu:Sca	1964	pr	435	n	bl	n	n	0	all/unsp	nev any	st
ESAKI	4		m	0	0	all	-	all	As:Jap	1961	CC	245	n	bl	y	n	0	cig+/-ot	nev cigs	st
ESAKI	5		f	0	0	all	-	all	As:Jap	1961	CC	245	n	bl	y	n	0	cig+/-ot	nev cigs	st
FAN	1		m	0	0	all	-	all	As:Chi	1990	CC	403	n	ot	y	n	0	cig+/-ot	nev cigs	st
FAN	2		f	0	0	all	-	all	As:Chi	1990	CC	403	n	ot	y	n	0	cig+/-ot	nev cigs	st
GAO	6	x	m	0	0	all	-	all	As:Chi	1984	CC	1405	n	ot	n	n	0	cig+/-ot	nev cigs	st
GAO	16	x	f	0	0	all	-	all	As:Chi	1984	CC	1405	n	ot	n	n	0	cig+/-ot	nev cigs	st
GAO2	6	x	m	0	0	all	-	all	As:Jap	1988	CC	282	n	bl	n	n	0	cig+/-ot	nev cigs	st
GARCIA	3		c	0	0	all	-	all	NAmer	1992	CC	416	n	bl	n	y	0	cig+/-ot	nev cigs	st
GARDIN	7		c	0	0	all	-	all	Eu:UK	1988	CC	143	n	V	y	n	0	all/unsp	nev any	st
GARSHI	17	x	m	0	0	all	-	all	NAmer	1981	CC	1081	o	bl	y	n	0	all/unsp	nev any	st
GENG	1		m	0	0	all	-	all	As:Chi	1985	CC	292	n	ot	*	n	0	cig+/-ot	nev any	st
GENG	2		f	0	0	all	-	all	As:Chi	1985	CC	292	n	ot	*	n	0	cig+/-ot	nev any	st
GER	17	x	c	0	0	all	-	all	As:oth	1990	CC	141	n	ot	y	n	0	all/unsp	nev any	st
GODLEY	5		m	0	0	all	-	all	NAmer	1966	CC	1986	n	bl	y	n	1	cig+/-ot	nev cigs	ot
GODLEY	6		f	0	0	all	-	all	NAmer	1966	CC	1986	n	bl	y	n	1	cig+/-ot	nev cigs	ot
GOLLED	21	x	m	35	99	all	-	all	Eu:UK	1952	CC	443	n	V	y	n	0	cig+/-ot	nev any	st
GOODMA	3		m	0	0	w+o	-	all	NAmer	1983	CC	326	n	bl	y	y	0	cig+/-ot	nev any	st
GOODMA	7		f	0	0	w+o	-	all	NAmer	1983	CC	326	n	bl	y	y	0	cig+/-ot	nev any	st
GRAHAM	22	x	m	0	0	wh	-	all	NAmer	1956	CC	685	n	bl	n	n	0	all/unsp	nev any	st
GREGOR	3		m	0	0	all	-	all	Eu:UK	1976	CC	104	n	V	n	y	0	cig+/-ot	nev cigs	st
GREGOR	7		f	0	0	all	-	all	Eu:UK	1976	CC	104	n	V	n	y	0	cig+/-ot	nev cigs	st
GSELL	8		m	0	0	all	-	all	Eu:wst	1937	CC	150	n	bl	n	y	0	all/unsp	nev any	st
HAENSZ	50	x	f	0	0	all	-	not alv	NAmer	1955	CC	158	n	bl	n	y	0	all/unsp	nev any	st
HAMMO2	18	x	m	0	0	all	6	all	NAmer	1967	pr	450	o	bl	n	n	0	all/unsp	nev any	st
HAMMON	129	x	m	0	0	wh	0	all	NAmer	1952	pr	448	n	bl	n	n	0	all/unsp	nev any	st
HANSEN	3		m	0	0	all	0	all	Eu:Sca	1968	pr	105	o	bl	y	n	2	all/unsp	nev any	ot
HEGMAN	1		c	0	0	all	-	all	NAmer	1989	CC	282	n	bl	y	y	0	all/unsp	nev any	st
HEIN	7		m	0	0	all	0	all	Eu:Sca	1970	pr	144	n	bl	n	n	0	all/unsp	nev any	st
HENNEK	3		m	0	0	all	0	all	NAmer	1982	pr	169	n	bl	n	n	0	all/unsp	nev any	st
HINDS	26	x	f	0	0	o	-	all	NAmer	1968	CC	292	n	bl	n	n	0	all/unsp	nev any	st
HIRAYA	147		m	0	0	all	0	all	As:Jap	1965	pr	1917	n	bl	n	n	1	cig+/-ot	nev any	ot
HIRAYA	150		f	0	0	all	0	all	As:Jap	1965	pr	1917	n	bl	n	n	1	cig+/-ot	nev any	ot
HITOSU	6	x	m	0	0	all	-	all	As:Jap	1960	CC	216	n	bl	y	n	0	all/unsp	nev any	st
HITOSU	12	x	f	0	0	all	-	all	As:Jap	1960	CC	216	n	bl	y	n	0	all/unsp	nev any	st
HOLE	15	x	m	0	0	all	0	all	Eu:UK	1972	pr	225	n	V	n	n	0	all/unsp	nev any	st
HOROWI	1		m	0	0	all	-	all	NAmer	1956	CC	236	n	V	n	n	0	cig+/-ot	nev any	st
HOROWI	2		f	0	0	all	-	all	NAmer	1956	CC	236	n	V	n	n	0	cig+/-ot	nev any	st
HORWIT	1		f	0	0	all	-	all	NAmer	1977	CC	112	n	bl	n	n	0	cig+/-ot	nev cigs	st
HU	15		m	0	0	all	-	all	As:Chi	1985	CC	227	n	ot	n	y	0	cig+/-ot	nev any	st
HU	16		f	0	0	all	-	all	As:Chi	1985	CC	227	n	ot	n	y	0	cig+/-ot	nev any	st
HU2	9		m	0	0	all	-	all	As:Chi	1977	CC	523	n	ot	y	n	0	cig+/-ot	nev cigs	st
HU2	10		f	0	0	all	-	all	As:Chi	1977	CC	523	n	ot	y	n	0	cig+/-ot	nev cigs	st
HUANG	1		c	0	0	all	-	all	As:Chi	1990	CC	135	n	ot	y	n	0	all/unsp	nev any	st
HUMBLE	14		m	0	0	w-hi	-	all	NAmer	1980	CC	521	n	bl	y	n	1	cig+/-ot	nev cigs	ot
HUMBLE	16		m	0	0	hi	-	all	NAmer	1980	CC	521	n	bl	y	n	1	cig+/-ot	nev cigs	ot
HUMBLE	18		f	0	0	w-hi	-	all	NAmer	1980	CC	521	n	bl	y	n	1	cig+/-ot	nev cigs	ot
HUMBLE	20		f	0	0	hi	-	all	NAmer	1980	CC	521	n	bl	y	n	1	cig+/-ot	nev cigs	ot
JAHN	3	x	f	0	0	all	-	all	Eu:Ger	1988	CC	1004	n	bl	n	n	0	cig+/-ot	nev any	st

Table 1A1 - 4

IESLC - Meta-analysis of Ever Smoking, Any product (or Cigarettes if Any not available)
All LC types
Least adjusted

REF	NRR	X	SEX	AGEL	AGEH	RACE	YF	LC TYPE	LOC	START	ST	NLC	R	VB	P	H	AD	PRODUCT	DENOM	De
JAIN	6	x	m	0	0	all	-	all	NAmer	1981	CC	845	n	V	y	n	0	cig+/-ot	nev cigs	st
JAIN	1	x	f	0	0	all	-	all	NAmer	1981	CC	845	n	V	y	n	0	cig+/-ot	nev cigs	st
JARUP	3	x	m	0	0	all	-	all	Eu:Sca	1928	CC	102	o	bl	y	n	0	all/unsp	nev any	st
JARVHO	3		m	0	0	all	-	all	Eu:Sca	1983	CC	147	n	bl	n	n	0	all/unsp	nev any	st
JARVHO	7		f	0	0	all	-	all	Eu:Sca	1983	CC	147	n	bl	n	n	0	all/unsp	nev any	st
JEDRYC	63	x	m	0	0	all	-	all	Eu:est	1980	CC	1630	n	bl	y	n	0	cig+/-ot	nev any	st
JEDRYC	68	x	f	0	0	all	-	all	Eu:est	1980	CC	1630	n	bl	y	n	0	cig+/-ot	nev any	st
JIANG	1		m	0	0	all	-	all	As:Chi	1984	CC	125	n	ot	n	n	0	all/unsp	nev any	st
JIANG	2		f	0	0	all	-	all	As:Chi	1984	CC	125	n	ot	n	n	0	all/unsp	nev any	st
JOLY	14		m	0	0	all	-	all	SCAmer	1978	CC	826	n	bl	n	n	0	all/unsp	nev any	st
JOLY	1		f	0	0	all	-	all	SCAmer	1978	CC	826	n	bl	n	n	0	cig+/-ot	nev any	st
JUSSAW	3	x	m	0	0	all	-	all	As:Ind	1964	CC	792	n	V	n	n	0	all/unsp	nev any	st
KAISE2	72		m	35	99	all	9	all	NAmer	1979	pr	318	n	bl	n	n	1	cig only	nev any	st
KAISE2	64		f	35	99	all	9	all	NAmer	1979	pr	318	n	bl	n	n	1	cig only	nev any	st
KAISER	13		m	0	0	all	0	all	NAmer	1964	pr	714	n	bl	n	n	2	cig+/-ot	nev cigs	ot
KAISER	10		f	0	0	all	0	all	NAmer	1964	pr	714	n	bl	n	n	2	cig+/-ot	nev cigs	ot
KATSOU	27	x	f	0	0	all	-	all	Eu:bal	1987	CC	101	n	bl	n	n	0	all/unsp	nev any	st
KAUFMA	8	x	c	0	0	all	-	all	NAmer	1981	CC	881	n	bl	n	n	0	cig+/-ot	nev cigs	st
KELLER	3		m	0	0	wh	-	all	NAmer	1985	CC	15038	n	bl	n	n	0	all/unsp	nev any	st
KELLER	11		m	0	0	nonw	-	all	NAmer	1985	CC	15038	n	bl	n	n	0	all/unsp	nev any	st
KELLER	7		f	0	0	wh	-	all	NAmer	1985	CC	15038	n	bl	n	n	0	all/unsp	nev any	st
KELLER	15		f	0	0	nonw	-	all	NAmer	1985	CC	15038	n	bl	n	n	0	all/unsp	nev any	st
KHUDER	4		m	0	0	all	-	all	NAmer	1985	CC	482	n	bl	n	y	0	cig+/-ot	nev cigs	st
KIHARA	31		c	0	0	jap	-	all	As:Jap	1991	CC	440	n	bl	n	n	0	all/unsp	nev any	st
KINLEN	6	x	m	0	0	all	0	all	Eu:UK	1967	pr	718	n	V	n	n	0	all/unsp	nev any	st
KJUUS	10		m	0	0	all	-	all	Eu:Sca	1979	CC	176	n	bl	n	n	0	all/unsp	nev any	st
KNEKT	76	x	m	20	69	all	21	all	Eu:Sca	1966	pr	515	n	bl	n	n	0	all/unsp	nev any	st
KO	1		f	0	0	all	-	all	As:oth	1992	CC	117	n	ot	n	y	3	cig+/-ot	nev cigs	or
KOHLME	1	x	c	0	0	all	-	all	Eu:Ger	1990	CC	239	n	bl	n	n	0	all/unsp	nev any	st
KOO	1		f	0	0	all	-	all	As:HK	1981	CC	200	n	bl	n	n	0	all/unsp	nev any	st
KOULUM	1		m	0	0	all	-	all	Eu:Sca	1936	CC	812	n	bl	n	n	0	all/unsp	nev any	st
KREUZE	14		f	1	45	all	-	all	Eu:Ger	1990	CC	2260	n	bl	n	n	0	all/unsp	nev any	st
KREUZE	16		f	55	69	all	-	all	Eu:Ger	1990	CC	2260	n	bl	n	n	0	all/unsp	nev any	st
KREYBE	24	x	m	0	0	all	-	all	Eu:Sca	1948	CC	300	n	bl	n	y	0	all/unsp	nev any	st
KREYBE	39	x	f	0	0	all	-	all	Eu:Sca	1948	CC	300	n	bl	n	y	0	all/unsp	nev any	st
KUBIK	28		m	0	0	all	0	all	Eu:est	1965	pr	108	n	bl	n	n	0	all/unsp	nev any	st
LAMTH	6		f	0	0	ch	-	all	As:HK	1983	CC	445	n	bl	n	n	0	all/unsp	nev any	or
LAMWK	1		f	0	0	ch	-	all	As:HK	1981	CC	163	n	bl	n	n	0	all/unsp	nev any	st
LAMWK2	9		m	0	0	all	-	q+s+l+a	As:HK	1976	CC	480	n	bl	n	n	0	all/unsp	nev any	st
LAMWK2	10		f	0	0	all	-	q+s+l+a	As:HK	1976	CC	480	n	bl	n	n	0	all/unsp	nev any	st
LANGE	34	x	m	0	0	all	0	all	Eu:Sca	1976	pr	268	n	bl	n	n	0	all/unsp	nev any	st
LANGE	31	x	f	0	0	all	0	all	Eu:Sca	1976	pr	268	n	bl	n	n	0	all/unsp	nev any	st
LAUSSM	10	x	m	0	0	all	-	all	Eu:Ger	1982	CC	432	n	bl	n	n	0	all/unsp	nev any	st
LEI	1		m	0	0	all	-	all	As:Chi	1986	CC	792	n	ot	y	n	0	all/unsp	nev any	st
LEI	2		f	0	0	all	-	all	As:Chi	1986	CC	792	n	ot	y	n	0	all/unsp	nev any	st
LEMARC	3		c	0	0	w+o	-	all	NAmer	1992	CC	341	n	bl	n	y	0	all/unsp	nev any	st
LETOUR	1		c	0	0	all	-	all	NAmer	1983	CC	738	n	V	y	y	0	cig+/-ot	nev cigs	st
LEVIN	32		m	0	0	all	-	all	NAmer	1938	CC	475	n	bl	n	n	1	all/unsp	nev any	st
LIDDEL	5		m	0	0	all	18	all	NAmer	1970	pr	304	m	V	n	n	1	cig+/-ot	nev cigs	ot
LIU	2		c	0	0	all	-	all	As:Chi	1980	CC	229	n	ot	*	n	2	all/unsp	nev any	or
LIU2	1	x	m	0	0	all	-	all	As:Chi	1983	CC	316	n	ot	n	n	0	all/unsp	nev any	st
LIU2	3	x	f	0	0	all	-	all	As:Chi	1983	CC	316	n	ot	n	n	0	all/unsp	nev any	st
LIU3	1	x	m	0	0	all	-	all	As:Chi	1985	CC	110	n	ot	n	n	0	all/unsp	nev any	st
LIU4	11		m	0	0	all	-	all	As:Chi	1986	CC	1000-00	n	ot	y	n	2	all/unsp	nev any	ot
LIU4	12		f	0	0	all	-	all	As:Chi	1986	CC	1000-00	n	ot	y	n	2	all/unsp	nev any	ot
LIU5	1		c	0	0	all	-	all	As:Chi	1978	CC	111	n	ot	y	n	0	all/unsp	nev any	st
LOMBA2	1		f	0	0	all	-	all	NAmer	1960	CC	225	n	bl	n	n	0	cig+/-ot	nev cigs	st
LOMBAR	12		m	0	0	all	-	all	NAmer	1951	CC	1040	n	bl	n	n	0	all/unsp	nev any	st
LUBIN2	45	x	m	0	0	all	-	all	Eu:mul	1976	CC	7804	n	bl	n	y	0	all/unsp	nev any	st
LUBIN2	101	x	f	0	0	all	-	all	Eu:mul	1976	CC	7804	n	bl	n	y	0	all/unsp	nev any	st
LUO	1	x	c	0	0	all	-	all	As:Chi	1990	CC	102	n	ot	n	y	0	cig+/-ot	nev cigs	st
MACLEN	71	x	m	0	0	ch	-	all	As:oth	1972	CC	233	n	bl	n	n	0	cig+/-ot	nev cigs	st
MACLEN	72	x	f	0	0	ch	-	all	As:oth	1972	CC	233	n	bl	n	n	0	cig+/-ot	nev cigs	st
MAGNUS	1	x	m	0	0	all	0	all	Eu:Sca	1953	pr	203	o	bl	y	n	0	all/unsp	nev any	st
MARSH	2	x	m	0	0	all	-	all	NAmer	1979	CC	150	n	bl	y	n	0	all/unsp	nev any	st
MARSH	4	x	f	0	0	all	-	all	NAmer	1979	CC	150	n	bl	y	n	0	all/unsp	nev any	st
MARSH2	2	x	c	0	0	all	-	all	NAmer	1979	CC	114	n	bl	y	n	0	all/unsp	nev any	st
MARTIS	4		m	0	0	all	-	all	Eu:UK	1972	CC	201	n	V	n	n	0	cig+/-ot	nev cigs	st
MASTRA	1	x	m	0	0	all	-	all	Eu:wst	1973	CC	309	n	bl	n	n	0	all/unsp	nev any	st
MATOS	26	x	m	0	0	all	-	all	SCAmer	1994	CC	200	n	bl	n	n	0	cig+/-ot	nev any	st

Table 1A1 - 4

IESLC - Meta-analysis of Ever Smoking, Any product (or Cigarettes if Any not available)
All LC types
Least adjusted

REF	NRR	X	SEX	AGEL	AGEH	RACE	YF	LC TYPE	LOC	START	ST	NLC	R	VB	P	H	AD	PRODUCT	DENOM	De
MATSUD	10		m	0	0	all	-	all	As:Jap	1965	CC	179	n	bl	n	n	0	cig+/-ot	nev cigs	st
MCCONN	1		m	0	0	all	-	all	Eu:UK	1946	CC	100	n	V	n	y	0	all/unsp	nev any	st
MCCONN	2		f	0	0	all	-	all	Eu:UK	1946	CC	100	n	V	n	y	0	all/unsp	nev any	st
MCDUFF	1		m	0	0	all	-	all	NAmer	1979	CC	165	n	V	y	n	0	cig+/-ot	nev cigs	st
MCLAUG	1		m	0	0	all	-	all	As:Chi	1972	CC	316	o	ot	y	n	0	all/unsp	nev any	st
MIGRAN	26	x	m	0	0	all	0	all	Eu:UK	1964	pr	259	n	V	n	n	0	all/unsp	nev any	st
MIGRAN	41	x	f	0	0	all	0	all	Eu:UK	1964	pr	259	n	V	n	n	0	all/unsp	nev any	st
MILLER	1	x	f	0	0	all	-	all	NAmer	1972	CC	168	n	bl	y	n	0	cig+/-ot	nev any	st
MILLS	3		m	0	0	wh	-	all	NAmer	1940	CC	444	n	bl	y	n	1	all/unsp	nev any	ot
MRFITR	6		m	0	0	all	0	all	NAmer	1973	pr	119	n	bl	n	n	0	cig+/-ot	nev cigs	ot
NAM	69	x	m	0	0	all	-	all	NAmer	1986	CC	1199	n	bl	y	n	0	cig+/-ot	nev cigs	ot
NAM	85	x	f	0	0	all	-	all	NAmer	1986	CC	1199	n	bl	y	n	0	cig+/-ot	nev cigs	ot
NOTAN2	1	x	m	0	0	all	-	all	As:Ind	1963	CC	683	n	V	n	n	0	all/unsp	nev any	st
NOU	11		m	30	64	all	-	all	Eu:Sca	1971	CC	273	n	bl	y	n	0	all/unsp	nev any	st
NOU	12		f	30	64	all	-	all	Eu:Sca	1971	CC	273	n	bl	y	n	0	all/unsp	nev any	st
ODRISC	3		c	0	0	all	-	all	Eu:UK	1992	CC	446	n	V	n	n	0	all/unsp	nev any	st
ORMOS	4		m	0	0	all	-	all	Eu:est	1947	CC	119	n	bl	y	y	0	cig+/-ot	nev any	st
ORMOS	26		f	0	0	all	-	all	Eu:est	1947	CC	119	n	bl	y	y	0	cig+/-ot	nev any	st
OSANN	17	x	m	0	0	all	-	all	NAmer	1984	CC	1986	n	bl	n	n	0	cig+/-ot	nev cigs	st
OSANN	21	x	f	0	0	all	-	all	NAmer	1984	CC	1986	n	bl	n	n	0	cig+/-ot	nev cigs	st
PARKIN	29	x	m	0	0	bl	-	all	Africa	1963	CC	877	n	V	y	n	0	all/unsp	nev any	st
PASTOR	5	x	m	0	0	all	-	all	Eu:wst	1976	CC	204	n	bl	y	n	0	all/unsp	nev any	st
PAWLEG	1	x	m	0	0	all	-	all	Eu:est	1992	CC	176	n	bl	n	y	0	all/unsp	nev any	st
PERNU	2		m	0	0	all	-	all	Eu:Sca	1944	CC	1606	n	bl	n	n	0	all/unsp	nev any	st
PERNU	1		f	0	0	all	-	all	Eu:Sca	1944	CC	1606	n	bl	n	n	0	all/unsp	nev any	st
PERSH2	5	x	c	0	0	all	-	all	Eu:Sca	1980	CC	1022	n	bl	y	n	0	all/unsp	nev any	st
PETO	5		m	0	0	all	0	all	Eu:UK	1954	pr	103	n	V	n	n	0	all/unsp	nev any	st
PEZZO2	10		m	0	0	all	-	all	SCAmer	1992	CC	367	n	bl	n	y	0	cig+/-ot	nev cigs	st
PEZZOT	25		m	0	0	all	-	all	SCAmer	1987	CC	215	n	bl	n	y	0	cig only	nev cigs	st
PIKE	4		m	0	0	w-hi	-	all	NAmer	1972	CC	731	n	bl	y	n	0	all/unsp	nev any	st
PIKE	8		f	0	0	w-hi	-	all	NAmer	1972	CC	731	n	bl	y	n	0	all/unsp	nev any	st
POFFIJ	1		c	0	0	all	-	all	Eu:mul	1990	CC	971	n	bl	n	n	0	all/unsp	nev any	st
POLEDN	3	x	c	0	0	all	-	all	NAmer	1978	CC	209	n	bl	y	n	0	cig+/-ot	nev cigs	st
QIAO2	3	x	m	0	0	all	0	all	As:Chi	1992	pr	241	m	ot	n	n	0	all/unsp	nev any	st
RACHTA	3	x	f	0	0	all	-	all	Eu:est	1991	CC	118	n	bl	n	y	0	cig+/-ot	nev cigs	st
RADZIK	1		c	0	0	all	-	all	Eu:est	1986	CC	189	n	bl	n	n	0	all/unsp	nev any	st
RANDIG	23		m	0	0	all	-	all	Eu:Ger	1951	CC	448	n	bl	n	n	0	all/unsp	nev any	st
RANDIG	24		f	0	0	all	-	all	Eu:Ger	1951	CC	448	n	bl	n	n	0	all/unsp	nev any	st
REN	1		m	0	0	all	-	all	As:Chi	1980	CC	244	n	ot	*	n	0	all/unsp	nev any	st
REN	2		f	0	0	all	-	all	As:Chi	1980	CC	244	n	ot	*	n	0	all/unsp	nev any	st
RONCO	1		m	0	0	all	-	all	Eu:wst	1976	CC	126	n	bl	y	n	0	all/unsp	nev any	st
ROTHSC	1	x	c	0	0	all	-	all	NAmer	1971	CC	284	n	bl	y	n	0	all/unsp	nev any	st
SADOWS	7	x	m	0	0	wh	-	all	NAmer	1938	CC	477	n	bl	n	n	0	all/unsp	nev any	st
SANKAR	1	x	m	0	0	all	-	all	As:Ind	1990	CC	281	n	V	n	n	0	all/unsp	nev any	st
SCHWAR	1		m	0	0	wh	-	all	NAmer	1984	CC	5588	n	bl	y	y	0	cig+/-ot	nev cigs	st
SCHWAR	2		m	0	0	bl	-	all	NAmer	1984	CC	5588	n	bl	y	y	0	cig+/-ot	nev cigs	st
SCHWAR	3		f	0	0	wh	-	all	NAmer	1984	CC	5588	n	bl	y	y	0	cig+/-ot	nev cigs	st
SCHWAR	4		f	0	0	bl	-	all	NAmer	1984	CC	5588	n	bl	y	y	0	cig+/-ot	nev cigs	st
SEGI	1		m	0	0	all	-	all	As:Jap	1948	CC	159	n	bl	n	n	0	all/unsp	nev any	ot
SEOW	1	x	f	0	0	ch	-	q+s+l+a	As:oth	1997	CC	153	n	bl	n	y	0	cig+/-ot	nev cigs	st
SHAW	12		c	0	0	wh	-	all	NAmer	1988	CC	335	n	V	n	y	0	all/unsp	nev any	st
SIEMIA	9	x	m	0	0	all	-	all	NAmer	1979	CC	857	n	V	y	y	0	cig+/-ot	nev cigs	st
SIMARA	5	x	m	0	0	all	-	all	As:oth	1971	CC	115	n	bl	n	n	0	cig+/-ot	nev cigs	st
SIMARA	6	x	f	0	0	all	-	all	As:oth	1971	CC	115	n	bl	n	n	0	cig+/-ot	nev cigs	st
SOBUE	91	x	m	0	0	all	-	q+s+l+a	As:Jap	1986	CC	1376	n	bl	n	y	0	cig+/-ot	nev cigs	st
SOBUE	95	x	f	0	0	all	-	q+s+l+a	As:Jap	1986	CC	1376	n	bl	n	y	0	cig+/-ot	nev cigs	st
SPEIZE	8		f	0	0	all	0	all	NAmer	1976	pr	593	n	bl	n	y	0	cig+/-ot	nev cigs	st
SPITZ	3		c	0	0	b+hi	-	all	NAmer	1992	CC	177	n	bl	n	y	0	cig+/-ot	nev cigs	st
STASZE	1		m	0	0	all	-	all	Eu:est	1954	CC	281	n	bl	n	y	0	all/unsp	nev any	st
STASZE	5		f	0	0	all	-	all	Eu:est	1954	CC	281	n	bl	n	y	0	all/unsp	nev any	st
STAYNE	1		m	0	0	all	-	all	NAmer	1969	CC	420	n	bl	n	n	0	all/unsp	nev any	st
STOCKS	31	x	m	0	0	all	-	all	Eu:UK	1952	CC	2932	n	V	y	n	0	all/unsp	nev any	st
STOCKS	50		f	0	0	all	-	all	Eu:UK	1952	CC	2932	n	V	y	n	1	cig+/-ot	nev any	ot
STOCKW	6		c	0	0	all	-	all	NAmer	1981	CC	22161	n	bl	n	n	0	all/unsp	nev any	st
STUCKE	3		m	0	0	all	-	all	Eu:wst	1989	CC	247	n	bl	n	y	0	all/unsp	nev any	ot
SUN	1		c	0	0	all	-	all	As:Chi	1992	CC	207	n	ot	n	y	0	all/unsp	nev any	st
SUZUK2	18	x	c	0	0	all	-	all	SCAmer	1991	CC	123	n	bl	n	y	0	all/unsp	nev any	st
SVENSS	56	x	f	0	0	all	-	all	Eu:Sca	1983	CC	210	n	bl	n	n	0	all/unsp	nev any	st
TANG	3		c	0	0	all	-	not s	NAmer	1992	CC	119	n	bl	n	y	0	cig+/-ot	nev cigs	st
TENKAN	22		m	0	0	all	17	all	Eu:Sca	1962	pr	242	n	bl	n	n	1	all/unsp	nev any	ot
TIZZAN	1		m	0	0	all	-	all	Eu:wst	1959	CC	1358	n	bl	n	n	0	all/unsp	nev any	st
TIZZAN	12		f	0	0	all	-	all	Eu:wst	1959	CC	1358	n	bl	n	n	0	all/unsp	nev any	st

Table 1A1 - 4

IESLC - Meta-analysis of Ever Smoking, Any product (or Cigarettes if Any not available)
All LC types
Least adjusted

REF	NRR	X	SEX	AGEL	AGEH	RACE	YF	LC TYPE	LOC	START	ST	NLC	R	VB	P	H	AD	PRODUCT	DENOM	De
TOKARS	3	x	m	0	0	all	-	all	Eu:est	1966	ot	162	o	bl	n	y	0	all/unsp	nev any	st
TOKARS	5	x	f	0	0	all	-	all	Eu:est	1966	ot	162	o	bl	n	y	0	all/unsp	nev any	st
TOUSEY	16	x	m	0	0	all	-	all	NAmer	1993	CC	507	n	bl	y	y	0	all/unsp	nev any	st
TOUSEY	26		f	0	0	all	-	all	NAmer	1993	CC	507	n	bl	y	y	0	all/unsp	nev any	st
TSUGAN	27		m	0	0	all	-	q+a	As:Jap	1976	CC	134	n	bl	n	y	0	all/unsp	nev any	st
TULINI	15	x	m	0	0	all	0	all	Eu:Sca	1967	pr	472	n	bl	n	n	1	all/unsp	nev any	ot
TULINI	21	x	f	0	0	all	0	all	Eu:Sca	1967	pr	472	n	bl	n	n	1	all/unsp	nev any	ot
TVERDA	22		m	0	0	all	0	all	Eu:Sca	1972	pr	238	n	bl	n	n	2	cig+/-ot	nev cigs	ot
WAKAI	13	x	m	0	0	all	-	all	As:Jap	1988	CC	333	n	bl	n	y	0	all/unsp	nev any	st
WAKAI	31	x	f	0	0	all	-	all	As:Jap	1988	CC	333	n	bl	n	y	0	all/unsp	nev any	st
WANG	1	x	m	0	0	all	-	all	As:Chi	1990	CC	390	n	ot	*	y	0	all/unsp	nev any	or
WANG	2	x	f	0	0	all	-	all	As:Chi	1990	CC	390	n	ot	*	y	0	all/unsp	nev any	or
WANG2	8	x	c	0	0	all	-	all	As:Chi	1980	CC	103	n	ot	n	n	0	cig+/-ot	nev cigs	st
WANG3	1		c	0	0	all	-	all	As:Chi	1981	CC	293	n	ot	*	n	0	all/unsp	nev any	st
WANG4	1	x	m	0	0	all	-	all	As:Chi	1976	CC	1170	n	ot	y	n	0	all/unsp	nev any	st
WICKLU	1		m	0	0	wh	-	all	NAmer	1968	CC	155	n	bl	y	n	0	cig+/-ot	nev+3	or
WIGLE	15	x	m	0	0	all	-	all	NAmer	1971	CC	728	n	V	n	n	0	all/unsp	nev any	st
WIGLE	18	x	f	0	0	all	-	all	NAmer	1971	CC	728	n	V	n	n	0	all/unsp	nev any	st
WILKIN	1	x	m	0	0	all	-	all	Eu:UK	1992	CC	271	n	V	n	n	0	cig+/-ot	nev cigs	st
WILKIN	2	x	f	0	0	all	-	all	Eu:UK	1992	CC	271	n	V	n	n	0	cig+/-ot	nev cigs	st
WU	37	x	f	0	0	wh	-	q+a	NAmer	1981	CC	220	n	bl	n	y	0	all/unsp	nev any	st
WUNSCH	1	x	m	0	0	all	-	all	SCAmer	1990	CC	398	n	bl	y	n	0	cig+/-ot	nev any	st
WUNSCH	7	x	f	0	0	all	-	all	SCAmer	1990	CC	398	n	bl	y	n	0	cig+/-ot	nev any	st
WUWILL	6	x	f	0	0	all	-	all	As:Chi	1985	CC	965	n	ot	n	n	0	cig+/-ot	nev cigs	st
WYNDE2	21		m	0	0	all	-	all	NAmer	1962	CC	404	n	bl	n	y	0	all/unsp	nev any	st
WYNDE3	49		m	0	0	all	-	all	NAmer	1966	CC	350	n	bl	n	y	0	all/unsp	nev any	st
WYNDE3	138		f	0	0	all	-	all	NAmer	1966	CC	350	n	bl	n	y	0	all/unsp	nev any	st
WYNDE4	48		m	0	0	all	-	all	NAmer	1948	CC	684	n	bl	y	n	0	all/unsp	nev any	st
WYNDE4	62		f	0	0	all	-	all	NAmer	1948	CC	684	n	bl	y	n	2	all/unsp	nev any	ot
WYNDE6	72		m	0	0	all	-	all	NAmer	1969	CC	4423	n	bl	n	y	0	all/unsp	nev any	st
WYNDE6	252		f	0	0	all	-	all	NAmer	1969	CC	4423	n	bl	n	y	0	cig+/-ot	nev cigs	st
XIANGZ	8	x	m	0	0	all	0	all	As:Chi	1976	pr	983	m	ot	n	n	0	all/unsp	nev any	st
XU	1	x	m	0	0	all	-	all	As:Chi	1985	CC	729	n	ot	n	n	0	all/unsp	nev any	st
XU2	1	x	c	0	0	all	-	all	As:Chi	1987	CC	610	o	ot	y	n	0	all/unsp	nev any	st
XU3	1	x	m	0	0	all	-	all	As:Chi	1981	CC	135	n	ot	n	n	0	all/unsp	nev any	st
XU3	3	x	f	0	0	all	-	all	As:Chi	1981	CC	135	n	ot	n	n	0	all/unsp	nev any	st
XU4	1		c	0	0	all	-	all	As:Chi	1981	CC	206	n	ot	*	n	0	all/unsp	nev any	st
YAMAGU	5	x	c	0	0	all	-	all	As:Jap	1989	CC	144	n	bl	n	y	0	all/unsp	nev any	st
YONG	2		c	0	0	all	0	all	NAmer	1971	pr	216	n	bl	n	n	1	cig+/-ot	nev cigs	ot
YUAN	1		m	0	0	all	0	all	As:Chi	1986	pr	142	n	ot	n	n	2	cig+/-ot	nev cigs	ot
ZHANG	1	x	c	0	0	all	-	all	As:Chi	1988	CC	100	n	ot	n	n	0	all/unsp	nev any	st
ZHENG	15		m	0	0	all	-	all	As:Chi	1982	CC	540	n	ot	*	y	0	cig+/-ot	nev cigs	st
ZHENG	24		f	0	0	all	-	all	As:Chi	1982	CC	540	n	ot	*	y	0	cig+/-ot	nev cigs	st
ZHOU	2		m	0	0	all	-	all	As:Chi	1978	CC	1360	n	ot	n	n	0	all/unsp	nev any	st
ZHOU	3		f	0	0	all	-	all	As:Chi	1978	CC	1360	n	ot	n	n	0	all/unsp	nev any	st

Cigarette type is all/unspec for all RRs
except for the following:

REF	NRR	CIGTYPE
ALDERS	12	MC only
DEAN3	124	MC only

Table 1A1 - 5

IESLC - Meta-analysis of Ever Smoking, Any product (or Cigarettes if Any not available)
All LC types
Least adjusted

REF	NRR	SEX	AD	Number Exposed Case	Number Exposed Cont	Non-exposed Case	Non-exposed Cont	RR	95.00%CI
ABELIN	1	m	0	116	341	2	183	31.13	(7.60- 127.40)
*ABRAHA	7	m	0	269	10351	10	3365	8.74	(4.66- 16.42)
*ABRAHA	8	f	0	62	5256	28	11589	4.88	(3.13- 7.62)
Subtotal ABRAHA								5.93	(4.12- 8.53)
AGUDO	8	f	0	23	23	80	183	2.29	(1.21- 4.32)
*AKIBA	3	m	0	393	207682	18	35833	3.77	(2.35- 6.04)
*AKIBA	7	f	0	83	65179	116	359850	3.95	(2.98- 5.24)
Subtotal AKIBA								3.90	(3.06- 4.97)
ALDERS	62	m	0	799	676	15	133	10.48	(6.09- 18.05)
ALDERS	12	f	0	530	371	75	243	4.63	(3.46- 6.19)
Subtotal ALDERS								5.55	(4.30- 7.18)
*AMANDU	3	m	0	126	111395	6	25350	4.78	(2.11- 10.84)
AMES	4	m	0	297	251	15	62	4.89	(2.72- 8.81)
*ANDERS	3	f	0	297	96164	46	195158	13.10	(9.61- 17.87)
*ARCHER	6	m	0	140	36269	6	9842	6.33	(2.80- 14.33)
ARMADA	29	m	0	321	261	4	64	19.68	(7.07- 54.75)
AUSTIN	3	c	0	161	237	5	88	11.96	(4.75- 30.09)
AUVINE	1	c	0	473	288	44	229	8.55	(6.00- 12.18)
AXELSO	1	c	0	90	86	62	371	6.26	(4.20- 9.34)
AXELSS	1	m	0	292	344	16	160	8.49	(4.96- 14.52)
AXELSS	11	f	0	110	109	18	154	8.63	(4.95- 15.05)
Subtotal AXELSS								8.56	(5.82- 12.59)
BAND	1	m	2	-	-	-	-	9.96	(7.38- 13.44)
BARBON	106	m	0	733	567	22	188	11.05	(7.01- 17.41)
BECHER	1	m	0	143	238	3	54	10.82	(3.32- 35.23)
BECHER	2	f	0	38	44	10	52	4.49	(2.01- 10.03)
Subtotal BECHER								5.93	(3.05- 11.53)
*BENSHL	18	m	1	-	-	-	-	5.90	(2.62- 13.31)
*BEST	22	m	0	221	24776	1	2854	25.46	(3.57- 181.46)
*BEST	18	f	1	-	-	-	-	2.24	(0.59- 8.44)
Subtotal BEST								4.81	(1.60- 14.47)
BLOHMK	3	m	0	762	587	126	301	3.10	(2.45- 3.92)
BLOT4	1	m	0	327	245	8	87	14.51	(6.91- 30.51)
BOFFET	32	m	0	5504	5505	117	1750	14.95	(12.36- 18.10)
*BOUCOT	9	m	0	121	44937	0	7551	40.83~	(2.54- 656.51)
BRESLO	37	m	0	486	451	7	42	6.47	(2.88- 14.54)
BRESLO	38	f	0	13	11	12	14	1.38	(0.45- 4.20)
Subtotal BRESLO								3.79	(1.97- 7.29)
*BRETT	10	m	0	144	47930	6	6530	3.27	(1.45- 7.40)
BROCKM	1	m	0	87	81	2	2	1.07	(0.15- 7.80)
BROCKM	2	f	0	24	54	4	18	2.00	(0.61- 6.54)
Subtotal BROCKM								1.70	(0.61- 4.70)
BROSS	12	m	0	902	784	38	170	5.15	(3.57- 7.41)
BROWN2	2	m	2	-	-	-	-	9.10	(8.30- 10.00)
BROWN2	1	f	2	-	-	-	-	12.70	(11.50- 13.90)
Subtotal BROWN2								10.72	(10.03- 11.46)
BUFFLE	1	m	0	470	419	5	47	10.54	(4.15- 26.76)
BUFFLE	5	f	0	419	284	41	198	7.12	(4.93- 10.30)
Subtotal BUFFLE								7.51	(5.33- 10.58)
CARPEN	7	c	0	328	469	15	241	11.24	(6.55- 19.29)
CASCO2	1	c	0	149	212	6	98	11.48	(4.90- 26.87)
CASCOR	1	c	0	365	362	22	295	13.52	(8.56- 21.35)
*CEDERL	107	m	2	-	-	-	-	5.92	(3.85- 9.12)
*CEDERL	112	f	2	-	-	-	-	4.18	(2.94- 5.93)
Subtotal CEDERL								4.80	(3.66- 6.30)
CHAN	9	m	0	206	161	2	43	27.51	(6.57- 115.26)
CHAN	10	f	0	105	50	84	139	3.48	(2.26- 5.35)
Subtotal CHAN								4.13	(2.73- 6.25)
*CHANG	6	m	0	78	1506	5	502	5.20	(2.12- 12.77)
*CHANG	12	f	0	42	1183	11	1139	3.68	(1.90- 7.10)
Subtotal CHANG								4.15	(2.44- 7.06)
CHATZI	4	c	0	255	365	27	129	3.34	(2.14- 5.21)
CHEN2	1	m	0	121	97	9	33	4.57	(2.09- 10.02)
CHEN2	2	f	0	38	30	25	33	1.67	(0.82- 3.39)
Subtotal CHEN2								2.62	(1.55- 4.44)
CHEN3	1	c	0	182	156	72	98	1.59	(1.09- 2.30)
CHIAZZ	2	m	0	139	209	4	47	7.81	(2.75- 22.18)
CHOI	1	m	0	267	465	13	95	4.20	(2.31- 7.64)
CHOI	5	f	0	19	26	76	164	1.58	(0.82- 3.02)
Subtotal CHOI								2.68	(1.72- 4.16)
*CHOW	7	m	0	206	202852	6	62913	10.65	(4.73- 23.98)

Table 1A1 - 5

IESLC - Meta-analysis of Ever Smoking, Any product (or Cigarettes if Any not available)
All LC types
Least adjusted

REF	NRR	SEX	AD	Number Exposed Case	Number Exposed Cont	Non-exposed Case	Non-exposed Cont	RR	95.00%CI
*CHYOU	9	m	0	214	5554	13	2406	7.13	(4.08- 12.46)
COMSTO	34	m	0	153	244	4	69	10.82	(3.87- 30.24)
COMSTO	46	f	0	88	87	13	115	8.95	(4.69- 17.06)
Subtotal COMSTO								9.44	(5.46- 16.31)
COOKSO	5	c	0	189	39	45	61	6.57	(3.92- 11.02)
CORREA	33	c	0	1202	886	51	388	10.32	(7.61- 14.00)
*CPSI	187	m	1	-	-	-	-	9.18	(7.36- 11.45)
*CPSI	274	f	1	-	-	-	-	2.79	(2.22- 3.51)
Subtotal CPSI								5.17	(4.41- 6.06)
*CPSII	104	m	1	-	-	-	-	12.83	(10.28- 16.01)
*CPSII	79	f	1	-	-	-	-	8.16	(6.93- 9.62)
Subtotal CPSII								9.58	(8.40- 10.93)
DAMBER	5	m	0	537	364	42	208	7.31	(5.11- 10.44)
DARBY	15	m	0	664	1724	3	384	49.30	(15.77- 154.07)
DARBY	16	f	0	292	548	23	529	12.26	(7.89- 19.05)
Subtotal DARBY								14.69	(9.74- 22.16)
DAVEYS	5	m	0	90	144	3	23	4.79	(1.40- 16.42)
DAVEYS	6	f	0	0	3	16	83	0.72~	(0.04- 14.66)
Subtotal DAVEYS								3.65	(1.17- 11.42)
DEAN	7	m	0	591	574	12	61	5.23	(2.79- 9.82)
DEAN2	3	m	0	769	688	33	112	3.79	(2.54- 5.67)
DEAN2	7	f	0	64	30	88	121	2.93	(1.76- 4.90)
Subtotal DEAN2								3.44	(2.51- 4.72)
DEAN3	47	m	0	591	2053	25	510	5.87	(3.89- 8.86)
DEAN3	124	f	0	109	1420	41	1538	2.88	(2.00- 4.15)
Subtotal DEAN3								3.94	(3.00- 5.19)
*DEKLER	6	m	2	-	-	-	-	20.29	(2.84- 145.18)
DESTE2	13	c	0	300	212	20	108	7.64	(4.60- 12.71)
DESTEF	5	m	0	470	334	27	163	8.50	(5.52- 13.07)
*DOCKER	3	c	4	-	-	-	-	4.29	(1.66- 11.06)
DOLL	6	m	0	1350	1296	7	61	9.08	(4.14- 19.92)
DOLL	12	f	0	68	49	40	59	2.05	(1.19- 3.53)
Subtotal DOLL								3.32	(2.12- 5.19)
*DOLL2	56	m	1	-	-	-	-	7.66	(4.86- 12.07)
DORANT	10	c	0	470	2033	14	1090	18.00	(10.52- 30.78)
DORGAN	6	m	0	721	455	15	93	9.82	(5.62- 17.16)
DORGAN	30	m	0	266	135	3	35	22.99	(6.94- 76.10)
DORGAN	53	f	0	757	229	103	244	7.83	(5.96- 10.30)
DORGAN	76	f	0	79	27	7	20	8.36	(3.18- 21.95)
Subtotal DORGAN								8.52	(6.75- 10.76)
*DORN	196	m	1	-	-	-	-	7.04	(5.60- 8.84)
DOSEME	17	m	0	1068	536	142	293	4.11	(3.28- 5.15)
DROSTE	3	m	0	471	443	7	93	14.13	(6.48- 30.78)
DU	1	m	0	538	-	28	-	3.53	(2.44- 5.11)
DU	2	f	0	191	-	92	-	1.93	(1.30- 2.87)
Subtotal DU								2.66	(2.03- 3.49)
*DUNN	6	m	0	137	52634	2	14160	18.43	(4.56- 74.42)
EBELIN	1	m	0	101	142	12	117	6.93	(3.63- 13.24)
*ENGELA	155	m	0	111	9235	7	2683	4.61	(2.15- 9.88)
*ENGELA	162	f	0	13	3262	13	10708	3.28	(1.52- 7.07)
Subtotal ENGELA								3.89	(2.27- 6.69)
ESAKI	4	m	0	155	143	16	28	1.90	(0.99- 3.65)
ESAKI	5	f	0	34	19	40	55	2.46	(1.23- 4.92)
Subtotal ESAKI								2.14	(1.33- 3.45)
FAN	1	m	0	216	498	36	236	2.84	(1.93- 4.18)
FAN	2	f	0	82	97	69	320	3.92	(2.65- 5.81)
Subtotal FAN								3.33	(2.53- 4.38)
GAO	6	m	0	671	558	62	202	3.92	(2.89- 5.32)
GAO	16	f	0	237	130	435	605	2.54	(1.98- 3.25)
Subtotal GAO								3.01	(2.48- 3.65)
GAO2	6	m	0	269	226	13	56	5.13	(2.73- 9.62)
GARCIA	3	c	0	395	307	21	139	8.52	(5.26- 13.80)
GARDIN	7	c	0	138	102	5	41	11.09	(4.23- 29.06)
GARSHI	17	m	0	1040	1596	41	363	5.77	(4.14- 8.04)
GENG	1	m	0	92	68	7	31	5.99	(2.49- 14.42)
GENG	2	f	0	126	75	67	118	2.96	(1.96- 4.48)
Subtotal GENG								3.36	(2.31- 4.89)
GER	17	c	0	90	318	51	246	1.37	(0.93- 2.00)
GODLEY	5	m	1	-	-	-	-	6.84	(5.60- 8.35)
GODLEY	6	f	1	-	-	-	-	5.54	(4.29- 7.15)
Subtotal GODLEY								6.31	(5.39- 7.39)

Table 1A1 - 5

IESLC - Meta-analysis of Ever Smoking, Any product (or Cigarettes if Any not available)
All LC types
Least adjusted

REF	NRR	SEX	AD	Number Exposed Case	Number Exposed Cont	Non-exposed Case	Non-exposed Cont	RR	95.00%CI
GOLLED	21	m	0	380	1966	15	490	6.31	(3.73- 10.68)
GOODMA	3	m	0	216	398	10	199	10.80	(5.60- 20.82)
GOODMA	7	f	0	81	91	19	177	8.29	(4.74- 14.52)
Subtotal GOODMA								9.27	(6.05- 14.19)
GRAHAM	22	m	0	667	1651	18	346	7.77	(4.79- 12.58)
GREGOR	3	m	0	72	98	10	14	1.03	(0.43- 2.45)
GREGOR	7	f	0	21	42	1	22	11.00	(1.39- 87.29)
Subtotal GREGOR								1.46	(0.66- 3.26)
GSELL	8	m	0	148	121	2	29	17.74	(4.15- 75.83)
HAENSZ	50	f	0	76	103	81	236	2.15	(1.46- 3.17)
*HAMMO2	18	m	0	180	10199	1	1457	25.71	(3.61- 183.40)
*HAMMON	129	m	0	425	510108	15	115884	6.44	(3.85- 10.77)
*HANSEN	3	m	2	-	-	-	-	1.53	(0.71- 3.91)
HEGMAN	1	c	0	255	1202	27	2080	16.34	(10.92- 24.45)
*HEIN	7	m	0	143	4471	1	457	14.62	(2.05- 104.23)
*HENNEK	3	m	0	146	11112	23	10919	6.24	(4.02- 9.67)
HINDS	26	f	0	167	592	124	1812	4.12	(3.21- 5.29)
*HIRAYA	147	m	1	-	-	-	-	4.36	(3.53- 5.39)
*HIRAYA	150	f	1	-	-	-	-	2.36	(1.90- 2.94)
Subtotal HIRAYA								3.24	(2.78- 3.77)
HITOSU	6	m	0	142	1787	7	242	2.75	(1.27- 5.94)
HITOSU	12	f	0	34	500	33	1893	3.90	(2.39- 6.36)
Subtotal HITOSU								3.53	(2.33- 5.33)
*HOLE	15	m	0	187	5866	7	1189	5.41	(2.55- 11.49)
HOROWI	1	m	0	182	525	19	196	3.58	(2.17- 5.90)
HOROWI	2	f	0	21	382	14	463	1.82	(0.91- 3.62)
Subtotal HOROWI								2.83	(1.89- 4.25)
HORWIT	1	f	0	97	92	11	118	11.31	(5.73- 22.34)
HU	15	m	0	120	94	41	67	2.09	(1.30- 3.35)
HU	16	f	0	26	18	40	48	1.73	(0.83- 3.61)
Subtotal HU								1.98	(1.33- 2.94)
HU2	9	m	0	294	228	49	115	3.03	(2.08- 4.41)
HU2	10	f	0	108	80	72	100	1.88	(1.23- 2.85)
Subtotal HU2								2.44	(1.85- 3.23)
HUANG	1	c	0	98	77	37	58	2.00	(1.20- 3.32)
HUMBLE	14	m	1	-	-	-	-	12.10	(5.12- 28.60)
HUMBLE	16	m	1	-	-	-	-	11.88	(2.65- 53.30)
HUMBLE	18	f	1	-	-	-	-	11.36	(5.32- 24.23)
HUMBLE	20	f	1	-	-	-	-	15.40	(4.87- 48.74)
Subtotal HUMBLE								12.28	(7.58- 19.90)
JAHN	3	f	0	112	67	53	98	3.09	(1.97- 4.85)
JAIN	6	m	0	391	277	12	85	10.00	(5.36- 18.66)
JAIN	1	f	0	390	196	52	214	8.19	(5.78- 11.60)
Subtotal JAIN								8.59	(6.34- 11.64)
JARUP	3	m	0	91	52	11	42	6.68	(3.17- 14.09)
JARVHO	3	m	0	99	57	1	16	27.79	(3.59- 215.09)
JARVHO	7	f	0	41	15	6	21	9.57	(3.24- 28.26)
Subtotal JARVHO								12.08	(4.64- 31.46)
JEDRYC	63	m	0	852	656	49	219	5.80	(4.19- 8.04)
JEDRYC	68	f	0	120	32	78	166	7.98	(4.97- 12.82)
Subtotal JEDRYC								6.43	(4.92- 8.41)
JIANG	1	m	0	93	83	7	17	2.72	(1.08- 6.89)
JIANG	2	f	0	11	6	14	19	2.49	(0.74- 8.35)
Subtotal JIANG								2.63	(1.26- 5.50)
JOLY	14	m	0	595	888	12	218	12.17	(6.75- 21.97)
JOLY	1	f	0	166	123	52	283	7.34	(5.04- 10.70)
Subtotal JOLY								8.50	(6.19- 11.68)
JUSSAW	3	m	0	643	168	149	624	16.03	(12.53- 20.51)
*KAISE2	72	m	1	-	-	-	-	5.40	(3.05- 9.57)
*KAISE2	64	f	1	-	-	-	-	10.09	(5.29- 19.27)
Subtotal KAISE2								7.11	(4.63- 10.90)
*KAISER	13	m	2	-	-	-	-	17.63	(11.98- 25.96)
*KAISER	10	f	2	-	-	-	-	5.63	(3.89- 8.14)
Subtotal KAISER								9.70	(7.43- 12.67)
KATSOU	27	f	0	53	22	48	67	3.36	(1.81- 6.25)
KAUFMA	8	c	0	846	1645	35	925	13.59	(9.60- 19.24)
KELLER	3	m	0	8066	2517	323	1017	10.09	(8.83- 11.52)
KELLER	11	m	0	1493	340	38	117	13.52	(9.20- 19.86)
KELLER	7	f	0	3998	1269	469	1860	12.49	(11.09- 14.08)
KELLER	15	f	0	584	214	67	232	9.45	(6.91- 12.93)
Subtotal KELLER								11.30	(10.40- 12.29)

Table 1A1 - 5

IESLC - Meta-analysis of Ever Smoking, Any product (or Cigarettes if Any not available)
All LC types
Least adjusted

REF	NRR	SEX	AD	Number Exposed Case	Number Exposed Cont	Non-exposed Case	Non-exposed Cont	RR	95.00%CI
KHUDER	4	m	0	459	785	23	309	7.86	(5.06- 12.19)
KIHARA	31	c	0	338	232	102	237	3.39	(2.54- 4.51)
*KINLEN	6	m	0	711	12722	7	1333	10.64	(5.07- 22.36)
KJUUS	10	m	0	174	152	2	24	13.74	(3.19- 59.08)
*KNEKT	76	m	0	111	51798	6	17814	6.36	(2.80- 14.46)
KO	1	f	3	-	-	-	-	4.20	(1.10- 15.60)
KOHLME	1	c	0	228	236	11	193	16.95	(8.99- 31.96)
KOO	1	f	0	112	63	88	137	2.77	(1.84- 4.16)
KOULUM	1	m	0	807	246	5	54	35.43	(14.02- 89.55)
KREUZE	14	f	0	62	42	6	38	9.35	(3.63- 24.08)
KREUZE	16	f	0	205	101	95	177	3.78	(2.68- 5.34)
Subtotal KREUZE								4.21	(3.04- 5.81)
KREYBE	24	m	0	252	3514	6	644	7.70	(3.41- 17.37)
KREYBE	39	f	0	12	328	30	657	0.80	(0.40- 1.59)
Subtotal KREYBE								2.04	(1.21- 3.44)
*KUBIK	28	m	0	106	8051	2	4271	28.12	(6.94- 113.84)
LAMTH	6	f	0	242	106	202	337	3.81	(2.86- 5.08)
LAMWK	1	f	0	88	41	75	144	4.12	(2.59- 6.55)
LAMWK2	9	m	0	244	161	23	43	2.83	(1.64- 4.88)
LAMWK2	10	f	0	75	50	65	139	3.21	(2.02- 5.10)
Subtotal LAMWK2								3.04	(2.14- 4.33)
*LANGE	34	m	0	195	5790	5	721	4.86	(2.01- 11.76)
*LANGE	31	f	0	61	5544	7	2159	3.39	(1.55- 7.41)
Subtotal LANGE								3.97	(2.21- 7.13)
LAUSSM	10	m	0	347	188	85	226	4.91	(3.61- 6.66)
LEI	1	m	0	443	361	41	123	3.68	(2.52- 5.38)
LEI	2	f	0	123	61	85	147	3.49	(2.32- 5.24)
Subtotal LEI								3.59	(2.72- 4.74)
LEMARC	3	c	0	309	288	32	168	5.63	(3.74- 8.49)
LETOUR	1	c	0	714	514	24	224	12.96	(8.38- 20.05)
LEVIN	32	m	1	-	-	-	-	4.86	(3.41- 6.92)
*LIDDEL	5	m	1	-	-	-	-	3.61	(2.27- 5.73)
LIU	2	c	2	-	-	-	-	1.92	(1.40- 2.64)
LIU2	1	m	0	212	180	12	44	4.32	(2.21- 8.43)
LIU2	3	f	0	54	23	38	69	4.26	(2.27- 7.99)
Subtotal LIU2								4.29	(2.71- 6.78)
LIU3	1	m	0	52	205	4	19	1.20	(0.39- 3.69)
LIU4	11	m	2	-	-	-	-	2.76	(2.69- 2.83)
LIU4	12	f	2	-	-	-	-	2.86	(2.77- 2.95)
Subtotal LIU4								2.80	(2.74- 2.85)
LIU5	1	c	0	85	70	26	41	1.91	(1.07- 3.44)
LOMBA2	1	f	0	149	353	76	239	1.33	(0.96- 1.83)
LOMBAR	12	m	0	1026	928	14	112	8.84	(5.04- 15.53)
LUBIN2	45	m	0	6729	10841	190	2617	8.55	(7.36- 9.94)
LUBIN2	101	f	0	549	561	336	1188	3.46	(2.92- 4.10)
Subtotal LUBIN2								5.73	(5.12- 6.41)
LUO	1	c	0	65	146	37	160	1.93	(1.21- 3.06)
MACLEN	71	m	0	142	119	5	15	3.58	(1.26- 10.14)
MACLEN	72	f	0	45	57	41	109	2.10	(1.23- 3.57)
Subtotal MACLEN								2.34	(1.46- 3.76)
*MAGNUS	1	m	0	189	3439	11	1086	5.43	(2.97- 9.93)
MARSH	2	m	0	98	155	2	31	9.80	(2.29- 41.87)
MARSH	4	f	0	42	64	8	71	5.82	(2.54- 13.33)
Subtotal MARSH								6.62	(3.22- 13.59)
MARSH2	2	c	0	102	149	12	56	3.19	(1.63- 6.26)
MARTIS	4	m	0	197	176	4	25	7.00	(2.39- 20.49)
MASTRA	1	m	0	303	265	6	44	8.38	(3.52- 19.99)
MATOS	26	m	0	188	283	11	110	6.64	(3.48- 12.68)
MATSUD	10	m	0	170	3314	3	1255	21.46	(6.84- 67.33)
MCCONN	1	m	0	88	174	5	12	1.21	(0.41- 3.55)
MCCONN	2	f	0	3	3	4	11	2.75	(0.38- 19.67)
Subtotal MCCONN								1.46	(0.57- 3.76)
MCDUFF	1	m	0	159	134	6	31	6.13	(2.48- 15.14)
MCLAUG	1	m	0	294	1082	22	270	3.33	(2.12- 5.25)
*MIGRAN	26	m	0	206	6719	4	867	6.65	(2.48- 17.83)
*MIGRAN	41	f	0	31	4086	4	3814	7.23	(2.56- 20.47)
Subtotal MIGRAN								6.92	(3.38- 14.16)
MILLER	1	f	0	140	1607	28	3638	11.32	(7.51- 17.06)
MILLS	3	m	1	-	-	-	-	1.33	(1.09- 1.63)
*MRFITR	6	m	0	119	11007	0	1859	40.37~	(2.51- 648.95)
NAM	69	m	0	610	1075	30	520	9.84	(6.72- 14.40)

Table 1A1 - 5

IESLC - Meta-analysis of Ever Smoking, Any product (or Cigarettes if Any not available)
All LC types
Least adjusted

REF	NRR	SEX	AD	Number Exposed Case	Number Exposed Cont	Non-exposed Case	Non-exposed Cont	RR	95.00%CI
NAM	85	f	0	292	496	52	885	10.02	(7.31- 13.73)
Subtotal NAM								9.94	(7.80- 12.68)
NOTAN2	1	m	0	549	735	134	544	3.03	(2.44- 3.77)
NOU	11	m	0	74	247	6	122	6.09	(2.58- 14.39)
NOU	12	f	0	10	92	4	261	7.09	(2.17- 23.17)
Subtotal NOU								6.42	(3.20- 12.87)
ODRISC	3	c	0	440	996	6	664	48.89	(21.71- 110.07)
ORMOS	4	m	0	87	1034	7	777	9.34	(4.30- 20.28)
ORMOS	26	f	0	1	234	23	1044	0.19	(0.03- 1.44)
Subtotal ORMOS								5.65	(2.74- 11.64)
OSANN	17	m	0	1108	1018	45	833	20.15	(14.75- 27.52)
OSANN	21	f	0	737	563	96	1093	14.90	(11.77- 18.87)
Subtotal OSANN								16.63	(13.78- 20.07)
PARKIN	29	m	0	401	1007	107	1248	4.64	(3.69- 5.84)
PASTOR	5	m	0	194	262	10	89	6.59	(3.34- 13.00)
PAWLEG	1	m	0	172	249	4	92	15.89	(5.73- 44.05)
PERNU	2	m	0	1380	438	97	275	8.93	(6.92- 11.53)
PERNU	1	f	0	19	89	110	971	1.88	(1.11- 3.21)
Subtotal PERNU								6.68	(5.31- 8.41)
PERSH2	5	c	0	844	924	178	1164	5.97	(4.97- 7.17)
*PETO	5	m	0	101	2423	2	295	6.15	(1.52- 24.79)
PEZZO2	10	m	0	361	469	6	117	15.01	(6.53- 34.48)
PEZZOT	25	m	0	211	317	4	116	19.30	(7.02- 53.10)
PIKE	4	m	0	514	375	18	69	5.25	(3.08- 8.98)
PIKE	8	f	0	163	90	36	96	4.83	(3.04- 7.66)
Subtotal PIKE								5.01	(3.53- 7.10)
POFFIJ	1	c	0	913	918	58	452	7.75	(5.81- 10.34)
POLEDN	3	c	0	196	271	12	139	8.38	(4.52- 15.54)
*QIAO2	3	m	0	231	6917	10	709	2.37	(1.26- 4.44)
RACHTA	3	f	0	85	43	33	98	5.87	(3.43- 10.06)
RADZIK	1	c	0	180	198	9	13	1.31	(0.55- 3.15)
RANDIG	23	m	0	410	359	5	22	5.03	(1.88- 13.41)
RANDIG	24	f	0	16	39	17	92	2.22	(1.02- 4.84)
Subtotal RANDIG								3.04	(1.65- 5.60)
REN	1	m	0	106	84	12	34	3.58	(1.74- 7.33)
REN	2	f	0	78	20	48	50	4.06	(2.16- 7.64)
Subtotal REN								3.84	(2.39- 6.17)
RONCO	1	m	0	120	306	6	78	5.10	(2.16- 12.01)
ROTHSC	1	c	0	271	222	13	62	5.82	(3.12- 10.86)
SADOWS	7	m	0	459	534	18	81	3.87	(2.29- 6.54)
SANKAR	1	m	0	251	439	28	767	15.66	(10.42- 23.55)
SCHWAR	1	m	0	2648	1019	119	376	8.21	(6.60- 10.22)
SCHWAR	2	m	0	863	275	50	104	6.53	(4.54- 9.39)
SCHWAR	3	f	0	1351	637	182	855	9.96	(8.28- 12.00)
SCHWAR	4	f	0	335	179	40	247	11.56	(7.90- 16.90)
Subtotal SCHWAR								9.05	(7.99- 10.25)
SEGI	1	m	0	140	1742	18	382	1.71	(1.03- 2.82)
SEOW	1	f	0	61	15	92	125	5.53	(2.96- 10.33)
SHAW	12	c	0	324	266	11	107	11.85	(6.24- 22.50)
SIEMIA	9	m	0	844	428	13	105	15.93	(8.85- 28.67)
SIMARA	5	m	0	33	264	27	433	2.00	(1.18- 3.41)
SIMARA	6	f	0	17	67	38	349	2.33	(1.24- 4.37)
Subtotal SIMARA								2.13	(1.42- 3.20)
SOBUE	91	m	0	1023	1013	34	128	3.80	(2.58- 5.60)
SOBUE	95	f	0	127	232	167	857	2.81	(2.14- 3.69)
Subtotal SOBUE								3.10	(2.48- 3.88)
*SPEIZE	8	f	0	535	1012074	58	776300	7.08	(5.40- 9.28)
SPITZ	3	c	0	170	169	7	128	18.39	(8.35- 40.53)
STASZE	1	m	0	255	754	5	158	10.69	(4.34- 26.33)
STASZE	5	f	0	6	153	15	1660	4.34	(1.66- 11.35)
Subtotal STASZE								7.01	(3.63- 13.53)
STAYNE	1	m	0	362	567	58	333	3.67	(2.69- 4.99)
STOCKS	31	m	0	2632	6477	45	638	5.76	(4.24- 7.82)
STOCKS	50	f	1	-	-	-	-	3.04	(2.35- 3.93)
Subtotal STOCKS								3.96	(3.25- 4.82)
STOCKW	6	c	0	19370	7069	2791	10641	10.45	(9.94- 10.98)
STUCKE	3	m	0	247	203	0	51	125.27~	(7.68-2042.38)
SUN	1	c	0	140	173	67	191	2.31	(1.62- 3.30)
SUZUK2	18	c	0	112	70	11	53	7.71	(3.77- 15.76)
SVENSS	56	f	0	172	89	38	120	6.10	(3.91- 9.53)
TANG	3	c	0	110	59	9	39	8.08	(3.66- 17.82)

Table 1A1 - 5

IESLC - Meta-analysis of Ever Smoking, Any product (or Cigarettes if Any not available)
All LC types
Least adjusted

REF	NRR	SEX	AD	Number Exposed Case	Number Exposed Cont	Non-exposed Case	Non-exposed Cont	RR	95.00%CI
*TENKAN	22	m	1	-	-	-	-	14.64	(6.29- 34.07)
TIZZAN	1	m	0	1036	911	180	305	1.93	(1.57- 2.36)
TIZZAN	12	f	0	25	28	25	114	4.07	(2.04- 8.13)
Subtotal TIZZAN								2.05	(1.68- 2.49)
TOKARS	3	m	0	147	243	1	53	32.06	(4.39- 234.30)
TOKARS	5	f	0	1	2	13	40	1.54	(0.13- 18.38)
Subtotal TOKARS								9.77	(2.07- 46.11)
TOUSEY	16	m	0	297	437	4	130	22.09	(8.08- 60.39)
TOUSEY	26	f	0	193	214	13	226	15.68	(8.67- 28.34)
Subtotal TOUSEY								17.12	(10.28- 28.52)
TSUGAN	27	m	0	73	71	18	22	1.26	(0.62- 2.54)
*TULINI	15	m	1	-	-	-	-	8.06	(4.38- 14.84)
*TULINI	21	f	1	-	-	-	-	14.95	(8.30- 26.95)
Subtotal TULINI								11.10	(7.26- 16.95)
*TVERDA	22	m	2	-	-	-	-	4.58	(2.97- 7.06)
WAKAI	13	m	0	235	424	10	65	3.60	(1.82- 7.14)
WAKAI	31	f	0	38	31	50	145	3.55	(2.00- 6.30)
Subtotal WAKAI								3.57	(2.30- 5.55)
WANG	1	m	0	262	-	29	-	3.47	(2.10- 5.80)
WANG	2	f	0	17	-	82	-	4.00	(1.30- 12.00)
Subtotal WANG								3.56	(2.24- 5.64)
WANG2	8	c	0	60	99	11	43	2.37	(1.14- 4.94)
WANG3	1	c	0	235	172	58	121	2.85	(1.97- 4.13)
WANG4	1	m	0	1043	18164	127	2374	1.07	(0.89- 1.30)
WICKLU	1	m	0	-	-	-	-	4.60	(2.80- 7.60)
WIGLE	15	m	0	598	798	15	204	10.19	(5.97- 17.40)
WIGLE	18	f	0	79	235	36	439	4.10	(2.68- 6.27)
Subtotal WIGLE								5.83	(4.18- 8.13)
WILKIN	1	m	0	173	372	2	108	25.11	(6.13- 102.89)
WILKIN	2	f	0	84	109	12	89	5.72	(2.93- 11.13)
Subtotal WILKIN								7.49	(4.10- 13.68)
WU	37	f	0	189	128	31	92	4.38	(2.75- 6.97)
WUNSCH	1	m	0	290	441	14	99	4.65	(2.61- 8.30)
WUNSCH	7	f	0	60	98	29	208	4.39	(2.65- 7.27)
Subtotal WUNSCH								4.50	(3.08- 6.58)
WUWILL	6	f	0	539	351	417	601	2.21	(1.84- 2.66)
WYNDE2	21	m	0	396	616	8	105	8.44	(4.07- 17.51)
WYNDE3	49	m	0	275	332	9	88	8.10	(4.00- 16.38)
WYNDE3	138	f	0	46	56	20	76	3.12	(1.67- 5.85)
Subtotal WYNDE3								4.76	(2.98- 7.61)
WYNDE4	48	m	0	632	665	12	115	9.11	(4.98- 16.67)
WYNDE4	62	f	2	-	-	-	-	2.87	(1.48- 5.55)
Subtotal WYNDE4								5.38	(3.45- 8.41)
WYNDE6	72	m	0	2823	1996	87	617	10.03	(7.96- 12.65)
WYNDE6	252	f	0	1354	701	159	856	10.40	(8.58- 12.60)
Subtotal WYNDE6								10.25	(8.84- 11.88)
*XIANGZ	8	m	0	907	13037	25	974	2.71	(1.83- 4.01)
XU	1	m	0	627	552	102	236	2.63	(2.03- 3.40)
XU2	1	c	0	501	582	82	377	3.96	(3.03- 5.17)
XU3	1	m	0	92	68	7	31	5.99	(2.49- 14.42)
XU3	3	f	0	23	11	13	25	4.02	(1.51- 10.74)
Subtotal XU3								5.02	(2.61- 9.66)
XU4	1	c	0	161	113	45	93	2.94	(1.92- 4.52)
YAMAGU	5	c	0	120	409	24	267	3.26	(2.05- 5.19)
*YONG	2	c	1	-	-	-	-	6.74	(4.47- 10.18)
*YUAN	1	m	2	-	-	-	-	6.50	(3.64- 11.60)
ZHANG	1	c	0	72	102	28	98	2.47	(1.47- 4.14)
ZHENG	15	m	0	279	218	33	94	3.65	(2.36- 5.63)
ZHENG	24	f	0	76	44	152	184	2.09	(1.36- 3.21)
Subtotal ZHENG								2.75	(2.03- 3.73)
ZHOU	2	m	0	740	41	275	36	2.36	(1.48- 3.77)
ZHOU	3	f	0	112	7	231	32	2.22	(0.95- 5.18)
Subtotal ZHOU								2.33	(1.54- 3.51)
Partial Totals				132841	2758155	14876	1778656		

*prospective study ~ With 0.5 adjustment for zero

Table 1A1 - 5

IESLC - Meta-analysis of Ever Smoking, Any product (or Cigarettes if Any not available)
All LC types
Least adjusted

REF	NRR	SEX	AD	Ys	Ws	Qs	Ps
ABELIN	1	m	0	3.44	1.93	7.71	0.0000
*ABRAHA	7	m	0	2.17	9.68	5.11	0.0000
*ABRAHA	8	f	0	1.59	19.39	0.40	0.0000
Subtotal ABRAHA				1.78	29.07	5.51	
AGUDO	8	f	0	0.83	9.53	3.60	0.0106
*AKIBA	3	m	0	1.33	17.22	0.23	0.0000
*AKIBA	7	f	0	1.37	48.42	0.23	0.0000
Subtotal AKIBA				1.36	65.65	0.46	
ALDERS	62	m	0	2.35	13.00	10.70	0.0000
ALDERS	12	f	0	1.53	45.39	0.37	0.0000
Subtotal ALDERS				1.71	58.39	11.07	
*AMANDU	3	m	0	1.56	5.73	0.09	0.0002
AMES	4	m	0	1.59	11.09	0.23	0.0000
*ANDERS	3	f	0	2.57	39.86	50.96	0.0000
*ARCHER	6	m	0	1.85	5.76	0.94	0.0000
ARMADA	29	m	0	2.98	3.67	8.67	0.0000
AUSTIN	3	c	0	2.48	4.51	4.87	0.0000
AUVINE	1	c	0	2.15	30.60	15.15	0.0000
AXELSO	1	c	0	1.83	24.06	3.71	0.0000
AXELSS	1	m	0	2.14	13.32	6.46	0.0000
AXELSS	11	f	0	2.16	12.45	6.34	0.0000
Subtotal AXELSS				2.15	25.77	12.80	
BAND	1	m	2	2.30	42.76	31.37	0.0000
BARBON	106	m	0	2.40	18.55	17.10	0.0000
BECHER	1	m	0	2.38	2.75	2.43	0.0001
BECHER	2	f	0	1.50	5.94	0.02	0.0003
Subtotal BECHER				1.78	8.70	2.45	
*BENSHL	18	m	1	1.77	5.82	0.64	0.0000
*BEST	22	m	0	3.24	1.00	3.21	0.0012
*BEST	18	f	1	0.81	2.17	0.88	0.2348
Subtotal BEST				1.57	3.17	4.09	
BLOHMK	3	m	0	1.13	70.05	6.75	0.0000
BLOT4	1	m	0	2.68	6.96	10.59	0.0000
BOFFET	32	m	0	2.71	105.47	168.21	0.0000
*BOUCOT	9	m	0	3.71	0.50	2.56	0.0089
BRESLO	37	m	0	1.87	5.85	1.05	0.0000
BRESLO	38	f	0	0.32	3.10	3.89	0.5717
Subtotal BRESLO				1.33	8.95	4.95	
*BRETT	10	m	0	1.18	5.77	0.38	0.0044
BROCKM	1	m	0	0.07	0.98	1.83	0.9437
BROCKM	2	f	0	0.69	2.73	1.53	0.2517
Subtotal BROCKM				0.53	3.71	3.37	
BROSS	12	m	0	1.64	28.92	1.11	0.0000
BROWN2	2	m	2	2.21	442.58	259.81	0.0000
BROWN2	1	f	2	2.54	427.71	517.06	0.0000
Subtotal BROWN2				2.37	870.29	776.87	
BUFFLE	1	m	0	2.36	4.43	3.70	0.0000
BUFFLE	5	f	0	1.96	28.29	7.69	0.0000
Subtotal BUFFLE				2.02	32.72	11.39	
CARPEN	7	c	0	2.42	13.16	12.56	0.0000
CASCO2	1	c	0	2.44	5.31	5.29	0.0000
CASCOR	1	c	0	2.60	18.40	24.85	0.0000
*CEDERL	107	m	2	1.78	20.66	2.34	0.0000
*CEDERL	112	f	2	1.43	31.21	0.00	0.0000
Subtotal CEDERL				1.57	51.88	2.34	
CHAN	9	m	0	3.31	1.87	6.56	0.0000
CHAN	10	f	0	1.25	20.57	0.79	0.0000
Subtotal CHAN				1.42	22.44	7.36	
*CHANG	6	m	0	1.65	4.76	0.20	0.0003
*CHANG	12	f	0	1.30	8.85	0.17	0.0001
Subtotal CHANG				1.42	13.61	0.38	
CHATZI	4	c	0	1.21	19.44	1.09	0.0000
CHEN2	1	m	0	1.52	6.25	0.04	0.0001
CHEN2	2	f	0	0.51	7.70	6.63	0.1539
Subtotal CHEN2				0.97	13.95	6.67	
CHEN3	1	c	0	0.46	27.78	26.66	0.0148
CHIAZZ	2	m	0	2.06	3.53	1.33	0.0001
CHOI	1	m	0	1.43	10.71	0.00	0.0000
CHOI	5	f	0	0.46	9.06	8.82	0.1703
Subtotal CHOI				0.99	19.78	8.82	
*CHOW	7	m	0	2.37	5.83	4.97	0.0000

Table 1A1 - 5

IESLC - Meta-analysis of Ever Smoking, Any product (or Cigarettes if Any not available)
All LC types
Least adjusted

REF	NRR	SEX	AD	Ys	Ws	Qs	Ps
*CHYOU	9	m	0	1.96	12.35	3.37	0.0000
COMSTO	34	m	0	2.38	3.63	3.20	0.0000
COMSTO	46	f	0	2.19	9.22	5.18	0.0000
Subtotal COMSTO				2.25	12.85	8.38	
COOKSO	5	c	0	1.88	14.38	2.79	0.0000
CORREA	33	c	0	2.33	41.42	32.96	0.0000
*CPSI	187	m	1	2.22	78.68	47.25	0.0000
*CPSI	274	f	1	1.03	73.22	12.67	0.0000
Subtotal CPSI				1.64	151.90	59.92	
*CPSII	104	m	1	2.55	78.29	96.41	0.0000
*CPSII	79	f	1	2.10	142.84	61.68	0.0000
Subtotal CPSII				2.26	221.13	158.10	
DAMBER	5	m	0	1.99	30.10	8.99	0.0000
DARBY	15	m	0	3.90	2.96	17.84	0.0000
DARBY	16	f	0	2.51	19.76	22.36	0.0000
Subtotal DARBY				2.69	22.71	40.20	
DAVEYS	5	m	0	1.57	2.53	0.04	0.0126
DAVEYS	6	f	0	-0.32	0.42	1.32	0.8327
Subtotal DAVEYS				1.30	2.96	1.36	
DEAN	7	m	0	1.66	9.69	0.44	0.0000
DEAN2	3	m	0	1.33	23.82	0.28	0.0000
DEAN2	7	f	0	1.08	14.58	1.95	0.0000
Subtotal DEAN2				1.24	38.40	2.23	
DEAN3	47	m	0	1.77	22.66	2.44	0.0000
DEAN3	124	f	0	1.06	28.64	4.23	0.0000
Subtotal DEAN3				1.37	51.29	6.67	
*DEKLER	6	m	2	3.01	0.99	2.44	0.0027
DESTE2	13	c	0	2.03	14.86	5.20	0.0000
DESTEF	5	m	0	2.14	20.71	10.07	0.0000
*DOCKER	3	c	4	1.46	4.27	0.00	0.0026
DOLL	6	m	0	2.21	6.22	3.63	0.0000
DOLL	12	f	0	0.72	12.98	6.83	0.0099
Subtotal DOLL				1.20	19.20	10.46	
*DOLL2	56	m	1	2.04	18.57	6.55	0.0000
DORANT	10	c	0	2.89	13.34	27.98	0.0000
DORGAN	6	m	0	2.28	12.35	8.77	0.0000
DORGAN	30	m	0	3.13	2.68	7.68	0.0000
DORGAN	53	f	0	2.06	51.30	19.46	0.0000
DORGAN	76	f	0	2.12	4.12	1.91	0.0000
Subtotal DORGAN				2.14	70.44	37.83	
*DORN	196	m	1	1.95	73.73	19.14	0.0000
DOSEME	17	m	0	1.41	75.43	0.06	0.0000
DROSTE	3	m	0	2.65	6.33	9.20	0.0000
DU	1	m	0	1.26	28.12	0.92	0.0000
DU	2	f	0	0.66	24.50	15.08	0.0011
Subtotal DU				0.98	52.62	16.00	
*DUNN	6	m	0	2.91	1.97	4.27	0.0000
EBELIN	1	m	0	1.94	9.19	2.25	0.0000
*ENGELA	155	m	0	1.53	6.61	0.05	0.0001
*ENGELA	162	f	0	1.19	6.52	0.42	0.0024
Subtotal ENGELA				1.36	13.12	0.47	
ESAKI	4	m	0	0.64	8.96	5.76	0.0554
ESAKI	5	f	0	0.90	7.99	2.34	0.0109
Subtotal ESAKI				0.76	16.94	8.10	
FAN	1	m	0	1.04	25.87	4.08	0.0000
FAN	2	f	0	1.37	24.92	0.14	0.0000
Subtotal FAN				1.20	50.80	4.22	
GAO	6	m	0	1.37	41.05	0.24	0.0000
GAO	16	f	0	0.93	63.04	16.50	0.0000
Subtotal GAO				1.10	104.09	16.75	
GAO2	6	m	0	1.63	9.72	0.36	0.0000
GARCIA	3	c	0	2.14	16.50	8.08	0.0000
GARDIN	7	c	0	2.41	4.14	3.85	0.0000
GARSHI	17	m	0	1.75	34.80	3.35	0.0000
GENG	1	m	0	1.79	4.98	0.60	0.0001
GENG	2	f	0	1.08	22.39	2.86	0.0000
Subtotal GENG				1.21	27.37	3.46	
GER	17	c	0	0.31	26.37	33.72	0.1100
GODLEY	5	m	1	1.92	96.28	22.25	0.0000
GODLEY	6	f	1	1.71	58.89	4.29	0.0000
Subtotal GODLEY				1.84	155.17	26.54	

Table 1A1 - 5

IESLC - Meta-analysis of Ever Smoking, Any product (or Cigarettes if Any not available)
All LC types
Least adjusted

REF	NRR	SEX	AD	Ys	Ws	Qs	Ps
GOLLED	21	m	0	1.84	13.92	2.23	0.0000
GOODMA	3	m	0	2.38	8.92	7.83	0.0000
GOODMA	7	f	0	2.12	12.25	5.55	0.0000
Subtotal GOODMA				2.23	21.17	13.39	
GRAHAM	22	m	0	2.05	16.52	6.10	0.0000
GREGOR	3	m	0	0.03	5.11	10.22	0.9492
GREGOR	7	f	0	2.40	0.90	0.82	0.0233
Subtotal GREGOR				0.38	6.01	11.04	
GSELL	8	m	0	2.88	1.82	3.74	0.0001
HAENSZ	50	f	0	0.77	25.35	11.61	0.0001
*HAMMO2	18	m	0	3.25	1.00	3.24	0.0012
*HAMMON	129	m	0	1.86	14.49	2.56	0.0000
*HANSEN	3	m	2	0.43	5.28	5.46	0.3285
HEGMAN	1	c	0	2.79	23.66	43.22	0.0000
*HEIN	7	m	0	2.68	1.00	1.53	0.0074
*HENNEK	3	m	0	1.83	19.94	3.01	0.0000
HINDS	26	f	0	1.42	61.37	0.04	0.0000
*HIRAYA	147	m	1	1.47	85.78	0.08	0.0000
*HIRAYA	150	f	1	0.86	80.63	27.44	0.0000
Subtotal HIRAYA				1.18	166.40	27.52	
HITOSU	6	m	0	1.01	6.47	1.20	0.0102
HITOSU	12	f	0	1.36	16.07	0.11	0.0000
Subtotal HITOSU				1.26	22.53	1.31	
*HOLE	15	m	0	1.69	6.79	0.41	0.0000
HOROWI	1	m	0	1.27	15.35	0.43	0.0000
HOROWI	2	f	0	0.60	8.08	5.76	0.0894
Subtotal HOROWI				1.04	23.43	6.19	
HORWIT	1	f	0	2.43	8.29	8.02	0.0000
HU	15	m	0	0.74	17.16	8.57	0.0023
HU	16	f	0	0.55	7.15	5.69	0.1413
Subtotal HU				0.68	24.31	14.26	
HU2	9	m	0	1.11	27.11	3.04	0.0000
HU2	10	f	0	0.63	21.91	14.50	0.0033
Subtotal HU2				0.89	49.01	17.53	
HUANG	1	c	0	0.69	14.82	8.37	0.0078
HUMBLE	14	m	1	2.49	5.19	5.74	0.0000
HUMBLE	16	m	1	2.47	1.71	1.82	0.0012
HUMBLE	18	f	1	2.43	6.68	6.53	0.0000
HUMBLE	20	f	1	2.73	2.90	4.84	0.0000
Subtotal HUMBLE				2.51	16.48	18.92	
JAHN	3	f	0	1.13	18.89	1.86	0.0000
JAIN	6	m	0	2.30	9.88	7.31	0.0000
JAIN	1	f	0	2.10	31.68	13.83	0.0000
Subtotal JAIN				2.15	41.55	21.14	
JARUP	3	m	0	1.90	6.90	1.44	0.0000
JARVHO	3	m	0	3.32	0.92	3.25	0.0015
JARVHO	7	f	0	2.26	3.28	2.18	0.0000
Subtotal JARVHO				2.49	4.19	5.43	
JEDRYC	63	m	0	1.76	36.14	3.62	0.0000
JEDRYC	68	f	0	2.08	17.12	6.90	0.0000
Subtotal JEDRYC				1.86	53.25	10.52	
JIANG	1	m	0	1.00	4.45	0.87	0.0346
JIANG	2	f	0	0.91	2.62	0.74	0.1401
Subtotal JIANG				0.97	7.08	1.60	
JOLY	14	m	0	2.50	11.02	12.32	0.0000
JOLY	1	f	0	1.99	27.09	8.25	0.0000
Subtotal JOLY				2.14	38.11	20.57	
JUSSAW	3	m	0	2.77	63.20	112.19	0.0000
*KAISE2	72	m	1	1.69	11.75	0.70	0.0000
*KAISE2	64	f	1	2.31	9.19	6.95	0.0000
Subtotal KAISE2				1.96	20.95	7.65	
*KAISER	13	m	2	2.87	25.69	52.36	0.0000
*KAISER	10	f	2	1.73	28.18	2.31	0.0000
Subtotal KAISER				2.27	53.88	54.67	
KATSOU	27	f	0	1.21	9.99	0.53	0.0001
KAUFMA	8	c	0	2.61	31.80	43.34	0.0000
KELLER	3	m	0	2.31	217.37	164.32	0.0000
KELLER	11	m	0	2.60	25.99	35.10	0.0000
KELLER	7	f	0	2.53	269.69	316.43	0.0000
KELLER	15	f	0	2.25	39.03	25.22	0.0000
Subtotal KELLER				2.43	552.08	541.07	

Table 1A1 - 5

IESLC - Meta-analysis of Ever Smoking, Any product (or Cigarettes if Any not available)
All LC types
Least adjusted

REF	NRR	SEX	AD	Ys	Ws	Qs	Ps
KHUDER	4	m	0	2.06	19.93	7.64	0.0000
KIHARA	31	c	0	1.22	46.97	2.33	0.0000
*KINLEN	6	m	0	2.36	6.97	5.94	0.0000
KJUUS	10	m	0	2.62	1.81	2.50	0.0004
*KNEKT	76	m	0	1.85	5.69	0.95	0.0000
KO	1	f	3	1.44	2.18	0.00	0.0339
KOHLME	1	c	0	2.83	9.55	18.40	0.0000
KOO	1	f	0	1.02	23.01	4.14	0.0000
KOULUM	1	m	0	3.57	4.47	20.18	0.0000
KREUZE	14	f	0	2.24	4.29	2.70	0.0000
KREUZE	16	f	0	1.33	32.30	0.40	0.0000
Subtotal KREUZE				1.44	36.60	3.11	
KREYBE	24	m	0	2.04	5.80	2.08	0.0000
KREYBE	39	f	0	-0.22	8.25	22.83	0.5245
Subtotal KREYBE				0.71	14.05	24.91	
*KUBIK	28	m	0	3.34	1.96	7.05	0.0000
LAMTH	6	f	0	1.34	46.55	0.51	0.0000
LAMWK	1	f	0	1.42	17.85	0.01	0.0000
LAMWK2	9	m	0	1.04	12.98	2.08	0.0002
LAMWK2	10	f	0	1.17	17.89	1.37	0.0000
Subtotal LAMWK2				1.11	30.86	3.45	
*LANGE	34	m	0	1.58	4.91	0.09	0.0005
*LANGE	31	f	0	1.22	6.30	0.31	0.0022
Subtotal LANGE				1.38	11.22	0.40	
LAUSSM	10	m	0	1.59	41.00	0.91	0.0000
LEI	1	m	0	1.30	26.63	0.51	0.0000
LEI	2	f	0	1.25	23.21	0.86	0.0000
Subtotal LEI				1.28	49.84	1.38	
LEMARC	3	c	0	1.73	22.77	1.87	0.0000
LETOUR	1	c	0	2.56	20.21	25.36	0.0000
LEVIN	32	m	1	1.58	30.68	0.59	0.0000
*LIDDEL	5	m	1	1.28	17.92	0.45	0.0000
LIU	2	c	2	0.65	38.19	23.82	0.0001
LIU2	1	m	0	1.46	8.60	0.00	0.0000
LIU2	3	f	0	1.45	9.73	0.00	0.0000
Subtotal LIU2				1.46	18.32	0.00	
LIU3	1	m	0	0.19	3.06	4.83	0.7444
LIU4	11	m	2	1.02	5969.41	1087.70	0.0000
LIU4	12	f	2	1.05	3876.64	593.49	0.0000
Subtotal LIU4				1.03	9846.05	1681.20	
LIU5	1	c	0	0.65	11.25	7.06	0.0293
LOMBA2	1	f	0	0.28	37.19	49.95	0.0841
LOMBAR	12	m	0	2.18	12.13	6.60	0.0000
LUBIN2	45	m	0	2.15	169.89	84.14	0.0000
LUBIN2	101	f	0	1.24	134.74	5.43	0.0000
Subtotal LUBIN2				1.75	304.63	89.58	
LUO	1	c	0	0.66	18.01	11.16	0.0054
MACLEN	71	m	0	1.28	3.54	0.10	0.0163
MACLEN	72	f	0	0.74	13.64	6.70	0.0062
Subtotal MACLEN				0.85	17.18	6.79	
*MAGNUS	1	m	0	1.69	10.53	0.65	0.0000
MARSH	2	m	0	2.28	1.82	1.29	0.0021
MARSH	4	f	0	1.76	5.60	0.57	0.0000
Subtotal MARSH				1.89	7.42	1.86	
MARSH2	2	c	0	1.16	8.50	0.67	0.0007
MARTIS	4	m	0	1.95	3.32	0.84	0.0004
MASTRA	1	m	0	2.13	5.09	2.38	0.0000
MATOS	26	m	0	1.89	9.19	1.87	0.0000
MATSUD	10	m	0	3.07	2.94	7.75	0.0000
MCCONN	1	m	0	0.19	3.33	5.19	0.7237
MCCONN	2	f	0	1.01	0.99	0.18	0.3136
Subtotal MCCONN				0.38	4.32	5.37	
MCDUFF	1	m	0	1.81	4.70	0.65	0.0001
MCLAUG	1	m	0	1.20	18.70	1.06	0.0000
*MIGRAN	26	m	0	1.89	3.94	0.81	0.0002
*MIGRAN	41	f	0	1.98	3.55	1.02	0.0002
Subtotal MIGRAN				1.93	7.49	1.83	
MILLER	1	f	0	2.43	22.85	22.15	0.0000
MILLS	3	m	1	0.29	96.62	128.58	0.0046
*MRFITR	6	m	0	3.70	0.50	2.54	0.0091
NAM	69	m	0	2.29	26.44	18.83	0.0000

Table 1A1 - 5

IESLC - Meta-analysis of Ever Smoking, Any product (or Cigarettes if Any not available)
All LC types
Least adjusted

REF	NRR	SEX	AD	Ys	Ws	Qs	Ps
NAM	85	f	0	2.30	38.76	28.83	0.0000
Subtotal NAM				2.30	65.19	47.66	
NOTAN2	1	m	0	1.11	80.11	8.87	0.0000
NOU	11	m	0	1.81	5.20	0.69	0.0000
NOU	12	f	0	1.96	2.74	0.73	0.0012
Subtotal NOU				1.86	7.94	1.42	
ODRISC	3	c	0	3.89	5.83	34.94	0.0000
ORMOS	4	m	0	2.23	6.39	4.01	0.0000
ORMOS	26	f	0	-1.64	0.95	9.06	0.1093
Subtotal ORMOS				1.73	7.34	13.07	
OSANN	17	m	0	3.00	39.51	96.28	0.0000
OSANN	21	f	0	2.70	69.13	109.68	0.0000
Subtotal OSANN				2.81	108.65	205.96	
PARKIN	29	m	0	1.54	73.35	0.64	0.0000
PASTOR	5	m	0	1.89	8.32	1.64	0.0000
PAWLEG	1	m	0	2.77	3.69	6.47	0.0000
PERNU	2	m	0	2.19	58.99	32.97	0.0000
PERNU	1	f	0	0.63	13.52	8.83	0.0198
Subtotal PERNU				1.90	72.50	41.80	
PERSH2	5	c	0	1.79	114.36	13.63	0.0000
*PETO	5	m	0	1.82	1.98	0.28	0.0107
PEZZO2	10	m	0	2.71	5.55	8.91	0.0000
PEZZOT	25	m	0	2.96	3.75	8.65	0.0000
PIKE	4	m	0	1.66	13.39	0.63	0.0000
PIKE	8	f	0	1.57	18.04	0.32	0.0000
Subtotal PIKE				1.61	31.43	0.95	
POFFIJ	1	c	0	2.05	46.21	16.95	0.0000
POLEDN	3	c	0	2.13	10.07	4.70	0.0000
*QIAO2	3	m	0	0.86	9.73	3.27	0.0072
RACHTA	3	f	0	1.77	13.24	1.42	0.0000
RADZIK	1	c	0	0.27	5.03	6.89	0.5411
RANDIG	23	m	0	1.61	3.99	0.12	0.0013
RANDIG	24	f	0	0.80	6.34	2.63	0.0447
Subtotal RANDIG				1.11	10.32	2.75	
REN	1	m	0	1.27	7.46	0.21	0.0005
REN	2	f	0	1.40	9.65	0.02	0.0000
Subtotal REN				1.35	17.11	0.23	
RONCO	1	m	0	1.63	5.23	0.18	0.0002
ROTHSC	1	c	0	1.76	9.88	1.01	0.0000
SADOWS	7	m	0	1.35	13.90	0.11	0.0000
SANKAR	1	m	0	2.75	23.11	39.60	0.0000
SCHWAR	1	m	0	2.11	80.50	35.42	0.0000
SCHWAR	2	m	0	1.88	29.06	5.47	0.0000
SCHWAR	3	f	0	2.30	111.43	81.81	0.0000
SCHWAR	4	f	0	2.45	26.58	26.86	0.0000
Subtotal SCHWAR				2.20	247.58	149.56	
SEGI	1	m	0	0.53	15.18	12.52	0.0375
SEOW	1	f	0	1.71	9.81	0.70	0.0000
SHAW	12	c	0	2.47	9.34	9.91	0.0000
SIEMIA	9	m	0	2.77	11.12	19.54	0.0000
SIMARA	5	m	0	0.70	13.62	7.59	0.0103
SIMARA	6	f	0	0.85	9.72	3.45	0.0084
Subtotal SIMARA				0.76	23.33	11.04	
SOBUE	91	m	0	1.34	25.52	0.29	0.0000
SOBUE	95	f	0	1.03	51.71	8.66	0.0000
Subtotal SOBUE				1.13	77.23	8.95	
*SPEIZE	8	f	0	1.96	52.33	13.85	0.0000
SPITZ	3	c	0	2.91	6.16	13.30	0.0000
STASZE	1	m	0	2.37	4.73	4.06	0.0000
STASZE	5	f	0	1.47	4.16	0.00	0.0028
Subtotal STASZE				1.95	8.88	4.06	
STAYNE	1	m	0	1.30	40.37	0.83	0.0000
STOCKS	31	m	0	1.75	41.11	3.93	0.0000
STOCKS	50	f	1	1.11	58.11	6.34	0.0000
Subtotal STOCKS				1.38	99.22	10.26	
STOCKW	6	c	0	2.35	1549.52	1266.93	0.0000
STUCKE	3	m	0	4.83	0.49	5.66	0.0007
SUN	1	c	0	0.84	30.23	11.11	0.0000
SUZUK2	18	c	0	2.04	7.52	2.71	0.0000
SVENSS	56	f	0	1.81	19.34	2.60	0.0000
TANG	3	c	0	2.09	6.14	2.57	0.0000

Table 1A1 - 5

IESLC - Meta-analysis of Ever Smoking, Any product (or Cigarettes if Any not available)
All LC types
Least adjusted

REF	NRR	SEX	AD	Ys	Ws	Qs	Ps
*TENKAN	22	m	1	2.68	5.38	8.30	0.0000
TIZZAN	1	m	0	0.66	91.77	56.72	0.0000
TIZZAN	12	f	0	1.40	8.03	0.01	0.0001
Subtotal TIZZAN				0.72	99.80	56.73	
TOKARS	3	m	0	3.47	0.97	3.98	0.0006
TOKARS	5	f	0	0.43	0.62	0.64	0.7336
Subtotal TOKARS				2.28	1.60	4.62	
TOUSEY	16	m	0	3.10	3.80	10.37	0.0000
TOUSEY	26	f	0	2.75	10.96	18.82	0.0000
Subtotal TOUSEY				2.84	14.76	29.20	
TSUGAN	27	m	0	0.23	7.76	11.44	0.5244
*TULINI	15	m	1	2.09	10.32	4.29	0.0000
*TULINI	21	f	1	2.70	11.08	17.66	0.0000
Subtotal TULINI				2.41	21.40	21.95	
*TVERDA	22	m	2	1.52	20.49	0.13	0.0000
WAKAI	13	m	0	1.28	8.20	0.21	0.0002
WAKAI	31	f	0	1.27	11.70	0.35	0.0000
Subtotal WAKAI				1.27	19.90	0.56	
WANG	1	m	0	1.24	14.89	0.58	0.0000
WANG	2	f	0	1.39	3.11	0.01	0.0145
Subtotal WANG				1.27	18.00	0.59	
WANG2	8	c	0	0.86	7.10	2.38	0.0216
WANG3	1	c	0	1.05	28.11	4.38	0.0000
WANG4	1	m	0	0.07	107.42	202.00	0.4630
WICKLU	1	m	0	1.53	15.41	0.11	0.0000
WIGLE	15	m	0	2.32	13.42	10.38	0.0000
WIGLE	18	f	0	1.41	21.29	0.02	0.0000
Subtotal WIGLE				1.76	34.71	10.40	
WILKIN	1	m	0	3.22	1.93	6.13	0.0000
WILKIN	2	f	0	1.74	8.65	0.78	0.0000
Subtotal WILKIN				2.01	10.58	6.91	
WU	37	f	0	1.48	17.78	0.02	0.0000
WUNSCH	1	m	0	1.54	11.46	0.10	0.0000
WUNSCH	7	f	0	1.48	15.11	0.02	0.0000
Subtotal WUNSCH				1.50	26.58	0.12	
WUWILL	6	f	0	0.79	114.07	47.85	0.0000
WYNDE2	21	m	0	2.13	7.21	3.44	0.0000
WYNDE3	49	m	0	2.09	7.74	3.27	0.0000
WYNDE3	138	f	0	1.14	9.73	0.90	0.0004
Subtotal WYNDE3				1.56	17.48	4.17	
WYNDE4	48	m	0	2.21	10.51	6.19	0.0000
WYNDE4	62	f	2	1.05	8.80	1.32	0.0018
Subtotal WYNDE4				1.68	19.31	7.51	
WYNDE6	72	m	0	2.31	71.58	53.38	0.0000
WYNDE6	252	f	0	2.34	103.92	84.10	0.0000
Subtotal WYNDE6				2.33	175.50	137.47	
*XIANGZ	8	m	0	1.00	25.00	4.95	0.0000
XU	1	m	0	0.97	57.31	12.98	0.0000
XU2	1	c	0	1.38	53.87	0.24	0.0000
XU3	1	m	0	1.79	4.98	0.60	0.0001
XU3	3	f	0	1.39	3.98	0.01	0.0055
Subtotal XU3				1.61	8.96	0.61	
XU4	1	c	0	1.08	20.82	2.73	0.0000
YAMAGU	5	c	0	1.18	17.80	1.19	0.0000
*YONG	2	c	1	1.91	22.68	4.93	0.0000
*YUAN	1	m	2	1.87	11.44	2.11	0.0000
ZHANG	1	c	0	0.90	14.37	4.15	0.0006
ZHENG	15	m	0	1.29	20.36	0.45	0.0000
ZHENG	24	f	0	0.74	20.88	10.36	0.0008
Subtotal ZHENG				1.01	41.24	10.81	
ZHOU	2	m	0	0.86	17.50	5.93	0.0003
ZHOU	3	f	0	0.80	5.34	2.23	0.0660
Subtotal ZHOU				0.84	22.83	8.16	

Table 1A1 - 5

IESLC - Meta-analysis of Ever Smoking, Any product (or Cigarettes if Any not available)
All LC types
Least adjusted

	N	331
	NS	236
	Wt	19662.90
Het	Chi	7709.92
Het	df	330
Het	P	***
Fixed	RR	4.23
	RRl	4.17
	RRu	4.29
	P	+++
Random	RR	5.48
	RRl	5.06
	RRu	5.93
	P	+++
Asymm	P	***

Table 1A1 - 6

IESLC - Meta-analysis of Ever Smoking, Any product (or Cigarettes if Any not available)
All LC types
Least adjusted

	Sex: combined	male	female	Total
N	46	174	111	331
NS	46	170	106	322
Wt	2489.29	10000.22	7173.39	19662.90
Het Chi	767.25	3320.87	2433.55	7709.92
Het df	45	173	110	330
Het P	***	***	***	***
Fixed RR	8.05	3.79	3.95	4.23
RRl	7.74	3.71	3.85	4.17
RRu	8.37	3.86	4.04	4.29
P	+++	+++	+++	+++
Random RR	5.79	6.32	4.36	5.48
RRl	4.67	5.61	3.79	5.06
RRu	7.17	7.11	5.02	5.93
P	+++	+++	+++	+++
Between Chi				1188.24
Between df				2
Between P				***
Btwn(F) P				***
Btwn(R) P				***

	Lung cancer type: all	other	Total
N	320	11	331
NS	228	8	236
Wt	19458.89	204.01	19662.90
Het Chi	7665.57	34.94	7709.92
Het df	319	10	330
Het P	***	***	***
Fixed RR	4.24	3.42	4.23
RRl	4.18	2.98	4.17
RRu	4.30	3.92	4.29
P	+++	+++	+++
Random RR	5.56	3.67	5.48
RRl	5.12	2.80	5.06
RRu	6.03	4.80	5.93
P	+++	+++	+++
Between Chi			9.40
Between df			1
Between P			**
Btwn(F) P			N.S.
Btwn(R) P			**

	Location: NAmer	UK	Scand	othEur	China	Japan	othAs	other	Total
N	116	30	32	51	51	18	19	14	331
NS	81	20	23	39	35	12	14	12	236
Wt	5613.78	390.73	502.07	1094.04	10962.85	469.00	405.77	224.67	19662.90
Het Chi	1207.69	150.94	123.24	472.46	189.20	49.61	237.20	28.60	7709.92
Het df	115	29	31	50	50	17	18	13	330
Het P	***	***	***	***	***	***	***	**	***
Fixed RR	8.83	5.01	6.20	5.56	2.77	3.25	4.23	6.30	4.23
RRl	8.60	4.54	5.68	5.24	2.72	2.96	3.83	5.53	4.17
RRu	9.06	5.53	6.77	5.90	2.82	3.55	4.66	7.19	4.29
P	+++	+++	+++	+++	+++	+++	+++	+++	+++
Random RR	7.55	5.88	6.09	6.13	2.66	3.21	3.81	7.07	5.48
RRl	6.83	4.58	4.99	4.96	2.48	2.70	2.62	5.69	5.06
RRu	8.34	7.56	7.42	7.57	2.86	3.82	5.54	8.79	5.93
P	+++	+++	+++	+++	+++	+++	+++	+++	+++
Between Chi									5250.98
Between df									7
Between P									***
Btwn(F) P									***
Btwn(R) P									***

Table 1A1 - 6

IESLC - Meta-analysis of Ever Smoking, Any product (or Cigarettes if Any not available)
All LC types
Least adjusted

Detailed Country in "other Europe"

	multi	Germany	othWest	East	Balkans	Total
N	4	17	13	14	3	51
NS	3	12	12	9	3	39
Wt	456.31	234.69	174.11	124.07	104.86	1094.04
Het Chi	133.70	71.89	143.82	41.71	0.90	472.46
Het df	3	16	12	13	2	50
Het P	***	***	***	***	N.S.	***
Fixed RR	7.37	4.58	3.96	6.18	3.88	5.56
RRl	6.73	4.03	3.41	5.19	3.20	5.24
RRu	8.08	5.20	4.59	7.37	4.70	5.90
P	+++	+++	+++	+++	+++	+++
Random RR	7.65	5.23	8.80	6.25	3.88	6.13
RRl	4.07	3.79	4.70	4.33	3.20	4.96
RRu	14.36	7.22	16.49	9.01	4.70	7.57
P	+++	+++	+++	+++	+++	+++
Between Chi						80.44
Between df						4
Between P						***
Btwn(F) P						(*)
Btwn(R) P						*

Detailed Country in "other Asia"

	India	HongKong	other	Total
N	3	7	9	19
NS	3	5	6	14
Wt	166.42	140.70	98.65	405.77
Het Chi	114.34	10.74	21.68	237.20
Het df	2	6	8	18
Het P	***	(*)	**	***
Fixed RR	7.17	3.52	2.25	4.23
RRl	6.16	2.99	1.85	3.83
RRu	8.34	4.15	2.74	4.66
P	+++	+++	+++	+++
Random RR	9.08	3.56	2.50	3.81
RRl	2.71	2.81	1.77	2.62
RRu	30.40	4.50	3.53	5.54
P	+++	+++	+++	+++
Between Chi				90.44
Between df				2
Between P				***
Btwn(F) P				*
Btwn(R) P				(*)

Detailed other continent

	SCAmer	Auslia	Africa	Total
N	10	1	3	14
NS	8	1	3	12
Wt	126.26	0.99	97.42	224.67
Het Chi	16.04	0.00	1.48	28.60
Het df	9	0	2	13
Het P	(*)	N.S.	N.S.	**
Fixed RR	7.53	20.29	4.95	6.30
RRl	6.33	2.84	4.06	5.53
RRu	8.97	145.07	6.03	7.19
P	+++	++	+++	+++
Random RR	7.74	20.29	4.95	7.07
RRl	6.07	2.84	4.06	5.69
RRu	9.86	145.07	6.03	8.79
P	+++	++	+++	+++
Between Chi				11.09
Between df				2
Between P				**
Btwn(F) P				(*)
Btwn(R) P				**

Table 1A1 - 6

IESLC - Meta-analysis of Ever Smoking, Any product (or Cigarettes if Any not available)
All LC types
Least adjusted

	Start year of study <1960	1960-69	1970-79	1980-89	1990+	Total
N	54	53	72	113	39	331
NS	39	38	52	75	32	236
Wt	942.32	1364.28	1417.05	15405.00	534.26	19662.90
Het Chi	489.79	470.95	622.40	5730.46	289.24	7709.92
Het df	53	52	71	112	38	330
Het P	***	***	***	***	***	***
Fixed RR	3.96	5.25	4.57	4.11	5.09	4.23
RRl	3.72	4.98	4.34	4.05	4.68	4.17
RRu	4.22	5.53	4.81	4.18	5.54	4.29
P	+++	+++	+++	+++	+++	+++
Random RR	4.59	5.42	5.17	5.86	6.23	5.48
RRl	3.70	4.55	4.37	5.11	4.87	5.06
RRu	5.71	6.46	6.12	6.73	7.97	5.93
P	+++	+++	+++	+++	+++	+++
Between Chi						107.08
Between df						4
Between P						***
Btwn(F) P						N.S.
Btwn(R) P						N.S.

	Study type (1) CC	other	Total
N	264	67	331
NS	185	51	236
Wt	18370.92	1291.98	19662.90
Het Chi	7143.76	387.29	7709.92
Het df	263	66	330
Het P	***	***	***
Fixed RR	4.12	6.06	4.23
RRl	4.06	5.74	4.17
RRu	4.18	6.40	4.29
P	+++	+++	+++
Random RR	5.29	6.28	5.48
RRl	4.84	5.40	5.06
RRu	5.79	7.30	5.93
P	+++	+++	+++
Between Chi			178.87
Between df			1
Between P			***
Btwn(F) P			**
Btwn(R) P			(*)

	Study type (2) CC	prosp	other	Total
N	264	61	6	331
NS	185	47	4	236
Wt	18370.92	1253.10	38.88	19662.90
Het Chi	7143.76	364.21	13.90	7709.92
Het df	263	60	5	330
Het P	***	***	*	***
Fixed RR	4.12	5.97	9.78	4.23
RRl	4.06	5.65	7.14	4.17
RRu	4.18	6.31	13.39	4.29
P	+++	+++	+++	+++
Random RR	5.29	6.08	9.53	5.48
RRl	4.84	5.21	5.20	5.06
RRu	5.79	7.10	17.49	5.93
P	+++	+++	+++	+++
Between Chi				188.04
Between df				2
Between P				***
Btwn(F) P				*
Btwn(R) P				(*)

Table 1A1 - 6

IESLC - Meta-analysis of Ever Smoking, Any product (or Cigarettes if Any not available)
All LC types
Least adjusted

	Study size (number of LC cases) 100-249	250-499	500-999	1000+	Total
N	116	88	64	63	331
NS	96	63	43	34	236
Wt	1024.51	1310.54	1573.81	15754.05	19662.90
Het Chi	512.48	599.24	647.12	5856.28	7709.92
Het df	115	87	63	62	330
Het P	***	***	***	***	***
Fixed RR	3.77	4.74	5.12	4.14	4.23
RRl	3.54	4.49	4.87	4.08	4.17
RRu	4.00	5.00	5.38	4.21	4.29
P	+++	+++	+++	+++	+++
Random RR	4.42	5.68	6.19	6.19	5.48
RRl	3.84	4.89	5.25	5.20	5.06
RRu	5.08	6.60	7.30	7.38	5.93
P	+++	+++	+++	+++	+++
Between Chi					94.80
Between df					3
Between P					***
Btwn(F) P					N.S.
Btwn(R) P					**

	Risky occupational population no	mining	othRisky	Total
N	312	7	12	331
NS	218	7	11	236
Wt	19444.00	76.23	142.68	19662.90
Het Chi	7667.01	10.01	28.73	7709.92
Het df	311	6	11	330
Het P	***	N.S.	**	***
Fixed RR	4.23	3.55	4.73	4.23
RRl	4.17	2.83	4.02	4.17
RRu	4.29	4.44	5.58	4.29
P	+++	+++	+++	+++
Random RR	5.50	3.79	5.46	5.48
RRl	5.06	2.77	3.90	5.06
RRu	5.97	5.18	7.63	5.93
P	+++	+++	+++	+++
Between Chi				4.17
Between df				2
Between P				N.S.
Btwn(F) P				N.S.
Btwn(R) P				(*)

	National cigarette tobacco type Virginia	blended	other	Total
N	51	227	53	331
NS	37	162	37	236
Wt	864.47	7807.04	10991.40	19662.90
Het Chi	364.32	2575.23	202.71	7709.92
Het df	50	226	52	330
Het P	***	***	***	***
Fixed RR	5.88	7.43	2.76	4.23
RRl	5.50	7.27	2.71	4.17
RRu	6.29	7.60	2.81	4.29
P	+++	+++	+++	+++
Random RR	6.38	6.27	2.63	5.48
RRl	5.24	5.75	2.44	5.06
RRu	7.78	6.82	2.83	5.93
P	+++	+++	+++	+++
Between Chi				4567.65
Between df				2
Between P				***
Btwn(F) P				***
Btwn(R) P				***

Table 1A1 - 6

IESLC - Meta-analysis of Ever Smoking, Any product (or Cigarettes if Any not available)
All LC types
Least adjusted

	Any proxy use No/nk	Yes	Total
N	230	101	331
NS	169	67	236
Wt	7471.33	12191.57	19662.90
Het Chi	3012.48	1973.63	7709.92
Het df	229	100	330
Het P	***	***	***
Fixed RR	6.80	3.16	4.23
RRl	6.65	3.10	4.17
RRu	6.96	3.22	4.29
P	+++	+++	+++
Random RR	5.45	5.45	5.48
RRl	4.97	4.88	5.06
RRu	5.98	6.08	5.93
P	+++	+++	+++
Between Chi			2723.81
Between df			1
Between P			***
Btwn(F) P			***
Btwn(R) P			N.S.

	Full histological confirmation No	Yes	Total
N	246	85	331
NS	177	59	236
Wt	17096.50	2566.40	19662.90
Het Chi	5856.75	862.70	7709.92
Het df	245	84	330
Het P	***	***	***
Fixed RR	3.88	7.55	4.23
RRl	3.82	7.26	4.17
RRu	3.94	7.85	4.29
P	+++	+++	+++
Random RR	5.26	6.18	5.48
RRl	4.81	5.36	5.06
RRu	5.74	7.13	5.93
P	+++	+++	+++
Between Chi			990.47
Between df			1
Between P			***
Btwn(F) P			***
Btwn(R) P			(*)

	Number of adjustment variables (1) 0	1	2+/+nk	Total
N	288	26	17	331
NS	209	17	13	239
Wt	7621.30	1085.10	10956.50	19662.90
Het Chi	3463.49	454.29	1648.03	7709.92
Het df	287	25	16	330
Het P	***	***	***	***
Fixed RR	6.26	5.16	3.16	4.23
RRl	6.12	4.86	3.10	4.17
RRu	6.40	5.47	3.22	4.29
P	+++	+++	+++	+++
Random RR	5.44	6.13	5.20	5.48
RRl	4.99	4.66	3.95	5.06
RRu	5.93	8.06	6.83	5.93
P	+++	+++	+++	+++
Between Chi				2144.10
Between df				2
Between P				***
Btwn(F) P				***
Btwn(R) P				N.S.

Table 1A1 - 6

IESLC - Meta-analysis of Ever Smoking, Any product (or Cigarettes if Any not available)
All LC types
Least adjusted

	Number of adjustment variables (2) 0	1	2	3-5	6+/+nk	Total
N	288	26	15	2		331
NS	209	17	11	2		239
Wt	7621.30	1085.10	10950.05	6.46		19662.90
Het Chi	3463.49	454.29	1647.45	0.00		7709.92
Het df	287	25	14	1		330
Het P	***	***	***	N.S.		***
Fixed RR	6.26	5.16	3.16	4.26		4.23
RRl	6.12	4.86	3.10	1.97		4.17
RRu	6.40	5.47	3.22	9.21		4.29
P	+++	+++	+++	+++		+++
Random RR	5.44	6.13	5.27	4.26		5.48
RRl	4.99	4.66	3.97	1.97		5.06
RRu	5.93	8.06	7.00	9.21		5.93
P	+++	+++	+++	+++		+++
Between Chi						2144.68
Between df						3
Between P						***
Btwn(F) P						***
Btwn(R) P						N.S.

	Product all/unsp	cig+/-ot	cig only	Total
N	206	116	9	331
NS	154	81	8	243
Wt	15724.80	3706.63	231.48	19662.90
Het Chi	5190.47	1499.71	84.67	7709.92
Het df	205	115	8	330
Het P	***	***	***	***
Fixed RR	3.79	6.49	7.29	4.23
RRl	3.73	6.29	6.41	4.17
RRu	3.85	6.70	8.29	4.29
P	+++	+++	+++	+++
Random RR	5.39	5.56	6.08	5.48
RRl	4.88	4.90	3.80	5.06
RRu	5.95	6.32	9.71	5.93
P	+++	+++	+++	+++
Between Chi				935.07
Between df				2
Between P				***
Btwn(F) P				***
Btwn(R) P				N.S.

	Denominator nev any	nev cigs	Total
N	237	94	331
NS	171	68	239
Wt	16531.10	3131.81	19662.90
Het Chi	5555.56	1246.53	7709.92
Het df	236	93	330
Het P	***	***	***
Fixed RR	3.85	6.93	4.23
RRl	3.79	6.69	4.17
RRu	3.91	7.18	4.29
P	+++	+++	+++
Random RR	5.30	5.91	5.48
RRl	4.84	5.13	5.06
RRu	5.81	6.80	5.93
P	+++	+++	+++
Between Chi			907.83
Between df			1
Between P			***
Btwn(F) P			***
Btwn(R) P			N.S.

Table 1A1 - 6

IESLC - Meta-analysis of Ever Smoking, Any product (or Cigarettes if Any not available)
All LC types
Least adjusted

	Derivation of RR/CI			
	Orig	StdCalc	Other	Total
N	10	276	45	331
NS	7	201	32	240
Wt	1043.24	7428.99	11190.67	19662.90
Het Chi	281.34	3353.60	1109.23	7709.92
Het df	9	275	44	330
Het P	***	***	***	***
Fixed RR	8.66	6.31	3.03	4.23
RRl	8.15	6.17	2.98	4.17
RRu	9.21	6.46	3.09	4.29
P	+++	+++	+++	+++
Random RR	4.23	5.47	5.65	5.48
RRl	2.76	5.01	4.90	5.06
RRu	6.49	5.97	6.53	5.93
P	+++	+++	+++	+++
Between Chi				2965.75
Between df				2
Between P				***
Btwn(F) P				***
Btwn(R) P				N.S.

	Study LIU4		
	LIU4	others	Total
N	2	329	331
NS	1	235	236
Wt	9846.05	9816.85	19662.90
Het Chi	2.98	4345.51	7709.92
Het df	1	328	330
Het P	(*)	***	***
Fixed RR	2.80	6.40	4.23
RRl	2.74	6.27	4.17
RRu	2.85	6.53	4.29
P	+++	+++	+++
Random RR	2.81	5.50	5.48
RRl	2.71	5.08	5.06
RRu	2.91	5.96	5.93
P	+++	+++	+++
Between Chi			3361.43
Between df			1
Between P			***
Btwn(F) P			***
Btwn(R) P			***

Table 1A1 - 7

IESLC - Meta-analysis of Ever Smoking, Any product (or Cigarettes if Any not available)
All LC types
Excluded studies (and stage at which they were excluded)

1	BOUCHA BUELL LAURIL MZILEN
2	BYERS2 ENSTRO HIRAY2 KANELL LIAW LICKIN MRFIT MURATA NOTANI RESTRE RIMING SAARIK SEGI2 SHIMIZ SITAS SOBUE2 TANG2 WALD WARSIN WATSON WU2 WYNDE5 WYNDE8 WYNDER
3	BERRIN CHEN TAO ULMER
6	BLOT1 BLOT2 BLOT3 HIRAOK JONES MOLLO PERSHA SCHWA2 VANDER
8	BROWN1 BYERS1 SUZUKI
10	BENHAM GILLIS ISHIMA LUBIN OSANN2 PISANI PRESCO QIAO ROOTS SIMONA VEIERO VUTUC WYNDE7

Table 1A1 - 8
Potentially overlapping studies

REF	REFGP	PRINC	OVERLAP/LINK
LUBIN2	LUBIN2	1	Lubin-combined
CHAN	CHAN	1	Chan/LamWK2
LAMWK2	LAMWK2	1	Chan/LamWK2
LAMTH	LAMTH	1	KOO/LAMTH/LAMWK
KOO	KOO	1	KOO/LAMTH/LAMWK
LAMWK	LAMWK	1	KOO/LAMTH/LAMWK
CASCOR	CASCOR	1	Roots/Cascor 1
TVERDA	TVERDA	1	VEIERO/TVERDAL
AKIBA	AKIBA	1	AKIBA/ISHIMA
HEIN	PRESCO	1	Subset of PRESCO
LANGE	PRESCO	1	Subset of PRESCO
BROSS	BYERS1	1	GRAHAM/BROSS/BYERS1
GRAHAM	BYERS1	1	GRAHAM/BROSS/BYERS1
CHYOU	CHYOU	1	GOODMA/CHYOU
GOODMA	GOODMA	1	GOODMA/CHYOU
HOLE	TANG2	1	Subset of TANG2
BENSHL	TANG2	1	Subset of TANG2
KAISER	KAISER	1	KAISER/OSANN2
MRFITR	MRFIT	2	Subset of MRFIT
WYNDE6	WYNDE6	1	WYNDE5/6/7/8
CPSI	CPSI	1	CPSI overall
MATSUD	MATSUD	1	SOBUE2/MATSUD
BOFFET	BOFFET	1	BOFFET-combined
JAHN	BOFFET	2	Subset of BOFFET
XIANGZ	XIANGZ	1	LUBIN/XIANGZ/QIAO

Table 1A1 - 9
Most adjusted - insufficient data for metaanalysis

REF	NRR	SEX	AGEL	AGEH	RACE	YF	LC TYPE	LOC	START	ST	NLC	R	VB	P	H	AD	PRODUCT	DENOM	De
DOLL2	43	f	0	0	all	11	all	Eu:UK	1951	pr	920	n	V	n	n	1	all/unsp	nev any	st
TAO	1	m	0	0	all	-	all	As:Chi	1988	CC	723	n	ot	n	n	5	all/unsp	nev any	or
TAO	2	f	0	0	all	-	all	As:Chi	1988	CC	723	n	ot	n	n	5	all/unsp	nev any	or
ULMER	1	c	0	0	all	-	all	Eu:Ger	1971	CC	726	n	bl	y	n	0	all/unsp	nev any	or

REF	NRR	RR	SIG	RRDATA comment
DOLL2	43	4.33		0
TAO	1	3.95		0
TAO	2	4.93		0
ULMER	1	12.72		0

Least adjusted - insufficient data for meta-analysis: as for adjusted plus the following

REF	NRR	SEX	AGEL	AGEH	RACE	YF	LC TYPE	LOC	START	ST	NLC	R	VB	P	H	AD	PRODUCT	DENOM	De
CORREA	59	m	0	0	wh	-	all	NAmer	1979	CC	1359	n	bl	y	n	0	cig+/-ot	nev cigs	or
CORREA	61	m	0	0	bl	-	all	NAmer	1979	CC	1359	n	bl	y	n	0	cig+/-ot	nev cigs	or
CORREA	60	f	0	0	wh	-	all	NAmer	1979	CC	1359	n	bl	y	n	0	cig+/-ot	nev cigs	or
CORREA	62	f	0	0	bl	-	all	NAmer	1979	CC	1359	n	bl	y	n	0	cig+/-ot	nev cigs	or
LIU	1	c	0	0	all	-	all	As:Chi	1980	CC	229	n	ot	*	n	0	all/unsp	nev any	or

REF	NRR	RR	SIG	RRDATA comment
CORREA	59	17.20		0
CORREA	61	13.10		0
CORREA	60	8.60		0

Table 1A1 - 9

IESLC - Meta-analysis of Ever Smoking, Any product (or Cigarettes if Any not available)
All LC types
Least adjusted - insufficient data for meta-analysis: as for adjusted plus the following

REF	NRR	RR	SIG	RRDATA comment
CORREA	62	22.00		0
LIU	1	2.45		0

Table 1A2 -

IESLC - Meta-analysis of Ever Smoking, Cigarettes (or Any Product if Cigarettes not available)
All LC types

This analysis is restricted to results for:
1) Non-dose-response data
2) Ever smokers
3) Results complete enough for use in metaanalysis

Within each study, results are then selected (in the following order of preference, within each sex) for:
4) PRODUCT: cigarettes regardless of other products, cigarettes only, all/unspec
5) CIGTYPE: all/unspecified, MC regardless of HR, MC only
6) DENOM: never smoked anything, never smoked cigarettes, (never +1 = +long term ex, +2 = +amount unknown, +3 = never cigs+long term ex)
7) Followup period (YF, prospective studies): whole study (coded as 0) or longest available
8) LCtype: all or nearest available, at least Squamous and Adeno. (q = squamous, s = small, l = large, a = adeno, mix = mixed, alv = alveolar)
9) Race: all or nearest available, otherwise by race (wh or w = white, bl or b = black, hi = hispanic ch = chinese, jap = japanese, haw = hawaiian, w+o = white + oriental, sca = scandinavian, as = asian)
10) For overlapping studies: principal rather than subsidiary studies
Finally by Age: whole study (coded as 0) if available, otherwise by widest available age group
and then for single sex results (m, f) in preference to combined sex results (c).

Results adjusted (AD) for the most potential confounders are then chosen in Sections -1 to -3
(and those which actually differ from the adjusted results in Table 1A1 - 1 are marked 'x' in Section -1)
and results adjusted for the least confounders in Sections -4 to -6. (Those least adjusted results which
actually differ from the most adjusted as marked 'x' in column X in Section -4)
(Results adjusted for an unknown number of confounder(s) are coded as 20.)

Section -7 shows excluded studies, together with the stage (as above) at which no qualifying
results were found.

Section -8 lists the potentially overlapping studies which have been included (1=principal, 2=subsidiary).

Section -9 lists any results which would have been included in preference except that they had data not complete
enough for use in meta-analysis, with their significance (yes/no), if known, and any further comment as entered
on the database.

In addition to those mentioned above, the following fields, levels and abbreviations are used:
* or nk = not known, n = no, y = yes, ot = other
nev = never
all/unspec = all or unspecified, cig+/-ot = cigarettes irrespective of other products (cigar, pipe etc)
MC = manufactured cigarettes, HR = hand-rolled cigarettes
REF: 6-character study reference
NRR: number of the RR on the database within the study
ST : study type (CC = case control, pr or prosp = prospective)
NLC: number of lung cancer cases in whole study
R : risky occupational population (n = no, m = mining, o = other risky)
VB : national cigarette type (V = at least 75% Virginia, bl = at least 75% blended, ot = other)
P : any proxy use
H : full histological confirmation
De : derivation of RR/CI (or = original, st = standard method, ot = other method of estimation)

Table 1A2 - 1

IESLC - Meta-analysis of Ever Smoking, Cigarettes (or Any Product if Cigarettes not available)
All LC types
Most adjusted

REF	NRR	1A1	SEX	AGEL	AGEH	RACE	YF	LC TYPE	LOC	START	ST	NLC	R	VB	P	H	AD	PRODUCT	DENOM	De
ABELIN	45	x	m	0	0	all	-	all	Eu:wst	1941	CC	118	n	bl	y	n	1	cig+/-ot	nev any	st
ABRAHA	7		m	0	0	all	0	q+s+a	Eu:est	1975	pr	571	n	bl	n	n	0	all/unsp	nev any	ot
ABRAHA	8		f	0	0	all	0	q+s+a	Eu:est	1975	pr	571	n	bl	n	n	0	all/unsp	nev any	ot
AGUDO	1		f	0	0	all	-	all	Eu:wst	1989	CC	103	n	bl	n	n	3	cig only	nev any	or
AKIBA	11		m	0	0	all	0	all	As:Jap	1963	pr	610	n	bl	n	n	5	cig+/-ot	nev cigs	ot
AKIBA	15		f	0	0	all	0	all	As:Jap	1963	pr	610	n	bl	n	n	5	cig+/-ot	nev cigs	ot
ALDERS	68	x	m	0	0	all	-	all	Eu:UK	1977	CC	1448	n	V	n	n	1	cig+/-ot	nev any	ot
ALDERS	6		f	0	0	all	-	all	Eu:UK	1977	CC	1448	n	V	n	n	1	cig only	nev any	ot
AMANDU	7		m	0	0	wh	0	all	NAmer	1959	pr	132	m	bl	n	n	2	cig+/-ot	nev cigs	ot
AMES	4		m	0	0	wh	-	all	NAmer	1959	ot	317	m	bl	n	n	0	all/unsp	nev any	st
ANDERS	3		f	0	0	all	0	all	NAmer	1986	pr	343	n	bl	n	n	0	cig+/-ot	nev cigs	st
ARCHER	6		m	0	0	wh	0	all	NAmer	1950	pr	146	m	bl	n	n	0	cig+/-ot	nev cigs	st
ARMADA	4	x	m	0	0	all	-	all	Eu:wst	1986	CC	325	n	bl	n	y	0	cig+/-ot	nev any	st
AUSTIN	7		c	0	0	all	-	all	NAmer	1970	CC	166	o	bl	y	n	3	cig+/-ot	nev cigs	ot
AUVINE	19		c	0	0	all	-	all	Eu:Sca	1986	CC	517	n	bl	y	n	2	cig+/-ot	nev cigs	ot
AXELSO	1		c	0	0	all	-	all	Eu:Sca	1960	CC	152	n	bl	y	n	0	all/unsp	nev any	st
AXELSS	8		m	0	0	sca	-	all	Eu:Sca	1989	CC	436	n	bl	n	n	6	all/unsp	nev any	ot
AXELSS	11		f	0	0	sca	-	all	Eu:Sca	1989	CC	436	n	bl	n	n	0	all/unsp	nev any	st
BAND	1		m	0	0	all	-	all	NAmer	1983	CC	2831	n	V	y	y	2	cig only	nev any	ot
BARBON	131		m	0	0	all	-	all	Eu:wst	1979	CC	755	n	bl	y	y	3	all/unsp	nev any	ot
BECHER	21	x	m	0	0	all	-	all	Eu:Ger	1985	CC	194	n	bl	n	y	2	cig+/-ot	nev any	ot
BECHER	16	x	f	0	0	all	-	all	Eu:Ger	1985	CC	194	n	bl	n	y	0	cig+/-ot	nev any	st
BENSHL	15	x	m	0	0	all	0	all	Eu:UK	1967	pr	486	n	V	n	n	1	cig+/-ot	nev any	ot
BEST	23	x	m	55	79	all	3	all	NAmer	1955	pr	381	n	V	n	n	0	cig+/-ot	nev any	st
BEST	18		f	0	0	all	0	all	NAmer	1955	pr	381	n	V	n	n	1	cig only	nev any	ot
BLOHMK	3		m	0	0	all	-	all	Eu:Ger	1978	CC	888	n	bl	n	y	0	all/unsp	nev any	st
BLOT4	1		m	0	0	wh	-	all	NAmer	1974	CC	335	n	bl	y	n	0	cig+/-ot	nev cigs	st
BOFFET	27	x	m	0	0	all	-	all	Eu:mul	1988	CC	5621	n	bl	y	n	2	cig+/-ot	nev any	ot
BOUCOT	122	x	m	0	0	all	0	all	NAmer	1951	pr	121	n	bl	n	n	2	cig+/-ot	nev any	ot
BRESLO	17	x	m	0	0	all	-	all	NAmer	1949	CC	518	n	bl	n	y	0	cig+/-ot	nev+1	st
BRESLO	23	x	f	0	0	all	-	all	NAmer	1949	CC	518	n	bl	n	y	0	cig+/-ot	nev+1	st
BRETT	10		m	0	0	all	0	all	Eu:UK	1960	pr	150	n	V	n	n	0	cig+/-ot	nev cigs	st
BROCKM	1		m	0	0	wh	-	all	Eu:Ger	1990	CC	117	n	bl	n	y	0	cig+/-ot	nev cigs	st
BROCKM	2		f	0	0	wh	-	all	Eu:Ger	1990	CC	117	n	bl	n	y	0	cig+/-ot	nev cigs	st
BROSS	13	x	m	0	0	wh	-	all	NAmer	1960	CC	974	n	bl	n	n	0	cig+/-ot	nev any	st
BROWN2	2		m	0	0	wh	-	all	NAmer	1984	CC	14596	n	bl	n	y	2	cig+/-ot	nev cigs	or
BROWN2	1		f	0	0	wh	-	all	NAmer	1984	CC	14596	n	bl	n	y	2	cig+/-ot	nev cigs	or
BUFFLE	2	x	m	0	0	wh	-	all	NAmer	1976	CC	943	n	bl	y	n	0	cig+/-ot	nev any	st
BUFFLE	6	x	f	0	0	wh	-	all	NAmer	1976	CC	943	n	bl	y	n	0	cig+/-ot	nev any	st
CARPEN	12		c	0	0	w+b	-	all	NAmer	1991	CC	356	n	bl	n	n	3	cig+/-ot	nev cigs	ot
CASCO2	1		c	0	0	wh	-	all	Eu:Ger	1991	CC	155	n	bl	n	n	0	all/unsp	nev any	st
CASCOR	1		c	0	0	wh	-	all	Eu:Ger	1985	CC	389	n	bl	n	y	0	all/unsp	nev any	st
CEDERL	107		m	0	0	all	16	all	Eu:Sca	1963	pr	491	n	bl	n	n	2	all/unsp	nev any	ot
CEDERL	112		f	0	0	all	0	all	Eu:Sca	1963	pr	491	n	bl	n	n	2	all/unsp	nev any	ot
CHAN	5	x	m	0	0	all	-	all	As:HK	1976	CC	397	n	bl	n	n	0	cig+/-ot	nev any	st
CHAN	6	x	f	0	0	all	-	all	As:HK	1976	CC	397	n	bl	n	n	0	cig+/-ot	nev any	st
CHANG	6		m	0	0	all	0	all	NAmer	1972	pr	136	n	bl	n	n	0	cig+/-ot	nev cigs	st
CHANG	12		f	0	0	all	0	all	NAmer	1972	pr	136	n	bl	n	n	0	cig+/-ot	nev cigs	st
CHATZI	4		c	0	0	all	-	all	Eu:bal	1987	CC	282	n	bl	n	y	0	all/unsp	nev any	st
CHEN2	1		m	0	0	all	-	all	As:Chi	1983	CC	193	n	ot	y	n	0	all/unsp	nev any	st
CHEN2	2		f	0	0	all	-	all	As:Chi	1983	CC	193	n	ot	y	n	0	all/unsp	nev any	st
CHEN3	1		c	0	0	all	-	all	As:Chi	1981	CC	254	n	ot	y	n	0	all/unsp	nev any	st
CHIAZZ	3		m	0	0	all	-	all	NAmer	1940	CC	144	o	bl	y	n	11	cig+/-ot	nev cigs	or
CHOI	1		m	0	0	all	-	all	As:oth	1985	CC	375	n	bl	n	n	0	cig+/-ot	nev cigs	st
CHOI	5		f	0	0	all	-	all	As:oth	1985	CC	375	n	bl	n	n	0	cig+/-ot	nev cigs	st
CHOW	54	x	m	0	0	wh	0	all	NAmer	1966	pr	219	n	bl	n	n	2	cig+/-ot	nev any	ot
CHYOU	7		m	0	0	jap	0	all	NAmer	1965	pr	227	n	bl	n	y	1	cig+/-ot	nev cigs	ot
COMSTO	33	x	m	0	0	all	-	all	NAmer	1975	ot	258	n	bl	n	n	0	cig+/-ot	nev any	st
COMSTO	45	x	f	0	0	all	-	all	NAmer	1975	ot	258	n	bl	n	n	0	cig+/-ot	nev any	st
COOKSO	4	x	c	0	0	bl	-	all	Africa	1961	CC	234	n	V	n	y	0	cig+/-ot	nev any	st
CORREA	34		c	0	0	all	-	all	NAmer	1979	CC	1359	n	bl	y	n	1	cig+/-ot	nev cigs	or
CPSI	187		m	35	84	all	6	all	NAmer	1959	pr	5138	n	bl	n	n	1	cig+/-ot	nev any	ot
CPSI	274		f	40	74	all	6	all	NAmer	1959	pr	5138	n	bl	n	n	1	cig+/-ot	nev cigs	ot
CPSII	104		m	35	99	all	4	all	NAmer	1982	pr	3229	n	bl	n	n	1	cig only	nev any	ot
CPSII	79		f	0	0	all	4	all	NAmer	1982	pr	3229	n	bl	n	n	1	cig+/-ot	nev cigs	ot
DAMBER	37	x	m	0	0	all	-	all	Eu:Sca	1972	CC	579	n	bl	y	n	1	cig+/-ot	nev any	ot
DARBY	15		m	0	0	wh	-	all	Eu:UK	1988	CC	982	n	V	n	n	0	all/unsp	nev any	st
DARBY	16		f	0	0	wh	-	all	Eu:UK	1988	CC	982	n	V	n	n	0	all/unsp	nev any	st
DAVEYS	5		m	0	0	all	-	all	Eu:Ger	1930	CC	109	n	bl	y	n	0	all/unsp	nev any	st
DAVEYS	6		f	0	0	all	-	all	Eu:Ger	1930	CC	109	n	bl	y	n	0	all/unsp	nev any	ot
DEAN	8	x	m	0	0	wh	-	all	Africa	1947	CC	603	n	V	y	n	0	cig+/-ot	nev any	st
DEAN2	12	x	m	0	0	all	-	all	Eu:UK	1960	CC	954	n	V	y	n	0	cig+/-ot	nev any	st

Table 1A2 - 1

IESLC - Meta-analysis of Ever Smoking, Cigarettes (or Any Product if Cigarettes not available)
All LC types
Most adjusted

REF	NRR	1A1	SEX	AGEL	AGEH	RACE	YF	LC TYPE	LOC	START	ST	NLC	R	VB	P	H	AD	PRODUCT	DENOM	De
DEAN2	20	x	f	0	0	all	-	all	Eu:UK	1960	CC	954	n	V	y	n	0	cig+/-ot	nev any	st
DEAN3	241	x	m	0	0	all	-	all	Eu:UK	1969	CC	766	n	V	y	n	1	cig only	nev any	ot
DEAN3	126		f	0	0	all	-	all	Eu:UK	1969	CC	766	n	V	y	n	3	cig only	nev any	ot
DEKLER	6		m	0	0	all	0	all	Auslia	1961	pr	138	m	V	n	n	2	all/unsp	nev any	ot
DESTE2	15	x	m	0	0	all	-	all	SCAmer	1993	CC	463	n	bl	n	n	0	all/unsp	nev any	st
DESTEF	13	x	m	0	0	all	-	all	SCAmer	1988	CC	497	n	bl	n	y	4	cig+/-ot	nev any	or
DOCKER	3		c	0	0	wh	0	all	NAmer	1974	pr	120	n	bl	n	n	4	cig+/-ot	nev cigs	ot
DOLL	20	x	m	0	0	all	-	all	Eu:UK	1948	CC	1465	n	V	n	n	0	cig+/-ot	nev any	st
DOLL	12		f	0	0	all	-	all	Eu:UK	1948	CC	1465	n	V	n	n	0	all/unsp	nev any	st
DOLL2	88	x	m	0	0	all	10	all	Eu:UK	1951	pr	920	n	V	n	n	1	cig+/-ot	nev any	ot
DORANT	10		c	0	0	all	0	all	Eu:wst	1986	ot	550	n	bl	n	y	0	all/unsp	nev any	st
DORGAN	107	x	m	0	0	wh	-	all	NAmer	1980	CC	2026	n	bl	y	y	2	cig+/-ot	nev any	or
DORGAN	95	x	f	0	0	all	-	all	NAmer	1980	CC	2026	n	bl	y	y	3	cig+/-ot	nev any	or
DORN	413	x	m	0	0	wh	5	all	NAmer	1954	pr	5097	n	bl	n	n	1	cig+/-ot	nev any	ot
DOSEME	1		m	0	0	all	-	all	Eu:bal	1979	CC	1210	n	bl	n	n	2	cig+/-ot	nev cigs	or
DROSTE	7		m	0	0	all	-	all	Eu:wst	1995	CC	478	n	bl	n	y	4	all/unsp	nev any	ot
DU	1		m	0	0	all	-	all	As:Chi	1985	CC	849	n	ot	y	n	0	all/unsp	nev any	or
DU	2		f	0	0	all	-	all	As:Chi	1985	CC	849	n	ot	y	n	0	all/unsp	nev any	or
DUNN	6		m	0	0	all	0	all	NAmer	1954	pr	139	o	bl	n	n	0	cig+/-ot	nev cigs	st
EBELIN	1		m	0	0	all	-	all	Eu:Ger	1980	CC	130	n	bl	n	n	0	all/unsp	nev any	st
ENGELA	36	x	m	0	0	all	0	all	Eu:Sca	1964	pr	435	n	bl	n	n	7	cig+/-ot	nev cigs	ot
ENGELA	49	x	f	0	0	all	0	all	Eu:Sca	1964	pr	435	n	bl	n	n	5	cig+/-ot	nev cigs	ot
ESAKI	4		m	0	0	all	-	all	As:Jap	1961	CC	245	n	bl	y	n	0	cig+/-ot	nev cigs	st
ESAKI	5		f	0	0	all	-	all	As:Jap	1961	CC	245	n	bl	y	n	0	cig+/-ot	nev cigs	st
FAN	1		m	0	0	all	-	all	As:Chi	1990	CC	403	n	ot	y	n	0	cig+/-ot	nev cigs	st
FAN	2		f	0	0	all	-	all	As:Chi	1990	CC	403	n	ot	y	n	0	cig+/-ot	nev cigs	st
GAO	1		m	0	0	all	-	all	As:Chi	1984	CC	1405	n	ot	n	n	2	cig+/-ot	nev cigs	or
GAO	11		f	0	0	all	-	all	As:Chi	1984	CC	1405	n	ot	n	n	2	cig+/-ot	nev cigs	or
GAO2	10		m	0	0	all	-	all	As:Jap	1988	CC	282	n	bl	n	n	1	cig+/-ot	nev cigs	ot
GARCIA	3		c	0	0	all	-	all	NAmer	1992	CC	416	n	bl	n	y	0	cig+/-ot	nev cigs	st
GARDIN	7		c	0	0	all	-	all	Eu:UK	1988	CC	143	n	V	y	n	0	all/unsp	nev any	st
GARSHI	25		m	0	0	all	-	all	NAmer	1981	CC	1081	o	bl	y	n	1	all/unsp	nev any	st
GENG	1		m	0	0	all	-	all	As:Chi	1985	CC	292	n	ot	*	n	0	cig+/-ot	nev any	st
GENG	2		f	0	0	all	-	all	As:Chi	1985	CC	292	n	ot	*	n	0	cig+/-ot	nev any	st
GER	21		c	0	0	all	-	all	As:oth	1990	CC	141	n	ot	y	n	14	all/unsp	nev any	ot
GODLEY	5		m	0	0	all	-	all	NAmer	1966	CC	1986	n	bl	y	n	1	cig+/-ot	nev cigs	ot
GODLEY	6		f	0	0	all	-	all	NAmer	1966	CC	1986	n	bl	y	n	1	cig+/-ot	nev cigs	ot
GOLLED	7		m	35	99	all	-	all	Eu:UK	1952	CC	443	n	V	y	n	1	cig+/-ot	nev any	ot
GOODMA	3		m	0	0	w+o	-	all	NAmer	1983	CC	326	n	bl	y	y	0	cig+/-ot	nev any	st
GOODMA	7		f	0	0	w+o	-	all	NAmer	1983	CC	326	n	bl	y	y	0	cig+/-ot	nev any	st
GRAHAM	23	x	m	0	0	wh	-	all	NAmer	1956	CC	685	n	bl	n	n	1	cig+/-ot	nev any	ot
GREGOR	3		m	0	0	all	-	all	Eu:UK	1976	CC	104	n	V	n	y	0	cig+/-ot	nev cigs	st
GREGOR	7		f	0	0	all	-	all	Eu:UK	1976	CC	104	n	V	n	y	0	cig+/-ot	nev cigs	st
GSELL	6	x	m	0	0	all	-	all	Eu:wst	1937	CC	150	n	bl	n	y	0	cig+/-ot	nev any	st
HAENSZ	56	x	f	0	0	all	-	not alv	NAmer	1955	CC	158	n	bl	n	y	0	cig+/-ot	nev any	st
HAMMO2	2	x	m	0	0	all	6	all	NAmer	1967	pr	450	o	bl	n	n	1	cig+/-ot	nev any	st
HAMMON	116	x	m	0	0	wh	0	all	NAmer	1952	pr	448	n	bl	n	n	1	cig+/-ot	nev any	ot
HANSEN	3		m	0	0	all	0	all	Eu:Sca	1968	pr	105	o	bl	y	n	2	all/unsp	nev any	ot
HEGMAN	1		c	0	0	all	-	all	NAmer	1989	CC	282	n	bl	y	y	0	all/unsp	nev any	st
HEIN	7		m	0	0	all	0	all	Eu:Sca	1970	pr	144	n	bl	n	n	0	all/unsp	nev any	st
HENNEK	3		m	0	0	all	0	all	NAmer	1982	pr	169	n	bl	n	n	0	all/unsp	nev any	st
HINDS	22		f	0	0	o	-	all	NAmer	1968	CC	292	n	bl	n	n	3	all/unsp	nev any	st
HIRAYA	147		m	0	0	all	0	all	As:Jap	1965	pr	1917	n	bl	n	n	1	cig+/-ot	nev any	ot
HIRAYA	150		f	0	0	all	0	all	As:Jap	1965	pr	1917	n	bl	n	n	1	cig+/-ot	nev any	ot
HITOSU	38		m	0	0	all	-	all	As:Jap	1960	CC	216	n	bl	y	n	1	all/unsp	nev any	st
HITOSU	62		f	0	0	all	-	all	As:Jap	1960	CC	216	n	bl	y	n	1	all/unsp	nev any	st
HOLE	8		m	0	0	all	0	all	Eu:UK	1972	pr	225	n	V	n	n	1	all/unsp	nev any	ot
HOROWI	1		m	0	0	all	-	all	NAmer	1956	CC	236	n	V	n	n	0	cig+/-ot	nev any	st
HOROWI	2		f	0	0	all	-	all	NAmer	1956	CC	236	n	V	n	n	0	cig+/-ot	nev any	st
HORWIT	1		f	0	0	all	-	all	NAmer	1977	CC	112	n	bl	n	n	0	cig+/-ot	nev cigs	st
HU	15		m	0	0	all	-	all	As:Chi	1985	CC	227	n	ot	n	y	0	cig+/-ot	nev any	st
HU	16		f	0	0	all	-	all	As:Chi	1985	CC	227	n	ot	n	y	0	cig+/-ot	nev any	st
HU2	9		m	0	0	all	-	all	As:Chi	1977	CC	523	n	ot	y	n	0	cig+/-ot	nev cigs	st
HU2	10		f	0	0	all	-	all	As:Chi	1977	CC	523	n	ot	y	n	0	cig+/-ot	nev cigs	st
HUANG	1		c	0	0	all	-	all	As:Chi	1990	CC	135	n	ot	y	n	0	all/unsp	nev any	st
HUMBLE	14		m	0	0	w-hi	-	all	NAmer	1980	CC	521	n	bl	y	n	1	cig+/-ot	nev cigs	ot
HUMBLE	16		m	0	0	hi	-	all	NAmer	1980	CC	521	n	bl	y	n	1	cig+/-ot	nev cigs	ot
HUMBLE	18		f	0	0	w-hi	-	all	NAmer	1980	CC	521	n	bl	y	n	1	cig+/-ot	nev cigs	ot
HUMBLE	20		f	0	0	hi	-	all	NAmer	1980	CC	521	n	bl	y	n	1	cig+/-ot	nev cigs	ot
JAHN	22		f	0	0	all	-	all	Eu:Ger	1988	CC	1004	n	bl	n	n	2	cig+/-ot	nev any	ot
JAIN	46		m	0	0	all	-	all	NAmer	1981	CC	845	n	V	y	n	2	cig+/-ot	nev cigs	or
JAIN	41		f	0	0	all	-	all	NAmer	1981	CC	845	n	V	y	n	2	cig+/-ot	nev cigs	or

Table 1A2 - 1

IESLC - Meta-analysis of Ever Smoking, Cigarettes (or Any Product if Cigarettes not available)
All LC types
Most adjusted

REF	NRR	1A1	SEX	AGEL	AGEH	RACE	YF	LC TYPE	LOC	START	ST	NLC	R	VB	P	H	AD	PRODUCT	DENOM	De
JARUP	6		m	0	0	all	-	all	Eu:Sca	1928	CC	102	o	bl	y	n	2	all/unsp	nev any	ot
JARVHO	3		m	0	0	all	-	all	Eu:Sca	1983	CC	147	n	bl	n	n	0	all/unsp	nev any	st
JARVHO	7		f	0	0	all	-	all	Eu:Sca	1983	CC	147	n	bl	n	n	0	all/unsp	nev any	st
JEDRYC	58		m	0	0	all	-	all	Eu:est	1980	CC	1630	n	bl	y	n	4	cig+/-ot	nev any	ot
JEDRYC	59		f	0	0	all	-	all	Eu:est	1980	CC	1630	n	bl	y	n	4	cig+/-ot	nev any	ot
JIANG	1		m	0	0	all	-	all	As:Chi	1984	CC	125	n	ot	n	n	0	all/unsp	nev any	st
JIANG	2		f	0	0	all	-	all	As:Chi	1984	CC	125	n	ot	n	n	0	all/unsp	nev any	st
JOLY	2	x	m	0	0	all	-	all	SCAmer	1978	CC	826	n	bl	n	n	0	cig+/-ot	nev any	st
JOLY	1		f	0	0	all	-	all	SCAmer	1978	CC	826	n	bl	n	n	0	cig+/-ot	nev any	st
JUSSAW	31	x	m	0	0	all	-	all	As:Ind	1964	CC	792	n	V	n	n	2	cig only	nev any	st
KAISE2	72		m	35	99	all	9	all	NAmer	1979	pr	318	n	bl	n	n	1	cig only	nev any	st
KAISE2	64		f	35	99	all	9	all	NAmer	1979	pr	318	n	bl	n	n	1	cig only	nev any	st
KAISER	13		m	0	0	all	0	all	NAmer	1964	pr	714	n	bl	n	n	2	cig+/-ot	nev cigs	ot
KAISER	10		f	0	0	all	0	all	NAmer	1964	pr	714	n	bl	n	n	2	cig+/-ot	nev cigs	ot
KATSOU	29		f	0	0	all	-	all	Eu:bal	1987	CC	101	n	bl	n	n	1	all/unsp	nev any	ot
KAUFMA	17		c	0	0	all	-	all	NAmer	1981	CC	881	n	bl	n	n	6	cig+/-ot	nev cigs	ot
KELLER	3		m	0	0	wh	-	all	NAmer	1985	CC	15038	n	bl	n	n	0	all/unsp	nev any	st
KELLER	11		m	0	0	nonw	-	all	NAmer	1985	CC	15038	n	bl	n	n	0	all/unsp	nev any	st
KELLER	7		f	0	0	wh	-	all	NAmer	1985	CC	15038	n	bl	n	n	0	all/unsp	nev any	st
KELLER	15		f	0	0	nonw	-	all	NAmer	1985	CC	15038	n	bl	n	n	0	all/unsp	nev any	st
KHUDER	4		m	0	0	all	-	all	NAmer	1985	CC	482	n	bl	n	y	0	cig+/-ot	nev cigs	st
KIHARA	31		c	0	0	jap	-	all	As:Jap	1991	CC	440	n	bl	n	n	0	all/unsp	nev any	st
KINLEN	17		m	0	0	all	0	all	Eu:UK	1967	pr	718	n	V	n	n	2	all/unsp	nev any	ot
KJUUS	3	x	m	0	0	all	-	all	Eu:Sca	1979	CC	176	n	bl	n	n	0	cig only	nev any	st
KNEKT	87		m	20	69	all	21	all	Eu:Sca	1966	pr	515	n	bl	n	n	1	all/unsp	nev any	ot
KO	1		f	0	0	all	-	all	As:oth	1992	CC	117	n	ot	n	y	3	cig+/-ot	nev cigs	or
KOHLME	2		c	0	0	all	-	all	Eu:Ger	1990	CC	239	n	bl	n	n	4	all/unsp	nev any	or
KOO	1		f	0	0	all	-	all	As:HK	1981	CC	200	n	bl	n	n	0	all/unsp	nev any	st
KOULUM	2	x	m	0	0	all	-	all	Eu:Sca	1936	CC	812	n	bl	n	n	0	cig only	nev any	st
KREUZE	60	x	f	1	45	all	-	all	Eu:Ger	1990	CC	2260	n	bl	n	n	3	cig+/-ot	nev any	ot
KREUZE	62	x	f	55	69	all	-	all	Eu:Ger	1990	CC	2260	n	bl	n	n	3	cig+/-ot	nev any	ot
KREYBE	12		m	0	0	all	-	all	Eu:Sca	1948	CC	300	n	bl	n	y	1	all/unsp	nev any	ot
KREYBE	30		f	0	0	all	-	all	Eu:Sca	1948	CC	300	n	bl	n	y	1	all/unsp	nev any	ot
KUBIK	27	x	m	0	0	all	0	all	Eu:est	1965	pr	108	n	bl	n	n	0	cig+/-ot	nev any	st
LAMTH	6		f	0	0	ch	-	all	As:HK	1983	CC	445	n	bl	n	n	0	all/unsp	nev any	or
LAMWK	1		f	0	0	ch	-	all	As:HK	1981	CC	163	n	bl	n	n	0	all/unsp	nev any	st
LAMWK2	9		m	0	0	all	-	q+s+l+a	As:HK	1976	CC	480	n	bl	n	n	0	all/unsp	nev any	st
LAMWK2	10		f	0	0	all	-	q+s+l+a	As:HK	1976	CC	480	n	bl	n	n	0	all/unsp	nev any	st
LANGE	40		m	0	0	all	0	all	Eu:Sca	1976	pr	268	n	bl	n	n	1	all/unsp	nev any	ot
LANGE	37		f	0	0	all	0	all	Eu:Sca	1976	pr	268	n	bl	n	n	1	all/unsp	nev any	ot
LAUSSM	11		m	0	0	all	-	all	Eu:Ger	1982	CC	432	n	bl	n	n	3	all/unsp	nev any	or
LEI	1		m	0	0	all	-	all	As:Chi	1986	CC	792	n	ot	y	n	0	all/unsp	nev any	st
LEI	2		f	0	0	all	-	all	As:Chi	1986	CC	792	n	ot	y	n	0	all/unsp	nev any	st
LEMARC	3		c	0	0	w+o	-	all	NAmer	1992	CC	341	n	bl	n	y	0	all/unsp	nev any	st
LETOUR	1		c	0	0	all	-	all	NAmer	1983	CC	738	n	V	y	y	0	cig+/-ot	nev cigs	st
LEVIN	30	x	m	0	0	all	-	all	NAmer	1938	CC	475	n	bl	n	n	1	cig+/-ot	nev any	st
LIDDEL	5		m	0	0	all	18	all	NAmer	1970	pr	304	m	V	n	n	1	cig+/-ot	nev cigs	ot
LIU	2		c	0	0	all	-	all	As:Chi	1980	CC	229	n	ot	*	n	2	all/unsp	nev any	or
LIU2	2		m	0	0	all	-	all	As:Chi	1983	CC	316	n	ot	n	n	3	all/unsp	nev any	ot
LIU2	4		f	0	0	all	-	all	As:Chi	1983	CC	316	n	ot	n	n	3	all/unsp	nev any	ot
LIU3	2		m	0	0	all	-	all	As:Chi	1985	CC	110	n	ot	n	n	2	all/unsp	nev any	or
LIU4	10	x	m	35	69	all	-	all	As:Chi	1986	CC	1000-00	n	ot	y	n	2	cig only	nev any	ot
LIU4	12		f	0	0	all	-	all	As:Chi	1986	CC	1000-00	n	ot	y	n	2	all/unsp	nev any	ot
LIU5	1		c	0	0	all	-	all	As:Chi	1978	CC	111	n	ot	y	n	0	all/unsp	nev any	st
LOMBA2	1		f	0	0	all	-	all	NAmer	1960	CC	225	n	bl	n	n	0	cig+/-ot	nev cigs	st
LOMBAR	2	x	m	0	0	all	-	all	NAmer	1951	CC	1040	n	bl	n	n	0	cig+/-ot	nev any	st
LUBIN2	48	x	m	0	0	all	-	all	Eu:mul	1976	CC	7804	n	bl	n	y	2	cig+/-ot	nev any	ot
LUBIN2	98	x	f	0	0	all	-	all	Eu:mul	1976	CC	7804	n	bl	n	y	1	cig only	nev any	ot
LUO	7		c	0	0	all	-	all	As:Chi	1990	CC	102	n	ot	n	y	20	cig+/-ot	nev cigs	or
MACLEN	73		c	0	0	ch	-	all	As:oth	1972	CC	233	n	bl	n	n	2	cig+/-ot	nev cigs	ot
MAGNUS	5		m	0	0	all	0	all	Eu:Sca	1953	pr	203	o	bl	y	n	3	all/unsp	nev any	ot
MARSH	1	x	m	0	0	all	-	all	NAmer	1979	CC	150	n	bl	y	n	0	cig+/-ot	nev any	st
MARSH	3	x	f	0	0	all	-	all	NAmer	1979	CC	150	n	bl	y	n	0	cig+/-ot	nev any	st
MARSH2	5		m	0	0	all	-	all	NAmer	1979	CC	114	n	bl	y	n	1	all/unsp	nev any	or
MARSH2	6		f	0	0	all	-	all	NAmer	1979	CC	114	n	bl	y	n	1	all/unsp	nev any	ot
MARTIS	4		m	0	0	all	-	all	Eu:UK	1972	CC	201	n	V	n	n	0	cig+/-ot	nev cigs	st
MASTRA	2		m	0	0	all	-	all	Eu:wst	1973	CC	309	n	bl	n	n	2	all/unsp	nev any	st
MATOS	27		m	0	0	all	-	all	SCAmer	1994	CC	200	n	bl	n	n	2	cig+/-ot	nev any	or
MATSUD	10		m	0	0	all	-	all	As:Jap	1965	CC	179	n	bl	n	n	0	cig+/-ot	nev cigs	st
MCCONN	1		m	0	0	all	-	all	Eu:UK	1946	CC	100	n	V	n	y	0	all/unsp	nev any	st

Table 1A2 - 1

IESLC - Meta-analysis of Ever Smoking, Cigarettes (or Any Product if Cigarettes not available)
All LC types
Most adjusted

REF	NRR	1A1	SEX	AGEL	AGEH	RACE	YF	LC TYPE	LOC	START	ST	NLC	R	VB	P	H	AD	PRODUCT	DENOM	De
MCCONN	2		f	0	0	all	-	all	Eu:UK	1946	CC	100	n	V	n	y	0	all/unsp	nev any	st
MCDUFF	1		m	0	0	all	-	all	NAmer	1979	CC	165	n	V	y	n	0	cig+/-ot	nev cigs	st
MCLAUG	1		m	0	0	all	-	all	As:Chi	1972	CC	316	o	ot	y	n	0	all/unsp	nev any	st
MIGRAN	27		m	0	0	all	0	all	Eu:UK	1964	pr	259	n	V	n	n	2	all/unsp	nev any	ot
MIGRAN	42		f	0	0	all	0	all	Eu:UK	1964	pr	259	n	V	n	n	2	all/unsp	nev any	ot
MILLER	2		f	0	0	all	-	all	NAmer	1972	CC	168	n	bl	y	n	1	cig+/-ot	nev any	ot
MILLS	1	x	m	0	0	wh	-	all	NAmer	1940	CC	444	n	bl	y	n	1	cig only	nev any	ot
MRFITR	6		m	0	0	all	0	all	NAmer	1973	pr	119	n	bl	n	n	0	cig+/-ot	nev cigs	ot
NAM	77		m	0	0	all	-	all	NAmer	1986	CC	1199	n	bl	y	n	1	cig+/-ot	nev cigs	ot
NAM	93		f	0	0	all	-	all	NAmer	1986	CC	1199	n	bl	y	n	1	cig+/-ot	nev cigs	ot
NOTAN2	19	x	m	0	0	all	-	all	As:Ind	1963	CC	683	n	V	n	n	2	cig only	nev any	ot
NOU	11		m	30	64	all	-	all	Eu:Sca	1971	CC	273	n	bl	y	n	0	all/unsp	nev any	st
NOU	12		f	30	64	all	-	all	Eu:Sca	1971	CC	273	n	bl	y	n	0	all/unsp	nev any	st
ODRISC	3		c	0	0	all	-	all	Eu:UK	1992	CC	446	n	V	n	n	0	all/unsp	nev any	st
ORMOS	4		m	0	0	all	-	all	Eu:est	1947	CC	119	n	bl	y	y	0	cig+/-ot	nev any	st
ORMOS	26		f	0	0	all	-	all	Eu:est	1947	CC	119	n	bl	y	y	0	cig+/-ot	nev any	st
OSANN	41		m	0	0	all	-	all	NAmer	1984	CC	1986	n	bl	n	n	2	cig+/-ot	nev cigs	or
OSANN	42		f	0	0	all	-	all	NAmer	1984	CC	1986	n	bl	n	n	2	cig+/-ot	nev cigs	or
PARKIN	31	x	m	0	0	bl	-	all	Africa	1963	CC	877	n	V	y	n	0	cig+/-ot	nev any	st
PASTOR	10		m	0	0	all	-	all	Eu:wst	1976	CC	204	n	bl	y	n	1	all/unsp	nev any	or
PAWLEG	2		m	0	0	all	-	all	Eu:est	1992	CC	176	n	bl	n	y	6	all/unsp	nev any	ot
PERNU	8	x	m	0	0	all	-	all	Eu:Sca	1944	CC	1606	n	bl	n	n	0	cig only	nev any	st
PERNU	4	x	f	0	0	all	-	all	Eu:Sca	1944	CC	1606	n	bl	n	n	0	cig only	nev any	st
PERSH2	11		c	0	0	all	-	all	Eu:Sca	1980	CC	1022	n	bl	y	n	4	all/unsp	nev any	ot
PETO	5		m	0	0	all	0	all	Eu:UK	1954	pr	103	n	V	n	n	0	all/unsp	nev any	st
PEZZO2	10		m	0	0	all	-	all	SCAmer	1992	CC	367	n	bl	n	y	0	cig+/-ot	nev cigs	st
PEZZOT	25		m	0	0	all	-	all	SCAmer	1987	CC	215	n	bl	n	y	0	cig only	nev cigs	st
PIKE	4		m	0	0	w-hi	-	all	NAmer	1972	CC	731	n	bl	y	n	0	all/unsp	nev any	st
PIKE	8		f	0	0	w-hi	-	all	NAmer	1972	CC	731	n	bl	y	n	0	all/unsp	nev any	st
POFFIJ	1		c	0	0	all	-	all	Eu:mul	1990	CC	971	n	bl	n	n	0	all/unsp	nev any	st
POLEDN	1		c	0	0	all	-	all	NAmer	1978	CC	209	n	bl	y	n	1	cig+/-ot	nev cigs	or
QIAO2	9	x	m	0	0	all	0	all	As:Chi	1992	pr	241	m	ot	n	n	0	cig+/-ot	nev any	st
RACHTA	15		f	0	0	all	-	all	Eu:est	1991	CC	118	n	bl	n	y	4	cig+/-ot	nev cigs	ot
RADZIK	1		c	0	0	all	-	all	Eu:est	1986	CC	189	n	bl	n	n	0	all/unsp	nev any	st
RANDIG	9	x	m	0	0	all	-	all	Eu:Ger	1951	CC	448	n	bl	n	n	0	cig+/-ot	nev any	st
RANDIG	10	x	f	0	0	all	-	all	Eu:Ger	1951	CC	448	n	bl	n	n	0	cig+/-ot	nev any	st
REN	1		m	0	0	all	-	all	As:Chi	1980	CC	244	n	ot	*	n	0	all/unsp	nev any	st
REN	2		f	0	0	all	-	all	As:Chi	1980	CC	244	n	ot	*	n	0	all/unsp	nev any	st
RONCO	3	x	m	0	0	all	-	all	Eu:wst	1976	CC	126	n	bl	y	n	2	cig only	nev any	ot
ROTHSC	2		c	0	0	all	-	all	NAmer	1971	CC	284	n	bl	y	n	1	all/unsp	nev any	st
SADOWS	4	x	m	0	0	wh	-	all	NAmer	1938	CC	477	n	bl	n	n	0	cig+/-ot	nev any	st
SANKAR	2		m	0	0	all	-	all	As:Ind	1990	CC	281	n	V	n	n	3	all/unsp	nev any	ot
SCHWAR	1		m	0	0	wh	-	all	NAmer	1984	CC	5588	n	bl	y	y	0	cig+/-ot	nev cigs	st
SCHWAR	2		m	0	0	bl	-	all	NAmer	1984	CC	5588	n	bl	y	y	0	cig+/-ot	nev cigs	st
SCHWAR	3		f	0	0	wh	-	all	NAmer	1984	CC	5588	n	bl	y	y	0	cig+/-ot	nev cigs	st
SCHWAR	4		f	0	0	bl	-	all	NAmer	1984	CC	5588	n	bl	y	y	0	cig+/-ot	nev cigs	st
SEGI	1		m	0	0	all	-	all	As:Jap	1948	CC	159	n	bl	n	n	0	all/unsp	nev any	ot
SEOW	6		f	0	0	ch	-	q+s+l+a	As:oth	1997	CC	153	n	bl	n	y	1	cig+/-ot	nev cigs	st
SHAW	12		c	0	0	wh	-	all	NAmer	1988	CC	335	n	V	n	y	0	all/unsp	nev any	st
SIEMIA	5		m	0	0	all	-	all	NAmer	1979	CC	857	n	V	y	y	7	cig+/-ot	nev cigs	or
SIMARA	3		m	0	0	all	-	all	As:oth	1971	CC	115	n	bl	n	n	6	cig+/-ot	nev cigs	ot
SIMARA	4		f	0	0	all	-	all	As:oth	1971	CC	115	n	bl	n	n	6	cig+/-ot	nev cigs	ot
SOBUE	105		m	0	0	all	-	q+s+l+a	As:Jap	1986	CC	1376	n	bl	n	y	1	cig+/-ot	nev cigs	ot
SOBUE	115		f	0	0	all	-	q+s+l+a	As:Jap	1986	CC	1376	n	bl	n	y	1	cig+/-ot	nev cigs	ot
SPEIZE	8		f	0	0	all	0	all	NAmer	1976	pr	593	n	bl	n	y	0	cig+/-ot	nev cigs	st
SPITZ	3		c	0	0	b+hi	-	all	NAmer	1992	CC	177	n	bl	n	y	0	cig+/-ot	nev cigs	st
STASZE	7	x	m	0	0	all	-	all	Eu:est	1954	CC	281	n	bl	n	y	0	cig+/-ot	nev any	st
STASZE	5		f	0	0	all	-	all	Eu:est	1954	CC	281	n	bl	n	y	0	all/unsp	nev any	st
STAYNE	1		m	0	0	all	-	all	NAmer	1969	CC	420	n	bl	n	n	0	all/unsp	nev any	st
STOCKS	46	x	m	0	0	all	-	all	Eu:UK	1952	CC	2932	n	V	y	n	2	cig+/-ot	nev any	st
STOCKS	50		f	0	0	all	-	all	Eu:UK	1952	CC	2932	n	V	y	n	1	cig+/-ot	nev any	ot
STOCKW	8	x	c	0	0	all	-	all	NAmer	1981	CC	22161	n	bl	n	n	0	cig+/-ot	nev any	st
STUCKE	3		m	0	0	all	-	all	Eu:wst	1989	CC	247	n	bl	n	y	0	all/unsp	nev any	ot
SUN	1		c	0	0	all	-	all	As:Chi	1992	CC	207	n	ot	n	y	0	all/unsp	nev any	st
SUZUK2	7	x	c	0	0	all	-	all	SCAmer	1991	CC	123	n	bl	n	y	3	cig only	nev any	or
SVENSS	71		f	0	0	all	-	all	Eu:Sca	1983	CC	210	n	bl	n	n	1	all/unsp	nev any	ot
TANG	3		c	0	0	all	-	not s	NAmer	1992	CC	119	n	bl	n	y	0	cig+/-ot	nev cigs	st
TENKAN	22		m	0	0	all	17	all	Eu:Sca	1962	pr	242	n	bl	n	n	1	all/unsp	nev any	ot
TIZZAN	2	x	m	0	0	all	-	all	Eu:wst	1959	CC	1358	n	bl	n	n	0	cig only	nev any	st
TIZZAN	22	x	f	0	0	all	-	all	Eu:wst	1959	CC	1358	n	bl	n	n	0	cig only	nev any	st
TOKARS	6		c	0	0	all	-	all	Eu:est	1966	ot	162	o	bl	n	y	3	all/unsp	nev any	or
TOUSEY	10	x	m	0	0	all	-	all	NAmer	1993	CC	507	n	bl	y	y	3	cig+/-ot	nev any	or

Table 1A2 - 1

IESLC - Meta-analysis of Ever Smoking, Cigarettes (or Any Product if Cigarettes not available)
All LC types
Most adjusted

REF	NRR	1A1	SEX	AGEL	AGEH	RACE	YF	LC TYPE	LOC	START	ST	NLC	R	VB	P	H	AD	PRODUCT	DENOM	De
TOUSEY	13	x	f	0	0	all	-	all	NAmer	1993	CC	507	n	bl	y	y	3	cig+/-ot	nev any	or
TSUGAN	27		m	0	0	all	-	q+a	As:Jap	1976	CC	134	n	bl	n	y	0	all/unsp	nev any	st
TULINI	38		m	0	0	all	0	all	Eu:Sca	1967	pr	472	n	bl	n	n	3	all/unsp	nev any	ot
TULINI	44		f	0	0	all	0	all	Eu:Sca	1967	pr	472	n	bl	n	n	3	all/unsp	nev any	ot
TVERDA	22		m	0	0	all	0	all	Eu:Sca	1972	pr	238	n	bl	n	n	2	cig+/-ot	nev cigs	ot
WAKAI	72		m	0	0	all	-	all	As:Jap	1988	CC	333	n	bl	n	y	2	all/unsp	nev any	ot
WAKAI	78		f	0	0	all	-	all	As:Jap	1988	CC	333	n	bl	n	y	2	all/unsp	nev any	ot
WANG	5		c	0	0	all	-	all	As:Chi	1990	CC	390	n	ot	*	y	6	all/unsp	nev any	or
WANG2	16		c	0	0	all	-	all	As:Chi	1980	CC	103	n	ot	n	n	4	cig+/-ot	nev cigs	ot
WANG3	1		c	0	0	all	-	all	As:Chi	1981	CC	293	n	ot	*	n	0	all/unsp	nev any	st
WANG4	2		m	0	0	all	-	all	As:Chi	1976	CC	1170	n	ot	y	n	2	all/unsp	nev any	st
WICKLU	1		m	0	0	wh	-	all	NAmer	1968	CC	155	n	bl	y	n	0	cig+/-ot	nev+3	or
WIGLE	13	x	m	0	0	all	-	all	NAmer	1971	CC	728	n	V	n	n	0	cig only	nev any	st
WIGLE	16	x	f	0	0	all	-	all	NAmer	1971	CC	728	n	V	n	n	0	cig only	nev any	st
WILKIN	3		c	0	0	all	-	all	Eu:UK	1992	CC	271	n	V	n	n	4	cig+/-ot	nev cigs	ot
WU	45		f	0	0	wh	-	q+a	NAmer	1981	CC	220	n	bl	n	y	2	all/unsp	nev any	ot
WUNSCH	4		m	0	0	all	-	all	SCAmer	1990	CC	398	n	bl	y	n	1	cig+/-ot	nev any	or
WUNSCH	10		f	0	0	all	-	all	SCAmer	1990	CC	398	n	bl	y	n	1	cig+/-ot	nev any	or
WUWILL	8		f	0	0	all	-	all	As:Chi	1985	CC	965	n	ot	n	n	3	cig+/-ot	nev cigs	or
WYNDE2	16	x	m	0	0	all	-	all	NAmer	1962	CC	404	n	bl	n	y	0	cig+/-ot	nev any	st
WYNDE3	48	x	m	0	0	all	-	all	NAmer	1966	CC	350	n	bl	n	y	0	cig+/-ot	nev any	st
WYNDE3	83	x	f	0	0	all	-	all	NAmer	1966	CC	350	n	bl	n	y	0	cig+/-ot	nev any	st
WYNDE4	48		m	0	0	all	-	all	NAmer	1948	CC	684	n	bl	y	n	0	all/unsp	nev any	st
WYNDE4	62		f	0	0	all	-	all	NAmer	1948	CC	684	n	bl	y	n	2	all/unsp	nev any	ot
WYNDE6	81	x	m	0	0	all	-	all	NAmer	1969	CC	4423	n	bl	n	y	0	cig+/-ot	nev any	st
WYNDE6	252		f	0	0	all	-	all	NAmer	1969	CC	4423	n	bl	n	y	0	cig+/-ot	nev cigs	st
XIANGZ	14	x	m	0	0	all	0	all	As:Chi	1976	pr	983	m	ot	n	n	2	cig+/-ot	nev any	ot
XU	2		m	0	0	all	-	all	As:Chi	1985	CC	729	n	ot	n	n	2	all/unsp	nev any	or
XU2	2		c	0	0	all	-	all	As:Chi	1987	CC	610	o	ot	y	n	7	all/unsp	nev any	ot
XU3	2		m	0	0	all	-	all	As:Chi	1981	CC	135	n	ot	n	n	1	all/unsp	nev any	or
XU3	4		f	0	0	all	-	all	As:Chi	1981	CC	135	n	ot	n	n	1	all/unsp	nev any	or
XU4	1		c	0	0	all	-	all	As:Chi	1981	CC	206	n	ot	*	n	0	all/unsp	nev any	st
YAMAGU	11		c	0	0	all	-	all	As:Jap	1989	CC	144	n	bl	n	y	1	all/unsp	nev any	ot
YONG	2		c	0	0	all	0	all	NAmer	1971	pr	216	n	bl	n	n	1	cig+/-ot	nev cigs	ot
YUAN	1		m	0	0	all	0	all	As:Chi	1986	pr	142	n	ot	n	n	2	cig+/-ot	nev cigs	ot
ZHANG	2		m	0	0	all	-	all	As:Chi	1988	CC	100	n	ot	n	n	7	all/unsp	nev any	or
ZHANG	3		f	0	0	all	-	all	As:Chi	1988	CC	100	n	ot	n	n	7	all/unsp	nev any	or
ZHENG	15		m	0	0	all	-	all	As:Chi	1982	CC	540	n	ot	*	y	0	cig+/-ot	nev cigs	st
ZHENG	24		f	0	0	all	-	all	As:Chi	1982	CC	540	n	ot	*	y	0	cig+/-ot	nev cigs	st
ZHOU	2		m	0	0	all	-	all	As:Chi	1978	CC	1360	n	ot	n	n	0	all/unsp	nev any	st
ZHOU	3		f	0	0	all	-	all	As:Chi	1978	CC	1360	n	ot	n	n	0	all/unsp	nev any	st

Cigarette type is all/unspec for all RRs
except for the following:

REF	NRR	CIGTYPE
ALDERS	6	MC only
DEAN3	241	MC only
DEAN3	126	MC only
DESTEF	13	MC only
JUSSAW	31	MC only
NOTAN2	19	MC only
PERNU	8	MC only
PERNU	4	MC only
SUZUK2	7	MC only

Table 1A2 - 2

IESLC - Meta-analysis of Ever Smoking, Cigarettes (or Any Product if Cigarettes not available)
All LC types
Most adjusted

REF	NRR	SEX	AD	Number Exposed Case	Number Exposed Cont	Non-exposed Case	Non-exposed Cont	RR	95.00%CI
ABELIN	45	m	1	-	-	-	-	39.29	(9.04- 170.68)
*ABRAHA	7	m	0	269	10351	10	3365	8.74	(4.66- 16.42)
*ABRAHA	8	f	0	62	5256	28	11589	4.88	(3.13- 7.62)
Subtotal ABRAHA								5.93	(4.12- 8.53)
AGUDO	1	f	3	-	-	-	-	3.10	(1.42- 6.75)
*AKIBA	11	m	5	-	-	-	-	4.75	(3.07- 7.34)
*AKIBA	15	f	5	-	-	-	-	3.16	(2.37- 4.21)
Subtotal AKIBA								3.58	(2.81- 4.54)
ALDERS	68	m	1	-	-	-	-	10.00	(5.81- 17.22)
ALDERS	6	f	1	-	-	-	-	4.75	(3.55- 6.35)
Subtotal ALDERS								5.61	(4.34- 7.24)
*AMANDU	7	m	2	-	-	-	-	5.89	(2.27- 15.28)
AMES	4	m	0	297	251	15	62	4.89	(2.72- 8.81)
*ANDERS	3	f	0	297	96164	46	195158	13.10	(9.61- 17.87)
*ARCHER	6	m	0	140	36269	6	9842	6.33	(2.80- 14.33)
ARMADA	4	m	0	317	254	4	64	19.97	(7.18- 55.57)
AUSTIN	7	c	3	-	-	-	-	11.12	(3.95- 31.28)
AUVINE	19	c	2	-	-	-	-	13.84	(7.90- 24.25)
AXELSO	1	c	0	90	86	62	371	6.26	(4.20- 9.34)
AXELSS	8	m	6	-	-	-	-	8.02	(4.62- 13.94)
AXELSS	11	f	0	110	109	18	154	8.63	(4.95- 15.05)
Subtotal AXELSS								8.32	(5.62- 12.31)
BAND	1	m	2	-	-	-	-	9.96	(7.38- 13.44)
BARBON	131	m	3	-	-	-	-	11.13	(7.02- 17.64)
BECHER	21	m	2	-	-	-	-	9.03	(3.09- 26.33)
BECHER	16	f	0	38	44	10	52	4.49	(2.01- 10.03)
Subtotal BECHER								5.78	(3.04- 10.99)
*BENSHL	15	m	1	-	-	-	-	8.02	(4.29- 15.01)
*BEST	23	m	0	212	21711	1	2854	27.87	(3.91- 198.68)
*BEST	18	f	1	-	-	-	-	2.24	(0.59- 8.44)
Subtotal BEST								4.95	(1.65- 14.89)
BLOHMK	3	m	0	762	587	126	301	3.10	(2.45- 3.92)
BLOT4	1	m	0	327	245	8	87	14.51	(6.91- 30.51)
BOFFET	27	m	2	-	-	-	-	14.43	(11.91- 17.49)
*BOUCOT	122	m	2	-	-	-	-	51.72	(3.22- 831.81)
BRESLO	17	m	0	471	383	7	42	7.38	(3.28- 16.61)
BRESLO	23	f	0	13	11	12	14	1.38	(0.45- 4.20)
Subtotal BRESLO								4.12	(2.14- 7.94)
*BRETT	10	m	0	144	47930	6	6530	3.27	(1.45- 7.40)
BROCKM	1	m	0	87	81	2	2	1.07	(0.15- 7.80)
BROCKM	2	f	0	24	54	4	18	2.00	(0.61- 6.54)
Subtotal BROCKM								1.70	(0.61- 4.70)
BROSS	13	m	0	831	612	38	170	6.07	(4.21- 8.77)
BROWN2	2	m	2	-	-	-	-	9.10	(8.30- 10.00)
BROWN2	1	f	2	-	-	-	-	12.70	(11.50- 13.90)
Subtotal BROWN2								10.72	(10.03- 11.46)
BUFFLE	2	m	0	461	373	5	47	11.62	(4.57- 29.50)
BUFFLE	6	f	0	419	284	41	198	7.12	(4.93- 10.30)
Subtotal BUFFLE								7.61	(5.40- 10.72)
CARPEN	12	c	3	-	-	-	-	14.88	(8.46- 26.18)
CASCO2	1	c	0	149	212	6	98	11.48	(4.90- 26.87)
CASCOR	1	c	0	365	362	22	295	13.52	(8.56- 21.35)
*CEDERL	107	m	2	-	-	-	-	5.92	(3.85- 9.12)
*CEDERL	112	f	2	-	-	-	-	4.18	(2.94- 5.93)
Subtotal CEDERL								4.80	(3.66- 6.30)
CHAN	5	m	0	206	161	2	43	27.51	(6.57- 115.26)
CHAN	6	f	0	105	50	84	139	3.48	(2.26- 5.35)
Subtotal CHAN								4.13	(2.73- 6.25)
*CHANG	6	m	0	78	1506	5	502	5.20	(2.12- 12.77)
*CHANG	12	f	0	42	1183	11	1139	3.68	(1.90- 7.10)
Subtotal CHANG								4.15	(2.44- 7.06)
CHATZI	4	c	0	255	365	27	129	3.34	(2.14- 5.21)
CHEN2	1	m	0	121	97	9	33	4.57	(2.09- 10.02)
CHEN2	2	f	0	38	30	25	33	1.67	(0.82- 3.39)
Subtotal CHEN2								2.62	(1.55- 4.44)
CHEN3	1	c	0	182	156	72	98	1.59	(1.09- 2.30)
CHIAZZ	3	m	11	-	-	-	-	26.17	(3.32- 206.50)
CHOI	1	m	0	267	465	13	95	4.20	(2.31- 7.64)
CHOI	5	f	0	19	26	76	164	1.58	(0.82- 3.02)
Subtotal CHOI								2.68	(1.72- 4.16)
*CHOW	54	m	2	-	-	-	-	11.58	(5.09- 26.34)

Table 1A2 - 2

IESLC - Meta-analysis of Ever Smoking, Cigarettes (or Any Product if Cigarettes not available)
All LC types
Most adjusted

REF	NRR	SEX	AD	Number Exposed Case	Number Exposed Cont	Non-exposed Case	Non-exposed Cont	RR	95.00%CI
*CHYOU	7	m	1	-	-	-	-	8.35	(4.76- 14.64)
COMSTO	33	m	0	151	229	4	69	11.37	(4.07- 31.82)
COMSTO	45	f	0	88	87	13	115	8.95	(4.69- 17.06)
Subtotal COMSTO								9.58	(5.54- 16.54)
COOKSO	4	c	0	184	38	45	61	6.56	(3.90- 11.04)
CORREA	34	c	1	-	-	-	-	11.40	(8.70- 15.00)
*CPSI	187	m	1	-	-	-	-	9.18	(7.36- 11.45)
*CPSI	274	f	1	-	-	-	-	2.79	(2.22- 3.51)
Subtotal CPSI								5.17	(4.41- 6.06)
*CPSII	104	m	1	-	-	-	-	12.83	(10.28- 16.01)
*CPSII	79	f	1	-	-	-	-	8.16	(6.93- 9.62)
Subtotal CPSII								9.58	(8.40- 10.93)
DAMBER	37	m	1	-	-	-	-	7.78	(5.29- 11.44)
DARBY	15	m	0	664	1724	3	384	49.30	(15.77- 154.07)
DARBY	16	f	0	292	548	23	529	12.26	(7.89- 19.05)
Subtotal DARBY								14.69	(9.74- 22.16)
DAVEYS	5	m	0	90	144	3	23	4.79	(1.40- 16.42)
DAVEYS	6	f	0	0	3	16	83	0.72~	(0.04- 14.66)
Subtotal DAVEYS								3.65	(1.17- 11.42)
DEAN	8	m	0	540	500	12	61	5.49	(2.92- 10.32)
DEAN2	12	m	0	686	556	33	112	4.19	(2.80- 6.27)
DEAN2	20	f	0	63	29	88	121	2.99	(1.78- 5.02)
Subtotal DEAN2								3.69	(2.68- 5.07)
DEAN3	241	m	1	-	-	-	-	6.11	(3.99- 9.34)
DEAN3	126	f	3	-	-	-	-	4.63	(3.03- 7.09)
Subtotal DEAN3								5.32	(3.94- 7.18)
*DEKLER	6	m	2	-	-	-	-	20.29	(2.84- 145.18)
DESTE2	15	m	0	432	314	31	151	6.70	(4.43- 10.13)
DESTEF	13	m	4	-	-	-	-	6.10	(3.70- 10.00)
*DOCKER	3	c	4	-	-	-	-	4.29	(1.66- 11.06)
DOLL	20	m	0	504	467	7	61	9.40	(4.26- 20.77)
DOLL	12	f	0	68	49	40	59	2.05	(1.19- 3.53)
Subtotal DOLL								3.34	(2.13- 5.23)
*DOLL2	88	m	1	-	-	-	-	11.58	(3.70- 36.24)
DORANT	10	c	0	470	2033	14	1090	18.00	(10.52- 30.78)
DORGAN	107	m	2	-	-	-	-	11.60	(6.50- 20.70)
DORGAN	95	f	3	-	-	-	-	8.50	(6.70- 10.80)
Subtotal DORGAN								8.89	(7.13- 11.09)
*DORN	413	m	1	-	-	-	-	8.83	(6.64- 11.75)
DOSEME	1	m	2	-	-	-	-	3.30	(2.60- 4.40)
DROSTE	7	m	4	-	-	-	-	8.62	(3.80- 19.56)
DU	1	m	0	538	-	28	-	3.53	(2.44- 5.11)
DU	2	f	0	191	-	92	-	1.93	(1.30- 2.87)
Subtotal DU								2.66	(2.03- 3.49)
*DUNN	6	m	0	137	52634	2	14160	18.43	(4.56- 74.42)
EBELIN	1	m	0	101	142	12	117	6.93	(3.63- 13.24)
*ENGELA	36	m	7	-	-	-	-	1.67	(1.03- 2.71)
*ENGELA	49	f	5	-	-	-	-	6.05	(3.08- 11.90)
Subtotal ENGELA								2.58	(1.74- 3.83)
ESAKI	4	m	0	155	143	16	28	1.90	(0.99- 3.65)
ESAKI	5	f	0	34	19	40	55	2.46	(1.23- 4.92)
Subtotal ESAKI								2.14	(1.33- 3.45)
FAN	1	m	0	216	498	36	236	2.84	(1.93- 4.18)
FAN	2	f	0	82	97	69	320	3.92	(2.65- 5.81)
Subtotal FAN								3.33	(2.53- 4.38)
GAO	1	m	2	-	-	-	-	3.90	(2.90- 5.40)
GAO	11	f	2	-	-	-	-	3.30	(2.50- 4.20)
Subtotal GAO								3.53	(2.90- 4.31)
GAO2	10	m	1	-	-	-	-	5.17	(2.76- 9.69)
GARCIA	3	c	0	395	307	21	139	8.52	(5.26- 13.80)
GARDIN	7	c	0	138	102	5	41	11.09	(4.23- 29.06)
GARSHI	25	m	1	-	-	-	-	5.81	(4.17- 8.10)
GENG	1	m	0	92	68	7	31	5.99	(2.49- 14.42)
GENG	2	f	0	126	75	67	118	2.96	(1.96- 4.48)
Subtotal GENG								3.36	(2.31- 4.89)
GER	21	c	14	-	-	-	-	1.84	(1.06- 3.20)
GODLEY	5	m	1	-	-	-	-	6.84	(5.60- 8.35)
GODLEY	6	f	1	-	-	-	-	5.54	(4.29- 7.15)
Subtotal GODLEY								6.31	(5.39- 7.39)
GOLLED	7	m	1	-	-	-	-	7.51	(4.44- 12.71)
GOODMA	3	m	0	216	398	10	199	10.80	(5.60- 20.82)

Table 1A2 - 2

IESLC - Meta-analysis of Ever Smoking, Cigarettes (or Any Product if Cigarettes not available)
All LC types
Most adjusted

REF	NRR	SEX	AD	Number Exposed Case	Number Exposed Cont	Non-exposed Case	Non-exposed Cont	RR	95.00%CI
GOODMA	7	f	0	81	91	19	177	8.29	(4.74- 14.52)
Subtotal GOODMA								9.27	(6.05- 14.19)
GRAHAM	23	m	1	-	-	-	-	8.31	(5.19- 13.30)
GREGOR	3	m	0	72	98	10	14	1.03	(0.43- 2.45)
GREGOR	7	f	0	21	42	1	22	11.00	(1.39- 87.29)
Subtotal GREGOR								1.46	(0.66- 3.26)
GSELL	6	m	0	60	42	2	29	20.71	(4.69- 91.56)
HAENSZ	56	f	0	74	103	81	236	2.09	(1.42- 3.09)
*HAMMO2	2	m	1	-	-	-	-	24.92	(3.49- 177.78)
*HAMMON	116	m	1	-	-	-	-	8.93	(5.33- 14.96)
*HANSEN	3	m	2	-	-	-	-	1.53	(0.71- 3.91)
HEGMAN	1	c	0	255	1202	27	2080	16.34	(10.92- 24.45)
*HEIN	7	m	0	143	4471	1	457	14.62	(2.05- 104.23)
*HENNEK	3	m	0	146	11112	23	10919	6.24	(4.02- 9.67)
HINDS	22	f	3	-	-	-	-	5.65	(4.14- 7.72)
*HIRAYA	147	m	1	-	-	-	-	4.36	(3.53- 5.39)
*HIRAYA	150	f	1	-	-	-	-	2.36	(1.90- 2.94)
Subtotal HIRAYA								3.24	(2.78- 3.77)
HITOSU	38	m	1	-	-	-	-	2.91	(1.34- 6.34)
HITOSU	62	f	1	-	-	-	-	3.40	(2.05- 5.64)
Subtotal HITOSU								3.25	(2.12- 4.96)
*HOLE	8	m	1	-	-	-	-	6.44	(3.03- 13.69)
HOROWI	1	m	0	182	525	19	196	3.58	(2.17- 5.90)
HOROWI	2	f	0	21	382	14	463	1.82	(0.91- 3.62)
Subtotal HOROWI								2.83	(1.89- 4.25)
HORWIT	1	f	0	97	92	11	118	11.31	(5.73- 22.34)
HU	15	m	0	120	94	41	67	2.09	(1.30- 3.35)
HU	16	f	0	26	18	40	48	1.73	(0.83- 3.61)
Subtotal HU								1.98	(1.33- 2.94)
HU2	9	m	0	294	228	49	115	3.03	(2.08- 4.41)
HU2	10	f	0	108	80	72	100	1.88	(1.23- 2.85)
Subtotal HU2								2.44	(1.85- 3.23)
HUANG	1	c	0	98	77	37	58	2.00	(1.20- 3.32)
HUMBLE	14	m	1	-	-	-	-	12.10	(5.12- 28.60)
HUMBLE	16	m	1	-	-	-	-	11.88	(2.65- 53.30)
HUMBLE	18	f	1	-	-	-	-	11.36	(5.32- 24.23)
HUMBLE	20	f	1	-	-	-	-	15.40	(4.87- 48.74)
Subtotal HUMBLE								12.28	(7.58- 19.90)
JAHN	22	f	2	-	-	-	-	3.30	(1.99- 5.49)
JAIN	46	m	2	-	-	-	-	8.30	(4.53- 17.00)
JAIN	41	f	2	-	-	-	-	9.20	(5.95- 15.10)
Subtotal JAIN								8.89	(6.08- 13.01)
JARUP	6	m	2	-	-	-	-	7.54	(2.80- 20.33)
JARVHO	3	m	0	99	57	1	16	27.79	(3.59- 215.09)
JARVHO	7	f	0	41	15	6	21	9.57	(3.24- 28.26)
Subtotal JARVHO								12.08	(4.64- 31.46)
JEDRYC	58	m	4	-	-	-	-	5.46	(3.85- 7.73)
JEDRYC	59	f	4	-	-	-	-	4.54	(2.56- 8.05)
Subtotal JEDRYC								5.19	(3.86- 7.00)
JIANG	1	m	0	93	83	7	17	2.72	(1.08- 6.89)
JIANG	2	f	0	11	6	14	19	2.49	(0.74- 8.35)
Subtotal JIANG								2.63	(1.26- 5.50)
JOLY	2	m	0	552	709	12	218	14.14	(7.83- 25.56)
JOLY	1	f	0	166	123	52	283	7.34	(5.04- 10.70)
Subtotal JOLY								8.87	(6.46- 12.19)
JUSSAW	31	m	2	-	-	-	-	8.64	(4.61- 17.88)
*KAISE2	72	m	1	-	-	-	-	5.40	(3.05- 9.57)
*KAISE2	64	f	1	-	-	-	-	10.09	(5.29- 19.27)
Subtotal KAISE2								7.11	(4.63- 10.90)
*KAISER	13	m	2	-	-	-	-	17.63	(11.98- 25.96)
*KAISER	10	f	2	-	-	-	-	5.63	(3.89- 8.14)
Subtotal KAISER								9.70	(7.43- 12.67)
KATSOU	29	f	1	-	-	-	-	3.30	(1.77- 6.15)
KAUFMA	17	c	6	-	-	-	-	12.38	(8.59- 17.85)
KELLER	3	m	0	8066	2517	323	1017	10.09	(8.83- 11.52)
KELLER	11	m	0	1493	340	38	117	13.52	(9.20- 19.86)
KELLER	7	f	0	3998	1269	469	1860	12.49	(11.09- 14.08)
KELLER	15	f	0	584	214	67	232	9.45	(6.91- 12.93)
Subtotal KELLER								11.30	(10.40- 12.29)
KHUDER	4	m	0	459	785	23	309	7.86	(5.06- 12.19)
KIHARA	31	c	0	338	232	102	237	3.39	(2.54- 4.51)

Table 1A2 - 2

IESLC - Meta-analysis of Ever Smoking, Cigarettes (or Any Product if Cigarettes not available)
All LC types
Most adjusted

REF	NRR	SEX	AD	Number Exposed Case	Number Exposed Cont	Non-exposed Case	Non-exposed Cont	RR	95.00%CI
*KINLEN	17	m	2	-	-	-	-	10.99	(5.24- 23.06)
KJUUS	3	m	0	151	127	2	24	14.27	(3.31- 61.54)
*KNEKT	87	m	1	-	-	-	-	6.42	(2.82- 14.62)
KO	1	f	3	-	-	-	-	4.20	(1.10- 15.60)
KOHLME	2	c	4	-	-	-	-	16.40	(6.90- 38.42)
KOO	1	f	0	112	63	88	137	2.77	(1.84- 4.16)
KOULUM	2	m	0	625	229	5	54	29.48	(11.65- 74.60)
KREUZE	60	f	3	-	-	-	-	9.21	(3.45- 24.53)
KREUZE	62	f	3	-	-	-	-	4.05	(2.81- 5.86)
Subtotal KREUZE								4.48	(3.18- 6.32)
KREYBE	12	m	1	-	-	-	-	6.61	(2.93- 14.92)
KREYBE	30	f	1	-	-	-	-	1.43	(0.71- 2.86)
Subtotal KREYBE								2.73	(1.61- 4.64)
*KUBIK	27	m	0	106	7829	2	4271	28.91	(7.14- 117.06)
LAMTH	6	f	0	242	106	202	337	3.81	(2.86- 5.08)
LAMWK	1	f	0	88	41	75	144	4.12	(2.59- 6.55)
LAMWK2	9	m	0	244	161	23	43	2.83	(1.64- 4.88)
LAMWK2	10	f	0	75	50	65	139	3.21	(2.02- 5.10)
Subtotal LAMWK2								3.04	(2.14- 4.33)
*LANGE	40	m	1	-	-	-	-	4.74	(1.77- 12.67)
*LANGE	37	f	1	-	-	-	-	4.93	(2.48- 9.81)
Subtotal LANGE								4.87	(2.77- 8.55)
LAUSSM	11	m	3	-	-	-	-	5.70	(4.10- 7.80)
LEI	1	m	0	443	361	41	123	3.68	(2.52- 5.38)
LEI	2	f	0	123	61	85	147	3.49	(2.32- 5.24)
Subtotal LEI								3.59	(2.72- 4.74)
LEMARC	3	c	0	309	288	32	168	5.63	(3.74- 8.49)
LETOUR	1	c	0	714	514	24	224	12.96	(8.38- 20.05)
LEVIN	30	m	1	-	-	-	-	6.97	(4.87- 9.97)
*LIDDEL	5	m	1	-	-	-	-	3.61	(2.27- 5.73)
LIU	2	c	2	-	-	-	-	1.92	(1.40- 2.64)
LIU2	2	m	3	-	-	-	-	5.19	(2.03- 13.25)
LIU2	4	f	3	-	-	-	-	4.65	(2.18- 9.93)
Subtotal LIU2								4.86	(2.69- 8.76)
LIU3	2	m	2	-	-	-	-	1.26	(0.30- 5.26)
LIU4	10	m	2	-	-	-	-	3.88	(3.78- 3.98)
LIU4	12	f	2	-	-	-	-	2.86	(2.77- 2.95)
Subtotal LIU4								3.43	(3.37- 3.50)
LIU5	1	c	0	85	70	26	41	1.91	(1.07- 3.44)
LOMBA2	1	f	0	149	353	76	239	1.33	(0.96- 1.83)
LOMBAR	2	m	0	978	782	14	112	10.01	(5.70- 17.58)
LUBIN2	48	m	2	-	-	-	-	8.74	(7.49- 10.19)
LUBIN2	98	f	1	-	-	-	-	3.90	(3.29- 4.62)
Subtotal LUBIN2								6.07	(5.42- 6.81)
LUO	7	c	20	-	-	-	-	2.70	(1.50- 5.00)
MACLEN	73	c	2	-	-	-	-	2.67	(1.66- 4.29)
*MAGNUS	5	m	3	-	-	-	-	4.13	(1.94- 8.77)
MARSH	1	m	0	98	150	2	31	10.13	(2.37- 43.27)
MARSH	3	f	0	42	64	8	71	5.82	(2.54- 13.33)
Subtotal MARSH								6.67	(3.25- 13.70)
MARSH2	5	m	1	-	-	-	-	1.89	(0.70- 5.14)
MARSH2	6	f	1	-	-	-	-	5.28	(1.89- 14.72)
Subtotal MARSH2								3.11	(1.52- 6.36)
MARTIS	4	m	0	197	176	4	25	7.00	(2.39- 20.49)
MASTRA	2	m	2	-	-	-	-	8.14	(3.32- 20.00)
MATOS	27	m	2	-	-	-	-	6.80	(3.50- 13.10)
MATSUD	10	m	0	170	3314	3	1255	21.46	(6.84- 67.33)
MCCONN	1	m	0	88	174	5	12	1.21	(0.41- 3.55)
MCCONN	2	f	0	3	3	4	11	2.75	(0.38- 19.67)
Subtotal MCCONN								1.46	(0.57- 3.76)
MCDUFF	1	m	0	159	134	6	31	6.13	(2.48- 15.14)
MCLAUG	1	m	0	294	1082	22	270	3.33	(2.12- 5.25)
*MIGRAN	27	m	2	-	-	-	-	3.61	(1.34- 9.72)
*MIGRAN	42	f	2	-	-	-	-	4.62	(1.63- 13.09)
Subtotal MIGRAN								4.06	(1.98- 8.32)
MILLER	2	f	1	-	-	-	-	4.99	(2.06- 12.10)
MILLS	1	m	1	-	-	-	-	1.27	(1.01- 1.61)
*MRFITR	6	m	0	119	11007	0	1859	40.37~	(2.51- 648.95)
NAM	77	m	1	-	-	-	-	8.71	(5.87- 12.93)
NAM	93	f	1	-	-	-	-	8.88	(6.35- 12.40)
Subtotal NAM								8.81	(6.82- 11.37)

Table 1A2 - 2

IESLC - Meta-analysis of Ever Smoking, Cigarettes (or Any Product if Cigarettes not available)
All LC types
Most adjusted

REF	NRR	SEX	AD	Number Exposed Case	Number Exposed Cont	Non-exposed Case	Non-exposed Cont	RR	95.00%CI
NOTAN2	19	m	2	-	-	-	-	2.36	(1.68- 3.31)
NOU	11	m	0	74	247	6	122	6.09	(2.58- 14.39)
NOU	12	f	0	10	92	4	261	7.09	(2.17- 23.17)
Subtotal NOU								6.42	(3.20- 12.87)
ODRISC	3	c	0	440	996	6	664	48.89	(21.71- 110.07)
ORMOS	4	m	0	87	1034	7	777	9.34	(4.30- 20.28)
ORMOS	26	f	0	1	234	23	1044	0.19	(0.03- 1.44)
Subtotal ORMOS								5.65	(2.74- 11.64)
OSANN	41	m	2	-	-	-	-	19.70	(14.40- 26.80)
OSANN	42	f	2	-	-	-	-	15.00	(11.80- 19.10)
Subtotal OSANN								16.62	(13.74- 20.10)
PARKIN	31	m	0	372	933	107	1248	4.65	(3.69- 5.86)
PASTOR	10	m	1	-	-	-	-	6.81	(3.38- 13.70)
PAWLEG	2	m	6	-	-	-	-	12.26	(4.07- 36.95)
PERNU	8	m	0	706	216	97	275	9.27	(7.02- 12.23)
PERNU	4	f	0	7	24	110	971	2.57	(1.08- 6.11)
Subtotal PERNU								8.22	(6.32- 10.71)
PERSH2	11	c	4	-	-	-	-	6.55	(5.46- 7.86)
*PETO	5	m	0	101	2423	2	295	6.15	(1.52- 24.79)
PEZZO2	10	m	0	361	469	6	117	15.01	(6.53- 34.48)
PEZZOT	25	m	0	211	317	4	116	19.30	(7.02- 53.10)
PIKE	4	m	0	514	375	18	69	5.25	(3.08- 8.98)
PIKE	8	f	0	163	90	36	96	4.83	(3.04- 7.66)
Subtotal PIKE								5.01	(3.53- 7.10)
POFFIJ	1	c	0	913	918	58	452	7.75	(5.81- 10.34)
POLEDN	1	c	1	-	-	-	-	9.24	(5.23- 16.33)
*QIAO2	9	m	0	197	6360	10	709	2.20	(1.17- 4.13)
RACHTA	15	f	4	-	-	-	-	8.21	(3.96- 17.05)
RADZIK	1	c	0	180	198	9	13	1.31	(0.55- 3.15)
RANDIG	9	m	0	277	245	5	22	4.97	(1.86- 13.34)
RANDIG	10	f	0	16	39	17	92	2.22	(1.02- 4.84)
Subtotal RANDIG								3.03	(1.64- 5.58)
REN	1	m	0	106	84	12	34	3.58	(1.74- 7.33)
REN	2	f	0	78	20	48	50	4.06	(2.16- 7.64)
Subtotal REN								3.84	(2.39- 6.17)
RONCO	3	m	2	-	-	-	-	5.43	(2.27- 12.96)
ROTHSC	2	c	1	-	-	-	-	5.55	(2.97- 10.37)
SADOWS	4	m	0	421	446	18	81	4.25	(2.51- 7.20)
SANKAR	2	m	3	-	-	-	-	13.62	(9.00- 20.62)
SCHWAR	1	m	0	2648	1019	119	376	8.21	(6.60- 10.22)
SCHWAR	2	m	0	863	275	50	104	6.53	(4.54- 9.39)
SCHWAR	3	f	0	1351	637	182	855	9.96	(8.28- 12.00)
SCHWAR	4	f	0	335	179	40	247	11.56	(7.90- 16.90)
Subtotal SCHWAR								9.05	(7.99- 10.25)
SEGI	1	m	0	140	1742	18	382	1.71	(1.03- 2.82)
SEOW	6	f	1	-	-	-	-	5.25	(2.80- 9.84)
SHAW	12	c	0	324	266	11	107	11.85	(6.24- 22.50)
SIEMIA	5	m	7	-	-	-	-	12.10	(6.60- 22.30)
SIMARA	3	m	6	-	-	-	-	1.65	(0.97- 2.81)
SIMARA	4	f	6	-	-	-	-	1.63	(0.87- 3.06)
Subtotal SIMARA								1.64	(1.09- 2.46)
SOBUE	105	m	1	-	-	-	-	3.72	(2.57- 5.38)
SOBUE	115	f	1	-	-	-	-	2.51	(1.89- 3.33)
Subtotal SOBUE								2.90	(2.32- 3.64)
*SPEIZE	8	f	0	535	1012074	58	776300	7.08	(5.40- 9.28)
SPITZ	3	c	0	170	169	7	128	18.39	(8.35- 40.53)
STASZE	7	m	0	251	653	5	158	12.15	(4.93- 29.94)
STASZE	5	f	0	6	153	15	1660	4.34	(1.66- 11.35)
Subtotal STASZE								7.50	(3.89- 14.48)
STAYNE	1	m	0	362	567	58	333	3.67	(2.69- 4.99)
STOCKS	46	m	2	-	-	-	-	6.94	(4.93- 9.77)
STOCKS	50	f	1	-	-	-	-	3.04	(2.35- 3.93)
Subtotal STOCKS								4.10	(3.33- 5.03)
STOCKW	8	c	0	18655	6414	2791	10641	11.09	(10.54- 11.66)
STUCKE	3	m	0	247	203	0	51	125.27~	(7.68-2042.38)
SUN	1	c	0	140	173	67	191	2.31	(1.62- 3.30)
SUZUK2	7	c	3	-	-	-	-	11.00	(3.40- 36.00)
SVENSS	71	f	1	-	-	-	-	6.18	(3.79- 10.07)
TANG	3	c	0	110	59	9	39	8.08	(3.66- 17.82)
*TENKAN	22	m	1	-	-	-	-	14.64	(6.29- 34.07)
TIZZAN	2	m	0	994	836	180	305	2.01	(1.64- 2.48)

Table 1A2 - 2

IESLC - Meta-analysis of Ever Smoking, Cigarettes (or Any Product if Cigarettes not available)
All LC types
Most adjusted

REF	NRR	SEX	AD	Number Exposed Case	Number Exposed Cont	Non-exposed Case	Non-exposed Cont	RR	95.00%CI
TIZZAN	22	f	0	25	28	25	114	4.07	(2.04- 8.13)
Subtotal TIZZAN								2.13	(1.75- 2.60)
TOKARS	6	c	3	-	-	-	-	6.60	(3.20- 13.70)
TOUSEY	10	m	3	-	-	-	-	21.40	(7.80- 59.00)
TOUSEY	13	f	3	-	-	-	-	16.80	(9.20- 30.70)
Subtotal TOUSEY								17.90	(10.67- 30.04)
TSUGAN	27	m	0	73	71	18	22	1.26	(0.62- 2.54)
*TULINI	38	m	3	-	-	-	-	7.71	(4.19- 14.18)
*TULINI	44	f	3	-	-	-	-	13.01	(7.24- 23.40)
Subtotal TULINI								10.12	(6.63- 15.44)
*TVERDA	22	m	2	-	-	-	-	4.58	(2.97- 7.06)
WAKAI	72	m	2	-	-	-	-	3.67	(1.84- 7.32)
WAKAI	78	f	2	-	-	-	-	4.49	(2.35- 8.59)
Subtotal WAKAI								4.09	(2.55- 6.55)
WANG	5	c	6	-	-	-	-	2.88	(1.74- 4.77)
WANG2	16	c	4	-	-	-	-	2.29	(1.12- 4.70)
WANG3	1	c	0	235	172	58	121	2.85	(1.97- 4.13)
WANG4	2	m	2	-	-	-	-	1.16	(0.96- 1.42)
WICKLU	1	m	0	-	-	-	-	4.60	(2.80- 7.60)
WIGLE	13	m	0	543	632	15	204	11.68	(6.83- 19.99)
WIGLE	16	f	0	78	235	36	439	4.05	(2.64- 6.19)
Subtotal WIGLE								6.09	(4.37- 8.51)
WILKIN	3	c	4	-	-	-	-	7.83	(4.45- 13.78)
WU	45	f	2	-	-	-	-	3.03	(1.81- 5.07)
WUNSCH	4	m	1	-	-	-	-	4.75	(2.66- 8.50)
WUNSCH	10	f	1	-	-	-	-	4.43	(2.62- 7.47)
Subtotal WUNSCH								4.57	(3.10- 6.74)
WUWILL	8	f	3	-	-	-	-	2.30	(1.90- 2.80)
WYNDE2	16	m	0	382	512	8	105	9.79	(4.71- 20.34)
WYNDE3	48	m	0	261	264	9	88	9.67	(4.77- 19.60)
WYNDE3	83	f	0	46	56	20	76	3.12	(1.67- 5.85)
Subtotal WYNDE3								5.14	(3.21- 8.22)
WYNDE4	48	m	0	632	665	12	115	9.11	(4.98- 16.67)
WYNDE4	62	f	2	-	-	-	-	2.87	(1.48- 5.55)
Subtotal WYNDE4								5.38	(3.45- 8.41)
WYNDE6	81	m	0	2765	1797	87	617	10.91	(8.65- 13.76)
WYNDE6	252	f	0	1354	701	159	856	10.40	(8.58- 12.60)
Subtotal WYNDE6								10.60	(9.14- 12.30)
*XIANGZ	14	m	2	-	-	-	-	1.79	(1.21- 2.65)
XU	2	m	2	-	-	-	-	2.70	(2.10- 3.50)
XU2	2	c	7	-	-	-	-	3.80	(2.84- 5.07)
XU3	2	m	1	-	-	-	-	5.99	(2.65- 13.50)
XU3	4	f	1	-	-	-	-	3.86	(1.39- 10.70)
Subtotal XU3								5.05	(2.67- 9.54)
XU4	1	c	0	161	113	45	93	2.94	(1.92- 4.52)
YAMAGU	11	c	1	-	-	-	-	3.97	(2.12- 7.40)
*YONG	2	c	1	-	-	-	-	6.74	(4.47- 10.18)
*YUAN	1	m	2	-	-	-	-	6.50	(3.64- 11.60)
ZHANG	2	m	7	-	-	-	-	4.00	(1.61- 9.91)
ZHANG	3	f	7	-	-	-	-	3.75	(1.80- 10.76)
Subtotal ZHANG								3.87	(2.05- 7.32)
ZHENG	15	m	0	279	218	33	94	3.65	(2.36- 5.63)
ZHENG	24	f	0	76	44	152	184	2.09	(1.36- 3.21)
Subtotal ZHENG								2.75	(2.03- 3.73)
ZHOU	2	m	0	740	41	275	36	2.36	(1.48- 3.77)
ZHOU	3	f	0	112	7	231	32	2.22	(0.95- 5.18)
Subtotal ZHOU								2.33	(1.54- 3.51)
Partial Totals				77291	1385753	9309	1083845		

*prospective study ~ With 0.5 adjustment for zero

Table 1A2 - 2

IESLC - Meta-analysis of Ever Smoking, Cigarettes (or Any Product if Cigarettes not available)
All LC types
Most adjusted

REF	NRR	SEX	AD	Ys	Ws	Qs	Ps
ABELIN	45	m	1	3.67	1.78	7.98	0.0000
*ABRAHA	7	m	0	2.17	9.68	3.65	0.0000
*ABRAHA	8	f	0	1.59	19.39	0.02	0.0000
Subtotal ABRAHA				1.78	29.07	3.67	
AGUDO	1	f	3	1.13	6.32	1.13	0.0044
*AKIBA	11	m	5	1.56	20.22	0.00	0.0000
*AKIBA	15	f	5	1.15	46.54	7.57	0.0000
Subtotal AKIBA				1.27	66.77	7.58	
ALDERS	68	m	1	2.30	13.02	7.29	0.0000
ALDERS	6	f	1	1.56	45.44	0.00	0.0000
Subtotal ALDERS				1.72	58.46	7.30	
*AMANDU	7	m	2	1.77	4.23	0.20	0.0003
AMES	4	m	0	1.59	11.09	0.01	0.0000
*ANDERS	3	f	0	2.57	39.86	41.37	0.0000
*ARCHER	6	m	0	1.85	5.76	0.49	0.0000
ARMADA	4	m	0	2.99	3.67	7.61	0.0000
AUSTIN	7	c	3	2.41	3.59	2.62	0.0000
AUVINE	19	c	2	2.63	12.22	14.08	0.0000
AXELSO	1	c	0	1.83	24.06	1.89	0.0000
AXELSS	8	m	6	2.08	12.60	3.51	0.0000
AXELSS	11	f	0	2.16	12.45	4.51	0.0000
Subtotal AXELSS				2.12	25.05	8.02	
BAND	1	m	2	2.30	42.76	23.71	0.0000
BARBON	131	m	3	2.41	18.10	13.25	0.0000
BECHER	21	m	2	2.20	3.35	1.40	0.0001
BECHER	16	f	0	1.50	5.94	0.02	0.0003
Subtotal BECHER				1.75	9.29	1.42	
*BENSHL	15	m	1	2.08	9.80	2.73	0.0000
*BEST	23	m	0	3.33	1.00	3.13	0.0009
*BEST	18	f	1	0.81	2.17	1.21	0.2348
Subtotal BEST				1.60	3.17	4.34	
BLOHMK	3	m	0	1.13	70.05	12.49	0.0000
BLOT4	1	m	0	2.68	6.96	8.75	0.0000
BOFFET	27	m	2	2.67	104.07	129.46	0.0000
*BOUCOT	122	m	2	3.95	0.50	2.85	0.0054
BRESLO	17	m	0	2.00	5.83	1.15	0.0000
BRESLO	23	f	0	0.32	3.10	4.71	0.5717
Subtotal BRESLO				1.42	8.93	5.86	
*BRETT	10	m	0	1.18	5.77	0.79	0.0044
BROCKM	1	m	0	0.07	0.98	2.15	0.9437
BROCKM	2	f	0	0.69	2.73	2.03	0.2517
Subtotal BROCKM				0.53	3.71	4.17	
BROSS	13	m	0	1.80	28.54	1.79	0.0000
BROWN2	2	m	2	2.21	442.58	189.46	0.0000
BROWN2	1	f	2	2.54	427.71	417.17	0.0000
Subtotal BROWN2				2.37	870.29	606.64	
BUFFLE	2	m	0	2.45	4.42	3.57	0.0000
BUFFLE	6	f	0	1.96	28.29	4.75	0.0000
Subtotal BUFFLE				2.03	32.71	8.32	
CARPEN	12	c	3	2.70	12.04	15.81	0.0000
CASCO2	1	c	0	2.44	5.31	4.17	0.0000
CASCOR	1	c	0	2.60	18.40	20.29	0.0000
*CEDERL	107	m	2	1.78	20.66	1.04	0.0000
*CEDERL	112	f	2	1.43	31.21	0.48	0.0000
Subtotal CEDERL				1.57	51.88	1.52	
CHAN	5	m	0	3.31	1.87	5.80	0.0000
CHAN	6	f	0	1.25	20.57	1.96	0.0000
Subtotal CHAN				1.42	22.44	7.76	
*CHANG	6	m	0	1.65	4.76	0.04	0.0003
*CHANG	12	f	0	1.30	8.85	0.56	0.0001
Subtotal CHANG				1.42	13.61	0.61	
CHATZI	4	c	0	1.21	19.44	2.36	0.0000
CHEN2	1	m	0	1.52	6.25	0.01	0.0001
CHEN2	2	f	0	0.51	7.70	8.32	0.1539
Subtotal CHEN2				0.97	13.95	8.33	
CHEN3	1	c	0	0.46	27.78	33.10	0.0148
CHIAZZ	3	m	11	3.26	0.90	2.64	0.0019
CHOI	1	m	0	1.43	10.71	0.15	0.0000
CHOI	5	f	0	0.46	9.06	10.94	0.1703
Subtotal CHOI				0.99	19.78	11.09	
*CHOW	54	m	2	2.45	5.69	4.56	0.0000

Table 1A2 - 2

IESLC - Meta-analysis of Ever Smoking, Cigarettes (or Any Product if Cigarettes not available)
All LC types
Most adjusted

REF	NRR	SEX	AD	Ys	Ws	Qs	Ps
*CHYOU	7	m	1	2.12	12.17	3.93	0.0000
COMSTO	33	m	0	2.43	3.63	2.79	0.0000
COMSTO	45	f	0	2.19	9.22	3.75	0.0000
Subtotal COMSTO				2.26	12.85	6.54	
COOKSO	4	c	0	1.88	14.21	1.52	0.0000
CORREA	34	c	1	2.43	51.78	40.07	0.0000
*CPSI	187	m	1	2.22	78.68	34.59	0.0000
*CPSI	274	f	1	1.03	73.22	20.41	0.0000
Subtotal CPSI				1.64	151.90	55.00	
*CPSII	104	m	1	2.55	78.29	77.95	0.0000
*CPSII	79	f	1	2.10	142.84	42.47	0.0000
Subtotal CPSII				2.26	221.13	120.41	
DAMBER	37	m	1	2.05	25.83	6.39	0.0000
DARBY	15	m	0	3.90	2.96	16.25	0.0000
DARBY	16	f	0	2.51	19.76	17.90	0.0000
Subtotal DARBY				2.69	22.71	34.16	
DAVEYS	5	m	0	1.57	2.53	0.00	0.0126
DAVEYS	6	f	0	-0.32	0.42	1.50	0.8327
Subtotal DAVEYS				1.30	2.96	1.50	
DEAN	8	m	0	1.70	9.65	0.21	0.0000
DEAN2	12	m	0	1.43	23.54	0.35	0.0000
DEAN2	20	f	0	1.09	14.29	3.02	0.0000
Subtotal DEAN2				1.30	37.83	3.37	
DEAN3	241	m	1	1.81	21.24	1.39	0.0000
DEAN3	126	f	3	1.53	21.26	0.01	0.0000
Subtotal DEAN3				1.67	42.50	1.40	
*DEKLER	6	m	2	3.01	0.99	2.11	0.0027
DESTE2	15	m	0	1.90	22.53	2.73	0.0000
DESTEF	13	m	4	1.81	15.54	1.01	0.0000
*DOCKER	3	c	4	1.46	4.27	0.04	0.0026
DOLL	20	m	0	2.24	6.12	2.89	0.0000
DOLL	12	f	0	0.72	12.98	9.10	0.0099
Subtotal DOLL				1.21	19.10	12.00	
*DOLL2	88	m	1	2.45	2.95	2.37	0.0000
DORANT	10	c	0	2.89	13.34	23.82	0.0000
DORGAN	107	m	2	2.45	11.45	9.21	0.0000
DORGAN	95	f	3	2.14	67.41	23.15	0.0000
Subtotal DORGAN				2.19	78.86	32.37	
*DORN	413	m	1	2.18	47.17	18.38	0.0000
DOSEME	1	m	2	1.19	55.52	7.20	0.0000
DROSTE	7	m	4	2.15	5.72	2.06	0.0000
DU	1	m	0	1.26	28.12	2.41	0.0000
DU	2	f	0	0.66	24.50	19.69	0.0011
Subtotal DU				0.98	52.62	22.10	
*DUNN	6	m	0	2.91	1.97	3.65	0.0000
EBELIN	1	m	0	1.94	9.19	1.34	0.0000
*ENGELA	36	m	7	0.51	16.42	17.80	0.0377
*ENGELA	49	f	5	1.80	8.41	0.51	0.0000
Subtotal ENGELA				0.95	24.83	18.31	
ESAKI	4	m	0	0.64	8.96	7.48	0.0554
ESAKI	5	f	0	0.90	7.99	3.41	0.0109
Subtotal ESAKI				0.76	16.94	10.89	
FAN	1	m	0	1.04	25.87	6.70	0.0000
FAN	2	f	0	1.37	24.92	0.88	0.0000
Subtotal FAN				1.20	50.80	7.58	
GAO	1	m	2	1.36	39.76	1.48	0.0000
GAO	11	f	2	1.19	57.09	7.40	0.0000
Subtotal GAO				1.26	96.85	8.88	
GAO2	10	m	1	1.64	9.74	0.08	0.0000
GARCIA	3	c	0	2.14	16.50	5.71	0.0000
GARDIN	7	c	0	2.41	4.14	3.01	0.0000
GARSHI	25	m	1	1.76	34.86	1.47	0.0000
GENG	1	m	0	1.79	4.98	0.28	0.0001
GENG	2	f	0	1.08	22.39	4.93	0.0000
Subtotal GENG				1.21	27.37	5.21	
GER	21	c	14	0.61	12.59	11.22	0.0305
GODLEY	5	m	1	1.92	96.28	13.10	0.0000
GODLEY	6	f	1	1.71	58.89	1.47	0.0000
Subtotal GODLEY				1.84	155.17	14.57	
GOLLED	7	m	1	2.02	13.89	2.97	0.0000
GOODMA	3	m	0	2.38	8.92	6.08	0.0000

Table 1A2 - 2

IESLC - Meta-analysis of Ever Smoking, Cigarettes (or Any Product if Cigarettes not available)
All LC types
Most adjusted

REF	NRR	SEX	AD	Ys	Ws	Qs	Ps
GOODMA	7	f	0	2.12	12.25	3.86	0.0000
Subtotal GOODMA				2.23	21.17	9.94	
GRAHAM	23	m	1	2.12	17.35	5.51	0.0000
GREGOR	3	m	0	0.03	5.11	11.91	0.9492
GREGOR	7	f	0	2.40	0.90	0.64	0.0233
Subtotal GREGOR				0.38	6.01	12.54	
GSELL	6	m	0	3.03	1.74	3.79	0.0001
HAENSZ	56	f	0	0.74	25.12	16.70	0.0002
*HAMMO2	2	m	1	3.22	0.99	2.75	0.0013
*HAMMON	116	m	1	2.19	14.43	5.83	0.0000
*HANSEN	3	m	2	0.43	5.28	6.73	0.3285
HEGMAN	1	c	0	2.79	23.66	36.36	0.0000
*HEIN	7	m	0	2.68	1.00	1.27	0.0074
*HENNEK	3	m	0	1.83	19.94	1.53	0.0000
HINDS	22	f	3	1.73	39.57	1.25	0.0000
*HIRAYA	147	m	1	1.47	85.78	0.57	0.0000
*HIRAYA	150	f	1	0.86	80.63	38.98	0.0000
Subtotal HIRAYA				1.18	166.40	39.55	
HITOSU	38	m	1	1.07	6.36	1.50	0.0071
HITOSU	62	f	1	1.22	15.00	1.64	0.0000
Subtotal HITOSU				1.18	21.36	3.14	
*HOLE	8	m	1	1.86	6.76	0.64	0.0000
HOROWI	1	m	0	1.27	15.35	1.20	0.0000
HOROWI	2	f	0	0.60	8.08	7.38	0.0894
Subtotal HOROWI				1.04	23.43	8.59	
HORWIT	1	f	0	2.43	8.29	6.30	0.0000
HU	15	m	0	0.74	17.16	11.50	0.0023
HU	16	f	0	0.55	7.15	7.21	0.1413
Subtotal HU				0.68	24.31	18.71	
HU2	9	m	0	1.11	27.11	5.41	0.0000
HU2	10	f	0	0.63	21.91	18.76	0.0033
Subtotal HU2				0.89	49.01	24.17	
HUANG	1	c	0	0.69	14.82	11.05	0.0078
HUMBLE	14	m	1	2.49	5.19	4.58	0.0000
HUMBLE	16	m	1	2.47	1.71	1.45	0.0012
HUMBLE	18	f	1	2.43	6.68	5.13	0.0000
HUMBLE	20	f	1	2.73	2.90	4.04	0.0000
Subtotal HUMBLE				2.51	16.48	15.19	
JAHN	22	f	2	1.19	14.92	1.93	0.0000
JAIN	46	m	2	2.12	8.79	2.78	0.0000
JAIN	41	f	2	2.22	17.72	7.84	0.0000
Subtotal JAIN				2.19	26.50	10.62	
JARUP	6	m	2	2.02	3.91	0.85	0.0001
JARVHO	3	m	0	3.32	0.92	2.88	0.0015
JARVHO	7	f	0	2.26	3.28	1.62	0.0000
Subtotal JARVHO				2.49	4.19	4.50	
JEDRYC	58	m	4	1.70	31.63	0.65	0.0000
JEDRYC	59	f	4	1.51	11.71	0.02	0.0000
Subtotal JEDRYC				1.65	43.33	0.67	
JIANG	1	m	0	1.00	4.45	1.36	0.0346
JIANG	2	f	0	0.91	2.62	1.08	0.1401
Subtotal JIANG				0.97	7.08	2.44	
JOLY	2	m	0	2.65	10.97	13.16	0.0000
JOLY	1	f	0	1.99	27.09	5.24	0.0000
Subtotal JOLY				2.18	38.06	18.41	
JUSSAW	31	m	2	2.16	8.36	3.04	0.0000
*KAISE2	72	m	1	1.69	11.75	0.21	0.0000
*KAISE2	64	f	1	2.31	9.19	5.28	0.0000
Subtotal KAISE2				1.96	20.95	5.48	
*KAISER	13	m	2	2.87	25.69	44.47	0.0000
*KAISER	10	f	2	1.73	28.18	0.85	0.0000
Subtotal KAISER				2.27	53.88	45.33	
KATSOU	29	f	1	1.19	9.91	1.28	0.0002
KAUFMA	17	c	6	2.52	28.72	26.59	0.0000
KELLER	3	m	0	2.31	217.37	124.75	0.0000
KELLER	11	m	0	2.60	25.99	28.67	0.0000
KELLER	7	f	0	2.53	269.69	254.43	0.0000
KELLER	15	f	0	2.25	39.03	18.69	0.0000
Subtotal KELLER				2.43	552.08	426.53	
KHUDER	4	m	0	2.06	19.93	5.13	0.0000
KIHARA	31	c	0	1.22	46.97	5.26	0.0000

Table 1A2 - 2

IESLC - Meta-analysis of Ever Smoking, Cigarettes (or Any Product if Cigarettes not available)
All LC types
Most adjusted

REF	NRR	SEX	AD	Ys	Ws	Qs	Ps
*KINLEN	17	m	2	2.40	7.00	4.97	0.0000
KJUUS	3	m	0	2.66	1.80	2.19	0.0004
*KNEKT	87	m	1	1.86	5.67	0.53	0.0000
KO	1	f	3	1.44	2.18	0.03	0.0339
KOHLME	2	c	4	2.80	5.21	8.06	0.0000
KOO	1	f	0	1.02	23.01	6.61	0.0000
KOULUM	2	m	0	3.38	4.45	14.91	0.0000
KREUZE	60	f	3	2.22	3.99	1.77	0.0000
KREUZE	62	f	3	1.40	28.45	0.69	0.0000
Subtotal KREUZE				1.50	32.44	2.46	
KREYBE	12	m	1	1.89	5.80	0.65	0.0000
KREYBE	30	f	1	0.36	7.92	11.33	0.3143
Subtotal KREYBE				1.01	13.71	11.98	
*KUBIK	27	m	0	3.36	1.96	6.44	0.0000
LAMTH	6	f	0	1.34	46.55	2.19	0.0000
LAMWK	1	f	0	1.42	17.85	0.34	0.0000
LAMWK2	9	m	0	1.04	12.98	3.41	0.0002
LAMWK2	10	f	0	1.17	17.89	2.70	0.0000
Subtotal LAMWK2				1.11	30.86	6.11	
*LANGE	40	m	1	1.56	3.97	0.00	0.0019
*LANGE	37	f	1	1.60	8.13	0.01	0.0000
Subtotal LANGE				1.58	12.09	0.01	
LAUSSM	11	m	3	1.74	37.15	1.29	0.0000
LEI	1	m	0	1.30	26.63	1.67	0.0000
LEI	2	f	0	1.25	23.21	2.16	0.0000
Subtotal LEI				1.28	49.84	3.83	
LEMARC	3	c	0	1.73	22.77	0.69	0.0000
LETOUR	1	c	0	2.56	20.21	20.55	0.0000
LEVIN	30	m	1	1.94	29.93	4.50	0.0000
*LIDDEL	5	m	1	1.28	17.92	1.31	0.0000
LIU	2	c	2	0.65	38.19	31.05	0.0001
LIU2	2	m	3	1.65	4.37	0.04	0.0006
LIU2	4	f	3	1.54	6.68	0.00	0.0001
Subtotal LIU2				1.58	11.05	0.04	
LIU3	2	m	2	0.23	1.87	3.28	0.7518
LIU4	10	m	2	1.36	5780.52	226.97	0.0000
LIU4	12	f	2	1.05	3876.64	981.48	0.0000
Subtotal LIU4				1.23	9657.16	1208.46	
LIU5	1	c	0	0.65	11.25	9.20	0.0293
LOMBA2	1	f	0	0.28	37.19	60.06	0.0841
LOMBAR	2	m	0	2.30	12.10	6.79	0.0000
LUBIN2	48	m	2	2.17	162.15	61.11	0.0000
LUBIN2	98	f	1	1.36	133.31	4.97	0.0000
Subtotal LUBIN2				1.80	295.46	66.08	
LUO	7	c	20	0.99	10.60	3.33	0.0012
MACLEN	73	c	2	0.98	17.04	5.58	0.0001
*MAGNUS	5	m	3	1.42	6.75	0.12	0.0002
MARSH	1	m	0	2.32	1.82	1.06	0.0018
MARSH	3	f	0	1.76	5.60	0.24	0.0000
Subtotal MARSH				1.90	7.42	1.30	
MARSH2	5	m	1	0.64	3.87	3.25	0.2107
MARSH2	6	f	1	1.66	3.65	0.04	0.0015
Subtotal MARSH2				1.14	7.51	3.30	
MARTIS	4	m	0	1.95	3.32	0.51	0.0004
MASTRA	2	m	2	2.10	4.76	1.40	0.0000
MATOS	27	m	2	1.92	8.82	1.16	0.0000
MATSUD	10	m	0	3.07	2.94	6.72	0.0000
MCCONN	1	m	0	0.19	3.33	6.16	0.7237
MCCONN	2	f	0	1.01	0.99	0.29	0.3136
Subtotal MCCONN				0.38	4.32	6.45	
MCDUFF	1	m	0	1.81	4.70	0.32	0.0001
MCLAUG	1	m	0	1.20	18.70	2.29	0.0000
*MIGRAN	27	m	2	1.28	3.91	0.29	0.0111
*MIGRAN	42	f	2	1.53	3.54	0.00	0.0040
Subtotal MIGRAN				1.40	7.45	0.29	
MILLER	2	f	1	1.61	4.90	0.01	0.0004
MILLS	1	m	1	0.24	71.48	123.00	0.0406
*MRFITR	6	m	0	3.70	0.50	2.29	0.0091
NAM	77	m	1	2.16	24.64	9.18	0.0000
NAM	93	f	1	2.18	34.31	13.61	0.0000
Subtotal NAM				2.18	58.95	22.79	

Table 1A2 - 2

IESLC - Meta-analysis of Ever Smoking, Cigarettes (or Any Product if Cigarettes not available)
All LC types
Most adjusted

REF	NRR	SEX	AD	Ys	Ws	Qs	Ps
NOTAN2	19	m	2	0.86	33.41	16.15	0.0000
NOU	11	m	0	1.81	5.20	0.33	0.0000
NOU	12	f	0	1.96	2.74	0.45	0.0012
Subtotal NOU				1.86	7.94	0.78	
ODRISC	3	c	0	3.89	5.83	31.82	0.0000
ORMOS	4	m	0	2.23	6.39	2.95	0.0000
ORMOS	26	f	0	-1.64	0.95	9.73	0.1093
Subtotal ORMOS				1.73	7.34	12.68	
OSANN	41	m	2	2.98	39.82	81.05	0.0000
OSANN	42	f	2	2.71	66.25	88.24	0.0000
Subtotal OSANN				2.81	106.08	169.29	
PARKIN	31	m	0	1.54	71.91	0.02	0.0000
PASTOR	10	m	1	1.92	7.85	1.04	0.0000
PAWLEG	2	m	6	2.51	3.16	2.86	0.0000
PERNU	8	m	0	2.23	50.02	22.62	0.0000
PERNU	4	f	0	0.95	5.14	1.90	0.0321
Subtotal PERNU				2.11	55.16	24.52	
PERSH2	11	c	4	1.88	115.76	12.26	0.0000
*PETO	5	m	0	1.82	1.98	0.14	0.0107
PEZZO2	10	m	0	2.71	5.55	7.40	0.0000
PEZZOT	25	m	0	2.96	3.75	7.42	0.0000
PIKE	4	m	0	1.66	13.39	0.15	0.0000
PIKE	8	f	0	1.57	18.04	0.01	0.0000
Subtotal PIKE				1.61	31.43	0.16	
POFFIJ	1	c	0	2.05	46.21	11.27	0.0000
POLEDN	1	c	1	2.22	11.85	5.31	0.0000
*QIAO2	9	m	0	0.79	9.66	5.69	0.0145
RACHTA	15	f	4	2.11	7.21	2.19	0.0000
RADZIK	1	c	0	0.27	5.03	8.27	0.5411
RANDIG	9	m	0	1.60	3.95	0.01	0.0014
RANDIG	10	f	0	0.80	6.34	3.62	0.0447
Subtotal RANDIG				1.11	10.29	3.63	
REN	1	m	0	1.27	7.46	0.58	0.0005
REN	2	f	0	1.40	9.65	0.22	0.0000
Subtotal REN				1.35	17.11	0.81	
RONCO	3	m	2	1.69	5.06	0.10	0.0001
ROTHSC	2	c	1	1.71	9.83	0.25	0.0000
SADOWS	4	m	0	1.45	13.79	0.16	0.0000
SANKAR	2	m	3	2.61	22.36	25.00	0.0000
SCHWAR	1	m	0	2.11	80.50	24.48	0.0000
SCHWAR	2	m	0	1.88	29.06	3.01	0.0000
SCHWAR	3	f	0	2.30	111.43	61.84	0.0000
SCHWAR	4	f	0	2.45	26.58	21.21	0.0000
Subtotal SCHWAR				2.20	247.58	110.54	
SEGI	1	m	0	0.53	15.18	15.79	0.0375
SEOW	6	f	1	1.66	9.73	0.11	0.0000
SHAW	12	c	0	2.47	9.34	7.87	0.0000
SIEMIA	5	m	7	2.49	10.37	9.14	0.0000
SIMARA	3	m	6	0.50	13.58	15.07	0.0650
SIMARA	4	f	6	0.49	9.71	11.03	0.1278
Subtotal SIMARA				0.50	23.30	26.09	
SOBUE	105	m	1	1.31	28.15	1.63	0.0000
SOBUE	115	f	1	0.92	47.90	19.24	0.0000
Subtotal SOBUE				1.07	76.05	20.86	
*SPEIZE	8	f	0	1.96	52.33	8.48	0.0000
SPITZ	3	c	0	2.91	6.16	11.35	0.0000
STASZE	7	m	0	2.50	4.72	4.20	0.0000
STASZE	5	f	0	1.47	4.16	0.03	0.0028
Subtotal STASZE				2.02	8.88	4.23	
STAYNE	1	m	0	1.30	40.37	2.63	0.0000
STOCKS	46	m	2	1.94	32.85	4.83	0.0000
STOCKS	50	f	1	1.11	58.11	11.36	0.0000
Subtotal STOCKS				1.41	90.96	16.19	
STOCKW	8	c	0	2.41	1511.07	1096.77	0.0000
STUCKE	3	m	0	4.83	0.49	5.29	0.0007
SUN	1	c	0	0.84	30.23	15.58	0.0000
SUZUK2	7	c	3	2.40	2.76	1.97	0.0001
SVENSS	71	f	1	1.82	16.09	1.15	0.0000
TANG	3	c	0	2.09	6.14	1.76	0.0000
*TENKAN	22	m	1	2.68	5.38	6.87	0.0000
TIZZAN	2	m	0	0.70	90.61	66.01	0.0000

Table 1A2 - 2

IESLC - Meta-analysis of Ever Smoking, Cigarettes (or Any Product if Cigarettes not available)
All LC types
Most adjusted

REF	NRR	SEX	AD	Ys	Ws	Qs	Ps
TIZZAN	22	f	0	1.40	8.03	0.18	0.0001
Subtotal TIZZAN				0.76	98.64	66.19	
TOKARS	6	c	3	1.89	7.27	0.81	0.0000
TOUSEY	10	m	3	3.06	3.75	8.55	0.0000
TOUSEY	13	f	3	2.82	10.58	17.00	0.0000
Subtotal TOUSEY				2.88	14.33	25.55	
TSUGAN	27	m	0	0.23	7.76	13.64	0.5244
*TULINI	38	m	3	2.04	10.34	2.47	0.0000
*TULINI	44	f	3	2.57	11.17	11.43	0.0000
Subtotal TULINI				2.31	21.50	13.90	
*TVERDA	22	m	2	1.52	20.49	0.02	0.0000
WAKAI	72	m	2	1.30	8.06	0.52	0.0002
WAKAI	78	f	2	1.50	9.15	0.02	0.0000
Subtotal WAKAI				1.41	17.20	0.54	
WANG	5	c	6	1.06	15.11	3.72	0.0000
WANG2	16	c	4	0.83	7.47	3.93	0.0235
WANG3	1	c	0	1.05	28.11	7.21	0.0000
WANG4	2	m	2	0.15	100.26	198.08	0.1372
WICKLU	1	m	0	1.53	15.41	0.01	0.0000
WIGLE	13	m	0	2.46	13.33	10.90	0.0000
WIGLE	16	f	0	1.40	21.22	0.52	0.0000
Subtotal WIGLE				1.81	34.55	11.42	
WILKIN	3	c	4	2.06	12.03	3.05	0.0000
WU	45	f	2	1.11	14.48	2.87	0.0000
WUNSCH	4	m	1	1.56	11.39	0.00	0.0000
WUNSCH	10	f	1	1.49	14.00	0.06	0.0000
Subtotal WUNSCH				1.52	25.38	0.06	
WUWILL	8	f	3	0.83	102.19	53.14	0.0000
WYNDE2	16	m	0	2.28	7.19	3.81	0.0000
WYNDE3	48	m	0	2.27	7.69	3.93	0.0000
WYNDE3	83	f	0	1.14	9.73	1.68	0.0004
Subtotal WYNDE3				1.64	17.42	5.61	
WYNDE4	48	m	0	2.21	10.51	4.51	0.0000
WYNDE4	62	f	2	1.05	8.80	2.20	0.0018
Subtotal WYNDE4				1.68	19.31	6.71	
WYNDE6	81	m	0	2.39	71.26	49.79	0.0000
WYNDE6	252	f	0	2.34	103.92	64.48	0.0000
Subtotal WYNDE6				2.36	175.18	114.27	
*XIANGZ	14	m	2	0.58	25.00	23.61	0.0036
XU	2	m	2	0.99	58.89	18.52	0.0000
XU2	2	c	7	1.34	45.75	2.19	0.0000
XU3	2	m	1	1.79	5.80	0.32	0.0000
XU3	4	f	1	1.35	3.69	0.15	0.0095
Subtotal XU3				1.62	9.49	0.48	
XU4	1	c	0	1.08	20.82	4.68	0.0000
YAMAGU	11	c	1	1.38	9.83	0.30	0.0000
*YONG	2	c	1	1.91	22.68	2.84	0.0000
*YUAN	1	m	2	1.87	11.44	1.16	0.0000
ZHANG	2	m	7	1.39	4.65	0.13	0.0028
ZHANG	3	f	7	1.32	4.81	0.26	0.0038
Subtotal ZHANG				1.35	9.46	0.39	
ZHENG	15	m	0	1.29	20.36	1.38	0.0000
ZHENG	24	f	0	0.74	20.88	13.91	0.0008
Subtotal ZHENG				1.01	41.24	15.30	
ZHOU	2	m	0	0.86	17.50	8.43	0.0003
ZHOU	3	f	0	0.80	5.34	3.07	0.0660
Subtotal ZHOU				0.84	22.83	11.50	

Table 1A2 - 2

IESLC - Meta-analysis of Ever Smoking, Cigarettes (or Any Product if Cigarettes not available)
All LC types
Most adjusted

	N	327
	NS	236
	Wt	19006.39
Het	Chi	6320.10
Het	df	326
Het	P	***
Fixed	RR	4.73
	RRl	4.66
	RRu	4.80
	P	+++
Random	RR	5.49
	RRl	5.10
	RRu	5.92
	P	+++
Asymm	P	**

Table 1A2 - 3

IESLC - Meta-analysis of Ever Smoking, Cigarettes (or Any Product if Cigarettes not available)
All LC types
Most adjusted

	Sex: combined	male	female	Total
N	47	172	108	327
NS	47	169	104	320
Wt	2408.39	9566.95	7031.04	19006.39
Het Chi	727.83	2169.03	2358.27	6320.10
Het df	46	171	107	326
Het P	***	***	***	***
Fixed RR	8.55	4.64	3.97	4.73
RRl	8.21	4.55	3.87	4.66
RRu	8.90	4.73	4.06	4.80
P	+++	+++	+++	+++
Random RR	6.02	6.15	4.42	5.49
RRl	4.87	5.56	3.83	5.10
RRu	7.45	6.81	5.10	5.92
P	+++	+++	+++	+++
Between Chi				1064.97
Between df				2
Between P				***
Btwn(F) P				***
Btwn(R) P				***

	Lung cancer type: all	other	Total
N	316	11	327
NS	228	8	236
Wt	18807.16	199.23	19006.39
Het Chi	6254.06	35.90	6320.10
Het df	315	10	326
Het P	***	***	***
Fixed RR	4.75	3.21	4.73
RRl	4.68	2.80	4.66
RRu	4.82	3.69	4.80
P	+++	+++	+++
Random RR	5.59	3.47	5.49
RRl	5.18	2.63	5.10
RRu	6.03	4.57	5.92
P	+++	+++	+++
Between Chi			30.14
Between df			1
Between P			***
Btwn(F) P			N.S.
Btwn(R) P			**

	Location: NAmer	UK	Scand	othEur	China	Japan	othAs	other	Total
N	115	29	32	50	51	18	18	14	327
NS	81	20	23	39	35	12	14	12	236
Wt	5464.23	362.80	465.05	1030.25	10718.29	457.15	289.45	219.17	19006.39
Het Chi	1117.43	141.51	114.21	425.58	455.89	52.89	91.52	28.46	6320.10
Het df	114	28	31	49	50	17	17	13	326
Het P	***	***	***	***	***	***	***	**	***
Fixed RR	9.16	5.33	6.31	5.62	3.33	3.19	3.43	6.17	4.73
RRl	8.92	4.81	5.76	5.28	3.27	2.91	3.06	5.41	4.66
RRu	9.41	5.91	6.91	5.97	3.40	3.49	3.85	7.05	4.80
P	+++	+++	+++	+++	+++	+++	+++	+++	+++
Random RR	7.63	6.03	6.26	6.05	2.77	3.21	3.49	6.97	5.49
RRl	6.92	4.67	5.13	4.91	2.51	2.68	2.64	5.58	5.10
RRu	8.42	7.78	7.64	7.44	3.06	3.85	4.63	8.70	5.92
P	+++	+++	+++	+++	+++	+++	+++	+++	+++
Between Chi									3892.60
Between df									7
Between P									***
Btwn(F) P									***
Btwn(R) P									***

Table 1A2 - 3

IESLC - Meta-analysis of Ever Smoking, Cigarettes (or Any Product if Cigarettes not available)
All LC types
Most adjusted

	Detailed Country in "other Europe" multi	Germany	othWest	East	Balkans	Total
N	4	17	13	13	3	50
NS	3	12	12	9	3	39
Wt	445.74	218.92	167.48	113.25	84.86	1030.25
Het Chi	105.30	62.72	129.90	37.35	0.00	425.58
Het df	3	16	12	12	2	49
Het P	***	***	***	***	N.S.	***
Fixed RR	7.62	4.66	4.07	5.77	3.31	5.62
RRl	6.95	4.09	3.50	4.80	2.67	5.28
RRu	8.36	5.33	4.74	6.94	4.09	5.97
P	+++	+++	+++	+++	+++	+++
Random RR	7.85	5.25	8.96	5.91	3.31	6.05
RRl	4.46	3.83	4.84	4.12	2.67	4.91
RRu	13.82	7.18	16.60	8.46	4.09	7.44
P	+++	+++	+++	+++	+++	+++
Between Chi						90.30
Between df						4
Between P						***
Btwn(F) P						*
Btwn(R) P						***

	Detailed Country in "other Asia" India	HongKong	other	Total
N	3	7	8	18
NS	3	5	6	14
Wt	64.13	140.70	84.62	289.45
Het Chi	43.73	10.74	16.02	91.52
Het df	2	6	7	17
Het P	***	(*)	*	***
Fixed RR	5.15	3.52	2.42	3.43
RRl	4.03	2.99	1.95	3.06
RRu	6.58	4.15	2.99	3.85
P	+++	+++	+++	+++
Random RR	6.46	3.56	2.46	3.49
RRl	1.90	2.81	1.76	2.64
RRu	21.92	4.50	3.43	4.63
P	++	+++	+++	+++
Between Chi				21.03
Between df				2
Between P				***
Btwn(F) P				N.S.
Btwn(R) P				N.S.

	Detailed other continent SCAmer	Auslia	Africa	Total
N	10	1	3	14
NS	8	1	3	12
Wt	122.40	0.99	95.77	219.17
Het Chi	17.99	0.00	1.51	28.46
Het df	9	0	2	13
Het P	*	N.S.	N.S.	**
Fixed RR	7.24	20.29	4.98	6.17
RRl	6.06	2.84	4.07	5.41
RRu	8.64	145.07	6.08	7.05
P	+++	++	+++	+++
Random RR	7.62	20.29	4.98	6.97
RRl	5.84	2.84	4.07	5.58
RRu	9.94	145.07	6.08	8.70
P	+++	++	+++	+++
Between Chi				8.95
Between df				2
Between P				*
Btwn(F) P				N.S.
Btwn(R) P				*

Table 1A2 - 3

IESLC - Meta-analysis of Ever Smoking, Cigarettes (or Any Product if Cigarettes not available)
All LC types
Most adjusted

	Start year of study <1960	1960-69	1970-79	1980-89	1990+	Total
N	54	52	72	112	37	327
NS	39	38	52	75	32	236
Wt	836.18	1250.26	1367.12	15057.96	494.87	19006.39
Het Chi	466.54	412.22	606.98	4575.51	227.79	6320.10
Het df	53	51	71	111	36	326
Het P	***	***	***	***	***	***
Fixed RR	4.17	5.16	4.59	4.72	5.33	4.73
RRl	3.90	4.88	4.35	4.65	4.88	4.66
RRu	4.47	5.46	4.84	4.80	5.82	4.80
P	+++	+++	+++	+++	+++	+++
Random RR	4.93	5.35	5.11	5.86	6.23	5.49
RRl	3.93	4.51	4.31	5.16	4.93	5.10
RRu	6.18	6.34	6.05	6.65	7.88	5.92
P	+++	+++	+++	+++	+++	+++
Between Chi						31.05
Between df						4
Between P						***
Btwn(F) P						N.S.
Btwn(R) P						N.S.

	Study type (1) CC	other	Total
N	261	66	327
NS	185	51	236
Wt	17738.92	1267.46	19006.39
Het Chi	5801.41	446.01	6320.10
Het df	260	65	326
Het P	***	***	***
Fixed RR	4.65	5.96	4.73
RRl	4.58	5.64	4.66
RRu	4.72	6.30	4.80
P	+++	+++	+++
Random RR	5.32	6.26	5.49
RRl	4.89	5.33	5.10
RRu	5.79	7.37	5.92
P	+++	+++	+++
Between Chi			72.67
Between df			1
Between P			***
Btwn(F) P			(*)
Btwn(R) P			(*)

	Study type (2) CC	prosp	other	Total
N	261	61	5	327
NS	185	47	4	236
Wt	17738.92	1222.92	44.55	19006.39
Het Chi	5801.41	425.87	11.41	6320.10
Het df	260	60	4	326
Het P	***	***	*	***
Fixed RR	4.65	5.87	9.21	4.73
RRl	4.58	5.55	6.87	4.66
RRu	4.72	6.21	12.35	4.80
P	+++	+++	+++	+++
Random RR	5.32	6.08	8.98	5.49
RRl	4.89	5.14	5.39	5.10
RRu	5.79	7.19	14.98	5.92
P	+++	+++	+++	+++
Between Chi				81.40
Between df				2
Between P				***
Btwn(F) P				N.S.
Btwn(R) P				(*)

Table 1A2 - 3

IESLC - Meta-analysis of Ever Smoking, Cigarettes (or Any Product if Cigarettes not available)
All LC types
Most adjusted

	Study size (number of LC cases) 100-249	250-499	500-999	1000+	Total
N	116	86	64	61	327
NS	96	63	43	34	236
Wt	938.45	1261.33	1386.31	15420.30	19006.39
Het Chi	453.65	563.27	553.58	4692.46	6320.10
Het df	115	85	63	60	326
Het P	***	***	***	***	***
Fixed RR	3.74	4.94	4.94	4.76	4.73
RRl	3.51	4.68	4.69	4.69	4.66
RRu	3.99	5.22	5.21	4.84	4.80
P	+++	+++	+++	+++	+++
Random RR	4.43	5.73	6.19	6.24	5.49
RRl	3.86	4.94	5.26	5.31	5.10
RRu	5.08	6.65	7.29	7.34	5.92
P	+++	+++	+++	+++	+++
Between Chi					57.14
Between df					3
Between P					***
Btwn(F) P					N.S.
Btwn(R) P					**

	Risky occupational population no	mining	othRisky	Total
N	309	7	11	327
NS	218	7	11	236
Wt	18801.76	74.66	129.97	19006.39
Het Chi	6260.50	19.59	26.04	6320.10
Het df	308	6	10	326
Het P	***	**	**	***
Fixed RR	4.74	3.08	4.62	4.73
RRl	4.67	2.45	3.89	4.66
RRu	4.81	3.86	5.49	4.80
P	+++	+++	+++	+++
Random RR	5.53	3.75	5.27	5.49
RRl	5.12	2.38	3.75	5.10
RRu	5.97	5.91	7.42	5.92
P	+++	+++	+++	+++
Between Chi				13.97
Between df				2
Between P				***
Btwn(F) P				N.S.
Btwn(R) P				N.S.

	National cigarette tobacco type Virginia	blended	other	Total
N	50	224	53	327
NS	37	162	37	236
Wt	716.64	7556.69	10733.06	19006.39
Het Chi	266.95	2505.28	460.45	6320.10
Het df	49	223	52	326
Het P	***	***	***	***
Fixed RR	5.72	7.65	3.33	4.73
RRl	5.31	7.48	3.27	4.66
RRu	6.15	7.82	3.39	4.80
P	+++	+++	+++	+++
Random RR	6.26	6.31	2.76	5.49
RRl	5.20	5.79	2.50	5.10
RRu	7.55	6.87	3.04	5.92
P	+++	+++	+++	+++
Between Chi				3087.42
Between df				2
Between P				***
Btwn(F) P				***
Btwn(R) P				***

Table 1A2 - 3

IESLC - Meta-analysis of Ever Smoking, Cigarettes (or Any Product if Cigarettes not available)
All LC types
Most adjusted

	Any proxy use No/nk	Yes	Total
N	227	100	327
NS	169	67	236
Wt	7144.39	11862.00	19006.39
Het Chi	2902.67	1672.15	6320.10
Het df	226	99	326
Het P	***	***	***
Fixed RR	6.99	3.74	4.73
RRl	6.83	3.67	4.66
RRu	7.15	3.81	4.80
P	+++	+++	+++
Random RR	5.51	5.42	5.49
RRl	5.02	4.88	5.10
RRu	6.06	6.03	5.92
P	+++	+++	+++
Between Chi			1745.27
Between df			1
Between P			***
Btwn(F) P			***
Btwn(R) P			N.S.

	Full histological confirmation No	Yes	Total
N	246	81	327
NS	177	59	236
Wt	16480.12	2526.27	19006.39
Het Chi	4831.93	799.74	6320.10
Het df	245	80	326
Het P	***	***	***
Fixed RR	4.39	7.69	4.73
RRl	4.32	7.40	4.66
RRu	4.46	8.00	4.80
P	+++	+++	+++
Random RR	5.28	6.19	5.49
RRl	4.85	5.37	5.10
RRu	5.74	7.14	5.92
P	+++	+++	+++
Between Chi			688.43
Between df			1
Between P			***
Btwn(F) P			***
Btwn(R) P			(*)

	Number of adjustment variables (1) 0	1	2+/+nk	Total
N	165	64	98	327
NS	117	47	79	243
Wt	4673.94	1712.42	12620.03	19006.39
Het Chi	1964.14	564.41	2605.35	6320.10
Het df	164	63	97	326
Het P	***	***	***	***
Fixed RR	7.15	5.33	3.99	4.73
RRl	6.95	5.08	3.92	4.66
RRu	7.36	5.59	4.06	4.80
P	+++	+++	+++	+++
Random RR	5.33	5.77	5.58	5.49
RRl	4.75	4.94	4.94	5.10
RRu	5.98	6.73	6.31	5.92
P	+++	+++	+++	+++
Between Chi				1186.20
Between df				2
Between P				***
Btwn(F) P				***
Btwn(R) P				N.S.

Table 1A2 - 3

IESLC - Meta-analysis of Ever Smoking, Cigarettes (or Any Product if Cigarettes not available)
All LC types
Most adjusted

	Number of adjustment variables (2) 0	1	2	3-5	6+/+nk	Total
N	165	64	49	35	14	327
NS	117	47	40	29	12	245
Wt	4673.94	1712.42	11711.05	720.01	188.97	19006.39
Het Chi	1964.14	564.41	2214.01	205.04	104.60	6320.10
Het df	164	63	48	34	13	326
Het P	***	***	***	***	***	***
Fixed RR	7.15	5.33	3.91	5.54	4.03	4.73
RRl	6.95	5.08	3.84	5.15	3.49	4.66
RRu	7.36	5.59	3.98	5.96	4.64	4.80
P	+++	+++	+++	+++	+++	+++
Random RR	5.33	5.77	5.49	6.50	4.08	5.49
RRl	4.75	4.94	4.63	5.33	2.64	5.10
RRu	5.98	6.73	6.51	7.92	6.29	5.92
P	+++	+++	+++	+++	+++	+++
Between Chi						1267.90
Between df						4
Between P						***
Btwn(F) P						***
Btwn(R) P						N.S.

	Product all/unsp	cig+/-ot	cig only	Total
N	127	175	25	327
NS	97	126	20	243
Wt	6329.34	6205.35	6471.69	19006.39
Het Chi	1783.03	2104.34	396.88	6320.10
Het df	126	174	24	326
Het P	***	***	***	***
Fixed RR	3.61	7.55	3.94	4.73
RRl	3.52	7.36	3.84	4.66
RRu	3.70	7.74	4.04	4.80
P	+++	+++	+++	+++
Random RR	4.81	6.04	5.43	5.49
RRl	4.23	5.47	4.27	5.10
RRu	5.47	6.66	6.91	5.92
P	+++	+++	+++	+++
Between Chi				2035.84
Between df				2
Between P				***
Btwn(F) P				***
Btwn(R) P				*

	Denominator nev any	nev cigs	Total
N	233	94	327
NS	170	69	239
Wt	15940.95	3065.43	19006.39
Het Chi	4538.68	1238.43	6320.10
Het df	232	93	326
Het P	***	***	***
Fixed RR	4.39	6.95	4.73
RRl	4.32	6.71	4.66
RRu	4.46	7.21	4.80
P	+++	+++	+++
Random RR	5.33	5.88	5.49
RRl	4.89	5.10	5.10
RRu	5.82	6.78	5.92
P	+++	+++	+++
Between Chi			542.98
Between df			1
Between P			***
Btwn(F) P			***
Btwn(R) P			N.S.

Table 1A2 - 3

IESLC - Meta-analysis of Ever Smoking, Cigarettes (or Any Product if Cigarettes not available)
All LC types
Most adjusted

	Derivation of RR/CI			
	Orig	StdCalc	Other	Total
N	43	169	115	327
NS	33	122	88	243
Wt	1806.05	4827.94	12372.39	19006.39
Het Chi	748.14	2200.31	1728.66	6320.10
Het df	42	168	114	326
Het P	***	***	***	***
Fixed RR	7.24	7.00	3.81	4.73
RRl	6.92	6.81	3.75	4.66
RRu	7.58	7.20	3.88	4.80
P	+++	+++	+++	+++
Random RR	5.72	5.43	5.47	5.49
RRl	4.58	4.83	4.96	5.10
RRu	7.14	6.10	6.03	5.92
P	+++	+++	+++	+++
Between Chi				1642.98
Between df				2
Between P				***
Btwn(F) P				***
Btwn(R) P				N.S.

	Study LIU4		
	LIU4	others	Total
N	2	325	327
NS	1	235	236
Wt	9657.16	9349.22	19006.39
Het Chi	215.88	4086.37	6320.10
Het df	1	324	326
Het P	***	***	***
Fixed RR	3.43	6.59	4.73
RRl	3.37	6.46	4.66
RRu	3.50	6.72	4.80
P	+++	+++	+++
Random RR	3.33	5.53	5.49
RRl	2.47	5.11	5.10
RRu	4.49	5.99	5.92
P	+++	+++	+++
Between Chi			2017.85
Between df			1
Between P			***
Btwn(F) P			***
Btwn(R) P			**

Table 1A2 - 4

IESLC - Meta-analysis of Ever Smoking, Cigarettes (or Any Product if Cigarettes not available)
All LC types
Least adjusted

REF	NRR	X	SEX	AGEL	AGEH	RACE	YF	LC TYPE	LOC	START	ST	NLC	R	VB	P	H	AD	PRODUCT	DENOM	De
ABELIN	2	x	m	0	0	all	-	all	Eu:wst	1941	CC	118	n	bl	y	n	0	cig+/-ot	nev any	st
ABRAHA	7		m	0	0	all	0	q+s+a	Eu:est	1975	pr	571	n	bl	n	n	0	all/unsp	nev any	ot
ABRAHA	8		f	0	0	all	0	q+s+a	Eu:est	1975	pr	571	n	bl	n	n	0	all/unsp	nev any	ot
AGUDO	8	x	f	0	0	all	-	all	Eu:wst	1989	CC	103	n	bl	n	n	0	cig only	nev any	st
AKIBA	3	x	m	0	0	all	0	all	As:Jap	1963	pr	610	n	bl	n	n	0	cig+/-ot	nev cigs	st
AKIBA	7	x	f	0	0	all	0	all	As:Jap	1963	pr	610	n	bl	n	n	0	cig+/-ot	nev cigs	st
ALDERS	61	x	m	0	0	all	-	all	Eu:UK	1977	CC	1448	n	V	n	n	0	cig+/-ot	nev any	st
ALDERS	12	x	f	0	0	all	-	all	Eu:UK	1977	CC	1448	n	V	n	n	0	cig only	nev any	st
AMANDU	3	x	m	0	0	wh	0	all	NAmer	1959	pr	132	m	bl	n	n	0	cig+/-ot	nev cigs	st
AMES	4		m	0	0	wh	-	all	NAmer	1959	ot	317	m	bl	n	n	0	all/unsp	nev any	st
ANDERS	3		f	0	0	all	0	all	NAmer	1986	pr	343	n	bl	n	n	0	cig+/-ot	nev cigs	st
ARCHER	6		m	0	0	wh	0	all	NAmer	1950	pr	146	m	bl	n	n	0	cig+/-ot	nev cigs	st
ARMADA	4		m	0	0	all	-	all	Eu:wst	1986	CC	325	n	bl	n	y	0	cig+/-ot	nev any	st
AUSTIN	3	x	c	0	0	all	-	all	NAmer	1970	CC	166	o	bl	y	n	0	cig+/-ot	nev cigs	st
AUVINE	1	x	c	0	0	all	-	all	Eu:Sca	1986	CC	517	n	bl	y	n	0	cig+/-ot	nev cigs	st
AXELSO	1		c	0	0	all	-	all	Eu:Sca	1960	CC	152	n	bl	y	n	0	all/unsp	nev any	st
AXELSS	1	x	m	0	0	sca	-	all	Eu:Sca	1989	CC	436	n	bl	n	n	0	all/unsp	nev any	st
AXELSS	11		f	0	0	sca	-	all	Eu:Sca	1989	CC	436	n	bl	n	n	0	all/unsp	nev any	st
BAND	1		m	0	0	all	-	all	NAmer	1983	CC	2831	n	V	y	y	2	cig only	nev any	ot
BARBON	106	x	m	0	0	all	-	all	Eu:wst	1979	CC	755	n	bl	y	y	0	all/unsp	nev any	st
BECHER	15	x	m	0	0	all	-	all	Eu:Ger	1985	CC	194	n	bl	n	y	0	cig+/-ot	nev any	st
BECHER	16		f	0	0	all	-	all	Eu:Ger	1985	CC	194	n	bl	n	y	0	cig+/-ot	nev any	st
BENSHL	15		m	0	0	all	0	all	Eu:UK	1967	pr	486	n	V	n	n	1	cig+/-ot	nev any	ot
BEST	23		m	55	79	all	3	all	NAmer	1955	pr	381	n	V	n	n	0	cig+/-ot	nev any	st
BEST	18		f	0	0	all	0	all	NAmer	1955	pr	381	n	V	n	n	1	cig only	nev any	ot
BLOHMK	3		m	0	0	all	-	all	Eu:Ger	1978	CC	888	n	bl	n	y	0	all/unsp	nev any	st
BLOT4	1		m	0	0	wh	-	all	NAmer	1974	CC	335	n	bl	y	n	0	cig+/-ot	nev cigs	st
BOFFET	7	x	m	0	0	all	-	all	Eu:mul	1988	CC	5621	n	bl	y	n	0	cig+/-ot	nev any	st
BOUCOT	58	x	m	0	0	all	0	all	NAmer	1951	pr	121	n	bl	n	n	0	cig+/-ot	nev any	ot
BRESLO	17		m	0	0	all	-	all	NAmer	1949	CC	518	n	bl	n	y	0	cig+/-ot	nev+1	st
BRESLO	23		f	0	0	all	-	all	NAmer	1949	CC	518	n	bl	n	y	0	cig+/-ot	nev+1	st
BRETT	10		m	0	0	all	0	all	Eu:UK	1960	pr	150	n	V	n	n	0	cig+/-ot	nev cigs	st
BROCKM	1		m	0	0	wh	-	all	Eu:Ger	1990	CC	117	n	bl	n	y	0	cig+/-ot	nev cigs	st
BROCKM	2		f	0	0	wh	-	all	Eu:Ger	1990	CC	117	n	bl	n	y	0	cig+/-ot	nev cigs	st
BROSS	13		m	0	0	wh	-	all	NAmer	1960	CC	974	n	bl	n	n	0	cig+/-ot	nev any	st
BROWN2	2		m	0	0	wh	-	all	NAmer	1984	CC	14596	n	bl	n	y	2	cig+/-ot	nev cigs	or
BROWN2	1		f	0	0	wh	-	all	NAmer	1984	CC	14596	n	bl	n	y	2	cig+/-ot	nev cigs	or
BUFFLE	2		m	0	0	wh	-	all	NAmer	1976	CC	943	n	bl	y	n	0	cig+/-ot	nev any	st
BUFFLE	6		f	0	0	wh	-	all	NAmer	1976	CC	943	n	bl	y	n	0	cig+/-ot	nev any	st
CARPEN	7	x	c	0	0	w+b	-	all	NAmer	1991	CC	356	n	bl	n	n	0	cig+/-ot	nev cigs	st
CASCO2	1		c	0	0	wh	-	all	Eu:Ger	1991	CC	155	n	bl	n	n	0	all/unsp	nev any	st
CASCOR	1		c	0	0	wh	-	all	Eu:Ger	1985	CC	389	n	bl	n	y	0	all/unsp	nev any	st
CEDERL	107		m	0	0	all	16	all	Eu:Sca	1963	pr	491	n	bl	n	n	2	all/unsp	nev any	ot
CEDERL	112		f	0	0	all	0	all	Eu:Sca	1963	pr	491	n	bl	n	n	2	all/unsp	nev any	ot
CHAN	5		m	0	0	all	-	all	As:HK	1976	CC	397	n	bl	n	n	0	cig+/-ot	nev any	st
CHAN	6		f	0	0	all	-	all	As:HK	1976	CC	397	n	bl	n	n	0	cig+/-ot	nev any	st
CHANG	6		m	0	0	all	0	all	NAmer	1972	pr	136	n	bl	n	n	0	cig+/-ot	nev cigs	st
CHANG	12		f	0	0	all	0	all	NAmer	1972	pr	136	n	bl	n	n	0	cig+/-ot	nev cigs	st
CHATZI	4		c	0	0	all	-	all	Eu:bal	1987	CC	282	n	bl	n	y	0	all/unsp	nev any	st
CHEN2	1		m	0	0	all	-	all	As:Chi	1983	CC	193	n	ot	y	n	0	all/unsp	nev any	st
CHEN2	2		f	0	0	all	-	all	As:Chi	1983	CC	193	n	ot	y	n	0	all/unsp	nev any	st
CHEN3	1		c	0	0	all	-	all	As:Chi	1981	CC	254	n	ot	y	n	0	all/unsp	nev any	st
CHIAZZ	2	x	m	0	0	all	-	all	NAmer	1940	CC	144	o	bl	y	n	0	cig+/-ot	nev cigs	st
CHOI	1		m	0	0	all	-	all	As:oth	1985	CC	375	n	bl	n	n	0	cig+/-ot	nev cigs	st
CHOI	5		f	0	0	all	-	all	As:oth	1985	CC	375	n	bl	n	n	0	cig+/-ot	nev cigs	st
CHOW	6	x	m	0	0	wh	0	all	NAmer	1966	pr	219	n	bl	n	n	0	cig+/-ot	nev any	st
CHYOU	9	x	m	0	0	jap	0	all	NAmer	1965	pr	227	n	bl	n	y	0	cig+/-ot	nev cigs	st
COMSTO	33		m	0	0	all	-	all	NAmer	1975	ot	258	n	bl	n	n	0	cig+/-ot	nev any	st
COMSTO	45		f	0	0	all	-	all	NAmer	1975	ot	258	n	bl	n	n	0	cig+/-ot	nev any	st
COOKSO	4		c	0	0	bl	-	all	Africa	1961	CC	234	n	V	n	y	0	cig+/-ot	nev any	st
CORREA	33	x	c	0	0	all	-	all	NAmer	1979	CC	1359	n	bl	y	n	0	cig+/-ot	nev cigs	st
CPSI	187		m	35	84	all	6	all	NAmer	1959	pr	5138	n	bl	n	n	1	cig+/-ot	nev any	ot
CPSI	274		f	40	74	all	6	all	NAmer	1959	pr	5138	n	bl	n	n	1	cig+/-ot	nev cigs	ot
CPSII	104		m	35	99	all	4	all	NAmer	1982	pr	3229	n	bl	n	n	1	cig only	nev any	ot
CPSII	79		f	0	0	all	4	all	NAmer	1982	pr	3229	n	bl	n	n	1	cig+/-ot	nev cigs	ot
DAMBER	26	x	m	0	0	all	-	all	Eu:Sca	1972	CC	579	n	bl	y	n	0	cig+/-ot	nev any	st
DARBY	15		m	0	0	wh	-	all	Eu:UK	1988	CC	982	n	V	n	n	0	all/unsp	nev any	st
DARBY	16		f	0	0	wh	-	all	Eu:UK	1988	CC	982	n	V	n	n	0	all/unsp	nev any	st
DAVEYS	5		m	0	0	all	-	all	Eu:Ger	1930	CC	109	n	bl	y	n	0	all/unsp	nev any	st
DAVEYS	6		f	0	0	all	-	all	Eu:Ger	1930	CC	109	n	bl	y	n	0	all/unsp	nev any	ot
DEAN	8		m	0	0	wh	-	all	Africa	1947	CC	603	n	V	y	n	0	cig+/-ot	nev any	st
DEAN2	12		m	0	0	all	-	all	Eu:UK	1960	CC	954	n	V	y	n	0	cig+/-ot	nev any	st

Table 1A2 - 4

IESLC - Meta-analysis of Ever Smoking, Cigarettes (or Any Product if Cigarettes not available)
All LC types
Least adjusted

REF	NRR	X	SEX	AGEL	AGEH	RACE	YF	LC TYPE	LOC	START	ST	NLC	R	VB	P	H	AD	PRODUCT	DENOM	De
DEAN2	20		f	0	0	all	-	all	Eu:UK	1960	CC	954	n	V	y	n	0	cig+/-ot	nev any	st
DEAN3	240	x	m	0	0	all	-	all	Eu:UK	1969	CC	766	n	V	y	n	0	cig only	nev any	st
DEAN3	124	x	f	0	0	all	-	all	Eu:UK	1969	CC	766	n	V	y	n	0	cig only	nev any	st
DEKLER	6		m	0	0	all	0	all	Auslia	1961	pr	138	m	V	n	n	2	all/unsp	nev any	ot
DESTE2	15		m	0	0	all	-	all	SCAmer	1993	CC	463	n	bl	n	n	0	all/unsp	nev any	st
DESTEF	11	x	m	0	0	all	-	all	SCAmer	1988	CC	497	n	bl	n	y	0	cig+/-ot	nev any	st
DOCKER	3		c	0	0	wh	0	all	NAmer	1974	pr	120	n	bl	n	n	4	cig+/-ot	nev cigs	ot
DOLL	20		m	0	0	all	-	all	Eu:UK	1948	CC	1465	n	V	n	n	0	cig+/-ot	nev any	st
DOLL	12		f	0	0	all	-	all	Eu:UK	1948	CC	1465	n	V	n	n	0	all/unsp	nev any	st
DOLL2	88		m	0	0	all	10	all	Eu:UK	1951	pr	920	n	V	n	n	1	cig+/-ot	nev any	ot
DORANT	10		c	0	0	all	0	all	Eu:wst	1986	ot	550	n	bl	n	y	0	all/unsp	nev any	st
DORGAN	7	x	m	0	0	wh	-	all	NAmer	1980	CC	2026	n	bl	y	y	0	cig+/-ot	nev any	st
DORGAN	31	x	m	0	0	bl	-	all	NAmer	1980	CC	2026	n	bl	y	y	0	cig+/-ot	nev any	st
DORGAN	95		f	0	0	all	-	all	NAmer	1980	CC	2026	n	bl	y	y	3	cig+/-ot	nev any	or
DORN	413		m	0	0	wh	5	all	NAmer	1954	pr	5097	n	bl	n	n	1	cig+/-ot	nev any	ot
DOSEME	17	x	m	0	0	all	-	all	Eu:bal	1979	CC	1210	n	bl	n	n	0	cig+/-ot	nev cigs	st
DROSTE	3	x	m	0	0	all	-	all	Eu:wst	1995	CC	478	n	bl	n	y	0	all/unsp	nev any	st
DU	1		m	0	0	all	-	all	As:Chi	1985	CC	849	n	ot	y	n	0	all/unsp	nev any	or
DU	2		f	0	0	all	-	all	As:Chi	1985	CC	849	n	ot	y	n	0	all/unsp	nev any	or
DUNN	6		m	0	0	all	0	all	NAmer	1954	pr	139	o	bl	n	n	0	cig+/-ot	nev cigs	st
EBELIN	1		m	0	0	all	-	all	Eu:Ger	1980	CC	130	n	bl	n	n	0	all/unsp	nev any	st
ENGELA	8	x	m	0	0	all	0	all	Eu:Sca	1964	pr	435	n	bl	n	n	0	cig+/-ot	nev cigs	st
ENGELA	22	x	f	0	0	all	0	all	Eu:Sca	1964	pr	435	n	bl	n	n	0	cig+/-ot	nev cigs	st
ESAKI	4		m	0	0	all	-	all	As:Jap	1961	CC	245	n	bl	y	n	0	cig+/-ot	nev cigs	st
ESAKI	5		f	0	0	all	-	all	As:Jap	1961	CC	245	n	bl	y	n	0	cig+/-ot	nev cigs	st
FAN	1		m	0	0	all	-	all	As:Chi	1990	CC	403	n	ot	y	n	0	cig+/-ot	nev cigs	st
FAN	2		f	0	0	all	-	all	As:Chi	1990	CC	403	n	ot	y	n	0	cig+/-ot	nev cigs	st
GAO	6	x	m	0	0	all	-	all	As:Chi	1984	CC	1405	n	ot	n	n	0	cig+/-ot	nev cigs	st
GAO	16	x	f	0	0	all	-	all	As:Chi	1984	CC	1405	n	ot	n	n	0	cig+/-ot	nev cigs	st
GAO2	6	x	m	0	0	all	-	all	As:Jap	1988	CC	282	n	bl	n	n	0	cig+/-ot	nev cigs	st
GARCIA	3		c	0	0	all	-	all	NAmer	1992	CC	416	n	bl	n	y	0	cig+/-ot	nev cigs	st
GARDIN	7		c	0	0	all	-	all	Eu:UK	1988	CC	143	n	V	y	n	0	all/unsp	nev any	st
GARSHI	17	x	m	0	0	all	-	all	NAmer	1981	CC	1081	o	bl	y	n	0	all/unsp	nev any	st
GENG	1		m	0	0	all	-	all	As:Chi	1985	CC	292	n	ot	*	n	0	cig+/-ot	nev any	st
GENG	2		f	0	0	all	-	all	As:Chi	1985	CC	292	n	ot	*	n	0	cig+/-ot	nev any	st
GER	17	x	c	0	0	all	-	all	As:oth	1990	CC	141	n	ot	y	n	0	all/unsp	nev any	st
GODLEY	5		m	0	0	all	-	all	NAmer	1966	CC	1986	n	bl	y	n	1	cig+/-ot	nev cigs	ot
GODLEY	6		f	0	0	all	-	all	NAmer	1966	CC	1986	n	bl	y	n	1	cig+/-ot	nev cigs	ot
GOLLED	21	x	m	35	99	all	-	all	Eu:UK	1952	CC	443	n	V	y	n	0	cig+/-ot	nev any	st
GOODMA	3		m	0	0	w+o	-	all	NAmer	1983	CC	326	n	bl	y	y	0	cig+/-ot	nev any	st
GOODMA	7		f	0	0	w+o	-	all	NAmer	1983	CC	326	n	bl	y	y	0	cig+/-ot	nev any	st
GRAHAM	12	x	m	0	0	wh	-	all	NAmer	1956	CC	685	n	bl	n	n	0	cig+/-ot	nev any	st
GREGOR	3		m	0	0	all	-	all	Eu:UK	1976	CC	104	n	V	n	y	0	cig+/-ot	nev cigs	st
GREGOR	7		f	0	0	all	-	all	Eu:UK	1976	CC	104	n	V	n	y	0	cig+/-ot	nev cigs	st
GSELL	6		m	0	0	all	-	all	Eu:wst	1937	CC	150	n	bl	n	y	0	cig+/-ot	nev any	st
HAENSZ	56		f	0	0	all	-	not alv	NAmer	1955	CC	158	n	bl	n	y	0	cig+/-ot	nev any	st
HAMMO2	16	x	m	0	0	all	6	all	NAmer	1967	pr	450	o	bl	n	n	0	cig+/-ot	nev any	st
HAMMON	128	x	m	0	0	wh	0	all	NAmer	1952	pr	448	n	bl	n	n	0	cig+/-ot	nev any	st
HANSEN	3		m	0	0	all	0	all	Eu:Sca	1968	pr	105	o	bl	y	n	2	all/unsp	nev any	ot
HEGMAN	1		c	0	0	all	-	all	NAmer	1989	CC	282	n	bl	y	y	0	all/unsp	nev any	st
HEIN	7		m	0	0	all	0	all	Eu:Sca	1970	pr	144	n	bl	n	n	0	all/unsp	nev any	st
HENNEK	3		m	0	0	all	0	all	NAmer	1982	pr	169	n	bl	n	n	0	all/unsp	nev any	st
HINDS	26	x	f	0	0	o	-	all	NAmer	1968	CC	292	n	bl	n	n	0	all/unsp	nev any	st
HIRAYA	147		m	0	0	all	0	all	As:Jap	1965	pr	1917	n	bl	n	n	1	cig+/-ot	nev any	ot
HIRAYA	150		f	0	0	all	0	all	As:Jap	1965	pr	1917	n	bl	n	n	1	cig+/-ot	nev any	ot
HITOSU	6	x	m	0	0	all	-	all	As:Jap	1960	CC	216	n	bl	y	n	0	all/unsp	nev any	st
HITOSU	12	x	f	0	0	all	-	all	As:Jap	1960	CC	216	n	bl	y	n	0	all/unsp	nev any	st
HOLE	15	x	m	0	0	all	0	all	Eu:UK	1972	pr	225	n	V	n	n	0	all/unsp	nev any	st
HOROWI	1		m	0	0	all	-	all	NAmer	1956	CC	236	n	V	n	n	0	cig+/-ot	nev any	st
HOROWI	2		f	0	0	all	-	all	NAmer	1956	CC	236	n	V	n	n	0	cig+/-ot	nev any	st
HORWIT	1		f	0	0	all	-	all	NAmer	1977	CC	112	n	bl	n	n	0	cig+/-ot	nev cigs	st
HU	15		m	0	0	all	-	all	As:Chi	1985	CC	227	n	ot	n	y	0	cig+/-ot	nev any	st
HU	16		f	0	0	all	-	all	As:Chi	1985	CC	227	n	ot	n	y	0	cig+/-ot	nev any	st
HU2	9		m	0	0	all	-	all	As:Chi	1977	CC	523	n	ot	y	n	0	cig+/-ot	nev cigs	st
HU2	10		f	0	0	all	-	all	As:Chi	1977	CC	523	n	ot	y	n	0	cig+/-ot	nev cigs	st
HUANG	1		c	0	0	all	-	all	As:Chi	1990	CC	135	n	ot	y	n	0	all/unsp	nev any	st
HUMBLE	14		m	0	0	w-hi	-	all	NAmer	1980	CC	521	n	bl	y	n	1	cig+/-ot	nev cigs	ot
HUMBLE	16		m	0	0	hi	-	all	NAmer	1980	CC	521	n	bl	y	n	1	cig+/-ot	nev cigs	ot
HUMBLE	18		f	0	0	w-hi	-	all	NAmer	1980	CC	521	n	bl	y	n	1	cig+/-ot	nev cigs	ot
HUMBLE	20		f	0	0	hi	-	all	NAmer	1980	CC	521	n	bl	y	n	1	cig+/-ot	nev cigs	ot
JAHN	3	x	f	0	0	all	-	all	Eu:Ger	1988	CC	1004	n	bl	n	n	0	cig+/-ot	nev any	st
JAIN	6	x	m	0	0	all	-	all	NAmer	1981	CC	845	n	V	y	n	0	cig+/-ot	nev cigs	st

Table 1A2 - 4

IESLC - Meta-analysis of Ever Smoking, Cigarettes (or Any Product if Cigarettes not available)
All LC types
Least adjusted

REF	NRR	X	SEX	AGEL	AGEH	RACE	YF	LC TYPE	LOC	START	ST	NLC	R	VB	P	H	AD	PRODUCT	DENOM	De
JAIN	1	x	f	0	0	all	-	all	NAmer	1981	CC	845	n	V	y	n	0	cig+/-ot	nev cigs	st
JARUP	3	x	m	0	0	all	-	all	Eu:Sca	1928	CC	102	o	bl	y	n	0	all/unsp	nev any	st
JARVHO	3		m	0	0	all	-	all	Eu:Sca	1983	CC	147	n	bl	n	n	0	all/unsp	nev any	st
JARVHO	7		f	0	0	all	-	all	Eu:Sca	1983	CC	147	n	bl	n	n	0	all/unsp	nev any	st
JEDRYC	63	x	m	0	0	all	-	all	Eu:est	1980	CC	1630	n	bl	y	n	0	cig+/-ot	nev any	st
JEDRYC	68	x	f	0	0	all	-	all	Eu:est	1980	CC	1630	n	bl	y	n	0	cig+/-ot	nev any	st
JIANG	1		m	0	0	all	-	all	As:Chi	1984	CC	125	n	ot	n	n	0	all/unsp	nev any	st
JIANG	2		f	0	0	all	-	all	As:Chi	1984	CC	125	n	ot	n	n	0	all/unsp	nev any	st
JOLY	2		m	0	0	all	-	all	SCAmer	1978	CC	826	n	bl	n	n	0	cig+/-ot	nev any	st
JOLY	1		f	0	0	all	-	all	SCAmer	1978	CC	826	n	bl	n	n	0	cig+/-ot	nev any	st
JUSSAW	2	x	m	0	0	all	-	all	As:Ind	1964	CC	792	n	V	n	n	0	cig only	nev any	st
KAISE2	72		m	35	99	all	9	all	NAmer	1979	pr	318	n	bl	n	n	1	cig only	nev any	st
KAISE2	64		f	35	99	all	9	all	NAmer	1979	pr	318	n	bl	n	n	1	cig only	nev any	st
KAISER	13		m	0	0	all	0	all	NAmer	1964	pr	714	n	bl	n	n	2	cig+/-ot	nev cigs	ot
KAISER	10		f	0	0	all	0	all	NAmer	1964	pr	714	n	bl	n	n	2	cig+/-ot	nev cigs	ot
KATSOU	27	x	f	0	0	all	-	all	Eu:bal	1987	CC	101	n	bl	n	n	0	all/unsp	nev any	st
KAUFMA	8	x	c	0	0	all	-	all	NAmer	1981	CC	881	n	bl	n	n	0	cig+/-ot	nev cigs	st
KELLER	3		m	0	0	wh	-	all	NAmer	1985	CC	15038	n	bl	n	n	0	all/unsp	nev any	st
KELLER	11		m	0	0	nonw	-	all	NAmer	1985	CC	15038	n	bl	n	n	0	all/unsp	nev any	st
KELLER	7		f	0	0	wh	-	all	NAmer	1985	CC	15038	n	bl	n	n	0	all/unsp	nev any	st
KELLER	15		f	0	0	nonw	-	all	NAmer	1985	CC	15038	n	bl	n	n	0	all/unsp	nev any	st
KHUDER	4		m	0	0	all	-	all	NAmer	1985	CC	482	n	bl	n	y	0	cig+/-ot	nev cigs	st
KIHARA	31		c	0	0	jap	-	all	As:Jap	1991	CC	440	n	bl	n	n	0	all/unsp	nev any	st
KINLEN	6	x	m	0	0	all	0	all	Eu:UK	1967	pr	718	n	V	n	n	0	all/unsp	nev any	st
KJUUS	3		m	0	0	all	-	all	Eu:Sca	1979	CC	176	n	bl	n	n	0	cig only	nev any	st
KNEKT	76	x	m	20	69	all	21	all	Eu:Sca	1966	pr	515	n	bl	n	n	0	all/unsp	nev any	st
KO	1		f	0	0	all	-	all	As:oth	1992	CC	117	n	ot	n	y	3	cig+/-ot	nev cigs	or
KOHLME	1	x	c	0	0	all	-	all	Eu:Ger	1990	CC	239	n	bl	n	n	0	all/unsp	nev any	st
KOO	1		f	0	0	all	-	all	As:HK	1981	CC	200	n	bl	n	n	0	all/unsp	nev any	st
KOULUM	2		m	0	0	all	-	all	Eu:Sca	1936	CC	812	n	bl	n	n	0	cig only	nev any	st
KREUZE	60		f	1	45	all	-	all	Eu:Ger	1990	CC	2260	n	bl	n	n	3	cig+/-ot	nev any	ot
KREUZE	62		f	55	69	all	-	all	Eu:Ger	1990	CC	2260	n	bl	n	n	3	cig+/-ot	nev any	ot
KREYBE	24	x	m	0	0	all	-	all	Eu:Sca	1948	CC	300	n	bl	n	y	0	all/unsp	nev any	st
KREYBE	39	x	f	0	0	all	-	all	Eu:Sca	1948	CC	300	n	bl	n	y	0	all/unsp	nev any	st
KUBIK	27		m	0	0	all	0	all	Eu:est	1965	pr	108	n	bl	n	n	0	cig+/-ot	nev any	st
LAMTH	6		f	0	0	ch	-	all	As:HK	1983	CC	445	n	bl	n	n	0	all/unsp	nev any	or
LAMWK	1		f	0	0	ch	-	all	As:HK	1981	CC	163	n	bl	n	n	0	all/unsp	nev any	st
LAMWK2	9		m	0	0	all	-	q+s+l+a	As:HK	1976	CC	480	n	bl	n	n	0	all/unsp	nev any	st
LAMWK2	10		f	0	0	all	-	q+s+l+a	As:HK	1976	CC	480	n	bl	n	n	0	all/unsp	nev any	st
LANGE	34	x	m	0	0	all	0	all	Eu:Sca	1976	pr	268	n	bl	n	n	0	all/unsp	nev any	st
LANGE	31	x	f	0	0	all	0	all	Eu:Sca	1976	pr	268	n	bl	n	n	0	all/unsp	nev any	st
LAUSSM	10	x	m	0	0	all	-	all	Eu:Ger	1982	CC	432	n	bl	n	n	0	all/unsp	nev any	st
LEI	1		m	0	0	all	-	all	As:Chi	1986	CC	792	n	ot	y	n	0	all/unsp	nev any	st
LEI	2		f	0	0	all	-	all	As:Chi	1986	CC	792	n	ot	y	n	0	all/unsp	nev any	st
LEMARC	3		c	0	0	w+o	-	all	NAmer	1992	CC	341	n	bl	n	y	0	all/unsp	nev any	st
LETOUR	1		c	0	0	all	-	all	NAmer	1983	CC	738	n	V	y	y	0	cig+/-ot	nev cigs	st
LEVIN	30		m	0	0	all	-	all	NAmer	1938	CC	475	n	bl	n	n	1	cig+/-ot	nev any	st
LIDDEL	5		m	0	0	all	18	all	NAmer	1970	pr	304	m	V	n	n	1	cig+/-ot	nev cigs	ot
LIU	2		c	0	0	all	-	all	As:Chi	1980	CC	229	n	ot	*	n	2	all/unsp	nev any	or
LIU2	1	x	m	0	0	all	-	all	As:Chi	1983	CC	316	n	ot	n	n	0	all/unsp	nev any	st
LIU2	3	x	f	0	0	all	-	all	As:Chi	1983	CC	316	n	ot	n	n	0	all/unsp	nev any	st
LIU3	1	x	m	0	0	all	-	all	As:Chi	1985	CC	110	n	ot	n	n	0	all/unsp	nev any	st
LIU4	10		m	35	69	all	-	all	As:Chi	1986	CC	1000-00	n	ot	y	n	2	cig only	nev any	ot
LIU4	12		f	0	0	all	-	all	As:Chi	1986	CC	1000-00	n	ot	y	n	2	all/unsp	nev any	ot
LIU5	1		c	0	0	all	-	all	As:Chi	1978	CC	111	n	ot	y	n	0	all/unsp	nev any	st
LOMBA2	1		f	0	0	all	-	all	NAmer	1960	CC	225	n	bl	n	n	0	cig+/-ot	nev cigs	st
LOMBAR	2		m	0	0	all	-	all	NAmer	1951	CC	1040	n	bl	n	n	0	cig+/-ot	nev any	st
LUBIN2	47	x	m	0	0	all	-	all	Eu:mul	1976	CC	7804	n	bl	n	y	0	cig+/-ot	nev any	st
LUBIN2	97	x	f	0	0	all	-	all	Eu:mul	1976	CC	7804	n	bl	n	y	0	cig only	nev any	st
LUO	1	x	c	0	0	all	-	all	As:Chi	1990	CC	102	n	ot	n	y	0	cig+/-ot	nev cigs	st
MACLEN	71	x	m	0	0	ch	-	all	As:oth	1972	CC	233	n	bl	n	n	0	cig+/-ot	nev cigs	st
MACLEN	72	x	f	0	0	ch	-	all	As:oth	1972	CC	233	n	bl	n	n	0	cig+/-ot	nev cigs	st
MAGNUS	1	x	m	0	0	all	0	all	Eu:Sca	1953	pr	203	o	bl	y	n	0	all/unsp	nev any	st
MARSH	1		m	0	0	all	-	all	NAmer	1979	CC	150	n	bl	y	n	0	cig+/-ot	nev any	st
MARSH	3		f	0	0	all	-	all	NAmer	1979	CC	150	n	bl	y	n	0	cig+/-ot	nev any	st
MARSH2	1	x	c	0	0	all	-	all	NAmer	1979	CC	114	n	bl	y	n	0	cig+/-ot	nev any	st
MARTIS	4		m	0	0	all	-	all	Eu:UK	1972	CC	201	n	V	n	n	0	cig+/-ot	nev cigs	st
MASTRA	1	x	m	0	0	all	-	all	Eu:wst	1973	CC	309	n	bl	n	n	0	all/unsp	nev any	st
MATOS	26	x	m	0	0	all	-	all	SCAmer	1994	CC	200	n	bl	n	n	0	cig+/-ot	nev any	st
MATSUD	10		m	0	0	all	-	all	As:Jap	1965	CC	179	n	bl	n	n	0	cig+/-ot	nev cigs	st

Table 1A2 - 4

IESLC - Meta-analysis of Ever Smoking, Cigarettes (or Any Product if Cigarettes not available)
All LC types
Least adjusted

REF	NRR	X	SEX	AGEL	AGEH	RACE	YF	LC TYPE	LOC	START	ST	NLC	R	VB	P	H	AD	PRODUCT	DENOM	De
MCCONN	1		m	0	0	all	-	all	Eu:UK	1946	CC	100	n	V	n	y	0	all/unsp	nev any	st
MCCONN	2		f	0	0	all	-	all	Eu:UK	1946	CC	100	n	V	n	y	0	all/unsp	nev any	st
MCDUFF	1		m	0	0	all	-	all	NAmer	1979	CC	165	n	V	y	n	0	cig+/-ot	nev cigs	st
MCLAUG	1		m	0	0	all	-	all	As:Chi	1972	CC	316	o	ot	y	n	0	all/unsp	nev any	st
MIGRAN	26	x	m	0	0	all	0	all	Eu:UK	1964	pr	259	n	V	n	n	0	all/unsp	nev any	st
MIGRAN	41	x	f	0	0	all	0	all	Eu:UK	1964	pr	259	n	V	n	n	0	all/unsp	nev any	st
MILLER	1	x	f	0	0	all	-	all	NAmer	1972	CC	168	n	bl	y	n	0	cig+/-ot	nev any	st
MILLS	1		m	0	0	wh	-	all	NAmer	1940	CC	444	n	bl	y	n	1	cig only	nev any	ot
MRFITR	6		m	0	0	all	0	all	NAmer	1973	pr	119	n	bl	n	n	0	cig+/-ot	nev cigs	ot
NAM	69	x	m	0	0	all	-	all	NAmer	1986	CC	1199	n	bl	y	n	0	cig+/-ot	nev cigs	ot
NAM	85	x	f	0	0	all	-	all	NAmer	1986	CC	1199	n	bl	y	n	0	cig+/-ot	nev cigs	ot
NOTAN2	7	x	m	0	0	all	-	all	As:Ind	1963	CC	683	n	V	n	n	0	cig only	nev any	st
NOU	11		m	30	64	all	-	all	Eu:Sca	1971	CC	273	n	bl	y	n	0	all/unsp	nev any	st
NOU	12		f	30	64	all	-	all	Eu:Sca	1971	CC	273	n	bl	y	n	0	all/unsp	nev any	st
ODRISC	3		c	0	0	all	-	all	Eu:UK	1992	CC	446	n	V	n	n	0	all/unsp	nev any	st
ORMOS	4		m	0	0	all	-	all	Eu:est	1947	CC	119	n	bl	y	y	0	cig+/-ot	nev any	st
ORMOS	26		f	0	0	all	-	all	Eu:est	1947	CC	119	n	bl	y	y	0	cig+/-ot	nev any	st
OSANN	17	x	m	0	0	all	-	all	NAmer	1984	CC	1986	n	bl	n	n	0	cig+/-ot	nev cigs	st
OSANN	21	x	f	0	0	all	-	all	NAmer	1984	CC	1986	n	bl	n	n	0	cig+/-ot	nev cigs	st
PARKIN	31		m	0	0	bl	-	all	Africa	1963	CC	877	n	V	y	n	0	cig+/-ot	nev any	st
PASTOR	5	x	m	0	0	all	-	all	Eu:wst	1976	CC	204	n	bl	y	n	0	all/unsp	nev any	st
PAWLEG	1	x	m	0	0	all	-	all	Eu:est	1992	CC	176	n	bl	n	y	0	all/unsp	nev any	st
PERNU	8		m	0	0	all	-	all	Eu:Sca	1944	CC	1606	n	bl	n	n	0	cig only	nev any	st
PERNU	4		f	0	0	all	-	all	Eu:Sca	1944	CC	1606	n	bl	n	n	0	cig only	nev any	st
PERSH2	5	x	c	0	0	all	-	all	Eu:Sca	1980	CC	1022	n	bl	y	n	0	all/unsp	nev any	st
PETO	5		m	0	0	all	0	all	Eu:UK	1954	pr	103	n	V	n	n	0	all/unsp	nev any	st
PEZZO2	10		m	0	0	all	-	all	SCAmer	1992	CC	367	n	bl	n	y	0	cig+/-ot	nev cigs	st
PEZZOT	25		m	0	0	all	-	all	SCAmer	1987	CC	215	n	bl	n	y	0	cig only	nev cigs	st
PIKE	4		m	0	0	w-hi	-	all	NAmer	1972	CC	731	n	bl	y	n	0	all/unsp	nev any	st
PIKE	8		f	0	0	w-hi	-	all	NAmer	1972	CC	731	n	bl	y	n	0	all/unsp	nev any	st
POFFIJ	1		c	0	0	all	-	all	Eu:mul	1990	CC	971	n	bl	n	n	0	all/unsp	nev any	st
POLEDN	3	x	c	0	0	all	-	all	NAmer	1978	CC	209	n	bl	y	n	0	cig+/-ot	nev cigs	st
QIAO2	9		m	0	0	all	0	all	As:Chi	1992	pr	241	m	ot	n	n	0	cig+/-ot	nev any	st
RACHTA	3	x	f	0	0	all	-	all	Eu:est	1991	CC	118	n	bl	n	y	0	cig+/-ot	nev cigs	st
RADZIK	1		c	0	0	all	-	all	Eu:est	1986	CC	189	n	bl	n	n	0	all/unsp	nev any	st
RANDIG	9		m	0	0	all	-	all	Eu:Ger	1951	CC	448	n	bl	n	n	0	cig+/-ot	nev any	st
RANDIG	10		f	0	0	all	-	all	Eu:Ger	1951	CC	448	n	bl	n	n	0	cig+/-ot	nev any	st
REN	1		m	0	0	all	-	all	As:Chi	1980	CC	244	n	ot	*	n	0	all/unsp	nev any	st
REN	2		f	0	0	all	-	all	As:Chi	1980	CC	244	n	ot	*	n	0	all/unsp	nev any	st
RONCO	2	x	m	0	0	all	-	all	Eu:wst	1976	CC	126	n	bl	y	n	0	cig only	nev any	st
ROTHSC	1	x	c	0	0	all	-	all	NAmer	1971	CC	284	n	bl	y	n	0	all/unsp	nev any	st
SADOWS	4		m	0	0	wh	-	all	NAmer	1938	CC	477	n	bl	n	n	0	cig+/-ot	nev any	st
SANKAR	1	x	m	0	0	all	-	all	As:Ind	1990	CC	281	n	V	n	n	0	all/unsp	nev any	st
SCHWAR	1		m	0	0	wh	-	all	NAmer	1984	CC	5588	n	bl	y	y	0	cig+/-ot	nev cigs	st
SCHWAR	2		m	0	0	bl	-	all	NAmer	1984	CC	5588	n	bl	y	y	0	cig+/-ot	nev cigs	st
SCHWAR	3		f	0	0	wh	-	all	NAmer	1984	CC	5588	n	bl	y	y	0	cig+/-ot	nev cigs	st
SCHWAR	4		f	0	0	bl	-	all	NAmer	1984	CC	5588	n	bl	y	y	0	cig+/-ot	nev cigs	st
SEGI	1		m	0	0	all	-	all	As:Jap	1948	CC	159	n	bl	n	n	0	all/unsp	nev any	ot
SEOW	1	x	f	0	0	ch	-	q+s+l+a	As:oth	1997	CC	153	n	bl	n	y	0	cig+/-ot	nev cigs	st
SHAW	12		c	0	0	wh	-	all	NAmer	1988	CC	335	n	V	n	y	0	all/unsp	nev any	st
SIEMIA	9	x	m	0	0	all	-	all	NAmer	1979	CC	857	n	V	y	y	0	cig+/-ot	nev cigs	st
SIMARA	5	x	m	0	0	all	-	all	As:oth	1971	CC	115	n	bl	n	n	0	cig+/-ot	nev cigs	st
SIMARA	6	x	f	0	0	all	-	all	As:oth	1971	CC	115	n	bl	n	n	0	cig+/-ot	nev cigs	st
SOBUE	91	x	m	0	0	all	-	q+s+l+a	As:Jap	1986	CC	1376	n	bl	n	y	0	cig+/-ot	nev cigs	st
SOBUE	95	x	f	0	0	all	-	q+s+l+a	As:Jap	1986	CC	1376	n	bl	n	y	0	cig+/-ot	nev cigs	st
SPEIZE	8		f	0	0	all	0	all	NAmer	1976	pr	593	n	bl	n	y	0	cig+/-ot	nev cigs	st
SPITZ	3		c	0	0	b+hi	-	all	NAmer	1992	CC	177	n	bl	n	y	0	cig+/-ot	nev cigs	st
STASZE	7		m	0	0	all	-	all	Eu:est	1954	CC	281	n	bl	n	y	0	cig+/-ot	nev any	st
STASZE	5		f	0	0	all	-	all	Eu:est	1954	CC	281	n	bl	n	y	0	all/unsp	nev any	st
STAYNE	1		m	0	0	all	-	all	NAmer	1969	CC	420	n	bl	n	n	0	all/unsp	nev any	st
STOCKS	30	x	m	0	0	all	-	all	Eu:UK	1952	CC	2932	n	V	y	n	0	cig+/-ot	nev any	st
STOCKS	50		f	0	0	all	-	all	Eu:UK	1952	CC	2932	n	V	y	n	1	cig+/-ot	nev any	ot
STOCKW	8		c	0	0	all	-	all	NAmer	1981	CC	22161	n	bl	n	n	0	cig+/-ot	nev any	st
STUCKE	3		m	0	0	all	-	all	Eu:wst	1989	CC	247	n	bl	n	y	0	all/unsp	nev any	ot
SUN	1		c	0	0	all	-	all	As:Chi	1992	CC	207	n	ot	n	y	0	all/unsp	nev any	st
SUZUK2	3	x	c	0	0	all	-	all	SCAmer	1991	CC	123	n	bl	n	y	0	cig only	nev any	st
SVENSS	56	x	f	0	0	all	-	all	Eu:Sca	1983	CC	210	n	bl	n	n	0	all/unsp	nev any	st
TANG	3		c	0	0	all	-	not s	NAmer	1992	CC	119	n	bl	n	y	0	cig+/-ot	nev cigs	st
TENKAN	22		m	0	0	all	17	all	Eu:Sca	1962	pr	242	n	bl	n	n	1	all/unsp	nev any	ot
TIZZAN	2		m	0	0	all	-	all	Eu:wst	1959	CC	1358	n	bl	n	n	0	cig only	nev any	st
TIZZAN	22		f	0	0	all	-	all	Eu:wst	1959	CC	1358	n	bl	n	n	0	cig only	nev any	st
TOKARS	3	x	m	0	0	all	-	all	Eu:est	1966	ot	162	o	bl	n	y	0	all/unsp	nev any	st

Table 1A2 - 4

IESLC - Meta-analysis of Ever Smoking, Cigarettes (or Any Product if Cigarettes not available)
All LC types
Least adjusted

REF	NRR	X	SEX	AGEL	AGEH	RACE	YF	LC TYPE	LOC	START	ST	NLC	R	VB	P	H	AD	PRODUCT	DENOM	De
TOKARS	5	x	f	0	0	all	-	all	Eu:est	1966	ot	162	o	bl	n	y	0	all/unsp	nev any	st
TOUSEY	2	x	m	0	0	all	-	all	NAmer	1993	CC	507	n	bl	y	y	0	cig+/-ot	nev any	st
TOUSEY	6	x	f	0	0	all	-	all	NAmer	1993	CC	507	n	bl	y	y	0	cig+/-ot	nev any	st
TSUGAN	27		m	0	0	all	-	q+a	As:Jap	1976	CC	134	n	bl	n	y	0	all/unsp	nev any	st
TULINI	15	x	m	0	0	all	0	all	Eu:Sca	1967	pr	472	n	bl	n	n	1	all/unsp	nev any	ot
TULINI	21	x	f	0	0	all	0	all	Eu:Sca	1967	pr	472	n	bl	n	n	1	all/unsp	nev any	ot
TVERDA	22		m	0	0	all	0	all	Eu:Sca	1972	pr	238	n	bl	n	n	2	cig+/-ot	nev cigs	ot
WAKAI	13	x	m	0	0	all	-	all	As:Jap	1988	CC	333	n	bl	n	y	0	all/unsp	nev any	st
WAKAI	31	x	f	0	0	all	-	all	As:Jap	1988	CC	333	n	bl	n	y	0	all/unsp	nev any	st
WANG	1	x	m	0	0	all	-	all	As:Chi	1990	CC	390	n	ot	*	y	0	all/unsp	nev any	or
WANG	2	x	f	0	0	all	-	all	As:Chi	1990	CC	390	n	ot	*	y	0	all/unsp	nev any	or
WANG2	8	x	c	0	0	all	-	all	As:Chi	1980	CC	103	n	ot	n	n	0	cig+/-ot	nev cigs	st
WANG3	1		c	0	0	all	-	all	As:Chi	1981	CC	293	n	ot	*	n	0	all/unsp	nev any	st
WANG4	1	x	m	0	0	all	-	all	As:Chi	1976	CC	1170	n	ot	y	n	0	all/unsp	nev any	st
WICKLU	1		m	0	0	wh	-	all	NAmer	1968	CC	155	n	bl	y	n	0	cig+/-ot	nev+3	or
WIGLE	13		m	0	0	all	-	all	NAmer	1971	CC	728	n	V	n	n	0	cig only	nev any	st
WIGLE	16		f	0	0	all	-	all	NAmer	1971	CC	728	n	V	n	n	0	cig only	nev any	st
WILKIN	1	x	m	0	0	all	-	all	Eu:UK	1992	CC	271	n	V	n	n	0	cig+/-ot	nev cigs	st
WILKIN	2	x	f	0	0	all	-	all	Eu:UK	1992	CC	271	n	V	n	n	0	cig+/-ot	nev cigs	st
WU	37	x	f	0	0	wh	-	q+a	NAmer	1981	CC	220	n	bl	n	y	0	all/unsp	nev any	st
WUNSCH	1	x	m	0	0	all	-	all	SCAmer	1990	CC	398	n	bl	y	n	0	cig+/-ot	nev any	st
WUNSCH	7	x	f	0	0	all	-	all	SCAmer	1990	CC	398	n	bl	y	n	0	cig+/-ot	nev any	st
WUWILL	6	x	f	0	0	all	-	all	As:Chi	1985	CC	965	n	ot	n	n	0	cig+/-ot	nev cigs	st
WYNDE2	16		m	0	0	all	-	all	NAmer	1962	CC	404	n	bl	n	y	0	cig+/-ot	nev any	st
WYNDE3	48		m	0	0	all	-	all	NAmer	1966	CC	350	n	bl	n	y	0	cig+/-ot	nev any	st
WYNDE3	83		f	0	0	all	-	all	NAmer	1966	CC	350	n	bl	n	y	0	cig+/-ot	nev any	st
WYNDE4	48		m	0	0	all	-	all	NAmer	1948	CC	684	n	bl	y	n	0	all/unsp	nev any	st
WYNDE4	62		f	0	0	all	-	all	NAmer	1948	CC	684	n	bl	y	n	2	all/unsp	nev any	ot
WYNDE6	81		m	0	0	all	-	all	NAmer	1969	CC	4423	n	bl	n	y	0	cig+/-ot	nev any	st
WYNDE6	252		f	0	0	all	-	all	NAmer	1969	CC	4423	n	bl	n	y	0	cig+/-ot	nev cigs	st
XIANGZ	6	x	m	0	0	all	0	all	As:Chi	1976	pr	983	m	ot	n	n	0	cig+/-ot	nev any	st
XU	1	x	m	0	0	all	-	all	As:Chi	1985	CC	729	n	ot	n	n	0	all/unsp	nev any	st
XU2	1	x	c	0	0	all	-	all	As:Chi	1987	CC	610	o	ot	y	n	0	all/unsp	nev any	st
XU3	1	x	m	0	0	all	-	all	As:Chi	1981	CC	135	n	ot	n	n	0	all/unsp	nev any	st
XU3	3	x	f	0	0	all	-	all	As:Chi	1981	CC	135	n	ot	n	n	0	all/unsp	nev any	st
XU4	1		c	0	0	all	-	all	As:Chi	1981	CC	206	n	ot	*	n	0	all/unsp	nev any	st
YAMAGU	5	x	c	0	0	all	-	all	As:Jap	1989	CC	144	n	bl	n	y	0	all/unsp	nev any	st
YONG	2		c	0	0	all	0	all	NAmer	1971	pr	216	n	bl	n	n	1	cig+/-ot	nev cigs	ot
YUAN	1		m	0	0	all	0	all	As:Chi	1986	pr	142	n	ot	n	n	2	cig+/-ot	nev cigs	ot
ZHANG	1	x	c	0	0	all	-	all	As:Chi	1988	CC	100	n	ot	n	n	0	all/unsp	nev any	st
ZHENG	15		m	0	0	all	-	all	As:Chi	1982	CC	540	n	ot	*	y	0	cig+/-ot	nev cigs	st
ZHENG	24		f	0	0	all	-	all	As:Chi	1982	CC	540	n	ot	*	y	0	cig+/-ot	nev cigs	st
ZHOU	2		m	0	0	all	-	all	As:Chi	1978	CC	1360	n	ot	n	n	0	all/unsp	nev any	st
ZHOU	3		f	0	0	all	-	all	As:Chi	1978	CC	1360	n	ot	n	n	0	all/unsp	nev any	st

Cigarette type is all/unspec for all RRs
except for the following:

REF	NRR	CIGTYPE
ALDERS	12	MC only
DEAN3	240	MC only
DEAN3	124	MC only
DESTEF	11	MC only
JUSSAW	2	MC only
NOTAN2	7	MC only
PERNU	8	MC only
PERNU	4	MC only
SUZUK2	3	MC only

Table 1A2 - 5

IESLC - Meta-analysis of Ever Smoking, Cigarettes (or Any Product if Cigarettes not available)
All LC types
Least adjusted

REF	NRR	SEX	AD	Number Exposed Case	Number Exposed Cont	Non-exposed Case	Non-exposed Cont	RR	95.00%CI
ABELIN	2	m	0	47	154	2	183	27.93	(6.67- 116.83)
*ABRAHA	7	m	0	269	10351	10	3365	8.74	(4.66- 16.42)
*ABRAHA	8	f	0	62	5256	28	11589	4.88	(3.13- 7.62)
Subtotal ABRAHA								5.93	(4.12- 8.53)
AGUDO	8	f	0	23	23	80	183	2.29	(1.21- 4.32)
*AKIBA	3	m	0	393	207682	18	35833	3.77	(2.35- 6.04)
*AKIBA	7	f	0	83	65179	116	359850	3.95	(2.98- 5.24)
Subtotal AKIBA								3.90	(3.06- 4.97)
ALDERS	61	m	0	782	641	15	133	10.82	(6.28- 18.64)
ALDERS	12	f	0	530	371	75	243	4.63	(3.46- 6.19)
Subtotal ALDERS								5.59	(4.33- 7.23)
*AMANDU	3	m	0	126	111395	6	25350	4.78	(2.11- 10.84)
AMES	4	m	0	297	251	15	62	4.89	(2.72- 8.81)
*ANDERS	3	f	0	297	96164	46	195158	13.10	(9.61- 17.87)
*ARCHER	6	m	0	140	36269	6	9842	6.33	(2.80- 14.33)
ARMADA	4	m	0	317	254	4	64	19.97	(7.18- 55.57)
AUSTIN	3	c	0	161	237	5	88	11.96	(4.75- 30.09)
AUVINE	1	c	0	473	288	44	229	8.55	(6.00- 12.18)
AXELSO	1	c	0	90	86	62	371	6.26	(4.20- 9.34)
AXELSS	1	m	0	292	344	16	160	8.49	(4.96- 14.52)
AXELSS	11	f	0	110	109	18	154	8.63	(4.95- 15.05)
Subtotal AXELSS								8.56	(5.82- 12.59)
BAND	1	m	2	-	-	-	-	9.96	(7.38- 13.44)
BARBON	106	m	0	733	567	22	188	11.05	(7.01- 17.41)
BECHER	15	m	0	137	217	3	54	11.36	(3.48- 37.06)
BECHER	16	f	0	38	44	10	52	4.49	(2.01- 10.03)
Subtotal BECHER								6.02	(3.10- 11.71)
*BENSHL	15	m	1	-	-	-	-	8.02	(4.29- 15.01)
*BEST	23	m	0	212	21711	1	2854	27.87	(3.91- 198.68)
*BEST	18	f	1	-	-	-	-	2.24	(0.59- 8.44)
Subtotal BEST								4.95	(1.65- 14.89)
BLOHMK	3	m	0	762	587	126	301	3.10	(2.45- 3.92)
BLOT4	1	m	0	327	245	8	87	14.51	(6.91- 30.51)
BOFFET	7	m	0	5386	5239	117	1750	15.38	(12.70- 18.61)
*BOUCOT	58	m	0	117	35761	0	7551	49.62~	(3.09- 797.95)
BRESLO	17	m	0	471	383	7	42	7.38	(3.28- 16.61)
BRESLO	23	f	0	13	11	12	14	1.38	(0.45- 4.20)
Subtotal BRESLO								4.12	(2.14- 7.94)
*BRETT	10	m	0	144	47930	6	6530	3.27	(1.45- 7.40)
BROCKM	1	m	0	87	81	2	2	1.07	(0.15- 7.80)
BROCKM	2	f	0	24	54	4	18	2.00	(0.61- 6.54)
Subtotal BROCKM								1.70	(0.61- 4.70)
BROSS	13	m	0	831	612	38	170	6.07	(4.21- 8.77)
BROWN2	2	m	2	-	-	-	-	9.10	(8.30- 10.00)
BROWN2	1	f	2	-	-	-	-	12.70	(11.50- 13.90)
Subtotal BROWN2								10.72	(10.03- 11.46)
BUFFLE	2	m	0	461	373	5	47	11.62	(4.57- 29.50)
BUFFLE	6	f	0	419	284	41	198	7.12	(4.93- 10.30)
Subtotal BUFFLE								7.61	(5.40- 10.72)
CARPEN	7	c	0	328	469	15	241	11.24	(6.55- 19.29)
CASCO2	1	c	0	149	212	6	98	11.48	(4.90- 26.87)
CASCOR	1	c	0	365	362	22	295	13.52	(8.56- 21.35)
*CEDERL	107	m	2	-	-	-	-	5.92	(3.85- 9.12)
*CEDERL	112	f	2	-	-	-	-	4.18	(2.94- 5.93)
Subtotal CEDERL								4.80	(3.66- 6.30)
CHAN	5	m	0	206	161	2	43	27.51	(6.57- 115.26)
CHAN	6	f	0	105	50	84	139	3.48	(2.26- 5.35)
Subtotal CHAN								4.13	(2.73- 6.25)
*CHANG	6	m	0	78	1506	5	502	5.20	(2.12- 12.77)
*CHANG	12	f	0	42	1183	11	1139	3.68	(1.90- 7.10)
Subtotal CHANG								4.15	(2.44- 7.06)
CHATZI	4	c	0	255	365	27	129	3.34	(2.14- 5.21)
CHEN2	1	m	0	121	97	9	33	4.57	(2.09- 10.02)
CHEN2	2	f	0	38	30	25	33	1.67	(0.82- 3.39)
Subtotal CHEN2								2.62	(1.55- 4.44)
CHEN3	1	c	0	182	156	72	98	1.59	(1.09- 2.30)
CHIAZZ	2	m	0	139	209	4	47	7.81	(2.75- 22.18)
CHOI	1	m	0	267	465	13	95	4.20	(2.31- 7.64)
CHOI	5	f	0	19	26	76	164	1.58	(0.82- 3.02)
Subtotal CHOI								2.68	(1.72- 4.16)
*CHOW	6	m	0	201	189175	6	62913	11.14	(4.95- 25.09)

Table 1A2 - 5

IESLC - Meta-analysis of Ever Smoking, Cigarettes (or Any Product if Cigarettes not available)
All LC types
Least adjusted

REF	NRR	SEX	AD	Number Exposed Case	Number Exposed Cont	Non-exposed Case	Non-exposed Cont	RR	95.00%CI
*CHYOU	9	m	0	214	5554	13	2406	7.13	(4.08- 12.46)
COMSTO	33	m	0	151	229	4	69	11.37	(4.07- 31.82)
COMSTO	45	f	0	88	87	13	115	8.95	(4.69- 17.06)
Subtotal COMSTO								9.58	(5.54- 16.54)
COOKSO	4	c	0	184	38	45	61	6.56	(3.90- 11.04)
CORREA	33	c	0	1202	886	51	388	10.32	(7.61- 14.00)
*CPSI	187	m	1	-	-	-	-	9.18	(7.36- 11.45)
*CPSI	274	f	1	-	-	-	-	2.79	(2.22- 3.51)
Subtotal CPSI								5.17	(4.41- 6.06)
*CPSII	104	m	1	-	-	-	-	12.83	(10.28- 16.01)
*CPSII	79	f	1	-	-	-	-	8.16	(6.93- 9.62)
Subtotal CPSII								9.58	(8.40- 10.93)
DAMBER	26	m	0	332	215	42	208	7.65	(5.26- 11.11)
DARBY	15	m	0	664	1724	3	384	49.30	(15.77- 154.07)
DARBY	16	f	0	292	548	23	529	12.26	(7.89- 19.05)
Subtotal DARBY								14.69	(9.74- 22.16)
DAVEYS	5	m	0	90	144	3	23	4.79	(1.40- 16.42)
DAVEYS	6	f	0	0	3	16	83	0.72~	(0.04- 14.66)
Subtotal DAVEYS								3.65	(1.17- 11.42)
DEAN	8	m	0	540	500	12	61	5.49	(2.92- 10.32)
DEAN2	12	m	0	686	556	33	112	4.19	(2.80- 6.27)
DEAN2	20	f	0	63	29	88	121	2.99	(1.78- 5.02)
Subtotal DEAN2								3.69	(2.68- 5.07)
DEAN3	240	m	0	399	1227	24	510	6.91	(4.52- 10.57)
DEAN3	124	f	0	109	1420	41	1538	2.88	(2.00- 4.15)
Subtotal DEAN3								4.18	(3.17- 5.52)
*DEKLER	6	m	2	-	-	-	-	20.29	(2.84- 145.18)
DESTE2	15	m	0	432	314	31	151	6.70	(4.43- 10.13)
DESTEF	11	m	0	108	113	27	163	5.77	(3.55- 9.37)
*DOCKER	3	c	4	-	-	-	-	4.29	(1.66- 11.06)
DOLL	20	m	0	504	467	7	61	9.40	(4.26- 20.77)
DOLL	12	f	0	68	49	40	59	2.05	(1.19- 3.53)
Subtotal DOLL								3.34	(2.13- 5.23)
*DOLL2	88	m	1	-	-	-	-	11.58	(3.70- 36.24)
DORANT	10	c	0	470	2033	14	1090	18.00	(10.52- 30.78)
DORGAN	7	m	0	699	400	15	93	10.83	(6.19- 18.95)
DORGAN	31	m	0	264	116	3	35	26.55	(8.00- 88.08)
DORGAN	95	f	3	-	-	-	-	8.50	(6.70- 10.80)
Subtotal DORGAN								9.15	(7.37- 11.35)
*DORN	413	m	1	-	-	-	-	8.83	(6.64- 11.75)
DOSEME	17	m	0	1068	536	142	293	4.11	(3.28- 5.15)
DROSTE	3	m	0	471	443	7	93	14.13	(6.48- 30.78)
DU	1	m	0	538	-	28	-	3.53	(2.44- 5.11)
DU	2	f	0	191	-	92	-	1.93	(1.30- 2.87)
Subtotal DU								2.66	(2.03- 3.49)
*DUNN	6	m	0	137	52634	2	14160	18.43	(4.56- 74.42)
EBELIN	1	m	0	101	142	12	117	6.93	(3.63- 13.24)
*ENGELA	8	m	0	306	168817	27	58716	3.94	(2.66- 5.84)
*ENGELA	22	f	0	71	104832	31	207789	4.54	(2.98- 6.92)
Subtotal ENGELA								4.21	(3.16- 5.61)
ESAKI	4	m	0	155	143	16	28	1.90	(0.99- 3.65)
ESAKI	5	f	0	34	19	40	55	2.46	(1.23- 4.92)
Subtotal ESAKI								2.14	(1.33- 3.45)
FAN	1	m	0	216	498	36	236	2.84	(1.93- 4.18)
FAN	2	f	0	82	97	69	320	3.92	(2.65- 5.81)
Subtotal FAN								3.33	(2.53- 4.38)
GAO	6	m	0	671	558	62	202	3.92	(2.89- 5.32)
GAO	16	f	0	237	130	435	605	2.54	(1.98- 3.25)
Subtotal GAO								3.01	(2.48- 3.65)
GAO2	6	m	0	269	226	13	56	5.13	(2.73- 9.62)
GARCIA	3	c	0	395	307	21	139	8.52	(5.26- 13.80)
GARDIN	7	c	0	138	102	5	41	11.09	(4.23- 29.06)
GARSHI	17	m	0	1040	1596	41	363	5.77	(4.14- 8.04)
GENG	1	m	0	92	68	7	31	5.99	(2.49- 14.42)
GENG	2	f	0	126	75	67	118	2.96	(1.96- 4.48)
Subtotal GENG								3.36	(2.31- 4.89)
GER	17	c	0	90	318	51	246	1.37	(0.93- 2.00)
GODLEY	5	m	1	-	-	-	-	6.84	(5.60- 8.35)
GODLEY	6	f	1	-	-	-	-	5.54	(4.29- 7.15)
Subtotal GODLEY								6.31	(5.39- 7.39)
GOLLED	21	m	0	380	1966	15	490	6.31	(3.73- 10.68)

Table 1A2 - 5

IESLC - Meta-analysis of Ever Smoking, Cigarettes (or Any Product if Cigarettes not available)
All LC types
Least adjusted

REF	NRR	SEX	AD	Number Exposed Case	Number Exposed Cont	Non-exposed Case	Non-exposed Cont	RR	95.00%CI
GOODMA	3	m	0	216	398	10	199	10.80	(5.60- 20.82)
GOODMA	7	f	0	81	91	19	177	8.29	(4.74- 14.52)
Subtotal GOODMA								9.27	(6.05- 14.19)
GRAHAM	12	m	0	618	1284	18	346	9.25	(5.71- 15.00)
GREGOR	3	m	0	72	98	10	14	1.03	(0.43- 2.45)
GREGOR	7	f	0	21	42	1	22	11.00	(1.39- 87.29)
Subtotal GREGOR								1.46	(0.66- 3.26)
GSELL	6	m	0	60	42	2	29	20.71	(4.69- 91.56)
HAENSZ	56	f	0	74	103	81	236	2.09	(1.42- 3.09)
*HAMMO2	16	m	0	179	9590	1	1457	27.20	(3.81- 193.97)
*HAMMON	128	m	0	397	382338	15	115884	8.02	(4.79- 13.43)
*HANSEN	3	m	2	-	-	-	-	1.53	(0.71- 3.91)
HEGMAN	1	c	0	255	1202	27	2080	16.34	(10.92- 24.45)
*HEIN	7	m	0	143	4471	1	457	14.62	(2.05- 104.23)
*HENNEK	3	m	0	146	11112	23	10919	6.24	(4.02- 9.67)
HINDS	26	f	0	167	592	124	1812	4.12	(3.21- 5.29)
*HIRAYA	147	m	1	-	-	-	-	4.36	(3.53- 5.39)
*HIRAYA	150	f	1	-	-	-	-	2.36	(1.90- 2.94)
Subtotal HIRAYA								3.24	(2.78- 3.77)
HITOSU	6	m	0	142	1787	7	242	2.75	(1.27- 5.94)
HITOSU	12	f	0	34	500	33	1893	3.90	(2.39- 6.36)
Subtotal HITOSU								3.53	(2.33- 5.33)
*HOLE	15	m	0	187	5866	7	1189	5.41	(2.55- 11.49)
HOROWI	1	m	0	182	525	19	196	3.58	(2.17- 5.90)
HOROWI	2	f	0	21	382	14	463	1.82	(0.91- 3.62)
Subtotal HOROWI								2.83	(1.89- 4.25)
HORWIT	1	f	0	97	92	11	118	11.31	(5.73- 22.34)
HU	15	m	0	120	94	41	67	2.09	(1.30- 3.35)
HU	16	f	0	26	18	40	48	1.73	(0.83- 3.61)
Subtotal HU								1.98	(1.33- 2.94)
HU2	9	m	0	294	228	49	115	3.03	(2.08- 4.41)
HU2	10	f	0	108	80	72	100	1.88	(1.23- 2.85)
Subtotal HU2								2.44	(1.85- 3.23)
HUANG	1	c	0	98	77	37	58	2.00	(1.20- 3.32)
HUMBLE	14	m	1	-	-	-	-	12.10	(5.12- 28.60)
HUMBLE	16	m	1	-	-	-	-	11.88	(2.65- 53.30)
HUMBLE	18	f	1	-	-	-	-	11.36	(5.32- 24.23)
HUMBLE	20	f	1	-	-	-	-	15.40	(4.87- 48.74)
Subtotal HUMBLE								12.28	(7.58- 19.90)
JAHN	3	f	0	112	67	53	98	3.09	(1.97- 4.85)
JAIN	6	m	0	391	277	12	85	10.00	(5.36- 18.66)
JAIN	1	f	0	390	196	52	214	8.19	(5.78- 11.60)
Subtotal JAIN								8.59	(6.34- 11.64)
JARUP	3	m	0	91	52	11	42	6.68	(3.17- 14.09)
JARVHO	3	m	0	99	57	1	16	27.79	(3.59- 215.09)
JARVHO	7	f	0	41	15	6	21	9.57	(3.24- 28.26)
Subtotal JARVHO								12.08	(4.64- 31.46)
JEDRYC	63	m	0	852	656	49	219	5.80	(4.19- 8.04)
JEDRYC	68	f	0	120	32	78	166	7.98	(4.97- 12.82)
Subtotal JEDRYC								6.43	(4.92- 8.41)
JIANG	1	m	0	93	83	7	17	2.72	(1.08- 6.89)
JIANG	2	f	0	11	6	14	19	2.49	(0.74- 8.35)
Subtotal JIANG								2.63	(1.26- 5.50)
JOLY	2	m	0	552	709	12	218	14.14	(7.83- 25.56)
JOLY	1	f	0	166	123	52	283	7.34	(5.04- 10.70)
Subtotal JOLY								8.87	(6.46- 12.19)
JUSSAW	2	m	0	126	77	149	624	6.85	(4.90- 9.58)
*KAISE2	72	m	1	-	-	-	-	5.40	(3.05- 9.57)
*KAISE2	64	f	1	-	-	-	-	10.09	(5.29- 19.27)
Subtotal KAISE2								7.11	(4.63- 10.90)
*KAISER	13	m	2	-	-	-	-	17.63	(11.98- 25.96)
*KAISER	10	f	2	-	-	-	-	5.63	(3.89- 8.14)
Subtotal KAISER								9.70	(7.43- 12.67)
KATSOU	27	f	0	53	22	48	67	3.36	(1.81- 6.25)
KAUFMA	8	c	0	846	1645	35	925	13.59	(9.60- 19.24)
KELLER	3	m	0	8066	2517	323	1017	10.09	(8.83- 11.52)
KELLER	11	m	0	1493	340	38	117	13.52	(9.20- 19.86)
KELLER	7	f	0	3998	1269	469	1860	12.49	(11.09- 14.08)
KELLER	15	f	0	584	214	67	232	9.45	(6.91- 12.93)
Subtotal KELLER								11.30	(10.40- 12.29)
KHUDER	4	m	0	459	785	23	309	7.86	(5.06- 12.19)

Table 1A2 - 5

IESLC - Meta-analysis of Ever Smoking, Cigarettes (or Any Product if Cigarettes not available)
All LC types
Least adjusted

REF	NRR	SEX	AD	Number Exposed Case	Number Exposed Cont	Non-exposed Case	Non-exposed Cont	RR	95.00%CI
KIHARA	31	c	0	338	232	102	237	3.39	(2.54- 4.51)
*KINLEN	6	m	0	711	12722	7	1333	10.64	(5.07- 22.36)
KJUUS	3	m	0	151	127	2	24	14.27	(3.31- 61.54)
*KNEKT	76	m	0	111	51798	6	17814	6.36	(2.80- 14.46)
KO	1	f	3	-	-	-	-	4.20	(1.10- 15.60)
KOHLME	1	c	0	228	236	11	193	16.95	(8.99- 31.96)
KOO	1	f	0	112	63	88	137	2.77	(1.84- 4.16)
KOULUM	2	m	0	625	229	5	54	29.48	(11.65- 74.60)
KREUZE	60	f	3	-	-	-	-	9.21	(3.45- 24.53)
KREUZE	62	f	3	-	-	-	-	4.05	(2.81- 5.86)
Subtotal KREUZE								4.48	(3.18- 6.32)
KREYBE	24	m	0	252	3514	6	644	7.70	(3.41- 17.37)
KREYBE	39	f	0	12	328	30	657	0.80	(0.40- 1.59)
Subtotal KREYBE								2.04	(1.21- 3.44)
*KUBIK	27	m	0	106	7829	2	4271	28.91	(7.14- 117.06)
LAMTH	6	f	0	242	106	202	337	3.81	(2.86- 5.08)
LAMWK	1	f	0	88	41	75	144	4.12	(2.59- 6.55)
LAMWK2	9	m	0	244	161	23	43	2.83	(1.64- 4.88)
LAMWK2	10	f	0	75	50	65	139	3.21	(2.02- 5.10)
Subtotal LAMWK2								3.04	(2.14- 4.33)
*LANGE	34	m	0	195	5790	5	721	4.86	(2.01- 11.76)
*LANGE	31	f	0	61	5544	7	2159	3.39	(1.55- 7.41)
Subtotal LANGE								3.97	(2.21- 7.13)
LAUSSM	10	m	0	347	188	85	226	4.91	(3.61- 6.66)
LEI	1	m	0	443	361	41	123	3.68	(2.52- 5.38)
LEI	2	f	0	123	61	85	147	3.49	(2.32- 5.24)
Subtotal LEI								3.59	(2.72- 4.74)
LEMARC	3	c	0	309	288	32	168	5.63	(3.74- 8.49)
LETOUR	1	c	0	714	514	24	224	12.96	(8.38- 20.05)
LEVIN	30	m	1	-	-	-	-	6.97	(4.87- 9.97)
*LIDDEL	5	m	1	-	-	-	-	3.61	(2.27- 5.73)
LIU	2	c	2	-	-	-	-	1.92	(1.40- 2.64)
LIU2	1	m	0	212	180	12	44	4.32	(2.21- 8.43)
LIU2	3	f	0	54	23	38	69	4.26	(2.27- 7.99)
Subtotal LIU2								4.29	(2.71- 6.78)
LIU3	1	m	0	52	205	4	19	1.20	(0.39- 3.69)
LIU4	10	m	2	-	-	-	-	3.88	(3.78- 3.98)
LIU4	12	f	2	-	-	-	-	2.86	(2.77- 2.95)
Subtotal LIU4								3.43	(3.37- 3.50)
LIU5	1	c	0	85	70	26	41	1.91	(1.07- 3.44)
LOMBA2	1	f	0	149	353	76	239	1.33	(0.96- 1.83)
LOMBAR	2	m	0	978	782	14	112	10.01	(5.70- 17.58)
LUBIN2	47	m	0	6630	10435	190	2617	8.75	(7.53- 10.17)
LUBIN2	97	f	0	548	559	336	1188	3.47	(2.93- 4.10)
Subtotal LUBIN2								5.81	(5.19- 6.50)
LUO	1	c	0	65	146	37	160	1.93	(1.21- 3.06)
MACLEN	71	m	0	142	119	5	15	3.58	(1.26- 10.14)
MACLEN	72	f	0	45	57	41	109	2.10	(1.23- 3.57)
Subtotal MACLEN								2.34	(1.46- 3.76)
*MAGNUS	1	m	0	189	3439	11	1086	5.43	(2.97- 9.93)
MARSH	1	m	0	98	150	2	31	10.13	(2.37- 43.27)
MARSH	3	f	0	42	64	8	71	5.82	(2.54- 13.33)
Subtotal MARSH								6.67	(3.25- 13.70)
MARSH2	1	c	0	102	145	12	56	3.28	(1.67- 6.43)
MARTIS	4	m	0	197	176	4	25	7.00	(2.39- 20.49)
MASTRA	1	m	0	303	265	6	44	8.38	(3.52- 19.99)
MATOS	26	m	0	188	283	11	110	6.64	(3.48- 12.68)
MATSUD	10	m	0	170	3314	3	1255	21.46	(6.84- 67.33)
MCCONN	1	m	0	88	174	5	12	1.21	(0.41- 3.55)
MCCONN	2	f	0	3	3	4	11	2.75	(0.38- 19.67)
Subtotal MCCONN								1.46	(0.57- 3.76)
MCDUFF	1	m	0	159	134	6	31	6.13	(2.48- 15.14)
MCLAUG	1	m	0	294	1082	22	270	3.33	(2.12- 5.25)
*MIGRAN	26	m	0	206	6719	4	867	6.65	(2.48- 17.83)
*MIGRAN	41	f	0	31	4086	4	3814	7.23	(2.56- 20.47)
Subtotal MIGRAN								6.92	(3.38- 14.16)
MILLER	1	f	0	140	1607	28	3638	11.32	(7.51- 17.06)
MILLS	1	m	1	-	-	-	-	1.27	(1.01- 1.61)
*MRFITR	6	m	0	119	11007	0	1859	40.37~	(2.51- 648.95)
NAM	69	m	0	610	1075	30	520	9.84	(6.72- 14.40)
NAM	85	f	0	292	496	52	885	10.02	(7.31- 13.73)

Table 1A2 - 5

IESLC - Meta-analysis of Ever Smoking, Cigarettes (or Any Product if Cigarettes not available)
All LC types
Least adjusted

REF	NRR	SEX	AD	Number Exposed Case	Cont	Non-exposed Case	Cont	RR	95.00%CI
Subtotal NAM								9.94	(7.80- 12.68)
NOTAN2	7	m	0	78	129	134	544	2.45	(1.75- 3.44)
NOU	11	m	0	74	247	6	122	6.09	(2.58- 14.39)
NOU	12	f	0	10	92	4	261	7.09	(2.17- 23.17)
Subtotal NOU								6.42	(3.20- 12.87)
ODRISC	3	c	0	440	996	6	664	48.89	(21.71- 110.07)
ORMOS	4	m	0	87	1034	7	777	9.34	(4.30- 20.28)
ORMOS	26	f	0	1	234	23	1044	0.19	(0.03- 1.44)
Subtotal ORMOS								5.65	(2.74- 11.64)
OSANN	17	m	0	1108	1018	45	833	20.15	(14.75- 27.52)
OSANN	21	f	0	737	563	96	1093	14.90	(11.77- 18.87)
Subtotal OSANN								16.63	(13.78- 20.07)
PARKIN	31	m	0	372	933	107	1248	4.65	(3.69- 5.86)
PASTOR	5	m	0	194	262	10	89	6.59	(3.34- 13.00)
PAWLEG	1	m	0	172	249	4	92	15.89	(5.73- 44.05)
PERNU	8	m	0	706	216	97	275	9.27	(7.02- 12.23)
PERNU	4	f	0	7	24	110	971	2.57	(1.08- 6.11)
Subtotal PERNU								8.22	(6.32- 10.71)
PERSH2	5	c	0	844	924	178	1164	5.97	(4.97- 7.17)
*PETO	5	m	0	101	2423	2	295	6.15	(1.52- 24.79)
PEZZO2	10	m	0	361	469	6	117	15.01	(6.53- 34.48)
PEZZOT	25	m	0	211	317	4	116	19.30	(7.02- 53.10)
PIKE	4	m	0	514	375	18	69	5.25	(3.08- 8.98)
PIKE	8	f	0	163	90	36	96	4.83	(3.04- 7.66)
Subtotal PIKE								5.01	(3.53- 7.10)
POFFIJ	1	c	0	913	918	58	452	7.75	(5.81- 10.34)
POLEDN	3	c	0	196	271	12	139	8.38	(4.52- 15.54)
*QIAO2	9	m	0	197	6360	10	709	2.20	(1.17- 4.13)
RACHTA	3	f	0	85	43	33	98	5.87	(3.43- 10.06)
RADZIK	1	c	0	180	198	9	13	1.31	(0.55- 3.15)
RANDIG	9	m	0	277	245	5	22	4.97	(1.86- 13.34)
RANDIG	10	f	0	16	39	17	92	2.22	(1.02- 4.84)
Subtotal RANDIG								3.03	(1.64- 5.58)
REN	1	m	0	106	84	12	34	3.58	(1.74- 7.33)
REN	2	f	0	78	20	48	50	4.06	(2.16- 7.64)
Subtotal REN								3.84	(2.39- 6.17)
RONCO	2	m	0	116	274	6	78	5.50	(2.33- 12.98)
ROTHSC	1	c	0	271	222	13	62	5.82	(3.12- 10.86)
SADOWS	4	m	0	421	446	18	81	4.25	(2.51- 7.20)
SANKAR	1	m	0	251	439	28	767	15.66	(10.42- 23.55)
SCHWAR	1	m	0	2648	1019	119	376	8.21	(6.60- 10.22)
SCHWAR	2	m	0	863	275	50	104	6.53	(4.54- 9.39)
SCHWAR	3	f	0	1351	637	182	855	9.96	(8.28- 12.00)
SCHWAR	4	f	0	335	179	40	247	11.56	(7.90- 16.90)
Subtotal SCHWAR								9.05	(7.99- 10.25)
SEGI	1	m	0	140	1742	18	382	1.71	(1.03- 2.82)
SEOW	1	f	0	61	15	92	125	5.53	(2.96- 10.33)
SHAW	12	c	0	324	266	11	107	11.85	(6.24- 22.50)
SIEMIA	9	m	0	844	428	13	105	15.93	(8.85- 28.67)
SIMARA	5	m	0	33	264	27	433	2.00	(1.18- 3.41)
SIMARA	6	f	0	17	67	38	349	2.33	(1.24- 4.37)
Subtotal SIMARA								2.13	(1.42- 3.20)
SOBUE	91	m	0	1023	1013	34	128	3.80	(2.58- 5.60)
SOBUE	95	f	0	127	232	167	857	2.81	(2.14- 3.69)
Subtotal SOBUE								3.10	(2.48- 3.88)
*SPEIZE	8	f	0	535	1012074	58	776300	7.08	(5.40- 9.28)
SPITZ	3	c	0	170	169	7	128	18.39	(8.35- 40.53)
STASZE	7	m	0	251	653	5	158	12.15	(4.93- 29.94)
STASZE	5	f	0	6	153	15	1660	4.34	(1.66- 11.35)
Subtotal STASZE								7.50	(3.89- 14.48)
STAYNE	1	m	0	362	567	58	333	3.67	(2.69- 4.99)
STOCKS	30	m	0	2421	5483	45	638	6.26	(4.61- 8.50)
STOCKS	50	f	1	-	-	-	-	3.04	(2.35- 3.93)
Subtotal STOCKS								4.10	(3.37- 4.99)
STOCKW	8	c	0	18655	6414	2791	10641	11.09	(10.54- 11.66)
STUCKE	3	m	0	247	203	0	51	125.27~	(7.68-2042.38)
SUN	1	c	0	140	173	67	191	2.31	(1.62- 3.30)
SUZUK2	3	c	0	82	63	11	53	6.27	(3.03- 12.98)
SVENSS	56	f	0	172	89	38	120	6.10	(3.91- 9.53)
TANG	3	c	0	110	59	9	39	8.08	(3.66- 17.82)
*TENKAN	22	m	1	-	-	-	-	14.64	(6.29- 34.07)

Table 1A2 - 5

IESLC - Meta-analysis of Ever Smoking, Cigarettes (or Any Product if Cigarettes not available)
All LC types
Least adjusted

REF	NRR	SEX	AD	Number Exposed Case	Number Exposed Cont	Non-exposed Case	Non-exposed Cont	RR	95.00%CI
TIZZAN	2	m	0	994	836	180	305	2.01	(1.64- 2.48)
TIZZAN	22	f	0	25	28	25	114	4.07	(2.04- 8.13)
Subtotal TIZZAN								2.13	(1.75- 2.60)
TOKARS	3	m	0	147	243	1	53	32.06	(4.39- 234.30)
TOKARS	5	f	0	1	2	13	40	1.54	(0.13- 18.38)
Subtotal TOKARS								9.77	(2.07- 46.11)
TOUSEY	2	m	0	293	389	4	130	24.48	(8.95- 66.97)
TOUSEY	6	f	0	192	212	13	226	15.74	(8.71- 28.46)
Subtotal TOUSEY								17.64	(10.59- 29.38)
TSUGAN	27	m	0	73	71	18	22	1.26	(0.62- 2.54)
*TULINI	15	m	1	-	-	-	-	8.06	(4.38- 14.84)
*TULINI	21	f	1	-	-	-	-	14.95	(8.30- 26.95)
Subtotal TULINI								11.10	(7.26- 16.95)
*TVERDA	22	m	2	-	-	-	-	4.58	(2.97- 7.06)
WAKAI	13	m	0	235	424	10	65	3.60	(1.82- 7.14)
WAKAI	31	f	0	38	31	50	145	3.55	(2.00- 6.30)
Subtotal WAKAI								3.57	(2.30- 5.55)
WANG	1	m	0	262	-	29	-	3.47	(2.10- 5.80)
WANG	2	f	0	17	-	82	-	4.00	(1.30- 12.00)
Subtotal WANG								3.56	(2.24- 5.64)
WANG2	8	c	0	60	99	11	43	2.37	(1.14- 4.94)
WANG3	1	c	0	235	172	58	121	2.85	(1.97- 4.13)
WANG4	1	m	0	1043	18164	127	2374	1.07	(0.89- 1.30)
WICKLU	1	m	0	-	-	-	-	4.60	(2.80- 7.60)
WIGLE	13	m	0	543	632	15	204	11.68	(6.83- 19.99)
WIGLE	16	f	0	78	235	36	439	4.05	(2.64- 6.19)
Subtotal WIGLE								6.09	(4.37- 8.51)
WILKIN	1	m	0	173	372	2	108	25.11	(6.13- 102.89)
WILKIN	2	f	0	84	109	12	89	5.72	(2.93- 11.13)
Subtotal WILKIN								7.49	(4.10- 13.68)
WU	37	f	0	189	128	31	92	4.38	(2.75- 6.97)
WUNSCH	1	m	0	290	441	14	99	4.65	(2.61- 8.30)
WUNSCH	7	f	0	60	98	29	208	4.39	(2.65- 7.27)
Subtotal WUNSCH								4.50	(3.08- 6.58)
WUWILL	6	f	0	539	351	417	601	2.21	(1.84- 2.66)
WYNDE2	16	m	0	382	512	8	105	9.79	(4.71- 20.34)
WYNDE3	48	m	0	261	264	9	88	9.67	(4.77- 19.60)
WYNDE3	83	f	0	46	56	20	76	3.12	(1.67- 5.85)
Subtotal WYNDE3								5.14	(3.21- 8.22)
WYNDE4	48	m	0	632	665	12	115	9.11	(4.98- 16.67)
WYNDE4	62	f	2	-	-	-	-	2.87	(1.48- 5.55)
Subtotal WYNDE4								5.38	(3.45- 8.41)
WYNDE6	81	m	0	2765	1797	87	617	10.91	(8.65- 13.76)
WYNDE6	252	f	0	1354	701	159	856	10.40	(8.58- 12.60)
Subtotal WYNDE6								10.60	(9.14- 12.30)
*XIANGZ	6	m	0	526	10580	25	974	1.94	(1.30- 2.88)
XU	1	m	0	627	552	102	236	2.63	(2.03- 3.40)
XU2	1	c	0	501	582	82	377	3.96	(3.03- 5.17)
XU3	1	m	0	92	68	7	31	5.99	(2.49- 14.42)
XU3	3	f	0	23	11	13	25	4.02	(1.51- 10.74)
Subtotal XU3								5.02	(2.61- 9.66)
XU4	1	c	0	161	113	45	93	2.94	(1.92- 4.52)
YAMAGU	5	c	0	120	409	24	267	3.26	(2.05- 5.19)
*YONG	2	c	1	-	-	-	-	6.74	(4.47- 10.18)
*YUAN	1	m	2	-	-	-	-	6.50	(3.64- 11.60)
ZHANG	1	c	0	72	102	28	98	2.47	(1.47- 4.14)
ZHENG	15	m	0	279	218	33	94	3.65	(2.36- 5.63)
ZHENG	24	f	0	76	44	152	184	2.09	(1.36- 3.21)
Subtotal ZHENG								2.75	(2.03- 3.73)
ZHOU	2	m	0	740	41	275	36	2.36	(1.48- 3.77)
ZHOU	3	f	0	112	7	231	32	2.22	(0.95- 5.18)
Subtotal ZHOU								2.33	(1.54- 3.51)
Partial Totals				126019	2853412	14713	2031334		

*prospective study ~ With 0.5 adjustment for zero

Table 1A2 - 5

IESLC - Meta-analysis of Ever Smoking, Cigarettes (or Any Product if Cigarettes not available)
All LC types
Least adjusted

REF	NRR	SEX	AD	Ys	Ws	Qs	Ps
ABELIN	2	m	0	3.33	1.88	5.92	0.0000
*ABRAHA	7	m	0	2.17	9.68	3.68	0.0000
*ABRAHA	8	f	0	1.59	19.39	0.02	0.0000
Subtotal ABRAHA				1.78	29.07	3.70	
AGUDO	8	f	0	0.83	9.53	5.01	0.0106
*AKIBA	3	m	0	1.33	17.22	0.88	0.0000
*AKIBA	7	f	0	1.37	48.42	1.54	0.0000
Subtotal AKIBA				1.36	65.65	2.42	
ALDERS	61	m	0	2.38	12.98	8.92	0.0000
ALDERS	12	f	0	1.53	45.39	0.02	0.0000
Subtotal ALDERS				1.72	58.37	8.94	
*AMANDU	3	m	0	1.56	5.73	0.00	0.0002
AMES	4	m	0	1.59	11.09	0.01	0.0000
*ANDERS	3	f	0	2.57	39.86	41.52	0.0000
*ARCHER	6	m	0	1.85	5.76	0.50	0.0000
ARMADA	4	m	0	2.99	3.67	7.62	0.0000
AUSTIN	3	c	0	2.48	4.51	3.89	0.0000
AUVINE	1	c	0	2.15	30.60	10.78	0.0000
AXELSO	1	c	0	1.83	24.06	1.92	0.0000
AXELSS	1	m	0	2.14	13.32	4.58	0.0000
AXELSS	11	f	0	2.16	12.45	4.54	0.0000
Subtotal AXELSS				2.15	25.77	9.12	
BAND	1	m	2	2.30	42.76	23.82	0.0000
BARBON	106	m	0	2.40	18.55	13.40	0.0000
BECHER	15	m	0	2.43	2.75	2.12	0.0001
BECHER	16	f	0	1.50	5.94	0.01	0.0003
Subtotal BECHER				1.80	8.69	2.14	
*BENSHL	15	m	1	2.08	9.80	2.75	0.0000
*BEST	23	m	0	3.33	1.00	3.14	0.0009
*BEST	18	f	1	0.81	2.17	1.21	0.2348
Subtotal BEST				1.60	3.17	4.35	
BLOHMK	3	m	0	1.13	70.05	12.38	0.0000
BLOT4	1	m	0	2.68	6.96	8.78	0.0000
BOFFET	7	m	0	2.73	105.32	146.81	0.0000
*BOUCOT	58	m	0	3.90	0.50	2.76	0.0059
BRESLO	17	m	0	2.00	5.83	1.16	0.0000
BRESLO	23	f	0	0.32	3.10	4.70	0.5717
Subtotal BRESLO				1.42	8.93	5.86	
*BRETT	10	m	0	1.18	5.77	0.78	0.0044
BROCKM	1	m	0	0.07	0.98	2.14	0.9437
BROCKM	2	f	0	0.69	2.73	2.02	0.2517
Subtotal BROCKM				0.53	3.71	4.16	
BROSS	13	m	0	1.80	28.54	1.81	0.0000
BROWN2	2	m	2	2.21	442.58	190.50	0.0000
BROWN2	1	f	2	2.54	427.71	418.69	0.0000
Subtotal BROWN2				2.37	870.29	609.19	
BUFFLE	2	m	0	2.45	4.42	3.58	0.0000
BUFFLE	6	f	0	1.96	28.29	4.79	0.0000
Subtotal BUFFLE				2.03	32.71	8.37	
CARPEN	7	c	0	2.42	13.16	9.89	0.0000
CASCO2	1	c	0	2.44	5.31	4.19	0.0000
CASCOR	1	c	0	2.60	18.40	20.36	0.0000
*CEDERL	107	m	2	1.78	20.66	1.06	0.0000
*CEDERL	112	f	2	1.43	31.21	0.46	0.0000
Subtotal CEDERL				1.57	51.88	1.52	
CHAN	5	m	0	3.31	1.87	5.81	0.0000
CHAN	6	f	0	1.25	20.57	1.93	0.0000
Subtotal CHAN				1.42	22.44	7.75	
*CHANG	6	m	0	1.65	4.76	0.04	0.0003
*CHANG	12	f	0	1.30	8.85	0.55	0.0001
Subtotal CHANG				1.42	13.61	0.60	
CHATZI	4	c	0	1.21	19.44	2.34	0.0000
CHEN2	1	m	0	1.52	6.25	0.01	0.0001
CHEN2	2	f	0	0.51	7.70	8.29	0.1539
Subtotal CHEN2				0.97	13.95	8.30	
CHEN3	1	c	0	0.46	27.78	32.99	0.0148
CHIAZZ	2	m	0	2.06	3.53	0.90	0.0001
CHOI	1	m	0	1.43	10.71	0.15	0.0000
CHOI	5	f	0	0.46	9.06	10.90	0.1703
Subtotal CHOI				0.99	19.78	11.05	
*CHOW	6	m	0	2.41	5.83	4.29	0.0000

Table 1A2 - 5

IESLC - Meta-analysis of Ever Smoking, Cigarettes (or Any Product if Cigarettes not available)
All LC types
Least adjusted

REF	NRR	SEX	AD	Ys	Ws	Qs	Ps
*CHYOU	9	m	0	1.96	12.35	2.10	0.0000
COMSTO	33	m	0	2.43	3.63	2.81	0.0000
COMSTO	45	f	0	2.19	9.22	3.77	0.0000
Subtotal COMSTO				2.26	12.85	6.57	
COOKSO	4	c	0	1.88	14.21	1.54	0.0000
CORREA	33	c	0	2.33	41.42	25.33	0.0000
*CPSI	187	m	1	2.22	78.68	34.77	0.0000
*CPSI	274	f	1	1.03	73.22	20.27	0.0000
Subtotal CPSI				1.64	151.90	55.04	
*CPSII	104	m	1	2.55	78.29	78.23	0.0000
*CPSII	79	f	1	2.10	142.84	42.75	0.0000
Subtotal CPSII				2.26	221.13	120.97	
DAMBER	26	m	0	2.03	27.56	6.41	0.0000
DARBY	15	m	0	3.90	2.96	16.28	0.0000
DARBY	16	f	0	2.51	19.76	17.97	0.0000
Subtotal DARBY				2.69	22.71	34.25	
DAVEYS	5	m	0	1.57	2.53	0.00	0.0126
DAVEYS	6	f	0	-0.32	0.42	1.49	0.8327
Subtotal DAVEYS				1.30	2.96	1.49	
DEAN	8	m	0	1.70	9.65	0.22	0.0000
DEAN2	12	m	0	1.43	23.54	0.34	0.0000
DEAN2	20	f	0	1.09	14.29	3.00	0.0000
Subtotal DEAN2				1.30	37.83	3.34	
DEAN3	240	m	0	1.93	21.30	3.09	0.0000
DEAN3	124	f	0	1.06	28.64	7.01	0.0000
Subtotal DEAN3				1.43	49.94	10.09	
*DEKLER	6	m	2	3.01	0.99	2.11	0.0027
DESTE2	15	m	0	1.90	22.53	2.76	0.0000
DESTEF	11	m	0	1.75	16.32	0.66	0.0000
*DOCKER	3	c	4	1.46	4.27	0.04	0.0026
DOLL	20	m	0	2.24	6.12	2.91	0.0000
DOLL	12	f	0	0.72	12.98	9.07	0.0099
Subtotal DOLL				1.21	19.10	11.97	
*DOLL2	88	m	1	2.45	2.95	2.37	0.0000
DORANT	10	c	0	2.89	13.34	23.89	0.0000
DORGAN	7	m	0	2.38	12.29	8.48	0.0000
DORGAN	31	m	0	3.28	2.67	7.97	0.0000
DORGAN	95	f	3	2.14	67.41	23.30	0.0000
Subtotal DORGAN				2.21	82.37	39.74	
*DORN	413	m	1	2.18	47.17	18.48	0.0000
DOSEME	17	m	0	1.41	75.43	1.45	0.0000
DROSTE	3	m	0	2.65	6.33	7.60	0.0000
DU	1	m	0	1.26	28.12	2.38	0.0000
DU	2	f	0	0.66	24.50	19.61	0.0011
Subtotal DU				0.98	52.62	21.99	
*DUNN	6	m	0	2.91	1.97	3.66	0.0000
EBELIN	1	m	0	1.94	9.19	1.36	0.0000
*ENGELA	8	m	0	1.37	24.82	0.81	0.0000
*ENGELA	22	f	0	1.51	21.59	0.03	0.0000
Subtotal ENGELA				1.44	46.41	0.84	
ESAKI	4	m	0	0.64	8.96	7.45	0.0554
ESAKI	5	f	0	0.90	7.99	3.39	0.0109
Subtotal ESAKI				0.76	16.94	10.84	
FAN	1	m	0	1.04	25.87	6.66	0.0000
FAN	2	f	0	1.37	24.92	0.86	0.0000
Subtotal FAN				1.20	50.80	7.52	
GAO	6	m	0	1.37	41.05	1.43	0.0000
GAO	16	f	0	0.93	63.04	24.37	0.0000
Subtotal GAO				1.10	104.09	25.80	
GAO2	6	m	0	1.63	9.72	0.07	0.0000
GARCIA	3	c	0	2.14	16.50	5.74	0.0000
GARDIN	7	c	0	2.41	4.14	3.02	0.0000
GARSHI	17	m	0	1.75	34.80	1.40	0.0000
GENG	1	m	0	1.79	4.98	0.28	0.0001
GENG	2	f	0	1.08	22.39	4.89	0.0000
Subtotal GENG				1.21	27.37	5.17	
GER	17	c	0	0.31	26.37	40.60	0.1100
GODLEY	5	m	1	1.92	96.28	13.22	0.0000
GODLEY	6	f	1	1.71	58.89	1.50	0.0000
Subtotal GODLEY				1.84	155.17	14.73	
GOLLED	21	m	0	1.84	13.92	1.18	0.0000

Table 1A2 - 5

IESLC - Meta-analysis of Ever Smoking, Cigarettes (or Any Product if Cigarettes not available)
All LC types
Least adjusted

REF	NRR	SEX	AD	Ys	Ws	Qs	Ps
GOODMA	3	m	0	2.38	8.92	6.10	0.0000
GOODMA	7	f	0	2.12	12.25	3.89	0.0000
Subtotal GOODMA				2.23	21.17	9.99	
GRAHAM	12	m	0	2.22	16.44	7.44	0.0000
GREGOR	3	m	0	0.03	5.11	11.88	0.9492
GREGOR	7	f	0	2.40	0.90	0.64	0.0233
Subtotal GREGOR				0.38	6.01	12.52	
GSELL	6	m	0	3.03	1.74	3.80	0.0001
HAENSZ	56	f	0	0.74	25.12	16.62	0.0002
*HAMMO2	16	m	0	3.30	1.00	3.05	0.0010
*HAMMON	128	m	0	2.08	14.46	4.06	0.0000
*HANSEN	3	m	2	0.43	5.28	6.70	0.3285
HEGMAN	1	c	0	2.79	23.66	36.47	0.0000
*HEIN	7	m	0	2.68	1.00	1.27	0.0074
*HENNEK	3	m	0	1.83	19.94	1.55	0.0000
HINDS	26	f	0	1.42	61.37	1.13	0.0000
*HIRAYA	147	m	1	1.47	85.78	0.55	0.0000
*HIRAYA	150	f	1	0.86	80.63	38.78	0.0000
Subtotal HIRAYA				1.18	166.40	39.33	
HITOSU	6	m	0	1.01	6.47	1.90	0.0102
HITOSU	12	f	0	1.36	16.07	0.59	0.0000
Subtotal HITOSU				1.26	22.53	2.48	
*HOLE	15	m	0	1.69	6.79	0.13	0.0000
HOROWI	1	m	0	1.27	15.35	1.19	0.0000
HOROWI	2	f	0	0.60	8.08	7.36	0.0894
Subtotal HOROWI				1.04	23.43	8.54	
HORWIT	1	f	0	2.43	8.29	6.33	0.0000
HU	15	m	0	0.74	17.16	11.45	0.0023
HU	16	f	0	0.55	7.15	7.18	0.1413
Subtotal HU				0.68	24.31	18.63	
HU2	9	m	0	1.11	27.11	5.36	0.0000
HU2	10	f	0	0.63	21.91	18.69	0.0033
Subtotal HU2				0.89	49.01	24.05	
HUANG	1	c	0	0.69	14.82	11.00	0.0078
HUMBLE	14	m	1	2.49	5.19	4.60	0.0000
HUMBLE	16	m	1	2.47	1.71	1.45	0.0012
HUMBLE	18	f	1	2.43	6.68	5.15	0.0000
HUMBLE	20	f	1	2.73	2.90	4.05	0.0000
Subtotal HUMBLE				2.51	16.48	15.25	
JAHN	3	f	0	1.13	18.89	3.39	0.0000
JAIN	6	m	0	2.30	9.88	5.56	0.0000
JAIN	1	f	0	2.10	31.68	9.60	0.0000
Subtotal JAIN				2.15	41.55	15.16	
JARUP	3	m	0	1.90	6.90	0.83	0.0000
JARVHO	3	m	0	3.32	0.92	2.88	0.0015
JARVHO	7	f	0	2.26	3.28	1.63	0.0000
Subtotal JARVHO				2.49	4.19	4.51	
JEDRYC	63	m	0	1.76	36.14	1.54	0.0000
JEDRYC	68	f	0	2.08	17.12	4.71	0.0000
Subtotal JEDRYC				1.86	53.25	6.25	
JIANG	1	m	0	1.00	4.45	1.35	0.0346
JIANG	2	f	0	0.91	2.62	1.08	0.1401
Subtotal JIANG				0.97	7.08	2.43	
JOLY	2	m	0	2.65	10.97	13.21	0.0000
JOLY	1	f	0	1.99	27.09	5.29	0.0000
Subtotal JOLY				2.18	38.06	18.49	
JUSSAW	2	m	0	1.92	34.20	4.75	0.0000
*KAISE2	72	m	1	1.69	11.75	0.21	0.0000
*KAISE2	64	f	1	2.31	9.19	5.30	0.0000
Subtotal KAISE2				1.96	20.95	5.51	
*KAISER	13	m	2	2.87	25.69	44.59	0.0000
*KAISER	10	f	2	1.73	28.18	0.87	0.0000
Subtotal KAISER				2.27	53.88	45.47	
KATSOU	27	f	0	1.21	9.99	1.15	0.0001
KAUFMA	8	c	0	2.61	31.80	35.55	0.0000
KELLER	3	m	0	2.31	217.37	125.34	0.0000
KELLER	11	m	0	2.60	25.99	28.76	0.0000
KELLER	7	f	0	2.53	269.69	255.37	0.0000
KELLER	15	f	0	2.25	39.03	18.79	0.0000
Subtotal KELLER				2.43	552.08	428.26	
KHUDER	4	m	0	2.06	19.93	5.16	0.0000

Table 1A2 - 5

IESLC - Meta-analysis of Ever Smoking, Cigarettes (or Any Product if Cigarettes not available)
All LC types
Least adjusted

REF	NRR	SEX	AD	Ys	Ws	Qs	Ps
KIHARA	31	c	0	1.22	46.97	5.20	0.0000
*KINLEN	6	m	0	2.36	6.97	4.60	0.0000
KJUUS	3	m	0	2.66	1.80	2.20	0.0004
*KNEKT	76	m	0	1.85	5.69	0.51	0.0000
KO	1	f	3	1.44	2.18	0.03	0.0339
KOHLME	1	c	0	2.83	9.55	15.60	0.0000
KOO	1	f	0	1.02	23.01	6.57	0.0000
KOULUM	2	m	0	3.38	4.45	14.94	0.0000
KREUZE	60	f	3	2.22	3.99	1.78	0.0000
KREUZE	62	f	3	1.40	28.45	0.67	0.0000
Subtotal KREUZE				1.50	32.44	2.45	
KREYBE	24	m	0	2.04	5.80	1.38	0.0000
KREYBE	39	f	0	-0.22	8.25	25.95	0.5245
Subtotal KREYBE				0.71	14.05	27.34	
*KUBIK	27	m	0	3.36	1.96	6.45	0.0000
LAMTH	6	f	0	1.34	46.55	2.15	0.0000
LAMWK	1	f	0	1.42	17.85	0.33	0.0000
LAMWK2	9	m	0	1.04	12.98	3.39	0.0002
LAMWK2	10	f	0	1.17	17.89	2.67	0.0000
Subtotal LAMWK2				1.11	30.86	6.06	
*LANGE	34	m	0	1.58	4.91	0.00	0.0005
*LANGE	31	f	0	1.22	6.30	0.69	0.0022
Subtotal LANGE				1.38	11.22	0.69	
LAUSSM	10	m	0	1.59	41.00	0.06	0.0000
LEI	1	m	0	1.30	26.63	1.65	0.0000
LEI	2	f	0	1.25	23.21	2.13	0.0000
Subtotal LEI				1.28	49.84	3.78	
LEMARC	3	c	0	1.73	22.77	0.71	0.0000
LETOUR	1	c	0	2.56	20.21	20.62	0.0000
LEVIN	30	m	1	1.94	29.93	4.54	0.0000
*LIDDEL	5	m	1	1.28	17.92	1.29	0.0000
LIU	2	c	2	0.65	38.19	30.93	0.0001
LIU2	1	m	0	1.46	8.60	0.07	0.0000
LIU2	3	f	0	1.45	9.73	0.10	0.0000
Subtotal LIU2				1.46	18.32	0.17	
LIU3	1	m	0	0.19	3.06	5.71	0.7444
LIU4	10	m	2	1.36	5780.52	222.89	0.0000
LIU4	12	f	2	1.05	3876.64	974.51	0.0000
Subtotal LIU4				1.23	9657.16	1197.41	
LIU5	1	c	0	0.65	11.25	9.16	0.0293
LOMBA2	1	f	0	0.28	37.19	59.89	0.0841
LOMBAR	2	m	0	2.30	12.10	6.82	0.0000
LUBIN2	47	m	0	2.17	169.72	64.61	0.0000
LUBIN2	97	f	0	1.24	134.56	12.86	0.0000
Subtotal LUBIN2				1.76	304.28	77.47	
LUO	1	c	0	0.66	18.01	14.50	0.0054
MACLEN	71	m	0	1.28	3.54	0.27	0.0163
MACLEN	72	f	0	0.74	13.64	8.97	0.0062
Subtotal MACLEN				0.85	17.18	9.24	
*MAGNUS	1	m	0	1.69	10.53	0.20	0.0000
MARSH	1	m	0	2.32	1.82	1.06	0.0018
MARSH	3	f	0	1.76	5.60	0.25	0.0000
Subtotal MARSH				1.90	7.42	1.31	
MARSH2	1	c	0	1.19	8.48	1.12	0.0005
MARTIS	4	m	0	1.95	3.32	0.51	0.0004
MASTRA	1	m	0	2.13	5.09	1.68	0.0000
MATOS	26	m	0	1.89	9.19	1.07	0.0000
MATSUD	10	m	0	3.07	2.94	6.74	0.0000
MCCONN	1	m	0	0.19	3.33	6.14	0.7237
MCCONN	2	f	0	1.01	0.99	0.29	0.3136
Subtotal MCCONN				0.38	4.32	6.43	
MCDUFF	1	m	0	1.81	4.70	0.32	0.0001
MCLAUG	1	m	0	1.20	18.70	2.26	0.0000
*MIGRAN	26	m	0	1.89	3.94	0.46	0.0002
*MIGRAN	41	f	0	1.98	3.55	0.65	0.0002
Subtotal MIGRAN				1.93	7.49	1.11	
MILLER	1	f	0	2.43	22.85	17.47	0.0000
MILLS	1	m	1	0.24	71.48	122.67	0.0406
*MRFITR	6	m	0	3.70	0.50	2.29	0.0091
NAM	69	m	0	2.29	26.44	14.24	0.0000
NAM	85	f	0	2.30	38.76	21.94	0.0000

Table 1A2 - 5

IESLC - Meta-analysis of Ever Smoking, Cigarettes (or Any Product if Cigarettes not available)
All LC types
Least adjusted

REF	NRR	SEX	AD	Ys	Ws	Qs	Ps
Subtotal NAM				2.30	65.19	36.17	
NOTAN2	7	m	0	0.90	33.47	14.33	0.0000
NOU	11	m	0	1.81	5.20	0.34	0.0000
NOU	12	f	0	1.96	2.74	0.45	0.0012
Subtotal NOU				1.86	7.94	0.79	
ODRISC	3	c	0	3.89	5.83	31.86	0.0000
ORMOS	4	m	0	2.23	6.39	2.97	0.0000
ORMOS	26	f	0	-1.64	0.95	9.72	0.1093
Subtotal ORMOS				1.73	7.34	12.69	
OSANN	17	m	0	3.00	39.51	83.18	0.0000
OSANN	21	f	0	2.70	69.13	91.34	0.0000
Subtotal OSANN				2.81	108.65	174.52	
PARKIN	31	m	0	1.54	71.91	0.02	0.0000
PASTOR	5	m	0	1.89	8.32	0.92	0.0000
PAWLEG	1	m	0	2.77	3.69	5.44	0.0000
PERNU	8	m	0	2.23	50.02	22.74	0.0000
PERNU	4	f	0	0.95	5.14	1.89	0.0321
Subtotal PERNU				2.11	55.16	24.63	
PERSH2	5	c	0	1.79	114.36	6.32	0.0000
*PETO	5	m	0	1.82	1.98	0.14	0.0107
PEZZO2	10	m	0	2.71	5.55	7.43	0.0000
PEZZOT	25	m	0	2.96	3.75	7.44	0.0000
PIKE	4	m	0	1.66	13.39	0.15	0.0000
PIKE	8	f	0	1.57	18.04	0.01	0.0000
Subtotal PIKE				1.61	31.43	0.16	
POFFIJ	1	c	0	2.05	46.21	11.35	0.0000
POLEDN	3	c	0	2.13	10.07	3.31	0.0000
*QIAO2	9	m	0	0.79	9.66	5.66	0.0145
RACHTA	3	f	0	1.77	13.24	0.63	0.0000
RADZIK	1	c	0	0.27	5.03	8.25	0.5411
RANDIG	9	m	0	1.60	3.95	0.01	0.0014
RANDIG	10	f	0	0.80	6.34	3.61	0.0447
Subtotal RANDIG				1.11	10.29	3.62	
REN	1	m	0	1.27	7.46	0.58	0.0005
REN	2	f	0	1.40	9.65	0.22	0.0000
Subtotal REN				1.35	17.11	0.80	
RONCO	2	m	0	1.71	5.21	0.12	0.0001
ROTHSC	1	c	0	1.76	9.88	0.43	0.0000
SADOWS	4	m	0	1.45	13.79	0.15	0.0000
SANKAR	1	m	0	2.75	23.11	33.22	0.0000
SCHWAR	1	m	0	2.11	80.50	24.64	0.0000
SCHWAR	2	m	0	1.88	29.06	3.05	0.0000
SCHWAR	3	f	0	2.30	111.43	62.13	0.0000
SCHWAR	4	f	0	2.45	26.58	21.29	0.0000
Subtotal SCHWAR				2.20	247.58	111.12	
SEGI	1	m	0	0.53	15.18	15.74	0.0375
SEOW	1	f	0	1.71	9.81	0.24	0.0000
SHAW	12	c	0	2.47	9.34	7.90	0.0000
SIEMIA	9	m	0	2.77	11.12	16.43	0.0000
SIMARA	5	m	0	0.70	13.62	9.99	0.0103
SIMARA	6	f	0	0.85	9.72	4.85	0.0084
Subtotal SIMARA				0.76	23.33	14.84	
SOBUE	91	m	0	1.34	25.52	1.20	0.0000
SOBUE	95	f	0	1.03	51.71	13.94	0.0000
Subtotal SOBUE				1.13	77.23	15.14	
*SPEIZE	8	f	0	1.96	52.33	8.56	0.0000
SPITZ	3	c	0	2.91	6.16	11.38	0.0000
STASZE	7	m	0	2.50	4.72	4.21	0.0000
STASZE	5	f	0	1.47	4.16	0.03	0.0028
Subtotal STASZE				2.02	8.88	4.24	
STAYNE	1	m	0	1.30	40.37	2.59	0.0000
STOCKS	30	m	0	1.83	41.01	3.26	0.0000
STOCKS	50	f	1	1.11	58.11	11.27	0.0000
Subtotal STOCKS				1.41	99.12	14.53	
STOCKW	8	c	0	2.41	1511.07	1101.39	0.0000
STUCKE	3	m	0	4.83	0.49	5.30	0.0007
SUN	1	c	0	0.84	30.23	15.51	0.0000
SUZUK2	3	c	0	1.84	7.25	0.58	0.0000
SVENSS	56	f	0	1.81	19.34	1.27	0.0000
TANG	3	c	0	2.09	6.14	1.77	0.0000
*TENKAN	22	m	1	2.68	5.38	6.89	0.0000

Table 1A2 - 5

IESLC - Meta-analysis of Ever Smoking, Cigarettes (or Any Product if Cigarettes not available)
All LC types
Least adjusted

REF	NRR	SEX	AD	Ys	Ws	Qs	Ps
TIZZAN	2	m	0	0.70	90.61	65.73	0.0000
TIZZAN	22	f	0	1.40	8.03	0.18	0.0001
Subtotal TIZZAN				0.76	98.64	65.91	
TOKARS	3	m	0	3.47	0.97	3.56	0.0006
TOKARS	5	f	0	0.43	0.62	0.79	0.7336
Subtotal TOKARS				2.28	1.60	4.35	
TOUSEY	2	m	0	3.20	3.79	10.27	0.0000
TOUSEY	6	f	0	2.76	10.96	15.89	0.0000
Subtotal TOUSEY				2.87	14.75	26.16	
TSUGAN	27	m	0	0.23	7.76	13.61	0.5244
*TULINI	15	m	1	2.09	10.32	2.95	0.0000
*TULINI	21	f	1	2.70	11.08	14.71	0.0000
Subtotal TULINI				2.41	21.40	17.67	
*TVERDA	22	m	2	1.52	20.49	0.02	0.0000
WAKAI	13	m	0	1.28	8.20	0.60	0.0002
WAKAI	31	f	0	1.27	11.70	0.94	0.0000
Subtotal WAKAI				1.27	19.90	1.54	
WANG	1	m	0	1.24	14.89	1.41	0.0000
WANG	2	f	0	1.39	3.11	0.09	0.0145
Subtotal WANG				1.27	18.00	1.50	
WANG2	8	c	0	0.86	7.10	3.37	0.0216
WANG3	1	c	0	1.05	28.11	7.16	0.0000
WANG4	1	m	0	0.07	107.42	235.74	0.4630
WICKLU	1	m	0	1.53	15.41	0.01	0.0000
WIGLE	13	m	0	2.46	13.33	10.95	0.0000
WIGLE	16	f	0	1.40	21.22	0.50	0.0000
Subtotal WIGLE				1.81	34.55	11.45	
WILKIN	1	m	0	3.22	1.93	5.39	0.0000
WILKIN	2	f	0	1.74	8.65	0.32	0.0000
Subtotal WILKIN				2.01	10.58	5.71	
WU	37	f	0	1.48	17.78	0.10	0.0000
WUNSCH	1	m	0	1.54	11.46	0.00	0.0000
WUNSCH	7	f	0	1.48	15.11	0.08	0.0000
Subtotal WUNSCH				1.50	26.58	0.08	
WUWILL	6	f	0	0.79	114.07	65.50	0.0000
WYNDE2	16	m	0	2.28	7.19	3.83	0.0000
WYNDE3	48	m	0	2.27	7.69	3.95	0.0000
WYNDE3	83	f	0	1.14	9.73	1.67	0.0004
Subtotal WYNDE3				1.64	17.42	5.61	
WYNDE4	48	m	0	2.21	10.51	4.54	0.0000
WYNDE4	62	f	2	1.05	8.80	2.18	0.0018
Subtotal WYNDE4				1.68	19.31	6.72	
WYNDE6	81	m	0	2.39	71.26	50.00	0.0000
WYNDE6	252	f	0	2.34	103.92	64.77	0.0000
Subtotal WYNDE6				2.36	175.18	114.78	
*XIANGZ	6	m	0	0.66	24.52	19.47	0.0011
XU	1	m	0	0.97	57.31	19.68	0.0000
XU2	1	c	0	1.38	53.87	1.68	0.0000
XU3	1	m	0	1.79	4.98	0.28	0.0001
XU3	3	f	0	1.39	3.98	0.10	0.0055
Subtotal XU3				1.61	8.96	0.39	
XU4	1	c	0	1.08	20.82	4.64	0.0000
YAMAGU	5	c	0	1.18	17.80	2.43	0.0000
*YONG	2	c	1	1.91	22.68	2.87	0.0000
*YUAN	1	m	2	1.87	11.44	1.17	0.0000
ZHANG	1	c	0	0.90	14.37	6.03	0.0006
ZHENG	15	m	0	1.29	20.36	1.36	0.0000
ZHENG	24	f	0	0.74	20.88	13.85	0.0008
Subtotal ZHENG				1.01	41.24	15.22	
ZHOU	2	m	0	0.86	17.50	8.39	0.0003
ZHOU	3	f	0	0.80	5.34	3.05	0.0660
Subtotal ZHOU				0.84	22.83	11.44	

Table 1A2 - 5

IESLC - Meta-analysis of Ever Smoking, Cigarettes (or Any Product if Cigarettes not available)
All LC types
Least adjusted

	N	330
	NS	236
	Wt	19312.28
Het	Chi	6506.72
Het	df	329
Het	P	***
Fixed	RR	4.72
	RRl	4.66
	RRu	4.79
	P	+++
Random	RR	5.50
	RRl	5.11
	RRu	5.93
	P	+++
Asymm	P	**

Table 1A2 - 6

IESLC - Meta-analysis of Ever Smoking, Cigarettes (or Any Product if Cigarettes not available)
All LC types
Least adjusted

		Sex		
	combined	male	female	Total
N	45	175	110	330
NS	45	171	106	322
Wt	2435.53	9689.61	7187.14	19312.28
Het Chi	814.31	2246.59	2440.73	6506.72
Het df	44	174	109	329
Het P	***	***	***	***
Fixed RR	8.32	4.67	3.96	4.72
RRl	8.00	4.57	3.87	4.66
RRu	8.66	4.76	4.05	4.79
P	+++	+++	+++	+++
Random RR	5.74	6.31	4.38	5.50
RRl	4.58	5.70	3.80	5.11
RRu	7.20	6.98	5.04	5.93
P	+++	+++	+++	+++
Between Chi				1005.10
Between df				2
Between P				***
Btwn(F) P				***
Btwn(R) P				***

Table 1A2 - 7

IESLC - Meta-analysis of Ever Smoking, Cigarettes (or Any Product if Cigarettes not available)
All LC types
Excluded studies (and stage at which they were excluded)

1	BOUCHA BUELL LAURIL MZILEN
2	BYERS2 ENSTRO HIRAY2 KANELL LIAW LICKIN MRFIT MURATA NOTANI RESTRE RIMING SAARIK SEGI2 SHIMIZ SITAS SOBUE2 TANG2 WALD WARSIN WATSON WU2 WYNDE5 WYNDE8 WYNDER
3	BERRIN CHEN TAO ULMER
6	BLOT1 BLOT2 BLOT3 HIRAOK JONES MOLLO PERSHA SCHWA2 VANDER
8	BROWN1 BYERS1 SUZUKI
10	BENHAM GILLIS ISHIMA LUBIN OSANN2 PISANI PRESCO QIAO ROOTS SIMONA VEIERO VUTUC WYNDE7

Table 1A2 - 8
Potentially overlapping studies

REF	REFGP	PRINC	OVERLAP/LINK
LUBIN2	LUBIN2	1	Lubin-combined
CHAN	CHAN	1	Chan/LamWK2
LAMWK2	LAMWK2	1	Chan/LamWK2
LAMTH	LAMTH	1	KOO/LAMTH/LAMWK
KOO	KOO	1	KOO/LAMTH/LAMWK
LAMWK	LAMWK	1	KOO/LAMTH/LAMWK
CASCOR	CASCOR	1	Roots/Cascor 1
TVERDA	TVERDA	1	VEIERO/TVERDAL
AKIBA	AKIBA	1	AKIBA/ISHIMA
HEIN	PRESCO	1	Subset of PRESCO
LANGE	PRESCO	1	Subset of PRESCO
BROSS	BYERS1	1	GRAHAM/BROSS/BYERS1
GRAHAM	BYERS1	1	GRAHAM/BROSS/BYERS1
CHYOU	CHYOU	1	GOODMA/CHYOU
GOODMA	GOODMA	1	GOODMA/CHYOU
HOLE	TANG2	1	Subset of TANG2
BENSHL	TANG2	1	Subset of TANG2
KAISER	KAISER	1	KAISER/OSANN2
MRFITR	MRFIT	2	Subset of MRFIT
WYNDE6	WYNDE6	1	WYNDE5/6/7/8
CPSI	CPSI	1	CPSI overall
MATSUD	MATSUD	1	SOBUE2/MATSUD
BOFFET	BOFFET	1	BOFFET-combined
JAHN	BOFFET	2	Subset of BOFFET
XIANGZ	XIANGZ	1	LUBIN/XIANGZ/QIAO

Table 1A2 - 9
Most adjusted - insufficient data for metaanalysis

REF	NRR	SEX	AGEL	AGEH	RACE	YF	LC TYPE	LOC	START	ST	NLC	R	VB	P	H	AD	PRODUCT	DENOM	De
DOLL2	43	f	0	0	all	11	all	Eu:UK	1951	pr	920	n	V	n	n	1	all/unsp	nev any	st
TAO	1	m	0	0	all	-	all	As:Chi	1988	CC	723	n	ot	n	n	5	all/unsp	nev any	or
TAO	2	f	0	0	all	-	all	As:Chi	1988	CC	723	n	ot	n	n	5	all/unsp	nev any	or
ULMER	1	c	0	0	all	-	all	Eu:Ger	1971	CC	726	n	bl	y	n	0	all/unsp	nev any	or

REF	NRR	RR	SIG	RRDATA comment
DOLL2	43	4.33		0
TAO	1	3.95		0
TAO	2	4.93		0
ULMER	1	12.72		0

Least adjusted - insufficient data for meta-analysis: as for adjusted plus the following

REF	NRR	SEX	AGEL	AGEH	RACE	YF	LC TYPE	LOC	START	ST	NLC	R	VB	P	H	AD	PRODUCT	DENOM	De
CORREA	59	m	0	0	wh	-	all	NAmer	1979	CC	1359	n	bl	y	n	0	cig+/-ot	nev cigs	or
CORREA	61	m	0	0	bl	-	all	NAmer	1979	CC	1359	n	bl	y	n	0	cig+/-ot	nev cigs	or
CORREA	60	f	0	0	wh	-	all	NAmer	1979	CC	1359	n	bl	y	n	0	cig+/-ot	nev cigs	or
CORREA	62	f	0	0	bl	-	all	NAmer	1979	CC	1359	n	bl	y	n	0	cig+/-ot	nev cigs	or
LIU	1	c	0	0	all	-	all	As:Chi	1980	CC	229	n	ot	*	n	0	all/unsp	nev any	or

REF	NRR	RR	SIG	RRDATA comment
CORREA	59	17.20		0
CORREA	61	13.10		0
CORREA	60	8.60		0

Table 1A2 - 9

IESLC - Meta-analysis of Ever Smoking, Cigarettes (or Any Product if Cigarettes not available)
All LC types
Least adjusted - insufficient data for meta-analysis: as for adjusted plus the following

REF	NRR	RR	SIG	RRDATA comment
CORREA	62	22.00		0
LIU	1	2.45		0

Table 1A3 -

IESLC - Meta-analysis of Ever Smoking, Cigarettes only
All LC types

This analysis is restricted to results for:
1) Non-dose-response data
2) Ever smokers
3) Results complete enough for use in metaanalysis

Within each study, results are then selected (in the following order of preference, within each sex) for:
4) PRODUCT: cigarettes only
5) CIGTYPE: all/unspecified, MC regardless of HR, MC only
6) DENOM: never smoked anything, never smoked cigarettes, (never +1 = +long term ex, +2 = +amount unknown,
+3 = never cigs+long term ex)
7) Followup period (YF, prospective studies): whole study (coded as 0) or longest available
8) LCtype: all or nearest available, at least Squamous and Adeno. (q = squamous, s = small,
l = large, a = adeno, mix = mixed, alv = alveolar)
9) Race: all or nearest available, otherwise by race (wh or w = white, bl or b = black, hi = hispanic
ch = chinese, jap = japanese, haw = hawaiian, w+o = white + oriental, sca = scandinavian, as = asian)
10) For overlapping studies: principal rather than subsidiary studies
Finally by Age: whole study (coded as 0) if available, otherwise by widest available age group
and then for single sex results (m, f) in preference to combined sex results (c).

Results adjusted (AD) for the most potential confounders are then chosen in Sections -1 to -3
(and those which actually differ from the adjusted results in Table 1A1 - 1 are marked 'x' in Section -1)
and results adjusted for the least confounders in Sections -4 to -6. (Those least adjusted results which
actually differ from the most adjusted as marked 'x' in column X in Section -4)
(Results adjusted for an unknown number of confounder(s) are coded as 20.)

Section -7 shows excluded studies, together with the stage (as above) at which no qualifying
results were found.

Section -8 lists the potentially overlapping studies which have been included (1=principal, 2=subsidiary).

Section -9 lists any results which would have been included in preference except that they had data not complete
enough for use in meta-analysis, with their significance (yes/no), if known, and any further comment as entered
on the database.

In addition to those mentioned above, the following fields, levels and abbreviations are used:
* or nk = not known, n = no, y = yes, ot = other
nev = never
all/unspec = all or unspecified, MC = manufactured cigarettes, HR = hand-rolled cigarettes
REF: 6-character study reference
NRR: number of the RR on the database within the study
ST : study type (CC = case control, pr or prosp = prospective)
NLC: number of lung cancer cases in whole study
R : risky occupational population (n = no, m = mining, o = other risky)
VB : national cigarette type (V = at least 75% Virginia, bl = at least 75% blended, ot = other)
P : any proxy use
H : full histological confirmation
De : derivation of RR/CI (or = original, st = standard method, ot = other method of estimation)

Table 1A3 - 1

IESLC - Meta-analysis of Ever Smoking, Cigarettes only
All LC types
Most adjusted

REF	NRR	1A1	SEX	AGEL	AGEH	RACE	YF	LC TYPE	LOC	START	ST	NLC	R	VB	P	H	AD	PRODUCT	DENOM	De
ABELIN	47	x	m	0	0	all	-	all	Eu:wst	1941	CC	118	n	bl	y	n	1	cig only	nev any	st
AGUDO	1		f	0	0	all	-	all	Eu:wst	1989	CC	103	n	bl	n	n	3	cig only	nev any	or
ALDERS	67	x	m	0	0	all	-	all	Eu:UK	1977	CC	1448	n	V	n	n	1	cig only	nev any	ot
ALDERS	6		f	0	0	all	-	all	Eu:UK	1977	CC	1448	n	V	n	n	1	cig only	nev any	ot
ARMADA	1	x	m	0	0	all	-	all	Eu:wst	1986	CC	325	n	bl	n	y	0	cig only	nev any	st
BAND	1		m	0	0	all	-	all	NAmer	1983	CC	2831	n	V	y	y	2	cig only	nev any	ot
BEST	1	x	m	0	0	all	0	all	NAmer	1955	pr	381	n	V	n	n	1	cig only	nev any	ot
BEST	18		f	0	0	all	0	all	NAmer	1955	pr	381	n	V	n	n	1	cig only	nev any	ot
BOFFET	3	x	m	0	0	all	-	all	Eu:mul	1988	CC	5621	n	bl	y	n	2	cig only	nev any	or
BOUCOT	113	x	m	0	0	all	0	all	NAmer	1951	pr	121	n	bl	n	n	2	cig only	nev any	ot
BRESLO	18	x	m	0	0	all	-	all	NAmer	1949	CC	518	n	bl	n	y	0	cig only	nev+1	st
BRESLO	24	x	f	0	0	all	-	all	NAmer	1949	CC	518	n	bl	n	y	0	cig only	nev+1	st
CHOW	15	x	m	0	0	wh	0	all	NAmer	1966	pr	219	n	bl	n	n	0	cig only	nev any	st
CPSI	73	x	m	0	0	wh	0	all	NAmer	1959	pr	5138	n	bl	n	n	1	cig only	nev any	st
CPSI	149	x	f	0	0	wh	0	all	NAmer	1959	pr	5138	n	bl	n	n	1	cig only	nev any	st
CPSII	104		m	35	99	all	4	all	NAmer	1982	pr	3229	n	bl	n	n	1	cig only	nev any	ot
DAMBER	35	x	m	0	0	all	-	all	Eu:Sca	1972	CC	579	n	bl	y	n	1	cig only	nev any	ot
DEAN	4	x	m	0	0	wh	-	all	Africa	1947	CC	603	n	V	y	n	0	cig only	nev any	st
DEAN2	9	x	m	0	0	all	-	all	Eu:UK	1960	CC	954	n	V	y	n	0	cig only	nev any	st
DEAN2	17	x	f	0	0	all	-	all	Eu:UK	1960	CC	954	n	V	y	n	0	cig only	nev any	st
DEAN3	241	x	m	0	0	all	-	all	Eu:UK	1969	CC	766	n	V	y	n	1	cig only	nev any	ot
DEAN3	126		f	0	0	all	-	all	Eu:UK	1969	CC	766	n	V	y	n	3	cig only	nev any	ot
DOLL	13	x	m	0	0	all	-	all	Eu:UK	1948	CC	1465	n	V	n	n	0	cig only	nev any	st
DOLL2	62	x	m	0	0	all	0	all	Eu:UK	1951	pr	920	n	V	n	n	1	cig only	nev any	ot
DORN	1	x	m	0	0	wh	0	all	NAmer	1954	pr	5097	n	bl	n	n	2	cig only	nev any	or
GOLLED	6	x	m	35	99	all	-	all	Eu:UK	1952	CC	443	n	V	y	n	1	cig only	nev any	ot
GRAHAM	3	x	m	0	0	wh	-	all	NAmer	1956	CC	685	n	bl	n	n	0	cig only	nev any	st
HAMMON	115	x	m	0	0	wh	0	all	NAmer	1952	pr	448	n	bl	n	n	1	cig only	nev any	ot
JOLY	48	x	m	0	0	all	-	all	SCAmer	1978	CC	826	n	bl	n	n	0	cig only	nev any	st
JUSSAW	31	x	m	0	0	all	-	all	As:Ind	1964	CC	792	n	V	n	n	2	cig only	nev any	st
KAISE2	72		m	35	99	all	9	all	NAmer	1979	pr	318	n	bl	n	n	1	cig only	nev any	st
KAISE2	64		f	35	99	all	9	all	NAmer	1979	pr	318	n	bl	n	n	1	cig only	nev any	st
KJUUS	3	x	m	0	0	all	-	all	Eu:Sca	1979	CC	176	n	bl	n	n	0	cig only	nev any	st
KOULUM	2	x	m	0	0	all	-	all	Eu:Sca	1936	CC	812	n	bl	n	n	0	cig only	nev any	st
LIU4	10	x	m	35	69	all	-	all	As:Chi	1986	CC	1000-00	n	ot	y	n	2	cig only	nev any	ot
LOMBAR	10	x	m	0	0	all	-	all	NAmer	1951	CC	1040	n	bl	n	n	0	cig only	nev any	st
LUBIN2	18	x	m	0	0	all	-	all	Eu:mul	1976	CC	7804	n	bl	n	y	2	cig only	nev any	ot
LUBIN2	98	x	f	0	0	all	-	all	Eu:mul	1976	CC	7804	n	bl	n	y	1	cig only	nev any	ot
MCCONN	16	x	c	0	0	all	-	all	Eu:UK	1946	CC	100	n	V	n	y	0	cig only	nev any	st
MILLS	1	x	m	0	0	wh	-	all	NAmer	1940	CC	444	n	bl	y	n	1	cig only	nev any	ot
NOTAN2	19	x	m	0	0	all	-	all	As:Ind	1963	CC	683	n	V	n	n	2	cig only	nev any	ot
PERNU	8	x	m	0	0	all	-	all	Eu:Sca	1944	CC	1606	n	bl	n	n	0	cig only	nev any	st
PERNU	4	x	f	0	0	all	-	all	Eu:Sca	1944	CC	1606	n	bl	n	n	0	cig only	nev any	st
PEZZOT	25		m	0	0	all	-	all	SCAmer	1987	CC	215	n	bl	n	y	0	cig only	nev cigs	st
RONCO	3	x	m	0	0	all	-	all	Eu:wst	1976	CC	126	n	bl	y	n	2	cig only	nev any	ot
SADOWS	28	x	m	0	0	wh	-	all	NAmer	1938	CC	477	n	bl	n	n	1	cig only	nev any	ot
STASZE	2	x	m	0	0	all	-	all	Eu:est	1954	CC	281	n	bl	n	y	0	cig only	nev any	st
SUZUK2	7	x	c	0	0	all	-	all	SCAmer	1991	CC	123	n	bl	n	y	3	cig only	nev any	or
TIZZAN	2	x	m	0	0	all	-	all	Eu:wst	1959	CC	1358	n	bl	n	n	0	cig only	nev any	st
TIZZAN	22	x	f	0	0	all	-	all	Eu:wst	1959	CC	1358	n	bl	n	n	0	cig only	nev any	st
WIGLE	13	x	m	0	0	all	-	all	NAmer	1971	CC	728	n	V	n	n	0	cig only	nev any	st
WIGLE	16	x	f	0	0	all	-	all	NAmer	1971	CC	728	n	V	n	n	0	cig only	nev any	st
WYNDE7	39	x	m	0	0	all	-	all	NAmer	1977	CC	2085	n	bl	n	y	0	cig only	nev any	st
XIANGZ	9	x	m	0	0	all	0	all	As:Chi	1976	pr	983	m	ot	n	n	2	cig only	nev any	ot

Cigarette type is all/unspec for all RRs
except for the following:

REF	NRR	CIGTYPE
ALDERS	6	MC only
DEAN3	241	MC only
DEAN3	126	MC only
JUSSAW	31	MC only
NOTAN2	19	MC only
PERNU	8	MC only
PERNU	4	MC only
SUZUK2	7	MC only

Table 1A3 - 2

IESLC - Meta-analysis of Ever Smoking, Cigarettes only
All LC types
Most adjusted

REF	NRR	SEX	AD	Number Exposed Case	Cont	Non-exposed Case	Cont	RR	95.00%CI
ABELIN	47	m	1	-	-	-	-	53.81	(11.36- 254.76)
AGUDO	1	f	3	-	-	-	-	3.10	(1.42- 6.75)
ALDERS	67	m	1	-	-	-	-	10.38	(6.00- 17.93)
ALDERS	6	f	1	-	-	-	-	4.75	(3.55- 6.35)
Subtotal ALDERS								5.64	(4.36- 7.29)
ARMADA	1	m	0	245	197	4	64	19.90	(7.12- 55.59)
BAND	1	m	2	-	-	-	-	9.96	(7.38- 13.44)
*BEST	1	m	1	-	-	-	-	14.20	(6.70- 30.10)
*BEST	18	f	1	-	-	-	-	2.24	(0.59- 8.44)
Subtotal BEST								9.09	(4.72- 17.48)
BOFFET	3	m	2	-	-	-	-	14.90	(12.30- 18.10)
*BOUCOT	113	m	2	-	-	-	-	53.14	(3.30- 856.06)
BRESLO	18	m	0	316	229	7	42	8.28	(3.65- 18.76)
BRESLO	24	f	0	13	11	12	14	1.38	(0.45- 4.20)
Subtotal BRESLO								4.42	(2.28- 8.54)
*CHOW	15	m	0	57	57162	6	62913	10.46	(4.51- 24.25)
*CPSI	73	m	1	-	-	-	-	10.07	(8.77- 11.56)
*CPSI	149	f	1	-	-	-	-	3.20	(2.81- 3.65)
Subtotal CPSI								5.50	(5.00- 6.05)
*CPSII	104	m	1	-	-	-	-	12.83	(10.28- 16.01)
DAMBER	35	m	1	-	-	-	-	7.07	(4.67- 10.68)
DEAN	4	m	0	403	385	12	61	5.32	(2.82- 10.04)
DEAN2	9	m	0	629	508	33	112	4.20	(2.80- 6.30)
DEAN2	17	f	0	62	29	88	121	2.94	(1.75- 4.94)
Subtotal DEAN2								3.67	(2.67- 5.05)
DEAN3	241	m	1	-	-	-	-	6.11	(3.99- 9.34)
DEAN3	126	f	3	-	-	-	-	4.63	(3.03- 7.09)
Subtotal DEAN3								5.32	(3.94- 7.18)
DOLL	13	m	0	1004	899	7	61	9.73	(4.43- 21.39)
*DOLL2	62	m	1	-	-	-	-	8.78	(5.55- 13.88)
*DORN	1	m	2	-	-	-	-	8.40	(7.50- 9.40)
GOLLED	6	m	1	-	-	-	-	7.65	(4.52- 12.94)
GRAHAM	3	m	0	474	951	18	346	9.58	(5.89- 15.58)
*HAMMON	115	m	1	-	-	-	-	9.94	(5.90- 16.73)
JOLY	48	m	0	379	499	12	218	13.80	(7.60- 25.05)
JUSSAW	31	m	2	-	-	-	-	8.64	(4.61- 17.88)
*KAISE2	72	m	1	-	-	-	-	5.40	(3.05- 9.57)
*KAISE2	64	f	1	-	-	-	-	10.09	(5.29- 19.27)
Subtotal KAISE2								7.11	(4.63- 10.90)
KJUUS	3	m	0	151	127	2	24	14.27	(3.31- 61.54)
KOULUM	2	m	0	625	229	5	54	29.48	(11.65- 74.60)
LIU4	10	m	2	-	-	-	-	3.88	(3.78- 3.98)
LOMBAR	10	m	0	486	302	14	112	12.87	(7.25- 22.85)
LUBIN2	18	m	2	-	-	-	-	8.87	(7.60- 10.35)
LUBIN2	98	f	1	-	-	-	-	3.90	(3.29- 4.62)
Subtotal LUBIN2								6.11	(5.45- 6.85)
MCCONN	16	c	0	68	138	9	23	1.26	(0.55- 2.87)
MILLS	1	m	1	-	-	-	-	1.27	(1.01- 1.61)
NOTAN2	19	m	2	-	-	-	-	2.36	(1.68- 3.31)
PERNU	8	m	0	706	216	97	275	9.27	(7.02- 12.23)
PERNU	4	f	0	7	24	110	971	2.57	(1.08- 6.11)
Subtotal PERNU								8.22	(6.32- 10.71)
PEZZOT	25	m	0	211	317	4	116	19.30	(7.02- 53.10)
RONCO	3	m	2	-	-	-	-	5.43	(2.27- 12.96)
SADOWS	28	m	1	-	-	-	-	4.46	(2.28- 8.72)
STASZE	2	m	0	218	552	5	158	12.48	(5.05- 30.82)
SUZUK2	7	c	3	-	-	-	-	11.00	(3.40- 36.00)
TIZZAN	2	m	0	994	836	180	305	2.01	(1.64- 2.48)
TIZZAN	22	f	0	25	28	25	114	4.07	(2.04- 8.13)
Subtotal TIZZAN								2.13	(1.75- 2.60)
WIGLE	13	m	0	543	632	15	204	11.68	(6.83- 19.99)
WIGLE	16	f	0	78	235	36	439	4.05	(2.64- 6.19)
Subtotal WIGLE								6.09	(4.37- 8.51)
WYNDE7	39	m	0	1645	2108	64	918	11.19	(8.62- 14.54)
*XIANGZ	9	m	2	-	-	-	-	1.70	(1.14- 2.54)
Partial Totals				9339	66614	765	67665		

*prospective study

Table 1A3 - 2

IESLC - Meta-analysis of Ever Smoking, Cigarettes only
All LC types
Most adjusted

REF	NRR	SEX	AD	Ys	Ws	Qs	Ps
ABELIN	47	m	1	3.99	1.59	10.01	0.0000
AGUDO	1	f	3	1.13	6.32	0.75	0.0044
ALDERS	67	m	1	2.34	12.82	9.57	0.0000
ALDERS	6	f	1	1.56	45.44	0.31	0.0000
Subtotal ALDERS				1.73	58.26	9.88	
ARMADA	1	m	0	2.99	3.64	8.35	0.0000
BAND	1	m	2	2.30	42.76	28.95	0.0000
*BEST	1	m	1	2.65	6.81	9.44	0.0000
*BEST	18	f	1	0.81	2.17	0.97	0.2348
Subtotal BEST				2.21	8.98	10.41	
BOFFET	3	m	2	2.70	102.96	154.67	0.0000
*BOUCOT	113	m	2	3.97	0.50	3.10	0.0051
BRESLO	18	m	0	2.11	5.74	2.34	0.0000
BRESLO	24	f	0	0.32	3.10	4.13	0.5717
Subtotal BRESLO				1.49	8.84	6.47	
*CHOW	15	m	0	2.35	5.43	4.12	0.0000
*CPSI	73	m	1	2.31	201.40	140.03	0.0000
*CPSI	149	f	1	1.16	224.63	21.95	0.0000
Subtotal CPSI				1.71	426.03	161.98	
*CPSII	104	m	1	2.55	78.29	90.65	0.0000
DAMBER	35	m	1	1.96	22.46	5.18	0.0000
DEAN	4	m	0	1.67	9.54	0.37	0.0000
DEAN2	9	m	0	1.44	23.37	0.04	0.0000
DEAN2	17	f	0	1.08	14.24	2.25	0.0000
Subtotal DEAN2				1.30	37.61	2.29	
DEAN3	241	m	1	1.81	21.24	2.37	0.0000
DEAN3	126	f	3	1.53	21.26	0.07	0.0000
Subtotal DEAN3				1.67	42.50	2.44	
DOLL	13	m	0	2.28	6.20	3.96	0.0000
*DOLL2	62	m	1	2.17	18.29	8.88	0.0000
*DORN	1	m	2	2.13	301.36	128.30	0.0000
GOLLED	6	m	1	2.03	13.89	4.34	0.0000
GRAHAM	3	m	0	2.26	16.23	9.98	0.0000
*HAMMON	115	m	1	2.30	14.15	9.53	0.0000
JOLY	48	m	0	2.62	10.80	14.26	0.0000
JUSSAW	31	m	2	2.16	8.36	3.87	0.0000
*KAISE2	72	m	1	1.69	11.75	0.52	0.0000
*KAISE2	64	f	1	2.31	9.19	6.42	0.0000
Subtotal KAISE2				1.96	20.95	6.94	
KJUUS	3	m	0	2.66	1.80	2.51	0.0004
KOULUM	2	m	0	3.38	4.45	16.21	0.0000
LIU4	10	m	2	1.36	5780.52	83.10	0.0000
LOMBAR	10	m	0	2.56	11.67	13.59	0.0000
LUBIN2	18	m	2	2.18	161.10	80.51	0.0000
LUBIN2	98	f	1	1.36	133.31	1.76	0.0000
Subtotal LUBIN2				1.81	294.41	82.27	
MCCONN	16	c	0	0.23	5.66	8.78	0.5832
MILLS	1	m	1	0.24	71.48	108.76	0.0406
NOTAN2	19	m	2	0.86	33.41	12.72	0.0000
PERNU	8	m	0	2.23	50.02	28.19	0.0000
PERNU	4	f	0	0.95	5.14	1.44	0.0321
Subtotal PERNU				2.11	55.16	29.63	
PEZZOT	25	m	0	2.96	3.75	8.27	0.0000
RONCO	3	m	2	1.69	5.06	0.24	0.0001
SADOWS	28	m	1	1.50	8.54	0.00	0.0000
STASZE	2	m	0	2.52	4.70	5.17	0.0000
SUZUK2	7	c	3	2.40	2.76	2.35	0.0001
TIZZAN	2	m	0	0.70	90.61	54.46	0.0000
TIZZAN	22	f	0	1.40	8.03	0.04	0.0001
Subtotal TIZZAN				0.76	98.64	54.50	
WIGLE	13	m	0	2.46	13.33	12.87	0.0000
WIGLE	16	f	0	1.40	21.22	0.13	0.0000
Subtotal WIGLE				1.81	34.55	13.00	
WYNDE7	39	m	0	2.42	56.19	49.61	0.0000
*XIANGZ	9	m	2	0.53	23.94	21.38	0.0094

Table 1A3 - 2

IESLC - Meta-analysis of Ever Smoking, Cigarettes only
All LC types
Most adjusted

	N	54
	NS	43
	Wt	7762.63
Het	Chi	1201.78
Het	df	53
Het	P	***
Fixed	RR	4.37
	RRl	4.28
	RRu	4.47
	P	+++
Random	RR	6.45
	RRl	5.41
	RRu	7.70
	P	+++
Asymm	P	**

Table 1A3 - 3

IESLC - Meta-analysis of Ever Smoking, Cigarettes only
All LC types
Most adjusted

	Sex: combined	male	female	Total
N	2	40	12	54
NS	2	40	12	54
Wt	8.42	7260.15	494.06	7762.63
Het Chi	8.72	1149.19	23.57	1201.78
Het df	1	39	11	53
Het P	**	***	*	***
Fixed RR	2.56	4.43	3.64	4.37
RRl	1.30	4.33	3.33	4.28
RRu	5.03	4.54	3.98	4.47
P	++	+++	+++	+++
Random RR	3.57	7.90	3.84	6.45
RRl	0.43	6.32	3.23	5.41
RRu	29.77	9.88	4.58	7.70
P	N.S.	+++	+++	+++
Between Chi				20.31
Between df				2
Between P				***
Btwn(F) P				N.S.
Btwn(R) P				***

	Lung cancer type: all	other	Total
N	54		54
NS	43		43
Wt	7762.63		7762.63
Het Chi	1201.78		1201.78
Het df	53		53
Het P	***		***
Fixed RR	4.37		4.37
RRl	4.28		4.28
RRu	4.47		4.47
P	+++		+++
Random RR	6.45		6.45
RRl	5.41		5.41
RRu	7.70		7.70
P	+++		+++
Between Chi			
Between df			
Between P			N.S.
Btwn(F) P			N.S.
Btwn(R) P			N.S.

	Location: NAmer	UK	Scand	othEur	China	Japan	othAs	other	Total
N	21	10	5	10	2		2	4	54
NS	16	7	4	8	2		2	4	43
Wt	1105.94	182.41	83.87	517.33	5804.46		41.78	26.86	7762.63
Het Chi	462.52	33.88	15.83	261.84	16.24		11.27	6.56	1201.78
Het df	20	9	4	9	1		1	3	53
Het P	***	***	**	***	***		***	(*)	***
Fixed RR	6.57	5.30	8.55	6.04	3.87		3.06	10.07	4.37
RRl	6.19	4.58	6.90	5.55	3.77		2.26	6.90	4.28
RRu	6.97	6.13	10.59	6.59	3.97		4.14	14.70	4.47
P	+++	+++	+++	+++	+++		+++	+++	+++
Random RR	7.02	5.31	8.68	7.06	2.63		4.36	10.58	6.45
RRl	5.06	3.96	5.01	4.10	1.17		1.23	5.83	5.41
RRu	9.73	7.14	15.04	12.15	5.90		15.53	19.22	7.70
P	+++	+++	+++	+++	+		+	+++	+++
Between Chi									393.65
Between df									6
Between P									***
Btwn(F) P									**
Btwn(R) P									(*)

Table 1A3 - 3

IESLC - Meta-analysis of Ever Smoking, Cigarettes only
All LC types
Most adjusted

Detailed Country in "other Europe"

	multi	Germany	othWest	East	Balkans	Total
N	3		6	1		10
NS	2		5	1		8
Wt	397.37		115.26	4.70		517.33
Het Chi	109.78		40.06	0.00		261.84
Het df	2		5	0		9
Het P	***		***	N.S.		***
Fixed RR	7.70		2.55	12.48		6.04
RRl	6.98		2.12	5.05		5.55
RRu	8.50		3.06	30.82		6.59
P	+++		+++	+++		+++
Random RR	8.01		6.11	12.48		7.06
RRl	3.84		2.72	5.05		4.10
RRu	16.70		13.73	30.82		12.15
P	+++		+++	+++		+++
Between Chi						112.00
Between df						2
Between P						***
Btwn(F) P						N.S.
Btwn(R) P						N.S.

Detailed Country in "other Asia"

	India	HongKong	other	Total
N	2			2
NS	2			2
Wt	41.78			41.78
Het Chi	11.27			11.27
Het df	1			1
Het P	***			***
Fixed RR	3.06			3.06
RRl	2.26			2.26
RRu	4.14			4.14
P	+++			+++
Random RR	4.36			4.36
RRl	1.23			1.23
RRu	15.53			15.53
P	+			+
Between Chi				
Between df				
Between P				N.S.
Btwn(F) P				N.S.
Btwn(R) P				N.S.

Detailed other continent

	SCAmer	Auslia	Africa	Total
N	3		1	4
NS	3		1	4
Wt	17.32		9.54	26.86
Het Chi	0.54		0.00	6.56
Het df	2		0	3
Het P	N.S.		N.S.	(*)
Fixed RR	14.31		5.32	10.07
RRl	8.94		2.82	6.90
RRu	22.92		10.04	14.70
P	+++		+++	+++
Random RR	14.31		5.32	10.58
RRl	8.94		2.82	5.83
RRu	22.92		10.04	19.22
P	+++		+++	+++
Between Chi				6.02
Between df				1
Between P				*
Btwn(F) P				*
Btwn(R) P				*

Table 1A3 - 3

IESLC - Meta-analysis of Ever Smoking, Cigarettes only
All LC types
Most adjusted

	Start year of study					
	<1960	1960-69	1970-79	1980-89	1990+	Total
N	25	7	14	7	1	54
NS	20	5	10	7	1	43
Wt	1085.89	127.31	528.42	6018.25	2.76	7762.63
Het Chi	535.10	24.88	135.79	342.78	0.00	1201.78
Het df	24	6	13	6	0	53
Het P	***	***	***	***	N.S.	***
Fixed RR	5.52	4.09	6.29	4.07	11.00	4.37
RRl	5.20	3.44	5.77	3.97	3.38	4.28
RRu	5.86	4.87	6.85	4.17	35.79	4.47
P	+++	+++	+++	+++	+++	+++
Random RR	6.21	4.64	6.65	9.54	11.00	6.45
RRl	4.47	3.21	4.87	4.88	3.38	5.41
RRu	8.64	6.72	9.10	18.65	35.79	7.70
P	+++	+++	+++	+++	+++	+++
Between Chi						163.23
Between df						4
Between P						***
Btwn(F) P						N.S.
Btwn(R) P						N.S.

	Study type (1)		
	CC	other	Total
N	41	13	54
NS	33	10	43
Wt	6864.72	897.91	7762.63
Het Chi	732.81	264.41	1201.78
Het df	40	12	53
Het P	***	***	***
Fixed RR	4.12	6.85	4.37
RRl	4.03	6.42	4.28
RRu	4.22	7.32	4.47
P	+++	+++	+++
Random RR	6.26	7.16	6.45
RRl	5.10	4.92	5.41
RRu	7.68	10.40	7.70
P	+++	+++	+++
Between Chi			204.56
Between df			1
Between P			***
Btwn(F) P			**
Btwn(R) P			N.S.

	Study type (2)			
	CC	prosp	other	Total
N	41	13		54
NS	33	10		43
Wt	6864.72	897.91		7762.63
Het Chi	732.81	264.41		1201.78
Het df	40	12		53
Het P	***	***		***
Fixed RR	4.12	6.85		4.37
RRl	4.03	6.42		4.28
RRu	4.22	7.32		4.47
P	+++	+++		+++
Random RR	6.26	7.16		6.45
RRl	5.10	4.92		5.41
RRu	7.68	10.40		7.70
P	+++	+++		+++
Between Chi				204.56
Between df				1
Between P				***
Btwn(F) P				**
Btwn(R) P				N.S.

Table 1A3 - 3

IESLC - Meta-analysis of Ever Smoking, Cigarettes only
All LC types
Most adjusted

	Study size (number of LC cases) 100-249	250-499	500-999	1000+	Total
N	9	10	17	18	54
NS	9	8	13	13	43
Wt	32.88	146.32	270.99	7312.45	7762.63
Het Chi	35.70	141.76	112.60	891.09	1201.78
Het df	8	9	16	17	53
Het P	***	***	***	***	***
Fixed RR	6.31	3.29	4.97	4.37	4.37
RRl	4.49	2.80	4.41	4.27	4.28
RRu	8.89	3.87	5.60	4.47	4.47
P	+++	+++	+++	+++	+++
Random RR	8.77	6.67	5.68	6.84	6.45
RRl	4.09	3.20	4.10	5.19	5.41
RRu	18.83	13.87	7.86	9.02	7.70
P	+++	+++	+++	+++	+++
Between Chi					20.63
Between df					3
Between P					***
Btwn(F) P					N.S.
Btwn(R) P					N.S.

	Risky occupational population no	mining	othRisky	Total
N	53	1		54
NS	42	1		43
Wt	7738.69	23.94		7762.63
Het Chi	1180.33	0.00		1201.78
Het df	52	0		53
Het P	***	N.S.		***
Fixed RR	4.39	1.70		4.37
RRl	4.29	1.14		4.28
RRu	4.49	2.54		4.47
P	+++	++		+++
Random RR	6.64	1.70		6.45
RRl	5.56	1.14		5.41
RRu	7.93	2.54		7.70
P	+++	++		+++
Between Chi				21.45
Between df				1
Between P				***
Btwn(F) P				N.S.
Btwn(R) P				***

	National cigarette tobacco type Virginia	blended	other	Total
N	18	34	2	54
NS	13	28	2	43
Wt	320.02	1638.15	5804.46	7762.63
Het Chi	92.28	736.89	16.24	1201.78
Het df	17	33	1	53
Het P	***	***	***	***
Fixed RR	5.53	6.47	3.87	4.37
RRl	4.96	6.16	3.77	4.28
RRu	6.17	6.79	3.97	4.47
P	+++	+++	+++	+++
Random RR	5.65	7.55	2.63	6.45
RRl	4.32	5.82	1.17	5.41
RRu	7.39	9.81	5.90	7.70
P	+++	+++	+	+++
Between Chi				356.38
Between df				2
Between P				***
Btwn(F) P				***
Btwn(R) P				*

Table 1A3 - 3

IESLC - Meta-analysis of Ever Smoking, Cigarettes only
All LC types
Most adjusted

	Any proxy use: No/nk	Yes	Total
N	41	13	54
NS	32	11	43
Wt	1632.27	6130.36	7762.63
Het Chi	586.27	343.03	1201.78
Het df	40	12	53
Het P	***	***	***
Fixed RR	6.29	3.97	4.37
RRl	5.99	3.87	4.28
RRu	6.60	4.07	4.47
P	+++	+++	+++
Random RR	6.79	5.70	6.45
RRl	5.49	3.79	5.41
RRu	8.40	8.57	7.70
P	+++	+++	+++
Between Chi			272.48
Between df			1
Between P			***
Btwn(F) P			***
Btwn(R) P			N.S.

	Full histological confirmation: No	Yes	Total
N	43	11	54
NS	34	9	43
Wt	7339.92	422.71	7762.63
Het Chi	996.22	107.32	1201.78
Het df	42	10	53
Het P	***	***	***
Fixed RR	4.26	6.99	4.37
RRl	4.16	6.35	4.28
RRu	4.36	7.69	4.47
P	+++	+++	+++
Random RR	6.28	7.28	6.45
RRl	5.15	4.90	5.41
RRu	7.66	10.81	7.70
P	+++	+++	+++
Between Chi			98.24
Between df			1
Between P			***
Btwn(F) P			*
Btwn(R) P			N.S.

	Number of adjustment variables (1): 0	1	2+/+nk	Total
N	23	18	13	54
NS	18	14	13	45
Wt	374.87	897.45	6490.32	7762.63
Het Chi	229.17	400.65	510.01	1201.78
Het df	22	17	12	53
Het P	***	***	***	***
Fixed RR	5.57	5.25	4.21	4.37
RRl	5.03	4.92	4.10	4.28
RRu	6.16	5.61	4.31	4.47
P	+++	+++	+++	+++
Random RR	6.91	6.54	5.89	6.45
RRl	4.84	4.59	4.06	5.41
RRu	9.87	9.33	8.56	7.70
P	+++	+++	+++	+++
Between Chi				61.95
Between df				2
Between P				***
Btwn(F) P				N.S.
Btwn(R) P				N.S.

Table 1A3 - 3

IESLC - Meta-analysis of Ever Smoking, Cigarettes only
All LC types
Most adjusted

	Number of adjustment variables (2) 0	1	2	3-5	6+/+nk	Total
N	23	18	10	3		54
NS	18	14	10	3		45
Wt	374.87	897.45	6459.97	30.34		7762.63
Het Chi	229.17	400.65	506.68	3.08		1201.78
Het df	22	17	9	2		53
Het P	***	***	***	N.S.		***
Fixed RR	5.57	5.25	4.20	4.61		4.37
RRl	5.03	4.92	4.10	3.23		4.28
RRu	6.16	5.61	4.31	6.58		4.47
P	+++	+++	+++	+++		+++
Random RR	6.91	6.54	6.15	4.72		6.45
RRl	4.84	4.59	4.02	2.82		5.41
RRu	9.87	9.33	9.42	7.89		7.70
P	+++	+++	+++	+++		+++
Between Chi						62.20
Between df						3
Between P						***
Btwn(F) P						N.S.
Btwn(R) P						N.S.

	Product all/unsp	cig+/-ot	cig only	Total
N			54	54
NS			43	43
Wt			7762.63	7762.63
Het Chi			1201.78	1201.78
Het df			53	53
Het P			***	***
Fixed RR			4.37	4.37
RRl			4.28	4.28
RRu			4.47	4.47
P			+++	+++
Random RR			6.45	6.45
RRl			5.41	5.41
RRu			7.70	7.70
P			+++	+++
Between Chi				
Between df				
Between P				N.S.
Btwn(F) P				N.S.
Btwn(R) P				N.S.

	Denominator nev any	nev cigs	Total
N	53	1	54
NS	42	1	43
Wt	7758.88	3.75	7762.63
Het Chi	1193.51	0.00	1201.78
Het df	52	0	53
Het P	***	N.S.	***
Fixed RR	4.37	19.30	4.37
RRl	4.27	7.02	4.28
RRu	4.47	53.10	4.47
P	+++	+++	+++
Random RR	6.36	19.30	6.45
RRl	5.33	7.02	5.41
RRu	7.59	53.10	7.70
P	+++	+++	+++
Between Chi			8.27
Between df			1
Between P			**
Btwn(F) P			N.S.
Btwn(R) P			*

Table 1A3 - 3

IESLC - Meta-analysis of Ever Smoking, Cigarettes only
All LC types
Most adjusted

	Derivation of RR/CI			
	Orig	StdCalc	Other	Total
N	4	29	21	54
NS	4	22	17	43
Wt	413.40	831.80	6517.43	7762.63
Het Chi	33.39	381.78	438.52	1201.78
Het df	3	28	20	53
Het P	***	***	***	***
Fixed RR	9.56	5.62	4.03	4.37
RRl	8.68	5.25	3.94	4.28
RRu	10.53	6.01	4.13	4.47
P	+++	+++	+++	+++
Random RR	8.71	7.13	5.51	6.45
RRl	5.29	5.34	4.24	5.41
RRu	14.34	9.53	7.17	7.70
P	+++	+++	+++	+++
Between Chi				348.09
Between df				2
Between P				***
Btwn(F) P				***
Btwn(R) P				N.S.

	Study LIU4		
	LIU4	others	Total
N	1	53	54
NS	1	42	43
Wt	5780.52	1982.11	7762.63
Het Chi	0.00	876.35	1201.78
Het df	0	52	53
Het P	N.S.	***	***
Fixed RR	3.88	6.21	4.37
RRl	3.78	5.94	4.28
RRu	3.98	6.48	4.47
P	+++	+++	+++
Random RR	3.88	6.58	6.45
RRl	3.78	5.39	5.41
RRu	3.98	8.05	7.70
P	+++	+++	+++
Between Chi			325.44
Between df			1
Between P			***
Btwn(F) P			***
Btwn(R) P			***

Table 1A3 - 4

IESLC - Meta-analysis of Ever Smoking, Cigarettes only
All LC types
Least adjusted

REF	NRR	X	SEX	AGEL	AGEH	RACE	YF	LC TYPE	LOC	START	ST	NLC	R	VB	P	H	AD	PRODUCT	DENOM	De
ABELIN	4	x	m	0	0	all	-	all	Eu:wst	1941	CC	118	n	bl	y	n	0	cig only	nev any	st
AGUDO	8	x	f	0	0	all	-	all	Eu:wst	1989	CC	103	n	bl	n	n	0	cig only	nev any	st
ALDERS	60	x	m	0	0	all	-	all	Eu:UK	1977	CC	1448	n	V	n	n	0	cig only	nev any	st
ALDERS	12	x	f	0	0	all	-	all	Eu:UK	1977	CC	1448	n	V	n	n	0	cig only	nev any	st
ARMADA	1		m	0	0	all	-	all	Eu:wst	1986	CC	325	n	bl	n	y	0	cig only	nev any	st
BAND	1		m	0	0	all	-	all	NAmer	1983	CC	2831	n	V	y	y	2	cig only	nev any	ot
BEST	1		m	0	0	all	0	all	NAmer	1955	pr	381	n	V	n	n	1	cig only	nev any	ot
BEST	18		f	0	0	all	0	all	NAmer	1955	pr	381	n	V	n	n	1	cig only	nev any	ot
BOFFET	6	x	m	0	0	all	-	all	Eu:mul	1988	CC	5621	n	bl	y	n	0	cig only	nev any	st
BOUCOT	1	x	m	0	0	all	0	all	NAmer	1951	pr	121	n	bl	n	n	0	cig only	nev any	ot
BRESLO	18		m	0	0	all	-	all	NAmer	1949	CC	518	n	bl	n	y	0	cig only	nev+1	st
BRESLO	24		f	0	0	all	-	all	NAmer	1949	CC	518	n	bl	n	y	0	cig only	nev+1	st
CHOW	15		m	0	0	wh	0	all	NAmer	1966	pr	219	n	bl	n	n	0	cig only	nev any	st
CPSI	73		m	0	0	wh	0	all	NAmer	1959	pr	5138	n	bl	n	n	1	cig only	nev any	st
CPSI	149		f	0	0	wh	0	all	NAmer	1959	pr	5138	n	bl	n	n	1	cig only	nev any	st
CPSII	104		m	35	99	all	4	all	NAmer	1982	pr	3229	n	bl	n	n	1	cig only	nev any	ot
DAMBER	1	x	m	0	0	all	-	all	Eu:Sca	1972	CC	579	n	bl	y	n	0	cig only	nev any	st
DEAN	4		m	0	0	wh	-	all	Africa	1947	CC	603	n	V	y	n	0	cig only	nev any	st
DEAN2	9		m	0	0	all	-	all	Eu:UK	1960	CC	954	n	V	y	n	0	cig only	nev any	st
DEAN2	17		f	0	0	all	-	all	Eu:UK	1960	CC	954	n	V	y	n	0	cig only	nev any	st
DEAN3	240	x	m	0	0	all	-	all	Eu:UK	1969	CC	766	n	V	y	n	0	cig only	nev any	st
DEAN3	124	x	f	0	0	all	-	all	Eu:UK	1969	CC	766	n	V	y	n	0	cig only	nev any	st
DOLL	13		m	0	0	all	-	all	Eu:UK	1948	CC	1465	n	V	n	n	0	cig only	nev any	st
DOLL2	62		m	0	0	all	0	all	Eu:UK	1951	pr	920	n	V	n	n	1	cig only	nev any	ot
DORN	1		m	0	0	wh	0	all	NAmer	1954	pr	5097	n	bl	n	n	2	cig only	nev any	or
GOLLED	20	x	m	35	99	all	-	all	Eu:UK	1952	CC	443	n	V	y	n	0	cig only	nev any	st
GRAHAM	3		m	0	0	wh	-	all	NAmer	1956	CC	685	n	bl	n	n	0	cig only	nev any	st
HAMMON	127	x	m	0	0	wh	0	all	NAmer	1952	pr	448	n	bl	n	n	0	cig only	nev any	st
JOLY	48		m	0	0	all	-	all	SCAmer	1978	CC	826	n	bl	n	n	0	cig only	nev any	st
JUSSAW	2	x	m	0	0	all	-	all	As:Ind	1964	CC	792	n	V	n	n	0	cig only	nev any	st
KAISE2	72		m	35	99	all	9	all	NAmer	1979	pr	318	n	bl	n	n	1	cig only	nev any	st
KAISE2	64		f	35	99	all	9	all	NAmer	1979	pr	318	n	bl	n	n	1	cig only	nev any	st
KJUUS	3		m	0	0	all	-	all	Eu:Sca	1979	CC	176	n	bl	n	n	0	cig only	nev any	st
KOULUM	2		m	0	0	all	-	all	Eu:Sca	1936	CC	812	n	bl	n	n	0	cig only	nev any	st
LIU4	10		m	35	69	all	-	all	As:Chi	1986	CC	1000-00	n	ot	y	n	2	cig only	nev any	ot
LOMBAR	10		m	0	0	all	-	all	NAmer	1951	CC	1040	n	bl	n	n	0	cig only	nev any	st
LUBIN2	17	x	m	0	0	all	-	all	Eu:mul	1976	CC	7804	n	bl	n	y	0	cig only	nev any	st
LUBIN2	97	x	f	0	0	all	-	all	Eu:mul	1976	CC	7804	n	bl	n	y	0	cig only	nev any	st
MCCONN	16		c	0	0	all	-	all	Eu:UK	1946	CC	100	n	V	n	y	0	cig only	nev any	st
MILLS	1		m	0	0	wh	-	all	NAmer	1940	CC	444	n	bl	y	n	1	cig only	nev any	ot
NOTAN2	7	x	m	0	0	all	-	all	As:Ind	1963	CC	683	n	V	n	n	0	cig only	nev any	st
PERNU	8		m	0	0	all	-	all	Eu:Sca	1944	CC	1606	n	bl	n	n	0	cig only	nev any	st
PERNU	4		f	0	0	all	-	all	Eu:Sca	1944	CC	1606	n	bl	n	n	0	cig only	nev any	st
PEZZOT	25		m	0	0	all	-	all	SCAmer	1987	CC	215	n	bl	n	y	0	cig only	nev cigs	st
RONCO	2	x	m	0	0	all	-	all	Eu:wst	1976	CC	126	n	bl	y	n	0	cig only	nev any	st
SADOWS	1	x	m	0	0	wh	-	all	NAmer	1938	CC	477	n	bl	n	n	0	cig only	nev any	st
STASZE	2		m	0	0	all	-	all	Eu:est	1954	CC	281	n	bl	n	y	0	cig only	nev any	st
SUZUK2	3	x	c	0	0	all	-	all	SCAmer	1991	CC	123	n	bl	n	y	0	cig only	nev any	st
TIZZAN	2		m	0	0	all	-	all	Eu:wst	1959	CC	1358	n	bl	n	n	0	cig only	nev any	st
TIZZAN	22		f	0	0	all	-	all	Eu:wst	1959	CC	1358	n	bl	n	n	0	cig only	nev any	st
WIGLE	13		m	0	0	all	-	all	NAmer	1971	CC	728	n	V	n	n	0	cig only	nev any	st
WIGLE	16		f	0	0	all	-	all	NAmer	1971	CC	728	n	V	n	n	0	cig only	nev any	st
WYNDE7	39		m	0	0	all	-	all	NAmer	1977	CC	2085	n	bl	n	y	0	cig only	nev any	st
XIANGZ	1	x	m	0	0	all	0	all	As:Chi	1976	pr	983	m	ot	n	n	0	cig only	nev any	st

Cigarette type is all/unspec for all RRs
except for the following:

REF	NRR	CIGTYPE
ALDERS	12	MC only
DEAN3	240	MC only
DEAN3	124	MC only
JUSSAW	2	MC only
NOTAN2	7	MC only
PERNU	8	MC only
PERNU	4	MC only
SUZUK2	3	MC only

Table 1A3 - 5

IESLC - Meta-analysis of Ever Smoking, Cigarettes only
All LC types
Least adjusted

REF	NRR	SEX	AD	Number Exposed Case	Number Exposed Cont	Non-exposed Case	Non-exposed Cont	RR	95.00%CI
ABELIN	4	m	0	30	90	2	183	30.50	(7.13- 130.47)
AGUDO	8	f	0	23	23	80	183	2.29	(1.21- 4.32)
ALDERS	60	m	0	576	462	15	133	11.05	(6.39- 19.12)
ALDERS	12	f	0	530	371	75	243	4.63	(3.46- 6.19)
Subtotal ALDERS								5.61	(4.34- 7.25)
ARMADA	1	m	0	245	197	4	64	19.90	(7.12- 55.59)
BAND	1	m	2	-	-	-	-	9.96	(7.38- 13.44)
*BEST	1	m	1	-	-	-	-	14.20	(6.70- 30.10)
*BEST	18	f	1	-	-	-	-	2.24	(0.59- 8.44)
Subtotal BEST								9.09	(4.72- 17.48)
BOFFET	6	m	0	4204	3930	117	1750	16.00	(13.20- 19.39)
*BOUCOT	1	m	0	93	28154	0	7551	50.16~	(3.11- 807.74)
BRESLO	18	m	0	316	229	7	42	8.28	(3.65- 18.76)
BRESLO	24	f	0	13	11	12	14	1.38	(0.45- 4.20)
Subtotal BRESLO								4.42	(2.28- 8.54)
*CHOW	15	m	0	57	57162	6	62913	10.46	(4.51- 24.25)
*CPSI	73	m	1	-	-	-	-	10.07	(8.77- 11.56)
*CPSI	149	f	1	-	-	-	-	3.20	(2.81- 3.65)
Subtotal CPSI								5.50	(5.00- 6.05)
*CPSII	104	m	1	-	-	-	-	12.83	(10.28- 16.01)
DAMBER	1	m	0	198	140	42	208	7.00	(4.71- 10.41)
DEAN	4	m	0	403	385	12	61	5.32	(2.82- 10.04)
DEAN2	9	m	0	629	508	33	112	4.20	(2.80- 6.30)
DEAN2	17	f	0	62	29	88	121	2.94	(1.75- 4.94)
Subtotal DEAN2								3.67	(2.67- 5.05)
DEAN3	240	m	0	399	1227	24	510	6.91	(4.52- 10.57)
DEAN3	124	f	0	109	1420	41	1538	2.88	(2.00- 4.15)
Subtotal DEAN3								4.18	(3.17- 5.52)
DOLL	13	m	0	1004	899	7	61	9.73	(4.43- 21.39)
*DOLL2	62	m	1	-	-	-	-	8.78	(5.55- 13.88)
*DORN	1	m	2	-	-	-	-	8.40	(7.50- 9.40)
GOLLED	20	m	0	344	1791	15	490	6.27	(3.71- 10.62)
GRAHAM	3	m	0	474	951	18	346	9.58	(5.89- 15.58)
*HAMMON	127	m	0	249	225565	15	115884	8.53	(5.06- 14.36)
JOLY	48	m	0	379	499	12	218	13.80	(7.60- 25.05)
JUSSAW	2	m	0	126	77	149	624	6.85	(4.90- 9.58)
*KAISE2	72	m	1	-	-	-	-	5.40	(3.05- 9.57)
*KAISE2	64	f	1	-	-	-	-	10.09	(5.29- 19.27)
Subtotal KAISE2								7.11	(4.63- 10.90)
KJUUS	3	m	0	151	127	2	24	14.27	(3.31- 61.54)
KOULUM	2	m	0	625	229	5	54	29.48	(11.65- 74.60)
LIU4	10	m	2	-	-	-	-	3.88	(3.78- 3.98)
LOMBAR	10	m	0	486	302	14	112	12.87	(7.25- 22.85)
LUBIN2	17	m	0	6042	9343	190	2617	8.91	(7.66- 10.36)
LUBIN2	97	f	0	548	559	336	1188	3.47	(2.93- 4.10)
Subtotal LUBIN2								5.86	(5.24- 6.56)
MCCONN	16	c	0	68	138	9	23	1.26	(0.55- 2.87)
MILLS	1	m	1	-	-	-	-	1.27	(1.01- 1.61)
NOTAN2	7	m	0	78	129	134	544	2.45	(1.75- 3.44)
PERNU	8	m	0	706	216	97	275	9.27	(7.02- 12.23)
PERNU	4	f	0	7	24	110	971	2.57	(1.08- 6.11)
Subtotal PERNU								8.22	(6.32- 10.71)
PEZZOT	25	m	0	211	317	4	116	19.30	(7.02- 53.10)
RONCO	2	m	0	116	274	6	78	5.50	(2.33- 12.98)
SADOWS	1	m	0	273	328	18	81	3.75	(2.19- 6.40)
STASZE	2	m	0	218	552	5	158	12.48	(5.05- 30.82)
SUZUK2	3	c	0	82	63	11	53	6.27	(3.03- 12.98)
TIZZAN	2	m	0	994	836	180	305	2.01	(1.64- 2.48)
TIZZAN	22	f	0	25	28	25	114	4.07	(2.04- 8.13)
Subtotal TIZZAN								2.13	(1.75- 2.60)
WIGLE	13	m	0	543	632	15	204	11.68	(6.83- 19.99)
WIGLE	16	f	0	78	235	36	439	4.05	(2.64- 6.19)
Subtotal WIGLE								6.09	(4.37- 8.51)
WYNDE7	39	m	0	1645	2108	64	918	11.19	(8.62- 14.54)
*XIANGZ	1	m	0	289	8011	25	974	1.41	(0.94- 2.10)
Partial Totals				23648	348571	2060	202497		

*prospective study

~ With 0.5 adjustment for zero

Table 1A3 - 5

IESLC - Meta-analysis of Ever Smoking, Cigarettes only
All LC types
Least adjusted

REF	NRR	SEX	AD	Ys	Ws	Qs	Ps
ABELIN	4	m	0	3.42	1.82	6.87	0.0000
AGUDO	8	f	0	0.83	9.53	3.98	0.0106
ALDERS	60	m	0	2.40	12.81	11.06	0.0000
ALDERS	12	f	0	1.53	45.39	0.16	0.0000
Subtotal ALDERS				1.72	58.20	11.21	
ARMADA	1	m	0	2.99	3.64	8.37	0.0000
BAND	1	m	2	2.30	42.76	29.09	0.0000
*BEST	1	m	1	2.65	6.81	9.47	0.0000
*BEST	18	f	1	0.81	2.17	0.97	0.2348
Subtotal BEST				2.21	8.98	10.44	
BOFFET	6	m	0	2.77	104.05	175.55	0.0000
*BOUCOT	1	m	0	3.92	0.50	2.96	0.0058
BRESLO	18	m	0	2.11	5.74	2.35	0.0000
BRESLO	24	f	0	0.32	3.10	4.12	0.5717
Subtotal BRESLO				1.49	8.84	6.47	
*CHOW	15	m	0	2.35	5.43	4.14	0.0000
*CPSI	73	m	1	2.31	201.40	140.71	0.0000
*CPSI	149	f	1	1.16	224.63	21.66	0.0000
Subtotal CPSI				1.71	426.03	162.38	
*CPSII	104	m	1	2.55	78.29	91.00	0.0000
DAMBER	1	m	0	1.95	24.50	5.48	0.0000
DEAN	4	m	0	1.67	9.54	0.37	0.0000
DEAN2	9	m	0	1.44	23.37	0.03	0.0000
DEAN2	17	f	0	1.08	14.24	2.23	0.0000
Subtotal DEAN2				1.30	37.61	2.26	
DEAN3	240	m	0	1.93	21.30	4.49	0.0000
DEAN3	124	f	0	1.06	28.64	4.96	0.0000
Subtotal DEAN3				1.43	49.94	9.45	
DOLL	13	m	0	2.28	6.20	3.98	0.0000
*DOLL2	62	m	1	2.17	18.29	8.93	0.0000
*DORN	1	m	2	2.13	301.36	129.11	0.0000
GOLLED	20	m	0	1.84	13.86	1.82	0.0000
GRAHAM	3	m	0	2.26	16.23	10.03	0.0000
*HAMMON	127	m	0	2.14	14.15	6.35	0.0000
JOLY	48	m	0	2.62	10.80	14.31	0.0000
JUSSAW	2	m	0	1.92	34.20	6.96	0.0000
*KAISE2	72	m	1	1.69	11.75	0.53	0.0000
*KAISE2	64	f	1	2.31	9.19	6.45	0.0000
Subtotal KAISE2				1.96	20.95	6.99	
KJUUS	3	m	0	2.66	1.80	2.52	0.0004
KOULUM	2	m	0	3.38	4.45	16.25	0.0000
LIU4	10	m	2	1.36	5780.52	80.30	0.0000
LOMBAR	10	m	0	2.56	11.67	13.64	0.0000
LUBIN2	17	m	0	2.19	168.98	85.95	0.0000
LUBIN2	97	f	0	1.24	134.56	7.16	0.0000
Subtotal LUBIN2				1.77	303.54	93.10	
MCCONN	16	c	0	0.23	5.66	8.75	0.5832
MILLS	1	m	1	0.24	71.48	108.40	0.0406
NOTAN2	7	m	0	0.90	33.47	11.09	0.0000
PERNU	8	m	0	2.23	50.02	28.34	0.0000
PERNU	4	f	0	0.95	5.14	1.43	0.0321
Subtotal PERNU				2.11	55.16	29.77	
PEZZOT	25	m	0	2.96	3.75	8.29	0.0000
RONCO	2	m	0	1.71	5.21	0.28	0.0001
SADOWS	1	m	0	1.32	13.40	0.31	0.0000
STASZE	2	m	0	2.52	4.70	5.19	0.0000
SUZUK2	3	c	0	1.84	7.25	0.95	0.0000
TIZZAN	2	m	0	0.70	90.61	54.17	0.0000
TIZZAN	22	f	0	1.40	8.03	0.04	0.0001
Subtotal TIZZAN				0.76	98.64	54.21	
WIGLE	13	m	0	2.46	13.33	12.93	0.0000
WIGLE	16	f	0	1.40	21.22	0.12	0.0000
Subtotal WIGLE				1.81	34.55	13.05	
WYNDE7	39	m	0	2.42	56.19	49.82	0.0000
*XIANGZ	1	m	0	0.34	23.64	30.36	0.0980

Table 1A3 - 5

IESLC - Meta-analysis of Ever Smoking, Cigarettes only
All LC types
Least adjusted

	N	54
	NS	43
	Wt	7820.78
Het	Chi	1244.81
Het	df	53
Het	P	***
Fixed	RR	4.37
	RRl	4.27
	RRu	4.46
	P	+++
Random	RR	6.18
	RRl	5.19
	RRu	7.37
	P	+++
Asymm	P	**

Table 1A3 - 6

IESLC - Meta-analysis of Ever Smoking, Cigarettes only
All LC types
Least adjusted

		combined	Sex male	female	Total
	N	2	40	12	54
	NS	2	40	12	54
	Wt	12.92	7302.03	505.84	7820.78
Het	Chi	8.20	1179.57	22.86	1244.81
Het	df	1	39	11	53
Het	P	**	***	*	***
Fixed	RR	3.10	4.44	3.42	4.37
	RRl	1.80	4.34	3.13	4.27
	RRu	5.35	4.55	3.73	4.46
	P	+++	+++	+++	+++
Random	RR	2.84	7.71	3.50	6.18
	RRl	0.59	6.17	2.96	5.19
	RRu	13.71	9.64	4.14	7.37
	P	N.S.	+++	+++	+++
Between	Chi				34.18
Between	df				2
Between	P				***
Btwn(F)	P				N.S.
Btwn(R)	P				***

Table 1A3 - 7

IESLC - Meta-analysis of Ever Smoking, Cigarettes only
All LC types
Excluded studies (and stage at which they were excluded)

1	BOUCHA BUELL LAURIL MZILEN
2	BYERS2 ENSTRO HIRAY2 KANELL LIAW LICKIN MRFIT MURATA NOTANI RESTRE RIMING SAARIK SEGI2 SHIMIZ SITAS SOBUE2 TANG2 WALD WARSIN WATSON WU2 WYNDE5 WYNDE8 WYNDER
3	BERRIN CHEN TAO ULMER
4	ABRAHA AKIBA AMANDU AMES ANDERS ARCHER AUSTIN AUVINE AXELSO AXELSS BARBON BECHER BENSHL BLOHMK BLOT1 BLOT2 BLOT3 BLOT4 BRETT BROCKM BROSS BROWN1 BROWN2 BUFFLE BYERS1 CARPEN CASCO2 CASCOR CEDERL CHAN CHANG CHATZI CHEN2 CHEN3 CHIAZZ CHOI CHYOU COMSTO COOKSO CORREA DARBY DAVEYS DEKLER DESTE2 DESTEF DOCKER DORANT DORGAN DOSEME DROSTE DU DUNN EBELIN ENGELA ESAKI FAN GAO GAO2 GARCIA GARDIN GARSHI GENG GER GILLIS GODLEY GOODMA GREGOR GSELL HAENSZ HAMMO2 HANSEN HEGMAN HEIN HENNEK HINDS HIRAOK HIRAYA HITOSU HOLE HOROWI HORWIT HU HU2 HUANG HUMBLE ISHIMA JAHN JAIN JARUP JARVHO JEDRYC JIANG JONES KAISER KATSOU KAUFMA KELLER KHUDER KIHARA KINLEN KNEKT KO KOHLME KOO KREUZE KREYBE KUBIK LAMTH LAMWK LAMWK2 LANGE LAUSSM LEI LEMARC LETOUR LEVIN LIDDEL LIU LIU2 LIU3 LIU5 LOMBA2 LUO MACLEN MAGNUS MARSH MARSH2 MARTIS MASTRA MATOS MATSUD MCDUFF MCLAUG MIGRAN MILLER MOLLO MRFITR NAM NOU ODRISC ORMOS OSANN OSANN2 PARKIN PASTOR PAWLEG PERSH2 PERSHA PETO PEZZO2 PIKE PISANI POFFIJ POLEDN PRESCO QIAO2 RACHTA RADZIK RANDIG REN ROOTS ROTHSC SANKAR SCHWAR SEGI SEOW SHAW SIEMIA SIMARA SIMONA SOBUE SPEIZE SPITZ STAYNE STOCKS STOCKW STUCKE SUN SUZUKI SVENSS TANG TENKAN TOKARS TOUSEY TSUGAN TULINI TVERDA VANDER VEIERO VUTUC WAKAI WANG WANG2 WANG3 WANG4 WICKLU WILKIN WU WUNSCH WUWILL WYNDE2 WYNDE3 WYNDE4 WYNDE6 XU XU2 XU3 XU4 YAMAGU YONG YUAN ZHANG ZHENG ZHOU
6	SCHWA2
10	BENHAM LUBIN QIAO

Table 1A3 - 8
Potentially overlapping studies

REF	REFGP	PRINC	OVERLAP/LINK
LUBIN2	LUBIN2	1	Lubin-combined
GRAHAM	BYERS1	1	GRAHAM/BROSS/BYERS1
WYNDE7	WYNDE6	2	WYNDE5/6/7/8
CPSI	CPSI	1	CPSI overall
BOFFET	BOFFET	1	BOFFET-combined
XIANGZ	XIANGZ	1	LUBIN/XIANGZ/QIAO

Table 1A4 -

IESLC - Meta-analysis of Ever Smoking, Any product (or Cigarettes if Any not available), Age <56
All LC types

This analysis is restricted to results for:
1) Non-dose-response data
2) Ever smokers
3) Age <56
4) Results complete enough for use in metaanalysis

Within each study, results are then selected (in the following order of preference, within each sex) for:
5) PRODUCT: all/unspec, cigarettes regardless of other products, cigarettes only
6) CIGTYPE: all/unspecified, MC regardless of HR, MC only
7) DENOM: never smoked anything, never smoked cigarettes, (never +1 = +long term ex, +2 = +amount unknown,
+3 = never cigs+long term ex)
8) Followup period (YF, prospective studies): whole study (coded as 0) or longest available
9) LCtype: all or nearest available, at least Squamous and Adeno. (q = squamous, s = small,
l = large, a = adeno, mix = mixed, alv = alveolar)
10) Race: all or nearest available, otherwise by race (wh or w = white, bl or b = black, hi = hispanic
ch = chinese, jap = japanese, haw = hawaiian, w+o = white + oriental, sca = scandinavian, as = asian)
11) For overlapping studies: principal rather than subsidiary studies
Finally by Age: whole study (actual age shown) if available, otherwise by widest available age group
and then for single sex results (m, f) in preference to combined sex results (c).

Results adjusted (AD) for the most potential confounders are then chosen in Sections -1 to -3
(and those which actually differ from the adjusted results in Table 1A1 - 1 are marked 'x' in Section -1)
and results adjusted for the least confounders in Sections -4 to -6. (Those least adjusted results which
actually differ from the most adjusted as marked 'x' in column X in Section -4)
(Results adjusted for an unknown number of confounder(s) are coded as 20.)

Section -7 shows excluded studies, together with the stage (as above) at which no qualifying
results were found.

Section -8 lists the potentially overlapping studies which have been included (1=principal, 2=subsidiary).

Section -9 lists any results which would have been included in preference except that they had data not complete
enough for use in meta-analysis, with their significance (yes/no), if known, and any further comment as entered
on the database.

In addition to those mentioned above, the following fields, levels and abbreviations are used:
* or nk = not known, n = no, y = yes, ot = other
nev = never
all/unspec = all or unspecified, cig+/-ot = cigarettes irrespective of other products (cigar, pipe etc)
MC = manufactured cigarettes, HR = hand-rolled cigarettes
REF: 6-character study reference
NRR: number of the RR on the database within the study
ST : study type (CC = case control, pr or prosp = prospective)
NLC: number of lung cancer cases in whole study
R : risky occupational population (n = no, m = mining, o = other risky)
VB : national cigarette type (V = at least 75% Virginia, bl = at least 75% blended, ot = other)
P : any proxy use
H : full histological confirmation
De : derivation of RR/CI (or = original, st = standard method, ot = other method of estimation)

Table 1A4 - 1

IESLC - Meta-analysis of Ever Smoking, Any product (or Cigarettes if Any not available), Age <56
All LC types
Most adjusted

REF	NRR	1A1	SEX	AGEL	AGEH	RACE	YF	LC TYPE	LOC	START	ST	NLC	R	VB	P	H	AD	PRODUCT	DENOM	De
BENHAM	68	x	m	1	49	all	-	not mix	Eu:wst	1976	CC	1625	n	bl	n	y	0	cig only	nev any	st
COMSTO	57	x	c	25	44	all	-	all	NAmer	1975	ot	258	n	bl	n	n	0	cig+/-ot	nev cigs	st
CPSI	163	x	m	35	54	all	6	all	NAmer	1959	pr	5138	n	bl	n	n	1	cig+/-ot	nev any	ot
CPSI	250	x	f	40	54	all	6	all	NAmer	1959	pr	5138	n	bl	n	n	1	cig+/-ot	nev cigs	ot
DEAN3	43	x	m	35	44	all	-	all	Eu:UK	1969	CC	766	n	V	y	n	0	all/unsp	nev any	st
DEAN3	44	x	m	45	54	all	-	all	Eu:UK	1969	CC	766	n	V	y	n	0	all/unsp	nev any	st
DEAN3	120	x	f	35	44	all	-	all	Eu:UK	1969	CC	766	n	V	y	n	0	cig only	nev any	st
DEAN3	121	x	f	45	54	all	-	all	Eu:UK	1969	CC	766	n	V	y	n	0	cig only	nev any	st
HAMMON	4	x	m	50	54	wh	0	all	NAmer	1952	pr	448	n	bl	n	n	0	all/unsp	nev any	st
HINDS	4	x	f	35	49	ch	-	all	NAmer	1968	CC	292	n	bl	n	n	0	all/unsp	nev any	st
HINDS	1	x	f	35	49	jap	-	all	NAmer	1968	CC	292	n	bl	n	n	0	all/unsp	nev any	st
HINDS	7	x	f	35	49	haw	-	all	NAmer	1968	CC	292	n	bl	n	n	0	all/unsp	nev any	st
HITOSU	24	x	m	1	49	all	-	all	As:Jap	1960	CC	216	n	bl	y	n	0	all/unsp	nev any	st
HITOSU	44	x	f	1	49	all	-	all	As:Jap	1960	CC	216	n	bl	y	n	0	all/unsp	nev any	st
KREUZE	13	x	m	1	45	all	-	all	Eu:Ger	1990	CC	2260	n	bl	n	n	0	all/unsp	nev any	st
KREUZE	14		f	1	45	all	-	all	Eu:Ger	1990	CC	2260	n	bl	n	n	0	all/unsp	nev any	st
KUBIK	9	x	m	40	54	all	4	all	Eu:est	1965	pr	108	n	bl	n	n	0	all/unsp	nev any	ot
LEVIN	18	x	m	35	44	all	-	all	NAmer	1938	CC	475	n	bl	n	n	0	all/unsp	nev any	st
LEVIN	19	x	m	45	54	all	-	all	NAmer	1938	CC	475	n	bl	n	n	0	all/unsp	nev any	st
NAM	5	x	m	25	54	all	-	all	NAmer	1986	CC	1199	n	bl	y	n	0	cig+/-ot	nev cigs	st
NAM	37	x	f	25	54	all	-	all	NAmer	1986	CC	1199	n	bl	y	n	0	cig+/-ot	nev cigs	st
PRESCO	38	x	m	20	49	all	0	all	Eu:Sca	1964	pr	867	n	bl	n	n	0	all/unsp	nev any	ot
PRESCO	37	x	f	20	49	all	0	all	Eu:Sca	1964	pr	867	n	bl	n	n	0	all/unsp	nev any	ot
SCHWAR	6	x	m	40	54	wh	-	all	NAmer	1984	CC	5588	n	bl	y	y	0	cig+/-ot	nev cigs	st
SCHWAR	5	x	m	40	54	bl	-	all	NAmer	1984	CC	5588	n	bl	y	y	0	cig+/-ot	nev cigs	st
SCHWAR	14	x	f	40	54	wh	-	all	NAmer	1984	CC	5588	n	bl	y	y	0	cig+/-ot	nev cigs	st
SCHWAR	13	x	f	40	54	bl	-	all	NAmer	1984	CC	5588	n	bl	y	y	0	cig+/-ot	nev cigs	st
SPEIZE	8		f	30	55	all	0	all	NAmer	1976	pr	593	n	bl	n	y	0	cig+/-ot	nev cigs	st
STOCKS	33	x	m	35	44	all	-	all	Eu:UK	1952	CC	2932	n	V	y	n	0	all/unsp	nev any	st
STOCKS	35	x	m	45	54	all	-	all	Eu:UK	1952	CC	2932	n	V	y	n	0	all/unsp	nev any	st
TSUGAN	27		m	30	49	all	-	q+a	As:Jap	1976	CC	134	n	bl	n	y	0	all/unsp	nev any	st
TVERDA	22		m	35	49	all	0	all	Eu:Sca	1972	pr	238	n	bl	n	n	2	cig+/-ot	nev cigs	ot
VUTUC	10	x	m	1	39	all	-	all	Eu:wst	1976	CC	1877	n	bl	n	n	0	cig+/-ot	nev cigs	st
VUTUC	33	x	m	41	50	all	-	all	Eu:wst	1976	CC	1877	n	bl	n	n	0	cig+/-ot	nev cigs	st
WUWILL	1	x	f	30	49	all	-	all	As:Chi	1985	CC	965	n	ot	n	n	0	cig+/-ot	nev cigs	st
WUWILL	2	x	f	50	54	all	-	all	As:Chi	1985	CC	965	n	ot	n	n	0	cig+/-ot	nev cigs	st
WYNDE6	427	x	m	1	54	wh	-	q+s+a	NAmer	1969	CC	4423	n	bl	n	y	0	cig+/-ot	nev cigs	st
WYNDE6	429	x	f	1	54	wh	-	q+s+a	NAmer	1969	CC	4423	n	bl	n	y	0	cig+/-ot	nev cigs	st

Cigarette type is all/unspec for all RRs
except for the following:

REF	NRR	CIGTYPE
DEAN3	120	MC only
DEAN3	121	MC only

Table 1A4 - 2

IESLC - Meta-analysis of Ever Smoking, Any product (or Cigarettes if Any not available), Age <56
All LC types
Most adjusted

REF	NRR	SEX	AD	Number Exposed Case	Number Exposed Cont	Non-exposed Case	Non-exposed Cont	RR	95.00%CI
BENHAM	68	m	0	217	304	9	103	8.17	(4.04- 16.50)
COMSTO	57	c	0	27	39	1	17	11.77	(1.48- 93.79)
*CPSI	163	m	1	-	-	-	-	7.63	(4.93- 11.79)
*CPSI	250	f	1	-	-	-	-	3.50	(2.45- 4.99)
Subtotal CPSI								4.78	(3.63- 6.30)
DEAN3	43	m	0	13	563	1	187	4.32	(0.56- 33.23)
DEAN3	44	m	0	84	650	1	145	18.74	(2.59- 135.69)
DEAN3	120	f	0	6	472	1	328	4.17	(0.50- 34.80)
DEAN3	121	f	0	23	469	4	326	4.00	(1.37- 11.67)
Subtotal DEAN3								5.21	(2.36- 11.52)
*HAMMON	4	m	0	87	186658	1	33255	15.50	(2.16- 111.27)
HINDS	4	f	0	1	12	4	42	0.88	(0.09- 8.58)
HINDS	1	f	0	9	139	6	350	3.78	(1.32- 10.81)
HINDS	7	f	0	26	118	2	139	15.31	(3.56- 65.87)
Subtotal HINDS								4.81	(2.16- 10.68)
HITOSU	24	m	0	8	993	1	118	0.95	(0.12- 7.67)
HITOSU	44	f	0	4	245	7	1108	2.58	(0.75- 8.90)
Subtotal HITOSU								1.99	(0.69- 5.77)
KREUZE	13	m	0	177	146	6	54	10.91	(4.56- 26.08)
KREUZE	14	f	0	62	42	6	38	9.35	(3.63- 24.08)
Subtotal KREUZE								10.16	(5.35- 19.29)
*KUBIK	9	m	0	11	4445	0	2420	12.52~	(0.74- 212.42)
LEVIN	18	m	0	30	208	1	51	7.36	(0.98- 55.22)
LEVIN	19	m	0	114	389	6	122	5.96	(2.56- 13.88)
Subtotal LEVIN								6.15	(2.82- 13.41)
NAM	5	m	0	56	448	1	176	22.00	(3.02- 160.15)
NAM	37	f	0	40	129	2	93	14.42	(3.40- 61.16)
Subtotal NAM								16.69	(5.19- 53.68)
*PRESCO	38	m	0	18	49324	0	10629	7.97~	(0.48- 132.30)
*PRESCO	37	f	0	7	33202	0	13953	6.30~	(0.36- 110.37)
Subtotal PRESCO								7.11	(0.96- 52.76)
SCHWAR	6	m	0	282	178	7	73	16.52	(7.44- 36.69)
SCHWAR	5	m	0	146	39	6	7	4.37	(1.39- 13.74)
SCHWAR	14	f	0	206	108	11	79	13.70	(6.99- 26.83)
SCHWAR	13	f	0	69	28	5	41	20.21	(7.24- 56.44)
Subtotal SCHWAR								13.19	(8.61- 20.21)
*SPEIZE	8	f	0	535	1012074	58	776300	7.08	(5.40- 9.28)
STOCKS	33	m	0	159	1182	2	163	10.96	(2.69- 44.64)
STOCKS	35	m	0	749	1875	4	149	14.88	(5.49- 40.31)
Subtotal STOCKS								13.43	(5.96- 30.28)
TSUGAN	27	m	0	73	71	18	22	1.26	(0.62- 2.54)
*TVERDA	22	m	2	-	-	-	-	4.58	(2.97- 7.06)
VUTUC	10	m	0	12	22	1	9	4.91	(0.55- 43.53)
VUTUC	33	m	0	97	157	11	64	3.59	(1.81- 7.15)
Subtotal VUTUC								3.70	(1.92- 7.13)
WUWILL	1	f	0	68	39	132	124	1.64	(1.03- 2.60)
WUWILL	2	f	0	121	69	82	127	2.72	(1.81- 4.07)
Subtotal WUWILL								2.18	(1.61- 2.96)
WYNDE6	427	m	0	309	177	17	150	15.40	(9.03- 26.29)
WYNDE6	429	f	0	224	115	15	183	23.76	(13.41- 42.12)
Subtotal WYNDE6								18.85	(12.75- 27.86)
Partial Totals				4070	1295129	429	841145		

*prospective study ~ With 0.5 adjustment for zero

Table 1A4 - 2

IESLC - Meta-analysis of Ever Smoking, Any product (or Cigarettes if Any not available), Age <56
All LC types
Most adjusted

REF	NRR	SEX	AD	Ys	Ws	Qs	Ps
BENHAM	68	m	0	2.10	7.77	0.92	0.0000
COMSTO	57	c	0	2.47	0.89	0.45	0.0199
*CPSI	163	m	1	2.03	20.21	1.54	0.0000
*CPSI	250	f	1	1.25	30.37	7.69	0.0000
Subtotal CPSI				1.56	50.58	9.23	
DEAN3	43	m	0	1.46	0.92	0.08	0.1600
DEAN3	44	m	0	2.93	0.98	1.35	0.0037
DEAN3	120	f	0	1.43	0.85	0.09	0.1872
DEAN3	121	f	0	1.39	3.35	0.46	0.0112
Subtotal DEAN3				1.65	6.10	1.98	
*HAMMON	4	m	0	2.74	0.99	0.96	0.0064
HINDS	4	f	0	-0.13	0.74	2.63	0.9087
HINDS	1	f	0	1.33	3.47	0.63	0.0132
HINDS	7	f	0	2.73	1.80	1.71	0.0002
Subtotal HINDS				1.57	6.02	4.97	
HITOSU	24	m	0	-0.05	0.88	2.88	0.9621
HITOSU	44	f	0	0.95	2.51	1.64	0.1323
Subtotal HITOSU				0.69	3.40	4.51	
KREUZE	13	m	0	2.39	5.06	2.03	0.0000
KREUZE	14	f	0	2.24	4.29	0.99	0.0000
Subtotal KREUZE				2.32	9.35	3.02	
*KUBIK	9	m	0	2.53	0.48	0.29	0.0801
LEVIN	18	m	0	2.00	0.95	0.05	0.0523
LEVIN	19	m	0	1.78	5.37	0.00	0.0000
Subtotal LEVIN				1.82	6.32	0.06	
NAM	5	m	0	3.09	0.97	1.74	0.0023
NAM	37	f	0	2.67	1.84	1.53	0.0003
Subtotal NAM				2.81	2.81	3.27	
*PRESCO	38	m	0	2.08	0.49	0.05	0.1474
*PRESCO	37	f	0	1.84	0.47	0.00	0.2075
Subtotal PRESCO				1.96	0.96	0.05	
SCHWAR	6	m	0	2.80	6.03	6.64	0.0000
SCHWAR	5	m	0	1.47	2.92	0.23	0.0117
SCHWAR	14	f	0	2.62	8.50	6.30	0.0000
SCHWAR	13	f	0	3.01	3.64	5.69	0.0000
Subtotal SCHWAR				2.58	21.10	18.86	
*SPEIZE	8	f	0	1.96	52.33	2.11	0.0000
STOCKS	33	m	0	2.39	1.95	0.79	0.0008
STOCKS	35	m	0	2.70	3.87	3.45	0.0000
Subtotal STOCKS				2.60	5.82	4.24	
TSUGAN	27	m	0	0.23	7.76	18.12	0.5244
*TVERDA	22	m	2	1.52	20.49	1.12	0.0000
VUTUC	10	m	0	1.59	0.81	0.02	0.1530
VUTUC	33	m	0	1.28	8.12	1.84	0.0003
Subtotal VUTUC				1.31	8.92	1.86	
WUWILL	1	f	0	0.49	17.86	28.47	0.0370
WUWILL	2	f	0	1.00	23.35	13.37	0.0000
Subtotal WUWILL				0.78	41.21	41.84	
WYNDE6	427	m	0	2.73	13.45	12.88	0.0000
WYNDE6	429	f	0	3.17	11.72	23.38	0.0000
Subtotal WYNDE6				2.94	25.17	36.26	

	N	38
	NS	20
	Wt	278.47
Het	Chi	154.13
Het	df	37
Het	P	***
Fixed	RR	5.79
	RRl	5.15
	RRu	6.51
	P	+++
Random	RR	6.57
	RRl	4.94
	RRu	8.74
	P	+++
Asymm	P	N.S.

Table 1A4 - 3

IESLC - Meta-analysis of Ever Smoking, Any product (or Cigarettes if Any not available), Age <56

All LC types

Most adjusted

	Sex combined	male	female	Total
N	1	21	16	38
NS	1	16	11	28
Wt	0.89	110.47	167.11	278.47
Het Chi	0.00	54.42	94.87	154.13
Het df	0	20	15	37
Het P	N.S.	***	***	***
Fixed RR	11.77	6.74	5.21	5.79
RRl	1.48	5.59	4.48	5.15
RRu	93.79	8.12	6.07	6.51
P	+	+++	+++	+++
Random RR	11.77	7.04	5.96	6.57
RRl	1.48	4.90	3.77	4.94
RRu	93.79	10.11	9.40	8.74
P	+	+++	+++	+++
Between Chi				4.84
Between df				2
Between P				(*)
Btwn(F) P				N.S.
Btwn(R) P				N.S.

Table 1A4 - 4

IESLC - Meta-analysis of Ever Smoking, Any product (or Cigarettes if Any not available), Age <56
All LC types
Least adjusted

REF	NRR	X	SEX	AGEL	AGEH	RACE	YF	LC TYPE	LOC	START	ST	NLC	R	VB	P	H	AD	PRODUCT	DENOM	De
BENHAM	68		m	1	49	all	-	not mix	Eu:wst	1976	CC	1625	n	bl	n	y	0	cig only	nev any	st
COMSTO	57		c	25	44	all	-	all	NAmer	1975	ot	258	n	bl	n	n	0	cig+/-ot	nev cigs	st
CPSI	163		m	35	54	all	6	all	NAmer	1959	pr	5138	n	bl	n	n	1	cig+/-ot	nev any	ot
CPSI	250		f	40	54	all	6	all	NAmer	1959	pr	5138	n	bl	n	n	1	cig+/-ot	nev cigs	ot
DEAN3	43		m	35	44	all	-	all	Eu:UK	1969	CC	766	n	V	y	n	0	all/unsp	nev any	st
DEAN3	44		m	45	54	all	-	all	Eu:UK	1969	CC	766	n	V	y	n	0	all/unsp	nev any	st
DEAN3	120		f	35	44	all	-	all	Eu:UK	1969	CC	766	n	V	y	n	0	cig only	nev any	st
DEAN3	121		f	45	54	all	-	all	Eu:UK	1969	CC	766	n	V	y	n	0	cig only	nev any	st
HAMMON	4		m	50	54	wh	0	all	NAmer	1952	pr	448	n	bl	n	n	0	all/unsp	nev any	st
HINDS	4		f	35	49	ch	-	all	NAmer	1968	CC	292	n	bl	n	n	0	all/unsp	nev any	st
HINDS	1		f	35	49	jap	-	all	NAmer	1968	CC	292	n	bl	n	n	0	all/unsp	nev any	st
HINDS	7		f	35	49	haw	-	all	NAmer	1968	CC	292	n	bl	n	n	0	all/unsp	nev any	st
HITOSU	24		m	1	49	all	-	all	As:Jap	1960	CC	216	n	bl	y	n	0	all/unsp	nev any	st
HITOSU	44		f	1	49	all	-	all	As:Jap	1960	CC	216	n	bl	y	n	0	all/unsp	nev any	st
KREUZE	13		m	1	45	all	-	all	Eu:Ger	1990	CC	2260	n	bl	n	n	0	all/unsp	nev any	st
KREUZE	14		f	1	45	all	-	all	Eu:Ger	1990	CC	2260	n	bl	n	n	0	all/unsp	nev any	st
KUBIK	9		m	40	54	all	4	all	Eu:est	1965	pr	108	n	bl	n	n	0	all/unsp	nev any	ot
LEVIN	18		m	35	44	all	-	all	NAmer	1938	CC	475	n	bl	n	n	0	all/unsp	nev any	st
LEVIN	19		m	45	54	all	-	all	NAmer	1938	CC	475	n	bl	n	n	0	all/unsp	nev any	st
NAM	5		m	25	54	all	-	all	NAmer	1986	CC	1199	n	bl	y	n	0	cig+/-ot	nev cigs	st
NAM	37		f	25	54	all	-	all	NAmer	1986	CC	1199	n	bl	y	n	0	cig+/-ot	nev cigs	st
PRESCO	38		m	20	49	all	0	all	Eu:Sca	1964	pr	867	n	bl	n	n	0	all/unsp	nev any	ot
PRESCO	37		f	20	49	all	0	all	Eu:Sca	1964	pr	867	n	bl	n	n	0	all/unsp	nev any	ot
SCHWAR	6		m	40	54	wh	-	all	NAmer	1984	CC	5588	n	bl	y	y	0	cig+/-ot	nev cigs	st
SCHWAR	5		m	40	54	bl	-	all	NAmer	1984	CC	5588	n	bl	y	y	0	cig+/-ot	nev cigs	st
SCHWAR	14		f	40	54	wh	-	all	NAmer	1984	CC	5588	n	bl	y	y	0	cig+/-ot	nev cigs	st
SCHWAR	13		f	40	54	bl	-	all	NAmer	1984	CC	5588	n	bl	y	y	0	cig+/-ot	nev cigs	st
SPEIZE	8		f	30	55	all	0	all	NAmer	1976	pr	593	n	bl	n	y	0	cig+/-ot	nev cigs	st
STOCKS	33		m	35	44	all	-	all	Eu:UK	1952	CC	2932	n	V	y	n	0	all/unsp	nev any	st
STOCKS	35		m	45	54	all	-	all	Eu:UK	1952	CC	2932	n	V	y	n	0	all/unsp	nev any	st
TSUGAN	27		m	30	49	all	-	q+a	As:Jap	1976	CC	134	n	bl	n	y	0	all/unsp	nev any	st
TVERDA	22		m	35	49	all	0	all	Eu:Sca	1972	pr	238	n	bl	n	n	2	cig+/-ot	nev cigs	ot
VUTUC	10		m	1	39	all	-	all	Eu:wst	1976	CC	1877	n	bl	n	n	0	cig+/-ot	nev cigs	st
VUTUC	33		m	41	50	all	-	all	Eu:wst	1976	CC	1877	n	bl	n	n	0	cig+/-ot	nev cigs	st
WUWILL	1		f	30	49	all	-	all	As:Chi	1985	CC	965	n	ot	n	n	0	cig+/-ot	nev cigs	st
WUWILL	2		f	50	54	all	-	all	As:Chi	1985	CC	965	n	ot	n	n	0	cig+/-ot	nev cigs	st
WYNDE6	427		m	1	54	wh	-	q+s+a	NAmer	1969	CC	4423	n	bl	n	y	0	cig+/-ot	nev cigs	st
WYNDE6	429		f	1	54	wh	-	q+s+a	NAmer	1969	CC	4423	n	bl	n	y	0	cig+/-ot	nev cigs	st

Cigarette type is all/unspec for all RRs
except for the following:

REF	NRR	CIGTYPE
DEAN3	120	MC only
DEAN3	121	MC only

Table 1A4 - 5

IESLC - Meta-analysis of Ever Smoking, Any product (or Cigarettes if Any not available), Age <56
All LC types
Least adjusted

REF	NRR	SEX	AD	Number Exposed Case	Number Exposed Cont	Non-exposed Case	Non-exposed Cont	RR	95.00%CI
BENHAM	68	m	0	217	304	9	103	8.17	(4.04- 16.50)
COMSTO	57	c	0	27	39	1	17	11.77	(1.48- 93.79)
*CPSI	163	m	1	-	-	-	-	7.63	(4.93- 11.79)
*CPSI	250	f	1	-	-	-	-	3.50	(2.45- 4.99)
Subtotal CPSI								4.78	(3.63- 6.30)
DEAN3	43	m	0	13	563	1	187	4.32	(0.56- 33.23)
DEAN3	44	m	0	84	650	1	145	18.74	(2.59- 135.69)
DEAN3	120	f	0	6	472	1	328	4.17	(0.50- 34.80)
DEAN3	121	f	0	23	469	4	326	4.00	(1.37- 11.67)
Subtotal DEAN3								5.21	(2.36- 11.52)
*HAMMON	4	m	0	87	186658	1	33255	15.50	(2.16- 111.27)
HINDS	4	f	0	1	12	4	42	0.88	(0.09- 8.58)
HINDS	1	f	0	9	139	6	350	3.78	(1.32- 10.81)
HINDS	7	f	0	26	118	2	139	15.31	(3.56- 65.87)
Subtotal HINDS								4.81	(2.16- 10.68)
HITOSU	24	m	0	8	993	1	118	0.95	(0.12- 7.67)
HITOSU	44	f	0	4	245	7	1108	2.58	(0.75- 8.90)
Subtotal HITOSU								1.99	(0.69- 5.77)
KREUZE	13	m	0	177	146	6	54	10.91	(4.56- 26.08)
KREUZE	14	f	0	62	42	6	38	9.35	(3.63- 24.08)
Subtotal KREUZE								10.16	(5.35- 19.29)
*KUBIK	9	m	0	11	4445	0	2420	12.52~	(0.74- 212.42)
LEVIN	18	m	0	30	208	1	51	7.36	(0.98- 55.22)
LEVIN	19	m	0	114	389	6	122	5.96	(2.56- 13.88)
Subtotal LEVIN								6.15	(2.82- 13.41)
NAM	5	m	0	56	448	1	176	22.00	(3.02- 160.15)
NAM	37	f	0	40	129	2	93	14.42	(3.40- 61.16)
Subtotal NAM								16.69	(5.19- 53.68)
*PRESCO	38	m	0	18	49324	0	10629	7.97~	(0.48- 132.30)
*PRESCO	37	f	0	7	33202	0	13953	6.30~	(0.36- 110.37)
Subtotal PRESCO								7.11	(0.96- 52.76)
SCHWAR	6	m	0	282	178	7	73	16.52	(7.44- 36.69)
SCHWAR	5	m	0	146	39	6	7	4.37	(1.39- 13.74)
SCHWAR	14	f	0	206	108	11	79	13.70	(6.99- 26.83)
SCHWAR	13	f	0	69	28	5	41	20.21	(7.24- 56.44)
Subtotal SCHWAR								13.19	(8.61- 20.21)
*SPEIZE	8	f	0	535	1012074	58	776300	7.08	(5.40- 9.28)
STOCKS	33	m	0	159	1182	2	163	10.96	(2.69- 44.64)
STOCKS	35	m	0	749	1875	4	149	14.88	(5.49- 40.31)
Subtotal STOCKS								13.43	(5.96- 30.28)
TSUGAN	27	m	0	73	71	18	22	1.26	(0.62- 2.54)
*TVERDA	22	m	2	-	-	-	-	4.58	(2.97- 7.06)
VUTUC	10	m	0	12	22	1	9	4.91	(0.55- 43.53)
VUTUC	33	m	0	97	157	11	64	3.59	(1.81- 7.15)
Subtotal VUTUC								3.70	(1.92- 7.13)
WUWILL	1	f	0	68	39	132	124	1.64	(1.03- 2.60)
WUWILL	2	f	0	121	69	82	127	2.72	(1.81- 4.07)
Subtotal WUWILL								2.18	(1.61- 2.96)
WYNDE6	427	m	0	309	177	17	150	15.40	(9.03- 26.29)
WYNDE6	429	f	0	224	115	15	183	23.76	(13.41- 42.12)
Subtotal WYNDE6								18.85	(12.75- 27.86)
Partial Totals				4070	1295129	429	841145		

*prospective study ~ With 0.5 adjustment for zero

Table 1A4 - 5

IESLC - Meta-analysis of Ever Smoking, Any product (or Cigarettes if Any not available), Age <56
All LC types
Least adjusted

REF	NRR	SEX	AD	Ys	Ws	Qs	Ps
BENHAM	68	m	0	2.10	7.77	0.92	0.0000
COMSTO	57	c	0	2.47	0.89	0.45	0.0199
*CPSI	163	m	1	2.03	20.21	1.54	0.0000
*CPSI	250	f	1	1.25	30.37	7.69	0.0000
Subtotal CPSI				1.56	50.58	9.23	
DEAN3	43	m	0	1.46	0.92	0.08	0.1600
DEAN3	44	m	0	2.93	0.98	1.35	0.0037
DEAN3	120	f	0	1.43	0.85	0.09	0.1872
DEAN3	121	f	0	1.39	3.35	0.46	0.0112
Subtotal DEAN3				1.65	6.10	1.98	
*HAMMON	4	m	0	2.74	0.99	0.96	0.0064
HINDS	4	f	0	-0.13	0.74	2.63	0.9087
HINDS	1	f	0	1.33	3.47	0.63	0.0132
HINDS	7	f	0	2.73	1.80	1.71	0.0002
Subtotal HINDS				1.57	6.02	4.97	
HITOSU	24	m	0	-0.05	0.88	2.88	0.9621
HITOSU	44	f	0	0.95	2.51	1.64	0.1323
Subtotal HITOSU				0.69	3.40	4.51	
KREUZE	13	m	0	2.39	5.06	2.03	0.0000
KREUZE	14	f	0	2.24	4.29	0.99	0.0000
Subtotal KREUZE				2.32	9.35	3.02	
*KUBIK	9	m	0	2.53	0.48	0.29	0.0801
LEVIN	18	m	0	2.00	0.95	0.05	0.0523
LEVIN	19	m	0	1.78	5.37	0.00	0.0000
Subtotal LEVIN				1.82	6.32	0.06	
NAM	5	m	0	3.09	0.97	1.74	0.0023
NAM	37	f	0	2.67	1.84	1.53	0.0003
Subtotal NAM				2.81	2.81	3.27	
*PRESCO	38	m	0	2.08	0.49	0.05	0.1474
*PRESCO	37	f	0	1.84	0.47	0.00	0.2075
Subtotal PRESCO				1.96	0.96	0.05	
SCHWAR	6	m	0	2.80	6.03	6.64	0.0000
SCHWAR	5	m	0	1.47	2.92	0.23	0.0117
SCHWAR	14	f	0	2.62	8.50	6.30	0.0000
SCHWAR	13	f	0	3.01	3.64	5.69	0.0000
Subtotal SCHWAR				2.58	21.10	18.86	
*SPEIZE	8	f	0	1.96	52.33	2.11	0.0000
STOCKS	33	m	0	2.39	1.95	0.79	0.0008
STOCKS	35	m	0	2.70	3.87	3.45	0.0000
Subtotal STOCKS				2.60	5.82	4.24	
TSUGAN	27	m	0	0.23	7.76	18.12	0.5244
*TVERDA	22	m	2	1.52	20.49	1.12	0.0000
VUTUC	10	m	0	1.59	0.81	0.02	0.1530
VUTUC	33	m	0	1.28	8.12	1.84	0.0003
Subtotal VUTUC				1.31	8.92	1.86	
WUWILL	1	f	0	0.49	17.86	28.47	0.0370
WUWILL	2	f	0	1.00	23.35	13.37	0.0000
Subtotal WUWILL				0.78	41.21	41.84	
WYNDE6	427	m	0	2.73	13.45	12.88	0.0000
WYNDE6	429	f	0	3.17	11.72	23.38	0.0000
Subtotal WYNDE6				2.94	25.17	36.26	

	N	38
	NS	20
	Wt	278.47
Het	Chi	154.13
Het	df	37
Het	P	***
Fixed	RR	5.79
	RRl	5.15
	RRu	6.51
	P	+++
Random	RR	6.57
	RRl	4.94
	RRu	8.74
	P	+++
Asymm	P	N.S.

Table 1A4 - 6

IESLC - Meta-analysis of Ever Smoking, Any product (or Cigarettes if Any not available), Age <56
All LC types
Least adjusted

	Sex combined	male	female	Total
N	1	21	16	38
NS	1	16	11	28
Wt	0.89	110.47	167.11	278.47
Het Chi	0.00	54.42	94.87	154.13
Het df	0	20	15	37
Het P	N.S.	***	***	***
Fixed RR	11.77	6.74	5.21	5.79
RRl	1.48	5.59	4.48	5.15
RRu	93.79	8.12	6.07	6.51
P	+	+++	+++	+++
Random RR	11.77	7.04	5.96	6.57
RRl	1.48	4.90	3.77	4.94
RRu	93.79	10.11	9.40	8.74
P	+	+++	+++	+++
Between Chi				4.84
Between df				2
Between P				(*)
Btwn(F) P				N.S.
Btwn(R) P				N.S.

Table 1A4 - 7

IESLC - Meta-analysis of Ever Smoking, Any product (or Cigarettes if Any not available), Age <56
All LC types
Excluded studies (and stage at which they were excluded)

1	BOUCHA BUELL LAURIL MZILEN
2	BYERS2 ENSTRO HIRAY2 KANELL LIAW LICKIN MRFIT MURATA NOTANI RESTRE RIMING SAARIK SEGI2 SHIMIZ SITAS SOBUE2 TANG2 WALD WARSIN WATSON WU2 WYNDE5 WYNDE8 WYNDER
3	ABELIN ABRAHA AGUDO AKIBA ALDERS AMANDU AMES ANDERS ARCHER ARMADA AUSTIN AUVINE AXELSO AXELSS BAND BARBON BECHER BENSHL BERRIN BEST BLOHMK BLOT1 BLOT2 BLOT3 BLOT4 BOFFET BOUCOT BRESLO BRETT BROCKM BROSS BROWN1 BROWN2 BUFFLE BYERS1 CARPEN CASCO2 CASCOR CEDERL CHAN CHANG CHATZI CHEN CHEN2 CHEN3 CHIAZZ CHOI CHOW CHYOU COOKSO CORREA CPSII DAMBER DARBY DAVEYS DEAN DEAN2 DEKLER DESTE2 DESTEF DOCKER DOLL DOLL2 DORANT DORGAN DORN DOSEME DROSTE DU DUNN EBELIN ENGELA ESAKI FAN GAO GAO2 GARCIA GARDIN GARSHI GENG GER GILLIS GODLEY GOLLED GOODMA GRAHAM GREGOR GSELL HAENSZ HAMMO2 HANSEN HEGMAN HEIN HENNEK HIRAOK HIRAYA HOLE HOROWI HORWIT HU HU2 HUANG HUMBLE ISHIMA JAHN JAIN JARUP JARVHO JEDRYC JIANG JOLY JONES JUSSAW KAISE2 KAISER KATSOU KAUFMA KELLER KHUDER KIHARA KINLEN KJUUS KNEKT KO KOHLME KOO KOULUM KREYBE LAMTH LAMWK LAMWK2 LANGE LAUSSM LEI LEMARC LETOUR LIDDEL LIU LIU2 LIU3 LIU4 LIU5 LOMBA2 LOMBAR LUBIN LUBIN2 LUO MACLEN MAGNUS MARSH MARSH2 MARTIS MASTRA MATOS MATSUD MCCONN MCDUFF MCLAUG MIGRAN MILLER MILLS MOLLO MRFITR NOTAN2 NOU ODRISC ORMOS OSANN OSANN2 PARKIN PASTOR PAWLEG PERNU PERSH2 PERSHA PETO PEZZO2 PEZZOT PIKE PISANI POFFIJ POLEDN QIAO QIAO2 RACHTA RADZIK RANDIG REN RONCO ROOTS ROTHSC SANKAR SCHWA2 SEGI SEOW SHAW SIEMIA SIMARA SIMONA SOBUE SPITZ STASZE STAYNE STOCKW STUCKE SUN SUZUK2 SUZUKI SVENSS TANG TAO TENKAN TIZZAN TOKARS TOUSEY TULINI ULMER VANDER VEIERO WAKAI WANG WANG2 WANG3 WANG4 WICKLU WIGLE WILKIN WU WUNSCH WYNDE2 WYNDE3 WYNDE7 XIANGZ XU XU2 XU3 XU4 YAMAGU YONG YUAN ZHANG ZHENG ZHOU
4	SADOWS
9	WYNDE4

Table 1A4 - 8
Potentially overlapping studies

REF	REFGP	PRINC	. OVERLAP/LINK
VUTUC	LUBIN2	2	Subset of Lubin2
BENHAM	LUBIN2	2	Subset of Lubin2
TVERDA	TVERDA	1	VEIERO/TVERDAL
PRESCO	PRESCO	2	PRESCO-combined
WYNDE6	WYNDE6	1	WYNDE5/6/7/8
CPSI	CPSI	1	CPSI overall

Table 1A4 - 9
Most adjusted - insufficient data for metaanalysis

REF	NRR	SEX	AGEL	AGEH	RACE	YF	LC TYPE	LOC	START	ST	NLC	R	VB	P	H	AD	PRODUCT	DENOM	De
SADOWS	11	m	1	49	wh	-	all	NAmer	1938	CC	477	n	bl	n	n	0	all/unsp	nev any	st

REF	NRR	RR	SIG	RRDATA comment
SADOWS	11	3.80		0

Table 1A5 -

IESLC - Meta-analysis of Ever Smoking, Any product (or Cigarettes if Any not available), Age 50-70
All LC types

This analysis is restricted to results for:
1) Non-dose-response data
2) Ever smokers
3) Maximum age range 50-70
4) Results complete enough for use in metaanalysis

Within each study, results are then selected (in the following order of preference, within each sex) for:
5) PRODUCT: all/unspec, cigarettes regardless of other products, cigarettes only
6) CIGTYPE: all/unspecified, MC regardless of HR, MC only
7) DENOM: never smoked anything, never smoked cigarettes, (never +1 = +long term ex, +2 = +amount unknown,
+3 = never cigs+long term ex)
8) Followup period (YF, prospective studies): whole study (coded as 0) or longest available
9) LCtype: all or nearest available, at least Squamous and Adeno. (q = squamous, s = small,
l = large, a = adeno, mix = mixed, alv = alveolar)
10) Race: all or nearest available, otherwise by race (wh or w = white, bl or b = black, hi = hispanic
ch = chinese, jap = japanese, haw = hawaiian, w+o = white + oriental, sca = scandinavian, as = asian)
11) For overlapping studies: principal rather than subsidiary studies
Finally by Age: whole study (actual age shown) if available, otherwise by widest available age group
and then for single sex results (m, f) in preference to combined sex results (c).

Results adjusted (AD) for the most potential confounders are then chosen in Sections -1 to -3
(and those which actually differ from the adjusted results in Table 1A1 - 1 are marked 'x' in Section -1)
and results adjusted for the least confounders in Sections -4 to -6. (Those least adjusted results which
actually differ from the most adjusted as marked 'x' in column X in Section -4)
(Results adjusted for an unknown number of confounder(s) are coded as 20.)

Section -7 shows excluded studies, together with the stage (as above) at which no qualifying
results were found.

Section -8 lists the potentially overlapping studies which have been included (1=principal, 2=subsidiary).

Section -9 lists any results which would have been included in preference except that they had data not complete
enough for use in meta-analysis, with their significance (yes/no), if known, and any further comment as entered
on the database.

In addition to those mentioned above, the following fields, levels and abbreviations are used:
* or nk = not known, n = no, y = yes, ot = other
nev = never
all/unspec = all or unspecified, cig+/-ot = cigarettes irrespective of other products (cigar, pipe etc)
MC = manufactured cigarettes, HR = hand-rolled cigarettes
REF: 6-character study reference
NRR: number of the RR on the database within the study
ST : study type (CC = case control, pr or prosp = prospective)
NLC: number of lung cancer cases in whole study
R : risky occupational population (n = no, m = mining, o = other risky)
VB : national cigarette type (V = at least 75% Virginia, bl = at least 75% blended, ot = other)
P : any proxy use
H : full histological confirmation
De : derivation of RR/CI (or = original, st = standard method, ot = other method of estimation)

Table 1A5 - 1

IESLC - Meta-analysis of Ever Smoking, Any product (or Cigarettes if Any not available), Age 50-70
All LC types
Most adjusted

REF	NRR	1A1	SEX	AGEL	AGEH	RACE	YF	LC TYPE	LOC	START	ST	NLC	R	VB	P	H	AD	PRODUCT	DENOM	De
ANDERS	3		f	55	69	all	0	all	NAmer	1986	pr	343	n	bl	n	n	0	cig+/-ot	nev cigs	st
BENHAM	69	x	m	50	59	all	-	not mix	Eu:wst	1976	CC	1625	n	bl	n	y	0	cig only	nev any	st
BENHAM	70	x	m	60	69	all	-	not mix	Eu:wst	1976	CC	1625	n	bl	n	y	0	cig only	nev any	st
BUFFLE	59	x	f	60	69	w-hi	-	all	NAmer	1976	CC	943	n	bl	y	n	0	cig+/-ot	nev cigs	st
CPSI	171	x	m	55	69	all	6	all	NAmer	1959	pr	5138	n	bl	n	n	1	cig+/-ot	nev any	ot
CPSI	113	x	f	55	64	wh	0	all	NAmer	1959	pr	5138	n	bl	n	n	0	cig only	nev any	st
DAMBER	23	x	m	60	69	all	-	all	Eu:Sca	1972	CC	579	n	bl	y	n	0	all/unsp	nev any	st
DEAN3	45	x	m	55	64	all	-	all	Eu:UK	1969	CC	766	n	V	y	n	0	all/unsp	nev any	st
DEAN3	122	x	f	55	64	all	-	all	Eu:UK	1969	CC	766	n	V	y	n	0	cig only	nev any	st
DORANT	10		c	55	69	all	0	all	Eu:wst	1986	ot	550	n	bl	n	y	0	all/unsp	nev any	st
DORN	412	x	m	55	64	wh	5	all	NAmer	1954	pr	5097	n	bl	n	n	1	cig+/-ot	nev any	ot
HAMMON	117		m	50	69	wh	0	all	NAmer	1952	pr	448	n	bl	n	n	1	all/unsp	nev any	ot
HINDS	5	x	f	50	64	ch	-	all	NAmer	1968	CC	292	n	bl	n	n	0	all/unsp	nev any	st
HINDS	2	x	f	50	64	jap	-	all	NAmer	1968	CC	292	n	bl	n	n	0	all/unsp	nev any	st
HINDS	8	x	f	50	64	haw	-	all	NAmer	1968	CC	292	n	bl	n	n	0	all/unsp	nev any	st
HITOSU	50	x	f	50	59	all	-	all	As:Jap	1960	CC	216	n	bl	y	n	0	all/unsp	nev any	st
KIHARA	1	x	m	50	69	jap	-	all	As:Jap	1991	CC	440	n	bl	n	n	0	all/unsp	nev any	st
KREUZE	15	x	m	55	69	all	-	all	Eu:Ger	1990	CC	2260	n	bl	n	n	0	all/unsp	nev any	st
KREUZE	16		f	55	69	all	-	all	Eu:Ger	1990	CC	2260	n	bl	n	n	0	all/unsp	nev any	st
KUBIK	13	x	m	55	64	all	4	all	Eu:est	1965	pr	108	n	bl	n	n	0	all/unsp	nev any	st
LEVIN	20	x	m	55	64	all	-	all	NAmer	1938	CC	475	n	bl	n	n	0	all/unsp	nev any	st
NAM	13	x	m	55	64	all	-	all	NAmer	1986	CC	1199	n	bl	y	n	0	cig+/-ot	nev cigs	st
NAM	45	x	f	55	64	all	-	all	NAmer	1986	CC	1199	n	bl	y	n	0	cig+/-ot	nev cigs	st
PRESCO	40	x	m	50	64	all	0	all	Eu:Sca	1964	pr	867	n	bl	n	n	0	all/unsp	nev any	st
PRESCO	39	x	f	50	64	all	0	all	Eu:Sca	1964	pr	867	n	bl	n	n	0	all/unsp	nev any	st
STOCKS	37	x	m	55	64	all	-	all	Eu:UK	1952	CC	2932	n	V	y	n	0	all/unsp	nev any	st
VUTUC	17	x	m	51	60	all	-	all	Eu:wst	1976	CC	1877	n	bl	n	n	0	cig+/-ot	nev cigs	st
WUWILL	2	x	f	50	54	all	-	all	As:Chi	1985	CC	965	n	ot	n	n	0	cig+/-ot	nev cigs	st
WUWILL	3	x	f	55	59	all	-	all	As:Chi	1985	CC	965	n	ot	n	n	0	cig+/-ot	nev cigs	st
WUWILL	4	x	f	60	64	all	-	all	As:Chi	1985	CC	965	n	ot	n	n	0	cig+/-ot	nev cigs	st
WUWILL	5	x	f	65	69	all	-	all	As:Chi	1985	CC	965	n	ot	n	n	0	cig+/-ot	nev cigs	st

Cigarette type is all/unspec for all RRs
except for the following:

REF	NRR	CIGTYPE
DEAN3	122	MC only

Table 1A5 - 2

IESLC - Meta-analysis of Ever Smoking, Any product (or Cigarettes if Any not available), Age 50-70
All LC types
Most adjusted

REF	NRR	SEX	AD	Number Exposed Case	Number Exposed Cont	Non-exposed Case	Non-exposed Cont	RR	95.00%CI
*ANDERS	3	f	0	297	96164	46	195158	13.10	(9.61- 17.87)
BENHAM	69	m	0	421	538	5	185	28.95	(11.80- 71.03)
BENHAM	70	m	0	377	347	12	140	12.68	(6.91- 23.27)
Subtotal BENHAM								16.43	(9.94- 27.17)
BUFFLE	59	f	0	90	69	8	38	6.20	(2.72- 14.13)
*CPSI	171	m	1	-	-	-	-	10.38	(7.60- 14.18)
*CPSI	113	f	0	260	682162	144	1366561	3.62	(2.95- 4.43)
Subtotal CPSI								4.96	(4.18- 5.88)
DAMBER	23	m	0	170	119	8	57	10.18	(4.68- 22.12)
DEAN3	45	m	0	209	454	7	96	6.31	(2.88- 13.84)
DEAN3	122	f	0	39	283	9	310	4.75	(2.26- 9.97)
Subtotal DEAN3								5.43	(3.17- 9.31)
DORANT	10	c	0	470	2033	14	1090	18.00	(10.52- 30.78)
*DORN	412	m	1	-	-	-	-	9.23	(6.53- 13.03)
*HAMMON	117	m	1	-	-	-	-	6.80	(4.06- 11.37)
HINDS	5	f	0	6	12	10	67	3.35	(1.03- 10.95)
HINDS	2	f	0	23	149	24	589	3.79	(2.08- 6.90)
HINDS	8	f	0	52	96	7	149	11.53	(5.03- 26.43)
Subtotal HINDS								5.16	(3.29- 8.09)
HITOSU	50	f	0	7	159	8	478	2.63	(0.94- 7.37)
KIHARA	1	m	0	358	184	24	73	5.92	(3.61- 9.70)
KREUZE	15	m	0	1686	1358	23	403	21.75	(14.20- 33.31)
KREUZE	16	f	0	205	101	95	177	3.78	(2.68- 5.34)
Subtotal KREUZE								7.56	(5.78- 9.88)
*KUBIK	13	m	0	49	3606	1	1851	25.15	(3.48- 182.00)
LEVIN	20	m	0	196	431	16	186	5.29	(3.09- 9.05)
NAM	13	m	0	191	173	5	42	9.27	(3.59- 23.97)
NAM	45	f	0	75	85	10	69	6.09	(2.93- 12.66)
Subtotal NAM								7.12	(3.99- 12.72)
*PRESCO	40	m	0	261	110947	3	12426	9.74	(3.12- 30.40)
*PRESCO	39	f	0	77	53905	5	19328	5.52	(2.24- 13.64)
Subtotal PRESCO								6.88	(3.39- 13.97)
STOCKS	37	m	0	1066	1997	21	153	3.89	(2.45- 6.17)
VUTUC	17	m	0	229	432	8	138	9.14	(4.40- 18.98)
WUWILL	2	f	0	121	69	82	127	2.72	(1.81- 4.07)
WUWILL	3	f	0	143	104	89	137	2.12	(1.47- 3.06)
WUWILL	4	f	0	125	75	59	116	3.28	(2.14- 5.01)
WUWILL	5	f	0	82	64	55	97	2.26	(1.42- 3.60)
Subtotal WUWILL								2.53	(2.06- 3.11)
Partial Totals				7285	956116	798	1600241		

*prospective study

REF	NRR	SEX	AD	Ys	Ws	Qs	Ps
*ANDERS	3	f	0	2.57	39.86	26.86	0.0000
BENHAM	69	m	0	3.37	4.77	12.42	0.0000
BENHAM	70	m	0	2.54	10.42	6.46	0.0000
Subtotal BENHAM				2.80	15.19	18.88	
BUFFLE	59	f	0	1.82	5.65	0.03	0.0000
*CPSI	171	m	1	2.34	39.50	13.65	0.0000
*CPSI	113	f	0	1.29	92.69	20.16	0.0000
Subtotal CPSI				1.60	132.19	33.81	
DAMBER	23	m	0	2.32	6.38	2.06	0.0000
DEAN3	45	m	0	1.84	6.24	0.05	0.0000
DEAN3	122	f	0	1.56	6.97	0.26	0.0000
Subtotal DEAN3				1.69	13.21	0.31	
DORANT	10	c	0	2.89	13.34	17.29	0.0000
*DORN	412	m	1	2.22	32.20	7.13	0.0000
*HAMMON	117	m	1	1.92	14.49	0.39	0.0000
HINDS	5	f	0	1.21	2.74	0.81	0.0454
HINDS	2	f	0	1.33	10.69	1.89	0.0000
HINDS	8	f	0	2.44	5.58	2.68	0.0000
Subtotal HINDS				1.64	19.01	5.37	
HITOSU	50	f	0	0.97	3.62	2.23	0.0657
KIHARA	1	m	0	1.78	15.72	0.01	0.0000
KREUZE	15	m	0	3.08	21.15	37.28	0.0000
KREUZE	16	f	0	1.33	32.30	5.75	0.0000
Subtotal KREUZE				2.02	53.45	43.03	
*KUBIK	13	m	0	3.22	0.98	2.13	0.0014
LEVIN	20	m	0	1.67	13.28	0.10	0.0000
NAM	13	m	0	2.23	4.26	0.96	0.0000

Table 1A5 - 2

IESLC - Meta-analysis of Ever Smoking, Any product (or Cigarettes if Any not available), Age 50-70
All LC types
Most adjusted

REF	NRR	SEX	AD	Ys	Ws	Qs	Ps
NAM	45	f	0	1.81	7.16	0.02	0.0000
Subtotal NAM				1.96	11.42	0.98	
*PRESCO	40	m	0	2.28	2.97	0.82	0.0001
*PRESCO	39	f	0	1.71	4.70	0.01	0.0002
Subtotal PRESCO				1.93	7.66	0.83	
STOCKS	37	m	0	1.36	17.99	2.79	0.0000
VUTUC	17	m	0	2.21	7.20	1.53	0.0000
WUWILL	2	f	0	1.00	23.35	13.23	0.0000
WUWILL	3	f	0	0.75	28.45	28.58	0.0001
WUWILL	4	f	0	1.19	21.32	6.81	0.0000
WUWILL	5	f	0	0.82	17.76	15.58	0.0006
Subtotal WUWILL				0.93	90.88	64.21	

	N	31
	NS	20
	Wt	513.72
Het	Chi	229.97
Het	df	30
Het	P	***
Fixed	RR	5.77
	RRl	5.29
	RRu	6.29
	P	+++
Random	RR	6.46
	RRl	4.99
	RRu	8.35
	P	+++
Asymm	P	N.S.

Table 1A5 - 3

IESLC - Meta-analysis of Ever Smoking, Any product (or Cigarettes if Any not available), Age 50-70
All LC types
Most adjusted

	Sex<br>combined	male	female	Total
N	1	15	15	31
NS	1	14	10	25
Wt	13.34	197.53	302.85	513.72
Het Chi	0.00	47.28	88.74	229.97
Het df	0	14	14	30
Het P	N.S.	***	***	***
Fixed RR	18.00	9.07	4.08	5.77
RRl	10.52	7.89	3.65	5.29
RRu	30.78	10.43	4.57	6.29
P	+++	+++	+++	+++
Random RR	18.00	9.17	4.21	6.46
RRl	10.52	6.93	3.07	4.99
RRu	30.78	12.13	5.78	8.35
P	+++	+++	+++	+++
Between Chi				93.94
Between df				2
Between P				***
Btwn(F) P				***
Btwn(R) P				***

Table 1A5 - 4

IESLC - Meta-analysis of Ever Smoking, Any product (or Cigarettes if Any not available), Age 50-70
All LC types
Least adjusted

REF	NRR	X	SEX	AGEL	AGEH	RACE	YF	LC TYPE	LOC	START	ST	NLC	R	VB	P	H	AD	PRODUCT	DENOM	De
ANDERS	3		f	55	69	all	0	all	NAmer	1986	pr	343	n	bl	n	n	0	cig+/-ot	nev cigs	st
BENHAM	69		m	50	59	all	-	not mix	Eu:wst	1976	CC	1625	n	bl	n	y	0	cig only	nev any	st
BENHAM	70		m	60	69	all	-	not mix	Eu:wst	1976	CC	1625	n	bl	n	y	0	cig only	nev any	st
BUFFLE	59		f	60	69	w-hi	-	all	NAmer	1976	CC	943	n	bl	y	n	0	cig+/-ot	nev cigs	st
CPSI	171		m	55	69	all	6	all	NAmer	1959	pr	5138	n	bl	n	n	1	cig+/-ot	nev any	ot
CPSI	113		f	55	64	wh	0	all	NAmer	1959	pr	5138	n	bl	n	n	0	cig only	nev any	st
DAMBER	23		m	60	69	all	-	all	Eu:Sca	1972	CC	579	n	bl	y	n	0	all/unsp	nev any	st
DEAN3	45		m	55	64	all	-	all	Eu:UK	1969	CC	766	n	V	y	n	0	all/unsp	nev any	st
DEAN3	122		f	55	64	all	-	all	Eu:UK	1969	CC	766	n	V	y	n	0	cig only	nev any	st
DORANT	10		c	55	69	all	0	all	Eu:wst	1986	ot	550	n	bl	n	y	0	all/unsp	nev any	st
DORN	412		m	55	64	wh	5	all	NAmer	1954	pr	5097	n	bl	n	n	1	cig+/-ot	nev any	ot
HAMMON	129	x	m	50	69	wh	0	all	NAmer	1952	pr	448	n	bl	n	n	0	all/unsp	nev any	st
HINDS	5		f	50	64	ch	-	all	NAmer	1968	CC	292	n	bl	n	n	0	all/unsp	nev any	st
HINDS	2		f	50	64	jap	-	all	NAmer	1968	CC	292	n	bl	n	n	0	all/unsp	nev any	st
HINDS	8		f	50	64	haw	-	all	NAmer	1968	CC	292	n	bl	n	n	0	all/unsp	nev any	st
HITOSU	50		f	50	59	all	-	all	As:Jap	1960	CC	216	n	bl	y	n	0	all/unsp	nev any	st
KIHARA	1		m	50	69	jap	-	all	As:Jap	1991	CC	440	n	bl	n	n	0	all/unsp	nev any	st
KREUZE	15		m	55	69	all	-	all	Eu:Ger	1990	CC	2260	n	bl	n	n	0	all/unsp	nev any	st
KREUZE	16		f	55	69	all	-	all	Eu:Ger	1990	CC	2260	n	bl	n	n	0	all/unsp	nev any	st
KUBIK	13		m	55	64	all	4	all	Eu:est	1965	pr	108	n	bl	n	n	0	all/unsp	nev any	st
LEVIN	20		m	55	64	all	-	all	NAmer	1938	CC	475	n	bl	n	n	0	all/unsp	nev any	st
NAM	13		m	55	64	all	-	all	NAmer	1986	CC	1199	n	bl	y	n	0	cig+/-ot	nev cigs	st
NAM	45		f	55	64	all	-	all	NAmer	1986	CC	1199	n	bl	y	n	0	cig+/-ot	nev cigs	st
PRESCO	40		m	50	64	all	0	all	Eu:Sca	1964	pr	867	n	bl	n	n	0	all/unsp	nev any	st
PRESCO	39		f	50	64	all	0	all	Eu:Sca	1964	pr	867	n	bl	n	n	0	all/unsp	nev any	st
STOCKS	37		m	55	64	all	-	all	Eu:UK	1952	CC	2932	n	V	y	n	0	all/unsp	nev any	st
VUTUC	17		m	51	60	all	-	all	Eu:wst	1976	CC	1877	n	bl	n	n	0	cig+/-ot	nev cigs	st
WUWILL	2		f	50	54	all	-	all	As:Chi	1985	CC	965	n	ot	n	n	0	cig+/-ot	nev cigs	st
WUWILL	3		f	55	59	all	-	all	As:Chi	1985	CC	965	n	ot	n	n	0	cig+/-ot	nev cigs	st
WUWILL	4		f	60	64	all	-	all	As:Chi	1985	CC	965	n	ot	n	n	0	cig+/-ot	nev cigs	st
WUWILL	5		f	65	69	all	-	all	As:Chi	1985	CC	965	n	ot	n	n	0	cig+/-ot	nev cigs	st

Cigarette type is all/unspec for all RRs
except for the following:

REF	NRR	CIGTYPE
DEAN3	122	MC only

Table 1A5 - 5

IESLC - Meta-analysis of Ever Smoking, Any product (or Cigarettes if Any not available), Age 50-70
All LC types
Least adjusted

REF	NRR	SEX	AD	Number Exposed Case	Number Exposed Cont	Non-exposed Case	Non-exposed Cont	RR	95.00%CI
*ANDERS	3	f	0	297	96164	46	195158	13.10	(9.61- 17.87)
BENHAM	69	m	0	421	538	5	185	28.95	(11.80- 71.03)
BENHAM	70	m	0	377	347	12	140	12.68	(6.91- 23.27)
Subtotal BENHAM								16.43	(9.94- 27.17)
BUFFLE	59	f	0	90	69	8	38	6.20	(2.72- 14.13)
*CPSI	171	m	1	-	-	-	-	10.38	(7.60- 14.18)
*CPSI	113	f	0	260	682162	144	1366561	3.62	(2.95- 4.43)
Subtotal CPSI								4.96	(4.18- 5.88)
DAMBER	23	m	0	170	119	8	57	10.18	(4.68- 22.12)
DEAN3	45	m	0	209	454	7	96	6.31	(2.88- 13.84)
DEAN3	122	f	0	39	283	9	310	4.75	(2.26- 9.97)
Subtotal DEAN3								5.43	(3.17- 9.31)
DORANT	10	c	0	470	2033	14	1090	18.00	(10.52- 30.78)
*DORN	412	m	1	-	-	-	-	9.23	(6.53- 13.03)
*HAMMON	129	m	0	425	510108	15	115884	6.44	(3.85- 10.77)
HINDS	5	f	0	6	12	10	67	3.35	(1.03- 10.95)
HINDS	2	f	0	23	149	24	589	3.79	(2.08- 6.90)
HINDS	8	f	0	52	96	7	149	11.53	(5.03- 26.43)
Subtotal HINDS								5.16	(3.29- 8.09)
HITOSU	50	f	0	7	159	8	478	2.63	(0.94- 7.37)
KIHARA	1	m	0	358	184	24	73	5.92	(3.61- 9.70)
KREUZE	15	m	0	1686	1358	23	403	21.75	(14.20- 33.31)
KREUZE	16	f	0	205	101	95	177	3.78	(2.68- 5.34)
Subtotal KREUZE								7.56	(5.78- 9.88)
*KUBIK	13	m	0	49	3606	1	1851	25.15	(3.48- 182.00)
LEVIN	20	m	0	196	431	16	186	5.29	(3.09- 9.05)
NAM	13	m	0	191	173	5	42	9.27	(3.59- 23.97)
NAM	45	f	0	75	85	10	69	6.09	(2.93- 12.66)
Subtotal NAM								7.12	(3.99- 12.72)
*PRESCO	40	m	0	261	110947	3	12426	9.74	(3.12- 30.40)
*PRESCO	39	f	0	77	53905	5	19328	5.52	(2.24- 13.64)
Subtotal PRESCO								6.88	(3.39- 13.97)
STOCKS	37	m	0	1066	1997	21	153	3.89	(2.45- 6.17)
VUTUC	17	m	0	229	432	8	138	9.14	(4.40- 18.98)
WUWILL	2	f	0	121	69	82	127	2.72	(1.81- 4.07)
WUWILL	3	f	0	143	104	89	137	2.12	(1.47- 3.06)
WUWILL	4	f	0	125	75	59	116	3.28	(2.14- 5.01)
WUWILL	5	f	0	82	64	55	97	2.26	(1.42- 3.60)
Subtotal WUWILL								2.53	(2.06- 3.11)
Partial Totals				7710	1466224	813	1716125		

*prospective study

REF	NRR	SEX	AD	Ys	Ws	Qs	Ps
*ANDERS	3	f	0	2.57	39.86	26.96	0.0000
BENHAM	69	m	0	3.37	4.77	12.45	0.0000
BENHAM	70	m	0	2.54	10.42	6.49	0.0000
Subtotal BENHAM				2.80	15.19	18.93	
BUFFLE	59	f	0	1.82	5.65	0.03	0.0000
*CPSI	171	m	1	2.34	39.50	13.72	0.0000
*CPSI	113	f	0	1.29	92.69	20.02	0.0000
Subtotal CPSI				1.60	132.19	33.75	
DAMBER	23	m	0	2.32	6.38	2.07	0.0000
DEAN3	45	m	0	1.84	6.24	0.05	0.0000
DEAN3	122	f	0	1.56	6.97	0.26	0.0000
Subtotal DEAN3				1.69	13.21	0.31	
DORANT	10	c	0	2.89	13.34	17.33	0.0000
*DORN	412	m	1	2.22	32.20	7.17	0.0000
*HAMMON	129	m	0	1.86	14.49	0.18	0.0000
HINDS	5	f	0	1.21	2.74	0.80	0.0454
HINDS	2	f	0	1.33	10.69	1.87	0.0000
HINDS	8	f	0	2.44	5.58	2.69	0.0000
Subtotal HINDS				1.64	19.01	5.37	
HITOSU	50	f	0	0.97	3.62	2.22	0.0657
KIHARA	1	m	0	1.78	15.72	0.01	0.0000
KREUZE	15	m	0	3.08	21.15	37.37	0.0000
KREUZE	16	f	0	1.33	32.30	5.71	0.0000
Subtotal KREUZE				2.02	53.45	43.08	
*KUBIK	13	m	0	3.22	0.98	2.13	0.0014
LEVIN	20	m	0	1.67	13.28	0.10	0.0000
NAM	13	m	0	2.23	4.26	0.97	0.0000

Table 1A5 - 5

IESLC - Meta-analysis of Ever Smoking, Any product (or Cigarettes if Any not available), Age 50-70
All LC types
Least adjusted

REF	NRR	SEX	AD	Ys	Ws	Qs	Ps
NAM	45	f	0	1.81	7.16	0.02	0.0000
Subtotal NAM				1.96	11.42	0.99	
*PRESCO	40	m	0	2.28	2.97	0.82	0.0001
*PRESCO	39	f	0	1.71	4.70	0.01	0.0002
Subtotal PRESCO				1.93	7.66	0.83	
STOCKS	37	m	0	1.36	17.99	2.77	0.0000
VUTUC	17	m	0	2.21	7.20	1.54	0.0000
WUWILL	2	f	0	1.00	23.35	13.18	0.0000
WUWILL	3	f	0	0.75	28.45	28.49	0.0001
WUWILL	4	f	0	1.19	21.32	6.77	0.0000
WUWILL	5	f	0	0.82	17.76	15.53	0.0006
Subtotal WUWILL				0.93	90.88	63.97	

	N	31
	NS	20
	Wt	513.72
Het	Chi	229.75
Het	df	30
Het	P	***
Fixed	RR	5.76
	RRl	5.28
	RRu	6.28
	P	+++
Random	RR	6.44
	RRl	4.98
	RRu	8.34
	P	+++
Asymm	P	N.S.

Table 1A5 - 6

IESLC - Meta-analysis of Ever Smoking, Any product (or Cigarettes if Any not available), Age 50-70
All LC types
Least adjusted

		Sex		
	combined	male	female	Total
N	1	15	15	31
NS	1	14	10	25
Wt	13.34	197.53	302.85	513.72
Het Chi	0.00	47.78	88.74	229.75
Het df	0	14	14	30
Het P	N.S.	***	***	***
Fixed RR	18.00	9.03	4.08	5.76
RRl	10.52	7.86	3.65	5.28
RRu	30.78	10.38	4.57	6.28
P	+++	+++	+++	+++
Random RR	18.00	9.13	4.21	6.44
RRl	10.52	6.89	3.07	4.98
RRu	30.78	12.10	5.78	8.34
P	+++	+++	+++	+++
Between Chi				93.22
Between df				2
Between P				***
Btwn(F) P				***
Btwn(R) P				***

Table 1A5 - 7

IESLC - Meta-analysis of Ever Smoking, Any product (or Cigarettes if Any not available), Age 50-70
All LC types
Excluded studies (and stage at which they were excluded)

1	BOUCHA BUELL LAURIL MZILEN
2	BYERS2 ENSTRO HIRAY2 KANELL LIAW LICKIN MRFIT MURATA NOTANI RESTRE RIMING SAARIK SEGI2 SHIMIZ SITAS SOBUE2 TANG2 WALD WARSIN WATSON WU2 WYNDE5 WYNDE8 WYNDER
3	ABELIN ABRAHA AGUDO AKIBA ALDERS AMANDU AMES ARCHER ARMADA AUSTIN AUVINE AXELSO AXELSS BAND BARBON BECHER BENSHL BERRIN BEST BLOHMK BLOT1 BLOT2 BLOT3 BLOT4 BOFFET BOUCOT BRESLO BRETT BROCKM BROSS BROWN1 BYERS1 CARPEN CASCO2 CASCOR CEDERL CHAN CHANG CHATZI CHEN CHEN2 CHEN3 CHIAZZ CHOI CHOW CHYOU COMSTO COOKSO CORREA CPSII DARBY DAVEYS DEAN DEAN2 DEKLER DESTE2 DESTEF DOCKER DOLL DOLL2 DORGAN DOSEME DROSTE DU DUNN EBELIN ENGELA ESAKI FAN GAO GAO2 GARCIA GARDIN GARSHI GENG GER GILLIS GODLEY GOLLED GOODMA GRAHAM GREGOR GSELL HAENSZ HAMMO2 HANSEN HEGMAN HEIN HENNEK HIRAOK HIRAYA HOLE HOROWI HORWIT HU HU2 HUANG HUMBLE ISHIMA JAHN JAIN JARUP JARVHO JEDRYC JIANG JOLY JONES JUSSAW KAISE2 KAISER KATSOU KAUFMA KELLER KHUDER KINLEN KJUUS KNEKT KO KOHLME KOO KOULUM KREYBE LAMTH LAMWK LAMWK2 LANGE LAUSSM LEI LEMARC LETOUR LIDDEL LIU LIU2 LIU3 LIU4 LIU5 LOMBA2 LOMBAR LUBIN LUBIN2 LUO MACLEN MAGNUS MARSH MARSH2 MARTIS MASTRA MATOS MATSUD MCCONN MCDUFF MCLAUG MIGRAN MILLER MILLS MOLLO MRFITR NOTAN2 NOU ODRISC ORMOS OSANN OSANN2 PARKIN PASTOR PAWLEG PERNU PERSH2 PERSHA PETO PEZZO2 PEZZOT PIKE PISANI POFFIJ POLEDN QIAO QIAO2 RACHTA RADZIK RANDIG REN RONCO ROOTS ROTHSC SANKAR SCHWA2 SCHWAR SEGI SEOW SHAW SIEMIA SIMARA SIMONA SOBUE SPEIZE SPITZ STASZE STAYNE STOCKW STUCKE SUN SUZUK2 SUZUKI SVENSS TANG TAO TENKAN TIZZAN TOKARS TOUSEY TSUGAN TULINI TVERDA ULMER VANDER VEIERO WAKAI WANG WANG2 WANG3 WANG4 WICKLU WIGLE WILKIN WU WUNSCH WYNDE2 WYNDE3 WYNDE6 WYNDE7 XIANGZ XU XU2 XU3 XU4 YAMAGU YONG YUAN ZHANG ZHENG ZHOU
4	BROWN2 SADOWS
9	WYNDE4

Table 1A5 - 8
Potentially overlapping studies

REF	REFGP	PRINC	. OVERLAP/LINK
VUTUC	LUBIN2	2	Subset of Lubin2
BENHAM	LUBIN2	2	Subset of Lubin2
PRESCO	PRESCO	2	PRESCO-combined
CPSI	CPSI	1	CPSI overall

Table 1A5 - 9
Most adjusted - insufficient data for metaanalysis

REF	NRR	SEX	AGEL	AGEH	RACE	YF	LC TYPE	LOC	START	ST	NLC	R	VB	P	H	AD	PRODUCT	DENOM	De
BROWN2	55	m	60	69	wh	-	all	NAmer	1984	CC	14596	n	bl	n	y	1	cig+/-ot	nev cigs	or
BROWN2	52	f	60	69	wh	-	all	NAmer	1984	CC	14596	n	bl	n	y	1	cig+/-ot	nev cigs	or
SADOWS	15	m	50	59	wh	-	all	NAmer	1938	CC	477	n	bl	n	n	0	all/unsp	nev any	st
SADOWS	19	m	60	69	wh	-	all	NAmer	1938	CC	477	n	bl	n	n	0	all/unsp	nev any	st

REF	NRR	RR	SIG	RRDATA comment
BROWN2	55	10.30		0
BROWN2	52	14.30		0
SADOWS	15	3.53		0
SADOWS	19	13.63		0

Table 1A6 -

IESLC - Meta-analysis of Ever Smoking, Any product (or Cigarettes if Any not available), Age 65+
All LC types

This analysis is restricted to results for:
1) Non-dose-response data
2) Ever smokers
3) Age 65+
4) Results complete enough for use in metaanalysis

Within each study, results are then selected (in the following order of preference, within each sex) for:
5) PRODUCT: all/unspec, cigarettes regardless of other products, cigarettes only
6) CIGTYPE: all/unspecified, MC regardless of HR, MC only
7) DENOM: never smoked anything, never smoked cigarettes, (never +1 = +long term ex, +2 = +amount unknown,
+3 = never cigs+long term ex)
8) Followup period (YF, prospective studies): whole study (coded as 0) or longest available
9) LCtype: all or nearest available, at least Squamous and Adeno. (q = squamous, s = small,
l = large, a = adeno, mix = mixed, alv = alveolar)
10) Race: all or nearest available, otherwise by race (wh or w = white, bl or b = black, hi = hispanic
ch = chinese, jap = japanese, haw = hawaiian, w+o = white + oriental, sca = scandinavian, as = asian)
11) For overlapping studies: principal rather than subsidiary studies
Finally by Age: whole study (actual age shown) if available, otherwise by widest available age group
and then for single sex results (m, f) in preference to combined sex results (c).

Results adjusted (AD) for the most potential confounders are then chosen in Sections -1 to -3
(and those which actually differ from the adjusted results in Table 1A1 - 1 are marked 'x' in Section -1)
and results adjusted for the least confounders in Sections -4 to -6. (Those least adjusted results which
actually differ from the most adjusted as marked 'x' in column X in Section -4)
(Results adjusted for an unknown number of confounder(s) are coded as 20.)

Section -7 shows excluded studies, together with the stage (as above) at which no qualifying
results were found.

Section -8 lists the potentially overlapping studies which have been included (1=principal, 2=subsidiary).

Section -9 lists any results which would have been included in preference except that they had data not complete
enough for use in meta-analysis, with their significance (yes/no), if known, and any further comment as entered
on the database.

In addition to those mentioned above, the following fields, levels and abbreviations are used:
* or nk = not known, n = no, y = yes, ot = other
nev = never
all/unspec = all or unspecified, cig+/-ot = cigarettes irrespective of other products (cigar, pipe etc)
MC = manufactured cigarettes, HR = hand-rolled cigarettes
REF: 6-character study reference
NRR: number of the RR on the database within the study
ST : study type (CC = case control, pr or prosp = prospective)
NLC: number of lung cancer cases in whole study
R : risky occupational population (n = no, m = mining, o = other risky)
VB : national cigarette type (V = at least 75% Virginia, bl = at least 75% blended, ot = other)
P : any proxy use
H : full histological confirmation
De : derivation of RR/CI (or = original, st = standard method, ot = other method of estimation)

Table 1A6 - 1

IESLC - Meta-analysis of Ever Smoking, Any product (or Cigarettes if Any not available), Age 65+
All LC types
Most adjusted

REF	NRR	1A1	SEX	AGEL	AGEH	RACE	YF	LC TYPE	LOC	START	ST	NLC	R	VB	P	H	AD	PRODUCT	DENOM	De
BENHAM	71	x	m	70	99	all	-	not mix	Eu:wst	1976	CC	1625	n	bl	n	y	0	cig only	nev any	st
BUFFLE	60	x	f	70	79	w-hi	-	all	NAmer	1976	CC	943	n	bl	y	n	0	cig+/-ot	nev cigs	st
COMSTO	59	x	c	65	99	all	-	all	NAmer	1975	ot	258	n	bl	n	n	0	cig+/-ot	nev cigs	st
CPSI	179	x	m	70	84	all	6	all	NAmer	1959	pr	5138	n	bl	n	n	1	cig+/-ot	nev any	ot
CPSI	114	x	f	65	74	wh	0	all	NAmer	1959	pr	5138	n	bl	n	n	0	cig only	nev any	st
CPSI	115	x	f	75	84	wh	0	all	NAmer	1959	pr	5138	n	bl	n	n	0	cig only	nev any	st
DAMBER	24	x	m	70	99	all	-	all	Eu:Sca	1972	CC	579	n	bl	y	n	0	all/unsp	nev any	st
DEAN3	46	x	m	65	99	all	-	all	Eu:UK	1969	CC	766	n	V	y	n	0	all/unsp	nev any	st
DEAN3	123	x	f	65	99	all	-	all	Eu:UK	1969	CC	766	n	V	y	n	0	cig only	nev any	st
DORN	195	x	m	65	84	wh	8	all	NAmer	1954	pr	5097	n	bl	n	n	1	all/unsp	nev any	ot
GARSHI	9	x	m	65	82	all	-	all	NAmer	1981	CC	1081	o	bl	y	n	0	all/unsp	nev any	st
GODLEY	2	x	m	65	79	all	-	all	NAmer	1966	CC	1986	n	bl	y	n	1	cig+/-ot	nev cigs	ot
GODLEY	4	x	f	65	79	all	-	all	NAmer	1966	CC	1986	n	bl	y	n	1	cig+/-ot	nev cigs	ot
HAMMON	16	x	m	65	69	wh	0	all	NAmer	1952	pr	448	n	bl	n	n	0	all/unsp	nev any	st
HINDS	6	x	f	65	99	ch	-	all	NAmer	1968	CC	292	n	bl	n	n	0	all/unsp	nev any	st
HINDS	3	x	f	65	99	jap	-	all	NAmer	1968	CC	292	n	bl	n	n	0	all/unsp	nev any	st
HINDS	9	x	f	65	99	haw	-	all	NAmer	1968	CC	292	n	bl	n	n	0	all/unsp	nev any	st
HUMBLE	56	x	m	65	84	w-hi	-	not alv	NAmer	1980	CC	521	n	bl	y	n	0	cig+/-ot	nev cigs	st
HUMBLE	60	x	m	65	84	hi	-	not alv	NAmer	1980	CC	521	n	bl	y	n	0	cig+/-ot	nev cigs	st
HUMBLE	58	x	f	65	84	w-hi	-	not alv	NAmer	1980	CC	521	n	bl	y	n	0	cig+/-ot	nev cigs	st
HUMBLE	62	x	f	65	84	hi	-	not alv	NAmer	1980	CC	521	n	bl	y	n	0	cig+/-ot	nev cigs	st
KAISE2	40	x	m	65	74	all	9	all	NAmer	1979	pr	318	n	bl	n	n	0	cig only	nev any	st
KAISE2	48	x	m	75	99	all	9	all	NAmer	1979	pr	318	n	bl	n	n	0	cig only	nev any	st
KAISE2	16	x	f	65	74	all	9	all	NAmer	1979	pr	318	n	bl	n	n	0	cig only	nev any	st
KAISE2	24	x	f	75	99	all	9	all	NAmer	1979	pr	318	n	bl	n	n	0	cig only	nev any	st
LEVIN	21	x	m	65	74	all	-	all	NAmer	1938	CC	475	n	bl	n	n	0	all/unsp	nev any	st
LEVIN	22	x	m	75	99	all	-	all	NAmer	1938	CC	475	n	bl	n	n	0	all/unsp	nev any	st
LIU4	5	x	m	70	99	all	-	all	As:Chi	1986	CC	1000-00	n	ot	y	n	2	all/unsp	nev any	ot
LIU4	6	x	f	70	99	all	-	all	As:Chi	1986	CC	1000-00	n	ot	y	n	2	all/unsp	nev any	ot
NAM	21	x	m	65	79	all	-	all	NAmer	1986	CC	1199	n	bl	y	n	0	cig+/-ot	nev cigs	st
NAM	29	x	m	80	99	all	-	all	NAmer	1986	CC	1199	n	bl	y	n	0	cig+/-ot	nev cigs	st
NAM	53	x	f	65	79	all	-	all	NAmer	1986	CC	1199	n	bl	y	n	0	cig+/-ot	nev cigs	st
NAM	61	x	f	80	99	all	-	all	NAmer	1986	CC	1199	n	bl	y	n	0	cig+/-ot	nev cigs	st
PRESCO	42	x	m	65	99	all	0	all	Eu:Sca	1964	pr	867	n	bl	n	n	0	all/unsp	nev any	st
PRESCO	41	x	f	65	99	all	0	all	Eu:Sca	1964	pr	867	n	bl	n	n	0	all/unsp	nev any	st
STOCKS	39	x	m	65	74	all	-	all	Eu:UK	1952	CC	2932	n	V	y	n	0	all/unsp	nev any	st
WUWILL	5	x	f	65	69	all	-	all	As:Chi	1985	CC	965	n	ot	n	n	0	cig+/-ot	nev cigs	st

Cigarette type is all/unspec for all RRs
except for the following:

REF	NRR	CIGTYPE
DEAN3	123	MC only

Table 1A6 - 2

IESLC - Meta-analysis of Ever Smoking, Any product (or Cigarettes if Any not available), Age 65+
All LC types
Most adjusted

REF	NRR	SEX	AD	Number Exposed Case	Number Exposed Cont	Non-exposed Case	Non-exposed Cont	RR	95.00%CI
BENHAM	71	m	0	169	203	7	95	11.30	(5.11- 25.00)
BUFFLE	60	f	0	41	18	3	30	22.78	(6.15- 84.40)
COMSTO	59	c	0	46	58	6	44	5.82	(2.28- 14.84)
*CPSI	179	m	1	-	-	-	-	8.67	(5.54- 13.57)
*CPSI	114	f	0	153	211823	178	957682	3.89	(3.13- 4.82)
*CPSI	115	f	0	31	39527	163	392909	1.89	(1.29- 2.78)
Subtotal CPSI								3.78	(3.18- 4.50)
DAMBER	24	m	0	261	160	23	123	8.72	(5.36- 14.19)
DEAN3	46	m	0	285	396	16	82	3.69	(2.11- 6.44)
DEAN3	123	f	0	41	196	27	574	4.45	(2.66- 7.42)
Subtotal DEAN3								4.08	(2.80- 5.95)
*DORN	195	m	1	-	-	-	-	5.84	(4.42- 7.71)
GARSHI	9	m	0	771	1130	32	294	6.27	(4.30- 9.13)
GODLEY	2	m	1	-	-	-	-	6.17	(4.88- 7.79)
GODLEY	4	f	1	-	-	-	-	5.21	(3.65- 7.43)
Subtotal GODLEY								5.86	(4.82- 7.13)
*HAMMON	16	m	0	83	78713	9	21892	2.56	(1.29- 5.10)
HINDS	6	f	0	4	8	24	58	1.21	(0.33- 4.39)
HINDS	3	f	0	26	29	38	326	7.69	(4.11- 14.40)
HINDS	9	f	0	20	29	9	92	7.05	(2.89- 17.18)
Subtotal HINDS								5.83	(3.62- 9.39)
HUMBLE	56	m	0	128	157	6	60	8.15	(3.41- 19.48)
HUMBLE	60	m	0	55	58	2	21	9.96	(2.23- 44.47)
HUMBLE	58	f	0	52	33	11	52	7.45	(3.40- 16.30)
HUMBLE	62	f	0	19	11	4	34	14.68	(4.10- 52.52)
Subtotal HUMBLE								8.80	(5.34- 14.49)
*KAISE2	40	m	0	25	11767	5	11466	4.87	(1.87- 12.72)
*KAISE2	48	m	0	14	3401	4	4486	4.62	(1.52- 14.01)
*KAISE2	16	f	0	26	13883	2	24159	22.62	(5.37- 95.30)
*KAISE2	24	f	0	9	3362	3	12285	10.96	(2.97- 40.47)
Subtotal KAISE2								7.24	(4.05- 12.94)
LEVIN	21	m	0	87	317	13	168	3.55	(1.92- 6.54)
LEVIN	22	m	0	11	99	1	86	9.56	(1.21- 75.53)
Subtotal LEVIN								3.84	(2.14- 6.91)
LIU4	5	m	2	-	-	-	-	2.47	(2.34- 2.61)
LIU4	6	f	2	-	-	-	-	2.50	(2.33- 2.68)
Subtotal LIU4								2.48	(2.38- 2.59)
NAM	21	m	0	296	311	18	130	6.87	(4.09- 11.54)
NAM	29	m	0	67	143	6	172	13.43	(5.66- 31.87)
NAM	53	f	0	142	199	18	251	9.95	(5.89- 16.81)
NAM	61	f	0	35	83	22	472	9.05	(5.06- 16.19)
Subtotal NAM								8.93	(6.66- 11.97)
*PRESCO	42	m	0	378	57442	3	6229	13.66	(4.39- 42.54)
*PRESCO	41	f	0	105	34709	9	19748	6.64	(3.36- 13.11)
Subtotal PRESCO								8.03	(4.48- 14.40)
STOCKS	39	m	0	658	1423	18	173	4.44	(2.71- 7.28)
WUWILL	5	f	0	82	64	55	97	2.26	(1.42- 3.60)
Partial Totals				4120	459752	735	1454290		

*prospective study

REF	NRR	SEX	AD	Ys	Ws	Qs	Ps
BENHAM	71	m	0	2.42	6.09	11.35	0.0000
BUFFLE	60	f	0	3.13	2.24	9.56	0.0000
COMSTO	59	c	0	1.76	4.38	2.15	0.0002
*CPSI	179	m	1	2.16	19.15	23.19	0.0000
*CPSI	114	f	0	1.36	82.32	7.32	0.0000
*CPSI	115	f	0	0.64	26.07	4.65	0.0011
Subtotal CPSI				1.33	127.53	35.16	
DAMBER	24	m	0	2.17	16.21	19.86	0.0000
DEAN3	46	m	0	1.31	12.39	0.75	0.0000
DEAN3	123	f	0	1.49	14.65	2.75	0.0000
Subtotal DEAN3				1.41	27.03	3.50	
*DORN	195	m	1	1.76	49.64	24.71	0.0000
GARSHI	9	m	0	1.84	27.15	16.36	0.0000
GODLEY	2	m	1	1.82	70.25	40.62	0.0000
GODLEY	4	f	1	1.65	30.41	10.64	0.0000
Subtotal GODLEY				1.77	100.66	51.26	
*HAMMON	16	m	0	0.94	8.12	0.11	0.0073
HINDS	6	f	0	0.19	2.30	1.74	0.7739
HINDS	3	f	0	2.04	9.77	9.40	0.0000

Table 1A6 - 2

IESLC - Meta-analysis of Ever Smoking, Any product (or Cigarettes if Any not available), Age 65+
All LC types
Most adjusted

REF	NRR	SEX	AD	Ys	Ws	Qs	Ps
HINDS	9	f	0	1.95	4.84	3.87	0.0000
Subtotal HINDS				1.76	16.92	15.02	
HUMBLE	56	m	0	2.10	5.06	5.47	0.0000
HUMBLE	60	m	0	2.30	1.72	2.63	0.0026
HUMBLE	58	f	0	2.01	6.26	5.64	0.0000
HUMBLE	62	f	0	2.69	2.36	6.26	0.0000
Subtotal HUMBLE				2.17	15.41	20.00	
*KAISE2	40	m	0	1.58	4.17	1.15	0.0012
*KAISE2	48	m	0	1.53	3.12	0.69	0.0069
*KAISE2	16	f	0	3.12	1.86	7.88	0.0000
*KAISE2	24	f	0	2.39	2.25	4.01	0.0003
Subtotal KAISE2				1.98	11.40	13.73	
LEVIN	21	m	0	1.27	10.25	0.44	0.0001
LEVIN	22	m	0	2.26	0.90	1.29	0.0324
Subtotal LEVIN				1.35	11.15	1.73	
LIU4	5	m	2	0.90	1288.59	30.96	0.0000
LIU4	6	f	2	0.92	784.55	16.03	0.0000
Subtotal LIU4				0.91	2073.14	46.99	
NAM	21	m	0	1.93	14.32	10.80	0.0000
NAM	29	m	0	2.60	5.14	12.17	0.0000
NAM	53	f	0	2.30	13.97	21.42	0.0000
NAM	61	f	0	2.20	11.34	14.82	0.0000
Subtotal NAM				2.19	44.77	59.21	
*PRESCO	42	m	0	2.61	2.98	7.21	0.0000
*PRESCO	41	f	0	1.89	8.29	5.76	0.0000
Subtotal PRESCO				2.08	11.27	12.97	
STOCKS	39	m	0	1.49	15.73	2.94	0.0000
WUWILL	5	f	0	0.82	17.76	1.06	0.0006

	N	37
	NS	19
	Wt	2586.60
Het	Chi	347.67
Het	df	36
Het	P	***
Fixed	RR	2.88
	RRl	2.78
	RRu	3.00
	P	+++
Random	RR	5.48
	RRl	4.59
	RRu	6.55
	P	+++
Asymm	P	***

Table 1A6 - 3

IESLC - Meta-analysis of Ever Smoking, Any product (or Cigarettes if Any not available), Age 65+
All LC types
Most adjusted

	Sex			
	combined	male	female	Total
N	1	19	17	37
NS	1	15	11	27
Wt	4.38	1560.97	1021.24	2586.60
Het Chi	0.00	212.69	132.74	347.67
Het df	0	18	16	36
Het P	N.S.	***	***	***
Fixed RR	5.82	2.89	2.86	2.88
RRl	2.28	2.75	2.69	2.78
RRu	14.84	3.04	3.04	3.00
P	+++	+++	+++	+++
Random RR	5.82	5.95	5.34	5.48
RRl	2.28	4.28	3.92	4.59
RRu	14.84	8.26	7.27	6.55
P	+++	+++	+++	+++
Between Chi				2.24
Between df				2
Between P				N.S.
Btwn(F) P				N.S.
Btwn(R) P				N.S.

	Study LIU4		
	LIU4	others	Total
N	2	35	37
NS	1	18	19
Wt	2073.14	513.46	2586.60
Het Chi	0.07	111.23	347.67
Het df	1	34	36
Het P	N.S.	***	***
Fixed RR	2.48	5.29	2.88
RRl	2.38	4.86	2.78
RRu	2.59	5.77	3.00
P	+++	+++	+++
Random RR	2.48	5.89	5.48
RRl	2.38	4.93	4.59
RRu	2.59	7.05	6.55
P	+++	+++	+++
Between Chi			236.37
Between df			1
Between P			***
Btwn(F) P			***
Btwn(R) P			***

Table 1A6 - 4

IESLC - Meta-analysis of Ever Smoking, Any product (or Cigarettes if Any not available), Age 65+
All LC types
Least adjusted

REF	NRR	X	SEX	AGEL	AGEH	RACE	YF	LC TYPE	LOC	START	ST	NLC	R	VB	P	H	AD	PRODUCT	DENOM	De
BENHAM	71		m	70	99	all	-	not mix	Eu:wst	1976	CC	1625	n	bl	n	y	0	cig only	nev any	st
BUFFLE	60		f	70	79	w-hi	-	all	NAmer	1976	CC	943	n	bl	y	n	0	cig+/-ot	nev cigs	st
COMSTO	59		c	65	99	all	-	all	NAmer	1975	ot	258	n	bl	n	n	0	cig+/-ot	nev cigs	st
CPSI	179		m	70	84	all	6	all	NAmer	1959	pr	5138	n	bl	n	n	1	cig+/-ot	nev any	ot
CPSI	114		f	65	74	wh	0	all	NAmer	1959	pr	5138	n	bl	n	n	0	cig only	nev any	st
CPSI	115		f	75	84	wh	0	all	NAmer	1959	pr	5138	n	bl	n	n	0	cig only	nev any	st
DAMBER	24		m	70	99	all	-	all	Eu:Sca	1972	CC	579	n	bl	y	n	0	all/unsp	nev any	st
DEAN3	46		m	65	99	all	-	all	Eu:UK	1969	CC	766	n	V	y	n	0	all/unsp	nev any	st
DEAN3	123		f	65	99	all	-	all	Eu:UK	1969	CC	766	n	V	y	n	0	cig only	nev any	st
DORN	195		m	65	84	wh	8	all	NAmer	1954	pr	5097	n	bl	n	n	1	all/unsp	nev any	ot
GARSHI	9		m	65	82	all	-	all	NAmer	1981	CC	1081	o	bl	y	n	0	all/unsp	nev any	st
GODLEY	2		m	65	79	all	-	all	NAmer	1966	CC	1986	n	bl	y	n	1	cig+/-ot	nev cigs	ot
GODLEY	4		f	65	79	all	-	all	NAmer	1966	CC	1986	n	bl	y	n	1	cig+/-ot	nev cigs	ot
HAMMON	16		m	65	69	wh	0	all	NAmer	1952	pr	448	n	bl	n	n	0	all/unsp	nev any	st
HINDS	6		f	65	99	ch	-	all	NAmer	1968	CC	292	n	bl	n	n	0	all/unsp	nev any	st
HINDS	3		f	65	99	jap	-	all	NAmer	1968	CC	292	n	bl	n	n	0	all/unsp	nev any	st
HINDS	9		f	65	99	haw	-	all	NAmer	1968	CC	292	n	bl	n	n	0	all/unsp	nev any	st
HUMBLE	56		m	65	84	w-hi	-	not alv	NAmer	1980	CC	521	n	bl	y	n	0	cig+/-ot	nev cigs	st
HUMBLE	60		m	65	84	hi	-	not alv	NAmer	1980	CC	521	n	bl	y	n	0	cig+/-ot	nev cigs	st
HUMBLE	58		f	65	84	w-hi	-	not alv	NAmer	1980	CC	521	n	bl	y	n	0	cig+/-ot	nev cigs	st
HUMBLE	62		f	65	84	hi	-	not alv	NAmer	1980	CC	521	n	bl	y	n	0	cig+/-ot	nev cigs	st
KAISE2	40		m	65	74	all	9	all	NAmer	1979	pr	318	n	bl	n	n	0	cig only	nev any	st
KAISE2	48		m	75	99	all	9	all	NAmer	1979	pr	318	n	bl	n	n	0	cig only	nev any	st
KAISE2	16		f	65	74	all	9	all	NAmer	1979	pr	318	n	bl	n	n	0	cig only	nev any	st
KAISE2	24		f	75	99	all	9	all	NAmer	1979	pr	318	n	bl	n	n	0	cig only	nev any	st
LEVIN	21		m	65	74	all	-	all	NAmer	1938	CC	475	n	bl	n	n	0	all/unsp	nev any	st
LEVIN	22		m	75	99	all	-	all	NAmer	1938	CC	475	n	bl	n	n	0	all/unsp	nev any	st
LIU4	5		m	70	99	all	-	all	As:Chi	1986	CC	1000-00	n	ot	y	n	2	all/unsp	nev any	ot
LIU4	6		f	70	99	all	-	all	As:Chi	1986	CC	1000-00	n	ot	y	n	2	all/unsp	nev any	ot
NAM	21		m	65	79	all	-	all	NAmer	1986	CC	1199	n	bl	y	n	0	cig+/-ot	nev cigs	st
NAM	29		m	80	99	all	-	all	NAmer	1986	CC	1199	n	bl	y	n	0	cig+/-ot	nev cigs	st
NAM	53		f	65	79	all	-	all	NAmer	1986	CC	1199	n	bl	y	n	0	cig+/-ot	nev cigs	st
NAM	61		f	80	99	all	-	all	NAmer	1986	CC	1199	n	bl	y	n	0	cig+/-ot	nev cigs	st
PRESCO	42		m	65	99	all	0	all	Eu:Sca	1964	pr	867	n	bl	n	n	0	all/unsp	nev any	st
PRESCO	41		f	65	99	all	0	all	Eu:Sca	1964	pr	867	n	bl	n	n	0	all/unsp	nev any	st
STOCKS	39		m	65	74	all	-	all	Eu:UK	1952	CC	2932	n	V	y	n	0	all/unsp	nev any	st
WUWILL	5		f	65	69	all	-	all	As:Chi	1985	CC	965	n	ot	n	n	0	cig+/-ot	nev cigs	st

Cigarette type is all/unspec for all RRs
except for the following:

REF	NRR	CIGTYPE
DEAN3	123	MC only

Table 1A6 - 5

IESLC - Meta-analysis of Ever Smoking, Any product (or Cigarettes if Any not available), Age 65+
All LC types
Least adjusted

REF	NRR	SEX	AD	Number Exposed Case	Number Exposed Cont	Non-exposed Case	Non-exposed Cont	RR	95.00%CI
BENHAM	71	m	0	169	203	7	95	11.30	(5.11- 25.00)
BUFFLE	60	f	0	41	18	3	30	22.78	(6.15- 84.40)
COMSTO	59	c	0	46	58	6	44	5.82	(2.28- 14.84)
*CPSI	179	m	1	-	-	-	-	8.67	(5.54- 13.57)
*CPSI	114	f	0	153	211823	178	957682	3.89	(3.13- 4.82)
*CPSI	115	f	0	31	39527	163	392909	1.89	(1.29- 2.78)
Subtotal CPSI								3.78	(3.18- 4.50)
DAMBER	24	m	0	261	160	23	123	8.72	(5.36- 14.19)
DEAN3	46	m	0	285	396	16	82	3.69	(2.11- 6.44)
DEAN3	123	f	0	41	196	27	574	4.45	(2.66- 7.42)
Subtotal DEAN3								4.08	(2.80- 5.95)
*DORN	195	m	1	-	-	-	-	5.84	(4.42- 7.71)
GARSHI	9	m	0	771	1130	32	294	6.27	(4.30- 9.13)
GODLEY	2	m	1	-	-	-	-	6.17	(4.88- 7.79)
GODLEY	4	f	1	-	-	-	-	5.21	(3.65- 7.43)
Subtotal GODLEY								5.86	(4.82- 7.13)
*HAMMON	16	m	0	83	78713	9	21892	2.56	(1.29- 5.10)
HINDS	6	f	0	4	8	24	58	1.21	(0.33- 4.39)
HINDS	3	f	0	26	29	38	326	7.69	(4.11- 14.40)
HINDS	9	f	0	20	29	9	92	7.05	(2.89- 17.18)
Subtotal HINDS								5.83	(3.62- 9.39)
HUMBLE	56	m	0	128	157	6	60	8.15	(3.41- 19.48)
HUMBLE	60	m	0	55	58	2	21	9.96	(2.23- 44.47)
HUMBLE	58	f	0	52	33	11	52	7.45	(3.40- 16.30)
HUMBLE	62	f	0	19	11	4	34	14.68	(4.10- 52.52)
Subtotal HUMBLE								8.80	(5.34- 14.49)
*KAISE2	40	m	0	25	11767	5	11466	4.87	(1.87- 12.72)
*KAISE2	48	m	0	14	3401	4	4486	4.62	(1.52- 14.01)
*KAISE2	16	f	0	26	13883	2	24159	22.62	(5.37- 95.30)
*KAISE2	24	f	0	9	3362	3	12285	10.96	(2.97- 40.47)
Subtotal KAISE2								7.24	(4.05- 12.94)
LEVIN	21	m	0	87	317	13	168	3.55	(1.92- 6.54)
LEVIN	22	m	0	11	99	1	86	9.56	(1.21- 75.53)
Subtotal LEVIN								3.84	(2.14- 6.91)
LIU4	5	m	2	-	-	-	-	2.47	(2.34- 2.61)
LIU4	6	f	2	-	-	-	-	2.50	(2.33- 2.68)
Subtotal LIU4								2.48	(2.38- 2.59)
NAM	21	m	0	296	311	18	130	6.87	(4.09- 11.54)
NAM	29	m	0	67	143	6	172	13.43	(5.66- 31.87)
NAM	53	f	0	142	199	18	251	9.95	(5.89- 16.81)
NAM	61	f	0	35	83	22	472	9.05	(5.06- 16.19)
Subtotal NAM								8.93	(6.66- 11.97)
*PRESCO	42	m	0	378	57442	3	6229	13.66	(4.39- 42.54)
*PRESCO	41	f	0	105	34709	9	19748	6.64	(3.36- 13.11)
Subtotal PRESCO								8.03	(4.48- 14.40)
STOCKS	39	m	0	658	1423	18	173	4.44	(2.71- 7.28)
WUWILL	5	f	0	82	64	55	97	2.26	(1.42- 3.60)
Partial Totals				4120	459752	735	1454290		

*prospective study

REF	NRR	SEX	AD	Ys	Ws	Qs	Ps
BENHAM	71	m	0	2.42	6.09	11.35	0.0000
BUFFLE	60	f	0	3.13	2.24	9.56	0.0000
COMSTO	59	c	0	1.76	4.38	2.15	0.0002
*CPSI	179	m	1	2.16	19.15	23.19	0.0000
*CPSI	114	f	0	1.36	82.32	7.32	0.0000
*CPSI	115	f	0	0.64	26.07	4.65	0.0011
Subtotal CPSI				1.33	127.53	35.16	
DAMBER	24	m	0	2.17	16.21	19.86	0.0000
DEAN3	46	m	0	1.31	12.39	0.75	0.0000
DEAN3	123	f	0	1.49	14.65	2.75	0.0000
Subtotal DEAN3				1.41	27.03	3.50	
*DORN	195	m	1	1.76	49.64	24.71	0.0000
GARSHI	9	m	0	1.84	27.15	16.36	0.0000
GODLEY	2	m	1	1.82	70.25	40.62	0.0000
GODLEY	4	f	1	1.65	30.41	10.64	0.0000
Subtotal GODLEY				1.77	100.66	51.26	
*HAMMON	16	m	0	0.94	8.12	0.11	0.0073
HINDS	6	f	0	0.19	2.30	1.74	0.7739
HINDS	3	f	0	2.04	9.77	9.40	0.0000

Table 1A6 - 5

IESLC - Meta-analysis of Ever Smoking, Any product (or Cigarettes if Any not available), Age 65+
All LC types
Least adjusted

REF	NRR	SEX	AD	Ys	Ws	Qs	Ps
HINDS	9	f	0	1.95	4.84	3.87	0.0000
Subtotal HINDS				1.76	16.92	15.02	
HUMBLE	56	m	0	2.10	5.06	5.47	0.0000
HUMBLE	60	m	0	2.30	1.72	2.63	0.0026
HUMBLE	58	f	0	2.01	6.26	5.64	0.0000
HUMBLE	62	f	0	2.69	2.36	6.26	0.0000
Subtotal HUMBLE				2.17	15.41	20.00	
*KAISE2	40	m	0	1.58	4.17	1.15	0.0012
*KAISE2	48	m	0	1.53	3.12	0.69	0.0069
*KAISE2	16	f	0	3.12	1.86	7.88	0.0000
*KAISE2	24	f	0	2.39	2.25	4.01	0.0003
Subtotal KAISE2				1.98	11.40	13.73	
LEVIN	21	m	0	1.27	10.25	0.44	0.0001
LEVIN	22	m	0	2.26	0.90	1.29	0.0324
Subtotal LEVIN				1.35	11.15	1.73	
LIU4	5	m	2	0.90	1288.59	30.96	0.0000
LIU4	6	f	2	0.92	784.55	16.03	0.0000
Subtotal LIU4				0.91	2073.14	46.99	
NAM	21	m	0	1.93	14.32	10.80	0.0000
NAM	29	m	0	2.60	5.14	12.17	0.0000
NAM	53	f	0	2.30	13.97	21.42	0.0000
NAM	61	f	0	2.20	11.34	14.82	0.0000
Subtotal NAM				2.19	44.77	59.21	
*PRESCO	42	m	0	2.61	2.98	7.21	0.0000
*PRESCO	41	f	0	1.89	8.29	5.76	0.0000
Subtotal PRESCO				2.08	11.27	12.97	
STOCKS	39	m	0	1.49	15.73	2.94	0.0000
WUWILL	5	f	0	0.82	17.76	1.06	0.0006

	N	37
	NS	19
	Wt	2586.60
Het	Chi	347.67
Het	df	36
Het	P	***
Fixed	RR	2.88
	RRl	2.78
	RRu	3.00
	P	+++
Random	RR	5.48
	RRl	4.59
	RRu	6.55
	P	+++
Asymm	P	***

Table 1A6 - 6

IESLC - Meta-analysis of Ever Smoking, Any product (or Cigarettes if Any not available), Age 65+
All LC types
Least adjusted

		combined	Sex male	female	Total
	N	1	19	17	37
	NS	1	15	11	27
	Wt	4.38	1560.97	1021.24	2586.60
Het	Chi	0.00	212.69	132.74	347.67
Het	df	0	18	16	36
Het	P	N.S.	***	***	***
Fixed	RR	5.82	2.89	2.86	2.88
	RRl	2.28	2.75	2.69	2.78
	RRu	14.84	3.04	3.04	3.00
	P	+++	+++	+++	+++
Random	RR	5.82	5.95	5.34	5.48
	RRl	2.28	4.28	3.92	4.59
	RRu	14.84	8.26	7.27	6.55
	P	+++	+++	+++	+++
Between	Chi				2.24
Between	df				2
Between	P				N.S.
Btwn(F)	P				N.S.
Btwn(R)	P				N.S.

Table 1A6 - 7

IESLC - Meta-analysis of Ever Smoking, Any product (or Cigarettes if Any not available), Age 65+
All LC types
Excluded studies (and stage at which they were excluded)

1 BOUCHA BUELL LAURIL MZILEN

2 BYERS2 ENSTRO HIRAY2 KANELL LIAW LICKIN MRFIT MURATA NOTANI RESTRE RIMING SAARIK SEGI2 SHIMIZ SITAS SOBUE2 TANG2 WALD WARSIN WATSON WU2 WYNDE5 WYNDE8 WYNDER

3 ABELIN ABRAHA AGUDO AKIBA ALDERS AMANDU AMES ANDERS ARCHER ARMADA AUSTIN AUVINE AXELSO AXELSS BAND BARBON BECHER BENSHL BERRIN BEST BLOHMK BLOT1 BLOT2 BLOT3 BLOT4 BOFFET BOUCOT BRESLO BRETT BROCKM BROSS BROWN1 BYERS1 CARPEN CASCO2 CASCOR CEDERL CHAN CHANG CHATZI CHEN CHEN2 CHEN3 CHIAZZ CHOI CHOW CHYOU COOKSO CORREA CPSII DARBY DAVEYS DEAN DEAN2 DEKLER DESTE2 DESTEF DOCKER DOLL DOLL2 DORANT DOSEME DROSTE DU DUNN EBELIN ENGELA ESAKI FAN GAO GAO2 GARCIA GARDIN GENG GER GILLIS GOLLED GOODMA GRAHAM GREGOR GSELL HAENSZ HAMMO2 HANSEN HEGMAN HEIN HENNEK HIRAOK HIRAYA HITOSU HOLE HOROWI HORWIT HU HU2 HUANG ISHIMA JAHN JAIN JARUP JARVHO JEDRYC JIANG JOLY JONES JUSSAW KAISER KATSOU KAUFMA KELLER KHUDER KIHARA KINLEN KJUUS KNEKT KO KOHLME KOO KOULUM KREUZE KREYBE KUBIK LAMTH LAMWK LAMWK2 LANGE LAUSSM LEI LEMARC LETOUR LIDDEL LIU LIU2 LIU3 LIU5 LOMBA2 LOMBAR LUBIN LUBIN2 LUO MACLEN MAGNUS MARSH MARSH2 MARTIS MASTRA MATOS MATSUD MCCONN MCDUFF MCLAUG MIGRAN MILLER MILLS MOLLO MRFITR NOTAN2 NOU ODRISC ORMOS OSANN OSANN2 PARKIN PASTOR PAWLEG PERNU PERSH2 PETO PEZZO2 PEZZOT PIKE PISANI POFFIJ POLEDN QIAO QIAO2 RACHTA RADZIK RANDIG REN RONCO ROOTS ROTHSC SANKAR SCHWA2 SCHWAR SEGI SEOW SHAW SIEMIA SIMARA SIMONA SOBUE SPEIZE SPITZ STASZE STAYNE STOCKW STUCKE SUN SUZUK2 SUZUKI SVENSS TANG TAO TENKAN TIZZAN TOKARS TOUSEY TSUGAN TULINI TVERDA ULMER VANDER VEIERO VUTUC WAKAI WANG WANG2 WANG3 WANG4 WICKLU WIGLE WILKIN WU WUNSCH WYNDE2 WYNDE3 WYNDE6 WYNDE7 XIANGZ XU XU2 XU3 XU4 YAMAGU YONG YUAN ZHANG ZHENG ZHOU

4 BROWN2 SADOWS

7 PERSHA

9 DORGAN WYNDE4

Table 1A6 - 8
Potentially overlapping studies

REF	REFGP	PRINC	. OVERLAP/LINK
BENHAM	LUBIN2	2	Subset of Lubin2
PRESCO	PRESCO	2	PRESCO-combined
CPSI	CPSI	1	CPSI overall

Table 1A6 - 9
Most adjusted - insufficient data for metaanalysis

REF	NRR	SEX	AGEL	AGEH	RACE	YF	LC TYPE	LOC	START	ST	NLC	R	VB	P	H	AD	PRODUCT	DENOM	De
BROWN2	56	m	70	99	wh	-	all	NAmer	1984	CC	14596	n	bl	n	y	1	cig+/-ot	nev cigs	or
BROWN2	53	f	70	99	wh	-	all	NAmer	1984	CC	14596	n	bl	n	y	1	cig+/-ot	nev cigs	or
SADOWS	23	m	70	99	wh	-	all	NAmer	1938	CC	477	n	bl	n	n	0	all/unsp	nev any	st

REF	NRR	RR	SIG	RRDATA comment
BROWN2	56	8.20		0
BROWN2	53	10.60		0
SADOWS	23	1.50		0

Table 1A7 -

IESLC - Meta-analysis of Ever Smoking, Cigarettes (or Any Product if Cigarettes not available), Age <56
All LC types

This analysis is restricted to results for:
1) Non-dose-response data
2) Ever smokers
3) Age <56
4) Results complete enough for use in metaanalysis

Within each study, results are then selected (in the following order of preference, within each sex) for:
5) PRODUCT: cigarettes regardless of other products, cigarettes only, all/unspec
6) CIGTYPE: all/unspecified, MC regardless of HR, MC only
7) DENOM: never smoked anything, never smoked cigarettes, (never +1 = +long term ex, +2 = +amount unknown,
+3 = never cigs+long term ex)
8) Followup period (YF, prospective studies): whole study (coded as 0) or longest available
9) LCtype: all or nearest available, at least Squamous and Adeno. (q = squamous, s = small,
l = large, a = adeno, mix = mixed, alv = alveolar)
10) Race: all or nearest available, otherwise by race (wh or w = white, bl or b = black, hi = hispanic
ch = chinese, jap = japanese, haw = hawaiian, w+o = white + oriental, sca = scandinavian, as = asian)
11) For overlapping studies: principal rather than subsidiary studies
Finally by Age: whole study (actual age shown) if available, otherwise by widest available age group
and then for single sex results (m, f) in preference to combined sex results (c).

Results adjusted (AD) for the most potential confounders are then chosen in Sections -1 to -3
(and those which actually differ from the adjusted results in Table 1A2 - 1 are marked 'x' in Section -1)
and results adjusted for the least confounders in Sections -4 to -6. (Those least adjusted results which
actually differ from the most adjusted as marked 'x' in column X in Section -4)
(Results adjusted for an unknown number of confounder(s) are coded as 20.)

Section -7 shows excluded studies, together with the stage (as above) at which no qualifying
results were found.

Section -8 lists the potentially overlapping studies which have been included (1=principal, 2=subsidiary).

Section -9 lists any results which would have been included in preference except that they had data not complete
enough for use in meta-analysis, with their significance (yes/no), if known, and any further comment as entered
on the database.

In addition to those mentioned above, the following fields, levels and abbreviations are used:
* or nk = not known, n = no, y = yes, ot = other
nev = never
all/unspec = all or unspecified, cig+/-ot = cigarettes irrespective of other products (cigar, pipe etc)
MC = manufactured cigarettes, HR = hand-rolled cigarettes
REF: 6-character study reference
NRR: number of the RR on the database within the study
ST : study type (CC = case control, pr or prosp = prospective)
NLC: number of lung cancer cases in whole study
R : risky occupational population (n = no, m = mining, o = other risky)
VB : national cigarette type (V = at least 75% Virginia, bl = at least 75% blended, ot = other)
P : any proxy use
H : full histological confirmation
De : derivation of RR/CI (or = original, st = standard method, ot = other method of estimation)

Table 1A7 - 1

IESLC - Meta-analysis of Ever Smoking, Cigarettes (or Any Product if Cigarettes not available), Age <56
All LC types
Most adjusted

REF	NRR	1A2	SEX	AGEL	AGEH	RACE	YF	LC TYPE	LOC	START	ST	NLC	R	VB	P	H	AD	PRODUCT	DENOM	De
BENHAM	68	x	m	1	49	all	-	not mix	Eu:wst	1976	CC	1625	n	bl	n	y	0	cig only	nev any	st
COMSTO	57	x	c	25	44	all	-	all	NAmer	1975	ot	258	n	bl	n	n	0	cig+/-ot	nev cigs	st
CPSI	163	x	m	35	54	all	6	all	NAmer	1959	pr	5138	n	bl	n	n	1	cig+/-ot	nev any	ot
CPSI	250	x	f	40	54	all	6	all	NAmer	1959	pr	5138	n	bl	n	n	1	cig+/-ot	nev cigs	ot
DEAN3	43	x	m	35	44	all	-	all	Eu:UK	1969	CC	766	n	V	y	n	0	all/unsp	nev any	st
DEAN3	44	x	m	45	54	all	-	all	Eu:UK	1969	CC	766	n	V	y	n	0	all/unsp	nev any	st
DEAN3	120	x	f	35	44	all	-	all	Eu:UK	1969	CC	766	n	V	y	n	0	cig only	nev any	st
DEAN3	121	x	f	45	54	all	-	all	Eu:UK	1969	CC	766	n	V	y	n	0	cig only	nev any	st
HAMMON	1	x	m	50	54	wh	0	all	NAmer	1952	pr	448	n	bl	n	n	0	cig+/-ot	nev any	st
HINDS	4	x	f	35	49	ch	-	all	NAmer	1968	CC	292	n	bl	n	n	0	all/unsp	nev any	st
HINDS	1	x	f	35	49	jap	-	all	NAmer	1968	CC	292	n	bl	n	n	0	all/unsp	nev any	st
HINDS	7	x	f	35	49	haw	-	all	NAmer	1968	CC	292	n	bl	n	n	0	all/unsp	nev any	st
HITOSU	24	x	m	1	49	all	-	all	As:Jap	1960	CC	216	n	bl	y	n	0	all/unsp	nev any	st
HITOSU	44	x	f	1	49	all	-	all	As:Jap	1960	CC	216	n	bl	y	n	0	all/unsp	nev any	st
KREUZE	59	x	m	1	45	all	-	all	Eu:Ger	1990	CC	2260	n	bl	n	n	3	cig+/-ot	nev any	ot
KREUZE	60		f	1	45	all	-	all	Eu:Ger	1990	CC	2260	n	bl	n	n	3	cig+/-ot	nev any	ot
KUBIK	4	x	m	40	54	all	4	all	Eu:est	1965	pr	108	n	bl	n	n	0	cig+/-ot	nev any	ot
LEVIN	9	x	m	35	44	all	-	all	NAmer	1938	CC	475	n	bl	n	n	0	cig+/-ot	nev any	st
LEVIN	10	x	m	45	54	all	-	all	NAmer	1938	CC	475	n	bl	n	n	0	cig+/-ot	nev any	st
NAM	5	x	m	25	54	all	-	all	NAmer	1986	CC	1199	n	bl	y	n	0	cig+/-ot	nev cigs	st
NAM	37	x	f	25	54	all	-	all	NAmer	1986	CC	1199	n	bl	y	n	0	cig+/-ot	nev cigs	st
PRESCO	38	x	m	20	49	all	0	all	Eu:Sca	1964	pr	867	n	bl	n	n	0	all/unsp	nev any	ot
PRESCO	37	x	f	20	49	all	0	all	Eu:Sca	1964	pr	867	n	bl	n	n	0	all/unsp	nev any	ot
SCHWAR	6	x	m	40	54	wh	-	all	NAmer	1984	CC	5588	n	bl	y	y	0	cig+/-ot	nev cigs	st
SCHWAR	5	x	m	40	54	bl	-	all	NAmer	1984	CC	5588	n	bl	y	y	0	cig+/-ot	nev cigs	st
SCHWAR	14	x	f	40	54	wh	-	all	NAmer	1984	CC	5588	n	bl	y	y	0	cig+/-ot	nev cigs	st
SCHWAR	13	x	f	40	54	bl	-	all	NAmer	1984	CC	5588	n	bl	y	y	0	cig+/-ot	nev cigs	st
SPEIZE	8		f	30	55	all	0	all	NAmer	1976	pr	593	n	bl	n	y	0	cig+/-ot	nev cigs	st
STOCKS	32	x	m	35	44	all	-	all	Eu:UK	1952	CC	2932	n	V	y	n	0	cig+/-ot	nev any	st
STOCKS	34	x	m	45	54	all	-	all	Eu:UK	1952	CC	2932	n	V	y	n	0	cig+/-ot	nev any	st
TSUGAN	27		m	30	49	all	-	q+a	As:Jap	1976	CC	134	n	bl	n	y	0	all/unsp	nev any	st
TVERDA	22		m	35	49	all	0	all	Eu:Sca	1972	pr	238	n	bl	n	n	2	cig+/-ot	nev cigs	ot
VUTUC	10	x	m	1	39	all	-	all	Eu:wst	1976	CC	1877	n	bl	n	n	0	cig+/-ot	nev cigs	st
VUTUC	33	x	m	41	50	all	-	all	Eu:wst	1976	CC	1877	n	bl	n	n	0	cig+/-ot	nev cigs	st
WUWILL	1	x	f	30	49	all	-	all	As:Chi	1985	CC	965	n	ot	n	n	0	cig+/-ot	nev cigs	st
WUWILL	2	x	f	50	54	all	-	all	As:Chi	1985	CC	965	n	ot	n	n	0	cig+/-ot	nev cigs	st
WYNDE6	427	x	m	1	54	wh	-	q+s+a	NAmer	1969	CC	4423	n	bl	n	y	0	cig+/-ot	nev cigs	st
WYNDE6	429	x	f	1	54	wh	-	q+s+a	NAmer	1969	CC	4423	n	bl	n	y	0	cig+/-ot	nev cigs	st

Cigarette type is all/unspec for all RRs
except for the following:

REF	NRR	CIGTYPE
DEAN3	120	MC only
DEAN3	121	MC only

Table 1A7 - 2

IESLC - Meta-analysis of Ever Smoking, Cigarettes (or Any Product if Cigarettes not available), Age <56
All LC types
Most adjusted

REF	NRR	SEX	AD	Number Exposed Case	Number Exposed Cont	Non-exposed Case	Non-exposed Cont	RR	95.00%CI
BENHAM	68	m	0	217	304	9	103	8.17	(4.04- 16.50)
COMSTO	57	c	0	27	39	1	17	11.77	(1.48- 93.79)
*CPSI	163	m	1	-	-	-	-	7.63	(4.93- 11.79)
*CPSI	250	f	1	-	-	-	-	3.50	(2.45- 4.99)
Subtotal CPSI								4.78	(3.63- 6.30)
DEAN3	43	m	0	13	563	1	187	4.32	(0.56- 33.23)
DEAN3	44	m	0	84	650	1	145	18.74	(2.59- 135.69)
DEAN3	120	f	0	6	472	1	328	4.17	(0.50- 34.80)
DEAN3	121	f	0	23	469	4	326	4.00	(1.37- 11.67)
Subtotal DEAN3								5.21	(2.36- 11.52)
*HAMMON	1	m	0	83	146141	1	33255	18.89	(2.63- 135.66)
HINDS	4	f	0	1	12	4	42	0.88	(0.09- 8.58)
HINDS	1	f	0	9	139	6	350	3.78	(1.32- 10.81)
HINDS	7	f	0	26	118	2	139	15.31	(3.56- 65.87)
Subtotal HINDS								4.81	(2.16- 10.68)
HITOSU	24	m	0	8	993	1	118	0.95	(0.12- 7.67)
HITOSU	44	f	0	4	245	7	1108	2.58	(0.75- 8.90)
Subtotal HITOSU								1.99	(0.69- 5.77)
KREUZE	59	m	3	-	-	-	-	11.38	(4.71- 27.48)
KREUZE	60	f	3	-	-	-	-	9.21	(3.45- 24.53)
Subtotal KREUZE								10.35	(5.37- 19.95)
*KUBIK	4	m	0	11	4373	0	2420	12.73~	(0.75- 215.92)
LEVIN	9	m	0	30	181	1	51	8.45	(1.13- 63.50)
LEVIN	10	m	0	109	318	6	122	6.97	(2.98- 16.27)
Subtotal LEVIN								7.17	(3.28- 15.68)
NAM	5	m	0	56	448	1	176	22.00	(3.02- 160.15)
NAM	37	f	0	40	129	2	93	14.42	(3.40- 61.16)
Subtotal NAM								16.69	(5.19- 53.68)
*PRESCO	38	m	0	18	49324	0	10629	7.97~	(0.48- 132.30)
*PRESCO	37	f	0	7	33202	0	13953	6.30~	(0.36- 110.37)
Subtotal PRESCO								7.11	(0.96- 52.76)
SCHWAR	6	m	0	282	178	7	73	16.52	(7.44- 36.69)
SCHWAR	5	m	0	146	39	6	7	4.37	(1.39- 13.74)
SCHWAR	14	f	0	206	108	11	79	13.70	(6.99- 26.83)
SCHWAR	13	f	0	69	28	5	41	20.21	(7.24- 56.44)
Subtotal SCHWAR								13.19	(8.61- 20.21)
*SPEIZE	8	f	0	535	1012074	58	776300	7.08	(5.40- 9.28)
STOCKS	32	m	0	159	1099	2	163	11.79	(2.90- 48.02)
STOCKS	34	m	0	721	1737	4	149	15.46	(5.71- 41.90)
Subtotal STOCKS								14.12	(6.26- 31.83)
TSUGAN	27	m	0	73	71	18	22	1.26	(0.62- 2.54)
*TVERDA	22	m	2	-	-	-	-	4.58	(2.97- 7.06)
VUTUC	10	m	0	12	22	1	9	4.91	(0.55- 43.53)
VUTUC	33	m	0	97	157	11	64	3.59	(1.81- 7.15)
Subtotal VUTUC								3.70	(1.92- 7.13)
WUWILL	1	f	0	68	39	132	124	1.64	(1.03- 2.60)
WUWILL	2	f	0	121	69	82	127	2.72	(1.81- 4.07)
Subtotal WUWILL								2.18	(1.61- 2.96)
WYNDE6	427	m	0	309	177	17	150	15.40	(9.03- 26.29)
WYNDE6	429	f	0	224	115	15	183	23.76	(13.41- 42.12)
Subtotal WYNDE6								18.85	(12.75- 27.86)
Partial Totals				3794	1254033	417	841053		

*prospective study ~ With 0.5 adjustment for zero

Table 1A7 - 2

IESLC - Meta-analysis of Ever Smoking, Cigarettes (or Any Product if Cigarettes not available), Age <56
All LC types
Most adjusted

REF	NRR	SEX	AD	Ys	Ws	Qs	Ps
BENHAM	68	m	0	2.10	7.77	0.90	0.0000
COMSTO	57	c	0	2.47	0.89	0.44	0.0199
*CPSI	163	m	1	2.03	20.21	1.49	0.0000
*CPSI	250	f	1	1.25	30.37	7.84	0.0000
Subtotal CPSI				1.56	50.58	9.33	
DEAN3	43	m	0	1.46	0.92	0.08	0.1600
DEAN3	44	m	0	2.93	0.98	1.34	0.0037
DEAN3	120	f	0	1.43	0.85	0.09	0.1872
DEAN3	121	f	0	1.39	3.35	0.47	0.0112
Subtotal DEAN3				1.65	6.10	1.99	
*HAMMON	1	m	0	2.94	0.99	1.37	0.0035
HINDS	4	f	0	-0.13	0.74	2.64	0.9087
HINDS	1	f	0	1.33	3.47	0.65	0.0132
HINDS	7	f	0	2.73	1.80	1.69	0.0002
Subtotal HINDS				1.57	6.02	4.98	
HITOSU	24	m	0	-0.05	0.88	2.89	0.9621
HITOSU	44	f	0	0.95	2.51	1.66	0.1323
Subtotal HITOSU				0.69	3.40	4.55	
KREUZE	59	m	3	2.43	4.94	2.22	0.0000
KREUZE	60	f	3	2.22	3.99	0.84	0.0000
Subtotal KREUZE				2.34	8.93	3.07	
*KUBIK	4	m	0	2.54	0.48	0.29	0.0782
LEVIN	9	m	0	2.13	0.94	0.13	0.0380
LEVIN	10	m	0	1.94	5.34	0.17	0.0000
Subtotal LEVIN				1.97	6.29	0.31	
NAM	5	m	0	3.09	0.97	1.72	0.0023
NAM	37	f	0	2.67	1.84	1.52	0.0003
Subtotal NAM				2.81	2.81	3.24	
*PRESCO	38	m	0	2.08	0.49	0.05	0.1474
*PRESCO	37	f	0	1.84	0.47	0.00	0.2075
Subtotal PRESCO				1.96	0.96	0.05	
SCHWAR	6	m	0	2.80	6.03	6.57	0.0000
SCHWAR	5	m	0	1.47	2.92	0.24	0.0117
SCHWAR	14	f	0	2.62	8.50	6.23	0.0000
SCHWAR	13	f	0	3.01	3.64	5.65	0.0000
Subtotal SCHWAR				2.58	21.10	18.69	
*SPEIZE	8	f	0	1.96	52.33	2.00	0.0000
STOCKS	32	m	0	2.47	1.95	0.97	0.0006
STOCKS	34	m	0	2.74	3.87	3.69	0.0000
Subtotal STOCKS				2.65	5.81	4.67	
TSUGAN	27	m	0	0.23	7.76	18.24	0.5244
*TVERDA	22	m	2	1.52	20.49	1.17	0.0000
VUTUC	10	m	0	1.59	0.81	0.02	0.1530
VUTUC	33	m	0	1.28	8.12	1.88	0.0003
Subtotal VUTUC				1.31	8.92	1.90	
WUWILL	1	f	0	0.49	17.86	28.70	0.0370
WUWILL	2	f	0	1.00	23.35	13.55	0.0000
Subtotal WUWILL				0.78	41.21	42.25	
WYNDE6	427	m	0	2.73	13.45	12.75	0.0000
WYNDE6	429	f	0	3.17	11.72	23.22	0.0000
Subtotal WYNDE6				2.94	25.17	35.96	

	N	38
	NS	20
	Wt	278.02
Het	Chi	155.40
Het	df	37
Het	P	***
Fixed	RR	5.82
	RRl	5.17
	RRu	6.54
	P	+++
Random	RR	6.66
	RRl	5.00
	RRu	8.87
	P	+++
Asymm	P	N.S.

Table 1A7 - 3

IESLC - Meta-analysis of Ever Smoking, Cigarettes (or Any Product if Cigarettes not available), Age <56
All LC types
Most adjusted

	combined	Sex male	female	Total
N	1	21	16	38
NS	1	16	11	28
Wt	0.89	110.32	166.81	278.02
Het Chi	0.00	55.31	94.70	155.40
Het df	0	20	15	37
Het P	N.S.	***	***	***
Fixed RR	11.77	6.84	5.21	5.82
RRl	1.48	5.68	4.47	5.17
RRu	93.79	8.24	6.06	6.54
P	+	+++	+++	+++
Random RR	11.77	7.22	5.95	6.66
RRl	1.48	5.01	3.77	5.00
RRu	93.79	10.41	9.39	8.87
P	+	+++	+++	+++
Between Chi				5.39
Between df				2
Between P				(*)
Btwn(F) P				N.S.
Btwn(R) P				N.S.

Table 1A7 - 4

IESLC - Meta-analysis of Ever Smoking, Cigarettes (or Any Product if Cigarettes not available), Age <56
All LC types
Least adjusted

REF	NRR	X	SEX	AGEL	AGEH	RACE	YF	LC TYPE	LOC	START	ST	NLC	R	VB	P	H	AD	PRODUCT	DENOM	De
BENHAM	68		m	1	49	all	-	not mix	Eu:wst	1976	CC	1625	n	bl	n	y	0	cig only	nev any	st
COMSTO	57		c	25	44	all	-	all	NAmer	1975	ot	258	n	bl	n	n	0	cig+/-ot	nev cigs	st
CPSI	163		m	35	54	all	6	all	NAmer	1959	pr	5138	n	bl	n	n	1	cig+/-ot	nev any	ot
CPSI	250		f	40	54	all	6	all	NAmer	1959	pr	5138	n	bl	n	n	1	cig+/-ot	nev cigs	ot
DEAN3	43		m	35	44	all	-	all	Eu:UK	1969	CC	766	n	V	y	n	0	all/unsp	nev any	st
DEAN3	44		m	45	54	all	-	all	Eu:UK	1969	CC	766	n	V	y	n	0	all/unsp	nev any	st
DEAN3	120		f	35	44	all	-	all	Eu:UK	1969	CC	766	n	V	y	n	0	cig only	nev any	st
DEAN3	121		f	45	54	all	-	all	Eu:UK	1969	CC	766	n	V	y	n	0	cig only	nev any	st
HAMMON	1		m	50	54	wh	0	all	NAmer	1952	pr	448	n	bl	n	n	0	cig+/-ot	nev any	st
HINDS	4		f	35	49	ch	-	all	NAmer	1968	CC	292	n	bl	n	n	0	all/unsp	nev any	st
HINDS	1		f	35	49	jap	-	all	NAmer	1968	CC	292	n	bl	n	n	0	all/unsp	nev any	st
HINDS	7		f	35	49	haw	-	all	NAmer	1968	CC	292	n	bl	n	n	0	all/unsp	nev any	st
HITOSU	24		m	1	49	all	-	all	As:Jap	1960	CC	216	n	bl	y	n	0	all/unsp	nev any	st
HITOSU	44		f	1	49	all	-	all	As:Jap	1960	CC	216	n	bl	y	n	0	all/unsp	nev any	st
KREUZE	59		m	1	45	all	-	all	Eu:Ger	1990	CC	2260	n	bl	n	n	3	cig+/-ot	nev any	ot
KREUZE	60		f	1	45	all	-	all	Eu:Ger	1990	CC	2260	n	bl	n	n	3	cig+/-ot	nev any	ot
KUBIK	4		m	40	54	all	4	all	Eu:est	1965	pr	108	n	bl	n	n	0	cig+/-ot	nev any	ot
LEVIN	9		m	35	44	all	-	all	NAmer	1938	CC	475	n	bl	n	n	0	cig+/-ot	nev any	st
LEVIN	10		m	45	54	all	-	all	NAmer	1938	CC	475	n	bl	n	n	0	cig+/-ot	nev any	st
NAM	5		m	25	54	all	-	all	NAmer	1986	CC	1199	n	bl	y	n	0	cig+/-ot	nev cigs	st
NAM	37		f	25	54	all	-	all	NAmer	1986	CC	1199	n	bl	y	n	0	cig+/-ot	nev cigs	st
PRESCO	38		m	20	49	all	0	all	Eu:Sca	1964	pr	867	n	bl	n	n	0	all/unsp	nev any	ot
PRESCO	37		f	20	49	all	0	all	Eu:Sca	1964	pr	867	n	bl	n	n	0	all/unsp	nev any	ot
SCHWAR	6		m	40	54	wh	-	all	NAmer	1984	CC	5588	n	bl	y	y	0	cig+/-ot	nev cigs	st
SCHWAR	5		m	40	54	bl	-	all	NAmer	1984	CC	5588	n	bl	y	y	0	cig+/-ot	nev cigs	st
SCHWAR	14		f	40	54	wh	-	all	NAmer	1984	CC	5588	n	bl	y	y	0	cig+/-ot	nev cigs	st
SCHWAR	13		f	40	54	bl	-	all	NAmer	1984	CC	5588	n	bl	y	y	0	cig+/-ot	nev cigs	st
SPEIZE	8		f	30	55	all	0	all	NAmer	1976	pr	593	n	bl	n	y	0	cig+/-ot	nev cigs	st
STOCKS	32		m	35	44	all	-	all	Eu:UK	1952	CC	2932	n	V	y	n	0	cig+/-ot	nev any	st
STOCKS	34		m	45	54	all	-	all	Eu:UK	1952	CC	2932	n	V	y	n	0	cig+/-ot	nev any	st
TSUGAN	27		m	30	49	all	-	q+a	As:Jap	1976	CC	134	n	bl	n	y	0	all/unsp	nev any	st
TVERDA	22		m	35	49	all	0	all	Eu:Sca	1972	pr	238	n	bl	n	n	2	cig+/-ot	nev cigs	ot
VUTUC	10		m	1	39	all	-	all	Eu:wst	1976	CC	1877	n	bl	n	n	0	cig+/-ot	nev cigs	st
VUTUC	33		m	41	50	all	-	all	Eu:wst	1976	CC	1877	n	bl	n	n	0	cig+/-ot	nev cigs	st
WUWILL	1		f	30	49	all	-	all	As:Chi	1985	CC	965	n	ot	n	n	0	cig+/-ot	nev cigs	st
WUWILL	2		f	50	54	all	-	all	As:Chi	1985	CC	965	n	ot	n	n	0	cig+/-ot	nev cigs	st
WYNDE6	427		m	1	54	wh	-	q+s+a	NAmer	1969	CC	4423	n	bl	n	y	0	cig+/-ot	nev cigs	st
WYNDE6	429		f	1	54	wh	-	q+s+a	NAmer	1969	CC	4423	n	bl	n	y	0	cig+/-ot	nev cigs	st

Cigarette type is all/unspec for all RRs
except for the following:

REF	NRR	CIGTYPE
DEAN3	120	MC only
DEAN3	121	MC only

Table 1A7 - 5

IESLC - Meta-analysis of Ever Smoking, Cigarettes (or Any Product if Cigarettes not available), Age <56
All LC types
Least adjusted

REF	NRR	SEX	AD	Number Exposed Case	Number Exposed Cont	Non-exposed Case	Non-exposed Cont	RR	95.00%CI
BENHAM	68	m	0	217	304	9	103	8.17	(4.04- 16.50)
COMSTO	57	c	0	27	39	1	17	11.77	(1.48- 93.79)
*CPSI	163	m	1	-	-	-	-	7.63	(4.93- 11.79)
*CPSI	250	f	1	-	-	-	-	3.50	(2.45- 4.99)
Subtotal CPSI								4.78	(3.63- 6.30)
DEAN3	43	m	0	13	563	1	187	4.32	(0.56- 33.23)
DEAN3	44	m	0	84	650	1	145	18.74	(2.59- 135.69)
DEAN3	120	f	0	6	472	1	328	4.17	(0.50- 34.80)
DEAN3	121	f	0	23	469	4	326	4.00	(1.37- 11.67)
Subtotal DEAN3								5.21	(2.36- 11.52)
*HAMMON	1	m	0	83	146141	1	33255	18.89	(2.63- 135.66)
HINDS	4	f	0	1	12	4	42	0.88	(0.09- 8.58)
HINDS	1	f	0	9	139	6	350	3.78	(1.32- 10.81)
HINDS	7	f	0	26	118	2	139	15.31	(3.56- 65.87)
Subtotal HINDS								4.81	(2.16- 10.68)
HITOSU	24	m	0	8	993	1	118	0.95	(0.12- 7.67)
HITOSU	44	f	0	4	245	7	1108	2.58	(0.75- 8.90)
Subtotal HITOSU								1.99	(0.69- 5.77)
KREUZE	59	m	3	-	-	-	-	11.38	(4.71- 27.48)
KREUZE	60	f	3	-	-	-	-	9.21	(3.45- 24.53)
Subtotal KREUZE								10.35	(5.37- 19.95)
*KUBIK	4	m	0	11	4373	0	2420	12.73~	(0.75- 215.92)
LEVIN	9	m	0	30	181	1	51	8.45	(1.13- 63.50)
LEVIN	10	m	0	109	318	6	122	6.97	(2.98- 16.27)
Subtotal LEVIN								7.17	(3.28- 15.68)
NAM	5	m	0	56	448	1	176	22.00	(3.02- 160.15)
NAM	37	f	0	40	129	2	93	14.42	(3.40- 61.16)
Subtotal NAM								16.69	(5.19- 53.68)
*PRESCO	38	m	0	18	49324	0	10629	7.97~	(0.48- 132.30)
*PRESCO	37	f	0	7	33202	0	13953	6.30~	(0.36- 110.37)
Subtotal PRESCO								7.11	(0.96- 52.76)
SCHWAR	6	m	0	282	178	7	73	16.52	(7.44- 36.69)
SCHWAR	5	m	0	146	39	6	7	4.37	(1.39- 13.74)
SCHWAR	14	f	0	206	108	11	79	13.70	(6.99- 26.83)
SCHWAR	13	f	0	69	28	5	41	20.21	(7.24- 56.44)
Subtotal SCHWAR								13.19	(8.61- 20.21)
*SPEIZE	8	f	0	535	1012074	58	776300	7.08	(5.40- 9.28)
STOCKS	32	m	0	159	1099	2	163	11.79	(2.90- 48.02)
STOCKS	34	m	0	721	1737	4	149	15.46	(5.71- 41.90)
Subtotal STOCKS								14.12	(6.26- 31.83)
TSUGAN	27	m	0	73	71	18	22	1.26	(0.62- 2.54)
*TVERDA	22	m	2	-	-	-	-	4.58	(2.97- 7.06)
VUTUC	10	m	0	12	22	1	9	4.91	(0.55- 43.53)
VUTUC	33	m	0	97	157	11	64	3.59	(1.81- 7.15)
Subtotal VUTUC								3.70	(1.92- 7.13)
WUWILL	1	f	0	68	39	132	124	1.64	(1.03- 2.60)
WUWILL	2	f	0	121	69	82	127	2.72	(1.81- 4.07)
Subtotal WUWILL								2.18	(1.61- 2.96)
WYNDE6	427	m	0	309	177	17	150	15.40	(9.03- 26.29)
WYNDE6	429	f	0	224	115	15	183	23.76	(13.41- 42.12)
Subtotal WYNDE6								18.85	(12.75- 27.86)
Partial Totals				3794	1254033	417	841053		

*prospective study ~ With 0.5 adjustment for zero

Table 1A7 - 5

IESLC - Meta-analysis of Ever Smoking, Cigarettes (or Any Product if Cigarettes not available), Age <56
All LC types
Least adjusted

REF	NRR	SEX	AD	Ys	Ws	Qs	Ps
BENHAM	68	m	0	2.10	7.77	0.90	0.0000
COMSTO	57	c	0	2.47	0.89	0.44	0.0199
*CPSI	163	m	1	2.03	20.21	1.49	0.0000
*CPSI	250	f	1	1.25	30.37	7.84	0.0000
Subtotal CPSI				1.56	50.58	9.33	
DEAN3	43	m	0	1.46	0.92	0.08	0.1600
DEAN3	44	m	0	2.93	0.98	1.34	0.0037
DEAN3	120	f	0	1.43	0.85	0.09	0.1872
DEAN3	121	f	0	1.39	3.35	0.47	0.0112
Subtotal DEAN3				1.65	6.10	1.99	
*HAMMON	1	m	0	2.94	0.99	1.37	0.0035
HINDS	4	f	0	-0.13	0.74	2.64	0.9087
HINDS	1	f	0	1.33	3.47	0.65	0.0132
HINDS	7	f	0	2.73	1.80	1.69	0.0002
Subtotal HINDS				1.57	6.02	4.98	
HITOSU	24	m	0	-0.05	0.88	2.89	0.9621
HITOSU	44	f	0	0.95	2.51	1.66	0.1323
Subtotal HITOSU				0.69	3.40	4.55	
KREUZE	59	m	3	2.43	4.94	2.22	0.0000
KREUZE	60	f	3	2.22	3.99	0.84	0.0000
Subtotal KREUZE				2.34	8.93	3.07	
*KUBIK	4	m	0	2.54	0.48	0.29	0.0782
LEVIN	9	m	0	2.13	0.94	0.13	0.0380
LEVIN	10	m	0	1.94	5.34	0.17	0.0000
Subtotal LEVIN				1.97	6.29	0.31	
NAM	5	m	0	3.09	0.97	1.72	0.0023
NAM	37	f	0	2.67	1.84	1.52	0.0003
Subtotal NAM				2.81	2.81	3.24	
*PRESCO	38	m	0	2.08	0.49	0.05	0.1474
*PRESCO	37	f	0	1.84	0.47	0.00	0.2075
Subtotal PRESCO				1.96	0.96	0.05	
SCHWAR	6	m	0	2.80	6.03	6.57	0.0000
SCHWAR	5	m	0	1.47	2.92	0.24	0.0117
SCHWAR	14	f	0	2.62	8.50	6.23	0.0000
SCHWAR	13	f	0	3.01	3.64	5.65	0.0000
Subtotal SCHWAR				2.58	21.10	18.69	
*SPEIZE	8	f	0	1.96	52.33	2.00	0.0000
STOCKS	32	m	0	2.47	1.95	0.97	0.0006
STOCKS	34	m	0	2.74	3.87	3.69	0.0000
Subtotal STOCKS				2.65	5.81	4.67	
TSUGAN	27	m	0	0.23	7.76	18.24	0.5244
*TVERDA	22	m	2	1.52	20.49	1.17	0.0000
VUTUC	10	m	0	1.59	0.81	0.02	0.1530
VUTUC	33	m	0	1.28	8.12	1.88	0.0003
Subtotal VUTUC				1.31	8.92	1.90	
WUWILL	1	f	0	0.49	17.86	28.70	0.0370
WUWILL	2	f	0	1.00	23.35	13.55	0.0000
Subtotal WUWILL				0.78	41.21	42.25	
WYNDE6	427	m	0	2.73	13.45	12.75	0.0000
WYNDE6	429	f	0	3.17	11.72	23.22	0.0000
Subtotal WYNDE6				2.94	25.17	35.96	

	N	38
	NS	20
	Wt	278.02
Het	Chi	155.40
Het	df	37
Het	P	***
Fixed	RR	5.82
	RRl	5.17
	RRu	6.54
	P	+++
Random	RR	6.66
	RRl	5.00
	RRu	8.87
	P	+++
Asymm	P	N.S.

Table 1A7 - 6

IESLC - Meta-analysis of Ever Smoking, Cigarettes (or Any Product if Cigarettes not available), Age <56
All LC types
Least adjusted

	Sex combined	male	female	Total
N	1	21	16	38
NS	1	16	11	28
Wt	0.89	110.32	166.81	278.02
Het Chi	0.00	55.31	94.70	155.40
Het df	0	20	15	37
Het P	N.S.	***	***	***
Fixed RR	11.77	6.84	5.21	5.82
RRl	1.48	5.68	4.47	5.17
RRu	93.79	8.24	6.06	6.54
P	+	+++	+++	+++
Random RR	11.77	7.22	5.95	6.66
RRl	1.48	5.01	3.77	5.00
RRu	93.79	10.41	9.39	8.87
P	+	+++	+++	+++
Between Chi				5.39
Between df				2
Between P				(*)
Btwn(F) P				N.S.
Btwn(R) P				N.S.

Table 1A7 - 7

IESLC - Meta-analysis of Ever Smoking, Cigarettes (or Any Product if Cigarettes not available), Age <56
All LC types
Excluded studies (and stage at which they were excluded)

1	BOUCHA BUELL LAURIL MZILEN
2	BYERS2 ENSTRO HIRAY2 KANELL LIAW LICKIN MRFIT MURATA NOTANI RESTRE RIMING SAARIK SEGI2 SHIMIZ SITAS SOBUE2 TANG2 WALD WARSIN WATSON WU2 WYNDE5 WYNDE8 WYNDER
3	ABELIN ABRAHA AGUDO AKIBA ALDERS AMANDU AMES ANDERS ARCHER ARMADA AUSTIN AUVINE AXELSO AXELSS BAND BARBON BECHER BENSHL BERRIN BEST BLOHMK BLOT1 BLOT2 BLOT3 BLOT4 BOFFET BOUCOT BRESLO BRETT BROCKM BROSS BROWN1 BROWN2 BUFFLE BYERS1 CARPEN CASCO2 CASCOR CEDERL CHAN CHANG CHATZI CHEN CHEN2 CHEN3 CHIAZZ CHOI CHOW CHYOU COOKSO CORREA CPSII DAMBER DARBY DAVEYS DEAN DEAN2 DEKLER DESTE2 DESTEF DOCKER DOLL DOLL2 DORANT DORGAN DORN DOSEME DROSTE DU DUNN EBELIN ENGELA ESAKI FAN GAO GAO2 GARCIA GARDIN GARSHI GENG GER GILLIS GODLEY GOLLED GOODMA GRAHAM GREGOR GSELL HAENSZ HAMMO2 HANSEN HEGMAN HEIN HENNEK HIRAOK HIRAYA HOLE HOROWI HORWIT HU HU2 HUANG HUMBLE ISHIMA JAHN JAIN JARUP JARVHO JEDRYC JIANG JOLY JONES JUSSAW KAISE2 KAISER KATSOU KAUFMA KELLER KHUDER KIHARA KINLEN KJUUS KNEKT KO KOHLME KOO KOULUM KREYBE LAMTH LAMWK LAMWK2 LANGE LAUSSM LEI LEMARC LETOUR LIDDEL LIU LIU2 LIU3 LIU4 LIU5 LOMBA2 LOMBAR LUBIN LUBIN2 LUO MACLEN MAGNUS MARSH MARSH2 MARTIS MASTRA MATOS MATSUD MCCONN MCDUFF MCLAUG MIGRAN MILLER MILLS MOLLO MRFITR NOTAN2 NOU ODRISC ORMOS OSANN OSANN2 PARKIN PASTOR PAWLEG PERNU PERSH2 PERSHA PETO PEZZO2 PEZZOT PIKE PISANI POFFIJ POLEDN QIAO QIAO2 RACHTA RADZIK RANDIG REN RONCO ROOTS ROTHSC SANKAR SCHWA2 SEGI SEOW SHAW SIEMIA SIMARA SIMONA SOBUE SPITZ STASZE STAYNE STOCKW STUCKE SUN SUZUK2 SUZUKI SVENSS TANG TAO TENKAN TIZZAN TOKARS TOUSEY TULINI ULMER VANDER VEIERO WAKAI WANG WANG2 WANG3 WANG4 WICKLU WIGLE WILKIN WU WUNSCH WYNDE2 WYNDE3 WYNDE7 XIANGZ XU XU2 XU3 XU4 YAMAGU YONG YUAN ZHANG ZHENG ZHOU
4	SADOWS
9	WYNDE4

Table 1A7 - 8
Potentially overlapping studies

REF	REFGP	PRINC	. OVERLAP/LINK
VUTUC	LUBIN2	2	Subset of Lubin2
BENHAM	LUBIN2	2	Subset of Lubin2
TVERDA	TVERDA	1	VEIERO/TVERDAL
PRESCO	PRESCO	2	PRESCO-combined
WYNDE6	WYNDE6	1	WYNDE5/6/7/8
CPSI	CPSI	1	CPSI overall

Table 1A7 - 9
Most adjusted - insufficient data for metaanalysis

REF	NRR	SEX	AGEL	AGEH	RACE	YF	LC TYPE	LOC	START	ST	NLC	R	VB	P	H	AD	PRODUCT	DENOM	De
SADOWS	8	m	1	49	wh	-	all	NAmer	1938	CC	477	n	bl	n	n	0	cig only	nev any	st

REF	NRR	RR	SIG	RRDATA comment
SADOWS	8	3.50		0

Table 1A8 -

IESLC - Meta-analysis of Ever Smoking, Cigarettes (or Any Product if Cigarettes not available), Age 50-70
All LC types

This analysis is restricted to results for:
1) Non-dose-response data
2) Ever smokers
3) Maximum age range 50-70
4) Results complete enough for use in metaanalysis

Within each study, results are then selected (in the following order of preference, within each sex) for:
5) PRODUCT: cigarettes regardless of other products, cigarettes only, all/unspec
6) CIGTYPE: all/unspecified, MC regardless of HR, MC only
7) DENOM: never smoked anything, never smoked cigarettes, (never +1 = +long term ex, +2 = +amount unknown,
+3 = never cigs+long term ex)
8) Followup period (YF, prospective studies): whole study (coded as 0) or longest available
9) LCtype: all or nearest available, at least Squamous and Adeno. (q = squamous, s = small,
l = large, a = adeno, mix = mixed, alv = alveolar)
10) Race: all or nearest available, otherwise by race (wh or w = white, bl or b = black, hi = hispanic
ch = chinese, jap = japanese, haw = hawaiian, w+o = white + oriental, sca = scandinavian, as = asian)
11) For overlapping studies: principal rather than subsidiary studies
Finally by Age: whole study (actual age shown) if available, otherwise by widest available age group
and then for single sex results (m, f) in preference to combined sex results (c).

Results adjusted (AD) for the most potential confounders are then chosen in Sections -1 to -3
(and those which actually differ from the adjusted results in Table 1A2 - 1 are marked 'x' in Section -1)
and results adjusted for the least confounders in Sections -4 to -6. (Those least adjusted results which
actually differ from the most adjusted as marked 'x' in column X in Section -4)
(Results adjusted for an unknown number of confounder(s) are coded as 20.)

Section -7 shows excluded studies, together with the stage (as above) at which no qualifying
results were found.

Section -8 lists the potentially overlapping studies which have been included (1=principal, 2=subsidiary).

Section -9 lists any results which would have been included in preference except that they had data not complete
enough for use in meta-analysis, with their significance (yes/no), if known, and any further comment as entered
on the database.

In addition to those mentioned above, the following fields, levels and abbreviations are used:
* or nk = not known, n = no, y = yes, ot = other
nev = never
all/unspec = all or unspecified, cig+/-ot = cigarettes irrespective of other products (cigar, pipe etc)
MC = manufactured cigarettes, HR = hand-rolled cigarettes
REF: 6-character study reference
NRR: number of the RR on the database within the study
ST : study type (CC = case control, pr or prosp = prospective)
NLC: number of lung cancer cases in whole study
R : risky occupational population (n = no, m = mining, o = other risky)
VB : national cigarette type (V = at least 75% Virginia, bl = at least 75% blended, ot = other)
P : any proxy use
H : full histological confirmation
De : derivation of RR/CI (or = original, st = standard method, ot = other method of estimation)

Table 1A8 - 1

IESLC - Meta-analysis of Ever Smoking, Cigarettes (or Any Product if Cigarettes not available), Age 50-70
All LC types
Most adjusted

REF	NRR	1A2	SEX	AGEL	AGEH	RACE	YF	LC TYPE	LOC	START	ST	NLC	R	VB	P	H	AD	PRODUCT	DENOM	De
ANDERS	3		f	55	69	all	0	all	NAmer	1986	pr	343	n	bl	n	n	0	cig+/-ot	nev cigs	st
BENHAM	69	x	m	50	59	all	-	not mix	Eu:wst	1976	CC	1625	n	bl	n	y	0	cig only	nev any	st
BENHAM	70	x	m	60	69	all	-	not mix	Eu:wst	1976	CC	1625	n	bl	n	y	0	cig only	nev any	st
BUFFLE	59	x	f	60	69	w-hi	-	all	NAmer	1976	CC	943	n	bl	y	n	0	cig+/-ot	nev cigs	st
CPSI	171	x	m	55	69	all	6	all	NAmer	1959	pr	5138	n	bl	n	n	1	cig+/-ot	nev any	ot
CPSI	113	x	f	55	64	wh	0	all	NAmer	1959	pr	5138	n	bl	n	n	0	cig only	nev any	st
DAMBER	23	x	m	60	69	all	-	all	Eu:Sca	1972	CC	579	n	bl	y	n	0	all/unsp	nev any	st
DEAN3	45	x	m	55	64	all	-	all	Eu:UK	1969	CC	766	n	V	y	n	0	all/unsp	nev any	st
DEAN3	122	x	f	55	64	all	-	all	Eu:UK	1969	CC	766	n	V	y	n	0	cig only	nev any	st
DORANT	10		c	55	69	all	0	all	Eu:wst	1986	ot	550	n	bl	n	y	0	all/unsp	nev any	st
DORN	412	x	m	55	64	wh	5	all	NAmer	1954	pr	5097	n	bl	n	n	1	cig+/-ot	nev any	ot
HAMMON	116		m	50	69	wh	0	all	NAmer	1952	pr	448	n	bl	n	n	1	cig+/-ot	nev any	ot
HINDS	5	x	f	50	64	ch	-	all	NAmer	1968	CC	292	n	bl	n	n	0	all/unsp	nev any	st
HINDS	2	x	f	50	64	jap	-	all	NAmer	1968	CC	292	n	bl	n	n	0	all/unsp	nev any	st
HINDS	8	x	f	50	64	haw	-	all	NAmer	1968	CC	292	n	bl	n	n	0	all/unsp	nev any	st
HITOSU	50	x	f	50	59	all	-	all	As:Jap	1960	CC	216	n	bl	y	n	0	all/unsp	nev any	st
KIHARA	1	x	m	50	69	jap	-	all	As:Jap	1991	CC	440	n	bl	n	n	0	all/unsp	nev any	st
KREUZE	61	x	m	55	69	all	-	all	Eu:Ger	1990	CC	2260	n	bl	n	n	3	cig+/-ot	nev any	ot
KREUZE	62		f	55	69	all	-	all	Eu:Ger	1990	CC	2260	n	bl	n	n	3	cig+/-ot	nev any	ot
KUBIK	5	x	m	55	64	all	4	all	Eu:est	1965	pr	108	n	bl	n	n	0	cig+/-ot	nev any	st
LEVIN	11	x	m	55	64	all	-	all	NAmer	1938	CC	475	n	bl	n	n	0	cig+/-ot	nev any	st
NAM	13	x	m	55	64	all	-	all	NAmer	1986	CC	1199	n	bl	y	n	0	cig+/-ot	nev cigs	st
NAM	45	x	f	55	64	all	-	all	NAmer	1986	CC	1199	n	bl	y	n	0	cig+/-ot	nev cigs	st
PRESCO	40	x	m	50	64	all	0	all	Eu:Sca	1964	pr	867	n	bl	n	n	0	all/unsp	nev any	st
PRESCO	39	x	f	50	64	all	0	all	Eu:Sca	1964	pr	867	n	bl	n	n	0	all/unsp	nev any	st
STOCKS	36	x	m	55	64	all	-	all	Eu:UK	1952	CC	2932	n	V	y	n	0	cig+/-ot	nev any	st
VUTUC	17	x	m	51	60	all	-	all	Eu:wst	1976	CC	1877	n	bl	n	n	0	cig+/-ot	nev cigs	st
WUWILL	2	x	f	50	54	all	-	all	As:Chi	1985	CC	965	n	ot	n	n	0	cig+/-ot	nev cigs	st
WUWILL	3	x	f	55	59	all	-	all	As:Chi	1985	CC	965	n	ot	n	n	0	cig+/-ot	nev cigs	st
WUWILL	4	x	f	60	64	all	-	all	As:Chi	1985	CC	965	n	ot	n	n	0	cig+/-ot	nev cigs	st
WUWILL	5	x	f	65	69	all	-	all	As:Chi	1985	CC	965	n	ot	n	n	0	cig+/-ot	nev cigs	st

Cigarette type is all/unspec for all RRs
except for the following:

REF	NRR	CIGTYPE
DEAN3	122	MC only

Table 1A8 - 2

IESLC - Meta-analysis of Ever Smoking, Cigarettes (or Any Product if Cigarettes not available), Age 50-70
All LC types
Most adjusted

REF	NRR	SEX	AD	Number Exposed Case	Cont	Non-exposed Case	Cont	RR	95.00%CI
*ANDERS	3	f	0	297	96164	46	195158	13.10	(9.61- 17.87)
BENHAM	69	m	0	421	538	5	185	28.95	(11.80- 71.03)
BENHAM	70	m	0	377	347	12	140	12.68	(6.91- 23.27)
Subtotal BENHAM								16.43	(9.94- 27.17)
BUFFLE	59	f	0	90	69	8	38	6.20	(2.72- 14.13)
*CPSI	171	m	1	-	-	-	-	10.38	(7.60- 14.18)
*CPSI	113	f	0	260	682162	144	1366561	3.62	(2.95- 4.43)
Subtotal CPSI								4.96	(4.18- 5.88)
DAMBER	23	m	0	170	119	8	57	10.18	(4.68- 22.12)
DEAN3	45	m	0	209	454	7	96	6.31	(2.88- 13.84)
DEAN3	122	f	0	39	283	9	310	4.75	(2.26- 9.97)
Subtotal DEAN3								5.43	(3.17- 9.31)
DORANT	10	c	0	470	2033	14	1090	18.00	(10.52- 30.78)
*DORN	412	m	1	-	-	-	-	9.23	(6.53- 13.03)
*HAMMON	116	m	1	-	-	-	-	8.93	(5.33- 14.96)
HINDS	5	f	0	6	12	10	67	3.35	(1.03- 10.95)
HINDS	2	f	0	23	149	24	589	3.79	(2.08- 6.90)
HINDS	8	f	0	52	96	7	149	11.53	(5.03- 26.43)
Subtotal HINDS								5.16	(3.29- 8.09)
HITOSU	50	f	0	7	159	8	478	2.63	(0.94- 7.37)
KIHARA	1	m	0	358	184	24	73	5.92	(3.61- 9.70)
KREUZE	61	m	3	-	-	-	-	20.67	(13.28- 32.16)
KREUZE	62	f	3	-	-	-	-	4.05	(2.81- 5.86)
Subtotal KREUZE								7.88	(5.94- 10.46)
*KUBIK	5	m	0	49	3456	1	1851	26.24	(3.63- 189.90)
LEVIN	11	m	0	182	304	16	186	6.96	(4.04- 11.97)
NAM	13	m	0	191	173	5	42	9.27	(3.59- 23.97)
NAM	45	f	0	75	85	10	69	6.09	(2.93- 12.66)
Subtotal NAM								7.12	(3.99- 12.72)
*PRESCO	40	m	0	261	110947	3	12426	9.74	(3.12- 30.40)
*PRESCO	39	f	0	77	53905	5	19328	5.52	(2.24- 13.64)
Subtotal PRESCO								6.88	(3.39- 13.97)
STOCKS	36	m	0	991	1678	21	153	4.30	(2.71- 6.84)
VUTUC	17	m	0	229	432	8	138	9.14	(4.40- 18.98)
WUWILL	2	f	0	121	69	82	127	2.72	(1.81- 4.07)
WUWILL	3	f	0	143	104	89	137	2.12	(1.47- 3.06)
WUWILL	4	f	0	125	75	59	116	3.28	(2.14- 5.01)
WUWILL	5	f	0	82	64	55	97	2.26	(1.42- 3.60)
Subtotal WUWILL								2.53	(2.06- 3.11)
Partial Totals				5305	954061	680	1599661		

*prospective study

REF	NRR	SEX	AD	Ys	Ws	Qs	Ps
*ANDERS	3	f	0	2.57	39.86	25.58	0.0000
BENHAM	69	m	0	3.37	4.77	12.12	0.0000
BENHAM	70	m	0	2.54	10.42	6.14	0.0000
Subtotal BENHAM				2.80	15.19	18.26	
BUFFLE	59	f	0	1.82	5.65	0.02	0.0000
*CPSI	171	m	1	2.34	39.50	12.76	0.0000
*CPSI	113	f	0	1.29	92.69	21.89	0.0000
Subtotal CPSI				1.60	132.19	34.65	
DAMBER	23	m	0	2.32	6.38	1.92	0.0000
DEAN3	45	m	0	1.84	6.24	0.03	0.0000
DEAN3	122	f	0	1.56	6.97	0.32	0.0000
Subtotal DEAN3				1.69	13.21	0.35	
DORANT	10	c	0	2.89	13.34	16.69	0.0000
*DORN	412	m	1	2.22	32.20	6.54	0.0000
*HAMMON	116	m	1	2.19	14.43	2.52	0.0000
HINDS	5	f	0	1.21	2.74	0.87	0.0454
HINDS	2	f	0	1.33	10.69	2.07	0.0000
HINDS	8	f	0	2.44	5.58	2.53	0.0000
Subtotal HINDS				1.64	19.01	5.46	
HITOSU	50	f	0	0.97	3.62	2.34	0.0657
KIHARA	1	m	0	1.78	15.72	0.00	0.0000
KREUZE	61	m	3	3.03	19.64	31.04	0.0000
KREUZE	62	f	3	1.40	28.45	3.96	0.0000
Subtotal KREUZE				2.06	48.09	34.99	
*KUBIK	5	m	0	3.27	0.98	2.19	0.0012
LEVIN	11	m	0	1.94	13.04	0.37	0.0000
NAM	13	m	0	2.23	4.26	0.88	0.0000

Table 1A8 - 2

IESLC - Meta-analysis of Ever Smoking, Cigarettes (or Any Product if Cigarettes not available), Age 50-70
All LC types
Most adjusted

REF	NRR	SEX	AD	Ys	Ws	Qs	Ps
NAM	45	f	0	1.81	7.16	0.01	0.0000
Subtotal NAM				1.96	11.42	0.89	
*PRESCO	40	m	0	2.28	2.97	0.76	0.0001
*PRESCO	39	f	0	1.71	4.70	0.02	0.0002
Subtotal PRESCO				1.93	7.66	0.78	
STOCKS	36	m	0	1.46	17.93	1.75	0.0000
VUTUC	17	m	0	2.21	7.20	1.40	0.0000
WUWILL	2	f	0	1.00	23.35	13.93	0.0000
WUWILL	3	f	0	0.75	28.45	29.71	0.0001
WUWILL	4	f	0	1.19	21.32	7.29	0.0000
WUWILL	5	f	0	0.82	17.76	16.24	0.0006
Subtotal WUWILL				0.93	90.88	67.18	

	N	31
	NS	20
	Wt	508.00
Het	Chi	223.90
Het	df	30
Het	P	***
Fixed	RR	5.88
	RRl	5.39
	RRu	6.41
	P	+++
Random	RR	6.61
	RRl	5.12
	RRu	8.54
	P	+++
Asymm	P	N.S.

Table 1A8 - 3

IESLC - Meta-analysis of Ever Smoking, Cigarettes (or Any Product if Cigarettes not available), Age 50-70
All LC types
Most adjusted

	Sex combined	male	female	Total
N	1	15	15	31
NS	1	14	10	25
Wt	13.34	195.68	298.99	508.00
Het Chi	0.00	37.17	88.54	223.90
Het df	0	14	14	30
Het P	N.S.	***	***	***
Fixed RR	18.00	9.41	4.11	5.88
RRl	10.52	8.18	3.67	5.39
RRu	30.78	10.83	4.61	6.41
P	+++	+++	+++	+++
Random RR	18.00	9.55	4.23	6.61
RRl	10.52	7.44	3.08	5.12
RRu	30.78	12.26	5.82	8.54
P	+++	+++	+++	+++
Between Chi				98.20
Between df				2
Between P				***
Btwn(F) P				***
Btwn(R) P				***

Table 1A8 - 4

IESLC - Meta-analysis of Ever Smoking, Cigarettes (or Any Product if Cigarettes not available), Age 50-70
All LC types
Least adjusted

REF	NRR	X	SEX	AGEL	AGEH	RACE	YF	LC TYPE	LOC	START	ST	NLC	R	VB	P	H	AD	PRODUCT	DENOM	De
ANDERS	3		f	55	69	all	0	all	NAmer	1986	pr	343	n	bl	n	n	0	cig+/-ot	nev cigs	st
BENHAM	69		m	50	59	all	-	not mix	Eu:wst	1976	CC	1625	n	bl	n	y	0	cig only	nev any	st
BENHAM	70		m	60	69	all	-	not mix	Eu:wst	1976	CC	1625	n	bl	n	y	0	cig only	nev any	st
BUFFLE	59		f	60	69	w-hi	-	all	NAmer	1976	CC	943	n	bl	y	n	0	cig+/-ot	nev cigs	st
CPSI	171		m	55	69	all	6	all	NAmer	1959	pr	5138	n	bl	n	n	1	cig+/-ot	nev any	ot
CPSI	113		f	55	64	wh	0	all	NAmer	1959	pr	5138	n	bl	n	n	0	cig only	nev any	st
DAMBER	23		m	60	69	all	-	all	Eu:Sca	1972	CC	579	n	bl	y	n	0	all/unsp	nev any	st
DEAN3	45		m	55	64	all	-	all	Eu:UK	1969	CC	766	n	V	y	n	0	all/unsp	nev any	st
DEAN3	122		f	55	64	all	-	all	Eu:UK	1969	CC	766	n	V	y	n	0	cig only	nev any	st
DORANT	10		c	55	69	all	0	all	Eu:wst	1986	ot	550	n	bl	n	y	0	all/unsp	nev any	st
DORN	412		m	55	64	wh	5	all	NAmer	1954	pr	5097	n	bl	n	n	1	cig+/-ot	nev any	ot
HAMMON	128	x	m	50	69	wh	0	all	NAmer	1952	pr	448	n	bl	n	n	0	cig+/-ot	nev any	st
HINDS	5		f	50	64	ch	-	all	NAmer	1968	CC	292	n	bl	n	n	0	all/unsp	nev any	st
HINDS	2		f	50	64	jap	-	all	NAmer	1968	CC	292	n	bl	n	n	0	all/unsp	nev any	st
HINDS	8		f	50	64	haw	-	all	NAmer	1968	CC	292	n	bl	n	n	0	all/unsp	nev any	st
HITOSU	50		f	50	59	all	-	all	As:Jap	1960	CC	216	n	bl	y	n	0	all/unsp	nev any	st
KIHARA	1		m	50	69	jap	-	all	As:Jap	1991	CC	440	n	bl	n	n	0	all/unsp	nev any	st
KREUZE	61		m	55	69	all	-	all	Eu:Ger	1990	CC	2260	n	bl	n	n	3	cig+/-ot	nev any	ot
KREUZE	62		f	55	69	all	-	all	Eu:Ger	1990	CC	2260	n	bl	n	n	3	cig+/-ot	nev any	ot
KUBIK	5		m	55	64	all	4	all	Eu:est	1965	pr	108	n	bl	n	n	0	cig+/-ot	nev any	st
LEVIN	11		m	55	64	all	-	all	NAmer	1938	CC	475	n	bl	n	n	0	cig+/-ot	nev any	st
NAM	13		m	55	64	all	-	all	NAmer	1986	CC	1199	n	bl	y	n	0	cig+/-ot	nev cigs	st
NAM	45		f	55	64	all	-	all	NAmer	1986	CC	1199	n	bl	y	n	0	cig+/-ot	nev cigs	st
PRESCO	40		m	50	64	all	0	all	Eu:Sca	1964	pr	867	n	bl	n	n	0	all/unsp	nev any	st
PRESCO	39		f	50	64	all	0	all	Eu:Sca	1964	pr	867	n	bl	n	n	0	all/unsp	nev any	st
STOCKS	36		m	55	64	all	-	all	Eu:UK	1952	CC	2932	n	V	y	n	0	cig+/-ot	nev any	st
VUTUC	17		m	51	60	all	-	all	Eu:wst	1976	CC	1877	n	bl	n	n	0	cig+/-ot	nev cigs	st
WUWILL	2		f	50	54	all	-	all	As:Chi	1985	CC	965	n	ot	n	n	0	cig+/-ot	nev cigs	st
WUWILL	3		f	55	59	all	-	all	As:Chi	1985	CC	965	n	ot	n	n	0	cig+/-ot	nev cigs	st
WUWILL	4		f	60	64	all	-	all	As:Chi	1985	CC	965	n	ot	n	n	0	cig+/-ot	nev cigs	st
WUWILL	5		f	65	69	all	-	all	As:Chi	1985	CC	965	n	ot	n	n	0	cig+/-ot	nev cigs	st

Cigarette type is all/unspec for all RRs
except for the following:

REF	NRR	CIGTYPE
DEAN3	122	MC only

Table 1A8 - 5

IESLC - Meta-analysis of Ever Smoking, Cigarettes (or Any Product if Cigarettes not available), Age 50-70
All LC types
Least adjusted

REF	NRR	SEX	AD	Number Exposed Case	Cont	Non-exposed Case	Cont	RR	95.00%CI
*ANDERS	3	f	0	297	96164	46	195158	13.10	(9.61- 17.87)
BENHAM	69	m	0	421	538	5	185	28.95	(11.80- 71.03)
BENHAM	70	m	0	377	347	12	140	12.68	(6.91- 23.27)
Subtotal BENHAM								16.43	(9.94- 27.17)
BUFFLE	59	f	0	90	69	8	38	6.20	(2.72- 14.13)
*CPSI	171	m	1	-	-	-	-	10.38	(7.60- 14.18)
*CPSI	113	f	0	260	682162	144	1366561	3.62	(2.95- 4.43)
Subtotal CPSI								4.96	(4.18- 5.88)
DAMBER	23	m	0	170	119	8	57	10.18	(4.68- 22.12)
DEAN3	45	m	0	209	454	7	96	6.31	(2.88- 13.84)
DEAN3	122	f	0	39	283	9	310	4.75	(2.26- 9.97)
Subtotal DEAN3								5.43	(3.17- 9.31)
DORANT	10	c	0	470	2033	14	1090	18.00	(10.52- 30.78)
*DORN	412	m	1	-	-	-	-	9.23	(6.53- 13.03)
*HAMMON	128	m	0	397	382338	15	115884	8.02	(4.79- 13.43)
HINDS	5	f	0	6	12	10	67	3.35	(1.03- 10.95)
HINDS	2	f	0	23	149	24	589	3.79	(2.08- 6.90)
HINDS	8	f	0	52	96	7	149	11.53	(5.03- 26.43)
Subtotal HINDS								5.16	(3.29- 8.09)
HITOSU	50	f	0	7	159	8	478	2.63	(0.94- 7.37)
KIHARA	1	m	0	358	184	24	73	5.92	(3.61- 9.70)
KREUZE	61	m	3	-	-	-	-	20.67	(13.28- 32.16)
KREUZE	62	f	3	-	-	-	-	4.05	(2.81- 5.86)
Subtotal KREUZE								7.88	(5.94- 10.46)
*KUBIK	5	m	0	49	3456	1	1851	26.24	(3.63- 189.90)
LEVIN	11	m	0	182	304	16	186	6.96	(4.04- 11.97)
NAM	13	m	0	191	173	5	42	9.27	(3.59- 23.97)
NAM	45	f	0	75	85	10	69	6.09	(2.93- 12.66)
Subtotal NAM								7.12	(3.99- 12.72)
*PRESCO	40	m	0	261	110947	3	12426	9.74	(3.12- 30.40)
*PRESCO	39	f	0	77	53905	5	19328	5.52	(2.24- 13.64)
Subtotal PRESCO								6.88	(3.39- 13.97)
STOCKS	36	m	0	991	1678	21	153	4.30	(2.71- 6.84)
VUTUC	17	m	0	229	432	8	138	9.14	(4.40- 18.98)
WUWILL	2	f	0	121	69	82	127	2.72	(1.81- 4.07)
WUWILL	3	f	0	143	104	89	137	2.12	(1.47- 3.06)
WUWILL	4	f	0	125	75	59	116	3.28	(2.14- 5.01)
WUWILL	5	f	0	82	64	55	97	2.26	(1.42- 3.60)
Subtotal WUWILL								2.53	(2.06- 3.11)
Partial Totals				5702	1336399	695	1715545		

*prospective study

REF	NRR	SEX	AD	Ys	Ws	Qs	Ps
*ANDERS	3	f	0	2.57	39.86	25.78	0.0000
BENHAM	69	m	0	3.37	4.77	12.17	0.0000
BENHAM	70	m	0	2.54	10.42	6.19	0.0000
Subtotal BENHAM				2.80	15.19	18.36	
BUFFLE	59	f	0	1.82	5.65	0.02	0.0000
*CPSI	171	m	1	2.34	39.50	12.89	0.0000
*CPSI	113	f	0	1.29	92.69	21.62	0.0000
Subtotal CPSI				1.60	132.19	34.51	
DAMBER	23	m	0	2.32	6.38	1.94	0.0000
DEAN3	45	m	0	1.84	6.24	0.03	0.0000
DEAN3	122	f	0	1.56	6.97	0.31	0.0000
Subtotal DEAN3				1.69	13.21	0.34	
DORANT	10	c	0	2.89	13.34	16.78	0.0000
*DORN	412	m	1	2.22	32.20	6.63	0.0000
*HAMMON	128	m	0	2.08	14.46	1.42	0.0000
HINDS	5	f	0	1.21	2.74	0.86	0.0454
HINDS	2	f	0	1.33	10.69	2.04	0.0000
HINDS	8	f	0	2.44	5.58	2.55	0.0000
Subtotal HINDS				1.64	19.01	5.45	
HITOSU	50	f	0	0.97	3.62	2.33	0.0657
KIHARA	1	m	0	1.78	15.72	0.00	0.0000
KREUZE	61	m	3	3.03	19.64	31.19	0.0000
KREUZE	62	f	3	1.40	28.45	3.89	0.0000
Subtotal KREUZE				2.06	48.09	35.08	
*KUBIK	5	m	0	3.27	0.98	2.20	0.0012
LEVIN	11	m	0	1.94	13.04	0.38	0.0000
NAM	13	m	0	2.23	4.26	0.90	0.0000

Table 1A8 - 5

IESLC - Meta-analysis of Ever Smoking, Cigarettes (or Any Product if Cigarettes not available), Age 50-70
All LC types
Least adjusted

REF	NRR	SEX	AD	Ys	Ws	Qs	Ps
NAM	45	f	0	1.81	7.16	0.01	0.0000
Subtotal NAM				1.96	11.42	0.91	
*PRESCO	40	m	0	2.28	2.97	0.77	0.0001
*PRESCO	39	f	0	1.71	4.70	0.02	0.0002
Subtotal PRESCO				1.93	7.66	0.78	
STOCKS	36	m	0	1.46	17.93	1.72	0.0000
VUTUC	17	m	0	2.21	7.20	1.42	0.0000
WUWILL	2	f	0	1.00	23.35	13.82	0.0000
WUWILL	3	f	0	0.75	28.45	29.53	0.0001
WUWILL	4	f	0	1.19	21.32	7.21	0.0000
WUWILL	5	f	0	0.82	17.76	16.14	0.0006
Subtotal WUWILL				0.93	90.88	66.72	

	N	31
	NS	20
	Wt	508.03
Het	Chi	222.78
Het	df	30
Het	P	***
Fixed	RR	5.86
	RRl	5.37
	RRu	6.40
	P	+++
Random	RR	6.59
	RRl	5.10
	RRu	8.50
	P	+++
Asymm	P	N.S.

Table 1A8 - 6

IESLC - Meta-analysis of Ever Smoking, Cigarettes (or Any Product if Cigarettes not available), Age 50-70
All LC types
Least adjusted

	combined	Sex male	female	Total
N	1	15	15	31
NS	1	14	10	25
Wt	13.34	195.71	298.99	508.03
Het Chi	0.00	37.49	88.54	222.78
Het df	0	14	14	30
Het P	N.S.	***	***	***
Fixed RR	18.00	9.34	4.11	5.86
RRl	10.52	8.12	3.67	5.37
RRu	30.78	10.74	4.61	6.40
P	+++	+++	+++	+++
Random RR	18.00	9.47	4.23	6.59
RRl	10.52	7.37	3.08	5.10
RRu	30.78	12.17	5.82	8.50
P	+++	+++	+++	+++
Between Chi				96.75
Between df				2
Between P				***
Btwn(F) P				***
Btwn(R) P				***

Table 1A8 - 7

IESLC - Meta-analysis of Ever Smoking, Cigarettes (or Any Product if Cigarettes not available), Age 50-70
All LC types
Excluded studies (and stage at which they were excluded)

```
1      BOUCHA  BUELL LAURIL MZILEN

2      BYERS2 ENSTRO HIRAY2 KANELL   LIAW LICKIN  MRFIT MURATA NOTANI RESTRE RIMING SAARIK  SEGI2 SHIMIZ  SITAS SOBUE2
        TANG2   WALD WARSIN WATSON    WU2 WYNDE5 WYNDE8 WYNDER

3      ABELIN ABRAHA  AGUDO  AKIBA ALDERS AMANDU   AMES ARCHER ARMADA AUSTIN AUVINE AXELSO AXELSS   BAND BARBON BECHER
       BENSHL BERRIN   BEST BLOHMK  BLOT1  BLOT2  BLOT3  BLOT4 BOFFET BOUCOT BRESLO  BRETT BROCKM  BROSS BROWN1 BYERS1
       CARPEN CASCO2 CASCOR CEDERL   CHAN  CHANG CHATZI   CHEN  CHEN2  CHEN3 CHIAZZ   CHOI   CHOW  CHYOU COMSTO COOKSO
       CORREA  CPSII  DARBY DAVEYS   DEAN  DEAN2 DEKLER DESTE2 DESTEF DOCKER   DOLL  DOLL2 DORGAN DOSEME DROSTE     DU
         DUNN EBELIN ENGELA  ESAKI    FAN    GAO   GAO2 GARCIA GARDIN GARSHI   GENG    GER GILLIS GODLEY GOLLED GOODMA
       GRAHAM GREGOR  GSELL HAENSZ HAMMO2 HANSEN HEGMAN   HEIN HENNEK HIRAOK HIRAYA   HOLE HOROWI HORWIT     HU    HU2
        HUANG HUMBLE ISHIMA   JAHN   JAIN  JARUP JARVHO JEDRYC  JIANG   JOLY  JONES JUSSAW KAISE2 KAISER KATSOU KAUFMA
       KELLER KHUDER KINLEN  KJUUS  KNEKT     KO KOHLME    KOO KOULUM KREYBE  LAMTH  LAMWK LAMWK2  LANGE LAUSSM    LEI
       LEMARC LETOUR LIDDEL    LIU   LIU2   LIU3   LIU4   LIU5 LOMBA2 LOMBAR  LUBIN LUBIN2    LUO MACLEN MAGNUS  MARSH
       MARSH2 MARTIS MASTRA  MATOS MATSUD MCCONN MCDUFF MCLAUG MIGRAN MILLER  MILLS  MOLLO MRFITR NOTAN2    NOU ODRISC
        ORMOS  OSANN OSANN2 PARKIN PASTOR PAWLEG  PERNU PERSH2 PERSHA   PETO PEZZO2 PEZZOT   PIKE PISANI POFFIJ POLEDN
         QIAO  QIAO2 RACHTA RADZIK RANDIG    REN  RONCO  ROOTS ROTHSC SANKAR SCHWA2 SCHWAR   SEGI   SEOW   SHAW SIEMIA
       SIMARA SIMONA  SOBUE SPEIZE  SPITZ STASZE STAYNE STOCKW STUCKE    SUN SUZUK2 SUZUKI SVENSS   TANG    TAO TENKAN
       TIZZAN TOKARS TOUSEY TSUGAN TULINI TVERDA  ULMER VANDER VEIERO  WAKAI   WANG  WANG2  WANG3  WANG4 WICKLU  WIGLE
       WILKIN     WU WUNSCH WYNDE2 WYNDE3 WYNDE6 WYNDE7 XIANGZ     XU    XU2    XU3    XU4 YAMAGU   YONG   YUAN  ZHANG
        ZHENG   ZHOU

4      BROWN2 SADOWS

9      WYNDE4
```

Table 1A8 - 8
Potentially overlapping studies

REF	REFGP	PRINC	OVERLAP/LINK
VUTUC	LUBIN2	2	Subset of Lubin2
BENHAM	LUBIN2	2	Subset of Lubin2
PRESCO	PRESCO	2	PRESCO-combined
CPSI	CPSI	1	CPSI overall

Table 1A8 - 9
Most adjusted - insufficient data for metaanalysis

REF	NRR	SEX	AGEL	AGEH	RACE	YF	LC TYPE	LOC	START	ST	NLC	R	VB	P	H	AD	PRODUCT	DENOM	De
BROWN2	55	m	60	69	wh	-	all	NAmer	1984	CC	14596	n	bl	n	y	1	cig+/-ot	nev cigs	or
BROWN2	52	f	60	69	wh	-	all	NAmer	1984	CC	14596	n	bl	n	y	1	cig+/-ot	nev cigs	or
SADOWS	12	m	50	59	wh	-	all	NAmer	1938	CC	477	n	bl	n	n	0	cig only	nev any	st
SADOWS	16	m	60	69	wh	-	all	NAmer	1938	CC	477	n	bl	n	n	0	cig only	nev any	st

REF	NRR	RR	SIG	RRDATA comment
BROWN2	55	10.30		0
BROWN2	52	14.30		0
SADOWS	12	3.56		0
SADOWS	16	18.52		0

Table 1A9 -

IESLC - Meta-analysis of Ever Smoking, Cigarettes (or Any Product if Cigarettes not available), Age 65+
All LC types

This analysis is restricted to results for:
1) Non-dose-response data
2) Ever smokers
3) Age 65+
4) Results complete enough for use in metaanalysis

Within each study, results are then selected (in the following order of preference, within each sex) for:
5) PRODUCT: cigarettes regardless of other products, cigarettes only, all/unspec
6) CIGTYPE: all/unspecified, MC regardless of HR, MC only
7) DENOM: never smoked anything, never smoked cigarettes, (never +1 = +long term ex, +2 = +amount unknown,
+3 = never cigs+long term ex)
8) Followup period (YF, prospective studies): whole study (coded as 0) or longest available
9) LCtype: all or nearest available, at least Squamous and Adeno. (q = squamous, s = small,
l = large, a = adeno, mix = mixed, alv = alveolar)
10) Race: all or nearest available, otherwise by race (wh or w = white, bl or b = black, hi = hispanic
ch = chinese, jap = japanese, haw = hawaiian, w+o = white + oriental, sca = scandinavian, as = asian)
11) For overlapping studies: principal rather than subsidiary studies
Finally by Age: whole study (actual age shown) if available, otherwise by widest available age group
and then for single sex results (m, f) in preference to combined sex results (c).

Results adjusted (AD) for the most potential confounders are then chosen in Sections -1 to -3
(and those which actually differ from the adjusted results in Table 1A2 - 1 are marked 'x' in Section -1)
and results adjusted for the least confounders in Sections -4 to -6. (Those least adjusted results which
actually differ from the most adjusted as marked 'x' in column X in Section -4)
(Results adjusted for an unknown number of confounder(s) are coded as 20.)

Section -7 shows excluded studies, together with the stage (as above) at which no qualifying
results were found.

Section -8 lists the potentially overlapping studies which have been included (1=principal, 2=subsidiary).

Section -9 lists any results which would have been included in preference except that they had data not complete
enough for use in meta-analysis, with their significance (yes/no), if known, and any further comment as entered
on the database.

In addition to those mentioned above, the following fields, levels and abbreviations are used:
* or nk = not known, n = no, y = yes, ot = other
nev = never
all/unspec = all or unspecified, cig+/-ot = cigarettes irrespective of other products (cigar, pipe etc)
MC = manufactured cigarettes, HR = hand-rolled cigarettes
REF: 6-character study reference
NRR: number of the RR on the database within the study
ST : study type (CC = case control, pr or prosp = prospective)
NLC: number of lung cancer cases in whole study
R : risky occupational population (n = no, m = mining, o = other risky)
VB : national cigarette type (V = at least 75% Virginia, bl = at least 75% blended, ot = other)
P : any proxy use
H : full histological confirmation
De : derivation of RR/CI (or = original, st = standard method, ot = other method of estimation)

Table 1A9 - 1

IESLC - Meta-analysis of Ever Smoking, Cigarettes (or Any Product if Cigarettes not available), Age 65+
All LC types
Most adjusted

REF	NRR	1A2	SEX	AGEL	AGEH	RACE	YF	LC TYPE	LOC	START	ST	NLC	R	VB	P	H	AD	PRODUCT	DENOM	De
BENHAM	71	x	m	70	99	all	-	not mix	Eu:wst	1976	CC	1625	n	bl	n	y	0	cig only	nev any	st
BUFFLE	60	x	f	70	79	w-hi	-	all	NAmer	1976	CC	943	n	bl	y	n	0	cig+/-ot	nev cigs	st
COMSTO	59	x	c	65	99	all	-	all	NAmer	1975	ot	258	n	bl	n	n	0	cig+/-ot	nev cigs	st
CPSI	179	x	m	70	84	all	6	all	NAmer	1959	pr	5138	n	bl	n	n	1	cig+/-ot	nev any	ot
CPSI	114	x	f	65	74	wh	0	all	NAmer	1959	pr	5138	n	bl	n	n	0	cig only	nev any	st
CPSI	115	x	f	75	84	wh	0	all	NAmer	1959	pr	5138	n	bl	n	n	0	cig only	nev any	st
DAMBER	24	x	m	70	99	all	-	all	Eu:Sca	1972	CC	579	n	bl	y	n	0	all/unsp	nev any	st
DEAN3	46	x	m	65	99	all	-	all	Eu:UK	1969	CC	766	n	V	y	n	0	all/unsp	nev any	st
DEAN3	123	x	f	65	99	all	-	all	Eu:UK	1969	CC	766	n	V	y	n	0	cig only	nev any	st
DORN	195	x	m	65	84	wh	8	all	NAmer	1954	pr	5097	n	bl	n	n	1	all/unsp	nev any	ot
GARSHI	9	x	m	65	82	all	-	all	NAmer	1981	CC	1081	o	bl	y	n	0	all/unsp	nev any	st
GODLEY	2	x	m	65	79	all	-	all	NAmer	1966	CC	1986	n	bl	y	n	1	cig+/-ot	nev cigs	ot
GODLEY	4	x	f	65	79	all	-	all	NAmer	1966	CC	1986	n	bl	y	n	1	cig+/-ot	nev cigs	ot
HAMMON	13	x	m	65	69	wh	0	all	NAmer	1952	pr	448	n	bl	n	n	0	cig+/-ot	nev any	st
HINDS	6	x	f	65	99	ch	-	all	NAmer	1968	CC	292	n	bl	n	n	0	all/unsp	nev any	st
HINDS	3	x	f	65	99	jap	-	all	NAmer	1968	CC	292	n	bl	n	n	0	all/unsp	nev any	st
HINDS	9	x	f	65	99	haw	-	all	NAmer	1968	CC	292	n	bl	n	n	0	all/unsp	nev any	st
HUMBLE	56	x	m	65	84	w-hi	-	not alv	NAmer	1980	CC	521	n	bl	y	n	0	cig+/-ot	nev cigs	st
HUMBLE	60	x	m	65	84	hi	-	not alv	NAmer	1980	CC	521	n	bl	y	n	0	cig+/-ot	nev cigs	st
HUMBLE	58	x	f	65	84	w-hi	-	not alv	NAmer	1980	CC	521	n	bl	y	n	0	cig+/-ot	nev cigs	st
HUMBLE	62	x	f	65	84	hi	-	not alv	NAmer	1980	CC	521	n	bl	y	n	0	cig+/-ot	nev cigs	st
KAISE2	40	x	m	65	74	all	9	all	NAmer	1979	pr	318	n	bl	n	n	0	cig only	nev any	st
KAISE2	48	x	m	75	99	all	9	all	NAmer	1979	pr	318	n	bl	n	n	0	cig only	nev any	st
KAISE2	16	x	f	65	74	all	9	all	NAmer	1979	pr	318	n	bl	n	n	0	cig only	nev any	st
KAISE2	24	x	f	75	99	all	9	all	NAmer	1979	pr	318	n	bl	n	n	0	cig only	nev any	st
LEVIN	12	x	m	65	74	all	-	all	NAmer	1938	CC	475	n	bl	n	n	0	cig+/-ot	nev any	st
LEVIN	13	x	m	75	99	all	-	all	NAmer	1938	CC	475	n	bl	n	n	0	cig+/-ot	nev any	st
LIU4	5	x	m	70	99	all	-	all	As:Chi	1986	CC	1000-00	n	ot	y	n	2	all/unsp	nev any	ot
LIU4	6	x	f	70	99	all	-	all	As:Chi	1986	CC	1000-00	n	ot	y	n	2	all/unsp	nev any	ot
NAM	21	x	m	65	79	all	-	all	NAmer	1986	CC	1199	n	bl	y	n	0	cig+/-ot	nev cigs	st
NAM	29	x	m	80	99	all	-	all	NAmer	1986	CC	1199	n	bl	y	n	0	cig+/-ot	nev cigs	st
NAM	53	x	f	65	79	all	-	all	NAmer	1986	CC	1199	n	bl	y	n	0	cig+/-ot	nev cigs	st
NAM	61	x	f	80	99	all	-	all	NAmer	1986	CC	1199	n	bl	y	n	0	cig+/-ot	nev cigs	st
PRESCO	42	x	m	65	99	all	0	all	Eu:Sca	1964	pr	867	n	bl	n	n	0	all/unsp	nev any	st
PRESCO	41	x	f	65	99	all	0	all	Eu:Sca	1964	pr	867	n	bl	n	n	0	all/unsp	nev any	st
STOCKS	38	x	m	65	74	all	-	all	Eu:UK	1952	CC	2932	n	V	y	n	0	cig+/-ot	nev any	st
WUWILL	5	x	f	65	69	all	-	all	As:Chi	1985	CC	965	n	ot	n	n	0	cig+/-ot	nev cigs	st

Cigarette type is all/unspec for all RRs
except for the following:

REF	NRR	CIGTYPE
DEAN3	123	MC only

Table 1A9 - 2

IESLC - Meta-analysis of Ever Smoking, Cigarettes (or Any Product if Cigarettes not available), Age 65+
All LC types
Most adjusted

REF	NRR	SEX	AD	Number Exposed Case	Number Exposed Cont	Non-exposed Case	Non-exposed Cont	RR	95.00%CI
BENHAM	71	m	0	169	203	7	95	11.30	(5.11- 25.00)
BUFFLE	60	f	0	41	18	3	30	22.78	(6.15- 84.40)
COMSTO	59	c	0	46	58	6	44	5.82	(2.28- 14.84)
*CPSI	179	m	1	-	-	-	-	8.67	(5.54- 13.57)
*CPSI	114	f	0	153	211823	178	957682	3.89	(3.13- 4.82)
*CPSI	115	f	0	31	39527	163	392909	1.89	(1.29- 2.78)
Subtotal CPSI								3.78	(3.18- 4.50)
DAMBER	24	m	0	261	160	23	123	8.72	(5.36- 14.19)
DEAN3	46	m	0	285	396	16	82	3.69	(2.11- 6.44)
DEAN3	123	f	0	41	196	27	574	4.45	(2.66- 7.42)
Subtotal DEAN3								4.08	(2.80- 5.95)
*DORN	195	m	1	-	-	-	-	5.84	(4.42- 7.71)
GARSHI	9	m	0	771	1130	32	294	6.27	(4.30- 9.13)
GODLEY	2	m	1	-	-	-	-	6.17	(4.88- 7.79)
GODLEY	4	f	1	-	-	-	-	5.21	(3.65- 7.43)
Subtotal GODLEY								5.86	(4.82- 7.13)
*HAMMON	13	m	0	70	40544	9	21892	4.20	(2.10- 8.41)
HINDS	6	f	0	4	8	24	58	1.21	(0.33- 4.39)
HINDS	3	f	0	26	29	38	326	7.69	(4.11- 14.40)
HINDS	9	f	0	20	29	9	92	7.05	(2.89- 17.18)
Subtotal HINDS								5.83	(3.62- 9.39)
HUMBLE	56	m	0	128	157	6	60	8.15	(3.41- 19.48)
HUMBLE	60	m	0	55	58	2	21	9.96	(2.23- 44.47)
HUMBLE	58	f	0	52	33	11	52	7.45	(3.40- 16.30)
HUMBLE	62	f	0	19	11	4	34	14.68	(4.10- 52.52)
Subtotal HUMBLE								8.80	(5.34- 14.49)
*KAISE2	40	m	0	25	11767	5	11466	4.87	(1.87- 12.72)
*KAISE2	48	m	0	14	3401	4	4486	4.62	(1.52- 14.01)
*KAISE2	16	f	0	26	13883	2	24159	22.62	(5.37- 95.30)
*KAISE2	24	f	0	9	3362	3	12285	10.96	(2.97- 40.47)
Subtotal KAISE2								7.24	(4.05- 12.94)
LEVIN	12	m	0	72	151	13	168	6.16	(3.28- 11.57)
LEVIN	13	m	0	7	26	1	86	23.15	(2.72- 196.93)
Subtotal LEVIN								6.85	(3.74- 12.54)
LIU4	5	m	2	-	-	-	-	2.47	(2.34- 2.61)
LIU4	6	f	2	-	-	-	-	2.50	(2.33- 2.68)
Subtotal LIU4								2.48	(2.38- 2.59)
NAM	21	m	0	296	311	18	130	6.87	(4.09- 11.54)
NAM	29	m	0	67	143	6	172	13.43	(5.66- 31.87)
NAM	53	f	0	142	199	18	251	9.95	(5.89- 16.81)
NAM	61	f	0	35	83	22	472	9.05	(5.06- 16.19)
Subtotal NAM								8.93	(6.66- 11.97)
*PRESCO	42	m	0	378	57442	3	6229	13.66	(4.39- 42.54)
*PRESCO	41	f	0	105	34709	9	19748	6.64	(3.36- 13.11)
Subtotal PRESCO								8.03	(4.48- 14.40)
STOCKS	38	m	0	550	969	18	173	5.46	(3.32- 8.96)
WUWILL	5	f	0	82	64	55	97	2.26	(1.42- 3.60)
Partial Totals				3980	420890	735	1454290		

*prospective study

REF	NRR	SEX	AD	Ys	Ws	Qs	Ps
BENHAM	71	m	0	2.42	6.09	11.27	0.0000
BUFFLE	60	f	0	3.13	2.24	9.52	0.0000
COMSTO	59	c	0	1.76	4.38	2.12	0.0002
*CPSI	179	m	1	2.16	19.15	22.98	0.0000
*CPSI	114	f	0	1.36	82.32	7.08	0.0000
*CPSI	115	f	0	0.64	26.07	4.76	0.0011
Subtotal CPSI				1.33	127.53	34.82	
DAMBER	24	m	0	2.17	16.21	19.68	0.0000
DEAN3	46	m	0	1.31	12.39	0.72	0.0000
DEAN3	123	f	0	1.49	14.65	2.68	0.0000
Subtotal DEAN3				1.41	27.03	3.40	
*DORN	195	m	1	1.76	49.64	24.36	0.0000
GARSHI	9	m	0	1.84	27.15	16.15	0.0000
GODLEY	2	m	1	1.82	70.25	40.09	0.0000
GODLEY	4	f	1	1.65	30.41	10.46	0.0000
Subtotal GODLEY				1.77	100.66	50.55	
*HAMMON	13	m	0	1.44	7.98	1.10	0.0001
HINDS	6	f	0	0.19	2.30	1.76	0.7739
HINDS	3	f	0	2.04	9.77	9.31	0.0000

Table 1A9 - 2

IESLC - Meta-analysis of Ever Smoking, Cigarettes (or Any Product if Cigarettes not available), Age 65+
All LC types
Most adjusted

REF	NRR	SEX	AD	Ys	Ws	Qs	Ps
HINDS	9	f	0	1.95	4.84	3.83	0.0000
Subtotal HINDS				1.76	16.92	14.90	
HUMBLE	56	m	0	2.10	5.06	5.41	0.0000
HUMBLE	60	m	0	2.30	1.72	2.61	0.0026
HUMBLE	58	f	0	2.01	6.26	5.58	0.0000
HUMBLE	62	f	0	2.69	2.36	6.22	0.0000
Subtotal HUMBLE				2.17	15.41	19.83	
*KAISE2	40	m	0	1.58	4.17	1.12	0.0012
*KAISE2	48	m	0	1.53	3.12	0.67	0.0069
*KAISE2	16	f	0	3.12	1.86	7.84	0.0000
*KAISE2	24	f	0	2.39	2.25	3.98	0.0003
Subtotal KAISE2				1.98	11.40	13.63	
LEVIN	12	m	0	1.82	9.67	5.50	0.0000
LEVIN	13	m	0	3.14	0.84	3.62	0.0040
Subtotal LEVIN				1.92	10.51	9.12	
LIU4	5	m	2	0.90	1288.59	33.00	0.0000
LIU4	6	f	2	0.92	784.55	17.17	0.0000
Subtotal LIU4				0.91	2073.14	50.17	
NAM	21	m	0	1.93	14.32	10.68	0.0000
NAM	29	m	0	2.60	5.14	12.09	0.0000
NAM	53	f	0	2.30	13.97	21.24	0.0000
NAM	61	f	0	2.20	11.34	14.69	0.0000
Subtotal NAM				2.19	44.77	58.70	
*PRESCO	42	m	0	2.61	2.98	7.16	0.0000
*PRESCO	41	f	0	1.89	8.29	5.69	0.0000
Subtotal PRESCO				2.08	11.27	12.85	
STOCKS	38	m	0	1.70	15.58	6.23	0.0000
WUWILL	5	f	0	0.82	17.76	1.10	0.0006

	N	37
	NS	19
	Wt	2585.66
Het	Chi	359.49
Het	df	36
Het	P	***
Fixed	RR	2.90
	RRl	2.79
	RRu	3.01
	P	+++
Random	RR	5.73
	RRl	4.78
	RRu	6.86
	P	+++
Asymm	P	***

Table 1A9 - 3

IESLC - Meta-analysis of Ever Smoking, Cigarettes (or Any Product if Cigarettes not available), Age 65+
All LC types
Most adjusted

	combined	Sex male	female	Total
N	1	19	17	37
NS	1	15	11	27
Wt	4.38	1560.03	1021.24	2585.66
Het Chi	0.00	224.38	132.74	359.49
Het df	0	18	16	36
Het P	N.S.	***	***	***
Fixed RR	5.82	2.92	2.86	2.90
RRl	2.28	2.78	2.69	2.79
RRu	14.84	3.07	3.04	3.01
P	+++	+++	+++	+++
Random RR	5.82	6.50	5.34	5.73
RRl	2.28	4.64	3.92	4.78
RRu	14.84	9.10	7.27	6.86
P	+++	+++	+++	+++
Between Chi				2.37
Between df				2
Between P				N.S.
Btwn(F) P				N.S.
Btwn(R) P				N.S.

	Study LIU4 LIU4	others	Total
N	2	35	37
NS	1	18	19
Wt	2073.14	512.52	2585.66
Het Chi	0.07	106.65	359.49
Het df	1	34	36
Het P	N.S.	***	***
Fixed RR	2.48	5.44	2.90
RRl	2.38	4.99	2.79
RRu	2.59	5.93	3.01
P	+++	+++	+++
Random RR	2.48	6.15	5.73
RRl	2.38	5.16	4.78
RRu	2.59	7.33	6.86
P	+++	+++	+++
Between Chi			252.77
Between df			1
Between P			***
Btwn(F) P			***
Btwn(R) P			***

Table 1A9 - 4

IESLC - Meta-analysis of Ever Smoking, Cigarettes (or Any Product if Cigarettes not available), Age 65+
All LC types
Least adjusted

REF	NRR	X	SEX	AGEL	AGEH	RACE	YF	LC TYPE	LOC	START	ST	NLC	R	VB	P	H	AD	PRODUCT	DENOM	De
BENHAM	71		m	70	99	all	-	not mix	Eu:wst	1976	CC	1625	n	bl	n	y	0	cig only	nev any	st
BUFFLE	60		f	70	79	w-hi	-	all	NAmer	1976	CC	943	n	bl	y	n	0	cig+/-ot	nev cigs	st
COMSTO	59		c	65	99	all	-	all	NAmer	1975	ot	258	n	bl	n	n	0	cig+/-ot	nev cigs	st
CPSI	179		m	70	84	all	6	all	NAmer	1959	pr	5138	n	bl	n	n	1	cig+/-ot	nev any	ot
CPSI	114		f	65	74	wh	0	all	NAmer	1959	pr	5138	n	bl	n	n	0	cig only	nev any	st
CPSI	115		f	75	84	wh	0	all	NAmer	1959	pr	5138	n	bl	n	n	0	cig only	nev any	st
DAMBER	24		m	70	99	all	-	all	Eu:Sca	1972	CC	579	n	bl	y	n	0	all/unsp	nev any	st
DEAN3	46		m	65	99	all	-	all	Eu:UK	1969	CC	766	n	V	y	n	0	all/unsp	nev any	st
DEAN3	123		f	65	99	all	-	all	Eu:UK	1969	CC	766	n	V	y	n	0	cig only	nev any	st
DORN	195		m	65	84	wh	8	all	NAmer	1954	pr	5097	n	bl	n	n	1	all/unsp	nev any	ot
GARSHI	9		m	65	82	all	-	all	NAmer	1981	CC	1081	o	bl	y	n	0	all/unsp	nev any	st
GODLEY	2		m	65	79	all	-	all	NAmer	1966	CC	1986	n	bl	y	n	1	cig+/-ot	nev cigs	ot
GODLEY	4		f	65	79	all	-	all	NAmer	1966	CC	1986	n	bl	y	n	1	cig+/-ot	nev cigs	ot
HAMMON	13		m	65	69	wh	0	all	NAmer	1952	pr	448	n	bl	n	n	0	cig+/-ot	nev any	st
HINDS	6		f	65	99	ch	-	all	NAmer	1968	CC	292	n	bl	n	n	0	all/unsp	nev any	st
HINDS	3		f	65	99	jap	-	all	NAmer	1968	CC	292	n	bl	n	n	0	all/unsp	nev any	st
HINDS	9		f	65	99	haw	-	all	NAmer	1968	CC	292	n	bl	n	n	0	all/unsp	nev any	st
HUMBLE	56		m	65	84	w-hi	-	not alv	NAmer	1980	CC	521	n	bl	y	n	0	cig+/-ot	nev cigs	st
HUMBLE	60		m	65	84	hi	-	not alv	NAmer	1980	CC	521	n	bl	y	n	0	cig+/-ot	nev cigs	st
HUMBLE	58		f	65	84	w-hi	-	not alv	NAmer	1980	CC	521	n	bl	y	n	0	cig+/-ot	nev cigs	st
HUMBLE	62		f	65	84	hi	-	not alv	NAmer	1980	CC	521	n	bl	y	n	0	cig+/-ot	nev cigs	st
KAISE2	40		m	65	74	all	9	all	NAmer	1979	pr	318	n	bl	n	n	0	cig only	nev any	st
KAISE2	48		m	75	99	all	9	all	NAmer	1979	pr	318	n	bl	n	n	0	cig only	nev any	st
KAISE2	16		f	65	74	all	9	all	NAmer	1979	pr	318	n	bl	n	n	0	cig only	nev any	st
KAISE2	24		f	75	99	all	9	all	NAmer	1979	pr	318	n	bl	n	n	0	cig only	nev any	st
LEVIN	12		m	65	74	all	-	all	NAmer	1938	CC	475	n	bl	n	n	0	cig+/-ot	nev any	st
LEVIN	13		m	75	99	all	-	all	NAmer	1938	CC	475	n	bl	n	n	0	cig+/-ot	nev any	st
LIU4	5		m	70	99	all	-	all	As:Chi	1986	CC	1000-00	n	ot	y	n	2	all/unsp	nev any	ot
LIU4	6		f	70	99	all	-	all	As:Chi	1986	CC	1000-00	n	ot	y	n	2	all/unsp	nev any	ot
NAM	21		m	65	79	all	-	all	NAmer	1986	CC	1199	n	bl	y	n	0	cig+/-ot	nev cigs	st
NAM	29		m	80	99	all	-	all	NAmer	1986	CC	1199	n	bl	y	n	0	cig+/-ot	nev cigs	st
NAM	53		f	65	79	all	-	all	NAmer	1986	CC	1199	n	bl	y	n	0	cig+/-ot	nev cigs	st
NAM	61		f	80	99	all	-	all	NAmer	1986	CC	1199	n	bl	y	n	0	cig+/-ot	nev cigs	st
PRESCO	42		m	65	99	all	0	all	Eu:Sca	1964	pr	867	n	bl	n	n	0	all/unsp	nev any	st
PRESCO	41		f	65	99	all	0	all	Eu:Sca	1964	pr	867	n	bl	n	n	0	all/unsp	nev any	st
STOCKS	38		m	65	74	all	-	all	Eu:UK	1952	CC	2932	n	V	y	n	0	cig+/-ot	nev any	st
WUWILL	5		f	65	69	all	-	all	As:Chi	1985	CC	965	n	ot	n	n	0	cig+/-ot	nev cigs	st

Cigarette type is all/unspec for all RRs
except for the following:

REF	NRR	CIGTYPE
DEAN3	123	MC only

Table 1A9 - 5

IESLC - Meta-analysis of Ever Smoking, Cigarettes (or Any Product if Cigarettes not available), Age 65+
All LC types
Least adjusted

REF	NRR	SEX	AD	Number Exposed Case	Number Exposed Cont	Non-exposed Case	Non-exposed Cont	RR	95.00%CI
BENHAM	71	m	0	169	203	7	95	11.30	(5.11- 25.00)
BUFFLE	60	f	0	41	18	3	30	22.78	(6.15- 84.40)
COMSTO	59	c	0	46	58	6	44	5.82	(2.28- 14.84)
*CPSI	179	m	1	-	-	-	-	8.67	(5.54- 13.57)
*CPSI	114	f	0	153	211823	178	957682	3.89	(3.13- 4.82)
*CPSI	115	f	0	31	39527	163	392909	1.89	(1.29- 2.78)
Subtotal CPSI								3.78	(3.18- 4.50)
DAMBER	24	m	0	261	160	23	123	8.72	(5.36- 14.19)
DEAN3	46	m	0	285	396	16	82	3.69	(2.11- 6.44)
DEAN3	123	f	0	41	196	27	574	4.45	(2.66- 7.42)
Subtotal DEAN3								4.08	(2.80- 5.95)
*DORN	195	m	1	-	-	-	-	5.84	(4.42- 7.71)
GARSHI	9	m	0	771	1130	32	294	6.27	(4.30- 9.13)
GODLEY	2	m	1	-	-	-	-	6.17	(4.88- 7.79)
GODLEY	4	f	1	-	-	-	-	5.21	(3.65- 7.43)
Subtotal GODLEY								5.86	(4.82- 7.13)
*HAMMON	13	m	0	70	40544	9	21892	4.20	(2.10- 8.41)
HINDS	6	f	0	4	8	24	58	1.21	(0.33- 4.39)
HINDS	3	f	0	26	29	38	326	7.69	(4.11- 14.40)
HINDS	9	f	0	20	29	9	92	7.05	(2.89- 17.18)
Subtotal HINDS								5.83	(3.62- 9.39)
HUMBLE	56	m	0	128	157	6	60	8.15	(3.41- 19.48)
HUMBLE	60	m	0	55	58	2	21	9.96	(2.23- 44.47)
HUMBLE	58	f	0	52	33	11	52	7.45	(3.40- 16.30)
HUMBLE	62	f	0	19	11	4	34	14.68	(4.10- 52.52)
Subtotal HUMBLE								8.80	(5.34- 14.49)
*KAISE2	40	m	0	25	11767	5	11466	4.87	(1.87- 12.72)
*KAISE2	48	m	0	14	3401	4	4486	4.62	(1.52- 14.01)
*KAISE2	16	f	0	26	13883	2	24159	22.62	(5.37- 95.30)
*KAISE2	24	f	0	9	3362	3	12285	10.96	(2.97- 40.47)
Subtotal KAISE2								7.24	(4.05- 12.94)
LEVIN	12	m	0	72	151	13	168	6.16	(3.28- 11.57)
LEVIN	13	m	0	7	26	1	86	23.15	(2.72- 196.93)
Subtotal LEVIN								6.85	(3.74- 12.54)
LIU4	5	m	2	-	-	-	-	2.47	(2.34- 2.61)
LIU4	6	f	2	-	-	-	-	2.50	(2.33- 2.68)
Subtotal LIU4								2.48	(2.38- 2.59)
NAM	21	m	0	296	311	18	130	6.87	(4.09- 11.54)
NAM	29	m	0	67	143	6	172	13.43	(5.66- 31.87)
NAM	53	f	0	142	199	18	251	9.95	(5.89- 16.81)
NAM	61	f	0	35	83	22	472	9.05	(5.06- 16.19)
Subtotal NAM								8.93	(6.66- 11.97)
*PRESCO	42	m	0	378	57442	3	6229	13.66	(4.39- 42.54)
*PRESCO	41	f	0	105	34709	9	19748	6.64	(3.36- 13.11)
Subtotal PRESCO								8.03	(4.48- 14.40)
STOCKS	38	m	0	550	969	18	173	5.46	(3.32- 8.96)
WUWILL	5	f	0	82	64	55	97	2.26	(1.42- 3.60)
Partial Totals				3980	420890	735	1454290		

*prospective study

REF	NRR	SEX	AD	Ys	Ws	Qs	Ps
BENHAM	71	m	0	2.42	6.09	11.27	0.0000
BUFFLE	60	f	0	3.13	2.24	9.52	0.0000
COMSTO	59	c	0	1.76	4.38	2.12	0.0002
*CPSI	179	m	1	2.16	19.15	22.98	0.0000
*CPSI	114	f	0	1.36	82.32	7.08	0.0000
*CPSI	115	f	0	0.64	26.07	4.76	0.0011
Subtotal CPSI				1.33	127.53	34.82	
DAMBER	24	m	0	2.17	16.21	19.68	0.0000
DEAN3	46	m	0	1.31	12.39	0.72	0.0000
DEAN3	123	f	0	1.49	14.65	2.68	0.0000
Subtotal DEAN3				1.41	27.03	3.40	
*DORN	195	m	1	1.76	49.64	24.36	0.0000
GARSHI	9	m	0	1.84	27.15	16.15	0.0000
GODLEY	2	m	1	1.82	70.25	40.09	0.0000
GODLEY	4	f	1	1.65	30.41	10.46	0.0000
Subtotal GODLEY				1.77	100.66	50.55	
*HAMMON	13	m	0	1.44	7.98	1.10	0.0001
HINDS	6	f	0	0.19	2.30	1.76	0.7739
HINDS	3	f	0	2.04	9.77	9.31	0.0000

Table 1A9 - 5

IESLC - Meta-analysis of Ever Smoking, Cigarettes (or Any Product if Cigarettes not available), Age 65+
All LC types
Least adjusted

REF	NRR	SEX	AD	Ys	Ws	Qs	Ps
HINDS	9	f	0	1.95	4.84	3.83	0.0000
Subtotal HINDS				1.76	16.92	14.90	
HUMBLE	56	m	0	2.10	5.06	5.41	0.0000
HUMBLE	60	m	0	2.30	1.72	2.61	0.0026
HUMBLE	58	f	0	2.01	6.26	5.58	0.0000
HUMBLE	62	f	0	2.69	2.36	6.22	0.0000
Subtotal HUMBLE				2.17	15.41	19.83	
*KAISE2	40	m	0	1.58	4.17	1.12	0.0012
*KAISE2	48	m	0	1.53	3.12	0.67	0.0069
*KAISE2	16	f	0	3.12	1.86	7.84	0.0000
*KAISE2	24	f	0	2.39	2.25	3.98	0.0003
Subtotal KAISE2				1.98	11.40	13.63	
LEVIN	12	m	0	1.82	9.67	5.50	0.0000
LEVIN	13	m	0	3.14	0.84	3.62	0.0040
Subtotal LEVIN				1.92	10.51	9.12	
LIU4	5	m	2	0.90	1288.59	33.00	0.0000
LIU4	6	f	2	0.92	784.55	17.17	0.0000
Subtotal LIU4				0.91	2073.14	50.17	
NAM	21	m	0	1.93	14.32	10.68	0.0000
NAM	29	m	0	2.60	5.14	12.09	0.0000
NAM	53	f	0	2.30	13.97	21.24	0.0000
NAM	61	f	0	2.20	11.34	14.69	0.0000
Subtotal NAM				2.19	44.77	58.70	
*PRESCO	42	m	0	2.61	2.98	7.16	0.0000
*PRESCO	41	f	0	1.89	8.29	5.69	0.0000
Subtotal PRESCO				2.08	11.27	12.85	
STOCKS	38	m	0	1.70	15.58	6.23	0.0000
WUWILL	5	f	0	0.82	17.76	1.10	0.0006

	N	37
	NS	19
	Wt	2585.66
Het	Chi	359.49
Het	df	36
Het	P	***
Fixed	RR	2.90
	RRl	2.79
	RRu	3.01
	P	+++
Random	RR	5.73
	RRl	4.78
	RRu	6.86
	P	+++
Asymm	P	***

Table 1A9 - 6

IESLC - Meta-analysis of Ever Smoking, Cigarettes (or Any Product if Cigarettes not available), Age 65+
All LC types
Least adjusted

	Sex combined	male	female	Total
N	1	19	17	37
NS	1	15	11	27
Wt	4.38	1560.03	1021.24	2585.66
Het Chi	0.00	224.38	132.74	359.49
Het df	0	18	16	36
Het P	N.S.	***	***	***
Fixed RR	5.82	2.92	2.86	2.90
RRl	2.28	2.78	2.69	2.79
RRu	14.84	3.07	3.04	3.01
P	+++	+++	+++	+++
Random RR	5.82	6.50	5.34	5.73
RRl	2.28	4.64	3.92	4.78
RRu	14.84	9.10	7.27	6.86
P	+++	+++	+++	+++
Between Chi				2.37
Between df				2
Between P				N.S.
Btwn(F) P				N.S.
Btwn(R) P				N.S.

Table 1A9 - 7

IESLC - Meta-analysis of Ever Smoking, Cigarettes (or Any Product if Cigarettes not available), Age 65+
All LC types
Excluded studies (and stage at which they were excluded)

1 BOUCHA BUELL LAURIL MZILEN

2 BYERS2 ENSTRO HIRAY2 KANELL LIAW LICKIN MRFIT MURATA NOTANI RESTRE RIMING SAARIK SEGI2 SHIMIZ SITAS SOBUE2 TANG2 WALD WARSIN WATSON WU2 WYNDE5 WYNDE8 WYNDER

3 ABELIN ABRAHA AGUDO AKIBA ALDERS AMANDU AMES ANDERS ARCHER ARMADA AUSTIN AUVINE AXELSO AXELSS BAND BARBON BECHER BENSHL BERRIN BEST BLOHMK BLOT1 BLOT2 BLOT3 BLOT4 BOFFET BOUCOT BRESLO BRETT BROCKM BROSS BROWN1 BYERS1 CARPEN CASCO2 CASCOR CEDERL CHAN CHANG CHATZI CHEN CHEN2 CHEN3 CHIAZZ CHOI CHOW CHYOU COOKSO CORREA CPSII DARBY DAVEYS DEAN DEAN2 DEKLER DESTE2 DESTEF DOCKER DOLL DOLL2 DORANT DOSEME DROSTE DU DUNN EBELIN ENGELA ESAKI FAN GAO GAO2 GARCIA GARDIN GENG GER GILLIS GOLLED GOODMA GRAHAM GREGOR GSELL HAENSZ HAMMO2 HANSEN HEGMAN HEIN HENNEK HIRAOK HIRAYA HITOSU HOLE HOROWI HORWIT HU HU2 HUANG ISHIMA JAHN JAIN JARUP JARVHO JEDRYC JIANG JOLY JONES JUSSAW KAISER KATSOU KAUFMA KELLER KHUDER KIHARA KINLEN KJUUS KNEKT KO KOHLME KOO KOULUM KREUZE KREYBE KUBIK LAMTH LAMWK LAMWK2 LANGE LAUSSM LEI LEMARC LETOUR LIDDEL LIU LIU2 LIU3 LIU5 LOMBA2 LOMBAR LUBIN LUBIN2 LUO MACLEN MAGNUS MARSH MARSH2 MARTIS MASTRA MATOS MATSUD MCCONN MCDUFF MCLAUG MIGRAN MILLER MILLS MOLLO MRFITR NOTAN2 NOU ODRISC ORMOS OSANN OSANN2 PARKIN PASTOR PAWLEG PERNU PERSH2 PETO PEZZO2 PEZZOT PIKE PISANI POFFIJ POLEDN QIAO QIAO2 RACHTA RADZIK RANDIG REN RONCO ROOTS ROTHSC SANKAR SCHWA2 SCHWAR SEGI SEOW SHAW SIEMIA SIMARA SIMONA SOBUE SPEIZE SPITZ STASZE STAYNE STOCKW STUCKE SUN SUZUK2 SUZUKI SVENSS TANG TAO TENKAN TIZZAN TOKARS TOUSEY TSUGAN TULINI TVERDA ULMER VANDER VEIERO VUTUC WAKAI WANG WANG2 WANG3 WANG4 WICKLU WIGLE WILKIN WU WUNSCH WYNDE2 WYNDE3 WYNDE6 WYNDE7 XIANGZ XU XU2 XU3 XU4 YAMAGU YONG YUAN ZHANG ZHENG ZHOU

4 BROWN2 SADOWS

7 PERSHA

9 DORGAN WYNDE4

Table 1A9 - 8

Potentially overlapping studies

REF	REFGP	PRINC	.	OVERLAP/LINK
BENHAM	LUBIN2	2		Subset of Lubin2
PRESCO	PRESCO	2		PRESCO-combined
CPSI	CPSI	1		CPSI overall

Table 1A9 - 9

Most adjusted - insufficient data for metaanalysis

REF	NRR	SEX	AGEL	AGEH	RACE	YF	LC TYPE	LOC	START	ST	NLC	R	VB	P	H	AD	PRODUCT	DENOM	De
BROWN2	56	m	70	99	wh	-	all	NAmer	1984	CC	14596	n	bl	n	y	1	cig+/-ot	nev cigs	or
BROWN2	53	f	70	99	wh	-	all	NAmer	1984	CC	14596	n	bl	n	y	1	cig+/-ot	nev cigs	or
SADOWS	20	m	70	99	wh	-	all	NAmer	1938	CC	477	n	bl	n	n	0	cig only	nev any	st

REF	NRR	RR	SIG	RRDATA comment
BROWN2	56	8.20		0
BROWN2	53	10.60		0
SADOWS	20	2.66		0

Table 1A10 -

IESLC - Meta-analysis of Ever Smoking, Cigarettes only, Age <56
All LC types

This analysis is restricted to results for:
1) Non-dose-response data
2) Ever smokers
3) Age <56
4) Results complete enough for use in metaanalysis

Within each study, results are then selected (in the following order of preference, within each sex) for:
5) PRODUCT: cigarettes only
6) CIGTYPE: all/unspecified, MC regardless of HR, MC only
7) DENOM: never smoked anything, never smoked cigarettes, (never +1 = +long term ex, +2 = +amount unknown,
+3 = never cigs+long term ex)
8) Followup period (YF, prospective studies): whole study (coded as 0) or longest available
9) LCtype: all or nearest available, at least Squamous and Adeno. (q = squamous, s = small,
l = large, a = adeno, mix = mixed, alv = alveolar)
10) Race: all or nearest available, otherwise by race (wh or w = white, bl or b = black, hi = hispanic
ch = chinese, jap = japanese, haw = hawaiian, w+o = white + oriental, sca = scandinavian, as = asian)
11) For overlapping studies: principal rather than subsidiary studies
Finally by Age: whole study (actual age shown) if available, otherwise by widest available age group
and then for single sex results (m, f) in preference to combined sex results (c).

Results adjusted (AD) for the most potential confounders are then chosen in Sections -1 to -3
(and those which actually differ from the adjusted results in Table 1A3 - 1 are marked 'x' in Section -1)
and results adjusted for the least confounders in Sections -4 to -6. (Those least adjusted results which
actually differ from the most adjusted as marked 'x' in column X in Section -4)
(Results adjusted for an unknown number of confounder(s) are coded as 20.)

Section -7 shows excluded studies, together with the stage (as above) at which no qualifying
results were found.

Section -8 lists the potentially overlapping studies which have been included (1=principal, 2=subsidiary).

Section -9 lists any results which would have been included in preference except that they had data not complete
enough for use in meta-analysis, with their significance (yes/no), if known, and any further comment as entered
on the database.

In addition to those mentioned above, the following fields, levels and abbreviations are used:
* or nk = not known, n = no, y = yes, ot = other
nev = never
all/unspec = all or unspecified, MC = manufactured cigarettes, HR = hand-rolled cigarettes
REF: 6-character study reference
NRR: number of the RR on the database within the study
ST : study type (CC = case control, pr or prosp = prospective)
NLC: number of lung cancer cases in whole study
R : risky occupational population (n = no, m = mining, o = other risky)
VB : national cigarette type (V = at least 75% Virginia, bl = at least 75% blended, ot = other)
P : any proxy use
H : full histological confirmation
De : derivation of RR/CI (or = original, st = standard method, ot = other method of estimation)

Table 1A10 - 1

IESLC - Meta-analysis of Ever Smoking, Cigarettes only, Age <56
All LC types
Most adjusted

REF	NRR	1A3	SEX	AGEL	AGEH	RACE	YF	LC TYPE	LOC	START	ST	NLC	R	VB	P	H	AD	PRODUCT	DENOM	De
BENHAM	68	x	m	1	49	all	-	not mix	Eu:wst	1976	CC	1625	n	bl	n	y	0	cig only	nev any	st
CPSI	36	x	m	40	54	wh	0	all	NAmer	1959	pr	5138	n	bl	n	n	0	cig only	nev any	st
CPSI	112	x	f	40	54	wh	0	all	NAmer	1959	pr	5138	n	bl	n	n	0	cig only	nev any	st
DEAN3	120	x	f	35	44	all	-	all	Eu:UK	1969	CC	766	n	V	y	n	0	cig only	nev any	st
DEAN3	121	x	f	45	54	all	-	all	Eu:UK	1969	CC	766	n	V	y	n	0	cig only	nev any	st
HAMMON	20	x	m	50	54	wh	0	all	NAmer	1952	pr	448	n	bl	n	n	0	cig only	nev any	ot

Cigarette type is all/unspec for all RRs
except for the following:

REF	NRR	CIGTYPE
DEAN3	120	MC only
DEAN3	121	MC only

Table 1A10 - 2

IESLC - Meta-analysis of Ever Smoking, Cigarettes only, Age <56
All LC types
Most adjusted

REF	NRR	SEX	AD	Number Exposed Case	Cont	Non-exposed Case	Cont	RR	95.00%CI
BENHAM	68	m	0	217	304	9	103	8.17	(4.04- 16.50)
*CPSI	36	m	0	427	740861	14	255642	10.52	(6.18- 17.92)
*CPSI	112	f	0	185	936087	47	1160026	4.88	(3.54- 6.72)
Subtotal CPSI								5.98	(4.55- 7.87)
DEAN3	120	f	0	6	472	1	328	4.17	(0.50- 34.80)
DEAN3	121	f	0	23	469	4	326	4.00	(1.37- 11.67)
Subtotal DEAN3								4.03	(1.55- 10.49)
*HAMMON	20	m	0	-	-	-	-	22.11	(3.06- 159.93)
Partial Totals				858	1678193	75	1416425		

*prospective study

REF	NRR	SEX	AD	Ys	Ws	Qs	Ps
BENHAM	68	m	0	2.10	7.77	0.61	0.0000
*CPSI	36	m	0	2.35	13.56	3.85	0.0000
*CPSI	112	f	0	1.58	37.48	2.09	0.0000
Subtotal CPSI				1.79	51.04	5.94	
DEAN3	120	f	0	1.43	0.85	0.13	0.1872
DEAN3	121	f	0	1.39	3.35	0.63	0.0112
Subtotal DEAN3				1.39	4.20	0.77	
*HAMMON	20	m	0	3.10	0.98	1.60	0.0022

	N	6
	NS	4
	Wt	63.99
Het	Chi	8.91
Het	df	5
Het	P	N.S.
Fixed	RR	6.18
	RRl	4.83
	RRu	7.89
	P	+++
Random	RR	6.81
	RRl	4.48
	RRu	10.36
	P	+++
Asymm	P	N.S.

Table 1A10 - 3

IESLC - Meta-analysis of Ever Smoking, Cigarettes only, Age <56
All LC types
Most adjusted

	combined	Sex male	female	Total
N		3	3	6
NS		3	2	5
Wt		22.31	41.68	63.99
Het Chi		0.97	0.14	8.91
Het df		2	2	5
Het P		N.S.	N.S.	N.S.
Fixed RR		9.96	4.78	6.18
RRl		6.57	3.53	4.83
RRu		15.08	6.48	7.89
P		+++	+++	+++
Random RR		9.96	4.78	6.81
RRl		6.57	3.53	4.48
RRu		15.08	6.48	10.36
P		+++	+++	+++
Between Chi				7.80
Between df				1
Between P				**
Btwn(F) P				**
Btwn(R) P				**

Too few RRs for analysis by factor

Table 1A10 - 4

IESLC - Meta-analysis of Ever Smoking, Cigarettes only, Age <56
All LC types
Least adjusted

REF	NRR	X	SEX	AGEL	AGEH	RACE	YF	LC TYPE	LOC	START	ST	NLC	R	VB	P	H	AD	PRODUCT	DENOM	De
BENHAM	68		m	1	49	all	-	not mix	Eu:wst	1976	CC	1625	n	bl	n	y	0	cig only	nev any	st
CPSI	36		m	40	54	wh	0	all	NAmer	1959	pr	5138	n	bl	n	n	0	cig only	nev any	st
CPSI	112		f	40	54	wh	0	all	NAmer	1959	pr	5138	n	bl	n	n	0	cig only	nev any	st
DEAN3	120		f	35	44	all	-	all	Eu:UK	1969	CC	766	n	V	y	n	0	cig only	nev any	st
DEAN3	121		f	45	54	all	-	all	Eu:UK	1969	CC	766	n	V	y	n	0	cig only	nev any	st
HAMMON	20		m	50	54	wh	0	all	NAmer	1952	pr	448	n	bl	n	n	0	cig only	nev any	ot

Cigarette type is all/unspec for all RRs
except for the following:

REF	NRR	CIGTYPE
DEAN3	120	MC only
DEAN3	121	MC only

Table 1A10 - 5

IESLC - Meta-analysis of Ever Smoking, Cigarettes only, Age <56
All LC types
Least adjusted

REF	NRR	SEX	AD	Number Exposed Case	Number Exposed Cont	Non-exposed Case	Non-exposed Cont	RR	95.00%CI
BENHAM	68	m	0	217	304	9	103	8.17	(4.04- 16.50)
*CPSI	36	m	0	427	740861	14	255642	10.52	(6.18- 17.92)
*CPSI	112	f	0	185	936087	47	1160026	4.88	(3.54- 6.72)
Subtotal CPSI								5.98	(4.55- 7.87)
DEAN3	120	f	0	6	472	1	328	4.17	(0.50- 34.80)
DEAN3	121	f	0	23	469	4	326	4.00	(1.37- 11.67)
Subtotal DEAN3								4.03	(1.55- 10.49)
*HAMMON	20	m	0	-	-	-	-	22.11	(3.06- 159.93)
Partial Totals				858	1678193	75	1416425		

*prospective study

REF	NRR	SEX	AD	Ys	Ws	Qs	Ps
BENHAM	68	m	0	2.10	7.77	0.61	0.0000
*CPSI	36	m	0	2.35	13.56	3.85	0.0000
*CPSI	112	f	0	1.58	37.48	2.09	0.0000
Subtotal CPSI				1.79	51.04	5.94	
DEAN3	120	f	0	1.43	0.85	0.13	0.1872
DEAN3	121	f	0	1.39	3.35	0.63	0.0112
Subtotal DEAN3				1.39	4.20	0.77	
*HAMMON	20	m	0	3.10	0.98	1.60	0.0022

	N	6
	NS	4
	Wt	63.99
Het	Chi	8.91
Het	df	5
Het	P	N.S.
Fixed	RR	6.18
	RRl	4.83
	RRu	7.89
	P	+++
Random	RR	6.81
	RRl	4.48
	RRu	10.36
	P	+++
Asymm	P	N.S.

Table 1A10 - 6

IESLC - Meta-analysis of Ever Smoking, Cigarettes only, Age <56
All LC types
Least adjusted

	Sex combined	male	female	Total
N		3	3	6
NS		3	2	5
Wt		22.31	41.68	63.99
Het Chi		0.97	0.14	8.91
Het df		2	2	5
Het P		N.S.	N.S.	N.S.
Fixed RR		9.96	4.78	6.18
RRl		6.57	3.53	4.83
RRu		15.08	6.48	7.89
P		+++	+++	+++
Random RR		9.96	4.78	6.81
RRl		6.57	3.53	4.48
RRu		15.08	6.48	10.36
P		+++	+++	+++
Between Chi				7.80
Between df				1
Between P				**
Btwn(F) P				**
Btwn(R) P				**

Table 1A10 - 7

IESLC - Meta-analysis of Ever Smoking, Cigarettes only, Age <56
All LC types
Excluded studies (and stage at which they were excluded)

1 BOUCHA BUELL LAURIL MZILEN

2 BYERS2 ENSTRO HIRAY2 KANELL LIAW LICKIN MRFIT MURATA NOTANI RESTRE RIMING SAARIK SEGI2 SHIMIZ SITAS SOBUE2 TANG2 WALD WARSIN WATSON WU2 WYNDE5 WYNDE8 WYNDER

3 ABELIN ABRAHA AGUDO AKIBA ALDERS AMANDU AMES ANDERS ARCHER ARMADA AUSTIN AUVINE AXELSO AXELSS BAND BARBON BECHER BENSHL BERRIN BEST BLOHMK BLOT1 BLOT2 BLOT3 BLOT4 BOFFET BOUCOT BRESLO BRETT BROCKM BROSS BROWN1 BROWN2 BUFFLE BYERS1 CARPEN CASCO2 CASCOR CEDERL CHAN CHANG CHATZI CHEN CHEN2 CHEN3 CHIAZZ CHOI CHOW CHYOU COOKSO CORREA CPSII DAMBER DARBY DAVEYS DEAN DEAN2 DEKLER DESTE2 DESTEF DOCKER DOLL DOLL2 DORANT DORGAN DORN DOSEME DROSTE DU DUNN EBELIN ENGELA ESAKI FAN GAO GAO2 GARCIA GARDIN GARSHI GENG GER GILLIS GODLEY GOLLED GOODMA GRAHAM GREGOR GSELL HAENSZ HAMMO2 HANSEN HEGMAN HEIN HENNEK HIRAOK HIRAYA HOLE HOROWI HORWIT HU HU2 HUANG HUMBLE ISHIMA JAHN JAIN JARUP JARVHO JEDRYC JIANG JOLY JONES JUSSAW KAISE2 KAISER KATSOU KAUFMA KELLER KHUDER KIHARA KINLEN KJUUS KNEKT KO KOHLME KOO KOULUM KREYBE LAMTH LAMWK LAMWK2 LANGE LAUSSM LEI LEMARC LETOUR LIDDEL LIU LIU2 LIU3 LIU4 LIU5 LOMBA2 LOMBAR LUBIN LUBIN2 LUO MACLEN MAGNUS MARSH MARSH2 MARTIS MASTRA MATOS MATSUD MCCONN MCDUFF MCLAUG MIGRAN MILLER MILLS MOLLO MRFITR NOTAN2 NOU ODRISC ORMOS OSANN OSANN2 PARKIN PASTOR PAWLEG PERNU PERSH2 PERSHA PETO PEZZO2 PEZZOT PIKE PISANI POFFIJ POLEDN QIAO QIAO2 RACHTA RADZIK RANDIG REN RONCO ROOTS ROTHSC SANKAR SCHWA2 SEGI SEOW SHAW SIEMIA SIMARA SIMONA SOBUE SPITZ STASZE STAYNE STOCKW STUCKE SUN SUZUK2 SUZUKI SVENSS TANG TAO TENKAN TIZZAN TOKARS TOUSEY TULINI ULMER VANDER VEIERO WAKAI WANG WANG2 WANG3 WANG4 WICKLU WIGLE WILKIN WU WUNSCH WYNDE2 WYNDE3 WYNDE7 XIANGZ XU XU2 XU3 XU4 YAMAGU YONG YUAN ZHANG ZHENG ZHOU

4 SADOWS

5 COMSTO HINDS HITOSU KREUZE KUBIK LEVIN NAM PRESCO SCHWAR SPEIZE STOCKS TSUGAN TVERDA VUTUC WUWILL WYNDE4 WYNDE6

Table 1A10 - 8

Potentially overlapping studies

REF	REFGP	PRINC	.	OVERLAP/LINK
BENHAM	LUBIN2	2		Subset of Lubin2
CPSI	CPSI	1		CPSI overall

Table 1A10 - 9

Most adjusted - insufficient data for metaanalysis

REF	NRR	SEX	AGEL	AGEH	RACE	YF	LC TYPE	LOC	START	ST	NLC	R	VB	P	H	AD	PRODUCT	DENOM	De
SADOWS	8	m	1	49	wh	-	all	NAmer	1938	CC	477	n	bl	n	n	0	cig only	nev any	st

REF	NRR	RR	SIG	RRDATA comment
SADOWS	8	3.50		0

Table 1A11 -

IESLC - Meta-analysis of Ever Smoking, Cigarettes only, Age 50-70
All LC types

This analysis is restricted to results for:
1) Non-dose-response data
2) Ever smokers
3) Maximum age range 50-70
4) Results complete enough for use in metaanalysis

Within each study, results are then selected (in the following order of preference, within each sex) for:
5) PRODUCT: cigarettes only
6) CIGTYPE: all/unspecified, MC regardless of HR, MC only
7) DENOM: never smoked anything, never smoked cigarettes, (never +1 = +long term ex, +2 = +amount unknown, +3 = never cigs+long term ex)
8) Followup period (YF, prospective studies): whole study (coded as 0) or longest available
9) LCtype: all or nearest available, at least Squamous and Adeno. (q = squamous, s = small, l = large, a = adeno, mix = mixed, alv = alveolar)
10) Race: all or nearest available, otherwise by race (wh or w = white, bl or b = black, hi = hispanic ch = chinese, jap = japanese, haw = hawaiian, w+o = white + oriental, sca = scandinavian, as = asian)
11) For overlapping studies: principal rather than subsidiary studies
Finally by Age: whole study (actual age shown) if available, otherwise by widest available age group and then for single sex results (m, f) in preference to combined sex results (c).

Results adjusted (AD) for the most potential confounders are then chosen in Sections -1 to -3 (and those which actually differ from the adjusted results in Table 1A3 - 1 are marked 'x' in Section -1) and results adjusted for the least confounders in Sections -4 to -6. (Those least adjusted results which actually differ from the most adjusted as marked 'x' in column X in Section -4)
(Results adjusted for an unknown number of confounder(s) are coded as 20.)

Section -7 shows excluded studies, together with the stage (as above) at which no qualifying results were found.

Section -8 lists the potentially overlapping studies which have been included (1=principal, 2=subsidiary).

Section -9 lists any results which would have been included in preference except that they had data not complete enough for use in meta-analysis, with their significance (yes/no), if known, and any further comment as entered on the database.

In addition to those mentioned above, the following fields, levels and abbreviations are used:
* or nk = not known, n = no, y = yes, ot = other
nev = never
all/unspec = all or unspecified, MC = manufactured cigarettes, HR = hand-rolled cigarettes
REF: 6-character study reference
NRR: number of the RR on the database within the study
ST : study type (CC = case control, pr or prosp = prospective)
NLC: number of lung cancer cases in whole study
R : risky occupational population (n = no, m = mining, o = other risky)
VB : national cigarette type (V = at least 75% Virginia, bl = at least 75% blended, ot = other)
P : any proxy use
H : full histological confirmation
De : derivation of RR/CI (or = original, st = standard method, ot = other method of estimation)

Table 1A11 - 1

IESLC - Meta-analysis of Ever Smoking, Cigarettes only, Age 50-70
All LC types
Most adjusted

REF	NRR	1A3	SEX	AGEL	AGEH	RACE	YF	LC TYPE	LOC	START	ST	NLC	R	VB	P	H	AD	PRODUCT	DENOM	De
BENHAM	69	x	m	50	59	all	-	not mix	Eu:wst	1976	CC	1625	n	bl	n	y	0	cig only	nev any	st
BENHAM	70	x	m	60	69	all	-	not mix	Eu:wst	1976	CC	1625	n	bl	n	y	0	cig only	nev any	st
CPSI	37	x	m	55	64	wh	0	all	NAmer	1959	pr	5138	n	bl	n	n	0	cig only	nev any	st
CPSI	113	x	f	55	64	wh	0	all	NAmer	1959	pr	5138	n	bl	n	n	0	cig only	nev any	st
DEAN3	122	x	f	55	64	all	-	all	Eu:UK	1969	CC	766	n	V	y	n	0	cig only	nev any	st
HAMMON	115		m	50	69	wh	0	all	NAmer	1952	pr	448	n	bl	n	n	1	cig only	nev any	ot

Cigarette type is all/unspec for all RRs
except for the following:

REF	NRR	CIGTYPE
DEAN3	122	MC only

Table 1A11 - 2

IESLC - Meta-analysis of Ever Smoking, Cigarettes only, Age 50-70
All LC types
Most adjusted

REF	NRR	SEX	AD	Number Exposed Case	Number Exposed Cont	Non-exposed Case	Non-exposed Cont	RR	95.00%CI
BENHAM	69	m	0	421	538	5	185	28.95	(11.80- 71.03)
BENHAM	70	m	0	377	347	12	140	12.68	(6.91- 23.27)
Subtotal BENHAM								16.43	(9.94- 27.17)
*CPSI	37	m	0	1598	903675	51	352725	12.23	(9.25- 16.16)
*CPSI	113	f	0	260	682162	144	1366561	3.62	(2.95- 4.43)
Subtotal CPSI								5.53	(4.69- 6.51)
DEAN3	122	f	0	39	283	9	310	4.75	(2.26- 9.97)
*HAMMON	115	m	1	-	-	-	-	9.94	(5.90- 16.73)
Partial Totals				2695	1587005	221	1719921		

*prospective study

REF	NRR	SEX	AD	Ys	Ws	Qs	Ps
BENHAM	69	m	0	3.37	4.77	11.06	0.0000
BENHAM	70	m	0	2.54	10.42	5.06	0.0000
Subtotal BENHAM				2.80	15.19	16.12	
*CPSI	37	m	0	2.50	49.43	21.61	0.0000
*CPSI	113	f	0	1.29	92.69	28.77	0.0000
Subtotal CPSI				1.71	142.12	50.38	
DEAN3	122	f	0	1.56	6.97	0.57	0.0000
*HAMMON	115	m	1	2.30	14.15	2.91	0.0000

	N	6
	NS	4
	Wt	178.42
Het	Chi	69.98
Het	df	5
Het	P	***
Fixed	RR	6.31
	RRl	5.45
	RRu	7.31
	P	+++
Random	RR	9.22
	RRl	4.83
	RRu	17.60
	P	+++
Asymm	P	N.S.

Table 1A11 - 3

IESLC - Meta-analysis of Ever Smoking, Cigarettes only, Age 50-70
All LC types
Most adjusted

	combined	Sex: male	female	Total
N		4	2	6
NS		3	2	5
Wt		78.76	99.66	178.42
Het Chi		4.13	0.48	69.98
Het df		3	1	5
Het P		N.S.	N.S.	***
Fixed RR		12.47	3.69	6.31
RRl		10.00	3.03	5.45
RRu		15.56	4.49	7.31
P		+++	+++	+++
Random RR		12.74	3.69	9.22
RRl		9.49	3.03	4.83
RRu		17.09	4.49	17.60
P		+++	+++	+++
Between Chi				65.37
Between df				1
Between P				***
Btwn(F) P				**
Btwn(R) P				***

Too few RRs for analysis by factor

Table 1A11 - 4

IESLC - Meta-analysis of Ever Smoking, Cigarettes only, Age 50-70
All LC types
Least adjusted

REF	NRR	X	SEX	AGEL	AGEH	RACE	YF	LC TYPE	LOC	START	ST	NLC	R	VB	P	H	AD	PRODUCT	DENOM	De
BENHAM	69		m	50	59	all	-	not mix	Eu:wst	1976	CC	1625	n	bl	n	y	0	cig only	nev any	st
BENHAM	70		m	60	69	all	-	not mix	Eu:wst	1976	CC	1625	n	bl	n	y	0	cig only	nev any	st
CPSI	37		m	55	64	wh	0	all	NAmer	1959	pr	5138	n	bl	n	n	0	cig only	nev any	st
CPSI	113		f	55	64	wh	0	all	NAmer	1959	pr	5138	n	bl	n	n	0	cig only	nev any	st
DEAN3	122		f	55	64	all	-	all	Eu:UK	1969	CC	766	n	V	y	n	0	cig only	nev any	st
HAMMON	127	x	m	50	69	wh	0	all	NAmer	1952	pr	448	n	bl	n	n	0	cig only	nev any	st

Cigarette type is all/unspec for all RRs
except for the following:

REF	NRR	CIGTYPE
DEAN3	122	MC only

Table 1A11 - 5

IESLC - Meta-analysis of Ever Smoking, Cigarettes only, Age 50-70
All LC types
Least adjusted

REF	NRR	SEX	AD	Number Exposed Case	Cont	Non-exposed Case	Cont	RR	95.00%CI
BENHAM	69	m	0	421	538	5	185	28.95	(11.80- 71.03)
BENHAM	70	m	0	377	347	12	140	12.68	(6.91- 23.27)
Subtotal BENHAM								16.43	(9.94- 27.17)
*CPSI	37	m	0	1598	903675	51	352725	12.23	(9.25- 16.16)
*CPSI	113	f	0	260	682162	144	1366561	3.62	(2.95- 4.43)
Subtotal CPSI								5.53	(4.69- 6.51)
DEAN3	122	f	0	39	283	9	310	4.75	(2.26- 9.97)
*HAMMON	127	m	0	249	225565	15	115884	8.53	(5.06- 14.36)
Totals				2944	1812570	236	1835805		

*prospective study

REF	NRR	SEX	AD	Ys	Ws	Qs	Ps
BENHAM	69	m	0	3.37	4.77	11.24	0.0000
BENHAM	70	m	0	2.54	10.42	5.24	0.0000
Subtotal BENHAM				2.80	15.19	16.48	
*CPSI	37	m	0	2.50	49.43	22.41	0.0000
*CPSI	113	f	0	1.29	92.69	27.53	0.0000
Subtotal CPSI				1.71	142.12	49.94	
DEAN3	122	f	0	1.56	6.97	0.52	0.0000
*HAMMON	127	m	0	2.14	14.15	1.38	0.0000

	N	6
	NS	4
	Wt	178.43
Het	Chi	68.32
Het	df	5
Het	P	***
Fixed	RR	6.24
	RRl	5.39
	RRu	7.22
	P	+++
Random	RR	8.97
	RRl	4.74
	RRu	17.00
	P	+++
Asymm	P	N.S.

Table 1A11 - 6

IESLC - Meta-analysis of Ever Smoking, Cigarettes only, Age 50-70
All LC types
Least adjusted

Sex combined	male	female	Total
N	4	2	6
NS	3	2	5
Wt	78.77	99.66	178.43
Het Chi	5.39	0.48	68.32
Het df	3	1	5
Het P	N.S.	N.S.	***
Fixed RR	12.13	3.69	6.24
RRl	9.73	3.03	5.39
RRu	15.13	4.49	7.22
P	+++	+++	+++
Random RR	12.47	3.69	8.97
RRl	8.81	3.03	4.74
RRu	17.67	4.49	17.00
P	+++	+++	+++
Between Chi			62.45
Between df			1
Between P			***
Btwn(F) P			**
Btwn(R) P			***

Table 1A11 - 7

IESLC - Meta-analysis of Ever Smoking, Cigarettes only, Age 50-70
All LC types
Excluded studies (and stage at which they were excluded)

1 BOUCHA BUELL LAURIL MZILEN

2 BYERS2 ENSTRO HIRAY2 KANELL LIAW LICKIN MRFIT MURATA NOTANI RESTRE RIMING SAARIK SEGI2 SHIMIZ SITAS SOBUE2 TANG2 WALD WARSIN WATSON WU2 WYNDE5 WYNDE8 WYNDER

3 ABELIN ABRAHA AGUDO AKIBA ALDERS AMANDU AMES ARCHER ARMADA AUSTIN AUVINE AXELSO AXELSS BAND BARBON BECHER BENSHL BERRIN BEST BLOHMK BLOT1 BLOT2 BLOT3 BLOT4 BOFFET BOUCOT BRESLO BRETT BROCKM BROSS BROWN1 BYERS1 CARPEN CASCO2 CASCOR CEDERL CHAN CHANG CHATZI CHEN CHEN2 CHEN3 CHIAZZ CHOI CHOW CHYOU COMSTO COOKSO CORREA CPSII DARBY DAVEYS DEAN DEAN2 DEKLER DESTE2 DESTEF DOCKER DOLL DOLL2 DORGAN DOSEME DROSTE DU DUNN EBELIN ENGELA ESAKI FAN GAO GAO2 GARCIA GARDIN GARSHI GENG GER GILLIS GODLEY GOLLED GOODMA GRAHAM GREGOR GSELL HAENSZ HAMMO2 HANSEN HEGMAN HEIN HENNEK HIRAOK HIRAYA HOLE HOROWI HORWIT HU HU2 HUANG HUMBLE ISHIMA JAHN JAIN JARUP JARVHO JEDRYC JIANG JOLY JONES JUSSAW KAISE2 KAISER KATSOU KAUFMA KELLER KHUDER KINLEN KJUUS KNEKT KO KOHLME KOO KOULUM KREYBE LAMTH LAMWK LAMWK2 LANGE LAUSSM LEI LEMARC LETOUR LIDDEL LIU LIU2 LIU3 LIU4 LIU5 LOMBA2 LOMBAR LUBIN LUBIN2 LUO MACLEN MAGNUS MARSH MARSH2 MARTIS MASTRA MATOS MATSUD MCCONN MCDUFF MCLAUG MIGRAN MILLER MILLS MOLLO MRFITR NOTAN2 NOU ODRISC ORMOS OSANN OSANN2 PARKIN PASTOR PAWLEG PERNU PERSH2 PERSHA PETO PEZZO2 PEZZOT PIKE PISANI POFFIJ POLEDN QIAO QIAO2 RACHTA RADZIK RANDIG REN RONCO ROOTS ROTHSC SANKAR SCHWA2 SCHWAR SEGI SEOW SHAW SIEMIA SIMARA SIMONA SOBUE SPEIZE SPITZ STASZE STAYNE STOCKW STUCKE SUN SUZUK2 SUZUKI SVENSS TANG TAO TENKAN TIZZAN TOKARS TOUSEY TSUGAN TULINI TVERDA ULMER VANDER VEIERO WAKAI WANG WANG2 WANG3 WANG4 WICKLU WIGLE WILKIN WU WUNSCH WYNDE2 WYNDE3 WYNDE6 WYNDE7 XIANGZ XU XU2 XU3 XU4 YAMAGU YONG YUAN ZHANG ZHENG ZHOU

4 BROWN2 SADOWS

5 ANDERS BUFFLE DAMBER DORANT DORN HINDS HITOSU KIHARA KREUZE KUBIK LEVIN NAM PRESCO STOCKS VUTUC WUWILL WYNDE4

Table 1A11 - 8
Potentially overlapping studies

REF	REFGP	PRINC	.	OVERLAP/LINK
BENHAM	LUBIN2	2		Subset of Lubin2
CPSI	CPSI	1		CPSI overall

Table 1A11 - 9
Most adjusted - insufficient data for metaanalysis

REF	NRR	SEX	AGEL	AGEH	RACE	YF	LC TYPE	LOC	START	ST	NLC	R	VB	P	H	AD	PRODUCT	DENOM	De
SADOWS	12	m	50	59	wh	-	all	NAmer	1938	CC	477	n	bl	n	n	0	cig only	nev any	st
SADOWS	16	m	60	69	wh	-	all	NAmer	1938	CC	477	n	bl	n	n	0	cig only	nev any	st

REF	NRR	RR	SIG	RRDATA comment
SADOWS	12	3.56		0
SADOWS	16	18.52		0

Table 1A12 -

IESLC - Meta-analysis of Ever Smoking, Cigarettes only, Age 65+
All LC types

This analysis is restricted to results for:
1) Non-dose-response data
2) Ever smokers
3) Age 65+
4) Results complete enough for use in metaanalysis

Within each study, results are then selected (in the following order of preference, within each sex) for:
5) PRODUCT: cigarettes only
6) CIGTYPE: all/unspecified, MC regardless of HR, MC only
7) DENOM: never smoked anything, never smoked cigarettes, (never +1 = +long term ex, +2 = +amount unknown,
+3 = never cigs+long term ex)
8) Followup period (YF, prospective studies): whole study (coded as 0) or longest available
9) LCtype: all or nearest available, at least Squamous and Adeno. (q = squamous, s = small,
l = large, a = adeno, mix = mixed, alv = alveolar)
10) Race: all or nearest available, otherwise by race (wh or w = white, bl or b = black, hi = hispanic
ch = chinese, jap = japanese, haw = hawaiian, w+o = white + oriental, sca = scandinavian, as = asian)
11) For overlapping studies: principal rather than subsidiary studies
Finally by Age: whole study (actual age shown) if available, otherwise by widest available age group
and then for single sex results (m, f) in preference to combined sex results (c).

Results adjusted (AD) for the most potential confounders are then chosen in Sections -1 to -3
(and those which actually differ from the adjusted results in Table 1A3 - 1 are marked 'x' in Section -1)
and results adjusted for the least confounders in Sections -4 to -6. (Those least adjusted results which
actually differ from the most adjusted as marked 'x' in column X in Section -4)
(Results adjusted for an unknown number of confounder(s) are coded as 20.)

Section -7 shows excluded studies, together with the stage (as above) at which no qualifying
results were found.

Section -8 lists the potentially overlapping studies which have been included (1=principal, 2=subsidiary).

Section -9 lists any results which would have been included in preference except that they had data not complete
enough for use in meta-analysis, with their significance (yes/no), if known, and any further comment as entered
on the database.

In addition to those mentioned above, the following fields, levels and abbreviations are used:
* or nk = not known, n = no, y = yes, ot = other
nev = never
all/unspec = all or unspecified, MC = manufactured cigarettes, HR = hand-rolled cigarettes
REF: 6-character study reference
NRR: number of the RR on the database within the study
ST : study type (CC = case control, pr or prosp = prospective)
NLC: number of lung cancer cases in whole study
R : risky occupational population (n = no, m = mining, o = other risky)
VB : national cigarette type (V = at least 75% Virginia, bl = at least 75% blended, ot = other)
P : any proxy use
H : full histological confirmation
De : derivation of RR/CI (or = original, st = standard method, ot = other method of estimation)

Table 1A12 - 1

IESLC - Meta-analysis of Ever Smoking, Cigarettes only, Age 65+
All LC types
Most adjusted

REF	NRR	1A3	SEX	AGEL	AGEH	RACE	YF	LC TYPE	LOC	START	ST	NLC	R	VB	P	H	AD	PRODUCT	DENOM	De
BENHAM	71	x	m	70	99	all	-	not mix	Eu:wst	1976	CC	1625	n	bl	n	y	0	cig only	nev any	st
CPSI	38	x	m	65	74	wh	0	all	NAmer	1959	pr	5138	n	bl	n	n	0	cig only	nev any	st
CPSI	39	x	m	75	84	wh	0	all	NAmer	1959	pr	5138	n	bl	n	n	0	cig only	nev any	st
CPSI	114	x	f	65	74	wh	0	all	NAmer	1959	pr	5138	n	bl	n	n	0	cig only	nev any	st
CPSI	115	x	f	75	84	wh	0	all	NAmer	1959	pr	5138	n	bl	n	n	0	cig only	nev any	st
DEAN3	123	x	f	65	99	all	-	all	Eu:UK	1969	CC	766	n	V	y	n	0	cig only	nev any	st
HAMMON	44	x	m	65	69	wh	0	all	NAmer	1952	pr	448	n	bl	n	n	0	cig only	nev any	ot
KAISE2	40	x	m	65	74	all	9	all	NAmer	1979	pr	318	n	bl	n	n	0	cig only	nev any	st
KAISE2	48	x	m	75	99	all	9	all	NAmer	1979	pr	318	n	bl	n	n	0	cig only	nev any	st
KAISE2	16	x	f	65	74	all	9	all	NAmer	1979	pr	318	n	bl	n	n	0	cig only	nev any	st
KAISE2	24	x	f	75	99	all	9	all	NAmer	1979	pr	318	n	bl	n	n	0	cig only	nev any	st

Cigarette type is all/unspec for all RRs
except for the following:

REF	NRR	CIGTYPE
DEAN3	123	MC only

Table 1A12 - 2

IESLC - Meta-analysis of Ever Smoking, Cigarettes only, Age 65+
All LC types
Most adjusted

REF	NRR	SEX	AD	Number Exposed Case	Number Exposed Cont	Non-exposed Case	Non-exposed Cont	RR	95.00%CI
BENHAM	71	m	0	169	203	7	95	11.30	(5.11- 25.00)
*CPSI	38	m	0	1358	380329	73	226284	11.07	(8.75- 14.01)
*CPSI	39	m	0	398	76882	58	91417	8.16	(6.20- 10.75)
*CPSI	114	f	0	153	211823	178	957682	3.89	(3.13- 4.82)
*CPSI	115	f	0	31	39527	163	392909	1.89	(1.29- 2.78)
Subtotal CPSI								5.80	(5.09- 6.60)
DEAN3	123	f	0	41	196	27	574	4.45	(2.66- 7.42)
*HAMMON	44	m	0	-	-	-	-	6.00	(2.90- 12.40)
*KAISE2	40	m	0	25	11767	5	11466	4.87	(1.87- 12.72)
*KAISE2	48	m	0	14	3401	4	4486	4.62	(1.52- 14.01)
*KAISE2	16	f	0	26	13883	2	24159	22.62	(5.37- 95.30)
*KAISE2	24	f	0	9	3362	3	12285	10.96	(2.97- 40.47)
Subtotal KAISE2								7.24	(4.05- 12.94)
Partial Totals				2224	741373	520	1721357		

*prospective study

REF	NRR	SEX	AD	Ys	Ws	Qs	Ps
BENHAM	71	m	0	2.42	6.09	2.62	0.0000
*CPSI	38	m	0	2.40	69.31	28.00	0.0000
*CPSI	39	m	0	2.10	50.68	5.54	0.0000
*CPSI	114	f	0	1.36	82.32	13.91	0.0000
*CPSI	115	f	0	0.64	26.07	33.38	0.0011
Subtotal CPSI				1.76	228.38	80.83	
DEAN3	123	f	0	1.49	14.65	1.12	0.0000
*HAMMON	44	m	0	1.79	7.28	0.00	0.0000
*KAISE2	40	m	0	1.58	4.17	0.14	0.0012
*KAISE2	48	m	0	1.53	3.12	0.18	0.0069
*KAISE2	16	f	0	3.12	1.86	3.39	0.0000
*KAISE2	24	f	0	2.39	2.25	0.88	0.0003
Subtotal KAISE2				1.98	11.40	4.59	

	N	11
	NS	5
	Wt	267.79
Het	Chi	89.16
Het	df	10
Het	P	***
Fixed	RR	5.86
	RRl	5.20
	RRu	6.61
	P	+++
Random	RR	6.11
	RRl	4.01
	RRu	9.33
	P	+++
Asymm	P	N.S.

Table 1A12 - 3

IESLC - Meta-analysis of Ever Smoking, Cigarettes only, Age 65+
All LC types
Most adjusted

Sex combined	male	female	Total
N	6	5	11
NS	4	3	7
Wt	140.65	127.14	267.79
Het Chi	7.85	21.00	89.16
Het df	5	4	10
Het P	N.S.	***	***
Fixed RR	9.20	3.56	5.86
RRl	7.80	2.99	5.20
RRu	10.86	4.23	6.61
P	+++	+++	+++
Random RR	8.57	4.42	6.11
RRl	6.62	2.56	4.01
RRu	11.09	7.62	9.33
P	+++	+++	+++
Between Chi			60.31
Between df			1
Between P			***
Btwn(F) P			**
Btwn(R) P			*

Table 1A12 - 4

IESLC - Meta-analysis of Ever Smoking, Cigarettes only, Age 65+
All LC types
Least adjusted

REF	NRR	X	SEX	AGEL	AGEH	RACE	YF	LC TYPE	LOC	START	ST	NLC	R	VB	P	H	AD	PRODUCT	DENOM	De
BENHAM	71		m	70	99	all	-	not mix	Eu:wst	1976	CC	1625	n	bl	n	y	0	cig only	nev any	st
CPSI	38		m	65	74	wh	0	all	NAmer	1959	pr	5138	n	bl	n	n	0	cig only	nev any	st
CPSI	39		m	75	84	wh	0	all	NAmer	1959	pr	5138	n	bl	n	n	0	cig only	nev any	st
CPSI	114		f	65	74	wh	0	all	NAmer	1959	pr	5138	n	bl	n	n	0	cig only	nev any	st
CPSI	115		f	75	84	wh	0	all	NAmer	1959	pr	5138	n	bl	n	n	0	cig only	nev any	st
DEAN3	123		f	65	99	all	-	all	Eu:UK	1969	CC	766	n	V	y	n	0	cig only	nev any	st
HAMMON	44		m	65	69	wh	0	all	NAmer	1952	pr	448	n	bl	n	n	0	cig only	nev any	ot
KAISE2	40		m	65	74	all	9	all	NAmer	1979	pr	318	n	bl	n	n	0	cig only	nev any	st
KAISE2	48		m	75	99	all	9	all	NAmer	1979	pr	318	n	bl	n	n	0	cig only	nev any	st
KAISE2	16		f	65	74	all	9	all	NAmer	1979	pr	318	n	bl	n	n	0	cig only	nev any	st
KAISE2	24		f	75	99	all	9	all	NAmer	1979	pr	318	n	bl	n	n	0	cig only	nev any	st

Cigarette type is all/unspec for all RRs
except for the following:

REF	NRR	CIGTYPE
DEAN3	123	MC only

Table 1A12 - 5

IESLC - Meta-analysis of Ever Smoking, Cigarettes only, Age 65+
All LC types
Least adjusted

REF	NRR	SEX	AD	Number Exposed Case	Cont	Non-exposed Case	Cont	RR	95.00%CI
BENHAM	71	m	0	169	203	7	95	11.30	(5.11- 25.00)
*CPSI	38	m	0	1358	380329	73	226284	11.07	(8.75- 14.01)
*CPSI	39	m	0	398	76882	58	91417	8.16	(6.20- 10.75)
*CPSI	114	f	0	153	211823	178	957682	3.89	(3.13- 4.82)
*CPSI	115	f	0	31	39527	163	392909	1.89	(1.29- 2.78)
Subtotal CPSI								5.80	(5.09- 6.60)
DEAN3	123	f	0	41	196	27	574	4.45	(2.66- 7.42)
*HAMMON	44	m	0	-	-	-	-	6.00	(2.90- 12.40)
*KAISE2	40	m	0	25	11767	5	11466	4.87	(1.87- 12.72)
*KAISE2	48	m	0	14	3401	4	4486	4.62	(1.52- 14.01)
*KAISE2	16	f	0	26	13883	2	24159	22.62	(5.37- 95.30)
*KAISE2	24	f	0	9	3362	3	12285	10.96	(2.97- 40.47)
Subtotal KAISE2								7.24	(4.05- 12.94)
Partial Totals				2224	741373	520	1721357		

*prospective study

REF	NRR	SEX	AD	Ys	Ws	Qs	Ps
BENHAM	71	m	0	2.42	6.09	2.62	0.0000
*CPSI	38	m	0	2.40	69.31	28.00	0.0000
*CPSI	39	m	0	2.10	50.68	5.54	0.0000
*CPSI	114	f	0	1.36	82.32	13.91	0.0000
*CPSI	115	f	0	0.64	26.07	33.38	0.0011
Subtotal CPSI				1.76	228.38	80.83	
DEAN3	123	f	0	1.49	14.65	1.12	0.0000
*HAMMON	44	m	0	1.79	7.28	0.00	0.0000
*KAISE2	40	m	0	1.58	4.17	0.14	0.0012
*KAISE2	48	m	0	1.53	3.12	0.18	0.0069
*KAISE2	16	f	0	3.12	1.86	3.39	0.0000
*KAISE2	24	f	0	2.39	2.25	0.88	0.0003
Subtotal KAISE2				1.98	11.40	4.59	

	N	11
	NS	5
	Wt	267.79
Het	Chi	89.16
Het	df	10
Het	P	***
Fixed	RR	5.86
	RRl	5.20
	RRu	6.61
	P	+++
Random	RR	6.11
	RRl	4.01
	RRu	9.33
	P	+++
Asymm	P	N.S.

Table 1A12 - 6

IESLC - Meta-analysis of Ever Smoking, Cigarettes only, Age 65+
All LC types
Least adjusted

	Sex combined male	female	Total
N	6	5	11
NS	4	3	7
Wt	140.65	127.14	267.79
Het Chi	7.85	21.00	89.16
Het df	5	4	10
Het P	N.S.	***	***
Fixed RR	9.20	3.56	5.86
RRl	7.80	2.99	5.20
RRu	10.86	4.23	6.61
P	+++	+++	+++
Random RR	8.57	4.42	6.11
RRl	6.62	2.56	4.01
RRu	11.09	7.62	9.33
P	+++	+++	+++
Between Chi			60.31
Between df			1
Between P			***
Btwn(F) P			**
Btwn(R) P			*

Table 1A12 - 7

IESLC - Meta-analysis of Ever Smoking, Cigarettes only, Age 65+
All LC types
Excluded studies (and stage at which they were excluded)

1 BOUCHA BUELL LAURIL MZILEN

2 BYERS2 ENSTRO HIRAY2 KANELL LIAW LICKIN MRFIT MURATA NOTANI RESTRE RIMING SAARIK SEGI2 SHIMIZ SITAS SOBUE2 TANG2 WALD WARSIN WATSON WU2 WYNDE5 WYNDE8 WYNDER

3 ABELIN ABRAHA AGUDO AKIBA ALDERS AMANDU AMES ANDERS ARCHER ARMADA AUSTIN AUVINE AXELSO AXELSS BAND BARBON BECHER BENSHL BERRIN BEST BLOHMK BLOT1 BLOT2 BLOT3 BLOT4 BOFFET BOUCOT BRESLO BRETT BROCKM BROSS BROWN1 BYERS1 CARPEN CASCO2 CASCOR CEDERL CHAN CHANG CHATZI CHEN CHEN2 CHEN3 CHIAZZ CHOI CHOW CHYOU COOKSO CORREA CPSII DARBY DAVEYS DEAN DEAN2 DEKLER DESTE2 DESTEF DOCKER DOLL DOLL2 DORANT DOSEME DROSTE DU DUNN EBELIN ENGELA ESAKI FAN GAO GAO2 GARCIA GARDIN GENG GER GILLIS GOLLED GOODMA GRAHAM GREGOR GSELL HAENSZ HAMMO2 HANSEN HEGMAN HEIN HENNEK HIRAOK HIRAYA HITOSU HOLE HOROWI HORWIT HU HU2 HUANG ISHIMA JAHN JAIN JARUP JARVHO JEDRYC JIANG JOLY JONES JUSSAW KAISER KATSOU KAUFMA KELLER KHUDER KIHARA KINLEN KJUUS KNEKT KO KOHLME KOO KOULUM KREUZE KREYBE KUBIK LAMTH LAMWK LAMWK2 LANGE LAUSSM LEI LEMARC LETOUR LIDDEL LIU LIU2 LIU3 LIU5 LOMBA2 LOMBAR LUBIN LUBIN2 LUO MACLEN MAGNUS MARSH MARSH2 MARTIS MASTRA MATOS MATSUD MCCONN MCDUFF MCLAUG MIGRAN MILLER MILLS MOLLO MRFITR NOTAN2 NOU ODRISC ORMOS OSANN OSANN2 PARKIN PASTOR PAWLEG PERNU PERSH2 PETO PEZZO2 PEZZOT PIKE PISANI POFFIJ POLEDN QIAO QIAO2 RACHTA RADZIK RANDIG REN RONCO ROOTS ROTHSC SANKAR SCHWA2 SCHWAR SEGI SEOW SHAW SIEMIA SIMARA SIMONA SOBUE SPEIZE SPITZ STASZE STAYNE STOCKW STUCKE SUN SUZUK2 SUZUKI SVENSS TANG TAO TENKAN TIZZAN TOKARS TOUSEY TSUGAN TULINI TVERDA ULMER VANDER VEIERO VUTUC WAKAI WANG WANG2 WANG3 WANG4 WICKLU WIGLE WILKIN WU WUNSCH WYNDE2 WYNDE3 WYNDE6 WYNDE7 XIANGZ XU XU2 XU3 XU4 YAMAGU YONG YUAN ZHANG ZHENG ZHOU

4 BROWN2 SADOWS

5 BUFFLE COMSTO DAMBER DORGAN DORN GARSHI GODLEY HINDS HUMBLE LEVIN LIU4 NAM PERSHA PRESCO STOCKS WUWILL WYNDE4

Table 1A12 - 8
Potentially overlapping studies

REF	REFGP	PRINC	.	OVERLAP/LINK
BENHAM	LUBIN2	2		Subset of Lubin2
CPSI	CPSI	1		CPSI overall

Table 1A12 - 9
Most adjusted - insufficient data for metaanalysis

REF	NRR	SEX	AGEL	AGEH	RACE	YF	LC TYPE	LOC	START	ST	NLC	R	VB	P	H	AD	PRODUCT	DENOM	De
SADOWS	20	m	70	99	wh	-	all	NAmer	1938	CC	477	n	bl	n	n	0	cig only	nev any	st

REF	NRR	RR	SIG	RRDATA comment
SADOWS	20	2.66		0
