# Supplementary material for: Systematic review with meta-analysis of the epidemiological evidence in the 1900s relating smoking to lung cancer
Source: BMC Cancer. 2012 Sep 3;12:385. doi: 10.1186/1471-2407-12-385 (PMC3505152; doi:10.1186/1471-2407-12-385)
Supplement: Additional file 5 — Detailed Analysis Tables (Individual file names as described in Additional file 1: Methods, Table1). [file 1471-2407-12-385-S5.zip › PDF/2D.pdf]

Table 2D1 -

IESLC - Meta-analysis of Ex Smoking, Any product (or Cigarettes if Any not available)
Squamous

This analysis is restricted to results for:
1) Non-dose-response data
2) Ex smokers
3) Results complete enough for use in metaanalysis

Within each study, results are then selected (in the following order of preference, within each sex) for:
4) PRODUCT: all/unspec, cigarettes regardless of other products, cigarettes only
5) CIGTYPE: all/unspecified, MC regardless of HR, MC only
6) DENOM: never smoked anything, never smoked cigarettes, (never +1 = +long term ex, +2 = +amount unknown,
+3 = never cigs+long term ex)
7) Followup period (YF, prospective studies): whole study (coded as 0) or longest available
8) LCtype: squamous or nearest available, but not adeno. (q = squamous, s = small,
a = adeno, KI = Kreyberg I, u = undifferentiated)
9) Race: all or nearest available, otherwise by race (wh or w = white, bl or b = black, hi = hispanic
ch = chinese, jap = japanese, haw = hawaiian, w+o = white + oriental, sca = scandinavian, as = asian)
10) For overlapping studies: principal rather than subsidiary studies
Finally by Age: whole study (coded as 0) if available, otherwise by widest available age group
and then for single sex results (m, f) in preference to combined sex results (c).

Results adjusted (AD) for the most potential confounders are then chosen in Sections -1 to -3
and results adjusted for the least confounders in Sections -4 to -6. (Those least adjusted results which
actually differ from the most adjusted as marked 'x' in column X in Section -4)
(Results adjusted for an unknown number of confounder(s) are coded as 20.)

Section -7 shows excluded studies, together with the stage (as above) at which no qualifying
results were found.

Section -8 lists the potentially overlapping studies which have been included (1=principal, 2=subsidiary).

Section -9 lists any results which would have been included in preference except that they had data not complete
enough for use in meta-analysis, with their significance (yes/no), if known, and any further comment as entered
on the database.

In addition to those mentioned above, the following fields, levels and abbreviations are used:
* or nk = not known, n = no, y = yes, ot = other
nev = never
all/unspec = all or unspecified, cig+/-ot = cigarettes irrespective of other products (cigar, pipe etc)
MC = manufactured cigarettes, HR = hand-rolled cigarettes
REF: 6-character study reference
NRR: number of the RR on the database within the study
ST : study type (CC = case control, pr or prosp = prospective)
NLC: number of lung cancer cases in whole study
R : risky occupational population (n = no, m = mining, o = other risky)
VB : national cigarette type (V = at least 75% Virginia, bl = at least 75% blended, ot = other)
P : any proxy use
H : full histological confirmation
De : derivation of RR/CI (or = original, st = standard method, ot = other method of estimation)

Table 2D1 - 1

IESLC - Meta-analysis of Ex Smoking, Any product (or Cigarettes if Any not available)
Squamous
Most adjusted

REF	NRR	SEX	AGEL	AGEH	RACE	YF	LC TYPE	LOC	START	ST	NLC	R	VB	P	H	AD	PRODUCT	DENOM	De
BARBON	16	m	0	0	all	-	q	Eu:wst	1979	CC	755	n	bl	y	y	1	all/unsp	nev any	or
BROWN2	26	m	0	0	wh	-	q	NAmer	1984	CC	14596	n	bl	n	y	2	cig+/-ot	nev cigs	or
BROWN2	25	f	0	0	wh	-	q	NAmer	1984	CC	14596	n	bl	n	y	2	cig+/-ot	nev cigs	or
BUFFLE	64	f	0	0	w-hi	-	q	NAmer	1976	CC	943	n	bl	y	n	0	cig+/-ot	nev cigs	st
COMSTO	19	m	0	0	all	-	q	NAmer	1975	ot	258	n	bl	n	n	0	cig+/-ot	nev cigs	st
COMSTO	27	f	0	0	all	-	q	NAmer	1975	ot	258	n	bl	n	n	0	cig+/-ot	nev cigs	ot
CORREA	39	c	0	0	all	-	q+s	NAmer	1979	CC	1359	n	bl	y	n	1	cig+/-ot	nev cigs	or
ENGELA	55	m	0	0	all	0	q	Eu:Sca	1964	pr	435	n	bl	n	n	7	cig+/-ot	nev cigs	or
HAENSZ	21	f	0	0	all	-	q+u	NAmer	1955	CC	158	n	bl	n	y	0	cig+/-ot	nev any	st
HAMMON	151	m	0	0	wh	0	not a	NAmer	1952	pr	448	n	bl	n	n	1	cig only	nev any	ot
JAHN	12	m	0	0	all	-	q	Eu:Ger	1988	CC	1004	n	bl	n	n	0	cig+/-ot	nev any	st
JAIN	28	m	0	0	all	-	q	NAmer	1981	CC	845	n	V	y	n	0	cig+/-ot	nev cigs	st
JAIN	23	f	0	0	all	-	q	NAmer	1981	CC	845	n	V	y	n	0	cig+/-ot	nev cigs	st
JEDRYC	23	m	0	0	all	-	q	Eu:est	1980	CC	1630	n	bl	y	n	0	cig+/-ot	nev any	st
KATSOU	19	f	0	0	all	-	KI	Eu:bal	1987	CC	101	n	bl	n	n	1	all/unsp	nev any	or
KHUDER	8	m	0	0	all	-	q	NAmer	1985	CC	482	n	bl	n	y	0	cig+/-ot	nev cigs	or
KIHARA	10	c	0	0	jap	-	q	As:Jap	1991	CC	440	n	bl	n	n	0	all/unsp	nev any	st
LUBIN2	257	m	0	0	all	-	q	Eu:mul	1976	CC	7804	n	bl	n	y	0	cig+/-ot	nev any	st
LUBIN2	269	f	0	0	all	-	q	Eu:mul	1976	CC	7804	n	bl	n	y	0	cig+/-ot	nev any	st
MATOS	41	m	0	0	all	-	q	SCAmer	1994	CC	200	n	bl	n	n	2	cig+/-ot	nev any	or
OSANN	27	m	0	0	all	-	q	NAmer	1984	CC	1986	n	bl	n	n	2	cig+/-ot	nev cigs	or
OSANN	28	f	0	0	all	-	q	NAmer	1984	CC	1986	n	bl	n	n	2	cig+/-ot	nev cigs	or
OSANN2	27	f	0	0	all	-	KI	NAmer	1964	ot	217	n	bl	n	y	1	cig+/-ot	nev cigs	or
SOBUE	33	m	0	0	all	-	q	As:Jap	1986	CC	1376	n	bl	n	y	1	cig+/-ot	nev cigs	or
SOBUE	43	f	0	0	all	-	q	As:Jap	1986	CC	1376	n	bl	n	y	1	cig+/-ot	nev cigs	or
SVENSS	2	f	0	0	all	-	q	Eu:Sca	1983	CC	210	n	bl	n	n	1	all/unsp	nev any	or
TSUGAN	12	m	0	0	all	-	q	As:Jap	1976	CC	134	n	bl	n	y	0	all/unsp	nev any	ot
WAKAI	9	m	0	0	all	-	q	As:Jap	1988	CC	333	n	bl	n	y	1	all/unsp	nev any	or
WAKAI	27	f	0	0	all	-	q	As:Jap	1988	CC	333	n	bl	n	y	1	all/unsp	nev any	or
WU	15	f	0	0	wh	-	q	NAmer	1981	CC	220	n	bl	n	y	2	all/unsp	nev any	or
WYNDE3	1	m	0	0	all	-	KI	NAmer	1966	CC	350	n	bl	n	y	0	all/unsp	nev any	st
WYNDE6	3	m	0	0	all	-	KI	NAmer	1969	CC	4423	n	bl	n	y	0	cig+/-ot	nev any	st
WYNDE6	192	f	0	0	all	-	KI	NAmer	1969	CC	4423	n	bl	n	y	0	cig+/-ot	nev cigs	st

Cigarette type is all/unspec for all RRs

Table 2D1 - 2

IESLC - Meta-analysis of Ex Smoking, Any product (or Cigarettes if Any not available)
Squamous
Most adjusted

REF	NRR	SEX	AD	Number Exposed Case	Number Exposed Cont	Non-exposed Case	Non-exposed Cont	RR	95.00%CI
BARBON	16	m	1	-	-	-	-	8.80	(3.70- 21.00)
BROWN2	26	m	2	-	-	-	-	8.70	(7.40- 10.20)
BROWN2	25	f	2	-	-	-	-	19.20	(15.20- 24.20)
Subtotal BROWN2								11.23	(9.84- 12.82)
BUFFLE	64	f	0	19	56	3	112	12.67	(3.60- 44.62)
COMSTO	19	m	0	17	129	2	84	5.53	(1.25- 24.58)
COMSTO	27	f	0	1	35	0	115	9.76~	(0.39- 244.93)
Subtotal COMSTO								6.12	(1.58- 23.67)
CORREA	39	c	1	-	-	-	-	15.50	(9.30- 26.00)
*ENGELA	55	m	7	-	-	-	-	4.10	(1.20- 14.00)
HAENSZ	21	f	0	3	9	44	236	1.79	(0.47- 6.87)
*HAMMON	151	m	1	-	-	-	-	1.23	(0.60- 2.52)
JAHN	12	m	0	190	402	3	138	21.74	(6.84- 69.13)
JAIN	28	m	0	47	159	2	85	12.56	(2.98- 52.99)
JAIN	23	f	0	22	97	6	214	8.09	(3.18- 20.59)
Subtotal JAIN								9.22	(4.21- 20.18)
JEDRYC	23	m	0	45	312	6	289	6.95	(2.92- 16.53)
KATSOU	19	f	1	-	-	-	-	4.70	(1.05- 21.14)
KHUDER	8	m	0	64	-	9	-	6.70	(3.20- 14.10)
KIHARA	10	c	0	21	70	5	237	14.22	(5.17- 39.09)
LUBIN2	257	m	0	1082	4228	54	2616	12.40	(9.39- 16.36)
LUBIN2	269	f	0	45	157	72	1180	4.70	(3.12- 7.06)
Subtotal LUBIN2								9.12	(7.25- 11.47)
MATOS	41	m	2	-	-	-	-	3.60	(1.00- 12.90)
OSANN	27	m	2	-	-	-	-	22.90	(11.00- 47.30)
OSANN	28	f	2	-	-	-	-	13.50	(6.80- 27.00)
Subtotal OSANN								17.32	(10.50- 28.59)
OSANN2	27	f	1	-	-	-	-	12.60	(1.40- 113.00)
SOBUE	33	m	1	-	-	-	-	13.10	(5.20- 33.40)
SOBUE	43	f	1	-	-	-	-	5.60	(2.30- 13.80)
Subtotal SOBUE								8.43	(4.42- 16.07)
SVENSS	2	f	1	-	-	-	-	4.00	(1.00- 16.90)
TSUGAN	12	m	0	2	2	0	5	11.00~	(0.37- 324.52)
WAKAI	9	m	1	-	-	-	-	6.16	(1.42- 26.70)
WAKAI	27	f	1	-	-	-	-	9.76	(0.85- 112.00)
Subtotal WAKAI								6.96	(1.98- 24.47)
WU	15	f	2	-	-	-	-	7.70	(0.80- 70.30)
WYNDE3	1	m	0	36	125	3	88	8.45	(2.52- 28.30)
WYNDE6	3	m	0	680	1056	29	617	13.70	(9.33- 20.13)
WYNDE6	192	f	0	161	325	40	856	10.60	(7.33- 15.33)
Subtotal WYNDE6								11.99	(9.18- 15.65)
Partial Totals				2435	7162	278	6872		

*prospective study ~ With 0.5 adjustment for zero

REF	NRR	SEX	AD	Ys	Ws	Qs	Ps
BARBON	16	m	1	2.17	5.10	0.12	0.0000
BROWN2	26	m	2	2.16	149.21	3.99	0.0000
BROWN2	25	f	2	2.95	71.05	28.03	0.0000
Subtotal BROWN2				2.42	220.26	32.02	
BUFFLE	64	f	0	2.54	2.42	0.11	0.0001
COMSTO	19	m	0	1.71	1.73	0.66	0.0245
COMSTO	27	f	0	2.28	0.37	0.00	0.1658
Subtotal COMSTO				1.81	2.10	0.66	
CORREA	39	c	1	2.74	14.54	2.49	0.0000
*ENGELA	55	m	7	1.41	2.55	2.14	0.0244
HAENSZ	21	f	0	0.58	2.12	6.47	0.3974
*HAMMON	151	m	1	0.21	7.46	33.53	0.5718
JAHN	12	m	0	3.08	2.87	1.63	0.0000
JAIN	28	m	0	2.53	1.85	0.08	0.0006
JAIN	23	f	0	2.09	4.40	0.25	0.0000
Subtotal JAIN				2.22	6.26	0.32	
JEDRYC	23	m	0	1.94	5.11	0.77	0.0000
KATSOU	19	f	1	1.55	1.70	1.04	0.0433
KHUDER	8	m	0	1.90	6.99	1.26	0.0000
KIHARA	10	c	0	2.65	3.76	0.40	0.0000
LUBIN2	257	m	0	2.52	49.85	1.81	0.0000
LUBIN2	269	f	0	1.55	23.08	14.04	0.0000
Subtotal LUBIN2				2.21	72.93	15.85	
MATOS	41	m	2	1.28	2.35	2.57	0.0496
OSANN	27	m	2	3.13	7.22	4.67	0.0000

Table 2D1 - 2

IESLC - Meta-analysis of Ex Smoking, Any product (or Cigarettes if Any not available)
Squamous
Most adjusted

REF	NRR	SEX	AD	Ys	Ws	Qs	Ps
OSANN	28	f	2	2.60	8.08	0.61	0.0000
Subtotal OSANN				2.85	15.30	5.29	
OSANN2	27	f	1	2.53	0.80	0.03	0.0237
SOBUE	33	m	1	2.57	4.44	0.27	0.0000
SOBUE	43	f	1	1.72	4.79	1.75	0.0002
Subtotal SOBUE				2.13	9.23	2.01	
SVENSS	2	f	1	1.39	1.92	1.70	0.0546
TSUGAN	12	m	0	2.40	0.34	0.00	0.1649
WAKAI	9	m	1	1.82	1.78	0.46	0.0151
WAKAI	27	f	1	2.28	0.64	0.00	0.0673
Subtotal WAKAI				1.94	2.43	0.46	
WU	15	f	2	2.04	0.77	0.06	0.0738
WYNDE3	1	m	0	2.13	2.63	0.10	0.0005
WYNDE6	3	m	0	2.62	25.96	2.19	0.0000
WYNDE6	192	f	0	2.36	28.20	0.03	0.0000
Subtotal WYNDE6				2.48	54.16	2.22	

	N	33
	NS	25
	Wt	446.08
Het	Chi	113.24
Het	df	32
Het	P	***
Fixed	RR	10.25
	RRl	9.34
	RRu	11.24
	P	+++
Random	RR	8.74
	RRl	6.94
	RRu	11.01
	P	+++
Asymm	P	N.S.

Table 2D1 - 3

IESLC - Meta-analysis of Ex Smoking, Any product (or Cigarettes if Any not available)
Squamous
Most adjusted

	Sex			
	combined	male	female	Total
N	2	17	14	33
NS	2	17	14	33
Wt	18.30	277.44	150.35	446.08
Het Chi	0.02	53.67	51.59	113.24
Het df	1	16	13	32
Het P	N.S.	***	***	***
Fixed RR	15.23	9.31	11.66	10.25
RRl	9.63	8.27	9.94	9.34
RRu	24.08	10.47	13.68	11.24
P	+++	+++	+++	+++
Random RR	15.23	8.41	8.03	8.74
RRl	9.63	6.18	5.18	6.94
RRu	24.08	11.45	12.45	11.01
P	+++	+++	+++	+++
Between Chi				7.96
Between df				2
Between P				*
Btwn(F) P				N.S.
Btwn(R) P				(*)

	Lung cancer type					
	q	q+s	q+u	KI	not a	Total
N	25	1	1	5	1	33
NS	18	1	1	4	1	25
Wt	362.67	14.54	2.12	59.29	7.46	446.08
Het Chi	67.24	0.00	0.00	2.60	0.00	113.24
Het df	24	0	0	4	0	32
Het P	***	N.S.	N.S.	N.S.	N.S.	***
Fixed RR	10.44	15.50	1.79	11.50	1.23	10.25
RRl	9.42	9.27	0.47	8.91	0.60	9.34
RRu	11.57	25.92	6.87	14.83	2.52	11.24
P	+++	+++	N.S.	+++	N.S.	+++
Random RR	9.52	15.50	1.79	11.50	1.23	8.74
RRl	7.45	9.27	0.47	8.91	0.60	6.94
RRu	12.16	25.92	6.87	14.83	2.52	11.01
P	+++	+++	N.S.	+++	N.S.	+++
Between Chi						43.40
Between df						4
Between P						***
Btwn(F) P						**
Btwn(R) P						***

	Location								
	NAmer	UK	Scand	othEur	China	Japan	othAs	other	Total
N	18		2	6		6		1	33
NS	13		2	5		4		1	25
Wt	335.80		4.47	87.71		15.75		2.35	446.08
Het Chi	83.53		0.00	18.16		2.74		0.00	113.24
Het df	17		1	5		5		0	32
Het P	***		N.S.	**		N.S.		N.S.	***
Fixed RR	10.83		4.06	9.10		9.32		3.60	10.25
RRl	9.73		1.61	7.38		5.69		1.00	9.34
RRu	12.05		10.25	11.22		15.28		12.93	11.24
P	+++		++	+++		+++		+	+++
Random RR	9.43		4.06	8.42		9.32		3.60	8.74
RRl	6.84		1.61	5.03		5.69		1.00	6.94
RRu	13.01		10.25	14.10		15.28		12.93	11.01
P	+++		++	+++		+++		+	+++
Between Chi									8.80
Between df									4
Between P									(*)
Btwn(F) P									N.S.
Btwn(R) P									N.S.

Table 2D1 - 3

IESLC - Meta-analysis of Ex Smoking, Any product (or Cigarettes if Any not available)
Squamous
Most adjusted

Detailed Country in "other Europe"

	multi	Germany	othWest	East	Balkans	Total
N	2	1	1	1	1	6
NS	1	1	1	1	1	5
Wt	72.93	2.87	5.10	5.11	1.70	87.71
Het Chi	14.86	0.00	0.00	0.00	0.00	18.16
Het df	1	0	0	0	0	5
Het P	***	N.S.	N.S.	N.S.	N.S.	**
Fixed RR	9.12	21.74	8.80	6.95	4.70	9.10
RRl	7.25	6.84	3.69	2.92	1.05	7.38
RRu	11.47	69.13	20.96	16.53	21.09	11.22
P	+++	+++	+++	+++	+	+++
Random RR	7.72	21.74	8.80	6.95	4.70	8.42
RRl	2.98	6.84	3.69	2.92	1.05	5.03
RRu	19.99	69.13	20.96	16.53	21.09	14.10
P	+++	+++	+++	+++	+	+++
Between Chi						3.30
Between df						4
Between P						N.S.
Btwn(F) P						N.S.
Btwn(R) P						N.S.

Detailed Country in "other Asia"

	India	HongKong	other	Total
N				
NS				
Wt				
Het Chi				
Het df				
Het P				N.S.
Fixed RR				
RRl				
RRu				
P				+++
Random RR				
RRl				
RRu				
P				+++
Between Chi				
Between df				
Between P				N.S.
Btwn(F) P				N.S.
Btwn(R) P				N.S.

Detailed other continent

	SCAmer	Auslia	Africa	Total
N	1			1
NS	1			1
Wt	2.35			2.35
Het Chi	0.00			0.00
Het df	0			0
Het P	N.S.			N.S.
Fixed RR	3.60			3.60
RRl	1.00			1.00
RRu	12.93			12.93
P	+			+
Random RR	3.60			3.60
RRl	1.00			1.00
RRu	12.93			12.93
P	+			+
Between Chi				
Between df				
Between P				N.S.
Btwn(F) P				N.S.
Btwn(R) P				N.S.

Table 2D1 - 3

IESLC - Meta-analysis of Ex Smoking, Any product (or Cigarettes if Any not available)
Squamous
Most adjusted

	Start year of study <1960	1960-69	1970-79	1980-89	1990+	Total
N	2	5	8	16	2	33
NS	2	4	6	11	2	25
Wt	9.58	60.13	97.42	272.84	6.11	446.08
Het Chi	0.23	3.93	19.08	45.28	2.73	113.24
Het df	1	4	7	15	1	32
Het P	N.S.	N.S.	**	***	(*)	***
Fixed RR	1.34	11.29	9.85	10.97	8.38	10.25
RRl	0.71	8.77	8.08	9.75	3.79	9.34
RRu	2.52	14.54	12.02	12.36	18.53	11.24
P	N.S.	+++	+++	+++	+++	+++
Random RR	1.34	11.29	9.39	10.35	7.58	8.74
RRl	0.71	8.77	6.00	7.57	1.98	6.94
RRu	2.52	14.54	14.68	14.15	29.00	11.01
P	N.S.	+++	+++	+++	++	+++
Between Chi						42.00
Between df						4
Between P						***
Btwn(F) P						**
Btwn(R) P						***

	Study type (1) CC	other	Total
N	28	5	33
NS	21	4	25
Wt	433.18	12.90	446.08
Het Chi	76.05	8.18	113.24
Het df	27	4	32
Het P	***	(*)	***
Fixed RR	10.71	2.34	10.25
RRl	9.74	1.35	9.34
RRu	11.76	4.03	11.24
P	+++	++	+++
Random RR	9.94	3.54	8.74
RRl	8.05	1.38	6.94
RRu	12.28	9.09	11.01
P	+++	++	+++
Between Chi			29.01
Between df			1
Between P			***
Btwn(F) P			**
Btwn(R) P			*

	Study type (2) CC	prosp	other	Total
N	28	2	3	33
NS	21	2	2	25
Wt	433.18	10.01	2.90	446.08
Het Chi	76.05	2.75	0.40	113.24
Het df	27	1	2	32
Het P	***	(*)	N.S.	***
Fixed RR	10.71	1.67	7.46	10.25
RRl	9.74	0.90	2.36	9.34
RRu	11.76	3.10	23.61	11.24
P	+++	N.S.	+++	+++
Random RR	9.94	2.02	7.46	8.74
RRl	8.05	0.63	2.36	6.94
RRu	12.28	6.44	23.61	11.01
P	+++	N.S.	+++	+++
Between Chi				34.04
Between df				2
Between P				***
Btwn(F) P				**
Btwn(R) P				*

Table 2D1 - 3

IESLC - Meta-analysis of Ex Smoking, Any product (or Cigarettes if Any not available)

Squamous

Most adjusted

	Study size (number of LC cases) 100-249	250-499	500-999	1000+	Total
N	7	9	4	13	33
NS	7	7	3	8	25
Wt	10.00	27.91	13.78	394.40	446.08
Het Chi	3.17	20.55	0.49	59.57	113.24
Het df	6	8	3	12	32
Het P	N.S.	**	N.S.	***	***
Fixed RR	4.03	4.59	9.58	11.13	10.25
RRl	2.17	3.17	5.65	10.08	9.34
RRu	7.49	6.65	16.25	12.28	11.24
P	+++	+++	+++	+++	+++
Random RR	4.03	5.48	9.58	11.53	8.74
RRl	2.17	2.84	5.65	8.87	6.94
RRu	7.49	10.58	16.25	14.99	11.01
P	+++	+++	+++	+++	+++
Between Chi					29.47
Between df					3
Between P					***
Btwn(F) P					*
Btwn(R) P					**

	Risky occupational population no	mining	othRisky	Total
N	33			33
NS	25			25
Wt	446.08			446.08
Het Chi	113.24			113.24
Het df	32			32
Het P	***			***
Fixed RR	10.25			10.25
RRl	9.34			9.34
RRu	11.24			11.24
P	+++			+++
Random RR	8.74			8.74
RRl	6.94			6.94
RRu	11.01			11.01
P	+++			+++
Between Chi				
Between df				
Between P				N.S.
Btwn(F) P				N.S.
Btwn(R) P				N.S.

	National cigarette tobacco type Virginia	blended	other	Total
N	2	31		33
NS	1	24		25
Wt	6.26	439.83		446.08
Het Chi	0.25	112.92		113.24
Het df	1	30		32
Het P	N.S.	***		***
Fixed RR	9.22	10.26		10.25
RRl	4.21	9.35		9.34
RRu	20.18	11.27		11.24
P	+++	+++		+++
Random RR	9.22	8.68		8.74
RRl	4.21	6.83		6.94
RRu	20.18	11.04		11.01
P	+++	+++		+++
Between Chi				0.07
Between df				1
Between P				N.S.
Btwn(F) P				N.S.
Btwn(R) P				N.S.

Table 2D1 - 3

IESLC - Meta-analysis of Ex Smoking, Any product (or Cigarettes if Any not available)
Squamous
Most adjusted

	Any proxy use No/nk	Yes	Total
N	27	6	33
NS	20	5	25
Wt	412.65	33.43	446.08
Het Chi	109.41	3.52	113.24
Het df	26	5	32
Het P	***	N.S.	***
Fixed RR	10.17	11.24	10.25
RRl	9.23	8.01	9.34
RRu	11.20	15.78	11.24
P	+++	+++	+++
Random RR	8.33	11.24	8.74
RRl	6.38	8.01	6.94
RRu	10.89	15.78	11.01
P	+++	+++	+++
Between Chi			0.31
Between df			1
Between P			N.S.
Btwn(F) P			N.S.
Btwn(R) P			N.S.

	Full histological confirmation No	Yes	Total
N	16	17	33
NS	13	12	25
Wt	68.35	377.74	446.08
Het Chi	51.31	60.37	113.24
Het df	15	16	32
Het P	***	***	***
Fixed RR	8.91	10.51	10.25
RRl	7.03	9.50	9.34
RRu	11.30	11.62	11.24
P	+++	+++	+++
Random RR	8.11	9.28	8.74
RRl	5.05	7.08	6.94
RRu	13.01	12.15	11.01
P	+++	+++	+++
Between Chi			1.56
Between df			1
Between P			N.S.
Btwn(F) P			N.S.
Btwn(R) P			N.S.

	Number of adjustment variables (1) 0	1	2+/+nk	Total
N	16	10	7	33
NS	12	8	5	25
Wt	161.68	43.18	241.22	446.08
Het Chi	29.49	35.24	39.83	113.24
Het df	15	9	6	32
Het P	*	***	***	***
Fixed RR	9.81	7.03	11.28	10.25
RRl	8.41	5.21	9.94	9.34
RRu	11.45	9.47	12.80	11.24
P	+++	+++	+++	+++
Random RR	9.11	6.43	11.09	8.74
RRl	6.95	3.31	6.78	6.94
RRu	11.94	12.47	18.13	11.01
P	+++	+++	+++	+++
Between Chi				8.68
Between df				2
Between P				*
Btwn(F) P				N.S.
Btwn(R) P				N.S.

Table 2D1 - 3

IESLC - Meta-analysis of Ex Smoking, Any product (or Cigarettes if Any not available)
Squamous
Most adjusted

Number of adjustment variables (2)

	0	1	2	3-5	6+/+nk	Total
N	16	10	6		1	33
NS	12	8	4		1	25
Wt	161.68	43.18	238.68		2.55	446.08
Het Chi	29.49	35.24	37.19		0.00	113.24
Het df	15	9	5		0	32
Het P	*	***	***		N.S.	***
Fixed RR	9.81	7.03	11.40		4.10	10.25
RRl	8.41	5.21	10.05		1.20	9.34
RRu	11.45	9.47	12.95		14.00	11.24
P	+++	+++	+++		+	+++
Random RR	9.11	6.43	12.33		4.10	8.74
RRl	6.95	3.31	7.38		1.20	6.94
RRu	11.94	12.47	20.59		14.00	11.01
P	+++	+++	+++		+	+++
Between Chi						11.32
Between df						3
Between P						*
Btwn(F) P						N.S.
Btwn(R) P						N.S.

Product

	all/unsp	cig+/-ot	cig only	Total
N	9	23	1	33
NS	8	16	1	25
Wt	18.64	419.98	7.46	446.08
Het Chi	2.88	74.87	0.00	113.24
Het df	8	22	0	32
Het P	N.S.	***	N.S.	***
Fixed RR	8.13	10.75	1.23	10.25
RRl	5.16	9.77	0.60	9.34
RRu	12.79	11.83	2.52	11.24
P	+++	+++	N.S.	+++
Random RR	8.13	9.99	1.23	8.74
RRl	5.16	7.93	0.60	6.94
RRu	12.79	12.58	2.52	11.01
P	+++	+++	N.S.	+++
Between Chi				35.50
Between df				2
Between P				***
Btwn(F) P				**
Btwn(R) P				***

Denominator

	nev any	nev cigs	Total
N	17	16	33
NS	15	11	26
Wt	137.45	308.64	446.08
Het Chi	61.55	43.98	113.24
Het df	16	15	32
Het P	***	***	***
Fixed RR	8.41	11.19	10.25
RRl	7.12	10.00	9.34
RRu	9.94	12.51	11.24
P	+++	+++	+++
Random RR	6.63	11.13	8.74
RRl	4.39	8.48	6.94
RRu	10.02	14.60	11.01
P	+++	+++	+++
Between Chi			7.71
Between df			1
Between P			**
Btwn(F) P			N.S.
Btwn(R) P			*

Table 2D1 - 3

IESLC - Meta-analysis of Ex Smoking, Any product (or Cigarettes if Any not available)
Squamous
Most adjusted

	Derivation of RR/CI			
	Orig	StdCalc	Other	Total
N	17	13	3	33
NS	13	10	3	26
Wt	283.93	153.99	8.17	446.08
Het Chi	49.81	28.42	2.92	113.24
Het df	16	12	2	32
Het P	***	**	N.S.	***
Fixed RR	10.99	9.98	1.48	10.25
RRl	9.78	8.52	0.74	9.34
RRu	12.34	11.69	2.93	11.24
P	+++	+++	N.S.	+++
Random RR	9.83	9.29	2.43	8.74
RRl	7.24	6.89	0.56	6.94
RRu	13.36	12.54	10.62	11.01
P	+++	+++	N.S.	+++
Between Chi				32.10
Between df				2
Between P				***
Btwn(F) P				**
Btwn(R) P				N.S.

Table 2D1 - 4

IESLC - Meta-analysis of Ex Smoking, Any product (or Cigarettes if Any not available)
Squamous
Least adjusted

REF	NRR	X	SEX	AGEL	AGEH	RACE	YF	LC TYPE	LOC	START	ST	NLC	R	VB	P	H	AD	PRODUCT	DENOM	De
BARBON	15	x	m	0	0	all	-	q	Eu:wst	1979	CC	755	n	bl	y	y	0	all/unsp	nev any	st
BROWN2	26		m	0	0	wh	-	q	NAmer	1984	CC	14596	n	bl	n	y	2	cig+/-ot	nev cigs	or
BROWN2	25		f	0	0	wh	-	q	NAmer	1984	CC	14596	n	bl	n	y	2	cig+/-ot	nev cigs	or
BUFFLE	64		f	0	0	w-hi	-	q	NAmer	1976	CC	943	n	bl	y	n	0	cig+/-ot	nev cigs	st
COMSTO	19		m	0	0	all	-	q	NAmer	1975	ot	258	n	bl	n	n	0	cig+/-ot	nev cigs	st
COMSTO	27		f	0	0	all	-	q	NAmer	1975	ot	258	n	bl	n	n	0	cig+/-ot	nev cigs	ot
CORREA	39		c	0	0	all	-	q+s	NAmer	1979	CC	1359	n	bl	y	n	1	cig+/-ot	nev cigs	or
ENGELA	55		m	0	0	all	0	q	Eu:Sca	1964	pr	435	n	bl	n	n	7	cig+/-ot	nev cigs	or
HAENSZ	21		f	0	0	all	-	q+u	NAmer	1955	CC	158	n	bl	n	y	0	cig+/-ot	nev any	st
HAMMON	151		m	0	0	wh	0	not a	NAmer	1952	pr	448	n	bl	n	n	1	cig only	nev any	ot
JAHN	12		m	0	0	all	-	q	Eu:Ger	1988	CC	1004	n	bl	n	n	0	cig+/-ot	nev any	st
JAIN	28		m	0	0	all	-	q	NAmer	1981	CC	845	n	V	y	n	0	cig+/-ot	nev cigs	st
JAIN	23		f	0	0	all	-	q	NAmer	1981	CC	845	n	V	y	n	0	cig+/-ot	nev cigs	st
JEDRYC	23		m	0	0	all	-	q	Eu:est	1980	CC	1630	n	bl	y	n	0	cig+/-ot	nev any	st
KATSOU	23	x	f	0	0	all	-	KI	Eu:bal	1987	CC	101	n	bl	n	n	0	all/unsp	nev any	st
KHUDER	8		m	0	0	all	-	q	NAmer	1985	CC	482	n	bl	n	y	0	cig+/-ot	nev cigs	or
KIHARA	10		c	0	0	jap	-	q	As:Jap	1991	CC	440	n	bl	n	n	0	all/unsp	nev any	st
LUBIN2	257		m	0	0	all	-	q	Eu:mul	1976	CC	7804	n	bl	n	y	0	cig+/-ot	nev any	st
LUBIN2	269		f	0	0	all	-	q	Eu:mul	1976	CC	7804	n	bl	n	y	0	cig+/-ot	nev any	st
MATOS	40	x	m	0	0	all	-	q	SCAmer	1994	CC	200	n	bl	n	n	0	cig+/-ot	nev any	st
OSANN	2	x	m	0	0	all	-	q	NAmer	1984	CC	1986	n	bl	n	n	0	cig+/-ot	nev cigs	st
OSANN	6	x	f	0	0	all	-	q	NAmer	1984	CC	1986	n	bl	n	n	0	cig+/-ot	nev cigs	st
OSANN2	9	x	f	0	0	all	-	KI	NAmer	1964	ot	217	n	bl	n	y	0	cig+/-ot	nev cigs	st
SOBUE	1	x	m	0	0	all	-	q	As:Jap	1986	CC	1376	n	bl	n	y	0	cig+/-ot	nev cigs	st
SOBUE	17	x	f	0	0	all	-	q	As:Jap	1986	CC	1376	n	bl	n	y	0	cig+/-ot	nev cigs	st
SVENSS	22	x	f	0	0	all	-	q	Eu:Sca	1983	CC	210	n	bl	n	n	0	all/unsp	nev any	st
TSUGAN	12		m	0	0	all	-	q	As:Jap	1976	CC	134	n	bl	n	y	0	all/unsp	nev any	ot
WAKAI	3	x	m	0	0	all	-	q	As:Jap	1988	CC	333	n	bl	n	y	0	all/unsp	nev any	st
WAKAI	21	x	f	0	0	all	-	q	As:Jap	1988	CC	333	n	bl	n	y	0	all/unsp	nev any	st
WU	10	x	f	0	0	wh	-	q	NAmer	1981	CC	220	n	bl	n	y	0	all/unsp	nev any	st
WYNDE3	1		m	0	0	all	-	KI	NAmer	1966	CC	350	n	bl	n	y	0	all/unsp	nev any	st
WYNDE6	3		m	0	0	all	-	KI	NAmer	1969	CC	4423	n	bl	n	y	0	cig+/-ot	nev any	st
WYNDE6	192		f	0	0	all	-	KI	NAmer	1969	CC	4423	n	bl	n	y	0	cig+/-ot	nev cigs	st

Cigarette type is all/unspec for all RRs

Table 2D1 - 5

IESLC - Meta-analysis of Ex Smoking, Any product (or Cigarettes if Any not available)
Squamous
Least adjusted

REF	NRR	SEX	AD	Number Exposed Case	Cont	Non-exposed Case	Cont	RR	95.00%CI
BARBON	15	m	0	58	205	6	188	8.87	(3.74- 21.02)
BROWN2	26	m	2	-	-	-	-	8.70	(7.40- 10.20)
BROWN2	25	f	2	-	-	-	-	19.20	(15.20- 24.20)
Subtotal BROWN2								11.23	(9.84- 12.82)
BUFFLE	64	f	0	19	56	3	112	12.67	(3.60- 44.62)
COMSTO	19	m	0	17	129	2	84	5.53	(1.25- 24.58)
COMSTO	27	f	0	1	35	0	115	9.76~	(0.39- 244.93)
Subtotal COMSTO								6.12	(1.58- 23.67)
CORREA	39	c	1	-	-	-	-	15.50	(9.30- 26.00)
*ENGELA	55	m	7	-	-	-	-	4.10	(1.20- 14.00)
HAENSZ	21	f	0	3	9	44	236	1.79	(0.47- 6.87)
*HAMMON	151	m	1	-	-	-	-	1.23	(0.60- 2.52)
JAHN	12	m	0	190	402	3	138	21.74	(6.84- 69.13)
JAIN	28	m	0	47	159	2	85	12.56	(2.98- 52.99)
JAIN	23	f	0	22	97	6	214	8.09	(3.18- 20.59)
Subtotal JAIN								9.22	(4.21- 20.18)
JEDRYC	23	m	0	45	312	6	289	6.95	(2.92- 16.53)
KATSOU	23	f	0	4	4	14	67	4.79	(1.07- 21.47)
KHUDER	8	m	0	64	-	9	-	6.70	(3.20- 14.10)
KIHARA	10	c	0	21	70	5	237	14.22	(5.17- 39.09)
LUBIN2	257	m	0	1082	4228	54	2616	12.40	(9.39- 16.36)
LUBIN2	269	f	0	45	157	72	1180	4.70	(3.12- 7.06)
Subtotal LUBIN2								9.12	(7.25- 11.47)
MATOS	40	m	0	14	151	3	110	3.40	(0.95- 12.12)
OSANN	2	m	0	105	477	8	833	22.92	(11.07- 47.45)
OSANN	6	f	0	29	196	12	1093	13.48	(6.76- 26.86)
Subtotal OSANN								17.33	(10.50- 28.58)
OSANN2	9	f	0	7	19	7	58	3.05	(0.95- 9.82)
SOBUE	1	m	0	121	363	3	128	14.22	(4.44- 45.51)
SOBUE	17	f	0	7	64	14	857	6.70	(2.61- 17.18)
Subtotal SOBUE								9.02	(4.34- 18.77)
SVENSS	22	f	0	6	36	5	120	4.00	(1.15- 13.88)
TSUGAN	12	m	0	2	2	0	5	11.00~	(0.37- 324.52)
WAKAI	3	m	0	27	140	2	65	6.27	(1.45- 27.16)
WAKAI	21	f	0	1	5	3	145	9.67	(0.85- 110.07)
Subtotal WAKAI								7.03	(2.00- 24.69)
WU	10	f	0	8	18	2	30	6.67	(1.27- 34.92)
WYNDE3	1	m	0	36	125	3	88	8.45	(2.52- 28.30)
WYNDE6	3	m	0	680	1056	29	617	13.70	(9.33- 20.13)
WYNDE6	192	f	0	161	325	40	856	10.60	(7.33- 15.33)
Subtotal WYNDE6								11.99	(9.18- 15.65)
Partial Totals				2822	8840	357	10566		

*prospective study ~ With 0.5 adjustment for zero

REF	NRR	SEX	AD	Ys	Ws	Qs	Ps
BARBON	15	m	0	2.18	5.15	0.10	0.0000
BROWN2	26	m	2	2.16	149.21	3.60	0.0000
BROWN2	25	f	2	2.95	71.05	28.76	0.0000
Subtotal BROWN2				2.42	220.26	32.36	
BUFFLE	64	f	0	2.54	2.42	0.12	0.0001
COMSTO	19	m	0	1.71	1.73	0.64	0.0245
COMSTO	27	f	0	2.28	0.37	0.00	0.1658
Subtotal COMSTO				1.81	2.10	0.64	
CORREA	39	c	1	2.74	14.54	2.59	0.0000
*ENGELA	55	m	7	1.41	2.55	2.10	0.0244
HAENSZ	21	f	0	0.58	2.12	6.41	0.3974
*HAMMON	151	m	1	0.21	7.46	33.27	0.5718
JAHN	12	m	0	3.08	2.87	1.66	0.0000
JAIN	28	m	0	2.53	1.85	0.08	0.0006
JAIN	23	f	0	2.09	4.40	0.23	0.0000
Subtotal JAIN				2.22	6.26	0.31	
JEDRYC	23	m	0	1.94	5.11	0.74	0.0000
KATSOU	23	f	0	1.57	1.71	0.97	0.0409
KHUDER	8	m	0	1.90	6.99	1.21	0.0000
KIHARA	10	c	0	2.65	3.76	0.42	0.0000
LUBIN2	257	m	0	2.52	49.85	1.97	0.0000
LUBIN2	269	f	0	1.55	23.08	13.74	0.0000
Subtotal LUBIN2				2.21	72.93	15.71	
MATOS	40	m	0	1.22	2.38	2.85	0.0592
OSANN	2	m	0	3.13	7.26	4.80	0.0000

Table 2D1 - 5

IESLC - Meta-analysis of Ex Smoking, Any product (or Cigarettes if Any not available)
Squamous
Least adjusted

REF	NRR	SEX	AD	Ys	Ws	Qs	Ps
OSANN	6	f	0	2.60	8.08	0.64	0.0000
Subtotal OSANN				2.85	15.33	5.44	
OSANN2	9	f	0	1.12	2.81	4.07	0.0613
SOBUE	1	m	0	2.65	2.84	0.32	0.0000
SOBUE	17	f	0	1.90	4.33	0.75	0.0001
Subtotal SOBUE				2.20	7.17	1.07	
SVENSS	22	f	0	1.39	2.48	2.16	0.0289
TSUGAN	12	m	0	2.40	0.34	0.00	0.1649
WAKAI	3	m	0	1.84	1.79	0.42	0.0141
WAKAI	21	f	0	2.27	0.65	0.00	0.0675
Subtotal WAKAI				1.95	2.44	0.42	
WU	10	f	0	1.90	1.40	0.25	0.0247
WYNDE3	1	m	0	2.13	2.63	0.09	0.0005
WYNDE6	3	m	0	2.62	25.96	2.32	0.0000
WYNDE6	192	f	0	2.36	28.20	0.05	0.0000
Subtotal WYNDE6				2.48	54.16	2.37	

	N	33
	NS	25
	Wt	447.35
Het	Chi	117.33
Het	df	32
Het	P	***
Fixed	RR	10.16
	RRl	9.26
	RRu	11.15
	P	+++
Random	RR	8.47
	RRl	6.71
	RRu	10.68
	P	+++
Asymm	P	N.S.

Table 2D1 - 6

IESLC - Meta-analysis of Ex Smoking, Any product (or Cigarettes if Any not available)
Squamous
Least adjusted

	Sex			
	combined	male	female	Total
N	2	17	14	33
NS	2	17	14	33
Wt	18.30	275.95	153.10	447.35
Het Chi	0.02	53.96	56.19	117.33
Het df	1	16	13	32
Het P	N.S.	***	***	***
Fixed RR	15.23	9.29	11.38	10.16
RRl	9.63	8.26	9.71	9.26
RRu	24.08	10.46	13.33	11.15
P	+++	+++	+++	+++
Random RR	15.23	8.38	7.42	8.47
RRl	9.63	6.13	4.79	6.71
RRu	24.08	11.44	11.49	10.68
P	+++	+++	+++	+++
Between Chi				7.15
Between df				2
Between P				*
Btwn(F) P				N.S.
Btwn(R) P				(*)

	Lung cancer type					
	q	q+s	q+u	KI	not a	Total
N	25	1	1	5	1	33
NS	18	1	1	4	1	25
Wt	361.92	14.54	2.12	61.31	7.46	447.35
Het Chi	67.32	0.00	0.00	7.26	0.00	117.33
Het df	24	0	0	4	0	32
Het P	***	N.S.	N.S.	N.S.	N.S.	***
Fixed RR	10.43	15.50	1.79	10.81	1.23	10.16
RRl	9.41	9.27	0.47	8.42	0.60	9.26
RRu	11.56	25.92	6.87	13.89	2.52	11.15
P	+++	+++	N.S.	+++	N.S.	+++
Random RR	9.50	15.50	1.79	9.48	1.23	8.47
RRl	7.44	9.27	0.47	6.24	0.60	6.71
RRu	12.14	25.92	6.87	14.42	2.52	10.68
P	+++	+++	N.S.	+++	N.S.	+++
Between Chi						42.75
Between df						4
Between P						***
Btwn(F) P						*
Btwn(R) P						***

	Location								
	NAmer	UK	Scand	othEur	China	Japan	othAs	other	Total
N	18		2	6		6		1	33
NS	13		2	5		4		1	25
Wt	338.48		5.03	87.77		13.70		2.38	447.35
Het Chi	88.24		0.00	18.12		1.90		0.00	117.33
Het df	17		1	5		5		0	32
Het P	***		N.S.	**		N.S.		N.S.	***
Fixed RR	10.70		4.05	9.10		9.83		3.40	10.16
RRl	9.62		1.69	7.39		5.79		0.95	9.26
RRu	11.90		9.71	11.22		16.69		12.12	11.15
P	+++		++	+++		+++		(+)	+++
Random RR	8.87		4.05	8.44		9.83		3.40	8.47
RRl	6.43		1.69	5.05		5.79		0.95	6.71
RRu	12.24		9.71	14.12		16.69		12.12	10.68
P	+++		++	+++		+++		(+)	+++
Between Chi									9.07
Between df									4
Between P									(*)
Btwn(F) P									N.S.
Btwn(R) P									N.S.

Table 2D1 - 6

IESLC - Meta-analysis of Ex Smoking, Any product (or Cigarettes if Any not available)
Squamous
Least adjusted

	Detailed Country in "other Europe"					
	multi	Germany	othWest	East	Balkans	Total
N	2	1	1	1	1	6
NS	1	1	1	1	1	5
Wt	72.93	2.87	5.15	5.11	1.71	87.77
Het Chi	14.86	0.00	0.00	0.00	0.00	18.12
Het df	1	0	0	0	0	5
Het P	***	N.S.	N.S.	N.S.	N.S.	**
Fixed RR	9.12	21.74	8.87	6.95	4.79	9.10
RRl	7.25	6.84	3.74	2.92	1.07	7.39
RRu	11.47	69.13	21.02	16.53	21.47	11.22
P	+++	+++	+++	+++	+	+++
Random RR	7.72	21.74	8.87	6.95	4.79	8.44
RRl	2.98	6.84	3.74	2.92	1.07	5.05
RRu	19.99	69.13	21.02	16.53	21.47	14.12
P	+++	+++	+++	+++	+	+++
Between Chi						3.26
Between df						4
Between P						N.S.
Btwn(F) P						N.S.
Btwn(R) P						N.S.

	Detailed Country in "other Asia"			
	India	HongKong	other	Total
N				
NS				
Wt				
Het Chi				
Het df				
Het P				N.S.
Fixed RR				
RRl				
RRu				
P				+++
Random RR				
RRl				
RRu				
P				+++
Between Chi				
Between df				
Between P				N.S.
Btwn(F) P				N.S.
Btwn(R) P				N.S.

	Detailed other continent			
	SCAmer	Auslia	Africa	Total
N	1			1
NS	1			1
Wt	2.38			2.38
Het Chi	0.00			0.00
Het df	0			0
Het P	N.S.			N.S.
Fixed RR	3.40			3.40
RRl	0.95			0.95
RRu	12.12			12.12
P	(+)			(+)
Random RR	3.40			3.40
RRl	0.95			0.95
RRu	12.12			12.12
P	(+)			(+)
Between Chi				
Between df				
Between P				N.S.
Btwn(F) P				N.S.
Btwn(R) P				N.S.

Table 2D1 - 6

IESLC - Meta-analysis of Ex Smoking, Any product (or Cigarettes if Any not available)
Squamous
Least adjusted

	Start year of study					
	<1960	1960-69	1970-79	1980-89	1990+	Total
N	2	5	8	16	2	33
NS	2	4	6	11	2	25
Wt	9.58	62.15	97.47	272.01	6.14	447.35
Het Chi	0.23	8.50	19.07	44.98	2.98	117.33
Het df	1	4	7	15	1	32
Het P	N.S.	(*)	**	***	(*)	***
Fixed RR	1.34	10.63	9.86	10.98	8.17	10.16
RRl	0.71	8.29	8.08	9.75	3.70	9.26
RRu	2.52	13.62	12.02	12.36	18.02	11.15
P	N.S.	+++	+++	+++	+++	+++
Random RR	1.34	8.91	9.40	10.35	7.34	8.47
RRl	0.71	5.68	6.01	7.58	1.81	6.71
RRu	2.52	13.97	14.69	14.15	29.71	10.68
P	N.S.	+++	+++	+++	++	+++
Between Chi						41.57
Between df						4
Between P						***
Btwn(F) P						*
Btwn(R) P						***

	Study type (1)		
	CC	other	Total
N	28	5	33
NS	21	4	25
Wt	432.43	14.92	447.35
Het Chi	76.08	6.10	117.33
Het df	27	4	32
Het P	***	N.S.	***
Fixed RR	10.71	2.25	10.16
RRl	9.74	1.35	9.26
RRu	11.76	3.73	11.15
P	+++	++	+++
Random RR	9.93	2.69	8.47
RRl	8.04	1.34	6.71
RRu	12.26	5.39	10.68
P	+++	++	+++
Between Chi			35.15
Between df			1
Between P			***
Btwn(F) P			***
Btwn(R) P			***

	Study type (2)			
	CC	prosp	other	Total
N	28	2	3	33
NS	21	2	2	25
Wt	432.43	10.01	4.91	447.35
Het Chi	76.08	2.75	0.68	117.33
Het df	27	1	2	32
Het P	***	(*)	N.S.	***
Fixed RR	10.71	1.67	4.11	10.16
RRl	9.74	0.90	1.70	9.26
RRu	11.76	3.10	9.95	11.15
P	+++	N.S.	++	+++
Random RR	9.93	2.02	4.11	8.47
RRl	8.04	0.63	1.70	6.71
RRu	12.26	6.44	9.95	10.68
P	+++	N.S.	++	+++
Between Chi				37.82
Between df				2
Between P				***
Btwn(F) P				**
Btwn(R) P				**

Table 2D1 - 6

IESLC - Meta-analysis of Ex Smoking, Any product (or Cigarettes if Any not available)
Squamous
Least adjusted

	Study size (number of LC cases) 100-249	250-499	500-999	1000+	Total
N	7	9	4	13	33
NS	7	7	3	8	25
Wt	13.24	27.91	13.83	392.37	447.35
Het Chi	2.24	20.56	0.48	58.50	117.33
Het df	6	8	3	12	32
Het P	N.S.	**	N.S.	***	***
Fixed RR	3.57	4.59	9.60	11.16	10.16
RRl	2.08	3.17	5.67	10.11	9.26
RRu	6.12	6.66	16.27	12.32	11.15
P	+++	+++	+++	+++	+++
Random RR	3.57	5.48	9.60	11.67	8.47
RRl	2.08	2.84	5.67	8.97	6.71
RRu	6.12	10.59	16.27	15.18	10.68
P	+++	+++	+++	+++	+++
Between Chi					35.55
Between df					3
Between P					***
Btwn(F) P					*
Btwn(R) P					***

	Risky occupational population no	mining	othRisky	Total
N	33			33
NS	25			25
Wt	447.35			447.35
Het Chi	117.33			117.33
Het df	32			32
Het P	***			***
Fixed RR	10.16			10.16
RRl	9.26			9.26
RRu	11.15			11.15
P	+++			+++
Random RR	8.47			8.47
RRl	6.71			6.71
RRu	10.68			10.68
P	+++			+++
Between Chi				
Between df				
Between P				N.S.
Btwn(F) P				N.S.
Btwn(R) P				N.S.

	National cigarette tobacco type Virginia	blended	other	Total
N	2	31		33
NS	1	24		25
Wt	6.26	441.09		447.35
Het Chi	0.25	117.02		117.33
Het df	1	30		32
Het P	N.S.	***		***
Fixed RR	9.22	10.18		10.16
RRl	4.21	9.27		9.26
RRu	20.18	11.17		11.15
P	+++	+++		+++
Random RR	9.22	8.40		8.47
RRl	4.21	6.60		6.71
RRu	20.18	10.69		10.68
P	+++	+++		+++
Between Chi				0.06
Between df				1
Between P				N.S.
Btwn(F) P				N.S.
Btwn(R) P				N.S.

Table 2D1 - 6

IESLC - Meta-analysis of Ex Smoking, Any product (or Cigarettes if Any not available)
Squamous
Least adjusted

	Any proxy use No/nk	Yes	Total
N	27	6	33
NS	20	5	25
Wt	413.87	33.48	447.35
Het Chi	113.44	3.51	117.33
Het df	26	5	32
Het P	***	N.S.	***
Fixed RR	10.08	11.25	10.16
RRl	9.15	8.02	9.26
RRu	11.10	15.79	11.15
P	+++	+++	+++
Random RR	8.01	11.25	8.47
RRl	6.12	8.02	6.71
RRu	10.48	15.79	10.68
P	+++	+++	+++
Between Chi			0.38
Between df			1
Between P			N.S.
Btwn(F) P			N.S.
Btwn(R) P			N.S.

	Full histological confirmation No	Yes	Total
N	16	17	33
NS	13	12	25
Wt	68.96	378.39	447.35
Het Chi	51.94	63.81	117.33
Het df	15	16	32
Het P	***	***	***
Fixed RR	8.84	10.42	10.16
RRl	6.98	9.42	9.26
RRu	11.20	11.53	11.15
P	+++	+++	+++
Random RR	8.05	8.87	8.47
RRl	5.02	6.74	6.71
RRu	12.91	11.66	10.68
P	+++	+++	+++
Between Chi			1.58
Between df			1
Between P			N.S.
Btwn(F) P			N.S.
Btwn(R) P			N.S.

	Number of adjustment variables (1) 0	1	2+/+nk	Total
N	28	2	3	33
NS	21	2	2	25
Wt	202.55	22.00	222.80	447.35
Het Chi	46.51	31.66	32.71	117.33
Het df	27	1	2	32
Het P	*	***	***	***
Fixed RR	9.67	6.56	11.10	10.16
RRl	8.42	4.32	9.74	9.26
RRu	11.10	9.97	12.66	11.15
P	+++	+++	+++	+++
Random RR	8.86	4.42	10.42	8.47
RRl	7.11	0.37	5.15	6.71
RRu	11.03	52.97	21.07	10.68
P	+++	N.S.	+++	+++
Between Chi				6.45
Between df				2
Between P				*
Btwn(F) P				N.S.
Btwn(R) P				N.S.

Table 2D1 - 6

IESLC - Meta-analysis of Ex Smoking, Any product (or Cigarettes if Any not available)
Squamous
Least adjusted

	Number of adjustment variables (2) 0	1	2	3-5	6+/+nk	Total
N	28	2	2		1	33
NS	21	2	1		1	25
Wt	202.55	22.00	220.26		2.55	447.35
Het Chi	46.51	31.66	30.16		0.00	117.33
Het df	27	1	1		0	32
Het P	*	***	***		N.S.	***
Fixed RR	9.67	6.56	11.23		4.10	10.16
RRl	8.42	4.32	9.84		1.20	9.26
RRu	11.10	9.97	12.82		14.00	11.15
P	+++	+++	+++		+	+++
Random RR	8.86	4.42	12.86		4.10	8.47
RRl	7.11	0.37	5.92		1.20	6.71
RRu	11.03	52.97	27.94		14.00	10.68
P	+++	N.S.	+++		+	+++
Between Chi						9.01
Between df						3
Between P						*
Btwn(F) P						N.S.
Btwn(R) P						N.S.

	Product all/unsp	cig+/-ot	cig only	Total
N	9	23	1	33
NS	8	16	1	25
Wt	19.90	419.99	7.46	447.35
Het Chi	3.16	78.63	0.00	117.33
Het df	8	22	0	32
Het P	N.S.	***	N.S.	***
Fixed RR	7.91	10.68	1.23	10.16
RRl	5.10	9.70	0.60	9.26
RRu	12.27	11.75	2.52	11.15
P	+++	+++	N.S.	+++
Random RR	7.91	9.67	1.23	8.47
RRl	5.10	7.64	0.60	6.71
RRu	12.27	12.24	2.52	10.68
P	+++	+++	N.S.	+++
Between Chi				35.54
Between df				2
Between P				***
Btwn(F) P				**
Btwn(R) P				***

	Denominator nev any	nev cigs	Total
N	17	16	33
NS	15	11	26
Wt	138.73	308.62	447.35
Het Chi	62.13	47.62	117.33
Het df	16	15	32
Het P	***	***	***
Fixed RR	8.37	11.09	10.16
RRl	7.09	9.92	9.26
RRu	9.88	12.40	11.15
P	+++	+++	+++
Random RR	6.58	10.64	8.47
RRl	4.37	8.03	6.71
RRu	9.89	14.08	10.68
P	+++	+++	+++
Between Chi			7.58
Between df			1
Between P			**
Btwn(F) P			N.S.
Btwn(R) P			(*)

Table 2D1 - 6

IESLC - Meta-analysis of Ex Smoking, Any product (or Cigarettes if Any not available)
Squamous
Least adjusted

	Derivation of RR/CI			
	Orig	StdCalc	Other	Total
N	5	25	3	33
NS	4	19	3	26
Wt	244.33	194.86	8.17	447.35
Het Chi	36.11	45.53	2.92	117.33
Het df	4	24	2	32
Het P	***	**	N.S.	***
Fixed RR	11.16	9.79	1.48	10.16
RRl	9.85	8.51	0.74	9.26
RRu	12.65	11.27	2.93	11.15
P	+++	+++	N.S.	+++
Random RR	10.64	8.92	2.43	8.47
RRl	6.43	7.06	0.56	6.71
RRu	17.58	11.27	10.62	10.68
P	+++	+++	N.S.	+++
Between Chi				32.77
Between df				2
Between P				***
Btwn(F) P				**
Btwn(R) P				N.S.

Table 2D1 - 7

IESLC - Meta-analysis of Ex Smoking, Any product (or Cigarettes if Any not available)
Squamous
Excluded studies (and stage at which they were excluded)

1 BOUCHA BUELL LAURIL MZILEN

2 ABELIN ABRAHA ALDERS AUVINE AXELSO BAND BERRIN BLOT1 BLOT2 BLOT3 BLOT4 BRESLO BROCKM BROWN1 BYERS1 BYERS2 CASCO2 CASCOR CHAN CHATZI CHEN CHEN2 CHEN3 CHIAZZ COOKSO DAVEYS DEAN DOSEME DU DUNN EBELIN ENSTRO ESAKI FAN GENG GER GODLEY GOLLED GSELL HANSEN HEGMAN HINDS HIRAOK HIRAY2 HOROWI HORWIT HU HU2 HUANG ISHIMA JARUP JIANG JONES JUSSAW KANELL KO KOHLME KOULUM KREYBE LAMTH LAMWK LAMWK2 LAUSSM LEI LETOUR LEVIN LIAW LICKIN LIU LIU2 LIU3 LIU4 LIU5 LOMBA2 LUO MAGNUS MARSH MARSH2 MARTIS MASTRA MATSUD MCCONN MCDUFF MCLAUG MILLER MILLS MOLLO MRFIT MURATA NOTAN2 NOTANI NOU ORMOS PASTOR PAWLEG PERNU PERSHA PIKE POFFIJ POLEDN QIAO RADZIK RANDIG REN RIMING RONCO ROOTS ROTHSC SAARIK SADOWS SANKAR SCHWA2 SEGI SEGI2 SEOW SHIMIZ SIEMIA SIMARA SITAS SOBUE2 STASZE STAYNE STOCKS SUN TANG2 TAO ULMER VANDER WALD WANG WANG3 WANG4 WARSIN WATSON WICKLU WILKIN WU2 WUWILL WYNDE2 WYNDE4 WYNDE5 WYNDE8 WYNDER XIANGZ XU XU2 XU3 XU4 YUAN ZHANG ZHENG ZHOU

3 RESTRE

4 BOFFET

8 AGUDO AKIBA AMANDU AMES ANDERS ARCHER ARMADA AUSTIN AXELSS BECHER BENSHL BEST BLOHMK BOUCOT BRETT BROSS CARPEN CEDERL CHANG CHOI CHOW CHYOU CPSI CPSII DAMBER DARBY DEAN2 DEAN3 DEKLER DESTE2 DESTEF DOCKER DOLL DOLL2 DORANT DORGAN DORN DROSTE GAO GAO2 GARCIA GARDIN GARSHI GILLIS GOODMA GRAHAM GREGOR HAMMO2 HEIN HENNEK HIRAYA HITOSU HOLE HUMBLE JARVHO JOLY KAISE2 KAISER KAUFMA KELLER KINLEN KJUUS KNEKT KOO KREUZE KUBIK LANGE LEMARC LIDDEL LOMBAR LUBIN MACLEN MIGRAN MRFITR NAM ODRISC PARKIN PERSH2 PETO PEZZO2 PEZZOT PISANI PRESCO QIAO2 RACHTA SCHWAR SHAW SIMONA SPEIZE SPITZ STOCKW STUCKE SUZUK2 SUZUKI TANG TENKAN TIZZAN TOKARS TOUSEY TULINI TVERDA VEIERO WANG2 WIGLE WUNSCH YAMAGU YONG

10 BENHAM VUTUC WYNDE7

Table 2D1 - 8
Potentially overlapping studies

REF	REFGP	PRINC	. OVERLAP/LINK
LUBIN2	LUBIN2	1	Lubin-combined
OSANN2	KAISER	2	KAISER/OSANN2
WYNDE6	WYNDE6	1	WYNDE5/6/7/8
JAHN	BOFFET	2	Subset of BOFFET

Table 2D2 -

IESLC - Meta-analysis of Ex Smoking, Cigarettes (or Any Product if Cigarettes not available)
Squamous

This analysis is restricted to results for:
1) Non-dose-response data
2) Ex smokers
3) Results complete enough for use in metaanalysis

Within each study, results are then selected (in the following order of preference, within each sex) for:
4) PRODUCT: cigarettes regardless of other products, cigarettes only, all/unspec
5) CIGTYPE: all/unspecified, MC regardless of HR, MC only
6) DENOM: never smoked anything, never smoked cigarettes, (never +1 = +long term ex, +2 = +amount unknown,
 +3 = never cigs+long term ex)
7) Followup period (YF, prospective studies): whole study (coded as 0) or longest available
8) LCtype: squamous or nearest available, but not adeno. (q = squamous, s = small,
 a = adeno, KI = Kreyberg I, u = undifferentiated)
9) Race: all or nearest available, otherwise by race (wh or w = white, bl or b = black, hi = hispanic
 ch = chinese, jap = japanese, haw = hawaiian, w+o = white + oriental, sca = scandinavian, as = asian)
10) For overlapping studies: principal rather than subsidiary studies
Finally by Age: whole study (coded as 0) if available, otherwise by widest available age group
and then for single sex results (m, f) in preference to combined sex results (c).

Results adjusted (AD) for the most potential confounders are then chosen in Sections -1 to -3
and results adjusted for the least confounders in Sections -4 to -6. (Those least adjusted results which
actually differ from the most adjusted as marked 'x' in column X in Section -4)
(Results adjusted for an unknown number of confounder(s) are coded as 20.)

Section -7 shows excluded studies, together with the stage (as above) at which no qualifying
results were found.

Section -8 lists the potentially overlapping studies which have been included (1=principal, 2=subsidiary).

Section -9 lists any results which would have been included in preference except that they had data not complete
enough for use in meta-analysis, with their significance (yes/no), if known, and any further comment as entered
on the database.

In addition to those mentioned above, the following fields, levels and abbreviations are used:
* or nk = not known, n = no, y = yes, ot = other
nev = never
all/unspec = all or unspecified, cig+/-ot = cigarettes irrespective of other products (cigar, pipe etc)
MC = manufactured cigarettes, HR = hand-rolled cigarettes
REF: 6-character study reference
NRR: number of the RR on the database within the study
ST : study type (CC = case control, pr or prosp = prospective)
NLC: number of lung cancer cases in whole study
R : risky occupational population (n = no, m = mining, o = other risky)
VB : national cigarette type (V = at least 75% Virginia, bl = at least 75% blended, ot = other)
P : any proxy use
H : full histological confirmation
De : derivation of RR/CI (or = original, st = standard method, ot = other method of estimation)

Table 2D2 - 1

IESLC - Meta-analysis of Ex Smoking, Cigarettes (or Any Product if Cigarettes not available)
Squamous
Most adjusted

REF	NRR	SEX	AGEL	AGEH	RACE	YF	LC TYPE	LOC	START	ST	NLC	R	VB	P	H	AD	PRODUCT	DENOM	De
BARBON	16	m	0	0	all	-	q	Eu:wst	1979	CC	755	n	bl	y	y	1	all/unsp	nev any	or
BROWN2	26	m	0	0	wh	-	q	NAmer	1984	CC	14596	n	bl	n	y	2	cig+/-ot	nev cigs	or
BROWN2	25	f	0	0	wh	-	q	NAmer	1984	CC	14596	n	bl	n	y	2	cig+/-ot	nev cigs	or
BUFFLE	64	f	0	0	w-hi	-	q	NAmer	1976	CC	943	n	bl	y	n	0	cig+/-ot	nev cigs	st
COMSTO	19	m	0	0	all	-	q	NAmer	1975	ot	258	n	bl	n	n	0	cig+/-ot	nev cigs	st
COMSTO	27	f	0	0	all	-	q	NAmer	1975	ot	258	n	bl	n	n	0	cig+/-ot	nev cigs	ot
CORREA	39	c	0	0	all	-	q+s	NAmer	1979	CC	1359	n	bl	y	n	1	cig+/-ot	nev cigs	or
ENGELA	55	m	0	0	all	0	q	Eu:Sca	1964	pr	435	n	bl	n	n	7	cig+/-ot	nev cigs	or
HAENSZ	21	f	0	0	all	-	q+u	NAmer	1955	CC	158	n	bl	n	y	0	cig+/-ot	nev any	st
HAMMON	151	m	0	0	wh	0	not a	NAmer	1952	pr	448	n	bl	n	n	1	cig only	nev any	ot
JAHN	12	m	0	0	all	-	q	Eu:Ger	1988	CC	1004	n	bl	n	n	0	cig+/-ot	nev any	st
JAIN	28	m	0	0	all	-	q	NAmer	1981	CC	845	n	V	y	n	0	cig+/-ot	nev cigs	st
JAIN	23	f	0	0	all	-	q	NAmer	1981	CC	845	n	V	y	n	0	cig+/-ot	nev cigs	st
JEDRYC	23	m	0	0	all	-	q	Eu:est	1980	CC	1630	n	bl	y	n	0	cig+/-ot	nev any	st
KATSOU	19	f	0	0	all	-	KI	Eu:bal	1987	CC	101	n	bl	n	n	1	all/unsp	nev any	or
KHUDER	8	m	0	0	all	-	q	NAmer	1985	CC	482	n	bl	n	y	0	cig+/-ot	nev cigs	or
KIHARA	10	c	0	0	jap	-	q	As:Jap	1991	CC	440	n	bl	n	n	0	all/unsp	nev any	st
LUBIN2	257	m	0	0	all	-	q	Eu:mul	1976	CC	7804	n	bl	n	y	0	cig+/-ot	nev any	st
LUBIN2	269	f	0	0	all	-	q	Eu:mul	1976	CC	7804	n	bl	n	y	0	cig+/-ot	nev any	st
MATOS	41	m	0	0	all	-	q	SCAmer	1994	CC	200	n	bl	n	n	2	cig+/-ot	nev any	or
OSANN	27	m	0	0	all	-	q	NAmer	1984	CC	1986	n	bl	n	n	2	cig+/-ot	nev cigs	or
OSANN	28	f	0	0	all	-	q	NAmer	1984	CC	1986	n	bl	n	n	2	cig+/-ot	nev cigs	or
OSANN2	27	f	0	0	all	-	KI	NAmer	1964	ot	217	n	bl	n	y	1	cig+/-ot	nev cigs	or
SOBUE	33	m	0	0	all	-	q	As:Jap	1986	CC	1376	n	bl	n	y	1	cig+/-ot	nev cigs	or
SOBUE	43	f	0	0	all	-	q	As:Jap	1986	CC	1376	n	bl	n	y	1	cig+/-ot	nev cigs	or
SVENSS	2	f	0	0	all	-	q	Eu:Sca	1983	CC	210	n	bl	n	n	1	all/unsp	nev any	or
TSUGAN	12	m	0	0	all	-	q	As:Jap	1976	CC	134	n	bl	n	y	0	all/unsp	nev any	ot
WAKAI	9	m	0	0	all	-	q	As:Jap	1988	CC	333	n	bl	n	y	1	all/unsp	nev any	or
WAKAI	27	f	0	0	all	-	q	As:Jap	1988	CC	333	n	bl	n	y	1	all/unsp	nev any	or
WU	15	f	0	0	wh	-	q	NAmer	1981	CC	220	n	bl	n	y	2	all/unsp	nev any	or
WYNDE3	1	m	0	0	all	-	KI	NAmer	1966	CC	350	n	bl	n	y	0	all/unsp	nev any	st
WYNDE6	3	m	0	0	all	-	KI	NAmer	1969	CC	4423	n	bl	n	y	0	cig+/-ot	nev any	st
WYNDE6	192	f	0	0	all	-	KI	NAmer	1969	CC	4423	n	bl	n	y	0	cig+/-ot	nev cigs	st

Cigarette type is all/unspec for all RRs

Table 2D2 - 2

IESLC - Meta-analysis of Ex Smoking, Cigarettes (or Any Product if Cigarettes not available)
Squamous
Most adjusted

REF	NRR	SEX	AD	Number Exposed Case	Cont	Non-exposed Case	Cont	RR	95.00%CI
BARBON	16	m	1	-	-	-	-	8.80	(3.70- 21.00)
BROWN2	26	m	2	-	-	-	-	8.70	(7.40- 10.20)
BROWN2	25	f	2	-	-	-	-	19.20	(15.20- 24.20)
Subtotal BROWN2								11.23	(9.84- 12.82)
BUFFLE	64	f	0	19	56	3	112	12.67	(3.60- 44.62)
COMSTO	19	m	0	17	129	2	84	5.53	(1.25- 24.58)
COMSTO	27	f	0	1	35	0	115	9.76~	(0.39- 244.93)
Subtotal COMSTO								6.12	(1.58- 23.67)
CORREA	39	c	1	-	-	-	-	15.50	(9.30- 26.00)
*ENGELA	55	m	7	-	-	-	-	4.10	(1.20- 14.00)
HAENSZ	21	f	0	3	9	44	236	1.79	(0.47- 6.87)
*HAMMON	151	m	1	-	-	-	-	1.23	(0.60- 2.52)
JAHN	12	m	0	190	402	3	138	21.74	(6.84- 69.13)
JAIN	28	m	0	47	159	2	85	12.56	(2.98- 52.99)
JAIN	23	f	0	22	97	6	214	8.09	(3.18- 20.59)
Subtotal JAIN								9.22	(4.21- 20.18)
JEDRYC	23	m	0	45	312	6	289	6.95	(2.92- 16.53)
KATSOU	19	f	1	-	-	-	-	4.70	(1.05- 21.14)
KHUDER	8	m	0	64	-	9	-	6.70	(3.20- 14.10)
KIHARA	10	c	0	21	70	5	237	14.22	(5.17- 39.09)
LUBIN2	257	m	0	1082	4228	54	2616	12.40	(9.39- 16.36)
LUBIN2	269	f	0	45	157	72	1180	4.70	(3.12- 7.06)
Subtotal LUBIN2								9.12	(7.25- 11.47)
MATOS	41	m	2	-	-	-	-	3.60	(1.00- 12.90)
OSANN	27	m	2	-	-	-	-	22.90	(11.00- 47.30)
OSANN	28	f	2	-	-	-	-	13.50	(6.80- 27.00)
Subtotal OSANN								17.32	(10.50- 28.59)
OSANN2	27	f	1	-	-	-	-	12.60	(1.40- 113.00)
SOBUE	33	m	1	-	-	-	-	13.10	(5.20- 33.40)
SOBUE	43	f	1	-	-	-	-	5.60	(2.30- 13.80)
Subtotal SOBUE								8.43	(4.42- 16.07)
SVENSS	2	f	1	-	-	-	-	4.00	(1.00- 16.90)
TSUGAN	12	m	0	2	2	0	5	11.00~	(0.37- 324.52)
WAKAI	9	m	1	-	-	-	-	6.16	(1.42- 26.70)
WAKAI	27	f	1	-	-	-	-	9.76	(0.85- 112.00)
Subtotal WAKAI								6.96	(1.98- 24.47)
WU	15	f	2	-	-	-	-	7.70	(0.80- 70.30)
WYNDE3	1	m	0	36	125	3	88	8.45	(2.52- 28.30)
WYNDE6	3	m	0	680	1056	29	617	13.70	(9.33- 20.13)
WYNDE6	192	f	0	161	325	40	856	10.60	(7.33- 15.33)
Subtotal WYNDE6								11.99	(9.18- 15.65)
Partial Totals				2435	7162	278	6872		

*prospective study ~ With 0.5 adjustment for zero

REF	NRR	SEX	AD	Ys	Ws	Qs	Ps
BARBON	16	m	1	2.17	5.10	0.12	0.0000
BROWN2	26	m	2	2.16	149.21	3.99	0.0000
BROWN2	25	f	2	2.95	71.05	28.03	0.0000
Subtotal BROWN2				2.42	220.26	32.02	
BUFFLE	64	f	0	2.54	2.42	0.11	0.0001
COMSTO	19	m	0	1.71	1.73	0.66	0.0245
COMSTO	27	f	0	2.28	0.37	0.00	0.1658
Subtotal COMSTO				1.81	2.10	0.66	
CORREA	39	c	1	2.74	14.54	2.49	0.0000
*ENGELA	55	m	7	1.41	2.55	2.14	0.0244
HAENSZ	21	f	0	0.58	2.12	6.47	0.3974
*HAMMON	151	m	1	0.21	7.46	33.53	0.5718
JAHN	12	m	0	3.08	2.87	1.63	0.0000
JAIN	28	m	0	2.53	1.85	0.08	0.0006
JAIN	23	f	0	2.09	4.40	0.25	0.0000
Subtotal JAIN				2.22	6.26	0.32	
JEDRYC	23	m	0	1.94	5.11	0.77	0.0000
KATSOU	19	f	1	1.55	1.70	1.04	0.0433
KHUDER	8	m	0	1.90	6.99	1.26	0.0000
KIHARA	10	c	0	2.65	3.76	0.40	0.0000
LUBIN2	257	m	0	2.52	49.85	1.81	0.0000
LUBIN2	269	f	0	1.55	23.08	14.04	0.0000
Subtotal LUBIN2				2.21	72.93	15.85	
MATOS	41	m	2	1.28	2.35	2.57	0.0496
OSANN	27	m	2	3.13	7.22	4.67	0.0000

Table 2D2 - 2

IESLC - Meta-analysis of Ex Smoking, Cigarettes (or Any Product if Cigarettes not available)
Squamous
Most adjusted

REF	NRR	SEX	AD	Ys	Ws	Qs	Ps
OSANN	28	f	2	2.60	8.08	0.61	0.0000
Subtotal OSANN				2.85	15.30	5.29	
OSANN2	27	f	1	2.53	0.80	0.03	0.0237
SOBUE	33	m	1	2.57	4.44	0.27	0.0000
SOBUE	43	f	1	1.72	4.79	1.75	0.0002
Subtotal SOBUE				2.13	9.23	2.01	
SVENSS	2	f	1	1.39	1.92	1.70	0.0546
TSUGAN	12	m	0	2.40	0.34	0.00	0.1649
WAKAI	9	m	1	1.82	1.78	0.46	0.0151
WAKAI	27	f	1	2.28	0.64	0.00	0.0673
Subtotal WAKAI				1.94	2.43	0.46	
WU	15	f	2	2.04	0.77	0.06	0.0738
WYNDE3	1	m	0	2.13	2.63	0.10	0.0005
WYNDE6	3	m	0	2.62	25.96	2.19	0.0000
WYNDE6	192	f	0	2.36	28.20	0.03	0.0000
Subtotal WYNDE6				2.48	54.16	2.22	

	N	33
	NS	25
	Wt	446.08
Het	Chi	113.24
Het	df	32
Het	P	***
Fixed	RR	10.25
	RRl	9.34
	RRu	11.24
	P	+++
Random	RR	8.74
	RRl	6.94
	RRu	11.01
	P	+++
Asymm	P	N.S.

Table 2D2 - 3

IESLC - Meta-analysis of Ex Smoking, Cigarettes (or Any Product if Cigarettes not available)
Squamous
Most adjusted

		Sex		
	combined	male	female	Total
N	2	17	14	33
NS	2	17	14	33
Wt	18.30	277.44	150.35	446.08
Het Chi	0.02	53.67	51.59	113.24
Het df	1	16	13	32
Het P	N.S.	***	***	***
Fixed RR	15.23	9.31	11.66	10.25
RRl	9.63	8.27	9.94	9.34
RRu	24.08	10.47	13.68	11.24
P	+++	+++	+++	+++
Random RR	15.23	8.41	8.03	8.74
RRl	9.63	6.18	5.18	6.94
RRu	24.08	11.45	12.45	11.01
P	+++	+++	+++	+++
Between Chi				7.96
Between df				2
Between P				*
Btwn(F) P				N.S.
Btwn(R) P				(*)

		Lung cancer type				
	q	q+s	q+u	KI	not a	Total
N	25	1	1	5	1	33
NS	18	1	1	4	1	25
Wt	362.67	14.54	2.12	59.29	7.46	446.08
Het Chi	67.24	0.00	0.00	2.60	0.00	113.24
Het df	24	0	0	4	0	32
Het P	***	N.S.	N.S.	N.S.	N.S.	***
Fixed RR	10.44	15.50	1.79	11.50	1.23	10.25
RRl	9.42	9.27	0.47	8.91	0.60	9.34
RRu	11.57	25.92	6.87	14.83	2.52	11.24
P	+++	+++	N.S.	+++	N.S.	+++
Random RR	9.52	15.50	1.79	11.50	1.23	8.74
RRl	7.45	9.27	0.47	8.91	0.60	6.94
RRu	12.16	25.92	6.87	14.83	2.52	11.01
P	+++	+++	N.S.	+++	N.S.	+++
Between Chi						43.40
Between df						4
Between P						***
Btwn(F) P						**
Btwn(R) P						***

				Location					
	NAmer	UK	Scand	othEur	China	Japan	othAs	other	Total
N	18		2	6		6		1	33
NS	13		2	5		4		1	25
Wt	335.80		4.47	87.71		15.75		2.35	446.08
Het Chi	83.53		0.00	18.16		2.74		0.00	113.24
Het df	17		1	5		5		0	32
Het P	***		N.S.	**		N.S.		N.S.	***
Fixed RR	10.83		4.06	9.10		9.32		3.60	10.25
RRl	9.73		1.61	7.38		5.69		1.00	9.34
RRu	12.05		10.25	11.22		15.28		12.93	11.24
P	+++		++	+++		+++		+	+++
Random RR	9.43		4.06	8.42		9.32		3.60	8.74
RRl	6.84		1.61	5.03		5.69		1.00	6.94
RRu	13.01		10.25	14.10		15.28		12.93	11.01
P	+++		++	+++		+++		+	+++
Between Chi									8.80
Between df									4
Between P									(*)
Btwn(F) P									N.S.
Btwn(R) P									N.S.

Table 2D2 - 3

IESLC - Meta-analysis of Ex Smoking, Cigarettes (or Any Product if Cigarettes not available)
Squamous
Most adjusted

	Detailed Country in "other Europe"					
	multi	Germany	othWest	East	Balkans	Total
N	2	1	1	1	1	6
NS	1	1	1	1	1	5
Wt	72.93	2.87	5.10	5.11	1.70	87.71
Het Chi	14.86	0.00	0.00	0.00	0.00	18.16
Het df	1	0	0	0	0	5
Het P	***	N.S.	N.S.	N.S.	N.S.	**
Fixed RR	9.12	21.74	8.80	6.95	4.70	9.10
RRl	7.25	6.84	3.69	2.92	1.05	7.38
RRu	11.47	69.13	20.96	16.53	21.09	11.22
P	+++	+++	+++	+++	+	+++
Random RR	7.72	21.74	8.80	6.95	4.70	8.42
RRl	2.98	6.84	3.69	2.92	1.05	5.03
RRu	19.99	69.13	20.96	16.53	21.09	14.10
P	+++	+++	+++	+++	+	+++
Between Chi						3.30
Between df						4
Between P						N.S.
Btwn(F) P						N.S.
Btwn(R) P						N.S.

	Detailed Country in "other Asia"			
	India	HongKong	other	Total
N				
NS				
Wt				
Het Chi				
Het df				
Het P				N.S.
Fixed RR				
RRl				
RRu				
P				+++
Random RR				
RRl				
RRu				
P				+++
Between Chi				
Between df				
Between P				N.S.
Btwn(F) P				N.S.
Btwn(R) P				N.S.

	Detailed other continent			
	SCAmer	Auslia	Africa	Total
N	1			1
NS	1			1
Wt	2.35			2.35
Het Chi	0.00			0.00
Het df	0			0
Het P	N.S.			N.S.
Fixed RR	3.60			3.60
RRl	1.00			1.00
RRu	12.93			12.93
P	+			+
Random RR	3.60			3.60
RRl	1.00			1.00
RRu	12.93			12.93
P	+			+
Between Chi				
Between df				
Between P				N.S.
Btwn(F) P				N.S.
Btwn(R) P				N.S.

Table 2D2 - 3

IESLC - Meta-analysis of Ex Smoking, Cigarettes (or Any Product if Cigarettes not available)
Squamous
Most adjusted

	<1960	1960-69	1970-79	1980-89	1990+	Total
	Start year of study					
N	2	5	8	16	2	33
NS	2	4	6	11	2	25
Wt	9.58	60.13	97.42	272.84	6.11	446.08
Het Chi	0.23	3.93	19.08	45.28	2.73	113.24
Het df	1	4	7	15	1	32
Het P	N.S.	N.S.	**	***	(*)	***
Fixed RR	1.34	11.29	9.85	10.97	8.38	10.25
RRl	0.71	8.77	8.08	9.75	3.79	9.34
RRu	2.52	14.54	12.02	12.36	18.53	11.24
P	N.S.	+++	+++	+++	+++	+++
Random RR	1.34	11.29	9.39	10.35	7.58	8.74
RRl	0.71	8.77	6.00	7.57	1.98	6.94
RRu	2.52	14.54	14.68	14.15	29.00	11.01
P	N.S.	+++	+++	+++	++	+++
Between Chi						42.00
Between df						4
Between P						***
Btwn(F) P						**
Btwn(R) P						***

	CC	other	Total
	Study type (1)		
N	28	5	33
NS	21	4	25
Wt	433.18	12.90	446.08
Het Chi	76.05	8.18	113.24
Het df	27	4	32
Het P	***	(*)	***
Fixed RR	10.71	2.34	10.25
RRl	9.74	1.35	9.34
RRu	11.76	4.03	11.24
P	+++	++	+++
Random RR	9.94	3.54	8.74
RRl	8.05	1.38	6.94
RRu	12.28	9.09	11.01
P	+++	++	+++
Between Chi			29.01
Between df			1
Between P			***
Btwn(F) P			**
Btwn(R) P			*

	CC	prosp	other	Total
	Study type (2)			
N	28	2	3	33
NS	21	2	2	25
Wt	433.18	10.01	2.90	446.08
Het Chi	76.05	2.75	0.40	113.24
Het df	27	1	2	32
Het P	***	(*)	N.S.	***
Fixed RR	10.71	1.67	7.46	10.25
RRl	9.74	0.90	2.36	9.34
RRu	11.76	3.10	23.61	11.24
P	+++	N.S.	+++	+++
Random RR	9.94	2.02	7.46	8.74
RRl	8.05	0.63	2.36	6.94
RRu	12.28	6.44	23.61	11.01
P	+++	N.S.	+++	+++
Between Chi				34.04
Between df				2
Between P				***
Btwn(F) P				**
Btwn(R) P				*

Table 2D2 - 3

IESLC - Meta-analysis of Ex Smoking, Cigarettes (or Any Product if Cigarettes not available)
Squamous
Most adjusted

	Study size (number of LC cases) 100-249	250-499	500-999	1000+	Total
N	7	9	4	13	33
NS	7	7	3	8	25
Wt	10.00	27.91	13.78	394.40	446.08
Het Chi	3.17	20.55	0.49	59.57	113.24
Het df	6	8	3	12	32
Het P	N.S.	**	N.S.	***	***
Fixed RR	4.03	4.59	9.58	11.13	10.25
RRl	2.17	3.17	5.65	10.08	9.34
RRu	7.49	6.65	16.25	12.28	11.24
P	+++	+++	+++	+++	+++
Random RR	4.03	5.48	9.58	11.53	8.74
RRl	2.17	2.84	5.65	8.87	6.94
RRu	7.49	10.58	16.25	14.99	11.01
P	+++	+++	+++	+++	+++
Between Chi					29.47
Between df					3
Between P					***
Btwn(F) P					*
Btwn(R) P					**

	Risky occupational population no	mining	othRisky	Total
N	33			33
NS	25			25
Wt	446.08			446.08
Het Chi	113.24			113.24
Het df	32			32
Het P	***			***
Fixed RR	10.25			10.25
RRl	9.34			9.34
RRu	11.24			11.24
P	+++			+++
Random RR	8.74			8.74
RRl	6.94			6.94
RRu	11.01			11.01
P	+++			+++
Between Chi				
Between df				
Between P				N.S.
Btwn(F) P				N.S.
Btwn(R) P				N.S.

	National cigarette tobacco type Virginia	blended	other	Total
N	2	31		33
NS	1	24		25
Wt	6.26	439.83		446.08
Het Chi	0.25	112.92		113.24
Het df	1	30		32
Het P	N.S.	***		***
Fixed RR	9.22	10.26		10.25
RRl	4.21	9.35		9.34
RRu	20.18	11.27		11.24
P	+++	+++		+++
Random RR	9.22	8.68		8.74
RRl	4.21	6.83		6.94
RRu	20.18	11.04		11.01
P	+++	+++		+++
Between Chi				0.07
Between df				1
Between P				N.S.
Btwn(F) P				N.S.
Btwn(R) P				N.S.

Table 2D2 - 3

IESLC - Meta-analysis of Ex Smoking, Cigarettes (or Any Product if Cigarettes not available)
Squamous
Most adjusted

	Any proxy use No/nk	Yes	Total
N	27	6	33
NS	20	5	25
Wt	412.65	33.43	446.08
Het Chi	109.41	3.52	113.24
Het df	26	5	32
Het P	***	N.S.	***
Fixed RR	10.17	11.24	10.25
RRl	9.23	8.01	9.34
RRu	11.20	15.78	11.24
P	+++	+++	+++
Random RR	8.33	11.24	8.74
RRl	6.38	8.01	6.94
RRu	10.89	15.78	11.01
P	+++	+++	+++
Between Chi			0.31
Between df			1
Between P			N.S.
Btwn(F) P			N.S.
Btwn(R) P			N.S.

	Full histological confirmation No	Yes	Total
N	16	17	33
NS	13	12	25
Wt	68.35	377.74	446.08
Het Chi	51.31	60.37	113.24
Het df	15	16	32
Het P	***	***	***
Fixed RR	8.91	10.51	10.25
RRl	7.03	9.50	9.34
RRu	11.30	11.62	11.24
P	+++	+++	+++
Random RR	8.11	9.28	8.74
RRl	5.05	7.08	6.94
RRu	13.01	12.15	11.01
P	+++	+++	+++
Between Chi			1.56
Between df			1
Between P			N.S.
Btwn(F) P			N.S.
Btwn(R) P			N.S.

	Number of adjustment variables (1) 0	1	2+/+nk	Total
N	16	10	7	33
NS	12	8	5	25
Wt	161.68	43.18	241.22	446.08
Het Chi	29.49	35.24	39.83	113.24
Het df	15	9	6	32
Het P	*	***	***	***
Fixed RR	9.81	7.03	11.28	10.25
RRl	8.41	5.21	9.94	9.34
RRu	11.45	9.47	12.80	11.24
P	+++	+++	+++	+++
Random RR	9.11	6.43	11.09	8.74
RRl	6.95	3.31	6.78	6.94
RRu	11.94	12.47	18.13	11.01
P	+++	+++	+++	+++
Between Chi				8.68
Between df				2
Between P				*
Btwn(F) P				N.S.
Btwn(R) P				N.S.

Table 2D2 - 3

IESLC - Meta-analysis of Ex Smoking, Cigarettes (or Any Product if Cigarettes not available)
Squamous
Most adjusted

	Number of adjustment variables (2) 0	1	2	3-5	6+/+nk	Total
N	16	10	6		1	33
NS	12	8	4		1	25
Wt	161.68	43.18	238.68		2.55	446.08
Het Chi	29.49	35.24	37.19		0.00	113.24
Het df	15	9	5		0	32
Het P	*	***	***		N.S.	***
Fixed RR	9.81	7.03	11.40		4.10	10.25
RRl	8.41	5.21	10.05		1.20	9.34
RRu	11.45	9.47	12.95		14.00	11.24
P	+++	+++	+++		+	+++
Random RR	9.11	6.43	12.33		4.10	8.74
RRl	6.95	3.31	7.38		1.20	6.94
RRu	11.94	12.47	20.59		14.00	11.01
P	+++	+++	+++		+	+++
Between Chi						11.32
Between df						3
Between P						*
Btwn(F) P						N.S.
Btwn(R) P						N.S.

	Product all/unsp	cig+/-ot	cig only	Total
N	9	23	1	33
NS	8	16	1	25
Wt	18.64	419.98	7.46	446.08
Het Chi	2.88	74.87	0.00	113.24
Het df	8	22	0	32
Het P	N.S.	***	N.S.	***
Fixed RR	8.13	10.75	1.23	10.25
RRl	5.16	9.77	0.60	9.34
RRu	12.79	11.83	2.52	11.24
P	+++	+++	N.S.	+++
Random RR	8.13	9.99	1.23	8.74
RRl	5.16	7.93	0.60	6.94
RRu	12.79	12.58	2.52	11.01
P	+++	+++	N.S.	+++
Between Chi				35.50
Between df				2
Between P				***
Btwn(F) P				**
Btwn(R) P				***

	Denominator nev any	nev cigs	Total
N	17	16	33
NS	15	11	26
Wt	137.45	308.64	446.08
Het Chi	61.55	43.98	113.24
Het df	16	15	32
Het P	***	***	***
Fixed RR	8.41	11.19	10.25
RRl	7.12	10.00	9.34
RRu	9.94	12.51	11.24
P	+++	+++	+++
Random RR	6.63	11.13	8.74
RRl	4.39	8.48	6.94
RRu	10.02	14.60	11.01
P	+++	+++	+++
Between Chi			7.71
Between df			1
Between P			**
Btwn(F) P			N.S.
Btwn(R) P			*

Table 2D2 - 3

IESLC - Meta-analysis of Ex Smoking, Cigarettes (or Any Product if Cigarettes not available)

Squamous
Most adjusted

		Derivation of RR/CI			
		Orig	StdCalc	Other	Total
	N	17	13	3	33
	NS	13	10	3	26
	Wt	283.93	153.99	8.17	446.08
Het	Chi	49.81	28.42	2.92	113.24
Het	df	16	12	2	32
Het	P	***	**	N.S.	***
Fixed	RR	10.99	9.98	1.48	10.25
	RRl	9.78	8.52	0.74	9.34
	RRu	12.34	11.69	2.93	11.24
	P	+++	+++	N.S.	+++
Random	RR	9.83	9.29	2.43	8.74
	RRl	7.24	6.89	0.56	6.94
	RRu	13.36	12.54	10.62	11.01
	P	+++	+++	N.S.	+++
Between	Chi				32.10
Between	df				2
Between	P				***
Btwn(F)	P				**
Btwn(R)	P				N.S.

Table 2D2 - 4

IESLC - Meta-analysis of Ex Smoking, Cigarettes (or Any Product if Cigarettes not available)
Squamous
Least adjusted

REF	NRR	X	SEX	AGEL	AGEH	RACE	YF	LC TYPE	LOC	START	ST	NLC	R	VB	P	H	AD	PRODUCT	DENOM	De
BARBON	15	x	m	0	0	all	-	q	Eu:wst	1979	CC	755	n	bl	y	y	0	all/unsp	nev any	st
BROWN2	26		m	0	0	wh	-	q	NAmer	1984	CC	14596	n	bl	n	y	2	cig+/-ot	nev cigs	or
BROWN2	25		f	0	0	wh	-	q	NAmer	1984	CC	14596	n	bl	n	y	2	cig+/-ot	nev cigs	or
BUFFLE	64		f	0	0	w-hi	-	q	NAmer	1976	CC	943	n	bl	y	n	0	cig+/-ot	nev cigs	st
COMSTO	19		m	0	0	all	-	q	NAmer	1975	ot	258	n	bl	n	n	0	cig+/-ot	nev cigs	st
COMSTO	27		f	0	0	all	-	q	NAmer	1975	ot	258	n	bl	n	n	0	cig+/-ot	nev cigs	ot
CORREA	39		c	0	0	all	-	q+s	NAmer	1979	CC	1359	n	bl	y	n	1	cig+/-ot	nev cigs	or
ENGELA	55		m	0	0	all	0	q	Eu:Sca	1964	pr	435	n	bl	n	n	7	cig+/-ot	nev cigs	or
HAENSZ	21		f	0	0	all	-	q+u	NAmer	1955	CC	158	n	bl	n	y	0	cig+/-ot	nev any	st
HAMMON	151		m	0	0	wh	0	not a	NAmer	1952	pr	448	n	bl	n	n	1	cig only	nev any	ot
JAHN	12		m	0	0	all	-	q	Eu:Ger	1988	CC	1004	n	bl	n	n	0	cig+/-ot	nev any	st
JAIN	28		m	0	0	all	-	q	NAmer	1981	CC	845	n	V	y	n	0	cig+/-ot	nev cigs	st
JAIN	23		f	0	0	all	-	q	NAmer	1981	CC	845	n	V	y	n	0	cig+/-ot	nev cigs	st
JEDRYC	23		m	0	0	all	-	q	Eu:est	1980	CC	1630	n	bl	y	n	0	cig+/-ot	nev any	st
KATSOU	23	x	f	0	0	all	-	KI	Eu:bal	1987	CC	101	n	bl	n	n	0	all/unsp	nev any	st
KHUDER	8		m	0	0	all	-	q	NAmer	1985	CC	482	n	bl	n	y	0	cig+/-ot	nev cigs	or
KIHARA	10		c	0	0	jap	-	q	As:Jap	1991	CC	440	n	bl	n	n	0	all/unsp	nev any	st
LUBIN2	257		m	0	0	all	-	q	Eu:mul	1976	CC	7804	n	bl	n	y	0	cig+/-ot	nev any	st
LUBIN2	269		f	0	0	all	-	q	Eu:mul	1976	CC	7804	n	bl	n	y	0	cig+/-ot	nev any	st
MATOS	40	x	m	0	0	all	-	q	SCAmer	1994	CC	200	n	bl	n	n	0	cig+/-ot	nev any	st
OSANN	2	x	m	0	0	all	-	q	NAmer	1984	CC	1986	n	bl	n	n	0	cig+/-ot	nev cigs	st
OSANN	6	x	f	0	0	all	-	q	NAmer	1984	CC	1986	n	bl	n	n	0	cig+/-ot	nev cigs	st
OSANN2	9	x	f	0	0	all	-	KI	NAmer	1964	ot	217	n	bl	n	y	0	cig+/-ot	nev cigs	st
SOBUE	1	x	m	0	0	all	-	q	As:Jap	1986	CC	1376	n	bl	n	y	0	cig+/-ot	nev cigs	st
SOBUE	17	x	f	0	0	all	-	q	As:Jap	1986	CC	1376	n	bl	n	y	0	cig+/-ot	nev cigs	st
SVENSS	22	x	f	0	0	all	-	q	Eu:Sca	1983	CC	210	n	bl	n	n	0	all/unsp	nev any	st
TSUGAN	12		m	0	0	all	-	q	As:Jap	1976	CC	134	n	bl	n	y	0	all/unsp	nev any	ot
WAKAI	3	x	m	0	0	all	-	q	As:Jap	1988	CC	333	n	bl	n	y	0	all/unsp	nev any	st
WAKAI	21	x	f	0	0	all	-	q	As:Jap	1988	CC	333	n	bl	n	y	0	all/unsp	nev any	st
WU	10	x	f	0	0	wh	-	q	NAmer	1981	CC	220	n	bl	n	y	0	all/unsp	nev any	st
WYNDE3	1		m	0	0	all	-	KI	NAmer	1966	CC	350	n	bl	n	y	0	all/unsp	nev any	st
WYNDE6	3		m	0	0	all	-	KI	NAmer	1969	CC	4423	n	bl	n	y	0	cig+/-ot	nev any	st
WYNDE6	192		f	0	0	all	-	KI	NAmer	1969	CC	4423	n	bl	n	y	0	cig+/-ot	nev cigs	st

Cigarette type is all/unspec for all RRs

Table 2D2 - 5

IESLC - Meta-analysis of Ex Smoking, Cigarettes (or Any Product if Cigarettes not available)
Squamous
Least adjusted

REF	NRR	SEX	AD	Number Exposed Case	Number Exposed Cont	Non-exposed Case	Non-exposed Cont	RR	95.00%CI
BARBON	15	m	0	58	205	6	188	8.87	(3.74- 21.02)
BROWN2	26	m	2	-	-	-	-	8.70	(7.40- 10.20)
BROWN2	25	f	2	-	-	-	-	19.20	(15.20- 24.20)
Subtotal BROWN2								11.23	(9.84- 12.82)
BUFFLE	64	f	0	19	56	3	112	12.67	(3.60- 44.62)
COMSTO	19	m	0	17	129	2	84	5.53	(1.25- 24.58)
COMSTO	27	f	0	1	35	0	115	9.76~	(0.39- 244.93)
Subtotal COMSTO								6.12	(1.58- 23.67)
CORREA	39	c	1	-	-	-	-	15.50	(9.30- 26.00)
*ENGELA	55	m	7	-	-	-	-	4.10	(1.20- 14.00)
HAENSZ	21	f	0	3	9	44	236	1.79	(0.47- 6.87)
*HAMMON	151	m	1	-	-	-	-	1.23	(0.60- 2.52)
JAHN	12	m	0	190	402	3	138	21.74	(6.84- 69.13)
JAIN	28	m	0	47	159	2	85	12.56	(2.98- 52.99)
JAIN	23	f	0	22	97	6	214	8.09	(3.18- 20.59)
Subtotal JAIN								9.22	(4.21- 20.18)
JEDRYC	23	m	0	45	312	6	289	6.95	(2.92- 16.53)
KATSOU	23	f	0	4	4	14	67	4.79	(1.07- 21.47)
KHUDER	8	m	0	64	-	9	-	6.70	(3.20- 14.10)
KIHARA	10	c	0	21	70	5	237	14.22	(5.17- 39.09)
LUBIN2	257	m	0	1082	4228	54	2616	12.40	(9.39- 16.36)
LUBIN2	269	f	0	45	157	72	1180	4.70	(3.12- 7.06)
Subtotal LUBIN2								9.12	(7.25- 11.47)
MATOS	40	m	0	14	151	3	110	3.40	(0.95- 12.12)
OSANN	2	m	0	105	477	8	833	22.92	(11.07- 47.45)
OSANN	6	f	0	29	196	12	1093	13.48	(6.76- 26.86)
Subtotal OSANN								17.33	(10.50- 28.58)
OSANN2	9	f	0	7	19	7	58	3.05	(0.95- 9.82)
SOBUE	1	m	0	121	363	3	128	14.22	(4.44- 45.51)
SOBUE	17	f	0	7	64	14	857	6.70	(2.61- 17.18)
Subtotal SOBUE								9.02	(4.34- 18.77)
SVENSS	22	f	0	6	36	5	120	4.00	(1.15- 13.88)
TSUGAN	12	m	0	2	2	0	5	11.00~	(0.37- 324.52)
WAKAI	3	m	0	27	140	2	65	6.27	(1.45- 27.16)
WAKAI	21	f	0	1	5	3	145	9.67	(0.85- 110.07)
Subtotal WAKAI								7.03	(2.00- 24.69)
WU	10	f	0	8	18	2	30	6.67	(1.27- 34.92)
WYNDE3	1	m	0	36	125	3	88	8.45	(2.52- 28.30)
WYNDE6	3	m	0	680	1056	29	617	13.70	(9.33- 20.13)
WYNDE6	192	f	0	161	325	40	856	10.60	(7.33- 15.33)
Subtotal WYNDE6								11.99	(9.18- 15.65)
Partial Totals				2822	8840	357	10566		

*prospective study ~ With 0.5 adjustment for zero

REF	NRR	SEX	AD	Ys	Ws	Qs	Ps
BARBON	15	m	0	2.18	5.15	0.10	0.0000
BROWN2	26	m	2	2.16	149.21	3.60	0.0000
BROWN2	25	f	2	2.95	71.05	28.76	0.0000
Subtotal BROWN2				2.42	220.26	32.36	
BUFFLE	64	f	0	2.54	2.42	0.12	0.0001
COMSTO	19	m	0	1.71	1.73	0.64	0.0245
COMSTO	27	f	0	2.28	0.37	0.00	0.1658
Subtotal COMSTO				1.81	2.10	0.64	
CORREA	39	c	1	2.74	14.54	2.59	0.0000
*ENGELA	55	m	7	1.41	2.55	2.10	0.0244
HAENSZ	21	f	0	0.58	2.12	6.41	0.3974
*HAMMON	151	m	1	0.21	7.46	33.27	0.5718
JAHN	12	m	0	3.08	2.87	1.66	0.0000
JAIN	28	m	0	2.53	1.85	0.08	0.0006
JAIN	23	f	0	2.09	4.40	0.23	0.0000
Subtotal JAIN				2.22	6.26	0.31	
JEDRYC	23	m	0	1.94	5.11	0.74	0.0000
KATSOU	23	f	0	1.57	1.71	0.97	0.0409
KHUDER	8	m	0	1.90	6.99	1.21	0.0000
KIHARA	10	c	0	2.65	3.76	0.42	0.0000
LUBIN2	257	m	0	2.52	49.85	1.97	0.0000
LUBIN2	269	f	0	1.55	23.08	13.74	0.0000
Subtotal LUBIN2				2.21	72.93	15.71	
MATOS	40	m	0	1.22	2.38	2.85	0.0592
OSANN	2	m	0	3.13	7.26	4.80	0.0000

Table 2D2 - 5

IESLC - Meta-analysis of Ex Smoking, Cigarettes (or Any Product if Cigarettes not available)
Squamous
Least adjusted

REF	NRR	SEX	AD	Ys	Ws	Qs	Ps
OSANN	6	f	0	2.60	8.08	0.64	0.0000
Subtotal OSANN				2.85	15.33	5.44	
OSANN2	9	f	0	1.12	2.81	4.07	0.0613
SOBUE	1	m	0	2.65	2.84	0.32	0.0000
SOBUE	17	f	0	1.90	4.33	0.75	0.0001
Subtotal SOBUE				2.20	7.17	1.07	
SVENSS	22	f	0	1.39	2.48	2.16	0.0289
TSUGAN	12	m	0	2.40	0.34	0.00	0.1649
WAKAI	3	m	0	1.84	1.79	0.42	0.0141
WAKAI	21	f	0	2.27	0.65	0.00	0.0675
Subtotal WAKAI				1.95	2.44	0.42	
WU	10	f	0	1.90	1.40	0.25	0.0247
WYNDE3	1	m	0	2.13	2.63	0.09	0.0005
WYNDE6	3	m	0	2.62	25.96	2.32	0.0000
WYNDE6	192	f	0	2.36	28.20	0.05	0.0000
Subtotal WYNDE6				2.48	54.16	2.37	

	N	33
	NS	25
	Wt	447.35
Het	Chi	117.33
Het	df	32
Het	P	***
Fixed	RR	10.16
	RRl	9.26
	RRu	11.15
	P	+++
Random	RR	8.47
	RRl	6.71
	RRu	10.68
	P	+++
Asymm	P	N.S.

Table 2D2 - 6

IESLC - Meta-analysis of Ex Smoking, Cigarettes (or Any Product if Cigarettes not available)
Squamous
Least adjusted

	combined	Sex male	female	Total
N	2	17	14	33
NS	2	17	14	33
Wt	18.30	275.95	153.10	447.35
Het Chi	0.02	53.96	56.19	117.33
Het df	1	16	13	32
Het P	N.S.	***	***	***
Fixed RR	15.23	9.29	11.38	10.16
RRl	9.63	8.26	9.71	9.26
RRu	24.08	10.46	13.33	11.15
P	+++	+++	+++	+++
Random RR	15.23	8.38	7.42	8.47
RRl	9.63	6.13	4.79	6.71
RRu	24.08	11.44	11.49	10.68
P	+++	+++	+++	+++
Between Chi				7.15
Between df				2
Between P				*
Btwn(F) P				N.S.
Btwn(R) P				(*)

Table 2D2 - 7

IESLC - Meta-analysis of Ex Smoking, Cigarettes (or Any Product if Cigarettes not available)
Squamous
Excluded studies (and stage at which they were excluded)

1 BOUCHA BUELL LAURIL MZILEN

2 ABELIN ABRAHA ALDERS AUVINE AXELSO BAND BERRIN BLOT1 BLOT2 BLOT3 BLOT4 BRESLO BROCKM BROWN1 BYERS1 BYERS2
CASCO2 CASCOR CHAN CHATZI CHEN CHEN2 CHEN3 CHIAZZ COOKSO DAVEYS DEAN DOSEME DU DUNN EBELIN ENSTRO
ESAKI FAN GENG GER GODLEY GOLLED GSELL HANSEN HEGMAN HINDS HIRAOK HIRAY2 HOROWI HORWIT HU HU2
HUANG ISHIMA JARUP JIANG JONES JUSSAW KANELL KO KOHLME KOULUM KREYBE LAMTH LAMWK LAMWK2 LAUSSM LEI
LETOUR LEVIN LIAW LICKIN LIU LIU2 LIU3 LIU4 LIU5 LOMBA2 LUO MAGNUS MARSH MARSH2 MARTIS MASTRA
MATSUD MCCONN MCDUFF MCLAUG MILLER MILLS MOLLO MRFIT MURATA NOTAN2 NOTANI NOU ORMOS PASTOR PAWLEG PERNU
PERSHA PIKE POFFIJ POLEDN QIAO RADZIK RANDIG REN RIMING RONCO ROOTS ROTHSC SAARIK SADOWS SANKAR SCHWA2
SEGI SEGI2 SEOW SHIMIZ SIEMIA SIMARA SITAS SOBUE2 STASZE STAYNE STOCKS SUN TANG2 TAO ULMER VANDER
WALD WANG WANG3 WANG4 WARSIN WATSON WICKLU WILKIN WU2 WUWILL WYNDE2 WYNDE4 WYNDE5 WYNDE8 WYNDER XIANGZ
XU XU2 XU3 XU4 YUAN ZHANG ZHENG ZHOU

3 RESTRE

4 BOFFET

8 AGUDO AKIBA AMANDU AMES ANDERS ARCHER ARMADA AUSTIN AXELSS BECHER BENSHL BEST BLOHMK BOUCOT BRETT BROSS
CARPEN CEDERL CHANG CHOI CHOW CHYOU CPSI CPSII DAMBER DARBY DEAN2 DEAN3 DEKLER DESTE2 DESTEF DOCKER
DOLL DOLL2 DORANT DORGAN DORN DROSTE GAO GAO2 GARCIA GARDIN GARSHI GILLIS GOODMA GRAHAM GREGOR HAMMO2
HEIN HENNEK HIRAYA HITOSU HOLE HUMBLE JARVHO JOLY KAISE2 KAISER KAUFMA KELLER KINLEN KJUUS KNEKT KOO
KREUZE KUBIK LANGE LEMARC LIDDEL LOMBAR LUBIN MACLEN MIGRAN MRFITR NAM ODRISC PARKIN PERSH2 PETO PEZZO2
PEZZOT PISANI PRESCO QIAO2 RACHTA SCHWAR SHAW SIMONA SPEIZE SPITZ STOCKW STUCKE SUZUK2 SUZUKI TANG TENKAN
TIZZAN TOKARS TOUSEY TULINI TVERDA VEIERO WANG2 WIGLE WUNSCH YAMAGU YONG

10 BENHAM VUTUC WYNDE7

Table 2D2 - 8
Potentially overlapping studies

REF	REFGP	PRINC	OVERLAP/LINK
LUBIN2	LUBIN2	1	Lubin-combined
OSANN2	KAISER	2	KAISER/OSANN2
WYNDE6	WYNDE6	1	WYNDE5/6/7/8
JAHN	BOFFET	2	Subset of BOFFET

Table 2D3 -

IESLC - Meta-analysis of Ex Smoking, Cigarettes only
Squamous

This analysis is restricted to results for:
1) Non-dose-response data
2) Ex smokers
3) Results complete enough for use in metaanalysis

Within each study, results are then selected (in the following order of preference, within each sex) for:
4) PRODUCT: cigarettes only
5) CIGTYPE: all/unspecified, MC regardless of HR, MC only
6) DENOM: never smoked anything, never smoked cigarettes, (never +1 = +long term ex, +2 = +amount unknown, +3 = never cigs+long term ex)
7) Followup period (YF, prospective studies): whole study (coded as 0) or longest available
8) LCtype: squamous or nearest available, but not adeno. (q = squamous, s = small, a = adeno, KI = Kreyberg I, u = undifferentiated)
9) Race: all or nearest available, otherwise by race (wh or w = white, bl or b = black, hi = hispanic ch = chinese, jap = japanese, haw = hawaiian, w+o = white + oriental, sca = scandinavian, as = asian)
10) For overlapping studies: principal rather than subsidiary studies
Finally by Age: whole study (coded as 0) if available, otherwise by widest available age group
and then for single sex results (m, f) in preference to combined sex results (c).

Results adjusted (AD) for the most potential confounders are then chosen in Sections -1 to -3
and results adjusted for the least confounders in Sections -4 to -6. (Those least adjusted results which actually differ from the most adjusted as marked 'x' in column X in Section -4)
(Results adjusted for an unknown number of confounder(s) are coded as 20.)

Section -7 shows excluded studies, together with the stage (as above) at which no qualifying results were found.

Section -8 lists the potentially overlapping studies which have been included (1=principal, 2=subsidiary).

Section -9 lists any results which would have been included in preference except that they had data not complete enough for use in meta-analysis, with their significance (yes/no), if known, and any further comment as entered on the database.

In addition to those mentioned above, the following fields, levels and abbreviations are used:
* or nk = not known, n = no, y = yes, ot = other
nev = never
all/unspec = all or unspecified, MC = manufactured cigarettes, HR = hand-rolled cigarettes
REF: 6-character study reference
NRR: number of the RR on the database within the study
ST : study type (CC = case control, pr or prosp = prospective)
NLC: number of lung cancer cases in whole study
R : risky occupational population (n = no, m = mining, o = other risky)
VB : national cigarette type (V = at least 75% Virginia, bl = at least 75% blended, ot = other)
P : any proxy use
H : full histological confirmation
De : derivation of RR/CI (or = original, st = standard method, ot = other method of estimation)

Table 2D3 - 1

IESLC - Meta-analysis of Ex Smoking, Cigarettes only
Squamous
Most adjusted

REF	NRR	SEX	AGEL	AGEH	RACE	YF	LC TYPE	LOC	START	ST	NLC	R	VB	P	H	AD	PRODUCT	DENOM	De
BENHAM	19	m	0	0	all	-	KI	Eu:wst	1976	CC	1625	n	bl	n	y	0	cig only	nev any	st
HAMMON	151	m	0	0	wh	0	not a	NAmer	1952	pr	448	n	bl	n	n	1	cig only	nev any	ot
WYNDE7	27	m	0	0	all	-	KI	NAmer	1977	CC	2085	n	bl	n	y	0	cig only	nev any	st

Cigarette type is all/unspec for all RRs

Table 2D3 - 2

IESLC - Meta-analysis of Ex Smoking, Cigarettes only
Squamous
Most adjusted

REF	NRR	SEX	AD	Number Exposed Case	Cont	Non-exposed Case	Cont	RR	95.00%CI
BENHAM	19	m	0	285	451	24	481	12.66	(8.19- 19.59)
*HAMMON	151	m	1	-	-	-	-	1.23	(0.60- 2.52)
WYNDE7	27	m	0	334	1115	22	918	12.50	(8.05- 19.41)
Partial Totals				619	1566	46	1399		

*prospective study

REF	NRR	SEX	AD	Ys	Ws	Qs	Ps
BENHAM	19	m	0	2.54	20.21	2.79	0.0000
*HAMMON	151	m	1	0.21	7.46	28.66	0.5718
WYNDE7	27	m	0	2.53	19.83	2.55	0.0000

	N	3
	NS	3
	Wt	47.50
Het	Chi	34.01
Het	df	2
Het	P	***
Fixed	RR	8.73
	RRl	6.57
	RRu	11.61
	P	+++
Random	RR	6.01
	RRl	1.77
	RRu	20.37
	P	++
Asymm	P	**

Table 2D3 - 3

IESLC - Meta-analysis of Ex Smoking, Cigarettes only
Squamous
Most adjusted

	Sex combined	male	female	Total
N		3		3
NS		3		3
Wt		47.50		47.50
Het Chi		34.01		34.01
Het df		2		2
Het P		***		***
Fixed RR		8.73		8.73
RRl		6.57		6.57
RRu		11.61		11.61
P		+++		+++
Random RR		6.01		6.01
RRl		1.77		1.77
RRu		20.37		20.37
P		++		++
Between Chi				
Between df				
Between P				N.S.
Btwn(F) P				N.S.
Btwn(R) P				N.S.

Too few RRs for analysis by factor

Table 2D3 - 4

IESLC - Meta-analysis of Ex Smoking, Cigarettes only
Squamous
Least adjusted

REF	NRR	X	SEX	AGEL	AGEH	RACE	YF	LC TYPE	LOC	START	ST	NLC	R	VB	P	H	AD	PRODUCT	DENOM	De
BENHAM	19		m	0	0	all	-	KI	Eu:wst	1976	CC	1625	n	bl	n	y	0	cig only	nev any	st
HAMMON	151		m	0	0	wh	0	not a	NAmer	1952	pr	448	n	bl	n	n	1	cig only	nev any	ot
WYNDE7	27		m	0	0	all	-	KI	NAmer	1977	CC	2085	n	bl	n	y	0	cig only	nev any	st

Cigarette type is all/unspec for all RRs

Table 2D3 - 5

IESLC - Meta-analysis of Ex Smoking, Cigarettes only
Squamous
Least adjusted

REF	NRR	SEX	AD	Number Exposed Case	Cont	Non-exposed Case	Cont	RR	95.00%CI
BENHAM	19	m	0	285	451	24	481	12.66	(8.19- 19.59)
*HAMMON	151	m	1	-	-	-	-	1.23	(0.60- 2.52)
WYNDE7	27	m	0	334	1115	22	918	12.50	(8.05- 19.41)
Partial Totals				619	1566	46	1399		

*prospective study

REF	NRR	SEX	AD	Ys	Ws	Qs	Ps
BENHAM	19	m	0	2.54	20.21	2.79	0.0000
*HAMMON	151	m	1	0.21	7.46	28.66	0.5718
WYNDE7	27	m	0	2.53	19.83	2.55	0.0000

	N	3
	NS	3
	Wt	47.50
Het	Chi	34.01
Het	df	2
Het	P	***
Fixed	RR	8.73
	RRl	6.57
	RRu	11.61
	P	+++
Random	RR	6.01
	RRl	1.77
	RRu	20.37
	P	++
Asymm	P	**

Table 2D3 - 6

IESLC - Meta-analysis of Ex Smoking, Cigarettes only
Squamous
Least adjusted

	Sex combined	male	female	Total
N		3		3
NS		3		3
Wt		47.50		47.50
Het Chi		34.01		34.01
Het df		2		2
Het P		***		***
Fixed RR		8.73		8.73
RRl		6.57		6.57
RRu		11.61		11.61
P		+++		+++
Random RR		6.01		6.01
RRl		1.77		1.77
RRu		20.37		20.37
P		++		++
Between Chi				
Between df				
Between P				N.S.
Btwn(F) P				N.S.
Btwn(R) P				N.S.

Table 2D3 - 7

IESLC - Meta-analysis of Ex Smoking, Cigarettes only
Squamous
Excluded studies (and stage at which they were excluded)

1	BOUCHA BUELL LAURIL MZILEN
2	ABELIN ABRAHA ALDERS AUVINE AXELSO BAND BERRIN BLOT1 BLOT2 BLOT3 BLOT4 BRESLO BROCKM BROWN1 BYERS1 BYERS2 CASCO2 CASCOR CHAN CHATZI CHEN CHEN2 CHEN3 CHIAZZ COOKSO DAVEYS DEAN DOSEME DU DUNN EBELIN ENSTRO ESAKI FAN GENG GER GODLEY GOLLED GSELL HANSEN HEGMAN HINDS HIRAOK HIRAY2 HOROWI HORWIT HU HU2 HUANG ISHIMA JARUP JIANG JONES JUSSAW KANELL KO KOHLME KOULUM KREYBE LAMTH LAMWK LAMWK2 LAUSSM LEI LETOUR LEVIN LIAW LICKIN LIU LIU2 LIU3 LIU4 LIU5 LOMBA2 LUO MAGNUS MARSH MARSH2 MARTIS MASTRA MATSUD MCCONN MCDUFF MCLAUG MILLER MILLS MOLLO MRFIT MURATA NOTAN2 NOTANI NOU ORMOS PASTOR PAWLEG PERNU PERSHA PIKE POFFIJ POLEDN QIAO RADZIK RANDIG REN RIMING RONCO ROOTS ROTHSC SAARIK SADOWS SANKAR SCHWA2 SEGI SEGI2 SEOW SHIMIZ SIEMIA SIMARA SITAS SOBUE2 STASZE STAYNE STOCKS SUN TANG2 TAO ULMER VANDER WALD WANG WANG3 WANG4 WARSIN WATSON WICKLU WILKIN WU2 WUWILL WYNDE2 WYNDE4 WYNDE5 WYNDE8 WYNDER XIANGZ XU XU2 XU3 XU4 YUAN ZHANG ZHENG ZHOU
3	RESTRE
4	AKIBA AMANDU AMES ANDERS ARCHER ARMADA AUSTIN AXELSS BARBON BECHER BENSHL BLOHMK BOFFET BRETT BROSS BROWN2 BUFFLE CARPEN CEDERL CHANG CHOI CHYOU COMSTO CORREA DARBY DEAN2 DEKLER DESTE2 DESTEF DOCKER DOLL DORANT DORGAN DROSTE ENGELA GAO GAO2 GARCIA GARDIN GARSHI GILLIS GOODMA GREGOR HAENSZ HAMMO2 HEIN HENNEK HIRAYA HITOSU HOLE HUMBLE JAHN JAIN JARVHO JEDRYC JOLY KAISER KATSOU KAUFMA KELLER KHUDER KIHARA KINLEN KJUUS KNEKT KOO KREUZE KUBIK LANGE LEMARC LIDDEL LUBIN MACLEN MATOS MIGRAN MRFITR NAM ODRISC OSANN OSANN2 PARKIN PERSH2 PETO PEZZO2 PISANI PRESCO QIAO2 RACHTA SCHWAR SHAW SIMONA SOBUE SPEIZE SPITZ STOCKW STUCKE SUZUK2 SUZUKI SVENSS TANG TENKAN TIZZAN TOKARS TOUSEY TSUGAN TULINI VEIERO VUTUC WAKAI WANG2 WU WUNSCH WYNDE3 WYNDE6 YAMAGU YONG
8	AGUDO BEST BOUCOT CHOW CPSI CPSII DAMBER DEAN3 DOLL2 DORN GRAHAM KAISE2 LOMBAR LUBIN2 PEZZOT TVERDA WIGLE

Table 2D3 - 8
Potentially overlapping studies

REF	REFGP	PRINC	OVERLAP/LINK
BENHAM	LUBIN2	2	Subset of Lubin2
WYNDE7	WYNDE6	2	WYNDE5/6/7/8
