# Supplementary material for: Systematic review with meta-analysis of the epidemiological evidence in the 1900s relating smoking to lung cancer
Source: BMC Cancer. 2012 Sep 3;12:385. doi: 10.1186/1471-2407-12-385 (PMC3505152; doi:10.1186/1471-2407-12-385)
Supplement: Additional file 5 — Detailed Analysis Tables (Individual file names as described in Additional file 1: Methods, Table1). [file 1471-2407-12-385-S5.zip › PDF/3D.pdf]

Table 3D1 -

IESLC - Meta-analysis of Ex Smoking, Any product (or Cigarettes if Any not available)
Adenocarcinoma

This analysis is restricted to results for:
1) Non-dose-response data
2) Ex smokers
3) Results complete enough for use in metaanalysis

Within each study, results are then selected (in the following order of preference, within each sex) for:
4) PRODUCT: all/unspec, cigarettes regardless of other products, cigarettes only
5) CIGTYPE: all/unspecified, MC regardless of HR, MC only
6) DENOM: never smoked anything, never smoked cigarettes, (never +1 = +long term ex, +2 = +amount unknown,
 +3 = never cigs+long term ex)
7) Followup period (YF, prospective studies): whole study (coded as 0) or longest available
8) LCtype: adeno or nearest available, but not squamous. (q = squamous, s = small,
 a = adeno, l = large, KII = Kreyberg II, al = alveolar, br = bronchiolar, u = undifferentiated)
9) Race: all or nearest available, otherwise by race (wh or w = white, bl or b = black, hi = hispanic
 ch = chinese, jap = japanese, haw = hawaiian, w+o = white + oriental, sca = scandinavian, as = asian)
10) For overlapping studies: principal rather than subsidiary studies
Finally by Age: whole study (coded as 0) if available, otherwise by widest available age group
and then for single sex results (m, f) in preference to combined sex results (c).

Results adjusted (AD) for the most potential confounders are then chosen in Sections -1 to -3
and results adjusted for the least confounders in Sections -4 to -6. (Those least adjusted results which
actually differ from the most adjusted as marked 'x' in column X in Section -4)
(Results adjusted for an unknown number of confounder(s) are coded as 20.)

Section -7 shows excluded studies, together with the stage (as above) at which no qualifying
results were found.

Section -8 lists the potentially overlapping studies which have been included (1=principal, 2=subsidiary).

Section -9 lists any results which would have been included in preference except that they had data not complete
enough for use in meta-analysis, with their significance (yes/no), if known, and any further comment as entered
on the database.

In addition to those mentioned above, the following fields, levels and abbreviations are used:
* or nk = not known, n = no, y = yes, ot = other
nev = never
all/unspec = all or unspecified, cig+/-ot = cigarettes irrespective of other products (cigar, pipe etc)
MC = manufactured cigarettes, HR = hand-rolled cigarettes
REF: 6-character study reference
NRR: number of the RR on the database within the study
ST : study type (CC = case control, pr or prosp = prospective)
NLC: number of lung cancer cases in whole study
R : risky occupational population (n = no, m = mining, o = other risky)
VB : national cigarette type (V = at least 75% Virginia, bl = at least 75% blended, ot = other)
P : any proxy use
H : full histological confirmation
De : derivation of RR/CI (or = original, st = standard method, ot = other method of estimation)

Table 3D1 - 1

IESLC - Meta-analysis of Ex Smoking, Any product (or Cigarettes if Any not available)
Adenocarcinoma
Most adjusted

REF	NRR	SEX	AGEL	AGEH	RACE	YF	LC TYPE	LOC	START	ST	NLC	R	VB	P	H	AD	PRODUCT	DENOM	De
BARBON	99	m	0	0	all	-	a	Eu:wst	1979	CC	755	n	bl	y	y	1	all/unsp	nev any	or
BROWN2	24	m	0	0	wh	-	a	NAmer	1984	CC	14596	n	bl	n	y	2	cig+/-ot	nev cigs	or
BROWN2	23	f	0	0	wh	-	a	NAmer	1984	CC	14596	n	bl	n	y	2	cig+/-ot	nev cigs	or
BUFFLE	72	f	0	0	w-hi	-	a	NAmer	1976	CC	943	n	bl	y	n	0	cig+/-ot	nev cigs	st
COMSTO	20	m	0	0	all	-	a	NAmer	1975	ot	258	n	bl	n	n	0	cig+/-ot	nev cigs	st
COMSTO	28	f	0	0	all	-	a	NAmer	1975	ot	258	n	bl	n	n	0	cig+/-ot	nev cigs	st
CORREA	40	c	0	0	all	-	a	NAmer	1979	CC	1359	n	bl	y	n	1	cig+/-ot	nev cigs	or
ENGELA	69	m	0	0	all	0	a	Eu:Sca	1964	pr	435	n	bl	n	n	7	cig+/-ot	nev cigs	or
HAENSZ	38	f	0	0	all	-	a	NAmer	1955	CC	158	n	bl	n	y	0	cig+/-ot	nev any	st
JAHN	13	m	0	0	all	-	a	Eu:Ger	1988	CC	1004	n	bl	n	n	0	cig+/-ot	nev any	st
JAIN	27	m	0	0	all	-	a	NAmer	1981	CC	845	n	V	y	n	0	cig+/-ot	nev cigs	st
JAIN	22	f	0	0	all	-	a	NAmer	1981	CC	845	n	V	y	n	0	cig+/-ot	nev cigs	st
JEDRYC	27	m	0	0	all	-	a	Eu:est	1980	CC	1630	n	bl	y	n	0	cig+/-ot	nev any	st
KATSOU	11	f	0	0	all	-	a	Eu:bal	1987	CC	101	n	bl	n	n	1	all/unsp	nev any	or
KHUDER	11	m	0	0	all	-	a	NAmer	1985	CC	482	n	bl	n	y	0	cig+/-ot	nev cigs	or
KIHARA	13	c	0	0	jap	-	a	As:Jap	1991	CC	440	n	bl	n	n	0	all/unsp	nev any	st
LUBIN2	260	m	0	0	all	-	a	Eu:mul	1976	CC	7804	n	bl	n	y	0	cig+/-ot	nev any	st
LUBIN2	272	f	0	0	all	-	a	Eu:mul	1976	CC	7804	n	bl	n	y	0	cig+/-ot	nev any	st
MATOS	55	m	0	0	all	-	a	SCAmer	1994	CC	200	n	bl	n	n	2	cig+/-ot	nev any	or
OSANN	31	m	0	0	all	-	a	NAmer	1984	CC	1986	n	bl	n	n	2	cig+/-ot	nev cigs	or
OSANN	32	f	0	0	all	-	a	NAmer	1984	CC	1986	n	bl	n	n	2	cig+/-ot	nev cigs	or
OSANN2	33	f	0	0	all	-	KII	NAmer	1964	ot	217	n	bl	n	y	1	cig+/-ot	nev cigs	or
SOBUE	35	m	0	0	all	-	a	As:Jap	1986	CC	1376	n	bl	n	y	1	cig+/-ot	nev cigs	or
SOBUE	45	f	0	0	all	-	a	As:Jap	1986	CC	1376	n	bl	n	y	1	cig+/-ot	nev cigs	or
SUZUKI	9	m	0	0	all	-	a	As:Jap	1978	CC	238	n	bl	n	y	2	cig+/-ot	nev any	or
SUZUKI	13	f	0	0	all	-	a	As:Jap	1978	CC	238	n	bl	n	y	2	cig+/-ot	nev any	or
SVENSS	4	f	0	0	all	-	a	Eu:Sca	1983	CC	210	n	bl	n	n	1	all/unsp	nev any	or
TSUGAN	1	m	0	0	all	-	a	As:Jap	1976	CC	134	n	bl	n	y	0	all/unsp	nev any	st
WAKAI	11	m	0	0	all	-	a	As:Jap	1988	CC	333	n	bl	n	y	1	all/unsp	nev any	or
WAKAI	29	f	0	0	all	-	a	As:Jap	1988	CC	333	n	bl	n	y	1	all/unsp	nev any	or
WU	6	f	0	0	wh	-	a	NAmer	1981	CC	220	n	bl	n	y	2	all/unsp	nev any	or
WYNDE3	21	m	0	0	all	-	KII	NAmer	1966	CC	350	n	bl	n	y	0	all/unsp	nev any	st
WYNDE6	6	m	0	0	all	-	KII	NAmer	1969	CC	4423	n	bl	n	y	0	cig+/-ot	nev any	st
WYNDE6	195	f	0	0	all	-	KII	NAmer	1969	CC	4423	n	bl	n	y	0	cig+/-ot	nev cigs	st

Cigarette type is all/unspec for all RRs

Table 3D1 - 2

IESLC - Meta-analysis of Ex Smoking, Any product (or Cigarettes if Any not available)
Adenocarcinoma
Most adjusted

REF	NRR	SEX	AD	Number Exposed Case	Number Exposed Cont	Non-exposed Case	Non-exposed Cont	RR	95.00%CI
BARBON	99	m	1	-	-	-	-	5.50	(2.40- 12.60)
BROWN2	24	m	2	-	-	-	-	7.40	(6.20- 8.80)
BROWN2	23	f	2	-	-	-	-	7.20	(6.20- 8.50)
Subtotal BROWN2								7.29	(6.48- 8.20)
BUFFLE	72	f	0	20	56	7	112	5.71	(2.28- 14.32)
COMSTO	20	m	0	13	129	2	84	4.23	(0.93- 19.23)
COMSTO	28	f	0	6	35	8	115	2.46	(0.80- 7.58)
Subtotal COMSTO								2.99	(1.21- 7.36)
CORREA	40	c	1	-	-	-	-	3.70	(2.10- 6.50)
*ENGELA	69	m	7	-	-	-	-	1.10	(0.40- 3.10)
HAENSZ	38	f	0	2	9	37	236	1.42	(0.29- 6.82)
JAHN	13	m	0	127	402	8	138	5.45	(2.60- 11.42)
JAIN	27	m	0	30	159	4	85	4.01	(1.37- 11.76)
JAIN	22	f	0	17	97	24	214	1.56	(0.80- 3.04)
Subtotal JAIN								2.03	(1.15- 3.57)
JEDRYC	27	m	0	21	312	7	289	2.78	(1.16- 6.63)
KATSOU	11	f	1	-	-	-	-	1.81	(0.37- 8.70)
KHUDER	11	m	0	63	-	7	-	8.00	(3.50- 18.20)
KIHARA	13	c	0	27	70	78	237	1.17	(0.70- 1.96)
LUBIN2	260	m	0	212	4228	57	2616	2.30	(1.71- 3.10)
LUBIN2	272	f	0	17	157	138	1180	0.93	(0.54- 1.57)
Subtotal LUBIN2								1.85	(1.43- 2.40)
MATOS	55	m	2	-	-	-	-	4.80	(1.80- 12.90)
OSANN	31	m	2	-	-	-	-	13.10	(7.40- 23.20)
OSANN	32	f	2	-	-	-	-	5.80	(3.80- 9.00)
Subtotal OSANN								7.79	(5.53- 11.00)
OSANN2	33	f	1	-	-	-	-	1.70	(0.50- 5.30)
SOBUE	35	m	1	-	-	-	-	1.50	(0.90- 2.40)
SOBUE	45	f	1	-	-	-	-	1.70	(1.00- 3.00)
Subtotal SOBUE								1.59	(1.10- 2.29)
SUZUKI	9	m	2	-	-	-	-	3.20	(1.52- 6.63)
SUZUKI	13	f	2	-	-	-	-	1.60	(0.48- 5.51)
Subtotal SUZUKI								2.66	(1.42- 5.00)
SVENSS	4	f	1	-	-	-	-	1.80	(0.80- 4.30)
TSUGAN	1	m	0	8	6	18	17	1.26	(0.36- 4.39)
WAKAI	11	m	1	-	-	-	-	1.40	(0.59- 3.31)
WAKAI	29	f	1	-	-	-	-	2.69	(0.68- 10.60)
Subtotal WAKAI								1.68	(0.81- 3.50)
WU	6	f	2	-	-	-	-	1.20	(0.60- 2.30)
WYNDE3	21	m	0	12	125	6	88	1.41	(0.51- 3.89)
WYNDE6	6	m	0	408	1056	58	617	4.11	(3.07- 5.51)
WYNDE6	195	f	0	171	325	119	856	3.78	(2.90- 4.94)
Subtotal WYNDE6								3.93	(3.23- 4.78)
Partial Totals				1154	7166	578	6884		

*prospective study

REF	NRR	SEX	AD	Ys	Ws	Qs	Ps
BARBON	99	m	1	1.70	5.59	0.29	0.0001
BROWN2	24	m	2	2.00	125.29	34.59	0.0000
BROWN2	23	f	2	1.97	154.35	38.29	0.0000
Subtotal BROWN2				1.99	279.64	72.88	
BUFFLE	72	f	0	1.74	4.55	0.32	0.0002
COMSTO	20	m	0	1.44	1.68	0.00	0.0618
COMSTO	28	f	0	0.90	3.04	1.00	0.1158
Subtotal COMSTO				1.09	4.72	1.00	
CORREA	40	c	1	1.31	12.04	0.34	0.0000
*ENGELA	69	m	7	0.10	3.66	6.99	0.8552
HAENSZ	38	f	0	0.35	1.56	1.98	0.6634
JAHN	13	m	0	1.70	7.01	0.34	0.0000
JAIN	27	m	0	1.39	3.32	0.03	0.0114
JAIN	22	f	0	0.45	8.66	9.18	0.1889
Subtotal JAIN				0.71	11.98	9.21	
JEDRYC	27	m	0	1.02	5.07	1.05	0.0213
KATSOU	11	f	1	0.59	1.54	1.20	0.4614
KHUDER	11	m	0	2.08	5.65	2.06	0.0000
KIHARA	13	c	0	0.16	14.63	25.39	0.5439
LUBIN2	260	m	0	0.83	43.71	18.05	0.0000
LUBIN2	272	f	0	-0.08	13.65	32.91	0.7760
Subtotal LUBIN2				0.62	57.35	50.96	
MATOS	55	m	2	1.57	3.96	0.03	0.0018

Table 3D1 - 2

IESLC - Meta-analysis of Ex Smoking, Any product (or Cigarettes if Any not available)
Adenocarcinoma
Most adjusted

REF	NRR	SEX	AD	Ys	Ws	Qs	Ps
OSANN	31	m	2	2.57	11.77	14.15	0.0000
OSANN	32	f	2	1.76	20.67	1.64	0.0000
Subtotal OSANN				2.05	32.44	15.79	
OSANN2	33	f	1	0.53	2.76	2.46	0.3783
SOBUE	35	m	1	0.41	15.97	18.31	0.1051
SOBUE	45	f	1	0.53	12.73	11.38	0.0583
Subtotal SOBUE				0.46	28.70	29.69	
SUZUKI	9	m	2	1.16	7.08	0.69	0.0020
SUZUKI	13	f	2	0.47	2.58	2.61	0.4503
Subtotal SUZUKI				0.98	9.66	3.30	
SVENSS	4	f	1	0.59	5.43	4.29	0.1707
TSUGAN	1	m	0	0.23	2.46	3.82	0.7175
WAKAI	11	m	1	0.34	5.17	6.71	0.4444
WAKAI	29	f	1	0.99	2.04	0.48	0.1579
Subtotal WAKAI				0.52	7.20	7.19	
WU	6	f	2	0.18	8.51	14.24	0.5948
WYNDE3	21	m	0	0.34	3.71	4.77	0.5097
WYNDE6	6	m	0	1.41	44.92	0.18	0.0000
WYNDE6	195	f	0	1.33	54.06	1.14	0.0000
Subtotal WYNDE6				1.37	98.99	1.31	

	N	34
	NS	25
	Wt	618.83
Het	Chi	260.90
Het	df	33
Het	P	***
Fixed	RR	4.38
	RRl	4.04
	RRu	4.73
	P	+++
Random	RR	2.85
	RRl	2.20
	RRu	3.70
	P	+++
Asymm	P	***

Table 3D1 - 3

IESLC - Meta-analysis of Ex Smoking, Any product (or Cigarettes if Any not available)
Adenocarcinoma
Most adjusted

	Sex: combined	male	female	Total
N	2	17	15	34
NS	2	17	15	34
Wt	26.66	296.03	296.13	618.83
Het Chi	8.73	111.36	122.96	260.90
Het df	1	16	14	33
Het P	**	***	***	***
Fixed RR	1.97	4.59	4.48	4.38
RRl	1.35	4.10	4.00	4.04
RRu	2.88	5.15	5.02	4.73
P	+++	+++	+++	+++
Random RR	2.07	3.45	2.39	2.85
RRl	0.67	2.40	1.55	2.20
RRu	6.38	4.96	3.69	3.70
P	N.S.	+++	+++	+++
Between Chi				17.85
Between df				2
Between P				***
Btwn(F) P				N.S.
Btwn(R) P				N.S.

	Lung cancer type: a	a+l	a+al+br	KII	not q+u	not q+s	Total
N	30			4			34
NS	22			3			25
Wt	513.37			105.46			618.83
Het Chi	251.76			5.66			260.90
Het df	29			3			33
Het P	***			N.S.			***
Fixed RR	4.53			3.71			4.38
RRl	4.15			3.06			4.04
RRu	4.94			4.49			4.73
P	+++			+++			+++
Random RR	2.85			3.40			2.85
RRl	2.11			2.45			2.20
RRu	3.85			4.71			3.70
P	+++			+++			+++
Between Chi							3.49
Between df							1
Between P							(*)
Btwn(F) P							N.S.
Btwn(R) P							N.S.

	Location: NAmer	UK	Scand	othEur	China	Japan	othAs	other	Total
N	17		2	6		8		1	34
NS	12		2	5		5		1	25
Wt	466.54		9.10	76.57		62.66		3.96	618.83
Het Chi	94.05		0.53	21.02		5.72		0.00	260.90
Het df	16		1	5		7		0	33
Het P	***		N.S.	***		N.S.		N.S.	***
Fixed RR	5.69		1.48	2.27		1.60		4.80	4.38
RRl	5.20		0.77	1.82		1.25		1.79	4.04
RRu	6.23		2.83	2.84		2.05		12.85	4.73
P	+++		N.S.	+++		+++		++	+++
Random RR	4.08		1.48	2.58		1.60		4.80	2.85
RRl	3.08		0.77	1.47		1.25		1.79	2.20
RRu	5.39		2.83	4.52		2.05		12.85	3.70
P	+++		N.S.	+++		+++		++	+++
Between Chi									139.58
Between df									4
Between P									***
Btwn(F) P									***
Btwn(R) P									***

Table 3D1 - 3

IESLC - Meta-analysis of Ex Smoking, Any product (or Cigarettes if Any not available)
Adenocarcinoma
Most adjusted

Detailed Country in "other Europe"

	multi	Germany	othWest	East	Balkans	Total
N	2	1	1	1	1	6
NS	1	1	1	1	1	5
Wt	57.35	7.01	5.59	5.07	1.54	76.57
Het Chi	8.62	0.00	0.00	0.00	0.00	21.02
Het df	1	0	0	0	0	5
Het P	**	N.S.	N.S.	N.S.	N.S.	***
Fixed RR	1.85	5.45	5.50	2.78	1.81	2.27
RRl	1.43	2.60	2.40	1.16	0.37	1.82
RRu	2.40	11.42	12.60	6.63	8.78	2.84
P	+++	+++	+++	+	N.S.	+++
Random RR	1.50	5.45	5.50	2.78	1.81	2.58
RRl	0.62	2.60	2.40	1.16	0.37	1.47
RRu	3.66	11.42	12.60	6.63	8.78	4.52
P	N.S.	+++	+++	+	N.S.	+++
Between Chi						12.40
Between df						4
Between P						*
Btwn(F) P						N.S.
Btwn(R) P						N.S.

Detailed Country in "other Asia"

	India	HongKong	other	Total
N				
NS				
Wt				
Het Chi				
Het df				
Het P				N.S.
Fixed RR				
RRl				
RRu				
P				+
Random RR				
RRl				
RRu				
P				+
Between Chi				
Between df				
Between P				N.S.
Btwn(F) P				N.S.
Btwn(R) P				N.S.

Detailed other continent

	SCAmer	Auslia	Africa	Total
N	1			1
NS	1			1
Wt	3.96			3.96
Het Chi	0.00			0.00
Het df	0			0
Het P	N.S.			N.S.
Fixed RR	4.80			4.80
RRl	1.79			1.79
RRu	12.85			12.85
P	++			++
Random RR	4.80			4.80
RRl	1.79			1.79
RRu	12.85			12.85
P	++			++
Between Chi				
Between df				
Between P				N.S.
Btwn(F) P				N.S.
Btwn(R) P				N.S.

Table 3D1 - 3

IESLC - Meta-analysis of Ex Smoking, Any product (or Cigarettes if Any not available)
Adenocarcinoma
Most adjusted

	Start year of study <1960	1960-69	1970-79	1980-89	1990+	Total
N	1	5	10	16	2	34
NS	1	4	7	11	2	25
Wt	1.56	109.12	96.37	393.19	18.59	618.83
Het Chi	0.00	10.89	24.55	131.64	6.20	260.90
Het df	0	4	9	15	1	33
Het P	N.S.	*	**	***	*	***
Fixed RR	1.42	3.56	2.38	5.67	1.58	4.38
RRl	0.29	2.95	1.95	5.13	1.00	4.04
RRu	6.82	4.29	2.91	6.26	2.49	4.73
P	N.S.	+++	+++	+++	+	+++
Random RR	1.42	2.80	2.59	3.37	2.22	2.85
RRl	0.29	1.86	1.75	2.35	0.56	2.20
RRu	6.82	4.21	3.84	4.85	8.79	3.70
P	N.S.	+++	+++	+++	N.S.	+++
Between Chi						87.63
Between df						4
Between P						***
Btwn(F) P						*
Btwn(R) P						N.S.

	Study type (1) CC	other	Total
N	30	4	34
NS	22	3	25
Wt	607.69	11.14	618.83
Het Chi	250.30	2.41	260.90
Het df	29	3	33
Het P	***	N.S.	***
Fixed RR	4.44	1.87	4.38
RRl	4.10	1.04	4.04
RRu	4.81	3.36	4.73
P	+++	+	+++
Random RR	2.96	1.87	2.85
RRl	2.26	1.04	2.20
RRu	3.87	3.36	3.70
P	+++	+	+++
Between Chi			8.19
Between df			1
Between P			**
Btwn(F) P			N.S.
Btwn(R) P			N.S.

	Study type (2) CC	prosp	other	Total
N	30	1	3	34
NS	22	1	2	25
Wt	607.69	3.66	7.47	618.83
Het Chi	250.30	0.00	0.87	260.90
Het df	29	0	2	33
Het P	***	N.S.	N.S.	***
Fixed RR	4.44	1.10	2.43	4.38
RRl	4.10	0.40	1.18	4.04
RRu	4.81	3.06	4.97	4.73
P	+++	N.S.	+	+++
Random RR	2.96	1.10	2.43	2.85
RRl	2.26	0.40	1.18	2.20
RRu	3.87	3.06	4.97	3.70
P	+++	N.S.	+	+++
Between Chi				9.73
Between df				2
Between P				**
Btwn(F) P				N.S.
Btwn(R) P				N.S.

Table 3D1 - 3

IESLC - Meta-analysis of Ex Smoking, Any product (or Cigarettes if Any not available)
Adenocarcinoma
Most adjusted

	Study size (number of LC cases) 100-249	250-499	500-999	1000+	Total
N	9	8	4	13	34
NS	8	6	3	8	25
Wt	35.88	39.58	22.12	521.24	618.83
Het Chi	7.78	18.52	7.79	163.06	260.90
Het df	8	7	3	12	33
Het P	N.S.	**	(*)	***	***
Fixed RR	1.95	1.86	3.23	5.00	4.38
RRl	1.40	1.36	2.13	4.59	4.04
RRu	2.70	2.54	4.90	5.45	4.73
P	+++	+++	+++	+++	+++
Random RR	1.95	2.10	3.58	3.69	2.85
RRl	1.40	1.21	1.79	2.60	2.20
RRu	2.70	3.64	7.13	5.22	3.70
P	+++	++	+++	+++	+++
Between Chi					63.75
Between df					3
Between P					***
Btwn(F) P					*
Btwn(R) P					*

	Risky occupational population no	mining	othRisky	Total
N	34			34
NS	25			25
Wt	618.83			618.83
Het Chi	260.90			260.90
Het df	33			33
Het P	***			***
Fixed RR	4.38			4.38
RRl	4.04			4.04
RRu	4.73			4.73
P	+++			+++
Random RR	2.85			2.85
RRl	2.20			2.20
RRu	3.70			3.70
P	+++			+++
Between Chi				
Between df				
Between P				N.S.
Btwn(F) P				N.S.
Btwn(R) P				N.S.

	National cigarette tobacco type Virginia	blended	other	Total
N	2	32		34
NS	1	24		25
Wt	11.98	606.85		618.83
Het Chi	2.13	251.56		260.90
Het df	1	31		33
Het P	N.S.	***		***
Fixed RR	2.03	4.44		4.38
RRl	1.15	4.10		4.04
RRu	3.57	4.81		4.73
P	+	+++		+++
Random RR	2.27	2.89		2.85
RRl	0.92	2.22		2.20
RRu	5.59	3.77		3.70
P	(+)	+++		+++
Between Chi				7.22
Between df				1
Between P				**
Btwn(F) P				N.S.
Btwn(R) P				N.S.

Table 3D1 - 3

IESLC - Meta-analysis of Ex Smoking, Any product (or Cigarettes if Any not available)
Adenocarcinoma
Most adjusted

	Any proxy use No/nk	Yes	Total
N	28	6	34
NS	20	5	25
Wt	579.60	39.23	618.83
Het Chi	249.49	8.10	260.90
Het df	27	5	33
Het P	***	N.S.	***
Fixed RR	4.46	3.30	4.38
RRl	4.11	2.42	4.04
RRu	4.84	4.52	4.73
P	+++	+++	+++
Random RR	2.73	3.39	2.85
RRl	2.04	2.25	2.20
RRu	3.65	5.11	3.70
P	+++	+++	+++
Between Chi			3.31
Between df			1
Between P			(*)
Btwn(F) P			N.S.
Btwn(R) P			N.S.

	Full histological confirmation No	Yes	Total
N	15	19	34
NS	12	13	25
Wt	107.03	511.79	618.83
Het Chi	60.56	193.84	260.90
Het df	14	18	33
Het P	***	***	***
Fixed RR	3.50	4.59	4.38
RRl	2.89	4.21	4.04
RRu	4.23	5.00	4.73
P	+++	+++	+++
Random RR	3.24	2.60	2.85
RRl	2.13	1.85	2.20
RRu	4.94	3.65	3.70
P	+++	+++	+++
Between Chi			6.50
Between df			1
Between P			*
Btwn(F) P			N.S.
Btwn(R) P			N.S.

	Number of adjustment variables (1) 0	1	2+/+nk	Total
N	16	9	9	34
NS	12	7	6	25
Wt	217.68	63.26	337.88	618.83
Het Chi	60.42	12.67	54.46	260.90
Het df	15	8	8	33
Het P	***	N.S.	***	***
Fixed RR	2.82	2.13	6.64	4.38
RRl	2.47	1.66	5.97	4.04
RRu	3.22	2.73	7.39	4.73
P	+++	+++	+++	+++
Random RR	2.61	2.17	4.42	2.85
RRl	1.91	1.56	3.05	2.20
RRu	3.58	3.03	6.40	3.70
P	+++	+++	+++	+++
Between Chi				133.36
Between df				2
Between P				***
Btwn(F) P				***
Btwn(R) P				*

Table 3D1 - 3

IESLC - Meta-analysis of Ex Smoking, Any product (or Cigarettes if Any not available)
Adenocarcinoma
Most adjusted

	Number of adjustment variables (2) 0	1	2	3-5	6+/+nk	Total
N	16	9	8		1	34
NS	12	7	5		1	25
Wt	217.68	63.26	334.21		3.66	618.83
Het Chi	60.42	12.67	42.49		0.00	260.90
Het df	15	8	7		0	33
Het P	***	N.S.	***		N.S.	***
Fixed RR	2.82	2.13	6.77		1.10	4.38
RRl	2.47	1.66	6.08		0.40	4.04
RRu	3.22	2.73	7.54		3.06	4.73
P	+++	+++	+++		N.S.	+++
Random RR	2.61	2.17	5.04		1.10	2.85
RRl	1.91	1.56	3.55		0.40	2.20
RRu	3.58	3.03	7.16		3.06	3.70
P	+++	+++	+++		N.S.	+++
Between Chi						145.33
Between df						3
Between P						***
Btwn(F) P						***
Btwn(R) P						***

	Product all/unsp	cig+/-ot	cig only	Total
N	9	25		34
NS	8	17		25
Wt	49.08	569.75		618.83
Het Chi	11.58	195.44		260.90
Het df	8	24		33
Het P	N.S.	***		***
Fixed RR	1.60	4.77		4.38
RRl	1.21	4.40		4.04
RRu	2.12	5.18		4.73
P	+++	+++		+++
Random RR	1.68	3.35		2.85
RRl	1.17	2.54		2.20
RRu	2.40	4.41		3.70
P	++	+++		+++
Between Chi				53.88
Between df				1
Between P				***
Btwn(F) P				**
Btwn(R) P				**

	Denominator nev any	nev cigs	Total
N	18	16	34
NS	15	11	26
Wt	178.62	440.21	618.83
Het Chi	53.25	115.19	260.90
Het df	17	15	33
Het P	***	***	***
Fixed RR	2.38	5.60	4.38
RRl	2.06	5.10	4.04
RRu	2.76	6.15	4.73
P	+++	+++	+++
Random RR	2.17	3.83	2.85
RRl	1.61	2.78	2.20
RRu	2.94	5.27	3.70
P	+++	+++	+++
Between Chi			92.47
Between df			1
Between P			***
Btwn(F) P			***
Btwn(R) P			*

Table 3D1 - 3

IESLC - Meta-analysis of Ex Smoking, Any product (or Cigarettes if Any not available)
Adenocarcinoma
Most adjusted

	Derivation of RR/CI			
	Orig	StdCalc	Other	Total
N	19	15		34
NS	14	11		25
Wt	406.79	212.03		618.83
Het Chi	136.77	54.12		260.90
Het df	18	14		33
Het P	***	***		***
Fixed RR	5.58	2.75		4.38
RRl	5.06	2.40		4.04
RRu	6.15	3.14		4.73
P	+++	+++		+++
Random RR	3.25	2.43		2.85
RRl	2.33	1.77		2.20
RRu	4.53	3.33		3.70
P	+++	+++		+++
Between Chi				70.02
Between df				1
Between P				***
Btwn(F) P				**
Btwn(R) P				N.S.

Table 3D1 - 4

IESLC - Meta-analysis of Ex Smoking, Any product (or Cigarettes if Any not available)
Adenocarcinoma
Least adjusted

REF	NRR	X	SEX	AGEL	AGEH	RACE	YF	LC TYPE	LOC	START	ST	NLC	R	VB	P	H	AD	PRODUCT	DENOM	De
BARBON	43	x	m	0	0	all	-	a	Eu:wst	1979	CC	755	n	bl	y	y	0	all/unsp	nev any	st
BROWN2	24		m	0	0	wh	-	a	NAmer	1984	CC	14596	n	bl	n	y	2	cig+/-ot	nev cigs	or
BROWN2	23		f	0	0	wh	-	a	NAmer	1984	CC	14596	n	bl	n	y	2	cig+/-ot	nev cigs	or
BUFFLE	72		f	0	0	w-hi	-	a	NAmer	1976	CC	943	n	bl	y	n	0	cig+/-ot	nev cigs	st
COMSTO	20		m	0	0	all	-	a	NAmer	1975	ot	258	n	bl	n	n	0	cig+/-ot	nev cigs	st
COMSTO	28		f	0	0	all	-	a	NAmer	1975	ot	258	n	bl	n	n	0	cig+/-ot	nev cigs	st
CORREA	40		c	0	0	all	-	a	NAmer	1979	CC	1359	n	bl	y	n	1	cig+/-ot	nev cigs	or
ENGELA	69		m	0	0	all	0	a	Eu:Sca	1964	pr	435	n	bl	n	n	7	cig+/-ot	nev cigs	or
HAENSZ	38		f	0	0	all	-	a	NAmer	1955	CC	158	n	bl	n	y	0	cig+/-ot	nev any	st
JAHN	13		m	0	0	all	-	a	Eu:Ger	1988	CC	1004	n	bl	n	n	0	cig+/-ot	nev any	st
JAIN	27		m	0	0	all	-	a	NAmer	1981	CC	845	n	V	y	n	0	cig+/-ot	nev cigs	st
JAIN	22		f	0	0	all	-	a	NAmer	1981	CC	845	n	V	y	n	0	cig+/-ot	nev cigs	st
JEDRYC	27		m	0	0	all	-	a	Eu:est	1980	CC	1630	n	bl	y	n	0	cig+/-ot	nev any	st
KATSOU	15	x	f	0	0	all	-	a	Eu:bal	1987	CC	101	n	bl	n	n	0	all/unsp	nev any	st
KHUDER	11		m	0	0	all	-	a	NAmer	1985	CC	482	n	bl	n	y	0	cig+/-ot	nev cigs	or
KIHARA	13		c	0	0	jap	-	a	As:Jap	1991	CC	440	n	bl	n	n	0	all/unsp	nev any	st
LUBIN2	260		m	0	0	all	-	a	Eu:mul	1976	CC	7804	n	bl	n	y	0	cig+/-ot	nev any	st
LUBIN2	272		f	0	0	all	-	a	Eu:mul	1976	CC	7804	n	bl	n	y	0	cig+/-ot	nev any	st
MATOS	54	x	m	0	0	all	-	a	SCAmer	1994	CC	200	n	bl	n	n	0	cig+/-ot	nev any	st
OSANN	3	x	m	0	0	all	-	a	NAmer	1984	CC	1986	n	bl	n	n	0	cig+/-ot	nev cigs	st
OSANN	7	x	f	0	0	all	-	a	NAmer	1984	CC	1986	n	bl	n	n	0	cig+/-ot	nev cigs	st
OSANN2	15	x	f	0	0	all	-	KII	NAmer	1964	ot	217	n	bl	n	y	0	cig+/-ot	nev cigs	st
SOBUE	5	x	m	0	0	all	-	a	As:Jap	1986	CC	1376	n	bl	n	y	0	cig+/-ot	nev cigs	st
SOBUE	21	x	f	0	0	all	-	a	As:Jap	1986	CC	1376	n	bl	n	y	0	cig+/-ot	nev cigs	st
SUZUKI	1	x	m	0	0	all	-	a	As:Jap	1978	CC	238	n	bl	n	y	0	cig+/-ot	nev any	st
SUZUKI	5	x	f	0	0	all	-	a	As:Jap	1978	CC	238	n	bl	n	y	0	cig+/-ot	nev any	st
SVENSS	24	x	f	0	0	all	-	a	Eu:Sca	1983	CC	210	n	bl	n	n	0	all/unsp	nev any	st
TSUGAN	1		m	0	0	all	-	a	As:Jap	1976	CC	134	n	bl	n	y	0	all/unsp	nev any	st
WAKAI	5	x	m	0	0	all	-	a	As:Jap	1988	CC	333	n	bl	n	y	0	all/unsp	nev any	st
WAKAI	23	x	f	0	0	all	-	a	As:Jap	1988	CC	333	n	bl	n	y	0	all/unsp	nev any	st
WU	1	x	f	0	0	wh	-	a	NAmer	1981	CC	220	n	bl	n	y	0	all/unsp	nev any	st
WYNDE3	21		m	0	0	all	-	KII	NAmer	1966	CC	350	n	bl	n	y	0	all/unsp	nev any	st
WYNDE6	6		m	0	0	all	-	KII	NAmer	1969	CC	4423	n	bl	n	y	0	cig+/-ot	nev any	st
WYNDE6	195		f	0	0	all	-	KII	NAmer	1969	CC	4423	n	bl	n	y	0	cig+/-ot	nev cigs	st

Cigarette type is all/unspec for all RRs

Table 3D1 - 5

IESLC - Meta-analysis of Ex Smoking, Any product (or Cigarettes if Any not available)
Adenocarcinoma
Least adjusted

REF	NRR	SEX	AD	Number Exposed Case	Number Exposed Cont	Non-exposed Case	Non-exposed Cont	RR	95.00%CI
BARBON	43	m	0	42	205	7	188	5.50	(2.41- 12.55)
BROWN2	24	m	2	-	-	-	-	7.40	(6.20- 8.80)
BROWN2	23	f	2	-	-	-	-	7.20	(6.20- 8.50)
Subtotal BROWN2								7.29	(6.48- 8.20)
BUFFLE	72	f	0	20	56	7	112	5.71	(2.28- 14.32)
COMSTO	20	m	0	13	129	2	84	4.23	(0.93- 19.23)
COMSTO	28	f	0	6	35	8	115	2.46	(0.80- 7.58)
Subtotal COMSTO								2.99	(1.21- 7.36)
CORREA	40	c	1	-	-	-	-	3.70	(2.10- 6.50)
*ENGELA	69	m	7	-	-	-	-	1.10	(0.40- 3.10)
HAENSZ	38	f	0	2	9	37	236	1.42	(0.29- 6.82)
JAHN	13	m	0	127	402	8	138	5.45	(2.60- 11.42)
JAIN	27	m	0	30	159	4	85	4.01	(1.37- 11.76)
JAIN	22	f	0	17	97	24	214	1.56	(0.80- 3.04)
Subtotal JAIN								2.03	(1.15- 3.57)
JEDRYC	27	m	0	21	312	7	289	2.78	(1.16- 6.63)
KATSOU	15	f	0	3	4	30	67	1.68	(0.35- 7.95)
KHUDER	11	m	0	63	-	7	-	8.00	(3.50- 18.20)
KIHARA	13	c	0	27	70	78	237	1.17	(0.70- 1.96)
LUBIN2	260	m	0	212	4228	57	2616	2.30	(1.71- 3.10)
LUBIN2	272	f	0	17	157	138	1180	0.93	(0.54- 1.57)
Subtotal LUBIN2								1.85	(1.43- 2.40)
MATOS	54	m	0	33	151	5	110	4.81	(1.82- 12.71)
OSANN	3	m	0	102	477	14	833	12.72	(7.20- 22.49)
OSANN	7	f	0	50	196	47	1093	5.93	(3.87- 9.09)
Subtotal OSANN								7.80	(5.54- 10.97)
OSANN2	15	f	0	11	12	22	43	1.79	(0.68- 4.71)
SOBUE	5	m	0	117	363	27	128	1.53	(0.96- 2.43)
SOBUE	21	f	0	20	64	137	857	1.95	(1.15- 3.33)
Subtotal SOBUE								1.70	(1.20- 2.41)
SUZUKI	1	m	0	25	55	14	99	3.21	(1.54- 6.69)
SUZUKI	5	f	0	5	7	55	133	1.73	(0.53- 5.68)
Subtotal SUZUKI								2.71	(1.45- 5.06)
SVENSS	24	f	0	12	36	22	120	1.82	(0.82- 4.03)
TSUGAN	1	m	0	8	6	18	17	1.26	(0.36- 4.39)
WAKAI	5	m	0	23	140	8	65	1.33	(0.57- 3.14)
WAKAI	23	f	0	4	5	46	145	2.52	(0.65- 9.79)
Subtotal WAKAI								1.60	(0.78- 3.30)
WU	1	f	0	21	37	29	62	1.21	(0.61- 2.43)
WYNDE3	21	m	0	12	125	6	88	1.41	(0.51- 3.89)
WYNDE6	6	m	0	408	1056	58	617	4.11	(3.07- 5.51)
WYNDE6	195	f	0	171	325	119	856	3.78	(2.90- 4.94)
Subtotal WYNDE6								3.93	(3.23- 4.78)
Partial Totals				1622	8918	1041	10827		

*prospective study

REF	NRR	SEX	AD	Ys	Ws	Qs	Ps
BARBON	43	m	0	1.71	5.65	0.30	0.0001
BROWN2	24	m	2	2.00	125.29	34.90	0.0000
BROWN2	23	f	2	1.97	154.35	38.65	0.0000
Subtotal BROWN2				1.99	279.64	73.55	
BUFFLE	72	f	0	1.74	4.55	0.33	0.0002
COMSTO	20	m	0	1.44	1.68	0.00	0.0618
COMSTO	28	f	0	0.90	3.04	0.99	0.1158
Subtotal COMSTO				1.09	4.72	1.00	
CORREA	40	c	1	1.31	12.04	0.33	0.0000
*ENGELA	69	m	7	0.10	3.66	6.96	0.8552
HAENSZ	38	f	0	0.35	1.56	1.97	0.6634
JAHN	13	m	0	1.70	7.01	0.35	0.0000
JAIN	27	m	0	1.39	3.32	0.02	0.0114
JAIN	22	f	0	0.45	8.66	9.14	0.1889
Subtotal JAIN				0.71	11.98	9.16	
JEDRYC	27	m	0	1.02	5.07	1.03	0.0213
KATSOU	15	f	0	0.52	1.58	1.45	0.5163
KHUDER	11	m	0	2.08	5.65	2.07	0.0000
KIHARA	13	c	0	0.16	14.63	25.29	0.5439
LUBIN2	260	m	0	0.83	43.71	17.91	0.0000
LUBIN2	272	f	0	-0.08	13.65	32.81	0.7760
Subtotal LUBIN2				0.62	57.35	50.73	
MATOS	54	m	0	1.57	4.06	0.04	0.0015

Table 3D1 - 5

IESLC - Meta-analysis of Ex Smoking, Any product (or Cigarettes if Any not available)
Adenocarcinoma
Least adjusted

REF	NRR	SEX	AD	Ys	Ws	Qs	Ps
OSANN	3	m	0	2.54	11.83	13.54	0.0000
OSANN	7	f	0	1.78	21.14	1.99	0.0000
Subtotal OSANN				2.05	32.97	15.53	
OSANN2	15	f	0	0.58	4.12	3.26	0.2368
SOBUE	5	m	0	0.42	17.81	19.62	0.0736
SOBUE	21	f	0	0.67	13.50	8.71	0.0138
Subtotal SOBUE				0.53	31.31	28.33	
SUZUKI	1	m	0	1.17	7.16	0.67	0.0018
SUZUKI	5	f	0	0.55	2.71	2.33	0.3680
Subtotal SUZUKI				1.00	9.87	3.00	
SVENSS	24	f	0	0.60	6.06	4.65	0.1410
TSUGAN	1	m	0	0.23	2.46	3.81	0.7175
WAKAI	5	m	0	0.29	5.24	7.35	0.5087
WAKAI	23	f	0	0.92	2.09	0.63	0.1812
Subtotal WAKAI				0.47	7.32	7.98	
WU	1	f	0	0.19	7.98	13.08	0.5847
WYNDE3	21	m	0	0.34	3.71	4.75	0.5097
WYNDE6	6	m	0	1.41	44.92	0.16	0.0000
WYNDE6	195	f	0	1.33	54.06	1.10	0.0000
Subtotal WYNDE6				1.37	98.99	1.26	

	N	34
	NS	25
	Wt	623.97
Het	Chi	260.24
Het	df	33
Het	P	***
Fixed	RR	4.37
	RRl	4.04
	RRu	4.72
	P	+++
Random	RR	2.86
	RRl	2.22
	RRu	3.70
	P	+++
Asymm	P	***

Table 3D1 - 6

IESLC - Meta-analysis of Ex Smoking, Any product (or Cigarettes if Any not available)
Adenocarcinoma
Least adjusted

	combined	Sex<br>male	female	Total
N	2	17	15	34
NS	2	17	15	34
Wt	26.66	298.24	299.06	623.97
Het Chi	8.73	112.96	120.87	260.24
Het df	1	16	14	33
Het P	**	***	***	***
Fixed RR	1.97	4.56	4.49	4.37
RRl	1.35	4.07	4.01	4.04
RRu	2.88	5.10	5.03	4.72
P	+++	+++	+++	+++
Random RR	2.07	3.43	2.42	2.86
RRl	0.67	2.38	1.58	2.22
RRu	6.38	4.94	3.70	3.70
P	N.S.	+++	+++	+++
Between Chi				17.68
Between df				2
Between P				***
Btwn(F) P				N.S.
Btwn(R) P				N.S.

	a	a+l	Lung cancer type<br>a+al+br	KII	not q+u	not q+s	Total
N	30			4			34
NS	22			3			25
Wt	517.15			106.82			623.97
Het Chi	250.31			6.15			260.24
Het df	29			3			33
Het P	***			N.S.			***
Fixed RR	4.52			3.68			4.37
RRl	4.15			3.04			4.04
RRu	4.93			4.45			4.72
P	+++			+++			+++
Random RR	2.86			3.32			2.86
RRl	2.13			2.37			2.22
RRu	3.86			4.64			3.70
P	+++			+++			+++
Between Chi							3.78
Between df							1
Between P							(*)
Btwn(F) P							N.S.
Btwn(R) P							N.S.

	NAmer	UK	Scand	Location<br>othEur	China	Japan	othAs	other	Total
N	17		2	6		8		1	34
NS	12		2	5		5		1	25
Wt	467.91		9.73	76.67		65.59		4.06	623.97
Het Chi	93.48		0.58	21.15		6.18		0.00	260.24
Het df	16		1	5		7		0	33
Het P	***		N.S.	***		N.S.		N.S.	***
Fixed RR	5.69		1.50	2.27		1.65		4.81	4.37
RRl	5.20		0.80	1.82		1.29		1.82	4.04
RRu	6.23		2.82	2.84		2.10		12.71	4.72
P	+++		N.S.	+++		+++		++	+++
Random RR	4.07		1.50	2.56		1.65		4.81	2.86
RRl	3.08		0.80	1.46		1.29		1.82	2.22
RRu	5.36		2.82	4.49		2.10		12.71	3.70
P	+++		N.S.	++		+++		++	+++
Between Chi									138.85
Between df									4
Between P									***
Btwn(F) P									***
Btwn(R) P									***

Table 3D1 - 6

IESLC - Meta-analysis of Ex Smoking, Any product (or Cigarettes if Any not available)
Adenocarcinoma
Least adjusted

Detailed Country in "other Europe"

	multi	Germany	othWest	East	Balkans	Total
N	2	1	1	1	1	6
NS	1	1	1	1	1	5
Wt	57.35	7.01	5.65	5.07	1.58	76.67
Het Chi	8.62	0.00	0.00	0.00	0.00	21.15
Het df	1	0	0	0	0	5
Het P	**	N.S.	N.S.	N.S.	N.S.	***
Fixed RR	1.85	5.45	5.50	2.78	1.68	2.27
RRl	1.43	2.60	2.41	1.16	0.35	1.82
RRu	2.40	11.42	12.55	6.63	7.95	2.84
P	+++	+++	+++	+	N.S.	+++
Random RR	1.50	5.45	5.50	2.78	1.68	2.56
RRl	0.62	2.60	2.41	1.16	0.35	1.46
RRu	3.66	11.42	12.55	6.63	7.95	4.49
P	N.S.	+++	+++	+	N.S.	++
Between Chi						12.53
Between df						4
Between P						*
Btwn(F) P						N.S.
Btwn(R) P						N.S.

Detailed Country in "other Asia"

	India	HongKong	other	Total
N				
NS				
Wt				
Het Chi				
Het df				
Het P				N.S.
Fixed RR				
RRl				
RRu				
P				+
Random RR				
RRl				
RRu				
P				+
Between Chi				
Between df				
Between P				N.S.
Btwn(F) P				N.S.
Btwn(R) P				N.S.

Detailed other continent

	SCAmer	Auslia	Africa	Total
N	1			1
NS	1			1
Wt	4.06			4.06
Het Chi	0.00			0.00
Het df	0			0
Het P	N.S.			N.S.
Fixed RR	4.81			4.81
RRl	1.82			1.82
RRu	12.71			12.71
P	++			++
Random RR	4.81			4.81
RRl	1.82			1.82
RRu	12.71			12.71
P	++			++
Between Chi				
Between df				
Between P				N.S.
Btwn(F) P				N.S.
Btwn(R) P				N.S.

Table 3D1 - 6

IESLC - Meta-analysis of Ex Smoking, Any product (or Cigarettes if Any not available)
Adenocarcinoma
Least adjusted

	Start year of study <1960	1960-69	1970-79	1980-89	1990+	Total
N	1	5	10	16	2	34
NS	1	4	7	11	2	25
Wt	1.56	110.48	96.65	396.59	18.69	623.97
Het Chi	0.00	11.31	24.50	130.93	6.34	260.24
Het df	0	4	9	15	1	33
Het P	N.S.	*	**	***	*	***
Fixed RR	1.42	3.53	2.39	5.65	1.59	4.37
RRl	0.29	2.93	1.96	5.12	1.01	4.04
RRu	6.82	4.26	2.92	6.23	2.51	4.72
P	N.S.	+++	+++	+++	+	+++
Random RR	1.42	2.76	2.60	3.39	2.23	2.86
RRl	0.29	1.84	1.76	2.37	0.56	2.22
RRu	6.82	4.15	3.85	4.85	8.84	3.70
P	N.S.	+++	+++	+++	N.S.	+++
Between Chi						87.16
Between df						4
Between P						***
Btwn(F) P						*
Btwn(R) P						N.S.

	Study type (1) CC	other	Total
N	30	4	34
NS	22	3	25
Wt	611.47	12.50	623.97
Het Chi	248.84	2.39	260.24
Het df	29	3	33
Het P	***	N.S.	***
Fixed RR	4.44	1.88	4.37
RRl	4.10	1.08	4.04
RRu	4.81	3.28	4.72
P	+++	+	+++
Random RR	2.97	1.88	2.86
RRl	2.27	1.08	2.22
RRu	3.88	3.28	3.70
P	+++	+	+++
Between Chi			9.01
Between df			1
Between P			**
Btwn(F) P			N.S.
Btwn(R) P			N.S.

	Study type (2) CC	prosp	other	Total
N	30	1	3	34
NS	22	1	2	25
Wt	611.47	3.66	8.83	623.97
Het Chi	248.84	0.00	0.89	260.24
Het df	29	0	2	33
Het P	***	N.S.	N.S.	***
Fixed RR	4.44	1.10	2.35	4.37
RRl	4.10	0.40	1.22	4.04
RRu	4.81	3.06	4.55	4.72
P	+++	N.S.	+	+++
Random RR	2.97	1.10	2.35	2.86
RRl	2.27	0.40	1.22	2.22
RRu	3.88	3.06	4.55	3.70
P	+++	N.S.	+	+++
Between Chi				10.51
Between df				2
Between P				**
Btwn(F) P				N.S.
Btwn(R) P				N.S.

Table 3D1 - 6

IESLC - Meta-analysis of Ex Smoking, Any product (or Cigarettes if Any not available)
Adenocarcinoma
Least adjusted

	Study size (number of LC cases)				
	100-249	250-499	500-999	1000+	Total
N	9	8	4	13	34
NS	8	6	3	8	25
Wt	37.70	39.70	22.18	524.38	623.97
Het Chi	7.65	18.59	7.81	161.59	260.24
Het df	8	7	3	12	33
Het P	N.S.	**	(*)	***	***
Fixed RR	1.98	1.84	3.24	5.00	4.37
RRl	1.44	1.35	2.13	4.59	4.04
RRu	2.72	2.52	4.91	5.44	4.72
P	+++	+++	+++	+++	+++
Random RR	1.98	2.07	3.58	3.73	2.86
RRl	1.44	1.20	1.79	2.64	2.22
RRu	2.72	3.59	7.14	5.26	3.70
P	+++	++	+++	+++	+++
Between Chi					64.60
Between df					3
Between P					***
Btwn(F) P					*
Btwn(R) P					*

	Risky occupational population			
	no	mining	othRisky	Total
N	34			34
NS	25			25
Wt	623.97			623.97
Het Chi	260.24			260.24
Het df	33			33
Het P	***			***
Fixed RR	4.37			4.37
RRl	4.04			4.04
RRu	4.72			4.72
P	+++			+++
Random RR	2.86			2.86
RRl	2.22			2.22
RRu	3.70			3.70
P	+++			+++
Between Chi				
Between df				
Between P				N.S.
Btwn(F) P				N.S.
Btwn(R) P				N.S.

	National cigarette tobacco type			
	Virginia	blended	other	Total
N	2	32		34
NS	1	24		25
Wt	11.98	611.99		623.97
Het Chi	2.13	250.94		260.24
Het df	1	31		33
Het P	N.S.	***		***
Fixed RR	2.03	4.43		4.37
RRl	1.15	4.09		4.04
RRu	3.57	4.80		4.72
P	+	+++		+++
Random RR	2.27	2.90		2.86
RRl	0.92	2.23		2.22
RRu	5.59	3.77		3.70
P	(+)	+++		+++
Between Chi				7.17
Between df				1
Between P				**
Btwn(F) P				N.S.
Btwn(R) P				N.S.

Table 3D1 - 6

IESLC - Meta-analysis of Ex Smoking, Any product (or Cigarettes if Any not available)
Adenocarcinoma
Least adjusted

	Any proxy use No/nk	Yes	Total
N	28	6	34
NS	20	5	25
Wt	584.68	39.29	623.97
Het Chi	248.88	8.12	260.24
Het df	27	5	33
Het P	***	N.S.	***
Fixed RR	4.45	3.31	4.37
RRl	4.10	2.42	4.04
RRu	4.82	4.52	4.72
P	+++	+++	+++
Random RR	2.74	3.39	2.86
RRl	2.05	2.25	2.22
RRu	3.66	5.12	3.70
P	+++	+++	+++
Between Chi			3.24
Between df			1
Between P			(*)
Btwn(F) P			N.S.
Btwn(R) P			N.S.

	Full histological confirmation No	Yes	Total
N	15	19	34
NS	12	13	25
Wt	108.35	515.62	623.97
Het Chi	60.79	192.99	260.24
Het df	14	18	33
Het P	***	***	***
Fixed RR	3.50	4.57	4.37
RRl	2.90	4.20	4.04
RRu	4.22	4.99	4.72
P	+++	+++	+++
Random RR	3.23	2.62	2.86
RRl	2.13	1.87	2.22
RRu	4.91	3.66	3.70
P	+++	+++	+++
Between Chi			6.46
Between df			1
Between P			*
Btwn(F) P			N.S.
Btwn(R) P			N.S.

	Number of adjustment variables (1) 0	1	2+/+nk	Total
N	30	1	3	34
NS	22	1	2	25
Wt	328.63	12.04	283.31	623.97
Het Chi	122.84	0.00	12.99	260.24
Het df	29	0	2	33
Het P	***	N.S.	**	***
Fixed RR	2.88	3.70	7.11	4.37
RRl	2.59	2.10	6.33	4.04
RRu	3.21	6.51	7.99	4.72
P	+++	+++	+++	+++
Random RR	2.68	3.70	5.96	2.86
RRl	2.09	2.10	4.08	2.22
RRu	3.43	6.51	8.71	3.70
P	+++	+++	+++	+++
Between Chi				124.41
Between df				2
Between P				***
Btwn(F) P				***
Btwn(R) P				**

Table 3D1 - 6

IESLC - Meta-analysis of Ex Smoking, Any product (or Cigarettes if Any not available)
Adenocarcinoma
Least adjusted

	Number of adjustment variables (2) 0	1	2	3-5	6+/+nk	Total
N	30	1	2		1	34
NS	22	1	1		1	25
Wt	328.63	12.04	279.64		3.66	623.97
Het Chi	122.84	0.00	0.05		0.00	260.24
Het df	29	0	1		0	33
Het P	***	N.S.	N.S.		N.S.	***
Fixed RR	2.88	3.70	7.29		1.10	4.37
RRl	2.59	2.10	6.48		0.40	4.04
RRu	3.21	6.51	8.20		3.06	4.72
P	+++	+++	+++		N.S.	+++
Random RR	2.68	3.70	7.29		1.10	2.86
RRl	2.09	2.10	6.48		0.40	2.22
RRu	3.43	6.51	8.20		3.06	3.70
P	+++	+++	+++		N.S.	+++
Between Chi						137.35
Between df						3
Between P						***
Btwn(F) P						***
Btwn(R) P						***

	Product all/unsp	cig+/-ot	cig only	Total
N	9	25		34
NS	8	17		25
Wt	49.41	574.56		623.97
Het Chi	11.56	194.64		260.24
Het df	8	24		33
Het P	N.S.	***		***
Fixed RR	1.60	4.76		4.37
RRl	1.21	4.39		4.04
RRu	2.11	5.16		4.72
P	+++	+++		+++
Random RR	1.67	3.37		2.86
RRl	1.17	2.56		2.22
RRu	2.38	4.43		3.70
P	++	+++		+++
Between Chi				54.04
Between df				1
Between P				***
Btwn(F) P				**
Btwn(R) P				**

	Denominator nev any	nev cigs	Total
N	18	16	34
NS	15	11	26
Wt	179.27	444.70	623.97
Het Chi	53.26	115.29	260.24
Het df	17	15	33
Het P	***	***	***
Fixed RR	2.39	5.57	4.37
RRl	2.06	5.07	4.04
RRu	2.76	6.11	4.72
P	+++	+++	+++
Random RR	2.17	3.85	2.86
RRl	1.61	2.81	2.22
RRu	2.93	5.28	3.70
P	+++	+++	+++
Between Chi			91.69
Between df			1
Between P			***
Btwn(F) P			***
Btwn(R) P			**

Table 3D1 - 6

IESLC - Meta-analysis of Ex Smoking, Any product (or Cigarettes if Any not available)
Adenocarcinoma
Least adjusted

	Derivation of RR/CI Orig	StdCalc	Other	Total
N	5	29		34
NS	4	21		25
Wt	301.00	322.97		623.97
Het Chi	18.03	116.85		260.24
Het df	4	28		33
Het P	**	***		***
Fixed RR	6.94	2.83		4.37
RRl	6.20	2.54		4.04
RRu	7.78	3.16		4.72
P	+++	+++		+++
Random RR	5.63	2.58		2.86
RRl	4.04	2.01		2.22
RRu	7.84	3.31		3.70
P	+++	+++		+++
Between Chi				125.36
Between df				1
Between P				***
Btwn(F) P				***
Btwn(R) P				***

Table 3D1 - 7

IESLC - Meta-analysis of Ex Smoking, Any product (or Cigarettes if Any not available)
Adenocarcinoma
Excluded studies (and stage at which they were excluded)

1 BOUCHA BUELL LAURIL MZILEN

2 ABELIN ABRAHA ALDERS AUVINE AXELSO BAND BERRIN BLOT1 BLOT2 BLOT3 BLOT4 BRESLO BROCKM BROWN1 BYERS1 BYERS2
CASCO2 CASCOR CHAN CHATZI CHEN CHEN2 CHEN3 CHIAZZ COOKSO DAVEYS DEAN DOSEME DU DUNN EBELIN ENSTRO
ESAKI FAN GENG GER GODLEY GOLLED GSELL HANSEN HEGMAN HINDS HIRAOK HIRAY2 HOROWI HORWIT HU HU2
HUANG ISHIMA JARUP JIANG JONES JUSSAW KANELL KO KOHLME KOULUM KREYBE LAMTH LAMWK LAMWK2 LAUSSM LEI
LETOUR LEVIN LIAW LICKIN LIU LIU2 LIU3 LIU4 LIU5 LOMBA2 LUO MAGNUS MARSH MARSH2 MARTIS MASTRA
MATSUD MCCONN MCDUFF MCLAUG MILLER MILLS MOLLO MRFIT MURATA NOTAN2 NOTANI NOU ORMOS PASTOR PAWLEG PERNU
PERSHA PIKE POFFIJ POLEDN QIAO RADZIK RANDIG REN RIMING RONCO ROOTS ROTHSC SAARIK SADOWS SANKAR SCHWA2
SEGI SEGI2 SEOW SHIMIZ SIEMIA SIMARA SITAS SOBUE2 STASZE STAYNE STOCKS SUN TANG2 TAO ULMER VANDER
WALD WANG WANG3 WANG4 WARSIN WATSON WICKLU WILKIN WU2 WUWILL WYNDE2 WYNDE4 WYNDE5 WYNDE8 WYNDER XIANGZ
XU XU2 XU3 XU4 YUAN ZHANG ZHENG ZHOU

3 RESTRE

4 BOFFET

8 AGUDO AKIBA AMANDU AMES ANDERS ARCHER ARMADA AUSTIN AXELSS BECHER BENSHL BEST BLOHMK BOUCOT BRETT BROSS
CARPEN CEDERL CHANG CHOI CHOW CHYOU CPSI CPSII DAMBER DARBY DEAN2 DEAN3 DEKLER DESTE2 DESTEF DOCKER
DOLL DOLL2 DORANT DORGAN DORN DROSTE GAO GAO2 GARCIA GARDIN GARSHI GILLIS GOODMA GRAHAM GREGOR HAMMO2
HAMMON HEIN HENNEK HIRAYA HITOSU HOLE HUMBLE JARVHO JOLY KAISE2 KAISER KAUFMA KELLER KINLEN KJUUS KNEKT
KOO KREUZE KUBIK LANGE LEMARC LIDDEL LOMBAR LUBIN MACLEN MIGRAN MRFITR NAM ODRISC PARKIN PERSH2 PETO
PEZZO2 PEZZOT PISANI PRESCO QIAO2 RACHTA SCHWAR SHAW SIMONA SPEIZE SPITZ STOCKW STUCKE SUZUK2 TANG TENKAN
TIZZAN TOKARS TOUSEY TULINI TVERDA VEIERO WANG2 WIGLE WUNSCH YAMAGU YONG

10 BENHAM VUTUC WYNDE7

Table 3D1 - 8
Potentially overlapping studies

REF	REFGP	PRINC	. OVERLAP/LINK
LUBIN2	LUBIN2	1	Lubin-combined
OSANN2	KAISER	2	KAISER/OSANN2
WYNDE6	WYNDE6	1	WYNDE5/6/7/8
JAHN	BOFFET	2	Subset of BOFFET

Table 3D2 -

IESLC - Meta-analysis of Ex Smoking, Cigarettes (or Any Product if Cigarettes not available)
Adenocarcinoma

This analysis is restricted to results for:
1) Non-dose-response data
2) Ex smokers
3) Results complete enough for use in metaanalysis

Within each study, results are then selected (in the following order of preference, within each sex) for:
4) PRODUCT: cigarettes regardless of other products, cigarettes only, all/unspec
5) CIGTYPE: all/unspecified, MC regardless of HR, MC only
6) DENOM: never smoked anything, never smoked cigarettes, (never +1 = +long term ex, +2 = +amount unknown,
+3 = never cigs+long term ex)
7) Followup period (YF, prospective studies): whole study (coded as 0) or longest available
8) LCtype: adeno or nearest available, but not squamous. (q = squamous, s = small,
a = adeno, l = large, KII = Kreyberg II, al = alveolar, br = bronchiolar, u = undifferentiated)
9) Race: all or nearest available, otherwise by race (wh or w = white, bl or b = black, hi = hispanic
ch = chinese, jap = japanese, haw = hawaiian, w+o = white + oriental, sca = scandinavian, as = asian)
10) For overlapping studies: principal rather than subsidiary studies
Finally by Age: whole study (coded as 0) if available, otherwise by widest available age group
and then for single sex results (m, f) in preference to combined sex results (c).

Results adjusted (AD) for the most potential confounders are then chosen in Sections -1 to -3
and results adjusted for the least confounders in Sections -4 to -6. (Those least adjusted results which
actually differ from the most adjusted as marked 'x' in column X in Section -4)
(Results adjusted for an unknown number of confounder(s) are coded as 20.)

Section -7 shows excluded studies, together with the stage (as above) at which no qualifying
results were found.

Section -8 lists the potentially overlapping studies which have been included (1=principal, 2=subsidiary).

Section -9 lists any results which would have been included in preference except that they had data not complete
enough for use in meta-analysis, with their significance (yes/no), if known, and any further comment as entered
on the database.

In addition to those mentioned above, the following fields, levels and abbreviations are used:
* or nk = not known, n = no, y = yes, ot = other
nev = never
all/unspec = all or unspecified, cig+/-ot = cigarettes irrespective of other products (cigar, pipe etc)
MC = manufactured cigarettes, HR = hand-rolled cigarettes
REF: 6-character study reference
NRR: number of the RR on the database within the study
ST : study type (CC = case control, pr or prosp = prospective)
NLC: number of lung cancer cases in whole study
R : risky occupational population (n = no, m = mining, o = other risky)
VB : national cigarette type (V = at least 75% Virginia, bl = at least 75% blended, ot = other)
P : any proxy use
H : full histological confirmation
De : derivation of RR/CI (or = original, st = standard method, ot = other method of estimation)

Table 3D2 - 1

IESLC - Meta-analysis of Ex Smoking, Cigarettes (or Any Product if Cigarettes not available)
Adenocarcinoma
Most adjusted

REF	NRR	SEX	AGEL	AGEH	RACE	YF	LC TYPE	LOC	START	ST	NLC	R	VB	P	H	AD	PRODUCT	DENOM	De
BARBON	99	m	0	0	all	-	a	Eu:wst	1979	CC	755	n	bl	y	y	1	all/unsp	nev any	or
BROWN2	24	m	0	0	wh	-	a	NAmer	1984	CC	14596	n	bl	n	y	2	cig+/-ot	nev cigs	or
BROWN2	23	f	0	0	wh	-	a	NAmer	1984	CC	14596	n	bl	n	y	2	cig+/-ot	nev cigs	or
BUFFLE	72	f	0	0	w-hi	-	a	NAmer	1976	CC	943	n	bl	y	n	0	cig+/-ot	nev cigs	st
COMSTO	20	m	0	0	all	-	a	NAmer	1975	ot	258	n	bl	n	n	0	cig+/-ot	nev cigs	st
COMSTO	28	f	0	0	all	-	a	NAmer	1975	ot	258	n	bl	n	n	0	cig+/-ot	nev cigs	st
CORREA	40	c	0	0	all	-	a	NAmer	1979	CC	1359	n	bl	y	n	1	cig+/-ot	nev cigs	or
ENGELA	69	m	0	0	all	0	a	Eu:Sca	1964	pr	435	n	bl	n	n	7	cig+/-ot	nev cigs	or
HAENSZ	38	f	0	0	all	-	a	NAmer	1955	CC	158	n	bl	n	y	0	cig+/-ot	nev any	st
JAHN	13	m	0	0	all	-	a	Eu:Ger	1988	CC	1004	n	bl	n	n	0	cig+/-ot	nev any	st
JAIN	27	m	0	0	all	-	a	NAmer	1981	CC	845	n	V	y	n	0	cig+/-ot	nev cigs	st
JAIN	22	f	0	0	all	-	a	NAmer	1981	CC	845	n	V	y	n	0	cig+/-ot	nev cigs	st
JEDRYC	27	m	0	0	all	-	a	Eu:est	1980	CC	1630	n	bl	y	n	0	cig+/-ot	nev any	st
KATSOU	11	f	0	0	all	-	a	Eu:bal	1987	CC	101	n	bl	n	n	1	all/unsp	nev any	or
KHUDER	11	m	0	0	all	-	a	NAmer	1985	CC	482	n	bl	n	y	0	cig+/-ot	nev cigs	or
KIHARA	13	c	0	0	jap	-	a	As:Jap	1991	CC	440	n	bl	n	n	0	all/unsp	nev any	st
LUBIN2	260	m	0	0	all	-	a	Eu:mul	1976	CC	7804	n	bl	n	y	0	cig+/-ot	nev any	st
LUBIN2	272	f	0	0	all	-	a	Eu:mul	1976	CC	7804	n	bl	n	y	0	cig+/-ot	nev any	st
MATOS	55	m	0	0	all	-	a	SCAmer	1994	CC	200	n	bl	n	n	2	cig+/-ot	nev any	or
OSANN	31	m	0	0	all	-	a	NAmer	1984	CC	1986	n	bl	n	n	2	cig+/-ot	nev cigs	or
OSANN	32	f	0	0	all	-	a	NAmer	1984	CC	1986	n	bl	n	n	2	cig+/-ot	nev cigs	or
OSANN2	33	f	0	0	all	-	KII	NAmer	1964	ot	217	n	bl	n	y	1	cig+/-ot	nev cigs	or
SOBUE	35	m	0	0	all	-	a	As:Jap	1986	CC	1376	n	bl	n	y	1	cig+/-ot	nev cigs	or
SOBUE	45	f	0	0	all	-	a	As:Jap	1986	CC	1376	n	bl	n	y	1	cig+/-ot	nev cigs	or
SUZUKI	9	m	0	0	all	-	a	As:Jap	1978	CC	238	n	bl	n	y	2	cig+/-ot	nev any	or
SUZUKI	13	f	0	0	all	-	a	As:Jap	1978	CC	238	n	bl	n	y	2	cig+/-ot	nev any	or
SVENSS	4	f	0	0	all	-	a	Eu:Sca	1983	CC	210	n	bl	n	n	1	all/unsp	nev any	or
TSUGAN	1	m	0	0	all	-	a	As:Jap	1976	CC	134	n	bl	n	y	0	all/unsp	nev any	st
WAKAI	11	m	0	0	all	-	a	As:Jap	1988	CC	333	n	bl	n	y	1	all/unsp	nev any	or
WAKAI	29	f	0	0	all	-	a	As:Jap	1988	CC	333	n	bl	n	y	1	all/unsp	nev any	or
WU	6	f	0	0	wh	-	a	NAmer	1981	CC	220	n	bl	n	y	2	all/unsp	nev any	or
WYNDE3	21	m	0	0	all	-	KII	NAmer	1966	CC	350	n	bl	n	y	0	all/unsp	nev any	st
WYNDE6	6	m	0	0	all	-	KII	NAmer	1969	CC	4423	n	bl	n	y	0	cig+/-ot	nev any	st
WYNDE6	195	f	0	0	all	-	KII	NAmer	1969	CC	4423	n	bl	n	y	0	cig+/-ot	nev cigs	st

Cigarette type is all/unspec for all RRs

Table 3D2 - 2

IESLC - Meta-analysis of Ex Smoking, Cigarettes (or Any Product if Cigarettes not available)
Adenocarcinoma
Most adjusted

REF	NRR	SEX	AD	Number Exposed Case	Number Exposed Cont	Non-exposed Case	Non-exposed Cont	RR	95.00%CI
BARBON	99	m	1	-	-	-	-	5.50	(2.40- 12.60)
BROWN2	24	m	2	-	-	-	-	7.40	(6.20- 8.80)
BROWN2	23	f	2	-	-	-	-	7.20	(6.20- 8.50)
Subtotal BROWN2								7.29	(6.48- 8.20)
BUFFLE	72	f	0	20	56	7	112	5.71	(2.28- 14.32)
COMSTO	20	m	0	13	129	2	84	4.23	(0.93- 19.23)
COMSTO	28	f	0	6	35	8	115	2.46	(0.80- 7.58)
Subtotal COMSTO								2.99	(1.21- 7.36)
CORREA	40	c	1	-	-	-	-	3.70	(2.10- 6.50)
*ENGELA	69	m	7	-	-	-	-	1.10	(0.40- 3.10)
HAENSZ	38	f	0	2	9	37	236	1.42	(0.29- 6.82)
JAHN	13	m	0	127	402	8	138	5.45	(2.60- 11.42)
JAIN	27	m	0	30	159	4	85	4.01	(1.37- 11.76)
JAIN	22	f	0	17	97	24	214	1.56	(0.80- 3.04)
Subtotal JAIN								2.03	(1.15- 3.57)
JEDRYC	27	m	0	21	312	7	289	2.78	(1.16- 6.63)
KATSOU	11	f	1	-	-	-	-	1.81	(0.37- 8.70)
KHUDER	11	m	0	63	-	7	-	8.00	(3.50- 18.20)
KIHARA	13	c	0	27	70	78	237	1.17	(0.70- 1.96)
LUBIN2	260	m	0	212	4228	57	2616	2.30	(1.71- 3.10)
LUBIN2	272	f	0	17	157	138	1180	0.93	(0.54- 1.57)
Subtotal LUBIN2								1.85	(1.43- 2.40)
MATOS	55	m	2	-	-	-	-	4.80	(1.80- 12.90)
OSANN	31	m	2	-	-	-	-	13.10	(7.40- 23.20)
OSANN	32	f	2	-	-	-	-	5.80	(3.80- 9.00)
Subtotal OSANN								7.79	(5.53- 11.00)
OSANN2	33	f	1	-	-	-	-	1.70	(0.50- 5.30)
SOBUE	35	m	1	-	-	-	-	1.50	(0.90- 2.40)
SOBUE	45	f	1	-	-	-	-	1.70	(1.00- 3.00)
Subtotal SOBUE								1.59	(1.10- 2.29)
SUZUKI	9	m	2	-	-	-	-	3.20	(1.52- 6.63)
SUZUKI	13	f	2	-	-	-	-	1.60	(0.48- 5.51)
Subtotal SUZUKI								2.66	(1.42- 5.00)
SVENSS	4	f	1	-	-	-	-	1.80	(0.80- 4.30)
TSUGAN	1	m	0	8	6	18	17	1.26	(0.36- 4.39)
WAKAI	11	m	1	-	-	-	-	1.40	(0.59- 3.31)
WAKAI	29	f	1	-	-	-	-	2.69	(0.68- 10.60)
Subtotal WAKAI								1.68	(0.81- 3.50)
WU	6	f	2	-	-	-	-	1.20	(0.60- 2.30)
WYNDE3	21	m	0	12	125	6	88	1.41	(0.51- 3.89)
WYNDE6	6	m	0	408	1056	58	617	4.11	(3.07- 5.51)
WYNDE6	195	f	0	171	325	119	856	3.78	(2.90- 4.94)
Subtotal WYNDE6								3.93	(3.23- 4.78)
Partial Totals				1154	7166	578	6884		

*prospective study

REF	NRR	SEX	AD	Ys	Ws	Qs	Ps
BARBON	99	m	1	1.70	5.59	0.29	0.0001
BROWN2	24	m	2	2.00	125.29	34.59	0.0000
BROWN2	23	f	2	1.97	154.35	38.29	0.0000
Subtotal BROWN2				1.99	279.64	72.88	
BUFFLE	72	f	0	1.74	4.55	0.32	0.0002
COMSTO	20	m	0	1.44	1.68	0.00	0.0618
COMSTO	28	f	0	0.90	3.04	1.00	0.1158
Subtotal COMSTO				1.09	4.72	1.00	
CORREA	40	c	1	1.31	12.04	0.34	0.0000
*ENGELA	69	m	7	0.10	3.66	6.99	0.8552
HAENSZ	38	f	0	0.35	1.56	1.98	0.6634
JAHN	13	m	0	1.70	7.01	0.34	0.0000
JAIN	27	m	0	1.39	3.32	0.03	0.0114
JAIN	22	f	0	0.45	8.66	9.18	0.1889
Subtotal JAIN				0.71	11.98	9.21	
JEDRYC	27	m	0	1.02	5.07	1.05	0.0213
KATSOU	11	f	1	0.59	1.54	1.20	0.4614
KHUDER	11	m	0	2.08	5.65	2.06	0.0000
KIHARA	13	c	0	0.16	14.63	25.39	0.5439
LUBIN2	260	m	0	0.83	43.71	18.05	0.0000
LUBIN2	272	f	0	-0.08	13.65	32.91	0.7760
Subtotal LUBIN2				0.62	57.35	50.96	
MATOS	55	m	2	1.57	3.96	0.03	0.0018

Table 3D2 - 2

IESLC - Meta-analysis of Ex Smoking, Cigarettes (or Any Product if Cigarettes not available)
Adenocarcinoma
Most adjusted

REF	NRR	SEX	AD	Ys	Ws	Qs	Ps
OSANN	31	m	2	2.57	11.77	14.15	0.0000
OSANN	32	f	2	1.76	20.67	1.64	0.0000
Subtotal OSANN				2.05	32.44	15.79	
OSANN2	33	f	1	0.53	2.76	2.46	0.3783
SOBUE	35	m	1	0.41	15.97	18.31	0.1051
SOBUE	45	f	1	0.53	12.73	11.38	0.0583
Subtotal SOBUE				0.46	28.70	29.69	
SUZUKI	9	m	2	1.16	7.08	0.69	0.0020
SUZUKI	13	f	2	0.47	2.58	2.61	0.4503
Subtotal SUZUKI				0.98	9.66	3.30	
SVENSS	4	f	1	0.59	5.43	4.29	0.1707
TSUGAN	1	m	0	0.23	2.46	3.82	0.7175
WAKAI	11	m	1	0.34	5.17	6.71	0.4444
WAKAI	29	f	1	0.99	2.04	0.48	0.1579
Subtotal WAKAI				0.52	7.20	7.19	
WU	6	f	2	0.18	8.51	14.24	0.5948
WYNDE3	21	m	0	0.34	3.71	4.77	0.5097
WYNDE6	6	m	0	1.41	44.92	0.18	0.0000
WYNDE6	195	f	0	1.33	54.06	1.14	0.0000
Subtotal WYNDE6				1.37	98.99	1.31	

	N	34
	NS	25
	Wt	618.83
Het	Chi	260.90
Het	df	33
Het	P	***
Fixed	RR	4.38
	RRl	4.04
	RRu	4.73
	P	+++
Random	RR	2.85
	RRl	2.20
	RRu	3.70
	P	+++
Asymm	P	***

Table 3D2 - 3

IESLC - Meta-analysis of Ex Smoking, Cigarettes (or Any Product if Cigarettes not available)
Adenocarcinoma
Most adjusted

	Sex: combined	male	female	Total
N	2	17	15	34
NS	2	17	15	34
Wt	26.66	296.03	296.13	618.83
Het Chi	8.73	111.36	122.96	260.90
Het df	1	16	14	33
Het P	**	***	***	***
Fixed RR	1.97	4.59	4.48	4.38
RRl	1.35	4.10	4.00	4.04
RRu	2.88	5.15	5.02	4.73
P	+++	+++	+++	+++
Random RR	2.07	3.45	2.39	2.85
RRl	0.67	2.40	1.55	2.20
RRu	6.38	4.96	3.69	3.70
P	N.S.	+++	+++	+++
Between Chi				17.85
Between df				2
Between P				***
Btwn(F) P				N.S.
Btwn(R) P				N.S.

	Lung cancer type: a	a+l	a+al+br	KII	not q+u	not q+s	Total
N	30			4			34
NS	22			3			25
Wt	513.37			105.46			618.83
Het Chi	251.76			5.66			260.90
Het df	29			3			33
Het P	***			N.S.			***
Fixed RR	4.53			3.71			4.38
RRl	4.15			3.06			4.04
RRu	4.94			4.49			4.73
P	+++			+++			+++
Random RR	2.85			3.40			2.85
RRl	2.11			2.45			2.20
RRu	3.85			4.71			3.70
P	+++			+++			+++
Between Chi							3.49
Between df							1
Between P							(*)
Btwn(F) P							N.S.
Btwn(R) P							N.S.

	Location: NAmer	UK	Scand	othEur	China	Japan	othAs	other	Total
N	17		2	6		8		1	34
NS	12		2	5		5		1	25
Wt	466.54		9.10	76.57		62.66		3.96	618.83
Het Chi	94.05		0.53	21.02		5.72		0.00	260.90
Het df	16		1	5		7		0	33
Het P	***		N.S.	***		N.S.		N.S.	***
Fixed RR	5.69		1.48	2.27		1.60		4.80	4.38
RRl	5.20		0.77	1.82		1.25		1.79	4.04
RRu	6.23		2.83	2.84		2.05		12.85	4.73
P	+++		N.S.	+++		+++		++	+++
Random RR	4.08		1.48	2.58		1.60		4.80	2.85
RRl	3.08		0.77	1.47		1.25		1.79	2.20
RRu	5.39		2.83	4.52		2.05		12.85	3.70
P	+++		N.S.	+++		+++		++	+++
Between Chi									139.58
Between df									4
Between P									***
Btwn(F) P									***
Btwn(R) P									***

Table 3D2 - 3

IESLC - Meta-analysis of Ex Smoking, Cigarettes (or Any Product if Cigarettes not available)
Adenocarcinoma
Most adjusted

Detailed Country in "other Europe"

	multi	Germany	othWest	East	Balkans	Total
N	2	1	1	1	1	6
NS	1	1	1	1	1	5
Wt	57.35	7.01	5.59	5.07	1.54	76.57
Het Chi	8.62	0.00	0.00	0.00	0.00	21.02
Het df	1	0	0	0	0	5
Het P	**	N.S.	N.S.	N.S.	N.S.	***
Fixed RR	1.85	5.45	5.50	2.78	1.81	2.27
RRl	1.43	2.60	2.40	1.16	0.37	1.82
RRu	2.40	11.42	12.60	6.63	8.78	2.84
P	+++	+++	+++	+	N.S.	+++
Random RR	1.50	5.45	5.50	2.78	1.81	2.58
RRl	0.62	2.60	2.40	1.16	0.37	1.47
RRu	3.66	11.42	12.60	6.63	8.78	4.52
P	N.S.	+++	+++	+	N.S.	+++
Between Chi						12.40
Between df						4
Between P						*
Btwn(F) P						N.S.
Btwn(R) P						N.S.

Detailed Country in "other Asia"

	India	HongKong	other	Total
N				
NS				
Wt				
Het Chi				
Het df				
Het P				N.S.
Fixed RR				
RRl				
RRu				
P				+
Random RR				
RRl				
RRu				
P				+
Between Chi				
Between df				
Between P				N.S.
Btwn(F) P				N.S.
Btwn(R) P				N.S.

Detailed other continent

	SCAmer	Auslia	Africa	Total
N	1			1
NS	1			1
Wt	3.96			3.96
Het Chi	0.00			0.00
Het df	0			0
Het P	N.S.			N.S.
Fixed RR	4.80			4.80
RRl	1.79			1.79
RRu	12.85			12.85
P	++			++
Random RR	4.80			4.80
RRl	1.79			1.79
RRu	12.85			12.85
P	++			++
Between Chi				
Between df				
Between P				N.S.
Btwn(F) P				N.S.
Btwn(R) P				N.S.

Table 3D2 - 3

IESLC - Meta-analysis of Ex Smoking, Cigarettes (or Any Product if Cigarettes not available)
Adenocarcinoma
Most adjusted

	Start year of study					
	<1960	1960-69	1970-79	1980-89	1990+	Total
N	1	5	10	16	2	34
NS	1	4	7	11	2	25
Wt	1.56	109.12	96.37	393.19	18.59	618.83
Het Chi	0.00	10.89	24.55	131.64	6.20	260.90
Het df	0	4	9	15	1	33
Het P	N.S.	*	**	***	*	***
Fixed RR	1.42	3.56	2.38	5.67	1.58	4.38
RRl	0.29	2.95	1.95	5.13	1.00	4.04
RRu	6.82	4.29	2.91	6.26	2.49	4.73
P	N.S.	+++	+++	+++	+	+++
Random RR	1.42	2.80	2.59	3.37	2.22	2.85
RRl	0.29	1.86	1.75	2.35	0.56	2.20
RRu	6.82	4.21	3.84	4.85	8.79	3.70
P	N.S.	+++	+++	+++	N.S.	+++
Between Chi						87.63
Between df						4
Between P						***
Btwn(F) P						*
Btwn(R) P						N.S.

	Study type (1)		
	CC	other	Total
N	30	4	34
NS	22	3	25
Wt	607.69	11.14	618.83
Het Chi	250.30	2.41	260.90
Het df	29	3	33
Het P	***	N.S.	***
Fixed RR	4.44	1.87	4.38
RRl	4.10	1.04	4.04
RRu	4.81	3.36	4.73
P	+++	+	+++
Random RR	2.96	1.87	2.85
RRl	2.26	1.04	2.20
RRu	3.87	3.36	3.70
P	+++	+	+++
Between Chi			8.19
Between df			1
Between P			**
Btwn(F) P			N.S.
Btwn(R) P			N.S.

	Study type (2)			
	CC	prosp	other	Total
N	30	1	3	34
NS	22	1	2	25
Wt	607.69	3.66	7.47	618.83
Het Chi	250.30	0.00	0.87	260.90
Het df	29	0	2	33
Het P	***	N.S.	N.S.	***
Fixed RR	4.44	1.10	2.43	4.38
RRl	4.10	0.40	1.18	4.04
RRu	4.81	3.06	4.97	4.73
P	+++	N.S.	+	+++
Random RR	2.96	1.10	2.43	2.85
RRl	2.26	0.40	1.18	2.20
RRu	3.87	3.06	4.97	3.70
P	+++	N.S.	+	+++
Between Chi				9.73
Between df				2
Between P				**
Btwn(F) P				N.S.
Btwn(R) P				N.S.

Table 3D2 - 3

IESLC - Meta-analysis of Ex Smoking, Cigarettes (or Any Product if Cigarettes not available)
Adenocarcinoma
Most adjusted

	Study size (number of LC cases) 100-249	250-499	500-999	1000+	Total
N	9	8	4	13	34
NS	8	6	3	8	25
Wt	35.88	39.58	22.12	521.24	618.83
Het Chi	7.78	18.52	7.79	163.06	260.90
Het df	8	7	3	12	33
Het P	N.S.	**	(*)	***	***
Fixed RR	1.95	1.86	3.23	5.00	4.38
RRl	1.40	1.36	2.13	4.59	4.04
RRu	2.70	2.54	4.90	5.45	4.73
P	+++	+++	+++	+++	+++
Random RR	1.95	2.10	3.58	3.69	2.85
RRl	1.40	1.21	1.79	2.60	2.20
RRu	2.70	3.64	7.13	5.22	3.70
P	+++	++	+++	+++	+++
Between Chi					63.75
Between df					3
Between P					***
Btwn(F) P					*
Btwn(R) P					*

	Risky occupational population no	mining	othRisky	Total
N	34			34
NS	25			25
Wt	618.83			618.83
Het Chi	260.90			260.90
Het df	33			33
Het P	***			***
Fixed RR	4.38			4.38
RRl	4.04			4.04
RRu	4.73			4.73
P	+++			+++
Random RR	2.85			2.85
RRl	2.20			2.20
RRu	3.70			3.70
P	+++			+++
Between Chi				
Between df				
Between P				N.S.
Btwn(F) P				N.S.
Btwn(R) P				N.S.

	National cigarette tobacco type Virginia	blended	other	Total
N	2	32		34
NS	1	24		25
Wt	11.98	606.85		618.83
Het Chi	2.13	251.56		260.90
Het df	1	31		33
Het P	N.S.	***		***
Fixed RR	2.03	4.44		4.38
RRl	1.15	4.10		4.04
RRu	3.57	4.81		4.73
P	+	+++		+++
Random RR	2.27	2.89		2.85
RRl	0.92	2.22		2.20
RRu	5.59	3.77		3.70
P	(+)	+++		+++
Between Chi				7.22
Between df				1
Between P				**
Btwn(F) P				N.S.
Btwn(R) P				N.S.

Table 3D2 - 3

IESLC - Meta-analysis of Ex Smoking, Cigarettes (or Any Product if Cigarettes not available)
Adenocarcinoma
Most adjusted

	Any proxy use No/nk	Yes	Total
N	28	6	34
NS	20	5	25
Wt	579.60	39.23	618.83
Het Chi	249.49	8.10	260.90
Het df	27	5	33
Het P	***	N.S.	***
Fixed RR	4.46	3.30	4.38
RRl	4.11	2.42	4.04
RRu	4.84	4.52	4.73
P	+++	+++	+++
Random RR	2.73	3.39	2.85
RRl	2.04	2.25	2.20
RRu	3.65	5.11	3.70
P	+++	+++	+++
Between Chi			3.31
Between df			1
Between P			(*)
Btwn(F) P			N.S.
Btwn(R) P			N.S.

	Full histological confirmation No	Yes	Total
N	15	19	34
NS	12	13	25
Wt	107.03	511.79	618.83
Het Chi	60.56	193.84	260.90
Het df	14	18	33
Het P	***	***	***
Fixed RR	3.50	4.59	4.38
RRl	2.89	4.21	4.04
RRu	4.23	5.00	4.73
P	+++	+++	+++
Random RR	3.24	2.60	2.85
RRl	2.13	1.85	2.20
RRu	4.94	3.65	3.70
P	+++	+++	+++
Between Chi			6.50
Between df			1
Between P			*
Btwn(F) P			N.S.
Btwn(R) P			N.S.

	Number of adjustment variables (1) 0	1	2+/+nk	Total
N	16	9	9	34
NS	12	7	6	25
Wt	217.68	63.26	337.88	618.83
Het Chi	60.42	12.67	54.46	260.90
Het df	15	8	8	33
Het P	***	N.S.	***	***
Fixed RR	2.82	2.13	6.64	4.38
RRl	2.47	1.66	5.97	4.04
RRu	3.22	2.73	7.39	4.73
P	+++	+++	+++	+++
Random RR	2.61	2.17	4.42	2.85
RRl	1.91	1.56	3.05	2.20
RRu	3.58	3.03	6.40	3.70
P	+++	+++	+++	+++
Between Chi				133.36
Between df				2
Between P				***
Btwn(F) P				***
Btwn(R) P				*

Table 3D2 - 3

IESLC - Meta-analysis of Ex Smoking, Cigarettes (or Any Product if Cigarettes not available)
Adenocarcinoma
Most adjusted

	Number of adjustment variables (2) 0	1	2	3-5	6+/+nk	Total
N	16	9	8		1	34
NS	12	7	5		1	25
Wt	217.68	63.26	334.21		3.66	618.83
Het Chi	60.42	12.67	42.49		0.00	260.90
Het df	15	8	7		0	33
Het P	***	N.S.	***		N.S.	***
Fixed RR	2.82	2.13	6.77		1.10	4.38
RRl	2.47	1.66	6.08		0.40	4.04
RRu	3.22	2.73	7.54		3.06	4.73
P	+++	+++	+++		N.S.	+++
Random RR	2.61	2.17	5.04		1.10	2.85
RRl	1.91	1.56	3.55		0.40	2.20
RRu	3.58	3.03	7.16		3.06	3.70
P	+++	+++	+++		N.S.	+++
Between Chi						145.33
Between df						3
Between P						***
Btwn(F) P						***
Btwn(R) P						***

	Product all/unsp	cig+/-ot	cig only	Total
N	9	25		34
NS	8	17		25
Wt	49.08	569.75		618.83
Het Chi	11.58	195.44		260.90
Het df	8	24		33
Het P	N.S.	***		***
Fixed RR	1.60	4.77		4.38
RRl	1.21	4.40		4.04
RRu	2.12	5.18		4.73
P	+++	+++		+++
Random RR	1.68	3.35		2.85
RRl	1.17	2.54		2.20
RRu	2.40	4.41		3.70
P	++	+++		+++
Between Chi				53.88
Between df				1
Between P				***
Btwn(F) P				**
Btwn(R) P				**

	Denominator nev any	nev cigs	Total
N	18	16	34
NS	15	11	26
Wt	178.62	440.21	618.83
Het Chi	53.25	115.19	260.90
Het df	17	15	33
Het P	***	***	***
Fixed RR	2.38	5.60	4.38
RRl	2.06	5.10	4.04
RRu	2.76	6.15	4.73
P	+++	+++	+++
Random RR	2.17	3.83	2.85
RRl	1.61	2.78	2.20
RRu	2.94	5.27	3.70
P	+++	+++	+++
Between Chi			92.47
Between df			1
Between P			***
Btwn(F) P			***
Btwn(R) P			*

Table 3D2 - 3

IESLC - Meta-analysis of Ex Smoking, Cigarettes (or Any Product if Cigarettes not available)
Adenocarcinoma
Most adjusted

		Derivation of RR/CI			
		Orig	StdCalc	Other	Total
	N	19	15		34
	NS	14	11		25
	Wt	406.79	212.03		618.83
Het	Chi	136.77	54.12		260.90
Het	df	18	14		33
Het	P	***	***		***
Fixed	RR	5.58	2.75		4.38
	RRl	5.06	2.40		4.04
	RRu	6.15	3.14		4.73
	P	+++	+++		+++
Random	RR	3.25	2.43		2.85
	RRl	2.33	1.77		2.20
	RRu	4.53	3.33		3.70
	P	+++	+++		+++
Between	Chi				70.02
Between	df				1
Between	P				***
Btwn(F)	P				**
Btwn(R)	P				N.S.

Table 3D2 - 4

IESLC - Meta-analysis of Ex Smoking, Cigarettes (or Any Product if Cigarettes not available)
Adenocarcinoma
Least adjusted

REF	NRR	X	SEX	AGEL	AGEH	RACE	YF	LC TYPE	LOC	START	ST	NLC	R	VB	P	H	AD	PRODUCT	DENOM	De
BARBON	43	x	m	0	0	all	-	a	Eu:wst	1979	CC	755	n	bl	y	y	0	all/unsp	nev any	st
BROWN2	24		m	0	0	wh	-	a	NAmer	1984	CC	14596	n	bl	n	y	2	cig+/-ot	nev cigs	or
BROWN2	23		f	0	0	wh	-	a	NAmer	1984	CC	14596	n	bl	n	y	2	cig+/-ot	nev cigs	or
BUFFLE	72		f	0	0	w-hi	-	a	NAmer	1976	CC	943	n	bl	y	n	0	cig+/-ot	nev cigs	st
COMSTO	20		m	0	0	all	-	a	NAmer	1975	ot	258	n	bl	n	n	0	cig+/-ot	nev cigs	st
COMSTO	28		f	0	0	all	-	a	NAmer	1975	ot	258	n	bl	n	n	0	cig+/-ot	nev cigs	st
CORREA	40		c	0	0	all	-	a	NAmer	1979	CC	1359	n	bl	y	n	1	cig+/-ot	nev cigs	or
ENGELA	69		m	0	0	all	0	a	Eu:Sca	1964	pr	435	n	bl	n	n	7	cig+/-ot	nev cigs	or
HAENSZ	38		f	0	0	all	-	a	NAmer	1955	CC	158	n	bl	n	y	0	cig+/-ot	nev any	st
JAHN	13		m	0	0	all	-	a	Eu:Ger	1988	CC	1004	n	bl	n	n	0	cig+/-ot	nev any	st
JAIN	27		m	0	0	all	-	a	NAmer	1981	CC	845	n	V	y	n	0	cig+/-ot	nev cigs	st
JAIN	22		f	0	0	all	-	a	NAmer	1981	CC	845	n	V	y	n	0	cig+/-ot	nev cigs	st
JEDRYC	27		m	0	0	all	-	a	Eu:est	1980	CC	1630	n	bl	y	n	0	cig+/-ot	nev any	st
KATSOU	15	x	f	0	0	all	-	a	Eu:bal	1987	CC	101	n	bl	n	n	0	all/unsp	nev any	st
KHUDER	11		m	0	0	all	-	a	NAmer	1985	CC	482	n	bl	n	y	0	cig+/-ot	nev cigs	or
KIHARA	13		c	0	0	jap	-	a	As:Jap	1991	CC	440	n	bl	n	n	0	all/unsp	nev any	st
LUBIN2	260		m	0	0	all	-	a	Eu:mul	1976	CC	7804	n	bl	n	y	0	cig+/-ot	nev any	st
LUBIN2	272		f	0	0	all	-	a	Eu:mul	1976	CC	7804	n	bl	n	y	0	cig+/-ot	nev any	st
MATOS	54	x	m	0	0	all	-	a	SCAmer	1994	CC	200	n	bl	n	n	0	cig+/-ot	nev any	st
OSANN	3	x	m	0	0	all	-	a	NAmer	1984	CC	1986	n	bl	n	n	0	cig+/-ot	nev cigs	st
OSANN	7	x	f	0	0	all	-	a	NAmer	1984	CC	1986	n	bl	n	n	0	cig+/-ot	nev cigs	st
OSANN2	15	x	f	0	0	all	-	KII	NAmer	1964	ot	217	n	bl	n	y	0	cig+/-ot	nev cigs	st
SOBUE	5	x	m	0	0	all	-	a	As:Jap	1986	CC	1376	n	bl	n	y	0	cig+/-ot	nev cigs	st
SOBUE	21	x	f	0	0	all	-	a	As:Jap	1986	CC	1376	n	bl	n	y	0	cig+/-ot	nev cigs	st
SUZUKI	1	x	m	0	0	all	-	a	As:Jap	1978	CC	238	n	bl	n	y	0	cig+/-ot	nev any	st
SUZUKI	5	x	f	0	0	all	-	a	As:Jap	1978	CC	238	n	bl	n	y	0	cig+/-ot	nev any	st
SVENSS	24	x	f	0	0	all	-	a	Eu:Sca	1983	CC	210	n	bl	n	n	0	all/unsp	nev any	st
TSUGAN	1		m	0	0	all	-	a	As:Jap	1976	CC	134	n	bl	n	y	0	all/unsp	nev any	st
WAKAI	5	x	m	0	0	all	-	a	As:Jap	1988	CC	333	n	bl	n	y	0	all/unsp	nev any	st
WAKAI	23	x	f	0	0	all	-	a	As:Jap	1988	CC	333	n	bl	n	y	0	all/unsp	nev any	st
WU	1	x	f	0	0	wh	-	a	NAmer	1981	CC	220	n	bl	n	y	0	all/unsp	nev any	st
WYNDE3	21		m	0	0	all	-	KII	NAmer	1966	CC	350	n	bl	n	y	0	all/unsp	nev any	st
WYNDE6	6		m	0	0	all	-	KII	NAmer	1969	CC	4423	n	bl	n	y	0	cig+/-ot	nev any	st
WYNDE6	195		f	0	0	all	-	KII	NAmer	1969	CC	4423	n	bl	n	y	0	cig+/-ot	nev cigs	st

Cigarette type is all/unspec for all RRs

Table 3D2 - 5

IESLC - Meta-analysis of Ex Smoking, Cigarettes (or Any Product if Cigarettes not available)
Adenocarcinoma
Least adjusted

REF	NRR	SEX	AD	Number Exposed Case	Number Exposed Cont	Non-exposed Case	Non-exposed Cont	RR	95.00%CI
BARBON	43	m	0	42	205	7	188	5.50	(2.41- 12.55)
BROWN2	24	m	2	-	-	-	-	7.40	(6.20- 8.80)
BROWN2	23	f	2	-	-	-	-	7.20	(6.20- 8.50)
Subtotal BROWN2								7.29	(6.48- 8.20)
BUFFLE	72	f	0	20	56	7	112	5.71	(2.28- 14.32)
COMSTO	20	m	0	13	129	2	84	4.23	(0.93- 19.23)
COMSTO	28	f	0	6	35	8	115	2.46	(0.80- 7.58)
Subtotal COMSTO								2.99	(1.21- 7.36)
CORREA	40	c	1	-	-	-	-	3.70	(2.10- 6.50)
*ENGELA	69	m	7	-	-	-	-	1.10	(0.40- 3.10)
HAENSZ	38	f	0	2	9	37	236	1.42	(0.29- 6.82)
JAHN	13	m	0	127	402	8	138	5.45	(2.60- 11.42)
JAIN	27	m	0	30	159	4	85	4.01	(1.37- 11.76)
JAIN	22	f	0	17	97	24	214	1.56	(0.80- 3.04)
Subtotal JAIN								2.03	(1.15- 3.57)
JEDRYC	27	m	0	21	312	7	289	2.78	(1.16- 6.63)
KATSOU	15	f	0	3	4	30	67	1.68	(0.35- 7.95)
KHUDER	11	m	0	63	-	7	-	8.00	(3.50- 18.20)
KIHARA	13	c	0	27	70	78	237	1.17	(0.70- 1.96)
LUBIN2	260	m	0	212	4228	57	2616	2.30	(1.71- 3.10)
LUBIN2	272	f	0	17	157	138	1180	0.93	(0.54- 1.57)
Subtotal LUBIN2								1.85	(1.43- 2.40)
MATOS	54	m	0	33	151	5	110	4.81	(1.82- 12.71)
OSANN	3	m	0	102	477	14	833	12.72	(7.20- 22.49)
OSANN	7	f	0	50	196	47	1093	5.93	(3.87- 9.09)
Subtotal OSANN								7.80	(5.54- 10.97)
OSANN2	15	f	0	11	12	22	43	1.79	(0.68- 4.71)
SOBUE	5	m	0	117	363	27	128	1.53	(0.96- 2.43)
SOBUE	21	f	0	20	64	137	857	1.95	(1.15- 3.33)
Subtotal SOBUE								1.70	(1.20- 2.41)
SUZUKI	1	m	0	25	55	14	99	3.21	(1.54- 6.69)
SUZUKI	5	f	0	5	7	55	133	1.73	(0.53- 5.68)
Subtotal SUZUKI								2.71	(1.45- 5.06)
SVENSS	24	f	0	12	36	22	120	1.82	(0.82- 4.03)
TSUGAN	1	m	0	8	6	18	17	1.26	(0.36- 4.39)
WAKAI	5	m	0	23	140	8	65	1.33	(0.57- 3.14)
WAKAI	23	f	0	4	5	46	145	2.52	(0.65- 9.79)
Subtotal WAKAI								1.60	(0.78- 3.30)
WU	1	f	0	21	37	29	62	1.21	(0.61- 2.43)
WYNDE3	21	m	0	12	125	6	88	1.41	(0.51- 3.89)
WYNDE6	6	m	0	408	1056	58	617	4.11	(3.07- 5.51)
WYNDE6	195	f	0	171	325	119	856	3.78	(2.90- 4.94)
Subtotal WYNDE6								3.93	(3.23- 4.78)
Partial Totals				1622	8918	1041	10827		

*prospective study

REF	NRR	SEX	AD	Ys	Ws	Qs	Ps
BARBON	43	m	0	1.71	5.65	0.30	0.0001
BROWN2	24	m	2	2.00	125.29	34.90	0.0000
BROWN2	23	f	2	1.97	154.35	38.65	0.0000
Subtotal BROWN2				1.99	279.64	73.55	
BUFFLE	72	f	0	1.74	4.55	0.33	0.0002
COMSTO	20	m	0	1.44	1.68	0.00	0.0618
COMSTO	28	f	0	0.90	3.04	0.99	0.1158
Subtotal COMSTO				1.09	4.72	1.00	
CORREA	40	c	1	1.31	12.04	0.33	0.0000
*ENGELA	69	m	7	0.10	3.66	6.96	0.8552
HAENSZ	38	f	0	0.35	1.56	1.97	0.6634
JAHN	13	m	0	1.70	7.01	0.35	0.0000
JAIN	27	m	0	1.39	3.32	0.02	0.0114
JAIN	22	f	0	0.45	8.66	9.14	0.1889
Subtotal JAIN				0.71	11.98	9.16	
JEDRYC	27	m	0	1.02	5.07	1.03	0.0213
KATSOU	15	f	0	0.52	1.58	1.45	0.5163
KHUDER	11	m	0	2.08	5.65	2.07	0.0000
KIHARA	13	c	0	0.16	14.63	25.29	0.5439
LUBIN2	260	m	0	0.83	43.71	17.91	0.0000
LUBIN2	272	f	0	-0.08	13.65	32.81	0.7760
Subtotal LUBIN2				0.62	57.35	50.73	
MATOS	54	m	0	1.57	4.06	0.04	0.0015

Table 3D2 - 5

IESLC - Meta-analysis of Ex Smoking, Cigarettes (or Any Product if Cigarettes not available)
Adenocarcinoma
Least adjusted

REF	NRR	SEX	AD	Ys	Ws	Qs	Ps
OSANN	3	m	0	2.54	11.83	13.54	0.0000
OSANN	7	f	0	1.78	21.14	1.99	0.0000
Subtotal OSANN				2.05	32.97	15.53	
OSANN2	15	f	0	0.58	4.12	3.26	0.2368
SOBUE	5	m	0	0.42	17.81	19.62	0.0736
SOBUE	21	f	0	0.67	13.50	8.71	0.0138
Subtotal SOBUE				0.53	31.31	28.33	
SUZUKI	1	m	0	1.17	7.16	0.67	0.0018
SUZUKI	5	f	0	0.55	2.71	2.33	0.3680
Subtotal SUZUKI				1.00	9.87	3.00	
SVENSS	24	f	0	0.60	6.06	4.65	0.1410
TSUGAN	1	m	0	0.23	2.46	3.81	0.7175
WAKAI	5	m	0	0.29	5.24	7.35	0.5087
WAKAI	23	f	0	0.92	2.09	0.63	0.1812
Subtotal WAKAI				0.47	7.32	7.98	
WU	1	f	0	0.19	7.98	13.08	0.5847
WYNDE3	21	m	0	0.34	3.71	4.75	0.5097
WYNDE6	6	m	0	1.41	44.92	0.16	0.0000
WYNDE6	195	f	0	1.33	54.06	1.10	0.0000
Subtotal WYNDE6				1.37	98.99	1.26	

	N	34
	NS	25
	Wt	623.97
Het	Chi	260.24
Het	df	33
Het	P	***
Fixed	RR	4.37
	RRl	4.04
	RRu	4.72
	P	+++
Random	RR	2.86
	RRl	2.22
	RRu	3.70
	P	+++
Asymm	P	***

Table 3D2 - 6

IESLC - Meta-analysis of Ex Smoking, Cigarettes (or Any Product if Cigarettes not available)
Adenocarcinoma
Least adjusted

	combined	Sex male	female	Total
N	2	17	15	34
NS	2	17	15	34
Wt	26.66	298.24	299.06	623.97
Het Chi	8.73	112.96	120.87	260.24
Het df	1	16	14	33
Het P	**	***	***	***
Fixed RR	1.97	4.56	4.49	4.37
RRl	1.35	4.07	4.01	4.04
RRu	2.88	5.10	5.03	4.72
P	+++	+++	+++	+++
Random RR	2.07	3.43	2.42	2.86
RRl	0.67	2.38	1.58	2.22
RRu	6.38	4.94	3.70	3.70
P	N.S.	+++	+++	+++
Between Chi				17.68
Between df				2
Between P				***
Btwn(F) P				N.S.
Btwn(R) P				N.S.

Table 3D2 - 7

IESLC - Meta-analysis of Ex Smoking, Cigarettes (or Any Product if Cigarettes not available)
Adenocarcinoma
Excluded studies (and stage at which they were excluded)

1 BOUCHA BUELL LAURIL MZILEN

2 ABELIN ABRAHA ALDERS AUVINE AXELSO BAND BERRIN BLOT1 BLOT2 BLOT3 BLOT4 BRESLO BROCKM BROWN1 BYERS1 BYERS2 CASCO2 CASCOR CHAN CHATZI CHEN CHEN2 CHEN3 CHIAZZ COOKSO DAVEYS DEAN DOSEME DU DUNN EBELIN ENSTRO ESAKI FAN GENG GER GODLEY GOLLED GSELL HANSEN HEGMAN HINDS HIRAOK HIRAY2 HOROWI HORWIT HU HU2 HUANG ISHIMA JARUP JIANG JONES JUSSAW KANELL KO KOHLME KOULUM KREYBE LAMTH LAMWK LAMWK2 LAUSSM LEI LETOUR LEVIN LIAW LICKIN LIU LIU2 LIU3 LIU4 LIU5 LOMBA2 LUO MAGNUS MARSH MARSH2 MARTIS MASTRA MATSUD MCCONN MCDUFF MCLAUG MILLER MILLS MOLLO MRFIT MURATA NOTAN2 NOTANI NOU ORMOS PASTOR PAWLEG PERNU PERSHA PIKE POFFIJ POLEDN QIAO RADZIK RANDIG REN RIMING RONCO ROOTS ROTHSC SAARIK SADOWS SANKAR SCHWA2 SEGI SEGI2 SEOW SHIMIZ SIEMIA SIMARA SITAS SOBUE2 STASZE STAYNE STOCKS SUN TANG2 TAO ULMER VANDER WALD WANG WANG3 WANG4 WARSIN WATSON WICKLU WILKIN WU2 WUWILL WYNDE2 WYNDE4 WYNDE5 WYNDE8 WYNDER XIANGZ XU XU2 XU3 XU4 YUAN ZHANG ZHENG ZHOU

3 RESTRE

4 BOFFET

8 AGUDO AKIBA AMANDU AMES ANDERS ARCHER ARMADA AUSTIN AXELSS BECHER BENSHL BEST BLOHMK BOUCOT BRETT BROSS CARPEN CEDERL CHANG CHOI CHOW CHYOU CPSI CPSII DAMBER DARBY DEAN2 DEAN3 DEKLER DESTE2 DESTEF DOCKER DOLL DOLL2 DORANT DORGAN DORN DROSTE GAO GAO2 GARCIA GARDIN GARSHI GILLIS GOODMA GRAHAM GREGOR HAMMO2 HAMMON HEIN HENNEK HIRAYA HITOSU HOLE HUMBLE JARVHO JOLY KAISE2 KAISER KAUFMA KELLER KINLEN KJUUS KNEKT KOO KREUZE KUBIK LANGE LEMARC LIDDEL LOMBAR LUBIN MACLEN MIGRAN MRFITR NAM ODRISC PARKIN PERSH2 PETO PEZZO2 PEZZOT PISANI PRESCO QIAO2 RACHTA SCHWAR SHAW SIMONA SPEIZE SPITZ STOCKW STUCKE SUZUK2 TANG TENKAN TIZZAN TOKARS TOUSEY TULINI TVERDA VEIERO WANG2 WIGLE WUNSCH YAMAGU YONG

10 BENHAM VUTUC WYNDE7

Table 3D2 - 8

Potentially overlapping studies

REF	REFGP	PRINC	OVERLAP/LINK
LUBIN2	LUBIN2	1	Lubin-combined
OSANN2	KAISER	2	KAISER/OSANN2
WYNDE6	WYNDE6	1	WYNDE5/6/7/8
JAHN	BOFFET	2	Subset of BOFFET

Table 3D3 -

IESLC - Meta-analysis of Ex Smoking, Cigarettes only
Adenocarcinoma

This analysis is restricted to results for:
1) Non-dose-response data
2) Ex smokers
3) Results complete enough for use in metaanalysis

Within each study, results are then selected (in the following order of preference, within each sex) for:
4) PRODUCT: cigarettes only
5) CIGTYPE: all/unspecified, MC regardless of HR, MC only
6) DENOM: never smoked anything, never smoked cigarettes, (never +1 = +long term ex, +2 = +amount unknown,
+3 = never cigs+long term ex)
7) Followup period (YF, prospective studies): whole study (coded as 0) or longest available
8) LCtype: adeno or nearest available, but not squamous. (q = squamous, s = small,
a = adeno, l = large, KII = Kreyberg II, al = alveolar, br = bronchiolar, u = undifferentiated)
9) Race: all or nearest available, otherwise by race (wh or w = white, bl or b = black, hi = hispanic
ch = chinese, jap = japanese, haw = hawaiian, w+o = white + oriental, sca = scandinavian, as = asian)
10) For overlapping studies: principal rather than subsidiary studies
Finally by Age: whole study (coded as 0) if available, otherwise by widest available age group
and then for single sex results (m, f) in preference to combined sex results (c).

Results adjusted (AD) for the most potential confounders are then chosen in Sections -1 to -3
and results adjusted for the least confounders in Sections -4 to -6. (Those least adjusted results which
actually differ from the most adjusted as marked 'x' in column X in Section -4)
(Results adjusted for an unknown number of confounder(s) are coded as 20.)

Section -7 shows excluded studies, together with the stage (as above) at which no qualifying
results were found.

Section -8 lists the potentially overlapping studies which have been included (1=principal, 2=subsidiary).

Section -9 lists any results which would have been included in preference except that they had data not complete
enough for use in meta-analysis, with their significance (yes/no), if known, and any further comment as entered
on the database.

In addition to those mentioned above, the following fields, levels and abbreviations are used:
* or nk = not known, n = no, y = yes, ot = other
nev = never
all/unspec = all or unspecified, MC = manufactured cigarettes, HR = hand-rolled cigarettes
REF: 6-character study reference
NRR: number of the RR on the database within the study
ST : study type (CC = case control, pr or prosp = prospective)
NLC: number of lung cancer cases in whole study
R : risky occupational population (n = no, m = mining, o = other risky)
VB : national cigarette type (V = at least 75% Virginia, bl = at least 75% blended, ot = other)
P : any proxy use
H : full histological confirmation
De : derivation of RR/CI (or = original, st = standard method, ot = other method of estimation)

Table 3D3 - 1

IESLC - Meta-analysis of Ex Smoking, Cigarettes only
Adenocarcinoma
Most adjusted

REF	NRR	SEX	AGEL	AGEH	RACE	YF	LC TYPE	LOC	START	ST	NLC	R	VB	P	H	AD	PRODUCT	DENOM	De
BENHAM	21	m	0	0	all	-	KII	Eu:wst	1976	CC	1625	n	bl	n	y	0	cig only	nev any	st
WYNDE7	37	m	0	0	all	-	KII	NAmer	1977	CC	2085	n	bl	n	y	0	cig only	nev any	st

Cigarette type is all/unspec for all RRs

Table 3D3 - 2

IESLC - Meta-analysis of Ex Smoking, Cigarettes only
Adenocarcinoma
Most adjusted

REF	NRR	SEX	AD	Number Exposed Case	Number Exposed Cont	Non-exposed Case	Non-exposed Cont	RR	95.00%CI
BENHAM	21	m	0	25	48	9	42	2.43	(1.02- 5.79)
WYNDE7	37	m	0	204	1115	42	918	4.00	(2.84- 5.64)
Totals				229	1163	51	960		

*prospective study

REF	NRR	SEX	AD	Ys	Ws	Qs	Ps
BENHAM	21	m	0	0.89	5.11	0.95	0.0447
WYNDE7	37	m	0	1.39	32.58	0.15	0.0000

	N	2
	NS	2
	Wt	37.68
Het	Chi	1.09
Het	df	1
Het	P	N.S.
Fixed	RR	3.74
	RRl	2.72
	RRu	5.14
	P	+++
Random	RR	3.68
	RRl	2.56
	RRu	5.30
	P	+++
Asymm	P	

Table 3D3 - 3

IESLC - Meta-analysis of Ex Smoking, Cigarettes only
Adenocarcinoma
Most adjusted

	Sex combined	male	female	Total
N		2		2
NS		2		2
Wt		37.68		37.68
Het Chi		1.09		1.09
Het df		1		1
Het P		N.S.		N.S.
Fixed RR		3.74		3.74
RRl		2.72		2.72
RRu		5.14		5.14
P		+++		+++
Random RR		3.68		3.68
RRl		2.56		2.56
RRu		5.30		5.30
P		+++		+++
Between Chi				
Between df				
Between P				N.S.
Btwn(F) P				N.S.
Btwn(R) P				N.S.

Too few RRs for analysis by factor

Table 3D3 - 4

IESLC - Meta-analysis of Ex Smoking, Cigarettes only
Adenocarcinoma
Least adjusted

REF	NRR	X	SEX	AGEL	AGEH	RACE	YF	LC TYPE	LOC	START	ST	NLC	R	VB	P	H	AD	PRODUCT	DENOM	De
BENHAM	21		m	0	0	all	-	KII	Eu:wst	1976	CC	1625	n	bl	n	y	0	cig only	nev any	st
WYNDE7	37		m	0	0	all	-	KII	NAmer	1977	CC	2085	n	bl	n	y	0	cig only	nev any	st

Cigarette type is all/unspec for all RRs

Table 3D3 - 5

IESLC - Meta-analysis of Ex Smoking, Cigarettes only
Adenocarcinoma
Least adjusted

REF	NRR	SEX	AD	Number Exposed Case	Number Exposed Cont	Non-exposed Case	Non-exposed Cont	RR	95.00%CI
BENHAM	21	m	0	25	48	9	42	2.43	(1.02- 5.79)
WYNDE7	37	m	0	204	1115	42	918	4.00	(2.84- 5.64)
Totals				229	1163	51	960		

*prospective study

REF	NRR	SEX	AD	Ys	Ws	Qs	Ps
BENHAM	21	m	0	0.89	5.11	0.95	0.0447
WYNDE7	37	m	0	1.39	32.58	0.15	0.0000

	N	2
	NS	2
	Wt	37.68
Het	Chi	1.09
Het	df	1
Het	P	N.S.
Fixed	RR	3.74
	RRl	2.72
	RRu	5.14
	P	+++
Random	RR	3.68
	RRl	2.56
	RRu	5.30
	P	+++
Asymm	P	

Table 3D3 - 6

IESLC - Meta-analysis of Ex Smoking, Cigarettes only
Adenocarcinoma
Least adjusted

	Sex combined	male	female	Total
N		2		2
NS		2		2
Wt		37.68		37.68
Het Chi		1.09		1.09
Het df		1		1
Het P		N.S.		N.S.
Fixed RR		3.74		3.74
RRl		2.72		2.72
RRu		5.14		5.14
P		+++		+++
Random RR		3.68		3.68
RRl		2.56		2.56
RRu		5.30		5.30
P		+++		+++
Between Chi				
Between df				
Between P				N.S.
Btwn(F) P				N.S.
Btwn(R) P				N.S.

Table 3D3 - 7

IESLC - Meta-analysis of Ex Smoking, Cigarettes only
Adenocarcinoma
Excluded studies (and stage at which they were excluded)

1 BOUCHA BUELL LAURIL MZILEN

2 ABELIN ABRAHA ALDERS AUVINE AXELSO BAND BERRIN BLOT1 BLOT2 BLOT3 BLOT4 BRESLO BROCKM BROWN1 BYERS1 BYERS2
CASCO2 CASCOR CHAN CHATZI CHEN CHEN2 CHEN3 CHIAZZ COOKSO DAVEYS DEAN DOSEME DU DUNN EBELIN ENSTRO
ESAKI FAN GENG GER GODLEY GOLLED GSELL HANSEN HEGMAN HINDS HIRAOK HIRAY2 HOROWI HORWIT HU HU2
HUANG ISHIMA JARUP JIANG JONES JUSSAW KANELL KO KOHLME KOULUM KREYBE LAMTH LAMWK LAMWK2 LAUSSM LEI
LETOUR LEVIN LIAW LICKIN LIU LIU2 LIU3 LIU4 LIU5 LOMBA2 LUO MAGNUS MARSH MARSH2 MARTIS MASTRA
MATSUD MCCONN MCDUFF MCLAUG MILLER MILLS MOLLO MRFIT MURATA NOTAN2 NOTANI NOU ORMOS PASTOR PAWLEG PERNU
PERSHA PIKE POFFIJ POLEDN QIAO RADZIK RANDIG REN RIMING RONCO ROOTS ROTHSC SAARIK SADOWS SANKAR SCHWA2
SEGI SEGI2 SEOW SHIMIZ SIEMIA SIMARA SITAS SOBUE2 STASZE STAYNE STOCKS SUN TANG2 TAO ULMER VANDER
WALD WANG WANG3 WANG4 WARSIN WATSON WICKLU WILKIN WU2 WUWILL WYNDE2 WYNDE4 WYNDE5 WYNDE8 WYNDER XIANGZ
XU XU2 XU3 XU4 YUAN ZHANG ZHENG ZHOU

3 RESTRE

4 AKIBA AMANDU AMES ANDERS ARCHER ARMADA AUSTIN AXELSS BARBON BECHER BENSHL BLOHMK BOFFET BRETT BROSS BROWN2
BUFFLE CARPEN CEDERL CHANG CHOI CHYOU COMSTO CORREA DARBY DEAN2 DEKLER DESTE2 DESTEF DOCKER DOLL DORANT
DORGAN DROSTE ENGELA GAO GAO2 GARCIA GARDIN GARSHI GILLIS GOODMA GREGOR HAENSZ HAMMO2 HEIN HENNEK HIRAYA
HITOSU HOLE HUMBLE JAHN JAIN JARVHO JEDRYC JOLY KAISER KATSOU KAUFMA KELLER KHUDER KIHARA KINLEN KJUUS
KNEKT KOO KREUZE KUBIK LANGE LEMARC LIDDEL LUBIN MACLEN MATOS MIGRAN MRFITR NAM ODRISC OSANN OSANN2
PARKIN PERSH2 PETO PEZZO2 PISANI PRESCO QIAO2 RACHTA SCHWAR SHAW SIMONA SOBUE SPEIZE SPITZ STOCKW STUCKE
SUZUK2 SUZUKI SVENSS TANG TENKAN TIZZAN TOKARS TOUSEY TSUGAN TULINI VEIERO VUTUC WAKAI WANG2 WU WUNSCH
WYNDE3 WYNDE6 YAMAGU YONG

8 AGUDO BEST BOUCOT CHOW CPSI CPSII DAMBER DEAN3 DOLL2 DORN GRAHAM HAMMON KAISE2 LOMBAR LUBIN2 PEZZOT
TVERDA WIGLE

Table 3D3 - 8

Potentially overlapping studies

REF	REFGP	PRINC	. OVERLAP/LINK
BENHAM	LUBIN2	2	Subset of Lubin2
WYNDE7	WYNDE6	2	WYNDE5/6/7/8
