# Supplementary material for: Systematic review with meta-analysis of the epidemiological evidence in the 1900s relating smoking to lung cancer
Source: BMC Cancer. 2012 Sep 3;12:385. doi: 10.1186/1471-2407-12-385 (PMC3505152; doi:10.1186/1471-2407-12-385)
Supplement: Additional file 5 — Detailed Analysis Tables (Individual file names as described in Additional file 1: Methods, Table1). [file 1471-2407-12-385-S5.zip › PDF/3H.pdf]

Table 3H1 -

IESLC - Meta-analysis of Ever Smoking by Age started, Overview
Adenocarcinoma, Any Product (or Cigarettes if Any not available)

This analysis is restricted to results for:
1) Ever smokers
2) Results by Age started
3) Categorical results by Age started
   Results by Age started are grouped under 2 schemes (S1, S2). Each scheme has a set of "key values". An interval is allocated to the category whose key value it includes, and intervals which include none or more than one of the key values are excluded. (Open-ended intervals are coded as 999)

S1	key value	maximum range
1	26	19+
2	18	15-25
3	14	1-17

S2	key value	maximum range
1	30	27+
2	26	23-29
3	22	19-25
4	18	15-21
5	14	11-17
6	10	1-13

4) Adenocarcinoma (or near equivalent)
5) Results complete enough for use in metaanalysis

Within each study, results are then selected (in the following order of preference, within each sex) for:
6) (not applicable)
7) PRODUCT: all/unspec, cigarettes regardless of other products, cigarettes only
8) CIGTYPE: all/unspecified, MC regardless of HR, MC only
9) (not applicable)
10) DENOM: never smoked anything, never smoked cigarettes, never any + low, never cigs + low
11) Followup period (YF, prospective studies): whole study (coded as 0) or longest available
12) LCtype: adeno or nearest available, but not squamous. (q = squamous, s = small,
          a = adeno, l = large, KII = Kreyberg II, al = alveolar, br = bronchiolar, u = undifferentiated)
13) Race: all or nearest available, otherwise by race (wh or w = white, bl or b = black, hi = hispanic
          ch = chinese, jap = japanese, haw = hawaiian, w+o = white + oriental, sca = scandinavian, as = asian)
14) For overlapping studies: principal rather than subsidiary studies
Finally by Age: whole study (coded as 0) if available, otherwise by widest available age group
and then for single sex results (m, f) in preference to results for both sexes combined (c).

Results adjusted (AD) for the most potential confounders are then chosen in Sections -1 to -3
and results adjusted for the least confounders in Sections -4 to -6. (Those least adjusted results which actually differ from the most adjusted are marked 'x' in column X in Section -4)

Section -7 shows excluded studies, together with the stage (as above) at which no qualifying results were found.

Section -8 lists the potentially overlapping studies which have been included (1=principal, 2=subsidiary).

Section -9 lists any results which would have been included in preference except that they had data not complete enough for use in meta-analysis, with their significance (yes/no), if known, and any further comment as entered on the database. It also lists as "gap" any categories for which no data were presented by the original authors.

In addition to those mentioned above, the following fields, levels and abbreviations are used:
* or nk = not known, n = no, y = yes, ot = other
nev = never
all/unspec = all or unspecified, cig+/-ot = cigarettes irrespective of other products (cigar, pipe etc)
MC = manufactured cigarettes, HR = hand-rolled cigarettes
exL, exH = range of exposure (low and high) in the smoking group, in terms of Age started
REF: 6-character study reference
NRR: number of the RR on the database within the study
ST : study type (CC = case control, pr or prosp = prospective)
NLC: number of lung cancer cases in whole study
R : risky occupational population (n = no, m = mining, o = other risky)
VB : national cigarette type (V = at least 75% Virginia, bl = at least 75% blended, ot = other)
P : any proxy use
H : full histological confirmation
De : derivation of RR/CI (or = original, st = standard method, ot = other method of estimation)

Table 3H1 - 1

IESLC - Meta-analysis of Ever Smoking by Age started, Overview
Adenocarcinoma, Any Product (or Cigarettes if Any not available)
Most adjusted

REF	NRR	SEX	AGEL	AGEH	RACE	YF	LC TYPE	LOC	START	ST	NLC	R	VB	P	H	AD	PRODUCT	exL	exH	S1	S2	DENOM	De
BARBON	736	m	0	0	all	-	a	Eu:wst	1979	CC	755	n	bl	y	y	1	all/unsp	20	999	1	0	nev any	or
BARBON	737	m	0	0	all	-	a	Eu:wst	1979	CC	755	n	bl	y	y	1	all/unsp	15	19	2	4	nev any	or
BARBON	738	m	0	0	all	-	a	Eu:wst	1979	CC	755	n	bl	y	y	1	all/unsp	1	14	3	0	nev any	or
BENHAM	521	m	0	0	all	-	KII	Eu:wst	1976	CC	1625	n	bl	n	y	0	cig only	20	999	1	0	nev any	st
BENHAM	522	m	0	0	all	-	KII	Eu:wst	1976	CC	1625	n	bl	n	y	0	cig only	1	19	0	0	nev any	st
HAENSZ	509	f	0	0	all	-	a	NAmer	1955	CC	158	n	bl	n	y	0	cig+/-ot	25	999	1	0	nev any	st
HAENSZ	510	f	0	0	all	-	a	NAmer	1955	CC	158	n	bl	n	y	0	cig+/-ot	1	24	0	0	nev any	st
JEDRYC	538	m	0	0	all	-	a	Eu:est	1980	CC	1630	n	bl	y	n	0	cig+/-ot	19	999	1	0	nev any	st
JEDRYC	539	m	0	0	all	-	a	Eu:est	1980	CC	1630	n	bl	y	n	0	cig+/-ot	17	18	2	4	nev any	st
JEDRYC	540	m	0	0	all	-	a	Eu:est	1980	CC	1630	n	bl	y	n	0	cig+/-ot	1	16	3	0	nev any	st
MATOS	656	m	0	0	all	-	a	SCAmer	1994	CC	200	n	bl	n	n	2	cig+/-ot	20	999	1	0	nev any	or
MATOS	657	m	0	0	all	-	a	SCAmer	1994	CC	200	n	bl	n	n	2	cig+/-ot	15	19	2	4	nev any	or
MATOS	658	m	0	0	all	-	a	SCAmer	1994	CC	200	n	bl	n	n	2	cig+/-ot	1	14	3	0	nev any	or
PEZZOT	552	m	0	0	all	-	a	SCAmer	1987	CC	215	n	bl	n	y	0	cig only	19	999	1	0	nev cigs	st
PEZZOT	553	m	0	0	all	-	a	SCAmer	1987	CC	215	n	bl	n	y	0	cig only	14	18	0	0	nev cigs	st
PEZZOT	554	m	0	0	all	-	a	SCAmer	1987	CC	215	n	bl	n	y	0	cig only	1	13	0	6	nev cigs	st
WYNDE6	613	m	0	0	wh	-	a	NAmer	1969	CC	4423	n	bl	n	y	0	cig+/-ot	21	999	1	0	nev cigs	st
WYNDE6	614	m	0	0	wh	-	a	NAmer	1969	CC	4423	n	bl	n	y	0	cig+/-ot	18	20	2	4	nev cigs	st
WYNDE6	615	m	0	0	wh	-	a	NAmer	1969	CC	4423	n	bl	n	y	0	cig+/-ot	1	17	3	0	nev cigs	st
WYNDE6	637	f	0	0	wh	-	a	NAmer	1969	CC	4423	n	bl	n	y	0	cig+/-ot	21	999	1	0	nev cigs	st
WYNDE6	638	f	0	0	wh	-	a	NAmer	1969	CC	4423	n	bl	n	y	0	cig+/-ot	18	20	2	4	nev cigs	st
WYNDE6	639	f	0	0	wh	-	a	NAmer	1969	CC	4423	n	bl	n	y	0	cig+/-ot	1	17	3	0	nev cigs	st
ZHENG	531	m	0	0	all	-	a	As:Chi	1982	CC	540	n	ot	*	y	0	cig+/-ot	30	999	0	1	nev cigs	or
ZHENG	532	m	0	0	all	-	a	As:Chi	1982	CC	540	n	ot	*	y	0	cig+/-ot	20	29	1	0	nev cigs	st
ZHENG	533	m	0	0	all	-	a	As:Chi	1982	CC	540	n	ot	*	y	0	cig+/-ot	1	19	0	0	nev cigs	st
ZHENG	538	f	0	0	all	-	a	As:Chi	1982	CC	540	n	ot	*	y	0	cig+/-ot	30	999	0	1	nev cigs	or
ZHENG	539	f	0	0	all	-	a	As:Chi	1982	CC	540	n	ot	*	y	0	cig+/-ot	1	29	0	0	nev cigs	st

Cigarette type is all/unspec for all RRs

In this overview table, subtotals and Qs values may be invalid and should be ignored

Table 3H1 - 2

IESLC - Meta-analysis of Ever Smoking by Age started, Overview
Adenocarcinoma, Any Product (or Cigarettes if Any not available)
Most adjusted

REF	NRR	SEX	AD	Number Exposed Case	Exposed Cont	Non-exposed Case	Non-exposed Cont	RR	95.00%CI
BARBON	736	m	1	45	-	7	-	5.70	(2.50- 12.90)
BARBON	737	m	1	77	-	7	-	6.00	(2.70- 13.30)
BARBON	738	m	1	29	-	7	-	33.40	(13.10- 84.90)
Subtotal BARBON								9.41	(5.78- 15.32)
BENHAM	521	m	0	41	64	9	42	2.99	(1.32- 6.79)
BENHAM	522	m	0	69	79	9	42	4.08	(1.85- 8.97)
Subtotal BENHAM								3.51	(1.99- 6.20)
HAENSZ	509	f	0	12	66	37	236	1.16	(0.57- 2.35)
HAENSZ	510	f	0	6	37	37	236	1.03	(0.41- 2.62)
Subtotal HAENSZ								1.11	(0.63- 1.95)
JEDRYC	538	m	0	48	502	7	289	3.95	(1.76- 8.84)
JEDRYC	539	m	0	29	221	7	289	5.42	(2.33- 12.60)
JEDRYC	540	m	0	12	111	7	289	4.46	(1.71- 11.63)
Subtotal JEDRYC								4.56	(2.77- 7.50)
MATOS	656	m	2	13	-	5	-	4.00	(1.40- 11.90)
MATOS	657	m	2	40	-	5	-	7.20	(2.70- 19.10)
MATOS	658	m	2	26	-	5	-	6.60	(2.40- 18.10)
Subtotal MATOS								5.86	(3.25- 10.54)
PEZZOT	552	m	0	18	105	3	116	6.63	(1.90- 23.15)
PEZZOT	553	m	0	33	145	3	116	8.80	(2.63- 29.42)
PEZZOT	554	m	0	9	67	3	116	5.19	(1.36- 19.85)
Subtotal PEZZOT								6.84	(3.30- 14.18)
WYNDE6	613	m	0	47	92	42	589	7.16	(4.47- 11.47)
WYNDE6	614	m	0	88	139	42	589	8.88	(5.88- 13.40)
WYNDE6	615	m	0	241	301	42	589	11.23	(7.87- 16.03)
WYNDE6	637	f	0	63	90	58	673	8.12	(5.34- 12.35)
WYNDE6	638	f	0	113	94	58	673	13.95	(9.51- 20.46)
WYNDE6	639	f	0	150	91	58	673	19.13	(13.16- 27.80)
Subtotal WYNDE6								11.24	(9.55- 13.22)
ZHENG	531	m	0	15	66	29	94	0.74	(0.37- 1.48)
ZHENG	532	m	0	64	109	29	94	1.90	(1.13- 3.20)
ZHENG	533	m	0	44	43	29	94	3.32	(1.84- 5.99)
ZHENG	538	f	0	7	16	119	184	0.68	(0.27- 1.69)
ZHENG	539	f	0	26	28	119	184	1.44	(0.80- 2.57)
Subtotal ZHENG								1.57	(1.19- 2.08)
Partial Totals				1365	2466	783	6207		

*prospective study

REF	NRR	SEX	AD	Ys	Ws	Qs	Ps
BARBON	736	m	1	1.74	5.71	0.02	0.0000
BARBON	737	m	1	1.79	6.04	0.00	0.0000
BARBON	738	m	1	3.51	4.40	12.82	0.0000
Subtotal BARBON				2.24	16.15	12.84	
BENHAM	521	m	0	1.10	5.72	2.85	0.0088
BENHAM	522	m	0	1.41	6.17	0.97	0.0005
Subtotal BENHAM				1.26	11.89	3.83	
HAENSZ	509	f	0	0.15	7.71	21.08	0.6808
HAENSZ	510	f	0	0.03	4.45	13.90	0.9433
Subtotal HAENSZ				0.11	12.15	34.97	
JEDRYC	538	m	0	1.37	5.91	1.09	0.0008
JEDRYC	539	m	0	1.69	5.40	0.07	0.0001
JEDRYC	540	m	0	1.50	4.19	0.39	0.0022
Subtotal JEDRYC				1.52	15.50	1.55	
MATOS	656	m	2	1.39	3.36	0.58	0.0111
MATOS	657	m	2	1.97	4.01	0.12	0.0001
MATOS	658	m	2	1.89	3.76	0.03	0.0003
Subtotal MATOS				1.77	11.13	0.73	
PEZZOT	552	m	0	1.89	2.46	0.02	0.0030
PEZZOT	553	m	0	2.17	2.64	0.37	0.0004
PEZZOT	554	m	0	1.65	2.14	0.05	0.0160
Subtotal PEZZOT				1.92	7.23	0.44	
WYNDE6	613	m	0	1.97	17.35	0.49	0.0000
WYNDE6	614	m	0	2.18	22.69	3.31	0.0000
WYNDE6	615	m	0	2.42	30.32	11.53	0.0000
WYNDE6	637	f	0	2.09	21.88	1.88	0.0000
WYNDE6	638	f	0	2.64	26.17	18.18	0.0000
WYNDE6	639	f	0	2.95	27.49	36.30	0.0000
Subtotal WYNDE6				2.42	145.89	71.69	
ZHENG	531	m	0	-0.31	7.88	34.99	0.3910
ZHENG	532	m	0	0.64	14.30	19.19	0.0149

Table 3H1 - 2

IESLC - Meta-analysis of Ever Smoking by Age started, Overview
Adenocarcinoma, Any Product (or Cigarettes if Any not available)
Most adjusted

REF	NRR	SEX	AD	Ys	Ws	Qs	Ps
ZHENG	533	m	0	1.20	10.98	3.99	0.0001
ZHENG	538	f	0	-0.39	4.56	21.93	0.4038
ZHENG	539	f	0	0.36	11.36	23.56	0.2228
Subtotal ZHENG				0.45	49.08	103.66	

N	27
NS	8

Table 3H1 - 3

IESLC - Meta-analysis of Ever Smoking by Age started, Overview
Adenocarcinoma, Any Product (or Cigarettes if Any not available)
Most adjusted

	Sex			
	combined	male	female	Total
N		20	7	27
NS		7	3	10

In this overview table, other than the "N" rows, entries in the "absent" and "Total" columns may be invalid and should be ignored

	Age started (broad categories)				
	absent	19+k26	15-25k18	1-17k14	Total
N	8	9	5	5	27
NS	4	8	4	4	20
Wt	50.17	84.38	64.32	70.16	269.02
Het Chi	29.81	37.83	7.21	15.03	229.69
Het df	7	8	4	4	26
Het P	***	***	N.S.	**	***
Fixed RR	1.86	4.34	9.74	13.62	6.06
RRl	1.41	3.51	7.63	10.78	5.38
RRu	2.45	5.38	12.43	17.22	6.83
P	+++	+++	+++	+++	+++
Random RR	2.01	3.92	8.84	12.34	4.60
RRl	1.11	2.39	6.14	7.23	3.17
RRu	3.65	6.42	12.73	21.08	6.67
P	+	+++	+++	+++	+++

	Age started (narrow categories)							
	absent	27+k30	23-29k26	19-25k22	15-21k18	11-17k14	1-13k10	Total
N	19	2			5		1	27
NS	8	1			4		1	13
Wt	190.13	12.44			64.32		2.14	269.02
Het Chi	150.97	0.02			7.21		0.00	229.69
Het df	18	1			4		0	26
Het P	***	N.S.			N.S.		N.S.	***
Fixed RR	5.95	0.71			9.74		5.19	6.06
RRl	5.16	0.41			7.63		1.36	5.38
RRu	6.86	1.24			12.43		19.85	6.83
P	+++	N.S.			+++		+	+++
Random RR	4.79	0.71			8.84		5.19	4.60
RRl	3.11	0.41			6.14		1.36	3.17
RRu	7.38	1.24			12.73		19.85	6.67
P	+++	N.S.			+++		+	+++

Table 3H1 - 3

IESLC - Meta-analysis of Ever Smoking by Age started, Overview
Adenocarcinoma, Any Product (or Cigarettes if Any not available)
Most adjusted

MALES

	Age started (broad categories) absent	19+k26	15-25k18	1-17k14	Total
N	5	7	4	4	20
NS	3	7	4	4	18
Wt	29.80	54.79	38.15	42.68	165.42
Het Chi	19.37	15.59	1.52	9.83	91.86
Het df	4	6	3	3	19
Het P	***	*	N.S.	*	***
Fixed RR	2.62	4.07	7.61	10.95	5.61
RRl	1.83	3.13	5.54	8.11	4.82
RRu	3.75	5.31	10.45	14.78	6.53
P	+++	+++	+++	+++	+++
Random RR	3.14	4.10	7.61	10.46	5.16
RRl	1.35	2.59	5.54	5.18	3.61
RRu	7.29	6.50	10.45	21.13	7.38
P	++	+++	+++	+++	+++

		Age started (narrow categories)						
	absent	27+k30	23-29k26	19-25k22	15-21k18	11-17k14	1-13k10	Total
N	14	1			4		1	20
NS	7	1			4		1	12
Wt	117.25	7.88			38.15		2.14	165.42
Het Chi	54.15	0.00			1.52		0.00	91.86
Het df	13	0			3		0	19
Het P	***	N.S.			N.S.		N.S.	***
Fixed RR	5.83	0.74			7.61		5.19	5.61
RRl	4.86	0.37			5.54		1.36	4.82
RRu	6.98	1.48			10.45		19.85	6.53
P	+++	N.S.			+++		+	+++
Random RR	5.51	0.74			7.61		5.19	5.16
RRl	3.70	0.37			5.54		1.36	3.61
RRu	8.20	1.48			10.45		19.85	7.38
P	+++	N.S.			+++		+	+++

FEMALES

	Age started (broad categories) absent	19+k26	15-25k18	1-17k14	Total
N	3	2	1	1	7
NS	2	2	1	1	4
Wt	20.37	29.58	26.17	27.49	103.61
Het Chi	1.89	21.59	0.00	0.00	135.24
Het df	2	1	0	0	6
Het P	N.S.	***	N.S.	N.S.	***
Fixed RR	1.13	4.89	13.95	19.13	6.86
RRl	0.73	3.41	9.51	13.16	5.66
RRu	1.74	7.01	20.46	27.80	8.32
P	N.S.	+++	+++	+++	+++
Random RR	1.13	3.14	13.95	19.13	3.22
RRl	0.73	0.47	9.51	13.16	1.24
RRu	1.74	21.12	20.46	27.80	8.39
P	N.S.	N.S.	+++	+++	+

Table 3H1 - 3

IESLC - Meta-analysis of Ever Smoking by Age started, Overview
Adenocarcinoma, Any Product (or Cigarettes if Any not available)
Most adjusted

FEMALES

	absent	27+k30	23-29k26	19-25k22	15-21k18	11-17k14	1-13k10	Total
		Age started (narrow categories)						
N	5	1			1			7
NS	3	1			1			4
Wt	72.88	4.56			26.17			103.61
Het Chi	96.70	0.00			0.00			135.24
Het df	4	0			0			6
Het P	***	N.S.			N.S.			***
Fixed RR	6.15	0.68			13.95			6.86
RRl	4.89	0.27			9.51			5.66
RRu	7.73	1.69			20.46			8.32
P	+++	N.S.			+++			+++
Random RR	3.17	0.68			13.95			3.22
RRl	0.96	0.27			9.51			1.24
RRu	10.43	1.69			20.46			8.39
P	(+)	N.S.			+++			+

Table 3H1 - 4

IESLC - Meta-analysis of Ever Smoking by Age started, Overview
Adenocarcinoma, Any Product (or Cigarettes if Any not available)
Least adjusted

REF	NRR	X	SEX	AGEL	AGEH	RACE	YF	LC TYPE	LOC	START	ST	NLC	R	VB	P	H	AD	PRODUCT	exL	exH	S1	S2	DENOM	De
BARBON	731	x	m	0	0	all	-	a	Eu:wst	1979	CC	755	n	bl	y	y	0	all/unsp	20	999	1	0	nev any	st
BARBON	732	x	m	0	0	all	-	a	Eu:wst	1979	CC	755	n	bl	y	y	0	all/unsp	15	19	2	4	nev any	st
BARBON	733	x	m	0	0	all	-	a	Eu:wst	1979	CC	755	n	bl	y	y	0	all/unsp	1	14	3	0	nev any	st
BENHAM	521		m	0	0	all	-	KII	Eu:wst	1976	CC	1625	n	bl	n	y	0	cig only	20	999	1	0	nev any	st
BENHAM	522		m	0	0	all	-	KII	Eu:wst	1976	CC	1625	n	bl	n	y	0	cig only	1	19	0	0	nev any	st
HAENSZ	509		f	0	0	all	-	a	NAmer	1955	CC	158	n	bl	n	y	0	cig+/-ot	25	999	1	0	nev any	st
HAENSZ	510		f	0	0	all	-	a	NAmer	1955	CC	158	n	bl	n	y	0	cig+/-ot	1	24	0	0	nev any	st
JEDRYC	538		m	0	0	all	-	a	Eu:est	1980	CC	1630	n	bl	y	n	0	cig+/-ot	19	999	1	0	nev any	st
JEDRYC	539		m	0	0	all	-	a	Eu:est	1980	CC	1630	n	bl	y	n	0	cig+/-ot	17	18	2	4	nev any	st
JEDRYC	540		m	0	0	all	-	a	Eu:est	1980	CC	1630	n	bl	y	n	0	cig+/-ot	1	16	3	0	nev any	st
MATOS	651	x	m	0	0	all	-	a	SCAmer	1994	CC	200	n	bl	n	n	0	cig+/-ot	20	999	1	0	nev any	st
MATOS	652	x	m	0	0	all	-	a	SCAmer	1994	CC	200	n	bl	n	n	0	cig+/-ot	15	19	2	4	nev any	st
MATOS	653	x	m	0	0	all	-	a	SCAmer	1994	CC	200	n	bl	n	n	0	cig+/-ot	1	14	3	0	nev any	st
PEZZOT	552		m	0	0	all	-	a	SCAmer	1987	CC	215	n	bl	n	y	0	cig only	19	999	1	0	nev cigs	st
PEZZOT	553		m	0	0	all	-	a	SCAmer	1987	CC	215	n	bl	n	y	0	cig only	14	18	0	0	nev cigs	st
PEZZOT	554		m	0	0	all	-	a	SCAmer	1987	CC	215	n	bl	n	y	0	cig only	1	13	0	6	nev cigs	st
WYNDE6	613		m	0	0	wh	-	a	NAmer	1969	CC	4423	n	bl	n	y	0	cig+/-ot	21	999	1	0	nev cigs	st
WYNDE6	614		m	0	0	wh	-	a	NAmer	1969	CC	4423	n	bl	n	y	0	cig+/-ot	18	20	2	4	nev cigs	st
WYNDE6	615		m	0	0	wh	-	a	NAmer	1969	CC	4423	n	bl	n	y	0	cig+/-ot	1	17	3	0	nev cigs	st
WYNDE6	637		f	0	0	wh	-	a	NAmer	1969	CC	4423	n	bl	n	y	0	cig+/-ot	21	999	1	0	nev cigs	st
WYNDE6	638		f	0	0	wh	-	a	NAmer	1969	CC	4423	n	bl	n	y	0	cig+/-ot	18	20	2	4	nev cigs	st
WYNDE6	639		f	0	0	wh	-	a	NAmer	1969	CC	4423	n	bl	n	y	0	cig+/-ot	1	17	3	0	nev cigs	st
ZHENG	531		m	0	0	all	-	a	As:Chi	1982	CC	540	n	ot	*	y	0	cig+/-ot	30	999	0	1	nev cigs	or
ZHENG	532		m	0	0	all	-	a	As:Chi	1982	CC	540	n	ot	*	y	0	cig+/-ot	20	29	1	0	nev cigs	st
ZHENG	533		m	0	0	all	-	a	As:Chi	1982	CC	540	n	ot	*	y	0	cig+/-ot	1	19	0	0	nev cigs	st
ZHENG	538		f	0	0	all	-	a	As:Chi	1982	CC	540	n	ot	*	y	0	cig+/-ot	30	999	0	1	nev cigs	or
ZHENG	539		f	0	0	all	-	a	As:Chi	1982	CC	540	n	ot	*	y	0	cig+/-ot	1	29	0	0	nev cigs	st

Cigarette type is all/unspec for all RRs

In this overview table, subtotals and Qs values may be invalid and should be ignored

Table 3H1 - 5

IESLC - Meta-analysis of Ever Smoking by Age started, Overview
Adenocarcinoma, Any Product (or Cigarettes if Any not available)
Least adjusted

REF	NRR	SEX	AD	Number Exposed Case	Cont	Non-exposed Case	Cont	RR	95.00%CI
BARBON	731	m	0	45	207	7	188	5.84	(2.57- 13.26)
BARBON	732	m	0	77	337	7	188	6.14	(2.77- 13.58)
BARBON	733	m	0	29	23	7	188	33.86	(13.33- 86.00)
Subtotal BARBON								9.61	(5.90- 15.63)
BENHAM	521	m	0	41	64	9	42	2.99	(1.32- 6.79)
BENHAM	522	m	0	69	79	9	42	4.08	(1.85- 8.97)
Subtotal BENHAM								3.51	(1.99- 6.20)
HAENSZ	509	f	0	12	66	37	236	1.16	(0.57- 2.35)
HAENSZ	510	f	0	6	37	37	236	1.03	(0.41- 2.62)
Subtotal HAENSZ								1.11	(0.63- 1.95)
JEDRYC	538	m	0	48	502	7	289	3.95	(1.76- 8.84)
JEDRYC	539	m	0	29	221	7	289	5.42	(2.33- 12.60)
JEDRYC	540	m	0	12	111	7	289	4.46	(1.71- 11.63)
Subtotal JEDRYC								4.56	(2.77- 7.50)
MATOS	651	m	0	13	73	5	110	3.92	(1.34- 11.46)
MATOS	652	m	0	40	120	5	110	7.33	(2.79- 19.25)
MATOS	653	m	0	26	90	5	110	6.36	(2.35- 17.22)
Subtotal MATOS								5.81	(3.24- 10.39)
PEZZOT	552	m	0	18	105	3	116	6.63	(1.90- 23.15)
PEZZOT	553	m	0	33	145	3	116	8.80	(2.63- 29.42)
PEZZOT	554	m	0	9	67	3	116	5.19	(1.36- 19.85)
Subtotal PEZZOT								6.84	(3.30- 14.18)
WYNDE6	613	m	0	47	92	42	589	7.16	(4.47- 11.47)
WYNDE6	614	m	0	88	139	42	589	8.88	(5.88- 13.40)
WYNDE6	615	m	0	241	301	42	589	11.23	(7.87- 16.03)
WYNDE6	637	f	0	63	90	58	673	8.12	(5.34- 12.35)
WYNDE6	638	f	0	113	94	58	673	13.95	(9.51- 20.46)
WYNDE6	639	f	0	150	91	58	673	19.13	(13.16- 27.80)
Subtotal WYNDE6								11.24	(9.55- 13.22)
ZHENG	531	m	0	15	66	29	94	0.74	(0.37- 1.48)
ZHENG	532	m	0	64	109	29	94	1.90	(1.13- 3.20)
ZHENG	533	m	0	44	43	29	94	3.32	(1.84- 5.99)
ZHENG	538	f	0	7	16	119	184	0.68	(0.27- 1.69)
ZHENG	539	f	0	26	28	119	184	1.44	(0.80- 2.57)
Subtotal ZHENG								1.57	(1.19- 2.08)
Totals				1365	3316	783	7101		

*prospective study

REF	NRR	SEX	AD	Ys	Ws	Qs	Ps
BARBON	731	m	0	1.76	5.71	0.01	0.0000
BARBON	732	m	0	1.81	6.09	0.00	0.0000
BARBON	733	m	0	3.52	4.42	13.08	0.0000
Subtotal BARBON				2.26	16.22	13.08	
BENHAM	521	m	0	1.10	5.72	2.86	0.0088
BENHAM	522	m	0	1.41	6.17	0.98	0.0005
Subtotal BENHAM				1.26	11.89	3.84	
HAENSZ	509	f	0	0.15	7.71	21.10	0.6808
HAENSZ	510	f	0	0.03	4.45	13.91	0.9433
Subtotal HAENSZ				0.11	12.15	35.01	
JEDRYC	538	m	0	1.37	5.91	1.09	0.0008
JEDRYC	539	m	0	1.69	5.40	0.07	0.0001
JEDRYC	540	m	0	1.50	4.19	0.39	0.0022
Subtotal JEDRYC				1.52	15.50	1.56	
MATOS	651	m	0	1.37	3.34	0.64	0.0126
MATOS	652	m	0	1.99	4.13	0.15	0.0001
MATOS	653	m	0	1.85	3.87	0.01	0.0003
Subtotal MATOS				1.76	11.33	0.79	
PEZZOT	552	m	0	1.89	2.46	0.02	0.0030
PEZZOT	553	m	0	2.17	2.64	0.36	0.0004
PEZZOT	554	m	0	1.65	2.14	0.05	0.0160
Subtotal PEZZOT				1.92	7.23	0.44	
WYNDE6	613	m	0	1.97	17.35	0.48	0.0000
WYNDE6	614	m	0	2.18	22.69	3.29	0.0000
WYNDE6	615	m	0	2.42	30.32	11.49	0.0000
WYNDE6	637	f	0	2.09	21.88	1.86	0.0000
WYNDE6	638	f	0	2.64	26.17	18.14	0.0000
WYNDE6	639	f	0	2.95	27.49	36.24	0.0000
Subtotal WYNDE6				2.42	145.89	71.51	
ZHENG	531	m	0	-0.31	7.88	35.02	0.3910
ZHENG	532	m	0	0.64	14.30	19.22	0.0149

Table 3H1 - 5

IESLC - Meta-analysis of Ever Smoking by Age started, Overview
Adenocarcinoma, Any Product (or Cigarettes if Any not available)
Least adjusted

REF	NRR	SEX	AD	Ys	Ws	Qs	Ps
ZHENG	533	m	0	1.20	10.98	4.00	0.0001
ZHENG	538	f	0	-0.39	4.56	21.95	0.4038
ZHENG	539	f	0	0.36	11.36	23.60	0.2228
Subtotal ZHENG				0.45	49.08	103.79	

N	27
NS	8

Table 3H1 - 6

IESLC - Meta-analysis of Ever Smoking by Age started, Overview
Adenocarcinoma, Any Product (or Cigarettes if Any not available)
Least adjusted

	Sex			
	combined	male	female	Total
N		20	7	27
NS		7	3	10

In this overview table, other than the "N" rows, entries in the "absent" and "Total" columns may be invalid and should be ignored

	Age started (broad categories)				
	absent	19+k26	15-25k18	1-17k14	Total
N	8	9	5	5	27
NS	4	8	4	4	20
Wt	50.17	84.36	64.47	70.29	269.29
Het Chi	29.81	37.92	7.06	15.43	230.02
Het df	7	8	4	4	26
Het P	***	***	N.S.	**	***
Fixed RR	1.86	4.35	9.76	13.60	6.07
RRl	1.41	3.51	7.65	10.76	5.38
RRu	2.45	5.38	12.46	17.18	6.84
P	+++	+++	+++	+++	+++
Random RR	2.01	3.92	8.90	12.28	4.61
RRl	1.11	2.39	6.21	7.15	3.17
RRu	3.65	6.43	12.76	21.08	6.68
P	+	+++	+++	+++	+++

	Age started (narrow categories)							
	absent	27+k30	23-29k26	19-25k22	15-21k18	11-17k14	1-13k10	Total
N	19	2			5		1	27
NS	8	1			4		1	13
Wt	190.24	12.44			64.47		2.14	269.29
Het Chi	151.27	0.02			7.06		0.00	230.02
Het df	18	1			4		0	26
Het P	***	N.S.			N.S.		N.S.	***
Fixed RR	5.95	0.71			9.76		5.19	6.07
RRl	5.16	0.41			7.65		1.36	5.38
RRu	6.86	1.24			12.46		19.85	6.84
P	+++	N.S.			+++		+	+++
Random RR	4.79	0.71			8.90		5.19	4.61
RRl	3.10	0.41			6.21		1.36	3.17
RRu	7.38	1.24			12.76		19.85	6.68
P	+++	N.S.			+++		+	+++

Table 3H1 - 6

IESLC - Meta-analysis of Ever Smoking by Age started, Overview
Adenocarcinoma, Any Product (or Cigarettes if Any not available)
Least adjusted

MALES

	Age started (broad categories)				
	absent	19+k26	15-25k18	1-17k14	Total
N	5	7	4	4	20
NS	3	7	4	4	18
Wt	29.80	54.78	38.31	42.80	165.68
Het Chi	19.37	15.69	1.45	10.17	92.24
Het df	4	6	3	3	19
Het P	***	*	N.S.	*	***
Fixed RR	2.62	4.08	7.65	10.92	5.62
RRl	1.83	3.13	5.57	8.09	4.82
RRu	3.75	5.32	10.50	14.74	6.54
P	+++	+++	+++	+++	+++
Random RR	3.14	4.11	7.65	10.40	5.17
RRl	1.35	2.59	5.57	5.11	3.62
RRu	7.29	6.52	10.50	21.19	7.39
P	++	+++	+++	+++	+++

		Age started (narrow categories)						
	absent	27+k30	23-29k26	19-25k22	15-21k18	11-17k14	1-13k10	Total
N	14	1			4		1	20
NS	7	1			4		1	12
Wt	117.36	7.88			38.31		2.14	165.68
Het Chi	54.45	0.00			1.45		0.00	92.24
Het df	13	0			3		0	19
Het P	***	N.S.			N.S.		N.S.	***
Fixed RR	5.83	0.74			7.65		5.19	5.62
RRl	4.86	0.37			5.57		1.36	4.82
RRu	6.99	1.48			10.50		19.85	6.54
P	+++	N.S.			+++		+	+++
Random RR	5.50	0.74			7.65		5.19	5.17
RRl	3.69	0.37			5.57		1.36	3.62
RRu	8.20	1.48			10.50		19.85	7.39
P	+++	N.S.			+++		+	+++

FEMALES

	Age started (broad categories)				
	absent	19+k26	15-25k18	1-17k14	Total
N	3	2	1	1	7
NS	2	2	1	1	4
Wt	20.37	29.58	26.17	27.49	103.61
Het Chi	1.89	21.59	0.00	0.00	135.24
Het df	2	1	0	0	6
Het P	N.S.	***	N.S.	N.S.	***
Fixed RR	1.13	4.89	13.95	19.13	6.86
RRl	0.73	3.41	9.51	13.16	5.66
RRu	1.74	7.01	20.46	27.80	8.32
P	N.S.	+++	+++	+++	+++
Random RR	1.13	3.14	13.95	19.13	3.22
RRl	0.73	0.47	9.51	13.16	1.24
RRu	1.74	21.12	20.46	27.80	8.39
P	N.S.	N.S.	+++	+++	+

Table 3H1 - 6

IESLC - Meta-analysis of Ever Smoking by Age started, Overview
Adenocarcinoma, Any Product (or Cigarettes if Any not available)
Least adjusted

FEMALES

	absent	Age started (narrow categories) 27+k30	23-29k26	19-25k22	15-21k18	11-17k14	1-13k10	Total
N	5	1			1			7
NS	3	1			1			4
Wt	72.88	4.56			26.17			103.61
Het Chi	96.70	0.00			0.00			135.24
Het df	4	0			0			6
Het P	***	N.S.			N.S.			***
Fixed RR	6.15	0.68			13.95			6.86
RRl	4.89	0.27			9.51			5.66
RRu	7.73	1.69			20.46			8.32
P	+++	N.S.			+++			+++
Random RR	3.17	0.68			13.95			3.22
RRl	0.96	0.27			9.51			1.24
RRu	10.43	1.69			20.46			8.39
P	(+)	N.S.			+++			+

Table 3H1 - 7

IESLC - Meta-analysis of Ever Smoking by Age started, Overview
Adenocarcinoma, Any Product (or Cigarettes if Any not available)
Excluded studies (and stage at which they were excluded)

1	AKIBA AMANDU AMES BECHER BENSHL BEST BLOT1 BROSS BROWN3 CARPEN CEDERL CHYOU CPSI CPSII DARBY DEAN2 DEAN3 DOLL2 ENGELA GAO2 GARCIA GILLIS GRAHAM GURSEL HAMMO2 HIRAYA HOLE HUMBLE JAHN JAIN KAISE2 KATSOU KAUFMA LAUSSM LIAW MCDUFF MIGRAN MRFITR PEZZO2 PISANI PRESCO QIAO SEGI2 SPEIZE SVENSS TVERDA WAKAI WATSON WIGLE WU WYNDE3 WYNDE8
2	AXELSS BOUCHA BOUCOT CHEN DESTEF DORGAN DOSEME FAN GARSHI GER HAMMON JUSSAW KOO KREUZE LEVIN MCCONN NOTAN2 OSANN2 RESTRE SADOWS VUTUC WANG2 WU2 WUWILL WYNDE2 XU ZHOU
3	GUO SPITZ STASZE ZHANG
4	AGUDO ARMADA AUVINE BOFFET BRESLO BUFFLE CHEN2 CHIAZZ CHOI CORREA DAMBER DOLL DORN GAO GENG HU HU2 JOLY KOULUM LETOUR LIU3 LIU4 LIU5 LUBIN LUBIN2 LUO PERNU QIAO2 RACHTA SOBUE SUZUK2 TIZZAN WYNDE7 YUAN
5	ALDERS
10	HEGMAN KHUDER

Table 3H1 - 8
Potentially overlapping studies

REF	REFGP	PRINC	.	OVERLAP/LINK
BENHAM	LUBIN2	2		Subset of Lubin2
WYNDE6	WYNDE6	1		WYNDE5/6/7/8

Table 3H2 -

IESLC - Meta-analysis of Ever Smoking, Age started, "Low"
Adenocarcinoma, Any Product (or Cigarettes if Any not available)

This analysis is restricted to results for:
1) Ever smokers
2) Results by Age started
3) Categorical results by Age started
4) Adenocarcinoma (or near equivalent)
5) Results complete enough for use in metaanalysis

Within each study, results are then selected (in the following order of preference, within each sex) for:
6) (not applicable)
7) PRODUCT: all/unspec, cigarettes regardless of other products, cigarettes only
8) CIGTYPE: all/unspecified, MC regardless of HR, MC only
9) (not applicable)
10) DENOM: never smoked anything, never smoked cigarettes, never any + low, never cigs + low
11) Followup period (YF, prospective studies): whole study (coded as 0) or longest available
12) LCtype: adeno or nearest available, but not squamous. (q = squamous, s = small,
a = adeno, l = large, KII = Kreyberg II, al = alveolar, br = bronchiolar, u = undifferentiated)
13) Race: all or nearest available, otherwise by race (wh or w = white, bl or b = black, hi = hispanic
ch = chinese, jap = japanese, haw = hawaiian, w+o = white + oriental, sca = scandinavian, as = asian)
14) Age started "low" in key scheme 1 (key value 26, maximum range 19+)
15) For overlapping studies: principal rather than subsidiary studies
Finally by Age: whole study (coded as 0) if available, otherwise by widest available age group
and then for single sex results (m, f) in preference to results for both sexes combined (c).

Results adjusted (AD) for the most potential confounders are then chosen in Sections -1 to -3
and results adjusted for the least confounders in Sections -4 to -6. (Those least adjusted results which
actually differ from the most adjusted are marked 'x' in column X in Section -4)

Section -7 shows excluded studies, together with the stage (as above) at which no qualifying
results were found.

Section -8 lists the potentially overlapping studies which have been included (1=principal, 2=subsidiary).

Section -9 lists any results which would have been included in preference except that they had data not complete
enough for use in meta-analysis, with their significance (yes/no), if known, and any further comment as entered
on the database. It also lists as "gap" any categories for which no data were presented by the original authors.

In addition to those mentioned above, the following fields, levels and abbreviations are used:
* or nk = not known, n = no, y = yes, ot = other
nev = never
all/unspec = all or unspecified, cig+/-ot = cigarettes irrespective of other products (cigar, pipe etc)
MC = manufactured cigarettes, HR = hand-rolled cigarettes
exL, exH = range of exposure (low and high) in the smoking group, in terms of Age started
REF: 6-character study reference
NRR: number of the RR on the database within the study
ST : study type (CC = case control, pr or prosp = prospective)
NLC: number of lung cancer cases in whole study
R : risky occupational population (n = no, m = mining, o = other risky)
VB : national cigarette type (V = at least 75% Virginia, bl = at least 75% blended, ot = other)
P : any proxy use
H : full histological confirmation
De : derivation of RR/CI (or = original, st = standard method, ot = other method of estimation)

Table 3H2 - 1

IESLC - Meta-analysis of Ever Smoking, Age started, "Low"
Adenocarcinoma, Any Product (or Cigarettes if Any not available)
Most adjusted

REF	NRR	SEX	AGEL	AGEH	RACE	YF	LC TYPE	LOC	START	ST	NLC	R	VB	P	H	AD	PRODUCT	exL	exH	DENOM	De
BARBON	736	m	0	0	all	-	a	Eu:wst	1979	CC	755	n	bl	y	y	1	all/unsp	20	999	nev any	or
BENHAM	521	m	0	0	all	-	KII	Eu:wst	1976	CC	1625	n	bl	n	y	0	cig only	20	999	nev any	st
HAENSZ	509	f	0	0	all	-	a	NAmer	1955	CC	158	n	bl	n	y	0	cig+/-ot	25	999	nev any	st
JEDRYC	538	m	0	0	all	-	a	Eu:est	1980	CC	1630	n	bl	y	n	0	cig+/-ot	19	999	nev any	st
MATOS	656	m	0	0	all	-	a	SCAmer	1994	CC	200	n	bl	n	n	2	cig+/-ot	20	999	nev any	or
PEZZOT	552	m	0	0	all	-	a	SCAmer	1987	CC	215	n	bl	n	y	0	cig only	19	999	nev cigs	st
WYNDE6	613	m	0	0	wh	-	a	NAmer	1969	CC	4423	n	bl	n	y	0	cig+/-ot	21	999	nev cigs	st
WYNDE6	637	f	0	0	wh	-	a	NAmer	1969	CC	4423	n	bl	n	y	0	cig+/-ot	21	999	nev cigs	st
ZHENG	532	m	0	0	all	-	a	As:Chi	1982	CC	540	n	ot	*	y	0	cig+/-ot	20	29	nev cigs	st

Cigarette type is all/unspec for all RRs

Table 3H2 - 2

IESLC - Meta-analysis of Ever Smoking, Age started, "Low"
Adenocarcinoma, Any Product (or Cigarettes if Any not available)
Most adjusted

REF	NRR	SEX	AD	Number Exposed Case	Cont	Non-exposed Case	Cont	RR	95.00%CI
BARBON	736	m	1	45	-	7	-	5.70	(2.50- 12.90)
BENHAM	521	m	0	41	64	9	42	2.99	(1.32- 6.79)
HAENSZ	509	f	0	12	66	37	236	1.16	(0.57- 2.35)
JEDRYC	538	m	0	48	502	7	289	3.95	(1.76- 8.84)
MATOS	656	m	2	13	-	5	-	4.00	(1.40- 11.90)
PEZZOT	552	m	0	18	105	3	116	6.63	(1.90- 23.15)
WYNDE6	613	m	0	47	92	42	589	7.16	(4.47- 11.47)
WYNDE6	637	f	0	63	90	58	673	8.12	(5.34- 12.35)
Subtotal WYNDE6								7.68	(5.62- 10.51)
ZHENG	532	m	0	64	109	29	94	1.90	(1.13- 3.20)
Partial Totals				351	1028	197	2039		

*prospective study

REF	NRR	SEX	AD	Ys	Ws	Qs	Ps
BARBON	736	m	1	1.74	5.71	0.42	0.0000
BENHAM	521	m	0	1.10	5.72	0.80	0.0088
HAENSZ	509	f	0	0.15	7.71	13.44	0.6808
JEDRYC	538	m	0	1.37	5.91	0.05	0.0008
MATOS	656	m	2	1.39	3.36	0.02	0.0111
PEZZOT	552	m	0	1.89	2.46	0.44	0.0030
WYNDE6	613	m	0	1.97	17.35	4.34	0.0000
WYNDE6	637	f	0	2.09	21.88	8.57	0.0000
Subtotal WYNDE6				2.04	39.22	12.91	
ZHENG	532	m	0	0.64	14.30	9.74	0.0149

	N	9
	NS	8
	Wt	84.38
Het	Chi	37.83
Het	df	8
Het	P	***
Fixed	RR	4.34
	RRl	3.51
	RRu	5.38
	P	+++
Random	RR	3.92
	RRl	2.39
	RRu	6.42
	P	+++
Asymm	P	N.S.

Table 3H2 - 3

IESLC - Meta-analysis of Ever Smoking, Age started, "Low"
Adenocarcinoma, Any Product (or Cigarettes if Any not available)
Most adjusted

	combined	Sex: male	female	Total
N		7	2	9
NS		7	2	9
Wt		54.79	29.58	84.38
Het Chi		15.59	21.59	37.83
Het df		6	1	8
Het P		*	***	***
Fixed RR		4.07	4.89	4.34
RRl		3.13	3.41	3.51
RRu		5.31	7.01	5.38
P		+++	+++	+++
Random RR		4.10	3.14	3.92
RRl		2.59	0.47	2.39
RRu		6.50	21.12	6.42
P		+++	N.S.	+++
Between Chi				0.64
Between df				1
Between P				N.S.
Btwn(F) P				N.S.
Btwn(R) P				N.S.

Too few RRs for analysis by factor

Table 3H2 - 4

IESLC - Meta-analysis of Ever Smoking, Age started, "Low"
Adenocarcinoma, Any Product (or Cigarettes if Any not available)
Least adjusted

REF	NRR	X	SEX	AGEL	AGEH	RACE	YF	LC TYPE	LOC	START	ST	NLC	R	VB	P	H	AD	PRODUCT	exL	exH	DENOM	De
BARBON	731	x	m	0	0	all	-	a	Eu:wst	1979	CC	755	n	bl	y	y	0	all/unsp	20	999	nev any	st
BENHAM	521		m	0	0	all	-	KII	Eu:wst	1976	CC	1625	n	bl	n	y	0	cig only	20	999	nev any	st
HAENSZ	509		f	0	0	all	-	a	NAmer	1955	CC	158	n	bl	n	y	0	cig+/-ot	25	999	nev any	st
JEDRYC	538		m	0	0	all	-	a	Eu:est	1980	CC	1630	n	bl	y	n	0	cig+/-ot	19	999	nev any	st
MATOS	651	x	m	0	0	all	-	a	SCAmer	1994	CC	200	n	bl	n	n	0	cig+/-ot	20	999	nev any	st
PEZZOT	552		m	0	0	all	-	a	SCAmer	1987	CC	215	n	bl	n	y	0	cig only	19	999	nev cigs	st
WYNDE6	613		m	0	0	wh	-	a	NAmer	1969	CC	4423	n	bl	n	y	0	cig+/-ot	21	999	nev cigs	st
WYNDE6	637		f	0	0	wh	-	a	NAmer	1969	CC	4423	n	bl	n	y	0	cig+/-ot	21	999	nev cigs	st
ZHENG	532		m	0	0	all	-	a	As:Chi	1982	CC	540	n	ot	*	y	0	cig+/-ot	20	29	nev cigs	st

Cigarette type is all/unspec for all RRs

Table 3H2 - 5

IESLC - Meta-analysis of Ever Smoking, Age started, "Low"
Adenocarcinoma, Any Product (or Cigarettes if Any not available)
Least adjusted

REF	NRR	SEX	AD	Number Exposed Case	Cont	Non-exposed Case	Cont	RR	95.00%CI
BARBON	731	m	0	45	207	7	188	5.84	(2.57- 13.26)
BENHAM	521	m	0	41	64	9	42	2.99	(1.32- 6.79)
HAENSZ	509	f	0	12	66	37	236	1.16	(0.57- 2.35)
JEDRYC	538	m	0	48	502	7	289	3.95	(1.76- 8.84)
MATOS	651	m	0	13	73	5	110	3.92	(1.34- 11.46)
PEZZOT	552	m	0	18	105	3	116	6.63	(1.90- 23.15)
WYNDE6	613	m	0	47	92	42	589	7.16	(4.47- 11.47)
WYNDE6	637	f	0	63	90	58	673	8.12	(5.34- 12.35)
Subtotal WYNDE6								7.68	(5.62- 10.51)
ZHENG	532	m	0	64	109	29	94	1.90	(1.13- 3.20)
Totals				351	1308	197	2337		

*prospective study

REF	NRR	SEX	AD	Ys	Ws	Qs	Ps
BARBON	731	m	0	1.76	5.71	0.50	0.0000
BENHAM	521	m	0	1.10	5.72	0.80	0.0088
HAENSZ	509	f	0	0.15	7.71	13.46	0.6808
JEDRYC	538	m	0	1.37	5.91	0.06	0.0008
MATOS	651	m	0	1.37	3.34	0.04	0.0126
PEZZOT	552	m	0	1.89	2.46	0.44	0.0030
WYNDE6	613	m	0	1.97	17.35	4.33	0.0000
WYNDE6	637	f	0	2.09	21.88	8.55	0.0000
Subtotal WYNDE6				2.04	39.22	12.87	
ZHENG	532	m	0	0.64	14.30	9.76	0.0149

	N	9
	NS	8
	Wt	84.36
Het	Chi	37.92
Het	df	8
Het	P	***
Fixed	RR	4.35
	RRl	3.51
	RRu	5.38
	P	+++
Random	RR	3.92
	RRl	2.39
	RRu	6.43
	P	+++
Asymm	P	N.S.

Table 3H2 - 6

IESLC - Meta-analysis of Ever Smoking, Age started, "Low"
Adenocarcinoma, Any Product (or Cigarettes if Any not available)
Least adjusted

	Sex<br>combined	male	female	Total
N		7	2	9
NS		7	2	9
Wt		54.78	29.58	84.36
Het Chi		15.69	21.59	37.92
Het df		6	1	8
Het P		*	***	***
Fixed RR		4.08	4.89	4.35
RRl		3.13	3.41	3.51
RRu		5.32	7.01	5.38
P		+++	+++	+++
Random RR		4.11	3.14	3.92
RRl		2.59	0.47	2.39
RRu		6.52	21.12	6.43
P		+++	N.S.	+++
Between Chi				0.63
Between df				1
Between P				N.S.
Btwn(F) P				N.S.
Btwn(R) P				N.S.

Table 3H2 - 7

IESLC - Meta-analysis of Ever Smoking, Age started, "Low"
Adenocarcinoma, Any Product (or Cigarettes if Any not available)
Excluded studies (and stage at which they were excluded)

1	AKIBA AMANDU AMES BECHER BENSHL BEST BLOT1 BROSS BROWN3 CARPEN CEDERL CHYOU CPSI CPSII DARBY DEAN2 DEAN3 DOLL2 ENGELA GAO2 GARCIA GILLIS GRAHAM GURSEL HAMMO2 HIRAYA HOLE HUMBLE JAHN JAIN KAISE2 KATSOU KAUFMA LAUSSM LIAW MCDUFF MIGRAN MRFITR PEZZO2 PISANI PRESCO QIAO SEGI2 SPEIZE SVENSS TVERDA WAKAI WATSON WIGLE WU WYNDE3 WYNDE8
2	AXELSS BOUCHA BOUCOT CHEN DESTEF DORGAN DOSEME FAN GARSHI GER HAMMON JUSSAW KOO KREUZE LEVIN MCCONN NOTAN2 OSANN2 RESTRE SADOWS VUTUC WANG2 WU2 WUWILL WYNDE2 XU ZHOU
3	GUO SPITZ STASZE ZHANG
4	AGUDO ARMADA AUVINE BOFFET BRESLO BUFFLE CHEN2 CHIAZZ CHOI CORREA DAMBER DOLL DORN GAO GENG HU HU2 JOLY KOULUM LETOUR LIU3 LIU4 LIU5 LUBIN LUBIN2 LUO PERNU QIAO2 RACHTA SOBUE SUZUK2 TIZZAN WYNDE7 YUAN
5	ALDERS
10	HEGMAN KHUDER

Table 3H2 - 8

Potentially overlapping studies

REF	REFGP	PRINC	. OVERLAP/LINK
BENHAM	LUBIN2	2	Subset of Lubin2
WYNDE6	WYNDE6	1	WYNDE5/6/7/8

Table 3H3 -

IESLC - Meta-analysis of Ever Smoking, Age started, "Mid"
Adenocarcinoma, Any Product (or Cigarettes if Any not available)

This analysis is restricted to results for:
1) Ever smokers
2) Results by Age started
3) Categorical results by Age started
4) Adenocarcinoma (or near equivalent)
5) Results complete enough for use in metaanalysis

Within each study, results are then selected (in the following order of preference, within each sex) for:
6) (not applicable)
7) PRODUCT: all/unspec, cigarettes regardless of other products, cigarettes only
8) CIGTYPE: all/unspecified, MC regardless of HR, MC only
9) (not applicable)
10) DENOM: never smoked anything, never smoked cigarettes, never any + low, never cigs + low
11) Followup period (YF, prospective studies): whole study (coded as 0) or longest available
12) LCtype: adeno or nearest available, but not squamous. (q = squamous, s = small,
a = adeno, l = large, KII = Kreyberg II, al = alveolar, br = bronchiolar, u = undifferentiated)
13) Race: all or nearest available, otherwise by race (wh or w = white, bl or b = black, hi = hispanic
ch = chinese, jap = japanese, haw = hawaiian, w+o = white + oriental, sca = scandinavian, as = asian)
14) Age started "mid" in key scheme 1 (key value 18, maximum range 15-25)
15) For overlapping studies: principal rather than subsidiary studies
Finally by Age: whole study (coded as 0) if available, otherwise by widest available age group
and then for single sex results (m, f) in preference to results for both sexes combined (c).

Results adjusted (AD) for the most potential confounders are then chosen in Sections -1 to -3
and results adjusted for the least confounders in Sections -4 to -6. (Those least adjusted results which
actually differ from the most adjusted are marked 'x' in column X in Section -4)

Section -7 shows excluded studies, together with the stage (as above) at which no qualifying
results were found.

Section -8 lists the potentially overlapping studies which have been included (1=principal, 2=subsidiary).

Section -9 lists any results which would have been included in preference except that they had data not complete
enough for use in meta-analysis, with their significance (yes/no), if known, and any further comment as entered
on the database. It also lists as "gap" any categories for which no data were presented by the original authors.

In addition to those mentioned above, the following fields, levels and abbreviations are used:
* or nk = not known, n = no, y = yes, ot = other
nev = never
all/unspec = all or unspecified, cig+/-ot = cigarettes irrespective of other products (cigar, pipe etc)
MC = manufactured cigarettes, HR = hand-rolled cigarettes
exL, exH = range of exposure (low and high) in the smoking group, in terms of Age started
REF: 6-character study reference
NRR: number of the RR on the database within the study
ST : study type (CC = case control, pr or prosp = prospective)
NLC: number of lung cancer cases in whole study
R : risky occupational population (n = no, m = mining, o = other risky)
VB : national cigarette type (V = at least 75% Virginia, bl = at least 75% blended, ot = other)
P : any proxy use
H : full histological confirmation
De : derivation of RR/CI (or = original, st = standard method, ot = other method of estimation)

Table 3H3 - 1

IESLC - Meta-analysis of Ever Smoking, Age started, "Mid"
Adenocarcinoma, Any Product (or Cigarettes if Any not available)
Most adjusted

REF	NRR	SEX	AGEL	AGEH	RACE	YF	LC TYPE	LOC	START	ST	NLC	R	VB	P	H	AD	PRODUCT	exL	exH	DENOM	De
BARBON	737	m	0	0	all	-	a	Eu:wst	1979	CC	755	n	bl	y	y	1	all/unsp	15	19	nev any	or
JEDRYC	539	m	0	0	all	-	a	Eu:est	1980	CC	1630	n	bl	y	n	0	cig+/-ot	17	18	nev any	st
MATOS	657	m	0	0	all	-	a	SCAmer	1994	CC	200	n	bl	n	n	2	cig+/-ot	15	19	nev any	or
WYNDE6	614	m	0	0	wh	-	a	NAmer	1969	CC	4423	n	bl	n	y	0	cig+/-ot	18	20	nev cigs	st
WYNDE6	638	f	0	0	wh	-	a	NAmer	1969	CC	4423	n	bl	n	y	0	cig+/-ot	18	20	nev cigs	st

Cigarette type is all/unspec for all RRs

Table 3H3 - 2

IESLC - Meta-analysis of Ever Smoking, Age started, "Mid"
Adenocarcinoma, Any Product (or Cigarettes if Any not available)
Most adjusted

REF	NRR	SEX	AD	Number Exposed Case	Exposed Cont	Non-exposed Case	Non-exposed Cont	RR	95.00%CI
BARBON	737	m	1	77	-	7	-	6.00	(2.70- 13.30)
JEDRYC	539	m	0	29	221	7	289	5.42	(2.33- 12.60)
MATOS	657	m	2	40	-	5	-	7.20	(2.70- 19.10)
WYNDE6	614	m	0	88	139	42	589	8.88	(5.88- 13.40)
WYNDE6	638	f	0	113	94	58	673	13.95	(9.51- 20.46)
Subtotal WYNDE6								11.31	(8.54- 14.97)
Partial Totals				347	454	119	1551		

*prospective study

REF	NRR	SEX	AD	Ys	Ws	Qs	Ps
BARBON	737	m	1	1.79	6.04	1.42	0.0000
JEDRYC	539	m	0	1.69	5.40	1.86	0.0001
MATOS	657	m	2	1.97	4.01	0.37	0.0001
WYNDE6	614	m	0	2.18	22.69	0.19	0.0000
WYNDE6	638	f	0	2.64	26.17	3.38	0.0000
Subtotal WYNDE6				2.43	48.86	3.57	

	N	5
	NS	4
	Wt	64.32
Het	Chi	7.21
Het	df	4
Het	P	N.S.
Fixed	RR	9.74
	RRl	7.63
	RRu	12.43
	P	+++
Random	RR	8.84
	RRl	6.14
	RRu	12.73
	P	+++
Asymm	P	N.S.

Table 3H3 - 3

IESLC - Meta-analysis of Ever Smoking, Age started, "Mid"
Adenocarcinoma, Any Product (or Cigarettes if Any not available)
Most adjusted

	Sex combined	male	female	Total
N		4	1	5
NS		4	1	4
Wt		38.15	26.17	64.32
Het Chi		1.52	0.00	7.21
Het df		3	0	4
Het P		N.S.	N.S.	N.S.
Fixed RR		7.61	13.95	9.74
RRl		5.54	9.51	7.63
RRu		10.45	20.46	12.43
P		+++	+++	+++
Random RR		7.61	13.95	8.84
RRl		5.54	9.51	6.14
RRu		10.45	20.46	12.73
P		+++	+++	+++
Between Chi				5.70
Between df				1
Between P				*
Btwn(F) P				*
Btwn(R) P				*

Too few RRs for analysis by factor

Table 3H3 - 4

IESLC - Meta-analysis of Ever Smoking, Age started, "Mid"
Adenocarcinoma, Any Product (or Cigarettes if Any not available)
Least adjusted

REF	NRR	X	SEX	AGEL	AGEH	RACE	YF	LC TYPE	LOC	START	ST	NLC	R	VB	P	H	AD	PRODUCT	exL	exH	DENOM	De
BARBON	732	x	m	0	0	all	-	a	Eu:wst	1979	CC	755	n	bl	y	y	0	all/unsp	15	19	nev any	st
JEDRYC	539		m	0	0	all	-	a	Eu:est	1980	CC	1630	n	bl	y	n	0	cig+/-ot	17	18	nev any	st
MATOS	652	x	m	0	0	all	-	a	SCAmer	1994	CC	200	n	bl	n	n	0	cig+/-ot	15	19	nev any	st
WYNDE6	614		m	0	0	wh	-	a	NAmer	1969	CC	4423	n	bl	n	y	0	cig+/-ot	18	20	nev cigs	st
WYNDE6	638		f	0	0	wh	-	a	NAmer	1969	CC	4423	n	bl	n	y	0	cig+/-ot	18	20	nev cigs	st

Cigarette type is all/unspec for all RRs

Table 3H3 - 5

IESLC - Meta-analysis of Ever Smoking, Age started, "Mid"
Adenocarcinoma, Any Product (or Cigarettes if Any not available)
Least adjusted

REF	NRR	SEX	AD	Number Exposed Case	Cont	Non-exposed Case	Cont	RR	95.00%CI
BARBON	732	m	0	77	337	7	188	6.14	(2.77- 13.58)
JEDRYC	539	m	0	29	221	7	289	5.42	(2.33- 12.60)
MATOS	652	m	0	40	120	5	110	7.33	(2.79- 19.25)
WYNDE6	614	m	0	88	139	42	589	8.88	(5.88- 13.40)
WYNDE6	638	f	0	113	94	58	673	13.95	(9.51- 20.46)
Subtotal WYNDE6								11.31	(8.54- 14.97)
Totals				347	911	119	1849		

*prospective study

REF	NRR	SEX	AD	Ys	Ws	Qs	Ps
BARBON	732	m	0	1.81	6.09	1.31	0.0000
JEDRYC	539	m	0	1.69	5.40	1.87	0.0001
MATOS	652	m	0	1.99	4.13	0.34	0.0001
WYNDE6	614	m	0	2.18	22.69	0.20	0.0000
WYNDE6	638	f	0	2.64	26.17	3.33	0.0000
Subtotal WYNDE6				2.43	48.86	3.54	

	N	5
	NS	4
	Wt	64.47
Het	Chi	7.06
Het	df	4
Het	P	N.S.
Fixed	RR	9.76
	RRl	7.65
	RRu	12.46
	P	+++
Random	RR	8.90
	RRl	6.21
	RRu	12.76
	P	+++
Asymm	P	N.S.

Table 3H3 - 6

IESLC - Meta-analysis of Ever Smoking, Age started, "Mid"
Adenocarcinoma, Any Product (or Cigarettes if Any not available)
Least adjusted

	Sex combined	male	female	Total
N		4	1	5
NS		4	1	4
Wt		38.31	26.17	64.47
Het Chi		1.45	0.00	7.06
Het df		3	0	4
Het P		N.S.	N.S.	N.S.
Fixed RR		7.65	13.95	9.76
RRl		5.57	9.51	7.65
RRu		10.50	20.46	12.46
P		+++	+++	+++
Random RR		7.65	13.95	8.90
RRl		5.57	9.51	6.21
RRu		10.50	20.46	12.76
P		+++	+++	+++
Between Chi				5.61
Between df				1
Between P				*
Btwn(F) P				*
Btwn(R) P				*

Table 3H3 - 7

IESLC - Meta-analysis of Ever Smoking, Age started, "Mid"
Adenocarcinoma, Any Product (or Cigarettes if Any not available)
Excluded studies (and stage at which they were excluded)

Stage	Studies
1	AKIBA AMANDU AMES BECHER BENSHL BEST BLOT1 BROSS BROWN3 CARPEN CEDERL CHYOU CPSI CPSII DARBY DEAN2 DEAN3 DOLL2 ENGELA GAO2 GARCIA GILLIS GRAHAM GURSEL HAMMO2 HIRAYA HOLE HUMBLE JAHN JAIN KAISE2 KATSOU KAUFMA LAUSSM LIAW MCDUFF MIGRAN MRFITR PEZZO2 PISANI PRESCO QIAO SEGI2 SPEIZE SVENSS TVERDA WAKAI WATSON WIGLE WU WYNDE3 WYNDE8
2	AXELSS BOUCHA BOUCOT CHEN DESTEF DORGAN DOSEME FAN GARSHI GER HAMMON JUSSAW KOO KREUZE LEVIN MCCONN NOTAN2 OSANN2 RESTRE SADOWS VUTUC WANG2 WU2 WUWILL WYNDE2 XU ZHOU
3	GUO SPITZ STASZE ZHANG
4	AGUDO ARMADA AUVINE BOFFET BRESLO BUFFLE CHEN2 CHIAZZ CHOI CORREA DAMBER DOLL DORN GAO GENG HU HU2 JOLY KOULUM LETOUR LIU3 LIU4 LIU5 LUBIN LUBIN2 LUO PERNU QIAO2 RACHTA SOBUE SUZUK2 TIZZAN WYNDE7 YUAN
5	ALDERS
10	HEGMAN KHUDER
14	BENHAM HAENSZ PEZZOT ZHENG

Table 3H3 - 8
Potentially overlapping studies

REF	REFGP	PRINC	.	OVERLAP/LINK
WYNDE6	WYNDE6	1		WYNDE5/6/7/8

Table 3H4 -

IESLC - Meta-analysis of Ever Smoking, Age started, "High"
Adenocarcinoma, Any Product (or Cigarettes if Any not available)

This analysis is restricted to results for:
1) Ever smokers
2) Results by Age started
3) Categorical results by Age started
4) Adenocarcinoma (or near equivalent)
5) Results complete enough for use in metaanalysis

Within each study, results are then selected (in the following order of preference, within each sex) for:
6) PRODUCT: all/unspec, cigarettes regardless of other products, cigarettes only
7) CIGTYPE: all/unspecified, MC regardless of HR, MC only
8) (not applicable)
9) DENOM: never smoked anything, never smoked cigarettes, never any + low, never cigs + low
10) Followup period (YF, prospective studies): whole study (coded as 0) or longest available
11) LCtype: adeno or nearest available, but not squamous. (q = squamous, s = small,
a = adeno, l = large, KII = Kreyberg II, al = alveolar, br = bronchiolar, u = undifferentiated)
12) Race: all or nearest available, otherwise by race (wh or w = white, bl or b = black, hi = hispanic
ch = chinese, jap = japanese, haw = hawaiian, w+o = white + oriental, sca = scandinavian, as = asian)
13) Age started "high" in key scheme 1 (key value 14, maximum range 1-17)
14) For overlapping studies: principal rather than subsidiary studies
Finally by Age: whole study (coded as 0) if available, otherwise by widest available age group
and then for single sex results (m, f) in preference to results for both sexes combined (c).

Results adjusted (AD) for the most potential confounders are then chosen in Sections -1 to -3
and results adjusted for the least confounders in Sections -4 to -6. (Those least adjusted results which
actually differ from the most adjusted are marked 'x' in column X in Section -4)

Section -7 shows excluded studies, together with the stage (as above) at which no qualifying
results were found.

Section -8 lists the potentially overlapping studies which have been included (1=principal, 2=subsidiary).

Section -9 lists any results which would have been included in preference except that they had data not complete
enough for use in meta-analysis, with their significance (yes/no), if known, and any further comment as entered
on the database. It also lists as "gap" any categories for which no data were presented by the original authors.

In addition to those mentioned above, the following fields, levels and abbreviations are used:
* or nk = not known, n = no, y = yes, ot = other
nev = never
all/unspec = all or unspecified, cig+/-ot = cigarettes irrespective of other products (cigar, pipe etc)
MC = manufactured cigarettes, HR = hand-rolled cigarettes
exL, exH = range of exposure (low and high) in the smoking group, in terms of Age started
REF: 6-character study reference
NRR: number of the RR on the database within the study
ST : study type (CC = case control, pr or prosp = prospective)
NLC: number of lung cancer cases in whole study
R : risky occupational population (n = no, m = mining, o = other risky)
VB : national cigarette type (V = at least 75% Virginia, bl = at least 75% blended, ot = other)
P : any proxy use
H : full histological confirmation
De : derivation of RR/CI (or = original, st = standard method, ot = other method of estimation)

Table 3H4 - 1

IESLC - Meta-analysis of Ever Smoking, Age started, "High"
Adenocarcinoma, Any Product (or Cigarettes if Any not available)
Most adjusted

REF	NRR	SEX	AGEL	AGEH	RACE	YF	LC TYPE	LOC	START	ST	NLC	R	VB	P	H	AD	PRODUCT	exL	exH	DENOM	De
BARBON	738	m	0	0	all	-	a	Eu:wst	1979	CC	755	n	bl	y	y	1	all/unsp	1	14	nev any	or
JEDRYC	540	m	0	0	all	-	a	Eu:est	1980	CC	1630	n	bl	y	n	0	cig+/-ot	1	16	nev any	st
MATOS	658	m	0	0	all	-	a	SCAmer	1994	CC	200	n	bl	n	n	2	cig+/-ot	1	14	nev any	or
WYNDE6	615	m	0	0	wh	-	a	NAmer	1969	CC	4423	n	bl	n	y	0	cig+/-ot	1	17	nev cigs	st
WYNDE6	639	f	0	0	wh	-	a	NAmer	1969	CC	4423	n	bl	n	y	0	cig+/-ot	1	17	nev cigs	st

Cigarette type is all/unspec for all RRs

Table 3H4 - 2

IESLC - Meta-analysis of Ever Smoking, Age started, "High"
Adenocarcinoma, Any Product (or Cigarettes if Any not available)
Most adjusted

REF	NRR	SEX	AD	Number Exposed Case	Number Exposed Cont	Non-exposed Case	Non-exposed Cont	RR	95.00%CI
BARBON	738	m	1	29	-	7	-	33.40	(13.10- 84.90)
JEDRYC	540	m	0	12	111	7	289	4.46	(1.71- 11.63)
MATOS	658	m	2	26	-	5	-	6.60	(2.40- 18.10)
WYNDE6	615	m	0	241	301	42	589	11.23	(7.87- 16.03)
WYNDE6	639	f	0	150	91	58	673	19.13	(13.16- 27.80)
Subtotal WYNDE6								14.46	(11.18- 18.72)
Partial Totals				458	503	119	1551		

*prospective study

REF	NRR	SEX	AD	Ys	Ws	Qs	Ps
BARBON	738	m	1	3.51	4.40	3.54	0.0000
JEDRYC	540	m	0	1.50	4.19	5.22	0.0022
MATOS	658	m	2	1.89	3.76	1.98	0.0003
WYNDE6	615	m	0	2.42	30.32	1.13	0.0000
WYNDE6	639	f	0	2.95	27.49	3.16	0.0000
Subtotal WYNDE6				2.67	57.81	4.30	

	N	5
	NS	4
	Wt	70.16
Het	Chi	15.03
Het	df	4
Het	P	**
Fixed	RR	13.62
	RRl	10.78
	RRu	17.22
	P	+++
Random	RR	12.34
	RRl	7.23
	RRu	21.08
	P	+++
Asymm	P	N.S.

Table 3H4 - 3

IESLC - Meta-analysis of Ever Smoking, Age started, "High"
Adenocarcinoma, Any Product (or Cigarettes if Any not available)
Most adjusted

	Sex combined	male	female	Total
N		4	1	5
NS		4	1	4
Wt		42.68	27.49	70.16
Het Chi		9.83	0.00	15.03
Het df		3	0	4
Het P		*	N.S.	**
Fixed RR		10.95	19.13	13.62
RRl		8.11	13.16	10.78
RRu		14.78	27.80	17.22
P		+++	+++	+++
Random RR		10.46	19.13	12.34
RRl		5.18	13.16	7.23
RRu		21.13	27.80	21.08
P		+++	+++	+++
Between Chi				5.20
Between df				1
Between P				*
Btwn(F) P				N.S.
Btwn(R) P				N.S.

Too few RRs for analysis by factor

Table 3H4 - 4

IESLC - Meta-analysis of Ever Smoking, Age started, "High"
Adenocarcinoma, Any Product (or Cigarettes if Any not available)
Least adjusted

REF	NRR	X	SEX	AGEL	AGEH	RACE	YF	LC TYPE	LOC	START	ST	NLC	R	VB	P	H	AD	PRODUCT	exL	exH	DENOM	De
BARBON	733	x	m	0	0	all	-	a	Eu:wst	1979	CC	755	n	bl	y	y	0	all/unsp	1	14	nev any	st
JEDRYC	540		m	0	0	all	-	a	Eu:est	1980	CC	1630	n	bl	y	n	0	cig+/-ot	1	16	nev any	st
MATOS	653	x	m	0	0	all	-	a	SCAmer	1994	CC	200	n	bl	n	n	0	cig+/-ot	1	14	nev any	st
WYNDE6	615		m	0	0	wh	-	a	NAmer	1969	CC	4423	n	bl	n	y	0	cig+/-ot	1	17	nev cigs	st
WYNDE6	639		f	0	0	wh	-	a	NAmer	1969	CC	4423	n	bl	n	y	0	cig+/-ot	1	17	nev cigs	st

Cigarette type is all/unspec for all RRs

Table 3H4 - 5

IESLC - Meta-analysis of Ever Smoking, Age started, "High"
Adenocarcinoma, Any Product (or Cigarettes if Any not available)
Least adjusted

REF	NRR	SEX	AD	Number Exposed Case	Cont	Non-exposed Case	Cont	RR	95.00%CI
BARBON	733	m	0	29	23	7	188	33.86	(13.33- 86.00)
JEDRYC	540	m	0	12	111	7	289	4.46	(1.71- 11.63)
MATOS	653	m	0	26	90	5	110	6.36	(2.35- 17.22)
WYNDE6	615	m	0	241	301	42	589	11.23	(7.87- 16.03)
WYNDE6	639	f	0	150	91	58	673	19.13	(13.16- 27.80)
Subtotal WYNDE6								14.46	(11.18- 18.72)
Totals				458	616	119	1849		

*prospective study

REF	NRR	SEX	AD	Ys	Ws	Qs	Ps
BARBON	733	m	0	3.52	4.42	3.68	0.0000
JEDRYC	540	m	0	1.50	4.19	5.20	0.0022
MATOS	653	m	0	1.85	3.87	2.24	0.0003
WYNDE6	615	m	0	2.42	30.32	1.11	0.0000
WYNDE6	639	f	0	2.95	27.49	3.20	0.0000
Subtotal WYNDE6				2.67	57.81	4.31	

	N	5
	NS	4
	Wt	70.29
Het	Chi	15.43
Het	df	4
Het	P	**
Fixed	RR	13.60
	RRl	10.76
	RRu	17.18
	P	+++
Random	RR	12.28
	RRl	7.15
	RRu	21.08
	P	+++
Asymm	P	N.S.

Table 3H4 - 6

IESLC - Meta-analysis of Ever Smoking, Age started, "High"
Adenocarcinoma, Any Product (or Cigarettes if Any not available)
Least adjusted

	Sex combined	male	female	Total
N		4	1	5
NS		4	1	4
Wt		42.80	27.49	70.29
Het Chi		10.17	0.00	15.43
Het df		3	0	4
Het P		*	N.S.	**
Fixed RR		10.92	19.13	13.60
RRl		8.09	13.16	10.76
RRu		14.74	27.80	17.18
P		+++	+++	+++
Random RR		10.40	19.13	12.28
RRl		5.11	13.16	7.15
RRu		21.19	27.80	21.08
P		+++	+++	+++
Between Chi				5.25
Between df				1
Between P				*
Btwn(F) P				N.S.
Btwn(R) P				N.S.

Table 3H4 - 7

IESLC - Meta-analysis of Ever Smoking, Age started, "High"
Adenocarcinoma, Any Product (or Cigarettes if Any not available)
Excluded studies (and stage at which they were excluded)

1	AKIBA AMANDU AMES BECHER BENSHL BEST BLOT1 BROSS BROWN3 CARPEN CEDERL CHYOU CPSI CPSII DARBY DEAN2 DEAN3 DOLL2 ENGELA GAO2 GARCIA GILLIS GRAHAM GURSEL HAMMO2 HIRAYA HOLE HUMBLE JAHN JAIN KAISE2 KATSOU KAUFMA LAUSSM LIAW MCDUFF MIGRAN MRFITR PEZZO2 PISANI PRESCO QIAO SEGI2 SPEIZE SVENSS TVERDA WAKAI WATSON WIGLE WU WYNDE3 WYNDE8
2	AXELSS BOUCHA BOUCOT CHEN DESTEF DORGAN DOSEME FAN GARSHI GER HAMMON JUSSAW KOO KREUZE LEVIN MCCONN NOTAN2 OSANN2 RESTRE SADOWS VUTUC WANG2 WU2 WUWILL WYNDE2 XU ZHOU
3	GUO SPITZ STASZE ZHANG
4	AGUDO ARMADA AUVINE BOFFET BRESLO BUFFLE CHEN2 CHIAZZ CHOI CORREA DAMBER DOLL DORN GAO GENG HU HU2 JOLY KOULUM LETOUR LIU3 LIU4 LIU5 LUBIN LUBIN2 LUO PERNU QIAO2 RACHTA SOBUE SUZUK2 TIZZAN WYNDE7 YUAN
5	ALDERS
10	HEGMAN KHUDER
14	BENHAM HAENSZ PEZZOT ZHENG

Table 3H4 - 8
Potentially overlapping studies

REF	REFGP	PRINC	.	OVERLAP/LINK
WYNDE6	WYNDE6	1		WYNDE5/6/7/8

Table 3H5 -

IESLC - Meta-analysis of Ever Smoking, Age started, "Highest vs lowest"
Adenocarcinoma, Any Product (or Cigarettes if Any not available)

This analysis is restricted to results for:
1) Ever smokers
2) Results by Age started
3) Categorical results by Age started
4) Denominator (unexposed) = "low"
5) Adenocarcinoma (or near equivalent)
6) Results complete enough for use in metaanalysis

Within each study, results are then selected (in the following order of preference, within each sex) for:
7) (not applicable)
8) PRODUCT: all/unspec, cigarettes regardless of other products, cigarettes only
9) CIGTYPE: all/unspecified, MC regardless of HR, MC only
10) Results with least adjustment for other aspects of smoking (ADOS)
11) The highest vs lowest category
12) Followup period (YF, prospective studies): whole study (coded as 0) or longest available
13) LCtype: adeno or nearest available, but not squamous. (q = squamous, s = small,
a = adeno, l = large, KII = Kreyberg II, al = alveolar, br = bronchiolar, u = undifferentiated)
14) Race: all or nearest available, otherwise by race (wh or w = white, bl or b = black, hi = hispanic
ch = chinese, jap = japanese, haw = hawaiian, w+o = white + oriental, sca = scandinavian, as = asian)
15) For overlapping studies: principal rather than subsidiary studies
Finally by Age: whole study (coded as 0) if available, otherwise by widest available age group
and then for single sex results (m, f) in preference to results for both sexes combined (c).

Results adjusted (AD) for the most potential confounders are then chosen in Sections -1 to -3
and results adjusted for the least confounders in Sections -4 to -6. (Those least adjusted results which
actually differ from the most adjusted are marked 'x' in column X in Section -4)

Section -7 shows excluded studies, together with the stage (as above) at which no qualifying
results were found.

Section -8 lists the potentially overlapping studies which have been included (1=principal, 2=subsidiary).

Section -9 lists any results which would have been included in preference except that they had data not complete
enough for use in meta-analysis, with their significance (yes/no), if known, and any further comment as entered
on the database. It also lists as "gap" any categories for which no data were presented by the original authors.

In addition to those mentioned above, the following fields, levels and abbreviations are used:
* or nk = not known, n = no, y = yes, ot = other
all/unspec = all or unspecified, cig+/-ot = cigarettes irrespective of other products (cigar, pipe etc)
MC = manufactured cigarettes, HR = hand-rolled cigarettes
exL, exH = range of exposure (low and high) in the "highest" group, in terms of Age started
unexL, unexH = range of exposure (low and high) in the "lowest" group, in terms of Age started
REF: 6-character study reference
NRR: number of the RR on the database within the study
ST : study type (CC = case control, pr or prosp = prospective)
NLC: number of lung cancer cases in whole study
R : risky occupational population (n = no, m = mining, o = other risky)
VB : national cigarette type (V = at least 75% Virginia, bl = at least 75% blended, ot = other)
P : any proxy use
H : full histological confirmation
De : derivation of RR/CI (or = original, st = standard method, ot = other method of estimation)

Table 3H5 - 1

IESLC - Meta-analysis of Ever Smoking, Age started, "Highest vs lowest"
Adenocarcinoma, Any Product (or Cigarettes if Any not available)
Most adjusted

REF	NRR	SEX	AGEL	AGEH	RACE	YF	LC TYPE	LOC	START	ST	NLC	R	VB	P	H	AD	ADOS	PRODUCT	exL	exH	unexL	unexH	De
BARBON	740	m	0	0	all	-	a	Eu:wst	1979	CC	755	n	bl	y	y	1	0	all/unsp	1	14	20	999	ot
BENHAM	523	m	0	0	all	-	KII	Eu:wst	1976	CC	1625	n	bl	n	y	0	0	cig only	1	19	20	999	st
HAENSZ	534	f	0	0	all	-	a	NAmer	1955	CC	158	n	bl	n	y	2	0	cig+/-ot	1	24	25	999	ot
HEGMAN	505	m	0	0	all	-	a	NAmer	1989	CC	282	n	bl	y	y	0	0	all/unsp	1	19	20	999	st
HEGMAN	510	f	0	0	all	-	a	NAmer	1989	CC	282	n	bl	y	y	0	0	all/unsp	1	25	26	999	ot
JEDRYC	542	m	0	0	all	-	a	Eu:est	1980	CC	1630	n	bl	y	n	0	0	cig+/-ot	1	16	19	999	st
KHUDER	528	m	0	0	all	-	a	NAmer	1985	CC	482	n	bl	n	y	5	3#	cig+/-ot	1	15	20	999	or
MATOS	660	m	0	0	all	-	a	SCAmer	1994	CC	200	n	bl	n	n	2	0	cig+/-ot	1	14	20	999	ot
PEZZOT	558	m	0	0	all	-	a	SCAmer	1987	CC	215	n	bl	n	y	2	0	cig only	1	13	19	999	ot
WYNDE6	617	m	0	0	wh	-	a	NAmer	1969	CC	4423	n	bl	n	y	0	0	cig+/-ot	1	17	21	999	st
WYNDE6	641	f	0	0	wh	-	a	NAmer	1969	CC	4423	n	bl	n	y	0	0	cig+/-ot	1	17	21	999	st
ZHENG	537	m	0	0	all	-	a	As:Chi	1982	CC	540	n	ot	*	y	1	0	cig+/-ot	1	19	30	999	ot
ZHENG	541	f	0	0	all	-	a	As:Chi	1982	CC	540	n	ot	*	y	1	0	cig+/-ot	1	29	30	999	ot

Comments on values in listings

KHUDER ADOS Duration of smoking (years), number of cigarettes per day and Quitted smoking

Cigarette type is all/unspec for all RRs

Table 3H5 - 2

IESLC - Meta-analysis of Ever Smoking, Age started, "Highest vs lowest"
Adenocarcinoma, Any Product (or Cigarettes if Any not available)
Most adjusted

REF	NRR	SEX	AD	Number Case	Exposed Cont	Non-exposed Case	Cont	RR	95.00%CI
BARBON	740	m	1	29	-	45	-	5.86	(3.10- 11.07)
BENHAM	523	m	0	69	79	41	64	1.36	(0.82- 2.27)
HAENSZ	534	f	2	6	-	12	-	0.88	(0.31- 2.53)
HEGMAN	505	m	0	45	716	6	289	3.03	(1.28- 7.17)
HEGMAN	510	f	0	32	169	0	28	10.93~	(0.65- 183.56)
Subtotal HEGMAN								3.38	(1.48- 7.71)
JEDRYC	542	m	0	12	111	48	502	1.13	(0.58- 2.20)
KHUDER	528	m	5	-	-	-	-	0.80	(0.40- 1.60)
MATOS	660	m	2	26	-	13	-	1.65	(0.80- 3.40)
PEZZOT	558	m	2	9	-	18	-	1.11	(0.41- 3.04)
WYNDE6	617	m	0	241	301	47	92	1.57	(1.06- 2.32)
WYNDE6	641	f	0	150	91	63	90	2.35	(1.56- 3.56)
Subtotal WYNDE6								1.90	(1.43- 2.52)
ZHENG	537	m	1	44	-	15	-	4.54	(2.25- 9.15)
ZHENG	541	f	1	26	-	7	-	2.00	(0.71- 5.64)
Subtotal ZHENG								3.51	(1.96- 6.27)
Partial Totals				689	1467	315	1065		

*prospective study ~ With 0.5 adjustment for zero

REF	NRR	SEX	AD	Ys	Ws	Qs	Ps
BARBON	740	m	1	1.77	9.48	12.26	0.0000
BENHAM	523	m	0	0.31	14.89	1.54	0.2317
HAENSZ	534	f	2	-0.13	3.49	2.01	0.8113
HEGMAN	505	m	0	1.11	5.16	1.17	0.0119
HEGMAN	510	f	0	2.39	0.48	1.50	0.0966
Subtotal HEGMAN				1.22	5.64	2.67	
JEDRYC	542	m	0	0.12	8.68	2.24	0.7175
KHUDER	528	m	5	-0.22	8.00	5.84	0.5281
MATOS	660	m	2	0.50	7.34	0.12	0.1749
PEZZOT	558	m	2	0.10	3.83	1.06	0.8382
WYNDE6	617	m	0	0.45	25.24	0.84	0.0240
WYNDE6	641	f	0	0.86	22.40	1.14	0.0001
Subtotal WYNDE6				0.64	47.64	1.97	
ZHENG	537	m	1	1.51	7.81	6.07	0.0000
ZHENG	541	f	1	0.69	3.58	0.01	0.1898
Subtotal ZHENG				1.26	11.39	6.08	

	N	13
	NS	10
	Wt	120.38
Het	Chi	35.80
Het	df	12
Het	P	***
Fixed	RR	1.88
	RRl	1.57
	RRu	2.25
	P	+++
Random	RR	1.89
	RRl	1.35
	RRu	2.64
	P	+++
Asymm	P	N.S.

Table 3H5 - 3

IESLC - Meta-analysis of Ever Smoking, Age started, "Highest vs lowest"
Adenocarcinoma, Any Product (or Cigarettes if Any not available)
Most adjusted

	Sex			
	combined	male	female	Total
N		9	4	13
NS		9	4	13
Wt		90.43	29.95	120.38
Het Chi		31.01	4.25	35.80
Het df		8	3	12
Het P		***	N.S.	***
Fixed RR		1.81	2.11	1.88
RRl		1.47	1.48	1.57
RRu		2.22	3.02	2.25
P		+++	+++	+++
Random RR		1.88	1.97	1.89
RRl		1.23	1.13	1.35
RRu		2.87	3.42	2.64
P		++	+	+++
Between Chi				0.54
Between df				1
Between P				N.S.
Btwn(F) P				N.S.
Btwn(R) P				N.S.

	Lung cancer type						
	a	a+l	a+al+br	KII	not q+u	not q+s	Total
N	12			1			13
NS	9			1			10
Wt	105.49			14.89			120.38
Het Chi	34.04			0.00			35.80
Het df	11			0			12
Het P	***			N.S.			***
Fixed RR	1.97			1.36			1.88
RRl	1.63			0.82			1.57
RRu	2.38			2.27			2.25
P	+++			N.S.			+++
Random RR	1.96			1.36			1.89
RRl	1.36			0.82			1.35
RRu	2.83			2.27			2.64
P	+++			N.S.			+++
Between Chi							1.75
Between df							1
Between P							N.S.
Btwn(F) P							N.S.
Btwn(R) P							N.S.

	Location								
	NAmer	UK	Scand	othEur	China	Japan	othAs	other	Total
N	6			3	2			2	13
NS	4			3	1			2	10
Wt	64.77			33.06	11.39			11.17	120.38
Het Chi	11.98			15.96	1.65			0.40	35.80
Het df	5			2	1			1	12
Het P	*			***	N.S.			N.S.	***
Fixed RR	1.72			1.97	3.51			1.44	1.88
RRl	1.35			1.40	1.96			0.80	1.57
RRu	2.20			2.77	6.27			2.59	2.25
P	+++			+++	+++			N.S.	+++
Random RR	1.66			2.07	3.30			1.44	1.89
RRl	1.06			0.78	1.51			0.80	1.35
RRu	2.59			5.52	7.23			2.59	2.64
P	+			N.S.	++			N.S.	+++
Between Chi									5.81
Between df									3
Between P									N.S.
Btwn(F) P									N.S.
Btwn(R) P									N.S.

Table 3H5 - 3

IESLC - Meta-analysis of Ever Smoking, Age started, "Highest vs lowest"
Adenocarcinoma, Any Product (or Cigarettes if Any not available)
Most adjusted

Detailed Country in "other Europe"

	multi	Germany	othWest	East	Balkans	Total
N			2	1		3
NS			2	1		3
Wt			24.37	8.68		33.06
Het Chi			12.32	0.00		15.96
Het df			1	0		2
Het P			***	N.S.		***
Fixed RR			2.40	1.13		1.97
RRl			1.62	0.58		1.40
RRu			3.58	2.20		2.77
P			+++	N.S.		+++
Random RR			2.79	1.13		2.07
RRl			0.67	0.58		0.78
RRu			11.64	2.20		5.52
P			N.S.	N.S.		N.S.
Between Chi						3.65
Between df						1
Between P						(*)
Btwn(F) P						N.S.
Btwn(R) P						N.S.

Detailed Country in "other Asia"

	India	HongKong	other	Total
N				
NS				
Wt				
Het Chi				
Het df				
Het P				N.S.
Fixed RR				
RRl				
RRu				
P				N.S.
Random RR				
RRl				
RRu				
P				N.S.
Between Chi				
Between df				
Between P				N.S.
Btwn(F) P				N.S.
Btwn(R) P				N.S.

Detailed other continent

	SCAmer	Total
N	2	2
NS	2	2
Wt	11.17	11.17
Het Chi	0.40	0.40
Het df	1	1
Het P	N.S.	N.S.
Fixed RR	1.44	1.44
RRl	0.80	0.80
RRu	2.59	2.59
P	N.S.	N.S.
Random RR	1.44	1.44
RRl	0.80	0.80
RRu	2.59	2.59
P	N.S.	N.S.
Between Chi		
Between df		
Between P		N.S.
Btwn(F) P		N.S.
Btwn(R) P		N.S.

Table 3H5 - 3

IESLC - Meta-analysis of Ever Smoking, Age started, "Highest vs lowest"
Adenocarcinoma, Any Product (or Cigarettes if Any not available)
Most adjusted

	Start year of study					
	<1960	1960-69	1970-79	1980-89	1990+	Total
N	1	2	2	7	1	13
NS	1	1	2	5	1	10
Wt	3.49	47.64	24.37	37.54	7.34	120.38
Het Chi	0.00	1.97	12.32	17.68	0.00	35.80
Het df	0	1	1	6	0	12
Het P	N.S.	N.S.	***	**	N.S.	***
Fixed RR	0.88	1.90	2.40	1.74	1.65	1.88
RRl	0.31	1.43	1.62	1.27	0.80	1.57
RRu	2.51	2.52	3.58	2.40	3.40	2.25
P	N.S.	+++	+++	+++	N.S.	+++
Random RR	0.88	1.91	2.79	1.85	1.65	1.89
RRl	0.31	1.28	0.67	1.03	0.80	1.35
RRu	2.51	2.84	11.64	3.33	3.40	2.64
P	N.S.	++	N.S.	+	N.S.	+++
Between Chi						3.83
Between df						4
Between P						N.S.
Btwn(F) P						N.S.
Btwn(R) P						N.S.

	Study type (1)		
	CC	other	Total
N	13		13
NS	10		10
Wt	120.38		120.38
Het Chi	35.80		35.80
Het df	12		12
Het P	***		***
Fixed RR	1.88		1.88
RRl	1.57		1.57
RRu	2.25		2.25
P	+++		+++
Random RR	1.89		1.89
RRl	1.35		1.35
RRu	2.64		2.64
P	+++		+++
Between Chi			
Between df			
Between P			N.S.
Btwn(F) P			N.S.
Btwn(R) P			N.S.

	Study type (2)			
	CC	prosp	other	Total
N	13			13
NS	10			10
Wt	120.38			120.38
Het Chi	35.80			35.80
Het df	12			12
Het P	***			***
Fixed RR	1.88			1.88
RRl	1.57			1.57
RRu	2.25			2.25
P	+++			+++
Random RR	1.89			1.89
RRl	1.35			1.35
RRu	2.64			2.64
P	+++			+++
Between Chi				
Between df				
Between P				N.S.
Btwn(F) P				N.S.
Btwn(R) P				N.S.

Table 3H5 - 3

IESLC - Meta-analysis of Ever Smoking, Age started, "Highest vs lowest"
Adenocarcinoma, Any Product (or Cigarettes if Any not available)
Most adjusted

	Study size (number of LC cases) 100-249	250-499	500-999	1000+	Total
N	3	3	3	4	13
NS	3	2	2	3	10
Wt	14.65	13.64	20.87	71.21	120.38
Het Chi	1.04	7.59	3.01	4.68	35.80
Het df	2	2	2	3	12
Het P	N.S.	*	N.S.	N.S.	***
Fixed RR	1.28	1.45	4.43	1.66	1.88
RRl	0.77	0.85	2.88	1.32	1.57
RRu	2.14	2.47	6.80	2.10	2.25
P	N.S.	N.S.	+++	+++	+++
Random RR	1.28	2.02	4.24	1.63	1.89
RRl	0.77	0.57	2.47	1.21	1.35
RRu	2.14	7.08	7.28	2.19	2.64
P	N.S.	N.S.	+++	++	+++
Between Chi					19.47
Between df					3
Between P					***
Btwn(F) P					(*)
Btwn(R) P					**

	Risky occupational population no	mining	othRisky	Total
N	13			13
NS	10			10
Wt	120.38			120.38
Het Chi	35.80			35.80
Het df	12			12
Het P	***			***
Fixed RR	1.88			1.88
RRl	1.57			1.57
RRu	2.25			2.25
P	+++			+++
Random RR	1.89			1.89
RRl	1.35			1.35
RRu	2.64			2.64
P	+++			+++
Between Chi				
Between df				
Between P				N.S.
Btwn(F) P				N.S.
Btwn(R) P				N.S.

	National cigarette tobacco type Virginia	blended	other	Total
N		11	2	13
NS		9	1	10
Wt		108.99	11.39	120.38
Het Chi		29.25	1.65	35.80
Het df		10	1	12
Het P		**	N.S.	***
Fixed RR		1.76	3.51	1.88
RRl		1.46	1.96	1.57
RRu		2.13	6.27	2.25
P		+++	+++	+++
Random RR		1.73	3.30	1.89
RRl		1.22	1.51	1.35
RRu		2.46	7.23	2.64
P		++	++	+++
Between Chi				4.90
Between df				1
Between P				*
Btwn(F) P				N.S.
Btwn(R) P				N.S.

Table 3H5 - 3

IESLC - Meta-analysis of Ever Smoking, Age started, "Highest vs lowest"
Adenocarcinoma, Any Product (or Cigarettes if Any not available)
Most adjusted

	Any proxy use No/nk	Yes	Total
N	9	4	13
NS	7	3	10
Wt	96.57	23.81	120.38
Het Chi	17.66	13.24	35.80
Het df	8	3	12
Het P	*	**	***
Fixed RR	1.70	2.82	1.88
RRl	1.39	1.89	1.57
RRu	2.08	4.22	2.25
P	+++	+++	+++
Random RR	1.64	3.07	1.89
RRl	1.19	1.18	1.35
RRu	2.27	8.01	2.64
P	++	+	+++
Between Chi			4.90
Between df			1
Between P			*
Btwn(F) P			N.S.
Btwn(R) P			N.S.

	Full histological confirmation No	Yes	Total
N	2	11	13
NS	2	8	10
Wt	16.02	104.36	120.38
Het Chi	0.57	33.15	35.80
Het df	1	10	12
Het P	N.S.	***	***
Fixed RR	1.34	1.98	1.88
RRl	0.82	1.63	1.57
RRu	2.19	2.40	2.25
P	N.S.	+++	+++
Random RR	1.34	2.02	1.89
RRl	0.82	1.37	1.35
RRu	2.19	2.97	2.64
P	N.S.	+++	+++
Between Chi			2.08
Between df			1
Between P			N.S.
Btwn(F) P			N.S.
Btwn(R) P			N.S.

	Number of adjustment variables (1) 0	1	2+/+nk	Total
N	6	3	4	13
NS	4	2	4	10
Wt	76.86	20.87	22.65	120.38
Het Chi	8.04	3.01	2.19	35.80
Het df	5	2	3	12
Het P	N.S.	N.S.	N.S.	***
Fixed RR	1.75	4.43	1.08	1.88
RRl	1.40	2.88	0.72	1.57
RRu	2.19	6.80	1.64	2.25
P	+++	+++	N.S.	+++
Random RR	1.76	4.24	1.08	1.89
RRl	1.29	2.47	0.72	1.35
RRu	2.40	7.28	1.64	2.64
P	+++	+++	N.S.	+++
Between Chi				22.56
Between df				2
Between P				***
Btwn(F) P				**
Btwn(R) P				***

Table 3H5 - 3

IESLC - Meta-analysis of Ever Smoking, Age started, "Highest vs lowest"
Adenocarcinoma, Any Product (or Cigarettes if Any not available)
Most adjusted

	Number of adjustment variables (2) 0	1	2	3-5	6+/+nk	Total
N	6	3	3	1		13
NS	4	2	3	1		10
Wt	76.86	20.87	14.65	8.00		120.38
Het Chi	8.04	3.01	1.04	0.00		35.80
Het df	5	2	2	0		12
Het P	N.S.	N.S.	N.S.	N.S.		***
Fixed RR	1.75	4.43	1.28	0.80		1.88
RRl	1.40	2.88	0.77	0.40		1.57
RRu	2.19	6.80	2.14	1.60		2.25
P	+++	+++	N.S.	N.S.		+++
Random RR	1.76	4.24	1.28	0.80		1.89
RRl	1.29	2.47	0.77	0.40		1.35
RRu	2.40	7.28	2.14	1.60		2.64
P	+++	+++	N.S.	N.S.		+++
Between Chi						23.71
Between df						3
Between P						***
Btwn(F) P						*
Btwn(R) P						***

	Product all/unsp	cig+/-ot	cig only	Total
N	3	8	2	13
NS	2	6	2	10
Wt	15.13	86.53	18.72	120.38
Het Chi	1.80	17.66	0.13	35.80
Het df	2	7	1	12
Het P	N.S.	*	N.S.	***
Fixed RR	4.77	1.73	1.31	1.88
RRl	2.88	1.40	0.83	1.57
RRu	7.90	2.13	2.06	2.25
P	+++	+++	N.S.	+++
Random RR	4.77	1.66	1.31	1.89
RRl	2.88	1.15	0.83	1.35
RRu	7.90	2.38	2.06	2.64
P	+++	++	N.S.	+++
Between Chi				16.21
Between df				2
Between P				***
Btwn(F) P				*
Btwn(R) P				***

	Derivation of RR/CI Orig	StdCalc	Other	Total
N	1	5	7	13
NS	1	4	6	11
Wt	8.00	76.38	36.01	120.38
Het Chi	0.00	6.41	18.26	35.80
Het df	0	4	6	12
Het P	N.S.	N.S.	**	***
Fixed RR	0.80	1.73	2.71	1.88
RRl	0.40	1.38	1.95	1.57
RRu	1.60	2.17	3.75	2.25
P	N.S.	+++	+++	+++
Random RR	0.80	1.72	2.40	1.89
RRl	0.40	1.28	1.30	1.35
RRu	1.60	2.32	4.42	2.64
P	N.S.	+++	++	+++
Between Chi				11.13
Between df				2
Between P				**
Btwn(F) P				N.S.
Btwn(R) P				(*)

Table 3H5 - 4

IESLC - Meta-analysis of Ever Smoking, Age started, "Highest vs lowest"
Adenocarcinoma, Any Product (or Cigarettes if Any not available)
Least adjusted

REF	NRR	X	SEX	AGEL	AGEH	RACE	YF	LC TYPE	LOC	START	ST	NLC	R	VB	P	H	AD	ADOS	PRODUCT	exL	exH	unexL	unexH	De
BARBON	735	x	m	0	0	all	-	a	Eu:wst	1979	CC	755	n	bl	y	y	0	0	all/unsp	1	14	20	999	st
BENHAM	523		m	0	0	all	-	KII	Eu:wst	1976	CC	1625	n	bl	n	y	0	0	cig only	1	19	20	999	st
HAENSZ	513	x	f	0	0	all	-	a	NAmer	1955	CC	158	n	bl	n	y	0	0	cig+/-ot	1	24	25	999	st
HEGMAN	505		m	0	0	all	-	a	NAmer	1989	CC	282	n	bl	y	y	0	0	all/unsp	1	19	20	999	st
HEGMAN	510		f	0	0	all	-	a	NAmer	1989	CC	282	n	bl	y	y	0	0	all/unsp	1	25	26	999	ot
JEDRYC	542		m	0	0	all	-	a	Eu:est	1980	CC	1630	n	bl	y	n	0	0	cig+/-ot	1	16	19	999	st
KHUDER	528		m	0	0	all	-	a	NAmer	1985	CC	482	n	bl	n	y	5	3#	cig+/-ot	1	15	20	999	or
MATOS	655	x	m	0	0	all	-	a	SCAmer	1994	CC	200	n	bl	n	n	0	0	cig+/-ot	1	14	20	999	st
PEZZOT	556	x	m	0	0	all	-	a	SCAmer	1987	CC	215	n	bl	n	y	0	0	cig only	1	13	19	999	st
WYNDE6	617		m	0	0	wh	-	a	NAmer	1969	CC	4423	n	bl	n	y	0	0	cig+/-ot	1	17	21	999	st
WYNDE6	641		f	0	0	wh	-	a	NAmer	1969	CC	4423	n	bl	n	y	0	0	cig+/-ot	1	17	21	999	st
ZHENG	535	x	m	0	0	all	-	a	As:Chi	1982	CC	540	n	ot	*	y	0	0	cig+/-ot	1	19	30	999	st
ZHENG	540	x	f	0	0	all	-	a	As:Chi	1982	CC	540	n	ot	*	y	0	0	cig+/-ot	1	29	30	999	st

Comments on values in listings

KHUDER ADOS Duration of smoking (years), number of cigarettes per day and Quitted smoking

Cigarette type is all/unspec for all RRs

Table 3H5 - 5

IESLC - Meta-analysis of Ever Smoking, Age started, "Highest vs lowest"
Adenocarcinoma, Any Product (or Cigarettes if Any not available)
Least adjusted

REF	NRR	SEX	AD	Number Exposed Case	Number Exposed Cont	Non-exposed Case	Non-exposed Cont	RR	95.00%CI
BARBON	735	m	0	29	23	45	207	5.80	(3.07- 10.95)
BENHAM	523	m	0	69	79	41	64	1.36	(0.82- 2.27)
HAENSZ	513	f	0	6	37	12	66	0.89	(0.31- 2.57)
HEGMAN	505	m	0	45	716	6	289	3.03	(1.28- 7.17)
HEGMAN	510	f	0	32	169	0	28	10.93~	(0.65- 183.56)
Subtotal HEGMAN								3.38	(1.48- 7.71)
JEDRYC	542	m	0	12	111	48	502	1.13	(0.58- 2.20)
KHUDER	528	m	5	-	-	-	-	0.80	(0.40- 1.60)
MATOS	655	m	0	26	90	13	73	1.62	(0.78- 3.38)
PEZZOT	556	m	0	9	15	18	32	1.07	(0.39- 2.92)
WYNDE6	617	m	0	241	301	47	92	1.57	(1.06- 2.32)
WYNDE6	641	f	0	150	91	63	90	2.35	(1.56- 3.56)
Subtotal WYNDE6								1.90	(1.43- 2.52)
ZHENG	535	m	0	44	43	15	66	4.50	(2.23- 9.07)
ZHENG	540	f	0	26	28	7	16	2.12	(0.75- 5.98)
Subtotal ZHENG								3.56	(1.99- 6.35)
Partial Totals				689	1703	315	1525		

*prospective study ~ With 0.5 adjustment for zero

REF	NRR	SEX	AD	Ys	Ws	Qs	Ps
BARBON	735	m	0	1.76	9.52	12.09	0.0000
BENHAM	523	m	0	0.31	14.89	1.54	0.2317
HAENSZ	513	f	0	-0.11	3.42	1.90	0.8324
HEGMAN	505	m	0	1.11	5.16	1.17	0.0119
HEGMAN	510	f	0	2.39	0.48	1.50	0.0966
Subtotal HEGMAN				1.22	5.64	2.67	
JEDRYC	542	m	0	0.12	8.68	2.24	0.7175
KHUDER	528	m	5	-0.22	8.00	5.83	0.5281
MATOS	655	m	0	0.48	7.13	0.15	0.1963
PEZZOT	556	m	0	0.06	3.78	1.21	0.9002
WYNDE6	617	m	0	0.45	25.24	0.83	0.0240
WYNDE6	641	f	0	0.86	22.40	1.14	0.0001
Subtotal WYNDE6				0.64	47.64	1.97	
ZHENG	535	m	0	1.50	7.82	5.97	0.0000
ZHENG	540	f	0	0.75	3.58	0.05	0.1546
Subtotal ZHENG				1.27	11.40	6.02	

	N	13
	NS	10
	Wt	120.11
Het	Chi	35.64
Het	df	12
Het	P	***
Fixed	RR	1.88
	RRl	1.57
	RRu	2.25
	P	+++
Random	RR	1.89
	RRl	1.35
	RRu	2.64
	P	+++
Asymm	P	N.S.

Table 3H5 - 6

IESLC - Meta-analysis of Ever Smoking, Age started, "Highest vs lowest"
Adenocarcinoma, Any Product (or Cigarettes if Any not available)
Least adjusted

Sex combined	male	female	Total
N	9	4	13
NS	9	4	13
Wt	90.23	29.88	120.11
Het Chi	30.89	4.11	35.64
Het df	8	3	12
Het P	***	N.S.	***
Fixed RR	1.80	2.13	1.88
RRl	1.47	1.49	1.57
RRu	2.22	3.05	2.25
P	+++	+++	+++
Random RR	1.86	2.01	1.89
RRl	1.22	1.17	1.35
RRu	2.85	3.44	2.64
P	++	+	+++
Between Chi			0.64
Between df			1
Between P			N.S.
Btwn(F) P			N.S.
Btwn(R) P			N.S.

Table 3H5 - 7

IESLC - Meta-analysis of Ever Smoking, Age started, "Highest vs lowest"
Adenocarcinoma, Any Product (or Cigarettes if Any not available)
Excluded studies (and stage at which they were excluded)

1 AKIBA AMANDU AMES BECHER BENSHL BEST BLOT1 BROSS BROWN3 CARPEN CEDERL CHYOU CPSI CPSII DARBY DEAN2 DEAN3 DOLL2 ENGELA GAO2 GARCIA GILLIS GRAHAM GURSEL HAMMO2 HIRAYA HOLE HUMBLE JAHN JAIN KAISE2 KATSOU KAUFMA LAUSSM LIAW MCDUFF MIGRAN MRFITR PEZZO2 PISANI PRESCO QIAO SEGI2 SPEIZE SVENSS TVERDA WAKAI WATSON WIGLE WU WYNDE3 WYNDE8

2 AXELSS BOUCHA BOUCOT CHEN DESTEF DORGAN DOSEME FAN GARSHI GER HAMMON JUSSAW KOO KREUZE LEVIN MCCONN NOTAN2 OSANN2 RESTRE SADOWS VUTUC WANG2 WU2 WUWILL WYNDE2 XU ZHOU

3 GUO SPITZ STASZE ZHANG

5 AGUDO ARMADA AUVINE BOFFET BRESLO BUFFLE CHEN2 CHIAZZ CHOI CORREA DAMBER DOLL DORN GAO GENG HU HU2 JOLY KOULUM LETOUR LIU3 LIU4 LIU5 LUBIN LUBIN2 LUO PERNU QIAO2 RACHTA SOBUE SUZUK2 TIZZAN WYNDE7 YUAN

6 ALDERS

Table 3H5 - 8
Potentially overlapping studies

REF	REFGP	PRINC	.	OVERLAP/LINK
BENHAM	LUBIN2	2		Subset of Lubin2
WYNDE6	WYNDE6	1		WYNDE5/6/7/8

Table 3H5 - 9
Most adjusted - insufficient data for meta-analysis

REF	NRR	SEX	AGEL	AGEH	RACE	YF	LC TYPE	LOC	START	ST	NLC	R	VB	P	H	AD	ADOS	PRODUCT	exL	exH	unexL	unexH	De
ALDERS	563	m	0	0	all	-	not q+s	Eu:UK	1977	CC	1448	n		V	n	n	2	1#cig only	1	14	25	999	ot
ALDERS	566	f	0	0	all	-	not q+s	Eu:UK	1977	CC	1448	n		V	n	n	2	1#cig only	1	14	25	999	ot

Comments on values in listings

ALDERS ADOS Number of cigs/day
ALDERS ADOS Number of cigs/day

REF	NRR	RR	SIG	RRDATA comment
ALDERS	563	0.53	n	0
ALDERS	566	1.92	n	0

Table 3H6 -

IESLC - Meta-analysis of Current Smoking by Age started, Overview
Adenocarcinoma, Any Product (or Cigarettes if Any not available)

This analysis is restricted to results for:
1) Current smokers
2) Results by Age started
3) Categorical results by Age started
Results by Age started are grouped under 2 schemes (S1, S2). Each scheme has a set of "key values". An interval is allocated to the category whose key value it includes, and intervals which include none or more than one of the key values are excluded. (Open-ended intervals are coded as 999)

S1	key value	maximum range
1	26	19+
2	18	15-25
3	14	1-17

S2	key value	maximum range
1	30	27+
2	26	23-29
3	22	19-25
4	18	15-21
5	14	11-17
6	10	1-13

4) Adenocarcinoma (or near equivalent)
5) Results complete enough for use in metaanalysis

Within each study, results are then selected (in the following order of preference, within each sex) for:
6) (not applicable)
7) PRODUCT: all/unspec, cigarettes regardless of other products, cigarettes only
8) CIGTYPE: all/unspecified, MC regardless of HR, MC only
9) (not applicable)
10) DENOM: never smoked anything, never smoked cigarettes, never any + low, never cigs + low
11) Followup period (YF, prospective studies): whole study (coded as 0) or longest available
12) LCtype: adeno or nearest available, but not squamous. (q = squamous, s = small,
a = adeno, l = large, KII = Kreyberg II, al = alveolar, br = bronchiolar, u = undifferentiated)
13) Race: all or nearest available, otherwise by race (wh or w = white, bl or b = black, hi = hispanic
ch = chinese, jap = japanese, haw = hawaiian, w+o = white + oriental, sca = scandinavian, as = asian)
14) For overlapping studies: principal rather than subsidiary studies
Finally by Age: whole study (coded as 0) if available, otherwise by widest available age group
and then for single sex results (m, f) in preference to results for both sexes combined (c).

Results adjusted (AD) for the most potential confounders are then chosen in Sections -1 to -3
and results adjusted for the least confounders in Sections -4 to -6. (Those least adjusted results which actually differ from the most adjusted are marked 'x' in column X in Section -4)

Section -7 shows excluded studies, together with the stage (as above) at which no qualifying results were found.

Section -8 lists the potentially overlapping studies which have been included (1=principal, 2=subsidiary).

Section -9 lists any results which would have been included in preference except that they had data not complete enough for use in meta-analysis, with their significance (yes/no), if known, and any further comment as entered on the database. It also lists as "gap" any categories for which no data were presented by the original authors.

In addition to those mentioned above, the following fields, levels and abbreviations are used:
* or nk = not known, n = no, y = yes, ot = other
nev = never
all/unspec = all or unspecified, cig+/-ot = cigarettes irrespective of other products (cigar, pipe etc)
MC = manufactured cigarettes, HR = hand-rolled cigarettes
exL, exH = range of exposure (low and high) in the smoking group, in terms of Age started
REF: 6-character study reference
NRR: number of the RR on the database within the study
ST : study type (CC = case control, pr or prosp = prospective)
NLC: number of lung cancer cases in whole study
R : risky occupational population (n = no, m = mining, o = other risky)
VB : national cigarette type (V = at least 75% Virginia, bl = at least 75% blended, ot = other)
P : any proxy use
H : full histological confirmation
De : derivation of RR/CI (or = original, st = standard method, ot = other method of estimation)

Table 3H6 - 1

IESLC - Meta-analysis of Current Smoking by Age started, Overview
Adenocarcinoma, Any Product (or Cigarettes if Any not available)
Most adjusted

REF	NRR	SEX	AGEL	AGEH	RACE	YF	LC TYPE	LOC	START	ST	NLC	R	VB	P	H	AD	PRODUCT	exL	exH	S1	S2	DENOM	De
ENGELA	527	m	0	0	all	0	a	Eu:Sca	1964	pr	435	n	bl	n	n	0	cig+/-ot	20	999	1	0	nev cigs	st
ENGELA	528	m	0	0	all	0	a	Eu:Sca	1964	pr	435	n	bl	n	n	0	cig+/-ot	1	19	0	0	nev cigs	st
SVENSS	525	f	0	0	all	-	a	Eu:Sca	1983	CC	210	n	bl	n	n	0	all/unsp	26	999	1	0	nev any	st
SVENSS	526	f	0	0	all	-	a	Eu:Sca	1983	CC	210	n	bl	n	n	0	all/unsp	19	25	0	3	nev any	st
SVENSS	527	f	0	0	all	-	a	Eu:Sca	1983	CC	210	n	bl	n	n	0	all/unsp	0	18	0	0	nev any	st
WAKAI	511	m	0	0	all	-	a	As:Jap	1988	CC	333	n	bl	n	y	0	cig+/-ot	30	999	0	1	nev any	st
WAKAI	512	m	0	0	all	-	a	As:Jap	1988	CC	333	n	bl	n	y	0	cig+/-ot	20	29	1	0	nev any	st
WAKAI	513	m	0	0	all	-	a	As:Jap	1988	CC	333	n	bl	n	y	0	cig+/-ot	1	19	0	0	nev any	st
WU	525	f	0	0	wh	-	a	NAmer	1981	CC	220	n	bl	n	y	2	all/unsp	25	999	1	0	nev any	or
WU	526	f	0	0	wh	-	a	NAmer	1981	CC	220	n	bl	n	y	2	all/unsp	19	24	0	3	nev any	or
WU	527	f	0	0	wh	-	a	NAmer	1981	CC	220	n	bl	n	y	2	all/unsp	0	18	0	0	nev any	or

Cigarette type is all/unspec for all RRs

In this overview table, subtotals and Qs values may be invalid and should be ignored

Table 3H6 - 2

IESLC - Meta-analysis of Current Smoking by Age started, Overview
Adenocarcinoma, Any Product (or Cigarettes if Any not available)
Most adjusted

REF	NRR	SEX	AD	Number Exposed Case	Cont	Non-exposed Case	Cont	RR	95.00%CI
*ENGELA	527	m	0	8	39957	7	58716	1.68	(0.61- 4.63)
*ENGELA	528	m	0	25	50732	7	58716	4.13	(1.79- 9.56)
Subtotal ENGELA								2.87	(1.50- 5.47)
SVENSS	525	f	0	10	18	22	120	3.03	(1.24- 7.43)
SVENSS	526	f	0	11	14	22	120	4.29	(1.72- 10.66)
SVENSS	527	f	0	17	21	22	120	4.42	(2.01- 9.68)
Subtotal SVENSS								3.90	(2.38- 6.40)
WAKAI	511	m	0	4	25	8	65	1.30	(0.36- 4.70)
WAKAI	512	m	0	52	183	8	65	2.31	(1.04- 5.12)
WAKAI	513	m	0	18	74	8	65	1.98	(0.81- 4.85)
Subtotal WAKAI								1.97	(1.15- 3.38)
WU	525	f	2	8	-	29	-	1.10	(0.40- 3.20)
WU	526	f	2	22	-	29	-	2.50	(1.00- 5.80)
WU	527	f	2	69	-	29	-	8.00	(3.60- 17.90)
Subtotal WU								3.30	(1.97- 5.52)
Partial Totals				244	91024	191	117987		

*prospective study

REF	NRR	SEX	AD	Ys	Ws	Qs	Ps
*ENGELA	527	m	0	0.52	3.73	1.22	0.3164
*ENGELA	528	m	0	1.42	5.47	0.59	0.0009
Subtotal ENGELA				1.05	9.20	1.81	
SVENSS	525	f	0	1.11	4.78	0.00	0.0154
SVENSS	526	f	0	1.46	4.63	0.62	0.0017
SVENSS	527	f	0	1.49	6.24	0.97	0.0002
Subtotal SVENSS				1.36	15.64	1.59	
WAKAI	511	m	0	0.26	2.32	1.59	0.6892
WAKAI	512	m	0	0.84	6.06	0.39	0.0395
WAKAI	513	m	0	0.68	4.77	0.80	0.1366
Subtotal WAKAI				0.68	13.16	2.78	
WU	525	f	2	0.10	3.55	3.52	0.8574
WU	526	f	2	0.92	4.97	0.15	0.0410
WU	527	f	2	2.08	5.97	5.84	0.0000
Subtotal WU				1.19	14.50	9.51	

N	11
NS	4

Table 3H6 - 3

IESLC - Meta-analysis of Current Smoking by Age started, Overview
Adenocarcinoma, Any Product (or Cigarettes if Any not available)
Most adjusted

	Sex			
	combined	male	female	Total
N		5	6	11
NS		2	2	4

In this overview table, other than the "N" rows, entries in the "absent" and "Total" columns may be invalid and should be ignored

	Age started (broad categories)				
	absent	19+k26	15-25k18	1-17k14	Total
N	7	4			11
NS	4	4			8
Wt	34.38	18.12			52.50
Het Chi	9.09	2.33			15.70
Het df	6	3			10
Het P	N.S.	N.S.			N.S.
Fixed RR	3.66	2.01			2.98
RRl	2.62	1.27			2.27
RRu	5.11	3.18			3.90
P	+++	++			+++
Random RR	3.56	2.01			2.88
RRl	2.35	1.27			2.05
RRu	5.39	3.18			4.06
P	+++	++			+++

		Age started (narrow categories)						
	absent	27+k30	23-29k26	19-25k22	15-21k18	11-17k14	1-13k10	Total
N	8	1		2				11
NS	4	1		2				7
Wt	40.58	2.32		9.60				52.50
Het Chi	13.31	0.00		0.70				15.70
Het df	7	0		1				10
Het P	(*)	N.S.		N.S.				N.S.
Fixed RR	3.06	1.30		3.24				2.98
RRl	2.25	0.36		1.72				2.27
RRu	4.16	4.70		6.10				3.90
P	+++	N.S.		+++				+++
Random RR	2.94	1.30		3.24				2.88
RRl	1.92	0.36		1.72				2.05
RRu	4.50	4.70		6.10				4.06
P	+++	N.S.		+++				+++

Table 3H6 - 3

IESLC - Meta-analysis of Current Smoking by Age started, Overview
Adenocarcinoma, Any Product (or Cigarettes if Any not available)
Most adjusted

MALES

		Age started (broad categories)				
		absent	19+k26	15-25k18	1-17k14	Total
	N	3	2			5
	NS	2	2			4
	Wt	12.57	9.79			22.36
Het	Chi	2.64	0.23			3.12
Het	df	2	1			4
Het	P	N.S.	N.S.			N.S.
Fixed	RR	2.52	2.04			2.30
	RRl	1.45	1.09			1.52
	RRu	4.38	3.83			3.48
	P	++	+			+++
Random	RR	2.45	2.04			2.30
	RRl	1.28	1.09			1.52
	RRu	4.68	3.83			3.48
	P	++	+			+++

				Age started (narrow categories)					
		absent	27+k30	23-29k26	19-25k22	15-21k18	11-17k14	1-13k10	Total
	N	4	1						5
	NS	2	1						2
	Wt	20.04	2.32						22.36
Het	Chi	2.27	0.00						3.12
Het	df	3	0						4
Het	P	N.S.	N.S.						N.S.
Fixed	RR	2.46	1.30						2.30
	RRl	1.59	0.36						1.52
	RRu	3.81	4.70						3.48
	P	+++	N.S.						+++
Random	RR	2.46	1.30						2.30
	RRl	1.59	0.36						1.52
	RRu	3.81	4.70						3.48
	P	+++	N.S.						+++

FEMALES

		Age started (broad categories)				
		absent	19+k26	15-25k18	1-17k14	Total
	N	4	2			6
	NS	2	2			4
	Wt	21.81	8.33			30.14
Het	Chi	3.71	2.09			10.01
Het	df	3	1			5
Het	P	N.S.	N.S.			(*)
Fixed	RR	4.54	1.97			3.60
	RRl	2.98	1.00			2.52
	RRu	6.90	3.88			5.14
	P	+++	(+)			+++
Random	RR	4.51	1.89			3.45
	RRl	2.83	0.70			2.08
	RRu	7.20	5.09			5.73
	P	+++	N.S.			+++

Table 3H6 - 3

IESLC - Meta-analysis of Current Smoking by Age started, Overview
Adenocarcinoma, Any Product (or Cigarettes if Any not available)
Most adjusted

FEMALES

		absent	27+k30	23-29k26	19-25k22	15-21k18	11-17k14	1-13k10	Total
			Age started (narrow categories)						
	N	4			2				6
	NS	2			2				4
	Wt	20.54			9.60				30.14
Het	Chi	9.16			0.70				10.01
Het	df	3			1				5
Het	P	*			N.S.				(*)
Fixed	RR	3.78			3.24				3.60
	RRl	2.45			1.72				2.52
	RRu	5.83			6.10				5.14
	P	+++			+++				+++
Random	RR	3.47			3.24				3.45
	RRl	1.62			1.72				2.08
	RRu	7.43			6.10				5.73
	P	++			+++				+++

Table 3H6 - 4

IESLC - Meta-analysis of Current Smoking by Age started, Overview
Adenocarcinoma, Any Product (or Cigarettes if Any not available)
Least adjusted

REF	NRR	X	SEX	AGEL	AGEH	RACE	YF	LC TYPE	LOC	START	ST	NLC	R	VB	P	H	AD	PRODUCT	exL	exH	S1	S2	DENOM	De
ENGELA	527		m	0	0	all	0	a	Eu:Sca	1964	pr	435	n	bl	n	n	0	cig+/-ot	20	999	1	0	nev cigs	st
ENGELA	528		m	0	0	all	0	a	Eu:Sca	1964	pr	435	n	bl	n	n	0	cig+/-ot	1	19	0	0	nev cigs	st
SVENSS	525		f	0	0	all	-	a	Eu:Sca	1983	CC	210	n	bl	n	n	0	all/unsp	26	999	1	0	nev any	st
SVENSS	526		f	0	0	all	-	a	Eu:Sca	1983	CC	210	n	bl	n	n	0	all/unsp	19	25	0	3	nev any	st
SVENSS	527		f	0	0	all	-	a	Eu:Sca	1983	CC	210	n	bl	n	n	0	all/unsp	0	18	0	0	nev any	st
WAKAI	511		m	0	0	all	-	a	As:Jap	1988	CC	333	n	bl	n	y	0	cig+/-ot	30	999	0	1	nev any	st
WAKAI	512		m	0	0	all	-	a	As:Jap	1988	CC	333	n	bl	n	y	0	cig+/-ot	20	29	1	0	nev any	st
WAKAI	513		m	0	0	all	-	a	As:Jap	1988	CC	333	n	bl	n	y	0	cig+/-ot	1	19	0	0	nev any	st
WU	501	x	f	0	0	wh	-	a	NAmer	1981	CC	220	n	bl	n	y	0	all/unsp	25	999	1	0	nev any	st
WU	502	x	f	0	0	wh	-	a	NAmer	1981	CC	220	n	bl	n	y	0	all/unsp	19	24	0	3	nev any	st
WU	503	x	f	0	0	wh	-	a	NAmer	1981	CC	220	n	bl	n	y	0	all/unsp	0	18	0	0	nev any	st

Cigarette type is all/unspec for all RRs

In this overview table, subtotals and Qs values may be invalid and should be ignored

Table 3H6 - 5

IESLC - Meta-analysis of Current Smoking by Age started, Overview
Adenocarcinoma, Any Product (or Cigarettes if Any not available)
Least adjusted

REF	NRR	SEX	AD	Number Case	Exposed Cont	Non-exposed Case	Cont	RR	95.00%CI
*ENGELA	527	m	0	8	39957	7	58716	1.68	(0.61- 4.63)
*ENGELA	528	m	0	25	50732	7	58716	4.13	(1.79- 9.56)
Subtotal ENGELA								2.87	(1.50- 5.47)
SVENSS	525	f	0	10	18	22	120	3.03	(1.24- 7.43)
SVENSS	526	f	0	11	14	22	120	4.29	(1.72- 10.66)
SVENSS	527	f	0	17	21	22	120	4.42	(2.01- 9.68)
Subtotal SVENSS								3.90	(2.38- 6.40)
WAKAI	511	m	0	4	25	8	65	1.30	(0.36- 4.70)
WAKAI	512	m	0	52	183	8	65	2.31	(1.04- 5.12)
WAKAI	513	m	0	18	74	8	65	1.98	(0.81- 4.85)
Subtotal WAKAI								1.97	(1.15- 3.38)
WU	501	f	0	8	14	29	62	1.22	(0.46- 3.24)
WU	502	f	0	22	15	29	62	3.14	(1.42- 6.91)
WU	503	f	0	69	21	29	62	7.02	(3.64- 13.56)
Subtotal WU								3.74	(2.38- 5.85)
Totals				244	91074	191	118173		

*prospective study

REF	NRR	SEX	AD	Ys	Ws	Qs	Ps
*ENGELA	527	m	0	0.52	3.73	1.44	0.3164
*ENGELA	528	m	0	1.42	5.47	0.43	0.0009
Subtotal ENGELA				1.05	9.20	1.87	
SVENSS	525	f	0	1.11	4.78	0.00	0.0154
SVENSS	526	f	0	1.46	4.63	0.46	0.0017
SVENSS	527	f	0	1.49	6.24	0.74	0.0002
Subtotal SVENSS				1.36	15.64	1.21	
WAKAI	511	m	0	0.26	2.32	1.79	0.6892
WAKAI	512	m	0	0.84	6.06	0.56	0.0395
WAKAI	513	m	0	0.68	4.77	1.00	0.1366
Subtotal WAKAI				0.68	13.16	3.35	
WU	501	f	0	0.20	4.05	3.57	0.6871
WU	502	f	0	1.14	6.15	0.00	0.0046
WU	503	f	0	1.95	8.87	5.81	0.0000
Subtotal WU				1.32	19.06	9.39	

N	11
NS	4

Table 3H6 - 6

IESLC - Meta-analysis of Current Smoking by Age started, Overview
Adenocarcinoma, Any Product (or Cigarettes if Any not available)
Least adjusted

	Sex: combined	male	female	Total
N		5	6	11
NS		2	2	4

In this overview table, other than the "N" rows, entries in the "absent" and "Total" columns may be invalid and should be ignored

	Age started (broad categories): absent	19+k26	15-25k18	1-17k14	Total
N	7	4			11
NS	4	4			8
Wt	38.45	18.62			57.07
Het Chi	8.53	2.04			15.82
Het df	6	3			10
Het P	N.S.	N.S.			N.S.
Fixed RR	3.86	2.02			3.13
RRl	2.81	1.28			2.41
RRu	5.30	3.19			4.05
P	+++	++			+++
Random RR	3.71	2.02			2.98
RRl	2.53	1.28			2.14
RRu	5.44	3.19			4.15
P	+++	++			+++

	absent	Age started (narrow categories): 27+k30	23-29k26	19-25k22	15-21k18	11-17k14	1-13k10	Total
N	8	1		2				11
NS	4	1		2				7
Wt	43.97	2.32		10.77				57.07
Het Chi	13.56	0.00		0.26				15.82
Het df	7	0		1				10
Het P	(*)	N.S.		N.S.				N.S.
Fixed RR	3.17	1.30		3.59				3.13
RRl	2.36	0.36		1.97				2.41
RRu	4.26	4.70		6.52				4.05
P	+++	N.S.		+++				+++
Random RR	2.97	1.30		3.59				2.98
RRl	1.96	0.36		1.97				2.14
RRu	4.50	4.70		6.52				4.15
P	+++	N.S.		+++				+++

Table 3H6 - 6

IESLC - Meta-analysis of Current Smoking by Age started, Overview
Adenocarcinoma, Any Product (or Cigarettes if Any not available)
Least adjusted

MALES

	Age started (broad categories) absent	19+k26	15-25k18	1-17k14	Total
N	3	2			5
NS	2	2			4
Wt	12.57	9.79			22.36
Het Chi	2.64	0.23			3.12
Het df	2	1			4
Het P	N.S.	N.S.			N.S.
Fixed RR	2.52	2.04			2.30
RRl	1.45	1.09			1.52
RRu	4.38	3.83			3.48
P	++	+			+++
Random RR	2.45	2.04			2.30
RRl	1.28	1.09			1.52
RRu	4.68	3.83			3.48
P	++	+			+++

	absent	Age started (narrow categories) 27+k30	23-29k26	19-25k22	15-21k18	11-17k14	1-13k10	Total
N	4	1						5
NS	2	1						2
Wt	20.04	2.32						22.36
Het Chi	2.27	0.00						3.12
Het df	3	0						4
Het P	N.S.	N.S.						N.S.
Fixed RR	2.46	1.30						2.30
RRl	1.59	0.36						1.52
RRu	3.81	4.70						3.48
P	+++	N.S.						+++
Random RR	2.46	1.30						2.30
RRl	1.59	0.36						1.52
RRu	3.81	4.70						3.48
P	+++	N.S.						+++

FEMALES

	Age started (broad categories) absent	19+k26	15-25k18	1-17k14	Total
N	4	2			6
NS	2	2			4
Wt	25.88	8.82			34.71
Het Chi	2.50	1.81			9.24
Het df	3	1			5
Het P	N.S.	N.S.			(*)
Fixed RR	4.75	2.00			3.81
RRl	3.23	1.03			2.73
RRu	6.98	3.86			5.31
P	+++	+			+++
Random RR	4.75	1.96			3.61
RRl	3.23	0.81			2.28
RRu	6.98	4.78			5.70
P	+++	N.S.			+++

Table 3H6 - 6

IESLC - Meta-analysis of Current Smoking by Age started, Overview
Adenocarcinoma, Any Product (or Cigarettes if Any not available)
Least adjusted

FEMALES

		absent	27+k30	23-29k26	19-25k22	15-21k18	11-17k14	1-13k10	Total
			Age started (narrow categories)						
	N	4			2				6
	NS	2			2				4
	Wt	23.94			10.77				34.71
Het	Chi	8.93			0.26				9.24
Het	df	3			1				5
Het	P	*			N.S.				(*)
Fixed	RR	3.91			3.59				3.81
	RRl	2.62			1.97				2.73
	RRu	5.84			6.52				5.31
	P	+++			+++				+++
Random	RR	3.48			3.59				3.61
	RRl	1.72			1.97				2.28
	RRu	7.04			6.52				5.70
	P	+++			+++				+++

Table 3H6 - 7

IESLC - Meta-analysis of Current Smoking by Age started, Overview
Adenocarcinoma, Any Product (or Cigarettes if Any not available)
Excluded studies (and stage at which they were excluded)

1	AGUDO ALDERS ARMADA AUVINE AXELSS BARBON BECHER BENHAM BLOT1 BOFFET BOUCHA BRESLO BROWN3 CARPEN CHEN CHEN2 CHIAZZ CHOI CHYOU CORREA DAMBER DARBY DESTEF DOLL DOLL2 DORGAN DOSEME FAN GAO GARCIA GARSHI GENG GER GRAHAM GUO GURSEL HAENSZ HAMMO2 HAMMON HEGMAN HU HU2 JAHN JAIN JEDRYC JOLY JUSSAW KHUDER KOO KOULUM KREUZE LAUSSM LETOUR LEVIN LIU3 LIU4 LIU5 LUBIN LUBIN2 LUO MCCONN NOTAN2 OSANN2 PERNU PEZZOT PRESCO QIAO QIAO2 RACHTA RESTRE SADOWS STASZE SUZUK2 TIZZAN TVERDA VUTUC WANG2 WIGLE WU2 WUWILL WYNDE2 WYNDE3 XU YUAN ZHANG ZHENG ZHOU
2	AKIBA AMANDU AMES BENSHL BEST BOUCOT BROSS BUFFLE CPSII DEAN2 GILLIS HUMBLE KAISE2 KATSOU KAUFMA PEZZO2 PISANI SPITZ WATSON WYNDE8
3	MCDUFF WYNDE6
4	CEDERL CPSI DEAN3 DORN GAO2 HIRAYA HOLE LIAW MATOS MIGRAN MRFITR SEGI2 SOBUE SPEIZE WYNDE7

Table 3H7 -

IESLC - Meta-analysis of Current Smoking, Age started, "Low"
Adenocarcinoma, Any Product (or Cigarettes if Any not available)

This analysis is restricted to results for:
1) Current smokers
2) Results by Age started
3) Categorical results by Age started
4) Adenocarcinoma (or near equivalent)
5) Results complete enough for use in metaanalysis

Within each study, results are then selected (in the following order of preference, within each sex) for:
6) (not applicable)
7) PRODUCT: all/unspec, cigarettes regardless of other products, cigarettes only
8) CIGTYPE: all/unspecified, MC regardless of HR, MC only
9) (not applicable)
10) DENOM: never smoked anything, never smoked cigarettes, never any + low, never cigs + low
11) Followup period (YF, prospective studies): whole study (coded as 0) or longest available
12) LCtype: adeno or nearest available, but not squamous. (q = squamous, s = small,
a = adeno, l = large, KII = Kreyberg II, al = alveolar, br = bronchiolar, u = undifferentiated)
13) Race: all or nearest available, otherwise by race (wh or w = white, bl or b = black, hi = hispanic
ch = chinese, jap = japanese, haw = hawaiian, w+o = white + oriental, sca = scandinavian, as = asian)
14) Age started "low" in key scheme 1 (key value 26, maximum range 19+)
15) For overlapping studies: principal rather than subsidiary studies
Finally by Age: whole study (coded as 0) if available, otherwise by widest available age group
and then for single sex results (m, f) in preference to results for both sexes combined (c).

Results adjusted (AD) for the most potential confounders are then chosen in Sections -1 to -3
and results adjusted for the least confounders in Sections -4 to -6. (Those least adjusted results which
actually differ from the most adjusted are marked 'x' in column X in Section -4)

Section -7 shows excluded studies, together with the stage (as above) at which no qualifying
results were found.

Section -8 lists the potentially overlapping studies which have been included (1=principal, 2=subsidiary).

Section -9 lists any results which would have been included in preference except that they had data not complete
enough for use in meta-analysis, with their significance (yes/no), if known, and any further comment as entered
on the database. It also lists as "gap" any categories for which no data were presented by the original authors.

In addition to those mentioned above, the following fields, levels and abbreviations are used:
* or nk = not known, n = no, y = yes, ot = other
nev = never
all/unspec = all or unspecified, cig+/-ot = cigarettes irrespective of other products (cigar, pipe etc)
MC = manufactured cigarettes, HR = hand-rolled cigarettes
exL, exH = range of exposure (low and high) in the smoking group, in terms of Age started
REF: 6-character study reference
NRR: number of the RR on the database within the study
ST : study type (CC = case control, pr or prosp = prospective)
NLC: number of lung cancer cases in whole study
R : risky occupational population (n = no, m = mining, o = other risky)
VB : national cigarette type (V = at least 75% Virginia, bl = at least 75% blended, ot = other)
P : any proxy use
H : full histological confirmation
De : derivation of RR/CI (or = original, st = standard method, ot = other method of estimation)

Table 3H7 - 1

IESLC - Meta-analysis of Current Smoking, Age started, "Low"
Adenocarcinoma, Any Product (or Cigarettes if Any not available)
Most adjusted

REF	NRR	SEX	AGEL	AGEH	RACE	YF	LC TYPE	LOC	START	ST	NLC	R	VB	P	H	AD	PRODUCT	exL	exH	DENOM	De
ENGELA	527	m	0	0	all	0	a	Eu:Sca	1964	pr	435	n	bl	n	n	0	cig+/-ot	20	999	nev cigs	st
SVENSS	525	f	0	0	all	-	a	Eu:Sca	1983	CC	210	n	bl	n	n	0	all/unsp	26	999	nev any	st
WAKAI	512	m	0	0	all	-	a	As:Jap	1988	CC	333	n	bl	n	y	0	cig+/-ot	20	29	nev any	st
WU	525	f	0	0	wh	-	a	NAmer	1981	CC	220	n	bl	n	y	2	all/unsp	25	999	nev any	or

Cigarette type is all/unspec for all RRs

Table 3H7 - 2

IESLC - Meta-analysis of Current Smoking, Age started, "Low"
Adenocarcinoma, Any Product (or Cigarettes if Any not available)
Most adjusted

REF	NRR	SEX	AD	Number Case	Exposed Cont	Non-exposed Case	Cont	RR	95.00%CI
*ENGELA	527	m	0	8	39957	7	58716	1.68	(0.61- 4.63)
SVENSS	525	f	0	10	18	22	120	3.03	(1.24- 7.43)
WAKAI	512	m	0	52	183	8	65	2.31	(1.04- 5.12)
WU	525	f	2	8	-	29	-	1.10	(0.40- 3.20)
Partial Totals				78	40158	66	58901		

*prospective study

REF	NRR	SEX	AD	Ys	Ws	Qs	Ps
*ENGELA	527	m	0	0.52	3.73	0.12	0.3164
SVENSS	525	f	0	1.11	4.78	0.81	0.0154
WAKAI	512	m	0	0.84	6.06	0.12	0.0395
WU	525	f	2	0.10	3.55	1.29	0.8574

	N	4
	NS	4
	Wt	18.12
Het	Chi	2.33
Het	df	3
Het	P	N.S.
Fixed	RR	2.01
	RRl	1.27
	RRu	3.18
	P	++
Random	RR	2.01
	RRl	1.27
	RRu	3.18
	P	++
Asymm	P	N.S.

Table 3H7 - 3

IESLC - Meta-analysis of Current Smoking, Age started, "Low"
Adenocarcinoma, Any Product (or Cigarettes if Any not available)
Most adjusted

	Sex combined	male	female	Total
N		2	2	4
NS		2	2	4
Wt		9.79	8.33	18.12
Het Chi		0.23	2.09	2.33
Het df		1	1	3
Het P		N.S.	N.S.	N.S.
Fixed RR		2.04	1.97	2.01
RRl		1.09	1.00	1.27
RRu		3.83	3.88	3.18
P		+	(+)	++
Random RR		2.04	1.89	2.01
RRl		1.09	0.70	1.27
RRu		3.83	5.09	3.18
P		+	N.S.	++
Between Chi				0.01
Between df				1
Between P				N.S.
Btwn(F) P				N.S.
Btwn(R) P				N.S.

Too few RRs for analysis by factor

Table 3H7 - 4

IESLC - Meta-analysis of Current Smoking, Age started, "Low"
Adenocarcinoma, Any Product (or Cigarettes if Any not available)
Least adjusted

REF	NRR	X	SEX	AGEL	AGEH	RACE	YF	LC TYPE	LOC	START	ST	NLC	R	VB	P	H	AD	PRODUCT	exL	exH	DENOM	De
ENGELA	527		m	0	0	all	0	a	Eu:Sca	1964	pr	435	n	bl	n	n	0	cig+/-ot	20	999	nev cigs	st
SVENSS	525		f	0	0	all	-	a	Eu:Sca	1983	CC	210	n	bl	n	n	0	all/unsp	26	999	nev any	st
WAKAI	512		m	0	0	all	-	a	As:Jap	1988	CC	333	n	bl	n	y	0	cig+/-ot	20	29	nev any	st
WU	501	x	f	0	0	wh	-	a	NAmer	1981	CC	220	n	bl	n	y	0	all/unsp	25	999	nev any	st

Cigarette type is all/unspec for all RRs

Table 3H7 - 5

IESLC - Meta-analysis of Current Smoking, Age started, "Low"
Adenocarcinoma, Any Product (or Cigarettes if Any not available)
Least adjusted

REF	NRR	SEX	AD	Number Case	Exposed Cont	Non-exposed Case	Cont	RR	95.00%CI
*ENGELA	527	m	0	8	39957	7	58716	1.68	(0.61- 4.63)
SVENSS	525	f	0	10	18	22	120	3.03	(1.24- 7.43)
WAKAI	512	m	0	52	183	8	65	2.31	(1.04- 5.12)
WU	501	f	0	8	14	29	62	1.22	(0.46- 3.24)
Totals				78	40172	66	58963		

*prospective study

REF	NRR	SEX	AD	Ys	Ws	Qs	Ps
*ENGELA	527	m	0	0.52	3.73	0.13	0.3164
SVENSS	525	f	0	1.11	4.78	0.78	0.0154
WAKAI	512	m	0	0.84	6.06	0.11	0.0395
WU	501	f	0	0.20	4.05	1.03	0.6871

	N	4
	NS	4
	Wt	18.62
Het	Chi	2.04
Het	df	3
Het	P	N.S.
Fixed	RR	2.02
	RRl	1.28
	RRu	3.19
	P	++
Random	RR	2.02
	RRl	1.28
	RRu	3.19
	P	++
Asymm	P	N.S.

Table 3H7 - 6

IESLC - Meta-analysis of Current Smoking, Age started, "Low"
Adenocarcinoma, Any Product (or Cigarettes if Any not available)
Least adjusted

	Sex combined	male	female	Total
N		2	2	4
NS		2	2	4
Wt		9.79	8.82	18.62
Het Chi		0.23	1.81	2.04
Het df		1	1	3
Het P		N.S.	N.S.	N.S.
Fixed RR		2.04	2.00	2.02
RRl		1.09	1.03	1.28
RRu		3.83	3.86	3.19
P		+	+	++
Random RR		2.04	1.96	2.02
RRl		1.09	0.81	1.28
RRu		3.83	4.78	3.19
P		+	N.S.	++
Between Chi				0.00
Between df				1
Between P				N.S.
Btwn(F) P				N.S.
Btwn(R) P				N.S.

Table 3H7 - 7

IESLC - Meta-analysis of Current Smoking, Age started, "Low"
Adenocarcinoma, Any Product (or Cigarettes if Any not available)
Excluded studies (and stage at which they were excluded)

1	AGUDO ALDERS ARMADA AUVINE AXELSS BARBON BECHER BENHAM BLOT1 BOFFET BOUCHA BRESLO BROWN3 CARPEN CHEN CHEN2 CHIAZZ CHOI CHYOU CORREA DAMBER DARBY DESTEF DOLL DOLL2 DORGAN DOSEME FAN GAO GARCIA GARSHI GENG GER GRAHAM GUO GURSEL HAENSZ HAMMO2 HAMMON HEGMAN HU HU2 JAHN JAIN JEDRYC JOLY JUSSAW KHUDER KOO KOULUM KREUZE LAUSSM LETOUR LEVIN LIU3 LIU4 LIU5 LUBIN LUBIN2 LUO MCCONN NOTAN2 OSANN2 PERNU PEZZOT PRESCO QIAO QIAO2 RACHTA RESTRE SADOWS STASZE SUZUK2 TIZZAN TVERDA VUTUC WANG2 WIGLE WU2 WUWILL WYNDE2 WYNDE3 XU YUAN ZHANG ZHENG ZHOU
2	AKIBA AMANDU AMES BENSHL BEST BOUCOT BROSS BUFFLE CPSII DEAN2 GILLIS HUMBLE KAISE2 KATSOU KAUFMA PEZZO2 PISANI SPITZ WATSON WYNDE8
3	MCDUFF WYNDE6
4	CEDERL CPSI DEAN3 DORN GAO2 HIRAYA HOLE LIAW MATOS MIGRAN MRFITR SEGI2 SOBUE SPEIZE WYNDE7

Table 3H8 -

IESLC - Meta-analysis of Current Smoking, Age started, "Mid"
Adenocarcinoma, Any Product (or Cigarettes if Any not available)

This analysis is restricted to results for:
1) Current smokers
2) Results by Age started
3) Categorical results by Age started
4) Adenocarcinoma (or near equivalent)
5) Results complete enough for use in metaanalysis

Within each study, results are then selected (in the following order of preference, within each sex) for:
6) (not applicable)
7) PRODUCT: all/unspec, cigarettes regardless of other products, cigarettes only
8) CIGTYPE: all/unspecified, MC regardless of HR, MC only
9) (not applicable)
10) DENOM: never smoked anything, never smoked cigarettes, never any + low, never cigs + low
11) Followup period (YF, prospective studies): whole study (coded as 0) or longest available
12) LCtype: adeno or nearest available, but not squamous. (q = squamous, s = small,
a = adeno, l = large, KII = Kreyberg II, al = alveolar, br = bronchiolar, u = undifferentiated)
13) Race: all or nearest available, otherwise by race (wh or w = white, bl or b = black, hi = hispanic
ch = chinese, jap = japanese, haw = hawaiian, w+o = white + oriental, sca = scandinavian, as = asian)
14) Age started "mid" in key scheme 1 (key value 18, maximum range 15-25)
15) For overlapping studies: principal rather than subsidiary studies
Finally by Age: whole study (coded as 0) if available, otherwise by widest available age group
and then for single sex results (m, f) in preference to results for both sexes combined (c).

Results adjusted (AD) for the most potential confounders are then chosen in Sections -1 to -3
and results adjusted for the least confounders in Sections -4 to -6. (Those least adjusted results which
actually differ from the most adjusted are marked 'x' in column X in Section -4)

Section -7 shows excluded studies, together with the stage (as above) at which no qualifying
results were found.

Section -8 lists the potentially overlapping studies which have been included (1=principal, 2=subsidiary).

Section -9 lists any results which would have been included in preference except that they had data not complete
enough for use in meta-analysis, with their significance (yes/no), if known, and any further comment as entered
on the database. It also lists as "gap" any categories for which no data were presented by the original authors.

In addition to those mentioned above, the following fields, levels and abbreviations are used:
* or nk = not known, n = no, y = yes, ot = other
nev = never
all/unspec = all or unspecified, cig+/-ot = cigarettes irrespective of other products (cigar, pipe etc)
MC = manufactured cigarettes, HR = hand-rolled cigarettes
exL, exH = range of exposure (low and high) in the smoking group, in terms of Age started
REF: 6-character study reference
NRR: number of the RR on the database within the study
ST : study type (CC = case control, pr or prosp = prospective)
NLC: number of lung cancer cases in whole study
R : risky occupational population (n = no, m = mining, o = other risky)
VB : national cigarette type (V = at least 75% Virginia, bl = at least 75% blended, ot = other)
P : any proxy use
H : full histological confirmation
De : derivation of RR/CI (or = original, st = standard method, ot = other method of estimation)

Table 3H8 - 0

No RRs selected for this analysis

Table 3H8 - 7

IESLC - Meta-analysis of Current Smoking, Age started, "Mid"
Adenocarcinoma, Any Product (or Cigarettes if Any not available)
Excluded studies (and stage at which they were excluded)

1 AGUDO ALDERS ARMADA AUVINE AXELSS BARBON BECHER BENHAM BLOT1 BOFFET BOUCHA BRESLO BROWN3 CARPEN CHEN CHEN2 CHIAZZ CHOI CHYOU CORREA DAMBER DARBY DESTEF DOLL DOLL2 DORGAN DOSEME FAN GAO GARCIA GARSHI GENG GER GRAHAM GUO GURSEL HAENSZ HAMMO2 HAMMON HEGMAN HU HU2 JAHN JAIN JEDRYC JOLY JUSSAW KHUDER KOO KOULUM KREUZE LAUSSM LETOUR LEVIN LIU3 LIU4 LIU5 LUBIN LUBIN2 LUO MCCONN NOTAN2 OSANN2 PERNU PEZZOT PRESCO QIAO QIAO2 RACHTA RESTRE SADOWS STASZE SUZUK2 TIZZAN TVERDA VUTUC WANG2 WIGLE WU2 WUWILL WYNDE2 WYNDE3 XU YUAN ZHANG ZHENG ZHOU

2 AKIBA AMANDU AMES BENSHL BEST BOUCOT BROSS BUFFLE CPSII DEAN2 GILLIS HUMBLE KAISE2 KATSOU KAUFMA PEZZO2 PISANI SPITZ WATSON WYNDE8

3 MCDUFF WYNDE6

4 CEDERL CPSI DEAN3 DORN GAO2 HIRAYA HOLE LIAW MATOS MIGRAN MRFITR SEGI2 SOBUE SPEIZE WYNDE7

14 ENGELA SVENSS WAKAI WU

Table 3H9 -

IESLC - Meta-analysis of Current Smoking, Age started, "High"
Adenocarcinoma, Any Product (or Cigarettes if Any not available)

This analysis is restricted to results for:
1) Current smokers
2) Results by Age started
3) Categorical results by Age started
4) Adenocarcinoma (or near equivalent)
5) Results complete enough for use in metaanalysis

Within each study, results are then selected (in the following order of preference, within each sex) for:
6) PRODUCT: all/unspec, cigarettes regardless of other products, cigarettes only
7) CIGTYPE: all/unspecified, MC regardless of HR, MC only
8) (not applicable)
9) DENOM: never smoked anything, never smoked cigarettes, never any + low, never cigs + low
10) Followup period (YF, prospective studies): whole study (coded as 0) or longest available
11) LCtype: adeno or nearest available, but not squamous. (q = squamous, s = small,
a = adeno, l = large, KII = Kreyberg II, al = alveolar, br = bronchiolar, u = undifferentiated)
12) Race: all or nearest available, otherwise by race (wh or w = white, bl or b = black, hi = hispanic
ch = chinese, jap = japanese, haw = hawaiian, w+o = white + oriental, sca = scandinavian, as = asian)
13) Age started "high" in key scheme 1 (key value 14, maximum range 1-17)
14) For overlapping studies: principal rather than subsidiary studies
Finally by Age: whole study (coded as 0) if available, otherwise by widest available age group
and then for single sex results (m, f) in preference to results for both sexes combined (c).

Results adjusted (AD) for the most potential confounders are then chosen in Sections -1 to -3
and results adjusted for the least confounders in Sections -4 to -6. (Those least adjusted results which
actually differ from the most adjusted are marked 'x' in column X in Section -4)

Section -7 shows excluded studies, together with the stage (as above) at which no qualifying
results were found.

Section -8 lists the potentially overlapping studies which have been included (1=principal, 2=subsidiary).

Section -9 lists any results which would have been included in preference except that they had data not complete
enough for use in meta-analysis, with their significance (yes/no), if known, and any further comment as entered
on the database. It also lists as "gap" any categories for which no data were presented by the original authors.

In addition to those mentioned above, the following fields, levels and abbreviations are used:
* or nk = not known, n = no, y = yes, ot = other
nev = never
all/unspec = all or unspecified, cig+/-ot = cigarettes irrespective of other products (cigar, pipe etc)
MC = manufactured cigarettes, HR = hand-rolled cigarettes
exL, exH = range of exposure (low and high) in the smoking group, in terms of Age started
REF: 6-character study reference
NRR: number of the RR on the database within the study
ST : study type (CC = case control, pr or prosp = prospective)
NLC: number of lung cancer cases in whole study
R : risky occupational population (n = no, m = mining, o = other risky)
VB : national cigarette type (V = at least 75% Virginia, bl = at least 75% blended, ot = other)
P : any proxy use
H : full histological confirmation
De : derivation of RR/CI (or = original, st = standard method, ot = other method of estimation)

Table 3H9 - 0

No RRs selected for this analysis

Table 3H9 - 7

IESLC - Meta-analysis of Current Smoking, Age started, "High"
Adenocarcinoma, Any Product (or Cigarettes if Any not available)
Excluded studies (and stage at which they were excluded)

1 AGUDO ALDERS ARMADA AUVINE AXELSS BARBON BECHER BENHAM BLOT1 BOFFET BOUCHA BRESLO BROWN3 CARPEN CHEN CHEN2 CHIAZZ CHOI CHYOU CORREA DAMBER DARBY DESTEF DOLL DOLL2 DORGAN DOSEME FAN GAO GARCIA GARSHI GENG GER GRAHAM GUO GURSEL HAENSZ HAMMO2 HAMMON HEGMAN HU HU2 JAHN JAIN JEDRYC JOLY JUSSAW KHUDER KOO KOULUM KREUZE LAUSSM LETOUR LEVIN LIU3 LIU4 LIU5 LUBIN LUBIN2 LUO MCCONN NOTAN2 OSANN2 PERNU PEZZOT PRESCO QIAO QIAO2 RACHTA RESTRE SADOWS STASZE SUZUK2 TIZZAN TVERDA VUTUC WANG2 WIGLE WU2 WUWILL WYNDE2 WYNDE3 XU YUAN ZHANG ZHENG ZHOU

2 AKIBA AMANDU AMES BENSHL BEST BOUCOT BROSS BUFFLE CPSII DEAN2 GILLIS HUMBLE KAISE2 KATSOU KAUFMA PEZZO2 PISANI SPITZ WATSON WYNDE8

3 MCDUFF WYNDE6

4 CEDERL CPSI DEAN3 DORN GAO2 HIRAYA HOLE LIAW MATOS MIGRAN MRFITR SEGI2 SOBUE SPEIZE WYNDE7

14 ENGELA SVENSS WAKAI WU

Table 3H10 -

IESLC - Meta-analysis of Current Smoking, Age started, "Highest vs lowest"
Adenocarcinoma, Any Product (or Cigarettes if Any not available)

This analysis is restricted to results for:
1) Current smokers
2) Results by Age started
3) Categorical results by Age started
4) Denominator (unexposed) = "low"
5) Adenocarcinoma (or near equivalent)
6) Results complete enough for use in metaanalysis

Within each study, results are then selected (in the following order of preference, within each sex) for:
7) (not applicable)
8) PRODUCT: all/unspec, cigarettes regardless of other products, cigarettes only
9) CIGTYPE: all/unspecified, MC regardless of HR, MC only
10) Results with least adjustment for other aspects of smoking (ADOS)
11) The highest vs lowest category
12) Followup period (YF, prospective studies): whole study (coded as 0) or longest available
13) LCtype: adeno or nearest available, but not squamous. (q = squamous, s = small,
a = adeno, l = large, KII = Kreyberg II, al = alveolar, br = bronchiolar, u = undifferentiated)
14) Race: all or nearest available, otherwise by race (wh or w = white, bl or b = black, hi = hispanic
ch = chinese, jap = japanese, haw = hawaiian, w+o = white + oriental, sca = scandinavian, as = asian)
15) For overlapping studies: principal rather than subsidiary studies
Finally by Age: whole study (coded as 0) if available, otherwise by widest available age group
and then for single sex results (m, f) in preference to results for both sexes combined (c).

Results adjusted (AD) for the most potential confounders are then chosen in Sections -1 to -3
and results adjusted for the least confounders in Sections -4 to -6. (Those least adjusted results which
actually differ from the most adjusted are marked 'x' in column X in Section -4)

Section -7 shows excluded studies, together with the stage (as above) at which no qualifying
results were found.

Section -8 lists the potentially overlapping studies which have been included (1=principal, 2=subsidiary).

Section -9 lists any results which would have been included in preference except that they had data not complete
enough for use in meta-analysis, with their significance (yes/no), if known, and any further comment as entered
on the database. It also lists as "gap" any categories for which no data were presented by the original authors.

In addition to those mentioned above, the following fields, levels and abbreviations are used:
* or nk = not known, n = no, y = yes, ot = other
all/unspec = all or unspecified, cig+/-ot = cigarettes irrespective of other products (cigar, pipe etc)
MC = manufactured cigarettes, HR = hand-rolled cigarettes
exL, exH = range of exposure (low and high) in the "highest" group, in terms of Age started
unexL, unexH = range of exposure (low and high) in the "lowest" group, in terms of Age started
REF: 6-character study reference
NRR: number of the RR on the database within the study
ST : study type (CC = case control, pr or prosp = prospective)
NLC: number of lung cancer cases in whole study
R : risky occupational population (n = no, m = mining, o = other risky)
VB : national cigarette type (V = at least 75% Virginia, bl = at least 75% blended, ot = other)
P : any proxy use
H : full histological confirmation
De : derivation of RR/CI (or = original, st = standard method, ot = other method of estimation)

Table 3H10 - 1

IESLC - Meta-analysis of Current Smoking, Age started, "Highest vs lowest"
Adenocarcinoma, Any Product (or Cigarettes if Any not available)
Most adjusted

REF	NRR	SEX	AGEL	AGEH	RACE	YF	LC TYPE	LOC	START	ST	NLC	R	VB	P	H	AD	ADOS	PRODUCT	exL	exH	unexL	unexH	De
ENGELA	529	m	0	0	all	0	a	Eu:Sca	1964	pr	435	n	bl	n	n	0	0	cig+/-ot	1	19	20	999	st
SVENSS	529	f	0	0	all	-	a	Eu:Sca	1983	CC	210	n	bl	n	n	0	0	all/unsp	0	18	26	999	st
WAKAI	515	m	0	0	all	-	a	As:Jap	1988	CC	333	n	bl	n	y	0	0	cig+/-ot	1	19	30	999	st
WU	529	f	0	0	wh	-	a	NAmer	1981	CC	220	n	bl	n	y	2	0	all/unsp	0	18	25	999	ot

Cigarette type is all/unspec for all RRs

Table 3H10 - 2

IESLC - Meta-analysis of Current Smoking, Age started, "Highest vs lowest"
Adenocarcinoma, Any Product (or Cigarettes if Any not available)
Most adjusted

REF	NRR	SEX	AD	Number Case	Exposed Cont	Non-exposed Case	Cont	RR	95.00%CI
*ENGELA	529	m	0	25	50732	8	39957	2.46	(1.11- 5.46)
SVENSS	529	f	0	17	21	10	18	1.46	(0.53- 3.97)
WAKAI	515	m	0	18	74	4	25	1.52	(0.47- 4.92)
WU	529	f	2	69	-	8	-	7.27	(2.31- 22.86)
Partial Totals				129	50827	30	40000		

*prospective study

REF	NRR	SEX	AD	Ys	Ws	Qs	Ps
*ENGELA	529	m	0	0.90	6.06	0.00	0.0266
SVENSS	529	f	0	0.38	3.82	1.00	0.4620
WAKAI	515	m	0	0.42	2.78	0.62	0.4845
WU	529	f	2	1.98	2.92	3.50	0.0007

	N	4
	NS	4
	Wt	15.59
Het	Chi	5.12
Het	df	3
Het	P	N.S.
Fixed	RR	2.43
	RRl	1.48
	RRu	4.00
	P	+++
Random	RR	2.46
	RRl	1.27
	RRu	4.77
	P	++
Asymm	P	N.S.

Table 3H10 - 3

IESLC - Meta-analysis of Current Smoking, Age started, "Highest vs lowest"
Adenocarcinoma, Any Product (or Cigarettes if Any not available)
Most adjusted

	combined	Sex: male	female	Total
N		2	2	4
NS		2	2	4
Wt		8.85	6.74	15.59
Het Chi		0.44	4.28	5.12
Het df		1	1	3
Het P		N.S.	*	N.S.
Fixed RR		2.11	2.93	2.43
RRl		1.09	1.38	1.48
RRu		4.09	6.23	4.00
P		+	++	+++
Random RR		2.11	3.17	2.46
RRl		1.09	0.66	1.27
RRu		4.09	15.33	4.77
P		+	N.S.	++
Between Chi				0.40
Between df				1
Between P				N.S.
Btwn(F) P				N.S.
Btwn(R) P				N.S.

Too few RRs for analysis by factor

Table 3H10 - 4

IESLC - Meta-analysis of Current Smoking, Age started, "Highest vs lowest"
Adenocarcinoma, Any Product (or Cigarettes if Any not available)
Least adjusted

REF	NRR	X	SEX	AGEL	AGEH	RACE	YF	LC TYPE	LOC	START	ST	NLC	R	VB	P	H	AD	ADOS	PRODUCT	exL	exH	unexL	unexH	De
ENGELA	529		m	0	0	all	0	a	Eu:Sca	1964	pr	435	n	bl	n	n	0	0	cig+/-ot	1	19	20	999	st
SVENSS	529		f	0	0	all	-	a	Eu:Sca	1983	CC	210	n	bl	n	n	0	0	all/unsp	0	18	26	999	st
WAKAI	515		m	0	0	all	-	a	As:Jap	1988	CC	333	n	bl	n	y	0	0	cig+/-ot	1	19	30	999	st
WU	505	x	f	0	0	wh	-	a	NAmer	1981	CC	220	n	bl	n	y	0	0	all/unsp	0	18	25	999	st

Cigarette type is all/unspec for all RRs

Table 3H10 - 5

IESLC - Meta-analysis of Current Smoking, Age started, "Highest vs lowest"
Adenocarcinoma, Any Product (or Cigarettes if Any not available)
Least adjusted

REF	NRR	SEX	AD	Number Exposed Case	Number Exposed Cont	Non-exposed Case	Non-exposed Cont	RR	95.00%CI
*ENGELA	529	m	0	25	50732	8	39957	2.46	(1.11- 5.46)
SVENSS	529	f	0	17	21	10	18	1.46	(0.53- 3.97)
WAKAI	515	m	0	18	74	4	25	1.52	(0.47- 4.92)
WU	505	f	0	69	21	8	14	5.75	(2.12- 15.58)
Totals				129	50848	30	40014		

*prospective study

REF	NRR	SEX	AD	Ys	Ws	Qs	Ps
*ENGELA	529	m	0	0.90	6.06	0.00	0.0266
SVENSS	529	f	0	0.38	3.82	1.03	0.4620
WAKAI	515	m	0	0.42	2.78	0.64	0.4845
WU	505	f	0	1.75	3.87	2.81	0.0006

	N	4
	NS	4
	Wt	16.53
Het	Chi	4.48
Het	df	3
Het	P	N.S.
Fixed	RR	2.45
	RRl	1.51
	RRu	3.97
	P	+++
Random	RR	2.43
	RRl	1.34
	RRu	4.42
	P	++
Asymm	P	N.S.

Table 3H10 - 6

IESLC - Meta-analysis of Current Smoking, Age started, "Highest vs lowest"
Adenocarcinoma, Any Product (or Cigarettes if Any not available)
Least adjusted

	combined	Sex: male	female	Total
N		2	2	4
NS		2	2	4
Wt		8.85	7.68	16.53
Het Chi		0.44	3.62	4.48
Het df		1	1	3
Het P		N.S.	(*)	N.S.
Fixed RR		2.11	2.91	2.45
RRl		1.09	1.43	1.51
RRu		4.09	5.90	3.97
P		+	++	+++
Random RR		2.11	2.90	2.43
RRl		1.09	0.75	1.34
RRu		4.09	11.13	4.42
P		+	N.S.	++
Between Chi				0.42
Between df				1
Between P				N.S.
Btwn(F) P				N.S.
Btwn(R) P				N.S.

Table 3H10 - 7

IESLC - Meta-analysis of Current Smoking, Age started, "Highest vs lowest"
Adenocarcinoma, Any Product (or Cigarettes if Any not available)
Excluded studies (and stage at which they were excluded)

1	AGUDO ALDERS ARMADA AUVINE AXELSS BARBON BECHER BENHAM BLOT1 BOFFET BOUCHA BRESLO BROWN3 CARPEN CHEN CHEN2 CHIAZZ CHOI CHYOU CORREA DAMBER DARBY DESTEF DOLL DOLL2 DORGAN DOSEME FAN GAO GARCIA GARSHI GENG GER GRAHAM GUO GURSEL HAENSZ HAMMO2 HAMMON HEGMAN HU HU2 JAHN JAIN JEDRYC JOLY JUSSAW KHUDER KOO KOULUM KREUZE LAUSSM LETOUR LEVIN LIU3 LIU4 LIU5 LUBIN LUBIN2 LUO MCCONN NOTAN2 OSANN2 PERNU PEZZOT PRESCO QIAO QIAO2 RACHTA RESTRE SADOWS STASZE SUZUK2 TIZZAN TVERDA VUTUC WANG2 WIGLE WU2 WUWILL WYNDE2 WYNDE3 XU YUAN ZHANG ZHENG ZHOU
2	AKIBA AMANDU AMES BENSHL BEST BOUCOT BROSS BUFFLE CPSII DEAN2 GILLIS HUMBLE KAISE2 KATSOU KAUFMA PEZZO2 PISANI SPITZ WATSON WYNDE8
3	MCDUFF WYNDE6
5	CEDERL CPSI DEAN3 DORN GAO2 HIRAYA HOLE LIAW MATOS MIGRAN MRFITR SEGI2 SOBUE SPEIZE WYNDE7

Table 3H11 -

IESLC - Meta-analysis of Ever/current Smoking by Age started, Overview
Adenocarcinoma, Any Product (or Cigarettes if Any not available)

This analysis is restricted to results for:
1) Ever/current smokers
2) Results by Age started
3) Categorical results by Age started
Results by Age started are grouped under 2 schemes (S1, S2). Each scheme has a set of "key values". An interval is allocated to the category whose key value it includes, and intervals which include none or more than one of the key values are excluded. (Open-ended intervals are coded as 999)

S1	key value	maximum range
1	26	19+
2	18	15-25
3	14	1-17

S2	key value	maximum range
1	30	27+
2	26	23-29
3	22	19-25
4	18	15-21
5	14	11-17
6	10	1-13

4) Adenocarcinoma (or near equivalent)
5) Results complete enough for use in metaanalysis

Within each study, results are then selected (in the following order of preference, within each sex) for:
6) SMKSTA: ever, current
7) PRODUCT: all/unspec, cigarettes regardless of other products, cigarettes only
8) CIGTYPE: all/unspecified, MC regardless of HR, MC only
9) (not applicable)
10) DENOM: never smoked anything, never smoked cigarettes, never any + low, never cigs + low
11) Followup period (YF, prospective studies): whole study (coded as 0) or longest available
12) LCtype: adeno or nearest available, but not squamous. (q = squamous, s = small,
a = adeno, l = large, KII = Kreyberg II, al = alveolar, br = bronchiolar, u = undifferentiated)
13) Race: all or nearest available, otherwise by race (wh or w = white, bl or b = black, hi = hispanic
ch = chinese, jap = japanese, haw = hawaiian, w+o = white + oriental, sca = scandinavian, as = asian)
14) For overlapping studies: principal rather than subsidiary studies
Finally by Age: whole study (coded as 0) if available, otherwise by widest available age group
and then for single sex results (m, f) in preference to results for both sexes combined (c).

Results adjusted (AD) for the most potential confounders are then chosen in Sections -1 to -3
and results adjusted for the least confounders in Sections -4 to -6. (Those least adjusted results which actually differ from the most adjusted are marked 'x' in column X in Section -4)

Section -7 shows excluded studies, together with the stage (as above) at which no qualifying results were found.

Section -8 lists the potentially overlapping studies which have been included (1=principal, 2=subsidiary).

Section -9 lists any results which would have been included in preference except that they had data not complete enough for use in meta-analysis, with their significance (yes/no), if known, and any further comment as entered on the database. It also lists as "gap" any categories for which no data were presented by the original authors.

In addition to those mentioned above, the following fields, levels and abbreviations are used:
* or nk = not known, n = no, y = yes, ot = other
ev = ever, cu = current, nev = never
all/unspec = all or unspecified, cig+/-ot = cigarettes irrespective of other products (cigar, pipe etc)
MC = manufactured cigarettes, HR = hand-rolled cigarettes
exL, exH = range of exposure (low and high) in the smoking group, in terms of Age started
REF: 6-character study reference
NRR: number of the RR on the database within the study
ST : study type (CC = case control, pr or prosp = prospective)
NLC: number of lung cancer cases in whole study
R : risky occupational population (n = no, m = mining, o = other risky)
VB : national cigarette type (V = at least 75% Virginia, bl = at least 75% blended, ot = other)
P : any proxy use
H : full histological confirmation
De : derivation of RR/CI (or = original, st = standard method, ot = other method of estimation)

Table 3H11 - 1

IESLC - Meta-analysis of Ever/current Smoking by Age started, Overview
Adenocarcinoma, Any Product (or Cigarettes if Any not available)
Most adjusted

REF	NRR	SEX	AGEL	AGEH	RACE	YF	LC TYPE	LOC	START	ST	NLC	R	VB	P	H	AD	SM	PRODUCT	exL	exH	S1	S2	DENOM	De
BARBON	736	m	0	0	all	-	a	Eu:wst	1979	CC	755	n	bl	y	y	1	ev	all/unsp	20	999	1	0	nev any	or
BARBON	737	m	0	0	all	-	a	Eu:wst	1979	CC	755	n	bl	y	y	1	ev	all/unsp	15	19	2	4	nev any	or
BARBON	738	m	0	0	all	-	a	Eu:wst	1979	CC	755	n	bl	y	y	1	ev	all/unsp	1	14	3	0	nev any	or
BENHAM	521	m	0	0	all	-	KII	Eu:wst	1976	CC	1625	n	bl	n	y	0	ev	cig only	20	999	1	0	nev any	st
BENHAM	522	m	0	0	all	-	KII	Eu:wst	1976	CC	1625	n	bl	n	y	0	ev	cig only	1	19	0	0	nev any	st
ENGELA	527	m	0	0	all	0	a	Eu:Sca	1964	pr	435	n	bl	n	n	0	cu	cig+/-ot	20	999	1	0	nev cigs	st
ENGELA	528	m	0	0	all	0	a	Eu:Sca	1964	pr	435	n	bl	n	n	0	cu	cig+/-ot	1	19	0	0	nev cigs	st
HAENSZ	509	f	0	0	all	-	a	NAmer	1955	CC	158	n	bl	n	y	0	ev	cig+/-ot	25	999	1	0	nev any	st
HAENSZ	510	f	0	0	all	-	a	NAmer	1955	CC	158	n	bl	n	y	0	ev	cig+/-ot	1	24	0	0	nev any	st
JEDRYC	538	m	0	0	all	-	a	Eu:est	1980	CC	1630	n	bl	y	n	0	ev	cig+/-ot	19	999	1	0	nev any	st
JEDRYC	539	m	0	0	all	-	a	Eu:est	1980	CC	1630	n	bl	y	n	0	ev	cig+/-ot	17	18	2	4	nev any	st
JEDRYC	540	m	0	0	all	-	a	Eu:est	1980	CC	1630	n	bl	y	n	0	ev	cig+/-ot	1	16	3	0	nev any	st
MATOS	656	m	0	0	all	-	a	SCAmer	1994	CC	200	n	bl	n	n	2	ev	cig+/-ot	20	999	1	0	nev any	or
MATOS	657	m	0	0	all	-	a	SCAmer	1994	CC	200	n	bl	n	n	2	ev	cig+/-ot	15	19	2	4	nev any	or
MATOS	658	m	0	0	all	-	a	SCAmer	1994	CC	200	n	bl	n	n	2	ev	cig+/-ot	1	14	3	0	nev any	or
PEZZOT	552	m	0	0	all	-	a	SCAmer	1987	CC	215	n	bl	n	y	0	ev	cig only	19	999	1	0	nev cigs	st
PEZZOT	553	m	0	0	all	-	a	SCAmer	1987	CC	215	n	bl	n	y	0	ev	cig only	14	18	0	0	nev cigs	st
PEZZOT	554	m	0	0	all	-	a	SCAmer	1987	CC	215	n	bl	n	y	0	ev	cig only	1	13	0	6	nev cigs	st
SVENSS	525	f	0	0	all	-	a	Eu:Sca	1983	CC	210	n	bl	n	n	0	cu	all/unsp	26	999	1	0	nev any	st
SVENSS	526	f	0	0	all	-	a	Eu:Sca	1983	CC	210	n	bl	n	n	0	cu	all/unsp	19	25	0	3	nev any	st
SVENSS	527	f	0	0	all	-	a	Eu:Sca	1983	CC	210	n	bl	n	n	0	cu	all/unsp	0	18	0	0	nev any	st
WAKAI	511	m	0	0	all	-	a	As:Jap	1988	CC	333	n	bl	n	y	0	cu	cig+/-ot	30	999	0	1	nev any	st
WAKAI	512	m	0	0	all	-	a	As:Jap	1988	CC	333	n	bl	n	y	0	cu	cig+/-ot	20	29	1	0	nev any	st
WAKAI	513	m	0	0	all	-	a	As:Jap	1988	CC	333	n	bl	n	y	0	cu	cig+/-ot	1	19	0	0	nev any	st
WU	525	f	0	0	wh	-	a	NAmer	1981	CC	220	n	bl	n	y	2	cu	all/unsp	25	999	1	0	nev any	or
WU	526	f	0	0	wh	-	a	NAmer	1981	CC	220	n	bl	n	y	2	cu	all/unsp	19	24	0	3	nev any	or
WU	527	f	0	0	wh	-	a	NAmer	1981	CC	220	n	bl	n	y	2	cu	all/unsp	0	18	0	0	nev any	or
WYNDE6	613	m	0	0	wh	-	a	NAmer	1969	CC	4423	n	bl	n	y	0	ev	cig+/-ot	21	999	1	0	nev cigs	st
WYNDE6	614	m	0	0	wh	-	a	NAmer	1969	CC	4423	n	bl	n	y	0	ev	cig+/-ot	18	20	2	4	nev cigs	st
WYNDE6	615	m	0	0	wh	-	a	NAmer	1969	CC	4423	n	bl	n	y	0	ev	cig+/-ot	1	17	3	0	nev cigs	st
WYNDE6	637	f	0	0	wh	-	a	NAmer	1969	CC	4423	n	bl	n	y	0	ev	cig+/-ot	21	999	1	0	nev cigs	st
WYNDE6	638	f	0	0	wh	-	a	NAmer	1969	CC	4423	n	bl	n	y	0	ev	cig+/-ot	18	20	2	4	nev cigs	st
WYNDE6	639	f	0	0	wh	-	a	NAmer	1969	CC	4423	n	bl	n	y	0	ev	cig+/-ot	1	17	3	0	nev cigs	st
ZHENG	531	m	0	0	all	-	a	As:Chi	1982	CC	540	n	ot	*	y	0	ev	cig+/-ot	30	999	0	1	nev cigs	or
ZHENG	532	m	0	0	all	-	a	As:Chi	1982	CC	540	n	ot	*	y	0	ev	cig+/-ot	20	29	1	0	nev cigs	st
ZHENG	533	m	0	0	all	-	a	As:Chi	1982	CC	540	n	ot	*	y	0	ev	cig+/-ot	1	19	0	0	nev cigs	st
ZHENG	538	f	0	0	all	-	a	As:Chi	1982	CC	540	n	ot	*	y	0	ev	cig+/-ot	30	999	0	1	nev cigs	or
ZHENG	539	f	0	0	all	-	a	As:Chi	1982	CC	540	n	ot	*	y	0	ev	cig+/-ot	1	29	0	0	nev cigs	st

Cigarette type is all/unspec for all RRs

In this overview table, subtotals and Qs values may be invalid and should be ignored

Table 3H11 - 2

IESLC - Meta-analysis of Ever/current Smoking by Age started, Overview
Adenocarcinoma, Any Product (or Cigarettes if Any not available)
Most adjusted

REF	NRR	SEX	AD	Number Exposed Case	Cont	Non-exposed Case	Cont	RR	95.00%CI
BARBON	736	m	1	45	-	7	-	5.70	(2.50- 12.90)
BARBON	737	m	1	77	-	7	-	6.00	(2.70- 13.30)
BARBON	738	m	1	29	-	7	-	33.40	(13.10- 84.90)
Subtotal BARBON								9.41	(5.78- 15.32)
BENHAM	521	m	0	41	64	9	42	2.99	(1.32- 6.79)
BENHAM	522	m	0	69	79	9	42	4.08	(1.85- 8.97)
Subtotal BENHAM								3.51	(1.99- 6.20)
*ENGELA	527	m	0	8	39957	7	58716	1.68	(0.61- 4.63)
*ENGELA	528	m	0	25	50732	7	58716	4.13	(1.79- 9.56)
Subtotal ENGELA								2.87	(1.50- 5.47)
HAENSZ	509	f	0	12	66	37	236	1.16	(0.57- 2.35)
HAENSZ	510	f	0	6	37	37	236	1.03	(0.41- 2.62)
Subtotal HAENSZ								1.11	(0.63- 1.95)
JEDRYC	538	m	0	48	502	7	289	3.95	(1.76- 8.84)
JEDRYC	539	m	0	29	221	7	289	5.42	(2.33- 12.60)
JEDRYC	540	m	0	12	111	7	289	4.46	(1.71- 11.63)
Subtotal JEDRYC								4.56	(2.77- 7.50)
MATOS	656	m	2	13	-	5	-	4.00	(1.40- 11.90)
MATOS	657	m	2	40	-	5	-	7.20	(2.70- 19.10)
MATOS	658	m	2	26	-	5	-	6.60	(2.40- 18.10)
Subtotal MATOS								5.86	(3.25- 10.54)
PEZZOT	552	m	0	18	105	3	116	6.63	(1.90- 23.15)
PEZZOT	553	m	0	33	145	3	116	8.80	(2.63- 29.42)
PEZZOT	554	m	0	9	67	3	116	5.19	(1.36- 19.85)
Subtotal PEZZOT								6.84	(3.30- 14.18)
SVENSS	525	f	0	10	18	22	120	3.03	(1.24- 7.43)
SVENSS	526	f	0	11	14	22	120	4.29	(1.72- 10.66)
SVENSS	527	f	0	17	21	22	120	4.42	(2.01- 9.68)
Subtotal SVENSS								3.90	(2.38- 6.40)
WAKAI	511	m	0	4	25	8	65	1.30	(0.36- 4.70)
WAKAI	512	m	0	52	183	8	65	2.31	(1.04- 5.12)
WAKAI	513	m	0	18	74	8	65	1.98	(0.81- 4.85)
Subtotal WAKAI								1.97	(1.15- 3.38)
WU	525	f	2	8	-	29	-	1.10	(0.40- 3.20)
WU	526	f	2	22	-	29	-	2.50	(1.00- 5.80)
WU	527	f	2	69	-	29	-	8.00	(3.60- 17.90)
Subtotal WU								3.30	(1.97- 5.52)
WYNDE6	613	m	0	47	92	42	589	7.16	(4.47- 11.47)
WYNDE6	614	m	0	88	139	42	589	8.88	(5.88- 13.40)
WYNDE6	615	m	0	241	301	42	589	11.23	(7.87- 16.03)
WYNDE6	637	f	0	63	90	58	673	8.12	(5.34- 12.35)
WYNDE6	638	f	0	113	94	58	673	13.95	(9.51- 20.46)
WYNDE6	639	f	0	150	91	58	673	19.13	(13.16- 27.80)
Subtotal WYNDE6								11.24	(9.55- 13.22)
ZHENG	531	m	0	15	66	29	94	0.74	(0.37- 1.48)
ZHENG	532	m	0	64	109	29	94	1.90	(1.13- 3.20)
ZHENG	533	m	0	44	43	29	94	3.32	(1.84- 5.99)
ZHENG	538	f	0	7	16	119	184	0.68	(0.27- 1.69)
ZHENG	539	f	0	26	28	119	184	1.44	(0.80- 2.57)
Subtotal ZHENG								1.57	(1.19- 2.08)
Partial Totals				1609	93490	974	124194		

*prospective study

Table 3H11 - 2

IESLC - Meta-analysis of Ever/current Smoking by Age started, Overview
Adenocarcinoma, Any Product (or Cigarettes if Any not available)
Most adjusted

REF	NRR	SEX	AD	Ys	Ws	Qs	Ps
BARBON	736	m	1	1.74	5.71	0.02	0.0000
BARBON	737	m	1	1.79	6.04	0.07	0.0000
BARBON	738	m	1	3.51	4.40	14.62	0.0000
Subtotal BARBON				2.24	16.15	14.71	
BENHAM	521	m	0	1.10	5.72	1.99	0.0088
BENHAM	522	m	0	1.41	6.17	0.49	0.0005
Subtotal BENHAM				1.26	11.89	2.48	
*ENGELA	527	m	0	0.52	3.73	5.09	0.3164
*ENGELA	528	m	0	1.42	5.47	0.39	0.0009
Subtotal ENGELA				1.05	9.20	5.48	
HAENSZ	509	f	0	0.15	7.71	18.22	0.6808
HAENSZ	510	f	0	0.03	4.45	12.13	0.9433
Subtotal HAENSZ				0.11	12.15	30.35	
JEDRYC	538	m	0	1.37	5.91	0.58	0.0008
JEDRYC	539	m	0	1.69	5.40	0.00	0.0001
JEDRYC	540	m	0	1.50	4.19	0.15	0.0022
Subtotal JEDRYC				1.52	15.50	0.73	
MATOS	656	m	2	1.39	3.36	0.30	0.0111
MATOS	657	m	2	1.97	4.01	0.33	0.0001
MATOS	658	m	2	1.89	3.76	0.15	0.0003
Subtotal MATOS				1.77	11.13	0.79	
PEZZOT	552	m	0	1.89	2.46	0.10	0.0030
PEZZOT	553	m	0	2.17	2.64	0.63	0.0004
PEZZOT	554	m	0	1.65	2.14	0.00	0.0160
Subtotal PEZZOT				1.92	7.23	0.74	
SVENSS	525	f	0	1.11	4.78	1.59	0.0154
SVENSS	526	f	0	1.46	4.63	0.25	0.0017
SVENSS	527	f	0	1.49	6.24	0.25	0.0002
Subtotal SVENSS				1.36	15.64	2.09	
WAKAI	511	m	0	0.26	2.32	4.71	0.6892
WAKAI	512	m	0	0.84	6.06	4.37	0.0395
WAKAI	513	m	0	0.68	4.77	4.82	0.1366
Subtotal WAKAI				0.68	13.16	13.89	
WU	525	f	2	0.10	3.55	8.99	0.8574
WU	526	f	2	0.92	4.97	2.94	0.0410
WU	527	f	2	2.08	5.97	0.93	0.0000
Subtotal WU				1.19	14.50	12.86	
WYNDE6	613	m	0	1.97	17.35	1.39	0.0000
WYNDE6	614	m	0	2.18	22.69	5.63	0.0000
WYNDE6	615	m	0	2.42	30.32	16.28	0.0000
WYNDE6	637	f	0	2.09	21.88	3.66	0.0000
WYNDE6	638	f	0	2.64	26.17	23.61	0.0000
WYNDE6	639	f	0	2.95	27.49	44.02	0.0000
Subtotal WYNDE6				2.42	145.89	94.59	
ZHENG	531	m	0	-0.31	7.88	31.24	0.3910
ZHENG	532	m	0	0.64	14.30	15.53	0.0149
ZHENG	533	m	0	1.20	10.98	2.60	0.0001
ZHENG	538	f	0	-0.39	4.56	19.67	0.4038
ZHENG	539	f	0	0.36	11.36	19.91	0.2228
Subtotal ZHENG				0.45	49.08	88.95	

N	38
NS	12

Table 3H11 - 3

IESLC - Meta-analysis of Ever/current Smoking by Age started, Overview
Adenocarcinoma, Any Product (or Cigarettes if Any not available)
Most adjusted

		Sex		
	combined	male	female	Total
N		25	13	38
NS		9	5	14

In this overview table, other than the "N" rows, entries in the "absent" and "Total" columns may be invalid and should be ignored

	Age started (broad categories)				
	absent	19+k26	15-25k18	1-17k14	Total
N	15	13	5	5	38
NS	8	12	4	4	28
Wt	84.55	102.50	64.32	70.16	321.53
Het Chi	48.24	49.04	7.21	15.03	267.63
Het df	14	12	4	4	37
Het P	***	***	N.S.	**	***
Fixed RR	2.45	3.79	9.74	13.62	5.40
RRl	1.98	3.12	7.63	10.78	4.84
RRu	3.03	4.60	12.43	17.22	6.02
P	+++	+++	+++	+++	+++
Random RR	2.56	3.21	8.84	12.34	3.99
RRl	1.70	2.12	6.14	7.23	2.94
RRu	3.84	4.87	12.73	21.08	5.43
P	+++	+++	+++	+++	+++

		Age started (narrow categories)						
	absent	27+k30	23-29k26	19-25k22	15-21k18	11-17k14	1-13k10	Total
N	27	3		2	5		1	38
NS	12	2		2	4		1	21
Wt	230.71	14.76		9.60	64.32		2.14	321.53
Het Chi	179.10	0.72		0.70	7.21		0.00	267.63
Het df	26	2		1	4		0	37
Het P	***	N.S.		N.S.	N.S.		N.S.	***
Fixed RR	5.29	0.78		3.24	9.74		5.19	5.40
RRl	4.65	0.47		1.72	7.63		1.36	4.84
RRu	6.02	1.31		6.10	12.43		19.85	6.02
P	+++	N.S.		+++	+++		+	+++
Random RR	4.13	0.78		3.24	8.84		5.19	3.99
RRl	2.90	0.47		1.72	6.14		1.36	2.94
RRu	5.89	1.31		6.10	12.73		19.85	5.43
P	+++	N.S.		+++	+++		+	+++

Table 3H11 - 3

IESLC - Meta-analysis of Ever/current Smoking by Age started, Overview
Adenocarcinoma, Any Product (or Cigarettes if Any not available)
Most adjusted

MALES

	Age started (broad categories) absent	19+k26	15-25k18	1-17k14	Total
N	8	9	4	4	25
NS	5	9	4	4	22
Wt	42.37	64.59	38.15	42.68	187.78
Het Chi	22.02	19.77	1.52	9.83	110.62
Het df	7	8	3	3	24
Het P	**	*	N.S.	*	***
Fixed RR	2.59	3.67	7.61	10.95	5.04
RRl	1.92	2.88	5.54	8.11	4.37
RRu	3.50	4.68	10.45	14.78	5.82
P	+++	+++	+++	+++	+++
Random RR	2.77	3.55	7.61	10.46	4.39
RRl	1.58	2.36	5.54	5.18	3.17
RRu	4.85	5.34	10.45	21.13	6.08
P	+++	+++	+++	+++	+++

Age started (narrow categories)

	absent	27+k30	23-29k26	19-25k22	15-21k18	11-17k14	1-13k10	Total
N	18	2			4		1	25
NS	9	2			4		1	16
Wt	137.29	10.20			38.15		2.14	187.78
Het Chi	69.17	0.58			1.52		0.00	110.62
Het df	17	1			3		0	24
Het P	***	N.S.			N.S.		N.S.	***
Fixed RR	5.14	0.84			7.61		5.19	5.04
RRl	4.35	0.45			5.54		1.36	4.37
RRu	6.07	1.55			10.45		19.85	5.82
P	+++	N.S.			+++		+	+++
Random RR	4.61	0.84			7.61		5.19	4.39
RRl	3.22	0.45			5.54		1.36	3.17
RRu	6.60	1.55			10.45		19.85	6.08
P	+++	N.S.			+++		+	+++

FEMALES

	Age started (broad categories) absent	19+k26	15-25k18	1-17k14	Total
N	7	4	1	1	13
NS	4	4	1	1	8
Wt	42.18	37.91	26.17	27.49	133.75
Het Chi	25.96	29.08	0.00	0.00	154.95
Het df	6	3	0	0	12
Het P	***	***	N.S.	N.S.	***
Fixed RR	2.32	4.00	13.95	19.13	5.93
RRl	1.71	2.91	9.51	13.16	5.01
RRu	3.13	5.50	20.46	27.80	7.03
P	+++	+++	+++	+++	+++
Random RR	2.35	2.46	13.95	19.13	3.29
RRl	1.24	0.80	9.51	13.16	1.74
RRu	4.46	7.54	20.46	27.80	6.24
P	++	N.S.	+++	+++	+++

Table 3H11 - 3

IESLC - Meta-analysis of Ever/current Smoking by Age started, Overview
Adenocarcinoma, Any Product (or Cigarettes if Any not available)
Most adjusted

FEMALES

	absent	Age started (narrow categories) 27+k30	23-29k26	19-25k22	15-21k18	11-17k14	1-13k10	Total
N	9	1		2	1			13
NS	5	1		2	1			8
Wt	93.42	4.56		9.60	26.17			133.75
Het Chi	109.64	0.00		0.70	0.00			154.95
Het df	8	0		1	0			12
Het P	***	N.S.		N.S.	N.S.			***
Fixed RR	5.52	0.68		3.24	13.95			5.93
RRl	4.51	0.27		1.72	9.51			5.01
RRu	6.77	1.69		6.10	20.46			7.03
P	+++	N.S.		+++	+++			+++
Random RR	3.27	0.68		3.24	13.95			3.29
RRl	1.48	0.27		1.72	9.51			1.74
RRu	7.21	1.69		6.10	20.46			6.24
P	++	N.S.		+++	+++			+++

Table 3H11 - 4

IESLC - Meta-analysis of Ever/current Smoking by Age started, Overview
Adenocarcinoma, Any Product (or Cigarettes if Any not available)
Least adjusted

REF	NRR	X	SEX	AGEL	AGEH	RACE	YF	LC TYPE	LOC	START	ST	NLC	R	VB	P	H	AD	SM	PRODUCT	exL	exH	S1	S2	DENOM	De
BARBON	731	x	m	0	0	all	-	a	Eu:wst	1979	CC	755	n	bl	y	y	0	ev	all/unsp	20	999	1	0	nev any	st
BARBON	732	x	m	0	0	all	-	a	Eu:wst	1979	CC	755	n	bl	y	y	0	ev	all/unsp	15	19	2	4	nev any	st
BARBON	733	x	m	0	0	all	-	a	Eu:wst	1979	CC	755	n	bl	y	y	0	ev	all/unsp	1	14	3	0	nev any	st
BENHAM	521		m	0	0	all	-	KII	Eu:wst	1976	CC	1625	n	bl	n	y	0	ev	cig only	20	999	1	0	nev any	st
BENHAM	522		m	0	0	all	-	KII	Eu:wst	1976	CC	1625	n	bl	n	y	0	ev	cig only	1	19	0	0	nev any	st
ENGELA	527		m	0	0	all	0	a	Eu:Sca	1964	pr	435	n	bl	n	n	0	cu	cig+/-ot	20	999	1	0	nev cigs	st
ENGELA	528		m	0	0	all	0	a	Eu:Sca	1964	pr	435	n	bl	n	n	0	cu	cig+/-ot	1	19	0	0	nev cigs	st
HAENSZ	509		f	0	0	all	-	a	NAmer	1955	CC	158	n	bl	n	y	0	ev	cig+/-ot	25	999	1	0	nev any	st
HAENSZ	510		f	0	0	all	-	a	NAmer	1955	CC	158	n	bl	n	y	0	ev	cig+/-ot	1	24	0	0	nev any	st
JEDRYC	538		m	0	0	all	-	a	Eu:est	1980	CC	1630	n	bl	y	n	0	ev	cig+/-ot	19	999	1	0	nev any	st
JEDRYC	539		m	0	0	all	-	a	Eu:est	1980	CC	1630	n	bl	y	n	0	ev	cig+/-ot	17	18	2	4	nev any	st
JEDRYC	540		m	0	0	all	-	a	Eu:est	1980	CC	1630	n	bl	y	n	0	ev	cig+/-ot	1	16	3	0	nev any	st
MATOS	651	x	m	0	0	all	-	a	SCAmer	1994	CC	200	n	bl	n	n	0	ev	cig+/-ot	20	999	1	0	nev any	st
MATOS	652	x	m	0	0	all	-	a	SCAmer	1994	CC	200	n	bl	n	n	0	ev	cig+/-ot	15	19	2	4	nev any	st
MATOS	653	x	m	0	0	all	-	a	SCAmer	1994	CC	200	n	bl	n	n	0	ev	cig+/-ot	1	14	3	0	nev any	st
PEZZOT	552		m	0	0	all	-	a	SCAmer	1987	CC	215	n	bl	n	y	0	ev	cig only	19	999	1	0	nev cigs	st
PEZZOT	553		m	0	0	all	-	a	SCAmer	1987	CC	215	n	bl	n	y	0	ev	cig only	14	18	0	0	nev cigs	st
PEZZOT	554		m	0	0	all	-	a	SCAmer	1987	CC	215	n	bl	n	y	0	ev	cig only	1	13	0	6	nev cigs	st
SVENSS	525		f	0	0	all	-	a	Eu:Sca	1983	CC	210	n	bl	n	n	0	cu	all/unsp	26	999	1	0	nev any	st
SVENSS	526		f	0	0	all	-	a	Eu:Sca	1983	CC	210	n	bl	n	n	0	cu	all/unsp	19	25	0	3	nev any	st
SVENSS	527		f	0	0	all	-	a	Eu:Sca	1983	CC	210	n	bl	n	n	0	cu	all/unsp	0	18	0	0	nev any	st
WAKAI	511		m	0	0	all	-	a	As:Jap	1988	CC	333	n	bl	n	y	0	cu	cig+/-ot	30	999	0	1	nev any	st
WAKAI	512		m	0	0	all	-	a	As:Jap	1988	CC	333	n	bl	n	y	0	cu	cig+/-ot	20	29	1	0	nev any	st
WAKAI	513		m	0	0	all	-	a	As:Jap	1988	CC	333	n	bl	n	y	0	cu	cig+/-ot	1	19	0	0	nev any	st
WU	501	x	f	0	0	wh	-	a	NAmer	1981	CC	220	n	bl	n	y	0	cu	all/unsp	25	999	1	0	nev any	st
WU	502	x	f	0	0	wh	-	a	NAmer	1981	CC	220	n	bl	n	y	0	cu	all/unsp	19	24	0	3	nev any	st
WU	503	x	f	0	0	wh	-	a	NAmer	1981	CC	220	n	bl	n	y	0	cu	all/unsp	0	18	0	0	nev any	st
WYNDE6	613		m	0	0	wh	-	a	NAmer	1969	CC	4423	n	bl	n	y	0	ev	cig+/-ot	21	999	1	0	nev cigs	st
WYNDE6	614		m	0	0	wh	-	a	NAmer	1969	CC	4423	n	bl	n	y	0	ev	cig+/-ot	18	20	2	4	nev cigs	st
WYNDE6	615		m	0	0	wh	-	a	NAmer	1969	CC	4423	n	bl	n	y	0	ev	cig+/-ot	1	17	3	0	nev cigs	st
WYNDE6	637		f	0	0	wh	-	a	NAmer	1969	CC	4423	n	bl	n	y	0	ev	cig+/-ot	21	999	1	0	nev cigs	st
WYNDE6	638		f	0	0	wh	-	a	NAmer	1969	CC	4423	n	bl	n	y	0	ev	cig+/-ot	18	20	2	4	nev cigs	st
WYNDE6	639		f	0	0	wh	-	a	NAmer	1969	CC	4423	n	bl	n	y	0	ev	cig+/-ot	1	17	3	0	nev cigs	st
ZHENG	531		m	0	0	all	-	a	As:Chi	1982	CC	540	n	ot	*	y	0	ev	cig+/-ot	30	999	0	1	nev cigs	or
ZHENG	532		m	0	0	all	-	a	As:Chi	1982	CC	540	n	ot	*	y	0	ev	cig+/-ot	20	29	1	0	nev cigs	st
ZHENG	533		m	0	0	all	-	a	As:Chi	1982	CC	540	n	ot	*	y	0	ev	cig+/-ot	1	19	0	0	nev cigs	st
ZHENG	538		f	0	0	all	-	a	As:Chi	1982	CC	540	n	ot	*	y	0	ev	cig+/-ot	30	999	0	1	nev cigs	or
ZHENG	539		f	0	0	all	-	a	As:Chi	1982	CC	540	n	ot	*	y	0	ev	cig+/-ot	1	29	0	0	nev cigs	st

Cigarette type is all/unspec for all RRs

In this overview table, subtotals and Qs values may be invalid and should be ignored

Table 3H11 - 5

IESLC - Meta-analysis of Ever/current Smoking by Age started, Overview
Adenocarcinoma, Any Product (or Cigarettes if Any not available)
Least adjusted

REF	NRR	SEX	AD	Number Exposed Case	Cont	Non-exposed Case	Cont	RR	95.00%CI
BARBON	731	m	0	45	207	7	188	5.84	(2.57- 13.26)
BARBON	732	m	0	77	337	7	188	6.14	(2.77- 13.58)
BARBON	733	m	0	29	23	7	188	33.86	(13.33- 86.00)
Subtotal BARBON								9.61	(5.90- 15.63)
BENHAM	521	m	0	41	64	9	42	2.99	(1.32- 6.79)
BENHAM	522	m	0	69	79	9	42	4.08	(1.85- 8.97)
Subtotal BENHAM								3.51	(1.99- 6.20)
*ENGELA	527	m	0	8	39957	7	58716	1.68	(0.61- 4.63)
*ENGELA	528	m	0	25	50732	7	58716	4.13	(1.79- 9.56)
Subtotal ENGELA								2.87	(1.50- 5.47)
HAENSZ	509	f	0	12	66	37	236	1.16	(0.57- 2.35)
HAENSZ	510	f	0	6	37	37	236	1.03	(0.41- 2.62)
Subtotal HAENSZ								1.11	(0.63- 1.95)
JEDRYC	538	m	0	48	502	7	289	3.95	(1.76- 8.84)
JEDRYC	539	m	0	29	221	7	289	5.42	(2.33- 12.60)
JEDRYC	540	m	0	12	111	7	289	4.46	(1.71- 11.63)
Subtotal JEDRYC								4.56	(2.77- 7.50)
MATOS	651	m	0	13	73	5	110	3.92	(1.34- 11.46)
MATOS	652	m	0	40	120	5	110	7.33	(2.79- 19.25)
MATOS	653	m	0	26	90	5	110	6.36	(2.35- 17.22)
Subtotal MATOS								5.81	(3.24- 10.39)
PEZZOT	552	m	0	18	105	3	116	6.63	(1.90- 23.15)
PEZZOT	553	m	0	33	145	3	116	8.80	(2.63- 29.42)
PEZZOT	554	m	0	9	67	3	116	5.19	(1.36- 19.85)
Subtotal PEZZOT								6.84	(3.30- 14.18)
SVENSS	525	f	0	10	18	22	120	3.03	(1.24- 7.43)
SVENSS	526	f	0	11	14	22	120	4.29	(1.72- 10.66)
SVENSS	527	f	0	17	21	22	120	4.42	(2.01- 9.68)
Subtotal SVENSS								3.90	(2.38- 6.40)
WAKAI	511	m	0	4	25	8	65	1.30	(0.36- 4.70)
WAKAI	512	m	0	52	183	8	65	2.31	(1.04- 5.12)
WAKAI	513	m	0	18	74	8	65	1.98	(0.81- 4.85)
Subtotal WAKAI								1.97	(1.15- 3.38)
WU	501	f	0	8	14	29	62	1.22	(0.46- 3.24)
WU	502	f	0	22	15	29	62	3.14	(1.42- 6.91)
WU	503	f	0	69	21	29	62	7.02	(3.64- 13.56)
Subtotal WU								3.74	(2.38- 5.85)
WYNDE6	613	m	0	47	92	42	589	7.16	(4.47- 11.47)
WYNDE6	614	m	0	88	139	42	589	8.88	(5.88- 13.40)
WYNDE6	615	m	0	241	301	42	589	11.23	(7.87- 16.03)
WYNDE6	637	f	0	63	90	58	673	8.12	(5.34- 12.35)
WYNDE6	638	f	0	113	94	58	673	13.95	(9.51- 20.46)
WYNDE6	639	f	0	150	91	58	673	19.13	(13.16- 27.80)
Subtotal WYNDE6								11.24	(9.55- 13.22)
ZHENG	531	m	0	15	66	29	94	0.74	(0.37- 1.48)
ZHENG	532	m	0	64	109	29	94	1.90	(1.13- 3.20)
ZHENG	533	m	0	44	43	29	94	3.32	(1.84- 5.99)
ZHENG	538	f	0	7	16	119	184	0.68	(0.27- 1.69)
ZHENG	539	f	0	26	28	119	184	1.44	(0.80- 2.57)
Subtotal ZHENG								1.57	(1.19- 2.08)
Totals				1609	94390	974	125274		

*prospective study

Table 3H11 - 5

IESLC - Meta-analysis of Ever/current Smoking by Age started, Overview
Adenocarcinoma, Any Product (or Cigarettes if Any not available)
Least adjusted

REF	NRR	SEX	AD	Ys	Ws	Qs	Ps
BARBON	731	m	0	1.76	5.71	0.03	0.0000
BARBON	732	m	0	1.81	6.09	0.10	0.0000
BARBON	733	m	0	3.52	4.42	14.90	0.0000
Subtotal BARBON				2.26	16.22	15.03	
BENHAM	521	m	0	1.10	5.72	2.00	0.0088
BENHAM	522	m	0	1.41	6.17	0.49	0.0005
Subtotal BENHAM				1.26	11.89	2.49	
*ENGELA	527	m	0	0.52	3.73	5.10	0.3164
*ENGELA	528	m	0	1.42	5.47	0.39	0.0009
Subtotal ENGELA				1.05	9.20	5.49	
HAENSZ	509	f	0	0.15	7.71	18.25	0.6808
HAENSZ	510	f	0	0.03	4.45	12.15	0.9433
Subtotal HAENSZ				0.11	12.15	30.40	
JEDRYC	538	m	0	1.37	5.91	0.58	0.0008
JEDRYC	539	m	0	1.69	5.40	0.00	0.0001
JEDRYC	540	m	0	1.50	4.19	0.15	0.0022
Subtotal JEDRYC				1.52	15.50	0.73	
MATOS	651	m	0	1.37	3.34	0.34	0.0126
MATOS	652	m	0	1.99	4.13	0.39	0.0001
MATOS	653	m	0	1.85	3.87	0.10	0.0003
Subtotal MATOS				1.76	11.33	0.83	
PEZZOT	552	m	0	1.89	2.46	0.10	0.0030
PEZZOT	553	m	0	2.17	2.64	0.63	0.0004
PEZZOT	554	m	0	1.65	2.14	0.00	0.0160
Subtotal PEZZOT				1.92	7.23	0.73	
SVENSS	525	f	0	1.11	4.78	1.60	0.0154
SVENSS	526	f	0	1.46	4.63	0.25	0.0017
SVENSS	527	f	0	1.49	6.24	0.25	0.0002
Subtotal SVENSS				1.36	15.64	2.10	
WAKAI	511	m	0	0.26	2.32	4.72	0.6892
WAKAI	512	m	0	0.84	6.06	4.38	0.0395
WAKAI	513	m	0	0.68	4.77	4.83	0.1366
Subtotal WAKAI				0.68	13.16	13.92	
WU	501	f	0	0.20	4.05	8.95	0.6871
WU	502	f	0	1.14	6.15	1.82	0.0046
WU	503	f	0	1.95	8.87	0.61	0.0000
Subtotal WU				1.32	19.06	11.38	
WYNDE6	613	m	0	1.97	17.35	1.38	0.0000
WYNDE6	614	m	0	2.18	22.69	5.60	0.0000
WYNDE6	615	m	0	2.42	30.32	16.23	0.0000
WYNDE6	637	f	0	2.09	21.88	3.64	0.0000
WYNDE6	638	f	0	2.64	26.17	23.54	0.0000
WYNDE6	639	f	0	2.95	27.49	43.93	0.0000
Subtotal WYNDE6				2.42	145.89	94.31	
ZHENG	531	m	0	-0.31	7.88	31.28	0.3910
ZHENG	532	m	0	0.64	14.30	15.57	0.0149
ZHENG	533	m	0	1.20	10.98	2.61	0.0001
ZHENG	538	f	0	-0.39	4.56	19.70	0.4038
ZHENG	539	f	0	0.36	11.36	19.95	0.2228
Subtotal ZHENG				0.45	49.08	89.10	

N	38
NS	12

Table 3H11 - 6

IESLC - Meta-analysis of Ever/current Smoking by Age started, Overview
Adenocarcinoma, Any Product (or Cigarettes if Any not available)
Least adjusted

	Sex			
	combined	male	female	Total
N		25	13	38
NS		9	5	14

In this overview table, other than the "N" rows, entries in the "absent" and "Total" columns may be invalid and should be ignored

	Age started (broad categories)				
	absent	19+k26	15-25k18	1-17k14	Total
N	15	13	5	5	38
NS	8	12	4	4	28
Wt	88.62	102.98	64.47	70.29	326.36
Het Chi	49.93	48.90	7.06	15.43	266.53
Het df	14	12	4	4	37
Het P	***	***	N.S.	**	***
Fixed RR	2.55	3.79	9.76	13.60	5.40
RRl	2.07	3.12	7.65	10.76	4.85
RRu	3.15	4.59	12.46	17.18	6.02
P	+++	+++	+++	+++	+++
Random RR	2.60	3.23	8.90	12.28	4.02
RRl	1.73	2.14	6.21	7.15	2.97
RRu	3.89	4.88	12.76	21.08	5.44
P	+++	+++	+++	+++	+++

		Age started (narrow categories)						
	absent	27+k30	23-29k26	19-25k22	15-21k18	11-17k14	1-13k10	Total
N	27	3		2	5		1	38
NS	12	2		2	4		1	21
Wt	234.21	14.76		10.77	64.47		2.14	326.36
Het Chi	179.03	0.72		0.26	7.06		0.00	266.53
Het df	26	2		1	4		0	37
Het P	***	N.S.		N.S.	N.S.		N.S.	***
Fixed RR	5.28	0.78		3.59	9.76		5.19	5.40
RRl	4.65	0.47		1.97	7.65		1.36	4.85
RRu	6.01	1.31		6.52	12.46		19.85	6.02
P	+++	N.S.		+++	+++		+	+++
Random RR	4.12	0.78		3.59	8.90		5.19	4.02
RRl	2.90	0.47		1.97	6.21		1.36	2.97
RRu	5.86	1.31		6.52	12.76		19.85	5.44
P	+++	N.S.		+++	+++		+	+++

Table 3H11 - 6

IESLC - Meta-analysis of Ever/current Smoking by Age started, Overview
Adenocarcinoma, Any Product (or Cigarettes if Any not available)
Least adjusted

MALES

	Age started (broad categories) absent	19+k26	15-25k18	1-17k14	Total
N	8	9	4	4	25
NS	5	9	4	4	22
Wt	42.37	64.57	38.31	42.80	188.04
Het Chi	22.02	19.89	1.45	10.17	111.05
Het df	7	8	3	3	24
Het P	**	*	N.S.	*	***
Fixed RR	2.59	3.67	7.65	10.92	5.05
RRl	1.92	2.88	5.57	8.09	4.38
RRu	3.50	4.69	10.50	14.74	5.83
P	+++	+++	+++	+++	+++
Random RR	2.77	3.55	7.65	10.40	4.40
RRl	1.58	2.36	5.57	5.11	3.18
RRu	4.85	5.35	10.50	21.19	6.10
P	+++	+++	+++	+++	+++

		Age started (narrow categories)						
	absent	27+k30	23-29k26	19-25k22	15-21k18	11-17k14	1-13k10	Total
N	18	2			4		1	25
NS	9	2			4		1	16
Wt	137.40	10.20			38.31		2.14	188.04
Het Chi	69.48	0.58			1.45		0.00	111.05
Het df	17	1			3		0	24
Het P	***	N.S.			N.S.		N.S.	***
Fixed RR	5.14	0.84			7.65		5.19	5.05
RRl	4.35	0.45			5.57		1.36	4.38
RRu	6.08	1.55			10.50		19.85	5.83
P	+++	N.S.			+++		+	+++
Random RR	4.61	0.84			7.65		5.19	4.40
RRl	3.22	0.45			5.57		1.36	3.18
RRu	6.60	1.55			10.50		19.85	6.10
P	+++	N.S.			+++		+	+++

FEMALES

	Age started (broad categories) absent	19+k26	15-25k18	1-17k14	Total
N	7	4	1	1	13
NS	4	4	1	1	8
Wt	46.25	38.41	26.17	27.49	138.31
Het Chi	27.90	28.85	0.00	0.00	153.47
Het df	6	3	0	0	12
Het P	***	***	N.S.	N.S.	***
Fixed RR	2.52	3.98	13.95	19.13	5.92
RRl	1.89	2.90	9.51	13.16	5.01
RRu	3.36	5.46	20.46	27.80	6.99
P	+++	+++	+++	+++	+++
Random RR	2.42	2.51	13.95	19.13	3.35
RRl	1.28	0.84	9.51	13.16	1.80
RRu	4.55	7.55	20.46	27.80	6.24
P	++	N.S.	+++	+++	+++

Table 3H11 - 6

IESLC - Meta-analysis of Ever/current Smoking by Age started, Overview
Adenocarcinoma, Any Product (or Cigarettes if Any not available)
Least adjusted

FEMALES

	absent	27+k30	23-29k26	19-25k22	15-21k18	11-17k14	1-13k10	Total
		Age started (narrow categories)						
N	9	1		2	1			13
NS	5	1		2	1			8
Wt	96.81	4.56		10.77	26.17			138.31
Het Chi	109.29	0.00		0.26	0.00			153.47
Het df	8	0		1	0			12
Het P	***	N.S.		N.S.	N.S.			***
Fixed RR	5.50	0.68		3.59	13.95			5.92
RRl	4.50	0.27		1.97	9.51			5.01
RRu	6.71	1.69		6.52	20.46			6.99
P	+++	N.S.		+++	+++			+++
Random RR	3.27	0.68		3.59	13.95			3.35
RRl	1.51	0.27		1.97	9.51			1.80
RRu	7.07	1.69		6.52	20.46			6.24
P	++	N.S.		+++	+++			+++

Table 3H11 - 7

IESLC - Meta-analysis of Ever/current Smoking by Age started, Overview
Adenocarcinoma, Any Product (or Cigarettes if Any not available)
Excluded studies (and stage at which they were excluded)

1	BECHER BLOT1 BROWN3 CARPEN CHYOU DARBY DOLL2 GARCIA GRAHAM GURSEL HAMMO2 JAHN JAIN LAUSSM PRESCO QIAO TVERDA WIGLE WYNDE3
2	AKIBA AMANDU AMES AXELSS BENSHL BEST BOUCHA BOUCOT BROSS CHEN CPSII DEAN2 DESTEF DORGAN DOSEME FAN GARSHI GER GILLIS HAMMON HUMBLE JUSSAW KAISE2 KATSOU KAUFMA KOO KREUZE LEVIN MCCONN NOTAN2 OSANN2 PEZZO2 PISANI RESTRE SADOWS VUTUC WANG2 WATSON WU2 WUWILL WYNDE2 WYNDE8 XU ZHOU
3	GUO MCDUFF SPITZ STASZE ZHANG
4	AGUDO ARMADA AUVINE BOFFET BRESLO BUFFLE CEDERL CHEN2 CHIAZZ CHOI CORREA CPSI DAMBER DEAN3 DOLL DORN GAO GAO2 GENG HIRAYA HOLE HU HU2 JOLY KOULUM LETOUR LIAW LIU3 LIU4 LIU5 LUBIN LUBIN2 LUO MIGRAN MRFITR PERNU QIAO2 RACHTA SEGI2 SOBUE SPEIZE SUZUK2 TIZZAN WYNDE7 YUAN
5	ALDERS
10	HEGMAN KHUDER

Table 3H11 - 8
Potentially overlapping studies

REF	REFGP	PRINC	.	OVERLAP/LINK
BENHAM	LUBIN2	2		Subset of Lubin2
WYNDE6	WYNDE6	1		WYNDE5/6/7/8

Table 3H12 -

IESLC - Meta-analysis of Ever/current Smoking, Age started, "Low"
Adenocarcinoma, Any Product (or Cigarettes if Any not available)

This analysis is restricted to results for:
1) Ever/current smokers
2) Results by Age started
3) Categorical results by Age started
4) Adenocarcinoma (or near equivalent)
5) Results complete enough for use in metaanalysis

Within each study, results are then selected (in the following order of preference, within each sex) for:
6) SMKSTA: ever, current
7) PRODUCT: all/unspec, cigarettes regardless of other products, cigarettes only
8) CIGTYPE: all/unspecified, MC regardless of HR, MC only
9) (not applicable)
10) DENOM: never smoked anything, never smoked cigarettes, never any + low, never cigs + low
11) Followup period (YF, prospective studies): whole study (coded as 0) or longest available
12) LCtype: adeno or nearest available, but not squamous. (q = squamous, s = small,
a = adeno, l = large, KII = Kreyberg II, al = alveolar, br = bronchiolar, u = undifferentiated)
13) Race: all or nearest available, otherwise by race (wh or w = white, bl or b = black, hi = hispanic
ch = chinese, jap = japanese, haw = hawaiian, w+o = white + oriental, sca = scandinavian, as = asian)
14) Age started "low" in key scheme 1 (key value 26, maximum range 19+)
15) For overlapping studies: principal rather than subsidiary studies
Finally by Age: whole study (coded as 0) if available, otherwise by widest available age group
and then for single sex results (m, f) in preference to results for both sexes combined (c).

Results adjusted (AD) for the most potential confounders are then chosen in Sections -1 to -3
and results adjusted for the least confounders in Sections -4 to -6. (Those least adjusted results which
actually differ from the most adjusted are marked 'x' in column X in Section -4)

Section -7 shows excluded studies, together with the stage (as above) at which no qualifying
results were found.

Section -8 lists the potentially overlapping studies which have been included (1=principal, 2=subsidiary).

Section -9 lists any results which would have been included in preference except that they had data not complete
enough for use in meta-analysis, with their significance (yes/no), if known, and any further comment as entered
on the database. It also lists as "gap" any categories for which no data were presented by the original authors.

In addition to those mentioned above, the following fields, levels and abbreviations are used:
* or nk = not known, n = no, y = yes, ot = other
ev = ever, cu = current, nev = never
all/unspec = all or unspecified, cig+/-ot = cigarettes irrespective of other products (cigar, pipe etc)
MC = manufactured cigarettes, HR = hand-rolled cigarettes
exL, exH = range of exposure (low and high) in the smoking group, in terms of Age started
REF: 6-character study reference
NRR: number of the RR on the database within the study
ST : study type (CC = case control, pr or prosp = prospective)
NLC: number of lung cancer cases in whole study
R : risky occupational population (n = no, m = mining, o = other risky)
VB : national cigarette type (V = at least 75% Virginia, bl = at least 75% blended, ot = other)
P : any proxy use
H : full histological confirmation
De : derivation of RR/CI (or = original, st = standard method, ot = other method of estimation)

Table 3H12 - 1

IESLC - Meta-analysis of Ever/current Smoking, Age started, "Low"
Adenocarcinoma, Any Product (or Cigarettes if Any not available)
Most adjusted

REF	NRR	SEX	AGEL	AGEH	RACE	YF	LC TYPE	LOC	START	ST	NLC	R	VB	P	H	AD	SM	PRODUCT	exL	exH	DENOM	De
BARBON	736	m	0	0	all	-	a	Eu:wst	1979	CC	755	n	bl	y	y	1	ev	all/unsp	20	999	nev any	or
BENHAM	521	m	0	0	all	-	KII	Eu:wst	1976	CC	1625	n	bl	n	y	0	ev	cig only	20	999	nev any	st
ENGELA	527	m	0	0	all	0	a	Eu:Sca	1964	pr	435	n	bl	n	n	0	cu	cig+/-ot	20	999	nev cigs	st
HAENSZ	509	f	0	0	all	-	a	NAmer	1955	CC	158	n	bl	n	y	0	ev	cig+/-ot	25	999	nev any	st
JEDRYC	538	m	0	0	all	-	a	Eu:est	1980	CC	1630	n	bl	y	n	0	ev	cig+/-ot	19	999	nev any	st
MATOS	656	m	0	0	all	-	a	SCAmer	1994	CC	200	n	bl	n	n	2	ev	cig+/-ot	20	999	nev any	or
PEZZOT	552	m	0	0	all	-	a	SCAmer	1987	CC	215	n	bl	n	y	0	ev	cig only	19	999	nev cigs	st
SVENSS	525	f	0	0	all	-	a	Eu:Sca	1983	CC	210	n	bl	n	n	0	cu	all/unsp	26	999	nev any	st
WAKAI	512	m	0	0	all	-	a	As:Jap	1988	CC	333	n	bl	n	y	0	cu	cig+/-ot	20	29	nev any	st
WU	525	f	0	0	wh	-	a	NAmer	1981	CC	220	n	bl	n	y	2	cu	all/unsp	25	999	nev any	or
WYNDE6	613	m	0	0	wh	-	a	NAmer	1969	CC	4423	n	bl	n	y	0	ev	cig+/-ot	21	999	nev cigs	st
WYNDE6	637	f	0	0	wh	-	a	NAmer	1969	CC	4423	n	bl	n	y	0	ev	cig+/-ot	21	999	nev cigs	st
ZHENG	532	m	0	0	all	-	a	As:Chi	1982	CC	540	n	ot	*	y	0	ev	cig+/-ot	20	29	nev cigs	st

Cigarette type is all/unspec for all RRs

Table 3H12 - 2

IESLC - Meta-analysis of Ever/current Smoking, Age started, "Low"
Adenocarcinoma, Any Product (or Cigarettes if Any not available)
Most adjusted

REF	NRR	SEX	AD	Number Exposed Case	Exposed Cont	Non-exposed Case	Non-exposed Cont	RR	95.00%CI
BARBON	736	m	1	45	-	7	-	5.70	(2.50- 12.90)
BENHAM	521	m	0	41	64	9	42	2.99	(1.32- 6.79)
*ENGELA	527	m	0	8	39957	7	58716	1.68	(0.61- 4.63)
HAENSZ	509	f	0	12	66	37	236	1.16	(0.57- 2.35)
JEDRYC	538	m	0	48	502	7	289	3.95	(1.76- 8.84)
MATOS	656	m	2	13	-	5	-	4.00	(1.40- 11.90)
PEZZOT	552	m	0	18	105	3	116	6.63	(1.90- 23.15)
SVENSS	525	f	0	10	18	22	120	3.03	(1.24- 7.43)
WAKAI	512	m	0	52	183	8	65	2.31	(1.04- 5.12)
WU	525	f	2	8	-	29	-	1.10	(0.40- 3.20)
WYNDE6	613	m	0	47	92	42	589	7.16	(4.47- 11.47)
WYNDE6	637	f	0	63	90	58	673	8.12	(5.34- 12.35)
Subtotal WYNDE6								7.68	(5.62- 10.51)
ZHENG	532	m	0	64	109	29	94	1.90	(1.13- 3.20)
Partial Totals				429	41186	263	60940		

*prospective study

REF	NRR	SEX	AD	Ys	Ws	Qs	Ps
BARBON	736	m	1	1.74	5.71	0.95	0.0000
BENHAM	521	m	0	1.10	5.72	0.32	0.0088
*ENGELA	527	m	0	0.52	3.73	2.47	0.3164
HAENSZ	509	f	0	0.15	7.71	10.81	0.6808
JEDRYC	538	m	0	1.37	5.91	0.01	0.0008
MATOS	656	m	2	1.39	3.36	0.01	0.0111
PEZZOT	552	m	0	1.89	2.46	0.77	0.0030
SVENSS	525	f	0	1.11	4.78	0.24	0.0154
WAKAI	512	m	0	0.84	6.06	1.49	0.0395
WU	525	f	2	0.10	3.55	5.44	0.8574
WYNDE6	613	m	0	1.97	17.35	7.03	0.0000
WYNDE6	637	f	0	2.09	21.88	12.71	0.0000
Subtotal WYNDE6				2.04	39.22	19.74	
ZHENG	532	m	0	0.64	14.30	6.79	0.0149

	N	13
	NS	12
	Wt	102.50
Het	Chi	49.04
Het	df	12
Het	P	***
Fixed	RR	3.79
	RRl	3.12
	RRu	4.60
	P	+++
Random	RR	3.21
	RRl	2.12
	RRu	4.87
	P	+++
Asymm	P	N.S.

Table 3H12 - 3

IESLC - Meta-analysis of Ever/current Smoking, Age started, "Low"
Adenocarcinoma, Any Product (or Cigarettes if Any not available)
Most adjusted

	combined	Sex: male	female	Total
N		9	4	13
NS		9	4	13
Wt		64.59	37.91	102.50
Het Chi		19.77	29.08	49.04
Het df		8	3	12
Het P		*	***	***
Fixed RR		3.67	4.00	3.79
RRl		2.88	2.91	3.12
RRu		4.68	5.50	4.60
P		+++	+++	+++
Random RR		3.55	2.46	3.21
RRl		2.36	0.80	2.12
RRu		5.34	7.54	4.87
P		+++	N.S.	+++
Between Chi				0.18
Between df				1
Between P				N.S.
Btwn(F) P				N.S.
Btwn(R) P				N.S.

	Lung cancer type: a	a+l	a+al+br	KII	not q+u	not q+s	Total
N	12			1			13
NS	11			1			12
Wt	96.78			5.72			102.50
Het Chi	48.70			0.00			49.04
Het df	11			0			12
Het P	***			N.S.			***
Fixed RR	3.84			2.99			3.79
RRl	3.15			1.32			3.12
RRu	4.69			6.79			4.60
P	+++			++			+++
Random RR	3.23			2.99			3.21
RRl	2.07			1.32			2.12
RRu	5.04			6.79			4.87
P	+++			++			+++
Between Chi							0.34
Between df							1
Between P							N.S.
Btwn(F) P							N.S.
Btwn(R) P							N.S.

	Location: NAmer	UK	Scand	othEur	China	Japan	othAs	other	Total
N	4		2	3	1	1		2	13
NS	3		2	3	1	1		2	12
Wt	50.48		8.51	17.34	14.30	6.06		5.81	102.50
Het Chi	32.00		0.73	1.20	0.00	0.00		0.36	49.04
Het df	3		1	2	0	0		1	12
Het P	***		N.S.	N.S.	N.S.	N.S.		N.S.	***
Fixed RR	5.02		2.34	4.06	1.90	2.31		4.95	3.79
RRl	3.81		1.19	2.54	1.13	1.04		2.20	3.12
RRu	6.62		4.58	6.51	3.20	5.12		11.17	4.60
P	+++		+	+++	+	+		+++	+++
Random RR	3.16		2.34	4.06	1.90	2.31		4.95	3.21
RRl	1.19		1.19	2.54	1.13	1.04		2.20	2.12
RRu	8.34		4.58	6.51	3.20	5.12		11.17	4.87
P	+		+	+++	+	+		+++	+++
Between Chi									14.75
Between df									5
Between P									*
Btwn(F) P									N.S.
Btwn(R) P									N.S.

Table 3H12 - 3

IESLC - Meta-analysis of Ever/current Smoking, Age started, "Low"
Adenocarcinoma, Any Product (or Cigarettes if Any not available)
Most adjusted

Detailed Country in "other Europe"

	multi	Germany	othWest	East	Balkans	Total
N			2	1		3
NS			2	1		3
Wt			11.42	5.91		17.34
Het Chi			1.19	0.00		1.20
Het df			1	0		2
Het P			N.S.	N.S.		N.S.
Fixed RR			4.13	3.95		4.06
RRl			2.31	1.76		2.54
RRu			7.37	8.84		6.51
P			+++	+++		+++
Random RR			4.13	3.95		4.06
RRl			2.19	1.76		2.54
RRu			7.77	8.84		6.51
P			+++	+++		+++
Between Chi						0.01
Between df						1
Between P						N.S.
Btwn(F) P						N.S.
Btwn(R) P						N.S.

Detailed Country in "other Asia"

	India	HongKong	other	Total
N				
NS				
Wt				
Het Chi				
Het df				
Het P				N.S.
Fixed RR				
RRl				
RRu				
P				+++
Random RR				
RRl				
RRu				
P				+++
Between Chi				
Between df				
Between P				N.S.
Btwn(F) P				N.S.
Btwn(R) P				N.S.

Detailed other continent

	SCAmer	Total
N	2	2
NS	2	2
Wt	5.81	5.81
Het Chi	0.36	0.36
Het df	1	1
Het P	N.S.	N.S.
Fixed RR	4.95	4.95
RRl	2.20	2.20
RRu	11.17	11.17
P	+++	+++
Random RR	4.95	4.95
RRl	2.20	2.20
RRu	11.17	11.17
P	+++	+++
Between Chi		
Between df		
Between P		N.S.
Btwn(F) P		N.S.
Btwn(R) P		N.S.

Table 3H12 - 3

IESLC - Meta-analysis of Ever/current Smoking, Age started, "Low"
Adenocarcinoma, Any Product (or Cigarettes if Any not available)
Most adjusted

	Start year of study					
	<1960	1960-69	1970-79	1980-89	1990+	Total
N	1	3	2	6	1	13
NS	1	2	2	6	1	12
Wt	7.71	42.96	11.42	37.06	3.36	102.50
Het Chi	0.00	8.04	1.19	7.20	0.00	49.04
Het df	0	2	1	5	0	12
Het P	N.S.	*	N.S.	N.S.	N.S.	***
Fixed RR	1.16	6.73	4.13	2.41	4.00	3.79
RRl	0.57	4.99	2.31	1.75	1.37	3.12
RRu	2.35	9.08	7.37	3.33	11.66	4.60
P	N.S.	+++	+++	+++	+	+++
Random RR	1.16	5.43	4.13	2.51	4.00	3.21
RRl	0.57	2.79	2.19	1.67	1.37	2.12
RRu	2.35	10.58	7.77	3.76	11.66	4.87
P	N.S.	+++	+++	+++	+	+++
Between Chi						32.61
Between df						4
Between P						***
Btwn(F) P						*
Btwn(R) P						*

	Study type (1)		
	CC	other	Total
N	12	1	13
NS	11	1	12
Wt	98.77	3.73	102.50
Het Chi	46.47	0.00	49.04
Het df	11	0	12
Het P	***	N.S.	***
Fixed RR	3.91	1.68	3.79
RRl	3.21	0.61	3.12
RRu	4.76	4.63	4.60
P	+++	N.S.	+++
Random RR	3.36	1.68	3.21
RRl	2.19	0.61	2.12
RRu	5.17	4.63	4.87
P	+++	N.S.	+++
Between Chi			2.57
Between df			1
Between P			N.S.
Btwn(F) P			N.S.
Btwn(R) P			N.S.

	Study type (2)			
	CC	prosp	other	Total
N	12	1		13
NS	11	1		12
Wt	98.77	3.73		102.50
Het Chi	46.47	0.00		49.04
Het df	11	0		12
Het P	***	N.S.		***
Fixed RR	3.91	1.68		3.79
RRl	3.21	0.61		3.12
RRu	4.76	4.63		4.60
P	+++	N.S.		+++
Random RR	3.36	1.68		3.21
RRl	2.19	0.61		2.12
RRu	5.17	4.63		4.87
P	+++	N.S.		+++
Between Chi				2.57
Between df				1
Between P				N.S.
Btwn(F) P				N.S.
Btwn(R) P				N.S.

Table 3H12 - 3

IESLC - Meta-analysis of Ever/current Smoking, Age started, "Low"
Adenocarcinoma, Any Product (or Cigarettes if Any not available)
Most adjusted

	Study size (number of LC cases)				
	100-249	250-499	500-999	1000+	Total
N	5	2	2	4	13
NS	5	2	2	3	12
Wt	21.85	9.79	20.01	50.85	102.50
Het Chi	9.48	0.23	4.91	6.16	49.04
Het df	4	1	1	3	12
Het P	(*)	N.S.	*	N.S.	***
Fixed RR	2.09	2.04	2.60	6.40	3.79
RRl	1.37	1.09	1.68	4.86	3.12
RRu	3.17	3.83	4.03	8.42	4.60
P	+++	+	+++	+++	+++
Random RR	2.34	2.04	3.14	5.77	3.21
RRl	1.20	1.09	1.08	3.78	2.12
RRu	4.55	3.83	9.16	8.81	4.87
P	+	+	+	+++	+++
Between Chi					28.25
Between df					3
Between P					***
Btwn(F) P					*
Btwn(R) P					*

	Risky occupational population			
	no	mining	othRisky	Total
N	13			13
NS	12			12
Wt	102.50			102.50
Het Chi	49.04			49.04
Het df	12			12
Het P	***			***
Fixed RR	3.79			3.79
RRl	3.12			3.12
RRu	4.60			4.60
P	+++			+++
Random RR	3.21			3.21
RRl	2.12			2.12
RRu	4.87			4.87
P	+++			+++
Between Chi				
Between df				
Between P				N.S.
Btwn(F) P				N.S.
Btwn(R) P				N.S.

	National cigarette tobacco type			
	Virginia	blended	other	Total
N		12	1	13
NS		11	1	12
Wt		88.20	14.30	102.50
Het Chi		41.15	0.00	49.04
Het df		11	0	12
Het P		***	N.S.	***
Fixed RR		4.24	1.90	3.79
RRl		3.44	1.13	3.12
RRu		5.22	3.20	4.60
P		+++	+	+++
Random RR		3.40	1.90	3.21
RRl		2.21	1.13	2.12
RRu		5.22	3.20	4.87
P		+++	+	+++
Between Chi				7.89
Between df				1
Between P				**
Btwn(F) P				N.S.
Btwn(R) P				(*)

Table 3H12 - 3

IESLC - Meta-analysis of Ever/current Smoking, Age started, "Low"
Adenocarcinoma, Any Product (or Cigarettes if Any not available)
Most adjusted

	Any proxy use No/nk	Yes	Total
N	11	2	13
NS	10	2	12
Wt	90.88	11.62	102.50
Het Chi	48.00	0.39	49.04
Het df	10	1	12
Het P	***	N.S.	***
Fixed RR	3.68	4.73	3.79
RRl	3.00	2.66	3.12
RRu	4.53	8.40	4.60
P	+++	+++	+++
Random RR	2.98	4.73	3.21
RRl	1.84	2.66	2.12
RRu	4.82	8.40	4.87
P	+++	+++	+++
Between Chi			0.64
Between df			1
Between P			N.S.
Btwn(F) P			N.S.
Btwn(R) P			N.S.

	Full histological confirmation No	Yes	Total
N	4	9	13
NS	4	8	12
Wt	17.78	84.72	102.50
Het Chi	1.97	46.14	49.04
Het df	3	8	12
Het P	N.S.	***	***
Fixed RR	3.08	3.96	3.79
RRl	1.94	3.20	3.12
RRu	4.90	4.90	4.60
P	+++	+++	+++
Random RR	3.08	3.27	3.21
RRl	1.94	1.90	2.12
RRu	4.90	5.63	4.87
P	+++	+++	+++
Between Chi			0.92
Between df			1
Between P			N.S.
Btwn(F) P			N.S.
Btwn(R) P			N.S.

	Number of adjustment variables (1) 0	1	2+/+nk	Total
N	10	1	2	13
NS	9	1	2	12
Wt	89.88	5.71	6.91	102.50
Het Chi	42.60	0.00	2.88	49.04
Het df	9	0	1	12
Het P	***	N.S.	(*)	***
Fixed RR	3.87	5.70	2.06	3.79
RRl	3.15	2.51	0.98	3.12
RRu	4.76	12.95	4.34	4.60
P	+++	+++	(+)	+++
Random RR	3.26	5.70	2.08	3.21
RRl	2.02	2.51	0.59	2.12
RRu	5.25	12.95	7.38	4.87
P	+++	+++	N.S.	+++
Between Chi				3.56
Between df				2
Between P				N.S.
Btwn(F) P				N.S.
Btwn(R) P				N.S.

Table 3H12 - 3

IESLC - Meta-analysis of Ever/current Smoking, Age started, "Low"
Adenocarcinoma, Any Product (or Cigarettes if Any not available)
Most adjusted

	Number of adjustment variables (2) 0	1	2	3-5	6+/+nk	Total
N	10	1	2			13
NS	9	1	2			12
Wt	89.88	5.71	6.91			102.50
Het Chi	42.60	0.00	2.88			49.04
Het df	9	0	1			12
Het P	***	N.S.	(*)			***
Fixed RR	3.87	5.70	2.06			3.79
RRl	3.15	2.51	0.98			3.12
RRu	4.76	12.95	4.34			4.60
P	+++	+++	(+)			+++
Random RR	3.26	5.70	2.08			3.21
RRl	2.02	2.51	0.59			2.12
RRu	5.25	12.95	7.38			4.87
P	+++	+++	N.S.			+++
Between Chi						3.56
Between df						2
Between P						N.S.
Btwn(F) P						N.S.
Btwn(R) P						N.S.

	Smoking status ever	current	Total
N	9	4	13
NS	8	4	12
Wt	84.38	18.12	102.50
Het Chi	37.83	2.33	49.04
Het df	8	3	12
Het P	***	N.S.	***
Fixed RR	4.34	2.01	3.79
RRl	3.51	1.27	3.12
RRu	5.38	3.18	4.60
P	+++	++	+++
Random RR	3.92	2.01	3.21
RRl	2.39	1.27	2.12
RRu	6.42	3.18	4.87
P	+++	++	+++
Between Chi			8.88
Between df			1
Between P			**
Btwn(F) P			N.S.
Btwn(R) P			(*)

	Product all/unsp	cig+/-ot	cig only	Total
N	3	8	2	13
NS	3	7	2	12
Wt	14.04	80.29	8.17	102.50
Het Chi	5.93	41.20	1.09	49.04
Het df	2	7	1	12
Het P	(*)	***	N.S.	***
Fixed RR	3.03	3.94	3.80	3.79
RRl	1.80	3.17	1.91	3.12
RRu	5.11	4.90	7.54	4.60
P	+++	+++	+++	+++
Random RR	2.79	3.17	3.85	3.21
RRl	1.12	1.81	1.86	2.12
RRu	6.93	5.56	7.95	4.87
P	+	+++	+++	+++
Between Chi				0.82
Between df				2
Between P				N.S.
Btwn(F) P				N.S.
Btwn(R) P				N.S.

Table 3H12 - 3

IESLC - Meta-analysis of Ever/current Smoking, Age started, "Low"
Adenocarcinoma, Any Product (or Cigarettes if Any not available)
Most adjusted

	Denominator nev any	nev cigs	Total
N	8	5	13
NS	8	4	12
Wt	42.79	59.71	102.50
Het Chi	13.13	25.37	49.04
Het df	7	4	12
Het P	(*)	***	***
Fixed RR	2.60	4.97	3.79
RRl	1.92	3.86	3.12
RRu	3.50	6.41	4.60
P	+++	+++	+++
Random RR	2.63	4.25	3.21
RRl	1.74	2.11	2.12
RRu	3.99	8.57	4.87
P	+++	+++	+++
Between Chi			10.53
Between df			1
Between P			**
Btwn(F) P			N.S.
Btwn(R) P			N.S.

	Derivation of RR/CI Orig	StdCalc	Other	Total
N	3	10		13
NS	3	9		12
Wt	12.62	89.88		102.50
Het Chi	6.12	42.60		49.04
Het df	2	9		12
Het P	*	***		***
Fixed RR	3.26	3.87		3.79
RRl	1.88	3.15		3.12
RRu	5.67	4.76		4.60
P	+++	+++		+++
Random RR	3.02	3.26		3.21
RRl	1.13	2.02		2.12
RRu	8.06	5.25		4.87
P	+	+++		+++
Between Chi				0.32
Between df				1
Between P				N.S.
Btwn(F) P				N.S.
Btwn(R) P				N.S.

Table 3H12 - 4

IESLC - Meta-analysis of Ever/current Smoking, Age started, "Low"
Adenocarcinoma, Any Product (or Cigarettes if Any not available)
Least adjusted

REF	NRR	X	SEX	AGEL	AGEH	RACE	YF	LC TYPE	LOC	START	ST	NLC	R	VB	P	H	AD	SM	PRODUCT	exL	exH	DENOM	De
BARBON	731	x	m	0	0	all	-	a	Eu:wst	1979	CC	755	n	bl	y	y	0	ev	all/unsp	20	999	nev any	st
BENHAM	521		m	0	0	all	-	KII	Eu:wst	1976	CC	1625	n	bl	n	y	0	ev	cig only	20	999	nev any	st
ENGELA	527		m	0	0	all	0	a	Eu:Sca	1964	pr	435	n	bl	n	n	0	cu	cig+/-ot	20	999	nev cigs	st
HAENSZ	509		f	0	0	all	-	a	NAmer	1955	CC	158	n	bl	n	y	0	ev	cig+/-ot	25	999	nev any	st
JEDRYC	538		m	0	0	all	-	a	Eu:est	1980	CC	1630	n	bl	y	n	0	ev	cig+/-ot	19	999	nev any	st
MATOS	651	x	m	0	0	all	-	a	SCAmer	1994	CC	200	n	bl	n	n	0	ev	cig+/-ot	20	999	nev any	st
PEZZOT	552		m	0	0	all	-	a	SCAmer	1987	CC	215	n	bl	n	y	0	ev	cig only	19	999	nev cigs	st
SVENSS	525		f	0	0	all	-	a	Eu:Sca	1983	CC	210	n	bl	n	n	0	cu	all/unsp	26	999	nev any	st
WAKAI	512		m	0	0	all	-	a	As:Jap	1988	CC	333	n	bl	n	y	0	cu	cig+/-ot	20	29	nev any	st
WU	501	x	f	0	0	wh	-	a	NAmer	1981	CC	220	n	bl	n	y	0	cu	all/unsp	25	999	nev any	st
WYNDE6	613		m	0	0	wh	-	a	NAmer	1969	CC	4423	n	bl	n	y	0	ev	cig+/-ot	21	999	nev cigs	st
WYNDE6	637		f	0	0	wh	-	a	NAmer	1969	CC	4423	n	bl	n	y	0	ev	cig+/-ot	21	999	nev cigs	st
ZHENG	532		m	0	0	all	-	a	As:Chi	1982	CC	540	n	ot	*	y	0	ev	cig+/-ot	20	29	nev cigs	st

Cigarette type is all/unspec for all RRs

Table 3H12 - 5

IESLC - Meta-analysis of Ever/current Smoking, Age started, "Low"
Adenocarcinoma, Any Product (or Cigarettes if Any not available)
Least adjusted

REF	NRR	SEX	AD	Number Exposed Case	Cont	Non-exposed Case	Cont	RR	95.00%CI
BARBON	731	m	0	45	207	7	188	5.84	(2.57- 13.26)
BENHAM	521	m	0	41	64	9	42	2.99	(1.32- 6.79)
*ENGELA	527	m	0	8	39957	7	58716	1.68	(0.61- 4.63)
HAENSZ	509	f	0	12	66	37	236	1.16	(0.57- 2.35)
JEDRYC	538	m	0	48	502	7	289	3.95	(1.76- 8.84)
MATOS	651	m	0	13	73	5	110	3.92	(1.34- 11.46)
PEZZOT	552	m	0	18	105	3	116	6.63	(1.90- 23.15)
SVENSS	525	f	0	10	18	22	120	3.03	(1.24- 7.43)
WAKAI	512	m	0	52	183	8	65	2.31	(1.04- 5.12)
WU	501	f	0	8	14	29	62	1.22	(0.46- 3.24)
WYNDE6	613	m	0	47	92	42	589	7.16	(4.47- 11.47)
WYNDE6	637	f	0	63	90	58	673	8.12	(5.34- 12.35)
Subtotal WYNDE6								7.68	(5.62- 10.51)
ZHENG	532	m	0	64	109	29	94	1.90	(1.13- 3.20)
Totals				429	41480	263	61300		

*prospective study

REF	NRR	SEX	AD	Ys	Ws	Qs	Ps
BARBON	731	m	0	1.76	5.71	1.07	0.0000
BENHAM	521	m	0	1.10	5.72	0.32	0.0088
*ENGELA	527	m	0	0.52	3.73	2.47	0.3164
HAENSZ	509	f	0	0.15	7.71	10.79	0.6808
JEDRYC	538	m	0	1.37	5.91	0.01	0.0008
MATOS	651	m	0	1.37	3.34	0.00	0.0126
PEZZOT	552	m	0	1.89	2.46	0.77	0.0030
SVENSS	525	f	0	1.11	4.78	0.24	0.0154
WAKAI	512	m	0	0.84	6.06	1.48	0.0395
WU	501	f	0	0.20	4.05	5.18	0.6871
WYNDE6	613	m	0	1.97	17.35	7.06	0.0000
WYNDE6	637	f	0	2.09	21.88	12.75	0.0000
Subtotal WYNDE6				2.04	39.22	19.81	
ZHENG	532	m	0	0.64	14.30	6.76	0.0149

	N	13
	NS	12
	Wt	102.98
Het	Chi	48.90
Het	df	12
Het	P	***
Fixed	RR	3.79
	RRl	3.12
	RRu	4.59
	P	+++
Random	RR	3.23
	RRl	2.14
	RRu	4.88
	P	+++
Asymm	P	N.S.

Table 3H12 - 6

IESLC - Meta-analysis of Ever/current Smoking, Age started, "Low"
Adenocarcinoma, Any Product (or Cigarettes if Any not available)
Least adjusted

	Sex combined	male	female	Total
N		9	4	13
NS		9	4	13
Wt		64.57	38.41	102.98
Het Chi		19.89	28.85	48.90
Het df		8	3	12
Het P		*	***	***
Fixed RR		3.67	3.98	3.79
RRl		2.88	2.90	3.12
RRu		4.69	5.46	4.59
P		+++	+++	+++
Random RR		3.55	2.51	3.23
RRl		2.36	0.84	2.14
RRu		5.35	7.55	4.88
P		+++	N.S.	+++
Between Chi				0.16
Between df				1
Between P				N.S.
Btwn(F) P				N.S.
Btwn(R) P				N.S.

Table 3H12 - 7

IESLC - Meta-analysis of Ever/current Smoking, Age started, "Low"
Adenocarcinoma, Any Product (or Cigarettes if Any not available)
Excluded studies (and stage at which they were excluded)

1	BECHER BLOT1 BROWN3 CARPEN CHYOU DARBY DOLL2 GARCIA GRAHAM GURSEL HAMMO2 JAHN JAIN LAUSSM PRESCO QIAO TVERDA WIGLE WYNDE3
2	AKIBA AMANDU AMES AXELSS BENSHL BEST BOUCHA BOUCOT BROSS CHEN CPSII DEAN2 DESTEF DORGAN DOSEME FAN GARSHI GER GILLIS HAMMON HUMBLE JUSSAW KAISE2 KATSOU KAUFMA KOO KREUZE LEVIN MCCONN NOTAN2 OSANN2 PEZZO2 PISANI RESTRE SADOWS VUTUC WANG2 WATSON WU2 WUWILL WYNDE2 WYNDE8 XU ZHOU
3	GUO MCDUFF SPITZ STASZE ZHANG
4	AGUDO ARMADA AUVINE BOFFET BRESLO BUFFLE CEDERL CHEN2 CHIAZZ CHOI CORREA CPSI DAMBER DEAN3 DOLL DORN GAO GAO2 GENG HIRAYA HOLE HU HU2 JOLY KOULUM LETOUR LIAW LIU3 LIU4 LIU5 LUBIN LUBIN2 LUO MIGRAN MRFITR PERNU QIAO2 RACHTA SEGI2 SOBUE SPEIZE SUZUK2 TIZZAN WYNDE7 YUAN
5	ALDERS
10	HEGMAN KHUDER

Table 3H12 - 8
Potentially overlapping studies

REF	REFGP	PRINC	.	OVERLAP/LINK
BENHAM	LUBIN2	2		Subset of Lubin2
WYNDE6	WYNDE6	1		WYNDE5/6/7/8

Table 3H13 -

IESLC - Meta-analysis of Ever/current Smoking, Age started, "Mid"
Adenocarcinoma, Any Product (or Cigarettes if Any not available)

This analysis is restricted to results for:
1) Ever/current smokers
2) Results by Age started
3) Categorical results by Age started
4) Adenocarcinoma (or near equivalent)
5) Results complete enough for use in metaanalysis

Within each study, results are then selected (in the following order of preference, within each sex) for:
6) SMKSTA: ever, current
7) PRODUCT: all/unspec, cigarettes regardless of other products, cigarettes only
8) CIGTYPE: all/unspecified, MC regardless of HR, MC only
9) (not applicable)
10) DENOM: never smoked anything, never smoked cigarettes, never any + low, never cigs + low
11) Followup period (YF, prospective studies): whole study (coded as 0) or longest available
12) LCtype: adeno or nearest available, but not squamous. (q = squamous, s = small,
a = adeno, l = large, KII = Kreyberg II, al = alveolar, br = bronchiolar, u = undifferentiated)
13) Race: all or nearest available, otherwise by race (wh or w = white, bl or b = black, hi = hispanic
ch = chinese, jap = japanese, haw = hawaiian, w+o = white + oriental, sca = scandinavian, as = asian)
14) Age started "mid" in key scheme 1 (key value 18, maximum range 15-25)
15) For overlapping studies: principal rather than subsidiary studies
Finally by Age: whole study (coded as 0) if available, otherwise by widest available age group
and then for single sex results (m, f) in preference to results for both sexes combined (c).

Results adjusted (AD) for the most potential confounders are then chosen in Sections -1 to -3
and results adjusted for the least confounders in Sections -4 to -6. (Those least adjusted results which
actually differ from the most adjusted are marked 'x' in column X in Section -4)

Section -7 shows excluded studies, together with the stage (as above) at which no qualifying
results were found.

Section -8 lists the potentially overlapping studies which have been included (1=principal, 2=subsidiary).

Section -9 lists any results which would have been included in preference except that they had data not complete
enough for use in meta-analysis, with their significance (yes/no), if known, and any further comment as entered
on the database. It also lists as "gap" any categories for which no data were presented by the original authors.

In addition to those mentioned above, the following fields, levels and abbreviations are used:
* or nk = not known, n = no, y = yes, ot = other
ev = ever, cu = current, nev = never
all/unspec = all or unspecified, cig+/-ot = cigarettes irrespective of other products (cigar, pipe etc)
MC = manufactured cigarettes, HR = hand-rolled cigarettes
exL, exH = range of exposure (low and high) in the smoking group, in terms of Age started
REF: 6-character study reference
NRR: number of the RR on the database within the study
ST : study type (CC = case control, pr or prosp = prospective)
NLC: number of lung cancer cases in whole study
R : risky occupational population (n = no, m = mining, o = other risky)
VB : national cigarette type (V = at least 75% Virginia, bl = at least 75% blended, ot = other)
P : any proxy use
H : full histological confirmation
De : derivation of RR/CI (or = original, st = standard method, ot = other method of estimation)

Table 3H13 - 1

IESLC - Meta-analysis of Ever/current Smoking, Age started, "Mid"
Adenocarcinoma, Any Product (or Cigarettes if Any not available)
Most adjusted

REF	NRR	SEX	AGEL	AGEH	RACE	YF	LC TYPE	LOC	START	ST	NLC	R	VB	P	H	AD	SM	PRODUCT	exL	exH	DENOM	De
BARBON	737	m	0	0	all	-	a	Eu:wst	1979	CC	755	n	bl	y	y	1	ev	all/unsp	15	19	nev any	or
JEDRYC	539	m	0	0	all	-	a	Eu:est	1980	CC	1630	n	bl	y	n	0	ev	cig+/-ot	17	18	nev any	st
MATOS	657	m	0	0	all	-	a	SCAmer	1994	CC	200	n	bl	n	n	2	ev	cig+/-ot	15	19	nev any	or
WYNDE6	614	m	0	0	wh	-	a	NAmer	1969	CC	4423	n	bl	n	y	0	ev	cig+/-ot	18	20	nev cigs	st
WYNDE6	638	f	0	0	wh	-	a	NAmer	1969	CC	4423	n	bl	n	y	0	ev	cig+/-ot	18	20	nev cigs	st

Cigarette type is all/unspec for all RRs

Table 3H13 - 2

IESLC - Meta-analysis of Ever/current Smoking, Age started, "Mid"
Adenocarcinoma, Any Product (or Cigarettes if Any not available)
Most adjusted

REF	NRR	SEX	AD	Number Exposed Case	Cont	Non-exposed Case	Cont	RR	95.00%CI
BARBON	737	m	1	77	-	7	-	6.00	(2.70- 13.30)
JEDRYC	539	m	0	29	221	7	289	5.42	(2.33- 12.60)
MATOS	657	m	2	40	-	5	-	7.20	(2.70- 19.10)
WYNDE6	614	m	0	88	139	42	589	8.88	(5.88- 13.40)
WYNDE6	638	f	0	113	94	58	673	13.95	(9.51- 20.46)
Subtotal WYNDE6								11.31	(8.54- 14.97)
Partial Totals				347	454	119	1551		

*prospective study

REF	NRR	SEX	AD	Ys	Ws	Qs	Ps
BARBON	737	m	1	1.79	6.04	1.42	0.0000
JEDRYC	539	m	0	1.69	5.40	1.86	0.0001
MATOS	657	m	2	1.97	4.01	0.37	0.0001
WYNDE6	614	m	0	2.18	22.69	0.19	0.0000
WYNDE6	638	f	0	2.64	26.17	3.38	0.0000
Subtotal WYNDE6				2.43	48.86	3.57	

	N	5
	NS	4
	Wt	64.32
Het	Chi	7.21
Het	df	4
Het	P	N.S.
Fixed	RR	9.74
	RRl	7.63
	RRu	12.43
	P	+++
Random	RR	8.84
	RRl	6.14
	RRu	12.73
	P	+++
Asymm	P	N.S.

Table 3H13 - 3

IESLC - Meta-analysis of Ever/current Smoking, Age started, "Mid"
Adenocarcinoma, Any Product (or Cigarettes if Any not available)
Most adjusted

	Sex combined	male	female	Total
N		4	1	5
NS		4	1	4
Wt		38.15	26.17	64.32
Het Chi		1.52	0.00	7.21
Het df		3	0	4
Het P		N.S.	N.S.	N.S.
Fixed RR		7.61	13.95	9.74
RRl		5.54	9.51	7.63
RRu		10.45	20.46	12.43
P		+++	+++	+++
Random RR		7.61	13.95	8.84
RRl		5.54	9.51	6.14
RRu		10.45	20.46	12.73
P		+++	+++	+++
Between Chi				5.70
Between df				1
Between P				*
Btwn(F) P				*
Btwn(R) P				*

Too few RRs for analysis by factor

Table 3H13 - 4

IESLC - Meta-analysis of Ever/current Smoking, Age started, "Mid"
Adenocarcinoma, Any Product (or Cigarettes if Any not available)
Least adjusted

REF	NRR	X	SEX	AGEL	AGEH	RACE	YF	LC TYPE	LOC	START	ST	NLC	R	VB	P	H	AD	SM	PRODUCT	exL	exH	DENOM	De
BARBON	732	x	m	0	0	all	-	a	Eu:wst	1979	CC	755	n	bl	y	y	0	ev	all/unsp	15	19	nev any	st
JEDRYC	539		m	0	0	all	-	a	Eu:est	1980	CC	1630	n	bl	y	n	0	ev	cig+/-ot	17	18	nev any	st
MATOS	652	x	m	0	0	all	-	a	SCAmer	1994	CC	200	n	bl	n	n	0	ev	cig+/-ot	15	19	nev any	st
WYNDE6	614		m	0	0	wh	-	a	NAmer	1969	CC	4423	n	bl	n	y	0	ev	cig+/-ot	18	20	nev cigs	st
WYNDE6	638		f	0	0	wh	-	a	NAmer	1969	CC	4423	n	bl	n	y	0	ev	cig+/-ot	18	20	nev cigs	st

Cigarette type is all/unspec for all RRs

Table 3H13 - 5

IESLC - Meta-analysis of Ever/current Smoking, Age started, "Mid"
Adenocarcinoma, Any Product (or Cigarettes if Any not available)
Least adjusted

REF	NRR	SEX	AD	Number Exposed Case	Exposed Cont	Non-exposed Case	Non-exposed Cont	RR	95.00%CI
BARBON	732	m	0	77	337	7	188	6.14	(2.77- 13.58)
JEDRYC	539	m	0	29	221	7	289	5.42	(2.33- 12.60)
MATOS	652	m	0	40	120	5	110	7.33	(2.79- 19.25)
WYNDE6	614	m	0	88	139	42	589	8.88	(5.88- 13.40)
WYNDE6	638	f	0	113	94	58	673	13.95	(9.51- 20.46)
Subtotal WYNDE6								11.31	(8.54- 14.97)
Totals				347	911	119	1849		

*prospective study

REF	NRR	SEX	AD	Ys	Ws	Qs	Ps
BARBON	732	m	0	1.81	6.09	1.31	0.0000
JEDRYC	539	m	0	1.69	5.40	1.87	0.0001
MATOS	652	m	0	1.99	4.13	0.34	0.0001
WYNDE6	614	m	0	2.18	22.69	0.20	0.0000
WYNDE6	638	f	0	2.64	26.17	3.33	0.0000
Subtotal WYNDE6				2.43	48.86	3.54	

	N	5
	NS	4
	Wt	64.47
Het	Chi	7.06
Het	df	4
Het	P	N.S.
Fixed	RR	9.76
	RRl	7.65
	RRu	12.46
	P	+++
Random	RR	8.90
	RRl	6.21
	RRu	12.76
	P	+++
Asymm	P	N.S.

Table 3H13 - 6

IESLC - Meta-analysis of Ever/current Smoking, Age started, "Mid"
Adenocarcinoma, Any Product (or Cigarettes if Any not available)
Least adjusted

	Sex combined	male	female	Total
N		4	1	5
NS		4	1	4
Wt		38.31	26.17	64.47
Het Chi		1.45	0.00	7.06
Het df		3	0	4
Het P		N.S.	N.S.	N.S.
Fixed RR		7.65	13.95	9.76
RRl		5.57	9.51	7.65
RRu		10.50	20.46	12.46
P		+++	+++	+++
Random RR		7.65	13.95	8.90
RRl		5.57	9.51	6.21
RRu		10.50	20.46	12.76
P		+++	+++	+++
Between Chi				5.61
Between df				1
Between P				*
Btwn(F) P				*
Btwn(R) P				*

Table 3H13 - 7

IESLC - Meta-analysis of Ever/current Smoking, Age started, "Mid"
Adenocarcinoma, Any Product (or Cigarettes if Any not available)
Excluded studies (and stage at which they were excluded)

1	BECHER BLOT1 BROWN3 CARPEN CHYOU DARBY DOLL2 GARCIA GRAHAM GURSEL HAMMO2 JAHN JAIN LAUSSM PRESCO QIAO TVERDA WIGLE WYNDE3
2	AKIBA AMANDU AMES AXELSS BENSHL BEST BOUCHA BOUCOT BROSS CHEN CPSII DEAN2 DESTEF DORGAN DOSEME FAN GARSHI GER GILLIS HAMMON HUMBLE JUSSAW KAISE2 KATSOU KAUFMA KOO KREUZE LEVIN MCCONN NOTAN2 OSANN2 PEZZO2 PISANI RESTRE SADOWS VUTUC WANG2 WATSON WU2 WUWILL WYNDE2 WYNDE8 XU ZHOU
3	GUO MCDUFF SPITZ STASZE ZHANG
4	AGUDO ARMADA AUVINE BOFFET BRESLO BUFFLE CEDERL CHEN2 CHIAZZ CHOI CORREA CPSI DAMBER DEAN3 DOLL DORN GAO GAO2 GENG HIRAYA HOLE HU HU2 JOLY KOULUM LETOUR LIAW LIU3 LIU4 LIU5 LUBIN LUBIN2 LUO MIGRAN MRFITR PERNU QIAO2 RACHTA SEGI2 SOBUE SPEIZE SUZUK2 TIZZAN WYNDE7 YUAN
5	ALDERS
10	HEGMAN KHUDER
14	BENHAM ENGELA HAENSZ PEZZOT SVENSS WAKAI WU ZHENG

Table 3H13 - 8
Potentially overlapping studies

REF	REFGP	PRINC	.	OVERLAP/LINK
WYNDE6	WYNDE6	1		WYNDE5/6/7/8

Table 3H14 -

IESLC - Meta-analysis of Ever/current Smoking, Age started, "High"
Adenocarcinoma, Any Product (or Cigarettes if Any not available)

This analysis is restricted to results for:
1) Ever/current smokers
2) Results by Age started
3) Categorical results by Age started
4) Adenocarcinoma (or near equivalent)
5) Results complete enough for use in metaanalysis

Within each study, results are then selected (in the following order of preference, within each sex) for:
6) PRODUCT: all/unspec, cigarettes regardless of other products, cigarettes only
7) CIGTYPE: all/unspecified, MC regardless of HR, MC only
8) (not applicable)
9) DENOM: never smoked anything, never smoked cigarettes, never any + low, never cigs + low
10) Followup period (YF, prospective studies): whole study (coded as 0) or longest available
11) LCtype: adeno or nearest available, but not squamous. (q = squamous, s = small,
a = adeno, l = large, KII = Kreyberg II, al = alveolar, br = bronchiolar, u = undifferentiated)
12) Race: all or nearest available, otherwise by race (wh or w = white, bl or b = black, hi = hispanic
ch = chinese, jap = japanese, haw = hawaiian, w+o = white + oriental, sca = scandinavian, as = asian)
13) Age started "high" in key scheme 1 (key value 14, maximum range 1-17)
14) For overlapping studies: principal rather than subsidiary studies
Finally by Age: whole study (coded as 0) if available, otherwise by widest available age group
and then for single sex results (m, f) in preference to results for both sexes combined (c).

Results adjusted (AD) for the most potential confounders are then chosen in Sections -1 to -3
and results adjusted for the least confounders in Sections -4 to -6. (Those least adjusted results which
actually differ from the most adjusted are marked 'x' in column X in Section -4)

Section -7 shows excluded studies, together with the stage (as above) at which no qualifying
results were found.

Section -8 lists the potentially overlapping studies which have been included (1=principal, 2=subsidiary).

Section -9 lists any results which would have been included in preference except that they had data not complete
enough for use in meta-analysis, with their significance (yes/no), if known, and any further comment as entered
on the database. It also lists as "gap" any categories for which no data were presented by the original authors.

In addition to those mentioned above, the following fields, levels and abbreviations are used:
* or nk = not known, n = no, y = yes, ot = other
ev = ever, cu = current, nev = never
all/unspec = all or unspecified, cig+/-ot = cigarettes irrespective of other products (cigar, pipe etc)
MC = manufactured cigarettes, HR = hand-rolled cigarettes
exL, exH = range of exposure (low and high) in the smoking group, in terms of Age started
REF: 6-character study reference
NRR: number of the RR on the database within the study
ST : study type (CC = case control, pr or prosp = prospective)
NLC: number of lung cancer cases in whole study
R : risky occupational population (n = no, m = mining, o = other risky)
VB : national cigarette type (V = at least 75% Virginia, bl = at least 75% blended, ot = other)
P : any proxy use
H : full histological confirmation
De : derivation of RR/CI (or = original, st = standard method, ot = other method of estimation)

Table 3H14 - 1

IESLC - Meta-analysis of Ever/current Smoking, Age started, "High"
Adenocarcinoma, Any Product (or Cigarettes if Any not available)
Most adjusted

REF	NRR	SEX	AGEL	AGEH	RACE	YF	LC TYPE	LOC	START	ST	NLC	R	VB	P	H	AD	SM	PRODUCT	exL	exH	DENOM	De
BARBON	738	m	0	0	all	-	a	Eu:wst	1979	CC	755	n	bl	y	y	1	ev	all/unsp	1	14	nev any	or
JEDRYC	540	m	0	0	all	-	a	Eu:est	1980	CC	1630	n	bl	y	n	0	ev	cig+/-ot	1	16	nev any	st
MATOS	658	m	0	0	all	-	a	SCAmer	1994	CC	200	n	bl	n	n	2	ev	cig+/-ot	1	14	nev any	or
WYNDE6	615	m	0	0	wh	-	a	NAmer	1969	CC	4423	n	bl	n	y	0	ev	cig+/-ot	1	17	nev cigs	st
WYNDE6	639	f	0	0	wh	-	a	NAmer	1969	CC	4423	n	bl	n	y	0	ev	cig+/-ot	1	17	nev cigs	st

Cigarette type is all/unspec for all RRs

Table 3H14 - 2

IESLC - Meta-analysis of Ever/current Smoking, Age started, "High"
Adenocarcinoma, Any Product (or Cigarettes if Any not available)
Most adjusted

REF	NRR	SEX	AD	Number Exposed Case	Exposed Cont	Non-exposed Case	Non-exposed Cont	RR	95.00%CI
BARBON	738	m	1	29	-	7	-	33.40	(13.10- 84.90)
JEDRYC	540	m	0	12	111	7	289	4.46	(1.71- 11.63)
MATOS	658	m	2	26	-	5	-	6.60	(2.40- 18.10)
WYNDE6	615	m	0	241	301	42	589	11.23	(7.87- 16.03)
WYNDE6	639	f	0	150	91	58	673	19.13	(13.16- 27.80)
Subtotal WYNDE6								14.46	(11.18- 18.72)
Partial Totals				458	503	119	1551		

*prospective study

REF	NRR	SEX	AD	Ys	Ws	Qs	Ps
BARBON	738	m	1	3.51	4.40	3.54	0.0000
JEDRYC	540	m	0	1.50	4.19	5.22	0.0022
MATOS	658	m	2	1.89	3.76	1.98	0.0003
WYNDE6	615	m	0	2.42	30.32	1.13	0.0000
WYNDE6	639	f	0	2.95	27.49	3.16	0.0000
Subtotal WYNDE6				2.67	57.81	4.30	

	N	5
	NS	4
	Wt	70.16
Het	Chi	15.03
Het	df	4
Het	P	**
Fixed	RR	13.62
	RRl	10.78
	RRu	17.22
	P	+++
Random	RR	12.34
	RRl	7.23
	RRu	21.08
	P	+++
Asymm	P	N.S.

Table 3H14 - 3

IESLC - Meta-analysis of Ever/current Smoking, Age started, "High"
Adenocarcinoma, Any Product (or Cigarettes if Any not available)
Most adjusted

	Sex combined	male	female	Total
N		4	1	5
NS		4	1	4
Wt		42.68	27.49	70.16
Het Chi		9.83	0.00	15.03
Het df		3	0	4
Het P		*	N.S.	**
Fixed RR		10.95	19.13	13.62
RRl		8.11	13.16	10.78
RRu		14.78	27.80	17.22
P		+++	+++	+++
Random RR		10.46	19.13	12.34
RRl		5.18	13.16	7.23
RRu		21.13	27.80	21.08
P		+++	+++	+++
Between Chi				5.20
Between df				1
Between P				*
Btwn(F) P				N.S.
Btwn(R) P				N.S.

Too few RRs for analysis by factor

Table 3H14 - 4

IESLC - Meta-analysis of Ever/current Smoking, Age started, "High"
Adenocarcinoma, Any Product (or Cigarettes if Any not available)
Least adjusted

REF	NRR	X	SEX	AGEL	AGEH	RACE	YF	LC TYPE	LOC	START	ST	NLC	R	VB	P	H	AD	SM	PRODUCT	exL	exH	DENOM	De
BARBON	733	x	m	0	0	all	-	a	Eu:wst	1979	CC	755	n	bl	y	y	0	ev	all/unsp	1	14	nev any	st
JEDRYC	540		m	0	0	all	-	a	Eu:est	1980	CC	1630	n	bl	y	n	0	ev	cig+/-ot	1	16	nev any	st
MATOS	653	x	m	0	0	all	-	a	SCAmer	1994	CC	200	n	bl	n	n	0	ev	cig+/-ot	1	14	nev any	st
WYNDE6	615		m	0	0	wh	-	a	NAmer	1969	CC	4423	n	bl	n	y	0	ev	cig+/-ot	1	17	nev cigs	st
WYNDE6	639		f	0	0	wh	-	a	NAmer	1969	CC	4423	n	bl	n	y	0	ev	cig+/-ot	1	17	nev cigs	st

Cigarette type is all/unspec for all RRs

Table 3H14 - 5

IESLC - Meta-analysis of Ever/current Smoking, Age started, "High"
Adenocarcinoma, Any Product (or Cigarettes if Any not available)
Least adjusted

REF	NRR	SEX	AD	Number Exposed Case	Exposed Cont	Non-exposed Case	Non-exposed Cont	RR	95.00%CI
BARBON	733	m	0	29	23	7	188	33.86	(13.33- 86.00)
JEDRYC	540	m	0	12	111	7	289	4.46	(1.71- 11.63)
MATOS	653	m	0	26	90	5	110	6.36	(2.35- 17.22)
WYNDE6	615	m	0	241	301	42	589	11.23	(7.87- 16.03)
WYNDE6	639	f	0	150	91	58	673	19.13	(13.16- 27.80)
Subtotal WYNDE6								14.46	(11.18- 18.72)
Totals				458	616	119	1849		

*prospective study

REF	NRR	SEX	AD	Ys	Ws	Qs	Ps
BARBON	733	m	0	3.52	4.42	3.68	0.0000
JEDRYC	540	m	0	1.50	4.19	5.20	0.0022
MATOS	653	m	0	1.85	3.87	2.24	0.0003
WYNDE6	615	m	0	2.42	30.32	1.11	0.0000
WYNDE6	639	f	0	2.95	27.49	3.20	0.0000
Subtotal WYNDE6				2.67	57.81	4.31	

N	5
NS	4
Wt	70.29
Het Chi	15.43
Het df	4
Het P	**
Fixed RR	13.60
RRl	10.76
RRu	17.18
P	+++
Random RR	12.28
RRl	7.15
RRu	21.08
P	+++
Asymm P	N.S.

Table 3H14 - 6

IESLC - Meta-analysis of Ever/current Smoking, Age started, "High"
Adenocarcinoma, Any Product (or Cigarettes if Any not available)
Least adjusted

Sex combined	male	female	Total
N	4	1	5
NS	4	1	4
Wt	42.80	27.49	70.29
Het Chi	10.17	0.00	15.43
Het df	3	0	4
Het P	*	N.S.	**
Fixed RR	10.92	19.13	13.60
RRl	8.09	13.16	10.76
RRu	14.74	27.80	17.18
P	+++	+++	+++
Random RR	10.40	19.13	12.28
RRl	5.11	13.16	7.15
RRu	21.19	27.80	21.08
P	+++	+++	+++
Between Chi			5.25
Between df			1
Between P			*
Btwn(F) P			N.S.
Btwn(R) P			N.S.

Table 3H14 - 7

IESLC - Meta-analysis of Ever/current Smoking, Age started, "High"
Adenocarcinoma, Any Product (or Cigarettes if Any not available)
Excluded studies (and stage at which they were excluded)

1	BECHER BLOT1 BROWN3 CARPEN CHYOU DARBY DOLL2 GARCIA GRAHAM GURSEL HAMMO2 JAHN JAIN LAUSSM PRESCO QIAO TVERDA WIGLE WYNDE3
2	AKIBA AMANDU AMES AXELSS BENSHL BEST BOUCHA BOUCOT BROSS CHEN CPSII DEAN2 DESTEF DORGAN DOSEME FAN GARSHI GER GILLIS HAMMON HUMBLE JUSSAW KAISE2 KATSOU KAUFMA KOO KREUZE LEVIN MCCONN NOTAN2 OSANN2 PEZZO2 PISANI RESTRE SADOWS VUTUC WANG2 WATSON WU2 WUWILL WYNDE2 WYNDE8 XU ZHOU
3	GUO MCDUFF SPITZ STASZE ZHANG
4	AGUDO ARMADA AUVINE BOFFET BRESLO BUFFLE CEDERL CHEN2 CHIAZZ CHOI CORREA CPSI DAMBER DEAN3 DOLL DORN GAO GAO2 GENG HIRAYA HOLE HU HU2 JOLY KOULUM LETOUR LIAW LIU3 LIU4 LIU5 LUBIN LUBIN2 LUO MIGRAN MRFITR PERNU QIAO2 RACHTA SEGI2 SOBUE SPEIZE SUZUK2 TIZZAN WYNDE7 YUAN
5	ALDERS
10	HEGMAN KHUDER
14	BENHAM ENGELA HAENSZ PEZZOT SVENSS WAKAI WU ZHENG

Table 3H14 - 8
Potentially overlapping studies

REF	REFGP	PRINC	.	OVERLAP/LINK
WYNDE6	WYNDE6	1		WYNDE5/6/7/8

Table 3H15 -

IESLC - Meta-analysis of Ever/current Smoking, Age started, "Highest vs lowest"
Adenocarcinoma, Any Product (or Cigarettes if Any not available)

This analysis is restricted to results for:
1) Ever/current smokers
2) Results by Age started
3) Categorical results by Age started
4) Denominator (unexposed) = "low"
5) Adenocarcinoma (or near equivalent)
6) Results complete enough for use in metaanalysis

Within each study, results are then selected (in the following order of preference, within each sex) for:
7) SMKSTA: ever, current
8) PRODUCT: all/unspec, cigarettes regardless of other products, cigarettes only
9) CIGTYPE: all/unspecified, MC regardless of HR, MC only
10) Results with least adjustment for other aspects of smoking (ADOS)
11) The highest vs lowest category
12) Followup period (YF, prospective studies): whole study (coded as 0) or longest available
13) LCtype: adeno or nearest available, but not squamous. (q = squamous, s = small,
a = adeno, l = large, KII = Kreyberg II, al = alveolar, br = bronchiolar, u = undifferentiated)
14) Race: all or nearest available, otherwise by race (wh or w = white, bl or b = black, hi = hispanic
ch = chinese, jap = japanese, haw = hawaiian, w+o = white + oriental, sca = scandinavian, as = asian)
15) For overlapping studies: principal rather than subsidiary studies
Finally by Age: whole study (coded as 0) if available, otherwise by widest available age group
and then for single sex results (m, f) in preference to results for both sexes combined (c).

Results adjusted (AD) for the most potential confounders are then chosen in Sections -1 to -3
and results adjusted for the least confounders in Sections -4 to -6. (Those least adjusted results which
actually differ from the most adjusted are marked 'x' in column X in Section -4)

Section -7 shows excluded studies, together with the stage (as above) at which no qualifying
results were found.

Section -8 lists the potentially overlapping studies which have been included (1=principal, 2=subsidiary).

Section -9 lists any results which would have been included in preference except that they had data not complete
enough for use in meta-analysis, with their significance (yes/no), if known, and any further comment as entered
on the database. It also lists as "gap" any categories for which no data were presented by the original authors.

In addition to those mentioned above, the following fields, levels and abbreviations are used:
* or nk = not known, n = no, y = yes, ot = other
all/unspec = all or unspecified, cig+/-ot = cigarettes irrespective of other products (cigar, pipe etc)
MC = manufactured cigarettes, HR = hand-rolled cigarettes
exL, exH = range of exposure (low and high) in the "highest" group, in terms of Age started
unexL, unexH = range of exposure (low and high) in the "lowest" group, in terms of Age started
REF: 6-character study reference
NRR: number of the RR on the database within the study
ST : study type (CC = case control, pr or prosp = prospective)
NLC: number of lung cancer cases in whole study
R : risky occupational population (n = no, m = mining, o = other risky)
VB : national cigarette type (V = at least 75% Virginia, bl = at least 75% blended, ot = other)
P : any proxy use
H : full histological confirmation
De : derivation of RR/CI (or = original, st = standard method, ot = other method of estimation)

Table 3H15 - 1

IESLC - Meta-analysis of Ever/current Smoking, Age started, "Highest vs lowest"
Adenocarcinoma, Any Product (or Cigarettes if Any not available)
Most adjusted

REF	NRR	SEX	AGEL	AGEH	RACE	YF	LC TYPE	LOC	START	ST	NLC	R	VB	P	H	AD	ADOS	SM	PRODUCT	exL	exH	unexL	unexH	De
BARBON	740	m	0	0	all	-	a	Eu:wst	1979	CC	755	n	bl	y	y	1	0	ev	all/unsp	1	14	20	999	ot
BENHAM	523	m	0	0	all	-	KII	Eu:wst	1976	CC	1625	n	bl	n	y	0	0	ev	cig only	1	19	20	999	st
ENGELA	529	m	0	0	all	0	a	Eu:Sca	1964	pr	435	n	bl	n	n	0	0	cu	cig+/-ot	1	19	20	999	st
HAENSZ	534	f	0	0	all	-	a	NAmer	1955	CC	158	n	bl	n	y	2	0	ev	cig+/-ot	1	24	25	999	ot
HEGMAN	505	m	0	0	all	-	a	NAmer	1989	CC	282	n	bl	y	y	0	0	ev	all/unsp	1	19	20	999	st
HEGMAN	510	f	0	0	all	-	a	NAmer	1989	CC	282	n	bl	y	y	0	0	ev	all/unsp	1	25	26	999	ot
JEDRYC	542	m	0	0	all	-	a	Eu:est	1980	CC	1630	n	bl	y	n	0	0	ev	cig+/-ot	1	16	19	999	st
KHUDER	528	m	0	0	all	-	a	NAmer	1985	CC	482	n	bl	n	y	5	3#	ev	cig+/-ot	1	15	20	999	or
MATOS	660	m	0	0	all	-	a	SCAmer	1994	CC	200	n	bl	n	n	2	0	ev	cig+/-ot	1	14	20	999	ot
PEZZOT	558	m	0	0	all	-	a	SCAmer	1987	CC	215	n	bl	n	y	2	0	ev	cig only	1	13	19	999	ot
SVENSS	529	f	0	0	all	-	a	Eu:Sca	1983	CC	210	n	bl	n	n	0	0	cu	all/unsp	0	18	26	999	st
WAKAI	515	m	0	0	all	-	a	As:Jap	1988	CC	333	n	bl	n	y	0	0	cu	cig+/-ot	1	19	30	999	st
WU	529	f	0	0	wh	-	a	NAmer	1981	CC	220	n	bl	n	y	2	0	cu	all/unsp	0	18	25	999	ot
WYNDE6	617	m	0	0	wh	-	a	NAmer	1969	CC	4423	n	bl	n	y	0	0	ev	cig+/-ot	1	17	21	999	st
WYNDE6	641	f	0	0	wh	-	a	NAmer	1969	CC	4423	n	bl	n	y	0	0	ev	cig+/-ot	1	17	21	999	st
ZHENG	537	m	0	0	all	-	a	As:Chi	1982	CC	540	n	ot	*	y	1	0	ev	cig+/-ot	1	19	30	999	ot
ZHENG	541	f	0	0	all	-	a	As:Chi	1982	CC	540	n	ot	*	y	1	0	ev	cig+/-ot	1	29	30	999	ot

Comments on values in listings

KHUDER ADOS Duration of smoking (years), number of cigarettes per day and Quitted smoking

Cigarette type is all/unspec for all RRs

Table 3H15 - 2

IESLC - Meta-analysis of Ever/current Smoking, Age started, "Highest vs lowest"
Adenocarcinoma, Any Product (or Cigarettes if Any not available)
Most adjusted

REF	NRR	SEX	AD	Number Exposed Case	Number Exposed Cont	Non-exposed Case	Non-exposed Cont	RR	95.00%CI
BARBON	740	m	1	29	-	45	-	5.86	(3.10- 11.07)
BENHAM	523	m	0	69	79	41	64	1.36	(0.82- 2.27)
*ENGELA	529	m	0	25	50732	8	39957	2.46	(1.11- 5.46)
HAENSZ	534	f	2	6	-	12	-	0.88	(0.31- 2.53)
HEGMAN	505	m	0	45	716	6	289	3.03	(1.28- 7.17)
HEGMAN	510	f	0	32	169	0	28	10.93~	(0.65- 183.56)
Subtotal HEGMAN								3.38	(1.48- 7.71)
JEDRYC	542	m	0	12	111	48	502	1.13	(0.58- 2.20)
KHUDER	528	m	5	-	-	-	-	0.80	(0.40- 1.60)
MATOS	660	m	2	26	-	13	-	1.65	(0.80- 3.40)
PEZZOT	558	m	2	9	-	18	-	1.11	(0.41- 3.04)
SVENSS	529	f	0	17	21	10	18	1.46	(0.53- 3.97)
WAKAI	515	m	0	18	74	4	25	1.52	(0.47- 4.92)
WU	529	f	2	69	-	8	-	7.27	(2.31- 22.86)
WYNDE6	617	m	0	241	301	47	92	1.57	(1.06- 2.32)
WYNDE6	641	f	0	150	91	63	90	2.35	(1.56- 3.56)
Subtotal WYNDE6								1.90	(1.43- 2.52)
ZHENG	537	m	1	44	-	15	-	4.54	(2.25- 9.15)
ZHENG	541	f	1	26	-	7	-	2.00	(0.71- 5.64)
Subtotal ZHENG								3.51	(1.96- 6.27)
Partial Totals				818	52294	345	41065		

*prospective study ~ With 0.5 adjustment for zero

REF	NRR	SEX	AD	Ys	Ws	Qs	Ps
BARBON	740	m	1	1.77	9.48	11.63	0.0000
BENHAM	523	m	0	0.31	14.89	1.83	0.2317
*ENGELA	529	m	0	0.90	6.06	0.35	0.0266
HAENSZ	534	f	2	-0.13	3.49	2.17	0.8113
HEGMAN	505	m	0	1.11	5.16	1.03	0.0119
HEGMAN	510	f	0	2.39	0.48	1.45	0.0966
Subtotal HEGMAN				1.22	5.64	2.48	
JEDRYC	542	m	0	0.12	8.68	2.51	0.7175
KHUDER	528	m	5	-0.22	8.00	6.25	0.5281
MATOS	660	m	2	0.50	7.34	0.19	0.1749
PEZZOT	558	m	2	0.10	3.83	1.19	0.8382
SVENSS	529	f	0	0.38	3.82	0.31	0.4620
WAKAI	515	m	0	0.42	2.78	0.16	0.4845
WU	529	f	2	1.98	2.92	5.12	0.0007
WYNDE6	617	m	0	0.45	25.24	1.13	0.0240
WYNDE6	641	f	0	0.86	22.40	0.86	0.0001
Subtotal WYNDE6				0.64	47.64	1.99	
ZHENG	537	m	1	1.51	7.81	5.67	0.0000
ZHENG	541	f	1	0.69	3.58	0.00	0.1898
Subtotal ZHENG				1.26	11.39	5.67	

	N	17
	NS	14
	Wt	135.97
Het	Chi	41.84
Het	df	16
Het	P	***
Fixed	RR	1.94
	RRl	1.64
	RRu	2.29
	P	+++
Random	RR	1.99
	RRl	1.48
	RRu	2.67
	P	+++
Asymm	P	N.S.

Table 3H15 - 3

IESLC - Meta-analysis of Ever/current Smoking, Age started, "Highest vs lowest"
Adenocarcinoma, Any Product (or Cigarettes if Any not available)
Most adjusted

	Sex			
	combined	male	female	Total
N		11	6	17
NS		11	6	17
Wt		99.28	36.69	135.97
Het Chi		31.65	9.12	41.84
Het df		10	5	16
Het P		***	N.S.	***
Fixed RR		1.83	2.24	1.94
RRl		1.51	1.62	1.64
RRu		2.23	3.10	2.29
P		+++	+++	+++
Random RR		1.89	2.24	1.99
RRl		1.31	1.30	1.48
RRu		2.74	3.85	2.67
P		+++	++	+++
Between Chi				1.08
Between df				1
Between P				N.S.
Btwn(F) P				N.S.
Btwn(R) P				N.S.

	Lung cancer type						
	a	a+l	a+al+br	KII	not q+u	not q+s	Total
N	16			1			17
NS	13			1			14
Wt	121.08			14.89			135.97
Het Chi	39.78			0.00			41.84
Het df	15			0			16
Het P	***			N.S.			***
Fixed RR	2.02			1.36			1.94
RRl	1.69			0.82			1.64
RRu	2.42			2.27			2.29
P	+++			N.S.			+++
Random RR	2.05			1.36			1.99
RRl	1.50			0.82			1.48
RRu	2.82			2.27			2.67
P	+++			N.S.			+++
Between Chi							2.06
Between df							1
Between P							N.S.
Btwn(F) P							N.S.
Btwn(R) P							N.S.

	Location								
	NAmer	UK	Scand	othEur	China	Japan	othAs	other	Total
N	7		2	3	2	1		2	17
NS	5		2	3	1	1		2	14
Wt	67.69		9.88	33.06	11.39	2.78		11.17	135.97
Het Chi	17.79		0.64	15.96	1.65	0.00		0.40	41.84
Het df	6		1	2	1	0		1	16
Het P	**		N.S.	***	N.S.	N.S.		N.S.	***
Fixed RR	1.83		2.01	1.97	3.51	1.52		1.44	1.94
RRl	1.44		1.08	1.40	1.96	0.47		0.80	1.64
RRu	2.32		3.75	2.77	6.27	4.92		2.59	2.29
P	+++		+	+++	+++	N.S.		N.S.	+++
Random RR	1.94		2.01	2.07	3.30	1.52		1.44	1.99
RRl	1.19		1.08	0.78	1.51	0.47		0.80	1.48
RRu	3.16		3.75	5.52	7.23	4.92		2.59	2.67
P	++		+	N.S.	++	N.S.		N.S.	+++
Between Chi									5.40
Between df									5
Between P									N.S.
Btwn(F) P									N.S.
Btwn(R) P									N.S.

Table 3H15 - 3

IESLC - Meta-analysis of Ever/current Smoking, Age started, "Highest vs lowest"
Adenocarcinoma, Any Product (or Cigarettes if Any not available)
Most adjusted

Detailed Country in "other Europe"

	multi	Germany	othWest	East	Balkans	Total
N			2	1		3
NS			2	1		3
Wt			24.37	8.68		33.06
Het Chi			12.32	0.00		15.96
Het df			1	0		2
Het P			***	N.S.		***
Fixed RR			2.40	1.13		1.97
RRl			1.62	0.58		1.40
RRu			3.58	2.20		2.77
P			+++	N.S.		+++
Random RR			2.79	1.13		2.07
RRl			0.67	0.58		0.78
RRu			11.64	2.20		5.52
P			N.S.	N.S.		N.S.
Between Chi						3.65
Between df						1
Between P						(*)
Btwn(F) P						N.S.
Btwn(R) P						N.S.

Detailed Country in "other Asia"

	India	HongKong	other	Total
N				
NS				
Wt				
Het Chi				
Het df				
Het P				N.S.
Fixed RR				
RRl				
RRu				
P				N.S.
Random RR				
RRl				
RRu				
P				N.S.
Between Chi				
Between df				
Between P				N.S.
Btwn(F) P				N.S.
Btwn(R) P				N.S.

Detailed other continent

	SCAmer	Total
N	2	2
NS	2	2
Wt	11.17	11.17
Het Chi	0.40	0.40
Het df	1	1
Het P	N.S.	N.S.
Fixed RR	1.44	1.44
RRl	0.80	0.80
RRu	2.59	2.59
P	N.S.	N.S.
Random RR	1.44	1.44
RRl	0.80	0.80
RRu	2.59	2.59
P	N.S.	N.S.
Between Chi		
Between df		
Between P		N.S.
Btwn(F) P		N.S.
Btwn(R) P		N.S.

Table 3H15 - 3

IESLC - Meta-analysis of Ever/current Smoking, Age started, "Highest vs lowest"
Adenocarcinoma, Any Product (or Cigarettes if Any not available)
Most adjusted

	Start year of study					
	<1960	1960-69	1970-79	1980-89	1990+	Total
N	1	3	2	10	1	17
NS	1	2	2	8	1	14
Wt	3.49	53.70	24.37	47.06	7.34	135.97
Het Chi	0.00	2.33	12.32	23.61	0.00	41.84
Het df	0	2	1	9	0	16
Het P	N.S.	N.S.	***	**	N.S.	***
Fixed RR	0.88	1.95	2.40	1.86	1.65	1.94
RRl	0.31	1.50	1.62	1.40	0.80	1.64
RRu	2.51	2.55	3.58	2.48	3.40	2.29
P	N.S.	+++	+++	+++	N.S.	+++
Random RR	0.88	1.97	2.79	2.00	1.65	1.99
RRl	0.31	1.46	0.67	1.23	0.80	1.48
RRu	2.51	2.64	11.64	3.26	3.40	2.67
P	N.S.	+++	N.S.	++	N.S.	+++
Between Chi						3.58
Between df						4
Between P						N.S.
Btwn(F) P						N.S.
Btwn(R) P						N.S.

	Study type (1)		
	CC	other	Total
N	16	1	17
NS	13	1	14
Wt	129.90	6.06	135.97
Het Chi	41.47	0.00	41.84
Het df	15	0	16
Het P	***	N.S.	***
Fixed RR	1.91	2.46	1.94
RRl	1.61	1.11	1.64
RRu	2.27	5.46	2.29
P	+++	+	+++
Random RR	1.96	2.46	1.99
RRl	1.43	1.11	1.48
RRu	2.68	5.46	2.67
P	+++	+	+++
Between Chi			0.36
Between df			1
Between P			N.S.
Btwn(F) P			N.S.
Btwn(R) P			N.S.

	Study type (2)			
	CC	prosp	other	Total
N	16	1		17
NS	13	1		14
Wt	129.90	6.06		135.97
Het Chi	41.47	0.00		41.84
Het df	15	0		16
Het P	***	N.S.		***
Fixed RR	1.91	2.46		1.94
RRl	1.61	1.11		1.64
RRu	2.27	5.46		2.29
P	+++	+		+++
Random RR	1.96	2.46		1.99
RRl	1.43	1.11		1.48
RRu	2.68	5.46		2.67
P	+++	+		+++
Between Chi				0.36
Between df				1
Between P				N.S.
Btwn(F) P				N.S.
Btwn(R) P				N.S.

Table 3H15 - 3

IESLC - Meta-analysis of Ever/current Smoking, Age started, "Highest vs lowest"
Adenocarcinoma, Any Product (or Cigarettes if Any not available)
Most adjusted

	Study size (number of LC cases) 100-249	250-499	500-999	1000+	Total
N	5	5	3	4	17
NS	5	4	2	3	14
Wt	21.40	22.49	20.87	71.21	135.97
Het Chi	8.47	8.80	3.01	4.68	41.84
Het df	4	4	2	3	16
Het P	(*)	(*)	N.S.	N.S.	***
Fixed RR	1.66	1.68	4.43	1.66	1.94
RRl	1.09	1.11	2.88	1.32	1.64
RRu	2.54	2.55	6.80	2.10	2.29
P	+	+	+++	+++	+++
Random RR	1.70	1.87	4.24	1.63	1.99
RRl	0.91	0.96	2.47	1.21	1.48
RRu	3.20	3.64	7.28	2.19	2.67
P	(+)	(+)	+++	++	+++
Between Chi					16.89
Between df					3
Between P					***
Btwn(F) P					(*)
Btwn(R) P					*

	Risky occupational population no	mining	othRisky	Total
N	17			17
NS	14			14
Wt	135.97			135.97
Het Chi	41.84			41.84
Het df	16			16
Het P	***			***
Fixed RR	1.94			1.94
RRl	1.64			1.64
RRu	2.29			2.29
P	+++			+++
Random RR	1.99			1.99
RRl	1.48			1.48
RRu	2.67			2.67
P	+++			+++
Between Chi				
Between df				
Between P				N.S.
Btwn(F) P				N.S.
Btwn(R) P				N.S.

	National cigarette tobacco type Virginia	blended	other	Total
N		15	2	17
NS		13	1	14
Wt		124.58	11.39	135.97
Het Chi		35.80	1.65	41.84
Het df		14	1	16
Het P		**	N.S.	***
Fixed RR		1.83	3.51	1.94
RRl		1.54	1.96	1.64
RRu		2.19	6.27	2.29
P		+++	+++	+++
Random RR		1.86	3.30	1.99
RRl		1.37	1.51	1.48
RRu		2.53	7.23	2.67
P		+++	++	+++
Between Chi				4.39
Between df				1
Between P				*
Btwn(F) P				N.S.
Btwn(R) P				N.S.

Table 3H15 - 3

IESLC - Meta-analysis of Ever/current Smoking, Age started, "Highest vs lowest"
Adenocarcinoma, Any Product (or Cigarettes if Any not available)
Most adjusted

	Any proxy use No/nk	Yes	Total
N	13	4	17
NS	11	3	14
Wt	112.16	23.81	135.97
Het Chi	24.50	13.24	41.84
Het df	12	3	16
Het P	*	**	***
Fixed RR	1.79	2.82	1.94
RRl	1.49	1.89	1.64
RRu	2.15	4.22	2.29
P	+++	+++	+++
Random RR	1.79	3.07	1.99
RRl	1.34	1.18	1.48
RRu	2.39	8.01	2.67
P	+++	+	+++
Between Chi			4.10
Between df			1
Between P			*
Btwn(F) P			N.S.
Btwn(R) P			N.S.

	Full histological confirmation No	Yes	Total
N	4	13	17
NS	4	10	14
Wt	25.90	110.07	135.97
Het Chi	2.20	38.21	41.84
Het df	3	12	16
Het P	N.S.	***	***
Fixed RR	1.57	2.04	1.94
RRl	1.07	1.69	1.64
RRu	2.30	2.45	2.29
P	+	+++	+++
Random RR	1.57	2.14	1.99
RRl	1.07	1.48	1.48
RRu	2.30	3.10	2.67
P	+	+++	+++
Between Chi			1.43
Between df			1
Between P			N.S.
Btwn(F) P			N.S.
Btwn(R) P			N.S.

	Number of adjustment variables (1) 0	1	2+/+nk	Total
N	9	3	5	17
NS	7	2	5	14
Wt	89.52	20.87	25.57	135.97
Het Chi	8.91	3.01	11.56	41.84
Het df	8	2	4	16
Het P	N.S.	N.S.	*	***
Fixed RR	1.77	4.43	1.35	1.94
RRl	1.44	2.88	0.92	1.64
RRu	2.18	6.80	1.99	2.29
P	+++	+++	N.S.	+++
Random RR	1.77	4.24	1.48	1.99
RRl	1.41	2.47	0.75	1.48
RRu	2.22	7.28	2.92	2.67
P	+++	+++	N.S.	+++
Between Chi				18.36
Between df				2
Between P				***
Btwn(F) P				*
Btwn(R) P				**

Table 3H15 - 3

IESLC - Meta-analysis of Ever/current Smoking, Age started, "Highest vs lowest"
Adenocarcinoma, Any Product (or Cigarettes if Any not available)
Most adjusted

	Number of adjustment variables (2)					
	0	1	2	3-5	6+/+nk	Total
N	9	3	4	1		17
NS	7	2	4	1		14
Wt	89.52	20.87	17.58	8.00		135.97
Het Chi	8.91	3.01	8.39	0.00		41.84
Het df	8	2	3	0		16
Het P	N.S.	N.S.	*	N.S.		***
Fixed RR	1.77	4.43	1.71	0.80		1.94
RRl	1.44	2.88	1.07	0.40		1.64
RRu	2.18	6.80	2.73	1.60		2.29
P	+++	+++	+	N.S.		+++
Random RR	1.77	4.24	1.79	0.80		1.99
RRl	1.41	2.47	0.80	0.40		1.48
RRu	2.22	7.28	4.01	1.60		2.67
P	+++	+++	N.S.	N.S.		+++
Between Chi						21.53
Between df						3
Between P						***
Btwn(F) P						*
Btwn(R) P						**

	Smoking status		
	ever	current	Total
N	13	4	17
NS	10	4	14
Wt	120.38	15.59	135.97
Het Chi	35.80	5.12	41.84
Het df	12	3	16
Het P	***	N.S.	***
Fixed RR	1.88	2.43	1.94
RRl	1.57	1.48	1.64
RRu	2.25	4.00	2.29
P	+++	+++	+++
Random RR	1.89	2.46	1.99
RRl	1.35	1.27	1.48
RRu	2.64	4.77	2.67
P	+++	++	+++
Between Chi			0.92
Between df			1
Between P			N.S.
Btwn(F) P			N.S.
Btwn(R) P			N.S.

	Product			
	all/unsp	cig+/-ot	cig only	Total
N	5	10	2	17
NS	4	8	2	14
Wt	21.87	95.38	18.72	135.97
Het Chi	7.19	18.43	0.13	41.84
Het df	4	9	1	16
Het P	N.S.	*	N.S.	***
Fixed RR	4.10	1.76	1.31	1.94
RRl	2.70	1.44	0.83	1.64
RRu	6.24	2.15	2.06	2.29
P	+++	+++	N.S.	+++
Random RR	3.96	1.72	1.31	1.99
RRl	2.14	1.26	0.83	1.48
RRu	7.34	2.34	2.06	2.67
P	+++	+++	N.S.	+++
Between Chi				16.09
Between df				2
Between P				***
Btwn(F) P				*
Btwn(R) P				*

Table 3H15 - 3

IESLC - Meta-analysis of Ever/current Smoking, Age started, "Highest vs lowest"
Adenocarcinoma, Any Product (or Cigarettes if Any not available)
Most adjusted

	Derivation of RR/CI			
	Orig	StdCalc	Other	Total
N	1	8	8	17
NS	1	7	7	15
Wt	8.00	89.04	38.93	135.97
Het Chi	0.00	7.30	20.90	41.84
Het df	0	7	7	16
Het P	N.S.	N.S.	**	***
Fixed RR	0.80	1.75	2.91	1.94
RRl	0.40	1.42	2.13	1.64
RRu	1.60	2.16	3.99	2.29
P	N.S.	+++	+++	+++
Random RR	0.80	1.75	2.72	1.99
RRl	0.40	1.41	1.52	1.48
RRu	1.60	2.17	4.86	2.67
P	N.S.	+++	+++	+++
Between Chi				13.64
Between df				2
Between P				**
Btwn(F) P				(*)
Btwn(R) P				*

Table 3H15 - 4

IESLC - Meta-analysis of Ever/current Smoking, Age started, "Highest vs lowest"
Adenocarcinoma, Any Product (or Cigarettes if Any not available)
Least adjusted

REF	NRR	X	SEX	AGEL	AGEH	RACE	YF	LC TYPE	LOC	START	ST	NLC	R	VB	P	H	AD	ADOS	SM	PRODUCT	exL	exH	unexL	unexH	De
BARBON	735	x	m	0	0	all	-	a	Eu:wst	1979	CC	755	n	bl	y	y	0	0	ev	all/unsp	1	14	20	999	st
BENHAM	523		m	0	0	all	-	KII	Eu:wst	1976	CC	1625	n	bl	n	y	0	0	ev	cig only	1	19	20	999	st
ENGELA	529		m	0	0	all	0	a	Eu:Sca	1964	pr	435	n	bl	n	n	0	0	cu	cig+/-ot	1	19	20	999	st
HAENSZ	513	x	f	0	0	all	-	a	NAmer	1955	CC	158	n	bl	n	y	0	0	ev	cig+/-ot	1	24	25	999	st
HEGMAN	505		m	0	0	all	-	a	NAmer	1989	CC	282	n	bl	y	y	0	0	ev	all/unsp	1	19	20	999	st
HEGMAN	510		f	0	0	all	-	a	NAmer	1989	CC	282	n	bl	y	y	0	0	ev	all/unsp	1	25	26	999	ot
JEDRYC	542		m	0	0	all	-	a	Eu:est	1980	CC	1630	n	bl	y	n	0	0	ev	cig+/-ot	1	16	19	999	st
KHUDER	528		m	0	0	all	-	a	NAmer	1985	CC	482	n	bl	n	y	5	3#	ev	cig+/-ot	1	15	20	999	or
MATOS	655	x	m	0	0	all	-	a	SCAmer	1994	CC	200	n	bl	n	n	0	0	ev	cig+/-ot	1	14	20	999	st
PEZZOT	556	x	m	0	0	all	-	a	SCAmer	1987	CC	215	n	bl	n	y	0	0	ev	cig only	1	13	19	999	st
SVENSS	529		f	0	0	all	-	a	Eu:Sca	1983	CC	210	n	bl	n	n	0	0	cu	all/unsp	0	18	26	999	st
WAKAI	515		m	0	0	all	-	a	As:Jap	1988	CC	333	n	bl	n	y	0	0	cu	cig+/-ot	1	19	30	999	st
WU	505	x	f	0	0	wh	-	a	NAmer	1981	CC	220	n	bl	n	y	0	0	cu	all/unsp	0	18	25	999	st
WYNDE6	617		m	0	0	wh	-	a	NAmer	1969	CC	4423	n	bl	n	y	0	0	ev	cig+/-ot	1	17	21	999	st
WYNDE6	641		f	0	0	wh	-	a	NAmer	1969	CC	4423	n	bl	n	y	0	0	ev	cig+/-ot	1	17	21	999	st
ZHENG	535	x	m	0	0	all	-	a	As:Chi	1982	CC	540	n	ot	*	y	0	0	ev	cig+/-ot	1	19	30	999	st
ZHENG	540	x	f	0	0	all	-	a	As:Chi	1982	CC	540	n	ot	*	y	0	0	ev	cig+/-ot	1	29	30	999	st

Comments on values in listings

KHUDER ADOS Duration of smoking (years), number of cigarettes per day and Quitted smoking

Cigarette type is all/unspec for all RRs

Table 3H15 - 5

IESLC - Meta-analysis of Ever/current Smoking, Age started, "Highest vs lowest"
Adenocarcinoma, Any Product (or Cigarettes if Any not available)
Least adjusted

REF	NRR	SEX	AD	Number Exposed Case	Cont	Non-exposed Case	Cont	RR	95.00%CI
BARBON	735	m	0	29	23	45	207	5.80	(3.07- 10.95)
BENHAM	523	m	0	69	79	41	64	1.36	(0.82- 2.27)
*ENGELA	529	m	0	25	50732	8	39957	2.46	(1.11- 5.46)
HAENSZ	513	f	0	6	37	12	66	0.89	(0.31- 2.57)
HEGMAN	505	m	0	45	716	6	289	3.03	(1.28- 7.17)
HEGMAN	510	f	0	32	169	0	28	10.93~	(0.65- 183.56)
Subtotal HEGMAN								3.38	(1.48- 7.71)
JEDRYC	542	m	0	12	111	48	502	1.13	(0.58- 2.20)
KHUDER	528	m	5	-	-	-	-	0.80	(0.40- 1.60)
MATOS	655	m	0	26	90	13	73	1.62	(0.78- 3.38)
PEZZOT	556	m	0	9	15	18	32	1.07	(0.39- 2.92)
SVENSS	529	f	0	17	21	10	18	1.46	(0.53- 3.97)
WAKAI	515	m	0	18	74	4	25	1.52	(0.47- 4.92)
WU	505	f	0	69	21	8	14	5.75	(2.12- 15.58)
WYNDE6	617	m	0	241	301	47	92	1.57	(1.06- 2.32)
WYNDE6	641	f	0	150	91	63	90	2.35	(1.56- 3.56)
Subtotal WYNDE6								1.90	(1.43- 2.52)
ZHENG	535	m	0	44	43	15	66	4.50	(2.23- 9.07)
ZHENG	540	f	0	26	28	7	16	2.12	(0.75- 5.98)
Subtotal ZHENG								3.56	(1.99- 6.35)
Partial Totals				818	52551	345	41539		

*prospective study ~ With 0.5 adjustment for zero

REF	NRR	SEX	AD	Ys	Ws	Qs	Ps
BARBON	735	m	0	1.76	9.52	11.41	0.0000
BENHAM	523	m	0	0.31	14.89	1.86	0.2317
*ENGELA	529	m	0	0.90	6.06	0.34	0.0266
HAENSZ	513	f	0	-0.11	3.42	2.07	0.8324
HEGMAN	505	m	0	1.11	5.16	1.02	0.0119
HEGMAN	510	f	0	2.39	0.48	1.44	0.0966
Subtotal HEGMAN				1.22	5.64	2.46	
JEDRYC	542	m	0	0.12	8.68	2.54	0.7175
KHUDER	528	m	5	-0.22	8.00	6.28	0.5281
MATOS	655	m	0	0.48	7.13	0.23	0.1963
PEZZOT	556	m	0	0.06	3.78	1.35	0.9002
SVENSS	529	f	0	0.38	3.82	0.31	0.4620
WAKAI	515	m	0	0.42	2.78	0.17	0.4845
WU	505	f	0	1.75	3.87	4.56	0.0006
WYNDE6	617	m	0	0.45	25.24	1.16	0.0240
WYNDE6	641	f	0	0.86	22.40	0.84	0.0001
Subtotal WYNDE6				0.64	47.64	1.99	
ZHENG	535	m	0	1.50	7.82	5.54	0.0000
ZHENG	540	f	0	0.75	3.58	0.03	0.1546
Subtotal ZHENG				1.27	11.40	5.57	

	N	17
	NS	14
	Wt	136.65
Het	Chi	41.14
Het	df	16
Het	P	***
Fixed	RR	1.94
	RRl	1.64
	RRu	2.30
	P	+++
Random	RR	1.98
	RRl	1.48
	RRu	2.65
	P	+++
Asymm	P	N.S.

Table 3H15 - 6

IESLC - Meta-analysis of Ever/current Smoking, Age started, "Highest vs lowest"
Adenocarcinoma, Any Product (or Cigarettes if Any not available)
Least adjusted

	combined	Sex male	female	Total
N		11	6	17
NS		11	6	17
Wt		99.08	37.57	136.65
Het Chi		31.54	8.32	41.14
Het df		10	5	16
Het P		***	N.S.	***
Fixed RR		1.83	2.27	1.94
RRl		1.50	1.65	1.64
RRu		2.23	3.13	2.30
P		+++	+++	+++
Random RR		1.88	2.24	1.98
RRl		1.30	1.36	1.48
RRu		2.72	3.71	2.65
P		+++	++	+++
Between Chi				1.29
Between df				1
Between P				N.S.
Btwn(F) P				N.S.
Btwn(R) P				N.S.

Table 3H15 - 7

IESLC - Meta-analysis of Ever/current Smoking, Age started, "Highest vs lowest"
Adenocarcinoma, Any Product (or Cigarettes if Any not available)
Excluded studies (and stage at which they were excluded)

1	BECHER BLOT1 BROWN3 CARPEN CHYOU DARBY DOLL2 GARCIA GRAHAM GURSEL HAMMO2 JAHN JAIN LAUSSM PRESCO QIAO TVERDA WIGLE WYNDE3
2	AKIBA AMANDU AMES AXELSS BENSHL BEST BOUCHA BOUCOT BROSS CHEN CPSII DEAN2 DESTEF DORGAN DOSEME FAN GARSHI GER GILLIS HAMMON HUMBLE JUSSAW KAISE2 KATSOU KAUFMA KOO KREUZE LEVIN MCCONN NOTAN2 OSANN2 PEZZO2 PISANI RESTRE SADOWS VUTUC WANG2 WATSON WU2 WUWILL WYNDE2 WYNDE8 XU ZHOU
3	GUO MCDUFF SPITZ STASZE ZHANG
5	AGUDO ARMADA AUVINE BOFFET BRESLO BUFFLE CEDERL CHEN2 CHIAZZ CHOI CORREA CPSI DAMBER DEAN3 DOLL DORN GAO GAO2 GENG HIRAYA HOLE HU HU2 JOLY KOULUM LETOUR LIAW LIU3 LIU4 LIU5 LUBIN LUBIN2 LUO MIGRAN MRFITR PERNU QIAO2 RACHTA SEGI2 SOBUE SPEIZE SUZUK2 TIZZAN WYNDE7 YUAN
6	ALDERS

Table 3H15 - 8
Potentially overlapping studies

REF	REFGP	PRINC	. OVERLAP/LINK
BENHAM	LUBIN2	2	Subset of Lubin2
WYNDE6	WYNDE6	1	WYNDE5/6/7/8

Table 3H15 - 9
Most adjusted - insufficient data for meta-analysis

REF	NRR	SEX	AGEL	AGEH	RACE	YF	LC TYPE	LOC	START	ST	NLC	R	VB	P	H	AD	ADOS	SM	PRODUCT	exL	exH	unexL	unexH	De
ALDERS	563	m	0	0	all	-	not q+s	Eu:UK	1977	CC	1448	n		V	n	n	2	1#	ev cig only	1	14	25	999	ot
ALDERS	566	f	0	0	all	-	not q+s	Eu:UK	1977	CC	1448	n		V	n	n	2	1#	ev cig only	1	14	25	999	ot

Comments on values in listings

ALDERS ADOS Number of cigs/day
ALDERS ADOS Number of cigs/day

REF	NRR	RR	SIG	RRDATA comment
ALDERS	563	0.53	n	0
ALDERS	566	1.92	n	0

Table 3H16 -

IESLC - Meta-analysis of Ever/current Smoking by Age started, Overview
Adenocarcinoma, Cigarettes (or Any Product if Cigarettes not available)

This analysis is restricted to results for:
1) Ever/current smokers
2) Results by Age started
3) Categorical results by Age started
Results by Age started are grouped under 2 schemes (S1, S2). Each scheme has a set of "key values". An interval is allocated to the category whose key value it includes, and intervals which include none or more than one of the key values are excluded. (Open-ended intervals are coded as 999)

S1	key value	maximum range
1	26	19+
2	18	15-25
3	14	1-17

S2	key value	maximum range
1	30	27+
2	26	23-29
3	22	19-25
4	18	15-21
5	14	11-17
6	10	1-13

4) Adenocarcinoma (or near equivalent)
5) Results complete enough for use in metaanalysis

Within each study, results are then selected (in the following order of preference, within each sex) for:
6) SMKSTA: ever, current
7) PRODUCT: cigarettes regardless of other products, cigarettes only, all/unspec
8) CIGTYPE: all/unspecified, MC regardless of HR, MC only
9) (not applicable)
10) DENOM: never smoked anything, never smoked cigarettes, never any + low, never cigs + low
11) Followup period (YF, prospective studies): whole study (coded as 0) or longest available
12) LCtype: adeno or nearest available, but not squamous. (q = squamous, s = small,
a = adeno, l = large, KII = Kreyberg II, al = alveolar, br = bronchiolar, u = undifferentiated)
13) Race: all or nearest available, otherwise by race (wh or w = white, bl or b = black, hi = hispanic
ch = chinese, jap = japanese, haw = hawaiian, w+o = white + oriental, sca = scandinavian, as = asian)
14) For overlapping studies: principal rather than subsidiary studies
Finally by Age: whole study (coded as 0) if available, otherwise by widest available age group
and then for single sex results (m, f) in preference to results for both sexes combined (c).

Results adjusted (AD) for the most potential confounders are then chosen in Sections -1 to -3
(and those which actually differ from the adjusted results in Table 3H11 - 1 are marked 'x' in Section -1)
and results adjusted for the least confounders in Sections -4 to -6. (Those least adjusted results which
actually differ from the most adjusted are marked 'x' in column X in Section -4)

Section -7 shows excluded studies, together with the stage (as above) at which no qualifying
results were found.

Section -8 lists the potentially overlapping studies which have been included (1=principal, 2=subsidiary).

Section -9 lists any results which would have been included in preference except that they had data not complete
enough for use in meta-analysis, with their significance (yes/no), if known, and any further comment as entered
on the database. It also lists as "gap" any categories for which no data were presented by the original authors.

In addition to those mentioned above, the following fields, levels and abbreviations are used:
* or nk = not known, n = no, y = yes, ot = other
ev = ever, cu = current, nev = never
all/unspec = all or unspecified, cig+/-ot = cigarettes irrespective of other products (cigar, pipe etc)
MC = manufactured cigarettes, HR = hand-rolled cigarettes
exL, exH = range of exposure (low and high) in the smoking group, in terms of Age started
REF: 6-character study reference
NRR: number of the RR on the database within the study
ST : study type (CC = case control, pr or prosp = prospective)
NLC: number of lung cancer cases in whole study
R : risky occupational population (n = no, m = mining, o = other risky)
VB : national cigarette type (V = at least 75% Virginia, bl = at least 75% blended, ot = other)
P : any proxy use
H : full histological confirmation
De : derivation of RR/CI (or = original, st = standard method, ot = other method of estimation)

Table 3H16 - 1

IESLC - Meta-analysis of Ever/current Smoking by Age started, Overview
Adenocarcinoma, Cigarettes (or Any Product if Cigarettes not available)
Most adjusted

REF	NRR	XX11	SEX	AGEL	AGEH	RACE	YF	LC TYPE	LOC	START	ST	NLC	R	VB	P	H	AD	SM	PRODUCT	exL	exH	S1	S2	DENOM	De
BARBON	736		m	0	0	all	-	a	Eu:wst	1979	CC	755	n	bl	y	y	1	ev	all/unsp	20	999	1	0	nev any	or
BARBON	737		m	0	0	all	-	a	Eu:wst	1979	CC	755	n	bl	y	y	1	ev	all/unsp	15	19	2	4	nev any	or
BARBON	738		m	0	0	all	-	a	Eu:wst	1979	CC	755	n	bl	y	y	1	ev	all/unsp	1	14	3	0	nev any	or
BENHAM	521		m	0	0	all	-	KII	Eu:wst	1976	CC	1625	n	bl	n	y	0	ev	cig only	20	999	1	0	nev any	st
BENHAM	522		m	0	0	all	-	KII	Eu:wst	1976	CC	1625	n	bl	n	y	0	ev	cig only	1	19	0	0	nev any	st
ENGELA	527		m	0	0	all	0	a	Eu:Sca	1964	pr	435	n	bl	n	n	0	cu	cig+/-ot	20	999	1	0	nev cigs	st
ENGELA	528		m	0	0	all	0	a	Eu:Sca	1964	pr	435	n	bl	n	n	0	cu	cig+/-ot	1	19	0	0	nev cigs	st
HAENSZ	509		f	0	0	all	-	a	NAmer	1955	CC	158	n	bl	n	y	0	ev	cig+/-ot	25	999	1	0	nev any	st
HAENSZ	510		f	0	0	all	-	a	NAmer	1955	CC	158	n	bl	n	y	0	ev	cig+/-ot	1	24	0	0	nev any	st
JEDRYC	538		m	0	0	all	-	a	Eu:est	1980	CC	1630	n	bl	y	n	0	ev	cig+/-ot	19	999	1	0	nev any	st
JEDRYC	539		m	0	0	all	-	a	Eu:est	1980	CC	1630	n	bl	y	n	0	ev	cig+/-ot	17	18	2	4	nev any	st
JEDRYC	540		m	0	0	all	-	a	Eu:est	1980	CC	1630	n	bl	y	n	0	ev	cig+/-ot	1	16	3	0	nev any	st
MATOS	656		m	0	0	all	-	a	SCAmer	1994	CC	200	n	bl	n	n	2	ev	cig+/-ot	20	999	1	0	nev any	or
MATOS	657		m	0	0	all	-	a	SCAmer	1994	CC	200	n	bl	n	n	2	ev	cig+/-ot	15	19	2	4	nev any	or
MATOS	658		m	0	0	all	-	a	SCAmer	1994	CC	200	n	bl	n	n	2	ev	cig+/-ot	1	14	3	0	nev any	or
PEZZOT	552		m	0	0	all	-	a	SCAmer	1987	CC	215	n	bl	n	y	0	ev	cig only	19	999	1	0	nev cigs	st
PEZZOT	553		m	0	0	all	-	a	SCAmer	1987	CC	215	n	bl	n	y	0	ev	cig only	14	18	0	0	nev cigs	st
PEZZOT	554		m	0	0	all	-	a	SCAmer	1987	CC	215	n	bl	n	y	0	ev	cig only	1	13	0	6	nev cigs	st
SVENSS	525		f	0	0	all	-	a	Eu:Sca	1983	CC	210	n	bl	n	n	0	cu	all/unsp	26	999	1	0	nev any	st
SVENSS	526		f	0	0	all	-	a	Eu:Sca	1983	CC	210	n	bl	n	n	0	cu	all/unsp	19	25	0	3	nev any	st
SVENSS	527		f	0	0	all	-	a	Eu:Sca	1983	CC	210	n	bl	n	n	0	cu	all/unsp	0	18	0	0	nev any	st
WAKAI	511		m	0	0	all	-	a	As:Jap	1988	CC	333	n	bl	n	y	0	cu	cig+/-ot	30	999	0	1	nev any	st
WAKAI	512		m	0	0	all	-	a	As:Jap	1988	CC	333	n	bl	n	y	0	cu	cig+/-ot	20	29	1	0	nev any	st
WAKAI	513		m	0	0	all	-	a	As:Jap	1988	CC	333	n	bl	n	y	0	cu	cig+/-ot	1	19	0	0	nev any	st
WU	525		f	0	0	wh	-	a	NAmer	1981	CC	220	n	bl	n	y	2	cu	all/unsp	25	999	1	0	nev any	or
WU	526		f	0	0	wh	-	a	NAmer	1981	CC	220	n	bl	n	y	2	cu	all/unsp	19	24	0	3	nev any	or
WU	527		f	0	0	wh	-	a	NAmer	1981	CC	220	n	bl	n	y	2	cu	all/unsp	0	18	0	0	nev any	or
WYNDE6	613		m	0	0	wh	-	a	NAmer	1969	CC	4423	n	bl	n	y	0	ev	cig+/-ot	21	999	1	0	nev cigs	st
WYNDE6	614		m	0	0	wh	-	a	NAmer	1969	CC	4423	n	bl	n	y	0	ev	cig+/-ot	18	20	2	4	nev cigs	st
WYNDE6	615		m	0	0	wh	-	a	NAmer	1969	CC	4423	n	bl	n	y	0	ev	cig+/-ot	1	17	3	0	nev cigs	st
WYNDE6	637		f	0	0	wh	-	a	NAmer	1969	CC	4423	n	bl	n	y	0	ev	cig+/-ot	21	999	1	0	nev cigs	st
WYNDE6	638		f	0	0	wh	-	a	NAmer	1969	CC	4423	n	bl	n	y	0	ev	cig+/-ot	18	20	2	4	nev cigs	st
WYNDE6	639		f	0	0	wh	-	a	NAmer	1969	CC	4423	n	bl	n	y	0	ev	cig+/-ot	1	17	3	0	nev cigs	st
ZHENG	531		m	0	0	all	-	a	As:Chi	1982	CC	540	n	ot	*	y	0	ev	cig+/-ot	30	999	0	1	nev cigs	or
ZHENG	532		m	0	0	all	-	a	As:Chi	1982	CC	540	n	ot	*	y	0	ev	cig+/-ot	20	29	1	0	nev cigs	st
ZHENG	533		m	0	0	all	-	a	As:Chi	1982	CC	540	n	ot	*	y	0	ev	cig+/-ot	1	19	0	0	nev cigs	st
ZHENG	538		f	0	0	all	-	a	As:Chi	1982	CC	540	n	ot	*	y	0	ev	cig+/-ot	30	999	0	1	nev cigs	or
ZHENG	539		f	0	0	all	-	a	As:Chi	1982	CC	540	n	ot	*	y	0	ev	cig+/-ot	1	29	0	0	nev cigs	st

Cigarette type is all/unspec for all RRs

In this overview table, subtotals and Qs values may be invalid and should be ignored

Table 3H16 - 2

IESLC - Meta-analysis of Ever/current Smoking by Age started, Overview
Adenocarcinoma, Cigarettes (or Any Product if Cigarettes not available)
Most adjusted

REF	NRR	SEX	AD	Number Exposed Case	Cont	Non-exposed Case	Cont	RR	95.00%CI
BARBON	736	m	1	45	-	7	-	5.70	(2.50- 12.90)
BARBON	737	m	1	77	-	7	-	6.00	(2.70- 13.30)
BARBON	738	m	1	29	-	7	-	33.40	(13.10- 84.90)
Subtotal BARBON								9.41	(5.78- 15.32)
BENHAM	521	m	0	41	64	9	42	2.99	(1.32- 6.79)
BENHAM	522	m	0	69	79	9	42	4.08	(1.85- 8.97)
Subtotal BENHAM								3.51	(1.99- 6.20)
*ENGELA	527	m	0	8	39957	7	58716	1.68	(0.61- 4.63)
*ENGELA	528	m	0	25	50732	7	58716	4.13	(1.79- 9.56)
Subtotal ENGELA								2.87	(1.50- 5.47)
HAENSZ	509	f	0	12	66	37	236	1.16	(0.57- 2.35)
HAENSZ	510	f	0	6	37	37	236	1.03	(0.41- 2.62)
Subtotal HAENSZ								1.11	(0.63- 1.95)
JEDRYC	538	m	0	48	502	7	289	3.95	(1.76- 8.84)
JEDRYC	539	m	0	29	221	7	289	5.42	(2.33- 12.60)
JEDRYC	540	m	0	12	111	7	289	4.46	(1.71- 11.63)
Subtotal JEDRYC								4.56	(2.77- 7.50)
MATOS	656	m	2	13	-	5	-	4.00	(1.40- 11.90)
MATOS	657	m	2	40	-	5	-	7.20	(2.70- 19.10)
MATOS	658	m	2	26	-	5	-	6.60	(2.40- 18.10)
Subtotal MATOS								5.86	(3.25- 10.54)
PEZZOT	552	m	0	18	105	3	116	6.63	(1.90- 23.15)
PEZZOT	553	m	0	33	145	3	116	8.80	(2.63- 29.42)
PEZZOT	554	m	0	9	67	3	116	5.19	(1.36- 19.85)
Subtotal PEZZOT								6.84	(3.30- 14.18)
SVENSS	525	f	0	10	18	22	120	3.03	(1.24- 7.43)
SVENSS	526	f	0	11	14	22	120	4.29	(1.72- 10.66)
SVENSS	527	f	0	17	21	22	120	4.42	(2.01- 9.68)
Subtotal SVENSS								3.90	(2.38- 6.40)
WAKAI	511	m	0	4	25	8	65	1.30	(0.36- 4.70)
WAKAI	512	m	0	52	183	8	65	2.31	(1.04- 5.12)
WAKAI	513	m	0	18	74	8	65	1.98	(0.81- 4.85)
Subtotal WAKAI								1.97	(1.15- 3.38)
WU	525	f	2	8	-	29	-	1.10	(0.40- 3.20)
WU	526	f	2	22	-	29	-	2.50	(1.00- 5.80)
WU	527	f	2	69	-	29	-	8.00	(3.60- 17.90)
Subtotal WU								3.30	(1.97- 5.52)
WYNDE6	613	m	0	47	92	42	589	7.16	(4.47- 11.47)
WYNDE6	614	m	0	88	139	42	589	8.88	(5.88- 13.40)
WYNDE6	615	m	0	241	301	42	589	11.23	(7.87- 16.03)
WYNDE6	637	f	0	63	90	58	673	8.12	(5.34- 12.35)
WYNDE6	638	f	0	113	94	58	673	13.95	(9.51- 20.46)
WYNDE6	639	f	0	150	91	58	673	19.13	(13.16- 27.80)
Subtotal WYNDE6								11.24	(9.55- 13.22)
ZHENG	531	m	0	15	66	29	94	0.74	(0.37- 1.48)
ZHENG	532	m	0	64	109	29	94	1.90	(1.13- 3.20)
ZHENG	533	m	0	44	43	29	94	3.32	(1.84- 5.99)
ZHENG	538	f	0	7	16	119	184	0.68	(0.27- 1.69)
ZHENG	539	f	0	26	28	119	184	1.44	(0.80- 2.57)
Subtotal ZHENG								1.57	(1.19- 2.08)
Partial Totals				1609	93490	974	124194		

*prospective study

Table 3H16 - 2

IESLC - Meta-analysis of Ever/current Smoking by Age started, Overview
Adenocarcinoma, Cigarettes (or Any Product if Cigarettes not available)
Most adjusted

REF	NRR	SEX	AD	Ys	Ws	Qs	Ps
BARBON	736	m	1	1.74	5.71	0.02	0.0000
BARBON	737	m	1	1.79	6.04	0.07	0.0000
BARBON	738	m	1	3.51	4.40	14.62	0.0000
Subtotal BARBON				2.24	16.15	14.71	
BENHAM	521	m	0	1.10	5.72	1.99	0.0088
BENHAM	522	m	0	1.41	6.17	0.49	0.0005
Subtotal BENHAM				1.26	11.89	2.48	
*ENGELA	527	m	0	0.52	3.73	5.09	0.3164
*ENGELA	528	m	0	1.42	5.47	0.39	0.0009
Subtotal ENGELA				1.05	9.20	5.48	
HAENSZ	509	f	0	0.15	7.71	18.22	0.6808
HAENSZ	510	f	0	0.03	4.45	12.13	0.9433
Subtotal HAENSZ				0.11	12.15	30.35	
JEDRYC	538	m	0	1.37	5.91	0.58	0.0008
JEDRYC	539	m	0	1.69	5.40	0.00	0.0001
JEDRYC	540	m	0	1.50	4.19	0.15	0.0022
Subtotal JEDRYC				1.52	15.50	0.73	
MATOS	656	m	2	1.39	3.36	0.30	0.0111
MATOS	657	m	2	1.97	4.01	0.33	0.0001
MATOS	658	m	2	1.89	3.76	0.15	0.0003
Subtotal MATOS				1.77	11.13	0.79	
PEZZOT	552	m	0	1.89	2.46	0.10	0.0030
PEZZOT	553	m	0	2.17	2.64	0.63	0.0004
PEZZOT	554	m	0	1.65	2.14	0.00	0.0160
Subtotal PEZZOT				1.92	7.23	0.74	
SVENSS	525	f	0	1.11	4.78	1.59	0.0154
SVENSS	526	f	0	1.46	4.63	0.25	0.0017
SVENSS	527	f	0	1.49	6.24	0.25	0.0002
Subtotal SVENSS				1.36	15.64	2.09	
WAKAI	511	m	0	0.26	2.32	4.71	0.6892
WAKAI	512	m	0	0.84	6.06	4.37	0.0395
WAKAI	513	m	0	0.68	4.77	4.82	0.1366
Subtotal WAKAI				0.68	13.16	13.89	
WU	525	f	2	0.10	3.55	8.99	0.8574
WU	526	f	2	0.92	4.97	2.94	0.0410
WU	527	f	2	2.08	5.97	0.93	0.0000
Subtotal WU				1.19	14.50	12.86	
WYNDE6	613	m	0	1.97	17.35	1.39	0.0000
WYNDE6	614	m	0	2.18	22.69	5.63	0.0000
WYNDE6	615	m	0	2.42	30.32	16.28	0.0000
WYNDE6	637	f	0	2.09	21.88	3.66	0.0000
WYNDE6	638	f	0	2.64	26.17	23.61	0.0000
WYNDE6	639	f	0	2.95	27.49	44.02	0.0000
Subtotal WYNDE6				2.42	145.89	94.59	
ZHENG	531	m	0	-0.31	7.88	31.24	0.3910
ZHENG	532	m	0	0.64	14.30	15.53	0.0149
ZHENG	533	m	0	1.20	10.98	2.60	0.0001
ZHENG	538	f	0	-0.39	4.56	19.67	0.4038
ZHENG	539	f	0	0.36	11.36	19.91	0.2228
Subtotal ZHENG				0.45	49.08	88.95	

N	38
NS	12

Table 3H16 - 3

IESLC - Meta-analysis of Ever/current Smoking by Age started, Overview
Adenocarcinoma, Cigarettes (or Any Product if Cigarettes not available)
Most adjusted

	Sex			
	combined	male	female	Total
N		25	13	38
NS		9	5	14

In this overview table, other than the "N" rows, entries in the "absent" and "Total" columns may be invalid and should be ignored

	Age started (broad categories)				
	absent	19+k26	15-25k18	1-17k14	Total
N	15	13	5	5	38
NS	8	12	4	4	28
Wt	84.55	102.50	64.32	70.16	321.53
Het Chi	48.24	49.04	7.21	15.03	267.63
Het df	14	12	4	4	37
Het P	***	***	N.S.	**	***
Fixed RR	2.45	3.79	9.74	13.62	5.40
RRl	1.98	3.12	7.63	10.78	4.84
RRu	3.03	4.60	12.43	17.22	6.02
P	+++	+++	+++	+++	+++
Random RR	2.56	3.21	8.84	12.34	3.99
RRl	1.70	2.12	6.14	7.23	2.94
RRu	3.84	4.87	12.73	21.08	5.43
P	+++	+++	+++	+++	+++

	Age started (narrow categories)							
	absent	27+k30	23-29k26	19-25k22	15-21k18	11-17k14	1-13k10	Total
N	27	3		2	5		1	38
NS	12	2		2	4		1	21
Wt	230.71	14.76		9.60	64.32		2.14	321.53
Het Chi	179.10	0.72		0.70	7.21		0.00	267.63
Het df	26	2		1	4		0	37
Het P	***	N.S.		N.S.	N.S.		N.S.	***
Fixed RR	5.29	0.78		3.24	9.74		5.19	5.40
RRl	4.65	0.47		1.72	7.63		1.36	4.84
RRu	6.02	1.31		6.10	12.43		19.85	6.02
P	+++	N.S.		+++	+++		+	+++
Random RR	4.13	0.78		3.24	8.84		5.19	3.99
RRl	2.90	0.47		1.72	6.14		1.36	2.94
RRu	5.89	1.31		6.10	12.73		19.85	5.43
P	+++	N.S.		+++	+++		+	+++

Table 3H16 - 3

IESLC - Meta-analysis of Ever/current Smoking by Age started, Overview
Adenocarcinoma, Cigarettes (or Any Product if Cigarettes not available)
Most adjusted

MALES

		Age started (broad categories)				
		absent	19+k26	15-25k18	1-17k14	Total
	N	8	9	4	4	25
	NS	5	9	4	4	22
	Wt	42.37	64.59	38.15	42.68	187.78
Het	Chi	22.02	19.77	1.52	9.83	110.62
Het	df	7	8	3	3	24
Het	P	**	*	N.S.	*	***
Fixed	RR	2.59	3.67	7.61	10.95	5.04
	RRl	1.92	2.88	5.54	8.11	4.37
	RRu	3.50	4.68	10.45	14.78	5.82
	P	+++	+++	+++	+++	+++
Random	RR	2.77	3.55	7.61	10.46	4.39
	RRl	1.58	2.36	5.54	5.18	3.17
	RRu	4.85	5.34	10.45	21.13	6.08
	P	+++	+++	+++	+++	+++

				Age started (narrow categories)					
		absent	27+k30	23-29k26	19-25k22	15-21k18	11-17k14	1-13k10	Total
	N	18	2			4		1	25
	NS	9	2			4		1	16
	Wt	137.29	10.20			38.15		2.14	187.78
Het	Chi	69.17	0.58			1.52		0.00	110.62
Het	df	17	1			3		0	24
Het	P	***	N.S.			N.S.		N.S.	***
Fixed	RR	5.14	0.84			7.61		5.19	5.04
	RRl	4.35	0.45			5.54		1.36	4.37
	RRu	6.07	1.55			10.45		19.85	5.82
	P	+++	N.S.			+++		+	+++
Random	RR	4.61	0.84			7.61		5.19	4.39
	RRl	3.22	0.45			5.54		1.36	3.17
	RRu	6.60	1.55			10.45		19.85	6.08
	P	+++	N.S.			+++		+	+++

FEMALES

		Age started (broad categories)				
		absent	19+k26	15-25k18	1-17k14	Total
	N	7	4	1	1	13
	NS	4	4	1	1	8
	Wt	42.18	37.91	26.17	27.49	133.75
Het	Chi	25.96	29.08	0.00	0.00	154.95
Het	df	6	3	0	0	12
Het	P	***	***	N.S.	N.S.	***
Fixed	RR	2.32	4.00	13.95	19.13	5.93
	RRl	1.71	2.91	9.51	13.16	5.01
	RRu	3.13	5.50	20.46	27.80	7.03
	P	+++	+++	+++	+++	+++
Random	RR	2.35	2.46	13.95	19.13	3.29
	RRl	1.24	0.80	9.51	13.16	1.74
	RRu	4.46	7.54	20.46	27.80	6.24
	P	++	N.S.	+++	+++	+++

Table 3H16 - 3

IESLC - Meta-analysis of Ever/current Smoking by Age started, Overview
Adenocarcinoma, Cigarettes (or Any Product if Cigarettes not available)
Most adjusted

FEMALES

		Age started (narrow categories)						
	absent	27+k30	23-29k26	19-25k22	15-21k18	11-17k14	1-13k10	Total
N	9	1		2	1			13
NS	5	1		2	1			8
Wt	93.42	4.56		9.60	26.17			133.75
Het Chi	109.64	0.00		0.70	0.00			154.95
Het df	8	0		1	0			12
Het P	***	N.S.		N.S.	N.S.			***
Fixed RR	5.52	0.68		3.24	13.95			5.93
RRl	4.51	0.27		1.72	9.51			5.01
RRu	6.77	1.69		6.10	20.46			7.03
P	+++	N.S.		+++	+++			+++
Random RR	3.27	0.68		3.24	13.95			3.29
RRl	1.48	0.27		1.72	9.51			1.74
RRu	7.21	1.69		6.10	20.46			6.24
P	++	N.S.		+++	+++			+++

Table 3H16 - 4

IESLC - Meta-analysis of Ever/current Smoking by Age started, Overview
Adenocarcinoma, Cigarettes (or Any Product if Cigarettes not available)
Least adjusted

REF	NRR	X	SEX	AGEL	AGEH	RACE	YF	LC TYPE	LOC	START	ST	NLC	R	VB	P	H	AD	SM	PRODUCT	exL	exH	S1	S2	DENOM	De
BARBON	731	x	m	0	0	all	-	a	Eu:wst	1979	CC	755	n	bl	y	y	0	ev	all/unsp	20	999	1	0	nev any	st
BARBON	732	x	m	0	0	all	-	a	Eu:wst	1979	CC	755	n	bl	y	y	0	ev	all/unsp	15	19	2	4	nev any	st
BARBON	733	x	m	0	0	all	-	a	Eu:wst	1979	CC	755	n	bl	y	y	0	ev	all/unsp	1	14	3	0	nev any	st
BENHAM	521		m	0	0	all	-	KII	Eu:wst	1976	CC	1625	n	bl	n	y	0	ev	cig only	20	999	1	0	nev any	st
BENHAM	522		m	0	0	all	-	KII	Eu:wst	1976	CC	1625	n	bl	n	y	0	ev	cig only	1	19	0	0	nev any	st
ENGELA	527		m	0	0	all	0	a	Eu:Sca	1964	pr	435	n	bl	n	n	0	cu	cig+/-ot	20	999	1	0	nev cigs	st
ENGELA	528		m	0	0	all	0	a	Eu:Sca	1964	pr	435	n	bl	n	n	0	cu	cig+/-ot	1	19	0	0	nev cigs	st
HAENSZ	509		f	0	0	all	-	a	NAmer	1955	CC	158	n	bl	n	y	0	ev	cig+/-ot	25	999	1	0	nev any	st
HAENSZ	510		f	0	0	all	-	a	NAmer	1955	CC	158	n	bl	n	y	0	ev	cig+/-ot	1	24	0	0	nev any	st
JEDRYC	538		m	0	0	all	-	a	Eu:est	1980	CC	1630	n	bl	y	n	0	ev	cig+/-ot	19	999	1	0	nev any	st
JEDRYC	539		m	0	0	all	-	a	Eu:est	1980	CC	1630	n	bl	y	n	0	ev	cig+/-ot	17	18	2	4	nev any	st
JEDRYC	540		m	0	0	all	-	a	Eu:est	1980	CC	1630	n	bl	y	n	0	ev	cig+/-ot	1	16	3	0	nev any	st
MATOS	651	x	m	0	0	all	-	a	SCAmer	1994	CC	200	n	bl	n	n	0	ev	cig+/-ot	20	999	1	0	nev any	st
MATOS	652	x	m	0	0	all	-	a	SCAmer	1994	CC	200	n	bl	n	n	0	ev	cig+/-ot	15	19	2	4	nev any	st
MATOS	653	x	m	0	0	all	-	a	SCAmer	1994	CC	200	n	bl	n	n	0	ev	cig+/-ot	1	14	3	0	nev any	st
PEZZOT	552		m	0	0	all	-	a	SCAmer	1987	CC	215	n	bl	n	y	0	ev	cig only	19	999	1	0	nev cigs	st
PEZZOT	553		m	0	0	all	-	a	SCAmer	1987	CC	215	n	bl	n	y	0	ev	cig only	14	18	0	0	nev cigs	st
PEZZOT	554		m	0	0	all	-	a	SCAmer	1987	CC	215	n	bl	n	y	0	ev	cig only	1	13	0	6	nev cigs	st
SVENSS	525		f	0	0	all	-	a	Eu:Sca	1983	CC	210	n	bl	n	n	0	cu	all/unsp	26	999	1	0	nev any	st
SVENSS	526		f	0	0	all	-	a	Eu:Sca	1983	CC	210	n	bl	n	n	0	cu	all/unsp	19	25	0	3	nev any	st
SVENSS	527		f	0	0	all	-	a	Eu:Sca	1983	CC	210	n	bl	n	n	0	cu	all/unsp	0	18	0	0	nev any	st
WAKAI	511		m	0	0	all	-	a	As:Jap	1988	CC	333	n	bl	n	y	0	cu	cig+/-ot	30	999	0	1	nev any	st
WAKAI	512		m	0	0	all	-	a	As:Jap	1988	CC	333	n	bl	n	y	0	cu	cig+/-ot	20	29	1	0	nev any	st
WAKAI	513		m	0	0	all	-	a	As:Jap	1988	CC	333	n	bl	n	y	0	cu	cig+/-ot	1	19	0	0	nev any	st
WU	501	x	f	0	0	wh	-	a	NAmer	1981	CC	220	n	bl	n	y	0	cu	all/unsp	25	999	1	0	nev any	st
WU	502	x	f	0	0	wh	-	a	NAmer	1981	CC	220	n	bl	n	y	0	cu	all/unsp	19	24	0	3	nev any	st
WU	503	x	f	0	0	wh	-	a	NAmer	1981	CC	220	n	bl	n	y	0	cu	all/unsp	0	18	0	0	nev any	st
WYNDE6	613		m	0	0	wh	-	a	NAmer	1969	CC	4423	n	bl	n	y	0	ev	cig+/-ot	21	999	1	0	nev cigs	st
WYNDE6	614		m	0	0	wh	-	a	NAmer	1969	CC	4423	n	bl	n	y	0	ev	cig+/-ot	18	20	2	4	nev cigs	st
WYNDE6	615		m	0	0	wh	-	a	NAmer	1969	CC	4423	n	bl	n	y	0	ev	cig+/-ot	1	17	3	0	nev cigs	st
WYNDE6	637		f	0	0	wh	-	a	NAmer	1969	CC	4423	n	bl	n	y	0	ev	cig+/-ot	21	999	1	0	nev cigs	st
WYNDE6	638		f	0	0	wh	-	a	NAmer	1969	CC	4423	n	bl	n	y	0	ev	cig+/-ot	18	20	2	4	nev cigs	st
WYNDE6	639		f	0	0	wh	-	a	NAmer	1969	CC	4423	n	bl	n	y	0	ev	cig+/-ot	1	17	3	0	nev cigs	st
ZHENG	531		m	0	0	all	-	a	As:Chi	1982	CC	540	n	ot	*	y	0	ev	cig+/-ot	30	999	0	1	nev cigs	or
ZHENG	532		m	0	0	all	-	a	As:Chi	1982	CC	540	n	ot	*	y	0	ev	cig+/-ot	20	29	1	0	nev cigs	st
ZHENG	533		m	0	0	all	-	a	As:Chi	1982	CC	540	n	ot	*	y	0	ev	cig+/-ot	1	19	0	0	nev cigs	st
ZHENG	538		f	0	0	all	-	a	As:Chi	1982	CC	540	n	ot	*	y	0	ev	cig+/-ot	30	999	0	1	nev cigs	or
ZHENG	539		f	0	0	all	-	a	As:Chi	1982	CC	540	n	ot	*	y	0	ev	cig+/-ot	1	29	0	0	nev cigs	st

Cigarette type is all/unspec for all RRs

In this overview table, subtotals and Qs values may be invalid and should be ignored

Table 3H16 - 5

IESLC - Meta-analysis of Ever/current Smoking by Age started, Overview
Adenocarcinoma, Cigarettes (or Any Product if Cigarettes not available)
Least adjusted

REF	NRR	SEX	AD	Number Exposed Case	Cont	Non-exposed Case	Cont	RR	95.00%CI
BARBON	731	m	0	45	207	7	188	5.84	(2.57- 13.26)
BARBON	732	m	0	77	337	7	188	6.14	(2.77- 13.58)
BARBON	733	m	0	29	23	7	188	33.86	(13.33- 86.00)
Subtotal BARBON								9.61	(5.90- 15.63)
BENHAM	521	m	0	41	64	9	42	2.99	(1.32- 6.79)
BENHAM	522	m	0	69	79	9	42	4.08	(1.85- 8.97)
Subtotal BENHAM								3.51	(1.99- 6.20)
*ENGELA	527	m	0	8	39957	7	58716	1.68	(0.61- 4.63)
*ENGELA	528	m	0	25	50732	7	58716	4.13	(1.79- 9.56)
Subtotal ENGELA								2.87	(1.50- 5.47)
HAENSZ	509	f	0	12	66	37	236	1.16	(0.57- 2.35)
HAENSZ	510	f	0	6	37	37	236	1.03	(0.41- 2.62)
Subtotal HAENSZ								1.11	(0.63- 1.95)
JEDRYC	538	m	0	48	502	7	289	3.95	(1.76- 8.84)
JEDRYC	539	m	0	29	221	7	289	5.42	(2.33- 12.60)
JEDRYC	540	m	0	12	111	7	289	4.46	(1.71- 11.63)
Subtotal JEDRYC								4.56	(2.77- 7.50)
MATOS	651	m	0	13	73	5	110	3.92	(1.34- 11.46)
MATOS	652	m	0	40	120	5	110	7.33	(2.79- 19.25)
MATOS	653	m	0	26	90	5	110	6.36	(2.35- 17.22)
Subtotal MATOS								5.81	(3.24- 10.39)
PEZZOT	552	m	0	18	105	3	116	6.63	(1.90- 23.15)
PEZZOT	553	m	0	33	145	3	116	8.80	(2.63- 29.42)
PEZZOT	554	m	0	9	67	3	116	5.19	(1.36- 19.85)
Subtotal PEZZOT								6.84	(3.30- 14.18)
SVENSS	525	f	0	10	18	22	120	3.03	(1.24- 7.43)
SVENSS	526	f	0	11	14	22	120	4.29	(1.72- 10.66)
SVENSS	527	f	0	17	21	22	120	4.42	(2.01- 9.68)
Subtotal SVENSS								3.90	(2.38- 6.40)
WAKAI	511	m	0	4	25	8	65	1.30	(0.36- 4.70)
WAKAI	512	m	0	52	183	8	65	2.31	(1.04- 5.12)
WAKAI	513	m	0	18	74	8	65	1.98	(0.81- 4.85)
Subtotal WAKAI								1.97	(1.15- 3.38)
WU	501	f	0	8	14	29	62	1.22	(0.46- 3.24)
WU	502	f	0	22	15	29	62	3.14	(1.42- 6.91)
WU	503	f	0	69	21	29	62	7.02	(3.64- 13.56)
Subtotal WU								3.74	(2.38- 5.85)
WYNDE6	613	m	0	47	92	42	589	7.16	(4.47- 11.47)
WYNDE6	614	m	0	88	139	42	589	8.88	(5.88- 13.40)
WYNDE6	615	m	0	241	301	42	589	11.23	(7.87- 16.03)
WYNDE6	637	f	0	63	90	58	673	8.12	(5.34- 12.35)
WYNDE6	638	f	0	113	94	58	673	13.95	(9.51- 20.46)
WYNDE6	639	f	0	150	91	58	673	19.13	(13.16- 27.80)
Subtotal WYNDE6								11.24	(9.55- 13.22)
ZHENG	531	m	0	15	66	29	94	0.74	(0.37- 1.48)
ZHENG	532	m	0	64	109	29	94	1.90	(1.13- 3.20)
ZHENG	533	m	0	44	43	29	94	3.32	(1.84- 5.99)
ZHENG	538	f	0	7	16	119	184	0.68	(0.27- 1.69)
ZHENG	539	f	0	26	28	119	184	1.44	(0.80- 2.57)
Subtotal ZHENG								1.57	(1.19- 2.08)
Totals				1609	94390	974	125274		

*prospective study

Table 3H16 - 5

IESLC - Meta-analysis of Ever/current Smoking by Age started, Overview
Adenocarcinoma, Cigarettes (or Any Product if Cigarettes not available)
Least adjusted

REF	NRR	SEX	AD	Ys	Ws	Qs	Ps
BARBON	731	m	0	1.76	5.71	0.03	0.0000
BARBON	732	m	0	1.81	6.09	0.10	0.0000
BARBON	733	m	0	3.52	4.42	14.90	0.0000
Subtotal BARBON				2.26	16.22	15.03	
BENHAM	521	m	0	1.10	5.72	2.00	0.0088
BENHAM	522	m	0	1.41	6.17	0.49	0.0005
Subtotal BENHAM				1.26	11.89	2.49	
*ENGELA	527	m	0	0.52	3.73	5.10	0.3164
*ENGELA	528	m	0	1.42	5.47	0.39	0.0009
Subtotal ENGELA				1.05	9.20	5.49	
HAENSZ	509	f	0	0.15	7.71	18.25	0.6808
HAENSZ	510	f	0	0.03	4.45	12.15	0.9433
Subtotal HAENSZ				0.11	12.15	30.40	
JEDRYC	538	m	0	1.37	5.91	0.58	0.0008
JEDRYC	539	m	0	1.69	5.40	0.00	0.0001
JEDRYC	540	m	0	1.50	4.19	0.15	0.0022
Subtotal JEDRYC				1.52	15.50	0.73	
MATOS	651	m	0	1.37	3.34	0.34	0.0126
MATOS	652	m	0	1.99	4.13	0.39	0.0001
MATOS	653	m	0	1.85	3.87	0.10	0.0003
Subtotal MATOS				1.76	11.33	0.83	
PEZZOT	552	m	0	1.89	2.46	0.10	0.0030
PEZZOT	553	m	0	2.17	2.64	0.63	0.0004
PEZZOT	554	m	0	1.65	2.14	0.00	0.0160
Subtotal PEZZOT				1.92	7.23	0.73	
SVENSS	525	f	0	1.11	4.78	1.60	0.0154
SVENSS	526	f	0	1.46	4.63	0.25	0.0017
SVENSS	527	f	0	1.49	6.24	0.25	0.0002
Subtotal SVENSS				1.36	15.64	2.10	
WAKAI	511	m	0	0.26	2.32	4.72	0.6892
WAKAI	512	m	0	0.84	6.06	4.38	0.0395
WAKAI	513	m	0	0.68	4.77	4.83	0.1366
Subtotal WAKAI				0.68	13.16	13.92	
WU	501	f	0	0.20	4.05	8.95	0.6871
WU	502	f	0	1.14	6.15	1.82	0.0046
WU	503	f	0	1.95	8.87	0.61	0.0000
Subtotal WU				1.32	19.06	11.38	
WYNDE6	613	m	0	1.97	17.35	1.38	0.0000
WYNDE6	614	m	0	2.18	22.69	5.60	0.0000
WYNDE6	615	m	0	2.42	30.32	16.23	0.0000
WYNDE6	637	f	0	2.09	21.88	3.64	0.0000
WYNDE6	638	f	0	2.64	26.17	23.54	0.0000
WYNDE6	639	f	0	2.95	27.49	43.93	0.0000
Subtotal WYNDE6				2.42	145.89	94.31	
ZHENG	531	m	0	-0.31	7.88	31.28	0.3910
ZHENG	532	m	0	0.64	14.30	15.57	0.0149
ZHENG	533	m	0	1.20	10.98	2.61	0.0001
ZHENG	538	f	0	-0.39	4.56	19.70	0.4038
ZHENG	539	f	0	0.36	11.36	19.95	0.2228
Subtotal ZHENG				0.45	49.08	89.10	

N	38
NS	12

Table 3H16 - 6

IESLC - Meta-analysis of Ever/current Smoking by Age started, Overview
Adenocarcinoma, Cigarettes (or Any Product if Cigarettes not available)
Least adjusted

	Sex			
	combined	male	female	Total
N		25	13	38
NS		9	5	14

In this overview table, other than the "N" rows, entries in the "absent" and "Total" columns may be invalid and should be ignored

	Age started (broad categories)				
	absent	19+k26	15-25k18	1-17k14	Total
N	15	13	5	5	38
NS	8	12	4	4	28
Wt	88.62	102.98	64.47	70.29	326.36
Het Chi	49.93	48.90	7.06	15.43	266.53
Het df	14	12	4	4	37
Het P	***	***	N.S.	**	***
Fixed RR	2.55	3.79	9.76	13.60	5.40
RRl	2.07	3.12	7.65	10.76	4.85
RRu	3.15	4.59	12.46	17.18	6.02
P	+++	+++	+++	+++	+++
Random RR	2.60	3.23	8.90	12.28	4.02
RRl	1.73	2.14	6.21	7.15	2.97
RRu	3.89	4.88	12.76	21.08	5.44
P	+++	+++	+++	+++	+++

		Age started (narrow categories)						
	absent	27+k30	23-29k26	19-25k22	15-21k18	11-17k14	1-13k10	Total
N	27	3		2	5		1	38
NS	12	2		2	4		1	21
Wt	234.21	14.76		10.77	64.47		2.14	326.36
Het Chi	179.03	0.72		0.26	7.06		0.00	266.53
Het df	26	2		1	4		0	37
Het P	***	N.S.		N.S.	N.S.		N.S.	***
Fixed RR	5.28	0.78		3.59	9.76		5.19	5.40
RRl	4.65	0.47		1.97	7.65		1.36	4.85
RRu	6.01	1.31		6.52	12.46		19.85	6.02
P	+++	N.S.		+++	+++		+	+++
Random RR	4.12	0.78		3.59	8.90		5.19	4.02
RRl	2.90	0.47		1.97	6.21		1.36	2.97
RRu	5.86	1.31		6.52	12.76		19.85	5.44
P	+++	N.S.		+++	+++		+	+++

Table 3H16 - 6

IESLC - Meta-analysis of Ever/current Smoking by Age started, Overview
Adenocarcinoma, Cigarettes (or Any Product if Cigarettes not available)
Least adjusted

MALES

		Age started (broad categories)				
		absent	19+k26	15-25k18	1-17k14	Total
	N	8	9	4	4	25
	NS	5	9	4	4	22
	Wt	42.37	64.57	38.31	42.80	188.04
Het	Chi	22.02	19.89	1.45	10.17	111.05
Het	df	7	8	3	3	24
Het	P	**	*	N.S.	*	***
Fixed	RR	2.59	3.67	7.65	10.92	5.05
	RRl	1.92	2.88	5.57	8.09	4.38
	RRu	3.50	4.69	10.50	14.74	5.83
	P	+++	+++	+++	+++	+++
Random	RR	2.77	3.55	7.65	10.40	4.40
	RRl	1.58	2.36	5.57	5.11	3.18
	RRu	4.85	5.35	10.50	21.19	6.10
	P	+++	+++	+++	+++	+++

				Age started (narrow categories)					
		absent	27+k30	23-29k26	19-25k22	15-21k18	11-17k14	1-13k10	Total
	N	18	2			4		1	25
	NS	9	2			4		1	16
	Wt	137.40	10.20			38.31		2.14	188.04
Het	Chi	69.48	0.58			1.45		0.00	111.05
Het	df	17	1			3		0	24
Het	P	***	N.S.			N.S.		N.S.	***
Fixed	RR	5.14	0.84			7.65		5.19	5.05
	RRl	4.35	0.45			5.57		1.36	4.38
	RRu	6.08	1.55			10.50		19.85	5.83
	P	+++	N.S.			+++		+	+++
Random	RR	4.61	0.84			7.65		5.19	4.40
	RRl	3.22	0.45			5.57		1.36	3.18
	RRu	6.60	1.55			10.50		19.85	6.10
	P	+++	N.S.			+++		+	+++

FEMALES

		Age started (broad categories)				
		absent	19+k26	15-25k18	1-17k14	Total
	N	7	4	1	1	13
	NS	4	4	1	1	8
	Wt	46.25	38.41	26.17	27.49	138.31
Het	Chi	27.90	28.85	0.00	0.00	153.47
Het	df	6	3	0	0	12
Het	P	***	***	N.S.	N.S.	***
Fixed	RR	2.52	3.98	13.95	19.13	5.92
	RRl	1.89	2.90	9.51	13.16	5.01
	RRu	3.36	5.46	20.46	27.80	6.99
	P	+++	+++	+++	+++	+++
Random	RR	2.42	2.51	13.95	19.13	3.35
	RRl	1.28	0.84	9.51	13.16	1.80
	RRu	4.55	7.55	20.46	27.80	6.24
	P	++	N.S.	+++	+++	+++

Table 3H16 - 6

IESLC - Meta-analysis of Ever/current Smoking by Age started, Overview
Adenocarcinoma, Cigarettes (or Any Product if Cigarettes not available)
Least adjusted

FEMALES

	absent	Age started (narrow categories) 27+k30	23-29k26	19-25k22	15-21k18	11-17k14	1-13k10	Total
N	9	1		2	1			13
NS	5	1		2	1			8
Wt	96.81	4.56		10.77	26.17			138.31
Het Chi	109.29	0.00		0.26	0.00			153.47
Het df	8	0		1	0			12
Het P	***	N.S.		N.S.	N.S.			***
Fixed RR	5.50	0.68		3.59	13.95			5.92
RRl	4.50	0.27		1.97	9.51			5.01
RRu	6.71	1.69		6.52	20.46			6.99
P	+++	N.S.		+++	+++			+++
Random RR	3.27	0.68		3.59	13.95			3.35
RRl	1.51	0.27		1.97	9.51			1.80
RRu	7.07	1.69		6.52	20.46			6.24
P	++	N.S.		+++	+++			+++

Table 3H16 - 7

IESLC - Meta-analysis of Ever/current Smoking by Age started, Overview
Adenocarcinoma, Cigarettes (or Any Product if Cigarettes not available)
Excluded studies (and stage at which they were excluded)

1	BECHER BLOT1 BROWN3 CARPEN CHYOU DARBY DOLL2 GARCIA GRAHAM GURSEL HAMMO2 JAHN JAIN LAUSSM PRESCO QIAO TVERDA WIGLE WYNDE3
2	AKIBA AMANDU AMES AXELSS BENSHL BEST BOUCHA BOUCOT BROSS CHEN CPSII DEAN2 DESTEF DORGAN DOSEME FAN GARSHI GER GILLIS HAMMON HUMBLE JUSSAW KAISE2 KATSOU KAUFMA KOO KREUZE LEVIN MCCONN NOTAN2 OSANN2 PEZZO2 PISANI RESTRE SADOWS VUTUC WANG2 WATSON WU2 WUWILL WYNDE2 WYNDE8 XU ZHOU
3	GUO MCDUFF SPITZ STASZE ZHANG
4	AGUDO ARMADA AUVINE BOFFET BRESLO BUFFLE CEDERL CHEN2 CHIAZZ CHOI CORREA CPSI DAMBER DEAN3 DOLL DORN GAO GAO2 GENG HIRAYA HOLE HU HU2 JOLY KOULUM LETOUR LIAW LIU3 LIU4 LIU5 LUBIN LUBIN2 LUO MIGRAN MRFITR PERNU QIAO2 RACHTA SEGI2 SOBUE SPEIZE SUZUK2 TIZZAN WYNDE7 YUAN
5	ALDERS
10	HEGMAN KHUDER

Table 3H16 - 8

Potentially overlapping studies

REF	REFGP	PRINC	.	OVERLAP/LINK
BENHAM	LUBIN2	2		Subset of Lubin2
WYNDE6	WYNDE6	1		WYNDE5/6/7/8

Table 3H17 -

IESLC - Meta-analysis of Ever/current Smoking, Age started, "Low"
Adenocarcinoma, Cigarettes (or Any Product if Cigarettes not available)

This analysis is restricted to results for:
1) Ever/current smokers
2) Results by Age started
3) Categorical results by Age started
4) Adenocarcinoma (or near equivalent)
5) Results complete enough for use in metaanalysis

Within each study, results are then selected (in the following order of preference, within each sex) for:
6) SMKSTA: ever, current
7) PRODUCT: cigarettes regardless of other products, cigarettes only, all/unspec
8) CIGTYPE: all/unspecified, MC regardless of HR, MC only
9) (not applicable)
10) DENOM: never smoked anything, never smoked cigarettes, never any + low, never cigs + low
11) Followup period (YF, prospective studies): whole study (coded as 0) or longest available
12) LCtype: adeno or nearest available, but not squamous. (q = squamous, s = small,
a = adeno, l = large, KII = Kreyberg II, al = alveolar, br = bronchiolar, u = undifferentiated)
13) Race: all or nearest available, otherwise by race (wh or w = white, bl or b = black, hi = hispanic
ch = chinese, jap = japanese, haw = hawaiian, w+o = white + oriental, sca = scandinavian, as = asian)
14) Age started "low" in key scheme 1 (key value 26, maximum range 19+)
15) For overlapping studies: principal rather than subsidiary studies
Finally by Age: whole study (coded as 0) if available, otherwise by widest available age group
and then for single sex results (m, f) in preference to results for both sexes combined (c).

Results adjusted (AD) for the most potential confounders are then chosen in Sections -1 to -3
(and those which actually differ from the adjusted results in Table 3H12 - 1 are marked 'x' in Section -1)
and results adjusted for the least confounders in Sections -4 to -6. (Those least adjusted results which
actually differ from the most adjusted are marked 'x' in column X in Section -4)

Section -7 shows excluded studies, together with the stage (as above) at which no qualifying
results were found.

Section -8 lists the potentially overlapping studies which have been included (1=principal, 2=subsidiary).

Section -9 lists any results which would have been included in preference except that they had data not complete
enough for use in meta-analysis, with their significance (yes/no), if known, and any further comment as entered
on the database. It also lists as "gap" any categories for which no data were presented by the original authors.

In addition to those mentioned above, the following fields, levels and abbreviations are used:
* or nk = not known, n = no, y = yes, ot = other
ev = ever, cu = current, nev = never
all/unspec = all or unspecified, cig+/-ot = cigarettes irrespective of other products (cigar, pipe etc)
MC = manufactured cigarettes, HR = hand-rolled cigarettes
exL, exH = range of exposure (low and high) in the smoking group, in terms of Age started
REF: 6-character study reference
NRR: number of the RR on the database within the study
ST : study type (CC = case control, pr or prosp = prospective)
NLC: number of lung cancer cases in whole study
R : risky occupational population (n = no, m = mining, o = other risky)
VB : national cigarette type (V = at least 75% Virginia, bl = at least 75% blended, ot = other)
P : any proxy use
H : full histological confirmation
De : derivation of RR/CI (or = original, st = standard method, ot = other method of estimation)

Table 3H17 - 1

IESLC - Meta-analysis of Ever/current Smoking, Age started, "Low"
Adenocarcinoma, Cigarettes (or Any Product if Cigarettes not available)
Most adjusted

REF	NRR	XX12	SEX	AGEL	AGEH	RACE	YF	LC TYPE	LOC	START	ST	NLC	R	VB	P	H	AD	SM	PRODUCT	exL	exH	DENOM	De
BARBON	736		m	0	0	all	-	a	Eu:wst	1979	CC	755	n	bl	y	y	1	ev	all/unsp	20	999	nev any	or
BENHAM	521		m	0	0	all	-	KII	Eu:wst	1976	CC	1625	n	bl	n	y	0	ev	cig only	20	999	nev any	st
ENGELA	527		m	0	0	all	0	a	Eu:Sca	1964	pr	435	n	bl	n	n	0	cu	cig+/-ot	20	999	nev cigs	st
HAENSZ	509		f	0	0	all	-	a	NAmer	1955	CC	158	n	bl	n	y	0	ev	cig+/-ot	25	999	nev any	st
JEDRYC	538		m	0	0	all	-	a	Eu:est	1980	CC	1630	n	bl	y	n	0	ev	cig+/-ot	19	999	nev any	st
MATOS	656		m	0	0	all	-	a	SCAmer	1994	CC	200	n	bl	n	n	2	ev	cig+/-ot	20	999	nev any	or
PEZZOT	552		m	0	0	all	-	a	SCAmer	1987	CC	215	n	bl	n	y	0	ev	cig only	19	999	nev cigs	st
SVENSS	525		f	0	0	all	-	a	Eu:Sca	1983	CC	210	n	bl	n	n	0	cu	all/unsp	26	999	nev any	st
WAKAI	512		m	0	0	all	-	a	As:Jap	1988	CC	333	n	bl	n	y	0	cu	cig+/-ot	20	29	nev any	st
WU	525		f	0	0	wh	-	a	NAmer	1981	CC	220	n	bl	n	y	2	cu	all/unsp	25	999	nev any	or
WYNDE6	613		m	0	0	wh	-	a	NAmer	1969	CC	4423	n	bl	n	y	0	ev	cig+/-ot	21	999	nev cigs	st
WYNDE6	637		f	0	0	wh	-	a	NAmer	1969	CC	4423	n	bl	n	y	0	ev	cig+/-ot	21	999	nev cigs	st
ZHENG	532		m	0	0	all	-	a	As:Chi	1982	CC	540	n	ot	*	y	0	ev	cig+/-ot	20	29	nev cigs	st

Cigarette type is all/unspec for all RRs

Table 3H17 - 2

IESLC - Meta-analysis of Ever/current Smoking, Age started, "Low"
Adenocarcinoma, Cigarettes (or Any Product if Cigarettes not available)
Most adjusted

REF	NRR	SEX	AD	Number Exposed Case	Number Exposed Cont	Non-exposed Case	Non-exposed Cont	RR	95.00%CI
BARBON	736	m	1	45	-	7	-	5.70	(2.50- 12.90)
BENHAM	521	m	0	41	64	9	42	2.99	(1.32- 6.79)
*ENGELA	527	m	0	8	39957	7	58716	1.68	(0.61- 4.63)
HAENSZ	509	f	0	12	66	37	236	1.16	(0.57- 2.35)
JEDRYC	538	m	0	48	502	7	289	3.95	(1.76- 8.84)
MATOS	656	m	2	13	-	5	-	4.00	(1.40- 11.90)
PEZZOT	552	m	0	18	105	3	116	6.63	(1.90- 23.15)
SVENSS	525	f	0	10	18	22	120	3.03	(1.24- 7.43)
WAKAI	512	m	0	52	183	8	65	2.31	(1.04- 5.12)
WU	525	f	2	8	-	29	-	1.10	(0.40- 3.20)
WYNDE6	613	m	0	47	92	42	589	7.16	(4.47- 11.47)
WYNDE6	637	f	0	63	90	58	673	8.12	(5.34- 12.35)
Subtotal WYNDE6								7.68	(5.62- 10.51)
ZHENG	532	m	0	64	109	29	94	1.90	(1.13- 3.20)
Partial Totals				429	41186	263	60940		

*prospective study

REF	NRR	SEX	AD	Ys	Ws	Qs	Ps
BARBON	736	m	1	1.74	5.71	0.95	0.0000
BENHAM	521	m	0	1.10	5.72	0.32	0.0088
*ENGELA	527	m	0	0.52	3.73	2.47	0.3164
HAENSZ	509	f	0	0.15	7.71	10.81	0.6808
JEDRYC	538	m	0	1.37	5.91	0.01	0.0008
MATOS	656	m	2	1.39	3.36	0.01	0.0111
PEZZOT	552	m	0	1.89	2.46	0.77	0.0030
SVENSS	525	f	0	1.11	4.78	0.24	0.0154
WAKAI	512	m	0	0.84	6.06	1.49	0.0395
WU	525	f	2	0.10	3.55	5.44	0.8574
WYNDE6	613	m	0	1.97	17.35	7.03	0.0000
WYNDE6	637	f	0	2.09	21.88	12.71	0.0000
Subtotal WYNDE6				2.04	39.22	19.74	
ZHENG	532	m	0	0.64	14.30	6.79	0.0149

	N	13
	NS	12
	Wt	102.50
Het	Chi	49.04
Het	df	12
Het	P	***
Fixed	RR	3.79
	RRl	3.12
	RRu	4.60
	P	+++
Random	RR	3.21
	RRl	2.12
	RRu	4.87
	P	+++
Asymm	P	N.S.

Table 3H17 - 3

IESLC - Meta-analysis of Ever/current Smoking, Age started, "Low"
Adenocarcinoma, Cigarettes (or Any Product if Cigarettes not available)
Most adjusted

	Sex combined	male	female	Total
N		9	4	13
NS		9	4	13
Wt		64.59	37.91	102.50
Het Chi		19.77	29.08	49.04
Het df		8	3	12
Het P		*	***	***
Fixed RR		3.67	4.00	3.79
RRl		2.88	2.91	3.12
RRu		4.68	5.50	4.60
P		+++	+++	+++
Random RR		3.55	2.46	3.21
RRl		2.36	0.80	2.12
RRu		5.34	7.54	4.87
P		+++	N.S.	+++
Between Chi				0.18
Between df				1
Between P				N.S.
Btwn(F) P				N.S.
Btwn(R) P				N.S.

Table 3H17 - 4

IESLC - Meta-analysis of Ever/current Smoking, Age started, "Low"
Adenocarcinoma, Cigarettes (or Any Product if Cigarettes not available)
Least adjusted

REF	NRR	X	SEX	AGEL	AGEH	RACE	YF	LC TYPE	LOC	START	ST	NLC	R	VB	P	H	AD	SM	PRODUCT	exL	exH	DENOM	De
BARBON	731	x	m	0	0	all	-	a	Eu:wst	1979	CC	755	n	bl	y	y	0	ev	all/unsp	20	999	nev any	st
BENHAM	521		m	0	0	all	-	KII	Eu:wst	1976	CC	1625	n	bl	n	y	0	ev	cig only	20	999	nev any	st
ENGELA	527		m	0	0	all	0	a	Eu:Sca	1964	pr	435	n	bl	n	n	0	cu	cig+/-ot	20	999	nev cigs	st
HAENSZ	509		f	0	0	all	-	a	NAmer	1955	CC	158	n	bl	n	y	0	ev	cig+/-ot	25	999	nev any	st
JEDRYC	538		m	0	0	all	-	a	Eu:est	1980	CC	1630	n	bl	y	n	0	ev	cig+/-ot	19	999	nev any	st
MATOS	651	x	m	0	0	all	-	a	SCAmer	1994	CC	200	n	bl	n	n	0	ev	cig+/-ot	20	999	nev any	st
PEZZOT	552		m	0	0	all	-	a	SCAmer	1987	CC	215	n	bl	n	y	0	ev	cig only	19	999	nev cigs	st
SVENSS	525		f	0	0	all	-	a	Eu:Sca	1983	CC	210	n	bl	n	n	0	cu	all/unsp	26	999	nev any	st
WAKAI	512		m	0	0	all	-	a	As:Jap	1988	CC	333	n	bl	n	y	0	cu	cig+/-ot	20	29	nev any	st
WU	501	x	f	0	0	wh	-	a	NAmer	1981	CC	220	n	bl	n	y	0	cu	all/unsp	25	999	nev any	st
WYNDE6	613		m	0	0	wh	-	a	NAmer	1969	CC	4423	n	bl	n	y	0	ev	cig+/-ot	21	999	nev cigs	st
WYNDE6	637		f	0	0	wh	-	a	NAmer	1969	CC	4423	n	bl	n	y	0	ev	cig+/-ot	21	999	nev cigs	st
ZHENG	532		m	0	0	all	-	a	As:Chi	1982	CC	540	n	ot	*	y	0	ev	cig+/-ot	20	29	nev cigs	st

Cigarette type is all/unspec for all RRs

Table 3H17 - 5

IESLC - Meta-analysis of Ever/current Smoking, Age started, "Low"
Adenocarcinoma, Cigarettes (or Any Product if Cigarettes not available)
Least adjusted

REF	NRR	SEX	AD	Number Exposed Case	Cont	Non-exposed Case	Cont	RR	95.00%CI
BARBON	731	m	0	45	207	7	188	5.84	(2.57- 13.26)
BENHAM	521	m	0	41	64	9	42	2.99	(1.32- 6.79)
*ENGELA	527	m	0	8	39957	7	58716	1.68	(0.61- 4.63)
HAENSZ	509	f	0	12	66	37	236	1.16	(0.57- 2.35)
JEDRYC	538	m	0	48	502	7	289	3.95	(1.76- 8.84)
MATOS	651	m	0	13	73	5	110	3.92	(1.34- 11.46)
PEZZOT	552	m	0	18	105	3	116	6.63	(1.90- 23.15)
SVENSS	525	f	0	10	18	22	120	3.03	(1.24- 7.43)
WAKAI	512	m	0	52	183	8	65	2.31	(1.04- 5.12)
WU	501	f	0	8	14	29	62	1.22	(0.46- 3.24)
WYNDE6	613	m	0	47	92	42	589	7.16	(4.47- 11.47)
WYNDE6	637	f	0	63	90	58	673	8.12	(5.34- 12.35)
Subtotal WYNDE6								7.68	(5.62- 10.51)
ZHENG	532	m	0	64	109	29	94	1.90	(1.13- 3.20)
Totals				429	41480	263	61300		

*prospective study

REF	NRR	SEX	AD	Ys	Ws	Qs	Ps
BARBON	731	m	0	1.76	5.71	1.07	0.0000
BENHAM	521	m	0	1.10	5.72	0.32	0.0088
*ENGELA	527	m	0	0.52	3.73	2.47	0.3164
HAENSZ	509	f	0	0.15	7.71	10.79	0.6808
JEDRYC	538	m	0	1.37	5.91	0.01	0.0008
MATOS	651	m	0	1.37	3.34	0.00	0.0126
PEZZOT	552	m	0	1.89	2.46	0.77	0.0030
SVENSS	525	f	0	1.11	4.78	0.24	0.0154
WAKAI	512	m	0	0.84	6.06	1.48	0.0395
WU	501	f	0	0.20	4.05	5.18	0.6871
WYNDE6	613	m	0	1.97	17.35	7.06	0.0000
WYNDE6	637	f	0	2.09	21.88	12.75	0.0000
Subtotal WYNDE6				2.04	39.22	19.81	
ZHENG	532	m	0	0.64	14.30	6.76	0.0149

	N	13
	NS	12
	Wt	102.98
Het	Chi	48.90
Het	df	12
Het	P	***
Fixed	RR	3.79
	RRl	3.12
	RRu	4.59
	P	+++
Random	RR	3.23
	RRl	2.14
	RRu	4.88
	P	+++
Asymm	P	N.S.

Table 3H17 - 6

IESLC - Meta-analysis of Ever/current Smoking, Age started, "Low"
Adenocarcinoma, Cigarettes (or Any Product if Cigarettes not available)
Least adjusted

	Sex<br>combined male	female	Total
N	9	4	13
NS	9	4	13
Wt	64.57	38.41	102.98
Het Chi	19.89	28.85	48.90
Het df	8	3	12
Het P	*	***	***
Fixed RR	3.67	3.98	3.79
RRl	2.88	2.90	3.12
RRu	4.69	5.46	4.59
P	+++	+++	+++
Random RR	3.55	2.51	3.23
RRl	2.36	0.84	2.14
RRu	5.35	7.55	4.88
P	+++	N.S.	+++
Between Chi			0.16
Between df			1
Between P			N.S.
Btwn(F) P			N.S.
Btwn(R) P			N.S.

Table 3H17 - 7

IESLC - Meta-analysis of Ever/current Smoking, Age started, "Low"
Adenocarcinoma, Cigarettes (or Any Product if Cigarettes not available)
Excluded studies (and stage at which they were excluded)

1	BECHER BLOT1 BROWN3 CARPEN CHYOU DARBY DOLL2 GARCIA GRAHAM GURSEL HAMMO2 JAHN JAIN LAUSSM PRESCO QIAO TVERDA WIGLE WYNDE3
2	AKIBA AMANDU AMES AXELSS BENSHL BEST BOUCHA BOUCOT BROSS CHEN CPSII DEAN2 DESTEF DORGAN DOSEME FAN GARSHI GER GILLIS HAMMON HUMBLE JUSSAW KAISE2 KATSOU KAUFMA KOO KREUZE LEVIN MCCONN NOTAN2 OSANN2 PEZZO2 PISANI RESTRE SADOWS VUTUC WANG2 WATSON WU2 WUWILL WYNDE2 WYNDE8 XU ZHOU
3	GUO MCDUFF SPITZ STASZE ZHANG
4	AGUDO ARMADA AUVINE BOFFET BRESLO BUFFLE CEDERL CHEN2 CHIAZZ CHOI CORREA CPSI DAMBER DEAN3 DOLL DORN GAO GAO2 GENG HIRAYA HOLE HU HU2 JOLY KOULUM LETOUR LIAW LIU3 LIU4 LIU5 LUBIN LUBIN2 LUO MIGRAN MRFITR PERNU QIAO2 RACHTA SEGI2 SOBUE SPEIZE SUZUK2 TIZZAN WYNDE7 YUAN
5	ALDERS
10	HEGMAN KHUDER

Table 3H17 - 8

Potentially overlapping studies

REF	REFGP	PRINC	.	OVERLAP/LINK
BENHAM	LUBIN2	2		Subset of Lubin2
WYNDE6	WYNDE6	1		WYNDE5/6/7/8

Table 3H18 -

IESLC - Meta-analysis of Ever/current Smoking, Age started, "Mid"
Adenocarcinoma, Cigarettes (or Any Product if Cigarettes not available)

This analysis is restricted to results for:
1) Ever/current smokers
2) Results by Age started
3) Categorical results by Age started
4) Adenocarcinoma (or near equivalent)
5) Results complete enough for use in metaanalysis

Within each study, results are then selected (in the following order of preference, within each sex) for:
6) SMKSTA: ever, current
7) PRODUCT: cigarettes regardless of other products, cigarettes only, all/unspec
8) CIGTYPE: all/unspecified, MC regardless of HR, MC only
9) (not applicable)
10) DENOM: never smoked anything, never smoked cigarettes, never any + low, never cigs + low
11) Followup period (YF, prospective studies): whole study (coded as 0) or longest available
12) LCtype: adeno or nearest available, but not squamous. (q = squamous, s = small,
a = adeno, l = large, KII = Kreyberg II, al = alveolar, br = bronchiolar, u = undifferentiated)
13) Race: all or nearest available, otherwise by race (wh or w = white, bl or b = black, hi = hispanic
ch = chinese, jap = japanese, haw = hawaiian, w+o = white + oriental, sca = scandinavian, as = asian)
14) Age started "mid" in key scheme 1 (key value 18, maximum range 15-25)
15) For overlapping studies: principal rather than subsidiary studies
Finally by Age: whole study (coded as 0) if available, otherwise by widest available age group
and then for single sex results (m, f) in preference to results for both sexes combined (c).

Results adjusted (AD) for the most potential confounders are then chosen in Sections -1 to -3
(and those which actually differ from the adjusted results in Table 3H13 - 1 are marked 'x' in Section -1)
and results adjusted for the least confounders in Sections -4 to -6. (Those least adjusted results which
actually differ from the most adjusted are marked 'x' in column X in Section -4)

Section -7 shows excluded studies, together with the stage (as above) at which no qualifying
results were found.

Section -8 lists the potentially overlapping studies which have been included (1=principal, 2=subsidiary).

Section -9 lists any results which would have been included in preference except that they had data not complete
enough for use in meta-analysis, with their significance (yes/no), if known, and any further comment as entered
on the database. It also lists as "gap" any categories for which no data were presented by the original authors.

In addition to those mentioned above, the following fields, levels and abbreviations are used:
* or nk = not known, n = no, y = yes, ot = other
ev = ever, cu = current, nev = never
all/unspec = all or unspecified, cig+/-ot = cigarettes irrespective of other products (cigar, pipe etc)
MC = manufactured cigarettes, HR = hand-rolled cigarettes
exL, exH = range of exposure (low and high) in the smoking group, in terms of Age started
REF: 6-character study reference
NRR: number of the RR on the database within the study
ST : study type (CC = case control, pr or prosp = prospective)
NLC: number of lung cancer cases in whole study
R : risky occupational population (n = no, m = mining, o = other risky)
VB : national cigarette type (V = at least 75% Virginia, bl = at least 75% blended, ot = other)
P : any proxy use
H : full histological confirmation
De : derivation of RR/CI (or = original, st = standard method, ot = other method of estimation)

Table 3H18 - 1

IESLC - Meta-analysis of Ever/current Smoking, Age started, "Mid"
Adenocarcinoma, Cigarettes (or Any Product if Cigarettes not available)
Most adjusted

REF	NRR	XX13	SEX	AGEL	AGEH	RACE	YF	LC TYPE	LOC	START	ST	NLC	R	VB	P	H	AD	SM	PRODUCT	exL	exH	DENOM	De
BARBON	737		m	0	0	all	-	a	Eu:wst	1979	CC	755	n	bl	y	y	1	ev	all/unsp	15	19	nev any	or
JEDRYC	539		m	0	0	all	-	a	Eu:est	1980	CC	1630	n	bl	y	n	0	ev	cig+/-ot	17	18	nev any	st
MATOS	657		m	0	0	all	-	a	SCAmer	1994	CC	200	n	bl	n	n	2	ev	cig+/-ot	15	19	nev any	or
WYNDE6	614		m	0	0	wh	-	a	NAmer	1969	CC	4423	n	bl	n	y	0	ev	cig+/-ot	18	20	nev cigs	st
WYNDE6	638		f	0	0	wh	-	a	NAmer	1969	CC	4423	n	bl	n	y	0	ev	cig+/-ot	18	20	nev cigs	st

Cigarette type is all/unspec for all RRs

Table 3H18 - 2

IESLC - Meta-analysis of Ever/current Smoking, Age started, "Mid"
Adenocarcinoma, Cigarettes (or Any Product if Cigarettes not available)
Most adjusted

REF	NRR	SEX	AD	Number Exposed Case	Cont	Non-exposed Case	Cont	RR	95.00%CI
BARBON	737	m	1	77	-	7	-	6.00	(2.70- 13.30)
JEDRYC	539	m	0	29	221	7	289	5.42	(2.33- 12.60)
MATOS	657	m	2	40	-	5	-	7.20	(2.70- 19.10)
WYNDE6	614	m	0	88	139	42	589	8.88	(5.88- 13.40)
WYNDE6	638	f	0	113	94	58	673	13.95	(9.51- 20.46)
Subtotal WYNDE6								11.31	(8.54- 14.97)
Partial Totals				347	454	119	1551		

*prospective study

REF	NRR	SEX	AD	Ys	Ws	Qs	Ps
BARBON	737	m	1	1.79	6.04	1.42	0.0000
JEDRYC	539	m	0	1.69	5.40	1.86	0.0001
MATOS	657	m	2	1.97	4.01	0.37	0.0001
WYNDE6	614	m	0	2.18	22.69	0.19	0.0000
WYNDE6	638	f	0	2.64	26.17	3.38	0.0000
Subtotal WYNDE6				2.43	48.86	3.57	

	N	5
	NS	4
	Wt	64.32
Het	Chi	7.21
Het	df	4
Het	P	N.S.
Fixed	RR	9.74
	RRl	7.63
	RRu	12.43
	P	+++
Random	RR	8.84
	RRl	6.14
	RRu	12.73
	P	+++
Asymm	P	N.S.

Table 3H18 - 3

IESLC - Meta-analysis of Ever/current Smoking, Age started, "Mid"
Adenocarcinoma, Cigarettes (or Any Product if Cigarettes not available)
Most adjusted

	Sex			
	combined	male	female	Total
N		4	1	5
NS		4	1	4
Wt		38.15	26.17	64.32
Het Chi		1.52	0.00	7.21
Het df		3	0	4
Het P		N.S.	N.S.	N.S.
Fixed RR		7.61	13.95	9.74
RRl		5.54	9.51	7.63
RRu		10.45	20.46	12.43
P		+++	+++	+++
Random RR		7.61	13.95	8.84
RRl		5.54	9.51	6.14
RRu		10.45	20.46	12.73
P		+++	+++	+++
Between Chi				5.70
Between df				1
Between P				*
Btwn(F) P				*
Btwn(R) P				*

Too few RRs for analysis by factor

Table 3H18 - 4

IESLC - Meta-analysis of Ever/current Smoking, Age started, "Mid"
Adenocarcinoma, Cigarettes (or Any Product if Cigarettes not available)
Least adjusted

REF	NRR	X	SEX	AGEL	AGEH	RACE	YF	LC TYPE	LOC	START	ST	NLC	R	VB	P	H	AD	SM	PRODUCT	exL	exH	DENOM	De
BARBON	732	x	m	0	0	all	-	a	Eu:wst	1979	CC	755	n	bl	y	y	0	ev	all/unsp	15	19	nev any	st
JEDRYC	539		m	0	0	all	-	a	Eu:est	1980	CC	1630	n	bl	y	n	0	ev	cig+/-ot	17	18	nev any	st
MATOS	652	x	m	0	0	all	-	a	SCAmer	1994	CC	200	n	bl	n	n	0	ev	cig+/-ot	15	19	nev any	st
WYNDE6	614		m	0	0	wh	-	a	NAmer	1969	CC	4423	n	bl	n	y	0	ev	cig+/-ot	18	20	nev cigs	st
WYNDE6	638		f	0	0	wh	-	a	NAmer	1969	CC	4423	n	bl	n	y	0	ev	cig+/-ot	18	20	nev cigs	st

Cigarette type is all/unspec for all RRs

Table 3H18 - 5

IESLC - Meta-analysis of Ever/current Smoking, Age started, "Mid"
Adenocarcinoma, Cigarettes (or Any Product if Cigarettes not available)
Least adjusted

REF	NRR	SEX	AD	Number Exposed Case	Cont	Non-exposed Case	Cont	RR	95.00%CI
BARBON	732	m	0	77	337	7	188	6.14	(2.77- 13.58)
JEDRYC	539	m	0	29	221	7	289	5.42	(2.33- 12.60)
MATOS	652	m	0	40	120	5	110	7.33	(2.79- 19.25)
WYNDE6	614	m	0	88	139	42	589	8.88	(5.88- 13.40)
WYNDE6	638	f	0	113	94	58	673	13.95	(9.51- 20.46)
Subtotal WYNDE6								11.31	(8.54- 14.97)
Totals				347	911	119	1849		

*prospective study

REF	NRR	SEX	AD	Ys	Ws	Qs	Ps
BARBON	732	m	0	1.81	6.09	1.31	0.0000
JEDRYC	539	m	0	1.69	5.40	1.87	0.0001
MATOS	652	m	0	1.99	4.13	0.34	0.0001
WYNDE6	614	m	0	2.18	22.69	0.20	0.0000
WYNDE6	638	f	0	2.64	26.17	3.33	0.0000
Subtotal WYNDE6				2.43	48.86	3.54	

	N	5
	NS	4
	Wt	64.47
Het	Chi	7.06
Het	df	4
Het	P	N.S.
Fixed	RR	9.76
	RRl	7.65
	RRu	12.46
	P	+++
Random	RR	8.90
	RRl	6.21
	RRu	12.76
	P	+++
Asymm	P	N.S.

Table 3H18 - 6

IESLC - Meta-analysis of Ever/current Smoking, Age started, "Mid"
Adenocarcinoma, Cigarettes (or Any Product if Cigarettes not available)
Least adjusted

	Sex combined	male	female	Total
N		4	1	5
NS		4	1	4
Wt		38.31	26.17	64.47
Het Chi		1.45	0.00	7.06
Het df		3	0	4
Het P		N.S.	N.S.	N.S.
Fixed RR		7.65	13.95	9.76
RRl		5.57	9.51	7.65
RRu		10.50	20.46	12.46
P		+++	+++	+++
Random RR		7.65	13.95	8.90
RRl		5.57	9.51	6.21
RRu		10.50	20.46	12.76
P		+++	+++	+++
Between Chi				5.61
Between df				1
Between P				*
Btwn(F) P				*
Btwn(R) P				*

Table 3H18 - 7

IESLC - Meta-analysis of Ever/current Smoking, Age started, "Mid"
Adenocarcinoma, Cigarettes (or Any Product if Cigarettes not available)
Excluded studies (and stage at which they were excluded)

1	BECHER BLOT1 BROWN3 CARPEN CHYOU DARBY DOLL2 GARCIA GRAHAM GURSEL HAMMO2 JAHN JAIN LAUSSM PRESCO QIAO TVERDA WIGLE WYNDE3
2	AKIBA AMANDU AMES AXELSS BENSHL BEST BOUCHA BOUCOT BROSS CHEN CPSII DEAN2 DESTEF DORGAN DOSEME FAN GARSHI GER GILLIS HAMMON HUMBLE JUSSAW KAISE2 KATSOU KAUFMA KOO KREUZE LEVIN MCCONN NOTAN2 OSANN2 PEZZO2 PISANI RESTRE SADOWS VUTUC WANG2 WATSON WU2 WUWILL WYNDE2 WYNDE8 XU ZHOU
3	GUO MCDUFF SPITZ STASZE ZHANG
4	AGUDO ARMADA AUVINE BOFFET BRESLO BUFFLE CEDERL CHEN2 CHIAZZ CHOI CORREA CPSI DAMBER DEAN3 DOLL DORN GAO GAO2 GENG HIRAYA HOLE HU HU2 JOLY KOULUM LETOUR LIAW LIU3 LIU4 LIU5 LUBIN LUBIN2 LUO MIGRAN MRFITR PERNU QIAO2 RACHTA SEGI2 SOBUE SPEIZE SUZUK2 TIZZAN WYNDE7 YUAN
5	ALDERS
10	HEGMAN KHUDER
14	BENHAM ENGELA HAENSZ PEZZOT SVENSS WAKAI WU ZHENG

Table 3H18 - 8
Potentially overlapping studies

REF	REFGP	PRINC	.	OVERLAP/LINK
WYNDE6	WYNDE6	1		WYNDE5/6/7/8

Table 3H19 -

IESLC - Meta-analysis of Ever/current Smoking, Age started, "High"
Adenocarcinoma, Cigarettes (or Any Product if Cigarettes not available)

This analysis is restricted to results for:
1) Ever/current smokers
2) Results by Age started
3) Categorical results by Age started
4) Adenocarcinoma (or near equivalent)
5) Results complete enough for use in metaanalysis

Within each study, results are then selected (in the following order of preference, within each sex) for:
6) PRODUCT: cigarettes regardless of other products, cigarettes only, all/unspec
7) CIGTYPE: all/unspecified, MC regardless of HR, MC only
8) (not applicable)
9) DENOM: never smoked anything, never smoked cigarettes, never any + low, never cigs + low
10) Followup period (YF, prospective studies): whole study (coded as 0) or longest available
11) LCtype: adeno or nearest available, but not squamous. (q = squamous, s = small,
a = adeno, l = large, KII = Kreyberg II, al = alveolar, br = bronchiolar, u = undifferentiated)
12) Race: all or nearest available, otherwise by race (wh or w = white, bl or b = black, hi = hispanic
ch = chinese, jap = japanese, haw = hawaiian, w+o = white + oriental, sca = scandinavian, as = asian)
13) Age started "high" in key scheme 1 (key value 14, maximum range 1-17)
14) For overlapping studies: principal rather than subsidiary studies
Finally by Age: whole study (coded as 0) if available, otherwise by widest available age group
and then for single sex results (m, f) in preference to results for both sexes combined (c).

Results adjusted (AD) for the most potential confounders are then chosen in Sections -1 to -3
(and those which actually differ from the adjusted results in Table 3H14 - 1 are marked 'x' in Section -1)
and results adjusted for the least confounders in Sections -4 to -6. (Those least adjusted results which
actually differ from the most adjusted are marked 'x' in column X in Section -4)

Section -7 shows excluded studies, together with the stage (as above) at which no qualifying
results were found.

Section -8 lists the potentially overlapping studies which have been included (1=principal, 2=subsidiary).

Section -9 lists any results which would have been included in preference except that they had data not complete
enough for use in meta-analysis, with their significance (yes/no), if known, and any further comment as entered
on the database. It also lists as "gap" any categories for which no data were presented by the original authors.

In addition to those mentioned above, the following fields, levels and abbreviations are used:
* or nk = not known, n = no, y = yes, ot = other
ev = ever, cu = current, nev = never
all/unspec = all or unspecified, cig+/-ot = cigarettes irrespective of other products (cigar, pipe etc)
MC = manufactured cigarettes, HR = hand-rolled cigarettes
exL, exH = range of exposure (low and high) in the smoking group, in terms of Age started
REF: 6-character study reference
NRR: number of the RR on the database within the study
ST : study type (CC = case control, pr or prosp = prospective)
NLC: number of lung cancer cases in whole study
R : risky occupational population (n = no, m = mining, o = other risky)
VB : national cigarette type (V = at least 75% Virginia, bl = at least 75% blended, ot = other)
P : any proxy use
H : full histological confirmation
De : derivation of RR/CI (or = original, st = standard method, ot = other method of estimation)

Table 3H19 - 1

IESLC - Meta-analysis of Ever/current Smoking, Age started, "High"
Adenocarcinoma, Cigarettes (or Any Product if Cigarettes not available)
Most adjusted

REF	NRR	XX14	SEX	AGEL	AGEH	RACE	YF	LC TYPE	LOC	START	ST	NLC	R	VB	P	H	AD	SM	PRODUCT	exL	exH	DENOM	De
BARBON	738		m	0	0	all	-	a	Eu:wst	1979	CC	755	n	bl	y	y	1	ev	all/unsp	1	14	nev any	or
JEDRYC	540		m	0	0	all	-	a	Eu:est	1980	CC	1630	n	bl	y	n	0	ev	cig+/-ot	1	16	nev any	st
MATOS	658		m	0	0	all	-	a	SCAmer	1994	CC	200	n	bl	n	n	2	ev	cig+/-ot	1	14	nev any	or
WYNDE6	615		m	0	0	wh	-	a	NAmer	1969	CC	4423	n	bl	n	y	0	ev	cig+/-ot	1	17	nev cigs	st
WYNDE6	639		f	0	0	wh	-	a	NAmer	1969	CC	4423	n	bl	n	y	0	ev	cig+/-ot	1	17	nev cigs	st

Cigarette type is all/unspec for all RRs

Table 3H19 - 2

IESLC - Meta-analysis of Ever/current Smoking, Age started, "High"
Adenocarcinoma, Cigarettes (or Any Product if Cigarettes not available)
Most adjusted

REF	NRR	SEX	AD	Number Exposed Case	Exposed Cont	Non-exposed Case	Non-exposed Cont	RR	95.00%CI
BARBON	738	m	1	29	-	7	-	33.40	(13.10- 84.90)
JEDRYC	540	m	0	12	111	7	289	4.46	(1.71- 11.63)
MATOS	658	m	2	26	-	5	-	6.60	(2.40- 18.10)
WYNDE6	615	m	0	241	301	42	589	11.23	(7.87- 16.03)
WYNDE6	639	f	0	150	91	58	673	19.13	(13.16- 27.80)
Subtotal WYNDE6								14.46	(11.18- 18.72)
Partial Totals				458	503	119	1551		

*prospective study

REF	NRR	SEX	AD	Ys	Ws	Qs	Ps
BARBON	738	m	1	3.51	4.40	3.54	0.0000
JEDRYC	540	m	0	1.50	4.19	5.22	0.0022
MATOS	658	m	2	1.89	3.76	1.98	0.0003
WYNDE6	615	m	0	2.42	30.32	1.13	0.0000
WYNDE6	639	f	0	2.95	27.49	3.16	0.0000
Subtotal WYNDE6				2.67	57.81	4.30	

	N	5
	NS	4
	Wt	70.16
Het	Chi	15.03
Het	df	4
Het	P	**
Fixed	RR	13.62
	RRl	10.78
	RRu	17.22
	P	+++
Random	RR	12.34
	RRl	7.23
	RRu	21.08
	P	+++
Asymm	P	N.S.

Table 3H19 - 3

IESLC - Meta-analysis of Ever/current Smoking, Age started, "High"
Adenocarcinoma, Cigarettes (or Any Product if Cigarettes not available)
Most adjusted

	Sex combined	male	female	Total
N		4	1	5
NS		4	1	4
Wt		42.68	27.49	70.16
Het Chi		9.83	0.00	15.03
Het df		3	0	4
Het P		*	N.S.	**
Fixed RR		10.95	19.13	13.62
RRl		8.11	13.16	10.78
RRu		14.78	27.80	17.22
P		+++	+++	+++
Random RR		10.46	19.13	12.34
RRl		5.18	13.16	7.23
RRu		21.13	27.80	21.08
P		+++	+++	+++
Between Chi				5.20
Between df				1
Between P				*
Btwn(F) P				N.S.
Btwn(R) P				N.S.

Too few RRs for analysis by factor

Table 3H19 - 4

IESLC - Meta-analysis of Ever/current Smoking, Age started, "High"
Adenocarcinoma, Cigarettes (or Any Product if Cigarettes not available)
Least adjusted

REF	NRR	X	SEX	AGEL	AGEH	RACE	YF	LC TYPE	LOC	START	ST	NLC	R	VB	P	H	AD	SM	PRODUCT	exL	exH	DENOM	De
BARBON	733	x	m	0	0	all	-	a	Eu:wst	1979	CC	755	n	bl	y	y	0	ev	all/unsp	1	14	nev any	st
JEDRYC	540		m	0	0	all	-	a	Eu:est	1980	CC	1630	n	bl	y	n	0	ev	cig+/-ot	1	16	nev any	st
MATOS	653	x	m	0	0	all	-	a	SCAmer	1994	CC	200	n	bl	n	n	0	ev	cig+/-ot	1	14	nev any	st
WYNDE6	615		m	0	0	wh	-	a	NAmer	1969	CC	4423	n	bl	n	y	0	ev	cig+/-ot	1	17	nev cigs	st
WYNDE6	639		f	0	0	wh	-	a	NAmer	1969	CC	4423	n	bl	n	y	0	ev	cig+/-ot	1	17	nev cigs	st

Cigarette type is all/unspec for all RRs

Table 3H19 - 5

IESLC - Meta-analysis of Ever/current Smoking, Age started, "High"
Adenocarcinoma, Cigarettes (or Any Product if Cigarettes not available)
Least adjusted

REF	NRR	SEX	AD	Number Exposed Case	Number Exposed Cont	Non-exposed Case	Non-exposed Cont	RR	95.00%CI
BARBON	733	m	0	29	23	7	188	33.86	(13.33- 86.00)
JEDRYC	540	m	0	12	111	7	289	4.46	(1.71- 11.63)
MATOS	653	m	0	26	90	5	110	6.36	(2.35- 17.22)
WYNDE6	615	m	0	241	301	42	589	11.23	(7.87- 16.03)
WYNDE6	639	f	0	150	91	58	673	19.13	(13.16- 27.80)
Subtotal WYNDE6								14.46	(11.18- 18.72)
Totals				458	616	119	1849		

*prospective study

REF	NRR	SEX	AD	Ys	Ws	Qs	Ps
BARBON	733	m	0	3.52	4.42	3.68	0.0000
JEDRYC	540	m	0	1.50	4.19	5.20	0.0022
MATOS	653	m	0	1.85	3.87	2.24	0.0003
WYNDE6	615	m	0	2.42	30.32	1.11	0.0000
WYNDE6	639	f	0	2.95	27.49	3.20	0.0000
Subtotal WYNDE6				2.67	57.81	4.31	

	N	5
	NS	4
	Wt	70.29
Het	Chi	15.43
Het	df	4
Het	P	**
Fixed	RR	13.60
	RRl	10.76
	RRu	17.18
	P	+++
Random	RR	12.28
	RRl	7.15
	RRu	21.08
	P	+++
Asymm	P	N.S.

Table 3H19 - 6

IESLC - Meta-analysis of Ever/current Smoking, Age started, "High"
Adenocarcinoma, Cigarettes (or Any Product if Cigarettes not available)
Least adjusted

	Sex combined	male	female	Total
N		4	1	5
NS		4	1	4
Wt		42.80	27.49	70.29
Het Chi		10.17	0.00	15.43
Het df		3	0	4
Het P		*	N.S.	**
Fixed RR		10.92	19.13	13.60
RRl		8.09	13.16	10.76
RRu		14.74	27.80	17.18
P		+++	+++	+++
Random RR		10.40	19.13	12.28
RRl		5.11	13.16	7.15
RRu		21.19	27.80	21.08
P		+++	+++	+++
Between Chi				5.25
Between df				1
Between P				*
Btwn(F) P				N.S.
Btwn(R) P				N.S.

Table 3H19 - 7

IESLC - Meta-analysis of Ever/current Smoking, Age started, "High"
Adenocarcinoma, Cigarettes (or Any Product if Cigarettes not available)
Excluded studies (and stage at which they were excluded)

1	BECHER BLOT1 BROWN3 CARPEN CHYOU DARBY DOLL2 GARCIA GRAHAM GURSEL HAMMO2 JAHN JAIN LAUSSM PRESCO QIAO TVERDA WIGLE WYNDE3
2	AKIBA AMANDU AMES AXELSS BENSHL BEST BOUCHA BOUCOT BROSS CHEN CPSII DEAN2 DESTEF DORGAN DOSEME FAN GARSHI GER GILLIS HAMMON HUMBLE JUSSAW KAISE2 KATSOU KAUFMA KOO KREUZE LEVIN MCCONN NOTAN2 OSANN2 PEZZO2 PISANI RESTRE SADOWS VUTUC WANG2 WATSON WU2 WUWILL WYNDE2 WYNDE8 XU ZHOU
3	GUO MCDUFF SPITZ STASZE ZHANG
4	AGUDO ARMADA AUVINE BOFFET BRESLO BUFFLE CEDERL CHEN2 CHIAZZ CHOI CORREA CPSI DAMBER DEAN3 DOLL DORN GAO GAO2 GENG HIRAYA HOLE HU HU2 JOLY KOULUM LETOUR LIAW LIU3 LIU4 LIU5 LUBIN LUBIN2 LUO MIGRAN MRFITR PERNU QIAO2 RACHTA SEGI2 SOBUE SPEIZE SUZUK2 TIZZAN WYNDE7 YUAN
5	ALDERS
10	HEGMAN KHUDER
14	BENHAM ENGELA HAENSZ PEZZOT SVENSS WAKAI WU ZHENG

Table 3H19 - 8
Potentially overlapping studies

REF	REFGP	PRINC	.	OVERLAP/LINK
WYNDE6	WYNDE6	1		WYNDE5/6/7/8

Table 3H20 -

IESLC - Meta-analysis of Ever/current Smoking, Age started, "Highest vs lowest"
Adenocarcinoma, Cigarettes (or Any Product if Cigarettes not available)

This analysis is restricted to results for:
1) Ever/current smokers
2) Results by Age started
3) Categorical results by Age started
4) Denominator (unexposed) = "low"
5) Adenocarcinoma (or near equivalent)
6) Results complete enough for use in metaanalysis

Within each study, results are then selected (in the following order of preference, within each sex) for:
7) SMKSTA: ever, current
8) PRODUCT: cigarettes regardless of other products, cigarettes only, all/unspec
9) CIGTYPE: all/unspecified, MC regardless of HR, MC only
10) Results with least adjustment for other aspects of smoking (ADOS)
11) The highest vs lowest category
12) Followup period (YF, prospective studies): whole study (coded as 0) or longest available
13) LCtype: adeno or nearest available, but not squamous. (q = squamous, s = small,
a = adeno, l = large, KII = Kreyberg II, al = alveolar, br = bronchiolar, u = undifferentiated)
14) Race: all or nearest available, otherwise by race (wh or w = white, bl or b = black, hi = hispanic
ch = chinese, jap = japanese, haw = hawaiian, w+o = white + oriental, sca = scandinavian, as = asian)
15) For overlapping studies: principal rather than subsidiary studies
Finally by Age: whole study (coded as 0) if available, otherwise by widest available age group
and then for single sex results (m, f) in preference to results for both sexes combined (c).

Results adjusted (AD) for the most potential confounders are then chosen in Sections -1 to -3
(and those which actually differ from the adjusted results in Table 3H15 - 1 are marked 'x' in Section -1)
and results adjusted for the least confounders in Sections -4 to -6. (Those least adjusted results which
actually differ from the most adjusted are marked 'x' in column X in Section -4)

Section -7 shows excluded studies, together with the stage (as above) at which no qualifying
results were found.

Section -8 lists the potentially overlapping studies which have been included (1=principal, 2=subsidiary).

Section -9 lists any results which would have been included in preference except that they had data not complete
enough for use in meta-analysis, with their significance (yes/no), if known, and any further comment as entered
on the database. It also lists as "gap" any categories for which no data were presented by the original authors.

In addition to those mentioned above, the following fields, levels and abbreviations are used:
* or nk = not known, n = no, y = yes, ot = other
all/unspec = all or unspecified, cig+/-ot = cigarettes irrespective of other products (cigar, pipe etc)
MC = manufactured cigarettes, HR = hand-rolled cigarettes
exL, exH = range of exposure (low and high) in the "highest" group, in terms of Age started
unexL, unexH = range of exposure (low and high) in the "lowest" group, in terms of Age started
REF: 6-character study reference
NRR: number of the RR on the database within the study
ST : study type (CC = case control, pr or prosp = prospective)
NLC: number of lung cancer cases in whole study
R : risky occupational population (n = no, m = mining, o = other risky)
VB : national cigarette type (V = at least 75% Virginia, bl = at least 75% blended, ot = other)
P : any proxy use
H : full histological confirmation
De : derivation of RR/CI (or = original, st = standard method, ot = other method of estimation)

Table 3H20 - 1

IESLC - Meta-analysis of Ever/current Smoking, Age started, "Highest vs lowest"
Adenocarcinoma, Cigarettes (or Any Product if Cigarettes not available)
Most adjusted

REF	NRR	XX15	SEX	AGEL	AGEH	RACE	YF	LC TYPE	LOC	START	ST	NLC	R	VB	P	H	AD	ADOS	SM	PRODUCT	exL	exH	unexL	unexH	De
BARBON	740		m	0	0	all	-	a	Eu:wst	1979	CC	755	n	bl	y	y	1	0	ev	all/unsp	1	14	20	999	ot
BENHAM	523		m	0	0	all	-	KII	Eu:wst	1976	CC	1625	n	bl	n	y	0	0	ev	cig only	1	19	20	999	st
ENGELA	529		m	0	0	all	0	a	Eu:Sca	1964	pr	435	n	bl	n	n	0	0	cu	cig+/-ot	1	19	20	999	st
HAENSZ	534		f	0	0	all	-	a	NAmer	1955	CC	158	n	bl	n	y	2	0	ev	cig+/-ot	1	24	25	999	ot
HEGMAN	505		m	0	0	all	-	a	NAmer	1989	CC	282	n	bl	y	y	0	0	ev	all/unsp	1	19	20	999	st
HEGMAN	510		f	0	0	all	-	a	NAmer	1989	CC	282	n	bl	y	y	0	0	ev	all/unsp	1	25	26	999	ot
JEDRYC	542		m	0	0	all	-	a	Eu:est	1980	CC	1630	n	bl	y	n	0	0	ev	cig+/-ot	1	16	19	999	st
KHUDER	528		m	0	0	all	-	a	NAmer	1985	CC	482	n	bl	n	y	5	3#	ev	cig+/-ot	1	15	20	999	or
MATOS	660		m	0	0	all	-	a	SCAmer	1994	CC	200	n	bl	n	n	2	0	ev	cig+/-ot	1	14	20	999	ot
PEZZOT	558		m	0	0	all	-	a	SCAmer	1987	CC	215	n	bl	n	y	2	0	ev	cig only	1	13	19	999	ot
SVENSS	529		f	0	0	all	-	a	Eu:Sca	1983	CC	210	n	bl	n	n	0	0	cu	all/unsp	0	18	26	999	st
WAKAI	515		m	0	0	all	-	a	As:Jap	1988	CC	333	n	bl	n	y	0	0	cu	cig+/-ot	1	19	30	999	st
WU	529		f	0	0	wh	-	a	NAmer	1981	CC	220	n	bl	n	y	2	0	cu	all/unsp	0	18	25	999	ot
WYNDE6	617		m	0	0	wh	-	a	NAmer	1969	CC	4423	n	bl	n	y	0	0	ev	cig+/-ot	1	17	21	999	st
WYNDE6	641		f	0	0	wh	-	a	NAmer	1969	CC	4423	n	bl	n	y	0	0	ev	cig+/-ot	1	17	21	999	st
ZHENG	537		m	0	0	all	-	a	As:Chi	1982	CC	540	n	ot	*	y	1	0	ev	cig+/-ot	1	19	30	999	ot
ZHENG	541		f	0	0	all	-	a	As:Chi	1982	CC	540	n	ot	*	y	1	0	ev	cig+/-ot	1	29	30	999	ot

Comments on values in listings

KHUDER ADOS Duration of smoking (years), number of cigarettes per day and Quitted smoking

Cigarette type is all/unspec for all RRs

Table 3H20 - 2

IESLC - Meta-analysis of Ever/current Smoking, Age started, "Highest vs lowest"
Adenocarcinoma, Cigarettes (or Any Product if Cigarettes not available)
Most adjusted

REF	NRR	SEX	AD	Number Case	Exposed Cont	Non-exposed Case	Cont	RR	95.00%CI
BARBON	740	m	1	29	-	45	-	5.86	(3.10- 11.07)
BENHAM	523	m	0	69	79	41	64	1.36	(0.82- 2.27)
*ENGELA	529	m	0	25	50732	8	39957	2.46	(1.11- 5.46)
HAENSZ	534	f	2	6	-	12	-	0.88	(0.31- 2.53)
HEGMAN	505	m	0	45	716	6	289	3.03	(1.28- 7.17)
HEGMAN	510	f	0	32	169	0	28	10.93~	(0.65- 183.56)
Subtotal HEGMAN								3.38	(1.48- 7.71)
JEDRYC	542	m	0	12	111	48	502	1.13	(0.58- 2.20)
KHUDER	528	m	5	-	-	-	-	0.80	(0.40- 1.60)
MATOS	660	m	2	26	-	13	-	1.65	(0.80- 3.40)
PEZZOT	558	m	2	9	-	18	-	1.11	(0.41- 3.04)
SVENSS	529	f	0	17	21	10	18	1.46	(0.53- 3.97)
WAKAI	515	m	0	18	74	4	25	1.52	(0.47- 4.92)
WU	529	f	2	69	-	8	-	7.27	(2.31- 22.86)
WYNDE6	617	m	0	241	301	47	92	1.57	(1.06- 2.32)
WYNDE6	641	f	0	150	91	63	90	2.35	(1.56- 3.56)
Subtotal WYNDE6								1.90	(1.43- 2.52)
ZHENG	537	m	1	44	-	15	-	4.54	(2.25- 9.15)
ZHENG	541	f	1	26	-	7	-	2.00	(0.71- 5.64)
Subtotal ZHENG								3.51	(1.96- 6.27)
Partial Totals				818	52294	345	41065		

*prospective study ~ With 0.5 adjustment for zero

REF	NRR	SEX	AD	Ys	Ws	Qs	Ps
BARBON	740	m	1	1.77	9.48	11.63	0.0000
BENHAM	523	m	0	0.31	14.89	1.83	0.2317
*ENGELA	529	m	0	0.90	6.06	0.35	0.0266
HAENSZ	534	f	2	-0.13	3.49	2.17	0.8113
HEGMAN	505	m	0	1.11	5.16	1.03	0.0119
HEGMAN	510	f	0	2.39	0.48	1.45	0.0966
Subtotal HEGMAN				1.22	5.64	2.48	
JEDRYC	542	m	0	0.12	8.68	2.51	0.7175
KHUDER	528	m	5	-0.22	8.00	6.25	0.5281
MATOS	660	m	2	0.50	7.34	0.19	0.1749
PEZZOT	558	m	2	0.10	3.83	1.19	0.8382
SVENSS	529	f	0	0.38	3.82	0.31	0.4620
WAKAI	515	m	0	0.42	2.78	0.16	0.4845
WU	529	f	2	1.98	2.92	5.12	0.0007
WYNDE6	617	m	0	0.45	25.24	1.13	0.0240
WYNDE6	641	f	0	0.86	22.40	0.86	0.0001
Subtotal WYNDE6				0.64	47.64	1.99	
ZHENG	537	m	1	1.51	7.81	5.67	0.0000
ZHENG	541	f	1	0.69	3.58	0.00	0.1898
Subtotal ZHENG				1.26	11.39	5.67	

	N	17
	NS	14
	Wt	135.97
Het	Chi	41.84
Het	df	16
Het	P	***
Fixed	RR	1.94
	RRl	1.64
	RRu	2.29
	P	+++
Random	RR	1.99
	RRl	1.48
	RRu	2.67
	P	+++
Asymm	P	N.S.

Table 3H20 - 3

IESLC - Meta-analysis of Ever/current Smoking, Age started, "Highest vs lowest"
Adenocarcinoma, Cigarettes (or Any Product if Cigarettes not available)
Most adjusted

	Sex			
	combined	male	female	Total
N		11	6	17
NS		11	6	17
Wt		99.28	36.69	135.97
Het Chi		31.65	9.12	41.84
Het df		10	5	16
Het P		***	N.S.	***
Fixed RR		1.83	2.24	1.94
RRl		1.51	1.62	1.64
RRu		2.23	3.10	2.29
P		+++	+++	+++
Random RR		1.89	2.24	1.99
RRl		1.31	1.30	1.48
RRu		2.74	3.85	2.67
P		+++	++	+++
Between Chi				1.08
Between df				1
Between P				N.S.
Btwn(F) P				N.S.
Btwn(R) P				N.S.

	Lung cancer type						
	a	a+l	a+al+br	KII	not q+u	not q+s	Total
N	16			1			17
NS	13			1			14
Wt	121.08			14.89			135.97
Het Chi	39.78			0.00			41.84
Het df	15			0			16
Het P	***			N.S.			***
Fixed RR	2.02			1.36			1.94
RRl	1.69			0.82			1.64
RRu	2.42			2.27			2.29
P	+++			N.S.			+++
Random RR	2.05			1.36			1.99
RRl	1.50			0.82			1.48
RRu	2.82			2.27			2.67
P	+++			N.S.			+++
Between Chi							2.06
Between df							1
Between P							N.S.
Btwn(F) P							N.S.
Btwn(R) P							N.S.

	Location								
	NAmer	UK	Scand	othEur	China	Japan	othAs	other	Total
N	7		2	3	2	1		2	17
NS	5		2	3	1	1		2	14
Wt	67.69		9.88	33.06	11.39	2.78		11.17	135.97
Het Chi	17.79		0.64	15.96	1.65	0.00		0.40	41.84
Het df	6		1	2	1	0		1	16
Het P	**		N.S.	***	N.S.	N.S.		N.S.	***
Fixed RR	1.83		2.01	1.97	3.51	1.52		1.44	1.94
RRl	1.44		1.08	1.40	1.96	0.47		0.80	1.64
RRu	2.32		3.75	2.77	6.27	4.92		2.59	2.29
P	+++		+	+++	+++	N.S.		N.S.	+++
Random RR	1.94		2.01	2.07	3.30	1.52		1.44	1.99
RRl	1.19		1.08	0.78	1.51	0.47		0.80	1.48
RRu	3.16		3.75	5.52	7.23	4.92		2.59	2.67
P	++		+	N.S.	++	N.S.		N.S.	+++
Between Chi									5.40
Between df									5
Between P									N.S.
Btwn(F) P									N.S.
Btwn(R) P									N.S.

Table 3H20 - 3

IESLC - Meta-analysis of Ever/current Smoking, Age started, "Highest vs lowest"
Adenocarcinoma, Cigarettes (or Any Product if Cigarettes not available)
Most adjusted

Detailed Country in "other Europe"

	multi	Germany	othWest	East	Balkans	Total
N			2	1		3
NS			2	1		3
Wt			24.37	8.68		33.06
Het Chi			12.32	0.00		15.96
Het df			1	0		2
Het P			***	N.S.		***
Fixed RR			2.40	1.13		1.97
RRl			1.62	0.58		1.40
RRu			3.58	2.20		2.77
P			+++	N.S.		+++
Random RR			2.79	1.13		2.07
RRl			0.67	0.58		0.78
RRu			11.64	2.20		5.52
P			N.S.	N.S.		N.S.
Between Chi						3.65
Between df						1
Between P						(*)
Btwn(F) P						N.S.
Btwn(R) P						N.S.

Detailed Country in "other Asia"

	India	HongKong	other	Total
N				
NS				
Wt				
Het Chi				
Het df				
Het P				N.S.
Fixed RR				
RRl				
RRu				
P				N.S.
Random RR				
RRl				
RRu				
P				N.S.
Between Chi				
Between df				
Between P				N.S.
Btwn(F) P				N.S.
Btwn(R) P				N.S.

Detailed other continent

	SCAmer	Total
N	2	2
NS	2	2
Wt	11.17	11.17
Het Chi	0.40	0.40
Het df	1	1
Het P	N.S.	N.S.
Fixed RR	1.44	1.44
RRl	0.80	0.80
RRu	2.59	2.59
P	N.S.	N.S.
Random RR	1.44	1.44
RRl	0.80	0.80
RRu	2.59	2.59
P	N.S.	N.S.
Between Chi		
Between df		
Between P		N.S.
Btwn(F) P		N.S.
Btwn(R) P		N.S.

Table 3H20 - 3

IESLC - Meta-analysis of Ever/current Smoking, Age started, "Highest vs lowest"
Adenocarcinoma, Cigarettes (or Any Product if Cigarettes not available)
Most adjusted

	Start year of study <1960	1960-69	1970-79	1980-89	1990+	Total
N	1	3	2	10	1	17
NS	1	2	2	8	1	14
Wt	3.49	53.70	24.37	47.06	7.34	135.97
Het Chi	0.00	2.33	12.32	23.61	0.00	41.84
Het df	0	2	1	9	0	16
Het P	N.S.	N.S.	***	**	N.S.	***
Fixed RR	0.88	1.95	2.40	1.86	1.65	1.94
RRl	0.31	1.50	1.62	1.40	0.80	1.64
RRu	2.51	2.55	3.58	2.48	3.40	2.29
P	N.S.	+++	+++	+++	N.S.	+++
Random RR	0.88	1.97	2.79	2.00	1.65	1.99
RRl	0.31	1.46	0.67	1.23	0.80	1.48
RRu	2.51	2.64	11.64	3.26	3.40	2.67
P	N.S.	+++	N.S.	++	N.S.	+++
Between Chi						3.58
Between df						4
Between P						N.S.
Btwn(F) P						N.S.
Btwn(R) P						N.S.

	Study type (1) CC	other	Total
N	16	1	17
NS	13	1	14
Wt	129.90	6.06	135.97
Het Chi	41.47	0.00	41.84
Het df	15	0	16
Het P	***	N.S.	***
Fixed RR	1.91	2.46	1.94
RRl	1.61	1.11	1.64
RRu	2.27	5.46	2.29
P	+++	+	+++
Random RR	1.96	2.46	1.99
RRl	1.43	1.11	1.48
RRu	2.68	5.46	2.67
P	+++	+	+++
Between Chi			0.36
Between df			1
Between P			N.S.
Btwn(F) P			N.S.
Btwn(R) P			N.S.

	Study type (2) CC	prosp	other	Total
N	16	1		17
NS	13	1		14
Wt	129.90	6.06		135.97
Het Chi	41.47	0.00		41.84
Het df	15	0		16
Het P	***	N.S.		***
Fixed RR	1.91	2.46		1.94
RRl	1.61	1.11		1.64
RRu	2.27	5.46		2.29
P	+++	+		+++
Random RR	1.96	2.46		1.99
RRl	1.43	1.11		1.48
RRu	2.68	5.46		2.67
P	+++	+		+++
Between Chi				0.36
Between df				1
Between P				N.S.
Btwn(F) P				N.S.
Btwn(R) P				N.S.

Table 3H20 - 3

IESLC - Meta-analysis of Ever/current Smoking, Age started, "Highest vs lowest"
Adenocarcinoma, Cigarettes (or Any Product if Cigarettes not available)
Most adjusted

	Study size (number of LC cases)				
	100-249	250-499	500-999	1000+	Total
N	5	5	3	4	17
NS	5	4	2	3	14
Wt	21.40	22.49	20.87	71.21	135.97
Het Chi	8.47	8.80	3.01	4.68	41.84
Het df	4	4	2	3	16
Het P	(*)	(*)	N.S.	N.S.	***
Fixed RR	1.66	1.68	4.43	1.66	1.94
RRl	1.09	1.11	2.88	1.32	1.64
RRu	2.54	2.55	6.80	2.10	2.29
P	+	+	+++	+++	+++
Random RR	1.70	1.87	4.24	1.63	1.99
RRl	0.91	0.96	2.47	1.21	1.48
RRu	3.20	3.64	7.28	2.19	2.67
P	(+)	(+)	+++	++	+++
Between Chi					16.89
Between df					3
Between P					***
Btwn(F) P					(*)
Btwn(R) P					*

	Risky occupational population			
	no	mining	othRisky	Total
N	17			17
NS	14			14
Wt	135.97			135.97
Het Chi	41.84			41.84
Het df	16			16
Het P	***			***
Fixed RR	1.94			1.94
RRl	1.64			1.64
RRu	2.29			2.29
P	+++			+++
Random RR	1.99			1.99
RRl	1.48			1.48
RRu	2.67			2.67
P	+++			+++
Between Chi				
Between df				
Between P				N.S.
Btwn(F) P				N.S.
Btwn(R) P				N.S.

	National cigarette tobacco type			
	Virginia	blended	other	Total
N		15	2	17
NS		13	1	14
Wt		124.58	11.39	135.97
Het Chi		35.80	1.65	41.84
Het df		14	1	16
Het P		**	N.S.	***
Fixed RR		1.83	3.51	1.94
RRl		1.54	1.96	1.64
RRu		2.19	6.27	2.29
P		+++	+++	+++
Random RR		1.86	3.30	1.99
RRl		1.37	1.51	1.48
RRu		2.53	7.23	2.67
P		+++	++	+++
Between Chi				4.39
Between df				1
Between P				*
Btwn(F) P				N.S.
Btwn(R) P				N.S.

Table 3H20 - 3

IESLC - Meta-analysis of Ever/current Smoking, Age started, "Highest vs lowest"
Adenocarcinoma, Cigarettes (or Any Product if Cigarettes not available)
Most adjusted

	Any proxy use No/nk	Yes	Total
N	13	4	17
NS	11	3	14
Wt	112.16	23.81	135.97
Het Chi	24.50	13.24	41.84
Het df	12	3	16
Het P	*	**	***
Fixed RR	1.79	2.82	1.94
RRl	1.49	1.89	1.64
RRu	2.15	4.22	2.29
P	+++	+++	+++
Random RR	1.79	3.07	1.99
RRl	1.34	1.18	1.48
RRu	2.39	8.01	2.67
P	+++	+	+++
Between Chi			4.10
Between df			1
Between P			*
Btwn(F) P			N.S.
Btwn(R) P			N.S.

	Full histological confirmation No	Yes	Total
N	4	13	17
NS	4	10	14
Wt	25.90	110.07	135.97
Het Chi	2.20	38.21	41.84
Het df	3	12	16
Het P	N.S.	***	***
Fixed RR	1.57	2.04	1.94
RRl	1.07	1.69	1.64
RRu	2.30	2.45	2.29
P	+	+++	+++
Random RR	1.57	2.14	1.99
RRl	1.07	1.48	1.48
RRu	2.30	3.10	2.67
P	+	+++	+++
Between Chi			1.43
Between df			1
Between P			N.S.
Btwn(F) P			N.S.
Btwn(R) P			N.S.

	Number of adjustment variables (1) 0	1	2+/+nk	Total
N	9	3	5	17
NS	7	2	5	14
Wt	89.52	20.87	25.57	135.97
Het Chi	8.91	3.01	11.56	41.84
Het df	8	2	4	16
Het P	N.S.	N.S.	*	***
Fixed RR	1.77	4.43	1.35	1.94
RRl	1.44	2.88	0.92	1.64
RRu	2.18	6.80	1.99	2.29
P	+++	+++	N.S.	+++
Random RR	1.77	4.24	1.48	1.99
RRl	1.41	2.47	0.75	1.48
RRu	2.22	7.28	2.92	2.67
P	+++	+++	N.S.	+++
Between Chi				18.36
Between df				2
Between P				***
Btwn(F) P				*
Btwn(R) P				**

Table 3H20 - 3

IESLC - Meta-analysis of Ever/current Smoking, Age started, "Highest vs lowest"
Adenocarcinoma, Cigarettes (or Any Product if Cigarettes not available)
Most adjusted

	Number of adjustment variables (2) 0	1	2	3-5	6+/+nk	Total
N	9	3	4	1		17
NS	7	2	4	1		14
Wt	89.52	20.87	17.58	8.00		135.97
Het Chi	8.91	3.01	8.39	0.00		41.84
Het df	8	2	3	0		16
Het P	N.S.	N.S.	*	N.S.		***
Fixed RR	1.77	4.43	1.71	0.80		1.94
RRl	1.44	2.88	1.07	0.40		1.64
RRu	2.18	6.80	2.73	1.60		2.29
P	+++	+++	+	N.S.		+++
Random RR	1.77	4.24	1.79	0.80		1.99
RRl	1.41	2.47	0.80	0.40		1.48
RRu	2.22	7.28	4.01	1.60		2.67
P	+++	+++	N.S.	N.S.		+++
Between Chi						21.53
Between df						3
Between P						***
Btwn(F) P						*
Btwn(R) P						**

	Smoking status ever	current	Total
N	13	4	17
NS	10	4	14
Wt	120.38	15.59	135.97
Het Chi	35.80	5.12	41.84
Het df	12	3	16
Het P	***	N.S.	***
Fixed RR	1.88	2.43	1.94
RRl	1.57	1.48	1.64
RRu	2.25	4.00	2.29
P	+++	+++	+++
Random RR	1.89	2.46	1.99
RRl	1.35	1.27	1.48
RRu	2.64	4.77	2.67
P	+++	++	+++
Between Chi			0.92
Between df			1
Between P			N.S.
Btwn(F) P			N.S.
Btwn(R) P			N.S.

	Product all/unsp	cig+/-ot	cig only	Total
N	5	10	2	17
NS	4	8	2	14
Wt	21.87	95.38	18.72	135.97
Het Chi	7.19	18.43	0.13	41.84
Het df	4	9	1	16
Het P	N.S.	*	N.S.	***
Fixed RR	4.10	1.76	1.31	1.94
RRl	2.70	1.44	0.83	1.64
RRu	6.24	2.15	2.06	2.29
P	+++	+++	N.S.	+++
Random RR	3.96	1.72	1.31	1.99
RRl	2.14	1.26	0.83	1.48
RRu	7.34	2.34	2.06	2.67
P	+++	+++	N.S.	+++
Between Chi				16.09
Between df				2
Between P				***
Btwn(F) P				*
Btwn(R) P				*

Table 3H20 - 3

IESLC - Meta-analysis of Ever/current Smoking, Age started, "Highest vs lowest"
Adenocarcinoma, Cigarettes (or Any Product if Cigarettes not available)
Most adjusted

	Derivation of RR/CI Orig	StdCalc	Other	Total
N	1	8	8	17
NS	1	7	7	15
Wt	8.00	89.04	38.93	135.97
Het Chi	0.00	7.30	20.90	41.84
Het df	0	7	7	16
Het P	N.S.	N.S.	**	***
Fixed RR	0.80	1.75	2.91	1.94
RRl	0.40	1.42	2.13	1.64
RRu	1.60	2.16	3.99	2.29
P	N.S.	+++	+++	+++
Random RR	0.80	1.75	2.72	1.99
RRl	0.40	1.41	1.52	1.48
RRu	1.60	2.17	4.86	2.67
P	N.S.	+++	+++	+++
Between Chi				13.64
Between df				2
Between P				**
Btwn(F) P				(*)
Btwn(R) P				*

Table 3H20 - 4

IESLC - Meta-analysis of Ever/current Smoking, Age started, "Highest vs lowest"
Adenocarcinoma, Cigarettes (or Any Product if Cigarettes not available)
Least adjusted

REF	NRR	X	SEX	AGEL	AGEH	RACE	YF	LC TYPE	LOC	START	ST	NLC	R	VB	P	H	AD	ADOS	SM	PRODUCT	exL	exH	unexL	unexH	De
BARBON	735	x	m	0	0	all	-	a	Eu:wst	1979	CC	755	n	bl	y	y	0	0	ev	all/unsp	1	14	20	999	st
BENHAM	523		m	0	0	all	-	KII	Eu:wst	1976	CC	1625	n	bl	n	y	0	0	ev	cig only	1	19	20	999	st
ENGELA	529		m	0	0	all	0	a	Eu:Sca	1964	pr	435	n	bl	n	n	0	0	cu	cig+/-ot	1	19	20	999	st
HAENSZ	513	x	f	0	0	all	-	a	NAmer	1955	CC	158	n	bl	n	y	0	0	ev	cig+/-ot	1	24	25	999	st
HEGMAN	505		m	0	0	all	-	a	NAmer	1989	CC	282	n	bl	y	y	0	0	ev	all/unsp	1	19	20	999	st
HEGMAN	510		f	0	0	all	-	a	NAmer	1989	CC	282	n	bl	y	y	0	0	ev	all/unsp	1	25	26	999	ot
JEDRYC	542		m	0	0	all	-	a	Eu:est	1980	CC	1630	n	bl	y	n	0	0	ev	cig+/-ot	1	16	19	999	st
KHUDER	528		m	0	0	all	-	a	NAmer	1985	CC	482	n	bl	n	y	5	3#	ev	cig+/-ot	1	15	20	999	or
MATOS	655	x	m	0	0	all	-	a	SCAmer	1994	CC	200	n	bl	n	n	0	0	ev	cig+/-ot	1	14	20	999	st
PEZZOT	556	x	m	0	0	all	-	a	SCAmer	1987	CC	215	n	bl	n	y	0	0	ev	cig only	1	13	19	999	st
SVENSS	529		f	0	0	all	-	a	Eu:Sca	1983	CC	210	n	bl	n	n	0	0	cu	all/unsp	0	18	26	999	st
WAKAI	515		m	0	0	all	-	a	As:Jap	1988	CC	333	n	bl	n	y	0	0	cu	cig+/-ot	1	19	30	999	st
WU	505	x	f	0	0	wh	-	a	NAmer	1981	CC	220	n	bl	n	y	0	0	cu	all/unsp	0	18	25	999	st
WYNDE6	617		m	0	0	wh	-	a	NAmer	1969	CC	4423	n	bl	n	y	0	0	ev	cig+/-ot	1	17	21	999	st
WYNDE6	641		f	0	0	wh	-	a	NAmer	1969	CC	4423	n	bl	n	y	0	0	ev	cig+/-ot	1	17	21	999	st
ZHENG	535	x	m	0	0	all	-	a	As:Chi	1982	CC	540	n	ot	*	y	0	0	ev	cig+/-ot	1	19	30	999	st
ZHENG	540	x	f	0	0	all	-	a	As:Chi	1982	CC	540	n	ot	*	y	0	0	ev	cig+/-ot	1	29	30	999	st

Comments on values in listings

KHUDER ADOS Duration of smoking (years), number of cigarettes per day and Quitted smoking

Cigarette type is all/unspec for all RRs

Table 3H20 - 5

IESLC - Meta-analysis of Ever/current Smoking, Age started, "Highest vs lowest"
Adenocarcinoma, Cigarettes (or Any Product if Cigarettes not available)
Least adjusted

REF	NRR	SEX	AD	Number Exposed Case	Cont	Non-exposed Case	Cont	RR	95.00%CI
BARBON	735	m	0	29	23	45	207	5.80	(3.07- 10.95)
BENHAM	523	m	0	69	79	41	64	1.36	(0.82- 2.27)
*ENGELA	529	m	0	25	50732	8	39957	2.46	(1.11- 5.46)
HAENSZ	513	f	0	6	37	12	66	0.89	(0.31- 2.57)
HEGMAN	505	m	0	45	716	6	289	3.03	(1.28- 7.17)
HEGMAN	510	f	0	32	169	0	28	10.93~	(0.65- 183.56)
Subtotal HEGMAN								3.38	(1.48- 7.71)
JEDRYC	542	m	0	12	111	48	502	1.13	(0.58- 2.20)
KHUDER	528	m	5	-	-	-	-	0.80	(0.40- 1.60)
MATOS	655	m	0	26	90	13	73	1.62	(0.78- 3.38)
PEZZOT	556	m	0	9	15	18	32	1.07	(0.39- 2.92)
SVENSS	529	f	0	17	21	10	18	1.46	(0.53- 3.97)
WAKAI	515	m	0	18	74	4	25	1.52	(0.47- 4.92)
WU	505	f	0	69	21	8	14	5.75	(2.12- 15.58)
WYNDE6	617	m	0	241	301	47	92	1.57	(1.06- 2.32)
WYNDE6	641	f	0	150	91	63	90	2.35	(1.56- 3.56)
Subtotal WYNDE6								1.90	(1.43- 2.52)
ZHENG	535	m	0	44	43	15	66	4.50	(2.23- 9.07)
ZHENG	540	f	0	26	28	7	16	2.12	(0.75- 5.98)
Subtotal ZHENG								3.56	(1.99- 6.35)
Partial Totals				818	52551	345	41539		

*prospective study ~ With 0.5 adjustment for zero

REF	NRR	SEX	AD	Ys	Ws	Qs	Ps
BARBON	735	m	0	1.76	9.52	11.41	0.0000
BENHAM	523	m	0	0.31	14.89	1.86	0.2317
*ENGELA	529	m	0	0.90	6.06	0.34	0.0266
HAENSZ	513	f	0	-0.11	3.42	2.07	0.8324
HEGMAN	505	m	0	1.11	5.16	1.02	0.0119
HEGMAN	510	f	0	2.39	0.48	1.44	0.0966
Subtotal HEGMAN				1.22	5.64	2.46	
JEDRYC	542	m	0	0.12	8.68	2.54	0.7175
KHUDER	528	m	5	-0.22	8.00	6.28	0.5281
MATOS	655	m	0	0.48	7.13	0.23	0.1963
PEZZOT	556	m	0	0.06	3.78	1.35	0.9002
SVENSS	529	f	0	0.38	3.82	0.31	0.4620
WAKAI	515	m	0	0.42	2.78	0.17	0.4845
WU	505	f	0	1.75	3.87	4.56	0.0006
WYNDE6	617	m	0	0.45	25.24	1.16	0.0240
WYNDE6	641	f	0	0.86	22.40	0.84	0.0001
Subtotal WYNDE6				0.64	47.64	1.99	
ZHENG	535	m	0	1.50	7.82	5.54	0.0000
ZHENG	540	f	0	0.75	3.58	0.03	0.1546
Subtotal ZHENG				1.27	11.40	5.57	

	N	17
	NS	14
	Wt	136.65
Het	Chi	41.14
Het	df	16
Het	P	***
Fixed	RR	1.94
	RRl	1.64
	RRu	2.30
	P	+++
Random	RR	1.98
	RRl	1.48
	RRu	2.65
	P	+++
Asymm	P	N.S.

Table 3H20 - 6

IESLC - Meta-analysis of Ever/current Smoking, Age started, "Highest vs lowest"
Adenocarcinoma, Cigarettes (or Any Product if Cigarettes not available)
Least adjusted

	Sex combined	male	female	Total
N		11	6	17
NS		11	6	17
Wt		99.08	37.57	136.65
Het Chi		31.54	8.32	41.14
Het df		10	5	16
Het P		***	N.S.	***
Fixed RR		1.83	2.27	1.94
RRl		1.50	1.65	1.64
RRu		2.23	3.13	2.30
P		+++	+++	+++
Random RR		1.88	2.24	1.98
RRl		1.30	1.36	1.48
RRu		2.72	3.71	2.65
P		+++	++	+++
Between Chi				1.29
Between df				1
Between P				N.S.
Btwn(F) P				N.S.
Btwn(R) P				N.S.

Table 3H20 - 7

IESLC - Meta-analysis of Ever/current Smoking, Age started, "Highest vs lowest"
Adenocarcinoma, Cigarettes (or Any Product if Cigarettes not available)
Excluded studies (and stage at which they were excluded)

1	BECHER BLOT1 BROWN3 CARPEN CHYOU DARBY DOLL2 GARCIA GRAHAM GURSEL HAMMO2 JAHN JAIN LAUSSM PRESCO QIAO TVERDA WIGLE WYNDE3
2	AKIBA AMANDU AMES AXELSS BENSHL BEST BOUCHA BOUCOT BROSS CHEN CPSII DEAN2 DESTEF DORGAN DOSEME FAN GARSHI GER GILLIS HAMMON HUMBLE JUSSAW KAISE2 KATSOU KAUFMA KOO KREUZE LEVIN MCCONN NOTAN2 OSANN2 PEZZO2 PISANI RESTRE SADOWS VUTUC WANG2 WATSON WU2 WUWILL WYNDE2 WYNDE8 XU ZHOU
3	GUO MCDUFF SPITZ STASZE ZHANG
5	AGUDO ARMADA AUVINE BOFFET BRESLO BUFFLE CEDERL CHEN2 CHIAZZ CHOI CORREA CPSI DAMBER DEAN3 DOLL DORN GAO GAO2 GENG HIRAYA HOLE HU HU2 JOLY KOULUM LETOUR LIAW LIU3 LIU4 LIU5 LUBIN LUBIN2 LUO MIGRAN MRFITR PERNU QIAO2 RACHTA SEGI2 SOBUE SPEIZE SUZUK2 TIZZAN WYNDE7 YUAN
6	ALDERS

Table 3H20 - 8
Potentially overlapping studies

REF	REFGP	PRINC	.	OVERLAP/LINK
BENHAM	LUBIN2	2		Subset of Lubin2
WYNDE6	WYNDE6	1		WYNDE5/6/7/8

Table 3H20 - 9
Most adjusted - insufficient data for meta-analysis

REF	NRR	SEX	AGEL	AGEH	RACE	YF	LC TYPE	LOC	START	ST	NLC	R	VB	P	H	AD	ADOS	SM	PRODUCT	exL	exH	unexL	unexH	De
ALDERS	563	m	0	0	all	-	not q+s	Eu:UK	1977	CC	1448	n	V	n	n	2		1#	ev cig only	1	14	25	999	ot
ALDERS	566	f	0	0	all	-	not q+s	Eu:UK	1977	CC	1448	n	V	n	n	2		1#	ev cig only	1	14	25	999	ot

Comments on values in listings

ALDERS ADOS Number of cigs/day
ALDERS ADOS Number of cigs/day

REF	NRR	RR	SIG	RRDATA comment
ALDERS	563	0.53	n	0
ALDERS	566	1.92	n	0

Table 3H21 -

IESLC - Meta-analysis of Ever/current Smoking by Age started, Overview
Adenocarcinoma, Cigarettes only

This analysis is restricted to results for:
1) Ever/current smokers
2) Results by Age started
3) Categorical results by Age started
Results by Age started are grouped under 2 schemes (S1, S2). Each scheme has a set of "key values". An interval is allocated to the category whose key value it includes, and intervals which include none or more than one of the key values are excluded. (Open-ended intervals are coded as 999)

S1	key value	maximum range
1	26	19+
2	18	15-25
3	14	1-17

S2	key value	maximum range
1	30	27+
2	26	23-29
3	22	19-25
4	18	15-21
5	14	11-17
6	10	1-13

4) Adenocarcinoma (or near equivalent)
5) Results complete enough for use in metaanalysis

Within each study, results are then selected (in the following order of preference, within each sex) for:
6) SMKSTA: ever, current
7) PRODUCT: cigarettes only
8) CIGTYPE: all/unspecified, MC regardless of HR, MC only
9) (not applicable)
10) DENOM: never smoked anything, never smoked cigarettes, never any + low, never cigs + low
11) Followup period (YF, prospective studies): whole study (coded as 0) or longest available
12) LCtype: adeno or nearest available, but not squamous. (q = squamous, s = small,
a = adeno, l = large, KII = Kreyberg II, al = alveolar, br = bronchiolar, u = undifferentiated)
13) Race: all or nearest available, otherwise by race (wh or w = white, bl or b = black, hi = hispanic
ch = chinese, jap = japanese, haw = hawaiian, w+o = white + oriental, sca = scandinavian, as = asian)
14) For overlapping studies: principal rather than subsidiary studies
Finally by Age: whole study (coded as 0) if available, otherwise by widest available age group
and then for single sex results (m, f) in preference to results for both sexes combined (c).

Results adjusted (AD) for the most potential confounders are then chosen in Sections -1 to -3
(and those which actually differ from the adjusted results in Table 3H11 - 1 are marked 'x' in Section -1)
and results adjusted for the least confounders in Sections -4 to -6. (Those least adjusted results which
actually differ from the most adjusted are marked 'x' in column X in Section -4)

Section -7 shows excluded studies, together with the stage (as above) at which no qualifying
results were found.

Section -8 lists the potentially overlapping studies which have been included (1=principal, 2=subsidiary).

Section -9 lists any results which would have been included in preference except that they had data not complete
enough for use in meta-analysis, with their significance (yes/no), if known, and any further comment as entered
on the database. It also lists as "gap" any categories for which no data were presented by the original authors.

In addition to those mentioned above, the following fields, levels and abbreviations are used:
* or nk = not known, n = no, y = yes, ot = other
ev = ever, cu = current, nev = never
all/unspec = all or unspecified, MC = manufactured cigarettes, HR = hand-rolled cigarettes
exL, exH = range of exposure (low and high) in the smoking group, in terms of Age started
REF: 6-character study reference
NRR: number of the RR on the database within the study
ST : study type (CC = case control, pr or prosp = prospective)
NLC: number of lung cancer cases in whole study
R : risky occupational population (n = no, m = mining, o = other risky)
VB : national cigarette type (V = at least 75% Virginia, bl = at least 75% blended, ot = other)
P : any proxy use
H : full histological confirmation
De : derivation of RR/CI (or = original, st = standard method, ot = other method of estimation)

Table 3H21 - 1

IESLC - Meta-analysis of Ever/current Smoking by Age started, Overview
Adenocarcinoma, Cigarettes only
Most adjusted

REF	NRR	XX11	SEX	AGEL	AGEH	RACE	YF	LC TYPE	LOC	START	ST	NLC	R	VB	P	H	AD	SM	PRODUCT	exL	exH	S1	S2	DENOM	De
BENHAM	521		m	0	0	all	-	KII	Eu:wst	1976	CC	1625	n	bl	n	y	0	ev	cig only	20	999	1	0	nev any	st
BENHAM	522		m	0	0	all	-	KII	Eu:wst	1976	CC	1625	n	bl	n	y	0	ev	cig only	1	19	0	0	nev any	st
PEZZOT	552		m	0	0	all	-	a	SCAmer	1987	CC	215	n	bl	n	y	0	ev	cig only	19	999	1	0	nev cigs	st
PEZZOT	553		m	0	0	all	-	a	SCAmer	1987	CC	215	n	bl	n	y	0	ev	cig only	14	18	0	0	nev cigs	st
PEZZOT	554		m	0	0	all	-	a	SCAmer	1987	CC	215	n	bl	n	y	0	ev	cig only	1	13	0	6	nev cigs	st

Cigarette type is all/unspec for all RRs

In this overview table, subtotals and Qs values may be invalid and should be ignored

Table 3H21 - 2

IESLC - Meta-analysis of Ever/current Smoking by Age started, Overview
Adenocarcinoma, Cigarettes only
Most adjusted

REF	NRR	SEX	AD	Number Exposed Case	Number Exposed Cont	Non-exposed Case	Non-exposed Cont	RR	95.00%CI
BENHAM	521	m	0	41	64	9	42	2.99	(1.32- 6.79)
BENHAM	522	m	0	69	79	9	42	4.08	(1.85- 8.97)
Subtotal BENHAM								3.51	(1.99- 6.20)
PEZZOT	552	m	0	18	105	3	116	6.63	(1.90- 23.15)
PEZZOT	553	m	0	33	145	3	116	8.80	(2.63- 29.42)
PEZZOT	554	m	0	9	67	3	116	5.19	(1.36- 19.85)
Subtotal PEZZOT								6.84	(3.30- 14.18)
Totals				170	460	27	432		

*prospective study

REF	NRR	SEX	AD	Ys	Ws	Qs	Ps
BENHAM	521	m	0	1.10	5.72	0.98	0.0088
BENHAM	522	m	0	1.41	6.17	0.07	0.0005
Subtotal BENHAM				1.26	11.89	1.04	
PEZZOT	552	m	0	1.89	2.46	0.36	0.0030
PEZZOT	553	m	0	2.17	2.64	1.17	0.0004
PEZZOT	554	m	0	1.65	2.14	0.04	0.0160
Subtotal PEZZOT				1.92	7.23	1.57	

N	5
NS	2

Table 3H21 - 3

IESLC - Meta-analysis of Ever/current Smoking by Age started, Overview
Adenocarcinoma, Cigarettes only
Most adjusted

	Sex			
	combined	male	female	Total
N		5		5
NS		2		2

In this overview table, other than the "N" rows, entries in the "absent" and "Total" columns may be invalid and should be ignored

	Age started (broad categories)				
	absent	19+k26	15-25k18	1-17k14	Total
N	3	2			5
NS	2	2			4
Wt	10.94	8.17			19.12
Het Chi	1.09	1.09			2.61
Het df	2	1			4
Het P	N.S.	N.S.			N.S.
Fixed RR	5.14	3.80			4.52
RRl	2.84	1.91			2.89
RRu	9.30	7.54			7.07
P	+++	+++			+++
Random RR	5.14	3.85			4.52
RRl	2.84	1.86			2.89
RRu	9.30	7.95			7.07
P	+++	+++			+++

		Age started (narrow categories)						
	absent	27+k30	23-29k26	19-25k22	15-21k18	11-17k14	1-13k10	Total
N	4						1	5
NS	2						1	2
Wt	16.98						2.14	19.12
Het Chi	2.57						0.00	2.61
Het df	3						0	4
Het P	N.S.						N.S.	N.S.
Fixed RR	4.44						5.19	4.52
RRl	2.76						1.36	2.89
RRu	7.14						19.85	7.07
P	+++						+	+++
Random RR	4.44						5.19	4.52
RRl	2.76						1.36	2.89
RRu	7.14						19.85	7.07
P	+++						+	+++

Table 3H21 - 3

IESLC - Meta-analysis of Ever/current Smoking by Age started, Overview
Adenocarcinoma, Cigarettes only
Most adjusted

MALES

	Age started (broad categories) absent	19+k26	15-25k18	1-17k14	Total
N	3	2			5
NS	2	2			4
Wt	10.94	8.17			19.12
Het Chi	1.09	1.09			2.61
Het df	2	1			4
Het P	N.S.	N.S.			N.S.
Fixed RR	5.14	3.80			4.52
RRl	2.84	1.91			2.89
RRu	9.30	7.54			7.07
P	+++	+++			+++
Random RR	5.14	3.85			4.52
RRl	2.84	1.86			2.89
RRu	9.30	7.95			7.07
P	+++	+++			+++

	absent	Age started (narrow categories) 27+k30	23-29k26	19-25k22	15-21k18	11-17k14	1-13k10	Total
N	4						1	5
NS	2						1	2
Wt	16.98						2.14	19.12
Het Chi	2.57						0.00	2.61
Het df	3						0	4
Het P	N.S.						N.S.	N.S.
Fixed RR	4.44						5.19	4.52
RRl	2.76						1.36	2.89
RRu	7.14						19.85	7.07
P	+++						+	+++
Random RR	4.44						5.19	4.52
RRl	2.76						1.36	2.89
RRu	7.14						19.85	7.07
P	+++						+	+++

Table 3H21 - 4

IESLC - Meta-analysis of Ever/current Smoking by Age started, Overview
Adenocarcinoma, Cigarettes only
Least adjusted

REF	NRR	X	SEX	AGEL	AGEH	RACE	YF	LC TYPE	LOC	START	ST	NLC	R	VB	P	H	AD	SM	PRODUCT	exL	exH	S1	S2	DENOM	De
BENHAM	521		m	0	0	all	-	KII	Eu:wst	1976	CC	1625	n	bl	n	y	0	ev	cig only	20	999	1	0	nev any	st
BENHAM	522		m	0	0	all	-	KII	Eu:wst	1976	CC	1625	n	bl	n	y	0	ev	cig only	1	19	0	0	nev any	st
PEZZOT	552		m	0	0	all	-	a	SCAmer	1987	CC	215	n	bl	n	y	0	ev	cig only	19	999	1	0	nev cigs	st
PEZZOT	553		m	0	0	all	-	a	SCAmer	1987	CC	215	n	bl	n	y	0	ev	cig only	14	18	0	0	nev cigs	st
PEZZOT	554		m	0	0	all	-	a	SCAmer	1987	CC	215	n	bl	n	y	0	ev	cig only	1	13	0	6	nev cigs	st

Cigarette type is all/unspec for all RRs

In this overview table, subtotals and Qs values may be invalid and should be ignored

Table 3H21 - 5

IESLC - Meta-analysis of Ever/current Smoking by Age started, Overview
Adenocarcinoma, Cigarettes only
Least adjusted

				Number Exposed		Non-exposed			
REF	NRR	SEX	AD	Case	Cont	Case	Cont	RR	95.00%CI
BENHAM	521	m	0	41	64	9	42	2.99	(1.32- 6.79)
BENHAM	522	m	0	69	79	9	42	4.08	(1.85- 8.97)
Subtotal BENHAM								3.51	(1.99- 6.20)
PEZZOT	552	m	0	18	105	3	116	6.63	(1.90- 23.15)
PEZZOT	553	m	0	33	145	3	116	8.80	(2.63- 29.42)
PEZZOT	554	m	0	9	67	3	116	5.19	(1.36- 19.85)
Subtotal PEZZOT								6.84	(3.30- 14.18)
Totals				170	460	27	432		

*prospective study

REF	NRR	SEX	AD	Ys	Ws	Qs	Ps
BENHAM	521	m	0	1.10	5.72	0.98	0.0088
BENHAM	522	m	0	1.41	6.17	0.07	0.0005
Subtotal BENHAM				1.26	11.89	1.04	
PEZZOT	552	m	0	1.89	2.46	0.36	0.0030
PEZZOT	553	m	0	2.17	2.64	1.17	0.0004
PEZZOT	554	m	0	1.65	2.14	0.04	0.0160
Subtotal PEZZOT				1.92	7.23	1.57	

N	5
NS	2

Table 3H21 - 6

IESLC - Meta-analysis of Ever/current Smoking by Age started, Overview
Adenocarcinoma, Cigarettes only
Least adjusted

	Sex combined	male	female	Total
N		5		5
NS		2		2

In this overview table, other than the "N" rows, entries in the "absent" and "Total" columns may be invalid and should be ignored

	Age started (broad categories) absent	19+k26	15-25k18	1-17k14	Total
N	3	2			5
NS	2	2			4
Wt	10.94	8.17			19.12
Het Chi	1.09	1.09			2.61
Het df	2	1			4
Het P	N.S.	N.S.			N.S.
Fixed RR	5.14	3.80			4.52
RRl	2.84	1.91			2.89
RRu	9.30	7.54			7.07
P	+++	+++			+++
Random RR	5.14	3.85			4.52
RRl	2.84	1.86			2.89
RRu	9.30	7.95			7.07
P	+++	+++			+++

	absent	Age started (narrow categories) 27+k30	23-29k26	19-25k22	15-21k18	11-17k14	1-13k10	Total
N	4						1	5
NS	2						1	2
Wt	16.98						2.14	19.12
Het Chi	2.57						0.00	2.61
Het df	3						0	4
Het P	N.S.						N.S.	N.S.
Fixed RR	4.44						5.19	4.52
RRl	2.76						1.36	2.89
RRu	7.14						19.85	7.07
P	+++						+	+++
Random RR	4.44						5.19	4.52
RRl	2.76						1.36	2.89
RRu	7.14						19.85	7.07
P	+++						+	+++

Table 3H21 - 6

IESLC - Meta-analysis of Ever/current Smoking by Age started, Overview
Adenocarcinoma, Cigarettes only
Least adjusted

MALES

	Age started (broad categories) absent	19+k26	15-25k18	1-17k14	Total
N	3	2			5
NS	2	2			4
Wt	10.94	8.17			19.12
Het Chi	1.09	1.09			2.61
Het df	2	1			4
Het P	N.S.	N.S.			N.S.
Fixed RR	5.14	3.80			4.52
RRl	2.84	1.91			2.89
RRu	9.30	7.54			7.07
P	+++	+++			+++
Random RR	5.14	3.85			4.52
RRl	2.84	1.86			2.89
RRu	9.30	7.95			7.07
P	+++	+++			+++

	absent	Age started (narrow categories) 27+k30	23-29k26	19-25k22	15-21k18	11-17k14	1-13k10	Total
N	4						1	5
NS	2						1	2
Wt	16.98						2.14	19.12
Het Chi	2.57						0.00	2.61
Het df	3						0	4
Het P	N.S.						N.S.	N.S.
Fixed RR	4.44						5.19	4.52
RRl	2.76						1.36	2.89
RRu	7.14						19.85	7.07
P	+++						+	+++
Random RR	4.44						5.19	4.52
RRl	2.76						1.36	2.89
RRu	7.14						19.85	7.07
P	+++						+	+++

Table 3H21 - 7

IESLC - Meta-analysis of Ever/current Smoking by Age started, Overview
Adenocarcinoma, Cigarettes only
Excluded studies (and stage at which they were excluded)

1	BECHER BLOT1 BROWN3 CARPEN CHYOU DARBY DOLL2 GARCIA GRAHAM GURSEL HAMMO2 JAHN JAIN LAUSSM PRESCO QIAO TVERDA WIGLE WYNDE3
2	AKIBA AMANDU AMES AXELSS BENSHL BEST BOUCHA BOUCOT BROSS CHEN CPSII DEAN2 DESTEF DORGAN DOSEME FAN GARSHI GER GILLIS HAMMON HUMBLE JUSSAW KAISE2 KATSOU KAUFMA KOO KREUZE LEVIN MCCONN NOTAN2 OSANN2 PEZZO2 PISANI RESTRE SADOWS VUTUC WANG2 WATSON WU2 WUWILL WYNDE2 WYNDE8 XU ZHOU
3	GUO MCDUFF SPITZ STASZE ZHANG
4	AGUDO ARMADA AUVINE BOFFET BRESLO BUFFLE CEDERL CHEN2 CHIAZZ CHOI CORREA CPSI DAMBER DEAN3 DOLL DORN GAO GAO2 GENG HIRAYA HOLE HU HU2 JOLY KOULUM LETOUR LIAW LIU3 LIU4 LIU5 LUBIN LUBIN2 LUO MIGRAN MRFITR PERNU QIAO2 RACHTA SEGI2 SOBUE SPEIZE SUZUK2 TIZZAN WYNDE7 YUAN
5	ALDERS
7	BARBON ENGELA HAENSZ HEGMAN JEDRYC KHUDER MATOS SVENSS WAKAI WU WYNDE6 ZHENG

Table 3H21 - 8

Potentially overlapping studies

REF	REFGP	PRINC	OVERLAP/LINK
BENHAM	LUBIN2	2	Subset of Lubin2

Table 3H22 -

IESLC - Meta-analysis of Ever/current Smoking, Age started, "Low"
Adenocarcinoma, Cigarettes only

This analysis is restricted to results for:
1) Ever/current smokers
2) Results by Age started
3) Categorical results by Age started
4) Adenocarcinoma (or near equivalent)
5) Results complete enough for use in metaanalysis

Within each study, results are then selected (in the following order of preference, within each sex) for:
6) SMKSTA: ever, current
7) PRODUCT: cigarettes only
8) CIGTYPE: all/unspecified, MC regardless of HR, MC only
9) (not applicable)
10) DENOM: never smoked anything, never smoked cigarettes, never any + low, never cigs + low
11) Followup period (YF, prospective studies): whole study (coded as 0) or longest available
12) LCtype: adeno or nearest available, but not squamous. (q = squamous, s = small,
a = adeno, l = large, KII = Kreyberg II, al = alveolar, br = bronchiolar, u = undifferentiated)
13) Race: all or nearest available, otherwise by race (wh or w = white, bl or b = black, hi = hispanic
ch = chinese, jap = japanese, haw = hawaiian, w+o = white + oriental, sca = scandinavian, as = asian)
14) Age started "low" in key scheme 1 (key value 26, maximum range 19+)
15) For overlapping studies: principal rather than subsidiary studies
Finally by Age: whole study (coded as 0) if available, otherwise by widest available age group
and then for single sex results (m, f) in preference to results for both sexes combined (c).

Results adjusted (AD) for the most potential confounders are then chosen in Sections -1 to -3
(and those which actually differ from the adjusted results in Table 3H12 - 1 are marked 'x' in Section -1)
and results adjusted for the least confounders in Sections -4 to -6. (Those least adjusted results which
actually differ from the most adjusted are marked 'x' in column X in Section -4)

Section -7 shows excluded studies, together with the stage (as above) at which no qualifying
results were found.

Section -8 lists the potentially overlapping studies which have been included (1=principal, 2=subsidiary).

Section -9 lists any results which would have been included in preference except that they had data not complete
enough for use in meta-analysis, with their significance (yes/no), if known, and any further comment as entered
on the database. It also lists as "gap" any categories for which no data were presented by the original authors.

In addition to those mentioned above, the following fields, levels and abbreviations are used:
* or nk = not known, n = no, y = yes, ot = other
ev = ever, cu = current, nev = never
all/unspec = all or unspecified, MC = manufactured cigarettes, HR = hand-rolled cigarettes
exL, exH = range of exposure (low and high) in the smoking group, in terms of Age started
REF: 6-character study reference
NRR: number of the RR on the database within the study
ST : study type (CC = case control, pr or prosp = prospective)
NLC: number of lung cancer cases in whole study
R : risky occupational population (n = no, m = mining, o = other risky)
VB : national cigarette type (V = at least 75% Virginia, bl = at least 75% blended, ot = other)
P : any proxy use
H : full histological confirmation
De : derivation of RR/CI (or = original, st = standard method, ot = other method of estimation)

Table 3H22 - 1

IESLC - Meta-analysis of Ever/current Smoking, Age started, "Low"
Adenocarcinoma, Cigarettes only
Most adjusted

REF	NRR	XX12	SEX	AGEL	AGEH	RACE	YF	LC TYPE	LOC	START	ST	NLC	R	VB	P	H	AD	SM	PRODUCT	exL	exH	DENOM	De
BENHAM	521		m	0	0	all	-	KII	Eu:wst	1976	CC	1625	n	bl	n	y	0	ev	cig only	20	999	nev any	st
PEZZOT	552		m	0	0	all	-	a	SCAmer	1987	CC	215	n	bl	n	y	0	ev	cig only	19	999	nev cigs	st

Cigarette type is all/unspec for all RRs

Table 3H22 - 2

IESLC - Meta-analysis of Ever/current Smoking, Age started, "Low"
Adenocarcinoma, Cigarettes only
Most adjusted

REF	NRR	SEX	AD	Number Case	Exposed Cont	Non-exposed Case	Cont	RR	95.00%CI
BENHAM	521	m	0	41	64	9	42	2.99	(1.32- 6.79)
PEZZOT	552	m	0	18	105	3	116	6.63	(1.90- 23.15)
Totals				59	169	12	158		

*prospective study

REF	NRR	SEX	AD	Ys	Ws	Qs	Ps
BENHAM	521	m	0	1.10	5.72	0.33	0.0088
PEZZOT	552	m	0	1.89	2.46	0.76	0.0030

	N	2
	NS	2
	Wt	8.17
Het	Chi	1.09
Het	df	1
Het	P	N.S.
Fixed	RR	3.80
	RRl	1.91
	RRu	7.54
	P	+++
Random	RR	3.85
	RRl	1.86
	RRu	7.95
	P	+++
Asymm	P	

Table 3H22 - 3

IESLC - Meta-analysis of Ever/current Smoking, Age started, "Low"
Adenocarcinoma, Cigarettes only
Most adjusted

	Sex: combined	male	female	Total
N		2		2
NS		2		2
Wt		8.17		8.17
Het Chi		1.09		1.09
Het df		1		1
Het P		N.S.		N.S.
Fixed RR		3.80		3.80
RRl		1.91		1.91
RRu		7.54		7.54
P		+++		+++
Random RR		3.85		3.85
RRl		1.86		1.86
RRu		7.95		7.95
P		+++		+++
Between Chi				
Between df				
Between P				N.S.
Btwn(F) P				N.S.
Btwn(R) P				N.S.

Too few RRs for analysis by factor

Table 3H22 - 4

IESLC - Meta-analysis of Ever/current Smoking, Age started, "Low"
Adenocarcinoma, Cigarettes only
Least adjusted

REF	NRR	X	SEX	AGEL	AGEH	RACE	YF	LC TYPE	LOC	START	ST	NLC	R	VB	P	H	AD	SM	PRODUCT	exL	exH	DENOM	De
BENHAM	521		m	0	0	all	-	KII	Eu:wst	1976	CC	1625	n	bl	n	y	0	ev	cig only	20	999	nev any	st
PEZZOT	552		m	0	0	all	-	a	SCAmer	1987	CC	215	n	bl	n	y	0	ev	cig only	19	999	nev cigs	st

Cigarette type is all/unspec for all RRs

Table 3H22 - 5

IESLC - Meta-analysis of Ever/current Smoking, Age started, "Low"
Adenocarcinoma, Cigarettes only
Least adjusted

REF	NRR	SEX	AD	Number Exposed Case	Cont	Non-exposed Case	Cont	RR	95.00%CI
BENHAM	521	m	0	41	64	9	42	2.99	(1.32- 6.79)
PEZZOT	552	m	0	18	105	3	116	6.63	(1.90- 23.15)
Totals				59	169	12	158		

*prospective study

REF	NRR	SEX	AD	Ys	Ws	Qs	Ps
BENHAM	521	m	0	1.10	5.72	0.33	0.0088
PEZZOT	552	m	0	1.89	2.46	0.76	0.0030

	N	2
	NS	2
	Wt	8.17
Het	Chi	1.09
Het	df	1
Het	P	N.S.
Fixed	RR	3.80
	RRl	1.91
	RRu	7.54
	P	+++
Random	RR	3.85
	RRl	1.86
	RRu	7.95
	P	+++
Asymm	P	

Table 3H22 - 6

IESLC - Meta-analysis of Ever/current Smoking, Age started, "Low"
Adenocarcinoma, Cigarettes only
Least adjusted

	Sex combined	male	female	Total
N		2		2
NS		2		2
Wt		8.17		8.17
Het Chi		1.09		1.09
Het df		1		1
Het P		N.S.		N.S.
Fixed RR		3.80		3.80
RRl		1.91		1.91
RRu		7.54		7.54
P		+++		+++
Random RR		3.85		3.85
RRl		1.86		1.86
RRu		7.95		7.95
P		+++		+++
Between Chi				
Between df				
Between P				N.S.
Btwn(F) P				N.S.
Btwn(R) P				N.S.

Table 3H22 - 7

IESLC - Meta-analysis of Ever/current Smoking, Age started, "Low"
Adenocarcinoma, Cigarettes only
Excluded studies (and stage at which they were excluded)

Stage	Studies
1	BECHER BLOT1 BROWN3 CARPEN CHYOU DARBY DOLL2 GARCIA GRAHAM GURSEL HAMMO2 JAHN JAIN LAUSSM PRESCO QIAO TVERDA WIGLE WYNDE3
2	AKIBA AMANDU AMES AXELSS BENSHL BEST BOUCHA BOUCOT BROSS CHEN CPSII DEAN2 DESTEF DORGAN DOSEME FAN GARSHI GER GILLIS HAMMON HUMBLE JUSSAW KAISE2 KATSOU KAUFMA KOO KREUZE LEVIN MCCONN NOTAN2 OSANN2 PEZZO2 PISANI RESTRE SADOWS VUTUC WANG2 WATSON WU2 WUWILL WYNDE2 WYNDE8 XU ZHOU
3	GUO MCDUFF SPITZ STASZE ZHANG
4	AGUDO ARMADA AUVINE BOFFET BRESLO BUFFLE CEDERL CHEN2 CHIAZZ CHOI CORREA CPSI DAMBER DEAN3 DOLL DORN GAO GAO2 GENG HIRAYA HOLE HU HU2 JOLY KOULUM LETOUR LIAW LIU3 LIU4 LIU5 LUBIN LUBIN2 LUO MIGRAN MRFITR PERNU QIAO2 RACHTA SEGI2 SOBUE SPEIZE SUZUK2 TIZZAN WYNDE7 YUAN
5	ALDERS
7	BARBON ENGELA HAENSZ HEGMAN JEDRYC KHUDER MATOS SVENSS WAKAI WU WYNDE6 ZHENG

Table 3H22 - 8

Potentially overlapping studies

REF	REFGP	PRINC	.	OVERLAP/LINK
BENHAM	LUBIN2	2		Subset of Lubin2

Table 3H23 -

IESLC - Meta-analysis of Ever/current Smoking, Age started, "Mid"
Adenocarcinoma, Cigarettes only

This analysis is restricted to results for:
1) Ever/current smokers
2) Results by Age started
3) Categorical results by Age started
4) Adenocarcinoma (or near equivalent)
5) Results complete enough for use in metaanalysis

Within each study, results are then selected (in the following order of preference, within each sex) for:
6) SMKSTA: ever, current
7) PRODUCT: cigarettes only
8) CIGTYPE: all/unspecified, MC regardless of HR, MC only
9) (not applicable)
10) DENOM: never smoked anything, never smoked cigarettes, never any + low, never cigs + low
11) Followup period (YF, prospective studies): whole study (coded as 0) or longest available
12) LCtype: adeno or nearest available, but not squamous. (q = squamous, s = small,
a = adeno, l = large, KII = Kreyberg II, al = alveolar, br = bronchiolar, u = undifferentiated)
13) Race: all or nearest available, otherwise by race (wh or w = white, bl or b = black, hi = hispanic
ch = chinese, jap = japanese, haw = hawaiian, w+o = white + oriental, sca = scandinavian, as = asian)
14) Age started "mid" in key scheme 1 (key value 18, maximum range 15-25)
15) For overlapping studies: principal rather than subsidiary studies
Finally by Age: whole study (coded as 0) if available, otherwise by widest available age group
and then for single sex results (m, f) in preference to results for both sexes combined (c).

Results adjusted (AD) for the most potential confounders are then chosen in Sections -1 to -3
(and those which actually differ from the adjusted results in Table 3H13 - 1 are marked 'x' in Section -1)
and results adjusted for the least confounders in Sections -4 to -6. (Those least adjusted results which
actually differ from the most adjusted are marked 'x' in column X in Section -4)

Section -7 shows excluded studies, together with the stage (as above) at which no qualifying
results were found.

Section -8 lists the potentially overlapping studies which have been included (1=principal, 2=subsidiary).

Section -9 lists any results which would have been included in preference except that they had data not complete
enough for use in meta-analysis, with their significance (yes/no), if known, and any further comment as entered
on the database. It also lists as "gap" any categories for which no data were presented by the original authors.

In addition to those mentioned above, the following fields, levels and abbreviations are used:
* or nk = not known, n = no, y = yes, ot = other
ev = ever, cu = current, nev = never
all/unspec = all or unspecified, MC = manufactured cigarettes, HR = hand-rolled cigarettes
exL, exH = range of exposure (low and high) in the smoking group, in terms of Age started
REF: 6-character study reference
NRR: number of the RR on the database within the study
ST : study type (CC = case control, pr or prosp = prospective)
NLC: number of lung cancer cases in whole study
R : risky occupational population (n = no, m = mining, o = other risky)
VB : national cigarette type (V = at least 75% Virginia, bl = at least 75% blended, ot = other)
P : any proxy use
H : full histological confirmation
De : derivation of RR/CI (or = original, st = standard method, ot = other method of estimation)

Table 3H23 - 0

No RRs selected for this analysis

Table 3H23 - 7

IESLC - Meta-analysis of Ever/current Smoking, Age started, "Mid"
Adenocarcinoma, Cigarettes only
Excluded studies (and stage at which they were excluded)

1	BECHER BLOT1 BROWN3 CARPEN CHYOU DARBY DOLL2 GARCIA GRAHAM GURSEL HAMMO2 JAHN JAIN LAUSSM PRESCO QIAO TVERDA WIGLE WYNDE3
2	AKIBA AMANDU AMES AXELSS BENSHL BEST BOUCHA BOUCOT BROSS CHEN CPSII DEAN2 DESTEF DORGAN DOSEME FAN GARSHI GER GILLIS HAMMON HUMBLE JUSSAW KAISE2 KATSOU KAUFMA KOO KREUZE LEVIN MCCONN NOTAN2 OSANN2 PEZZO2 PISANI RESTRE SADOWS VUTUC WANG2 WATSON WU2 WUWILL WYNDE2 WYNDE8 XU ZHOU
3	GUO MCDUFF SPITZ STASZE ZHANG
4	AGUDO ARMADA AUVINE BOFFET BRESLO BUFFLE CEDERL CHEN2 CHIAZZ CHOI CORREA CPSI DAMBER DEAN3 DOLL DORN GAO GAO2 GENG HIRAYA HOLE HU HU2 JOLY KOULUM LETOUR LIAW LIU3 LIU4 LIU5 LUBIN LUBIN2 LUO MIGRAN MRFITR PERNU QIAO2 RACHTA SEGI2 SOBUE SPEIZE SUZUK2 TIZZAN WYNDE7 YUAN
5	ALDERS
7	BARBON ENGELA HAENSZ HEGMAN JEDRYC KHUDER MATOS SVENSS WAKAI WU WYNDE6 ZHENG
14	BENHAM PEZZOT

Table 3H24 -

IESLC - Meta-analysis of Ever/current Smoking, Age started, "High"
Adenocarcinoma, Cigarettes only

This analysis is restricted to results for:
1) Ever/current smokers
2) Results by Age started
3) Categorical results by Age started
4) Adenocarcinoma (or near equivalent)
5) Results complete enough for use in metaanalysis

Within each study, results are then selected (in the following order of preference, within each sex) for:
6) PRODUCT: cigarettes only
7) CIGTYPE: all/unspecified, MC regardless of HR, MC only
8) (not applicable)
9) DENOM: never smoked anything, never smoked cigarettes, never any + low, never cigs + low
10) Followup period (YF, prospective studies): whole study (coded as 0) or longest available
11) LCtype: adeno or nearest available, but not squamous. (q = squamous, s = small,
a = adeno, l = large, KII = Kreyberg II, al = alveolar, br = bronchiolar, u = undifferentiated)
12) Race: all or nearest available, otherwise by race (wh or w = white, bl or b = black, hi = hispanic
ch = chinese, jap = japanese, haw = hawaiian, w+o = white + oriental, sca = scandinavian, as = asian)
13) Age started "high" in key scheme 1 (key value 14, maximum range 1-17)
14) For overlapping studies: principal rather than subsidiary studies
Finally by Age: whole study (coded as 0) if available, otherwise by widest available age group
and then for single sex results (m, f) in preference to results for both sexes combined (c).

Results adjusted (AD) for the most potential confounders are then chosen in Sections -1 to -3
(and those which actually differ from the adjusted results in Table 3H14 - 1 are marked 'x' in Section -1)
and results adjusted for the least confounders in Sections -4 to -6. (Those least adjusted results which
actually differ from the most adjusted are marked 'x' in column X in Section -4)

Section -7 shows excluded studies, together with the stage (as above) at which no qualifying
results were found.

Section -8 lists the potentially overlapping studies which have been included (1=principal, 2=subsidiary).

Section -9 lists any results which would have been included in preference except that they had data not complete
enough for use in meta-analysis, with their significance (yes/no), if known, and any further comment as entered
on the database. It also lists as "gap" any categories for which no data were presented by the original authors.

In addition to those mentioned above, the following fields, levels and abbreviations are used:
* or nk = not known, n = no, y = yes, ot = other
ev = ever, cu = current, nev = never
all/unspec = all or unspecified, MC = manufactured cigarettes, HR = hand-rolled cigarettes
exL, exH = range of exposure (low and high) in the smoking group, in terms of Age started
REF: 6-character study reference
NRR: number of the RR on the database within the study
ST : study type (CC = case control, pr or prosp = prospective)
NLC: number of lung cancer cases in whole study
R : risky occupational population (n = no, m = mining, o = other risky)
VB : national cigarette type (V = at least 75% Virginia, bl = at least 75% blended, ot = other)
P : any proxy use
H : full histological confirmation
De : derivation of RR/CI (or = original, st = standard method, ot = other method of estimation)

Table 3H24 - 0

No RRs selected for this analysis

Table 3H24 - 7

IESLC - Meta-analysis of Ever/current Smoking, Age started, "High"
Adenocarcinoma, Cigarettes only
Excluded studies (and stage at which they were excluded)

1 BECHER BLOT1 BROWN3 CARPEN CHYOU DARBY DOLL2 GARCIA GRAHAM GURSEL HAMMO2 JAHN JAIN LAUSSM PRESCO QIAO TVERDA WIGLE WYNDE3

2 AKIBA AMANDU AMES AXELSS BENSHL BEST BOUCHA BOUCOT BROSS CHEN CPSII DEAN2 DESTEF DORGAN DOSEME FAN GARSHI GER GILLIS HAMMON HUMBLE JUSSAW KAISE2 KATSOU KAUFMA KOO KREUZE LEVIN MCCONN NOTAN2 OSANN2 PEZZO2 PISANI RESTRE SADOWS VUTUC WANG2 WATSON WU2 WUWILL WYNDE2 WYNDE8 XU ZHOU

3 GUO MCDUFF SPITZ STASZE ZHANG

4 AGUDO ARMADA AUVINE BOFFET BRESLO BUFFLE CEDERL CHEN2 CHIAZZ CHOI CORREA CPSI DAMBER DEAN3 DOLL DORN GAO GAO2 GENG HIRAYA HOLE HU HU2 JOLY KOULUM LETOUR LIAW LIU3 LIU4 LIU5 LUBIN LUBIN2 LUO MIGRAN MRFITR PERNU QIAO2 RACHTA SEGI2 SOBUE SPEIZE SUZUK2 TIZZAN WYNDE7 YUAN

5 ALDERS

7 BARBON ENGELA HAENSZ HEGMAN JEDRYC KHUDER MATOS SVENSS WAKAI WU WYNDE6 ZHENG

14 BENHAM PEZZOT

Table 3H25 -

IESLC - Meta-analysis of Ever/current Smoking, Age started, "Highest vs lowest"
Adenocarcinoma, Cigarettes only

This analysis is restricted to results for:
1) Ever/current smokers
2) Results by Age started
3) Categorical results by Age started
4) Denominator (unexposed) = "low"
5) Adenocarcinoma (or near equivalent)
6) Results complete enough for use in metaanalysis

Within each study, results are then selected (in the following order of preference, within each sex) for:
7) SMKSTA: ever, current
8) PRODUCT: cigarettes only
9) CIGTYPE: all/unspecified, MC regardless of HR, MC only
10) Results with least adjustment for other aspects of smoking (ADOS)
11) The highest vs lowest category
12) Followup period (YF, prospective studies): whole study (coded as 0) or longest available
13) LCtype: adeno or nearest available, but not squamous. (q = squamous, s = small,
a = adeno, l = large, KII = Kreyberg II, al = alveolar, br = bronchiolar, u = undifferentiated)
14) Race: all or nearest available, otherwise by race (wh or w = white, bl or b = black, hi = hispanic
ch = chinese, jap = japanese, haw = hawaiian, w+o = white + oriental, sca = scandinavian, as = asian)
15) For overlapping studies: principal rather than subsidiary studies
Finally by Age: whole study (coded as 0) if available, otherwise by widest available age group
and then for single sex results (m, f) in preference to results for both sexes combined (c).

Results adjusted (AD) for the most potential confounders are then chosen in Sections -1 to -3
(and those which actually differ from the adjusted results in Table 3H15 - 1 are marked 'x' in Section -1)
and results adjusted for the least confounders in Sections -4 to -6. (Those least adjusted results which
actually differ from the most adjusted are marked 'x' in column X in Section -4)

Section -7 shows excluded studies, together with the stage (as above) at which no qualifying
results were found.

Section -8 lists the potentially overlapping studies which have been included (1=principal, 2=subsidiary).

Section -9 lists any results which would have been included in preference except that they had data not complete
enough for use in meta-analysis, with their significance (yes/no), if known, and any further comment as entered
on the database. It also lists as "gap" any categories for which no data were presented by the original authors.

In addition to those mentioned above, the following fields, levels and abbreviations are used:
* or nk = not known, n = no, y = yes, ot = other
all/unspec = all or unspecified, MC = manufactured cigarettes, HR = hand-rolled cigarettes
exL, exH = range of exposure (low and high) in the "highest" group, in terms of Age started
unexL, unexH = range of exposure (low and high) in the "lowest" group, in terms of Age started
REF: 6-character study reference
NRR: number of the RR on the database within the study
ST : study type (CC = case control, pr or prosp = prospective)
NLC: number of lung cancer cases in whole study
R : risky occupational population (n = no, m = mining, o = other risky)
VB : national cigarette type (V = at least 75% Virginia, bl = at least 75% blended, ot = other)
P : any proxy use
H : full histological confirmation
De : derivation of RR/CI (or = original, st = standard method, ot = other method of estimation)

Table 3H25 - 1

IESLC - Meta-analysis of Ever/current Smoking, Age started, "Highest vs lowest"
Adenocarcinoma, Cigarettes only
Most adjusted

REF	NRR	XX15	SEX	AGEL	AGEH	RACE	YF	LC TYPE	LOC	START	ST	NLC	R	VB	P	H	AD	ADOS	SM	PRODUCT	exL	exH	unexL	unexH	De
BENHAM	523		m	0	0	all	-	KII	Eu:wst	1976	CC	1625	n	bl	n	y	0	0	ev	cig only	1	19	20	999	st
PEZZOT	558		m	0	0	all	-	a	SCAmer	1987	CC	215	n	bl	n	y	2	0	ev	cig only	1	13	19	999	ot

Cigarette type is all/unspec for all RRs

Table 3H25 - 2

IESLC - Meta-analysis of Ever/current Smoking, Age started, "Highest vs lowest"
Adenocarcinoma, Cigarettes only
Most adjusted

REF	NRR	SEX	AD	Number Case	Exposed Cont	Non-exposed Case	Cont	RR	95.00%CI
BENHAM	523	m	0	69	79	41	64	1.36	(0.82- 2.27)
PEZZOT	558	m	2	9	-	18	-	1.11	(0.41- 3.04)
Partial Totals				78	79	59	64		

*prospective study

REF	NRR	SEX	AD	Ys	Ws	Qs	Ps
BENHAM	523	m	0	0.31	14.89	0.03	0.2317
PEZZOT	558	m	2	0.10	3.83	0.10	0.8382

	N	2
	NS	2
	Wt	18.72
Het	Chi	0.13
Het	df	1
Het	P	N.S.
Fixed	RR	1.31
	RRl	0.83
	RRu	2.06
	P	N.S.
Random	RR	1.31
	RRl	0.83
	RRu	2.06
	P	N.S.
Asymm	P	

Table 3H25 - 3

IESLC - Meta-analysis of Ever/current Smoking, Age started, "Highest vs lowest"
Adenocarcinoma, Cigarettes only
Most adjusted

	Sex: combined	Sex: male	Sex: female	Total
N		2		2
NS		2		2
Wt		18.72		18.72
Het Chi		0.13		0.13
Het df		1		1
Het P		N.S.		N.S.
Fixed RR		1.31		1.31
RRl		0.83		0.83
RRu		2.06		2.06
P		N.S.		N.S.
Random RR		1.31		1.31
RRl		0.83		0.83
RRu		2.06		2.06
P		N.S.		N.S.
Between Chi				
Between df				
Between P				N.S.
Btwn(F) P				N.S.
Btwn(R) P				N.S.

Too few RRs for analysis by factor

Table 3H25 - 4

IESLC - Meta-analysis of Ever/current Smoking, Age started, "Highest vs lowest"
Adenocarcinoma, Cigarettes only
Least adjusted

REF	NRR	X	SEX	AGEL	AGEH	RACE	YF	LC TYPE	LOC	START	ST	NLC	R	VB	P	H	AD	ADOS	SM	PRODUCT	exL	exH	unexL	unexH	De
BENHAM	523		m	0	0	all	-	KII	Eu:wst	1976	CC	1625	n	bl	n	y	0	0	ev	cig only	1	19	20	999	st
PEZZOT	556	x	m	0	0	all	-	a	SCAmer	1987	CC	215	n	bl	n	y	0	0	ev	cig only	1	13	19	999	st

Cigarette type is all/unspec for all RRs

Table 3H25 - 5

IESLC - Meta-analysis of Ever/current Smoking, Age started, "Highest vs lowest"
Adenocarcinoma, Cigarettes only
Least adjusted

REF	NRR	SEX	AD	Number Exposed Case	Cont	Non-exposed Case	Cont	RR	95.00%CI
BENHAM	523	m	0	69	79	41	64	1.36	(0.82- 2.27)
PEZZOT	556	m	0	9	15	18	32	1.07	(0.39- 2.92)
Totals				78	94	59	96		

*prospective study

REF	NRR	SEX	AD	Ys	Ws	Qs	Ps
BENHAM	523	m	0	0.31	14.89	0.04	0.2317
PEZZOT	556	m	0	0.06	3.78	0.14	0.9002

	N	2
	NS	2
	Wt	18.67
Het	Chi	0.18
Het	df	1
Het	P	N.S.
Fixed	RR	1.30
	RRl	0.82
	RRu	2.04
	P	N.S.
Random	RR	1.30
	RRl	0.82
	RRu	2.04
	P	N.S.
Asymm	P	

Table 3H25 - 6

IESLC - Meta-analysis of Ever/current Smoking, Age started, "Highest vs lowest"
Adenocarcinoma, Cigarettes only
Least adjusted

		Sex		
	combined	male	female	Total
N		2		2
NS		2		2
Wt		18.67		18.67
Het Chi		0.18		0.18
Het df		1		1
Het P		N.S.		N.S.
Fixed RR		1.30		1.30
RRl		0.82		0.82
RRu		2.04		2.04
P		N.S.		N.S.
Random RR		1.30		1.30
RRl		0.82		0.82
RRu		2.04		2.04
P		N.S.		N.S.
Between Chi				
Between df				
Between P				N.S.
Btwn(F) P				N.S.
Btwn(R) P				N.S.

Table 3H25 - 7

IESLC - Meta-analysis of Ever/current Smoking, Age started, "Highest vs lowest"
Adenocarcinoma, Cigarettes only
Excluded studies (and stage at which they were excluded)

1	BECHER BLOT1 BROWN3 CARPEN CHYOU DARBY DOLL2 GARCIA GRAHAM GURSEL HAMMO2 JAHN JAIN LAUSSM PRESCO QIAO TVERDA WIGLE WYNDE3
2	AKIBA AMANDU AMES AXELSS BENSHL BEST BOUCHA BOUCOT BROSS CHEN CPSII DEAN2 DESTEF DORGAN DOSEME FAN GARSHI GER GILLIS HAMMON HUMBLE JUSSAW KAISE2 KATSOU KAUFMA KOO KREUZE LEVIN MCCONN NOTAN2 OSANN2 PEZZO2 PISANI RESTRE SADOWS VUTUC WANG2 WATSON WU2 WUWILL WYNDE2 WYNDE8 XU ZHOU
3	GUO MCDUFF SPITZ STASZE ZHANG
5	AGUDO ARMADA AUVINE BOFFET BRESLO BUFFLE CEDERL CHEN2 CHIAZZ CHOI CORREA CPSI DAMBER DEAN3 DOLL DORN GAO GAO2 GENG HIRAYA HOLE HU HU2 JOLY KOULUM LETOUR LIAW LIU3 LIU4 LIU5 LUBIN LUBIN2 LUO MIGRAN MRFITR PERNU QIAO2 RACHTA SEGI2 SOBUE SPEIZE SUZUK2 TIZZAN WYNDE7 YUAN
6	ALDERS
8	BARBON ENGELA HAENSZ HEGMAN JEDRYC KHUDER MATOS SVENSS WAKAI WU WYNDE6 ZHENG

Table 3H25 - 8

Potentially overlapping studies

REF	REFGP	PRINC	.	OVERLAP/LINK
BENHAM	LUBIN2	2		Subset of Lubin2

Table 3H25 - 9

Most adjusted - insufficient data for meta-analysis

REF	NRR	SEX	AGEL	AGEH	RACE	YF	LC TYPE	LOC	START	ST	NLC	R	VB	P	H	AD	ADOS	SM	PRODUCT	exL	exH	unexL	unexH	De
ALDERS	563	m	0	0	all	-	not q+s	Eu:UK	1977	CC	1448	n		V	n	n	2	1#ev	cig only	1	14	25	999	ot
ALDERS	566	f	0	0	all	-	not q+s	Eu:UK	1977	CC	1448	n		V	n	n	2	1#ev	cig only	1	14	25	999	ot

Comments on values in listings

ALDERS ADOS Number of cigs/day
ALDERS ADOS Number of cigs/day

REF	NRR	RR	SIG	RRDATA comment
ALDERS	563	0.53	n	0
ALDERS	566	1.92	n	0
