# Supplementary material for: Systematic review with meta-analysis of the epidemiological evidence in the 1900s relating smoking to lung cancer
Source: BMC Cancer. 2012 Sep 3;12:385. doi: 10.1186/1471-2407-12-385 (PMC3505152; doi:10.1186/1471-2407-12-385)
Supplement: Additional file 5 — Detailed Analysis Tables (Individual file names as described in Additional file 1: Methods, Table1). [file 1471-2407-12-385-S5.zip › PDF/3B.pdf]

Table 3B1 -

IESLC - Meta-analysis of Current Smoking (vs never smoking), Any product (or Cigarettes if Any not available)
Adenocarcinoma

This analysis is restricted to results for:
1) Non-dose-response data
2) Current smokers
3) Results complete enough for use in metaanalysis

Within each study, results are then selected (in the following order of preference, within each sex) for:
4) PRODUCT: all/unspec, cigarettes regardless of other products, cigarettes only
5) CIGTYPE: all/unspecified, MC regardless of HR, MC only
6) DENOM: never smoked anything, never smoked cigarettes, (never +1 = +long term ex, +2 = +amount unknown,
+3 = never cigs+long term ex)
7) Followup period (YF, prospective studies): whole study (coded as 0) or longest available
8) LCtype: adeno or nearest available, but not squamous. (q = squamous, s = small,
a = adeno, l = large, KII = Kreyberg II, al = alveolar, br = bronchiolar, u = undifferentiated)
9) Race: all or nearest available, otherwise by race (wh or w = white, bl or b = black, hi = hispanic
ch = chinese, jap = japanese, haw = hawaiian, w+o = white + oriental, sca = scandinavian, as = asian)
10) For overlapping studies: principal rather than subsidiary studies
Finally by Age: whole study (coded as 0) if available, otherwise by widest available age group
and then for single sex results (m, f) in preference to combined sex results (c).

Results adjusted (AD) for the most potential confounders are then chosen in Sections -1 to -3
and results adjusted for the least confounders in Sections -4 to -6. (Those least adjusted results which
actually differ from the most adjusted as marked 'x' in column X in Section -4)
(Results adjusted for an unknown number of confounder(s) are coded as 20.)

Section -7 shows excluded studies, together with the stage (as above) at which no qualifying
results were found.

Section -8 lists the potentially overlapping studies which have been included (1=principal, 2=subsidiary).

Section -9 lists any results which would have been included in preference except that they had data not complete
enough for use in meta-analysis, with their significance (yes/no), if known, and any further comment as entered
on the database.

In addition to those mentioned above, the following fields, levels and abbreviations are used:
* or nk = not known, n = no, y = yes, ot = other
nev = never
all/unspec = all or unspecified, cig+/-ot = cigarettes irrespective of other products (cigar, pipe etc)
MC = manufactured cigarettes, HR = hand-rolled cigarettes
REF: 6-character study reference
NRR: number of the RR on the database within the study
ST : study type (CC = case control, pr or prosp = prospective)
NLC: number of lung cancer cases in whole study
R : risky occupational population (n = no, m = mining, o = other risky)
VB : national cigarette type (V = at least 75% Virginia, bl = at least 75% blended, ot = other)
P : any proxy use
H : full histological confirmation
De : derivation of RR/CI (or = original, st = standard method, ot = other method of estimation)

Table 3B1 - 1

IESLC - Meta-analysis of Current Smoking (vs never smoking), Any product (or Cigarettes if Any not available)
Adenocarcinoma
Most adjusted

REF	NRR	SEX	AGEL	AGEH	RACE	YF	LC TYPE	LOC	START	ST	NLC	R	VB	P	H	AD	PRODUCT	DENOM	De
BARBON	100	m	0	0	all	-	a	Eu:wst	1979	CC	755	n	bl	y	y	1	all/unsp	nev any	or
BOUCOT	147	m	0	0	all	0	a	NAmer	1951	pr	121	n	bl	n	n	2	cig only	nev any	ot
BROWN2	14	m	0	0	wh	-	a	NAmer	1984	CC	14596	n	bl	n	y	2	cig+/-ot	nev cigs	or
BROWN2	13	f	0	0	wh	-	a	NAmer	1984	CC	14596	n	bl	n	y	2	cig+/-ot	nev cigs	or
BUFFLE	71	f	0	0	w-hi	-	a	NAmer	1976	CC	943	n	bl	y	n	0	cig+/-ot	nev cigs	st
COMSTO	24	m	0	0	all	-	a	NAmer	1975	ot	258	n	bl	n	n	0	cig+/-ot	nev cigs	st
COMSTO	31	f	0	0	all	-	a	NAmer	1975	ot	258	n	bl	n	n	0	cig+/-ot	nev cigs	st
CORREA	44	c	0	0	all	-	a	NAmer	1979	CC	1359	n	bl	y	n	1	cig+/-ot	nev cigs	or
CPSI	404	m	0	0	all	2	a	NAmer	1959	pr	5138	n	bl	n	n	1	cig only	nev any	ot
CPSI	406	f	0	0	all	2	a	NAmer	1959	pr	5138	n	bl	n	n	1	cig only	nev any	ot
CPSII	115	m	0	0	all	2	a	NAmer	1982	pr	3229	n	bl	n	n	1	cig only	nev any	st
CPSII	118	f	0	0	all	2	a	NAmer	1982	pr	3229	n	bl	n	n	1	cig+/-ot	nev cigs	st
DORN	340	m	0	0	wh	8	a	NAmer	1954	pr	5097	n	bl	n	n	1	cig only	nev any	ot
ENGELA	70	m	0	0	all	0	a	Eu:Sca	1964	pr	435	n	bl	n	n	7	cig+/-ot	nev cigs	ot
HAENSZ	37	f	0	0	all	-	a	NAmer	1955	CC	158	n	bl	n	y	0	cig+/-ot	nev any	st
JAHN	8	m	0	0	all	-	a	Eu:Ger	1988	CC	1004	n	bl	n	n	0	cig+/-ot	nev any	st
JAIN	17	m	0	0	all	-	a	NAmer	1981	CC	845	n	V	y	n	0	cig+/-ot	nev cigs	st
JAIN	12	f	0	0	all	-	a	NAmer	1981	CC	845	n	V	y	n	0	cig+/-ot	nev cigs	st
JEDRYC	26	m	0	0	all	-	a	Eu:est	1980	CC	1630	n	bl	y	n	0	cig+/-ot	nev any	st
KATSOU	12	f	0	0	all	-	a	Eu:bal	1987	CC	101	n	bl	n	n	1	all/unsp	nev any	or
KHUDER	17	m	0	0	all	-	a	NAmer	1985	CC	482	n	bl	n	y	0	cig+/-ot	nev cigs	or
KIHARA	5	c	0	0	jap	-	a	As:Jap	1991	CC	440	n	bl	n	n	0	all/unsp	nev any	st
LUBIN2	252	m	0	0	all	-	a	Eu:mul	1976	CC	7804	n	bl	n	y	0	cig+/-ot	nev any	st
LUBIN2	264	f	0	0	all	-	a	Eu:mul	1976	CC	7804	n	bl	n	y	0	cig+/-ot	nev any	st
MATOS	53	m	0	0	all	-	a	SCAmer	1994	CC	200	n	bl	n	n	2	cig+/-ot	nev any	or
OSANN	39	m	0	0	all	-	a	NAmer	1984	CC	1986	n	bl	n	n	2	cig+/-ot	nev cigs	or
OSANN	40	f	0	0	all	-	a	NAmer	1984	CC	1986	n	bl	n	n	2	cig+/-ot	nev cigs	or
OSANN2	32	f	0	0	all	-	KII	NAmer	1964	ot	217	n	bl	n	y	1	cig+/-ot	nev cigs	or
SOBUE	36	m	0	0	all	-	a	As:Jap	1986	CC	1376	n	bl	n	y	1	cig+/-ot	nev cigs	or
SOBUE	46	f	0	0	all	-	a	As:Jap	1986	CC	1376	n	bl	n	y	1	cig+/-ot	nev cigs	or
SOBUE2	2	m	0	0	all	-	a	As:Jap	1965	CC	2083	n	bl	n	n	2	cig+/-ot	nev any	or
SOBUE2	6	f	0	0	all	-	a	As:Jap	1965	CC	2083	n	bl	n	n	2	cig+/-ot	nev any	or
SUZUKI	10	m	0	0	all	-	a	As:Jap	1978	CC	238	n	bl	n	y	2	cig+/-ot	nev any	or
SUZUKI	14	f	0	0	all	-	a	As:Jap	1978	CC	238	n	bl	n	y	2	cig+/-ot	nev any	or
SVENSS	99	f	0	0	all	-	a	Eu:Sca	1983	CC	210	n	bl	n	n	1	all/unsp	nev any	ot
TSUGAN	3	m	0	0	all	-	a	As:Jap	1976	CC	134	n	bl	n	y	0	all/unsp	nev any	st
TSUGAN	9	f	0	0	all	-	a	As:Jap	1976	CC	134	n	bl	n	y	0	all/unsp	nev any	or
WAKAI	12	m	0	0	all	-	a	As:Jap	1988	CC	333	n	bl	n	y	1	all/unsp	nev any	or
WAKAI	30	f	0	0	all	-	a	As:Jap	1988	CC	333	n	bl	n	y	1	all/unsp	nev any	or
WU	7	f	0	0	wh	-	a	NAmer	1981	CC	220	n	bl	n	y	2	all/unsp	nev any	or
WU2	1	f	0	0	all	-	a	NAmer	1983	CC	336	n	bl	n	y	2	all/unsp	nev any	or
WYNDE3	30	m	0	0	all	-	KII	NAmer	1966	CC	350	n	bl	n	y	0	all/unsp	nev any	st
WYNDE6	15	m	0	0	all	-	KII	NAmer	1969	CC	4423	n	bl	n	y	0	cig+/-ot	nev any	st
WYNDE6	204	f	0	0	all	-	KII	NAmer	1969	CC	4423	n	bl	n	y	0	cig+/-ot	nev cigs	st

Cigarette type is all/unspec for all RRs

Table 3B1 - 2

IESLC - Meta-analysis of Current Smoking (vs never smoking), Any product (or Cigarettes if Any not available)
Adenocarcinoma
Most adjusted

REF	NRR	SEX	AD	Number Exposed Case	Number Exposed Cont	Non-exposed Case	Non-exposed Cont	RR	95.00%CI
BARBON	100	m	1	-	-	-	-	7.90	(3.60- 17.40)
*BOUCOT	147	m	2	-	-	-	-	10.95	(0.65- 183.57)
BROWN2	14	m	2	-	-	-	-	9.10	(7.60- 10.80)
BROWN2	13	f	2	-	-	-	-	7.20	(6.20- 8.30)
Subtotal BROWN2								7.92	(7.08- 8.86)
BUFFLE	71	f	0	56	110	7	112	8.15	(3.56- 18.65)
COMSTO	24	m	0	30	100	2	84	12.60	(2.92- 54.28)
COMSTO	31	f	0	23	52	8	115	6.36	(2.67- 15.16)
Subtotal COMSTO								7.60	(3.60- 16.04)
CORREA	44	c	1	-	-	-	-	6.70	(4.30- 10.60)
*CPSI	404	m	1	-	-	-	-	4.58	(1.74- 12.05)
*CPSI	406	f	1	-	-	-	-	1.43	(0.47- 4.39)
Subtotal CPSI								2.78	(1.34- 5.78)
*CPSII	115	m	1	-	-	-	-	19.22	(6.46- 57.16)
*CPSII	118	f	1	-	-	-	-	8.23	(4.36- 15.54)
Subtotal CPSII								10.21	(5.89- 17.67)
*DORN	340	m	1	-	-	-	-	5.95	(3.85- 9.22)
*ENGELA	70	m	7	-	-	-	-	7.06	(2.69- 18.50)
HAENSZ	37	f	0	16	94	37	236	1.09	(0.58- 2.05)
JAHN	8	m	0	75	269	8	138	4.81	(2.26- 10.26)
JAIN	17	m	0	60	118	4	85	10.81	(3.78- 30.87)
JAIN	12	f	0	69	99	24	214	6.21	(3.69- 10.47)
Subtotal JAIN								6.93	(4.35- 11.07)
JEDRYC	26	m	0	68	516	7	289	5.44	(2.47- 12.00)
KATSOU	12	f	1	-	-	-	-	1.70	(0.75- 3.89)
KHUDER	17	m	0	92	-	7	-	8.20	(3.60- 18.40)
KIHARA	5	c	0	103	162	78	237	1.93	(1.35- 2.76)
LUBIN2	252	m	0	454	6209	57	2616	3.36	(2.54- 4.44)
LUBIN2	264	f	0	69	410	138	1180	1.44	(1.06- 1.96)
Subtotal LUBIN2								2.30	(1.87- 2.83)
MATOS	53	m	2	-	-	-	-	7.90	(3.00- 20.90)
OSANN	39	m	2	-	-	-	-	21.70	(12.50- 39.70)
OSANN	40	f	2	-	-	-	-	11.60	(8.20- 16.40)
Subtotal OSANN								13.69	(10.17- 18.43)
OSANN2	32	f	1	-	-	-	-	3.20	(1.50- 6.60)
SOBUE	36	m	1	-	-	-	-	1.90	(1.30- 3.00)
SOBUE	46	f	1	-	-	-	-	1.30	(0.90- 2.00)
Subtotal SOBUE								1.56	(1.17- 2.08)
SOBUE2	2	m	2	-	-	-	-	3.10	(2.40- 3.70)
SOBUE2	6	f	2	-	-	-	-	1.80	(1.40- 2.20)
Subtotal SOBUE2								2.39	(2.04- 2.79)
SUZUKI	10	m	2	-	-	-	-	5.00	(2.71- 9.27)
SUZUKI	14	f	2	-	-	-	-	2.40	(1.19- 4.86)
Subtotal SUZUKI								3.64	(2.29- 5.78)
SVENSS	99	f	1	-	-	-	-	3.78	(1.91- 7.48)
TSUGAN	3	m	0	45	50	18	17	0.85	(0.39- 1.85)
TSUGAN	9	f	0	6	10	33	30	0.55	(0.18- 1.68)
Subtotal TSUGAN								0.74	(0.39- 1.40)
WAKAI	12	m	1	-	-	-	-	2.18	(1.00- 4.76)
WAKAI	30	f	1	-	-	-	-	1.14	(0.49- 2.61)
Subtotal WAKAI								1.61	(0.91- 2.85)
WU	7	f	2	-	-	-	-	4.10	(2.30- 7.50)
WU2	1	f	2	-	-	-	-	4.50	(3.00- 6.90)
WYNDE3	30	m	0	56	207	6	88	3.97	(1.65- 9.55)
WYNDE6	15	m	0	651	741	58	617	9.35	(7.00- 12.48)
WYNDE6	204	f	0	472	376	119	856	9.03	(7.14- 11.42)
Subtotal WYNDE6								9.15	(7.63- 10.98)
Partial Totals				2345	9523	611	6914		

*prospective study

Table 3B1 - 2

IESLC - Meta-analysis of Current Smoking (vs never smoking), Any product (or Cigarettes if Any not available)
Adenocarcinoma
Most adjusted

REF	NRR	SEX	AD	Ys	Ws	Qs	Ps
BARBON	100	m	1	2.07	6.19	1.58	0.0000
*BOUCOT	147	m	2	2.39	0.48	0.33	0.0964
BROWN2	14	m	2	2.21	124.44	51.93	0.0000
BROWN2	13	f	2	1.97	180.58	30.62	0.0000
Subtotal BROWN2				2.07	305.02	82.55	
BUFFLE	71	f	0	2.10	5.59	1.60	0.0000
COMSTO	24	m	0	2.53	1.80	1.70	0.0007
COMSTO	31	f	0	1.85	5.09	0.42	0.0000
Subtotal COMSTO				2.03	6.89	2.12	
CORREA	44	c	1	1.90	18.88	2.18	0.0000
*CPSI	404	m	1	1.52	4.10	0.01	0.0021
*CPSI	406	f	1	0.36	3.08	4.47	0.5303
Subtotal CPSI				1.02	7.18	4.47	
*CPSII	115	m	1	2.96	3.23	6.28	0.0000
*CPSII	118	f	1	2.11	9.51	2.83	0.0000
Subtotal CPSII				2.32	12.75	9.11	
*DORN	340	m	1	1.78	20.15	0.99	0.0000
*ENGELA	70	m	7	1.95	4.13	0.64	0.0001
HAENSZ	37	f	0	0.08	9.58	20.98	0.7992
JAHN	8	m	0	1.57	6.70	0.00	0.0000
JAIN	17	m	0	2.38	3.49	2.33	0.0000
JAIN	12	f	0	1.83	14.10	0.99	0.0000
Subtotal JAIN				1.94	17.58	3.32	
JEDRYC	26	m	0	1.69	6.14	0.11	0.0000
KATSOU	12	f	1	0.53	5.67	6.04	0.2064
KHUDER	17	m	0	2.10	5.77	1.70	0.0000
KIHARA	5	c	0	0.66	30.38	24.81	0.0003
LUBIN2	252	m	0	1.21	49.29	6.09	0.0000
LUBIN2	264	f	0	0.36	39.96	57.38	0.0214
Subtotal LUBIN2				0.83	89.24	63.47	
MATOS	53	m	2	2.07	4.08	1.04	0.0000
OSANN	39	m	2	3.08	11.51	26.41	0.0000
OSANN	40	f	2	2.45	31.98	25.26	0.0000
Subtotal OSANN				2.62	43.49	51.67	
OSANN2	32	f	1	1.16	7.00	1.12	0.0021
SOBUE	36	m	1	0.64	21.97	18.61	0.0026
SOBUE	46	f	1	0.26	24.10	40.72	0.1978
Subtotal SOBUE				0.44	46.07	59.34	
SOBUE2	2	m	2	1.13	82.01	15.22	0.0000
SOBUE2	6	f	2	0.59	75.22	71.43	0.0000
Subtotal SOBUE2				0.87	157.22	86.65	
SUZUKI	10	m	2	1.61	10.16	0.02	0.0000
SUZUKI	14	f	2	0.88	7.76	3.66	0.0147
Subtotal SUZUKI				1.29	17.92	3.68	
SVENSS	99	f	1	1.33	8.25	0.45	0.0001
TSUGAN	3	m	0	-0.16	6.39	19.00	0.6813
TSUGAN	9	f	0	-0.61	3.03	14.24	0.2916
Subtotal TSUGAN				-0.31	9.41	33.23	
WAKAI	12	m	1	0.78	6.31	3.87	0.0502
WAKAI	30	f	1	0.13	5.49	11.25	0.7588
Subtotal WAKAI				0.48	11.80	15.12	
WU	7	f	2	1.41	11.00	0.25	0.0000
WU2	1	f	2	1.50	22.15	0.08	0.0000
WYNDE3	30	m	0	1.38	4.98	0.17	0.0021
WYNDE6	15	m	0	2.23	45.98	20.81	0.0000
WYNDE6	204	f	0	2.20	69.69	28.39	0.0000
Subtotal WYNDE6				2.21	115.67	49.19	

Table 3B1 - 2

IESLC - Meta-analysis of Current Smoking (vs never smoking), Any product (or Cigarettes if Any not available)
Adenocarcinoma
Most adjusted

	N	44
	NS	31
	Wt	1017.36
Het	Chi	527.97
Het	df	43
Het	P	***
Fixed	RR	4.77
	RRl	4.49
	RRu	5.07
	P	+++
Random	RR	4.21
	RRl	3.32
	RRu	5.34
	P	+++
Asymm	P	N.S.

Table 3B1 - 3

IESLC - Meta-analysis of Current Smoking (vs never smoking), Any product (or Cigarettes if Any not available)
Adenocarcinoma
Most adjusted

	Sex			
	combined	male	female	Total
N	2	22	20	44
NS	2	22	20	44
Wt	49.25	429.29	538.82	1017.36
Het Chi	18.00	169.26	318.73	527.97
Het df	1	21	19	43
Het P	***	***	***	***
Fixed RR	3.11	5.54	4.40	4.77
RRl	2.35	5.04	4.05	4.49
RRu	4.11	6.09	4.79	5.07
P	+++	+++	+++	+++
Random RR	3.57	5.56	3.20	4.21
RRl	1.06	4.06	2.18	3.32
RRu	12.07	7.61	4.71	5.34
P	+	+++	+++	+++
Between Chi				21.98
Between df				2
Between P				***
Btwn(F) P				N.S.
Btwn(R) P				(*)

	Lung cancer type						
	a	a+l	a+al+br	KII	not q+u	not q+s	Total
N	40			4			44
NS	28			3			31
Wt	889.71			127.65			1017.36
Het Chi	471.72			10.21			527.97
Het df	39			3			43
Het P	***			*			***
Fixed RR	4.40			8.36			4.77
RRl	4.12			7.03			4.49
RRu	4.70			9.95			5.07
P	+++			+++			+++
Random RR	4.05			6.86			4.21
RRl	3.15			4.62			3.32
RRu	5.22			10.19			5.34
P	+++			+++			+++
Between Chi							46.05
Between df							1
Between P							***
Btwn(F) P							(*)
Btwn(R) P							*

	Location								
	NAmer	UK	Scand	othEur	China	Japan	othAs	other	Total
N	24		2	6		11		1	44
NS	17		2	5		6		1	31
Wt	614.16		12.38	113.94		272.81		4.08	1017.36
Het Chi	96.60		1.07	31.70		40.77		0.00	527.97
Het df	23		1	5		10		0	43
Het P	***		N.S.	***		***		N.S.	***
Fixed RR	7.63		4.66	2.65		2.11		7.90	4.77
RRl	7.05		2.67	2.20		1.87		2.99	4.49
RRu	8.25		8.13	3.18		2.37		20.85	5.07
P	+++		+++	+++		+++		+++	+++
Random RR	6.72		4.69	3.30		1.87		7.90	4.21
RRl	5.50		2.62	1.93		1.42		2.99	3.32
RRu	8.21		8.40	5.65		2.47		20.85	5.34
P	+++		+++	+++		+++		+++	+++
Between Chi									357.82
Between df									4
Between P									***
Btwn(F) P									***
Btwn(R) P									***

Table 3B1 - 3

IESLC - Meta-analysis of Current Smoking (vs never smoking), Any product (or Cigarettes if Any not available)
Adenocarcinoma
Most adjusted

	Detailed Country in "other Europe"					
	multi	Germany	othWest	East	Balkans	Total
N	2	1	1	1	1	6
NS	1	1	1	1	1	5
Wt	89.24	6.70	6.19	6.14	5.67	113.94
Het Chi	15.82	0.00	0.00	0.00	0.00	31.70
Het df	1	0	0	0	0	5
Het P	***	N.S.	N.S.	N.S.	N.S.	***
Fixed RR	2.30	4.81	7.90	5.44	1.70	2.65
RRl	1.87	2.26	3.59	2.47	0.75	2.20
RRu	2.83	10.26	17.37	12.00	3.87	3.18
P	+++	+++	+++	+++	N.S.	+++
Random RR	2.20	4.81	7.90	5.44	1.70	3.30
RRl	0.96	2.26	3.59	2.47	0.75	1.93
RRu	5.05	10.26	17.37	12.00	3.87	5.65
P	(+)	+++	+++	+++	N.S.	+++
Between Chi						15.88
Between df						4
Between P						**
Btwn(F) P						N.S.
Btwn(R) P						*

	Detailed Country in "other Asia"			
	India	HongKong	other	Total
N				
NS				
Wt				
Het Chi				
Het df				
Het P				N.S.
Fixed RR				
RRl				
RRu				
P				+++
Random RR				
RRl				
RRu				
P				+++
Between Chi				
Between df				
Between P				N.S.
Btwn(F) P				N.S.
Btwn(R) P				N.S.

	Detailed other continent			
	SCAmer	Auslia	Africa	Total
N	1			1
NS	1			1
Wt	4.08			4.08
Het Chi	0.00			0.00
Het df	0			0
Het P	N.S.			N.S.
Fixed RR	7.90			7.90
RRl	2.99			2.99
RRu	20.85			20.85
P	+++			+++
Random RR	7.90			7.90
RRl	2.99			2.99
RRu	20.85			20.85
P	+++			+++
Between Chi				
Between df				
Between P				N.S.
Btwn(F) P				N.S.
Btwn(R) P				N.S.

Table 3B1 - 3

IESLC - Meta-analysis of Current Smoking (vs never smoking), Any product (or Cigarettes if Any not available)
Adenocarcinoma
Most adjusted

	Start year of study <1960	1960-69	1970-79	1980-89	1990+	Total
N	5	7	11	19	2	44
NS	4	5	7	13	2	31
Wt	37.39	289.01	154.13	502.38	34.45	1017.36
Het Chi	22.12	133.45	74.33	187.40	7.13	527.97
Het df	4	6	10	18	1	43
Het P	***	***	***	***	**	***
Fixed RR	3.35	4.22	2.99	6.38	2.28	4.77
RRl	2.43	3.76	2.55	5.84	1.63	4.49
RRu	4.62	4.74	3.50	6.96	3.19	5.07
P	+++	+++	+++	+++	+++	+++
Random RR	2.92	4.55	3.41	5.06	3.62	4.21
RRl	1.17	2.50	2.10	3.65	0.92	3.32
RRu	7.28	8.30	5.53	7.02	14.29	5.34
P	+	+++	+++	+++	(+)	+++
Between Chi						103.54
Between df						4
Between P						***
Btwn(F) P						(*)
Btwn(R) P						N.S.

	Study type (1) CC	other	Total
N	34	10	44
NS	24	7	31
Wt	958.78	58.58	1017.36
Het Chi	509.03	16.01	527.97
Het df	33	9	43
Het P	***	(*)	***
Fixed RR	4.71	5.93	4.77
RRl	4.42	4.59	4.49
RRu	5.01	7.66	5.07
P	+++	+++	+++
Random RR	3.88	5.95	4.21
RRl	2.96	4.07	3.32
RRu	5.07	8.69	5.34
P	+++	+++	+++
Between Chi			2.93
Between df			1
Between P			(*)
Btwn(F) P			N.S.
Btwn(R) P			(*)

	Study type (2) CC	prosp	other	Total
N	34	7	3	44
NS	24	5	2	31
Wt	958.78	44.69	13.89	1017.36
Het Chi	509.03	12.15	3.22	527.97
Het df	33	6	2	43
Het P	***	(*)	N.S.	***
Fixed RR	4.71	6.28	4.92	4.77
RRl	4.42	4.68	2.91	4.49
RRu	5.01	8.42	8.32	5.07
P	+++	+++	+++	+++
Random RR	3.88	6.22	5.32	4.21
RRl	2.96	3.84	2.63	3.32
RRu	5.07	10.07	10.74	5.34
P	+++	+++	+++	+++
Between Chi				3.57
Between df				2
Between P				N.S.
Btwn(F) P				N.S.
Btwn(R) P				N.S.

Table 3B1 - 3

IESLC - Meta-analysis of Current Smoking (vs never smoking), Any product (or Cigarettes if Any not available)
Adenocarcinoma
Most adjusted

	Study size (number of LC cases)				
	100-249	250-499	500-999	1000+	Total
N	11	9	4	20	44
NS	9	7	3	12	31
Wt	73.39	86.11	29.37	828.50	1017.36
Het Chi	37.81	30.80	1.01	404.12	527.97
Het df	10	8	3	19	43
Het P	***	***	N.S.	***	***
Fixed RR	2.54	3.20	7.35	5.18	4.77
RRl	2.02	2.59	5.12	4.84	4.49
RRu	3.19	3.95	10.55	5.54	5.07
P	+++	+++	+++	+++	+++
Random RR	2.45	3.83	7.35	4.97	4.21
RRl	1.54	2.41	5.12	3.54	3.32
RRu	3.90	6.09	10.55	6.97	5.34
P	+++	+++	+++	+++	+++
Between Chi					54.23
Between df					3
Between P					***
Btwn(F) P					N.S.
Btwn(R) P					**

	Risky occupational population			
	no	mining	othRisky	Total
N	44			44
NS	31			31
Wt	1017.36			1017.36
Het Chi	527.97			527.97
Het df	43			43
Het P	***			***
Fixed RR	4.77			4.77
RRl	4.49			4.49
RRu	5.07			5.07
P	+++			+++
Random RR	4.21			4.21
RRl	3.32			3.32
RRu	5.34			5.34
P	+++			+++
Between Chi				
Between df				
Between P				N.S.
Btwn(F) P				N.S.
Btwn(R) P				N.S.

	National cigarette tobacco type			
	Virginia	blended	other	Total
N	2	42		44
NS	1	30		31
Wt	17.58	999.78		1017.36
Het Chi	0.85	524.61		527.97
Het df	1	41		43
Het P	N.S.	***		***
Fixed RR	6.93	4.74		4.77
RRl	4.35	4.45		4.49
RRu	11.07	5.04		5.07
P	+++	+++		+++
Random RR	6.93	4.10		4.21
RRl	4.35	3.21		3.32
RRu	11.07	5.23		5.34
P	+++	+++		+++
Between Chi				2.51
Between df				1
Between P				N.S.
Btwn(F) P				N.S.
Btwn(R) P				(*)

Table 3B1 - 3

IESLC - Meta-analysis of Current Smoking (vs never smoking), Any product (or Cigarettes if Any not available)
Adenocarcinoma
Most adjusted

	Any proxy use		
	No/nk	Yes	Total
N	38	6	44
NS	26	5	31
Wt	962.98	54.38	1017.36
Het Chi	518.78	1.49	527.97
Het df	37	5	43
Het P	***	N.S.	***
Fixed RR	4.67	6.88	4.77
RRl	4.39	5.27	4.49
RRu	4.98	8.97	5.07
P	+++	+++	+++
Random RR	3.88	6.88	4.21
RRl	2.99	5.27	3.32
RRu	5.03	8.97	5.34
P	+++	+++	+++
Between Chi			7.71
Between df			1
Between P			**
Btwn(F) P			N.S.
Btwn(R) P			**

	Full histological confirmation		
	No	Yes	Total
N	23	21	44
NS	17	14	31
Wt	355.55	661.81	1017.36
Het Chi	184.15	326.35	527.97
Het df	22	20	43
Het P	***	***	***
Fixed RR	3.99	5.25	4.77
RRl	3.59	4.87	4.49
RRu	4.43	5.67	5.07
P	+++	+++	+++
Random RR	5.59	3.20	4.21
RRl	3.98	2.28	3.32
RRu	7.84	4.49	5.34
P	+++	+++	+++
Between Chi			17.48
Between df			1
Between P			***
Btwn(F) P			N.S.
Btwn(R) P			*

	Number of adjustment variables (1)			
	0	1	2+/+nk	Total
N	17	14	13	44
NS	12	10	9	31
Wt	307.94	143.93	565.49	1017.36
Het Chi	197.14	79.28	213.12	527.97
Het df	16	13	12	43
Het P	***	***	***	***
Fixed RR	4.28	3.25	5.58	4.77
RRl	3.83	2.76	5.13	4.49
RRu	4.79	3.83	6.05	5.07
P	+++	+++	+++	+++
Random RR	3.90	3.46	5.68	4.21
RRl	2.54	2.27	3.80	3.32
RRu	6.00	5.29	8.49	5.34
P	+++	+++	+++	+++
Between Chi				38.43
Between df				2
Between P				***
Btwn(F) P				N.S.
Btwn(R) P				N.S.

Table 3B1 - 3

IESLC - Meta-analysis of Current Smoking (vs never smoking), Any product (or Cigarettes if Any not available)
Adenocarcinoma
Most adjusted

	Number of adjustment variables (2) 0	1	2	3-5	6+/+nk	Total
N	17	14	12		1	44
NS	12	10	8		1	31
Wt	307.94	143.93	561.36		4.13	1017.36
Het Chi	197.14	79.28	212.89		0.00	527.97
Het df	16	13	11		0	43
Het P	***	***	***		N.S.	***
Fixed RR	4.28	3.25	5.57		7.06	4.77
RRl	3.83	2.76	5.12		2.69	4.49
RRu	4.79	3.83	6.05		18.51	5.07
P	+++	+++	+++		+++	+++
Random RR	3.90	3.46	5.60		7.06	4.21
RRl	2.54	2.27	3.68		2.69	3.32
RRu	6.00	5.29	8.50		18.51	5.34
P	+++	+++	+++		+++	+++
Between Chi						38.67
Between df						3
Between P						***
Btwn(F) P						N.S.
Btwn(R) P						N.S.

	Product all/unsp	cig+/-ot	cig only	Total
N	11	28	5	44
NS	9	19	4	32
Wt	109.83	876.49	31.04	1017.36
Het Chi	41.63	429.96	11.10	527.97
Het df	10	27	4	43
Het P	***	***	*	***
Fixed RR	2.61	5.11	5.69	4.77
RRl	2.16	4.79	4.00	4.49
RRu	3.14	5.46	8.09	5.07
P	+++	+++	+++	+++
Random RR	2.40	5.02	5.56	4.21
RRl	1.59	3.76	2.61	3.32
RRu	3.61	6.70	11.85	5.34
P	+++	+++	+++	+++
Between Chi				45.28
Between df				2
Between P				***
Btwn(F) P				N.S.
Btwn(R) P				*

	Denominator nev any	nev cigs	Total
N	27	17	44
NS	21	12	33
Wt	477.73	539.63	1017.36
Het Chi	189.32	147.85	527.97
Het df	26	16	43
Het P	***	***	***
Fixed RR	3.01	7.17	4.77
RRl	2.75	6.59	4.49
RRu	3.29	7.80	5.07
P	+++	+++	+++
Random RR	3.10	6.61	4.21
RRl	2.37	4.89	3.32
RRu	4.07	8.95	5.34
P	+++	+++	+++
Between Chi			190.80
Between df			1
Between P			***
Btwn(F) P			***
Btwn(R) P			***

Table 3B1 - 3

IESLC - Meta-analysis of Current Smoking (vs never smoking), Any product (or Cigarettes if Any not available)

Adenocarcinoma

Most adjusted

	Derivation of RR/CI			
	Orig	StdCalc	Other	Total
N	21	17	6	44
NS	15	12	5	32
Wt	665.29	311.89	40.19	1017.36
Het Chi	326.66	192.60	6.86	527.97
Het df	20	16	5	43
Het P	***	***	N.S.	***
Fixed RR	4.91	4.47	4.85	4.77
RRl	4.55	4.00	3.56	4.49
RRu	5.30	5.00	6.61	5.07
P	+++	+++	+++	+++
Random RR	3.90	4.64	4.59	4.21
RRl	2.77	3.05	3.06	3.32
RRu	5.48	7.07	6.89	5.34
P	+++	+++	+++	+++
Between Chi				1.86
Between df				2
Between P				N.S.
Btwn(F) P				N.S.
Btwn(R) P				N.S.

Table 3B1 - 4

IESLC - Meta-analysis of Current Smoking (vs never smoking), Any product (or Cigarettes if Any not available)
Adenocarcinoma
Least adjusted

REF	NRR	X	SEX	AGEL	AGEH	RACE	YF	LC TYPE	LOC	START	ST	NLC	R	VB	P	H	AD	PRODUCT	DENOM	De
BARBON	44	x	m	0	0	all	-	a	Eu:wst	1979	CC	755	n	bl	y	y	0	all/unsp	nev any	st
BOUCOT	72	x	m	0	0	all	0	a	NAmer	1951	pr	121	n	bl	n	n	0	cig only	nev any	ot
BROWN2	14		m	0	0	wh	-	a	NAmer	1984	CC	14596	n	bl	n	y	2	cig+/-ot	nev cigs	or
BROWN2	13		f	0	0	wh	-	a	NAmer	1984	CC	14596	n	bl	n	y	2	cig+/-ot	nev cigs	or
BUFFLE	71		f	0	0	w-hi	-	a	NAmer	1976	CC	943	n	bl	y	n	0	cig+/-ot	nev cigs	st
COMSTO	24		m	0	0	all	-	a	NAmer	1975	ot	258	n	bl	n	n	0	cig+/-ot	nev cigs	st
COMSTO	31		f	0	0	all	-	a	NAmer	1975	ot	258	n	bl	n	n	0	cig+/-ot	nev cigs	st
CORREA	44		c	0	0	all	-	a	NAmer	1979	CC	1359	n	bl	y	n	1	cig+/-ot	nev cigs	or
CPSI	404		m	0	0	all	2	a	NAmer	1959	pr	5138	n	bl	n	n	1	cig only	nev any	ot
CPSI	406		f	0	0	all	2	a	NAmer	1959	pr	5138	n	bl	n	n	1	cig only	nev any	ot
CPSII	115		m	0	0	all	2	a	NAmer	1982	pr	3229	n	bl	n	n	1	cig only	nev any	st
CPSII	118		f	0	0	all	2	a	NAmer	1982	pr	3229	n	bl	n	n	1	cig+/-ot	nev cigs	st
DORN	340		m	0	0	wh	8	a	NAmer	1954	pr	5097	n	bl	n	n	1	cig only	nev any	ot
ENGELA	70		m	0	0	all	0	a	Eu:Sca	1964	pr	435	n	bl	n	n	7	cig+/-ot	nev cigs	ot
HAENSZ	37		f	0	0	all	-	a	NAmer	1955	CC	158	n	bl	n	y	0	cig+/-ot	nev any	st
JAHN	8		m	0	0	all	-	a	Eu:Ger	1988	CC	1004	n	bl	n	n	0	cig+/-ot	nev any	st
JAIN	17		m	0	0	all	-	a	NAmer	1981	CC	845	n	V	y	n	0	cig+/-ot	nev cigs	st
JAIN	12		f	0	0	all	-	a	NAmer	1981	CC	845	n	V	y	n	0	cig+/-ot	nev cigs	st
JEDRYC	26		m	0	0	all	-	a	Eu:est	1980	CC	1630	n	bl	y	n	0	cig+/-ot	nev any	st
KATSOU	16	x	f	0	0	all	-	a	Eu:bal	1987	CC	101	n	bl	n	n	0	all/unsp	nev any	st
KHUDER	17		m	0	0	all	-	a	NAmer	1985	CC	482	n	bl	n	y	0	cig+/-ot	nev cigs	or
KIHARA	5		c	0	0	jap	-	a	As:Jap	1991	CC	440	n	bl	n	n	0	all/unsp	nev any	st
LUBIN2	252		m	0	0	all	-	a	Eu:mul	1976	CC	7804	n	bl	n	y	0	cig+/-ot	nev any	st
LUBIN2	264		f	0	0	all	-	a	Eu:mul	1976	CC	7804	n	bl	n	y	0	cig+/-ot	nev any	st
MATOS	52	x	m	0	0	all	-	a	SCAmer	1994	CC	200	n	bl	n	n	0	cig+/-ot	nev any	st
OSANN	11	x	m	0	0	all	-	a	NAmer	1984	CC	1986	n	bl	n	n	0	cig+/-ot	nev cigs	st
OSANN	15	x	f	0	0	all	-	a	NAmer	1984	CC	1986	n	bl	n	n	0	cig+/-ot	nev cigs	st
OSANN2	14	x	f	0	0	all	-	KII	NAmer	1964	ot	217	n	bl	n	y	0	cig+/-ot	nev cigs	st
SOBUE	6	x	m	0	0	all	-	a	As:Jap	1986	CC	1376	n	bl	n	y	0	cig+/-ot	nev cigs	st
SOBUE	22	x	f	0	0	all	-	a	As:Jap	1986	CC	1376	n	bl	n	y	0	cig+/-ot	nev cigs	st
SOBUE2	2		m	0	0	all	-	a	As:Jap	1965	CC	2083	n	bl	n	n	2	cig+/-ot	nev any	or
SOBUE2	6		f	0	0	all	-	a	As:Jap	1965	CC	2083	n	bl	n	n	2	cig+/-ot	nev any	or
SUZUKI	2	x	m	0	0	all	-	a	As:Jap	1978	CC	238	n	bl	n	y	0	cig+/-ot	nev any	st
SUZUKI	6	x	f	0	0	all	-	a	As:Jap	1978	CC	238	n	bl	n	y	0	cig+/-ot	nev any	st
SVENSS	64	x	f	0	0	all	-	a	Eu:Sca	1983	CC	210	n	bl	n	n	0	all/unsp	nev any	st
TSUGAN	3		m	0	0	all	-	a	As:Jap	1976	CC	134	n	bl	n	y	0	all/unsp	nev any	st
TSUGAN	9		f	0	0	all	-	a	As:Jap	1976	CC	134	n	bl	n	y	0	all/unsp	nev any	or
WAKAI	6	x	m	0	0	all	-	a	As:Jap	1988	CC	333	n	bl	n	y	0	all/unsp	nev any	st
WAKAI	24	x	f	0	0	all	-	a	As:Jap	1988	CC	333	n	bl	n	y	0	all/unsp	nev any	st
WU	2	x	f	0	0	wh	-	a	NAmer	1981	CC	220	n	bl	n	y	0	all/unsp	nev any	st
WU2	1		f	0	0	all	-	a	NAmer	1983	CC	336	n	bl	n	y	2	all/unsp	nev any	or
WYNDE3	30		m	0	0	all	-	KII	NAmer	1966	CC	350	n	bl	n	y	0	all/unsp	nev any	st
WYNDE6	15		m	0	0	all	-	KII	NAmer	1969	CC	4423	n	bl	n	y	0	cig+/-ot	nev any	st
WYNDE6	204		f	0	0	all	-	KII	NAmer	1969	CC	4423	n	bl	n	y	0	cig+/-ot	nev cigs	st

Cigarette type is all/unspec for all RRs

Table 3B1 - 5

IESLC - Meta-analysis of Current Smoking (vs never smoking), Any product (or Cigarettes if Any not available)
Adenocarcinoma
Least adjusted

REF	NRR	SEX	AD	Number Exposed Case	Number Exposed Cont	Non-exposed Case	Non-exposed Cont	RR	95.00%CI
BARBON	44	m	0	109	362	7	188	8.09	(3.69- 17.72)
*BOUCOT	72	m	0	14	22177	0	7551	9.87~	(0.59- 165.51)
BROWN2	14	m	2	-	-	-	-	9.10	(7.60- 10.80)
BROWN2	13	f	2	-	-	-	-	7.20	(6.20- 8.30)
Subtotal BROWN2								7.92	(7.08- 8.86)
BUFFLE	71	f	0	56	110	7	112	8.15	(3.56- 18.65)
COMSTO	24	m	0	30	100	2	84	12.60	(2.92- 54.28)
COMSTO	31	f	0	23	52	8	115	6.36	(2.67- 15.16)
Subtotal COMSTO								7.60	(3.60- 16.04)
CORREA	44	c	1	-	-	-	-	6.70	(4.30- 10.60)
*CPSI	404	m	1	-	-	-	-	4.58	(1.74- 12.05)
*CPSI	406	f	1	-	-	-	-	1.43	(0.47- 4.39)
Subtotal CPSI								2.78	(1.34- 5.78)
*CPSII	115	m	1	-	-	-	-	19.22	(6.46- 57.16)
*CPSII	118	f	1	-	-	-	-	8.23	(4.36- 15.54)
Subtotal CPSII								10.21	(5.89- 17.67)
*DORN	340	m	1	-	-	-	-	5.95	(3.85- 9.22)
*ENGELA	70	m	7	-	-	-	-	7.06	(2.69- 18.50)
HAENSZ	37	f	0	16	94	37	236	1.09	(0.58- 2.05)
JAHN	8	m	0	75	269	8	138	4.81	(2.26- 10.26)
JAIN	17	m	0	60	118	4	85	10.81	(3.78- 30.87)
JAIN	12	f	0	69	99	24	214	6.21	(3.69- 10.47)
Subtotal JAIN								6.93	(4.35- 11.07)
JEDRYC	26	m	0	68	516	7	289	5.44	(2.47- 12.00)
KATSOU	16	f	0	15	18	30	67	1.86	(0.83- 4.18)
KHUDER	17	m	0	92	-	7	-	8.20	(3.60- 18.40)
KIHARA	5	c	0	103	162	78	237	1.93	(1.35- 2.76)
LUBIN2	252	m	0	454	6209	57	2616	3.36	(2.54- 4.44)
LUBIN2	264	f	0	69	410	138	1180	1.44	(1.06- 1.96)
Subtotal LUBIN2								2.30	(1.87- 2.83)
MATOS	52	m	0	46	132	5	110	7.67	(2.94- 19.96)
OSANN	11	m	0	217	541	14	833	23.87	(13.75- 41.41)
OSANN	15	f	0	193	367	47	1093	12.23	(8.70- 17.18)
Subtotal OSANN								14.70	(11.01- 19.64)
OSANN2	14	f	0	50	28	22	43	3.49	(1.75- 6.97)
SOBUE	6	m	0	276	650	27	128	2.01	(1.30- 3.12)
SOBUE	22	f	0	38	168	137	857	1.41	(0.95- 2.10)
Subtotal SOBUE								1.66	(1.24- 2.22)
SOBUE2	2	m	2	-	-	-	-	3.10	(2.40- 3.70)
SOBUE2	6	f	2	-	-	-	-	1.80	(1.40- 2.20)
Subtotal SOBUE2								2.39	(2.04- 2.79)
SUZUKI	2	m	0	119	162	14	99	5.19	(2.83- 9.54)
SUZUKI	6	f	0	20	20	55	133	2.42	(1.21- 4.84)
Subtotal SUZUKI								3.73	(2.36- 5.89)
SVENSS	64	f	0	38	53	22	120	3.91	(2.11- 7.25)
TSUGAN	3	m	0	45	50	18	17	0.85	(0.39- 1.85)
TSUGAN	9	f	0	6	10	33	30	0.55	(0.18- 1.68)
Subtotal TSUGAN								0.74	(0.39- 1.40)
WAKAI	6	m	0	75	284	8	65	2.15	(0.99- 4.67)
WAKAI	24	f	0	9	26	46	145	1.09	(0.48- 2.50)
Subtotal WAKAI								1.56	(0.89- 2.75)
WU	2	f	0	99	50	29	62	4.23	(2.43- 7.39)
WU2	1	f	2	-	-	-	-	4.50	(3.00- 6.90)
WYNDE3	30	m	0	56	207	6	88	3.97	(1.65- 9.55)
WYNDE6	15	m	0	651	741	58	617	9.35	(7.00- 12.48)
WYNDE6	204	f	0	472	376	119	856	9.03	(7.14- 11.42)
Subtotal WYNDE6								9.15	(7.63- 10.98)
Partial Totals				3663	34561	1074	18408		

*prospective study ~ With 0.5 adjustment for zero

Table 3B1 - 5

IESLC - Meta-analysis of Current Smoking (vs never smoking), Any product (or Cigarettes if Any not available)
Adenocarcinoma
Least adjusted

REF	NRR	SEX	AD	Ys	Ws	Qs	Ps
BARBON	44	m	0	2.09	6.25	1.67	0.0000
*BOUCOT	72	m	0	2.29	0.48	0.25	0.1114
BROWN2	14	m	2	2.21	124.44	50.25	0.0000
BROWN2	13	f	2	1.97	180.58	29.07	0.0000
Subtotal BROWN2				2.07	305.02	79.32	
BUFFLE	71	f	0	2.10	5.59	1.54	0.0000
COMSTO	24	m	0	2.53	1.80	1.66	0.0007
COMSTO	31	f	0	1.85	5.09	0.39	0.0000
Subtotal COMSTO				2.03	6.89	2.05	
CORREA	44	c	1	1.90	18.88	2.05	0.0000
*CPSI	404	m	1	1.52	4.10	0.01	0.0021
*CPSI	406	f	1	0.36	3.08	4.54	0.5303
Subtotal CPSI				1.02	7.18	4.56	
*CPSII	115	m	1	2.96	3.23	6.18	0.0000
*CPSII	118	f	1	2.11	9.51	2.72	0.0000
Subtotal CPSII				2.32	12.75	8.91	
*DORN	340	m	1	1.78	20.15	0.89	0.0000
*ENGELA	70	m	7	1.95	4.13	0.60	0.0001
HAENSZ	37	f	0	0.08	9.58	21.28	0.7992
JAHN	8	m	0	1.57	6.70	0.00	0.0000
JAIN	17	m	0	2.38	3.49	2.27	0.0000
JAIN	12	f	0	1.83	14.10	0.91	0.0000
Subtotal JAIN				1.94	17.58	3.18	
JEDRYC	26	m	0	1.69	6.14	0.09	0.0000
KATSOU	16	f	0	0.62	5.87	5.31	0.1325
KHUDER	17	m	0	2.10	5.77	1.63	0.0000
KIHARA	5	c	0	0.66	30.38	25.39	0.0003
LUBIN2	252	m	0	1.21	49.29	6.46	0.0000
LUBIN2	264	f	0	0.36	39.96	58.39	0.0214
Subtotal LUBIN2				0.83	89.24	64.86	
MATOS	52	m	0	2.04	4.19	0.90	0.0000
OSANN	11	m	0	3.17	12.64	32.35	0.0000
OSANN	15	f	0	2.50	33.23	28.80	0.0000
Subtotal OSANN				2.69	45.87	61.15	
OSANN2	14	f	0	1.25	8.04	0.84	0.0004
SOBUE	6	m	0	0.70	20.00	15.25	0.0018
SOBUE	22	f	0	0.35	24.55	36.88	0.0855
Subtotal SOBUE				0.51	44.54	52.13	
SOBUE2	2	m	2	1.13	82.01	15.98	0.0000
SOBUE2	6	f	2	0.59	75.22	72.98	0.0000
Subtotal SOBUE2				0.87	157.22	88.96	
SUZUKI	2	m	0	1.65	10.41	0.06	0.0000
SUZUKI	6	f	0	0.88	7.96	3.79	0.0128
Subtotal SUZUKI				1.32	18.36	3.84	
SVENSS	64	f	0	1.36	10.10	0.44	0.0000
TSUGAN	3	m	0	-0.16	6.39	19.23	0.6813
TSUGAN	9	f	0	-0.61	3.03	14.37	0.2916
Subtotal TSUGAN				-0.31	9.41	33.60	
WAKAI	6	m	0	0.76	6.36	4.17	0.0542
WAKAI	24	f	0	0.09	5.61	12.38	0.8363
Subtotal WAKAI				0.45	11.97	16.55	
WU	2	f	0	1.44	12.39	0.21	0.0000
WU2	1	f	2	1.50	22.15	0.10	0.0000
WYNDE3	30	m	0	1.38	4.98	0.19	0.0021
WYNDE6	15	m	0	2.23	45.98	20.16	0.0000
WYNDE6	204	f	0	2.20	69.69	27.46	0.0000
Subtotal WYNDE6				2.21	115.67	47.61	

Table 3B1 - 5

IESLC - Meta-analysis of Current Smoking (vs never smoking), Any product (or Cigarettes if Any not available)
Adenocarcinoma
Least adjusted

	N	44
	NS	31
	Wt	1023.48
Het	Chi	530.13
Het	df	43
Het	P	***
Fixed	RR	4.82
	RRl	4.53
	RRu	5.12
	P	+++
Random	RR	4.27
	RRl	3.37
	RRu	5.40
	P	+++
Asymm	P	N.S.

Table 3B1 - 6

IESLC - Meta-analysis of Current Smoking (vs never smoking), Any product (or Cigarettes if Any not available)
Adenocarcinoma
Least adjusted

	Sex			
	combined	male	female	Total
N	2	22	20	44
NS	2	22	20	44
Wt	49.25	428.92	545.31	1023.48
Het Chi	18.00	170.18	318.85	530.13
Het df	1	21	19	43
Het P	***	***	***	***
Fixed RR	3.11	5.62	4.44	4.82
RRl	2.35	5.11	4.09	4.53
RRu	4.11	6.18	4.83	5.12
P	+++	+++	+++	+++
Random RR	3.57	5.62	3.26	4.27
RRl	1.06	4.10	2.23	3.37
RRu	12.07	7.70	4.78	5.40
P	+	+++	+++	+++
Between Chi				23.10
Between df				2
Between P				***
Btwn(F) P				N.S.
Btwn(R) P				(*)

	Lung cancer type						
	a	a+l	a+al+br	KII	not q+u	not q+s	Total
N	40			4			44
NS	28			3			31
Wt	894.79			128.69			1023.48
Het Chi	475.92			9.88			530.13
Het df	39			3			43
Het P	***			*			***
Fixed RR	4.45			8.34			4.82
RRl	4.17			7.02			4.53
RRu	4.76			9.92			5.12
P	+++			+++			+++
Random RR	4.10			6.93			4.27
RRl	3.18			4.72			3.37
RRu	5.28			10.18			5.40
P	+++			+++			+++
Between Chi							44.33
Between df							1
Between P							***
Btwn(F) P							(*)
Btwn(R) P							*

	Location								
	NAmer	UK	Scand	othEur	China	Japan	othAs	other	Total
N	24		2	6		11		1	44
NS	17		2	5		6		1	31
Wt	618.97		14.24	114.19		271.89		4.19	1023.48
Het Chi	101.90		1.02	31.70		40.06		0.00	530.13
Het df	23		1	5		10		0	43
Het P	***		N.S.	***		***		N.S.	***
Fixed RR	7.67		4.64	2.66		2.13		7.67	4.82
RRl	7.09		2.76	2.22		1.90		2.94	4.53
RRu	8.30		7.80	3.20		2.40		19.96	5.12
P	+++		+++	+++		+++		+++	+++
Random RR	6.78		4.66	3.35		1.91		7.67	4.27
RRl	5.53		2.75	1.96		1.44		2.94	3.37
RRu	8.32		7.89	5.73		2.51		19.96	5.40
P	+++		+++	+++		+++		+++	+++
Between Chi									355.45
Between df									4
Between P									***
Btwn(F) P									***
Btwn(R) P									***

Table 3B1 - 6

IESLC - Meta-analysis of Current Smoking (vs never smoking), Any product (or Cigarettes if Any not available)
Adenocarcinoma
Least adjusted

Detailed Country in "other Europe"

	multi	Germany	othWest	East	Balkans	Total
N	2	1	1	1	1	6
NS	1	1	1	1	1	5
Wt	89.24	6.70	6.25	6.14	5.87	114.19
Het Chi	15.82	0.00	0.00	0.00	0.00	31.70
Het df	1	0	0	0	0	5
Het P	***	N.S.	N.S.	N.S.	N.S.	***
Fixed RR	2.30	4.81	8.09	5.44	1.86	2.66
RRl	1.87	2.26	3.69	2.47	0.83	2.22
RRu	2.83	10.26	17.72	12.00	4.18	3.20
P	+++	+++	+++	+++	N.S.	+++
Random RR	2.20	4.81	8.09	5.44	1.86	3.35
RRl	0.96	2.26	3.69	2.47	0.83	1.96
RRu	5.05	10.26	17.72	12.00	4.18	5.73
P	(+)	+++	+++	+++	N.S.	+++
Between Chi						15.88
Between df						4
Between P						**
Btwn(F) P						N.S.
Btwn(R) P						(*)

Detailed Country in "other Asia"

	India	HongKong	other	Total
N				
NS				
Wt				
Het Chi				
Het df				
Het P				N.S.
Fixed RR				
RRl				
RRu				
P				+++
Random RR				
RRl				
RRu				
P				+++
Between Chi				
Between df				
Between P				N.S.
Btwn(F) P				N.S.
Btwn(R) P				N.S.

Detailed other continent

	SCAmer	Auslia	Africa	Total
N	1			1
NS	1			1
Wt	4.19			4.19
Het Chi	0.00			0.00
Het df	0			0
Het P	N.S.			N.S.
Fixed RR	7.67			7.67
RRl	2.94			2.94
RRu	19.96			19.96
P	+++			+++
Random RR	7.67			7.67
RRl	2.94			2.94
RRu	19.96			19.96
P	+++			+++
Between Chi				
Between df				
Between P				N.S.
Btwn(F) P				N.S.
Btwn(R) P				N.S.

Table 3B1 - 6

IESLC - Meta-analysis of Current Smoking (vs never smoking), Any product (or Cigarettes if Any not available)
Adenocarcinoma
Least adjusted

	Start year of study					
	<1960	1960-69	1970-79	1980-89	1990+	Total
N	5	7	11	19	2	44
NS	4	5	7	13	2	31
Wt	37.39	290.04	154.63	506.85	34.57	1023.48
Het Chi	22.01	133.20	75.14	184.30	7.00	530.13
Het df	4	6	10	18	1	43
Het P	***	***	***	***	**	***
Fixed RR	3.35	4.23	3.01	6.49	2.28	4.82
RRl	2.43	3.77	2.57	5.95	1.64	4.53
RRu	4.61	4.74	3.52	7.08	3.19	5.12
P	+++	+++	+++	+++	+++	+++
Random RR	2.89	4.60	3.43	5.17	3.57	4.27
RRl	1.16	2.53	2.12	3.75	0.93	3.37
RRu	7.21	8.36	5.57	7.14	13.68	5.40
P	+	+++	+++	+++	(+)	+++
Between Chi						108.49
Between df						4
Between P						***
Btwn(F) P						(*)
Btwn(R) P						N.S.

	Study type (1)		
	CC	other	Total
N	34	10	44
NS	24	7	31
Wt	963.86	59.62	1023.48
Het Chi	511.88	15.55	530.13
Het df	33	9	43
Het P	***	(*)	***
Fixed RR	4.76	5.93	4.82
RRl	4.47	4.60	4.53
RRu	5.07	7.64	5.12
P	+++	+++	+++
Random RR	3.93	5.97	4.27
RRl	3.01	4.13	3.37
RRu	5.14	8.64	5.40
P	+++	+++	+++
Between Chi			2.70
Between df			1
Between P			N.S.
Btwn(F) P			N.S.
Btwn(R) P			(*)

	Study type (2)			
	CC	prosp	other	Total
N	34	7	3	44
NS	24	5	2	31
Wt	963.86	44.69	14.93	1023.48
Het Chi	511.88	12.10	2.87	530.13
Het df	33	6	2	43
Het P	***	(*)	N.S.	***
Fixed RR	4.76	6.27	5.00	4.82
RRl	4.47	4.68	3.01	4.53
RRu	5.07	8.41	8.30	5.12
P	+++	+++	+++	+++
Random RR	3.93	6.21	5.34	4.27
RRl	3.01	3.84	2.81	3.37
RRu	5.14	10.03	10.15	5.40
P	+++	+++	+++	+++
Between Chi				3.28
Between df				2
Between P				N.S.
Btwn(F) P				N.S.
Btwn(R) P				N.S.

Table 3B1 - 6

IESLC - Meta-analysis of Current Smoking (vs never smoking), Any product (or Cigarettes if Any not available)
Adenocarcinoma
Least adjusted

	Study size (number of LC cases) 100-249	250-499	500-999	1000+	Total
N	11	9	4	20	44
NS	9	7	3	12	31
Wt	78.43	86.28	29.42	829.35	1023.48
Het Chi	39.38	31.53	1.03	404.68	530.13
Het df	10	8	3	19	43
Het P	***	***	N.S.	***	***
Fixed RR	2.67	3.18	7.39	5.24	4.82
RRl	2.14	2.57	5.15	4.90	4.53
RRu	3.33	3.92	10.60	5.61	5.12
P	+++	+++	+++	+++	+++
Random RR	2.52	3.81	7.39	5.05	4.27
RRl	1.59	2.38	5.15	3.60	3.37
RRu	4.00	6.08	10.60	7.08	5.40
P	+++	+++	+++	+++	+++
Between Chi					53.51
Between df					3
Between P					***
Btwn(F) P					N.S.
Btwn(R) P					**

	Risky occupational population no	mining	othRisky	Total
N	44			44
NS	31			31
Wt	1023.48			1023.48
Het Chi	530.13			530.13
Het df	43			43
Het P	***			***
Fixed RR	4.82			4.82
RRl	4.53			4.53
RRu	5.12			5.12
P	+++			+++
Random RR	4.27			4.27
RRl	3.37			3.37
RRu	5.40			5.40
P	+++			+++
Between Chi				
Between df				
Between P				N.S.
Btwn(F) P				N.S.
Btwn(R) P				N.S.

	National cigarette tobacco type Virginia	blended	other	Total
N	2	42		44
NS	1	30		31
Wt	17.58	1005.90		1023.48
Het Chi	0.85	526.91		530.13
Het df	1	41		43
Het P	N.S.	***		***
Fixed RR	6.93	4.79		4.82
RRl	4.35	4.50		4.53
RRu	11.07	5.10		5.12
P	+++	+++		+++
Random RR	6.93	4.15		4.27
RRl	4.35	3.25		3.37
RRu	11.07	5.29		5.40
P	+++	+++		+++
Between Chi				2.37
Between df				1
Between P				N.S.
Btwn(F) P				N.S.
Btwn(R) P				(*)

Table 3B1 - 6

IESLC - Meta-analysis of Current Smoking (vs never smoking), Any product (or Cigarettes if Any not available)
Adenocarcinoma
Least adjusted

	Any proxy use No/nk	Yes	Total
N	38	6	44
NS	26	5	31
Wt	969.04	54.44	1023.48
Het Chi	521.21	1.53	530.13
Het df	37	5	43
Het P	***	N.S.	***
Fixed RR	4.72	6.90	4.82
RRl	4.44	5.29	4.53
RRu	5.03	9.00	5.12
P	+++	+++	+++
Random RR	3.93	6.90	4.27
RRl	3.03	5.29	3.37
RRu	5.10	9.00	5.40
P	+++	+++	+++
Between Chi			7.39
Between df			1
Between P			**
Btwn(F) P			N.S.
Btwn(R) P			**

	Full histological confirmation No	Yes	Total
N	23	21	44
NS	17	14	31
Wt	360.10	663.38	1023.48
Het Chi	195.80	318.15	530.13
Het df	22	20	43
Het P	***	***	***
Fixed RR	4.06	5.29	4.82
RRl	3.67	4.90	4.53
RRu	4.51	5.71	5.12
P	+++	+++	+++
Random RR	5.65	3.24	4.27
RRl	4.00	2.32	3.37
RRu	7.99	4.53	5.40
P	+++	+++	+++
Between Chi			16.19
Between df			1
Between P			***
Btwn(F) P			N.S.
Btwn(R) P			*

	Number of adjustment variables (1) 0	1	2+/+nk	Total
N	32	6	6	44
NS	23	4	4	31
Wt	476.01	58.95	488.52	1023.48
Het Chi	337.83	12.02	165.50	530.13
Het df	31	5	5	43
Het P	***	*	***	***
Fixed RR	4.27	6.33	5.25	4.82
RRl	3.91	4.90	4.80	4.53
RRu	4.67	8.17	5.73	5.12
P	+++	+++	+++	+++
Random RR	3.93	6.16	4.66	4.27
RRl	2.86	3.97	2.67	3.37
RRu	5.40	9.56	8.14	5.40
P	+++	+++	+++	+++
Between Chi				14.78
Between df				2
Between P				***
Btwn(F) P				N.S.
Btwn(R) P				N.S.

Table 3B1 - 6

IESLC - Meta-analysis of Current Smoking (vs never smoking), Any product (or Cigarettes if Any not available)
Adenocarcinoma
Least adjusted

	Number of adjustment variables (2) 0	1	2	3-5	6+/+nk	Total
N	32	6	5		1	44
NS	23	4	3		1	31
Wt	476.01	58.95	484.39		4.13	1023.48
Het Chi	337.83	12.02	165.13		0.00	530.13
Het df	31	5	4		0	43
Het P	***	*	***		N.S.	***
Fixed RR	4.27	6.33	5.23		7.06	4.82
RRl	3.91	4.90	4.79		2.69	4.53
RRu	4.67	8.17	5.72		18.51	5.12
P	+++	+++	+++		+++	+++
Random RR	3.93	6.16	4.41		7.06	4.27
RRl	2.86	3.97	2.42		2.69	3.37
RRu	5.40	9.56	8.02		18.51	5.40
P	+++	+++	+++		+++	+++
Between Chi						15.15
Between df						3
Between P						**
Btwn(F) P						N.S.
Btwn(R) P						N.S.

	Product all/unsp	cig+/-ot	cig only	Total
N	11	28	5	44
NS	9	19	4	32
Wt	113.50	878.94	31.04	1023.48
Het Chi	43.33	430.34	11.04	530.13
Het df	10	27	4	43
Het P	***	***	*	***
Fixed RR	2.66	5.17	5.68	4.82
RRl	2.21	4.84	4.00	4.53
RRu	3.20	5.53	8.08	5.12
P	+++	+++	+++	+++
Random RR	2.43	5.10	5.53	4.27
RRl	1.62	3.82	2.60	3.37
RRu	3.66	6.80	11.75	5.40
P	+++	+++	+++	+++
Between Chi				45.42
Between df				2
Between P				***
Btwn(F) P				N.S.
Btwn(R) P				*

	Denominator nev any	nev cigs	Total
N	27	17	44
NS	21	12	33
Wt	481.96	541.52	1023.48
Het Chi	190.69	141.43	530.13
Het df	26	16	43
Het P	***	***	***
Fixed RR	3.02	7.30	4.82
RRl	2.77	6.71	4.53
RRu	3.31	7.94	5.12
P	+++	+++	+++
Random RR	3.12	6.77	4.27
RRl	2.38	5.04	3.37
RRu	4.08	9.10	5.40
P	+++	+++	+++
Between Chi			198.01
Between df			1
Between P			***
Btwn(F) P			***
Btwn(R) P			***

Table 3B1 - 6

IESLC - Meta-analysis of Current Smoking (vs never smoking), Any product (or Cigarettes if Any not available)
Adenocarcinoma
Least adjusted

		Derivation of RR/CI			
		Orig	StdCalc	Other	Total
	N	8	31	5	44
	NS	6	22	4	32
	Wt	512.07	479.47	31.94	1023.48
Het	Chi	182.93	333.24	6.15	530.13
Het	df	7	30	4	43
Het	P	***	***	N.S.	***
Fixed	RR	5.24	4.39	5.17	4.82
	RRl	4.80	4.02	3.65	4.53
	RRu	5.71	4.80	7.31	5.12
	P	+++	+++	+++	+++
Random	RR	4.18	4.28	4.74	4.27
	RRl	2.54	3.13	2.81	3.37
	RRu	6.86	5.87	7.98	5.40
	P	+++	+++	+++	+++
Between	Chi				7.82
Between	df				2
Between	P				*
Btwn(F)	P				N.S.
Btwn(R)	P				N.S.

Table 3B1 - 7

IESLC - Meta-analysis of Current Smoking (vs never smoking), Any product (or Cigarettes if Any not available)
Adenocarcinoma
Excluded studies (and stage at which they were excluded)

1	BOUCHA BUELL LAURIL MZILEN
2	ABELIN ABRAHA AUVINE AXELSO BAND BERRIN BLOT1 BLOT2 BLOT3 BLOT4 BRESLO BROCKM BROWN1 BYERS1 CASCO2 CASCOR CHAN CHATZI CHEN CHEN2 CHEN3 CHIAZZ COOKSO DAVEYS DEAN DOSEME DU DUNN EBELIN ESAKI FAN GENG GER GODLEY GOLLED GSELL HANSEN HEGMAN HINDS HIRAOK HOROWI HORWIT HU HU2 HUANG ISHIMA JARUP JIANG JONES JUSSAW KO KOHLME KOULUM KREYBE LAMTH LAMWK LAMWK2 LAUSSM LEI LETOUR LEVIN LIU LIU2 LIU3 LIU4 LIU5 LOMBA2 LUO MAGNUS MARSH MARSH2 MARTIS MASTRA MATSUD MCCONN MCDUFF MCLAUG MILLER MILLS MOLLO NOTAN2 NOU ORMOS PASTOR PAWLEG PERNU PERSHA PIKE POFFIJ POLEDN PRESCO QIAO RADZIK RANDIG REN RONCO ROOTS ROTHSC SADOWS SANKAR SCHWA2 SEGI SEOW SIEMIA SIMARA STASZE STOCKS SUN TAO ULMER VANDER WANG WANG3 WANG4 WICKLU WILKIN WUWILL WYNDE2 WYNDE4 XIANGZ XU XU2 XU3 XU4 YUAN ZHANG ZHENG ZHOU
3	RESTRE
4	BOFFET
5	RIMING TANG2 WYNDE5 WYNDE8
6	BYERS2 HIRAY2 LICKIN MRFIT MURATA NOTANI SAARIK SHIMIZ SITAS STAYNE WARSIN WATSON WYNDER
8	AGUDO AKIBA ALDERS AMANDU AMES ANDERS ARCHER ARMADA AUSTIN AXELSS BECHER BENSHL BEST BLOHMK BRETT BROSS CARPEN CEDERL CHANG CHOI CHOW CHYOU DAMBER DARBY DEAN2 DEAN3 DEKLER DESTE2 DESTEF DOCKER DOLL DOLL2 DORANT DORGAN DROSTE ENSTRO GAO GAO2 GARCIA GARDIN GARSHI GILLIS GOODMA GRAHAM GREGOR HAMMO2 HAMMON HEIN HENNEK HIRAYA HITOSU HOLE HUMBLE JARVHO JOLY KAISE2 KAISER KANELL KAUFMA KELLER KINLEN KJUUS KNEKT KOO KREUZE KUBIK LANGE LEMARC LIAW LIDDEL LOMBAR LUBIN MACLEN MIGRAN MRFITR NAM ODRISC PARKIN PERSH2 PETO PEZZO2 PEZZOT PISANI QIAO2 RACHTA SCHWAR SEGI2 SHAW SIMONA SPEIZE SPITZ STOCKW STUCKE SUZUK2 TANG TENKAN TIZZAN TOKARS TOUSEY TULINI TVERDA VEIERO WALD WANG2 WIGLE WUNSCH YAMAGU YONG
10	BENHAM VUTUC WYNDE7

Table 3B1 - 8
Potentially overlapping studies

REF	REFGP	PRINC	.	OVERLAP/LINK
LUBIN2	LUBIN2	1		Lubin-combined
OSANN2	KAISER	2		KAISER/OSANN2
WYNDE6	WYNDE6	1		WYNDE5/6/7/8
CPSI	CPSI	1		CPSI overall
SOBUE2	SOBUE2	1		SOBUE2/MATSUD
JAHN	BOFFET	2		Subset of BOFFET

Table 3B1 - 9
Most adjusted - insufficient data for metaanalysis

REF	NRR	SEX	AGEL	AGEH	RACE	YF	LC TYPE	LOC	START	ST	NLC	R	VB	P	H	AD	PRODUCT	DENOM	De
CHYOU	6	m	0	0	jap	0	a	NAmer	1965	pr	227	n	bl	n	y	1	cig+/-ot	nev cigs	or

REF	NRR	RR	SIG	RRDATA comment
CHYOU	6	6.80		0

Table 3B2 -

IESLC - Meta-analysis of Current Smoking (vs never smoking), Cigarettes (or Any Product if Cigarettes not available)
Adenocarcinoma

This analysis is restricted to results for:
1) Non-dose-response data
2) Current smokers
3) Results complete enough for use in metaanalysis

Within each study, results are then selected (in the following order of preference, within each sex) for:
4) PRODUCT: cigarettes regardless of other products, cigarettes only, all/unspec
5) CIGTYPE: all/unspecified, MC regardless of HR, MC only
6) DENOM: never smoked anything, never smoked cigarettes, (never +1 = +long term ex, +2 = +amount unknown, +3 = never cigs+long term ex)
7) Followup period (YF, prospective studies): whole study (coded as 0) or longest available
8) LCtype: adeno or nearest available, but not squamous. (q = squamous, s = small, a = adeno, l = large, KII = Kreyberg II, al = alveolar, br = bronchiolar, u = undifferentiated)
9) Race: all or nearest available, otherwise by race (wh or w = white, bl or b = black, hi = hispanic ch = chinese, jap = japanese, haw = hawaiian, w+o = white + oriental, sca = scandinavian, as = asian)
10) For overlapping studies: principal rather than subsidiary studies
Finally by Age: whole study (coded as 0) if available, otherwise by widest available age group
and then for single sex results (m, f) in preference to combined sex results (c).

Results adjusted (AD) for the most potential confounders are then chosen in Sections -1 to -3
(and those which actually differ from the adjusted results in Table 3B1 - 1 are marked 'x' in Section -1)
and results adjusted for the least confounders in Sections -4 to -6. (Those least adjusted results which
actually differ from the most adjusted as marked 'x' in column X in Section -4)
(Results adjusted for an unknown number of confounder(s) are coded as 20.)

Section -7 shows excluded studies, together with the stage (as above) at which no qualifying
results were found.

Section -8 lists the potentially overlapping studies which have been included (1=principal, 2=subsidiary).

Section -9 lists any results which would have been included in preference except that they had data not complete
enough for use in meta-analysis, with their significance (yes/no), if known, and any further comment as entered
on the database.

In addition to those mentioned above, the following fields, levels and abbreviations are used:
* or nk = not known, n = no, y = yes, ot = other
nev = never
all/unspec = all or unspecified, cig+/-ot = cigarettes irrespective of other products (cigar, pipe etc)
MC = manufactured cigarettes, HR = hand-rolled cigarettes
REF: 6-character study reference
NRR: number of the RR on the database within the study
ST : study type (CC = case control, pr or prosp = prospective)
NLC: number of lung cancer cases in whole study
R : risky occupational population (n = no, m = mining, o = other risky)
VB : national cigarette type (V = at least 75% Virginia, bl = at least 75% blended, ot = other)
P : any proxy use
H : full histological confirmation
De : derivation of RR/CI (or = original, st = standard method, ot = other method of estimation)

Table 3B2 - 1

IESLC - Meta-analysis of Current Smoking (vs never smoking), Cigarettes (or Any Product if Cigarettes not available)
Adenocarcinoma
Most adjusted

REF	NRR	3B1	SEX	AGEL	AGEH	RACE	YF	LC TYPE	LOC	START	ST	NLC	R	VB	P	H	AD	PRODUCT	DENOM	De
BARBON	100		m	0	0	all	-	a	Eu:wst	1979	CC	755	n	bl	y	y	1	all/unsp	nev any	or
BOUCOT	147		m	0	0	all	0	a	NAmer	1951	pr	121	n	bl	n	n	2	cig only	nev any	ot
BROWN2	14		m	0	0	wh	-	a	NAmer	1984	CC	14596	n	bl	n	y	2	cig+/-ot	nev cigs	or
BROWN2	13		f	0	0	wh	-	a	NAmer	1984	CC	14596	n	bl	n	y	2	cig+/-ot	nev cigs	or
BUFFLE	71		f	0	0	w-hi	-	a	NAmer	1976	CC	943	n	bl	y	n	0	cig+/-ot	nev cigs	st
COMSTO	24		m	0	0	all	-	a	NAmer	1975	ot	258	n	bl	n	n	0	cig+/-ot	nev cigs	st
COMSTO	31		f	0	0	all	-	a	NAmer	1975	ot	258	n	bl	n	n	0	cig+/-ot	nev cigs	st
CORREA	44		c	0	0	all	-	a	NAmer	1979	CC	1359	n	bl	y	n	1	cig+/-ot	nev cigs	or
CPSI	404		m	0	0	all	2	a	NAmer	1959	pr	5138	n	bl	n	n	1	cig only	nev any	ot
CPSI	406		f	0	0	all	2	a	NAmer	1959	pr	5138	n	bl	n	n	1	cig only	nev any	ot
CPSII	115		m	0	0	all	2	a	NAmer	1982	pr	3229	n	bl	n	n	1	cig only	nev any	st
CPSII	118		f	0	0	all	2	a	NAmer	1982	pr	3229	n	bl	n	n	1	cig+/-ot	nev cigs	st
DORN	340		m	0	0	wh	8	a	NAmer	1954	pr	5097	n	bl	n	n	1	cig only	nev any	ot
ENGELA	70		m	0	0	all	0	a	Eu:Sca	1964	pr	435	n	bl	n	n	7	cig+/-ot	nev cigs	ot
HAENSZ	37		f	0	0	all	-	a	NAmer	1955	CC	158	n	bl	n	y	0	cig+/-ot	nev any	st
JAHN	8		m	0	0	all	-	a	Eu:Ger	1988	CC	1004	n	bl	n	n	0	cig+/-ot	nev any	st
JAIN	17		m	0	0	all	-	a	NAmer	1981	CC	845	n	V	y	n	0	cig+/-ot	nev cigs	st
JAIN	12		f	0	0	all	-	a	NAmer	1981	CC	845	n	V	y	n	0	cig+/-ot	nev cigs	st
JEDRYC	26		m	0	0	all	-	a	Eu:est	1980	CC	1630	n	bl	y	n	0	cig+/-ot	nev any	st
KATSOU	12		f	0	0	all	-	a	Eu:bal	1987	CC	101	n	bl	n	n	1	all/unsp	nev any	or
KHUDER	17		m	0	0	all	-	a	NAmer	1985	CC	482	n	bl	n	y	0	cig+/-ot	nev cigs	or
KIHARA	5		c	0	0	jap	-	a	As:Jap	1991	CC	440	n	bl	n	n	0	all/unsp	nev any	st
LUBIN2	252		m	0	0	all	-	a	Eu:mul	1976	CC	7804	n	bl	n	y	0	cig+/-ot	nev any	st
LUBIN2	264		f	0	0	all	-	a	Eu:mul	1976	CC	7804	n	bl	n	y	0	cig+/-ot	nev any	st
MATOS	53		m	0	0	all	-	a	SCAmer	1994	CC	200	n	bl	n	n	2	cig+/-ot	nev any	or
OSANN	39		m	0	0	all	-	a	NAmer	1984	CC	1986	n	bl	n	n	2	cig+/-ot	nev cigs	or
OSANN	40		f	0	0	all	-	a	NAmer	1984	CC	1986	n	bl	n	n	2	cig+/-ot	nev cigs	or
OSANN2	32		f	0	0	all	-	KII	NAmer	1964	ot	217	n	bl	n	y	1	cig+/-ot	nev cigs	or
SOBUE	36		m	0	0	all	-	a	As:Jap	1986	CC	1376	n	bl	n	y	1	cig+/-ot	nev cigs	or
SOBUE	46		f	0	0	all	-	a	As:Jap	1986	CC	1376	n	bl	n	y	1	cig+/-ot	nev cigs	or
SOBUE2	2		m	0	0	all	-	a	As:Jap	1965	CC	2083	n	bl	n	n	2	cig+/-ot	nev any	or
SOBUE2	6		f	0	0	all	-	a	As:Jap	1965	CC	2083	n	bl	n	n	2	cig+/-ot	nev any	or
SUZUKI	10		m	0	0	all	-	a	As:Jap	1978	CC	238	n	bl	n	y	2	cig+/-ot	nev any	or
SUZUKI	14		f	0	0	all	-	a	As:Jap	1978	CC	238	n	bl	n	y	2	cig+/-ot	nev any	or
SVENSS	99		f	0	0	all	-	a	Eu:Sca	1983	CC	210	n	bl	n	n	1	all/unsp	nev any	ot
TSUGAN	3		m	0	0	all	-	a	As:Jap	1976	CC	134	n	bl	n	y	0	all/unsp	nev any	st
TSUGAN	9		f	0	0	all	-	a	As:Jap	1976	CC	134	n	bl	n	y	0	all/unsp	nev any	or
WAKAI	12		m	0	0	all	-	a	As:Jap	1988	CC	333	n	bl	n	y	1	all/unsp	nev any	or
WAKAI	30		f	0	0	all	-	a	As:Jap	1988	CC	333	n	bl	n	y	1	all/unsp	nev any	or
WU	7		f	0	0	wh	-	a	NAmer	1981	CC	220	n	bl	n	y	2	all/unsp	nev any	or
WU2	1		f	0	0	all	-	a	NAmer	1983	CC	336	n	bl	n	y	2	all/unsp	nev any	or
WYNDE3	30		m	0	0	all	-	KII	NAmer	1966	CC	350	n	bl	n	y	0	all/unsp	nev any	st
WYNDE6	15		m	0	0	all	-	KII	NAmer	1969	CC	4423	n	bl	n	y	0	cig+/-ot	nev any	st
WYNDE6	204		f	0	0	all	-	KII	NAmer	1969	CC	4423	n	bl	n	y	0	cig+/-ot	nev cigs	st

Cigarette type is all/unspec for all RRs

Table 3B2 - 2

IESLC - Meta-analysis of Current Smoking (vs never smoking), Cigarettes (or Any Product if Cigarettes not available)
Adenocarcinoma
Most adjusted

REF	NRR	SEX	AD	Number Exposed Case	Number Exposed Cont	Non-exposed Case	Non-exposed Cont	RR	95.00%CI
BARBON	100	m	1	-	-	-	-	7.90	(3.60- 17.40)
*BOUCOT	147	m	2	-	-	-	-	10.95	(0.65- 183.57)
BROWN2	14	m	2	-	-	-	-	9.10	(7.60- 10.80)
BROWN2	13	f	2	-	-	-	-	7.20	(6.20- 8.30)
Subtotal BROWN2								7.92	(7.08- 8.86)
BUFFLE	71	f	0	56	110	7	112	8.15	(3.56- 18.65)
COMSTO	24	m	0	30	100	2	84	12.60	(2.92- 54.28)
COMSTO	31	f	0	23	52	8	115	6.36	(2.67- 15.16)
Subtotal COMSTO								7.60	(3.60- 16.04)
CORREA	44	c	1	-	-	-	-	6.70	(4.30- 10.60)
*CPSI	404	m	1	-	-	-	-	4.58	(1.74- 12.05)
*CPSI	406	f	1	-	-	-	-	1.43	(0.47- 4.39)
Subtotal CPSI								2.78	(1.34- 5.78)
*CPSII	115	m	1	-	-	-	-	19.22	(6.46- 57.16)
*CPSII	118	f	1	-	-	-	-	8.23	(4.36- 15.54)
Subtotal CPSII								10.21	(5.89- 17.67)
*DORN	340	m	1	-	-	-	-	5.95	(3.85- 9.22)
*ENGELA	70	m	7	-	-	-	-	7.06	(2.69- 18.50)
HAENSZ	37	f	0	16	94	37	236	1.09	(0.58- 2.05)
JAHN	8	m	0	75	269	8	138	4.81	(2.26- 10.26)
JAIN	17	m	0	60	118	4	85	10.81	(3.78- 30.87)
JAIN	12	f	0	69	99	24	214	6.21	(3.69- 10.47)
Subtotal JAIN								6.93	(4.35- 11.07)
JEDRYC	26	m	0	68	516	7	289	5.44	(2.47- 12.00)
KATSOU	12	f	1	-	-	-	-	1.70	(0.75- 3.89)
KHUDER	17	m	0	92	-	7	-	8.20	(3.60- 18.40)
KIHARA	5	c	0	103	162	78	237	1.93	(1.35- 2.76)
LUBIN2	252	m	0	454	6209	57	2616	3.36	(2.54- 4.44)
LUBIN2	264	f	0	69	410	138	1180	1.44	(1.06- 1.96)
Subtotal LUBIN2								2.30	(1.87- 2.83)
MATOS	53	m	2	-	-	-	-	7.90	(3.00- 20.90)
OSANN	39	m	2	-	-	-	-	21.70	(12.50- 39.70)
OSANN	40	f	2	-	-	-	-	11.60	(8.20- 16.40)
Subtotal OSANN								13.69	(10.17- 18.43)
OSANN2	32	f	1	-	-	-	-	3.20	(1.50- 6.60)
SOBUE	36	m	1	-	-	-	-	1.90	(1.30- 3.00)
SOBUE	46	f	1	-	-	-	-	1.30	(0.90- 2.00)
Subtotal SOBUE								1.56	(1.17- 2.08)
SOBUE2	2	m	2	-	-	-	-	3.10	(2.40- 3.70)
SOBUE2	6	f	2	-	-	-	-	1.80	(1.40- 2.20)
Subtotal SOBUE2								2.39	(2.04- 2.79)
SUZUKI	10	m	2	-	-	-	-	5.00	(2.71- 9.27)
SUZUKI	14	f	2	-	-	-	-	2.40	(1.19- 4.86)
Subtotal SUZUKI								3.64	(2.29- 5.78)
SVENSS	99	f	1	-	-	-	-	3.78	(1.91- 7.48)
TSUGAN	3	m	0	45	50	18	17	0.85	(0.39- 1.85)
TSUGAN	9	f	0	6	10	33	30	0.55	(0.18- 1.68)
Subtotal TSUGAN								0.74	(0.39- 1.40)
WAKAI	12	m	1	-	-	-	-	2.18	(1.00- 4.76)
WAKAI	30	f	1	-	-	-	-	1.14	(0.49- 2.61)
Subtotal WAKAI								1.61	(0.91- 2.85)
WU	7	f	2	-	-	-	-	4.10	(2.30- 7.50)
WU2	1	f	2	-	-	-	-	4.50	(3.00- 6.90)
WYNDE3	30	m	0	56	207	6	88	3.97	(1.65- 9.55)
WYNDE6	15	m	0	651	741	58	617	9.35	(7.00- 12.48)
WYNDE6	204	f	0	472	376	119	856	9.03	(7.14- 11.42)
Subtotal WYNDE6								9.15	(7.63- 10.98)
Partial Totals				2345	9523	611	6914		

*prospective study

Table 3B2 - 2

IESLC - Meta-analysis of Current Smoking (vs never smoking), Cigarettes (or Any Product if Cigarettes not available)
Adenocarcinoma
Most adjusted

REF	NRR	SEX	AD	Ys	Ws	Qs	Ps
BARBON	100	m	1	2.07	6.19	1.58	0.0000
*BOUCOT	147	m	2	2.39	0.48	0.33	0.0964
BROWN2	14	m	2	2.21	124.44	51.93	0.0000
BROWN2	13	f	2	1.97	180.58	30.62	0.0000
Subtotal BROWN2				2.07	305.02	82.55	
BUFFLE	71	f	0	2.10	5.59	1.60	0.0000
COMSTO	24	m	0	2.53	1.80	1.70	0.0007
COMSTO	31	f	0	1.85	5.09	0.42	0.0000
Subtotal COMSTO				2.03	6.89	2.12	
CORREA	44	c	1	1.90	18.88	2.18	0.0000
*CPSI	404	m	1	1.52	4.10	0.01	0.0021
*CPSI	406	f	1	0.36	3.08	4.47	0.5303
Subtotal CPSI				1.02	7.18	4.47	
*CPSII	115	m	1	2.96	3.23	6.28	0.0000
*CPSII	118	f	1	2.11	9.51	2.83	0.0000
Subtotal CPSII				2.32	12.75	9.11	
*DORN	340	m	1	1.78	20.15	0.99	0.0000
*ENGELA	70	m	7	1.95	4.13	0.64	0.0001
HAENSZ	37	f	0	0.08	9.58	20.98	0.7992
JAHN	8	m	0	1.57	6.70	0.00	0.0000
JAIN	17	m	0	2.38	3.49	2.33	0.0000
JAIN	12	f	0	1.83	14.10	0.99	0.0000
Subtotal JAIN				1.94	17.58	3.32	
JEDRYC	26	m	0	1.69	6.14	0.11	0.0000
KATSOU	12	f	1	0.53	5.67	6.04	0.2064
KHUDER	17	m	0	2.10	5.77	1.70	0.0000
KIHARA	5	c	0	0.66	30.38	24.81	0.0003
LUBIN2	252	m	0	1.21	49.29	6.09	0.0000
LUBIN2	264	f	0	0.36	39.96	57.38	0.0214
Subtotal LUBIN2				0.83	89.24	63.47	
MATOS	53	m	2	2.07	4.08	1.04	0.0000
OSANN	39	m	2	3.08	11.51	26.41	0.0000
OSANN	40	f	2	2.45	31.98	25.26	0.0000
Subtotal OSANN				2.62	43.49	51.67	
OSANN2	32	f	1	1.16	7.00	1.12	0.0021
SOBUE	36	m	1	0.64	21.97	18.61	0.0026
SOBUE	46	f	1	0.26	24.10	40.72	0.1978
Subtotal SOBUE				0.44	46.07	59.34	
SOBUE2	2	m	2	1.13	82.01	15.22	0.0000
SOBUE2	6	f	2	0.59	75.22	71.43	0.0000
Subtotal SOBUE2				0.87	157.22	86.65	
SUZUKI	10	m	2	1.61	10.16	0.02	0.0000
SUZUKI	14	f	2	0.88	7.76	3.66	0.0147
Subtotal SUZUKI				1.29	17.92	3.68	
SVENSS	99	f	1	1.33	8.25	0.45	0.0001
TSUGAN	3	m	0	-0.16	6.39	19.00	0.6813
TSUGAN	9	f	0	-0.61	3.03	14.24	0.2916
Subtotal TSUGAN				-0.31	9.41	33.23	
WAKAI	12	m	1	0.78	6.31	3.87	0.0502
WAKAI	30	f	1	0.13	5.49	11.25	0.7588
Subtotal WAKAI				0.48	11.80	15.12	
WU	7	f	2	1.41	11.00	0.25	0.0000
WU2	1	f	2	1.50	22.15	0.08	0.0000
WYNDE3	30	m	0	1.38	4.98	0.17	0.0021
WYNDE6	15	m	0	2.23	45.98	20.81	0.0000
WYNDE6	204	f	0	2.20	69.69	28.39	0.0000
Subtotal WYNDE6				2.21	115.67	49.19	

Table 3B2 - 2

IESLC - Meta-analysis of Current Smoking (vs never smoking), Cigarettes (or Any Product if Cigarettes not available)
Adenocarcinoma
Most adjusted

	N	44
	NS	31
	Wt	1017.36
Het	Chi	527.97
Het	df	43
Het	P	***
Fixed	RR	4.77
	RRl	4.49
	RRu	5.07
	P	+++
Random	RR	4.21
	RRl	3.32
	RRu	5.34
	P	+++
Asymm	P	N.S.

Table 3B2 - 3

IESLC - Meta-analysis of Current Smoking (vs never smoking), Cigarettes (or Any Product if Cigarettes not available)
Adenocarcinoma
Most adjusted

	Sex			
	combined	male	female	Total
N	2	22	20	44
NS	2	22	20	44
Wt	49.25	429.29	538.82	1017.36
Het Chi	18.00	169.26	318.73	527.97
Het df	1	21	19	43
Het P	***	***	***	***
Fixed RR	3.11	5.54	4.40	4.77
RRl	2.35	5.04	4.05	4.49
RRu	4.11	6.09	4.79	5.07
P	+++	+++	+++	+++
Random RR	3.57	5.56	3.20	4.21
RRl	1.06	4.06	2.18	3.32
RRu	12.07	7.61	4.71	5.34
P	+	+++	+++	+++
Between Chi				21.98
Between df				2
Between P				***
Btwn(F) P				N.S.
Btwn(R) P				(*)

	Lung cancer type						
	a	a+l	a+al+br	KII	not q+u	not q+s	Total
N	40			4			44
NS	28			3			31
Wt	889.71			127.65			1017.36
Het Chi	471.72			10.21			527.97
Het df	39			3			43
Het P	***			*			***
Fixed RR	4.40			8.36			4.77
RRl	4.12			7.03			4.49
RRu	4.70			9.95			5.07
P	+++			+++			+++
Random RR	4.05			6.86			4.21
RRl	3.15			4.62			3.32
RRu	5.22			10.19			5.34
P	+++			+++			+++
Between Chi							46.05
Between df							1
Between P							***
Btwn(F) P							(*)
Btwn(R) P							*

	Location								
	NAmer	UK	Scand	othEur	China	Japan	othAs	other	Total
N	24		2	6		11		1	44
NS	17		2	5		6		1	31
Wt	614.16		12.38	113.94		272.81		4.08	1017.36
Het Chi	96.60		1.07	31.70		40.77		0.00	527.97
Het df	23		1	5		10		0	43
Het P	***		N.S.	***		***		N.S.	***
Fixed RR	7.63		4.66	2.65		2.11		7.90	4.77
RRl	7.05		2.67	2.20		1.87		2.99	4.49
RRu	8.25		8.13	3.18		2.37		20.85	5.07
P	+++		+++	+++		+++		+++	+++
Random RR	6.72		4.69	3.30		1.87		7.90	4.21
RRl	5.50		2.62	1.93		1.42		2.99	3.32
RRu	8.21		8.40	5.65		2.47		20.85	5.34
P	+++		+++	+++		+++		+++	+++
Between Chi									357.82
Between df									4
Between P									***
Btwn(F) P									***
Btwn(R) P									***

Table 3B2 - 3

IESLC - Meta-analysis of Current Smoking (vs never smoking), Cigarettes (or Any Product if Cigarettes not available)
Adenocarcinoma
Most adjusted

	Detailed Country in "other Europe"					
	multi	Germany	othWest	East	Balkans	Total
N	2	1	1	1	1	6
NS	1	1	1	1	1	5
Wt	89.24	6.70	6.19	6.14	5.67	113.94
Het Chi	15.82	0.00	0.00	0.00	0.00	31.70
Het df	1	0	0	0	0	5
Het P	***	N.S.	N.S.	N.S.	N.S.	***
Fixed RR	2.30	4.81	7.90	5.44	1.70	2.65
RRl	1.87	2.26	3.59	2.47	0.75	2.20
RRu	2.83	10.26	17.37	12.00	3.87	3.18
P	+++	+++	+++	+++	N.S.	+++
Random RR	2.20	4.81	7.90	5.44	1.70	3.30
RRl	0.96	2.26	3.59	2.47	0.75	1.93
RRu	5.05	10.26	17.37	12.00	3.87	5.65
P	(+)	+++	+++	+++	N.S.	+++
Between Chi						15.88
Between df						4
Between P						**
Btwn(F) P						N.S.
Btwn(R) P						*

	Detailed Country in "other Asia"			
	India	HongKong	other	Total
N				
NS				
Wt				
Het Chi				
Het df				
Het P				N.S.
Fixed RR				
RRl				
RRu				
P				+++
Random RR				
RRl				
RRu				
P				+++
Between Chi				
Between df				
Between P				N.S.
Btwn(F) P				N.S.
Btwn(R) P				N.S.

	Detailed other continent			
	SCAmer	Auslia	Africa	Total
N	1			1
NS	1			1
Wt	4.08			4.08
Het Chi	0.00			0.00
Het df	0			0
Het P	N.S.			N.S.
Fixed RR	7.90			7.90
RRl	2.99			2.99
RRu	20.85			20.85
P	+++			+++
Random RR	7.90			7.90
RRl	2.99			2.99
RRu	20.85			20.85
P	+++			+++
Between Chi				
Between df				
Between P				N.S.
Btwn(F) P				N.S.
Btwn(R) P				N.S.

Table 3B2 - 3

IESLC - Meta-analysis of Current Smoking (vs never smoking), Cigarettes (or Any Product if Cigarettes not available)
Adenocarcinoma
Most adjusted

		Start year of study				
	<1960	1960-69	1970-79	1980-89	1990+	Total
N	5	7	11	19	2	44
NS	4	5	7	13	2	31
Wt	37.39	289.01	154.13	502.38	34.45	1017.36
Het Chi	22.12	133.45	74.33	187.40	7.13	527.97
Het df	4	6	10	18	1	43
Het P	***	***	***	***	**	***
Fixed RR	3.35	4.22	2.99	6.38	2.28	4.77
RRl	2.43	3.76	2.55	5.84	1.63	4.49
RRu	4.62	4.74	3.50	6.96	3.19	5.07
P	+++	+++	+++	+++	+++	+++
Random RR	2.92	4.55	3.41	5.06	3.62	4.21
RRl	1.17	2.50	2.10	3.65	0.92	3.32
RRu	7.28	8.30	5.53	7.02	14.29	5.34
P	+	+++	+++	+++	(+)	+++
Between Chi						103.54
Between df						4
Between P						***
Btwn(F) P						(*)
Btwn(R) P						N.S.

	Study type (1)		
	CC	other	Total
N	34	10	44
NS	24	7	31
Wt	958.78	58.58	1017.36
Het Chi	509.03	16.01	527.97
Het df	33	9	43
Het P	***	(*)	***
Fixed RR	4.71	5.93	4.77
RRl	4.42	4.59	4.49
RRu	5.01	7.66	5.07
P	+++	+++	+++
Random RR	3.88	5.95	4.21
RRl	2.96	4.07	3.32
RRu	5.07	8.69	5.34
P	+++	+++	+++
Between Chi			2.93
Between df			1
Between P			(*)
Btwn(F) P			N.S.
Btwn(R) P			(*)

	Study type (2)			
	CC	prosp	other	Total
N	34	7	3	44
NS	24	5	2	31
Wt	958.78	44.69	13.89	1017.36
Het Chi	509.03	12.15	3.22	527.97
Het df	33	6	2	43
Het P	***	(*)	N.S.	***
Fixed RR	4.71	6.28	4.92	4.77
RRl	4.42	4.68	2.91	4.49
RRu	5.01	8.42	8.32	5.07
P	+++	+++	+++	+++
Random RR	3.88	6.22	5.32	4.21
RRl	2.96	3.84	2.63	3.32
RRu	5.07	10.07	10.74	5.34
P	+++	+++	+++	+++
Between Chi				3.57
Between df				2
Between P				N.S.
Btwn(F) P				N.S.
Btwn(R) P				N.S.

Table 3B2 - 3

IESLC - Meta-analysis of Current Smoking (vs never smoking), Cigarettes (or Any Product if Cigarettes not available)
Adenocarcinoma
Most adjusted

	Study size (number of LC cases) 100-249	250-499	500-999	1000+	Total
N	11	9	4	20	44
NS	9	7	3	12	31
Wt	73.39	86.11	29.37	828.50	1017.36
Het Chi	37.81	30.80	1.01	404.12	527.97
Het df	10	8	3	19	43
Het P	***	***	N.S.	***	***
Fixed RR	2.54	3.20	7.35	5.18	4.77
RRl	2.02	2.59	5.12	4.84	4.49
RRu	3.19	3.95	10.55	5.54	5.07
P	+++	+++	+++	+++	+++
Random RR	2.45	3.83	7.35	4.97	4.21
RRl	1.54	2.41	5.12	3.54	3.32
RRu	3.90	6.09	10.55	6.97	5.34
P	+++	+++	+++	+++	+++
Between Chi					54.23
Between df					3
Between P					***
Btwn(F) P					N.S.
Btwn(R) P					**

	Risky occupational population no	mining	othRisky	Total
N	44			44
NS	31			31
Wt	1017.36			1017.36
Het Chi	527.97			527.97
Het df	43			43
Het P	***			***
Fixed RR	4.77			4.77
RRl	4.49			4.49
RRu	5.07			5.07
P	+++			+++
Random RR	4.21			4.21
RRl	3.32			3.32
RRu	5.34			5.34
P	+++			+++
Between Chi				
Between df				
Between P				N.S.
Btwn(F) P				N.S.
Btwn(R) P				N.S.

	National cigarette tobacco type Virginia	blended	other	Total
N	2	42		44
NS	1	30		31
Wt	17.58	999.78		1017.36
Het Chi	0.85	524.61		527.97
Het df	1	41		43
Het P	N.S.	***		***
Fixed RR	6.93	4.74		4.77
RRl	4.35	4.45		4.49
RRu	11.07	5.04		5.07
P	+++	+++		+++
Random RR	6.93	4.10		4.21
RRl	4.35	3.21		3.32
RRu	11.07	5.23		5.34
P	+++	+++		+++
Between Chi				2.51
Between df				1
Between P				N.S.
Btwn(F) P				N.S.
Btwn(R) P				(*)

Table 3B2 - 3

IESLC - Meta-analysis of Current Smoking (vs never smoking), Cigarettes (or Any Product if Cigarettes not available)
Adenocarcinoma
Most adjusted

	Any proxy use No/nk	Yes	Total
N	38	6	44
NS	26	5	31
Wt	962.98	54.38	1017.36
Het Chi	518.78	1.49	527.97
Het df	37	5	43
Het P	***	N.S.	***
Fixed RR	4.67	6.88	4.77
RRl	4.39	5.27	4.49
RRu	4.98	8.97	5.07
P	+++	+++	+++
Random RR	3.88	6.88	4.21
RRl	2.99	5.27	3.32
RRu	5.03	8.97	5.34
P	+++	+++	+++
Between Chi			7.71
Between df			1
Between P			**
Btwn(F) P			N.S.
Btwn(R) P			**

	Full histological confirmation No	Yes	Total
N	23	21	44
NS	17	14	31
Wt	355.55	661.81	1017.36
Het Chi	184.15	326.35	527.97
Het df	22	20	43
Het P	***	***	***
Fixed RR	3.99	5.25	4.77
RRl	3.59	4.87	4.49
RRu	4.43	5.67	5.07
P	+++	+++	+++
Random RR	5.59	3.20	4.21
RRl	3.98	2.28	3.32
RRu	7.84	4.49	5.34
P	+++	+++	+++
Between Chi			17.48
Between df			1
Between P			***
Btwn(F) P			N.S.
Btwn(R) P			*

	Number of adjustment variables (1) 0	1	2+/+nk	Total
N	17	14	13	44
NS	12	10	9	31
Wt	307.94	143.93	565.49	1017.36
Het Chi	197.14	79.28	213.12	527.97
Het df	16	13	12	43
Het P	***	***	***	***
Fixed RR	4.28	3.25	5.58	4.77
RRl	3.83	2.76	5.13	4.49
RRu	4.79	3.83	6.05	5.07
P	+++	+++	+++	+++
Random RR	3.90	3.46	5.68	4.21
RRl	2.54	2.27	3.80	3.32
RRu	6.00	5.29	8.49	5.34
P	+++	+++	+++	+++
Between Chi				38.43
Between df				2
Between P				***
Btwn(F) P				N.S.
Btwn(R) P				N.S.

Table 3B2 - 3

IESLC - Meta-analysis of Current Smoking (vs never smoking), Cigarettes (or Any Product if Cigarettes not available)
Adenocarcinoma
Most adjusted

	Number of adjustment variables (2) 0	1	2	3-5	6+/+nk	Total
N	17	14	12		1	44
NS	12	10	8		1	31
Wt	307.94	143.93	561.36		4.13	1017.36
Het Chi	197.14	79.28	212.89		0.00	527.97
Het df	16	13	11		0	43
Het P	***	***	***		N.S.	***
Fixed RR	4.28	3.25	5.57		7.06	4.77
RRl	3.83	2.76	5.12		2.69	4.49
RRu	4.79	3.83	6.05		18.51	5.07
P	+++	+++	+++		+++	+++
Random RR	3.90	3.46	5.60		7.06	4.21
RRl	2.54	2.27	3.68		2.69	3.32
RRu	6.00	5.29	8.50		18.51	5.34
P	+++	+++	+++		+++	+++
Between Chi						38.67
Between df						3
Between P						***
Btwn(F) P						N.S.
Btwn(R) P						N.S.

	Product all/unsp	cig+/-ot	cig only	Total
N	11	28	5	44
NS	9	19	4	32
Wt	109.83	876.49	31.04	1017.36
Het Chi	41.63	429.96	11.10	527.97
Het df	10	27	4	43
Het P	***	***	*	***
Fixed RR	2.61	5.11	5.69	4.77
RRl	2.16	4.79	4.00	4.49
RRu	3.14	5.46	8.09	5.07
P	+++	+++	+++	+++
Random RR	2.40	5.02	5.56	4.21
RRl	1.59	3.76	2.61	3.32
RRu	3.61	6.70	11.85	5.34
P	+++	+++	+++	+++
Between Chi				45.28
Between df				2
Between P				***
Btwn(F) P				N.S.
Btwn(R) P				*

	Denominator nev any	nev cigs	Total
N	27	17	44
NS	21	12	33
Wt	477.73	539.63	1017.36
Het Chi	189.32	147.85	527.97
Het df	26	16	43
Het P	***	***	***
Fixed RR	3.01	7.17	4.77
RRl	2.75	6.59	4.49
RRu	3.29	7.80	5.07
P	+++	+++	+++
Random RR	3.10	6.61	4.21
RRl	2.37	4.89	3.32
RRu	4.07	8.95	5.34
P	+++	+++	+++
Between Chi			190.80
Between df			1
Between P			***
Btwn(F) P			***
Btwn(R) P			***

Table 3B2 - 3

IESLC - Meta-analysis of Current Smoking (vs never smoking), Cigarettes (or Any Product if Cigarettes not available)
Adenocarcinoma
Most adjusted

	Derivation of RR/CI			
	Orig	StdCalc	Other	Total
N	21	17	6	44
NS	15	12	5	32
Wt	665.29	311.89	40.19	1017.36
Het Chi	326.66	192.60	6.86	527.97
Het df	20	16	5	43
Het P	***	***	N.S.	***
Fixed RR	4.91	4.47	4.85	4.77
RRl	4.55	4.00	3.56	4.49
RRu	5.30	5.00	6.61	5.07
P	+++	+++	+++	+++
Random RR	3.90	4.64	4.59	4.21
RRl	2.77	3.05	3.06	3.32
RRu	5.48	7.07	6.89	5.34
P	+++	+++	+++	+++
Between Chi				1.86
Between df				2
Between P				N.S.
Btwn(F) P				N.S.
Btwn(R) P				N.S.

Table 3B2 - 4

IESLC - Meta-analysis of Current Smoking (vs never smoking), Cigarettes (or Any Product if Cigarettes not available)
Adenocarcinoma
Least adjusted

REF	NRR	X	SEX	AGEL	AGEH	RACE	YF	LC TYPE	LOC	START	ST	NLC	R	VB	P	H	AD	PRODUCT	DENOM	De
BARBON	44	x	m	0	0	all	-	a	Eu:wst	1979	CC	755	n	bl	y	y	0	all/unsp	nev any	st
BOUCOT	72	x	m	0	0	all	0	a	NAmer	1951	pr	121	n	bl	n	n	0	cig only	nev any	ot
BROWN2	14		m	0	0	wh	-	a	NAmer	1984	CC	14596	n	bl	n	y	2	cig+/-ot	nev cigs	or
BROWN2	13		f	0	0	wh	-	a	NAmer	1984	CC	14596	n	bl	n	y	2	cig+/-ot	nev cigs	or
BUFFLE	71		f	0	0	w-hi	-	a	NAmer	1976	CC	943	n	bl	y	n	0	cig+/-ot	nev cigs	st
COMSTO	24		m	0	0	all	-	a	NAmer	1975	ot	258	n	bl	n	n	0	cig+/-ot	nev cigs	st
COMSTO	31		f	0	0	all	-	a	NAmer	1975	ot	258	n	bl	n	n	0	cig+/-ot	nev cigs	st
CORREA	44		c	0	0	all	-	a	NAmer	1979	CC	1359	n	bl	y	n	1	cig+/-ot	nev cigs	or
CPSI	404		m	0	0	all	2	a	NAmer	1959	pr	5138	n	bl	n	n	1	cig only	nev any	ot
CPSI	406		f	0	0	all	2	a	NAmer	1959	pr	5138	n	bl	n	n	1	cig only	nev any	ot
CPSII	115		m	0	0	all	2	a	NAmer	1982	pr	3229	n	bl	n	n	1	cig only	nev any	st
CPSII	118		f	0	0	all	2	a	NAmer	1982	pr	3229	n	bl	n	n	1	cig+/-ot	nev cigs	st
DORN	340		m	0	0	wh	8	a	NAmer	1954	pr	5097	n	bl	n	n	1	cig only	nev any	ot
ENGELA	70		m	0	0	all	0	a	Eu:Sca	1964	pr	435	n	bl	n	n	7	cig+/-ot	nev cigs	ot
HAENSZ	37		f	0	0	all	-	a	NAmer	1955	CC	158	n	bl	n	y	0	cig+/-ot	nev any	st
JAHN	8		m	0	0	all	-	a	Eu:Ger	1988	CC	1004	n	bl	n	n	0	cig+/-ot	nev any	st
JAIN	17		m	0	0	all	-	a	NAmer	1981	CC	845	n	V	y	n	0	cig+/-ot	nev cigs	st
JAIN	12		f	0	0	all	-	a	NAmer	1981	CC	845	n	V	y	n	0	cig+/-ot	nev cigs	st
JEDRYC	26		m	0	0	all	-	a	Eu:est	1980	CC	1630	n	bl	y	n	0	cig+/-ot	nev any	st
KATSOU	16	x	f	0	0	all	-	a	Eu:bal	1987	CC	101	n	bl	n	n	0	all/unsp	nev any	st
KHUDER	17		m	0	0	all	-	a	NAmer	1985	CC	482	n	bl	n	y	0	cig+/-ot	nev cigs	or
KIHARA	5		c	0	0	jap	-	a	As:Jap	1991	CC	440	n	bl	n	n	0	all/unsp	nev any	st
LUBIN2	252		m	0	0	all	-	a	Eu:mul	1976	CC	7804	n	bl	n	y	0	cig+/-ot	nev any	st
LUBIN2	264		f	0	0	all	-	a	Eu:mul	1976	CC	7804	n	bl	n	y	0	cig+/-ot	nev any	st
MATOS	52	x	m	0	0	all	-	a	SCAmer	1994	CC	200	n	bl	n	n	0	cig+/-ot	nev any	st
OSANN	11	x	m	0	0	all	-	a	NAmer	1984	CC	1986	n	bl	n	n	0	cig+/-ot	nev cigs	st
OSANN	15	x	f	0	0	all	-	a	NAmer	1984	CC	1986	n	bl	n	n	0	cig+/-ot	nev cigs	st
OSANN2	14	x	f	0	0	all	-	KII	NAmer	1964	ot	217	n	bl	n	y	0	cig+/-ot	nev cigs	st
SOBUE	6	x	m	0	0	all	-	a	As:Jap	1986	CC	1376	n	bl	n	y	0	cig+/-ot	nev cigs	st
SOBUE	22	x	f	0	0	all	-	a	As:Jap	1986	CC	1376	n	bl	n	y	0	cig+/-ot	nev cigs	st
SOBUE2	2		m	0	0	all	-	a	As:Jap	1965	CC	2083	n	bl	n	n	2	cig+/-ot	nev any	or
SOBUE2	6		f	0	0	all	-	a	As:Jap	1965	CC	2083	n	bl	n	n	2	cig+/-ot	nev any	or
SUZUKI	2	x	m	0	0	all	-	a	As:Jap	1978	CC	238	n	bl	n	y	0	cig+/-ot	nev any	st
SUZUKI	6	x	f	0	0	all	-	a	As:Jap	1978	CC	238	n	bl	n	y	0	cig+/-ot	nev any	st
SVENSS	64	x	f	0	0	all	-	a	Eu:Sca	1983	CC	210	n	bl	n	n	0	all/unsp	nev any	st
TSUGAN	3		m	0	0	all	-	a	As:Jap	1976	CC	134	n	bl	n	y	0	all/unsp	nev any	st
TSUGAN	9		f	0	0	all	-	a	As:Jap	1976	CC	134	n	bl	n	y	0	all/unsp	nev any	or
WAKAI	6	x	m	0	0	all	-	a	As:Jap	1988	CC	333	n	bl	n	y	0	all/unsp	nev any	st
WAKAI	24	x	f	0	0	all	-	a	As:Jap	1988	CC	333	n	bl	n	y	0	all/unsp	nev any	st
WU	2	x	f	0	0	wh	-	a	NAmer	1981	CC	220	n	bl	n	y	0	all/unsp	nev any	st
WU2	1		f	0	0	all	-	a	NAmer	1983	CC	336	n	bl	n	y	2	all/unsp	nev any	or
WYNDE3	30		m	0	0	all	-	KII	NAmer	1966	CC	350	n	bl	n	y	0	all/unsp	nev any	st
WYNDE6	15		m	0	0	all	-	KII	NAmer	1969	CC	4423	n	bl	n	y	0	cig+/-ot	nev any	st
WYNDE6	204		f	0	0	all	-	KII	NAmer	1969	CC	4423	n	bl	n	y	0	cig+/-ot	nev cigs	st

Cigarette type is all/unspec for all RRs

Table 3B2 - 5

IESLC - Meta-analysis of Current Smoking (vs never smoking), Cigarettes (or Any Product if Cigarettes not available)
Adenocarcinoma
Least adjusted

REF	NRR	SEX	AD	Number Exposed Case	Number Exposed Cont	Non-exposed Case	Non-exposed Cont	RR	95.00%CI
BARBON	44	m	0	109	362	7	188	8.09	(3.69- 17.72)
*BOUCOT	72	m	0	14	22177	0	7551	9.87~	(0.59- 165.51)
BROWN2	14	m	2	-	-	-	-	9.10	(7.60- 10.80)
BROWN2	13	f	2	-	-	-	-	7.20	(6.20- 8.30)
Subtotal BROWN2								7.92	(7.08- 8.86)
BUFFLE	71	f	0	56	110	7	112	8.15	(3.56- 18.65)
COMSTO	24	m	0	30	100	2	84	12.60	(2.92- 54.28)
COMSTO	31	f	0	23	52	8	115	6.36	(2.67- 15.16)
Subtotal COMSTO								7.60	(3.60- 16.04)
CORREA	44	c	1	-	-	-	-	6.70	(4.30- 10.60)
*CPSI	404	m	1	-	-	-	-	4.58	(1.74- 12.05)
*CPSI	406	f	1	-	-	-	-	1.43	(0.47- 4.39)
Subtotal CPSI								2.78	(1.34- 5.78)
*CPSII	115	m	1	-	-	-	-	19.22	(6.46- 57.16)
*CPSII	118	f	1	-	-	-	-	8.23	(4.36- 15.54)
Subtotal CPSII								10.21	(5.89- 17.67)
*DORN	340	m	1	-	-	-	-	5.95	(3.85- 9.22)
*ENGELA	70	m	7	-	-	-	-	7.06	(2.69- 18.50)
HAENSZ	37	f	0	16	94	37	236	1.09	(0.58- 2.05)
JAHN	8	m	0	75	269	8	138	4.81	(2.26- 10.26)
JAIN	17	m	0	60	118	4	85	10.81	(3.78- 30.87)
JAIN	12	f	0	69	99	24	214	6.21	(3.69- 10.47)
Subtotal JAIN								6.93	(4.35- 11.07)
JEDRYC	26	m	0	68	516	7	289	5.44	(2.47- 12.00)
KATSOU	16	f	0	15	18	30	67	1.86	(0.83- 4.18)
KHUDER	17	m	0	92	-	7	-	8.20	(3.60- 18.40)
KIHARA	5	c	0	103	162	78	237	1.93	(1.35- 2.76)
LUBIN2	252	m	0	454	6209	57	2616	3.36	(2.54- 4.44)
LUBIN2	264	f	0	69	410	138	1180	1.44	(1.06- 1.96)
Subtotal LUBIN2								2.30	(1.87- 2.83)
MATOS	52	m	0	46	132	5	110	7.67	(2.94- 19.96)
OSANN	11	m	0	217	541	14	833	23.87	(13.75- 41.41)
OSANN	15	f	0	193	367	47	1093	12.23	(8.70- 17.18)
Subtotal OSANN								14.70	(11.01- 19.64)
OSANN2	14	f	0	50	28	22	43	3.49	(1.75- 6.97)
SOBUE	6	m	0	276	650	27	128	2.01	(1.30- 3.12)
SOBUE	22	f	0	38	168	137	857	1.41	(0.95- 2.10)
Subtotal SOBUE								1.66	(1.24- 2.22)
SOBUE2	2	m	2	-	-	-	-	3.10	(2.40- 3.70)
SOBUE2	6	f	2	-	-	-	-	1.80	(1.40- 2.20)
Subtotal SOBUE2								2.39	(2.04- 2.79)
SUZUKI	2	m	0	119	162	14	99	5.19	(2.83- 9.54)
SUZUKI	6	f	0	20	20	55	133	2.42	(1.21- 4.84)
Subtotal SUZUKI								3.73	(2.36- 5.89)
SVENSS	64	f	0	38	53	22	120	3.91	(2.11- 7.25)
TSUGAN	3	m	0	45	50	18	17	0.85	(0.39- 1.85)
TSUGAN	9	f	0	6	10	33	30	0.55	(0.18- 1.68)
Subtotal TSUGAN								0.74	(0.39- 1.40)
WAKAI	6	m	0	75	284	8	65	2.15	(0.99- 4.67)
WAKAI	24	f	0	9	26	46	145	1.09	(0.48- 2.50)
Subtotal WAKAI								1.56	(0.89- 2.75)
WU	2	f	0	99	50	29	62	4.23	(2.43- 7.39)
WU2	1	f	2	-	-	-	-	4.50	(3.00- 6.90)
WYNDE3	30	m	0	56	207	6	88	3.97	(1.65- 9.55)
WYNDE6	15	m	0	651	741	58	617	9.35	(7.00- 12.48)
WYNDE6	204	f	0	472	376	119	856	9.03	(7.14- 11.42)
Subtotal WYNDE6								9.15	(7.63- 10.98)
Partial Totals				3663	34561	1074	18408		

*prospective study ~ With 0.5 adjustment for zero

Table 3B2 - 5

IESLC - Meta-analysis of Current Smoking (vs never smoking), Cigarettes (or Any Product if Cigarettes not available)
Adenocarcinoma
Least adjusted

REF	NRR	SEX	AD	Ys	Ws	Qs	Ps
BARBON	44	m	0	2.09	6.25	1.67	0.0000
*BOUCOT	72	m	0	2.29	0.48	0.25	0.1114
BROWN2	14	m	2	2.21	124.44	50.25	0.0000
BROWN2	13	f	2	1.97	180.58	29.07	0.0000
Subtotal BROWN2				2.07	305.02	79.32	
BUFFLE	71	f	0	2.10	5.59	1.54	0.0000
COMSTO	24	m	0	2.53	1.80	1.66	0.0007
COMSTO	31	f	0	1.85	5.09	0.39	0.0000
Subtotal COMSTO				2.03	6.89	2.05	
CORREA	44	c	1	1.90	18.88	2.05	0.0000
*CPSI	404	m	1	1.52	4.10	0.01	0.0021
*CPSI	406	f	1	0.36	3.08	4.54	0.5303
Subtotal CPSI				1.02	7.18	4.56	
*CPSII	115	m	1	2.96	3.23	6.18	0.0000
*CPSII	118	f	1	2.11	9.51	2.72	0.0000
Subtotal CPSII				2.32	12.75	8.91	
*DORN	340	m	1	1.78	20.15	0.89	0.0000
*ENGELA	70	m	7	1.95	4.13	0.60	0.0001
HAENSZ	37	f	0	0.08	9.58	21.28	0.7992
JAHN	8	m	0	1.57	6.70	0.00	0.0000
JAIN	17	m	0	2.38	3.49	2.27	0.0000
JAIN	12	f	0	1.83	14.10	0.91	0.0000
Subtotal JAIN				1.94	17.58	3.18	
JEDRYC	26	m	0	1.69	6.14	0.09	0.0000
KATSOU	16	f	0	0.62	5.87	5.31	0.1325
KHUDER	17	m	0	2.10	5.77	1.63	0.0000
KIHARA	5	c	0	0.66	30.38	25.39	0.0003
LUBIN2	252	m	0	1.21	49.29	6.46	0.0000
LUBIN2	264	f	0	0.36	39.96	58.39	0.0214
Subtotal LUBIN2				0.83	89.24	64.86	
MATOS	52	m	0	2.04	4.19	0.90	0.0000
OSANN	11	m	0	3.17	12.64	32.35	0.0000
OSANN	15	f	0	2.50	33.23	28.80	0.0000
Subtotal OSANN				2.69	45.87	61.15	
OSANN2	14	f	0	1.25	8.04	0.84	0.0004
SOBUE	6	m	0	0.70	20.00	15.25	0.0018
SOBUE	22	f	0	0.35	24.55	36.88	0.0855
Subtotal SOBUE				0.51	44.54	52.13	
SOBUE2	2	m	2	1.13	82.01	15.98	0.0000
SOBUE2	6	f	2	0.59	75.22	72.98	0.0000
Subtotal SOBUE2				0.87	157.22	88.96	
SUZUKI	2	m	0	1.65	10.41	0.06	0.0000
SUZUKI	6	f	0	0.88	7.96	3.79	0.0128
Subtotal SUZUKI				1.32	18.36	3.84	
SVENSS	64	f	0	1.36	10.10	0.44	0.0000
TSUGAN	3	m	0	-0.16	6.39	19.23	0.6813
TSUGAN	9	f	0	-0.61	3.03	14.37	0.2916
Subtotal TSUGAN				-0.31	9.41	33.60	
WAKAI	6	m	0	0.76	6.36	4.17	0.0542
WAKAI	24	f	0	0.09	5.61	12.38	0.8363
Subtotal WAKAI				0.45	11.97	16.55	
WU	2	f	0	1.44	12.39	0.21	0.0000
WU2	1	f	2	1.50	22.15	0.10	0.0000
WYNDE3	30	m	0	1.38	4.98	0.19	0.0021
WYNDE6	15	m	0	2.23	45.98	20.16	0.0000
WYNDE6	204	f	0	2.20	69.69	27.46	0.0000
Subtotal WYNDE6				2.21	115.67	47.61	

Table 3B2 - 5

IESLC - Meta-analysis of Current Smoking (vs never smoking), Cigarettes (or Any Product if Cigarettes not available)
Adenocarcinoma
Least adjusted

	N	44
	NS	31
	Wt	1023.48
Het	Chi	530.13
Het	df	43
Het	P	***
Fixed	RR	4.82
	RRl	4.53
	RRu	5.12
	P	+++
Random	RR	4.27
	RRl	3.37
	RRu	5.40
	P	+++
Asymm	P	N.S.

Table 3B2 - 6

IESLC - Meta-analysis of Current Smoking (vs never smoking), Cigarettes (or Any Product if Cigarettes not available)
Adenocarcinoma
Least adjusted

	Sex combined	male	female	Total
N	2	22	20	44
NS	2	22	20	44
Wt	49.25	428.92	545.31	1023.48
Het Chi	18.00	170.18	318.85	530.13
Het df	1	21	19	43
Het P	***	***	***	***
Fixed RR	3.11	5.62	4.44	4.82
RRl	2.35	5.11	4.09	4.53
RRu	4.11	6.18	4.83	5.12
P	+++	+++	+++	+++
Random RR	3.57	5.62	3.26	4.27
RRl	1.06	4.10	2.23	3.37
RRu	12.07	7.70	4.78	5.40
P	+	+++	+++	+++
Between Chi				23.10
Between df				2
Between P				***
Btwn(F) P				N.S.
Btwn(R) P				(*)

Table 3B2 - 7

IESLC - Meta-analysis of Current Smoking (vs never smoking), Cigarettes (or Any Product if Cigarettes not available)
Adenocarcinoma
Excluded studies (and stage at which they were excluded)

Stage	Studies
1	BOUCHA BUELL LAURIL MZILEN
2	ABELIN ABRAHA AUVINE AXELSO BAND BERRIN BLOT1 BLOT2 BLOT3 BLOT4 BRESLO BROCKM BROWN1 BYERS1 CASCO2 CASCOR CHAN CHATZI CHEN CHEN2 CHEN3 CHIAZZ COOKSO DAVEYS DEAN DOSEME DU DUNN EBELIN ESAKI FAN GENG GER GODLEY GOLLED GSELL HANSEN HEGMAN HINDS HIRAOK HOROWI HORWIT HU HU2 HUANG ISHIMA JARUP JIANG JONES JUSSAW KO KOHLME KOULUM KREYBE LAMTH LAMWK LAMWK2 LAUSSM LEI LETOUR LEVIN LIU LIU2 LIU3 LIU4 LIU5 LOMBA2 LUO MAGNUS MARSH MARSH2 MARTIS MASTRA MATSUD MCCONN MCDUFF MCLAUG MILLER MILLS MOLLO NOTAN2 NOU ORMOS PASTOR PAWLEG PERNU PERSHA PIKE POFFIJ POLEDN PRESCO QIAO RADZIK RANDIG REN RONCO ROOTS ROTHSC SADOWS SANKAR SCHWA2 SEGI SEOW SIEMIA SIMARA STASZE STOCKS SUN TAO ULMER VANDER WANG WANG3 WANG4 WICKLU WILKIN WUWILL WYNDE2 WYNDE4 XIANGZ XU XU2 XU3 XU4 YUAN ZHANG ZHENG ZHOU
3	RESTRE
4	BOFFET
5	RIMING TANG2 WYNDE5 WYNDE8
6	BYERS2 HIRAY2 LICKIN MRFIT MURATA NOTANI SAARIK SHIMIZ SITAS STAYNE WARSIN WATSON WYNDER
8	AGUDO AKIBA ALDERS AMANDU AMES ANDERS ARCHER ARMADA AUSTIN AXELSS BECHER BENSHL BEST BLOHMK BRETT BROSS CARPEN CEDERL CHANG CHOI CHOW CHYOU DAMBER DARBY DEAN2 DEAN3 DEKLER DESTE2 DESTEF DOCKER DOLL DOLL2 DORANT DORGAN DROSTE ENSTRO GAO GAO2 GARCIA GARDIN GARSHI GILLIS GOODMA GRAHAM GREGOR HAMMO2 HAMMON HEIN HENNEK HIRAYA HITOSU HOLE HUMBLE JARVHO JOLY KAISE2 KAISER KANELL KAUFMA KELLER KINLEN KJUUS KNEKT KOO KREUZE KUBIK LANGE LEMARC LIAW LIDDEL LOMBAR LUBIN MACLEN MIGRAN MRFITR NAM ODRISC PARKIN PERSH2 PETO PEZZO2 PEZZOT PISANI QIAO2 RACHTA SCHWAR SEGI2 SHAW SIMONA SPEIZE SPITZ STOCKW STUCKE SUZUK2 TANG TENKAN TIZZAN TOKARS TOUSEY TULINI TVERDA VEIERO WALD WANG2 WIGLE WUNSCH YAMAGU YONG
10	BENHAM VUTUC WYNDE7

Table 3B2 - 8

Potentially overlapping studies

REF	REFGP	PRINC	.	OVERLAP/LINK
LUBIN2	LUBIN2	1		Lubin-combined
OSANN2	KAISER	2		KAISER/OSANN2
WYNDE6	WYNDE6	1		WYNDE5/6/7/8
CPSI	CPSI	1		CPSI overall
SOBUE2	SOBUE2	1		SOBUE2/MATSUD
JAHN	BOFFET	2		Subset of BOFFET

Table 3B2 - 9

Most adjusted - insufficient data for metaanalysis

REF	NRR	SEX	AGEL	AGEH	RACE	YF	LC TYPE	LOC	START	ST	NLC	R	VB	P	H	AD	PRODUCT	DENOM	De
CHYOU	6	m	0	0	jap	0	a	NAmer	1965	pr	227	n	bl	n	y	1	cig+/-ot	nev cigs	or

REF	NRR	RR	SIG	RRDATA comment
CHYOU	6	6.80		0

Table 3B3 -

IESLC - Meta-analysis of Current Smoking (vs never smoking), Cigarettes only
Adenocarcinoma

This analysis is restricted to results for:
1) Non-dose-response data
2) Current smokers
3) Results complete enough for use in metaanalysis

Within each study, results are then selected (in the following order of preference, within each sex) for:
4) PRODUCT: cigarettes only
5) CIGTYPE: all/unspecified, MC regardless of HR, MC only
6) DENOM: never smoked anything, never smoked cigarettes, (never +1 = +long term ex, +2 = +amount unknown,
+3 = never cigs+long term ex)
7) Followup period (YF, prospective studies): whole study (coded as 0) or longest available
8) LCtype: adeno or nearest available, but not squamous. (q = squamous, s = small,
a = adeno, l = large, KII = Kreyberg II, al = alveolar, br = bronchiolar, u = undifferentiated)
9) Race: all or nearest available, otherwise by race (wh or w = white, bl or b = black, hi = hispanic
ch = chinese, jap = japanese, haw = hawaiian, w+o = white + oriental, sca = scandinavian, as = asian)
10) For overlapping studies: principal rather than subsidiary studies
Finally by Age: whole study (coded as 0) if available, otherwise by widest available age group
and then for single sex results (m, f) in preference to combined sex results (c).

Results adjusted (AD) for the most potential confounders are then chosen in Sections -1 to -3
(and those which actually differ from the adjusted results in Table 3B1 - 1 are marked 'x' in Section -1)
and results adjusted for the least confounders in Sections -4 to -6. (Those least adjusted results which
actually differ from the most adjusted as marked 'x' in column X in Section -4)
(Results adjusted for an unknown number of confounder(s) are coded as 20.)

Section -7 shows excluded studies, together with the stage (as above) at which no qualifying
results were found.

Section -8 lists the potentially overlapping studies which have been included (1=principal, 2=subsidiary).

Section -9 lists any results which would have been included in preference except that they had data not complete
enough for use in meta-analysis, with their significance (yes/no), if known, and any further comment as entered
on the database.

In addition to those mentioned above, the following fields, levels and abbreviations are used:
* or nk = not known, n = no, y = yes, ot = other
nev = never
all/unspec = all or unspecified, MC = manufactured cigarettes, HR = hand-rolled cigarettes
REF: 6-character study reference
NRR: number of the RR on the database within the study
ST : study type (CC = case control, pr or prosp = prospective)
NLC: number of lung cancer cases in whole study
R : risky occupational population (n = no, m = mining, o = other risky)
VB : national cigarette type (V = at least 75% Virginia, bl = at least 75% blended, ot = other)
P : any proxy use
H : full histological confirmation
De : derivation of RR/CI (or = original, st = standard method, ot = other method of estimation)

Table 3B3 - 1

IESLC - Meta-analysis of Current Smoking (vs never smoking), Cigarettes only
Adenocarcinoma
Most adjusted

REF	NRR	3B1	SEX	AGEL	AGEH	RACE	YF	LC TYPE	LOC	START	ST	NLC	R	VB	P	H	AD	PRODUCT	DENOM	De
BENHAM	22	x	m	0	0	all	-	KII	Eu:wst	1976	CC	1625	n	bl	n	y	0	cig only	nev any	st
BOUCOT	147		m	0	0	all	0	a	NAmer	1951	pr	121	n	bl	n	n	2	cig only	nev any	ot
CPSI	404		m	0	0	all	2	a	NAmer	1959	pr	5138	n	bl	n	n	1	cig only	nev any	ot
CPSI	406		f	0	0	all	2	a	NAmer	1959	pr	5138	n	bl	n	n	1	cig only	nev any	ot
CPSII	115		m	0	0	all	2	a	NAmer	1982	pr	3229	n	bl	n	n	1	cig only	nev any	st
DORN	340		m	0	0	wh	8	a	NAmer	1954	pr	5097	n	bl	n	n	1	cig only	nev any	ot
WYNDE7	32	x	m	0	0	all	-	KII	NAmer	1977	CC	2085	n	bl	n	y	0	cig only	nev any	st

Cigarette type is all/unspec for all RRs

Table 3B3 - 2

IESLC - Meta-analysis of Current Smoking (vs never smoking), Cigarettes only
Adenocarcinoma
Most adjusted

REF	NRR	SEX	AD	Number Exposed Case	Number Exposed Cont	Non-exposed Case	Non-exposed Cont	RR	95.00%CI
BENHAM	22	m	0	85	95	9	42	4.18	(1.92- 9.08)
*BOUCOT	147	m	2	-	-	-	-	10.95	(0.65- 183.57)
*CPSI	404	m	1	-	-	-	-	4.58	(1.74- 12.05)
*CPSI	406	f	1	-	-	-	-	1.43	(0.47- 4.39)
Subtotal CPSI								2.78	(1.34- 5.78)
*CPSII	115	m	1	-	-	-	-	19.22	(6.46- 57.16)
*DORN	340	m	1	-	-	-	-	5.95	(3.85- 9.22)
WYNDE7	32	m	0	441	993	42	918	9.71	(6.99- 13.49)
Partial Totals				526	1088	51	960		

*prospective study

REF	NRR	SEX	AD	Ys	Ws	Qs	Ps
BENHAM	22	m	0	1.43	6.36	1.87	0.0003
*BOUCOT	147	m	2	2.39	0.48	0.09	0.0964
*CPSI	404	m	1	1.52	4.10	0.83	0.0021
*CPSI	406	f	1	0.36	3.08	8.02	0.5303
Subtotal CPSI				1.02	7.18	8.85	
*CPSII	115	m	1	2.96	3.23	3.13	0.0000
*DORN	340	m	1	1.78	20.15	0.72	0.0000
WYNDE7	32	m	0	2.27	35.49	3.21	0.0000

	N	7
	NS	6
	Wt	72.90
Het	Chi	17.87
Het	df	6
Het	P	**
Fixed	RR	7.18
	RRl	5.71
	RRu	9.04
	P	+++
Random	RR	6.05
	RRl	3.69
	RRu	9.92
	P	+++
Asymm	P	N.S.

Table 3B3 - 3

IESLC - Meta-analysis of Current Smoking (vs never smoking), Cigarettes only
Adenocarcinoma
Most adjusted

	Sex			
	combined	male	female	Total
N		6	1	7
NS		6	1	7
Wt		69.82	3.08	72.90
Het Chi		9.50	0.00	17.87
Het df		5	0	6
Het P		(*)	N.S.	**
Fixed RR		7.71	1.43	7.18
RRl		6.10	0.47	5.71
RRu		9.75	4.37	9.04
P		+++	N.S.	+++
Random RR		7.26	1.43	6.05
RRl		4.88	0.47	3.69
RRu		10.82	4.37	9.92
P		+++	N.S.	+++
Between Chi				8.37
Between df				1
Between P				**
Btwn(F) P				(*)
Btwn(R) P				**

Too few RRs for analysis by factor

Table 3B3 - 4

IESLC - Meta-analysis of Current Smoking (vs never smoking), Cigarettes only
Adenocarcinoma
Least adjusted

REF	NRR	X	SEX	AGEL	AGEH	RACE	YF	LC TYPE	LOC	START	ST	NLC	R	VB	P	H	AD	PRODUCT	DENOM	De
BENHAM	22		m	0	0	all	-	KII	Eu:wst	1976	CC	1625	n	bl	n	y	0	cig only	nev any	st
BOUCOT	72	x	m	0	0	all	0	a	NAmer	1951	pr	121	n	bl	n	n	0	cig only	nev any	ot
CPSI	404		m	0	0	all	2	a	NAmer	1959	pr	5138	n	bl	n	n	1	cig only	nev any	ot
CPSI	406		f	0	0	all	2	a	NAmer	1959	pr	5138	n	bl	n	n	1	cig only	nev any	ot
CPSII	115		m	0	0	all	2	a	NAmer	1982	pr	3229	n	bl	n	n	1	cig only	nev any	st
DORN	340		m	0	0	wh	8	a	NAmer	1954	pr	5097	n	bl	n	n	1	cig only	nev any	ot
WYNDE7	32		m	0	0	all	-	KII	NAmer	1977	CC	2085	n	bl	n	y	0	cig only	nev any	st

Cigarette type is all/unspec for all RRs

Table 3B3 - 5

IESLC - Meta-analysis of Current Smoking (vs never smoking), Cigarettes only
Adenocarcinoma
Least adjusted

REF	NRR	SEX	AD	Number Exposed Case	Number Exposed Cont	Non-exposed Case	Non-exposed Cont	RR	95.00%CI
BENHAM	22	m	0	85	95	9	42	4.18	(1.92- 9.08)
*BOUCOT	72	m	0	14	22177	0	7551	9.87~	(0.59- 165.51)
*CPSI	404	m	1	-	-	-	-	4.58	(1.74- 12.05)
*CPSI	406	f	1	-	-	-	-	1.43	(0.47- 4.39)
Subtotal CPSI								2.78	(1.34- 5.78)
*CPSII	115	m	1	-	-	-	-	19.22	(6.46- 57.16)
*DORN	340	m	1	-	-	-	-	5.95	(3.85- 9.22)
WYNDE7	32	m	0	441	993	42	918	9.71	(6.99- 13.49)
Partial Totals				540	23265	51	8511		

*prospective study ~ With 0.5 adjustment for zero

REF	NRR	SEX	AD	Ys	Ws	Qs	Ps
BENHAM	22	m	0	1.43	6.36	1.87	0.0003
*BOUCOT	72	m	0	2.29	0.48	0.05	0.1114
*CPSI	404	m	1	1.52	4.10	0.83	0.0021
*CPSI	406	f	1	0.36	3.08	8.01	0.5303
Subtotal CPSI				1.02	7.18	8.84	
*CPSII	115	m	1	2.96	3.23	3.13	0.0000
*DORN	340	m	1	1.78	20.15	0.71	0.0000
WYNDE7	32	m	0	2.27	35.49	3.23	0.0000

	N	7
	NS	6
	Wt	72.90
Het	Chi	17.83
Het	df	6
Het	P	**
Fixed	RR	7.18
	RRl	5.71
	RRu	9.03
	P	+++
Random	RR	6.03
	RRl	3.68
	RRu	9.89
	P	+++
Asymm	P	N.S.

Table 3B3 - 6

IESLC - Meta-analysis of Current Smoking (vs never smoking), Cigarettes only
Adenocarcinoma
Least adjusted

	Sex combined	male	female	Total
N		6	1	7
NS		6	1	7
Wt		69.82	3.08	72.90
Het Chi		9.47	0.00	17.83
Het df		5	0	6
Het P		(*)	N.S.	**
Fixed RR		7.71	1.43	7.18
RRl		6.10	0.47	5.71
RRu		9.75	4.37	9.03
P		+++	N.S.	+++
Random RR		7.25	1.43	6.03
RRl		4.87	0.47	3.68
RRu		10.79	4.37	9.89
P		+++	N.S.	+++
Between Chi				8.37
Between df				1
Between P				**
Btwn(F) P				(*)
Btwn(R) P				**

Table 3B3 - 7

IESLC - Meta-analysis of Current Smoking (vs never smoking), Cigarettes only
Adenocarcinoma
Excluded studies (and stage at which they were excluded)

1 BOUCHA BUELL LAURIL MZILEN

2 ABELIN ABRAHA AUVINE AXELSO BAND BERRIN BLOT1 BLOT2 BLOT3 BLOT4 BRESLO BROCKM BROWN1 BYERS1 CASCO2 CASCOR
CHAN CHATZI CHEN CHEN2 CHEN3 CHIAZZ COOKSO DAVEYS DEAN DOSEME DU DUNN EBELIN ESAKI FAN GENG
GER GODLEY GOLLED GSELL HANSEN HEGMAN HINDS HIRAOK HOROWI HORWIT HU HU2 HUANG ISHIMA JARUP JIANG
JONES JUSSAW KO KOHLME KOULUM KREYBE LAMTH LAMWK LAMWK2 LAUSSM LEI LETOUR LEVIN LIU LIU2 LIU3
LIU4 LIU5 LOMBA2 LUO MAGNUS MARSH MARSH2 MARTIS MASTRA MATSUD MCCONN MCDUFF MCLAUG MILLER MILLS MOLLO
NOTAN2 NOU ORMOS PASTOR PAWLEG PERNU PERSHA PIKE POFFIJ POLEDN PRESCO QIAO RADZIK RANDIG REN RONCO
ROOTS ROTHSC SADOWS SANKAR SCHWA2 SEGI SEOW SIEMIA SIMARA STASZE STOCKS SUN TAO ULMER VANDER WANG
WANG3 WANG4 WICKLU WILKIN WUWILL WYNDE2 WYNDE4 XIANGZ XU XU2 XU3 XU4 YUAN ZHANG ZHENG ZHOU

3 RESTRE

4 AKIBA AMANDU AMES ANDERS ARCHER ARMADA AUSTIN AXELSS BARBON BECHER BENSHL BLOHMK BOFFET BRETT BROSS BROWN2
BUFFLE BYERS2 CARPEN CHANG CHOI CHYOU COMSTO CORREA DARBY DEAN2 DEKLER DESTE2 DESTEF DOCKER DOLL DORANT
DORGAN DROSTE GAO GAO2 GARCIA GARSHI GILLIS GOODMA GREGOR HAENSZ HAMMO2 HENNEK HIRAYA HITOSU HOLE HUMBLE
JAHN JAIN JARVHO JEDRYC JOLY KAISER KANELL KATSOU KAUFMA KELLER KHUDER KIHARA KINLEN KJUUS KNEKT KOO
KREUZE KUBIK LEMARC LIAW LICKIN LIDDEL LUBIN MACLEN MATOS MRFIT MRFITR MURATA NAM NOTANI ODRISC OSANN
OSANN2 PARKIN PERSH2 PETO PEZZO2 PISANI QIAO2 RACHTA SAARIK SCHWAR SEGI2 SHAW SHIMIZ SIMONA SITAS SOBUE
SOBUE2 SPEIZE SPITZ STAYNE STOCKW STUCKE SUZUK2 SUZUKI SVENSS TANG TENKAN TIZZAN TOKARS TOUSEY TSUGAN TULINI
VUTUC WAKAI WANG2 WARSIN WATSON WU WU2 WUNSCH WYNDE3 WYNDE5 WYNDE6 WYNDE8 YAMAGU YONG

5 LANGE RIMING TANG2

6 HIRAY2 WYNDER

8 AGUDO ALDERS BEST CEDERL CHOW DAMBER DEAN3 DOLL2 ENGELA ENSTRO GARDIN GRAHAM HAMMON HEIN KAISE2 LOMBAR
LUBIN2 MIGRAN PEZZOT TVERDA VEIERO WALD WIGLE

Table 3B3 - 8
Potentially overlapping studies

REF	REFGP	PRINC	. OVERLAP/LINK
BENHAM	LUBIN2	2	Subset of Lubin2
WYNDE7	WYNDE6	2	WYNDE5/6/7/8
CPSI	CPSI	1	CPSI overall

Table 3B4 -

IESLC - Meta-analysis of Current Smoking, Any product (or Cigarettes if Any not available), Age <56
Adenocarcinoma

This analysis is restricted to results for:
1) Non-dose-response data
2) Current smokers
3) Age <56
4) Results complete enough for use in metaanalysis

Within each study, results are then selected (in the following order of preference, within each sex) for:
5) PRODUCT: all/unspec, cigarettes regardless of other products, cigarettes only
6) CIGTYPE: all/unspecified, MC regardless of HR, MC only
7) DENOM: never smoked anything, never smoked cigarettes, (never +1 = +long term ex, +2 = +amount unknown,
+3 = never cigs+long term ex)
8) Followup period (YF, prospective studies): whole study (coded as 0) or longest available
9) LCtype: all or nearest available, at least Squamous and Adeno. (q = squamous, s = small,
a = adeno, l = large, KII = Kreyberg II, al = alveolar, br = bronchiolar, u = undifferentiated)
10) Race: all or nearest available, otherwise by race (wh or w = white, bl or b = black, hi = hispanic
ch = chinese, jap = japanese, haw = hawaiian, w+o = white + oriental, sca = scandinavian, as = asian)
11) For overlapping studies: principal rather than subsidiary studies
Finally by Age: whole study (actual age shown) if available, otherwise by widest available age group
and then for single sex results (m, f) in preference to combined sex results (c).

Results adjusted (AD) for the most potential confounders are then chosen in Sections -1 to -3
(and those which actually differ from the adjusted results in Table 3B1 - 1 are marked 'x' in Section -1)
and results adjusted for the least confounders in Sections -4 to -6. (Those least adjusted results which
actually differ from the most adjusted as marked 'x' in column X in Section -4)
(Results adjusted for an unknown number of confounder(s) are coded as 20.)

Section -7 shows excluded studies, together with the stage (as above) at which no qualifying
results were found.

Section -8 lists the potentially overlapping studies which have been included (1=principal, 2=subsidiary).

Section -9 lists any results which would have been included in preference except that they had data not complete
enough for use in meta-analysis, with their significance (yes/no), if known, and any further comment as entered
on the database.

In addition to those mentioned above, the following fields, levels and abbreviations are used:
* or nk = not known, n = no, y = yes, ot = other
nev = never
all/unspec = all or unspecified, cig+/-ot = cigarettes irrespective of other products (cigar, pipe etc)
MC = manufactured cigarettes, HR = hand-rolled cigarettes
REF: 6-character study reference
NRR: number of the RR on the database within the study
ST : study type (CC = case control, pr or prosp = prospective)
NLC: number of lung cancer cases in whole study
R : risky occupational population (n = no, m = mining, o = other risky)
VB : national cigarette type (V = at least 75% Virginia, bl = at least 75% blended, ot = other)
P : any proxy use
H : full histological confirmation
De : derivation of RR/CI (or = original, st = standard method, ot = other method of estimation)

Table 3B4 - 1

IESLC - Meta-analysis of Current Smoking, Any product (or Cigarettes if Any not available), Age <56
Adenocarcinoma
Most adjusted

REF	NRR	3B1	SEX	AGEL	AGEH	RACE	YF	LC TYPE	LOC	START	ST	NLC	R	VB	P	H	AD	PRODUCT	DENOM	De
TSUGAN	3		m	30	49	all	-	a	As:Jap	1976	CC	134	n	bl	n	y	0	all/unsp	nev any	st
TSUGAN	9		f	30	49	all	-	a	As:Jap	1976	CC	134	n	bl	n	y	0	all/unsp	nev any	or
VUTUC	24	x	m	41	50	all	-	KII	Eu:wst	1976	CC	1877	n	bl	n	n	0	cig+/-ot	nev cigs	st

Cigarette type is all/unspec for all RRs

Table 3B4 - 2

IESLC - Meta-analysis of Current Smoking, Any product (or Cigarettes if Any not available), Age <56
Adenocarcinoma
Most adjusted

REF	NRR	SEX	AD	Number Exposed Case	Number Exposed Cont	Non-exposed Case	Non-exposed Cont	RR	95.00%CI
TSUGAN	3	m	0	45	50	18	17	0.85	(0.39- 1.85)
TSUGAN	9	f	0	6	10	33	30	0.55	(0.18- 1.68)
Subtotal TSUGAN								0.74	(0.39- 1.40)
VUTUC	24	m	0	5	104	4	64	0.77	(0.20- 2.97)
Totals				56	164	55	111		

*prospective study

REF	NRR	SEX	AD	Ys	Ws	Qs	Ps
TSUGAN	3	m	0	-0.16	6.39	0.12	0.6813
TSUGAN	9	f	0	-0.61	3.03	0.29	0.2916
Subtotal TSUGAN				-0.31	9.41	0.40	
VUTUC	24	m	0	-0.26	2.10	0.00	0.7035

	N	3
	NS	2
	Wt	11.52
Het	Chi	0.41
Het	df	2
Het	P	N.S.
Fixed	RR	0.74
	RRl	0.42
	RRu	1.32
	P	N.S.
Random	RR	0.74
	RRl	0.42
	RRu	1.32
	P	N.S.
Asymm	P	N.S.

Table 3B4 - 3

IESLC - Meta-analysis of Current Smoking, Any product (or Cigarettes if Any not available), Age <56
Adenocarcinoma
Most adjusted

	Sex combined	male	female	Total
N		2	1	3
NS		2	1	3
Wt		8.49	3.03	11.52
Het Chi		0.02	0.00	0.41
Het df		1	0	2
Het P		N.S.	N.S.	N.S.
Fixed RR		0.83	0.55	0.74
RRl		0.42	0.18	0.42
RRu		1.62	1.68	1.32
P		N.S.	N.S.	N.S.
Random RR		0.83	0.55	0.74
RRl		0.42	0.18	0.42
RRu		1.62	1.68	1.32
P		N.S.	N.S.	N.S.
Between Chi				0.39
Between df				1
Between P				N.S.
Btwn(F) P				N.S.
Btwn(R) P				N.S.

Too few RRs for analysis by factor

Table 3B4 - 4

IESLC - Meta-analysis of Current Smoking, Any product (or Cigarettes if Any not available), Age <56
Adenocarcinoma
Least adjusted

REF	NRR	X	SEX	AGEL	AGEH	RACE	YF	LC TYPE	LOC	START	ST	NLC	R	VB	P	H	AD	PRODUCT	DENOM	De
TSUGAN	3		m	30	49	all	-	a	As:Jap	1976	CC	134	n	bl	n	y	0	all/unsp	nev any	st
TSUGAN	9		f	30	49	all	-	a	As:Jap	1976	CC	134	n	bl	n	y	0	all/unsp	nev any	or
VUTUC	24		m	41	50	all	-	KII	Eu:wst	1976	CC	1877	n	bl	n	n	0	cig+/-ot	nev cigs	st

Cigarette type is all/unspec for all RRs

Table 3B4 - 5

IESLC - Meta-analysis of Current Smoking, Any product (or Cigarettes if Any not available), Age <56
Adenocarcinoma
Least adjusted

REF	NRR	SEX	AD	Number Exposed Case	Number Exposed Cont	Non-exposed Case	Non-exposed Cont	RR	95.00%CI
TSUGAN	3	m	0	45	50	18	17	0.85	(0.39- 1.85)
TSUGAN	9	f	0	6	10	33	30	0.55	(0.18- 1.68)
Subtotal TSUGAN								0.74	(0.39- 1.40)
VUTUC	24	m	0	5	104	4	64	0.77	(0.20- 2.97)
Totals				56	164	55	111		

*prospective study

REF	NRR	SEX	AD	Ys	Ws	Qs	Ps
TSUGAN	3	m	0	-0.16	6.39	0.12	0.6813
TSUGAN	9	f	0	-0.61	3.03	0.29	0.2916
Subtotal TSUGAN				-0.31	9.41	0.40	
VUTUC	24	m	0	-0.26	2.10	0.00	0.7035

	N	3
	NS	2
	Wt	11.52
Het	Chi	0.41
Het	df	2
Het	P	N.S.
Fixed	RR	0.74
	RRl	0.42
	RRu	1.32
	P	N.S.
Random	RR	0.74
	RRl	0.42
	RRu	1.32
	P	N.S.
Asymm	P	N.S.

Table 3B4 - 6

IESLC - Meta-analysis of Current Smoking, Any product (or Cigarettes if Any not available), Age <56
Adenocarcinoma
Least adjusted

	Sex			
	combined	male	female	Total
N		2	1	3
NS		2	1	3
Wt		8.49	3.03	11.52
Het Chi		0.02	0.00	0.41
Het df		1	0	2
Het P		N.S.	N.S.	N.S.
Fixed RR		0.83	0.55	0.74
RRl		0.42	0.18	0.42
RRu		1.62	1.68	1.32
P		N.S.	N.S.	N.S.
Random RR		0.83	0.55	0.74
RRl		0.42	0.18	0.42
RRu		1.62	1.68	1.32
P		N.S.	N.S.	N.S.
Between Chi				0.39
Between df				1
Between P				N.S.
Btwn(F) P				N.S.
Btwn(R) P				N.S.

Table 3B4 - 7

IESLC - Meta-analysis of Current Smoking, Any product (or Cigarettes if Any not available), Age <56
Adenocarcinoma
Excluded studies (and stage at which they were excluded)

1	BOUCHA BUELL LAURIL MZILEN
2	ABELIN ABRAHA AUVINE AXELSO BAND BERRIN BLOT1 BLOT2 BLOT3 BLOT4 BRESLO BROCKM BROWN1 BYERS1 CASCO2 CASCOR
	CHAN CHATZI CHEN CHEN2 CHEN3 CHIAZZ COOKSO DAVEYS DEAN DOSEME DU DUNN EBELIN ESAKI FAN GENG
	GER GODLEY GOLLED GSELL HANSEN HEGMAN HINDS HIRAOK HOROWI HORWIT HU HU2 HUANG ISHIMA JARUP JIANG
	JONES JUSSAW KO KOHLME KOULUM KREYBE LAMTH LAMWK LAMWK2 LAUSSM LEI LETOUR LEVIN LIU LIU2 LIU3
	LIU4 LIU5 LOMBA2 LUO MAGNUS MARSH MARSH2 MARTIS MASTRA MATSUD MCCONN MCDUFF MCLAUG MILLER MILLS MOLLO
	NOTAN2 NOU ORMOS PASTOR PAWLEG PERNU PERSHA PIKE POFFIJ POLEDN PRESCO QIAO RADZIK RANDIG REN RONCO
	ROOTS ROTHSC SADOWS SANKAR SCHWA2 SEGI SEOW SIEMIA SIMARA STASZE STOCKS SUN TAO ULMER VANDER WANG
	WANG3 WANG4 WICKLU WILKIN WUWILL WYNDE2 WYNDE4 XIANGZ XU XU2 XU3 XU4 YUAN ZHANG ZHENG ZHOU
3	AGUDO AKIBA ALDERS AMANDU AMES ANDERS ARCHER ARMADA AUSTIN AXELSS BARBON BECHER BENHAM BENSHL BLOHMK BOFFET
	BOUCOT BRETT BROSS BROWN2 BUFFLE BYERS2 CARPEN CEDERL CHANG CHOI CHOW CHYOU CORREA DAMBER DARBY DEAN2
	DEKLER DESTE2 DESTEF DOCKER DOLL DOLL2 DORANT DORGAN DORN DROSTE ENGELA ENSTRO GAO GAO2 GARCIA GARDIN
	GARSHI GILLIS GOODMA GRAHAM GREGOR HAENSZ HAMMO2 HAMMON HEIN HENNEK HIRAY2 HUMBLE JAHN JAIN JARVHO JEDRYC
	JOLY KAISER KATSOU KAUFMA KELLER KHUDER KIHARA KINLEN KJUUS KNEKT KOO LANGE LEMARC LIAW LICKIN LIDDEL
	LOMBAR LUBIN LUBIN2 MACLEN MATOS MIGRAN MRFIT MRFITR MURATA NOTANI ODRISC OSANN OSANN2 PARKIN PERSH2 PETO
	PEZZO2 PEZZOT PISANI QIAO2 RACHTA RESTRE RIMING SAARIK SCHWAR SHAW SHIMIZ SIMONA SITAS SOBUE SOBUE2 SPITZ
	STAYNE STOCKW STUCKE SUZUK2 SUZUKI SVENSS TANG TANG2 TENKAN TIZZAN TOKARS TOUSEY TULINI VEIERO WAKAI WALD
	WANG2 WARSIN WATSON WIGLE WU WU2 WUNSCH WYNDE3 WYNDE5 WYNDE6 WYNDE7 WYNDE8 WYNDER YAMAGU YONG
4	HIRAYA
6	KAISE2
7	HOLE
9	BEST COMSTO CPSI CPSII DEAN3 HITOSU KANELL KREUZE KUBIK NAM SEGI2 SPEIZE TVERDA

Table 3B4 - 8
Potentially overlapping studies

REF	REFGP	PRINC	.	OVERLAP/LINK
VUTUC	LUBIN2	2		Subset of Lubin2

Table 3B5 -

IESLC - Meta-analysis of Current Smoking, Any product (or Cigarettes if Any not available), Age 50-70
Adenocarcinoma

This analysis is restricted to results for:
1) Non-dose-response data
2) Current smokers
3) Maximum age range 50-70
4) Results complete enough for use in metaanalysis

Within each study, results are then selected (in the following order of preference, within each sex) for:
5) PRODUCT: all/unspec, cigarettes regardless of other products, cigarettes only
6) CIGTYPE: all/unspecified, MC regardless of HR, MC only
7) DENOM: never smoked anything, never smoked cigarettes, (never +1 = +long term ex, +2 = +amount unknown, +3 = never cigs+long term ex)
8) Followup period (YF, prospective studies): whole study (coded as 0) or longest available
9) LCtype: all or nearest available, at least Squamous and Adeno. (q = squamous, s = small, a = adeno, l = large, KII = Kreyberg II, al = alveolar, br = bronchiolar, u = undifferentiated)
10) Race: all or nearest available, otherwise by race (wh or w = white, bl or b = black, hi = hispanic ch = chinese, jap = japanese, haw = hawaiian, w+o = white + oriental, sca = scandinavian, as = asian)
11) For overlapping studies: principal rather than subsidiary studies
Finally by Age: whole study (actual age shown) if available, otherwise by widest available age group and then for single sex results (m, f) in preference to combined sex results (c).

Results adjusted (AD) for the most potential confounders are then chosen in Sections -1 to -3 (and those which actually differ from the adjusted results in Table 3B1 - 1 are marked 'x' in Section -1) and results adjusted for the least confounders in Sections -4 to -6. (Those least adjusted results which actually differ from the most adjusted as marked 'x' in column X in Section -4)
(Results adjusted for an unknown number of confounder(s) are coded as 20.)

Section -7 shows excluded studies, together with the stage (as above) at which no qualifying results were found.

Section -8 lists the potentially overlapping studies which have been included (1=principal, 2=subsidiary).

Section -9 lists any results which would have been included in preference except that they had data not complete enough for use in meta-analysis, with their significance (yes/no), if known, and any further comment as entered on the database.

In addition to those mentioned above, the following fields, levels and abbreviations are used:
* or nk = not known, n = no, y = yes, ot = other
nev = never
all/unspec = all or unspecified, cig+/-ot = cigarettes irrespective of other products (cigar, pipe etc)
MC = manufactured cigarettes, HR = hand-rolled cigarettes
REF: 6-character study reference
NRR: number of the RR on the database within the study
ST : study type (CC = case control, pr or prosp = prospective)
NLC: number of lung cancer cases in whole study
R : risky occupational population (n = no, m = mining, o = other risky)
VB : national cigarette type (V = at least 75% Virginia, bl = at least 75% blended, ot = other)
P : any proxy use
H : full histological confirmation
De : derivation of RR/CI (or = original, st = standard method, ot = other method of estimation)

Table 3B5 - 0

No RRs selected for this analysis

Table 3B5 - 7

IESLC - Meta-analysis of Current Smoking, Any product (or Cigarettes if Any not available), Age 50-70
Adenocarcinoma
Excluded studies (and stage at which they were excluded)

1 BOUCHA BUELL LAURIL MZILEN

2 ABELIN ABRAHA AUVINE AXELSO BAND BERRIN BLOT1 BLOT2 BLOT3 BLOT4 BRESLO BROCKM BROWN1 BYERS1 CASCO2 CASCOR CHAN CHATZI CHEN CHEN2 CHEN3 CHIAZZ COOKSO DAVEYS DEAN DOSEME DU DUNN EBELIN ESAKI FAN GENG GER GODLEY GOLLED GSELL HANSEN HEGMAN HINDS HIRAOK HOROWI HORWIT HU HU2 HUANG ISHIMA JARUP JIANG JONES JUSSAW KO KOHLME KOULUM KREYBE LAMTH LAMWK LAMWK2 LAUSSM LEI LETOUR LEVIN LIU LIU2 LIU3 LIU4 LIU5 LOMBA2 LUO MAGNUS MARSH MARSH2 MARTIS MASTRA MATSUD MCCONN MCDUFF MCLAUG MILLER MILLS MOLLO NOTAN2 NOU ORMOS PASTOR PAWLEG PERNU PERSHA PIKE POFFIJ POLEDN PRESCO QIAO RADZIK RANDIG REN RONCO ROOTS ROTHSC SADOWS SANKAR SCHWA2 SEGI SEOW SIEMIA SIMARA STASZE STOCKS SUN TAO ULMER VANDER WANG WANG3 WANG4 WICKLU WILKIN WUWILL WYNDE2 WYNDE4 XIANGZ XU XU2 XU3 XU4 YUAN ZHANG ZHENG ZHOU

3 AGUDO AKIBA ALDERS AMANDU AMES ARCHER ARMADA AUSTIN AXELSS BARBON BECHER BENHAM BENSHL BLOHMK BOFFET BOUCOT BRETT BROSS BROWN2 BUFFLE BYERS2 CARPEN CHANG CHOI CHOW CHYOU COMSTO CORREA DAMBER DARBY DEAN2 DEKLER DESTE2 DESTEF DOCKER DOLL DOLL2 DORGAN DROSTE ENGELA ENSTRO GAO GAO2 GARCIA GARDIN GARSHI GILLIS GOODMA GRAHAM GREGOR HAENSZ HAMMO2 HEIN HENNEK HIRAY2 HUMBLE JAHN JAIN JARVHO JEDRYC JOLY KAISER KATSOU KAUFMA KELLER KHUDER KIHARA KINLEN KJUUS KNEKT KOO LANGE LEMARC LIAW LICKIN LIDDEL LOMBAR LUBIN LUBIN2 MACLEN MATOS MIGRAN MRFIT MRFITR MURATA NOTANI ODRISC OSANN OSANN2 PARKIN PERSH2 PETO PEZZO2 PEZZOT PISANI QIAO2 RACHTA RESTRE RIMING SAARIK SCHWAR SHAW SHIMIZ SIMONA SITAS SOBUE SOBUE2 SPEIZE SPITZ STAYNE STOCKW STUCKE SUZUK2 SUZUKI SVENSS TANG TANG2 TENKAN TIZZAN TOKARS TOUSEY TSUGAN TULINI TVERDA VEIERO VUTUC WAKAI WALD WANG2 WARSIN WATSON WIGLE WU WU2 WUNSCH WYNDE3 WYNDE5 WYNDE6 WYNDE7 WYNDE8 WYNDER YAMAGU YONG

4 HIRAYA

6 KAISE2

7 HOLE

9 ANDERS BEST CEDERL CPSI CPSII DEAN3 DORANT DORN HAMMON HITOSU KANELL KREUZE KUBIK NAM SEGI2

Table 3B6 -

IESLC - Meta-analysis of Current Smoking, Any product (or Cigarettes if Any not available), Age 65+
Adenocarcinoma

This analysis is restricted to results for:
1) Non-dose-response data
2) Current smokers
3) Age 65+
4) Results complete enough for use in metaanalysis

Within each study, results are then selected (in the following order of preference, within each sex) for:
5) PRODUCT: all/unspec, cigarettes regardless of other products, cigarettes only
6) CIGTYPE: all/unspecified, MC regardless of HR, MC only
7) DENOM: never smoked anything, never smoked cigarettes, (never +1 = +long term ex, +2 = +amount unknown,
+3 = never cigs+long term ex)
8) Followup period (YF, prospective studies): whole study (coded as 0) or longest available
9) LCtype: all or nearest available, at least Squamous and Adeno. (q = squamous, s = small,
a = adeno, l = large, KII = Kreyberg II, al = alveolar, br = bronchiolar, u = undifferentiated)
10) Race: all or nearest available, otherwise by race (wh or w = white, bl or b = black, hi = hispanic
ch = chinese, jap = japanese, haw = hawaiian, w+o = white + oriental, sca = scandinavian, as = asian)
11) For overlapping studies: principal rather than subsidiary studies
Finally by Age: whole study (actual age shown) if available, otherwise by widest available age group
and then for single sex results (m, f) in preference to combined sex results (c).

Results adjusted (AD) for the most potential confounders are then chosen in Sections -1 to -3
(and those which actually differ from the adjusted results in Table 3B1 - 1 are marked 'x' in Section -1)
and results adjusted for the least confounders in Sections -4 to -6. (Those least adjusted results which
actually differ from the most adjusted as marked 'x' in column X in Section -4)
(Results adjusted for an unknown number of confounder(s) are coded as 20.)

Section -7 shows excluded studies, together with the stage (as above) at which no qualifying
results were found.

Section -8 lists the potentially overlapping studies which have been included (1=principal, 2=subsidiary).

Section -9 lists any results which would have been included in preference except that they had data not complete
enough for use in meta-analysis, with their significance (yes/no), if known, and any further comment as entered
on the database.

In addition to those mentioned above, the following fields, levels and abbreviations are used:
* or nk = not known, n = no, y = yes, ot = other
nev = never
all/unspec = all or unspecified, cig+/-ot = cigarettes irrespective of other products (cigar, pipe etc)
MC = manufactured cigarettes, HR = hand-rolled cigarettes
REF: 6-character study reference
NRR: number of the RR on the database within the study
ST : study type (CC = case control, pr or prosp = prospective)
NLC: number of lung cancer cases in whole study
R : risky occupational population (n = no, m = mining, o = other risky)
VB : national cigarette type (V = at least 75% Virginia, bl = at least 75% blended, ot = other)
P : any proxy use
H : full histological confirmation
De : derivation of RR/CI (or = original, st = standard method, ot = other method of estimation)

Table 3B6 - 0

No RRs selected for this analysis

Table 3B6 - 7

IESLC - Meta-analysis of Current Smoking, Any product (or Cigarettes if Any not available), Age 65+
Adenocarcinoma
Excluded studies (and stage at which they were excluded)

1 BOUCHA BUELL LAURIL MZILEN

2 ABELIN ABRAHA AUVINE AXELSO BAND BERRIN BLOT1 BLOT2 BLOT3 BLOT4 BRESLO BROCKM BROWN1 BYERS1 CASCO2 CASCOR CHAN
CHATZI CHEN CHEN2 CHEN3 CHIAZZ COOKSO DAVEYS DEAN DOSEME DU DUNN EBELIN ESAKI FAN GENG GER GODLEY
GOLLED GSELL HANSEN HEGMAN HINDS HIRAOK HOROWI HORWIT HU HU2 HUANG ISHIMA JARUP JIANG JONES JUSSAW KO
KOHLME KOULUM KREYBE LAMTH LAMWK LAMWK2 LAUSSM LEI LETOUR LEVIN LIU LIU2 LIU3 LIU4 LIU5 LOMBA2 LUO
MAGNUS MARSH MARSH2 MARTIS MASTRA MATSUD MCCONN MCDUFF MCLAUG MILLER MILLS MOLLO NOTAN2 NOU ORMOS PASTOR PAWLEG
PERNU PERSHA PIKE POFFIJ POLEDN PRESCO QIAO RADZIK RANDIG REN RONCO ROOTS ROTHSC SADOWS SANKAR SCHWA2 SEGI
SEOW SIEMIA SIMARA STASZE STOCKS SUN TAO ULMER VANDER WANG WANG3 WANG4 WICKLU WILKIN WUWILL WYNDE2 WYNDE4
XIANGZ XU XU2 XU3 XU4 YUAN ZHANG ZHENG ZHOU

3 AGUDO AKIBA ALDERS AMANDU AMES ANDERS ARCHER ARMADA AUSTIN AXELSS BARBON BECHER BENHAM BENSHL BLOHMK BOFFET BOUCOT
BRETT BROSS BROWN2 BUFFLE BYERS2 CARPEN CEDERL CHANG CHOI CHOW CHYOU CORREA DAMBER DARBY DEAN2 DEKLER DESTE2
DESTEF DOCKER DOLL DOLL2 DORANT DORGAN DROSTE ENGELA ENSTRO GAO GAO2 GARCIA GARDIN GILLIS GOODMA GRAHAM GREGOR
HAENSZ HAMMO2 HAMMON HEIN HENNEK HIRAY2 HITOSU HOLE JAHN JAIN JARVHO JEDRYC JOLY KAISER KATSOU KAUFMA KELLER
KHUDER KIHARA KINLEN KJUUS KNEKT KOO KREUZE KUBIK LANGE LEMARC LIAW LICKIN LIDDEL LOMBAR LUBIN LUBIN2 MACLEN
MATOS MIGRAN MRFIT MRFITR MURATA NOTANI ODRISC OSANN OSANN2 PARKIN PERSH2 PETO PEZZO2 PEZZOT PISANI QIAO2 RACHTA
RESTRE RIMING SAARIK SCHWAR SHAW SHIMIZ SIMONA SITAS SOBUE SOBUE2 SPEIZE SPITZ STAYNE STOCKW STUCKE SUZUK2 SUZUKI
SVENSS TANG TANG2 TIZZAN TOKARS TOUSEY TSUGAN TULINI TVERDA VEIERO VUTUC WAKAI WALD WANG2 WARSIN WATSON WIGLE
WU WU2 WUNSCH WYNDE3 WYNDE5 WYNDE6 WYNDE7 WYNDE8 WYNDER YAMAGU YONG

4 HIRAYA

7 TENKAN

9 BEST COMSTO CPSI CPSII DEAN3 DORN GARSHI HUMBLE KAISE2 KANELL NAM SEGI2

Table 3B7 -

IESLC - Meta-analysis of Current Smoking, Cigarettes (or Any Product if Cigarettes not available), Age <56
Adenocarcinoma

This analysis is restricted to results for:
1) Non-dose-response data
2) Current smokers
3) Age <56
4) Results complete enough for use in metaanalysis

Within each study, results are then selected (in the following order of preference, within each sex) for:
5) PRODUCT: cigarettes regardless of other products, cigarettes only, all/unspec
6) CIGTYPE: all/unspecified, MC regardless of HR, MC only
7) DENOM: never smoked anything, never smoked cigarettes, (never +1 = +long term ex, +2 = +amount unknown, +3 = never cigs+long term ex)
8) Followup period (YF, prospective studies): whole study (coded as 0) or longest available
9) LCtype: all or nearest available, at least Squamous and Adeno. (q = squamous, s = small, a = adeno, l = large, KII = Kreyberg II, al = alveolar, br = bronchiolar, u = undifferentiated)
10) Race: all or nearest available, otherwise by race (wh or w = white, bl or b = black, hi = hispanic ch = chinese, jap = japanese, haw = hawaiian, w+o = white + oriental, sca = scandinavian, as = asian)
11) For overlapping studies: principal rather than subsidiary studies
Finally by Age: whole study (actual age shown) if available, otherwise by widest available age group and then for single sex results (m, f) in preference to combined sex results (c).

Results adjusted (AD) for the most potential confounders are then chosen in Sections -1 to -3 (and those which actually differ from the adjusted results in Table 3B2 - 1 are marked 'x' in Section -1) and results adjusted for the least confounders in Sections -4 to -6. (Those least adjusted results which actually differ from the most adjusted as marked 'x' in column X in Section -4) (Results adjusted for an unknown number of confounder(s) are coded as 20.)

Section -7 shows excluded studies, together with the stage (as above) at which no qualifying results were found.

Section -8 lists the potentially overlapping studies which have been included (1=principal, 2=subsidiary).

Section -9 lists any results which would have been included in preference except that they had data not complete enough for use in meta-analysis, with their significance (yes/no), if known, and any further comment as entered on the database.

In addition to those mentioned above, the following fields, levels and abbreviations are used:
* or nk = not known, n = no, y = yes, ot = other
nev = never
all/unspec = all or unspecified, cig+/-ot = cigarettes irrespective of other products (cigar, pipe etc)
MC = manufactured cigarettes, HR = hand-rolled cigarettes
REF: 6-character study reference
NRR: number of the RR on the database within the study
ST : study type (CC = case control, pr or prosp = prospective)
NLC: number of lung cancer cases in whole study
R : risky occupational population (n = no, m = mining, o = other risky)
VB : national cigarette type (V = at least 75% Virginia, bl = at least 75% blended, ot = other)
P : any proxy use
H : full histological confirmation
De : derivation of RR/CI (or = original, st = standard method, ot = other method of estimation)

Table 3B7 - 1

IESLC - Meta-analysis of Current Smoking, Cigarettes (or Any Product if Cigarettes not available), Age <56
Adenocarcinoma
Most adjusted

REF	NRR	3B2	SEX	AGEL	AGEH	RACE	YF	LC TYPE	LOC	START	ST	NLC	R	VB	P	H	AD	PRODUCT	DENOM	De
TSUGAN	3		m	30	49	all	-	a	As:Jap	1976	CC	134	n	bl	n	y	0	all/unsp	nev any	st
TSUGAN	9		f	30	49	all	-	a	As:Jap	1976	CC	134	n	bl	n	y	0	all/unsp	nev any	or
VUTUC	24	x	m	41	50	all	-	KII	Eu:wst	1976	CC	1877	n	bl	n	n	0	cig+/-ot	nev cigs	st

Cigarette type is all/unspec for all RRs

Table 3B7 - 2

IESLC - Meta-analysis of Current Smoking, Cigarettes (or Any Product if Cigarettes not available), Age <56
Adenocarcinoma
Most adjusted

REF	NRR	SEX	AD	Number Exposed Case	Number Exposed Cont	Non-exposed Case	Non-exposed Cont	RR	95.00%CI
TSUGAN	3	m	0	45	50	18	17	0.85	(0.39- 1.85)
TSUGAN	9	f	0	6	10	33	30	0.55	(0.18- 1.68)
Subtotal TSUGAN								0.74	(0.39- 1.40)
VUTUC	24	m	0	5	104	4	64	0.77	(0.20- 2.97)
Totals				56	164	55	111		

*prospective study

REF	NRR	SEX	AD	Ys	Ws	Qs	Ps
TSUGAN	3	m	0	-0.16	6.39	0.12	0.6813
TSUGAN	9	f	0	-0.61	3.03	0.29	0.2916
Subtotal TSUGAN				-0.31	9.41	0.40	
VUTUC	24	m	0	-0.26	2.10	0.00	0.7035

	N	3
	NS	2
	Wt	11.52
Het	Chi	0.41
Het	df	2
Het	P	N.S.
Fixed	RR	0.74
	RRl	0.42
	RRu	1.32
	P	N.S.
Random	RR	0.74
	RRl	0.42
	RRu	1.32
	P	N.S.
Asymm	P	N.S.

Table 3B7 - 3

IESLC - Meta-analysis of Current Smoking, Cigarettes (or Any Product if Cigarettes not available), Age <56
Adenocarcinoma
Most adjusted

	combined	Sex male	female	Total
N		2	1	3
NS		2	1	3
Wt		8.49	3.03	11.52
Het Chi		0.02	0.00	0.41
Het df		1	0	2
Het P		N.S.	N.S.	N.S.
Fixed RR		0.83	0.55	0.74
RRl		0.42	0.18	0.42
RRu		1.62	1.68	1.32
P		N.S.	N.S.	N.S.
Random RR		0.83	0.55	0.74
RRl		0.42	0.18	0.42
RRu		1.62	1.68	1.32
P		N.S.	N.S.	N.S.
Between Chi				0.39
Between df				1
Between P				N.S.
Btwn(F) P				N.S.
Btwn(R) P				N.S.

Too few RRs for analysis by factor

Table 3B7 - 4

IESLC - Meta-analysis of Current Smoking, Cigarettes (or Any Product if Cigarettes not available), Age <56
Adenocarcinoma
Least adjusted

REF	NRR	X	SEX	AGEL	AGEH	RACE	YF	LC TYPE	LOC	START	ST	NLC	R	VB	P	H	AD	PRODUCT	DENOM	De
TSUGAN	3		m	30	49	all	-	a	As:Jap	1976	CC	134	n	bl	n	y	0	all/unsp	nev any	st
TSUGAN	9		f	30	49	all	-	a	As:Jap	1976	CC	134	n	bl	n	y	0	all/unsp	nev any	or
VUTUC	24		m	41	50	all	-	KII	Eu:wst	1976	CC	1877	n	bl	n	n	0	cig+/-ot	nev cigs	st

Cigarette type is all/unspec for all RRs

Table 3B7 - 5

IESLC - Meta-analysis of Current Smoking, Cigarettes (or Any Product if Cigarettes not available), Age <56
Adenocarcinoma
Least adjusted

REF	NRR	SEX	AD	Number Exposed Case	Cont	Non-exposed Case	Cont	RR	95.00%CI
TSUGAN	3	m	0	45	50	18	17	0.85	(0.39- 1.85)
TSUGAN	9	f	0	6	10	33	30	0.55	(0.18- 1.68)
Subtotal TSUGAN								0.74	(0.39- 1.40)
VUTUC	24	m	0	5	104	4	64	0.77	(0.20- 2.97)
Totals				56	164	55	111		

*prospective study

REF	NRR	SEX	AD	Ys	Ws	Qs	Ps
TSUGAN	3	m	0	-0.16	6.39	0.12	0.6813
TSUGAN	9	f	0	-0.61	3.03	0.29	0.2916
Subtotal TSUGAN				-0.31	9.41	0.40	
VUTUC	24	m	0	-0.26	2.10	0.00	0.7035

	N	3
	NS	2
	Wt	11.52
Het	Chi	0.41
Het	df	2
Het	P	N.S.
Fixed	RR	0.74
	RRl	0.42
	RRu	1.32
	P	N.S.
Random	RR	0.74
	RRl	0.42
	RRu	1.32
	P	N.S.
Asymm	P	N.S.

Table 3B7 - 6

IESLC - Meta-analysis of Current Smoking, Cigarettes (or Any Product if Cigarettes not available), Age <56
Adenocarcinoma
Least adjusted

	Sex			
	combined	male	female	Total
N		2	1	3
NS		2	1	3
Wt		8.49	3.03	11.52
Het Chi		0.02	0.00	0.41
Het df		1	0	2
Het P		N.S.	N.S.	N.S.
Fixed RR		0.83	0.55	0.74
RRl		0.42	0.18	0.42
RRu		1.62	1.68	1.32
P		N.S.	N.S.	N.S.
Random RR		0.83	0.55	0.74
RRl		0.42	0.18	0.42
RRu		1.62	1.68	1.32
P		N.S.	N.S.	N.S.
Between Chi				0.39
Between df				1
Between P				N.S.
Btwn(F) P				N.S.
Btwn(R) P				N.S.

Table 3B7 - 7

IESLC - Meta-analysis of Current Smoking, Cigarettes (or Any Product if Cigarettes not available), Age <56
Adenocarcinoma
Excluded studies (and stage at which they were excluded)

Stage	Studies
1	BOUCHA BUELL LAURIL MZILEN
2	ABELIN ABRAHA AUVINE AXELSO BAND BERRIN BLOT1 BLOT2 BLOT3 BLOT4 BRESLO BROCKM BROWN1 BYERS1 CASCO2 CASCOR CHAN CHATZI CHEN CHEN2 CHEN3 CHIAZZ COOKSO DAVEYS DEAN DOSEME DU DUNN EBELIN ESAKI FAN GENG GER GODLEY GOLLED GSELL HANSEN HEGMAN HINDS HIRAOK HOROWI HORWIT HU HU2 HUANG ISHIMA JARUP JIANG JONES JUSSAW KO KOHLME KOULUM KREYBE LAMTH LAMWK LAMWK2 LAUSSM LEI LETOUR LEVIN LIU LIU2 LIU3 LIU4 LIU5 LOMBA2 LUO MAGNUS MARSH MARSH2 MARTIS MASTRA MATSUD MCCONN MCDUFF MCLAUG MILLER MILLS MOLLO NOTAN2 NOU ORMOS PASTOR PAWLEG PERNU PERSHA PIKE POFFIJ POLEDN PRESCO QIAO RADZIK RANDIG REN RONCO ROOTS ROTHSC SADOWS SANKAR SCHWA2 SEGI SEOW SIEMIA SIMARA STASZE STOCKS SUN TAO ULMER VANDER WANG WANG3 WANG4 WICKLU WILKIN WUWILL WYNDE2 WYNDE4 XIANGZ XU XU2 XU3 XU4 YUAN ZHANG ZHENG ZHOU
3	AGUDO AKIBA ALDERS AMANDU AMES ANDERS ARCHER ARMADA AUSTIN AXELSS BARBON BECHER BENHAM BENSHL BLOHMK BOFFET BOUCOT BRETT BROSS BROWN2 BUFFLE BYERS2 CARPEN CEDERL CHANG CHOI CHOW CHYOU CORREA DAMBER DARBY DEAN2 DEKLER DESTE2 DESTEF DOCKER DOLL DOLL2 DORANT DORGAN DORN DROSTE ENGELA ENSTRO GAO GAO2 GARCIA GARDIN GARSHI GILLIS GOODMA GRAHAM GREGOR HAENSZ HAMMO2 HAMMON HEIN HENNEK HIRAY2 HUMBLE JAHN JAIN JARVHO JEDRYC JOLY KAISER KATSOU KAUFMA KELLER KHUDER KIHARA KINLEN KJUUS KNEKT KOO LANGE LEMARC LIAW LICKIN LIDDEL LOMBAR LUBIN LUBIN2 MACLEN MATOS MIGRAN MRFIT MRFITR MURATA NOTANI ODRISC OSANN OSANN2 PARKIN PERSH2 PETO PEZZO2 PEZZOT PISANI QIAO2 RACHTA RESTRE RIMING SAARIK SCHWAR SHAW SHIMIZ SIMONA SITAS SOBUE SOBUE2 SPITZ STAYNE STOCKW STUCKE SUZUK2 SUZUKI SVENSS TANG TANG2 TENKAN TIZZAN TOKARS TOUSEY TULINI VEIERO WAKAI WALD WANG2 WARSIN WATSON WIGLE WU WU2 WUNSCH WYNDE3 WYNDE5 WYNDE6 WYNDE7 WYNDE8 WYNDER YAMAGU YONG
4	HIRAYA
6	KAISE2
7	HOLE
9	BEST COMSTO CPSI CPSII DEAN3 HITOSU KANELL KREUZE KUBIK NAM SEGI2 SPEIZE TVERDA

Table 3B7 - 8
Potentially overlapping studies

REF	REFGP	PRINC	. OVERLAP/LINK
VUTUC	LUBIN2	2	Subset of Lubin2

Table 3B8 -

IESLC - Meta-analysis of Current Smoking, Cigarettes (or Any Product if Cigarettes not available), Age 50-70
Adenocarcinoma

This analysis is restricted to results for:
1) Non-dose-response data
2) Current smokers
3) Maximum age range 50-70
4) Results complete enough for use in metaanalysis

Within each study, results are then selected (in the following order of preference, within each sex) for:
5) PRODUCT: cigarettes regardless of other products, cigarettes only, all/unspec
6) CIGTYPE: all/unspecified, MC regardless of HR, MC only
7) DENOM: never smoked anything, never smoked cigarettes, (never +1 = +long term ex, +2 = +amount unknown,
+3 = never cigs+long term ex)
8) Followup period (YF, prospective studies): whole study (coded as 0) or longest available
9) LCtype: all or nearest available, at least Squamous and Adeno. (q = squamous, s = small,
a = adeno, l = large, KII = Kreyberg II, al = alveolar, br = bronchiolar, u = undifferentiated)
10) Race: all or nearest available, otherwise by race (wh or w = white, bl or b = black, hi = hispanic
ch = chinese, jap = japanese, haw = hawaiian, w+o = white + oriental, sca = scandinavian, as = asian)
11) For overlapping studies: principal rather than subsidiary studies
Finally by Age: whole study (actual age shown) if available, otherwise by widest available age group
and then for single sex results (m, f) in preference to combined sex results (c).

Results adjusted (AD) for the most potential confounders are then chosen in Sections -1 to -3
(and those which actually differ from the adjusted results in Table 3B2 - 1 are marked 'x' in Section -1)
and results adjusted for the least confounders in Sections -4 to -6. (Those least adjusted results which
actually differ from the most adjusted as marked 'x' in column X in Section -4)
(Results adjusted for an unknown number of confounder(s) are coded as 20.)

Section -7 shows excluded studies, together with the stage (as above) at which no qualifying
results were found.

Section -8 lists the potentially overlapping studies which have been included (1=principal, 2=subsidiary).

Section -9 lists any results which would have been included in preference except that they had data not complete
enough for use in meta-analysis, with their significance (yes/no), if known, and any further comment as entered
on the database.

In addition to those mentioned above, the following fields, levels and abbreviations are used:
* or nk = not known, n = no, y = yes, ot = other
nev = never
all/unspec = all or unspecified, cig+/-ot = cigarettes irrespective of other products (cigar, pipe etc)
MC = manufactured cigarettes, HR = hand-rolled cigarettes
REF: 6-character study reference
NRR: number of the RR on the database within the study
ST : study type (CC = case control, pr or prosp = prospective)
NLC: number of lung cancer cases in whole study
R : risky occupational population (n = no, m = mining, o = other risky)
VB : national cigarette type (V = at least 75% Virginia, bl = at least 75% blended, ot = other)
P : any proxy use
H : full histological confirmation
De : derivation of RR/CI (or = original, st = standard method, ot = other method of estimation)

Table 3B8 - 0

No RRs selected for this analysis

Table 3B8 - 7

IESLC - Meta-analysis of Current Smoking, Cigarettes (or Any Product if Cigarettes not available), Age 50-70
Adenocarcinoma
Excluded studies (and stage at which they were excluded)

1 BOUCHA BUELL LAURIL MZILEN

2 ABELIN ABRAHA AUVINE AXELSO BAND BERRIN BLOT1 BLOT2 BLOT3 BLOT4 BRESLO BROCKM BROWN1 BYERS1 CASCO2 CASCOR CHAN
CHATZI CHEN CHEN2 CHEN3 CHIAZZ COOKSO DAVEYS DEAN DOSEME DU DUNN EBELIN ESAKI FAN GENG GER GODLEY
GOLLED GSELL HANSEN HEGMAN HINDS HIRAOK HOROWI HORWIT HU HU2 HUANG ISHIMA JARUP JIANG JONES JUSSAW KO
KOHLME KOULUM KREYBE LAMTH LAMWK LAMWK2 LAUSSM LEI LETOUR LEVIN LIU LIU2 LIU3 LIU4 LIU5 LOMBA2 LUO
MAGNUS MARSH MARSH2 MARTIS MASTRA MATSUD MCCONN MCDUFF MCLAUG MILLER MILLS MOLLO NOTAN2 NOU ORMOS PASTOR PAWLEG
PERNU PERSHA PIKE POFFIJ POLEDN PRESCO QIAO RADZIK RANDIG REN RONCO ROOTS ROTHSC SADOWS SANKAR SCHWA2 SEGI
SEOW SIEMIA SIMARA STASZE STOCKS SUN TAO ULMER VANDER WANG WANG3 WANG4 WICKLU WILKIN WUWILL WYNDE2 WYNDE4
XIANGZ XU XU2 XU3 XU4 YUAN ZHANG ZHENG ZHOU

3 AGUDO AKIBA ALDERS AMANDU AMES ARCHER ARMADA AUSTIN AXELSS BARBON BECHER BENHAM BENSHL BLOHMK BOFFET BOUCOT BRETT
BROSS BROWN2 BUFFLE BYERS2 CARPEN CHANG CHOI CHOW CHYOU COMSTO CORREA DAMBER DARBY DEAN2 DEKLER DESTE2 DESTEF
DOCKER DOLL DOLL2 DORGAN DROSTE ENGELA ENSTRO GAO GAO2 GARCIA GARDIN GARSHI GILLIS GOODMA GRAHAM GREGOR HAENSZ
HAMMO2 HEIN HENNEK HIRAY2 HUMBLE JAHN JAIN JARVHO JEDRYC JOLY KAISER KATSOU KAUFMA KELLER KHUDER KIHARA KINLEN
KJUUS KNEKT KOO LANGE LEMARC LIAW LICKIN LIDDEL LOMBAR LUBIN LUBIN2 MACLEN MATOS MIGRAN MRFIT MRFITR MURATA
NOTANI ODRISC OSANN OSANN2 PARKIN PERSH2 PETO PEZZO2 PEZZOT PISANI QIAO2 RACHTA RESTRE RIMING SAARIK SCHWAR SHAW
SHIMIZ SIMONA SITAS SOBUE SOBUE2 SPEIZE SPITZ STAYNE STOCKW STUCKE SUZUK2 SUZUKI SVENSS TANG TANG2 TENKAN TIZZAN
TOKARS TOUSEY TSUGAN TULINI TVERDA VEIERO VUTUC WAKAI WALD WANG2 WARSIN WATSON WIGLE WU WU2 WUNSCH WYNDE3
WYNDE5 WYNDE6 WYNDE7 WYNDE8 WYNDER YAMAGU YONG

4 HIRAYA

6 KAISE2

7 HOLE

9 ANDERS BEST CEDERL CPSI CPSII DEAN3 DORANT DORN HAMMON HITOSU KANELL KREUZE KUBIK NAM SEGI2

Table 3B9 -

IESLC - Meta-analysis of Current Smoking, Cigarettes (or Any Product if Cigarettes not available), Age 65+
Adenocarcinoma

This analysis is restricted to results for:
1) Non-dose-response data
2) Current smokers
3) Age 65+
4) Results complete enough for use in metaanalysis

Within each study, results are then selected (in the following order of preference, within each sex) for:
5) PRODUCT: cigarettes regardless of other products, cigarettes only, all/unspec
6) CIGTYPE: all/unspecified, MC regardless of HR, MC only
7) DENOM: never smoked anything, never smoked cigarettes, (never +1 = +long term ex, +2 = +amount unknown, +3 = never cigs+long term ex)
8) Followup period (YF, prospective studies): whole study (coded as 0) or longest available
9) LCtype: all or nearest available, at least Squamous and Adeno. (q = squamous, s = small, a = adeno, l = large, KII = Kreyberg II, al = alveolar, br = bronchiolar, u = undifferentiated)
10) Race: all or nearest available, otherwise by race (wh or w = white, bl or b = black, hi = hispanic ch = chinese, jap = japanese, haw = hawaiian, w+o = white + oriental, sca = scandinavian, as = asian)
11) For overlapping studies: principal rather than subsidiary studies
Finally by Age: whole study (actual age shown) if available, otherwise by widest available age group and then for single sex results (m, f) in preference to combined sex results (c).

Results adjusted (AD) for the most potential confounders are then chosen in Sections -1 to -3 (and those which actually differ from the adjusted results in Table 3B2 - 1 are marked 'x' in Section -1) and results adjusted for the least confounders in Sections -4 to -6. (Those least adjusted results which actually differ from the most adjusted as marked 'x' in column X in Section -4)
(Results adjusted for an unknown number of confounder(s) are coded as 20.)

Section -7 shows excluded studies, together with the stage (as above) at which no qualifying results were found.

Section -8 lists the potentially overlapping studies which have been included (1=principal, 2=subsidiary).

Section -9 lists any results which would have been included in preference except that they had data not complete enough for use in meta-analysis, with their significance (yes/no), if known, and any further comment as entered on the database.

In addition to those mentioned above, the following fields, levels and abbreviations are used:
* or nk = not known, n = no, y = yes, ot = other
nev = never
all/unspec = all or unspecified, cig+/-ot = cigarettes irrespective of other products (cigar, pipe etc)
MC = manufactured cigarettes, HR = hand-rolled cigarettes
REF: 6-character study reference
NRR: number of the RR on the database within the study
ST : study type (CC = case control, pr or prosp = prospective)
NLC: number of lung cancer cases in whole study
R : risky occupational population (n = no, m = mining, o = other risky)
VB : national cigarette type (V = at least 75% Virginia, bl = at least 75% blended, ot = other)
P : any proxy use
H : full histological confirmation
De : derivation of RR/CI (or = original, st = standard method, ot = other method of estimation)

Table 3B9 - 0

No RRs selected for this analysis

Table 3B9 - 7

IESLC - Meta-analysis of Current Smoking, Cigarettes (or Any Product if Cigarettes not available), Age 65+
Adenocarcinoma
Excluded studies (and stage at which they were excluded)

1 BOUCHA BUELL LAURIL MZILEN

2 ABELIN ABRAHA AUVINE AXELSO BAND BERRIN BLOT1 BLOT2 BLOT3 BLOT4 BRESLO BROCKM BROWN1 BYERS1 CASCO2 CASCOR CHAN
CHATZI CHEN CHEN2 CHEN3 CHIAZZ COOKSO DAVEYS DEAN DOSEME DU DUNN EBELIN ESAKI FAN GENG GER GODLEY
GOLLED GSELL HANSEN HEGMAN HINDS HIRAOK HOROWI HORWIT HU HU2 HUANG ISHIMA JARUP JIANG JONES JUSSAW KO
KOHLME KOULUM KREYBE LAMTH LAMWK LAMWK2 LAUSSM LEI LETOUR LEVIN LIU LIU2 LIU3 LIU4 LIU5 LOMBA2 LUO
MAGNUS MARSH MARSH2 MARTIS MASTRA MATSUD MCCONN MCDUFF MCLAUG MILLER MILLS MOLLO NOTAN2 NOU ORMOS PASTOR PAWLEG
PERNU PERSHA PIKE POFFIJ POLEDN PRESCO QIAO RADZIK RANDIG REN RONCO ROOTS ROTHSC SADOWS SANKAR SCHWA2 SEGI
SEOW SIEMIA SIMARA STASZE STOCKS SUN TAO ULMER VANDER WANG WANG3 WANG4 WICKLU WILKIN WUWILL WYNDE2 WYNDE4
XIANGZ XU XU2 XU3 XU4 YUAN ZHANG ZHENG ZHOU

3 AGUDO AKIBA ALDERS AMANDU AMES ANDERS ARCHER ARMADA AUSTIN AXELSS BARBON BECHER BENHAM BENSHL BLOHMK BOFFET BOUCOT
BRETT BROSS BROWN2 BUFFLE BYERS2 CARPEN CEDERL CHANG CHOI CHOW CHYOU CORREA DAMBER DARBY DEAN2 DEKLER DESTE2
DESTEF DOCKER DOLL DOLL2 DORANT DORGAN DROSTE ENGELA ENSTRO GAO GAO2 GARCIA GARDIN GILLIS GOODMA GRAHAM GREGOR
HAENSZ HAMMO2 HAMMON HEIN HENNEK HIRAY2 HITOSU HOLE JAHN JAIN JARVHO JEDRYC JOLY KAISER KATSOU KAUFMA KELLER
KHUDER KIHARA KINLEN KJUUS KNEKT KOO KREUZE KUBIK LANGE LEMARC LIAW LICKIN LIDDEL LOMBAR LUBIN LUBIN2 MACLEN
MATOS MIGRAN MRFIT MRFITR MURATA NOTANI ODRISC OSANN OSANN2 PARKIN PERSH2 PETO PEZZO2 PEZZOT PISANI QIAO2 RACHTA
RESTRE RIMING SAARIK SCHWAR SHAW SHIMIZ SIMONA SITAS SOBUE SOBUE2 SPEIZE SPITZ STAYNE STOCKW STUCKE SUZUK2 SUZUKI
SVENSS TANG TANG2 TIZZAN TOKARS TOUSEY TSUGAN TULINI TVERDA VEIERO VUTUC WAKAI WALD WANG2 WARSIN WATSON WIGLE
WU WU2 WUNSCH WYNDE3 WYNDE5 WYNDE6 WYNDE7 WYNDE8 WYNDER YAMAGU YONG

4 HIRAYA

7 TENKAN

9 BEST COMSTO CPSI CPSII DEAN3 DORN GARSHI HUMBLE KAISE2 KANELL NAM SEGI2

Table 3B10 -

IESLC - Meta-analysis of Current Smoking, Cigarettes only, Age <56
Adenocarcinoma

This analysis is restricted to results for:
1) Non-dose-response data
2) Current smokers
3) Age <56
4) Results complete enough for use in metaanalysis

Within each study, results are then selected (in the following order of preference, within each sex) for:
5) PRODUCT: cigarettes only
6) CIGTYPE: all/unspecified, MC regardless of HR, MC only
7) DENOM: never smoked anything, never smoked cigarettes, (never +1 = +long term ex, +2 = +amount unknown, +3 = never cigs+long term ex)
8) Followup period (YF, prospective studies): whole study (coded as 0) or longest available
9) LCtype: all or nearest available, at least Squamous and Adeno. (q = squamous, s = small, a = adeno, l = large, KII = Kreyberg II, al = alveolar, br = bronchiolar, u = undifferentiated)
10) Race: all or nearest available, otherwise by race (wh or w = white, bl or b = black, hi = hispanic ch = chinese, jap = japanese, haw = hawaiian, w+o = white + oriental, sca = scandinavian, as = asian)
11) For overlapping studies: principal rather than subsidiary studies
Finally by Age: whole study (actual age shown) if available, otherwise by widest available age group and then for single sex results (m, f) in preference to combined sex results (c).

Results adjusted (AD) for the most potential confounders are then chosen in Sections -1 to -3 (and those which actually differ from the adjusted results in Table 3B3 - 1 are marked 'x' in Section -1) and results adjusted for the least confounders in Sections -4 to -6. (Those least adjusted results which actually differ from the most adjusted as marked 'x' in column X in Section -4)
(Results adjusted for an unknown number of confounder(s) are coded as 20.)

Section -7 shows excluded studies, together with the stage (as above) at which no qualifying results were found.

Section -8 lists the potentially overlapping studies which have been included (1=principal, 2=subsidiary).

Section -9 lists any results which would have been included in preference except that they had data not complete enough for use in meta-analysis, with their significance (yes/no), if known, and any further comment as entered on the database.

In addition to those mentioned above, the following fields, levels and abbreviations are used:
* or nk = not known, n = no, y = yes, ot = other
nev = never
all/unspec = all or unspecified, MC = manufactured cigarettes, HR = hand-rolled cigarettes
REF: 6-character study reference
NRR: number of the RR on the database within the study
ST : study type (CC = case control, pr or prosp = prospective)
NLC: number of lung cancer cases in whole study
R : risky occupational population (n = no, m = mining, o = other risky)
VB : national cigarette type (V = at least 75% Virginia, bl = at least 75% blended, ot = other)
P : any proxy use
H : full histological confirmation
De : derivation of RR/CI (or = original, st = standard method, ot = other method of estimation)

Table 3B10 - 0

No RRs selected for this analysis

Table 3B10 - 7

IESLC - Meta-analysis of Current Smoking, Cigarettes only, Age <56
Adenocarcinoma
Excluded studies (and stage at which they were excluded)

1 BOUCHA BUELL LAURIL MZILEN

2 ABELIN ABRAHA AUVINE AXELSO BAND BERRIN BLOT1 BLOT2 BLOT3 BLOT4 BRESLO BROCKM BROWN1 BYERS1 CASCO2 CASCOR CHAN
CHATZI CHEN CHEN2 CHEN3 CHIAZZ COOKSO DAVEYS DEAN DOSEME DU DUNN EBELIN ESAKI FAN GENG GER GODLEY
GOLLED GSELL HANSEN HEGMAN HINDS HIRAOK HOROWI HORWIT HU HU2 HUANG ISHIMA JARUP JIANG JONES JUSSAW KO
KOHLME KOULUM KREYBE LAMTH LAMWK LAMWK2 LAUSSM LEI LETOUR LEVIN LIU LIU2 LIU3 LIU4 LIU5 LOMBA2 LUO
MAGNUS MARSH MARSH2 MARTIS MASTRA MATSUD MCCONN MCDUFF MCLAUG MILLER MILLS MOLLO NOTAN2 NOU ORMOS PASTOR PAWLEG
PERNU PERSHA PIKE POFFIJ POLEDN PRESCO QIAO RADZIK RANDIG REN RONCO ROOTS ROTHSC SADOWS SANKAR SCHWA2 SEGI
SEOW SIEMIA SIMARA STASZE STOCKS SUN TAO ULMER VANDER WANG WANG3 WANG4 WICKLU WILKIN WUWILL WYNDE2 WYNDE4
XIANGZ XU XU2 XU3 XU4 YUAN ZHANG ZHENG ZHOU

3 AGUDO AKIBA ALDERS AMANDU AMES ANDERS ARCHER ARMADA AUSTIN AXELSS BARBON BECHER BENHAM BENSHL BLOHMK BOFFET BOUCOT
BRETT BROSS BROWN2 BUFFLE BYERS2 CARPEN CEDERL CHANG CHOI CHOW CHYOU CORREA DAMBER DARBY DEAN2 DEKLER DESTE2
DESTEF DOCKER DOLL DOLL2 DORANT DORGAN DORN DROSTE ENGELA ENSTRO GAO GAO2 GARCIA GARDIN GARSHI GILLIS GOODMA
GRAHAM GREGOR HAENSZ HAMMO2 HAMMON HEIN HENNEK HIRAY2 HUMBLE JAHN JAIN JARVHO JEDRYC JOLY KAISER KATSOU KAUFMA
KELLER KHUDER KIHARA KINLEN KJUUS KNEKT KOO LANGE LEMARC LIAW LICKIN LIDDEL LOMBAR LUBIN LUBIN2 MACLEN MATOS
MIGRAN MRFIT MRFITR MURATA NOTANI ODRISC OSANN OSANN2 PARKIN PERSH2 PETO PEZZO2 PEZZOT PISANI QIAO2 RACHTA RESTRE
RIMING SAARIK SCHWAR SHAW SHIMIZ SIMONA SITAS SOBUE SOBUE2 SPITZ STAYNE STOCKW STUCKE SUZUK2 SUZUKI SVENSS TANG
TANG2 TENKAN TIZZAN TOKARS TOUSEY TULINI VEIERO WAKAI WALD WANG2 WARSIN WATSON WIGLE WU WU2 WUNSCH WYNDE3
WYNDE5 WYNDE6 WYNDE7 WYNDE8 WYNDER YAMAGU YONG

4 HIRAYA

5 COMSTO HITOSU HOLE KANELL KREUZE KUBIK NAM SEGI2 SPEIZE TSUGAN VUTUC

6 KAISE2

9 BEST CPSI CPSII DEAN3 TVERDA

Table 3B11 -

IESLC - Meta-analysis of Current Smoking, Cigarettes only, Age 50-70
Adenocarcinoma

This analysis is restricted to results for:
1) Non-dose-response data
2) Current smokers
3) Maximum age range 50-70
4) Results complete enough for use in metaanalysis

Within each study, results are then selected (in the following order of preference, within each sex) for:
5) PRODUCT: cigarettes only
6) CIGTYPE: all/unspecified, MC regardless of HR, MC only
7) DENOM: never smoked anything, never smoked cigarettes, (never +1 = +long term ex, +2 = +amount unknown, +3 = never cigs+long term ex)
8) Followup period (YF, prospective studies): whole study (coded as 0) or longest available
9) LCtype: all or nearest available, at least Squamous and Adeno. (q = squamous, s = small, a = adeno, l = large, KII = Kreyberg II, al = alveolar, br = bronchiolar, u = undifferentiated)
10) Race: all or nearest available, otherwise by race (wh or w = white, bl or b = black, hi = hispanic ch = chinese, jap = japanese, haw = hawaiian, w+o = white + oriental, sca = scandinavian, as = asian)
11) For overlapping studies: principal rather than subsidiary studies
Finally by Age: whole study (actual age shown) if available, otherwise by widest available age group and then for single sex results (m, f) in preference to combined sex results (c).

Results adjusted (AD) for the most potential confounders are then chosen in Sections -1 to -3 (and those which actually differ from the adjusted results in Table 3B3 - 1 are marked 'x' in Section -1) and results adjusted for the least confounders in Sections -4 to -6. (Those least adjusted results which actually differ from the most adjusted as marked 'x' in column X in Section -4)
(Results adjusted for an unknown number of confounder(s) are coded as 20.)

Section -7 shows excluded studies, together with the stage (as above) at which no qualifying results were found.

Section -8 lists the potentially overlapping studies which have been included (1=principal, 2=subsidiary).

Section -9 lists any results which would have been included in preference except that they had data not complete enough for use in meta-analysis, with their significance (yes/no), if known, and any further comment as entered on the database.

In addition to those mentioned above, the following fields, levels and abbreviations are used:
* or nk = not known, n = no, y = yes, ot = other
nev = never
all/unspec = all or unspecified, MC = manufactured cigarettes, HR = hand-rolled cigarettes
REF: 6-character study reference
NRR: number of the RR on the database within the study
ST : study type (CC = case control, pr or prosp = prospective)
NLC: number of lung cancer cases in whole study
R : risky occupational population (n = no, m = mining, o = other risky)
VB : national cigarette type (V = at least 75% Virginia, bl = at least 75% blended, ot = other)
P : any proxy use
H : full histological confirmation
De : derivation of RR/CI (or = original, st = standard method, ot = other method of estimation)

Table 3B11 - 0

No RRs selected for this analysis

Table 3B11 - 7

IESLC - Meta-analysis of Current Smoking, Cigarettes only, Age 50-70
Adenocarcinoma
Excluded studies (and stage at which they were excluded)

1 BOUCHA BUELL LAURIL MZILEN

2 ABELIN ABRAHA AUVINE AXELSO BAND BERRIN BLOT1 BLOT2 BLOT3 BLOT4 BRESLO BROCKM BROWN1 BYERS1 CASCO2 CASCOR CHAN
CHATZI CHEN CHEN2 CHEN3 CHIAZZ COOKSO DAVEYS DEAN DOSEME DU DUNN EBELIN ESAKI FAN GENG GER GODLEY
GOLLED GSELL HANSEN HEGMAN HINDS HIRAOK HOROWI HORWIT HU HU2 HUANG ISHIMA JARUP JIANG JONES JUSSAW KO
KOHLME KOULUM KREYBE LAMTH LAMWK LAMWK2 LAUSSM LEI LETOUR LEVIN LIU LIU2 LIU3 LIU4 LIU5 LOMBA2 LUO
MAGNUS MARSH MARSH2 MARTIS MASTRA MATSUD MCCONN MCDUFF MCLAUG MILLER MILLS MOLLO NOTAN2 NOU ORMOS PASTOR PAWLEG
PERNU PERSHA PIKE POFFIJ POLEDN PRESCO QIAO RADZIK RANDIG REN RONCO ROOTS ROTHSC SADOWS SANKAR SCHWA2 SEGI
SEOW SIEMIA SIMARA STASZE STOCKS SUN TAO ULMER VANDER WANG WANG3 WANG4 WICKLU WILKIN WUWILL WYNDE2 WYNDE4
XIANGZ XU XU2 XU3 XU4 YUAN ZHANG ZHENG ZHOU

3 AGUDO AKIBA ALDERS AMANDU AMES ARCHER ARMADA AUSTIN AXELSS BARBON BECHER BENHAM BENSHL BLOHMK BOFFET BOUCOT BRETT
BROSS BROWN2 BUFFLE BYERS2 CARPEN CHANG CHOI CHOW CHYOU COMSTO CORREA DAMBER DARBY DEAN2 DEKLER DESTE2 DESTEF
DOCKER DOLL DOLL2 DORGAN DROSTE ENGELA ENSTRO GAO GAO2 GARCIA GARDIN GARSHI GILLIS GOODMA GRAHAM GREGOR HAENSZ
HAMMO2 HEIN HENNEK HIRAY2 HUMBLE JAHN JAIN JARVHO JEDRYC JOLY KAISER KATSOU KAUFMA KELLER KHUDER KIHARA KINLEN
KJUUS KNEKT KOO LANGE LEMARC LIAW LICKIN LIDDEL LOMBAR LUBIN LUBIN2 MACLEN MATOS MIGRAN MRFIT MRFITR MURATA
NOTANI ODRISC OSANN OSANN2 PARKIN PERSH2 PETO PEZZO2 PEZZOT PISANI QIAO2 RACHTA RESTRE RIMING SAARIK SCHWAR SHAW
SHIMIZ SIMONA SITAS SOBUE SOBUE2 SPEIZE SPITZ STAYNE STOCKW STUCKE SUZUK2 SUZUKI SVENSS TANG TANG2 TENKAN TIZZAN
TOKARS TOUSEY TSUGAN TULINI TVERDA VEIERO VUTUC WAKAI WALD WANG2 WARSIN WATSON WIGLE WU WU2 WUNSCH WYNDE3
WYNDE5 WYNDE6 WYNDE7 WYNDE8 WYNDER YAMAGU YONG

4 HIRAYA

5 ANDERS DORANT DORN HITOSU HOLE KANELL KREUZE KUBIK NAM SEGI2

6 KAISE2

9 BEST CEDERL CPSI CPSII DEAN3 HAMMON

Table 3B12 -

IESLC - Meta-analysis of Current Smoking, Cigarettes only, Age 65+
Adenocarcinoma

This analysis is restricted to results for:
1) Non-dose-response data
2) Current smokers
3) Age 65+
4) Results complete enough for use in metaanalysis

Within each study, results are then selected (in the following order of preference, within each sex) for:
5) PRODUCT: cigarettes only
6) CIGTYPE: all/unspecified, MC regardless of HR, MC only
7) DENOM: never smoked anything, never smoked cigarettes, (never +1 = +long term ex, +2 = +amount unknown,
+3 = never cigs+long term ex)
8) Followup period (YF, prospective studies): whole study (coded as 0) or longest available
9) LCtype: all or nearest available, at least Squamous and Adeno. (q = squamous, s = small,
a = adeno, l = large, KII = Kreyberg II, al = alveolar, br = bronchiolar, u = undifferentiated)
10) Race: all or nearest available, otherwise by race (wh or w = white, bl or b = black, hi = hispanic
ch = chinese, jap = japanese, haw = hawaiian, w+o = white + oriental, sca = scandinavian, as = asian)
11) For overlapping studies: principal rather than subsidiary studies
Finally by Age: whole study (actual age shown) if available, otherwise by widest available age group
and then for single sex results (m, f) in preference to combined sex results (c).

Results adjusted (AD) for the most potential confounders are then chosen in Sections -1 to -3
(and those which actually differ from the adjusted results in Table 3B3 - 1 are marked 'x' in Section -1)
and results adjusted for the least confounders in Sections -4 to -6. (Those least adjusted results which
actually differ from the most adjusted as marked 'x' in column X in Section -4)
(Results adjusted for an unknown number of confounder(s) are coded as 20.)

Section -7 shows excluded studies, together with the stage (as above) at which no qualifying
results were found.

Section -8 lists the potentially overlapping studies which have been included (1=principal, 2=subsidiary).

Section -9 lists any results which would have been included in preference except that they had data not complete
enough for use in meta-analysis, with their significance (yes/no), if known, and any further comment as entered
on the database.

In addition to those mentioned above, the following fields, levels and abbreviations are used:
* or nk = not known, n = no, y = yes, ot = other
nev = never
all/unspec = all or unspecified, MC = manufactured cigarettes, HR = hand-rolled cigarettes
REF: 6-character study reference
NRR: number of the RR on the database within the study
ST : study type (CC = case control, pr or prosp = prospective)
NLC: number of lung cancer cases in whole study
R : risky occupational population (n = no, m = mining, o = other risky)
VB : national cigarette type (V = at least 75% Virginia, bl = at least 75% blended, ot = other)
P : any proxy use
H : full histological confirmation
De : derivation of RR/CI (or = original, st = standard method, ot = other method of estimation)

Table 3B12 - 0

No RRs selected for this analysis

Table 3B12 - 7

IESLC - Meta-analysis of Current Smoking, Cigarettes only, Age 65+
Adenocarcinoma
Excluded studies (and stage at which they were excluded)

1 BOUCHA BUELL LAURIL MZILEN

2 ABELIN ABRAHA AUVINE AXELSO BAND BERRIN BLOT1 BLOT2 BLOT3 BLOT4 BRESLO BROCKM BROWN1 BYERS1 CASCO2 CASCOR CHAN
CHATZI CHEN CHEN2 CHEN3 CHIAZZ COOKSO DAVEYS DEAN DOSEME DU DUNN EBELIN ESAKI FAN GENG GER GODLEY
GOLLED GSELL HANSEN HEGMAN HINDS HIRAOK HOROWI HORWIT HU HU2 HUANG ISHIMA JARUP JIANG JONES JUSSAW KO
KOHLME KOULUM KREYBE LAMTH LAMWK LAMWK2 LAUSSM LEI LETOUR LEVIN LIU LIU2 LIU3 LIU4 LIU5 LOMBA2 LUO
MAGNUS MARSH MARSH2 MARTIS MASTRA MATSUD MCCONN MCDUFF MCLAUG MILLER MILLS MOLLO NOTAN2 NOU ORMOS PASTOR PAWLEG
PERNU PERSHA PIKE POFFIJ POLEDN PRESCO QIAO RADZIK RANDIG REN RONCO ROOTS ROTHSC SADOWS SANKAR SCHWA2 SEGI
SEOW SIEMIA SIMARA STASZE STOCKS SUN TAO ULMER VANDER WANG WANG3 WANG4 WICKLU WILKIN WUWILL WYNDE2 WYNDE4
XIANGZ XU XU2 XU3 XU4 YUAN ZHANG ZHENG ZHOU

3 AGUDO AKIBA ALDERS AMANDU AMES ANDERS ARCHER ARMADA AUSTIN AXELSS BARBON BECHER BENHAM BENSHL BLOHMK BOFFET BOUCOT
BRETT BROSS BROWN2 BUFFLE BYERS2 CARPEN CEDERL CHANG CHOI CHOW CHYOU CORREA DAMBER DARBY DEAN2 DEKLER DESTE2
DESTEF DOCKER DOLL DOLL2 DORANT DORGAN DROSTE ENGELA ENSTRO GAO GAO2 GARCIA GARDIN GILLIS GOODMA GRAHAM GREGOR
HAENSZ HAMMO2 HAMMON HEIN HENNEK HIRAY2 HITOSU HOLE JAHN JAIN JARVHO JEDRYC JOLY KAISER KATSOU KAUFMA KELLER
KHUDER KIHARA KINLEN KJUUS KNEKT KOO KREUZE KUBIK LANGE LEMARC LIAW LICKIN LIDDEL LOMBAR LUBIN LUBIN2 MACLEN
MATOS MIGRAN MRFIT MRFITR MURATA NOTANI ODRISC OSANN OSANN2 PARKIN PERSH2 PETO PEZZO2 PEZZOT PISANI QIAO2 RACHTA
RESTRE RIMING SAARIK SCHWAR SHAW SHIMIZ SIMONA SITAS SOBUE SOBUE2 SPEIZE SPITZ STAYNE STOCKW STUCKE SUZUK2 SUZUKI
SVENSS TANG TANG2 TIZZAN TOKARS TOUSEY TSUGAN TULINI TVERDA VEIERO VUTUC WAKAI WALD WANG2 WARSIN WATSON WIGLE
WU WU2 WUNSCH WYNDE3 WYNDE5 WYNDE6 WYNDE7 WYNDE8 WYNDER YAMAGU YONG

4 HIRAYA

5 COMSTO GARSHI HUMBLE KANELL NAM SEGI2 TENKAN

9 BEST CPSI CPSII DEAN3 DORN KAISE2

Table 3B13 -

IESLC - Meta-analysis of Current Smoking (vs non-current), Any product (or Cigarettes if Any not available)
Adenocarcinoma

This analysis is restricted to results for:
1) Non-dose-response data
2) Current smokers
3) Results complete enough for use in metaanalysis

Within each study, results are then selected (in the following order of preference, within each sex) for:
4) PRODUCT: all/unspec, cigarettes regardless of other products, cigarettes only
5) CIGTYPE: all/unspecified, MC regardless of HR, MC only
6) DENOM: non smoker of anything, non smoker of cigarettes
7) Followup period (YF, prospective studies): whole study (coded as 0) or longest available
8) LCtype: adeno or nearest available, but not squamous. (q = squamous, s = small,
a = adeno, l = large, KII = Kreyberg II, al = alveolar, br = bronchiolar, u = undifferentiated)
9) Race: all or nearest available, otherwise by race (wh or w = white, bl or b = black, hi = hispanic
ch = chinese, jap = japanese, haw = hawaiian, w+o = white + oriental, sca = scandinavian, as = asian)
10) For overlapping studies: principal rather than subsidiary studies
Finally by Age: whole study (coded as 0) if available, otherwise by widest available age group
and then for single sex results (m, f) in preference to combined sex results (c).

Results adjusted (AD) for the most potential confounders are then chosen in Sections -1 to -3
and results adjusted for the least confounders in Sections -4 to -6. (Those least adjusted results which
actually differ from the most adjusted as marked 'x' in column X in Section -4)
(Results adjusted for an unknown number of confounder(s) are coded as 20.)

Section -7 shows excluded studies, together with the stage (as above) at which no qualifying
results were found.

Section -8 lists the potentially overlapping studies which have been included (1=principal, 2=subsidiary).

Section -9 lists any results which would have been included in preference except that they had data not complete
enough for use in meta-analysis, with their significance (yes/no), if known, and any further comment as entered
on the database.

In addition to those mentioned above, the following fields, levels and abbreviations are used:
* or nk = not known, n = no, y = yes, ot = other
non = not current
all/unspec = all or unspecified, cig+/-ot = cigarettes irrespective of other products (cigar, pipe etc)
MC = manufactured cigarettes, HR = hand-rolled cigarettes
REF: 6-character study reference
NRR: number of the RR on the database within the study
ST : study type (CC = case control, pr or prosp = prospective)
NLC: number of lung cancer cases in whole study
R : risky occupational population (n = no, m = mining, o = other risky)
VB : national cigarette type (V = at least 75% Virginia, bl = at least 75% blended, ot = other)
P : any proxy use
H : full histological confirmation
De : derivation of RR/CI (or = original, st = standard method, ot = other method of estimation)

Table 3B13 - 1

IESLC - Meta-analysis of Current Smoking (vs non-current), Any product (or Cigarettes if Any not available)
Adenocarcinoma
Most adjusted

REF	NRR	SEX	AGEL	AGEH	RACE	YF	LC TYPE	LOC	START	ST	NLC	R	VB	P	H	AD	PRODUCT	DENOM	De
BARBON	125	m	0	0	all	-	a	Eu:wst	1979	CC	755	n	bl	y	y	1	all/unsp	non any	ot
BUFFLE	73	f	0	0	w-hi	-	a	NAmer	1976	CC	943	n	bl	y	n	0	cig+/-ot	non cigs	st
COMSTO	72	m	0	0	all	-	a	NAmer	1975	ot	258	n	bl	n	n	0	cig+/-ot	non cigs	st
COMSTO	84	f	0	0	all	-	a	NAmer	1975	ot	258	n	bl	n	n	0	cig+/-ot	non cigs	st
CORREA	67	c	0	0	all	-	a	NAmer	1979	CC	1359	n	bl	y	n	1	cig+/-ot	non cigs	ot
ENGELA	77	m	0	0	all	0	a	Eu:Sca	1964	pr	435	n	bl	n	n	7	cig+/-ot	non cigs	ot
HAENSZ	49	f	0	0	all	-	a	NAmer	1955	CC	158	n	bl	n	y	0	cig+/-ot	non cigs	st
JAHN	35	m	0	0	all	-	a	Eu:Ger	1988	CC	1004	n	bl	n	n	0	cig+/-ot	non cigs	st
JAIN	37	m	0	0	all	-	a	NAmer	1981	CC	845	n	V	y	n	0	cig+/-ot	non cigs	st
JAIN	32	f	0	0	all	-	a	NAmer	1981	CC	845	n	V	y	n	0	cig+/-ot	non cigs	st
JEDRYC	53	m	0	0	all	-	a	Eu:est	1980	CC	1630	n	bl	y	n	0	cig+/-ot	non any	st
KATSOU	34	f	0	0	all	-	a	Eu:bal	1987	CC	101	n	bl	n	n	1	all/unsp	non any	ot
KHUDER	32	m	0	0	all	-	a	NAmer	1985	CC	482	n	bl	n	y	0	cig+/-ot	non cigs	ot
KIHARA	21	c	0	0	jap	-	a	As:Jap	1991	CC	440	n	bl	n	n	0	all/unsp	non any	st
LUBIN2	256	m	0	0	all	-	a	Eu:mul	1976	CC	7804	n	bl	n	y	0	cig+/-ot	non any	st
LUBIN2	268	f	0	0	all	-	a	Eu:mul	1976	CC	7804	n	bl	n	y	0	cig+/-ot	non any	st
MATOS	89	m	0	0	all	-	a	SCAmer	1994	CC	200	n	bl	n	n	2	cig+/-ot	non any	ot
OSANN	79	m	0	0	all	-	a	NAmer	1984	CC	1986	n	bl	n	n	2	cig+/-ot	non cigs	ot
OSANN	80	f	0	0	all	-	a	NAmer	1984	CC	1986	n	bl	n	n	2	cig+/-ot	non cigs	ot
OSANN2	36	f	0	0	all	-	KII	NAmer	1964	ot	217	n	bl	n	y	1	cig+/-ot	non cigs	ot
SAARIK	3	c	0	0	wh	-	a	Eu:Sca	1988	CC	205	n	bl	n	y	0	all/unsp	non any	st
SEGI2	34	m	0	0	all	-	a	As:Jap	1962	CC	378	n	bl	n	n	1	cig+/-ot	non any	ot
SEGI2	36	f	0	0	all	-	a	As:Jap	1962	CC	378	n	bl	n	n	1	cig+/-ot	non any	ot
SHIMIZ	1	m	0	0	all	-	a	As:Jap	1977	CC	751	n	bl	y	n	2	all/unsp	non any	or
SHIMIZ	6	f	0	0	all	-	a	As:Jap	1977	CC	751	n	bl	y	n	2	all/unsp	non any	or
SOBUE	100	m	0	0	all	-	a	As:Jap	1986	CC	1376	n	bl	n	y	1	cig+/-ot	non cigs	ot
SOBUE	110	f	0	0	all	-	a	As:Jap	1986	CC	1376	n	bl	n	y	1	cig+/-ot	non cigs	ot
STAYNE	8	m	0	0	all	-	a	NAmer	1969	CC	420	n	bl	n	n	1	cig+/-ot	non cigs	st
SUZUKI	12	m	0	0	all	-	a	As:Jap	1978	CC	238	n	bl	n	y	2	cig+/-ot	non any	ot
SUZUKI	16	f	0	0	all	-	a	As:Jap	1978	CC	238	n	bl	n	y	2	cig+/-ot	non any	ot
SVENSS	94	f	0	0	all	-	a	Eu:Sca	1983	CC	210	n	bl	n	n	1	all/unsp	non any	ot
TSUGAN	7	m	0	0	all	-	a	As:Jap	1976	CC	134	n	bl	n	y	0	all/unsp	non any	st
WAKAI	77	m	0	0	all	-	a	As:Jap	1988	CC	333	n	bl	n	y	1	all/unsp	non any	ot
WAKAI	83	f	0	0	all	-	a	As:Jap	1988	CC	333	n	bl	n	y	1	all/unsp	non any	ot
WU	22	f	0	0	wh	-	a	NAmer	1981	CC	220	n	bl	n	y	2	all/unsp	non any	ot
WYNDE3	33	m	0	0	all	-	KII	NAmer	1966	CC	350	n	bl	n	y	0	all/unsp	non any	st
WYNDE6	150	m	0	0	all	-	KII	NAmer	1969	CC	4423	n	bl	n	y	0	cig+/-ot	non cigs	st
WYNDE6	258	f	0	0	all	-	KII	NAmer	1969	CC	4423	n	bl	n	y	0	cig+/-ot	non cigs	st

Cigarette type is all/unspec for all RRs

Table 3B13 - 2

IESLC - Meta-analysis of Current Smoking (vs non-current), Any product (or Cigarettes if Any not available)
Adenocarcinoma
Most adjusted

REF	NRR	SEX	AD	Number Exposed Case	Cont	Non-exposed Case	Cont	RR	95.00%CI
BARBON	125	m	1	-	-	-	-	2.38	(1.64- 3.44)
BUFFLE	73	f	0	56	110	27	168	3.17	(1.89- 5.32)
COMSTO	72	m	0	30	100	15	213	4.26	(2.19- 8.27)
COMSTO	84	f	0	23	52	14	150	4.74	(2.27- 9.89)
Subtotal COMSTO								4.47	(2.73- 7.31)
CORREA	67	c	1	-	-	-	-	3.74	(2.76- 5.08)
*ENGELA	77	m	7	-	-	-	-	6.60	(3.52- 12.35)
HAENSZ	49	f	0	16	94	39	245	1.07	(0.57- 2.00)
JAHN	35	m	0	75	269	137	570	1.16	(0.85- 1.59)
JAIN	37	m	0	60	118	34	244	3.65	(2.27- 5.87)
JAIN	32	f	0	69	99	41	311	5.29	(3.38- 8.27)
Subtotal JAIN								4.44	(3.21- 6.15)
JEDRYC	53	m	0	68	516	28	601	2.83	(1.79- 4.46)
KATSOU	34	f	1	-	-	-	-	1.63	(0.72- 3.68)
KHUDER	32	m	0	92	-	70	-	1.72	(1.17- 2.52)
KIHARA	21	c	0	103	162	105	307	1.86	(1.33- 2.59)
LUBIN2	256	m	0	454	6209	270	6844	1.85	(1.59- 2.16)
LUBIN2	268	f	0	69	410	155	1337	1.45	(1.07- 1.97)
Subtotal LUBIN2								1.76	(1.54- 2.02)
MATOS	89	m	2	-	-	-	-	2.46	(1.52- 3.98)
OSANN	79	m	2	-	-	-	-	3.44	(2.64- 4.49)
OSANN	80	f	2	-	-	-	-	6.65	(5.04- 8.77)
Subtotal OSANN								4.72	(3.89- 5.71)
OSANN2	36	f	1	-	-	-	-	2.85	(1.43- 5.69)
SAARIK	3	c	0	38	66	43	224	3.00	(1.79- 5.02)
SEGI2	34	m	1	-	-	-	-	1.18	(0.56- 2.47)
SEGI2	36	f	1	-	-	-	-	1.02	(0.44- 2.34)
Subtotal SEGI2								1.11	(0.64- 1.93)
SHIMIZ	1	m	2	-	-	-	-	1.90	(1.30- 3.00)
SHIMIZ	6	f	2	-	-	-	-	2.90	(1.70- 5.00)
Subtotal SHIMIZ								2.23	(1.60- 3.10)
SOBUE	100	m	1	-	-	-	-	1.47	(1.13- 1.91)
SOBUE	110	f	1	-	-	-	-	1.24	(0.84- 1.84)
Subtotal SOBUE								1.39	(1.12- 1.73)
STAYNE	8	m	1	-	-	-	-	3.10	(1.42- 6.75)
SUZUKI	12	m	2	-	-	-	-	2.78	(1.81- 4.27)
SUZUKI	16	f	2	-	-	-	-	2.33	(1.16- 4.68)
Subtotal SUZUKI								2.65	(1.84- 3.82)
SVENSS	94	f	1	-	-	-	-	3.16	(1.70- 5.85)
TSUGAN	7	m	0	45	50	26	23	0.80	(0.40- 1.59)
WAKAI	77	m	1	-	-	-	-	1.72	(1.09- 2.71)
WAKAI	83	f	1	-	-	-	-	1.08	(0.47- 2.48)
Subtotal WAKAI								1.54	(1.04- 2.30)
WU	22	f	2	-	-	-	-	3.71	(2.24- 6.16)
WYNDE3	33	m	0	56	207	18	213	3.20	(1.82- 5.63)
WYNDE6	150	m	0	651	741	486	1872	3.38	(2.93- 3.91)
WYNDE6	258	f	0	472	376	290	1181	5.11	(4.24- 6.16)
Subtotal WYNDE6								3.95	(3.52- 4.43)
Partial Totals				2377	9579	1798	14503		

*prospective study

REF	NRR	SEX	AD	Ys	Ws	Qs	Ps
BARBON	125	m	1	0.87	28.00	0.32	0.0000
BUFFLE	73	f	0	1.15	14.30	0.46	0.0000
COMSTO	72	m	0	1.45	8.72	1.98	0.0000
COMSTO	84	f	0	1.56	7.10	2.41	0.0000
Subtotal COMSTO				1.50	15.82	4.39	
CORREA	67	c	1	1.32	41.28	4.94	0.0000
*ENGELA	77	m	7	1.89	9.75	8.15	0.0000
HAENSZ	49	f	0	0.07	9.72	7.98	0.8345
JAHN	35	m	0	0.15	38.31	26.06	0.3583
JAIN	37	m	0	1.29	17.05	1.76	0.0000
JAIN	32	f	0	1.67	19.16	9.17	0.0000
Subtotal JAIN				1.49	36.21	10.93	
JEDRYC	53	m	0	1.04	18.51	0.08	0.0000
KATSOU	34	f	1	0.49	5.77	1.36	0.2404
KHUDER	32	m	0	0.54	26.10	4.85	0.0056
KIHARA	21	c	0	0.62	34.89	4.35	0.0003
LUBIN2	256	m	0	0.62	160.94	20.41	0.0000
LUBIN2	268	f	0	0.37	41.44	14.94	0.0164

Table 3B13 - 2

IESLC - Meta-analysis of Current Smoking (vs non-current), Any product (or Cigarettes if Any not available)
Adenocarcinoma
Most adjusted

REF	NRR	SEX	AD	Ys	Ws	Qs	Ps
Subtotal LUBIN2				0.57	202.38	35.36	
MATOS	89	m	2	0.90	16.58	0.09	0.0002
OSANN	79	m	2	1.24	54.48	3.75	0.0000
OSANN	80	f	2	1.89	50.08	42.52	0.0000
Subtotal OSANN				1.55	104.56	46.26	
OSANN2	36	f	1	1.05	8.06	0.04	0.0030
SAARIK	3	c	0	1.10	14.45	0.23	0.0000
SEGI2	34	m	1	0.17	6.98	4.55	0.6620
SEGI2	36	f	1	0.02	5.50	5.00	0.9630
Subtotal SEGI2				0.10	12.48	9.55	
SHIMIZ	1	m	2	0.64	21.97	2.41	0.0026
SHIMIZ	6	f	2	1.06	13.20	0.11	0.0001
Subtotal SHIMIZ				0.80	35.18	2.52	
SOBUE	100	m	1	0.39	55.77	19.28	0.0040
SOBUE	110	f	1	0.22	24.99	14.36	0.2822
Subtotal SOBUE				0.33	80.76	33.64	
STAYNE	8	m	1	1.13	6.32	0.16	0.0044
SUZUKI	12	m	2	1.02	20.86	0.05	0.0000
SUZUKI	16	f	2	0.85	7.90	0.13	0.0175
Subtotal SUZUKI				0.97	28.76	0.18	
SVENSS	94	f	1	1.15	10.06	0.32	0.0003
TSUGAN	7	m	0	-0.23	8.05	11.62	0.5177
WAKAI	77	m	1	0.54	18.52	3.44	0.0196
WAKAI	83	f	1	0.08	5.55	4.46	0.8561
Subtotal WAKAI				0.43	24.08	7.90	
WU	22	f	2	1.31	15.02	1.71	0.0000
WYNDE3	33	m	0	1.16	12.06	0.44	0.0001
WYNDE6	150	m	0	1.22	182.57	11.04	0.0000
WYNDE6	258	f	0	1.63	110.21	47.78	0.0000
Subtotal WYNDE6				1.37	292.78	58.82	

	N	38
	NS	28
	Wt	1150.25
Het	Chi	282.70
Het	df	37
Het	P	***
Fixed	RR	2.65
	RRl	2.50
	RRu	2.80
	P	+++
Random	RR	2.46
	RRl	2.07
	RRu	2.93
	P	+++
Asymm	P	N.S.

Table 3B13 - 3

IESLC - Meta-analysis of Current Smoking (vs non-current), Any product (or Cigarettes if Any not available)
Adenocarcinoma
Most adjusted

		Sex		
	combined	male	female	Total
N	3	19	16	38
NS	3	19	16	38
Wt	90.63	711.56	348.07	1150.25
Het Chi	9.36	110.40	134.07	282.70
Het df	2	18	15	37
Het P	**	***	***	***
Fixed RR	2.76	2.35	3.34	2.65
RRl	2.25	2.18	3.00	2.50
RRu	3.39	2.53	3.71	2.80
P	+++	+++	+++	+++
Random RR	2.74	2.31	2.57	2.46
RRl	1.72	1.88	1.82	2.07
RRu	4.37	2.83	3.62	2.93
P	+++	+++	+++	+++
Between Chi				28.87
Between df				2
Between P				***
Btwn(F) P				N.S.
Btwn(R) P				N.S.

Table 3B13 - 4

IESLC - Meta-analysis of Current Smoking (vs non-current), Any product (or Cigarettes if Any not available)
Adenocarcinoma
Least adjusted

REF	NRR	X	SEX	AGEL	AGEH	RACE	YF	LC TYPE	LOC	START	ST	NLC	R	VB	P	H	AD	PRODUCT	DENOM	De
BARBON	124	x	m	0	0	all	-	a	Eu:wst	1979	CC	755	n	bl	y	y	0	all/unsp	non any	st
BUFFLE	73		f	0	0	w-hi	-	a	NAmer	1976	CC	943	n	bl	y	n	0	cig+/-ot	non cigs	st
COMSTO	72		m	0	0	all	-	a	NAmer	1975	ot	258	n	bl	n	n	0	cig+/-ot	non cigs	st
COMSTO	84		f	0	0	all	-	a	NAmer	1975	ot	258	n	bl	n	n	0	cig+/-ot	non cigs	st
CORREA	67		c	0	0	all	-	a	NAmer	1979	CC	1359	n	bl	y	n	1	cig+/-ot	non cigs	ot
ENGELA	77		m	0	0	all	0	a	Eu:Sca	1964	pr	435	n	bl	n	n	7	cig+/-ot	non cigs	ot
HAENSZ	49		f	0	0	all	-	a	NAmer	1955	CC	158	n	bl	n	y	0	cig+/-ot	non cigs	st
JAHN	35		m	0	0	all	-	a	Eu:Ger	1988	CC	1004	n	bl	n	n	0	cig+/-ot	non cigs	st
JAIN	37		m	0	0	all	-	a	NAmer	1981	CC	845	n	V	y	n	0	cig+/-ot	non cigs	st
JAIN	32		f	0	0	all	-	a	NAmer	1981	CC	845	n	V	y	n	0	cig+/-ot	non cigs	st
JEDRYC	53		m	0	0	all	-	a	Eu:est	1980	CC	1630	n	bl	y	n	0	cig+/-ot	non any	st
KATSOU	32	x	f	0	0	all	-	a	Eu:bal	1987	CC	101	n	bl	n	n	0	all/unsp	non any	st
KHUDER	32		m	0	0	all	-	a	NAmer	1985	CC	482	n	bl	n	y	0	cig+/-ot	non cigs	ot
KIHARA	21		c	0	0	jap	-	a	As:Jap	1991	CC	440	n	bl	n	n	0	all/unsp	non any	st
LUBIN2	256		m	0	0	all	-	a	Eu:mul	1976	CC	7804	n	bl	n	y	0	cig+/-ot	non any	st
LUBIN2	268		f	0	0	all	-	a	Eu:mul	1976	CC	7804	n	bl	n	y	0	cig+/-ot	non any	st
MATOS	88	x	m	0	0	all	-	a	SCAmer	1994	CC	200	n	bl	n	n	0	cig+/-ot	non any	st
OSANN	67	x	m	0	0	all	-	a	NAmer	1984	CC	1986	n	bl	n	n	0	cig+/-ot	non cigs	st
OSANN	71	x	f	0	0	all	-	a	NAmer	1984	CC	1986	n	bl	n	n	0	cig+/-ot	non cigs	st
OSANN2	18	x	f	0	0	all	-	KII	NAmer	1964	ot	217	n	bl	n	y	0	cig+/-ot	non cigs	st
SAARIK	3		c	0	0	wh	-	a	Eu:Sca	1988	CC	205	n	bl	n	y	0	all/unsp	non any	st
SEGI2	33	x	m	0	0	all	-	a	As:Jap	1962	CC	378	n	bl	n	n	0	cig+/-ot	non any	st
SEGI2	35	x	f	0	0	all	-	a	As:Jap	1962	CC	378	n	bl	n	n	0	cig+/-ot	non any	st
SHIMIZ	1		m	0	0	all	-	a	As:Jap	1977	CC	751	n	bl	y	n	2	all/unsp	non any	or
SHIMIZ	6		f	0	0	all	-	a	As:Jap	1977	CC	751	n	bl	y	n	2	all/unsp	non any	or
SOBUE	8	x	m	0	0	all	-	a	As:Jap	1986	CC	1376	n	bl	n	y	0	cig+/-ot	non cigs	st
SOBUE	24	x	f	0	0	all	-	a	As:Jap	1986	CC	1376	n	bl	n	y	0	cig+/-ot	non cigs	st
STAYNE	8		m	0	0	all	-	a	NAmer	1969	CC	420	n	bl	n	n	1	cig+/-ot	non cigs	st
SUZUKI	4	x	m	0	0	all	-	a	As:Jap	1978	CC	238	n	bl	n	y	0	cig+/-ot	non any	st
SUZUKI	8	x	f	0	0	all	-	a	As:Jap	1978	CC	238	n	bl	n	y	0	cig+/-ot	non any	st
SVENSS	69	x	f	0	0	all	-	a	Eu:Sca	1983	CC	210	n	bl	n	n	0	all/unsp	non any	st
TSUGAN	7		m	0	0	all	-	a	As:Jap	1976	CC	134	n	bl	n	y	0	all/unsp	non any	st
WAKAI	18	x	m	0	0	all	-	a	As:Jap	1988	CC	333	n	bl	n	y	0	all/unsp	non any	st
WAKAI	36	x	f	0	0	all	-	a	As:Jap	1988	CC	333	n	bl	n	y	0	all/unsp	non any	st
WU	19	x	f	0	0	wh	-	a	NAmer	1981	CC	220	n	bl	n	y	0	all/unsp	non any	st
WYNDE3	33		m	0	0	all	-	KII	NAmer	1966	CC	350	n	bl	n	y	0	all/unsp	non any	st
WYNDE6	150		m	0	0	all	-	KII	NAmer	1969	CC	4423	n	bl	n	y	0	cig+/-ot	non cigs	st
WYNDE6	258		f	0	0	all	-	KII	NAmer	1969	CC	4423	n	bl	n	y	0	cig+/-ot	non cigs	st

Cigarette type is all/unspec for all RRs

Table 3B13 - 5

IESLC - Meta-analysis of Current Smoking (vs non-current), Any product (or Cigarettes if Any not available)
Adenocarcinoma
Least adjusted

REF	NRR	SEX	AD	Number Exposed Case	Exposed Cont	Non-exposed Case	Non-exposed Cont	RR	95.00%CI
BARBON	124	m	0	109	362	49	393	2.41	(1.67- 3.48)
BUFFLE	73	f	0	56	110	27	168	3.17	(1.89- 5.32)
COMSTO	72	m	0	30	100	15	213	4.26	(2.19- 8.27)
COMSTO	84	f	0	23	52	14	150	4.74	(2.27- 9.89)
Subtotal COMSTO								4.47	(2.73- 7.31)
CORREA	67	c	1	-	-	-	-	3.74	(2.76- 5.08)
*ENGELA	77	m	7	-	-	-	-	6.60	(3.52- 12.35)
HAENSZ	49	f	0	16	94	39	245	1.07	(0.57- 2.00)
JAHN	35	m	0	75	269	137	570	1.16	(0.85- 1.59)
JAIN	37	m	0	60	118	34	244	3.65	(2.27- 5.87)
JAIN	32	f	0	69	99	41	311	5.29	(3.38- 8.27)
Subtotal JAIN								4.44	(3.21- 6.15)
JEDRYC	53	m	0	68	516	28	601	2.83	(1.79- 4.46)
KATSOU	32	f	0	15	18	33	71	1.79	(0.81- 3.99)
KHUDER	32	m	0	92	-	70	-	1.72	(1.17- 2.52)
KIHARA	21	c	0	103	162	105	307	1.86	(1.33- 2.59)
LUBIN2	256	m	0	454	6209	270	6844	1.85	(1.59- 2.16)
LUBIN2	268	f	0	69	410	155	1337	1.45	(1.07- 1.97)
Subtotal LUBIN2								1.76	(1.54- 2.02)
MATOS	88	m	0	46	132	38	261	2.39	(1.48- 3.86)
OSANN	67	m	0	217	541	116	1310	4.53	(3.54- 5.80)
OSANN	71	f	0	193	367	97	1289	6.99	(5.33- 9.16)
Subtotal OSANN								5.52	(4.60- 6.62)
OSANN2	18	f	0	50	28	33	55	2.98	(1.58- 5.60)
SAARIK	3	c	0	38	66	43	224	3.00	(1.79- 5.02)
SEGI2	33	m	0	53	485	9	102	1.24	(0.59- 2.59)
SEGI2	35	f	0	9	34	32	128	1.06	(0.46- 2.43)
Subtotal SEGI2								1.16	(0.67- 2.01)
SHIMIZ	1	m	2	-	-	-	-	1.90	(1.30- 3.00)
SHIMIZ	6	f	2	-	-	-	-	2.90	(1.70- 5.00)
Subtotal SHIMIZ								2.23	(1.60- 3.10)
SOBUE	8	m	0	276	650	144	491	1.45	(1.15- 1.83)
SOBUE	24	f	0	38	168	157	921	1.33	(0.90- 1.96)
Subtotal SOBUE								1.42	(1.16- 1.73)
STAYNE	8	m	1	-	-	-	-	3.10	(1.42- 6.75)
SUZUKI	4	m	0	119	162	39	154	2.90	(1.90- 4.43)
SUZUKI	8	f	0	20	20	60	140	2.33	(1.17- 4.65)
Subtotal SUZUKI								2.73	(1.90- 3.92)
SVENSS	69	f	0	38	53	34	156	3.29	(1.88- 5.75)
TSUGAN	7	m	0	45	50	26	23	0.80	(0.40- 1.59)
WAKAI	18	m	0	75	284	31	205	1.75	(1.11- 2.75)
WAKAI	36	f	0	9	26	50	150	1.04	(0.46- 2.36)
Subtotal WAKAI								1.55	(1.04- 2.30)
WU	19	f	0	99	50	50	99	3.92	(2.42- 6.34)
WYNDE3	33	m	0	56	207	18	213	3.20	(1.82- 5.63)
WYNDE6	150	m	0	651	741	486	1872	3.38	(2.93- 3.91)
WYNDE6	258	f	0	472	376	290	1181	5.11	(4.24- 6.16)
Subtotal WYNDE6								3.95	(3.52- 4.43)
Partial Totals				3743	12959	2770	20428		

*prospective study

REF	NRR	SEX	AD	Ys	Ws	Qs	Ps
BARBON	124	m	0	0.88	28.66	0.34	0.0000
BUFFLE	73	f	0	1.15	14.30	0.38	0.0000
COMSTO	72	m	0	1.45	8.72	1.83	0.0000
COMSTO	84	f	0	1.56	7.10	2.27	0.0000
Subtotal COMSTO				1.50	15.82	4.10	
CORREA	67	c	1	1.32	41.28	4.45	0.0000
*ENGELA	77	m	7	1.89	9.75	7.83	0.0000
HAENSZ	49	f	0	0.07	9.72	8.30	0.8345
JAHN	35	m	0	0.15	38.31	27.18	0.3583
JAIN	37	m	0	1.29	17.05	1.57	0.0000
JAIN	32	f	0	1.67	19.16	8.71	0.0000
Subtotal JAIN				1.49	36.21	10.28	
JEDRYC	53	m	0	1.04	18.51	0.04	0.0000
KATSOU	32	f	0	0.58	6.00	0.99	0.1526
KHUDER	32	m	0	0.54	26.10	5.25	0.0056
KIHARA	21	c	0	0.62	34.89	4.80	0.0003
LUBIN2	256	m	0	0.62	160.94	22.48	0.0000
LUBIN2	268	f	0	0.37	41.44	15.83	0.0164

Table 3B13 - 5

IESLC - Meta-analysis of Current Smoking (vs non-current), Any product (or Cigarettes if Any not available)
Adenocarcinoma
Least adjusted

REF	NRR	SEX	AD	Ys	Ws	Qs	Ps
Subtotal LUBIN2				0.57	202.38	38.32	
MATOS	88	m	0	0.87	16.82	0.23	0.0003
OSANN	67	m	0	1.51	63.13	17.06	0.0000
OSANN	71	f	0	1.94	52.66	47.86	0.0000
Subtotal OSANN				1.71	115.78	64.92	
OSANN2	18	f	0	1.09	9.60	0.10	0.0007
SAARIK	3	c	0	1.10	14.45	0.17	0.0000
SEGI2	33	m	0	0.21	7.05	4.26	0.5701
SEGI2	35	f	0	0.06	5.57	4.85	0.8927
Subtotal SEGI2				0.14	12.62	9.11	
SHIMIZ	1	m	2	0.64	21.97	2.68	0.0026
SHIMIZ	6	f	2	1.06	13.20	0.07	0.0001
Subtotal SHIMIZ				0.80	35.18	2.75	
SOBUE	8	m	0	0.37	70.71	27.25	0.0019
SOBUE	24	f	0	0.28	25.17	12.62	0.1559
Subtotal SOBUE				0.35	95.88	39.87	
STAYNE	8	m	1	1.13	6.32	0.12	0.0044
SUZUKI	4	m	0	1.06	21.41	0.12	0.0000
SUZUKI	8	f	0	0.85	8.08	0.17	0.0160
Subtotal SUZUKI				1.01	29.49	0.28	
SVENSS	69	f	0	1.19	12.34	0.49	0.0000
TSUGAN	7	m	0	-0.23	8.05	11.96	0.5177
WAKAI	18	m	0	0.56	18.52	3.48	0.0164
WAKAI	36	f	0	0.04	5.67	5.15	0.9284
Subtotal WAKAI				0.44	24.20	8.63	
WU	19	f	0	1.37	16.61	2.34	0.0000
WYNDE3	33	m	0	1.16	12.06	0.36	0.0001
WYNDE6	150	m	0	1.22	182.57	9.51	0.0000
WYNDE6	258	f	0	1.63	110.21	45.26	0.0000
Subtotal WYNDE6				1.37	292.78	54.77	

	N	38
	NS	28
	Wt	1184.12
Het	Chi	308.38
Het	df	37
Het	P	***
Fixed	RR	2.69
	RRl	2.54
	RRu	2.85
	P	+++
Random	RR	2.51
	RRl	2.10
	RRu	3.00
	P	+++
Asymm	P	N.S.

Table 3B13 - 6

IESLC - Meta-analysis of Current Smoking (vs non-current), Any product (or Cigarettes if Any not available)
Adenocarcinoma
Least adjusted

		Sex		
	combined	male	female	Total
N	3	19	16	38
NS	3	19	16	38
Wt	90.63	736.65	356.84	1184.12
Het Chi	9.36	133.47	135.58	308.38
Het df	2	18	15	37
Het P	**	***	***	***
Fixed RR	2.76	2.40	3.41	2.69
RRl	2.25	2.23	3.07	2.54
RRu	3.39	2.58	3.78	2.85
P	+++	+++	+++	+++
Random RR	2.74	2.36	2.63	2.51
RRl	1.72	1.90	1.87	2.10
RRu	4.37	2.94	3.69	3.00
P	+++	+++	+++	+++
Between Chi				29.97
Between df				2
Between P				***
Btwn(F) P				N.S.
Btwn(R) P				N.S.

Table 3B13 - 7

IESLC - Meta-analysis of Current Smoking (vs non-current), Any product (or Cigarettes if Any not available)
Adenocarcinoma
Excluded studies (and stage at which they were excluded)

1	BOUCHA BUELL LAURIL MZILEN
2	ABELIN ABRAHA AUVINE AXELSO BAND BERRIN BLOT1 BLOT2 BLOT3 BLOT4 BRESLO BROCKM BROWN1 BYERS1 CASCO2 CASCOR CHAN CHATZI CHEN CHEN2 CHEN3 CHIAZZ COOKSO DAVEYS DEAN DOSEME DU DUNN EBELIN ESAKI FAN GENG GER GODLEY GOLLED GSELL HANSEN HEGMAN HINDS HIRAOK HOROWI HORWIT HU HU2 HUANG ISHIMA JARUP JIANG JONES JUSSAW KO KOHLME KOULUM KREYBE LAMTH LAMWK LAMWK2 LAUSSM LEI LETOUR LEVIN LIU LIU2 LIU3 LIU4 LIU5 LOMBA2 LUO MAGNUS MARSH MARSH2 MARTIS MASTRA MATSUD MCCONN MCDUFF MCLAUG MILLER MILLS MOLLO NOTAN2 NOU ORMOS PASTOR PAWLEG PERNU PERSHA PIKE POFFIJ POLEDN PRESCO QIAO RADZIK RANDIG REN RONCO ROOTS ROTHSC SADOWS SANKAR SCHWA2 SEGI SEOW SIEMIA SIMARA STASZE STOCKS SUN TAO ULMER VANDER WANG WANG3 WANG4 WICKLU WILKIN WUWILL WYNDE2 WYNDE4 XIANGZ XU XU2 XU3 XU4 YUAN ZHANG ZHENG ZHOU
3	RESTRE
4	BOFFET
5	RIMING TANG2 WYNDE5 WYNDE8
6	ALDERS BENHAM BEST BOUCOT BROWN2 DAMBER ENSTRO GILLIS GOODMA HAMMON KANELL LIAW LUBIN SOBUE2 WALD WU2 WYNDE7
8	AGUDO AKIBA AMANDU AMES ANDERS ARCHER ARMADA AUSTIN AXELSS BECHER BENSHL BLOHMK BRETT BROSS BYERS2 CARPEN CEDERL CHANG CHOI CHOW CHYOU CPSI CPSII DARBY DEAN2 DEAN3 DEKLER DESTE2 DESTEF DOCKER DOLL DOLL2 DORANT DORGAN DORN DROSTE GAO GAO2 GARCIA GARDIN GARSHI GRAHAM GREGOR HAMMO2 HEIN HENNEK HIRAY2 HIRAYA HITOSU HOLE HUMBLE JARVHO JOLY KAISE2 KAISER KAUFMA KELLER KINLEN KJUUS KNEKT KOO KREUZE KUBIK LANGE LEMARC LICKIN LIDDEL LOMBAR MACLEN MIGRAN MRFIT MRFITR MURATA NAM NOTANI ODRISC PARKIN PERSH2 PETO PEZZO2 PEZZOT PISANI QIAO2 RACHTA SCHWAR SHAW SIMONA SITAS SPEIZE SPITZ STOCKW STUCKE SUZUK2 TANG TENKAN TIZZAN TOKARS TOUSEY TULINI TVERDA VEIERO WANG2 WARSIN WATSON WIGLE WUNSCH WYNDER YAMAGU YONG
10	VUTUC

Table 3B13 - 8

Potentially overlapping studies

REF	REFGP	PRINC	. OVERLAP/LINK
LUBIN2	LUBIN2	1	Lubin-combined
OSANN2	KAISER	2	KAISER/OSANN2
WYNDE6	WYNDE6	1	WYNDE5/6/7/8
JAHN	BOFFET	2	Subset of BOFFET

Table 3B14 -

IESLC - Meta-analysis of Current Smoking (vs non-current), Cigarettes (or Any Product if Cigarettes not available)
Adenocarcinoma

This analysis is restricted to results for:
1) Non-dose-response data
2) Current smokers
3) Results complete enough for use in metaanalysis

Within each study, results are then selected (in the following order of preference, within each sex) for:
4) PRODUCT: cigarettes regardless of other products, cigarettes only, all/unspec
5) CIGTYPE: all/unspecified, MC regardless of HR, MC only
6) DENOM: non smoker of anything, non smoker of cigarettes
7) Followup period (YF, prospective studies): whole study (coded as 0) or longest available
8) LCtype: adeno or nearest available, but not squamous. (q = squamous, s = small,
a = adeno, l = large, KII = Kreyberg II, al = alveolar, br = bronchiolar, u = undifferentiated)
9) Race: all or nearest available, otherwise by race (wh or w = white, bl or b = black, hi = hispanic
ch = chinese, jap = japanese, haw = hawaiian, w+o = white + oriental, sca = scandinavian, as = asian)
10) For overlapping studies: principal rather than subsidiary studies
Finally by Age: whole study (coded as 0) if available, otherwise by widest available age group
and then for single sex results (m, f) in preference to combined sex results (c).

Results adjusted (AD) for the most potential confounders are then chosen in Sections -1 to -3
and results adjusted for the least confounders in Sections -4 to -6. (Those least adjusted results which
actually differ from the most adjusted as marked 'x' in column X in Section -4)
(Results adjusted for an unknown number of confounder(s) are coded as 20.)

Section -7 shows excluded studies, together with the stage (as above) at which no qualifying
results were found.

Section -8 lists the potentially overlapping studies which have been included (1=principal, 2=subsidiary).

Section -9 lists any results which would have been included in preference except that they had data not complete
enough for use in meta-analysis, with their significance (yes/no), if known, and any further comment as entered
on the database.

In addition to those mentioned above, the following fields, levels and abbreviations are used:
* or nk = not known, n = no, y = yes, ot = other
non = not current
all/unspec = all or unspecified, cig+/-ot = cigarettes irrespective of other products (cigar, pipe etc)
MC = manufactured cigarettes, HR = hand-rolled cigarettes
REF: 6-character study reference
NRR: number of the RR on the database within the study
ST : study type (CC = case control, pr or prosp = prospective)
NLC: number of lung cancer cases in whole study
R : risky occupational population (n = no, m = mining, o = other risky)
VB : national cigarette type (V = at least 75% Virginia, bl = at least 75% blended, ot = other)
P : any proxy use
H : full histological confirmation
De : derivation of RR/CI (or = original, st = standard method, ot = other method of estimation)

Table 3B14 - 1

IESLC - Meta-analysis of Current Smoking (vs non-current), Cigarettes (or Any Product if Cigarettes not available)
Adenocarcinoma
Most adjusted

REF	NRR	SEX	AGEL	AGEH	RACE	YF	LC TYPE	LOC	START	ST	NLC	R	VB	P	H	AD	PRODUCT	DENOM	De
BARBON	125	m	0	0	all	-	a	Eu:wst	1979	CC	755	n	bl	y	y	1	all/unsp	non any	ot
BUFFLE	73	f	0	0	w-hi	-	a	NAmer	1976	CC	943	n	bl	y	n	0	cig+/-ot	non cigs	st
COMSTO	72	m	0	0	all	-	a	NAmer	1975	ot	258	n	bl	n	n	0	cig+/-ot	non cigs	st
COMSTO	84	f	0	0	all	-	a	NAmer	1975	ot	258	n	bl	n	n	0	cig+/-ot	non cigs	st
CORREA	67	c	0	0	all	-	a	NAmer	1979	CC	1359	n	bl	y	n	1	cig+/-ot	non cigs	ot
ENGELA	77	m	0	0	all	0	a	Eu:Sca	1964	pr	435	n	bl	n	n	7	cig+/-ot	non cigs	ot
HAENSZ	49	f	0	0	all	-	a	NAmer	1955	CC	158	n	bl	n	y	0	cig+/-ot	non cigs	st
JAHN	35	m	0	0	all	-	a	Eu:Ger	1988	CC	1004	n	bl	n	n	0	cig+/-ot	non cigs	st
JAIN	37	m	0	0	all	-	a	NAmer	1981	CC	845	n	V	y	n	0	cig+/-ot	non cigs	st
JAIN	32	f	0	0	all	-	a	NAmer	1981	CC	845	n	V	y	n	0	cig+/-ot	non cigs	st
JEDRYC	53	m	0	0	all	-	a	Eu:est	1980	CC	1630	n	bl	y	n	0	cig+/-ot	non any	st
KATSOU	34	f	0	0	all	-	a	Eu:bal	1987	CC	101	n	bl	n	n	1	all/unsp	non any	ot
KHUDER	32	m	0	0	all	-	a	NAmer	1985	CC	482	n	bl	n	y	0	cig+/-ot	non cigs	ot
KIHARA	21	c	0	0	jap	-	a	As:Jap	1991	CC	440	n	bl	n	n	0	all/unsp	non any	st
LUBIN2	256	m	0	0	all	-	a	Eu:mul	1976	CC	7804	n	bl	n	y	0	cig+/-ot	non any	st
LUBIN2	268	f	0	0	all	-	a	Eu:mul	1976	CC	7804	n	bl	n	y	0	cig+/-ot	non any	st
MATOS	89	m	0	0	all	-	a	SCAmer	1994	CC	200	n	bl	n	n	2	cig+/-ot	non any	ot
OSANN	79	m	0	0	all	-	a	NAmer	1984	CC	1986	n	bl	n	n	2	cig+/-ot	non cigs	ot
OSANN	80	f	0	0	all	-	a	NAmer	1984	CC	1986	n	bl	n	n	2	cig+/-ot	non cigs	ot
OSANN2	36	f	0	0	all	-	KII	NAmer	1964	ot	217	n	bl	n	y	1	cig+/-ot	non cigs	ot
SAARIK	3	c	0	0	wh	-	a	Eu:Sca	1988	CC	205	n	bl	n	y	0	all/unsp	non any	st
SEGI2	34	m	0	0	all	-	a	As:Jap	1962	CC	378	n	bl	n	n	1	cig+/-ot	non any	ot
SEGI2	36	f	0	0	all	-	a	As:Jap	1962	CC	378	n	bl	n	n	1	cig+/-ot	non any	ot
SHIMIZ	1	m	0	0	all	-	a	As:Jap	1977	CC	751	n	bl	y	n	2	all/unsp	non any	or
SHIMIZ	6	f	0	0	all	-	a	As:Jap	1977	CC	751	n	bl	y	n	2	all/unsp	non any	or
SOBUE	100	m	0	0	all	-	a	As:Jap	1986	CC	1376	n	bl	n	y	1	cig+/-ot	non cigs	ot
SOBUE	110	f	0	0	all	-	a	As:Jap	1986	CC	1376	n	bl	n	y	1	cig+/-ot	non cigs	ot
STAYNE	8	m	0	0	all	-	a	NAmer	1969	CC	420	n	bl	n	n	1	cig+/-ot	non cigs	st
SUZUKI	12	m	0	0	all	-	a	As:Jap	1978	CC	238	n	bl	n	y	2	cig+/-ot	non any	ot
SUZUKI	16	f	0	0	all	-	a	As:Jap	1978	CC	238	n	bl	n	y	2	cig+/-ot	non any	ot
SVENSS	94	f	0	0	all	-	a	Eu:Sca	1983	CC	210	n	bl	n	n	1	all/unsp	non any	ot
TSUGAN	7	m	0	0	all	-	a	As:Jap	1976	CC	134	n	bl	n	y	0	all/unsp	non any	st
WAKAI	77	m	0	0	all	-	a	As:Jap	1988	CC	333	n	bl	n	y	1	all/unsp	non any	ot
WAKAI	83	f	0	0	all	-	a	As:Jap	1988	CC	333	n	bl	n	y	1	all/unsp	non any	ot
WU	22	f	0	0	wh	-	a	NAmer	1981	CC	220	n	bl	n	y	2	all/unsp	non any	ot
WYNDE3	33	m	0	0	all	-	KII	NAmer	1966	CC	350	n	bl	n	y	0	all/unsp	non any	st
WYNDE6	150	m	0	0	all	-	KII	NAmer	1969	CC	4423	n	bl	n	y	0	cig+/-ot	non cigs	st
WYNDE6	258	f	0	0	all	-	KII	NAmer	1969	CC	4423	n	bl	n	y	0	cig+/-ot	non cigs	st

Cigarette type is all/unspec for all RRs

Table 3B14 - 2

IESLC - Meta-analysis of Current Smoking (vs non-current), Cigarettes (or Any Product if Cigarettes not available)
Adenocarcinoma
Most adjusted

REF	NRR	SEX	AD	Number Exposed Case	Number Exposed Cont	Non-exposed Case	Non-exposed Cont	RR	95.00%CI
BARBON	125	m	1	-	-	-	-	2.38	(1.64- 3.44)
BUFFLE	73	f	0	56	110	27	168	3.17	(1.89- 5.32)
COMSTO	72	m	0	30	100	15	213	4.26	(2.19- 8.27)
COMSTO	84	f	0	23	52	14	150	4.74	(2.27- 9.89)
Subtotal COMSTO								4.47	(2.73- 7.31)
CORREA	67	c	1	-	-	-	-	3.74	(2.76- 5.08)
*ENGELA	77	m	7	-	-	-	-	6.60	(3.52- 12.35)
HAENSZ	49	f	0	16	94	39	245	1.07	(0.57- 2.00)
JAHN	35	m	0	75	269	137	570	1.16	(0.85- 1.59)
JAIN	37	m	0	60	118	34	244	3.65	(2.27- 5.87)
JAIN	32	f	0	69	99	41	311	5.29	(3.38- 8.27)
Subtotal JAIN								4.44	(3.21- 6.15)
JEDRYC	53	m	0	68	516	28	601	2.83	(1.79- 4.46)
KATSOU	34	f	1	-	-	-	-	1.63	(0.72- 3.68)
KHUDER	32	m	0	92	-	70	-	1.72	(1.17- 2.52)
KIHARA	21	c	0	103	162	105	307	1.86	(1.33- 2.59)
LUBIN2	256	m	0	454	6209	270	6844	1.85	(1.59- 2.16)
LUBIN2	268	f	0	69	410	155	1337	1.45	(1.07- 1.97)
Subtotal LUBIN2								1.76	(1.54- 2.02)
MATOS	89	m	2	-	-	-	-	2.46	(1.52- 3.98)
OSANN	79	m	2	-	-	-	-	3.44	(2.64- 4.49)
OSANN	80	f	2	-	-	-	-	6.65	(5.04- 8.77)
Subtotal OSANN								4.72	(3.89- 5.71)
OSANN2	36	f	1	-	-	-	-	2.85	(1.43- 5.69)
SAARIK	3	c	0	38	66	43	224	3.00	(1.79- 5.02)
SEGI2	34	m	1	-	-	-	-	1.18	(0.56- 2.47)
SEGI2	36	f	1	-	-	-	-	1.02	(0.44- 2.34)
Subtotal SEGI2								1.11	(0.64- 1.93)
SHIMIZ	1	m	2	-	-	-	-	1.90	(1.30- 3.00)
SHIMIZ	6	f	2	-	-	-	-	2.90	(1.70- 5.00)
Subtotal SHIMIZ								2.23	(1.60- 3.10)
SOBUE	100	m	1	-	-	-	-	1.47	(1.13- 1.91)
SOBUE	110	f	1	-	-	-	-	1.24	(0.84- 1.84)
Subtotal SOBUE								1.39	(1.12- 1.73)
STAYNE	8	m	1	-	-	-	-	3.10	(1.42- 6.75)
SUZUKI	12	m	2	-	-	-	-	2.78	(1.81- 4.27)
SUZUKI	16	f	2	-	-	-	-	2.33	(1.16- 4.68)
Subtotal SUZUKI								2.65	(1.84- 3.82)
SVENSS	94	f	1	-	-	-	-	3.16	(1.70- 5.85)
TSUGAN	7	m	0	45	50	26	23	0.80	(0.40- 1.59)
WAKAI	77	m	1	-	-	-	-	1.72	(1.09- 2.71)
WAKAI	83	f	1	-	-	-	-	1.08	(0.47- 2.48)
Subtotal WAKAI								1.54	(1.04- 2.30)
WU	22	f	2	-	-	-	-	3.71	(2.24- 6.16)
WYNDE3	33	m	0	56	207	18	213	3.20	(1.82- 5.63)
WYNDE6	150	m	0	651	741	486	1872	3.38	(2.93- 3.91)
WYNDE6	258	f	0	472	376	290	1181	5.11	(4.24- 6.16)
Subtotal WYNDE6								3.95	(3.52- 4.43)
Partial Totals				2377	9579	1798	14503		

*prospective study

REF	NRR	SEX	AD	Ys	Ws	Qs	Ps
BARBON	125	m	1	0.87	28.00	0.32	0.0000
BUFFLE	73	f	0	1.15	14.30	0.46	0.0000
COMSTO	72	m	0	1.45	8.72	1.98	0.0000
COMSTO	84	f	0	1.56	7.10	2.41	0.0000
Subtotal COMSTO				1.50	15.82	4.39	
CORREA	67	c	1	1.32	41.28	4.94	0.0000
*ENGELA	77	m	7	1.89	9.75	8.15	0.0000
HAENSZ	49	f	0	0.07	9.72	7.98	0.8345
JAHN	35	m	0	0.15	38.31	26.06	0.3583
JAIN	37	m	0	1.29	17.05	1.76	0.0000
JAIN	32	f	0	1.67	19.16	9.17	0.0000
Subtotal JAIN				1.49	36.21	10.93	
JEDRYC	53	m	0	1.04	18.51	0.08	0.0000
KATSOU	34	f	1	0.49	5.77	1.36	0.2404
KHUDER	32	m	0	0.54	26.10	4.85	0.0056
KIHARA	21	c	0	0.62	34.89	4.35	0.0003
LUBIN2	256	m	0	0.62	160.94	20.41	0.0000
LUBIN2	268	f	0	0.37	41.44	14.94	0.0164

Table 3B14 - 2

IESLC - Meta-analysis of Current Smoking (vs non-current), Cigarettes (or Any Product if Cigarettes not available)
Adenocarcinoma
Most adjusted

REF	NRR	SEX	AD	Ys	Ws	Qs	Ps
Subtotal LUBIN2				0.57	202.38	35.36	
MATOS	89	m	2	0.90	16.58	0.09	0.0002
OSANN	79	m	2	1.24	54.48	3.75	0.0000
OSANN	80	f	2	1.89	50.08	42.52	0.0000
Subtotal OSANN				1.55	104.56	46.26	
OSANN2	36	f	1	1.05	8.06	0.04	0.0030
SAARIK	3	c	0	1.10	14.45	0.23	0.0000
SEGI2	34	m	1	0.17	6.98	4.55	0.6620
SEGI2	36	f	1	0.02	5.50	5.00	0.9630
Subtotal SEGI2				0.10	12.48	9.55	
SHIMIZ	1	m	2	0.64	21.97	2.41	0.0026
SHIMIZ	6	f	2	1.06	13.20	0.11	0.0001
Subtotal SHIMIZ				0.80	35.18	2.52	
SOBUE	100	m	1	0.39	55.77	19.28	0.0040
SOBUE	110	f	1	0.22	24.99	14.36	0.2822
Subtotal SOBUE				0.33	80.76	33.64	
STAYNE	8	m	1	1.13	6.32	0.16	0.0044
SUZUKI	12	m	2	1.02	20.86	0.05	0.0000
SUZUKI	16	f	2	0.85	7.90	0.13	0.0175
Subtotal SUZUKI				0.97	28.76	0.18	
SVENSS	94	f	1	1.15	10.06	0.32	0.0003
TSUGAN	7	m	0	-0.23	8.05	11.62	0.5177
WAKAI	77	m	1	0.54	18.52	3.44	0.0196
WAKAI	83	f	1	0.08	5.55	4.46	0.8561
Subtotal WAKAI				0.43	24.08	7.90	
WU	22	f	2	1.31	15.02	1.71	0.0000
WYNDE3	33	m	0	1.16	12.06	0.44	0.0001
WYNDE6	150	m	0	1.22	182.57	11.04	0.0000
WYNDE6	258	f	0	1.63	110.21	47.78	0.0000
Subtotal WYNDE6				1.37	292.78	58.82	

	N	38
	NS	28
	Wt	1150.25
Het	Chi	282.70
Het	df	37
Het	P	***
Fixed	RR	2.65
	RRl	2.50
	RRu	2.80
	P	+++
Random	RR	2.46
	RRl	2.07
	RRu	2.93
	P	+++
Asymm	P	N.S.

Table 3B14 - 3

IESLC - Meta-analysis of Current Smoking (vs non-current), Cigarettes (or Any Product if Cigarettes not available)
Adenocarcinoma
Most adjusted

	Sex combined	male	female	Total
N	3	19	16	38
NS	3	19	16	38
Wt	90.63	711.56	348.07	1150.25
Het Chi	9.36	110.40	134.07	282.70
Het df	2	18	15	37
Het P	**	***	***	***
Fixed RR	2.76	2.35	3.34	2.65
RRl	2.25	2.18	3.00	2.50
RRu	3.39	2.53	3.71	2.80
P	+++	+++	+++	+++
Random RR	2.74	2.31	2.57	2.46
RRl	1.72	1.88	1.82	2.07
RRu	4.37	2.83	3.62	2.93
P	+++	+++	+++	+++
Between Chi				28.87
Between df				2
Between P				***
Btwn(F) P				N.S.
Btwn(R) P				N.S.

Table 3B14 - 4

IESLC - Meta-analysis of Current Smoking (vs non-current), Cigarettes (or Any Product if Cigarettes not available)
Adenocarcinoma
Least adjusted

REF	NRR	X	SEX	AGEL	AGEH	RACE	YF	LC TYPE	LOC	START	ST	NLC	R	VB	P	H	AD	PRODUCT	DENOM	De
BARBON	124	x	m	0	0	all	-	a	Eu:wst	1979	CC	755	n	bl	y	y	0	all/unsp	non any	st
BUFFLE	73		f	0	0	w-hi	-	a	NAmer	1976	CC	943	n	bl	y	n	0	cig+/-ot	non cigs	st
COMSTO	72		m	0	0	all	-	a	NAmer	1975	ot	258	n	bl	n	n	0	cig+/-ot	non cigs	st
COMSTO	84		f	0	0	all	-	a	NAmer	1975	ot	258	n	bl	n	n	0	cig+/-ot	non cigs	st
CORREA	67		c	0	0	all	-	a	NAmer	1979	CC	1359	n	bl	y	n	1	cig+/-ot	non cigs	ot
ENGELA	77		m	0	0	all	0	a	Eu:Sca	1964	pr	435	n	bl	n	n	7	cig+/-ot	non cigs	ot
HAENSZ	49		f	0	0	all	-	a	NAmer	1955	CC	158	n	bl	n	y	0	cig+/-ot	non cigs	st
JAHN	35		m	0	0	all	-	a	Eu:Ger	1988	CC	1004	n	bl	n	n	0	cig+/-ot	non cigs	st
JAIN	37		m	0	0	all	-	a	NAmer	1981	CC	845	n	V	y	n	0	cig+/-ot	non cigs	st
JAIN	32		f	0	0	all	-	a	NAmer	1981	CC	845	n	V	y	n	0	cig+/-ot	non cigs	st
JEDRYC	53		m	0	0	all	-	a	Eu:est	1980	CC	1630	n	bl	y	n	0	cig+/-ot	non any	st
KATSOU	32	x	f	0	0	all	-	a	Eu:bal	1987	CC	101	n	bl	n	n	0	all/unsp	non any	st
KHUDER	32		m	0	0	all	-	a	NAmer	1985	CC	482	n	bl	n	y	0	cig+/-ot	non cigs	ot
KIHARA	21		c	0	0	jap	-	a	As:Jap	1991	CC	440	n	bl	n	n	0	all/unsp	non any	st
LUBIN2	256		m	0	0	all	-	a	Eu:mul	1976	CC	7804	n	bl	n	y	0	cig+/-ot	non any	st
LUBIN2	268		f	0	0	all	-	a	Eu:mul	1976	CC	7804	n	bl	n	y	0	cig+/-ot	non any	st
MATOS	88	x	m	0	0	all	-	a	SCAmer	1994	CC	200	n	bl	n	n	0	cig+/-ot	non any	st
OSANN	67	x	m	0	0	all	-	a	NAmer	1984	CC	1986	n	bl	n	n	0	cig+/-ot	non cigs	st
OSANN	71	x	f	0	0	all	-	a	NAmer	1984	CC	1986	n	bl	n	n	0	cig+/-ot	non cigs	st
OSANN2	18	x	f	0	0	all	-	KII	NAmer	1964	ot	217	n	bl	n	y	0	cig+/-ot	non cigs	st
SAARIK	3		c	0	0	wh	-	a	Eu:Sca	1988	CC	205	n	bl	n	y	0	all/unsp	non any	st
SEGI2	33	x	m	0	0	all	-	a	As:Jap	1962	CC	378	n	bl	n	n	0	cig+/-ot	non any	st
SEGI2	35	x	f	0	0	all	-	a	As:Jap	1962	CC	378	n	bl	n	n	0	cig+/-ot	non any	st
SHIMIZ	1		m	0	0	all	-	a	As:Jap	1977	CC	751	n	bl	y	n	2	all/unsp	non any	or
SHIMIZ	6		f	0	0	all	-	a	As:Jap	1977	CC	751	n	bl	y	n	2	all/unsp	non any	or
SOBUE	8	x	m	0	0	all	-	a	As:Jap	1986	CC	1376	n	bl	n	y	0	cig+/-ot	non cigs	st
SOBUE	24	x	f	0	0	all	-	a	As:Jap	1986	CC	1376	n	bl	n	y	0	cig+/-ot	non cigs	st
STAYNE	8		m	0	0	all	-	a	NAmer	1969	CC	420	n	bl	n	n	1	cig+/-ot	non cigs	st
SUZUKI	4	x	m	0	0	all	-	a	As:Jap	1978	CC	238	n	bl	n	y	0	cig+/-ot	non any	st
SUZUKI	8	x	f	0	0	all	-	a	As:Jap	1978	CC	238	n	bl	n	y	0	cig+/-ot	non any	st
SVENSS	69	x	f	0	0	all	-	a	Eu:Sca	1983	CC	210	n	bl	n	n	0	all/unsp	non any	st
TSUGAN	7		m	0	0	all	-	a	As:Jap	1976	CC	134	n	bl	n	y	0	all/unsp	non any	st
WAKAI	18	x	m	0	0	all	-	a	As:Jap	1988	CC	333	n	bl	n	y	0	all/unsp	non any	st
WAKAI	36	x	f	0	0	all	-	a	As:Jap	1988	CC	333	n	bl	n	y	0	all/unsp	non any	st
WU	19	x	f	0	0	wh	-	a	NAmer	1981	CC	220	n	bl	n	y	0	all/unsp	non any	st
WYNDE3	33		m	0	0	all	-	KII	NAmer	1966	CC	350	n	bl	n	y	0	all/unsp	non any	st
WYNDE6	150		m	0	0	all	-	KII	NAmer	1969	CC	4423	n	bl	n	y	0	cig+/-ot	non cigs	st
WYNDE6	258		f	0	0	all	-	KII	NAmer	1969	CC	4423	n	bl	n	y	0	cig+/-ot	non cigs	st

Cigarette type is all/unspec for all RRs

Table 3B14 - 5

IESLC - Meta-analysis of Current Smoking (vs non-current), Cigarettes (or Any Product if Cigarettes not available)
Adenocarcinoma
Least adjusted

REF	NRR	SEX	AD	Number Exposed Case	Number Exposed Cont	Non-exposed Case	Non-exposed Cont	RR	95.00%CI
BARBON	124	m	0	109	362	49	393	2.41	(1.67- 3.48)
BUFFLE	73	f	0	56	110	27	168	3.17	(1.89- 5.32)
COMSTO	72	m	0	30	100	15	213	4.26	(2.19- 8.27)
COMSTO	84	f	0	23	52	14	150	4.74	(2.27- 9.89)
Subtotal COMSTO								4.47	(2.73- 7.31)
CORREA	67	c	1	-	-	-	-	3.74	(2.76- 5.08)
*ENGELA	77	m	7	-	-	-	-	6.60	(3.52- 12.35)
HAENSZ	49	f	0	16	94	39	245	1.07	(0.57- 2.00)
JAHN	35	m	0	75	269	137	570	1.16	(0.85- 1.59)
JAIN	37	m	0	60	118	34	244	3.65	(2.27- 5.87)
JAIN	32	f	0	69	99	41	311	5.29	(3.38- 8.27)
Subtotal JAIN								4.44	(3.21- 6.15)
JEDRYC	53	m	0	68	516	28	601	2.83	(1.79- 4.46)
KATSOU	32	f	0	15	18	33	71	1.79	(0.81- 3.99)
KHUDER	32	m	0	92	-	70	-	1.72	(1.17- 2.52)
KIHARA	21	c	0	103	162	105	307	1.86	(1.33- 2.59)
LUBIN2	256	m	0	454	6209	270	6844	1.85	(1.59- 2.16)
LUBIN2	268	f	0	69	410	155	1337	1.45	(1.07- 1.97)
Subtotal LUBIN2								1.76	(1.54- 2.02)
MATOS	88	m	0	46	132	38	261	2.39	(1.48- 3.86)
OSANN	67	m	0	217	541	116	1310	4.53	(3.54- 5.80)
OSANN	71	f	0	193	367	97	1289	6.99	(5.33- 9.16)
Subtotal OSANN								5.52	(4.60- 6.62)
OSANN2	18	f	0	50	28	33	55	2.98	(1.58- 5.60)
SAARIK	3	c	0	38	66	43	224	3.00	(1.79- 5.02)
SEGI2	33	m	0	53	485	9	102	1.24	(0.59- 2.59)
SEGI2	35	f	0	9	34	32	128	1.06	(0.46- 2.43)
Subtotal SEGI2								1.16	(0.67- 2.01)
SHIMIZ	1	m	2	-	-	-	-	1.90	(1.30- 3.00)
SHIMIZ	6	f	2	-	-	-	-	2.90	(1.70- 5.00)
Subtotal SHIMIZ								2.23	(1.60- 3.10)
SOBUE	8	m	0	276	650	144	491	1.45	(1.15- 1.83)
SOBUE	24	f	0	38	168	157	921	1.33	(0.90- 1.96)
Subtotal SOBUE								1.42	(1.16- 1.73)
STAYNE	8	m	1	-	-	-	-	3.10	(1.42- 6.75)
SUZUKI	4	m	0	119	162	39	154	2.90	(1.90- 4.43)
SUZUKI	8	f	0	20	20	60	140	2.33	(1.17- 4.65)
Subtotal SUZUKI								2.73	(1.90- 3.92)
SVENSS	69	f	0	38	53	34	156	3.29	(1.88- 5.75)
TSUGAN	7	m	0	45	50	26	23	0.80	(0.40- 1.59)
WAKAI	18	m	0	75	284	31	205	1.75	(1.11- 2.75)
WAKAI	36	f	0	9	26	50	150	1.04	(0.46- 2.36)
Subtotal WAKAI								1.55	(1.04- 2.30)
WU	19	f	0	99	50	50	99	3.92	(2.42- 6.34)
WYNDE3	33	m	0	56	207	18	213	3.20	(1.82- 5.63)
WYNDE6	150	m	0	651	741	486	1872	3.38	(2.93- 3.91)
WYNDE6	258	f	0	472	376	290	1181	5.11	(4.24- 6.16)
Subtotal WYNDE6								3.95	(3.52- 4.43)
Partial Totals				3743	12959	2770	20428		

*prospective study

REF	NRR	SEX	AD	Ys	Ws	Qs	Ps
BARBON	124	m	0	0.88	28.66	0.34	0.0000
BUFFLE	73	f	0	1.15	14.30	0.38	0.0000
COMSTO	72	m	0	1.45	8.72	1.83	0.0000
COMSTO	84	f	0	1.56	7.10	2.27	0.0000
Subtotal COMSTO				1.50	15.82	4.10	
CORREA	67	c	1	1.32	41.28	4.45	0.0000
*ENGELA	77	m	7	1.89	9.75	7.83	0.0000
HAENSZ	49	f	0	0.07	9.72	8.30	0.8345
JAHN	35	m	0	0.15	38.31	27.18	0.3583
JAIN	37	m	0	1.29	17.05	1.57	0.0000
JAIN	32	f	0	1.67	19.16	8.71	0.0000
Subtotal JAIN				1.49	36.21	10.28	
JEDRYC	53	m	0	1.04	18.51	0.04	0.0000
KATSOU	32	f	0	0.58	6.00	0.99	0.1526
KHUDER	32	m	0	0.54	26.10	5.25	0.0056
KIHARA	21	c	0	0.62	34.89	4.80	0.0003
LUBIN2	256	m	0	0.62	160.94	22.48	0.0000
LUBIN2	268	f	0	0.37	41.44	15.83	0.0164

Table 3B14 - 5

IESLC - Meta-analysis of Current Smoking (vs non-current), Cigarettes (or Any Product if Cigarettes not available)
Adenocarcinoma
Least adjusted

REF	NRR	SEX	AD	Ys	Ws	Qs	Ps
Subtotal LUBIN2				0.57	202.38	38.32	
MATOS	88	m	0	0.87	16.82	0.23	0.0003
OSANN	67	m	0	1.51	63.13	17.06	0.0000
OSANN	71	f	0	1.94	52.66	47.86	0.0000
Subtotal OSANN				1.71	115.78	64.92	
OSANN2	18	f	0	1.09	9.60	0.10	0.0007
SAARIK	3	c	0	1.10	14.45	0.17	0.0000
SEGI2	33	m	0	0.21	7.05	4.26	0.5701
SEGI2	35	f	0	0.06	5.57	4.85	0.8927
Subtotal SEGI2				0.14	12.62	9.11	
SHIMIZ	1	m	2	0.64	21.97	2.68	0.0026
SHIMIZ	6	f	2	1.06	13.20	0.07	0.0001
Subtotal SHIMIZ				0.80	35.18	2.75	
SOBUE	8	m	0	0.37	70.71	27.25	0.0019
SOBUE	24	f	0	0.28	25.17	12.62	0.1559
Subtotal SOBUE				0.35	95.88	39.87	
STAYNE	8	m	1	1.13	6.32	0.12	0.0044
SUZUKI	4	m	0	1.06	21.41	0.12	0.0000
SUZUKI	8	f	0	0.85	8.08	0.17	0.0160
Subtotal SUZUKI				1.01	29.49	0.28	
SVENSS	69	f	0	1.19	12.34	0.49	0.0000
TSUGAN	7	m	0	-0.23	8.05	11.96	0.5177
WAKAI	18	m	0	0.56	18.52	3.48	0.0164
WAKAI	36	f	0	0.04	5.67	5.15	0.9284
Subtotal WAKAI				0.44	24.20	8.63	
WU	19	f	0	1.37	16.61	2.34	0.0000
WYNDE3	33	m	0	1.16	12.06	0.36	0.0001
WYNDE6	150	m	0	1.22	182.57	9.51	0.0000
WYNDE6	258	f	0	1.63	110.21	45.26	0.0000
Subtotal WYNDE6				1.37	292.78	54.77	

	N	38
	NS	28
	Wt	1184.12
Het	Chi	308.38
Het	df	37
Het	P	***
Fixed	RR	2.69
	RRl	2.54
	RRu	2.85
	P	+++
Random	RR	2.51
	RRl	2.10
	RRu	3.00
	P	+++
Asymm	P	N.S.

Table 3B14 - 6

IESLC - Meta-analysis of Current Smoking (vs non-current), Cigarettes (or Any Product if Cigarettes not available)
Adenocarcinoma
Least adjusted

	combined	Sex male	female	Total
N	3	19	16	38
NS	3	19	16	38
Wt	90.63	736.65	356.84	1184.12
Het Chi	9.36	133.47	135.58	308.38
Het df	2	18	15	37
Het P	**	***	***	***
Fixed RR	2.76	2.40	3.41	2.69
RRl	2.25	2.23	3.07	2.54
RRu	3.39	2.58	3.78	2.85
P	+++	+++	+++	+++
Random RR	2.74	2.36	2.63	2.51
RRl	1.72	1.90	1.87	2.10
RRu	4.37	2.94	3.69	3.00
P	+++	+++	+++	+++
Between Chi				29.97
Between df				2
Between P				***
Btwn(F) P				N.S.
Btwn(R) P				N.S.

Table 3B14 - 7

IESLC - Meta-analysis of Current Smoking (vs non-current), Cigarettes (or Any Product if Cigarettes not available)
Adenocarcinoma
Excluded studies (and stage at which they were excluded)

Stage	Studies
1	BOUCHA BUELL LAURIL MZILEN
2	ABELIN ABRAHA AUVINE AXELSO BAND BERRIN BLOT1 BLOT2 BLOT3 BLOT4 BRESLO BROCKM BROWN1 BYERS1 CASCO2 CASCOR CHAN CHATZI CHEN CHEN2 CHEN3 CHIAZZ COOKSO DAVEYS DEAN DOSEME DU DUNN EBELIN ESAKI FAN GENG GER GODLEY GOLLED GSELL HANSEN HEGMAN HINDS HIRAOK HOROWI HORWIT HU HU2 HUANG ISHIMA JARUP JIANG JONES JUSSAW KO KOHLME KOULUM KREYBE LAMTH LAMWK LAMWK2 LAUSSM LEI LETOUR LEVIN LIU LIU2 LIU3 LIU4 LIU5 LOMBA2 LUO MAGNUS MARSH MARSH2 MARTIS MASTRA MATSUD MCCONN MCDUFF MCLAUG MILLER MILLS MOLLO NOTAN2 NOU ORMOS PASTOR PAWLEG PERNU PERSHA PIKE POFFIJ POLEDN PRESCO QIAO RADZIK RANDIG REN RONCO ROOTS ROTHSC SADOWS SANKAR SCHWA2 SEGI SEOW SIEMIA SIMARA STASZE STOCKS SUN TAO ULMER VANDER WANG WANG3 WANG4 WICKLU WILKIN WUWILL WYNDE2 WYNDE4 XIANGZ XU XU2 XU3 XU4 YUAN ZHANG ZHENG ZHOU
3	RESTRE
4	BOFFET
5	RIMING TANG2 WYNDE5 WYNDE8
6	ALDERS BENHAM BEST BOUCOT BROWN2 DAMBER ENSTRO GILLIS GOODMA HAMMON KANELL LIAW LUBIN SOBUE2 WALD WU2 WYNDE7
8	AGUDO AKIBA AMANDU AMES ANDERS ARCHER ARMADA AUSTIN AXELSS BECHER BENSHL BLOHMK BRETT BROSS BYERS2 CARPEN CEDERL CHANG CHOI CHOW CHYOU CPSI CPSII DARBY DEAN2 DEAN3 DEKLER DESTE2 DESTEF DOCKER DOLL DOLL2 DORANT DORGAN DORN DROSTE GAO GAO2 GARCIA GARDIN GARSHI GRAHAM GREGOR HAMMO2 HEIN HENNEK HIRAY2 HIRAYA HITOSU HOLE HUMBLE JARVHO JOLY KAISE2 KAISER KAUFMA KELLER KINLEN KJUUS KNEKT KOO KREUZE KUBIK LANGE LEMARC LICKIN LIDDEL LOMBAR MACLEN MIGRAN MRFIT MRFITR MURATA NAM NOTANI ODRISC PARKIN PERSH2 PETO PEZZO2 PEZZOT PISANI QIAO2 RACHTA SCHWAR SHAW SIMONA SITAS SPEIZE SPITZ STOCKW STUCKE SUZUK2 TANG TENKAN TIZZAN TOKARS TOUSEY TULINI TVERDA VEIERO WANG2 WARSIN WATSON WIGLE WUNSCH WYNDER YAMAGU YONG
10	VUTUC

Table 3B14 - 8
Potentially overlapping studies

REF	REFGP	PRINC	.	OVERLAP/LINK
LUBIN2	LUBIN2	1		Lubin-combined
OSANN2	KAISER	2		KAISER/OSANN2
WYNDE6	WYNDE6	1		WYNDE5/6/7/8
JAHN	BOFFET	2		Subset of BOFFET

Table 3B15 -

IESLC - Meta-analysis of Current Smoking (vs non-current), Cigarettes only
Adenocarcinoma

This analysis is restricted to results for:
1) Non-dose-response data
2) Current smokers
3) Results complete enough for use in metaanalysis

Within each study, results are then selected (in the following order of preference, within each sex) for:
4) PRODUCT: cigarettes only
5) CIGTYPE: all/unspecified, MC regardless of HR, MC only
6) DENOM: non smoker of anything, non smoker of cigarettes
7) Followup period (YF, prospective studies): whole study (coded as 0) or longest available
8) LCtype: adeno or nearest available, but not squamous. (q = squamous, s = small,
a = adeno, l = large, KII = Kreyberg II, al = alveolar, br = bronchiolar, u = undifferentiated)
9) Race: all or nearest available, otherwise by race (wh or w = white, bl or b = black, hi = hispanic
ch = chinese, jap = japanese, haw = hawaiian, w+o = white + oriental, sca = scandinavian, as = asian)
10) For overlapping studies: principal rather than subsidiary studies
Finally by Age: whole study (coded as 0) if available, otherwise by widest available age group
and then for single sex results (m, f) in preference to combined sex results (c).

Results adjusted (AD) for the most potential confounders are then chosen in Sections -1 to -3
and results adjusted for the least confounders in Sections -4 to -6. (Those least adjusted results which
actually differ from the most adjusted as marked 'x' in column X in Section -4)
(Results adjusted for an unknown number of confounder(s) are coded as 20.)

Section -7 shows excluded studies, together with the stage (as above) at which no qualifying
results were found.

Section -8 lists the potentially overlapping studies which have been included (1=principal, 2=subsidiary).

Section -9 lists any results which would have been included in preference except that they had data not complete
enough for use in meta-analysis, with their significance (yes/no), if known, and any further comment as entered
on the database.

In addition to those mentioned above, the following fields, levels and abbreviations are used:
* or nk = not known, n = no, y = yes, ot = other
non = not current
all/unspec = all or unspecified, MC = manufactured cigarettes, HR = hand-rolled cigarettes
REF: 6-character study reference
NRR: number of the RR on the database within the study
ST : study type (CC = case control, pr or prosp = prospective)
NLC: number of lung cancer cases in whole study
R : risky occupational population (n = no, m = mining, o = other risky)
VB : national cigarette type (V = at least 75% Virginia, bl = at least 75% blended, ot = other)
P : any proxy use
H : full histological confirmation
De : derivation of RR/CI (or = original, st = standard method, ot = other method of estimation)

Table 3B15 - 0

No RRs selected for this analysis

Table 3B15 - 7

IESLC - Meta-analysis of Current Smoking (vs non-current), Cigarettes only
Adenocarcinoma
Excluded studies (and stage at which they were excluded)

1 BOUCHA BUELL LAURIL MZILEN

2 ABELIN ABRAHA AUVINE AXELSO BAND BERRIN BLOT1 BLOT2 BLOT3 BLOT4 BRESLO BROCKM BROWN1 BYERS1 CASCO2 CASCOR CHAN CHATZI CHEN CHEN2 CHEN3 CHIAZZ COOKSO DAVEYS DEAN DOSEME DU DUNN EBELIN ESAKI FAN GENG GER GODLEY GOLLED GSELL HANSEN HEGMAN HINDS HIRAOK HOROWI HORWIT HU HU2 HUANG ISHIMA JARUP JIANG JONES JUSSAW KO KOHLME KOULUM KREYBE LAMTH LAMWK LAMWK2 LAUSSM LEI LETOUR LEVIN LIU LIU2 LIU3 LIU4 LIU5 LOMBA2 LUO MAGNUS MARSH MARSH2 MARTIS MASTRA MATSUD MCCONN MCDUFF MCLAUG MILLER MILLS MOLLO NOTAN2 NOU ORMOS PASTOR PAWLEG PERNU PERSHA PIKE POFFIJ POLEDN PRESCO QIAO RADZIK RANDIG REN RONCO ROOTS ROTHSC SADOWS SANKAR SCHWA2 SEGI SEOW SIEMIA SIMARA STASZE STOCKS SUN TAO ULMER VANDER WANG WANG3 WANG4 WICKLU WILKIN WUWILL WYNDE2 WYNDE4 XIANGZ XU XU2 XU3 XU4 YUAN ZHANG ZHENG ZHOU

3 RESTRE

4 AKIBA AMANDU AMES ANDERS ARCHER ARMADA AUSTIN AXELSS BARBON BECHER BENSHL BLOHMK BOFFET BRETT BROSS BROWN2 BUFFLE BYERS2 CARPEN CHANG CHOI CHYOU COMSTO CORREA DARBY DEAN2 DEKLER DESTE2 DESTEF DOCKER DOLL DORANT DORGAN DROSTE GAO GAO2 GARCIA GARSHI GILLIS GOODMA GREGOR HAENSZ HAMMO2 HENNEK HIRAYA HITOSU HOLE HUMBLE JAHN JAIN JARVHO JEDRYC JOLY KAISER KANELL KATSOU KAUFMA KELLER KHUDER KIHARA KINLEN KJUUS KNEKT KOO KREUZE KUBIK LEMARC LIAW LICKIN LIDDEL LUBIN MACLEN MATOS MRFIT MRFITR MURATA NAM NOTANI ODRISC OSANN OSANN2 PARKIN PERSH2 PETO PEZZO2 PISANI QIAO2 RACHTA SAARIK SCHWAR SEGI2 SHAW SHIMIZ SIMONA SITAS SOBUE SOBUE2 SPEIZE SPITZ STAYNE STOCKW STUCKE SUZUK2 SUZUKI SVENSS TANG TENKAN TIZZAN TOKARS TOUSEY TSUGAN TULINI VUTUC WAKAI WANG2 WARSIN WATSON WU WU2 WUNSCH WYNDE3 WYNDE5 WYNDE6 WYNDE8 YAMAGU YONG

5 LANGE RIMING TANG2

6 ALDERS BENHAM BEST BOUCOT CHOW DAMBER ENSTRO HAMMON WALD WYNDE7

8 AGUDO CEDERL CPSI CPSII DEAN3 DOLL2 DORN ENGELA GARDIN GRAHAM HEIN HIRAY2 KAISE2 LOMBAR LUBIN2 MIGRAN PEZZOT TVERDA VEIERO WIGLE WYNDER
