# Supplementary material for: Systematic review with meta-analysis of the epidemiological evidence in the 1900s relating smoking to lung cancer
Source: BMC Cancer. 2012 Sep 3;12:385. doi: 10.1186/1471-2407-12-385 (PMC3505152; doi:10.1186/1471-2407-12-385)
Supplement: Additional file 5 — Detailed Analysis Tables (Individual file names as described in Additional file 1: Methods, Table1). [file 1471-2407-12-385-S5.zip › PDF/2F.pdf]

Table 2F1 -

IESLC - Meta-analysis of Cigarette Smoking, only Filter vs only Plain (or nearest available)
Squamous

This analysis is restricted to results for:
1) Non-dose-response data
2) Results complete enough for use in metaanalysis

Within each study, results are then selected (in the following order of preference, within each sex) for:
3) CIGTYP: filter only/NOS, always, mainly, both, equally, ever
4) DENOM: plain only/NOS, always, mainly, ever
5) PRODUCT: cigarettes regardless of other products, cigarettes only (Note only study ALDERS has both product definitions available)
6) SMKSTA: ever, current. (Note only study MATOS has both ever and current available)
7) LCtype: all or nearest available, at least Squamous and Adeno. (q = squamous, s = small, l = large, a = adeno, mix = mixed, alv = alveolar)
8) Race: all or nearest available, otherwise by race (wh or w = white, bl or b = black, hi = hispanic ch = chinese, jap = japanese, haw = hawaiian, w+o = white + oriental, sca = scandinavian, as = asian)
9) Followup period (YF, prospective studies): whole study (coded as 0) or longest available
10) For overlapping studies: principal rather than subsidiary studies
Finally by Age: whole study (coded as 0) if available, otherwise by widest available age group
and then for single sex results (m, f) in preference to combined sex results (c).

Results adjusted (AD) for the most potential confounders are then chosen in Sections -1 to -3 and results adjusted for the least confounders in Sections -4 to -6. (Those least adjusted results which actually differ from the most adjusted as marked 'x' in column X in Section -4)
(Results adjusted for an unknown number of confounder(s) are coded as 20.)

Section -7 shows excluded studies, together with the stage (as above) at which no qualifying results were found.

Section -8 lists the potentially overlapping studies which have been included (1=principal, 2=subsidiary).

Section -9 lists any results which would have been included in preference except that they had data not complete enough for use in meta-analysis, with their significance (yes/no), if known, and any further comment as entered on the database.

In addition to those mentioned above, the following fields, levels and abbreviations are used:
* or nk = not known, n = no, y = yes, ot = other
ev = ever, cu = current, cig+/-ot = cigarettes irrespective of other products (cigar, pipe etc)
f = filter, p = plain, NOS = not otherwise specified
REF: 6-character study reference
NRR: number of the RR on the database within the study
ST : study type (CC = case control, pr or prosp = prospective)
NLC: number of lung cancer cases in whole study
R : risky occupational population (n = no, m = mining, o = other risky)
VB : national cigarette type (V = at least 75% Virginia, bl = at least 75% blended, ot = other)
P : any proxy use
H : full histological confirmation
De : derivation of RR/CI (or = original, st = standard method, ot = other method of estimation)

Table 2F1 - 1

IESLC - Meta-analysis of Cigarette Smoking, only Filter vs only Plain (or nearest available)
Squamous
Most adjusted

| REF | NRR | SEX | AGEL | AGEH | RACE | YF | LC TYPE | LOC | START | ST | NLC | R | VB | P | H | AD | SM | PRODUCT | CIGTYP | DENOM | De |
|---|---|---|---|---|---|---|---|---|---|---|---|---|---|---|---|---|---|---|---|---|---|
| LUBIN2 | 201 | m | 0 | 0 | all | - | q | Eu:mul | 1976 | CC | 7804 | n | bl | n | y | 3 | ev | cig+/-ot | only f | always p | ot |
| LUBIN2 | 233 | f | 0 | 0 | all | - | q | Eu:mul | 1976 | CC | 7804 | n | bl | n | y | 3 | ev | cig+/-ot | only f | always p | ot |
| MATOS | 51 | m | 0 | 0 | all | - | q | SCAmer | 1994 | CC | 200 | n | bl | n | n | 3 | ev | cig+/-ot | mainly f | mainly p | st |
| PEZZOT | 12 | m | 0 | 0 | all | - | q | SCAmer | 1987 | CC | 215 | n | bl | n | y | 4 | ev | cig only | only f | ever p | ot |
| SOBUE | 85 | m | 0 | 0 | all | - | q | As:Jap | 1986 | CC | 1376 | n | bl | n | y | 5 | cu | cig+/-ot | only f | p NOS | st |
| WAKAI | 68 | m | 0 | 0 | all | - | q | As:Jap | 1988 | CC | 333 | n | bl | n | y | 5 | cu | cig+/-ot | only f | p NOS | st |
| WYNDE3 | 90 | m | 0 | 0 | all | - | KI | NAmer | 1966 | CC | 350 | n | bl | n | y | 1 | ev | cig+/-ot | mainly f | always p | st |
| WYNDE5 | 15 | m | 0 | 0 | wh | - | KI | NAmer | 1969 | CC | 1365 | n | bl | n | y | 2 | cu | cig+/-ot | mainly f | always p | st |
| WYNDE5 | 14 | m | 0 | 0 | wh | - | KI | NAmer | 1969 | CC | 1365 | n | bl | n | y | 2 | cu | cig+/-ot | mainly f | always p | st |
| WYNDE5 | 23 | f | 0 | 0 | wh | - | KI | NAmer | 1969 | CC | 1365 | n | bl | n | y | 2 | cu | cig+/-ot | mainly f | always p | st |
| WYNDE5 | 22 | f | 0 | 0 | wh | - | KI | NAmer | 1969 | CC | 1365 | n | bl | n | y | 2 | cu | cig+/-ot | mainly f | always p | st |
| WYNDE6 | 310 | m | 0 | 0 | all | - | q | NAmer | 1969 | CC | 4423 | n | bl | n | y | 3 | cu | cig+/-ot | only f | always p | or |
| WYNDE6 | 316 | f | 0 | 0 | all | - | q | NAmer | 1969 | CC | 4423 | n | bl | n | y | 3 | cu | cig+/-ot | only f | always p | or |

Table 2F1 - 2

IESLC - Meta-analysis of Cigarette Smoking, only Filter vs only Plain (or nearest available)
Squamous
Most adjusted

| REF | NRR | SEX | AD | Number Exposed Case | Number Exposed Cont | Non-exposed Case | Non-exposed Cont | RR | 95.00%CI |
|---|---|---|---|---|---|---|---|---|---|
| LUBIN2 | 201 | m | 3 | - | - | - | - | 0.53 | ( 0.45- 0.62) |
| LUBIN2 | 233 | f | 3 | - | - | - | - | 0.15 | ( 0.09- 0.26) |
| Subtotal LUBIN2 | | | | | | | | 0.48 | ( 0.41- 0.56) |
| MATOS | 51 | m | 3 | - | - | - | - | 0.71 | ( 0.27- 1.67) |
| PEZZOT | 12 | m | 4 | - | - | - | - | 0.20 | ( 0.11- 0.37) |
| SOBUE | 85 | m | 5 | - | - | - | - | 0.45 | ( 0.25- 0.83) |
| WAKAI | 68 | m | 5 | - | - | - | - | 0.45 | ( 0.14- 1.52) |
| WYNDE3 | 90 | m | 1 | - | - | - | - | 0.56 | ( 0.34- 0.92) |
| WYNDE5 | 14 | m | 2 | - | - | - | - | 0.84 | ( 0.65- 1.09) |
| WYNDE5 | 15 | m | 2 | - | - | - | - | 0.79 | ( 0.61- 1.03) |
| WYNDE5 | 22 | f | 2 | - | - | - | - | 0.78 | ( 0.40- 1.49) |
| WYNDE5 | 23 | f | 2 | - | - | - | - | 0.73 | ( 0.38- 1.39) |
| Subtotal WYNDE5 | | | | | | | | 0.81 | ( 0.68- 0.96) |
| WYNDE6 | 310 | m | 3 | - | - | - | - | 0.80 | ( 0.50- 1.20) |
| WYNDE6 | 316 | f | 3 | - | - | - | - | 0.40 | ( 0.20- 0.80) |
| Subtotal WYNDE6 | | | | | | | | 0.66 | ( 0.45- 0.95) |
| Partial Totals | | | | 0 | 0 | 0 | 0 | | |

*prospective study

| REF | NRR | SEX | AD | Ys | Ws | Qs | Ps |
|---|---|---|---|---|---|---|---|
| LUBIN2 | 201 | m | 3 | -0.63 | 149.62 | 1.27 | 0.0000 |
| LUBIN2 | 233 | f | 3 | -1.90 | 13.65 | 25.04 | 0.0000 |
| Subtotal LUBIN2 | | | | -0.74 | 163.27 | 26.32 | |
| MATOS | 51 | m | 3 | -0.34 | 4.63 | 0.19 | 0.4613 |
| PEZZOT | 12 | m | 4 | -1.61 | 10.44 | 11.88 | 0.0000 |
| SOBUE | 85 | m | 5 | -0.80 | 10.67 | 0.70 | 0.0091 |
| WAKAI | 68 | m | 5 | -0.80 | 2.70 | 0.18 | 0.1894 |
| WYNDE3 | 90 | m | 1 | -0.58 | 15.51 | 0.02 | 0.0224 |
| WYNDE5 | 14 | m | 2 | -0.17 | 57.50 | 7.80 | 0.1861 |
| WYNDE5 | 15 | m | 2 | -0.24 | 55.99 | 5.28 | 0.0778 |
| WYNDE5 | 22 | f | 2 | -0.25 | 8.89 | 0.77 | 0.4589 |
| WYNDE5 | 23 | f | 2 | -0.31 | 9.14 | 0.47 | 0.3415 |
| Subtotal WYNDE5 | | | | -0.22 | 131.51 | 14.32 | |
| WYNDE6 | 310 | m | 3 | -0.22 | 20.05 | 2.05 | 0.3177 |
| WYNDE6 | 316 | f | 3 | -0.92 | 8.00 | 1.12 | 0.0096 |
| Subtotal WYNDE6 | | | | -0.42 | 28.04 | 3.16 | |

| | | |
|---|---|---|
| | N | 13 |
| | NS | 8 |
| | Wt | 366.77 |
| Het | Chi | 56.77 |
| Het | df | 12 |
| Het | P | *** |
| Fixed | RR | 0.58 |
| | RRl | 0.52 |
| | RRu | 0.64 |
| | P | --- |
| Random | RR | 0.52 |
| | RRl | 0.40 |
| | RRu | 0.68 |
| | P | --- |
| Asymm | P | N.S. |

Table 2F1 - 3

IESLC - Meta-analysis of Cigarette Smoking, only Filter vs only Plain (or nearest available)
Squamous
Most adjusted

| | Sex | | | |
|---|---|---|---|---|
| | combined | male | female | Total |
| N | | 9 | 4 | 13 |
| NS | | 8 | 3 | 11 |
| Wt | | 327.10 | 39.67 | 366.77 |
| Het Chi | | 28.50 | 20.31 | 56.77 |
| Het df | | 8 | 3 | 12 |
| Het P | | *** | *** | *** |
| Fixed RR | | 0.61 | 0.38 | 0.58 |
| RRl | | 0.55 | 0.28 | 0.52 |
| RRu | | 0.68 | 0.52 | 0.64 |
| P | | --- | --- | --- |
| Random RR | | 0.59 | 0.42 | 0.52 |
| RRl | | 0.45 | 0.19 | 0.40 |
| RRu | | 0.75 | 0.96 | 0.68 |
| P | | --- | - | --- |
| Between Chi | | | | 7.96 |
| Between df | | | | 1 |
| Between P | | | | ** |
| Btwn(F) P | | | | N.S. |
| Btwn(R) P | | | | N.S. |

| | All LC (or nearest) | | | | | |
|---|---|---|---|---|---|---|
| | q | q+s | q+u | KI | not a | Total |
| N | 8 | | | 5 | | 13 |
| NS | 6 | | | 2 | | 8 |
| Wt | 219.76 | | | 147.02 | | 366.77 |
| Het Chi | 34.21 | | | 2.06 | | 56.77 |
| Het df | 7 | | | 4 | | 12 |
| Het P | *** | | | N.S. | | *** |
| Fixed RR | 0.48 | | | 0.78 | | 0.58 |
| RRl | 0.42 | | | 0.66 | | 0.52 |
| RRu | 0.55 | | | 0.91 | | 0.64 |
| P | --- | | | -- | | --- |
| Random RR | 0.40 | | | 0.78 | | 0.52 |
| RRl | 0.27 | | | 0.66 | | 0.40 |
| RRu | 0.60 | | | 0.91 | | 0.68 |
| P | --- | | | -- | | --- |
| Between Chi | | | | | | 20.50 |
| Between df | | | | | | 1 |
| Between P | | | | | | *** |
| Btwn(F) P | | | | | | * |
| Btwn(R) P | | | | | | ** |

| | Location | | | | | | | | |
|---|---|---|---|---|---|---|---|---|---|
| | NAmer | UK | Scand | othEur | China | Japan | othAs | other | Total |
| N | 7 | | | 2 | | 2 | | 2 | 13 |
| NS | 3 | | | 1 | | 2 | | 2 | 8 |
| Wt | 175.06 | | | 163.27 | | 13.37 | | 15.07 | 366.77 |
| Het Chi | 5.47 | | | 19.93 | | 0.00 | | 5.15 | 56.77 |
| Het df | 6 | | | 1 | | 1 | | 1 | 12 |
| Het P | N.S. | | | *** | | N.S. | | * | *** |
| Fixed RR | 0.76 | | | 0.48 | | 0.45 | | 0.30 | 0.58 |
| RRl | 0.65 | | | 0.41 | | 0.26 | | 0.18 | 0.52 |
| RRu | 0.88 | | | 0.56 | | 0.77 | | 0.49 | 0.64 |
| P | --- | | | --- | | -- | | --- | --- |
| Random RR | 0.76 | | | 0.29 | | 0.45 | | 0.36 | 0.52 |
| RRl | 0.65 | | | 0.08 | | 0.26 | | 0.10 | 0.40 |
| RRu | 0.88 | | | 1.00 | | 0.77 | | 1.24 | 0.68 |
| P | --- | | | - | | -- | | N.S. | --- |
| Between Chi | | | | | | | | | 26.22 |
| Between df | | | | | | | | | 3 |
| Between P | | | | | | | | | *** |
| Btwn(F) P | | | | | | | | | N.S. |
| Btwn(R) P | | | | | | | | | (*) |

Table 2F1 - 3

IESLC - Meta-analysis of Cigarette Smoking, only Filter vs only Plain (or nearest available)
Squamous
Most adjusted

| | Detailed Country in "other Europe" | | | | | |
|---|---|---|---|---|---|---|
| | multi | Germany | othWest | East | Balkans | Total |
| N | 2 | | | | | 2 |
| NS | 1 | | | | | 1 |
| Wt | 163.27 | | | | | 163.27 |
| Het Chi | 19.93 | | | | | 19.93 |
| Het df | 1 | | | | | 1 |
| Het P | *** | | | | | *** |
| Fixed RR | 0.48 | | | | | 0.48 |
| RRl | 0.41 | | | | | 0.41 |
| RRu | 0.56 | | | | | 0.56 |
| P | --- | | | | | --- |
| Random RR | 0.29 | | | | | 0.29 |
| RRl | 0.08 | | | | | 0.08 |
| RRu | 1.00 | | | | | 1.00 |
| P | - | | | | | - |
| Between Chi | | | | | | |
| Between df | | | | | | |
| Between P | | | | | | N.S. |
| Btwn(F) P | | | | | | N.S. |
| Btwn(R) P | | | | | | N.S. |

| | Detailed Country in "other Asia" | | | |
|---|---|---|---|---|
| | India | HongKong | other | Total |
| N | | | | |
| NS | | | | |
| Wt | | | | |
| Het Chi | | | | |
| Het df | | | | |
| Het P | | | | |
| Fixed RR | | | | |
| RRl | | | | |
| RRu | | | | |
| P | | | | |
| Random RR | | | | |
| RRl | | | | |
| RRu | | | | |
| P | | | | |
| Between Chi | | | | |
| Between df | | | | |
| Between P | | | | N.S. |
| Btwn(F) P | | | | N.S. |
| Btwn(R) P | | | | N.S. |

| | Detailed other continent | | | |
|---|---|---|---|---|
| | SCAmer | Auslia | Africa | Total |
| N | 2 | | | 2 |
| NS | 2 | | | 2 |
| Wt | 15.07 | | | 15.07 |
| Het Chi | 5.15 | | | 5.15 |
| Het df | 1 | | | 1 |
| Het P | * | | | * |
| Fixed RR | 0.30 | | | 0.30 |
| RRl | 0.18 | | | 0.18 |
| RRu | 0.49 | | | 0.49 |
| P | --- | | | --- |
| Random RR | 0.36 | | | 0.36 |
| RRl | 0.10 | | | 0.10 |
| RRu | 1.24 | | | 1.24 |
| P | N.S. | | | N.S. |
| Between Chi | | | | |
| Between df | | | | |
| Between P | | | | N.S. |
| Btwn(F) P | | | | N.S. |
| Btwn(R) P | | | | N.S. |

Table 2F1 - 3

IESLC - Meta-analysis of Cigarette Smoking, only Filter vs only Plain (or nearest available)
Squamous
Most adjusted

| | Start year of study <1960 | 1960-69 | 1970-79 | 1980-89 | 1990+ | Total |
|---|---|---|---|---|---|---|
| N | | 7 | 2 | 3 | 1 | 13 |
| NS | | 3 | 1 | 3 | 1 | 8 |
| Wt | | 175.06 | 163.27 | 23.82 | 4.63 | 366.77 |
| Het Chi | | 5.47 | 19.93 | 3.86 | 0.00 | 56.77 |
| Het df | | 6 | 1 | 2 | 0 | 12 |
| Het P | | N.S. | *** | N.S. | N.S. | *** |
| Fixed RR | | 0.76 | 0.48 | 0.32 | 0.71 | 0.58 |
| RRl | | 0.65 | 0.41 | 0.21 | 0.29 | 0.52 |
| RRu | | 0.88 | 0.56 | 0.47 | 1.77 | 0.64 |
| P | | --- | --- | --- | N.S. | --- |
| Random RR | | 0.76 | 0.29 | 0.32 | 0.71 | 0.52 |
| RRl | | 0.65 | 0.08 | 0.18 | 0.29 | 0.40 |
| RRu | | 0.88 | 1.00 | 0.59 | 1.77 | 0.68 |
| P | | --- | - | --- | N.S. | --- |
| Between Chi | | | | | | 27.51 |
| Between df | | | | | | 3 |
| Between P | | | | | | *** |
| Btwn(F) P | | | | | | (*) |
| Btwn(R) P | | | | | | * |

| | Study type (1) CC | other | Total |
|---|---|---|---|
| N | 13 | | 13 |
| NS | 8 | | 8 |
| Wt | 366.77 | | 366.77 |
| Het Chi | 56.77 | | 56.77 |
| Het df | 12 | | 12 |
| Het P | *** | | *** |
| Fixed RR | 0.58 | | 0.58 |
| RRl | 0.52 | | 0.52 |
| RRu | 0.64 | | 0.64 |
| P | --- | | --- |
| Random RR | 0.52 | | 0.52 |
| RRl | 0.40 | | 0.40 |
| RRu | 0.68 | | 0.68 |
| P | --- | | --- |
| Between Chi | | | |
| Between df | | | |
| Between P | | | N.S. |
| Btwn(F) P | | | N.S. |
| Btwn(R) P | | | N.S. |

| | Study type (2) CC | prosp | other | Total |
|---|---|---|---|---|
| N | 13 | | | 13 |
| NS | 8 | | | 8 |
| Wt | 366.77 | | | 366.77 |
| Het Chi | 56.77 | | | 56.77 |
| Het df | 12 | | | 12 |
| Het P | *** | | | *** |
| Fixed RR | 0.58 | | | 0.58 |
| RRl | 0.52 | | | 0.52 |
| RRu | 0.64 | | | 0.64 |
| P | --- | | | --- |
| Random RR | 0.52 | | | 0.52 |
| RRl | 0.40 | | | 0.40 |
| RRu | 0.68 | | | 0.68 |
| P | --- | | | --- |
| Between Chi | | | | |
| Between df | | | | |
| Between P | | | | N.S. |
| Btwn(F) P | | | | N.S. |
| Btwn(R) P | | | | N.S. |

Table 2F1 - 3

IESLC - Meta-analysis of Cigarette Smoking, only Filter vs only Plain (or nearest available)

Squamous

Most adjusted

| | Study size (number of LC cases) | | | | |
|---|---|---|---|---|---|
| | 100-249 | 250-499 | 500-999 | 1000+ | Total |
| N | 2 | 2 | | 9 | 13 |
| NS | 2 | 2 | | 4 | 8 |
| Wt | 15.07 | 18.21 | | 333.49 | 366.77 |
| Het Chi | 5.15 | 0.11 | | 44.11 | 56.77 |
| Het df | 1 | 1 | | 8 | 12 |
| Het P | * | N.S. | | *** | *** |
| Fixed RR | 0.30 | 0.54 | | 0.60 | 0.58 |
| RRl | 0.18 | 0.34 | | 0.54 | 0.52 |
| RRu | 0.49 | 0.86 | | 0.67 | 0.64 |
| P | --- | -- | | --- | --- |
| Random RR | 0.36 | 0.54 | | 0.56 | 0.52 |
| RRl | 0.10 | 0.34 | | 0.42 | 0.40 |
| RRu | 1.24 | 0.86 | | 0.76 | 0.68 |
| P | N.S. | -- | | --- | --- |
| Between Chi | | | | | 7.40 |
| Between df | | | | | 2 |
| Between P | | | | | * |
| Btwn(F) P | | | | | N.S. |
| Btwn(R) P | | | | | N.S. |

| | Risky occupational population | | | |
|---|---|---|---|---|
| | no | mining | othRisky | Total |
| N | 13 | | | 13 |
| NS | 8 | | | 8 |
| Wt | 366.77 | | | 366.77 |
| Het Chi | 56.77 | | | 56.77 |
| Het df | 12 | | | 12 |
| Het P | *** | | | *** |
| Fixed RR | 0.58 | | | 0.58 |
| RRl | 0.52 | | | 0.52 |
| RRu | 0.64 | | | 0.64 |
| P | --- | | | --- |
| Random RR | 0.52 | | | 0.52 |
| RRl | 0.40 | | | 0.40 |
| RRu | 0.68 | | | 0.68 |
| P | --- | | | --- |
| Between Chi | | | | |
| Between df | | | | |
| Between P | | | | N.S. |
| Btwn(F) P | | | | N.S. |
| Btwn(R) P | | | | N.S. |

| | National cigarette tobacco type | | | |
|---|---|---|---|---|
| | Virginia | blended | other | Total |
| N | | 13 | | 13 |
| NS | | 8 | | 8 |
| Wt | | 366.77 | | 366.77 |
| Het Chi | | 56.77 | | 56.77 |
| Het df | | 12 | | 12 |
| Het P | | *** | | *** |
| Fixed RR | | 0.58 | | 0.58 |
| RRl | | 0.52 | | 0.52 |
| RRu | | 0.64 | | 0.64 |
| P | | --- | | --- |
| Random RR | | 0.52 | | 0.52 |
| RRl | | 0.40 | | 0.40 |
| RRu | | 0.68 | | 0.68 |
| P | | --- | | --- |
| Between Chi | | | | |
| Between df | | | | |
| Between P | | | | N.S. |
| Btwn(F) P | | | | N.S. |
| Btwn(R) P | | | | N.S. |

Table 2F1 - 3

IESLC - Meta-analysis of Cigarette Smoking, only Filter vs only Plain (or nearest available)
Squamous
Most adjusted

| | Any proxy use No/nk | Yes | Total |
|---|---|---|---|
| N | 13 | | 13 |
| NS | 8 | | 8 |
| Wt | 366.77 | | 366.77 |
| Het Chi | 56.77 | | 56.77 |
| Het df | 12 | | 12 |
| Het P | *** | | *** |
| Fixed RR | 0.58 | | 0.58 |
| RRl | 0.52 | | 0.52 |
| RRu | 0.64 | | 0.64 |
| P | --- | | --- |
| Random RR | 0.52 | | 0.52 |
| RRl | 0.40 | | 0.40 |
| RRu | 0.68 | | 0.68 |
| P | --- | | --- |
| Between Chi | | | |
| Between df | | | |
| Between P | | | N.S. |
| Btwn(F) P | | | N.S. |
| Btwn(R) P | | | N.S. |

| | Full histological confirmation No | Yes | Total |
|---|---|---|---|
| N | 1 | 12 | 13 |
| NS | 1 | 7 | 8 |
| Wt | 4.63 | 362.15 | 366.77 |
| Het Chi | 0.00 | 56.58 | 56.77 |
| Het df | 0 | 11 | 12 |
| Het P | N.S. | *** | *** |
| Fixed RR | 0.71 | 0.58 | 0.58 |
| RRl | 0.29 | 0.52 | 0.52 |
| RRu | 1.77 | 0.64 | 0.64 |
| P | N.S. | --- | --- |
| Random RR | 0.71 | 0.51 | 0.52 |
| RRl | 0.29 | 0.39 | 0.40 |
| RRu | 1.77 | 0.68 | 0.68 |
| P | N.S. | --- | --- |
| Between Chi | | | 0.19 |
| Between df | | | 1 |
| Between P | | | N.S. |
| Btwn(F) P | | | N.S. |
| Btwn(R) P | | | N.S. |

| | Number of adjustment variables (1) 0 | 1 | 2+/+nk | Total |
|---|---|---|---|---|
| N | | 1 | 12 | 13 |
| NS | | 1 | 7 | 8 |
| Wt | | 15.51 | 351.27 | 366.77 |
| Het Chi | | 0.00 | 56.74 | 56.77 |
| Het df | | 0 | 11 | 12 |
| Het P | | N.S. | *** | *** |
| Fixed RR | | 0.56 | 0.58 | 0.58 |
| RRl | | 0.34 | 0.52 | 0.52 |
| RRu | | 0.92 | 0.65 | 0.64 |
| P | | - | --- | --- |
| Random RR | | 0.56 | 0.52 | 0.52 |
| RRl | | 0.34 | 0.39 | 0.40 |
| RRu | | 0.92 | 0.69 | 0.68 |
| P | | - | --- | --- |
| Between Chi | | | | 0.02 |
| Between df | | | | 1 |
| Between P | | | | N.S. |
| Btwn(F) P | | | | N.S. |
| Btwn(R) P | | | | N.S. |

Table 2F1 - 3

IESLC - Meta-analysis of Cigarette Smoking, only Filter vs only Plain (or nearest available)
Squamous
Most adjusted

| | Number of adjustment variables (2) 0 | 1 | 2 | 3-5 | 6+/+nk | Total |
|---|---|---|---|---|---|---|
| N | | 1 | 4 | 8 | | 13 |
| NS | | 1 | 1 | 6 | | 8 |
| Wt | | 15.51 | 131.51 | 219.76 | | 366.77 |
| Het Chi | | 0.00 | 0.22 | 34.21 | | 56.77 |
| Het df | | 0 | 3 | 7 | | 12 |
| Het P | | N.S. | N.S. | *** | | *** |
| Fixed RR | | 0.56 | 0.81 | 0.48 | | 0.58 |
| RRl | | 0.34 | 0.68 | 0.42 | | 0.52 |
| RRu | | 0.92 | 0.96 | 0.55 | | 0.64 |
| P | | - | - | --- | | --- |
| Random RR | | 0.56 | 0.81 | 0.40 | | 0.52 |
| RRl | | 0.34 | 0.68 | 0.27 | | 0.40 |
| RRu | | 0.92 | 0.96 | 0.60 | | 0.68 |
| P | | - | - | --- | | --- |
| Between Chi | | | | | | 22.34 |
| Between df | | | | | | 2 |
| Between P | | | | | | *** |
| Btwn(F) P | | | | | | (*) |
| Btwn(R) P | | | | | | ** |

| | Smoking status ever | current | Total |
|---|---|---|---|
| N | 5 | 8 | 13 |
| NS | 4 | 4 | 8 |
| Wt | 193.85 | 172.93 | 366.77 |
| Het Chi | 28.84 | 7.64 | 56.77 |
| Het df | 4 | 7 | 12 |
| Het P | *** | N.S. | *** |
| Fixed RR | 0.47 | 0.75 | 0.58 |
| RRl | 0.40 | 0.64 | 0.52 |
| RRu | 0.54 | 0.87 | 0.64 |
| P | --- | --- | --- |
| Random RR | 0.36 | 0.74 | 0.52 |
| RRl | 0.20 | 0.63 | 0.40 |
| RRu | 0.63 | 0.87 | 0.68 |
| P | --- | --- | --- |
| Between Chi | | | 20.29 |
| Between df | | | 1 |
| Between P | | | *** |
| Btwn(F) P | | | * |
| Btwn(R) P | | | * |

| | Product cig+/-ot | cig only | Total |
|---|---|---|---|
| N | 12 | 1 | 13 |
| NS | 7 | 1 | 8 |
| Wt | 356.33 | 10.44 | 366.77 |
| Het Chi | 44.54 | 0.00 | 56.77 |
| Het df | 11 | 0 | 12 |
| Het P | *** | N.S. | *** |
| Fixed RR | 0.60 | 0.20 | 0.58 |
| RRl | 0.54 | 0.11 | 0.52 |
| RRu | 0.67 | 0.37 | 0.64 |
| P | --- | --- | --- |
| Random RR | 0.57 | 0.20 | 0.52 |
| RRl | 0.44 | 0.11 | 0.40 |
| RRu | 0.73 | 0.37 | 0.68 |
| P | --- | --- | --- |
| Between Chi | | | 12.23 |
| Between df | | | 1 |
| Between P | | | *** |
| Btwn(F) P | | | N.S. |
| Btwn(R) P | | | ** |

Table 2F1 - 3

IESLC - Meta-analysis of Cigarette Smoking, only Filter vs only Plain (or nearest available)
Squamous
Most adjusted

| | Cigarette type | | | | | | |
|---|---|---|---|---|---|---|---|
| | only f | always f | mainly f | equl p&f | both p&f | ever f | Total |
| N | 7 | | 6 | | | | 13 |
| NS | 5 | | 3 | | | | 8 |
| Wt | 215.13 | | 151.65 | | | | 366.77 |
| Het Chi | 33.47 | | 2.10 | | | | 56.77 |
| Het df | 6 | | 5 | | | | 12 |
| Het P | *** | | N.S. | | | | *** |
| Fixed RR | 0.47 | | 0.77 | | | | 0.58 |
| RRl | 0.42 | | 0.66 | | | | 0.52 |
| RRu | 0.54 | | 0.91 | | | | 0.64 |
| P | --- | | -- | | | | --- |
| Random RR | 0.38 | | 0.77 | | | | 0.52 |
| RRl | 0.24 | | 0.66 | | | | 0.40 |
| RRu | 0.59 | | 0.91 | | | | 0.68 |
| P | --- | | -- | | | | --- |
| Between Chi | | | | | | | 21.20 |
| Between df | | | | | | | 1 |
| Between P | | | | | | | *** |
| Btwn(F) P | | | | | | | * |
| Btwn(R) P | | | | | | | ** |

| | Denominator | | | | |
|---|---|---|---|---|---|
| | ever p | mainly p | p NOS | always p | Total |
| N | 1 | 1 | 2 | 9 | 13 |
| NS | 1 | 1 | 2 | 4 | 8 |
| Wt | 10.44 | 4.63 | 13.37 | 338.33 | 366.77 |
| Het Chi | 0.00 | 0.00 | 0.00 | 43.27 | 56.77 |
| Het df | 0 | 0 | 1 | 8 | 12 |
| Het P | N.S. | N.S. | N.S. | *** | *** |
| Fixed RR | 0.20 | 0.71 | 0.45 | 0.61 | 0.58 |
| RRl | 0.11 | 0.29 | 0.26 | 0.54 | 0.52 |
| RRu | 0.37 | 1.77 | 0.77 | 0.67 | 0.64 |
| P | --- | N.S. | -- | --- | --- |
| Random RR | 0.20 | 0.71 | 0.45 | 0.57 | 0.52 |
| RRl | 0.11 | 0.29 | 0.26 | 0.43 | 0.40 |
| RRu | 0.37 | 1.77 | 0.77 | 0.77 | 0.68 |
| P | --- | N.S. | -- | --- | --- |
| Between Chi | | | | | 13.49 |
| Between df | | | | | 3 |
| Between P | | | | | ** |
| Btwn(F) P | | | | | N.S. |
| Btwn(R) P | | | | | * |

| | Derivation of RR/CI | | | |
|---|---|---|---|---|
| | Orig | StdCalc | Other | Total |
| N | 2 | 8 | 3 | 13 |
| NS | 1 | 5 | 2 | 8 |
| Wt | 28.04 | 165.02 | 173.71 | 366.77 |
| Het Chi | 2.75 | 5.71 | 27.35 | 56.77 |
| Het df | 1 | 7 | 2 | 12 |
| Het P | (*) | N.S. | *** | *** |
| Fixed RR | 0.66 | 0.74 | 0.45 | 0.58 |
| RRl | 0.45 | 0.64 | 0.39 | 0.52 |
| RRu | 0.95 | 0.86 | 0.53 | 0.64 |
| P | - | --- | --- | --- |
| Random RR | 0.60 | 0.74 | 0.26 | 0.52 |
| RRl | 0.31 | 0.64 | 0.10 | 0.40 |
| RRu | 1.17 | 0.86 | 0.64 | 0.68 |
| P | N.S. | --- | -- | --- |
| Between Chi | | | | 20.96 |
| Between df | | | | 2 |
| Between P | | | | *** |
| Btwn(F) P | | | | (*) |
| Btwn(R) P | | | | (*) |

Table 2F1 - 4

IESLC - Meta-analysis of Cigarette Smoking, only Filter vs only Plain (or nearest available)
Squamous
Least adjusted

| REF | NRR | X | SEX | AGEL | AGEH | RACE | YF | LC TYPE | LOC | START | ST | NLC | R | VB | P | H | AD | SM | PRODUCT | CIGTYP | DENOM | De |
|---|---|---|---|---|---|---|---|---|---|---|---|---|---|---|---|---|---|---|---|---|---|---|
| LUBIN2 | 185 | x | m | 0 | 0 | all | - | q | Eu:mul | 1976 | CC | 7804 | n | bl | n | y | 0 | ev | cig+/-ot | only f | always p | st |
| LUBIN2 | 217 | x | f | 0 | 0 | all | - | q | Eu:mul | 1976 | CC | 7804 | n | bl | n | y | 0 | ev | cig+/-ot | only f | always p | st |
| MATOS | 73 | x | m | 0 | 0 | all | - | q | SCAmer | 1994 | CC | 200 | n | bl | n | n | 0 | ev | cig+/-ot | mainly f | mainly p | st |
| PEZZOT | 11 | x | m | 0 | 0 | all | - | q | SCAmer | 1987 | CC | 215 | n | bl | n | y | 2 | ev | cig only | only f | ever p | ot |
| SOBUE | 73 | x | m | 0 | 0 | all | - | q | As:Jap | 1986 | CC | 1376 | n | bl | n | y | 0 | cu | cig+/-ot | only f | p NOS | st |
| WAKAI | 67 | x | m | 0 | 0 | all | - | q | As:Jap | 1988 | CC | 333 | n | bl | n | y | 0 | cu | cig+/-ot | only f | p NOS | st |
| WYNDE3 | 89 | x | m | 0 | 0 | all | - | KI | NAmer | 1966 | CC | 350 | n | bl | n | y | 0 | ev | cig+/-ot | mainly f | always p | st |
| WYNDE5 | 12 | x | m | 0 | 0 | wh | - | KI | NAmer | 1969 | CC | 1365 | n | bl | n | y | 0 | cu | cig+/-ot | mainly f | always p | st |
| WYNDE5 | 20 | x | f | 0 | 0 | wh | - | KI | NAmer | 1969 | CC | 1365 | n | bl | n | y | 0 | cu | cig+/-ot | mainly f | always p | st |
| WYNDE6 | 298 | x | m | 0 | 0 | all | - | q | NAmer | 1969 | CC | 4423 | n | bl | n | y | 0 | cu | cig+/-ot | only f | always p | st |
| WYNDE6 | 304 | x | f | 0 | 0 | all | - | q | NAmer | 1969 | CC | 4423 | n | bl | n | y | 0 | cu | cig+/-ot | only f | always p | st |

Table 2F1 - 5

IESLC - Meta-analysis of Cigarette Smoking, only Filter vs only Plain (or nearest available)
Squamous
Least adjusted

| REF | NRR | SEX | AD | Number Exposed Case | Number Exposed Cont | Non-exposed Case | Non-exposed Cont | RR | 95.00%CI |
|---|---|---|---|---|---|---|---|---|---|
| LUBIN2 | 185 | m | 0 | 190 | 1018 | 1340 | 3810 | 0.53 | ( 0.45- 0.63) |
| LUBIN2 | 217 | f | 0 | 40 | 213 | 32 | 62 | 0.36 | ( 0.21- 0.63) |
| Subtotal LUBIN2 | | | | | | | | 0.51 | ( 0.44- 0.60) |
| MATOS | 73 | m | 0 | 38 | 229 | 9 | 46 | 0.85 | ( 0.38- 1.87) |
| PEZZOT | 11 | m | 2 | - | - | - | - | 0.20 | ( 0.11- 0.37) |
| SOBUE | 73 | m | 0 | 220 | 540 | 27 | 26 | 0.39 | ( 0.22- 0.69) |
| WAKAI | 67 | m | 0 | 81 | 271 | 5 | 9 | 0.54 | ( 0.18- 1.65) |
| WYNDE3 | 89 | m | 0 | 66 | 82 | 81 | 62 | 0.62 | ( 0.39- 0.98) |
| WYNDE5 | 12 | m | 0 | 143 | 879 | 150 | 704 | 0.76 | ( 0.59- 0.98) |
| WYNDE5 | 20 | f | 0 | 50 | 857 | 13 | 166 | 0.74 | ( 0.40- 1.40) |
| Subtotal WYNDE5 | | | | | | | | 0.76 | ( 0.60- 0.96) |
| WYNDE6 | 298 | m | 0 | 62 | 122 | 159 | 165 | 0.53 | ( 0.36- 0.77) |
| WYNDE6 | 304 | f | 0 | 57 | 158 | 33 | 30 | 0.33 | ( 0.18- 0.59) |
| Subtotal WYNDE6 | | | | | | | | 0.46 | ( 0.33- 0.63) |
| Partial Totals | | | | 947 | 4369 | 1849 | 5080 | | |

*prospective study

| REF | NRR | SEX | AD | Ys | Ws | Qs | Ps |
|---|---|---|---|---|---|---|---|
| LUBIN2 | 185 | m | 0 | -0.63 | 137.85 | 0.07 | 0.0000 |
| LUBIN2 | 217 | f | 0 | -1.01 | 12.97 | 2.07 | 0.0003 |
| Subtotal LUBIN2 | | | | -0.67 | 150.83 | 2.14 | |
| MATOS | 73 | m | 0 | -0.16 | 6.11 | 1.22 | 0.6838 |
| PEZZOT | 11 | m | 2 | -1.61 | 10.44 | 10.40 | 0.0000 |
| SOBUE | 73 | m | 0 | -0.94 | 12.21 | 1.28 | 0.0011 |
| WAKAI | 67 | m | 0 | -0.62 | 3.06 | 0.00 | 0.2785 |
| WYNDE3 | 89 | m | 0 | -0.48 | 17.91 | 0.29 | 0.0403 |
| WYNDE5 | 12 | m | 0 | -0.27 | 61.66 | 7.20 | 0.0341 |
| WYNDE5 | 20 | f | 0 | -0.29 | 9.60 | 0.97 | 0.3616 |
| Subtotal WYNDE5 | | | | -0.27 | 71.27 | 8.17 | |
| WYNDE6 | 298 | m | 0 | -0.64 | 27.27 | 0.02 | 0.0008 |
| WYNDE6 | 304 | f | 0 | -1.11 | 11.43 | 2.89 | 0.0002 |
| Subtotal WYNDE6 | | | | -0.78 | 38.69 | 2.92 | |

| | | |
|---|---|---|
| | N | 11 |
| | NS | 8 |
| | Wt | 310.52 |
| Het | Chi | 26.41 |
| Het | df | 10 |
| Het | P | ** |
| Fixed | RR | 0.54 |
| | RRl | 0.49 |
| | RRu | 0.61 |
| | P | --- |
| Random | RR | 0.50 |
| | RRl | 0.40 |
| | RRu | 0.63 |
| | P | --- |
| Asymm | P | N.S. |

Table 2F1 - 6

IESLC - Meta-analysis of Cigarette Smoking, only Filter vs only Plain (or nearest available)

Squamous

Least adjusted

| | Sex combined | male | female | Total |
|---|---|---|---|---|
| N | | 8 | 3 | 11 |
| NS | | 8 | 3 | 11 |
| Wt | | 276.52 | 34.01 | 310.52 |
| Het Chi | | 20.26 | 4.10 | 26.41 |
| Het df | | 7 | 2 | 10 |
| Het P | | ** | N.S. | ** |
| Fixed RR | | 0.56 | 0.43 | 0.54 |
| RRl | | 0.50 | 0.31 | 0.49 |
| RRu | | 0.63 | 0.60 | 0.61 |
| P | | --- | --- | --- |
| Random RR | | 0.53 | 0.44 | 0.50 |
| RRl | | 0.41 | 0.27 | 0.40 |
| RRu | | 0.68 | 0.71 | 0.63 |
| P | | --- | --- | --- |
| Between Chi | | | | 2.05 |
| Between df | | | | 1 |
| Between P | | | | N.S. |
| Btwn(F) P | | | | N.S. |
| Btwn(R) P | | | | N.S. |

Table 2F1 - 7

IESLC - Meta-analysis of Cigarette Smoking, only Filter vs only Plain (or nearest available)
Squamous
Excluded studies (and stage at which they were excluded)

| | | | | | | | | | | | | | | | | |
|---|---|---|---|---|---|---|---|---|---|---|---|---|---|---|---|---|
| 1 | BOUCHA | BUELL | LAURIL | MZILEN | | | | | | | | | | | | |
| 2 | BERRIN | CHEN | RESTRE | TAO | ULMER | | | | | | | | | | | |
| 3 | ABELIN | ABRAHA | AKIBA | AMANDU | AMES | ANDERS | ARCHER | AUSTIN | AUVINE | AXELSO | AXELSS | BAND | BARBON | BENSHL | BEST | BLOHMK |
| | BLOT1 | BLOT2 | BLOT3 | BLOT4 | BOFFET | BOUCOT | BRESLO | BRETT | BROCKM | BROWN1 | BROWN2 | BYERS1 | BYERS2 | CARPEN | CASCO2 | CASCOR |
| | CEDERL | CHAN | CHANG | CHATZI | CHEN2 | CHEN3 | CHIAZZ | CHOW | CHYOU | COMSTO | COOKSO | CPSI | DAMBER | DARBY | DAVEYS | DEAN |
| | DEKLER | DOCKER | DOLL2 | DORANT | DORGAN | DORN | DOSEME | DROSTE | DU | DUNN | EBELIN | ENSTRO | ESAKI | FAN | GAO | GAO2 |
| | GARCIA | GARDIN | GARSHI | GENG | GER | GILLIS | GODLEY | GOLLED | GOODMA | GRAHAM | GREGOR | GSELL | HAENSZ | HAMMO2 | HAMMON | HANSEN |
| | HEGMAN | HEIN | HENNEK | HINDS | HIRAOK | HIRAY2 | HIRAYA | HITOSU | HOLE | HOROWI | HORWIT | HU | HU2 | HUANG | HUMBLE | ISHIMA |
| | JAHN | JAIN | JARUP | JARVHO | JEDRYC | JIANG | JOLY | JONES | JUSSAW | KAISER | KANELL | KATSOU | KAUFMA | KELLER | KIHARA | KINLEN |
| | KJUUS | KNEKT | KO | KOHLME | KOO | KOULUM | KREUZE | KREYBE | KUBIK | LAMTH | LAMWK | LAMWK2 | LAUSSM | LEI | LEMARC | LETOUR |
| | LEVIN | LIAW | LICKIN | LIDDEL | LIU | LIU2 | LIU3 | LIU4 | LIU5 | LOMBA2 | LOMBAR | LUBIN | LUO | MAGNUS | MARSH | MARSH2 |
| | MARTIS | MASTRA | MATSUD | MCCONN | MCDUFF | MCLAUG | MILLER | MILLS | MOLLO | MRFIT | MURATA | NAM | NOTAN2 | NOTANI | NOU | ODRISC |
| | ORMOS | OSANN | OSANN2 | PARKIN | PASTOR | PAWLEG | PERNU | PERSH2 | PERSHA | PETO | PEZZO2 | PIKE | PISANI | POFFIJ | POLEDN | PRESCO |
| | QIAO | QIAO2 | RACHTA | RADZIK | RANDIG | REN | RONCO | ROOTS | ROTHSC | SAARIK | SADOWS | SANKAR | SCHWA2 | SCHWAR | SEGI | SEOW |
| | SHAW | SHIMIZ | SIEMIA | SIMARA | SIMONA | SITAS | SOBUE2 | SPEIZE | SPITZ | STASZE | STAYNE | STOCKS | STOCKW | STUCKE | SUN | SUZUK2 |
| | SUZUKI | SVENSS | TANG | TENKAN | TIZZAN | TOKARS | TOUSEY | TSUGAN | TULINI | TVERDA | VANDER | VEIERO | VUTUC | WALD | WANG | WANG2 |
| | WANG3 | WANG4 | WARSIN | WATSON | WICKLU | WIGLE | WILKIN | WU | WU2 | WUNSCH | WUWILL | WYNDE2 | WYNDE4 | WYNDE7 | WYNDE8 | WYNDER |
| | XIANGZ | XU | XU2 | XU3 | XU4 | YAMAGU | YONG | YUAN | ZHANG | ZHENG | ZHOU | | | | | |
| 7 | AGUDO | ALDERS | ARMADA | BECHER | BROSS | BUFFLE | CHOI | CORREA | CPSII | DEAN2 | DEAN3 | DESTE2 | DESTEF | DOLL | ENGELA | KAISE2 |
| | KHUDER | LANGE | MACLEN | MIGRAN | MRFITR | RIMING | SEGI2 | TANG2 | | | | | | | | |
| 10 | BENHAM | | | | | | | | | | | | | | | |

Table 2F1 - 8
Potentially overlapping studies

| REF | REFGP | PRINC | . OVERLAP/LINK |
|---|---|---|---|
| LUBIN2 | LUBIN2 | 1 | Lubin-combined |
| WYNDE5 | WYNDE6 | 2 | WYNDE5/6/7/8 |
| WYNDE6 | WYNDE6 | 1 | WYNDE5/6/7/8 |

Table 2F2 -

IESLC - Meta-analysis of Cigarette Smoking, ever Filter vs only Plain (or nearest available)
Squamous

This analysis is restricted to results for:
1) Non-dose-response data
2) Results complete enough for use in metaanalysis

Within each study, results are then selected (in the following order of preference, within each sex) for:
3) CIGTYP: filter ever, equally, both, mainly, always, only/NOS
4) DENOM: plain only/NOS, always, mainly, ever
5) PRODUCT: cigarettes regardless of other products, cigarettes only (Note only study ALDERS has both product definitions available)
6) SMKSTA: ever, current. (Note only study MATOS has both ever and current available)
7) LCtype: all or nearest available, at least Squamous and Adeno. (q = squamous, s = small,
l = large, a = adeno, mix = mixed, alv = alveolar)
8) Race: all or nearest available, otherwise by race (wh or w = white, bl or b = black, hi = hispanic
ch = chinese, jap = japanese, haw = hawaiian, w+o = white + oriental, sca = scandinavian, as = asian)
9) Followup period (YF, prospective studies): whole study (coded as 0) or longest available
10) For overlapping studies: principal rather than subsidiary studies
Finally by Age: whole study (coded as 0) if available, otherwise by widest available age group
and then for single sex results (m, f) in preference to combined sex results (c).

Results adjusted (AD) for the most potential confounders are then chosen in Sections -1 to -3
(and those which actually differ from the adjusted results in Table 2F1 - 1 are marked 'x' in Section -1)
and results adjusted for the least confounders in Sections -4 to -6. (Those least adjusted results which
actually differ from the most adjusted as marked 'x' in column X in Section -4)
(Results adjusted for an unknown number of confounder(s) are coded as 20.)

Section -7 shows excluded studies, together with the stage (as above) at which no qualifying
results were found.

Section -8 lists the potentially overlapping studies which have been included (1=principal, 2=subsidiary).

Section -9 lists any results which would have been included in preference except that they had data not complete
enough for use in meta-analysis, with their significance (yes/no), if known, and any further comment as entered
on the database.

In addition to those mentioned above, the following fields, levels and abbreviations are used:
* or nk = not known, n = no, y = yes, ot = other
ev = ever, cu = current, cig+/-ot = cigarettes irrespective of other products (cigar, pipe etc)
f = filter, p = plain, NOS = not otherwise specified
REF: 6-character study reference
NRR: number of the RR on the database within the study
ST : study type (CC = case control, pr or prosp = prospective)
NLC: number of lung cancer cases in whole study
R : risky occupational population (n = no, m = mining, o = other risky)
VB : national cigarette type (V = at least 75% Virginia, bl = at least 75% blended, ot = other)
P : any proxy use
H : full histological confirmation
De : derivation of RR/CI (or = original, st = standard method, ot = other method of estimation)

Table 2F2 - 1

IESLC - Meta-analysis of Cigarette Smoking, ever Filter vs only Plain (or nearest available)
Squamous
Most adjusted

| REF | NRR | 2F1 | SEX | AGEL | AGEH | RACE | YF | LC TYPE | LOC | START | ST | NLC | R | VB | P | H | AD | SM | PRODUCT | CIGTYP | DENOM | De |
|---|---|---|---|---|---|---|---|---|---|---|---|---|---|---|---|---|---|---|---|---|---|---|
| LUBIN2 | 209 | x | m | 0 | 0 | all | - | q | Eu:mul | 1976 | CC | 7804 | n | bl | n | y | 3 | ev | cig+/-ot | ever f | always p | ot |
| LUBIN2 | 241 | x | f | 0 | 0 | all | - | q | Eu:mul | 1976 | CC | 7804 | n | bl | n | y | 3 | ev | cig+/-ot | ever f | always p | ot |
| MATOS | 51 | | m | 0 | 0 | all | - | q | SCAmer | 1994 | CC | 200 | n | bl | n | n | 3 | ev | cig+/-ot | mainly f | mainly p | st |
| PEZZOT | 12 | | m | 0 | 0 | all | - | q | SCAmer | 1987 | CC | 215 | n | bl | n | y | 4 | ev | cig only | only f | ever p | ot |
| SOBUE | 85 | | m | 0 | 0 | all | - | q | As:Jap | 1986 | CC | 1376 | n | bl | n | y | 5 | cu | cig+/-ot | only f | p NOS | st |
| WAKAI | 68 | | m | 0 | 0 | all | - | q | As:Jap | 1988 | CC | 333 | n | bl | n | y | 5 | cu | cig+/-ot | only f | p NOS | st |
| WYNDE3 | 90 | | m | 0 | 0 | all | - | KI | NAmer | 1966 | CC | 350 | n | bl | n | y | 1 | ev | cig+/-ot | mainly f | always p | st |
| WYNDE5 | 1 | x | m | 0 | 0 | all | - | KI | NAmer | 1969 | CC | 1365 | n | bl | n | y | 0 | cu | cig+/-ot | ever f | always p | st |
| WYNDE5 | 4 | x | f | 0 | 0 | all | - | KI | NAmer | 1969 | CC | 1365 | n | bl | n | y | 0 | cu | cig+/-ot | ever f | always p | st |
| WYNDE6 | 312 | x | m | 0 | 0 | all | - | q | NAmer | 1969 | CC | 4423 | n | bl | n | y | 3 | cu | cig+/-ot | ever f | always p | ot |
| WYNDE6 | 318 | x | f | 0 | 0 | all | - | q | NAmer | 1969 | CC | 4423 | n | bl | n | y | 3 | cu | cig+/-ot | ever f | always p | ot |

Table 2F2 - 2

IESLC - Meta-analysis of Cigarette Smoking, ever Filter vs only Plain (or nearest available)
Squamous
Most adjusted

| REF | NRR | SEX | AD | Number Exposed Case | Number Exposed Cont | Non-exposed Case | Non-exposed Cont | RR | 95.00%CI |
|---|---|---|---|---|---|---|---|---|---|
| LUBIN2 | 209 | m | 3 | - | - | - | - | 0.93 | ( 0.86- 1.00) |
| LUBIN2 | 241 | f | 3 | - | - | - | - | 0.22 | ( 0.14- 0.33) |
| Subtotal LUBIN2 | | | | | | | | 0.89 | ( 0.83- 0.96) |
| MATOS | 51 | m | 3 | - | - | - | - | 0.71 | ( 0.27- 1.67) |
| PEZZOT | 12 | m | 4 | - | - | - | - | 0.20 | ( 0.11- 0.37) |
| SOBUE | 85 | m | 5 | - | - | - | - | 0.45 | ( 0.25- 0.83) |
| WAKAI | 68 | m | 5 | - | - | - | - | 0.45 | ( 0.14- 1.52) |
| WYNDE3 | 90 | m | 1 | - | - | - | - | 0.56 | ( 0.34- 0.92) |
| WYNDE5 | 1 | m | 0 | 278 | 629 | 200 | 398 | 0.88 | ( 0.71- 1.10) |
| WYNDE5 | 4 | f | 0 | 84 | 200 | 21 | 30 | 0.60 | ( 0.32- 1.11) |
| Subtotal WYNDE5 | | | | | | | | 0.84 | ( 0.68- 1.04) |
| WYNDE6 | 312 | m | 3 | - | - | - | - | 0.89 | ( 0.68- 1.16) |
| WYNDE6 | 318 | f | 3 | - | - | - | - | 0.55 | ( 0.31- 1.00) |
| Subtotal WYNDE6 | | | | | | | | 0.82 | ( 0.64- 1.04) |
| Partial Totals | | | | 362 | 829 | 221 | 428 | | |

*prospective study

| REF | NRR | SEX | AD | Ys | Ws | Qs | Ps |
|---|---|---|---|---|---|---|---|
| LUBIN2 | 209 | m | 3 | -0.07 | 675.49 | 5.70 | 0.0593 |
| LUBIN2 | 241 | f | 3 | -1.51 | 20.90 | 38.07 | 0.0000 |
| Subtotal LUBIN2 | | | | -0.12 | 696.39 | 43.77 | |
| MATOS | 51 | m | 3 | -0.34 | 4.63 | 0.15 | 0.4613 |
| PEZZOT | 12 | m | 4 | -1.61 | 10.44 | 21.81 | 0.0000 |
| SOBUE | 85 | m | 5 | -0.80 | 10.67 | 4.29 | 0.0091 |
| WAKAI | 68 | m | 5 | -0.80 | 2.70 | 1.09 | 0.1894 |
| WYNDE3 | 90 | m | 1 | -0.58 | 15.51 | 2.68 | 0.0224 |
| WYNDE5 | 1 | m | 0 | -0.13 | 78.74 | 0.10 | 0.2546 |
| WYNDE5 | 4 | f | 0 | -0.51 | 10.22 | 1.23 | 0.1025 |
| Subtotal WYNDE5 | | | | -0.17 | 88.96 | 1.33 | |
| WYNDE6 | 312 | m | 3 | -0.12 | 53.87 | 0.12 | 0.3924 |
| WYNDE6 | 318 | f | 3 | -0.60 | 11.20 | 2.10 | 0.0454 |
| Subtotal WYNDE6 | | | | -0.20 | 65.07 | 2.23 | |

| | | |
|---|---|---|
| | N | 11 |
| | NS | 8 |
| | Wt | 894.38 |
| Het | Chi | 77.33 |
| Het | df | 10 |
| Het | P | *** |
| Fixed | RR | 0.85 |
| | RRl | 0.79 |
| | RRu | 0.91 |
| | P | --- |
| Random | RR | 0.55 |
| | RRl | 0.41 |
| | RRu | 0.74 |
| | P | --- |
| Asymm | P | * |

Table 2F2 - 3

IESLC - Meta-analysis of Cigarette Smoking, ever Filter vs only Plain (or nearest available)
Squamous
Most adjusted

| | Sex combined | male | female | Total |
|---|---|---|---|---|
| N | | 8 | 3 | 11 |
| NS | | 8 | 3 | 11 |
| Wt | | 852.06 | 42.32 | 894.38 |
| Het Chi | | 34.36 | 9.75 | 77.33 |
| Het df | | 7 | 2 | 10 |
| Het P | | *** | ** | *** |
| Fixed RR | | 0.89 | 0.36 | 0.85 |
| RRl | | 0.83 | 0.26 | 0.79 |
| RRu | | 0.95 | 0.48 | 0.91 |
| P | | --- | --- | --- |
| Random RR | | 0.65 | 0.41 | 0.55 |
| RRl | | 0.50 | 0.20 | 0.41 |
| RRu | | 0.85 | 0.81 | 0.74 |
| P | | -- | - | --- |
| Between Chi | | | | 33.23 |
| Between df | | | | 1 |
| Between P | | | | *** |
| Btwn(F) P | | | | * |
| Btwn(R) P | | | | N.S. |

| | All LC (or nearest) q | q+s | q+u | KI | not a | Total |
|---|---|---|---|---|---|---|
| N | 8 | | | 3 | | 11 |
| NS | 6 | | | 2 | | 8 |
| Wt | 789.91 | | | 104.47 | | 894.38 |
| Het Chi | 73.26 | | | 3.52 | | 77.33 |
| Het df | 7 | | | 2 | | 10 |
| Het P | *** | | | N.S. | | *** |
| Fixed RR | 0.86 | | | 0.79 | | 0.85 |
| RRl | 0.80 | | | 0.65 | | 0.79 |
| RRu | 0.92 | | | 0.96 | | 0.91 |
| P | --- | | | - | | --- |
| Random RR | 0.49 | | | 0.72 | | 0.55 |
| RRl | 0.32 | | | 0.53 | | 0.41 |
| RRu | 0.77 | | | 1.00 | | 0.74 |
| P | -- | | | - | | --- |
| Between Chi | | | | | | 0.55 |
| Between df | | | | | | 1 |
| Between P | | | | | | N.S. |
| Btwn(F) P | | | | | | N.S. |
| Btwn(R) P | | | | | | N.S. |

| | Location NAmer | UK | Scand | othEur | China | Japan | othAs | other | Total |
|---|---|---|---|---|---|---|---|---|---|
| N | 5 | | | 2 | | 2 | | 2 | 11 |
| NS | 3 | | | 1 | | 2 | | 2 | 8 |
| Wt | 169.54 | | | 696.39 | | 13.37 | | 15.07 | 894.38 |
| Het Chi | 5.71 | | | 42.13 | | 0.00 | | 5.15 | 77.33 |
| Het df | 4 | | | 1 | | 1 | | 1 | 10 |
| Het P | N.S. | | | *** | | N.S. | | * | *** |
| Fixed RR | 0.80 | | | 0.89 | | 0.45 | | 0.30 | 0.85 |
| RRl | 0.69 | | | 0.83 | | 0.26 | | 0.18 | 0.79 |
| RRu | 0.93 | | | 0.96 | | 0.77 | | 0.49 | 0.91 |
| P | -- | | | -- | | -- | | --- | --- |
| Random RR | 0.77 | | | 0.46 | | 0.45 | | 0.36 | 0.55 |
| RRl | 0.63 | | | 0.11 | | 0.26 | | 0.10 | 0.41 |
| RRu | 0.94 | | | 1.89 | | 0.77 | | 1.24 | 0.74 |
| P | -- | | | N.S. | | -- | | N.S. | --- |
| Between Chi | | | | | | | | | 24.35 |
| Between df | | | | | | | | | 3 |
| Between P | | | | | | | | | *** |
| Btwn(F) P | | | | | | | | | N.S. |
| Btwn(R) P | | | | | | | | | N.S. |

Table 2F2 - 3

IESLC - Meta-analysis of Cigarette Smoking, ever Filter vs only Plain (or nearest available)
Squamous
Most adjusted

Detailed Country in "other Europe"

| | | multi | Germany | othWest | East | Balkans | Total |
|---|---|---|---|---|---|---|---|
| | N | 2 | | | | | 2 |
| | NS | 1 | | | | | 1 |
| | Wt | 696.39 | | | | | 696.39 |
| Het | Chi | 42.13 | | | | | 42.13 |
| Het | df | 1 | | | | | 1 |
| Het | P | *** | | | | | *** |
| Fixed | RR | 0.89 | | | | | 0.89 |
| | RRl | 0.83 | | | | | 0.83 |
| | RRu | 0.96 | | | | | 0.96 |
| | P | -- | | | | | -- |
| Random | RR | 0.46 | | | | | 0.46 |
| | RRl | 0.11 | | | | | 0.11 |
| | RRu | 1.89 | | | | | 1.89 |
| | P | N.S. | | | | | N.S. |
| Between | Chi | | | | | | |
| Between | df | | | | | | |
| Between | P | | | | | | N.S. |
| Btwn(F) | P | | | | | | N.S. |
| Btwn(R) | P | | | | | | N.S. |

Detailed Country in "other Asia"

| | | India | HongKong | other | Total |
|---|---|---|---|---|---|
| | N | | | | |
| | NS | | | | |
| | Wt | | | | |
| Het | Chi | | | | |
| Het | df | | | | |
| Het | P | | | | |
| Fixed | RR | | | | |
| | RRl | | | | |
| | RRu | | | | |
| | P | | | | |
| Random | RR | | | | |
| | RRl | | | | |
| | RRu | | | | |
| | P | | | | |
| Between | Chi | | | | |
| Between | df | | | | |
| Between | P | | | | N.S. |
| Btwn(F) | P | | | | N.S. |
| Btwn(R) | P | | | | N.S. |

Detailed other continent

| | | SCAmer | Auslia | Africa | Total |
|---|---|---|---|---|---|
| | N | 2 | | | 2 |
| | NS | 2 | | | 2 |
| | Wt | 15.07 | | | 15.07 |
| Het | Chi | 5.15 | | | 5.15 |
| Het | df | 1 | | | 1 |
| Het | P | * | | | * |
| Fixed | RR | 0.30 | | | 0.30 |
| | RRl | 0.18 | | | 0.18 |
| | RRu | 0.49 | | | 0.49 |
| | P | --- | | | --- |
| Random | RR | 0.36 | | | 0.36 |
| | RRl | 0.10 | | | 0.10 |
| | RRu | 1.24 | | | 1.24 |
| | P | N.S. | | | N.S. |
| Between | Chi | | | | |
| Between | df | | | | |
| Between | P | | | | N.S. |
| Btwn(F) | P | | | | N.S. |
| Btwn(R) | P | | | | N.S. |

Table 2F2 - 3

IESLC - Meta-analysis of Cigarette Smoking, ever Filter vs only Plain (or nearest available)
Squamous
Most adjusted

| | Start year of study <1960 | 1960-69 | 1970-79 | 1980-89 | 1990+ | Total |
|---|---|---|---|---|---|---|
| N | | 5 | 2 | 3 | 1 | 11 |
| NS | | 3 | 1 | 3 | 1 | 8 |
| Wt | | 169.54 | 696.39 | 23.82 | 4.63 | 894.38 |
| Het Chi | | 5.71 | 42.13 | 3.86 | 0.00 | 77.33 |
| Het df | | 4 | 1 | 2 | 0 | 10 |
| Het P | | N.S. | *** | N.S. | N.S. | *** |
| Fixed RR | | 0.80 | 0.89 | 0.32 | 0.71 | 0.85 |
| RRl | | 0.69 | 0.83 | 0.21 | 0.29 | 0.79 |
| RRu | | 0.93 | 0.96 | 0.47 | 1.77 | 0.91 |
| P | | -- | -- | --- | N.S. | --- |
| Random RR | | 0.77 | 0.46 | 0.32 | 0.71 | 0.55 |
| RRl | | 0.63 | 0.11 | 0.18 | 0.29 | 0.41 |
| RRu | | 0.94 | 1.89 | 0.59 | 1.77 | 0.74 |
| P | | -- | N.S. | --- | N.S. | --- |
| Between Chi | | | | | | 25.64 |
| Between df | | | | | | 3 |
| Between P | | | | | | *** |
| Btwn(F) P | | | | | | N.S. |
| Btwn(R) P | | | | | | (*) |

| | Study type (1) CC | other | Total |
|---|---|---|---|
| N | 11 | | 11 |
| NS | 8 | | 8 |
| Wt | 894.38 | | 894.38 |
| Het Chi | 77.33 | | 77.33 |
| Het df | 10 | | 10 |
| Het P | *** | | *** |
| Fixed RR | 0.85 | | 0.85 |
| RRl | 0.79 | | 0.79 |
| RRu | 0.91 | | 0.91 |
| P | --- | | --- |
| Random RR | 0.55 | | 0.55 |
| RRl | 0.41 | | 0.41 |
| RRu | 0.74 | | 0.74 |
| P | --- | | --- |
| Between Chi | | | |
| Between df | | | |
| Between P | | | N.S. |
| Btwn(F) P | | | N.S. |
| Btwn(R) P | | | N.S. |

| | Study type (2) CC | prosp | other | Total |
|---|---|---|---|---|
| N | 11 | | | 11 |
| NS | 8 | | | 8 |
| Wt | 894.38 | | | 894.38 |
| Het Chi | 77.33 | | | 77.33 |
| Het df | 10 | | | 10 |
| Het P | *** | | | *** |
| Fixed RR | 0.85 | | | 0.85 |
| RRl | 0.79 | | | 0.79 |
| RRu | 0.91 | | | 0.91 |
| P | --- | | | --- |
| Random RR | 0.55 | | | 0.55 |
| RRl | 0.41 | | | 0.41 |
| RRu | 0.74 | | | 0.74 |
| P | --- | | | --- |
| Between Chi | | | | |
| Between df | | | | |
| Between P | | | | N.S. |
| Btwn(F) P | | | | N.S. |
| Btwn(R) P | | | | N.S. |

Table 2F2 - 3

IESLC - Meta-analysis of Cigarette Smoking, ever Filter vs only Plain (or nearest available)
Squamous
Most adjusted

| | Study size (number of LC cases) | | | | |
|---|---|---|---|---|---|
| | 100-249 | 250-499 | 500-999 | 1000+ | Total |
| N | 2 | 2 | | 7 | 11 |
| NS | 2 | 2 | | 4 | 8 |
| Wt | 15.07 | 18.21 | | 861.10 | 894.38 |
| Het Chi | 5.15 | 0.11 | | 50.94 | 77.33 |
| Het df | 1 | 1 | | 6 | 10 |
| Het P | * | N.S. | | *** | *** |
| Fixed RR | 0.30 | 0.54 | | 0.87 | 0.85 |
| RRl | 0.18 | 0.34 | | 0.82 | 0.79 |
| RRu | 0.49 | 0.86 | | 0.93 | 0.91 |
| P | --- | -- | | --- | --- |
| Random RR | 0.36 | 0.54 | | 0.62 | 0.55 |
| RRl | 0.10 | 0.34 | | 0.45 | 0.41 |
| RRu | 1.24 | 0.86 | | 0.85 | 0.74 |
| P | N.S. | -- | | -- | --- |
| Between Chi | | | | | 21.13 |
| Between df | | | | | 2 |
| Between P | | | | | *** |
| Btwn(F) P | | | | | N.S. |
| Btwn(R) P | | | | | N.S. |

| | Risky occupational population | | | |
|---|---|---|---|---|
| | no | mining | othRisky | Total |
| N | 11 | | | 11 |
| NS | 8 | | | 8 |
| Wt | 894.38 | | | 894.38 |
| Het Chi | 77.33 | | | 77.33 |
| Het df | 10 | | | 10 |
| Het P | *** | | | *** |
| Fixed RR | 0.85 | | | 0.85 |
| RRl | 0.79 | | | 0.79 |
| RRu | 0.91 | | | 0.91 |
| P | --- | | | --- |
| Random RR | 0.55 | | | 0.55 |
| RRl | 0.41 | | | 0.41 |
| RRu | 0.74 | | | 0.74 |
| P | --- | | | --- |
| Between Chi | | | | |
| Between df | | | | |
| Between P | | | | N.S. |
| Btwn(F) P | | | | N.S. |
| Btwn(R) P | | | | N.S. |

| | National cigarette tobacco type | | | |
|---|---|---|---|---|
| | Virginia | blended | other | Total |
| N | | 11 | | 11 |
| NS | | 8 | | 8 |
| Wt | | 894.38 | | 894.38 |
| Het Chi | | 77.33 | | 77.33 |
| Het df | | 10 | | 10 |
| Het P | | *** | | *** |
| Fixed RR | | 0.85 | | 0.85 |
| RRl | | 0.79 | | 0.79 |
| RRu | | 0.91 | | 0.91 |
| P | | --- | | --- |
| Random RR | | 0.55 | | 0.55 |
| RRl | | 0.41 | | 0.41 |
| RRu | | 0.74 | | 0.74 |
| P | | --- | | --- |
| Between Chi | | | | |
| Between df | | | | |
| Between P | | | | N.S. |
| Btwn(F) P | | | | N.S. |
| Btwn(R) P | | | | N.S. |

Table 2F2 - 3

IESLC - Meta-analysis of Cigarette Smoking, ever Filter vs only Plain (or nearest available)
Squamous
Most adjusted

| | Any proxy use | | |
|---|---|---|---|
| | No/nk | Yes | Total |
| N | 11 | | 11 |
| NS | 8 | | 8 |
| Wt | 894.38 | | 894.38 |
| Het Chi | 77.33 | | 77.33 |
| Het df | 10 | | 10 |
| Het P | *** | | *** |
| Fixed RR | 0.85 | | 0.85 |
| RRl | 0.79 | | 0.79 |
| RRu | 0.91 | | 0.91 |
| P | --- | | --- |
| Random RR | 0.55 | | 0.55 |
| RRl | 0.41 | | 0.41 |
| RRu | 0.74 | | 0.74 |
| P | --- | | --- |
| Between Chi | | | |
| Between df | | | |
| Between P | | | N.S. |
| Btwn(F) P | | | N.S. |
| Btwn(R) P | | | N.S. |

| | Full histological confirmation | | |
|---|---|---|---|
| | No | Yes | Total |
| N | 1 | 10 | 11 |
| NS | 1 | 7 | 8 |
| Wt | 4.63 | 889.75 | 894.38 |
| Het Chi | 0.00 | 77.19 | 77.33 |
| Het df | 0 | 9 | 10 |
| Het P | N.S. | *** | *** |
| Fixed RR | 0.71 | 0.85 | 0.85 |
| RRl | 0.29 | 0.80 | 0.79 |
| RRu | 1.77 | 0.91 | 0.91 |
| P | N.S. | --- | --- |
| Random RR | 0.71 | 0.54 | 0.55 |
| RRl | 0.29 | 0.40 | 0.41 |
| RRu | 1.77 | 0.74 | 0.74 |
| P | N.S. | --- | --- |
| Between Chi | | | 0.15 |
| Between df | | | 1 |
| Between P | | | N.S. |
| Btwn(F) P | | | N.S. |
| Btwn(R) P | | | N.S. |

| | Number of adjustment variables (1) | | | |
|---|---|---|---|---|
| | 0 | 1 | 2+/+nk | Total |
| N | 2 | 1 | 8 | 11 |
| NS | 1 | 1 | 6 | 8 |
| Wt | 88.96 | 15.51 | 789.91 | 894.38 |
| Het Chi | 1.32 | 0.00 | 73.26 | 77.33 |
| Het df | 1 | 0 | 7 | 10 |
| Het P | N.S. | N.S. | *** | *** |
| Fixed RR | 0.84 | 0.56 | 0.86 | 0.85 |
| RRl | 0.68 | 0.34 | 0.80 | 0.79 |
| RRu | 1.04 | 0.92 | 0.92 | 0.91 |
| P | N.S. | - | --- | --- |
| Random RR | 0.81 | 0.56 | 0.49 | 0.55 |
| RRl | 0.60 | 0.34 | 0.32 | 0.41 |
| RRu | 1.10 | 0.92 | 0.77 | 0.74 |
| P | N.S. | - | -- | --- |
| Between Chi | | | | 2.75 |
| Between df | | | | 2 |
| Between P | | | | N.S. |
| Btwn(F) P | | | | N.S. |
| Btwn(R) P | | | | N.S. |

Table 2F2 - 3

IESLC - Meta-analysis of Cigarette Smoking, ever Filter vs only Plain (or nearest available)
Squamous
Most adjusted

| | Number of adjustment variables (2) 0 | 1 | 2 | 3-5 | 6+/+nk | Total |
|---|---|---|---|---|---|---|
| N | 2 | 1 | | 8 | | 11 |
| NS | 1 | 1 | | 6 | | 8 |
| Wt | 88.96 | 15.51 | | 789.91 | | 894.38 |
| Het Chi | 1.32 | 0.00 | | 73.26 | | 77.33 |
| Het df | 1 | 0 | | 7 | | 10 |
| Het P | N.S. | N.S. | | *** | | *** |
| Fixed RR | 0.84 | 0.56 | | 0.86 | | 0.85 |
| RRl | 0.68 | 0.34 | | 0.80 | | 0.79 |
| RRu | 1.04 | 0.92 | | 0.92 | | 0.91 |
| P | N.S. | - | | --- | | --- |
| Random RR | 0.81 | 0.56 | | 0.49 | | 0.55 |
| RRl | 0.60 | 0.34 | | 0.32 | | 0.41 |
| RRu | 1.10 | 0.92 | | 0.77 | | 0.74 |
| P | N.S. | - | | -- | | --- |
| Between Chi | | | | | | 2.75 |
| Between df | | | | | | 2 |
| Between P | | | | | | N.S. |
| Btwn(F) P | | | | | | N.S. |
| Btwn(R) P | | | | | | N.S. |

| | Smoking status ever | current | Total |
|---|---|---|---|
| N | 5 | 6 | 11 |
| NS | 4 | 4 | 8 |
| Wt | 726.97 | 167.41 | 894.38 |
| Het Chi | 68.22 | 8.15 | 77.33 |
| Het df | 4 | 5 | 10 |
| Het P | *** | N.S. | *** |
| Fixed RR | 0.86 | 0.79 | 0.85 |
| RRl | 0.80 | 0.68 | 0.79 |
| RRu | 0.93 | 0.92 | 0.91 |
| P | --- | -- | --- |
| Random RR | 0.44 | 0.73 | 0.55 |
| RRl | 0.21 | 0.58 | 0.41 |
| RRu | 0.93 | 0.91 | 0.74 |
| P | - | -- | --- |
| Between Chi | | | 0.97 |
| Between df | | | 1 |
| Between P | | | N.S. |
| Btwn(F) P | | | N.S. |
| Btwn(R) P | | | N.S. |

| | Product cig+/-ot | cig only | Total |
|---|---|---|---|
| N | 10 | 1 | 11 |
| NS | 7 | 1 | 8 |
| Wt | 883.93 | 10.44 | 894.38 |
| Het Chi | 55.27 | 0.00 | 77.33 |
| Het df | 9 | 0 | 10 |
| Het P | *** | N.S. | *** |
| Fixed RR | 0.86 | 0.20 | 0.85 |
| RRl | 0.81 | 0.11 | 0.79 |
| RRu | 0.92 | 0.37 | 0.91 |
| P | --- | --- | --- |
| Random RR | 0.61 | 0.20 | 0.55 |
| RRl | 0.46 | 0.11 | 0.41 |
| RRu | 0.80 | 0.37 | 0.74 |
| P | --- | --- | --- |
| Between Chi | | | 22.06 |
| Between df | | | 1 |
| Between P | | | *** |
| Btwn(F) P | | | (*) |
| Btwn(R) P | | | ** |

Table 2F2 - 3

IESLC - Meta-analysis of Cigarette Smoking, ever Filter vs only Plain (or nearest available)
Squamous
Most adjusted

| | Cigarette type | | | | | | |
|---|---|---|---|---|---|---|---|
| | only f | always f | mainly f | equl p&f | both p&f | ever f | Total |
| N | 3 | | 2 | | | 6 | 11 |
| NS | 3 | | 2 | | | 3 | 8 |
| Wt | 23.82 | | 20.14 | | | 850.43 | 894.38 |
| Het Chi | 3.86 | | 0.20 | | | 46.21 | 77.33 |
| Het df | 2 | | 1 | | | 5 | 10 |
| Het P | N.S. | | N.S. | | | *** | *** |
| Fixed RR | 0.32 | | 0.59 | | | 0.88 | 0.85 |
| RRl | 0.21 | | 0.38 | | | 0.82 | 0.79 |
| RRu | 0.47 | | 0.92 | | | 0.94 | 0.91 |
| P | --- | | - | | | --- | --- |
| Random RR | 0.32 | | 0.59 | | | 0.64 | 0.55 |
| RRl | 0.18 | | 0.38 | | | 0.46 | 0.41 |
| RRu | 0.59 | | 0.92 | | | 0.90 | 0.74 |
| P | --- | | - | | | -- | --- |
| Between Chi | | | | | | | 27.07 |
| Between df | | | | | | | 2 |
| Between P | | | | | | | *** |
| Btwn(F) P | | | | | | | N.S. |
| Btwn(R) P | | | | | | | N.S. |

| | Denominator | | | | |
|---|---|---|---|---|---|
| | ever p | mainly p | p NOS | always p | Total |
| N | 1 | 1 | 2 | 7 | 11 |
| NS | 1 | 1 | 2 | 4 | 8 |
| Wt | 10.44 | 4.63 | 13.37 | 865.93 | 894.38 |
| Het Chi | 0.00 | 0.00 | 0.00 | 49.32 | 77.33 |
| Het df | 0 | 0 | 1 | 6 | 10 |
| Het P | N.S. | N.S. | N.S. | *** | *** |
| Fixed RR | 0.20 | 0.71 | 0.45 | 0.87 | 0.85 |
| RRl | 0.11 | 0.29 | 0.26 | 0.82 | 0.79 |
| RRu | 0.37 | 1.77 | 0.77 | 0.93 | 0.91 |
| P | --- | N.S. | -- | --- | --- |
| Random RR | 0.20 | 0.71 | 0.45 | 0.63 | 0.55 |
| RRl | 0.11 | 0.29 | 0.26 | 0.47 | 0.41 |
| RRu | 0.37 | 1.77 | 0.77 | 0.86 | 0.74 |
| P | --- | N.S. | -- | -- | --- |
| Between Chi | | | | | 28.02 |
| Between df | | | | | 3 |
| Between P | | | | | *** |
| Btwn(F) P | | | | | N.S. |
| Btwn(R) P | | | | | ** |

| | Derivation of RR/CI | | | |
|---|---|---|---|---|
| | Orig | StdCalc | Other | Total |
| N | | 6 | 5 | 11 |
| NS | | 5 | 3 | 8 |
| Wt | | 122.47 | 771.91 | 894.38 |
| Het Chi | | 7.32 | 67.45 | 77.33 |
| Het df | | 5 | 4 | 10 |
| Het P | | N.S. | *** | *** |
| Fixed RR | | 0.74 | 0.87 | 0.85 |
| RRl | | 0.62 | 0.81 | 0.79 |
| RRu | | 0.89 | 0.93 | 0.91 |
| P | | --- | --- | --- |
| Random RR | | 0.66 | 0.48 | 0.55 |
| RRl | | 0.51 | 0.27 | 0.41 |
| RRu | | 0.86 | 0.84 | 0.74 |
| P | | -- | -- | --- |
| Between Chi | | | | 2.56 |
| Between df | | | | 1 |
| Between P | | | | N.S. |
| Btwn(F) P | | | | N.S. |
| Btwn(R) P | | | | N.S. |

Table 2F2 - 4

IESLC - Meta-analysis of Cigarette Smoking, ever Filter vs only Plain (or nearest available)
Squamous
Least adjusted

| REF | NRR | X | SEX | AGEL | AGEH | RACE | YF | LC TYPE | LOC | START | ST | NLC | R | VB | P | H | AD | SM | PRODUCT | CIGTYP | DENOM | De |
|---|---|---|---|---|---|---|---|---|---|---|---|---|---|---|---|---|---|---|---|---|---|---|
| LUBIN2 | 193 | x | m | 0 | 0 | all | - | q | Eu:mul | 1976 | CC | 7804 | n | bl | n | y | 0 | ev | cig+/-ot | ever f | always p | st |
| LUBIN2 | 225 | x | f | 0 | 0 | all | - | q | Eu:mul | 1976 | CC | 7804 | n | bl | n | y | 0 | ev | cig+/-ot | ever f | always p | st |
| MATOS | 73 | x | m | 0 | 0 | all | - | q | SCAmer | 1994 | CC | 200 | n | bl | n | n | 0 | ev | cig+/-ot | mainly f | mainly p | st |
| PEZZOT | 11 | x | m | 0 | 0 | all | - | q | SCAmer | 1987 | CC | 215 | n | bl | n | y | 2 | ev | cig only | only f | ever p | ot |
| SOBUE | 73 | x | m | 0 | 0 | all | - | q | As:Jap | 1986 | CC | 1376 | n | bl | n | y | 0 | cu | cig+/-ot | only f | p NOS | st |
| WAKAI | 67 | x | m | 0 | 0 | all | - | q | As:Jap | 1988 | CC | 333 | n | bl | n | y | 0 | cu | cig+/-ot | only f | p NOS | st |
| WYNDE3 | 89 | x | m | 0 | 0 | all | - | KI | NAmer | 1966 | CC | 350 | n | bl | n | y | 0 | ev | cig+/-ot | mainly f | always p | st |
| WYNDE5 | 1 | | m | 0 | 0 | all | - | KI | NAmer | 1969 | CC | 1365 | n | bl | n | y | 0 | cu | cig+/-ot | ever f | always p | st |
| WYNDE5 | 4 | | f | 0 | 0 | all | - | KI | NAmer | 1969 | CC | 1365 | n | bl | n | y | 0 | cu | cig+/-ot | ever f | always p | st |
| WYNDE6 | 300 | x | m | 0 | 0 | all | - | q | NAmer | 1969 | CC | 4423 | n | bl | n | y | 0 | cu | cig+/-ot | ever f | always p | st |
| WYNDE6 | 306 | x | f | 0 | 0 | all | - | q | NAmer | 1969 | CC | 4423 | n | bl | n | y | 0 | cu | cig+/-ot | ever f | always p | st |

Table 2F2 - 5

IESLC - Meta-analysis of Cigarette Smoking, ever Filter vs only Plain (or nearest available)
Squamous
Least adjusted

| REF | NRR | SEX | AD | Number Exposed Case | Number Exposed Cont | Non-exposed Case | Non-exposed Cont | RR | 95.00%CI |
|---|---|---|---|---|---|---|---|---|---|
| LUBIN2 | 193 | m | 0 | 2255 | 6627 | 1340 | 3810 | 0.97 | ( 0.89- 1.05) |
| LUBIN2 | 225 | f | 0 | 168 | 505 | 32 | 62 | 0.64 | ( 0.41- 1.02) |
| Subtotal LUBIN2 | | | | | | | | 0.96 | ( 0.89- 1.03) |
| MATOS | 73 | m | 0 | 38 | 229 | 9 | 46 | 0.85 | ( 0.38- 1.87) |
| PEZZOT | 11 | m | 2 | - | - | - | - | 0.20 | ( 0.11- 0.37) |
| SOBUE | 73 | m | 0 | 220 | 540 | 27 | 26 | 0.39 | ( 0.22- 0.69) |
| WAKAI | 67 | m | 0 | 81 | 271 | 5 | 9 | 0.54 | ( 0.18- 1.65) |
| WYNDE3 | 89 | m | 0 | 66 | 82 | 81 | 62 | 0.62 | ( 0.39- 0.98) |
| WYNDE5 | 1 | m | 0 | 278 | 629 | 200 | 398 | 0.88 | ( 0.71- 1.10) |
| WYNDE5 | 4 | f | 0 | 84 | 200 | 21 | 30 | 0.60 | ( 0.32- 1.11) |
| Subtotal WYNDE5 | | | | | | | | 0.84 | ( 0.68- 1.04) |
| WYNDE6 | 300 | m | 0 | 571 | 711 | 159 | 165 | 0.83 | ( 0.65- 1.06) |
| WYNDE6 | 306 | f | 0 | 248 | 437 | 33 | 30 | 0.52 | ( 0.31- 0.87) |
| Subtotal WYNDE6 | | | | | | | | 0.76 | ( 0.61- 0.95) |
| Partial Totals | | | | 4009 | 10231 | 1907 | 4638 | | |

*prospective study

| REF | NRR | SEX | AD | Ys | Ws | Qs | Ps |
|---|---|---|---|---|---|---|---|
| LUBIN2 | 193 | m | 0 | -0.03 | 623.79 | 4.94 | 0.4092 |
| LUBIN2 | 225 | f | 0 | -0.44 | 18.08 | 1.82 | 0.0618 |
| Subtotal LUBIN2 | | | | -0.04 | 641.87 | 6.76 | |
| MATOS | 73 | m | 0 | -0.16 | 6.11 | 0.01 | 0.6838 |
| PEZZOT | 11 | m | 2 | -1.61 | 10.44 | 23.10 | 0.0000 |
| SOBUE | 73 | m | 0 | -0.94 | 12.21 | 8.08 | 0.0011 |
| WAKAI | 67 | m | 0 | -0.62 | 3.06 | 0.76 | 0.2785 |
| WYNDE3 | 89 | m | 0 | -0.48 | 17.91 | 2.35 | 0.0403 |
| WYNDE5 | 1 | m | 0 | -0.13 | 78.74 | 0.00 | 0.2546 |
| WYNDE5 | 4 | f | 0 | -0.51 | 10.22 | 1.54 | 0.1025 |
| Subtotal WYNDE5 | | | | -0.17 | 88.96 | 1.55 | |
| WYNDE6 | 300 | m | 0 | -0.18 | 64.48 | 0.23 | 0.1433 |
| WYNDE6 | 306 | f | 0 | -0.66 | 14.29 | 4.16 | 0.0123 |
| Subtotal WYNDE6 | | | | -0.27 | 78.78 | 4.40 | |

| | | |
|---|---|---|
| | N | 11 |
| | NS | 8 |
| | Wt | 859.35 |
| Het | Chi | 47.01 |
| Het | df | 10 |
| Het | P | *** |
| Fixed | RR | 0.89 |
| | RRl | 0.83 |
| | RRu | 0.95 |
| | P | --- |
| Random | RR | 0.64 |
| | RRl | 0.50 |
| | RRu | 0.80 |
| | P | --- |
| Asymm | P | ** |

Table 2F2 - 6

IESLC - Meta-analysis of Cigarette Smoking, ever Filter vs only Plain (or nearest available)
Squamous
Least adjusted

| | Sex | | | |
|---|---|---|---|---|
| | combined | male | female | Total |
| N | | 8 | 3 | 11 |
| NS | | 8 | 3 | 11 |
| Wt | | 816.76 | 42.59 | 859.35 |
| Het Chi | | 39.11 | 0.40 | 47.01 |
| Het df | | 7 | 2 | 10 |
| Het P | | *** | N.S. | *** |
| Fixed RR | | 0.90 | 0.59 | 0.89 |
| RRl | | 0.84 | 0.44 | 0.83 |
| RRu | | 0.97 | 0.79 | 0.95 |
| P | | -- | --- | --- |
| Random RR | | 0.65 | 0.59 | 0.64 |
| RRl | | 0.50 | 0.44 | 0.50 |
| RRu | | 0.86 | 0.79 | 0.80 |
| P | | -- | --- | --- |
| Between Chi | | | | 7.50 |
| Between df | | | | 1 |
| Between P | | | | ** |
| Btwn(F) P | | | | N.S. |
| Btwn(R) P | | | | N.S. |

Table 2F2 - 7

IESLC - Meta-analysis of Cigarette Smoking, ever Filter vs only Plain (or nearest available)
Squamous
Excluded studies (and stage at which they were excluded)

| | |
|---|---|
| 1 | BOUCHA BUELL LAURIL MZILEN |
| 2 | BERRIN CHEN RESTRE TAO ULMER |
| 3 | ABELIN ABRAHA AKIBA AMANDU AMES ANDERS ARCHER AUSTIN AUVINE AXELSO AXELSS BAND BARBON BENSHL BEST BLOHMK BLOT1 BLOT2 BLOT3 BLOT4 BOFFET BOUCOT BRESLO BRETT BROCKM BROWN1 BROWN2 BYERS1 BYERS2 CARPEN CASCO2 CASCOR CEDERL CHAN CHANG CHATZI CHEN2 CHEN3 CHIAZZ CHOW CHYOU COMSTO COOKSO CPSI DAMBER DARBY DAVEYS DEAN DEKLER DOCKER DOLL2 DORANT DORGAN DORN DOSEME DROSTE DU DUNN EBELIN ENSTRO ESAKI FAN GAO GAO2 GARCIA GARDIN GARSHI GENG GER GILLIS GODLEY GOLLED GOODMA GRAHAM GREGOR GSELL HAENSZ HAMMO2 HAMMON HANSEN HEGMAN HEIN HENNEK HINDS HIRAOK HIRAY2 HIRAYA HITOSU HOLE HOROWI HORWIT HU HU2 HUANG HUMBLE ISHIMA JAHN JAIN JARUP JARVHO JEDRYC JIANG JOLY JONES JUSSAW KAISER KANELL KATSOU KAUFMA KELLER KIHARA KINLEN KJUUS KNEKT KO KOHLME KOO KOULUM KREUZE KREYBE KUBIK LAMTH LAMWK LAMWK2 LAUSSM LEI LEMARC LETOUR LEVIN LIAW LICKIN LIDDEL LIU LIU2 LIU3 LIU4 LIU5 LOMBA2 LOMBAR LUBIN LUO MAGNUS MARSH MARSH2 MARTIS MASTRA MATSUD MCCONN MCDUFF MCLAUG MILLER MILLS MOLLO MRFIT MURATA NAM NOTAN2 NOTANI NOU ODRISC ORMOS OSANN OSANN2 PARKIN PASTOR PAWLEG PERNU PERSH2 PERSHA PETO PEZZO2 PIKE PISANI POFFIJ POLEDN PRESCO QIAO QIAO2 RACHTA RADZIK RANDIG REN RONCO ROOTS ROTHSC SAARIK SADOWS SANKAR SCHWA2 SCHWAR SEGI SEOW SHAW SHIMIZ SIEMIA SIMARA SIMONA SITAS SOBUE2 SPEIZE SPITZ STASZE STAYNE STOCKS STOCKW STUCKE SUN SUZUK2 SUZUKI SVENSS TANG TENKAN TIZZAN TOKARS TOUSEY TSUGAN TULINI TVERDA VANDER VEIERO VUTUC WALD WANG WANG2 WANG3 WANG4 WARSIN WATSON WICKLU WIGLE WILKIN WU WU2 WUNSCH WUWILL WYNDE2 WYNDE4 WYNDE7 WYNDE8 WYNDER XIANGZ XU XU2 XU3 XU4 YAMAGU YONG YUAN ZHANG ZHENG ZHOU |
| 7 | AGUDO ALDERS ARMADA BECHER BROSS BUFFLE CHOI CORREA CPSII DEAN2 DEAN3 DESTE2 DESTEF DOLL ENGELA KAISE2 KHUDER LANGE MACLEN MIGRAN MRFITR RIMING SEGI2 TANG2 |
| 10 | BENHAM |

Table 2F2 - 8

Potentially overlapping studies

| REF | REFGP | PRINC | . | OVERLAP/LINK |
|---|---|---|---|---|
| LUBIN2 | LUBIN2 | 1 | | Lubin-combined |
| WYNDE5 | WYNDE6 | 2 | | WYNDE5/6/7/8 |
| WYNDE6 | WYNDE6 | 1 | | WYNDE5/6/7/8 |

Table 2F3 -

IESLC - Meta-analysis of Cigarette Smoking, only Filter vs ever Plain (or nearest available)
Squamous

This analysis is restricted to results for:
1) Non-dose-response data
2) Results complete enough for use in metaanalysis

Within each study, results are then selected (in the following order of preference, within each sex) for:
3) CIGTYP: filter only/NOS, always, mainly, both, equally, ever
4) DENOM: plain ever, mainly, always, only/NOS
5) PRODUCT: cigarettes regardless of other products, cigarettes only (Note only study ALDERS has both product definitions available)
6) SMKSTA: ever, current (Note only study MATOS has both ever and current available)
7) LCtype: all or nearest available, at least Squamous and Adeno. (q = squamous, s = small, l = large, a = adeno, mix = mixed, alv = alveolar)
8) Race: all or nearest available, otherwise by race (wh or w = white, bl or b = black, hi = hispanic ch = chinese, jap = japanese, haw = hawaiian, w+o = white + oriental, sca = scandinavian, as = asian)
9) Followup period (YF, prospective studies): whole study (coded as 0) or longest available
10) For overlapping studies: principal rather than subsidiary studies
Finally by Age: whole study (coded as 0) if available, otherwise by widest available age group
and then for single sex results (m, f) in preference to combined sex results (c).

Results adjusted (AD) for the most potential confounders are then chosen in Sections -1 to -3
(and those which actually differ from the adjusted results in Table 2F1 - 1 are marked 'x' in Section -1)
and results adjusted for the least confounders in Sections -4 to -6. (Those least adjusted results which
actually differ from the most adjusted as marked 'x' in column X in Section -4)
(Results adjusted for an unknown number of confounder(s) are coded as 20.)

Section -7 shows excluded studies, together with the stage (as above) at which no qualifying
results were found.

Section -8 lists the potentially overlapping studies which have been included (1=principal, 2=subsidiary).

Section -9 lists any results which would have been included in preference except that they had data not complete
enough for use in meta-analysis, with their significance (yes/no), if known, and any further comment as entered
on the database.

In addition to those mentioned above, the following fields, levels and abbreviations are used:
* or nk = not known, n = no, y = yes, ot = other
ev = ever, cu = current, cig+/-ot = cigarettes irrespective of other products (cigar, pipe etc)
f = filter, p = plain, NOS = not otherwise specified
REF: 6-character study reference
NRR: number of the RR on the database within the study
ST : study type (CC = case control, pr or prosp = prospective)
NLC: number of lung cancer cases in whole study
R  : risky occupational population (n = no, m = mining, o = other risky)
VB : national cigarette type (V = at least 75% Virginia, bl = at least 75% blended, ot = other)
P  : any proxy use
H  : full histological confirmation
De : derivation of RR/CI (or = original, st = standard method, ot = other method of estimation)

Table 2F3 - 1

IESLC - Meta-analysis of Cigarette Smoking, only Filter vs ever Plain (or nearest available)
Squamous
Most adjusted

| REF | NRR | 2F1 | SEX | AGEL | AGEH | RACE | YF | LC TYPE | LOC | START | ST | NLC | R | VB | P | H | AD | SM | PRODUCT | CIGTYP | DENOM | De |
|---|---|---|---|---|---|---|---|---|---|---|---|---|---|---|---|---|---|---|---|---|---|---|
| LUBIN2 | 213 | x | m | 0 | 0 | all | - | q | Eu:mul | 1976 | CC | 7804 | n | bl | n | y | 3 | ev | cig+/-ot | only f | ever p | ot |
| LUBIN2 | 245 | x | f | 0 | 0 | all | - | q | Eu:mul | 1976 | CC | 7804 | n | bl | n | y | 3 | ev | cig+/-ot | only f | ever p | ot |
| MATOS | 51 | | m | 0 | 0 | all | - | q | SCAmer | 1994 | CC | 200 | n | bl | n | n | 3 | ev | cig+/-ot | mainly f | mainly p | st |
| PEZZOT | 12 | | m | 0 | 0 | all | - | q | SCAmer | 1987 | CC | 215 | n | bl | n | y | 4 | ev | cig only | only f | ever p | ot |
| SOBUE | 85 | | m | 0 | 0 | all | - | q | As:Jap | 1986 | CC | 1376 | n | bl | n | y | 5 | cu | cig+/-ot | only f | p NOS | st |
| WAKAI | 68 | | m | 0 | 0 | all | - | q | As:Jap | 1988 | CC | 333 | n | bl | n | y | 5 | cu | cig+/-ot | only f | p NOS | st |
| WYNDE3 | 90 | | m | 0 | 0 | all | - | KI | NAmer | 1966 | CC | 350 | n | bl | n | y | 1 | ev | cig+/-ot | mainly f | always p | st |
| WYNDE5 | 15 | | m | 0 | 0 | wh | - | KI | NAmer | 1969 | CC | 1365 | n | bl | n | y | 2 | cu | cig+/-ot | mainly f | always p | st |
| WYNDE5 | 14 | | m | 0 | 0 | wh | - | KI | NAmer | 1969 | CC | 1365 | n | bl | n | y | 2 | cu | cig+/-ot | mainly f | always p | st |
| WYNDE5 | 23 | | f | 0 | 0 | wh | - | KI | NAmer | 1969 | CC | 1365 | n | bl | n | y | 2 | cu | cig+/-ot | mainly f | always p | st |
| WYNDE5 | 22 | | f | 0 | 0 | wh | - | KI | NAmer | 1969 | CC | 1365 | n | bl | n | y | 2 | cu | cig+/-ot | mainly f | always p | st |
| WYNDE6 | 311 | x | m | 0 | 0 | all | - | q | NAmer | 1969 | CC | 4423 | n | bl | n | y | 3 | cu | cig+/-ot | only f | ever p | ot |
| WYNDE6 | 317 | x | f | 0 | 0 | all | - | q | NAmer | 1969 | CC | 4423 | n | bl | n | y | 3 | cu | cig+/-ot | only f | ever p | ot |

Table 2F3 - 2

IESLC - Meta-analysis of Cigarette Smoking, only Filter vs ever Plain (or nearest available)
Squamous
Most adjusted

| REF | NRR | SEX | AD | Number Exposed Case | Number Exposed Cont | Non-exposed Case | Non-exposed Cont | RR | 95.00%CI |
|---|---|---|---|---|---|---|---|---|---|
| LUBIN2 | 213 | m | 3 | - | - | - | - | 0.93 | ( 0.86- 1.00) |
| LUBIN2 | 245 | f | 3 | - | - | - | - | 0.82 | ( 0.57- 1.17) |
| Subtotal LUBIN2 | | | | | | | | 0.93 | ( 0.86- 1.00) |
| MATOS | 51 | m | 3 | - | - | - | - | 0.71 | ( 0.27- 1.67) |
| PEZZOT | 12 | m | 4 | - | - | - | - | 0.20 | ( 0.11- 0.37) |
| SOBUE | 85 | m | 5 | - | - | - | - | 0.45 | ( 0.25- 0.83) |
| WAKAI | 68 | m | 5 | - | - | - | - | 0.45 | ( 0.14- 1.52) |
| WYNDE3 | 90 | m | 1 | - | - | - | - | 0.56 | ( 0.34- 0.92) |
| WYNDE5 | 14 | m | 2 | - | - | - | - | 0.84 | ( 0.65- 1.09) |
| WYNDE5 | 15 | m | 2 | - | - | - | - | 0.79 | ( 0.61- 1.03) |
| WYNDE5 | 22 | f | 2 | - | - | - | - | 0.78 | ( 0.40- 1.49) |
| WYNDE5 | 23 | f | 2 | - | - | - | - | 0.73 | ( 0.38- 1.39) |
| Subtotal WYNDE5 | | | | | | | | 0.81 | ( 0.68- 0.96) |
| WYNDE6 | 311 | m | 3 | - | - | - | - | 0.87 | ( 0.59- 1.27) |
| WYNDE6 | 317 | f | 3 | - | - | - | - | 0.63 | ( 0.41- 0.98) |
| Subtotal WYNDE6 | | | | | | | | 0.76 | ( 0.57- 1.01) |
| Partial Totals | | | | 0 | 0 | 0 | 0 | | |

*prospective study

| REF | NRR | SEX | AD | Ys | Ws | Qs | Ps |
|---|---|---|---|---|---|---|---|
| LUBIN2 | 213 | m | 3 | -0.07 | 675.49 | 3.53 | 0.0593 |
| LUBIN2 | 245 | f | 3 | -0.20 | 29.71 | 0.09 | 0.2794 |
| Subtotal LUBIN2 | | | | -0.08 | 705.21 | 3.61 | |
| MATOS | 51 | m | 3 | -0.34 | 4.63 | 0.18 | 0.4613 |
| PEZZOT | 12 | m | 4 | -1.61 | 10.44 | 22.40 | 0.0000 |
| SOBUE | 85 | m | 5 | -0.80 | 10.67 | 4.56 | 0.0091 |
| WAKAI | 68 | m | 5 | -0.80 | 2.70 | 1.15 | 0.1894 |
| WYNDE3 | 90 | m | 1 | -0.58 | 15.51 | 2.93 | 0.0224 |
| WYNDE5 | 14 | m | 2 | -0.17 | 57.50 | 0.05 | 0.1861 |
| WYNDE5 | 15 | m | 2 | -0.24 | 55.99 | 0.46 | 0.0778 |
| WYNDE5 | 22 | f | 2 | -0.25 | 8.89 | 0.10 | 0.4589 |
| WYNDE5 | 23 | f | 2 | -0.31 | 9.14 | 0.26 | 0.3415 |
| Subtotal WYNDE5 | | | | -0.22 | 131.51 | 0.87 | |
| WYNDE6 | 311 | m | 3 | -0.14 | 26.14 | 0.00 | 0.4764 |
| WYNDE6 | 317 | f | 3 | -0.46 | 20.24 | 2.04 | 0.0377 |
| Subtotal WYNDE6 | | | | -0.28 | 46.38 | 2.04 | |

| | | |
|---|---|---|
| | N | 13 |
| | NS | 8 |
| | Wt | 927.05 |
| Het | Chi | 37.75 |
| Het | df | 12 |
| Het | P | *** |
| Fixed | RR | 0.87 |
| | RRl | 0.81 |
| | RRu | 0.92 |
| | P | --- |
| Random | RR | 0.69 |
| | RRl | 0.57 |
| | RRu | 0.83 |
| | P | --- |
| Asymm | P | ** |

Table 2F3 - 3

IESLC - Meta-analysis of Cigarette Smoking, only Filter vs ever Plain (or nearest available)
Squamous
Most adjusted

| | combined | Sex<br>male | female | Total |
|---|---|---|---|---|
| N | | 9 | 4 | 13 |
| NS | | 8 | 3 | 11 |
| Wt | | 859.08 | 67.97 | 927.05 |
| Het Chi | | 35.14 | 0.86 | 37.75 |
| Het df | | 8 | 3 | 12 |
| Het P | | *** | N.S. | *** |
| Fixed RR | | 0.88 | 0.74 | 0.87 |
| RRl | | 0.82 | 0.58 | 0.81 |
| RRu | | 0.94 | 0.94 | 0.92 |
| P | | --- | - | --- |
| Random RR | | 0.66 | 0.74 | 0.69 |
| RRl | | 0.51 | 0.58 | 0.57 |
| RRu | | 0.84 | 0.94 | 0.83 |
| P | | --- | - | --- |
| Between Chi | | | | 1.75 |
| Between df | | | | 1 |
| Between P | | | | N.S. |
| Btwn(F) P | | | | N.S. |
| Btwn(R) P | | | | N.S. |

| | q | All LC (or nearest)<br>q+s | q+u | KI | not a | Total |
|---|---|---|---|---|---|---|
| N | 8 | | | 5 | | 13 |
| NS | 6 | | | 2 | | 8 |
| Wt | 780.03 | | | 147.02 | | 927.05 |
| Het Chi | 33.62 | | | 2.06 | | 37.75 |
| Het df | 7 | | | 4 | | 12 |
| Het P | *** | | | N.S. | | *** |
| Fixed RR | 0.88 | | | 0.78 | | 0.87 |
| RRl | 0.82 | | | 0.66 | | 0.81 |
| RRu | 0.95 | | | 0.91 | | 0.92 |
| P | --- | | | -- | | --- |
| Random RR | 0.62 | | | 0.78 | | 0.69 |
| RRl | 0.45 | | | 0.66 | | 0.57 |
| RRu | 0.86 | | | 0.91 | | 0.83 |
| P | -- | | | -- | | --- |
| Between Chi | | | | | | 2.07 |
| Between df | | | | | | 1 |
| Between P | | | | | | N.S. |
| Btwn(F) P | | | | | | N.S. |
| Btwn(R) P | | | | | | N.S. |

| | NAmer | UK | Scand | Location<br>othEur | China | Japan | othAs | other | Total |
|---|---|---|---|---|---|---|---|---|---|
| N | 7 | | | 2 | | 2 | | 2 | 13 |
| NS | 3 | | | 1 | | 2 | | 2 | 8 |
| Wt | 193.40 | | | 705.21 | | 13.37 | | 15.07 | 927.05 |
| Het Chi | 3.28 | | | 0.45 | | 0.00 | | 5.15 | 37.75 |
| Het df | 6 | | | 1 | | 1 | | 1 | 12 |
| Het P | N.S. | | | N.S. | | N.S. | | * | *** |
| Fixed RR | 0.77 | | | 0.93 | | 0.45 | | 0.30 | 0.87 |
| RRl | 0.67 | | | 0.86 | | 0.26 | | 0.18 | 0.81 |
| RRu | 0.89 | | | 1.00 | | 0.77 | | 0.49 | 0.92 |
| P | --- | | | - | | -- | | --- | --- |
| Random RR | 0.77 | | | 0.93 | | 0.45 | | 0.36 | 0.69 |
| RRl | 0.67 | | | 0.86 | | 0.26 | | 0.10 | 0.57 |
| RRu | 0.89 | | | 1.00 | | 0.77 | | 1.24 | 0.83 |
| P | --- | | | - | | -- | | N.S. | --- |
| Between Chi | | | | | | | | | 28.88 |
| Between df | | | | | | | | | 3 |
| Between P | | | | | | | | | *** |
| Btwn(F) P | | | | | | | | | ** |
| Btwn(R) P | | | | | | | | | ** |

Table 2F3 - 3

IESLC - Meta-analysis of Cigarette Smoking, only Filter vs ever Plain (or nearest available)
Squamous
Most adjusted

| | Detailed Country in "other Europe" multi | Germany | othWest | East | Balkans | Total |
|---|---|---|---|---|---|---|
| N | 2 | | | | | 2 |
| NS | 1 | | | | | 1 |
| Wt | 705.21 | | | | | 705.21 |
| Het Chi | 0.45 | | | | | 0.45 |
| Het df | 1 | | | | | 1 |
| Het P | N.S. | | | | | N.S. |
| Fixed RR | 0.93 | | | | | 0.93 |
| RRl | 0.86 | | | | | 0.86 |
| RRu | 1.00 | | | | | 1.00 |
| P | - | | | | | - |
| Random RR | 0.93 | | | | | 0.93 |
| RRl | 0.86 | | | | | 0.86 |
| RRu | 1.00 | | | | | 1.00 |
| P | - | | | | | - |
| Between Chi | | | | | | |
| Between df | | | | | | |
| Between P | | | | | | N.S. |
| Btwn(F) P | | | | | | N.S. |
| Btwn(R) P | | | | | | N.S. |

| | Detailed Country in "other Asia" India | HongKong | other | Total |
|---|---|---|---|---|
| N | | | | |
| NS | | | | |
| Wt | | | | |
| Het Chi | | | | |
| Het df | | | | |
| Het P | | | | |
| Fixed RR | | | | |
| RRl | | | | |
| RRu | | | | |
| P | | | | |
| Random RR | | | | |
| RRl | | | | |
| RRu | | | | |
| P | | | | |
| Between Chi | | | | |
| Between df | | | | |
| Between P | | | | N.S. |
| Btwn(F) P | | | | N.S. |
| Btwn(R) P | | | | N.S. |

| | Detailed other continent SCAmer | Auslia | Africa | Total |
|---|---|---|---|---|
| N | 2 | | | 2 |
| NS | 2 | | | 2 |
| Wt | 15.07 | | | 15.07 |
| Het Chi | 5.15 | | | 5.15 |
| Het df | 1 | | | 1 |
| Het P | * | | | * |
| Fixed RR | 0.30 | | | 0.30 |
| RRl | 0.18 | | | 0.18 |
| RRu | 0.49 | | | 0.49 |
| P | --- | | | --- |
| Random RR | 0.36 | | | 0.36 |
| RRl | 0.10 | | | 0.10 |
| RRu | 1.24 | | | 1.24 |
| P | N.S. | | | N.S. |
| Between Chi | | | | |
| Between df | | | | |
| Between P | | | | N.S. |
| Btwn(F) P | | | | N.S. |
| Btwn(R) P | | | | N.S. |

Table 2F3 - 3

IESLC - Meta-analysis of Cigarette Smoking, only Filter vs ever Plain (or nearest available)
Squamous
Most adjusted

| | Start year of study | | | | | |
|---|---|---|---|---|---|---|
| | <1960 | 1960-69 | 1970-79 | 1980-89 | 1990+ | Total |
| N | | 7 | 2 | 3 | 1 | 13 |
| NS | | 3 | 1 | 3 | 1 | 8 |
| Wt | | 193.40 | 705.21 | 23.82 | 4.63 | 927.05 |
| Het Chi | | 3.28 | 0.45 | 3.86 | 0.00 | 37.75 |
| Het df | | 6 | 1 | 2 | 0 | 12 |
| Het P | | N.S. | N.S. | N.S. | N.S. | *** |
| Fixed RR | | 0.77 | 0.93 | 0.32 | 0.71 | 0.87 |
| RRl | | 0.67 | 0.86 | 0.21 | 0.29 | 0.81 |
| RRu | | 0.89 | 1.00 | 0.47 | 1.77 | 0.92 |
| P | | --- | - | --- | N.S. | --- |
| Random RR | | 0.77 | 0.93 | 0.32 | 0.71 | 0.69 |
| RRl | | 0.67 | 0.86 | 0.18 | 0.29 | 0.57 |
| RRu | | 0.89 | 1.00 | 0.59 | 1.77 | 0.83 |
| P | | --- | - | --- | N.S. | --- |
| Between Chi | | | | | | 30.17 |
| Between df | | | | | | 3 |
| Between P | | | | | | *** |
| Btwn(F) P | | | | | | ** |
| Btwn(R) P | | | | | | ** |

| | Study type (1) | | |
|---|---|---|---|
| | CC | other | Total |
| N | 13 | | 13 |
| NS | 8 | | 8 |
| Wt | 927.05 | | 927.05 |
| Het Chi | 37.75 | | 37.75 |
| Het df | 12 | | 12 |
| Het P | *** | | *** |
| Fixed RR | 0.87 | | 0.87 |
| RRl | 0.81 | | 0.81 |
| RRu | 0.92 | | 0.92 |
| P | --- | | --- |
| Random RR | 0.69 | | 0.69 |
| RRl | 0.57 | | 0.57 |
| RRu | 0.83 | | 0.83 |
| P | --- | | --- |
| Between Chi | | | |
| Between df | | | |
| Between P | | | N.S. |
| Btwn(F) P | | | N.S. |
| Btwn(R) P | | | N.S. |

| | Study type (2) | | | |
|---|---|---|---|---|
| | CC | prosp | other | Total |
| N | 13 | | | 13 |
| NS | 8 | | | 8 |
| Wt | 927.05 | | | 927.05 |
| Het Chi | 37.75 | | | 37.75 |
| Het df | 12 | | | 12 |
| Het P | *** | | | *** |
| Fixed RR | 0.87 | | | 0.87 |
| RRl | 0.81 | | | 0.81 |
| RRu | 0.92 | | | 0.92 |
| P | --- | | | --- |
| Random RR | 0.69 | | | 0.69 |
| RRl | 0.57 | | | 0.57 |
| RRu | 0.83 | | | 0.83 |
| P | --- | | | --- |
| Between Chi | | | | |
| Between df | | | | |
| Between P | | | | N.S. |
| Btwn(F) P | | | | N.S. |
| Btwn(R) P | | | | N.S. |

Table 2F3 - 3

IESLC - Meta-analysis of Cigarette Smoking, only Filter vs ever Plain (or nearest available)

Squamous

Most adjusted

| | Study size (number of LC cases) | | | | |
|---|---|---|---|---|---|
| | 100-249 | 250-499 | 500-999 | 1000+ | Total |
| N | 2 | 2 | | 9 | 13 |
| NS | 2 | 2 | | 4 | 8 |
| Wt | 15.07 | 18.21 | | 893.77 | 927.05 |
| Het Chi | 5.15 | 0.11 | | 10.40 | 37.75 |
| Het df | 1 | 1 | | 8 | 12 |
| Het P | * | N.S. | | N.S. | *** |
| Fixed RR | 0.30 | 0.54 | | 0.89 | 0.87 |
| RRl | 0.18 | 0.34 | | 0.83 | 0.81 |
| RRu | 0.49 | 0.86 | | 0.95 | 0.92 |
| P | --- | -- | | --- | --- |
| Random RR | 0.36 | 0.54 | | 0.83 | 0.69 |
| RRl | 0.10 | 0.34 | | 0.74 | 0.57 |
| RRu | 1.24 | 0.86 | | 0.93 | 0.83 |
| P | N.S. | -- | | -- | --- |
| Between Chi | | | | | 22.10 |
| Between df | | | | | 2 |
| Between P | | | | | *** |
| Btwn(F) P | | | | | * |
| Btwn(R) P | | | | | (*) |

| | Risky occupational population | | | |
|---|---|---|---|---|
| | no | mining | othRisky | Total |
| N | 13 | | | 13 |
| NS | 8 | | | 8 |
| Wt | 927.05 | | | 927.05 |
| Het Chi | 37.75 | | | 37.75 |
| Het df | 12 | | | 12 |
| Het P | *** | | | *** |
| Fixed RR | 0.87 | | | 0.87 |
| RRl | 0.81 | | | 0.81 |
| RRu | 0.92 | | | 0.92 |
| P | --- | | | --- |
| Random RR | 0.69 | | | 0.69 |
| RRl | 0.57 | | | 0.57 |
| RRu | 0.83 | | | 0.83 |
| P | --- | | | --- |
| Between Chi | | | | |
| Between df | | | | |
| Between P | | | | N.S. |
| Btwn(F) P | | | | N.S. |
| Btwn(R) P | | | | N.S. |

| | National cigarette tobacco type | | | |
|---|---|---|---|---|
| | Virginia | blended | other | Total |
| N | | 13 | | 13 |
| NS | | 8 | | 8 |
| Wt | | 927.05 | | 927.05 |
| Het Chi | | 37.75 | | 37.75 |
| Het df | | 12 | | 12 |
| Het P | | *** | | *** |
| Fixed RR | | 0.87 | | 0.87 |
| RRl | | 0.81 | | 0.81 |
| RRu | | 0.92 | | 0.92 |
| P | | --- | | --- |
| Random RR | | 0.69 | | 0.69 |
| RRl | | 0.57 | | 0.57 |
| RRu | | 0.83 | | 0.83 |
| P | | --- | | --- |
| Between Chi | | | | |
| Between df | | | | |
| Between P | | | | N.S. |
| Btwn(F) P | | | | N.S. |
| Btwn(R) P | | | | N.S. |

Table 2F3 - 3

IESLC - Meta-analysis of Cigarette Smoking, only Filter vs ever Plain (or nearest available)
Squamous
Most adjusted

| | Any proxy use No/nk | Yes | Total |
|---|---|---|---|
| N | 13 | | 13 |
| NS | 8 | | 8 |
| Wt | 927.05 | | 927.05 |
| Het Chi | 37.75 | | 37.75 |
| Het df | 12 | | 12 |
| Het P | *** | | *** |
| Fixed RR | 0.87 | | 0.87 |
| RRl | 0.81 | | 0.81 |
| RRu | 0.92 | | 0.92 |
| P | --- | | --- |
| Random RR | 0.69 | | 0.69 |
| RRl | 0.57 | | 0.57 |
| RRu | 0.83 | | 0.83 |
| P | --- | | --- |
| Between Chi | | | |
| Between df | | | |
| Between P | | | N.S. |
| Btwn(F) P | | | N.S. |
| Btwn(R) P | | | N.S. |

| | Full histological confirmation No | Yes | Total |
|---|---|---|---|
| N | 1 | 12 | 13 |
| NS | 1 | 7 | 8 |
| Wt | 4.63 | 922.42 | 927.05 |
| Het Chi | 0.00 | 37.57 | 37.75 |
| Het df | 0 | 11 | 12 |
| Het P | N.S. | *** | *** |
| Fixed RR | 0.71 | 0.87 | 0.87 |
| RRl | 0.29 | 0.81 | 0.81 |
| RRu | 1.77 | 0.92 | 0.92 |
| P | N.S. | --- | --- |
| Random RR | 0.71 | 0.69 | 0.69 |
| RRl | 0.29 | 0.57 | 0.57 |
| RRu | 1.77 | 0.84 | 0.83 |
| P | N.S. | --- | --- |
| Between Chi | | | 0.18 |
| Between df | | | 1 |
| Between P | | | N.S. |
| Btwn(F) P | | | N.S. |
| Btwn(R) P | | | N.S. |

| | Number of adjustment variables (1) 0 | 1 | 2+/+nk | Total |
|---|---|---|---|---|
| N | | 1 | 12 | 13 |
| NS | | 1 | 7 | 8 |
| Wt | | 15.51 | 911.54 | 927.05 |
| Het Chi | | 0.00 | 34.77 | 37.75 |
| Het df | | 0 | 11 | 12 |
| Het P | | N.S. | *** | *** |
| Fixed RR | | 0.56 | 0.87 | 0.87 |
| RRl | | 0.34 | 0.82 | 0.81 |
| RRu | | 0.92 | 0.93 | 0.92 |
| P | | - | --- | --- |
| Random RR | | 0.56 | 0.70 | 0.69 |
| RRl | | 0.34 | 0.58 | 0.57 |
| RRu | | 0.92 | 0.85 | 0.83 |
| P | | - | --- | --- |
| Between Chi | | | | 2.98 |
| Between df | | | | 1 |
| Between P | | | | (*) |
| Btwn(F) P | | | | N.S. |
| Btwn(R) P | | | | N.S. |

Table 2F3 - 3

IESLC - Meta-analysis of Cigarette Smoking, only Filter vs ever Plain (or nearest available)
Squamous
Most adjusted

| | Number of adjustment variables (2) 0 | 1 | 2 | 3-5 | 6+/+nk | Total |
|---|---|---|---|---|---|---|
| N | | 1 | 4 | 8 | | 13 |
| NS | | 1 | 1 | 6 | | 8 |
| Wt | | 15.51 | 131.51 | 780.03 | | 927.05 |
| Het Chi | | 0.00 | 0.22 | 33.62 | | 37.75 |
| Het df | | 0 | 3 | 7 | | 12 |
| Het P | | N.S. | N.S. | *** | | *** |
| Fixed RR | | 0.56 | 0.81 | 0.88 | | 0.87 |
| RRl | | 0.34 | 0.68 | 0.82 | | 0.81 |
| RRu | | 0.92 | 0.96 | 0.95 | | 0.92 |
| P | | - | - | --- | | --- |
| Random RR | | 0.56 | 0.81 | 0.62 | | 0.69 |
| RRl | | 0.34 | 0.68 | 0.45 | | 0.57 |
| RRu | | 0.92 | 0.96 | 0.86 | | 0.83 |
| P | | - | - | -- | | --- |
| Between Chi | | | | | | 3.91 |
| Between df | | | | | | 2 |
| Between P | | | | | | N.S. |
| Btwn(F) P | | | | | | N.S. |
| Btwn(R) P | | | | | | N.S. |

| | Smoking status ever | current | Total |
|---|---|---|---|
| N | 5 | 8 | 13 |
| NS | 4 | 4 | 8 |
| Wt | 735.78 | 191.26 | 927.05 |
| Het Chi | 28.33 | 5.54 | 37.75 |
| Het df | 4 | 7 | 12 |
| Het P | *** | N.S. | *** |
| Fixed RR | 0.89 | 0.76 | 0.87 |
| RRl | 0.83 | 0.66 | 0.81 |
| RRu | 0.96 | 0.88 | 0.92 |
| P | -- | --- | --- |
| Random RR | 0.60 | 0.76 | 0.69 |
| RRl | 0.38 | 0.66 | 0.57 |
| RRu | 0.95 | 0.88 | 0.83 |
| P | - | --- | --- |
| Between Chi | | | 3.88 |
| Between df | | | 1 |
| Between P | | | * |
| Btwn(F) P | | | N.S. |
| Btwn(R) P | | | N.S. |

| | Product cig+/-ot | cig only | Total |
|---|---|---|---|
| N | 12 | 1 | 13 |
| NS | 7 | 1 | 8 |
| Wt | 916.60 | 10.44 | 927.05 |
| Het Chi | 15.10 | 0.00 | 37.75 |
| Het df | 11 | 0 | 12 |
| Het P | N.S. | N.S. | *** |
| Fixed RR | 0.88 | 0.20 | 0.87 |
| RRl | 0.82 | 0.11 | 0.81 |
| RRu | 0.94 | 0.37 | 0.92 |
| P | --- | --- | --- |
| Random RR | 0.80 | 0.20 | 0.69 |
| RRl | 0.71 | 0.11 | 0.57 |
| RRu | 0.90 | 0.37 | 0.83 |
| P | --- | --- | --- |
| Between Chi | | | 22.66 |
| Between df | | | 1 |
| Between P | | | *** |
| Btwn(F) P | | | ** |
| Btwn(R) P | | | *** |

Table 2F3 - 3

IESLC - Meta-analysis of Cigarette Smoking, only Filter vs ever Plain (or nearest available)
Squamous
Most adjusted

| | Cigarette type | | | | | | |
|---|---|---|---|---|---|---|---|
| | only f | always f | mainly f | equl p&f | both p&f | ever f | Total |
| N | 7 | | 6 | | | | 13 |
| NS | 5 | | 3 | | | | 8 |
| Wt | 775.40 | | 151.65 | | | | 927.05 |
| Het Chi | 33.40 | | 2.10 | | | | 37.75 |
| Het df | 6 | | 5 | | | | 12 |
| Het P | *** | | N.S. | | | | *** |
| Fixed RR | 0.88 | | 0.77 | | | | 0.87 |
| RRl | 0.82 | | 0.66 | | | | 0.81 |
| RRu | 0.95 | | 0.91 | | | | 0.92 |
| P | --- | | -- | | | | --- |
| Random RR | 0.61 | | 0.77 | | | | 0.69 |
| RRl | 0.43 | | 0.66 | | | | 0.57 |
| RRu | 0.86 | | 0.91 | | | | 0.83 |
| P | -- | | -- | | | | --- |
| Between Chi | | | | | | | 2.26 |
| Between df | | | | | | | 1 |
| Between P | | | | | | | N.S. |
| Btwn(F) P | | | | | | | N.S. |
| Btwn(R) P | | | | | | | N.S. |

| | Denominator | | | | |
|---|---|---|---|---|---|
| | ever p | mainly p | p NOS | always p | Total |
| N | 5 | 1 | 2 | 5 | 13 |
| NS | 3 | 1 | 2 | 2 | 8 |
| Wt | 762.03 | 4.63 | 13.37 | 147.02 | 927.05 |
| Het Chi | 27.19 | 0.00 | 0.00 | 2.06 | 37.75 |
| Het df | 4 | 0 | 1 | 4 | 12 |
| Het P | *** | N.S. | N.S. | N.S. | *** |
| Fixed RR | 0.89 | 0.71 | 0.45 | 0.78 | 0.87 |
| RRl | 0.83 | 0.29 | 0.26 | 0.66 | 0.81 |
| RRu | 0.96 | 1.77 | 0.77 | 0.91 | 0.92 |
| P | -- | N.S. | -- | -- | --- |
| Random RR | 0.66 | 0.71 | 0.45 | 0.78 | 0.69 |
| RRl | 0.45 | 0.29 | 0.26 | 0.66 | 0.57 |
| RRu | 0.96 | 1.77 | 0.77 | 0.91 | 0.83 |
| P | - | N.S. | -- | -- | --- |
| Between Chi | | | | | 8.50 |
| Between df | | | | | 3 |
| Between P | | | | | * |
| Btwn(F) P | | | | | N.S. |
| Btwn(R) P | | | | | N.S. |

| | Derivation of RR/CI | | | |
|---|---|---|---|---|
| | Orig | StdCalc | Other | Total |
| N | | 8 | 5 | 13 |
| NS | | 5 | 3 | 8 |
| Wt | | 165.02 | 762.03 | 927.05 |
| Het Chi | | 5.71 | 27.19 | 37.75 |
| Het df | | 7 | 4 | 12 |
| Het P | | N.S. | *** | *** |
| Fixed RR | | 0.74 | 0.89 | 0.87 |
| RRl | | 0.64 | 0.83 | 0.81 |
| RRu | | 0.86 | 0.96 | 0.92 |
| P | | --- | -- | --- |
| Random RR | | 0.74 | 0.66 | 0.69 |
| RRl | | 0.64 | 0.45 | 0.57 |
| RRu | | 0.86 | 0.96 | 0.83 |
| P | | --- | - | --- |
| Between Chi | | | | 4.85 |
| Between df | | | | 1 |
| Between P | | | | * |
| Btwn(F) P | | | | N.S. |
| Btwn(R) P | | | | N.S. |

Table 2F3 - 4

IESLC - Meta-analysis of Cigarette Smoking, only Filter vs ever Plain (or nearest available)
Squamous
Least adjusted

| REF | NRR | X | SEX | AGEL | AGEH | RACE | YF | LC TYPE | LOC | START | ST | NLC | R | VB | P | H | AD | SM | PRODUCT | CIGTYP | DENOM | De |
|---|---|---|---|---|---|---|---|---|---|---|---|---|---|---|---|---|---|---|---|---|---|---|
| LUBIN2 | 197 | x | m | 0 | 0 | all | - | q | Eu:mul | 1976 | CC | 7804 | n | bl | n | y | 0 | ev | cig+/-ot | only f | ever p | st |
| LUBIN2 | 229 | x | f | 0 | 0 | all | - | q | Eu:mul | 1976 | CC | 7804 | n | bl | n | y | 0 | ev | cig+/-ot | only f | ever p | st |
| MATOS | 73 | x | m | 0 | 0 | all | - | q | SCAmer | 1994 | CC | 200 | n | bl | n | n | 0 | ev | cig+/-ot | mainly f | mainly p | st |
| PEZZOT | 11 | x | m | 0 | 0 | all | - | q | SCAmer | 1987 | CC | 215 | n | bl | n | y | 2 | ev | cig only | only f | ever p | ot |
| SOBUE | 73 | x | m | 0 | 0 | all | - | q | As:Jap | 1986 | CC | 1376 | n | bl | n | y | 0 | cu | cig+/-ot | only f | p NOS | st |
| WAKAI | 67 | x | m | 0 | 0 | all | - | q | As:Jap | 1988 | CC | 333 | n | bl | n | y | 0 | cu | cig+/-ot | only f | p NOS | st |
| WYNDE3 | 89 | x | m | 0 | 0 | all | - | KI | NAmer | 1966 | CC | 350 | n | bl | n | y | 0 | ev | cig+/-ot | mainly f | always p | st |
| WYNDE5 | 12 | x | m | 0 | 0 | wh | - | KI | NAmer | 1969 | CC | 1365 | n | bl | n | y | 0 | cu | cig+/-ot | mainly f | always p | st |
| WYNDE5 | 20 | x | f | 0 | 0 | wh | - | KI | NAmer | 1969 | CC | 1365 | n | bl | n | y | 0 | cu | cig+/-ot | mainly f | always p | st |
| WYNDE6 | 299 | x | m | 0 | 0 | all | - | q | NAmer | 1969 | CC | 4423 | n | bl | n | y | 0 | cu | cig+/-ot | only f | ever p | st |
| WYNDE6 | 305 | x | f | 0 | 0 | all | - | q | NAmer | 1969 | CC | 4423 | n | bl | n | y | 0 | cu | cig+/-ot | only f | ever p | st |

Table 2F3 - 5

IESLC - Meta-analysis of Cigarette Smoking, only Filter vs ever Plain (or nearest available)
Squamous
Least adjusted

| REF | NRR | SEX | AD | Number Exposed Case | Number Exposed Cont | Non-exposed Case | Non-exposed Cont | RR | 95.00%CI |
|---|---|---|---|---|---|---|---|---|---|
| LUBIN2 | 197 | m | 0 | 190 | 1018 | 3405 | 9419 | 0.52 | ( 0.44- 0.61) |
| LUBIN2 | 229 | f | 0 | 40 | 213 | 159 | 354 | 0.42 | ( 0.28- 0.62) |
| Subtotal LUBIN2 | | | | | | | | 0.50 | ( 0.43- 0.58) |
| MATOS | 73 | m | 0 | 38 | 229 | 9 | 46 | 0.85 | ( 0.38- 1.87) |
| PEZZOT | 11 | m | 2 | - | - | - | - | 0.20 | ( 0.11- 0.37) |
| SOBUE | 73 | m | 0 | 220 | 540 | 27 | 26 | 0.39 | ( 0.22- 0.69) |
| WAKAI | 67 | m | 0 | 81 | 271 | 5 | 9 | 0.54 | ( 0.18- 1.65) |
| WYNDE3 | 89 | m | 0 | 66 | 82 | 81 | 62 | 0.62 | ( 0.39- 0.98) |
| WYNDE5 | 12 | m | 0 | 143 | 879 | 150 | 704 | 0.76 | ( 0.59- 0.98) |
| WYNDE5 | 20 | f | 0 | 50 | 857 | 13 | 166 | 0.74 | ( 0.40- 1.40) |
| Subtotal WYNDE5 | | | | | | | | 0.76 | ( 0.60- 0.96) |
| WYNDE6 | 299 | m | 0 | 62 | 122 | 668 | 754 | 0.57 | ( 0.42- 0.79) |
| WYNDE6 | 305 | f | 0 | 57 | 158 | 224 | 309 | 0.50 | ( 0.35- 0.70) |
| Subtotal WYNDE6 | | | | | | | | 0.54 | ( 0.42- 0.68) |
| Partial Totals | | | | 947 | 4369 | 4741 | 11849 | | |

*prospective study

| REF | NRR | SEX | AD | Ys | Ws | Qs | Ps |
|---|---|---|---|---|---|---|---|
| LUBIN2 | 197 | m | 0 | -0.66 | 150.48 | 0.36 | 0.0000 |
| LUBIN2 | 229 | f | 0 | -0.87 | 25.77 | 1.74 | 0.0000 |
| Subtotal LUBIN2 | | | | -0.69 | 176.25 | 2.11 | |
| MATOS | 73 | m | 0 | -0.16 | 6.11 | 1.22 | 0.6838 |
| PEZZOT | 11 | m | 2 | -1.61 | 10.44 | 10.39 | 0.0000 |
| SOBUE | 73 | m | 0 | -0.94 | 12.21 | 1.28 | 0.0011 |
| WAKAI | 67 | m | 0 | -0.62 | 3.06 | 0.00 | 0.2785 |
| WYNDE3 | 89 | m | 0 | -0.48 | 17.91 | 0.29 | 0.0403 |
| WYNDE5 | 12 | m | 0 | -0.27 | 61.66 | 7.22 | 0.0341 |
| WYNDE5 | 20 | f | 0 | -0.29 | 9.60 | 0.97 | 0.3616 |
| Subtotal WYNDE5 | | | | -0.27 | 71.27 | 8.18 | |
| WYNDE6 | 299 | m | 0 | -0.56 | 36.83 | 0.12 | 0.0007 |
| WYNDE6 | 305 | f | 0 | -0.70 | 31.67 | 0.23 | 0.0001 |
| Subtotal WYNDE6 | | | | -0.62 | 68.51 | 0.35 | |

| | | |
|---|---|---|
| | N | 11 |
| | NS | 8 |
| | Wt | 365.76 |
| Het | Chi | 23.83 |
| Het | df | 10 |
| Het | P | ** |
| Fixed | RR | 0.54 |
| | RRl | 0.49 |
| | RRu | 0.60 |
| | P | --- |
| Random | RR | 0.53 |
| | RRl | 0.44 |
| | RRu | 0.64 |
| | P | --- |
| Asymm | P | N.S. |

Table 2F3 - 6

IESLC - Meta-analysis of Cigarette Smoking, only Filter vs ever Plain (or nearest available)

Squamous

Least adjusted

| | Sex combined | male | female | Total |
|---|---|---|---|---|
| N | | 8 | 3 | 11 |
| NS | | 8 | 3 | 11 |
| Wt | | 298.72 | 67.04 | 365.76 |
| Het Chi | | 20.75 | 2.34 | 23.83 |
| Het df | | 7 | 2 | 10 |
| Het P | | ** | N.S. | ** |
| Fixed RR | | 0.55 | 0.49 | 0.54 |
| RRl | | 0.49 | 0.39 | 0.49 |
| RRu | | 0.62 | 0.63 | 0.60 |
| P | | --- | --- | --- |
| Random RR | | 0.53 | 0.50 | 0.53 |
| RRl | | 0.42 | 0.38 | 0.44 |
| RRu | | 0.68 | 0.65 | 0.64 |
| P | | --- | --- | --- |
| Between Chi | | | | 0.74 |
| Between df | | | | 1 |
| Between P | | | | N.S. |
| Btwn(F) P | | | | N.S. |
| Btwn(R) P | | | | N.S. |

Table 2F3 - 7

IESLC - Meta-analysis of Cigarette Smoking, only Filter vs ever Plain (or nearest available)
Squamous
Excluded studies (and stage at which they were excluded)

| | |
|---|---|
| 1 | BOUCHA BUELL LAURIL MZILEN |
| 2 | BERRIN CHEN RESTRE TAO ULMER |
| 3 | ABELIN ABRAHA AKIBA AMANDU AMES ANDERS ARCHER AUSTIN AUVINE AXELSO AXELSS BAND BARBON BENSHL BEST BLOHMK BLOT1 BLOT2 BLOT3 BLOT4 BOFFET BOUCOT BRESLO BRETT BROCKM BROWN1 BROWN2 BYERS1 BYERS2 CARPEN CASCO2 CASCOR CEDERL CHAN CHANG CHATZI CHEN2 CHEN3 CHIAZZ CHOW CHYOU COMSTO COOKSO CPSI DAMBER DARBY DAVEYS DEAN DEKLER DOCKER DOLL2 DORANT DORGAN DORN DOSEME DROSTE DU DUNN EBELIN ENSTRO ESAKI FAN GAO GAO2 GARCIA GARDIN GARSHI GENG GER GILLIS GODLEY GOLLED GOODMA GRAHAM GREGOR GSELL HAENSZ HAMMO2 HAMMON HANSEN HEGMAN HEIN HENNEK HINDS HIRAOK HIRAY2 HIRAYA HITOSU HOLE HOROWI HORWIT HU HU2 HUANG HUMBLE ISHIMA JAHN JAIN JARUP JARVHO JEDRYC JIANG JOLY JONES JUSSAW KAISER KANELL KATSOU KAUFMA KELLER KIHARA KINLEN KJUUS KNEKT KO KOHLME KOO KOULUM KREUZE KREYBE KUBIK LAMTH LAMWK LAMWK2 LAUSSM LEI LEMARC LETOUR LEVIN LIAW LICKIN LIDDEL LIU LIU2 LIU3 LIU4 LIU5 LOMBA2 LOMBAR LUBIN LUO MAGNUS MARSH MARSH2 MARTIS MASTRA MATSUD MCCONN MCDUFF MCLAUG MILLER MILLS MOLLO MRFIT MURATA NAM NOTAN2 NOTANI NOU ODRISC ORMOS OSANN OSANN2 PARKIN PASTOR PAWLEG PERNU PERSH2 PERSHA PETO PEZZO2 PIKE PISANI POFFIJ POLEDN PRESCO QIAO QIAO2 RACHTA RADZIK RANDIG REN RONCO ROOTS ROTHSC SAARIK SADOWS SANKAR SCHWA2 SCHWAR SEGI SEOW SHAW SHIMIZ SIEMIA SIMARA SIMONA SITAS SOBUE2 SPEIZE SPITZ STASZE STAYNE STOCKS STOCKW STUCKE SUN SUZUK2 SUZUKI SVENSS TANG TENKAN TIZZAN TOKARS TOUSEY TSUGAN TULINI TVERDA VANDER VEIERO VUTUC WALD WANG WANG2 WANG3 WANG4 WARSIN WATSON WICKLU WIGLE WILKIN WU WU2 WUNSCH WUWILL WYNDE2 WYNDE4 WYNDE7 WYNDE8 WYNDER XIANGZ XU XU2 XU3 XU4 YAMAGU YONG YUAN ZHANG ZHENG ZHOU |
| 7 | AGUDO ALDERS ARMADA BECHER BROSS BUFFLE CHOI CORREA CPSII DEAN2 DEAN3 DESTE2 DESTEF DOLL ENGELA KAISE2 KHUDER LANGE MACLEN MIGRAN MRFITR RIMING SEGI2 TANG2 |
| 10 | BENHAM |

Table 2F3 - 8
Potentially overlapping studies

| REF | REFGP | PRINC | . | OVERLAP/LINK |
|---|---|---|---|---|
| LUBIN2 | LUBIN2 | 1 | | Lubin-combined |
| WYNDE5 | WYNDE6 | 2 | | WYNDE5/6/7/8 |
| WYNDE6 | WYNDE6 | 1 | | WYNDE5/6/7/8 |

Table 2F4 -

IESLC - Meta-analysis of Cigarette Smoking, Hand-rolled vs Manufactured
Squamous

This analysis is restricted to results for:
1) Non-dose-response data
2) Results complete enough for use in metaanalysis

Within each study, results are then selected (in the following order of preference, within each sex) for:
3) CIGTYP: hand-rolled any, both, mainly, only
4) DENOM: manufactured only ever, only current, any, ever
5) PRODUCT: cigarettes regardless of other products, cigarettes only
6) SMKSTA: ever, current
7) LCtype: all or nearest available, at least Squamous and Adeno. (q = squamous, s = small,
l = large, a = adeno, mix = mixed, alv = alveolar)
8) Race: all or nearest available, otherwise by race (wh or w = white, bl or b = black, hi = hispanic
ch = chinese, jap = japanese, haw = hawaiian, w+o = white + oriental, sca = scandinavian, as = asian)
9) Followup period (YF, prospective studies): whole study (coded as 0) or longest available
10) For overlapping studies: principal rather than subsidiary studies
Finally by Age: whole study (coded as 0) if available, otherwise by widest available age group
and then for single sex results (m, f) in preference to combined sex results (c).

Results adjusted (AD) for the most potential confounders are then chosen in Sections -1 to -3
and results adjusted for the least confounders in Sections -4 to -6. (Those least adjusted results which
actually differ from the most adjusted as marked 'x' in column X in Section -4)
(Results adjusted for an unknown number of confounder(s) are coded as 20.)

Section -7 shows excluded studies, together with the stage (as above) at which no qualifying
results were found.

Section -8 lists the potentially overlapping studies which have been included (1=principal, 2=subsidiary).

Section -9 lists any results which would have been included in preference except that they had data not complete
enough for use in meta-analysis, with their significance (yes/no), if known, and any further comment as entered
on the database.

In addition to those mentioned above, the following fields, levels and abbreviations are used:
* or nk = not known, n = no, y = yes, ot = other
ev = ever, cu = current, cig+/-ot = cigarettes irrespective of other products (cigar, pipe etc)
m or mc = manufactured cigarettes, h or hr = hand-rolled cigarettes
REF: 6-character study reference
NRR: number of the RR on the database within the study
ST : study type (CC = case control, pr or prosp = prospective)
NLC: number of lung cancer cases in whole study
R : risky occupational population (n = no, m = mining, o = other risky)
VB : national cigarette type (V = at least 75% Virginia, bl = at least 75% blended, ot = other)
P : any proxy use
H : full histological confirmation
De : derivation of RR/CI (or = original, st = standard method, ot = other method of estimation)

Table 2F4 - 1

IESLC - Meta-analysis of Cigarette Smoking, Hand-rolled vs Manufactured
Squamous
Most adjusted

| REF | NRR | SEX | AGEL | AGEH | RACE | YF | LC TYPE | LOC | START | ST | NLC | R | VB | P | H | AD | SM | PRODUCT | CIGTYP | DENOM | De |
|---|---|---|---|---|---|---|---|---|---|---|---|---|---|---|---|---|---|---|---|---|---|
| ALDERS | 135 | m | 0 | 0 | all | - | q+s | Eu:UK | 1977 | CC | 1448 | n | V | n | n | 1 | ev | cig only | both m&h | only mc | ot |
| BENHAM | 118 | m | 0 | 0 | all | - | KI | Eu:wst | 1976 | CC | 1625 | n | bl | n | y | 7 | ev | cig only | both m&h | only mc | ot |
| DESTEF | 35 | m | 0 | 0 | all | - | q | SCAmer | 1988 | CC | 497 | n | bl | n | y | 6 | ev | cig+/-ot | both m&h | only mc | or |
| ENGELA | 188 | m | 0 | 0 | all | 0 | q | Eu:Sca | 1964 | pr | 435 | n | bl | n | n | 7 | cu | cig+/-ot | both m&h | only mc | ot |
| JUSSAW | 39 | m | 0 | 0 | all | - | KI | As:Ind | 1964 | CC | 792 | n | V | n | n | 0 | ev | cig only | only hr | only mc | st |

Table 2F4 - 2

IESLC - Meta-analysis of Cigarette Smoking, Hand-rolled vs Manufactured
Squamous
Most adjusted

| REF | NRR | SEX | AD | Number Exposed Case | Number Exposed Cont | Non-exposed Case | Non-exposed Cont | RR | 95.00%CI |
|---|---|---|---|---|---|---|---|---|---|
| ALDERS | 135 | m | 1 | - | - | - | - | 1.59 | ( 1.07- 2.37) |
| BENHAM | 118 | m | 7 | - | - | - | - | 1.28 | ( 0.99- 1.66) |
| DESTEF | 35 | m | 6 | - | - | - | - | 1.20 | ( 0.80- 1.80) |
| *ENGELA | 188 | m | 7 | - | - | - | - | 1.86 | ( 0.97- 3.54) |
| JUSSAW | 39 | m | 0 | 66 | 85 | 17 | 77 | 3.52 | ( 1.90- 6.51) |
| Partial Totals | | | | 66 | 85 | 17 | 77 | | |

*prospective study

| REF | NRR | SEX | AD | Ys | Ws | Qs | Ps |
|---|---|---|---|---|---|---|---|
| ALDERS | 135 | m | 1 | 0.46 | 24.30 | 0.14 | 0.0223 |
| BENHAM | 118 | m | 7 | 0.25 | 57.52 | 1.13 | 0.0612 |
| DESTEF | 35 | m | 6 | 0.18 | 23.37 | 0.98 | 0.3781 |
| *ENGELA | 188 | m | 7 | 0.62 | 9.17 | 0.50 | 0.0602 |
| JUSSAW | 39 | m | 0 | 1.26 | 10.13 | 7.68 | 0.0001 |

| | | |
|---|---|---|
| | N | 5 |
| | NS | 5 |
| | Wt | 124.48 |
| Het | Chi | 10.43 |
| Het | df | 4 |
| Het | P | * |
| Fixed | RR | 1.47 |
| | RRl | 1.24 |
| | RRu | 1.76 |
| | P | +++ |
| Random | RR | 1.62 |
| | RRl | 1.18 |
| | RRu | 2.21 |
| | P | ++ |
| Asymm | P | N.S. |

Table 2F4 - 3

IESLC - Meta-analysis of Cigarette Smoking, Hand-rolled vs Manufactured
Squamous
Most adjusted

| | Sex combined | male | female | Total |
|---|---|---|---|---|
| N | | 5 | | 5 |
| NS | | 5 | | 5 |
| Wt | | 124.48 | | 124.48 |
| Het Chi | | 10.43 | | 10.43 |
| Het df | | 4 | | 4 |
| Het P | | * | | * |
| Fixed RR | | 1.47 | | 1.47 |
| RRl | | 1.24 | | 1.24 |
| RRu | | 1.76 | | 1.76 |
| P | | +++ | | +++ |
| Random RR | | 1.62 | | 1.62 |
| RRl | | 1.18 | | 1.18 |
| RRu | | 2.21 | | 2.21 |
| P | | ++ | | ++ |
| Between Chi | | | | |
| Between df | | | | |
| Between P | | | | N.S. |
| Btwn(F) P | | | | N.S. |
| Btwn(R) P | | | | N.S. |

Too few RRs for analysis by factor

Table 2F4 - 4

IESLC - Meta-analysis of Cigarette Smoking, Hand-rolled vs Manufactured
Squamous
Least adjusted

| REF | NRR | X | SEX | AGEL | AGEH | RACE | YF | LC TYPE | LOC | START | ST | NLC | R | VB | P | H | AD | SM | PRODUCT | CIGTYP | DENOM | De |
|---|---|---|---|---|---|---|---|---|---|---|---|---|---|---|---|---|---|---|---|---|---|---|
| ALDERS | 143 | x | m | 0 | 0 | all | - | q+s | Eu:UK | 1977 | CC | 1448 | n | V | n | n | 0 | ev | cig only | both m&h | only mc | st |
| BENHAM | 116 | x | m | 0 | 0 | all | - | KI | Eu:wst | 1976 | CC | 1625 | n | bl | n | y | 0 | ev | cig only | both m&h | only mc | st |
| DESTEF | 35 | | m | 0 | 0 | all | - | q | SCAmer | 1988 | CC | 497 | n | bl | n | y | 6 | ev | cig+/-ot | both m&h | only mc | or |
| ENGELA | 188 | | m | 0 | 0 | all | 0 | q | Eu:Sca | 1964 | pr | 435 | n | bl | n | n | 7 | cu | cig+/-ot | both m&h | only mc | ot |
| JUSSAW | 39 | | m | 0 | 0 | all | - | KI | As:Ind | 1964 | CC | 792 | n | V | n | n | 0 | ev | cig only | only hr | only mc | st |

Table 2F4 - 5

IESLC - Meta-analysis of Cigarette Smoking, Hand-rolled vs Manufactured
Squamous
Least adjusted

| REF | NRR | SEX | AD | Number Exposed Case | Number Exposed Cont | Non-exposed Case | Non-exposed Cont | RR | 95.00%CI |
|---|---|---|---|---|---|---|---|---|---|
| ALDERS | 143 | m | 0 | 77 | 113 | 130 | 349 | 1.83 | ( 1.29- 2.60) |
| BENHAM | 116 | m | 0 | 244 | 209 | 830 | 1040 | 1.46 | ( 1.19- 1.80) |
| DESTEF | 35 | m | 6 | - | - | - | - | 1.20 | ( 0.80- 1.80) |
| *ENGELA | 188 | m | 7 | - | - | - | - | 1.86 | ( 0.97- 3.54) |
| JUSSAW | 39 | m | 0 | 66 | 85 | 17 | 77 | 3.52 | ( 1.90- 6.51) |
| Partial Totals | | | | 387 | 407 | 977 | 1466 | | |

*prospective study

| REF | NRR | SEX | AD | Ys | Ws | Qs | Ps |
|---|---|---|---|---|---|---|---|
| ALDERS | 143 | m | 0 | 0.60 | 30.87 | 0.62 | 0.0008 |
| BENHAM | 116 | m | 0 | 0.38 | 90.50 | 0.60 | 0.0003 |
| DESTEF | 35 | m | 6 | 0.18 | 23.37 | 1.83 | 0.3781 |
| *ENGELA | 188 | m | 7 | 0.62 | 9.17 | 0.23 | 0.0602 |
| JUSSAW | 39 | m | 0 | 1.26 | 10.13 | 6.41 | 0.0001 |

| | | |
|---|---|---|
| | N | 5 |
| | NS | 5 |
| | Wt | 164.04 |
| Het | Chi | 9.69 |
| Het | df | 4 |
| Het | P | * |
| Fixed | RR | 1.59 |
| | RRl | 1.36 |
| | RRu | 1.85 |
| | P | +++ |
| Random | RR | 1.71 |
| | RRl | 1.29 |
| | RRu | 2.26 |
| | P | +++ |
| Asymm | P | N.S. |

Table 2F4 - 6

IESLC - Meta-analysis of Cigarette Smoking, Hand-rolled vs Manufactured
Squamous
Least adjusted

| | Sex combined | male | female | Total |
|---|---|---|---|---|
| N | | 5 | | 5 |
| NS | | 5 | | 5 |
| Wt | | 164.04 | | 164.04 |
| Het Chi | | 9.69 | | 9.69 |
| Het df | | 4 | | 4 |
| Het P | | * | | * |
| Fixed RR | | 1.59 | | 1.59 |
| RRl | | 1.36 | | 1.36 |
| RRu | | 1.85 | | 1.85 |
| P | | +++ | | +++ |
| Random RR | | 1.71 | | 1.71 |
| RRl | | 1.29 | | 1.29 |
| RRu | | 2.26 | | 2.26 |
| P | | +++ | | +++ |
| Between Chi | | | | |
| Between df | | | | |
| Between P | | | | N.S. |
| Btwn(F) P | | | | N.S. |
| Btwn(R) P | | | | N.S. |

Table 2F4 - 7

IESLC - Meta-analysis of Cigarette Smoking, Hand-rolled vs Manufactured
Squamous
Excluded studies (and stage at which they were excluded)

| | | | | | | | | | | | | | | | | |
|---|---|---|---|---|---|---|---|---|---|---|---|---|---|---|---|---|
| 1 | BOUCHA | BUELL | LAURIL | MZILEN | | | | | | | | | | | | |
| 2 | BERRIN | CHEN | RESTRE | TAO | ULMER | | | | | | | | | | | |
| 3 | ABELIN | ABRAHA | AGUDO | AKIBA | AMANDU | AMES | ANDERS | ARCHER | ARMADA | AUSTIN | AUVINE | AXELSO | AXELSS | BAND | BARBON | BECHER |
| | BENSHL | BEST | BLOHMK | BLOT1 | BLOT2 | BLOT3 | BLOT4 | BOFFET | BOUCOT | BRESLO | BRETT | BROCKM | BROSS | BROWN1 | BROWN2 | BYERS1 |
| | BYERS2 | CARPEN | CASCO2 | CASCOR | CEDERL | CHANG | CHATZI | CHEN2 | CHEN3 | CHIAZZ | CHOI | CHOW | CHYOU | COMSTO | COOKSO | CORREA |
| | CPSI | CPSII | DAMBER | DARBY | DAVEYS | DEAN | DEAN2 | DEKLER | DOCKER | DOLL | DOLL2 | DORANT | DORGAN | DORN | DOSEME | DROSTE |
| | DU | DUNN | EBELIN | ENSTRO | ESAKI | FAN | GAO | GAO2 | GARCIA | GARSHI | GENG | GER | GILLIS | GODLEY | GOLLED | GOODMA |
| | GRAHAM | GREGOR | GSELL | HAENSZ | HAMMO2 | HAMMON | HANSEN | HEGMAN | HEIN | HENNEK | HINDS | HIRAOK | HIRAY2 | HIRAYA | HITOSU | HOLE |
| | HOROWI | HORWIT | HUANG | HUMBLE | ISHIMA | JAHN | JAIN | JARUP | JARVHO | JEDRYC | JIANG | JOLY | JONES | KAISE2 | KAISER | KANELL |
| | KATSOU | KAUFMA | KELLER | KHUDER | KIHARA | KINLEN | KJUUS | KNEKT | KO | KOHLME | KOO | KOULUM | KREUZE | KREYBE | KUBIK | LAMTH |
| | LAMWK | LAMWK2 | LANGE | LAUSSM | LEI | LEMARC | LETOUR | LEVIN | LIAW | LICKIN | LIDDEL | LIU | LIU2 | LIU3 | LIU4 | LIU5 |
| | LOMBA2 | LOMBAR | LUBIN | LUBIN2 | LUO | MAGNUS | MARSH | MARSH2 | MARTIS | MASTRA | MATOS | MATSUD | MCCONN | MCDUFF | MCLAUG | MILLER |
| | MILLS | MOLLO | MRFIT | MRFITR | MURATA | NAM | NOTANI | NOU | ODRISC | ORMOS | OSANN | OSANN2 | PARKIN | PASTOR | PAWLEG | PERSH2 |
| | PERSHA | PETO | PEZZO2 | PEZZOT | PIKE | PISANI | POFFIJ | POLEDN | PRESCO | QIAO | QIAO2 | RACHTA | RADZIK | RANDIG | REN | RIMING |
| | RONCO | ROOTS | ROTHSC | SAARIK | SADOWS | SANKAR | SCHWA2 | SCHWAR | SEGI | SEGI2 | SEOW | SHAW | SHIMIZ | SIEMIA | SIMARA | SIMONA |
| | SITAS | SOBUE | SOBUE2 | SPEIZE | SPITZ | STASZE | STAYNE | STOCKS | STOCKW | STUCKE | SUN | SUZUKI | SVENSS | TANG | TANG2 | TENKAN |
| | TIZZAN | TOKARS | TOUSEY | TSUGAN | TULINI | TVERDA | VANDER | VEIERO | VUTUC | WAKAI | WALD | WANG | WANG2 | WANG3 | WANG4 | WARSIN |
| | WATSON | WICKLU | WIGLE | WILKIN | WU | WU2 | WUNSCH | WUWILL | WYNDE2 | WYNDE3 | WYNDE4 | WYNDE5 | WYNDE6 | WYNDE7 | WYNDE8 | WYNDER |
| | XIANGZ | XU | XU2 | XU3 | XU4 | YAMAGU | YONG | YUAN | ZHANG | ZHENG | ZHOU | | | | | |
| 4 | BUFFLE | DEAN3 | | | | | | | | | | | | | | |
| 6 | CHAN | DESTE2 | GARDIN | HU | HU2 | MACLEN | MIGRAN | NOTAN2 | PERNU | SUZUK2 | | | | | | |

Table 2F4 - 8

Potentially overlapping studies

| REF | REFGP | PRINC | . OVERLAP/LINK |
|---|---|---|---|
| BENHAM | LUBIN2 | 2 | Subset of Lubin2 |

Table 2F5 -

IESLC - Meta-analysis of Cigarette Smoking, Menthol vs non-menthol
Squamous

This analysis is restricted to results for:
1) Non-dose-response data
2) Results complete enough for use in metaanalysis

Within each study, results are then selected (in the following order of preference, within each sex) for:
3) Cigarette type: menthol
4) Denominator: non-menthol
5) PRODUCT: cigarettes regardless of other products, cigarettes only
6) SMKSTA: ever, current
7) LCtype: all or nearest available, at least Squamous and Adeno. (q = squamous, s = small,
l = large, a = adeno, mix = mixed, alv = alveolar)
8) Race: all or nearest available, otherwise by race (wh or w = white, bl or b = black, hi = hispanic
ch = chinese, jap = japanese, haw = hawaiian, w+o = white + oriental, sca = scandinavian, as = asian)
9) Followup period (YF, prospective studies): whole study (coded as 0) or longest available
10) For overlapping studies: principal rather than subsidiary studies
Finally by Age: whole study (coded as 0) if available, otherwise by widest available age group
and then for single sex results (m, f) in preference to combined sex results (c).

Results adjusted (AD) for the most potential confounders are then chosen in Sections -1 to -3
and results adjusted for the least confounders in Sections -4 to -6. (Those least adjusted results which
actually differ from the most adjusted as marked 'x' in column X in Section -4)
(Results adjusted for an unknown number of confounder(s) are coded as 20.)

Section -7 shows excluded studies, together with the stage (as above) at which no qualifying
results were found.

Section -8 lists the potentially overlapping studies which have been included (1=principal, 2=subsidiary).

Section -9 lists any results which would have been included in preference except that they had data not complete
enough for use in meta-analysis, with their significance (yes/no), if known, and any further comment as entered
on the database.

In addition to those mentioned above, the following fields, levels and abbreviations are used:
* or nk = not known, n = no, y = yes, ot = other
ev = ever, cu = current, cig+/-ot = cigarettes irrespective of other products (cigar, pipe etc)
REF: 6-character study reference
NRR: number of the RR on the database within the study
ST : study type (CC = case control, pr or prosp = prospective)
NLC: number of lung cancer cases in whole study
R  : risky occupational population (n = no, m = mining, o = other risky)
VB : national cigarette type (V = at least 75% Virginia, bl = at least 75% blended, ot = other)
P  : any proxy use
H  : full histological confirmation
De : derivation of RR/CI (or = original, st = standard method, ot = other method of estimation)

Table 2F5 - 1

IESLC - Meta-analysis of Cigarette Smoking, Menthol vs non-menthol
Squamous
Most adjusted

| REF | NRR | SEX | AGEL | AGEH | RACE | YF | LC TYPE | LOC | START | ST | NLC | R | VB | P | H | AD | SM | PRODUCT | De |
|---|---|---|---|---|---|---|---|---|---|---|---|---|---|---|---|---|---|---|---|
| WYNDE8 | 5 | c | 0 | 0 | all | - | q | NAmer | 1985 | CC | 1044 | n | bl | n | y | 8 | cu | cig+/-ot | ot |

Table 2F5 - 2

IESLC - Meta-analysis of Cigarette Smoking, Menthol vs non-menthol
Squamous
Most adjusted

| REF | NRR | SEX | AD | Number Exposed Case | Cont | Non-exposed Case | Cont | RR | 95.00%CI |
|---|---|---|---|---|---|---|---|---|---|
| WYNDE8 | 5 | c | 8 | - | - | - | - | 1.04 | ( 0.75- 1.44) |
| Partial Totals | | | | 0 | 0 | 0 | 0 | | |

*prospective study

| REF | NRR | SEX | AD | Ys | Ws | Qs | Ps |
|---|---|---|---|---|---|---|---|
| WYNDE8 | 5 | c | 8 | 0.04 | 36.11 | 0.00 | 0.8137 |

| | | |
|---|---|---|
| | N | 1 |
| | NS | 1 |
| | Wt | 36.11 |
| Het | Chi | 0.00 |
| Het | df | 0 |
| Het | P | N.S. |
| Fixed | RR | 1.04 |
| | RRl | 0.75 |
| | RRu | 1.44 |
| | P | N.S. |
| Random | RR | 1.04 |
| | RRl | 0.75 |
| | RRu | 1.44 |
| | P | N.S. |
| Asymm | P | |

Table 2F5 - 3

IESLC - Meta-analysis of Cigarette Smoking, Menthol vs non-menthol
Squamous
Most adjusted

| | | Sex | | |
|---|---|---|---|---|
| | combined | male | female | Total |
| N | 1 | | | 1 |
| NS | 1 | | | 1 |
| Wt | 36.11 | | | 36.11 |
| Het Chi | 0.00 | | | 0.00 |
| Het df | 0 | | | 0 |
| Het P | N.S. | | | N.S. |
| Fixed RR | 1.04 | | | 1.04 |
| RRl | 0.75 | | | 0.75 |
| RRu | 1.44 | | | 1.44 |
| P | N.S. | | | N.S. |
| Random RR | 1.04 | | | 1.04 |
| RRl | 0.75 | | | 0.75 |
| RRu | 1.44 | | | 1.44 |
| P | N.S. | | | N.S. |
| Between Chi | | | | |
| Between df | | | | |
| Between P | | | | N.S. |
| Btwn(F) P | | | | N.S. |
| Btwn(R) P | | | | N.S. |

Too few RRs for analysis by factor

Table 2F5 - 4

IESLC - Meta-analysis of Cigarette Smoking, Menthol vs non-menthol
Squamous
Least adjusted

| REF | NRR | X | SEX | AGEL | AGEH | RACE | YF | LC TYPE | LOC | START | ST | NLC | R | VB | P | H | AD | SM | PRODUCT | De |
|---|---|---|---|---|---|---|---|---|---|---|---|---|---|---|---|---|---|---|---|---|
| WYNDE8 | 5 | | c | 0 | 0 | all | - | q | NAmer | 1985 | CC | 1044 | n | bl | n | y | 8 | cu | cig+/-ot | ot |

Table 2F5 - 5

IESLC - Meta-analysis of Cigarette Smoking, Menthol vs non-menthol
Squamous
Least adjusted

| REF | NRR | SEX | AD | Number Exposed Case | Cont | Non-exposed Case | Cont | RR | 95.00%CI |
|---|---|---|---|---|---|---|---|---|---|
| WYNDE8 | 5 | c | 8 | - | - | - | - | 1.04 | ( 0.75- 1.44) |
| Partial Totals | | | | 0 | 0 | 0 | 0 | | |

*prospective study

| REF | NRR | SEX | AD | Ys | Ws | Qs | Ps |
|---|---|---|---|---|---|---|---|
| WYNDE8 | 5 | c | 8 | 0.04 | 36.11 | 0.00 | 0.8137 |

| | | |
|---|---|---|
| | N | 1 |
| | NS | 1 |
| | Wt | 36.11 |
| Het | Chi | 0.00 |
| Het | df | 0 |
| Het | P | N.S. |
| Fixed | RR | 1.04 |
| | RRl | 0.75 |
| | RRu | 1.44 |
| | P | N.S. |
| Random | RR | 1.04 |
| | RRl | 0.75 |
| | RRu | 1.44 |
| | P | N.S. |
| Asymm | P | |

Table 2F5 - 6

IESLC - Meta-analysis of Cigarette Smoking, Menthol vs non-menthol
Squamous
Least adjusted

| | Sex combined | male | female | Total |
|---|---|---|---|---|
| N | 1 | | | 1 |
| NS | 1 | | | 1 |
| Wt | 36.11 | | | 36.11 |
| Het Chi | 0.00 | | | 0.00 |
| Het df | 0 | | | 0 |
| Het P | N.S. | | | N.S. |
| Fixed RR | 1.04 | | | 1.04 |
| RRl | 0.75 | | | 0.75 |
| RRu | 1.44 | | | 1.44 |
| P | N.S. | | | N.S. |
| Random RR | 1.04 | | | 1.04 |
| RRl | 0.75 | | | 0.75 |
| RRu | 1.44 | | | 1.44 |
| P | N.S. | | | N.S. |
| Between Chi | | | | |
| Between df | | | | |
| Between P | | | | N.S. |
| Btwn(F) P | | | | N.S. |
| Btwn(R) P | | | | N.S. |

Table 2F5 - 7

IESLC - Meta-analysis of Cigarette Smoking, Menthol vs non-menthol
Squamous
Excluded studies (and stage at which they were excluded)

| | | | | | | | | | | | | | | | | |
|---|---|---|---|---|---|---|---|---|---|---|---|---|---|---|---|---|
| 1 | BOUCHA | BUELL | LAURIL | MZILEN | | | | | | | | | | | | |
| 2 | BERRIN | CHEN | RESTRE | TAO | ULMER | | | | | | | | | | | |
| 3 | ABELIN | ABRAHA | AGUDO | AKIBA | ALDERS | AMANDU | AMES | ANDERS | ARCHER | ARMADA | AUSTIN | AUVINE | AXELSO | AXELSS | BAND | BARBON |
| | BECHER | BENHAM | BENSHL | BEST | BLOHMK | BLOT1 | BLOT2 | BLOT3 | BLOT4 | BOFFET | BOUCOT | BRESLO | BRETT | BROCKM | BROWN1 | BROWN2 |
| | BUFFLE | BYERS1 | BYERS2 | CASCO2 | CASCOR | CEDERL | CHAN | CHANG | CHATZI | CHEN2 | CHEN3 | CHIAZZ | CHOI | CHOW | CHYOU | COMSTO |
| | COOKSO | CORREA | CPSI | CPSII | DAMBER | DARBY | DAVEYS | DEAN | DEAN2 | DEAN3 | DEKLER | DESTE2 | DESTEF | DOCKER | DOLL | DOLL2 |
| | DORANT | DORGAN | DORN | DOSEME | DROSTE | DU | DUNN | EBELIN | ENGELA | ENSTRO | ESAKI | FAN | GAO | GAO2 | GARCIA | GARDIN |
| | GARSHI | GENG | GER | GILLIS | GODLEY | GOLLED | GOODMA | GRAHAM | GREGOR | GSELL | HAENSZ | HAMMO2 | HAMMON | HANSEN | HEGMAN | HEIN |
| | HENNEK | HINDS | HIRAOK | HIRAY2 | HIRAYA | HITOSU | HOLE | HOROWI | HORWIT | HU | HU2 | HUANG | HUMBLE | ISHIMA | JAHN | JAIN |
| | JARUP | JARVHO | JEDRYC | JIANG | JOLY | JONES | JUSSAW | KAISER | KANELL | KATSOU | KAUFMA | KELLER | KHUDER | KIHARA | KINLEN | KJUUS |
| | KNEKT | KO | KOHLME | KOO | KOULUM | KREUZE | KREYBE | KUBIK | LAMTH | LAMWK | LAMWK2 | LANGE | LAUSSM | LEI | LEMARC | LETOUR |
| | LEVIN | LIAW | LICKIN | LIDDEL | LIU | LIU2 | LIU3 | LIU4 | LIU5 | LOMBA2 | LOMBAR | LUBIN | LUBIN2 | LUO | MACLEN | MAGNUS |
| | MARSH | MARSH2 | MARTIS | MASTRA | MATOS | MATSUD | MCCONN | MCDUFF | MCLAUG | MIGRAN | MILLER | MILLS | MOLLO | MRFIT | MRFITR | MURATA |
| | NAM | NOTAN2 | NOTANI | NOU | ODRISC | ORMOS | OSANN | OSANN2 | PARKIN | PASTOR | PAWLEG | PERNU | PERSH2 | PERSHA | PETO | PEZZO2 |
| | PEZZOT | PIKE | PISANI | POFFIJ | POLEDN | PRESCO | QIAO | QIAO2 | RACHTA | RADZIK | RANDIG | REN | RIMING | RONCO | ROOTS | ROTHSC |
| | SAARIK | SADOWS | SANKAR | SCHWA2 | SCHWAR | SEGI | SEGI2 | SEOW | SHAW | SHIMIZ | SIEMIA | SIMARA | SIMONA | SITAS | SOBUE | SOBUE2 |
| | SPEIZE | SPITZ | STASZE | STAYNE | STOCKS | STOCKW | STUCKE | SUN | SUZUK2 | SUZUKI | SVENSS | TANG | TANG2 | TENKAN | TIZZAN | TOKARS |
| | TOUSEY | TSUGAN | TULINI | TVERDA | VANDER | VEIERO | VUTUC | WAKAI | WALD | WANG | WANG2 | WANG3 | WANG4 | WARSIN | WATSON | WICKLU |
| | WIGLE | WILKIN | WU | WU2 | WUNSCH | WUWILL | WYNDE2 | WYNDE3 | WYNDE4 | WYNDE5 | WYNDE6 | WYNDE7 | WYNDER | XIANGZ | XU | XU2 |
| | XU3 | XU4 | YAMAGU | YONG | YUAN | ZHANG | ZHENG | ZHOU | | | | | | | | |
| 4 | BROSS | | | | | | | | | | | | | | | |
| 7 | CARPEN | KAISE2 | | | | | | | | | | | | | | |

Table 2F5 - 8
Potentially overlapping studies

| REF | REFGP | PRINC | . | OVERLAP/LINK |
|---|---|---|---|---|
| WYNDE8 | WYNDE6 | 2 | | WYNDE5/6/7/8 |
