# Supplementary material for: Systematic review with meta-analysis of the epidemiological evidence in the 1900s relating smoking to lung cancer
Source: BMC Cancer. 2012 Sep 3;12:385. doi: 10.1186/1471-2407-12-385 (PMC3505152; doi:10.1186/1471-2407-12-385)
Supplement: Additional file 5 — Detailed Analysis Tables (Individual file names as described in Additional file 1: Methods, Table1). [file 1471-2407-12-385-S5.zip › PDF/1D.pdf]

Table 1D1 -

IESLC - Meta-analysis of Ex Smoking, Any product (or Cigarettes if Any not available)
All LC types

This analysis is restricted to results for:
1) Non-dose-response data
2) Ex smokers
3) Results complete enough for use in metaanalysis

Within each study, results are then selected (in the following order of preference, within each sex) for:
4) PRODUCT: all/unspec, cigarettes regardless of other products, cigarettes only
5) CIGTYPE: all/unspecified, MC regardless of HR, MC only
6) DENOM: never smoked anything, never smoked cigarettes, (never +1 = +long term ex, +2 = +amount unknown,
+3 = never cigs+long term ex)
7) Followup period (YF, prospective studies): whole study (coded as 0) or longest available
8) LCtype: all or nearest available, at least Squamous and Adeno. (q = squamous, s = small,
l = large, a = adeno, mix = mixed, alv = alveolar)
9) Race: all or nearest available, otherwise by race (wh or w = white, bl or b = black, hi = hispanic
ch = chinese, jap = japanese, haw = hawaiian, w+o = white + oriental, sca = scandinavian, as = asian)
10) For overlapping studies: principal rather than subsidiary studies
Finally by Age: whole study (coded as 0) if available, otherwise by widest available age group
and then for single sex results (m, f) in preference to combined sex results (c).

Results adjusted (AD) for the most potential confounders are then chosen in Sections -1 to -3
and results adjusted for the least confounders in Sections -4 to -6. (Those least adjusted results which
actually differ from the most adjusted as marked 'x' in column X in Section -4)
(Results adjusted for an unknown number of confounder(s) are coded as 20.)

Section -7 shows excluded studies, together with the stage (as above) at which no qualifying
results were found.

Section -8 lists the potentially overlapping studies which have been included (1=principal, 2=subsidiary).

Section -9 lists any results which would have been included in preference except that they had data not complete
enough for use in meta-analysis, with their significance (yes/no), if known, and any further comment as entered
on the database.

In addition to those mentioned above, the following fields, levels and abbreviations are used:
* or nk = not known, n = no, y = yes, ot = other
nev = never
all/unspec = all or unspecified, cig+/-ot = cigarettes irrespective of other products (cigar, pipe etc)
MC = manufactured cigarettes, HR = hand-rolled cigarettes
REF: 6-character study reference
NRR: number of the RR on the database within the study
ST : study type (CC = case control, pr or prosp = prospective)
NLC: number of lung cancer cases in whole study
R : risky occupational population (n = no, m = mining, o = other risky)
VB : national cigarette type (V = at least 75% Virginia, bl = at least 75% blended, ot = other)
P : any proxy use
H : full histological confirmation
De : derivation of RR/CI (or = original, st = standard method, ot = other method of estimation)

Table 1D1 - 1

IESLC - Meta-analysis of Ex Smoking, Any product (or Cigarettes if Any not available)
All LC types
Most adjusted

REF	NRR	SEX	AGEL	AGEH	RACE	YF	LC TYPE	LOC	START	ST	NLC	R	VB	P	H	AD	PRODUCT	DENOM	De
AGUDO	2	f	0	0	all	-	all	Eu:wst	1989	CC	103	n	bl	n	n	3	cig only	nev any	or
AKIBA	9	m	0	0	all	0	all	As:Jap	1963	pr	610	n	bl	n	n	5	cig+/-ot	nev cigs	or
AKIBA	13	f	0	0	all	0	all	As:Jap	1963	pr	610	n	bl	n	n	5	cig+/-ot	nev cigs	or
AMANDU	6	m	0	0	wh	0	all	NAmer	1959	pr	132	m	bl	n	n	2	cig+/-ot	nev cigs	ot
AMES	3	m	0	0	wh	-	all	NAmer	1959	ot	317	m	bl	n	n	0	all/unsp	nev any	st
ANDERS	1	f	0	0	all	0	all	NAmer	1986	pr	343	n	bl	n	n	0	cig+/-ot	nev cigs	st
ARCHER	4	m	0	0	wh	0	all	NAmer	1950	pr	146	m	bl	n	n	0	cig+/-ot	nev cigs	st
ARMADA	28	m	0	0	all	-	all	Eu:wst	1986	CC	325	n	bl	n	y	0	cig+/-ot	nev any	st
AUSTIN	5	c	0	0	all	-	all	NAmer	1970	CC	166	o	bl	y	n	3	cig+/-ot	nev cigs	or
AXELSS	4	m	0	0	sca	-	all	Eu:Sca	1989	CC	436	n	bl	n	n	0	all/unsp	nev any	st
AXELSS	9	f	0	0	sca	-	all	Eu:Sca	1989	CC	436	n	bl	n	n	0	all/unsp	nev any	st
BARBON	2	m	0	0	all	-	all	Eu:wst	1979	CC	755	n	bl	y	y	1	all/unsp	nev any	or
BECHER	5	m	0	0	all	-	all	Eu:Ger	1985	CC	194	n	bl	n	y	0	all/unsp	nev any	st
BECHER	6	f	0	0	all	-	all	Eu:Ger	1985	CC	194	n	bl	n	y	0	all/unsp	nev any	st
BENSHL	9	m	40	64	all	10	all	Eu:UK	1967	pr	486	n	V	n	n	1	all/unsp	nev any	ot
BEST	3	m	0	0	all	0	all	NAmer	1955	pr	381	n	V	n	n	1	cig only	nev any	ot
BLOHMK	2	m	0	0	all	-	all	Eu:Ger	1978	CC	888	n	bl	n	y	0	all/unsp	nev any	st
BOUCOT	115	m	0	0	all	0	all	NAmer	1951	pr	121	n	bl	n	n	2	cig only	nev any	ot
BRETT	9	m	0	0	all	0	all	Eu:UK	1960	pr	150	n	V	n	n	0	cig+/-ot	nev cigs	st
BROSS	6	m	0	0	wh	-	all	NAmer	1960	CC	974	n	bl	n	n	0	cig+/-ot	nev any	st
BROWN2	22	m	0	0	wh	-	all	NAmer	1984	CC	14596	n	bl	n	y	2	cig+/-ot	nev cigs	or
BROWN2	21	f	0	0	wh	-	all	NAmer	1984	CC	14596	n	bl	n	y	2	cig+/-ot	nev cigs	or
BUFFLE	4	m	0	0	wh	-	all	NAmer	1976	CC	943	n	bl	y	n	0	cig+/-ot	nev any	st
BUFFLE	8	f	0	0	wh	-	all	NAmer	1976	CC	943	n	bl	y	n	0	cig+/-ot	nev any	st
CARPEN	10	c	0	0	w+b	-	all	NAmer	1991	CC	356	n	bl	n	n	3	cig+/-ot	nev cigs	or
CEDERL	114	m	0	0	all	0	all	Eu:Sca	1963	pr	491	n	bl	n	n	2	all/unsp	nev any	or
CEDERL	74	f	0	0	all	0	all	Eu:Sca	1963	pr	491	n	bl	n	n	2	all/unsp	nev any	or
CHANG	1	m	0	0	all	0	all	NAmer	1972	pr	136	n	bl	n	n	0	cig+/-ot	nev cigs	st
CHANG	7	f	0	0	all	0	all	NAmer	1972	pr	136	n	bl	n	n	0	cig+/-ot	nev cigs	st
CHOI	2	m	0	0	all	-	all	As:oth	1985	CC	375	n	bl	n	n	0	cig+/-ot	nev cigs	st
CHOI	6	f	0	0	all	-	all	As:oth	1985	CC	375	n	bl	n	n	0	cig+/-ot	nev cigs	st
CHOW	26	m	0	0	wh	0	all	NAmer	1966	pr	219	n	bl	n	n	0	all/unsp	nev any	st
CHYOU	1	m	0	0	jap	0	all	NAmer	1965	pr	227	n	bl	n	y	1	cig+/-ot	nev cigs	or
COMSTO	2	m	0	0	all	-	all	NAmer	1975	ot	258	n	bl	n	n	0	cig+/-ot	nev any	st
COMSTO	7	f	0	0	all	-	all	NAmer	1975	ot	258	n	bl	n	n	0	cig+/-ot	nev any	st
CORREA	38	c	0	0	all	-	all	NAmer	1979	CC	1359	n	bl	y	n	1	cig+/-ot	nev cigs	or
CPSI	72	m	0	0	wh	0	all	NAmer	1959	pr	5138	n	bl	n	n	1	cig only	nev any	st
CPSI	280	f	40	74	all	6	all	NAmer	1959	pr	5138	n	bl	n	n	1	cig+/-ot	nev cigs	ot
CPSII	91	m	35	99	all	4	all	NAmer	1982	pr	3229	n	bl	n	n	1	cig only	nev any	or
CPSII	78	f	0	0	all	4	all	NAmer	1982	pr	3229	n	bl	n	n	1	cig+/-ot	nev cigs	ot
DAMBER	15	m	0	0	all	-	all	Eu:Sca	1972	CC	579	n	bl	y	n	1	all/unsp	nev any	ot
DARBY	5	m	0	0	wh	-	all	Eu:UK	1988	CC	982	n	V	n	n	0	all/unsp	nev any	st
DARBY	12	f	0	0	wh	-	all	Eu:UK	1988	CC	982	n	V	n	n	0	all/unsp	nev any	st
DEAN2	1	m	0	0	all	-	all	Eu:UK	1960	CC	954	n	V	y	n	0	all/unsp	nev any	st
DEAN2	5	f	0	0	all	-	all	Eu:UK	1960	CC	954	n	V	y	n	0	all/unsp	nev any	st
DEAN3	28	m	0	0	all	-	all	Eu:UK	1969	CC	766	n	V	y	n	3	all/unsp	nev any	ot
DEAN3	112	f	0	0	all	-	all	Eu:UK	1969	CC	766	n	V	y	n	3	cig only	nev any	ot
DEKLER	1	m	0	0	all	0	all	Auslia	1961	pr	138	m	V	n	n	2	all/unsp	nev any	or
DESTE2	3	c	0	0	all	-	all	SCAmer	1993	CC	463	n	bl	n	n	7	all/unsp	nev any	or
DESTEF	49	m	0	0	all	-	all	SCAmer	1988	CC	497	n	bl	n	y	4	all/unsp	nev any	ot
DOCKER	2	c	0	0	wh	0	all	NAmer	1974	pr	120	n	bl	n	n	4	cig+/-ot	nev cigs	or
DOLL	91	m	0	0	all	-	all	Eu:UK	1948	CC	1465	n	V	n	n	0	all/unsp	nev any	st
DOLL	94	f	0	0	all	-	all	Eu:UK	1948	CC	1465	n	V	n	n	0	all/unsp	nev any	st
DOLL2	55	m	0	0	all	0	all	Eu:UK	1951	pr	920	n	V	n	n	1	all/unsp	nev any	ot
DOLL2	9	f	0	0	all	22	all	Eu:UK	1951	pr	920	n	V	n	n	1	cig only	nev any	ot
DORANT	1	m	0	0	all	0	all	Eu:wst	1986	ot	550	n	bl	n	y	0	all/unsp	nev any	st
DORGAN	8	m	0	0	wh	-	all	NAmer	1980	CC	2026	n	bl	y	y	0	cig+/-ot	nev any	st
DORGAN	32	m	0	0	bl	-	all	NAmer	1980	CC	2026	n	bl	y	y	0	cig+/-ot	nev any	st
DORGAN	55	f	0	0	wh	-	all	NAmer	1980	CC	2026	n	bl	y	y	0	cig+/-ot	nev any	st
DORGAN	78	f	0	0	bl	-	all	NAmer	1980	CC	2026	n	bl	y	y	0	cig+/-ot	nev any	st
DORN	102	m	35	84	wh	8	all	NAmer	1954	pr	5097	n	bl	n	n	1	all/unsp	nev any	ot
DROSTE	5	m	0	0	all	-	all	Eu:wst	1995	CC	478	n	bl	n	y	4	all/unsp	nev any	or
ENGELA	157	m	0	0	all	12	all	Eu:Sca	1964	pr	435	n	bl	n	n	1	all/unsp	nev any	ot
ENGELA	43	f	0	0	all	0	all	Eu:Sca	1964	pr	435	n	bl	n	n	5	cig+/-ot	nev cigs	or
GAO	31	m	0	0	all	-	all	As:Chi	1984	CC	1405	n	ot	n	n	2	cig+/-ot	nev cigs	ot
GAO	32	f	0	0	all	-	all	As:Chi	1984	CC	1405	n	ot	n	n	2	cig+/-ot	nev cigs	ot
GAO2	9	m	0	0	all	-	all	As:Jap	1988	CC	282	n	bl	n	n	1	cig+/-ot	nev cigs	or
GARCIA	1	c	0	0	all	-	all	NAmer	1992	CC	416	n	bl	n	y	0	cig+/-ot	nev cigs	st
GARDIN	1	c	0	0	all	-	all	Eu:UK	1988	CC	143	n	V	y	n	0	all/unsp	nev any	st
GARSHI	30	m	0	0	all	-	all	NAmer	1981	CC	1081	o	bl	y	n	1	all/unsp	nev any	st
GOODMA	1	m	0	0	w+o	-	all	NAmer	1983	CC	326	n	bl	y	y	0	cig+/-ot	nev any	st
GOODMA	5	f	0	0	w+o	-	all	NAmer	1983	CC	326	n	bl	y	y	0	cig+/-ot	nev any	st

Table 1D1 - 1

IESLC - Meta-analysis of Ex Smoking, Any product (or Cigarettes if Any not available)
All LC types
Most adjusted

REF	NRR	SEX	AGEL	AGEH	RACE	YF	LC TYPE	LOC	START	ST	NLC	R	VB	P	H	AD	PRODUCT	DENOM	De
GRAHAM	26	m	0	0	wh	-	all	NAmer	1956	CC	685	n	bl	n	n	1	all/unsp	nev any	ot
GREGOR	1	m	0	0	all	-	all	Eu:UK	1976	CC	104	n	V	n	y	0	cig+/-ot	nev cigs	st
GREGOR	5	f	0	0	all	-	all	Eu:UK	1976	CC	104	n	V	n	y	0	cig+/-ot	nev cigs	st
HAENSZ	55	f	0	0	all	-	not alv	NAmer	1955	CC	158	n	bl	n	y	0	cig+/-ot	nev any	st
HAMMO2	12	m	0	0	all	0	all	NAmer	1967	pr	450	o	bl	n	n	1	cig+/-ot	nev any	ot
HEIN	6	m	0	0	all	0	all	Eu:Sca	1970	pr	144	n	bl	n	n	0	all/unsp	nev any	st
HENNEK	1	m	0	0	all	0	all	NAmer	1982	pr	169	n	bl	n	n	0	all/unsp	nev any	st
HIRAYA	146	m	0	0	all	0	all	As:Jap	1965	pr	1917	n	bl	n	n	1	cig+/-ot	nev any	ot
HIRAYA	149	f	0	0	all	0	all	As:Jap	1965	pr	1917	n	bl	n	n	1	cig+/-ot	nev any	ot
HITOSU	33	m	0	0	all	-	all	As:Jap	1960	CC	216	n	bl	y	n	1	all/unsp	nev any	st
HITOSU	58	f	0	0	all	-	all	As:Jap	1960	CC	216	n	bl	y	n	1	all/unsp	nev any	st
HOLE	7	m	0	0	all	0	all	Eu:UK	1972	pr	225	n	V	n	n	1	all/unsp	nev any	ot
HUMBLE	1	m	0	0	w-hi	-	all	NAmer	1980	CC	521	n	bl	y	n	1	cig+/-ot	nev cigs	or
HUMBLE	4	m	0	0	hi	-	all	NAmer	1980	CC	521	n	bl	y	n	1	cig+/-ot	nev cigs	or
HUMBLE	7	f	0	0	w-hi	-	all	NAmer	1980	CC	521	n	bl	y	n	1	cig+/-ot	nev cigs	or
HUMBLE	10	f	0	0	hi	-	all	NAmer	1980	CC	521	n	bl	y	n	1	cig+/-ot	nev cigs	or
JAHN	10	m	0	0	all	-	all	Eu:Ger	1988	CC	1004	n	bl	n	n	0	cig+/-ot	nev any	st
JAIN	55	m	0	0	all	-	all	NAmer	1981	CC	845	n	V	y	n	2	cig+/-ot	nev cigs	ot
JAIN	53	f	0	0	all	-	all	NAmer	1981	CC	845	n	V	y	n	2	cig+/-ot	nev cigs	ot
JARVHO	1	m	0	0	all	-	all	Eu:Sca	1983	CC	147	n	bl	n	n	0	all/unsp	nev any	st
JARVHO	5	f	0	0	all	-	all	Eu:Sca	1983	CC	147	n	bl	n	n	0	all/unsp	nev any	st
JEDRYC	64	m	0	0	all	-	all	Eu:est	1980	CC	1630	n	bl	y	n	0	cig+/-ot	nev any	st
JEDRYC	69	f	0	0	all	-	all	Eu:est	1980	CC	1630	n	bl	y	n	0	cig+/-ot	nev any	st
JOLY	22	m	0	0	all	-	all	SCAmer	1978	CC	826	n	bl	n	n	0	all/unsp	nev any	st
JOLY	19	f	0	0	all	-	all	SCAmer	1978	CC	826	n	bl	n	n	0	cig+/-ot	nev any	st
KAISE2	65	m	35	99	all	9	all	NAmer	1979	pr	318	n	bl	n	n	1	cig only	nev any	st
KAISE2	57	f	35	99	all	9	all	NAmer	1979	pr	318	n	bl	n	n	1	cig only	nev any	st
KAISER	5	m	0	0	all	0	all	NAmer	1964	pr	714	n	bl	n	n	2	cig+/-ot	nev cigs	or
KAISER	1	f	0	0	all	0	all	NAmer	1964	pr	714	n	bl	n	n	2	cig+/-ot	nev cigs	or
KATSOU	1	f	0	0	all	-	all	Eu:bal	1987	CC	101	n	bl	n	n	1	all/unsp	nev any	or
KAUFMA	10	c	0	0	all	-	all	NAmer	1981	CC	881	n	bl	n	n	6	cig+/-ot	nev cigs	or
KELLER	2	m	0	0	wh	-	all	NAmer	1985	CC	15038	n	bl	n	n	0	all/unsp	nev any	st
KELLER	10	m	0	0	nonw	-	all	NAmer	1985	CC	15038	n	bl	n	n	0	all/unsp	nev any	st
KELLER	6	f	0	0	wh	-	all	NAmer	1985	CC	15038	n	bl	n	n	0	all/unsp	nev any	st
KELLER	14	f	0	0	nonw	-	all	NAmer	1985	CC	15038	n	bl	n	n	0	all/unsp	nev any	st
KHUDER	13	m	0	0	all	-	all	NAmer	1985	CC	482	n	bl	n	y	0	cig+/-ot	nev cigs	or
KIHARA	15	c	0	0	jap	-	all	As:Jap	1991	CC	440	n	bl	n	n	0	all/unsp	nev any	st
KINLEN	12	m	0	0	all	0	all	Eu:UK	1967	pr	718	n	V	n	n	2	all/unsp	nev any	ot
KJUUS	2	m	0	0	all	-	all	Eu:Sca	1979	CC	176	n	bl	n	n	0	all/unsp	nev any	st
KNEKT	27	m	20	69	all	21	all	Eu:Sca	1966	pr	515	n	bl	n	n	1	all/unsp	nev any	or
KOO	8	f	0	0	all	-	all	As:HK	1981	CC	200	n	bl	n	n	0	all/unsp	nev any	st
KREUZE	17	m	1	45	all	-	all	Eu:Ger	1990	CC	2260	n	bl	n	n	3	all/unsp	nev any	or
KREUZE	28	m	55	69	all	-	all	Eu:Ger	1990	CC	2260	n	bl	n	n	3	all/unsp	nev any	or
KREUZE	23	f	1	45	all	-	all	Eu:Ger	1990	CC	2260	n	bl	n	n	3	all/unsp	nev any	or
KREUZE	34	f	55	69	all	-	all	Eu:Ger	1990	CC	2260	n	bl	n	n	3	all/unsp	nev any	or
KUBIK	11	m	0	0	all	0	all	Eu:est	1965	pr	108	n	bl	n	n	0	cig+/-ot	nev any	st
LANGE	14	m	0	0	all	0	all	Eu:Sca	1976	pr	268	n	bl	n	n	1	all/unsp	nev any	or
LANGE	10	f	0	0	all	0	all	Eu:Sca	1976	pr	268	n	bl	n	n	1	all/unsp	nev any	or
LEMARC	1	c	0	0	w+o	-	all	NAmer	1992	CC	341	n	bl	n	y	0	all/unsp	nev any	st
LIDDEL	1	m	0	0	all	18	all	NAmer	1970	pr	304	m	V	n	n	1	cig+/-ot	nev cigs	ot
LOMBAR	8	m	0	0	all	-	all	NAmer	1951	CC	1040	n	bl	n	n	0	cig+/-ot	nev any	st
LUBIN	39	m	0	0	all	-	all	As:Chi	1984	CC	427	m	ot	y	n	0	cig+/-ot	nev any	st
LUBIN2	36	m	0	0	all	-	all	Eu:mul	1976	CC	7804	n	bl	n	y	2	all/unsp	nev any	ot
LUBIN2	319	f	0	0	all	-	all	Eu:mul	1976	CC	7804	n	bl	n	y	0	cig+/-ot	nev any	st
MACLEN	7	m	0	0	ch	-	all	As:oth	1972	CC	233	n	bl	n	n	0	cig+/-ot	nev cigs	st
MACLEN	8	f	0	0	ch	-	all	As:oth	1972	CC	233	n	bl	n	n	0	cig+/-ot	nev cigs	st
MATOS	15	m	0	0	all	-	all	SCAmer	1994	CC	200	n	bl	n	n	2	cig+/-ot	nev any	or
MIGRAN	25	m	0	0	all	0	all	Eu:UK	1964	pr	259	n	V	n	n	2	all/unsp	nev any	ot
MIGRAN	40	f	0	0	all	0	all	Eu:UK	1964	pr	259	n	V	n	n	0	all/unsp	nev any	st
MRFITR	1	m	0	0	all	0	all	NAmer	1973	pr	119	n	bl	n	n	0	cig+/-ot	nev cigs	ot
NAM	73	m	0	0	all	-	all	NAmer	1986	CC	1199	n	bl	y	n	1	cig+/-ot	nev cigs	ot
NAM	89	f	0	0	all	-	all	NAmer	1986	CC	1199	n	bl	y	n	1	cig+/-ot	nev cigs	ot
ODRISC	2	c	0	0	all	-	all	Eu:UK	1992	CC	446	n	V	n	n	0	all/unsp	nev any	st
OSANN	25	m	0	0	all	-	all	NAmer	1984	CC	1986	n	bl	n	n	2	cig+/-ot	nev cigs	or
OSANN	26	f	0	0	all	-	all	NAmer	1984	CC	1986	n	bl	n	n	2	cig+/-ot	nev cigs	or
PARKIN	13	m	0	0	bl	-	all	Africa	1963	CC	877	n	V	y	n	6	all/unsp	nev any	or
PERSH2	7	c	0	0	all	-	all	Eu:Sca	1980	CC	1022	n	bl	y	n	4	all/unsp	nev any	ot
PETO	1	m	0	0	all	0	all	Eu:UK	1954	pr	103	n	V	n	n	0	all/unsp	nev any	st
PEZZO2	1	m	0	0	all	-	all	SCAmer	1992	CC	367	n	bl	n	y	0	cig+/-ot	nev cigs	st
PEZZOT	1	m	0	0	all	-	all	SCAmer	1987	CC	215	n	bl	n	y	0	cig only	nev cigs	st
QIAO2	13	m	0	0	all	0	all	As:Chi	1992	pr	241	m	ot	n	n	1	all/unsp	nev any	or
RACHTA	8	f	0	0	all	-	all	Eu:est	1991	CC	118	n	bl	n	y	1	cig+/-ot	nev cigs	or

Table 1D1 - 1

IESLC - Meta-analysis of Ex Smoking, Any product (or Cigarettes if Any not available)
All LC types
Most adjusted

REF	NRR	SEX	AGEL	AGEH	RACE	YF	LC TYPE	LOC	START	ST	NLC	R	VB	P	H	AD	PRODUCT	DENOM	De
SCHWAR	21	m	0	0	wh	-	all	NAmer	1984	CC	5588	n	bl	y	y	0	cig+/-ot	nev cigs	st
SCHWAR	22	m	0	0	bl	-	all	NAmer	1984	CC	5588	n	bl	y	y	0	cig+/-ot	nev cigs	st
SCHWAR	23	f	0	0	wh	-	all	NAmer	1984	CC	5588	n	bl	y	y	0	cig+/-ot	nev cigs	st
SCHWAR	24	f	0	0	bl	-	all	NAmer	1984	CC	5588	n	bl	y	y	0	cig+/-ot	nev cigs	st
SHAW	3	c	0	0	wh	-	all	NAmer	1988	CC	335	n	V	n	y	0	all/unsp	nev any	st
SOBUE	41	m	0	0	all	-	q+s+l+a	As:Jap	1986	CC	1376	n	bl	n	y	1	cig+/-ot	nev cigs	or
SOBUE	51	f	0	0	all	-	q+s+l+a	As:Jap	1986	CC	1376	n	bl	n	y	1	cig+/-ot	nev cigs	or
SPEIZE	7	f	0	0	all	0	all	NAmer	1976	pr	593	n	bl	n	y	0	cig+/-ot	nev cigs	st
SPITZ	1	c	0	0	b+hi	-	all	NAmer	1992	CC	177	n	bl	n	y	0	cig+/-ot	nev cigs	st
STOCKW	4	c	0	0	all	-	all	NAmer	1981	CC	22161	n	bl	n	n	0	cig+/-ot	nev any	st
STUCKE	1	m	0	0	all	-	all	Eu:wst	1989	CC	247	n	bl	n	y	0	all/unsp	nev any	ot
SUZUK2	5	c	0	0	all	-	all	SCAmer	1991	CC	123	n	bl	n	y	3	all/unsp	nev any	or
SVENSS	1	f	0	0	all	-	all	Eu:Sca	1983	CC	210	n	bl	n	n	1	all/unsp	nev any	or
TANG	2	c	0	0	all	-	not s	NAmer	1992	CC	119	n	bl	n	y	0	cig+/-ot	nev cigs	st
TENKAN	9	m	0	0	all	17	all	Eu:Sca	1962	pr	242	n	bl	n	n	1	all/unsp	nev any	ot
TIZZAN	6	m	0	0	all	-	all	Eu:wst	1959	CC	1358	n	bl	n	n	0	all/unsp	nev any	st
TIZZAN	14	f	0	0	all	-	all	Eu:wst	1959	CC	1358	n	bl	n	n	0	all/unsp	nev any	st
TOKARS	2	m	0	0	all	-	all	Eu:est	1966	ot	162	o	bl	n	y	0	all/unsp	nev any	st
TOUSEY	11	m	0	0	all	-	all	NAmer	1993	CC	507	n	bl	y	y	3	cig+/-ot	nev any	or
TOUSEY	14	f	0	0	all	-	all	NAmer	1993	CC	507	n	bl	y	y	3	cig+/-ot	nev any	or
TSUGAN	26	m	0	0	all	-	q+a	As:Jap	1976	CC	134	n	bl	n	y	0	all/unsp	nev any	st
TULINI	24	m	0	0	all	0	all	Eu:Sca	1967	pr	472	n	bl	n	n	3	all/unsp	nev any	or
TULINI	30	f	0	0	all	0	all	Eu:Sca	1967	pr	472	n	bl	n	n	3	all/unsp	nev any	or
TVERDA	1	m	0	0	all	0	all	Eu:Sca	1972	pr	238	n	bl	n	n	2	cig+/-ot	nev cigs	ot
TVERDA	18	f	0	0	all	0	all	Eu:Sca	1972	pr	238	n	bl	n	n	0	cig only	nev cigs	ot
WAKAI	7	m	0	0	all	-	all	As:Jap	1988	CC	333	n	bl	n	y	2	all/unsp	nev any	or
WAKAI	25	f	0	0	all	-	all	As:Jap	1988	CC	333	n	bl	n	y	2	all/unsp	nev any	or
WANG2	20	c	0	0	all	-	all	As:Chi	1980	CC	103	n	ot	n	n	4	cig+/-ot	nev cigs	ot
WIGLE	26	m	0	0	all	-	all	NAmer	1971	CC	728	n	V	n	n	1	all/unsp	nev any	ot
WIGLE	31	f	0	0	all	-	all	NAmer	1971	CC	728	n	V	n	n	1	all/unsp	nev any	ot
WU	41	f	0	0	wh	-	q+a	NAmer	1981	CC	220	n	bl	n	y	2	all/unsp	nev any	st
WUNSCH	6	m	0	0	all	-	all	SCAmer	1990	CC	398	n	bl	y	n	1	cig+/-ot	nev any	or
WUNSCH	12	f	0	0	all	-	all	SCAmer	1990	CC	398	n	bl	y	n	1	cig+/-ot	nev any	or
WYNDE3	41	m	0	0	all	-	all	NAmer	1966	CC	350	n	bl	n	y	0	all/unsp	nev any	st
WYNDE6	9	m	0	0	all	-	all	NAmer	1969	CC	4423	n	bl	n	y	0	cig+/-ot	nev any	st
WYNDE6	198	f	0	0	all	-	all	NAmer	1969	CC	4423	n	bl	n	y	0	cig+/-ot	nev cigs	st
YAMAGU	9	c	0	0	all	-	all	As:Jap	1989	CC	144	n	bl	n	y	1	all/unsp	nev any	or
YONG	1	c	0	0	all	0	all	NAmer	1971	pr	216	n	bl	n	n	1	cig+/-ot	nev cigs	or

Cigarette type is all/unspec for all RRs

except for the following:

REF	NRR	CIGTYPE
DEAN3	112	MC only

Table 1D1 - 2

IESLC - Meta-analysis of Ex Smoking, Any product (or Cigarettes if Any not available)
All LC types
Most adjusted

REF	NRR	SEX	AD	Number Exposed Case	Cont	Non-exposed Case	Cont	RR	95.00%CI
AGUDO	2	f	3	-	-	-	-	1.61	(0.37- 6.91)
*AKIBA	9	m	5	-	-	-	-	2.50	(1.50- 4.30)
*AKIBA	13	f	5	-	-	-	-	1.40	(0.70- 2.60)
Subtotal AKIBA								1.99	(1.32- 3.00)
*AMANDU	6	m	2	-	-	-	-	2.13	(0.62- 7.29)
AMES	3	m	0	147	115	15	62	5.28	(2.86- 9.77)
*ANDERS	1	f	0	85	54902	46	195158	6.57	(4.59- 9.40)
*ARCHER	4	m	0	18	3740	6	9842	7.89	(3.14- 19.87)
ARMADA	28	m	0	129	132	4	64	15.64	(5.53- 44.19)
AUSTIN	5	c	3	-	-	-	-	7.40	(2.60- 21.50)
AXELSS	4	m	0	98	214	16	160	4.58	(2.60- 8.07)
AXELSS	9	f	0	14	40	18	154	2.99	(1.37- 6.53)
Subtotal AXELSS								3.95	(2.50- 6.26)
BARBON	2	m	1	-	-	-	-	7.10	(4.40- 11.60)
BECHER	5	m	0	42	116	3	54	6.52	(1.93- 21.96)
BECHER	6	f	0	5	18	10	52	1.44	(0.44- 4.80)
Subtotal BECHER								3.04	(1.29- 7.14)
*BENSHL	9	m	1	-	-	-	-	2.95	(1.25- 6.95)
*BEST	3	m	1	-	-	-	-	6.06	(2.53- 14.51)
BLOHMK	2	m	0	343	274	126	301	2.99	(2.30- 3.88)
*BOUCOT	115	m	2	-	-	-	-	20.86	(1.20- 361.47)
*BRETT	9	m	0	9	10482	6	6530	0.93	(0.33- 2.62)
BROSS	6	m	0	212	146	38	170	6.50	(4.31- 9.79)
BROWN2	22	m	2	-	-	-	-	7.20	(6.50- 7.90)
BROWN2	21	f	2	-	-	-	-	11.60	(10.40- 13.00)
Subtotal BROWN2								8.85	(8.23- 9.53)
BUFFLE	4	m	0	204	154	5	47	12.45	(4.84- 32.05)
BUFFLE	8	f	0	106	101	41	198	5.07	(3.29- 7.81)
Subtotal BUFFLE								5.92	(4.00- 8.78)
CARPEN	10	c	3	-	-	-	-	7.00	(3.85- 12.73)
*CEDERL	114	m	2	-	-	-	-	1.25	(0.67- 2.34)
*CEDERL	74	f	2	-	-	-	-	1.08	(0.34- 3.44)
Subtotal CEDERL								1.21	(0.70- 2.10)
*CHANG	1	m	0	43	1087	5	502	3.97	(1.58- 9.97)
*CHANG	7	f	0	12	580	11	1139	2.14	(0.95- 4.83)
Subtotal CHANG								2.81	(1.53- 5.16)
CHOI	2	m	0	35	136	13	95	1.88	(0.94- 3.74)
CHOI	6	f	0	6	3	76	164	4.32	(1.05- 17.72)
Subtotal CHOI								2.21	(1.19- 4.10)
*CHOW	26	m	0	27	71657	6	62913	3.95	(1.63- 9.57)
*CHYOU	1	m	1	-	-	-	-	3.10	(1.60- 5.80)
COMSTO	2	m	0	46	129	4	69	6.15	(2.13- 17.80)
COMSTO	7	f	0	11	35	13	115	2.78	(1.14- 6.75)
Subtotal COMSTO								3.85	(1.95- 7.61)
CORREA	38	c	1	-	-	-	-	6.50	(4.70- 9.10)
*CPSI	72	m	1	-	-	-	-	3.74	(3.14- 4.46)
*CPSI	280	f	1	-	-	-	-	1.38	(0.81- 2.35)
Subtotal CPSI								3.39	(2.87- 4.01)
*CPSII	91	m	1	-	-	-	-	9.36	(7.43- 11.77)
*CPSII	78	f	1	-	-	-	-	4.84	(4.00- 5.86)
Subtotal CPSII								6.33	(5.47- 7.34)
DAMBER	15	m	1	-	-	-	-	4.04	(2.67- 6.11)
DARBY	5	m	0	285	1106	3	384	32.98	(10.51- 103.49)
DARBY	12	f	0	94	317	23	529	6.82	(4.23- 10.99)
Subtotal DARBY								8.61	(5.55- 13.37)
DEAN2	1	m	0	98	88	33	112	3.78	(2.33- 6.13)
DEAN2	5	f	0	5	2	88	121	3.44	(0.65- 18.13)
Subtotal DEAN2								3.75	(2.36- 5.97)
DEAN3	28	m	3	-	-	-	-	4.48	(2.75- 7.30)
DEAN3	112	f	3	-	-	-	-	1.17	(0.51- 2.69)
Subtotal DEAN3								3.18	(2.08- 4.84)
*DEKLER	1	m	2	-	-	-	-	10.70	(1.40- 81.90)
DESTE2	3	c	7	-	-	-	-	8.10	(4.50- 14.70)
DESTEF	49	m	4	-	-	-	-	6.20	(3.78- 10.15)
*DOCKER	2	c	4	-	-	-	-	2.54	(0.90- 7.18)
DOLL	91	m	0	70	124	7	61	4.92	(2.13- 11.34)
DOLL	94	f	0	10	8	40	59	1.84	(0.67- 5.08)
Subtotal DOLL								3.31	(1.74- 6.30)
*DOLL2	55	m	1	-	-	-	-	4.17	(2.61- 6.66)
*DOLL2	9	f	1	-	-	-	-	3.29	(0.88- 12.24)
Subtotal DOLL2								4.06	(2.61- 6.31)

Table 1D1 - 2

IESLC - Meta-analysis of Ex Smoking, Any product (or Cigarettes if Any not available)
All LC types
Most adjusted

REF	NRR	SEX	AD	Number Exposed Case	Number Exposed Cont	Non-exposed Case	Non-exposed Cont	RR	95.00%CI
DORANT	1	m	0	146	771	7	159	4.30	(1.98- 9.36)
DORGAN	8	m	0	236	230	15	93	6.36	(3.58- 11.30)
DORGAN	32	m	0	49	56	3	35	10.21	(2.95- 35.27)
DORGAN	55	f	0	146	110	103	244	3.14	(2.24- 4.41)
DORGAN	78	f	0	11	10	7	20	3.14	(0.93- 10.58)
Subtotal DORGAN								3.92	(2.98- 5.17)
*DORN	102	m	1	-	-	-	-	4.12	(3.19- 5.31)
DROSTE	5	m	4	-	-	-	-	4.20	(1.80- 9.80)
*ENGELA	157	m	1	-	-	-	-	2.80	(1.08- 7.20)
*ENGELA	43	f	5	-	-	-	-	2.00	(0.80- 4.90)
Subtotal ENGELA								2.35	(1.22- 4.52)
GAO	31	m	2	-	-	-	-	4.17	(2.85- 6.12)
GAO	32	f	2	-	-	-	-	4.24	(2.67- 6.75)
Subtotal GAO								4.20	(3.13- 5.64)
GAO2	9	m	1	-	-	-	-	3.56	(1.83- 6.91)
GARCIA	1	c	0	226	233	21	139	6.42	(3.92- 10.52)
GARDIN	1	c	0	41	44	5	41	7.64	(2.75- 21.22)
GARSHI	30	m	1	-	-	-	-	4.04	(2.84- 5.74)
GOODMA	1	m	0	68	229	10	199	5.91	(2.96- 11.79)
GOODMA	5	f	0	23	35	19	177	6.12	(3.02- 12.42)
Subtotal GOODMA								6.01	(3.67- 9.85)
GRAHAM	26	m	1	-	-	-	-	14.87	(8.94- 24.71)
GREGOR	1	m	0	23	45	10	14	0.72	(0.28- 1.86)
GREGOR	5	f	0	4	16	1	22	5.50	(0.56- 53.99)
Subtotal GREGOR								0.97	(0.40- 2.34)
HAENSZ	55	f	0	5	9	81	236	1.62	(0.53- 4.97)
*HAMMO2	12	m	1	-	-	-	-	6.46	(2.63- 15.85)
*HEIN	6	m	0	11	979	1	457	5.13	(0.66- 39.65)
*HENNEK	1	m	0	67	8674	23	10919	3.67	(2.29- 5.88)
*HIRAYA	146	m	1	-	-	-	-	1.71	(1.08- 2.72)
*HIRAYA	149	f	1	-	-	-	-	2.98	(1.14- 7.77)
Subtotal HIRAYA								1.90	(1.25- 2.88)
HITOSU	33	m	1	-	-	-	-	3.95	(1.63- 9.55)
HITOSU	58	f	1	-	-	-	-	6.72	(2.55- 17.68)
Subtotal HITOSU								5.03	(2.62- 9.66)
*HOLE	7	m	1	-	-	-	-	2.69	(1.16- 6.23)
HUMBLE	1	m	1	-	-	-	-	7.20	(3.00- 17.60)
HUMBLE	4	m	1	-	-	-	-	8.00	(1.90- 42.20)
HUMBLE	7	f	1	-	-	-	-	6.50	(2.80- 15.40)
HUMBLE	10	f	1	-	-	-	-	6.30	(1.50- 27.80)
Subtotal HUMBLE								6.88	(4.04- 11.71)
JAHN	10	m	0	455	402	18	138	8.68	(5.22- 14.44)
JAIN	55	m	2	-	-	-	-	4.16	(2.04- 8.46)
JAIN	53	f	2	-	-	-	-	3.54	(2.04- 6.13)
Subtotal JAIN								3.76	(2.43- 5.81)
JARVHO	1	m	0	26	28	1	16	14.86	(1.84- 120.07)
JARVHO	5	f	0	10	8	6	21	4.38	(1.19- 16.04)
Subtotal JARVHO								6.15	(2.04- 18.54)
JEDRYC	64	m	0	137	196	49	219	3.12	(2.14- 4.56)
JEDRYC	69	f	0	13	8	78	166	3.46	(1.38- 8.69)
Subtotal JEDRYC								3.17	(2.23- 4.50)
JOLY	22	m	0	108	223	12	218	8.80	(4.71- 16.44)
JOLY	19	f	0	34	27	52	283	6.85	(3.82- 12.31)
Subtotal JOLY								7.70	(5.02- 11.81)
*KAISE2	65	m	1	-	-	-	-	3.39	(1.77- 6.48)
*KAISE2	57	f	1	-	-	-	-	5.02	(2.17- 11.61)
Subtotal KAISE2								3.93	(2.35- 6.56)
*KAISER	5	m	2	-	-	-	-	4.65	(2.84- 7.64)
*KAISER	1	f	2	-	-	-	-	3.02	(1.83- 4.99)
Subtotal KAISER								3.76	(2.64- 5.35)
KATSOU	1	f	1	-	-	-	-	2.84	(0.81- 9.98)
KAUFMA	10	c	6	-	-	-	-	6.80	(4.60- 9.90)
KELLER	2	m	0	3003	1307	323	1017	7.23	(6.28- 8.33)
KELLER	10	m	0	440	128	38	117	10.58	(6.99- 16.04)
KELLER	6	f	0	1094	477	469	1860	9.10	(7.85- 10.54)
KELLER	14	f	0	130	79	67	232	5.70	(3.86- 8.42)
Subtotal KELLER								8.02	(7.28- 8.82)
KHUDER	13	m	0	184	-	23	-	7.50	(4.80- 11.90)
KIHARA	15	c	0	55	70	102	237	1.83	(1.20- 2.79)
*KINLEN	12	m	2	-	-	-	-	4.88	(2.26- 10.56)
KJUUS	2	m	0	39	75	2	24	6.24	(1.40- 27.78)

Table 1D1 - 2

IESLC - Meta-analysis of Ex Smoking, Any product (or Cigarettes if Any not available)
All LC types
Most adjusted

REF	NRR	SEX	AD	Number Exposed Case	Number Exposed Cont	Non-exposed Case	Non-exposed Cont	RR	95.00%CI
*KNEKT	27	m	1	-	-	-	-	2.60	(1.00- 6.40)
KOO	8	f	0	22	10	56	85	3.34	(1.47- 7.58)
KREUZE	17	m	3	-	-	-	-	1.70	(0.60- 5.20)
KREUZE	28	m	3	-	-	-	-	9.10	(5.90- 14.10)
KREUZE	23	f	3	-	-	-	-	2.50	(0.70- 9.00)
KREUZE	34	f	3	-	-	-	-	1.40	(0.80- 2.40)
Subtotal KREUZE								3.93	(2.87- 5.39)
*KUBIK	11	m	0	8	1487	2	4271	11.49	(2.44- 54.04)
*LANGE	14	m	1	-	-	-	-	2.10	(0.70- 6.00)
*LANGE	10	f	1	-	-	-	-	3.40	(1.10- 11.00)
Subtotal LANGE								2.63	(1.20- 5.76)
LEMARC	1	c	0	142	223	32	168	3.34	(2.17- 5.15)
*LIDDEL	1	m	1	-	-	-	-	1.40	(0.79- 2.51)
LOMBAR	8	m	0	126	172	14	112	5.86	(3.21- 10.69)
LUBIN	39	m	0	70	139	9	72	4.03	(1.90- 8.53)
LUBIN2	36	m	2	-	-	-	-	4.38	(3.71- 5.16)
LUBIN2	319	f	0	100	157	288	1180	2.61	(1.97- 3.46)
Subtotal LUBIN2								3.84	(3.33- 4.43)
MACLEN	7	m	0	5	11	5	15	1.36	(0.32- 5.89)
MACLEN	8	f	0	3	10	41	109	0.80	(0.21- 3.04)
Subtotal MACLEN								1.02	(0.38- 2.74)
MATOS	15	m	2	-	-	-	-	5.30	(2.60- 10.70)
*MIGRAN	25	m	2	-	-	-	-	2.55	(0.87- 7.50)
*MIGRAN	40	f	0	1	621	4	3814	1.54	(0.17- 13.71)
Subtotal MIGRAN								2.31	(0.88- 6.07)
*MRFITR	1	m	0	13	2813	0	1859	17.84~	(1.06- 300.00)
NAM	73	m	1	-	-	-	-	9.17	(6.12- 13.74)
NAM	89	f	1	-	-	-	-	8.27	(5.76- 11.87)
Subtotal NAM								8.66	(6.61- 11.34)
ODRISC	2	c	0	147	398	6	664	40.87	(17.90- 93.34)
OSANN	25	m	2	-	-	-	-	12.20	(8.80- 17.00)
OSANN	26	f	2	-	-	-	-	8.10	(6.00- 11.00)
Subtotal OSANN								9.77	(7.82- 12.22)
PARKIN	13	m	6	-	-	-	-	3.40	(1.90- 5.80)
PERSH2	7	c	4	-	-	-	-	2.61	(1.98- 3.44)
*PETO	1	m	0	2	387	2	295	0.76	(0.11- 5.38)
PEZZO2	1	m	0	128	271	6	117	9.21	(3.95- 21.48)
PEZZOT	1	m	0	66	188	4	116	10.18	(3.61- 28.67)
*QIAO2	13	m	1	-	-	-	-	1.33	(0.65- 2.71)
RACHTA	8	f	1	-	-	-	-	3.80	(1.21- 7.84)
SCHWAR	21	m	0	996	670	119	376	4.70	(3.74- 5.90)
SCHWAR	22	m	0	219	136	50	104	3.35	(2.25- 4.99)
SCHWAR	23	f	0	322	328	182	855	4.61	(3.69- 5.76)
SCHWAR	24	f	0	79	89	40	247	5.48	(3.49- 8.60)
Subtotal SCHWAR								4.54	(3.94- 5.22)
SHAW	3	c	0	112	169	11	107	6.45	(3.32- 12.53)
SOBUE	41	m	1	-	-	-	-	2.80	(1.90- 4.20)
SOBUE	51	f	1	-	-	-	-	2.10	(1.40- 3.20)
Subtotal SOBUE								2.44	(1.83- 3.25)
*SPEIZE	7	f	0	144	522081	58	776300	3.69	(2.72- 5.01)
SPITZ	1	c	0	67	80	7	128	15.31	(6.70- 35.02)
STOCKW	4	c	0	6185	3057	2791	10641	7.71	(7.26- 8.19)
STUCKE	1	m	0	178	135	0	51	135.69~	(8.30-2218.34)
SUZUK2	5	c	3	-	-	-	-	7.70	(2.20- 27.00)
SVENSS	1	f	1	-	-	-	-	2.60	(1.40- 5.10)
TANG	2	c	0	58	34	9	39	7.39	(3.19- 17.11)
*TENKAN	9	m	1	-	-	-	-	4.02	(1.50- 10.80)
TIZZAN	6	m	0	346	292	180	305	2.01	(1.58- 2.56)
TIZZAN	14	f	0	8	10	25	114	3.65	(1.31- 10.17)
Subtotal TIZZAN								2.07	(1.64- 2.62)
TOKARS	2	m	0	37	86	1	53	22.80	(3.04- 171.13)
TOUSEY	11	m	3	-	-	-	-	13.20	(4.80- 36.60)
TOUSEY	14	f	3	-	-	-	-	9.10	(4.80- 17.10)
Subtotal TOUSEY								10.10	(5.90- 17.31)
TSUGAN	26	m	0	10	8	18	22	1.53	(0.50- 4.68)
*TULINI	24	m	3	-	-	-	-	3.03	(1.54- 5.98)
*TULINI	30	f	3	-	-	-	-	3.69	(1.71- 7.99)
Subtotal TULINI								3.30	(1.98- 5.49)
*TVERDA	1	m	2	-	-	-	-	0.49	(0.24- 1.01)
*TVERDA	18	f	0	0	38953	3	157431	0.58~	(0.03- 11.18)
Subtotal TVERDA								0.49	(0.25- 0.99)

Table 1D1 - 2

IESLC - Meta-analysis of Ex Smoking, Any product (or Cigarettes if Any not available)
All LC types
Most adjusted

REF	NRR	SEX	AD	Number Exposed Case	Cont	Non-exposed Case	Cont	RR	95.00%CI
WAKAI	7	m	2	-	-	-	-	2.43	(1.16- 5.06)
WAKAI	25	f	2	-	-	-	-	5.33	(1.21- 23.50)
Subtotal WAKAI								2.84	(1.47- 5.49)
WANG2	20	c	4	-	-	-	-	2.00	(0.77- 5.20)
WIGLE	26	m	1	-	-	-	-	6.50	(3.71- 11.40)
WIGLE	31	f	1	-	-	-	-	2.10	(1.02- 4.33)
Subtotal WIGLE								4.25	(2.73- 6.62)
WU	41	f	2	-	-	-	-	1.40	(0.74- 2.66)
WUNSCH	6	m	1	-	-	-	-	3.28	(1.77- 6.07)
WUNSCH	12	f	1	-	-	-	-	3.51	(1.74- 7.10)
Subtotal WUNSCH								3.38	(2.13- 5.37)
WYNDE3	41	m	0	48	125	9	88	3.75	(1.75- 8.05)
WYNDE6	9	m	0	1088	1056	87	617	7.31	(5.75- 9.29)
WYNDE6	198	f	0	332	325	159	856	5.50	(4.38- 6.91)
Subtotal WYNDE6								6.29	(5.34- 7.43)
YAMAGU	9	c	1	-	-	-	-	2.90	(1.43- 5.90)
*YONG	1	c	1	-	-	-	-	4.10	(2.55- 6.60)
Partial Totals				20000	734696	6420	1257569		
*prospective study								~ With 0.5 adjustment for zero	

REF	NRR	SEX	AD	Ys	Ws	Qs	Ps
AGUDO	2	f	3	0.48	1.79	2.94	0.5236
*AKIBA	9	m	5	0.92	13.85	9.80	0.0006
*AKIBA	13	f	5	0.34	8.92	18.02	0.3148
Subtotal AKIBA				0.69	22.78	27.82	
*AMANDU	6	m	2	0.76	2.53	2.54	0.2291
AMES	3	m	0	1.66	10.17	0.09	0.0000
*ANDERS	1	f	0	1.88	29.87	0.47	0.0000
*ARCHER	4	m	0	2.07	4.51	0.43	0.0000
ARMADA	28	m	0	2.75	3.56	3.50	0.0000
AUSTIN	5	c	3	2.00	3.44	0.21	0.0002
AXELSS	4	m	0	1.52	11.96	0.67	0.0000
AXELSS	9	f	0	1.10	6.31	2.75	0.0059
Subtotal AXELSS				1.37	18.27	3.42	
BARBON	2	m	1	1.96	16.35	0.67	0.0000
BECHER	5	m	0	1.87	2.60	0.04	0.0025
BECHER	6	f	0	0.37	2.67	5.15	0.5481
Subtotal BECHER				1.11	5.27	5.19	
*BENSHL	9	m	1	1.08	5.22	2.38	0.0134
*BEST	3	m	1	1.80	5.04	0.01	0.0001
BLOHMK	2	m	0	1.10	56.10	24.59	0.0000
*BOUCOT	115	m	2	3.04	0.47	0.77	0.0370
*BRETT	9	m	0	-0.07	3.60	12.00	0.8976
BROSS	6	m	0	1.87	22.85	0.30	0.0000
BROWN2	22	m	2	1.97	403.85	18.96	0.0000
BROWN2	21	f	2	2.45	308.59	148.45	0.0000
Subtotal BROWN2				2.18	712.44	167.41	
BUFFLE	4	m	0	2.52	4.30	2.51	0.0000
BUFFLE	8	f	0	1.62	20.50	0.37	0.0000
Subtotal BUFFLE				1.78	24.80	2.88	
CARPEN	10	c	3	1.95	10.74	0.38	0.0000
*CEDERL	114	m	2	0.22	9.82	23.13	0.4843
*CEDERL	74	f	2	0.08	2.87	8.10	0.8963
Subtotal CEDERL				0.19	12.69	31.23	
*CHANG	1	m	0	1.38	4.54	0.65	0.0033
*CHANG	7	f	0	0.76	5.83	5.77	0.0659
Subtotal CHANG				1.03	10.36	6.42	
CHOI	2	m	0	0.63	8.11	10.27	0.0721
CHOI	6	f	0	1.46	1.93	0.17	0.0424
Subtotal CHOI				0.79	10.03	10.44	
*CHOW	26	m	0	1.37	4.91	0.72	0.0023
*CHYOU	1	m	1	1.13	9.26	3.63	0.0006
COMSTO	2	m	0	1.82	3.40	0.01	0.0008
COMSTO	7	f	0	1.02	4.88	2.63	0.0240
Subtotal COMSTO				1.35	8.28	2.65	
CORREA	38	c	1	1.87	35.20	0.46	0.0000
*CPSI	72	m	1	1.32	124.77	23.97	0.0000
*CPSI	280	f	1	0.32	13.54	27.90	0.2359
Subtotal CPSI				1.22	138.32	51.88	
*CPSII	91	m	1	2.24	72.61	16.66	0.0000

Table 1D1 - 2

IESLC - Meta-analysis of Ex Smoking, Any product (or Cigarettes if Any not available)
All LC types
Most adjusted

REF	NRR	SEX	AD	Ys	Ws	Qs	Ps
*CPSII	78	f	1	1.58	105.38	3.43	0.0000
Subtotal CPSII				1.85	177.99	20.09	
DAMBER	15	m	1	1.40	22.42	2.92	0.0000
DARBY	5	m	0	3.50	2.94	8.88	0.0000
DARBY	12	f	0	1.92	16.90	0.45	0.0000
Subtotal DARBY				2.15	19.84	9.33	
DEAN2	1	m	0	1.33	16.45	3.01	0.0000
DEAN2	5	f	0	1.23	1.39	0.38	0.1455
Subtotal DEAN2				1.32	17.84	3.39	
DEAN3	28	m	3	1.50	16.12	1.07	0.0000
DEAN3	112	f	3	0.16	5.56	14.23	0.7113
Subtotal DEAN3				1.16	21.68	15.30	
*DEKLER	1	m	2	2.37	0.93	0.35	0.0224
DESTE2	3	c	7	2.09	10.97	1.23	0.0000
DESTEF	49	m	4	1.82	15.75	0.07	0.0000
*DOCKER	2	c	4	0.93	3.56	2.43	0.0785
DOLL	91	m	0	1.59	5.51	0.15	0.0002
DOLL	94	f	0	0.61	3.75	4.92	0.2364
Subtotal DOLL				1.20	9.25	5.06	
*DOLL2	55	m	1	1.43	17.51	1.90	0.0000
*DOLL2	9	f	1	1.19	2.22	0.71	0.0762
Subtotal DOLL2				1.40	19.73	2.61	
DORANT	1	m	0	1.46	6.36	0.57	0.0002
DORGAN	8	m	0	1.85	11.63	0.10	0.0000
DORGAN	32	m	0	2.32	2.50	0.80	0.0002
DORGAN	55	f	0	1.15	33.62	12.58	0.0000
DORGAN	78	f	0	1.15	2.61	0.98	0.0645
Subtotal DORGAN				1.37	50.35	14.46	
*DORN	102	m	1	1.42	59.18	6.90	0.0000
DROSTE	5	m	4	1.44	5.35	0.56	0.0009
*ENGELA	157	m	1	1.03	4.27	2.26	0.0334
*ENGELA	43	f	5	0.69	4.68	5.30	0.1338
Subtotal ENGELA				0.85	8.95	7.56	
GAO	31	m	2	1.43	26.31	2.86	0.0000
GAO	32	f	2	1.44	17.86	1.75	0.0000
Subtotal GAO				1.43	44.17	4.60	
GAO2	9	m	1	1.27	8.70	2.07	0.0002
GARCIA	1	c	0	1.86	15.74	0.16	0.0000
GARDIN	1	c	0	2.03	3.68	0.28	0.0001
GARSHI	30	m	1	1.40	31.03	4.05	0.0000
GOODMA	1	m	0	1.78	8.06	0.00	0.0000
GOODMA	5	f	0	1.81	7.67	0.02	0.0000
Subtotal GOODMA				1.79	15.73	0.03	
GRAHAM	26	m	1	2.70	14.87	13.19	0.0000
GREGOR	1	m	0	-0.33	4.22	18.46	0.4919
GREGOR	5	f	0	1.70	0.74	0.00	0.1435
Subtotal GREGOR				-0.03	4.95	18.46	
HAENSZ	55	f	0	0.48	3.05	4.97	0.4002
*HAMMO2	12	m	1	1.87	4.76	0.06	0.0000
*HEIN	6	m	0	1.64	0.92	0.01	0.1167
*HENNEK	1	m	0	1.30	17.18	3.61	0.0000
*HIRAYA	146	m	1	0.54	18.01	26.85	0.0228
*HIRAYA	149	f	1	1.09	4.17	1.85	0.0257
Subtotal HIRAYA				0.64	22.18	28.69	
HITOSU	33	m	1	1.37	4.92	0.72	0.0023
HITOSU	58	f	1	1.91	4.10	0.09	0.0001
Subtotal HITOSU				1.62	9.01	0.81	
*HOLE	7	m	1	0.99	5.44	3.21	0.0210
HUMBLE	1	m	1	1.97	4.91	0.23	0.0000
HUMBLE	4	m	1	2.08	1.60	0.17	0.0086
HUMBLE	7	f	1	1.87	5.29	0.07	0.0000
HUMBLE	10	f	1	1.84	1.80	0.01	0.0135
Subtotal HUMBLE				1.93	13.60	0.48	
JAHN	10	m	0	2.16	14.82	2.41	0.0000
JAIN	55	m	2	1.43	7.59	0.84	0.0001
JAIN	53	f	2	1.26	12.69	3.09	0.0000
Subtotal JAIN				1.32	20.29	3.93	
JARVHO	1	m	0	2.70	0.88	0.78	0.0114
JARVHO	5	f	0	1.48	2.28	0.18	0.0260
Subtotal JARVHO				1.82	3.16	0.96	
JEDRYC	64	m	0	1.14	26.76	10.23	0.0000

Table 1D1 - 2

IESLC - Meta-analysis of Ex Smoking, Any product (or Cigarettes if Any not available)
All LC types
Most adjusted

REF	NRR	SEX	AD	Ys	Ws	Qs	Ps
JEDRYC	69	f	0	1.24	4.53	1.21	0.0083
Subtotal JEDRYC				1.15	31.29	11.44	
JOLY	22	m	0	2.17	9.84	1.71	0.0000
JOLY	19	f	0	1.92	11.21	0.31	0.0000
Subtotal JOLY				2.04	21.05	2.03	
*KAISE2	65	m	1	1.22	9.12	2.63	0.0002
*KAISE2	57	f	1	1.61	5.46	0.11	0.0002
Subtotal KAISE2				1.37	14.59	2.74	
*KAISER	5	m	2	1.54	15.69	0.76	0.0000
*KAISER	1	f	2	1.11	15.27	6.49	0.0000
Subtotal KAISER				1.32	30.96	7.26	
KATSOU	1	f	1	1.04	2.44	1.24	0.1033
KAUFMA	10	c	6	1.92	26.16	0.67	0.0000
KELLER	2	m	0	1.98	193.15	9.47	0.0000
KELLER	10	m	0	2.36	22.25	8.06	0.0000
KELLER	6	f	0	2.21	176.05	35.71	0.0000
KELLER	14	f	0	1.74	25.26	0.01	0.0000
Subtotal KELLER				2.08	416.70	53.25	
KHUDER	13	m	0	2.01	18.64	1.24	0.0000
KIHARA	15	c	0	0.60	21.51	28.72	0.0052
*KINLEN	12	m	2	1.59	6.46	0.19	0.0001
KJUUS	2	m	0	1.83	1.72	0.01	0.0163
*KNEKT	27	m	1	0.96	4.46	2.87	0.0436
KOO	8	f	0	1.21	5.71	1.74	0.0040
KREUZE	17	m	3	0.53	3.30	4.96	0.3354
KREUZE	28	m	3	2.21	20.24	4.12	0.0000
KREUZE	23	f	3	0.92	2.36	1.67	0.1596
KREUZE	34	f	3	0.34	12.73	25.71	0.2299
Subtotal KREUZE				1.37	38.63	36.45	
*KUBIK	11	m	0	2.44	1.60	0.75	0.0020
*LANGE	14	m	1	0.74	3.33	3.43	0.1758
*LANGE	10	f	1	1.22	2.90	0.83	0.0372
Subtotal LANGE				0.97	6.23	4.26	
LEMARC	1	c	0	1.21	20.52	6.22	0.0000
*LIDDEL	1	m	1	0.34	11.50	23.22	0.2539
LOMBAR	8	m	0	1.77	10.63	0.00	0.0000
LUBIN	39	m	0	1.39	6.83	0.90	0.0003
LUBIN2	36	m	2	1.48	141.18	11.10	0.0000
LUBIN2	319	f	0	0.96	48.33	30.79	0.0000
Subtotal LUBIN2				1.34	189.52	41.89	
MACLEN	7	m	0	0.31	1.79	3.76	0.6779
MACLEN	8	f	0	-0.23	2.14	8.43	0.7406
Subtotal MACLEN				0.02	3.94	12.18	
MATOS	15	m	2	1.67	7.68	0.06	0.0000
*MIGRAN	25	m	2	0.94	3.31	2.23	0.0885
*MIGRAN	40	f	0	0.43	0.80	1.41	0.7011
Subtotal MIGRAN				0.84	4.11	3.65	
*MRFITR	1	m	0	2.88	0.48	0.61	0.0454
NAM	73	m	1	2.22	23.49	4.94	0.0000
NAM	89	f	1	2.11	29.39	3.71	0.0000
Subtotal NAM				2.16	52.88	8.65	
ODRISC	2	c	0	3.71	5.63	21.49	0.0000
OSANN	25	m	2	2.50	35.44	19.62	0.0000
OSANN	26	f	2	2.09	41.82	4.68	0.0000
Subtotal OSANN				2.28	77.26	24.30	
PARKIN	13	m	6	1.22	12.34	3.51	0.0000
PERSH2	7	c	4	0.96	50.36	32.07	0.0000
*PETO	1	m	0	-0.27	1.01	4.14	0.7854
PEZZO2	1	m	0	2.22	5.36	1.15	0.0000
PEZZOT	1	m	0	2.32	3.58	1.14	0.0000
*QIAO2	13	m	1	0.29	7.54	16.34	0.4336
RACHTA	8	f	1	1.34	4.40	0.79	0.0051
SCHWAR	21	m	0	1.55	73.75	3.27	0.0000
SCHWAR	22	m	0	1.21	24.08	7.25	0.0000
SCHWAR	23	f	0	1.53	78.01	4.08	0.0000
SCHWAR	24	f	0	1.70	18.89	0.06	0.0000
Subtotal SCHWAR				1.51	194.73	14.66	
SHAW	3	c	0	1.86	8.69	0.10	0.0000
SOBUE	41	m	1	1.03	24.42	12.94	0.0000
SOBUE	51	f	1	0.74	22.48	23.19	0.0004
Subtotal SOBUE				0.89	46.91	36.12	

Table 1D1 - 2

IESLC - Meta-analysis of Ex Smoking, Any product (or Cigarettes if Any not available)
All LC types
Most adjusted

REF	NRR	SEX	AD	Ys	Ws	Qs	Ps
*SPEIZE	7	f	0	1.31	41.35	8.42	0.0000
SPITZ	1	c	0	2.73	5.61	5.30	0.0000
STOCKW	4	c	0	2.04	1062.62	86.67	0.0000
STUCKE	1	m	0	4.91	0.49	4.89	0.0006
SUZUK2	5	c	3	2.04	2.44	0.20	0.0014
SVENSS	1	f	1	0.96	9.19	5.91	0.0038
TANG	2	c	0	2.00	5.45	0.32	0.0000
*TENKAN	9	m	1	1.39	3.94	0.53	0.0057
TIZZAN	6	m	0	0.70	66.01	74.22	0.0000
TIZZAN	14	f	0	1.29	3.65	0.78	0.0134
Subtotal TIZZAN				0.73	69.66	75.01	
TOKARS	2	m	0	3.13	0.95	1.77	0.0024
TOUSEY	11	m	3	2.58	3.72	2.52	0.0000
TOUSEY	14	f	3	2.21	9.52	1.94	0.0000
Subtotal TOUSEY				2.31	13.24	4.46	
TSUGAN	26	m	0	0.42	3.07	5.46	0.4579
*TULINI	24	m	3	1.11	8.35	3.51	0.0014
*TULINI	30	f	3	1.31	6.46	1.32	0.0009
Subtotal TULINI				1.19	14.81	4.83	
*TVERDA	1	m	2	-0.71	7.44	45.42	0.0517
*TVERDA	18	f	0	-0.55	0.44	2.33	0.7164
Subtotal TVERDA				-0.70	7.88	47.75	
WAKAI	7	m	2	0.89	7.08	5.35	0.0181
WAKAI	25	f	2	1.67	1.75	0.01	0.0270
Subtotal WAKAI				1.04	8.83	5.37	
WANG2	20	c	4	0.69	4.21	4.77	0.1549
WIGLE	26	m	1	1.87	12.19	0.16	0.0000
WIGLE	31	f	1	0.74	7.35	7.58	0.0443
Subtotal WIGLE				1.45	19.54	7.74	
WU	41	f	2	0.34	9.39	18.95	0.3026
WUNSCH	6	m	1	1.19	10.12	3.28	0.0002
WUNSCH	12	f	1	1.26	7.77	1.96	0.0005
Subtotal WUNSCH				1.22	17.89	5.24	
WYNDE3	41	m	0	1.32	6.61	1.25	0.0007
WYNDE6	9	m	0	1.99	66.75	3.57	0.0000
WYNDE6	198	f	0	1.70	73.82	0.21	0.0000
Subtotal WYNDE6				1.84	140.57	3.78	
YAMAGU	9	c	1	1.06	7.65	3.67	0.0032
*YONG	1	c	1	1.41	16.99	2.04	0.0000

	N	182
	NS	124
	Wt	4738.71
Het	Chi	1262.58
Het	df	181
Het	P	***
Fixed	RR	5.80
	RRl	5.63
	RRu	5.96
	P	+++
Random	RR	4.30
	RRl	3.93
	RRu	4.71
	P	+++
Asymm	P	***

Table 1D1 - 3

IESLC - Meta-analysis of Ex Smoking, Any product (or Cigarettes if Any not available)
All LC types
Most adjusted

	Sex: combined	male	female	Total
N	20	100	62	182
NS	20	95	57	172
Wt	1321.19	2093.19	1324.33	4738.71
Het Chi	151.85	556.21	479.31	1262.58
Het df	19	99	61	181
Het P	***	***	***	***
Fixed RR	6.98	5.15	5.81	5.80
RRl	6.61	4.93	5.51	5.63
RRu	7.37	5.37	6.14	5.96
P	+++	+++	+++	+++
Random RR	5.58	4.48	3.58	4.30
RRl	4.23	3.98	3.00	3.93
RRu	7.36	5.06	4.29	4.71
P	+++	+++	+++	+++
Between Chi				75.21
Between df				2
Between P				***
Btwn(F) P				**
Btwn(R) P				*

	Lung cancer type: all	other	Total
N	176	6	182
NS	119	5	124
Wt	4670.85	67.86	4738.71
Het Chi	1195.97	11.70	1262.58
Het df	175	5	181
Het P	***	*	***
Fixed RR	5.87	2.37	5.80
RRl	5.71	1.87	5.63
RRu	6.04	3.01	5.96
P	+++	+++	+++
Random RR	4.39	2.36	4.30
RRl	4.01	1.57	3.93
RRu	4.81	3.55	4.71
P	+++	+++	+++
Between Chi			54.91
Between df			1
Between P			***
Btwn(F) P			**
Btwn(R) P			**

	Location: NAmer	UK	Scand	othEur	China	Japan	othAs	other	Total
N	80	21	21	24	5	14	5	12	182
NS	53	14	14	17	4	9	3	10	124
Wt	3665.65	128.45	165.00	448.57	62.75	150.64	19.68	97.97	4738.71
Het Chi	506.70	86.95	44.88	109.66	9.97	16.35	4.66	14.96	1262.58
Het df	79	20	20	23	4	13	4	11	181
Het P	***	***	**	***	*	N.S.	N.S.	N.S.	***
Fixed RR	6.79	4.14	2.67	3.57	3.46	2.35	2.13	5.77	5.80
RRl	6.57	3.48	2.29	3.26	2.70	2.01	1.37	4.73	5.63
RRu	7.01	4.92	3.11	3.92	4.44	2.76	3.32	7.03	5.96
P	+++	+++	+++	+++	+++	+++	+++	+++	+++
Random RR	5.44	3.72	2.62	3.96	3.09	2.40	2.12	5.84	4.30
RRl	4.91	2.51	2.01	3.07	2.02	1.99	1.29	4.60	3.93
RRu	6.03	5.50	3.42	5.12	4.74	2.89	3.47	7.41	4.71
P	+++	+++	+++	+++	+++	+++	++	+++	+++
Between Chi									468.44
Between df									7
Between P									***
Btwn(F) P									***
Btwn(R) P									***

Table 1D1 - 3

IESLC - Meta-analysis of Ex Smoking, Any product (or Cigarettes if Any not available)
All LC types
Most adjusted

	Detailed Country in "other Europe"					
	multi	Germany	othWest	East	Balkans	Total
N	2	8	8	5	1	24
NS	1	4	7	4	1	17
Wt	189.52	114.82	103.57	38.23	2.44	448.57
Het Chi	9.65	47.24	41.89	5.94	0.00	109.66
Het df	1	7	7	4	0	23
Het P	**	***	***	N.S.	N.S.	***
Fixed RR	3.84	3.77	2.97	3.59	2.84	3.57
RRl	3.33	3.14	2.45	2.61	0.81	3.26
RRu	4.43	4.52	3.60	4.93	9.97	3.92
P	+++	+++	+++	+++	N.S.	+++
Random RR	3.43	3.46	4.91	4.16	2.84	3.96
RRl	2.06	1.96	2.58	2.52	0.81	3.07
RRu	5.69	6.13	9.37	6.87	9.97	5.12
P	+++	+++	+++	+++	N.S.	+++
Between Chi						4.93
Between df						4
Between P						N.S.
Btwn(F) P						N.S.
Btwn(R) P						N.S.

	Detailed Country in "other Asia"			
	India	HongKong	other	Total
N		1	4	5
NS		1	2	3
Wt		5.71	13.97	19.68
Het Chi		0.00	3.04	4.66
Het df		0	3	4
Het P		N.S.	N.S.	N.S.
Fixed RR		3.34	1.77	2.13
RRl		1.47	1.05	1.37
RRu		7.58	3.00	3.32
P		++	+	+++
Random RR		3.34	1.77	2.12
RRl		1.47	1.04	1.29
RRu		7.58	3.02	3.47
P		++	+	++
Between Chi				1.62
Between df				1
Between P				N.S.
Btwn(F) P				N.S.
Btwn(R) P				N.S.

	Detailed other continent			
	SCAmer	Auslia	Africa	Total
N	10	1	1	12
NS	8	1	1	10
Wt	84.71	0.93	12.34	97.97
Het Chi	10.74	0.00	0.00	14.96
Het df	9	0	0	11
Het P	N.S.	N.S.	N.S.	N.S.
Fixed RR	6.19	10.70	3.40	5.77
RRl	5.00	1.40	1.95	4.73
RRu	7.66	81.84	5.94	7.03
P	+++	+	+++	+++
Random RR	6.21	10.70	3.40	5.84
RRl	4.90	1.40	1.95	4.60
RRu	7.86	81.84	5.94	7.41
P	+++	+	+++	+++
Between Chi				4.22
Between df				2
Between P				N.S.
Btwn(F) P				N.S.
Btwn(R) P				N.S.

Table 1D1 - 3

IESLC - Meta-analysis of Ex Smoking, Any product (or Cigarettes if Any not available)
All LC types
Most adjusted

	Start year of study					
	<1960	1960-69	1970-79	1980-89	1990+	Total
N	18	36	36	70	22	182
NS	14	25	25	43	17	124
Wt	348.41	393.49	529.68	3268.43	198.71	4738.71
Het Chi	83.35	114.50	129.43	476.79	120.77	1262.58
Het df	17	35	35	69	21	181
Het P	***	***	***	***	***	***
Fixed RR	3.55	4.06	3.81	6.91	4.73	5.80
RRl	3.19	3.68	3.50	6.68	4.11	5.63
RRu	3.94	4.48	4.14	7.15	5.43	5.96
P	+++	+++	+++	+++	+++	+++
Random RR	3.81	3.32	3.48	5.22	5.17	4.30
RRl	2.84	2.71	2.85	4.65	3.66	3.93
RRu	5.11	4.08	4.25	5.86	7.31	4.71
P	+++	+++	+++	+++	+++	+++
Between Chi						337.74
Between df						4
Between P						***
Btwn(F) P						***
Btwn(R) P						***

	Study type (1)		
	CC	other	Total
N	123	59	182
NS	80	44	124
Wt	3968.66	770.05	4738.71
Het Chi	886.67	219.51	1262.58
Het df	122	58	181
Het P	***	***	***
Fixed RR	6.28	3.84	5.80
RRl	6.09	3.58	5.63
RRu	6.48	4.12	5.96
P	+++	+++	+++
Random RR	4.88	3.18	4.30
RRl	4.41	2.70	3.93
RRu	5.41	3.74	4.71
P	+++	+++	+++
Between Chi			156.39
Between df			1
Between P			***
Btwn(F) P			***
Btwn(R) P			***

	Study type (2)			
	CC	prosp	other	Total
N	123	54	5	182
NS	80	40	4	124
Wt	3968.66	744.30	25.75	4738.71
Het Chi	886.67	214.08	4.13	1262.58
Het df	122	53	4	181
Het P	***	***	N.S.	***
Fixed RR	6.28	3.81	4.79	5.80
RRl	6.09	3.54	3.25	5.63
RRu	6.48	4.09	7.04	5.96
P	+++	+++	+++	+++
Random RR	4.88	3.06	4.79	4.30
RRl	4.41	2.58	3.23	3.93
RRu	5.41	3.63	7.11	4.71
P	+++	+++	+++	+++
Between Chi				157.69
Between df				2
Between P				***
Btwn(F) P				***
Btwn(R) P				***

Table 1D1 - 3

IESLC - Meta-analysis of Ex Smoking, Any product (or Cigarettes if Any not available)
All LC types
Most adjusted

	Study size (number of LC cases)				
	100-249	250-499	500-999	1000+	Total
N	51	45	37	49	182
NS	44	33	23	24	124
Wt	215.41	371.56	475.06	3676.68	4738.71
Het Chi	133.05	138.61	119.93	695.56	1262.58
Het df	50	44	36	48	181
Het P	***	***	***	***	***
Fixed RR	3.18	4.21	4.61	6.39	5.80
RRl	2.79	3.80	4.21	6.19	5.63
RRu	3.64	4.66	5.04	6.60	5.96
P	+++	+++	+++	+++	+++
Random RR	3.36	4.13	4.86	4.76	4.30
RRl	2.65	3.42	4.07	4.13	3.93
RRu	4.25	4.99	5.80	5.48	4.71
P	+++	+++	+++	+++	+++
Between Chi					175.42
Between df					3
Between P					***
Btwn(F) P					***
Btwn(R) P					*

	Risky occupational population			
	no	mining	othRisky	Total
N	171	7	4	182
NS	113	7	4	124
Wt	4654.53	44.00	40.19	4738.71
Het Chi	1212.29	21.40	4.26	1262.58
Het df	170	6	3	181
Het P	***	**	N.S.	***
Fixed RR	5.85	2.84	4.69	5.80
RRl	5.68	2.11	3.44	5.63
RRu	6.02	3.81	6.38	5.96
P	+++	+++	+++	+++
Random RR	4.32	3.17	5.46	4.30
RRl	3.94	1.74	3.33	3.93
RRu	4.74	5.76	8.96	4.71
P	+++	+++	+++	+++
Between Chi				24.63
Between df				2
Between P				***
Btwn(F) P				N.S.
Btwn(R) P				N.S.

	National cigarette tobacco type			
	Virginia	blended	other	Total
N	30	147	5	182
NS	21	99	4	124
Wt	206.77	4469.19	62.75	4738.71
Het Chi	109.95	1092.81	9.97	1262.58
Het df	29	146	4	181
Het P	***	***	*	***
Fixed RR	3.95	5.94	3.46	5.80
RRl	3.45	5.77	2.70	5.63
RRu	4.53	6.12	4.44	5.96
P	+++	+++	+++	+++
Random RR	3.79	4.45	3.09	4.30
RRl	2.85	4.04	2.02	3.93
RRu	5.04	4.91	4.74	4.71
P	+++	+++	+++	+++
Between Chi				49.85
Between df				2
Between P				***
Btwn(F) P				*
Btwn(R) P				N.S.

Table 1D1 - 3

IESLC - Meta-analysis of Ex Smoking, Any product (or Cigarettes if Any not available)
All LC types
Most adjusted

	Any proxy use No/nk	Yes	Total
N	141	41	182
NS	102	22	124
Wt	4073.74	664.97	4738.71
Het Chi	1125.95	95.09	1262.58
Het df	140	40	181
Het P	***	***	***
Fixed RR	6.02	4.60	5.80
RRl	5.84	4.26	5.63
RRu	6.21	4.96	5.96
P	+++	+++	+++
Random RR	4.12	4.79	4.30
RRl	3.70	4.20	3.93
RRu	4.59	5.47	4.71
P	+++	+++	+++
Between Chi			41.54
Between df			1
Between P			***
Btwn(F) P			*
Btwn(R) P			(*)

	Full histological confirmation No	Yes	Total
N	131	51	182
NS	88	36	124
Wt	3080.45	1658.27	4738.71
Het Chi	850.70	410.50	1262.58
Het df	130	50	181
Het P	***	***	***
Fixed RR	5.73	5.93	5.80
RRl	5.53	5.65	5.63
RRu	5.93	6.23	5.96
P	+++	+++	+++
Random RR	4.11	4.76	4.30
RRl	3.68	4.02	3.93
RRu	4.60	5.63	4.71
P	+++	+++	+++
Between Chi			1.38
Between df			1
Between P			N.S.
Btwn(F) P			N.S.
Btwn(R) P			N.S.

	Number of adjustment variables (1) 0	1	2+/+nk	Total
N	86	48	48	182
NS	60	34	34	128
Wt	2561.03	828.71	1348.97	4738.71
Het Chi	519.37	205.14	461.79	1262.58
Het df	85	47	47	181
Het P	***	***	***	***
Fixed RR	6.10	4.40	6.23	5.80
RRl	5.87	4.11	5.91	5.63
RRu	6.34	4.71	6.58	5.96
P	+++	+++	+++	+++
Random RR	4.84	3.96	3.81	4.30
RRl	4.26	3.37	3.11	3.93
RRu	5.50	4.65	4.66	4.71
P	+++	+++	+++	+++
Between Chi				76.28
Between df				2
Between P				***
Btwn(F) P				**
Btwn(R) P				(*)

Table 1D1 - 3

IESLC - Meta-analysis of Ex Smoking, Any product (or Cigarettes if Any not available)
All LC types
Most adjusted

	Number of adjustment variables (2) 0	1	2	3-5	6+/+nk	Total
N	86	48	23	22	3	182
NS	60	34	16	15	3	128
Wt	2561.03	828.71	1086.04	213.48	49.46	4738.71
Het Chi	519.37	205.14	288.12	85.80	5.37	1262.58
Het df	85	47	22	21	2	181
Het P	***	***	***	***	(*)	***
Fixed RR	6.10	4.40	6.98	3.54	5.95	5.80
RRl	5.87	4.11	6.58	3.09	4.50	5.63
RRu	6.34	4.71	7.41	4.05	7.86	5.96
P	+++	+++	+++	+++	+++	+++
Random RR	4.84	3.96	3.89	3.49	5.78	4.30
RRl	4.26	3.37	2.95	2.60	3.58	3.93
RRu	5.50	4.65	5.13	4.69	9.33	4.71
P	+++	+++	+++	+++	+++	+++
Between Chi						158.78
Between df						4
Between P						***
Btwn(F) P						***
Btwn(R) P						(*)

	Product all/unsp	cig+/-ot	cig only	Total
N	81	90	11	182
NS	61	61	10	132
Wt	1341.72	3165.92	231.07	4738.71
Het Chi	442.02	673.63	59.84	1262.58
Het df	80	89	10	181
Het P	***	***	***	***
Fixed RR	4.76	6.38	4.96	5.80
RRl	4.51	6.16	4.36	5.63
RRu	5.02	6.60	5.64	5.96
P	+++	+++	+++	+++
Random RR	4.09	4.49	4.18	4.30
RRl	3.53	3.98	2.63	3.93
RRu	4.74	5.06	6.66	4.71
P	+++	+++	+++	+++
Between Chi				87.09
Between df				2
Between P				***
Btwn(F) P				**
Btwn(R) P				N.S.

	Denominator nev any	nev cigs	Total
N	121	61	182
NS	86	42	128
Wt	3017.21	1721.50	4738.71
Het Chi	758.61	497.85	1262.58
Het df	120	60	181
Het P	***	***	***
Fixed RR	5.64	6.08	5.80
RRl	5.44	5.80	5.63
RRu	5.85	6.37	5.96
P	+++	+++	+++
Random RR	4.35	4.18	4.30
RRl	3.89	3.56	3.93
RRu	4.88	4.91	4.71
P	+++	+++	+++
Between Chi			6.11
Between df			1
Between P			*
Btwn(F) P			N.S.
Btwn(R) P			N.S.

Table 1D1 - 3

IESLC - Meta-analysis of Ex Smoking, Any product (or Cigarettes if Any not available)
All LC types
Most adjusted

	Derivation of RR/CI			
	Orig	StdCalc	Other	Total
N	55	89	38	182
NS	38	61	30	129
Wt	1319.78	2729.77	689.16	4738.71
Het Chi	399.73	566.89	181.23	1262.58
Het df	54	88	37	181
Het P	***	***	***	***
Fixed RR	6.76	5.88	4.09	5.80
RRl	6.40	5.66	3.80	5.63
RRu	7.13	6.10	4.41	5.96
P	+++	+++	+++	+++
Random RR	4.19	4.66	3.67	4.30
RRl	3.51	4.12	3.03	3.93
RRu	5.01	5.28	4.46	4.71
P	+++	+++	+++	+++
Between Chi				114.73
Between df				2
Between P				***
Btwn(F) P				***
Btwn(R) P				N.S.

Table 1D1 - 4

IESLC - Meta-analysis of Ex Smoking, Any product (or Cigarettes if Any not available)
All LC types
Least adjusted

REF	NRR	X	SEX	AGEL	AGEH	RACE	YF	LC TYPE	LOC	START	ST	NLC	R	VB	P	H	AD	PRODUCT	DENOM	De
AGUDO	9	x	f	0	0	all	-	all	Eu:wst	1989	CC	103	n	bl	n	n	0	cig only	nev any	st
AKIBA	1	x	m	0	0	all	0	all	As:Jap	1963	pr	610	n	bl	n	n	0	cig+/-ot	nev cigs	or
AKIBA	5	x	f	0	0	all	0	all	As:Jap	1963	pr	610	n	bl	n	n	0	cig+/-ot	nev cigs	or
AMANDU	2	x	m	0	0	wh	0	all	NAmer	1959	pr	132	m	bl	n	n	0	cig+/-ot	nev cigs	st
AMES	3		m	0	0	wh	-	all	NAmer	1959	ot	317	m	bl	n	n	0	all/unsp	nev any	st
ANDERS	1		f	0	0	all	0	all	NAmer	1986	pr	343	n	bl	n	n	0	cig+/-ot	nev cigs	st
ARCHER	4		m	0	0	wh	0	all	NAmer	1950	pr	146	m	bl	n	n	0	cig+/-ot	nev cigs	st
ARMADA	28		m	0	0	all	-	all	Eu:wst	1986	CC	325	n	bl	n	y	0	cig+/-ot	nev any	st
AUSTIN	1	x	c	0	0	all	-	all	NAmer	1970	CC	166	o	bl	y	n	0	cig+/-ot	nev cigs	st
AXELSS	4		m	0	0	sca	-	all	Eu:Sca	1989	CC	436	n	bl	n	n	0	all/unsp	nev any	st
AXELSS	9		f	0	0	sca	-	all	Eu:Sca	1989	CC	436	n	bl	n	n	0	all/unsp	nev any	st
BARBON	1	x	m	0	0	all	-	all	Eu:wst	1979	CC	755	n	bl	y	y	0	all/unsp	nev any	st
BECHER	5		m	0	0	all	-	all	Eu:Ger	1985	CC	194	n	bl	n	y	0	all/unsp	nev any	st
BECHER	6		f	0	0	all	-	all	Eu:Ger	1985	CC	194	n	bl	n	y	0	all/unsp	nev any	st
BENSHL	9		m	40	64	all	10	all	Eu:UK	1967	pr	486	n	V	n	n	1	all/unsp	nev any	ot
BEST	3		m	0	0	all	0	all	NAmer	1955	pr	381	n	V	n	n	1	cig only	nev any	ot
BLOHMK	2		m	0	0	all	-	all	Eu:Ger	1978	CC	888	n	bl	n	y	0	all/unsp	nev any	st
BOUCOT	3	x	m	0	0	all	0	all	NAmer	1951	pr	121	n	bl	n	n	0	cig only	nev any	ot
BRETT	9		m	0	0	all	0	all	Eu:UK	1960	pr	150	n	V	n	n	0	cig+/-ot	nev cigs	st
BROSS	6		m	0	0	wh	-	all	NAmer	1960	CC	974	n	bl	n	n	0	cig+/-ot	nev any	st
BROWN2	22		m	0	0	wh	-	all	NAmer	1984	CC	14596	n	bl	n	y	2	cig+/-ot	nev cigs	or
BROWN2	21		f	0	0	wh	-	all	NAmer	1984	CC	14596	n	bl	n	y	2	cig+/-ot	nev cigs	or
BUFFLE	4		m	0	0	wh	-	all	NAmer	1976	CC	943	n	bl	y	n	0	cig+/-ot	nev any	st
BUFFLE	8		f	0	0	wh	-	all	NAmer	1976	CC	943	n	bl	y	n	0	cig+/-ot	nev any	st
CARPEN	8	x	c	0	0	w+b	-	all	NAmer	1991	CC	356	n	bl	n	n	0	cig+/-ot	nev cigs	st
CEDERL	114		m	0	0	all	0	all	Eu:Sca	1963	pr	491	n	bl	n	n	2	all/unsp	nev any	or
CEDERL	74		f	0	0	all	0	all	Eu:Sca	1963	pr	491	n	bl	n	n	2	all/unsp	nev any	or
CHANG	1		m	0	0	all	0	all	NAmer	1972	pr	136	n	bl	n	n	0	cig+/-ot	nev cigs	st
CHANG	7		f	0	0	all	0	all	NAmer	1972	pr	136	n	bl	n	n	0	cig+/-ot	nev cigs	st
CHOI	2		m	0	0	all	-	all	As:oth	1985	CC	375	n	bl	n	n	0	cig+/-ot	nev cigs	st
CHOI	6		f	0	0	all	-	all	As:oth	1985	CC	375	n	bl	n	n	0	cig+/-ot	nev cigs	st
CHOW	26		m	0	0	wh	0	all	NAmer	1966	pr	219	n	bl	n	n	0	all/unsp	nev any	st
CHYOU	3	x	m	0	0	jap	0	all	NAmer	1965	pr	227	n	bl	n	y	0	cig+/-ot	nev cigs	st
COMSTO	2		m	0	0	all	-	all	NAmer	1975	ot	258	n	bl	n	n	0	cig+/-ot	nev any	st
COMSTO	7		f	0	0	all	-	all	NAmer	1975	ot	258	n	bl	n	n	0	cig+/-ot	nev any	st
CORREA	37	x	c	0	0	all	-	all	NAmer	1979	CC	1359	n	bl	y	n	0	cig+/-ot	nev cigs	st
CPSI	72		m	0	0	wh	0	all	NAmer	1959	pr	5138	n	bl	n	n	1	cig only	nev any	st
CPSI	280		f	40	74	all	6	all	NAmer	1959	pr	5138	n	bl	n	n	1	cig+/-ot	nev cigs	ot
CPSII	91		m	35	99	all	4	all	NAmer	1982	pr	3229	n	bl	n	n	1	cig only	nev any	or
CPSII	78		f	0	0	all	4	all	NAmer	1982	pr	3229	n	bl	n	n	1	cig+/-ot	nev cigs	ot
DAMBER	15		m	0	0	all	-	all	Eu:Sca	1972	CC	579	n	bl	y	n	1	all/unsp	nev any	ot
DARBY	5		m	0	0	wh	-	all	Eu:UK	1988	CC	982	n	V	n	n	0	all/unsp	nev any	st
DARBY	12		f	0	0	wh	-	all	Eu:UK	1988	CC	982	n	V	n	n	0	all/unsp	nev any	st
DEAN2	1		m	0	0	all	-	all	Eu:UK	1960	CC	954	n	V	y	n	0	all/unsp	nev any	st
DEAN2	5		f	0	0	all	-	all	Eu:UK	1960	CC	954	n	V	y	n	0	all/unsp	nev any	st
DEAN3	26	x	m	0	0	all	-	all	Eu:UK	1969	CC	766	n	V	y	n	0	all/unsp	nev any	st
DEAN3	110	x	f	0	0	all	-	all	Eu:UK	1969	CC	766	n	V	y	n	0	cig only	nev any	st
DEKLER	1		m	0	0	all	0	all	Auslia	1961	pr	138	m	V	n	n	2	all/unsp	nev any	or
DESTE2	1	x	c	0	0	all	-	all	SCAmer	1993	CC	463	n	bl	n	n	0	all/unsp	nev any	st
DESTEF	10	x	m	0	0	all	-	all	SCAmer	1988	CC	497	n	bl	n	y	0	all/unsp	nev any	st
DOCKER	2		c	0	0	wh	0	all	NAmer	1974	pr	120	n	bl	n	n	4	cig+/-ot	nev cigs	or
DOLL	91		m	0	0	all	-	all	Eu:UK	1948	CC	1465	n	V	n	n	0	all/unsp	nev any	st
DOLL	94		f	0	0	all	-	all	Eu:UK	1948	CC	1465	n	V	n	n	0	all/unsp	nev any	st
DOLL2	55		m	0	0	all	0	all	Eu:UK	1951	pr	920	n	V	n	n	1	all/unsp	nev any	ot
DOLL2	9		f	0	0	all	22	all	Eu:UK	1951	pr	920	n	V	n	n	1	cig only	nev any	ot
DORANT	1		m	0	0	all	0	all	Eu:wst	1986	ot	550	n	bl	n	y	0	all/unsp	nev any	st
DORGAN	8		m	0	0	wh	-	all	NAmer	1980	CC	2026	n	bl	y	y	0	cig+/-ot	nev any	st
DORGAN	32		m	0	0	bl	-	all	NAmer	1980	CC	2026	n	bl	y	y	0	cig+/-ot	nev any	st
DORGAN	55		f	0	0	wh	-	all	NAmer	1980	CC	2026	n	bl	y	y	0	cig+/-ot	nev any	st
DORGAN	78		f	0	0	bl	-	all	NAmer	1980	CC	2026	n	bl	y	y	0	cig+/-ot	nev any	st
DORN	102		m	35	84	wh	8	all	NAmer	1954	pr	5097	n	bl	n	n	1	all/unsp	nev any	ot
DROSTE	1	x	m	0	0	all	-	all	Eu:wst	1995	CC	478	n	bl	n	y	0	all/unsp	nev any	st
ENGELA	153	x	m	0	0	all	12	all	Eu:Sca	1964	pr	435	n	bl	n	n	0	all/unsp	nev any	st
ENGELA	15	x	f	0	0	all	0	all	Eu:Sca	1964	pr	435	n	bl	n	n	0	cig+/-ot	nev cigs	st
GAO	27	x	m	0	0	all	-	all	As:Chi	1984	CC	1405	n	ot	n	n	0	cig+/-ot	nev cigs	st
GAO	28	x	f	0	0	all	-	all	As:Chi	1984	CC	1405	n	ot	n	n	0	cig+/-ot	nev cigs	st
GAO2	5	x	m	0	0	all	-	all	As:Jap	1988	CC	282	n	bl	n	n	0	cig+/-ot	nev cigs	st
GARCIA	1		c	0	0	all	-	all	NAmer	1992	CC	416	n	bl	n	y	0	cig+/-ot	nev cigs	st
GARDIN	1		c	0	0	all	-	all	Eu:UK	1988	CC	143	n	V	y	n	0	all/unsp	nev any	st
GARSHI	22	x	m	0	0	all	-	all	NAmer	1981	CC	1081	o	bl	y	n	0	all/unsp	nev any	st
GOODMA	1		m	0	0	w+o	-	all	NAmer	1983	CC	326	n	bl	y	y	0	cig+/-ot	nev any	st
GOODMA	5		f	0	0	w+o	-	all	NAmer	1983	CC	326	n	bl	y	y	0	cig+/-ot	nev any	st

Table 1D1 - 4

IESLC - Meta-analysis of Ex Smoking, Any product (or Cigarettes if Any not available)
All LC types
Least adjusted

REF	NRR	X	SEX	AGEL	AGEH	RACE	YF	LC TYPE	LOC	START	ST	NLC	R	VB	P	H	AD	PRODUCT	DENOM	De
GRAHAM	21	x	m	0	0	wh	-	all	NAmer	1956	CC	685	n	bl	n	n	0	all/unsp	nev any	st
GREGOR	1		m	0	0	all	-	all	Eu:UK	1976	CC	104	n	V	n	y	0	cig+/-ot	nev cigs	st
GREGOR	5		f	0	0	all	-	all	Eu:UK	1976	CC	104	n	V	n	y	0	cig+/-ot	nev cigs	st
HAENSZ	55		f	0	0	all	-	not alv	NAmer	1955	CC	158	n	bl	n	y	0	cig+/-ot	nev any	st
HAMMO2	26	x	m	0	0	all	0	all	NAmer	1967	pr	450	o	bl	n	n	0	cig+/-ot	nev any	st
HEIN	6		m	0	0	all	0	all	Eu:Sca	1970	pr	144	n	bl	n	n	0	all/unsp	nev any	st
HENNEK	1		m	0	0	all	0	all	NAmer	1982	pr	169	n	bl	n	n	0	all/unsp	nev any	st
HIRAYA	146		m	0	0	all	0	all	As:Jap	1965	pr	1917	n	bl	n	n	1	cig+/-ot	nev any	ot
HIRAYA	149		f	0	0	all	0	all	As:Jap	1965	pr	1917	n	bl	n	n	1	cig+/-ot	nev any	ot
HITOSU	1	x	m	0	0	all	-	all	As:Jap	1960	CC	216	n	bl	y	n	0	all/unsp	nev any	st
HITOSU	8	x	f	0	0	all	-	all	As:Jap	1960	CC	216	n	bl	y	n	0	all/unsp	nev any	st
HOLE	14	x	m	0	0	all	0	all	Eu:UK	1972	pr	225	n	V	n	n	0	all/unsp	nev any	st
HUMBLE	1		m	0	0	w-hi	-	all	NAmer	1980	CC	521	n	bl	y	n	1	cig+/-ot	nev cigs	or
HUMBLE	4		m	0	0	hi	-	all	NAmer	1980	CC	521	n	bl	y	n	1	cig+/-ot	nev cigs	or
HUMBLE	7		f	0	0	w-hi	-	all	NAmer	1980	CC	521	n	bl	y	n	1	cig+/-ot	nev cigs	or
HUMBLE	10		f	0	0	hi	-	all	NAmer	1980	CC	521	n	bl	y	n	1	cig+/-ot	nev cigs	or
JAHN	10		m	0	0	all	-	all	Eu:Ger	1988	CC	1004	n	bl	n	n	0	cig+/-ot	nev any	st
JAIN	26	x	m	0	0	all	-	all	NAmer	1981	CC	845	n	V	y	n	0	cig+/-ot	nev cigs	st
JAIN	21	x	f	0	0	all	-	all	NAmer	1981	CC	845	n	V	y	n	0	cig+/-ot	nev cigs	st
JARVHO	1		m	0	0	all	-	all	Eu:Sca	1983	CC	147	n	bl	n	n	0	all/unsp	nev any	st
JARVHO	5		f	0	0	all	-	all	Eu:Sca	1983	CC	147	n	bl	n	n	0	all/unsp	nev any	st
JEDRYC	64		m	0	0	all	-	all	Eu:est	1980	CC	1630	n	bl	y	n	0	cig+/-ot	nev any	st
JEDRYC	69		f	0	0	all	-	all	Eu:est	1980	CC	1630	n	bl	y	n	0	cig+/-ot	nev any	st
JOLY	22		m	0	0	all	-	all	SCAmer	1978	CC	826	n	bl	n	n	0	all/unsp	nev any	st
JOLY	19		f	0	0	all	-	all	SCAmer	1978	CC	826	n	bl	n	n	0	cig+/-ot	nev any	st
KAISE2	65		m	35	99	all	9	all	NAmer	1979	pr	318	n	bl	n	n	1	cig only	nev any	st
KAISE2	57		f	35	99	all	9	all	NAmer	1979	pr	318	n	bl	n	n	1	cig only	nev any	st
KAISER	5		m	0	0	all	0	all	NAmer	1964	pr	714	n	bl	n	n	2	cig+/-ot	nev cigs	or
KAISER	1		f	0	0	all	0	all	NAmer	1964	pr	714	n	bl	n	n	2	cig+/-ot	nev cigs	or
KATSOU	5	x	f	0	0	all	-	all	Eu:bal	1987	CC	101	n	bl	n	n	0	all/unsp	nev any	st
KAUFMA	1	x	c	0	0	all	-	all	NAmer	1981	CC	881	n	bl	n	n	0	cig+/-ot	nev cigs	st
KELLER	2		m	0	0	wh	-	all	NAmer	1985	CC	15038	n	bl	n	n	0	all/unsp	nev any	st
KELLER	10		m	0	0	nonw	-	all	NAmer	1985	CC	15038	n	bl	n	n	0	all/unsp	nev any	st
KELLER	6		f	0	0	wh	-	all	NAmer	1985	CC	15038	n	bl	n	n	0	all/unsp	nev any	st
KELLER	14		f	0	0	nonw	-	all	NAmer	1985	CC	15038	n	bl	n	n	0	all/unsp	nev any	st
KHUDER	13		m	0	0	all	-	all	NAmer	1985	CC	482	n	bl	n	y	0	cig+/-ot	nev cigs	or
KIHARA	15		c	0	0	jap	-	all	As:Jap	1991	CC	440	n	bl	n	n	0	all/unsp	nev any	st
KINLEN	1	x	m	0	0	all	0	all	Eu:UK	1967	pr	718	n	V	n	n	0	all/unsp	nev any	st
KJUUS	2		m	0	0	all	-	all	Eu:Sca	1979	CC	176	n	bl	n	n	0	all/unsp	nev any	st
KNEKT	14	x	m	20	69	all	21	all	Eu:Sca	1966	pr	515	n	bl	n	n	0	all/unsp	nev any	st
KOO	8		f	0	0	all	-	all	As:HK	1981	CC	200	n	bl	n	n	0	all/unsp	nev any	st
KREUZE	1	x	m	1	45	all	-	all	Eu:Ger	1990	CC	2260	n	bl	n	n	0	all/unsp	nev any	st
KREUZE	3	x	m	55	69	all	-	all	Eu:Ger	1990	CC	2260	n	bl	n	n	0	all/unsp	nev any	st
KREUZE	2	x	f	1	45	all	-	all	Eu:Ger	1990	CC	2260	n	bl	n	n	0	all/unsp	nev any	st
KREUZE	4	x	f	55	69	all	-	all	Eu:Ger	1990	CC	2260	n	bl	n	n	0	all/unsp	nev any	st
KUBIK	11		m	0	0	all	0	all	Eu:est	1965	pr	108	n	bl	n	n	0	cig+/-ot	nev any	st
LANGE	5	x	m	0	0	all	0	all	Eu:Sca	1976	pr	268	n	bl	n	n	0	all/unsp	nev any	st
LANGE	1	x	f	0	0	all	0	all	Eu:Sca	1976	pr	268	n	bl	n	n	0	all/unsp	nev any	st
LEMARC	1		c	0	0	w+o	-	all	NAmer	1992	CC	341	n	bl	n	y	0	all/unsp	nev any	st
LIDDEL	1		m	0	0	all	18	all	NAmer	1970	pr	304	m	V	n	n	1	cig+/-ot	nev cigs	ot
LOMBAR	8		m	0	0	all	-	all	NAmer	1951	CC	1040	n	bl	n	n	0	cig+/-ot	nev any	st
LUBIN	39		m	0	0	all	-	all	As:Chi	1984	CC	427	m	ot	y	n	0	cig+/-ot	nev any	st
LUBIN2	35	x	m	0	0	all	-	all	Eu:mul	1976	CC	7804	n	bl	n	y	0	all/unsp	nev any	st
LUBIN2	319		f	0	0	all	-	all	Eu:mul	1976	CC	7804	n	bl	n	y	0	cig+/-ot	nev any	st
MACLEN	7		m	0	0	ch	-	all	As:oth	1972	CC	233	n	bl	n	n	0	cig+/-ot	nev cigs	st
MACLEN	8		f	0	0	ch	-	all	As:oth	1972	CC	233	n	bl	n	n	0	cig+/-ot	nev cigs	st
MATOS	14	x	m	0	0	all	-	all	SCAmer	1994	CC	200	n	bl	n	n	0	cig+/-ot	nev any	st
MIGRAN	24	x	m	0	0	all	0	all	Eu:UK	1964	pr	259	n	V	n	n	0	all/unsp	nev any	st
MIGRAN	40		f	0	0	all	0	all	Eu:UK	1964	pr	259	n	V	n	n	0	all/unsp	nev any	st
MRFITR	1		m	0	0	all	0	all	NAmer	1973	pr	119	n	bl	n	n	0	cig+/-ot	nev cigs	ot
NAM	65	x	m	0	0	all	-	all	NAmer	1986	CC	1199	n	bl	y	n	0	cig+/-ot	nev cigs	ot
NAM	81	x	f	0	0	all	-	all	NAmer	1986	CC	1199	n	bl	y	n	0	cig+/-ot	nev cigs	ot
ODRISC	2		c	0	0	all	-	all	Eu:UK	1992	CC	446	n	V	n	n	0	all/unsp	nev any	st
OSANN	1	x	m	0	0	all	-	all	NAmer	1984	CC	1986	n	bl	n	n	0	cig+/-ot	nev cigs	st
OSANN	5	x	f	0	0	all	-	all	NAmer	1984	CC	1986	n	bl	n	n	0	cig+/-ot	nev cigs	st
PARKIN	16	x	m	0	0	bl	-	all	Africa	1963	CC	877	n	V	y	n	0	all/unsp	nev any	st
PERSH2	1	x	c	0	0	all	-	all	Eu:Sca	1980	CC	1022	n	bl	y	n	0	all/unsp	nev any	st
PETO	1		m	0	0	all	0	all	Eu:UK	1954	pr	103	n	V	n	n	0	all/unsp	nev any	st
PEZZO2	1		m	0	0	all	-	all	SCAmer	1992	CC	367	n	bl	n	y	0	cig+/-ot	nev cigs	st
PEZZOT	1		m	0	0	all	-	all	SCAmer	1987	CC	215	n	bl	n	y	0	cig only	nev cigs	st
QIAO2	1	x	m	0	0	all	0	all	As:Chi	1992	pr	241	m	ot	n	n	0	all/unsp	nev any	st
RACHTA	1	x	f	0	0	all	-	all	Eu:est	1991	CC	118	n	bl	n	y	0	cig+/-ot	nev cigs	st

Table 1D1 - 4

IESLC - Meta-analysis of Ex Smoking, Any product (or Cigarettes if Any not available)
All LC types
Least adjusted

REF	NRR	X	SEX	AGEL	AGEH	RACE	YF	LC TYPE	LOC	START	ST	NLC	R	VB	P	H	AD	PRODUCT	DENOM	De
SCHWAR	21		m	0	0	wh	-	all	NAmer	1984	CC	5588	n	bl	y	y	0	cig+/-ot	nev cigs	st
SCHWAR	22		m	0	0	bl	-	all	NAmer	1984	CC	5588	n	bl	y	y	0	cig+/-ot	nev cigs	st
SCHWAR	23		f	0	0	wh	-	all	NAmer	1984	CC	5588	n	bl	y	y	0	cig+/-ot	nev cigs	st
SCHWAR	24		f	0	0	bl	-	all	NAmer	1984	CC	5588	n	bl	y	y	0	cig+/-ot	nev cigs	st
SHAW	3		c	0	0	wh	-	all	NAmer	1988	CC	335	n	V	n	y	0	all/unsp	nev any	st
SOBUE	89	x	m	0	0	all	-	q+s+l+a	As:Jap	1986	CC	1376	n	bl	n	y	0	cig+/-ot	nev cigs	st
SOBUE	93	x	f	0	0	all	-	q+s+l+a	As:Jap	1986	CC	1376	n	bl	n	y	0	cig+/-ot	nev cigs	st
SPEIZE	7		f	0	0	all	0	all	NAmer	1976	pr	593	n	bl	n	y	0	cig+/-ot	nev cigs	st
SPITZ	1		c	0	0	b+hi	-	all	NAmer	1992	CC	177	n	bl	n	y	0	cig+/-ot	nev cigs	st
STOCKW	4		c	0	0	all	-	all	NAmer	1981	CC	22161	n	bl	n	n	0	cig+/-ot	nev any	st
STUCKE	1		m	0	0	all	-	all	Eu:wst	1989	CC	247	n	bl	n	y	0	all/unsp	nev any	ot
SUZUK2	1	x	c	0	0	all	-	all	SCAmer	1991	CC	123	n	bl	n	y	0	all/unsp	nev any	st
SVENSS	21	x	f	0	0	all	-	all	Eu:Sca	1983	CC	210	n	bl	n	n	0	all/unsp	nev any	st
TANG	2		c	0	0	all	-	not s	NAmer	1992	CC	119	n	bl	n	y	0	cig+/-ot	nev cigs	st
TENKAN	9		m	0	0	all	17	all	Eu:Sca	1962	pr	242	n	bl	n	n	1	all/unsp	nev any	ot
TIZZAN	6		m	0	0	all	-	all	Eu:wst	1959	CC	1358	n	bl	n	n	0	all/unsp	nev any	st
TIZZAN	14		f	0	0	all	-	all	Eu:wst	1959	CC	1358	n	bl	n	n	0	all/unsp	nev any	st
TOKARS	2		m	0	0	all	-	all	Eu:est	1966	ot	162	o	bl	n	y	0	all/unsp	nev any	st
TOUSEY	3	x	m	0	0	all	-	all	NAmer	1993	CC	507	n	bl	y	y	0	cig+/-ot	nev any	st
TOUSEY	7	x	f	0	0	all	-	all	NAmer	1993	CC	507	n	bl	y	y	0	cig+/-ot	nev any	st
TSUGAN	26		m	0	0	all	-	q+a	As:Jap	1976	CC	134	n	bl	n	y	0	all/unsp	nev any	st
TULINI	1	x	m	0	0	all	0	all	Eu:Sca	1967	pr	472	n	bl	n	n	1	all/unsp	nev any	or
TULINI	7	x	f	0	0	all	0	all	Eu:Sca	1967	pr	472	n	bl	n	n	1	all/unsp	nev any	or
TVERDA	1		m	0	0	all	0	all	Eu:Sca	1972	pr	238	n	bl	n	n	2	cig+/-ot	nev cigs	ot
TVERDA	18		f	0	0	all	0	all	Eu:Sca	1972	pr	238	n	bl	n	n	0	cig only	nev cigs	ot
WAKAI	1	x	m	0	0	all	-	all	As:Jap	1988	CC	333	n	bl	n	y	0	all/unsp	nev any	st
WAKAI	19	x	f	0	0	all	-	all	As:Jap	1988	CC	333	n	bl	n	y	0	all/unsp	nev any	st
WANG2	19	x	c	0	0	all	-	all	As:Chi	1980	CC	103	n	ot	n	n	0	cig+/-ot	nev cigs	st
WIGLE	9	x	m	0	0	all	-	all	NAmer	1971	CC	728	n	V	n	n	0	all/unsp	nev any	st
WIGLE	12	x	f	0	0	all	-	all	NAmer	1971	CC	728	n	V	n	n	0	all/unsp	nev any	st
WU	33	x	f	0	0	wh	-	q+a	NAmer	1981	CC	220	n	bl	n	y	0	all/unsp	nev any	st
WUNSCH	3	x	m	0	0	all	-	all	SCAmer	1990	CC	398	n	bl	y	n	0	cig+/-ot	nev any	st
WUNSCH	9	x	f	0	0	all	-	all	SCAmer	1990	CC	398	n	bl	y	n	0	cig+/-ot	nev any	st
WYNDE3	41		m	0	0	all	-	all	NAmer	1966	CC	350	n	bl	n	y	0	all/unsp	nev any	st
WYNDE6	9		m	0	0	all	-	all	NAmer	1969	CC	4423	n	bl	n	y	0	cig+/-ot	nev any	st
WYNDE6	198		f	0	0	all	-	all	NAmer	1969	CC	4423	n	bl	n	y	0	cig+/-ot	nev cigs	st
YAMAGU	4	x	c	0	0	all	-	all	As:Jap	1989	CC	144	n	bl	n	y	0	all/unsp	nev any	st
YONG	1		c	0	0	all	0	all	NAmer	1971	pr	216	n	bl	n	n	1	cig+/-ot	nev cigs	or

Cigarette type is all/unspec for all RRs

except for the following:

REF	NRR	CIGTYPE
DEAN3	110	MC only

Table 1D1 - 5

IESLC - Meta-analysis of Ex Smoking, Any product (or Cigarettes if Any not available)
All LC types
Least adjusted

REF	NRR	SEX	AD	Number Exposed Case	Cont	Non-exposed Case	Cont	RR	95.00%CI
AGUDO	9	f	0	3	6	80	183	1.14	(0.28- 4.69)
*AKIBA	1	m	0	48	36303	18	35833	2.63	(1.53- 4.52)
*AKIBA	5	f	0	9	13942	116	359850	2.00	(1.02- 3.94)
Subtotal AKIBA								2.37	(1.55- 3.61)
*AMANDU	2	m	0	11	14687	6	25350	3.16	(1.17- 8.55)
AMES	3	m	0	147	115	15	62	5.28	(2.86- 9.77)
*ANDERS	1	f	0	85	54902	46	195158	6.57	(4.59- 9.40)
*ARCHER	4	m	0	18	3740	6	9842	7.89	(3.14- 19.87)
ARMADA	28	m	0	129	132	4	64	15.64	(5.53- 44.19)
AUSTIN	1	c	0	50	112	5	88	7.86	(3.01- 20.54)
AXELSS	4	m	0	98	214	16	160	4.58	(2.60- 8.07)
AXELSS	9	f	0	14	40	18	154	2.99	(1.37- 6.53)
Subtotal AXELSS								3.95	(2.50- 6.26)
BARBON	1	m	0	171	205	22	188	7.13	(4.38- 11.59)
BECHER	5	m	0	42	116	3	54	6.52	(1.93- 21.96)
BECHER	6	f	0	5	18	10	52	1.44	(0.44- 4.80)
Subtotal BECHER								3.04	(1.29- 7.14)
*BENSHL	9	m	1	-	-	-	-	2.95	(1.25- 6.95)
*BEST	3	m	1	-	-	-	-	6.06	(2.53- 14.51)
BLOHMK	2	m	0	343	274	126	301	2.99	(2.30- 3.88)
*BOUCOT	3	m	0	8	5977	0	7551	21.48~	(1.24- 372.01)
*BRETT	9	m	0	9	10482	6	6530	0.93	(0.33- 2.62)
BROSS	6	m	0	212	146	38	170	6.50	(4.31- 9.79)
BROWN2	22	m	2	-	-	-	-	7.20	(6.50- 7.90)
BROWN2	21	f	2	-	-	-	-	11.60	(10.40- 13.00)
Subtotal BROWN2								8.85	(8.23- 9.53)
BUFFLE	4	m	0	204	154	5	47	12.45	(4.84- 32.05)
BUFFLE	8	f	0	106	101	41	198	5.07	(3.29- 7.81)
Subtotal BUFFLE								5.92	(4.00- 8.78)
CARPEN	8	c	0	109	307	15	241	5.70	(3.24- 10.04)
*CEDERL	114	m	2	-	-	-	-	1.25	(0.67- 2.34)
*CEDERL	74	f	2	-	-	-	-	1.08	(0.34- 3.44)
Subtotal CEDERL								1.21	(0.70- 2.10)
*CHANG	1	m	0	43	1087	5	502	3.97	(1.58- 9.97)
*CHANG	7	f	0	12	580	11	1139	2.14	(0.95- 4.83)
Subtotal CHANG								2.81	(1.53- 5.16)
CHOI	2	m	0	35	136	13	95	1.88	(0.94- 3.74)
CHOI	6	f	0	6	3	76	164	4.32	(1.05- 17.72)
Subtotal CHOI								2.21	(1.19- 4.10)
*CHOW	26	m	0	27	71657	6	62913	3.95	(1.63- 9.57)
*CHYOU	3	m	0	33	2084	13	2406	2.93	(1.55- 5.55)
COMSTO	2	m	0	46	129	4	69	6.15	(2.13- 17.80)
COMSTO	7	f	0	11	35	13	115	2.78	(1.14- 6.75)
Subtotal COMSTO								3.85	(1.95- 7.61)
CORREA	37	c	0	258	315	51	388	6.23	(4.46- 8.71)
*CPSI	72	m	1	-	-	-	-	3.74	(3.14- 4.46)
*CPSI	280	f	1	-	-	-	-	1.38	(0.81- 2.35)
Subtotal CPSI								3.39	(2.87- 4.01)
*CPSII	91	m	1	-	-	-	-	9.36	(7.43- 11.77)
*CPSII	78	f	1	-	-	-	-	4.84	(4.00- 5.86)
Subtotal CPSII								6.33	(5.47- 7.34)
DAMBER	15	m	1	-	-	-	-	4.04	(2.67- 6.11)
DARBY	5	m	0	285	1106	3	384	32.98	(10.51- 103.49)
DARBY	12	f	0	94	317	23	529	6.82	(4.23- 10.99)
Subtotal DARBY								8.61	(5.55- 13.37)
DEAN2	1	m	0	98	88	33	112	3.78	(2.33- 6.13)
DEAN2	5	f	0	5	2	88	121	3.44	(0.65- 18.13)
Subtotal DEAN2								3.75	(2.36- 5.97)
DEAN3	26	m	0	89	417	25	510	4.35	(2.74- 6.91)
DEAN3	110	f	0	7	262	41	1538	1.00	(0.44- 2.26)
Subtotal DEAN3								3.04	(2.03- 4.54)
*DEKLER	1	m	2	-	-	-	-	10.70	(1.40- 81.90)
DESTE2	1	c	0	84	61	20	108	7.44	(4.16- 13.28)
DESTEF	10	m	0	108	108	27	163	6.04	(3.71- 9.82)
*DOCKER	2	c	4	-	-	-	-	2.54	(0.90- 7.18)
DOLL	91	m	0	70	124	7	61	4.92	(2.13- 11.34)
DOLL	94	f	0	10	8	40	59	1.84	(0.67- 5.08)
Subtotal DOLL								3.31	(1.74- 6.30)
*DOLL2	55	m	1	-	-	-	-	4.17	(2.61- 6.66)
*DOLL2	9	f	1	-	-	-	-	3.29	(0.88- 12.24)
Subtotal DOLL2								4.06	(2.61- 6.31)

Table 1D1 - 5

IESLC - Meta-analysis of Ex Smoking, Any product (or Cigarettes if Any not available)
All LC types
Least adjusted

REF	NRR	SEX	AD	Number Exposed Case	Cont	Non-exposed Case	Cont	RR	95.00%CI
DORANT	1	m	0	146	771	7	159	4.30	(1.98- 9.36)
DORGAN	8	m	0	236	230	15	93	6.36	(3.58- 11.30)
DORGAN	32	m	0	49	56	3	35	10.21	(2.95- 35.27)
DORGAN	55	f	0	146	110	103	244	3.14	(2.24- 4.41)
DORGAN	78	f	0	11	10	7	20	3.14	(0.93- 10.58)
Subtotal DORGAN								3.92	(2.98- 5.17)
*DORN	102	m	1	-	-	-	-	4.12	(3.19- 5.31)
DROSTE	1	m	0	92	176	7	93	6.94	(3.09- 15.59)
*ENGELA	153	m	0	11	2599	7	2683	1.62	(0.63- 4.18)
*ENGELA	15	f	0	6	24392	31	207789	1.65	(0.69- 3.95)
Subtotal ENGELA								1.64	(0.86- 3.11)
GAO	27	m	0	142	120	62	202	3.86	(2.65- 5.61)
GAO	28	f	0	67	30	435	605	3.11	(1.98- 4.86)
Subtotal GAO								3.53	(2.65- 4.70)
GAO2	5	m	0	85	109	13	56	3.36	(1.72- 6.54)
GARCIA	1	c	0	226	233	21	139	6.42	(3.92- 10.52)
GARDIN	1	c	0	41	44	5	41	7.64	(2.75- 21.22)
GARSHI	22	m	0	291	633	41	363	4.07	(2.86- 5.78)
GOODMA	1	m	0	68	229	10	199	5.91	(2.96- 11.79)
GOODMA	5	f	0	23	35	19	177	6.12	(3.02- 12.42)
Subtotal GOODMA								6.01	(3.67- 9.85)
GRAHAM	21	m	0	150	178	18	346	16.20	(9.62- 27.28)
GREGOR	1	m	0	23	45	10	14	0.72	(0.28- 1.86)
GREGOR	5	f	0	4	16	1	22	5.50	(0.56- 53.99)
Subtotal GREGOR								0.97	(0.40- 2.34)
HAENSZ	55	f	0	5	9	81	236	1.62	(0.53- 4.97)
*HAMMO2	26	m	0	90	2201	5	891	7.29	(2.97- 17.87)
*HEIN	6	m	0	11	979	1	457	5.13	(0.66- 39.65)
*HENNEK	1	m	0	67	8674	23	10919	3.67	(2.29- 5.88)
*HIRAYA	146	m	1	-	-	-	-	1.71	(1.08- 2.72)
*HIRAYA	149	f	1	-	-	-	-	2.98	(1.14- 7.77)
Subtotal HIRAYA								1.90	(1.25- 2.88)
HITOSU	1	m	0	25	190	7	242	4.55	(1.93- 10.74)
HITOSU	8	f	0	6	41	33	1893	8.39	(3.33- 21.13)
Subtotal HITOSU								6.05	(3.22- 11.34)
*HOLE	14	m	0	24	1736	7	1189	2.35	(1.02- 5.43)
HUMBLE	1	m	1	-	-	-	-	7.20	(3.00- 17.60)
HUMBLE	4	m	1	-	-	-	-	8.00	(1.90- 42.20)
HUMBLE	7	f	1	-	-	-	-	6.50	(2.80- 15.40)
HUMBLE	10	f	1	-	-	-	-	6.30	(1.50- 27.80)
Subtotal HUMBLE								6.88	(4.04- 11.71)
JAHN	10	m	0	455	402	18	138	8.68	(5.22- 14.44)
JAIN	26	m	0	126	159	12	85	5.61	(2.94- 10.73)
JAIN	21	f	0	85	97	52	214	3.61	(2.37- 5.49)
Subtotal JAIN								4.11	(2.89- 5.85)
JARVHO	1	m	0	26	28	1	16	14.86	(1.84- 120.07)
JARVHO	5	f	0	10	8	6	21	4.38	(1.19- 16.04)
Subtotal JARVHO								6.15	(2.04- 18.54)
JEDRYC	64	m	0	137	196	49	219	3.12	(2.14- 4.56)
JEDRYC	69	f	0	13	8	78	166	3.46	(1.38- 8.69)
Subtotal JEDRYC								3.17	(2.23- 4.50)
JOLY	22	m	0	108	223	12	218	8.80	(4.71- 16.44)
JOLY	19	f	0	34	27	52	283	6.85	(3.82- 12.31)
Subtotal JOLY								7.70	(5.02- 11.81)
*KAISE2	65	m	1	-	-	-	-	3.39	(1.77- 6.48)
*KAISE2	57	f	1	-	-	-	-	5.02	(2.17- 11.61)
Subtotal KAISE2								3.93	(2.35- 6.56)
*KAISER	5	m	2	-	-	-	-	4.65	(2.84- 7.64)
*KAISER	1	f	2	-	-	-	-	3.02	(1.83- 4.99)
Subtotal KAISER								3.76	(2.64- 5.35)
KATSOU	5	f	0	8	4	48	67	2.79	(0.79- 9.80)
KAUFMA	1	c	0	225	759	35	925	7.83	(5.42- 11.33)
KELLER	2	m	0	3003	1307	323	1017	7.23	(6.28- 8.33)
KELLER	10	m	0	440	128	38	117	10.58	(6.99- 16.04)
KELLER	6	f	0	1094	477	469	1860	9.10	(7.85- 10.54)
KELLER	14	f	0	130	79	67	232	5.70	(3.86- 8.42)
Subtotal KELLER								8.02	(7.28- 8.82)
KHUDER	13	m	0	184	-	23	-	7.50	(4.80- 11.90)
KIHARA	15	c	0	55	70	102	237	1.83	(1.20- 2.79)
*KINLEN	1	m	0	75	2843	7	1333	5.02	(2.32- 10.87)
KJUUS	2	m	0	39	75	2	24	6.24	(1.40- 27.78)

Table 1D1 - 5

IESLC - Meta-analysis of Ex Smoking, Any product (or Cigarettes if Any not available)
All LC types
Least adjusted

REF	NRR	SEX	AD	Number Exposed Case	Number Exposed Cont	Non-exposed Case	Non-exposed Cont	RR	95.00%CI
*KNEKT	14	m	0	18	15309	6	17814	3.49	(1.39- 8.79)
KOO	8	f	0	22	10	56	85	3.34	(1.47- 7.58)
KREUZE	1	m	0	9	47	6	54	1.72	(0.57- 5.20)
KREUZE	3	m	0	434	834	23	403	9.12	(5.90- 14.10)
KREUZE	2	f	0	7	19	6	38	2.33	(0.69- 7.92)
KREUZE	4	f	0	35	47	95	177	1.39	(0.84- 2.30)
Subtotal KREUZE								3.68	(2.71- 5.00)
*KUBIK	11	m	0	8	1487	2	4271	11.49	(2.44- 54.04)
*LANGE	5	m	0	21	1253	5	721	2.42	(0.92- 6.38)
*LANGE	1	f	0	8	1089	7	2159	2.27	(0.82- 6.23)
Subtotal LANGE								2.34	(1.16- 4.72)
LEMARC	1	c	0	142	223	32	168	3.34	(2.17- 5.15)
*LIDDEL	1	m	1	-	-	-	-	1.40	(0.79- 2.51)
LOMBAR	8	m	0	126	172	14	112	5.86	(3.21- 10.69)
LUBIN	39	m	0	70	139	9	72	4.03	(1.90- 8.53)
LUBIN2	35	m	0	1029	3097	190	2617	4.58	(3.89- 5.39)
LUBIN2	319	f	0	100	157	288	1180	2.61	(1.97- 3.46)
Subtotal LUBIN2								3.97	(3.45- 4.58)
MACLEN	7	m	0	5	11	5	15	1.36	(0.32- 5.89)
MACLEN	8	f	0	3	10	41	109	0.80	(0.21- 3.04)
Subtotal MACLEN								1.02	(0.38- 2.74)
MATOS	14	m	0	76	151	11	110	5.03	(2.55- 9.92)
*MIGRAN	24	m	0	24	1574	4	867	3.30	(1.15- 9.49)
*MIGRAN	40	f	0	1	621	4	3814	1.54	(0.17- 13.71)
Subtotal MIGRAN								2.86	(1.11- 7.40)
*MRFITR	1	m	0	13	2813	0	1859	17.84~	(1.06- 300.00)
NAM	65	m	0	369	486	30	520	13.16	(8.89- 19.48)
NAM	81	f	0	159	262	52	885	10.33	(7.34- 14.54)
Subtotal NAM								11.47	(8.86- 14.84)
ODRISC	2	c	0	147	398	6	664	40.87	(17.90- 93.34)
OSANN	1	m	0	317	477	45	833	12.30	(8.83- 17.14)
OSANN	5	f	0	140	196	96	1093	8.13	(6.02- 10.99)
Subtotal OSANN								9.80	(7.84- 12.25)
PARKIN	16	m	0	26	61	107	1248	4.97	(3.02- 8.19)
PERSH2	1	c	0	108	293	178	1164	2.41	(1.84- 3.16)
*PETO	1	m	0	2	387	2	295	0.76	(0.11- 5.38)
PEZZO2	1	m	0	128	271	6	117	9.21	(3.95- 21.48)
PEZZOT	1	m	0	66	188	4	116	10.18	(3.61- 28.67)
*QIAO2	1	m	0	33	816	10	709	2.87	(1.42- 5.78)
RACHTA	1	f	0	13	10	33	98	3.86	(1.55- 9.63)
SCHWAR	21	m	0	996	670	119	376	4.70	(3.74- 5.90)
SCHWAR	22	m	0	219	136	50	104	3.35	(2.25- 4.99)
SCHWAR	23	f	0	322	328	182	855	4.61	(3.69- 5.76)
SCHWAR	24	f	0	79	89	40	247	5.48	(3.49- 8.60)
Subtotal SCHWAR								4.54	(3.94- 5.22)
SHAW	3	c	0	112	169	11	107	6.45	(3.32- 12.53)
SOBUE	89	m	0	287	363	34	128	2.98	(1.98- 4.48)
SOBUE	93	f	0	32	64	167	857	2.57	(1.63- 4.05)
Subtotal SOBUE								2.79	(2.06- 3.78)
*SPEIZE	7	f	0	144	522081	58	776300	3.69	(2.72- 5.01)
SPITZ	1	c	0	67	80	7	128	15.31	(6.70- 35.02)
STOCKW	4	c	0	6185	3057	2791	10641	7.71	(7.26- 8.19)
STUCKE	1	m	0	178	135	0	51	135.69~	(8.30-2218.34)
SUZUK2	1	c	0	34	40	11	53	4.10	(1.85- 9.06)
SVENSS	21	f	0	30	36	38	120	2.63	(1.43- 4.83)
TANG	2	c	0	58	34	9	39	7.39	(3.19- 17.11)
*TENKAN	9	m	1	-	-	-	-	4.02	(1.50- 10.80)
TIZZAN	6	m	0	346	292	180	305	2.01	(1.58- 2.56)
TIZZAN	14	f	0	8	10	25	114	3.65	(1.31- 10.17)
Subtotal TIZZAN								2.07	(1.64- 2.62)
TOKARS	2	m	0	37	86	1	53	22.80	(3.04- 171.13)
TOUSEY	3	m	0	133	298	4	130	14.51	(5.25- 40.05)
TOUSEY	7	f	0	65	134	13	226	8.43	(4.48- 15.88)
Subtotal TOUSEY								9.81	(5.74- 16.79)
TSUGAN	26	m	0	10	8	18	22	1.53	(0.50- 4.68)
*TULINI	1	m	1	-	-	-	-	2.91	(1.47- 5.74)
*TULINI	7	f	1	-	-	-	-	3.73	(1.73- 8.07)
Subtotal TULINI								3.25	(1.95- 5.40)
*TVERDA	1	m	2	-	-	-	-	0.49	(0.24- 1.01)
*TVERDA	18	f	0	0	38953	3	157431	0.58~	(0.03- 11.18)
Subtotal TVERDA								0.49	(0.25- 0.99)

Table 1D1 - 5

IESLC - Meta-analysis of Ex Smoking, Any product (or Cigarettes if Any not available)
All LC types
Least adjusted

REF	NRR	SEX	AD	Number Case	Exposed Cont	Non-exposed Case	Cont	RR	95.00%CI
WAKAI	1	m	0	54	140	10	65	2.51	(1.20- 5.23)
WAKAI	19	f	0	5	5	50	145	2.90	(0.81- 10.44)
Subtotal WAKAI								2.60	(1.37- 4.92)
WANG2	19	c	0	11	21	11	43	2.05	(0.76- 5.48)
WIGLE	9	m	0	144	276	15	204	7.10	(4.05- 12.44)
WIGLE	12	f	0	11	66	36	439	2.03	(0.99- 4.19)
Subtotal WIGLE								4.43	(2.84- 6.91)
WU	33	f	0	29	55	31	92	1.56	(0.85- 2.87)
WUNSCH	3	m	0	100	200	14	99	3.54	(1.92- 6.50)
WUNSCH	9	f	0	17	36	29	208	3.39	(1.69- 6.79)
Subtotal WUNSCH								3.47	(2.19- 5.49)
WYNDE3	41	m	0	48	125	9	88	3.75	(1.75- 8.05)
WYNDE6	9	m	0	1088	1056	87	617	7.31	(5.75- 9.29)
WYNDE6	198	f	0	332	325	159	856	5.50	(4.38- 6.91)
Subtotal WYNDE6								6.29	(5.34- 7.43)
YAMAGU	4	c	0	44	162	24	267	3.02	(1.77- 5.16)
*YONG	1	c	1	-	-	-	-	4.10	(2.55- 6.60)
Partial Totals				26318	873666	9070	1945528		

*prospective study ~ With 0.5 adjustment for zero

REF	NRR	SEX	AD	Ys	Ws	Qs	Ps
AGUDO	9	f	0	0.13	1.93	5.11	0.8520
*AKIBA	1	m	0	0.97	13.10	8.25	0.0005
*AKIBA	5	f	0	0.69	8.36	9.51	0.0447
Subtotal AKIBA				0.86	21.46	17.75	
*AMANDU	2	m	0	1.15	3.88	1.44	0.0232
AMES	3	m	0	1.66	10.17	0.09	0.0000
*ANDERS	1	f	0	1.88	29.87	0.44	0.0000
*ARCHER	4	m	0	2.07	4.51	0.42	0.0000
ARMADA	28	m	0	2.75	3.56	3.48	0.0000
AUSTIN	1	c	0	2.06	4.16	0.38	0.0000
AXELSS	4	m	0	1.52	11.96	0.69	0.0000
AXELSS	9	f	0	1.10	6.31	2.79	0.0059
Subtotal AXELSS				1.37	18.27	3.47	
BARBON	1	m	0	1.96	16.26	0.67	0.0000
BECHER	5	m	0	1.87	2.60	0.03	0.0025
BECHER	6	f	0	0.37	2.67	5.18	0.5481
Subtotal BECHER				1.11	5.27	5.21	
*BENSHL	9	m	1	1.08	5.22	2.41	0.0134
*BEST	3	m	1	1.80	5.04	0.01	0.0001
BLOHMK	2	m	0	1.10	56.10	24.86	0.0000
*BOUCOT	3	m	0	3.07	0.47	0.81	0.0351
*BRETT	9	m	0	-0.07	3.60	12.05	0.8976
BROSS	6	m	0	1.87	22.85	0.28	0.0000
BROWN2	22	m	2	1.97	403.85	18.31	0.0000
BROWN2	21	f	2	2.45	308.59	146.87	0.0000
Subtotal BROWN2				2.18	712.44	165.18	
BUFFLE	4	m	0	2.52	4.30	2.49	0.0000
BUFFLE	8	f	0	1.62	20.50	0.39	0.0000
Subtotal BUFFLE				1.78	24.80	2.88	
CARPEN	8	c	0	1.74	12.01	0.00	0.0000
*CEDERL	114	m	2	0.22	9.82	23.24	0.4843
*CEDERL	74	f	2	0.08	2.87	8.14	0.8963
Subtotal CEDERL				0.19	12.69	31.38	
*CHANG	1	m	0	1.38	4.54	0.66	0.0033
*CHANG	7	f	0	0.76	5.83	5.82	0.0659
Subtotal CHANG				1.03	10.36	6.48	
CHOI	2	m	0	0.63	8.11	10.34	0.0721
CHOI	6	f	0	1.46	1.93	0.17	0.0424
Subtotal CHOI				0.79	10.03	10.51	
*CHOW	26	m	0	1.37	4.91	0.74	0.0023
*CHYOU	3	m	0	1.08	9.40	4.42	0.0010
COMSTO	2	m	0	1.82	3.40	0.01	0.0008
COMSTO	7	f	0	1.02	4.88	2.66	0.0240
Subtotal COMSTO				1.35	8.28	2.67	
CORREA	37	c	0	1.83	34.20	0.16	0.0000
*CPSI	72	m	1	1.32	124.77	24.38	0.0000
*CPSI	280	f	1	0.32	13.54	28.05	0.2359
Subtotal CPSI				1.22	138.32	52.43	
*CPSII	91	m	1	2.24	72.61	16.40	0.0000

Table 1D1 - 5

IESLC - Meta-analysis of Ex Smoking, Any product (or Cigarettes if Any not available)
All LC types
Least adjusted

REF	NRR	SEX	AD	Ys	Ws	Qs	Ps
*CPSII	78	f	1	1.58	105.38	3.58	0.0000
Subtotal CPSII				1.85	177.99	19.98	
DAMBER	15	m	1	1.40	22.42	2.99	0.0000
DARBY	5	m	0	3.50	2.94	8.84	0.0000
DARBY	12	f	0	1.92	16.90	0.43	0.0000
Subtotal DARBY				2.15	19.84	9.27	
DEAN2	1	m	0	1.33	16.45	3.06	0.0000
DEAN2	5	f	0	1.23	1.39	0.39	0.1455
Subtotal DEAN2				1.32	17.84	3.45	
DEAN3	26	m	0	1.47	17.99	1.51	0.0000
DEAN3	110	f	0	0.00	5.82	18.02	0.9957
Subtotal DEAN3				1.11	23.81	19.53	
*DEKLER	1	m	2	2.37	0.93	0.34	0.0224
DESTE2	1	c	0	2.01	11.42	0.69	0.0000
DESTEF	10	m	0	1.80	16.21	0.02	0.0000
*DOCKER	2	c	4	0.93	3.56	2.45	0.0785
DOLL	91	m	0	1.59	5.51	0.16	0.0002
DOLL	94	f	0	0.61	3.75	4.95	0.2364
Subtotal DOLL				1.20	9.25	5.10	
*DOLL2	55	m	1	1.43	17.51	1.94	0.0000
*DOLL2	9	f	1	1.19	2.22	0.72	0.0762
Subtotal DOLL2				1.40	19.73	2.67	
DORANT	1	m	0	1.46	6.36	0.58	0.0002
DORGAN	8	m	0	1.85	11.63	0.09	0.0000
DORGAN	32	m	0	2.32	2.50	0.79	0.0002
DORGAN	55	f	0	1.15	33.62	12.74	0.0000
DORGAN	78	f	0	1.15	2.61	0.99	0.0645
Subtotal DORGAN				1.37	50.35	14.61	
*DORN	102	m	1	1.42	59.18	7.05	0.0000
DROSTE	1	m	0	1.94	5.88	0.18	0.0000
*ENGELA	153	m	0	0.48	4.29	7.00	0.3162
*ENGELA	15	f	0	0.50	5.03	8.00	0.2622
Subtotal ENGELA				0.49	9.32	15.00	
GAO	27	m	0	1.35	27.43	4.65	0.0000
GAO	28	f	0	1.13	19.15	7.55	0.0000
Subtotal GAO				1.26	46.58	12.20	
GAO2	5	m	0	1.21	8.64	2.61	0.0004
GARCIA	1	c	0	1.86	15.74	0.15	0.0000
GARDIN	1	c	0	2.03	3.68	0.27	0.0001
GARSHI	22	m	0	1.40	31.09	3.97	0.0000
GOODMA	1	m	0	1.78	8.06	0.00	0.0000
GOODMA	5	f	0	1.81	7.67	0.02	0.0000
Subtotal GOODMA				1.79	15.73	0.02	
GRAHAM	21	m	0	2.78	14.14	14.82	0.0000
GREGOR	1	m	0	-0.33	4.22	18.52	0.4919
GREGOR	5	f	0	1.70	0.74	0.00	0.1435
Subtotal GREGOR				-0.03	4.95	18.53	
HAENSZ	55	f	0	0.48	3.05	5.00	0.4002
*HAMMO2	26	m	0	1.99	4.77	0.24	0.0000
*HEIN	6	m	0	1.64	0.92	0.01	0.1167
*HENNEK	1	m	0	1.30	17.18	3.66	0.0000
*HIRAYA	146	m	1	0.54	18.01	27.01	0.0228
*HIRAYA	149	f	1	1.09	4.17	1.87	0.0257
Subtotal HIRAYA				0.64	22.18	28.88	
HITOSU	1	m	0	1.51	5.20	0.32	0.0006
HITOSU	8	f	0	2.13	4.51	0.61	0.0000
Subtotal HITOSU				1.80	9.71	0.92	
*HOLE	14	m	0	0.85	5.46	4.50	0.0460
HUMBLE	1	m	1	1.97	4.91	0.22	0.0000
HUMBLE	4	m	1	2.08	1.60	0.16	0.0086
HUMBLE	7	f	1	1.87	5.29	0.06	0.0000
HUMBLE	10	f	1	1.84	1.80	0.01	0.0135
Subtotal HUMBLE				1.93	13.60	0.46	
JAHN	10	m	0	2.16	14.82	2.37	0.0000
JAIN	26	m	0	1.73	9.15	0.01	0.0000
JAIN	21	f	0	1.28	21.75	4.98	0.0000
Subtotal JAIN				1.41	30.90	4.99	
JARVHO	1	m	0	2.70	0.88	0.77	0.0114
JARVHO	5	f	0	1.48	2.28	0.19	0.0260
Subtotal JARVHO				1.82	3.16	0.96	
JEDRYC	64	m	0	1.14	26.76	10.35	0.0000

Table 1D1 - 5

IESLC - Meta-analysis of Ex Smoking, Any product (or Cigarettes if Any not available)
All LC types
Least adjusted

REF	NRR	SEX	AD	Ys	Ws	Qs	Ps
JEDRYC	69	f	0	1.24	4.53	1.23	0.0083
Subtotal JEDRYC				1.15	31.29	11.58	
JOLY	22	m	0	2.17	9.84	1.68	0.0000
JOLY	19	f	0	1.92	11.21	0.30	0.0000
Subtotal JOLY				2.04	21.05	1.98	
*KAISE2	65	m	1	1.22	9.12	2.66	0.0002
*KAISE2	57	f	1	1.61	5.46	0.12	0.0002
Subtotal KAISE2				1.37	14.59	2.78	
*KAISER	5	m	2	1.54	15.69	0.79	0.0000
*KAISER	1	f	2	1.11	15.27	6.57	0.0000
Subtotal KAISER				1.32	30.96	7.36	
KATSOU	5	f	0	1.03	2.43	1.31	0.1092
KAUFMA	1	c	0	2.06	28.24	2.50	0.0000
KELLER	2	m	0	1.98	193.15	9.15	0.0000
KELLER	10	m	0	2.36	22.25	7.96	0.0000
KELLER	6	f	0	2.21	176.05	35.12	0.0000
KELLER	14	f	0	1.74	25.26	0.01	0.0000
Subtotal KELLER				2.08	416.70	52.25	
KHUDER	13	m	0	2.01	18.64	1.20	0.0000
KIHARA	15	c	0	0.60	21.51	28.90	0.0052
*KINLEN	1	m	0	1.61	6.45	0.14	0.0000
KJUUS	2	m	0	1.83	1.72	0.01	0.0163
*KNEKT	14	m	0	1.25	4.50	1.18	0.0080
KOO	8	f	0	1.21	5.71	1.76	0.0040
KREUZE	1	m	0	0.54	3.15	4.66	0.3341
KREUZE	3	m	0	2.21	20.22	4.08	0.0000
KREUZE	2	f	0	0.85	2.57	2.15	0.1740
KREUZE	4	f	0	0.33	15.15	31.13	0.2025
Subtotal KREUZE				1.30	41.09	42.02	
*KUBIK	11	m	0	2.44	1.60	0.74	0.0020
*LANGE	5	m	0	0.88	4.07	3.15	0.0749
*LANGE	1	f	0	0.82	3.75	3.34	0.1131
Subtotal LANGE				0.85	7.83	6.48	
LEMARC	1	c	0	1.21	20.52	6.30	0.0000
*LIDDEL	1	m	1	0.34	11.50	23.34	0.2539
LOMBAR	8	m	0	1.77	10.63	0.00	0.0000
LUBIN	39	m	0	1.39	6.83	0.92	0.0003
LUBIN2	35	m	0	1.52	144.09	8.31	0.0000
LUBIN2	319	f	0	0.96	48.33	31.08	0.0000
Subtotal LUBIN2				1.38	192.43	39.40	
MACLEN	7	m	0	0.31	1.79	3.78	0.6779
MACLEN	8	f	0	-0.23	2.14	8.46	0.7406
Subtotal MACLEN				0.02	3.94	12.23	
MATOS	14	m	0	1.62	8.35	0.18	0.0000
*MIGRAN	24	m	0	1.20	3.45	1.10	0.0264
*MIGRAN	40	f	0	0.43	0.80	1.42	0.7011
Subtotal MIGRAN				1.05	4.25	2.53	
*MRFITR	1	m	0	2.88	0.48	0.61	0.0454
NAM	65	m	0	2.58	24.98	16.64	0.0000
NAM	81	f	0	2.33	32.82	10.81	0.0000
Subtotal NAM				2.44	57.81	27.45	
ODRISC	2	c	0	3.71	5.63	21.41	0.0000
OSANN	1	m	0	2.51	34.88	19.55	0.0000
OSANN	5	f	0	2.10	42.42	4.75	0.0000
Subtotal OSANN				2.28	77.29	24.30	
PARKIN	16	m	0	1.60	15.38	0.38	0.0000
PERSH2	1	c	0	0.88	52.22	40.56	0.0000
*PETO	1	m	0	-0.27	1.01	4.16	0.7854
PEZZO2	1	m	0	2.22	5.36	1.13	0.0000
PEZZOT	1	m	0	2.32	3.58	1.12	0.0000
*QIAO2	1	m	0	1.05	7.83	3.92	0.0032
RACHTA	1	f	0	1.35	4.60	0.77	0.0038
SCHWAR	21	m	0	1.55	73.75	3.38	0.0000
SCHWAR	22	m	0	1.21	24.08	7.35	0.0000
SCHWAR	23	f	0	1.53	78.01	4.22	0.0000
SCHWAR	24	f	0	1.70	18.89	0.07	0.0000
Subtotal SCHWAR				1.51	194.73	15.01	
SHAW	3	c	0	1.86	8.69	0.09	0.0000
SOBUE	89	m	0	1.09	23.01	10.34	0.0000
SOBUE	93	f	0	0.94	18.51	12.41	0.0001
Subtotal SOBUE				1.02	41.52	22.75	

Table 1D1 - 5

IESLC - Meta-analysis of Ex Smoking, Any product (or Cigarettes if Any not available)
All LC types
Least adjusted

REF	NRR	SEX	AD	Ys	Ws	Qs	Ps
*SPEIZE	7	f	0	1.31	41.35	8.56	0.0000
SPITZ	1	c	0	2.73	5.61	5.26	0.0000
STOCKW	4	c	0	2.04	1062.62	84.43	0.0000
STUCKE	1	m	0	4.91	0.49	4.88	0.0006
SUZUK2	1	c	0	1.41	6.09	0.75	0.0005
SVENSS	21	f	0	0.97	10.44	6.58	0.0018
TANG	2	c	0	2.00	5.45	0.31	0.0000
*TENKAN	9	m	1	1.39	3.94	0.54	0.0057
TIZZAN	6	m	0	0.70	66.01	74.74	0.0000
TIZZAN	14	f	0	1.29	3.65	0.80	0.0134
Subtotal TIZZAN				0.73	69.66	75.54	
TOKARS	2	m	0	3.13	0.95	1.76	0.0024
TOUSEY	3	m	0	2.67	3.72	3.11	0.0000
TOUSEY	7	f	0	2.13	9.60	1.32	0.0000
Subtotal TOUSEY				2.28	13.32	4.43	
TSUGAN	26	m	0	0.42	3.07	5.49	0.4579
*TULINI	1	m	1	1.07	8.28	3.98	0.0021
*TULINI	7	f	1	1.32	6.48	1.28	0.0008
Subtotal TULINI				1.18	14.76	5.26	
*TVERDA	1	m	2	-0.71	7.44	45.56	0.0517
*TVERDA	18	f	0	-0.55	0.44	2.34	0.7164
Subtotal TVERDA				-0.70	7.88	47.89	
WAKAI	1	m	0	0.92	7.09	5.03	0.0144
WAKAI	19	f	0	1.06	2.34	1.14	0.1032
Subtotal WAKAI				0.96	9.43	6.16	
WANG2	19	c	0	0.72	3.96	4.32	0.1540
WIGLE	9	m	0	1.96	12.17	0.48	0.0000
WIGLE	12	f	0	0.71	7.35	8.13	0.0546
Subtotal WIGLE				1.49	19.52	8.61	
WU	33	f	0	0.45	10.44	18.01	0.1480
WUNSCH	3	m	0	1.26	10.36	2.57	0.0000
WUNSCH	9	f	0	1.22	7.94	2.33	0.0006
Subtotal WUNSCH				1.24	18.30	4.90	
WYNDE3	41	m	0	1.32	6.61	1.27	0.0007
WYNDE6	9	m	0	1.99	66.75	3.46	0.0000
WYNDE6	198	f	0	1.70	73.82	0.24	0.0000
Subtotal WYNDE6				1.84	140.57	3.70	
YAMAGU	4	c	0	1.11	13.46	5.78	0.0000
*YONG	1	c	1	1.41	16.99	2.08	0.0000

	N	182
	NS	124
	Wt	4784.26
Het	Chi	1281.46
Het	df	181
Het	P	***
Fixed	RR	5.82
	RRl	5.66
	RRu	5.99
	P	+++
Random	RR	4.35
	RRl	3.97
	RRu	4.76
	P	+++
Asymm	P	***

Table 1D1 - 6

IESLC - Meta-analysis of Ex Smoking, Any product (or Cigarettes if Any not available)
All LC types
Least adjusted

	Sex: combined	male	female	Total
N	20	100	62	182
NS	20	95	57	172
Wt	1335.78	2106.32	1342.16	4784.26
Het Chi	167.57	557.58	492.92	1281.46
Het df	19	99	61	181
Het P	***	***	***	***
Fixed RR	6.91	5.23	5.80	5.82
RRl	6.55	5.01	5.50	5.66
RRu	7.29	5.46	6.12	5.99
P	+++	+++	+++	+++
Random RR	5.40	4.63	3.55	4.35
RRl	4.08	4.10	2.96	3.97
RRu	7.15	5.22	4.25	4.76
P	+++	+++	+++	+++
Between Chi				63.38
Between df				2
Between P				***
Btwn(F) P				*
Btwn(R) P				*

	Lung cancer type: all	other	Total
N	176	6	182
NS	119	5	124
Wt	4720.74	63.53	4784.26
Het Chi	1229.36	10.62	1281.46
Het df	175	5	181
Het P	***	(*)	***
Fixed RR	5.88	2.61	5.82
RRl	5.72	2.04	5.66
RRu	6.05	3.33	5.99
P	+++	+++	+++
Random RR	4.43	2.56	4.35
RRl	4.04	1.72	3.97
RRu	4.85	3.80	4.76
P	+++	+++	+++
Between Chi			41.48
Between df			1
Between P			***
Btwn(F) P			*
Btwn(R) P			**

	Location: NAmer	UK	Scand	othEur	China	Japan	othAs	other	Total
N	80	21	21	24	5	14	5	12	182
NS	53	14	14	17	4	9	3	10	124
Wt	3686.23	130.73	170.07	454.71	65.20	150.97	19.68	106.67	4784.26
Het Chi	524.32	89.94	46.97	118.87	2.03	16.46	4.66	11.48	1281.46
Het df	79	20	20	23	4	13	4	11	181
Het P	***	***	***	***	N.S.	N.S.	N.S.	N.S.	***
Fixed RR	6.81	4.10	2.55	3.63	3.38	2.55	2.13	5.77	5.82
RRl	6.60	3.45	2.20	3.31	2.65	2.17	1.37	4.77	5.66
RRu	7.04	4.86	2.97	3.98	4.30	2.99	3.32	6.98	5.99
P	+++	+++	+++	+++	+++	+++	+++	+++	+++
Random RR	5.53	3.70	2.52	4.02	3.38	2.59	2.12	5.77	4.35
RRl	4.98	2.49	1.93	3.10	2.65	2.15	1.29	4.75	3.97
RRu	6.13	5.49	3.29	5.23	4.30	3.12	3.47	7.02	4.76
P	+++	+++	+++	+++	+++	+++	++	+++	+++
Between Chi									466.72
Between df									7
Between P									***
Btwn(F) P									***
Btwn(R) P									***

Table 1D1 - 6

IESLC - Meta-analysis of Ex Smoking, Any product (or Cigarettes if Any not available)
All LC types
Least adjusted

	Detailed Country in "other Europe"					
	multi	Germany	othWest	East	Balkans	Total
N	2	8	8	5	1	24
NS	1	4	7	4	1	17
Wt	192.43	117.28	104.14	38.43	2.43	454.71
Het Chi	11.42	49.90	46.56	5.95	0.00	118.87
Het df	1	7	7	4	0	23
Het P	***	***	***	N.S.	N.S.	***
Fixed RR	3.97	3.68	3.04	3.60	2.79	3.63
RRl	3.45	3.07	2.51	2.62	0.79	3.31
RRu	4.58	4.41	3.68	4.93	9.80	3.98
P	+++	+++	+++	+++	N.S.	+++
Random RR	3.50	3.43	5.12	4.17	2.79	4.02
RRl	2.02	1.93	2.62	2.53	0.79	3.10
RRu	6.06	6.10	10.03	6.87	9.80	5.23
P	+++	+++	+++	+++	N.S.	+++
Between Chi						5.05
Between df						4
Between P						N.S.
Btwn(F) P						N.S.
Btwn(R) P						N.S.

	Detailed Country in "other Asia"			
	India	HongKong	other	Total
N		1	4	5
NS		1	2	3
Wt		5.71	13.97	19.68
Het Chi		0.00	3.04	4.66
Het df		0	3	4
Het P		N.S.	N.S.	N.S.
Fixed RR		3.34	1.77	2.13
RRl		1.47	1.05	1.37
RRu		7.58	3.00	3.32
P		++	+	+++
Random RR		3.34	1.77	2.12
RRl		1.47	1.04	1.29
RRu		7.58	3.02	3.47
P		++	+	++
Between Chi				1.62
Between df				1
Between P				N.S.
Btwn(F) P				N.S.
Btwn(R) P				N.S.

	Detailed other continent			
	SCAmer	Auslia	Africa	Total
N	10	1	1	12
NS	8	1	1	10
Wt	90.36	0.93	15.38	106.67
Het Chi	10.76	0.00	0.00	11.48
Het df	9	0	0	11
Het P	N.S.	N.S.	N.S.	N.S.
Fixed RR	5.88	10.70	4.97	5.77
RRl	4.78	1.40	3.02	4.77
RRu	7.23	81.84	8.19	6.98
P	+++	+	+++	+++
Random RR	5.88	10.70	4.97	5.77
RRl	4.68	1.40	3.02	4.75
RRu	7.39	81.84	8.19	7.02
P	+++	+	+++	+++
Between Chi				0.73
Between df				2
Between P				N.S.
Btwn(F) P				N.S.
Btwn(R) P				N.S.

Table 1D1 - 6

IESLC - Meta-analysis of Ex Smoking, Any product (or Cigarettes if Any not available)
All LC types
Least adjusted

	Start year of study <1960	1960-69	1970-79	1980-89	1990+	Total
N	18	36	36	70	22	182
NS	14	25	25	43	17	124
Wt	349.03	398.67	533.83	3294.01	208.72	4784.26
Het Chi	84.87	116.55	133.22	502.83	112.43	1281.46
Het df	17	35	35	69	21	181
Het P	***	***	***	***	***	***
Fixed RR	3.56	4.15	3.83	6.92	4.73	5.82
RRl	3.20	3.76	3.52	6.69	4.13	5.66
RRu	3.95	4.58	4.17	7.16	5.42	5.99
P	+++	+++	+++	+++	+++	+++
Random RR	3.88	3.42	3.46	5.26	5.24	4.35
RRl	2.89	2.79	2.83	4.67	3.79	3.97
RRu	5.20	4.19	4.23	5.91	7.25	4.76
P	+++	+++	+++	+++	+++	+++
Between Chi						331.56
Between df						4
Between P						***
Btwn(F) P						***
Btwn(R) P						***

	Study type (1) CC	other	Total
N	123	59	182
NS	80	44	124
Wt	4011.63	772.64	4784.26
Het Chi	918.61	210.47	1281.46
Het df	122	58	181
Het P	***	***	***
Fixed RR	6.29	3.87	5.82
RRl	6.10	3.61	5.66
RRu	6.49	4.16	5.99
P	+++	+++	+++
Random RR	4.92	3.24	4.35
RRl	4.44	2.76	3.97
RRu	5.45	3.80	4.76
P	+++	+++	+++
Between Chi			152.38
Between df			1
Between P			***
Btwn(F) P			***
Btwn(R) P			***

	Study type (2) CC	prosp	other	Total
N	123	54	5	182
NS	80	40	4	124
Wt	4011.63	746.88	25.75	4784.26
Het Chi	918.61	205.14	4.13	1281.46
Het df	122	53	4	181
Het P	***	***	N.S.	***
Fixed RR	6.29	3.85	4.79	5.82
RRl	6.10	3.58	3.25	5.66
RRu	6.49	4.13	7.04	5.99
P	+++	+++	+++	+++
Random RR	4.92	3.13	4.79	4.35
RRl	4.44	2.64	3.23	3.97
RRu	5.45	3.70	7.11	4.76
P	+++	+++	+++	+++
Between Chi				153.58
Between df				2
Between P				***
Btwn(F) P				***
Btwn(R) P				***

Table 1D1 - 6

IESLC - Meta-analysis of Ex Smoking, Any product (or Cigarettes if Any not available)
All LC types
Least adjusted

	Study size (number of LC cases) 100-249	250-499	500-999	1000+	Total
N	51	45	37	49	182
NS	44	33	23	24	124
Wt	231.15	377.30	490.87	3684.95	4784.26
Het Chi	128.77	143.20	120.29	716.07	1281.46
Het df	50	44	36	48	181
Het P	***	***	***	***	***
Fixed RR	3.29	4.15	4.75	6.41	5.82
RRl	2.89	3.76	4.35	6.21	5.66
RRu	3.74	4.60	5.19	6.62	5.99
P	+++	+++	+++	+++	+++
Random RR	3.42	4.05	5.06	4.80	4.35
RRl	2.73	3.35	4.25	4.16	3.97
RRu	4.28	4.90	6.03	5.53	4.76
P	+++	+++	+++	+++	+++
Between Chi					173.12
Between df					3
Between P					***
Btwn(F) P					***
Btwn(R) P					*

	Risky occupational population no	mining	othRisky	Total
N	171	7	4	182
NS	113	7	4	124
Wt	4697.64	45.65	40.97	4784.26
Het Chi	1244.38	15.85	4.98	1281.46
Het df	170	6	3	181
Het P	***	*	N.S.	***
Fixed RR	5.86	3.30	4.85	5.82
RRl	5.70	2.47	3.57	5.66
RRu	6.03	4.42	6.58	5.99
P	+++	+++	+++	+++
Random RR	4.34	3.66	5.97	4.35
RRl	3.96	2.22	3.48	3.97
RRu	4.76	6.03	10.24	4.76
P	+++	+++	+++	+++
Between Chi				16.24
Between df				2
Between P				***
Btwn(F) P				N.S.
Btwn(R) P				N.S.

	National cigarette tobacco type Virginia	blended	other	Total
N	30	147	5	182
NS	21	99	4	124
Wt	222.68	4496.38	65.20	4784.26
Het Chi	115.72	1113.41	2.03	1281.46
Het df	29	146	4	181
Het P	***	***	N.S.	***
Fixed RR	4.08	5.97	3.38	5.82
RRl	3.58	5.80	2.65	5.66
RRu	4.65	6.15	4.30	5.99
P	+++	+++	+++	+++
Random RR	3.90	4.48	3.38	4.35
RRl	2.94	4.06	2.65	3.97
RRu	5.18	4.94	4.30	4.76
P	+++	+++	+++	+++
Between Chi				50.30
Between df				2
Between P				***
Btwn(F) P				*
Btwn(R) P				(*)

Table 1D1 - 6

IESLC - Meta-analysis of Ex Smoking, Any product (or Cigarettes if Any not available)
All LC types
Least adjusted

	Any proxy use No/nk	Yes	Total
N	141	41	182
NS	102	22	124
Wt	4095.84	688.43	4784.26
Het Chi	1115.92	130.71	1281.46
Het df	140	40	181
Het P	***	***	***
Fixed RR	6.03	4.73	5.82
RRl	5.84	4.39	5.66
RRu	6.21	5.09	5.99
P	+++	+++	+++
Random RR	4.13	5.01	4.35
RRl	3.72	4.31	3.97
RRu	4.60	5.82	4.76
P	+++	+++	+++
Between Chi			34.83
Between df			1
Between P			***
Btwn(F) P			*
Btwn(R) P			*

	Full histological confirmation No	Yes	Total
N	131	51	182
NS	88	36	124
Wt	3116.05	1668.21	4784.26
Het Chi	883.85	396.02	1281.46
Het df	130	50	181
Het P	***	***	***
Fixed RR	5.74	5.97	5.82
RRl	5.54	5.69	5.66
RRu	5.95	6.26	5.99
P	+++	+++	+++
Random RR	4.17	4.77	4.35
RRl	3.73	4.05	3.97
RRu	4.67	5.62	4.76
P	+++	+++	+++
Between Chi			1.59
Between df			1
Between P			N.S.
Btwn(F) P			N.S.
Btwn(R) P			N.S.

	Number of adjustment variables (1) 0	1	2+/+nk	Total
N	150	23	9	182
NS	105	14	6	125
Wt	3490.79	525.45	768.03	4784.26
Het Chi	876.23	102.57	174.36	1281.46
Het df	149	22	8	181
Het P	***	***	***	***
Fixed RR	5.68	4.30	8.00	5.82
RRl	5.49	3.94	7.46	5.66
RRu	5.87	4.68	8.59	5.99
P	+++	+++	+++	+++
Random RR	4.53	3.81	3.12	4.35
RRl	4.10	3.05	1.94	3.97
RRu	4.99	4.75	5.01	4.76
P	+++	+++	+++	+++
Between Chi				128.30
Between df				2
Between P				***
Btwn(F) P				***
Btwn(R) P				N.S.

Table 1D1 - 6

IESLC - Meta-analysis of Ex Smoking, Any product (or Cigarettes if Any not available)
All LC types
Least adjusted

	Number of adjustment variables (2) 0	1	2	3-5	6+/+nk	Total
N	150	23	8	1		182
NS	105	14	5	1		125
Wt	3490.79	525.45	764.46	3.56		4784.26
Het Chi	876.23	102.57	169.64	0.00		1281.46
Het df	149	22	7	0		181
Het P	***	***	***	N.S.		***
Fixed RR	5.68	4.30	8.04	2.54		5.82
RRl	5.49	3.94	7.49	0.90		5.66
RRu	5.87	4.68	8.64	7.17		5.99
P	+++	+++	+++	(+)		+++
Random RR	4.53	3.81	3.18	2.54		4.35
RRl	4.10	3.05	1.94	0.90		3.97
RRu	4.99	4.75	5.22	7.17		4.76
P	+++	+++	+++	(+)		+++
Between Chi						133.01
Between df						3
Between P						***
Btwn(F) P						***
Btwn(R) P						N.S.

	Product all/unsp	cig+/-ot	cig only	Total
N	81	90	11	182
NS	61	61	10	132
Wt	1369.63	3183.16	231.47	4784.26
Het Chi	447.71	677.03	65.04	1281.46
Het df	80	89	10	181
Het P	***	***	***	***
Fixed RR	4.77	6.42	4.91	5.82
RRl	4.52	6.20	4.32	5.66
RRu	5.03	6.65	5.59	5.99
P	+++	+++	+++	+++
Random RR	4.14	4.56	3.99	4.35
RRl	3.58	4.05	2.47	3.97
RRu	4.79	5.13	6.45	4.76
P	+++	+++	+++	+++
Between Chi				91.68
Between df				2
Between P				***
Btwn(F) P				**
Btwn(R) P				N.S.

	Denominator nev any	nev cigs	Total
N	121	61	182
NS	86	42	128
Wt	3046.70	1737.57	4784.26
Het Chi	769.66	503.21	1281.46
Het df	120	60	181
Het P	***	***	***
Fixed RR	5.64	6.15	5.82
RRl	5.44	5.87	5.66
RRu	5.84	6.45	5.99
P	+++	+++	+++
Random RR	4.37	4.27	4.35
RRl	3.91	3.64	3.97
RRu	4.90	5.02	4.76
P	+++	+++	+++
Between Chi			8.59
Between df			1
Between P			**
Btwn(F) P			N.S.
Btwn(R) P			N.S.

Table 1D1 - 6

IESLC - Meta-analysis of Ex Smoking, Any product (or Cigarettes if Any not available)
All LC types
Least adjusted

	Derivation of RR/CI			
	Orig	StdCalc	Other	Total
N	19	144	19	182
NS	11	100	15	126
Wt	918.64	3530.36	335.26	4784.26
Het Chi	170.12	842.02	152.41	1281.46
Het df	18	143	18	181
Het P	***	***	***	***
Fixed RR	7.81	5.54	4.34	5.82
RRl	7.32	5.36	3.90	5.66
RRu	8.33	5.73	4.83	5.99
P	+++	+++	+++	+++
Random RR	4.47	4.44	3.63	4.35
RRl	3.40	4.02	2.50	3.97
RRu	5.89	4.89	5.29	4.76
P	+++	+++	+++	+++
Between Chi				116.91
Between df				2
Between P				***
Btwn(F) P				***
Btwn(R) P				N.S.

Table 1D1 - 7

IESLC - Meta-analysis of Ex Smoking, Any product (or Cigarettes if Any not available)
All LC types
Excluded studies (and stage at which they were excluded)

1	BOUCHA BUELL LAURIL MZILEN
2	ABELIN ABRAHA ALDERS AUVINE AXELSO BAND BERRIN BLOT1 BLOT2 BLOT3 BLOT4 BRESLO BROCKM BROWN1 BYERS1 BYERS2 CASCO2 CASCOR CHAN CHATZI CHEN CHEN2 CHEN3 CHIAZZ COOKSO DAVEYS DEAN DOSEME DU DUNN EBELIN ENSTRO ESAKI FAN GENG GER GODLEY GOLLED GSELL HANSEN HEGMAN HINDS HIRAOK HIRAY2 HOROWI HORWIT HU HU2 HUANG ISHIMA JARUP JIANG JONES JUSSAW KANELL KO KOHLME KOULUM KREYBE LAMTH LAMWK LAMWK2 LAUSSM LEI LETOUR LEVIN LIAW LICKIN LIU LIU2 LIU3 LIU4 LIU5 LOMBA2 LUO MAGNUS MARSH MARSH2 MARTIS MASTRA MATSUD MCCONN MCDUFF MCLAUG MILLER MILLS MOLLO MRFIT MURATA NOTAN2 NOTANI NOU ORMOS PASTOR PAWLEG PERNU PERSHA PIKE POFFIJ POLEDN QIAO RADZIK RANDIG REN RIMING RONCO ROOTS ROTHSC SAARIK SADOWS SANKAR SCHWA2 SEGI SEGI2 SEOW SHIMIZ SIEMIA SIMARA SITAS SOBUE2 STASZE STAYNE STOCKS SUN TANG2 TAO ULMER VANDER WALD WANG WANG3 WANG4 WARSIN WATSON WICKLU WILKIN WU2 WUWILL WYNDE2 WYNDE4 WYNDE5 WYNDE8 WYNDER XIANGZ XU XU2 XU3 XU4 YUAN ZHANG ZHENG ZHOU
3	RESTRE
4	BOFFET
8	HAMMON SUZUKI
10	BENHAM GILLIS OSANN2 PISANI PRESCO SIMONA VEIERO VUTUC WYNDE7

Table 1D1 - 8

Potentially overlapping studies

REF	REFGP	PRINC	.	OVERLAP/LINK
LUBIN2	LUBIN2	1		Lubin-combined
KOO	KOO	1		KOO/LAMTH/LAMWK
TVERDA	TVERDA	1		VEIERO/TVERDAL
AKIBA	AKIBA	1		AKIBA/ISHIMA
HEIN	PRESCO	1		Subset of PRESCO
LANGE	PRESCO	1		Subset of PRESCO
BROSS	BYERS1	1		GRAHAM/BROSS/BYERS1
GRAHAM	BYERS1	1		GRAHAM/BROSS/BYERS1
CHYOU	CHYOU	1		GOODMA/CHYOU
GOODMA	GOODMA	1		GOODMA/CHYOU
HOLE	TANG2	1		Subset of TANG2
BENSHL	TANG2	1		Subset of TANG2
KAISER	KAISER	1		KAISER/OSANN2
MRFITR	MRFIT	2		Subset of MRFIT
WYNDE6	WYNDE6	1		WYNDE5/6/7/8
CPSI	CPSI	1		CPSI overall
JAHN	BOFFET	2		Subset of BOFFET
LUBIN	XIANGZ	2		LUBIN/XIANGZ/QIAO

Table 1D1 - 9

Most adjusted - insufficient data for metaanalysis

REF	NRR	SEX	AGEL	AGEH	RACE	YF	LC TYPE	LOC	START	ST	NLC	R	VB	P	H	AD	PRODUCT	DENOM	De
DOLL2	44	f	0	0	all	11	all	Eu:UK	1951	pr	920	n	V	n	n	1	all/unsp	nev any	st
RESTRE	1	c	0	0	all	-	all	SCAmer	1978	CC	102	n	bl	n	n	2	cig+/-ot	nev cigs	ot

REF	NRR	RR	SIG	RRDATA comment
DOLL2	44	2.67		0
RESTRE	1	4.72	y	0

Table 1D2 -

IESLC - Meta-analysis of Ex Smoking, Cigarettes (or Any Product if Cigarettes not available)
All LC types

This analysis is restricted to results for:
1) Non-dose-response data
2) Ex smokers
3) Results complete enough for use in metaanalysis

Within each study, results are then selected (in the following order of preference, within each sex) for:
4) PRODUCT: cigarettes regardless of other products, cigarettes only, all/unspec
5) CIGTYPE: all/unspecified, MC regardless of HR, MC only
6) DENOM: never smoked anything, never smoked cigarettes, (never +1 = +long term ex, +2 = +amount unknown,
+3 = never cigs+long term ex)
7) Followup period (YF, prospective studies): whole study (coded as 0) or longest available
8) LCtype: all or nearest available, at least Squamous and Adeno. (q = squamous, s = small,
l = large, a = adeno, mix = mixed, alv = alveolar)
9) Race: all or nearest available, otherwise by race (wh or w = white, bl or b = black, hi = hispanic
ch = chinese, jap = japanese, haw = hawaiian, w+o = white + oriental, sca = scandinavian, as = asian)
10) For overlapping studies: principal rather than subsidiary studies
Finally by Age: whole study (coded as 0) if available, otherwise by widest available age group
and then for single sex results (m, f) in preference to combined sex results (c).

Results adjusted (AD) for the most potential confounders are then chosen in Sections -1 to -3
and results adjusted for the least confounders in Sections -4 to -6. (Those least adjusted results which
actually differ from the most adjusted as marked 'x' in column X in Section -4)
(Results adjusted for an unknown number of confounder(s) are coded as 20.)

Section -7 shows excluded studies, together with the stage (as above) at which no qualifying
results were found.

Section -8 lists the potentially overlapping studies which have been included (1=principal, 2=subsidiary).

Section -9 lists any results which would have been included in preference except that they had data not complete
enough for use in meta-analysis, with their significance (yes/no), if known, and any further comment as entered
on the database.

In addition to those mentioned above, the following fields, levels and abbreviations are used:
* or nk = not known, n = no, y = yes, ot = other
nev = never
all/unspec = all or unspecified, cig+/-ot = cigarettes irrespective of other products (cigar, pipe etc)
MC = manufactured cigarettes, HR = hand-rolled cigarettes
REF: 6-character study reference
NRR: number of the RR on the database within the study
ST : study type (CC = case control, pr or prosp = prospective)
NLC: number of lung cancer cases in whole study
R : risky occupational population (n = no, m = mining, o = other risky)
VB : national cigarette type (V = at least 75% Virginia, bl = at least 75% blended, ot = other)
P : any proxy use
H : full histological confirmation
De : derivation of RR/CI (or = original, st = standard method, ot = other method of estimation)

Table 1D2 - 1

IESLC - Meta-analysis of Ex Smoking, Cigarettes (or Any Product if Cigarettes not available)
All LC types
Most adjusted

REF	NRR	SEX	AGEL	AGEH	RACE	YF	LC TYPE	LOC	START	ST	NLC	R	VB	P	H	AD	PRODUCT	DENOM	De
AGUDO	2	f	0	0	all	-	all	Eu:wst	1989	CC	103	n	bl	n	n	3	cig only	nev any	or
AKIBA	9	m	0	0	all	0	all	As:Jap	1963	pr	610	n	bl	n	n	5	cig+/-ot	nev cigs	or
AKIBA	13	f	0	0	all	0	all	As:Jap	1963	pr	610	n	bl	n	n	5	cig+/-ot	nev cigs	or
AMANDU	6	m	0	0	wh	0	all	NAmer	1959	pr	132	m	bl	n	n	2	cig+/-ot	nev cigs	ot
AMES	3	m	0	0	wh	-	all	NAmer	1959	ot	317	m	bl	n	n	0	all/unsp	nev any	st
ANDERS	1	f	0	0	all	0	all	NAmer	1986	pr	343	n	bl	n	n	0	cig+/-ot	nev cigs	st
ARCHER	4	m	0	0	wh	0	all	NAmer	1950	pr	146	m	bl	n	n	0	cig+/-ot	nev cigs	st
ARMADA	28	m	0	0	all	-	all	Eu:wst	1986	CC	325	n	bl	n	y	0	cig+/-ot	nev any	st
AUSTIN	5	c	0	0	all	-	all	NAmer	1970	CC	166	o	bl	y	n	3	cig+/-ot	nev cigs	or
AXELSS	4	m	0	0	sca	-	all	Eu:Sca	1989	CC	436	n	bl	n	n	0	all/unsp	nev any	st
AXELSS	9	f	0	0	sca	-	all	Eu:Sca	1989	CC	436	n	bl	n	n	0	all/unsp	nev any	st
BARBON	2	m	0	0	all	-	all	Eu:wst	1979	CC	755	n	bl	y	y	1	all/unsp	nev any	or
BECHER	5	m	0	0	all	-	all	Eu:Ger	1985	CC	194	n	bl	n	y	0	all/unsp	nev any	st
BECHER	6	f	0	0	all	-	all	Eu:Ger	1985	CC	194	n	bl	n	y	0	all/unsp	nev any	st
BENSHL	27	m	0	0	all	0	all	Eu:UK	1967	pr	486	n	V	n	n	2	cig+/-ot	nev any	ot
BEST	3	m	0	0	all	0	all	NAmer	1955	pr	381	n	V	n	n	1	cig only	nev any	ot
BLOHMK	2	m	0	0	all	-	all	Eu:Ger	1978	CC	888	n	bl	n	y	0	all/unsp	nev any	st
BOUCOT	115	m	0	0	all	0	all	NAmer	1951	pr	121	n	bl	n	n	2	cig only	nev any	ot
BRETT	9	m	0	0	all	0	all	Eu:UK	1960	pr	150	n	V	n	n	0	cig+/-ot	nev cigs	st
BROSS	6	m	0	0	wh	-	all	NAmer	1960	CC	974	n	bl	n	n	0	cig+/-ot	nev any	st
BROWN2	22	m	0	0	wh	-	all	NAmer	1984	CC	14596	n	bl	n	y	2	cig+/-ot	nev cigs	or
BROWN2	21	f	0	0	wh	-	all	NAmer	1984	CC	14596	n	bl	n	y	2	cig+/-ot	nev cigs	or
BUFFLE	4	m	0	0	wh	-	all	NAmer	1976	CC	943	n	bl	y	n	0	cig+/-ot	nev any	st
BUFFLE	8	f	0	0	wh	-	all	NAmer	1976	CC	943	n	bl	y	n	0	cig+/-ot	nev any	st
CARPEN	10	c	0	0	w+b	-	all	NAmer	1991	CC	356	n	bl	n	n	3	cig+/-ot	nev cigs	or
CEDERL	114	m	0	0	all	0	all	Eu:Sca	1963	pr	491	n	bl	n	n	2	all/unsp	nev any	or
CEDERL	74	f	0	0	all	0	all	Eu:Sca	1963	pr	491	n	bl	n	n	2	all/unsp	nev any	or
CHANG	1	m	0	0	all	0	all	NAmer	1972	pr	136	n	bl	n	n	0	cig+/-ot	nev cigs	st
CHANG	7	f	0	0	all	0	all	NAmer	1972	pr	136	n	bl	n	n	0	cig+/-ot	nev cigs	st
CHOI	2	m	0	0	all	-	all	As:oth	1985	CC	375	n	bl	n	n	0	cig+/-ot	nev cigs	st
CHOI	6	f	0	0	all	-	all	As:oth	1985	CC	375	n	bl	n	n	0	cig+/-ot	nev cigs	st
CHOW	9	m	0	0	wh	0	all	NAmer	1966	pr	219	n	bl	n	n	2	cig+/-ot	nev any	ot
CHYOU	1	m	0	0	jap	0	all	NAmer	1965	pr	227	n	bl	n	y	1	cig+/-ot	nev cigs	or
COMSTO	2	m	0	0	all	-	all	NAmer	1975	ot	258	n	bl	n	n	0	cig+/-ot	nev any	st
COMSTO	7	f	0	0	all	-	all	NAmer	1975	ot	258	n	bl	n	n	0	cig+/-ot	nev any	st
CORREA	38	c	0	0	all	-	all	NAmer	1979	CC	1359	n	bl	y	n	1	cig+/-ot	nev cigs	or
CPSI	72	m	0	0	wh	0	all	NAmer	1959	pr	5138	n	bl	n	n	1	cig only	nev any	st
CPSI	280	f	40	74	all	6	all	NAmer	1959	pr	5138	n	bl	n	n	1	cig+/-ot	nev cigs	ot
CPSII	91	m	35	99	all	4	all	NAmer	1982	pr	3229	n	bl	n	n	1	cig only	nev any	or
CPSII	78	f	0	0	all	4	all	NAmer	1982	pr	3229	n	bl	n	n	1	cig+/-ot	nev cigs	ot
DAMBER	17	m	0	0	all	-	all	Eu:Sca	1972	CC	579	n	bl	y	n	1	cig only	nev any	ot
DARBY	5	m	0	0	wh	-	all	Eu:UK	1988	CC	982	n	V	n	n	0	all/unsp	nev any	st
DARBY	12	f	0	0	wh	-	all	Eu:UK	1988	CC	982	n	V	n	n	0	all/unsp	nev any	st
DEAN2	1	m	0	0	all	-	all	Eu:UK	1960	CC	954	n	V	y	n	0	all/unsp	nev any	st
DEAN2	5	f	0	0	all	-	all	Eu:UK	1960	CC	954	n	V	y	n	0	all/unsp	nev any	st
DEAN3	181	m	0	0	all	-	all	Eu:UK	1969	CC	766	n	V	y	n	1	cig only	nev any	ot
DEAN3	112	f	0	0	all	-	all	Eu:UK	1969	CC	766	n	V	y	n	3	cig only	nev any	ot
DEKLER	1	m	0	0	all	0	all	Auslia	1961	pr	138	m	V	n	n	2	all/unsp	nev any	or
DESTE2	3	c	0	0	all	-	all	SCAmer	1993	CC	463	n	bl	n	n	7	all/unsp	nev any	or
DESTEF	49	m	0	0	all	-	all	SCAmer	1988	CC	497	n	bl	n	y	4	all/unsp	nev any	ot
DOCKER	2	c	0	0	wh	0	all	NAmer	1974	pr	120	n	bl	n	n	4	cig+/-ot	nev cigs	or
DOLL	91	m	0	0	all	-	all	Eu:UK	1948	CC	1465	n	V	n	n	0	all/unsp	nev any	st
DOLL	94	f	0	0	all	-	all	Eu:UK	1948	CC	1465	n	V	n	n	0	all/unsp	nev any	st
DOLL2	90	m	0	0	all	10	all	Eu:UK	1951	pr	920	n	V	n	n	1	cig+/-ot	nev any	ot
DOLL2	9	f	0	0	all	22	all	Eu:UK	1951	pr	920	n	V	n	n	1	cig only	nev any	ot
DORANT	1	m	0	0	all	0	all	Eu:wst	1986	ot	550	n	bl	n	y	0	all/unsp	nev any	st
DORGAN	8	m	0	0	wh	-	all	NAmer	1980	CC	2026	n	bl	y	y	0	cig+/-ot	nev any	st
DORGAN	32	m	0	0	bl	-	all	NAmer	1980	CC	2026	n	bl	y	y	0	cig+/-ot	nev any	st
DORGAN	55	f	0	0	wh	-	all	NAmer	1980	CC	2026	n	bl	y	y	0	cig+/-ot	nev any	st
DORGAN	78	f	0	0	bl	-	all	NAmer	1980	CC	2026	n	bl	y	y	0	cig+/-ot	nev any	st
DORN	20	m	0	0	wh	15	all	NAmer	1954	pr	5097	n	bl	n	n	2	cig+/-ot	nev any	ot
DROSTE	5	m	0	0	all	-	all	Eu:wst	1995	CC	478	n	bl	n	y	4	all/unsp	nev any	or
ENGELA	29	m	0	0	all	0	all	Eu:Sca	1964	pr	435	n	bl	n	n	7	cig+/-ot	nev cigs	or
ENGELA	43	f	0	0	all	0	all	Eu:Sca	1964	pr	435	n	bl	n	n	5	cig+/-ot	nev cigs	or
GAO	31	m	0	0	all	-	all	As:Chi	1984	CC	1405	n	ot	n	n	2	cig+/-ot	nev cigs	ot
GAO	32	f	0	0	all	-	all	As:Chi	1984	CC	1405	n	ot	n	n	2	cig+/-ot	nev cigs	ot
GAO2	9	m	0	0	all	-	all	As:Jap	1988	CC	282	n	bl	n	n	1	cig+/-ot	nev cigs	or
GARCIA	1	c	0	0	all	-	all	NAmer	1992	CC	416	n	bl	n	y	0	cig+/-ot	nev cigs	st
GARDIN	1	c	0	0	all	-	all	Eu:UK	1988	CC	143	n	V	y	n	0	all/unsp	nev any	st
GARSHI	30	m	0	0	all	-	all	NAmer	1981	CC	1081	o	bl	y	n	1	all/unsp	nev any	st
GOODMA	1	m	0	0	w+o	-	all	NAmer	1983	CC	326	n	bl	y	y	0	cig+/-ot	nev any	st
GOODMA	5	f	0	0	w+o	-	all	NAmer	1983	CC	326	n	bl	y	y	0	cig+/-ot	nev any	st

Table 1D2 - 1

IESLC - Meta-analysis of Ex Smoking, Cigarettes (or Any Product if Cigarettes not available)
All LC types
Most adjusted

REF	NRR	SEX	AGEL	AGEH	RACE	YF	LC TYPE	LOC	START	ST	NLC	R	VB	P	H	AD	PRODUCT	DENOM	De
GRAHAM	8	m	0	0	wh	-	all	NAmer	1956	CC	685	n	bl	n	n	1	cig+/-ot	nev any	ot
GREGOR	1	m	0	0	all	-	all	Eu:UK	1976	CC	104	n	V	n	y	0	cig+/-ot	nev cigs	st
GREGOR	5	f	0	0	all	-	all	Eu:UK	1976	CC	104	n	V	n	y	0	cig+/-ot	nev cigs	st
HAENSZ	55	f	0	0	all	-	not alv	NAmer	1955	CC	158	n	bl	n	y	0	cig+/-ot	nev any	st
HAMMO2	12	m	0	0	all	0	all	NAmer	1967	pr	450	o	bl	n	n	1	cig+/-ot	nev any	ot
HEIN	6	m	0	0	all	0	all	Eu:Sca	1970	pr	144	n	bl	n	n	0	all/unsp	nev any	st
HENNEK	1	m	0	0	all	0	all	NAmer	1982	pr	169	n	bl	n	n	0	all/unsp	nev any	st
HIRAYA	146	m	0	0	all	0	all	As:Jap	1965	pr	1917	n	bl	n	n	1	cig+/-ot	nev any	ot
HIRAYA	149	f	0	0	all	0	all	As:Jap	1965	pr	1917	n	bl	n	n	1	cig+/-ot	nev any	ot
HITOSU	33	m	0	0	all	-	all	As:Jap	1960	CC	216	n	bl	y	n	1	all/unsp	nev any	st
HITOSU	58	f	0	0	all	-	all	As:Jap	1960	CC	216	n	bl	y	n	1	all/unsp	nev any	st
HOLE	7	m	0	0	all	0	all	Eu:UK	1972	pr	225	n	V	n	n	1	all/unsp	nev any	ot
HUMBLE	1	m	0	0	w-hi	-	all	NAmer	1980	CC	521	n	bl	y	n	1	cig+/-ot	nev cigs	or
HUMBLE	4	m	0	0	hi	-	all	NAmer	1980	CC	521	n	bl	y	n	1	cig+/-ot	nev cigs	or
HUMBLE	7	f	0	0	w-hi	-	all	NAmer	1980	CC	521	n	bl	y	n	1	cig+/-ot	nev cigs	or
HUMBLE	10	f	0	0	hi	-	all	NAmer	1980	CC	521	n	bl	y	n	1	cig+/-ot	nev cigs	or
JAHN	10	m	0	0	all	-	all	Eu:Ger	1988	CC	1004	n	bl	n	n	0	cig+/-ot	nev any	st
JAIN	55	m	0	0	all	-	all	NAmer	1981	CC	845	n	V	y	n	2	cig+/-ot	nev cigs	ot
JAIN	53	f	0	0	all	-	all	NAmer	1981	CC	845	n	V	y	n	2	cig+/-ot	nev cigs	ot
JARVHO	1	m	0	0	all	-	all	Eu:Sca	1983	CC	147	n	bl	n	n	0	all/unsp	nev any	st
JARVHO	5	f	0	0	all	-	all	Eu:Sca	1983	CC	147	n	bl	n	n	0	all/unsp	nev any	st
JEDRYC	64	m	0	0	all	-	all	Eu:est	1980	CC	1630	n	bl	y	n	0	cig+/-ot	nev any	st
JEDRYC	69	f	0	0	all	-	all	Eu:est	1980	CC	1630	n	bl	y	n	0	cig+/-ot	nev any	st
JOLY	20	m	0	0	all	-	all	SCAmer	1978	CC	826	n	bl	n	n	0	cig+/-ot	nev any	st
JOLY	19	f	0	0	all	-	all	SCAmer	1978	CC	826	n	bl	n	n	0	cig+/-ot	nev any	st
KAISE2	65	m	35	99	all	9	all	NAmer	1979	pr	318	n	bl	n	n	1	cig only	nev any	st
KAISE2	57	f	35	99	all	9	all	NAmer	1979	pr	318	n	bl	n	n	1	cig only	nev any	st
KAISER	5	m	0	0	all	0	all	NAmer	1964	pr	714	n	bl	n	n	2	cig+/-ot	nev cigs	or
KAISER	1	f	0	0	all	0	all	NAmer	1964	pr	714	n	bl	n	n	2	cig+/-ot	nev cigs	or
KATSOU	1	f	0	0	all	-	all	Eu:bal	1987	CC	101	n	bl	n	n	1	all/unsp	nev any	or
KAUFMA	10	c	0	0	all	-	all	NAmer	1981	CC	881	n	bl	n	n	6	cig+/-ot	nev cigs	or
KELLER	2	m	0	0	wh	-	all	NAmer	1985	CC	15038	n	bl	n	n	0	all/unsp	nev any	st
KELLER	10	m	0	0	nonw	-	all	NAmer	1985	CC	15038	n	bl	n	n	0	all/unsp	nev any	st
KELLER	6	f	0	0	wh	-	all	NAmer	1985	CC	15038	n	bl	n	n	0	all/unsp	nev any	st
KELLER	14	f	0	0	nonw	-	all	NAmer	1985	CC	15038	n	bl	n	n	0	all/unsp	nev any	st
KHUDER	13	m	0	0	all	-	all	NAmer	1985	CC	482	n	bl	n	y	0	cig+/-ot	nev cigs	or
KIHARA	15	c	0	0	jap	-	all	As:Jap	1991	CC	440	n	bl	n	n	0	all/unsp	nev any	st
KINLEN	12	m	0	0	all	0	all	Eu:UK	1967	pr	718	n	V	n	n	2	all/unsp	nev any	ot
KJUUS	2	m	0	0	all	-	all	Eu:Sca	1979	CC	176	n	bl	n	n	0	all/unsp	nev any	st
KNEKT	27	m	20	69	all	21	all	Eu:Sca	1966	pr	515	n	bl	n	n	1	all/unsp	nev any	or
KOO	8	f	0	0	all	-	all	As:HK	1981	CC	200	n	bl	n	n	0	all/unsp	nev any	st
KREUZE	17	m	1	45	all	-	all	Eu:Ger	1990	CC	2260	n	bl	n	n	3	all/unsp	nev any	or
KREUZE	28	m	55	69	all	-	all	Eu:Ger	1990	CC	2260	n	bl	n	n	3	all/unsp	nev any	or
KREUZE	23	f	1	45	all	-	all	Eu:Ger	1990	CC	2260	n	bl	n	n	3	all/unsp	nev any	or
KREUZE	34	f	55	69	all	-	all	Eu:Ger	1990	CC	2260	n	bl	n	n	3	all/unsp	nev any	or
KUBIK	11	m	0	0	all	0	all	Eu:est	1965	pr	108	n	bl	n	n	0	cig+/-ot	nev any	st
LANGE	14	m	0	0	all	0	all	Eu:Sca	1976	pr	268	n	bl	n	n	1	all/unsp	nev any	or
LANGE	10	f	0	0	all	0	all	Eu:Sca	1976	pr	268	n	bl	n	n	1	all/unsp	nev any	or
LEMARC	1	c	0	0	w+o	-	all	NAmer	1992	CC	341	n	bl	n	y	0	all/unsp	nev any	st
LIDDEL	1	m	0	0	all	18	all	NAmer	1970	pr	304	m	V	n	n	1	cig+/-ot	nev cigs	ot
LOMBAR	8	m	0	0	all	-	all	NAmer	1951	CC	1040	n	bl	n	n	0	cig+/-ot	nev any	st
LUBIN	39	m	0	0	all	-	all	As:Chi	1984	CC	427	m	ot	y	n	0	cig+/-ot	nev any	st
LUBIN2	38	m	0	0	all	-	all	Eu:mul	1976	CC	7804	n	bl	n	y	2	cig+/-ot	nev any	ot
LUBIN2	319	f	0	0	all	-	all	Eu:mul	1976	CC	7804	n	bl	n	y	0	cig+/-ot	nev any	st
MACLEN	7	m	0	0	ch	-	all	As:oth	1972	CC	233	n	bl	n	n	0	cig+/-ot	nev cigs	st
MACLEN	8	f	0	0	ch	-	all	As:oth	1972	CC	233	n	bl	n	n	0	cig+/-ot	nev cigs	st
MATOS	15	m	0	0	all	-	all	SCAmer	1994	CC	200	n	bl	n	n	2	cig+/-ot	nev any	or
MIGRAN	22	m	0	0	all	0	all	Eu:UK	1964	pr	259	n	V	n	n	2	cig+/-ot	nev any	ot
MIGRAN	39	f	0	0	all	0	all	Eu:UK	1964	pr	259	n	V	n	n	0	cig+/-ot	nev any	st
MRFITR	1	m	0	0	all	0	all	NAmer	1973	pr	119	n	bl	n	n	0	cig+/-ot	nev cigs	ot
NAM	73	m	0	0	all	-	all	NAmer	1986	CC	1199	n	bl	y	n	1	cig+/-ot	nev cigs	ot
NAM	89	f	0	0	all	-	all	NAmer	1986	CC	1199	n	bl	y	n	1	cig+/-ot	nev cigs	ot
ODRISC	2	c	0	0	all	-	all	Eu:UK	1992	CC	446	n	V	n	n	0	all/unsp	nev any	st
OSANN	25	m	0	0	all	-	all	NAmer	1984	CC	1986	n	bl	n	n	2	cig+/-ot	nev cigs	or
OSANN	26	f	0	0	all	-	all	NAmer	1984	CC	1986	n	bl	n	n	2	cig+/-ot	nev cigs	or
PARKIN	19	m	0	0	bl	-	all	Africa	1963	CC	877	n	V	y	n	0	cig+/-ot	nev any	st
PERSH2	7	c	0	0	all	-	all	Eu:Sca	1980	CC	1022	n	bl	y	n	4	all/unsp	nev any	ot
PETO	1	m	0	0	all	0	all	Eu:UK	1954	pr	103	n	V	n	n	0	all/unsp	nev any	st
PEZZO2	1	m	0	0	all	-	all	SCAmer	1992	CC	367	n	bl	n	y	0	cig+/-ot	nev cigs	st
PEZZOT	1	m	0	0	all	-	all	SCAmer	1987	CC	215	n	bl	n	y	0	cig only	nev cigs	st
QIAO2	7	m	0	0	all	0	all	As:Chi	1992	pr	241	m	ot	n	n	0	cig+/-ot	nev any	st
RACHTA	8	f	0	0	all	-	all	Eu:est	1991	CC	118	n	bl	n	y	1	cig+/-ot	nev cigs	or

Table 1D2 - 1

IESLC - Meta-analysis of Ex Smoking, Cigarettes (or Any Product if Cigarettes not available)
All LC types
Most adjusted

REF	NRR	SEX	AGEL	AGEH	RACE	YF	LC TYPE	LOC	START	ST	NLC	R	VB	P	H	AD	PRODUCT	DENOM	De
SCHWAR	21	m	0	0	wh	-	all	NAmer	1984	CC	5588	n	bl	y	y	0	cig+/-ot	nev cigs	st
SCHWAR	22	m	0	0	bl	-	all	NAmer	1984	CC	5588	n	bl	y	y	0	cig+/-ot	nev cigs	st
SCHWAR	23	f	0	0	wh	-	all	NAmer	1984	CC	5588	n	bl	y	y	0	cig+/-ot	nev cigs	st
SCHWAR	24	f	0	0	bl	-	all	NAmer	1984	CC	5588	n	bl	y	y	0	cig+/-ot	nev cigs	st
SHAW	3	c	0	0	wh	-	all	NAmer	1988	CC	335	n	V	n	y	0	all/unsp	nev any	st
SOBUE	41	m	0	0	all	-	q+s+l+a	As:Jap	1986	CC	1376	n	bl	n	y	1	cig+/-ot	nev cigs	or
SOBUE	51	f	0	0	all	-	q+s+l+a	As:Jap	1986	CC	1376	n	bl	n	y	1	cig+/-ot	nev cigs	or
SPEIZE	7	f	0	0	all	0	all	NAmer	1976	pr	593	n	bl	n	y	0	cig+/-ot	nev cigs	st
SPITZ	1	c	0	0	b+hi	-	all	NAmer	1992	CC	177	n	bl	n	y	0	cig+/-ot	nev cigs	st
STOCKW	4	c	0	0	all	-	all	NAmer	1981	CC	22161	n	bl	n	n	0	cig+/-ot	nev any	st
STUCKE	1	m	0	0	all	-	all	Eu:wst	1989	CC	247	n	bl	n	y	0	all/unsp	nev any	ot
SUZUK2	5	c	0	0	all	-	all	SCAmer	1991	CC	123	n	bl	n	y	3	all/unsp	nev any	or
SVENSS	1	f	0	0	all	-	all	Eu:Sca	1983	CC	210	n	bl	n	n	1	all/unsp	nev any	or
TANG	2	c	0	0	all	-	not s	NAmer	1992	CC	119	n	bl	n	y	0	cig+/-ot	nev cigs	st
TENKAN	9	m	0	0	all	17	all	Eu:Sca	1962	pr	242	n	bl	n	n	1	all/unsp	nev any	ot
TIZZAN	6	m	0	0	all	-	all	Eu:wst	1959	CC	1358	n	bl	n	n	0	all/unsp	nev any	st
TIZZAN	14	f	0	0	all	-	all	Eu:wst	1959	CC	1358	n	bl	n	n	0	all/unsp	nev any	st
TOKARS	2	m	0	0	all	-	all	Eu:est	1966	ot	162	o	bl	n	y	0	all/unsp	nev any	st
TOUSEY	11	m	0	0	all	-	all	NAmer	1993	CC	507	n	bl	y	y	3	cig+/-ot	nev any	or
TOUSEY	14	f	0	0	all	-	all	NAmer	1993	CC	507	n	bl	y	y	3	cig+/-ot	nev any	or
TSUGAN	26	m	0	0	all	-	q+a	As:Jap	1976	CC	134	n	bl	n	y	0	all/unsp	nev any	st
TULINI	24	m	0	0	all	0	all	Eu:Sca	1967	pr	472	n	bl	n	n	3	all/unsp	nev any	or
TULINI	30	f	0	0	all	0	all	Eu:Sca	1967	pr	472	n	bl	n	n	3	all/unsp	nev any	or
TVERDA	1	m	0	0	all	0	all	Eu:Sca	1972	pr	238	n	bl	n	n	2	cig+/-ot	nev cigs	ot
TVERDA	18	f	0	0	all	0	all	Eu:Sca	1972	pr	238	n	bl	n	n	0	cig only	nev cigs	ot
WAKAI	7	m	0	0	all	-	all	As:Jap	1988	CC	333	n	bl	n	y	2	all/unsp	nev any	or
WAKAI	25	f	0	0	all	-	all	As:Jap	1988	CC	333	n	bl	n	y	2	all/unsp	nev any	or
WANG2	20	c	0	0	all	-	all	As:Chi	1980	CC	103	n	ot	n	n	4	cig+/-ot	nev cigs	ot
WIGLE	7	m	0	0	all	-	all	NAmer	1971	CC	728	n	V	n	n	0	cig only	nev any	st
WIGLE	10	f	0	0	all	-	all	NAmer	1971	CC	728	n	V	n	n	0	cig only	nev any	st
WU	41	f	0	0	wh	-	q+a	NAmer	1981	CC	220	n	bl	n	y	2	all/unsp	nev any	st
WUNSCH	6	m	0	0	all	-	all	SCAmer	1990	CC	398	n	bl	y	n	1	cig+/-ot	nev any	or
WUNSCH	12	f	0	0	all	-	all	SCAmer	1990	CC	398	n	bl	y	n	1	cig+/-ot	nev any	or
WYNDE3	41	m	0	0	all	-	all	NAmer	1966	CC	350	n	bl	n	y	0	all/unsp	nev any	st
WYNDE6	9	m	0	0	all	-	all	NAmer	1969	CC	4423	n	bl	n	y	0	cig+/-ot	nev any	st
WYNDE6	198	f	0	0	all	-	all	NAmer	1969	CC	4423	n	bl	n	y	0	cig+/-ot	nev cigs	st
YAMAGU	9	c	0	0	all	-	all	As:Jap	1989	CC	144	n	bl	n	y	1	all/unsp	nev any	or
YONG	1	c	0	0	all	0	all	NAmer	1971	pr	216	n	bl	n	n	1	cig+/-ot	nev cigs	or

Cigarette type is all/unspec for all RRs

except for the following:

REF	NRR	CIGTYPE
DEAN3	181	MC only
DEAN3	112	MC only

Table 1D2 - 2

IESLC - Meta-analysis of Ex Smoking, Cigarettes (or Any Product if Cigarettes not available)
All LC types
Most adjusted

REF	NRR	SEX	AD	Number Exposed Case	Number Exposed Cont	Non-exposed Case	Non-exposed Cont	RR	95.00%CI
AGUDO	2	f	3	-	-	-	-	1.61	(0.37- 6.91)
*AKIBA	9	m	5	-	-	-	-	2.50	(1.50- 4.30)
*AKIBA	13	f	5	-	-	-	-	1.40	(0.70- 2.60)
Subtotal AKIBA								1.99	(1.32- 3.00)
*AMANDU	6	m	2	-	-	-	-	2.13	(0.62- 7.29)
AMES	3	m	0	147	115	15	62	5.28	(2.86- 9.77)
*ANDERS	1	f	0	85	54902	46	195158	6.57	(4.59- 9.40)
*ARCHER	4	m	0	18	3740	6	9842	7.89	(3.14- 19.87)
ARMADA	28	m	0	129	132	4	64	15.64	(5.53- 44.19)
AUSTIN	5	c	3	-	-	-	-	7.40	(2.60- 21.50)
AXELSS	4	m	0	98	214	16	160	4.58	(2.60- 8.07)
AXELSS	9	f	0	14	40	18	154	2.99	(1.37- 6.53)
Subtotal AXELSS								3.95	(2.50- 6.26)
BARBON	2	m	1	-	-	-	-	7.10	(4.40- 11.60)
BECHER	5	m	0	42	116	3	54	6.52	(1.93- 21.96)
BECHER	6	f	0	5	18	10	52	1.44	(0.44- 4.80)
Subtotal BECHER								3.04	(1.29- 7.14)
*BENSHL	27	m	2	-	-	-	-	3.07	(1.57- 6.03)
*BEST	3	m	1	-	-	-	-	6.06	(2.53- 14.51)
BLOHMK	2	m	0	343	274	126	301	2.99	(2.30- 3.88)
*BOUCOT	115	m	2	-	-	-	-	20.86	(1.20- 361.47)
*BRETT	9	m	0	9	10482	6	6530	0.93	(0.33- 2.62)
BROSS	6	m	0	212	146	38	170	6.50	(4.31- 9.79)
BROWN2	22	m	2	-	-	-	-	7.20	(6.50- 7.90)
BROWN2	21	f	2	-	-	-	-	11.60	(10.40- 13.00)
Subtotal BROWN2								8.85	(8.23- 9.53)
BUFFLE	4	m	0	204	154	5	47	12.45	(4.84- 32.05)
BUFFLE	8	f	0	106	101	41	198	5.07	(3.29- 7.81)
Subtotal BUFFLE								5.92	(4.00- 8.78)
CARPEN	10	c	3	-	-	-	-	7.00	(3.85- 12.73)
*CEDERL	114	m	2	-	-	-	-	1.25	(0.67- 2.34)
*CEDERL	74	f	2	-	-	-	-	1.08	(0.34- 3.44)
Subtotal CEDERL								1.21	(0.70- 2.10)
*CHANG	1	m	0	43	1087	5	502	3.97	(1.58- 9.97)
*CHANG	7	f	0	12	580	11	1139	2.14	(0.95- 4.83)
Subtotal CHANG								2.81	(1.53- 5.16)
CHOI	2	m	0	35	136	13	95	1.88	(0.94- 3.74)
CHOI	6	f	0	6	3	76	164	4.32	(1.05- 17.72)
Subtotal CHOI								2.21	(1.19- 4.10)
*CHOW	9	m	2	-	-	-	-	5.79	(2.48- 13.51)
*CHYOU	1	m	1	-	-	-	-	3.10	(1.60- 5.80)
COMSTO	2	m	0	46	129	4	69	6.15	(2.13- 17.80)
COMSTO	7	f	0	11	35	13	115	2.78	(1.14- 6.75)
Subtotal COMSTO								3.85	(1.95- 7.61)
CORREA	38	c	1	-	-	-	-	6.50	(4.70- 9.10)
*CPSI	72	m	1	-	-	-	-	3.74	(3.14- 4.46)
*CPSI	280	f	1	-	-	-	-	1.38	(0.81- 2.35)
Subtotal CPSI								3.39	(2.87- 4.01)
*CPSII	91	m	1	-	-	-	-	9.36	(7.43- 11.77)
*CPSII	78	f	1	-	-	-	-	4.84	(4.00- 5.86)
Subtotal CPSII								6.33	(5.47- 7.34)
DAMBER	17	m	1	-	-	-	-	3.15	(1.80- 5.49)
DARBY	5	m	0	285	1106	3	384	32.98	(10.51- 103.49)
DARBY	12	f	0	94	317	23	529	6.82	(4.23- 10.99)
Subtotal DARBY								8.61	(5.55- 13.37)
DEAN2	1	m	0	98	88	33	112	3.78	(2.33- 6.13)
DEAN2	5	f	0	5	2	88	121	3.44	(0.65- 18.13)
Subtotal DEAN2								3.75	(2.36- 5.97)
DEAN3	181	m	1	-	-	-	-	3.21	(1.96- 5.25)
DEAN3	112	f	3	-	-	-	-	1.17	(0.51- 2.69)
Subtotal DEAN3								2.47	(1.62- 3.77)
*DEKLER	1	m	2	-	-	-	-	10.70	(1.40- 81.90)
DESTE2	3	c	7	-	-	-	-	8.10	(4.50- 14.70)
DESTEF	49	m	4	-	-	-	-	6.20	(3.78- 10.15)
*DOCKER	2	c	4	-	-	-	-	2.54	(0.90- 7.18)
DOLL	91	m	0	70	124	7	61	4.92	(2.13- 11.34)
DOLL	94	f	0	10	8	40	59	1.84	(0.67- 5.08)
Subtotal DOLL								3.31	(1.74- 6.30)
*DOLL2	90	m	1	-	-	-	-	4.22	(1.23- 14.48)
*DOLL2	9	f	1	-	-	-	-	3.29	(0.88- 12.24)
Subtotal DOLL2								3.76	(1.53- 9.24)

Table 1D2 - 2

IESLC - Meta-analysis of Ex Smoking, Cigarettes (or Any Product if Cigarettes not available)
All LC types
Most adjusted

REF	NRR	SEX	AD	Number Exposed Case	Number Exposed Cont	Non-exposed Case	Non-exposed Cont	RR	95.00%CI
DORANT	1	m	0	146	771	7	159	4.30	(1.98- 9.36)
DORGAN	8	m	0	236	230	15	93	6.36	(3.58- 11.30)
DORGAN	32	m	0	49	56	3	35	10.21	(2.95- 35.27)
DORGAN	55	f	0	146	110	103	244	3.14	(2.24- 4.41)
DORGAN	78	f	0	11	10	7	20	3.14	(0.93- 10.58)
Subtotal DORGAN								3.92	(2.98- 5.17)
*DORN	20	m	2	-	-	-	-	3.97	(3.32- 4.75)
DROSTE	5	m	4	-	-	-	-	4.20	(1.80- 9.80)
*ENGELA	29	m	7	-	-	-	-	1.30	(0.80- 2.20)
*ENGELA	43	f	5	-	-	-	-	2.00	(0.80- 4.90)
Subtotal ENGELA								1.44	(0.93- 2.24)
GAO	31	m	2	-	-	-	-	4.17	(2.85- 6.12)
GAO	32	f	2	-	-	-	-	4.24	(2.67- 6.75)
Subtotal GAO								4.20	(3.13- 5.64)
GAO2	9	m	1	-	-	-	-	3.56	(1.83- 6.91)
GARCIA	1	c	0	226	233	21	139	6.42	(3.92- 10.52)
GARDIN	1	c	0	41	44	5	41	7.64	(2.75- 21.22)
GARSHI	30	m	1	-	-	-	-	4.04	(2.84- 5.74)
GOODMA	1	m	0	68	229	10	199	5.91	(2.96- 11.79)
GOODMA	5	f	0	23	35	19	177	6.12	(3.02- 12.42)
Subtotal GOODMA								6.01	(3.67- 9.85)
GRAHAM	8	m	1	-	-	-	-	14.87	(8.94- 24.71)
GREGOR	1	m	0	23	45	10	14	0.72	(0.28- 1.86)
GREGOR	5	f	0	4	16	1	22	5.50	(0.56- 53.99)
Subtotal GREGOR								0.97	(0.40- 2.34)
HAENSZ	55	f	0	5	9	81	236	1.62	(0.53- 4.97)
*HAMMO2	12	m	1	-	-	-	-	6.46	(2.63- 15.85)
*HEIN	6	m	0	11	979	1	457	5.13	(0.66- 39.65)
*HENNEK	1	m	0	67	8674	23	10919	3.67	(2.29- 5.88)
*HIRAYA	146	m	1	-	-	-	-	1.71	(1.08- 2.72)
*HIRAYA	149	f	1	-	-	-	-	2.98	(1.14- 7.77)
Subtotal HIRAYA								1.90	(1.25- 2.88)
HITOSU	33	m	1	-	-	-	-	3.95	(1.63- 9.55)
HITOSU	58	f	1	-	-	-	-	6.72	(2.55- 17.68)
Subtotal HITOSU								5.03	(2.62- 9.66)
*HOLE	7	m	1	-	-	-	-	2.69	(1.16- 6.23)
HUMBLE	1	m	1	-	-	-	-	7.20	(3.00- 17.60)
HUMBLE	4	m	1	-	-	-	-	8.00	(1.90- 42.20)
HUMBLE	7	f	1	-	-	-	-	6.50	(2.80- 15.40)
HUMBLE	10	f	1	-	-	-	-	6.30	(1.50- 27.80)
Subtotal HUMBLE								6.88	(4.04- 11.71)
JAHN	10	m	0	455	402	18	138	8.68	(5.22- 14.44)
JAIN	55	m	2	-	-	-	-	4.16	(2.04- 8.46)
JAIN	53	f	2	-	-	-	-	3.54	(2.04- 6.13)
Subtotal JAIN								3.76	(2.43- 5.81)
JARVHO	1	m	0	26	28	1	16	14.86	(1.84- 120.07)
JARVHO	5	f	0	10	8	6	21	4.38	(1.19- 16.04)
Subtotal JARVHO								6.15	(2.04- 18.54)
JEDRYC	64	m	0	137	196	49	219	3.12	(2.14- 4.56)
JEDRYC	69	f	0	13	8	78	166	3.46	(1.38- 8.69)
Subtotal JEDRYC								3.17	(2.23- 4.50)
JOLY	20	m	0	101	185	12	218	9.92	(5.28- 18.62)
JOLY	19	f	0	34	27	52	283	6.85	(3.82- 12.31)
Subtotal JOLY								8.13	(5.30- 12.49)
*KAISE2	65	m	1	-	-	-	-	3.39	(1.77- 6.48)
*KAISE2	57	f	1	-	-	-	-	5.02	(2.17- 11.61)
Subtotal KAISE2								3.93	(2.35- 6.56)
*KAISER	5	m	2	-	-	-	-	4.65	(2.84- 7.64)
*KAISER	1	f	2	-	-	-	-	3.02	(1.83- 4.99)
Subtotal KAISER								3.76	(2.64- 5.35)
KATSOU	1	f	1	-	-	-	-	2.84	(0.81- 9.98)
KAUFMA	10	c	6	-	-	-	-	6.80	(4.60- 9.90)
KELLER	2	m	0	3003	1307	323	1017	7.23	(6.28- 8.33)
KELLER	10	m	0	440	128	38	117	10.58	(6.99- 16.04)
KELLER	6	f	0	1094	477	469	1860	9.10	(7.85- 10.54)
KELLER	14	f	0	130	79	67	232	5.70	(3.86- 8.42)
Subtotal KELLER								8.02	(7.28- 8.82)
KHUDER	13	m	0	184	-	23	-	7.50	(4.80- 11.90)
KIHARA	15	c	0	55	70	102	237	1.83	(1.20- 2.79)
*KINLEN	12	m	2	-	-	-	-	4.88	(2.26- 10.56)
KJUUS	2	m	0	39	75	2	24	6.24	(1.40- 27.78)

Table 1D2 - 2

IESLC - Meta-analysis of Ex Smoking, Cigarettes (or Any Product if Cigarettes not available)
All LC types
Most adjusted

				Number Exposed		Non-exposed					
REF	NRR	SEX	AD	Case	Cont	Case	Cont	RR		95.00%CI	
*KNEKT	27	m	1	-	-	-	-	2.60	(	1.00-	6.40)
KOO	8	f	0	22	10	56	85	3.34	(	1.47-	7.58)
KREUZE	17	m	3	-	-	-	-	1.70	(	0.60-	5.20)
KREUZE	28	m	3	-	-	-	-	9.10	(	5.90-	14.10)
KREUZE	23	f	3	-	-	-	-	2.50	(	0.70-	9.00)
KREUZE	34	f	3	-	-	-	-	1.40	(	0.80-	2.40)
Subtotal KREUZE								3.93	(	2.87-	5.39)
*KUBIK	11	m	0	8	1487	2	4271	11.49	(	2.44-	54.04)
*LANGE	14	m	1	-	-	-	-	2.10	(	0.70-	6.00)
*LANGE	10	f	1	-	-	-	-	3.40	(	1.10-	11.00)
Subtotal LANGE								2.63	(	1.20-	5.76)
LEMARC	1	c	0	142	223	32	168	3.34	(	2.17-	5.15)
*LIDDEL	1	m	1	-	-	-	-	1.40	(	0.79-	2.51)
LOMBAR	8	m	0	126	172	14	112	5.86	(	3.21-	10.69)
LUBIN	39	m	0	70	139	9	72	4.03	(	1.90-	8.53)
LUBIN2	38	m	2	-	-	-	-	4.44	(	3.77-	5.24)
LUBIN2	319	f	0	100	157	288	1180	2.61	(	1.97-	3.46)
Subtotal LUBIN2								3.88	(	3.36-	4.47)
MACLEN	7	m	0	5	11	5	15	1.36	(	0.32-	5.89)
MACLEN	8	f	0	3	10	41	109	0.80	(	0.21-	3.04)
Subtotal MACLEN								1.02	(	0.38-	2.74)
MATOS	15	m	2	-	-	-	-	5.30	(	2.60-	10.70)
*MIGRAN	22	m	2	-	-	-	-	2.59	(	0.88-	7.60)
*MIGRAN	39	f	0	1	599	4	3814	1.59	(	0.18-	14.22)
Subtotal MIGRAN								2.36	(	0.90-	6.20)
*MRFITR	1	m	0	13	2813	0	1859	17.84~	(	1.06-	300.00)
NAM	73	m	1	-	-	-	-	9.17	(	6.12-	13.74)
NAM	89	f	1	-	-	-	-	8.27	(	5.76-	11.87)
Subtotal NAM								8.66	(	6.61-	11.34)
ODRISC	2	c	0	147	398	6	664	40.87	(	17.90-	93.34)
OSANN	25	m	2	-	-	-	-	12.20	(	8.80-	17.00)
OSANN	26	f	2	-	-	-	-	8.10	(	6.00-	11.00)
Subtotal OSANN								9.77	(	7.82-	12.22)
PARKIN	19	m	0	26	59	107	1248	5.14	(	3.11-	8.49)
PERSH2	7	c	4	-	-	-	-	2.61	(	1.98-	3.44)
*PETO	1	m	0	2	387	2	295	0.76	(	0.11-	5.38)
PEZZO2	1	m	0	128	271	6	117	9.21	(	3.95-	21.48)
PEZZOT	1	m	0	66	188	4	116	10.18	(	3.61-	28.67)
*QIAO2	7	m	0	41	961	10	709	3.02	(	1.53-	6.00)
RACHTA	8	f	1	-	-	-	-	3.80	(	1.21-	7.84)
SCHWAR	21	m	0	996	670	119	376	4.70	(	3.74-	5.90)
SCHWAR	22	m	0	219	136	50	104	3.35	(	2.25-	4.99)
SCHWAR	23	f	0	322	328	182	855	4.61	(	3.69-	5.76)
SCHWAR	24	f	0	79	89	40	247	5.48	(	3.49-	8.60)
Subtotal SCHWAR								4.54	(	3.94-	5.22)
SHAW	3	c	0	112	169	11	107	6.45	(	3.32-	12.53)
SOBUE	41	m	1	-	-	-	-	2.80	(	1.90-	4.20)
SOBUE	51	f	1	-	-	-	-	2.10	(	1.40-	3.20)
Subtotal SOBUE								2.44	(	1.83-	3.25)
*SPEIZE	7	f	0	144	522081	58	776300	3.69	(	2.72-	5.01)
SPITZ	1	c	0	67	80	7	128	15.31	(	6.70-	35.02)
STOCKW	4	c	0	6185	3057	2791	10641	7.71	(	7.26-	8.19)
STUCKE	1	m	0	178	135	0	51	135.69~	(	8.30-	2218.34)
SUZUK2	5	c	3	-	-	-	-	7.70	(	2.20-	27.00)
SVENSS	1	f	1	-	-	-	-	2.60	(	1.40-	5.10)
TANG	2	c	0	58	34	9	39	7.39	(	3.19-	17.11)
*TENKAN	9	m	1	-	-	-	-	4.02	(	1.50-	10.80)
TIZZAN	6	m	0	346	292	180	305	2.01	(	1.58-	2.56)
TIZZAN	14	f	0	8	10	25	114	3.65	(	1.31-	10.17)
Subtotal TIZZAN								2.07	(	1.64-	2.62)
TOKARS	2	m	0	37	86	1	53	22.80	(	3.04-	171.13)
TOUSEY	11	m	3	-	-	-	-	13.20	(	4.80-	36.60)
TOUSEY	14	f	3	-	-	-	-	9.10	(	4.80-	17.10)
Subtotal TOUSEY								10.10	(	5.90-	17.31)
TSUGAN	26	m	0	10	8	18	22	1.53	(	0.50-	4.68)
*TULINI	24	m	3	-	-	-	-	3.03	(	1.54-	5.98)
*TULINI	30	f	3	-	-	-	-	3.69	(	1.71-	7.99)
Subtotal TULINI								3.30	(	1.98-	5.49)
*TVERDA	1	m	2	-	-	-	-	0.49	(	0.24-	1.01)
*TVERDA	18	f	0	0	38953	3	157431	0.58~	(	0.03-	11.18)
Subtotal TVERDA								0.49	(	0.25-	0.99)

Table 1D2 - 2

IESLC - Meta-analysis of Ex Smoking, Cigarettes (or Any Product if Cigarettes not available)
All LC types
Most adjusted

REF	NRR	SEX	AD	Number Exposed Case	Number Exposed Cont	Non-exposed Case	Non-exposed Cont	RR	95.00%CI
WAKAI	7	m	2	-	-	-	-	2.43	(1.16- 5.06)
WAKAI	25	f	2	-	-	-	-	5.33	(1.21- 23.50)
Subtotal WAKAI								2.84	(1.47- 5.49)
WANG2	20	c	4	-	-	-	-	2.00	(0.77- 5.20)
WIGLE	7	m	0	128	217	15	204	8.02	(4.55- 14.16)
WIGLE	10	f	0	11	66	36	439	2.03	(0.99- 4.19)
Subtotal WIGLE								4.75	(3.04- 7.43)
WU	41	f	2	-	-	-	-	1.40	(0.74- 2.66)
WUNSCH	6	m	1	-	-	-	-	3.28	(1.77- 6.07)
WUNSCH	12	f	1	-	-	-	-	3.51	(1.74- 7.10)
Subtotal WUNSCH								3.38	(2.13- 5.37)
WYNDE3	41	m	0	48	125	9	88	3.75	(1.75- 8.05)
WYNDE6	9	m	0	1088	1056	87	617	7.31	(5.75- 9.29)
WYNDE6	198	f	0	332	325	159	856	5.50	(4.38- 6.91)
Subtotal WYNDE6								6.29	(5.34- 7.43)
YAMAGU	9	c	1	-	-	-	-	2.90	(1.43- 5.90)
*YONG	1	c	1	-	-	-	-	4.10	(2.55- 6.60)
Partial Totals				20172	664282	6582	1197256		

*prospective study ~ With 0.5 adjustment for zero

REF	NRR	SEX	AD	Ys	Ws	Qs	Ps
AGUDO	2	f	3	0.48	1.79	2.92	0.5236
*AKIBA	9	m	5	0.92	13.85	9.66	0.0006
*AKIBA	13	f	5	0.34	8.92	17.87	0.3148
Subtotal AKIBA				0.69	22.78	27.53	
*AMANDU	6	m	2	0.76	2.53	2.51	0.2291
AMES	3	m	0	1.66	10.17	0.08	0.0000
*ANDERS	1	f	0	1.88	29.87	0.51	0.0000
*ARCHER	4	m	0	2.07	4.51	0.45	0.0000
ARMADA	28	m	0	2.75	3.56	3.55	0.0000
AUSTIN	5	c	3	2.00	3.44	0.22	0.0002
AXELSS	4	m	0	1.52	11.96	0.63	0.0000
AXELSS	9	f	0	1.10	6.31	2.70	0.0059
Subtotal AXELSS				1.37	18.27	3.34	
BARBON	2	m	1	1.96	16.35	0.71	0.0000
BECHER	5	m	0	1.87	2.60	0.04	0.0025
BECHER	6	f	0	0.37	2.67	5.11	0.5481
Subtotal BECHER				1.11	5.27	5.15	
*BENSHL	27	m	2	1.12	8.49	3.37	0.0011
*BEST	3	m	1	1.80	5.04	0.01	0.0001
BLOHMK	2	m	0	1.10	56.10	24.15	0.0000
*BOUCOT	115	m	2	3.04	0.47	0.78	0.0370
*BRETT	9	m	0	-0.07	3.60	11.93	0.8976
BROSS	6	m	0	1.87	22.85	0.33	0.0000
BROWN2	22	m	2	1.97	403.85	20.01	0.0000
BROWN2	21	f	2	2.45	308.59	151.01	0.0000
Subtotal BROWN2				2.18	712.44	171.02	
BUFFLE	4	m	0	2.52	4.30	2.55	0.0000
BUFFLE	8	f	0	1.62	20.50	0.34	0.0000
Subtotal BUFFLE				1.78	24.80	2.89	
CARPEN	10	c	3	1.95	10.74	0.41	0.0000
*CEDERL	114	m	2	0.22	9.82	22.95	0.4843
*CEDERL	74	f	2	0.08	2.87	8.04	0.8963
Subtotal CEDERL				0.19	12.69	30.99	
*CHANG	1	m	0	1.38	4.54	0.63	0.0033
*CHANG	7	f	0	0.76	5.83	5.71	0.0659
Subtotal CHANG				1.03	10.36	6.33	
CHOI	2	m	0	0.63	8.11	10.16	0.0721
CHOI	6	f	0	1.46	1.93	0.16	0.0424
Subtotal CHOI				0.79	10.03	10.33	
*CHOW	9	m	2	1.76	5.35	0.00	0.0000
*CHYOU	1	m	1	1.13	9.26	3.56	0.0006
COMSTO	2	m	0	1.82	3.40	0.01	0.0008
COMSTO	7	f	0	1.02	4.88	2.59	0.0240
Subtotal COMSTO				1.35	8.28	2.61	
CORREA	38	c	1	1.87	35.20	0.51	0.0000
*CPSI	72	m	1	1.32	124.77	23.33	0.0000
*CPSI	280	f	1	0.32	13.54	27.67	0.2359
Subtotal CPSI				1.22	138.32	51.00	
*CPSII	91	m	1	2.24	72.61	17.08	0.0000

Table 1D2 - 2

IESLC - Meta-analysis of Ex Smoking, Cigarettes (or Any Product if Cigarettes not available)
All LC types
Most adjusted

REF	NRR	SEX	AD	Ys	Ws	Qs	Ps
*CPSII	78	f	1	1.58	105.38	3.21	0.0000
Subtotal CPSII				1.85	177.99	20.29	
DAMBER	17	m	1	1.15	12.36	4.51	0.0001
DARBY	5	m	0	3.50	2.94	8.94	0.0000
DARBY	12	f	0	1.92	16.90	0.48	0.0000
Subtotal DARBY				2.15	19.84	9.42	
DEAN2	1	m	0	1.33	16.45	2.93	0.0000
DEAN2	5	f	0	1.23	1.39	0.37	0.1455
Subtotal DEAN2				1.32	17.84	3.30	
DEAN3	181	m	1	1.17	15.83	5.42	0.0000
DEAN3	112	f	3	0.16	5.56	14.13	0.7113
Subtotal DEAN3				0.90	21.39	19.55	
*DEKLER	1	m	2	2.37	0.93	0.36	0.0224
DESTE2	3	c	7	2.09	10.97	1.27	0.0000
DESTEF	49	m	4	1.82	15.75	0.08	0.0000
*DOCKER	2	c	4	0.93	3.56	2.39	0.0785
DOLL	91	m	0	1.59	5.51	0.14	0.0002
DOLL	94	f	0	0.61	3.75	4.87	0.2364
Subtotal DOLL				1.20	9.25	5.00	
*DOLL2	90	m	1	1.44	2.53	0.25	0.0221
*DOLL2	9	f	1	1.19	2.22	0.70	0.0762
Subtotal DOLL2				1.32	4.74	0.94	
DORANT	1	m	0	1.46	6.36	0.54	0.0002
DORGAN	8	m	0	1.85	11.63	0.11	0.0000
DORGAN	32	m	0	2.32	2.50	0.82	0.0002
DORGAN	55	f	0	1.15	33.62	12.34	0.0000
DORGAN	78	f	0	1.15	2.61	0.96	0.0645
Subtotal DORGAN				1.37	50.35	14.23	
*DORN	20	m	2	1.38	119.77	16.64	0.0000
DROSTE	5	m	4	1.44	5.35	0.54	0.0009
*ENGELA	29	m	7	0.26	15.02	33.30	0.3093
*ENGELA	43	f	5	0.69	4.68	5.24	0.1338
Subtotal ENGELA				0.36	19.69	38.54	
GAO	31	m	2	1.43	26.31	2.75	0.0000
GAO	32	f	2	1.44	17.86	1.68	0.0000
Subtotal GAO				1.43	44.17	4.44	
GAO2	9	m	1	1.27	8.70	2.02	0.0002
GARCIA	1	c	0	1.86	15.74	0.18	0.0000
GARDIN	1	c	0	2.03	3.68	0.29	0.0001
GARSHI	30	m	1	1.40	31.03	3.92	0.0000
GOODMA	1	m	0	1.78	8.06	0.01	0.0000
GOODMA	5	f	0	1.81	7.67	0.03	0.0000
Subtotal GOODMA				1.79	15.73	0.03	
GRAHAM	8	m	1	2.70	14.87	13.36	0.0000
GREGOR	1	m	0	-0.33	4.22	18.35	0.4919
GREGOR	5	f	0	1.70	0.74	0.00	0.1435
Subtotal GREGOR				-0.03	4.95	18.35	
HAENSZ	55	f	0	0.48	3.05	4.92	0.4002
*HAMMO2	12	m	1	1.87	4.76	0.06	0.0000
*HEIN	6	m	0	1.64	0.92	0.01	0.1167
*HENNEK	1	m	0	1.30	17.18	3.51	0.0000
*HIRAYA	146	m	1	0.54	18.01	26.59	0.0228
*HIRAYA	149	f	1	1.09	4.17	1.81	0.0257
Subtotal HIRAYA				0.64	22.18	28.40	
HITOSU	33	m	1	1.37	4.92	0.70	0.0023
HITOSU	58	f	1	1.91	4.10	0.10	0.0001
Subtotal HITOSU				1.62	9.01	0.80	
*HOLE	7	m	1	0.99	5.44	3.16	0.0210
HUMBLE	1	m	1	1.97	4.91	0.24	0.0000
HUMBLE	4	m	1	2.08	1.60	0.17	0.0086
HUMBLE	7	f	1	1.87	5.29	0.08	0.0000
HUMBLE	10	f	1	1.84	1.80	0.01	0.0135
Subtotal HUMBLE				1.93	13.60	0.51	
JAHN	10	m	0	2.16	14.82	2.48	0.0000
JAIN	55	m	2	1.43	7.59	0.81	0.0001
JAIN	53	f	2	1.26	12.69	3.01	0.0000
Subtotal JAIN				1.32	20.29	3.82	
JARVHO	1	m	0	2.70	0.88	0.79	0.0114
JARVHO	5	f	0	1.48	2.28	0.17	0.0260
Subtotal JARVHO				1.82	3.16	0.96	
JEDRYC	64	m	0	1.14	26.76	10.03	0.0000

Table 1D2 - 2

IESLC - Meta-analysis of Ex Smoking, Cigarettes (or Any Product if Cigarettes not available)
All LC types
Most adjusted

REF	NRR	SEX	AD	Ys	Ws	Qs	Ps
JEDRYC	69	f	0	1.24	4.53	1.18	0.0083
Subtotal JEDRYC				1.15	31.29	11.21	
JOLY	20	m	0	2.29	9.69	2.86	0.0000
JOLY	19	f	0	1.92	11.21	0.34	0.0000
Subtotal JOLY				2.10	20.90	3.19	
*KAISE2	65	m	1	1.22	9.12	2.57	0.0002
*KAISE2	57	f	1	1.61	5.46	0.10	0.0002
Subtotal KAISE2				1.37	14.59	2.67	
*KAISER	5	m	2	1.54	15.69	0.72	0.0000
*KAISER	1	f	2	1.11	15.27	6.38	0.0000
Subtotal KAISER				1.32	30.96	7.10	
KATSOU	1	f	1	1.04	2.44	1.22	0.1033
KAUFMA	10	c	6	1.92	26.16	0.72	0.0000
KELLER	2	m	0	1.98	193.15	9.98	0.0000
KELLER	10	m	0	2.36	22.25	8.22	0.0000
KELLER	6	f	0	2.21	176.05	36.66	0.0000
KELLER	14	f	0	1.74	25.26	0.00	0.0000
Subtotal KELLER				2.08	416.70	54.87	
KHUDER	13	m	0	2.01	18.64	1.29	0.0000
KIHARA	15	c	0	0.60	21.51	28.42	0.0052
*KINLEN	12	m	2	1.59	6.46	0.18	0.0001
KJUUS	2	m	0	1.83	1.72	0.01	0.0163
*KNEKT	27	m	1	0.96	4.46	2.83	0.0436
KOO	8	f	0	1.21	5.71	1.70	0.0040
KREUZE	17	m	3	0.53	3.30	4.91	0.3354
KREUZE	28	m	3	2.21	20.24	4.22	0.0000
KREUZE	23	f	3	0.92	2.36	1.64	0.1596
KREUZE	34	f	3	0.34	12.73	25.49	0.2299
Subtotal KREUZE				1.37	38.63	36.27	
*KUBIK	11	m	0	2.44	1.60	0.76	0.0020
*LANGE	14	m	1	0.74	3.33	3.39	0.1758
*LANGE	10	f	1	1.22	2.90	0.81	0.0372
Subtotal LANGE				0.97	6.23	4.20	
LEMARC	1	c	0	1.21	20.52	6.09	0.0000
*LIDDEL	1	m	1	0.34	11.50	23.02	0.2539
LOMBAR	8	m	0	1.77	10.63	0.00	0.0000
LUBIN	39	m	0	1.39	6.83	0.88	0.0003
LUBIN2	38	m	2	1.49	141.75	9.64	0.0000
LUBIN2	319	f	0	0.96	48.33	30.34	0.0000
Subtotal LUBIN2				1.36	190.08	39.98	
MACLEN	7	m	0	0.31	1.79	3.73	0.6779
MACLEN	8	f	0	-0.23	2.14	8.38	0.7406
Subtotal MACLEN				0.02	3.94	12.10	
MATOS	15	m	2	1.67	7.68	0.05	0.0000
*MIGRAN	22	m	2	0.95	3.31	2.11	0.0836
*MIGRAN	39	f	0	0.46	0.80	1.33	0.6773
Subtotal MIGRAN				0.86	4.11	3.44	
*MRFITR	1	m	0	2.88	0.48	0.62	0.0454
NAM	73	m	1	2.22	23.49	5.07	0.0000
NAM	89	f	1	2.11	29.39	3.83	0.0000
Subtotal NAM				2.16	52.88	8.90	
ODRISC	2	c	0	3.71	5.63	21.62	0.0000
OSANN	25	m	2	2.50	35.44	19.93	0.0000
OSANN	26	f	2	2.09	41.82	4.85	0.0000
Subtotal OSANN				2.28	77.26	24.78	
PARKIN	19	m	0	1.64	15.25	0.20	0.0000
PERSH2	7	c	4	0.96	50.36	31.60	0.0000
*PETO	1	m	0	-0.27	1.01	4.12	0.7854
PEZZO2	1	m	0	2.22	5.36	1.18	0.0000
PEZZOT	1	m	0	2.32	3.58	1.16	0.0000
*QIAO2	7	m	0	1.11	8.20	3.41	0.0015
RACHTA	8	f	1	1.34	4.40	0.76	0.0051
SCHWAR	21	m	0	1.55	73.75	3.09	0.0000
SCHWAR	22	m	0	1.21	24.08	7.09	0.0000
SCHWAR	23	f	0	1.53	78.01	3.87	0.0000
SCHWAR	24	f	0	1.70	18.89	0.05	0.0000
Subtotal SCHWAR				1.51	194.73	14.10	
SHAW	3	c	0	1.86	8.69	0.11	0.0000
SOBUE	41	m	1	1.03	24.42	12.73	0.0000
SOBUE	51	f	1	0.74	22.48	22.92	0.0004
Subtotal SOBUE				0.89	46.91	35.64	

Table 1D2 - 2

IESLC - Meta-analysis of Ex Smoking, Cigarettes (or Any Product if Cigarettes not available)
All LC types
Most adjusted

REF	NRR	SEX	AD	Ys	Ws	Qs	Ps
*SPEIZE	7	f	0	1.31	41.35	8.20	0.0000
SPITZ	1	c	0	2.73	5.61	5.36	0.0000
STOCKW	4	c	0	2.04	1062.62	90.31	0.0000
STUCKE	1	m	0	4.91	0.49	4.91	0.0006
SUZUK2	5	c	3	2.04	2.44	0.21	0.0014
SVENSS	1	f	1	0.96	9.19	5.83	0.0038
TANG	2	c	0	2.00	5.45	0.34	0.0000
*TENKAN	9	m	1	1.39	3.94	0.51	0.0057
TIZZAN	6	m	0	0.70	66.01	73.39	0.0000
TIZZAN	14	f	0	1.29	3.65	0.76	0.0134
Subtotal TIZZAN				0.73	69.66	74.16	
TOKARS	2	m	0	3.13	0.95	1.79	0.0024
TOUSEY	11	m	3	2.58	3.72	2.56	0.0000
TOUSEY	14	f	3	2.21	9.52	1.99	0.0000
Subtotal TOUSEY				2.31	13.24	4.54	
TSUGAN	26	m	0	0.42	3.07	5.41	0.4579
*TULINI	24	m	3	1.11	8.35	3.45	0.0014
*TULINI	30	f	3	1.31	6.46	1.29	0.0009
Subtotal TULINI				1.19	14.81	4.74	
*TVERDA	1	m	2	-0.71	7.44	45.20	0.0517
*TVERDA	18	f	0	-0.55	0.44	2.32	0.7164
Subtotal TVERDA				-0.70	7.88	47.52	
WAKAI	7	m	2	0.89	7.08	5.28	0.0181
WAKAI	25	f	2	1.67	1.75	0.01	0.0270
Subtotal WAKAI				1.04	8.83	5.29	
WANG2	20	c	4	0.69	4.21	4.72	0.1549
WIGLE	7	m	0	2.08	11.91	1.30	0.0000
WIGLE	10	f	0	0.71	7.35	7.98	0.0546
Subtotal WIGLE				1.56	19.25	9.28	
WU	41	f	2	0.34	9.39	18.79	0.3026
WUNSCH	6	m	1	1.19	10.12	3.21	0.0002
WUNSCH	12	f	1	1.26	7.77	1.91	0.0005
Subtotal WUNSCH				1.22	17.89	5.12	
WYNDE3	41	m	0	1.32	6.61	1.21	0.0007
WYNDE6	9	m	0	1.99	66.75	3.76	0.0000
WYNDE6	198	f	0	1.70	73.82	0.16	0.0000
Subtotal WYNDE6				1.84	140.57	3.92	
YAMAGU	9	c	1	1.06	7.65	3.61	0.0032
*YONG	1	c	1	1.41	16.99	1.97	0.0000

	N	182
	NS	124
	Wt	4792.11
Het	Chi	1294.12
Het	df	181
Het	P	***
Fixed	RR	5.76
	RRl	5.60
	RRu	5.93
	P	+++
Random	RR	4.30
	RRl	3.93
	RRu	4.71
	P	+++
Asymm	P	***

Table 1D2 - 3

IESLC - Meta-analysis of Ex Smoking, Cigarettes (or Any Product if Cigarettes not available)
All LC types
Most adjusted

	combined	Sex male	female	Total
N	20	100	62	182
NS	20	95	57	172
Wt	1321.19	2146.60	1324.33	4792.11
Het Chi	151.85	581.32	479.72	1294.12
Het df	19	99	61	181
Het P	***	***	***	***
Fixed RR	6.98	5.09	5.81	5.76
RRl	6.61	4.88	5.51	5.60
RRu	7.37	5.31	6.14	5.93
P	+++	+++	+++	+++
Random RR	5.58	4.49	3.58	4.30
RRl	4.23	3.98	2.99	3.93
RRu	7.36	5.07	4.29	4.71
P	+++	+++	+++	+++
Between Chi				81.23
Between df				2
Between P				***
Btwn(F) P				**
Btwn(R) P				*

	Lung cancer type all	other	Total
N	176	6	182
NS	119	5	124
Wt	4724.25	67.86	4792.11
Het Chi	1228.25	11.70	1294.12
Het df	175	5	181
Het P	***	*	***
Fixed RR	5.84	2.37	5.76
RRl	5.67	1.87	5.60
RRu	6.01	3.01	5.93
P	+++	+++	+++
Random RR	4.40	2.36	4.30
RRl	4.01	1.57	3.93
RRu	4.82	3.55	4.71
P	+++	+++	+++
Between Chi			54.17
Between df			1
Between P			***
Btwn(F) P			**
Btwn(R) P			**

	NAmer	UK	Scand	Location othEur	China	Japan	othAs	other	Total
N	80	21	21	24	5	14	5	12	182
NS	53	14	14	17	4	9	3	10	124
Wt	3726.39	116.44	165.68	449.14	63.41	150.64	19.68	100.74	4792.11
Het Chi	525.70	87.61	47.09	110.48	2.63	16.35	4.66	12.47	1294.12
Het df	79	20	20	23	4	13	4	11	181
Het P	***	***	***	***	N.S.	N.S.	N.S.	N.S.	***
Fixed RR	6.73	3.93	2.39	3.59	3.81	2.35	2.13	6.12	5.76
RRl	6.52	3.28	2.05	3.27	2.98	2.01	1.37	5.03	5.60
RRu	6.95	4.71	2.78	3.94	4.88	2.76	3.32	7.43	5.93
P	+++	+++	+++	+++	+++	+++	+++	+++	+++
Random RR	5.47	3.63	2.42	3.97	3.81	2.40	2.12	6.14	4.30
RRl	4.93	2.40	1.85	3.07	2.98	1.99	1.29	4.97	3.93
RRu	6.06	5.48	3.17	5.13	4.88	2.89	3.47	7.59	4.71
P	+++	+++	+++	+++	+++	+++	++	+++	+++
Between Chi									487.13
Between df									7
Between P									***
Btwn(F) P									***
Btwn(R) P									***

Table 1D2 - 3

IESLC - Meta-analysis of Ex Smoking, Cigarettes (or Any Product if Cigarettes not available)
All LC types
Most adjusted

Detailed Country in "other Europe"

	multi	Germany	othWest	East	Balkans	Total
N	2	8	8	5	1	24
NS	1	4	7	4	1	17
Wt	190.08	114.82	103.57	38.23	2.44	449.14
Het Chi	10.18	47.24	41.89	5.94	0.00	110.48
Het df	1	7	7	4	0	23
Het P	**	***	***	N.S.	N.S.	***
Fixed RR	3.88	3.77	2.97	3.59	2.84	3.59
RRl	3.36	3.14	2.45	2.61	0.81	3.27
RRu	4.47	4.52	3.60	4.93	9.97	3.94
P	+++	+++	+++	+++	N.S.	+++
Random RR	3.45	3.46	4.91	4.16	2.84	3.97
RRl	2.05	1.96	2.58	2.52	0.81	3.07
RRu	5.80	6.13	9.37	6.87	9.97	5.13
P	+++	+++	+++	+++	N.S.	+++
Between Chi						5.23
Between df						4
Between P						N.S.
Btwn(F) P						N.S.
Btwn(R) P						N.S.

Detailed Country in "other Asia"

	India	HongKong	other	Total
N		1	4	5
NS		1	2	3
Wt		5.71	13.97	19.68
Het Chi		0.00	3.04	4.66
Het df		0	3	4
Het P		N.S.	N.S.	N.S.
Fixed RR		3.34	1.77	2.13
RRl		1.47	1.05	1.37
RRu		7.58	3.00	3.32
P		++	+	+++
Random RR		3.34	1.77	2.12
RRl		1.47	1.04	1.29
RRu		7.58	3.02	3.47
P		++	+	++
Between Chi				1.62
Between df				1
Between P				N.S.
Btwn(F) P				N.S.
Btwn(R) P				N.S.

Detailed other continent

	SCAmer	Auslia	Africa	Total
N	10	1	1	12
NS	8	1	1	10
Wt	84.56	0.93	15.25	100.74
Het Chi	11.66	0.00	0.00	12.47
Het df	9	0	0	11
Het P	N.S.	N.S.	N.S.	N.S.
Fixed RR	6.27	10.70	5.14	6.12
RRl	5.07	1.40	3.11	5.03
RRu	7.76	81.84	8.49	7.43
P	+++	+	+++	+++
Random RR	6.30	10.70	5.14	6.14
RRl	4.92	1.40	3.11	4.97
RRu	8.07	81.84	8.49	7.59
P	+++	+	+++	+++
Between Chi				0.80
Between df				2
Between P				N.S.
Btwn(F) P				N.S.
Btwn(R) P				N.S.

Table 1D2 - 3

IESLC - Meta-analysis of Ex Smoking, Cigarettes (or Any Product if Cigarettes not available)
All LC types
Most adjusted

	Start year of study <1960	1960-69	1970-79	1980-89	1990+	Total
N	18	36	36	70	22	182
NS	14	25	25	43	17	124
Wt	394.02	410.55	519.74	3268.43	199.37	4792.11
Het Chi	83.15	134.79	135.76	476.79	110.11	1294.12
Het df	17	35	35	69	21	181
Het P	***	***	***	***	***	***
Fixed RR	3.57	3.92	3.82	6.91	4.87	5.76
RRl	3.23	3.56	3.50	6.68	4.24	5.60
RRu	3.94	4.32	4.16	7.15	5.59	5.93
P	+++	+++	+++	+++	+++	+++
Random RR	3.79	3.27	3.47	5.22	5.36	4.30
RRl	2.83	2.64	2.83	4.65	3.85	3.93
RRu	5.06	4.05	4.27	5.86	7.46	4.71
P	+++	+++	+++	+++	+++	+++
Between Chi						353.53
Between df						4
Between P						***
Btwn(F) P						***
Btwn(R) P						***

	Study type (1) CC	other	Total
N	123	59	182
NS	80	44	124
Wt	3961.35	830.77	4792.11
Het Chi	890.28	229.20	1294.12
Het df	122	58	181
Het P	***	***	***
Fixed RR	6.29	3.80	5.76
RRl	6.10	3.55	5.60
RRu	6.49	4.07	5.93
P	+++	+++	+++
Random RR	4.89	3.18	4.30
RRl	4.41	2.70	3.93
RRu	5.42	3.74	4.71
P	+++	+++	+++
Between Chi			174.64
Between df			1
Between P			***
Btwn(F) P			***
Btwn(R) P			***

	Study type (2) CC	prosp	other	Total
N	123	54	5	182
NS	80	40	4	124
Wt	3961.35	805.02	25.75	4792.11
Het Chi	890.28	223.65	4.13	1294.12
Het df	122	53	4	181
Het P	***	***	N.S.	***
Fixed RR	6.29	3.77	4.79	5.76
RRl	6.10	3.52	3.25	5.60
RRu	6.49	4.04	7.04	5.93
P	+++	+++	+++	+++
Random RR	4.89	3.06	4.79	4.30
RRl	4.41	2.58	3.23	3.93
RRu	5.42	3.63	7.11	4.71
P	+++	+++	+++	+++
Between Chi				176.06
Between df				2
Between P				***
Btwn(F) P				***
Btwn(R) P				***

Table 1D2 - 3

IESLC - Meta-analysis of Ex Smoking, Cigarettes (or Any Product if Cigarettes not available)
All LC types
Most adjusted

	Study size (number of LC cases)				
	100-249	250-499	500-999	1000+	Total
N	51	45	37	49	182
NS	44	33	23	24	124
Wt	216.51	385.57	452.19	3737.84	4792.11
Het Chi	128.70	157.99	126.43	709.71	1294.12
Het df	50	44	36	48	181
Het P	***	***	***	***	***
Fixed RR	3.31	4.03	4.64	6.34	5.76
RRl	2.89	3.65	4.23	6.14	5.60
RRu	3.78	4.46	5.09	6.55	5.93
P	+++	+++	+++	+++	+++
Random RR	3.46	4.03	4.90	4.76	4.30
RRl	2.75	3.30	4.07	4.13	3.93
RRu	4.36	4.91	5.91	5.48	4.71
P	+++	+++	+++	+++	+++
Between Chi					171.28
Between df					3
Between P					***
Btwn(F) P					***
Btwn(R) P					(*)

	Risky occupational population			
	no	mining	othRisky	Total
N	171	7	4	182
NS	113	7	4	124
Wt	4707.26	44.66	40.19	4792.11
Het Chi	1257.21	16.23	4.26	1294.12
Het df	170	6	3	181
Het P	***	*	N.S.	***
Fixed RR	5.80	3.26	4.69	5.76
RRl	5.64	2.43	3.44	5.60
RRu	5.97	4.37	6.38	5.93
P	+++	+++	+++	+++
Random RR	4.30	3.57	5.46	4.30
RRl	3.92	2.13	3.33	3.93
RRu	4.73	5.99	8.96	4.71
P	+++	+++	+++	+++
Between Chi				16.42
Between df				2
Between P				***
Btwn(F) P				N.S.
Btwn(R) P				N.S.

	National cigarette tobacco type			
	Virginia	blended	other	Total
N	30	147	5	182
NS	21	99	4	124
Wt	197.38	4531.32	63.41	4792.11
Het Chi	114.36	1137.03	2.63	1294.12
Het df	29	146	4	181
Het P	***	***	N.S.	***
Fixed RR	3.98	5.89	3.81	5.76
RRl	3.46	5.72	2.98	5.60
RRu	4.57	6.06	4.88	5.93
P	+++	+++	+++	+++
Random RR	3.83	4.42	3.81	4.30
RRl	2.84	4.00	2.98	3.93
RRu	5.15	4.88	4.88	4.71
P	+++	+++	+++	+++
Between Chi				40.11
Between df				2
Between P				***
Btwn(F) P				(*)
Btwn(R) P				N.S.

Table 1D2 - 3

IESLC - Meta-analysis of Ex Smoking, Cigarettes (or Any Product if Cigarettes not available)
All LC types
Most adjusted

	Any proxy use No/nk	Yes	Total
N	141	41	182
NS	102	22	124
Wt	4134.58	657.53	4792.11
Het Chi	1156.84	97.58	1294.12
Het df	140	40	181
Het P	***	***	***
Fixed RR	5.98	4.59	5.76
RRl	5.80	4.25	5.60
RRu	6.16	4.95	5.93
P	+++	+++	+++
Random RR	4.13	4.77	4.30
RRl	3.71	4.17	3.93
RRu	4.60	5.46	4.71
P	+++	+++	+++
Between Chi			39.71
Between df			1
Between P			***
Btwn(F) P			*
Btwn(R) P			N.S.

	Full histological confirmation No	Yes	Total
N	131	51	182
NS	88	36	124
Wt	3133.28	1658.83	4792.11
Het Chi	882.40	409.40	1294.12
Het df	130	50	181
Het P	***	***	***
Fixed RR	5.67	5.94	5.76
RRl	5.48	5.66	5.60
RRu	5.87	6.23	5.93
P	+++	+++	+++
Random RR	4.12	4.76	4.30
RRl	3.68	4.02	3.93
RRu	4.61	5.63	4.71
P	+++	+++	+++
Between Chi			2.32
Between df			1
Between P			N.S.
Btwn(F) P			N.S.
Btwn(R) P			N.S.

	Number of adjustment variables (1) 0	1	2+/+nk	Total
N	89	43	50	182
NS	62	30	36	128
Wt	2598.67	723.75	1469.69	4792.11
Het Chi	533.54	189.05	516.43	1294.12
Het df	88	42	49	181
Het P	***	***	***	***
Fixed RR	6.08	4.47	5.95	5.76
RRl	5.85	4.16	5.65	5.60
RRu	6.31	4.81	6.26	5.93
P	+++	+++	+++	+++
Random RR	4.82	4.04	3.72	4.30
RRl	4.25	3.39	3.05	3.93
RRu	5.46	4.82	4.54	4.71
P	+++	+++	+++	+++
Between Chi				55.10
Between df				2
Between P				***
Btwn(F) P				*
Btwn(R) P				(*)

Table 1D2 - 3

IESLC - Meta-analysis of Ex Smoking, Cigarettes (or Any Product if Cigarettes not available)
All LC types
Most adjusted

	Number of adjustment variables (2) 0	1	2	3-5	6+/+nk	Total
N	89	43	26	21	3	182
NS	62	30	19	15	3	129
Wt	2598.67	723.75	1220.20	197.36	52.14	4792.11
Het Chi	533.54	189.05	326.00	84.83	31.36	1294.12
Het df	88	42	25	20	2	181
Het P	***	***	***	***	***	***
Fixed RR	6.08	4.47	6.57	3.47	4.38	5.76
RRl	5.85	4.16	6.21	3.02	3.34	5.60
RRu	6.31	4.81	6.95	3.99	5.75	5.93
P	+++	+++	+++	+++	+++	+++
Random RR	4.82	4.04	3.92	3.44	4.15	4.30
RRl	4.25	3.39	3.04	2.51	1.36	3.93
RRu	5.46	4.82	5.06	4.71	12.66	4.71
P	+++	+++	+++	+++	+	+++
Between Chi						129.34
Between df						4
Between P						***
Btwn(F) P						***
Btwn(R) P						N.S.

	Product all/unsp	cig+/-ot	cig only	Total
N	65	102	15	182
NS	47	69	12	128
Wt	1002.68	3510.93	278.50	4792.11
Het Chi	390.43	778.87	73.33	1294.12
Het df	64	101	14	181
Het P	***	***	***	***
Fixed RR	4.89	6.13	4.73	5.76
RRl	4.60	5.93	4.20	5.60
RRu	5.21	6.34	5.31	5.93
P	+++	+++	+++	+++
Random RR	4.11	4.47	4.01	4.30
RRl	3.43	3.99	2.82	3.93
RRu	4.91	5.00	5.72	4.71
P	+++	+++	+++	+++
Between Chi				51.49
Between df				2
Between P				***
Btwn(F) P				*
Btwn(R) P				N.S.

	Denominator nev any	nev cigs	Total
N	120	62	182
NS	85	42	127
Wt	3055.60	1736.52	4792.11
Het Chi	756.46	533.27	1294.12
Het df	119	61	181
Het P	***	***	***
Fixed RR	5.63	6.00	5.76
RRl	5.44	5.72	5.60
RRu	5.84	6.29	5.93
P	+++	+++	+++
Random RR	4.42	4.08	4.30
RRl	3.95	3.46	3.93
RRu	4.95	4.80	4.71
P	+++	+++	+++
Between Chi			4.39
Between df			1
Between P			*
Btwn(F) P			N.S.
Btwn(R) P			N.S.

Table 1D2 - 3

IESLC - Meta-analysis of Ex Smoking, Cigarettes (or Any Product if Cigarettes not available)
All LC types
Most adjusted

		Derivation of RR/CI			
		Orig	StdCalc	Other	Total
	N	54	92	36	182
	NS	36	63	29	128
	Wt	1314.92	2767.42	709.77	4792.11
Het	Chi	414.78	580.39	177.41	1294.12
Het	df	53	91	35	181
Het	P	***	***	***	***
Fixed	RR	6.74	5.86	4.05	5.76
	RRl	6.38	5.65	3.76	5.60
	RRu	7.11	6.08	4.35	5.93
	P	+++	+++	+++	+++
Random	RR	4.19	4.65	3.64	4.30
	RRl	3.48	4.12	2.98	3.93
	RRu	5.03	5.25	4.44	4.71
	P	+++	+++	+++	+++
Between	Chi				121.54
Between	df				2
Between	P				***
Btwn(F)	P				***
Btwn(R)	P				N.S.

Table 1D2 - 4

IESLC - Meta-analysis of Ex Smoking, Cigarettes (or Any Product if Cigarettes not available)
All LC types
Least adjusted

REF	NRR	X	SEX	AGEL	AGEH	RACE	YF	LC TYPE	LOC	START	ST	NLC	R	VB	P	H	AD	PRODUCT	DENOM	De
AGUDO	9	x	f	0	0	all	-	all	Eu:wst	1989	CC	103	n	bl	n	n	0	cig only	nev any	st
AKIBA	1	x	m	0	0	all	0	all	As:Jap	1963	pr	610	n	bl	n	n	0	cig+/-ot	nev cigs	or
AKIBA	5	x	f	0	0	all	0	all	As:Jap	1963	pr	610	n	bl	n	n	0	cig+/-ot	nev cigs	or
AMANDU	2	x	m	0	0	wh	0	all	NAmer	1959	pr	132	m	bl	n	n	0	cig+/-ot	nev cigs	st
AMES	3		m	0	0	wh	-	all	NAmer	1959	ot	317	m	bl	n	n	0	all/unsp	nev any	st
ANDERS	1		f	0	0	all	0	all	NAmer	1986	pr	343	n	bl	n	n	0	cig+/-ot	nev cigs	st
ARCHER	4		m	0	0	wh	0	all	NAmer	1950	pr	146	m	bl	n	n	0	cig+/-ot	nev cigs	st
ARMADA	28		m	0	0	all	-	all	Eu:wst	1986	CC	325	n	bl	n	y	0	cig+/-ot	nev any	st
AUSTIN	1	x	c	0	0	all	-	all	NAmer	1970	CC	166	o	bl	y	n	0	cig+/-ot	nev cigs	st
AXELSS	4		m	0	0	sca	-	all	Eu:Sca	1989	CC	436	n	bl	n	n	0	all/unsp	nev any	st
AXELSS	9		f	0	0	sca	-	all	Eu:Sca	1989	CC	436	n	bl	n	n	0	all/unsp	nev any	st
BARBON	1	x	m	0	0	all	-	all	Eu:wst	1979	CC	755	n	bl	y	y	0	all/unsp	nev any	st
BECHER	5		m	0	0	all	-	all	Eu:Ger	1985	CC	194	n	bl	n	y	0	all/unsp	nev any	st
BECHER	6		f	0	0	all	-	all	Eu:Ger	1985	CC	194	n	bl	n	y	0	all/unsp	nev any	st
BENSHL	1	x	m	0	0	all	0	all	Eu:UK	1967	pr	486	n	V	n	n	1	cig+/-ot	nev any	ot
BEST	3		m	0	0	all	0	all	NAmer	1955	pr	381	n	V	n	n	1	cig only	nev any	ot
BLOHMK	2		m	0	0	all	-	all	Eu:Ger	1978	CC	888	n	bl	n	y	0	all/unsp	nev any	st
BOUCOT	3	x	m	0	0	all	0	all	NAmer	1951	pr	121	n	bl	n	n	0	cig only	nev any	ot
BRETT	9		m	0	0	all	0	all	Eu:UK	1960	pr	150	n	V	n	n	0	cig+/-ot	nev cigs	st
BROSS	6		m	0	0	wh	-	all	NAmer	1960	CC	974	n	bl	n	n	0	cig+/-ot	nev any	st
BROWN2	22		m	0	0	wh	-	all	NAmer	1984	CC	14596	n	bl	n	y	2	cig+/-ot	nev cigs	or
BROWN2	21		f	0	0	wh	-	all	NAmer	1984	CC	14596	n	bl	n	y	2	cig+/-ot	nev cigs	or
BUFFLE	4		m	0	0	wh	-	all	NAmer	1976	CC	943	n	bl	y	n	0	cig+/-ot	nev any	st
BUFFLE	8		f	0	0	wh	-	all	NAmer	1976	CC	943	n	bl	y	n	0	cig+/-ot	nev any	st
CARPEN	8	x	c	0	0	w+b	-	all	NAmer	1991	CC	356	n	bl	n	n	0	cig+/-ot	nev cigs	st
CEDERL	114		m	0	0	all	0	all	Eu:Sca	1963	pr	491	n	bl	n	n	2	all/unsp	nev any	or
CEDERL	74		f	0	0	all	0	all	Eu:Sca	1963	pr	491	n	bl	n	n	2	all/unsp	nev any	or
CHANG	1		m	0	0	all	0	all	NAmer	1972	pr	136	n	bl	n	n	0	cig+/-ot	nev cigs	st
CHANG	7		f	0	0	all	0	all	NAmer	1972	pr	136	n	bl	n	n	0	cig+/-ot	nev cigs	st
CHOI	2		m	0	0	all	-	all	As:oth	1985	CC	375	n	bl	n	n	0	cig+/-ot	nev cigs	st
CHOI	6		f	0	0	all	-	all	As:oth	1985	CC	375	n	bl	n	n	0	cig+/-ot	nev cigs	st
CHOW	2	x	m	0	0	wh	0	all	NAmer	1966	pr	219	n	bl	n	n	0	cig+/-ot	nev any	st
CHYOU	3	x	m	0	0	jap	0	all	NAmer	1965	pr	227	n	bl	n	y	0	cig+/-ot	nev cigs	st
COMSTO	2		m	0	0	all	-	all	NAmer	1975	ot	258	n	bl	n	n	0	cig+/-ot	nev cigs	st
COMSTO	7		f	0	0	all	-	all	NAmer	1975	ot	258	n	bl	n	n	0	cig+/-ot	nev any	st
CORREA	37	x	c	0	0	all	-	all	NAmer	1979	CC	1359	n	bl	y	n	0	cig+/-ot	nev cigs	st
CPSI	72		m	0	0	wh	0	all	NAmer	1959	pr	5138	n	bl	n	n	1	cig only	nev any	st
CPSI	280		f	40	74	all	6	all	NAmer	1959	pr	5138	n	bl	n	n	1	cig+/-ot	nev cigs	ot
CPSII	91		m	35	99	all	4	all	NAmer	1982	pr	3229	n	bl	n	n	1	cig only	nev any	or
CPSII	78		f	0	0	all	4	all	NAmer	1982	pr	3229	n	bl	n	n	1	cig+/-ot	nev cigs	ot
DAMBER	17		m	0	0	all	-	all	Eu:Sca	1972	CC	579	n	bl	y	n	1	cig only	nev any	ot
DARBY	5		m	0	0	wh	-	all	Eu:UK	1988	CC	982	n	V	n	n	0	all/unsp	nev any	st
DARBY	12		f	0	0	wh	-	all	Eu:UK	1988	CC	982	n	V	n	n	0	all/unsp	nev any	st
DEAN2	1		m	0	0	all	-	all	Eu:UK	1960	CC	954	n	V	y	n	0	all/unsp	nev any	st
DEAN2	5		f	0	0	all	-	all	Eu:UK	1960	CC	954	n	V	y	n	0	all/unsp	nev any	st
DEAN3	180	x	m	0	0	all	-	all	Eu:UK	1969	CC	766	n	V	y	n	0	cig only	nev any	st
DEAN3	110	x	f	0	0	all	-	all	Eu:UK	1969	CC	766	n	V	y	n	0	cig only	nev any	st
DEKLER	1		m	0	0	all	0	all	Auslia	1961	pr	138	m	V	n	n	2	all/unsp	nev any	or
DESTE2	1	x	c	0	0	all	-	all	SCAmer	1993	CC	463	n	bl	n	n	0	all/unsp	nev any	st
DESTEF	10	x	m	0	0	all	-	all	SCAmer	1988	CC	497	n	bl	n	y	0	all/unsp	nev any	st
DOCKER	2		c	0	0	wh	0	all	NAmer	1974	pr	120	n	bl	n	n	4	cig+/-ot	nev cigs	or
DOLL	91		m	0	0	all	-	all	Eu:UK	1948	CC	1465	n	V	n	n	0	all/unsp	nev any	st
DOLL	94		f	0	0	all	-	all	Eu:UK	1948	CC	1465	n	V	n	n	0	all/unsp	nev any	st
DOLL2	90		m	0	0	all	10	all	Eu:UK	1951	pr	920	n	V	n	n	1	cig+/-ot	nev any	ot
DOLL2	9		f	0	0	all	22	all	Eu:UK	1951	pr	920	n	V	n	n	1	cig only	nev any	ot
DORANT	1		m	0	0	all	0	all	Eu:wst	1986	ot	550	n	bl	n	y	0	all/unsp	nev any	st
DORGAN	8		m	0	0	wh	-	all	NAmer	1980	CC	2026	n	bl	y	y	0	cig+/-ot	nev any	st
DORGAN	32		m	0	0	bl	-	all	NAmer	1980	CC	2026	n	bl	y	y	0	cig+/-ot	nev any	st
DORGAN	55		f	0	0	wh	-	all	NAmer	1980	CC	2026	n	bl	y	y	0	cig+/-ot	nev any	st
DORGAN	78		f	0	0	bl	-	all	NAmer	1980	CC	2026	n	bl	y	y	0	cig+/-ot	nev any	st
DORN	20		m	0	0	wh	15	all	NAmer	1954	pr	5097	n	bl	n	n	2	cig+/-ot	nev any	ot
DROSTE	1	x	m	0	0	all	-	all	Eu:wst	1995	CC	478	n	bl	n	y	0	all/unsp	nev any	st
ENGELA	1	x	m	0	0	all	0	all	Eu:Sca	1964	pr	435	n	bl	n	n	0	cig+/-ot	nev cigs	st
ENGELA	15	x	f	0	0	all	0	all	Eu:Sca	1964	pr	435	n	bl	n	n	0	cig+/-ot	nev cigs	st
GAO	27	x	m	0	0	all	-	all	As:Chi	1984	CC	1405	n	ot	n	n	0	cig+/-ot	nev cigs	st
GAO	28	x	f	0	0	all	-	all	As:Chi	1984	CC	1405	n	ot	n	n	0	cig+/-ot	nev cigs	st
GAO2	5	x	m	0	0	all	-	all	As:Jap	1988	CC	282	n	bl	n	n	0	cig+/-ot	nev cigs	st
GARCIA	1		c	0	0	all	-	all	NAmer	1992	CC	416	n	bl	n	y	0	cig+/-ot	nev cigs	st
GARDIN	1		c	0	0	all	-	all	Eu:UK	1988	CC	143	n	V	y	n	0	all/unsp	nev any	st
GARSHI	22	x	m	0	0	all	-	all	NAmer	1981	CC	1081	o	bl	y	n	0	all/unsp	nev any	st
GOODMA	1		m	0	0	w+o	-	all	NAmer	1983	CC	326	n	bl	y	y	0	cig+/-ot	nev any	st
GOODMA	5		f	0	0	w+o	-	all	NAmer	1983	CC	326	n	bl	y	y	0	cig+/-ot	nev any	st

Table 1D2 - 4

IESLC - Meta-analysis of Ex Smoking, Cigarettes (or Any Product if Cigarettes not available)
All LC types
Least adjusted

REF	NRR	X	SEX	AGEL	AGEH	RACE	YF	LC TYPE	LOC	START	ST	NLC	R	VB	P	H	AD	PRODUCT	DENOM	De
GRAHAM	7	x	m	0	0	wh	-	all	NAmer	1956	CC	685	n	bl	n	n	0	cig+/-ot	nev any	st
GREGOR	1		m	0	0	all	-	all	Eu:UK	1976	CC	104	n	V	n	y	0	cig+/-ot	nev cigs	st
GREGOR	5		f	0	0	all	-	all	Eu:UK	1976	CC	104	n	V	n	y	0	cig+/-ot	nev cigs	st
HAENSZ	55		f	0	0	all	-	not alv	NAmer	1955	CC	158	n	bl	n	y	0	cig+/-ot	nev any	st
HAMMO2	26	x	m	0	0	all	0	all	NAmer	1967	pr	450	o	bl	n	n	0	cig+/-ot	nev any	st
HEIN	6		m	0	0	all	0	all	Eu:Sca	1970	pr	144	n	bl	n	n	0	all/unsp	nev any	st
HENNEK	1		m	0	0	all	0	all	NAmer	1982	pr	169	n	bl	n	n	0	all/unsp	nev any	st
HIRAYA	146		m	0	0	all	0	all	As:Jap	1965	pr	1917	n	bl	n	n	1	cig+/-ot	nev any	ot
HIRAYA	149		f	0	0	all	0	all	As:Jap	1965	pr	1917	n	bl	n	n	1	cig+/-ot	nev any	ot
HITOSU	1	x	m	0	0	all	-	all	As:Jap	1960	CC	216	n	bl	y	n	0	all/unsp	nev any	st
HITOSU	8	x	f	0	0	all	-	all	As:Jap	1960	CC	216	n	bl	y	n	0	all/unsp	nev any	st
HOLE	14	x	m	0	0	all	0	all	Eu:UK	1972	pr	225	n	V	n	n	0	all/unsp	nev any	st
HUMBLE	1		m	0	0	w-hi	-	all	NAmer	1980	CC	521	n	bl	y	n	1	cig+/-ot	nev cigs	or
HUMBLE	4		m	0	0	hi	-	all	NAmer	1980	CC	521	n	bl	y	n	1	cig+/-ot	nev cigs	or
HUMBLE	7		f	0	0	w-hi	-	all	NAmer	1980	CC	521	n	bl	y	n	1	cig+/-ot	nev cigs	or
HUMBLE	10		f	0	0	hi	-	all	NAmer	1980	CC	521	n	bl	y	n	1	cig+/-ot	nev cigs	or
JAHN	10		m	0	0	all	-	all	Eu:Ger	1988	CC	1004	n	bl	n	n	0	cig+/-ot	nev any	st
JAIN	26	x	m	0	0	all	-	all	NAmer	1981	CC	845	n	V	y	n	0	cig+/-ot	nev cigs	st
JAIN	21	x	f	0	0	all	-	all	NAmer	1981	CC	845	n	V	y	n	0	cig+/-ot	nev cigs	st
JARVHO	1		m	0	0	all	-	all	Eu:Sca	1983	CC	147	n	bl	n	n	0	all/unsp	nev any	st
JARVHO	5		f	0	0	all	-	all	Eu:Sca	1983	CC	147	n	bl	n	n	0	all/unsp	nev any	st
JEDRYC	64		m	0	0	all	-	all	Eu:est	1980	CC	1630	n	bl	y	n	0	cig+/-ot	nev any	st
JEDRYC	69		f	0	0	all	-	all	Eu:est	1980	CC	1630	n	bl	y	n	0	cig+/-ot	nev any	st
JOLY	20		m	0	0	all	-	all	SCAmer	1978	CC	826	n	bl	n	n	0	cig+/-ot	nev any	st
JOLY	19		f	0	0	all	-	all	SCAmer	1978	CC	826	n	bl	n	n	0	cig+/-ot	nev any	st
KAISE2	65		m	35	99	all	9	all	NAmer	1979	pr	318	n	bl	n	n	1	cig only	nev any	st
KAISE2	57		f	35	99	all	9	all	NAmer	1979	pr	318	n	bl	n	n	1	cig only	nev any	st
KAISER	5		m	0	0	all	0	all	NAmer	1964	pr	714	n	bl	n	n	2	cig+/-ot	nev cigs	or
KAISER	1		f	0	0	all	0	all	NAmer	1964	pr	714	n	bl	n	n	2	cig+/-ot	nev cigs	or
KATSOU	5	x	f	0	0	all	-	all	Eu:bal	1987	CC	101	n	bl	n	n	0	all/unsp	nev any	st
KAUFMA	1	x	c	0	0	all	-	all	NAmer	1981	CC	881	n	bl	n	n	0	cig+/-ot	nev cigs	st
KELLER	2		m	0	0	wh	-	all	NAmer	1985	CC	15038	n	bl	n	n	0	all/unsp	nev any	st
KELLER	10		m	0	0	nonw	-	all	NAmer	1985	CC	15038	n	bl	n	n	0	all/unsp	nev any	st
KELLER	6		f	0	0	wh	-	all	NAmer	1985	CC	15038	n	bl	n	n	0	all/unsp	nev any	st
KELLER	14		f	0	0	nonw	-	all	NAmer	1985	CC	15038	n	bl	n	n	0	all/unsp	nev any	st
KHUDER	13		m	0	0	all	-	all	NAmer	1985	CC	482	n	bl	n	y	0	cig+/-ot	nev cigs	or
KIHARA	15		c	0	0	jap	-	all	As:Jap	1991	CC	440	n	bl	n	n	0	all/unsp	nev any	st
KINLEN	1	x	m	0	0	all	0	all	Eu:UK	1967	pr	718	n	V	n	n	0	all/unsp	nev any	st
KJUUS	2		m	0	0	all	-	all	Eu:Sca	1979	CC	176	n	bl	n	n	0	all/unsp	nev any	st
KNEKT	14	x	m	20	69	all	21	all	Eu:Sca	1966	pr	515	n	bl	n	n	0	all/unsp	nev any	st
KOO	8		f	0	0	all	-	all	As:HK	1981	CC	200	n	bl	n	n	0	all/unsp	nev any	st
KREUZE	1	x	m	1	45	all	-	all	Eu:Ger	1990	CC	2260	n	bl	n	n	0	all/unsp	nev any	st
KREUZE	3	x	m	55	69	all	-	all	Eu:Ger	1990	CC	2260	n	bl	n	n	0	all/unsp	nev any	st
KREUZE	2	x	f	1	45	all	-	all	Eu:Ger	1990	CC	2260	n	bl	n	n	0	all/unsp	nev any	st
KREUZE	4	x	f	55	69	all	-	all	Eu:Ger	1990	CC	2260	n	bl	n	n	0	all/unsp	nev any	st
KUBIK	11		m	0	0	all	0	all	Eu:est	1965	pr	108	n	bl	n	n	0	cig+/-ot	nev any	st
LANGE	5	x	m	0	0	all	0	all	Eu:Sca	1976	pr	268	n	bl	n	n	0	all/unsp	nev any	st
LANGE	1	x	f	0	0	all	0	all	Eu:Sca	1976	pr	268	n	bl	n	n	0	all/unsp	nev any	st
LEMARC	1		c	0	0	w+o	-	all	NAmer	1992	CC	341	n	bl	n	y	0	all/unsp	nev any	st
LIDDEL	1		m	0	0	all	18	all	NAmer	1970	pr	304	m	V	n	n	1	cig+/-ot	nev cigs	ot
LOMBAR	8		m	0	0	all	-	all	NAmer	1951	CC	1040	n	bl	n	n	0	cig+/-ot	nev any	st
LUBIN	39		m	0	0	all	-	all	As:Chi	1984	CC	427	m	ot	y	n	0	cig+/-ot	nev any	st
LUBIN2	37	x	m	0	0	all	-	all	Eu:mul	1976	CC	7804	n	bl	n	y	0	cig+/-ot	nev any	st
LUBIN2	319		f	0	0	all	-	all	Eu:mul	1976	CC	7804	n	bl	n	y	0	cig+/-ot	nev any	st
MACLEN	7		m	0	0	ch	-	all	As:oth	1972	CC	233	n	bl	n	n	0	cig+/-ot	nev cigs	st
MACLEN	8		f	0	0	ch	-	all	As:oth	1972	CC	233	n	bl	n	n	0	cig+/-ot	nev cigs	st
MATOS	14	x	m	0	0	all	-	all	SCAmer	1994	CC	200	n	bl	n	n	0	cig+/-ot	nev any	st
MIGRAN	21	x	m	0	0	all	0	all	Eu:UK	1964	pr	259	n	V	n	n	0	cig+/-ot	nev any	st
MIGRAN	39		f	0	0	all	0	all	Eu:UK	1964	pr	259	n	V	n	n	0	cig+/-ot	nev any	st
MRFITR	1		m	0	0	all	0	all	NAmer	1973	pr	119	n	bl	n	n	0	cig+/-ot	nev cigs	ot
NAM	65	x	m	0	0	all	-	all	NAmer	1986	CC	1199	n	bl	y	n	0	cig+/-ot	nev cigs	ot
NAM	81	x	f	0	0	all	-	all	NAmer	1986	CC	1199	n	bl	y	n	0	cig+/-ot	nev cigs	ot
ODRISC	2		c	0	0	all	-	all	Eu:UK	1992	CC	446	n	V	n	n	0	all/unsp	nev any	st
OSANN	1	x	m	0	0	all	-	all	NAmer	1984	CC	1986	n	bl	n	n	0	cig+/-ot	nev cigs	st
OSANN	5	x	f	0	0	all	-	all	NAmer	1984	CC	1986	n	bl	n	n	0	cig+/-ot	nev cigs	st
PARKIN	19		m	0	0	bl	-	all	Africa	1963	CC	877	n	V	y	n	0	cig+/-ot	nev any	st
PERSH2	1	x	c	0	0	all	-	all	Eu:Sca	1980	CC	1022	n	bl	y	n	0	all/unsp	nev any	st
PETO	1		m	0	0	all	0	all	Eu:UK	1954	pr	103	n	V	n	n	0	all/unsp	nev any	st
PEZZO2	1		m	0	0	all	-	all	SCAmer	1992	CC	367	n	bl	n	y	0	cig+/-ot	nev cigs	st
PEZZOT	1		m	0	0	all	-	all	SCAmer	1987	CC	215	n	bl	n	y	0	cig only	nev cigs	st
QIAO2	7		m	0	0	all	0	all	As:Chi	1992	pr	241	m	ot	n	n	0	cig+/-ot	nev any	st
RACHTA	1	x	f	0	0	all	-	all	Eu:est	1991	CC	118	n	bl	n	y	0	cig+/-ot	nev cigs	st

Table 1D2 - 4

IESLC - Meta-analysis of Ex Smoking, Cigarettes (or Any Product if Cigarettes not available)
All LC types
Least adjusted

REF	NRR	X	SEX	AGEL	AGEH	RACE	YF	LC TYPE	LOC	START	ST	NLC	R	VB	P	H	AD	PRODUCT	DENOM	De
SCHWAR	21		m	0	0	wh	-	all	NAmer	1984	CC	5588	n	bl	y	y	0	cig+/-ot	nev cigs	st
SCHWAR	22		m	0	0	bl	-	all	NAmer	1984	CC	5588	n	bl	y	y	0	cig+/-ot	nev cigs	st
SCHWAR	23		f	0	0	wh	-	all	NAmer	1984	CC	5588	n	bl	y	y	0	cig+/-ot	nev cigs	st
SCHWAR	24		f	0	0	bl	-	all	NAmer	1984	CC	5588	n	bl	y	y	0	cig+/-ot	nev cigs	st
SHAW	3		c	0	0	wh	-	all	NAmer	1988	CC	335	n	V	n	y	0	all/unsp	nev any	st
SOBUE	89	x	m	0	0	all	-	q+s+l+a	As:Jap	1986	CC	1376	n	bl	n	y	0	cig+/-ot	nev cigs	st
SOBUE	93	x	f	0	0	all	-	q+s+l+a	As:Jap	1986	CC	1376	n	bl	n	y	0	cig+/-ot	nev cigs	st
SPEIZE	7		f	0	0	all	0	all	NAmer	1976	pr	593	n	bl	n	y	0	cig+/-ot	nev cigs	st
SPITZ	1		c	0	0	b+hi	-	all	NAmer	1992	CC	177	n	bl	n	y	0	cig+/-ot	nev cigs	st
STOCKW	4		c	0	0	all	-	all	NAmer	1981	CC	22161	n	bl	n	n	0	cig+/-ot	nev any	st
STUCKE	1		m	0	0	all	-	all	Eu:wst	1989	CC	247	n	bl	n	y	0	all/unsp	nev any	ot
SUZUK2	1	x	c	0	0	all	-	all	SCAmer	1991	CC	123	n	bl	n	y	0	all/unsp	nev any	st
SVENSS	21	x	f	0	0	all	-	all	Eu:Sca	1983	CC	210	n	bl	n	n	0	all/unsp	nev any	st
TANG	2		c	0	0	all	-	not s	NAmer	1992	CC	119	n	bl	n	y	0	cig+/-ot	nev cigs	st
TENKAN	9		m	0	0	all	17	all	Eu:Sca	1962	pr	242	n	bl	n	n	1	all/unsp	nev any	ot
TIZZAN	6		m	0	0	all	-	all	Eu:wst	1959	CC	1358	n	bl	n	n	0	all/unsp	nev any	st
TIZZAN	14		f	0	0	all	-	all	Eu:wst	1959	CC	1358	n	bl	n	n	0	all/unsp	nev any	st
TOKARS	2		m	0	0	all	-	all	Eu:est	1966	ot	162	o	bl	n	y	0	all/unsp	nev any	st
TOUSEY	3	x	m	0	0	all	-	all	NAmer	1993	CC	507	n	bl	y	y	0	cig+/-ot	nev any	st
TOUSEY	7	x	f	0	0	all	-	all	NAmer	1993	CC	507	n	bl	y	y	0	cig+/-ot	nev any	st
TSUGAN	26		m	0	0	all	-	q+a	As:Jap	1976	CC	134	n	bl	n	y	0	all/unsp	nev any	st
TULINI	1	x	m	0	0	all	0	all	Eu:Sca	1967	pr	472	n	bl	n	n	1	all/unsp	nev any	or
TULINI	7	x	f	0	0	all	0	all	Eu:Sca	1967	pr	472	n	bl	n	n	1	all/unsp	nev any	or
TVERDA	1		m	0	0	all	0	all	Eu:Sca	1972	pr	238	n	bl	n	n	2	cig+/-ot	nev cigs	ot
TVERDA	18		f	0	0	all	0	all	Eu:Sca	1972	pr	238	n	bl	n	n	0	cig only	nev cigs	ot
WAKAI	1	x	m	0	0	all	-	all	As:Jap	1988	CC	333	n	bl	n	y	0	all/unsp	nev any	st
WAKAI	19	x	f	0	0	all	-	all	As:Jap	1988	CC	333	n	bl	n	y	0	all/unsp	nev any	st
WANG2	19	x	c	0	0	all	-	all	As:Chi	1980	CC	103	n	ot	n	n	0	cig+/-ot	nev cigs	st
WIGLE	7		m	0	0	all	-	all	NAmer	1971	CC	728	n	V	n	n	0	cig only	nev any	st
WIGLE	10		f	0	0	all	-	all	NAmer	1971	CC	728	n	V	n	n	0	cig only	nev any	st
WU	33	x	f	0	0	wh	-	q+a	NAmer	1981	CC	220	n	bl	n	y	0	all/unsp	nev any	st
WUNSCH	3	x	m	0	0	all	-	all	SCAmer	1990	CC	398	n	bl	y	n	0	cig+/-ot	nev any	st
WUNSCH	9	x	f	0	0	all	-	all	SCAmer	1990	CC	398	n	bl	y	n	0	cig+/-ot	nev any	st
WYNDE3	41		m	0	0	all	-	all	NAmer	1966	CC	350	n	bl	n	y	0	all/unsp	nev any	st
WYNDE6	9		m	0	0	all	-	all	NAmer	1969	CC	4423	n	bl	n	y	0	cig+/-ot	nev any	st
WYNDE6	198		f	0	0	all	-	all	NAmer	1969	CC	4423	n	bl	n	y	0	cig+/-ot	nev cigs	st
YAMAGU	4	x	c	0	0	all	-	all	As:Jap	1989	CC	144	n	bl	n	y	0	all/unsp	nev any	st
YONG	1		c	0	0	all	0	all	NAmer	1971	pr	216	n	bl	n	n	1	cig+/-ot	nev cigs	or

Cigarette type is all/unspec for all RRs

except for the following:

REF	NRR	CIGTYPE
DEAN3	180	MC only
DEAN3	110	MC only

Table 1D2 - 5

IESLC - Meta-analysis of Ex Smoking, Cigarettes (or Any Product if Cigarettes not available)
All LC types
Least adjusted

REF	NRR	SEX	AD	Number Exposed Case	Number Exposed Cont	Non-exposed Case	Non-exposed Cont	RR	95.00%CI
AGUDO	9	f	0	3	6	80	183	1.14	(0.28- 4.69)
*AKIBA	1	m	0	48	36303	18	35833	2.63	(1.53- 4.52)
*AKIBA	5	f	0	9	13942	116	359850	2.00	(1.02- 3.94)
Subtotal AKIBA								2.37	(1.55- 3.61)
*AMANDU	2	m	0	11	14687	6	25350	3.16	(1.17- 8.55)
AMES	3	m	0	147	115	15	62	5.28	(2.86- 9.77)
*ANDERS	1	f	0	85	54902	46	195158	6.57	(4.59- 9.40)
*ARCHER	4	m	0	18	3740	6	9842	7.89	(3.14- 19.87)
ARMADA	28	m	0	129	132	4	64	15.64	(5.53- 44.19)
AUSTIN	1	c	0	50	112	5	88	7.86	(3.01- 20.54)
AXELSS	4	m	0	98	214	16	160	4.58	(2.60- 8.07)
AXELSS	9	f	0	14	40	18	154	2.99	(1.37- 6.53)
Subtotal AXELSS								3.95	(2.50- 6.26)
BARBON	1	m	0	171	205	22	188	7.13	(4.38- 11.59)
BECHER	5	m	0	42	116	3	54	6.52	(1.93- 21.96)
BECHER	6	f	0	5	18	10	52	1.44	(0.44- 4.80)
Subtotal BECHER								3.04	(1.29- 7.14)
*BENSHL	1	m	1	-	-	-	-	2.92	(1.49- 5.71)
*BEST	3	m	1	-	-	-	-	6.06	(2.53- 14.51)
BLOHMK	2	m	0	343	274	126	301	2.99	(2.30- 3.88)
*BOUCOT	3	m	0	8	5977	0	7551	21.48~	(1.24- 372.01)
*BRETT	9	m	0	9	10482	6	6530	0.93	(0.33- 2.62)
BROSS	6	m	0	212	146	38	170	6.50	(4.31- 9.79)
BROWN2	22	m	2	-	-	-	-	7.20	(6.50- 7.90)
BROWN2	21	f	2	-	-	-	-	11.60	(10.40- 13.00)
Subtotal BROWN2								8.85	(8.23- 9.53)
BUFFLE	4	m	0	204	154	5	47	12.45	(4.84- 32.05)
BUFFLE	8	f	0	106	101	41	198	5.07	(3.29- 7.81)
Subtotal BUFFLE								5.92	(4.00- 8.78)
CARPEN	8	c	0	109	307	15	241	5.70	(3.24- 10.04)
*CEDERL	114	m	2	-	-	-	-	1.25	(0.67- 2.34)
*CEDERL	74	f	2	-	-	-	-	1.08	(0.34- 3.44)
Subtotal CEDERL								1.21	(0.70- 2.10)
*CHANG	1	m	0	43	1087	5	502	3.97	(1.58- 9.97)
*CHANG	7	f	0	12	580	11	1139	2.14	(0.95- 4.83)
Subtotal CHANG								2.81	(1.53- 5.16)
CHOI	2	m	0	35	136	13	95	1.88	(0.94- 3.74)
CHOI	6	f	0	6	3	76	164	4.32	(1.05- 17.72)
Subtotal CHOI								2.21	(1.19- 4.10)
*CHOW	2	m	0	63	107450	6	62913	6.15	(2.66- 14.20)
*CHYOU	3	m	0	33	2084	13	2406	2.93	(1.55- 5.55)
COMSTO	2	m	0	46	129	4	69	6.15	(2.13- 17.80)
COMSTO	7	f	0	11	35	13	115	2.78	(1.14- 6.75)
Subtotal COMSTO								3.85	(1.95- 7.61)
CORREA	37	c	0	258	315	51	388	6.23	(4.46- 8.71)
*CPSI	72	m	1	-	-	-	-	3.74	(3.14- 4.46)
*CPSI	280	f	1	-	-	-	-	1.38	(0.81- 2.35)
Subtotal CPSI								3.39	(2.87- 4.01)
*CPSII	91	m	1	-	-	-	-	9.36	(7.43- 11.77)
*CPSII	78	f	1	-	-	-	-	4.84	(4.00- 5.86)
Subtotal CPSII								6.33	(5.47- 7.34)
DAMBER	17	m	1	-	-	-	-	3.15	(1.80- 5.49)
DARBY	5	m	0	285	1106	3	384	32.98	(10.51- 103.49)
DARBY	12	f	0	94	317	23	529	6.82	(4.23- 10.99)
Subtotal DARBY								8.61	(5.55- 13.37)
DEAN2	1	m	0	98	88	33	112	3.78	(2.33- 6.13)
DEAN2	5	f	0	5	2	88	121	3.44	(0.65- 18.13)
Subtotal DEAN2								3.75	(2.36- 5.97)
DEAN3	180	m	0	62	297	24	510	4.44	(2.71- 7.26)
DEAN3	110	f	0	7	262	41	1538	1.00	(0.44- 2.26)
Subtotal DEAN3								2.97	(1.95- 4.53)
*DEKLER	1	m	2	-	-	-	-	10.70	(1.40- 81.90)
DESTE2	1	c	0	84	61	20	108	7.44	(4.16- 13.28)
DESTEF	10	m	0	108	108	27	163	6.04	(3.71- 9.82)
*DOCKER	2	c	4	-	-	-	-	2.54	(0.90- 7.18)
DOLL	91	m	0	70	124	7	61	4.92	(2.13- 11.34)
DOLL	94	f	0	10	8	40	59	1.84	(0.67- 5.08)
Subtotal DOLL								3.31	(1.74- 6.30)
*DOLL2	90	m	1	-	-	-	-	4.22	(1.23- 14.48)
*DOLL2	9	f	1	-	-	-	-	3.29	(0.88- 12.24)
Subtotal DOLL2								3.76	(1.53- 9.24)

Table 1D2 - 5

IESLC - Meta-analysis of Ex Smoking, Cigarettes (or Any Product if Cigarettes not available)
All LC types
Least adjusted

REF	NRR	SEX	AD	Number Exposed Case	Number Exposed Cont	Non-exposed Case	Non-exposed Cont	RR	95.00%CI
DORANT	1	m	0	146	771	7	159	4.30	(1.98- 9.36)
DORGAN	8	m	0	236	230	15	93	6.36	(3.58- 11.30)
DORGAN	32	m	0	49	56	3	35	10.21	(2.95- 35.27)
DORGAN	55	f	0	146	110	103	244	3.14	(2.24- 4.41)
DORGAN	78	f	0	11	10	7	20	3.14	(0.93- 10.58)
Subtotal DORGAN								3.92	(2.98- 5.17)
*DORN	20	m	2	-	-	-	-	3.97	(3.32- 4.75)
DROSTE	1	m	0	92	176	7	93	6.94	(3.09- 15.59)
*ENGELA	1	m	0	48	63903	27	58716	1.63	(1.02- 2.62)
*ENGELA	15	f	0	6	24392	31	207789	1.65	(0.69- 3.95)
Subtotal ENGELA								1.64	(1.08- 2.48)
GAO	27	m	0	142	120	62	202	3.86	(2.65- 5.61)
GAO	28	f	0	67	30	435	605	3.11	(1.98- 4.86)
Subtotal GAO								3.53	(2.65- 4.70)
GAO2	5	m	0	85	109	13	56	3.36	(1.72- 6.54)
GARCIA	1	c	0	226	233	21	139	6.42	(3.92- 10.52)
GARDIN	1	c	0	41	44	5	41	7.64	(2.75- 21.22)
GARSHI	22	m	0	291	633	41	363	4.07	(2.86- 5.78)
GOODMA	1	m	0	68	229	10	199	5.91	(2.96- 11.79)
GOODMA	5	f	0	23	35	19	177	6.12	(3.02- 12.42)
Subtotal GOODMA								6.01	(3.67- 9.85)
GRAHAM	7	m	0	150	178	18	346	16.20	(9.62- 27.28)
GREGOR	1	m	0	23	45	10	14	0.72	(0.28- 1.86)
GREGOR	5	f	0	4	16	1	22	5.50	(0.56- 53.99)
Subtotal GREGOR								0.97	(0.40- 2.34)
HAENSZ	55	f	0	5	9	81	236	1.62	(0.53- 4.97)
*HAMMO2	26	m	0	90	2201	5	891	7.29	(2.97- 17.87)
*HEIN	6	m	0	11	979	1	457	5.13	(0.66- 39.65)
*HENNEK	1	m	0	67	8674	23	10919	3.67	(2.29- 5.88)
*HIRAYA	146	m	1	-	-	-	-	1.71	(1.08- 2.72)
*HIRAYA	149	f	1	-	-	-	-	2.98	(1.14- 7.77)
Subtotal HIRAYA								1.90	(1.25- 2.88)
HITOSU	1	m	0	25	190	7	242	4.55	(1.93- 10.74)
HITOSU	8	f	0	6	41	33	1893	8.39	(3.33- 21.13)
Subtotal HITOSU								6.05	(3.22- 11.34)
*HOLE	14	m	0	24	1736	7	1189	2.35	(1.02- 5.43)
HUMBLE	1	m	1	-	-	-	-	7.20	(3.00- 17.60)
HUMBLE	4	m	1	-	-	-	-	8.00	(1.90- 42.20)
HUMBLE	7	f	1	-	-	-	-	6.50	(2.80- 15.40)
HUMBLE	10	f	1	-	-	-	-	6.30	(1.50- 27.80)
Subtotal HUMBLE								6.88	(4.04- 11.71)
JAHN	10	m	0	455	402	18	138	8.68	(5.22- 14.44)
JAIN	26	m	0	126	159	12	85	5.61	(2.94- 10.73)
JAIN	21	f	0	85	97	52	214	3.61	(2.37- 5.49)
Subtotal JAIN								4.11	(2.89- 5.85)
JARVHO	1	m	0	26	28	1	16	14.86	(1.84- 120.07)
JARVHO	5	f	0	10	8	6	21	4.38	(1.19- 16.04)
Subtotal JARVHO								6.15	(2.04- 18.54)
JEDRYC	64	m	0	137	196	49	219	3.12	(2.14- 4.56)
JEDRYC	69	f	0	13	8	78	166	3.46	(1.38- 8.69)
Subtotal JEDRYC								3.17	(2.23- 4.50)
JOLY	20	m	0	101	185	12	218	9.92	(5.28- 18.62)
JOLY	19	f	0	34	27	52	283	6.85	(3.82- 12.31)
Subtotal JOLY								8.13	(5.30- 12.49)
*KAISE2	65	m	1	-	-	-	-	3.39	(1.77- 6.48)
*KAISE2	57	f	1	-	-	-	-	5.02	(2.17- 11.61)
Subtotal KAISE2								3.93	(2.35- 6.56)
*KAISER	5	m	2	-	-	-	-	4.65	(2.84- 7.64)
*KAISER	1	f	2	-	-	-	-	3.02	(1.83- 4.99)
Subtotal KAISER								3.76	(2.64- 5.35)
KATSOU	5	f	0	8	4	48	67	2.79	(0.79- 9.80)
KAUFMA	1	c	0	225	759	35	925	7.83	(5.42- 11.33)
KELLER	2	m	0	3003	1307	323	1017	7.23	(6.28- 8.33)
KELLER	10	m	0	440	128	38	117	10.58	(6.99- 16.04)
KELLER	6	f	0	1094	477	469	1860	9.10	(7.85- 10.54)
KELLER	14	f	0	130	79	67	232	5.70	(3.86- 8.42)
Subtotal KELLER								8.02	(7.28- 8.82)
KHUDER	13	m	0	184	-	23	-	7.50	(4.80- 11.90)
KIHARA	15	c	0	55	70	102	237	1.83	(1.20- 2.79)
*KINLEN	1	m	0	75	2843	7	1333	5.02	(2.32- 10.87)
KJUUS	2	m	0	39	75	2	24	6.24	(1.40- 27.78)

Table 1D2 - 5

IESLC - Meta-analysis of Ex Smoking, Cigarettes (or Any Product if Cigarettes not available)
All LC types
Least adjusted

REF	NRR	SEX	AD	Number Exposed Case	Cont	Non-exposed Case	Cont	RR	95.00%CI
*KNEKT	14	m	0	18	15309	6	17814	3.49	(1.39- 8.79)
KOO	8	f	0	22	10	56	85	3.34	(1.47- 7.58)
KREUZE	1	m	0	9	47	6	54	1.72	(0.57- 5.20)
KREUZE	3	m	0	434	834	23	403	9.12	(5.90- 14.10)
KREUZE	2	f	0	7	19	6	38	2.33	(0.69- 7.92)
KREUZE	4	f	0	35	47	95	177	1.39	(0.84- 2.30)
Subtotal KREUZE								3.68	(2.71- 5.00)
*KUBIK	11	m	0	8	1487	2	4271	11.49	(2.44- 54.04)
*LANGE	5	m	0	21	1253	5	721	2.42	(0.92- 6.38)
*LANGE	1	f	0	8	1089	7	2159	2.27	(0.82- 6.23)
Subtotal LANGE								2.34	(1.16- 4.72)
LEMARC	1	c	0	142	223	32	168	3.34	(2.17- 5.15)
*LIDDEL	1	m	1	-	-	-	-	1.40	(0.79- 2.51)
LOMBAR	8	m	0	126	172	14	112	5.86	(3.21- 10.69)
LUBIN	39	m	0	70	139	9	72	4.03	(1.90- 8.53)
LUBIN2	37	m	0	1010	3002	190	2617	4.63	(3.93- 5.46)
LUBIN2	319	f	0	100	157	288	1180	2.61	(1.97- 3.46)
Subtotal LUBIN2								4.01	(3.48- 4.62)
MACLEN	7	m	0	5	11	5	15	1.36	(0.32- 5.89)
MACLEN	8	f	0	3	10	41	109	0.80	(0.21- 3.04)
Subtotal MACLEN								1.02	(0.38- 2.74)
MATOS	14	m	0	76	151	11	110	5.03	(2.55- 9.92)
*MIGRAN	21	m	0	23	1471	4	867	3.39	(1.18- 9.77)
*MIGRAN	39	f	0	1	599	4	3814	1.59	(0.18- 14.22)
Subtotal MIGRAN								2.94	(1.13- 7.62)
*MRFITR	1	m	0	13	2813	0	1859	17.84~	(1.06- 300.00)
NAM	65	m	0	369	486	30	520	13.16	(8.89- 19.48)
NAM	81	f	0	159	262	52	885	10.33	(7.34- 14.54)
Subtotal NAM								11.47	(8.86- 14.84)
ODRISC	2	c	0	147	398	6	664	40.87	(17.90- 93.34)
OSANN	1	m	0	317	477	45	833	12.30	(8.83- 17.14)
OSANN	5	f	0	140	196	96	1093	8.13	(6.02- 10.99)
Subtotal OSANN								9.80	(7.84- 12.25)
PARKIN	19	m	0	26	59	107	1248	5.14	(3.11- 8.49)
PERSH2	1	c	0	108	293	178	1164	2.41	(1.84- 3.16)
*PETO	1	m	0	2	387	2	295	0.76	(0.11- 5.38)
PEZZO2	1	m	0	128	271	6	117	9.21	(3.95- 21.48)
PEZZOT	1	m	0	66	188	4	116	10.18	(3.61- 28.67)
*QIAO2	7	m	0	41	961	10	709	3.02	(1.53- 6.00)
RACHTA	1	f	0	13	10	33	98	3.86	(1.55- 9.63)
SCHWAR	21	m	0	996	670	119	376	4.70	(3.74- 5.90)
SCHWAR	22	m	0	219	136	50	104	3.35	(2.25- 4.99)
SCHWAR	23	f	0	322	328	182	855	4.61	(3.69- 5.76)
SCHWAR	24	f	0	79	89	40	247	5.48	(3.49- 8.60)
Subtotal SCHWAR								4.54	(3.94- 5.22)
SHAW	3	c	0	112	169	11	107	6.45	(3.32- 12.53)
SOBUE	89	m	0	287	363	34	128	2.98	(1.98- 4.48)
SOBUE	93	f	0	32	64	167	857	2.57	(1.63- 4.05)
Subtotal SOBUE								2.79	(2.06- 3.78)
*SPEIZE	7	f	0	144	522081	58	776300	3.69	(2.72- 5.01)
SPITZ	1	c	0	67	80	7	128	15.31	(6.70- 35.02)
STOCKW	4	c	0	6185	3057	2791	10641	7.71	(7.26- 8.19)
STUCKE	1	m	0	178	135	0	51	135.69~	(8.30-2218.34)
SUZUK2	1	c	0	34	40	11	53	4.10	(1.85- 9.06)
SVENSS	21	f	0	30	36	38	120	2.63	(1.43- 4.83)
TANG	2	c	0	58	34	9	39	7.39	(3.19- 17.11)
*TENKAN	9	m	1	-	-	-	-	4.02	(1.50- 10.80)
TIZZAN	6	m	0	346	292	180	305	2.01	(1.58- 2.56)
TIZZAN	14	f	0	8	10	25	114	3.65	(1.31- 10.17)
Subtotal TIZZAN								2.07	(1.64- 2.62)
TOKARS	2	m	0	37	86	1	53	22.80	(3.04- 171.13)
TOUSEY	3	m	0	133	298	4	130	14.51	(5.25- 40.05)
TOUSEY	7	f	0	65	134	13	226	8.43	(4.48- 15.88)
Subtotal TOUSEY								9.81	(5.74- 16.79)
TSUGAN	26	m	0	10	8	18	22	1.53	(0.50- 4.68)
*TULINI	1	m	1	-	-	-	-	2.91	(1.47- 5.74)
*TULINI	7	f	1	-	-	-	-	3.73	(1.73- 8.07)
Subtotal TULINI								3.25	(1.95- 5.40)
*TVERDA	1	m	2	-	-	-	-	0.49	(0.24- 1.01)
*TVERDA	18	f	0	0	38953	3	157431	0.58~	(0.03- 11.18)
Subtotal TVERDA								0.49	(0.25- 0.99)

Table 1D2 - 5

IESLC - Meta-analysis of Ex Smoking, Cigarettes (or Any Product if Cigarettes not available)
All LC types
Least adjusted

REF	NRR	SEX	AD	Number Exposed Case	Number Exposed Cont	Non-exposed Case	Non-exposed Cont	RR	95.00%CI
WAKAI	1	m	0	54	140	10	65	2.51	(1.20- 5.23)
WAKAI	19	f	0	5	5	50	145	2.90	(0.81- 10.44)
Subtotal WAKAI								2.60	(1.37- 4.92)
WANG2	19	c	0	11	21	11	43	2.05	(0.76- 5.48)
WIGLE	7	m	0	128	217	15	204	8.02	(4.55- 14.16)
WIGLE	10	f	0	11	66	36	439	2.03	(0.99- 4.19)
Subtotal WIGLE								4.75	(3.04- 7.43)
WU	33	f	0	29	55	31	92	1.56	(0.85- 2.87)
WUNSCH	3	m	0	100	200	14	99	3.54	(1.92- 6.50)
WUNSCH	9	f	0	17	36	29	208	3.39	(1.69- 6.79)
Subtotal WUNSCH								3.47	(2.19- 5.49)
WYNDE3	41	m	0	48	125	9	88	3.75	(1.75- 8.05)
WYNDE6	9	m	0	1088	1056	87	617	7.31	(5.75- 9.29)
WYNDE6	198	f	0	332	325	159	856	5.50	(4.38- 6.91)
Subtotal WYNDE6								6.29	(5.34- 7.43)
YAMAGU	4	c	0	44	162	24	267	3.02	(1.77- 5.16)
*YONG	1	c	1	-	-	-	-	4.10	(2.55- 6.60)
Partial Totals				26329	970469	9089	2001561		

*prospective study ~ With 0.5 adjustment for zero

REF	NRR	SEX	AD	Ys	Ws	Qs	Ps
AGUDO	9	f	0	0.13	1.93	5.07	0.8520
*AKIBA	1	m	0	0.97	13.10	8.12	0.0005
*AKIBA	5	f	0	0.69	8.36	9.40	0.0447
Subtotal AKIBA				0.86	21.46	17.51	
*AMANDU	2	m	0	1.15	3.88	1.41	0.0232
AMES	3	m	0	1.66	10.17	0.08	0.0000
*ANDERS	1	f	0	1.88	29.87	0.48	0.0000
*ARCHER	4	m	0	2.07	4.51	0.44	0.0000
ARMADA	28	m	0	2.75	3.56	3.52	0.0000
AUSTIN	1	c	0	2.06	4.16	0.39	0.0000
AXELSS	4	m	0	1.52	11.96	0.65	0.0000
AXELSS	9	f	0	1.10	6.31	2.73	0.0059
Subtotal AXELSS				1.37	18.27	3.38	
BARBON	1	m	0	1.96	16.26	0.71	0.0000
BECHER	5	m	0	1.87	2.60	0.04	0.0025
BECHER	6	f	0	0.37	2.67	5.13	0.5481
Subtotal BECHER				1.11	5.27	5.17	
*BENSHL	1	m	1	1.07	8.51	3.98	0.0018
*BEST	3	m	1	1.80	5.04	0.01	0.0001
BLOHMK	2	m	0	1.10	56.10	24.40	0.0000
*BOUCOT	3	m	0	3.07	0.47	0.81	0.0351
*BRETT	9	m	0	-0.07	3.60	11.97	0.8976
BROSS	6	m	0	1.87	22.85	0.31	0.0000
BROWN2	22	m	2	1.97	403.85	19.40	0.0000
BROWN2	21	f	2	2.45	308.59	149.54	0.0000
Subtotal BROWN2				2.18	712.44	168.94	
BUFFLE	4	m	0	2.52	4.30	2.53	0.0000
BUFFLE	8	f	0	1.62	20.50	0.36	0.0000
Subtotal BUFFLE				1.78	24.80	2.88	
CARPEN	8	c	0	1.74	12.01	0.00	0.0000
*CEDERL	114	m	2	0.22	9.82	23.05	0.4843
*CEDERL	74	f	2	0.08	2.87	8.08	0.8963
Subtotal CEDERL				0.19	12.69	31.13	
*CHANG	1	m	0	1.38	4.54	0.64	0.0033
*CHANG	7	f	0	0.76	5.83	5.74	0.0659
Subtotal CHANG				1.03	10.36	6.39	
CHOI	2	m	0	0.63	8.11	10.23	0.0721
CHOI	6	f	0	1.46	1.93	0.16	0.0424
Subtotal CHOI				0.79	10.03	10.39	
*CHOW	2	m	0	1.82	5.48	0.02	0.0000
*CHYOU	3	m	0	1.08	9.40	4.34	0.0010
COMSTO	2	m	0	1.82	3.40	0.01	0.0008
COMSTO	7	f	0	1.02	4.88	2.62	0.0240
Subtotal COMSTO				1.35	8.28	2.63	
CORREA	37	c	0	1.83	34.20	0.19	0.0000
*CPSI	72	m	1	1.32	124.77	23.70	0.0000
*CPSI	280	f	1	0.32	13.54	27.80	0.2359
Subtotal CPSI				1.22	138.32	51.50	
*CPSII	91	m	1	2.24	72.61	16.84	0.0000

Table 1D2 - 5

IESLC - Meta-analysis of Ex Smoking, Cigarettes (or Any Product if Cigarettes not available)
All LC types
Least adjusted

REF	NRR	SEX	AD	Ys	Ws	Qs	Ps
*CPSII	78	f	1	1.58	105.38	3.34	0.0000
Subtotal CPSII				1.85	177.99	20.18	
DAMBER	17	m	1	1.15	12.36	4.56	0.0001
DARBY	5	m	0	3.50	2.94	8.91	0.0000
DARBY	12	f	0	1.92	16.90	0.46	0.0000
Subtotal DARBY				2.15	19.84	9.37	
DEAN2	1	m	0	1.33	16.45	2.97	0.0000
DEAN2	5	f	0	1.23	1.39	0.38	0.1455
Subtotal DEAN2				1.32	17.84	3.35	
DEAN3	180	m	0	1.49	15.84	1.11	0.0000
DEAN3	110	f	0	0.00	5.82	17.89	0.9957
Subtotal DEAN3				1.09	21.67	19.00	
*DEKLER	1	m	2	2.37	0.93	0.35	0.0224
DESTE2	1	c	0	2.01	11.42	0.72	0.0000
DESTEF	10	m	0	1.80	16.21	0.03	0.0000
*DOCKER	2	c	4	0.93	3.56	2.41	0.0785
DOLL	91	m	0	1.59	5.51	0.14	0.0002
DOLL	94	f	0	0.61	3.75	4.89	0.2364
Subtotal DOLL				1.20	9.25	5.04	
*DOLL2	90	m	1	1.44	2.53	0.25	0.0221
*DOLL2	9	f	1	1.19	2.22	0.71	0.0762
Subtotal DOLL2				1.32	4.74	0.96	
DORANT	1	m	0	1.46	6.36	0.56	0.0002
DORGAN	8	m	0	1.85	11.63	0.11	0.0000
DORGAN	32	m	0	2.32	2.50	0.81	0.0002
DORGAN	55	f	0	1.15	33.62	12.48	0.0000
DORGAN	78	f	0	1.15	2.61	0.97	0.0645
Subtotal DORGAN				1.37	50.35	14.36	
*DORN	20	m	2	1.38	119.77	16.94	0.0000
DROSTE	1	m	0	1.94	5.88	0.20	0.0000
*ENGELA	1	m	0	0.49	17.29	27.63	0.0413
*ENGELA	15	f	0	0.50	5.03	7.92	0.2622
Subtotal ENGELA				0.49	22.32	35.55	
GAO	27	m	0	1.35	27.43	4.51	0.0000
GAO	28	f	0	1.13	19.15	7.40	0.0000
Subtotal GAO				1.26	46.58	11.91	
GAO2	5	m	0	1.21	8.64	2.55	0.0004
GARCIA	1	c	0	1.86	15.74	0.17	0.0000
GARDIN	1	c	0	2.03	3.68	0.29	0.0001
GARSHI	22	m	0	1.40	31.09	3.84	0.0000
GOODMA	1	m	0	1.78	8.06	0.00	0.0000
GOODMA	5	f	0	1.81	7.67	0.02	0.0000
Subtotal GOODMA				1.79	15.73	0.03	
GRAHAM	7	m	0	2.78	14.14	15.00	0.0000
GREGOR	1	m	0	-0.33	4.22	18.41	0.4919
GREGOR	5	f	0	1.70	0.74	0.00	0.1435
Subtotal GREGOR				-0.03	4.95	18.42	
HAENSZ	55	f	0	0.48	3.05	4.95	0.4002
*HAMMO2	26	m	0	1.99	4.77	0.26	0.0000
*HEIN	6	m	0	1.64	0.92	0.01	0.1167
*HENNEK	1	m	0	1.30	17.18	3.57	0.0000
*HIRAYA	146	m	1	0.54	18.01	26.74	0.0228
*HIRAYA	149	f	1	1.09	4.17	1.83	0.0257
Subtotal HIRAYA				0.64	22.18	28.57	
HITOSU	1	m	0	1.51	5.20	0.30	0.0006
HITOSU	8	f	0	2.13	4.51	0.63	0.0000
Subtotal HITOSU				1.80	9.71	0.93	
*HOLE	14	m	0	0.85	5.46	4.44	0.0460
HUMBLE	1	m	1	1.97	4.91	0.24	0.0000
HUMBLE	4	m	1	2.08	1.60	0.17	0.0086
HUMBLE	7	f	1	1.87	5.29	0.07	0.0000
HUMBLE	10	f	1	1.84	1.80	0.01	0.0135
Subtotal HUMBLE				1.93	13.60	0.49	
JAHN	10	m	0	2.16	14.82	2.44	0.0000
JAIN	26	m	0	1.73	9.15	0.01	0.0000
JAIN	21	f	0	1.28	21.75	4.85	0.0000
Subtotal JAIN				1.41	30.90	4.86	
JARVHO	1	m	0	2.70	0.88	0.78	0.0114
JARVHO	5	f	0	1.48	2.28	0.18	0.0260
Subtotal JARVHO				1.82	3.16	0.96	
JEDRYC	64	m	0	1.14	26.76	10.15	0.0000

Table 1D2 - 5

IESLC - Meta-analysis of Ex Smoking, Cigarettes (or Any Product if Cigarettes not available)
All LC types
Least adjusted

REF	NRR	SEX	AD	Ys	Ws	Qs	Ps
JEDRYC	69	f	0	1.24	4.53	1.20	0.0083
Subtotal JEDRYC				1.15	31.29	11.34	
JOLY	20	m	0	2.29	9.69	2.82	0.0000
JOLY	19	f	0	1.92	11.21	0.32	0.0000
Subtotal JOLY				2.10	20.90	3.14	
*KAISE2	65	m	1	1.22	9.12	2.60	0.0002
*KAISE2	57	f	1	1.61	5.46	0.11	0.0002
Subtotal KAISE2				1.37	14.59	2.71	
*KAISER	5	m	2	1.54	15.69	0.75	0.0000
*KAISER	1	f	2	1.11	15.27	6.44	0.0000
Subtotal KAISER				1.32	30.96	7.19	
KATSOU	5	f	0	1.03	2.43	1.29	0.1092
KAUFMA	1	c	0	2.06	28.24	2.60	0.0000
KELLER	2	m	0	1.98	193.15	9.69	0.0000
KELLER	10	m	0	2.36	22.25	8.13	0.0000
KELLER	6	f	0	2.21	176.05	36.11	0.0000
KELLER	14	f	0	1.74	25.26	0.01	0.0000
Subtotal KELLER				2.08	416.70	53.93	
KHUDER	13	m	0	2.01	18.64	1.26	0.0000
KIHARA	15	c	0	0.60	21.51	28.59	0.0052
*KINLEN	1	m	0	1.61	6.45	0.13	0.0000
KJUUS	2	m	0	1.83	1.72	0.01	0.0163
*KNEKT	14	m	0	1.25	4.50	1.15	0.0080
KOO	8	f	0	1.21	5.71	1.72	0.0040
KREUZE	1	m	0	0.54	3.15	4.61	0.3341
KREUZE	3	m	0	2.21	20.22	4.19	0.0000
KREUZE	2	f	0	0.85	2.57	2.12	0.1740
KREUZE	4	f	0	0.33	15.15	30.86	0.2025
Subtotal KREUZE				1.30	41.09	41.79	
*KUBIK	11	m	0	2.44	1.60	0.76	0.0020
*LANGE	5	m	0	0.88	4.07	3.10	0.0749
*LANGE	1	f	0	0.82	3.75	3.29	0.1131
Subtotal LANGE				0.85	7.83	6.40	
LEMARC	1	c	0	1.21	20.52	6.16	0.0000
*LIDDEL	1	m	1	0.34	11.50	23.13	0.2539
LOMBAR	8	m	0	1.77	10.63	0.00	0.0000
LUBIN	39	m	0	1.39	6.83	0.89	0.0003
LUBIN2	37	m	0	1.53	143.50	7.04	0.0000
LUBIN2	319	f	0	0.96	48.33	30.60	0.0000
Subtotal LUBIN2				1.39	191.84	37.64	
MACLEN	7	m	0	0.31	1.79	3.74	0.6779
MACLEN	8	f	0	-0.23	2.14	8.41	0.7406
Subtotal MACLEN				0.02	3.94	12.15	
MATOS	14	m	0	1.62	8.35	0.16	0.0000
*MIGRAN	21	m	0	1.22	3.43	0.98	0.0238
*MIGRAN	39	f	0	0.46	0.80	1.33	0.6773
Subtotal MIGRAN				1.08	4.23	2.31	
*MRFITR	1	m	0	2.88	0.48	0.61	0.0454
NAM	65	m	0	2.58	24.98	16.90	0.0000
NAM	81	f	0	2.33	32.82	11.04	0.0000
Subtotal NAM				2.44	57.81	27.94	
ODRISC	2	c	0	3.71	5.63	21.55	0.0000
OSANN	1	m	0	2.51	34.88	19.87	0.0000
OSANN	5	f	0	2.10	42.42	4.93	0.0000
Subtotal OSANN				2.28	77.29	24.80	
PARKIN	19	m	0	1.64	15.25	0.21	0.0000
PERSH2	1	c	0	0.88	52.22	39.99	0.0000
*PETO	1	m	0	-0.27	1.01	4.13	0.7854
PEZZO2	1	m	0	2.22	5.36	1.16	0.0000
PEZZOT	1	m	0	2.32	3.58	1.15	0.0000
*QIAO2	7	m	0	1.11	8.20	3.44	0.0015
RACHTA	1	f	0	1.35	4.60	0.75	0.0038
SCHWAR	21	m	0	1.55	73.75	3.19	0.0000
SCHWAR	22	m	0	1.21	24.08	7.18	0.0000
SCHWAR	23	f	0	1.53	78.01	3.99	0.0000
SCHWAR	24	f	0	1.70	18.89	0.05	0.0000
Subtotal SCHWAR				1.51	194.73	14.42	
SHAW	3	c	0	1.86	8.69	0.10	0.0000
SOBUE	89	m	0	1.09	23.01	10.15	0.0000
SOBUE	93	f	0	0.94	18.51	12.22	0.0001
Subtotal SOBUE				1.02	41.52	22.37	

Table 1D2 - 5

IESLC - Meta-analysis of Ex Smoking, Cigarettes (or Any Product if Cigarettes not available)
All LC types
Least adjusted

REF	NRR	SEX	AD	Ys	Ws	Qs	Ps
*SPEIZE	7	f	0	1.31	41.35	8.33	0.0000
SPITZ	1	c	0	2.73	5.61	5.33	0.0000
STOCKW	4	c	0	2.04	1062.62	88.21	0.0000
STUCKE	1	m	0	4.91	0.49	4.90	0.0006
SUZUK2	1	c	0	1.41	6.09	0.72	0.0005
SVENSS	21	f	0	0.97	10.44	6.47	0.0018
TANG	2	c	0	2.00	5.45	0.33	0.0000
*TENKAN	9	m	1	1.39	3.94	0.52	0.0057
TIZZAN	6	m	0	0.70	66.01	73.87	0.0000
TIZZAN	14	f	0	1.29	3.65	0.78	0.0134
Subtotal TIZZAN				0.73	69.66	74.64	
TOKARS	2	m	0	3.13	0.95	1.78	0.0024
TOUSEY	3	m	0	2.67	3.72	3.15	0.0000
TOUSEY	7	f	0	2.13	9.60	1.37	0.0000
Subtotal TOUSEY				2.28	13.32	4.51	
TSUGAN	26	m	0	0.42	3.07	5.43	0.4579
*TULINI	1	m	1	1.07	8.28	3.91	0.0021
*TULINI	7	f	1	1.32	6.48	1.25	0.0008
Subtotal TULINI				1.18	14.76	5.15	
*TVERDA	1	m	2	-0.71	7.44	45.33	0.0517
*TVERDA	18	f	0	-0.55	0.44	2.32	0.7164
Subtotal TVERDA				-0.70	7.88	47.65	
WAKAI	1	m	0	0.92	7.09	4.95	0.0144
WAKAI	19	f	0	1.06	2.34	1.12	0.1032
Subtotal WAKAI				0.96	9.43	6.07	
WANG2	19	c	0	0.72	3.96	4.27	0.1540
WIGLE	7	m	0	2.08	11.91	1.28	0.0000
WIGLE	10	f	0	0.71	7.35	8.03	0.0546
Subtotal WIGLE				1.56	19.25	9.31	
WU	33	f	0	0.45	10.44	17.84	0.1480
WUNSCH	3	m	0	1.26	10.36	2.51	0.0000
WUNSCH	9	f	0	1.22	7.94	2.27	0.0006
Subtotal WUNSCH				1.24	18.30	4.78	
WYNDE3	41	m	0	1.32	6.61	1.23	0.0007
WYNDE6	9	m	0	1.99	66.75	3.65	0.0000
WYNDE6	198	f	0	1.70	73.82	0.19	0.0000
Subtotal WYNDE6				1.84	140.57	3.84	
YAMAGU	4	c	0	1.11	13.46	5.67	0.0000
*YONG	1	c	1	1.41	16.99	2.01	0.0000

	N	182
	NS	124
	Wt	4833.74
Het	Chi	1313.40
Het	df	181
Het	P	***
Fixed	RR	5.78
	RRl	5.62
	RRu	5.95
	P	+++
Random	RR	4.34
	RRl	3.97
	RRu	4.76
	P	+++
Asymm	P	***

Table 1D2 - 6

IESLC - Meta-analysis of Ex Smoking, Cigarettes (or Any Product if Cigarettes not available)
All LC types
Least adjusted

		combined	Sex male	female	Total
	N	20	100	62	182
	NS	20	95	57	172
	Wt	1335.78	2155.79	1342.16	4833.74
Het	Chi	167.57	583.60	492.85	1313.40
Het	df	19	99	61	181
Het	P	***	***	***	***
Fixed	RR	6.91	5.17	5.80	5.78
	RRl	6.55	4.95	5.50	5.62
	RRu	7.29	5.39	6.12	5.95
	P	+++	+++	+++	+++
Random	RR	5.40	4.63	3.55	4.34
	RRl	4.08	4.10	2.96	3.97
	RRu	7.15	5.22	4.25	4.76
	P	+++	+++	+++	+++
Between	Chi				69.37
Between	df				2
Between	P				***
Btwn(F)	P				**
Btwn(R)	P				*

Table 1D2 - 7

IESLC - Meta-analysis of Ex Smoking, Cigarettes (or Any Product if Cigarettes not available)
All LC types
Excluded studies (and stage at which they were excluded)

1	BOUCHA BUELL LAURIL MZILEN
2	ABELIN ABRAHA ALDERS AUVINE AXELSO BAND BERRIN BLOT1 BLOT2 BLOT3 BLOT4 BRESLO BROCKM BROWN1 BYERS1 BYERS2 CASCO2 CASCOR CHAN CHATZI CHEN CHEN2 CHEN3 CHIAZZ COOKSO DAVEYS DEAN DOSEME DU DUNN EBELIN ENSTRO ESAKI FAN GENG GER GODLEY GOLLED GSELL HANSEN HEGMAN HINDS HIRAOK HIRAY2 HOROWI HORWIT HU HU2 HUANG ISHIMA JARUP JIANG JONES JUSSAW KANELL KO KOHLME KOULUM KREYBE LAMTH LAMWK LAMWK2 LAUSSM LEI LETOUR LEVIN LIAW LICKIN LIU LIU2 LIU3 LIU4 LIU5 LOMBA2 LUO MAGNUS MARSH MARSH2 MARTIS MASTRA MATSUD MCCONN MCDUFF MCLAUG MILLER MILLS MOLLO MRFIT MURATA NOTAN2 NOTANI NOU ORMOS PASTOR PAWLEG PERNU PERSHA PIKE POFFIJ POLEDN QIAO RADZIK RANDIG REN RIMING RONCO ROOTS ROTHSC SAARIK SADOWS SANKAR SCHWA2 SEGI SEGI2 SEOW SHIMIZ SIEMIA SIMARA SITAS SOBUE2 STASZE STAYNE STOCKS SUN TANG2 TAO ULMER VANDER WALD WANG WANG3 WANG4 WARSIN WATSON WICKLU WILKIN WU2 WUWILL WYNDE2 WYNDE4 WYNDE5 WYNDE8 WYNDER XIANGZ XU XU2 XU3 XU4 YUAN ZHANG ZHENG ZHOU
3	RESTRE
4	BOFFET
8	HAMMON SUZUKI
10	BENHAM GILLIS OSANN2 PISANI PRESCO SIMONA VEIERO VUTUC WYNDE7

Table 1D2 - 8

Potentially overlapping studies

REF	REFGP	PRINC	.	OVERLAP/LINK
LUBIN2	LUBIN2	1		Lubin-combined
KOO	KOO	1		KOO/LAMTH/LAMWK
TVERDA	TVERDA	1		VEIERO/TVERDAL
AKIBA	AKIBA	1		AKIBA/ISHIMA
HEIN	PRESCO	1		Subset of PRESCO
LANGE	PRESCO	1		Subset of PRESCO
BROSS	BYERS1	1		GRAHAM/BROSS/BYERS1
GRAHAM	BYERS1	1		GRAHAM/BROSS/BYERS1
CHYOU	CHYOU	1		GOODMA/CHYOU
GOODMA	GOODMA	1		GOODMA/CHYOU
HOLE	TANG2	1		Subset of TANG2
BENSHL	TANG2	1		Subset of TANG2
KAISER	KAISER	1		KAISER/OSANN2
MRFITR	MRFIT	2		Subset of MRFIT
WYNDE6	WYNDE6	1		WYNDE5/6/7/8
CPSI	CPSI	1		CPSI overall
JAHN	BOFFET	2		Subset of BOFFET
LUBIN	XIANGZ	2		LUBIN/XIANGZ/QIAO

Table 1D2 - 9

Most adjusted - insufficient data for metaanalysis

REF	NRR	SEX	AGEL	AGEH	RACE	YF	LC TYPE	LOC	START	ST	NLC	R	VB	P	H	AD	PRODUCT	DENOM	De
RESTRE	1	c	0	0	all	-	all	SCAmer	1978	CC	102	n	bl	n	n	2	cig+/-ot	nev cigs	ot

REF	NRR	RR	SIG	RRDATA comment
RESTRE	1	4.72	y	0

Table 1D3 -

IESLC - Meta-analysis of Ex Smoking, Cigarettes only
All LC types

This analysis is restricted to results for:
1) Non-dose-response data
2) Ex smokers
3) Results complete enough for use in metaanalysis

Within each study, results are then selected (in the following order of preference, within each sex) for:
4) PRODUCT: cigarettes only
5) CIGTYPE: all/unspecified, MC regardless of HR, MC only
6) DENOM: never smoked anything, never smoked cigarettes, (never +1 = +long term ex, +2 = +amount unknown,
+3 = never cigs+long term ex)
7) Followup period (YF, prospective studies): whole study (coded as 0) or longest available
8) LCtype: all or nearest available, at least Squamous and Adeno. (q = squamous, s = small,
l = large, a = adeno, mix = mixed, alv = alveolar)
9) Race: all or nearest available, otherwise by race (wh or w = white, bl or b = black, hi = hispanic
ch = chinese, jap = japanese, haw = hawaiian, w+o = white + oriental, sca = scandinavian, as = asian)
10) For overlapping studies: principal rather than subsidiary studies
Finally by Age: whole study (coded as 0) if available, otherwise by widest available age group
and then for single sex results (m, f) in preference to combined sex results (c).

Results adjusted (AD) for the most potential confounders are then chosen in Sections -1 to -3
and results adjusted for the least confounders in Sections -4 to -6. (Those least adjusted results which
actually differ from the most adjusted as marked 'x' in column X in Section -4)
(Results adjusted for an unknown number of confounder(s) are coded as 20.)

Section -7 shows excluded studies, together with the stage (as above) at which no qualifying
results were found.

Section -8 lists the potentially overlapping studies which have been included (1=principal, 2=subsidiary).

Section -9 lists any results which would have been included in preference except that they had data not complete
enough for use in meta-analysis, with their significance (yes/no), if known, and any further comment as entered
on the database.

In addition to those mentioned above, the following fields, levels and abbreviations are used:
* or nk = not known, n = no, y = yes, ot = other
nev = never
all/unspec = all or unspecified, MC = manufactured cigarettes, HR = hand-rolled cigarettes
REF: 6-character study reference
NRR: number of the RR on the database within the study
ST : study type (CC = case control, pr or prosp = prospective)
NLC: number of lung cancer cases in whole study
R : risky occupational population (n = no, m = mining, o = other risky)
VB : national cigarette type (V = at least 75% Virginia, bl = at least 75% blended, ot = other)
P : any proxy use
H : full histological confirmation
De : derivation of RR/CI (or = original, st = standard method, ot = other method of estimation)

Table 1D3 - 1

IESLC - Meta-analysis of Ex Smoking, Cigarettes only
All LC types
Most adjusted

REF	NRR	SEX	AGEL	AGEH	RACE	YF	LC TYPE	LOC	START	ST	NLC	R	VB	P	H	AD	PRODUCT	DENOM	De
AGUDO	2	f	0	0	all	-	all	Eu:wst	1989	CC	103	n	bl	n	n	3	cig only	nev any	or
BEST	3	m	0	0	all	0	all	NAmer	1955	pr	381	n	V	n	n	1	cig only	nev any	ot
BOUCOT	115	m	0	0	all	0	all	NAmer	1951	pr	121	n	bl	n	n	2	cig only	nev any	ot
CHOW	17	m	0	0	wh	0	all	NAmer	1966	pr	219	n	bl	n	n	0	cig only	nev any	st
CPSI	72	m	0	0	wh	0	all	NAmer	1959	pr	5138	n	bl	n	n	1	cig only	nev any	st
CPSI	148	f	0	0	wh	0	all	NAmer	1959	pr	5138	n	bl	n	n	1	cig only	nev any	st
CPSII	91	m	35	99	all	4	all	NAmer	1982	pr	3229	n	bl	n	n	1	cig only	nev any	or
DAMBER	17	m	0	0	all	-	all	Eu:Sca	1972	CC	579	n	bl	y	n	1	cig only	nev any	ot
DEAN3	181	m	0	0	all	-	all	Eu:UK	1969	CC	766	n	V	y	n	1	cig only	nev any	ot
DEAN3	112	f	0	0	all	-	all	Eu:UK	1969	CC	766	n	V	y	n	3	cig only	nev any	ot
DOLL2	1	m	0	0	all	0	all	Eu:UK	1951	pr	920	n	V	n	n	1	cig only	nev any	ot
DOLL2	9	f	0	0	all	22	all	Eu:UK	1951	pr	920	n	V	n	n	1	cig only	nev any	ot
DORN	2	m	0	0	wh	0	all	NAmer	1954	pr	5097	n	bl	n	n	2	cig only	nev any	or
GRAHAM	1	m	0	0	wh	-	all	NAmer	1956	CC	685	n	bl	n	n	0	cig only	nev any	st
KAISE2	65	m	35	99	all	9	all	NAmer	1979	pr	318	n	bl	n	n	1	cig only	nev any	st
KAISE2	57	f	35	99	all	9	all	NAmer	1979	pr	318	n	bl	n	n	1	cig only	nev any	st
LOMBAR	4	m	0	0	all	-	all	NAmer	1951	CC	1040	n	bl	n	n	0	cig only	nev any	st
LUBIN2	10	m	0	0	all	-	all	Eu:mul	1976	CC	7804	n	bl	n	y	2	cig only	nev any	or
PEZZOT	1	m	0	0	all	-	all	SCAmer	1987	CC	215	n	bl	n	y	0	cig only	nev cigs	st
TVERDA	18	f	0	0	all	0	all	Eu:Sca	1972	pr	238	n	bl	n	n	0	cig only	nev cigs	ot
WIGLE	7	m	0	0	all	-	all	NAmer	1971	CC	728	n	V	n	n	0	cig only	nev any	st
WIGLE	10	f	0	0	all	-	all	NAmer	1971	CC	728	n	V	n	n	0	cig only	nev any	st
WYNDE7	6	m	0	0	all	-	all	NAmer	1977	CC	2085	n	bl	n	y	0	cig only	nev any	st

Cigarette type is all/unspec for all RRs

except for the following:

REF	NRR	CIGTYPE
DEAN3	181	MC only
DEAN3	112	MC only

Table 1D3 - 2

IESLC - Meta-analysis of Ex Smoking, Cigarettes only
All LC types
Most adjusted

REF	NRR	SEX	AD	Number Exposed Case	Number Exposed Cont	Non-exposed Case	Non-exposed Cont	RR	95.00%CI
AGUDO	2	f	3	-	-	-	-	1.61	(0.37- 6.91)
*BEST	3	m	1	-	-	-	-	6.06	(2.53- 14.51)
*BOUCOT	115	m	2	-	-	-	-	20.86	(1.20- 361.47)
*CHOW	17	m	0	12	31515	6	62913	3.99	(1.50- 10.64)
*CPSI	72	m	1	-	-	-	-	3.74	(3.14- 4.46)
*CPSI	148	f	1	-	-	-	-	1.04	(0.69- 1.58)
Subtotal CPSI								3.08	(2.62- 3.62)
*CPSII	91	m	1	-	-	-	-	9.36	(7.43- 11.77)
DAMBER	17	m	1	-	-	-	-	3.15	(1.80- 5.49)
DEAN3	181	m	1	-	-	-	-	3.21	(1.96- 5.25)
DEAN3	112	f	3	-	-	-	-	1.17	(0.51- 2.69)
Subtotal DEAN3								2.47	(1.62- 3.77)
*DOLL2	1	m	1	-	-	-	-	4.14	(2.56- 6.70)
*DOLL2	9	f	1	-	-	-	-	3.29	(0.88- 12.24)
Subtotal DOLL2								4.03	(2.56- 6.33)
*DORN	2	m	2	-	-	-	-	3.60	(3.10- 4.10)
GRAHAM	1	m	0	103	130	18	346	15.23	(8.88- 26.13)
*KAISE2	65	m	1	-	-	-	-	3.39	(1.77- 6.48)
*KAISE2	57	f	1	-	-	-	-	5.02	(2.17- 11.61)
Subtotal KAISE2								3.93	(2.35- 6.56)
LOMBAR	4	m	0	54	53	14	112	8.15	(4.16- 15.97)
LUBIN2	10	m	2	-	-	-	-	4.52	(3.80- 5.30)
PEZZOT	1	m	0	66	188	4	116	10.18	(3.61- 28.67)
*TVERDA	18	f	0	0	38953	3	157431	0.58~	(0.03- 11.18)
WIGLE	7	m	0	128	217	15	204	8.02	(4.55- 14.16)
WIGLE	10	f	0	11	66	36	439	2.03	(0.99- 4.19)
Subtotal WIGLE								4.75	(3.04- 7.43)
WYNDE7	6	m	0	538	1115	64	918	6.92	(5.26- 9.10)
Partial Totals				912	72237	160	222479		

*prospective study ~ With 0.5 adjustment for zero

REF	NRR	SEX	AD	Ys	Ws	Qs	Ps
AGUDO	2	f	3	0.48	1.79	1.79	0.5236
*BEST	3	m	1	1.80	5.04	0.54	0.0001
*BOUCOT	115	m	2	3.04	0.47	1.15	0.0370
*CHOW	17	m	0	1.38	4.00	0.03	0.0056
*CPSI	72	m	1	1.32	124.77	3.04	0.0000
*CPSI	148	f	1	0.04	22.39	46.16	0.8528
Subtotal CPSI				1.12	147.16	49.21	
*CPSII	91	m	1	2.24	72.61	42.07	0.0000
DAMBER	17	m	1	1.15	12.36	1.33	0.0001
DEAN3	181	m	1	1.17	15.83	1.51	0.0000
DEAN3	112	f	3	0.16	5.56	9.66	0.7113
Subtotal DEAN3				0.90	21.39	11.17	
*DOLL2	1	m	1	1.42	16.60	0.05	0.0000
*DOLL2	9	f	1	1.19	2.22	0.18	0.0762
Subtotal DOLL2				1.39	18.82	0.23	
*DORN	2	m	2	1.28	196.58	7.42	0.0000
GRAHAM	1	m	0	2.72	13.18	20.54	0.0000
*KAISE2	65	m	1	1.22	9.12	0.59	0.0002
*KAISE2	57	f	1	1.61	5.46	0.10	0.0002
Subtotal KAISE2				1.37	14.59	0.69	
LOMBAR	4	m	0	2.10	8.49	3.30	0.0000
LUBIN2	10	m	2	1.51	138.81	0.15	0.0000
PEZZOT	1	m	0	2.32	3.58	2.56	0.0000
*TVERDA	18	f	0	-0.55	0.44	1.79	0.7164
WIGLE	7	m	0	2.08	11.91	4.39	0.0000
WIGLE	10	f	0	0.71	7.35	4.31	0.0546
Subtotal WIGLE				1.56	19.25	8.70	
WYNDE7	6	m	0	1.93	51.36	10.84	0.0000

Table 1D3 - 2

IESLC - Meta-analysis of Ex Smoking, Cigarettes only
All LC types
Most adjusted

	N	23
	NS	18
	Wt	729.92
Het	Chi	163.50
Het	df	22
Het	P	***
Fixed	RR	4.37
	RRl	4.07
	RRu	4.70
	P	+++
Random	RR	4.28
	RRl	3.35
	RRu	5.46
	P	+++
Asymm	P	N.S.

Table 1D3 - 3

IESLC - Meta-analysis of Ex Smoking, Cigarettes only
All LC types
Most adjusted

	Sex			
	combined	male	female	Total
N		16	7	23
NS		16	7	23
Wt		684.72	45.20	729.92
Het Chi		96.19	13.76	163.50
Het df		15	6	22
Het P		***	*	***
Fixed RR		4.69	1.52	4.37
RRl		4.35	1.14	4.07
RRu		5.05	2.04	4.70
P		+++	++	+++
Random RR		5.43	1.81	4.28
RRl		4.30	1.07	3.35
RRu		6.84	3.07	5.46
P		+++	+	+++
Between Chi				53.55
Between df				1
Between P				***
Btwn(F) P				**
Btwn(R) P				***

	Lung cancer type		
	all	other	Total
N	23		23
NS	18		18
Wt	729.92		729.92
Het Chi	163.50		163.50
Het df	22		22
Het P	***		***
Fixed RR	4.37		4.37
RRl	4.07		4.07
RRu	4.70		4.70
P	+++		+++
Random RR	4.28		4.28
RRl	3.35		3.35
RRu	5.46		5.46
P	+++		+++
Between Chi			
Between df			
Between P			N.S.
Btwn(F) P			N.S.
Btwn(R) P			N.S.

	Location								
	NAmer	UK	Scand	othEur	China	Japan	othAs	other	Total
N	14	4	2	2				1	23
NS	11	2	2	2				1	18
Wt	532.73	40.20	12.79	140.61				3.58	729.92
Het Chi	144.17	6.69	1.22	1.89				0.00	163.50
Het df	13	3	1	1				0	22
Het P	***	(*)	N.S.	N.S.				N.S.	***
Fixed RR	4.48	3.11	2.97	4.46				10.18	4.37
RRl	4.11	2.28	1.72	3.78				3.61	4.07
RRu	4.88	4.23	5.14	5.26				28.67	4.70
P	+++	+++	+++	+++				+++	+++
Random RR	4.91	2.83	2.58	3.52				10.18	4.28
RRl	3.49	1.70	0.89	1.48				3.61	3.35
RRu	6.91	4.72	7.52	8.37				28.67	5.46
P	+++	+++	(+)	++				+++	+++
Between Chi									9.53
Between df									4
Between P									*
Btwn(F) P									N.S.
Btwn(R) P									N.S.

Table 1D3 - 3

IESLC - Meta-analysis of Ex Smoking, Cigarettes only
All LC types
Most adjusted

Detailed Country in "other Europe"

	multi	Germany	othWest	East	Balkans	Total
N	1		1			2
NS	1		1			2
Wt	138.81		1.79			140.61
Het Chi	0.00		0.00			1.89
Het df	0		0			1
Het P	N.S.		N.S.			N.S.
Fixed RR	4.52		1.61			4.46
RRl	3.83		0.37			3.78
RRu	5.34		6.96			5.26
P	+++		N.S.			+++
Random RR	4.52		1.61			3.52
RRl	3.83		0.37			1.48
RRu	5.34		6.96			8.37
P	+++		N.S.			++
Between Chi						1.89
Between df						1
Between P						N.S.
Btwn(F) P						N.S.
Btwn(R) P						N.S.

Detailed Country in "other Asia"

	India	HongKong	other	Total
N				
NS				
Wt				
Het Chi				
Het df				
Het P				
Fixed RR				
RRl				
RRu				
P				
Random RR				
RRl				
RRu				
P				
Between Chi				
Between df				
Between P				N.S.
Btwn(F) P				N.S.
Btwn(R) P				N.S.

Detailed other continent

	SCAmer	Auslia	Africa	Total
N	1			1
NS	1			1
Wt	3.58			3.58
Het Chi	0.00			0.00
Het df	0			0
Het P	N.S.			N.S.
Fixed RR	10.18			10.18
RRl	3.61			3.61
RRu	28.67			28.67
P	+++			+++
Random RR	10.18			10.18
RRl	3.61			3.61
RRu	28.67			28.67
P	+++			+++
Between Chi				
Between df				
Between P				N.S.
Btwn(F) P				N.S.
Btwn(R) P				N.S.

Table 1D3 - 3

IESLC - Meta-analysis of Ex Smoking, Cigarettes only
All LC types
Most adjusted

	Start year of study <1960	1960-69	1970-79	1980-89	1990+	Total
N	9	3	8	3		23
NS	7	2	6	3		18
Wt	389.74	25.39	236.81	77.98		729.92
Het Chi	70.78	4.97	21.21	5.48		163.50
Het df	8	2	7	2		22
Het P	***	(*)	**	(*)		***
Fixed RR	3.68	2.66	4.82	9.02		4.37
RRl	3.33	1.81	4.25	7.23		4.07
RRu	4.06	3.93	5.48	11.27		4.70
P	+++	+++	+++	+++		+++
Random RR	4.39	2.49	4.46	6.74		4.28
RRl	2.94	1.26	3.29	2.93		3.35
RRu	6.55	4.94	6.04	15.49		5.46
P	+++	++	+++	+++		+++
Between Chi						61.07
Between df						3
Between P						***
Btwn(F) P						*
Btwn(R) P						N.S.

	Study type (1) CC	other	Total
N	11	12	23
NS	9	9	18
Wt	270.22	459.69	729.92
Het Chi	55.43	100.24	163.50
Het df	10	11	22
Het P	***	***	***
Fixed RR	5.00	4.04	4.37
RRl	4.44	3.69	4.07
RRu	5.64	4.42	4.70
P	+++	+++	+++
Random RR	4.75	3.83	4.28
RRl	3.34	2.65	3.35
RRu	6.75	5.56	5.46
P	+++	+++	+++
Between Chi			7.84
Between df			1
Between P			**
Btwn(F) P			N.S.
Btwn(R) P			N.S.

	Study type (2) CC	prosp	other	Total
N	11	12		23
NS	9	9		18
Wt	270.22	459.69		729.92
Het Chi	55.43	100.24		163.50
Het df	10	11		22
Het P	***	***		***
Fixed RR	5.00	4.04		4.37
RRl	4.44	3.69		4.07
RRu	5.64	4.42		4.70
P	+++	+++		+++
Random RR	4.75	3.83		4.28
RRl	3.34	2.65		3.35
RRu	6.75	5.56		5.46
P	+++	+++		+++
Between Chi				7.84
Between df				1
Between P				**
Btwn(F) P				N.S.
Btwn(R) P				N.S.

Table 1D3 - 3

IESLC - Meta-analysis of Ex Smoking, Cigarettes only
All LC types
Most adjusted

	Study size (number of LC cases)				
	100-249	250-499	500-999	1000+	Total
N	5	3	8	7	23
NS	5	2	5	6	18
Wt	10.29	19.62	85.00	615.01	729.92
Het Chi	7.28	1.23	41.96	112.98	163.50
Het df	4	2	7	6	22
Het P	N.S.	N.S.	***	***	***
Fixed RR	4.69	4.39	4.39	4.36	4.37
RRl	2.55	2.82	3.55	4.03	4.07
RRu	8.64	6.83	5.42	4.72	4.70
P	+++	+++	+++	+++	+++
Random RR	4.28	4.39	3.91	4.40	4.28
RRl	1.70	2.82	2.28	3.04	3.35
RRu	10.78	6.83	6.71	6.37	5.46
P	++	+++	+++	+++	+++
Between Chi					0.05
Between df					3
Between P					N.S.
Btwn(F) P					N.S.
Btwn(R) P					N.S.

	Risky occupational population			
	no	mining	othRisky	Total
N	23			23
NS	18			18
Wt	729.92			729.92
Het Chi	163.50			163.50
Het df	22			22
Het P	***			***
Fixed RR	4.37			4.37
RRl	4.07			4.07
RRu	4.70			4.70
P	+++			+++
Random RR	4.28			4.28
RRl	3.35			3.35
RRu	5.46			5.46
P	+++			+++
Between Chi				
Between df				
Between P				N.S.
Btwn(F) P				N.S.
Btwn(R) P				N.S.

	National cigarette tobacco type			
	Virginia	blended	other	Total
N	7	16		23
NS	4	14		18
Wt	64.49	665.43		729.92
Het Chi	18.92	142.71		163.50
Het df	6	15		22
Het P	**	***		***
Fixed RR	3.71	4.44		4.37
RRl	2.91	4.12		4.07
RRu	4.74	4.79		4.70
P	+++	+++		+++
Random RR	3.49	4.66		4.28
RRl	2.20	3.47		3.35
RRu	5.52	6.25		5.46
P	+++	+++		+++
Between Chi				1.88
Between df				1
Between P				N.S.
Btwn(F) P				N.S.
Btwn(R) P				N.S.

Table 1D3 - 3

IESLC - Meta-analysis of Ex Smoking, Cigarettes only
All LC types
Most adjusted

	Any proxy use No/nk	Yes	Total
N	20	3	23
NS	16	2	18
Wt	696.18	33.74	729.92
Het Chi	150.63	4.65	163.50
Het df	19	2	22
Het P	***	(*)	***
Fixed RR	4.48	2.70	4.37
RRl	4.16	1.93	4.07
RRu	4.82	3.78	4.70
P	+++	+++	+++
Random RR	4.69	2.49	4.28
RRl	3.61	1.46	3.35
RRu	6.10	4.26	5.46
P	+++	+++	+++
Between Chi			8.22
Between df			1
Between P			**
Btwn(F) P			N.S.
Btwn(R) P			*

	Full histological confirmation No	Yes	Total
N	20	3	23
NS	15	3	18
Wt	536.16	193.76	729.92
Het Chi	148.13	8.51	163.50
Het df	19	2	22
Het P	***	*	***
Fixed RR	4.12	5.14	4.37
RRl	3.79	4.46	4.07
RRu	4.49	5.91	4.70
P	+++	+++	+++
Random RR	3.96	5.92	4.28
RRl	2.94	3.97	3.35
RRu	5.33	8.82	5.46
P	+++	+++	+++
Between Chi			6.86
Between df			1
Between P			**
Btwn(F) P			N.S.
Btwn(R) P			N.S.

	Number of adjustment variables (1) 0	1	2+/+nk	Total
N	8	10	5	23
NS	7	7	5	19
Wt	100.31	286.39	343.21	729.92
Het Chi	24.09	95.42	15.04	163.50
Het df	7	9	4	22
Het P	**	***	**	***
Fixed RR	7.11	4.27	3.87	4.37
RRl	5.84	3.80	3.48	4.07
RRu	8.64	4.79	4.30	4.70
P	+++	+++	+++	+++
Random RR	6.50	3.71	3.41	4.28
RRl	4.18	2.38	2.45	3.35
RRu	10.13	5.78	4.75	5.46
P	+++	+++	+++	+++
Between Chi				28.95
Between df				2
Between P				***
Btwn(F) P				N.S.
Btwn(R) P				(*)

Table 1D3 - 3

IESLC - Meta-analysis of Ex Smoking, Cigarettes only
All LC types
Most adjusted

	Number of adjustment variables (2) 0	1	2	3-5	6+/+nk	Total
N	8	10	3	2		23
NS	7	7	3	2		19
Wt	100.31	286.39	335.86	7.35		729.92
Het Chi	24.09	95.42	5.52	0.14		163.50
Het df	7	9	2	1		22
Het P	**	***	(*)	N.S.		***
Fixed RR	7.11	4.27	3.96	1.26		4.37
RRl	5.84	3.80	3.56	0.61		4.07
RRu	8.64	4.79	4.41	2.61		4.70
P	+++	+++	+++	N.S.		+++
Random RR	6.50	3.71	4.06	1.26		4.28
RRl	4.18	2.38	3.23	0.61		3.35
RRu	10.13	5.78	5.11	2.61		5.46
P	+++	+++	+++	N.S.		+++
Between Chi						38.34
Between df						3
Between P						***
Btwn(F) P						N.S.
Btwn(R) P						**

	Product all/unsp	cig+/-ot	cig only	Total
N			23	23
NS			18	18
Wt			729.92	729.92
Het Chi			163.50	163.50
Het df			22	22
Het P			***	***
Fixed RR			4.37	4.37
RRl			4.07	4.07
RRu			4.70	4.70
P			+++	+++
Random RR			4.28	4.28
RRl			3.35	3.35
RRu			5.46	5.46
P			+++	+++
Between Chi				
Between df				
Between P				N.S.
Btwn(F) P				N.S.
Btwn(R) P				N.S.

	Denominator nev any	nev cigs	Total
N	21	2	23
NS	16	2	18
Wt	725.90	4.02	729.92
Het Chi	159.14	3.21	163.50
Het df	20	1	22
Het P	***	(*)	***
Fixed RR	4.36	7.45	4.37
RRl	4.05	2.80	4.07
RRu	4.69	19.80	4.70
P	+++	+++	+++
Random RR	4.22	3.44	4.28
RRl	3.29	0.22	3.35
RRu	5.40	52.61	5.46
P	+++	N.S.	+++
Between Chi			1.15
Between df			1
Between P			N.S.
Btwn(F) P			N.S.
Btwn(R) P			N.S.

Table 1D3 - 3

IESLC - Meta-analysis of Ex Smoking, Cigarettes only
All LC types
Most adjusted

	Derivation of RR/CI			
	Orig	StdCalc	Other	Total
N	4	11	8	23
NS	4	8	6	18
Wt	409.79	261.62	58.51	729.92
Het Chi	50.47	95.81	11.66	163.50
Het df	3	10	7	22
Het P	***	***	N.S.	***
Fixed RR	4.59	4.31	3.31	4.37
RRl	4.17	3.82	2.56	4.07
RRu	5.06	4.87	4.27	4.70
P	+++	+++	+++	+++
Random RR	4.82	4.80	3.23	4.28
RRl	2.99	3.08	2.22	3.35
RRu	7.77	7.48	4.70	5.46
P	+++	+++	+++	+++
Between Chi				5.56
Between df				2
Between P				(*)
Btwn(F) P				N.S.
Btwn(R) P				N.S.

Table 1D3 - 4

IESLC - Meta-analysis of Ex Smoking, Cigarettes only
All LC types
Least adjusted

REF	NRR	X	SEX	AGEL	AGEH	RACE	YF	LC TYPE	LOC	START	ST	NLC	R	VB	P	H	AD	PRODUCT	DENOM	De
AGUDO	9	x	f	0	0	all	-	all	Eu:wst	1989	CC	103	n	bl	n	n	0	cig only	nev any	st
BEST	3		m	0	0	all	0	all	NAmer	1955	pr	381	n	V	n	n	1	cig only	nev any	ot
BOUCOT	3	x	m	0	0	all	0	all	NAmer	1951	pr	121	n	bl	n	n	0	cig only	nev any	ot
CHOW	17		m	0	0	wh	0	all	NAmer	1966	pr	219	n	bl	n	n	0	cig only	nev any	st
CPSI	72		m	0	0	wh	0	all	NAmer	1959	pr	5138	n	bl	n	n	1	cig only	nev any	st
CPSI	148		f	0	0	wh	0	all	NAmer	1959	pr	5138	n	bl	n	n	1	cig only	nev any	st
CPSII	91		m	35	99	all	4	all	NAmer	1982	pr	3229	n	bl	n	n	1	cig only	nev any	or
DAMBER	17		m	0	0	all	-	all	Eu:Sca	1972	CC	579	n	bl	y	n	1	cig only	nev any	ot
DEAN3	180	x	m	0	0	all	-	all	Eu:UK	1969	CC	766	n	V	y	n	0	cig only	nev any	st
DEAN3	110	x	f	0	0	all	-	all	Eu:UK	1969	CC	766	n	V	y	n	0	cig only	nev any	st
DOLL2	1		m	0	0	all	0	all	Eu:UK	1951	pr	920	n	V	n	n	1	cig only	nev any	ot
DOLL2	9		f	0	0	all	22	all	Eu:UK	1951	pr	920	n	V	n	n	1	cig only	nev any	ot
DORN	2		m	0	0	wh	0	all	NAmer	1954	pr	5097	n	bl	n	n	2	cig only	nev any	or
GRAHAM	1		m	0	0	wh	-	all	NAmer	1956	CC	685	n	bl	n	n	0	cig only	nev any	st
KAISE2	65		m	35	99	all	9	all	NAmer	1979	pr	318	n	bl	n	n	1	cig only	nev any	st
KAISE2	57		f	35	99	all	9	all	NAmer	1979	pr	318	n	bl	n	n	1	cig only	nev any	st
LOMBAR	4		m	0	0	all	-	all	NAmer	1951	CC	1040	n	bl	n	n	0	cig only	nev any	st
LUBIN2	9	x	m	0	0	all	-	all	Eu:mul	1976	CC	7804	n	bl	n	y	0	cig only	nev any	st
PEZZOT	1		m	0	0	all	-	all	SCAmer	1987	CC	215	n	bl	n	y	0	cig only	nev cigs	st
TVERDA	18		f	0	0	all	0	all	Eu:Sca	1972	pr	238	n	bl	n	n	0	cig only	nev cigs	ot
WIGLE	7		m	0	0	all	-	all	NAmer	1971	CC	728	n	V	n	n	0	cig only	nev any	st
WIGLE	10		f	0	0	all	-	all	NAmer	1971	CC	728	n	V	n	n	0	cig only	nev any	st
WYNDE7	6		m	0	0	all	-	all	NAmer	1977	CC	2085	n	bl	n	y	0	cig only	nev any	st

Cigarette type is all/unspec for all RRs

except for the following:

REF	NRR	CIGTYPE
DEAN3	180	MC only
DEAN3	110	MC only

Table 1D3 - 5

IESLC - Meta-analysis of Ex Smoking, Cigarettes only
All LC types
Least adjusted

REF	NRR	SEX	AD	Number Exposed Case	Number Exposed Cont	Non-exposed Case	Non-exposed Cont	RR	95.00%CI
AGUDO	9	f	0	3	6	80	183	1.14	(0.28- 4.69)
*BEST	3	m	1	-	-	-	-	6.06	(2.53- 14.51)
*BOUCOT	3	m	0	8	5977	0	7551	21.48~	(1.24- 372.01)
*CHOW	17	m	0	12	31515	6	62913	3.99	(1.50- 10.64)
*CPSI	72	m	1	-	-	-	-	3.74	(3.14- 4.46)
*CPSI	148	f	1	-	-	-	-	1.04	(0.69- 1.58)
Subtotal CPSI								3.08	(2.62- 3.62)
*CPSII	91	m	1	-	-	-	-	9.36	(7.43- 11.77)
DAMBER	17	m	1	-	-	-	-	3.15	(1.80- 5.49)
DEAN3	180	m	0	62	297	24	510	4.44	(2.71- 7.26)
DEAN3	110	f	0	7	262	41	1538	1.00	(0.44- 2.26)
Subtotal DEAN3								2.97	(1.95- 4.53)
*DOLL2	1	m	1	-	-	-	-	4.14	(2.56- 6.70)
*DOLL2	9	f	1	-	-	-	-	3.29	(0.88- 12.24)
Subtotal DOLL2								4.03	(2.56- 6.33)
*DORN	2	m	2	-	-	-	-	3.60	(3.10- 4.10)
GRAHAM	1	m	0	103	130	18	346	15.23	(8.88- 26.13)
*KAISE2	65	m	1	-	-	-	-	3.39	(1.77- 6.48)
*KAISE2	57	f	1	-	-	-	-	5.02	(2.17- 11.61)
Subtotal KAISE2								3.93	(2.35- 6.56)
LOMBAR	4	m	0	54	53	14	112	8.15	(4.16- 15.97)
LUBIN2	9	m	0	911	2662	190	2617	4.71	(4.00- 5.56)
PEZZOT	1	m	0	66	188	4	116	10.18	(3.61- 28.67)
*TVERDA	18	f	0	0	38953	3	157431	0.58~	(0.03- 11.18)
WIGLE	7	m	0	128	217	15	204	8.02	(4.55- 14.16)
WIGLE	10	f	0	11	66	36	439	2.03	(0.99- 4.19)
Subtotal WIGLE								4.75	(3.04- 7.43)
WYNDE7	6	m	0	538	1115	64	918	6.92	(5.26- 9.10)
Partial Totals				1903	81441	495	234878		

*prospective study ~ With 0.5 adjustment for zero

REF	NRR	SEX	AD	Ys	Ws	Qs	Ps
AGUDO	9	f	0	0.13	1.93	3.54	0.8520
*BEST	3	m	1	1.80	5.04	0.50	0.0001
*BOUCOT	3	m	0	3.07	0.47	1.18	0.0351
*CHOW	17	m	0	1.38	4.00	0.04	0.0056
*CPSI	72	m	1	1.32	124.77	3.54	0.0000
*CPSI	148	f	1	0.04	22.39	46.96	0.8528
Subtotal CPSI				1.12	147.16	50.50	
*CPSII	91	m	1	2.24	72.61	40.72	0.0000
DAMBER	17	m	1	1.15	12.36	1.43	0.0001
DEAN3	180	m	0	1.49	15.84	0.00	0.0000
DEAN3	110	f	0	0.00	5.82	12.85	0.9957
Subtotal DEAN3				1.09	21.67	12.85	
*DOLL2	1	m	1	1.42	16.60	0.07	0.0000
*DOLL2	9	f	1	1.19	2.22	0.20	0.0762
Subtotal DOLL2				1.39	18.82	0.27	
*DORN	2	m	2	1.28	196.58	8.39	0.0000
GRAHAM	1	m	0	2.72	13.18	20.13	0.0000
*KAISE2	65	m	1	1.22	9.12	0.65	0.0002
*KAISE2	57	f	1	1.61	5.46	0.09	0.0002
Subtotal KAISE2				1.37	14.59	0.74	
LOMBAR	4	m	0	2.10	8.49	3.17	0.0000
LUBIN2	9	m	0	1.55	140.48	0.56	0.0000
PEZZOT	1	m	0	2.32	3.58	2.49	0.0000
*TVERDA	18	f	0	-0.55	0.44	1.82	0.7164
WIGLE	7	m	0	2.08	11.91	4.21	0.0000
WIGLE	10	f	0	0.71	7.35	4.45	0.0546
Subtotal WIGLE				1.56	19.25	8.66	
WYNDE7	6	m	0	1.93	51.36	10.26	0.0000

Table 1D3 - 5

IESLC - Meta-analysis of Ex Smoking, Cigarettes only
All LC types
Least adjusted

	N	23
	NS	18
	Wt	732.00
Het	Chi	167.23
Het	df	22
Het	P	***
Fixed	RR	4.43
	RRl	4.12
	RRu	4.76
	P	+++
Random	RR	4.30
	RRl	3.36
	RRu	5.50
	P	+++
Asymm	P	N.S.

Table 1D3 - 6

IESLC - Meta-analysis of Ex Smoking, Cigarettes only
All LC types
Least adjusted

	combined	Sex male	female	Total
N		16	7	23
NS		16	7	23
Wt		686.39	45.60	732.00
Het Chi		93.66	14.49	167.23
Het df		15	6	22
Het P		***	*	***
Fixed RR		4.76	1.47	4.43
RRl		4.42	1.10	4.12
RRu		5.13	1.97	4.76
P		+++	++	+++
Random RR		5.56	1.71	4.30
RRl		4.43	1.00	3.36
RRu		6.99	2.93	5.50
P		+++	(+)	+++
Between Chi				59.08
Between df				1
Between P				***
Btwn(F) P				**
Btwn(R) P				***

Table 1D3 - 7

IESLC - Meta-analysis of Ex Smoking, Cigarettes only
All LC types
Excluded studies (and stage at which they were excluded)

1 BOUCHA BUELL LAURIL MZILEN

2 ABELIN ABRAHA ALDERS AUVINE AXELSO BAND BERRIN BLOT1 BLOT2 BLOT3 BLOT4 BRESLO BROCKM BROWN1 BYERS1 BYERS2
CASCO2 CASCOR CHAN CHATZI CHEN CHEN2 CHEN3 CHIAZZ COOKSO DAVEYS DEAN DOSEME DU DUNN EBELIN ENSTRO
ESAKI FAN GENG GER GODLEY GOLLED GSELL HANSEN HEGMAN HINDS HIRAOK HIRAY2 HOROWI HORWIT HU HU2
HUANG ISHIMA JARUP JIANG JONES JUSSAW KANELL KO KOHLME KOULUM KREYBE LAMTH LAMWK LAMWK2 LAUSSM LEI
LETOUR LEVIN LIAW LICKIN LIU LIU2 LIU3 LIU4 LIU5 LOMBA2 LUO MAGNUS MARSH MARSH2 MARTIS MASTRA
MATSUD MCCONN MCDUFF MCLAUG MILLER MILLS MOLLO MRFIT MURATA NOTAN2 NOTANI NOU ORMOS PASTOR PAWLEG PERNU
PERSHA PIKE POFFIJ POLEDN QIAO RADZIK RANDIG REN RIMING RONCO ROOTS ROTHSC SAARIK SADOWS SANKAR SCHWA2
SEGI SEGI2 SEOW SHIMIZ SIEMIA SIMARA SITAS SOBUE2 STASZE STAYNE STOCKS SUN TANG2 TAO ULMER VANDER
WALD WANG WANG3 WANG4 WARSIN WATSON WICKLU WILKIN WU2 WUWILL WYNDE2 WYNDE4 WYNDE5 WYNDE8 WYNDER XIANGZ
XU XU2 XU3 XU4 YUAN ZHANG ZHENG ZHOU

3 RESTRE

4 AKIBA AMANDU AMES ANDERS ARCHER ARMADA AUSTIN AXELSS BARBON BECHER BENSHL BLOHMK BOFFET BRETT BROSS BROWN2
BUFFLE CARPEN CEDERL CHANG CHOI CHYOU COMSTO CORREA DARBY DEAN2 DEKLER DESTE2 DESTEF DOCKER DOLL DORANT
DORGAN DROSTE ENGELA GAO GAO2 GARCIA GARDIN GARSHI GILLIS GOODMA GREGOR HAENSZ HAMMO2 HEIN HENNEK HIRAYA
HITOSU HOLE HUMBLE JAHN JAIN JARVHO JEDRYC JOLY KAISER KATSOU KAUFMA KELLER KHUDER KIHARA KINLEN KJUUS
KNEKT KOO KREUZE KUBIK LANGE LEMARC LIDDEL LUBIN MACLEN MATOS MIGRAN MRFITR NAM ODRISC OSANN OSANN2
PARKIN PERSH2 PETO PEZZO2 PISANI PRESCO QIAO2 RACHTA SCHWAR SHAW SIMONA SOBUE SPEIZE SPITZ STOCKW STUCKE
SUZUK2 SUZUKI SVENSS TANG TENKAN TIZZAN TOKARS TOUSEY TSUGAN TULINI VEIERO VUTUC WAKAI WANG2 WU WUNSCH
WYNDE3 WYNDE6 YAMAGU YONG

8 HAMMON

10 BENHAM

Table 1D3 - 8
Potentially overlapping studies

REF	REFGP	PRINC	. OVERLAP/LINK
LUBIN2	LUBIN2	1	Lubin-combined
TVERDA	TVERDA	1	VEIERO/TVERDAL
GRAHAM	BYERS1	1	GRAHAM/BROSS/BYERS1
WYNDE7	WYNDE6	2	WYNDE5/6/7/8
CPSI	CPSI	1	CPSI overall
